# The developmental transcriptome of the human heart

**DOI:** 10.1038/s41598-018-33837-6

**Published:** 2018-10-18

**Authors:** Eleftheria Pervolaraki, James Dachtler, Richard A. Anderson, Arun V. Holden

**Affiliations:** 1 School of Biomedical Sciences, University of Leeds, Leeds, LS2 9JT UK; 2 Department of Psychology, Durham University, Durham, DH1 3LE UK; 3 MRC Centre for Reproductive Health, University of Edinburgh, Edinburgh, EH16 4TJ UK

## Abstract

The human heart develops through complex mechanisms producing morphological and functional changes during gestation. We have recently demonstrated using diffusion tensor MRI that over the relatively short space of 40 days, between 100–140 days gestational age, the ventricular myocardium transforms from a disorganised tissue to the ordered structure characteristic of mature cardiac tissue. However, the genetic basis underpinning this maturation is unclear. Herein, we have used RNA-Seq to establish the developmentally-regulated transcriptome of gene expression in the developing human heart across three gestational ages in the first and second trimester. By comparing 9 weeks gestational age (WGA) with 12 WGA, we find 288 genes show significant differential expression. 305 genes were significantly altered comparing 12 and 16 WGA, and 806 genes differentially expressed between 9 and 16 WGA. Network analysis was used to identify genetic interactions, node properties and gene ontology categories. In summary, we present a comprehensive transcriptomic analysis of human heart development during early gestation, and identify differentially expressed genes during heart development between 9 and 16 weeks, overlapping the first and early second trimester.

## Introduction

In the developing human embryo, the heart is the first functional organ, with cell proliferation and differentiation establishing the different regions of the heart, the contractile atrial and ventricular chamber and the pacemaking and conducting system^1^. The canonical four chambered heart becomes recognisable after 8 weeks gestational age (WGA)^2^. Previously, diffusion tensor MRI has been used to reveal the developmental timeline of the human fetal heart^3,4^. The development of the human heart geometry and architecture occurs between 100 and 140 days gestational age (DGA; equivalent to 14–20 WGA)^3,4^, with fractional anisotropy significantly increasing linearly, whilst apparent diffusion coefficient decreases linearly^3^. Computational tractography of cardiac fibres can be carried out from 18 WGA^3^. Together, these findings show that within this relatively short period, myocyte orientation becomes more organised within the ventricular walls and septum, potentially explaining the maturation of ventricular blood flow and the slowing of the heart rate in late pregnancy^5,6^.

Previously, we examined the expression of two proteins that are co-expressed at myocyte gap junctions; connexin 40 (Cx40) and connexin 43 (Cx43). Cx40 is notable as it is the major ventricular gap junction protein^7^ and is required for the development of the Purkinje fibre network^8^. We found that both Cx40 and Cx43 were significantly upregulated between 10 and 19 WGA, providing one of the molecular mechanisms underpinning heart development^3^. However, it is implausible that these are the only proteins contributing to development.

Each developmental stage of the heart is characterised by changes in gene expression profiles^9^. The cardiac transcriptome regulatory network of the embryonic murine heart has previously been described^10,11^, providing information on transcription factor expression profiles and their role in cardiac pathogenesis. The existence of a ‘fetal gene programme’ is well established^12^ and analysis of individual genes relating to health and disease or metabolic processes has provided support for the concept, and insights into the development of pathologies^13^.

As yet, transcriptomic approaches have not been broadly applied to the human heart to specifically study gene regulation across early development. However, these methods have been applied to examine the genetic regulation of myocyte development, particularly for methylation and cardiac enhancers^14,15^. Further, several studies compare fetal gene expression as a grouped age to either adult or diseased tissue, or lab animal species (e.g. mice^15,16^). This has three potential problems. First, by not examining expression across developmental periods (i.e. across different trimesters), we lack information on the evolution of gene regulation across gestation. Second, comparisons between fetal (as a grouped or pooled sample) and adult tissue will likely dilute the ability to identify tightly temporally-regulated gene expression in gestation, which may constrain interpretations about developmental genetics. Third, the age of fetal tissue could be a significant confound. Few studies examine multiple time points within gestation. Comparisons of a single time point in development to adult (or younger but mature) time points or even to animal species could be spurious. For example, we previously observed significant upregulation of Cx40 between 8 and 19 WGA^3^. Thus, a comparison of 19 WGA fetal heart to adult tissue would draw very different conclusions regarding the importance of this gene compared to 8 WGA tissue.

Herein, we have applied transcriptomic methods (RNA-Seq) to human fetal hearts at three developmental time points; 9, 12 and 16 WGA. The importance of performing this study specifically with human tissue is two-fold. First, we have previously demonstrated that the developmental timeline of the human heart does not map to other lab-animal species (pigs)^4^. Second, clinical interventions (for example, fetal cardiovascular pharmacology) need to be led by the developmental expression timelines of different genes (e.g. ion channels), which are currently unknown. We compare differential gene expression between each time point and employ bioinformatics to understand gene ontology of fetal heart development.

## Results

**Figure 1:**
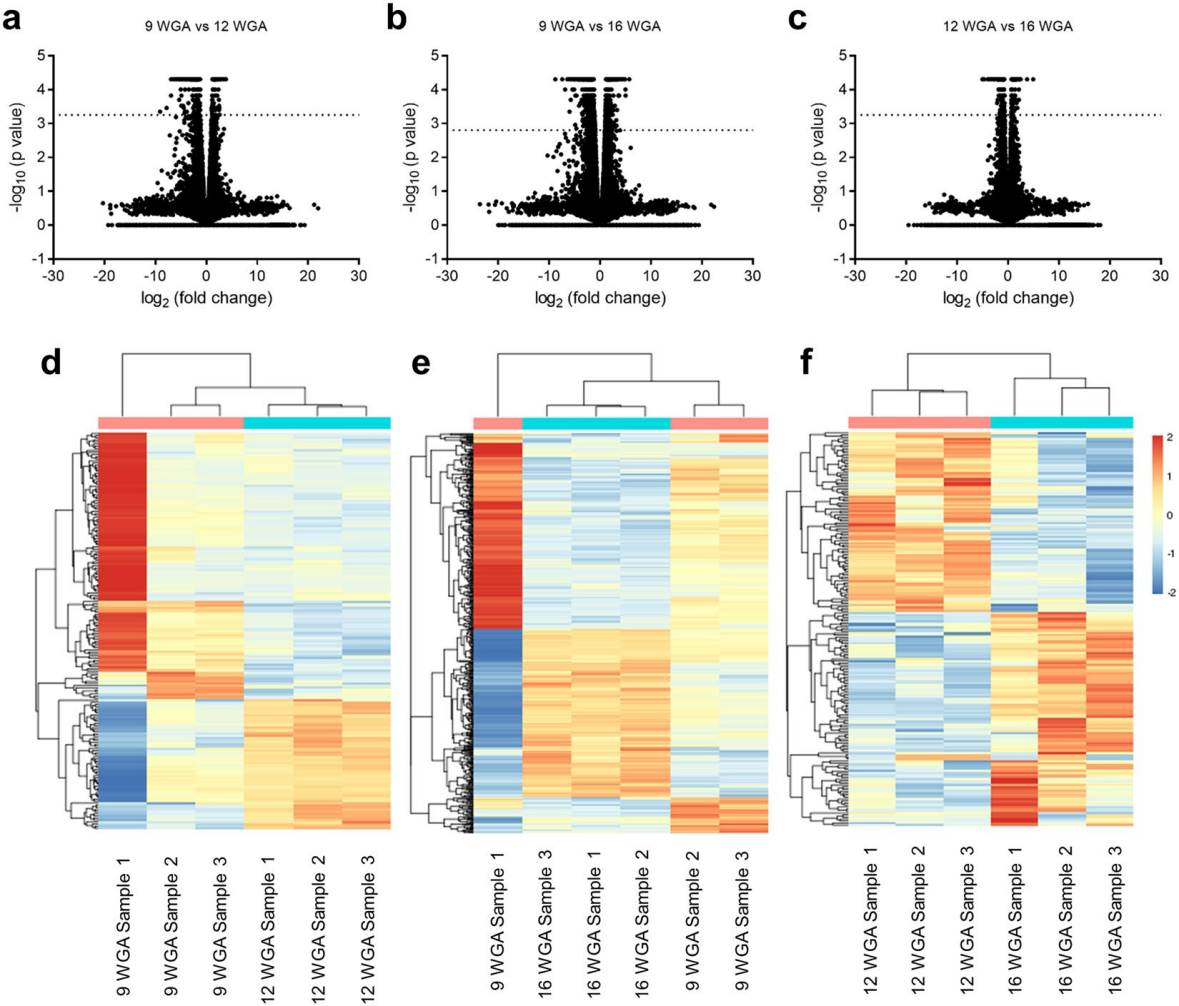
Differential gene expression during development. Volcano plots (log2 fold change against -log10 of the P value) of the ~57,000 transcripts comparing (**a**) 9 and 12 weeks gestation age (WGA), (**b**) 9 and 16 WGA and (**c**) 12 and 16 WGA. Differential expression was considered significant for genes above the line, representing the Bonferroni correct significance cut-off. (**d**–**f**) Heat map representation of gene expression (upregulation in red and down regulation in blue) of the individual samples comparing 9 and 12 WGA (**d**), 9 and 16 WGA (**e**) and 12 and 16 WGA (**f**). The denograms indicate the clustering of samples and time points (top) and genes (left).

To identify gene expression changes of the human fetal heart, we performed RNA-Seq on mRNA derived from hearts at 9, 12 and 16 weeks gestational age (WGA). Our RNA-Seq identified more than 57,000 transcripts at the three developmental stages (Fig. 1a–c). We then profiled differential gene expression comparing 9 and 12, 9 and 16 and 12 and 16 WGA (Fig. 1d–f). A total of 288 genes (Fig. 1d) were found to be differentially expressed in the fetal hearts at 9 WGA when compared to 12 WGA (Extended Data Table 1). Of these, 189 genes were found to be downregulated and 100 genes were found to be upregulated. A total of 806 genes (Fig. 1e) were found differentially expressed in the fetal hearts at 9 WGA when compared to 16 WGA (Extended Data Table 2). Of these, 444 genes were found to be downregulated and the rest were found to be upregulated. A total of 305 genes (Fig. 1f) were found to have significant differential expression at 12 WGA when compared to 16 WGA (Extended Data Table 3). Of these, 118 genes were found to be downregulated and the rest were found to be upregulated.

To predict the biological pathways involved in the development of the human heart, we used Gene Ontology (GO) tools (DAVID Bioinformatics Software and PANTHER). Genes were categorised according to their predicted functionality (Extended Data Fig. 1). Binding (GO: 0005488) and catalytic activity (GO: 0003824) are the predominant molecular functions (Extended Data Fig. 1a,d,g); cellular (GO: 0009987) and metabolic (GO: 0008152) processes the predominant biological processes (Extended Data Fig. 1b,e,h); cellular processes (Extended Data Fig. 1c,f,i) were predominantly matched to GO groups related to organelle morphogenesis.

To further explore the potential roles of the differentially expressed genes (DEGs), we undertook predictive network analysis to identify gene hubs (nodes) and potential gene-gene interactions (edges) based upon published interactions using Cytoscape and GeneMANIA^17,18^. We also identified 20 additional genes most likely to interact with our DEGs to make a functional network, and examined GO functions within that network. We took two approaches in our analysis. First, we explored the network of all significantly upregulated and downregulated DEGs, and subsequently, specifically examining significantly upregulated and downregulated DEGs at each time comparison (for full gene network and GO data, see Extended Data Tables 4–6). Between 9 and 12 WGA, we identified 300 nodes and 6054 edges, with *JUN* (Entrez: 3725) being the largest hub. Muscle structure development (GO: 0061061) and muscle organ development (GO: 0007517) were the leading GO terms. For upregulated DEGs, we identified 113 nodes and 1473 edges (Extended Data Fig. 2a), with the gene *MGP* (Entrez: 4256) exhibiting the greatest hub connectivity (Extended Data Table 4). Conversely, for downregulated DEGs, there were 207 nodes and 3234 edges (Extended Data Fig. 3a), with *JUN* (Entrez: 3725) being the largest hub gene and GO suggesting a switch in response to zinc (GO: 0071294) (Extended Data Table 4). For all DEGs between 9 and 16 weeks, 778 nodes and 35,912 edges were identified, with *CDH13* (Entrez: 1012) being the hub gene, and again muscle structure development (GO: 0061061) being the top GO term. 353 nodes and 9121 edges were identified for upregulated DEGs, with *SGCG* (Entrez: 6445) being the largest hub gene (Extended Data Fig. 2b and Table 5). Of the downregulated DEGs, there were 445 nodes and 13,244 edges. Again, *JUN* was the largest hub, with GO functions suggesting changes to the Wnt signalling pathway (Extended Data Fig. 3b and Table 5). Finally, between 12 and 16 WGA for all significant DEGs, we identified 302 nodes and 6949 nodes. The hub gene was *FMOD* (Entrez: 2331), with the top GO term being extracellular matrix (GO: 0031012). For upregulated DEGs, a network of 145 nodes and 2066 edges was found, with *MYOM1* (Entrez: 8736) being the largest hub (Extended Data Fig. 2c and Table 6). The downregulated DEG network consisted of 177 nodes and 3283 edges, with *HBB* (Entrez: 3043) being the hub gene (Extended Data Fig. 3c and Table 6).

**Figure 2:**
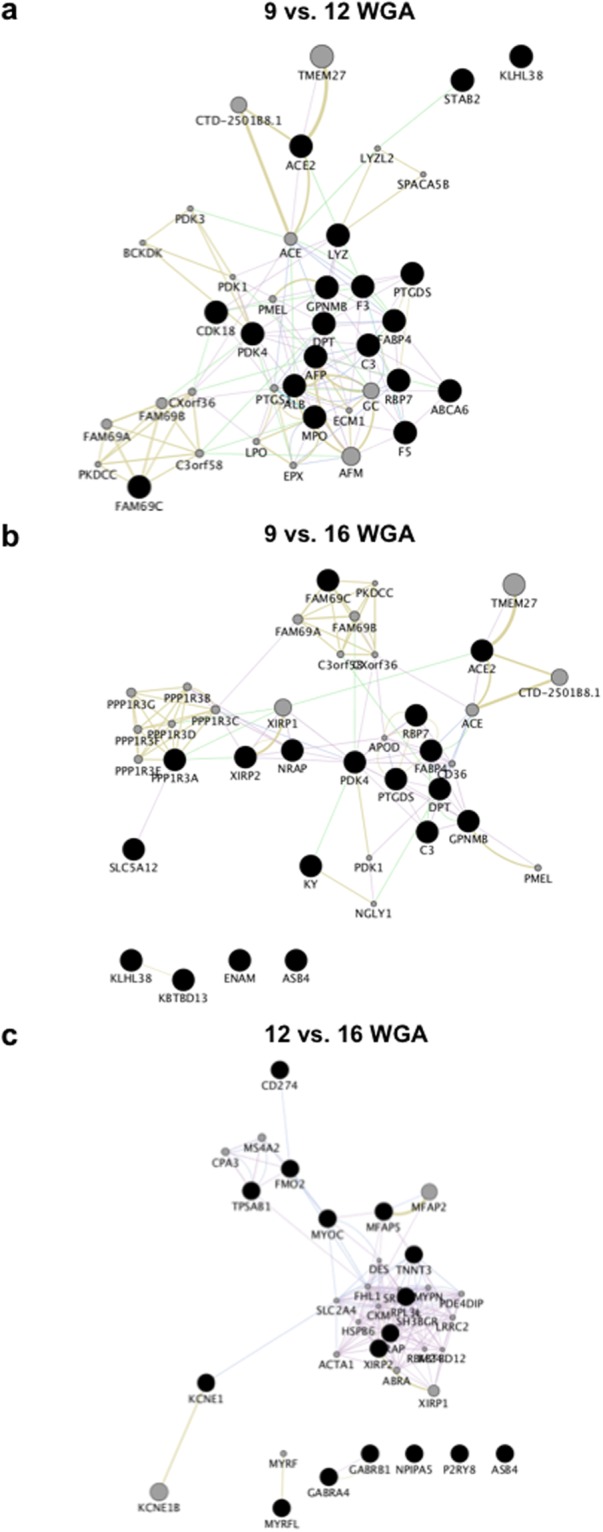
Predictive gene network analysis for significantly upregulated genes. The 20 genes with the greatest log2 fold increase between age comparisons (black circles; nodes) were used to identify functional connectivity within the network. Connectivity predictions (lines; edges between nodes) were facilitated by identifying 20 additional genes (grey circles) of known gene-gene or protein-protein interactions that best create a functional network to identify hub nodes. (**a**–**c**) Network analysis for 9 compared to 12 weeks gestation age (WGA), 9 compared to 16 WGA, and 12 compared to 16 WGA. Note for all analysis, some genes did not have known interactions to the networks, and hence sit unconnected to the network (e.g. bottom of **c**). Edges are colour coded to signify the predicted connections between nodes; co-expression: purple; co-localisation: blue; genetic interactions: green; shared protein domains: yellow; predicted: orange; physical interactions: pink; pathway: light blue.

**Figure 3:**
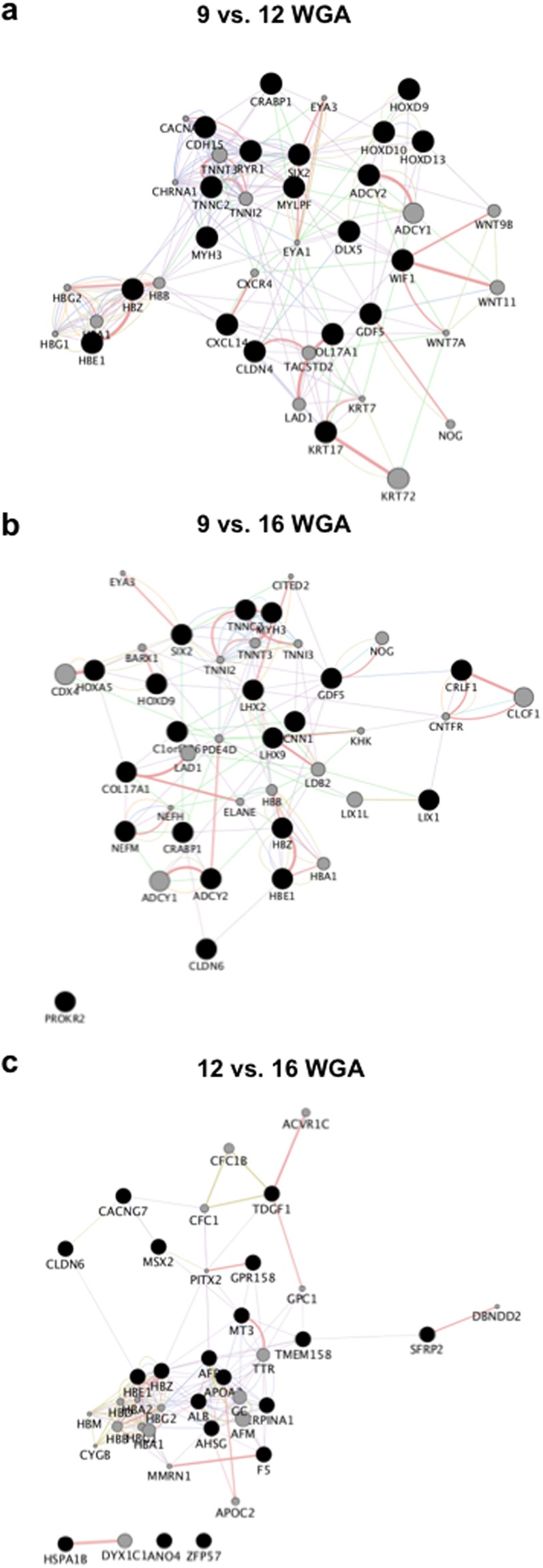
Predictive gene network analysis for significantly downregulated genes. We performed that same predictive gene network analysis as in Fig. 2, except the 20 genes with the greatest log2 fold decrease between age comparisons (black circles) were used. (**a**–**c**) Network analysis for 9 compared to 12 weeks gestation age (WGA), 9 compared to 16 WGA, and 12 compared to 16 WGA. The most significant gene ontology functions were analysed from the resultant network (see Main Text). Edges are colour coded to signify the predicted connections between nodes; co-expression: purple; co-localisation: blue; genetic interactions: green; shared protein domains: yellow; predicted: orange; physical interactions: pink; pathway: light blue.

Second, to constrain our interpretations, we then focused our network prediction analysis to the 20 significantly upregulated and downregulated DEGs with the highest log2 fold change. Between 9 and 12 WGA, our network analysis of upregulated DEGs finds functions related to acetyl-CoA as the most significant GO (q = 0.0075), centring on *PDK4* (Fig. 2a, Extended Data Table 7). Within the downregulated DEG network, *SIX2* (Entrez: 10736) was the hub gene, with GO function reductions linked to musculoskeletal movement (q < 0.0001) (Fig. 3a, Extended Data Table 7). Comparing 9 and 16 WGA, *PDK4* was again the hub gene, with glucose metabolic process being the major GO function (q = 0.009), although 4 genes were outside of the predictive network (Fig. 2b, Extended Data Table 8). Of the downregulated DEG network, the hub gene was *TNNT3* (Entrez: 7140), with GO suggesting changes in actin-mediated cell contraction (q < 0.0001) (Fig. 3b, Extended Data Table 8). Finally, for the comparison of DEGs between 12 and 16 WGA, our upregulated network predicts *FHL1* (Entrez: 2273) as the hub gene, with contractile fibre and myofibril being the most significant GO terms (q < 0.0001) (Fig. 2c, Extended Data Table 9). In the downregulated network, *HBZ* (Entrez: 3050) was the predicted hub gene, with the most significant GO functional change being blood microparticle (q < 0.0001) (Fig. 3c, Extended Data Table 9).

## Discussion

Together, this network analysis presents a preliminary characterisation of the transcriptional regulation of the human heart during the first and early second trimester. To facilitate investigation into our dataset, we provide R plots for all significant DEGs in Extended Data File 10. To provide an example of the potential of the dataset, we examined the transcripts that had the strongest expression difference between 9 and 16 WGA.

The gene which showed the largest log2 fold change between 9 and 16 WGA was nebulin related anchoring protein (*NRAP*, Entrez: 4892). *NRAP* is expressed at intercalated discs in cardiac muscle and is an actin-binding cytoskeletal protein^19,20^. During development, *NRAP* is involved in the myofibrillar assembly in the embryonic murine heart^21^. Interestingly, a patient with dilated cardiomyopathy with biventricular failure was recently found to have a homozygous truncating mutation (rs201084642) which introduced a stop codon to all *NRAP* isoforms^22^. Although relatively little is known about this gene, our study highlights its clear importance in embryonic development and its potential role in cardiac disease. Conversely, the two transcripts that exhibit the greatest downregulation between 9 and 16 WGA belong to the hemoglobin family (hemoglobin subunit zeta (*HBZ*), Entrez ID: 3050; hemoglobin subunit epsilon 1 (*HBE1*), Entrez ID: 3046). Hemoglobin Gower-1 contains two epsilon and two zeta chains, and is the exclusive embryonic hemoglobin. The strong downregulation of these transcripts is likely to reflect the change from embryonic to fetal hemoglobin forms (e.g. Hb F (α2γ2), Gower-2 (α2ε2), Portland-1 (ζ2γ2), and Portland-2 (ζ2β2)^23^. Nevertheless, our results corroborate previous descriptions of the change in hemoglobin forms during development^24,25^ and further this by highlighting this to be the strongest transcriptional change in the developing heart.

Our analysis furthers recent efforts to characterise the developing fetal heart. Whilst others have looked at development with a focus toward cardiac enhancers and methylation^14,15^, we have reported differential gene expression. Our use of RNA-Seq hold advantages over other genomic studies that have used microarrays^16,26^, given our methodology is not constrained by the limiting factors associated with microarrays. A notable recent RNA-Seq study found that 316 genes were specifically associated with the human fetal heart, and interestingly, 20 of those genes are known candidates for congestive heart failure^27^. However, as the dataset for this study originated from the ENCODE project, it is difficult to ascertain when in development these comparisons were made. Whilst we have made no association of our dataset to cardiovascular disease, our experimental approach would enable disease-related genes of interest to be examined across development to determine if down-regulation could identify a preceding cardiovascular condition.

An example of this, to highlight the potential of our dataset, can be assessed by exploring the role of ion channels in development. Comparing 9 and 16 WGA, the sodium voltage-gated channel beta subunit 2 (*SCN2B*) (Entrez ID: 6327) has ~2 fold increase in expression, and was the most significantly upregulated sodium channel subunit. *SCN2B* is particularly interesting, as it has several reported links to cardiac dysfunction. Brugada syndrome is associated with atrial and ventricular arrhythmia, and can lead to sudden cardiac death. Mutations in *SCN2B* have been characterised in human Brugada syndrome sufferers^28–30^, with the likely consequence of these mutations being to disrupt the ability of β2-subunits to chaperone voltage-gated sodium channel α-subunits, particularly Na_v_ 1.5, to the plasma membrane^31,32^. The underlying causes of Brugada syndrome, and sudden death syndromes generally, are poorly understood. By identifying timepoints in development where *SCN2B* expression occurs, we will be better placed to understand how mutations within a sodium channel subunit can alter myocyte function, which ultimately leads to Brugada syndrome.

A limitation of the current study, and generally those studies using human fetal tissue, is the limited quantities in which tissue can be obtained. Indeed, obtaining time matched tissue for the three time points has been the culmination of several years’ effort. However, low samples per group potentially risks false positives and negatives. Our analysis is likely robust for the genes with the greatest log2 fold change (e.g. *NRAP* has a log2 fold change of ~5), although additional studies with access to greater volumes of tissue will be able to further constrain the gene lists.

In summary, our transcriptomic analysis will present further opportunities to investigate new candidates for cardiac development. To facilitate this, the full transcript expression database is made available via the European Bioinformatics Institute (accession number E-MTAB-7031) for others to investigate.

## Materials and Methods

### Tissue Collection and Ethics

The acquisition of fetal tissue has been described elsewhere^3^. All samples were clinically assessed and found free of any visually identifiable abnormalities and dysmorphism. Storage and use of tissue was in premises licensed by the 2004 Human Tissues Act (UK) and all protocols had ethical committee approval.

The samples were acquired following elective termination of pregnancy, with informed written parental consent obtained from all subjects. All methods were carried out in accordance with relevant guidelines and regulations. All experimental protocols were approved by NHS Lothian, the University of Edinburgh Research Governance Hope and the University of Leeds ethical committee (Study Code LREC 08/S1101/1) and carried out in accordance with the relevant guidelines and regulations. The samples were obtained legally under the U.K. Abortion Act of 1967. In England, Wales and Scotland the Abortion Act 1967 allows a pregnancy to be terminated by a registered medical practitioner if the pregnancy has not exceeded its twenty-fourth week of gestation. All samples were collected after informed consent was secured by both parents (when possible) or just the mother (in the case of a single parent). All samples used in this study originated from abortions due to social reasons. Any samples from abortions performed due to medical reasons were not made available to the study. All mothers and fetuses were being medically examined throughout their pregnancy until the time of the elective termination. No defects were seen using ultrasound in utero during gestation. All fetuses appeared morphologically normal during regular checks with the midwife and upon medical termination of pregnancy as certified by the physician. Gestational age was measured by ultrasound scan before the procedure and confirmed by measuring foot length afterwards. 3 age matched hearts per time group (9 (63 ± 1 days; 1 male and 2 female), 12 (84 ± 1 days; 2 male and 1 female) and 16 (112 ± 1 days; 2 male and 1 female) weeks gestational age; the maximum number we could source over a two year period) were used in the study. Following tissue collection, samples were snap frozen, stored at −80 °C and transferred from Edinburgh to Leeds on dry ice, where they were subsequently stored at −80 °C until use.

### mRNA Preparation

Whole heart was homogenised by pestle and mortar in liquid nitrogen. 100 mg of the resulting powder was used to extract RNA. The extraction method was followed as outlined in the BioRad Aurum Total RNA Fatty and Fibrous Tissue Kit (cat no. 732–6830). Briefly, the sample was disrupted in PureZol, after which chloroform was added and the lysate centrifuged. RNA was extracted in the aqueous phase and an equal amount of ethanol was added. The mixture was transferred to a RNA binding column and centrifuged. Low stringency wash solution, DNase I, high stringency wash buffer followed by low stringency wash solution was used to purify the RNA, which was then eluted and stored at −80 °C until use. All RNA extraction were performed at the same time to eliminate the variable of interest, i.e. gestational age.

### RNA-Seq

Illumina compatible barcoded mRNA sequencing libraries were made from 100 ng of total RNA after polyA selection for mature mRNAs using the TruSeq Stranded mRNA Sample Preparation Kit’s high sample protocol (Illumina). Before pooling the libraries were visualised using a Tapestation (Agilent) to determine insert size, while their concentration was calculated using the Picogreen dsDNA quantification assay (Thermofisher Scientific). 2 nM of an equimolar pool of the libraries was sequenced on a single HiSeq3000 150 bp paired end lane with the data exported as a pair of fastq formatted data files for each sample. Sample quality was assessed prior to sequencing. RIN scores are provided in Table 1 alongside the number of reads obtained per sample.

**Table 1:** Sample quality and sequencing information for each sample.

Gestational Age (+/−1 day)	Sample	Sex	RIN Score	Mapped Reads
9 weeks	1	Female	8.3	21, 783, 318
	2	Male	8.2	24, 774, 584
	3	Female	8.8	25, 912, 645
12 weeks	1	Male	7.6	27, 595, 131
	2	Female	9.3	24, 869, 794
	3	Male	8.9	27, 020, 255
16 weeks	1	Male	8.6	25, 841, 584
	2	Male	9.2	25, 737, 663
	3	Female	9.0	23, 087, 778

The full transcript expression database has been deposited at the European Genome-Phenome Archive (EGA), hosted at the European Bioinformatics Institute (accession number E-MTAB-7031) for use by others, subject to reference of the current manuscript.

### Data Analysis and Quality Control

Low quality base calls and adaptor sequences where removed from the sequence data using Cutadapt^33^ with the resultant data checked for quality using FastQC^34^. Transcripts were excluded from any subsequent analysis if one point in the pairwise analysis did not have at least 5 reads for each of the samples. The false discovery rate (FDR) was set to a cut off of 0.05 for all transcripts. Specifically, we applied the Bonferroni correction for multiple comparisons, and only accepted genes with corrected significance (P value in tables) of α < 0.05. The data was then aligned to the human genome reference sequence (hg19) using the STAR aligner^35^ with reference to the hg19 annotation obtained from the UCSC Table Browser^36^. RNA expression values and differentially expressed genes were determined using the Cufflinks/CuffDiff analysis pipeline^37^, with the resultant data visualised with significance cut-off using the cummerbund R package^37,38^. These significant genes were then used in subsequent analyses. Genes that had a log2 fold change of ‘infinite’ were not included in the analysis, given one age comparison did not include a RNA value. Further information can be found in Extended Data.

To control for intra and inter time point variability, we further analysed the datasets. We undertook this given in Fig. 1d and e, 9 WGA sample 1 appears to have a different expression profile compared to 9 WGA samples 2 and 3. We have clustered the samples using the significantly differentially expressed genes (as identified by CuffDiff (see above)) across the whole dataset, and visualised the sample relationships as a dendrogram above a heat map of the differentially expressed genes (Extended Data Fig. 4a). It can be seen that sample variability by comparing within a time point is low, and that time points 12 WGA and 16 WGA cluster more closely together compared to 9 WGA. Although 9 WGA sample 1 does seem to differ from the other samples in the analysis, it was closely linked to the other samples within the time point. When the Cooks distance for each transcript within a sample is displayed as a boxplot (Extended Data, Fig. 4b), it can be seen that 9 WGA sample 1 is not unduly different from the other samples. The likely visual difference observed in Fig. 1d,e is driven by the very small variability between 9 WGA samples 2 and 3, and does not reflect unduly altered gene expression from 9 WGA sample 1.

### Gene Network Analysis

Gene ontology for all significant differentially expressed genes (significance as indicated by the Cufflinks/CuffDiff analysis) were analysed by DAVID (https://david.ncifcrf.gov/) and PANTHER (http://www.pantherdb.org/). The Java application Cytoscape and the plugin GeneMANIA^17,18^ were used to generate a predictive gene interaction network from the RNA transcripts that showed significant differential expression between the age comparisons. Network analysis was conducted by analysing the total upregulated and downregulated differentially expressed genes for each age comparison, followed by the 20 genes with the greatest log2 fold change. In both cases, the analysis method was the same. Transcripts were analysed if the transcript was characterised. Uncharacterised transcripts were excluded, but for the 20 gene network analysis, uncharacterised transcripts were not replaced with any further transcripts. 20 further genes were added into the network that best predicted interaction connectivity. GeneMANIA predicts network connectivity based upon published datasets covering co-expression, physical interactions, genetic interactions, shared protein domains, co-localisation, pathway and predicted. Networks were weighted by the automatically selected weighting method. Nodes and edges were derived from the resultant network. Hub genes were calculated by analysing the network as ‘undirected’, with the gene with the greatest number of interactions (degree) being considered the hub. This hub included all genes in the network, which might be a node suggested by the predictive analysis that best connects the other nodes. The size of the predicted nodes (grey circles) reflects the weighting scores of the individual gene-gene comparisons, with scores assigned to each gene pair reflecting how often paths that start at a given gene node end up in one of the query nodes and how heavily weighted those paths were^39^. In short, the larger the predict gene node, the most gene-gene weight has been associated with that predicted connection. Weighting scores reflective node size can be found in the Extended Data. The weight of the edges were calculated by Cytoscape, with graphical representation shown by line thickness, and numerical ‘weight’ found in Extended Data tables. Gene ontology functions were calculated from the network analysis from a false discovery rate corrected hypergeometric test for enrichment, with Q-values calculated using the Benjamini-Hochberg procedure. Categories are displayed up to a Q-value cut-off of 0.1.

## Electronic supplementary material

Supplementary Materials

Supplementary Dataset 1

Supplementary Dataset 2

Supplementary Dataset 3

Supplementary Dataset 4

Supplementary Dataset 5

Supplementary Dataset 6

Supplementary Dataset 7

Supplementary Dataset 8

Supplementary Dataset 9

Supplementary Dataset 10

## Data Availability

Genome data has been deposited at the European Genome-Phenome Archive (EGA) which is hosted at the EBI and the CRG, under accession number E-MTAB-7031, for use by the scientific community subject to reference of the current paper.
