# Supplementary material for: The developmental transcriptome of the human heart
Source: Sci Rep. 2018 Oct 18;8:15362. doi: 10.1038/s41598-018-33837-6 (PMC6194117; doi:10.1038/s41598-018-33837-6)
Supplement: Supplementary file 11 — Supplementary Dataset 10 [file 41598_2018_33837_MOESM11_ESM.pdf]

The developmental transcriptome of the human heart

Extended Data

Eleftheria Pervolaraki Ph.D[1*], James Dachtler Ph.D[1,2], Richard A. Anderson MBChB, Ph.D[3], Arun V. Holden Ph.D[1]

[1]School of Biomedical Sciences, University of Leeds, Leeds, LS2 9JT, UK.
[2]Department of Psychology, Durham University, Durham, DH1 3LE, UK.
[3]MRC Centre for Reproductive Health, University of Edinburgh, Edinburgh, EH16 4TJ, UK.

*Correspondence should be addressed to Dr. Eleftheria Pervolaraki, School of Biomedical Sciences, University of Leeds, Leeds, LS2 9JT, UK.
fbsepe@leeds.ac.uk, +44(0) 113 34 31869

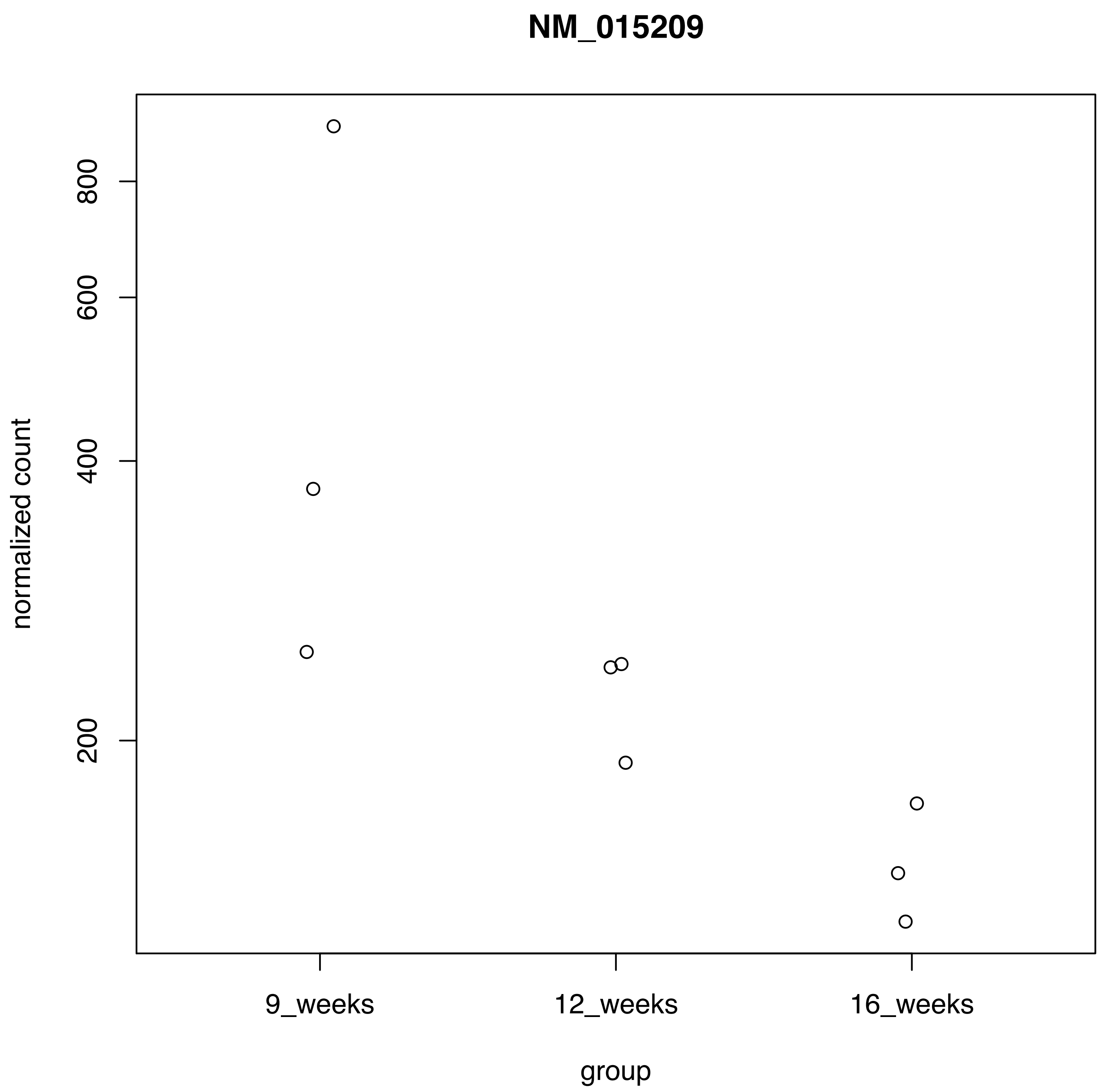
NM_015209
normalized count
800
600
400
200
9_weeks
12_weeks
16_weeks
group

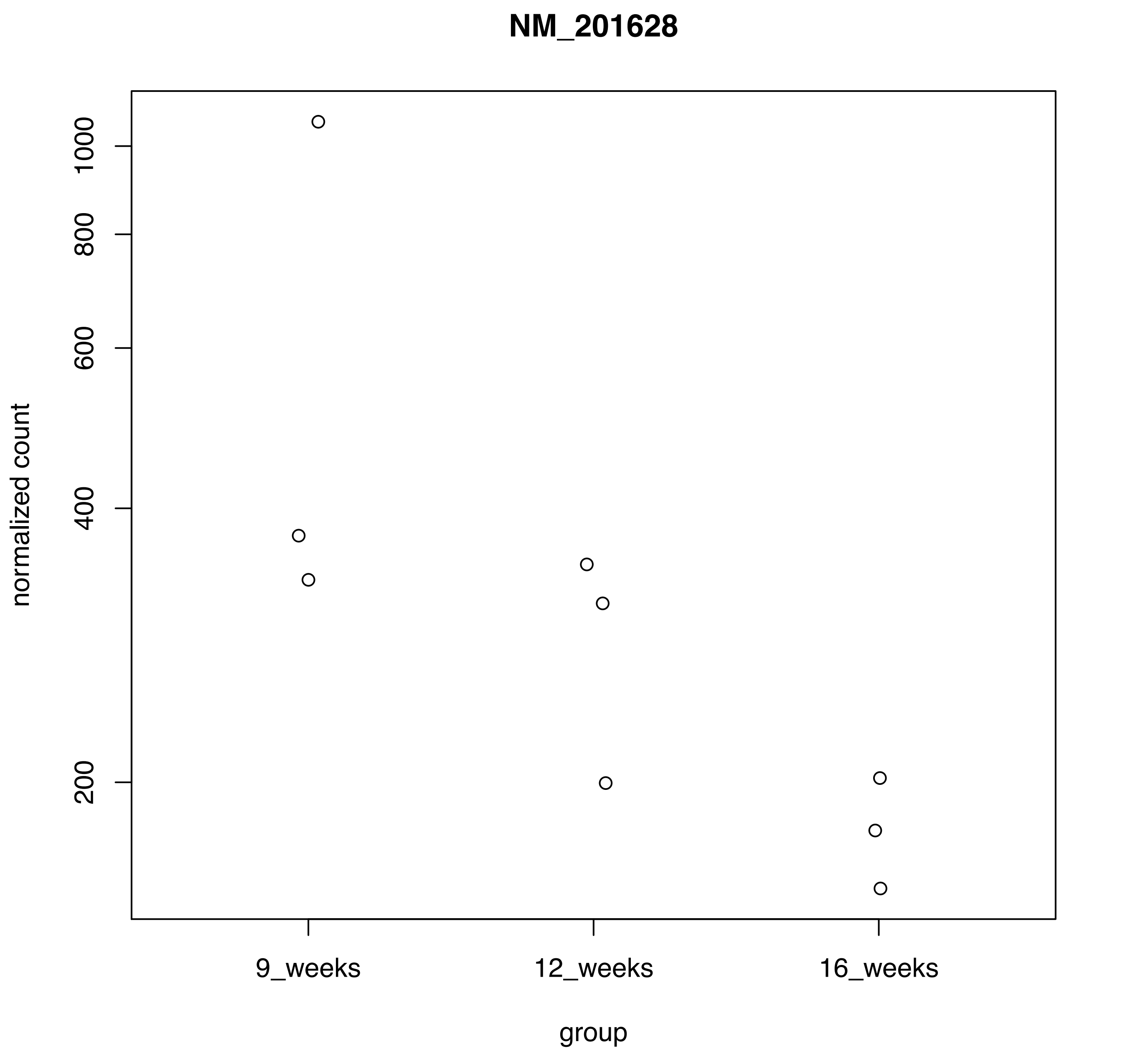

NM_201628
normalized count
1000
800
600
400
200
9_weeks
12_weeks
16_weeks
group

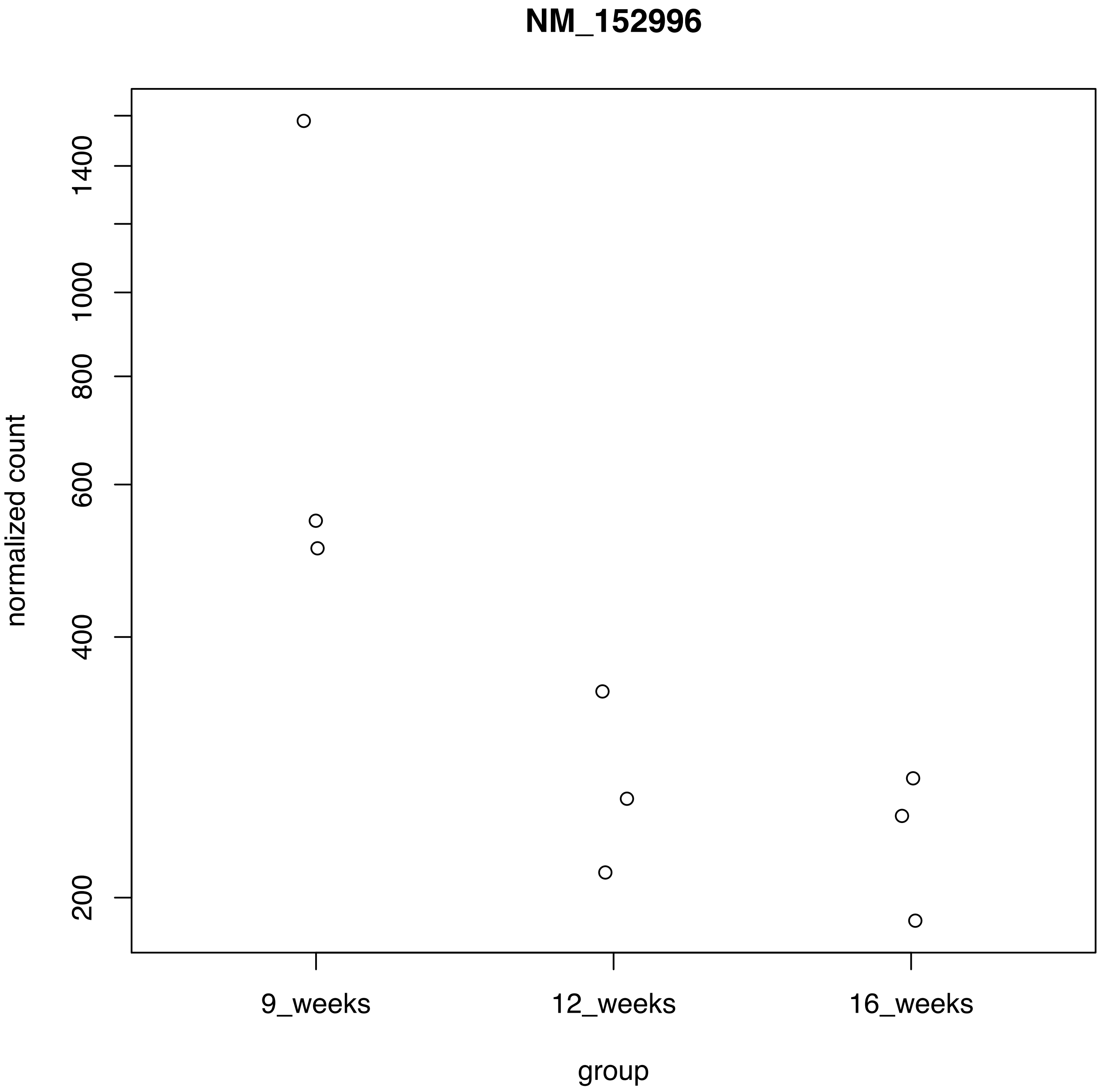

NM_152996
normalized count
1400
1000
800
600
400
200
9_weeks
12_weeks
16_weeks
group

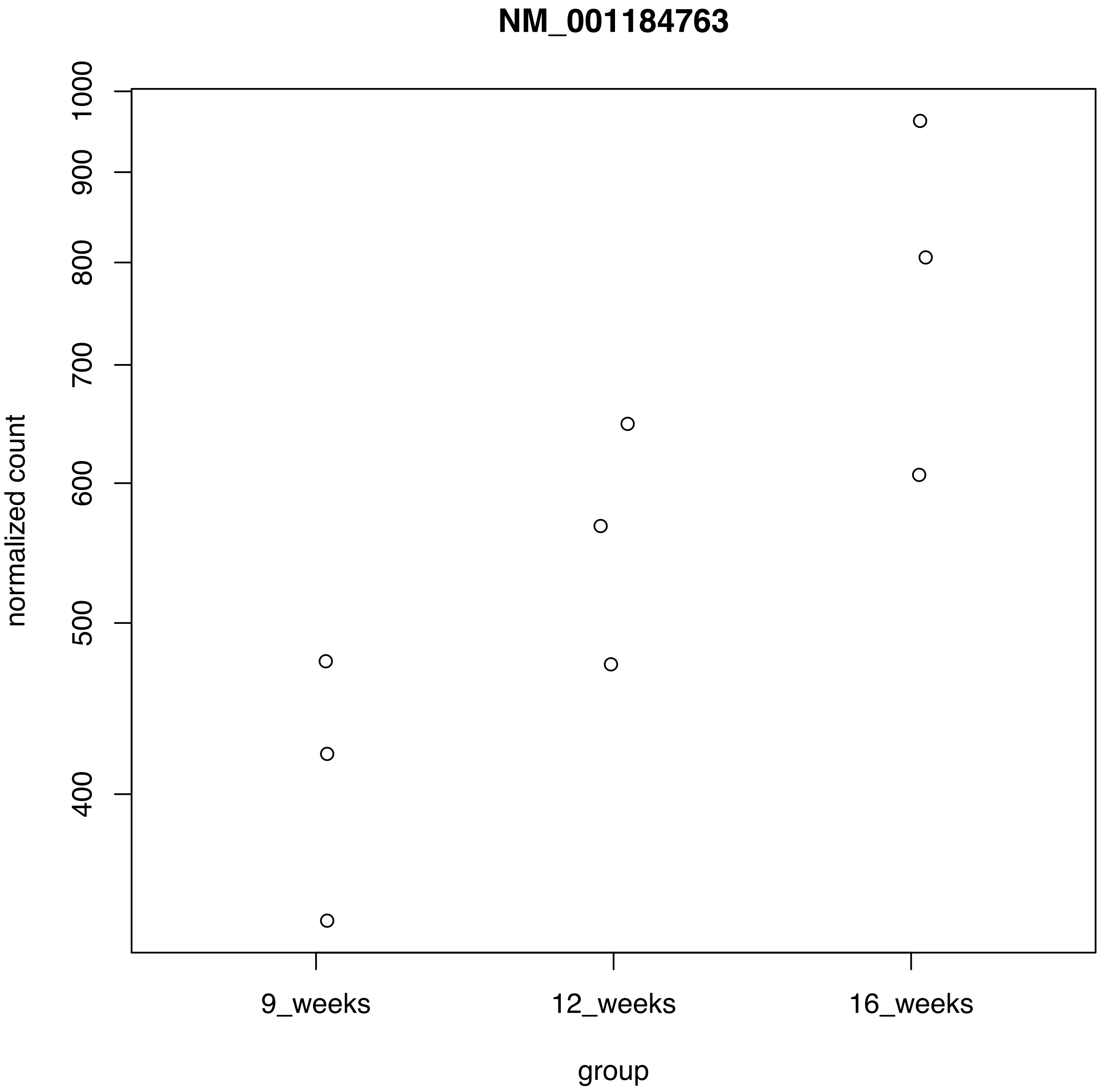
NM_001184763
normalized count
1000
900
800
700
600
500
400
9_weeks
12_weeks
16_weeks
group

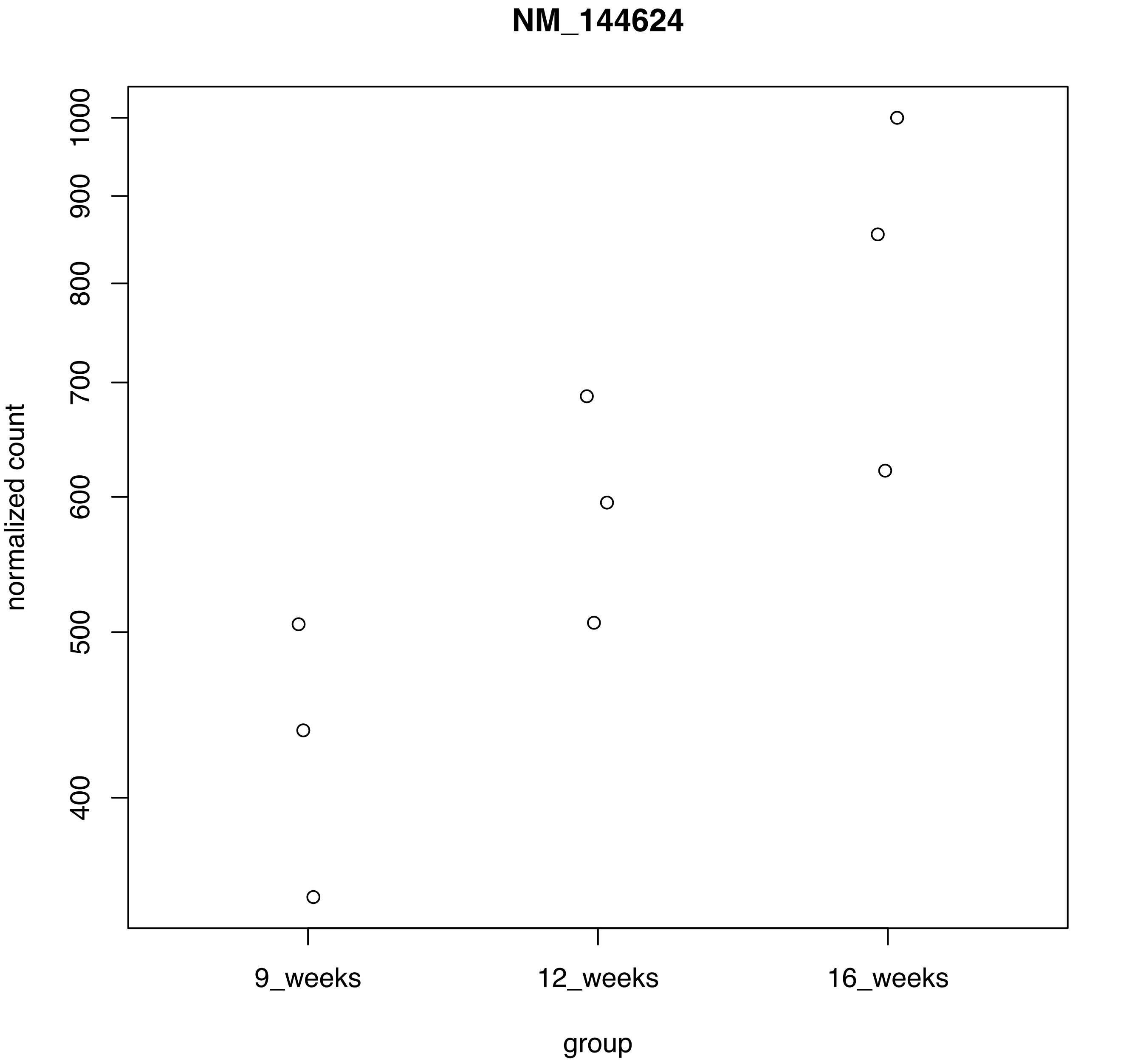
NM_144624
normalized count
1000
900
800
700
600
500
400
9_weeks
12_weeks
16_weeks
group

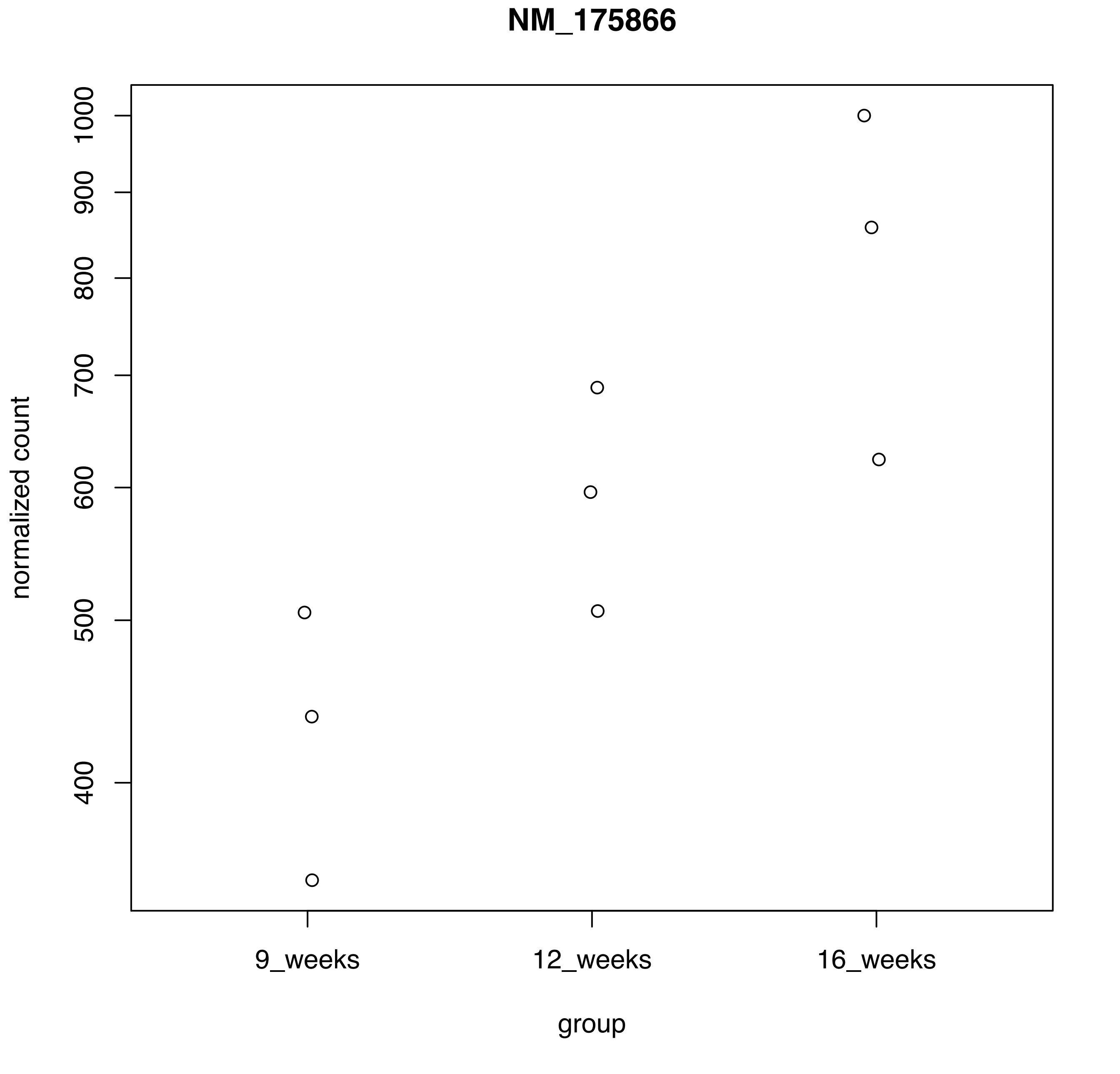
NM_175866
normalized count
1000
900
800
700
600
500
400
9_weeks
12_weeks
16_weeks
group

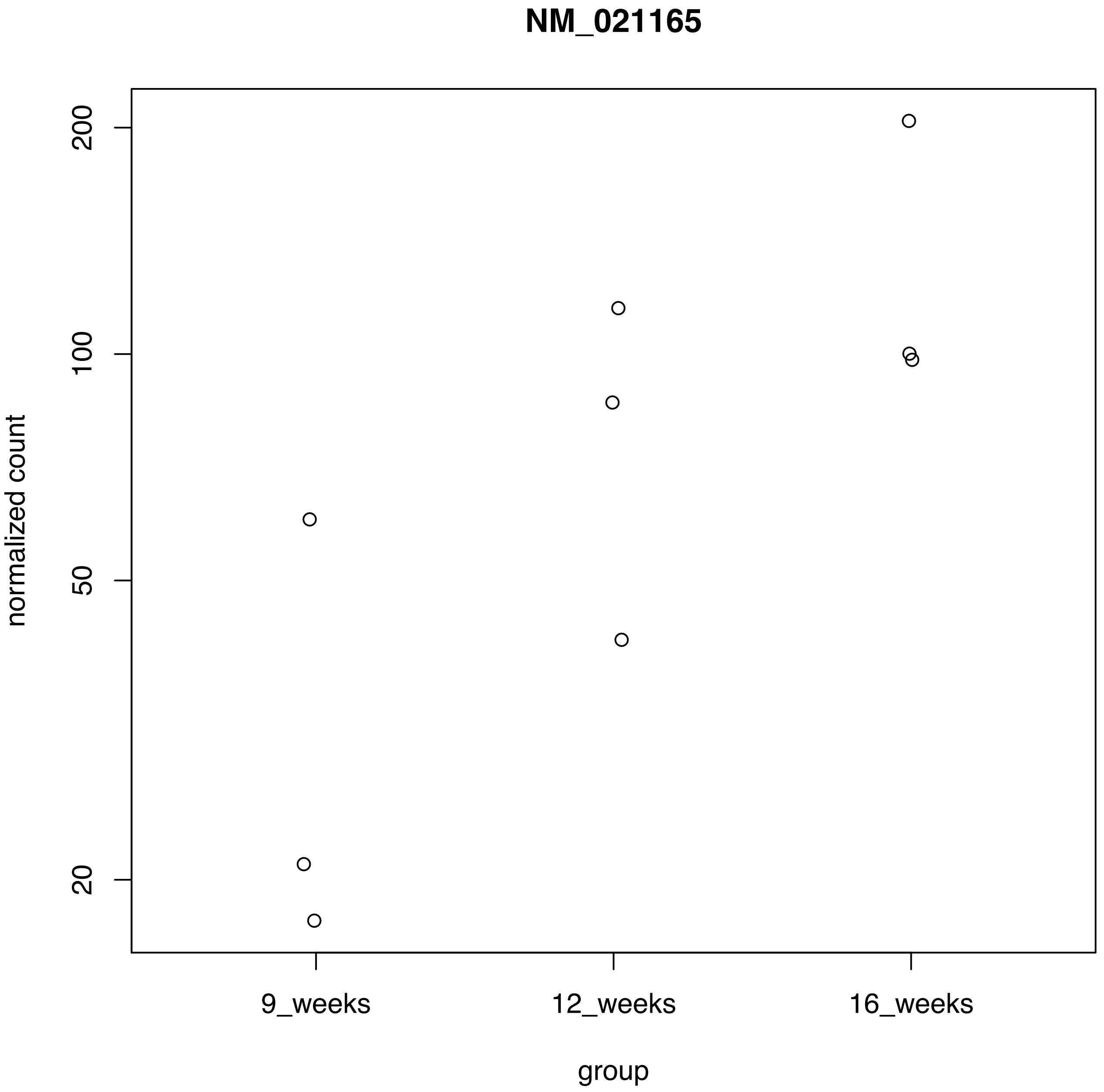

NM_021165
normalized count
200
100
50
20
9_weeks
12_weeks
16_weeks
group

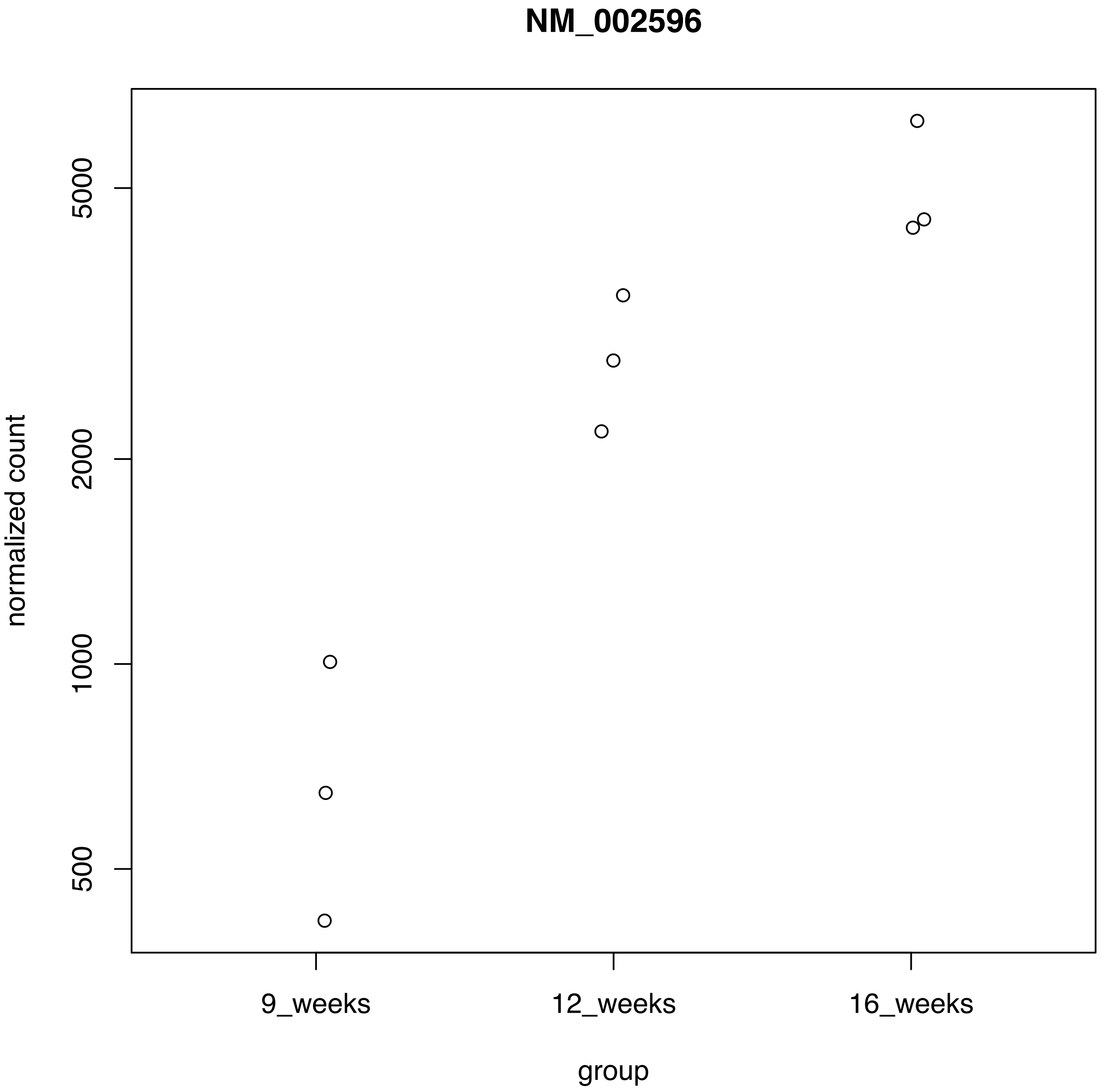
NM_002596
normalized count
5000
2000
1000
500
9_weeks
12_weeks
16_weeks
group

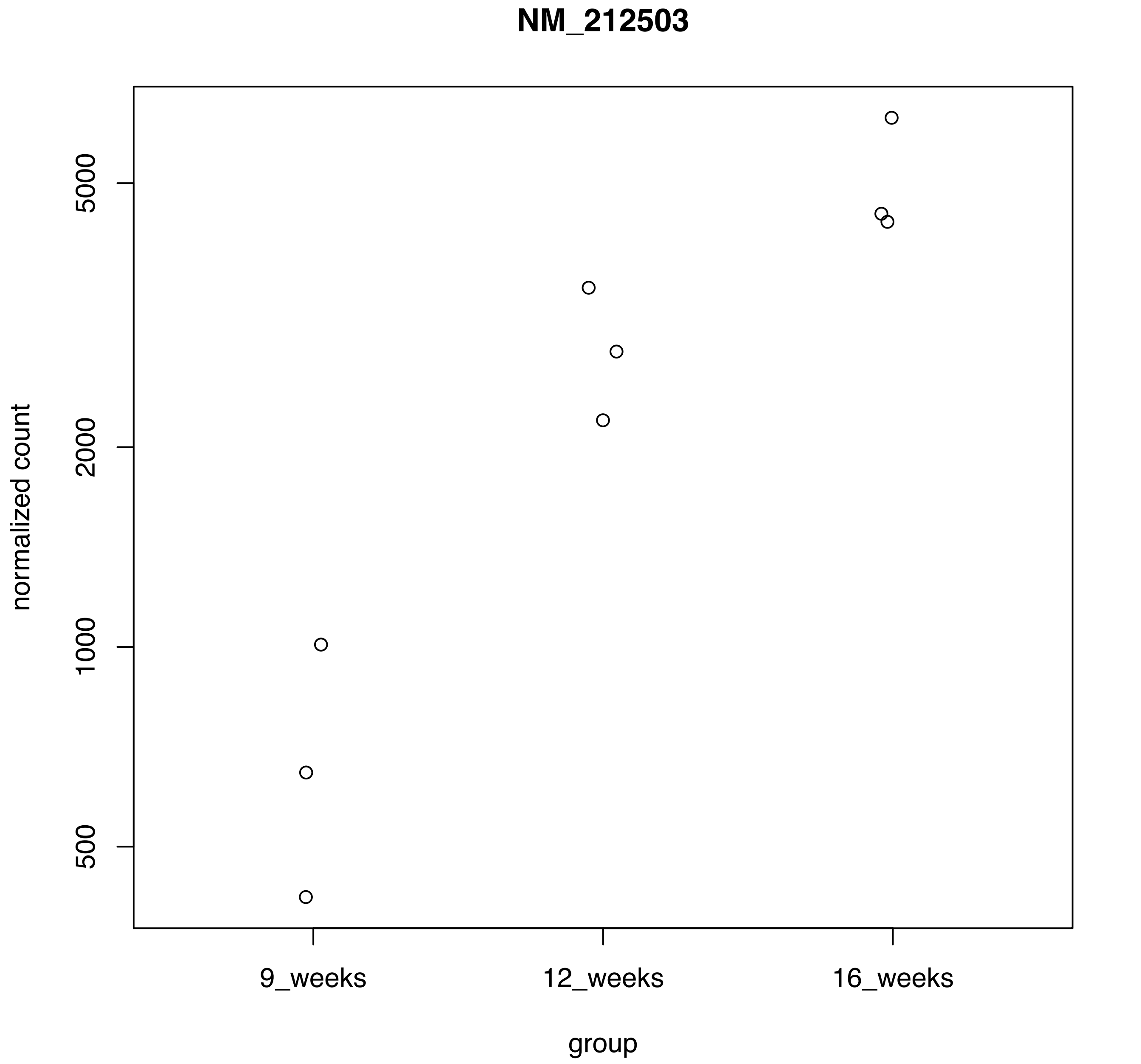
NM_212503
normalized count
5000
2000
1000
500
9_weeks
12_weeks
16_weeks
group

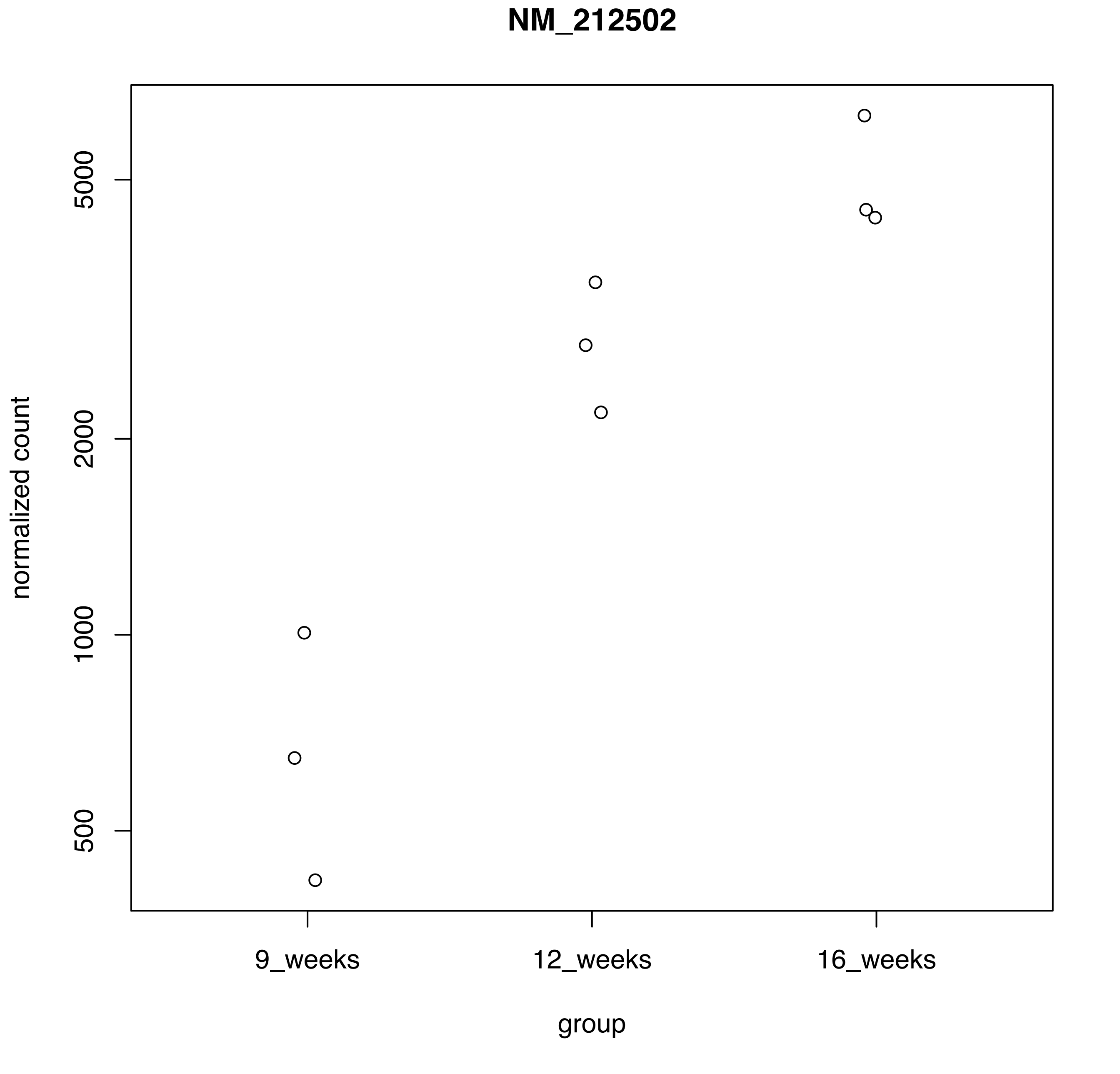

NM_212502
normalized count
5000
2000
1000
500
9_weeks
12_weeks
16_weeks
group

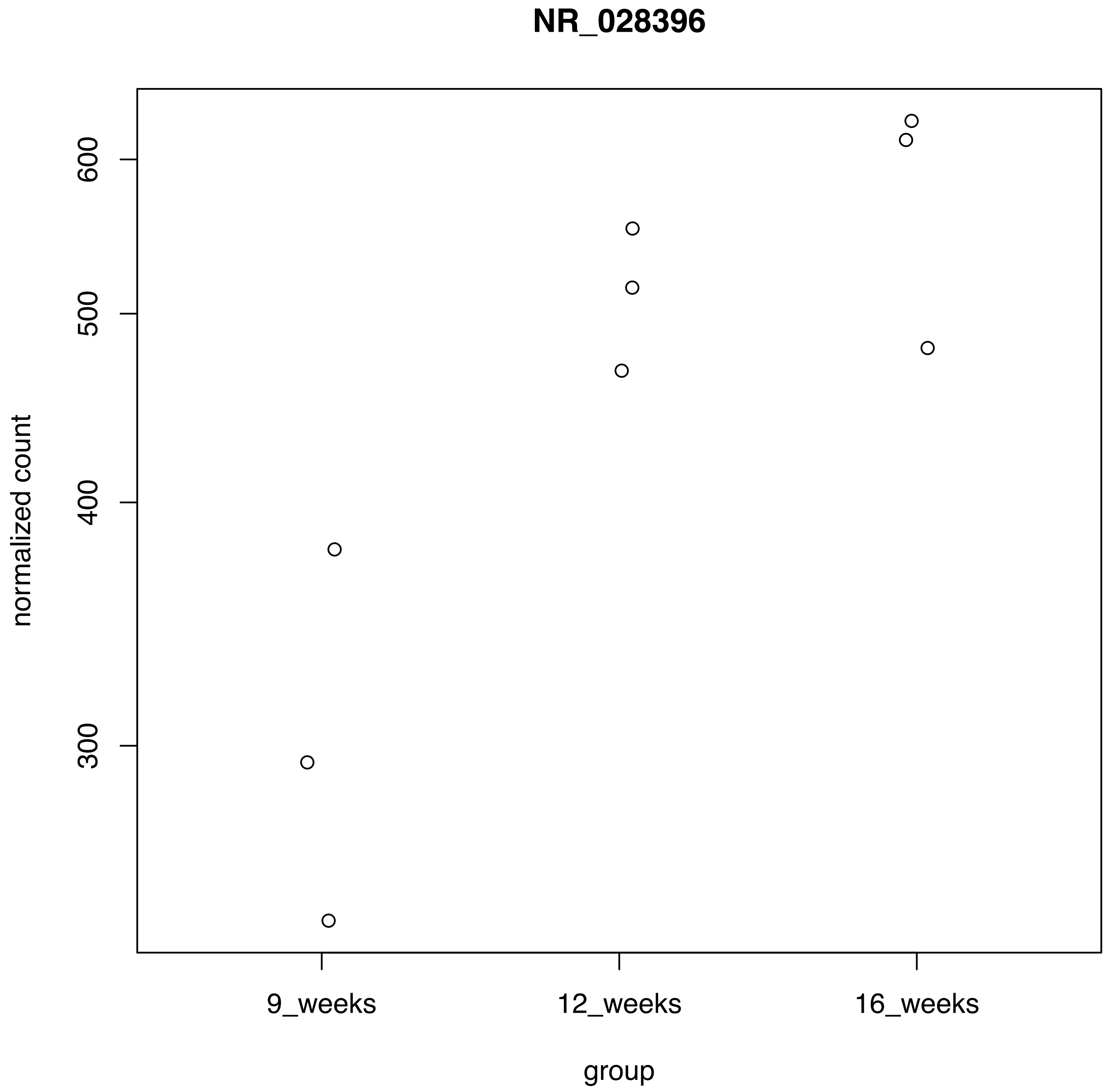

NR_028396
normalized count
600
500
400
300
9_weeks
12_weeks
16_weeks
group

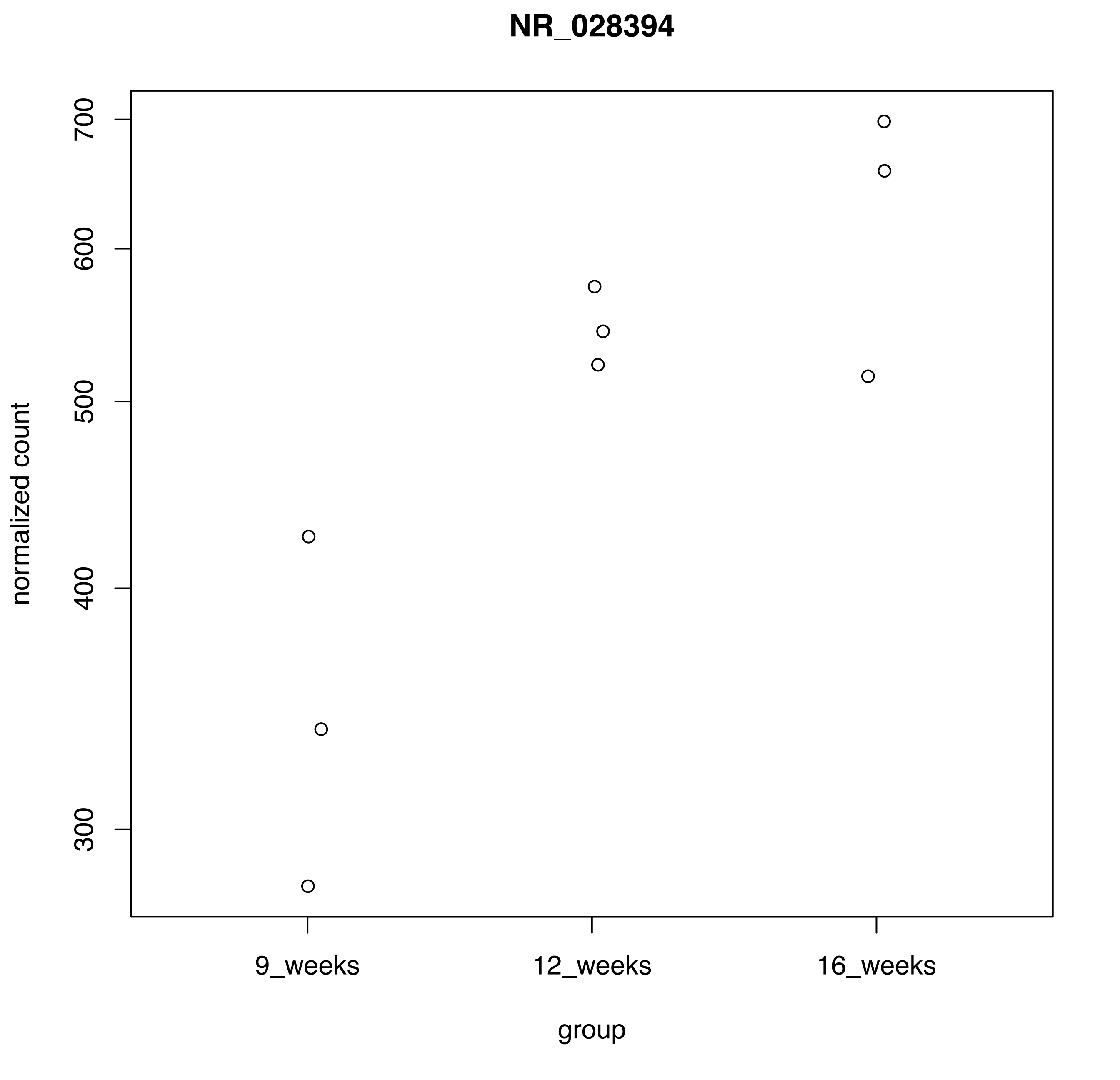
NR_028394
normalized count
700
600
500
400
300
9_weeks
12_weeks
16_weeks
group

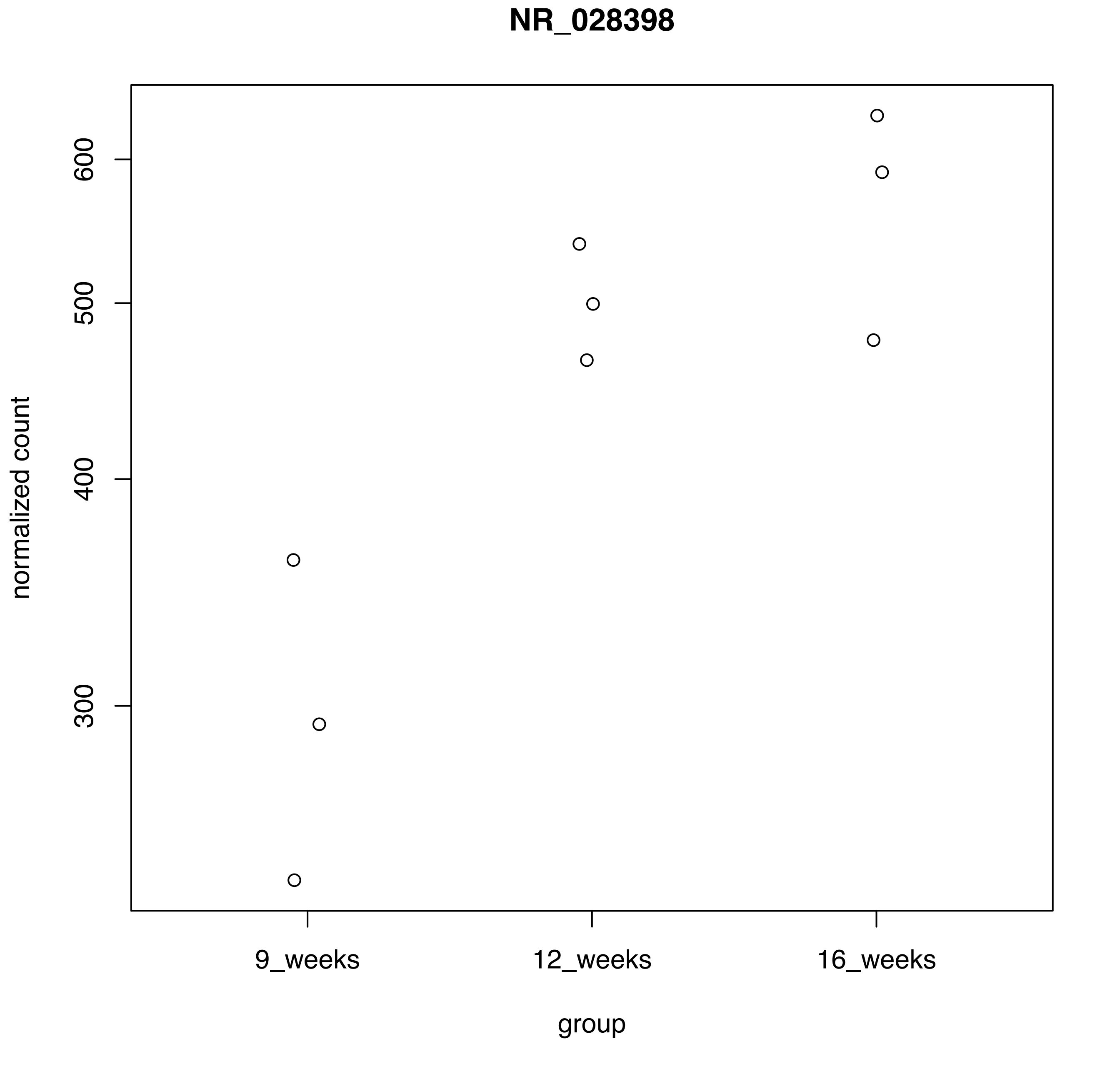
NR_028398
normalized count
600
500
400
300
9_weeks
12_weeks
16_weeks
group

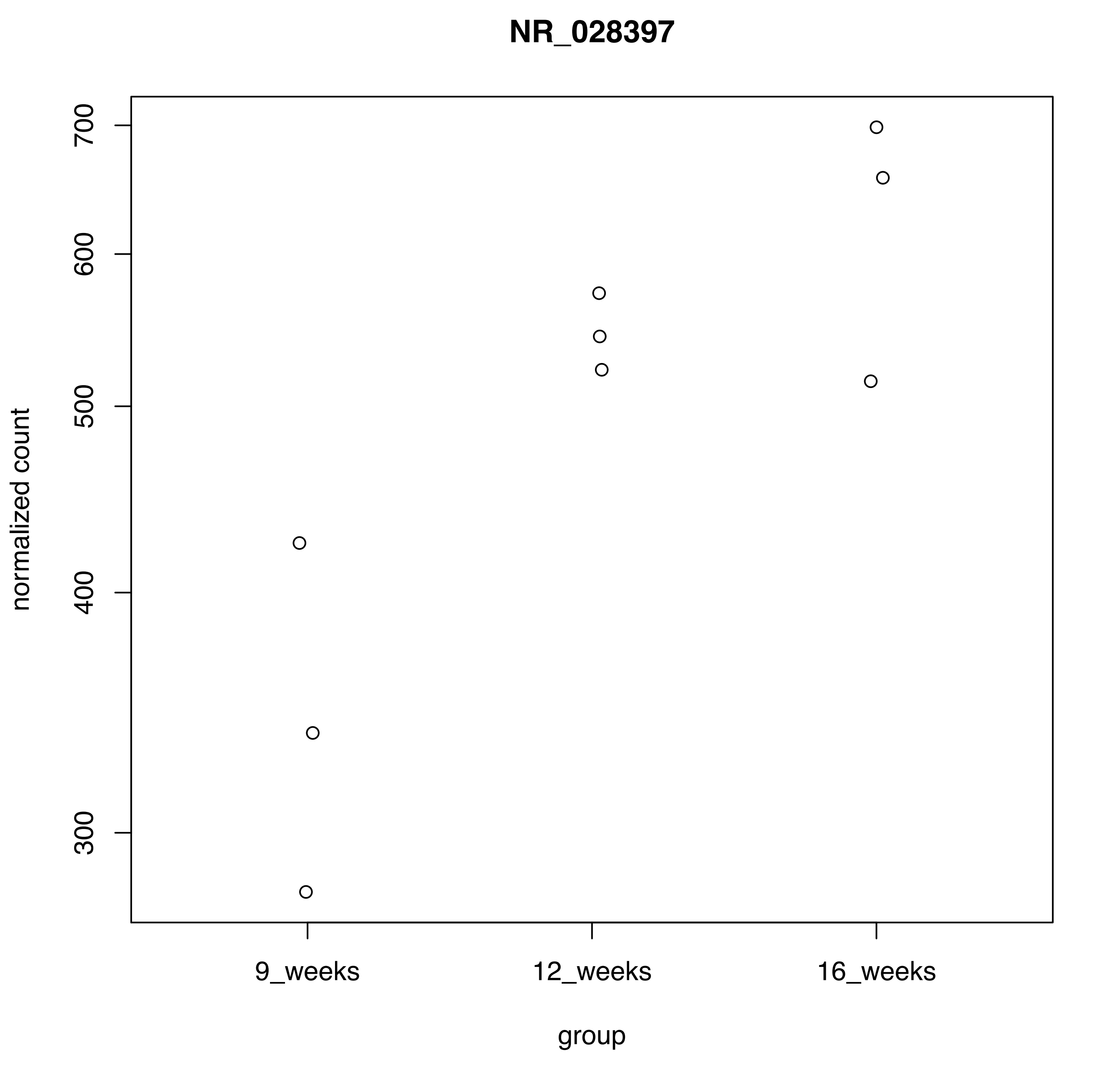
NR_028397
normalized count
700
600
500
400
300
9_weeks
12_weeks
16_weeks
group

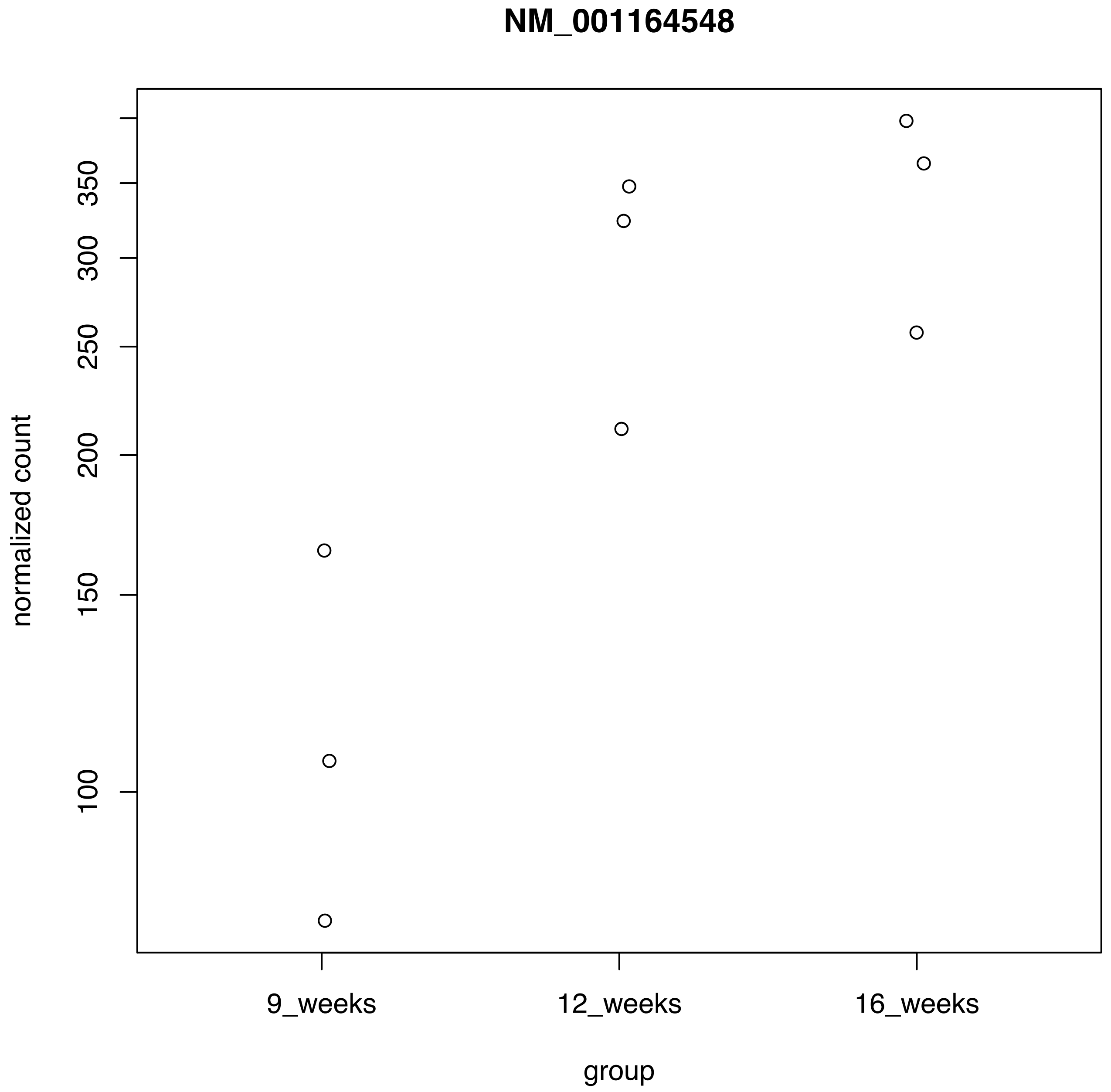
NM_001164548
normalized count
100
150
200
250
300
350
9_weeks
12_weeks
16_weeks
group

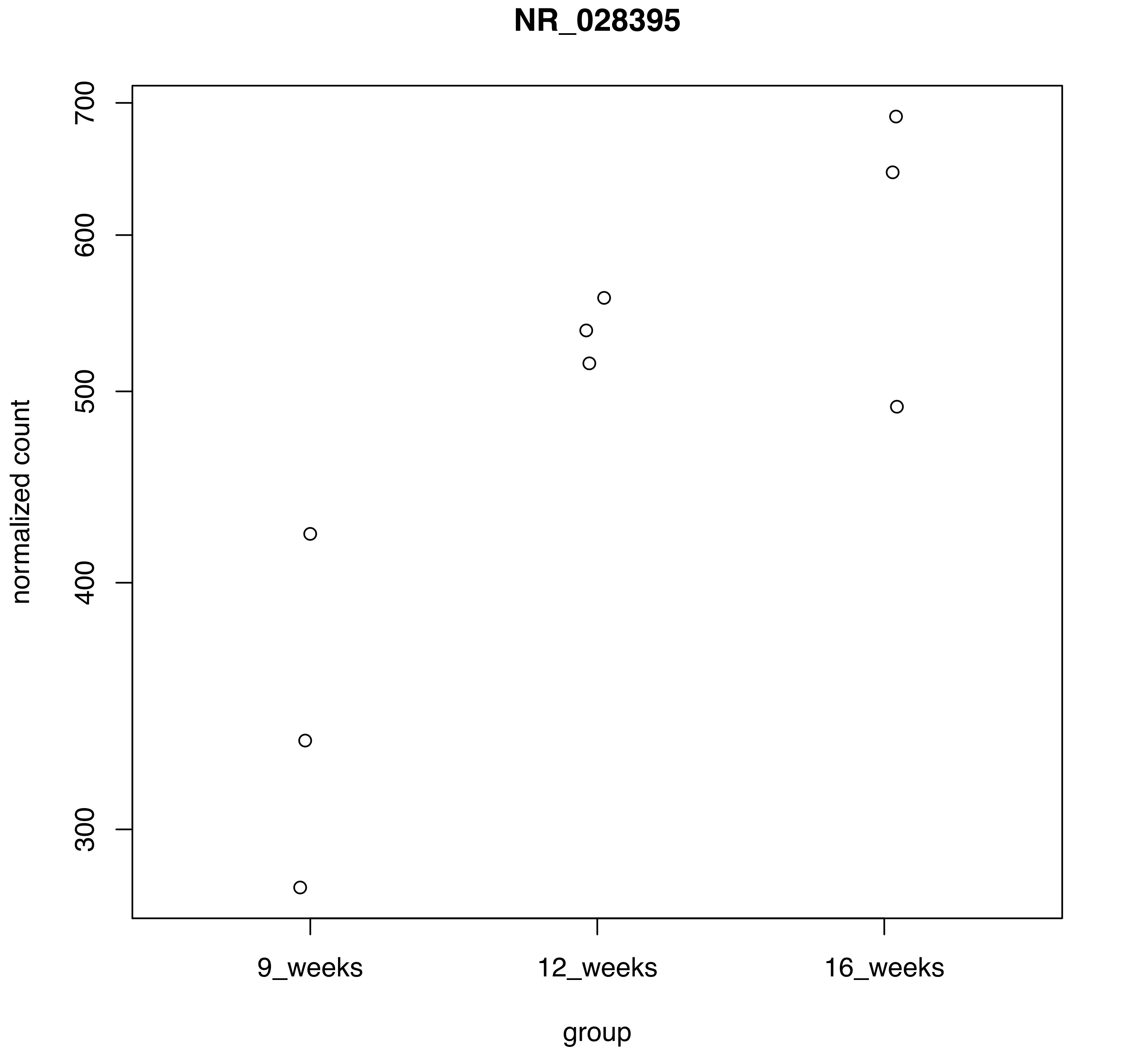
NR_028395
normalized count
300
400
500
600
700
9_weeks
12_weeks
16_weeks
group

NM_001164556

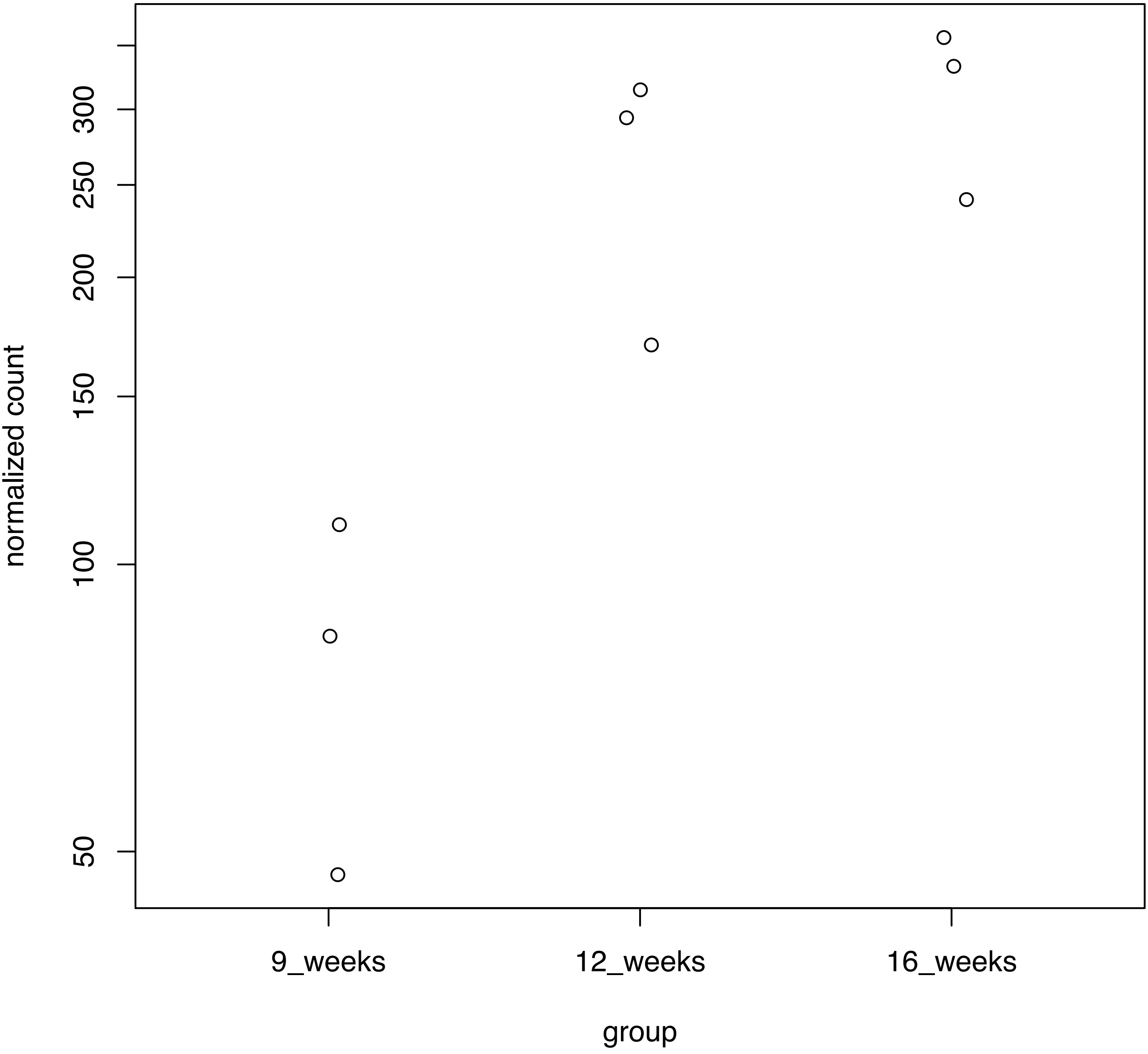

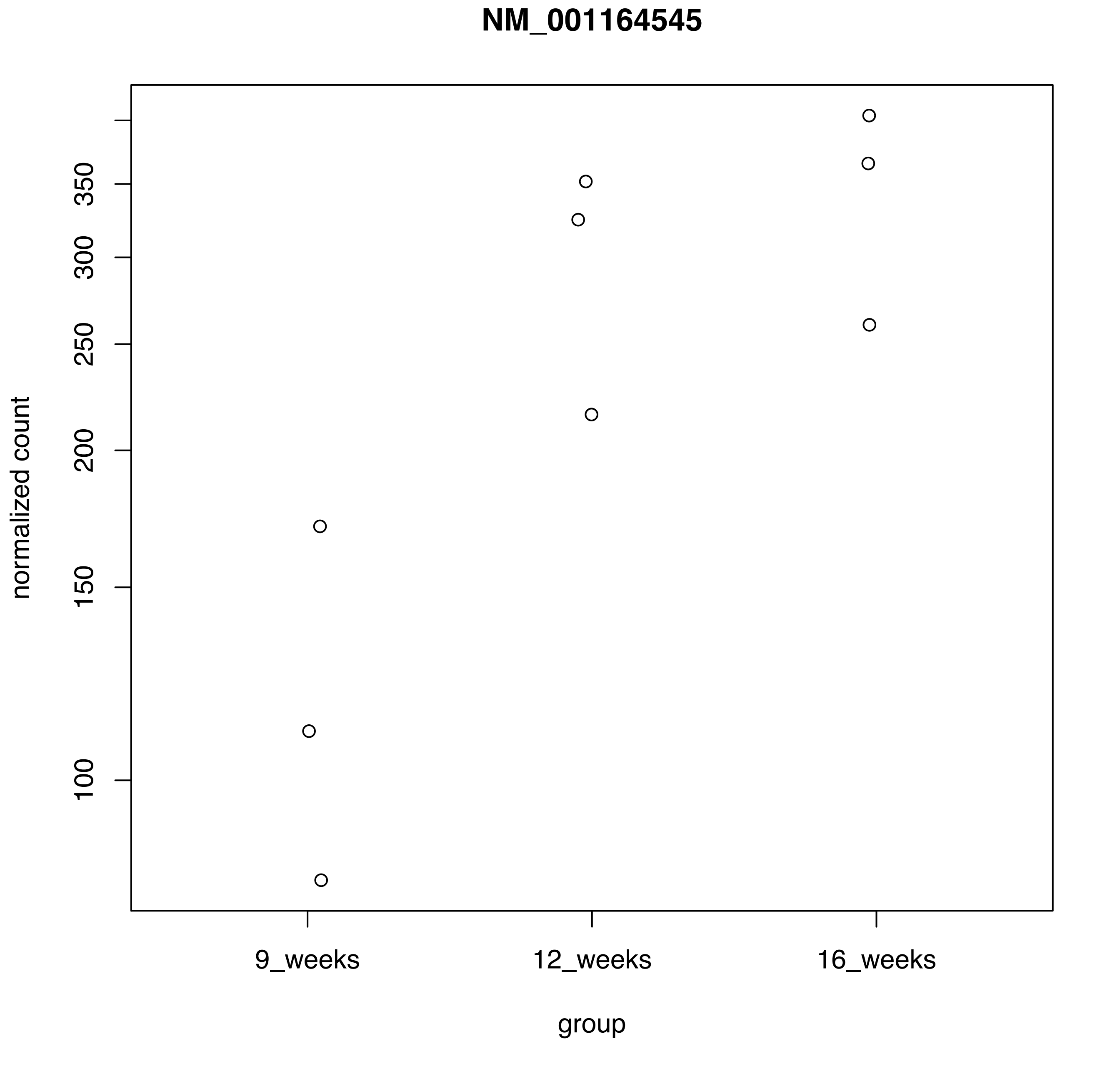
NM_001164545
normalized count
100
150
200
250
300
350
9_weeks
12_weeks
16_weeks
group

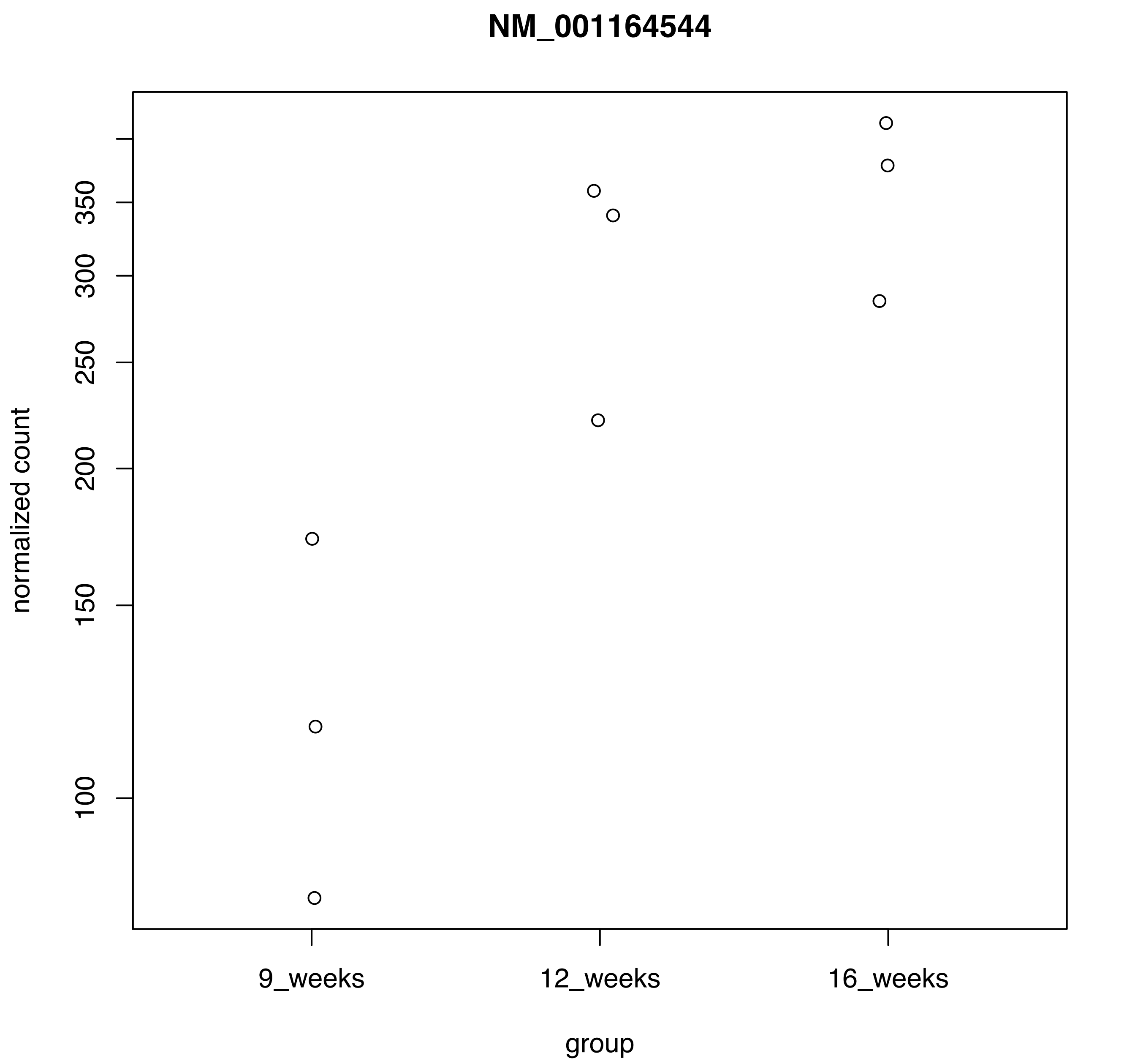

NM_001164544
normalized count
100
150
200
250
300
350
9_weeks
12_weeks
16_weeks
group

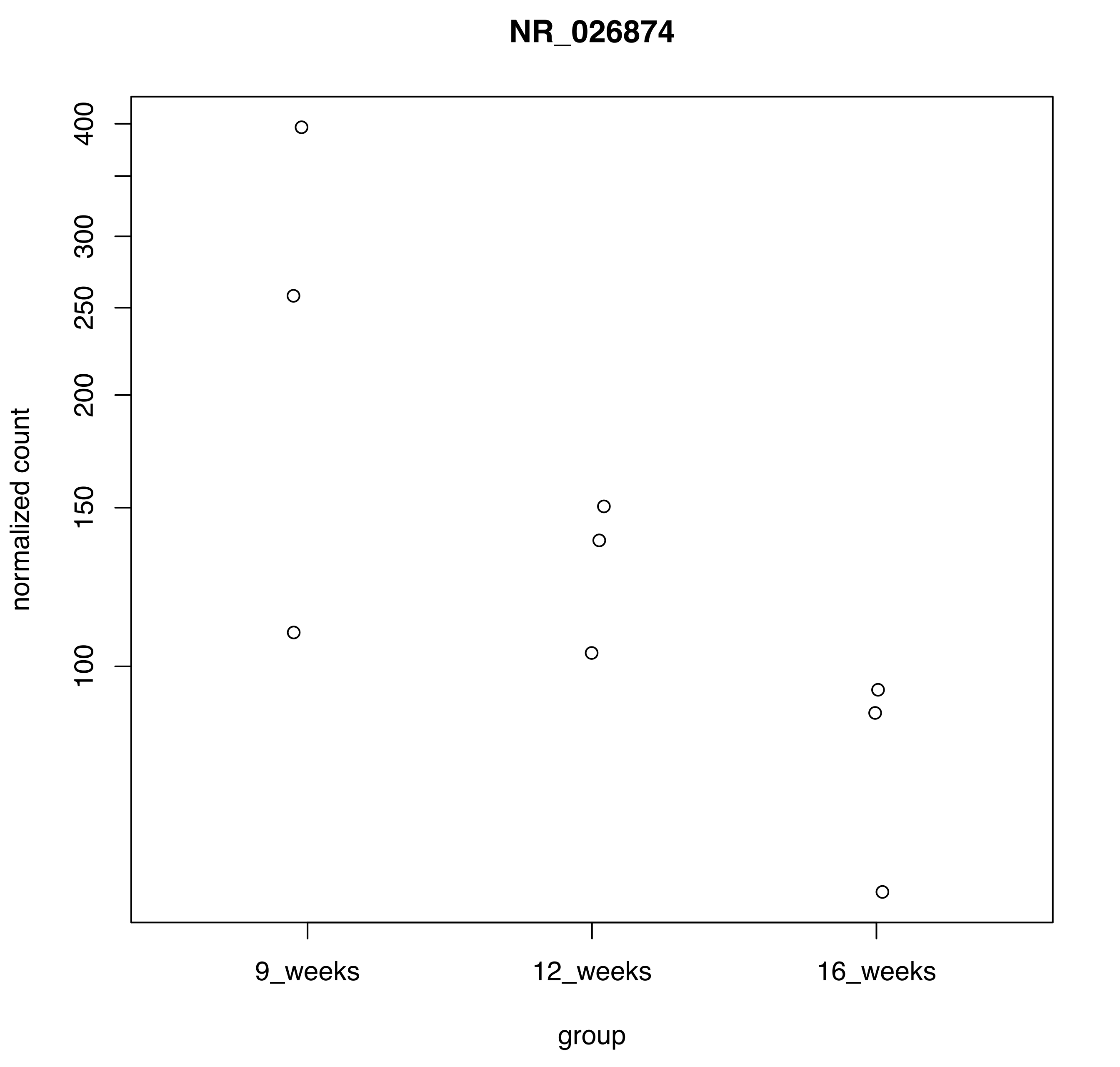
NR_026874
normalized count
400
300
250
200
150
100
9_weeks
12_weeks
16_weeks
group

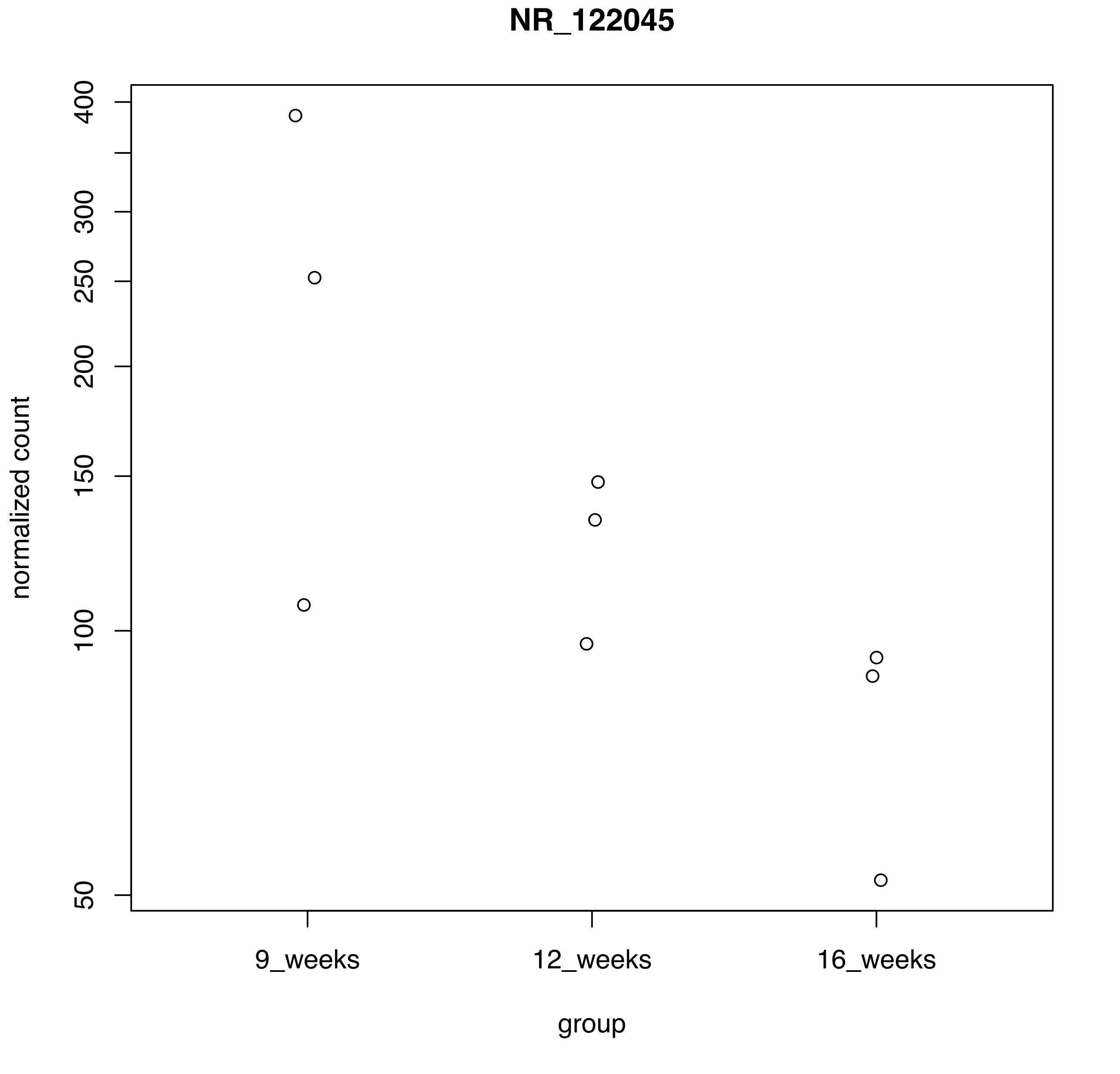

NR_122045
normalized count
400
300
250
200
150
100
50
9_weeks
12_weeks
16_weeks
group

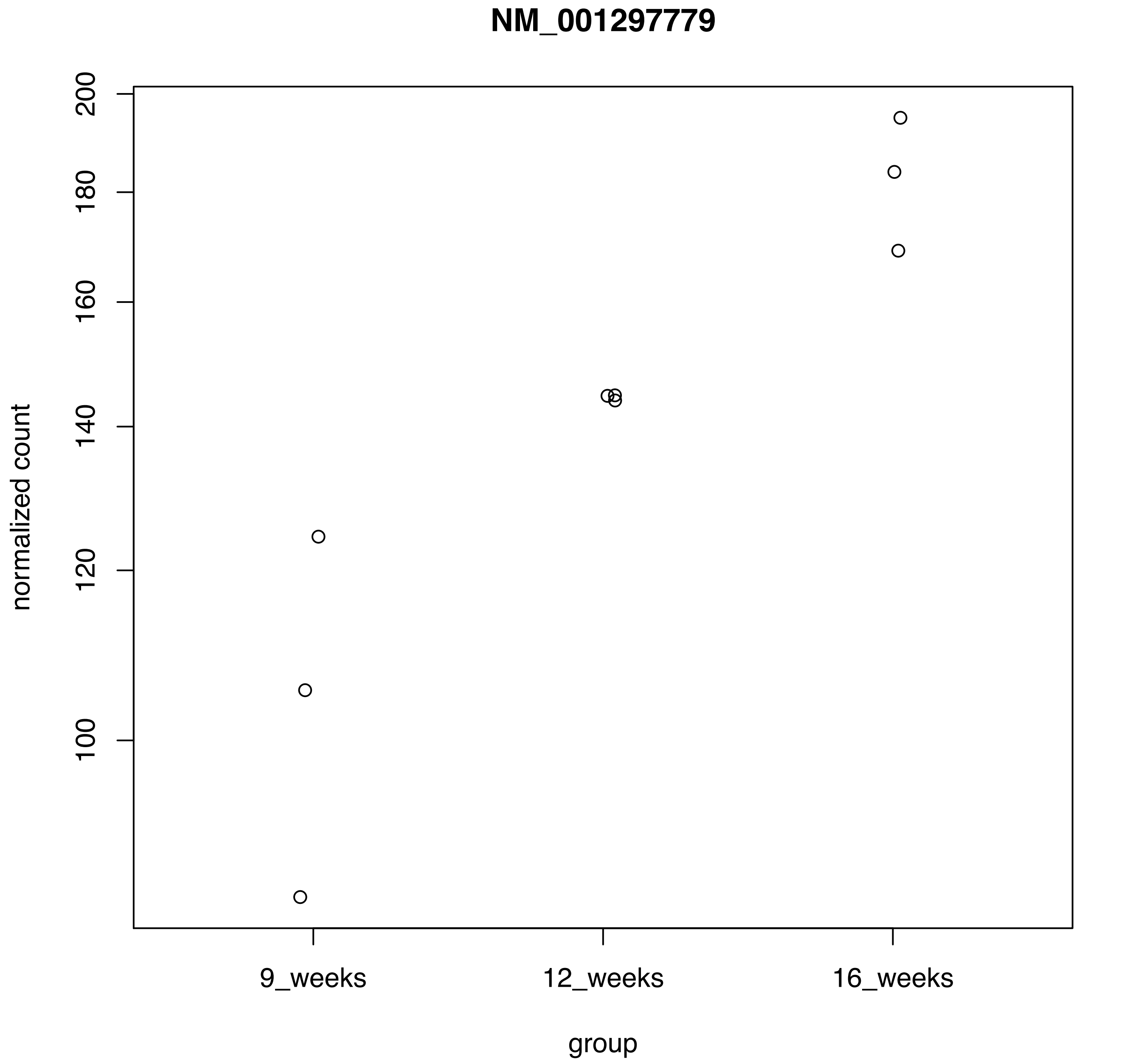
NM_001297779
normalized count
200
180
160
140
120
100
9_weeks
12_weeks
16_weeks
group

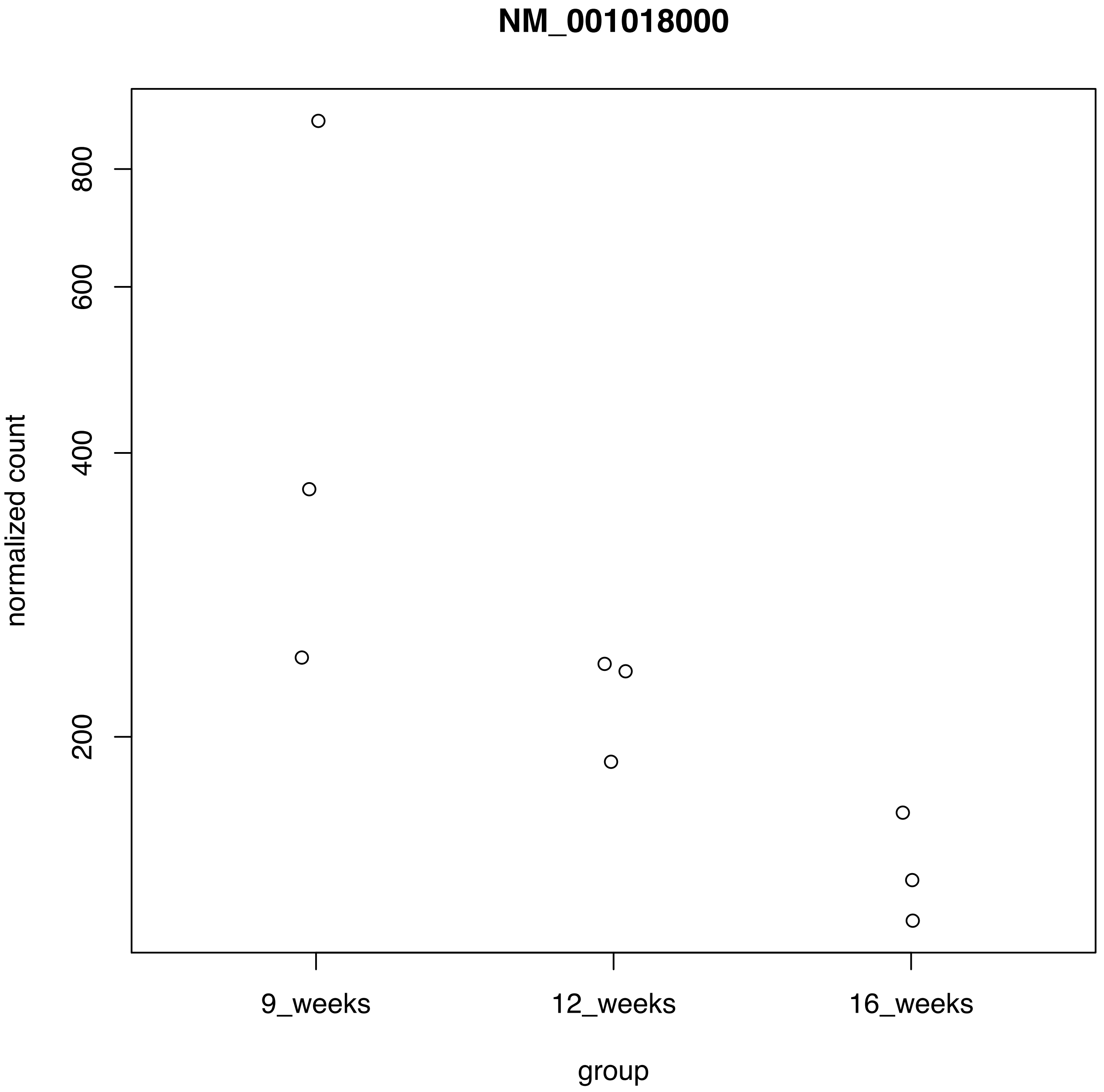
NM_001018000
normalized count
800
600
400
200
9_weeks
12_weeks
16_weeks
group

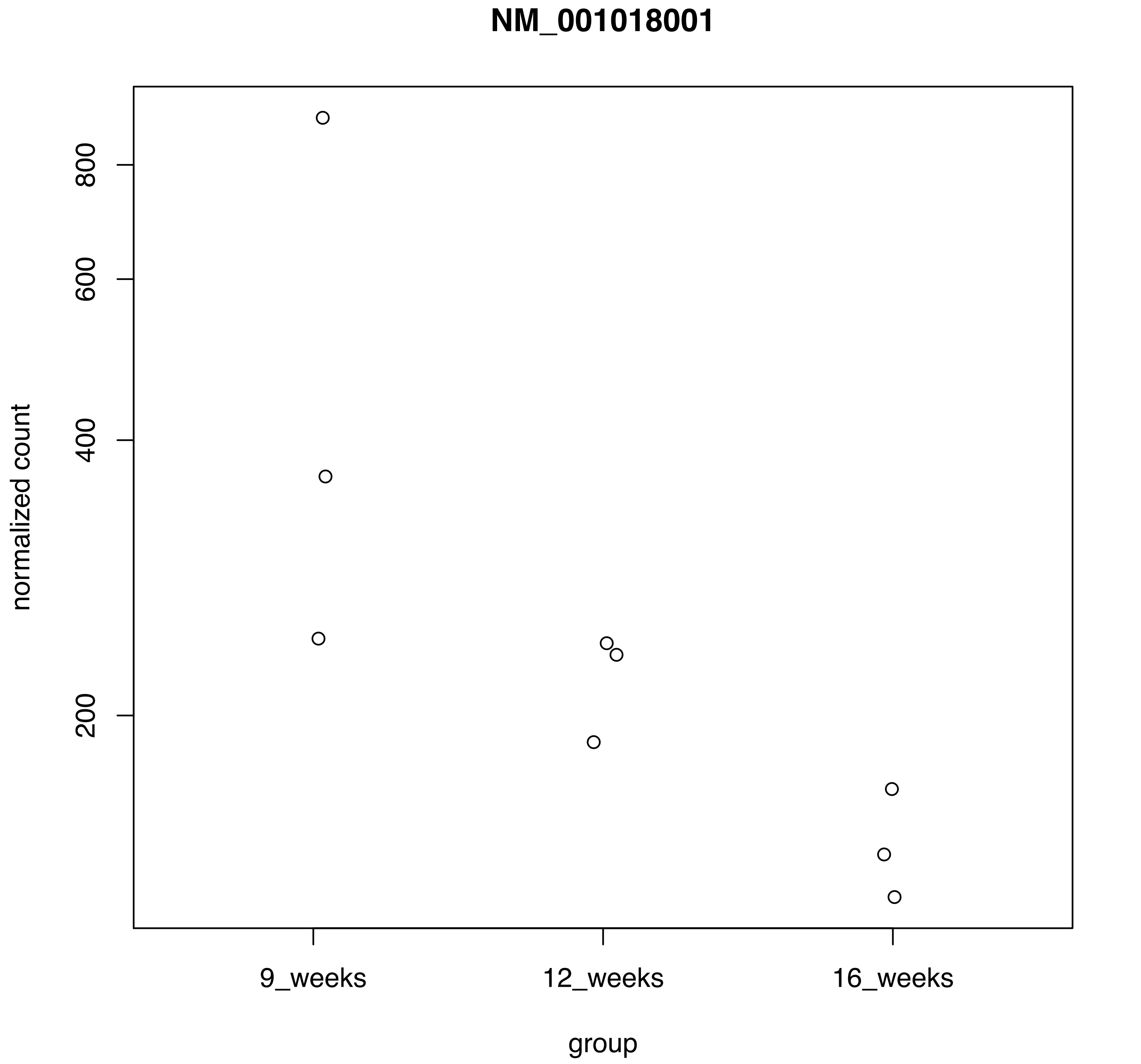

NM_001018001
normalized count
800
600
400
200
9_weeks
12_weeks
16_weeks
group

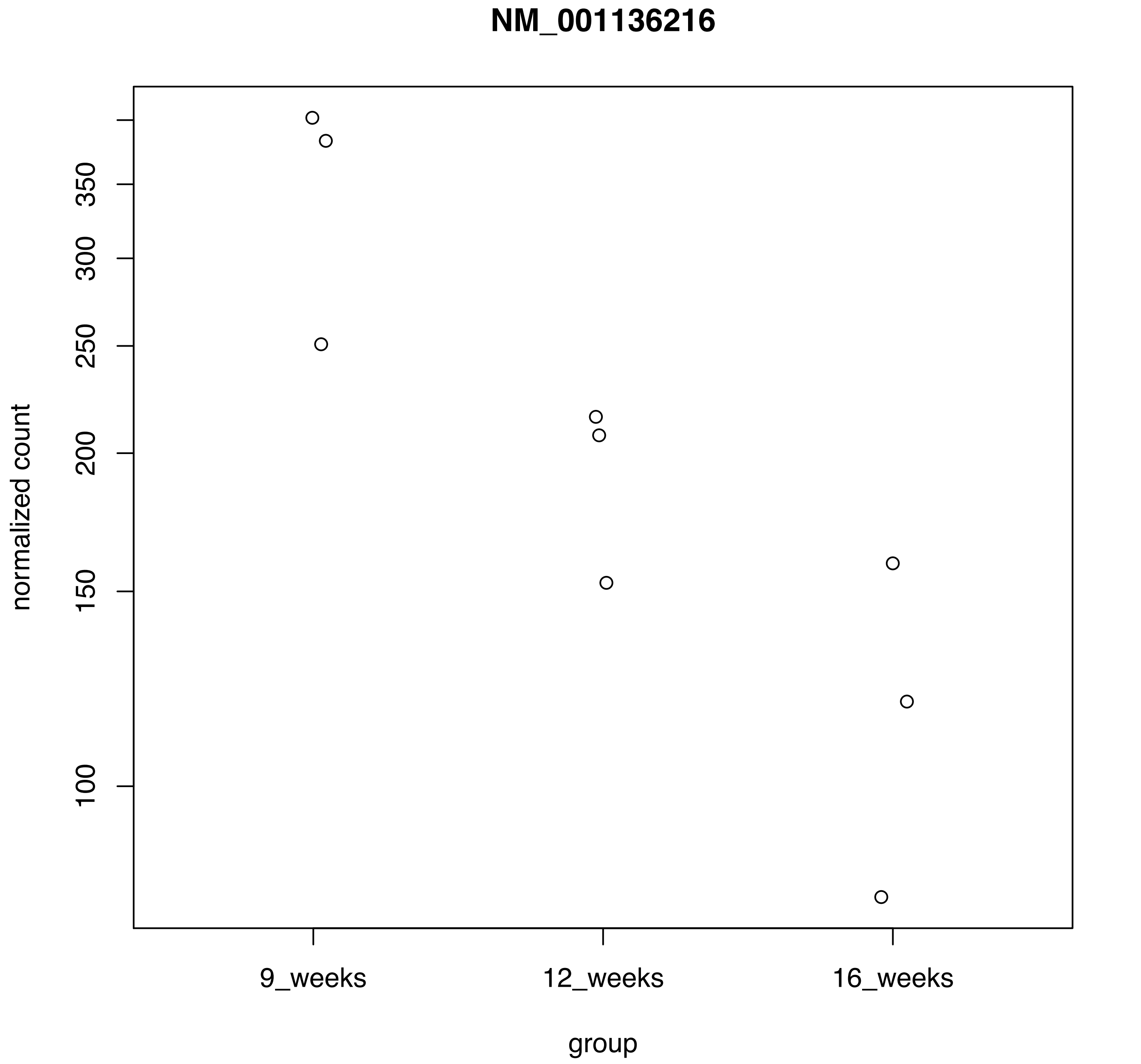

NM_001136216
normalized count
group
9_weeks
12_weeks
16_weeks
100
150
200
250
300
350

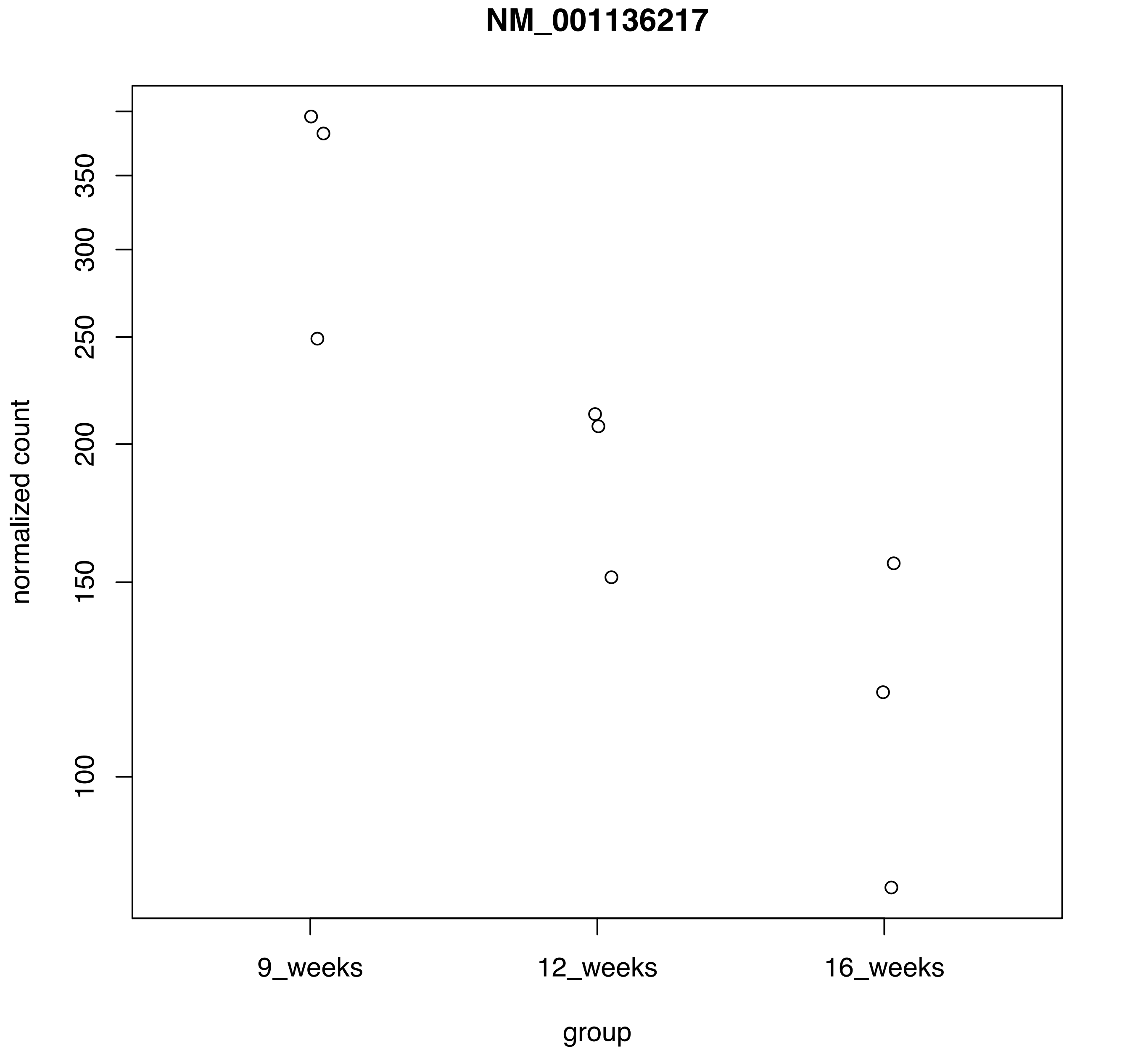

NM_001136217
normalized count
100
150
200
250
300
350
9_weeks
12_weeks
16_weeks
group

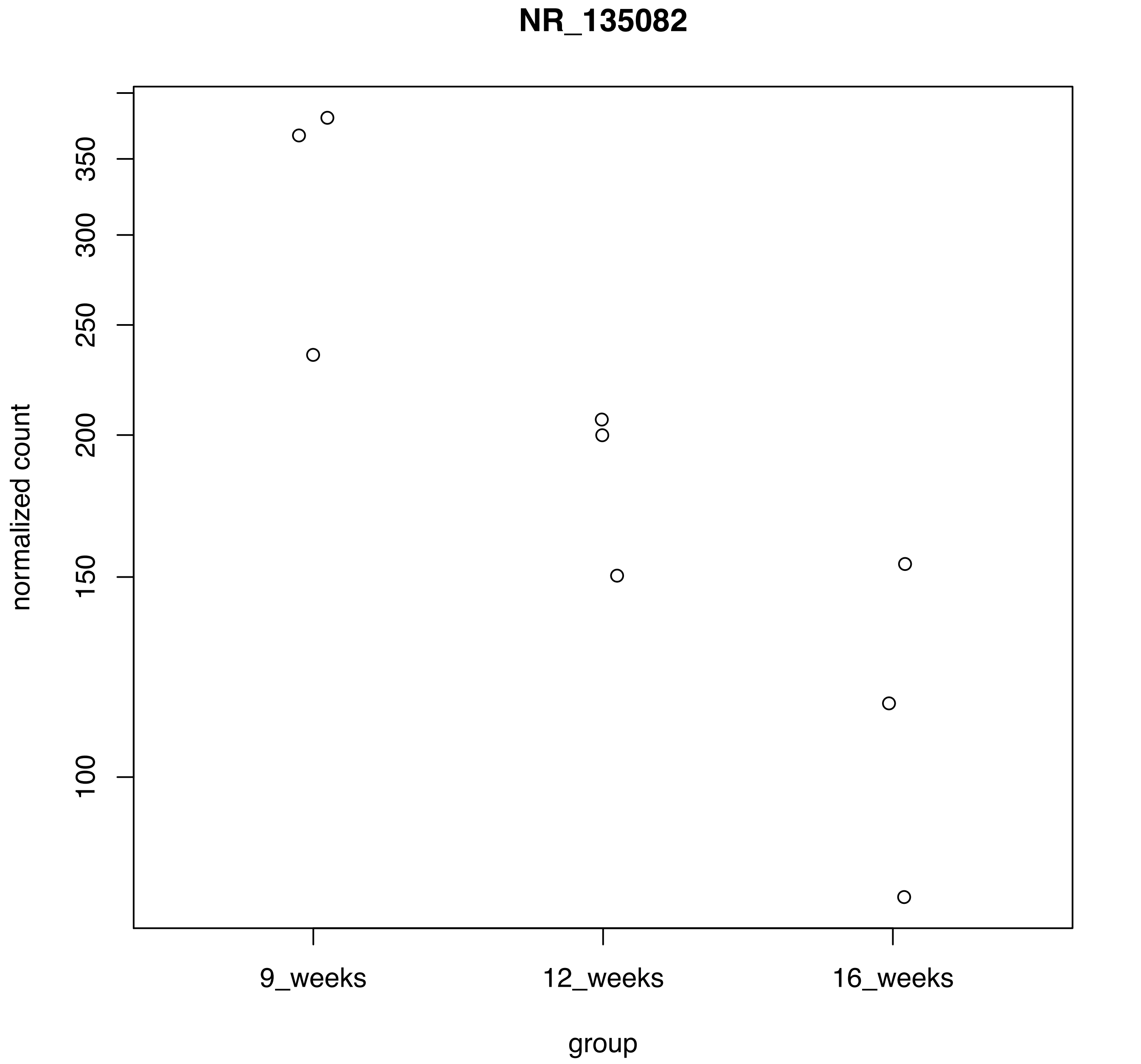

NR_135082
normalized count
100
150
200
250
300
350
9_weeks
12_weeks
16_weeks
group

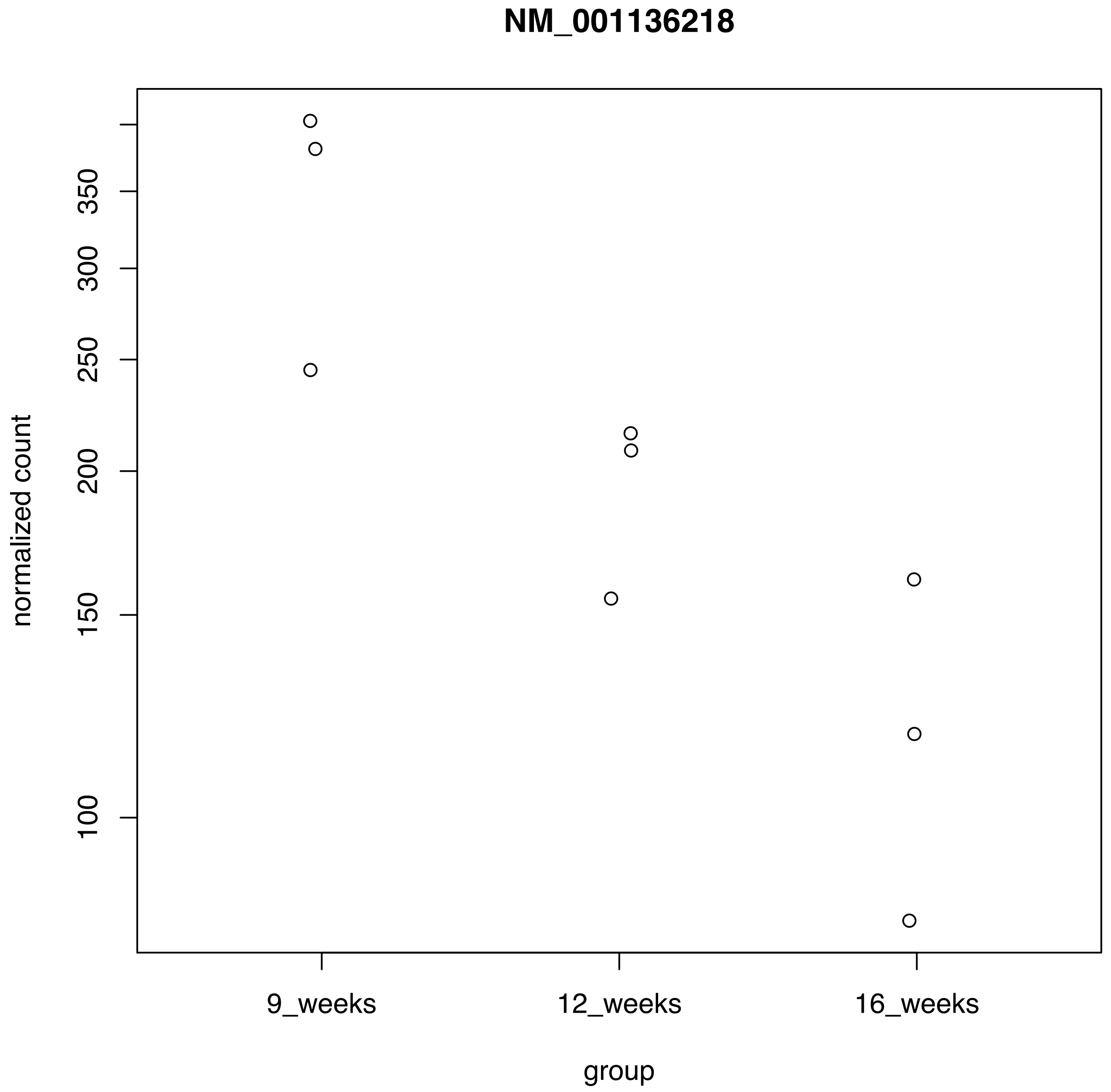
NM_001136218
normalized count
100
150
200
250
300
350
9_weeks
12_weeks
16_weeks
group

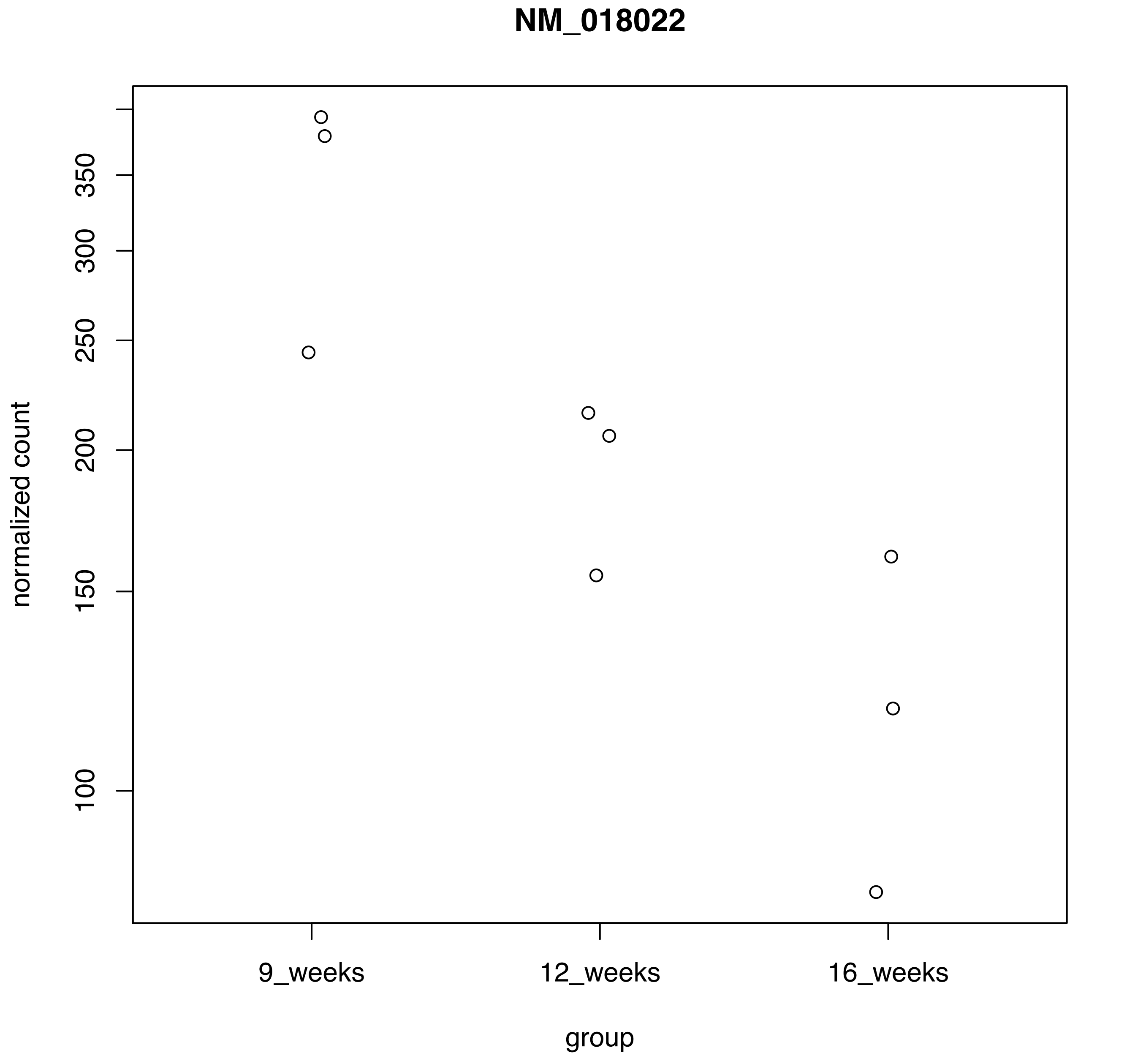

NM_018022
normalized count
350
300
250
200
150
100
9_weeks
12_weeks
16_weeks
group

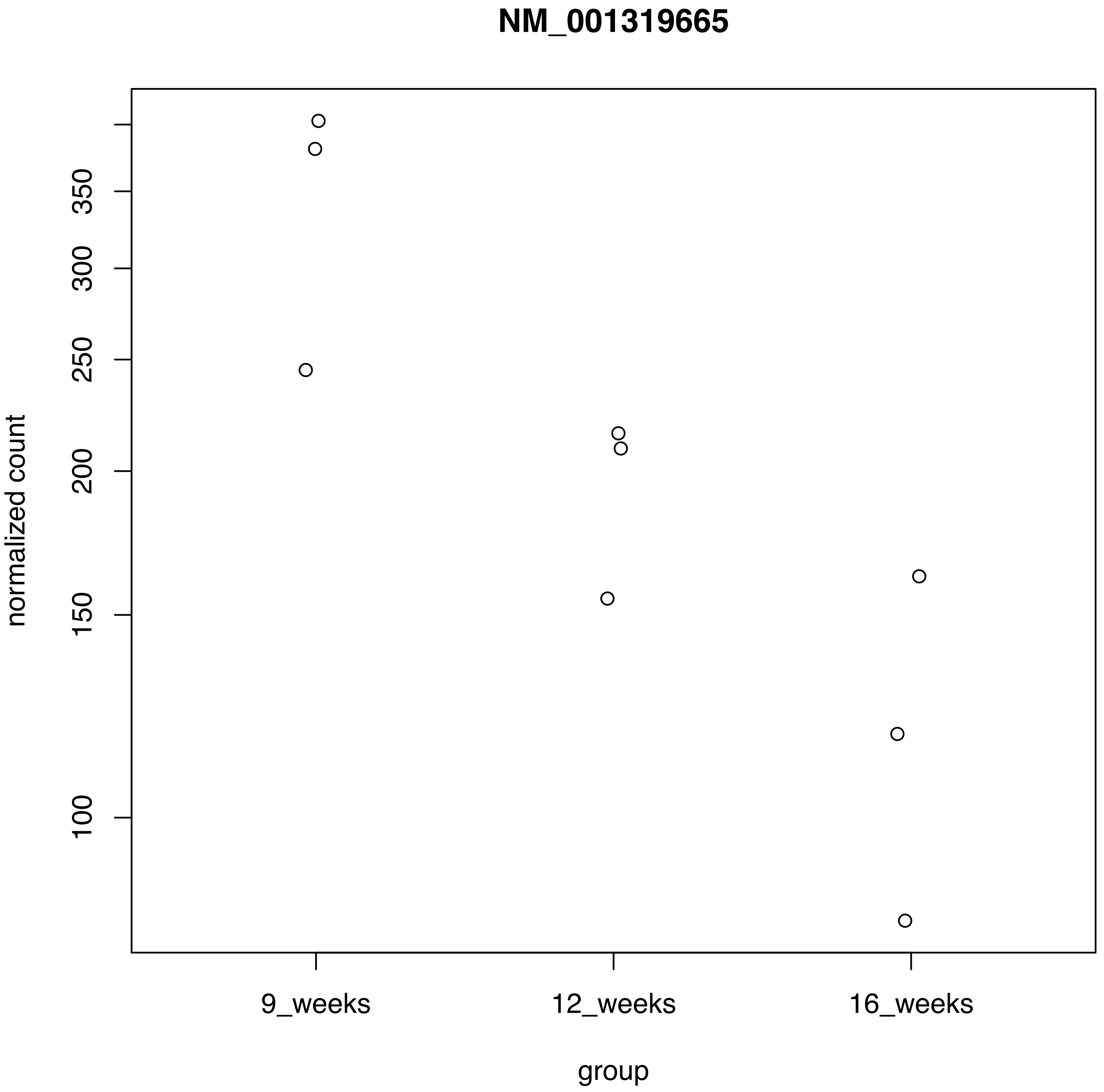
NM_001319665
normalized count
100
150
200
250
300
350
9_weeks
12_weeks
16_weeks
group

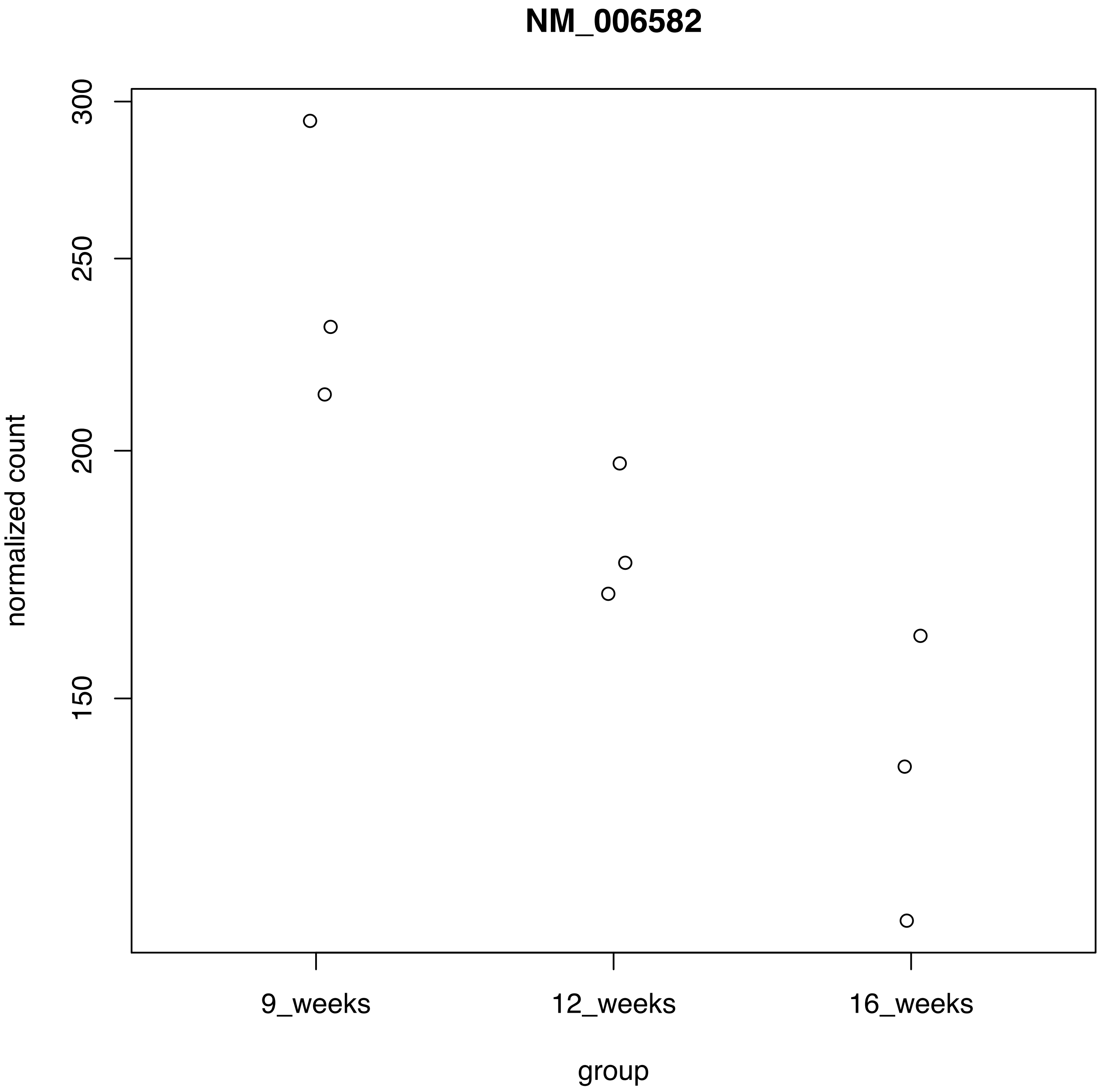
NM_006582
normalized count
300
250
200
150
9_weeks
12_weeks
16_weeks
group

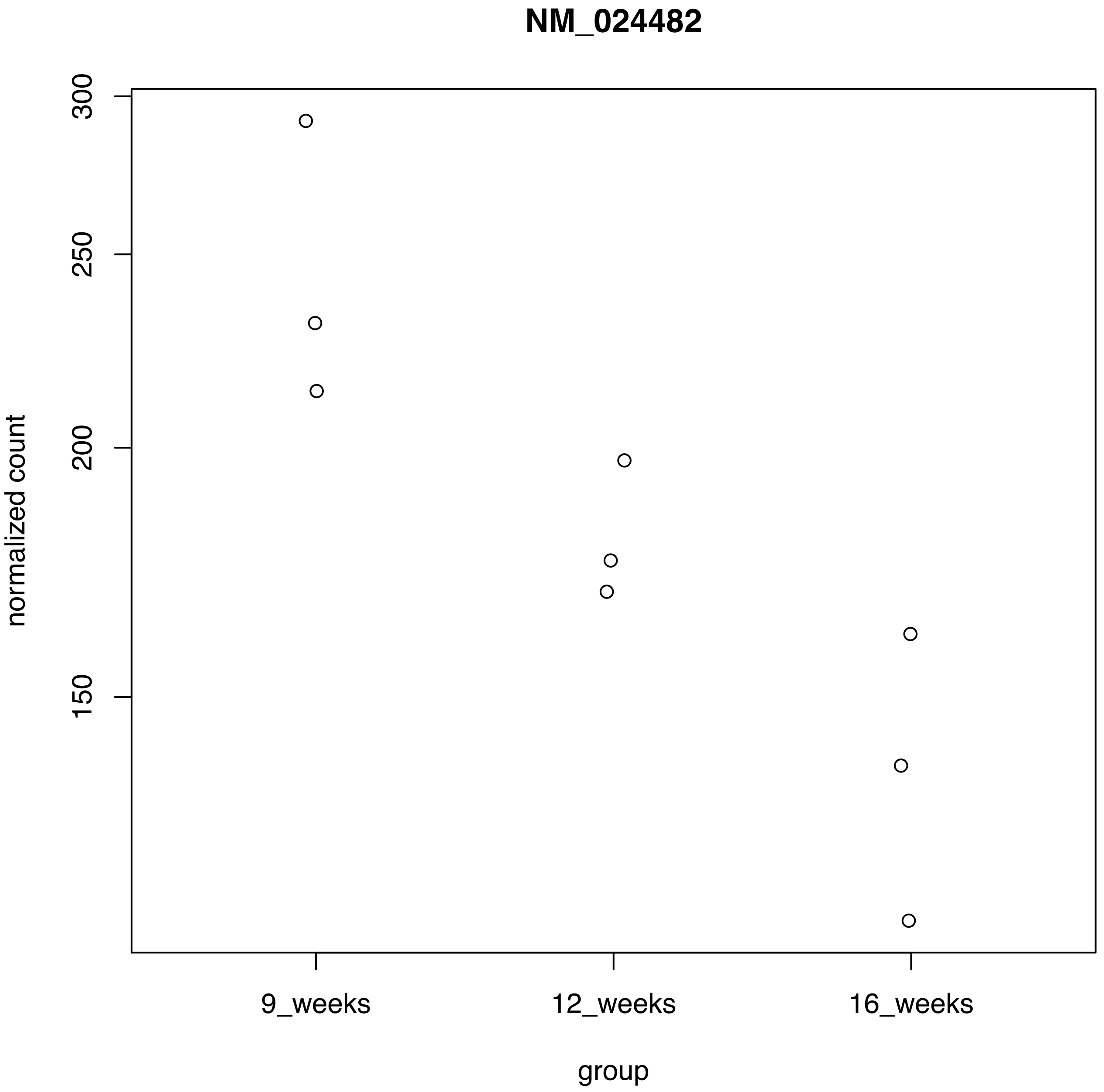
NM_024482
normalized count
300
250
200
150
9_weeks
12_weeks
16_weeks
group

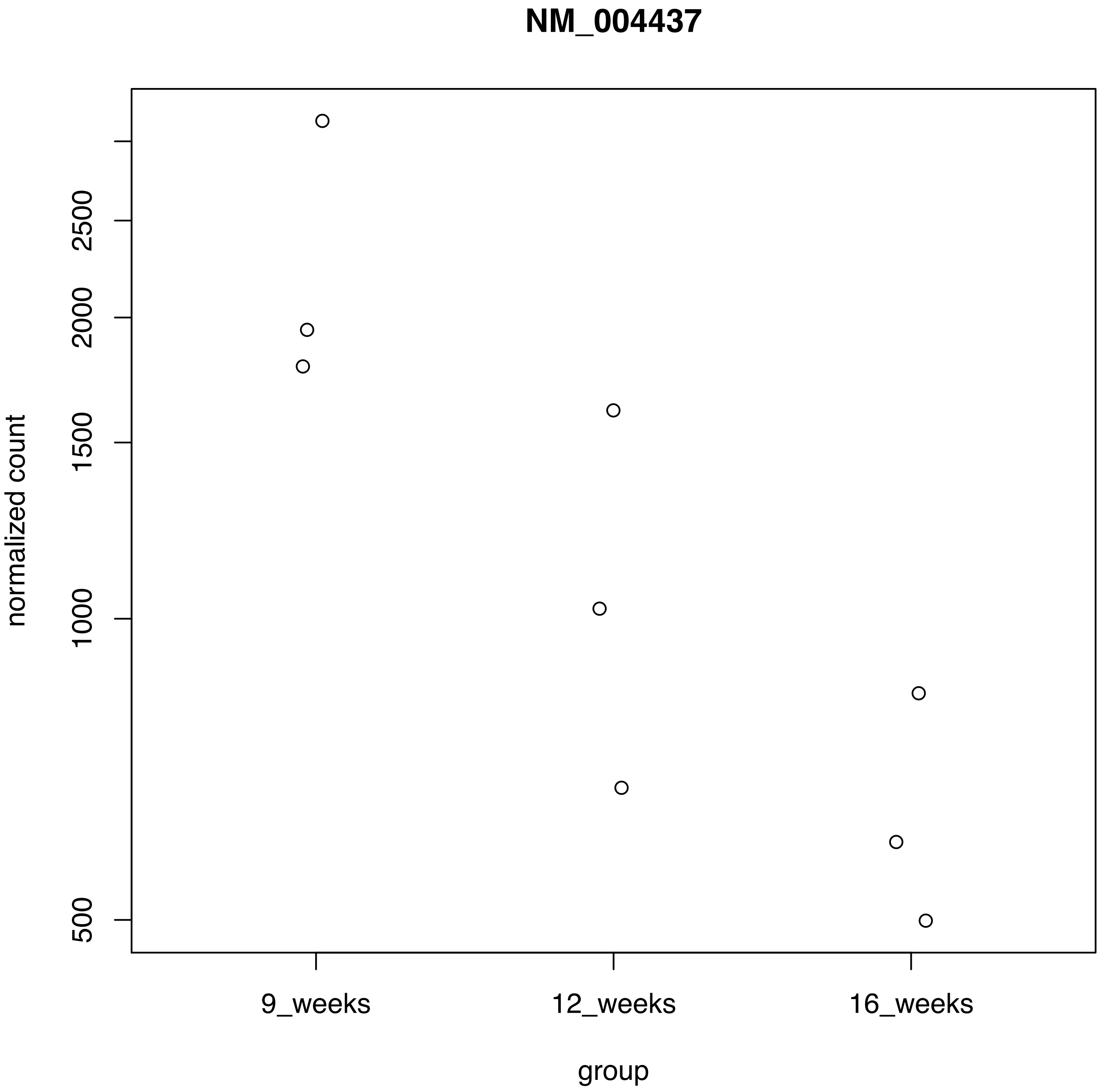

NM_004437
normalized count
2500
2000
1500
1000
500
9_weeks
12_weeks
16_weeks
group

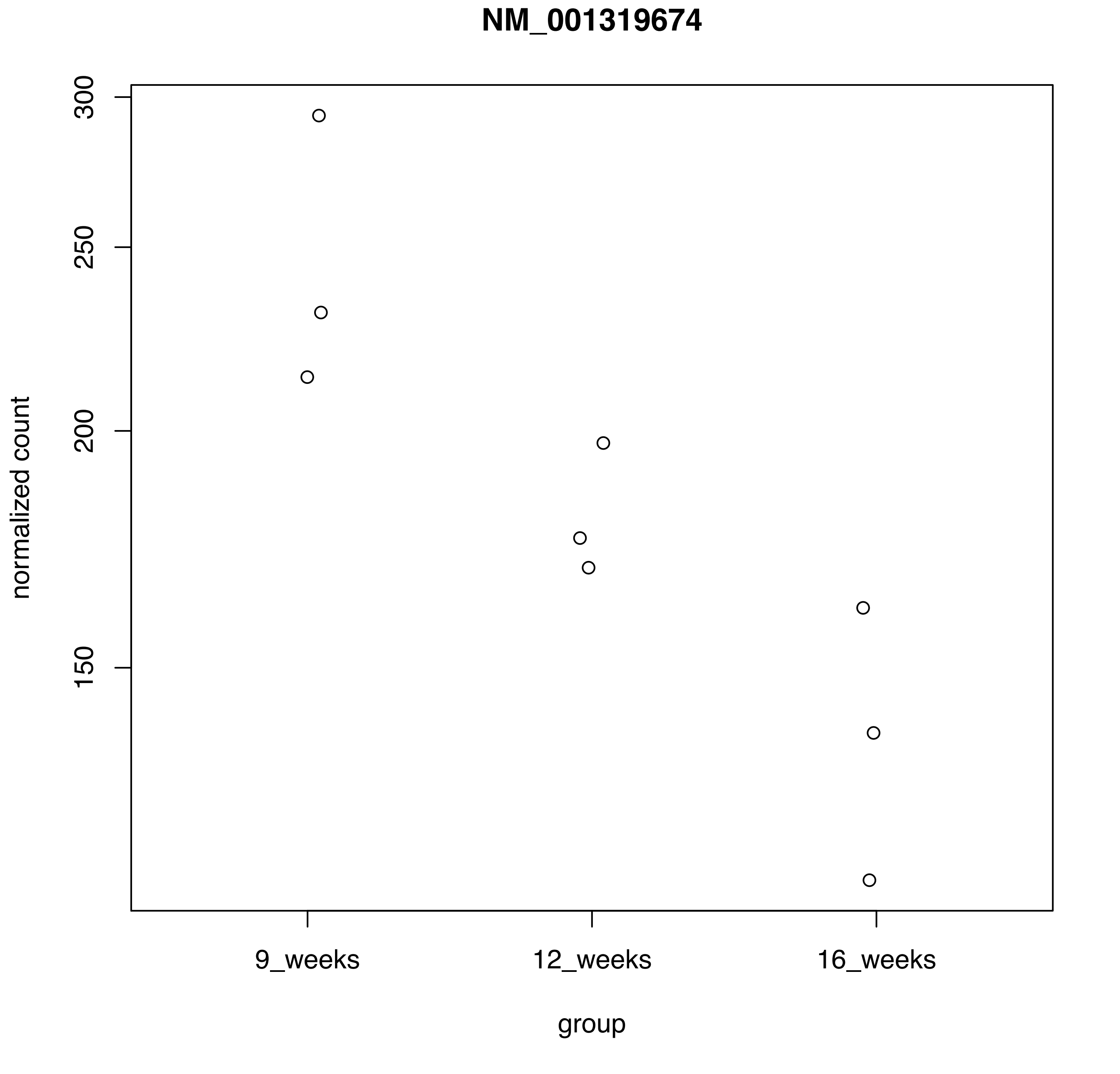
NM_001319674
normalized count
300
250
200
150
9_weeks
12_weeks
16_weeks
group

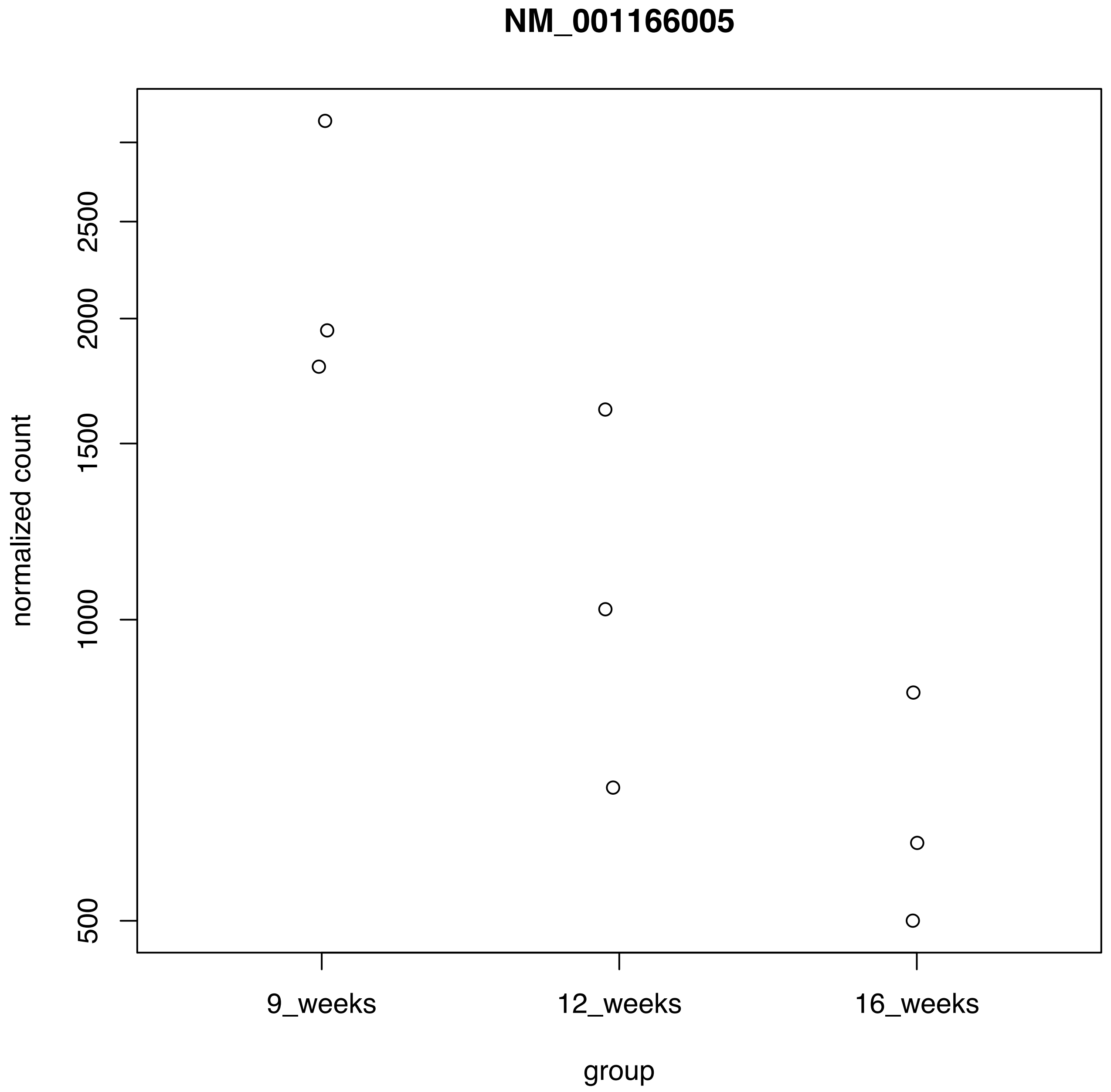
NM_001166005
normalized count
500
1000
1500
2000
2500
9_weeks
12_weeks
16_weeks
group

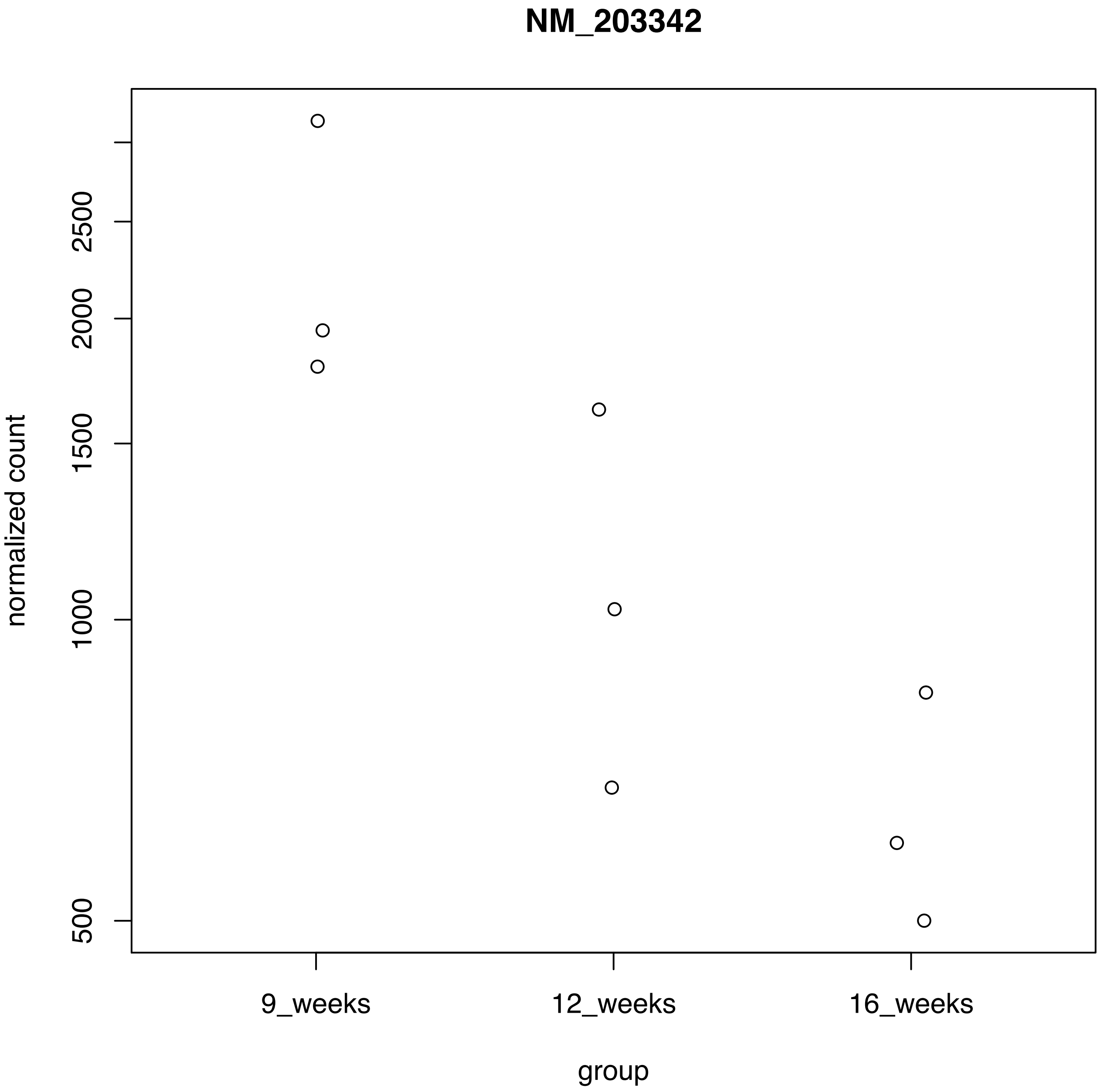
NM_203342
normalized count
500
1000
1500
2000
2500
9_weeks
12_weeks
16_weeks
group

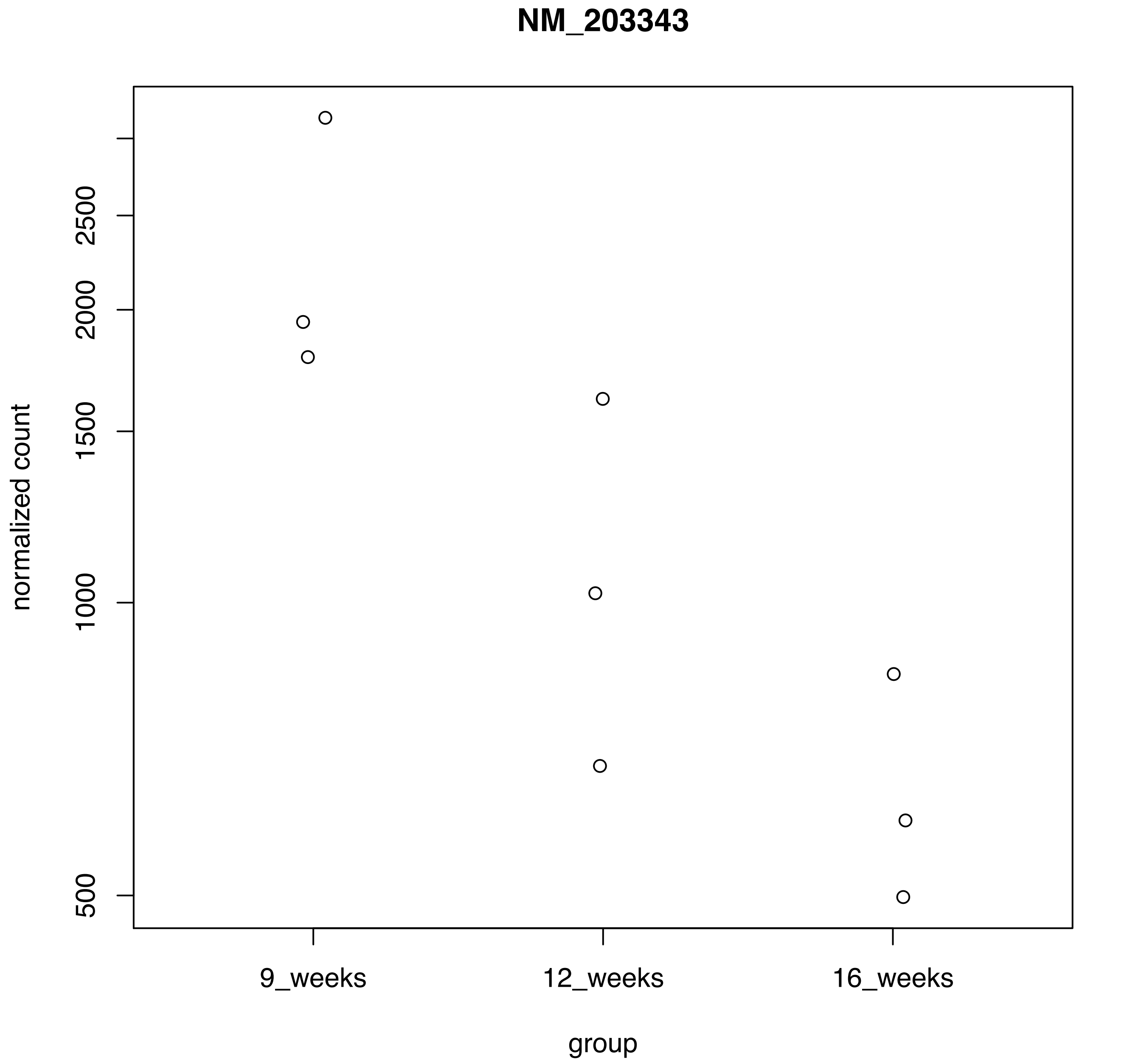

NM_203343
normalized count
2500
2000
1500
1000
500
9_weeks
12_weeks
16_weeks
group

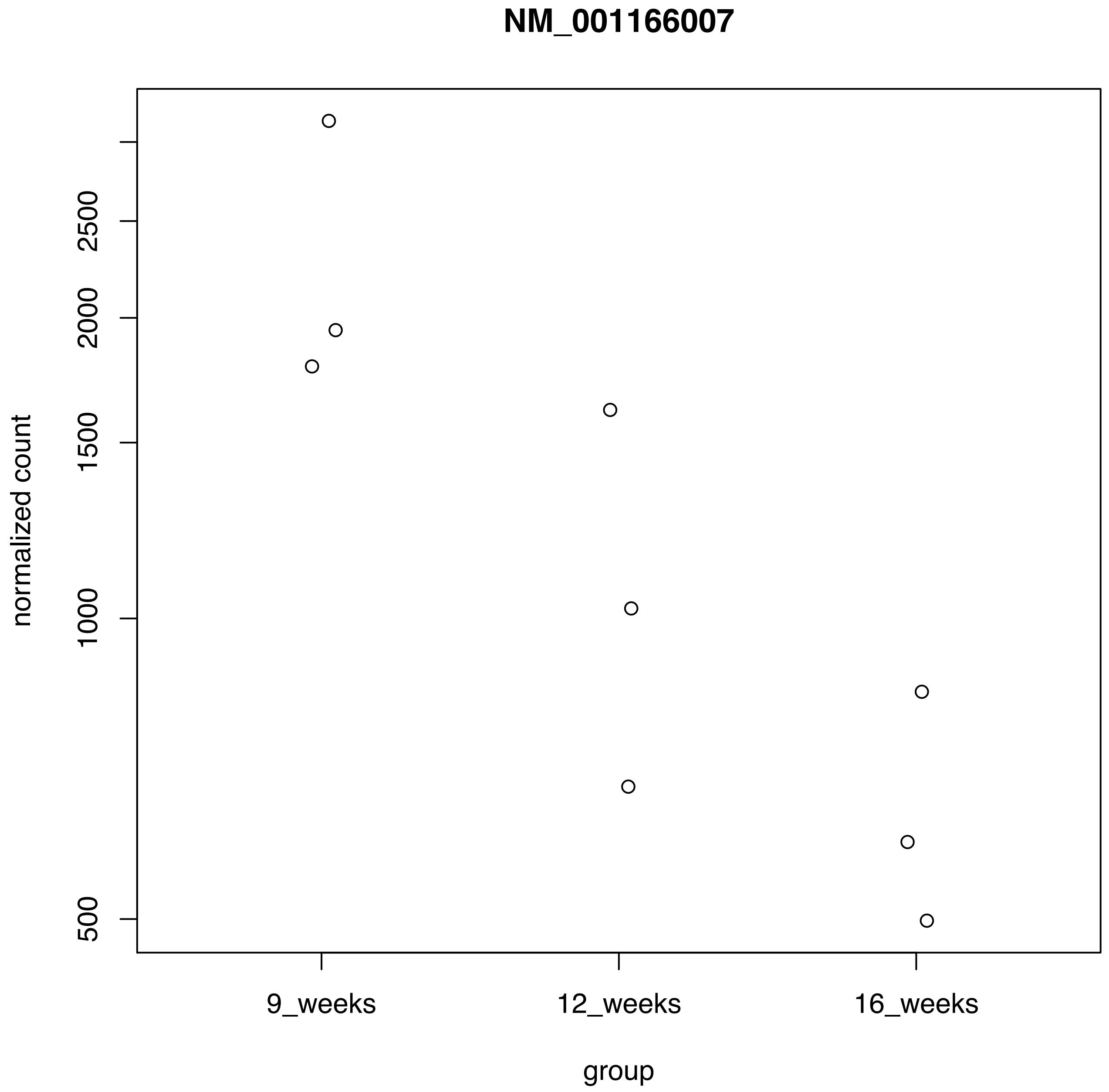

NM_001166007
normalized count
500
1000
1500
2000
2500
9_weeks
12_weeks
16_weeks
group

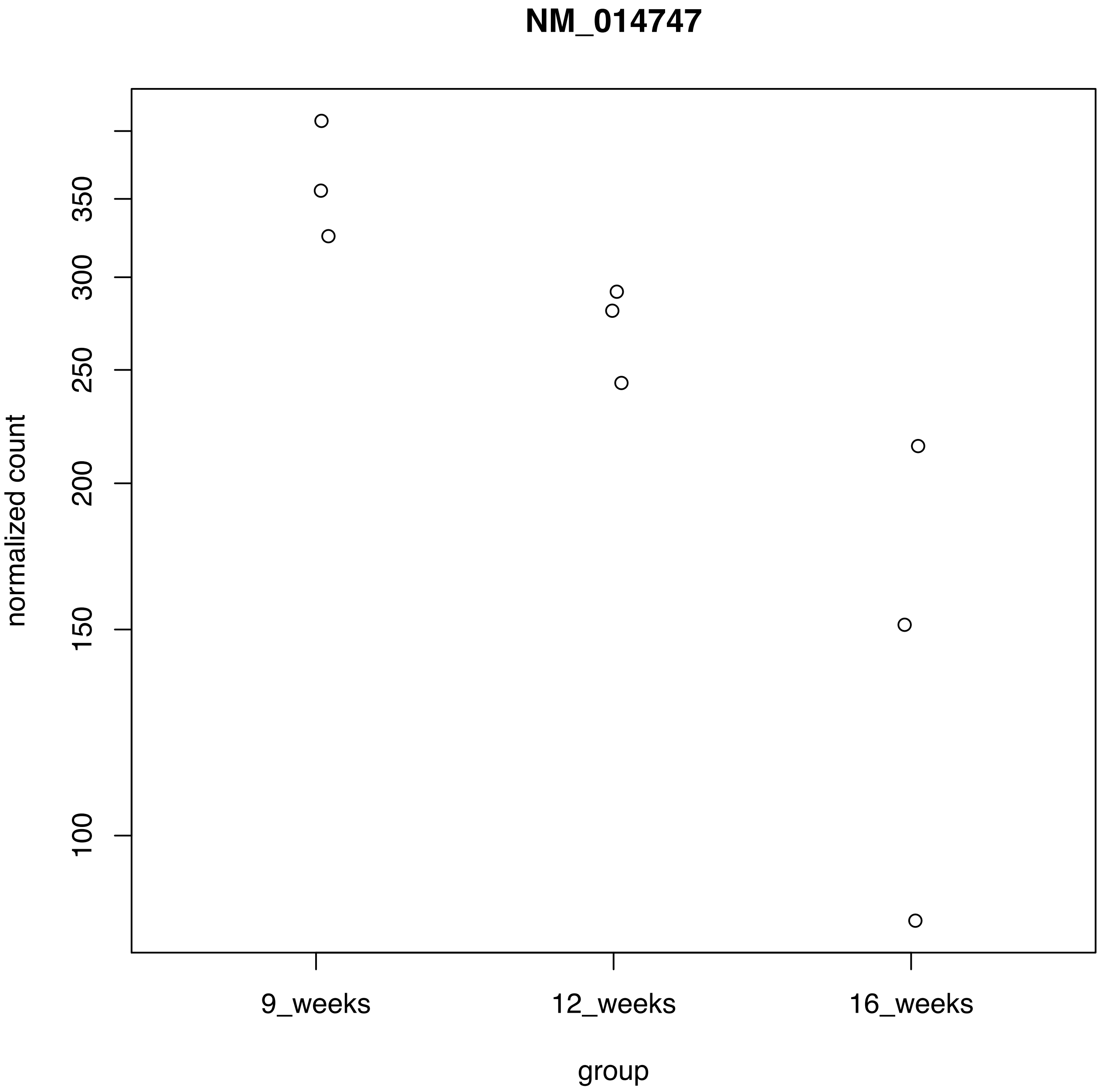
NM_014747
normalized count
100
150
200
250
300
350
9_weeks
12_weeks
16_weeks
group

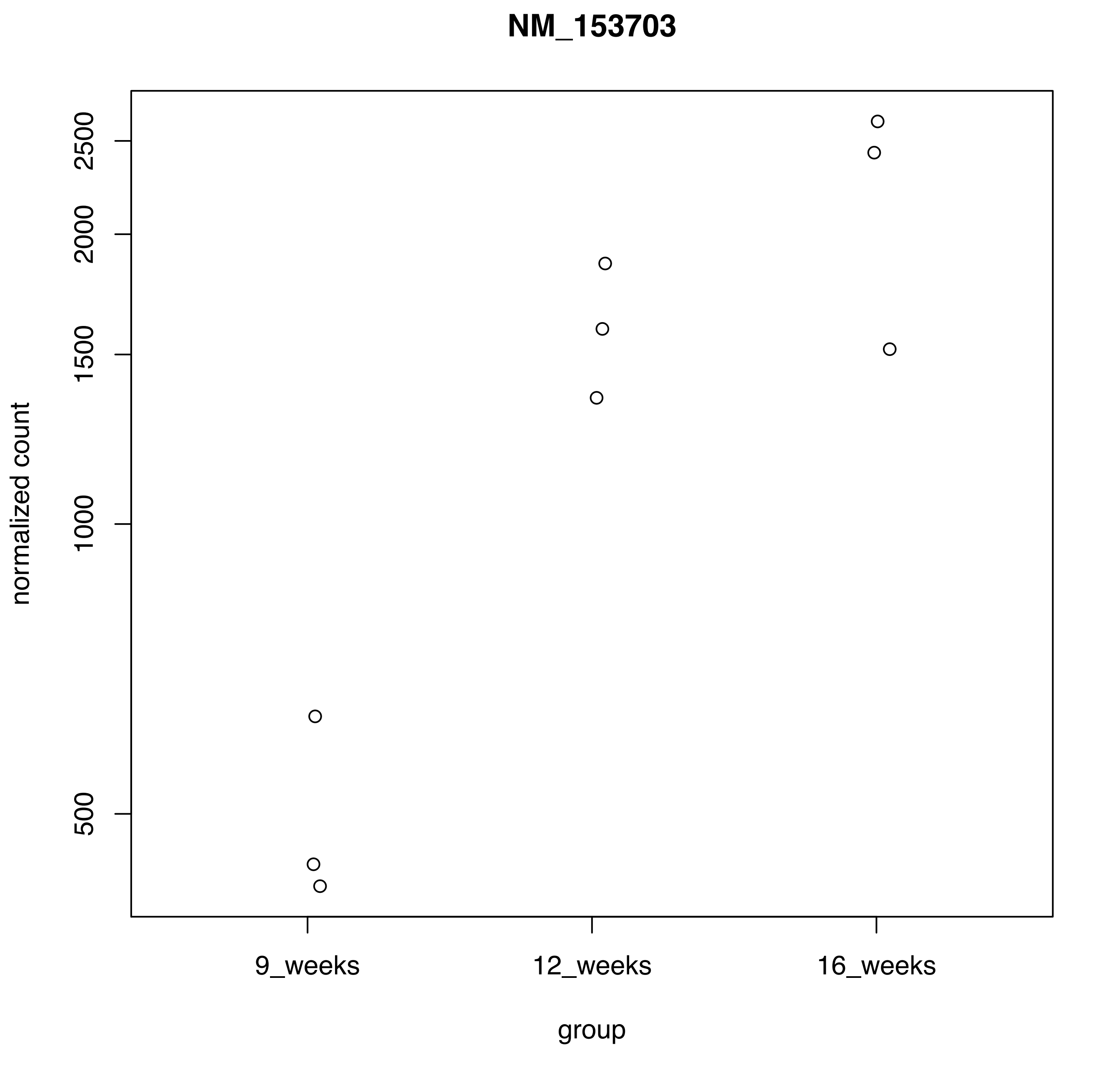
NM_153703
normalized count
2500
2000
1500
1000
500
9_weeks
12_weeks
16_weeks
group

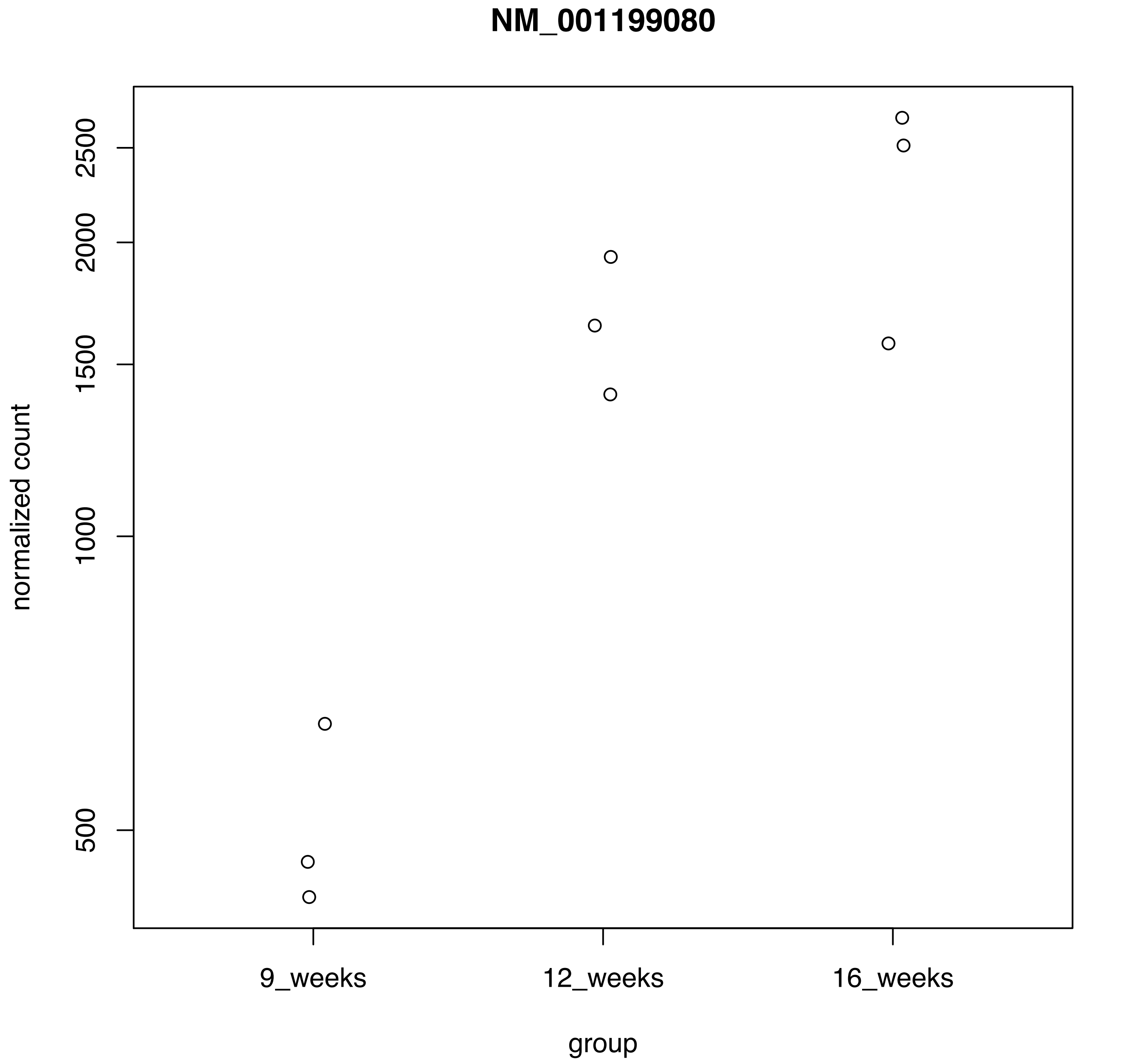

NM_001199080
normalized count
500
1000
1500
2000
2500
9_weeks
12_weeks
16_weeks
group

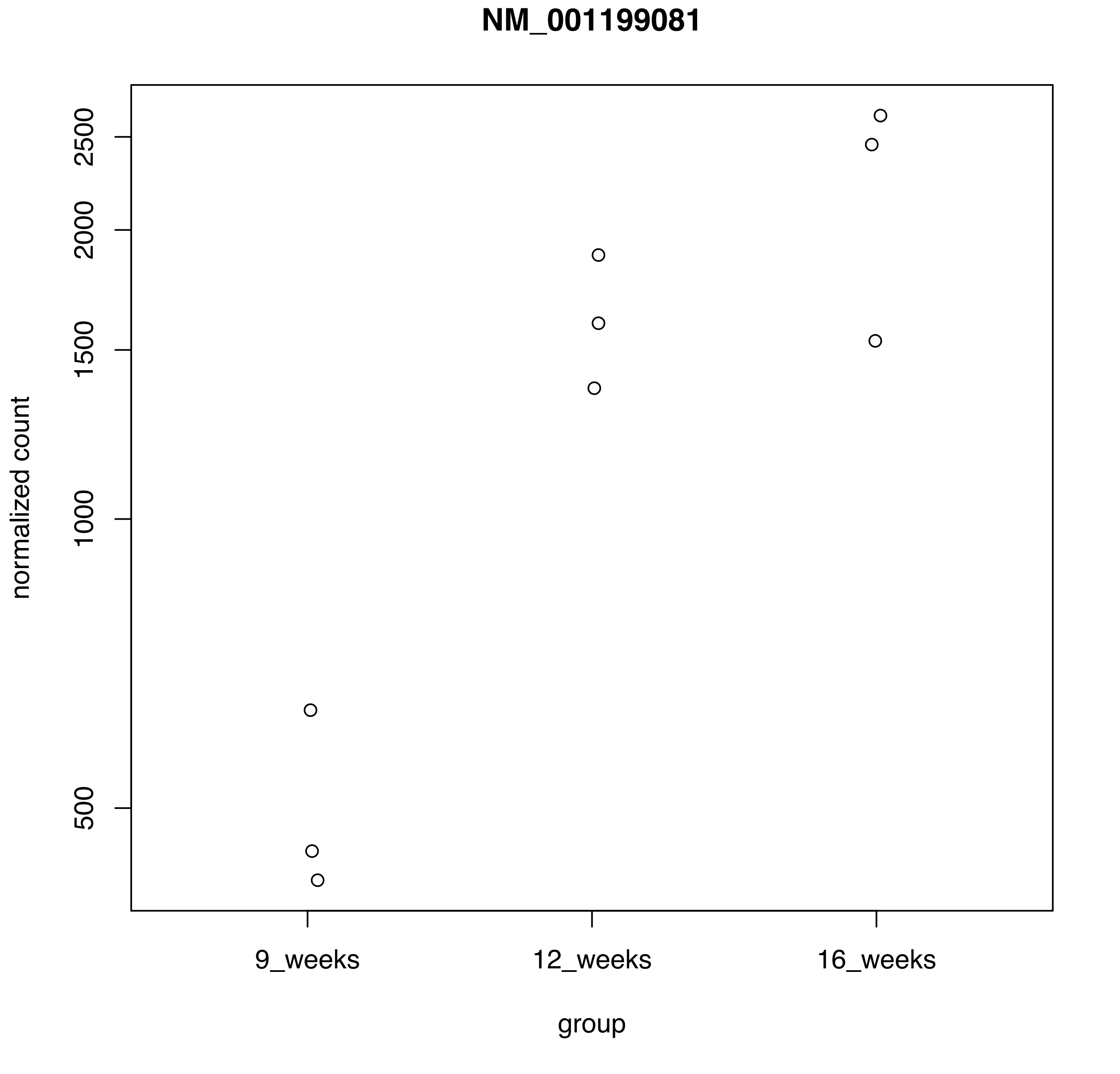
NM_001199081
normalized count
2500
2000
1500
1000
500
9_weeks
12_weeks
16_weeks
group

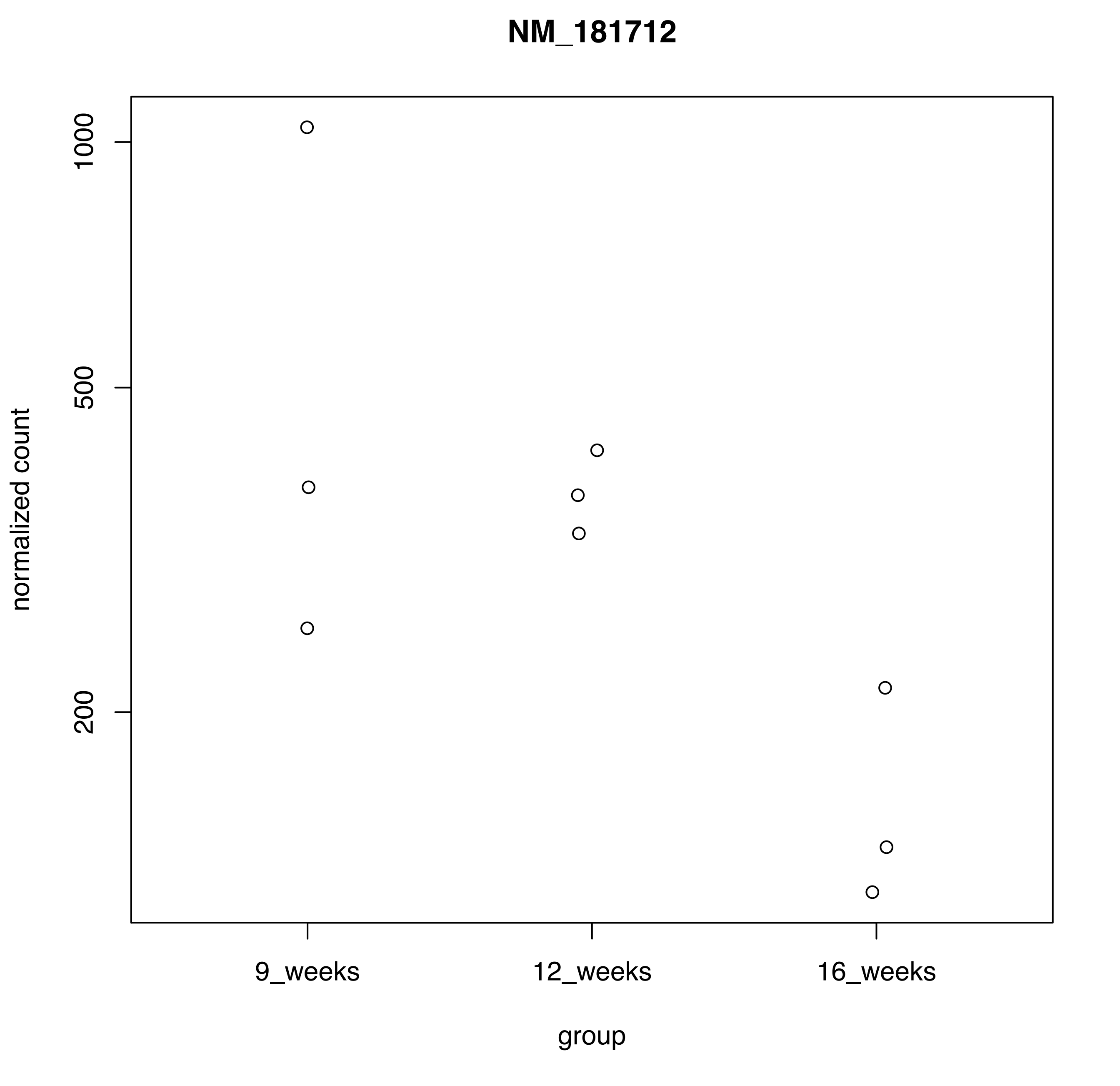
NM_181712
normalized count
1000
500
200
9_weeks
12_weeks
16_weeks
group

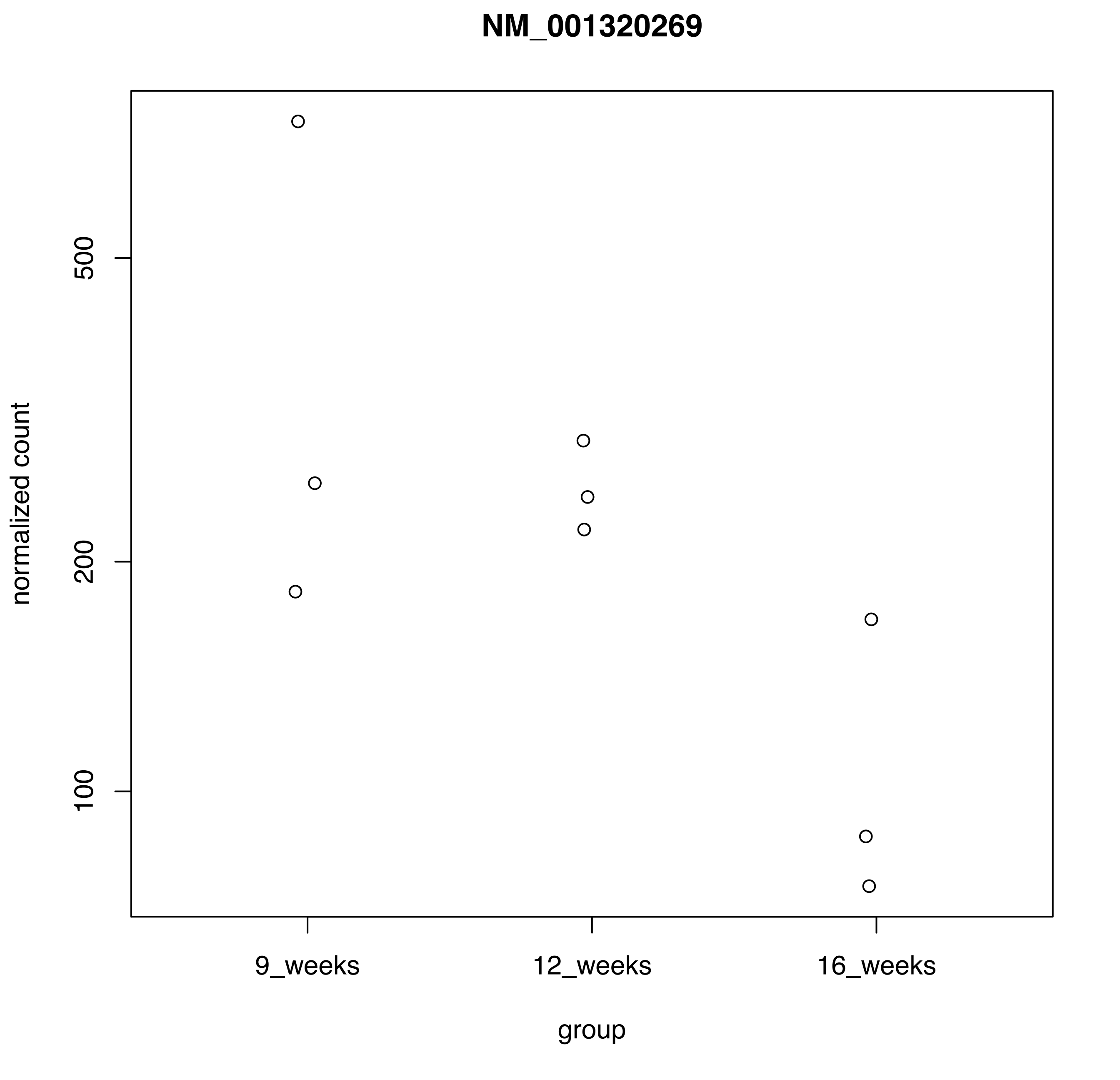
NM_001320269
normalized count
500
200
100
9_weeks
12_weeks
16_weeks
group

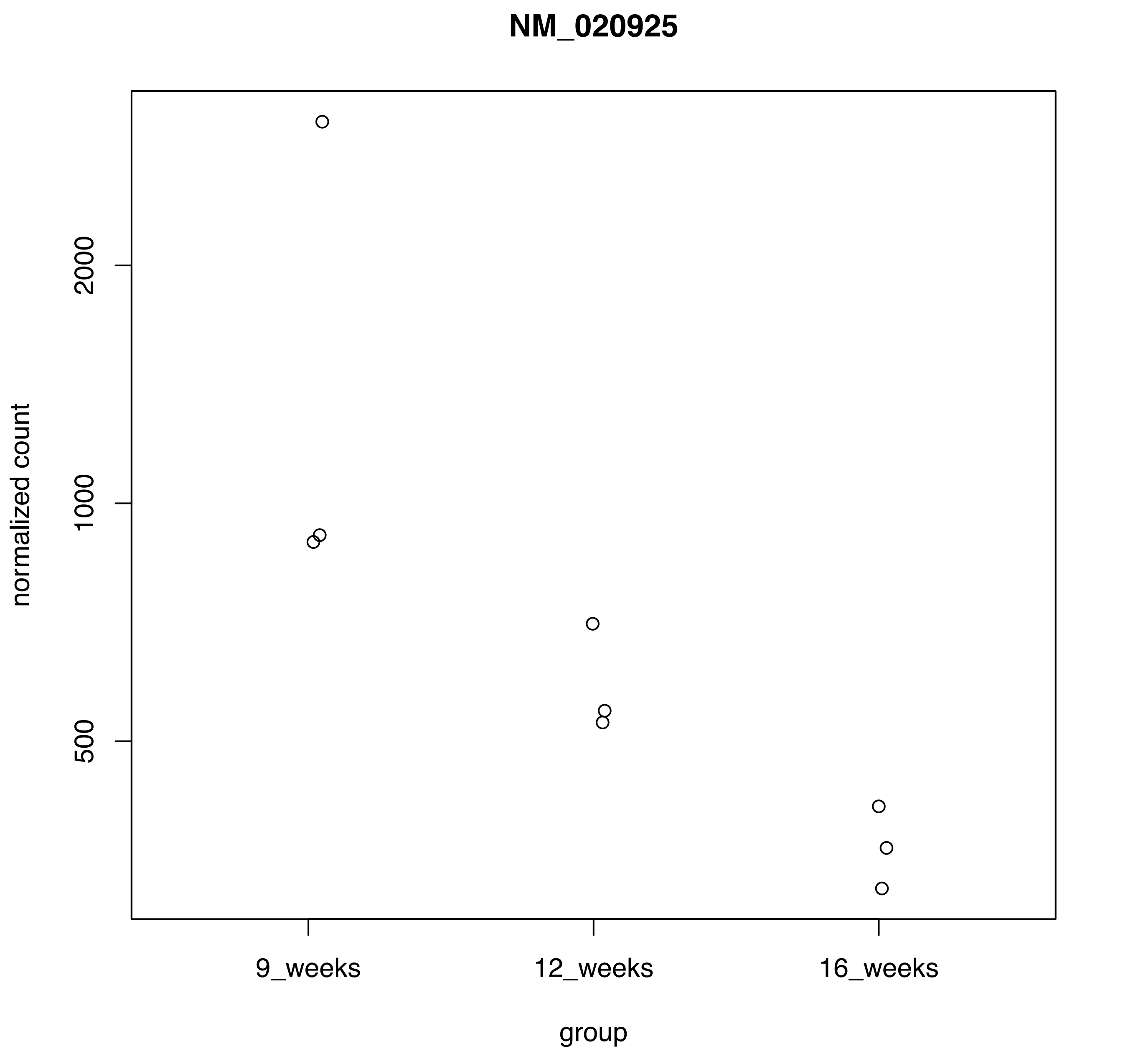
NM_020925
normalized count
2000
1000
500
9_weeks
12_weeks
16_weeks
group

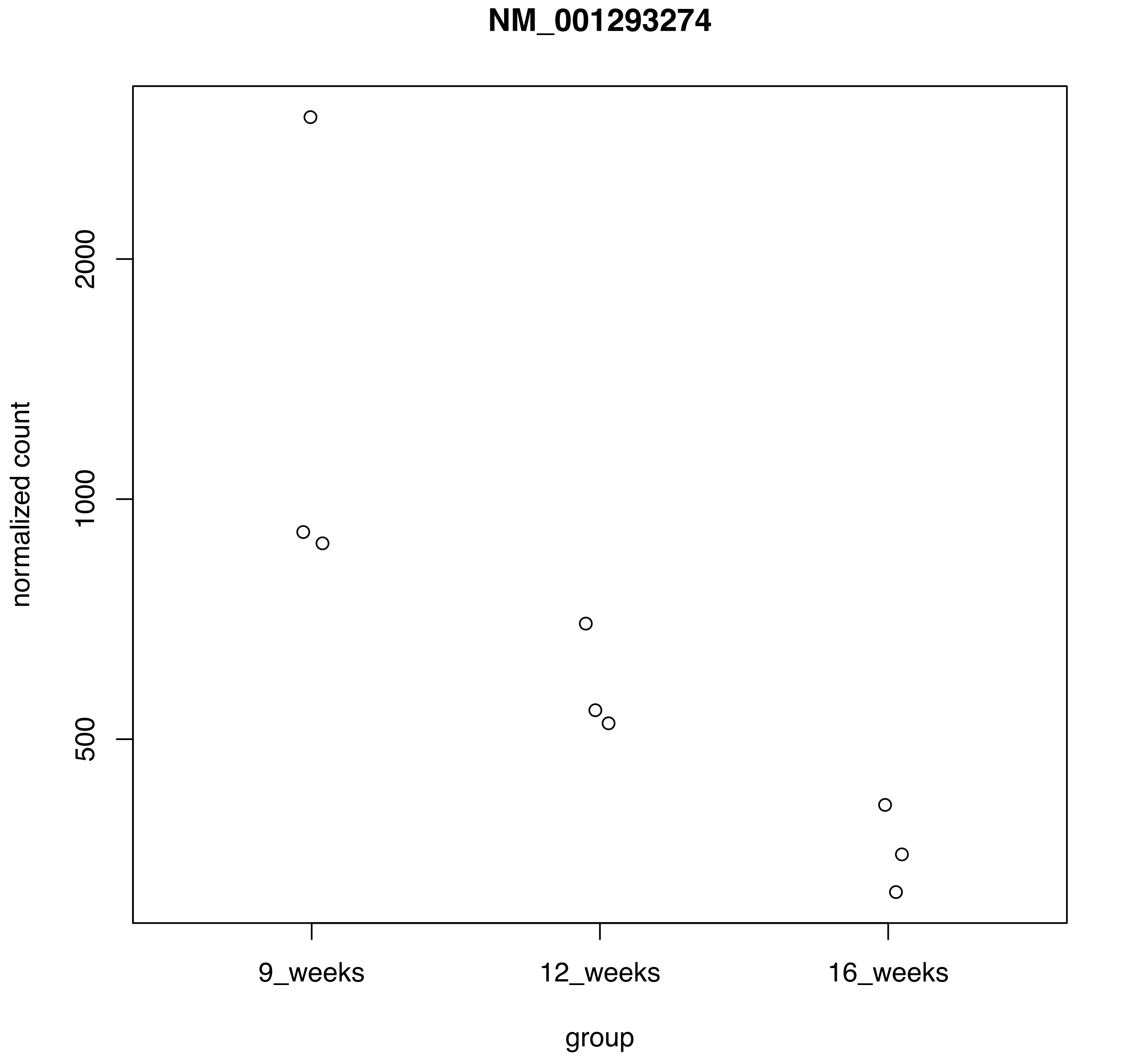
NM_001293274
2000
1000
500
normalized count
9_weeks
12_weeks
16_weeks
group

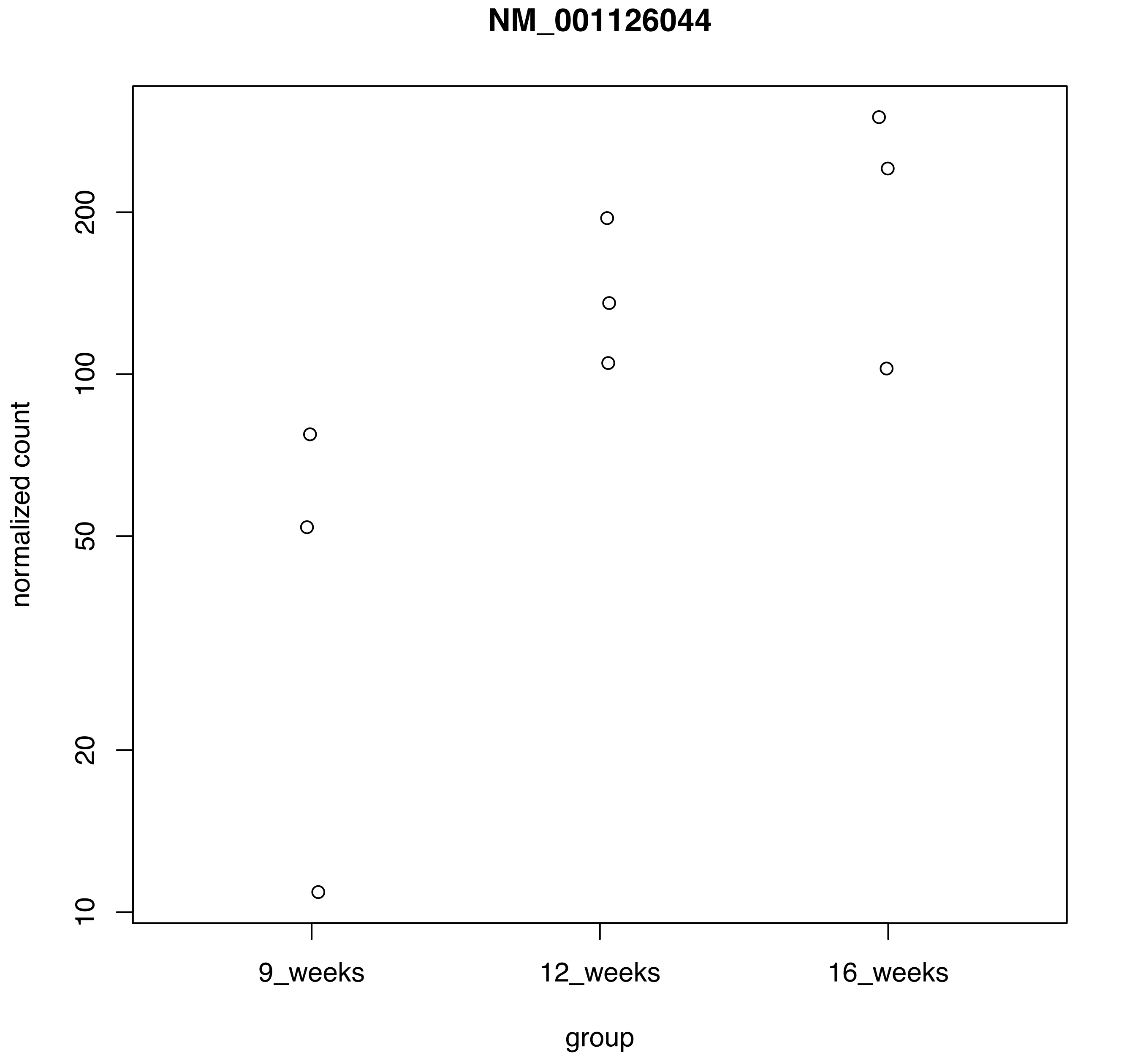

NM_001126044
normalized count
10
20
50
100
200
9_weeks
12_weeks
16_weeks
group

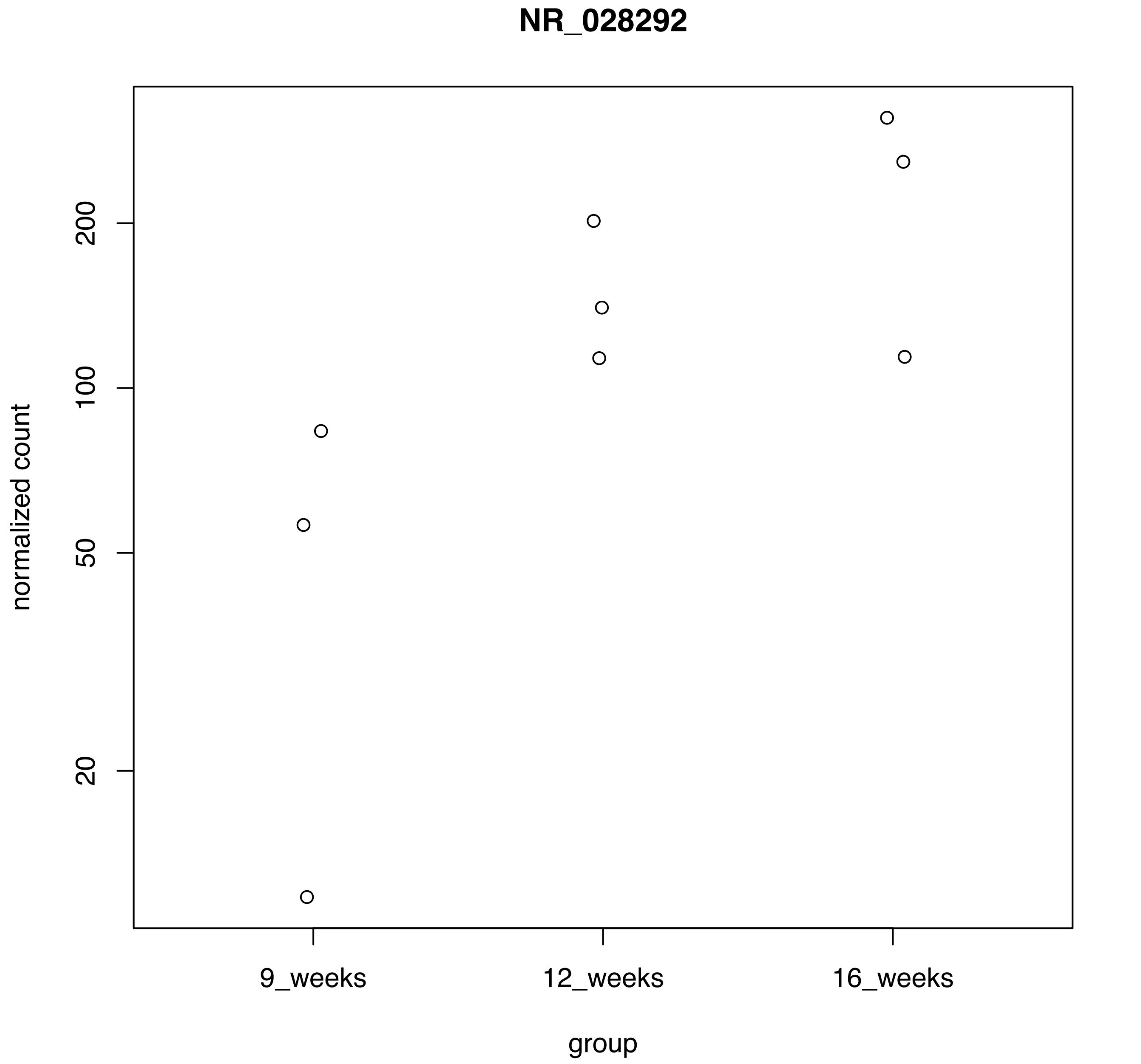

NR_028292
normalized count
20
50
100
200
9_weeks
12_weeks
16_weeks
group

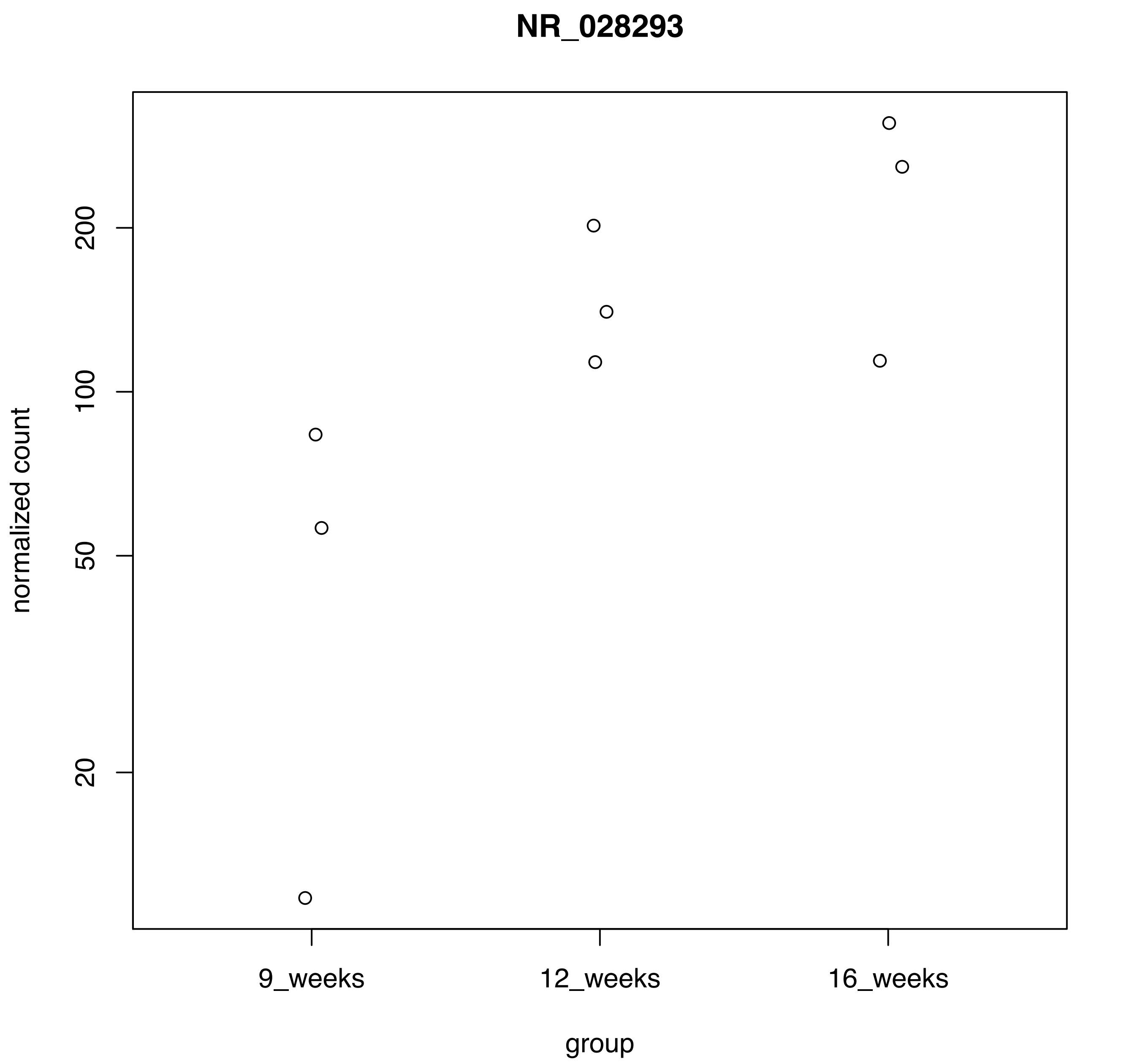
NR_028293
normalized count
200
100
50
20
9_weeks
12_weeks
16_weeks
group

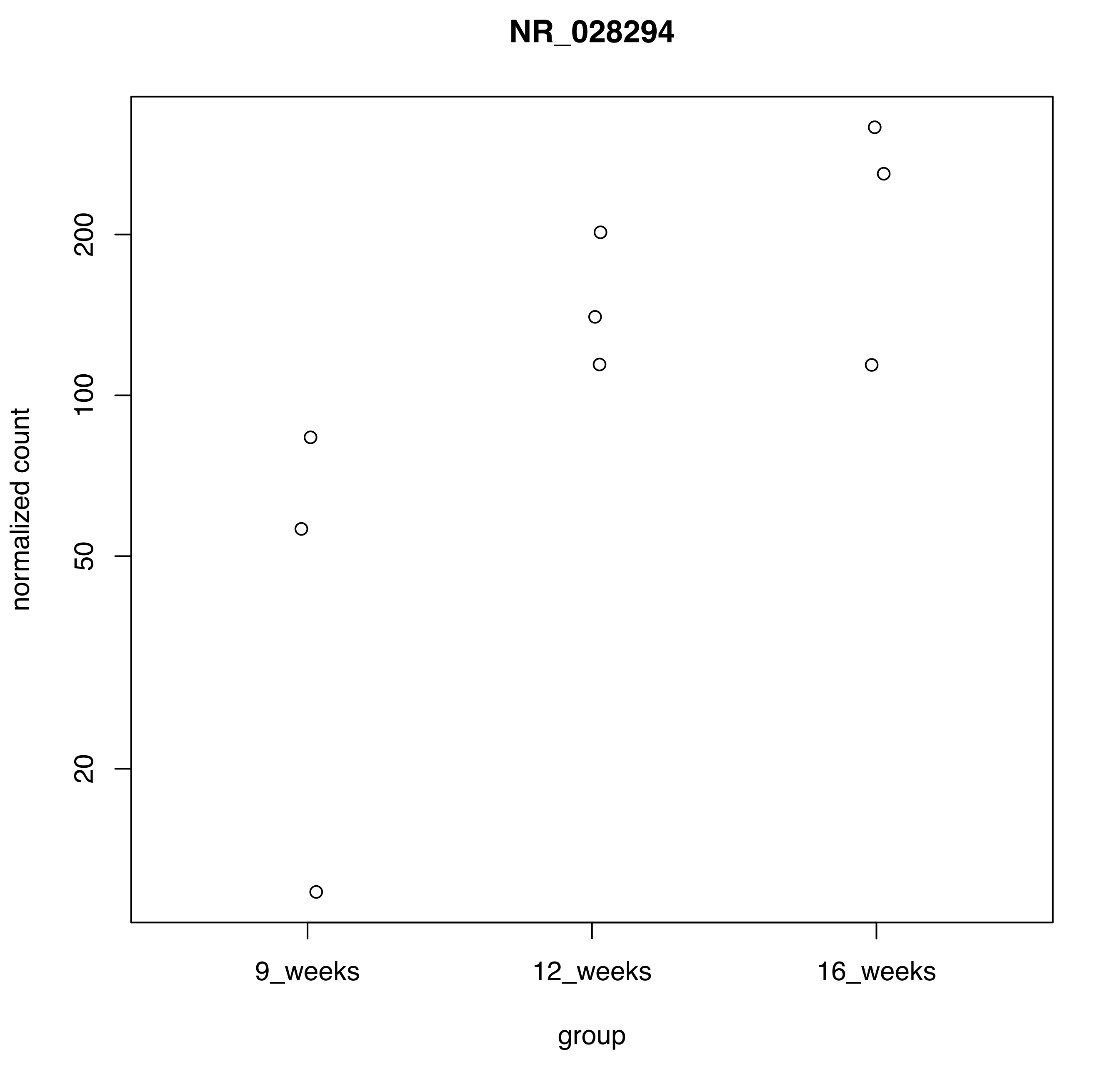
NR_028294
normalized count
200
100
50
20
9_weeks
12_weeks
16_weeks
group

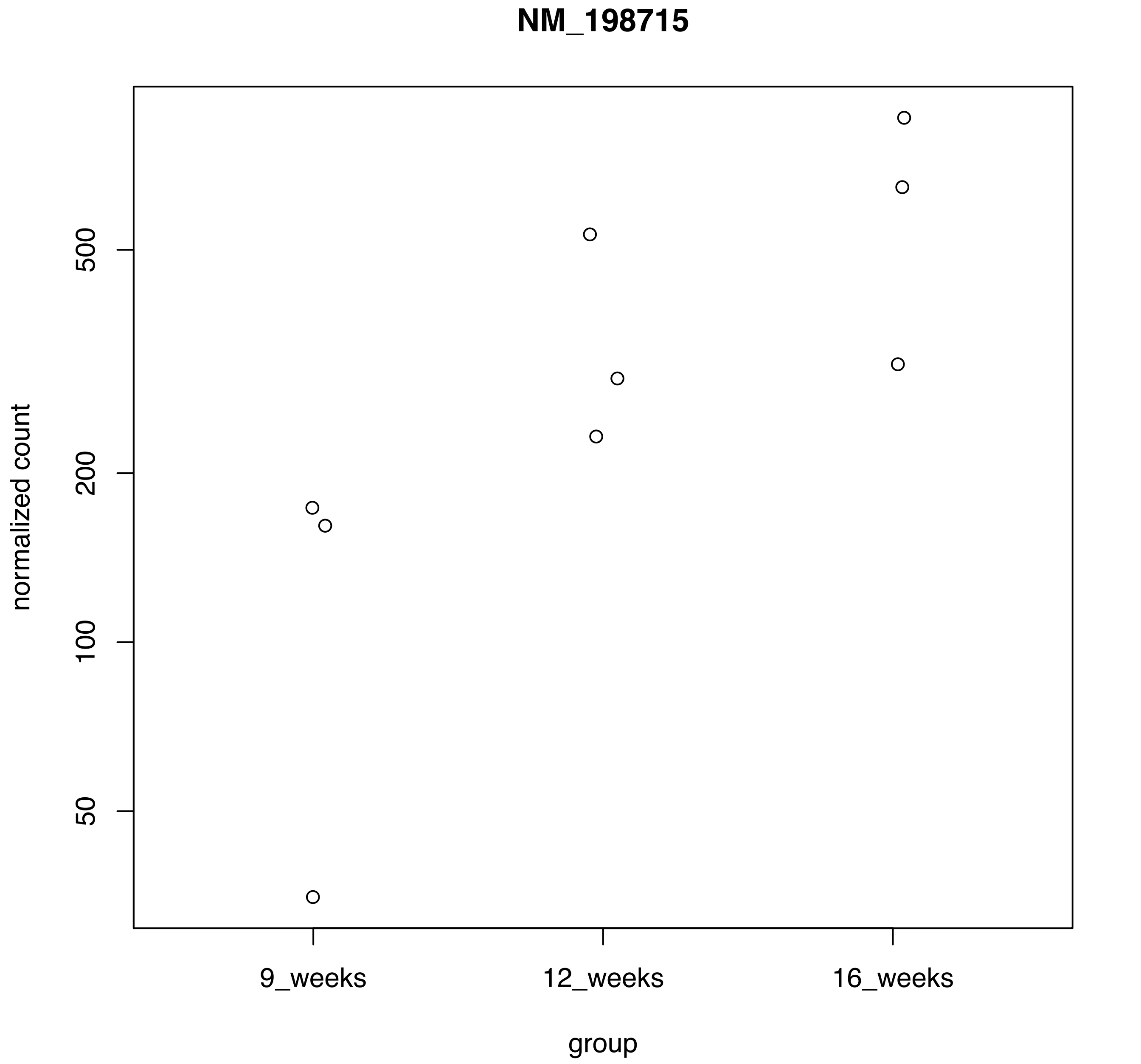
NM_198715
normalized count
500
200
100
50
9_weeks
12_weeks
16_weeks
group

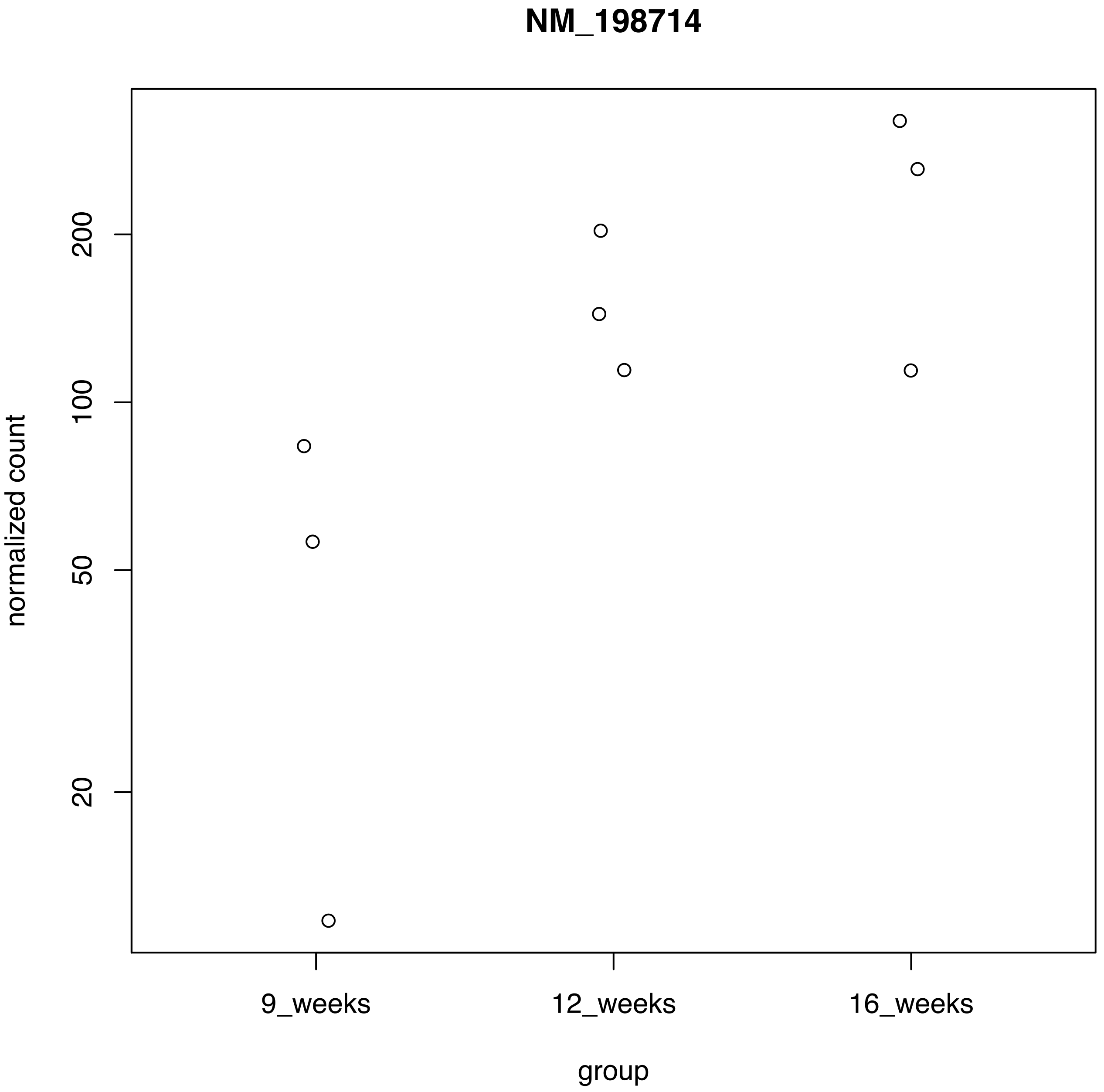
NM_198714
normalized count
200
100
50
20
9_weeks
12_weeks
16_weeks
group

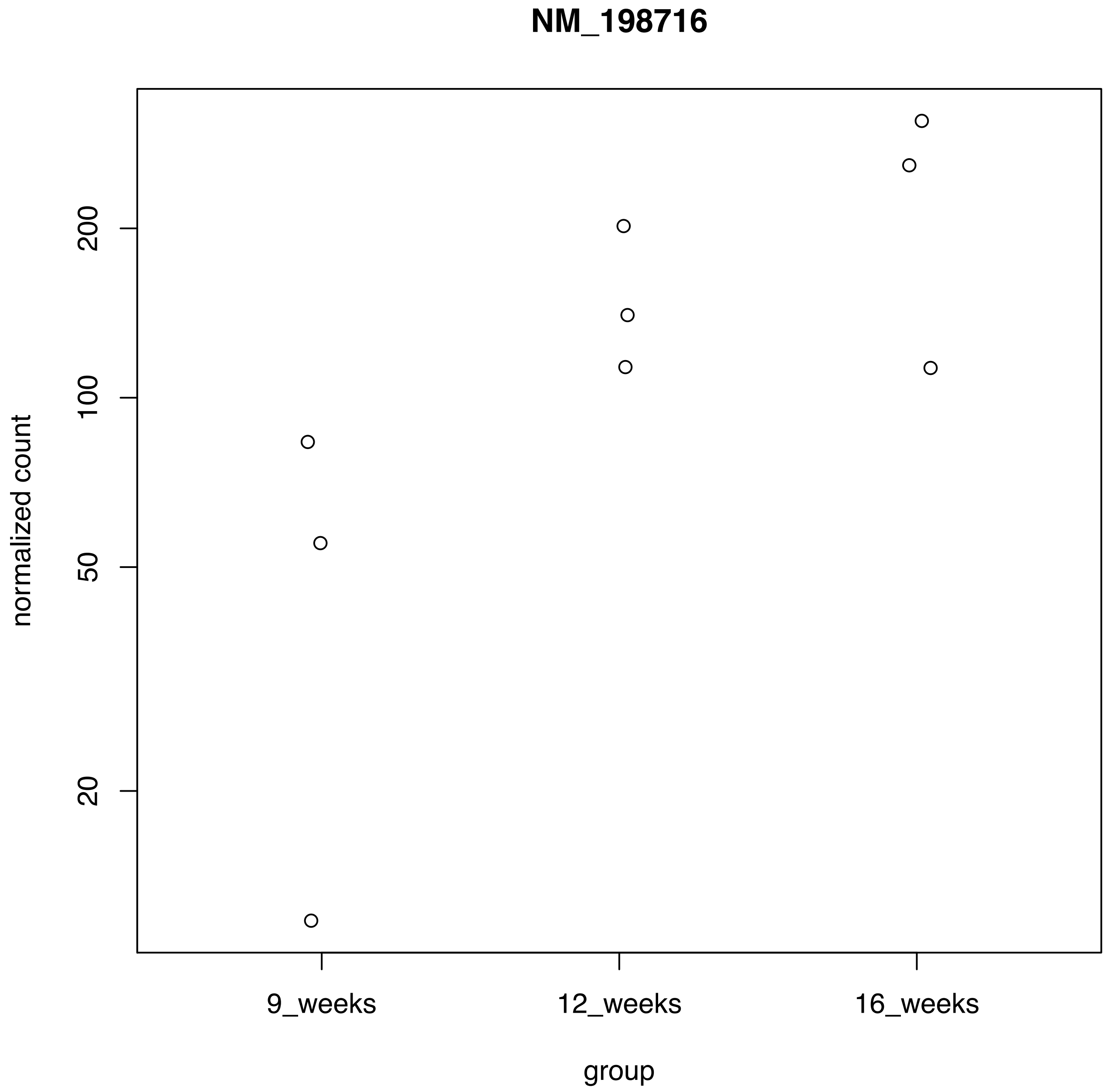
NM_198716
normalized count
200
100
50
20
9_weeks
12_weeks
16_weeks
group

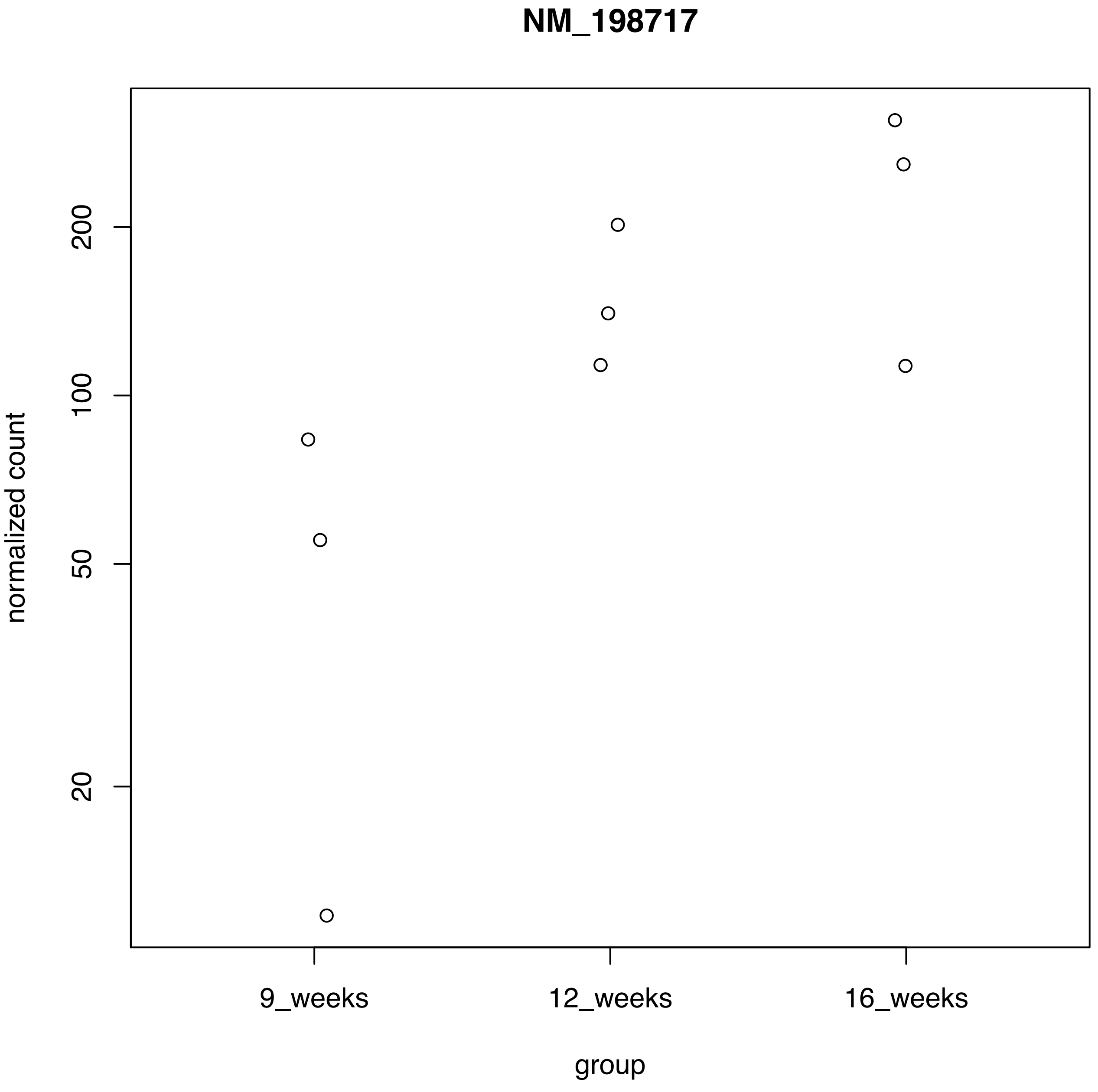

NM_198717
normalized count
20
50
100
200
9_weeks
12_weeks
16_weeks
group

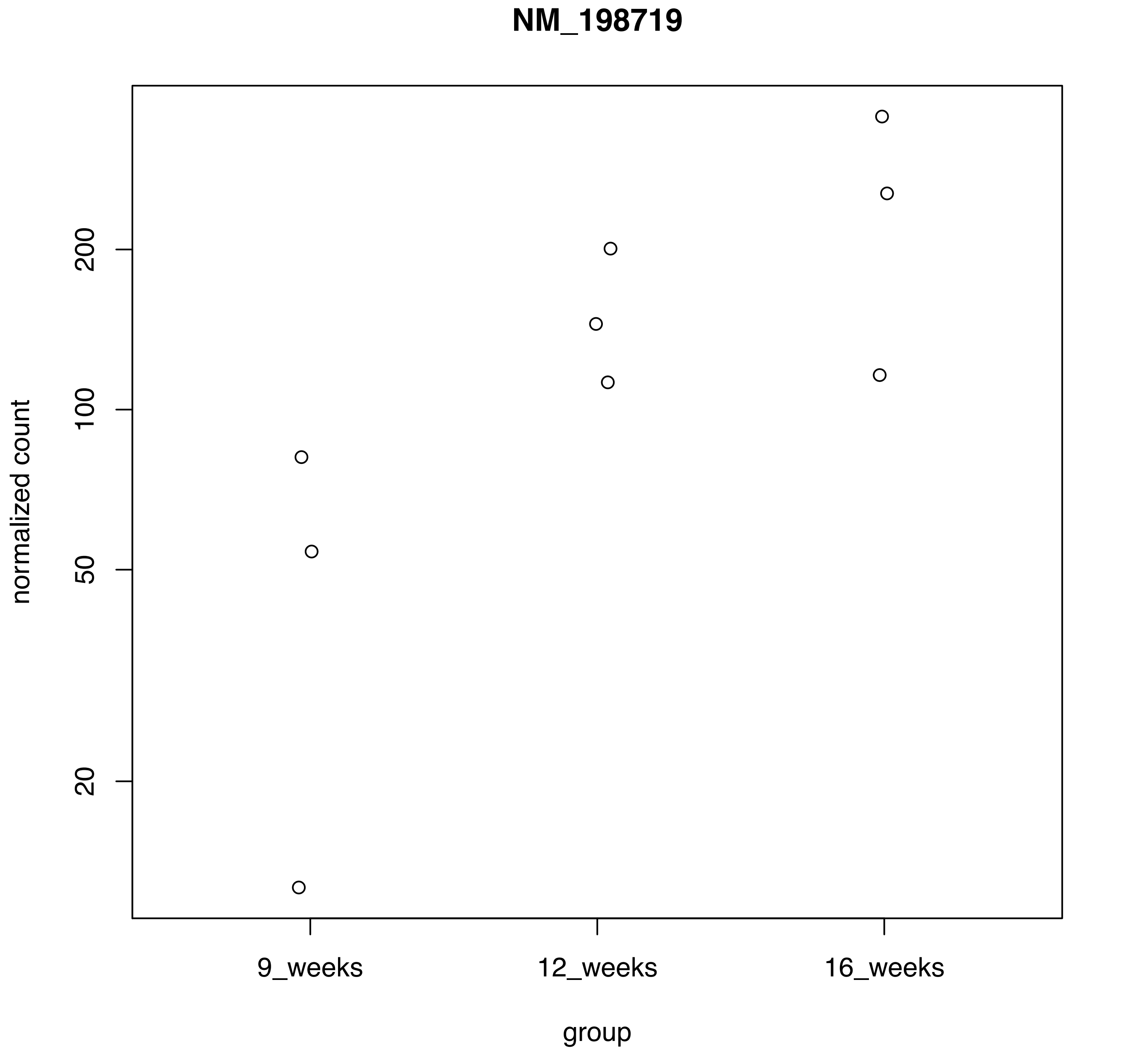
NM_198719
normalized count
200
100
50
20
9_weeks
12_weeks
16_weeks
group

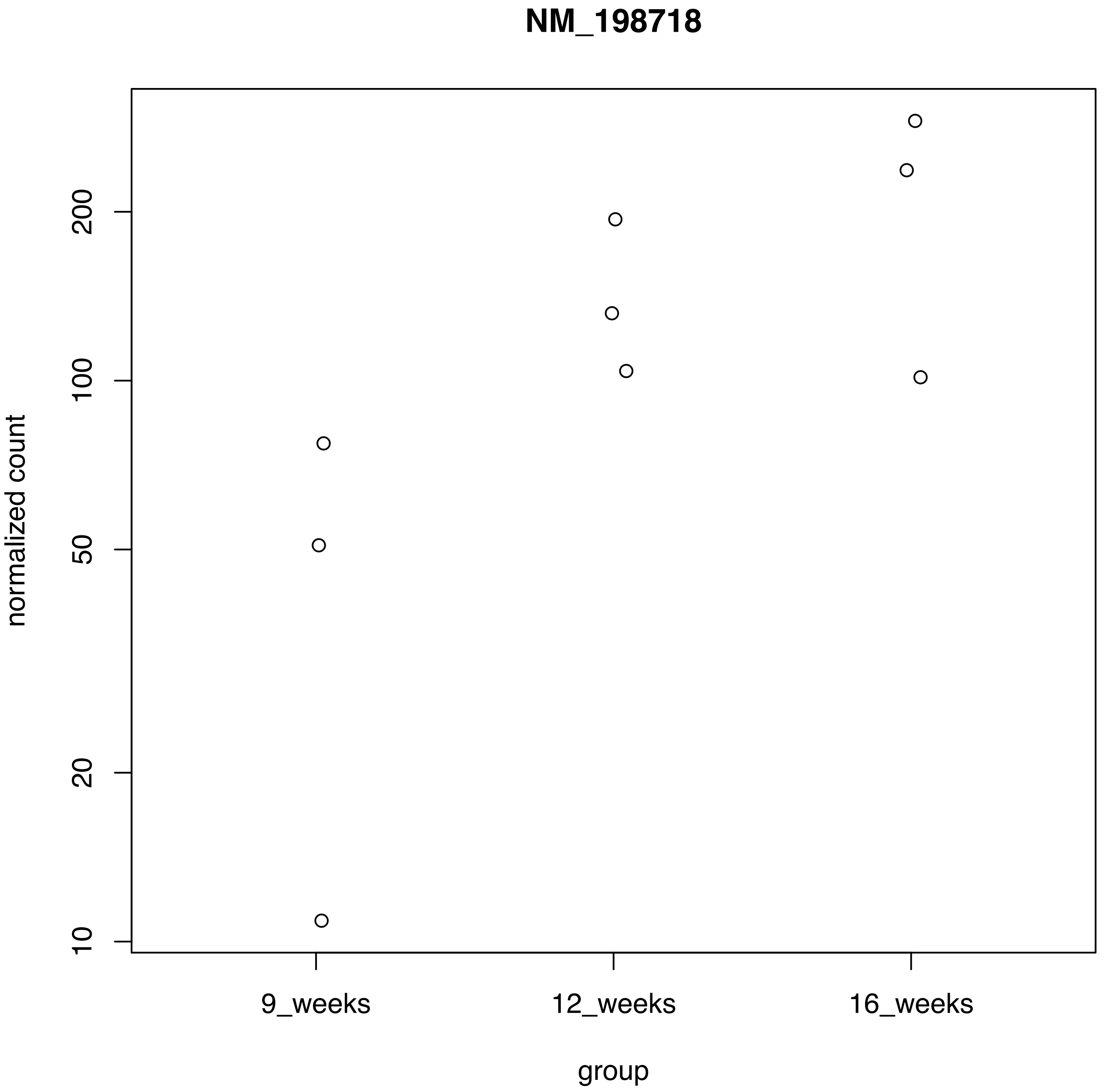

NM_198718
normalized count
10
20
50
100
200
9_weeks
12_weeks
16_weeks
group

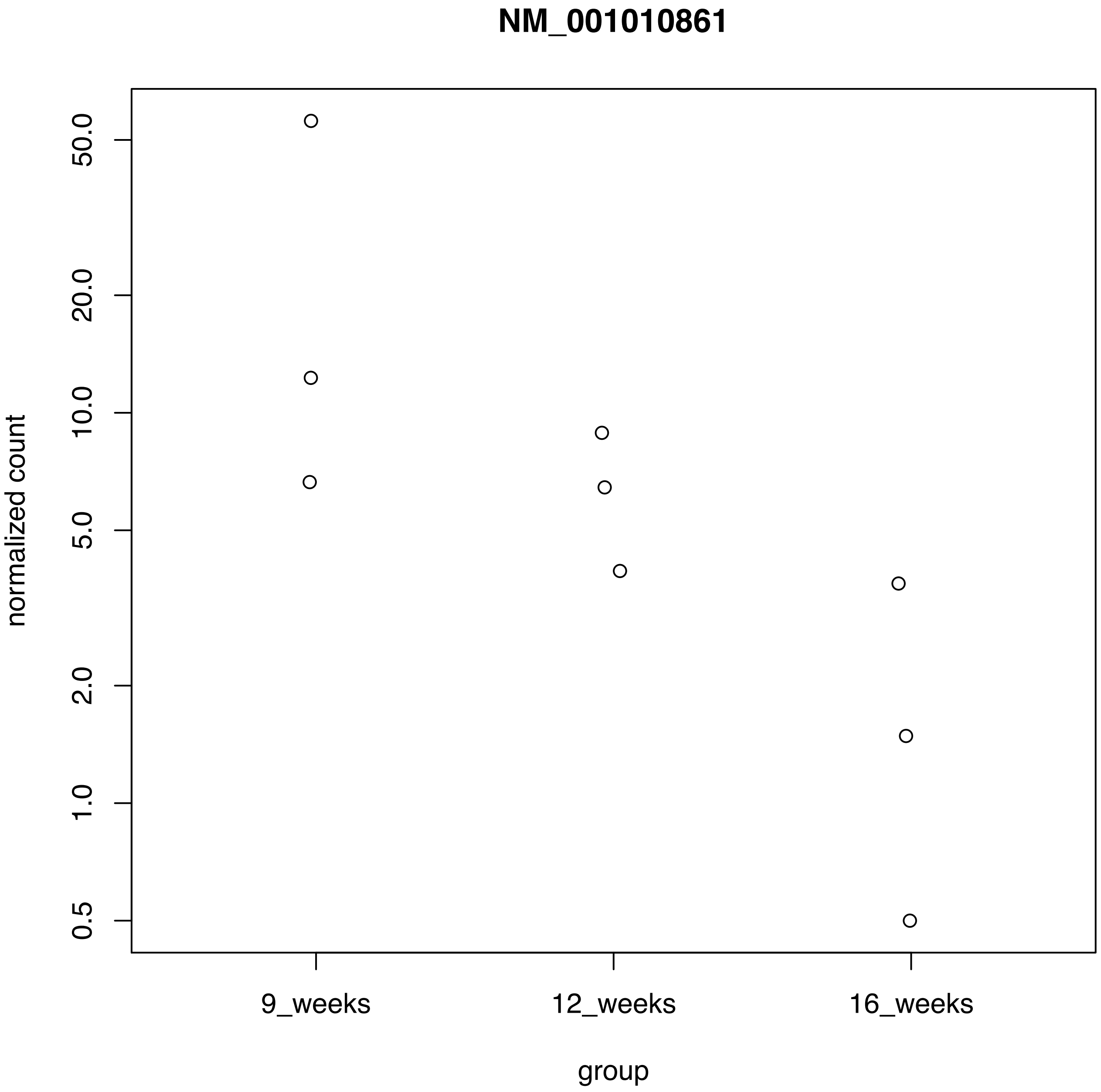
NM_001010861
normalized count
50.0
20.0
10.0
5.0
2.0
1.0
0.5
9_weeks
12_weeks
16_weeks
group

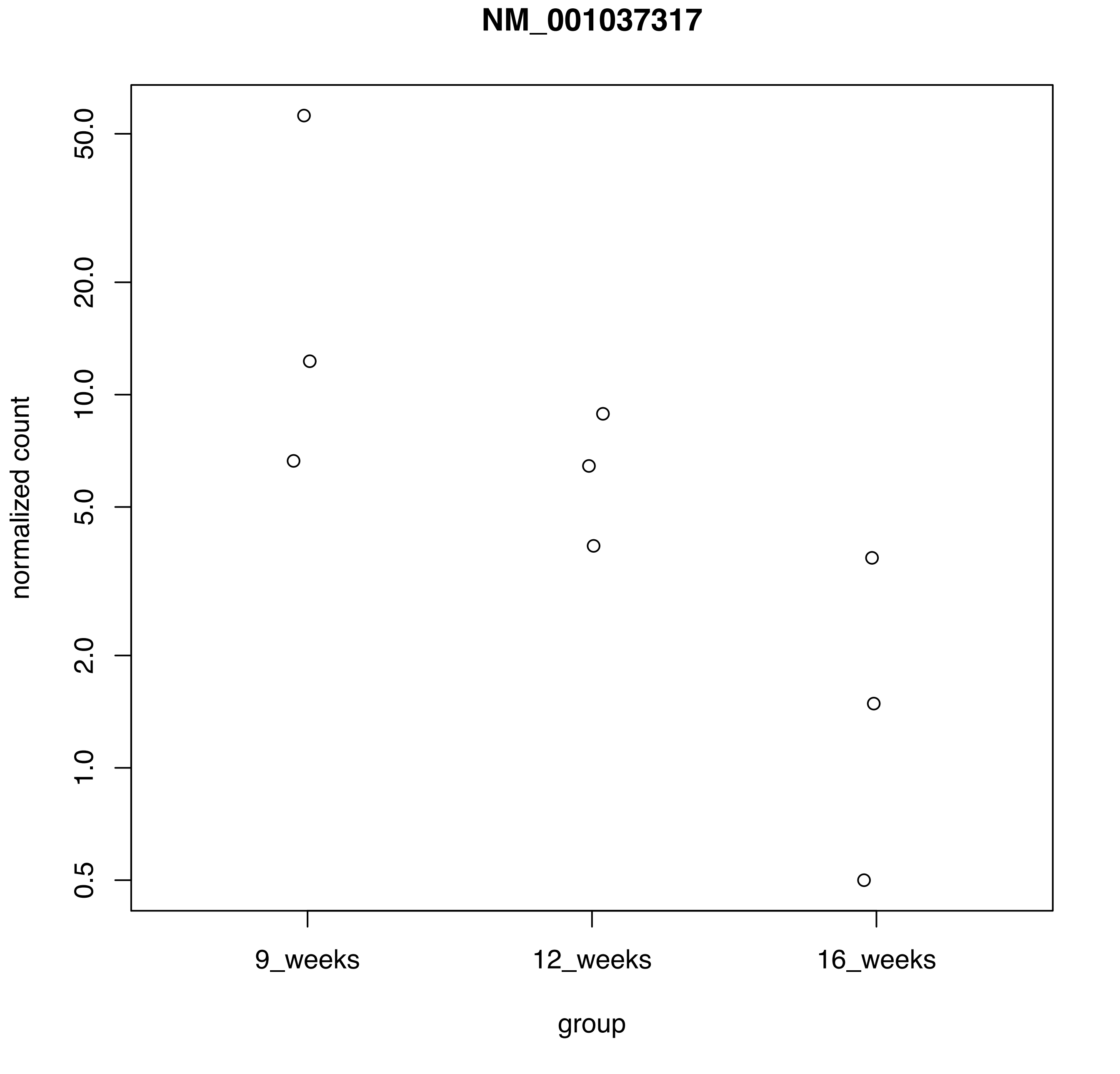

NM_001037317
normalized count
50.0
20.0
10.0
5.0
2.0
1.0
0.5
9_weeks
12_weeks
16_weeks
group

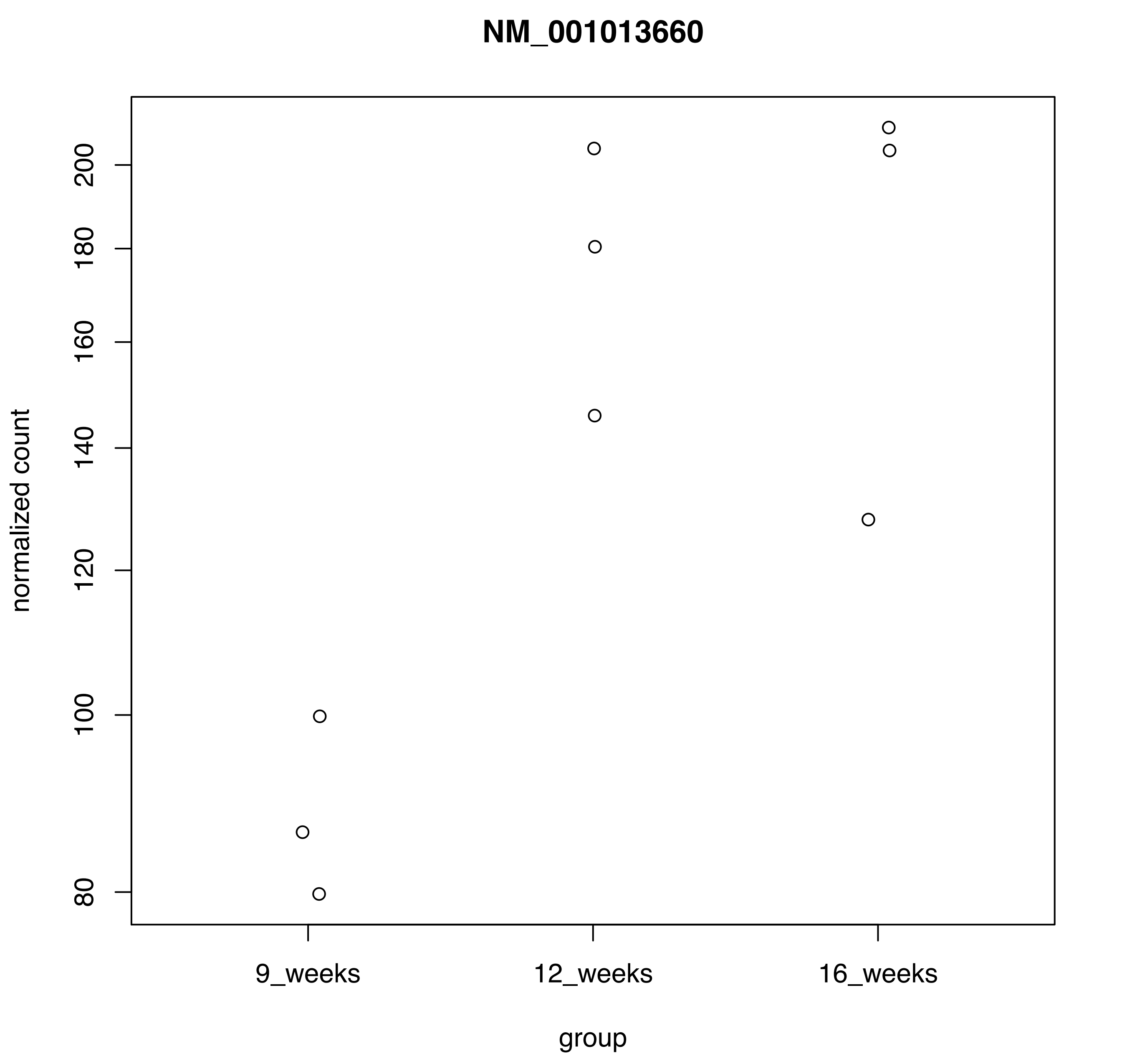
NM_001013660
normalized count
80
100
120
140
160
180
200
9_weeks
12_weeks
16_weeks
group

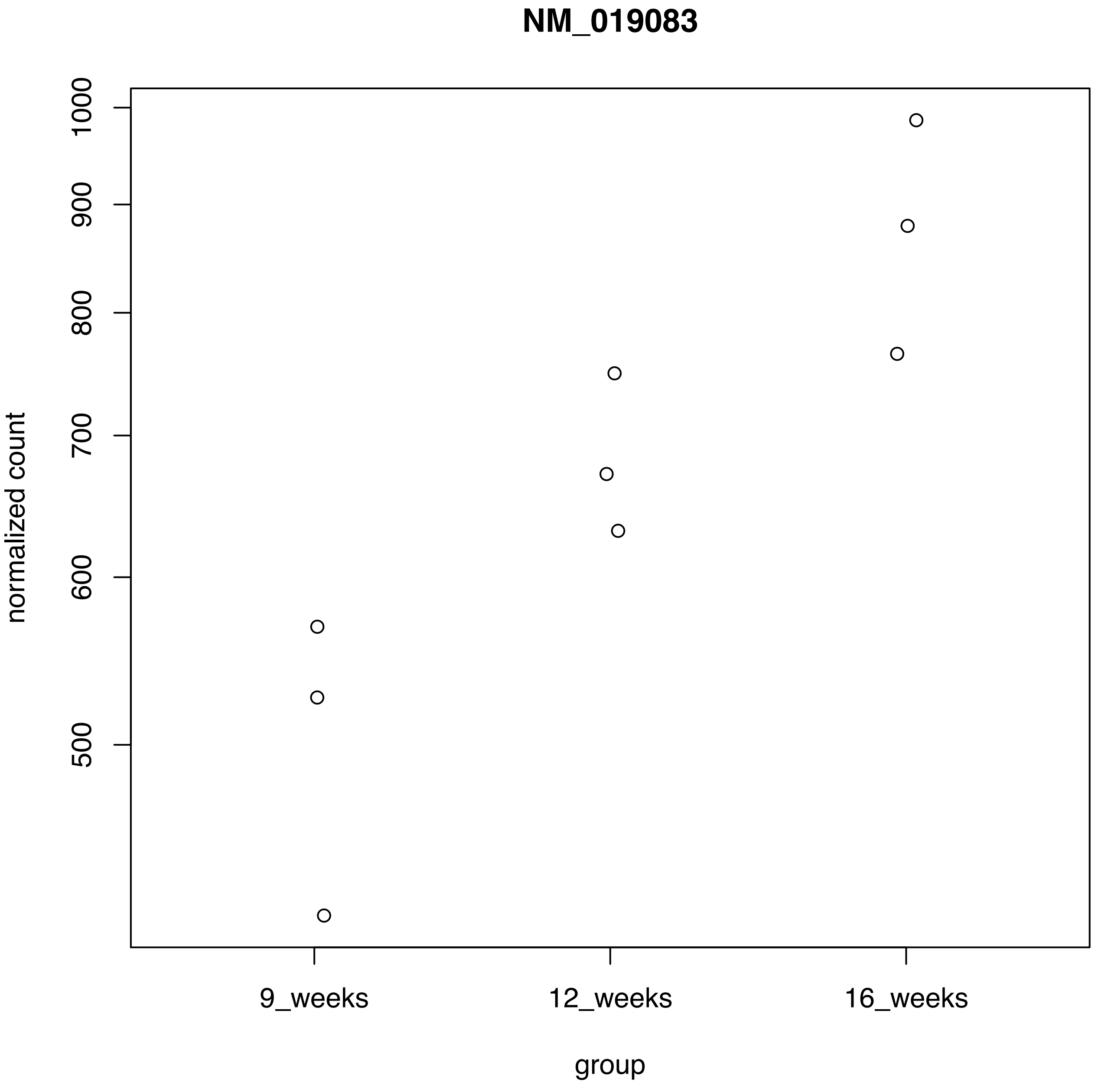
NM_019083
normalized count
1000
900
800
700
600
500
9_weeks
12_weeks
16_weeks
group

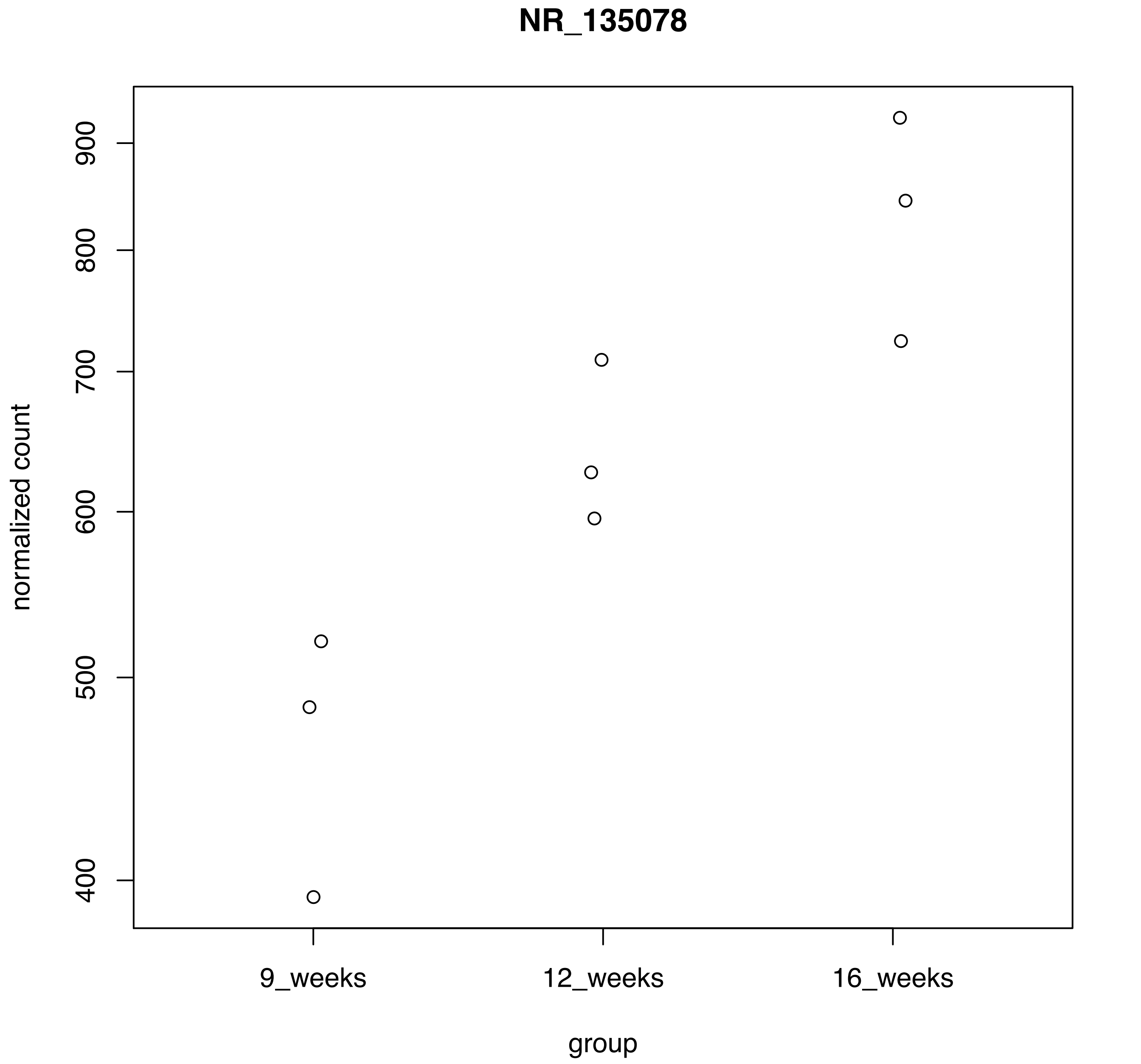
NR_135078
normalized count
900
800
700
600
500
400
9_weeks
12_weeks
16_weeks
group

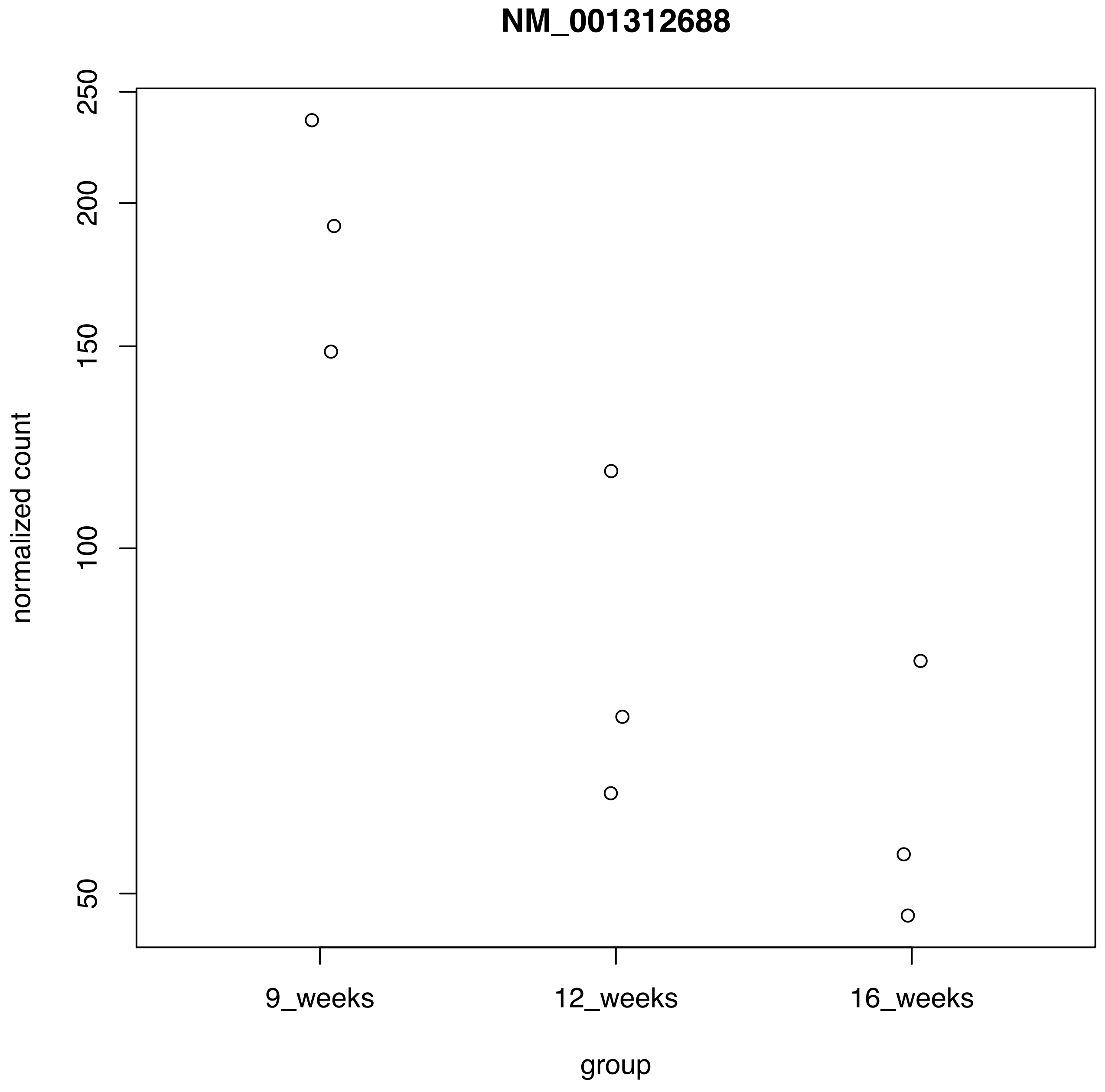
NM_001312688
normalized count
250
200
150
100
50
9_weeks
12_weeks
16_weeks
group

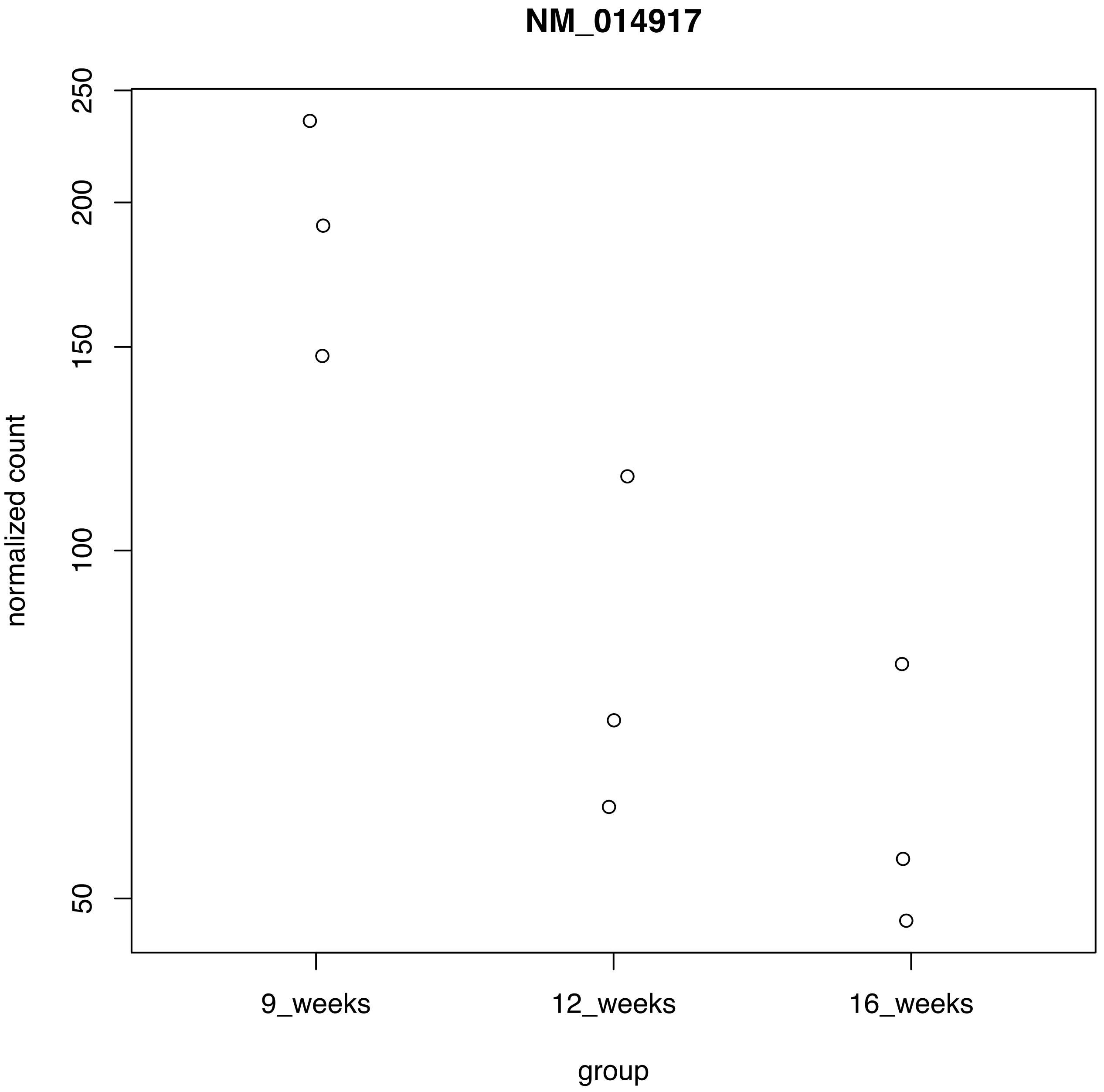
NM_014917
250
200
150
100
50
normalized count
9_weeks
12_weeks
16_weeks
group

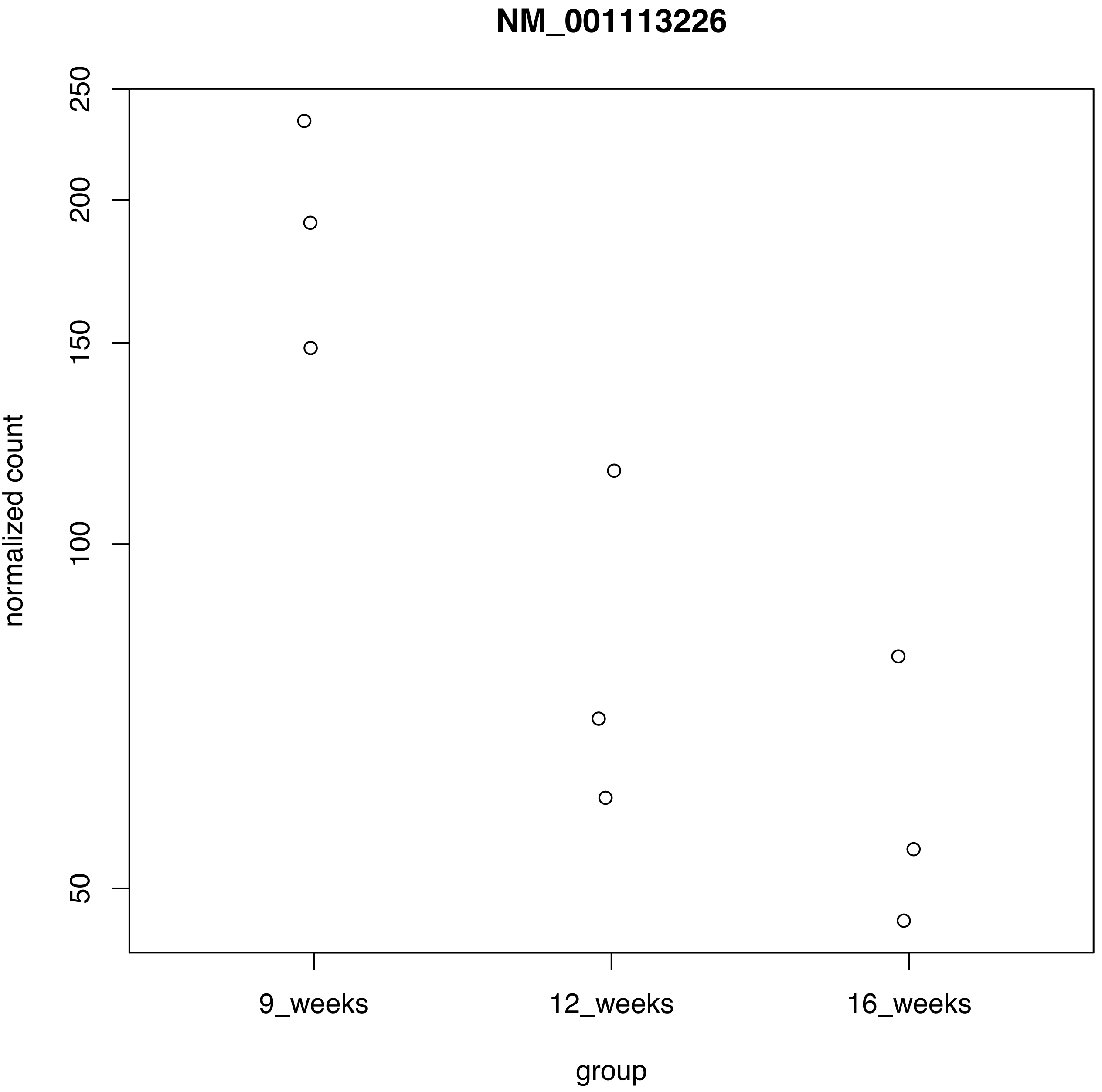
NM_001113226
normalized count
250
200
150
100
50
9_weeks
12_weeks
16_weeks
group

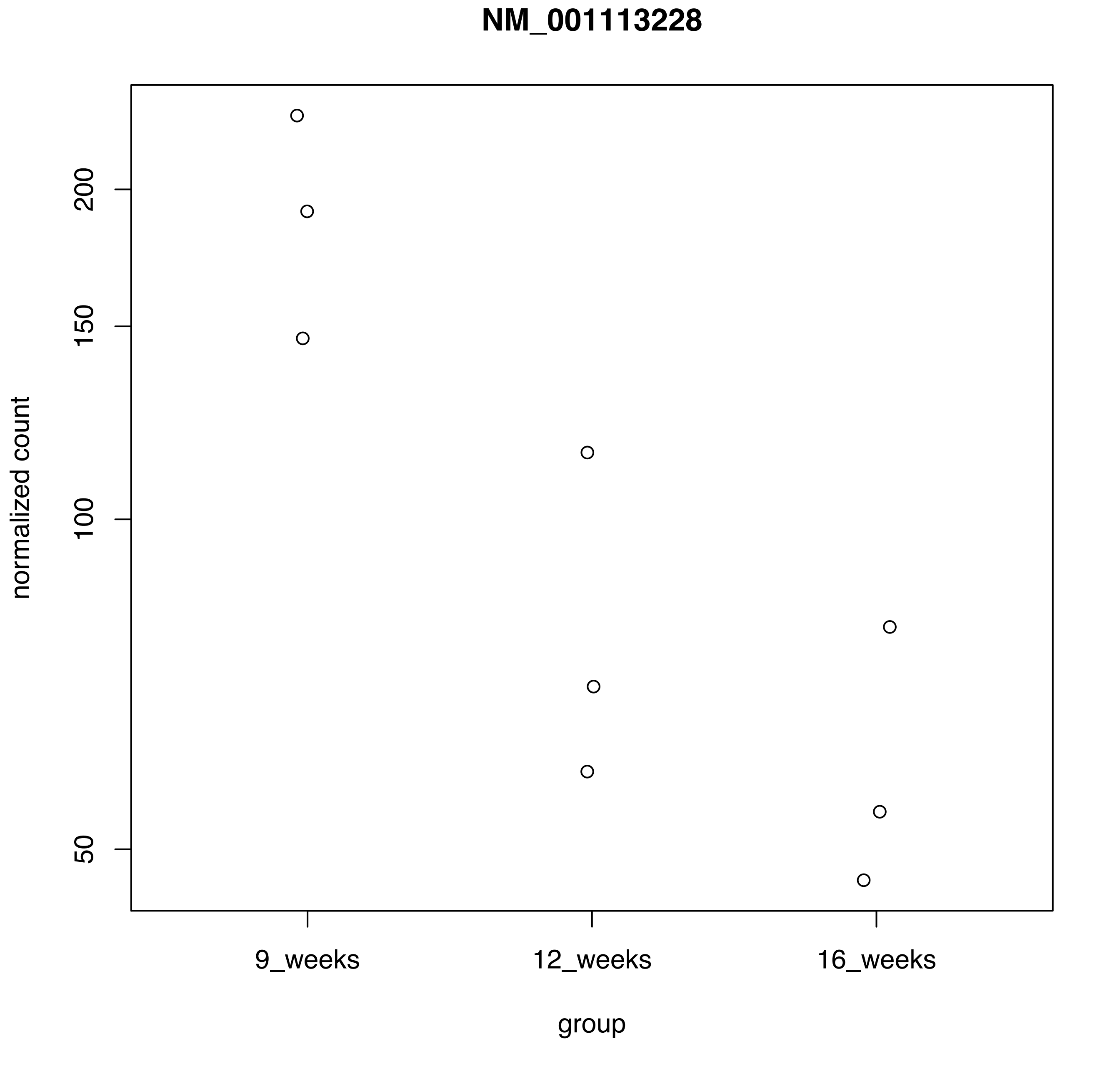
NM_001113228
normalized count
200
150
100
50
9_weeks
12_weeks
16_weeks
group

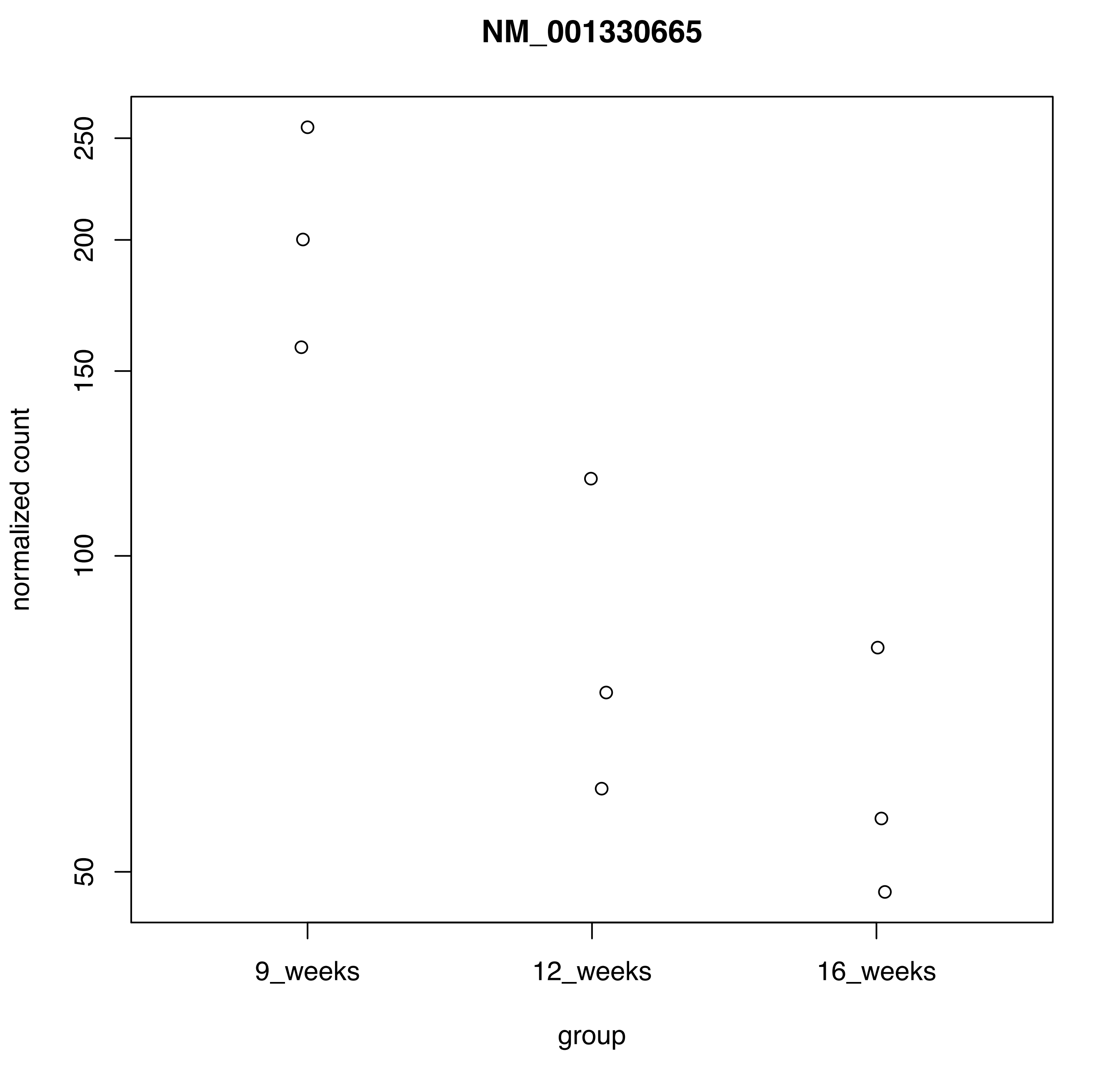

NM_001330665
normalized count
250
200
150
100
50
9_weeks
12_weeks
16_weeks
group

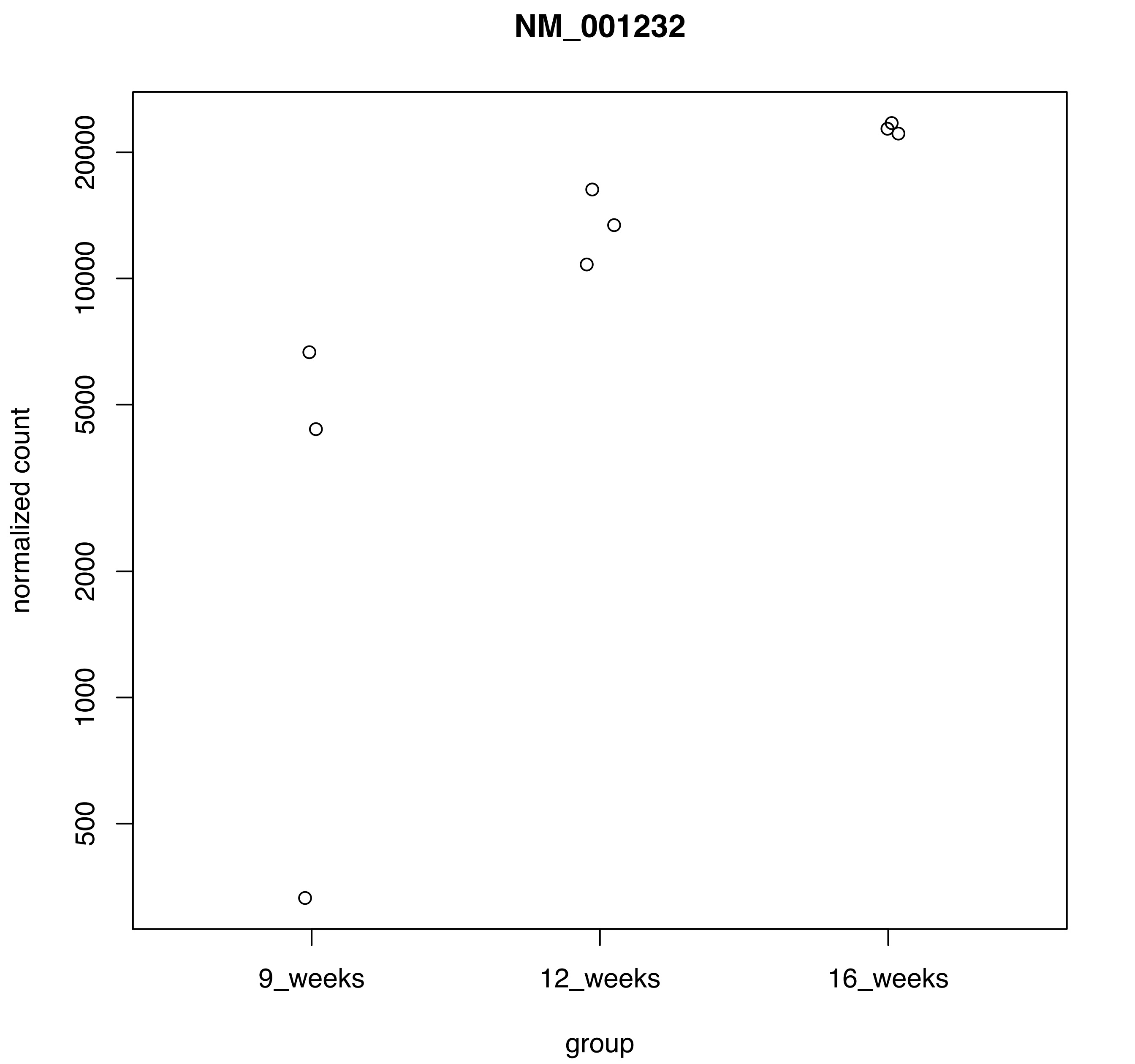
NM_001232
normalized count
20000
10000
5000
2000
1000
500
9_weeks
12_weeks
16_weeks
group

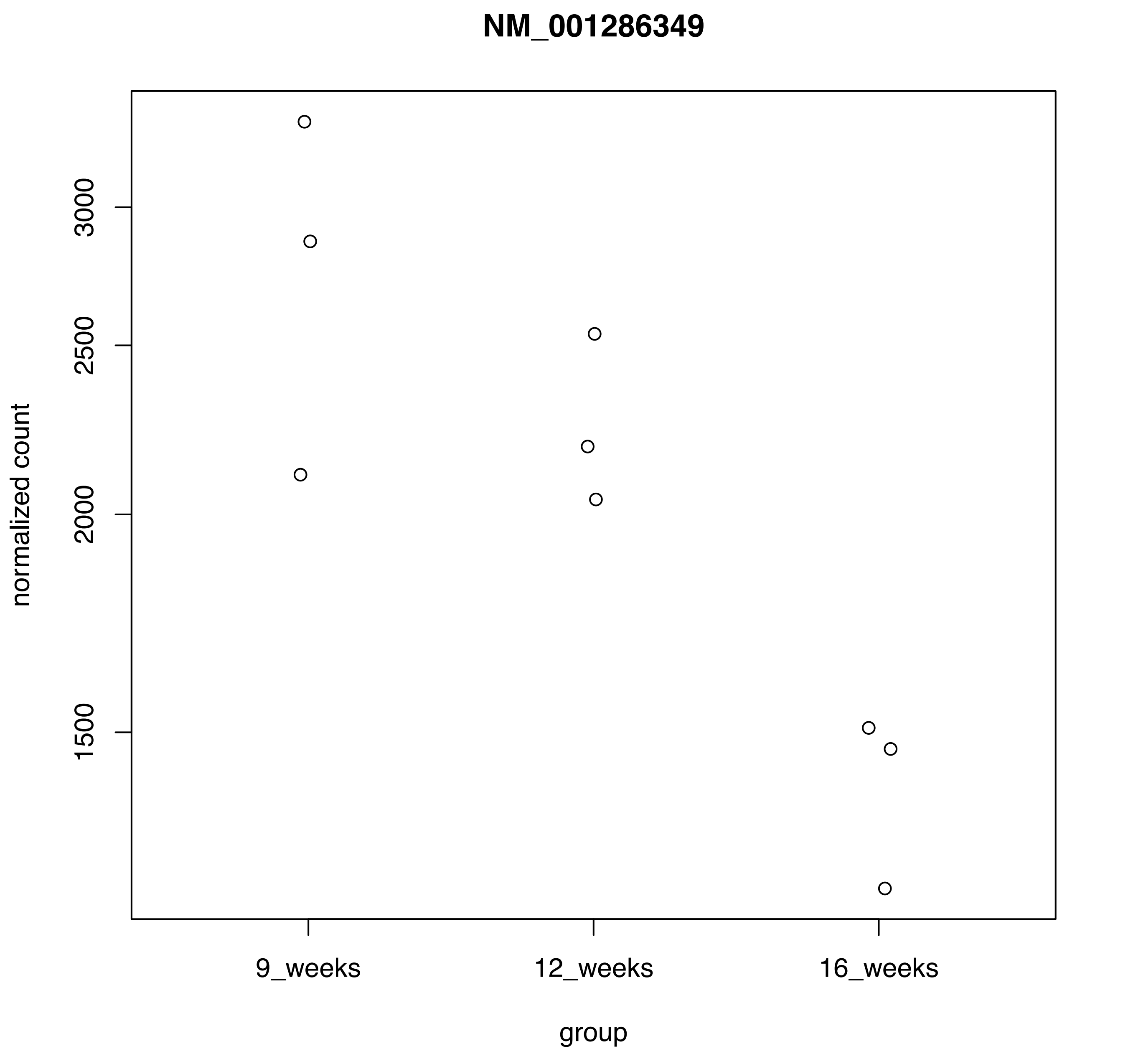
NM_001286349
normalized count
3000
2500
2000
1500
9_weeks
12_weeks
16_weeks
group

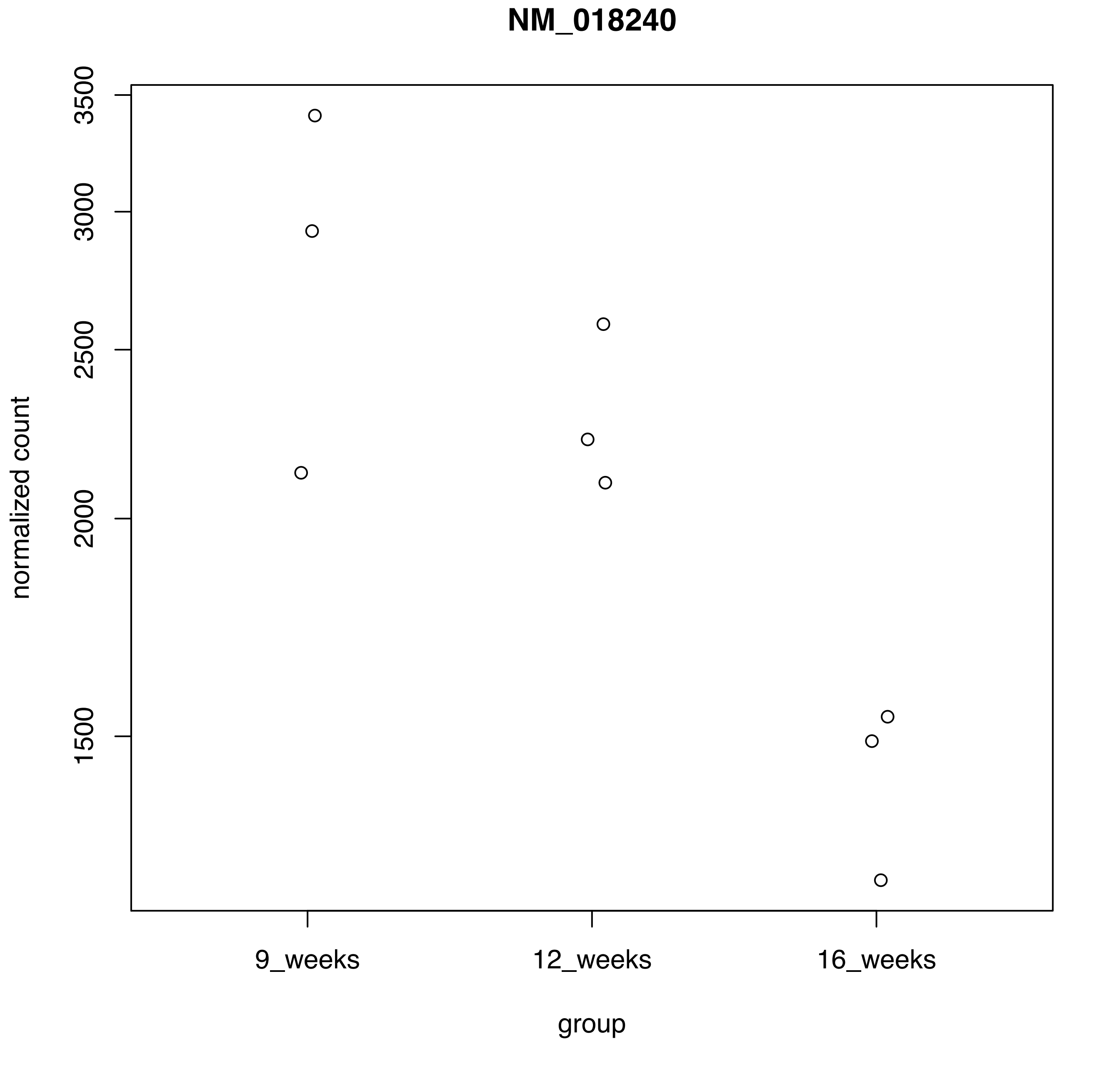
NM_018240
normalized count
3500
3000
2500
2000
1500
9_weeks
12_weeks
16_weeks
group

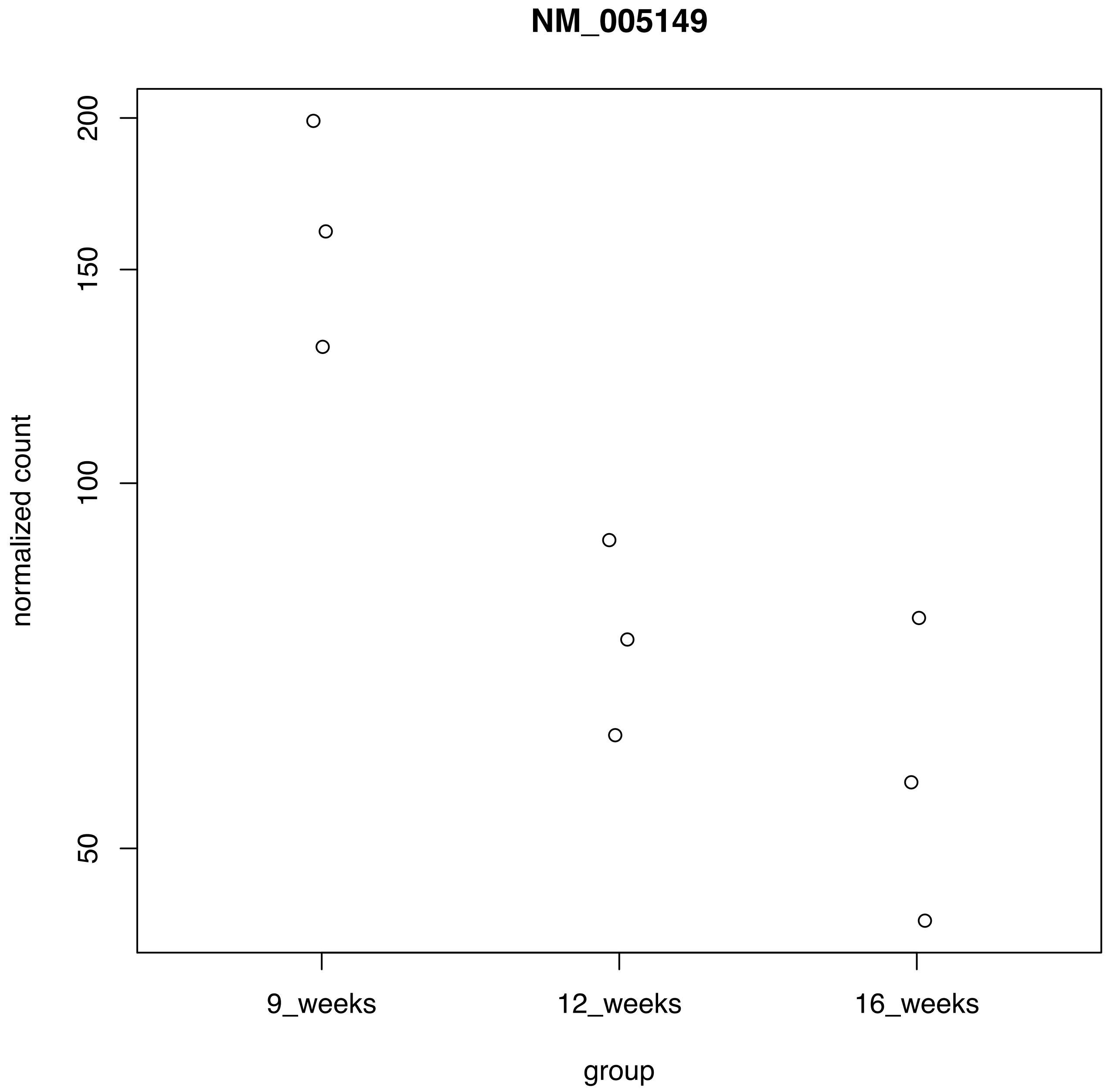
NM_005149
200
150
100
50
normalized count
9_weeks
12_weeks
16_weeks
group

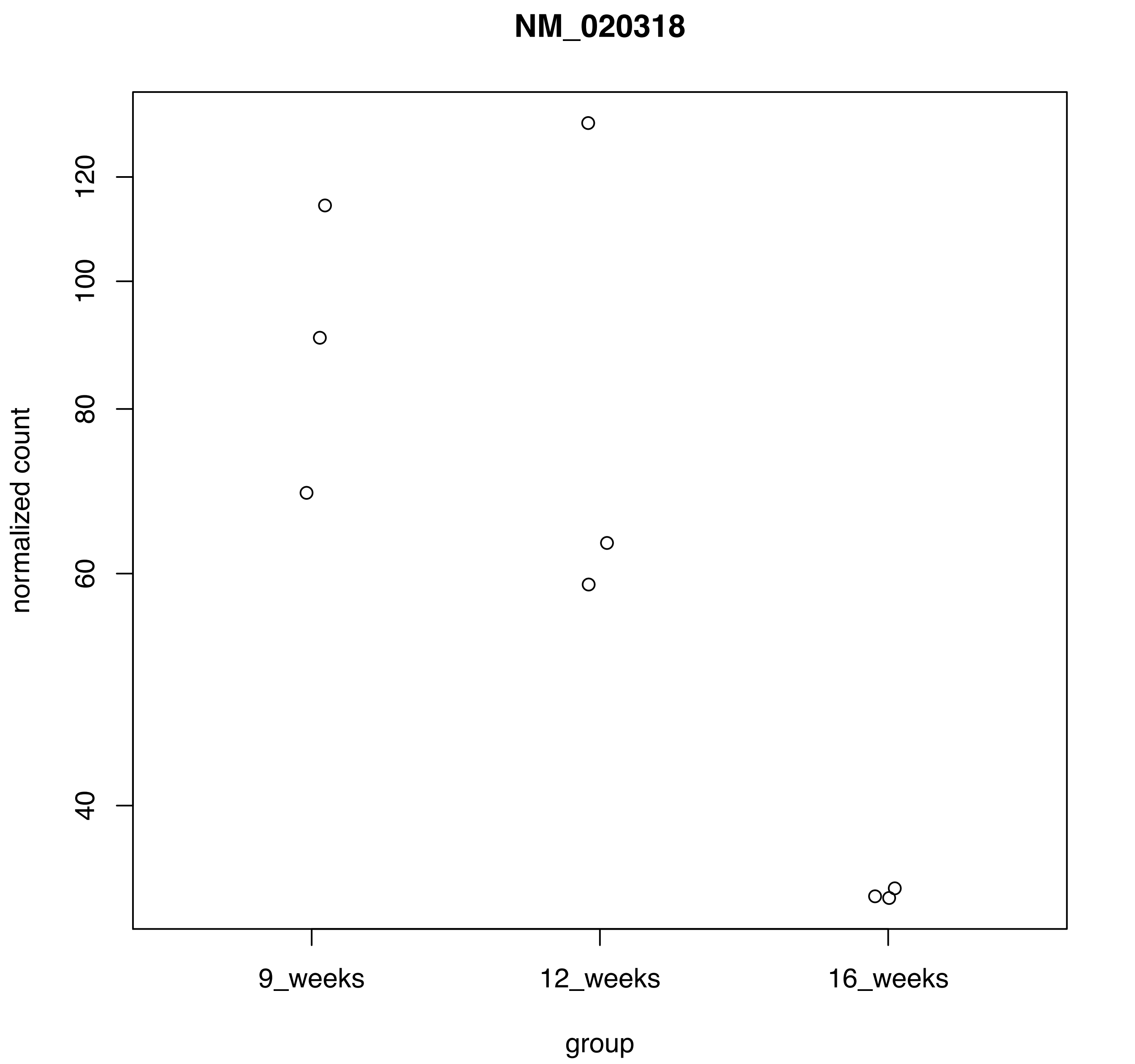

NM_020318
normalized count
40
60
80
100
120
9_weeks
12_weeks
16_weeks
group

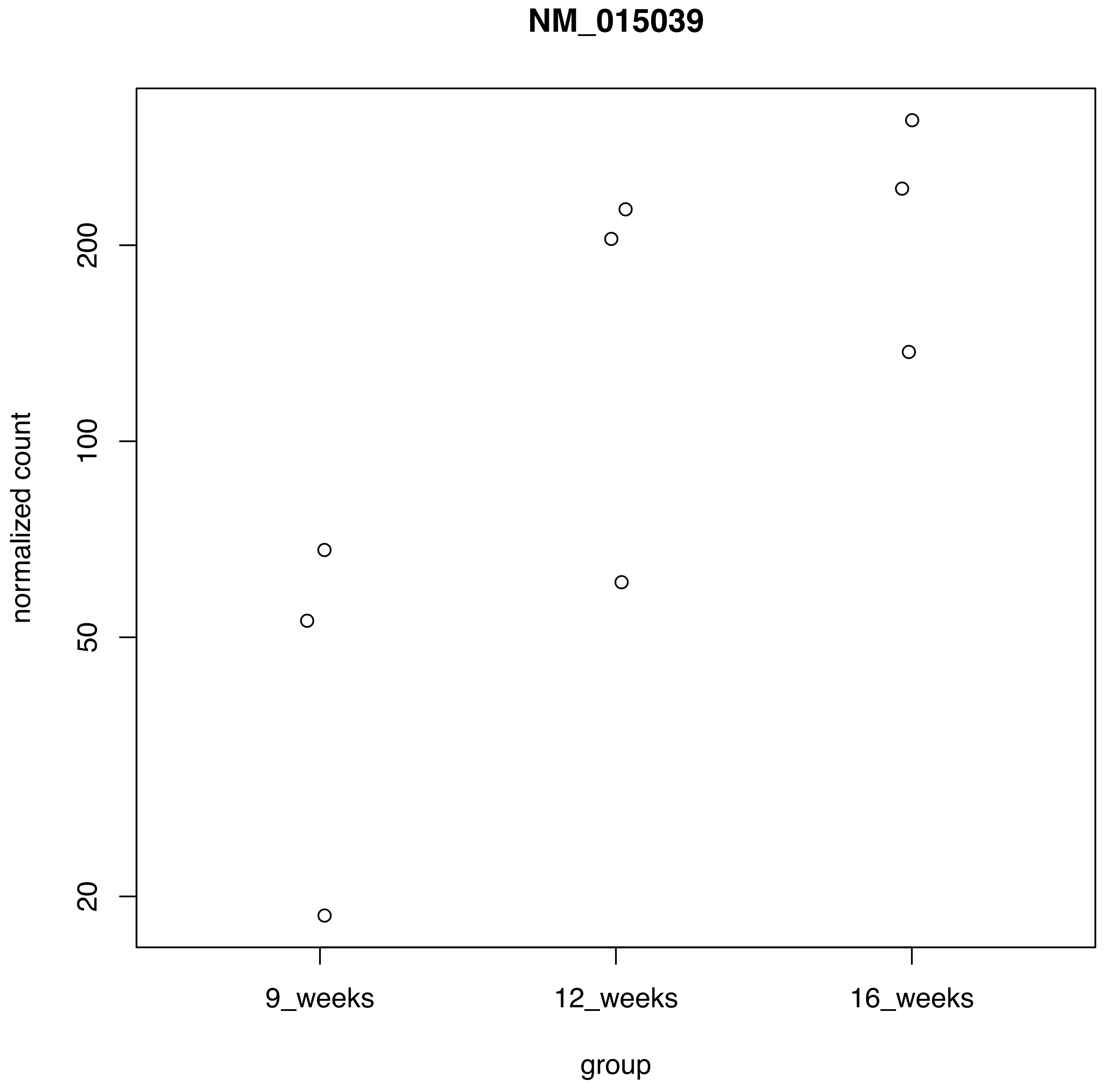

NM_015039
normalized count
20
50
100
200
9_weeks
12_weeks
16_weeks
group

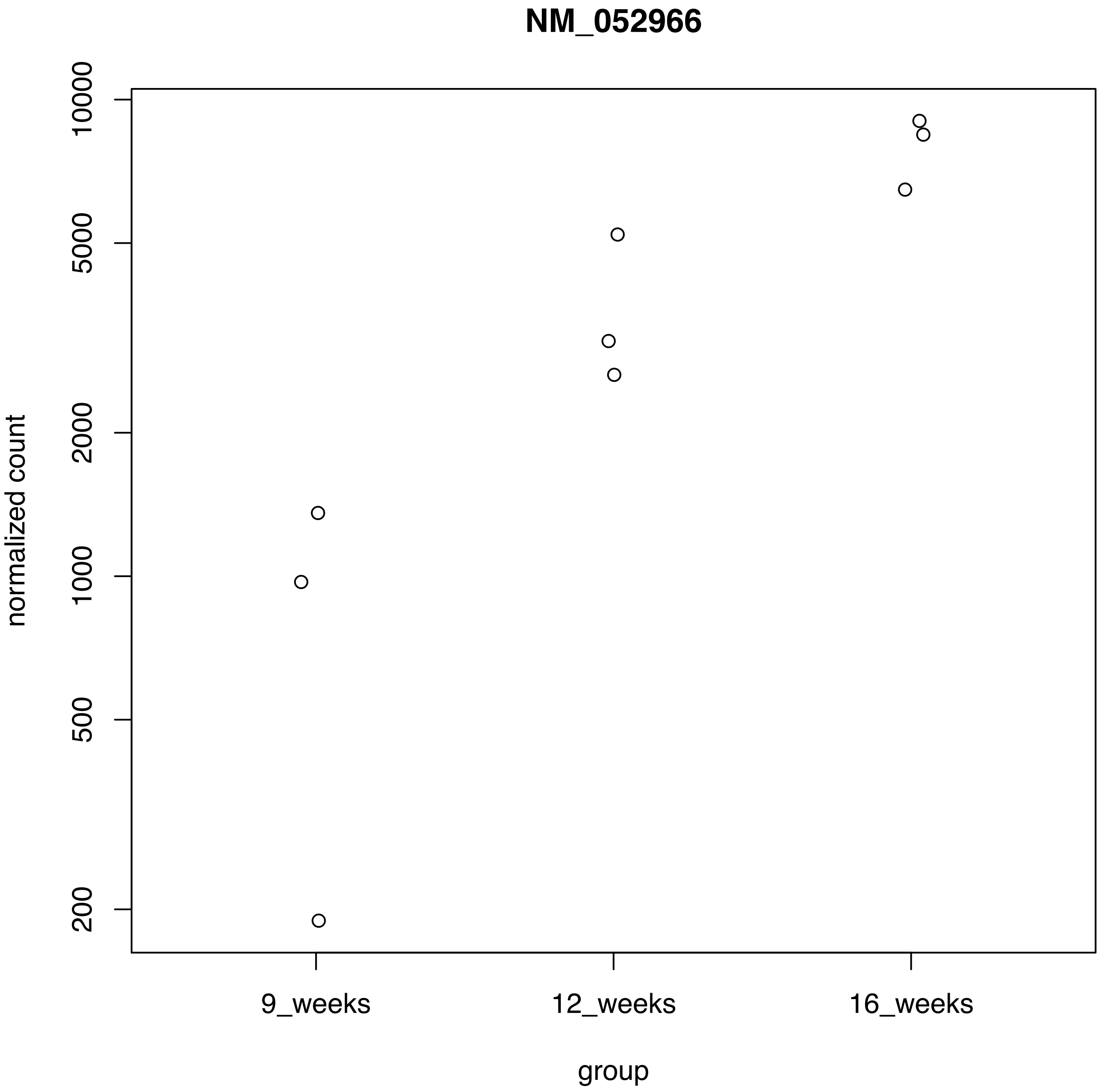
NM_052966
normalized count
10000
5000
2000
1000
500
200
9_weeks
12_weeks
16_weeks
group

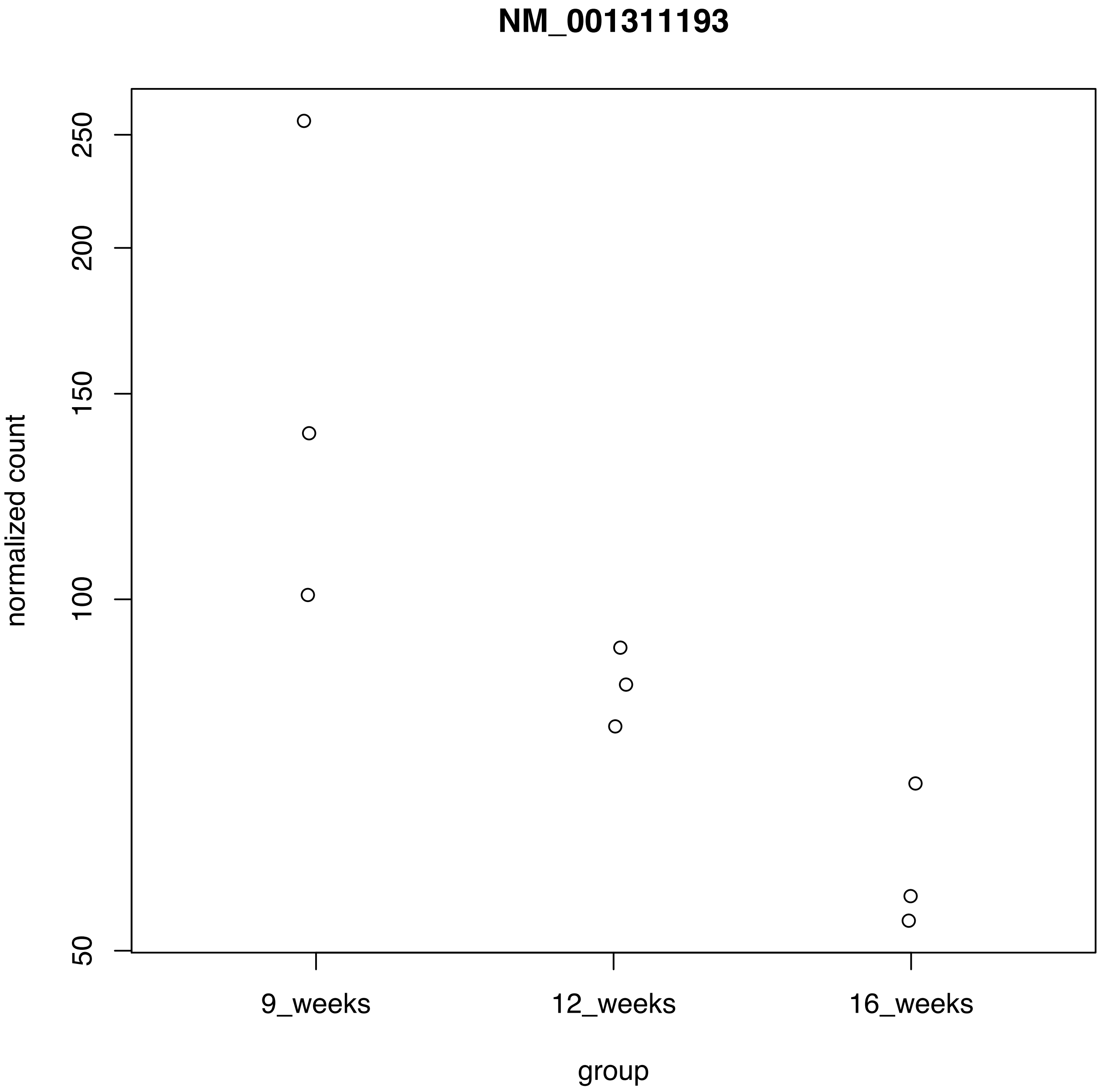
NM_001311193
normalized count
250
200
150
100
50
9_weeks
12_weeks
16_weeks
group

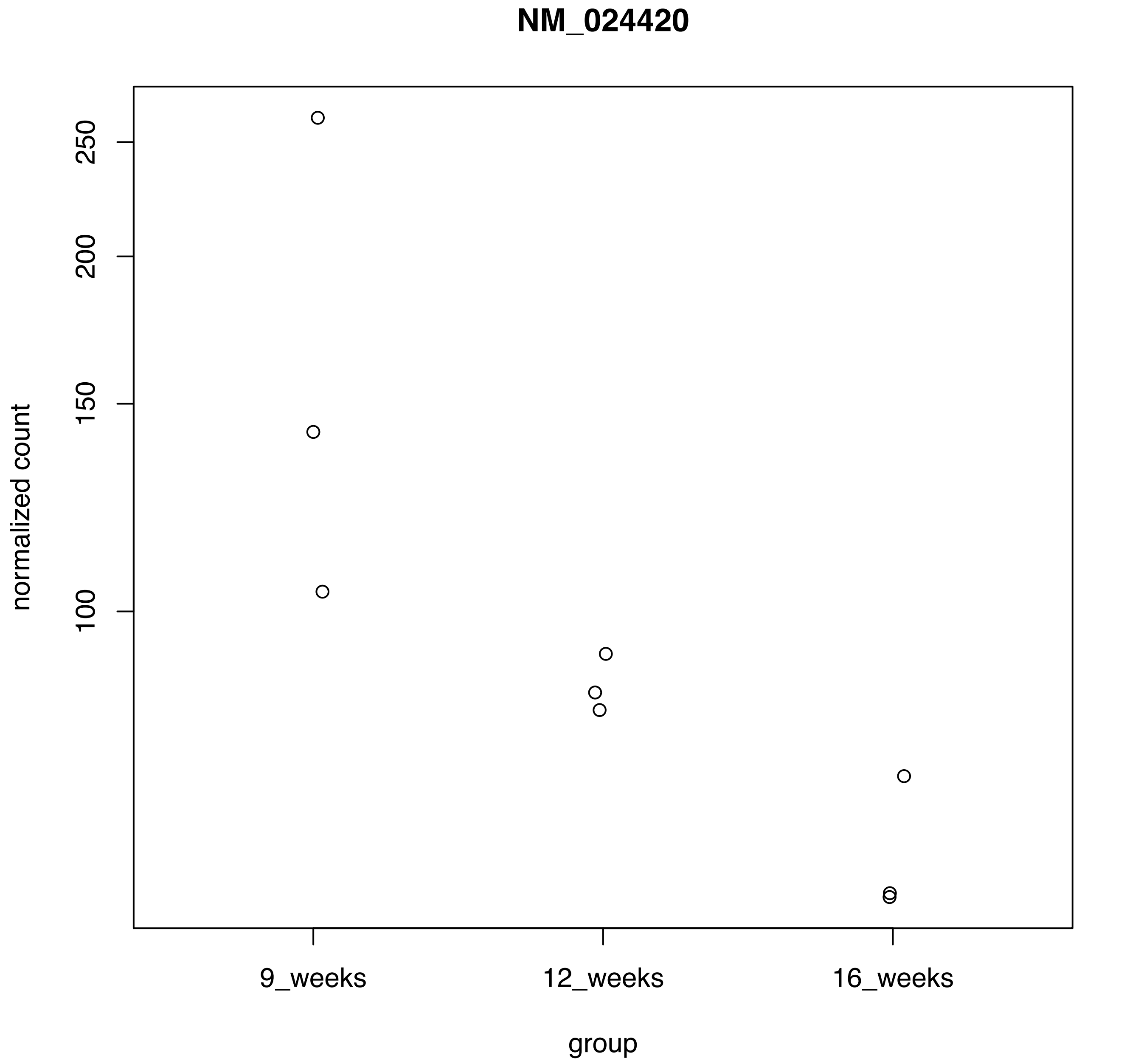
NM_024420
normalized count
100
150
200
250
9_weeks
12_weeks
16_weeks
group

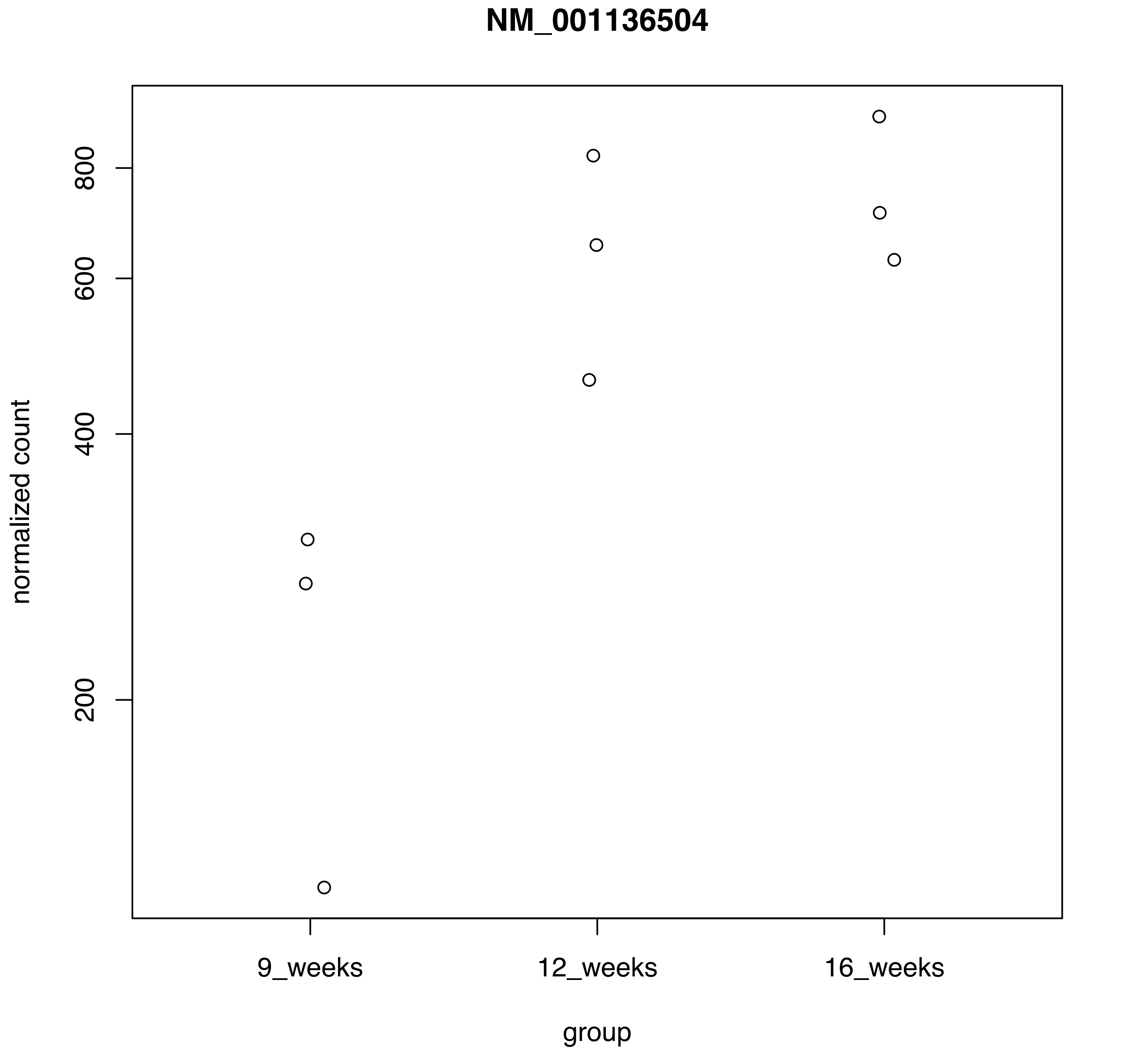

NM_001136504
normalized count
800
600
400
200
9_weeks
12_weeks
16_weeks
group

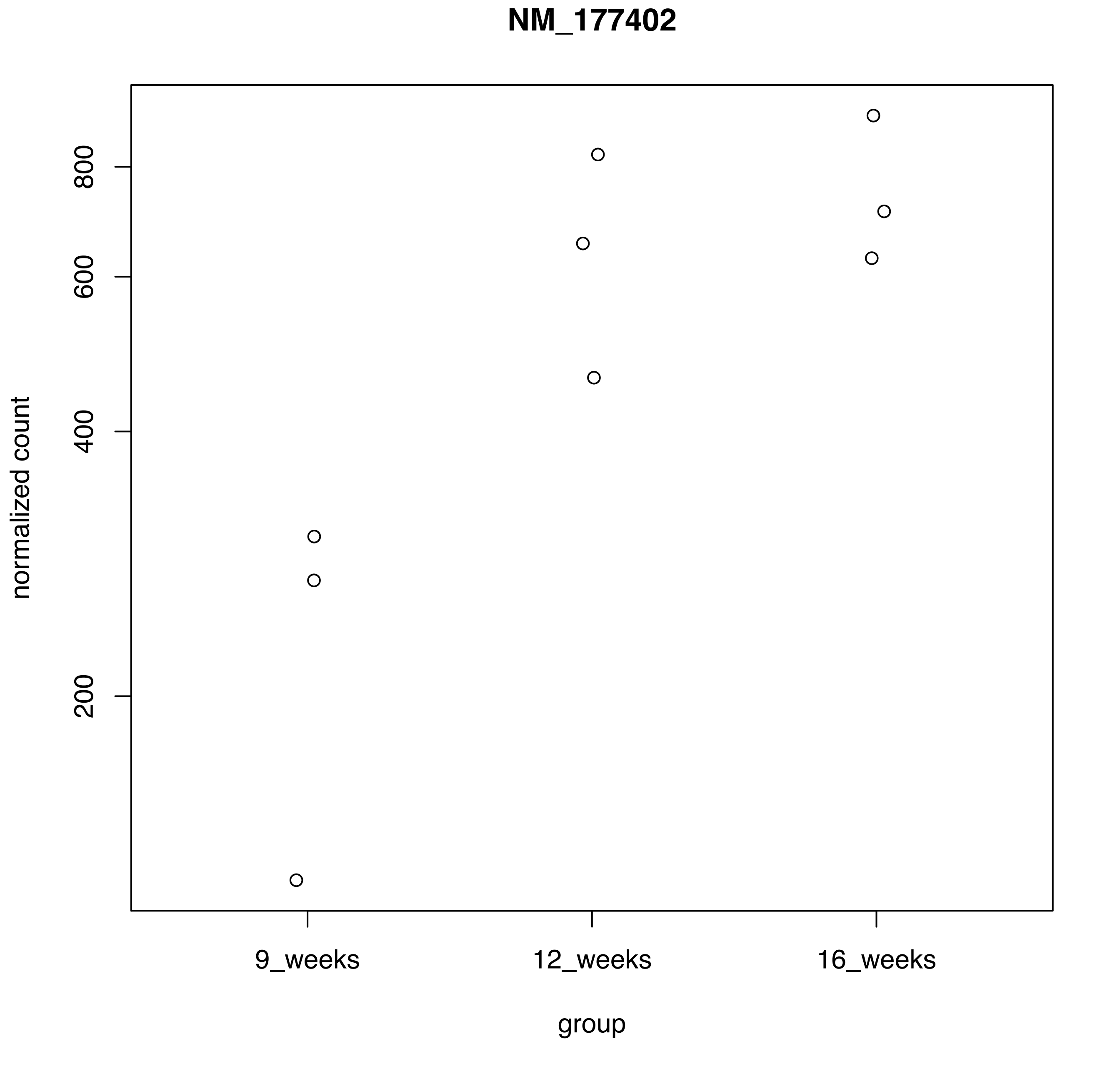
NM_177402
normalized count
800
600
400
200
9_weeks
12_weeks
16_weeks
group

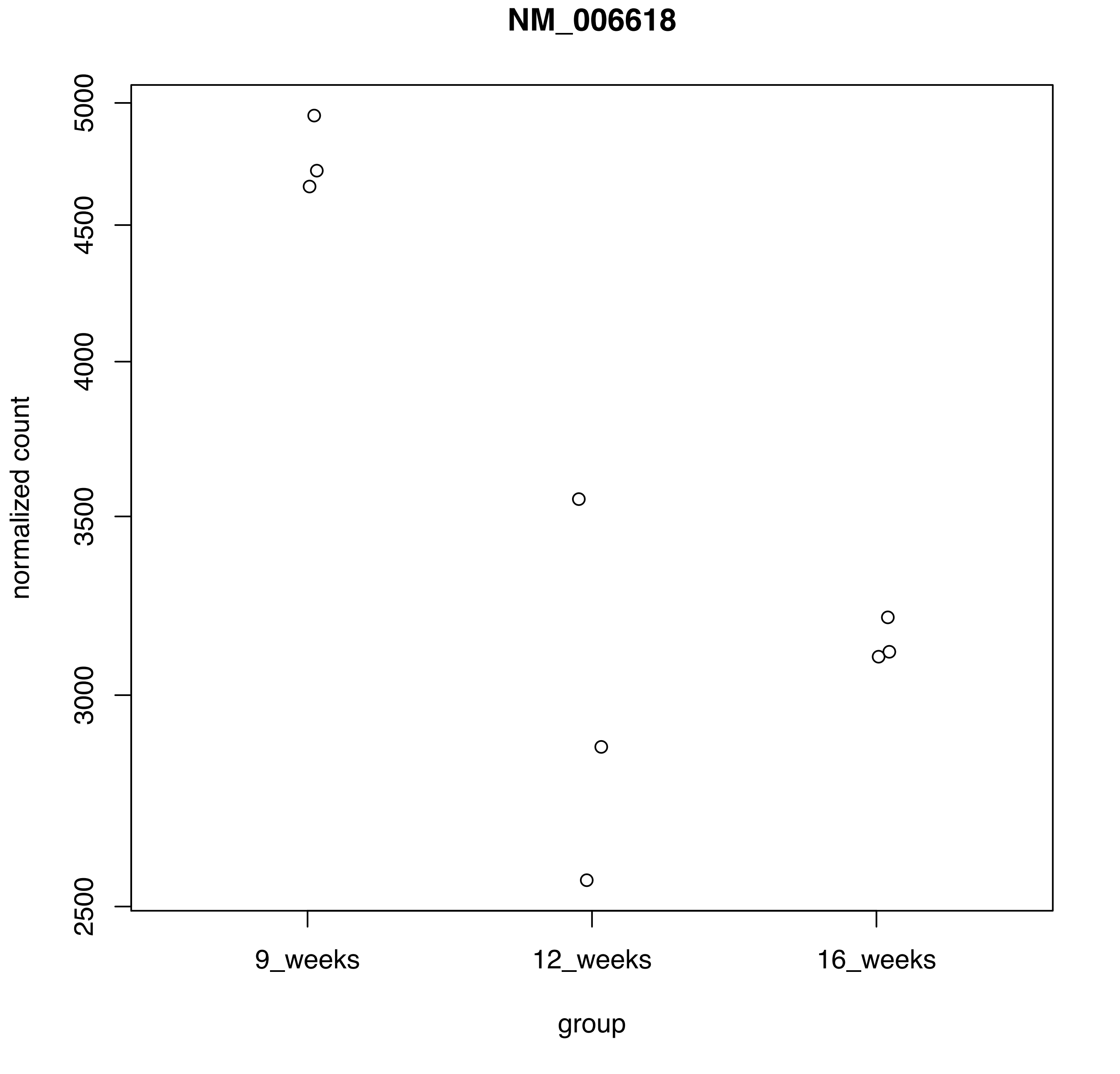
NM_006618
normalized count
5000
4500
4000
3500
3000
2500
9_weeks
12_weeks
16_weeks
group

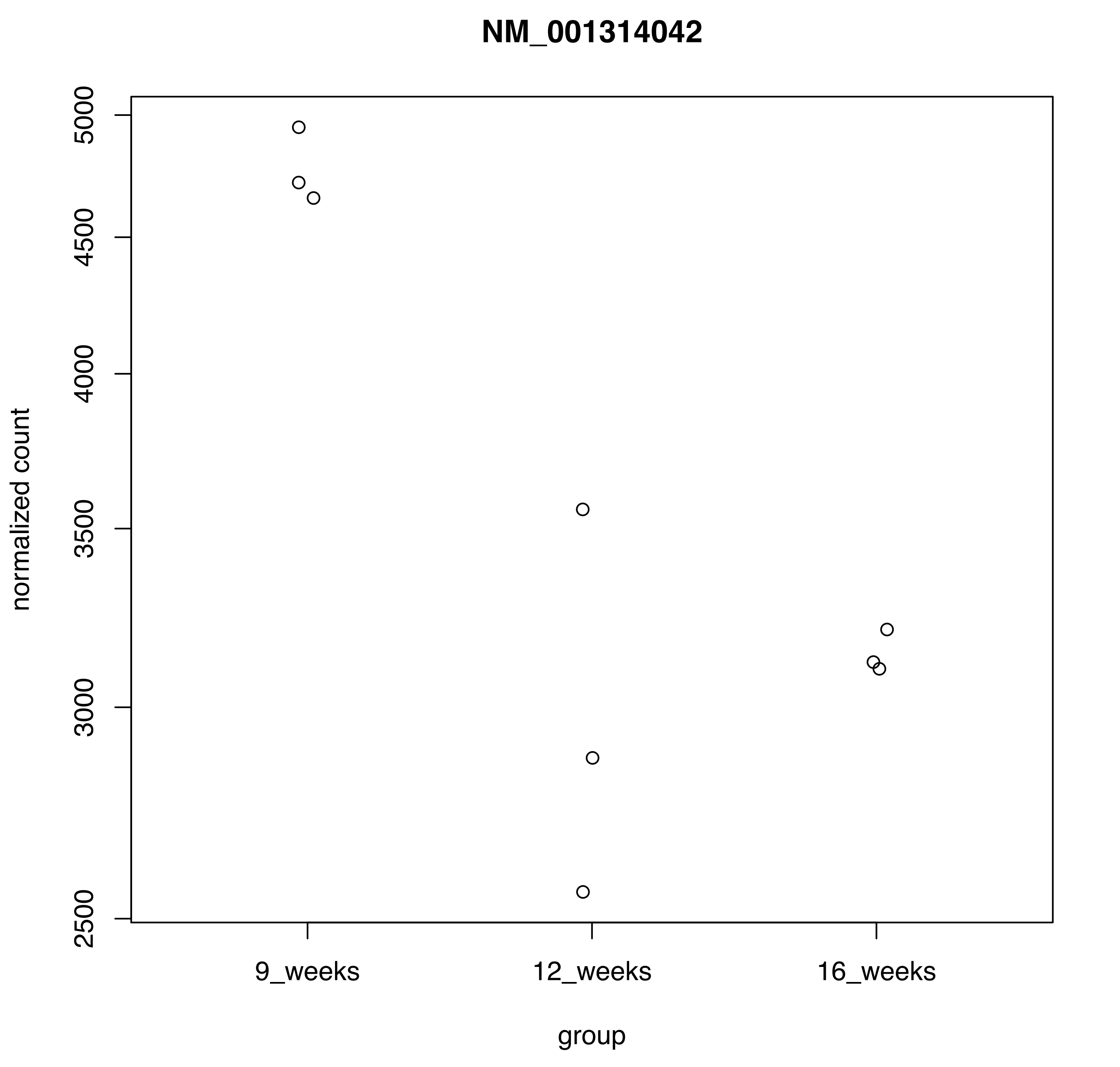
NM_001314042
normalized count
5000
4500
4000
3500
3000
2500
9_weeks
12_weeks
16_weeks
group

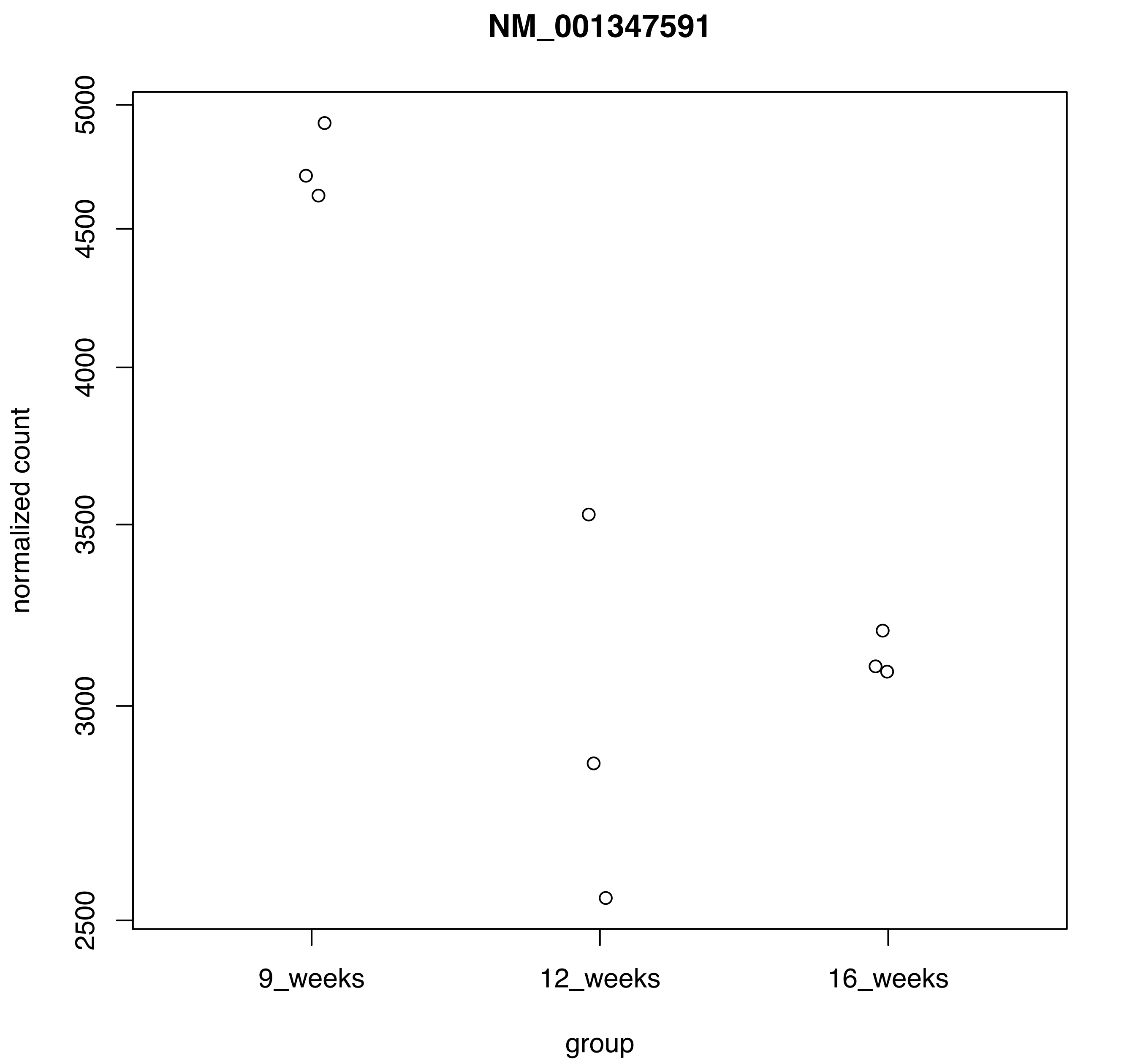
NM_001347591
normalized count
5000
4500
4000
3500
3000
2500
9_weeks
12_weeks
16_weeks
group

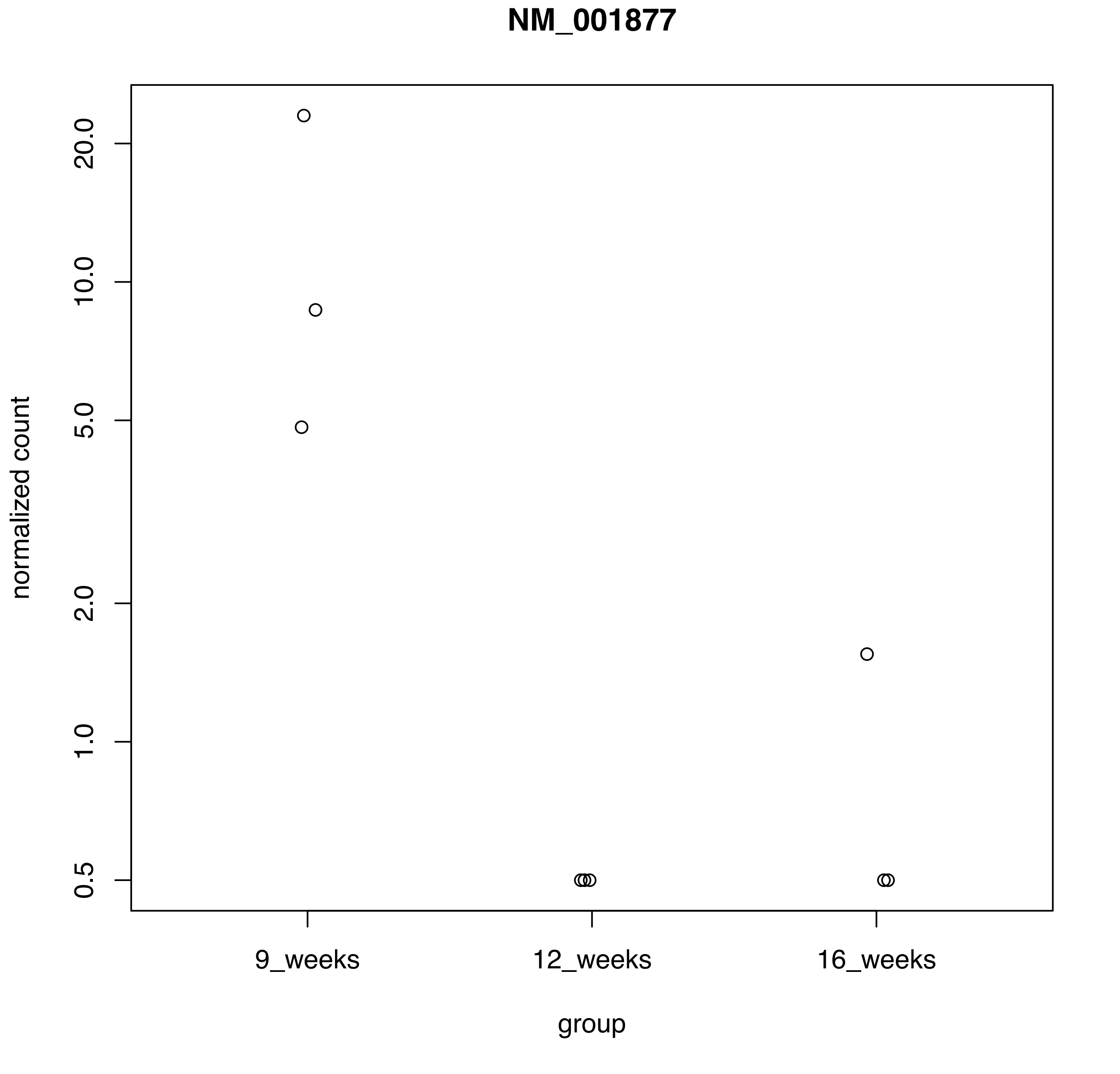
NM_001877
normalized count
20.0
10.0
5.0
2.0
1.0
0.5
9_weeks
12_weeks
16_weeks
group

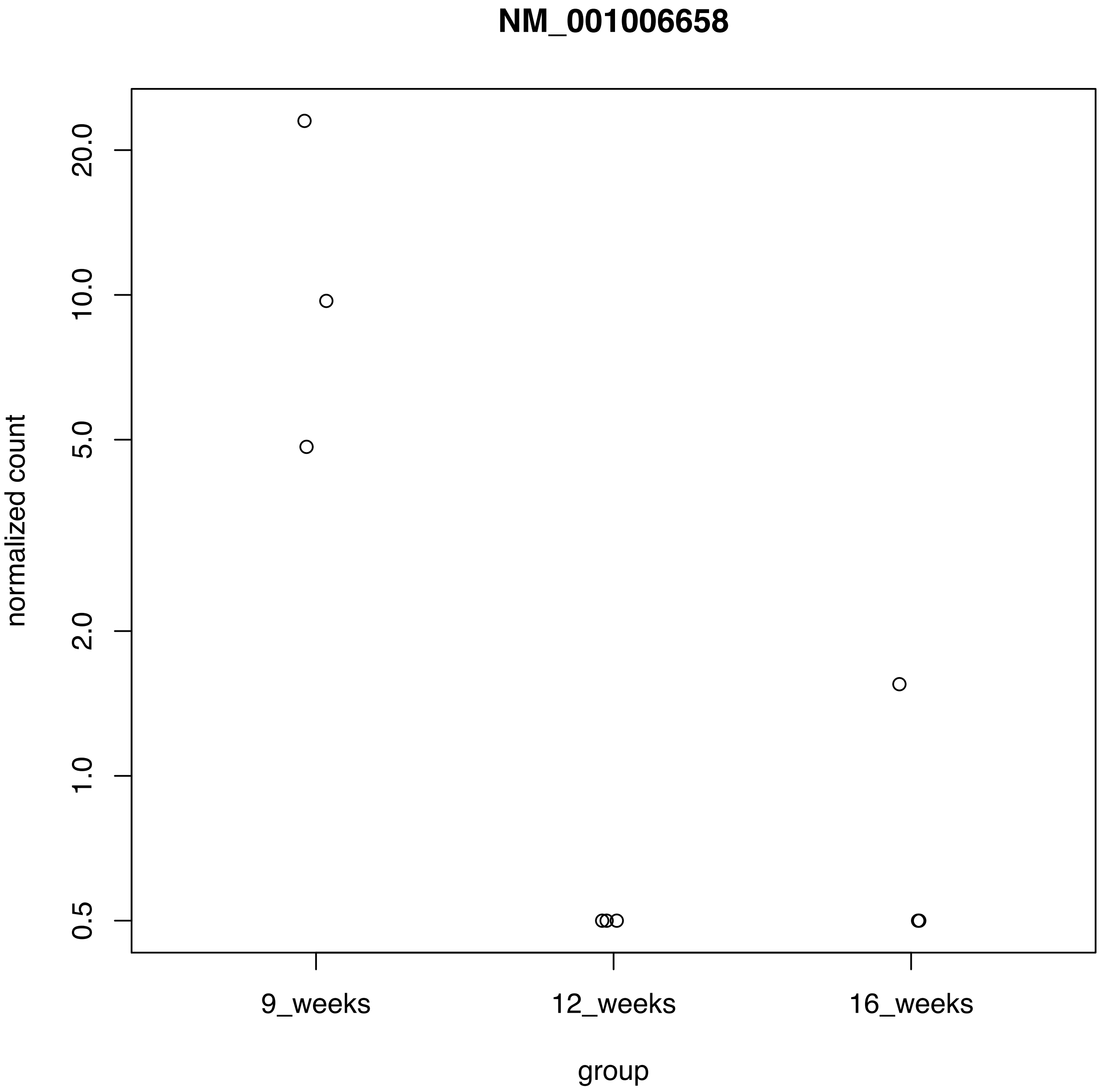
NM_001006658
normalized count
20.0
10.0
5.0
2.0
1.0
0.5
9_weeks
12_weeks
16_weeks
group

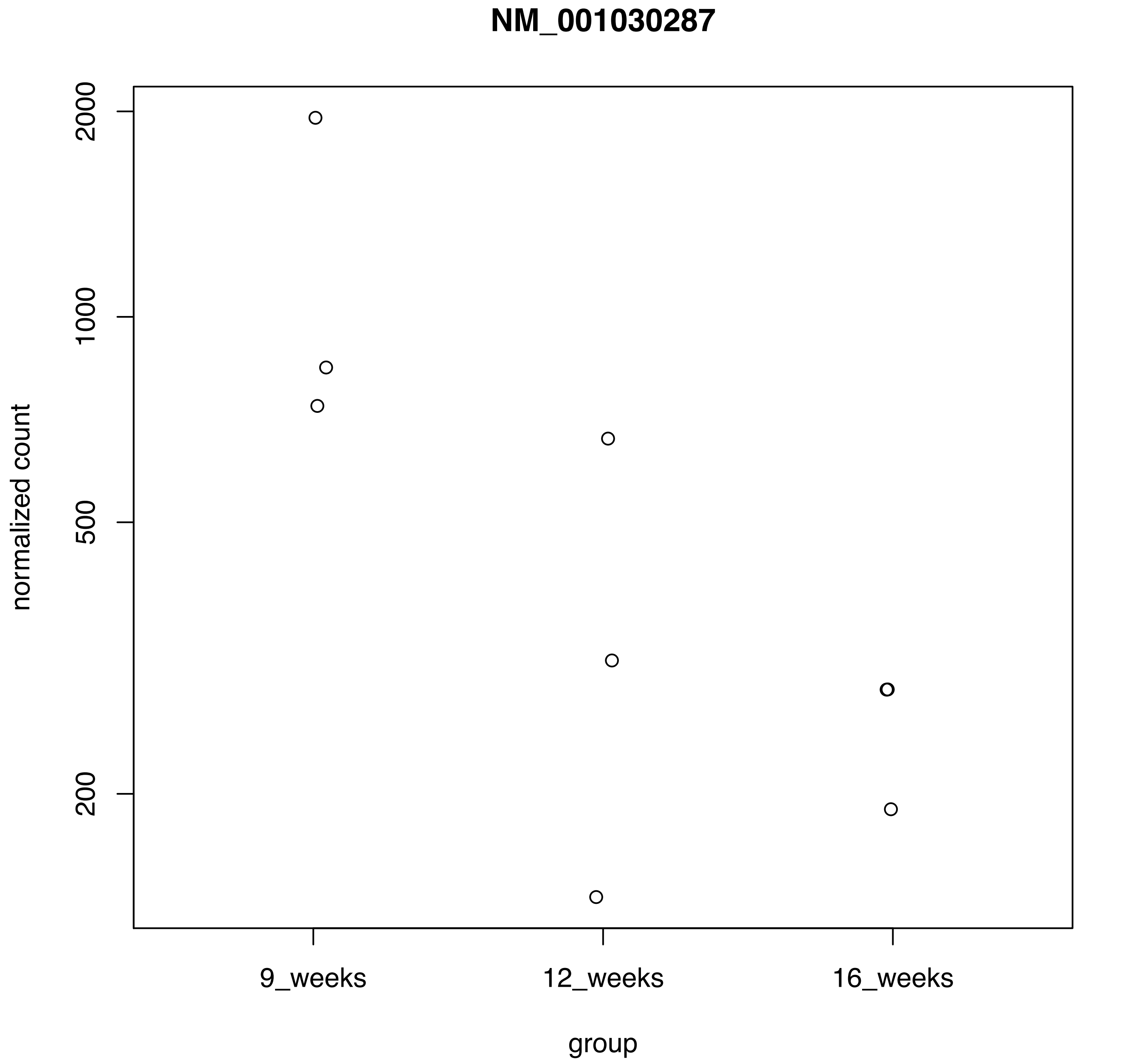

NM_001030287
2000
1000
500
200
normalized count
9_weeks
12_weeks
16_weeks
group

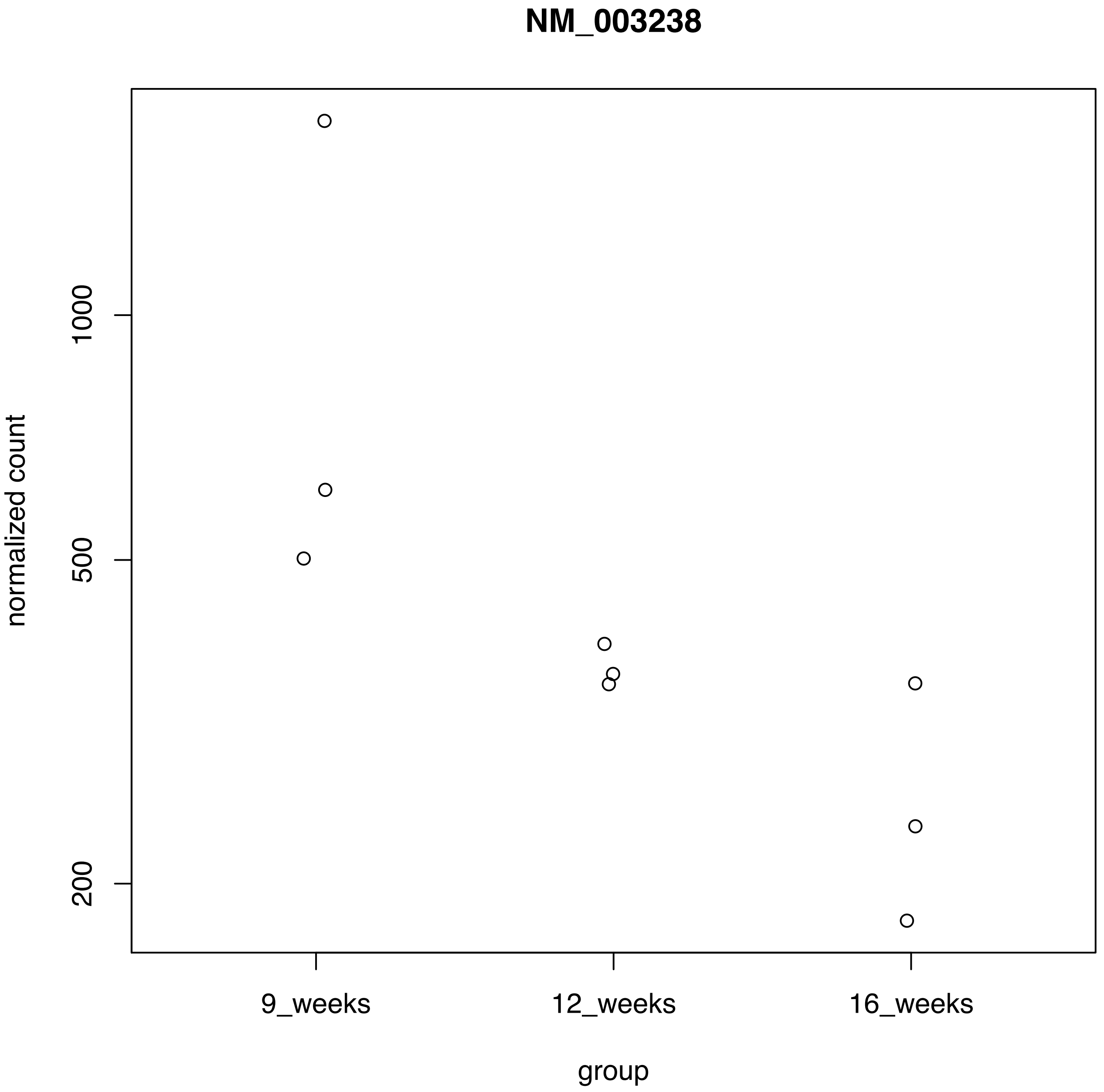
NM_003238
normalized count
1000
500
200
9_weeks
12_weeks
16_weeks
group

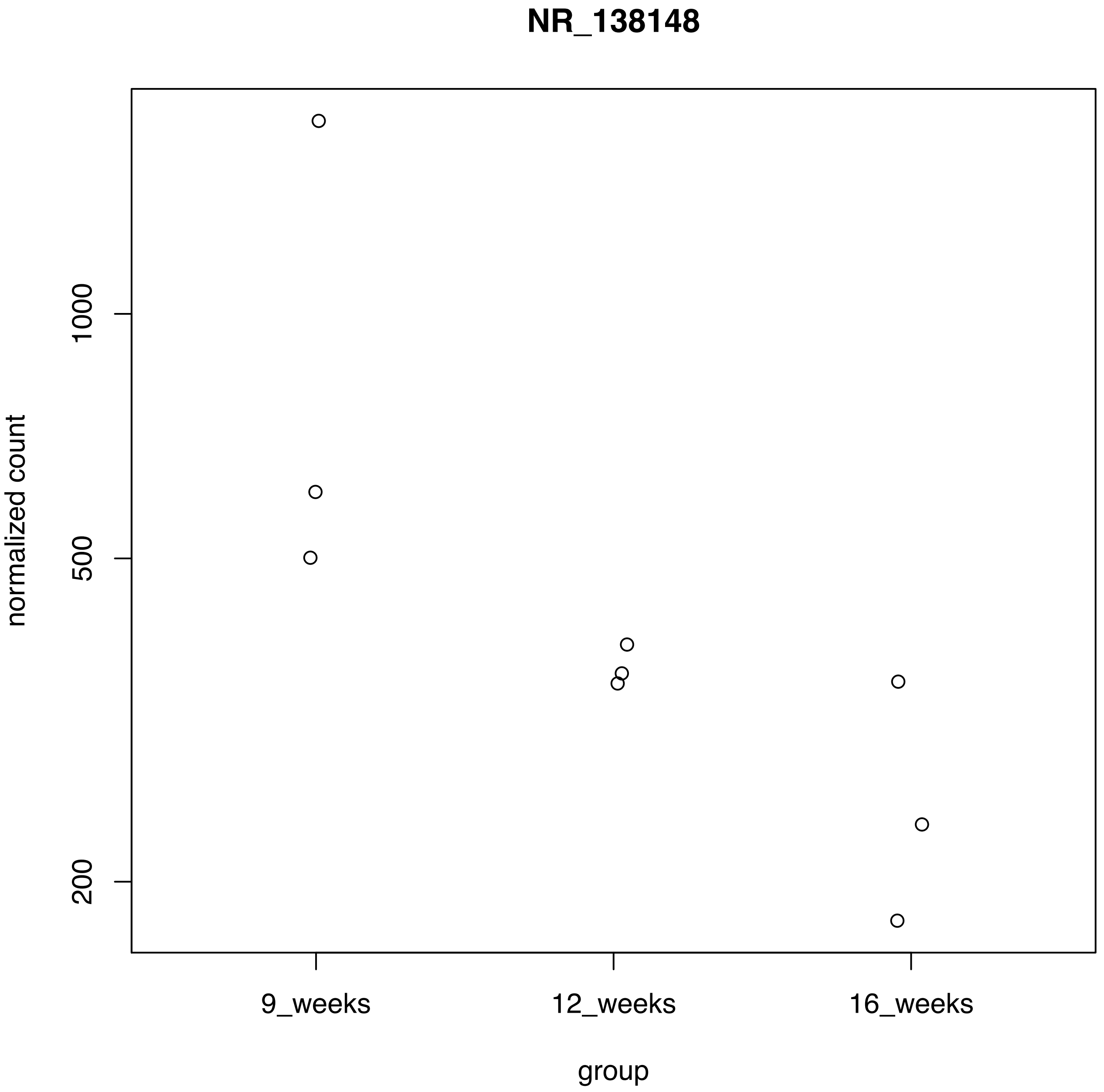
NR_138148
normalized count
1000
500
200
9_weeks
12_weeks
16_weeks
group

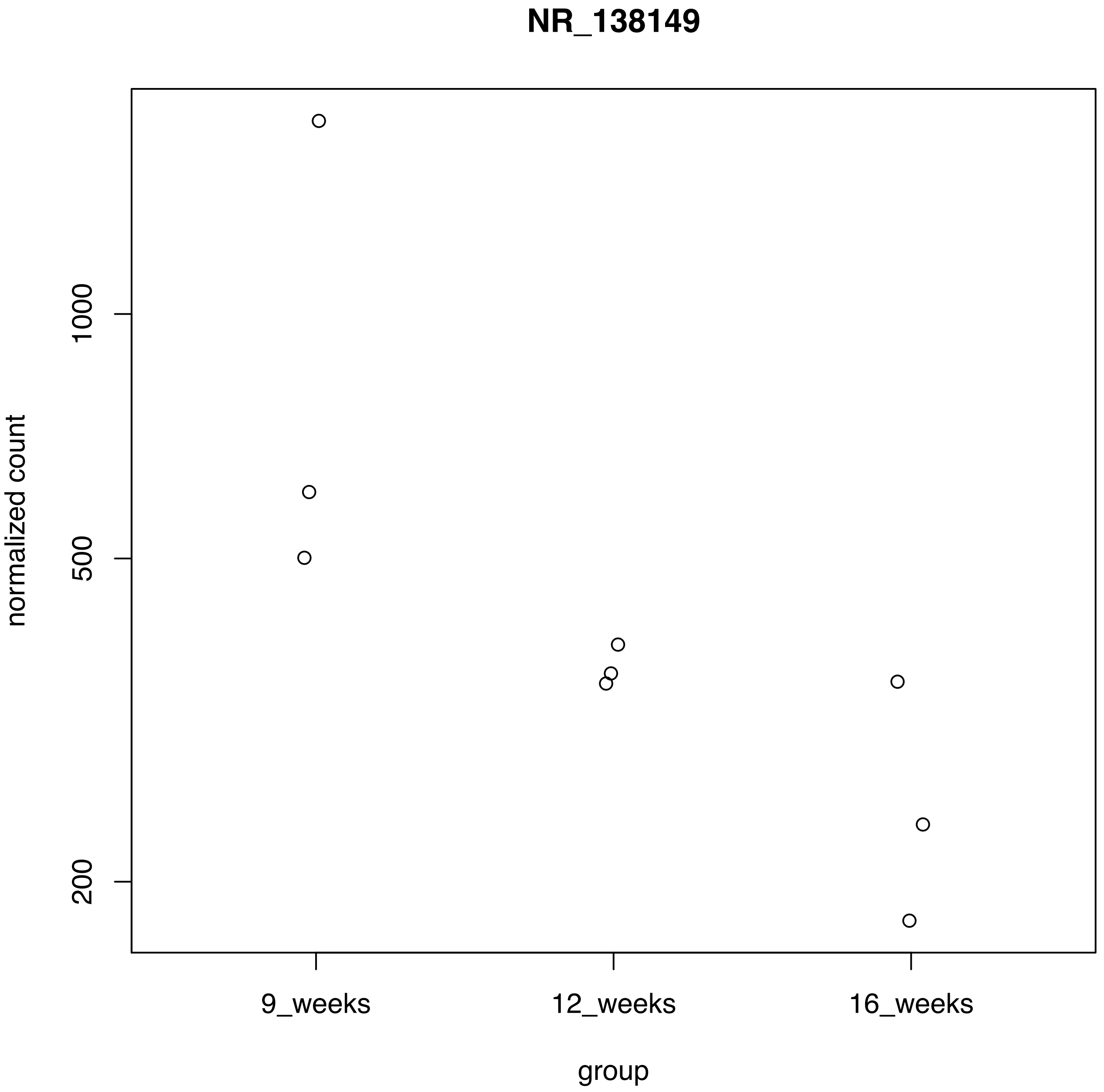
NR_138149
normalized count
1000
500
200
9_weeks
12_weeks
16_weeks
group

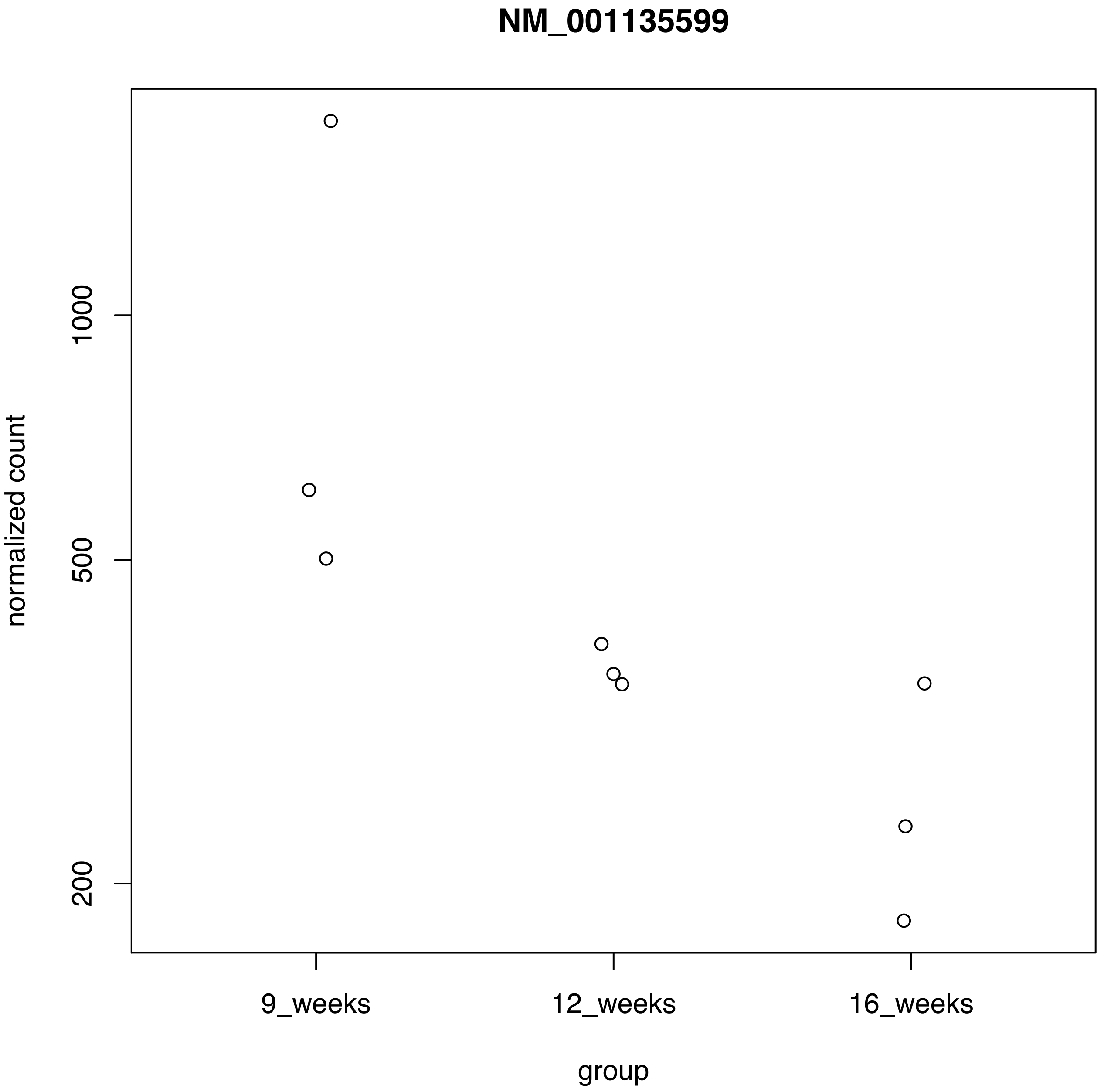
NM_001135599
normalized count
1000
500
200
9_weeks
12_weeks
16_weeks
group

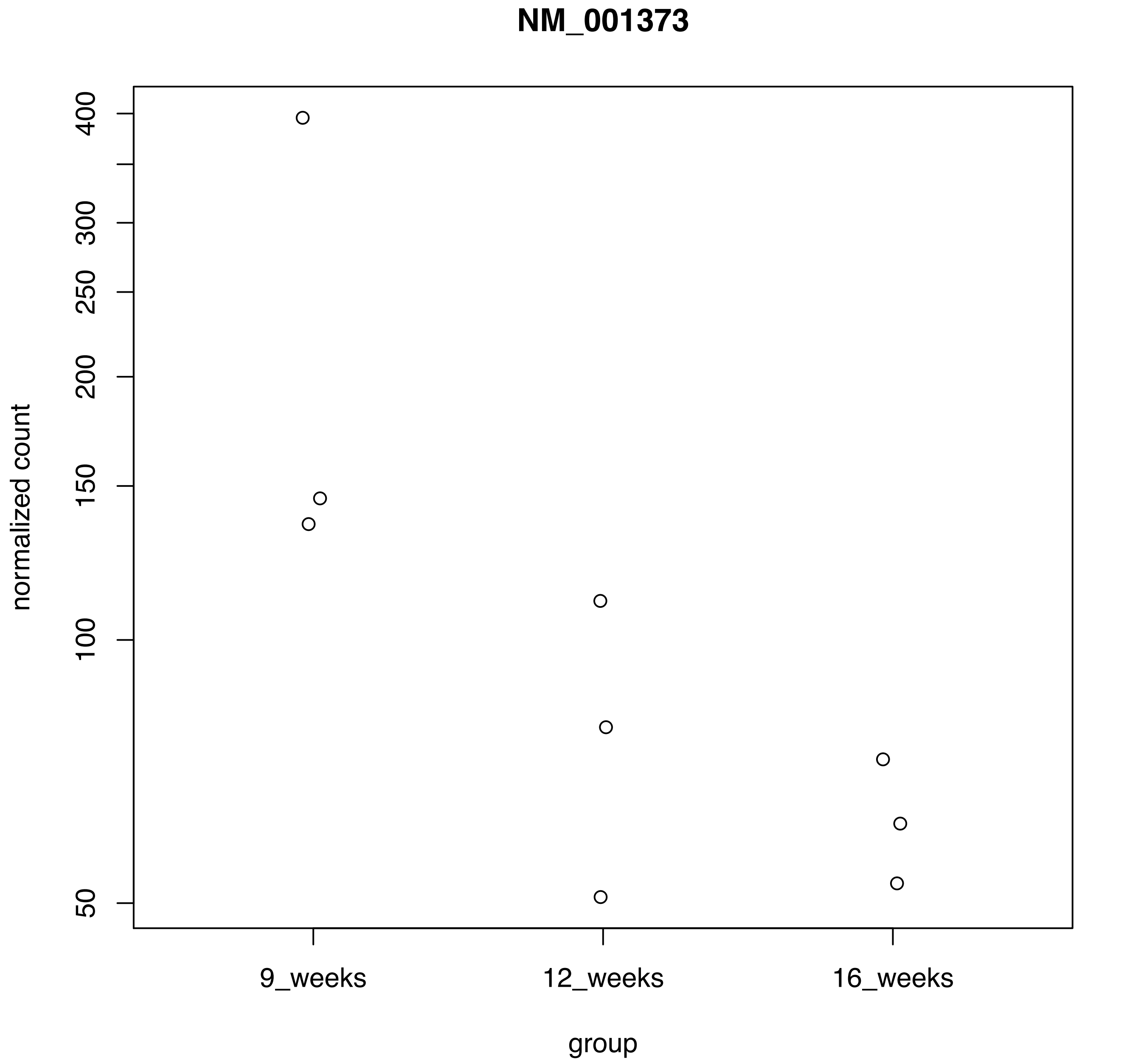
NM_001373
normalized count
400
300
250
200
150
100
50
9_weeks
12_weeks
16_weeks
group

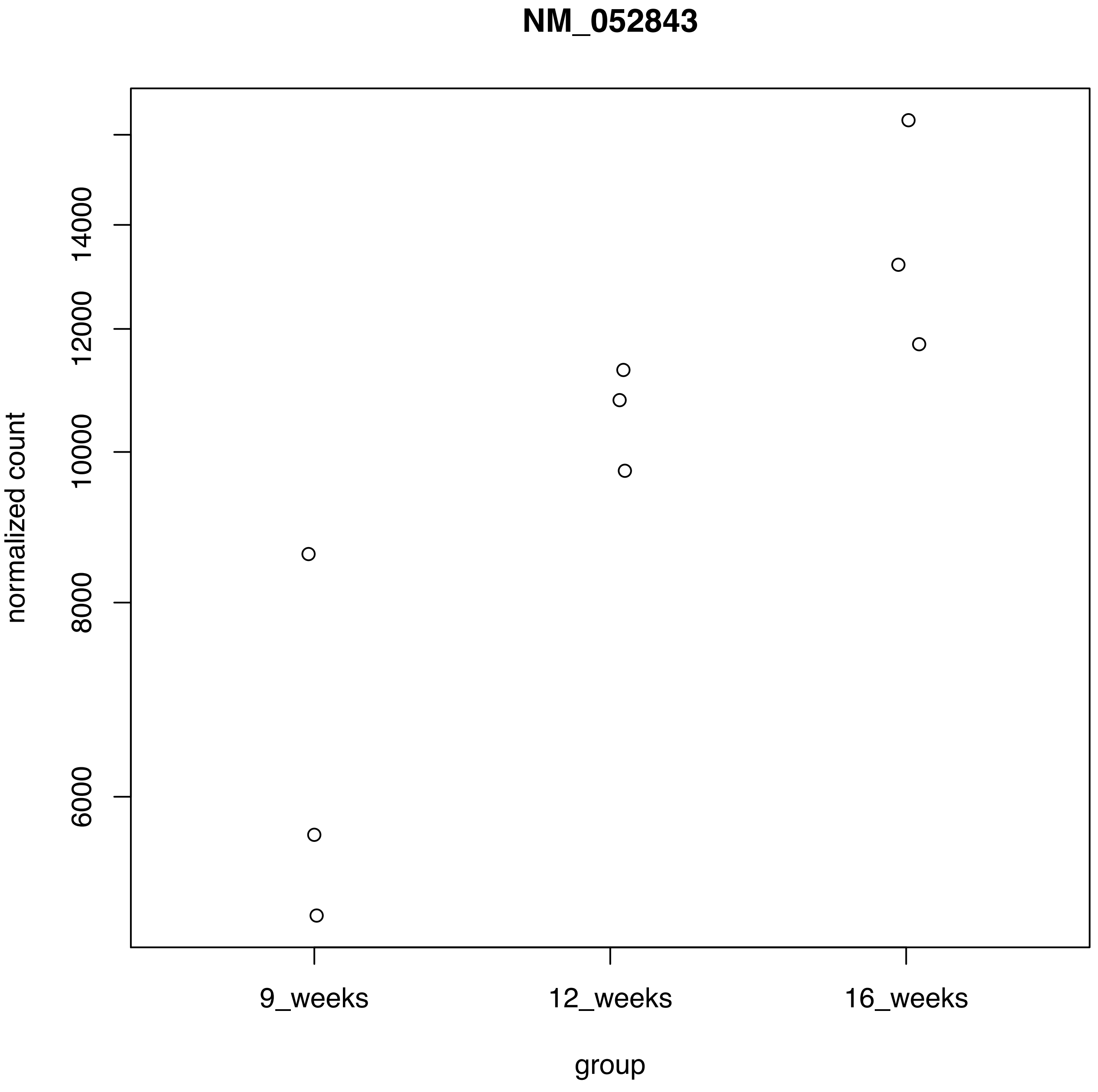
NM_052843
normalized count
6000
8000
10000
12000
14000
9_weeks
12_weeks
16_weeks
group

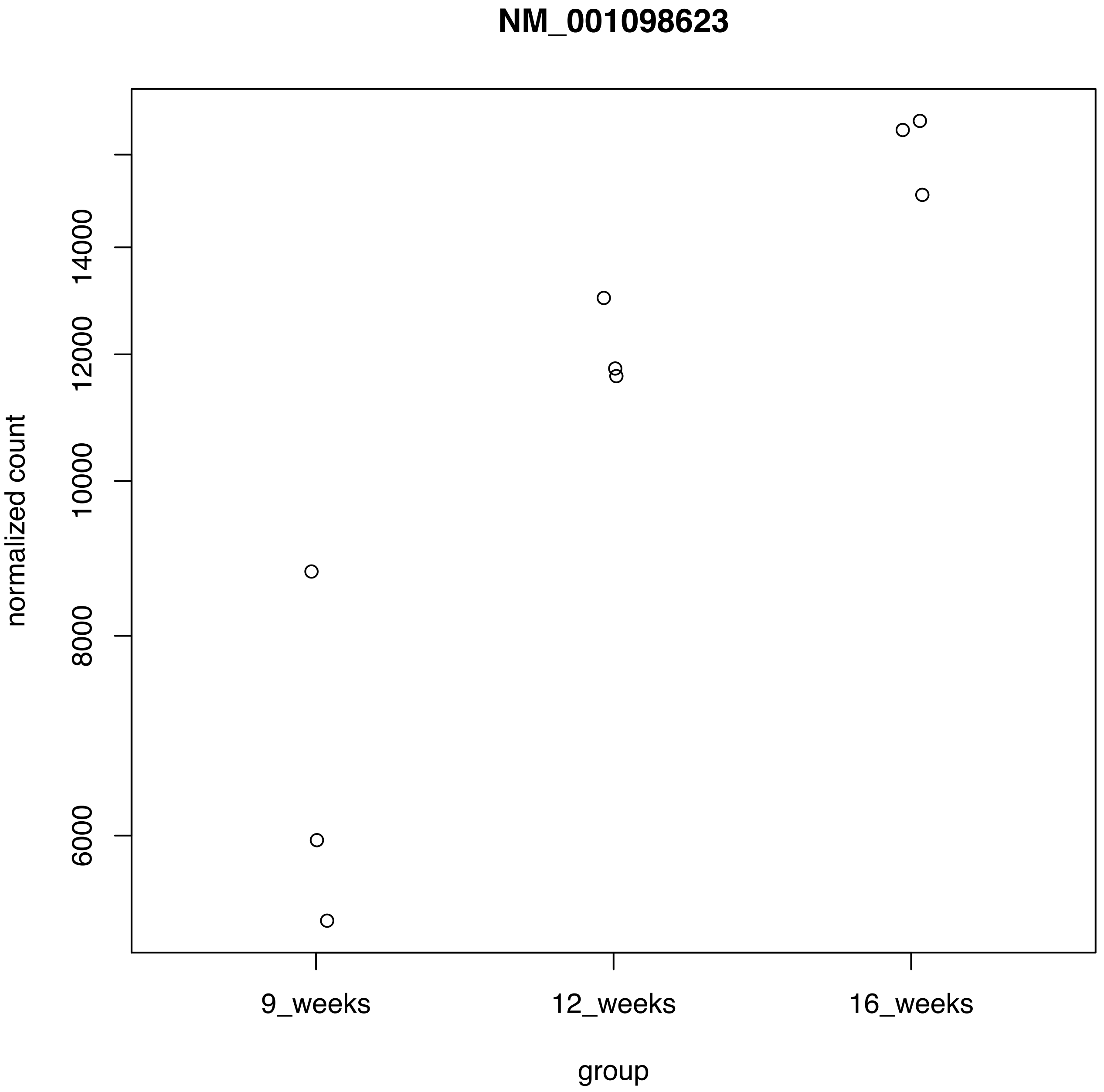
NM_001098623
normalized count
6000
8000
10000
12000
14000
9_weeks
12_weeks
16_weeks
group

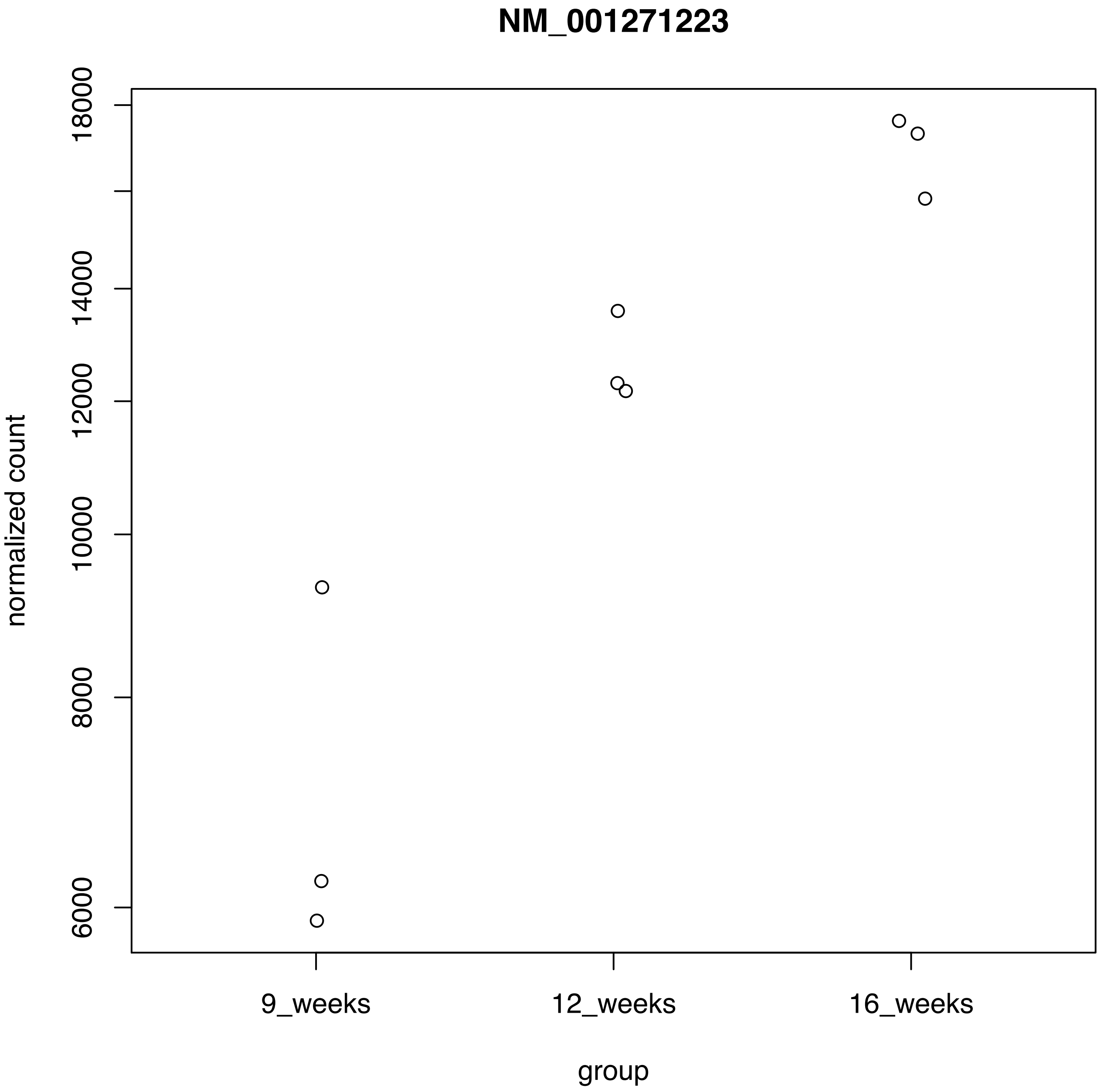
NM_001271223
normalized count
18000
14000
12000
10000
8000
6000
9_weeks
12_weeks
16_weeks
group

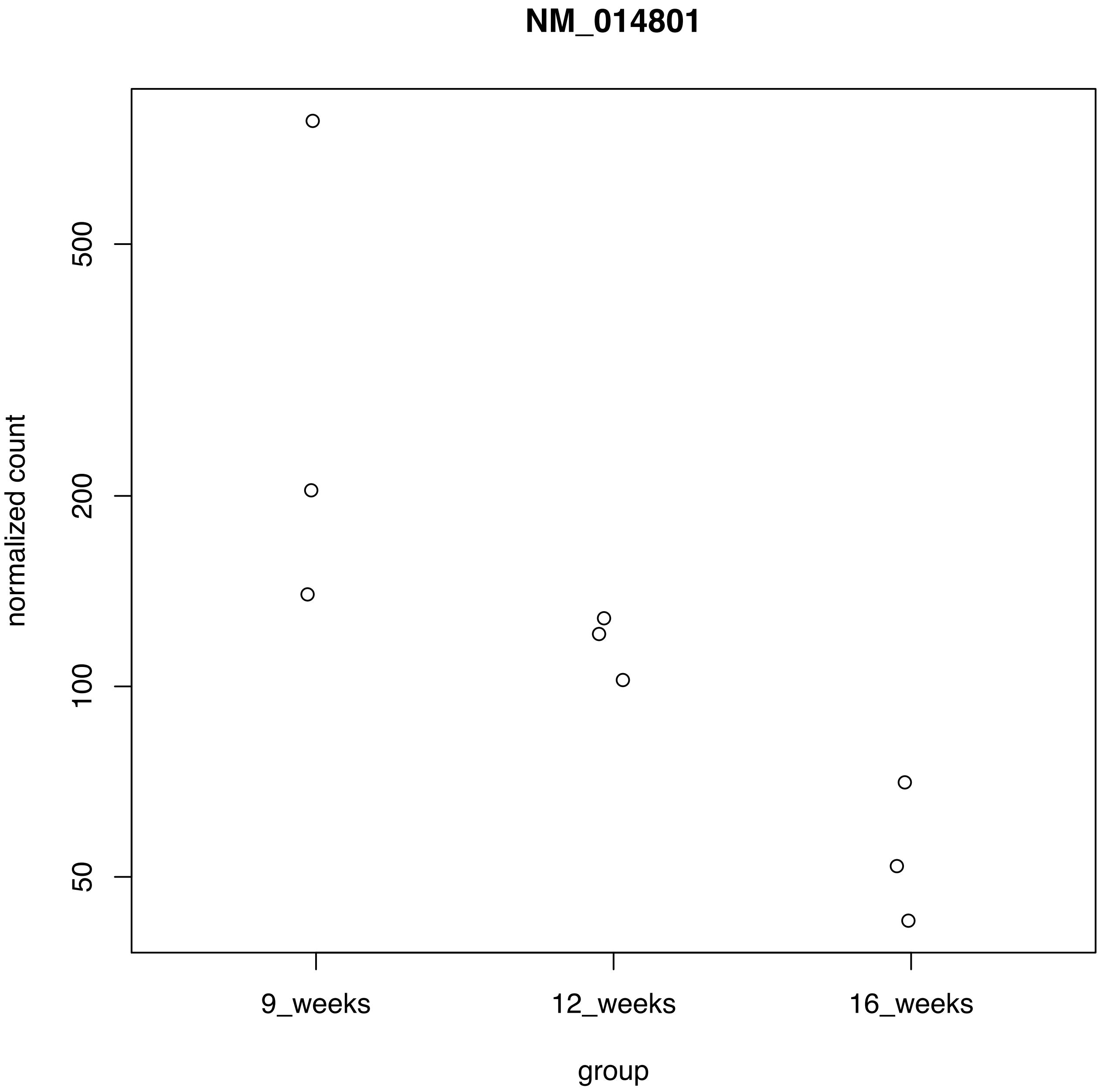
NM_014801
normalized count
500
200
100
50
9_weeks
12_weeks
16_weeks
group

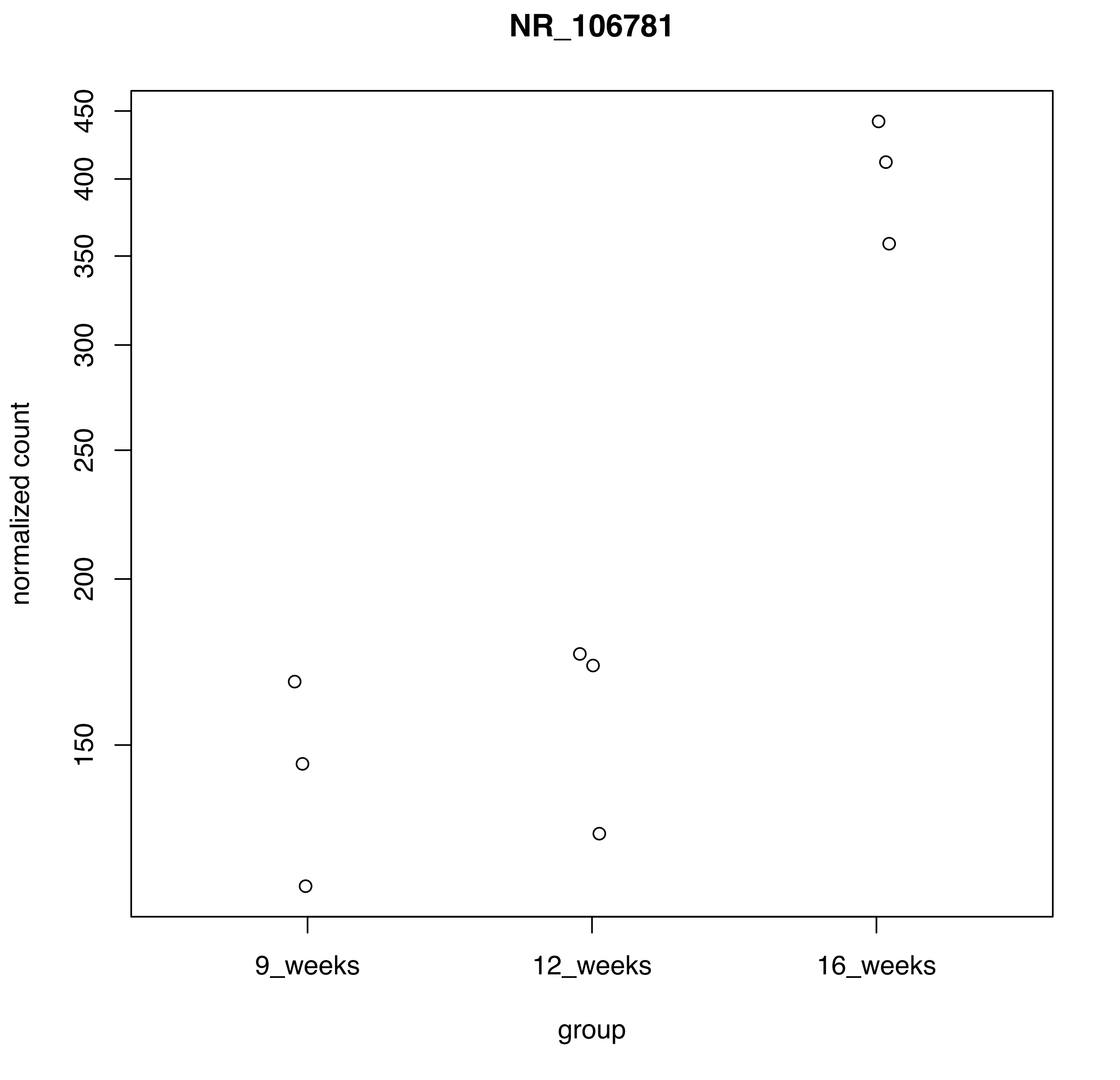

NR_106781
normalized count
450
400
350
300
250
200
150
9_weeks
12_weeks
16_weeks
group

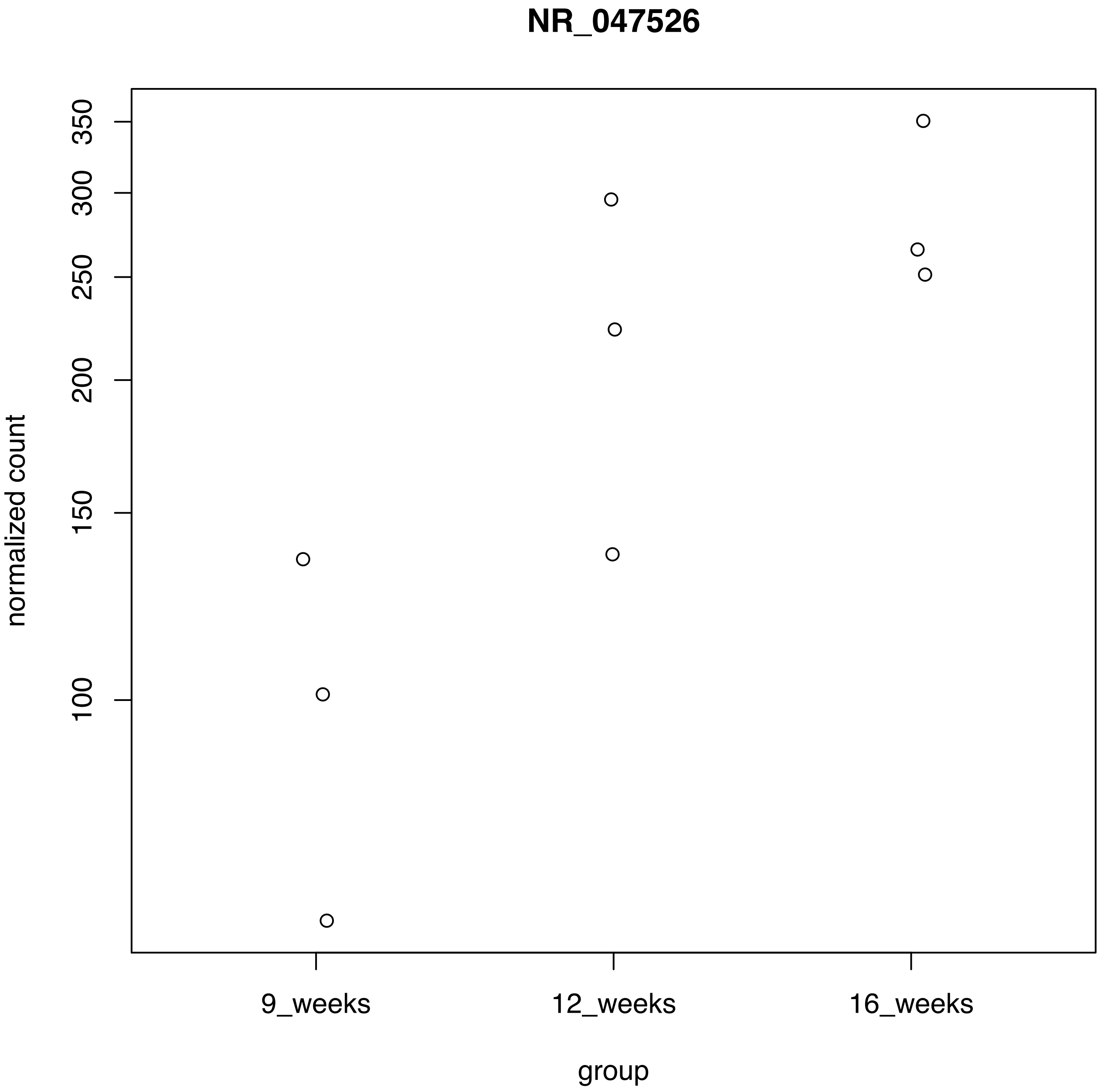

NR_047526
normalized count
350
300
250
200
150
100
9_weeks
12_weeks
16_weeks
group

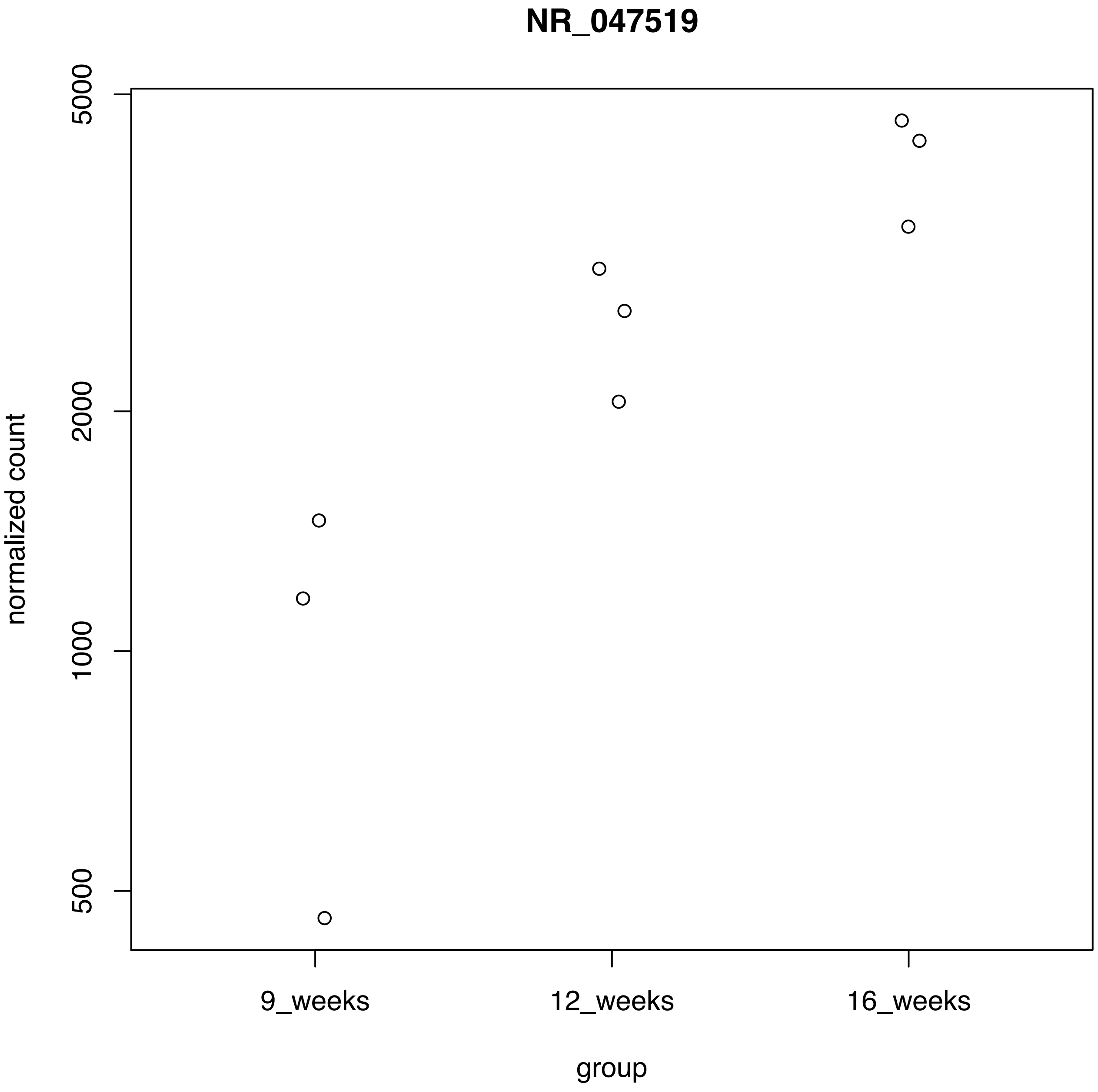
NR_047519
normalized count
5000
2000
1000
500
9_weeks
12_weeks
16_weeks
group

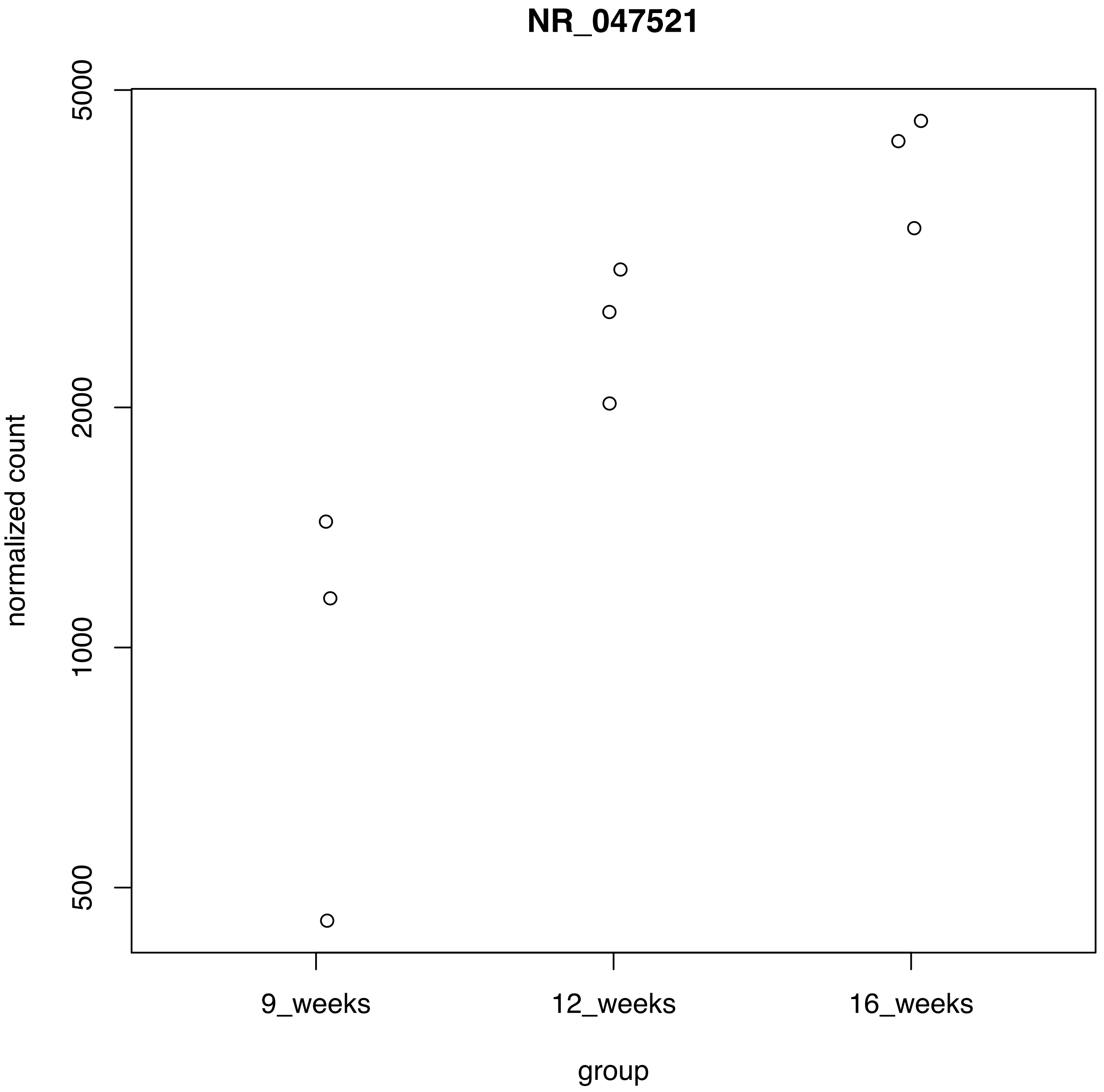
NR_047521
5000
2000
1000
500
normalized count
9_weeks
12_weeks
16_weeks
group

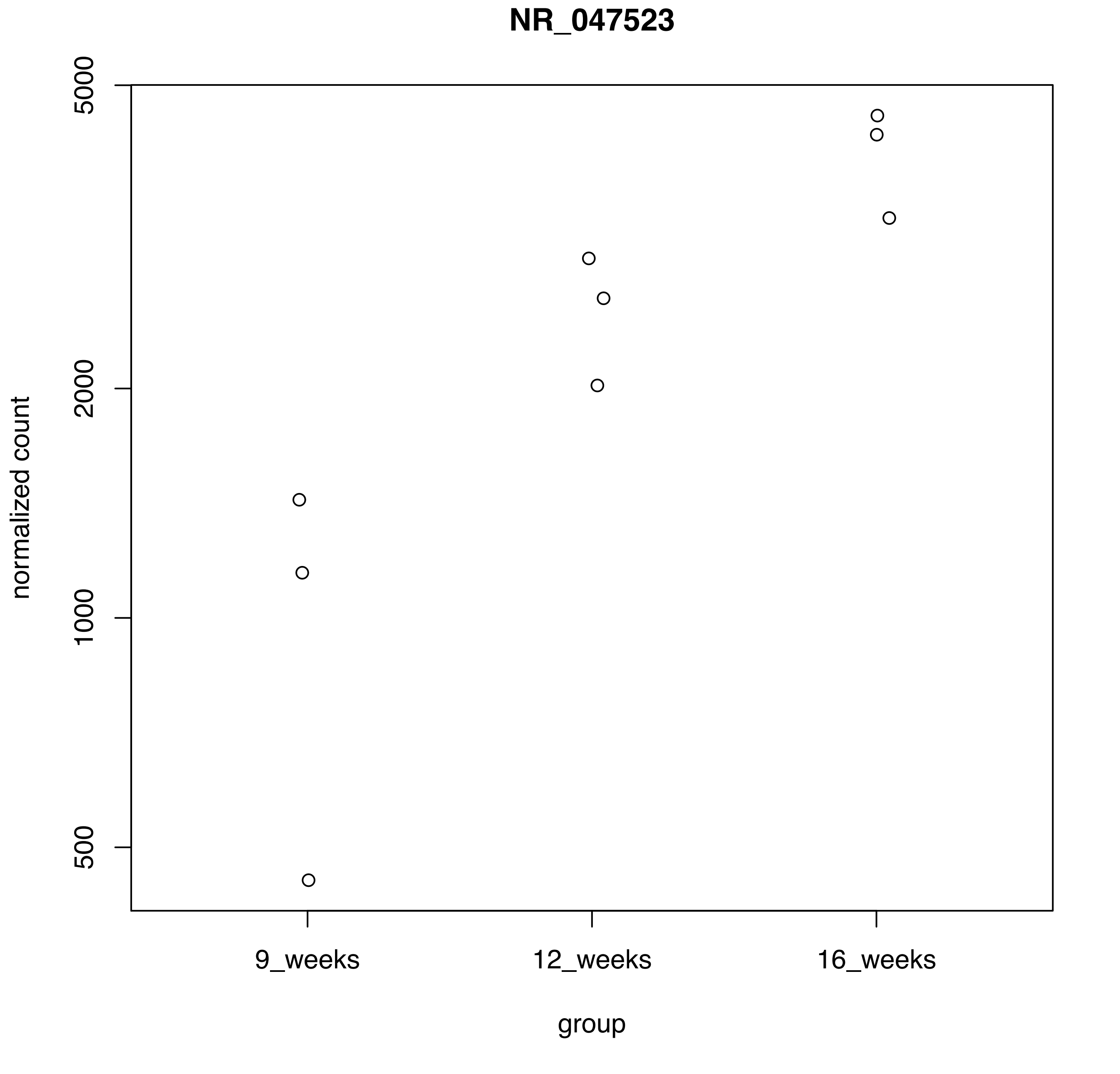
NR_047523
normalized count
5000
2000
1000
500
9_weeks
12_weeks
16_weeks
group

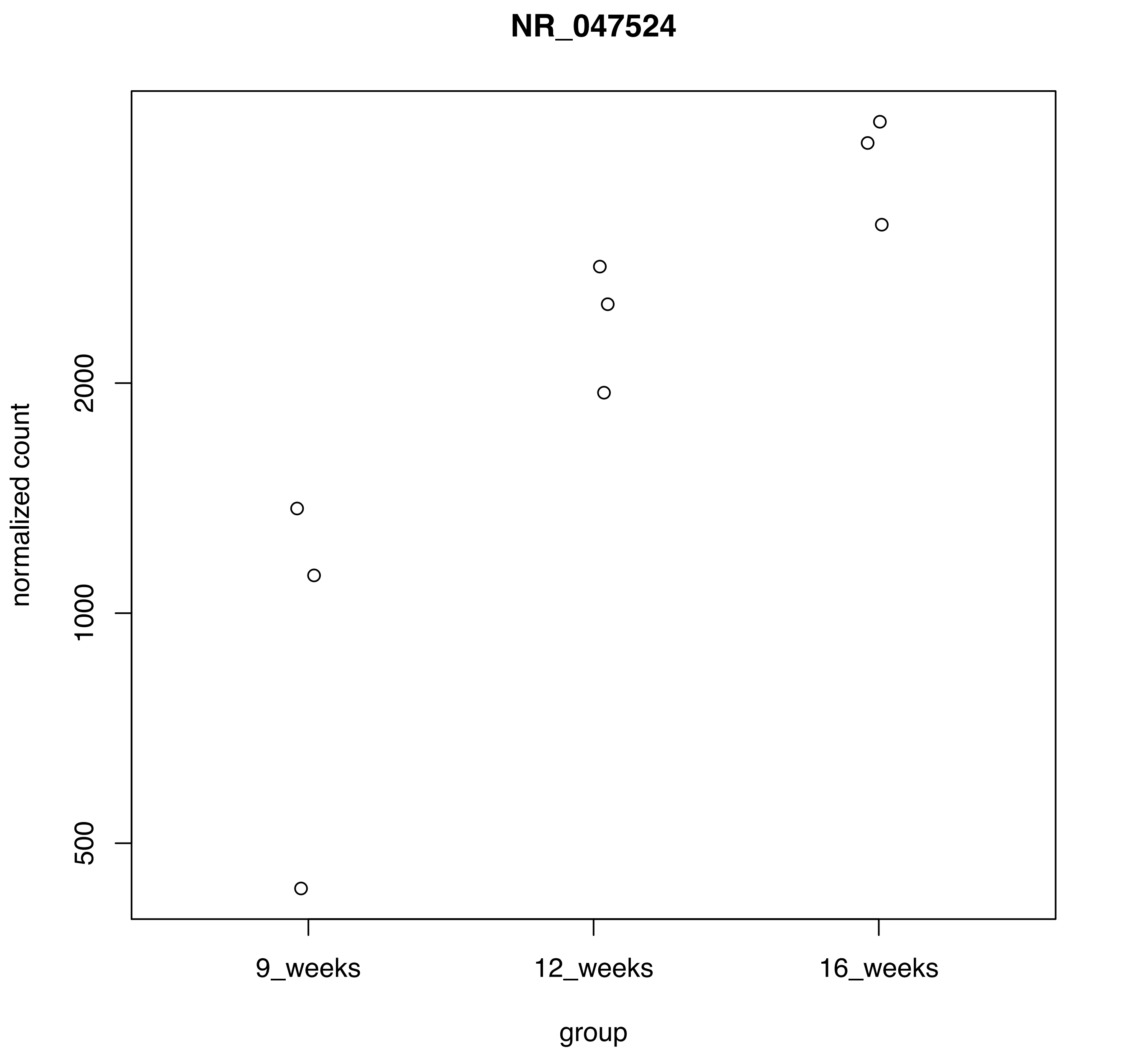
NR_047524
normalized count
2000
1000
500
9_weeks
12_weeks
16_weeks
group

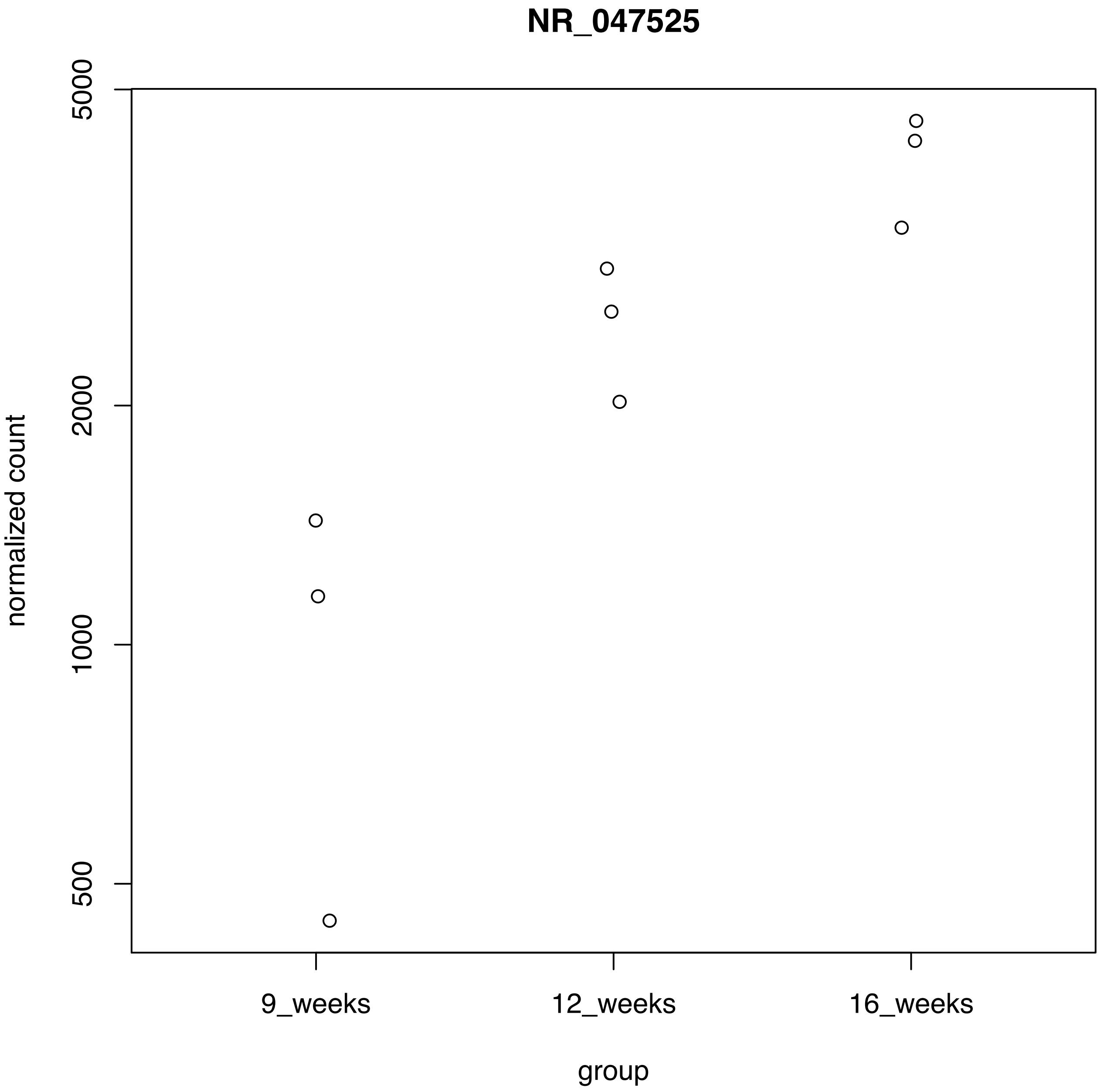
NR_047525
normalized count
5000
2000
1000
500
9_weeks
12_weeks
16_weeks
group

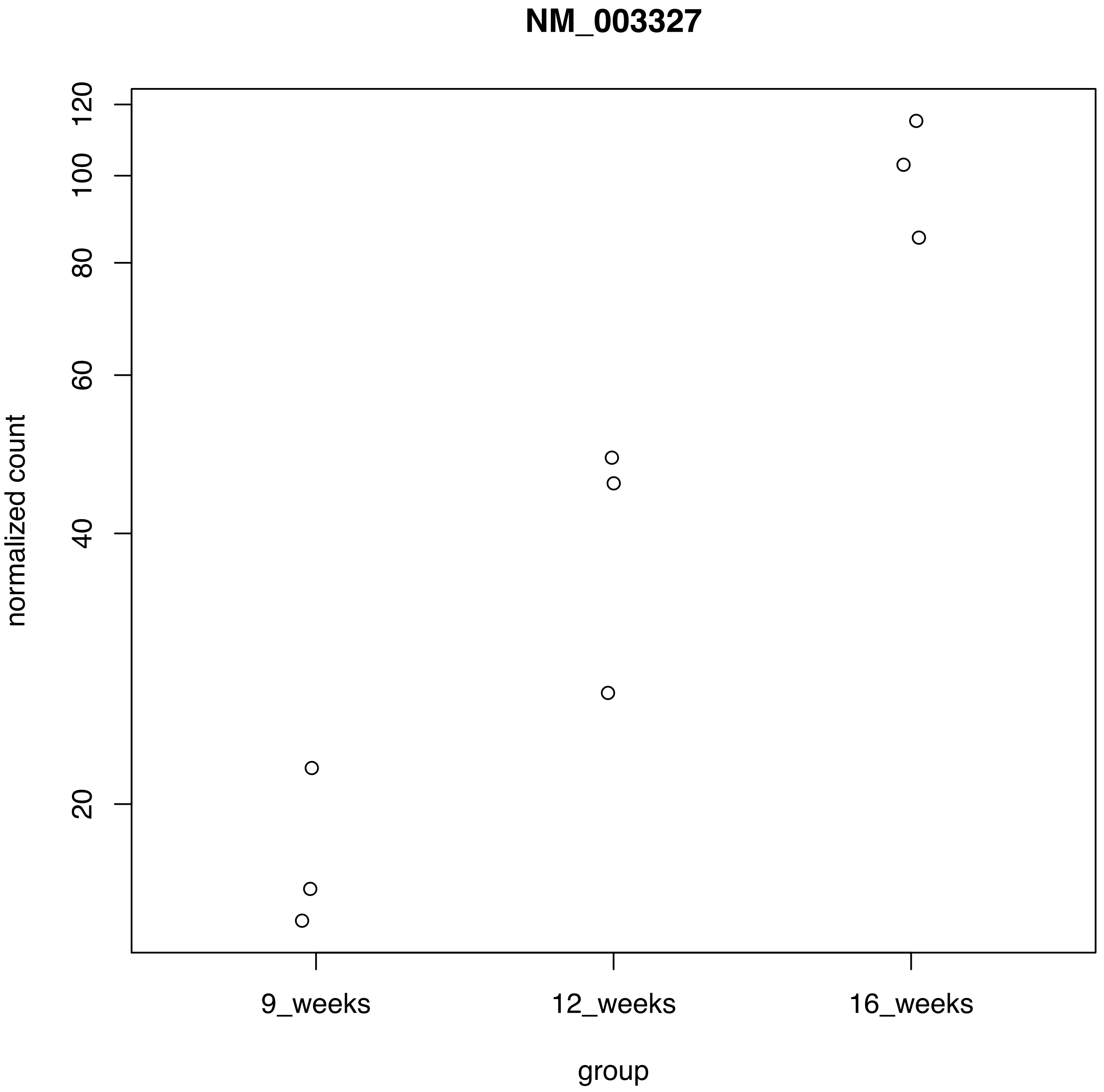

NM_003327
normalized count
120
100
80
60
40
20
9_weeks
12_weeks
16_weeks
group

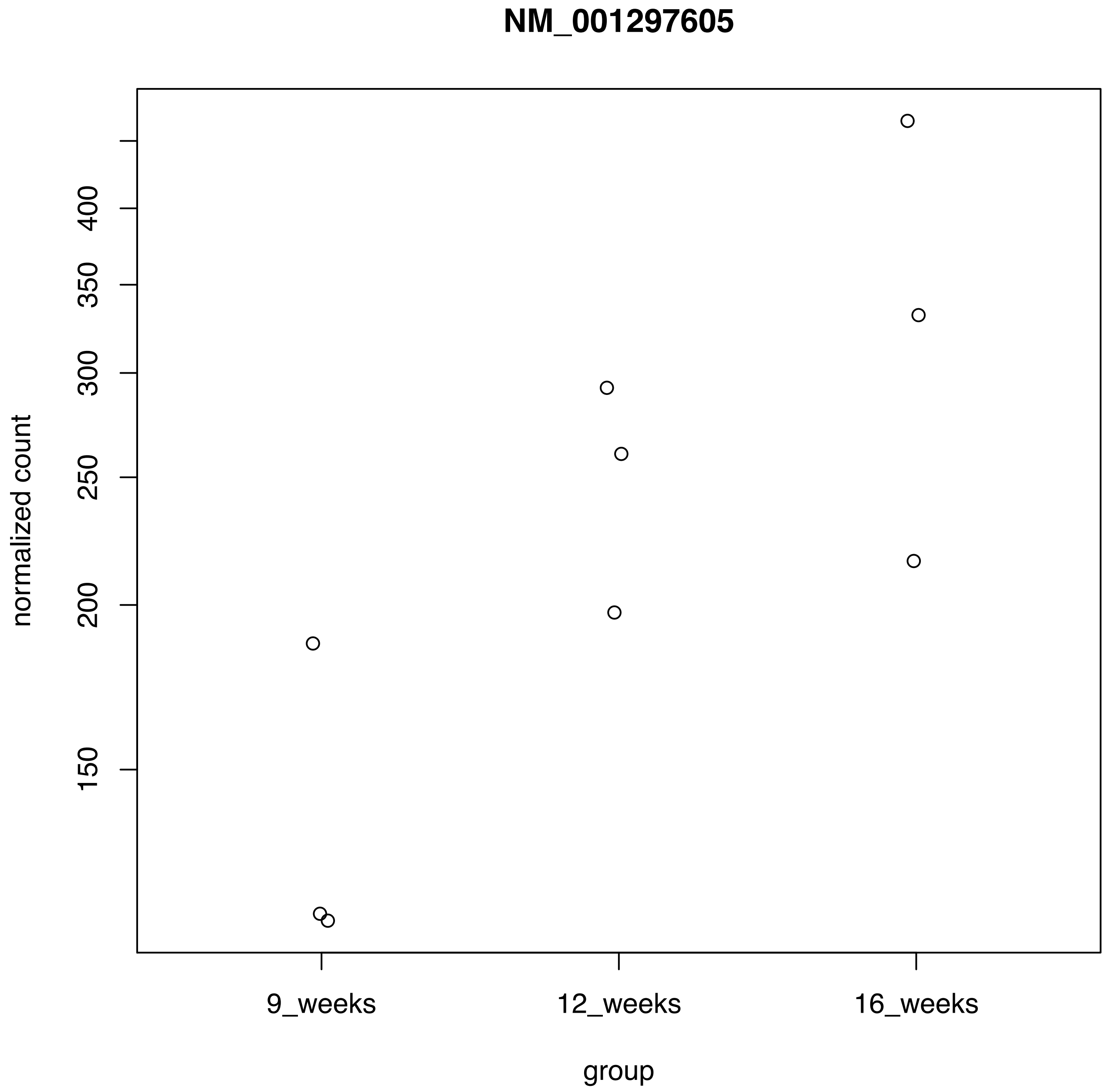
NM_001297605
normalized count
150
200
250
300
350
400
9_weeks
12_weeks
16_weeks
group

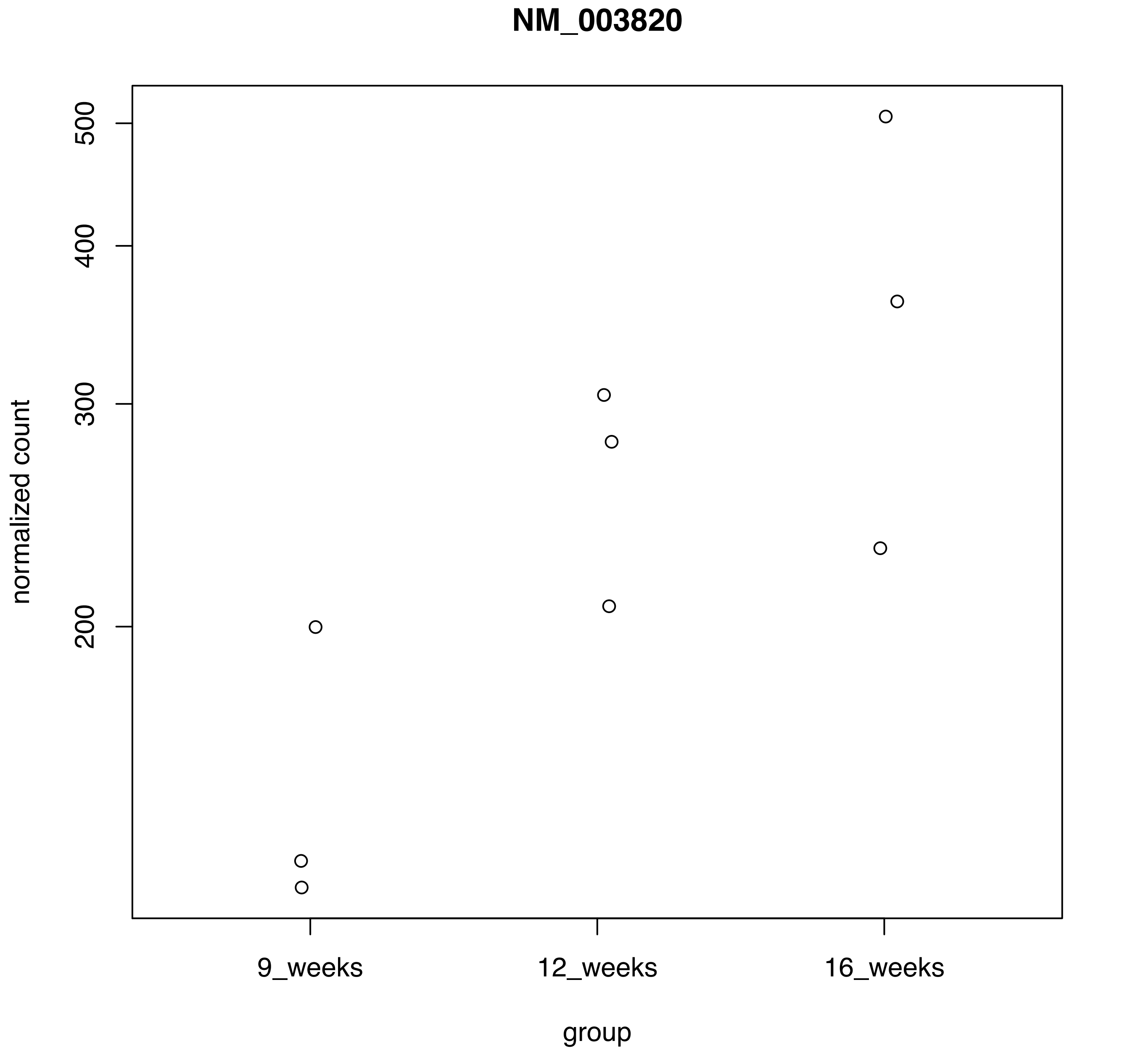
NM_003820
normalized count
500
400
300
200
9_weeks
12_weeks
16_weeks
group

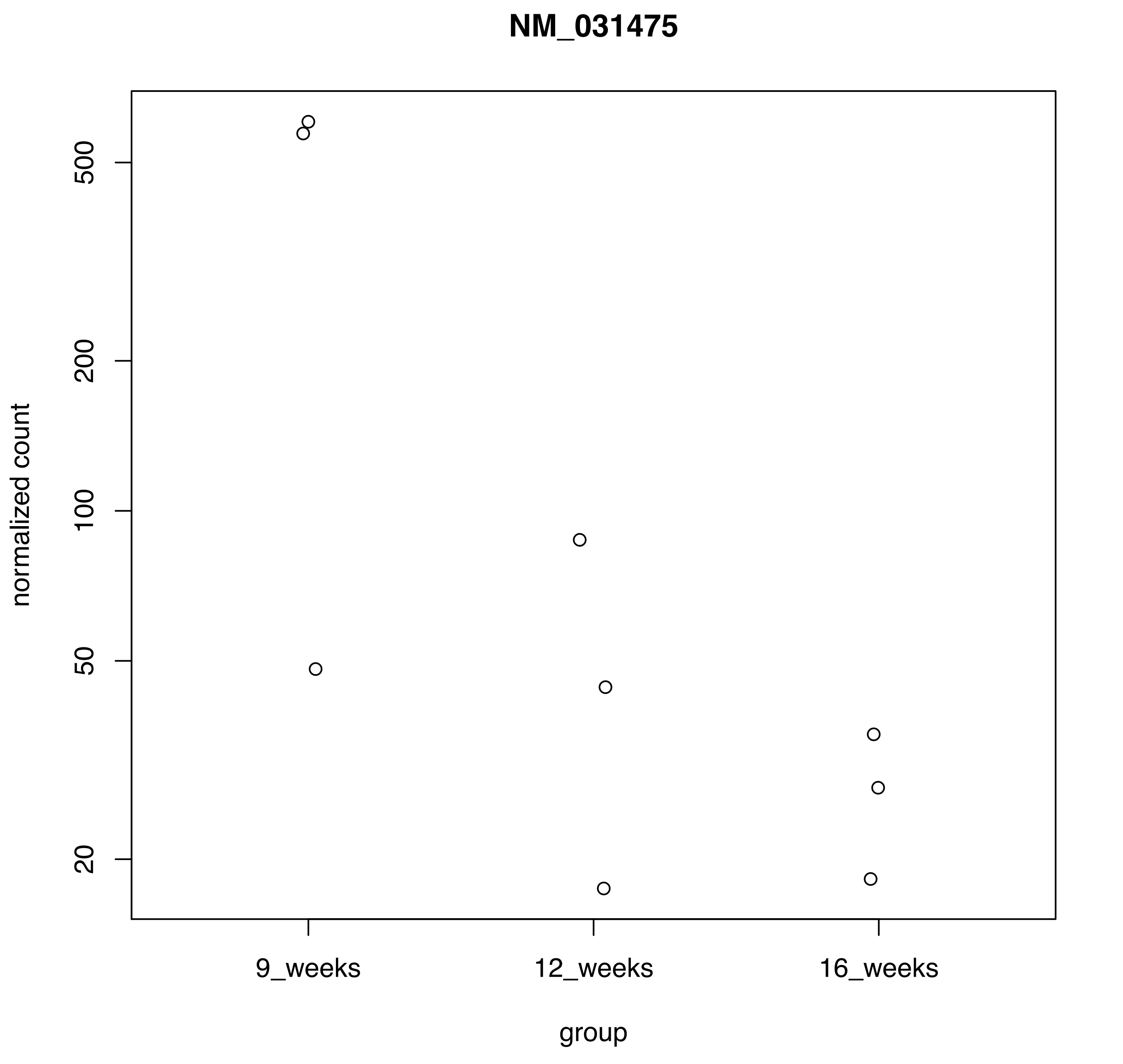
NM_031475
normalized count
500
200
100
50
20
9_weeks
12_weeks
16_weeks
group

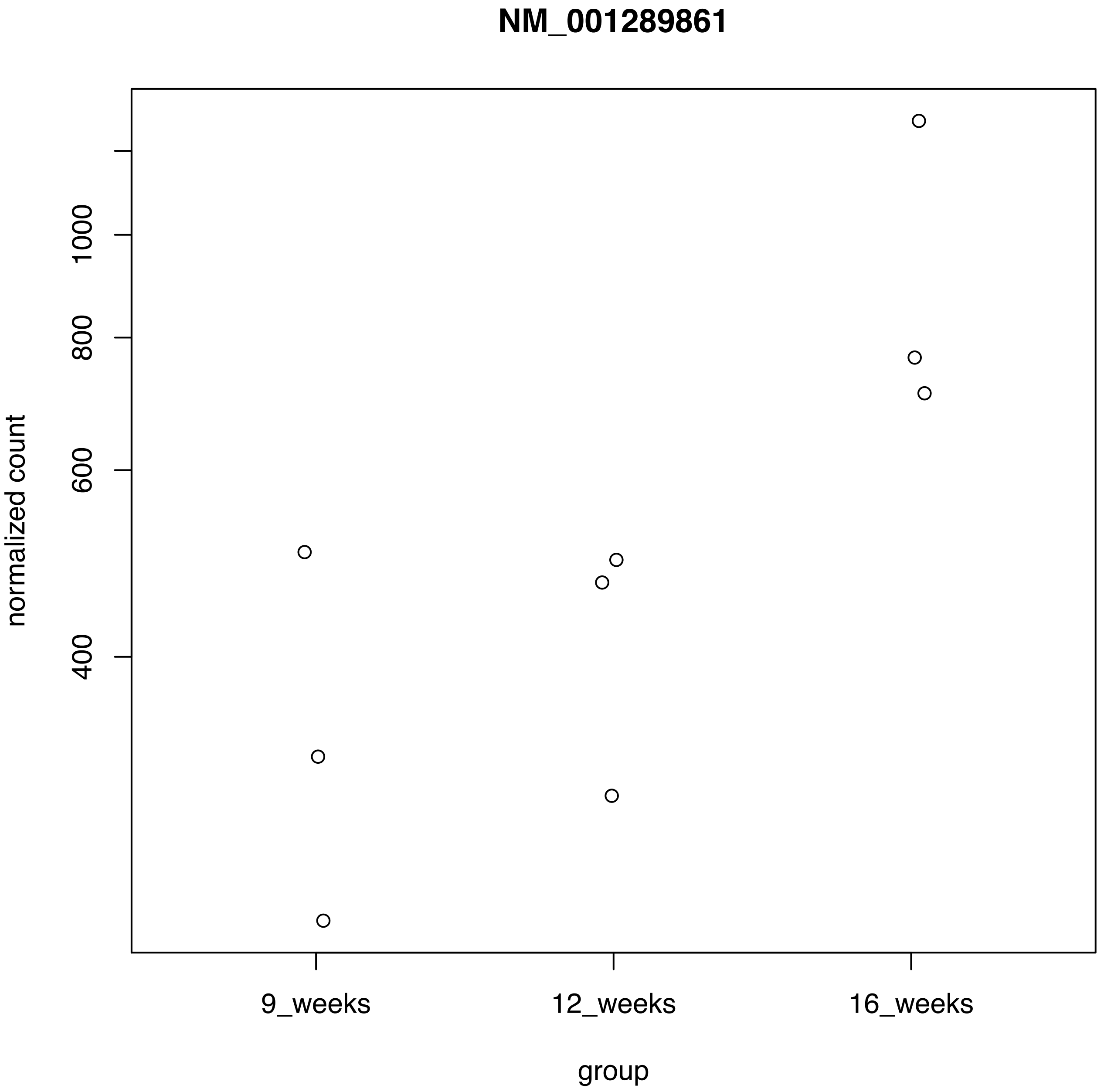

NM_001289861
normalized count
1000
800
600
400
9_weeks
12_weeks
16_weeks
group

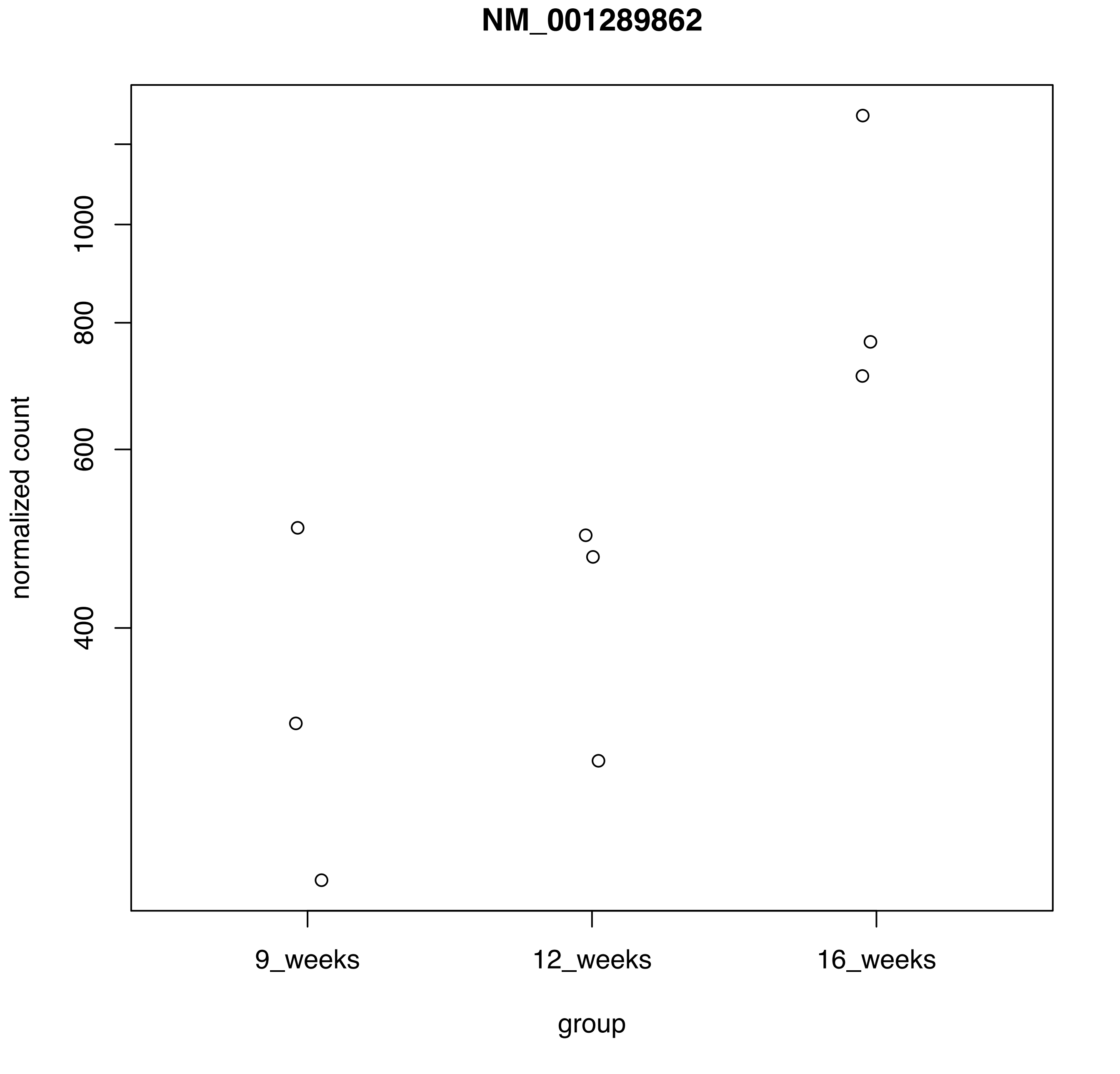
NM_001289862
normalized count
400
600
800
1000
9_weeks
12_weeks
16_weeks
group

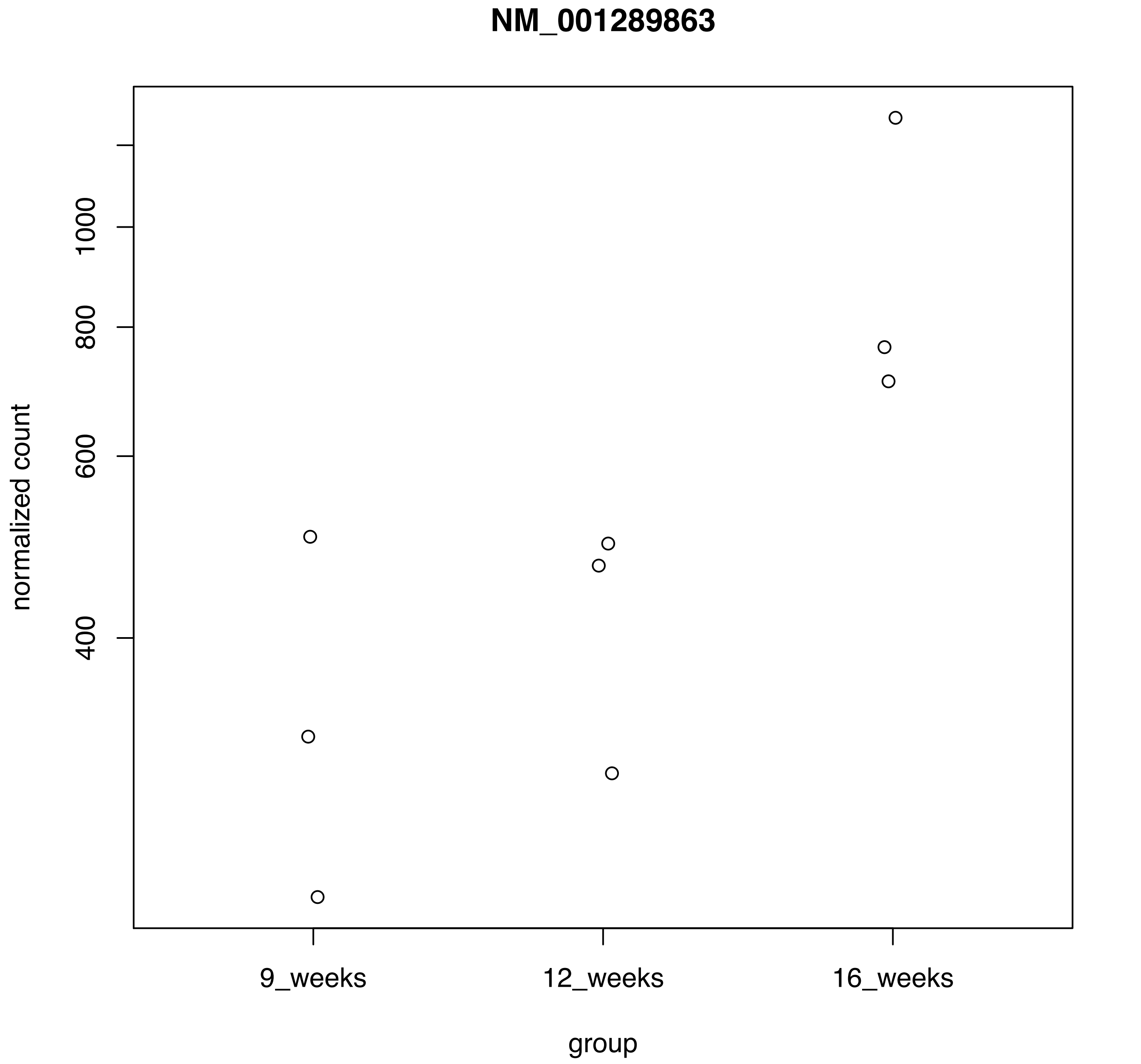
NM_001289863
normalized count
400
600
800
1000
9_weeks
12_weeks
16_weeks
group

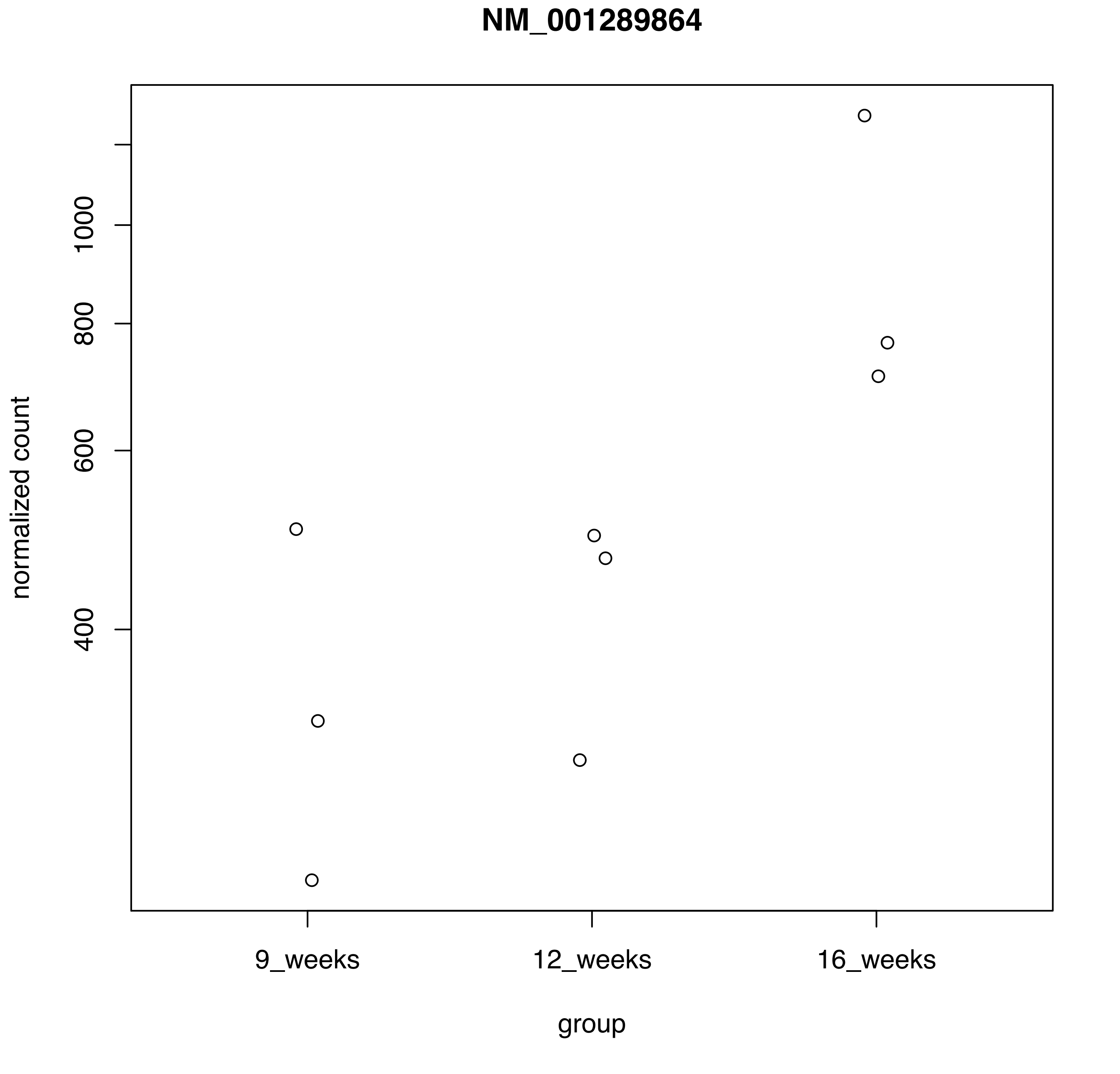
NM_001289864
normalized count
1000
800
600
400
9_weeks
12_weeks
16_weeks
group

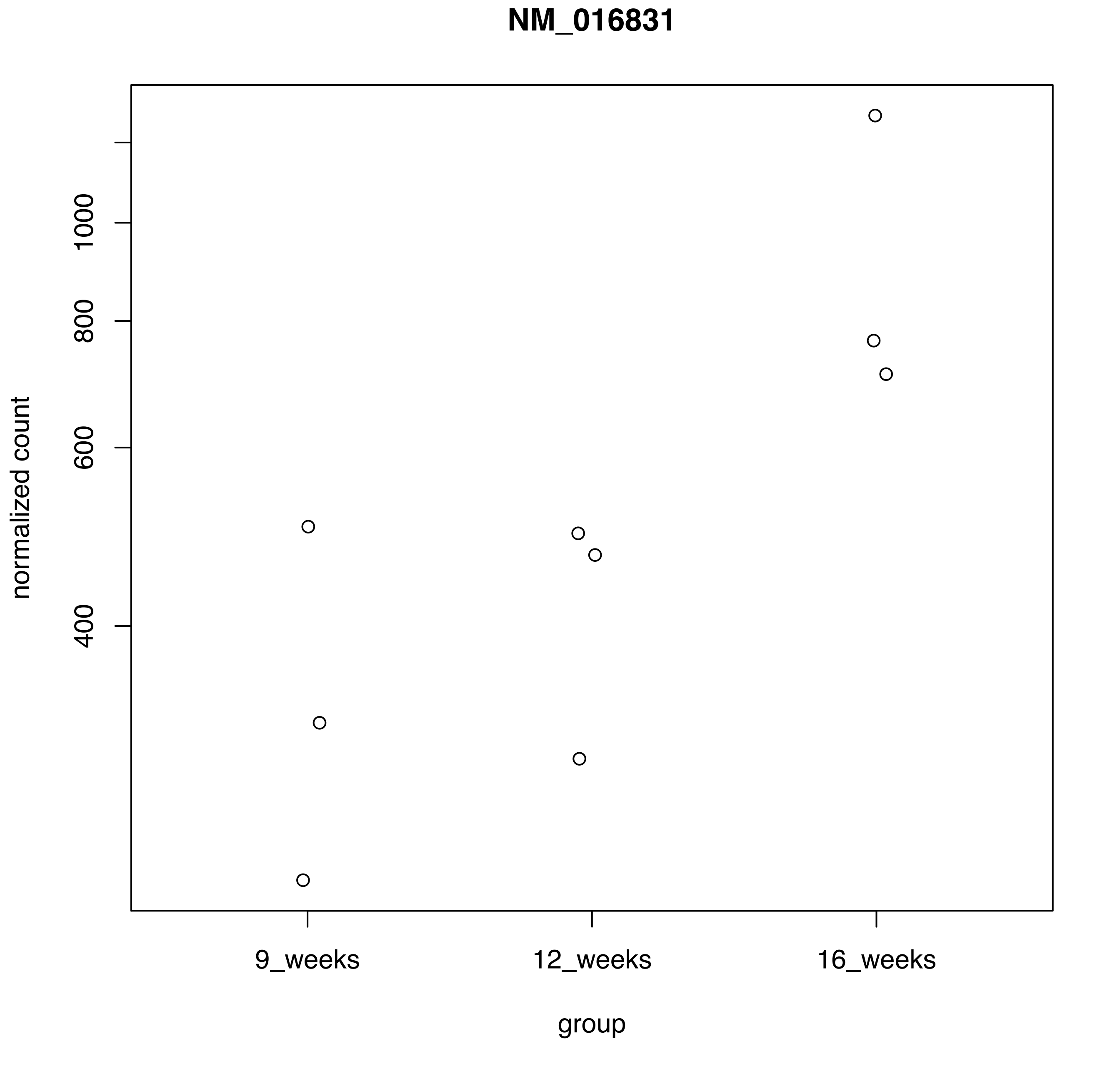
NM_016831
normalized count
400
600
800
1000
9_weeks
12_weeks
16_weeks
group

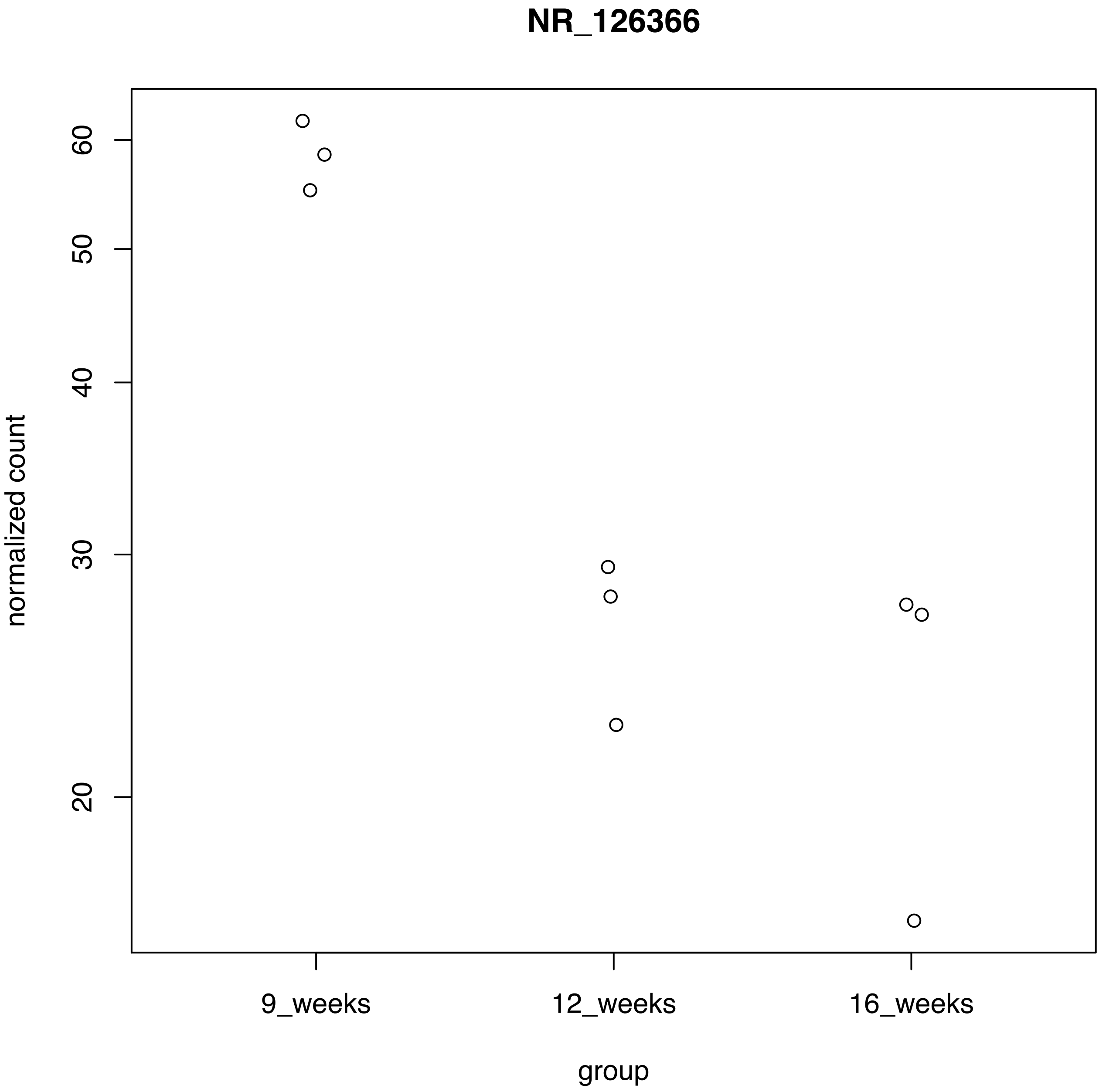
NR_126366
normalized count
60
50
40
30
20
9_weeks
12_weeks
16_weeks
group

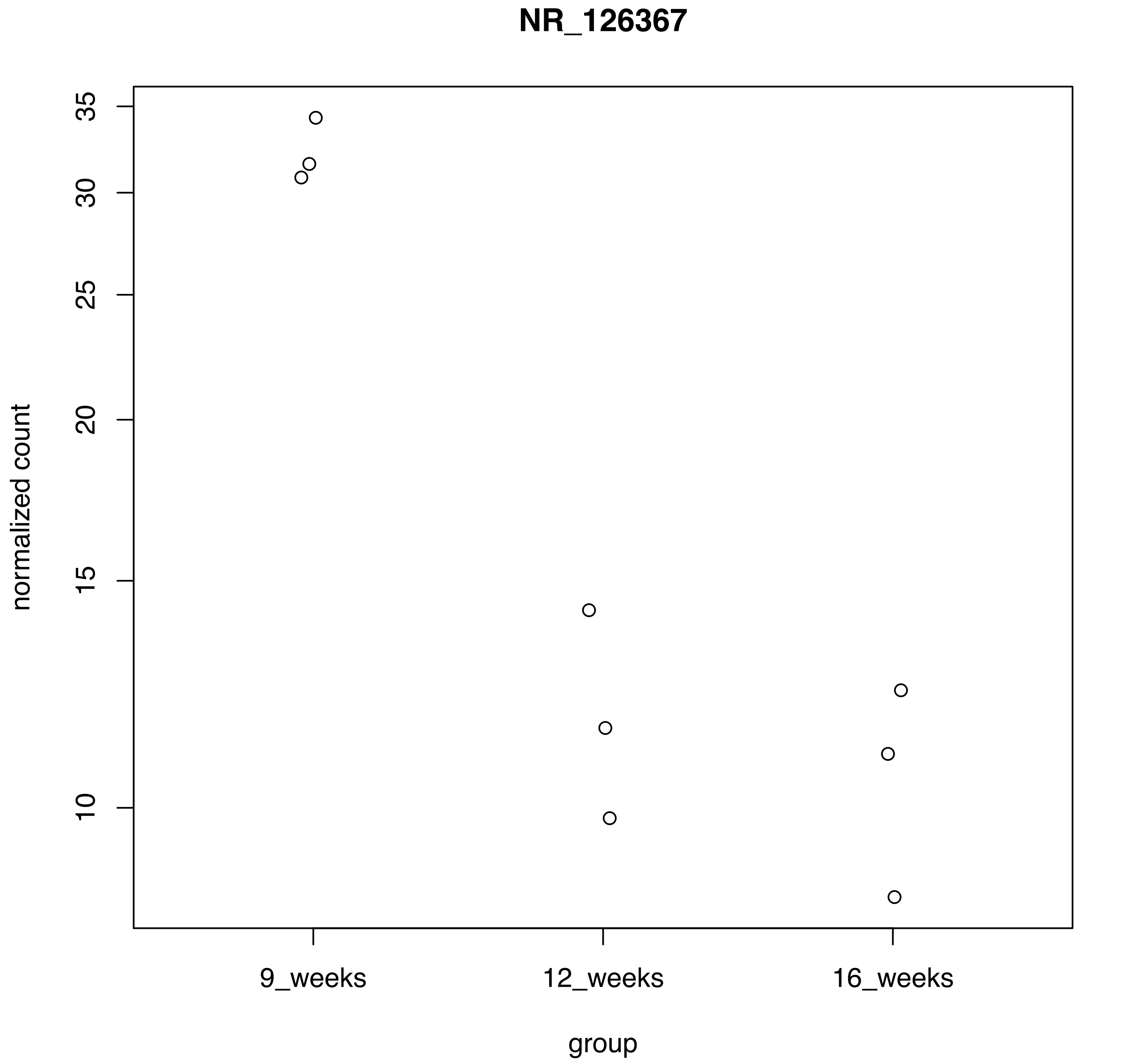

NR_126367
35
30
25
20
15
10
normalized count
9_weeks
12_weeks
16_weeks
group

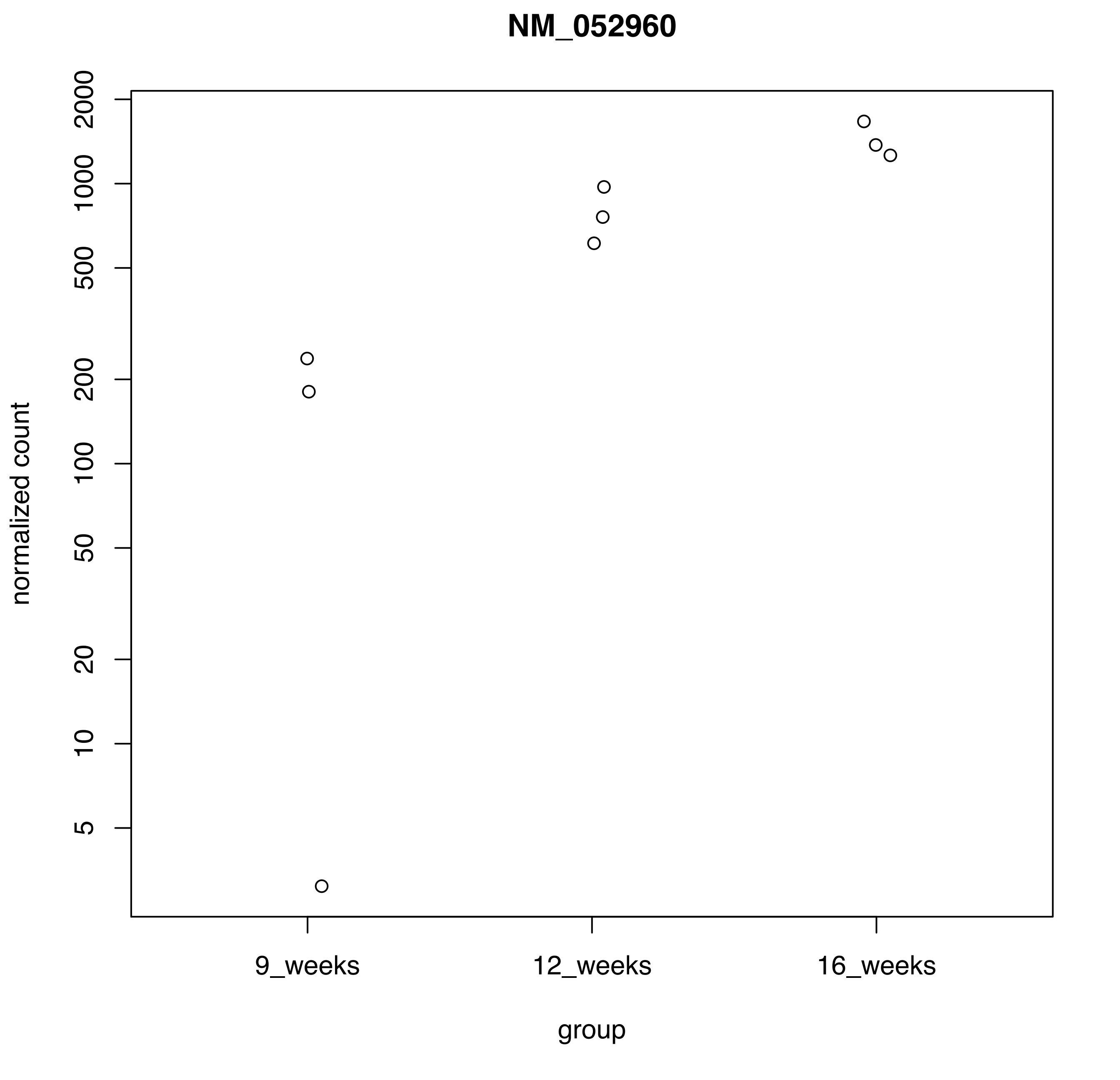
NM_052960
normalized count
2000
1000
500
200
100
50
20
10
5
9_weeks
12_weeks
16_weeks
group

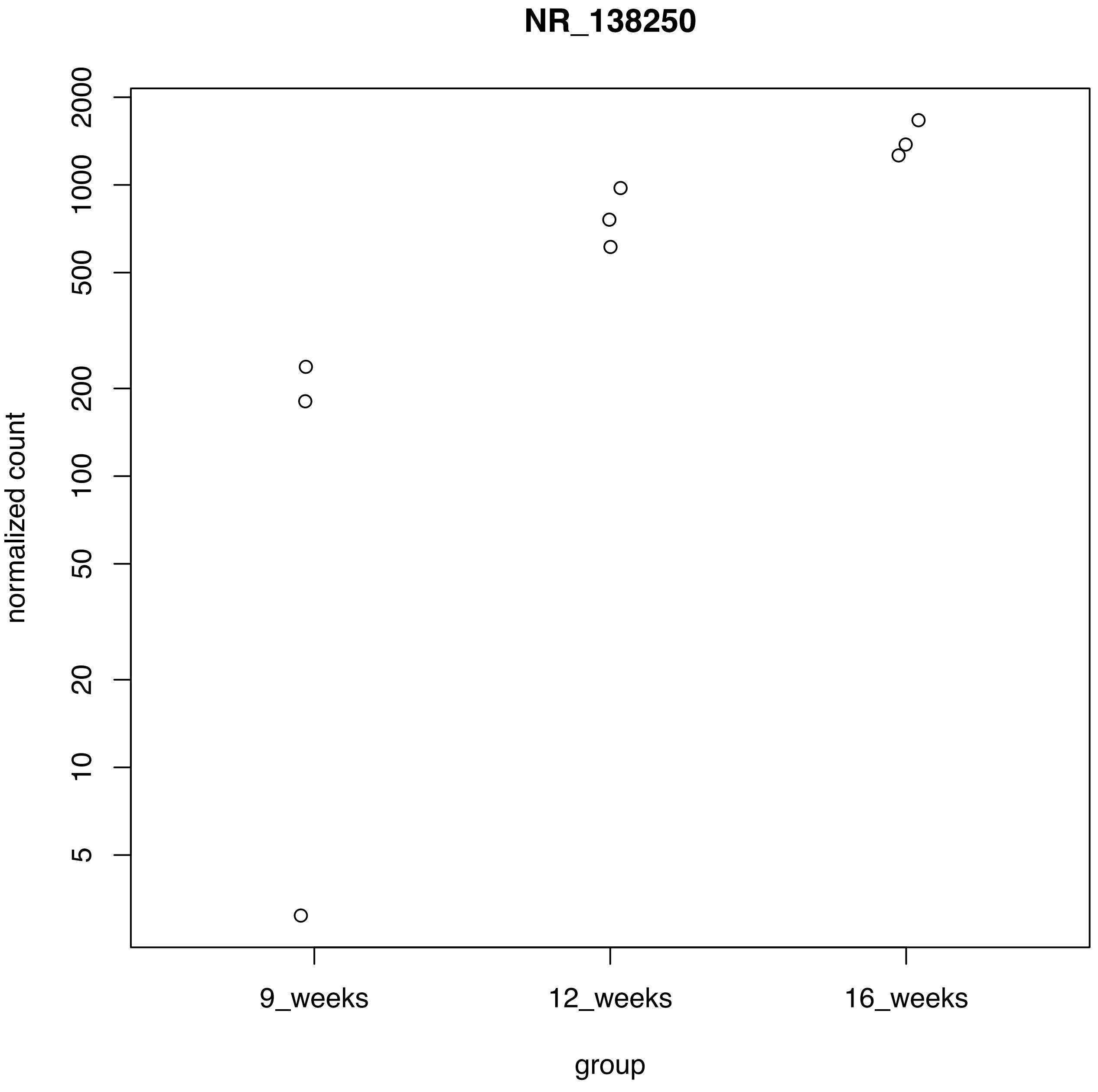
NR_138250
normalized count
2000
1000
500
200
100
50
20
10
5
9_weeks
12_weeks
16_weeks
group

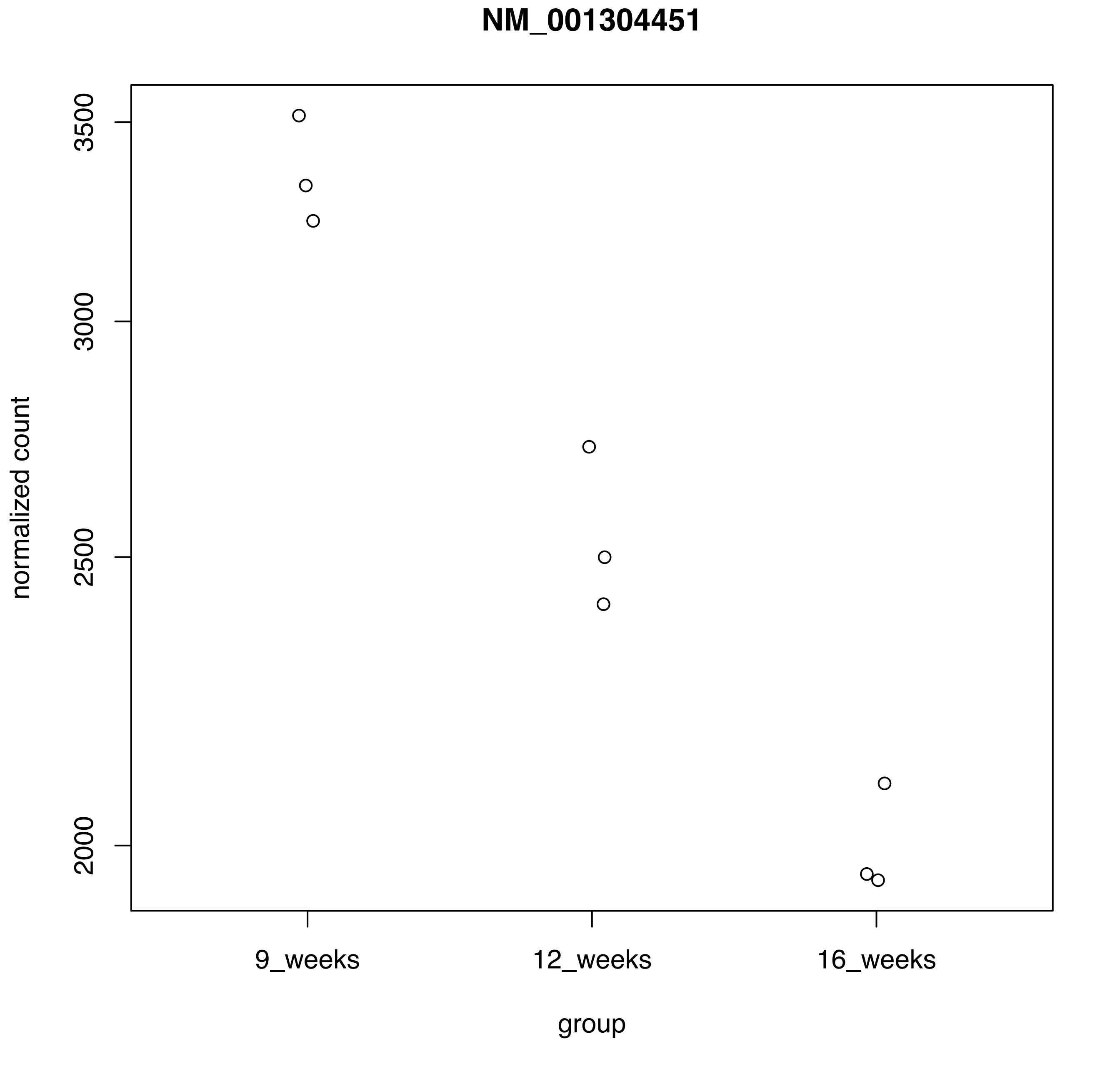

NM_001304451
normalized count
3500
3000
2500
2000
9_weeks
12_weeks
16_weeks
group

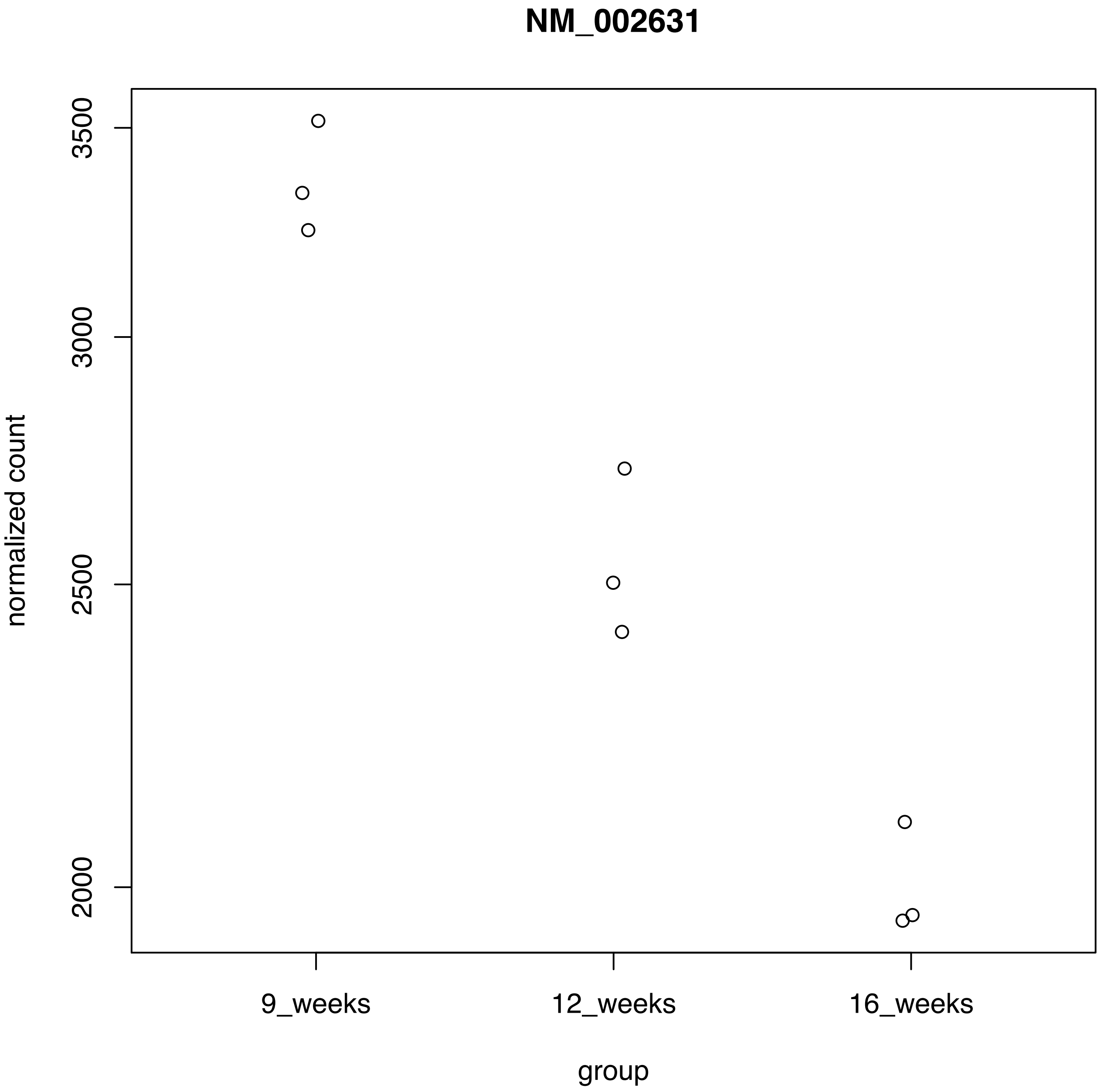
NM_002631
normalized count
3500
3000
2500
2000
9_weeks
12_weeks
16_weeks
group

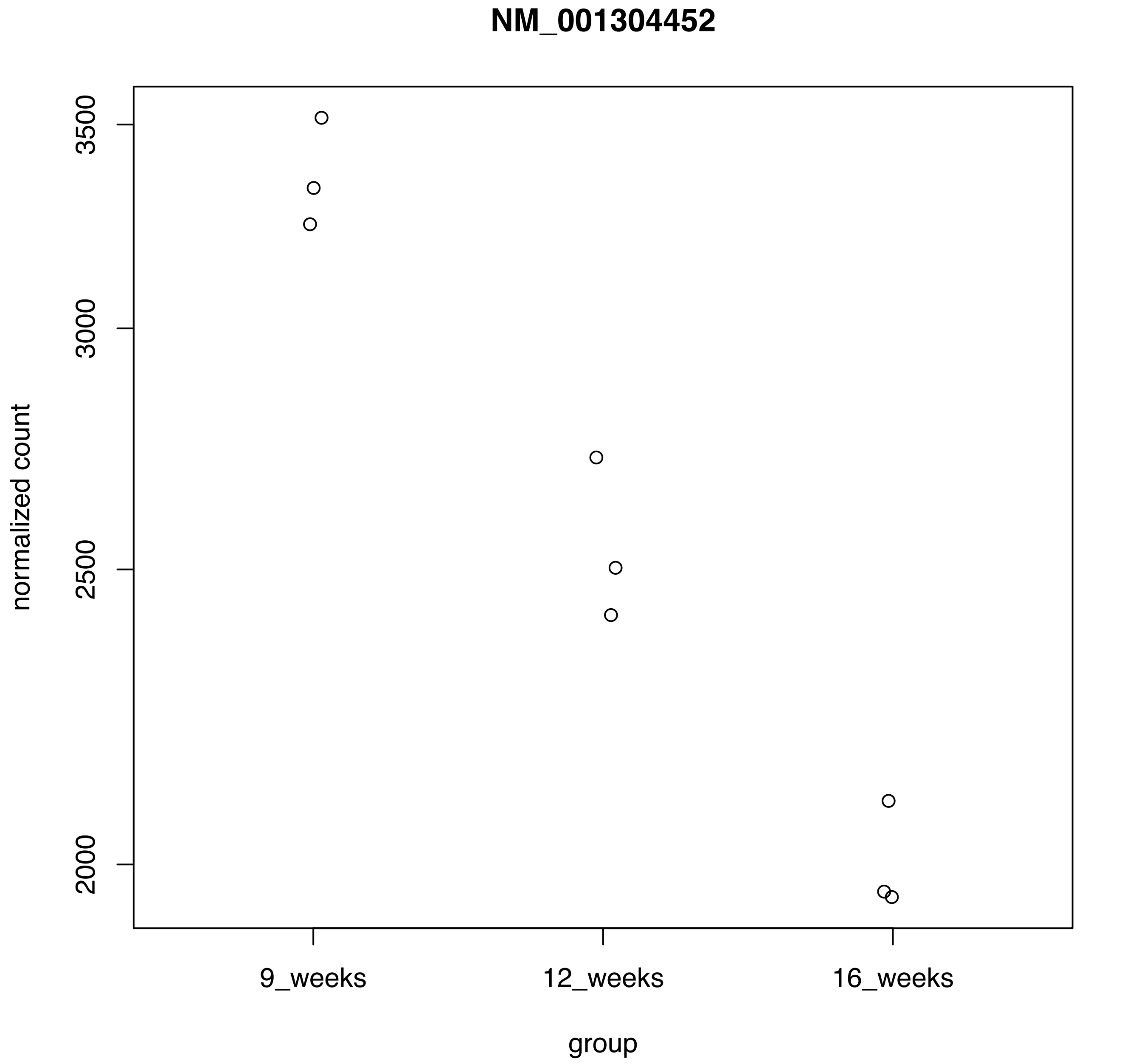

NM_001304452
normalized count
3500
3000
2500
2000
9_weeks
12_weeks
16_weeks
group

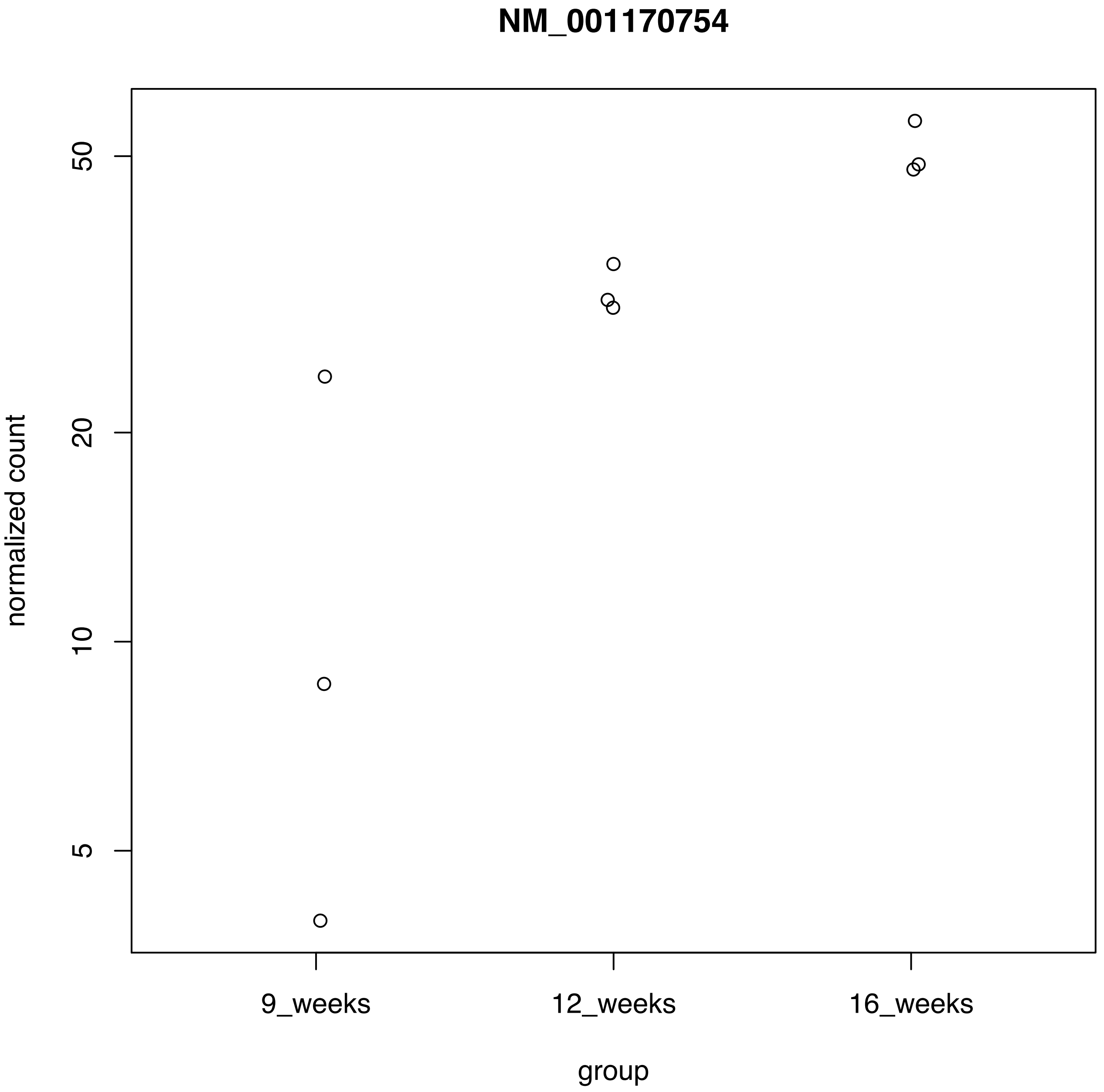
NM_001170754
normalized count
50
20
10
5
9_weeks
12_weeks
16_weeks
group

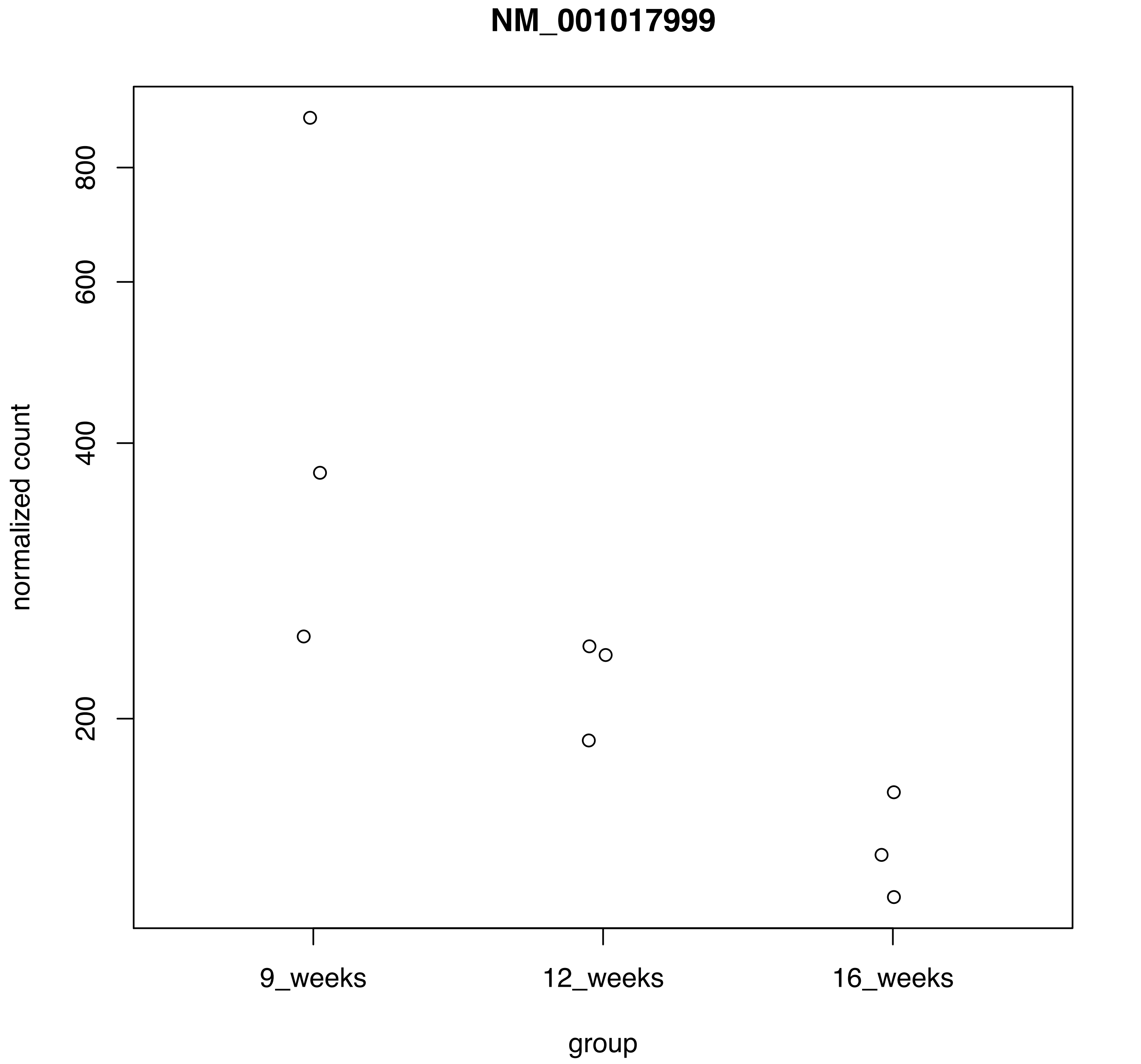

NM_001017999
normalized count
800
600
400
200
9_weeks
12_weeks
16_weeks
group

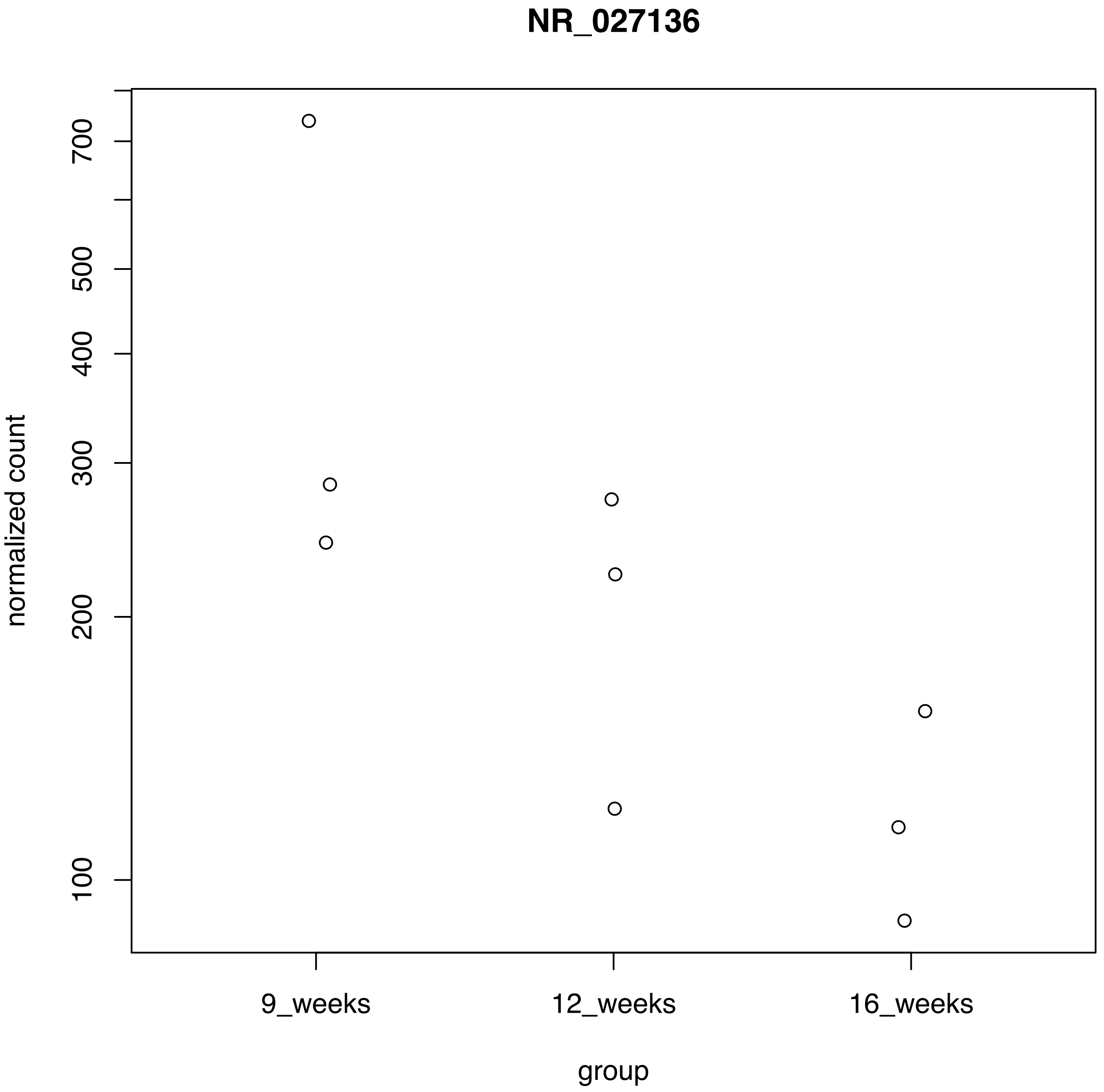
NR_027136
normalized count
700
500
400
300
200
100
9_weeks
12_weeks
16_weeks
group

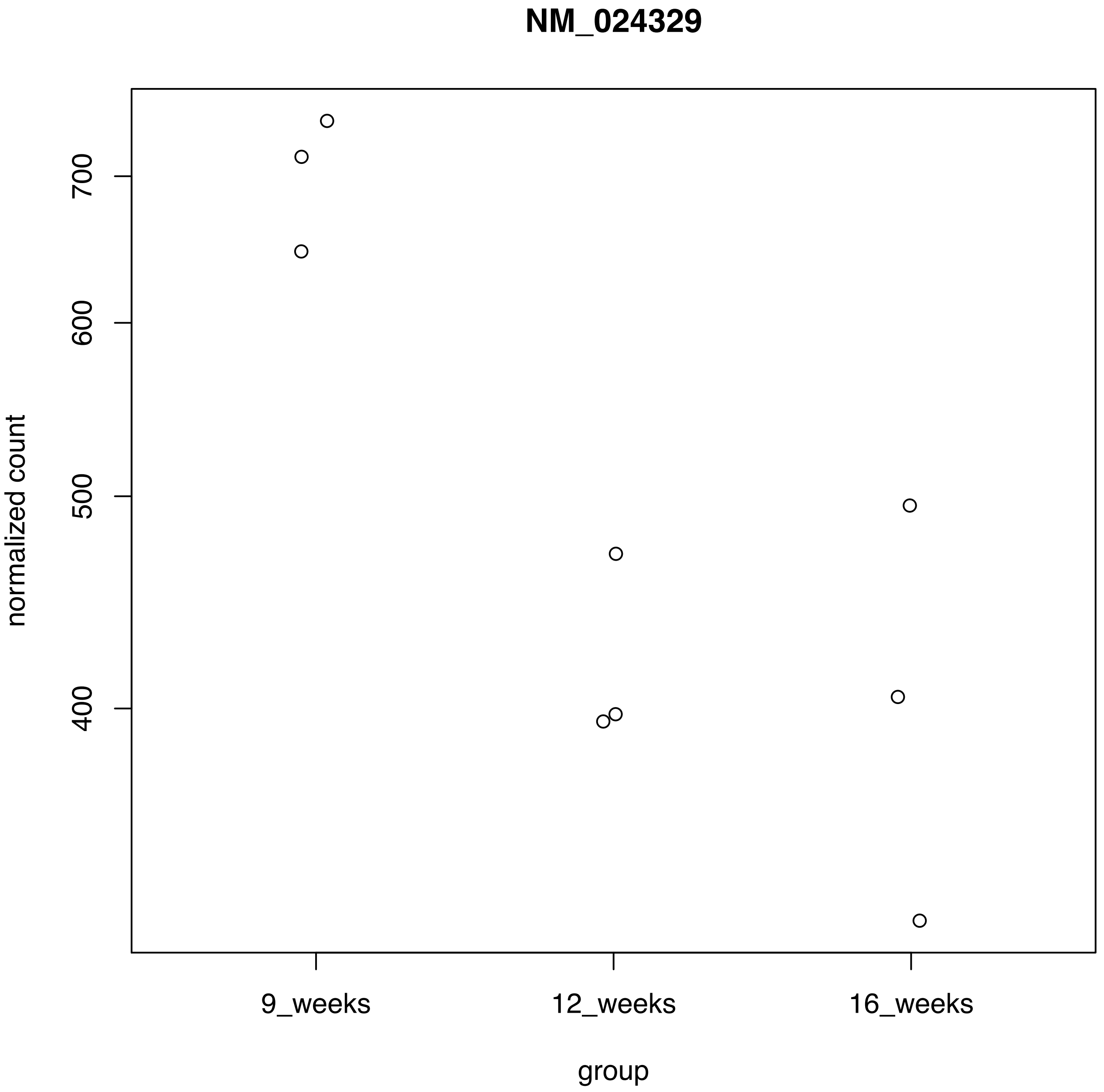
NM_024329
normalized count
700
600
500
400
9_weeks
12_weeks
16_weeks
group

NR_004408

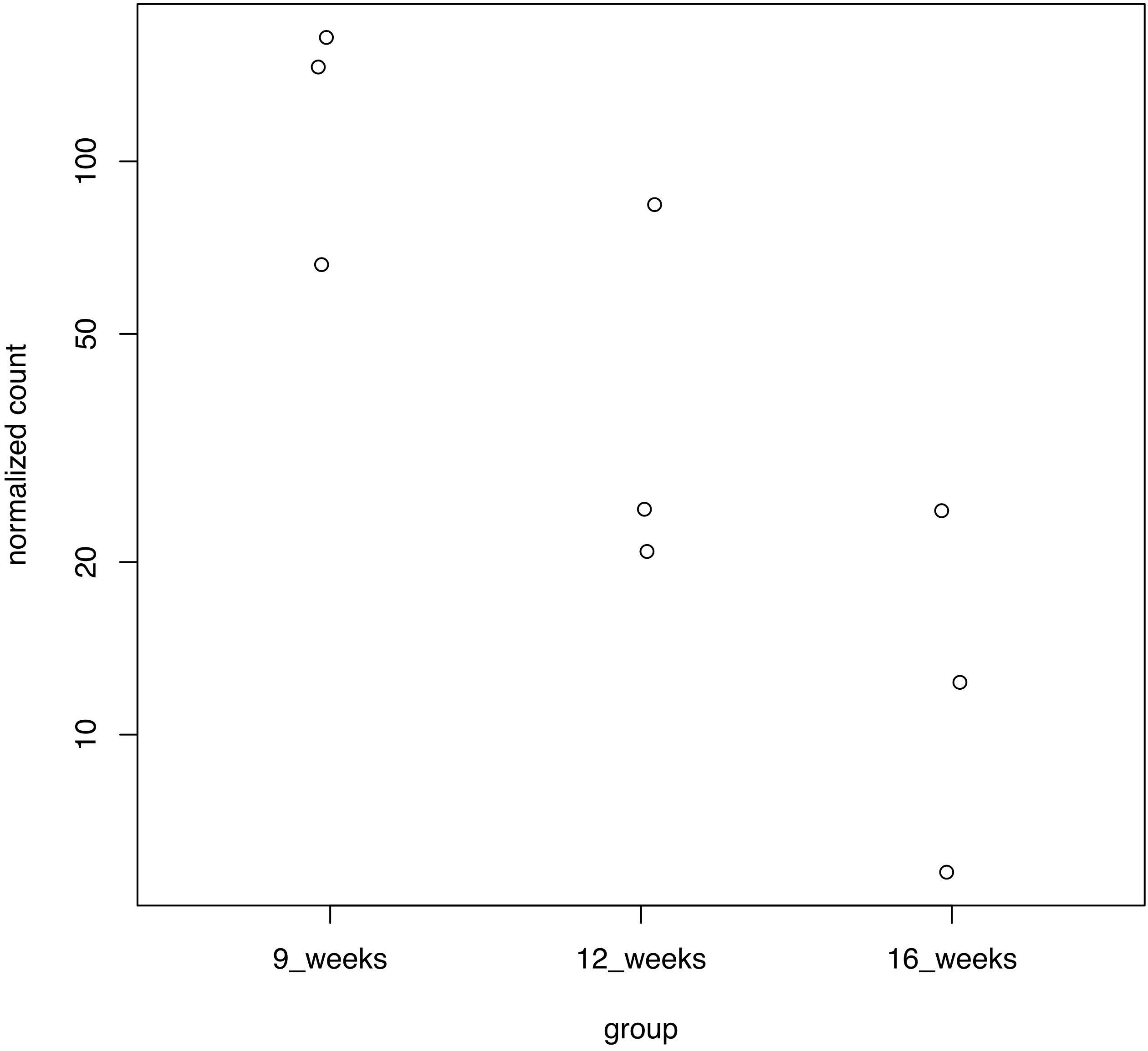

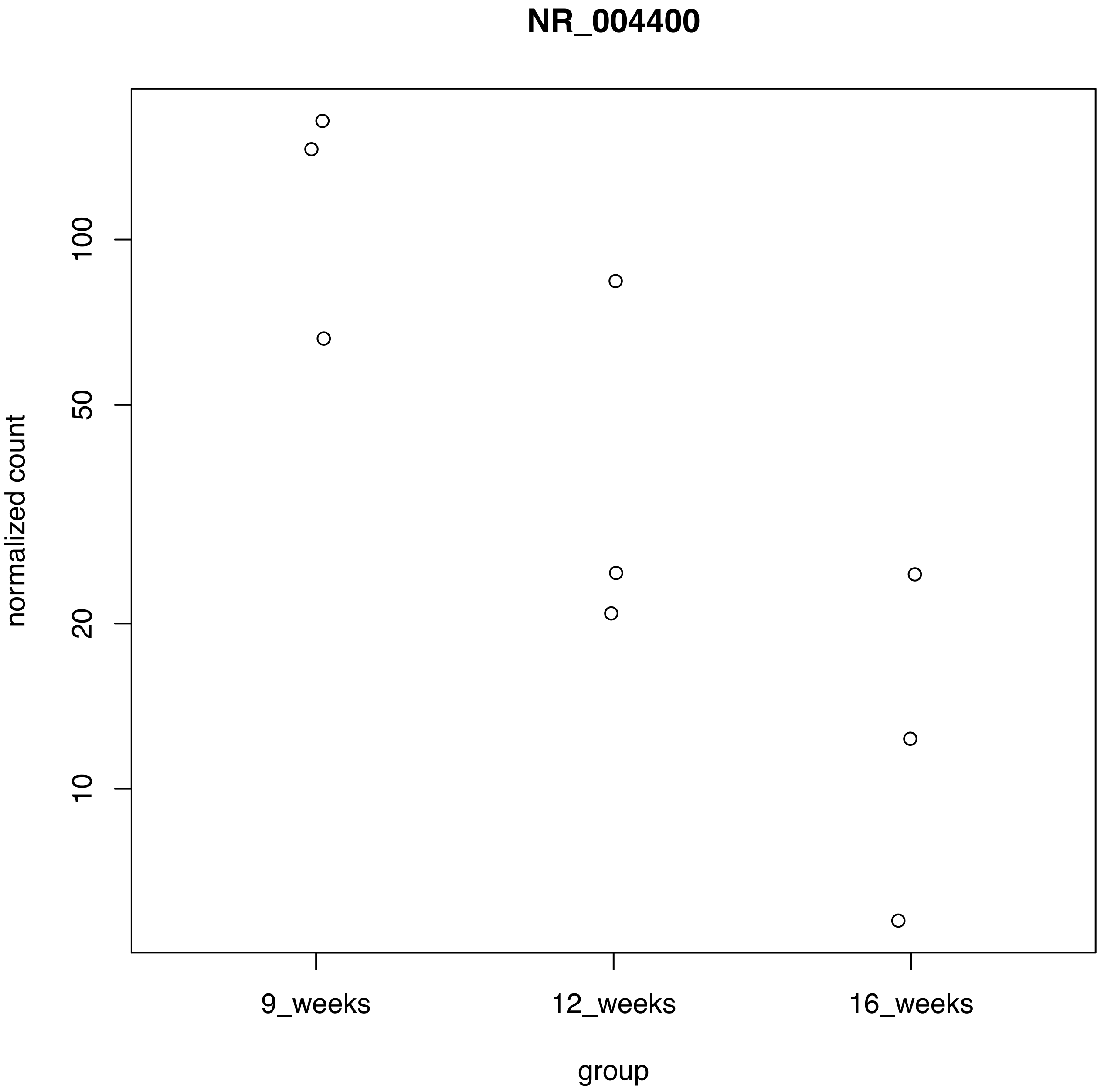

NR_004400
normalized count
100
50
20
10
9_weeks
12_weeks
16_weeks
group

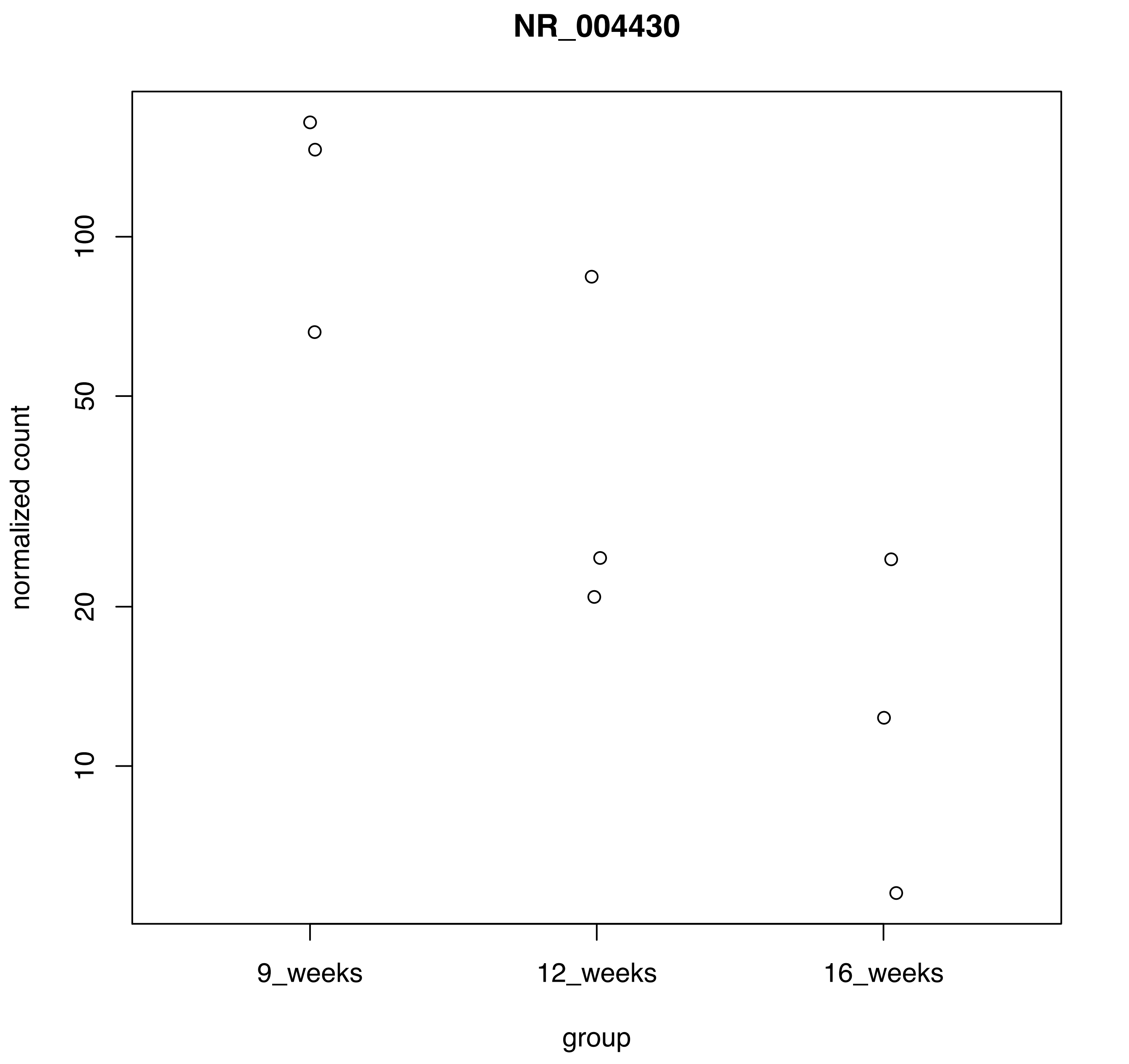
NR_004430
normalized count
100
50
20
10
9_weeks
12_weeks
16_weeks
group

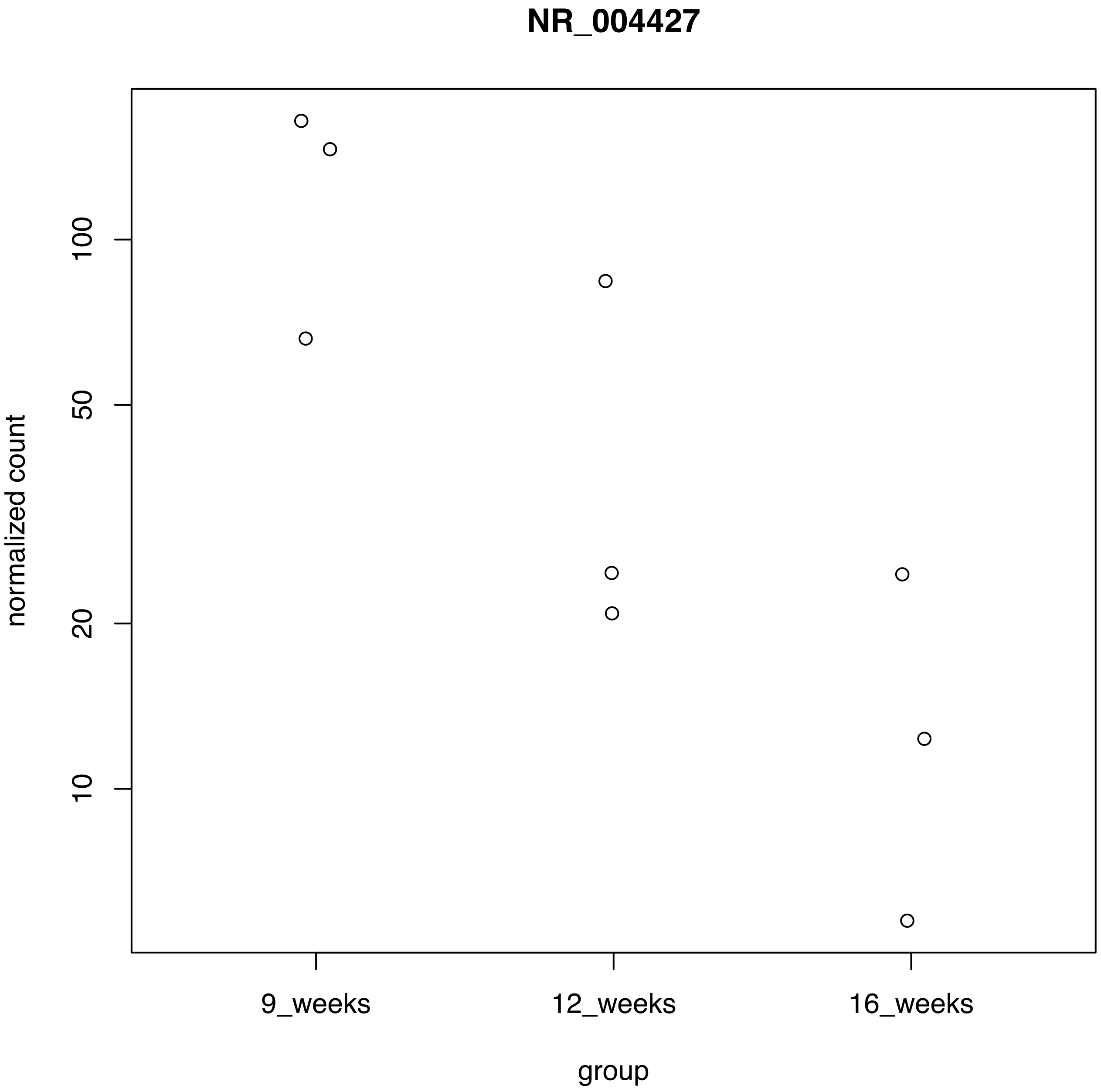

NR_004427
normalized count
100
50
20
10
9_weeks
12_weeks
16_weeks
group

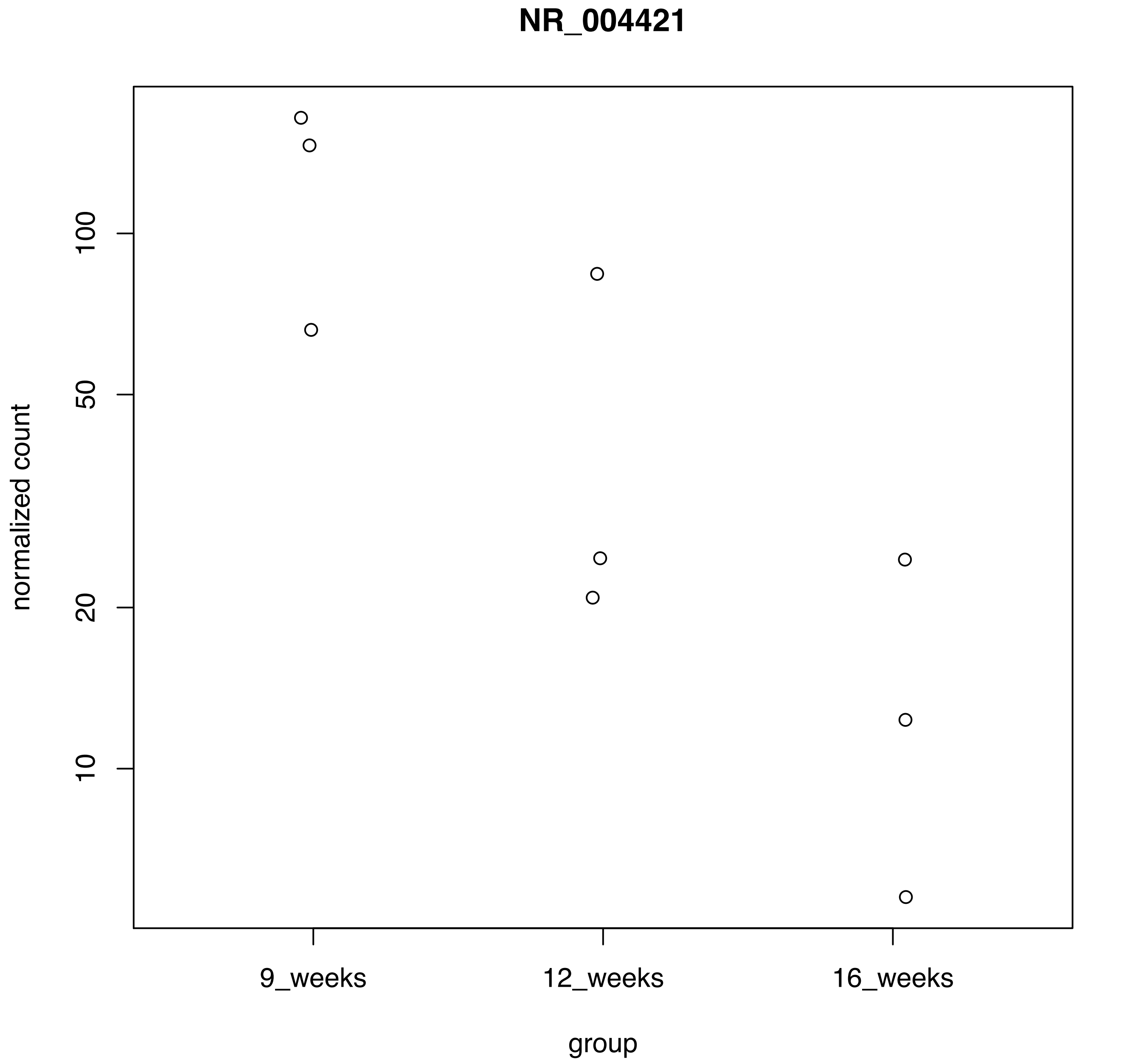

NR_004421
normalized count
100
50
20
10
9_weeks
12_weeks
16_weeks
group

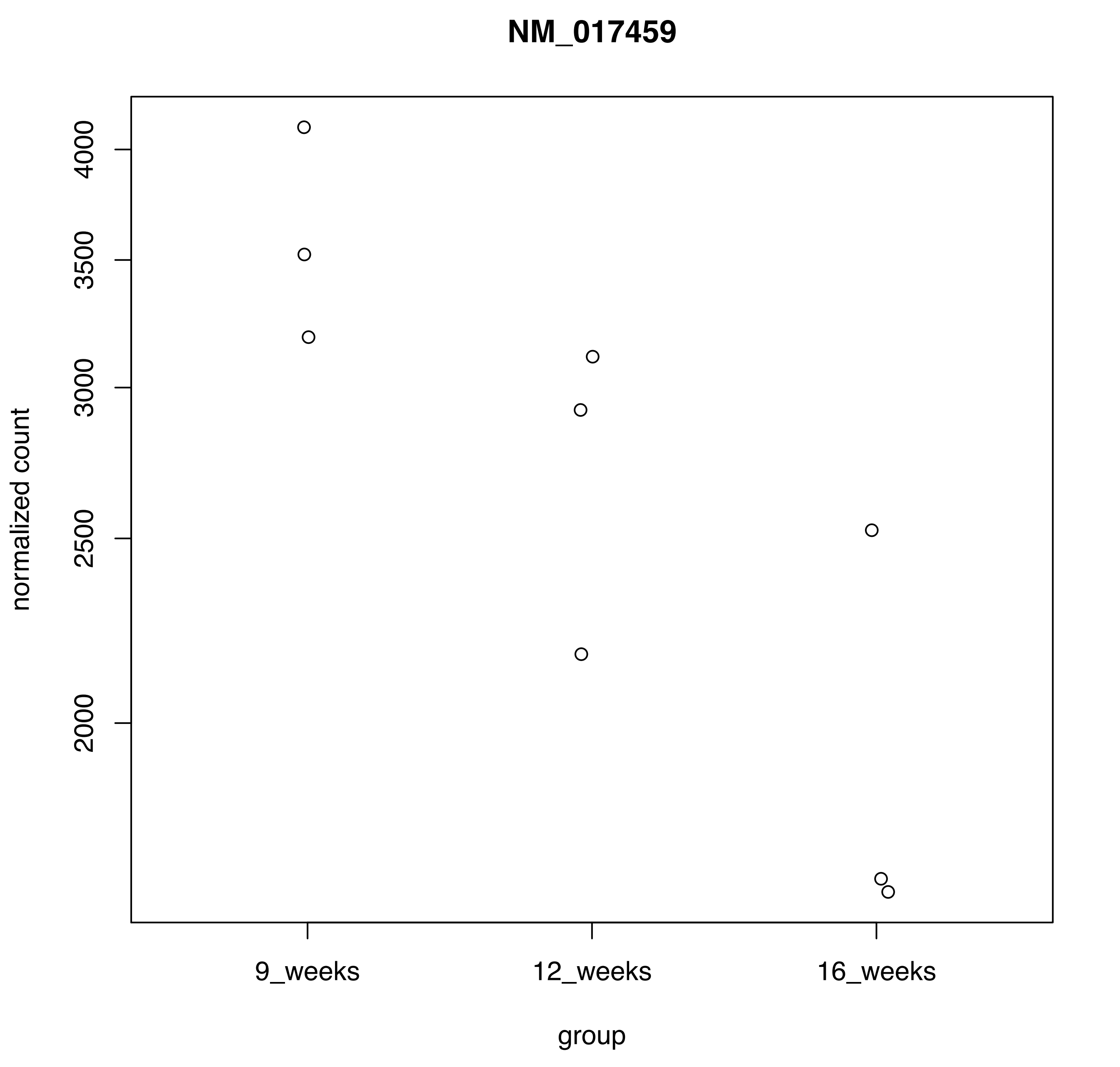

NM_017459
normalized count
2000
2500
3000
3500
4000
9_weeks
12_weeks
16_weeks
group

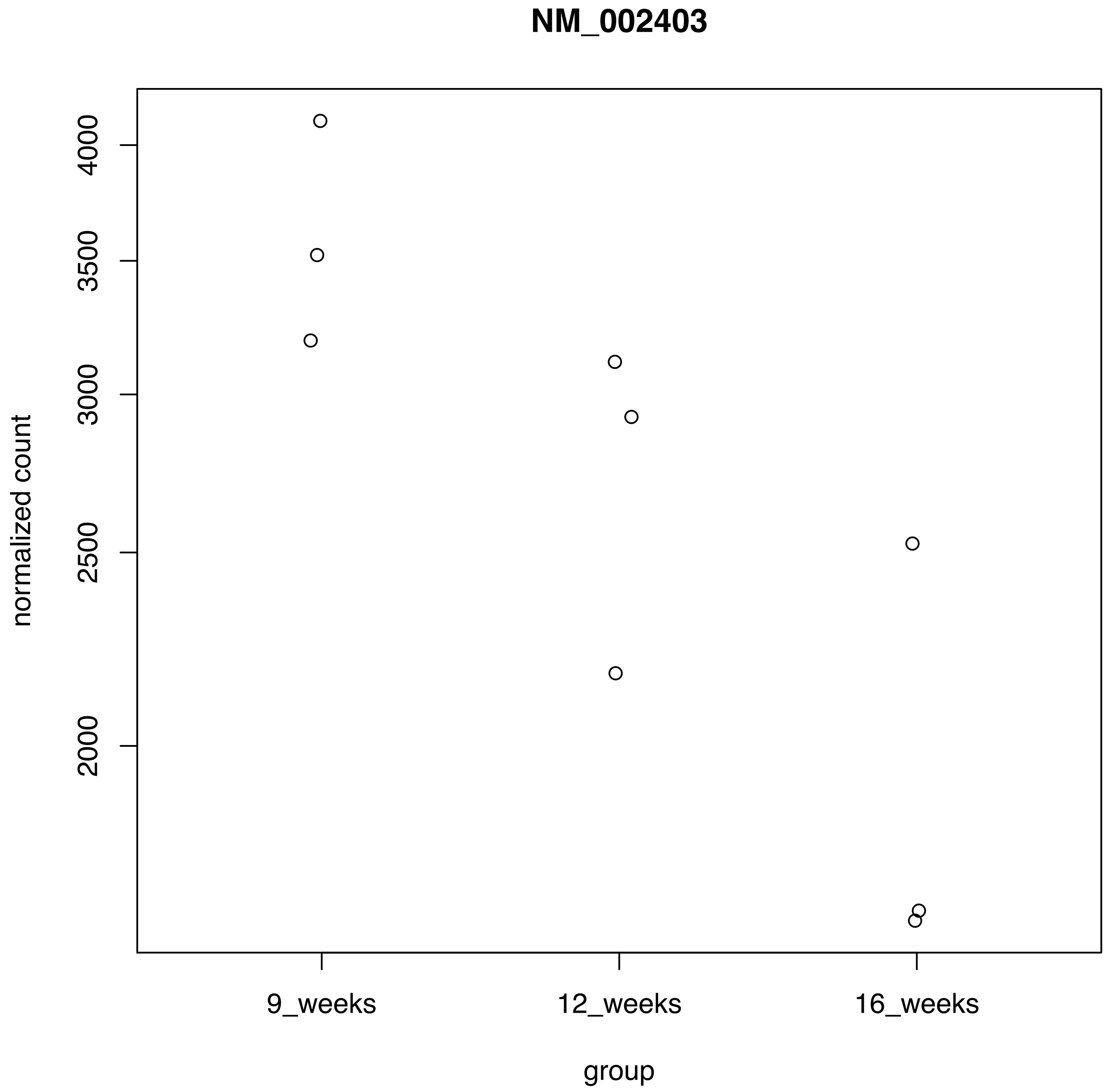
NM_002403
normalized count
4000
3500
3000
2500
2000
9_weeks
12_weeks
16_weeks
group

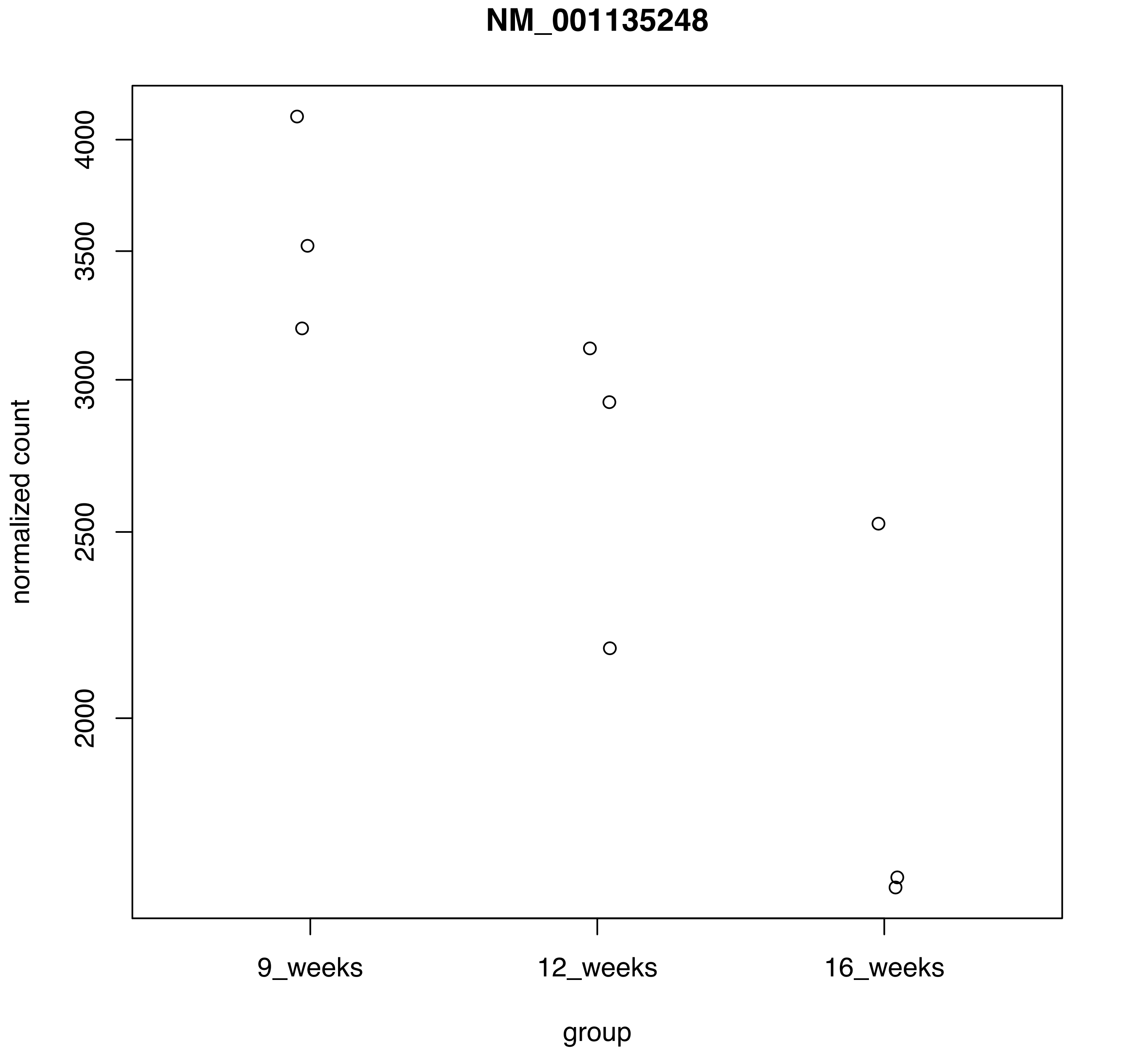
NM_001135248
normalized count
4000
3500
3000
2500
2000
9_weeks
12_weeks
16_weeks
group

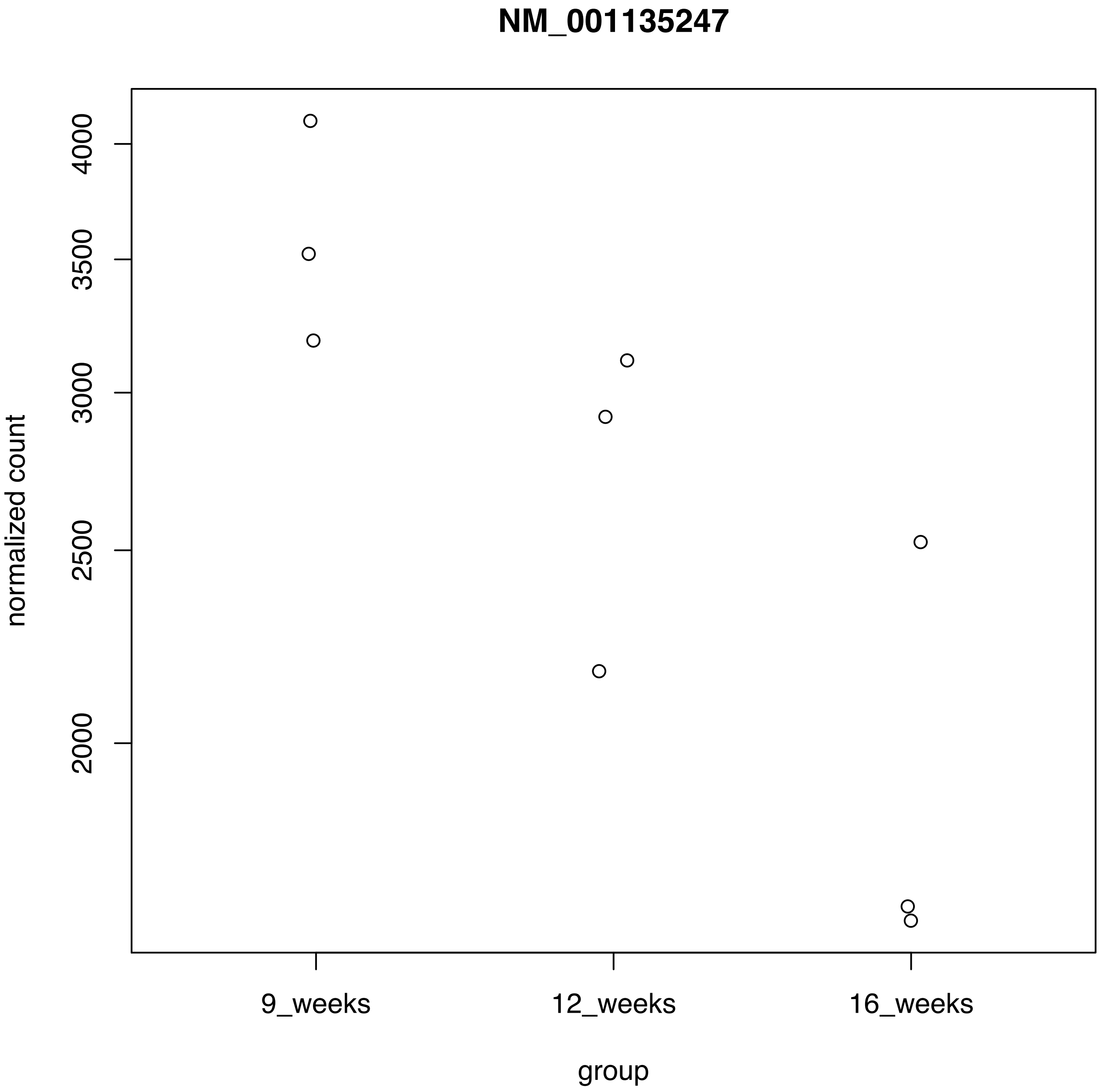
NM_001135247
normalized count
4000
3500
3000
2500
2000
9_weeks
12_weeks
16_weeks
group

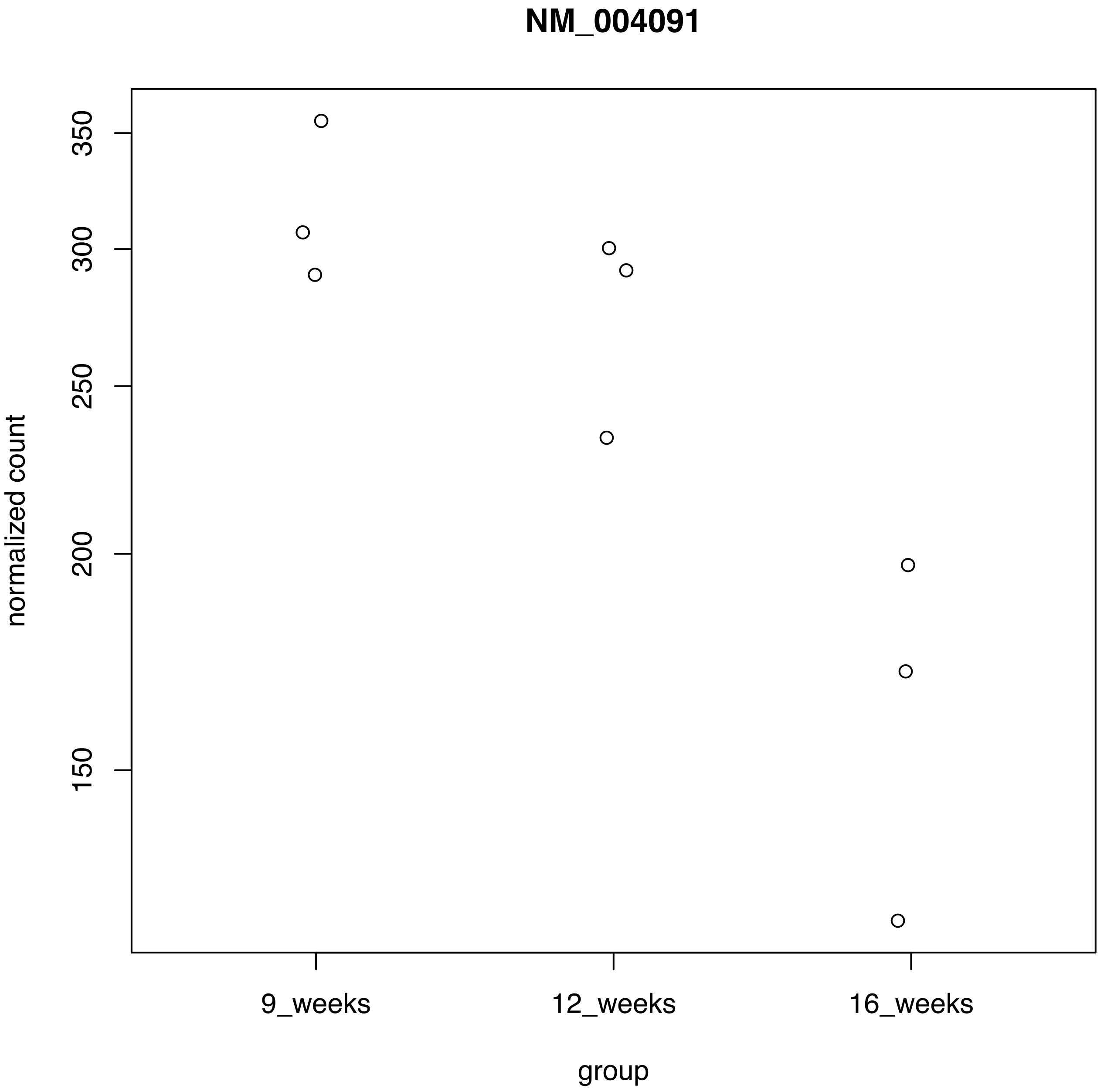
NM_004091
normalized count
350
300
250
200
150
9_weeks
12_weeks
16_weeks
group

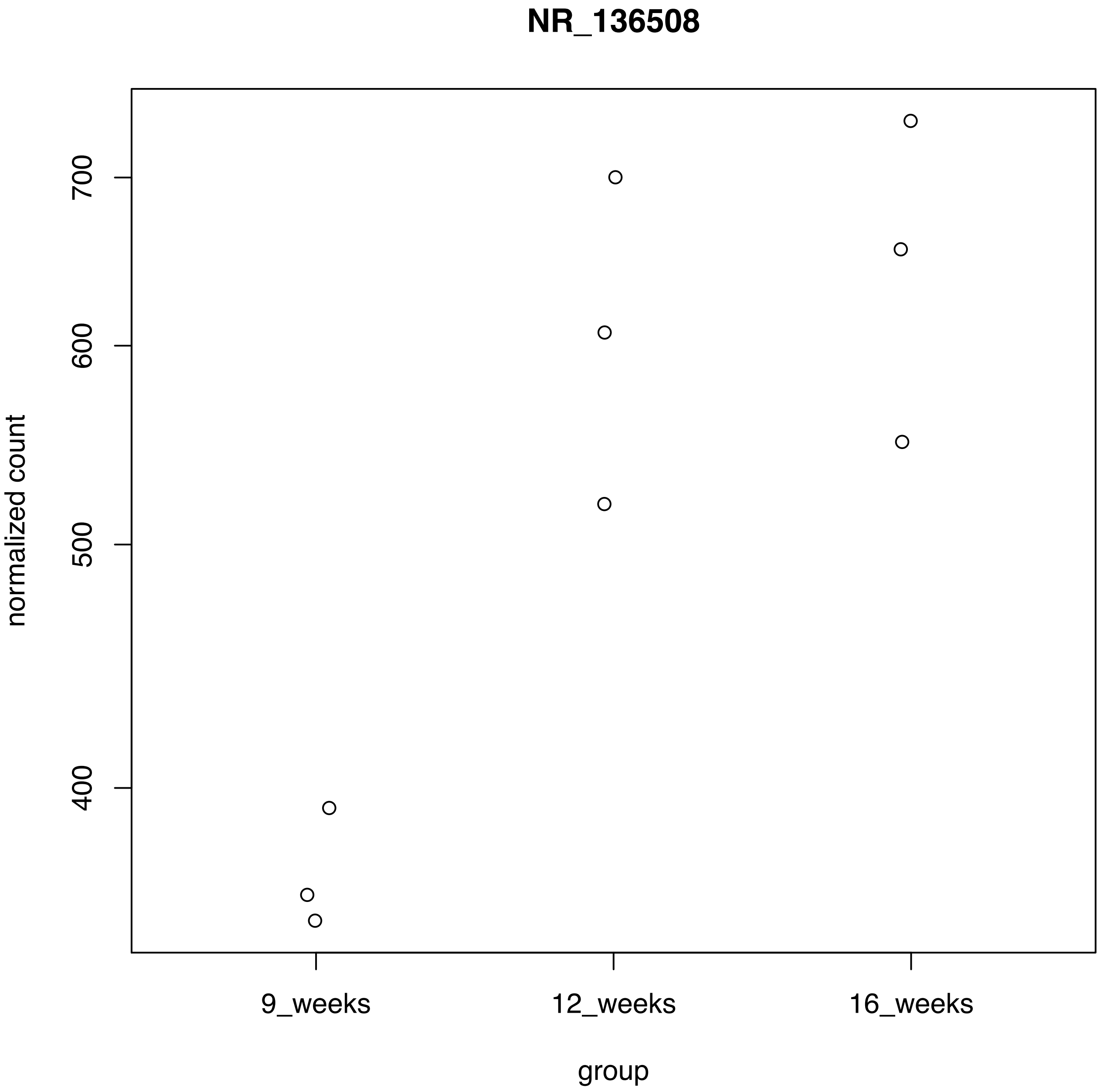
NR_136508
normalized count
400
500
600
700
9_weeks
12_weeks
16_weeks
group

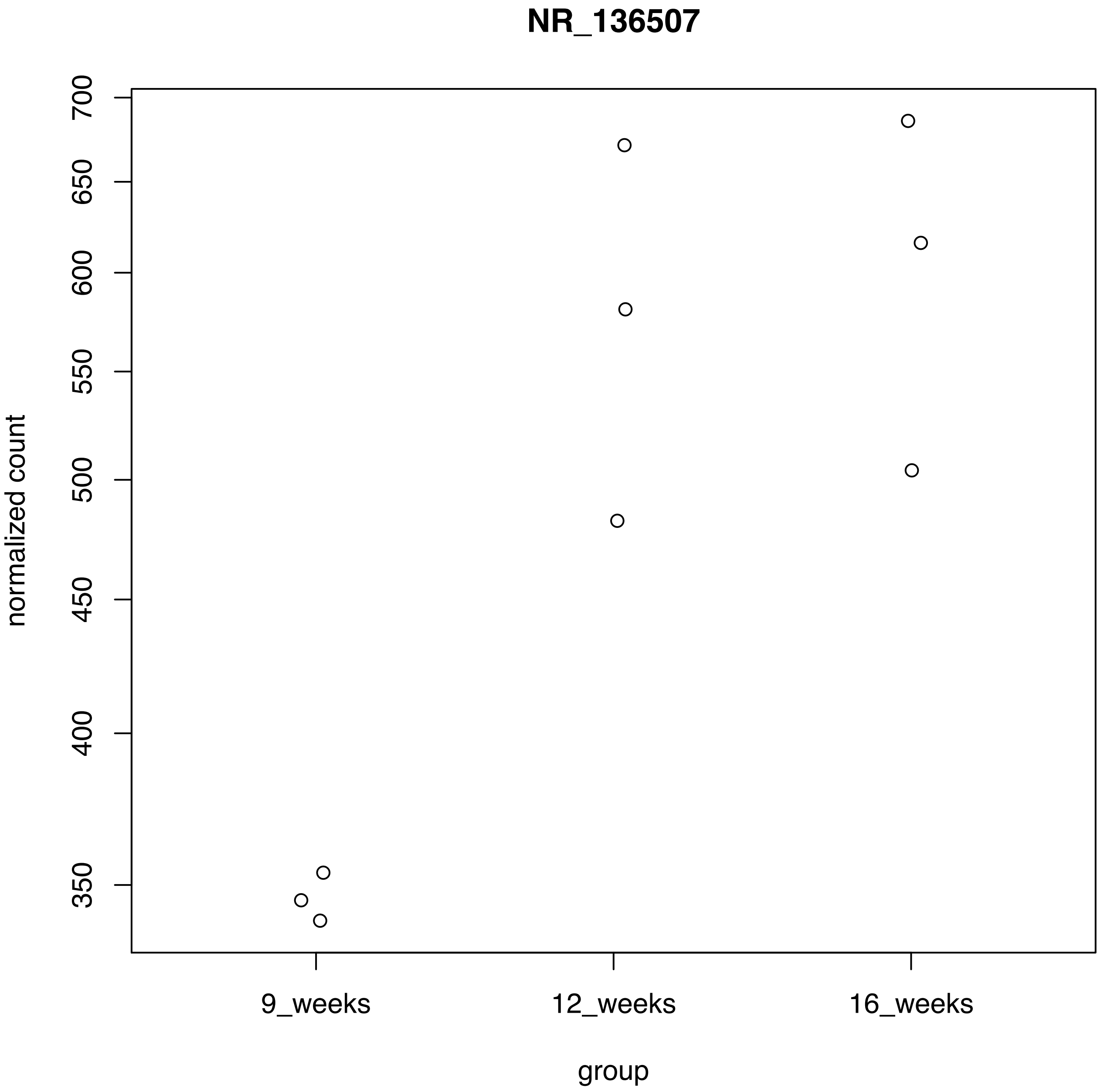
NR_136507
normalized count
700
650
600
550
500
450
400
350
9_weeks
12_weeks
16_weeks
group

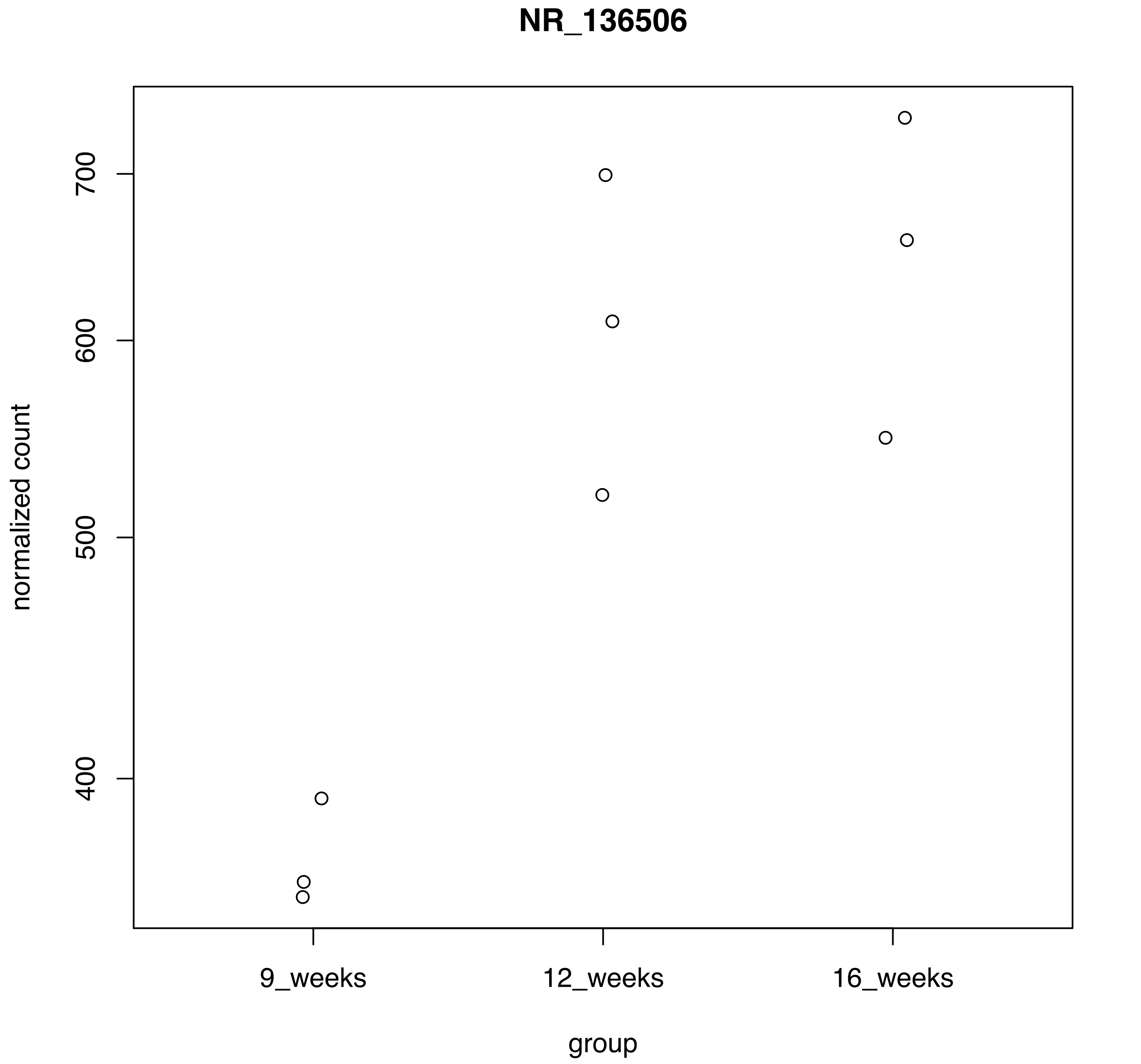

NR_136506
700
600
500
400
normalized count
9_weeks
12_weeks
16_weeks
group

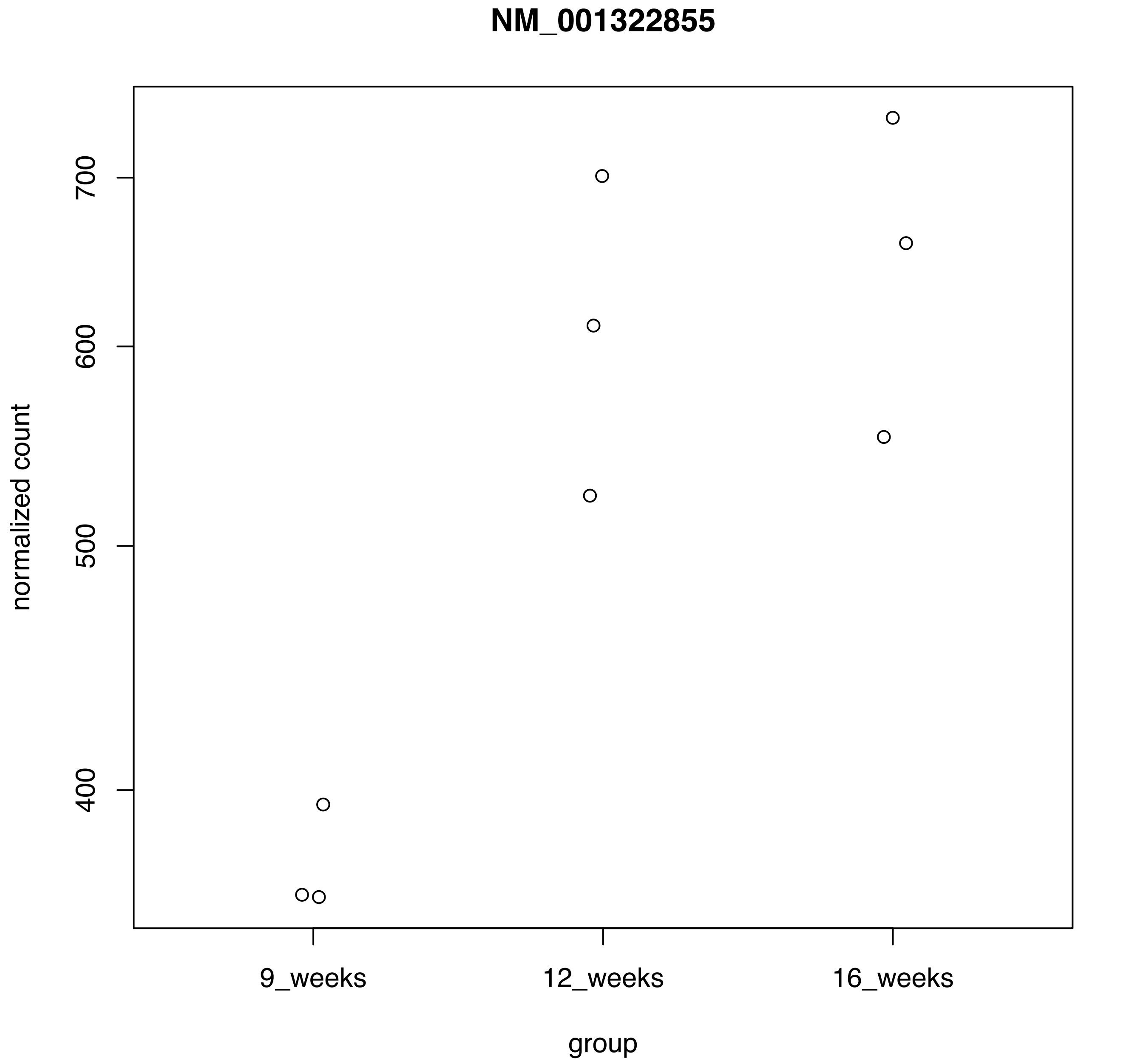
NM_001322855
normalized count
400
500
600
700
9_weeks
12_weeks
16_weeks
group

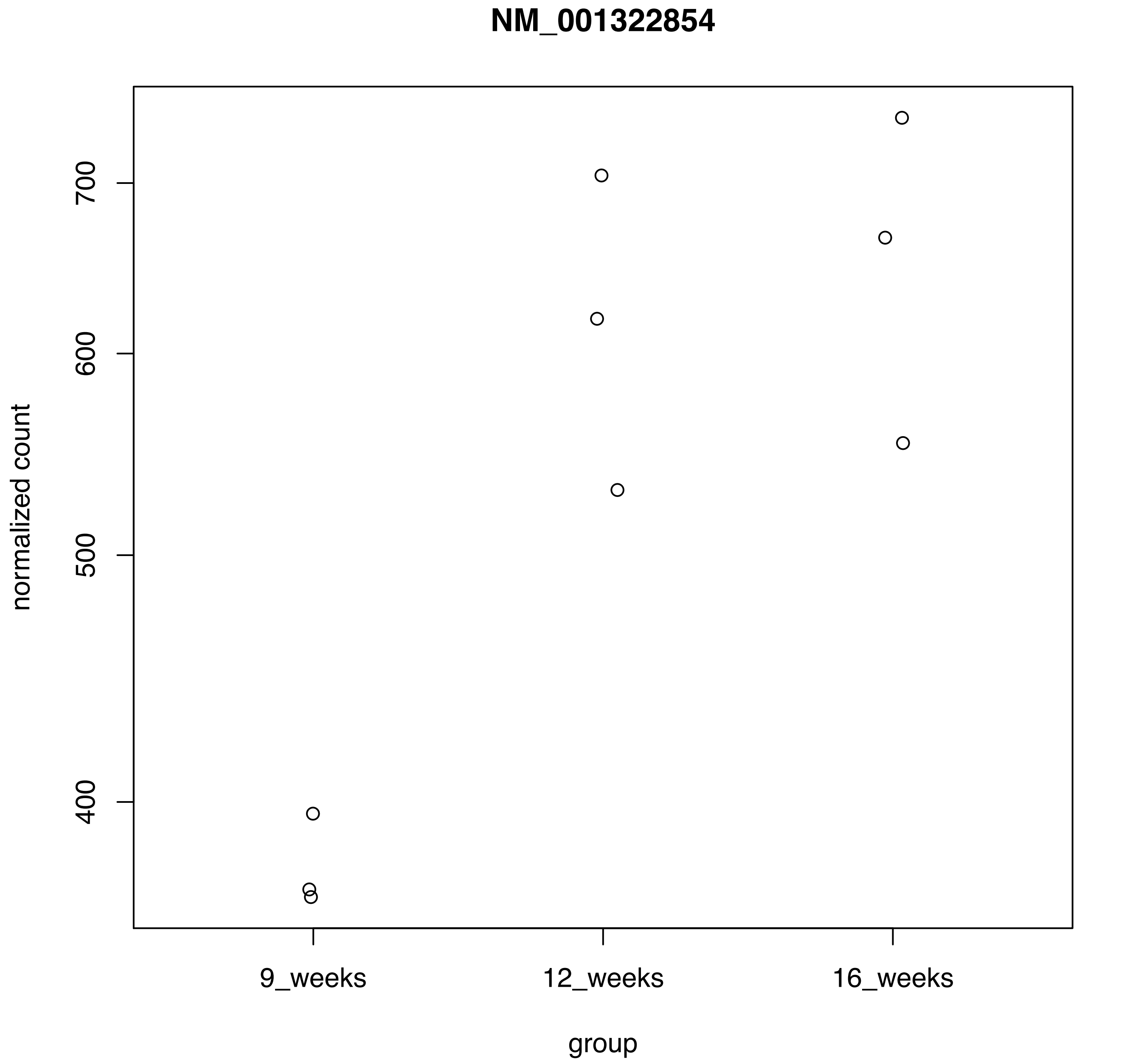

NM_001322854
normalized count
700
600
500
400
9_weeks
12_weeks
16_weeks
group

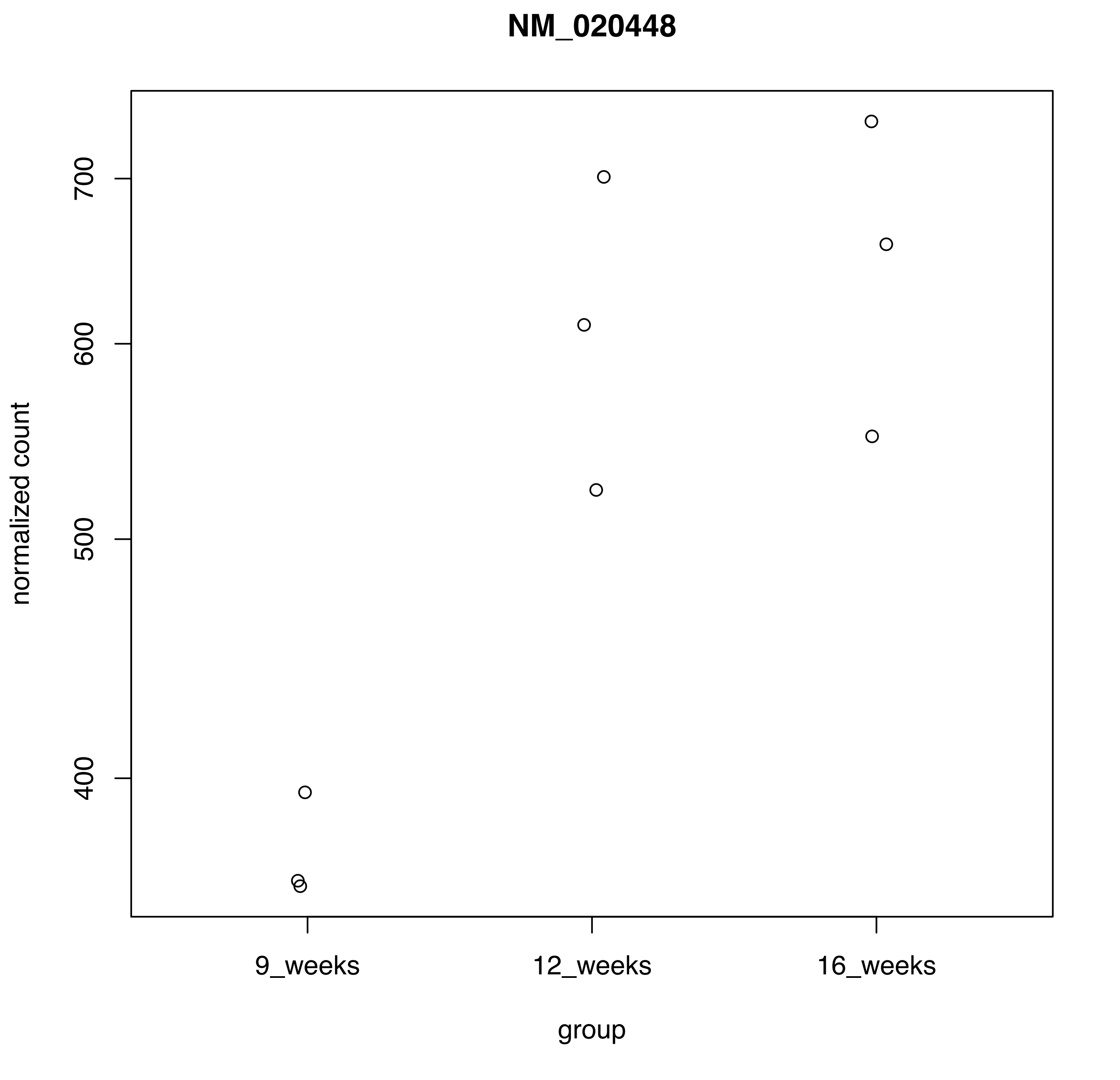
NM_020448
normalized count
700
600
500
400
9_weeks
12_weeks
16_weeks
group

NM_001322860

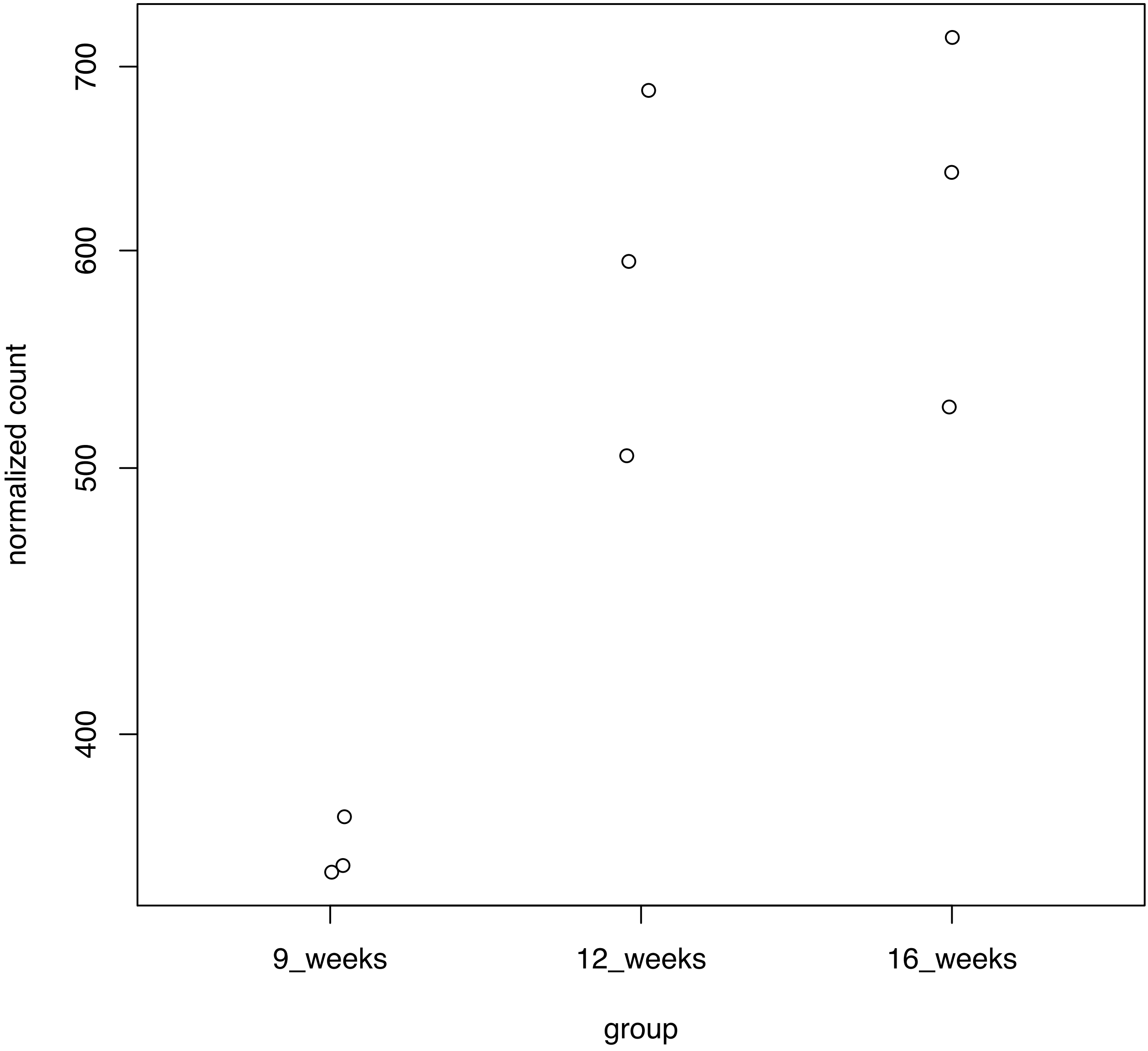

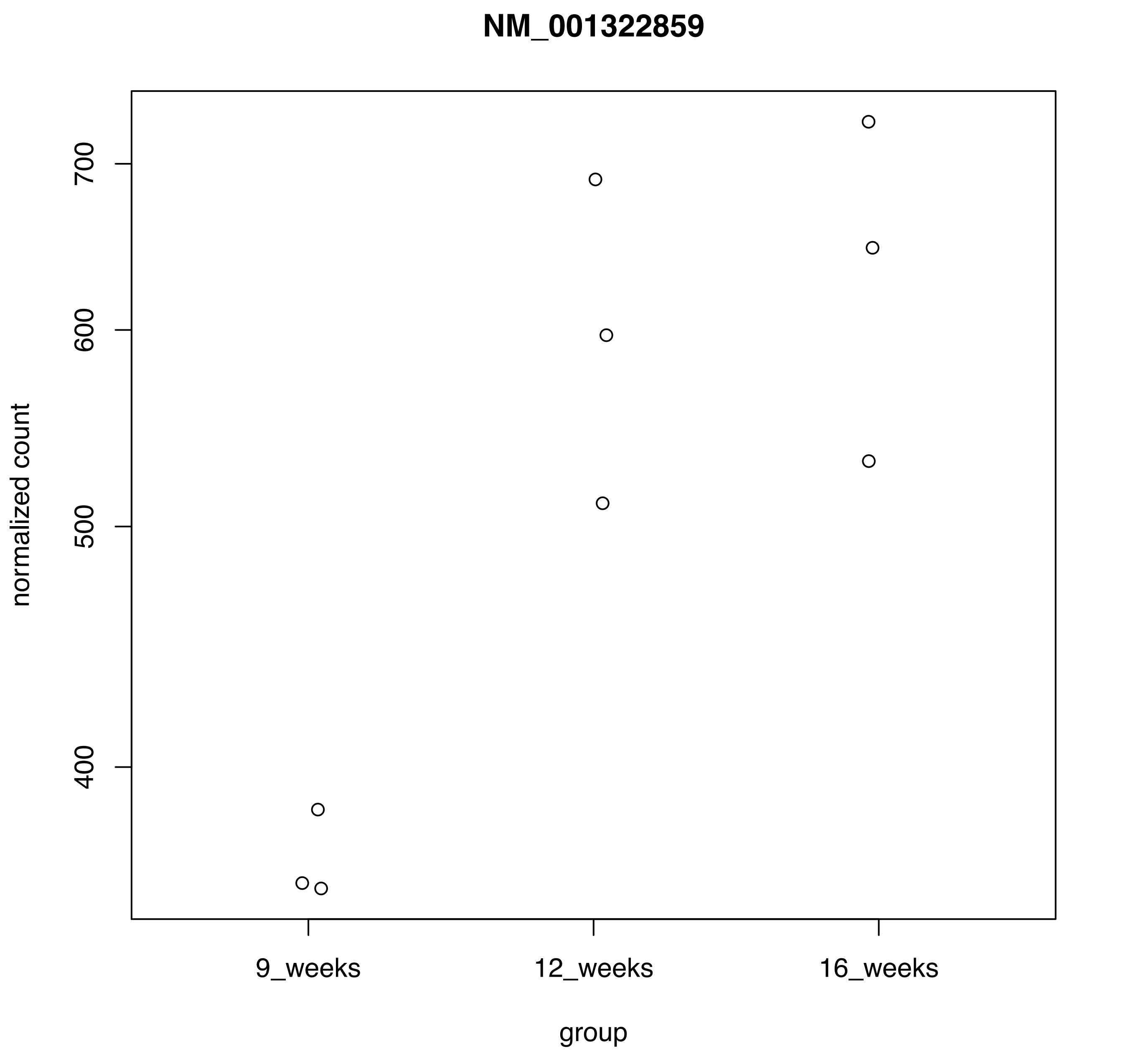
NM_001322859
normalized count
700
600
500
400
9_weeks
12_weeks
16_weeks
group

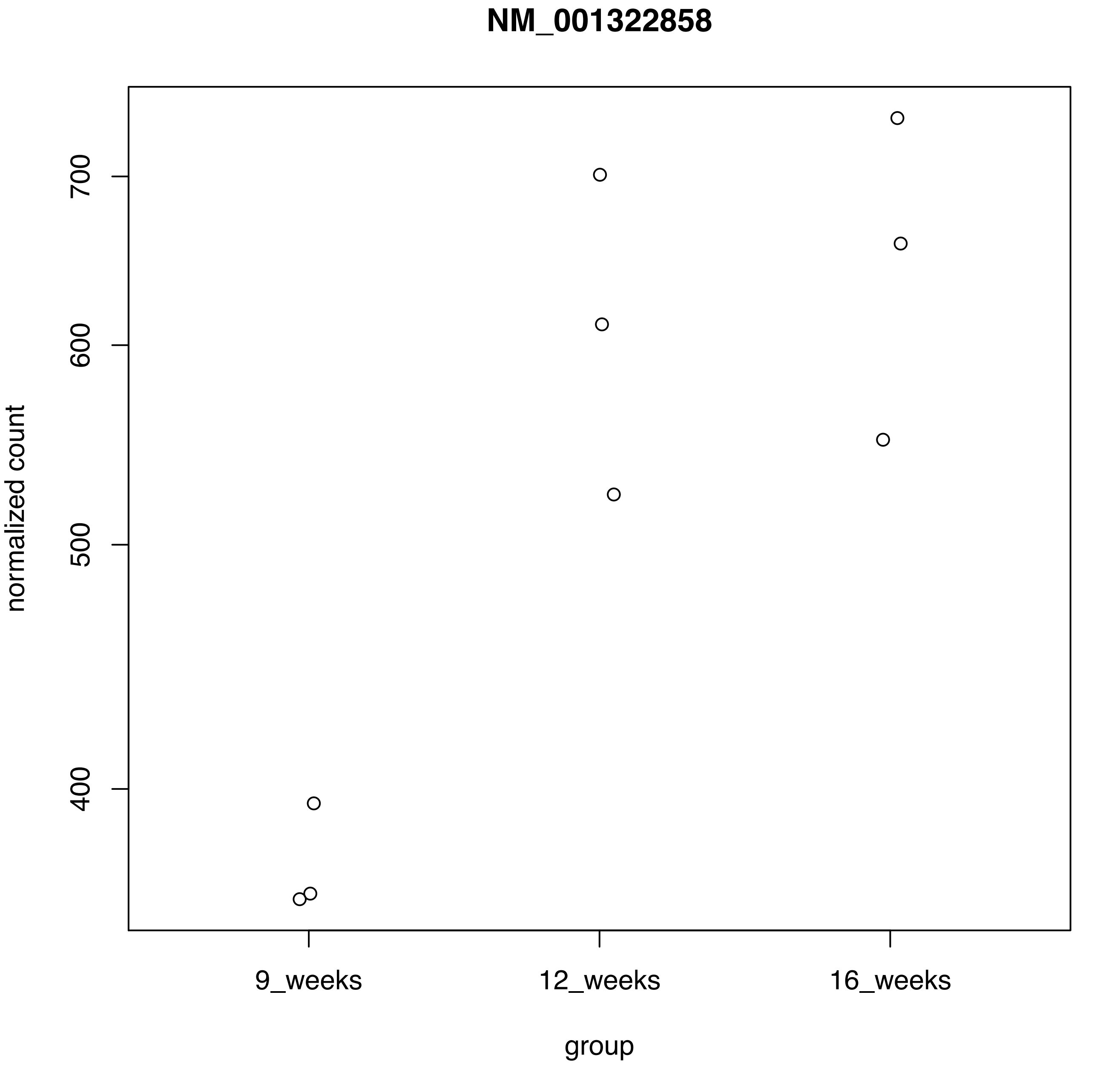
NM_001322858
normalized count
400
500
600
700
9_weeks
12_weeks
16_weeks
group

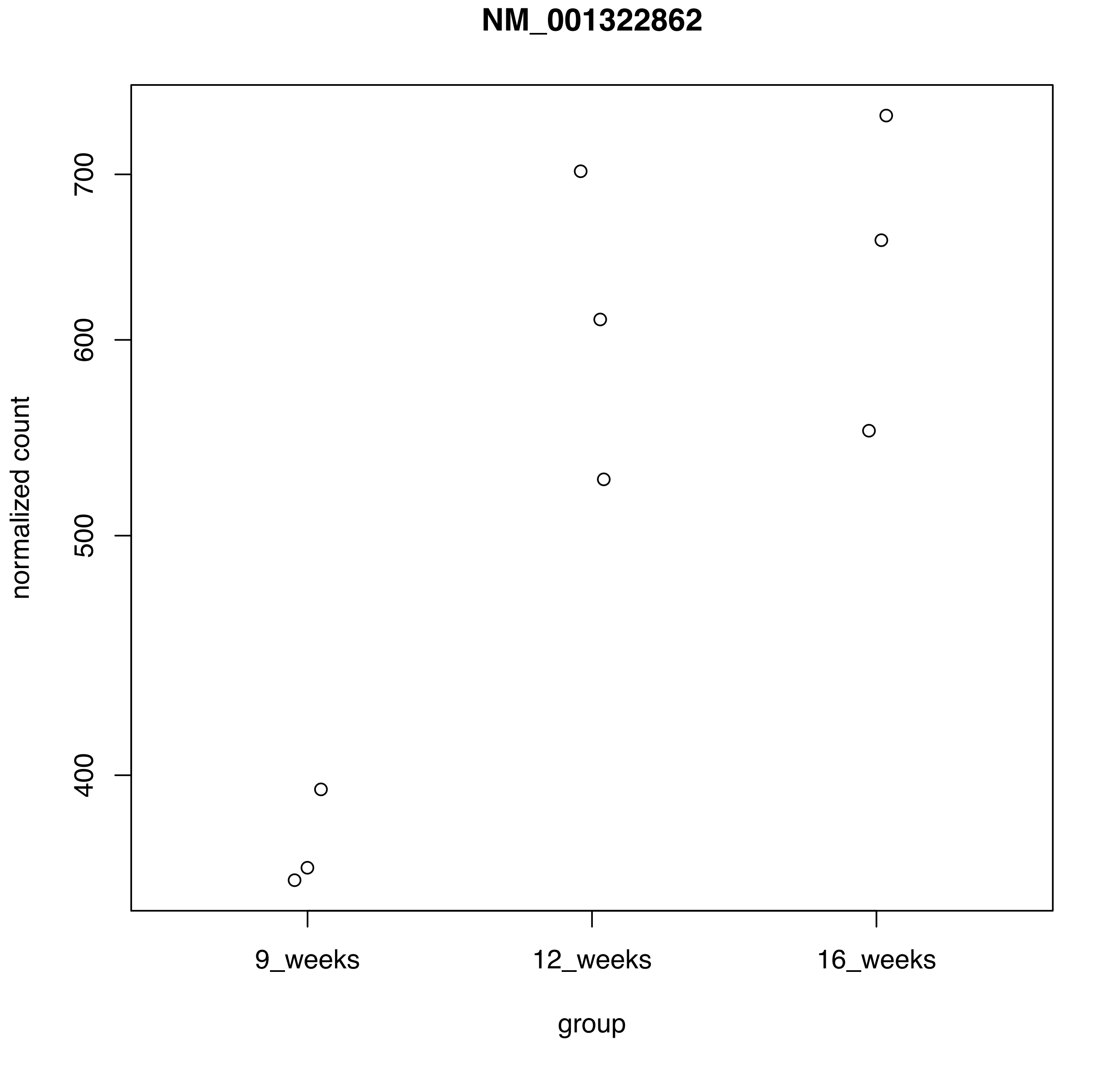
NM_001322862
normalized count
700
600
500
400
9_weeks
12_weeks
16_weeks
group

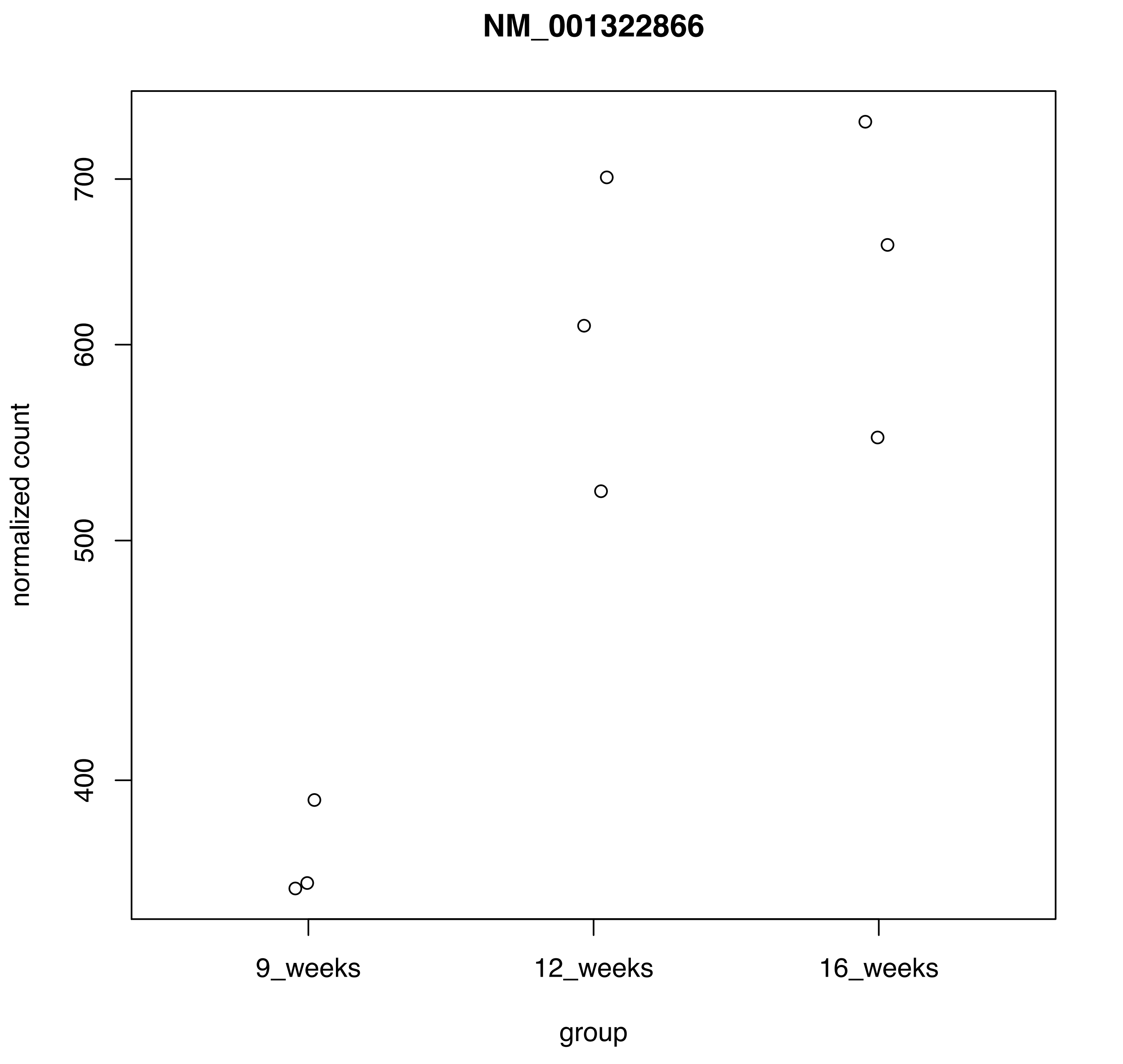
NM_001322866
normalized count
700
600
500
400
9_weeks
12_weeks
16_weeks
group

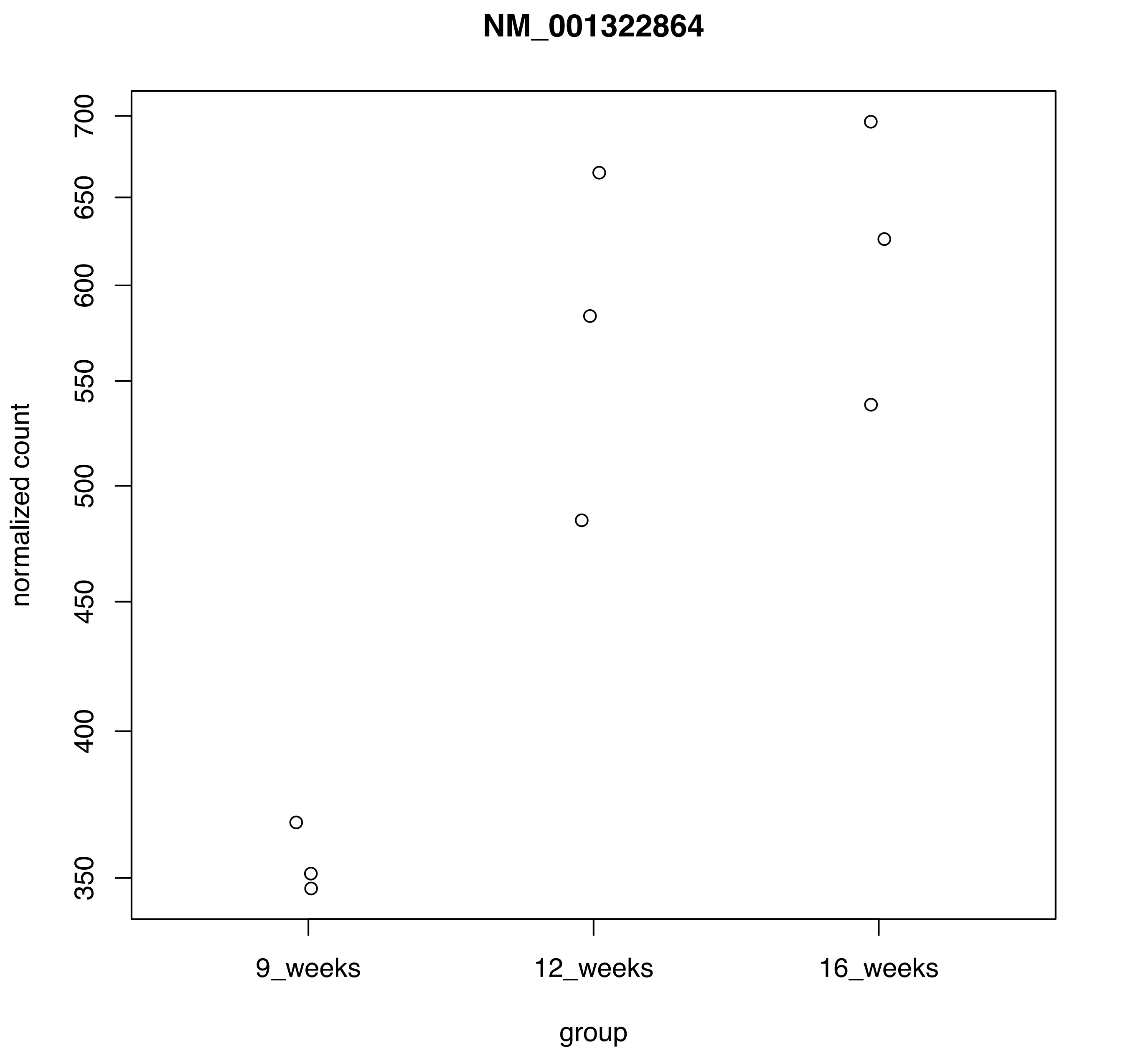
NM_001322864
normalized count
700
650
600
550
500
450
400
350
9_weeks
12_weeks
16_weeks
group

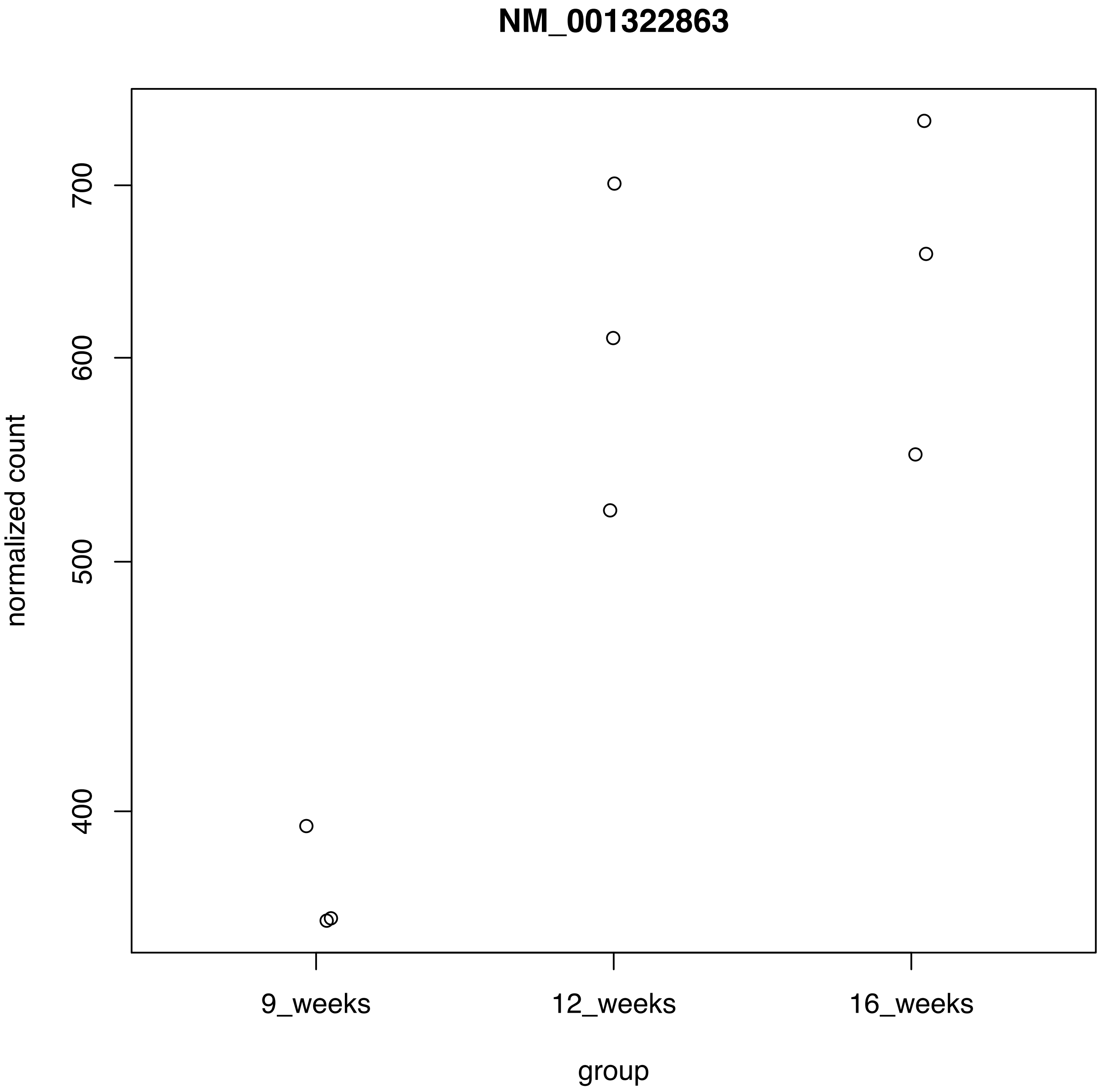

NM_001322863
normalized count
700
600
500
400
9_weeks
12_weeks
16_weeks
group

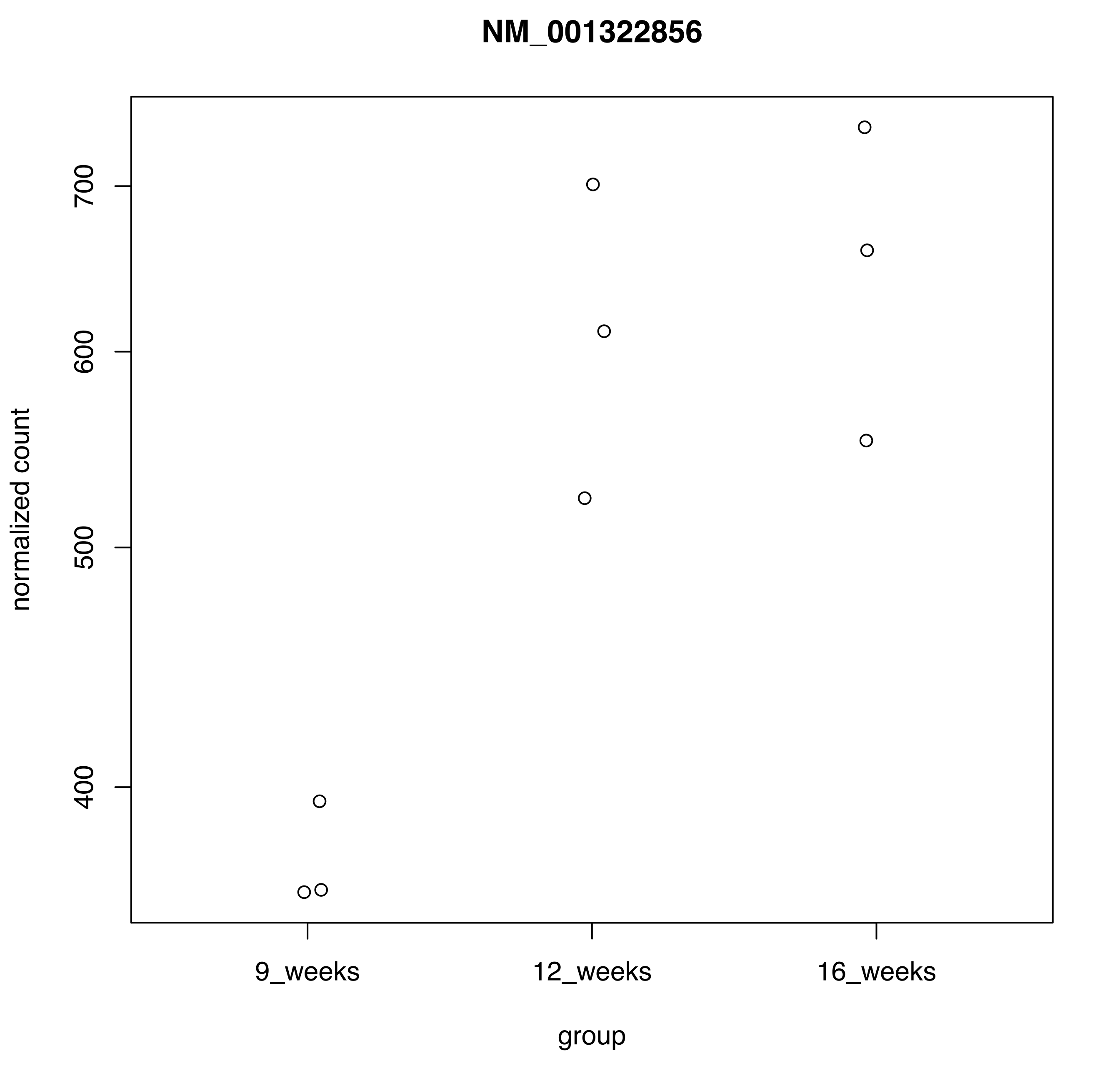
NM_001322856
normalized count
700
600
500
400
9_weeks
12_weeks
16_weeks
group

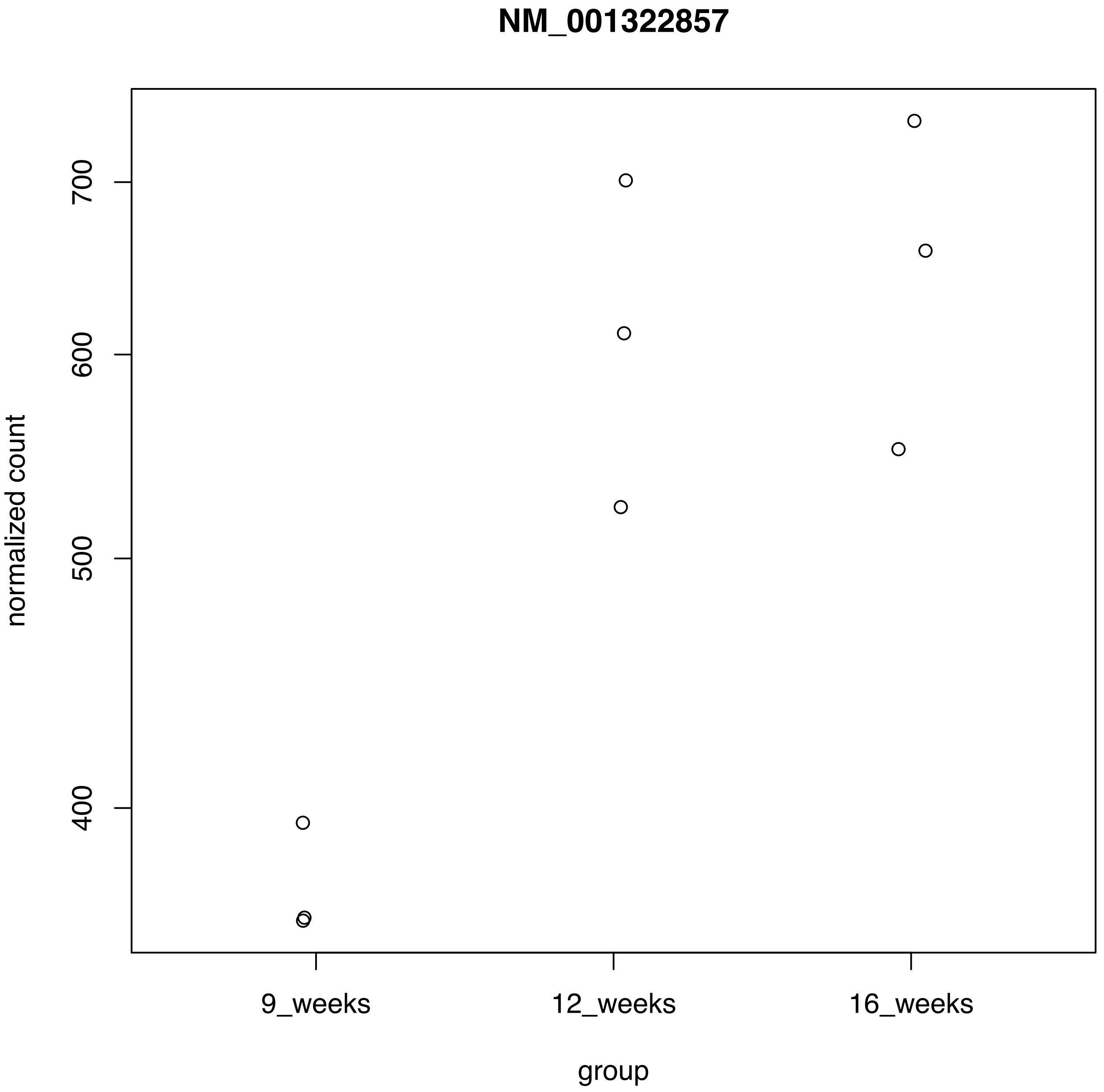
NM_001322857
normalized count
400
500
600
700
9_weeks
12_weeks
16_weeks
group

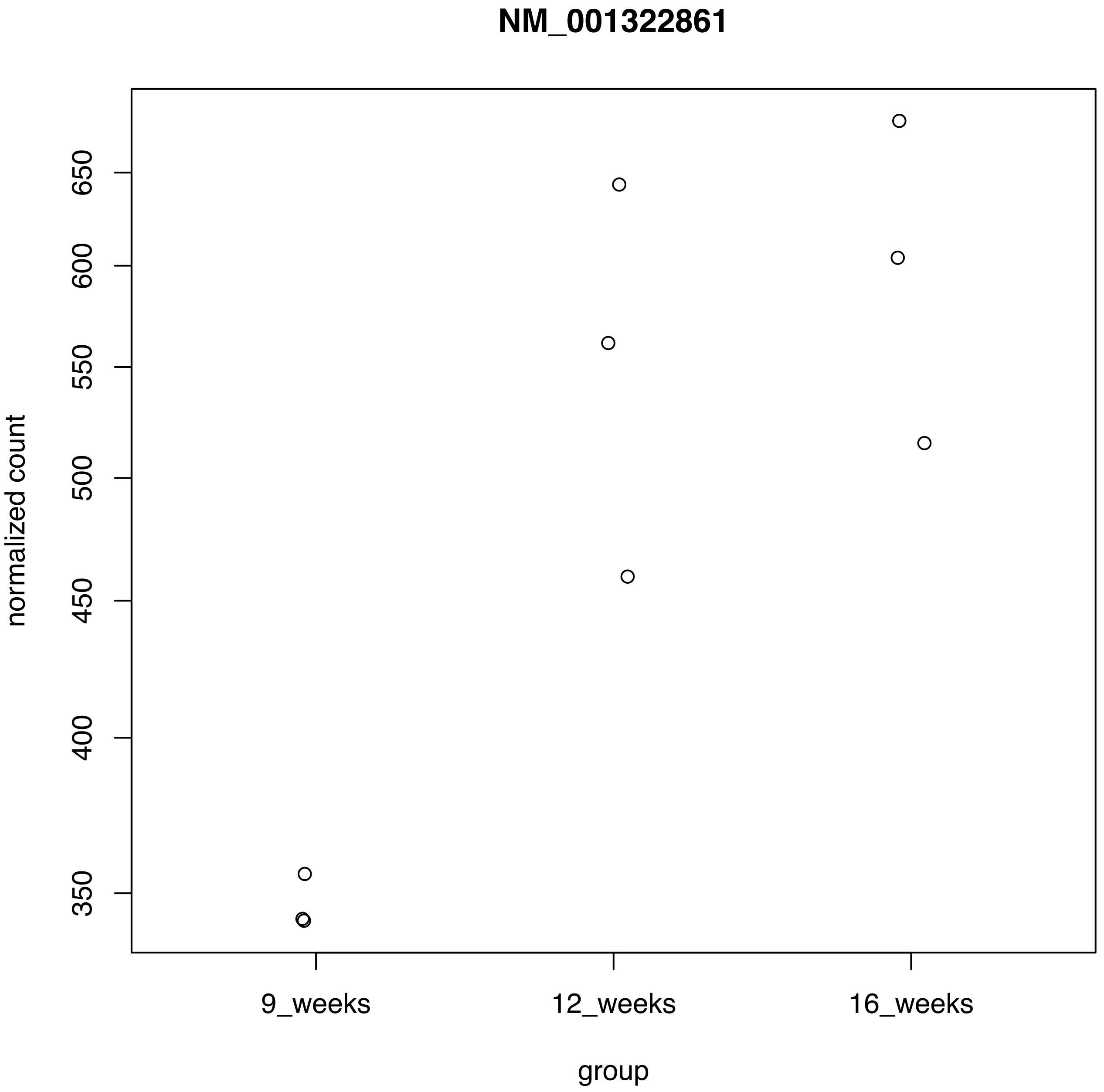
NM_001322861
normalized count
650
600
550
500
450
400
350
9_weeks
12_weeks
16_weeks
group

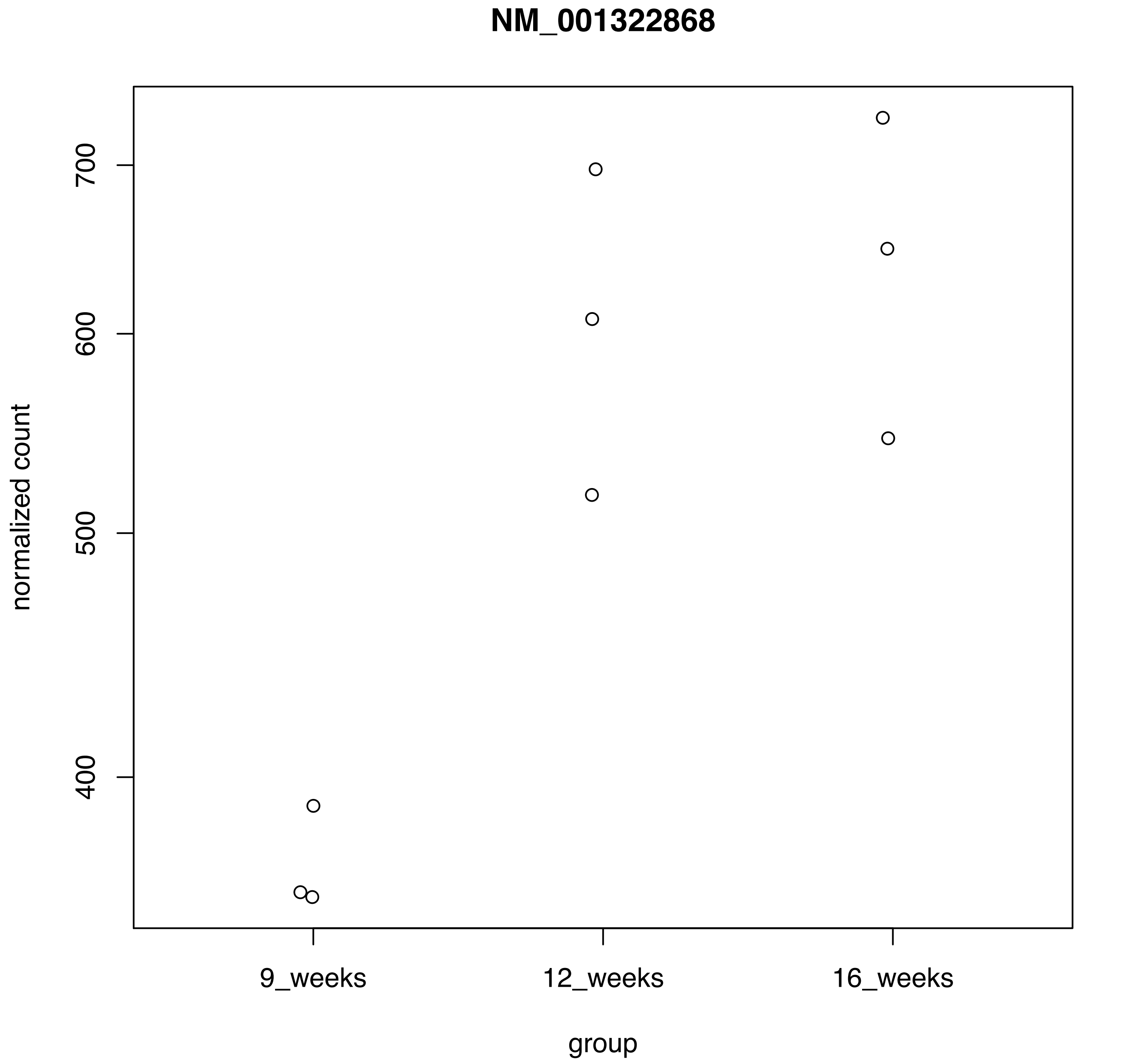
NM_001322868
normalized count
700
600
500
400
9_weeks
12_weeks
16_weeks
group

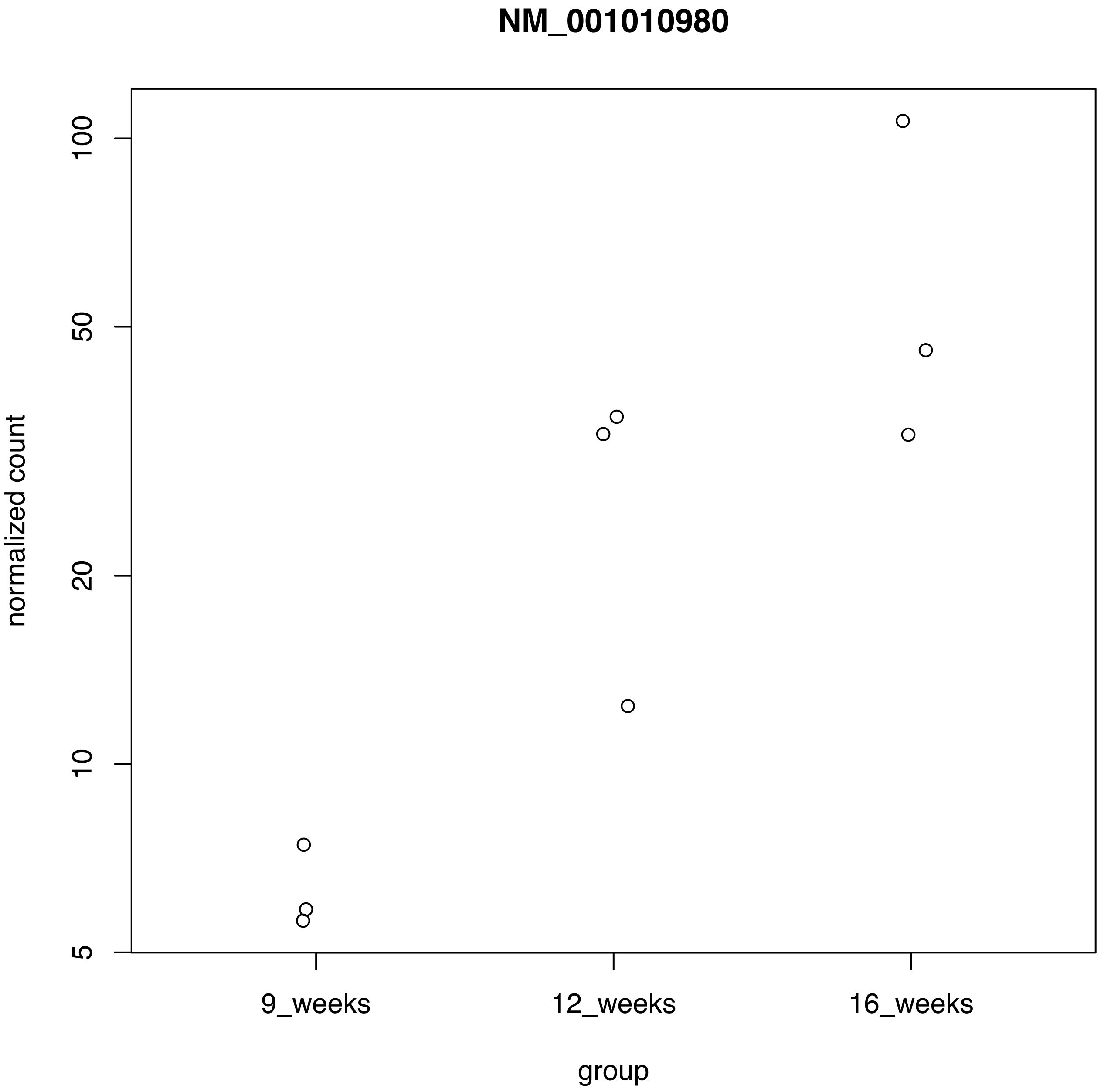
NM_001010980
normalized count
100
50
20
10
5
9_weeks
12_weeks
16_weeks
group

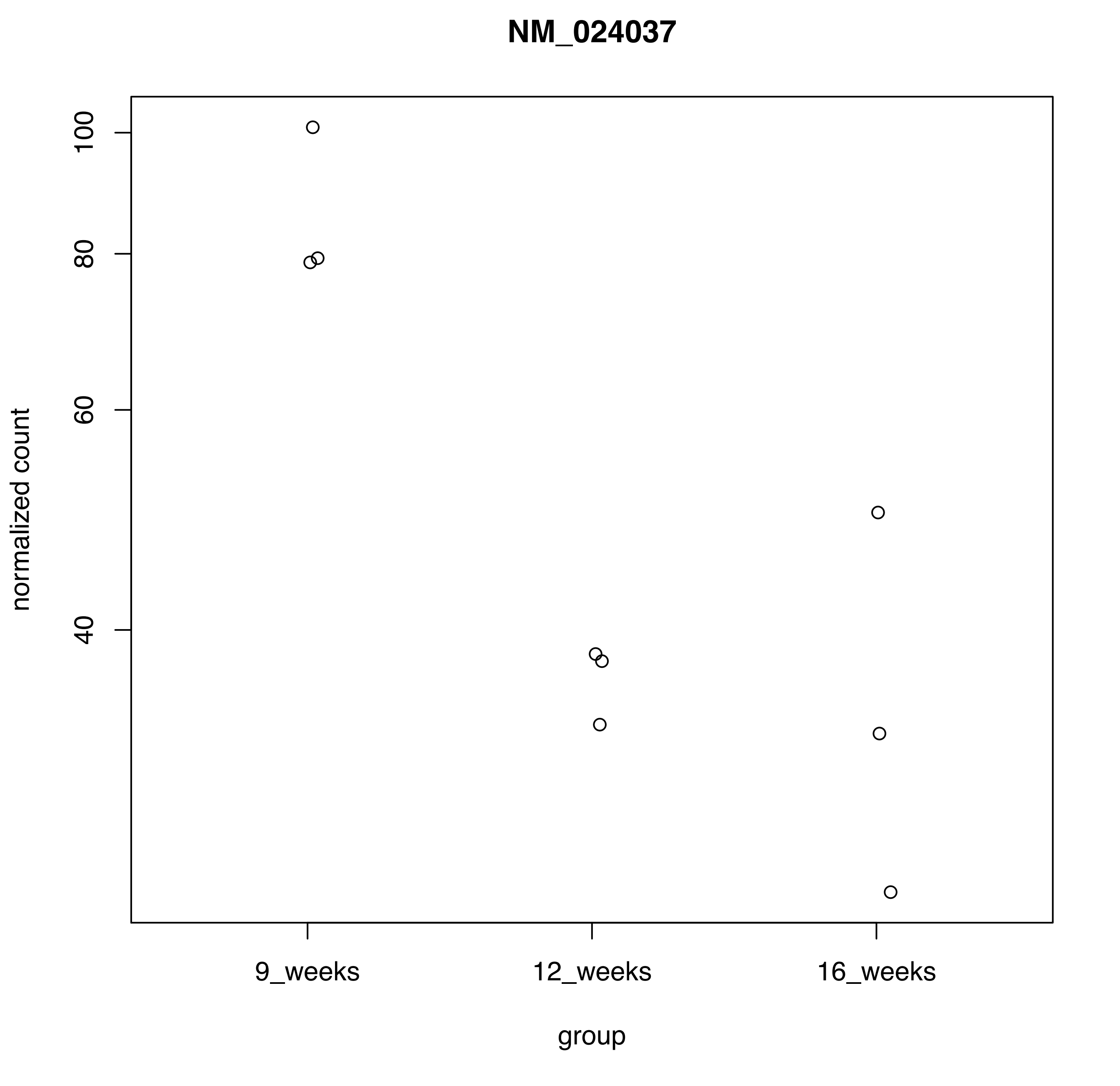

NM_024037
normalized count
100
80
60
40
9_weeks
12_weeks
16_weeks
group

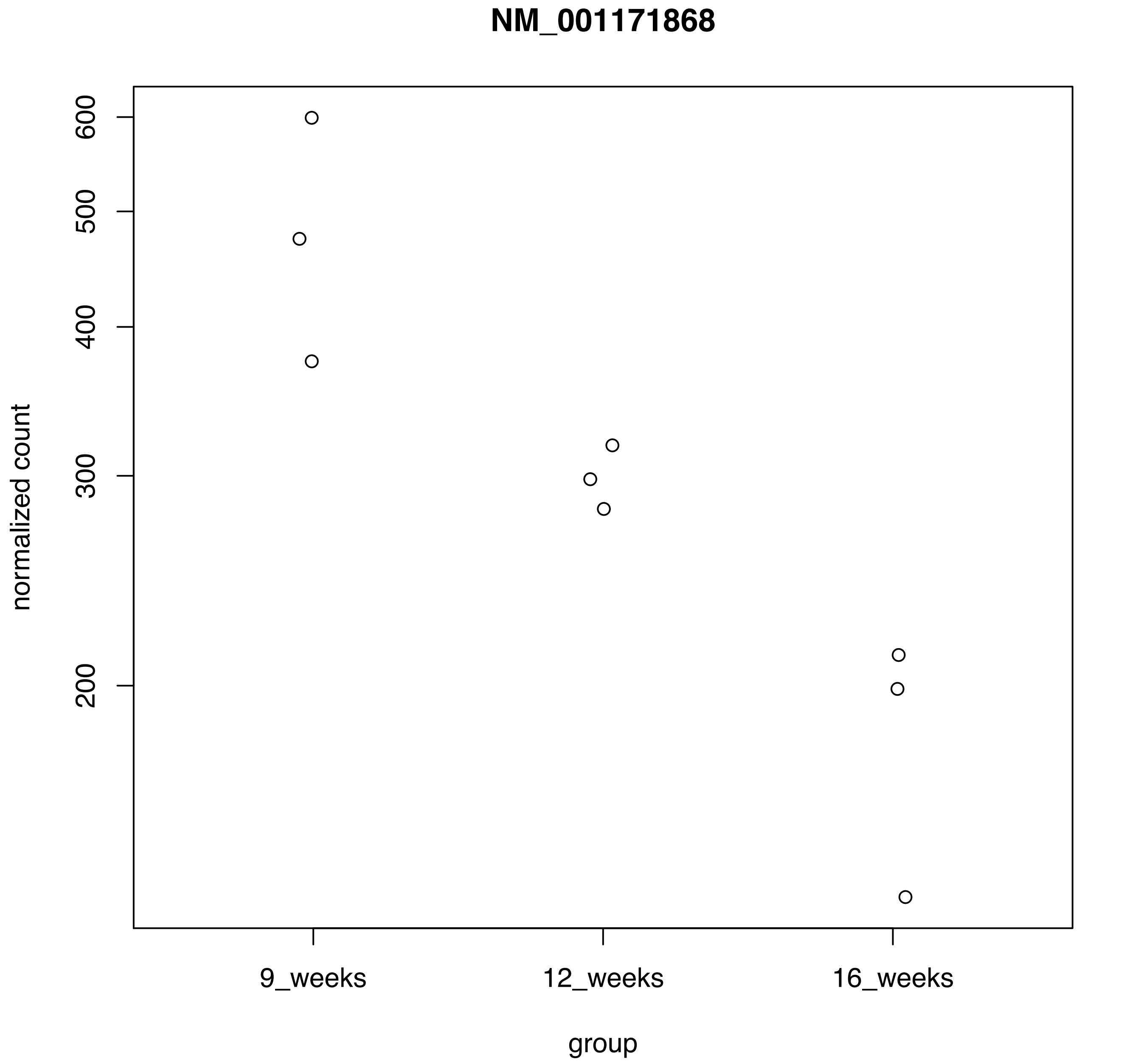

NM_001171868
normalized count
600
500
400
300
200
9_weeks
12_weeks
16_weeks
group

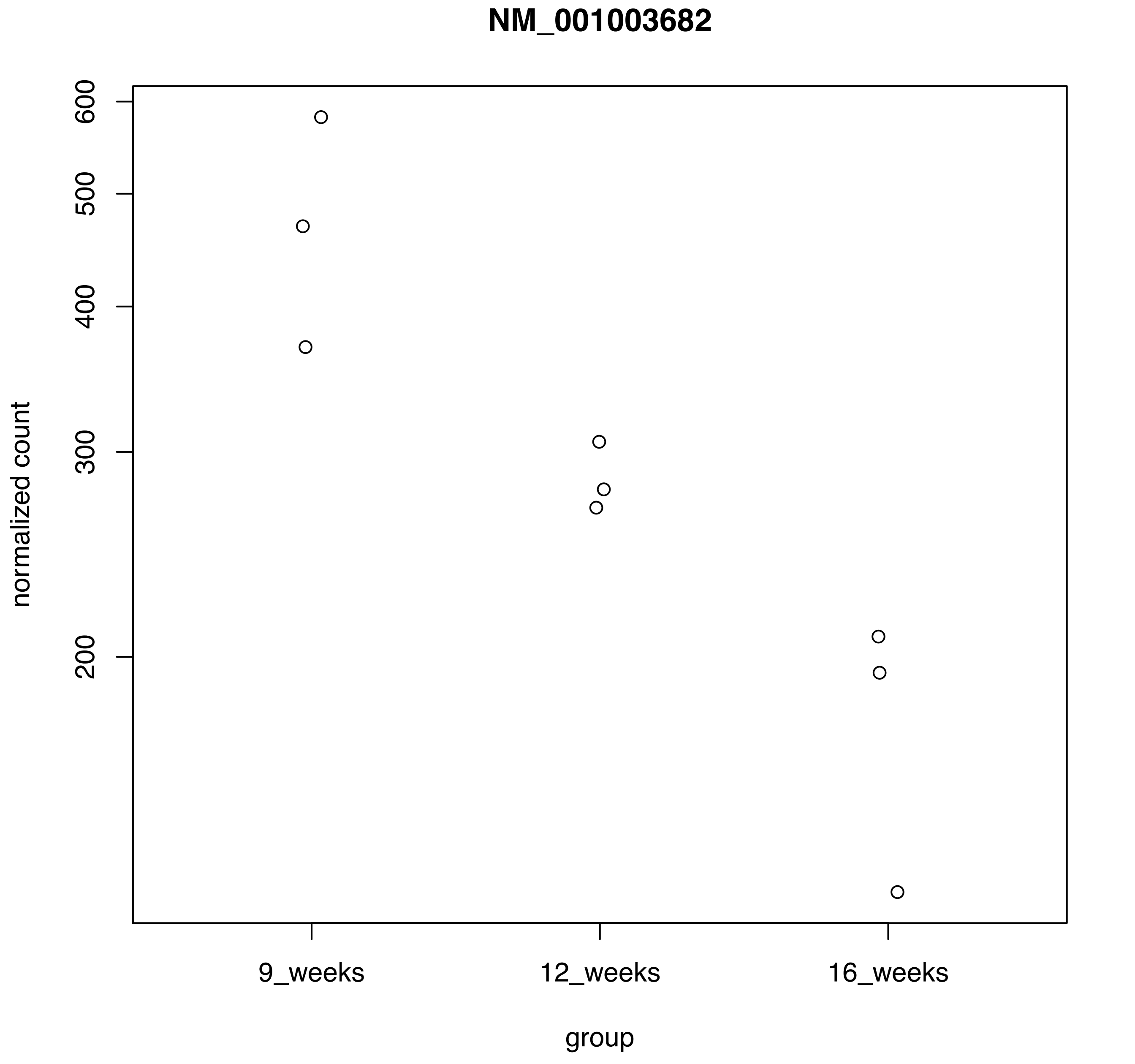

NM_001003682
600
500
400
300
200
normalized count
9_weeks
12_weeks
16_weeks
group

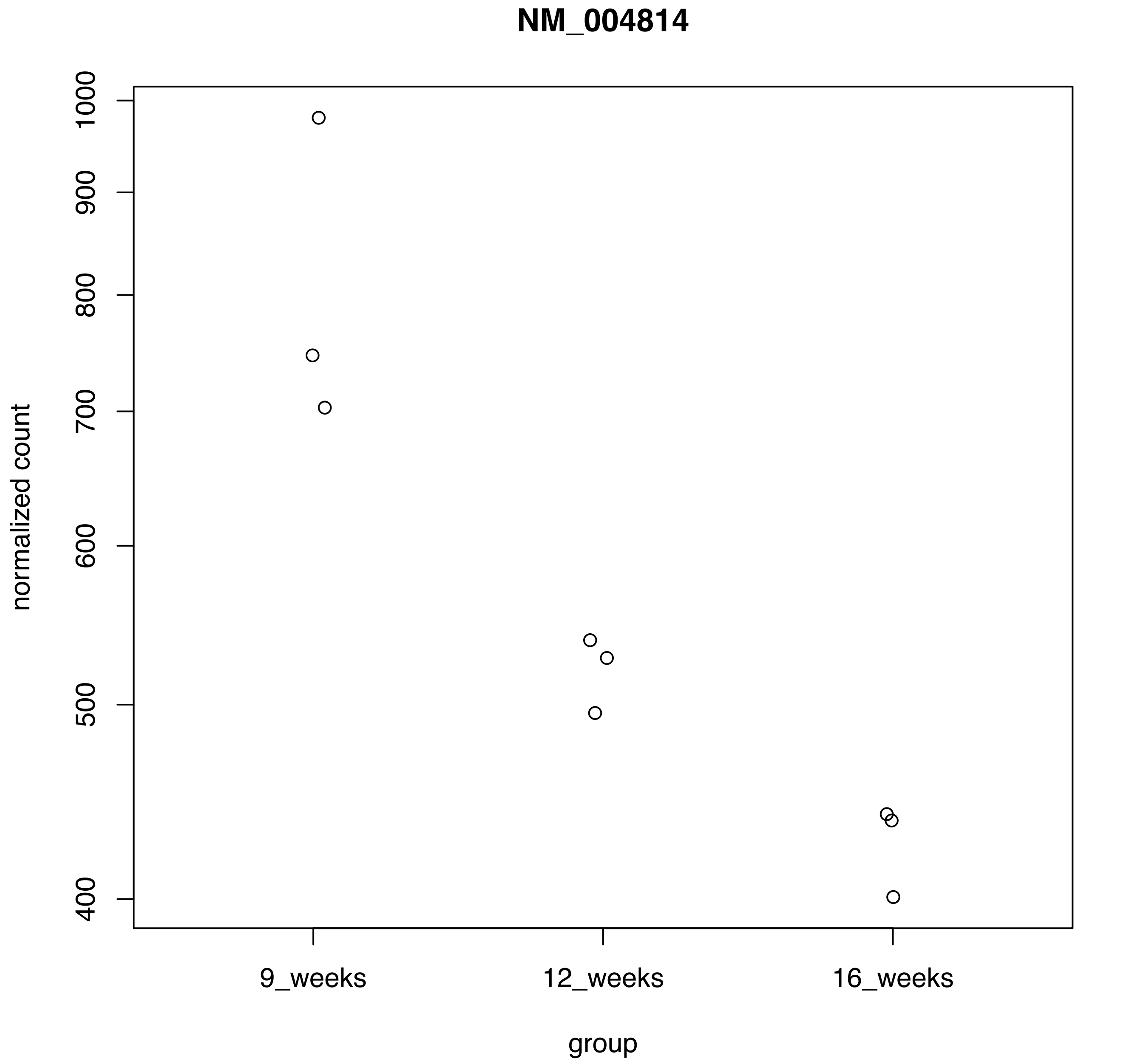
NM_004814
normalized count
1000
900
800
700
600
500
400
9_weeks
12_weeks
16_weeks
group

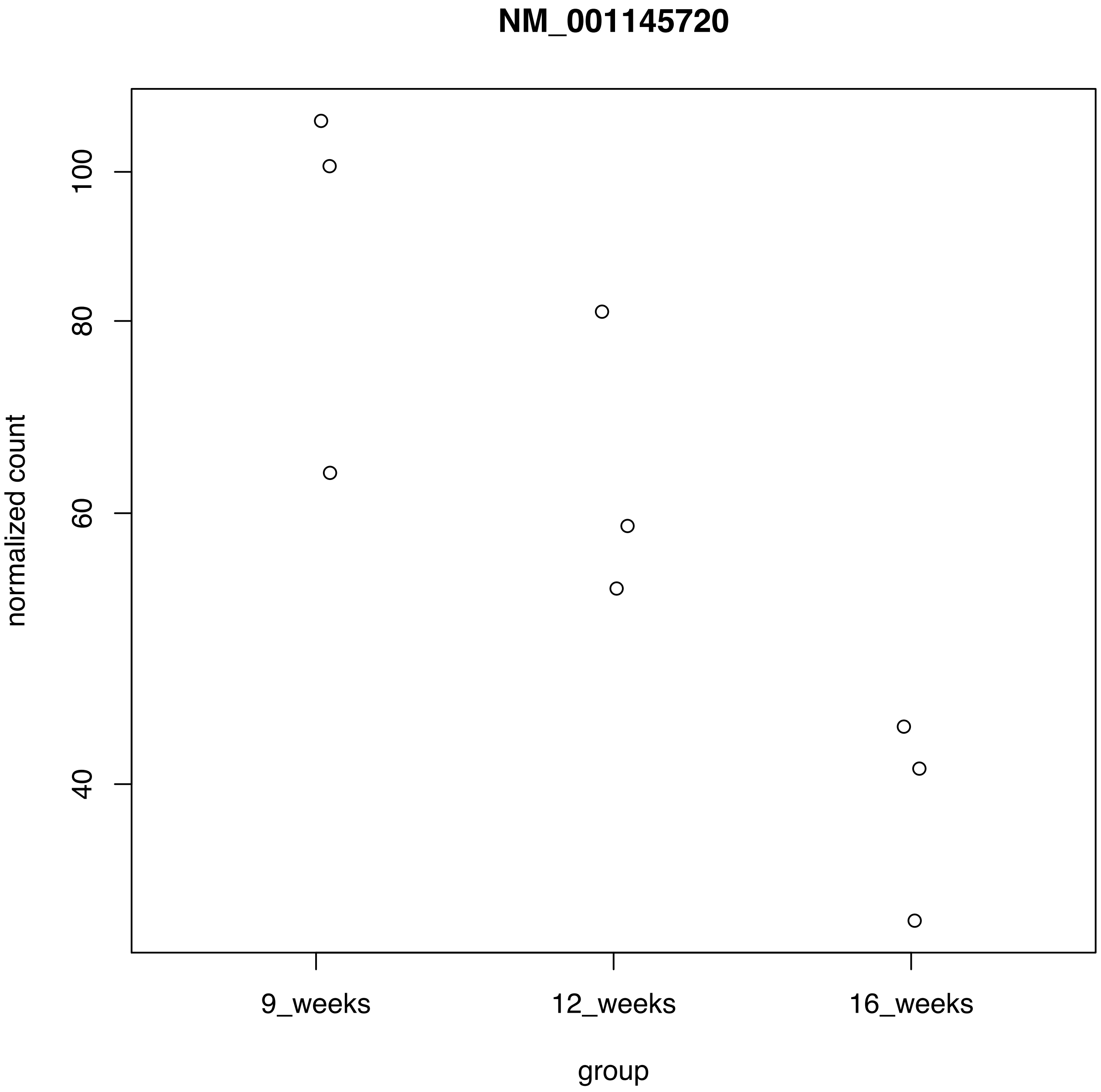
NM_001145720
normalized count
100
80
60
40
9_weeks
12_weeks
16_weeks
group

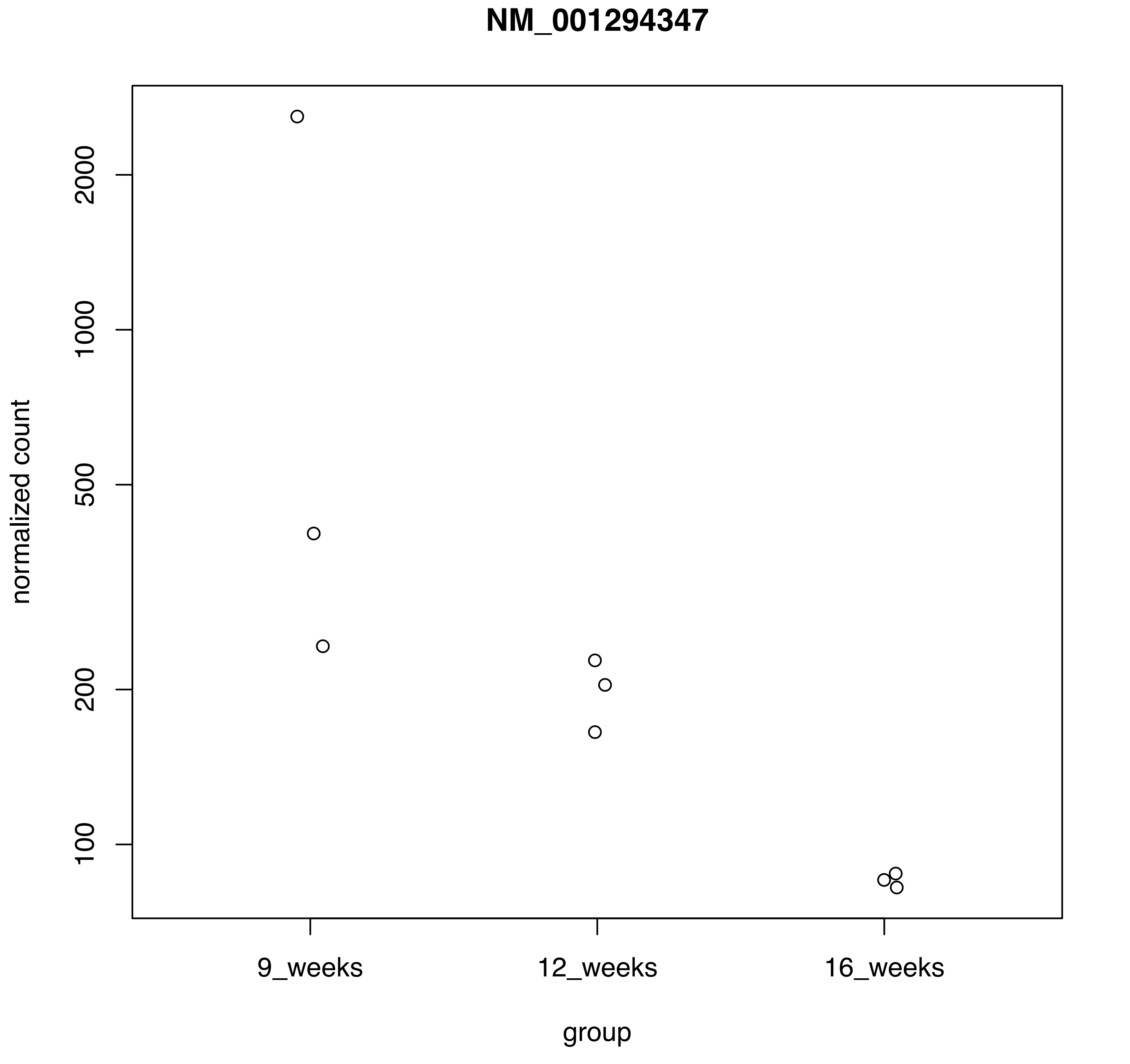
NM_001294347
normalized count
2000
1000
500
200
100
9_weeks
12_weeks
16_weeks
group

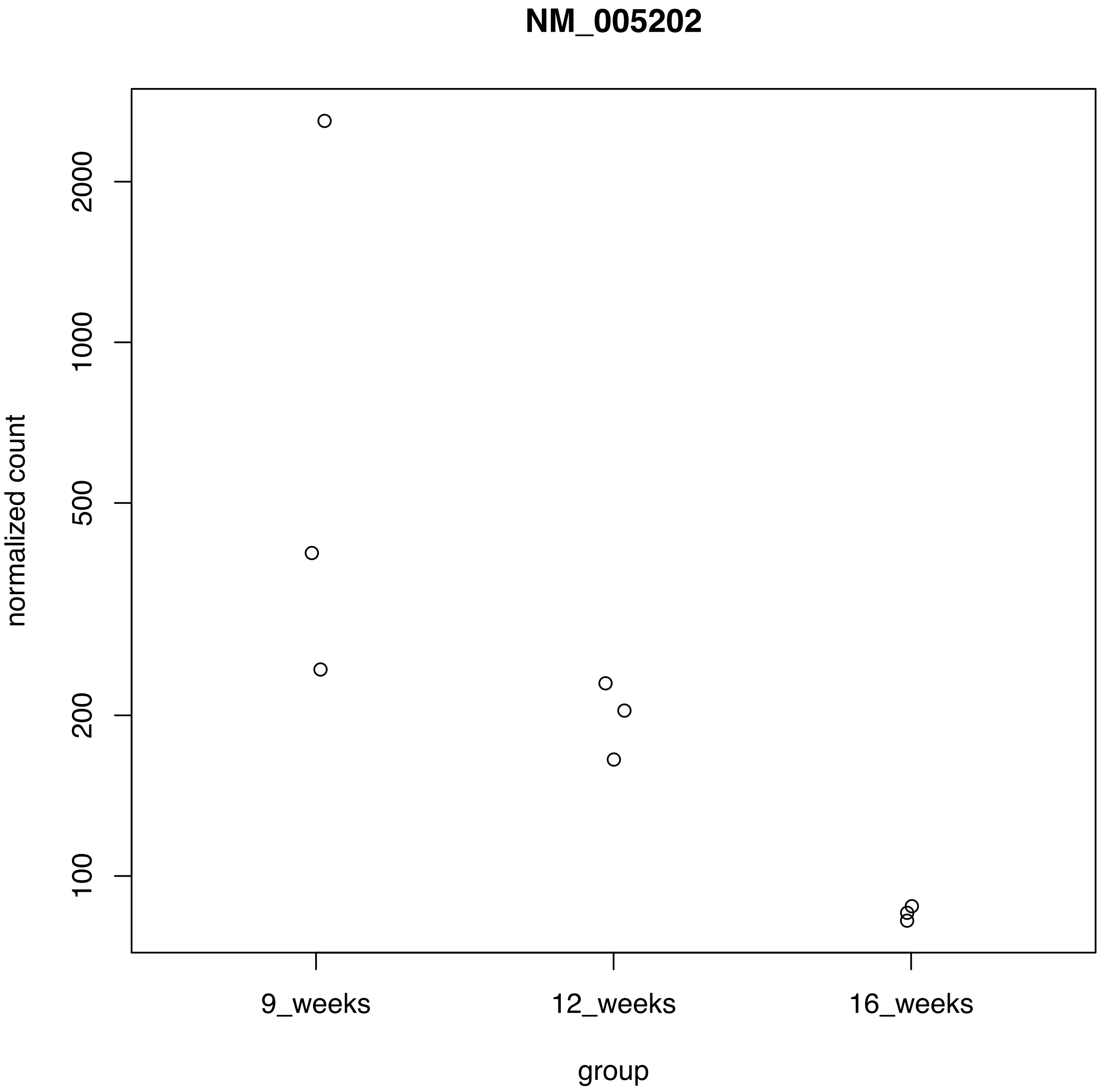
NM_005202
normalized count
2000
1000
500
200
100
9_weeks
12_weeks
16_weeks
group

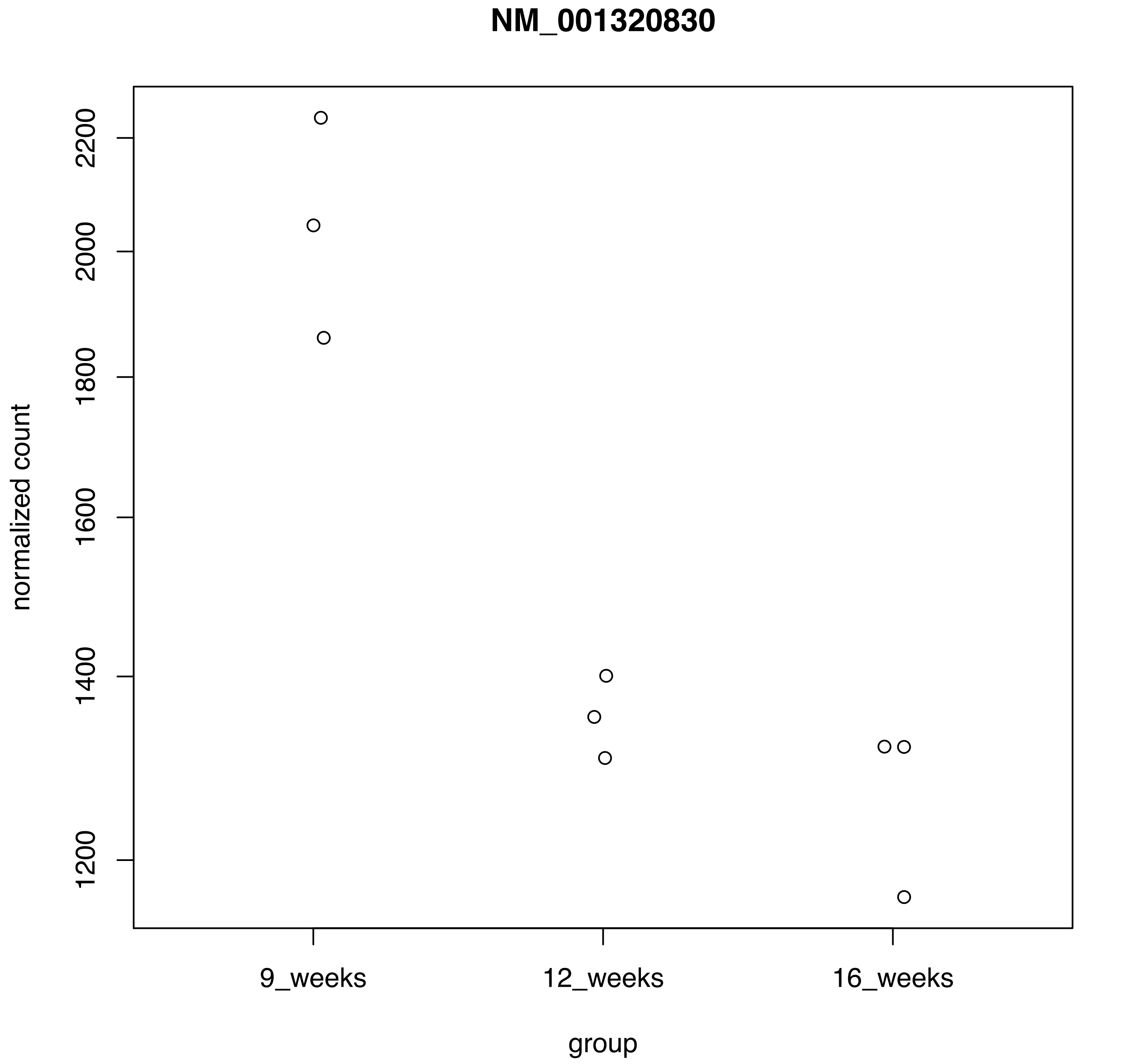
NM_001320830
normalized count
2200
2000
1800
1600
1400
1200
9_weeks
12_weeks
16_weeks
group

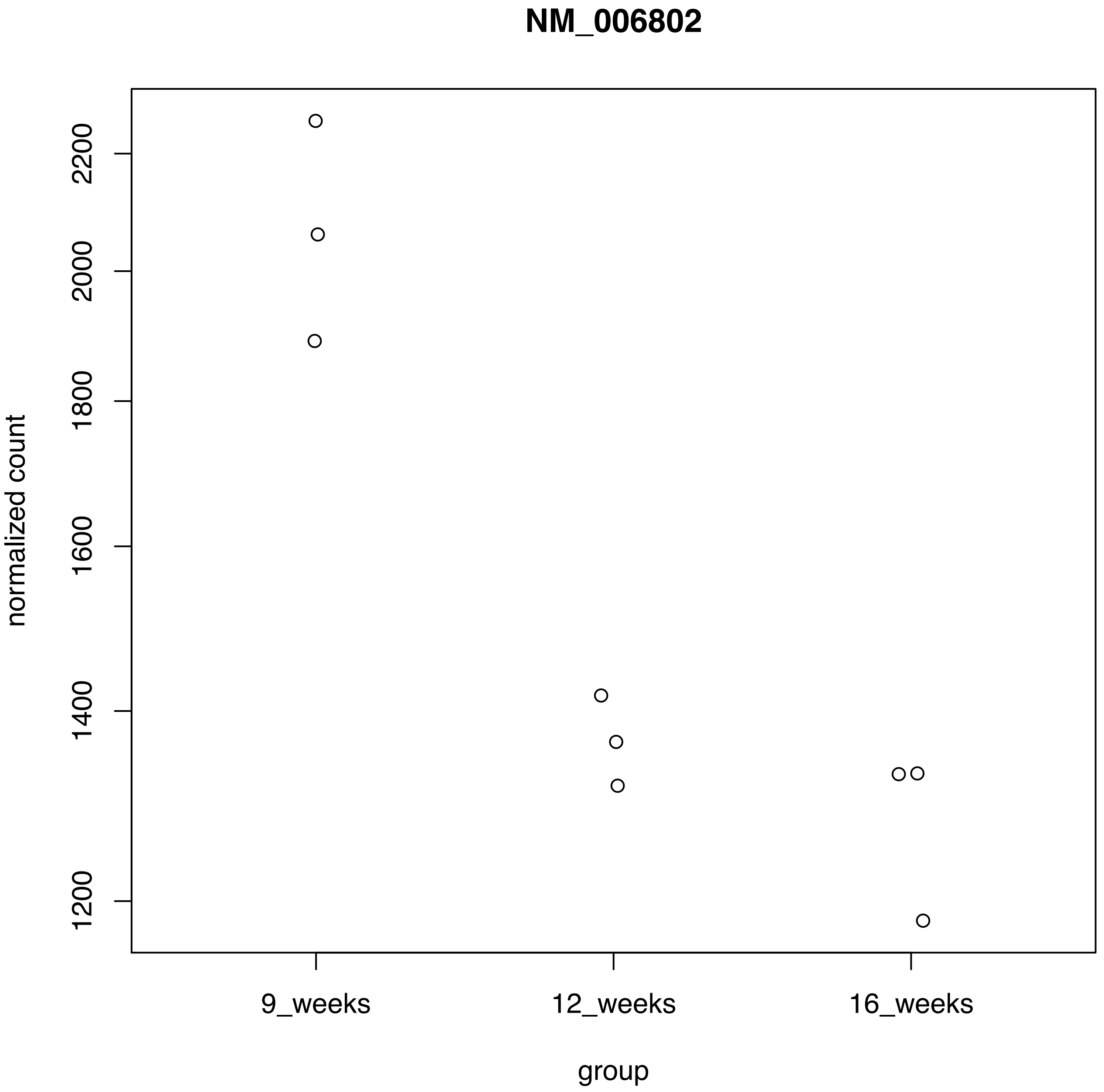

NM_006802
normalized count
2200
2000
1800
1600
1400
1200
9_weeks
12_weeks
16_weeks
group

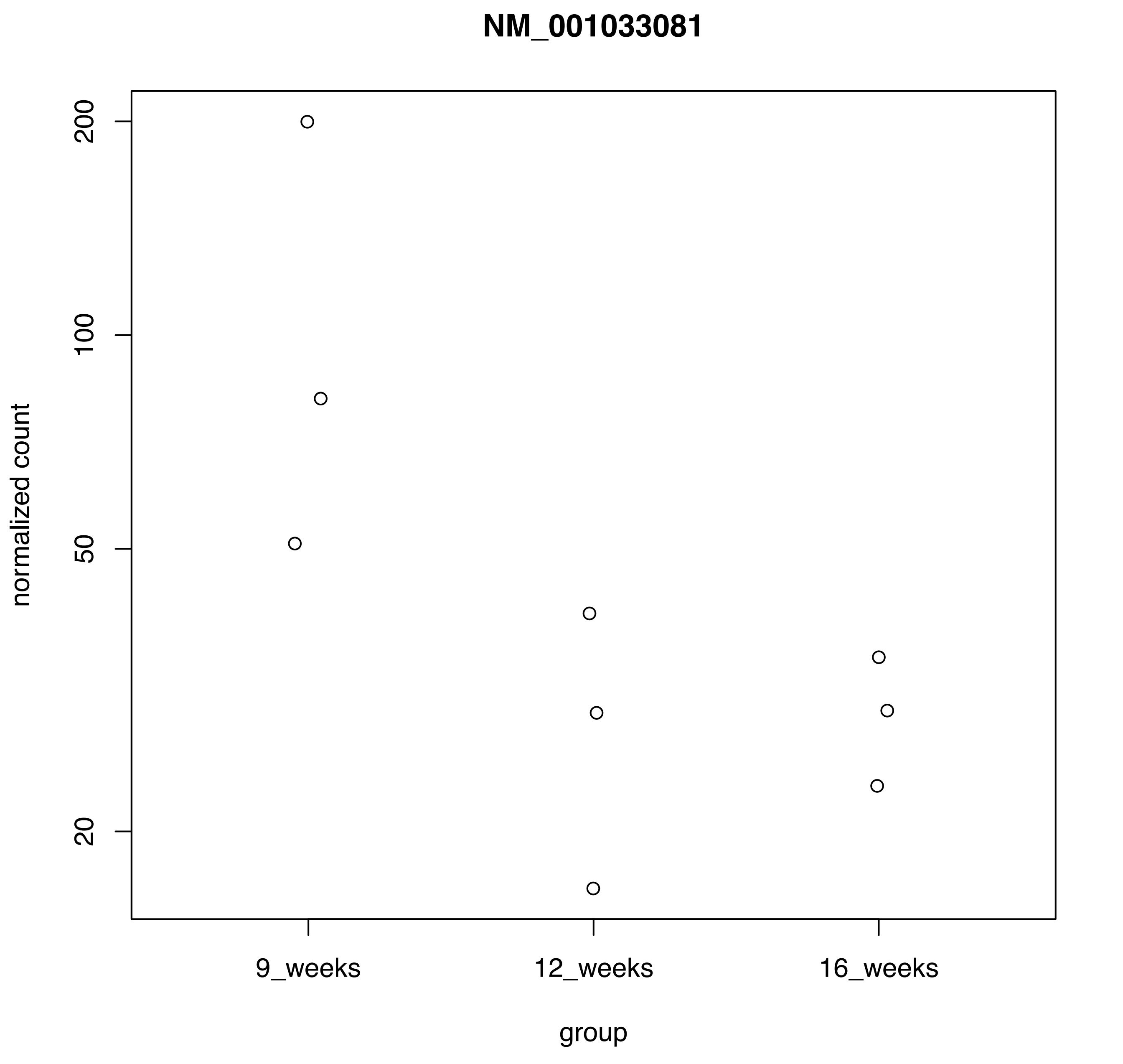

NM_001033081
200
100
50
20
normalized count
9_weeks
12_weeks
16_weeks
group

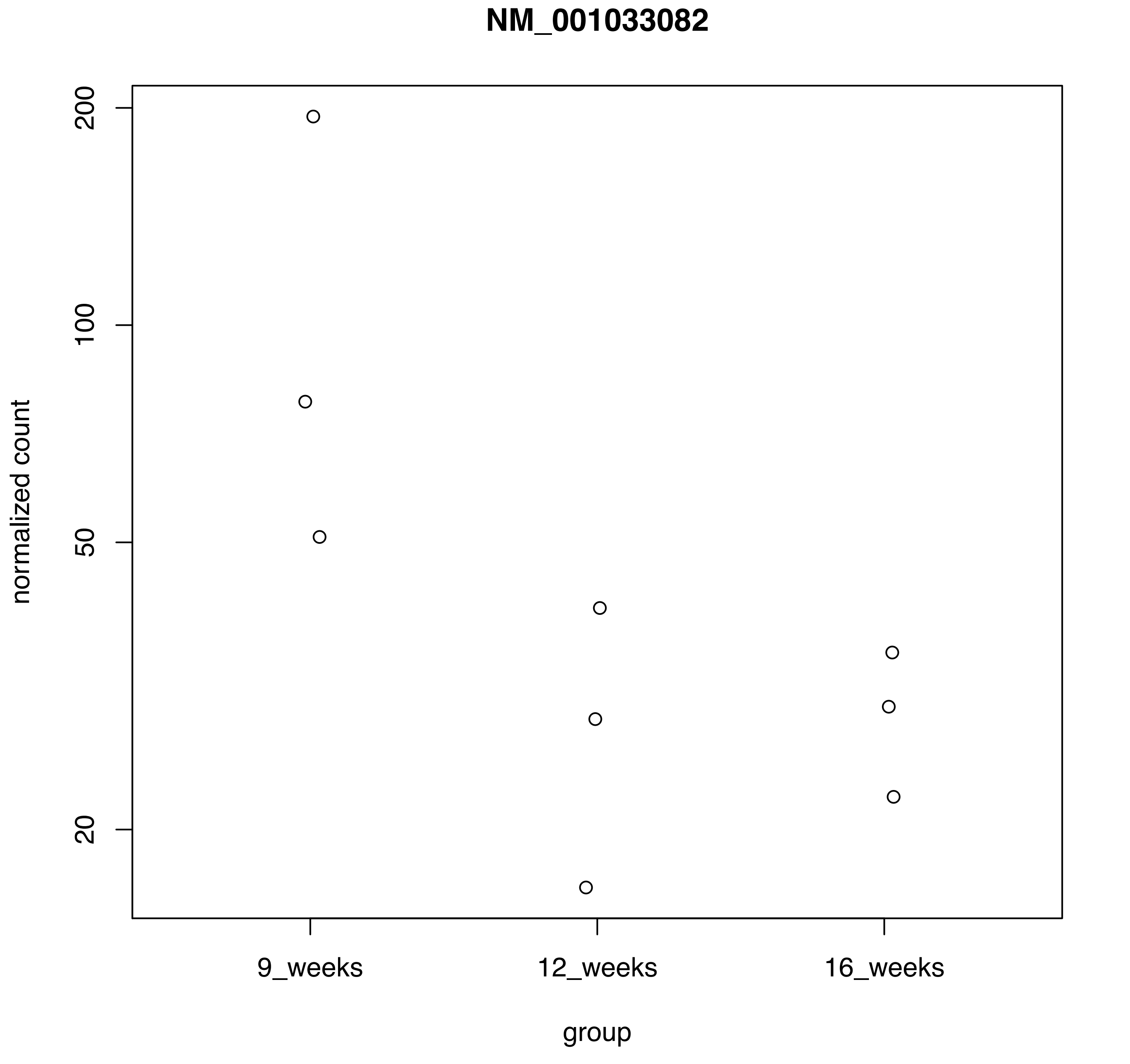
NM_001033082
normalized count
200
100
50
20
9_weeks
12_weeks
16_weeks
group

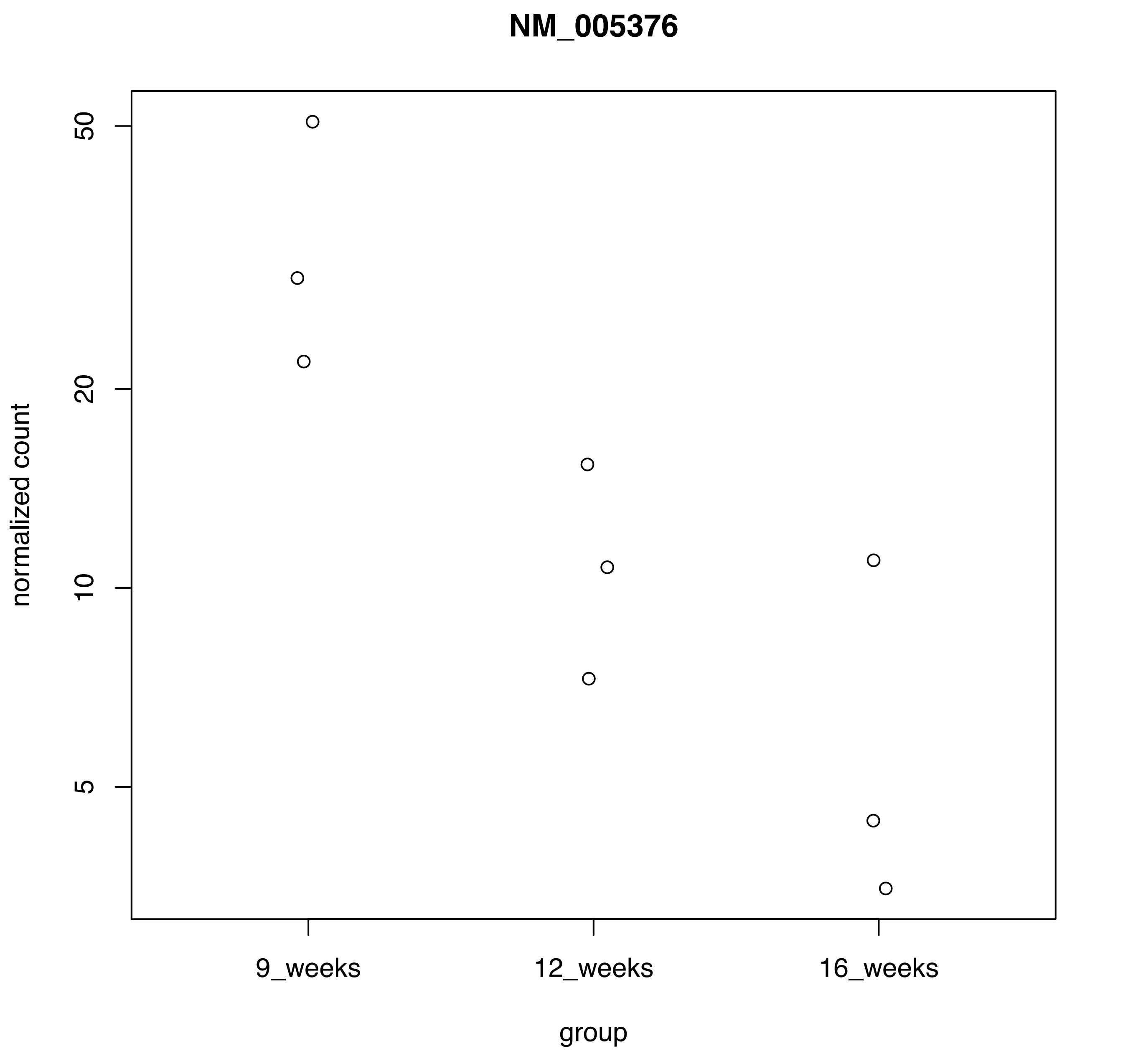
NM_005376
normalized count
50
20
10
5
9_weeks
12_weeks
16_weeks
group

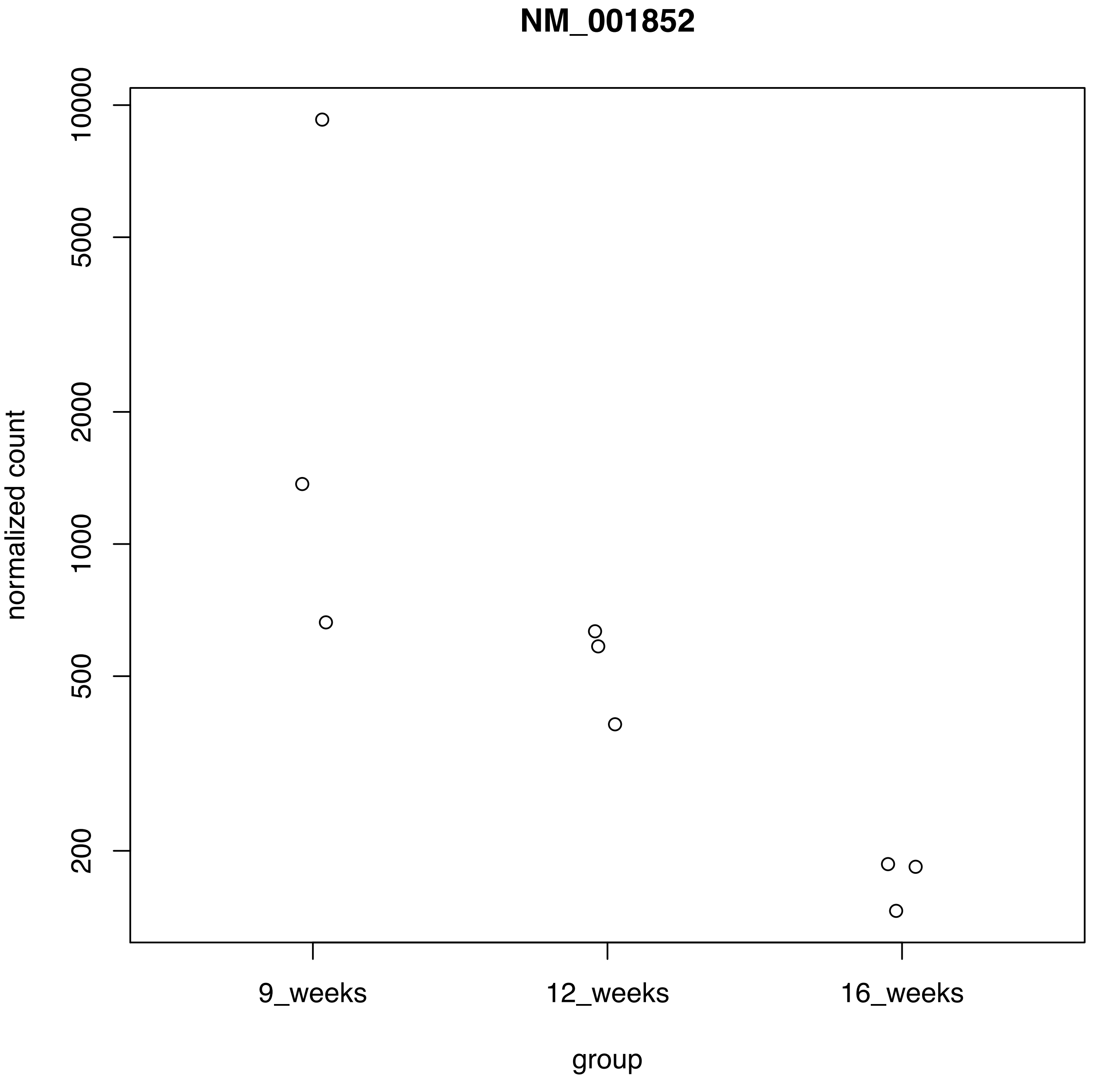
NM_001852
normalized count
10000
5000
2000
1000
500
200
9_weeks
12_weeks
16_weeks
group

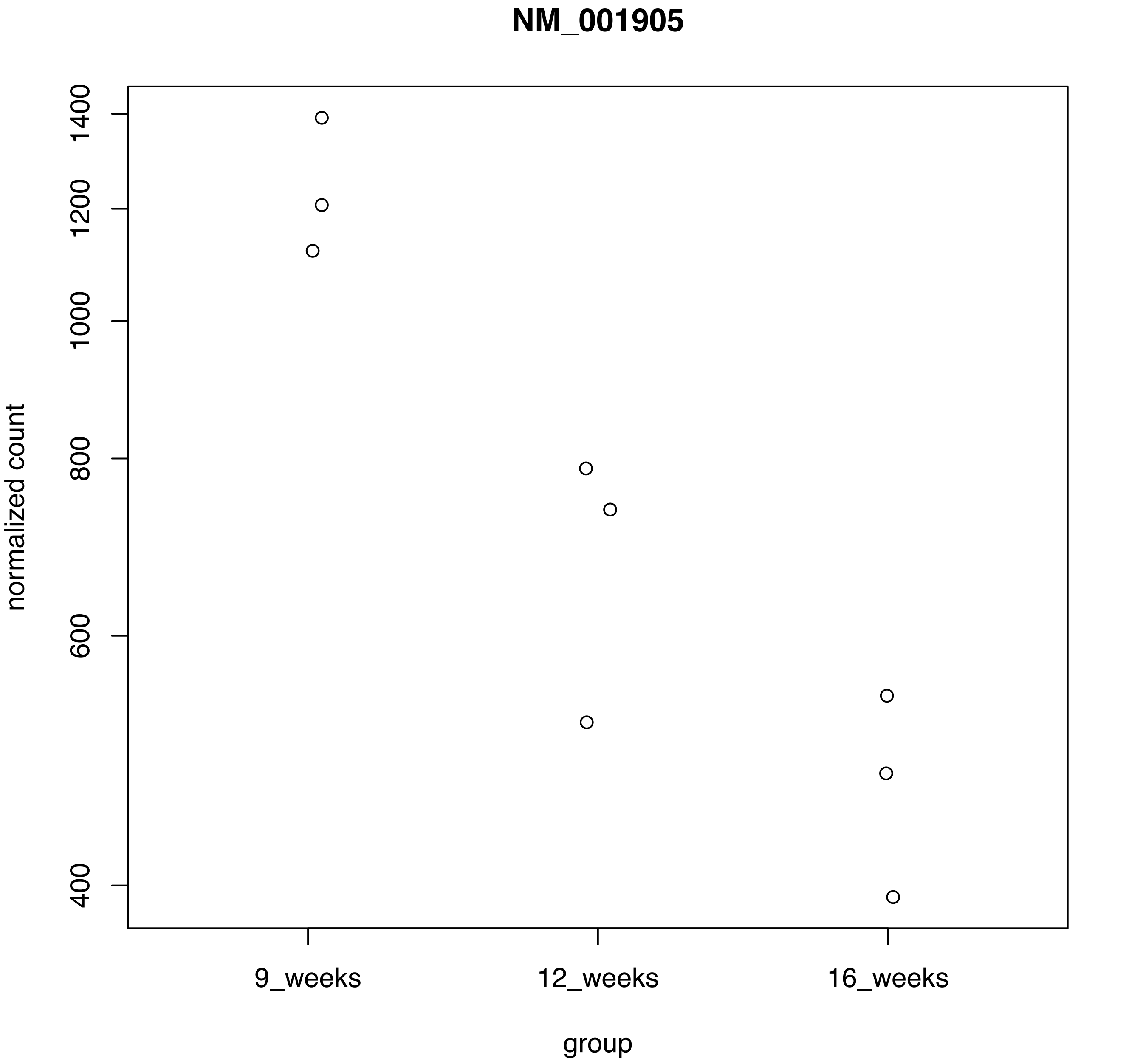
NM_001905
normalized count
1400
1200
1000
800
600
400
9_weeks
12_weeks
16_weeks
group

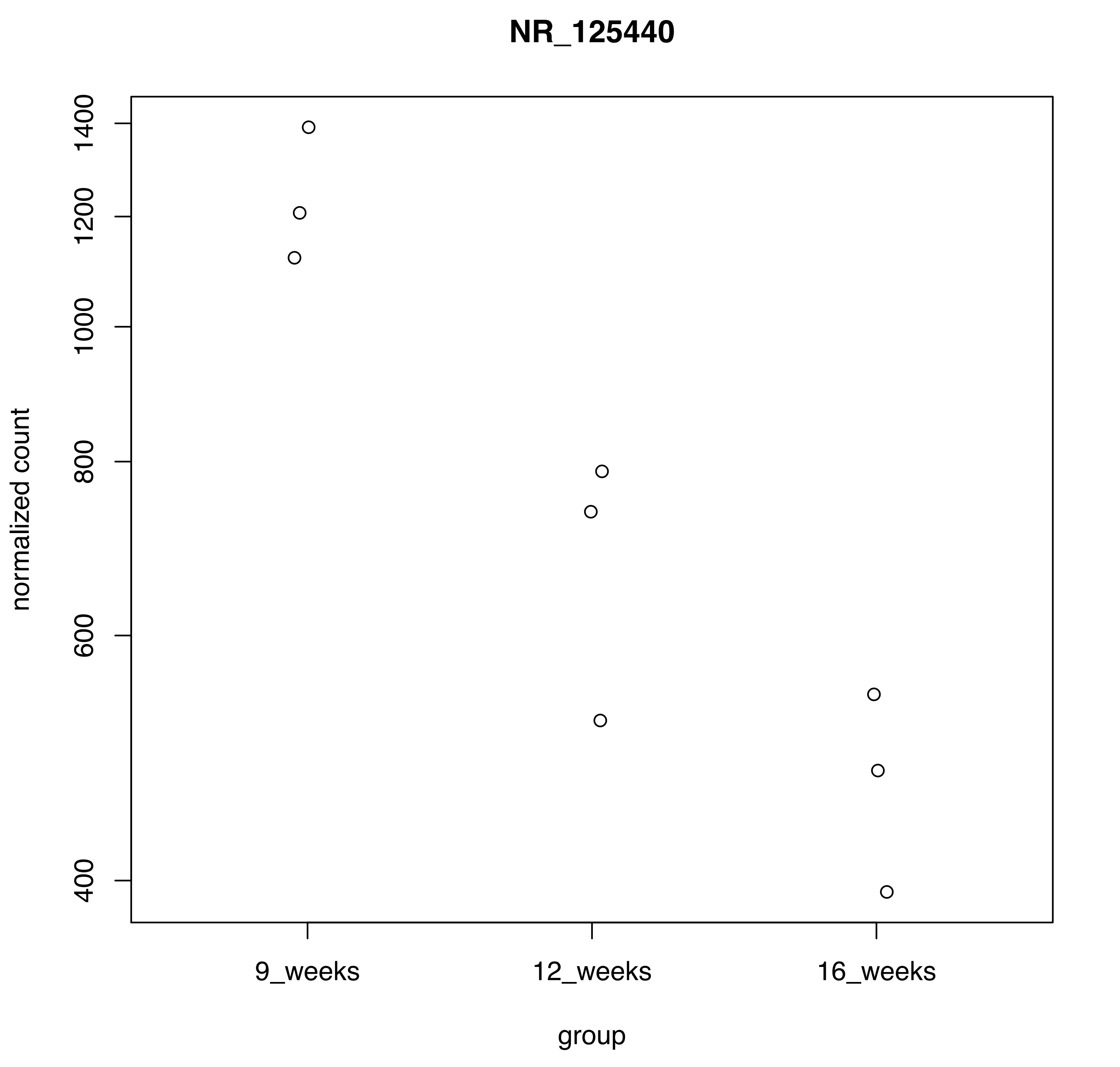
NR_125440
normalized count
1400
1200
1000
800
600
400
9_weeks
12_weeks
16_weeks
group

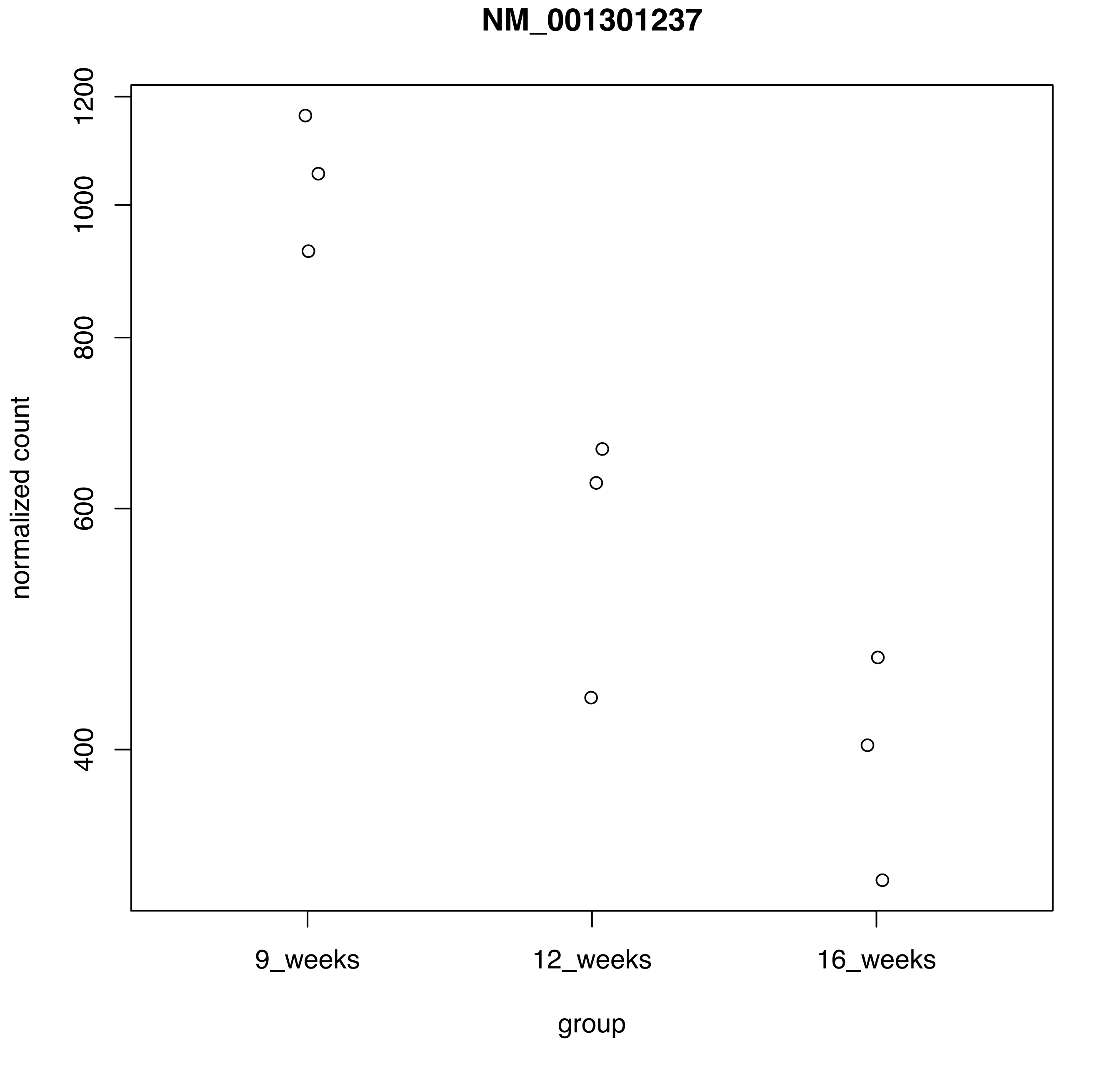
NM_001301237
normalized count
1200
1000
800
600
400
9_weeks
12_weeks
16_weeks
group

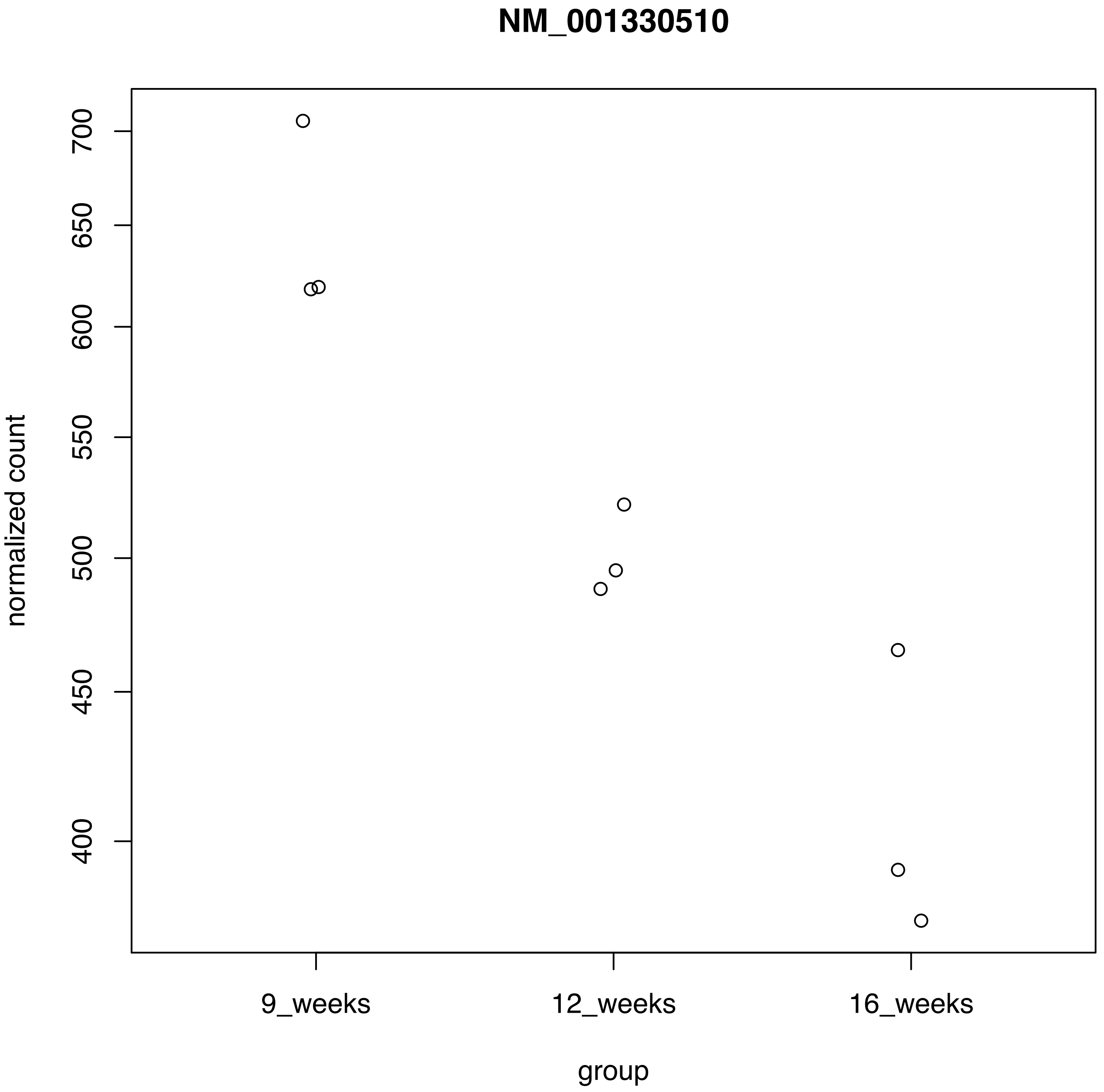
NM_001330510
normalized count
700
650
600
550
500
450
400
9_weeks
12_weeks
16_weeks
group

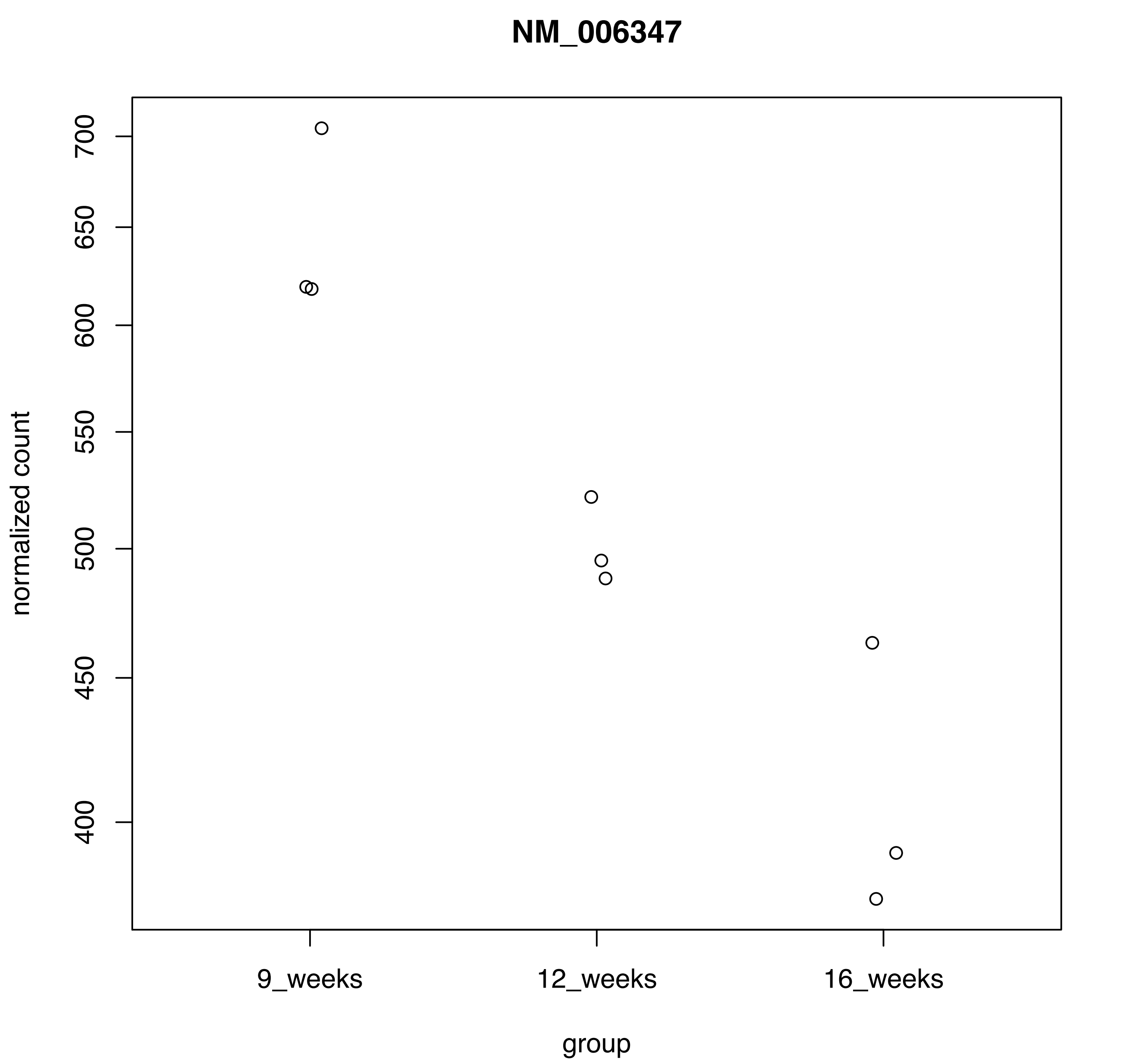

NM_006347
normalized count
700
650
600
550
500
450
400
9_weeks
12_weeks
16_weeks
group

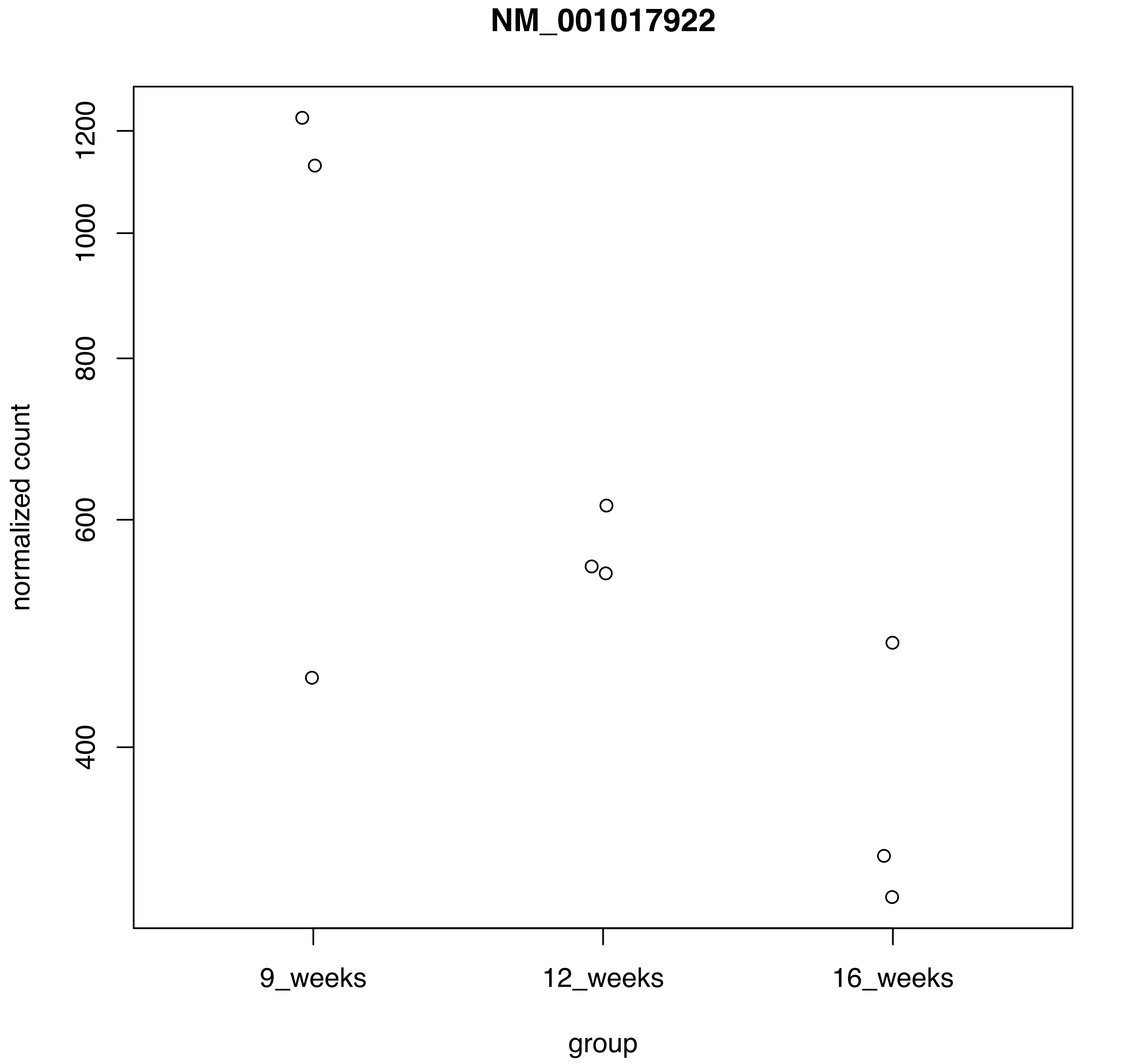

NM_001017922
normalized count
1200
1000
800
600
400
9_weeks
12_weeks
16_weeks
group

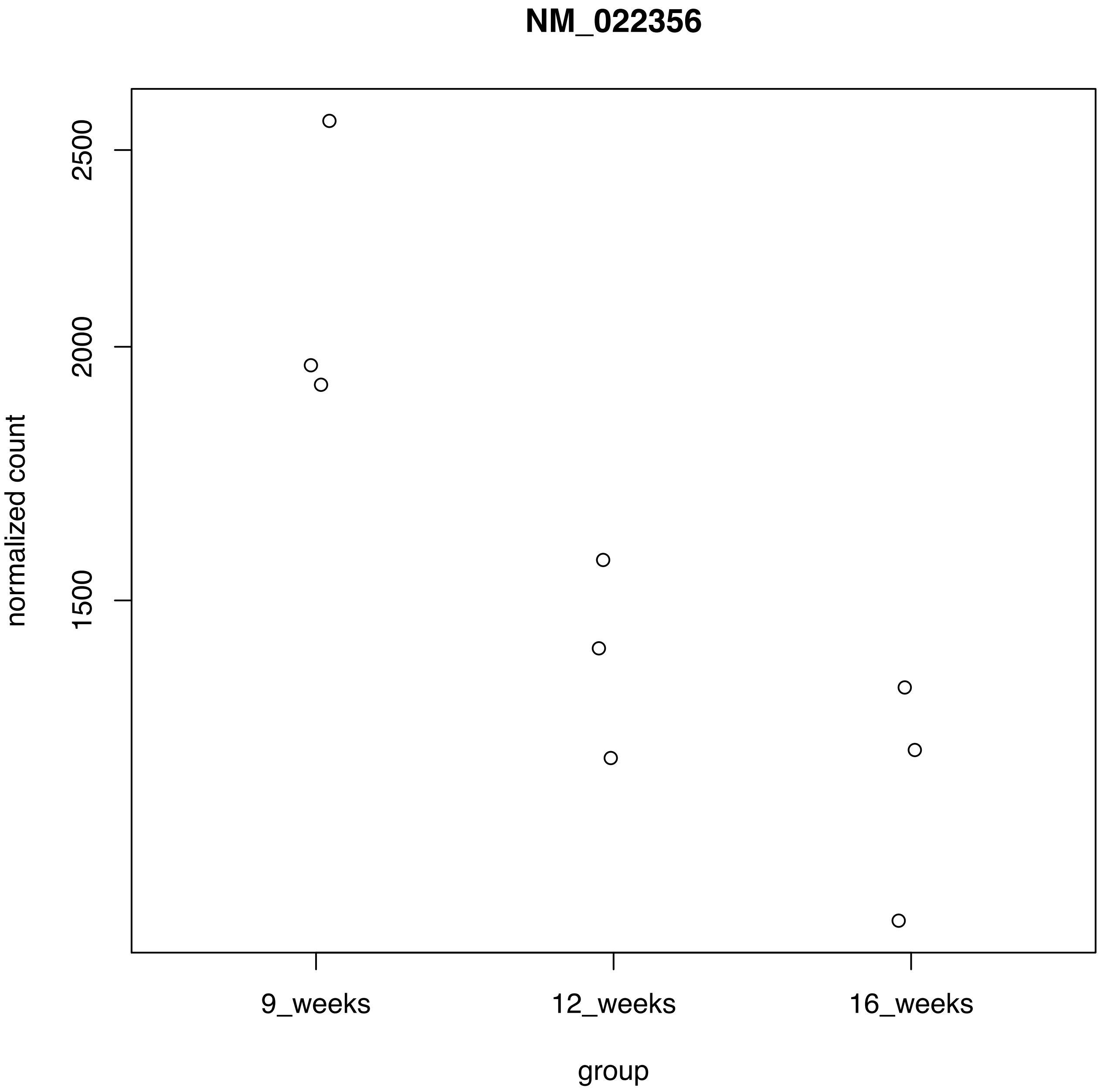

NM_022356
normalized count
2500
2000
1500
9_weeks
12_weeks
16_weeks
group

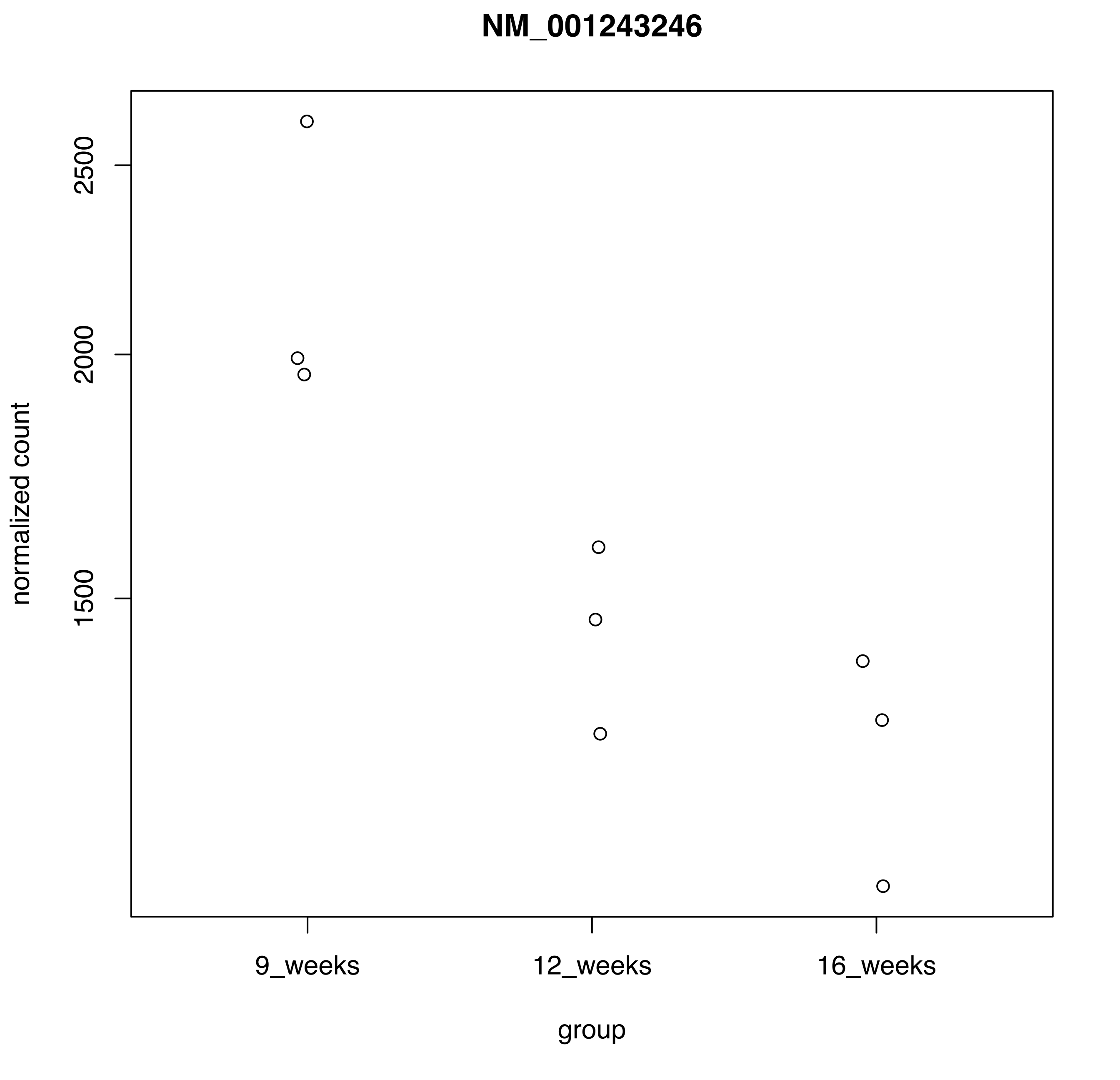
NM_001243246
normalized count
2500
2000
1500
9_weeks
12_weeks
16_weeks
group

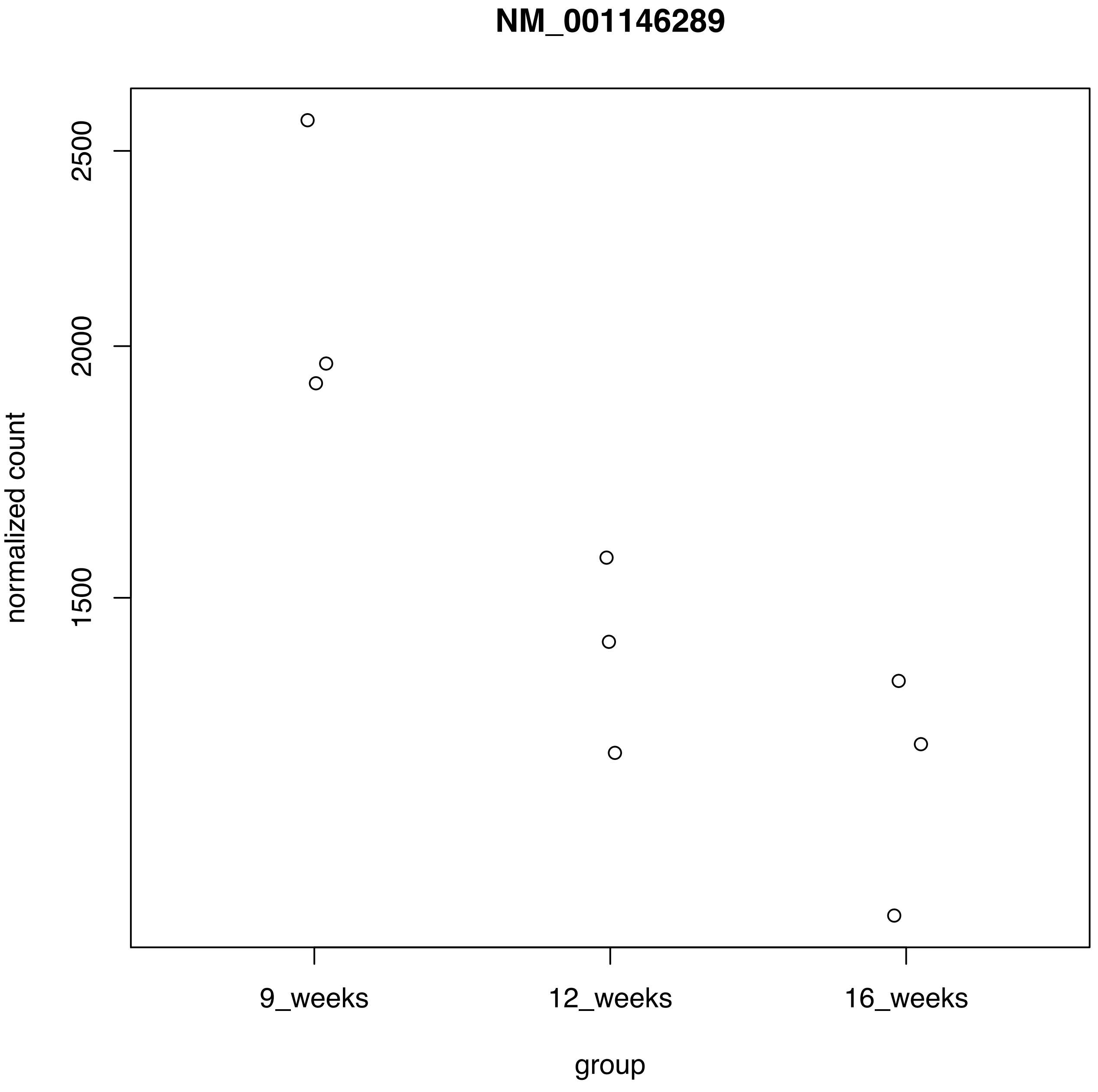
NM_001146289
normalized count
2500
2000
1500
9_weeks
12_weeks
16_weeks
group

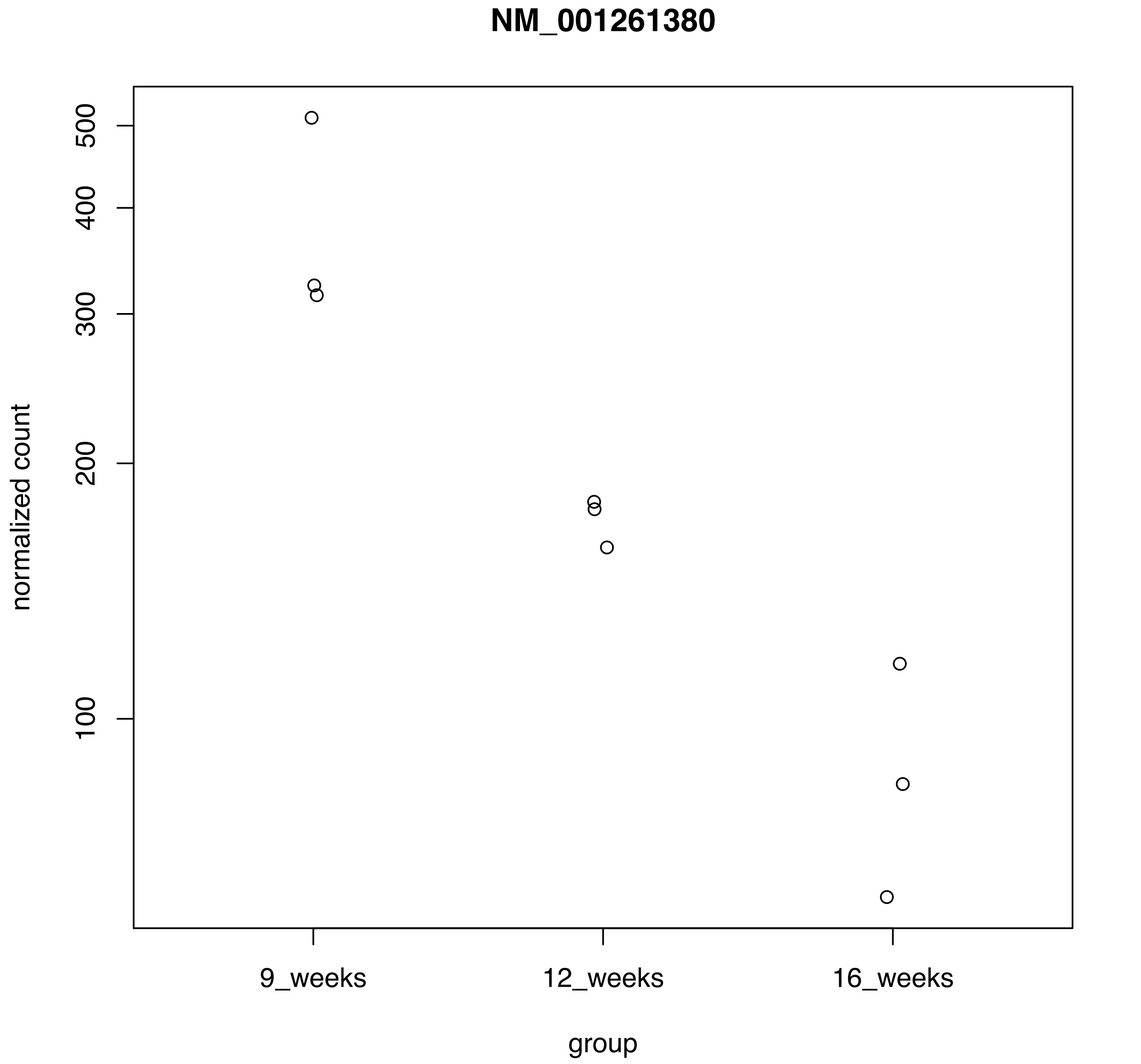
NM_001261380
normalized count
500
400
300
200
100
9_weeks
12_weeks
16_weeks
group

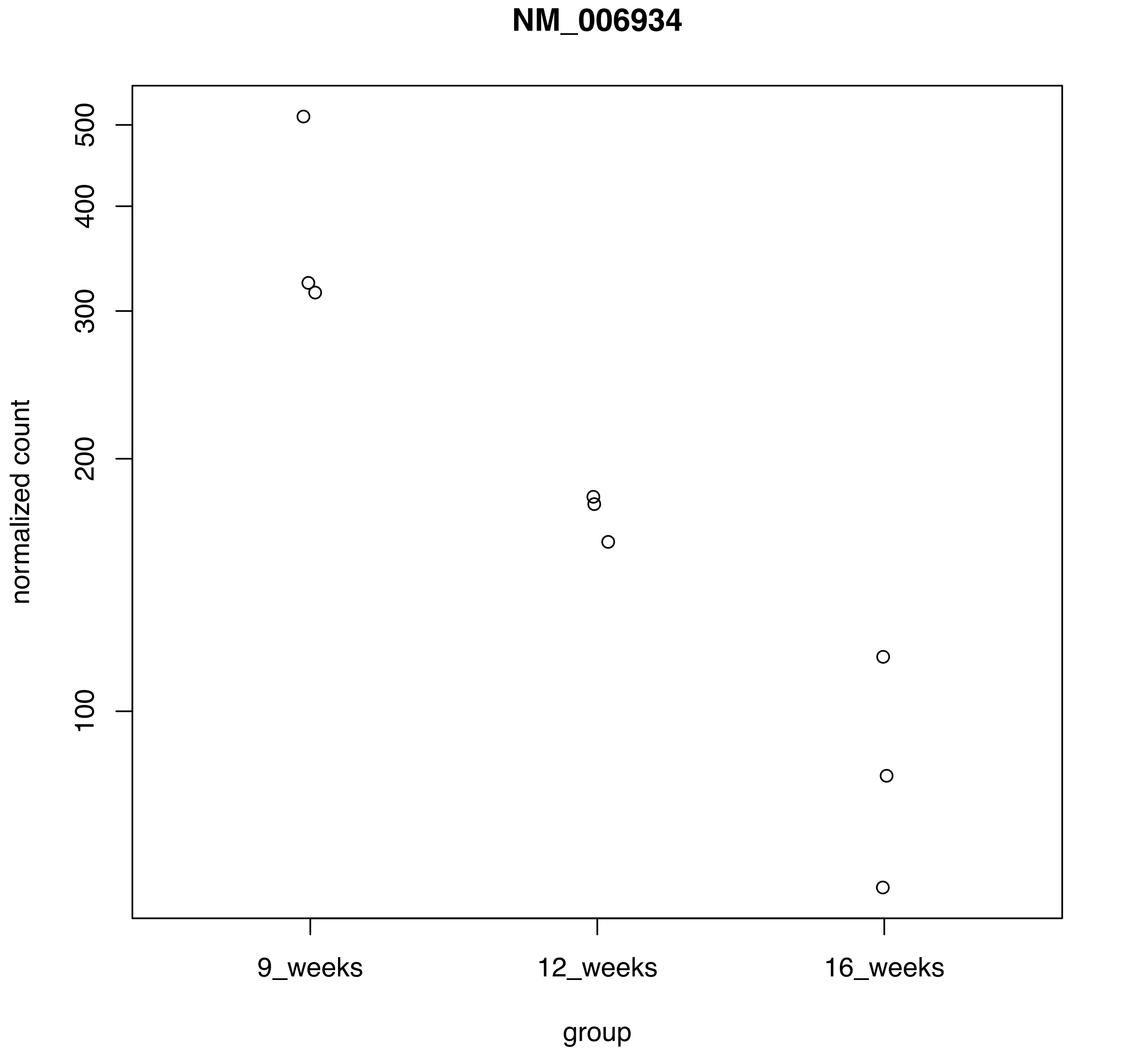

NM_006934
normalized count
500
400
300
200
100
9_weeks
12_weeks
16_weeks
group

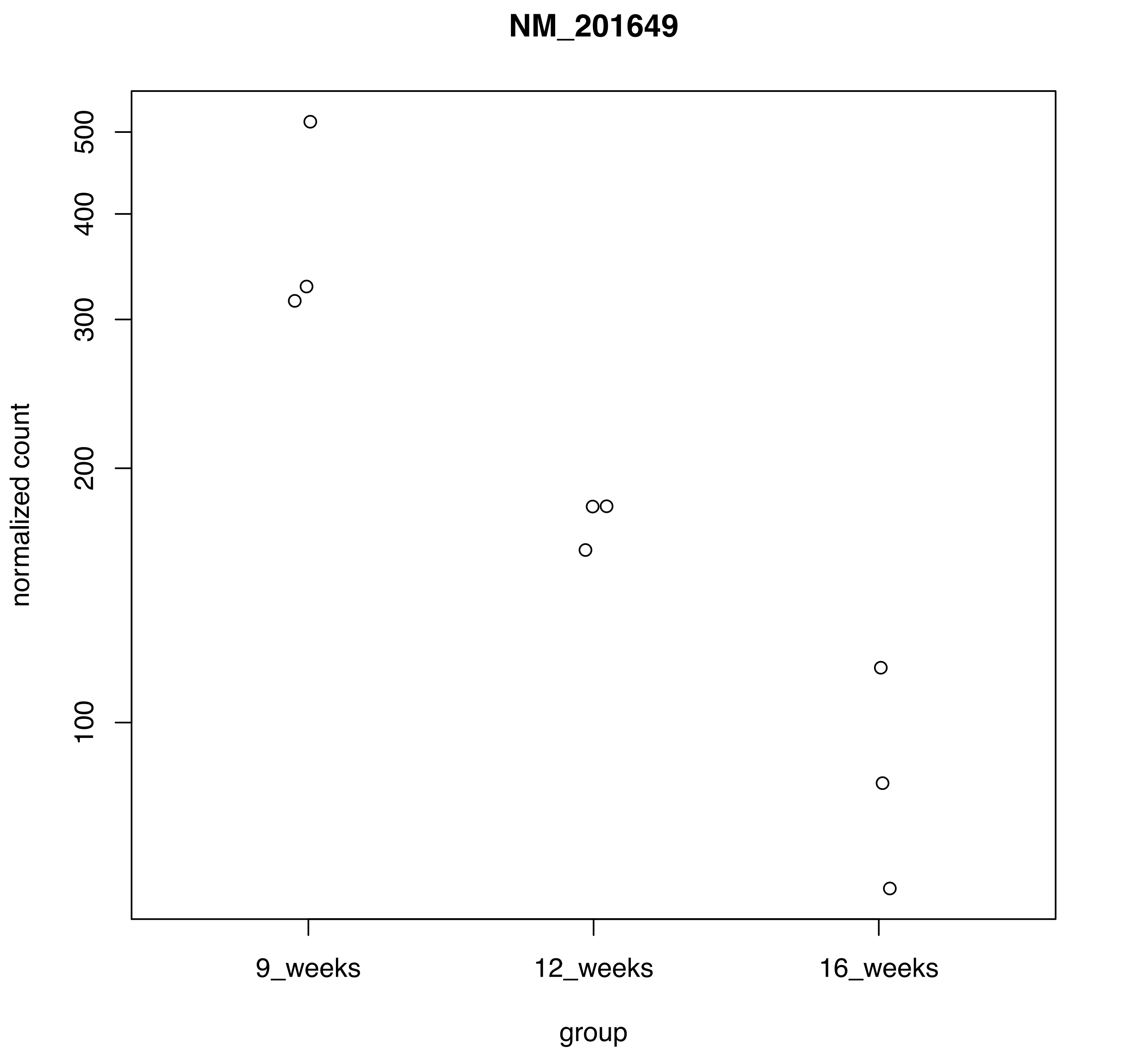
NM_201649
normalized count
500
400
300
200
100
9_weeks
12_weeks
16_weeks
group

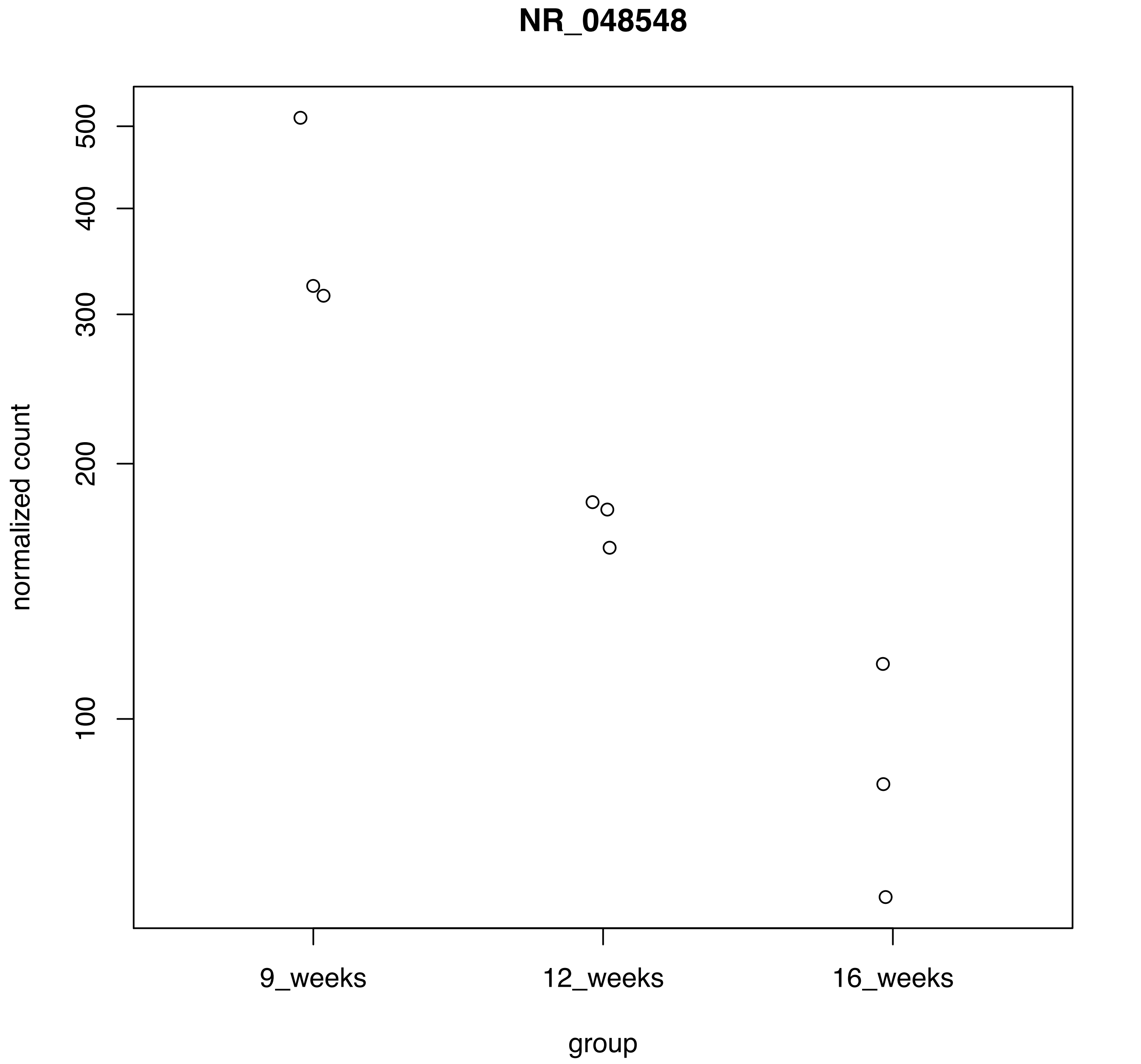

NR_048548
normalized count
500
400
300
200
100
9_weeks
12_weeks
16_weeks
group

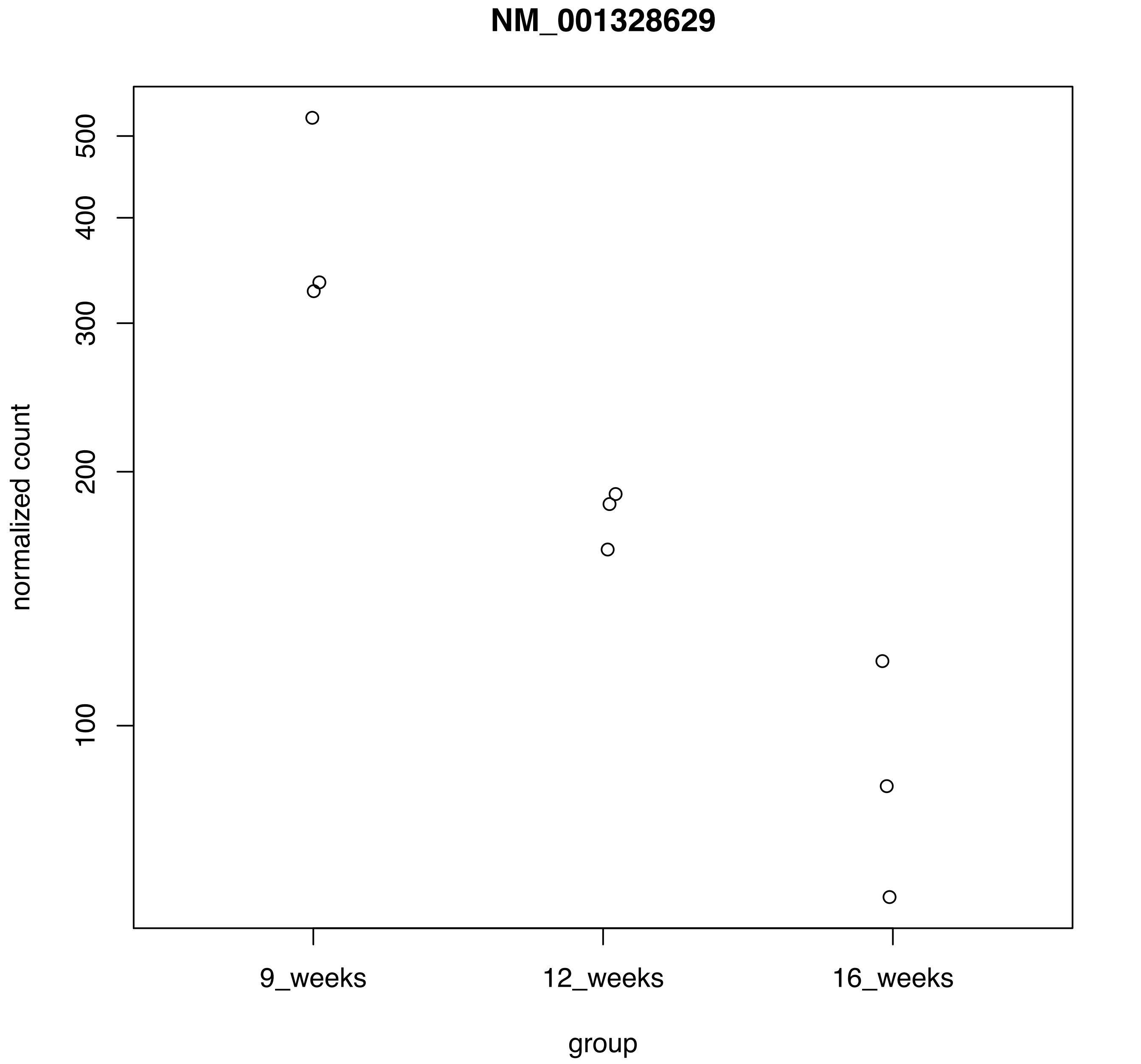
NM_001328629
normalized count
500
400
300
200
100
9_weeks
12_weeks
16_weeks
group

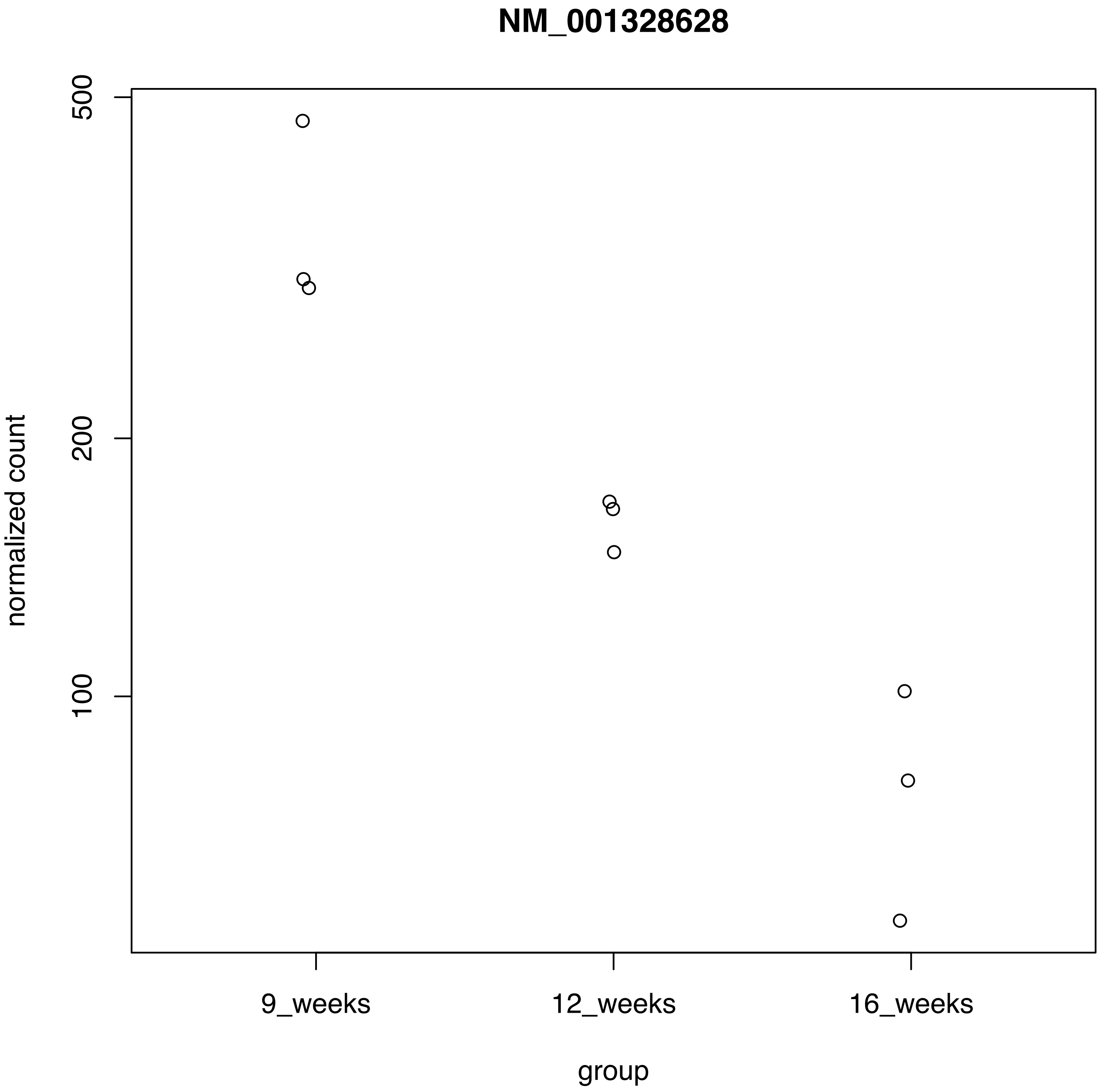
NM_001328628
normalized count
500
200
100
9_weeks
12_weeks
16_weeks
group

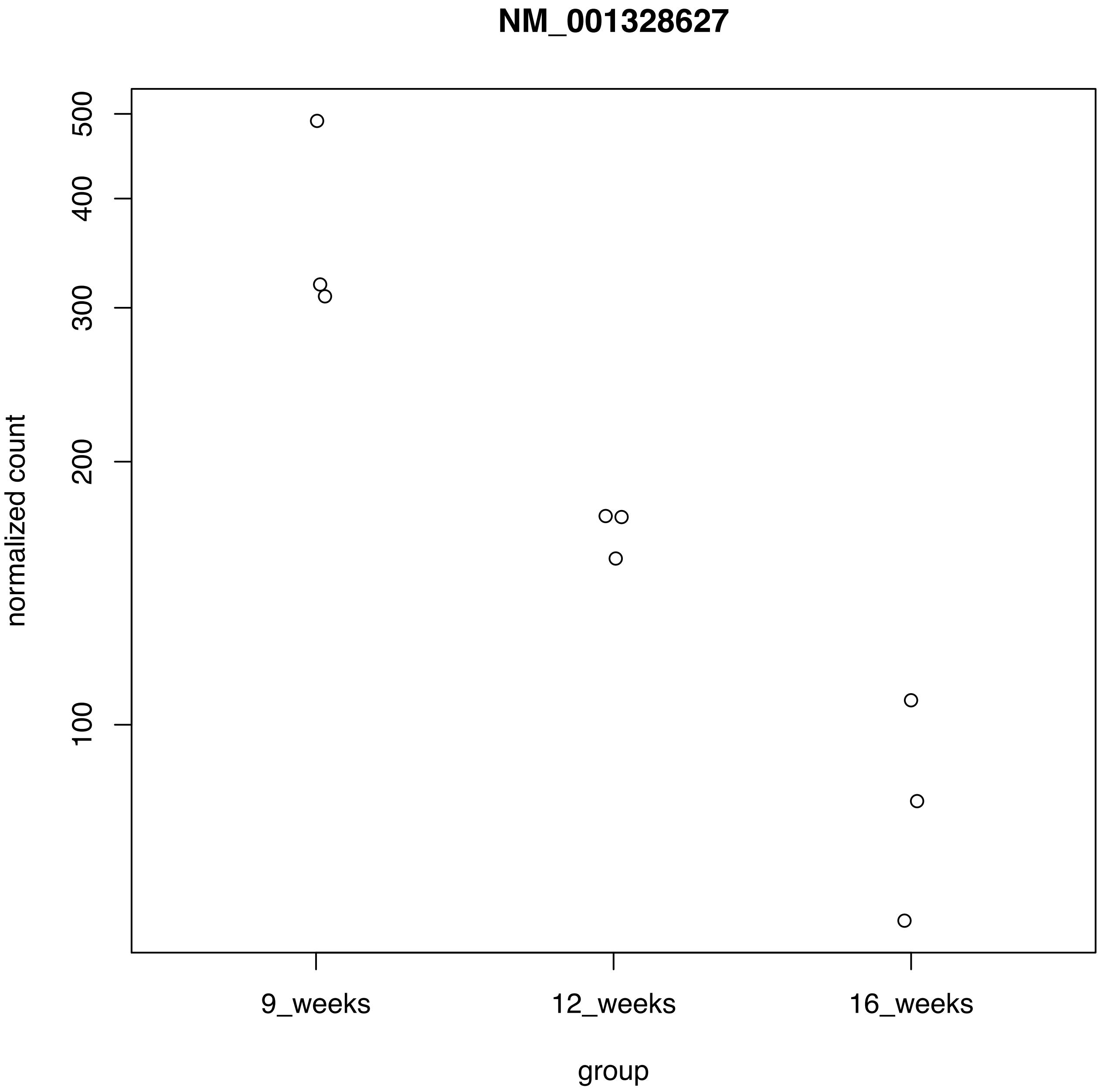

NM_001328627
normalized count
500
400
300
200
100
9_weeks
12_weeks
16_weeks
group

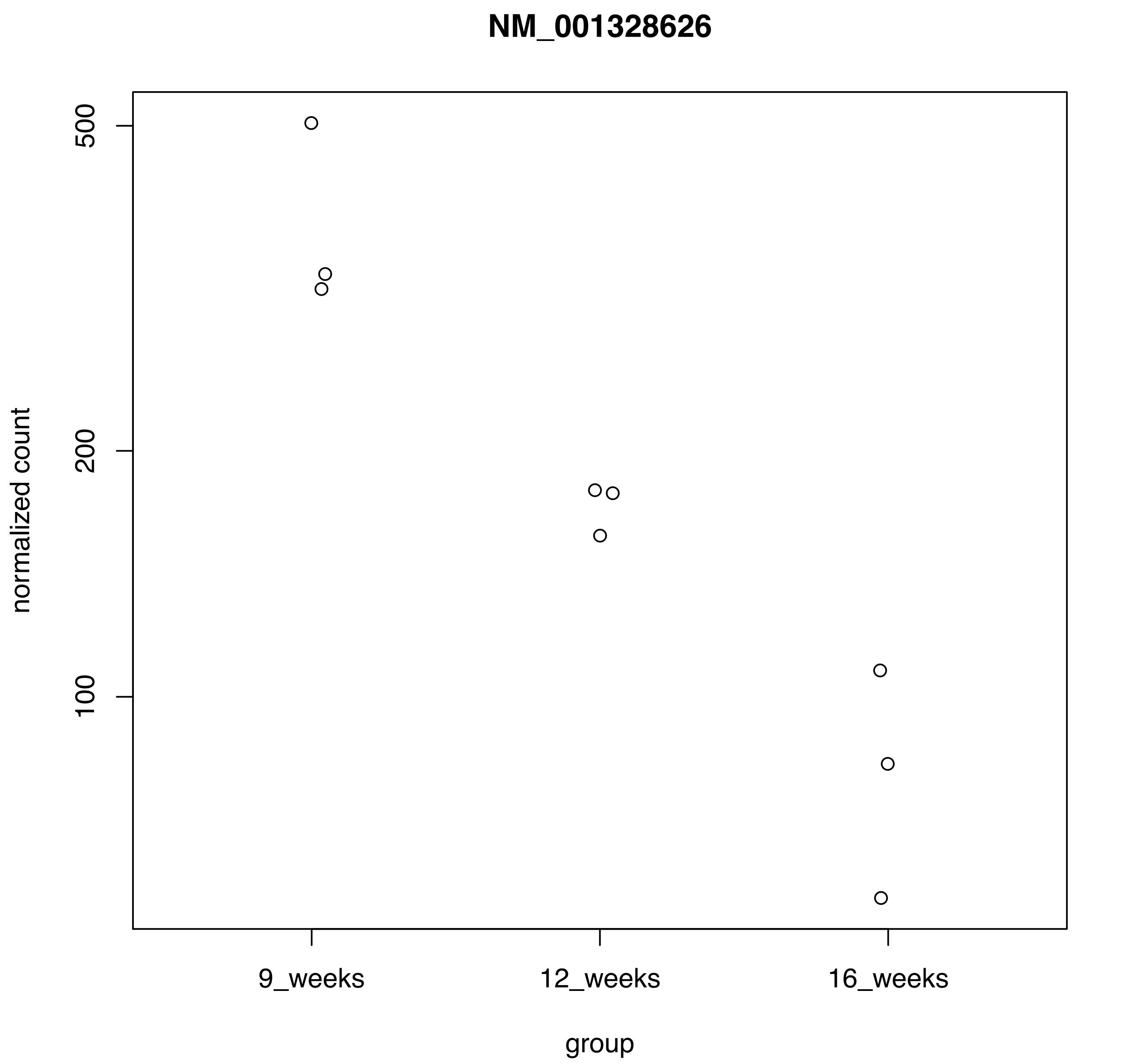
NM_001328626
normalized count
500
200
100
9_weeks
12_weeks
16_weeks
group

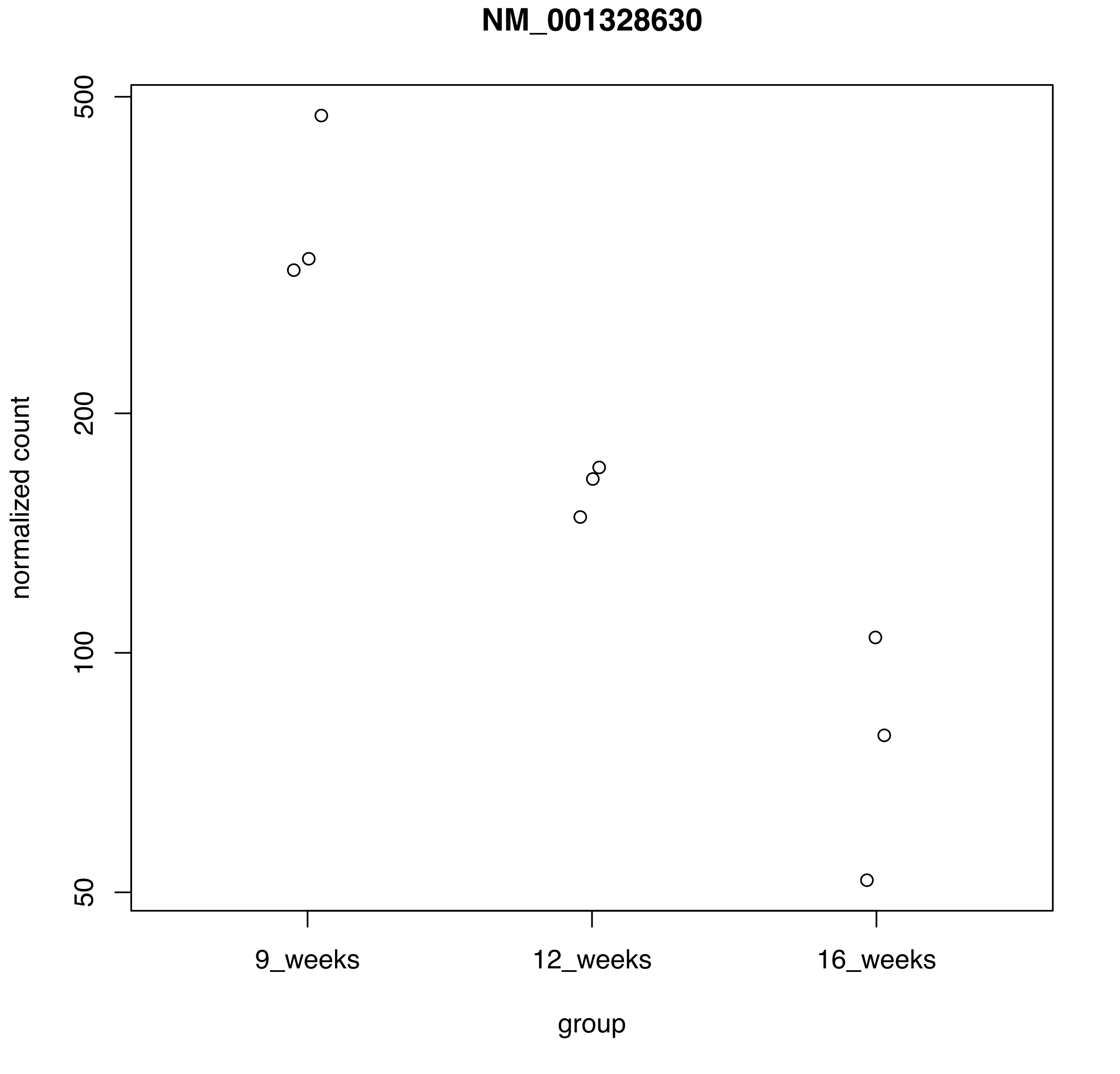

NM_001328630
normalized count
500
200
100
50
9_weeks
12_weeks
16_weeks
group

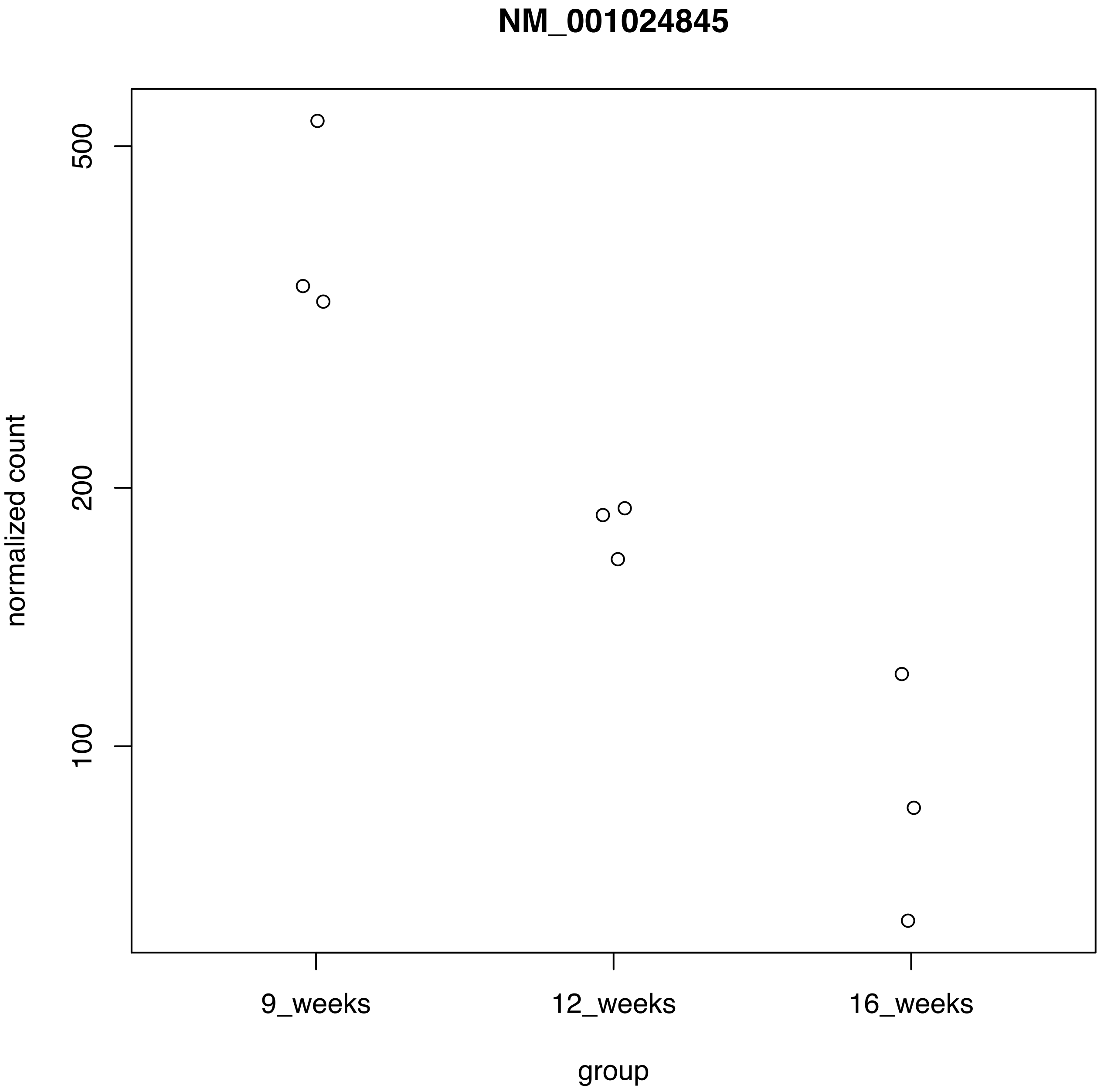
NM_001024845
normalized count
500
200
100
9_weeks
12_weeks
16_weeks
group

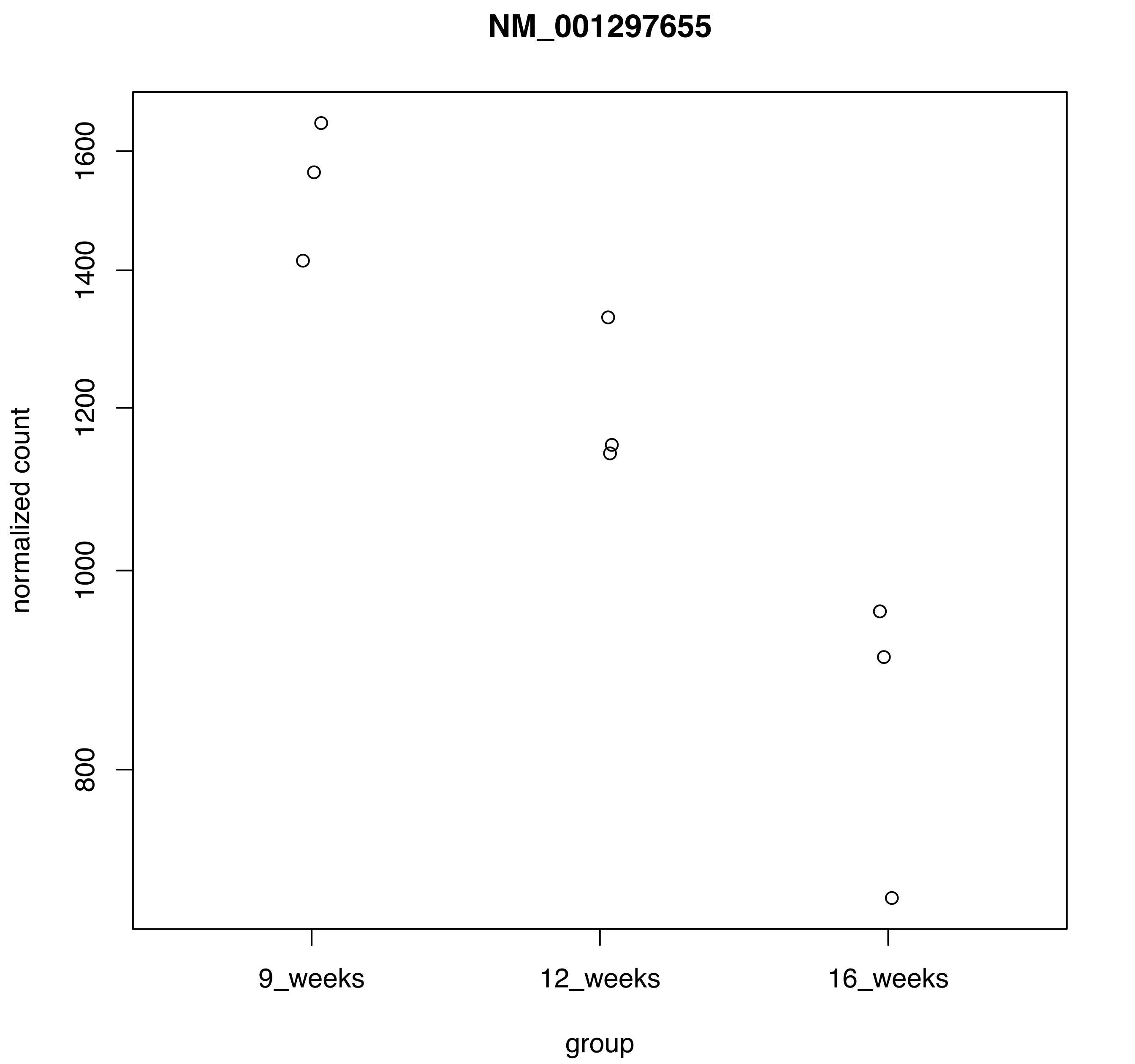
NM_001297655
normalized count
800
1000
1200
1400
1600
9_weeks
12_weeks
16_weeks
group

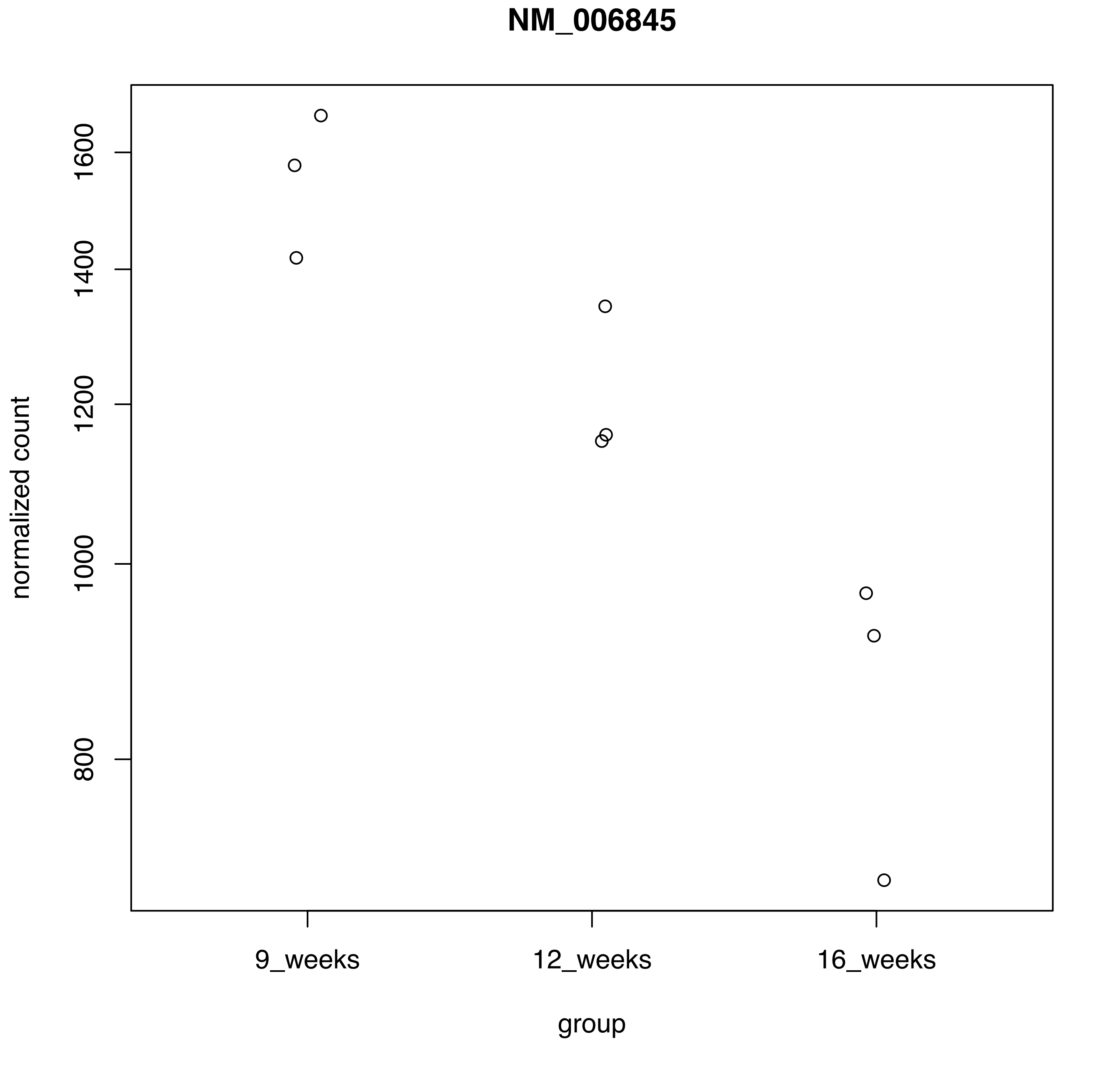
NM_006845
normalized count
1600
1400
1200
1000
800
9_weeks
12_weeks
16_weeks
group

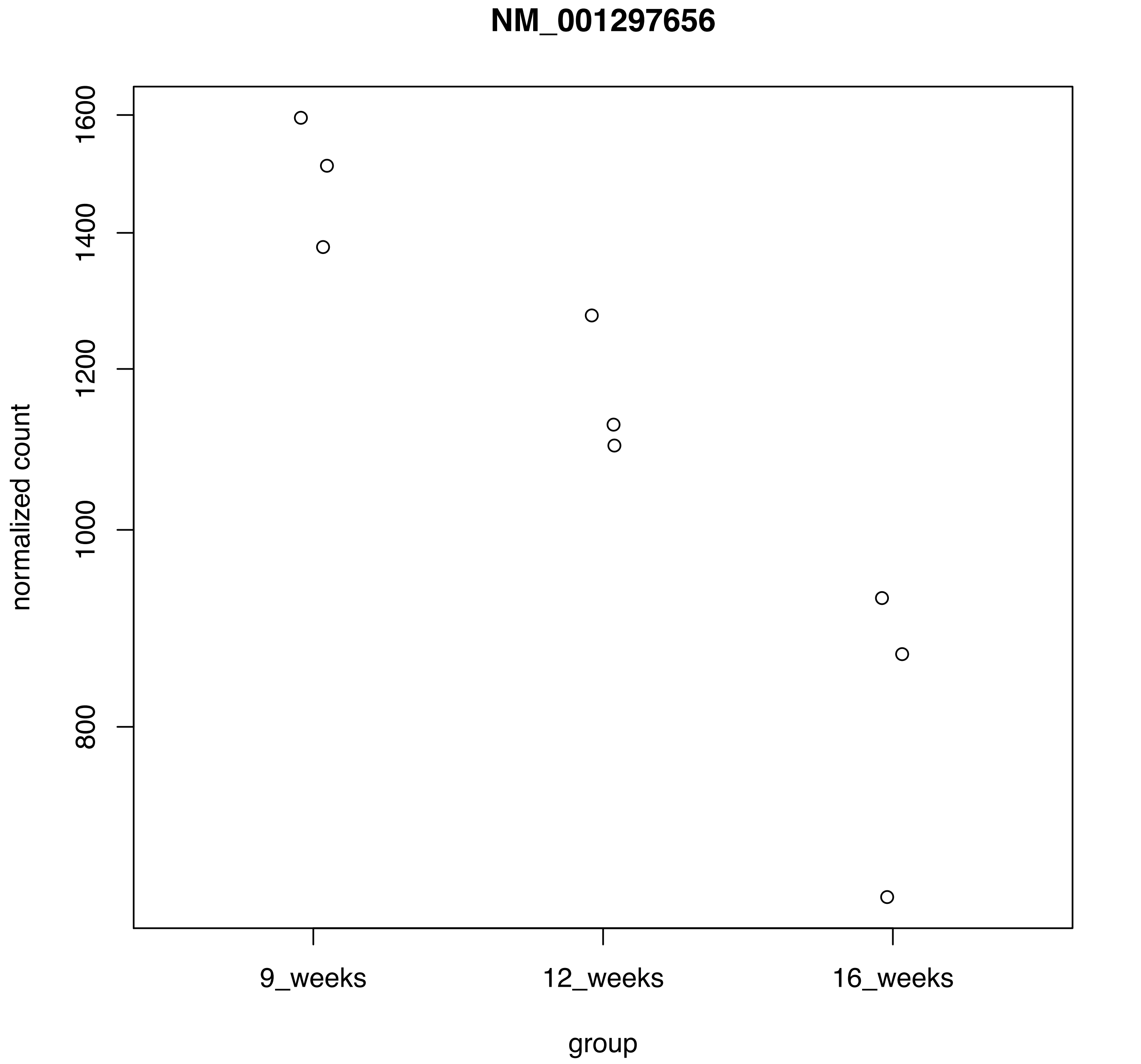

NM_001297656
normalized count
1600
1400
1200
1000
800
9_weeks
12_weeks
16_weeks
group

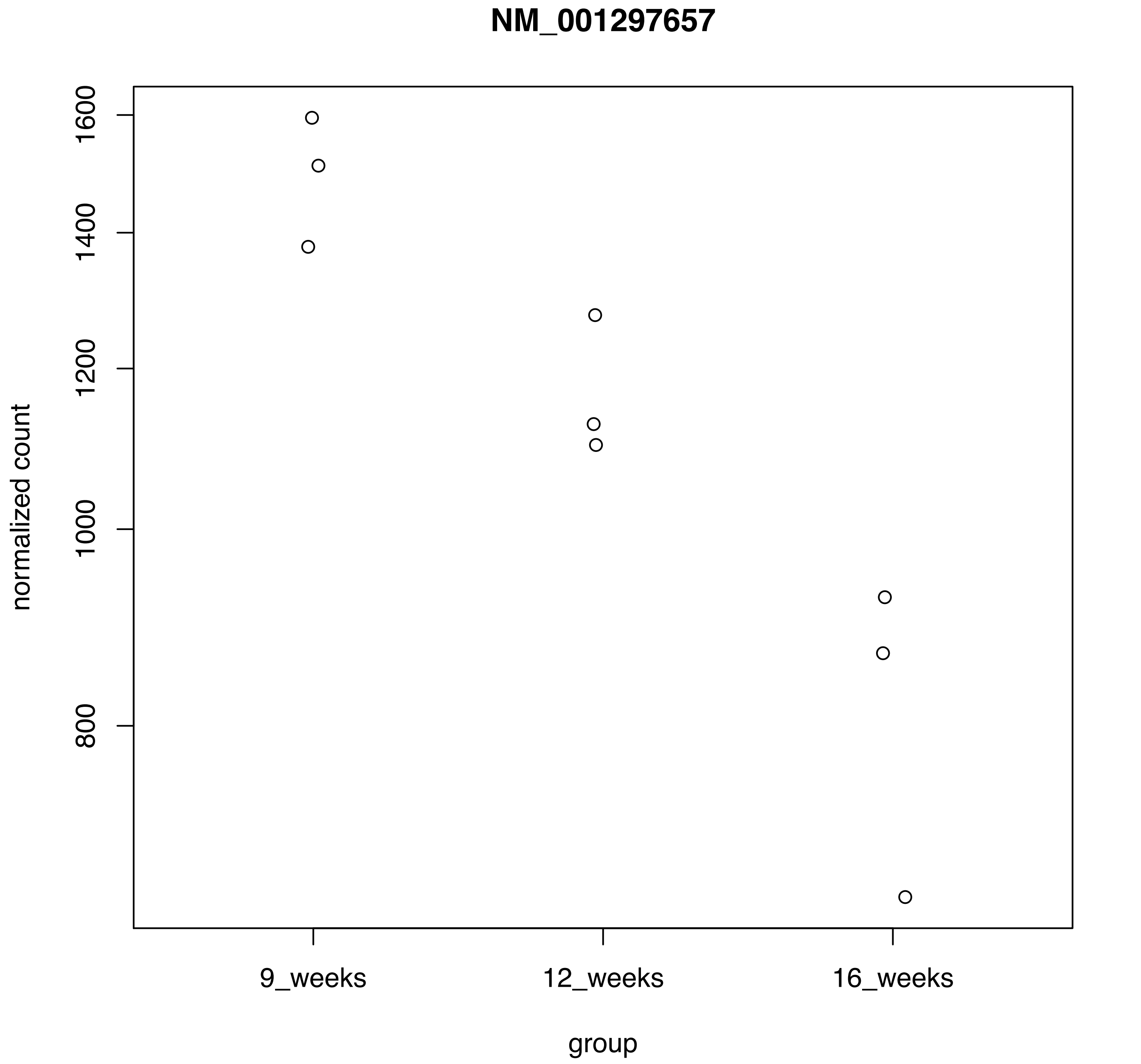

NM_001297657
normalized count
1600
1400
1200
1000
800
9_weeks
12_weeks
16_weeks
group

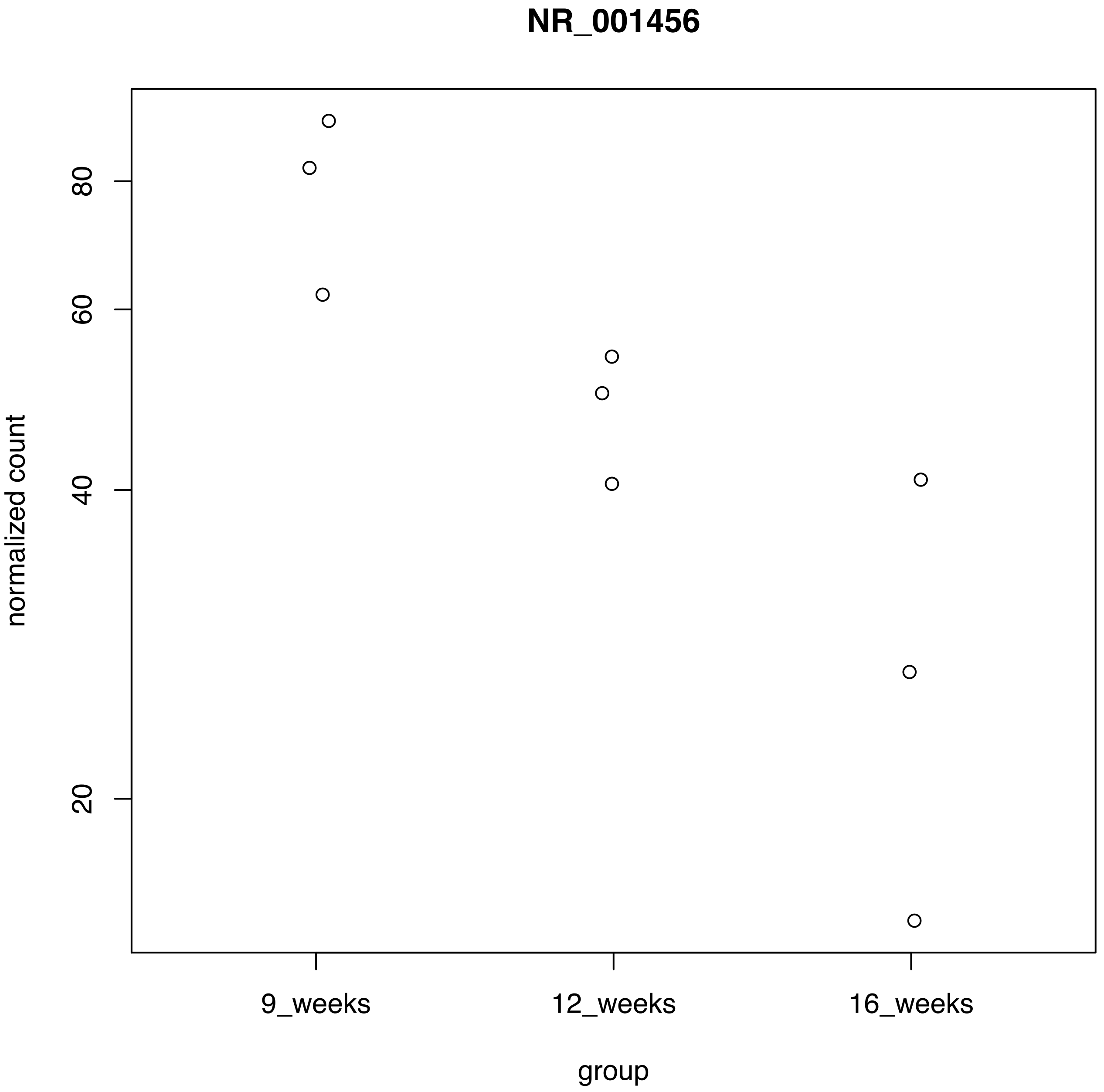

NR_001456
normalized count
80
60
40
20
9_weeks
12_weeks
16_weeks
group

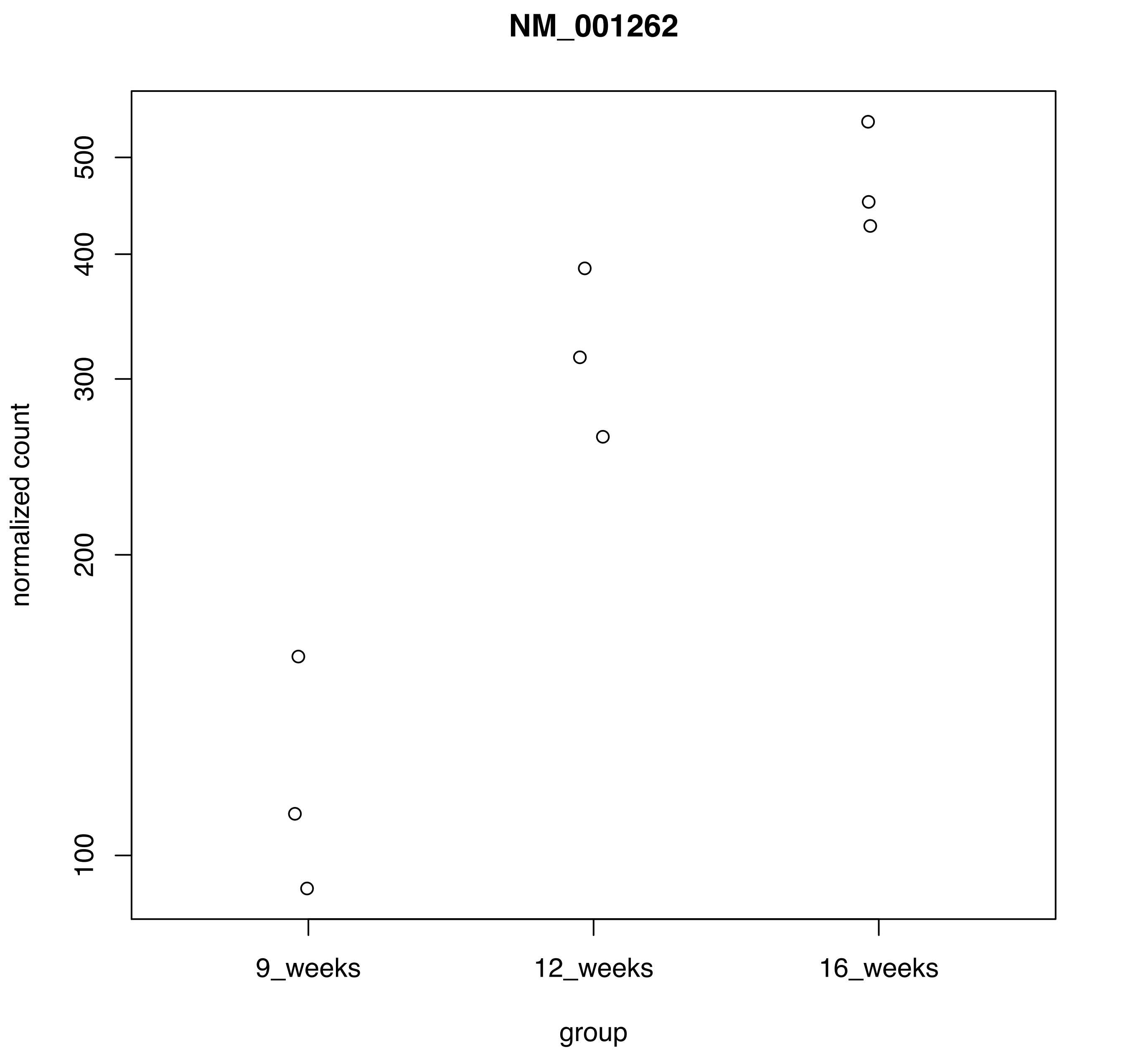

NM_001262
normalized count
500
400
300
200
100
9_weeks
12_weeks
16_weeks
group

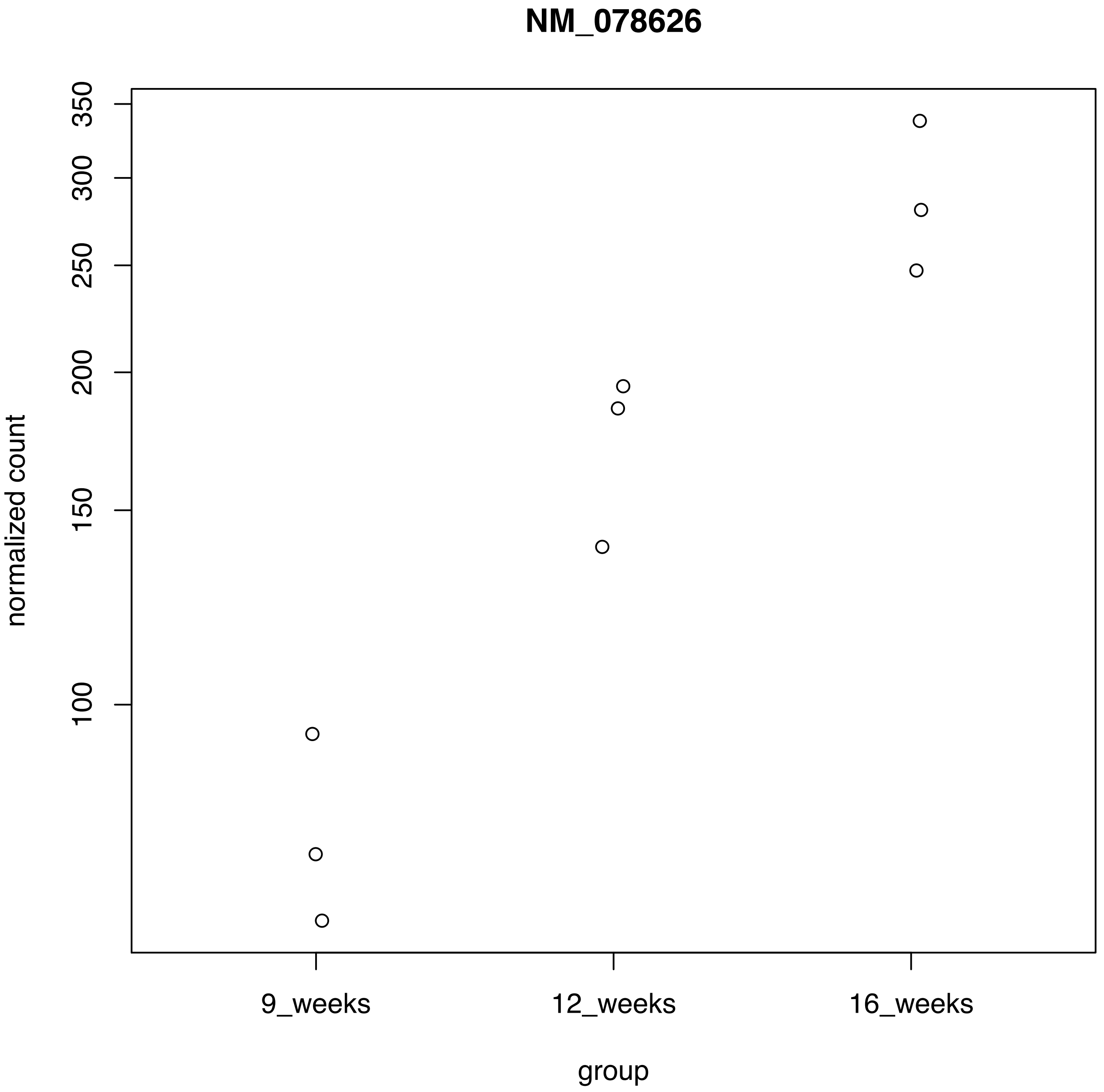
NM_078626
normalized count
350
300
250
200
150
100
9_weeks
12_weeks
16_weeks
group

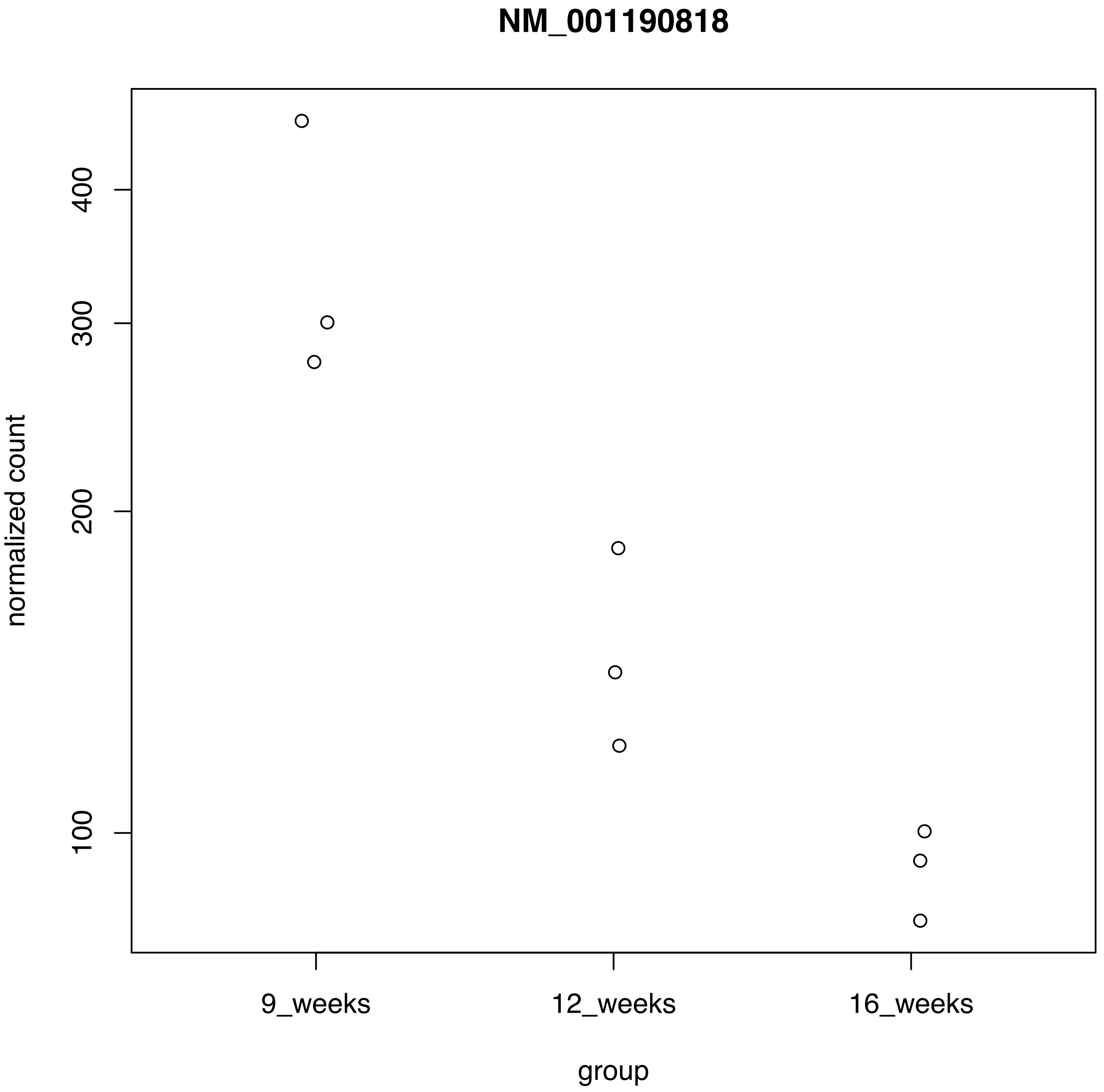
NM_001190818
normalized count
100
200
300
400
9_weeks
12_weeks
16_weeks
group

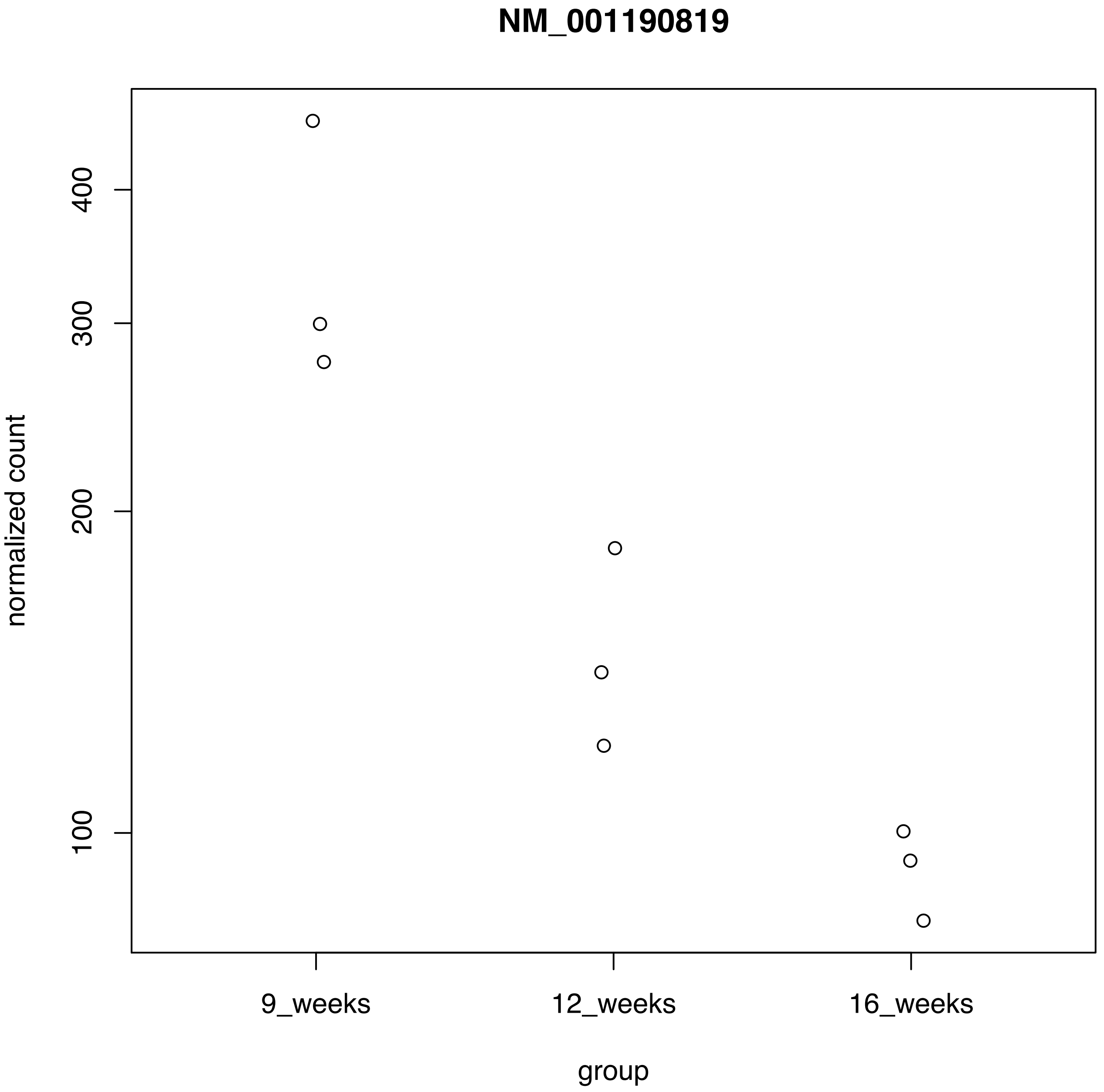
NM_001190819
normalized count
100
200
300
400
9_weeks
12_weeks
16_weeks
group

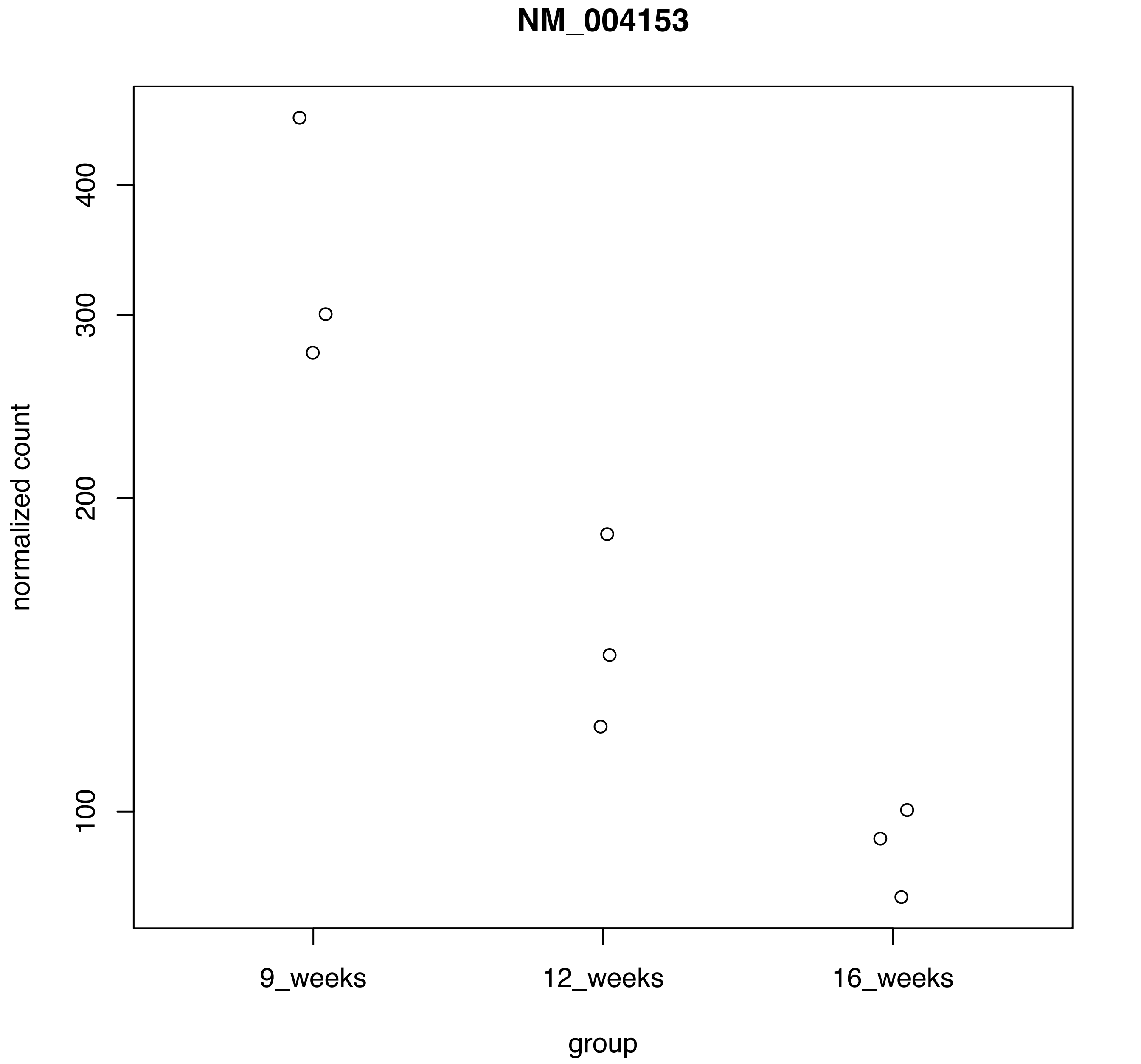
NM_004153
normalized count
400
300
200
100
9_weeks
12_weeks
16_weeks
group

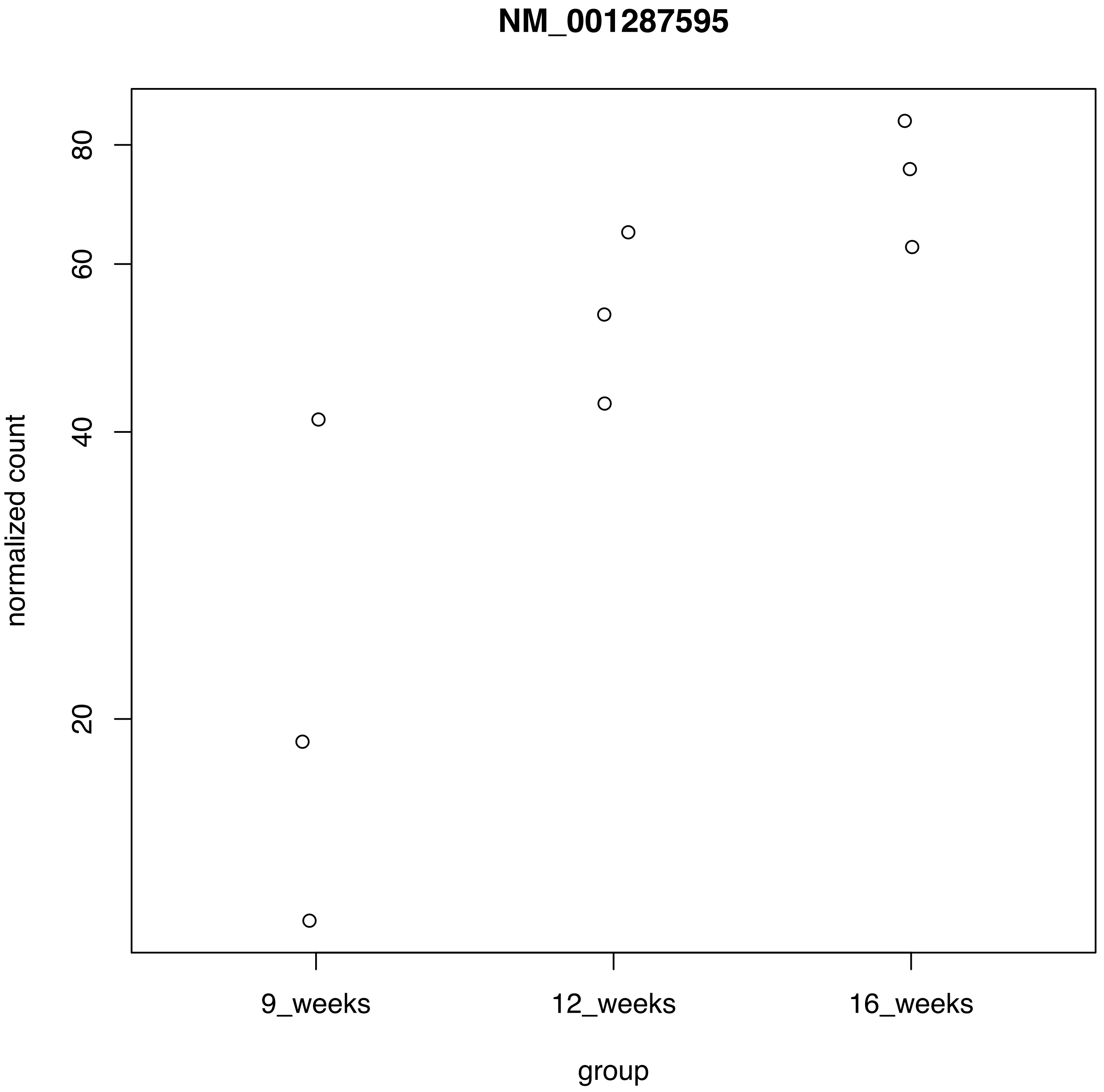
NM_001287595
normalized count
20
40
60
80
9_weeks
12_weeks
16_weeks
group

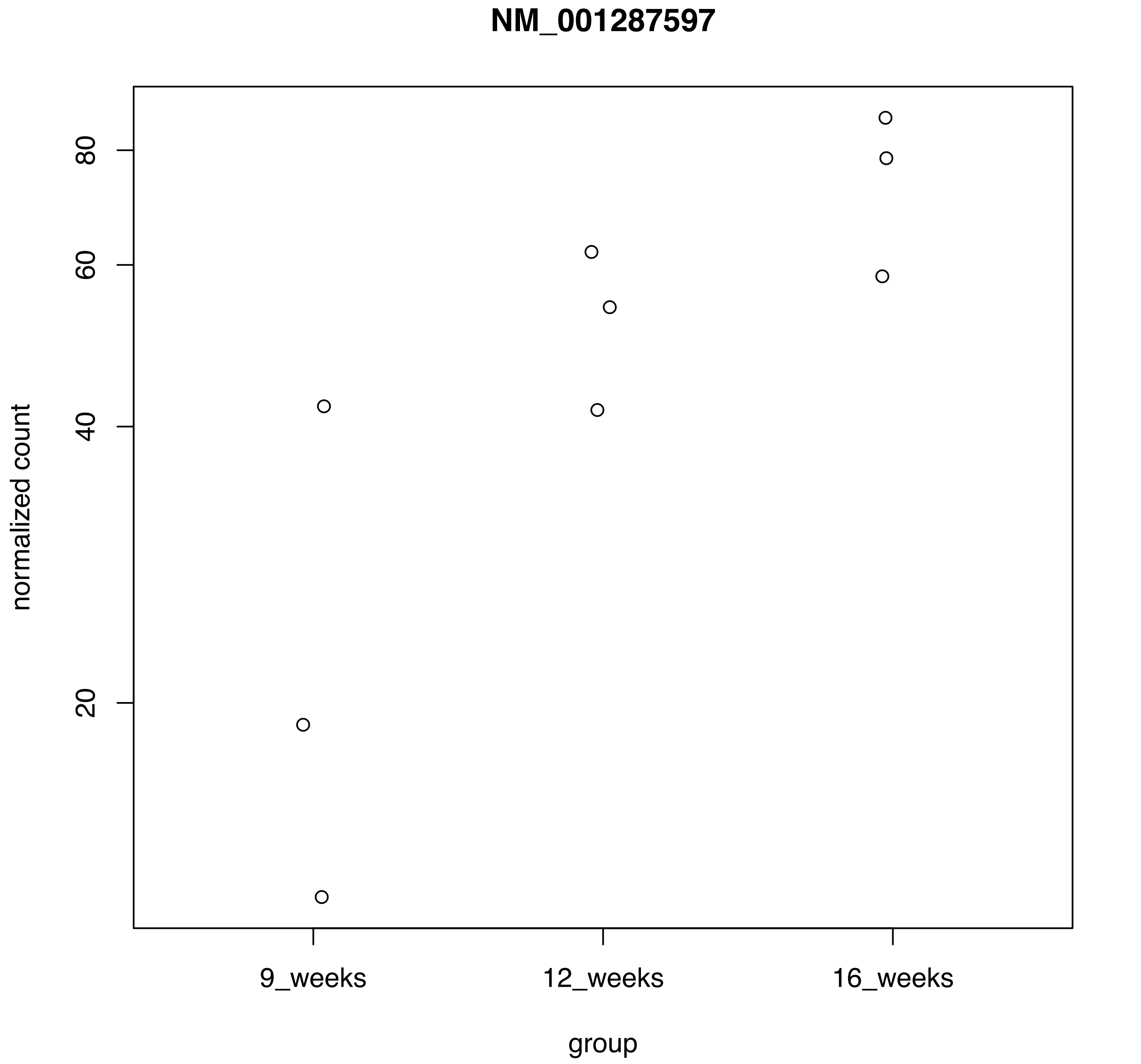

NM_001287597
normalized count
80
60
40
20
9_weeks
12_weeks
16_weeks
group

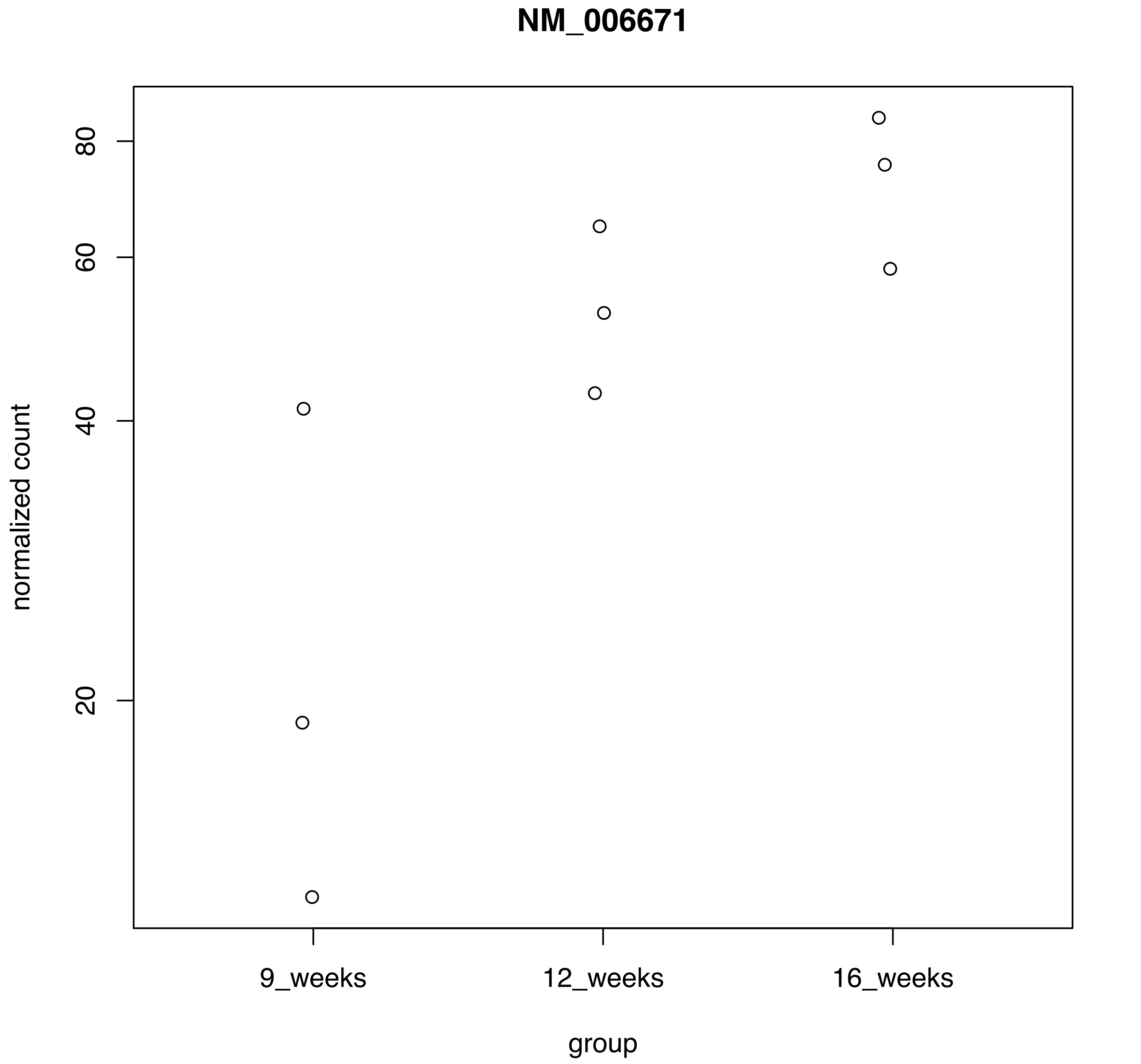
NM_006671
normalized count
80
60
40
20
9_weeks
12_weeks
16_weeks
group

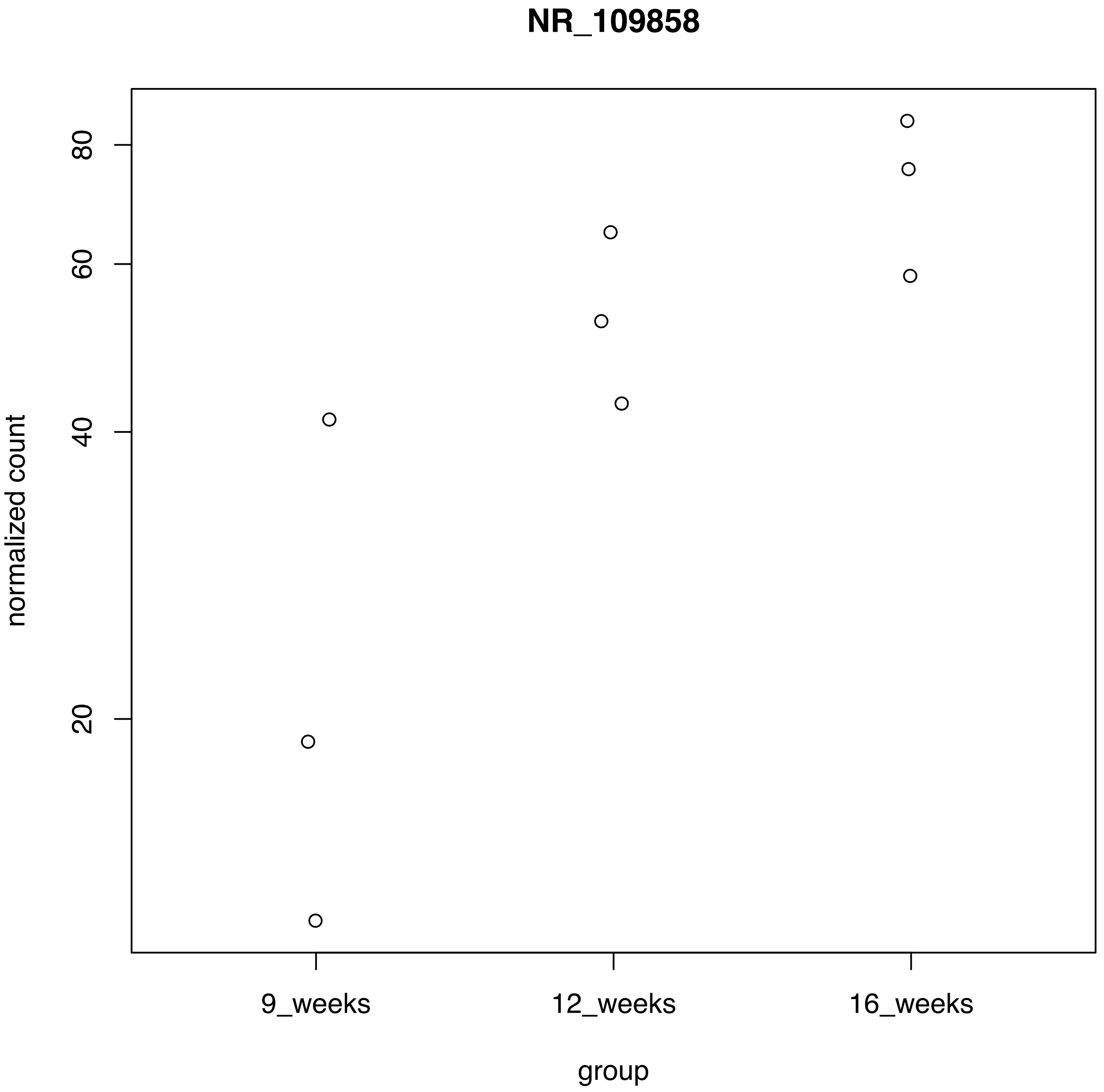
NR_109858
normalized count
80
60
40
20
9_weeks
12_weeks
16_weeks
group

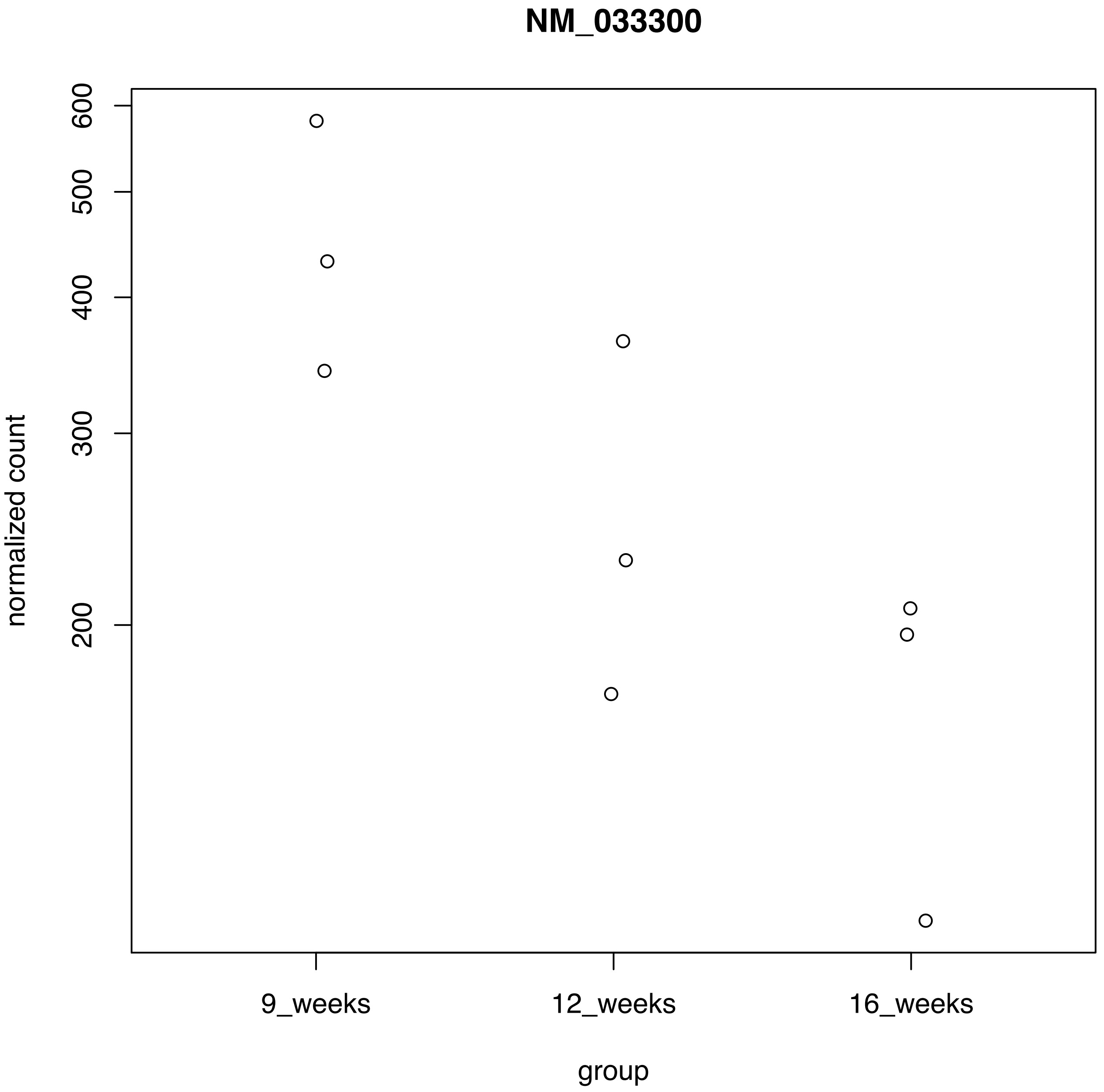
NM_033300
normalized count
600
500
400
300
200
9_weeks
12_weeks
16_weeks
group

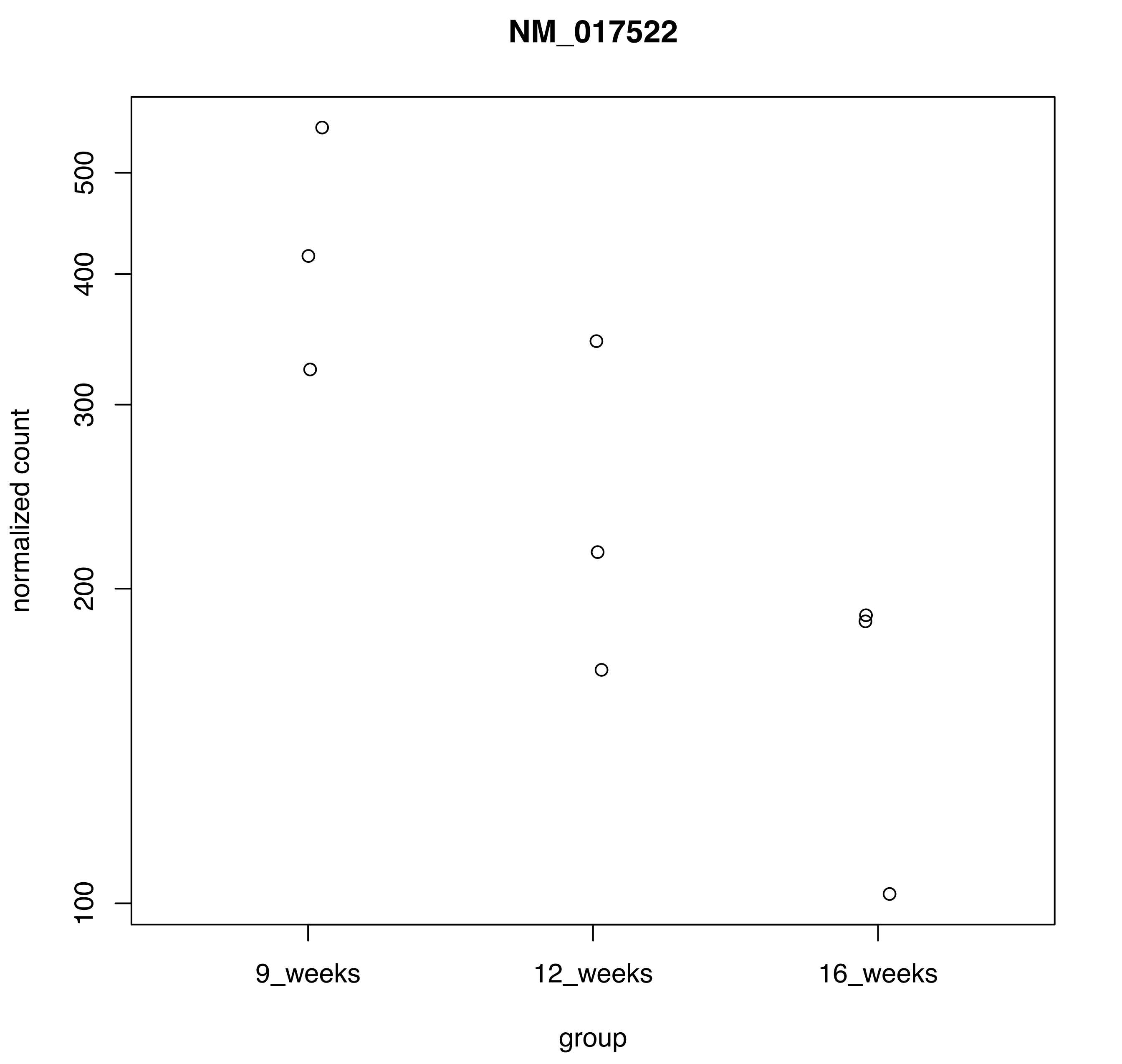
NM_017522
normalized count
100
200
300
400
500
9_weeks
12_weeks
16_weeks
group

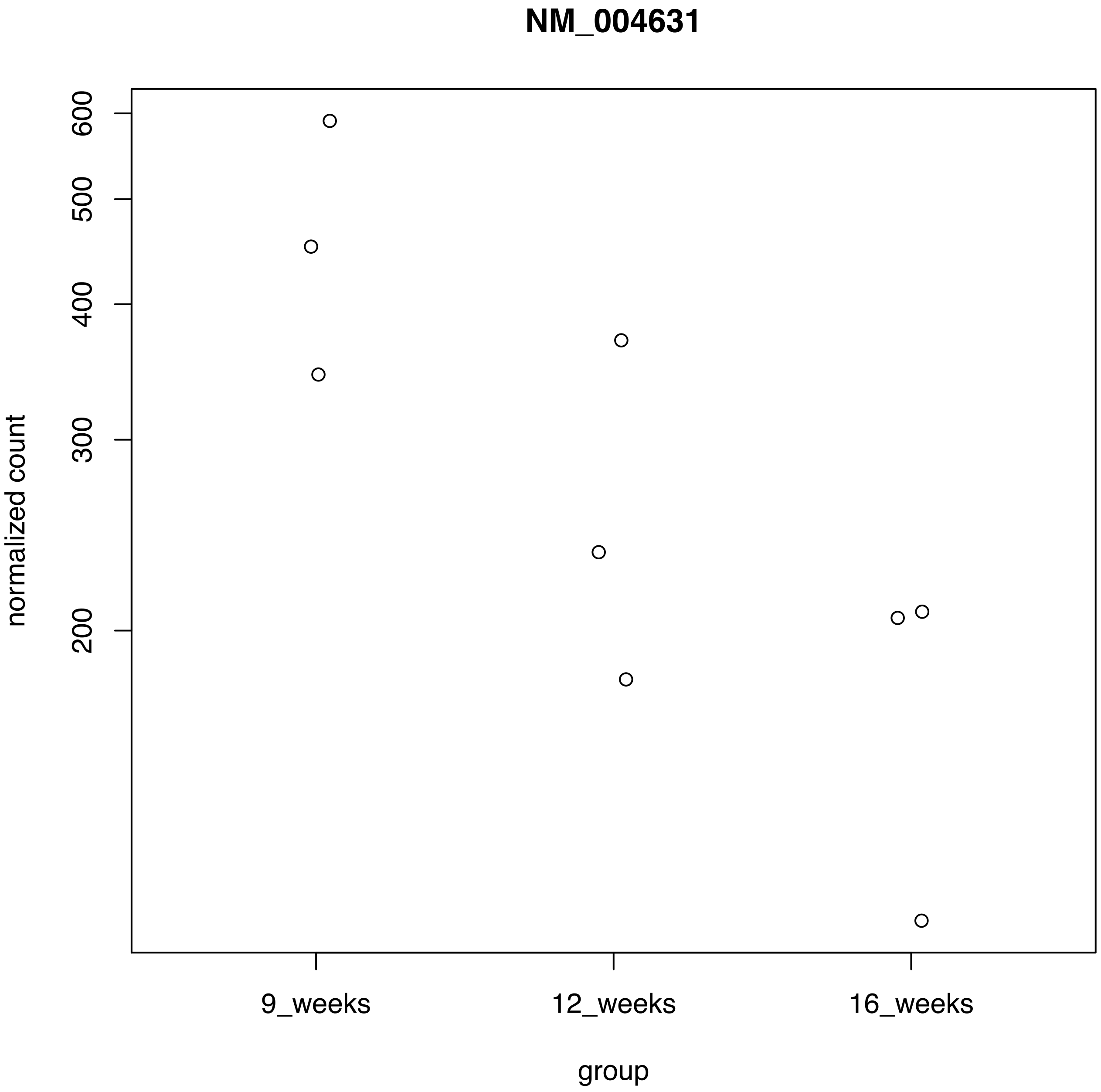
NM_004631
normalized count
600
500
400
300
200
9_weeks
12_weeks
16_weeks
group

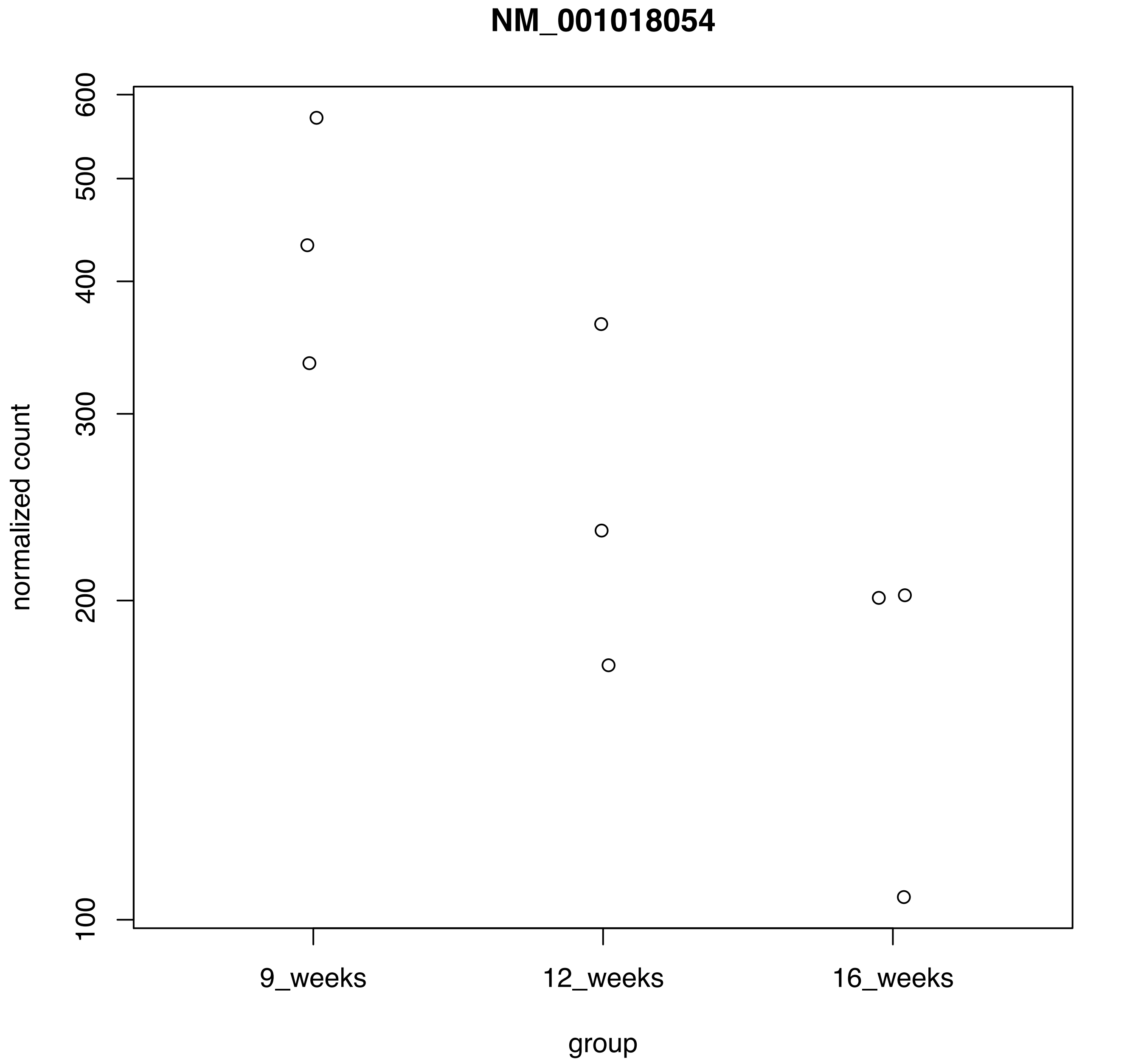
NM_001018054
normalized count
600
500
400
300
200
100
9_weeks
12_weeks
16_weeks
group

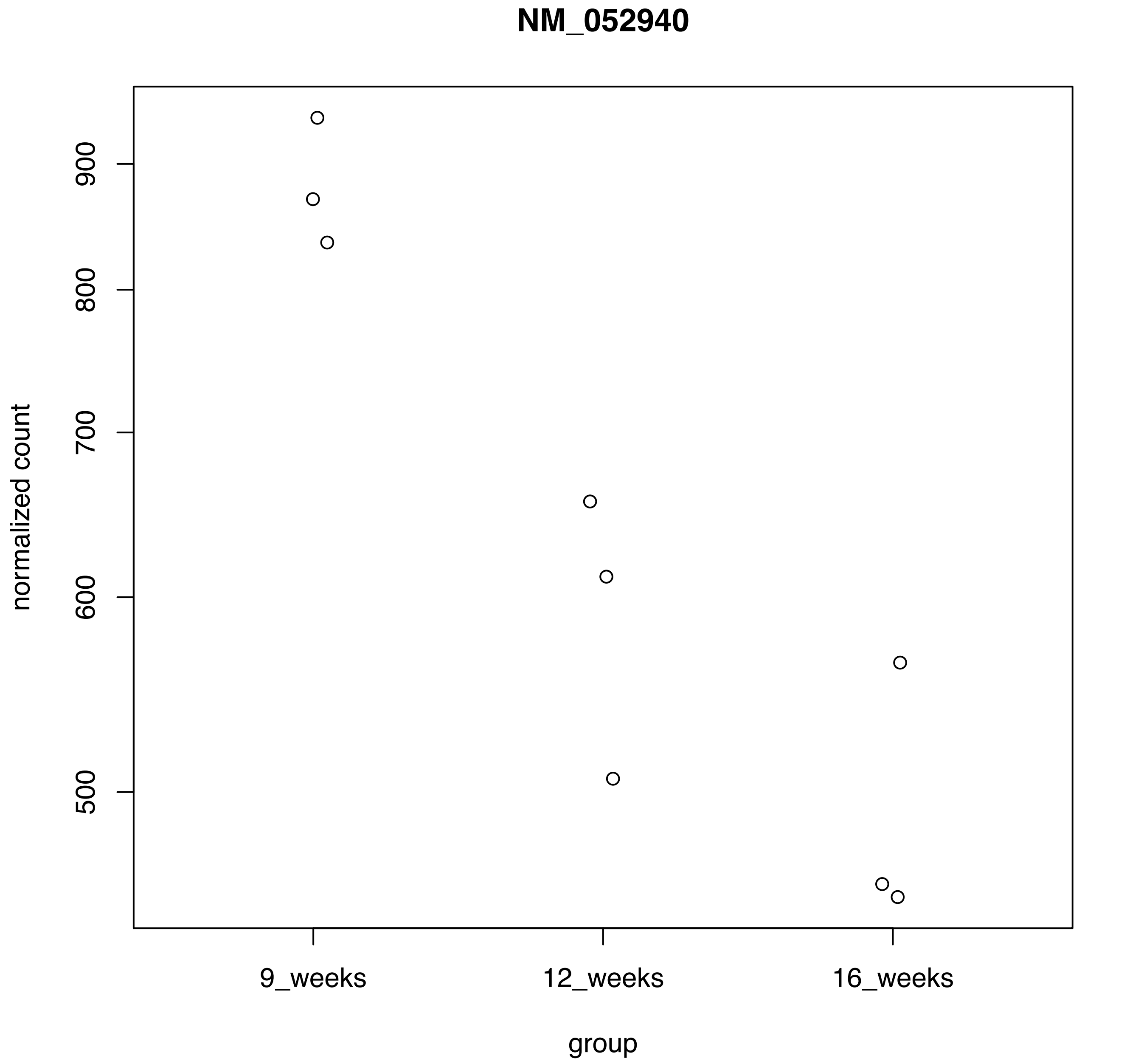
NM_052940
normalized count
900
800
700
600
500
9_weeks
12_weeks
16_weeks
group

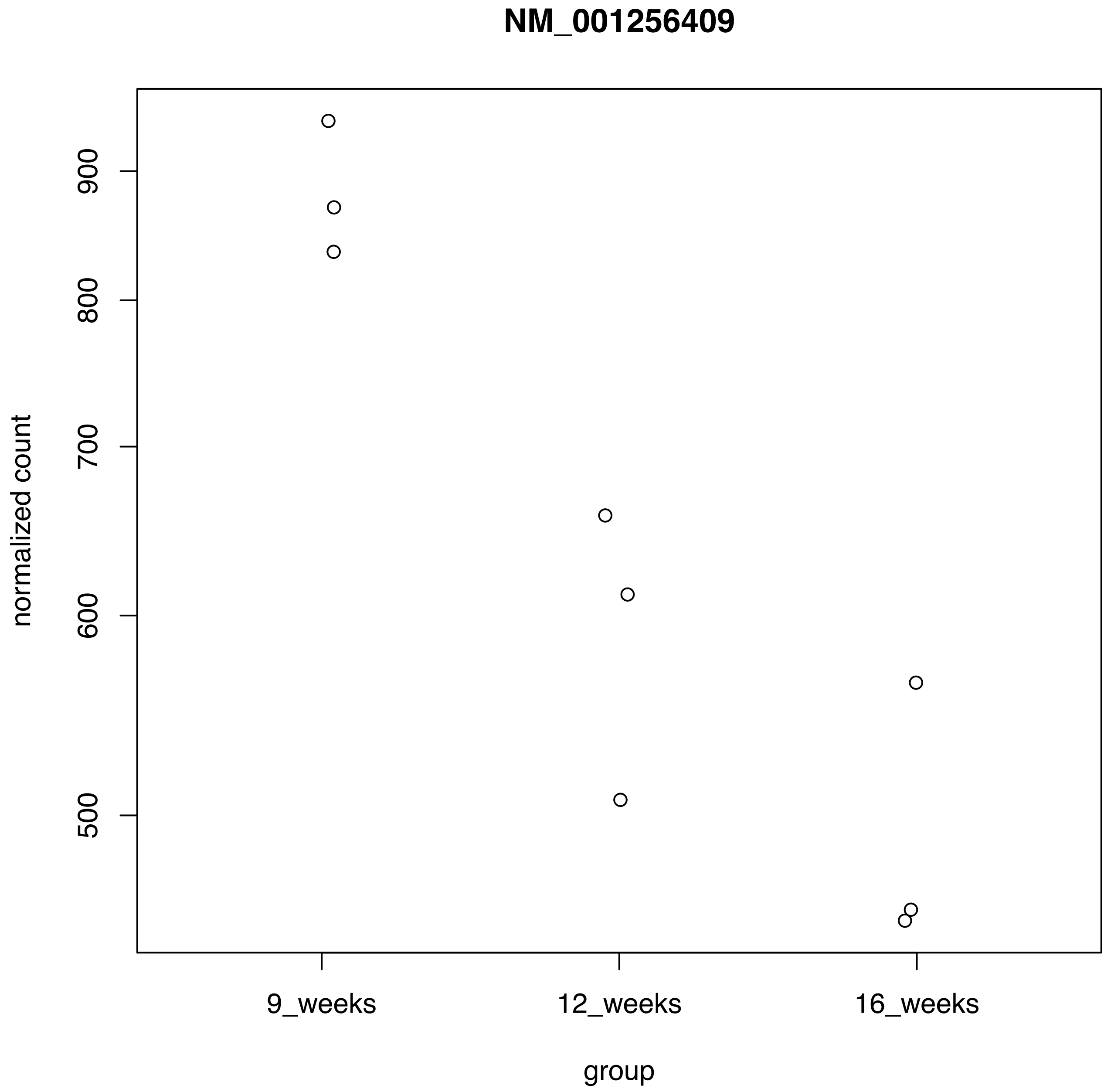

NM_001256409
normalized count
900
800
700
600
500
9_weeks
12_weeks
16_weeks
group

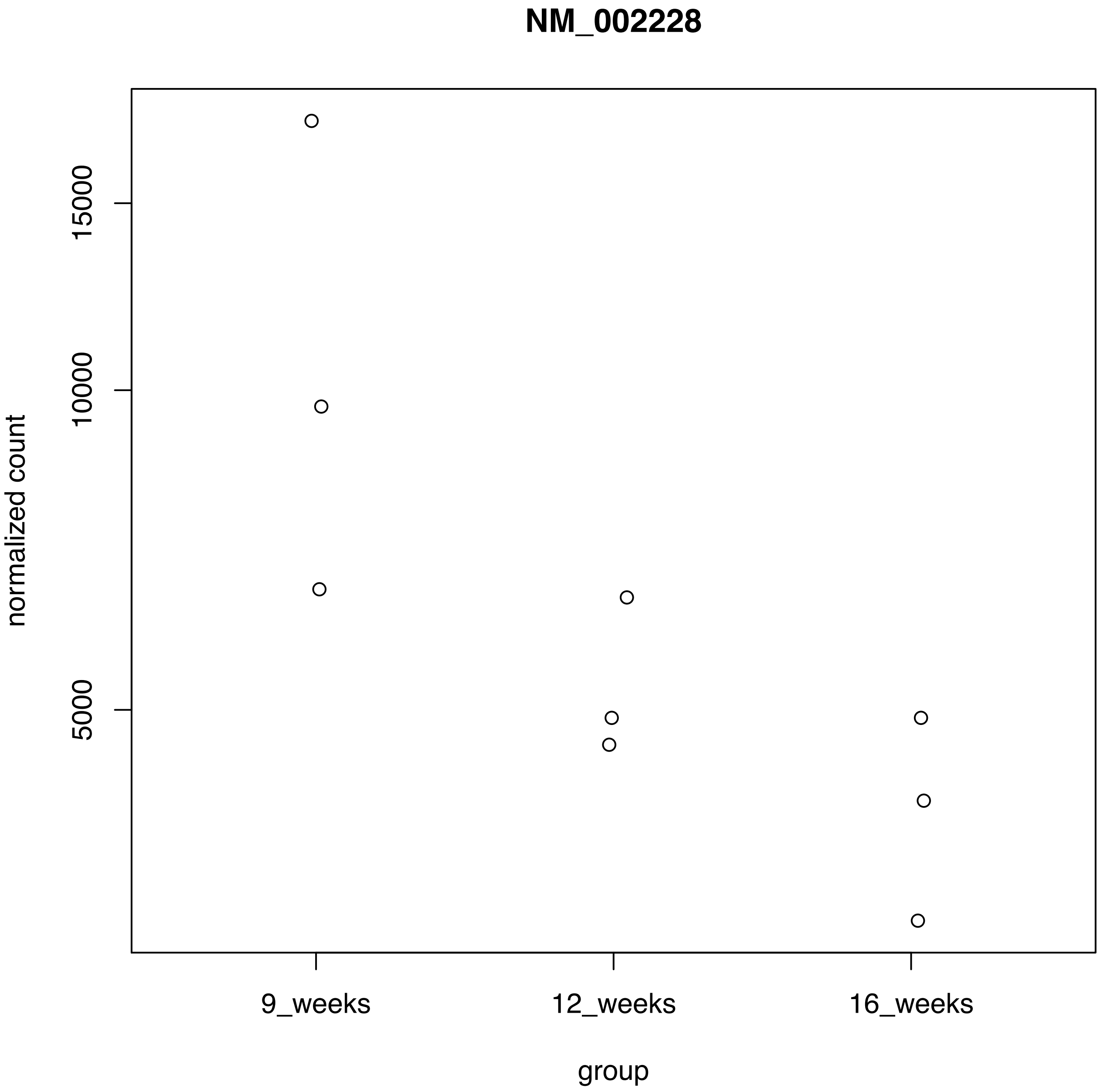
NM_002228
normalized count
15000
10000
5000
9_weeks
12_weeks
16_weeks
group

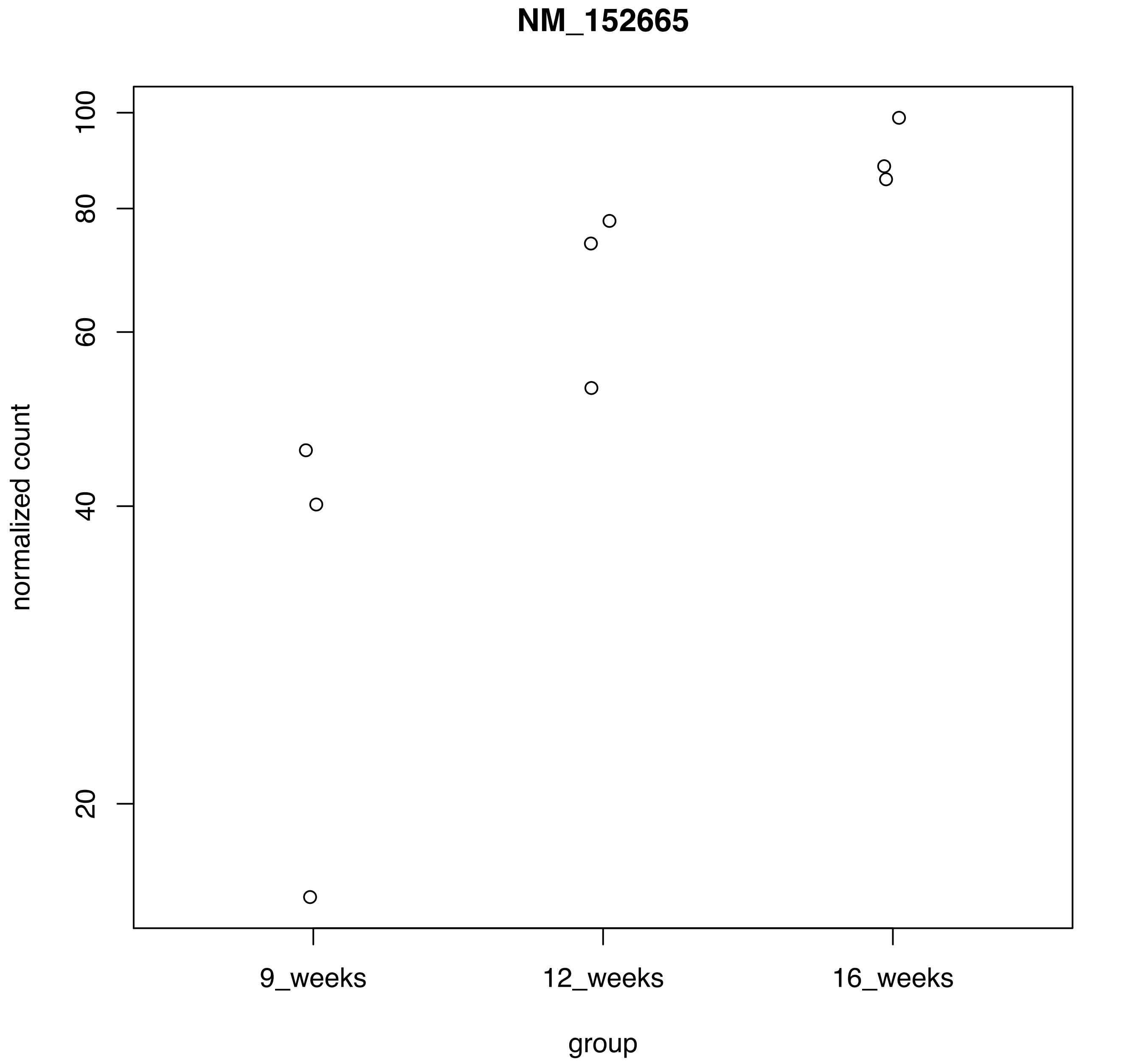

NM_152665
normalized count
100
80
60
40
20
9_weeks
12_weeks
16_weeks
group

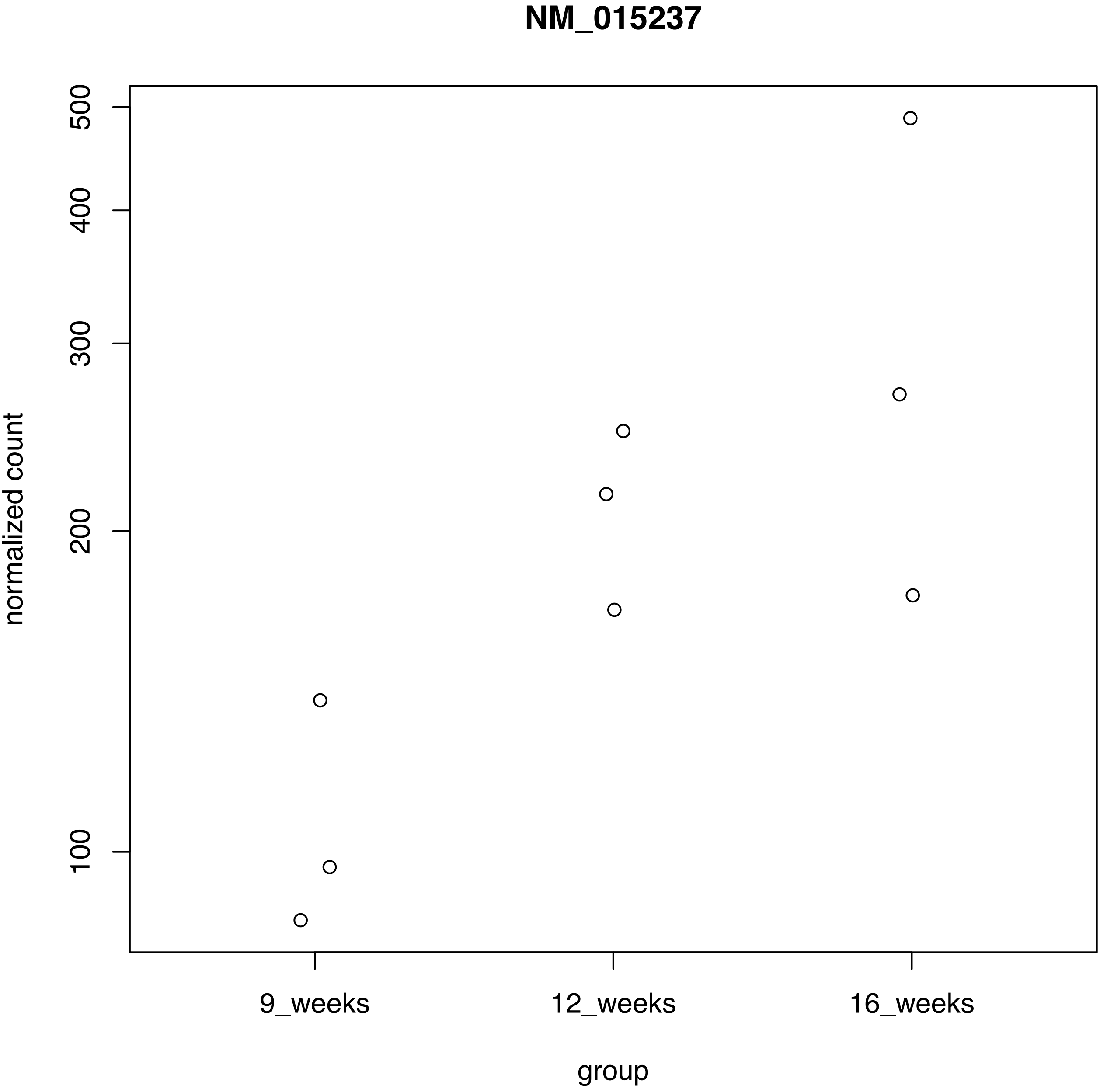
NM_015237
normalized count
100
200
300
400
500
9_weeks
12_weeks
16_weeks
group

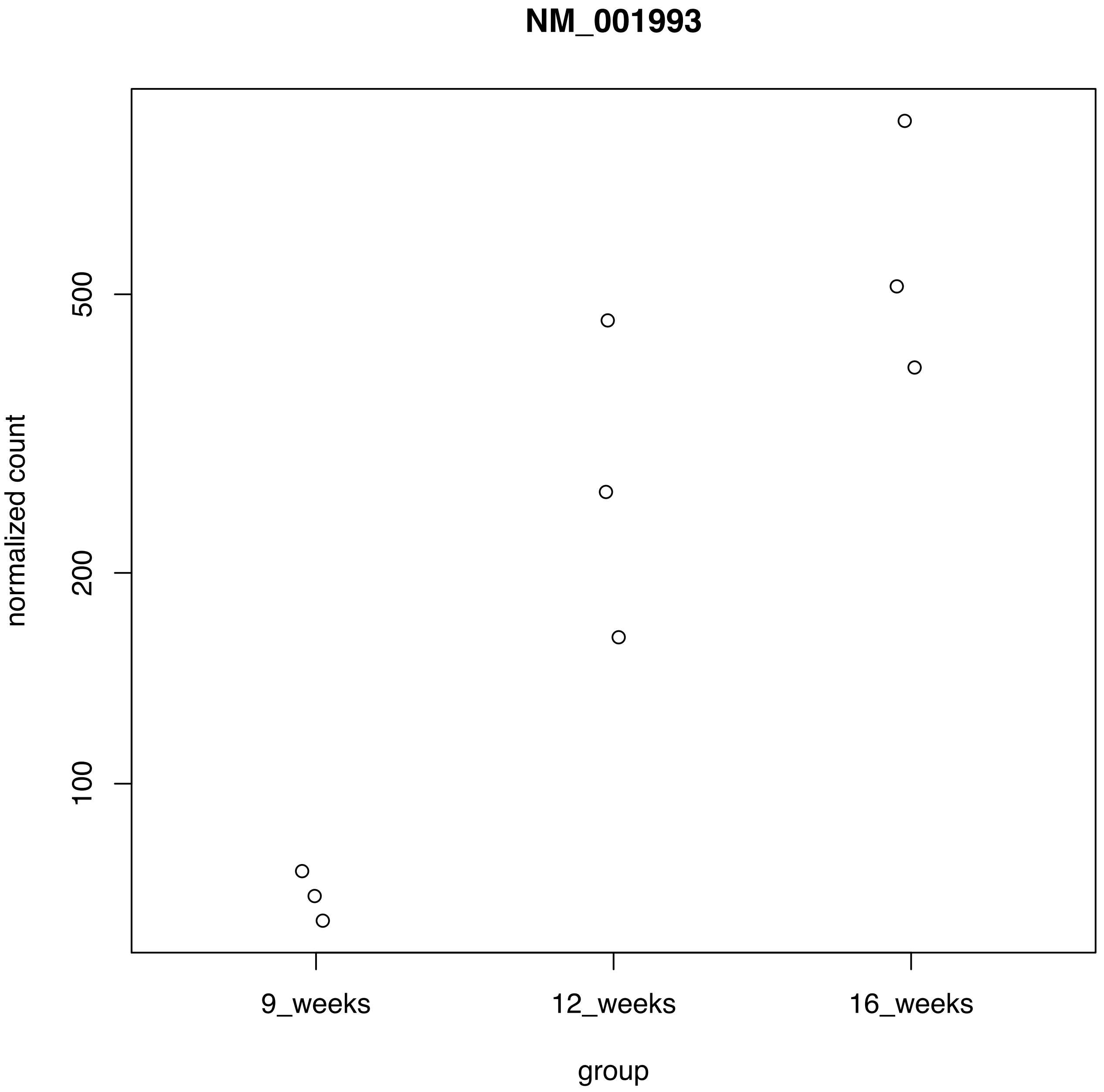
NM_001993
normalized count
500
200
100
9_weeks
12_weeks
16_weeks
group

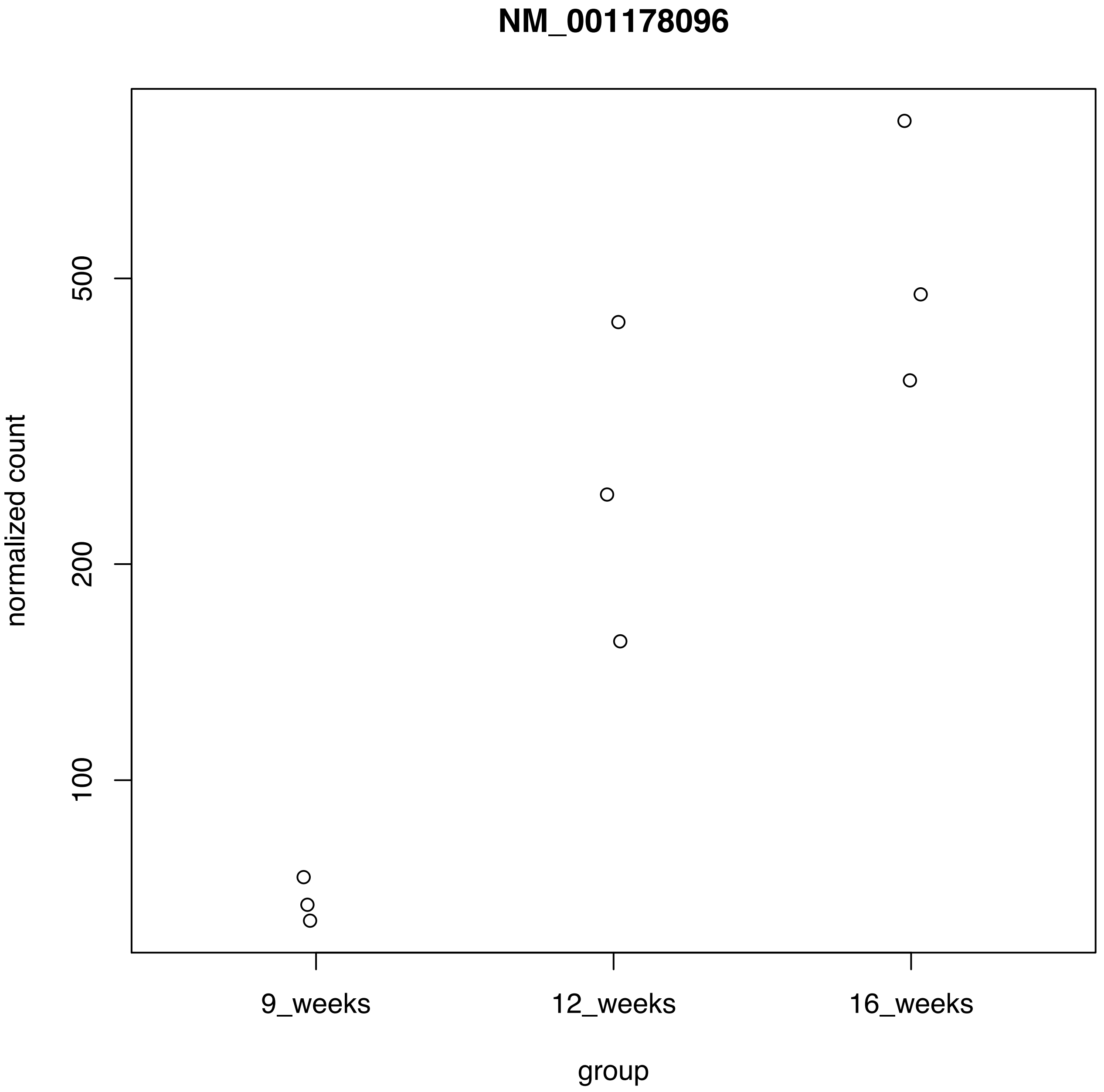

NM_001178096
normalized count
500
200
100
9_weeks
12_weeks
16_weeks
group

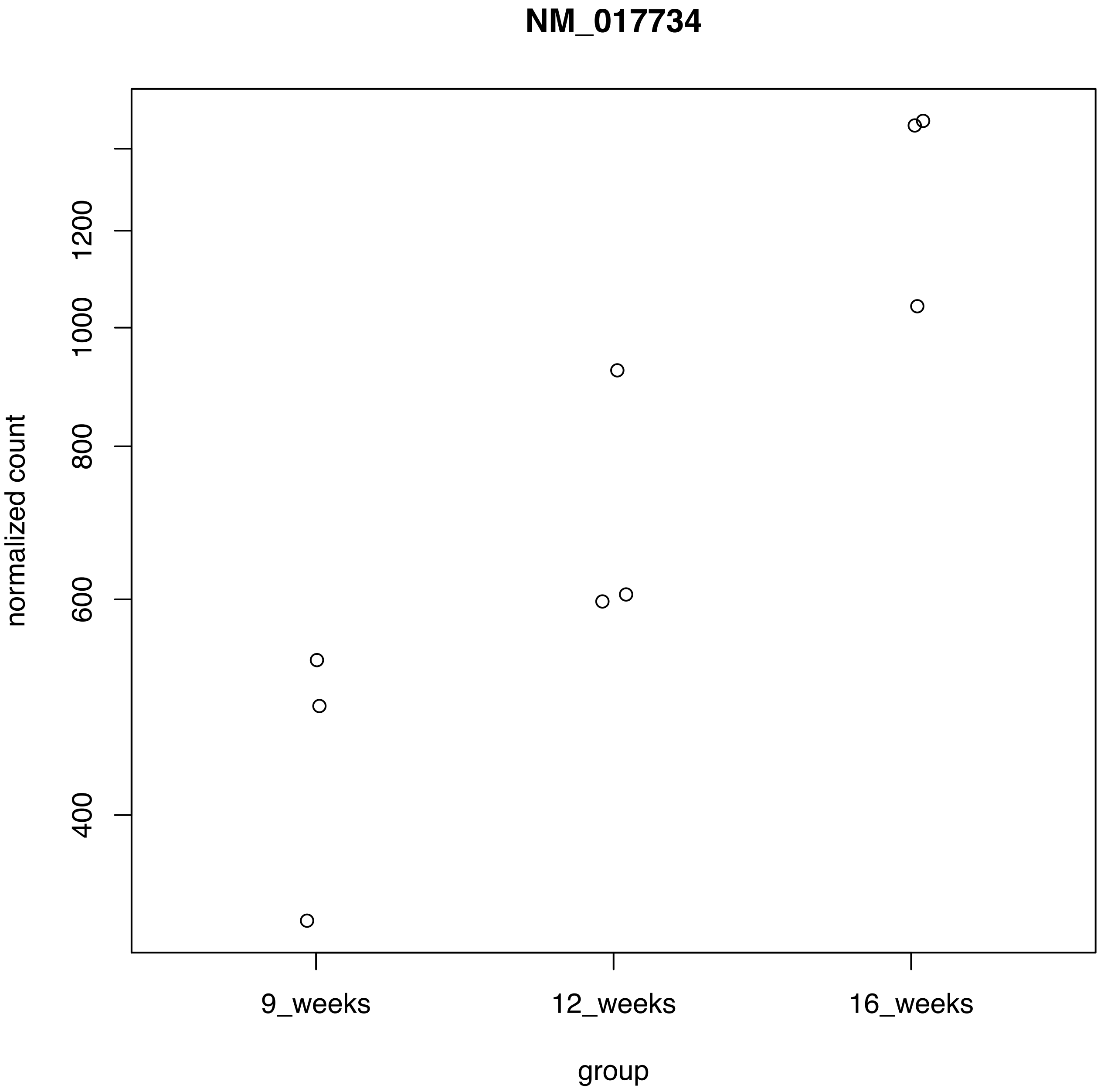
NM_017734
normalized count
400
600
800
1000
1200
9_weeks
12_weeks
16_weeks
group

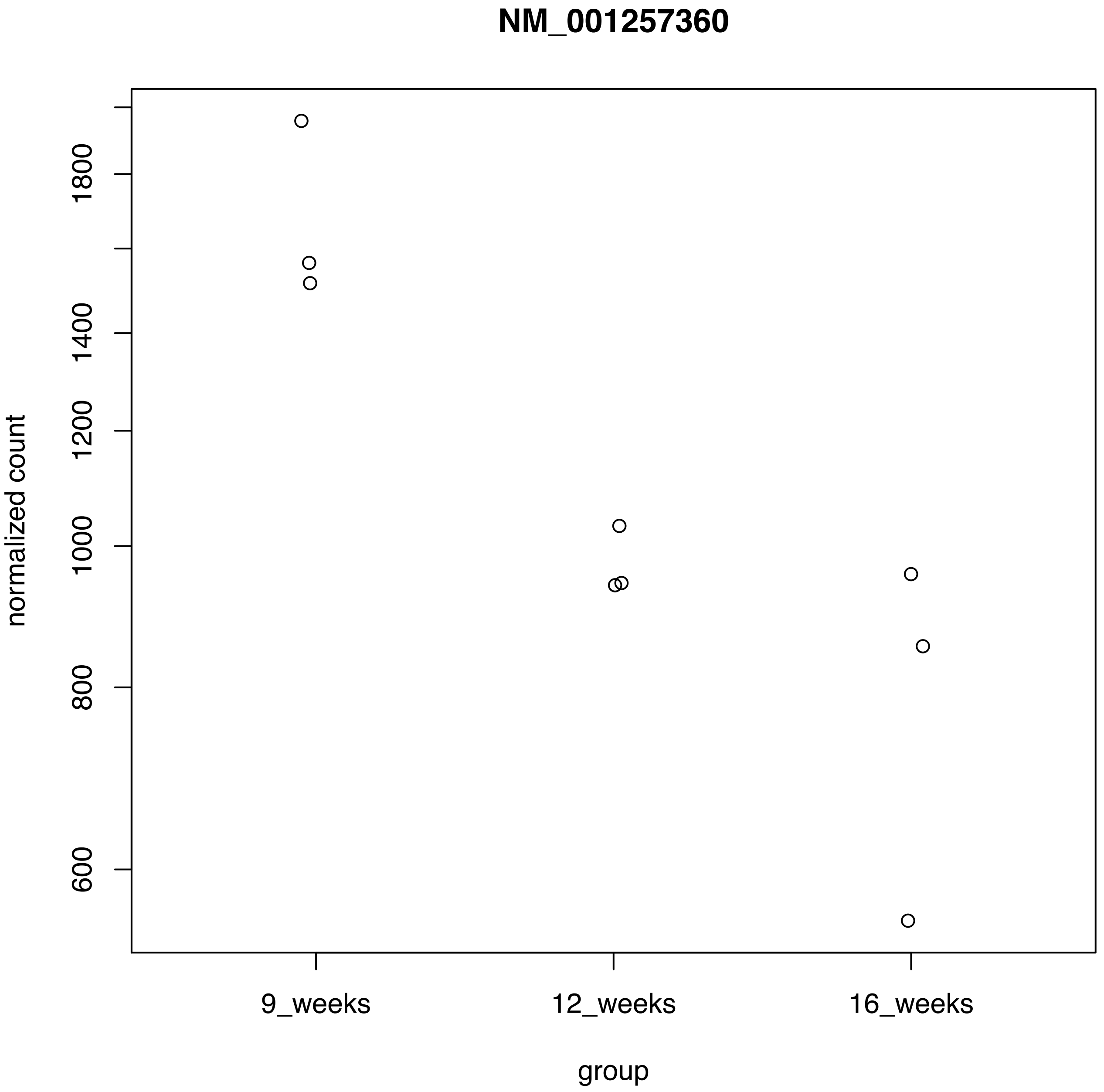
NM_001257360
normalized count
1800
1400
1200
1000
800
600
9_weeks
12_weeks
16_weeks
group

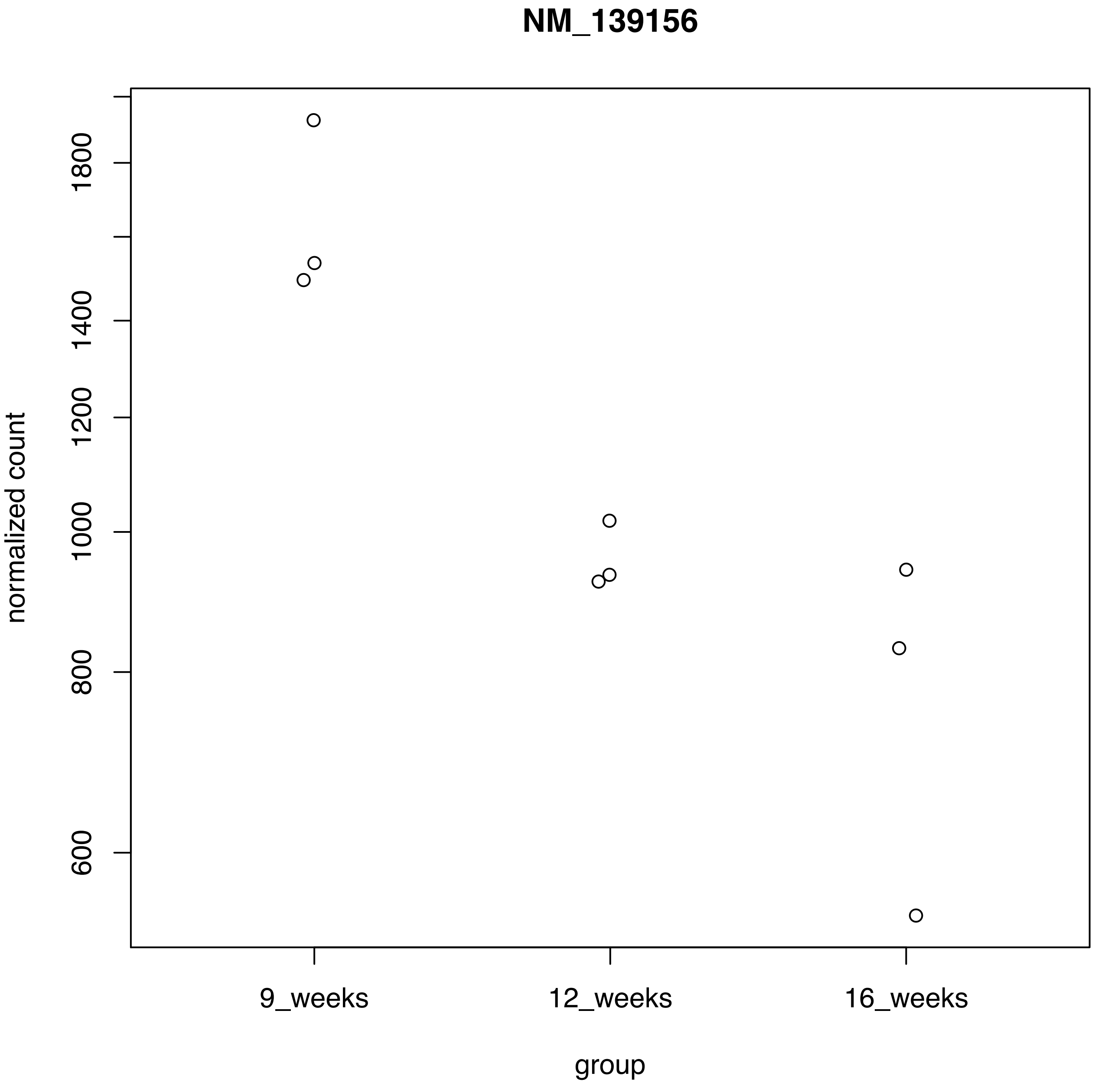
NM_139156
normalized count
1800
1400
1200
1000
800
600
9_weeks
12_weeks
16_weeks
group

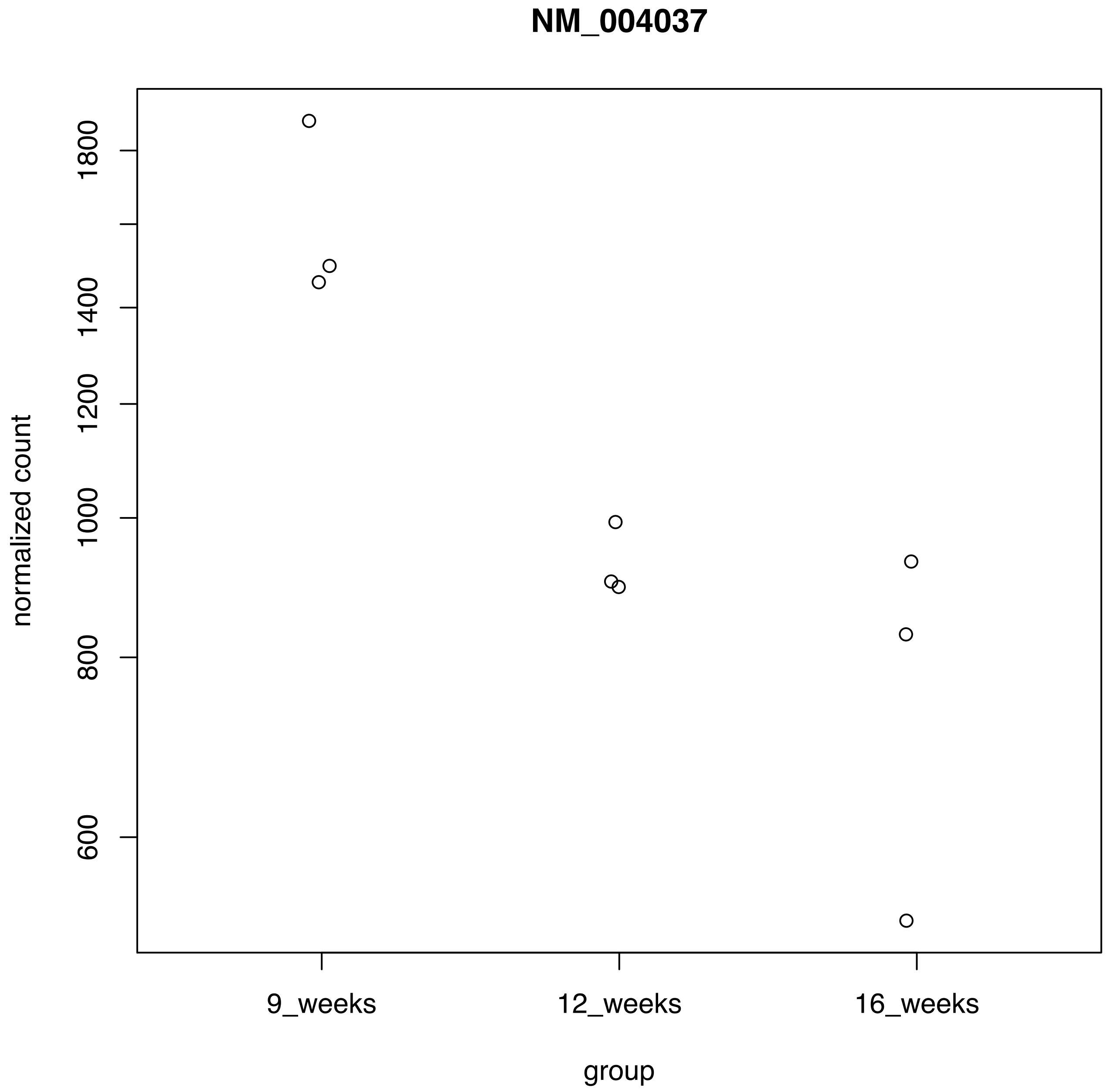

NM_004037
normalized count
1800
1400
1200
1000
800
600
9_weeks
12_weeks
16_weeks
group

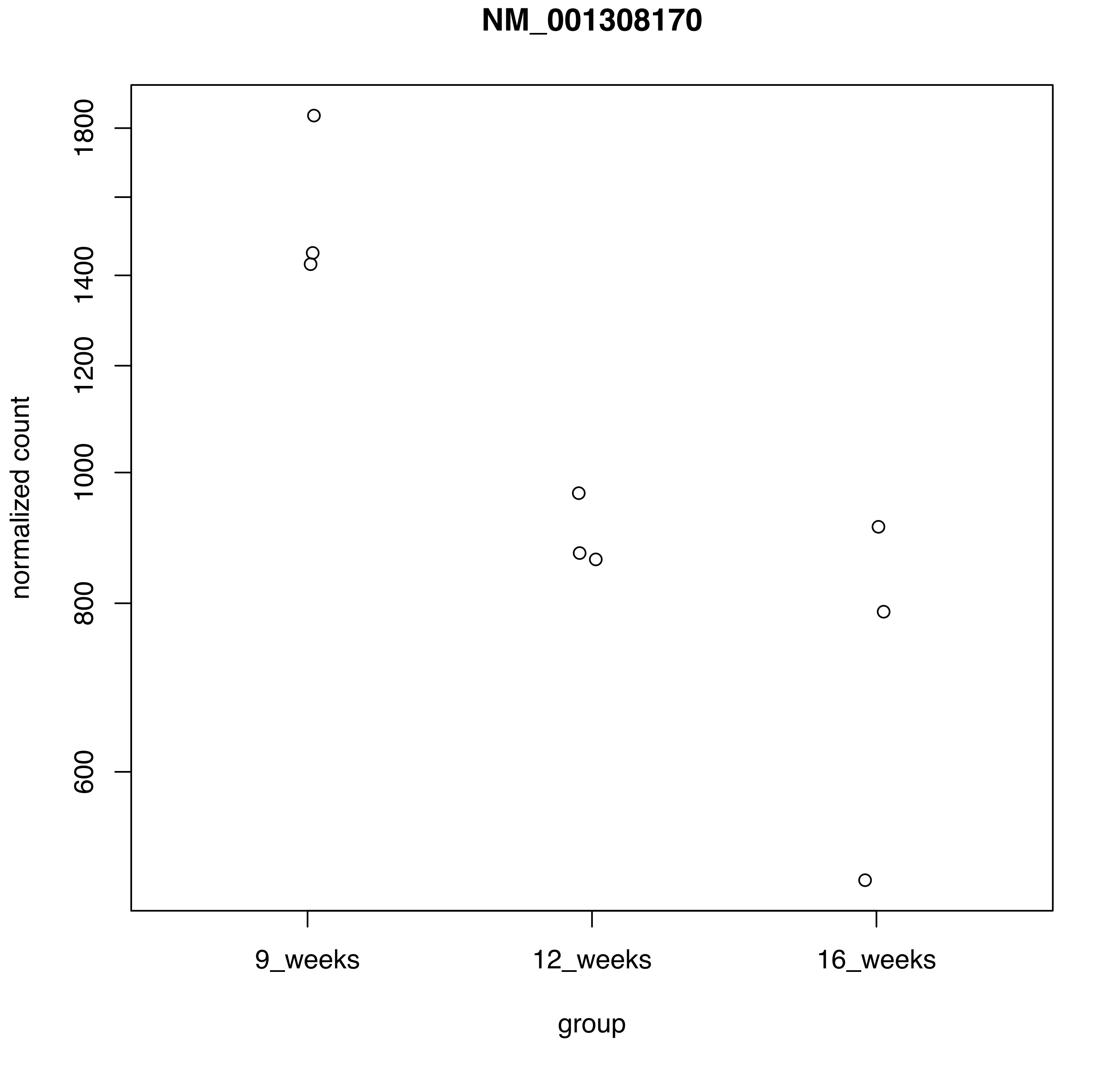

NM_001308170
normalized count
1800
1400
1200
1000
800
600
9_weeks
12_weeks
16_weeks
group

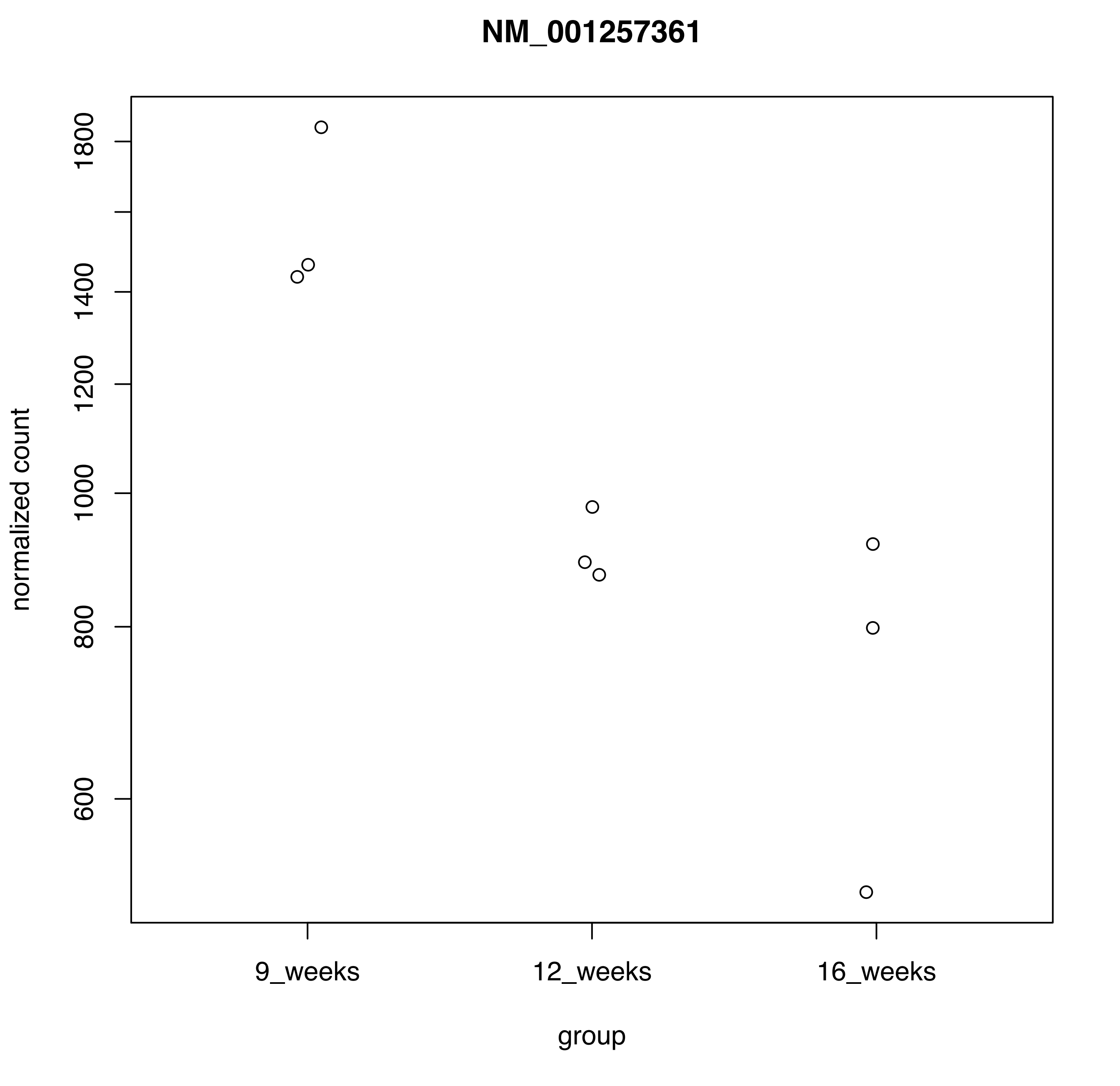
NM_001257361
normalized count
1800
1400
1200
1000
800
600
9_weeks
12_weeks
16_weeks
group

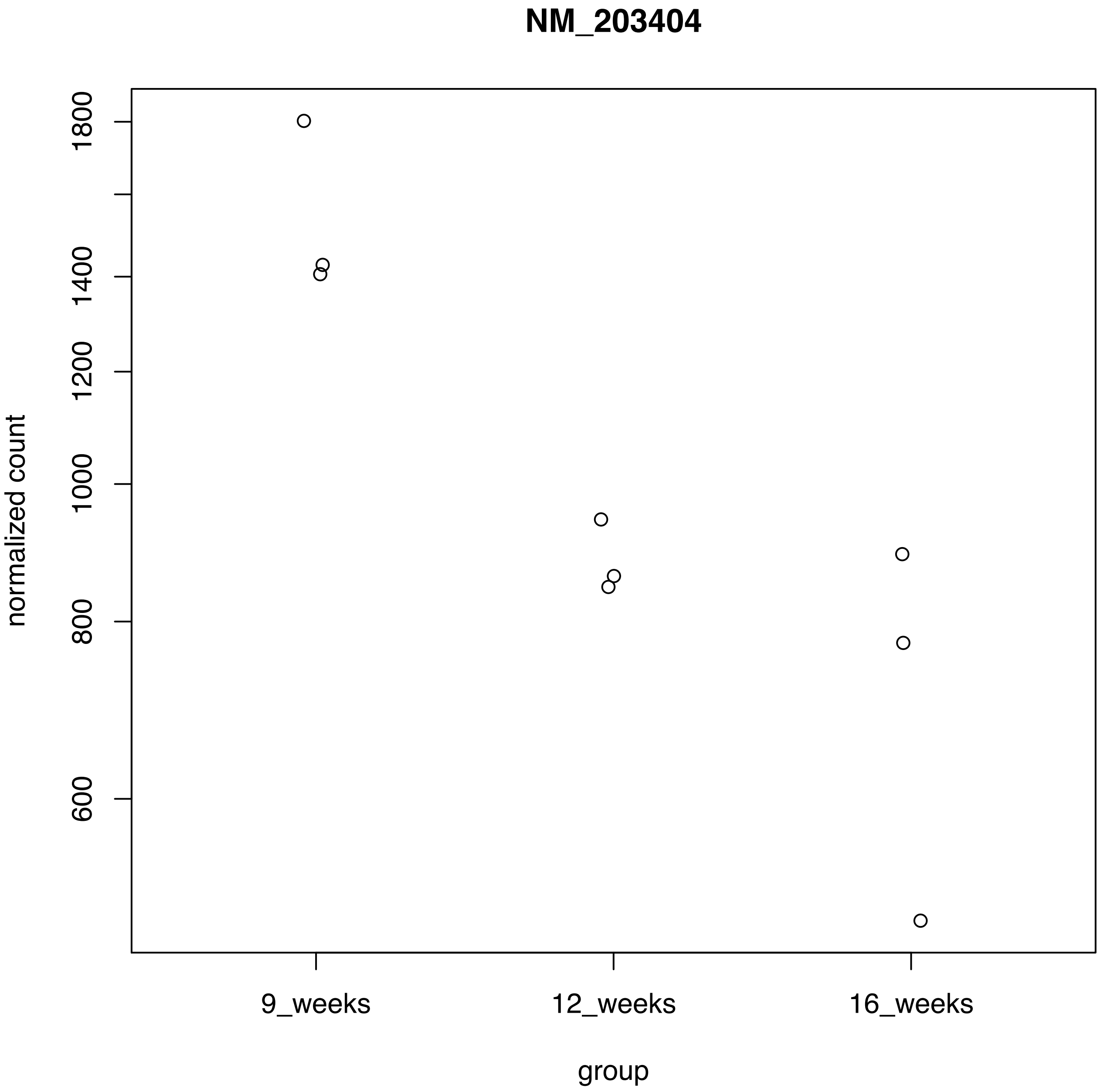

NM_203404
normalized count
1800
1400
1200
1000
800
600
9_weeks
12_weeks
16_weeks
group

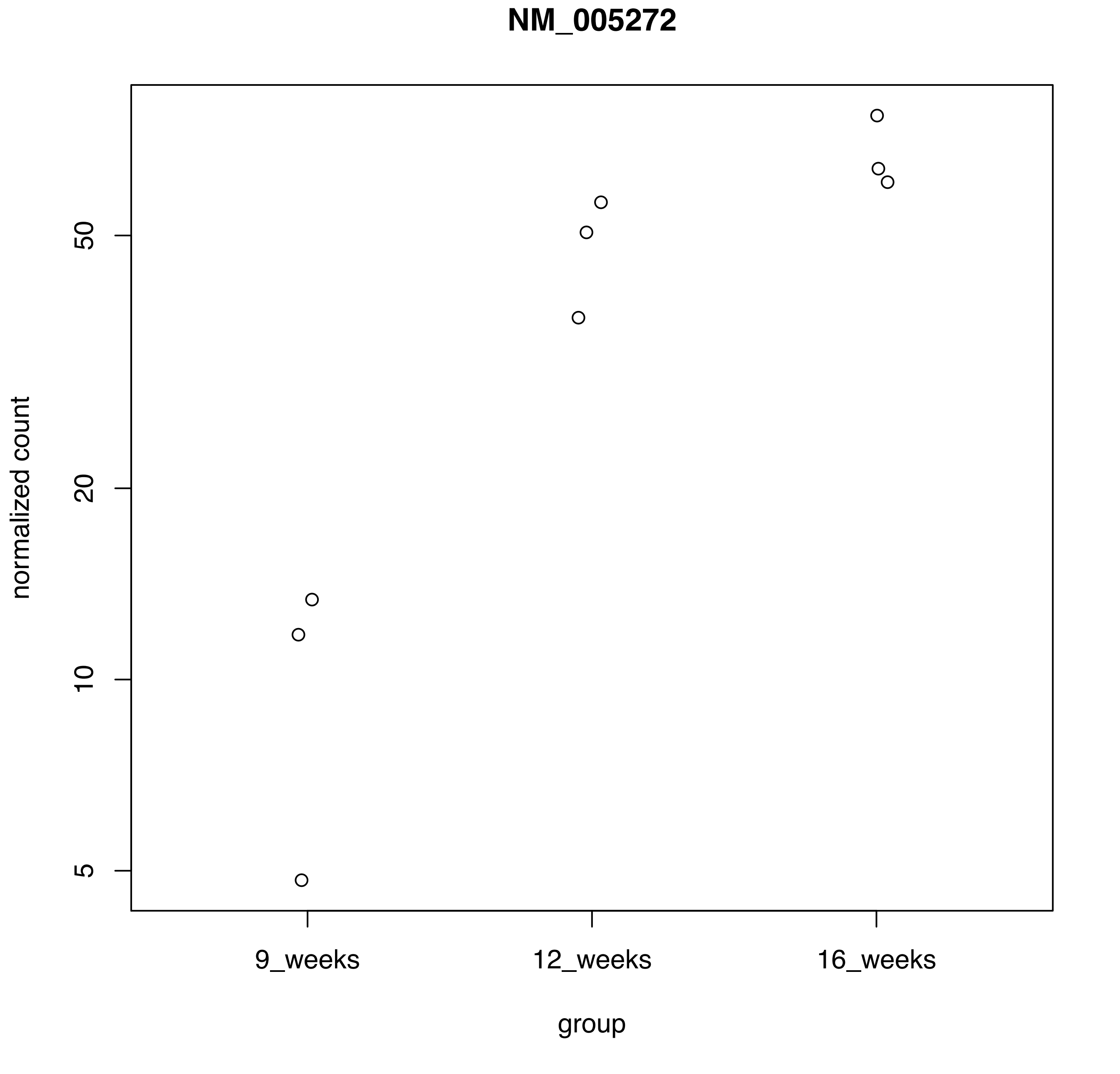

NM_005272
normalized count
5
10
20
50
9_weeks
12_weeks
16_weeks
group

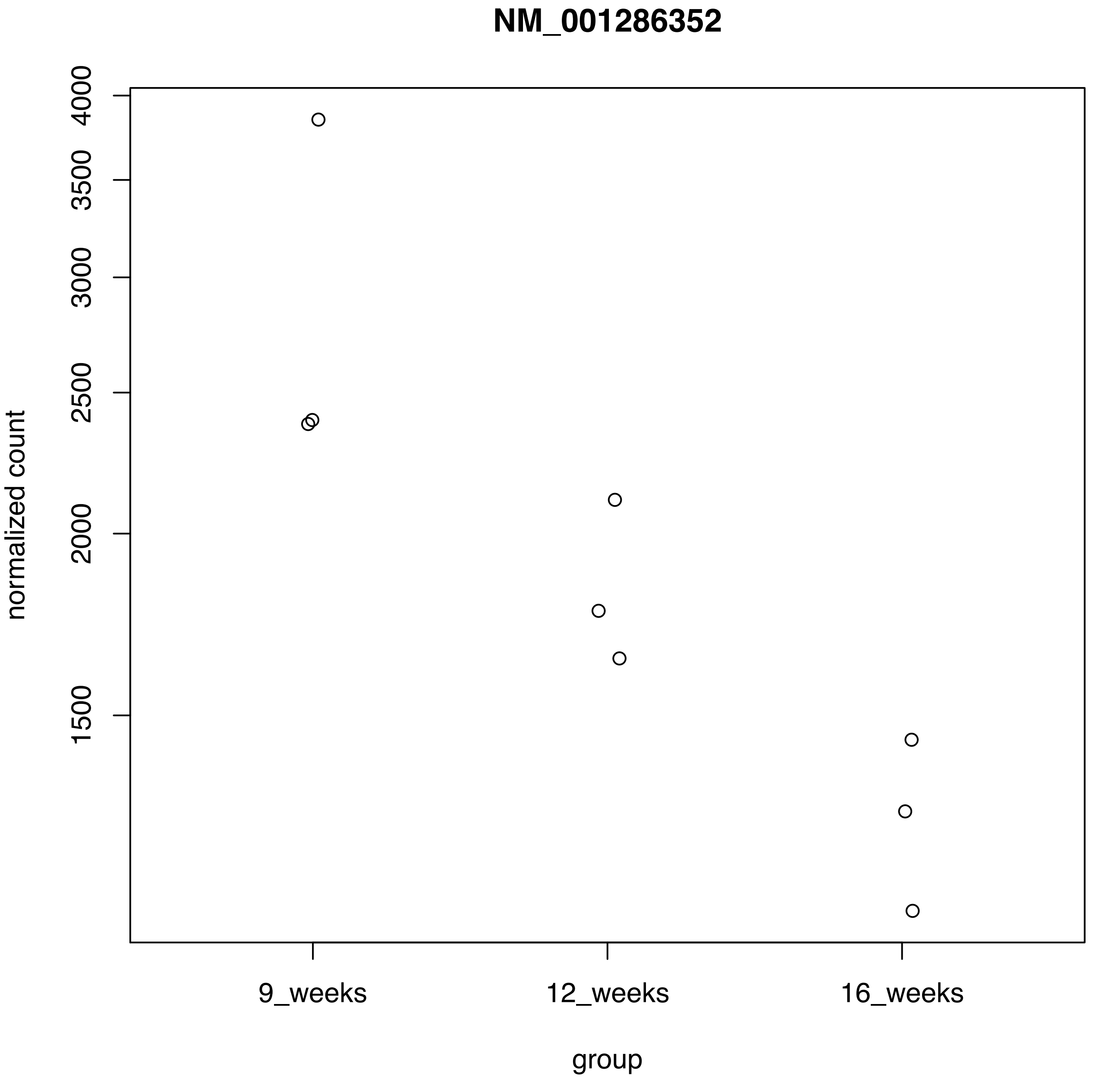
NM_001286352
normalized count
4000
3500
3000
2500
2000
1500
9_weeks
12_weeks
16_weeks
group

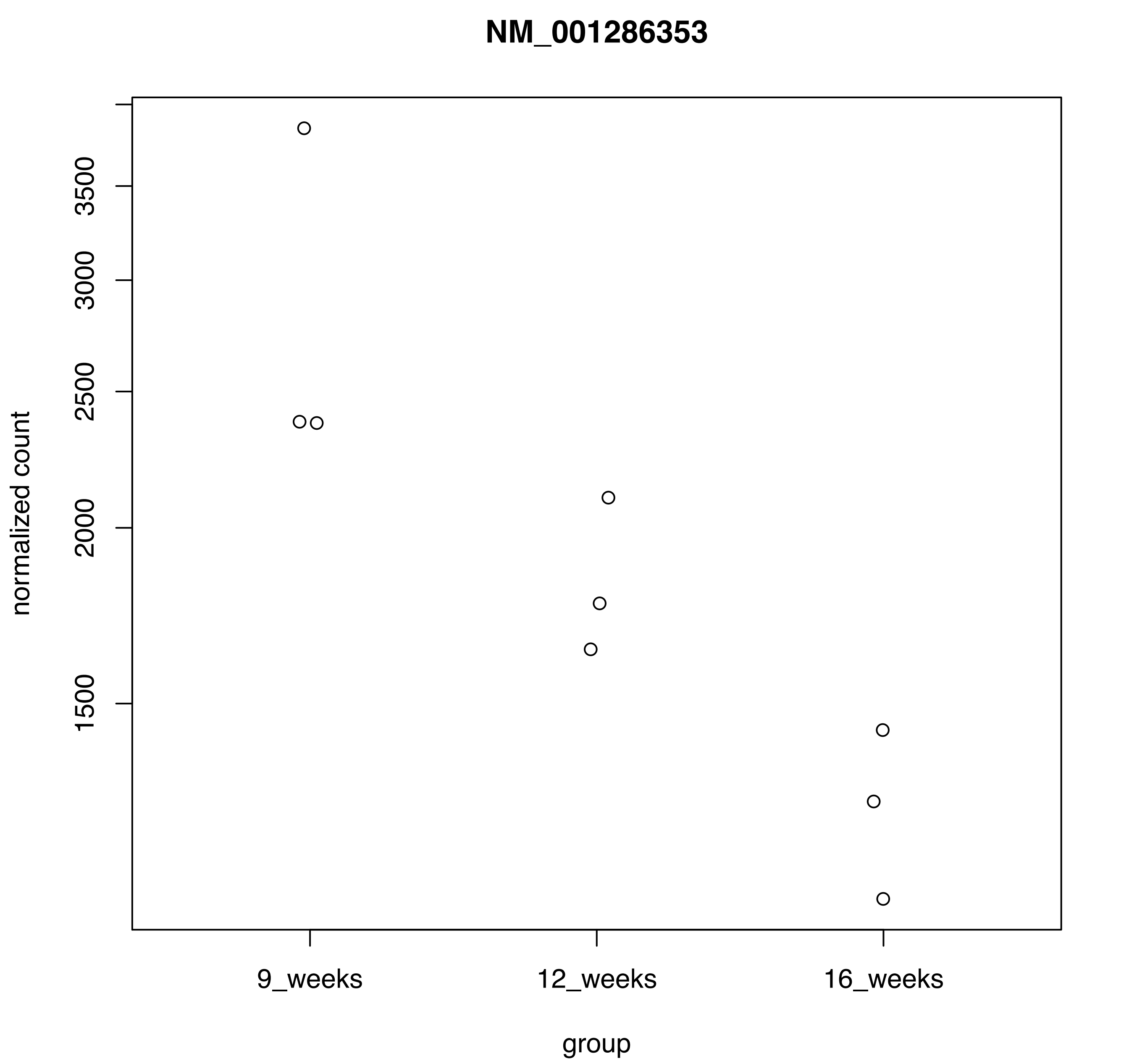

NM_001286353
normalized count
1500
2000
2500
3000
3500
9_weeks
12_weeks
16_weeks
group

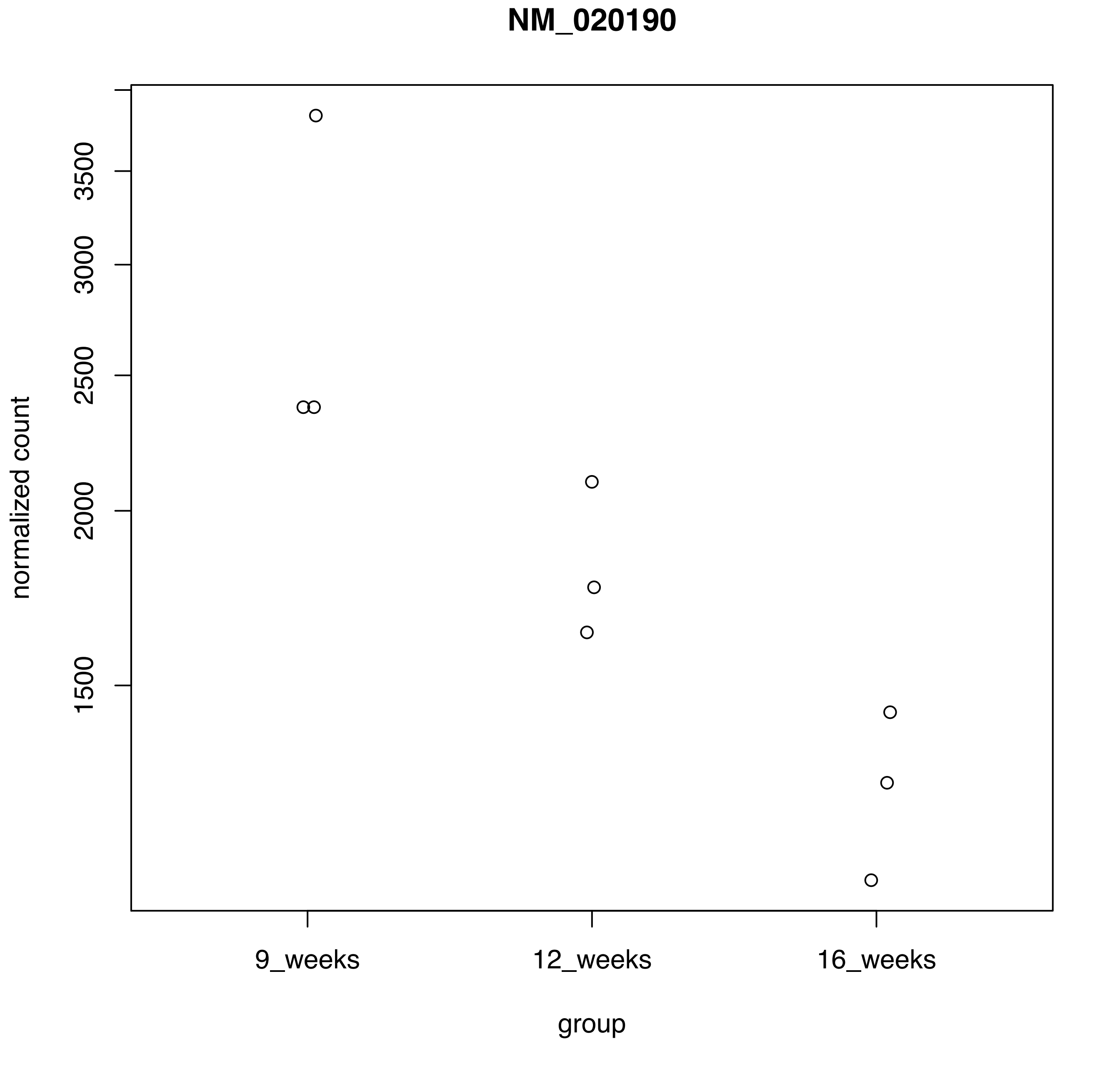

NM_020190
normalized count
1500
2000
2500
3000
3500
9_weeks
12_weeks
16_weeks
group

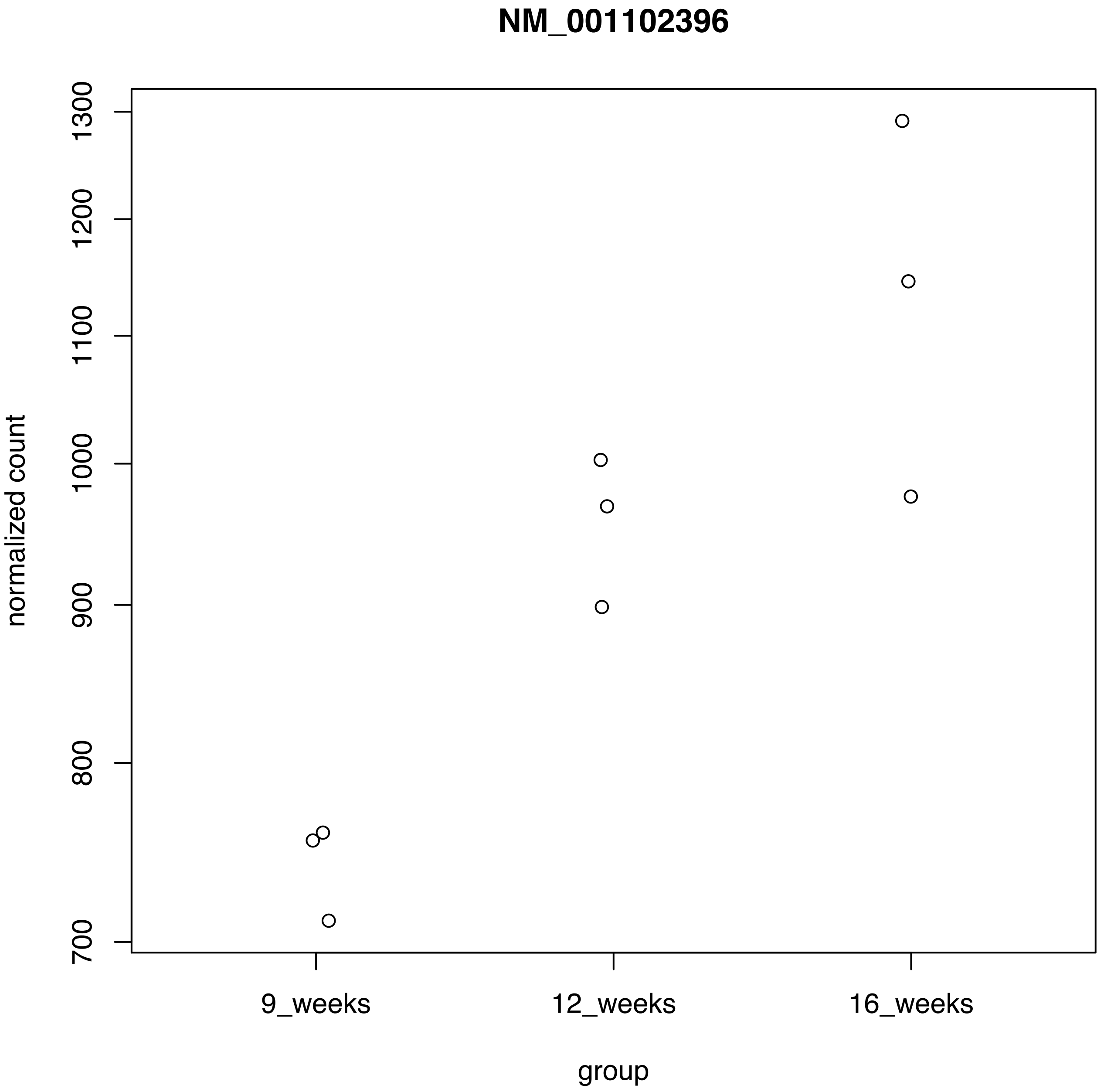
NM_001102396
normalized count
1300
1200
1100
1000
900
800
700
9_weeks
12_weeks
16_weeks
group

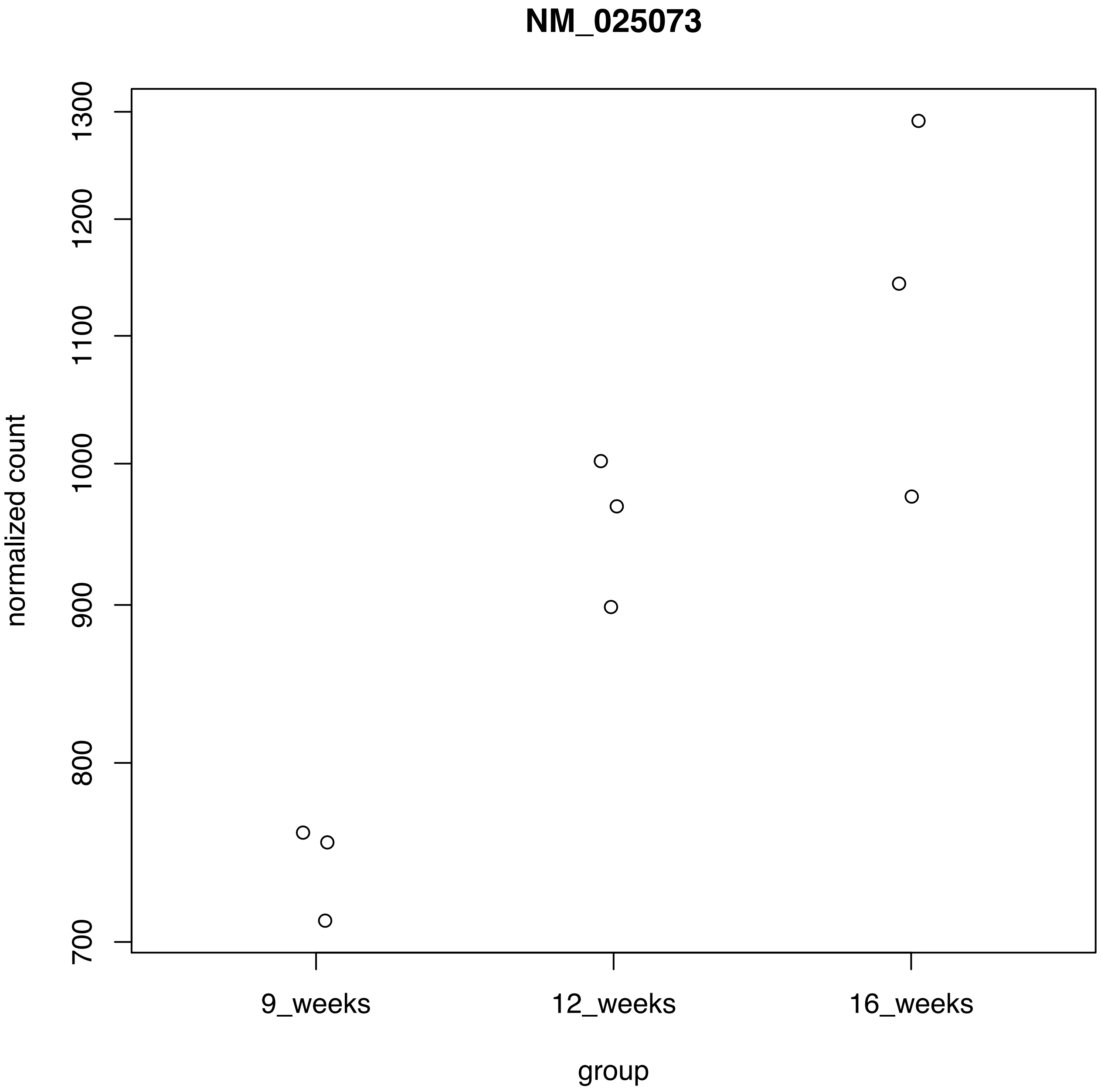

NM_025073
normalized count
1300
1200
1100
1000
900
800
700
9_weeks
12_weeks
16_weeks
group

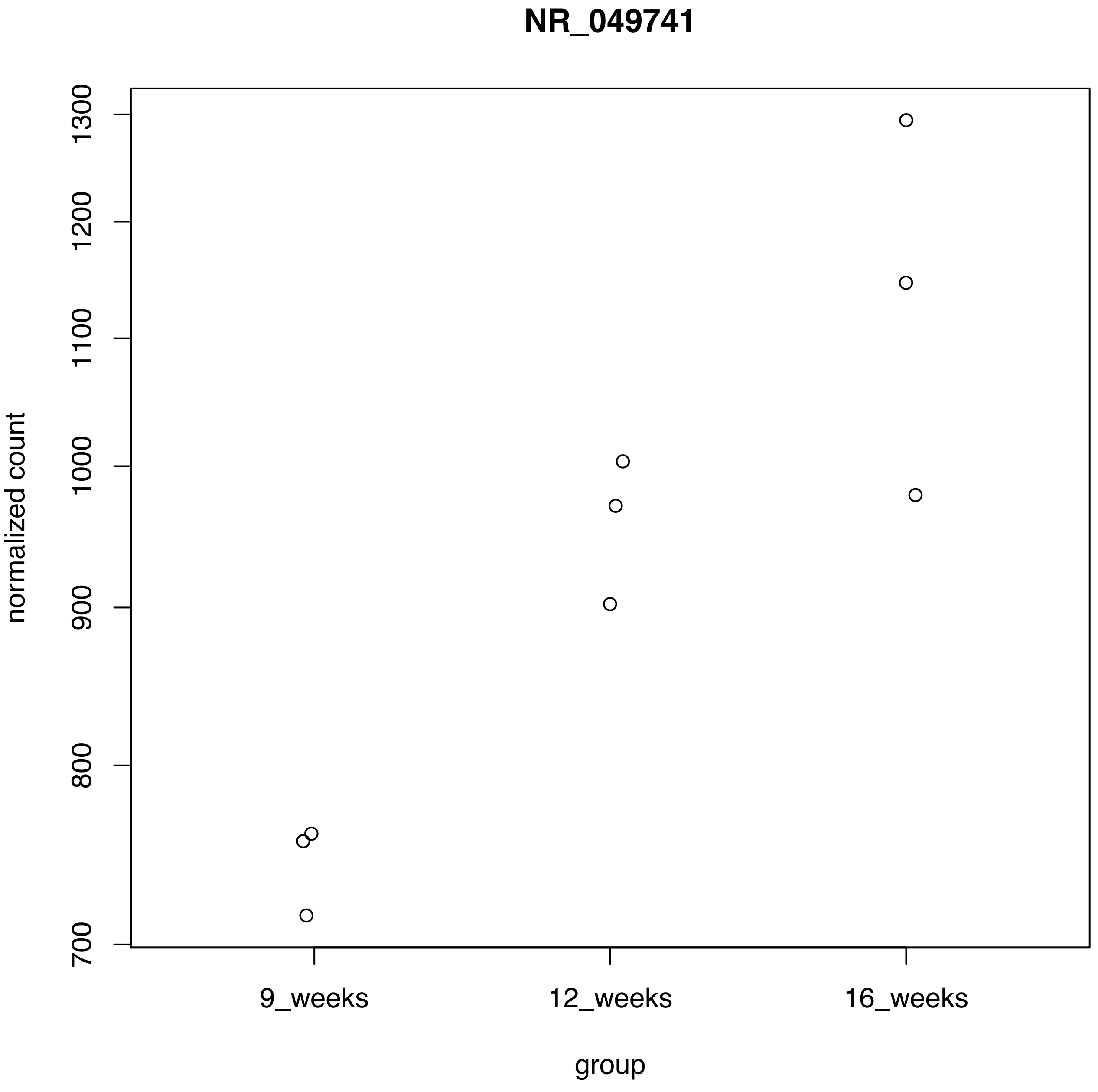

NR_049741
normalized count
700
800
900
1000
1100
1200
1300
9_weeks
12_weeks
16_weeks
group

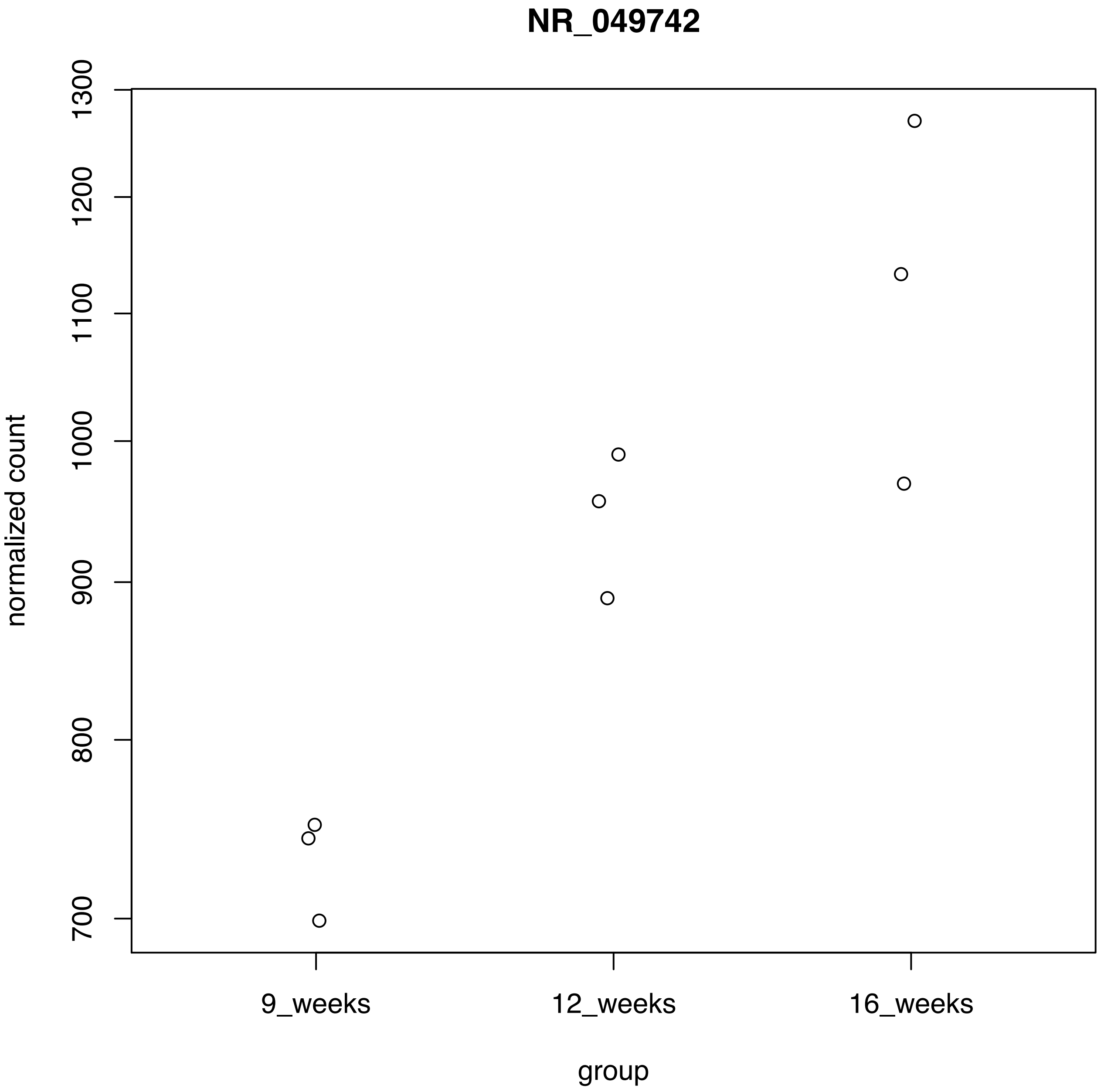
NR_049742
normalized count
1300
1200
1100
1000
900
800
700
group
9_weeks
12_weeks
16_weeks

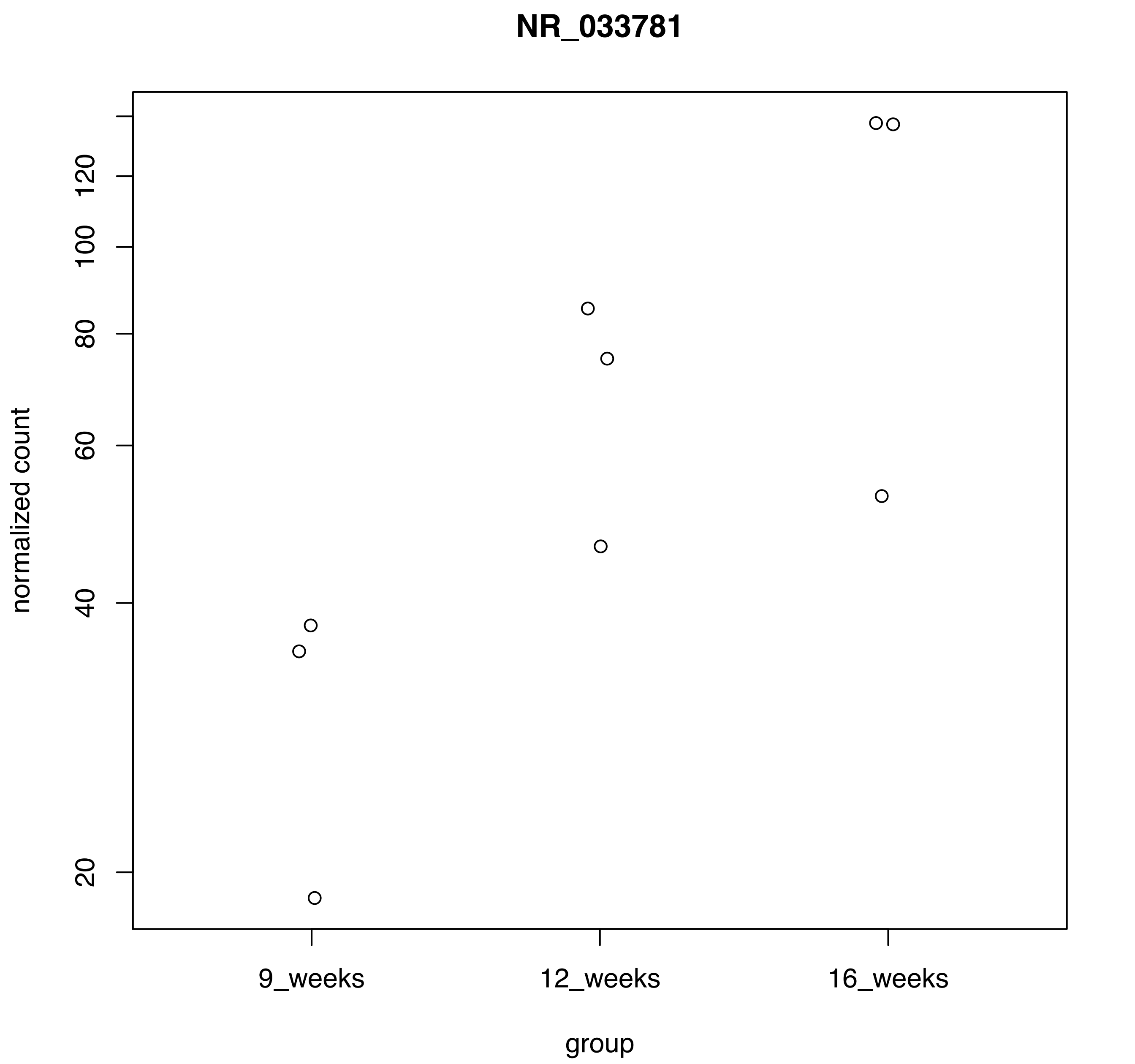

NR_033781
normalized count
20
40
60
80
100
120
9_weeks
12_weeks
16_weeks
group

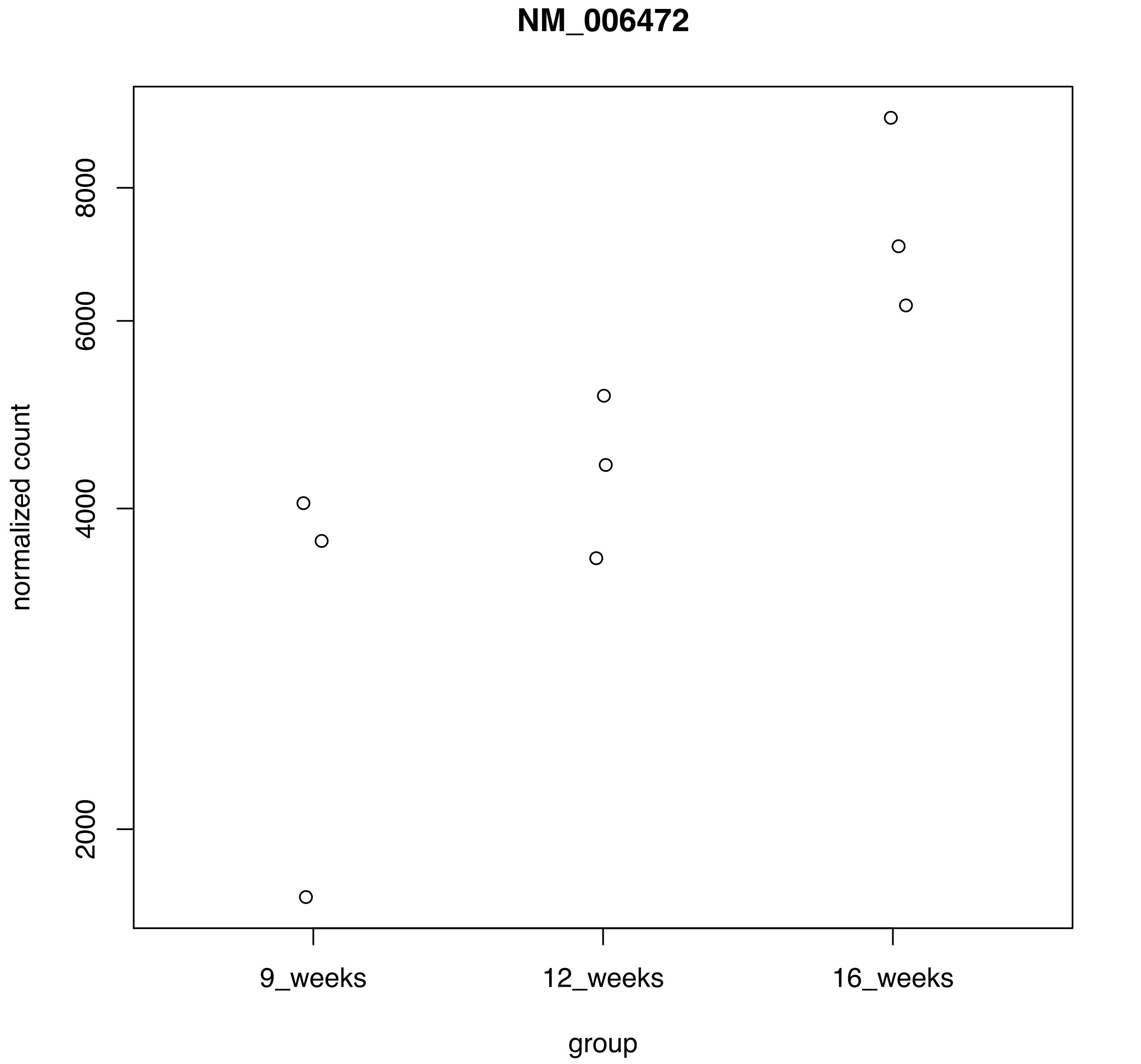
NM_006472
normalized count
8000
6000
4000
2000
9_weeks
12_weeks
16_weeks
group

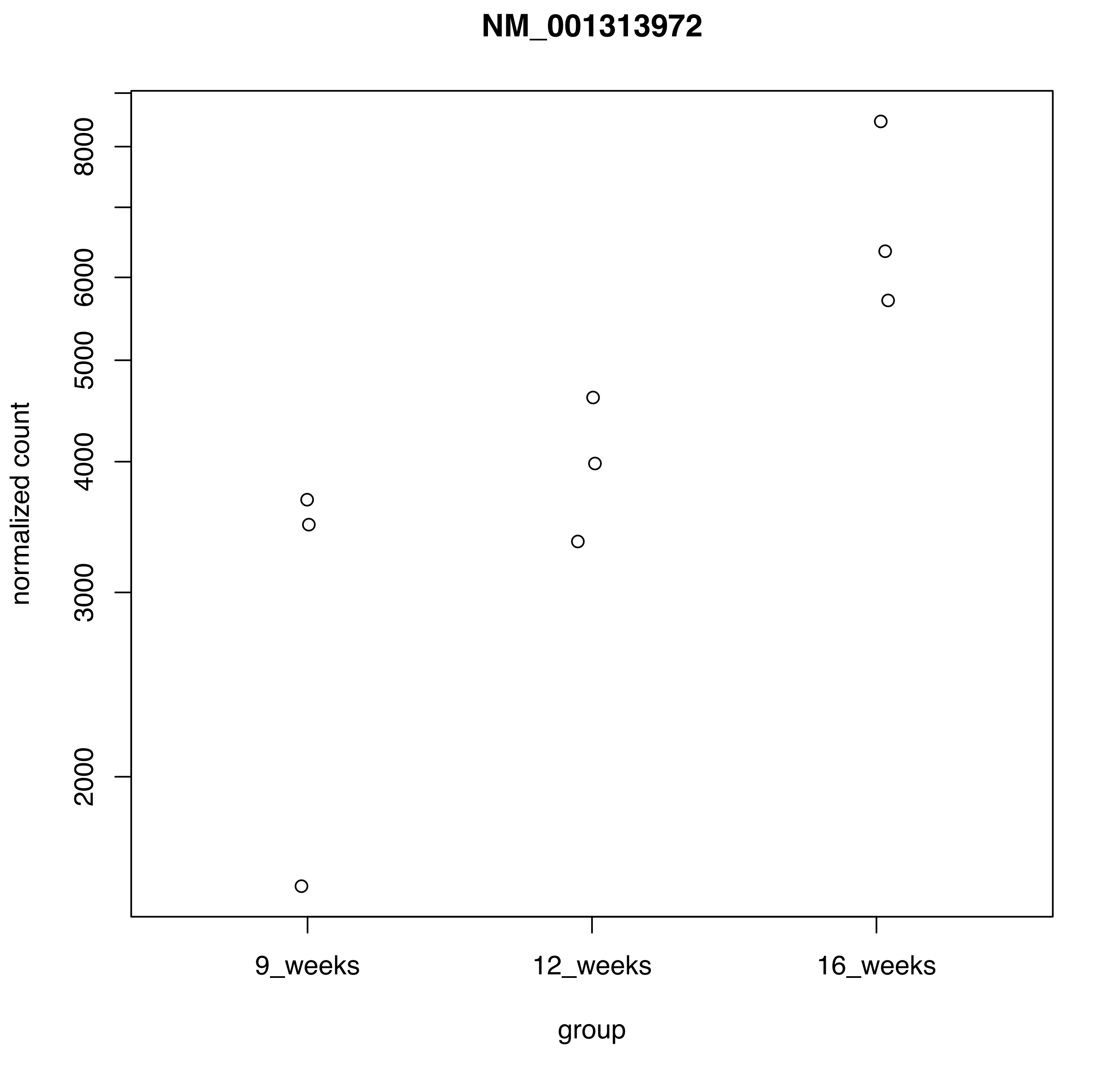
NM_001313972
normalized count
2000
3000
4000
5000
6000
8000
9_weeks
12_weeks
16_weeks
group

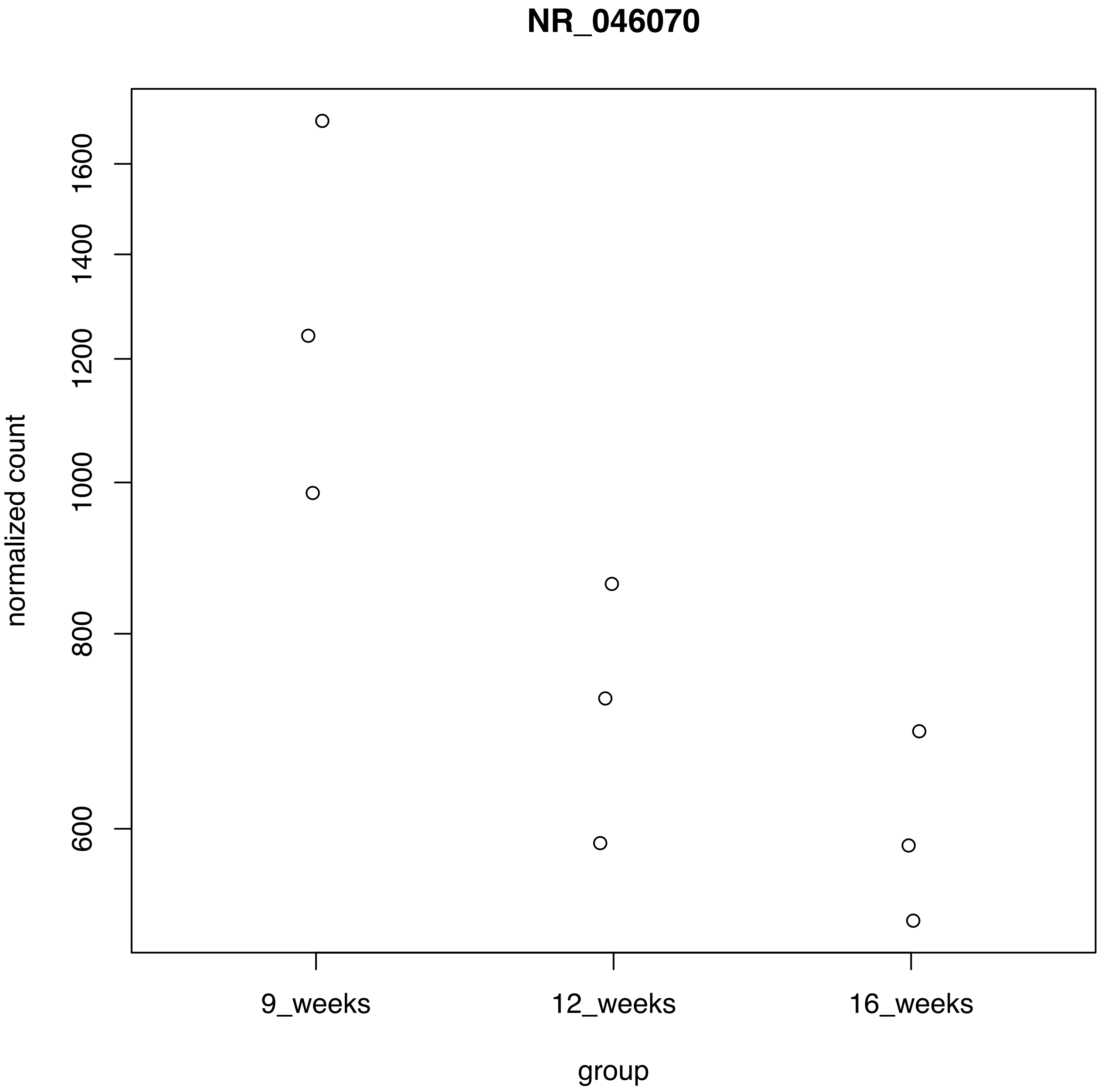
NR_046070
normalized count
1600
1400
1200
1000
800
600
9_weeks
12_weeks
16_weeks
group

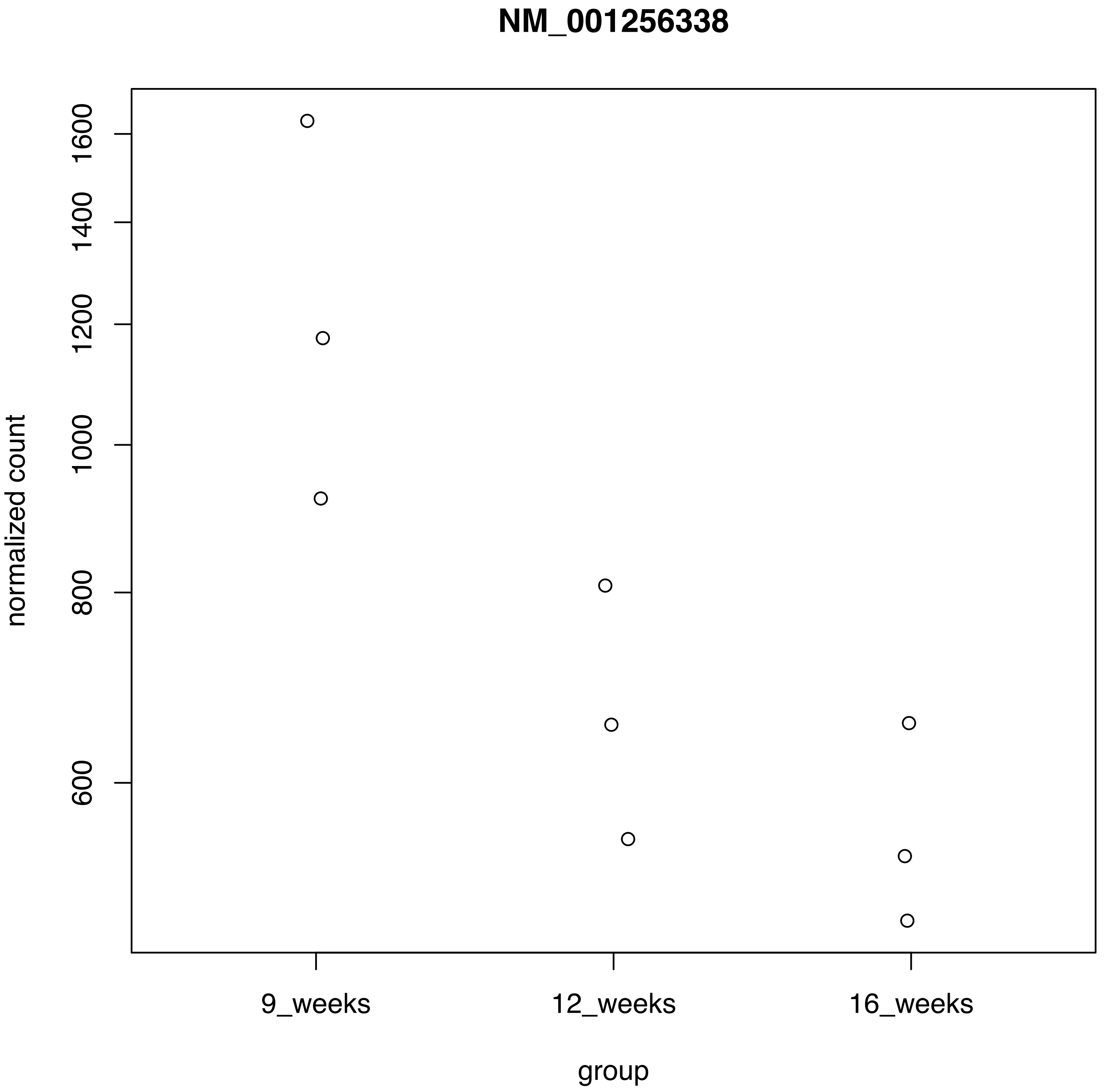
NM_001256338
normalized count
1600
1400
1200
1000
800
600
9_weeks
12_weeks
16_weeks
group

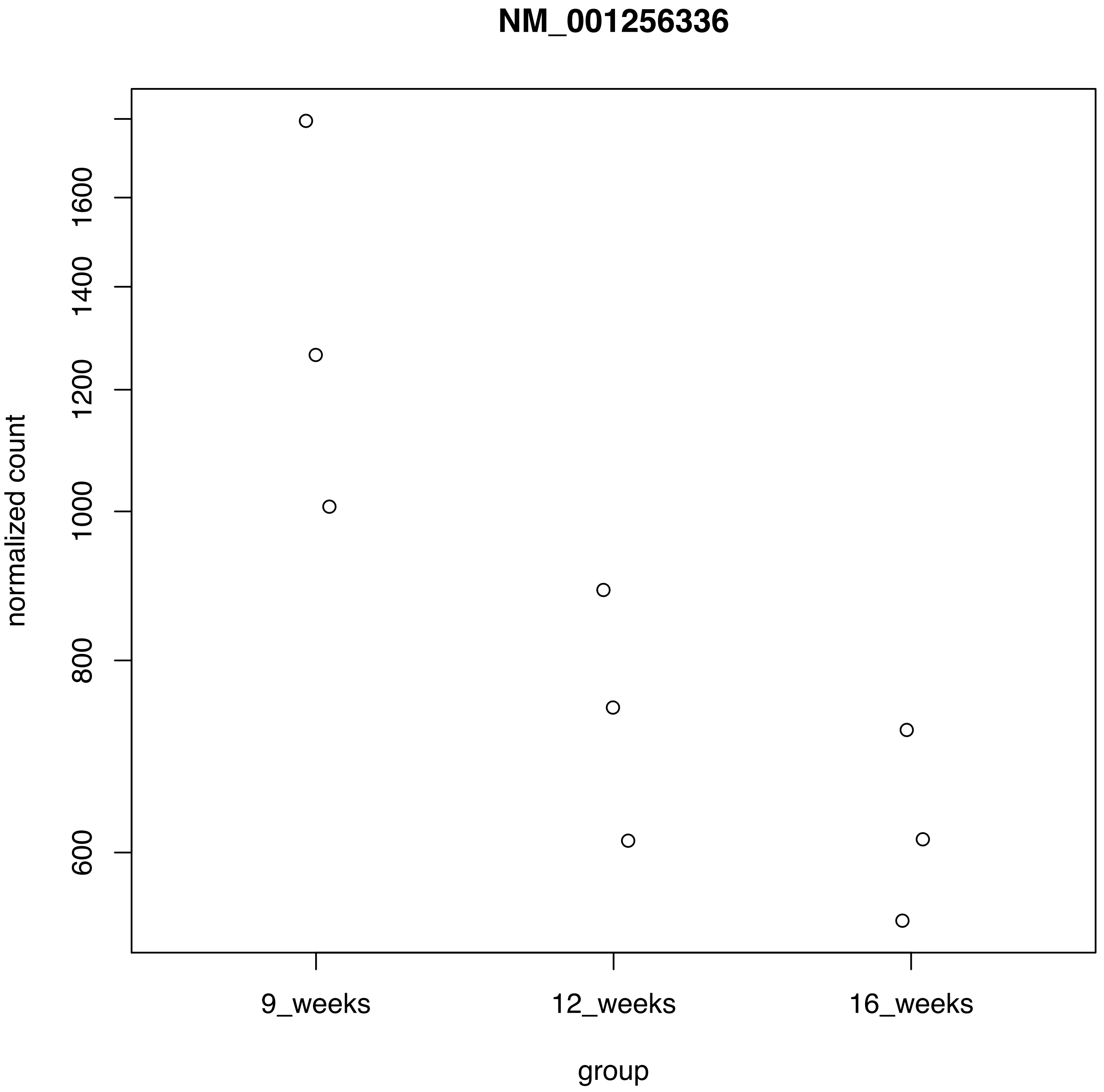
NM_001256336
normalized count
600
800
1000
1200
1400
1600
9_weeks
12_weeks
16_weeks
group

NM_001256337

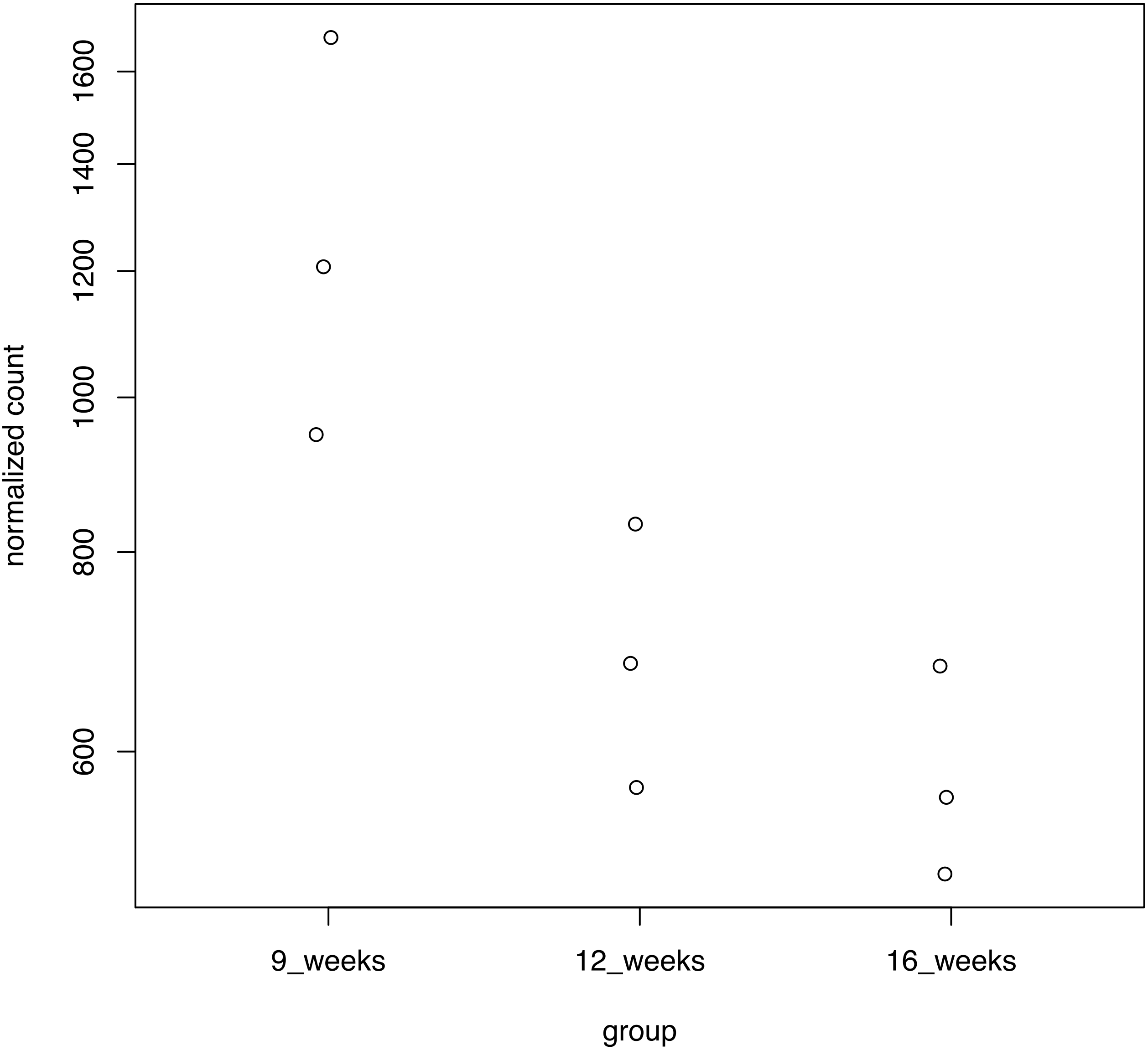

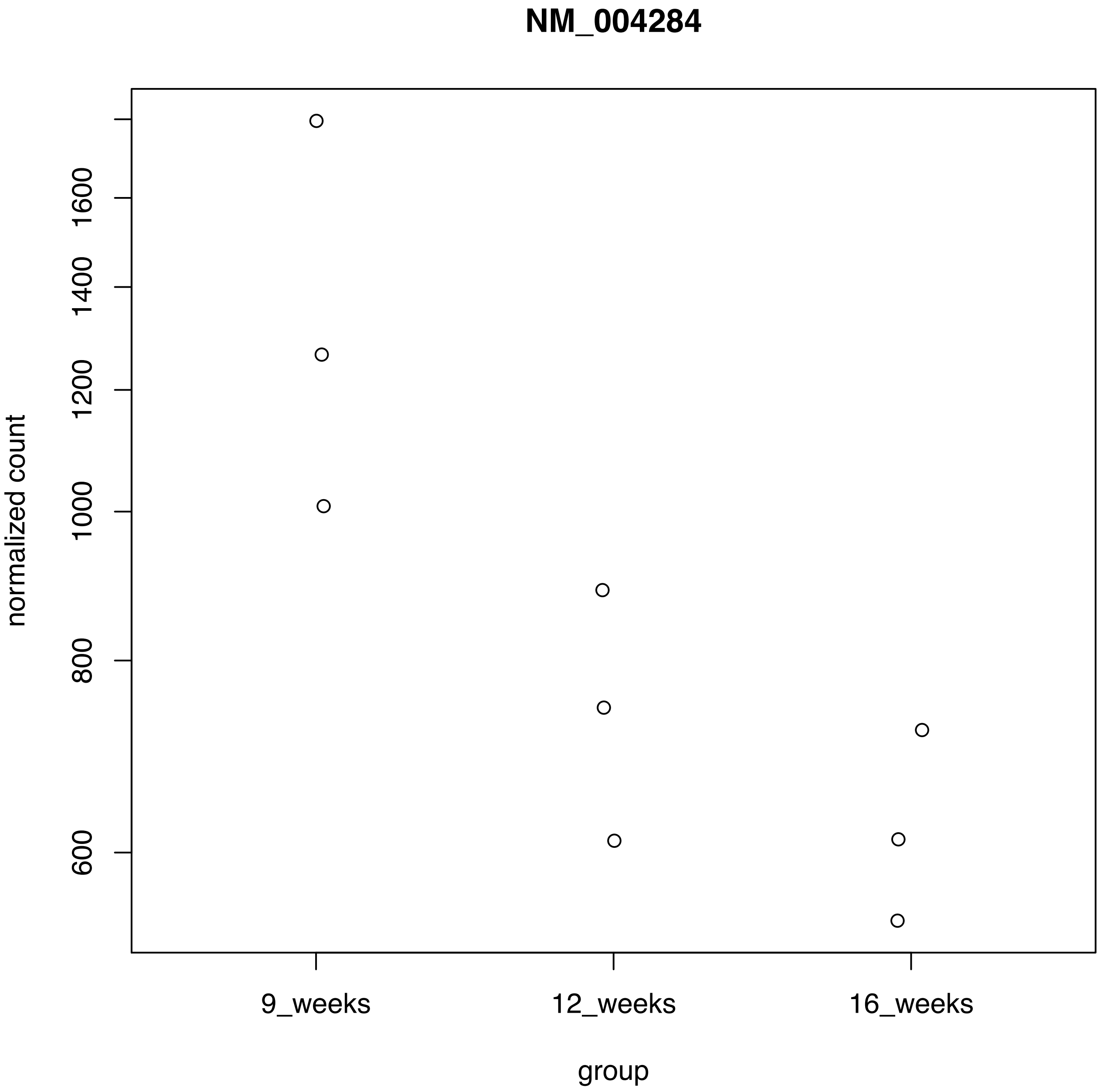
NM_004284
normalized count
1600
1400
1200
1000
800
600
9_weeks
12_weeks
16_weeks
group

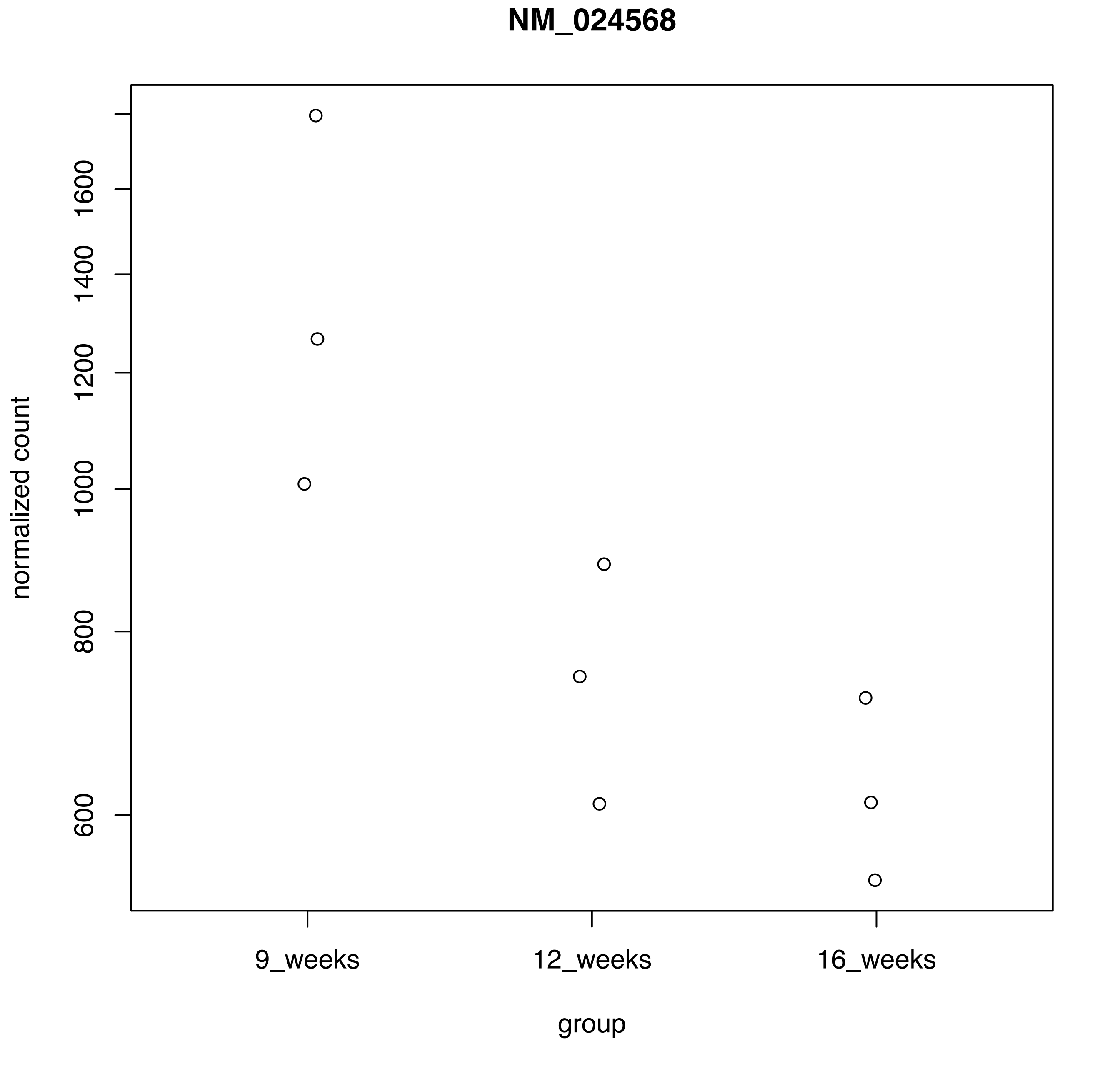
NM_024568
normalized count
1600
1400
1200
1000
800
600
9_weeks
12_weeks
16_weeks
group

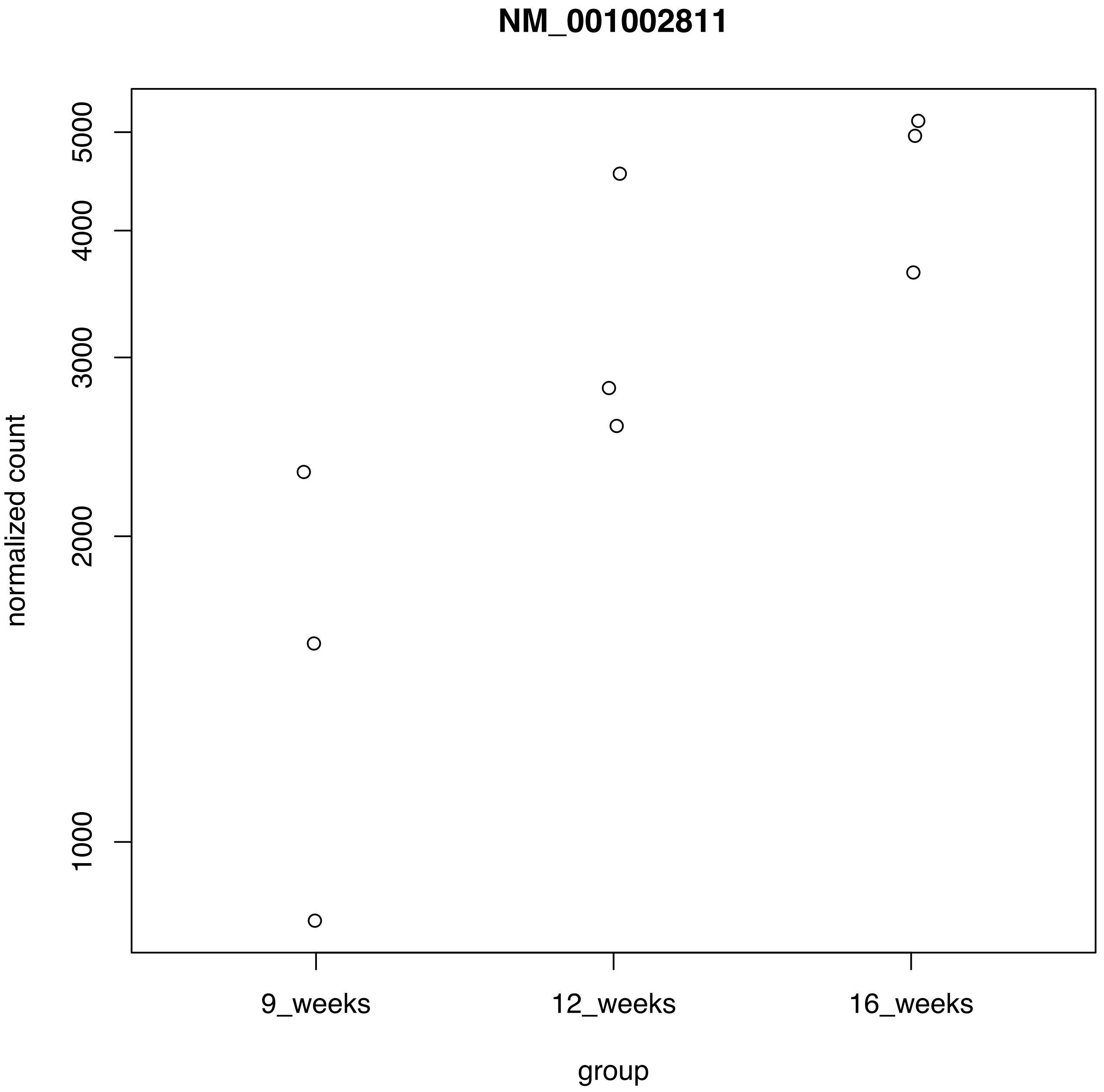

NM_001002811
normalized count
5000
4000
3000
2000
1000
9_weeks
12_weeks
16_weeks
group

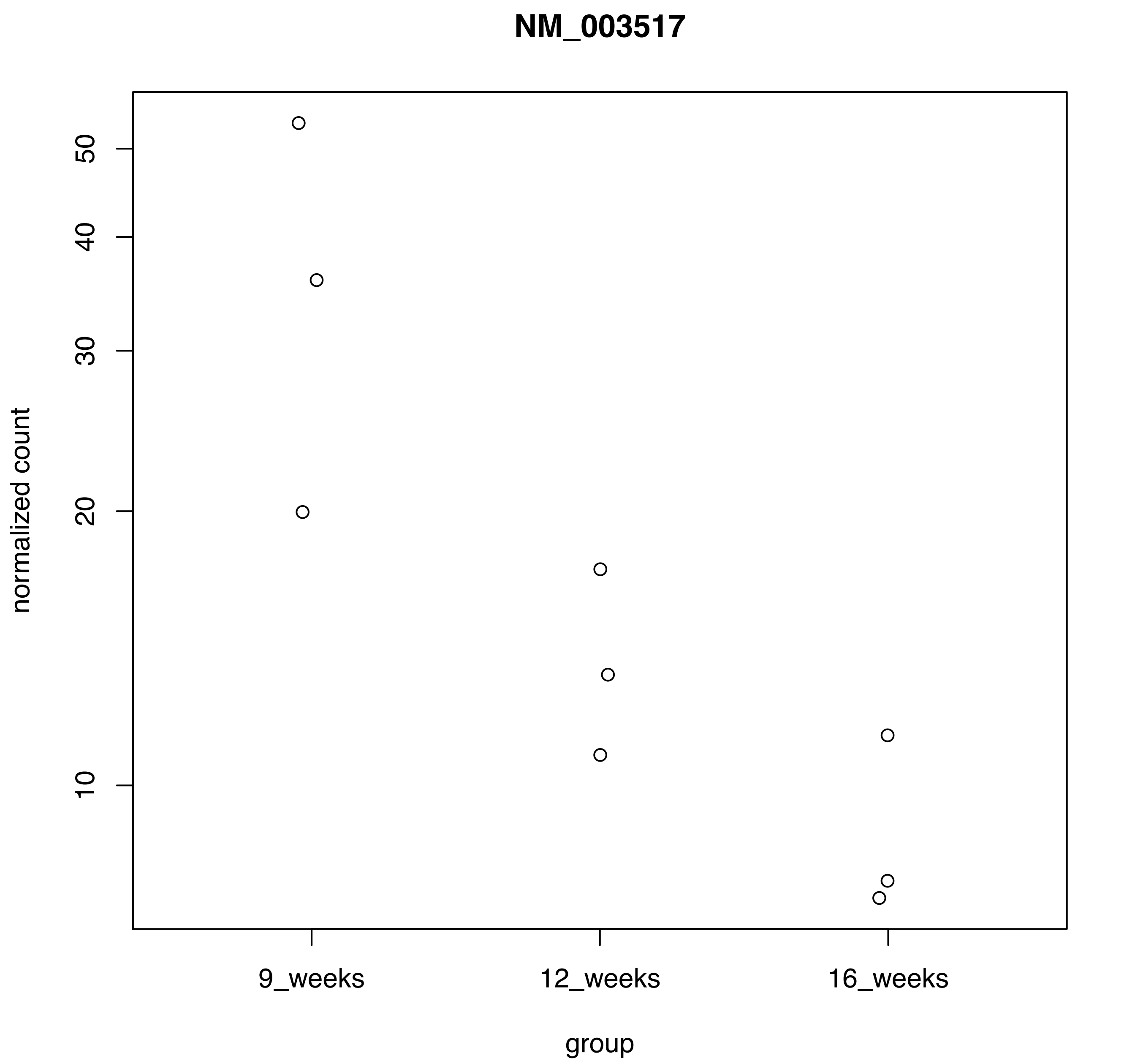
NM_003517
normalized count
10
20
30
40
50
9_weeks
12_weeks
16_weeks
group

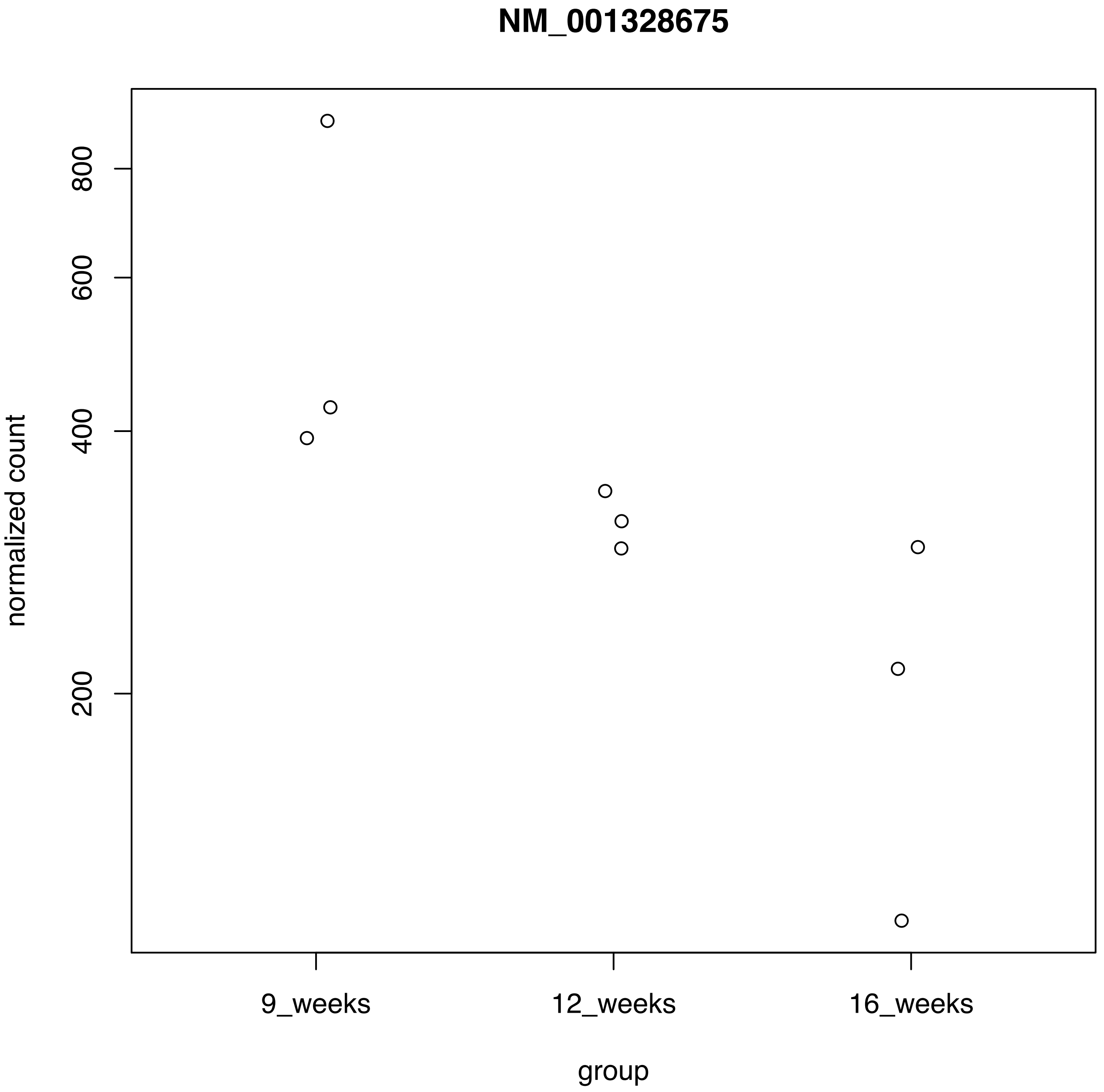
NM_001328675
800
600
400
200
normalized count
9_weeks
12_weeks
16_weeks
group

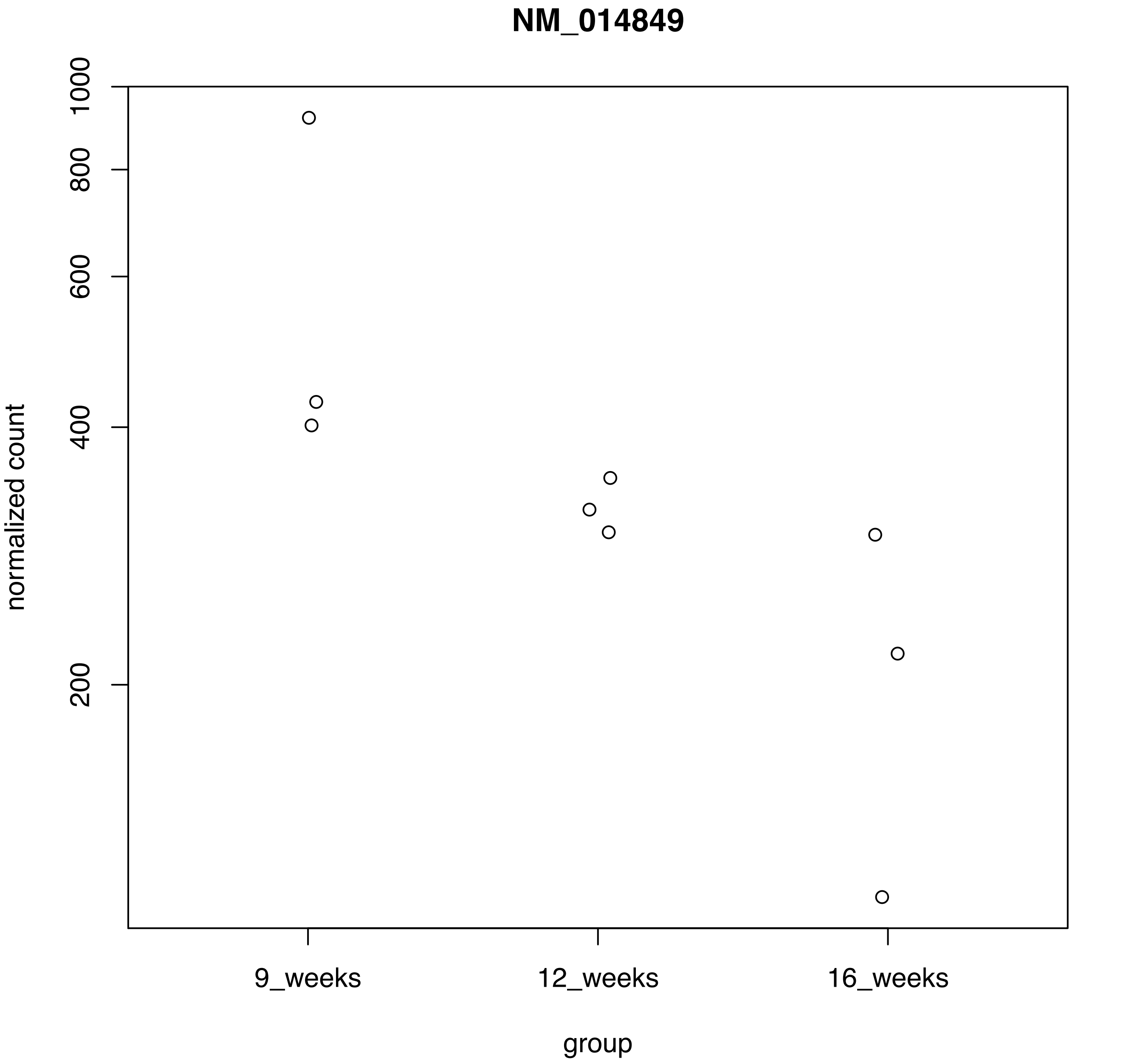
NM_014849
normalized count
1000
800
600
400
200
9_weeks
12_weeks
16_weeks
group

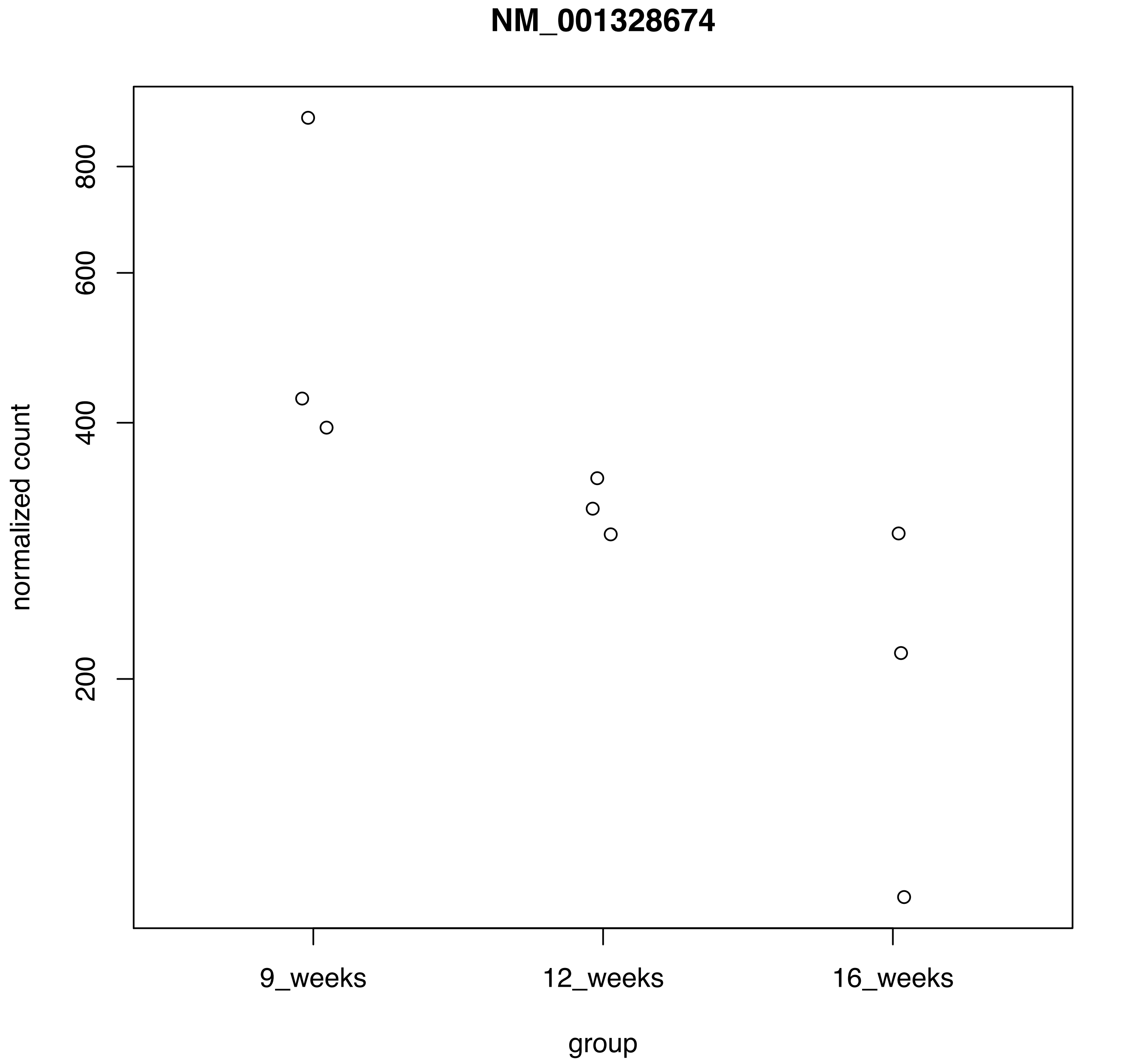

NM_001328674
800
600
400
200
normalized count
9_weeks
12_weeks
16_weeks
group

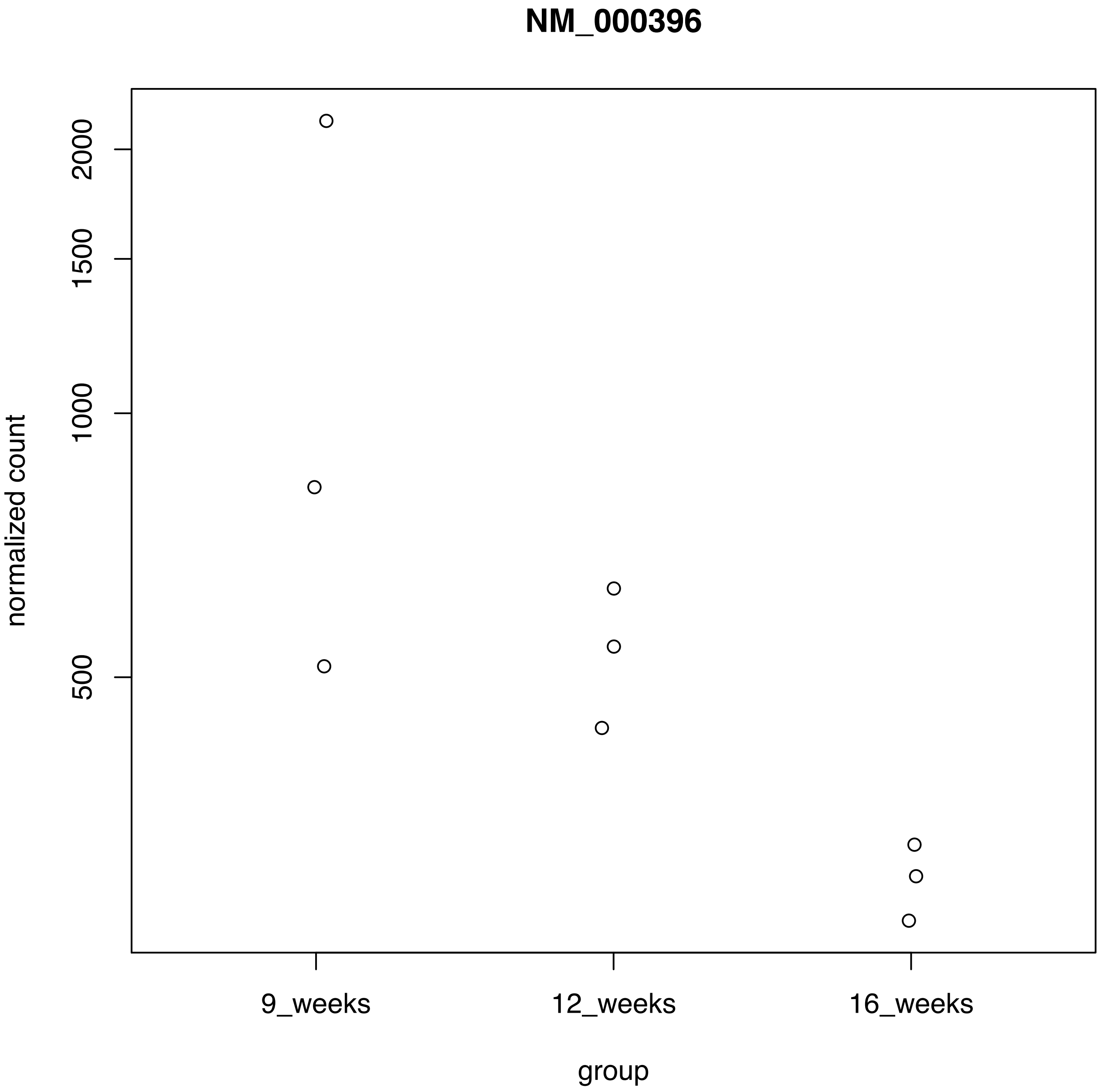
NM_000396
normalized count
2000
1500
1000
500
9_weeks
12_weeks
16_weeks
group

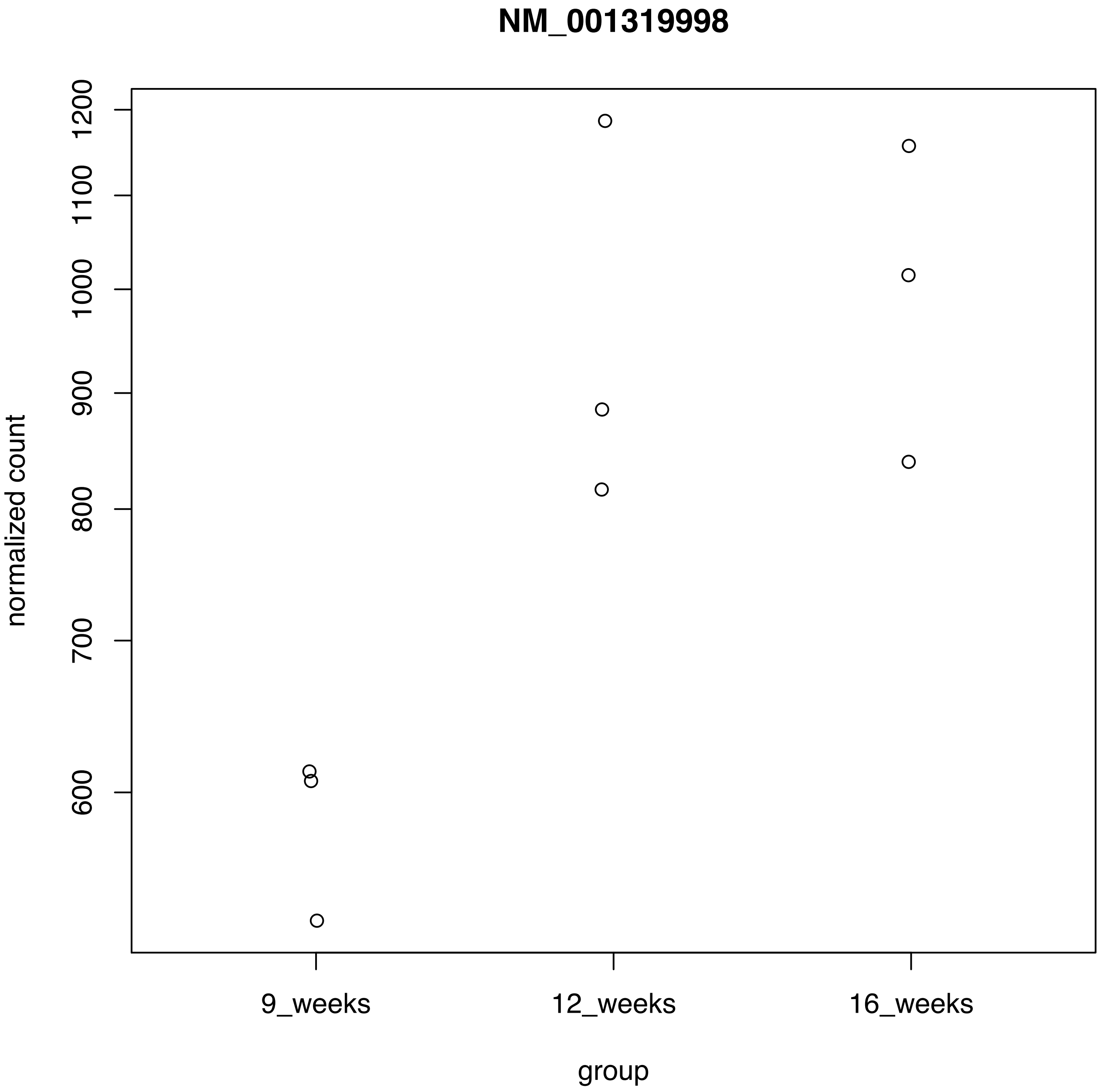
NM_001319998
normalized count
1200
1100
1000
900
800
700
600
9_weeks
12_weeks
16_weeks
group

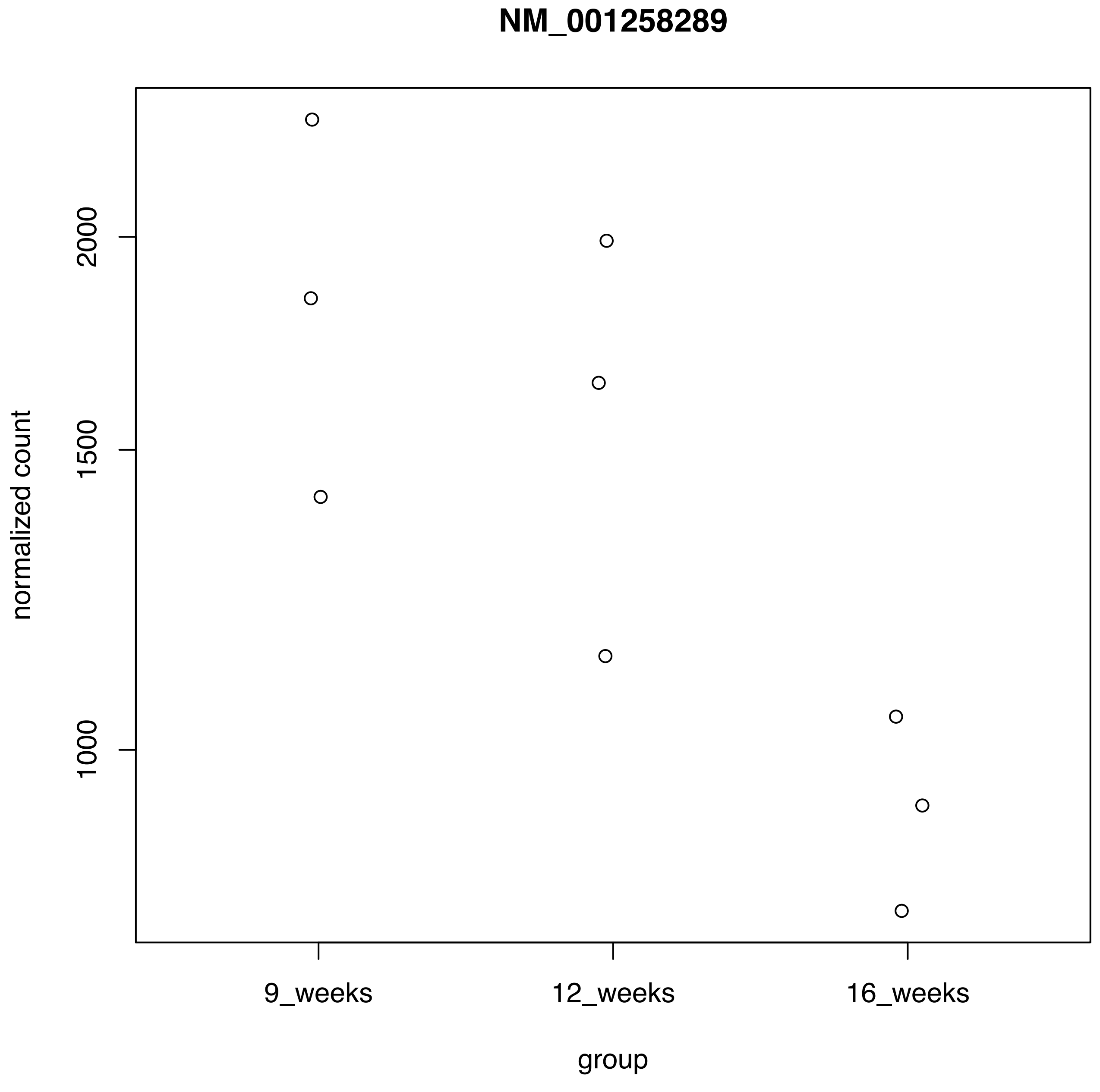
NM_001258289
normalized count
2000
1500
1000
9_weeks
12_weeks
16_weeks
group

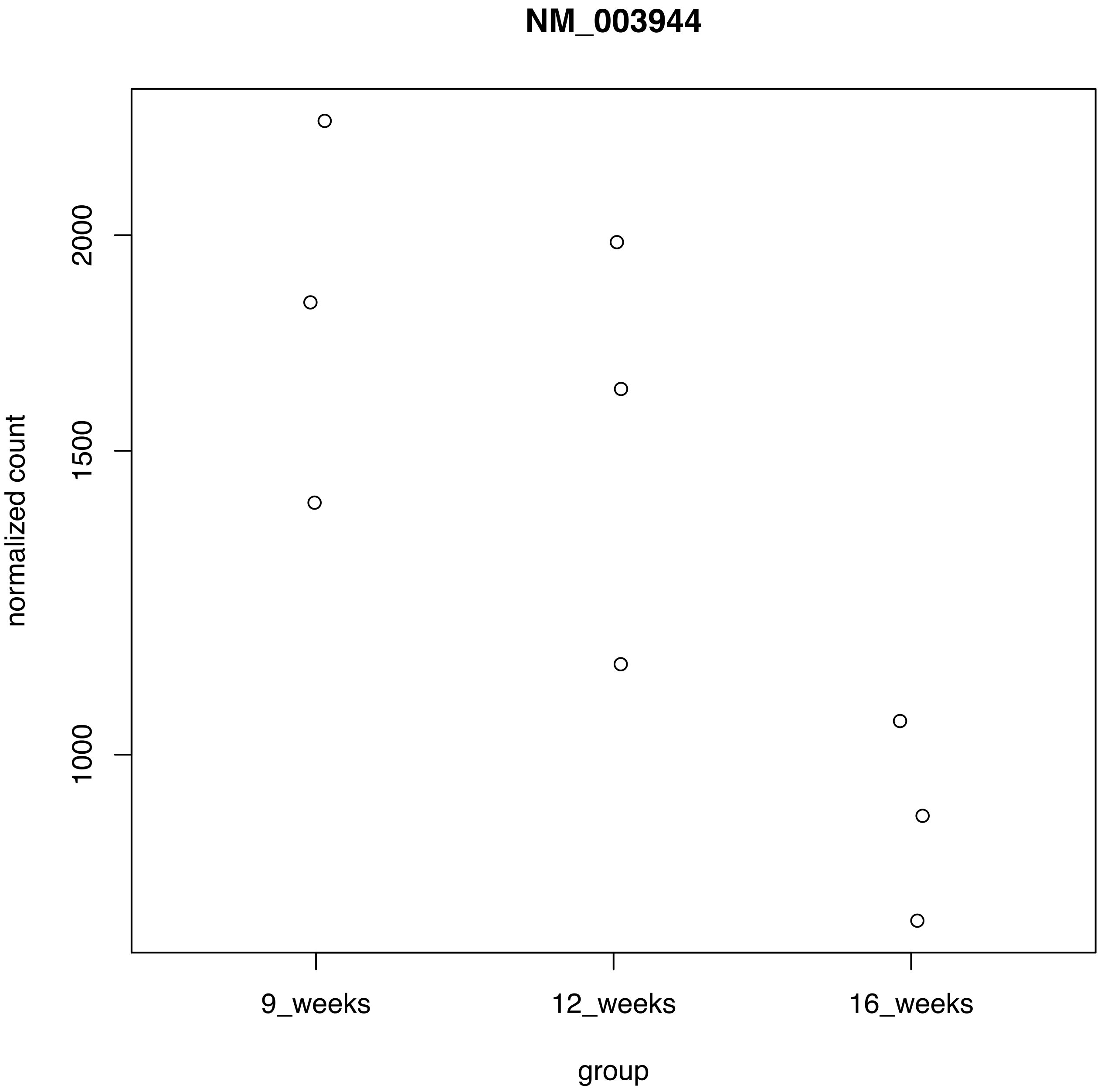
NM_003944
normalized count
2000
1500
1000
9_weeks
12_weeks
16_weeks
group

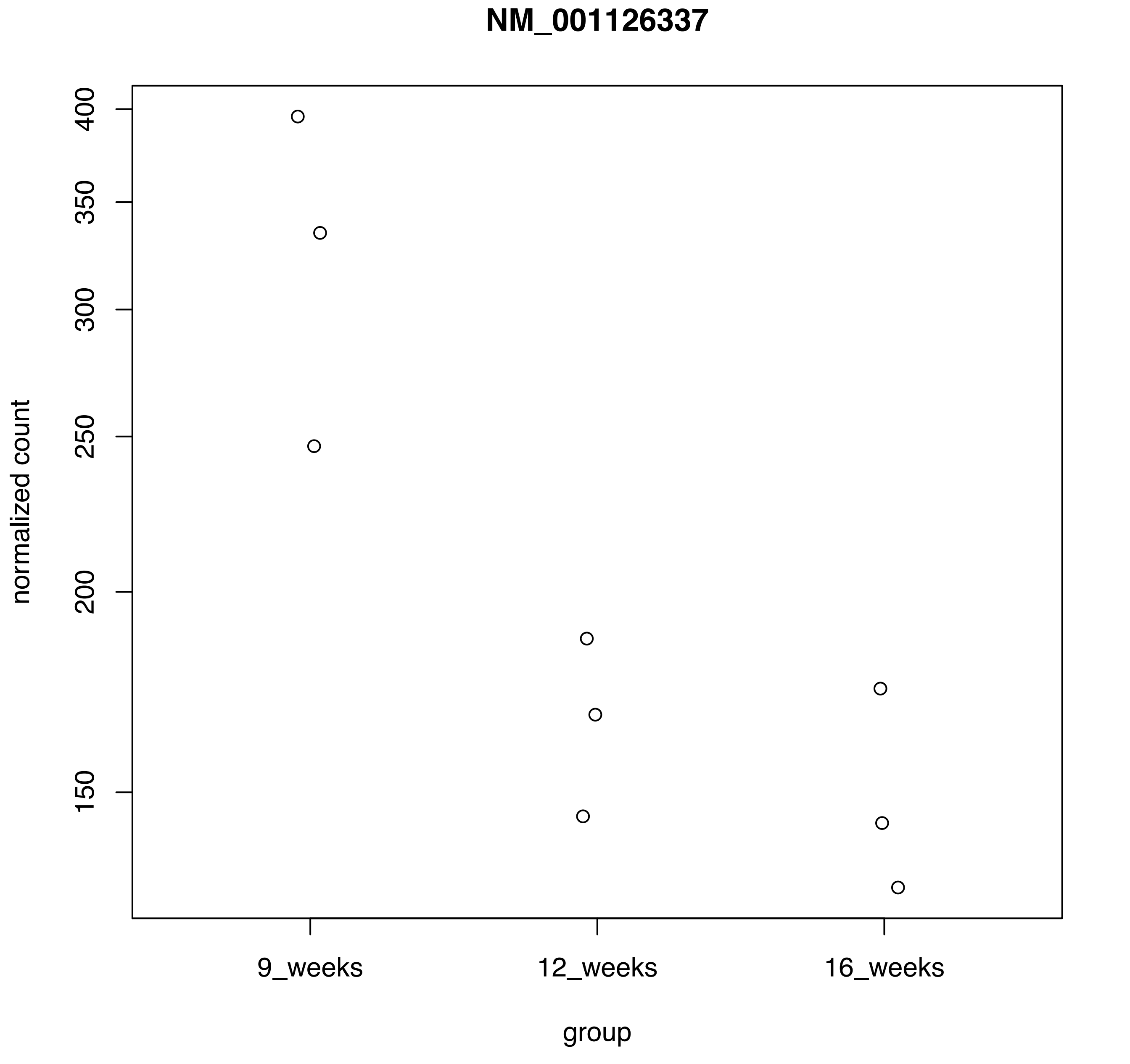

NM_001126337
normalized count
400
350
300
250
200
150
9_weeks
12_weeks
16_weeks
group

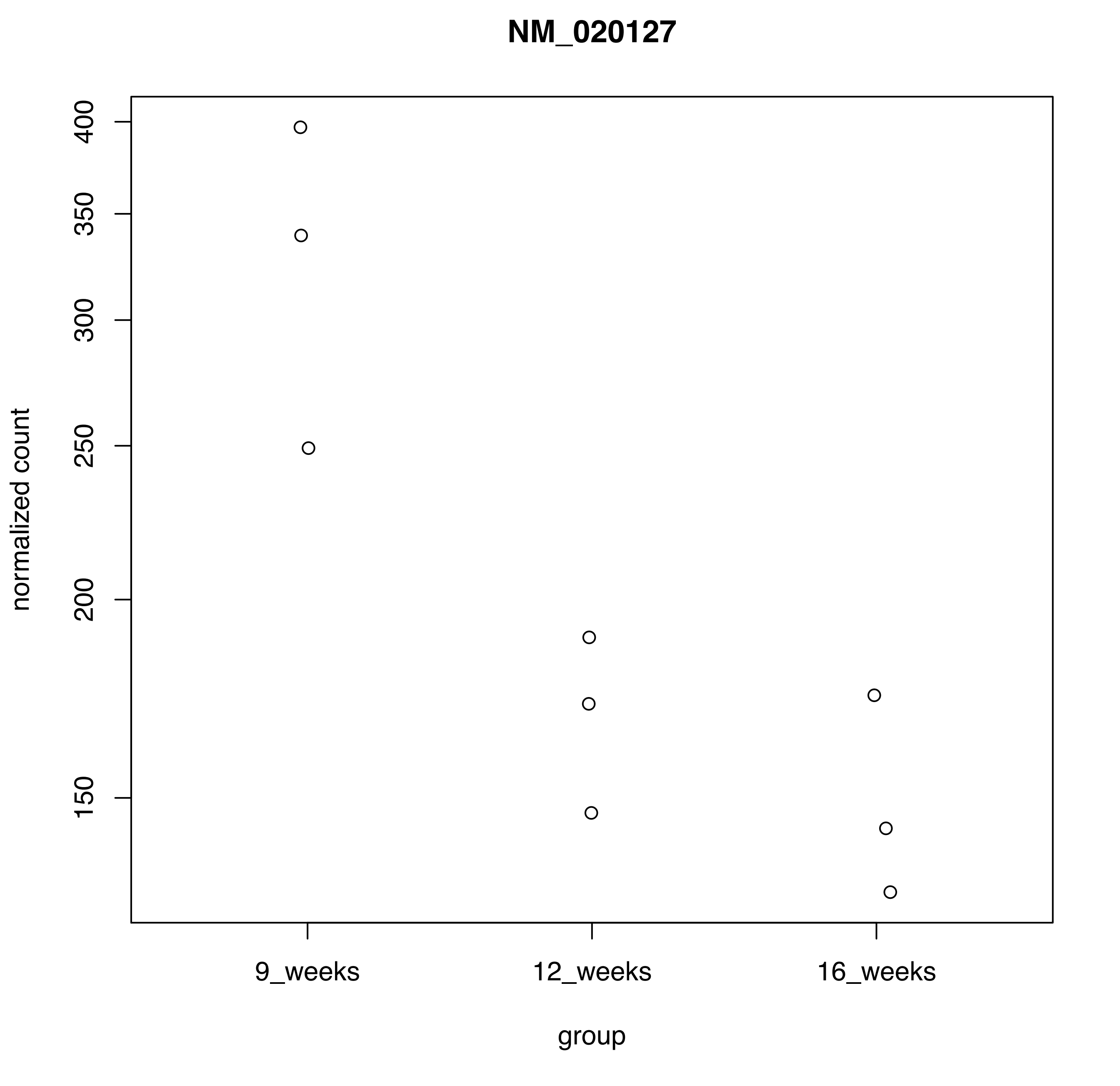

NM_020127
normalized count
400
350
300
250
200
150
9_weeks
12_weeks
16_weeks
group

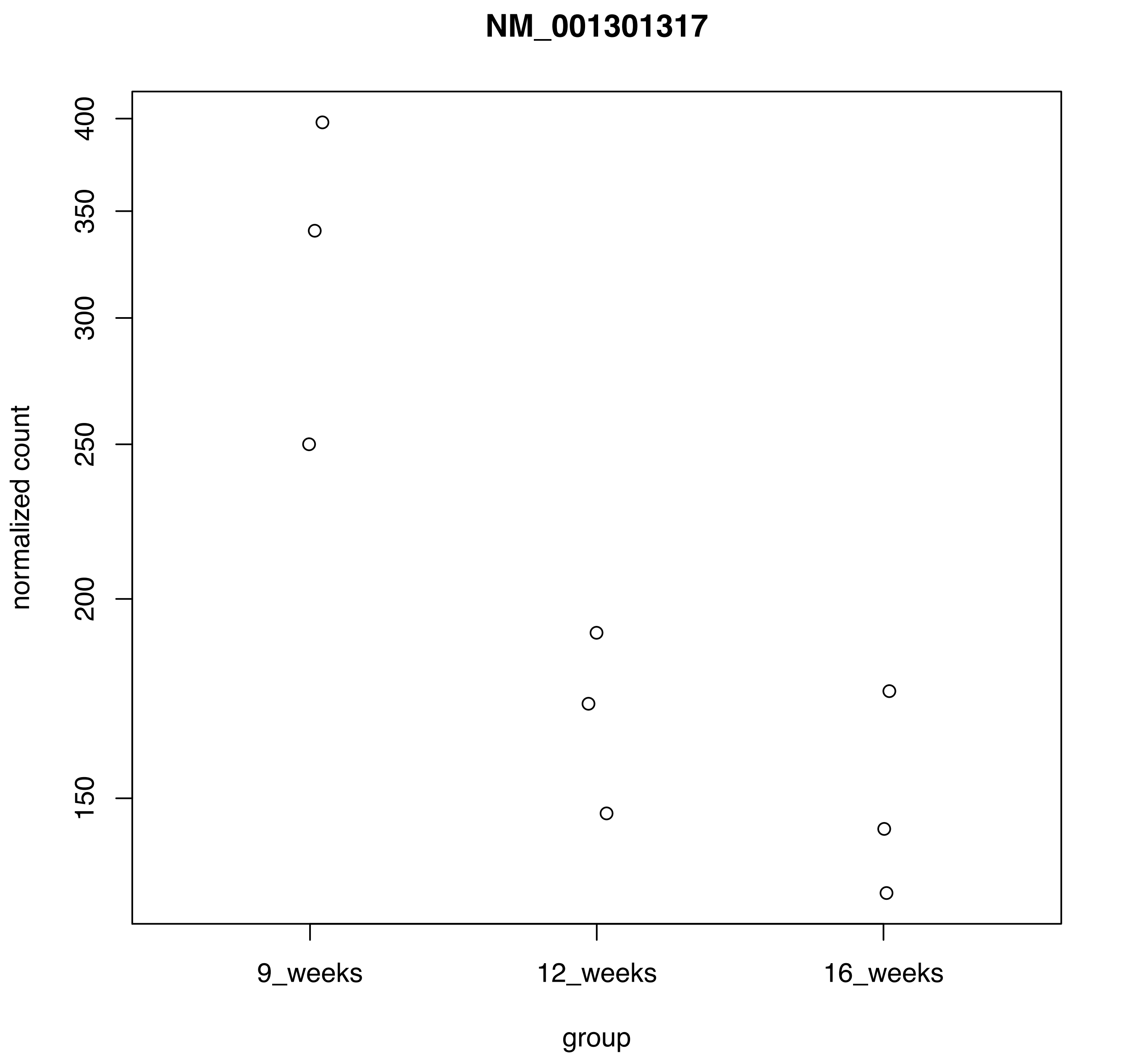
NM_001301317
normalized count
400
350
300
250
200
150
9_weeks
12_weeks
16_weeks
group

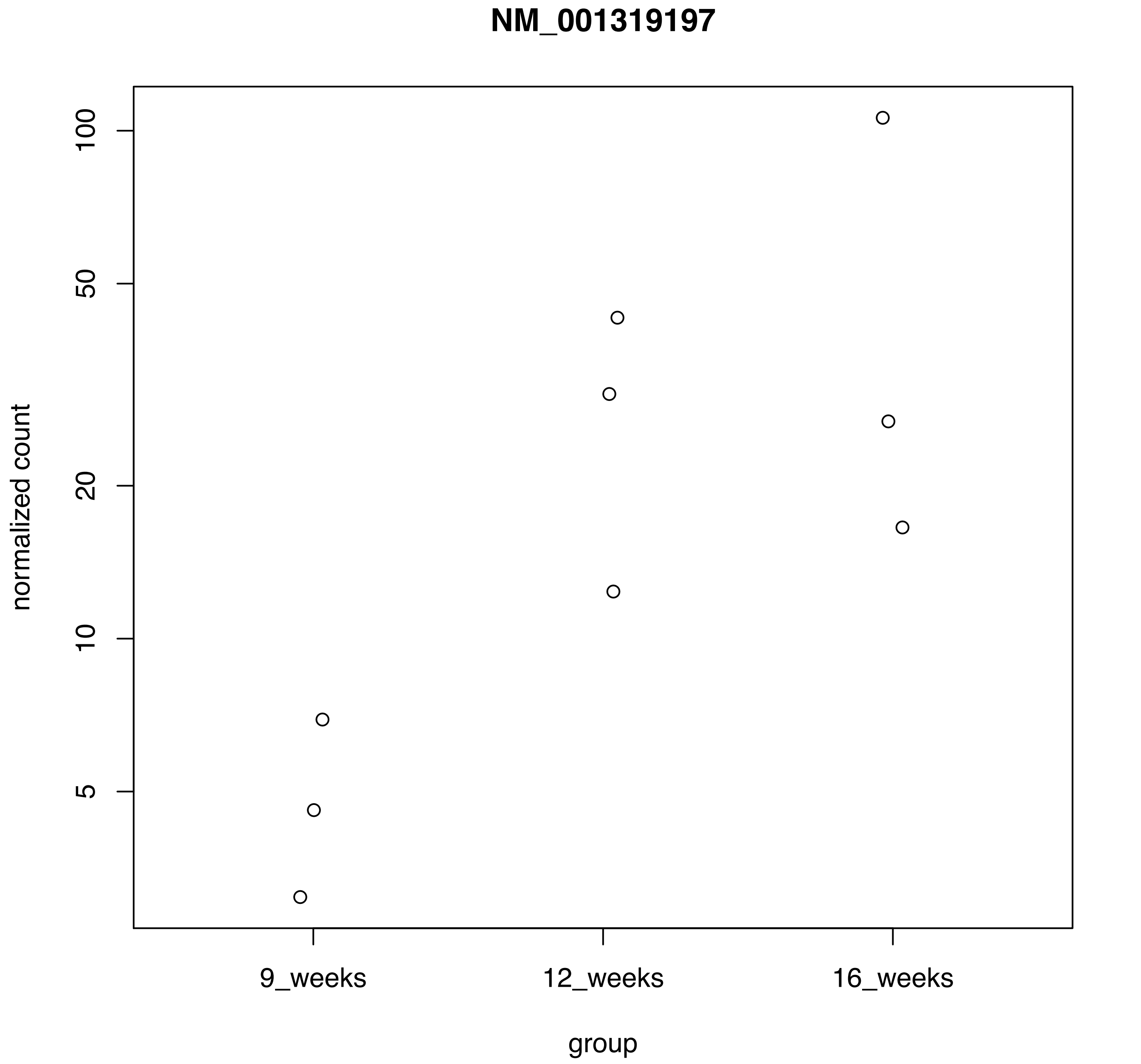
NM_001319197
normalized count
100
50
20
10
5
group
9_weeks
12_weeks
16_weeks

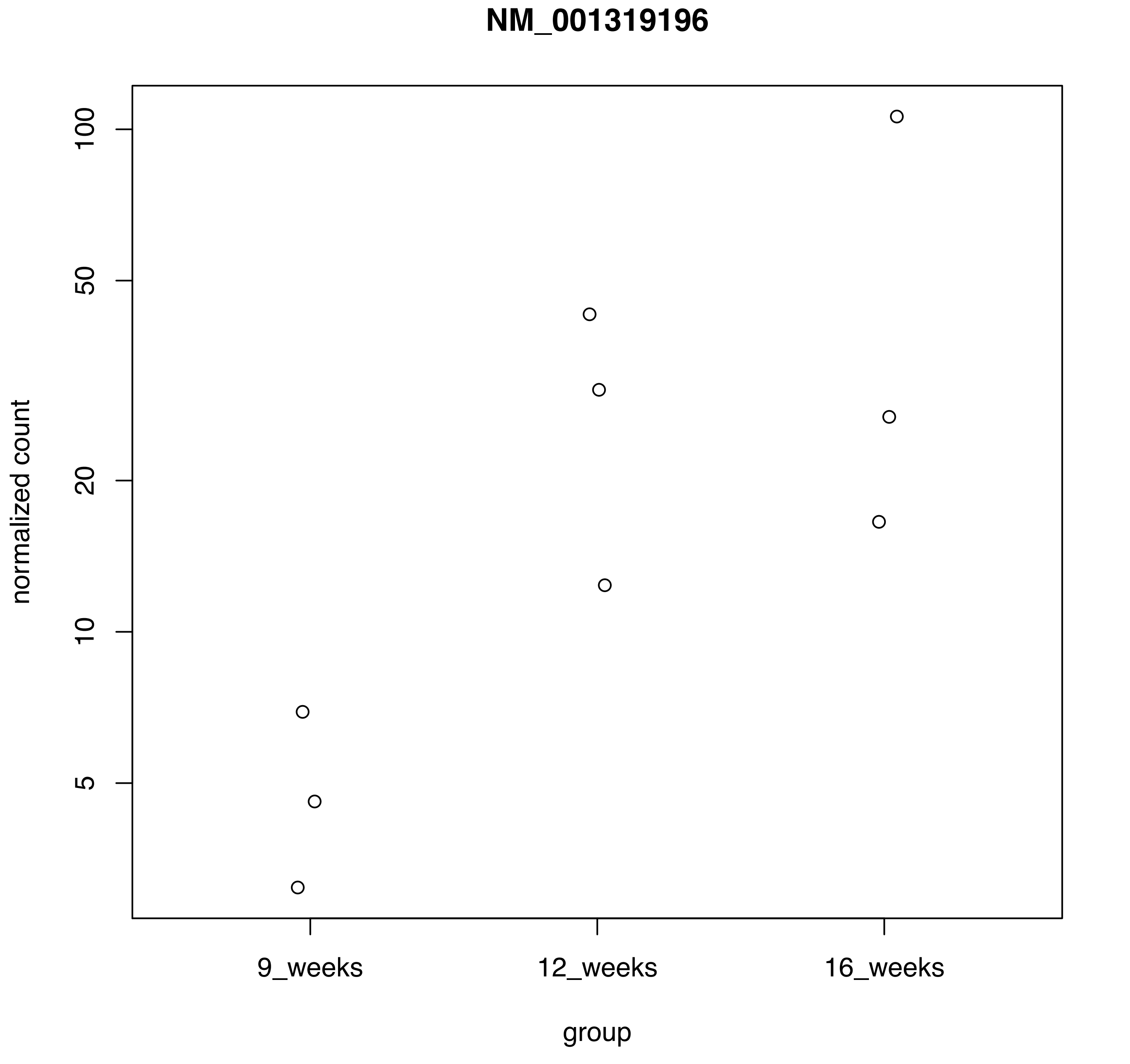
NM_001319196
normalized count
100
50
20
10
5
9_weeks
12_weeks
16_weeks
group

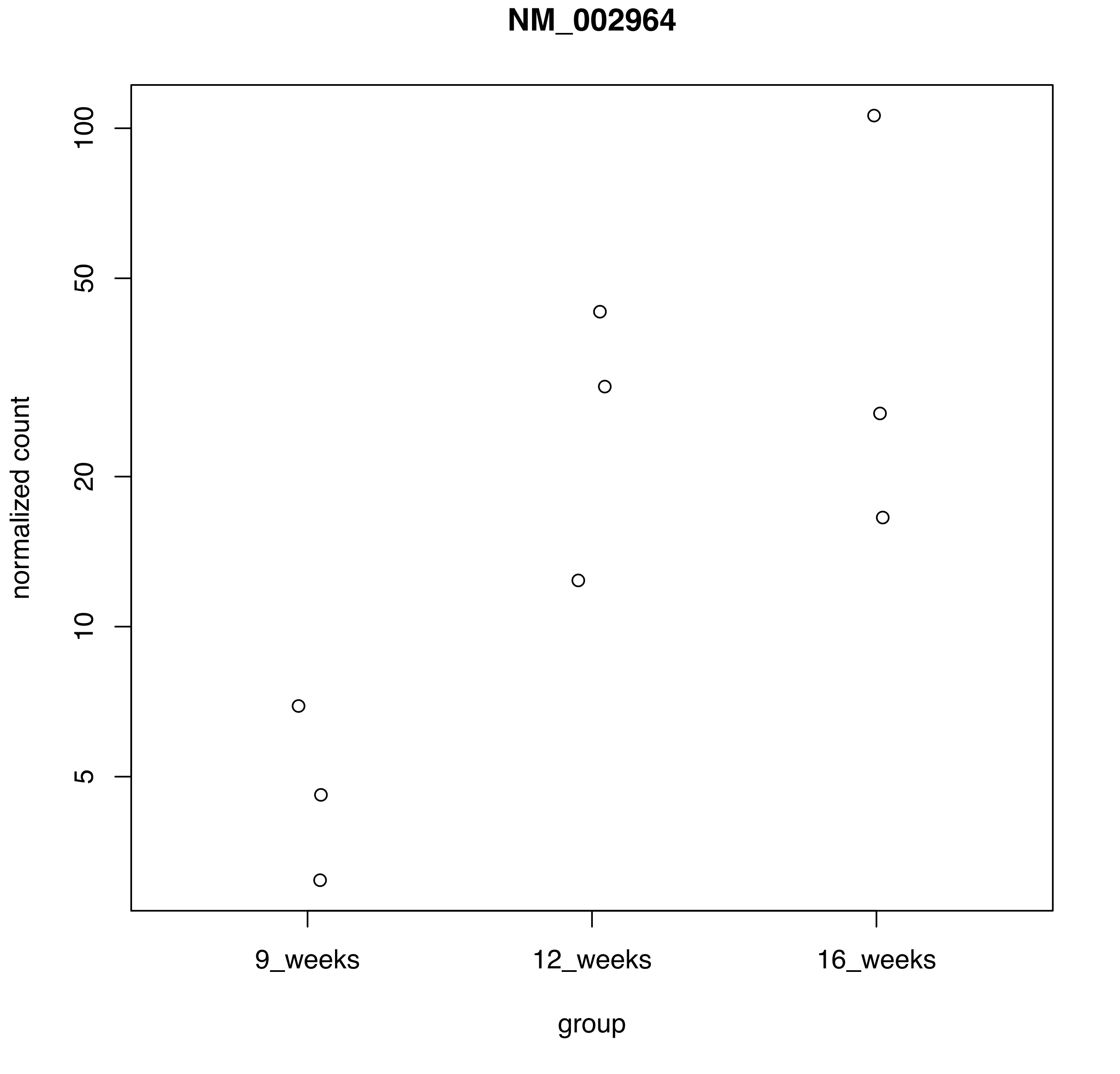

NM_002964
normalized count
100
50
20
10
5
9_weeks
12_weeks
16_weeks
group

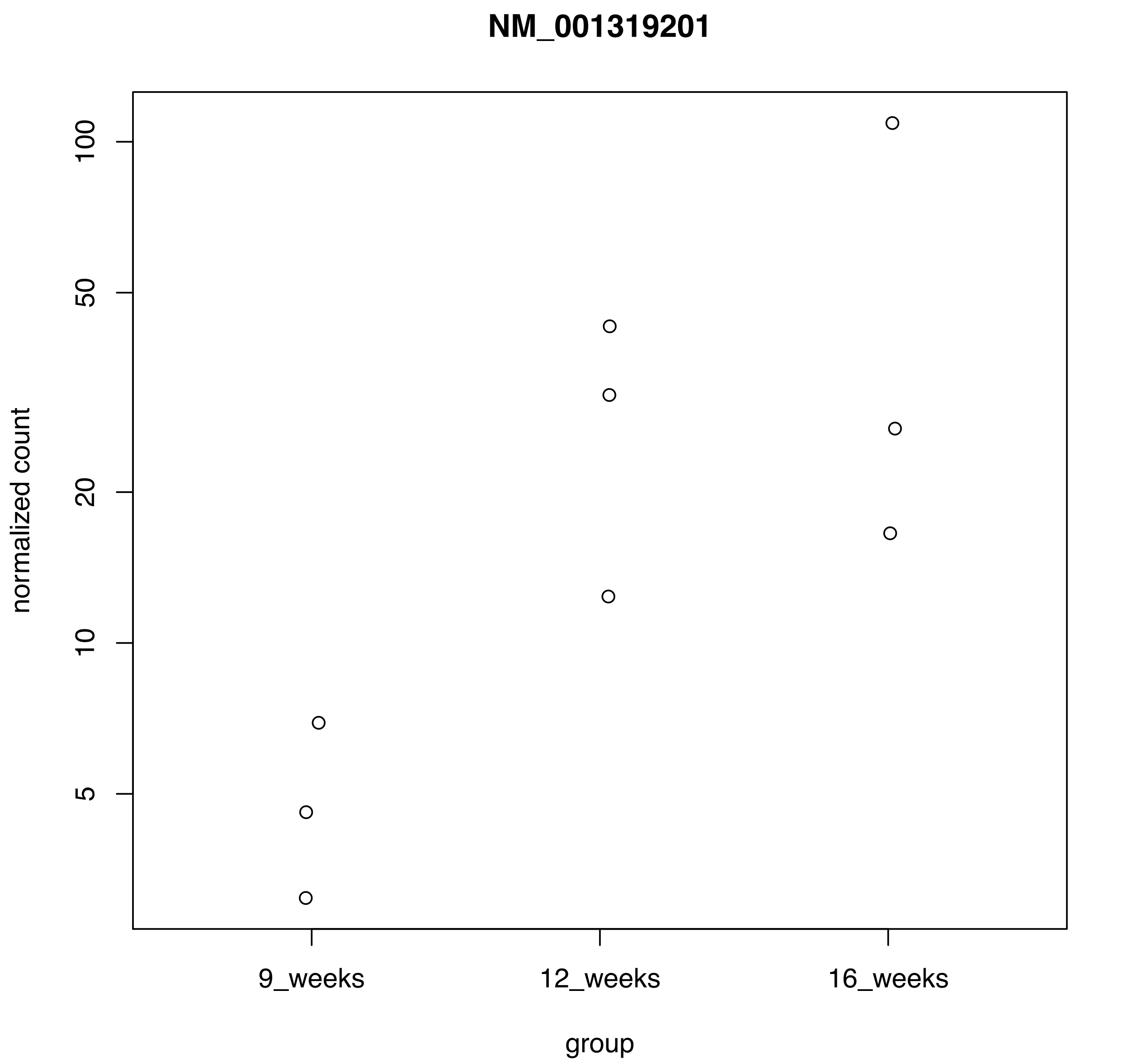
NM_001319201
normalized count
100
50
20
10
5
9_weeks
12_weeks
16_weeks
group

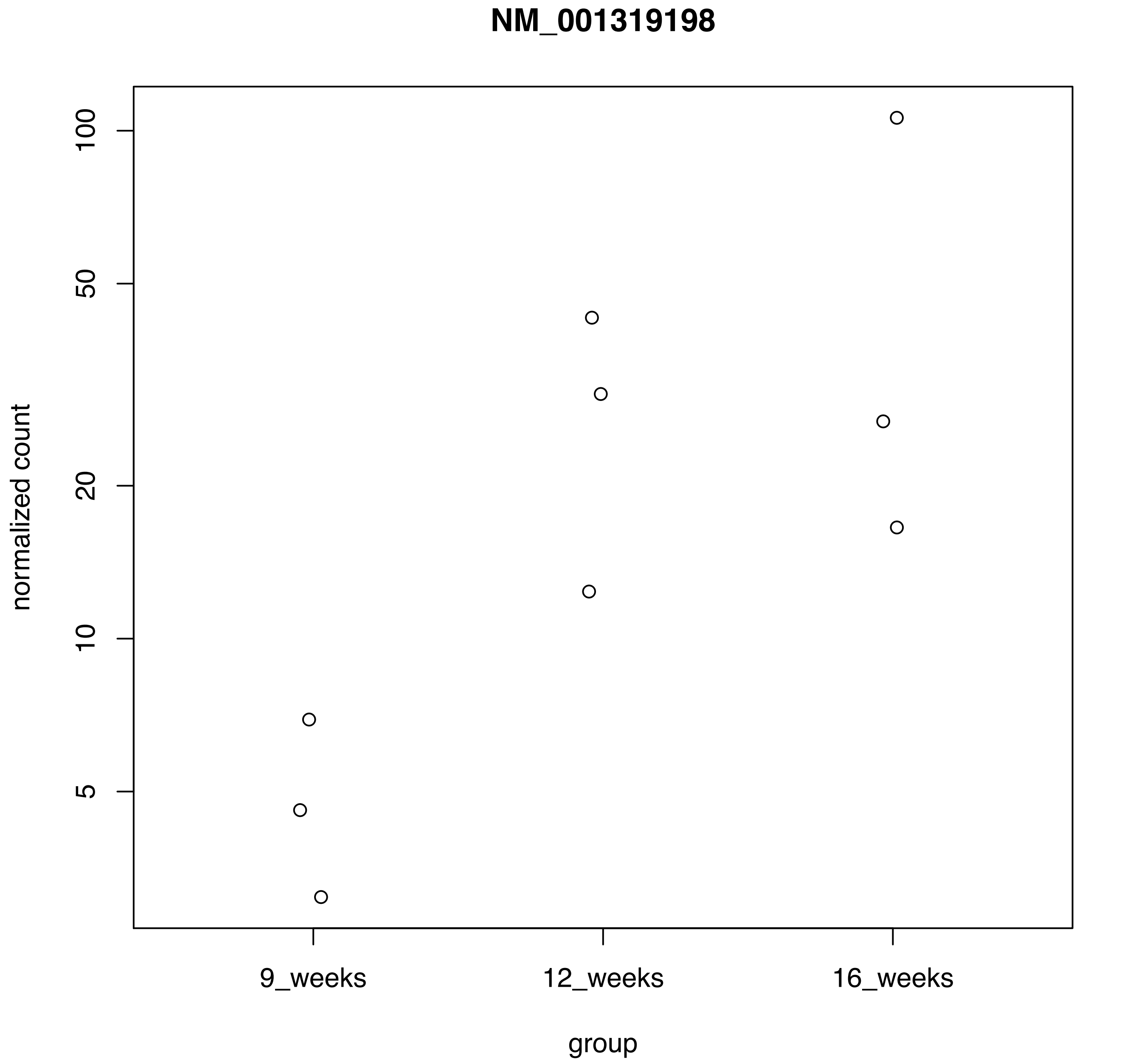
NM_001319198
normalized count
100
50
20
10
5
9_weeks
12_weeks
16_weeks
group

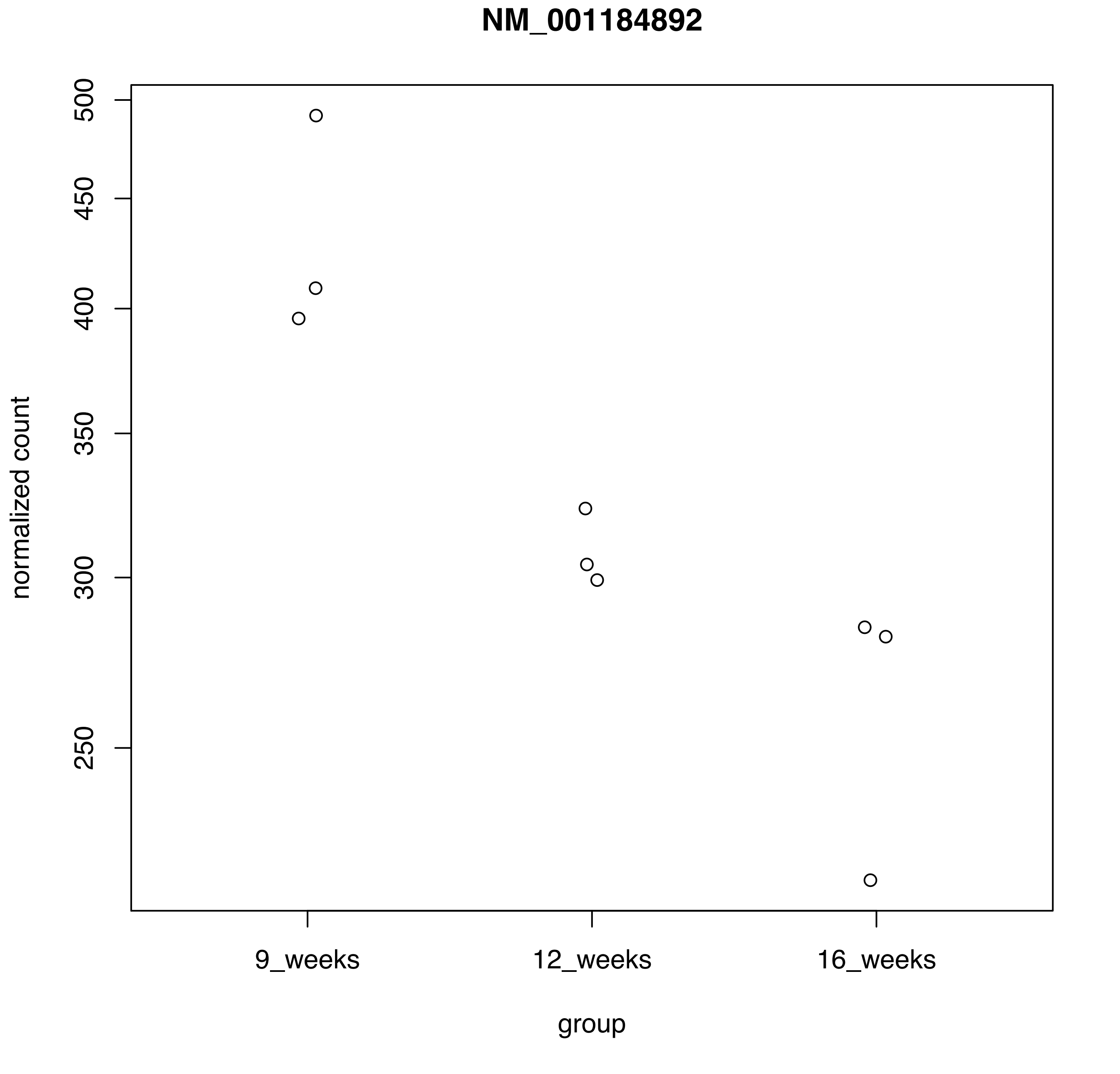
NM_001184892
normalized count
500
450
400
350
300
250
9_weeks
12_weeks
16_weeks
group

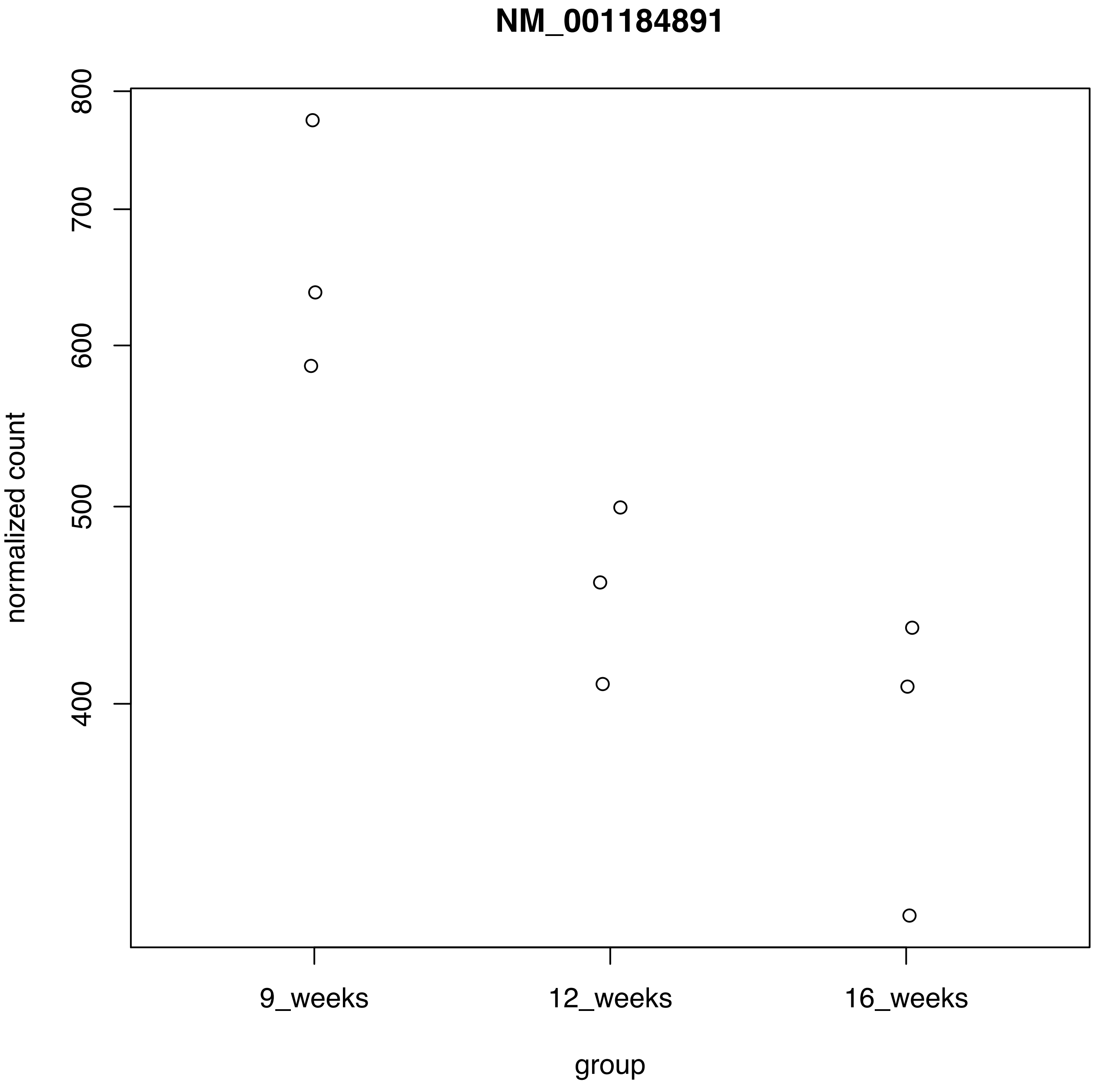
NM_001184891
normalized count
800
700
600
500
400
9_weeks
12_weeks
16_weeks
group

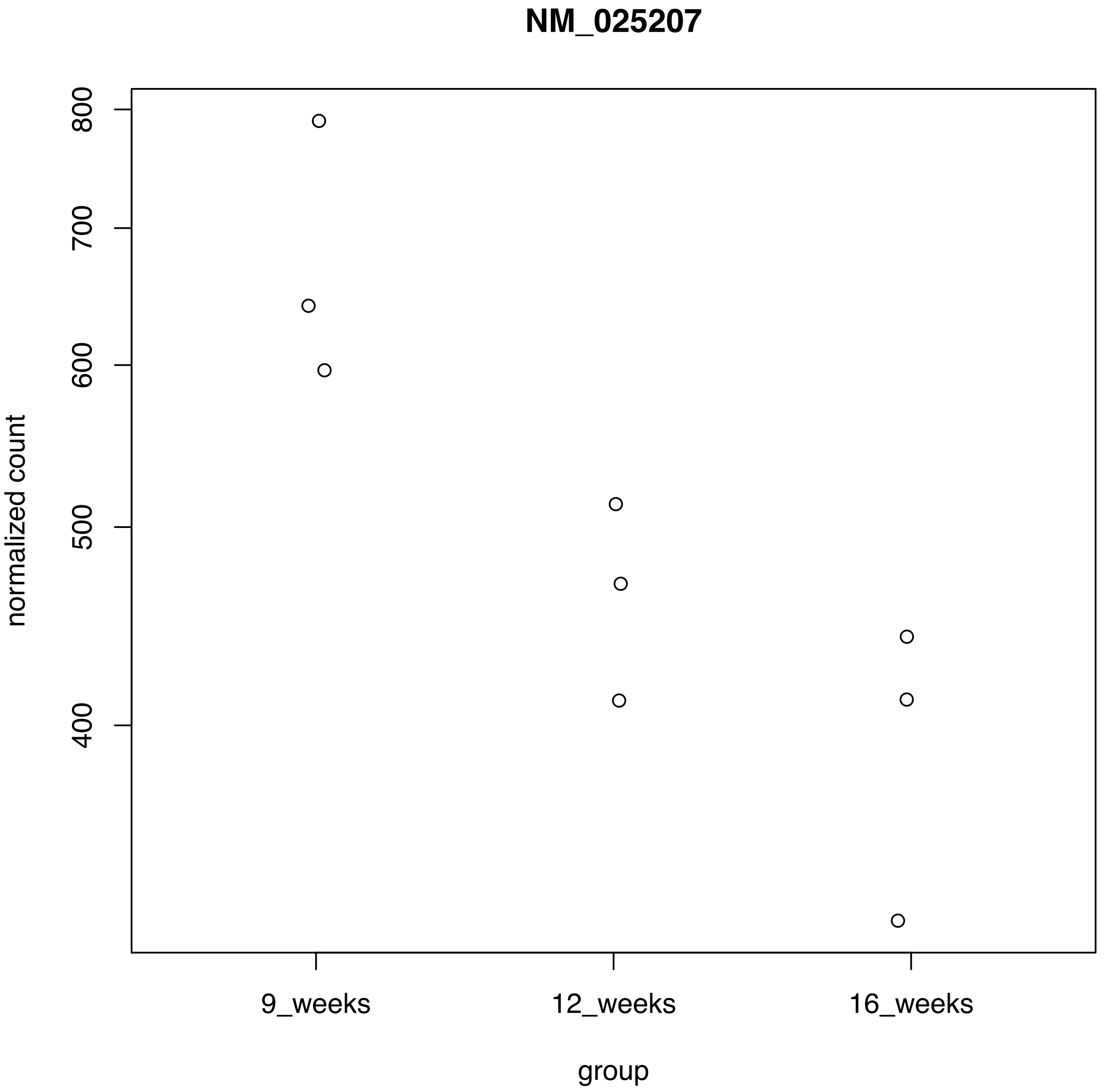
NM_025207
800
700
600
500
400
normalized count
9_weeks
12_weeks
16_weeks
group

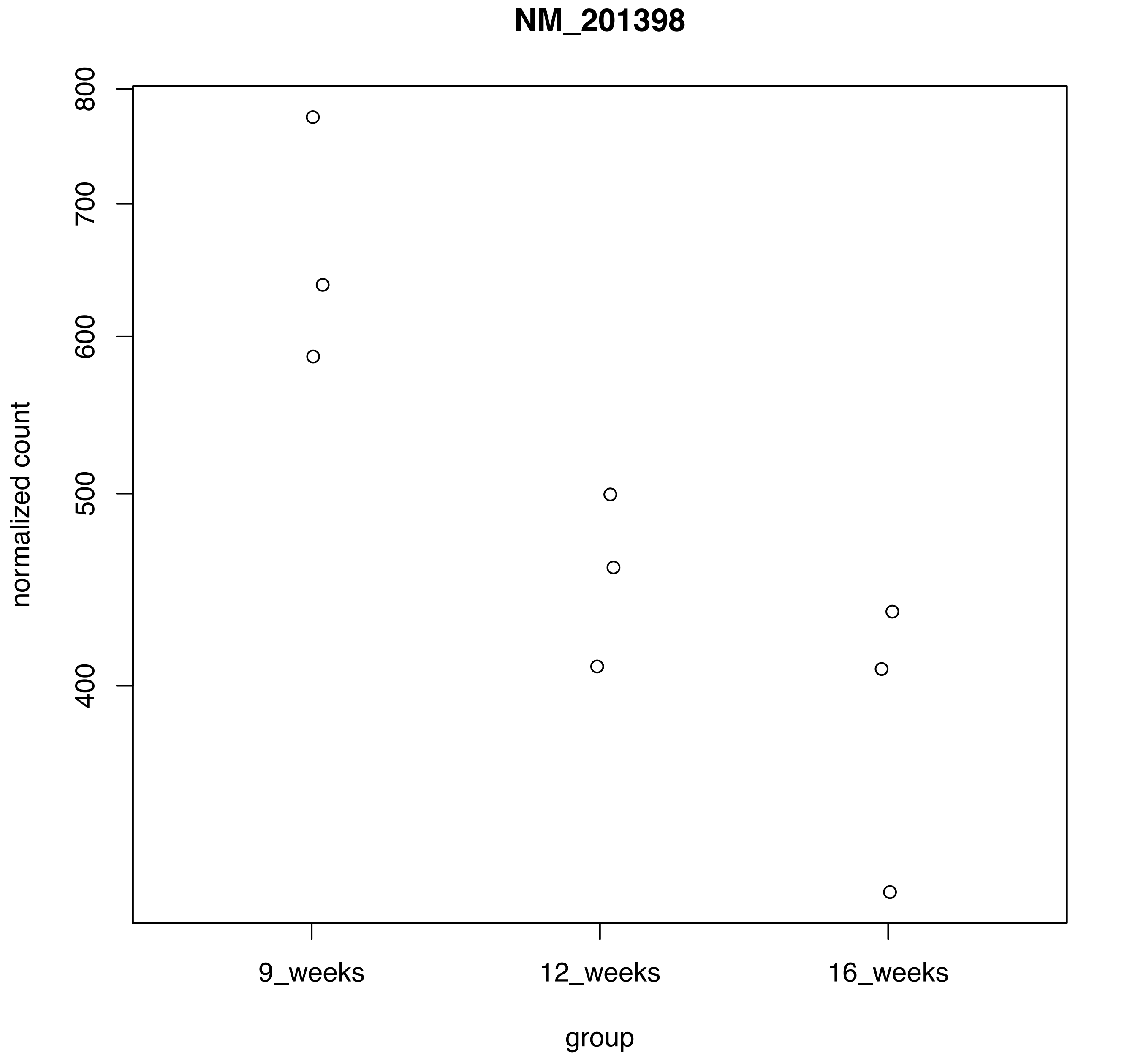

NM_201398
800
700
600
500
400
normalized count
9_weeks
12_weeks
16_weeks
group

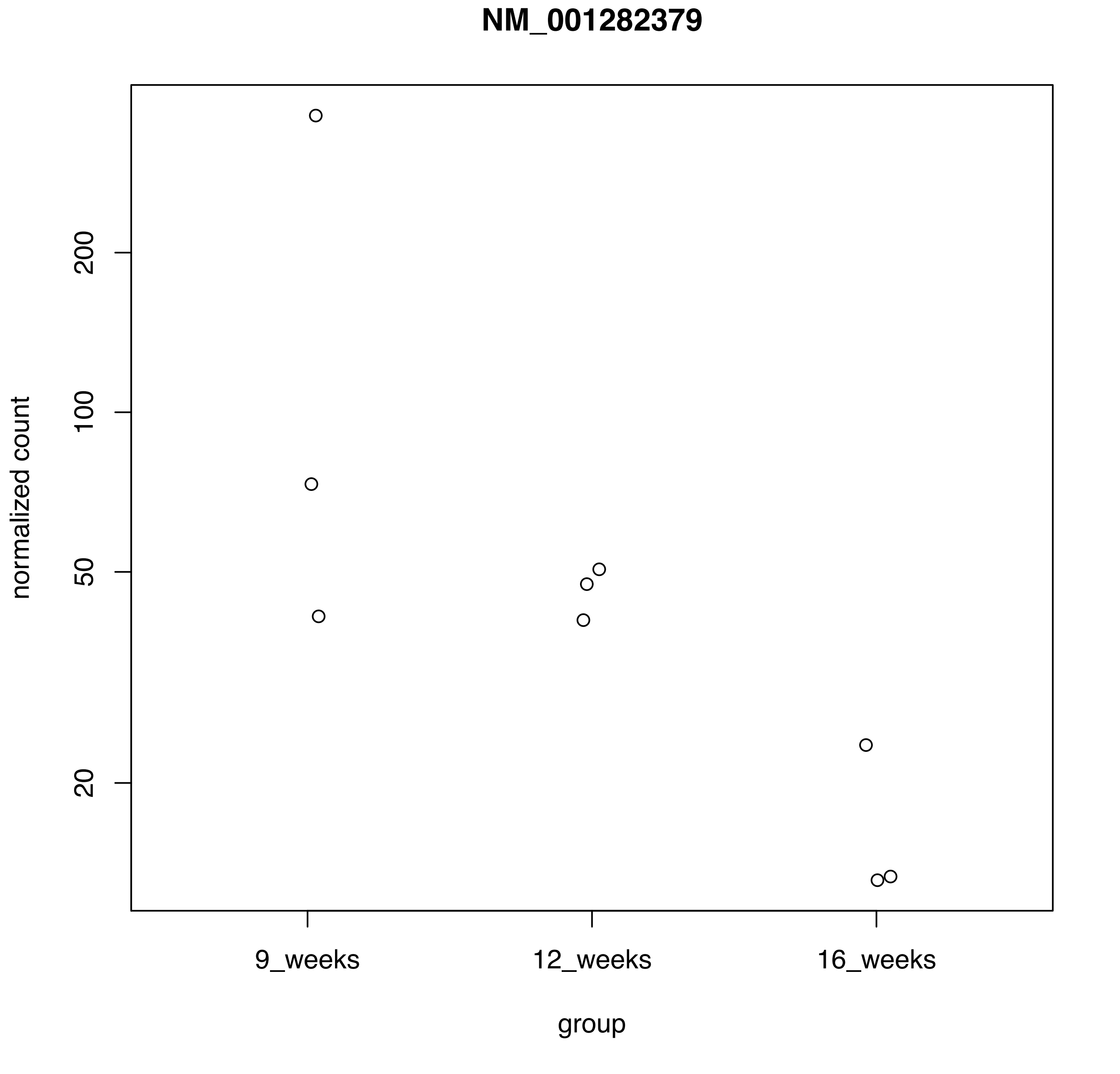

NM_001282379
normalized count
200
100
50
20
9_weeks
12_weeks
16_weeks
group

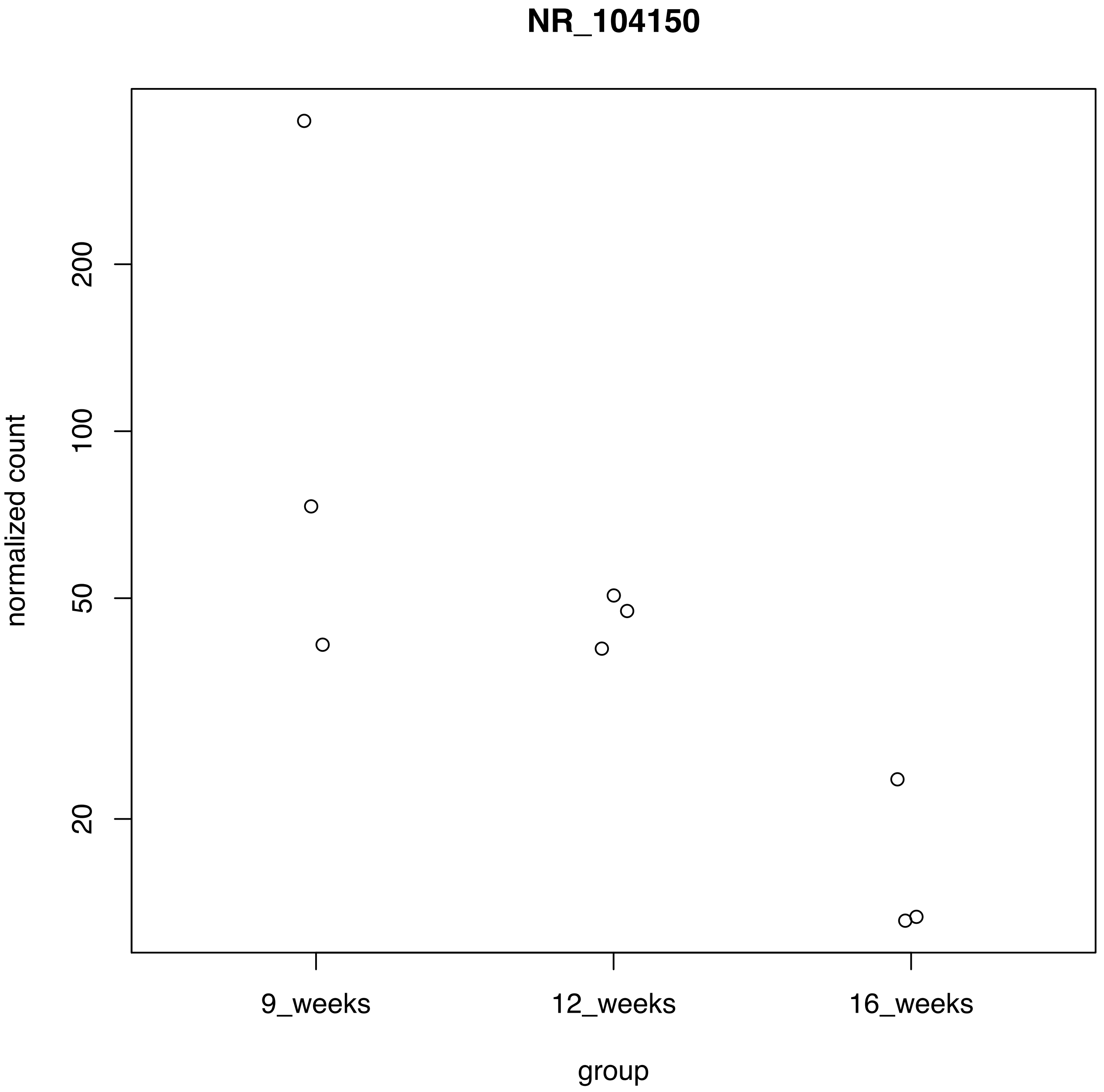
NR_104150
normalized count
200
100
50
20
9_weeks
12_weeks
16_weeks
group

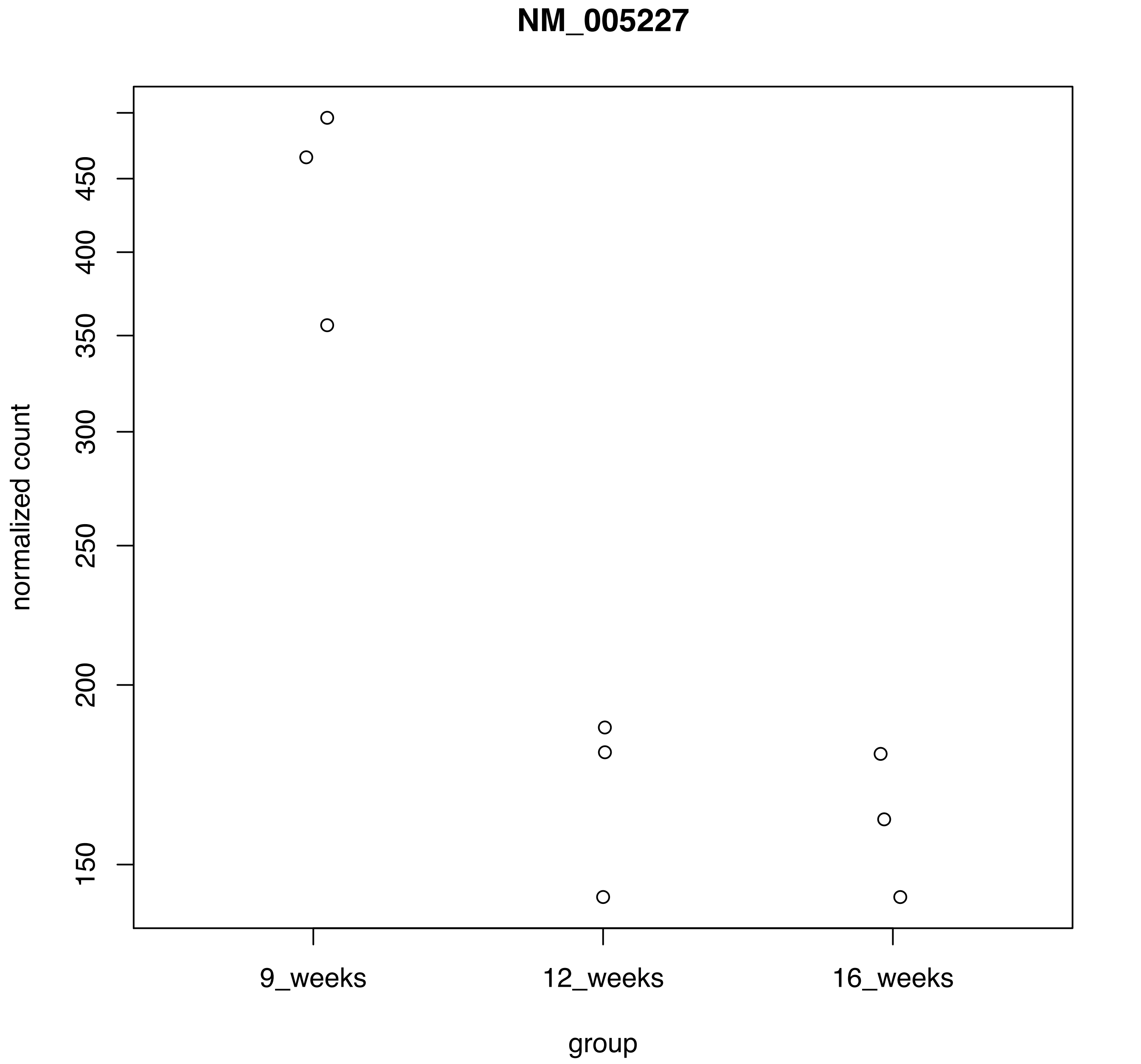

NM_005227
normalized count
150
200
250
300
350
400
450
9_weeks
12_weeks
16_weeks
group

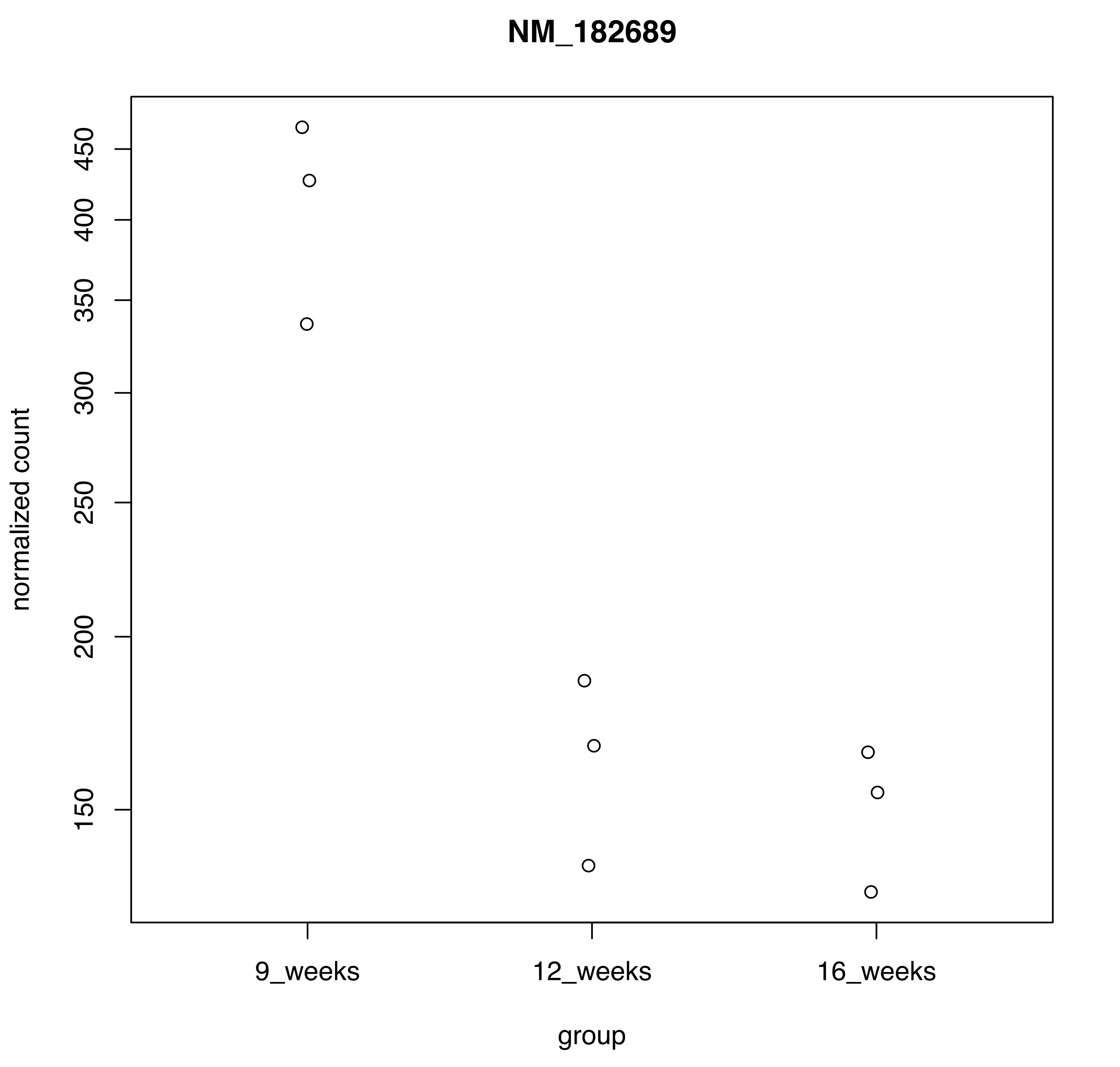
NM_182689
normalized count
450
400
350
300
250
200
150
9_weeks
12_weeks
16_weeks
group

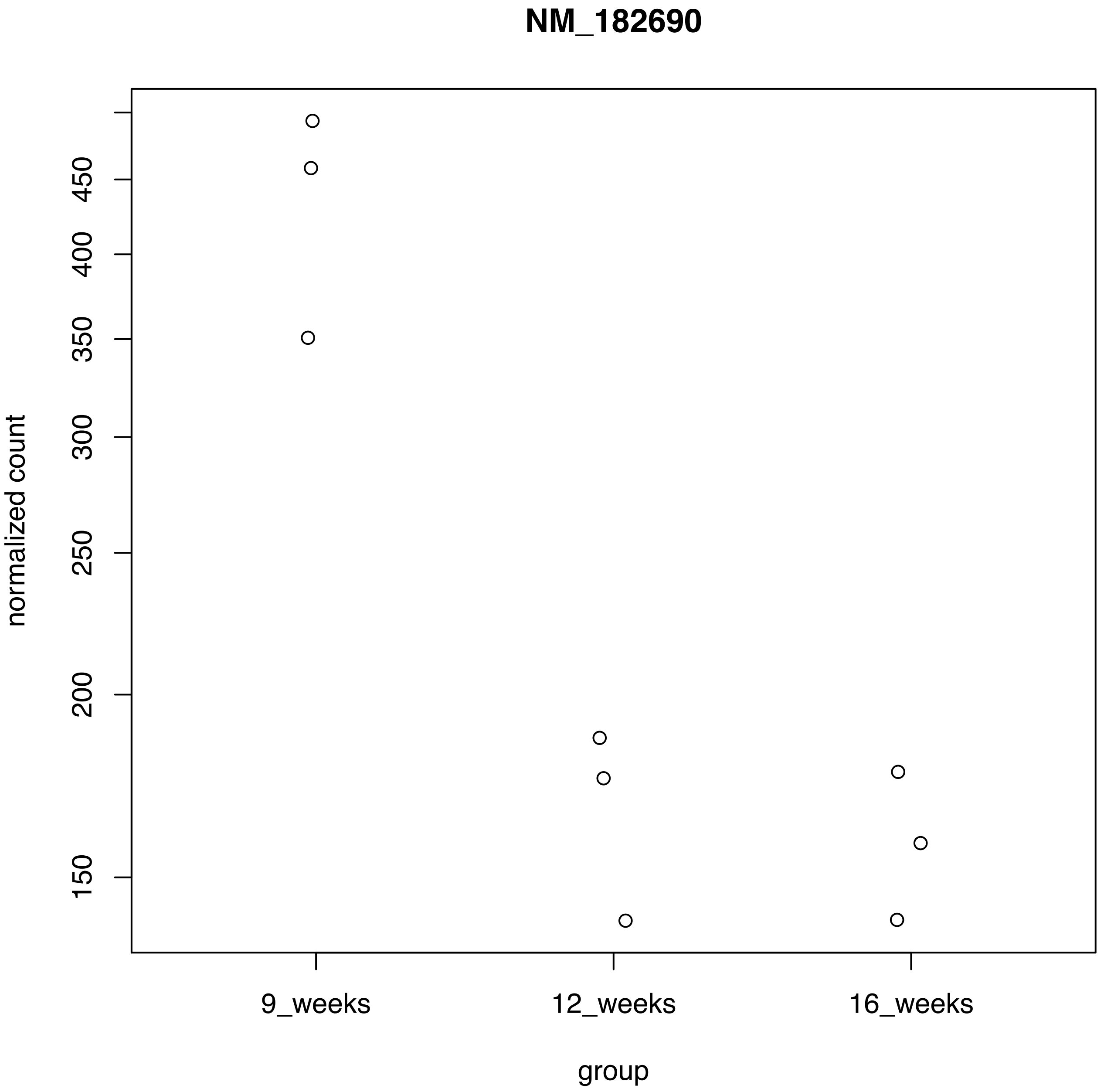
NM_182690
normalized count
450
400
350
300
250
200
150
9_weeks
12_weeks
16_weeks
group

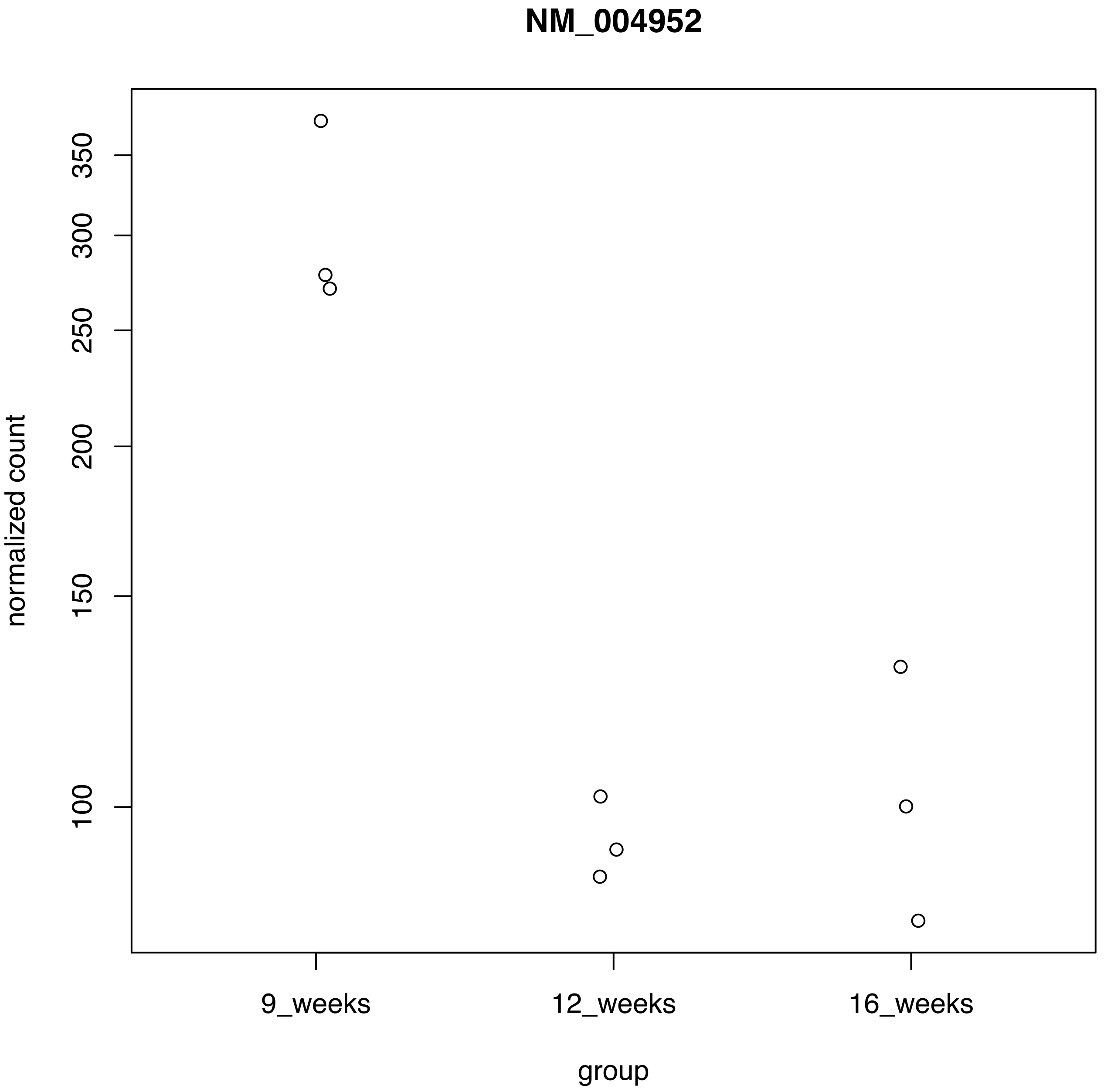
NM_004952
normalized count
350
300
250
200
150
100
9_weeks
12_weeks
16_weeks
group

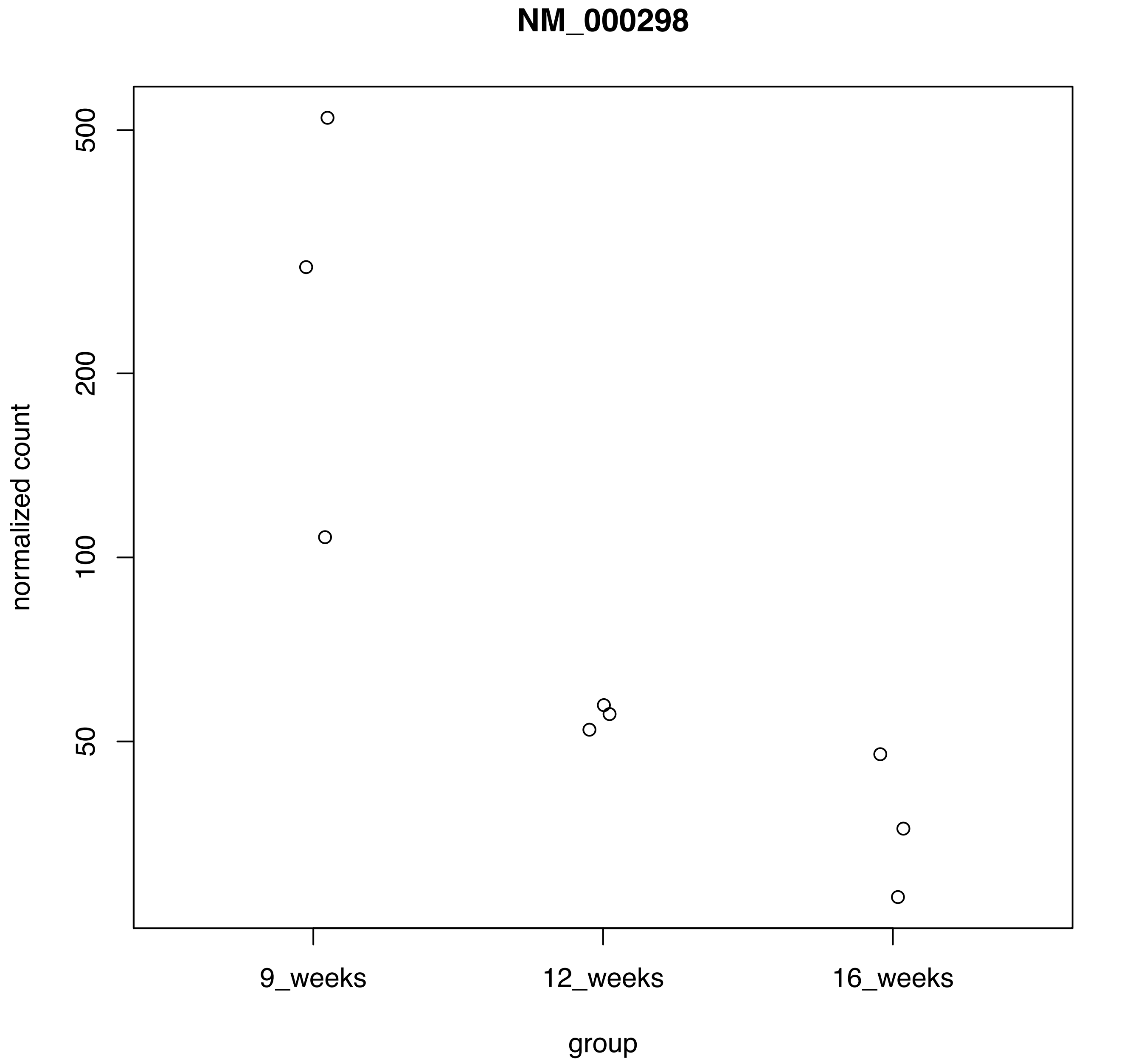
NM_000298
normalized count
500
200
100
50
9_weeks
12_weeks
16_weeks
group

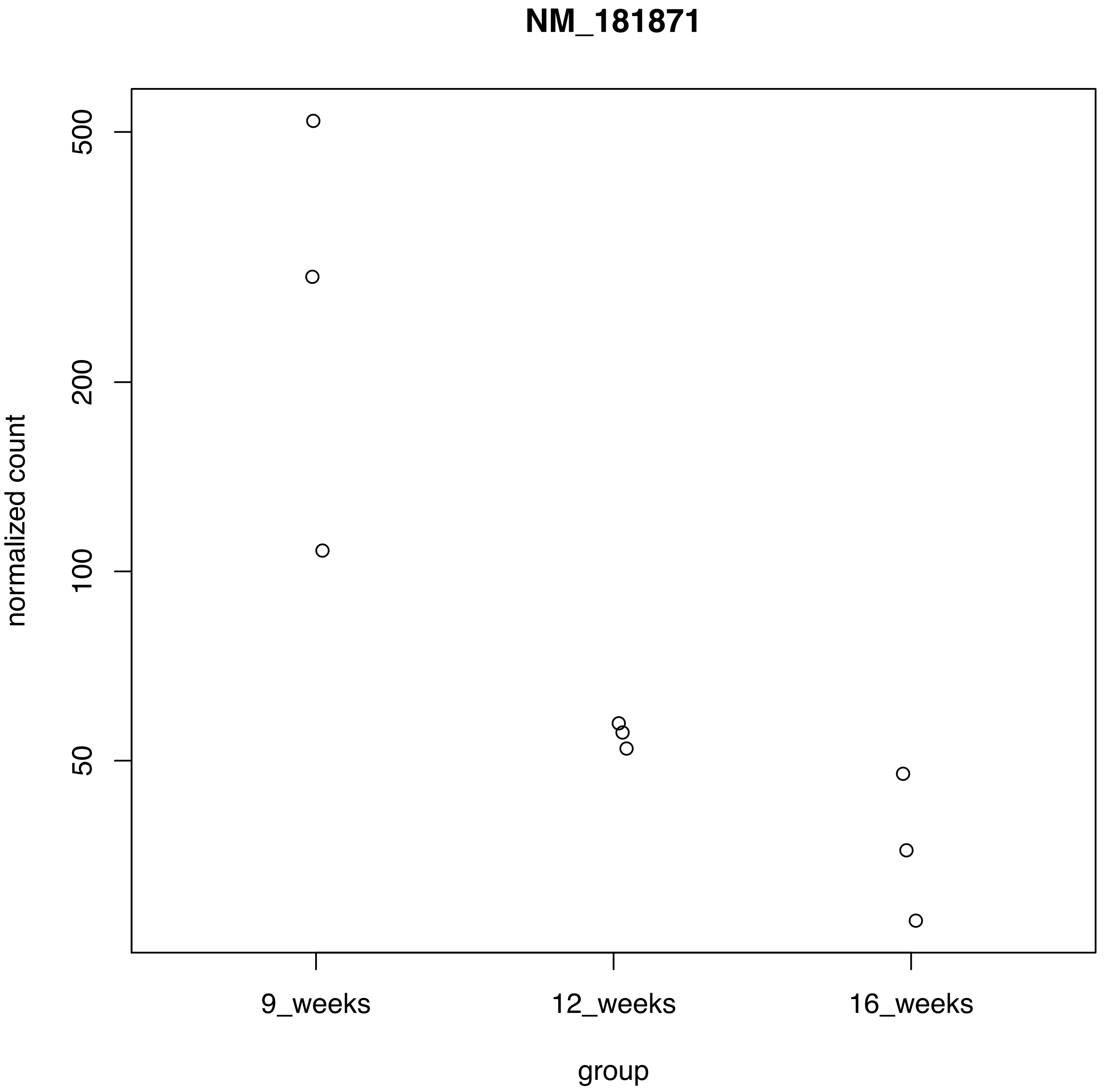
NM_181871
normalized count
500
200
100
50
9_weeks
12_weeks
16_weeks
group

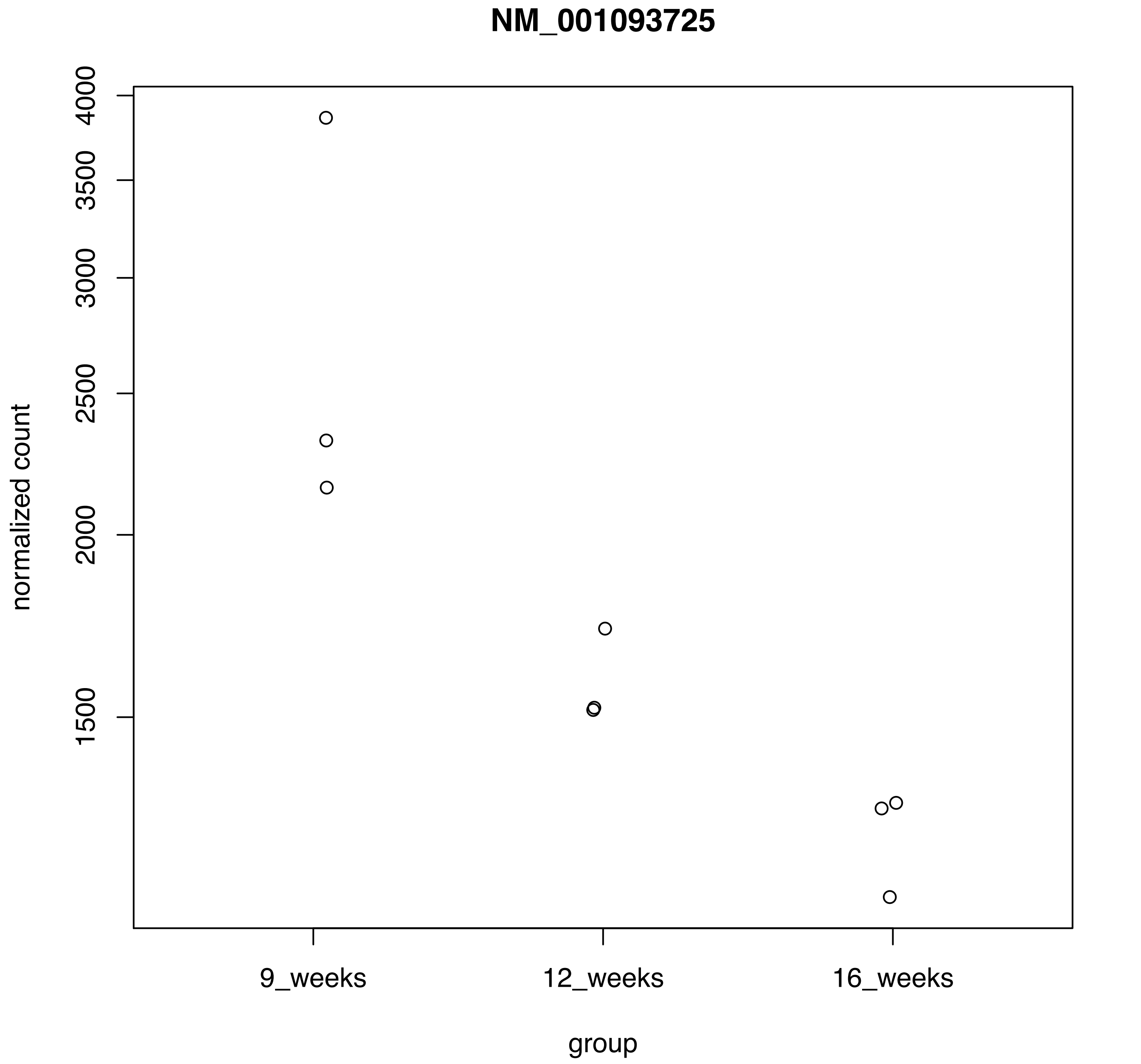

NM_001093725
normalized count
4000
3500
3000
2500
2000
1500
9_weeks
12_weeks
16_weeks
group

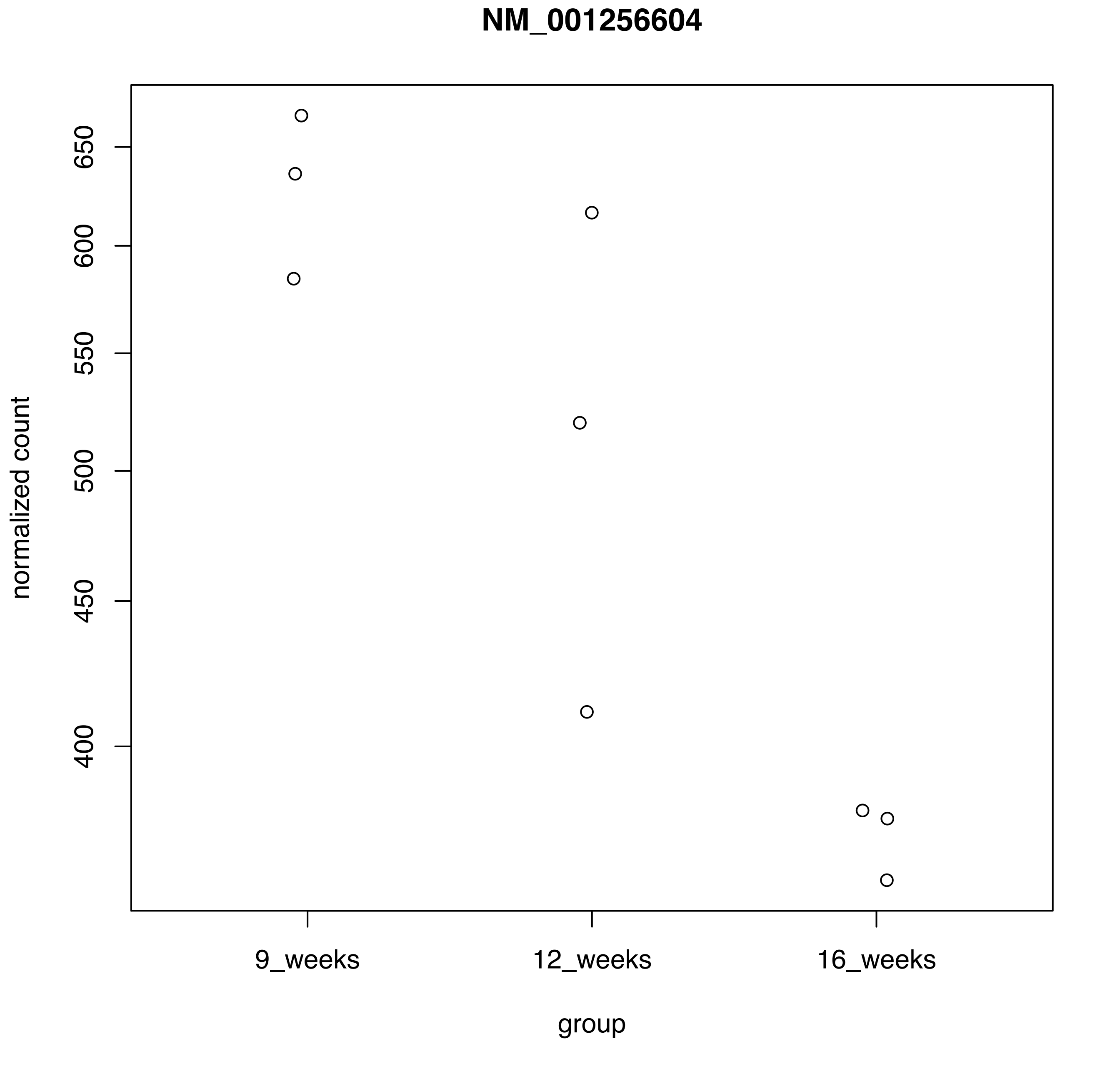

NM_001256604
normalized count
650
600
550
500
450
400
9_weeks
12_weeks
16_weeks
group

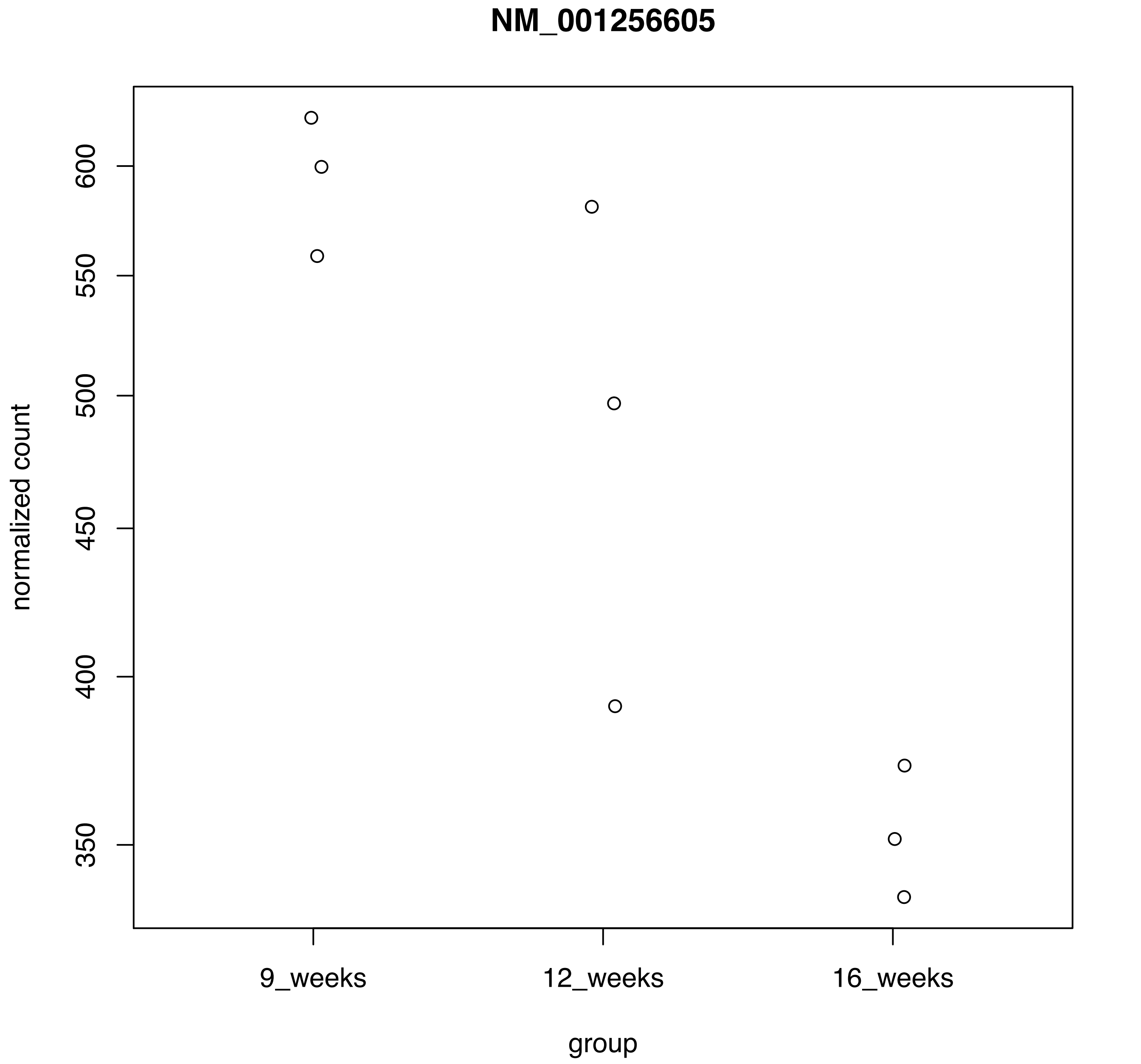
NM_001256605
normalized count
350
400
450
500
550
600
9_weeks
12_weeks
16_weeks
group

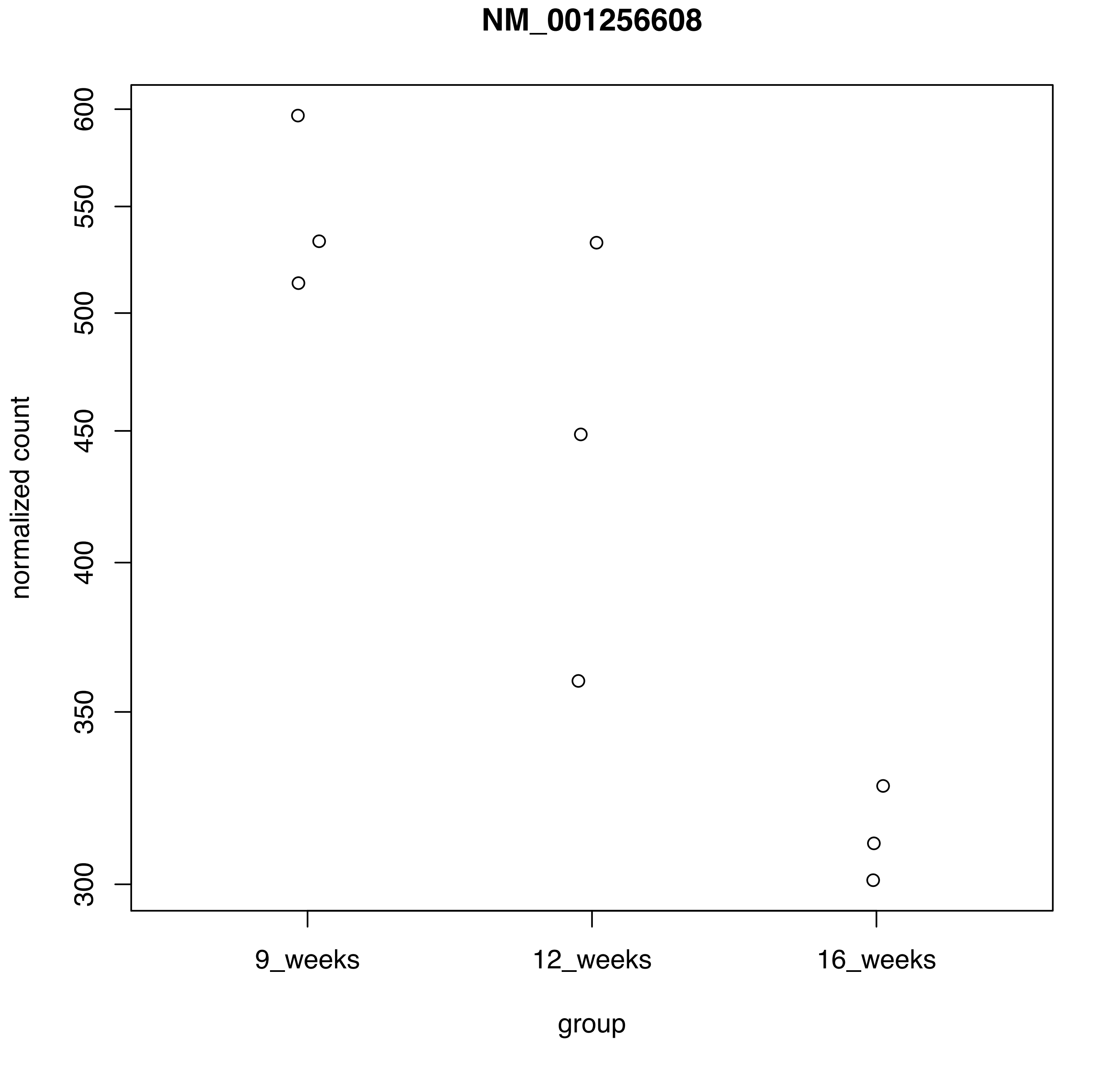
NM_001256608
normalized count
600
550
500
450
400
350
300
9_weeks
12_weeks
16_weeks
group

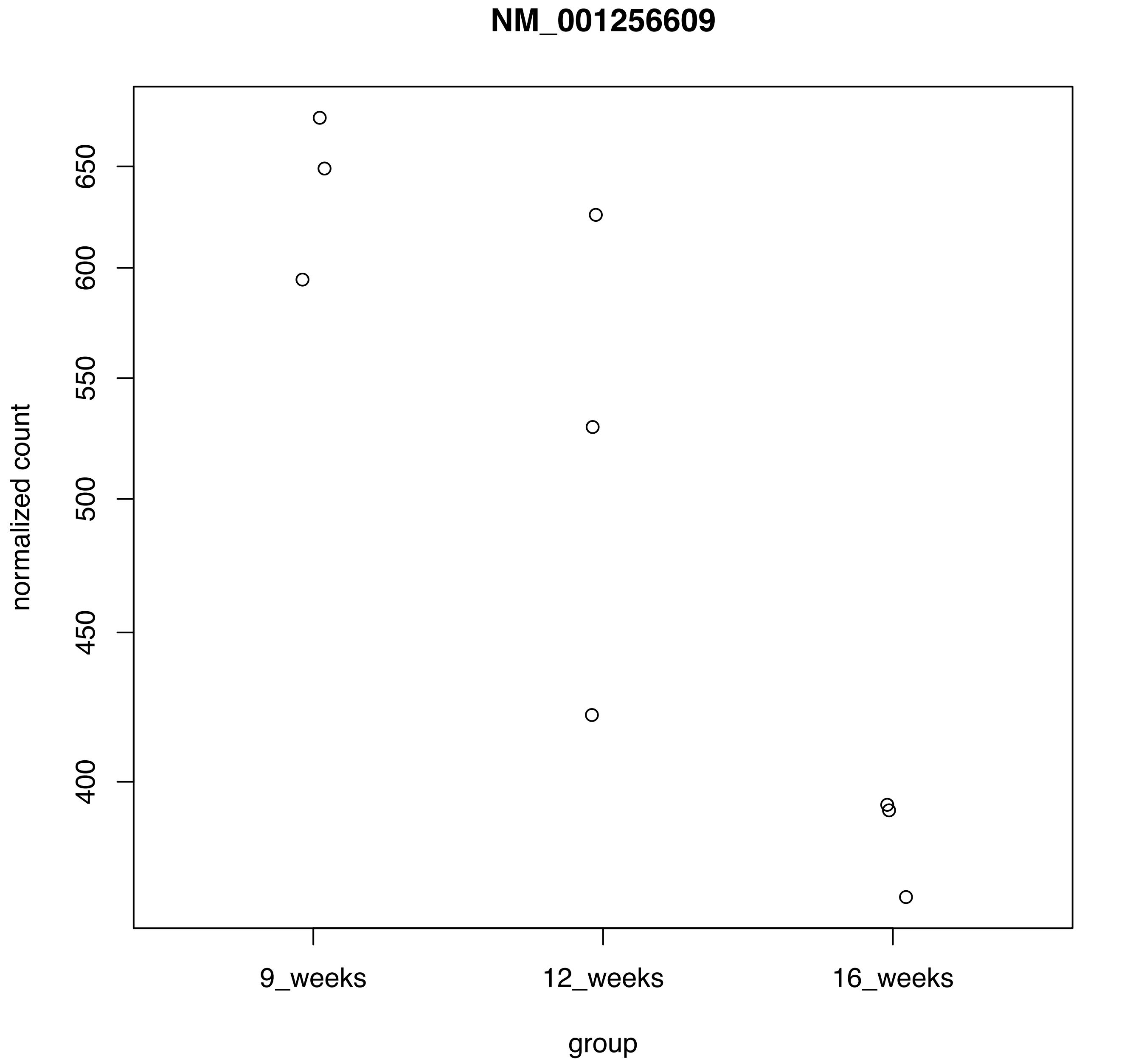
NM_001256609
normalized count
650
600
550
500
450
400
9_weeks
12_weeks
16_weeks
group

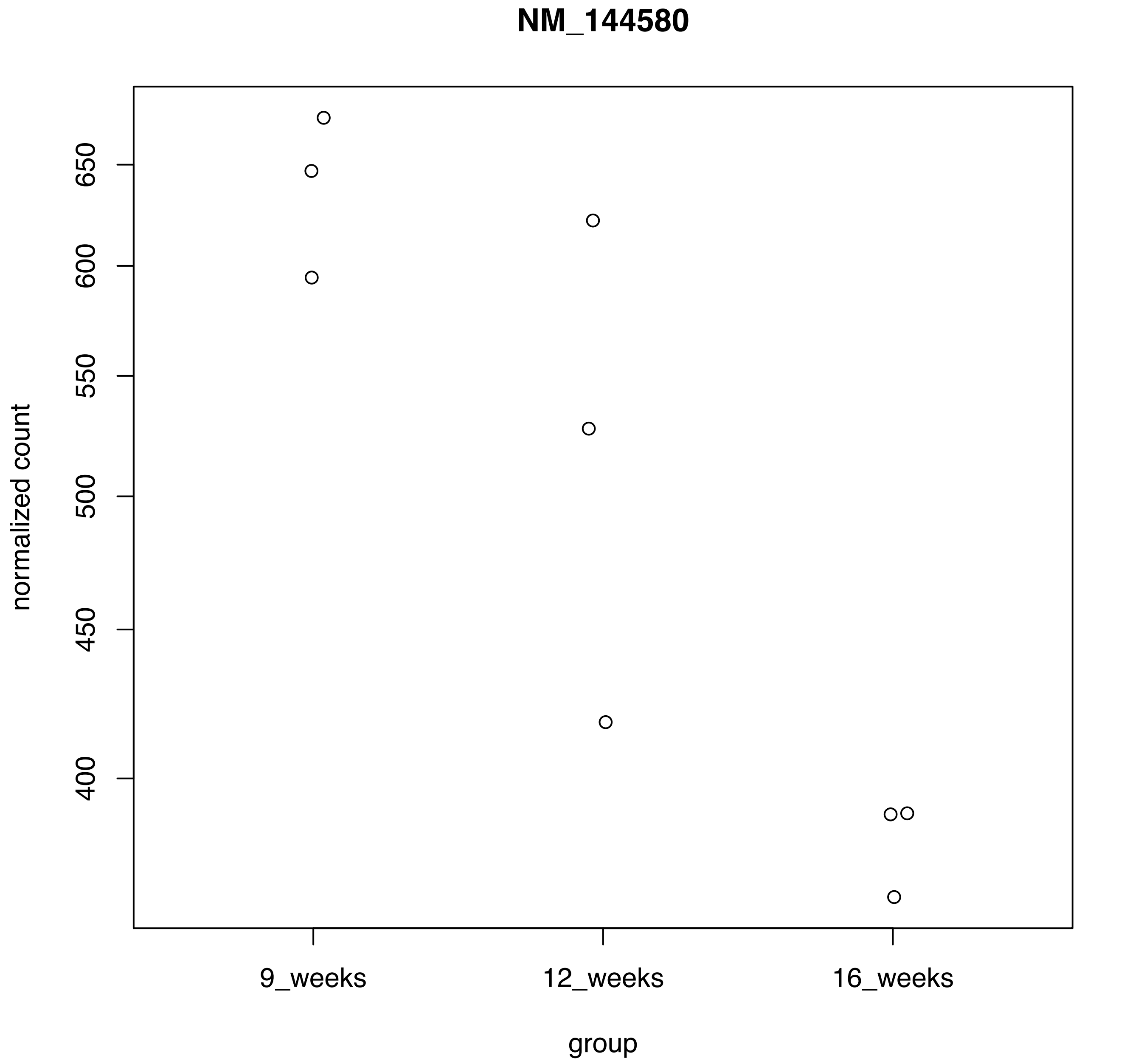
NM_144580
normalized count
650
600
550
500
450
400
9_weeks
12_weeks
16_weeks
group

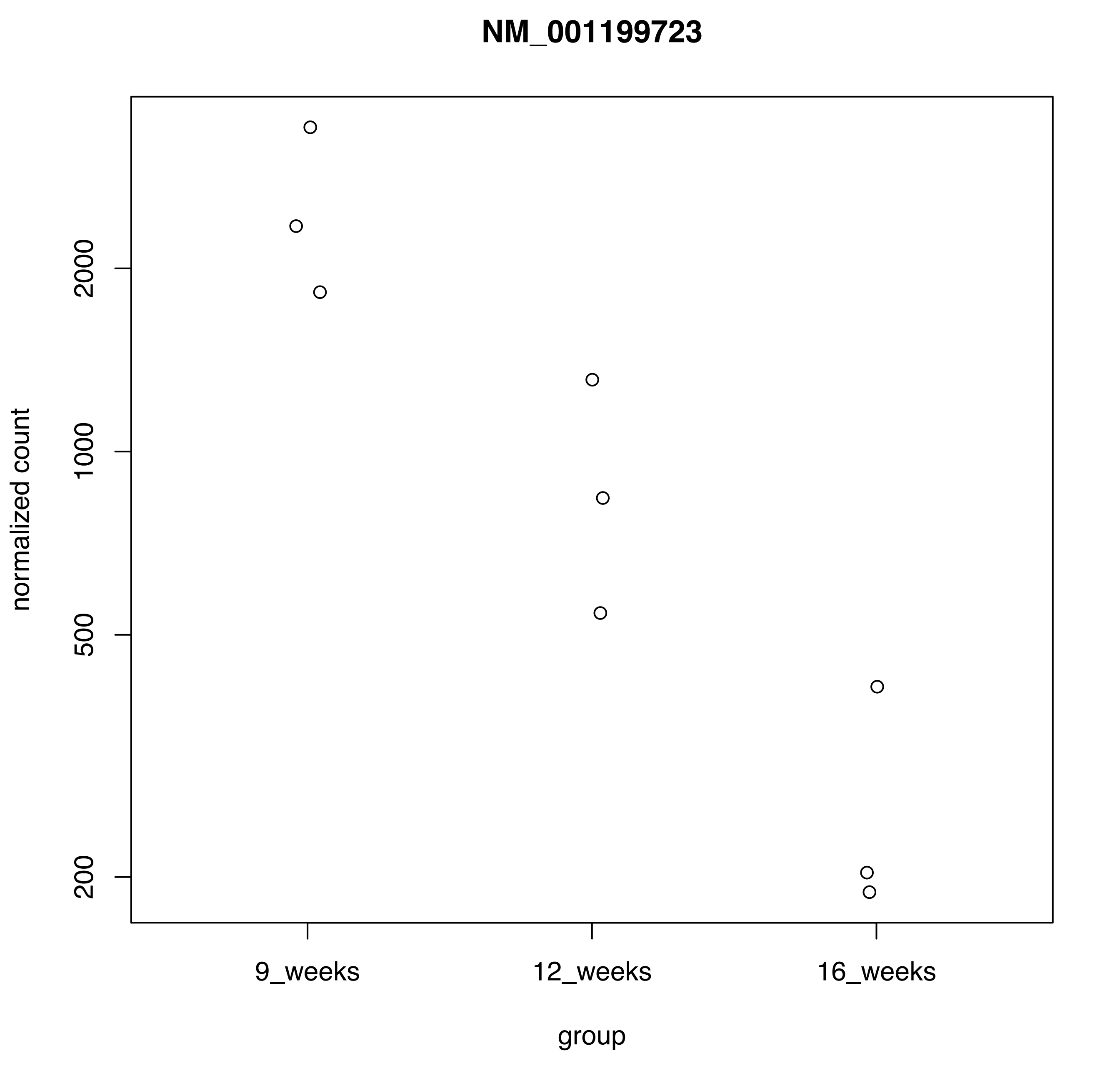
NM_001199723
normalized count
2000
1000
500
200
9_weeks
12_weeks
16_weeks
group

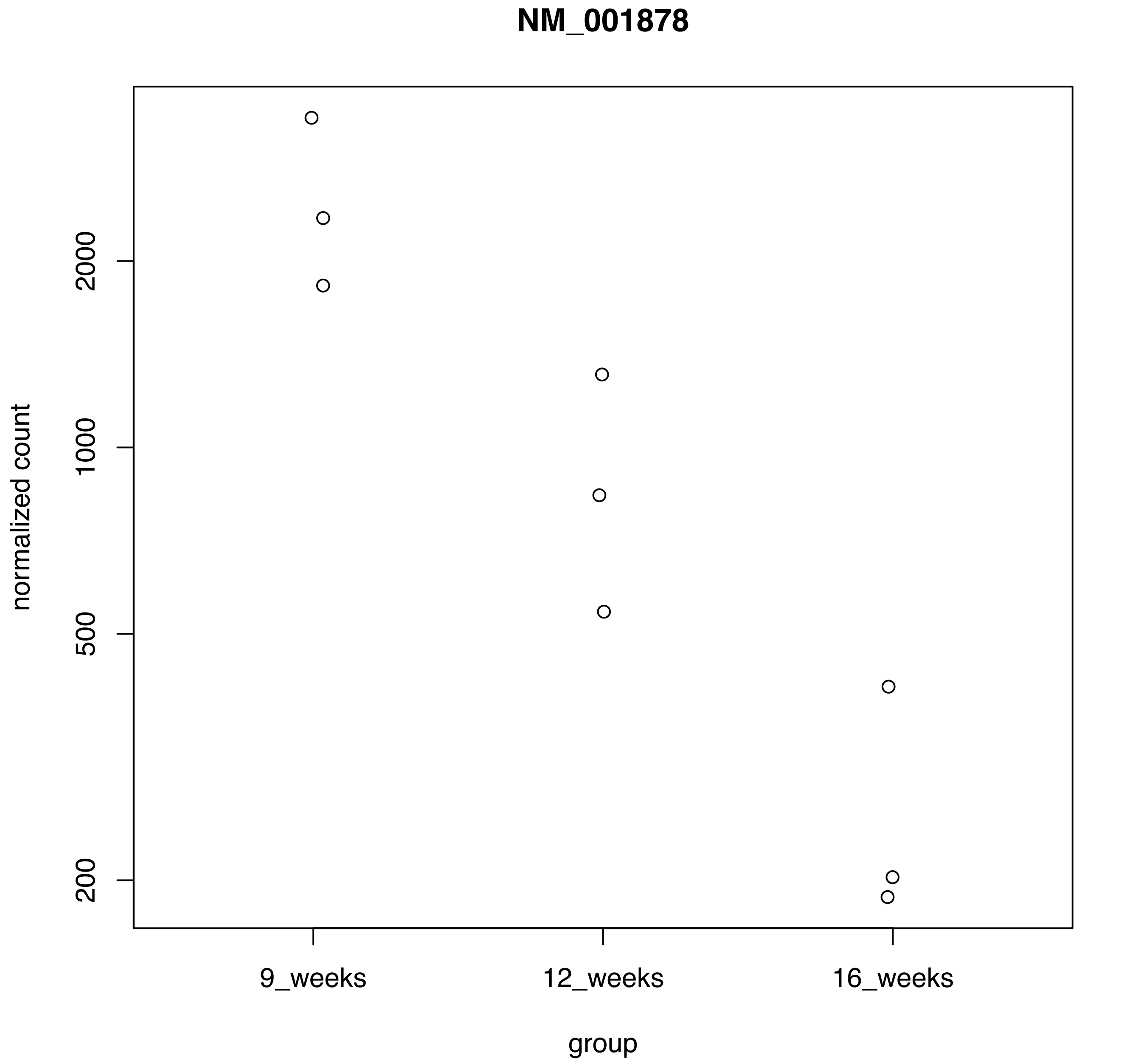
NM_001878
normalized count
2000
1000
500
200
9_weeks
12_weeks
16_weeks
group

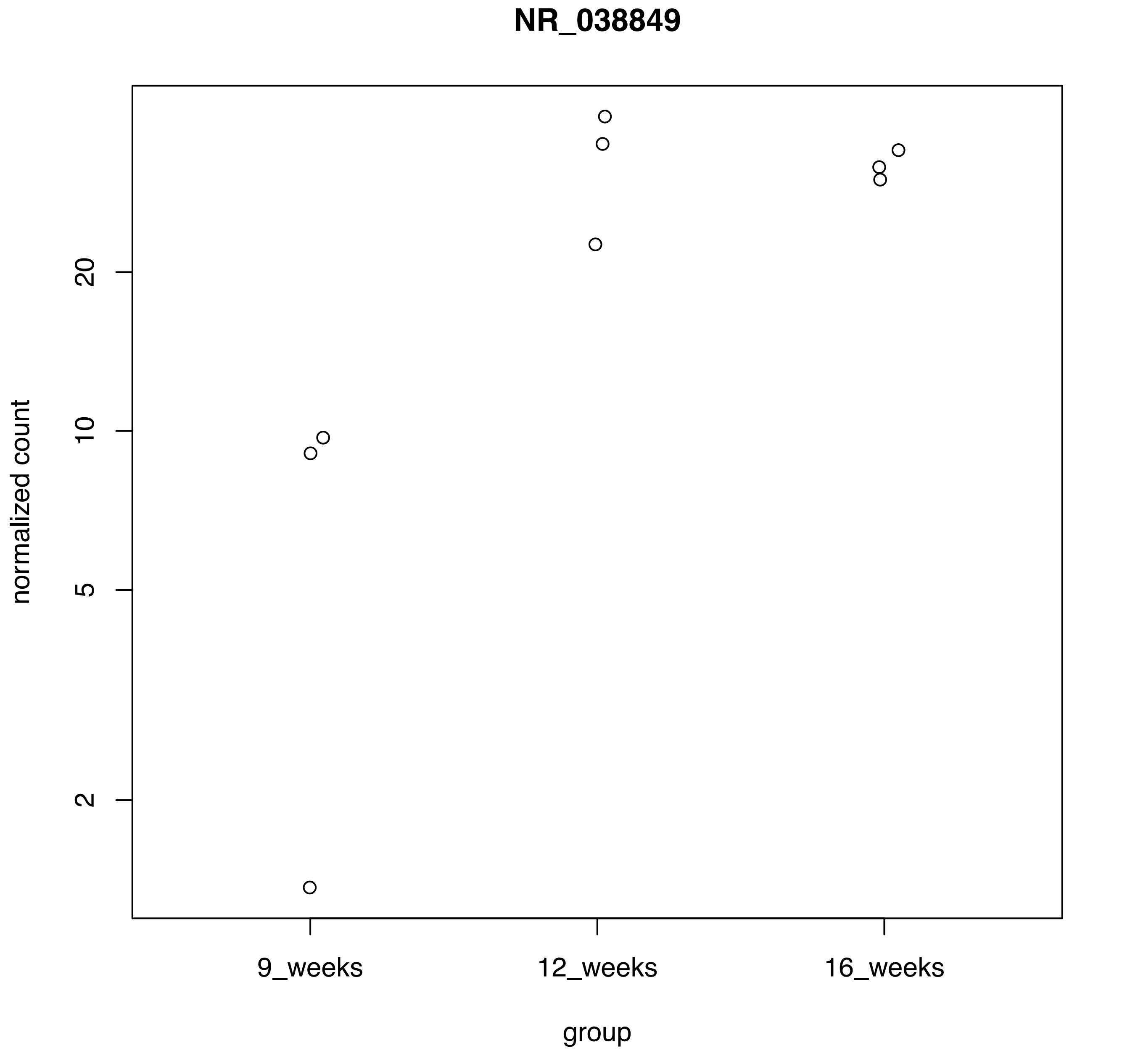
NR_038849
normalized count
20
10
5
2
9_weeks
12_weeks
16_weeks
group

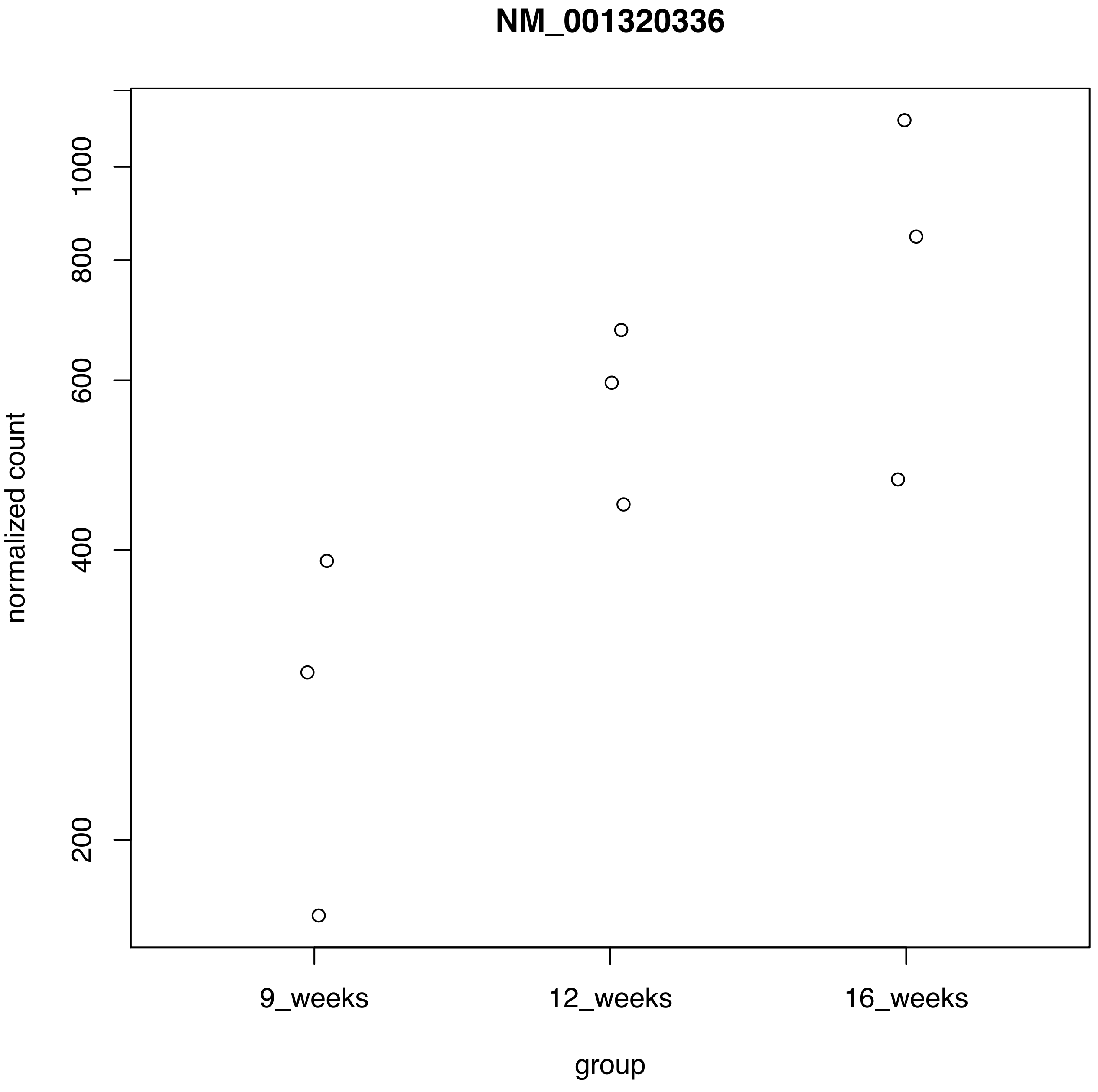

NM_001320336
normalized count
200
400
600
800
1000
9_weeks
12_weeks
16_weeks
group

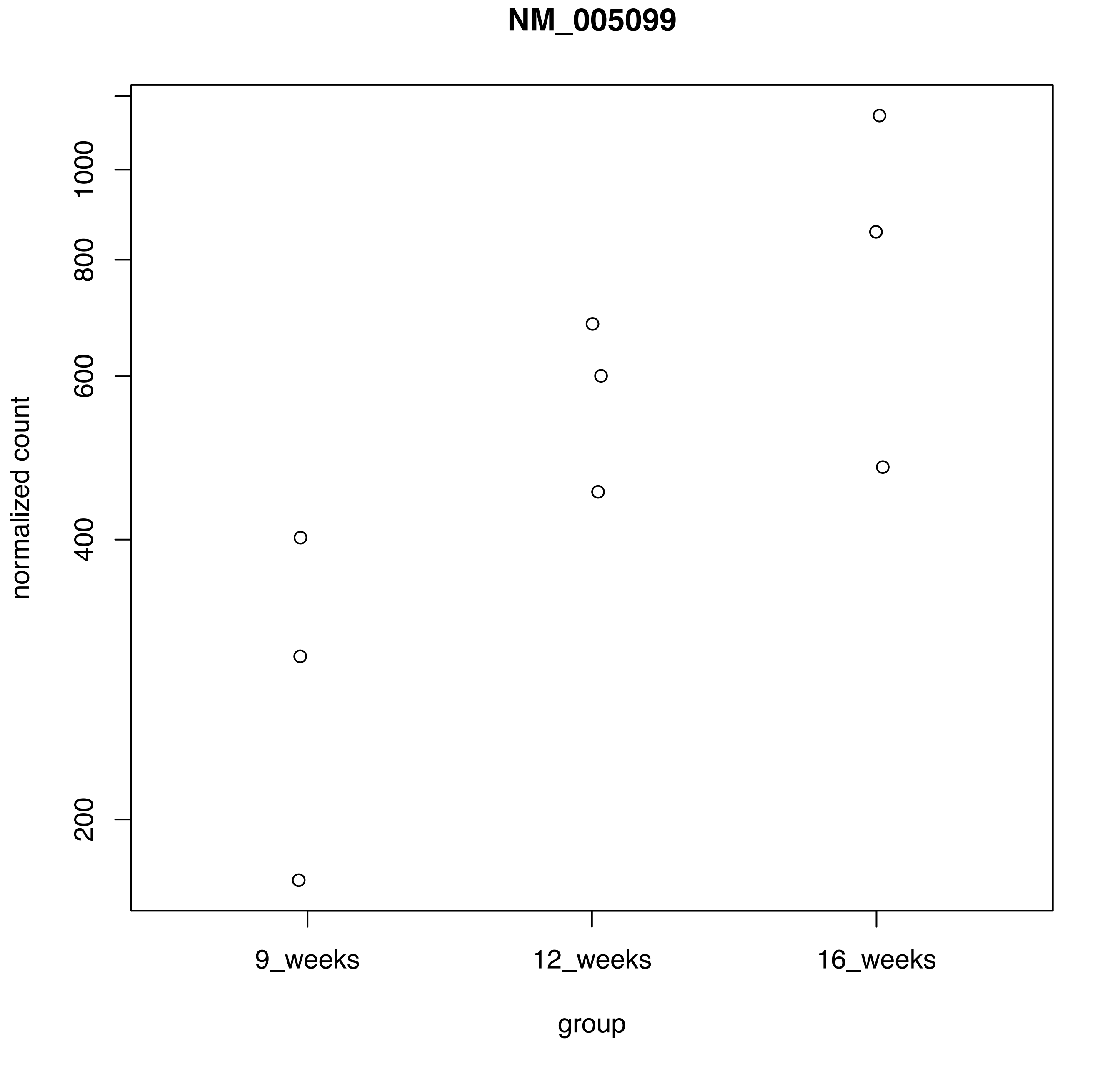

NM_005099
normalized count
200
400
600
800
1000
9_weeks
12_weeks
16_weeks
group

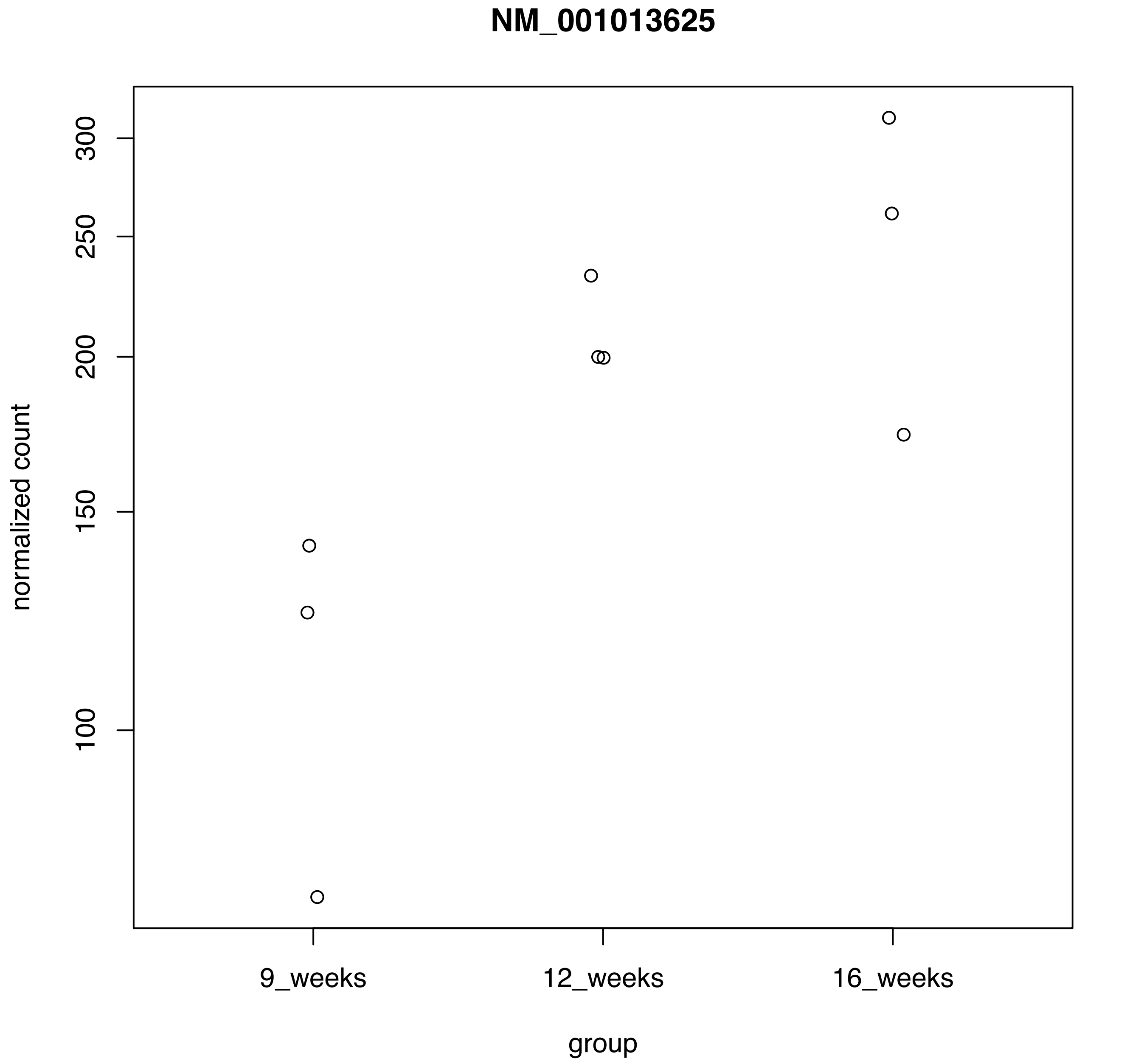
NM_001013625
normalized count
300
250
200
150
100
9_weeks
12_weeks
16_weeks
group

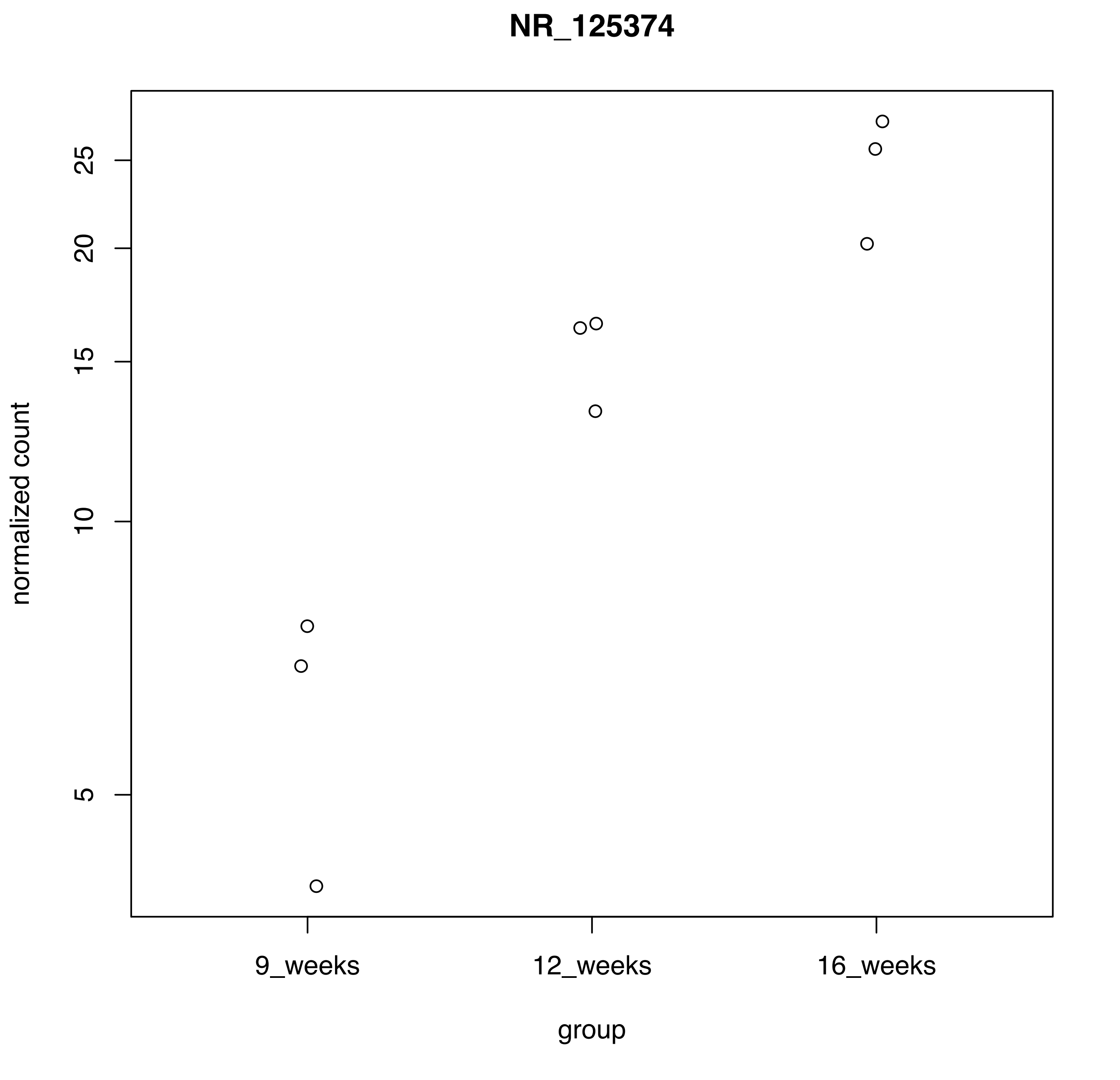

NR_125374
normalized count
25
20
15
10
5
9_weeks
12_weeks
16_weeks
group

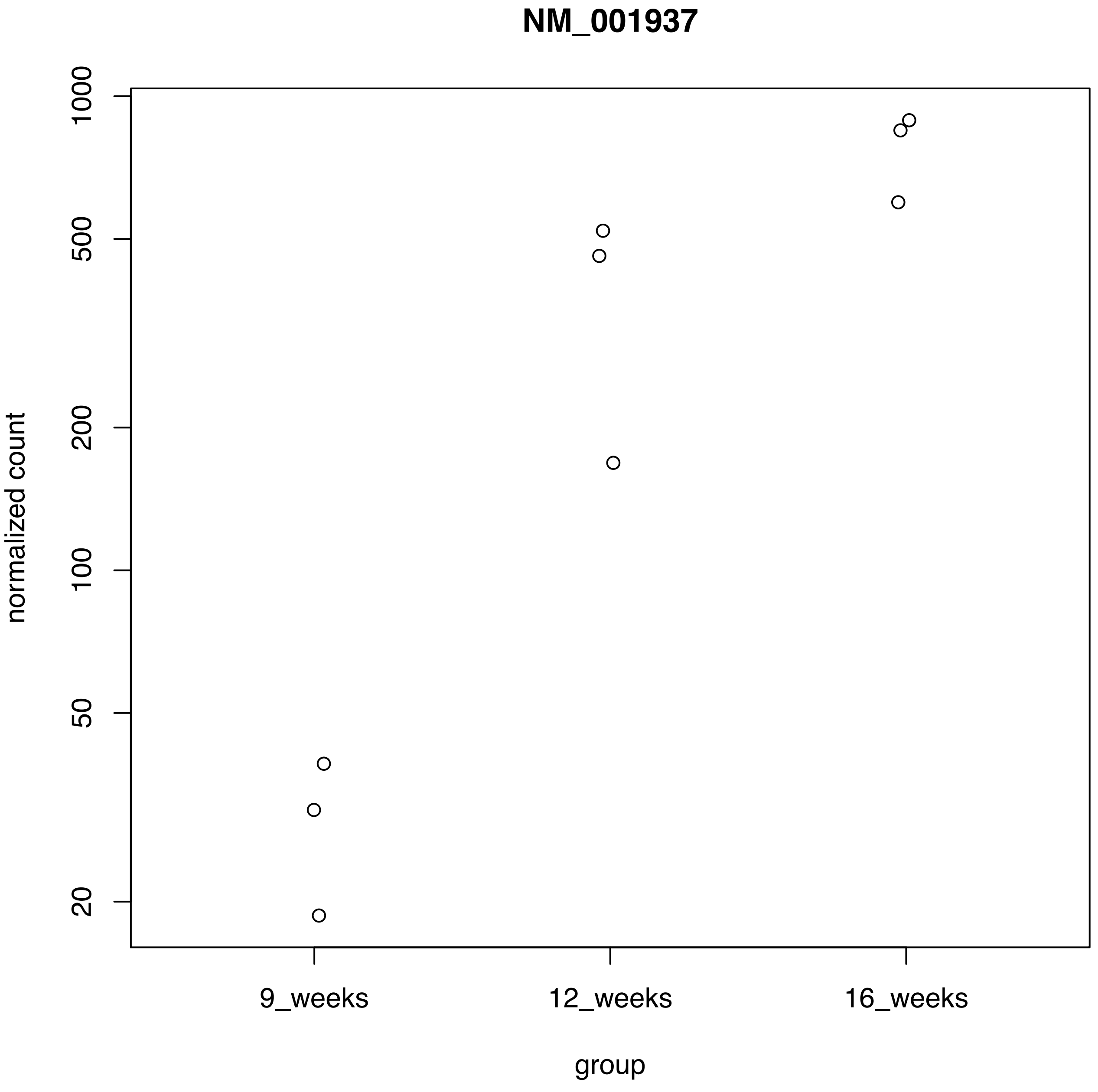

NM_001937
normalized count
1000
500
200
100
50
20
9_weeks
12_weeks
16_weeks
group

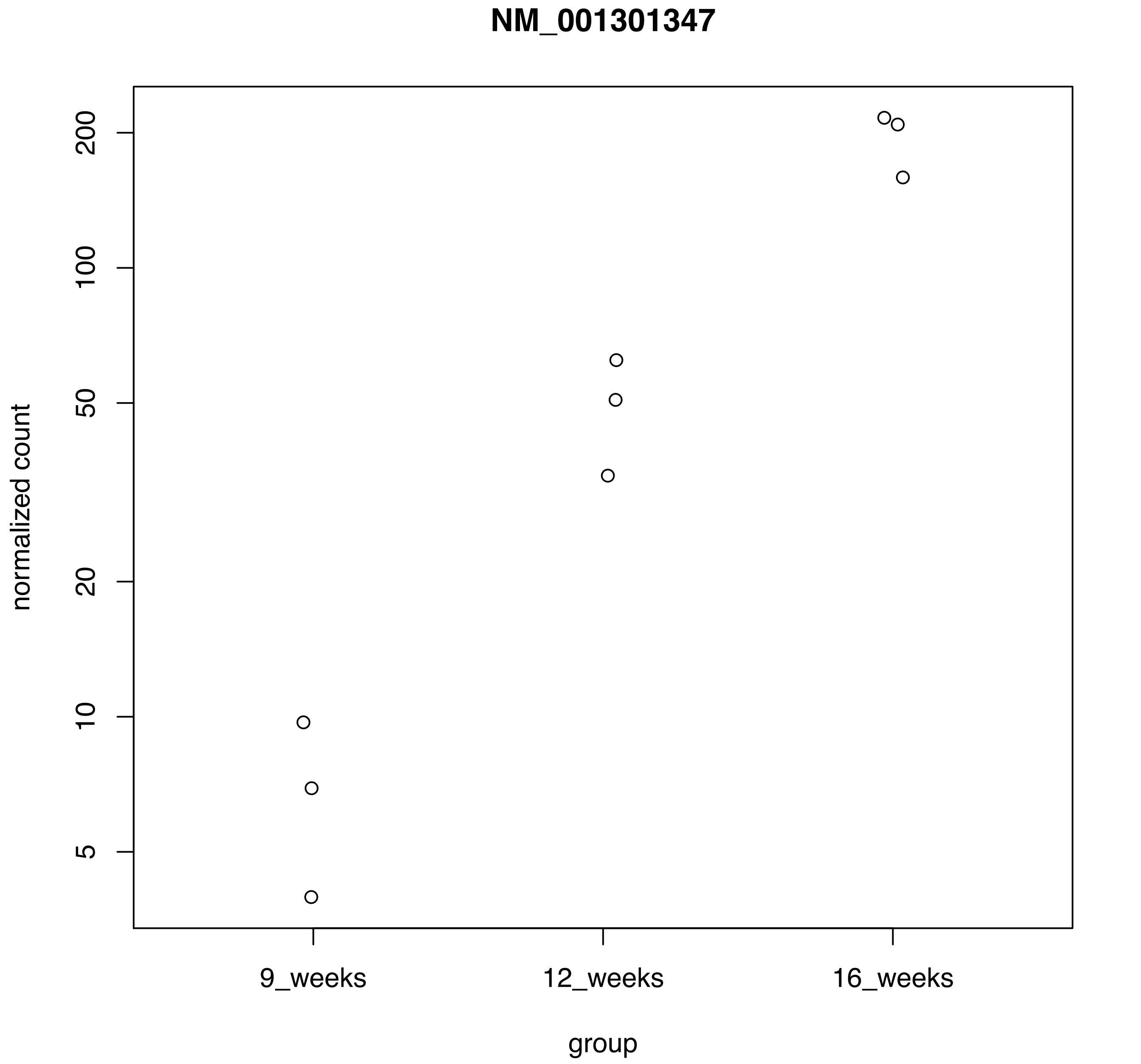

NM_001301347
normalized count
200
100
50
20
10
5
9_weeks
12_weeks
16_weeks
group

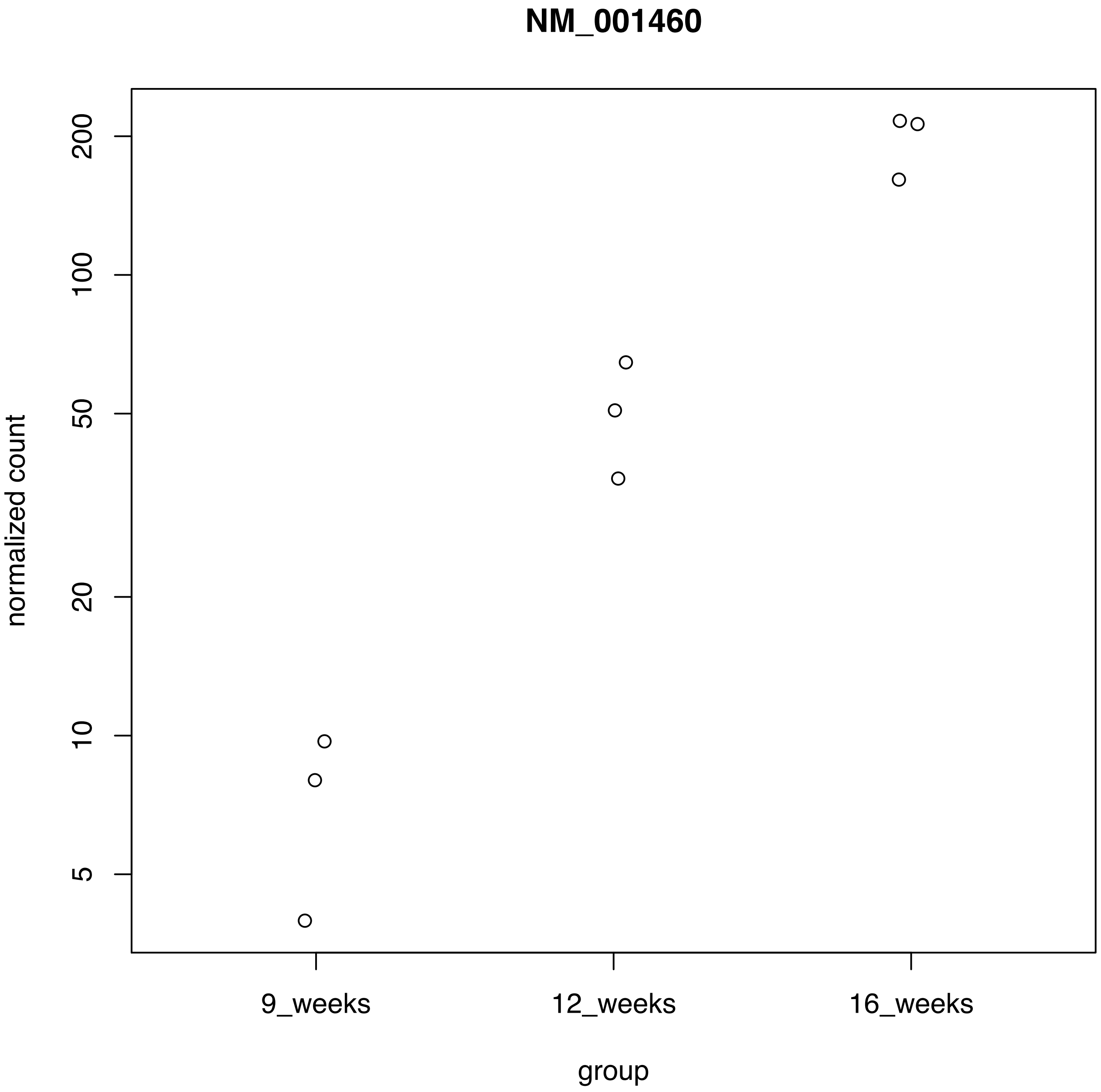
NM_001460
normalized count
200
100
50
20
10
5
9_weeks
12_weeks
16_weeks
group

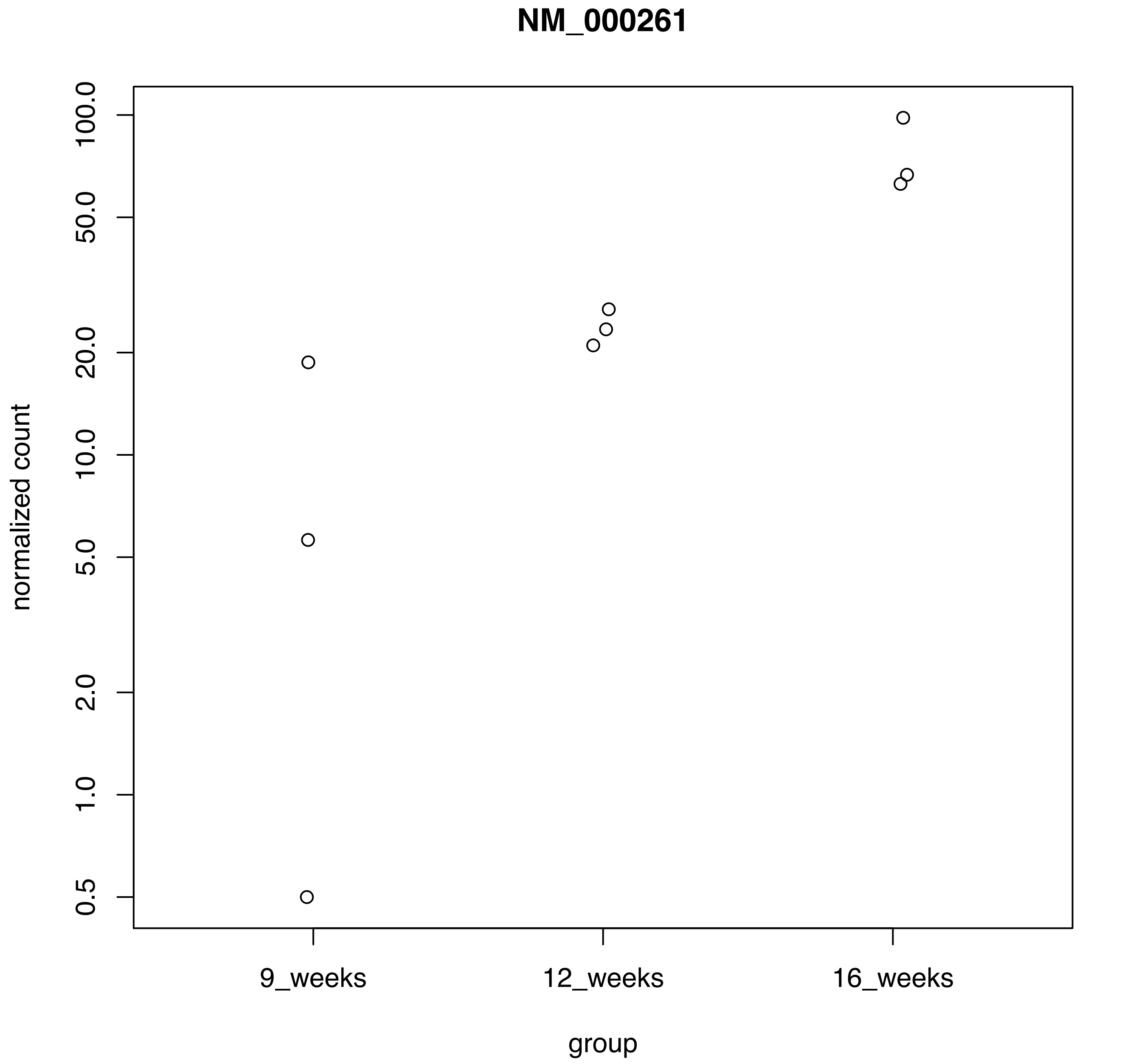
NM_000261
normalized count
100.0
50.0
20.0
10.0
5.0
2.0
1.0
0.5
9_weeks
12_weeks
16_weeks
group

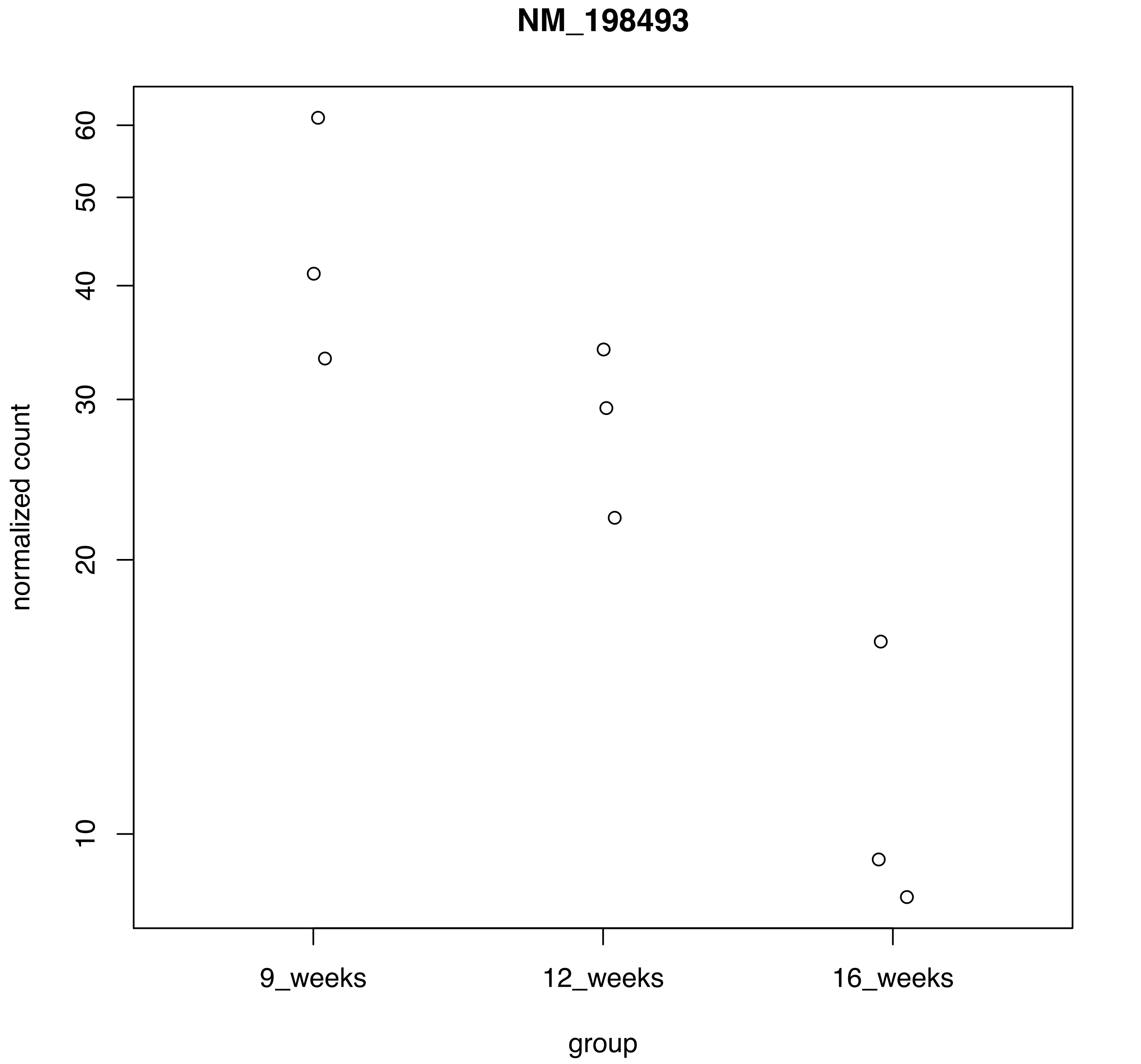

NM_198493
normalized count
60
50
40
30
20
10
9_weeks
12_weeks
16_weeks
group

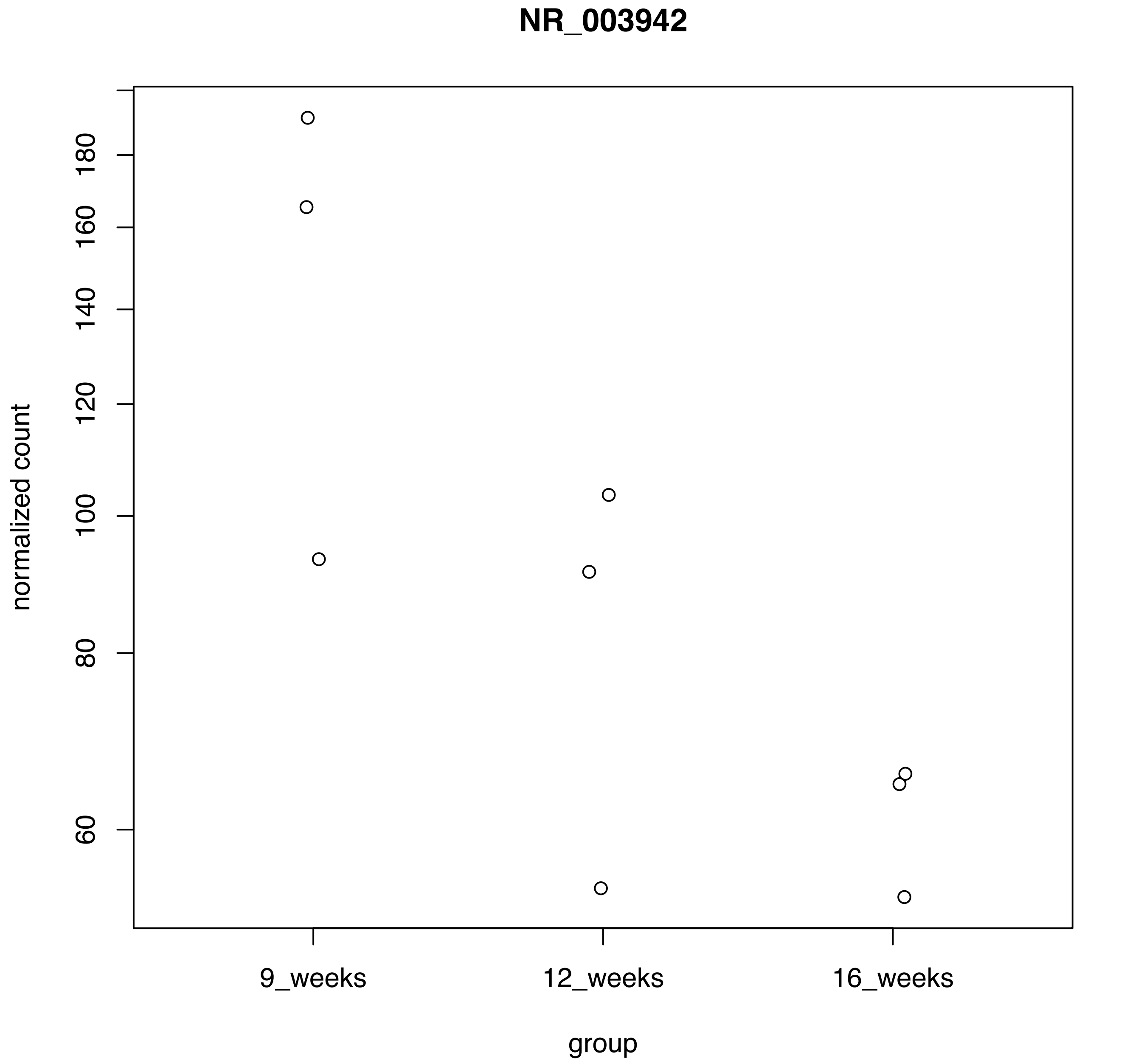
NR_003942
normalized count
60
80
100
120
140
160
180
9_weeks
12_weeks
16_weeks
group

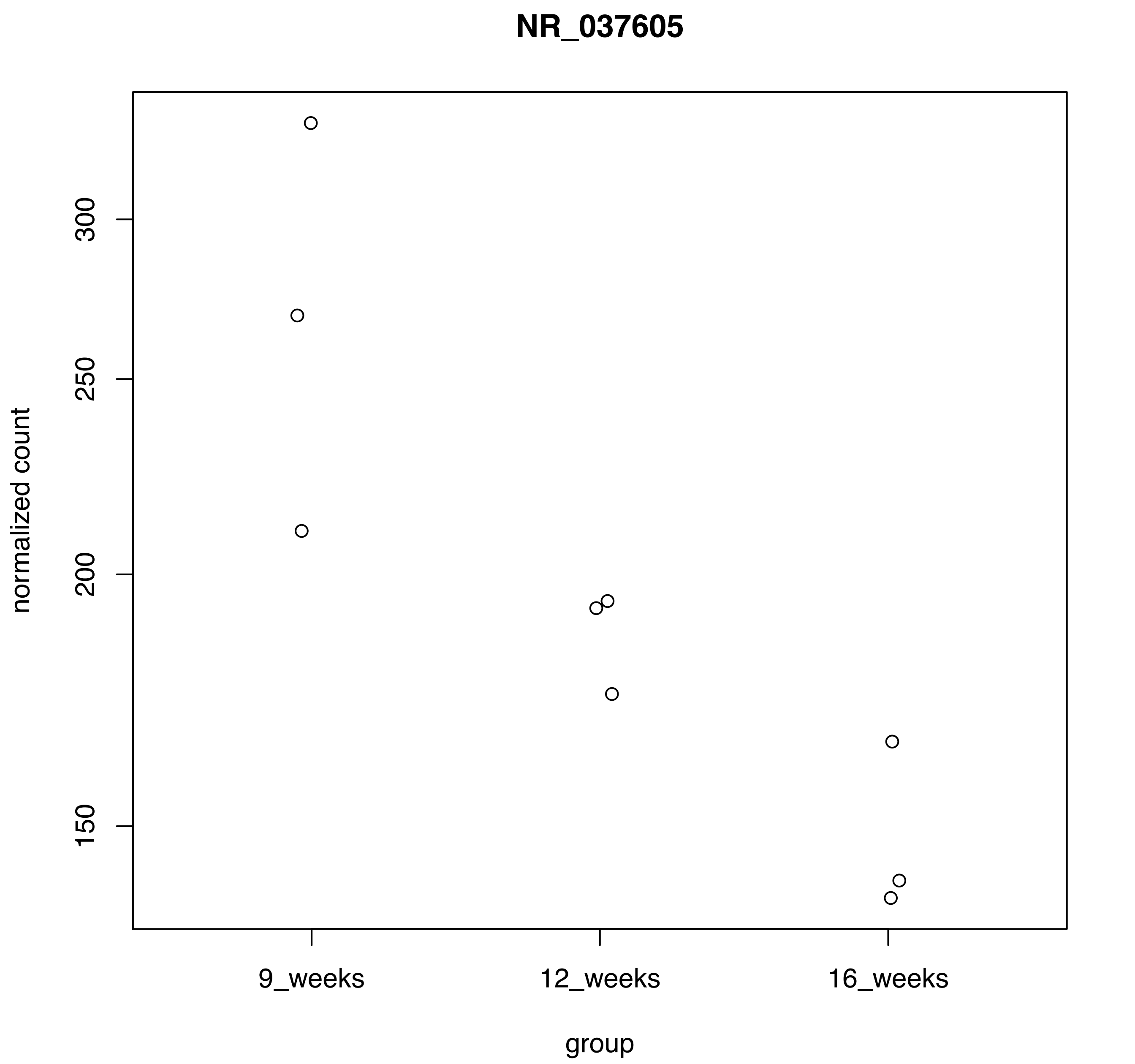

NR_037605
normalized count
300
250
200
150
9_weeks
12_weeks
16_weeks
group

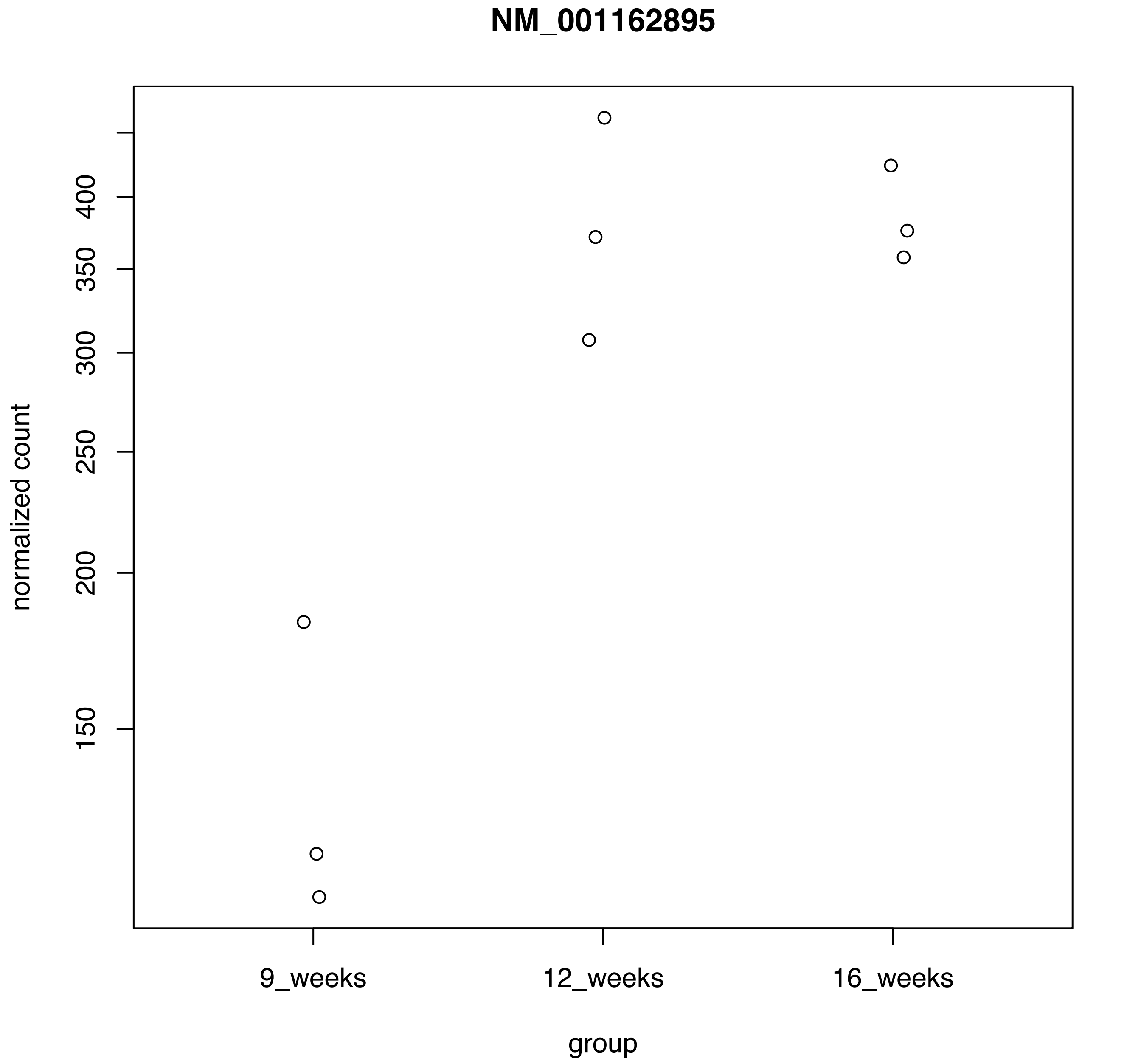
NM_001162895
normalized count
150
200
250
300
350
400
9_weeks
12_weeks
16_weeks
group

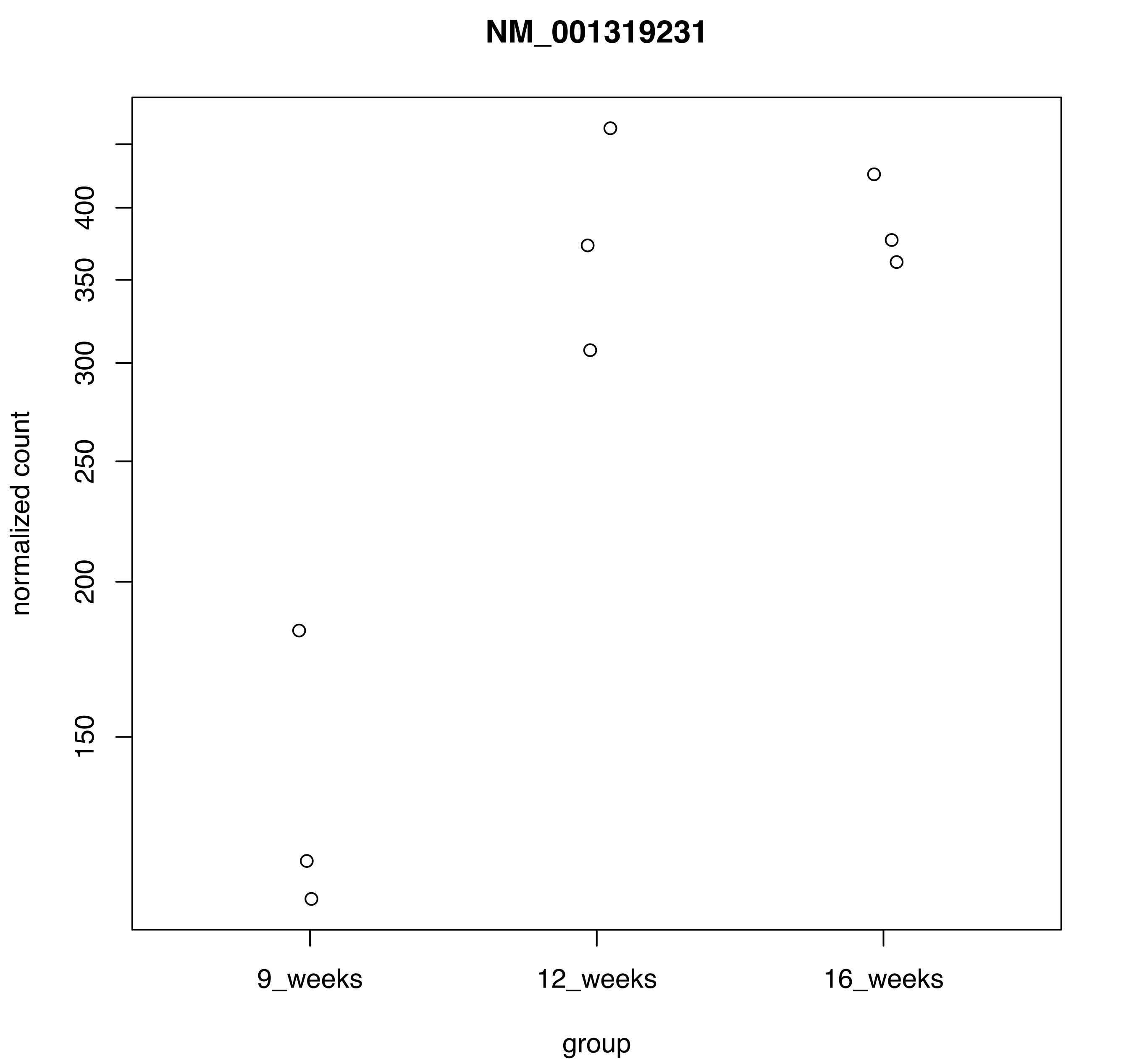
NM_001319231
normalized count
150
200
250
300
350
400
9_weeks
12_weeks
16_weeks
group

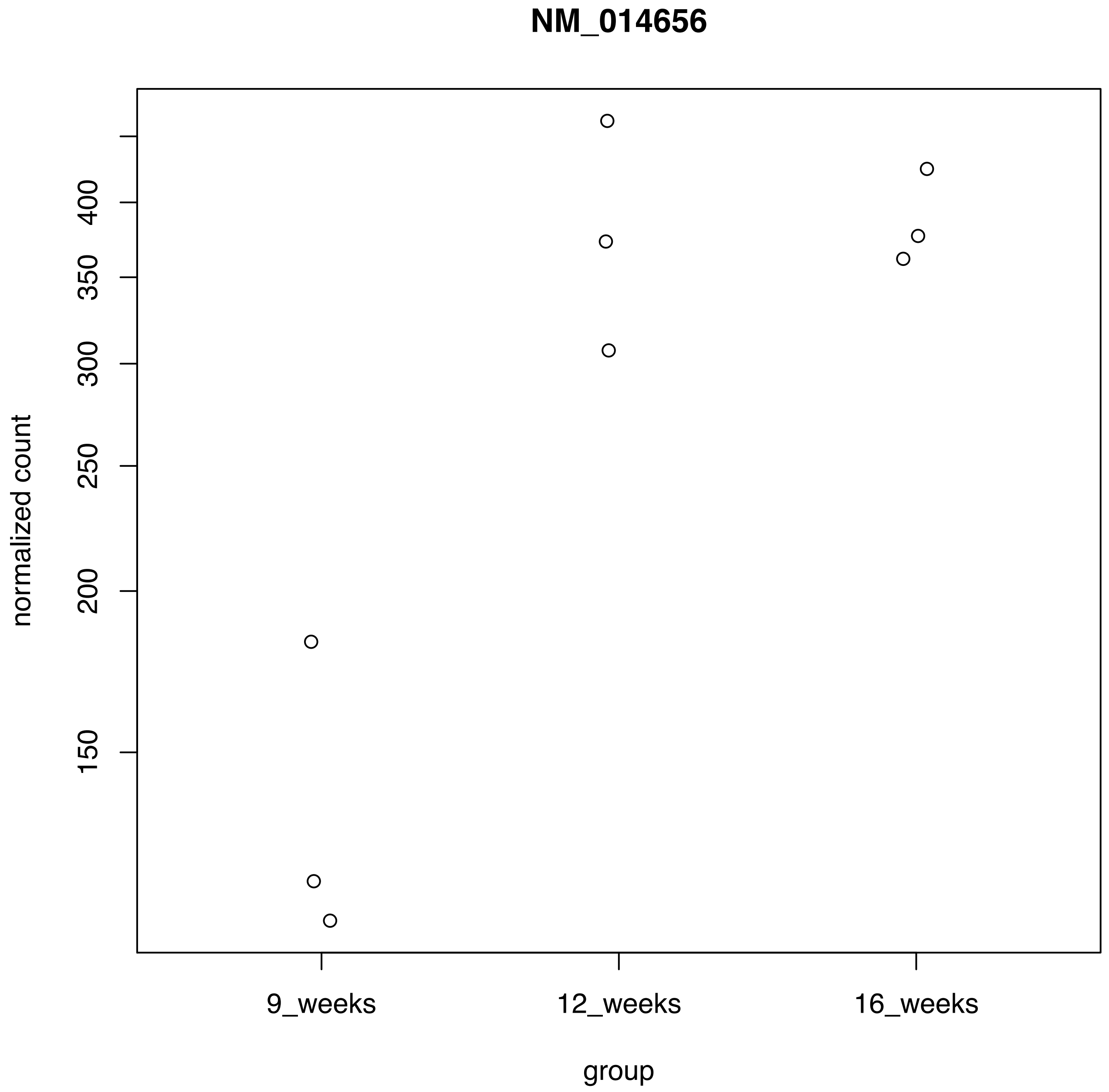

NM_014656
normalized count
150
200
250
300
350
400
9_weeks
12_weeks
16_weeks
group

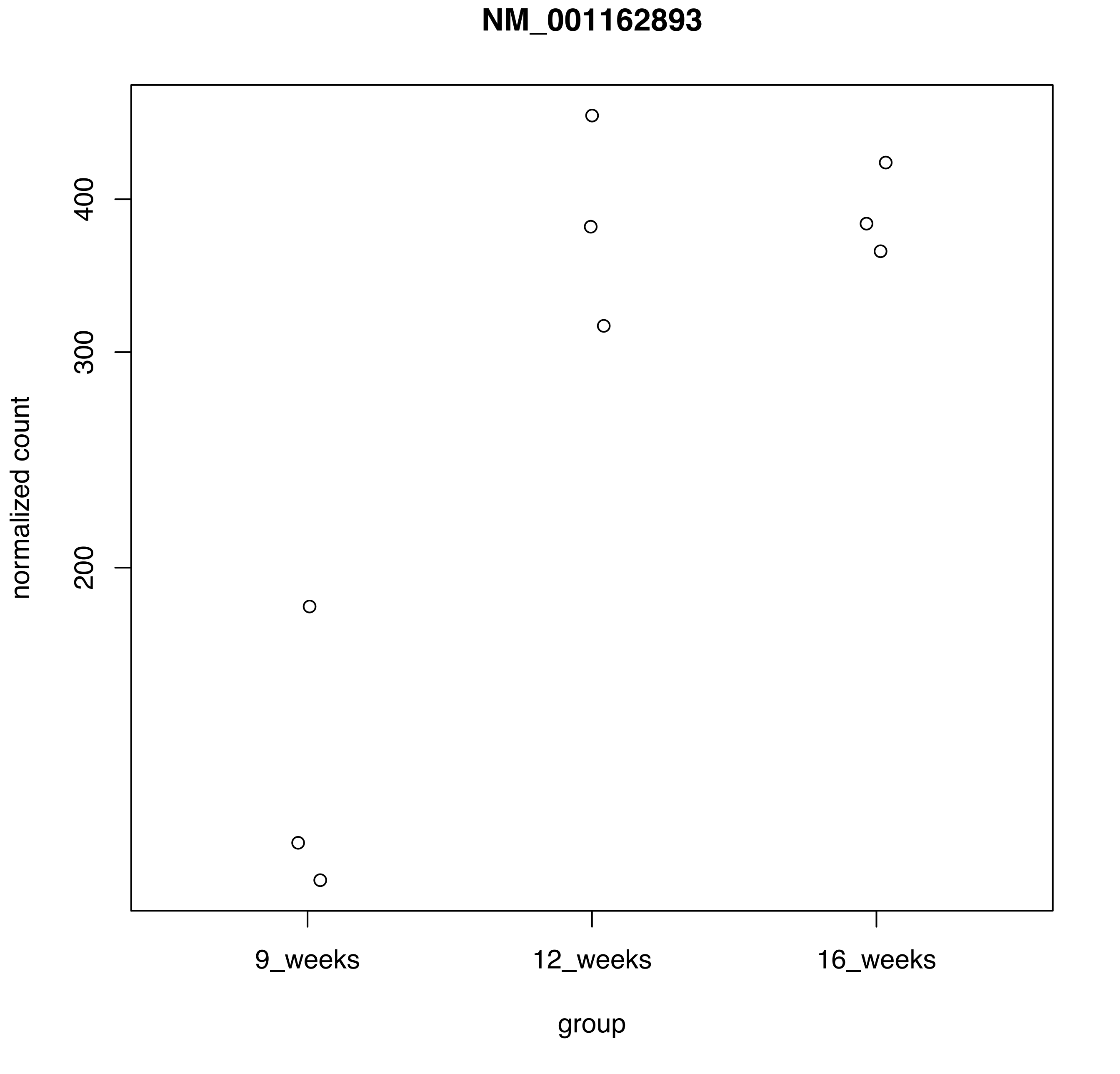
NM_001162893
normalized count
400
300
200
9_weeks
12_weeks
16_weeks
group

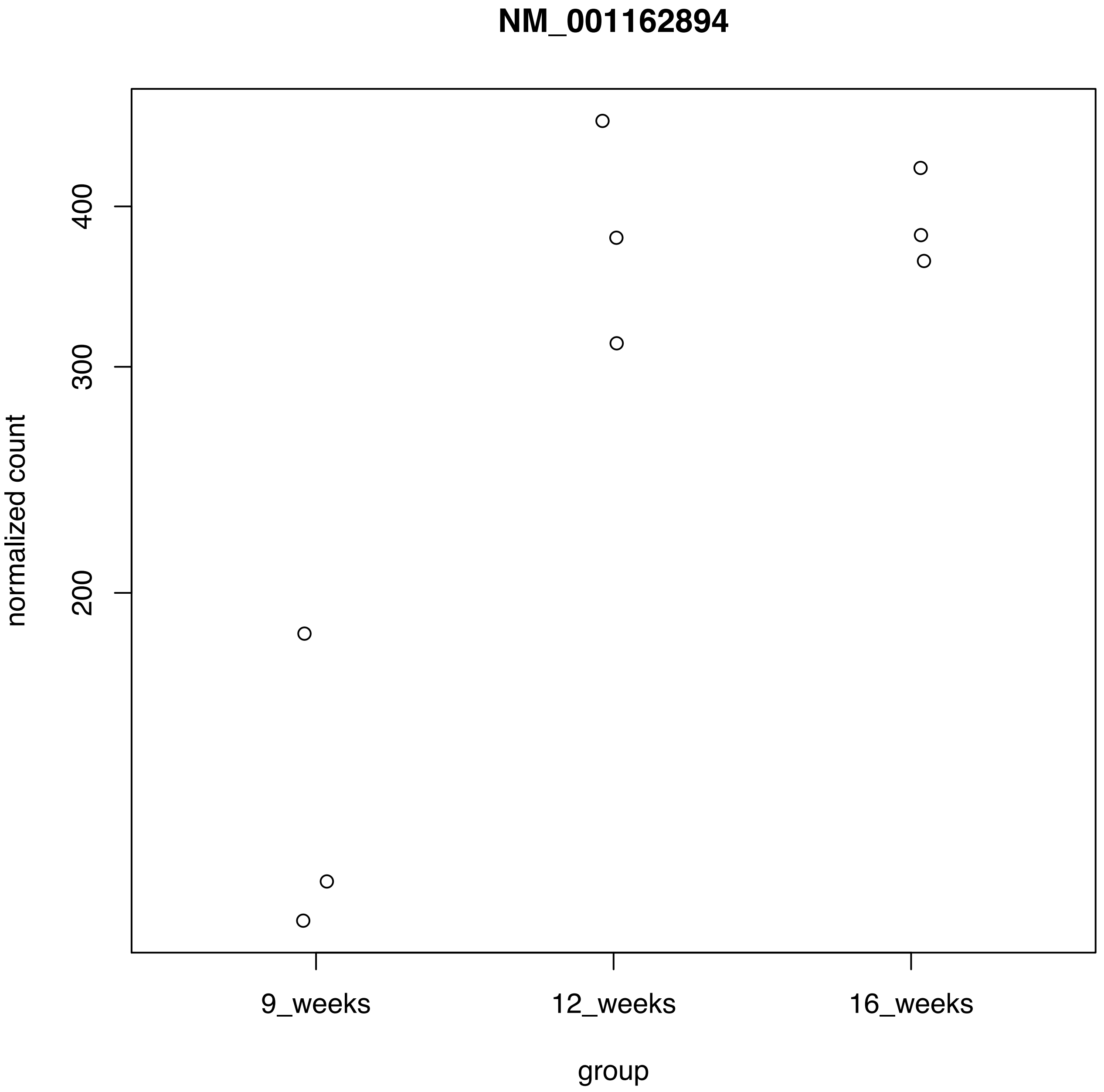
NM_001162894
normalized count
400
300
200
9_weeks
12_weeks
16_weeks
group

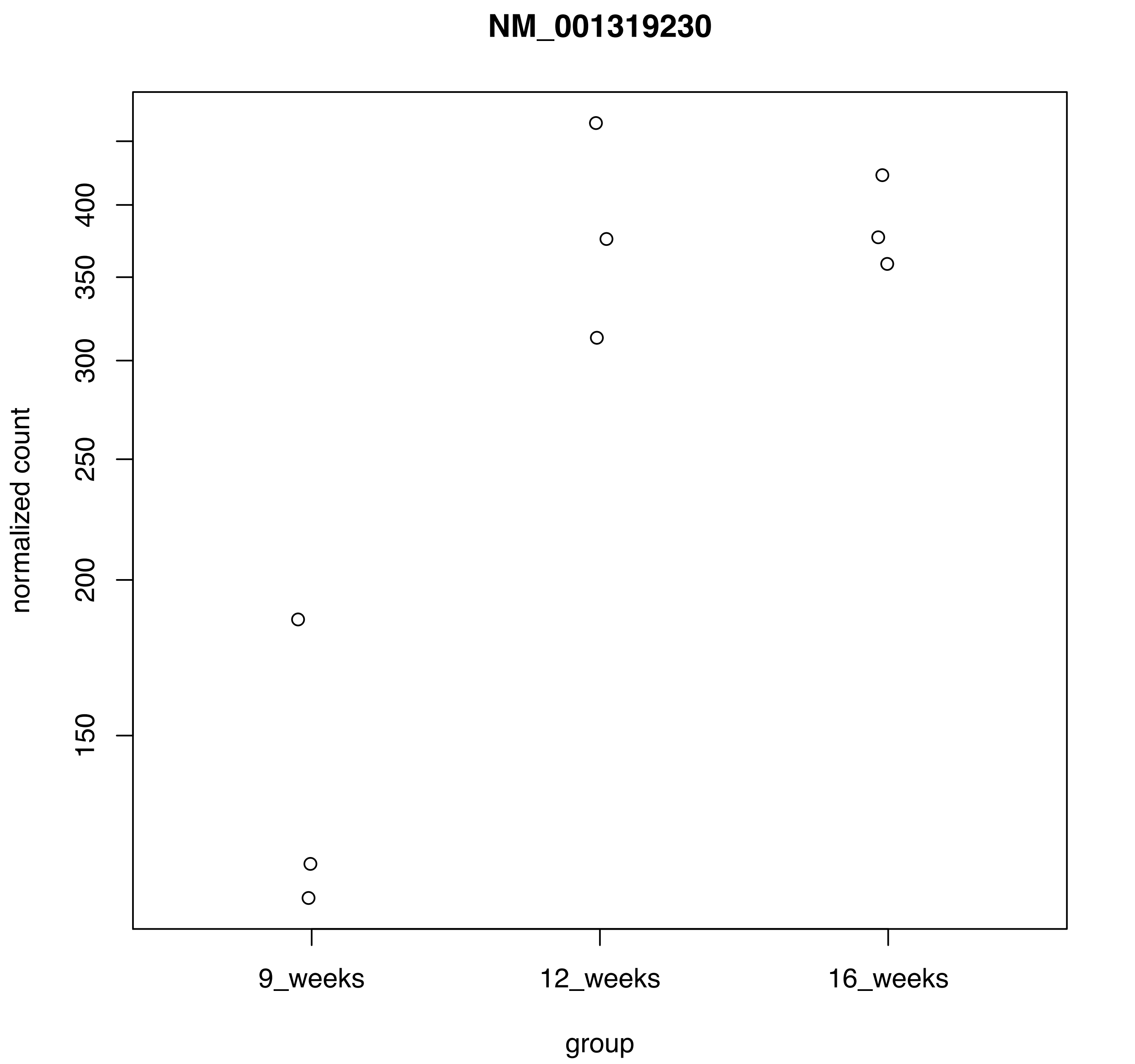
NM_001319230
normalized count
150
200
250
300
350
400
9_weeks
12_weeks
16_weeks
group

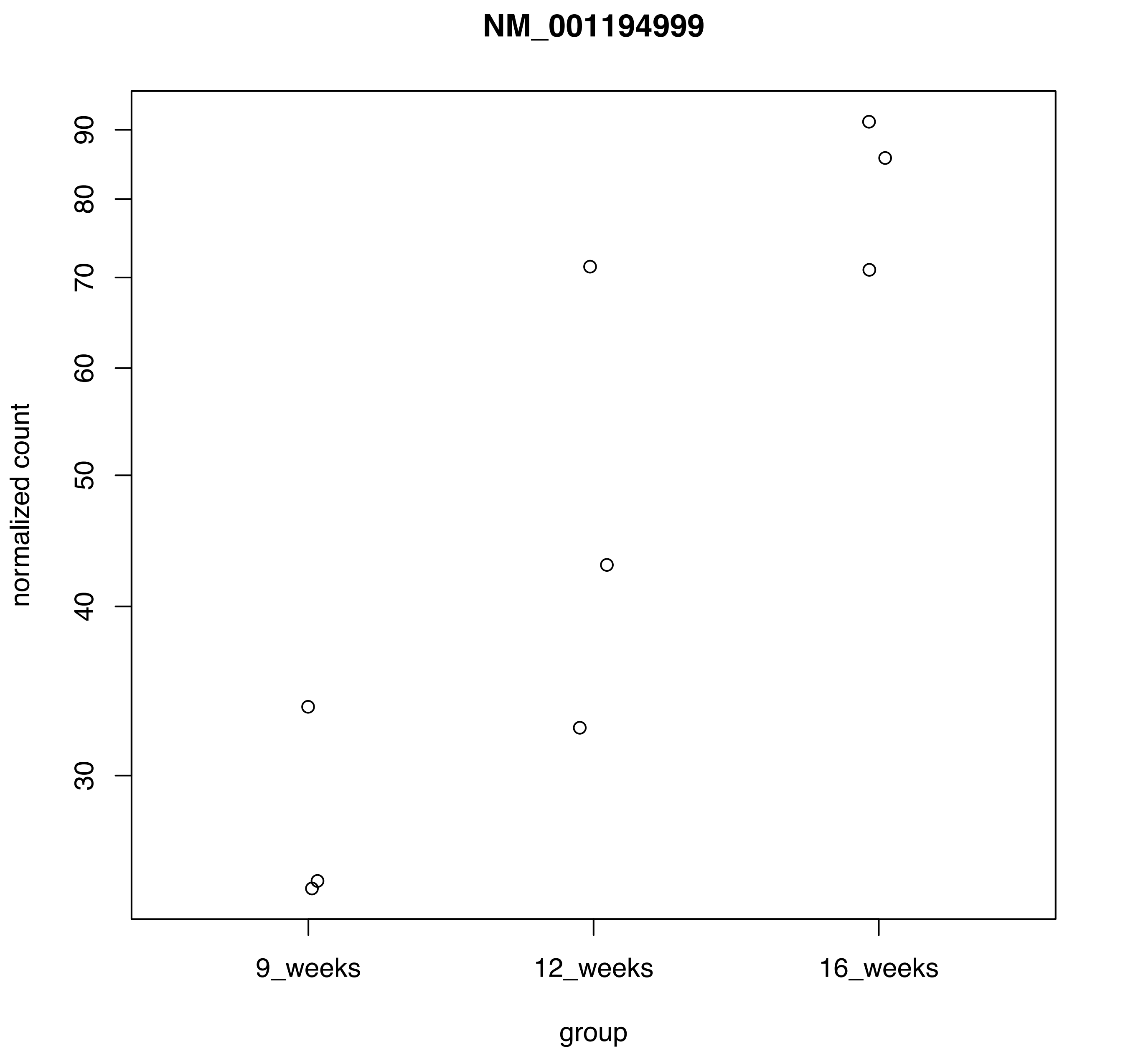
NM_001194999
normalized count
90
80
70
60
50
40
30
9_weeks
12_weeks
16_weeks
group

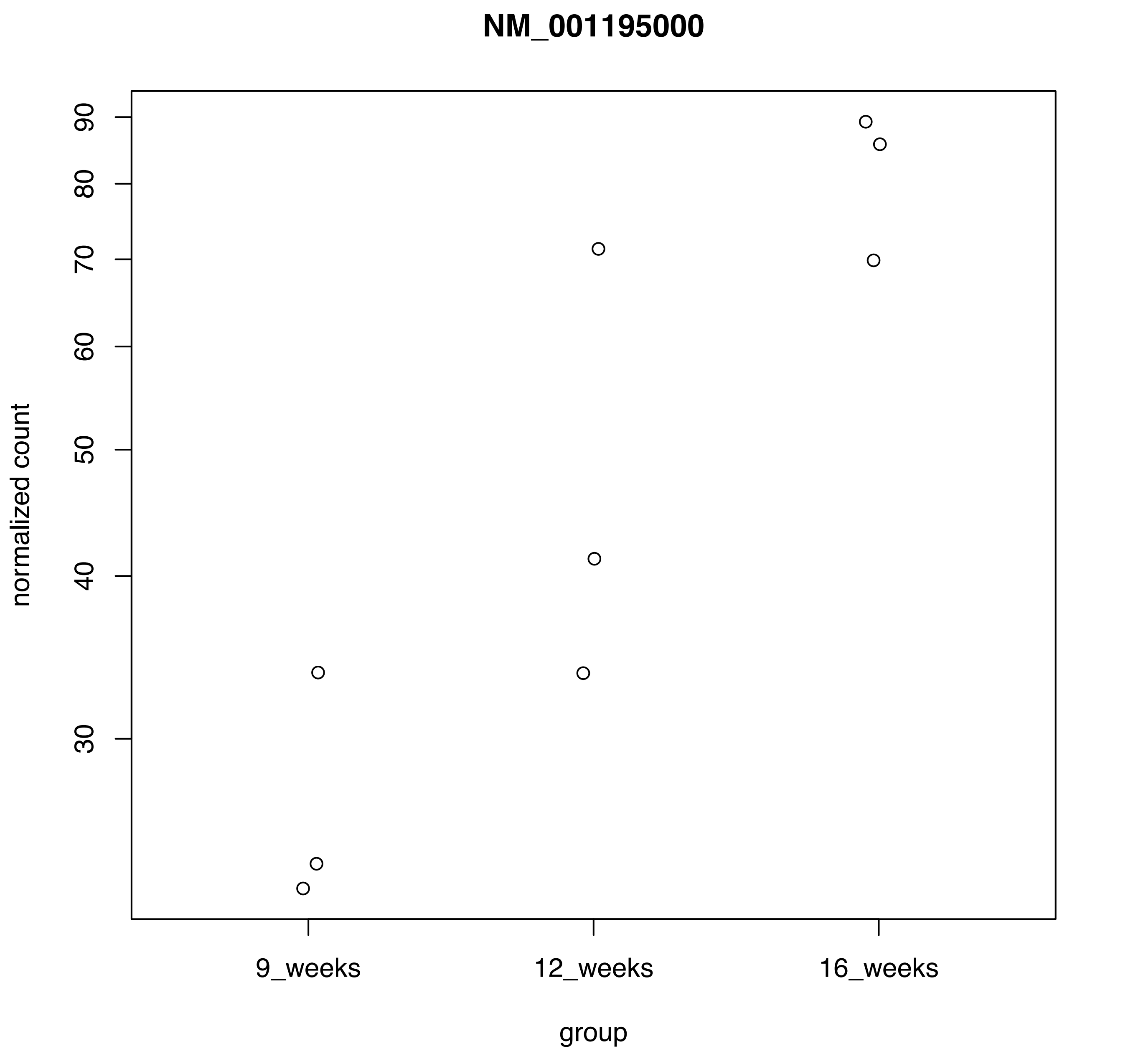

NM_001195000
normalized count
90
80
70
60
50
40
30
9_weeks
12_weeks
16_weeks
group

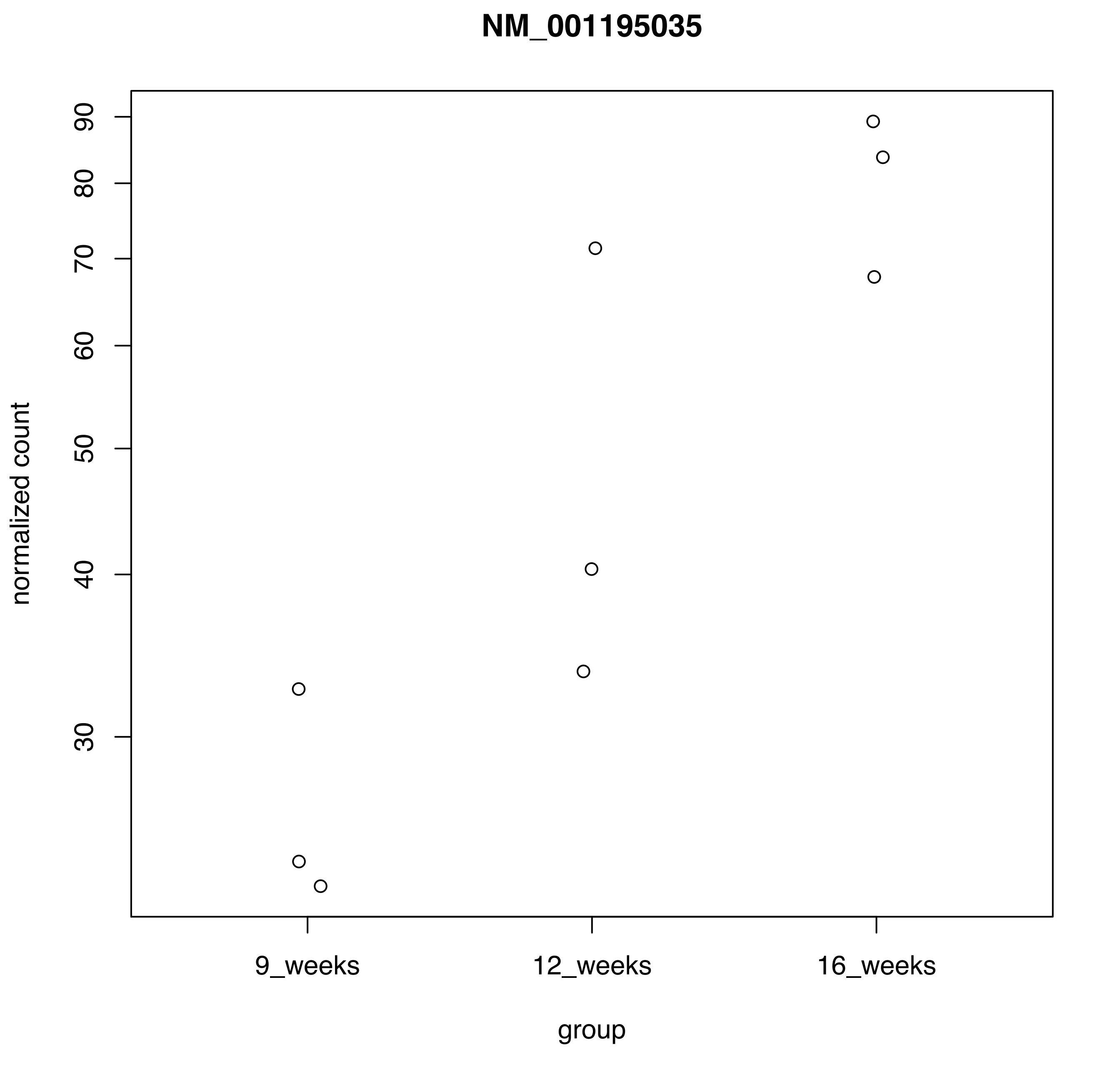

NM_001195035
normalized count
90
80
70
60
50
40
30
9_weeks
12_weeks
16_weeks
group

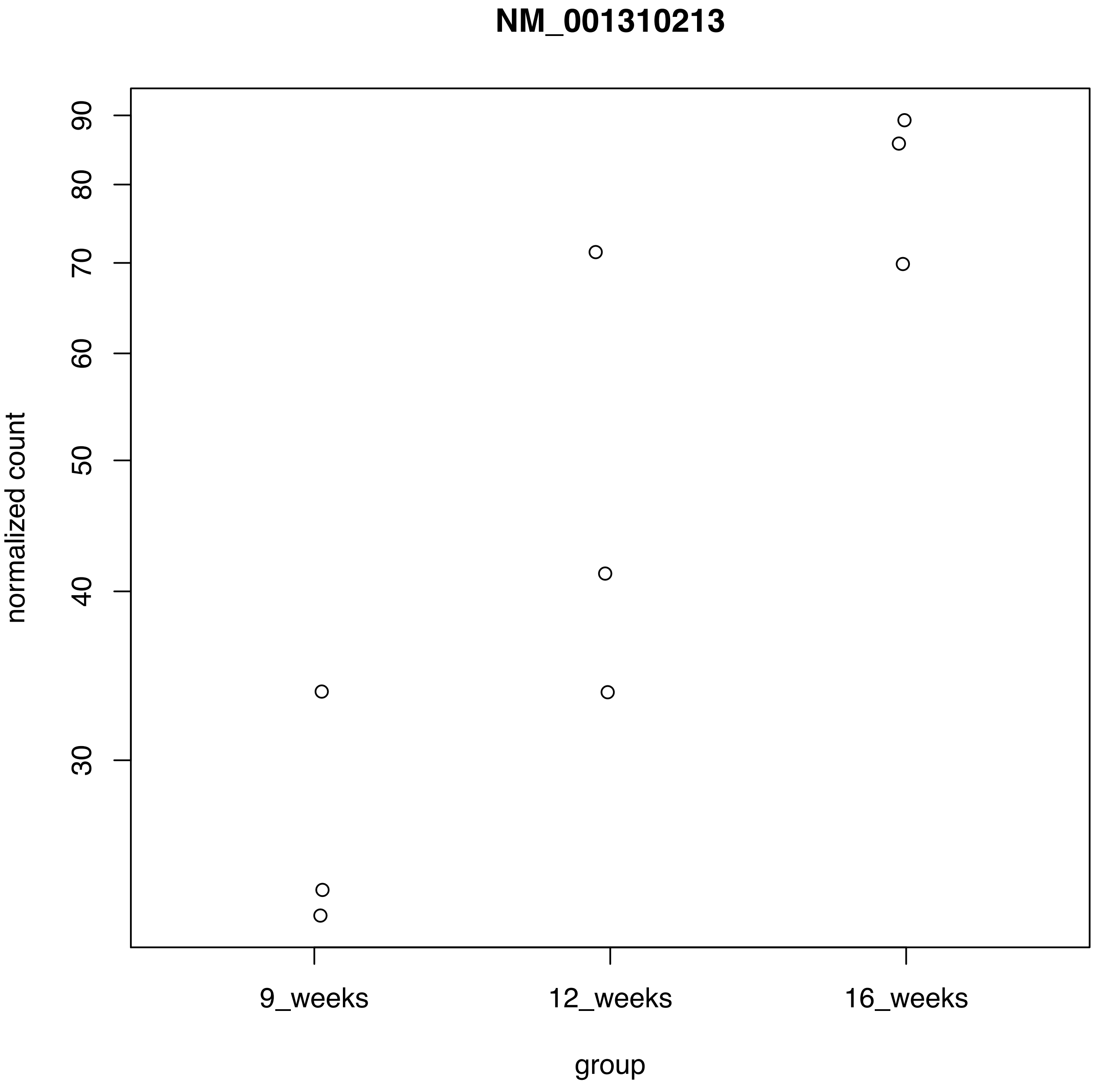
NM_001310213
normalized count
90
80
70
60
50
40
30
9_weeks
12_weeks
16_weeks
group

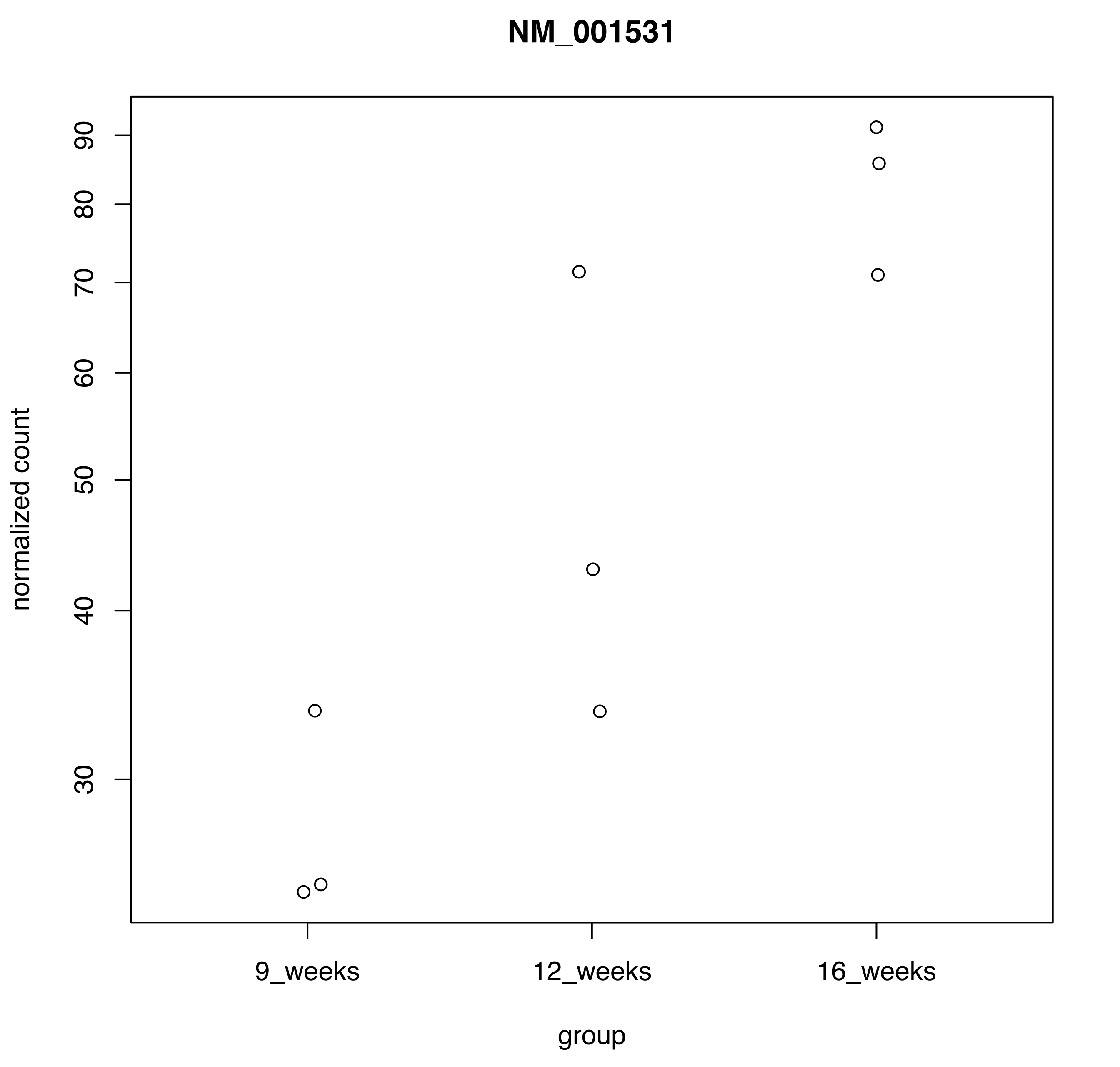
NM_001531
normalized count
90
80
70
60
50
40
30
9_weeks
12_weeks
16_weeks
group

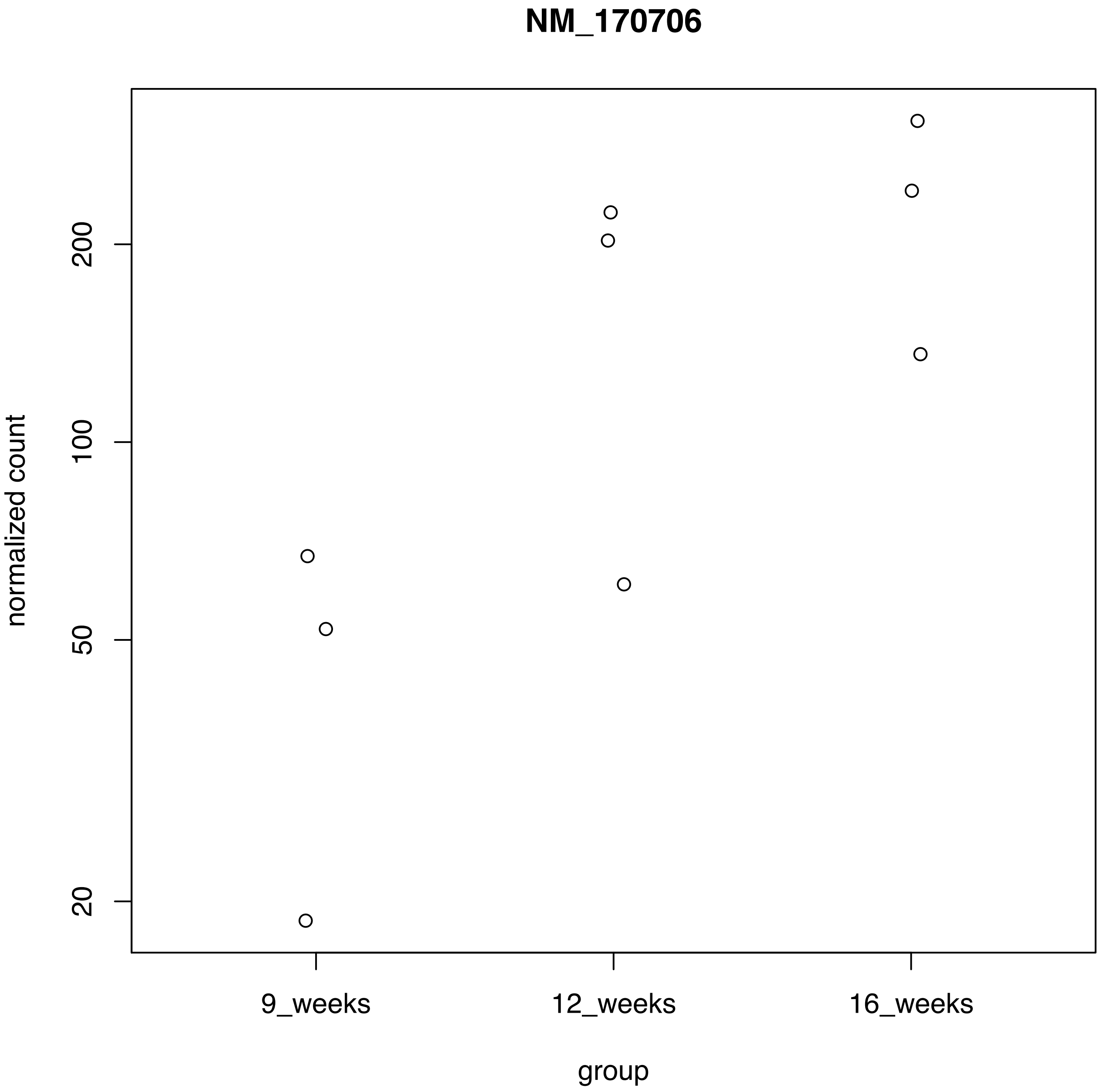
NM_170706
normalized count
200
100
50
20
9_weeks
12_weeks
16_weeks
group

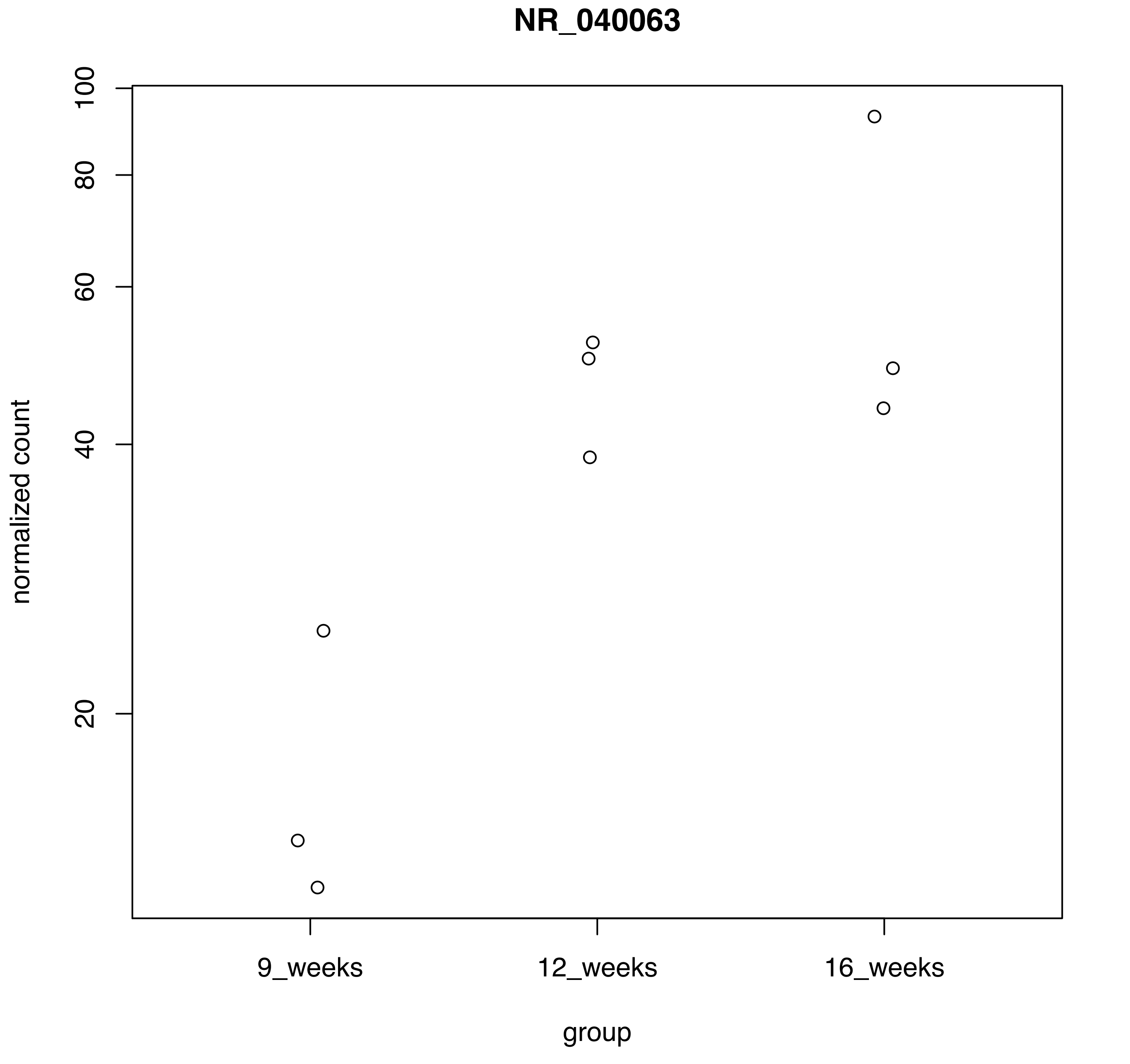
NR_040063
normalized count
100
80
60
40
20
9_weeks
12_weeks
16_weeks
group

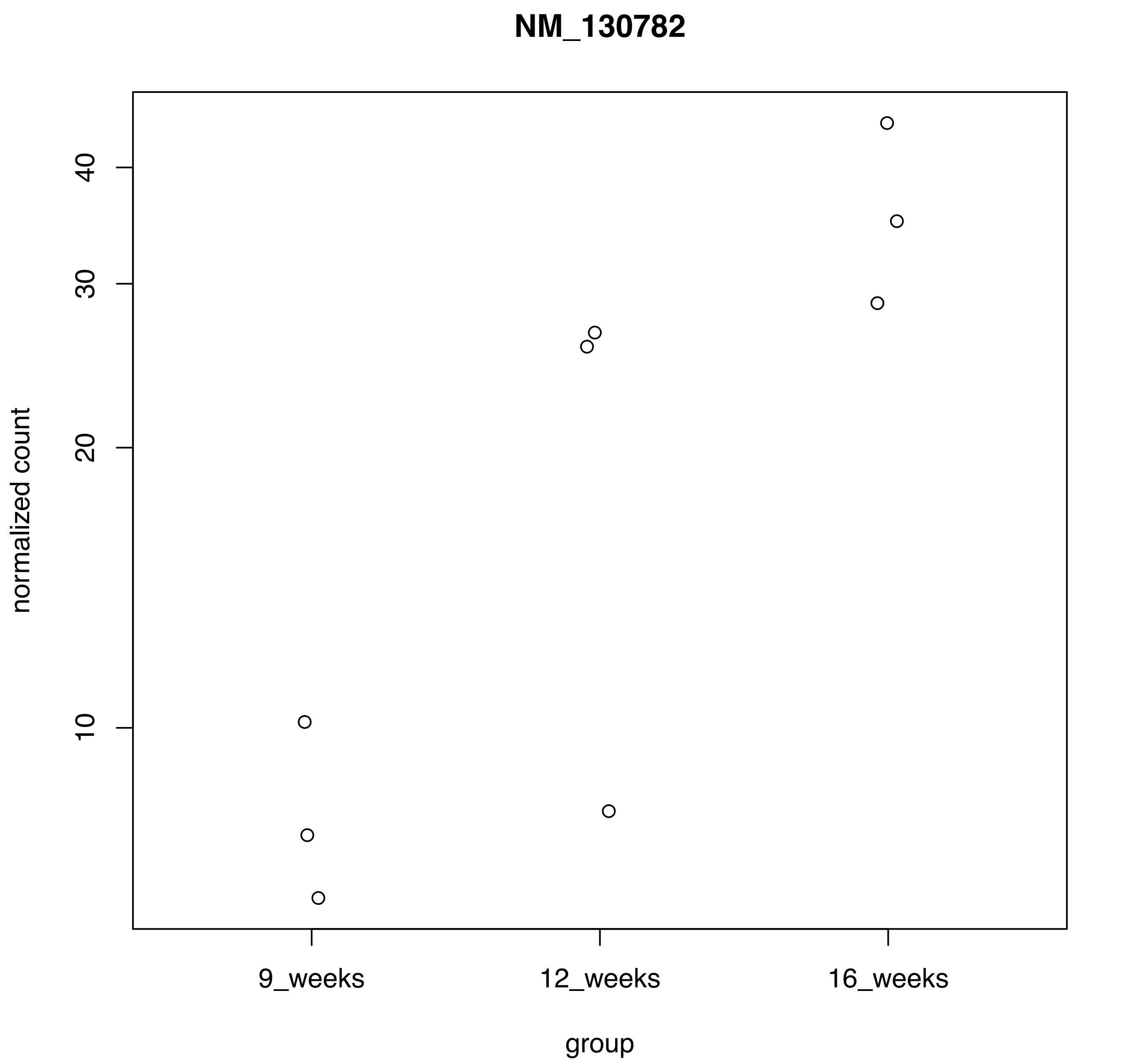

NM_130782
normalized count
10
20
30
40
9_weeks
12_weeks
16_weeks
group

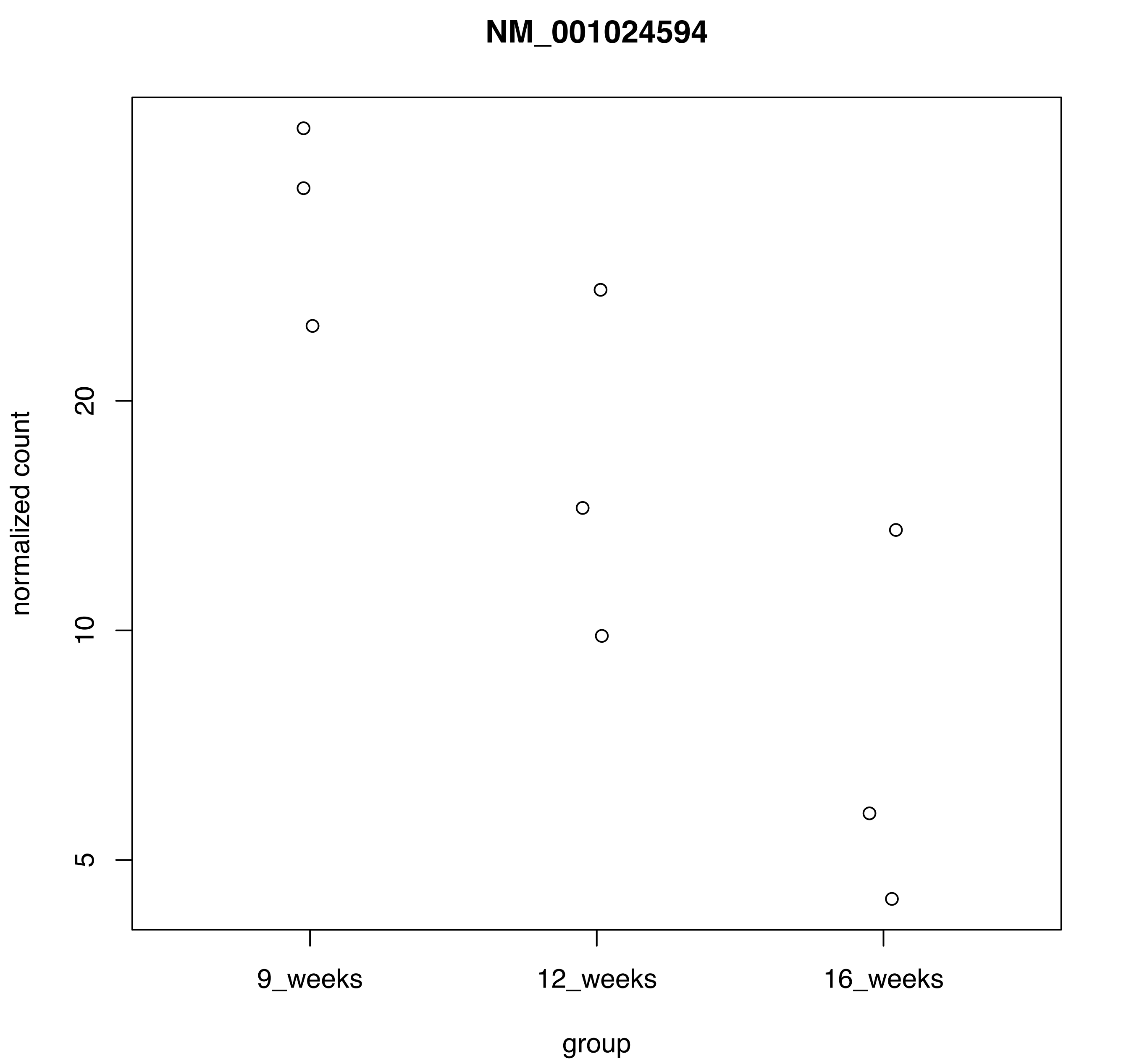

NM_001024594
normalized count
5
10
20
9_weeks
12_weeks
16_weeks
group

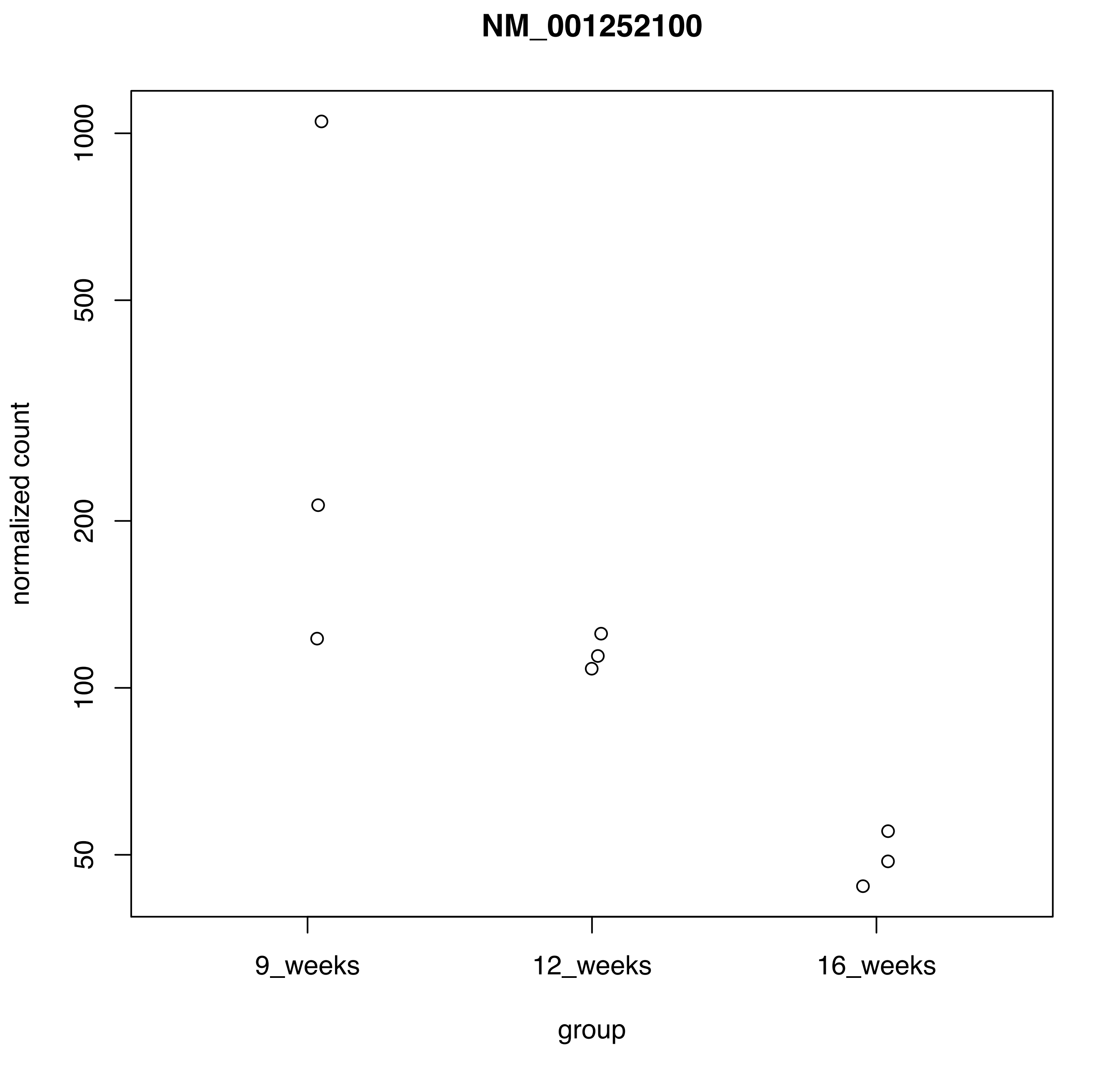
NM_001252100
normalized count
1000
500
200
100
50
9_weeks
12_weeks
16_weeks
group

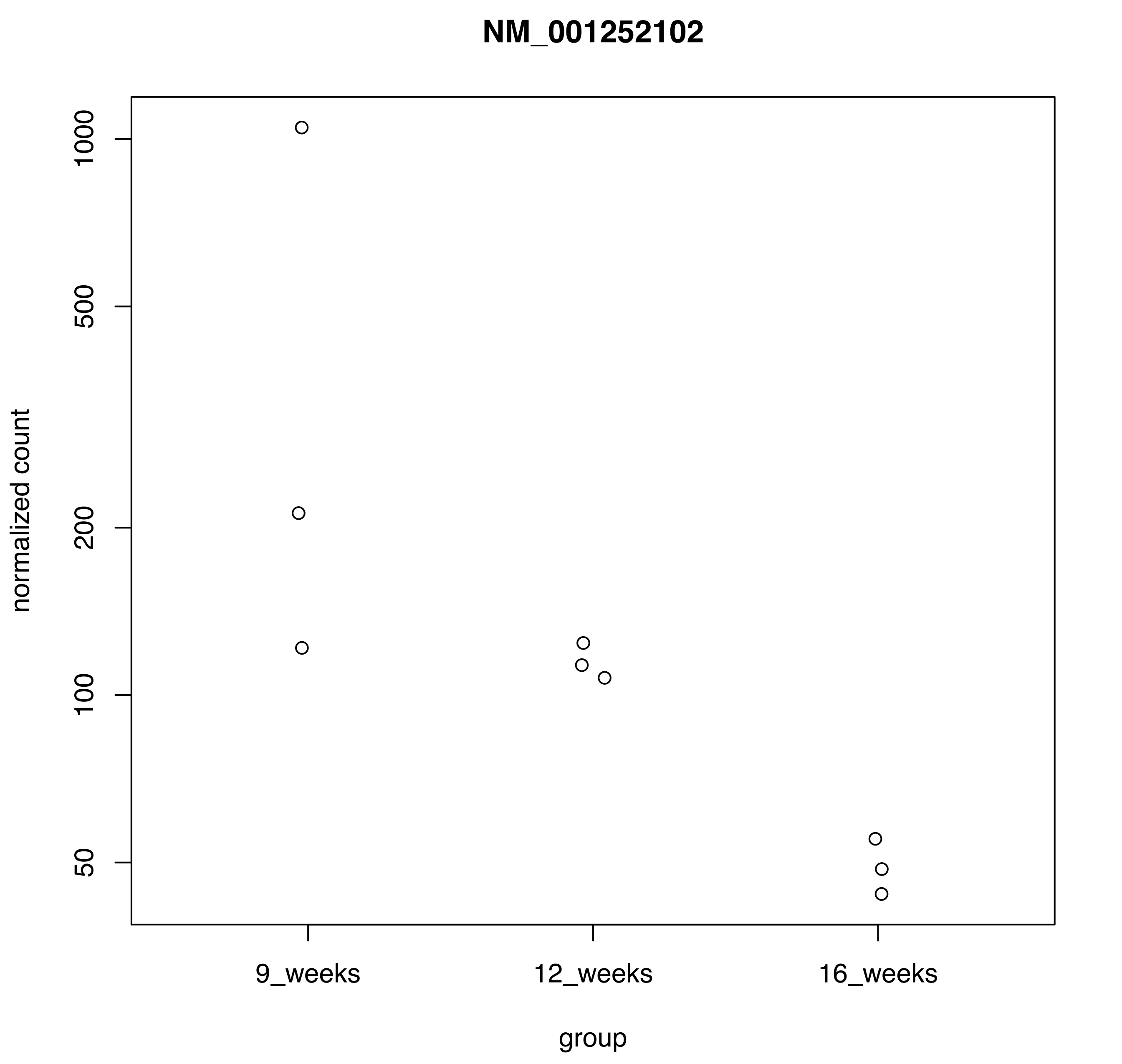

NM_001252102
normalized count
1000
500
200
100
50
9_weeks
12_weeks
16_weeks
group

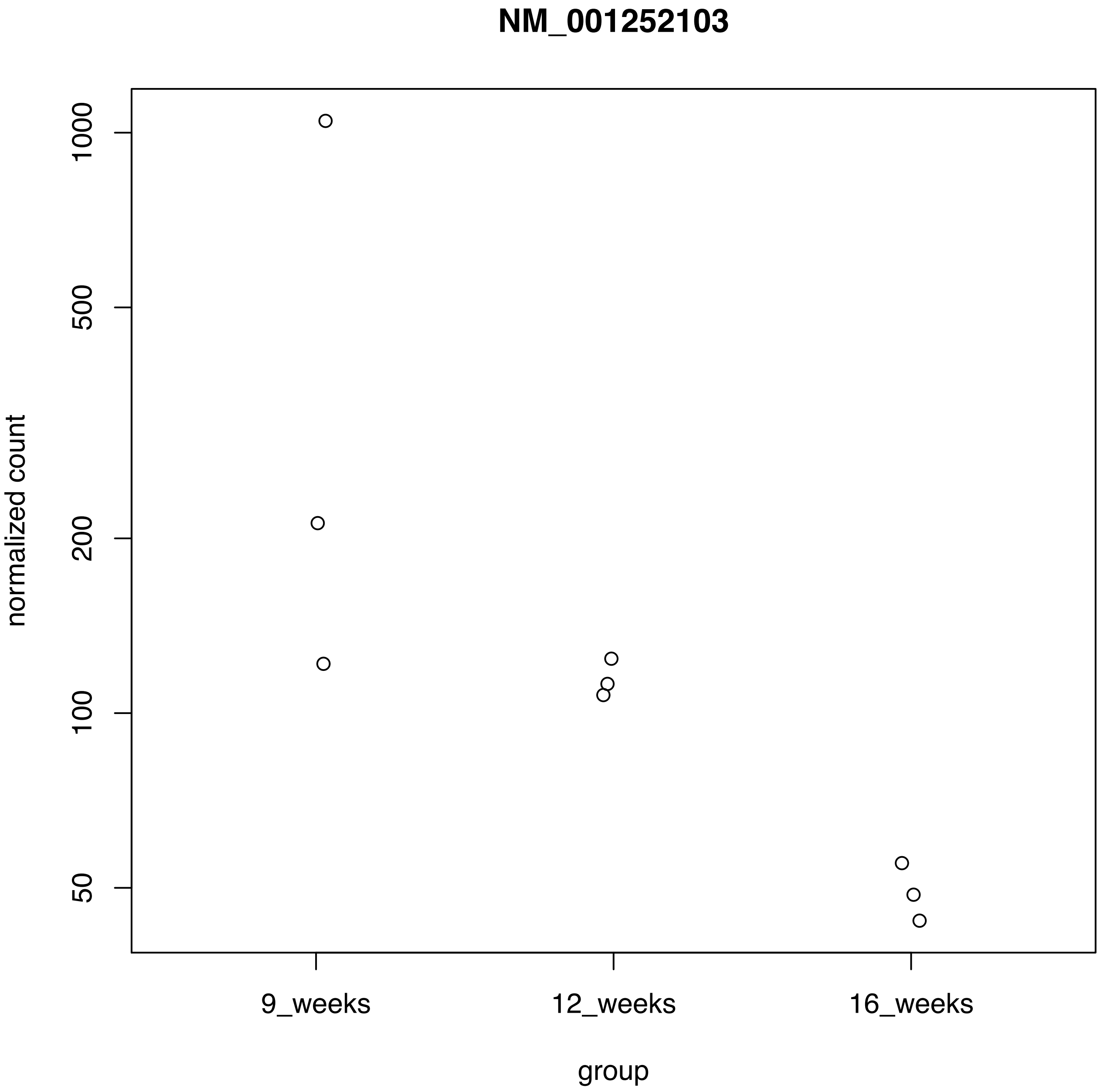
NM_001252103
normalized count
1000
500
200
100
50
9_weeks
12_weeks
16_weeks
group

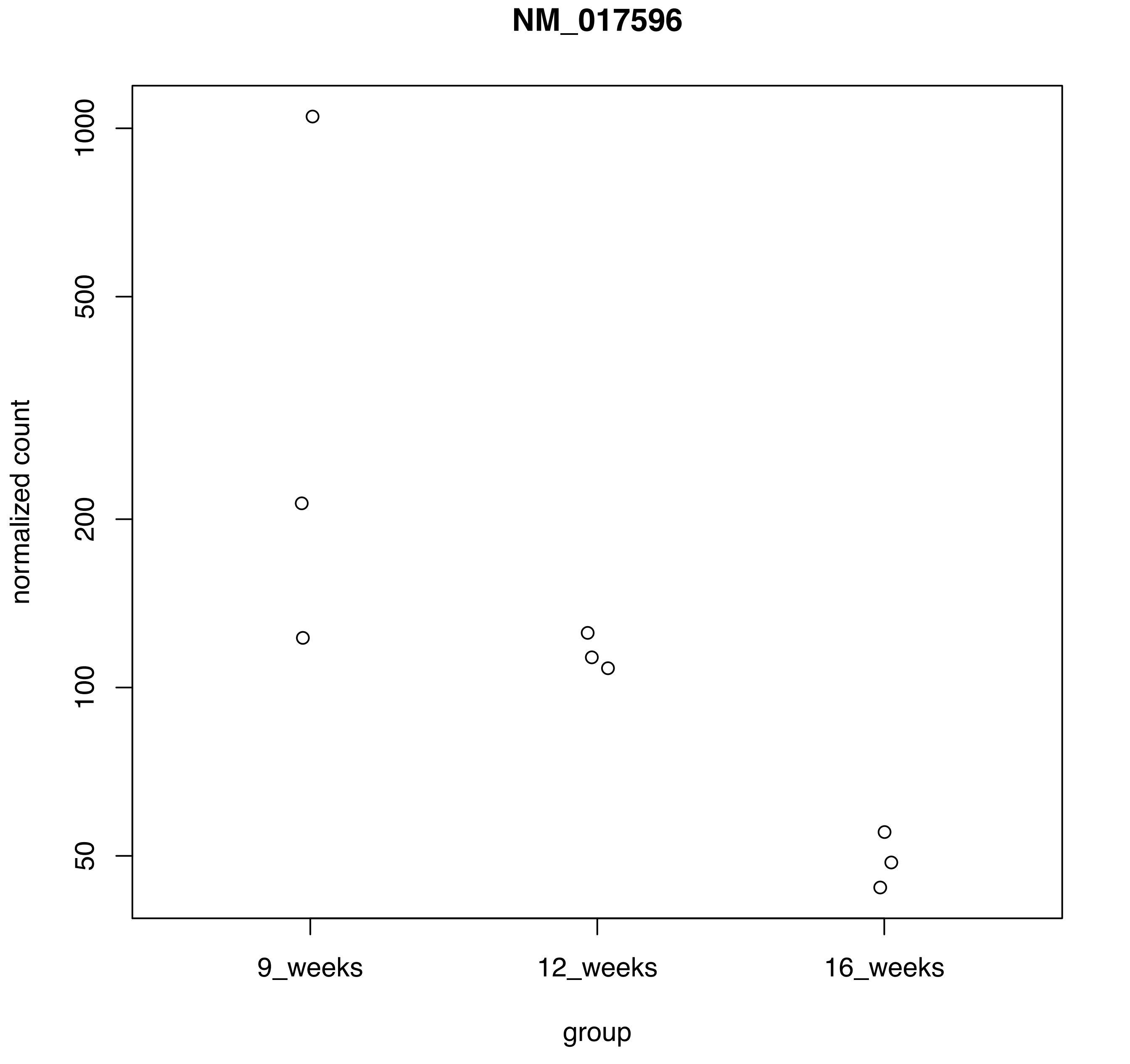
NM_017596
normalized count
1000
500
200
100
50
9_weeks
12_weeks
16_weeks
group

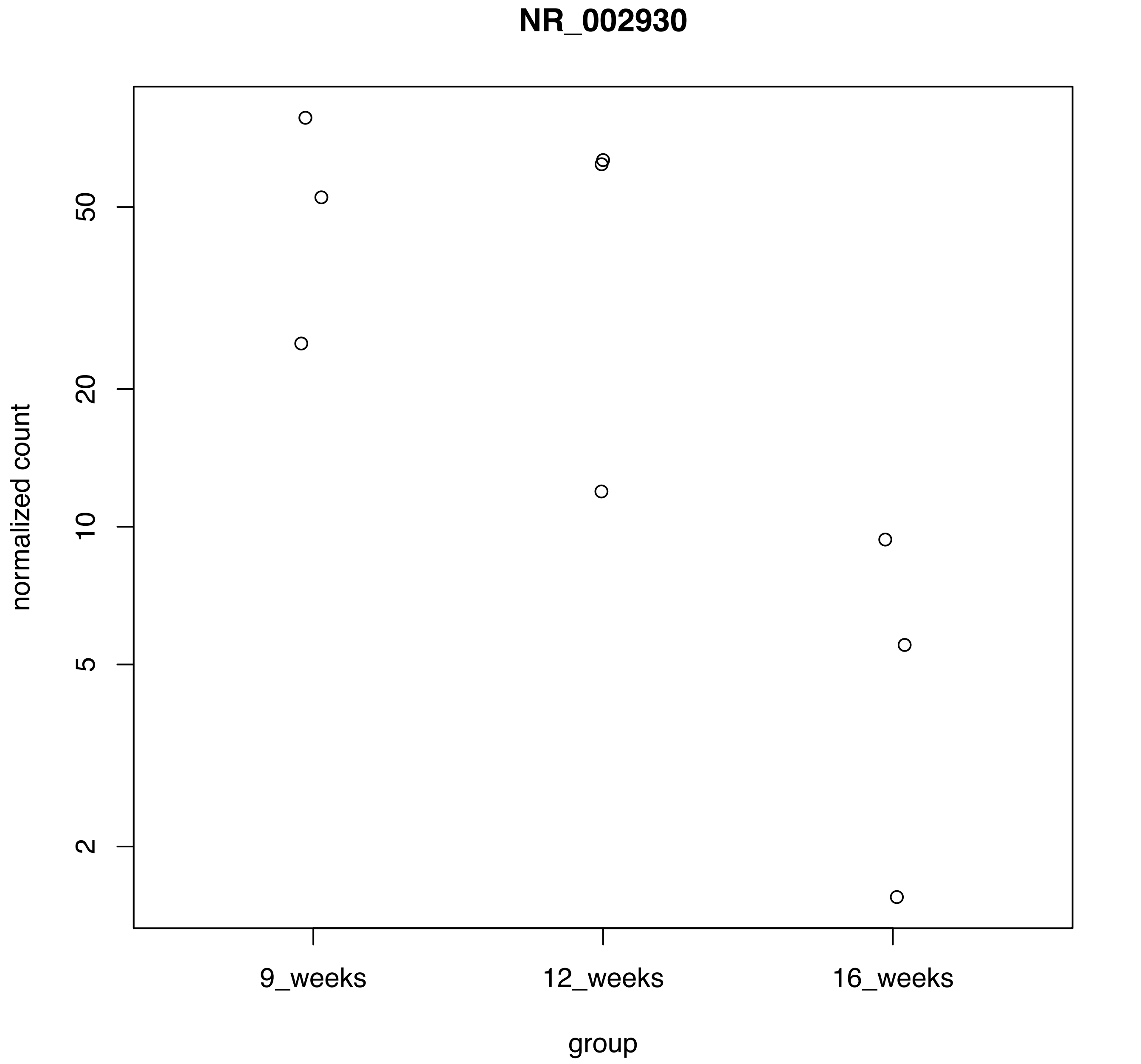
NR_002930
normalized count
50
20
10
5
2
9_weeks
12_weeks
16_weeks
group

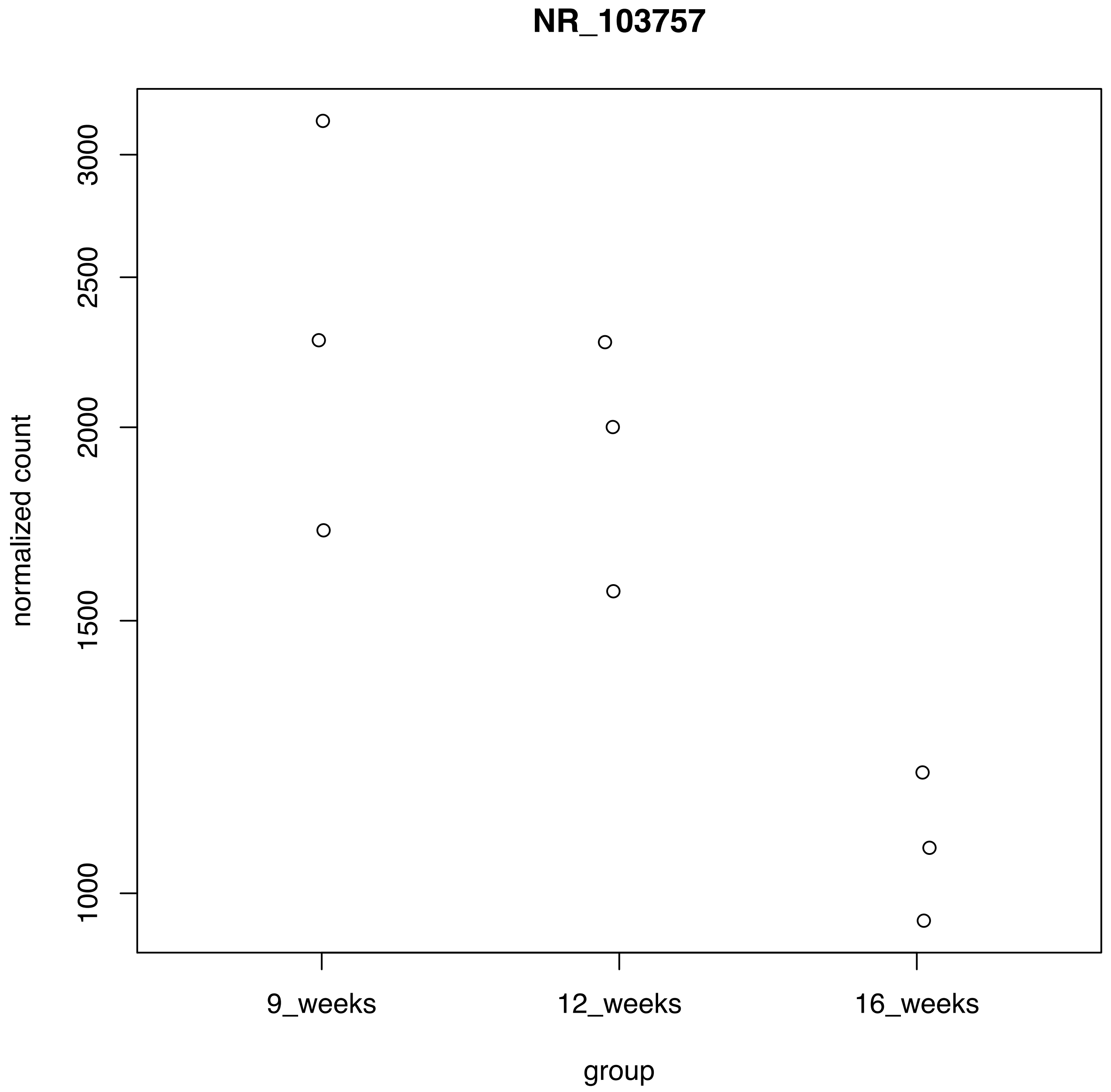
NR_103757
normalized count
3000
2500
2000
1500
1000
9_weeks
12_weeks
16_weeks
group

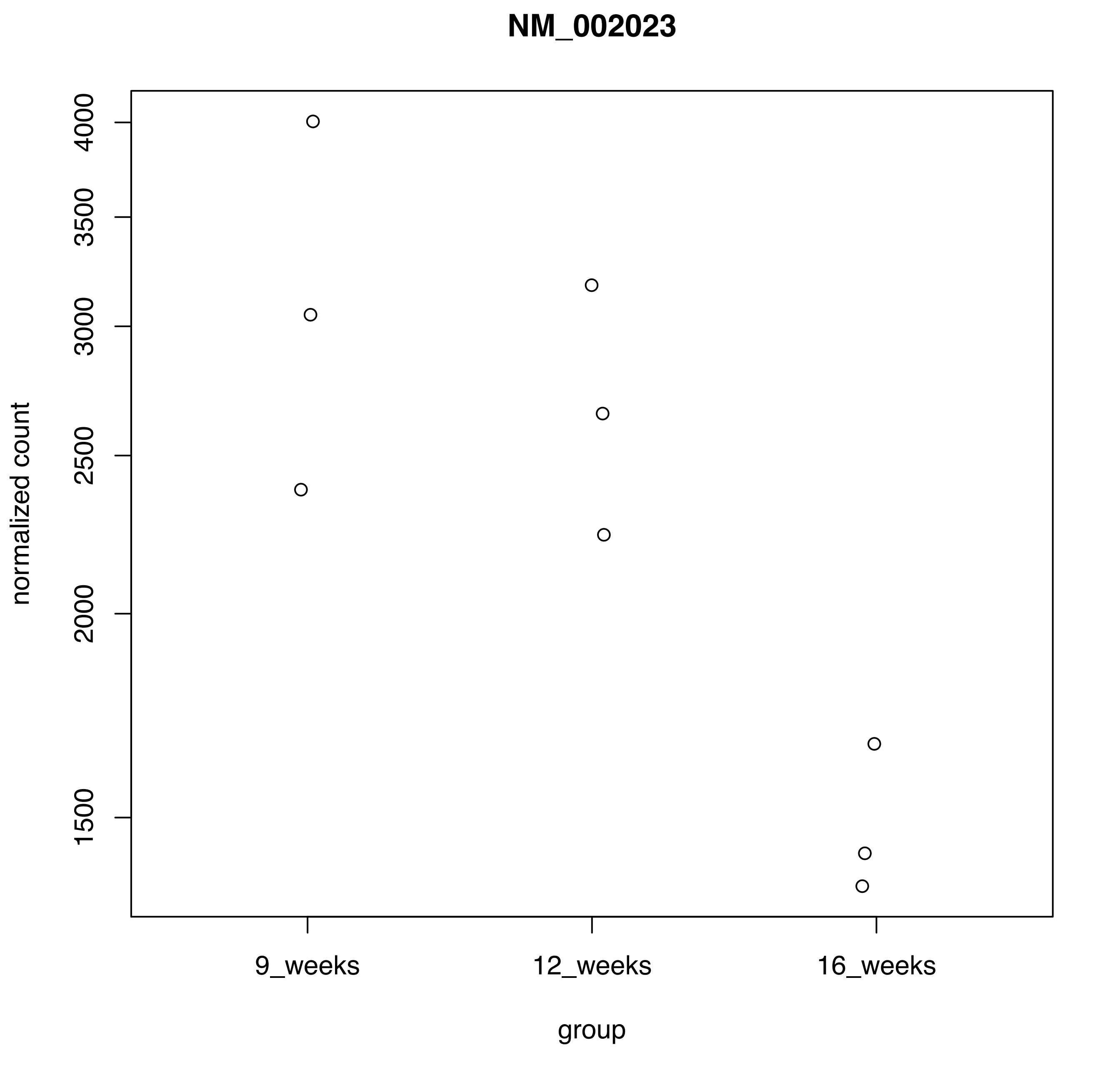

NM_002023
normalized count
4000
3500
3000
2500
2000
1500
9_weeks
12_weeks
16_weeks
group

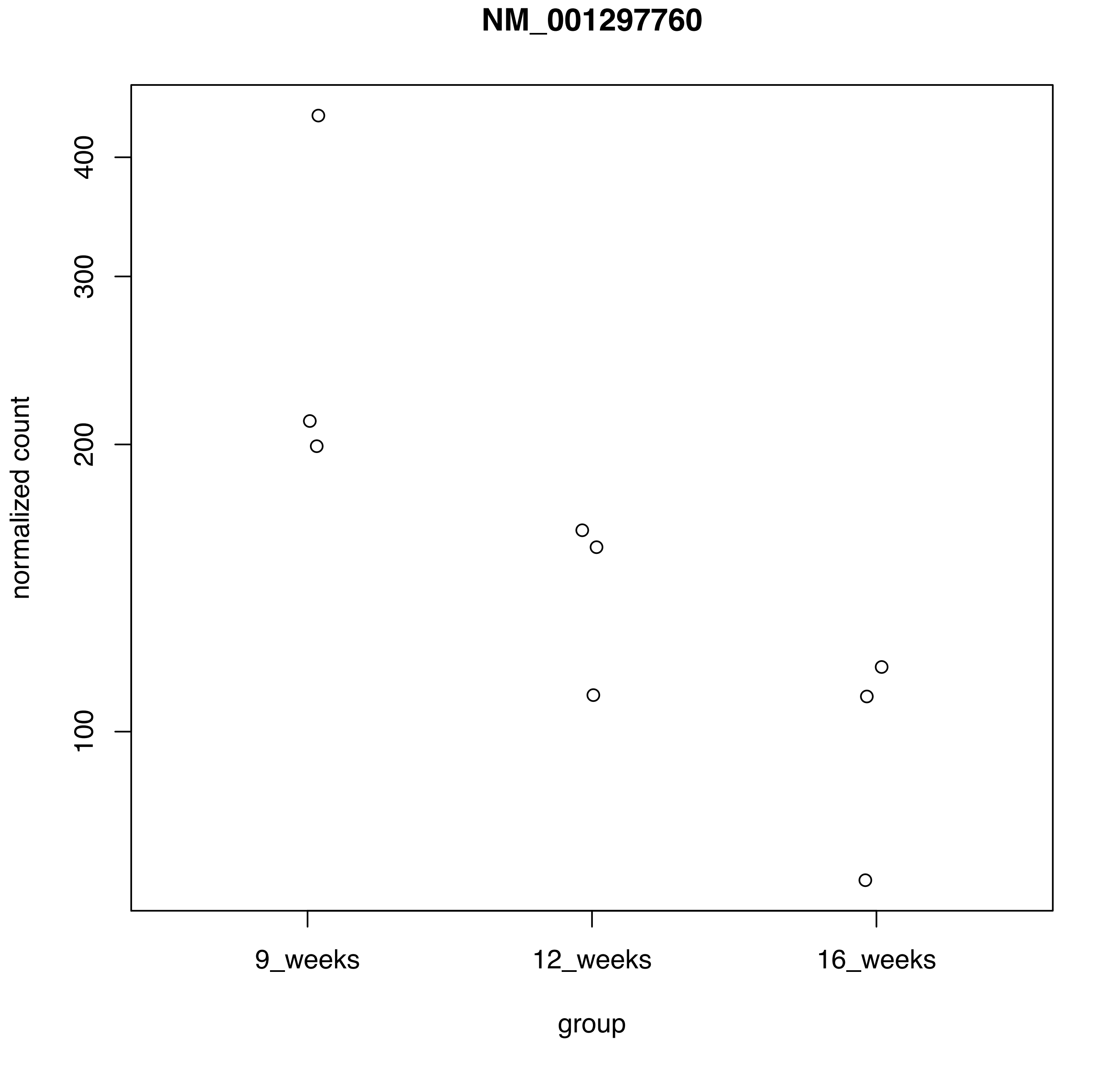

NM_001297760
normalized count
100
200
300
400
9_weeks
12_weeks
16_weeks
group

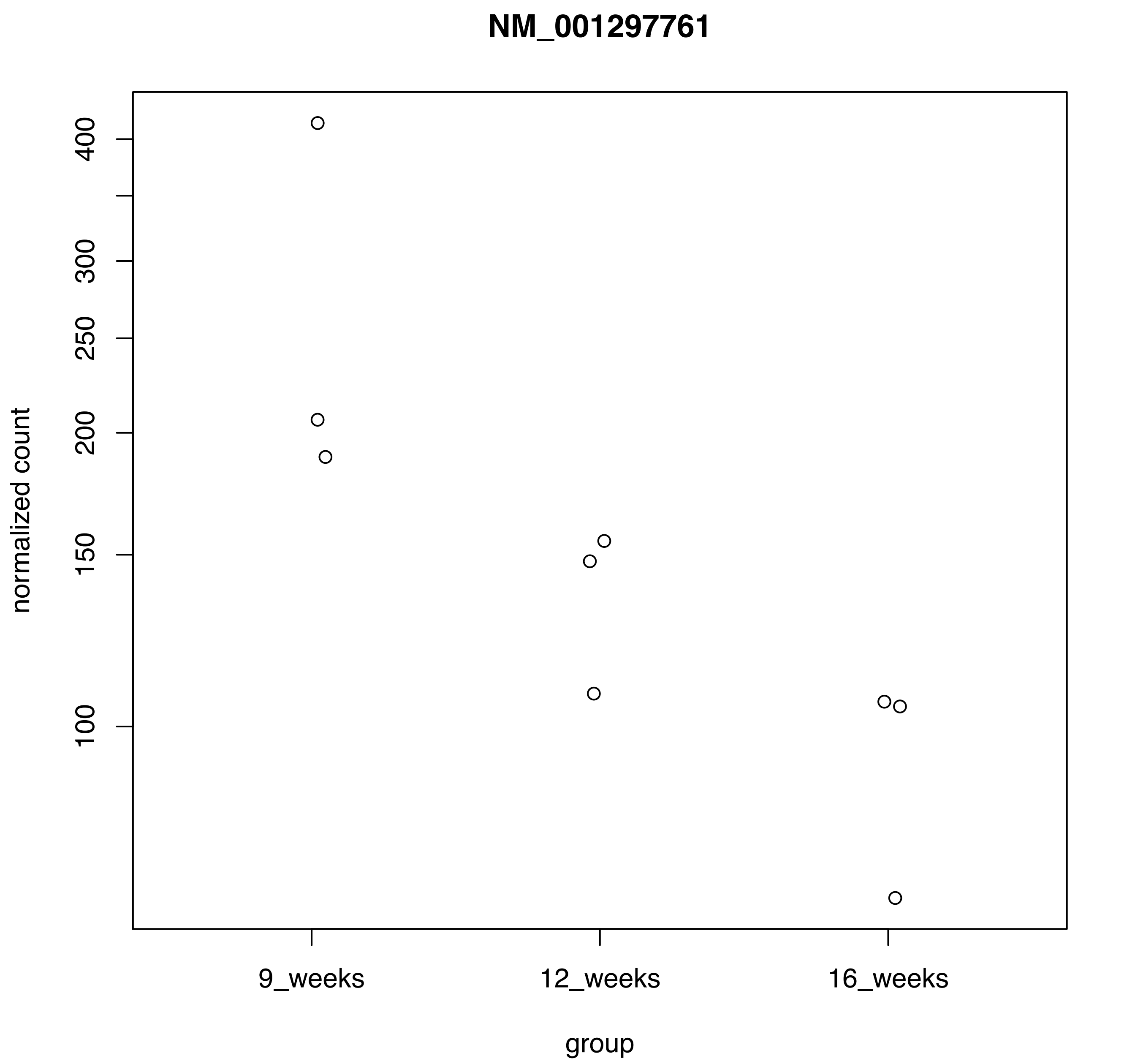
NM_001297761
normalized count
400
300
250
200
150
100
9_weeks
12_weeks
16_weeks
group

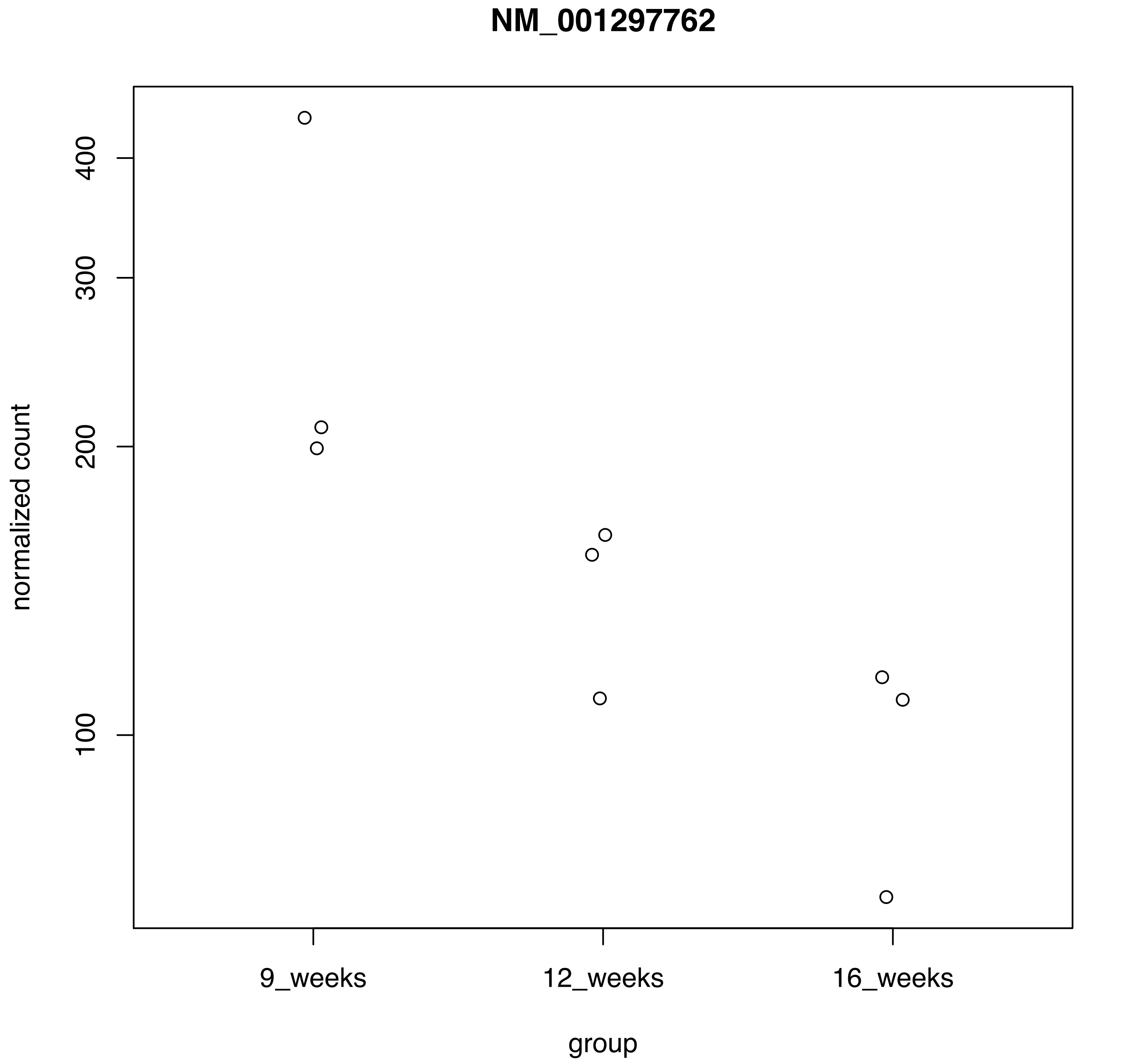

NM_001297762
normalized count
400
300
200
100
9_weeks
12_weeks
16_weeks
group

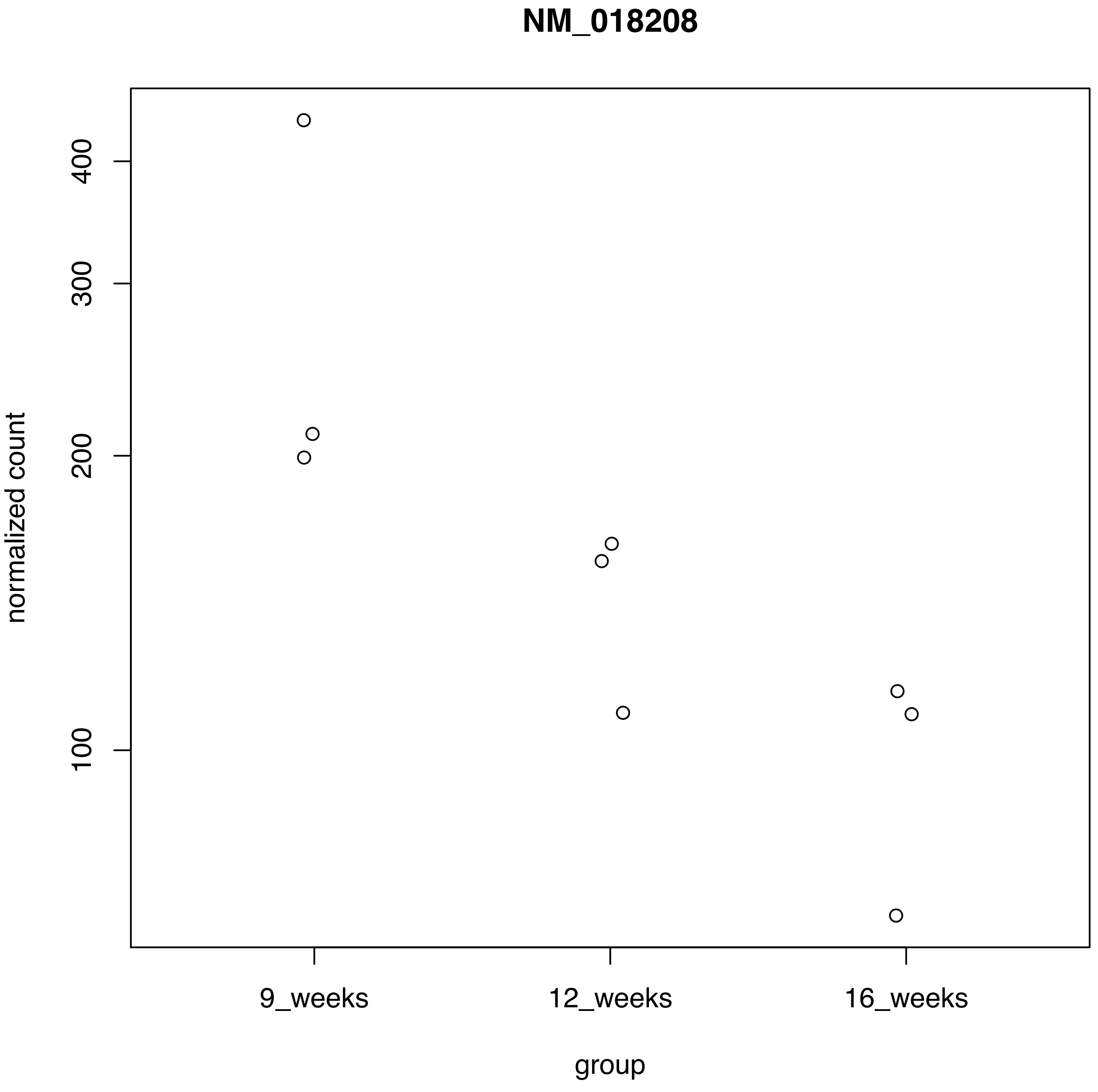
NM_018208
normalized count
400
300
200
100
9_weeks
12_weeks
16_weeks
group

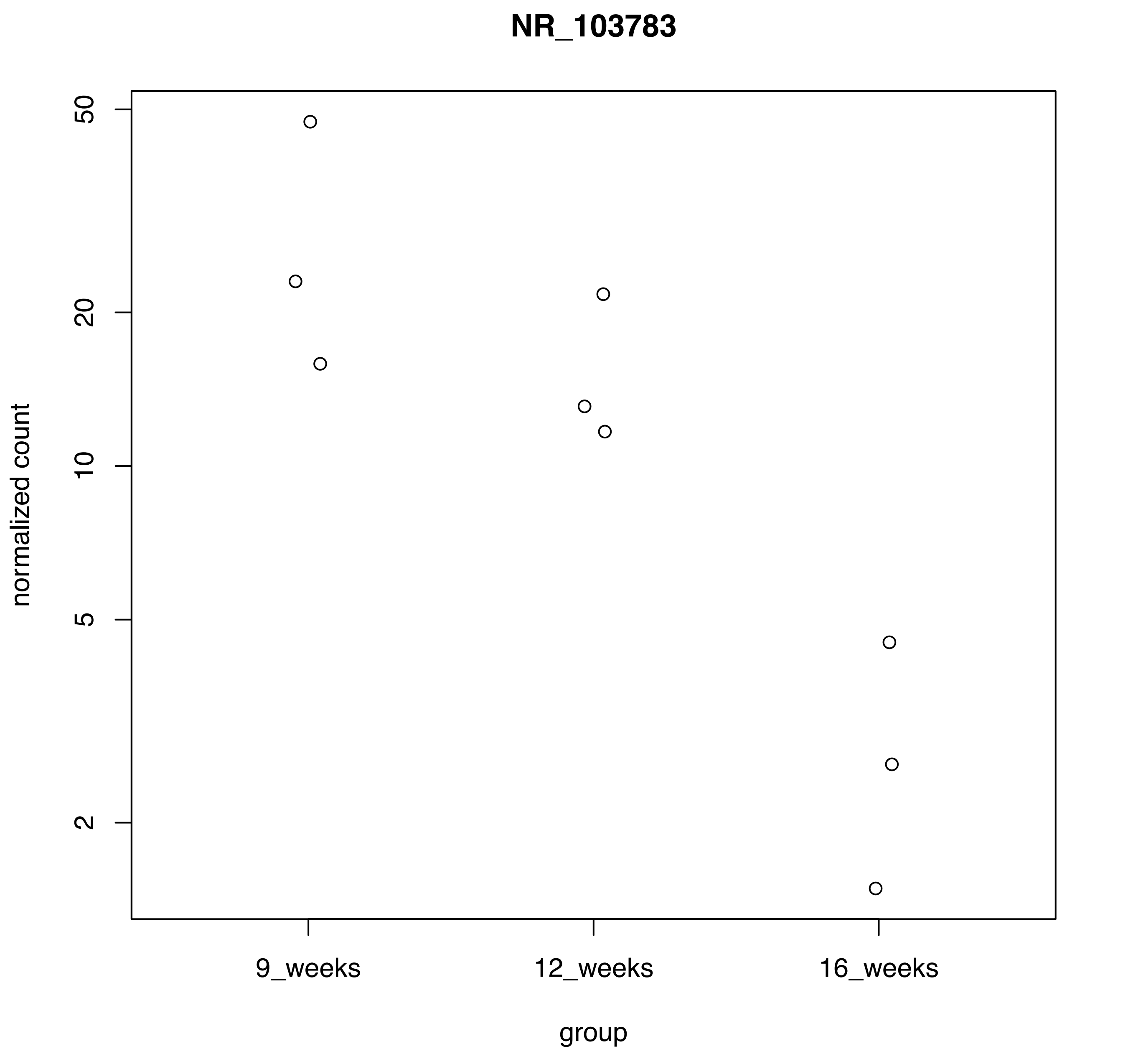
NR_103783
normalized count
50
20
10
5
2
9_weeks
12_weeks
16_weeks
group

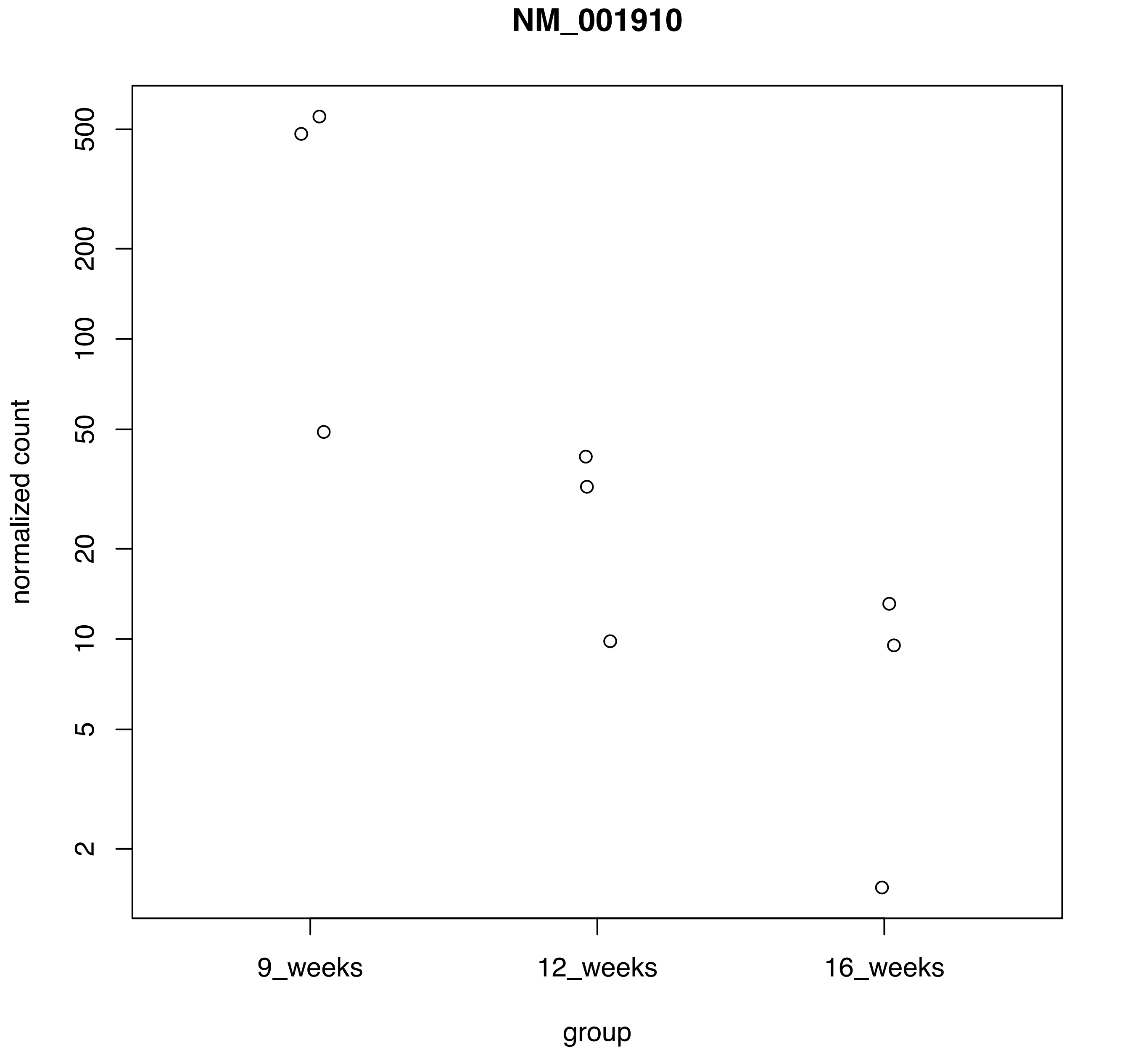
NM_001910
normalized count
500
200
100
50
20
10
5
2
9_weeks
12_weeks
16_weeks
group

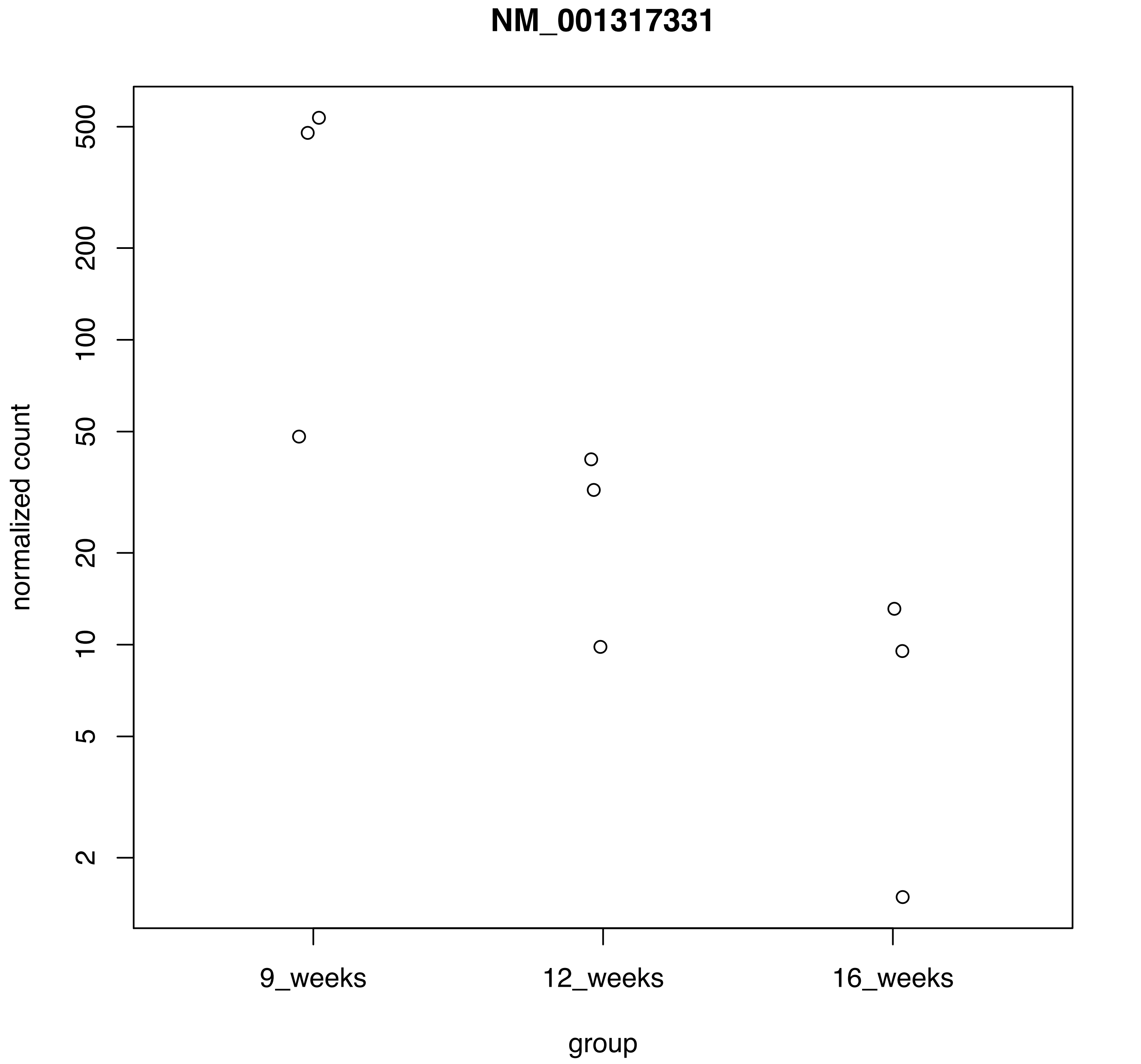
NM_001317331
normalized count
500
200
100
50
20
10
5
2
9_weeks
12_weeks
16_weeks
group

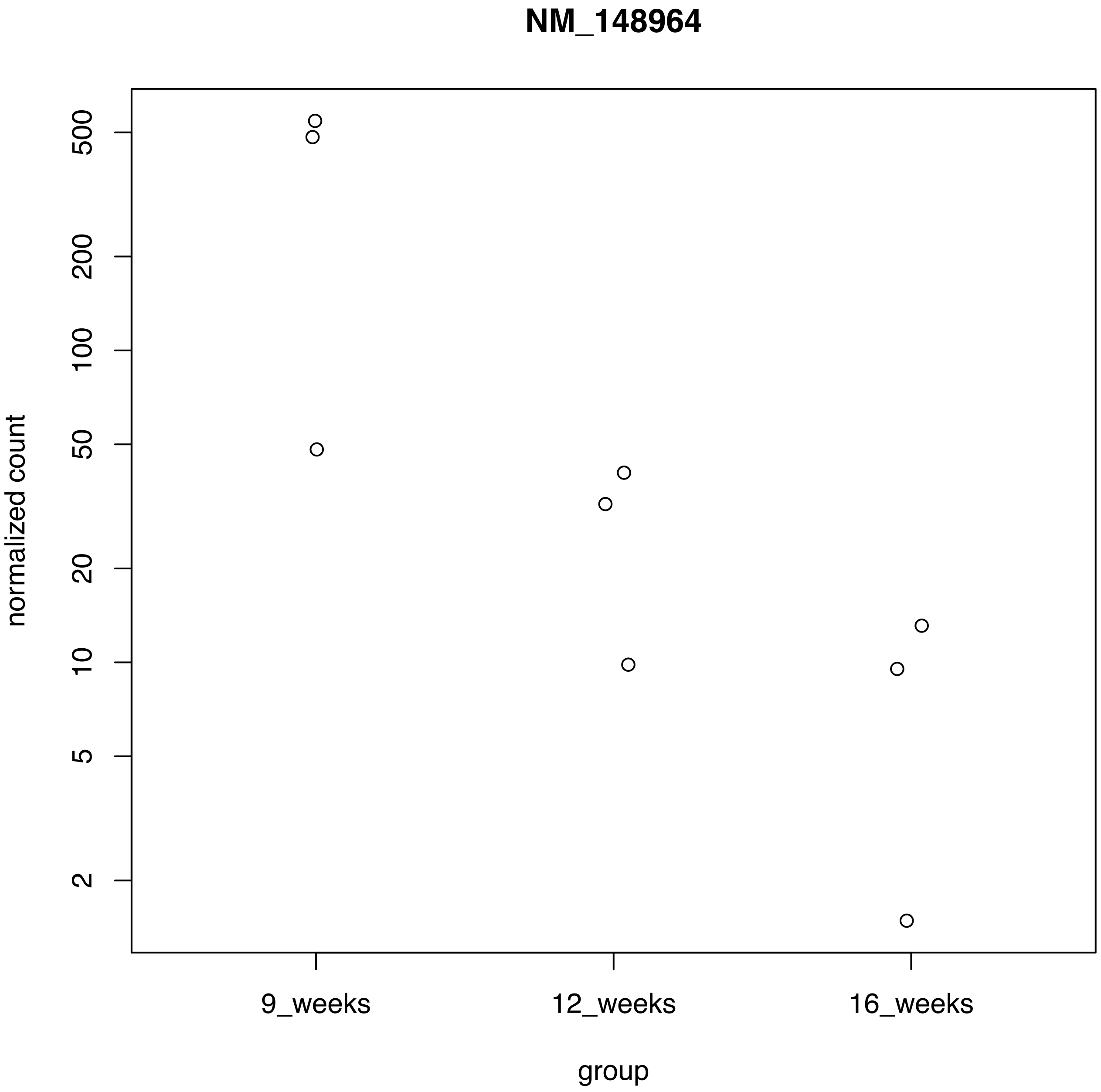

NM_148964
normalized count
500
200
100
50
20
10
5
2
9_weeks
12_weeks
16_weeks
group

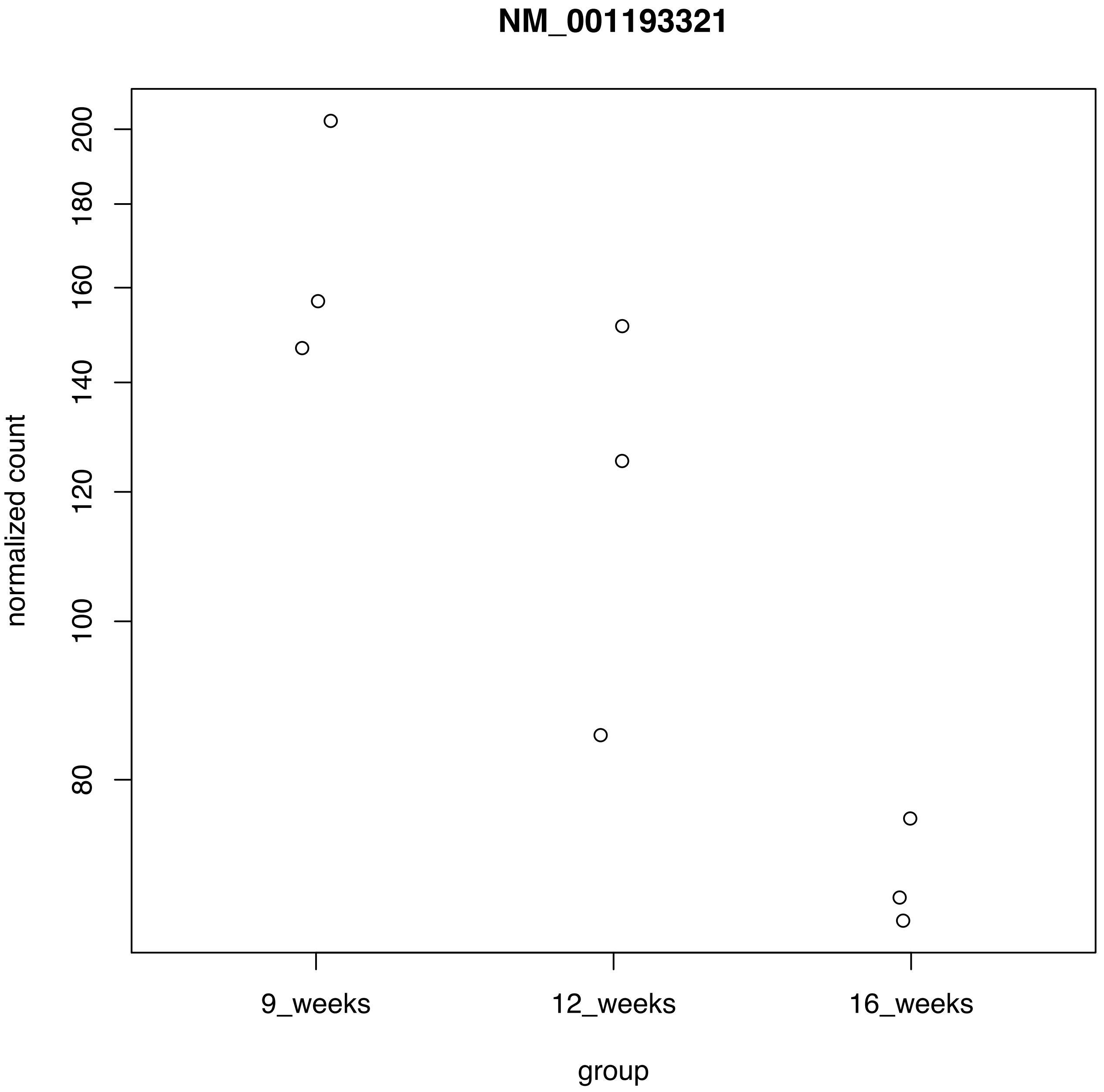
NM_001193321
normalized count
200
180
160
140
120
100
80
9_weeks
12_weeks
16_weeks
group

NM_001193322

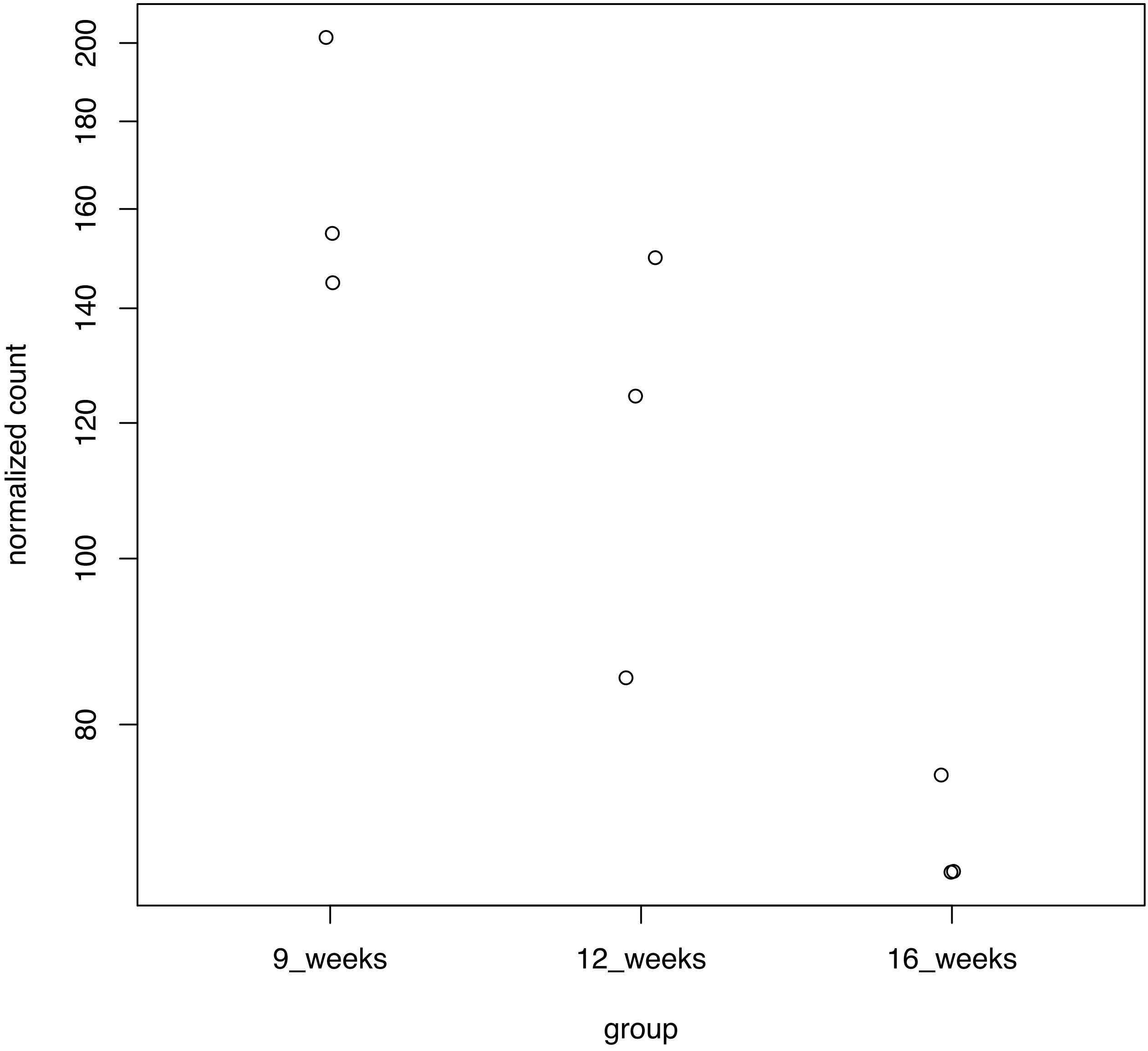

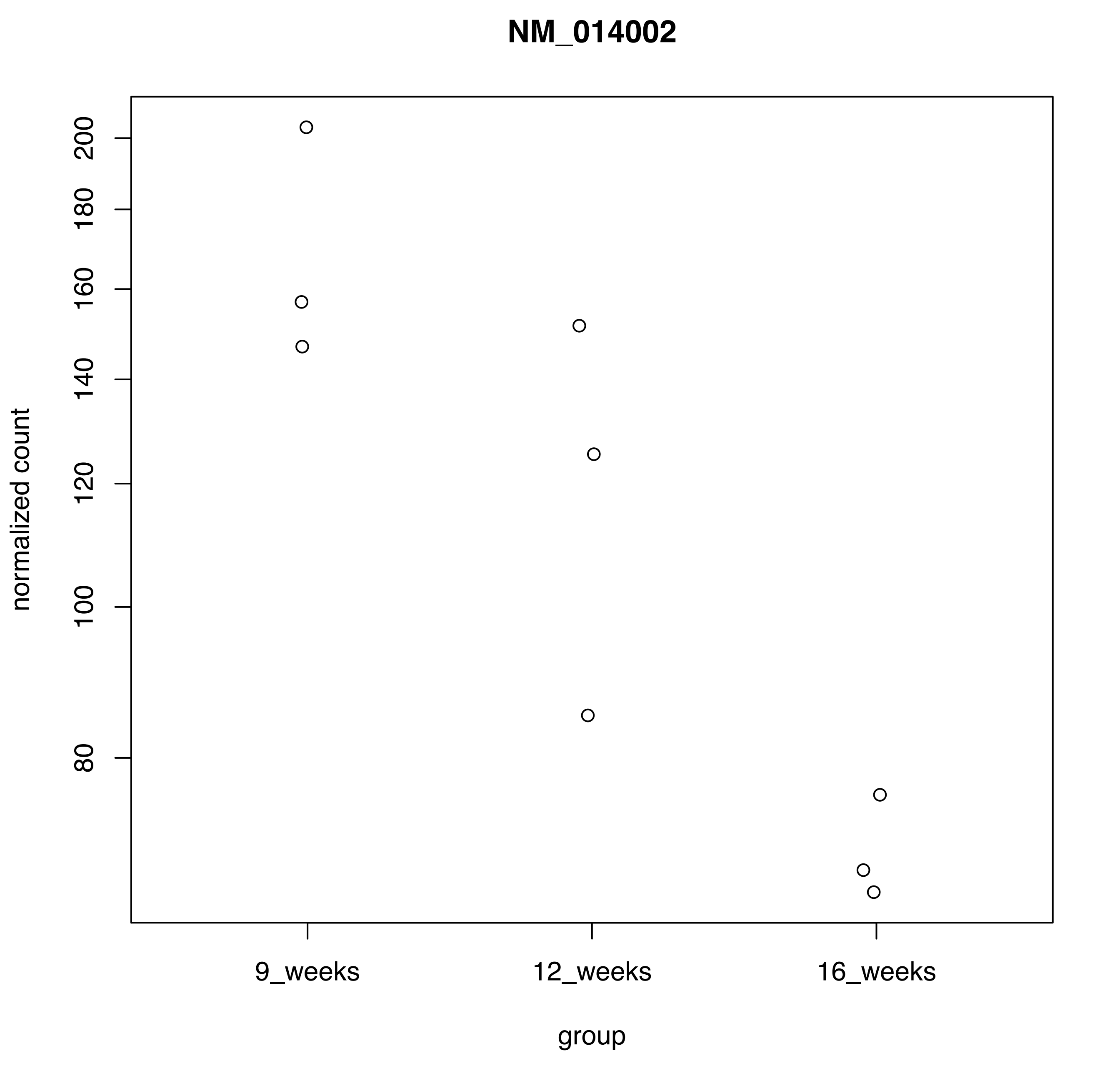
NM_014002
normalized count
200
180
160
140
120
100
80
9_weeks
12_weeks
16_weeks
group

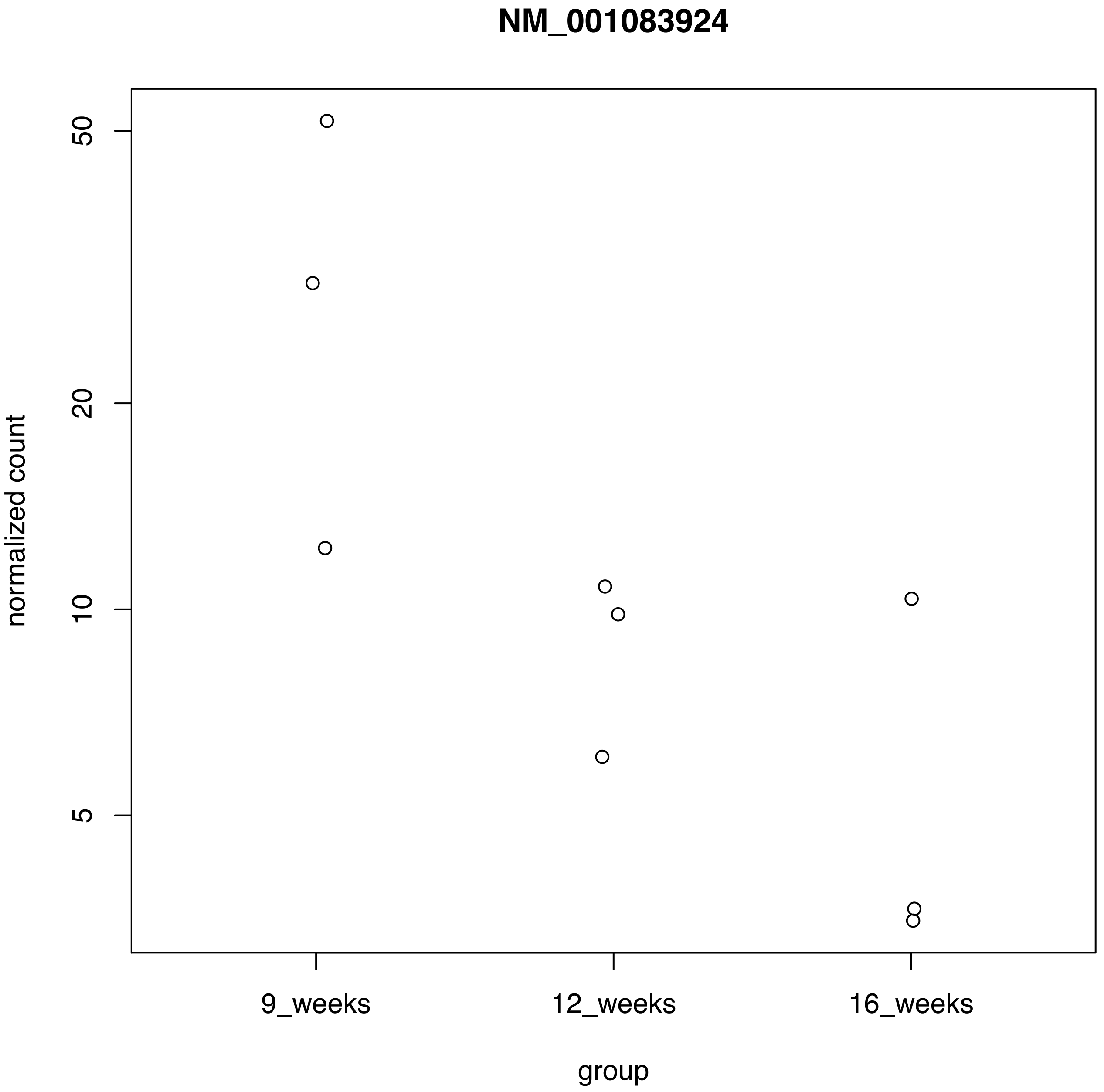
NM_001083924
normalized count
50
20
10
5
9_weeks
12_weeks
16_weeks
group

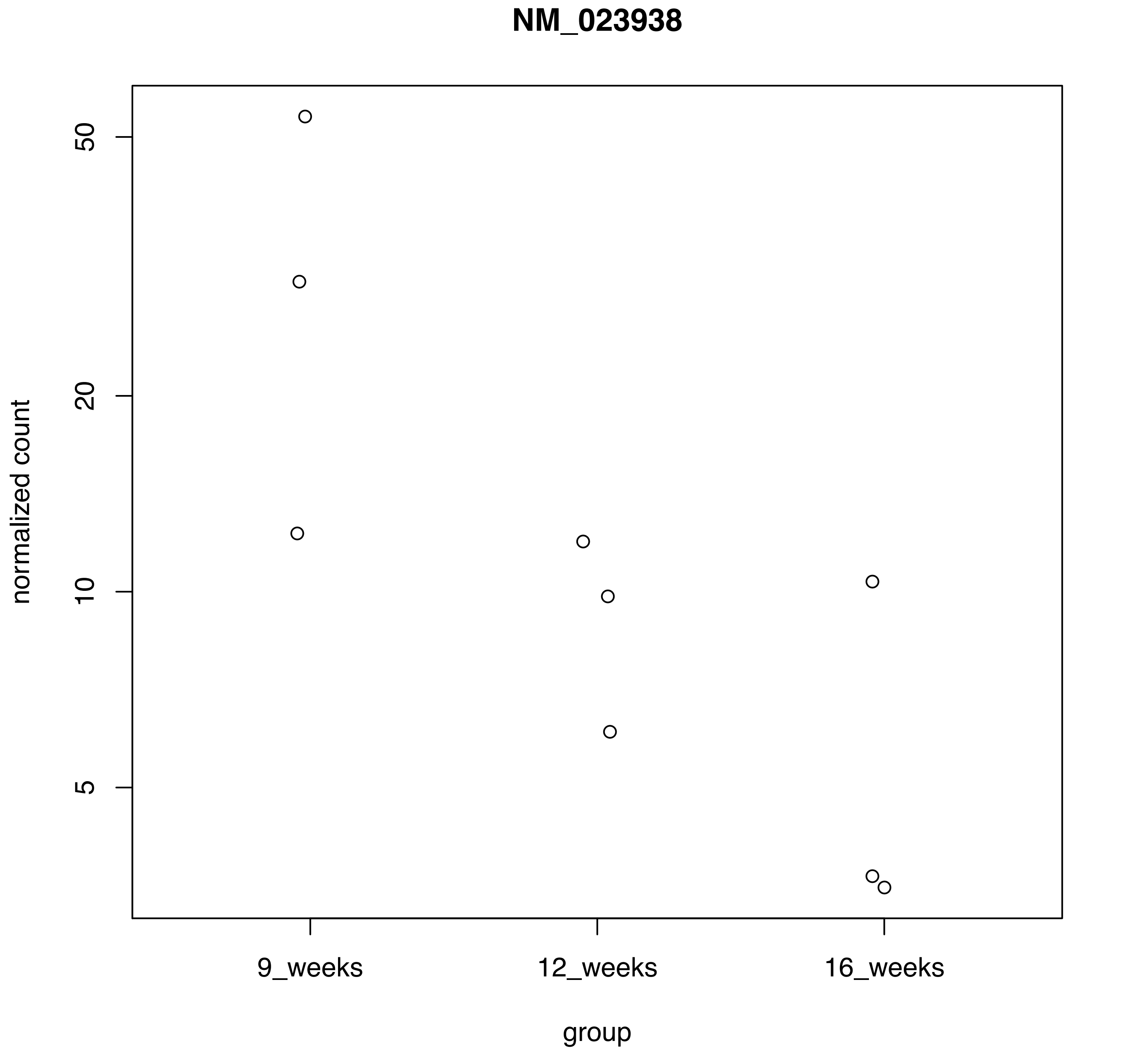

NM_023938
normalized count
50
20
10
5
9_weeks
12_weeks
16_weeks
group

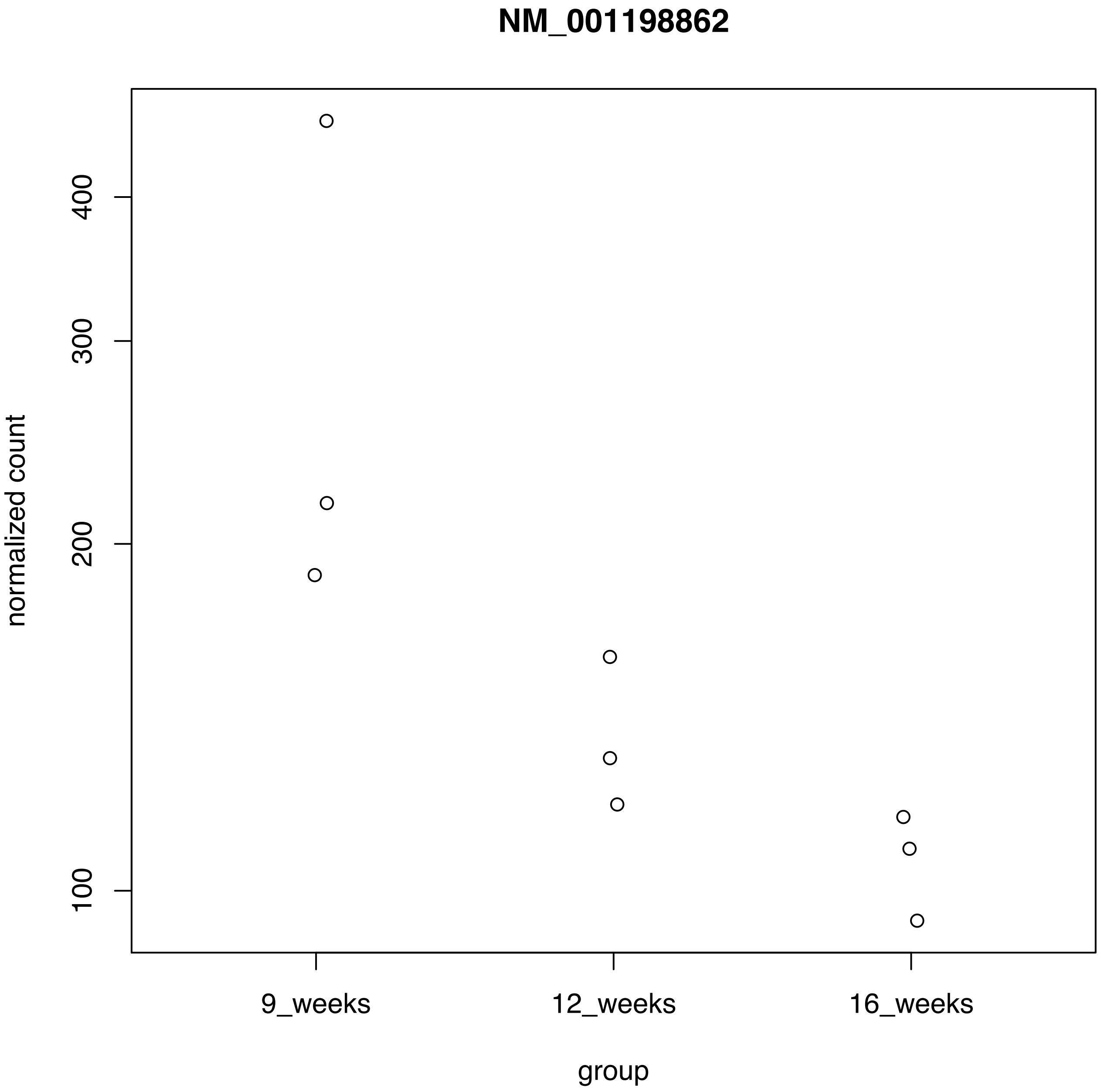
NM_001198862
normalized count
100
200
300
400
9_weeks
12_weeks
16_weeks
group

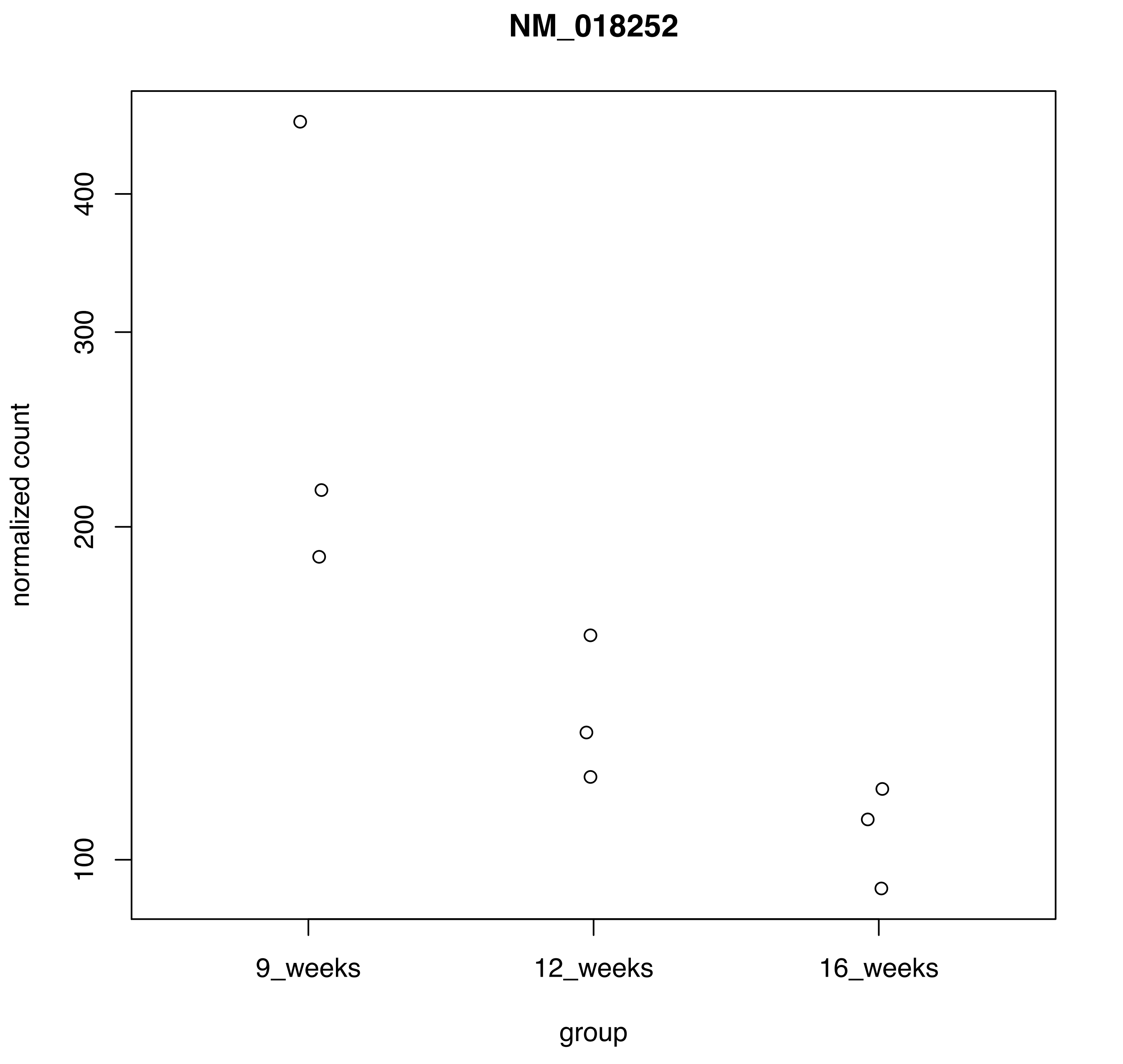
NM_018252
normalized count
100
200
300
400
9_weeks
12_weeks
16_weeks
group

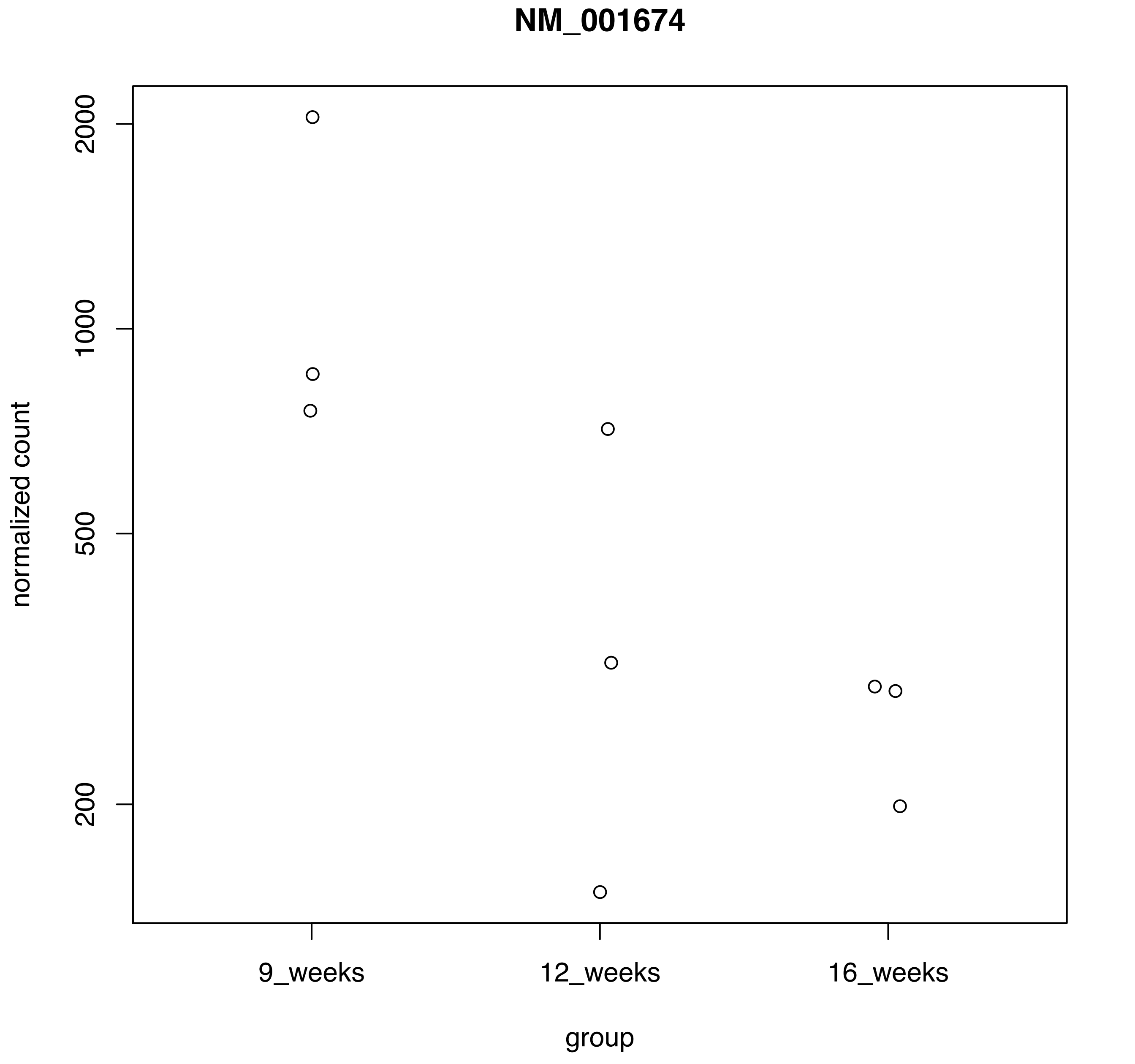

NM_001674
normalized count
2000
1000
500
200
9_weeks
12_weeks
16_weeks
group

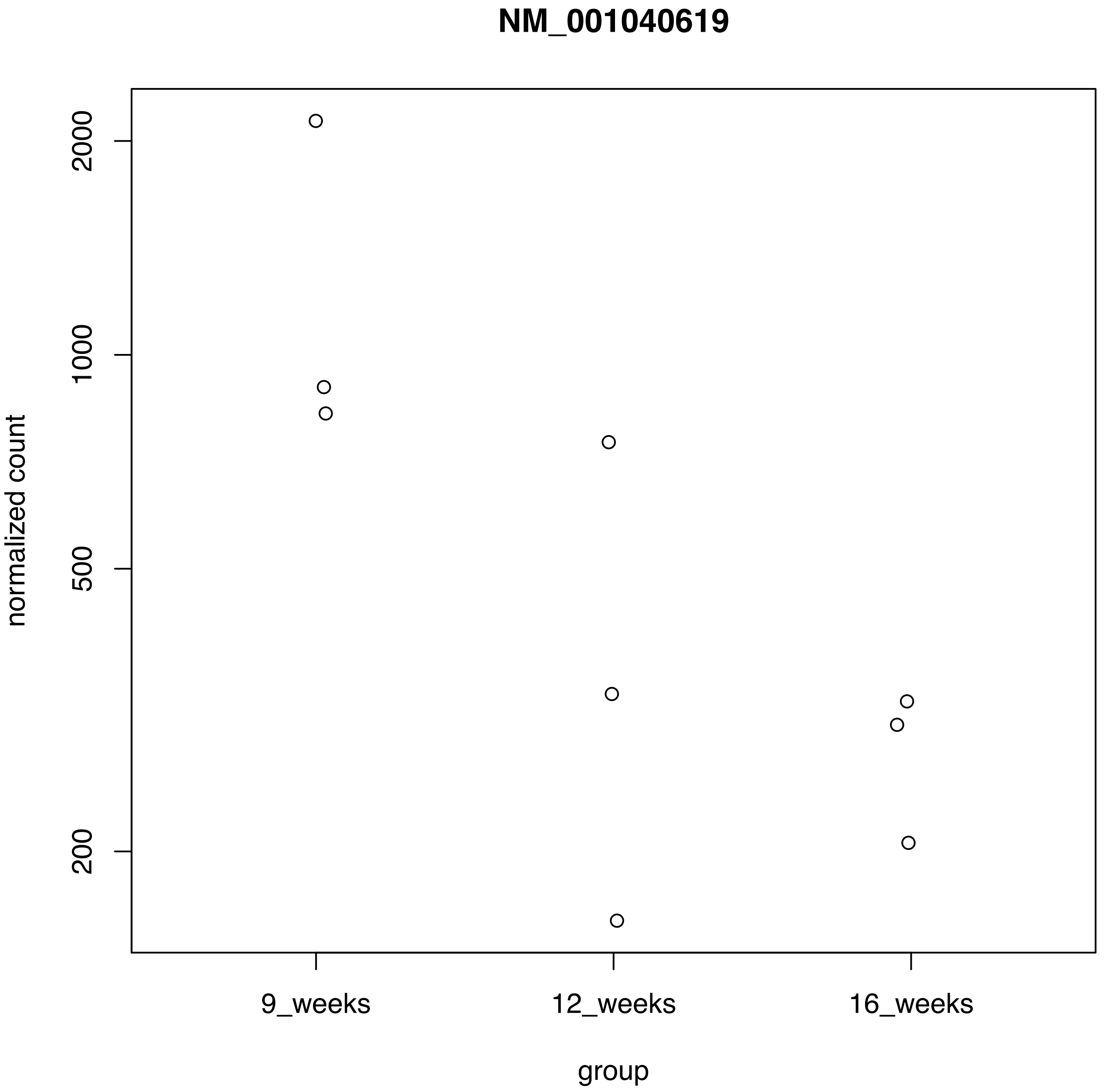

NM_001040619
normalized count
2000
1000
500
200
9_weeks
12_weeks
16_weeks
group

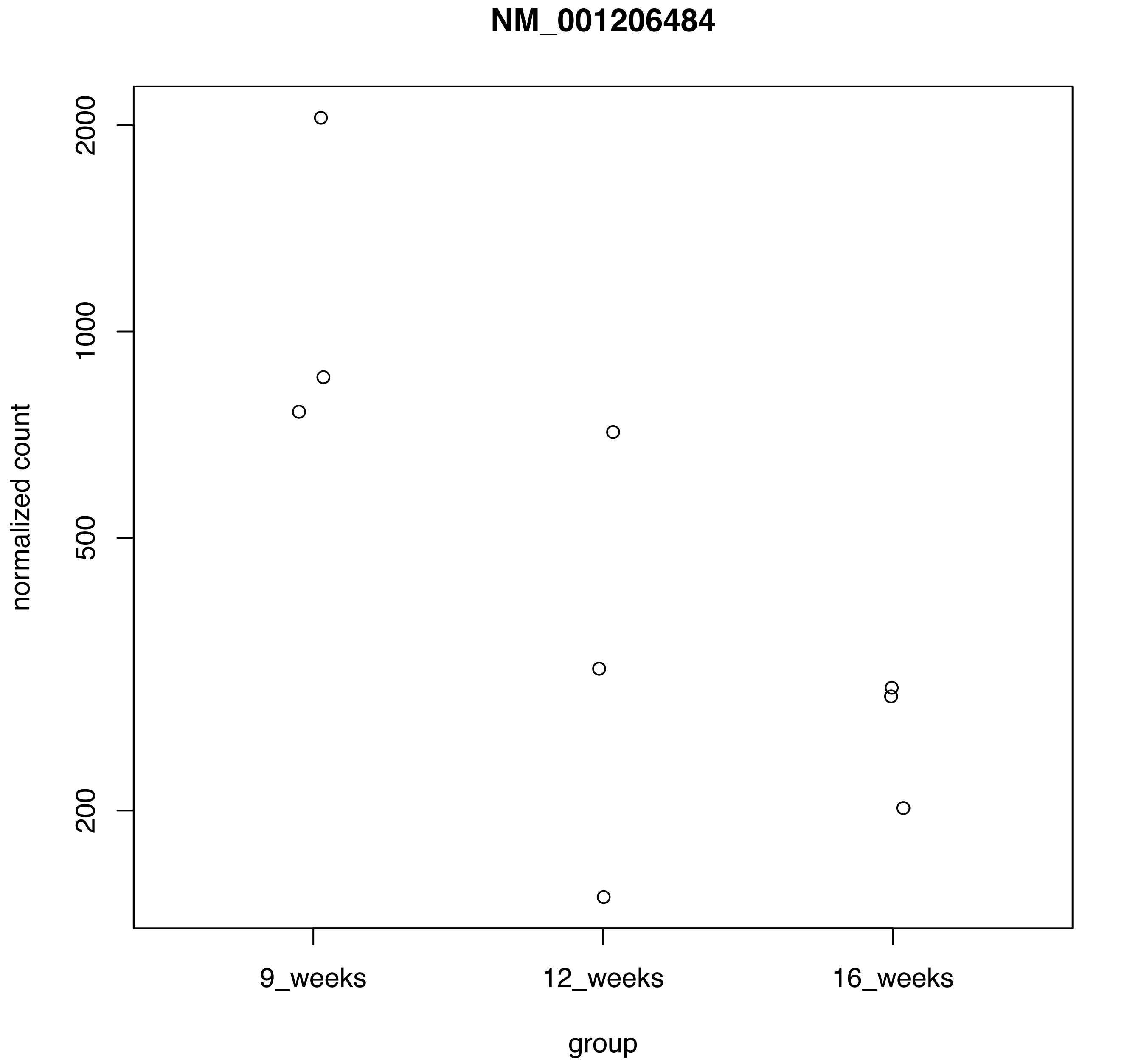
NM_001206484
normalized count
2000
1000
500
200
9_weeks
12_weeks
16_weeks
group

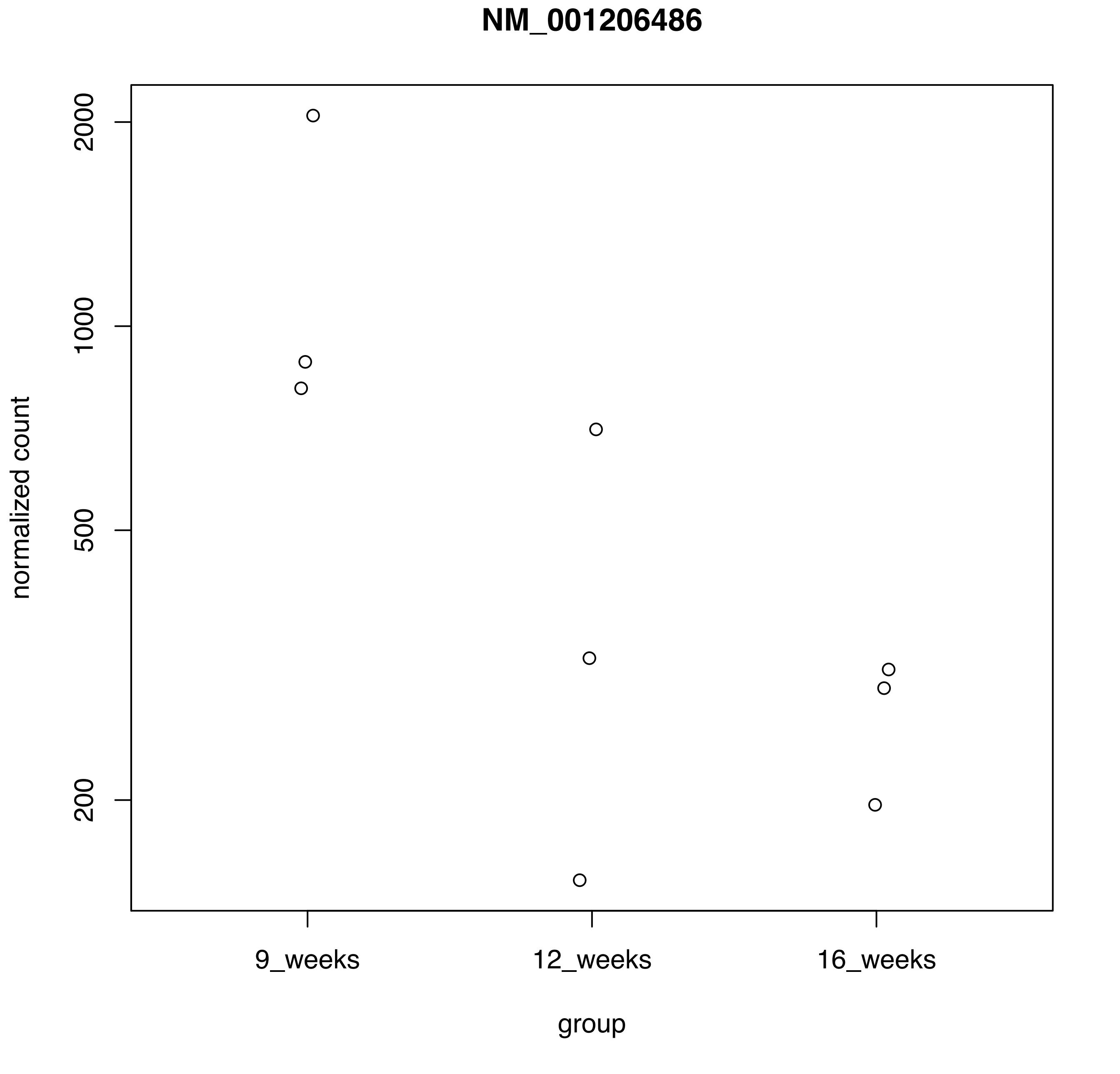
NM_001206486
2000
1000
500
200
normalized count
9_weeks
12_weeks
16_weeks
group

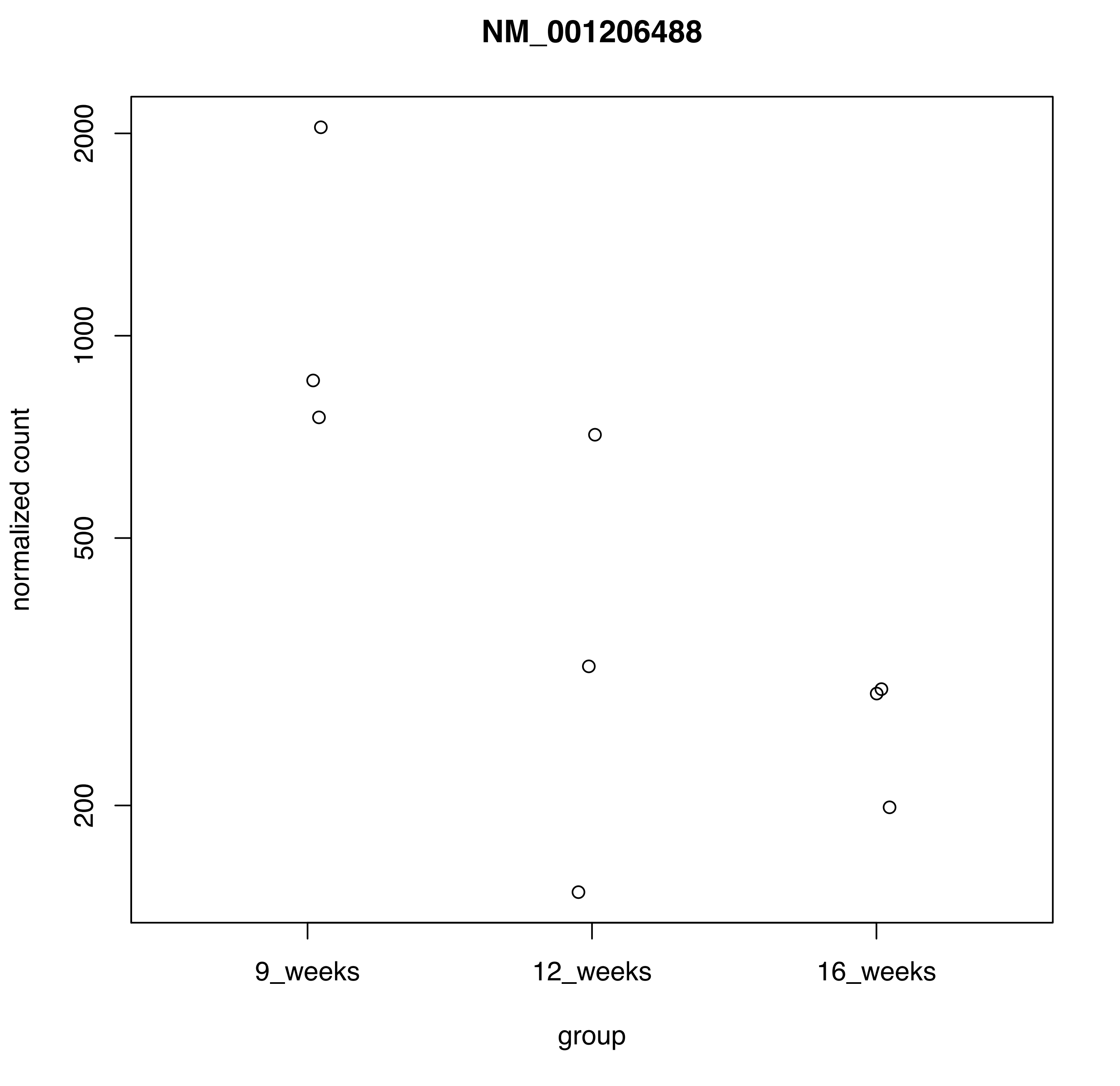

NM_001206488
normalized count
2000
1000
500
200
9_weeks
12_weeks
16_weeks
group

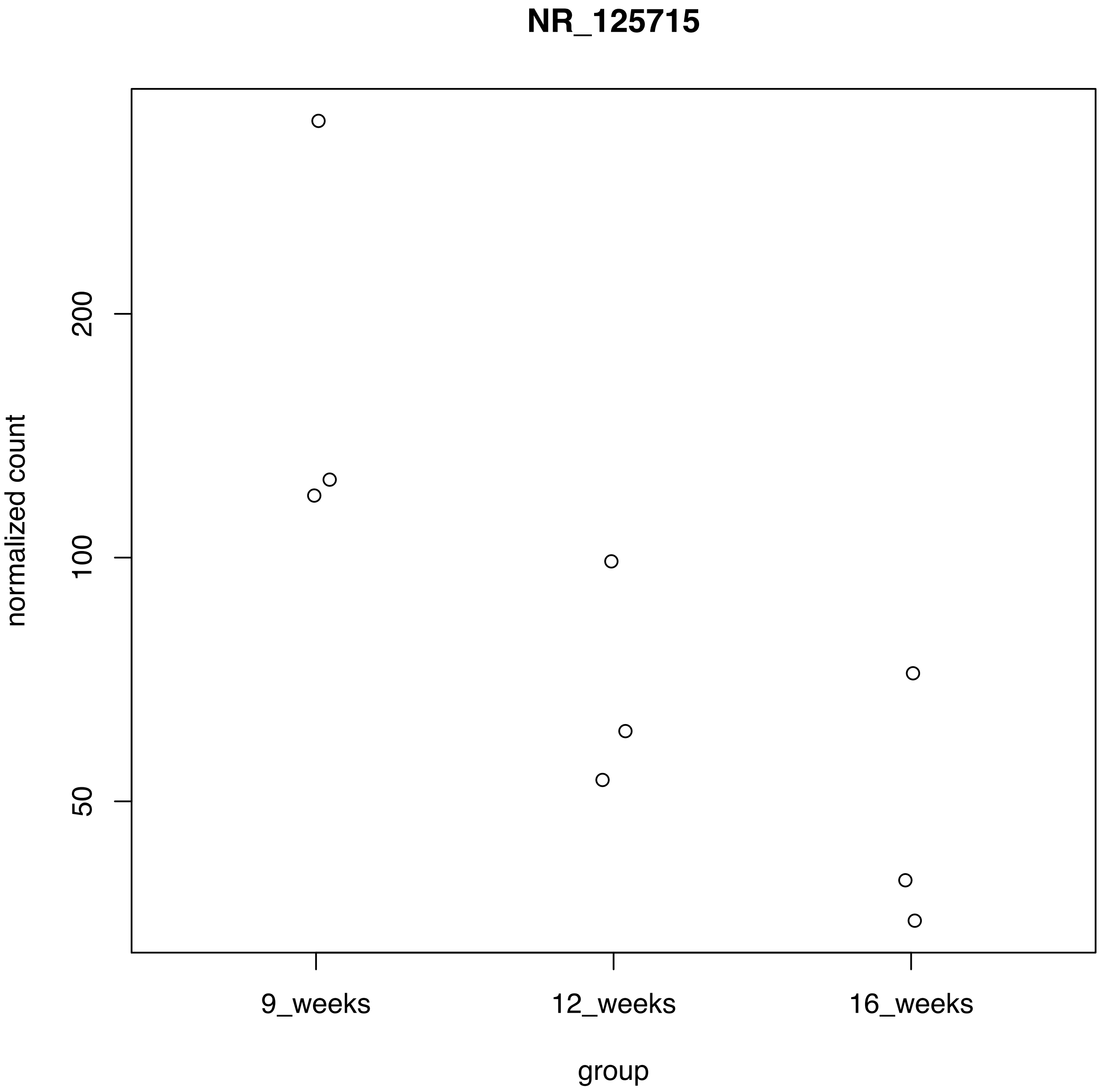

NR_125715
normalized count
200
100
50
9_weeks
12_weeks
16_weeks
group

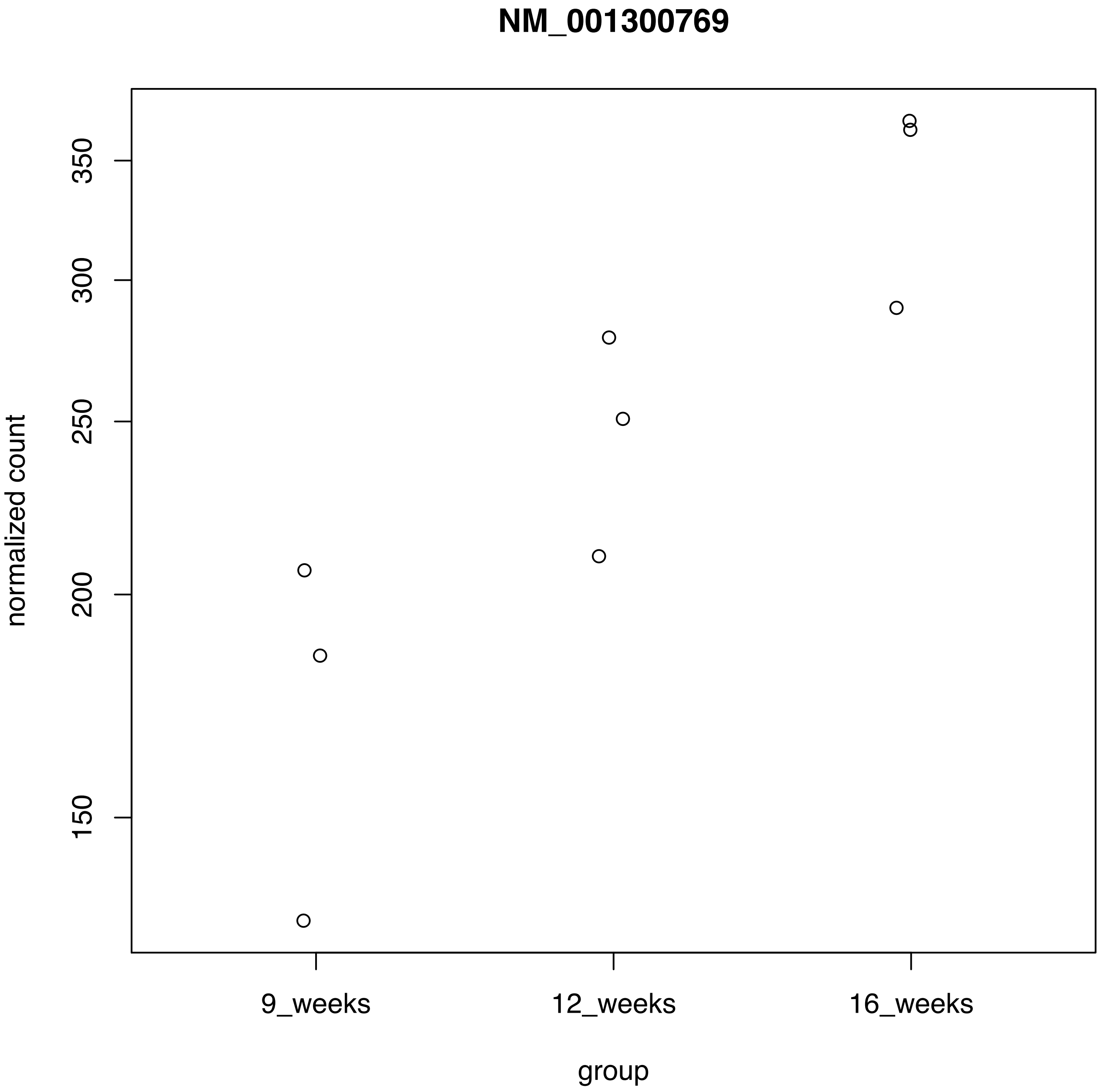

NM_001300769
normalized count
150
200
250
300
350
9_weeks
12_weeks
16_weeks
group

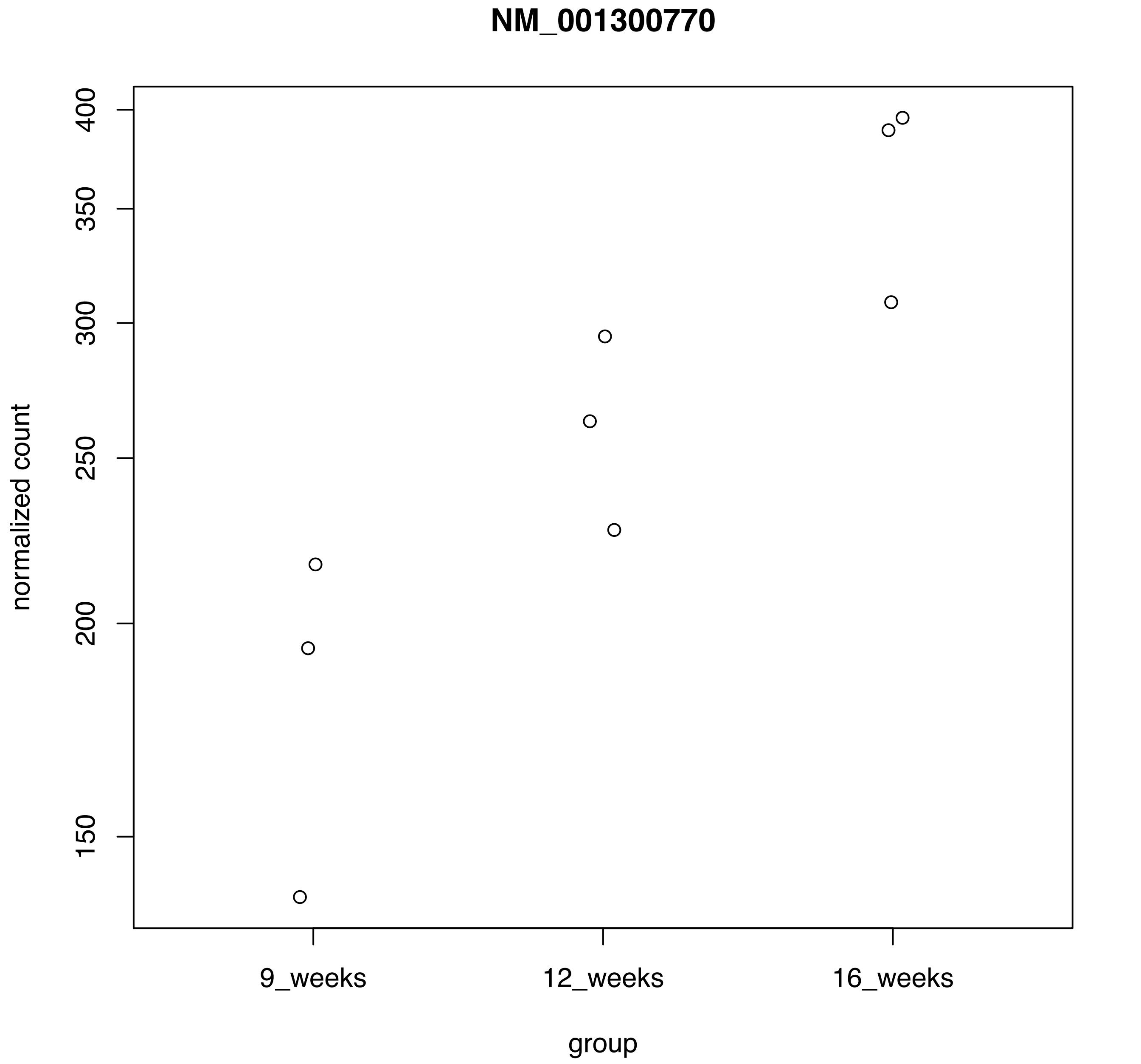

NM_001300770
normalized count
400
350
300
250
200
150
9_weeks
12_weeks
16_weeks
group

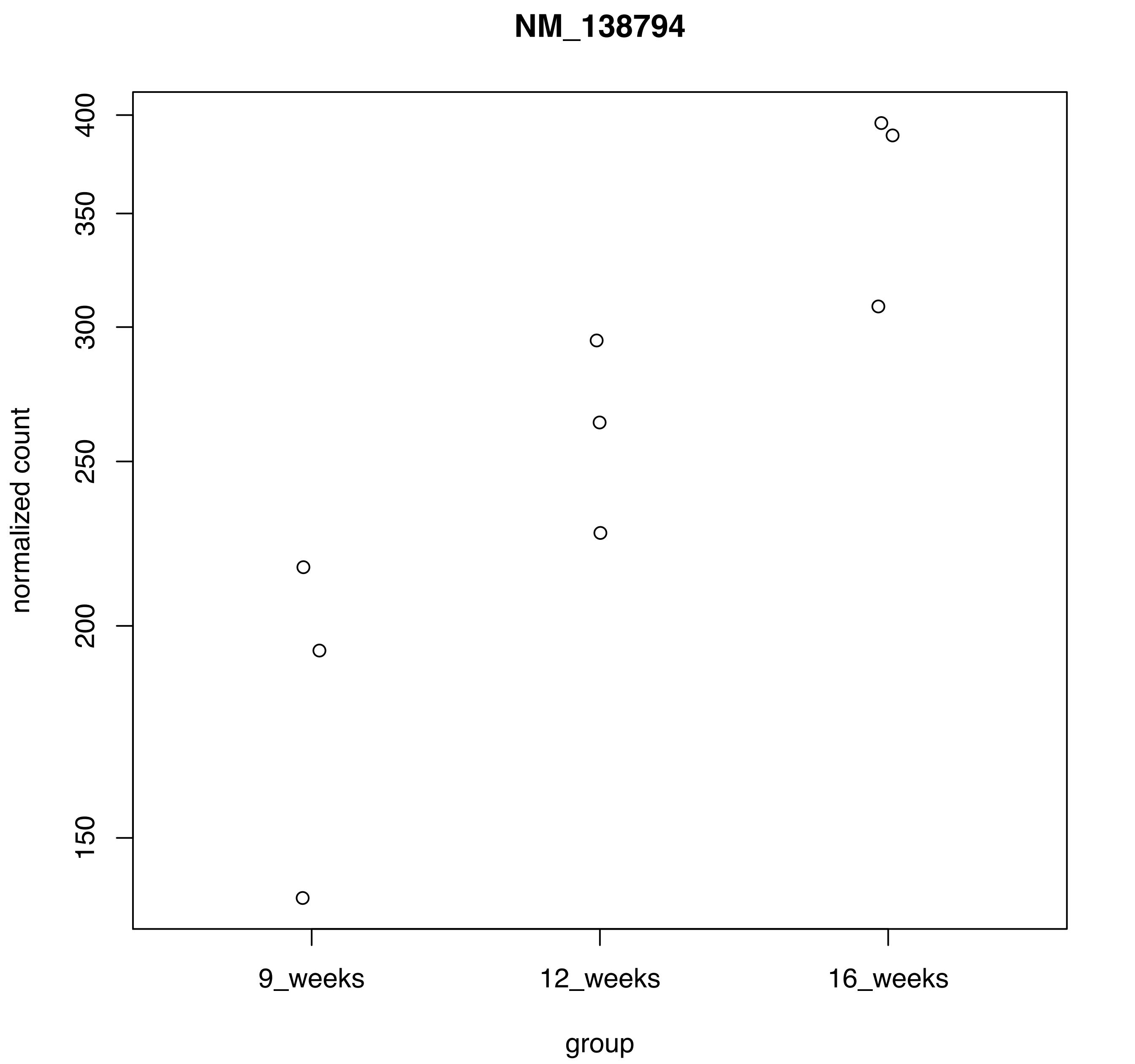
NM_138794
normalized count
400
350
300
250
200
150
9_weeks
12_weeks
16_weeks
group

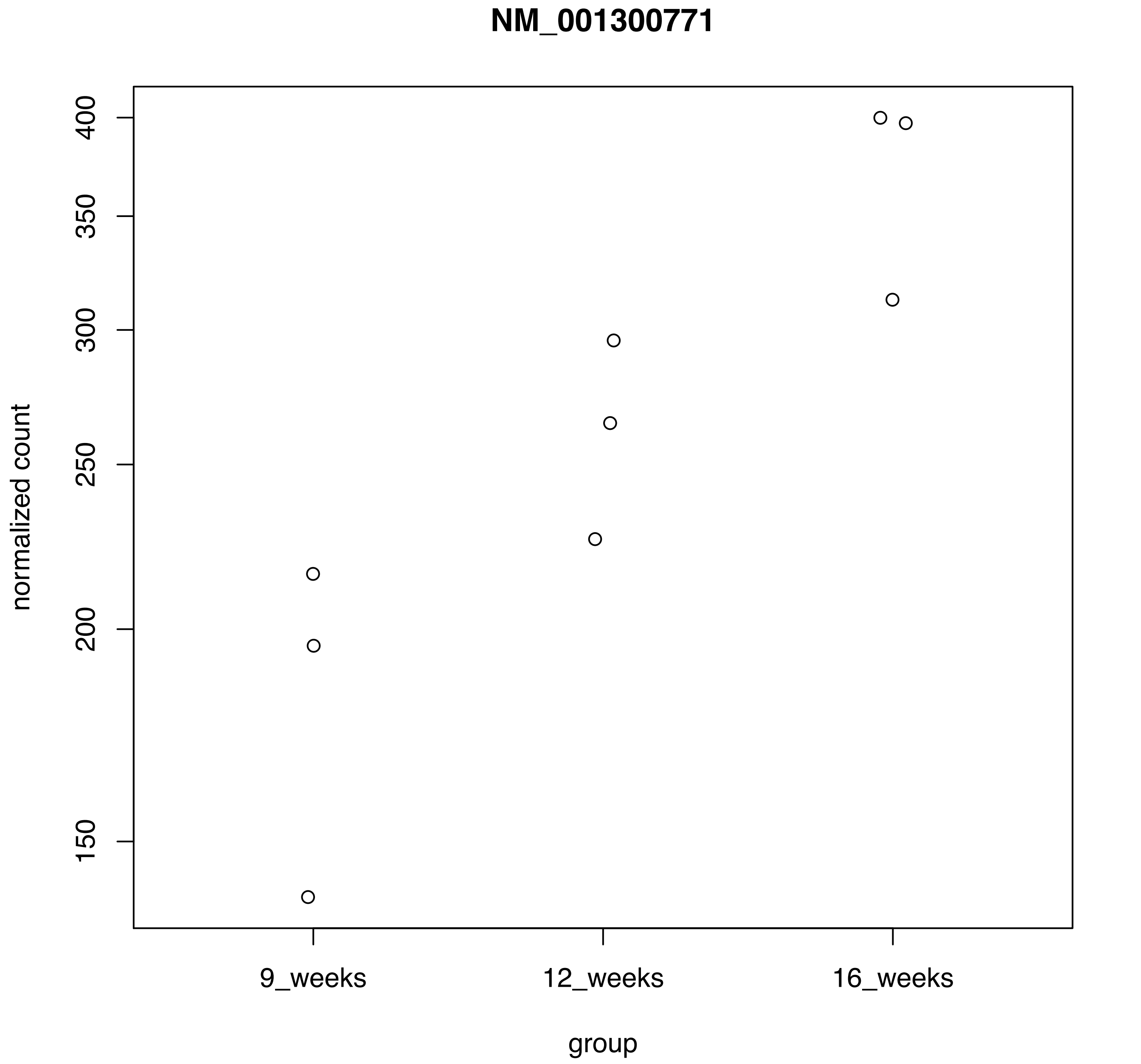
NM_001300771
normalized count
400
350
300
250
200
150
9_weeks
12_weeks
16_weeks
group

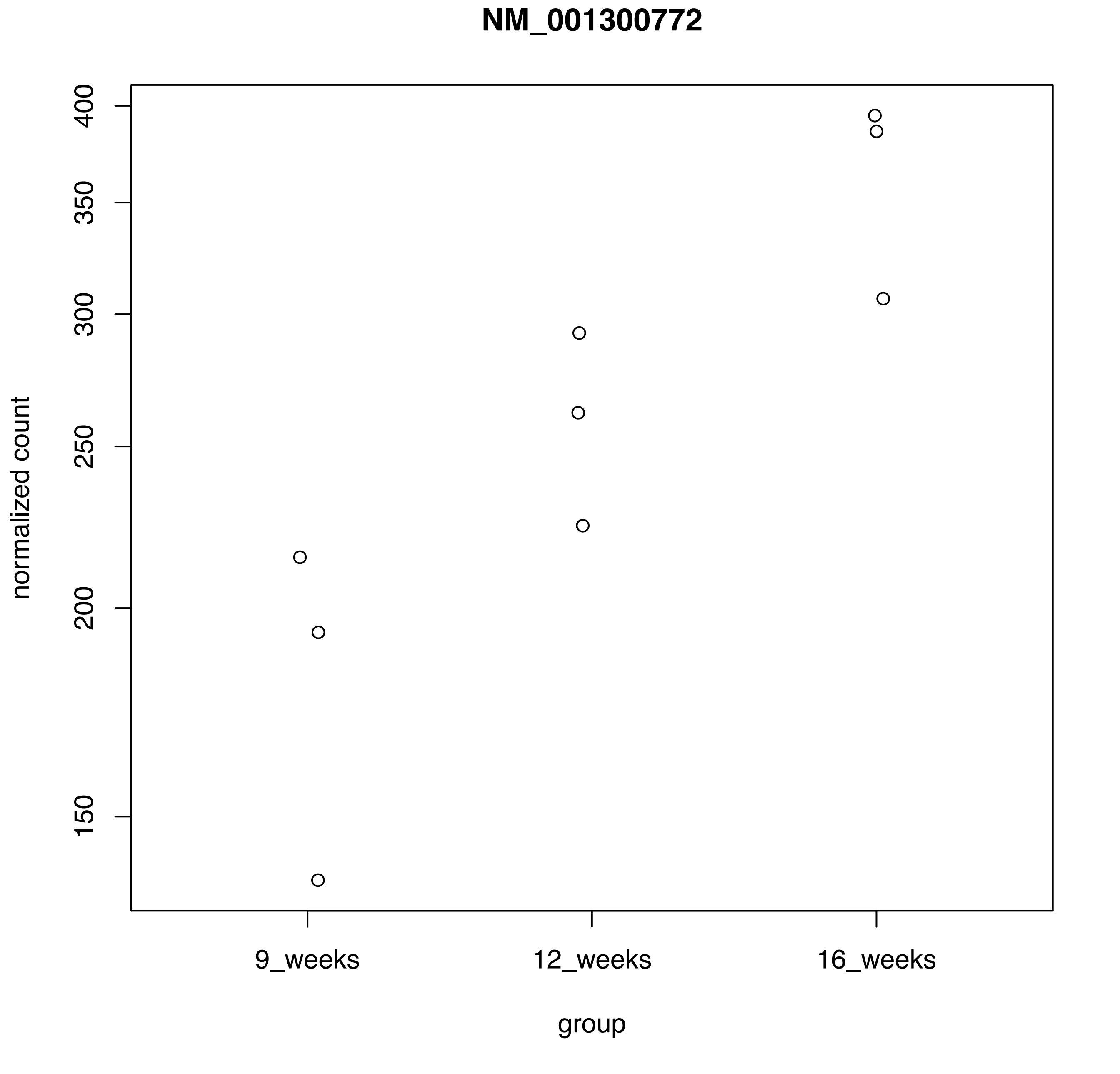

NM_001300772
normalized count
400
350
300
250
200
150
9_weeks
12_weeks
16_weeks
group

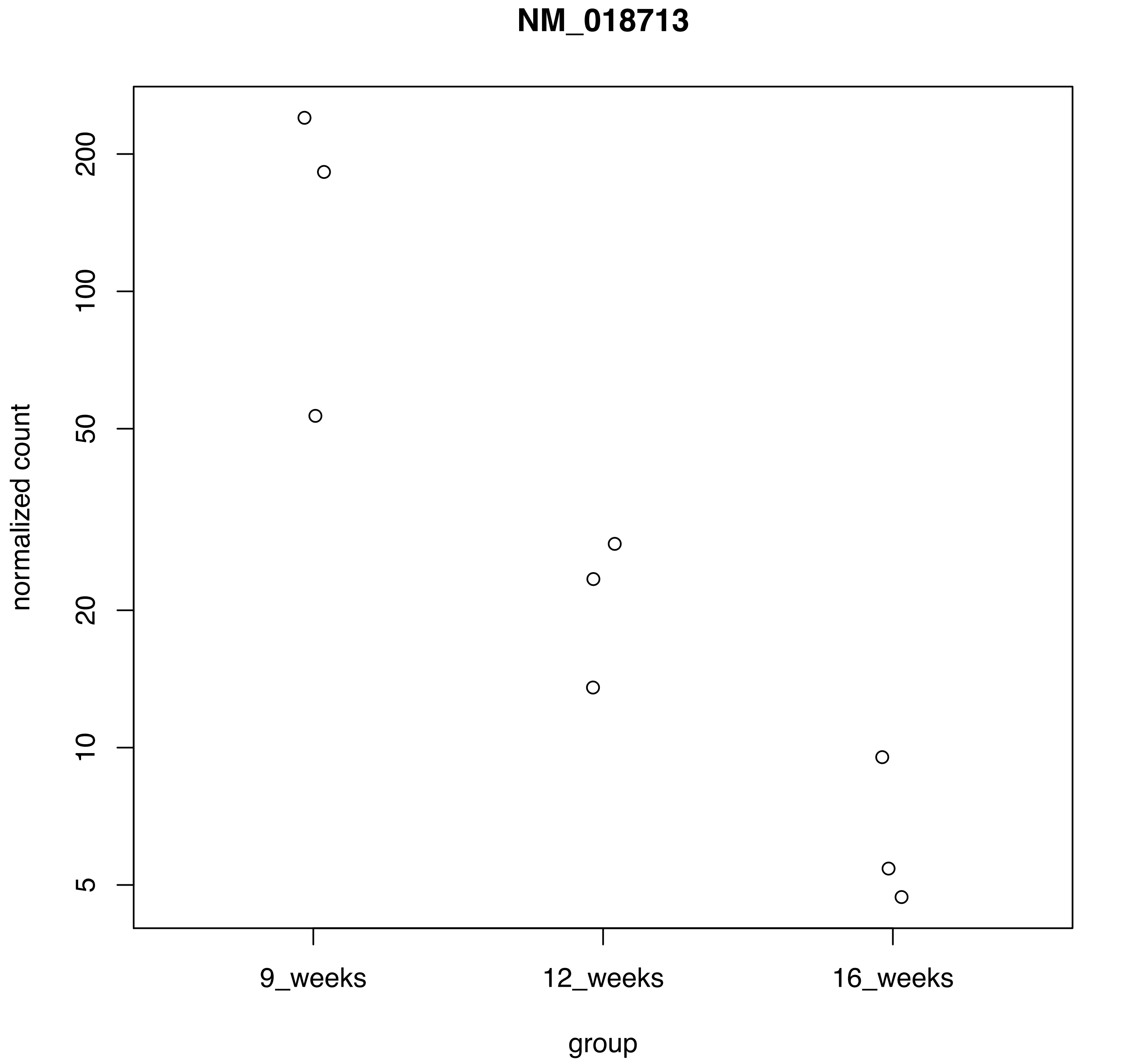

NM_018713
normalized count
200
100
50
20
10
5
9_weeks
12_weeks
16_weeks
group

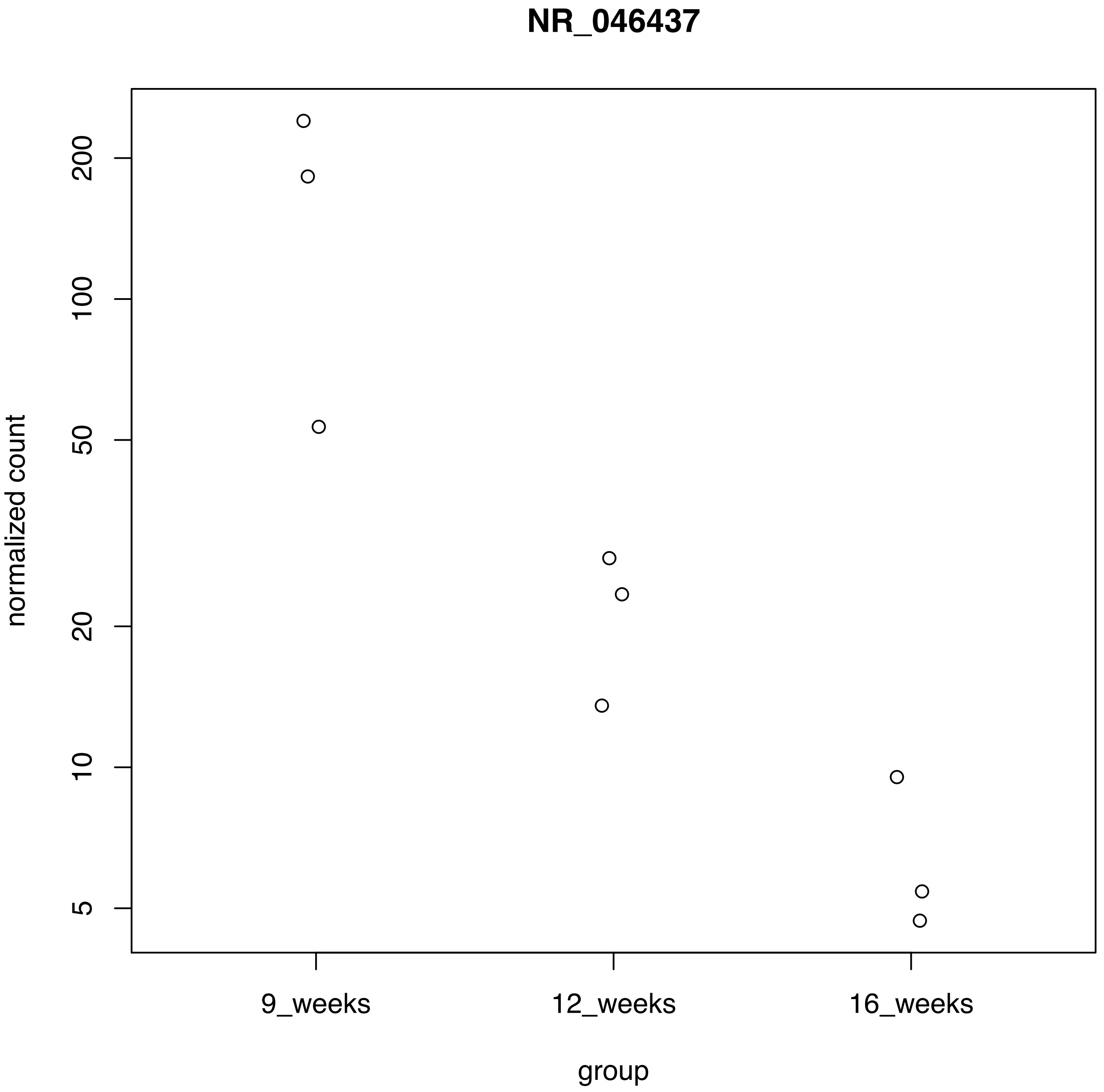
NR_046437
normalized count
200
100
50
20
10
5
9_weeks
12_weeks
16_weeks
group

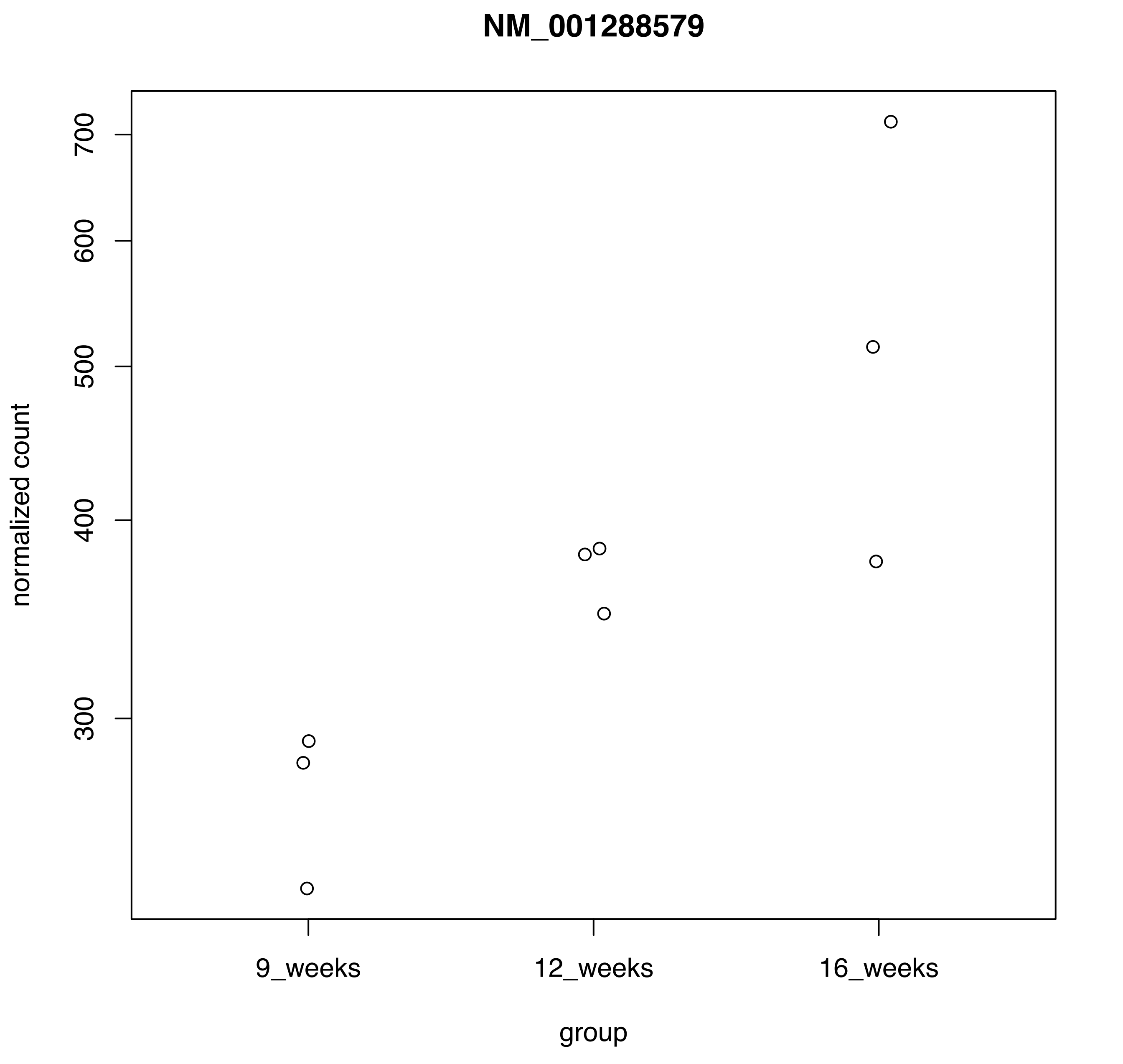

NM_001288579
normalized count
700
600
500
400
300
9_weeks
12_weeks
16_weeks
group

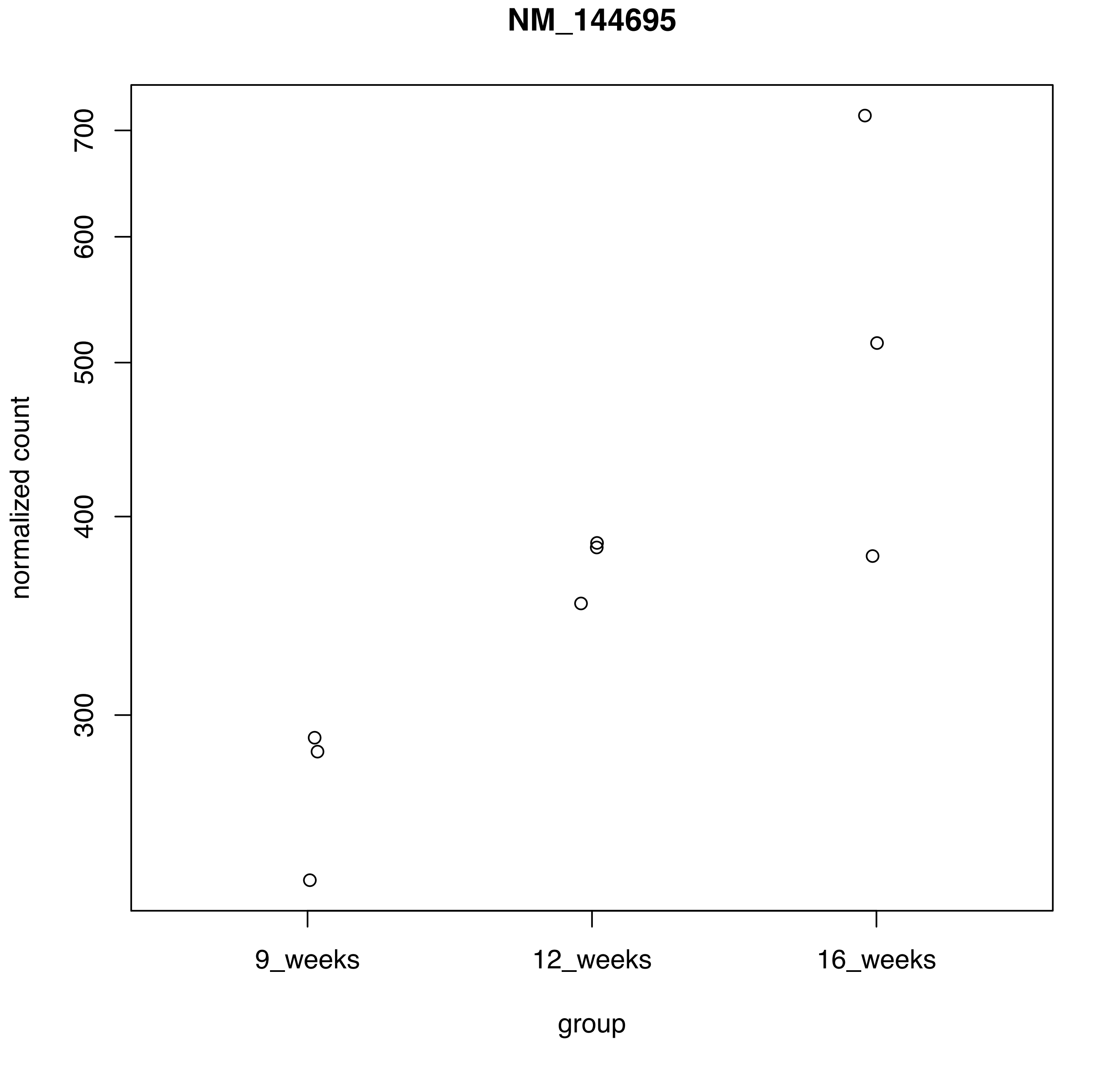

NM_144695
normalized count
700
600
500
400
300
9_weeks
12_weeks
16_weeks
group

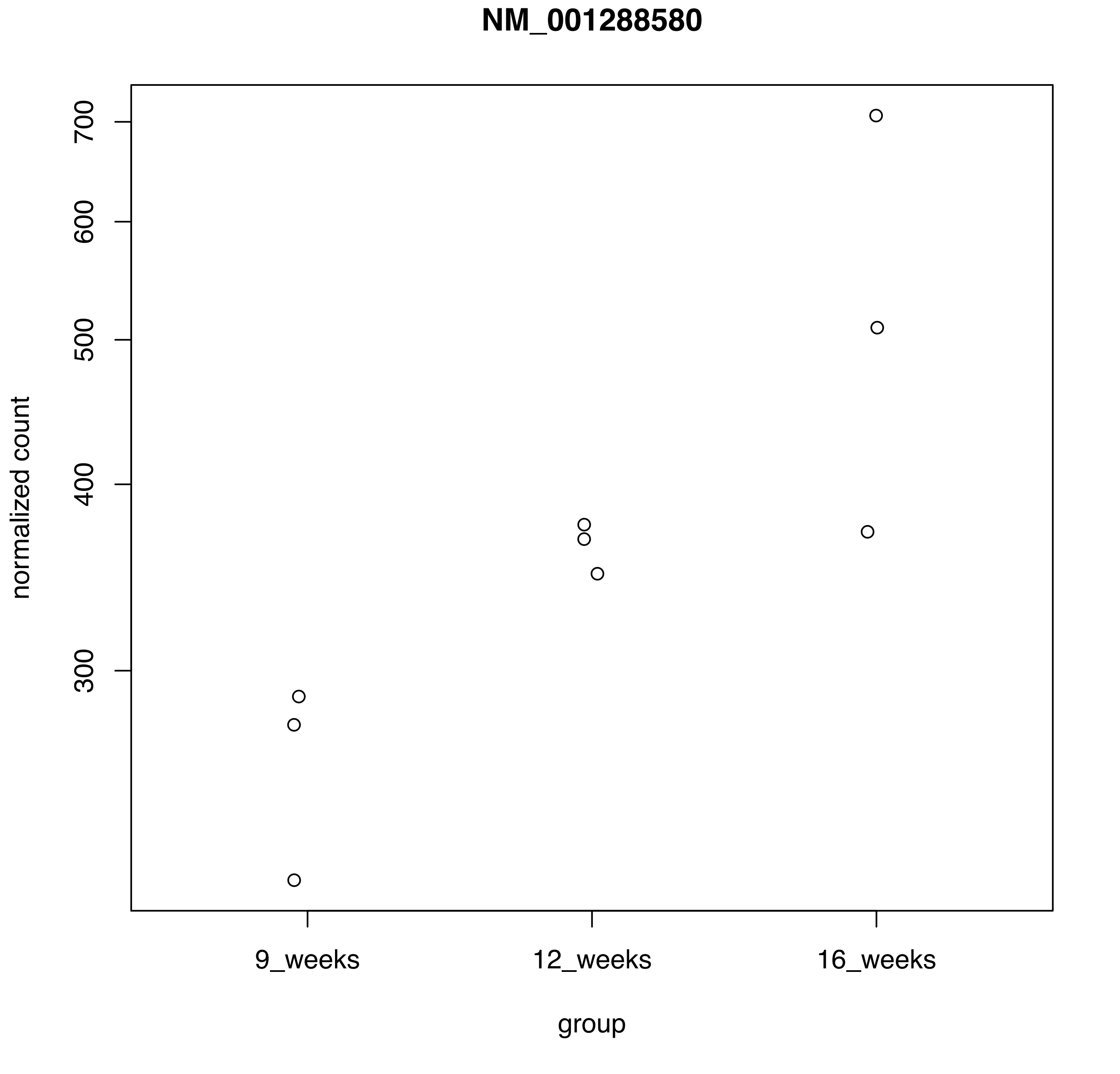
NM_001288580
normalized count
700
600
500
400
300
9_weeks
12_weeks
16_weeks
group

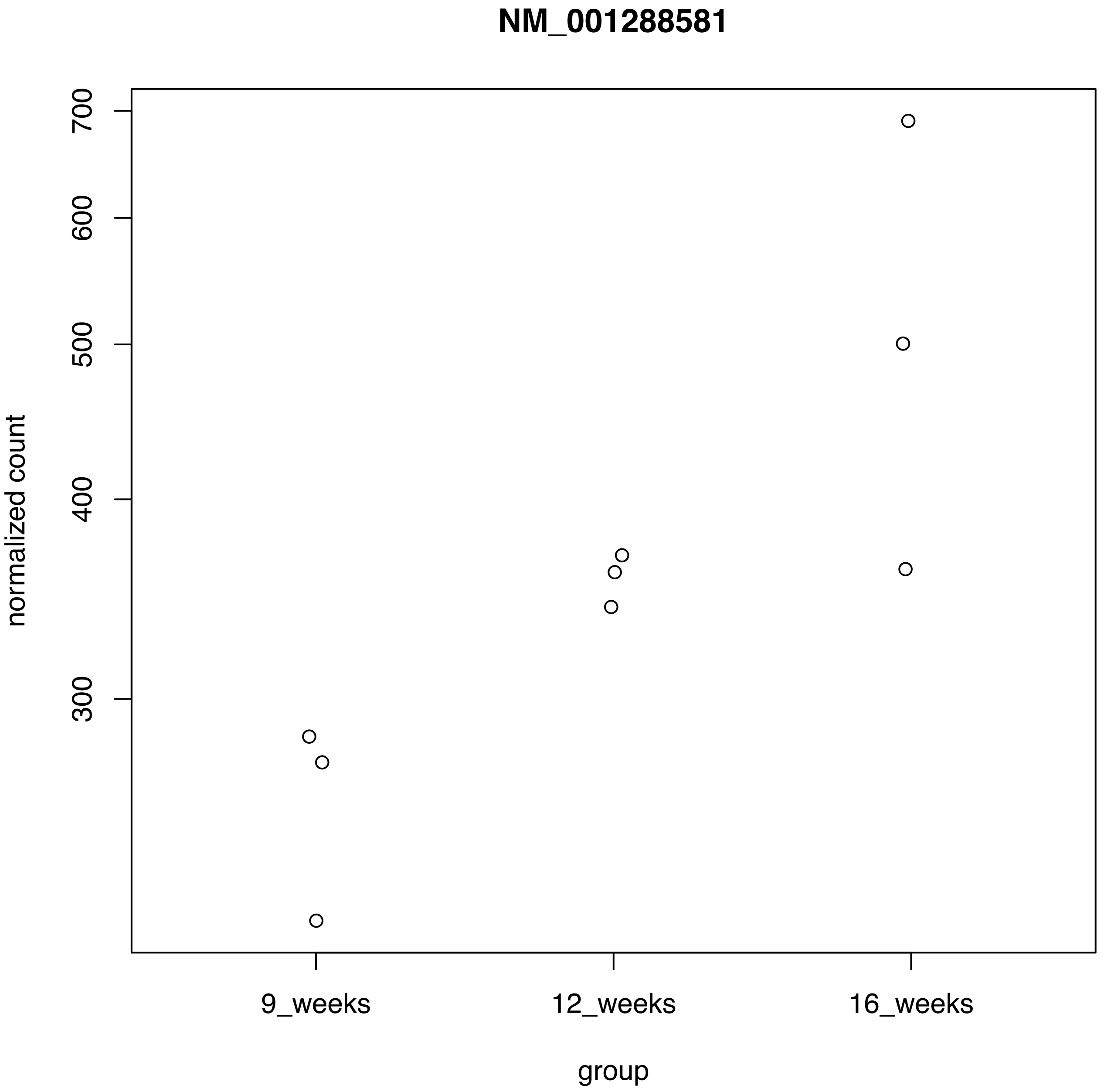

NM_001288581
700
600
500
400
300
normalized count
9_weeks
12_weeks
16_weeks
group

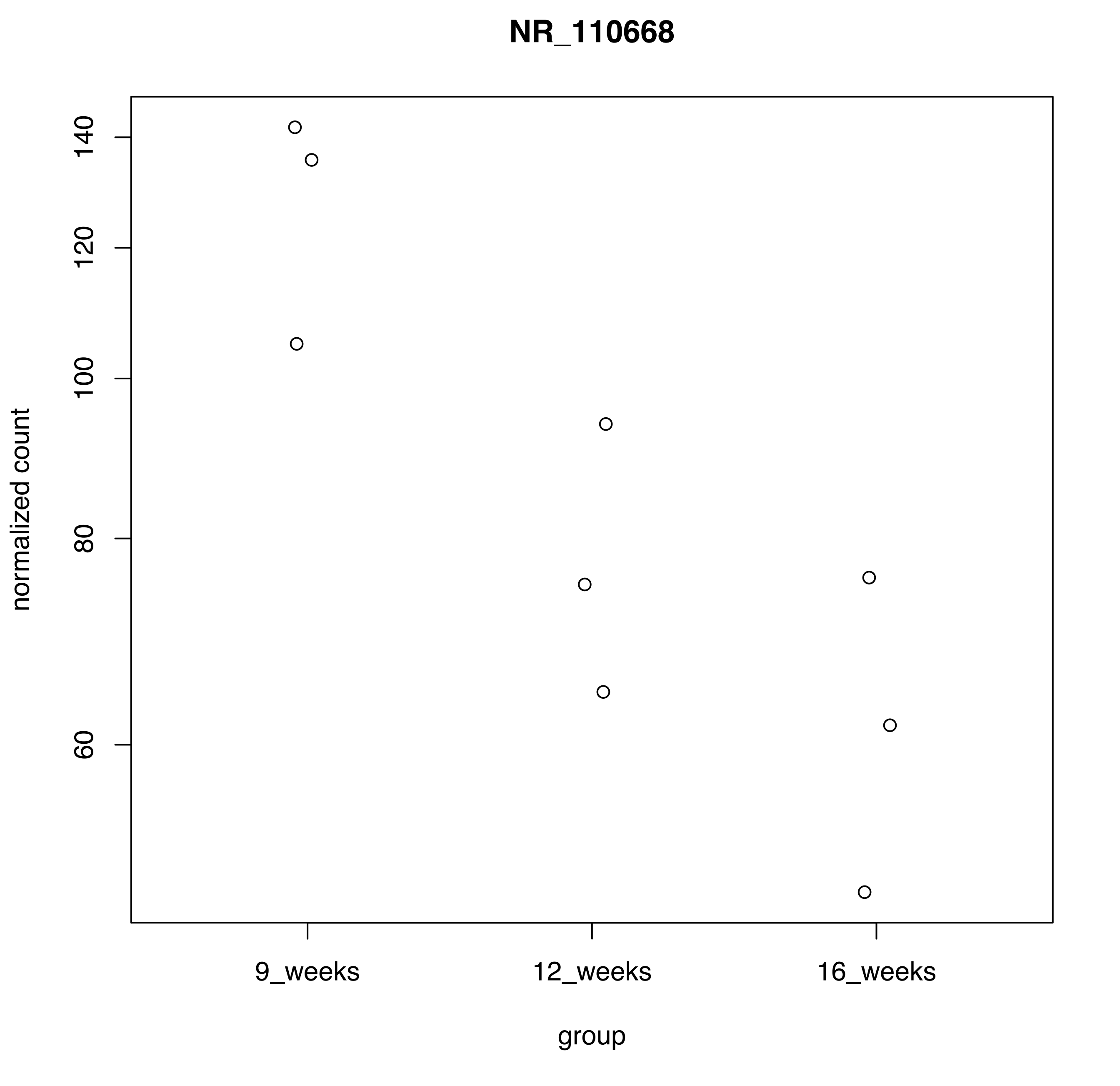

NR_110668
normalized count
140
120
100
80
60
9_weeks
12_weeks
16_weeks
group

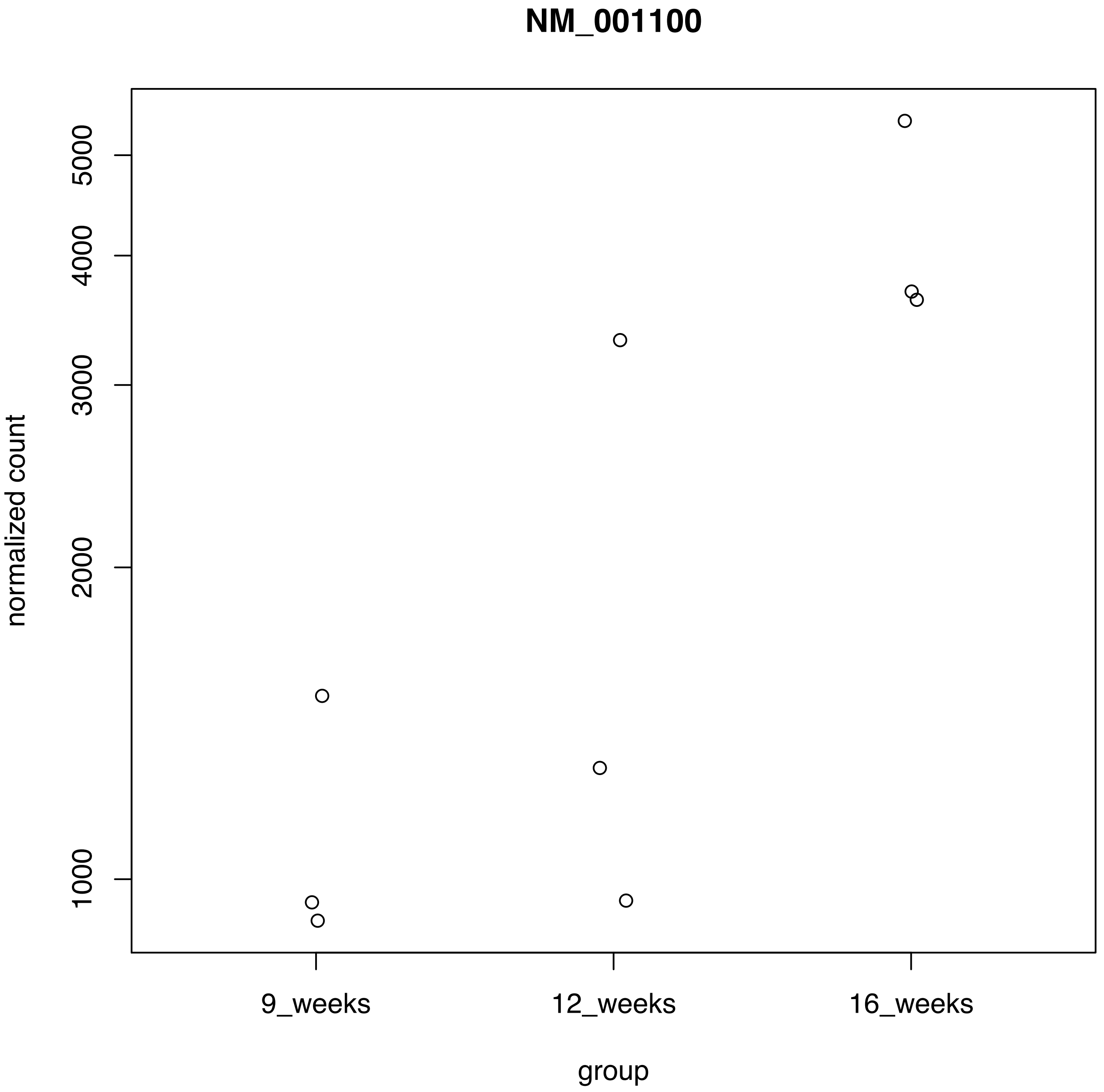
NM_001100
normalized count
5000
4000
3000
2000
1000
9_weeks
12_weeks
16_weeks
group

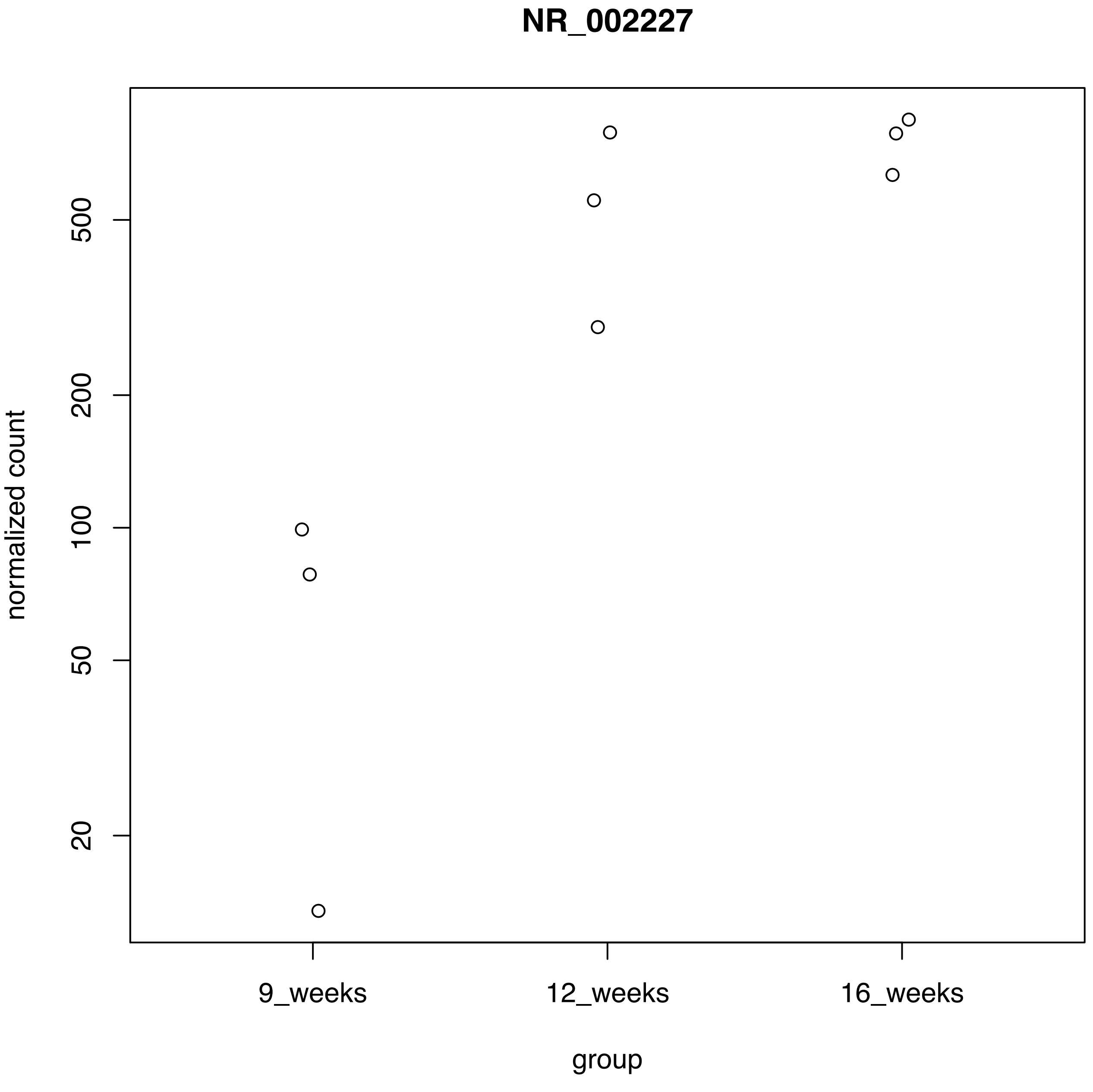
NR_002227
normalized count
500
200
100
50
20
9_weeks
12_weeks
16_weeks
group

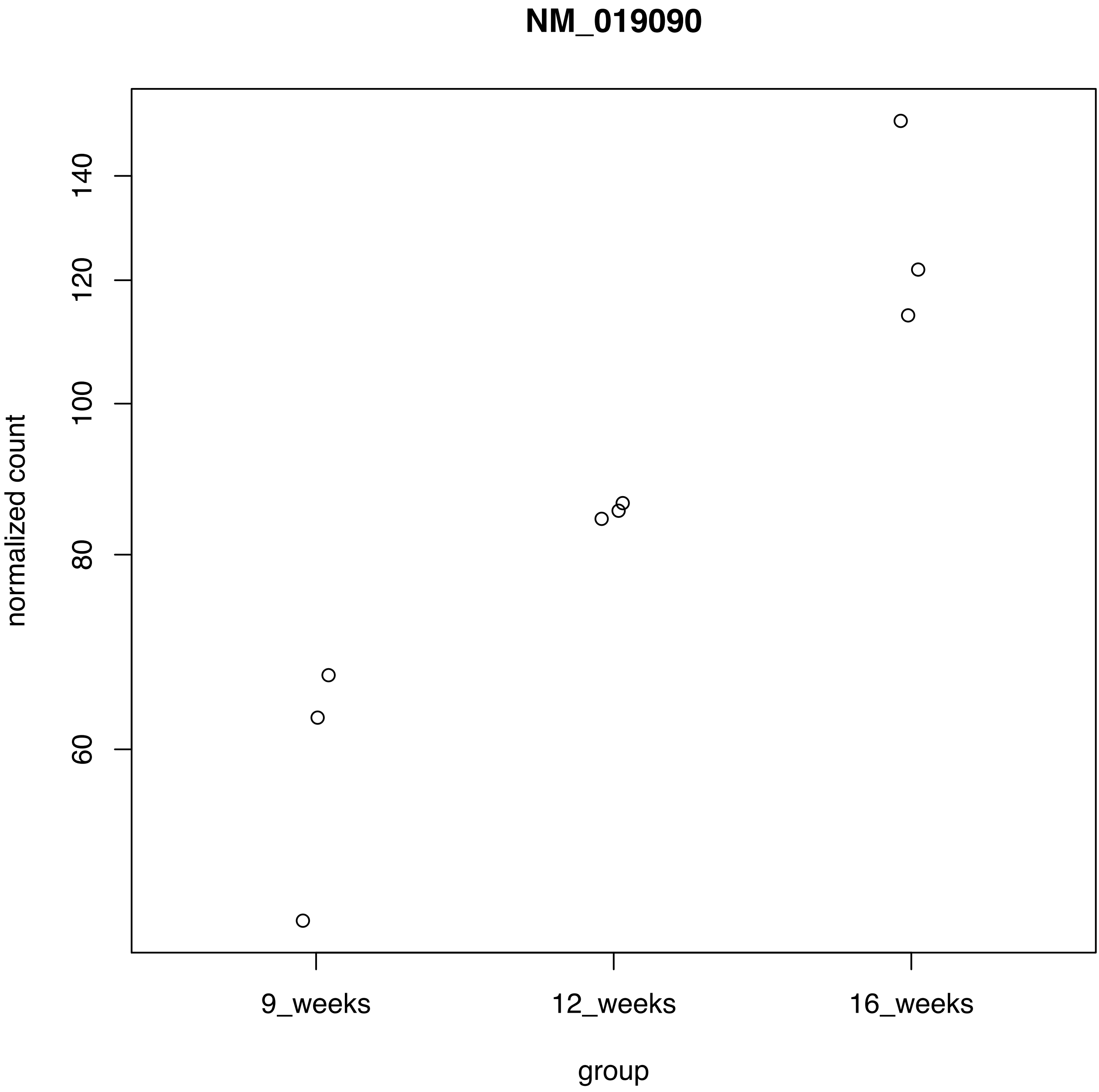
NM_019090
normalized count
60
80
100
120
140
9_weeks
12_weeks
16_weeks
group

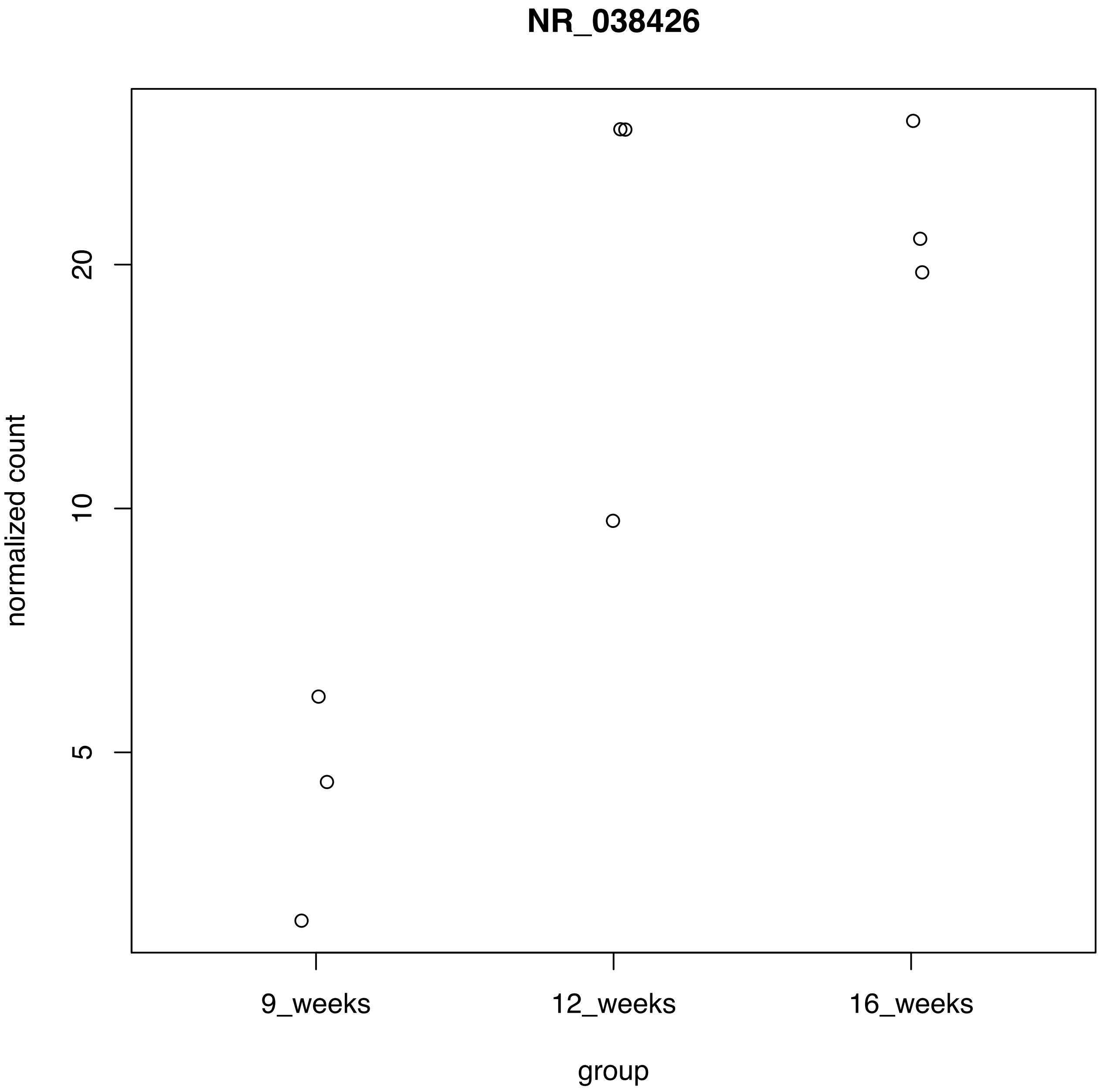
NR_038426
normalized count
20
10
5
9_weeks
12_weeks
16_weeks
group

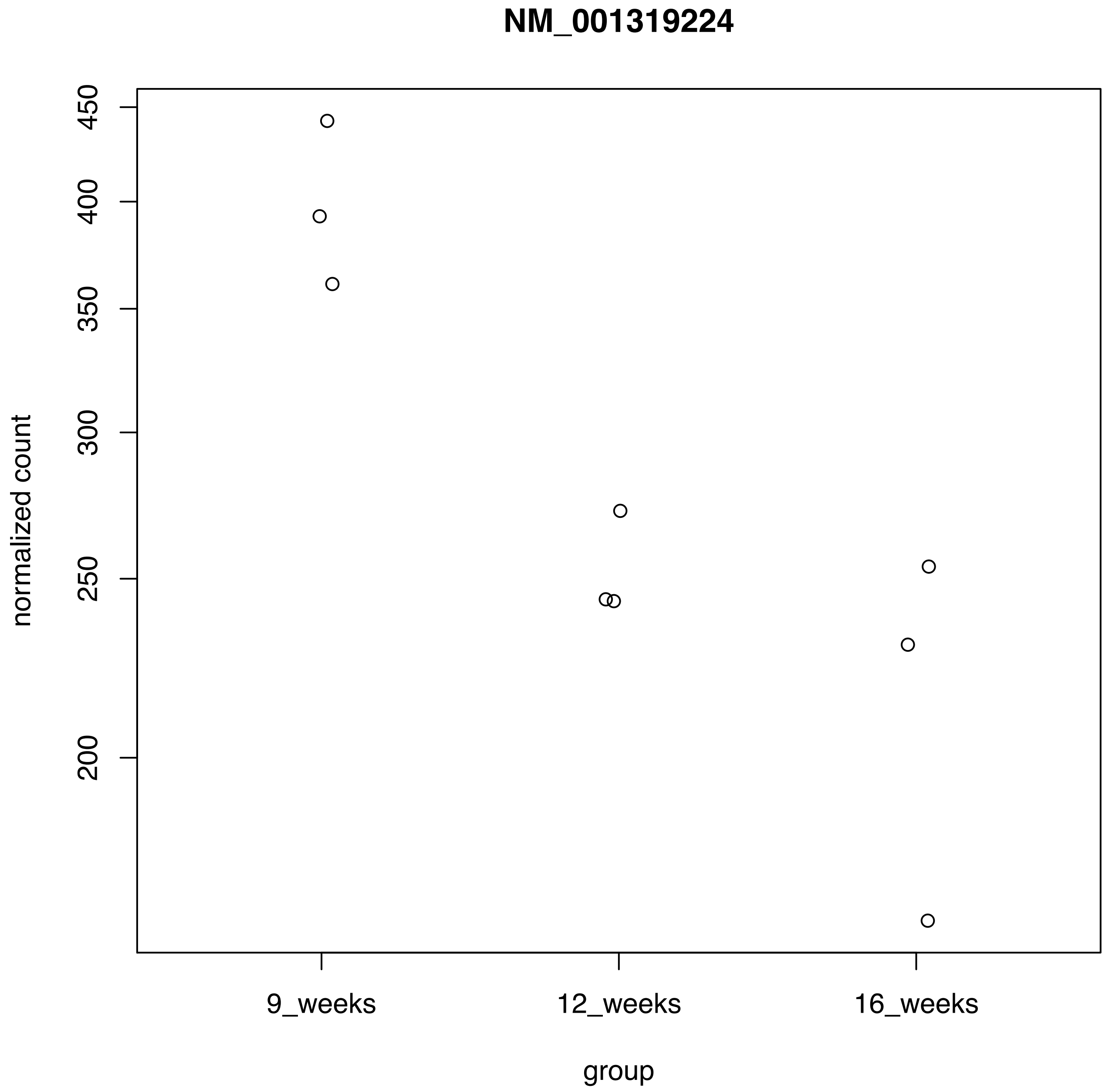
NM_001319224
normalized count
450
400
350
300
250
200
9_weeks
12_weeks
16_weeks
group

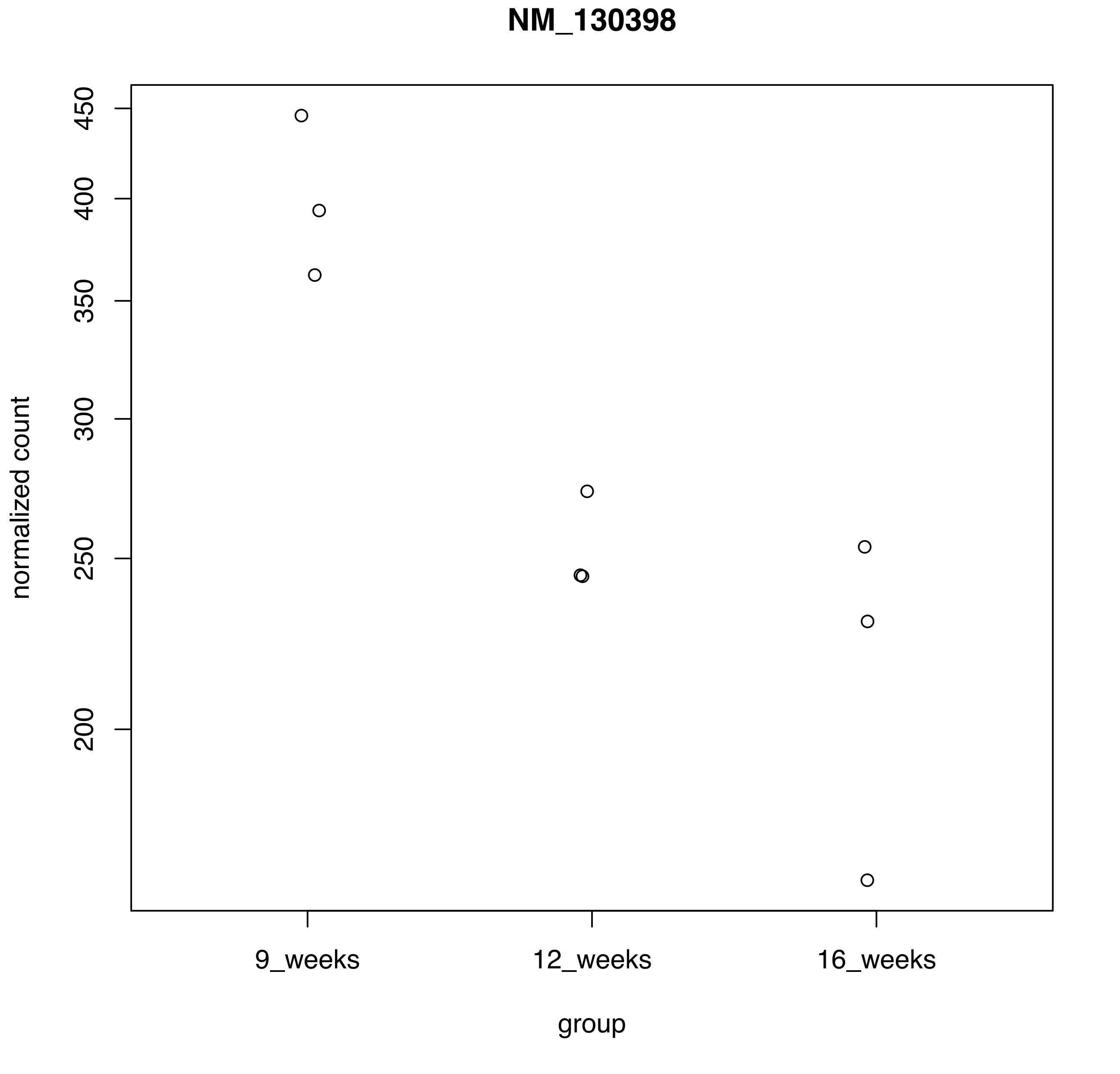

NM_130398
normalized count
450
400
350
300
250
200
9_weeks
12_weeks
16_weeks
group

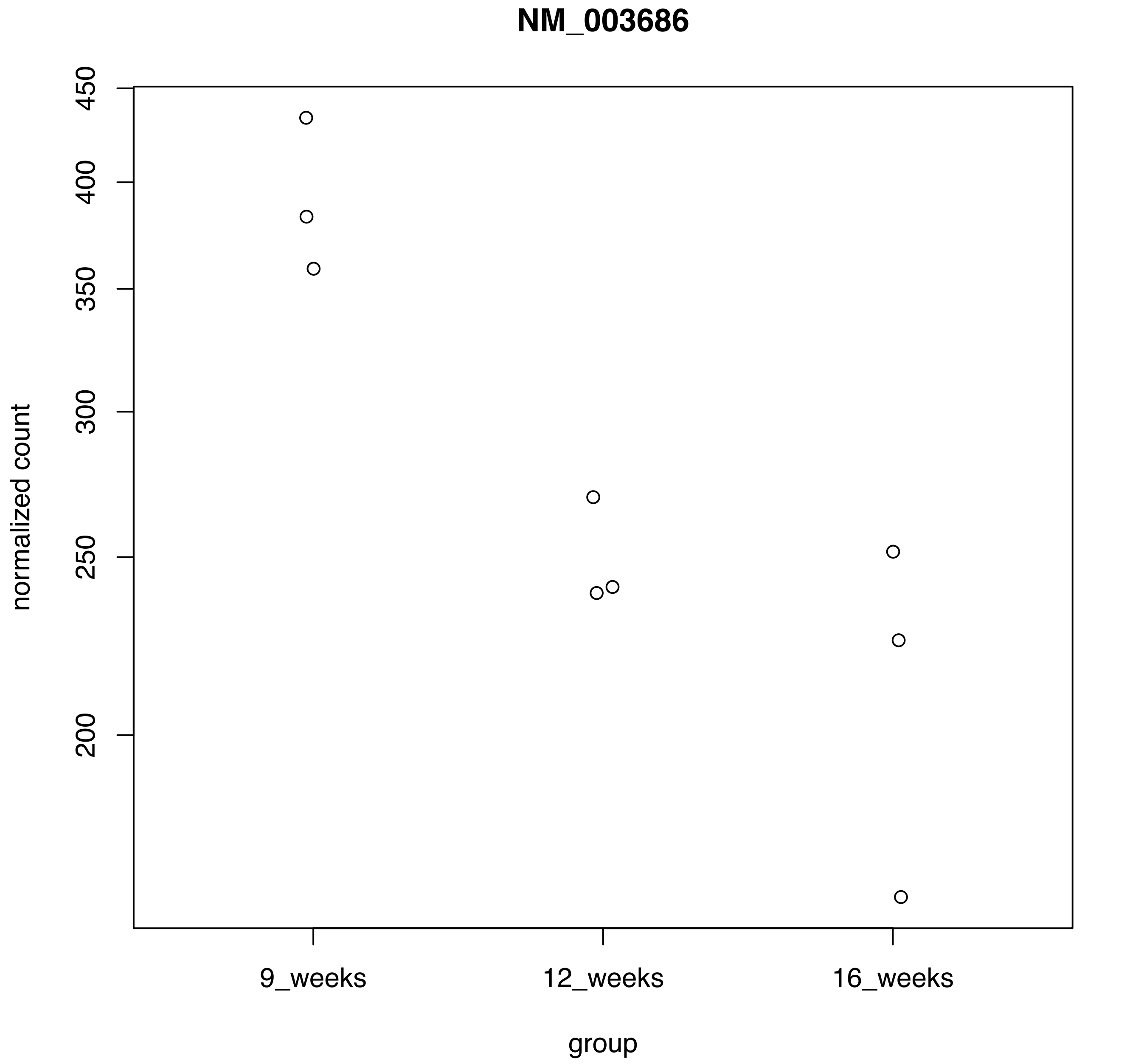
NM_003686
normalized count
450
400
350
300
250
200
9_weeks
12_weeks
16_weeks
group

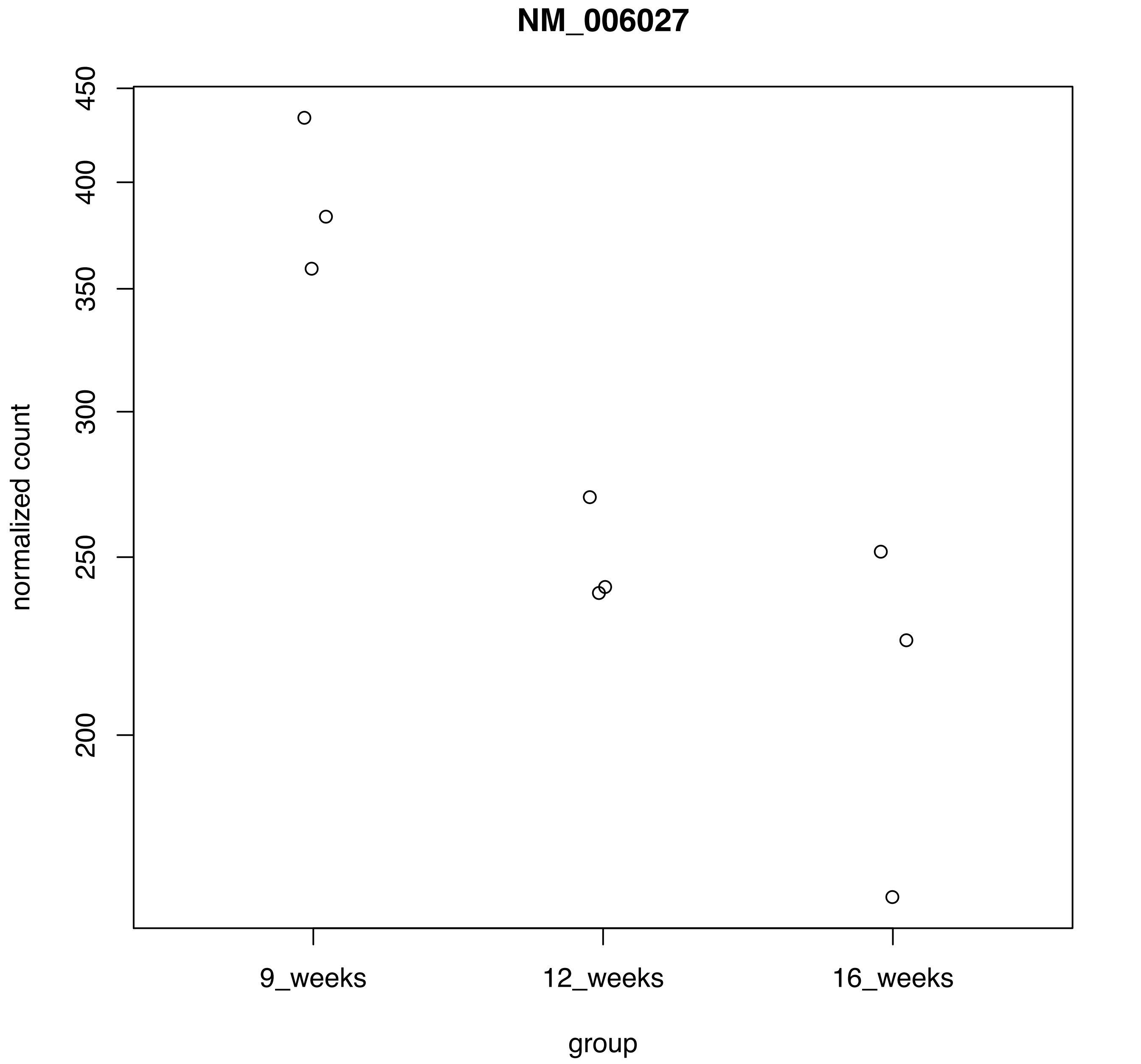
NM_006027
normalized count
450
400
350
300
250
200
9_weeks
12_weeks
16_weeks
group

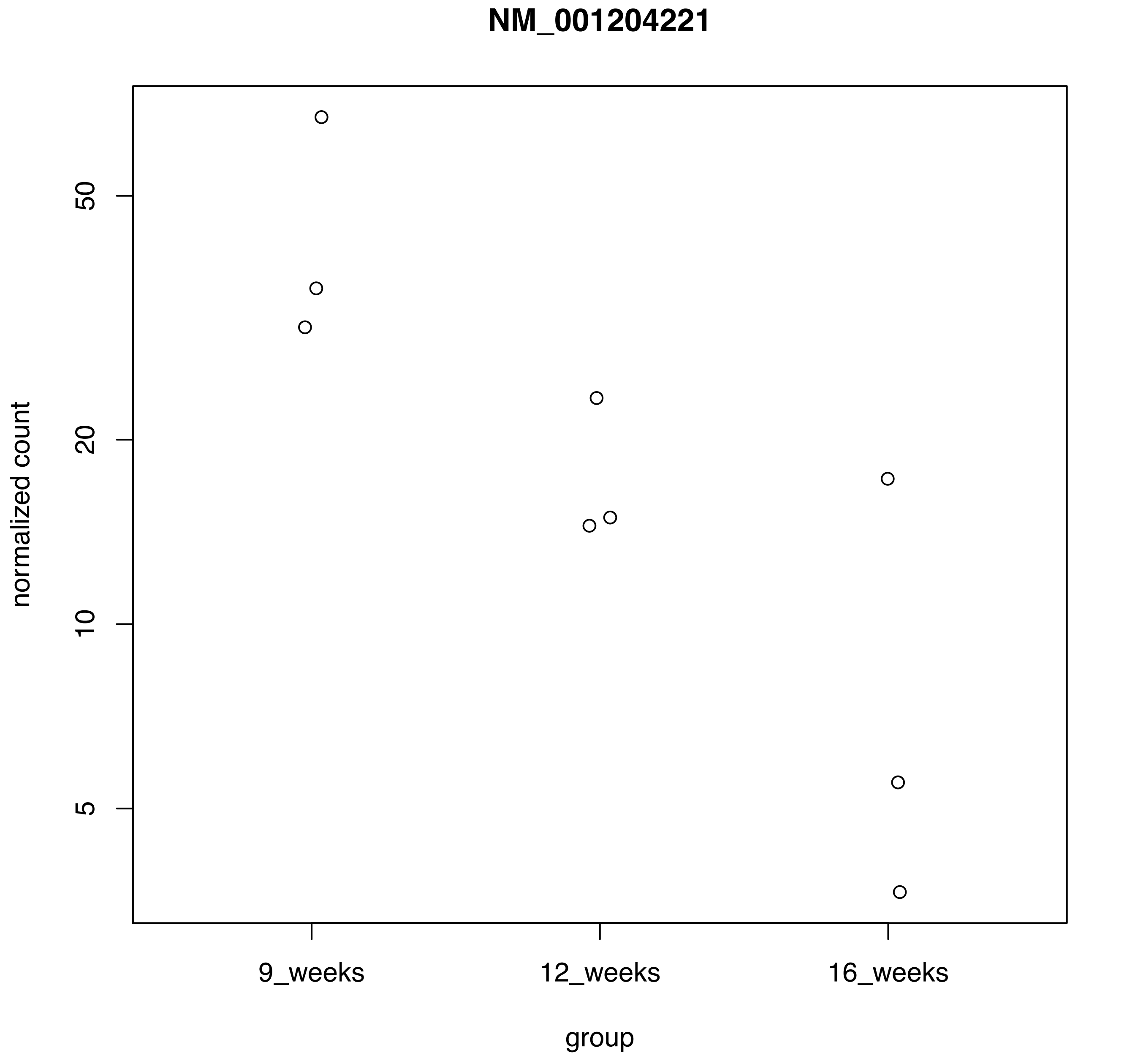

NM_001204221
normalized count
50
20
10
5
9_weeks
12_weeks
16_weeks
group

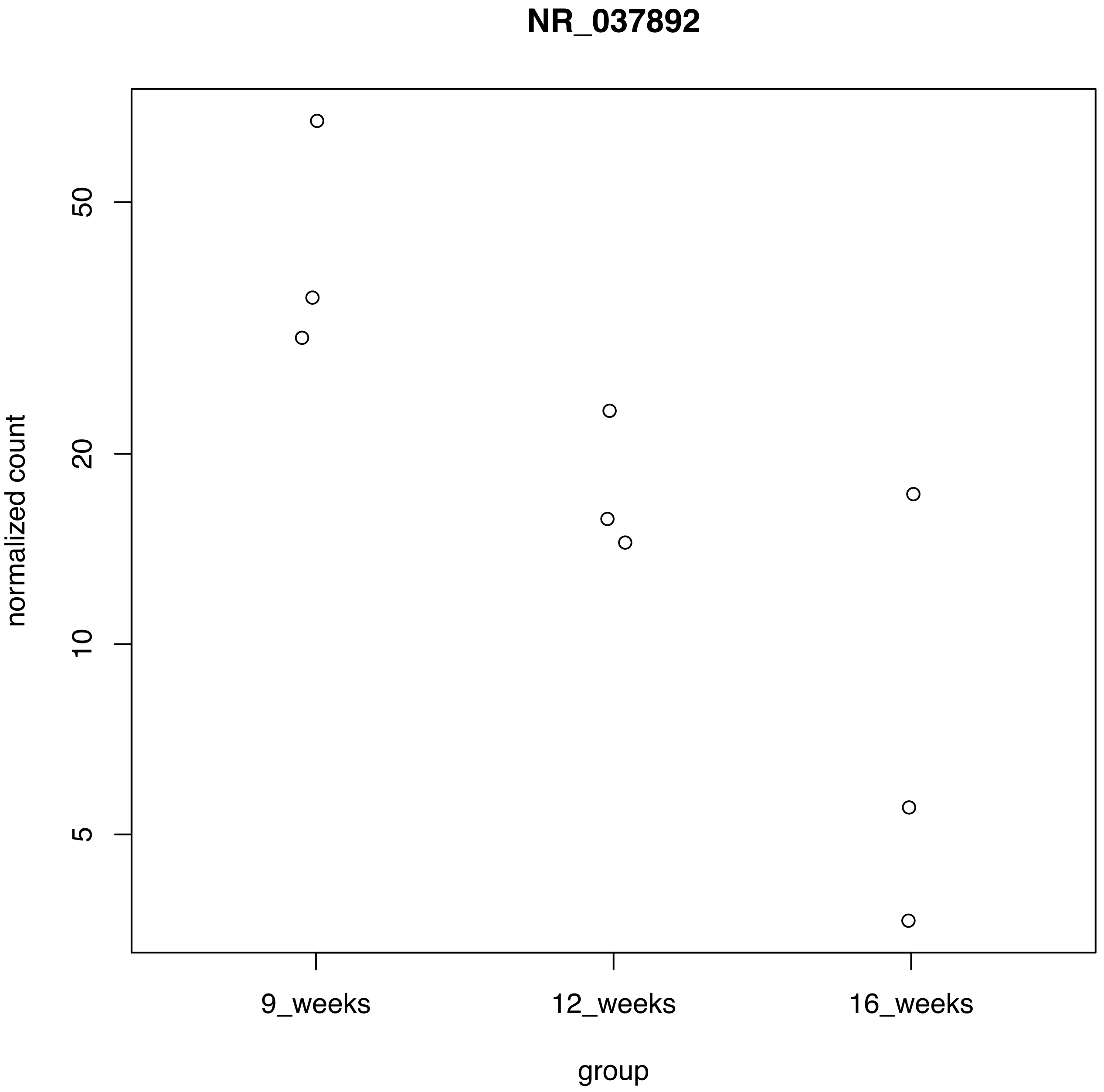
NR_037892
normalized count
50
20
10
5
9_weeks
12_weeks
16_weeks
group

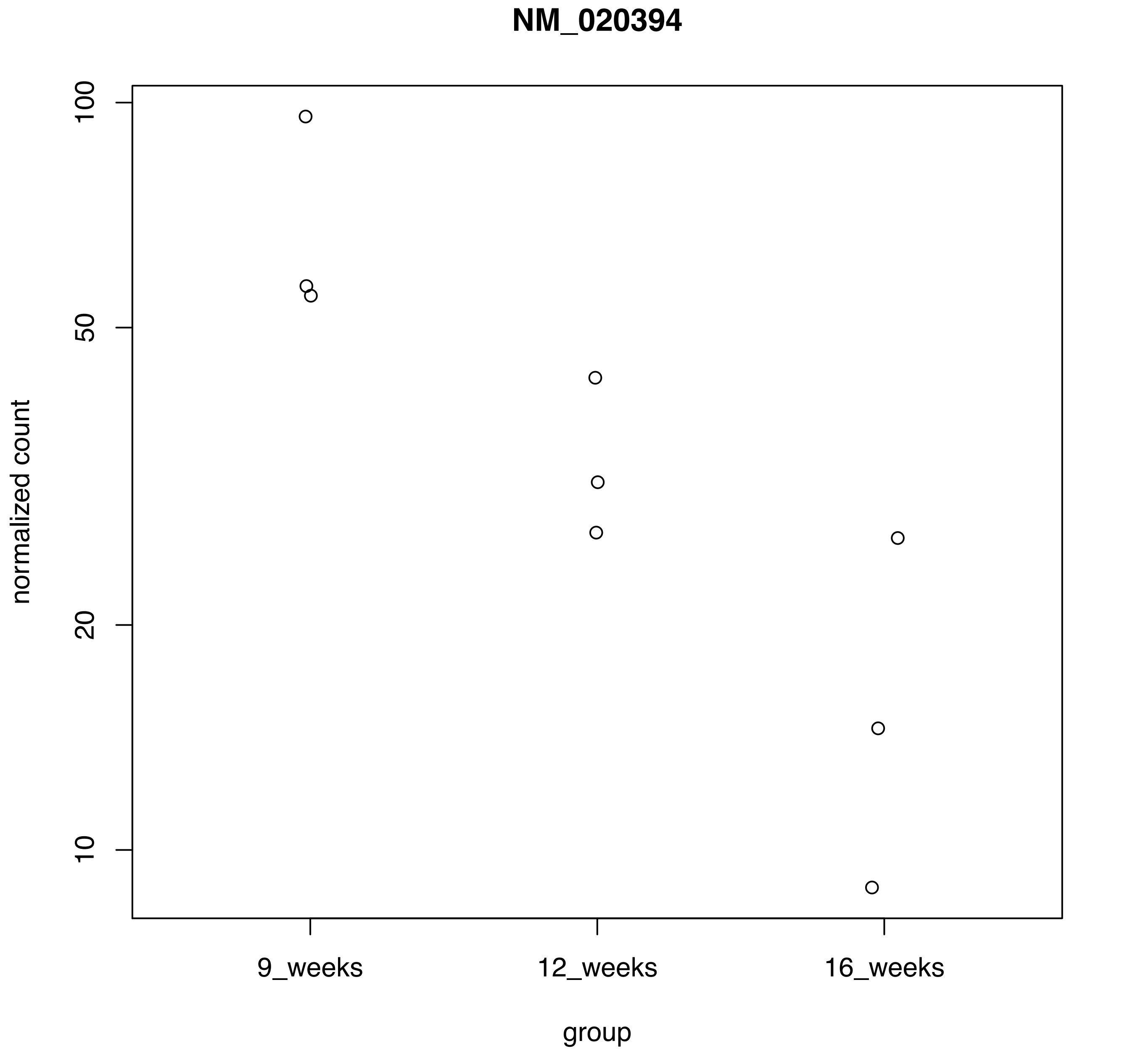

NM_020394
normalized count
100
50
20
10
9_weeks
12_weeks
16_weeks
group

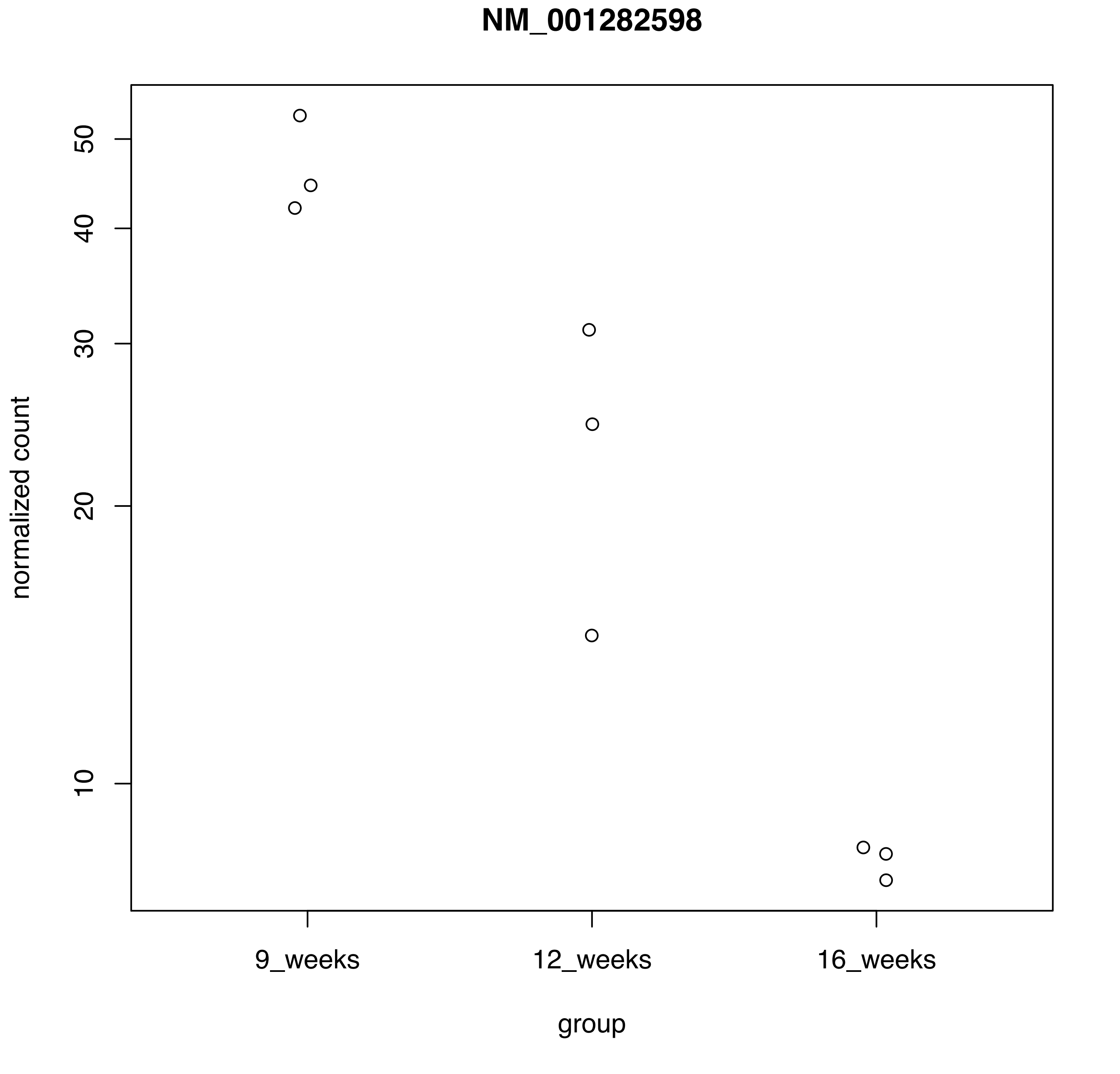
NM_001282598
normalized count
50
40
30
20
10
9_weeks
12_weeks
16_weeks
group

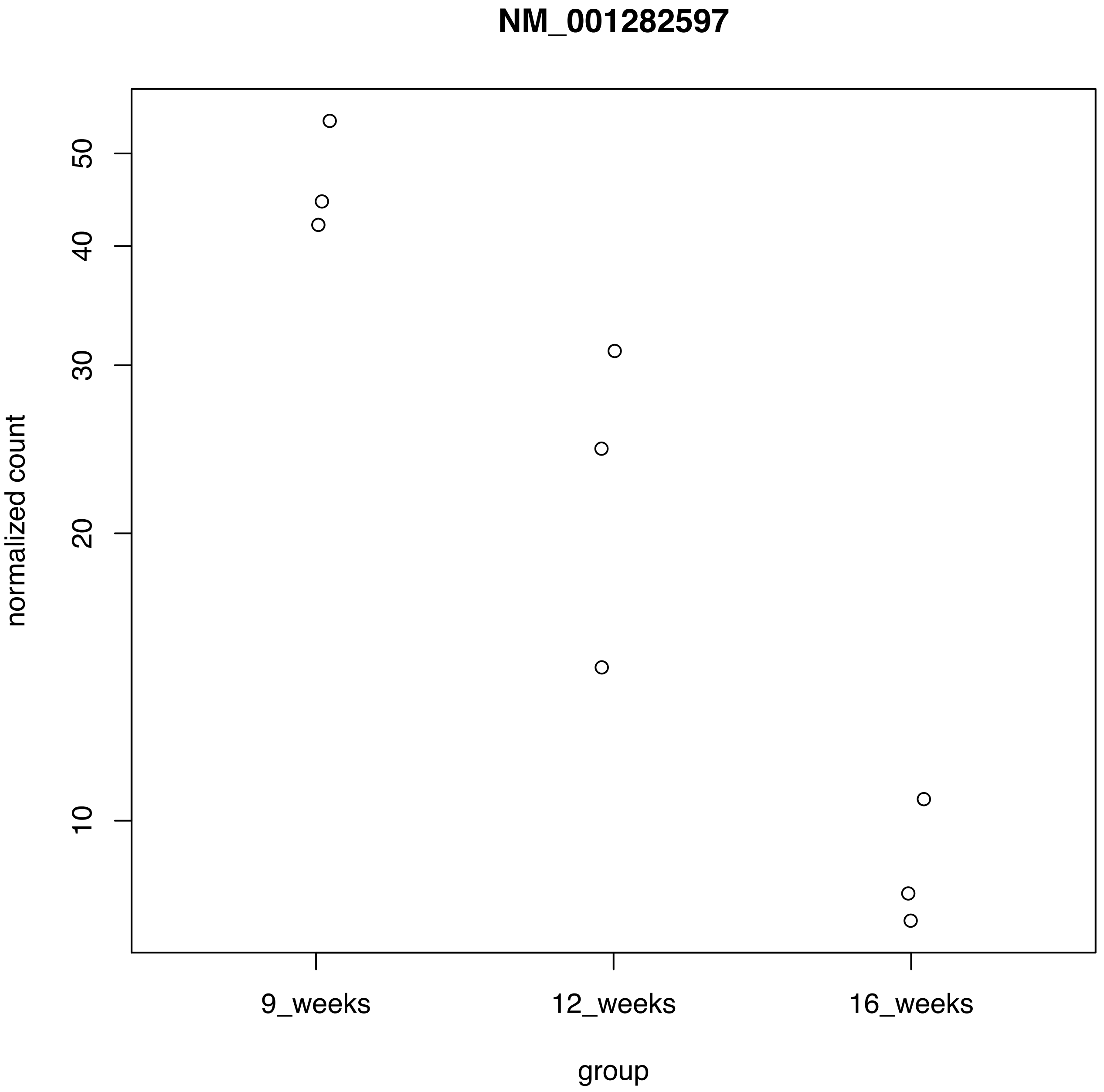
NM_001282597
normalized count
50
40
30
20
10
9_weeks
12_weeks
16_weeks
group

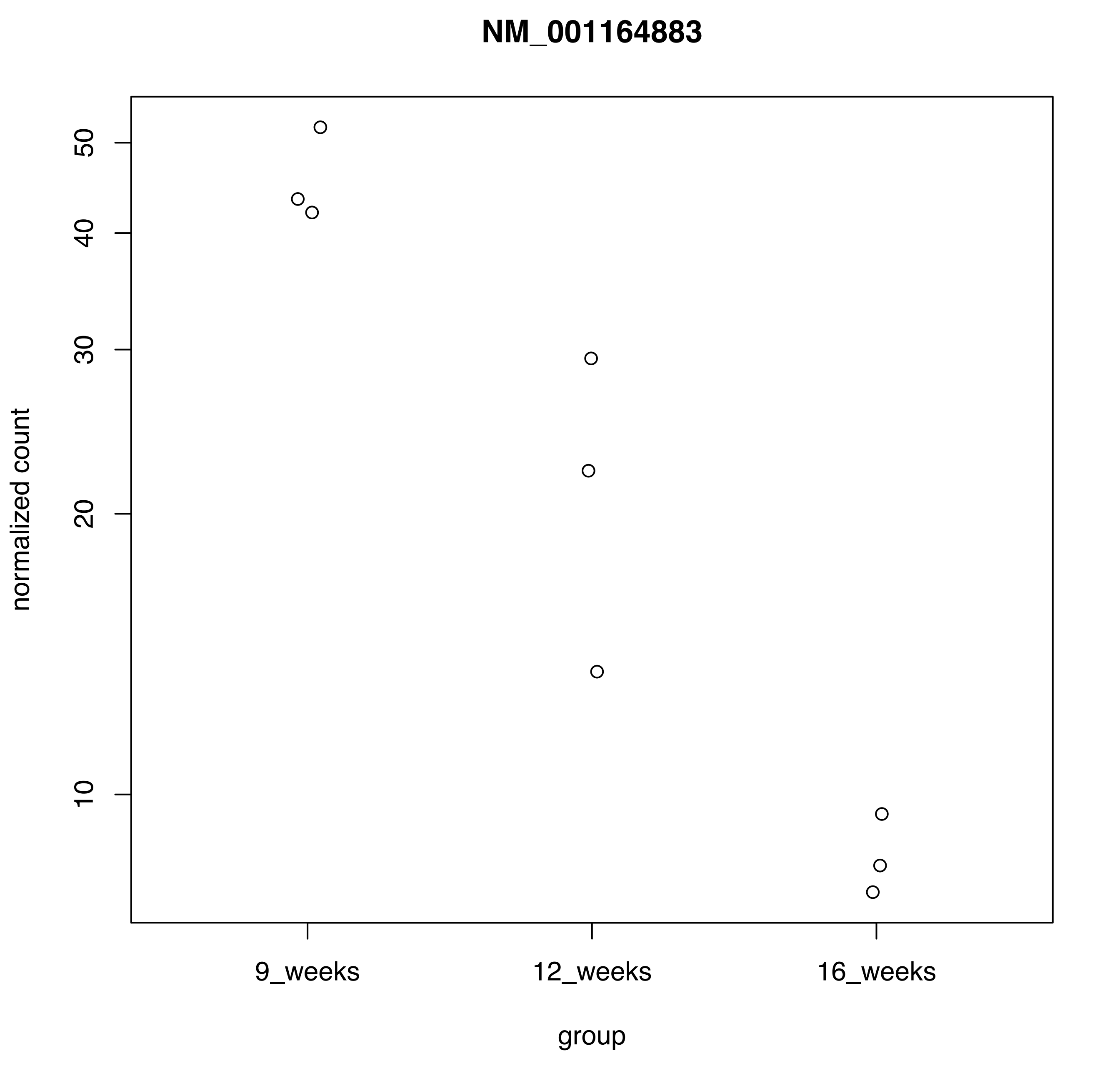
NM_001164883
normalized count
50
40
30
20
10
9_weeks
12_weeks
16_weeks
group

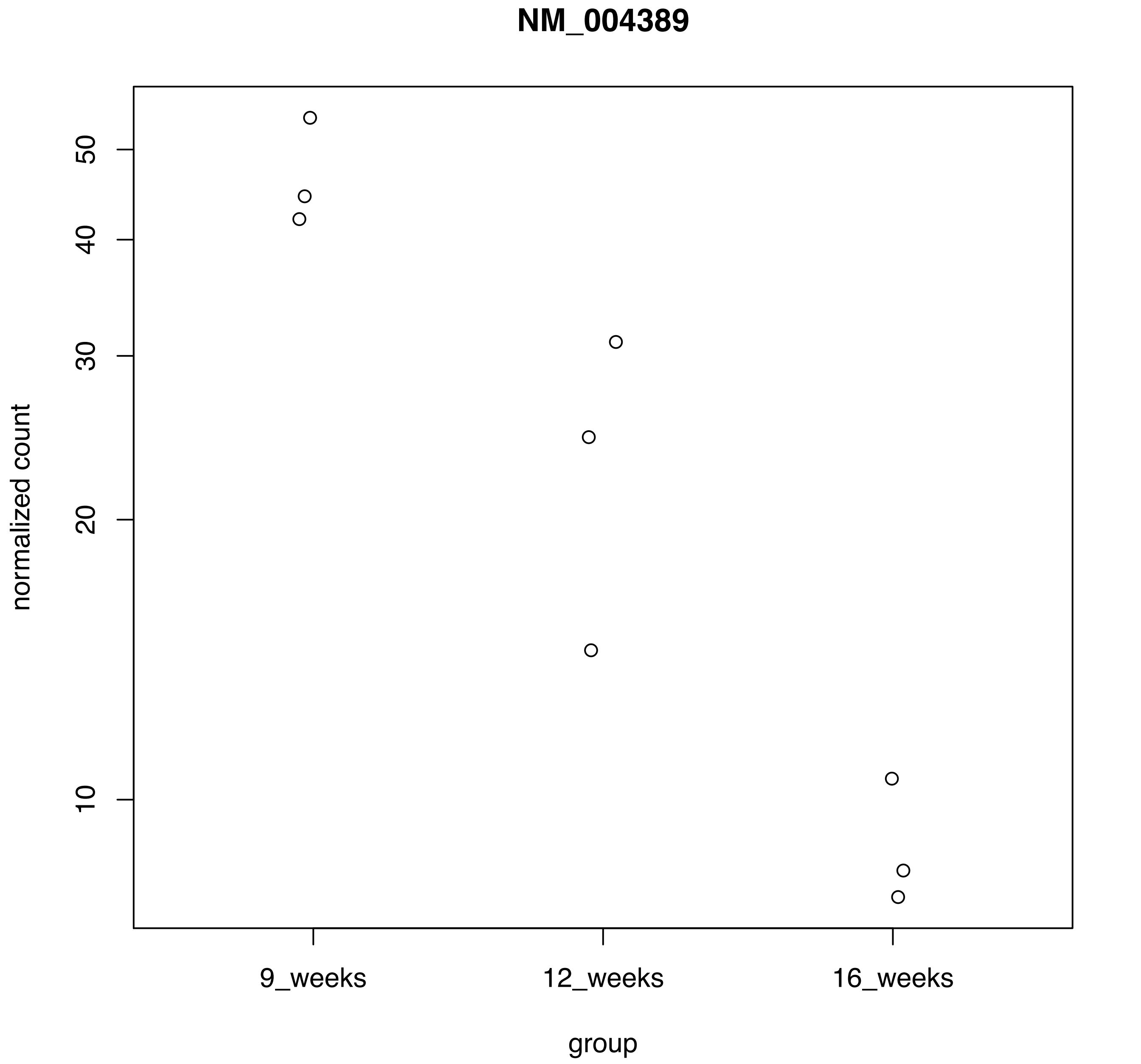

NM_004389
normalized count
10
20
30
40
50
9_weeks
12_weeks
16_weeks
group

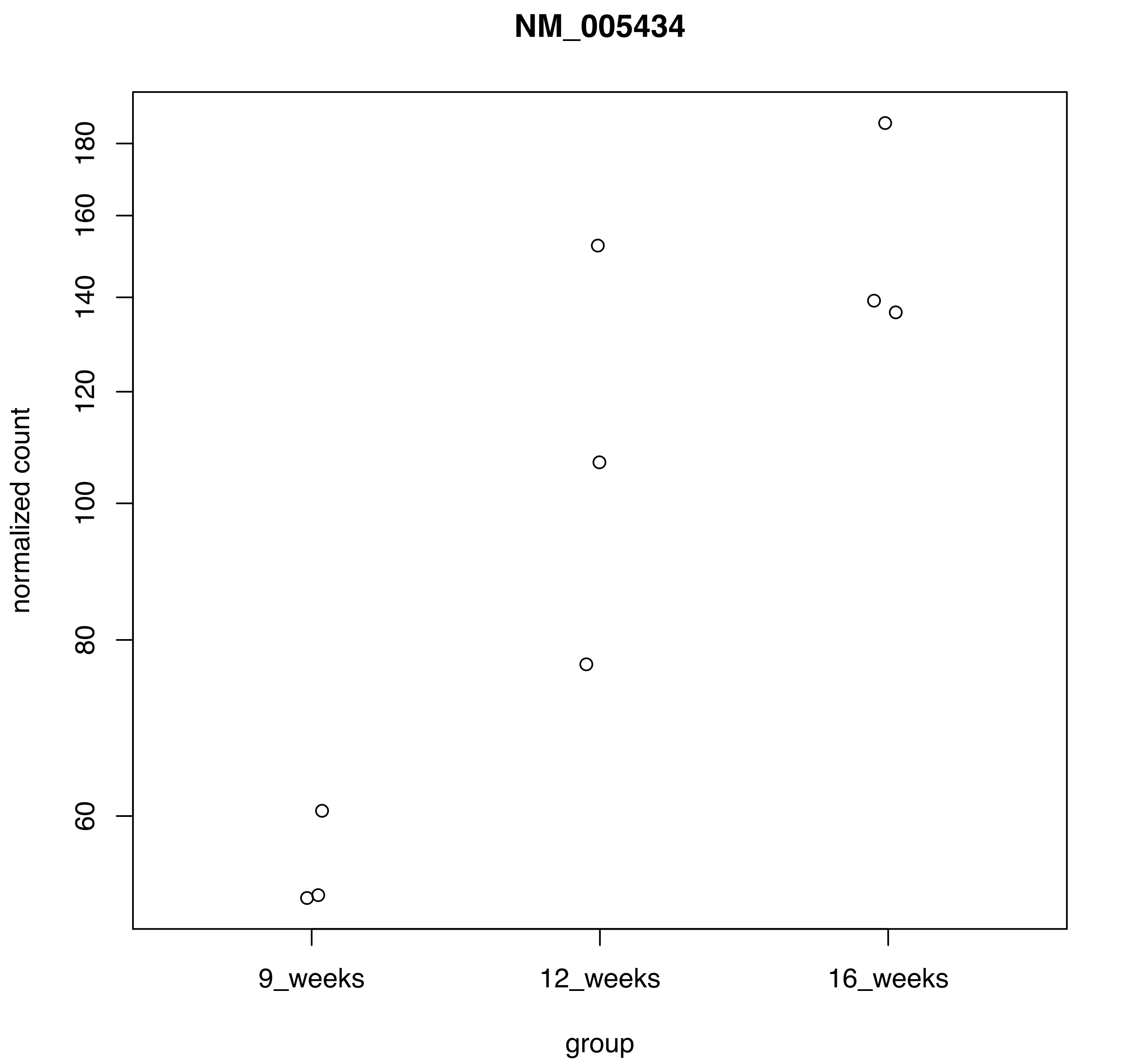

NM_005434
normalized count
60
80
100
120
140
160
180
9_weeks
12_weeks
16_weeks
group

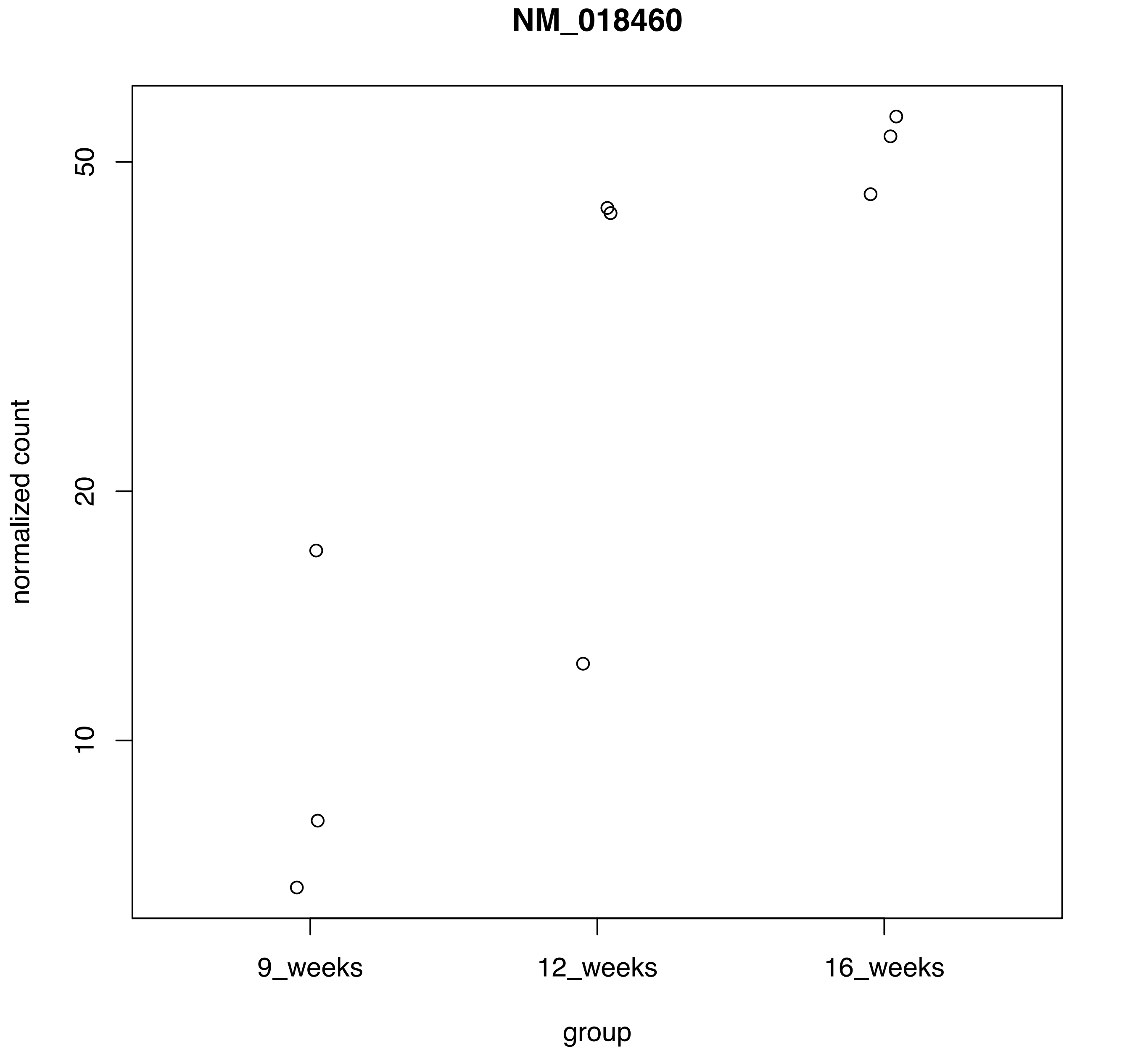

NM_018460
normalized count
50
20
10
9_weeks
12_weeks
16_weeks
group

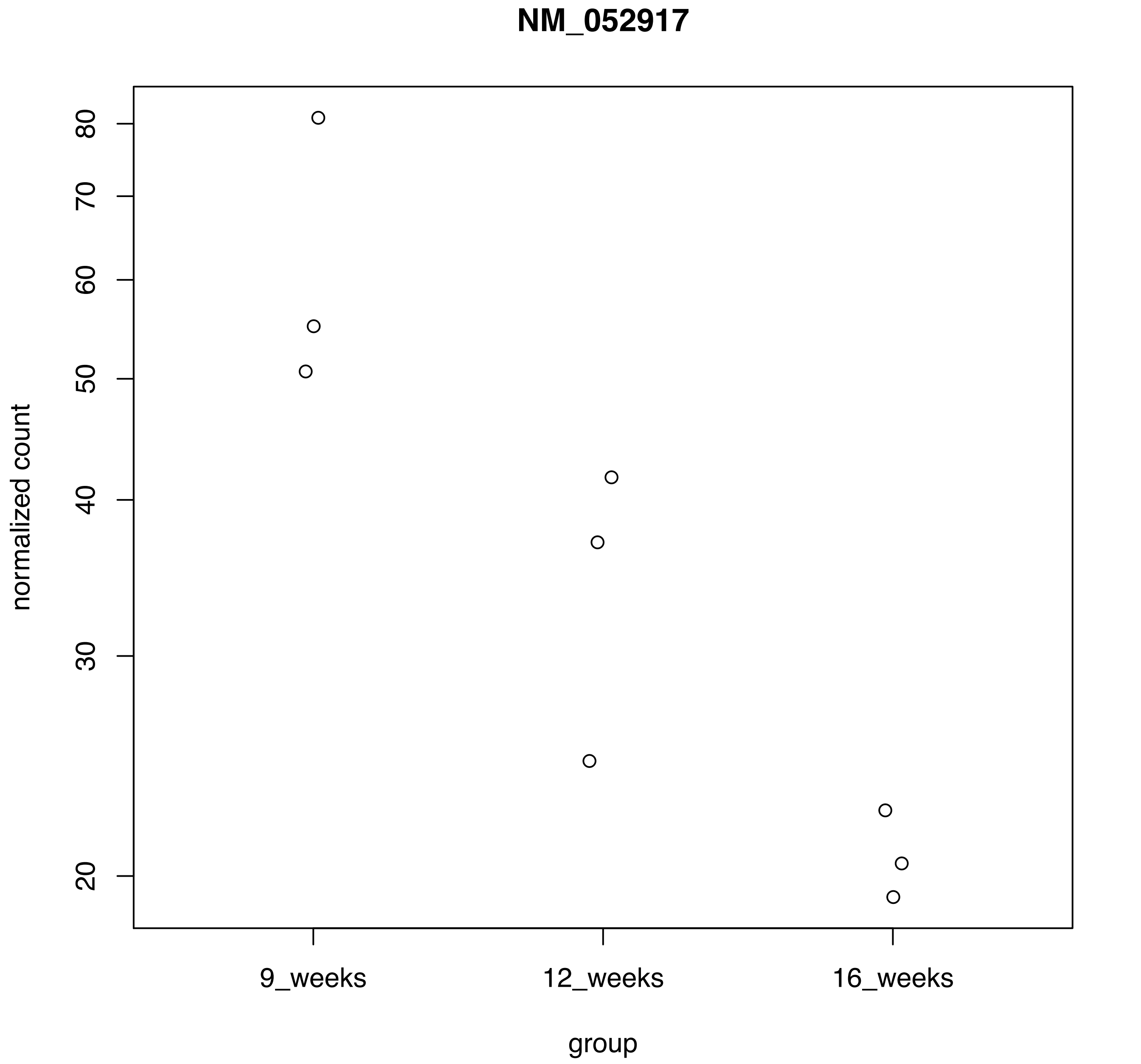
NM_052917
normalized count
20
30
40
50
60
70
80
9_weeks
12_weeks
16_weeks
group

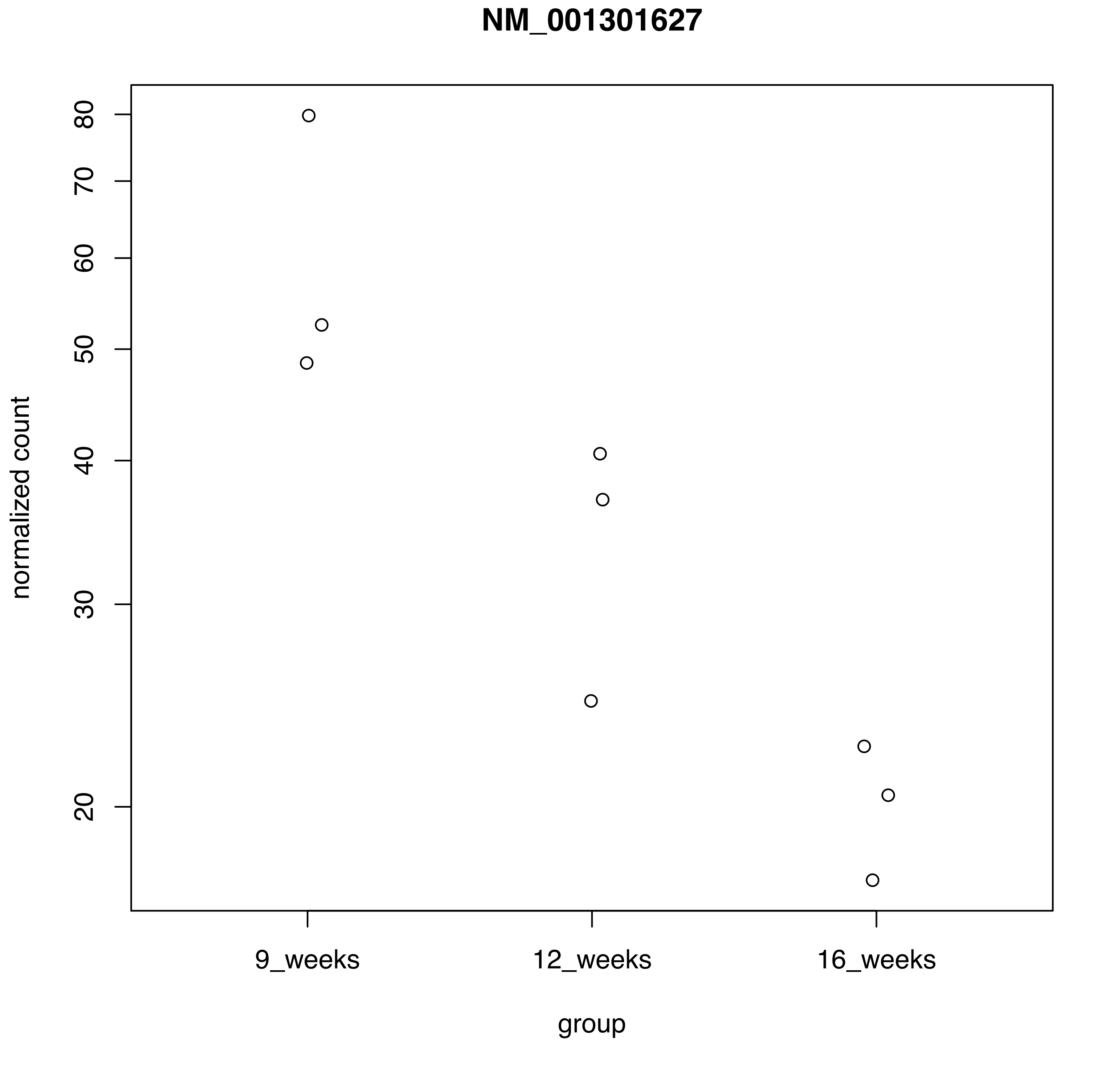

NM_001301627
normalized count
80
70
60
50
40
30
20
9_weeks
12_weeks
16_weeks
group

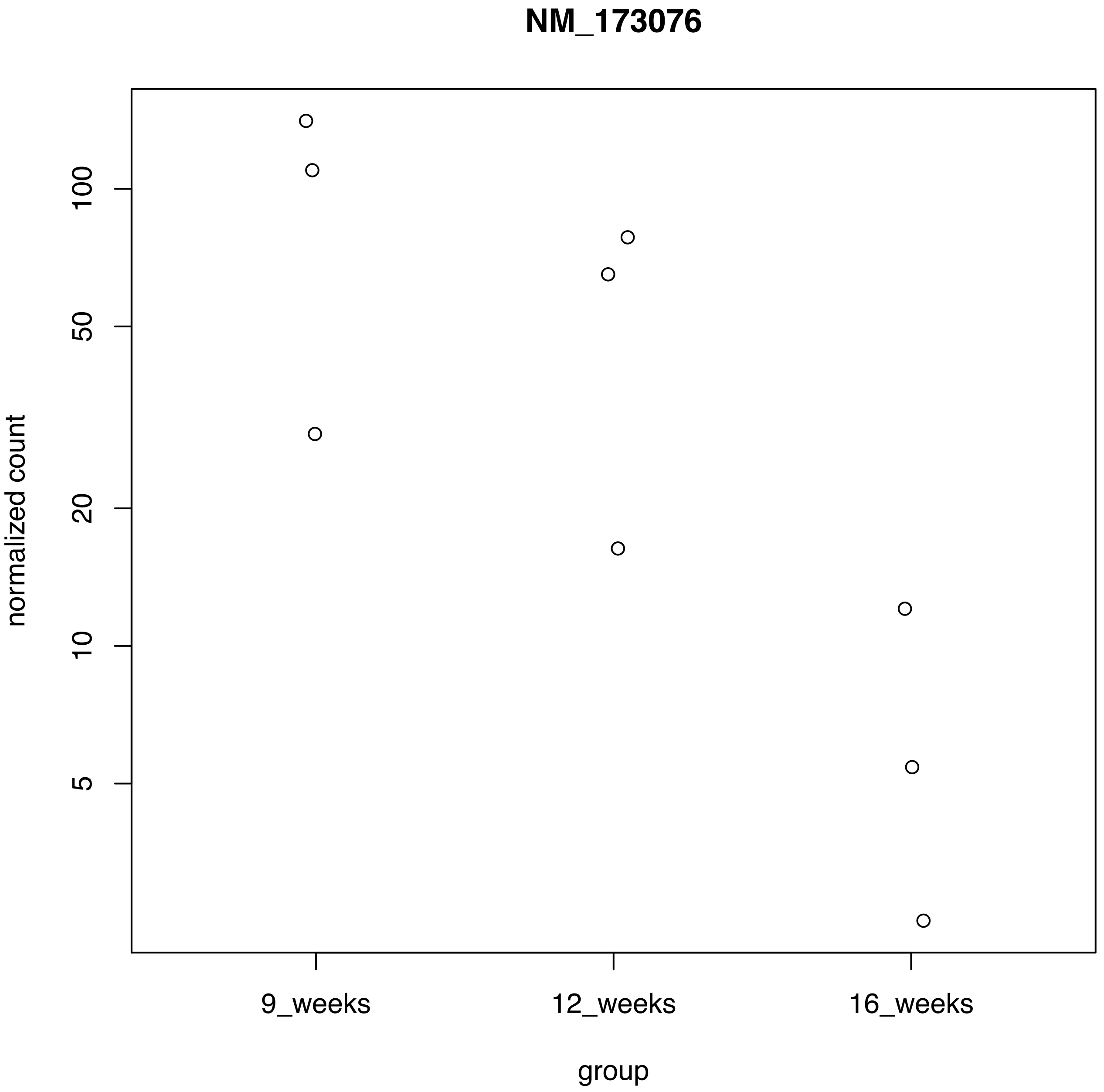
NM_173076
normalized count
100
50
20
10
5
9_weeks
12_weeks
16_weeks
group

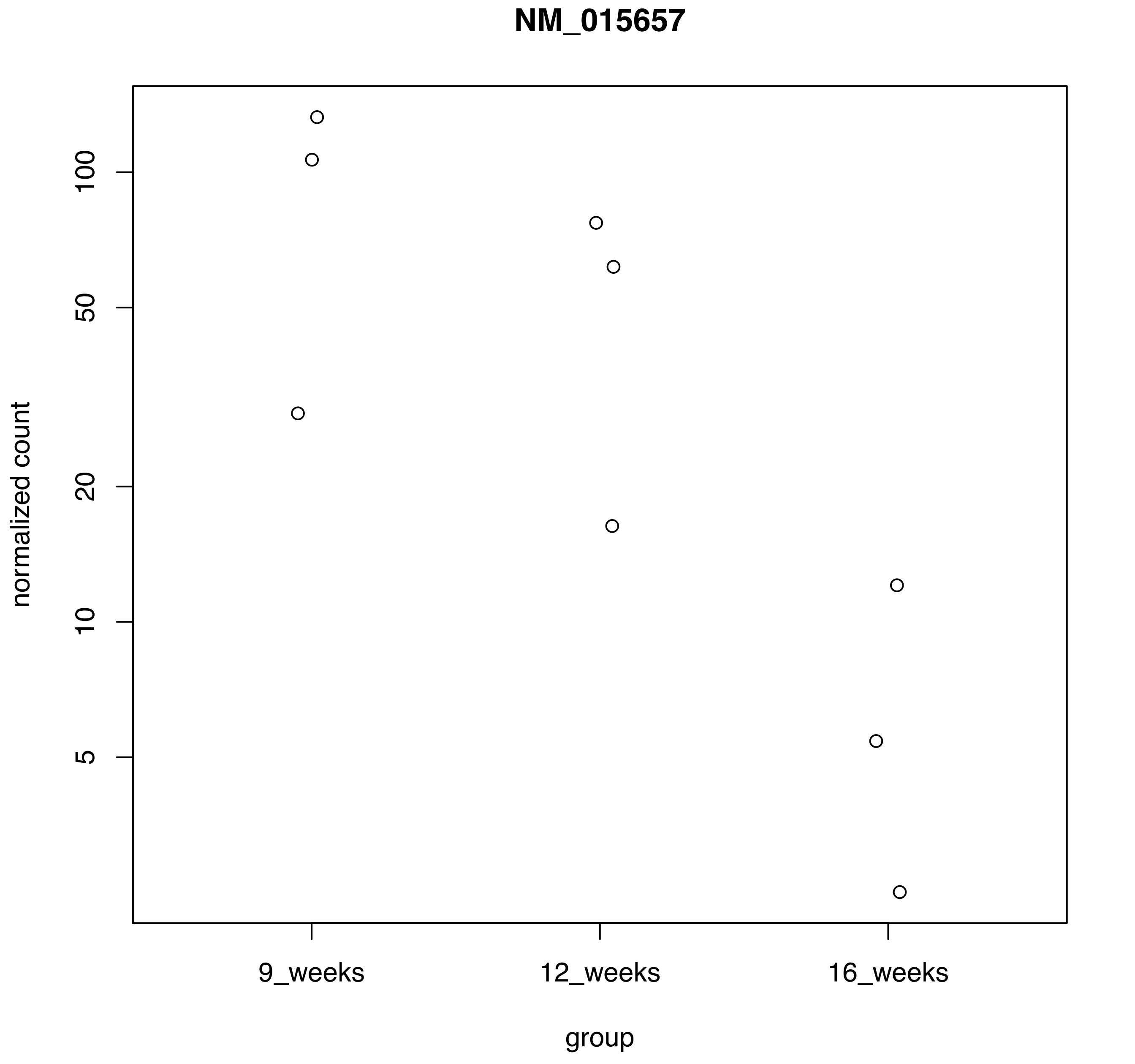
NM_015657
normalized count
100
50
20
10
5
9_weeks
12_weeks
16_weeks
group

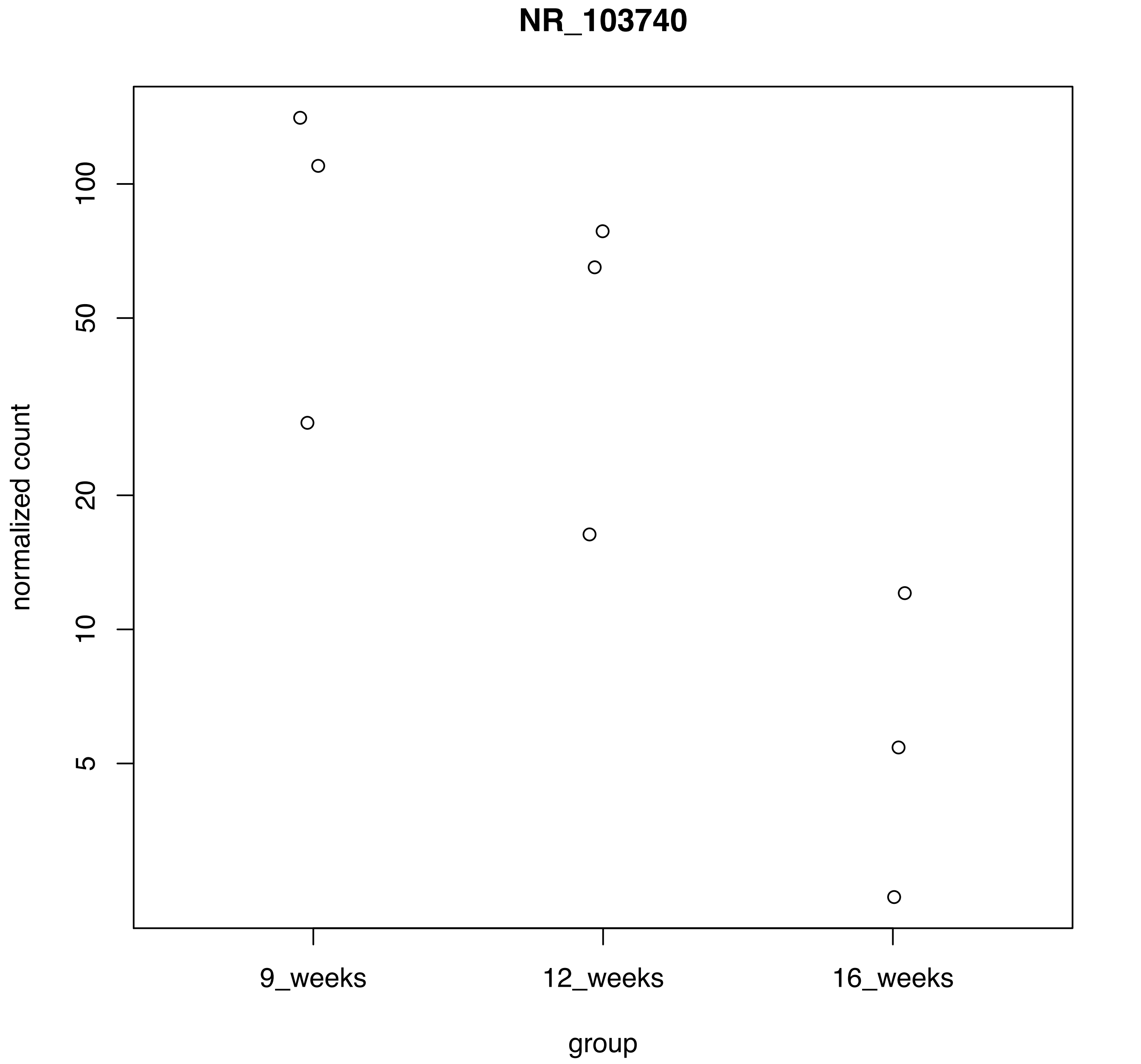
NR_103740
normalized count
100
50
20
10
5
9_weeks
12_weeks
16_weeks
group

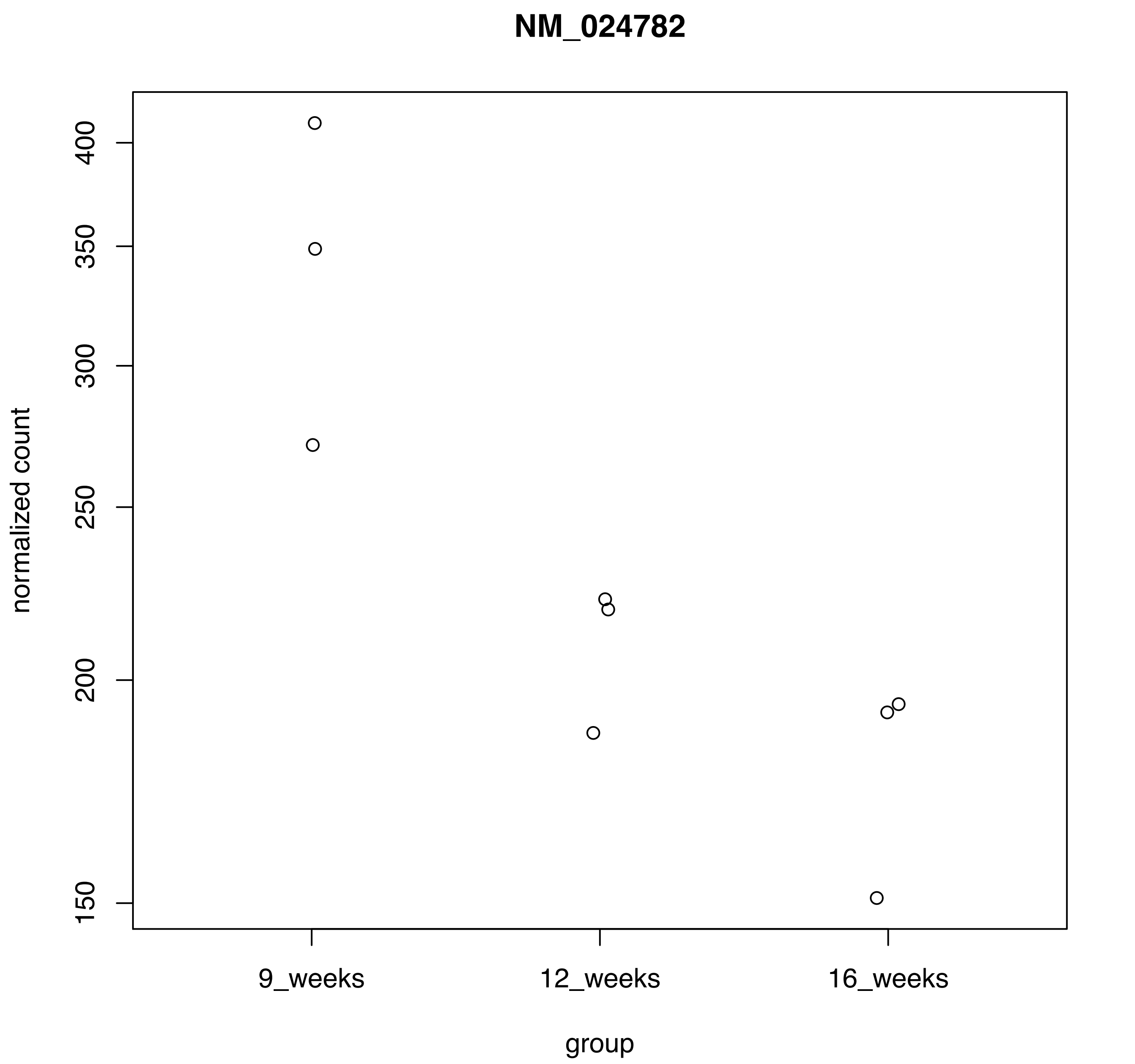
NM_024782
normalized count
150
200
250
300
350
400
9_weeks
12_weeks
16_weeks
group

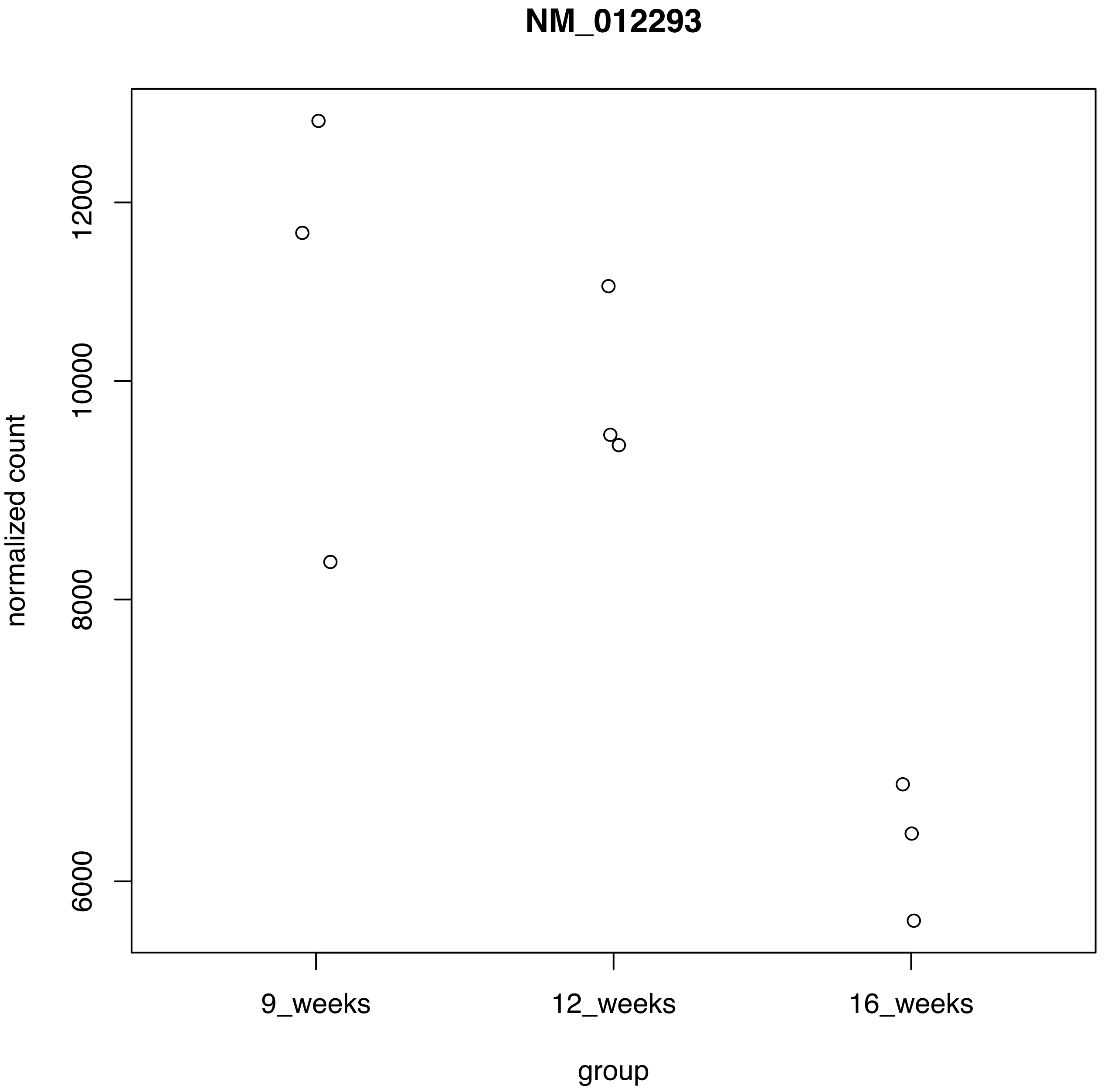

NM_012293
normalized count
12000
10000
8000
6000
9_weeks
12_weeks
16_weeks
group

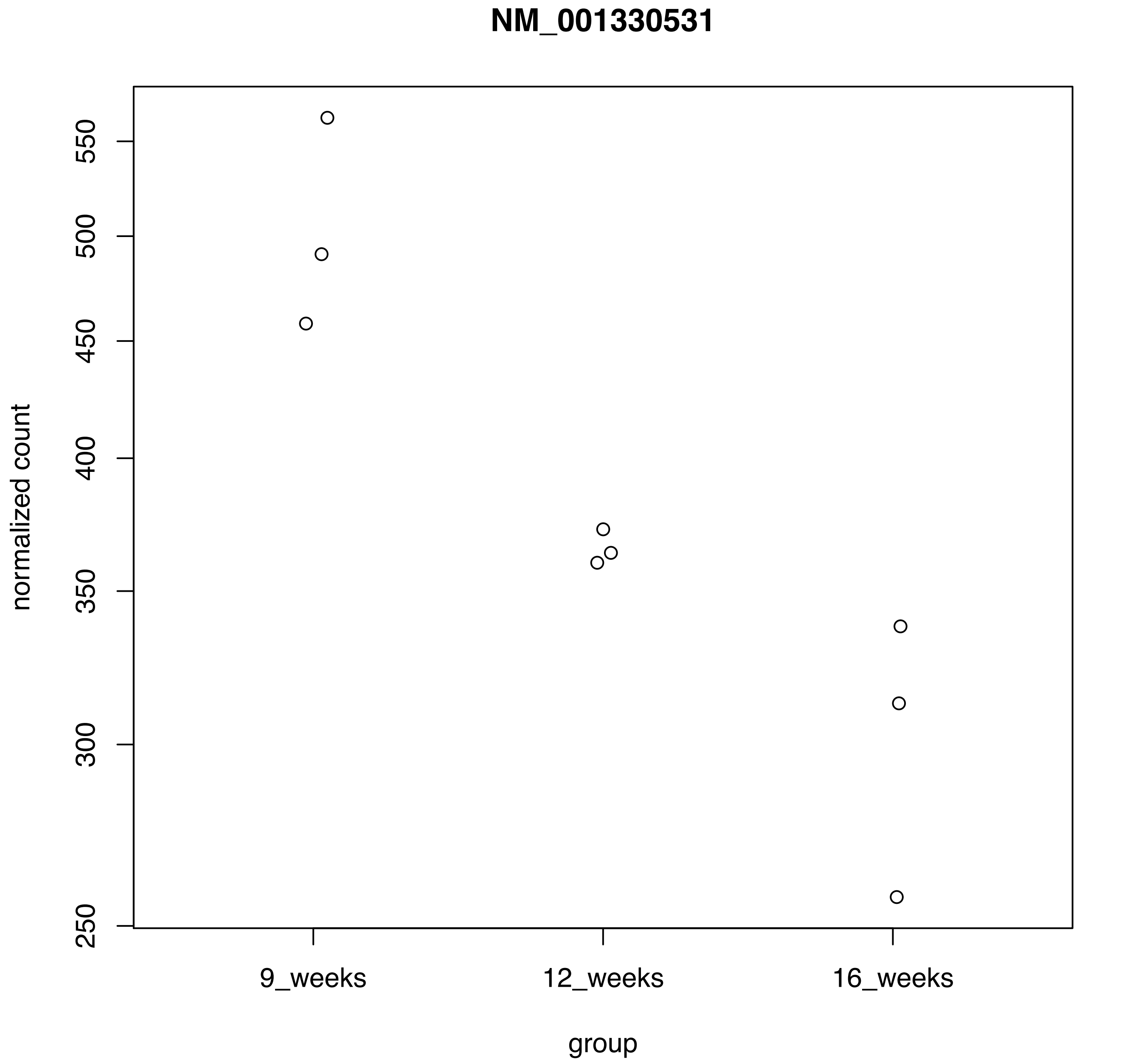

NM_001330531
normalized count
550
500
450
400
350
300
250
9_weeks
12_weeks
16_weeks
group

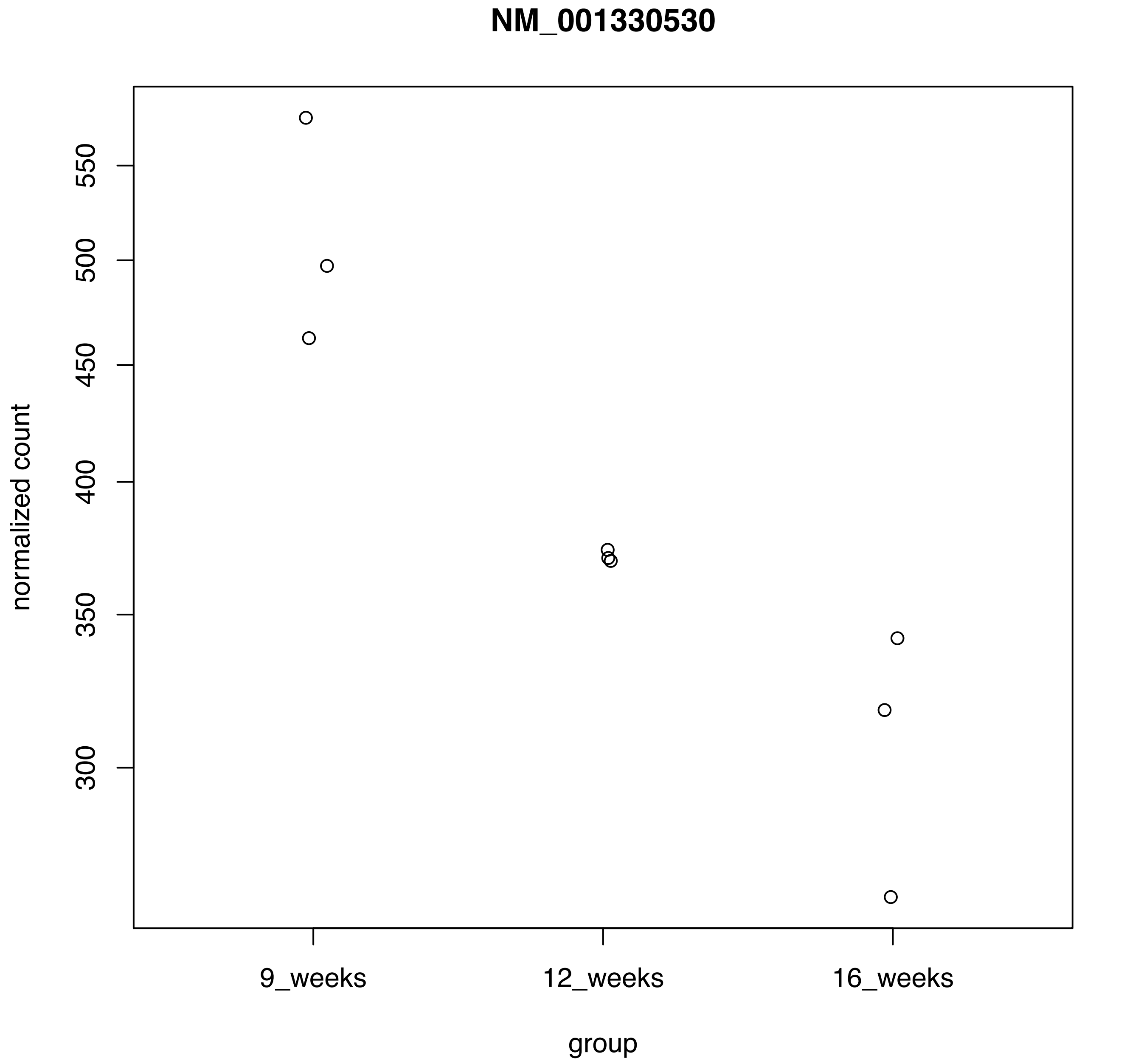

NM_001330530
normalized count
550
500
450
400
350
300
9_weeks
12_weeks
16_weeks
group

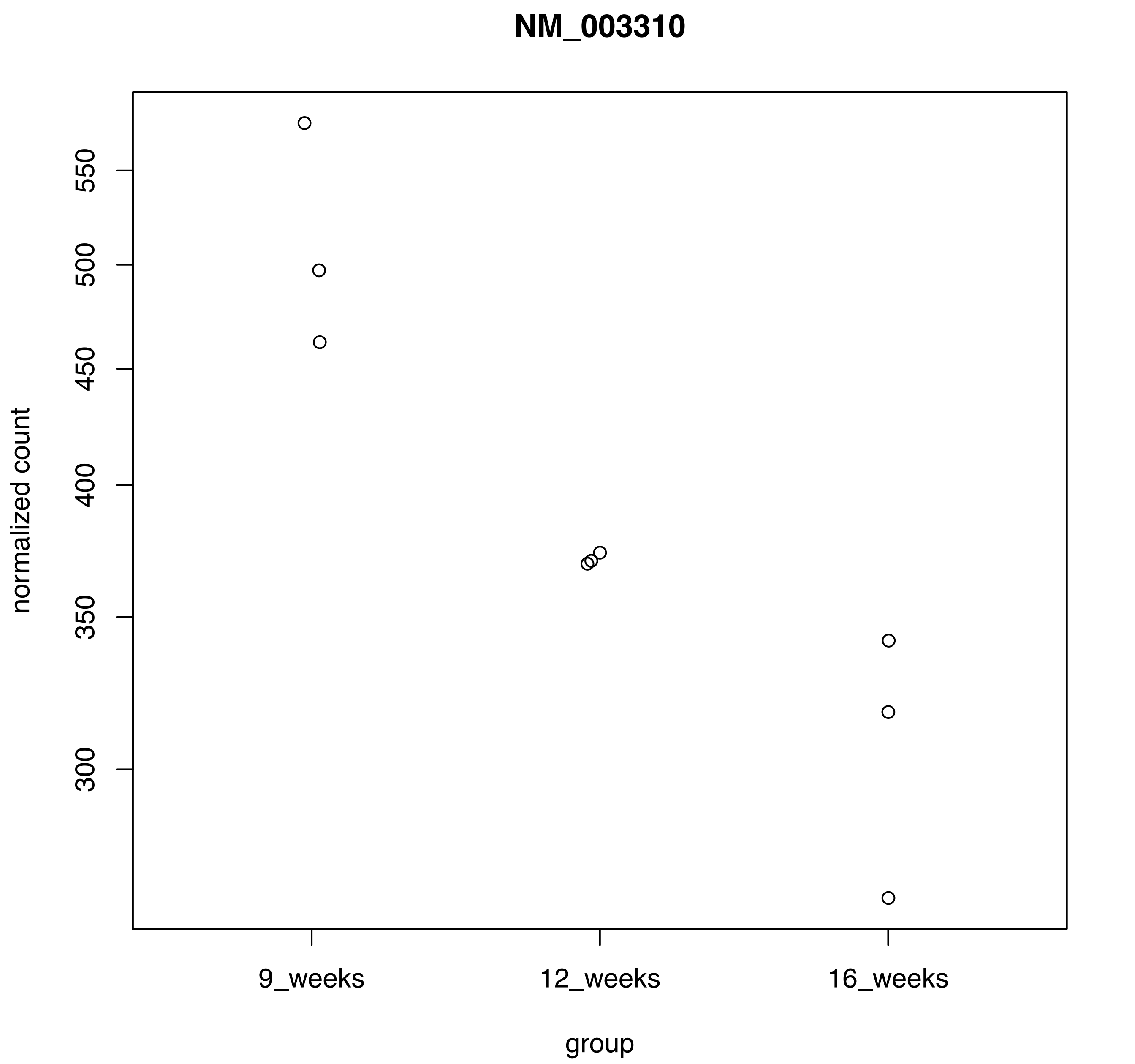
NM_003310
normalized count
550
500
450
400
350
300
9_weeks
12_weeks
16_weeks
group

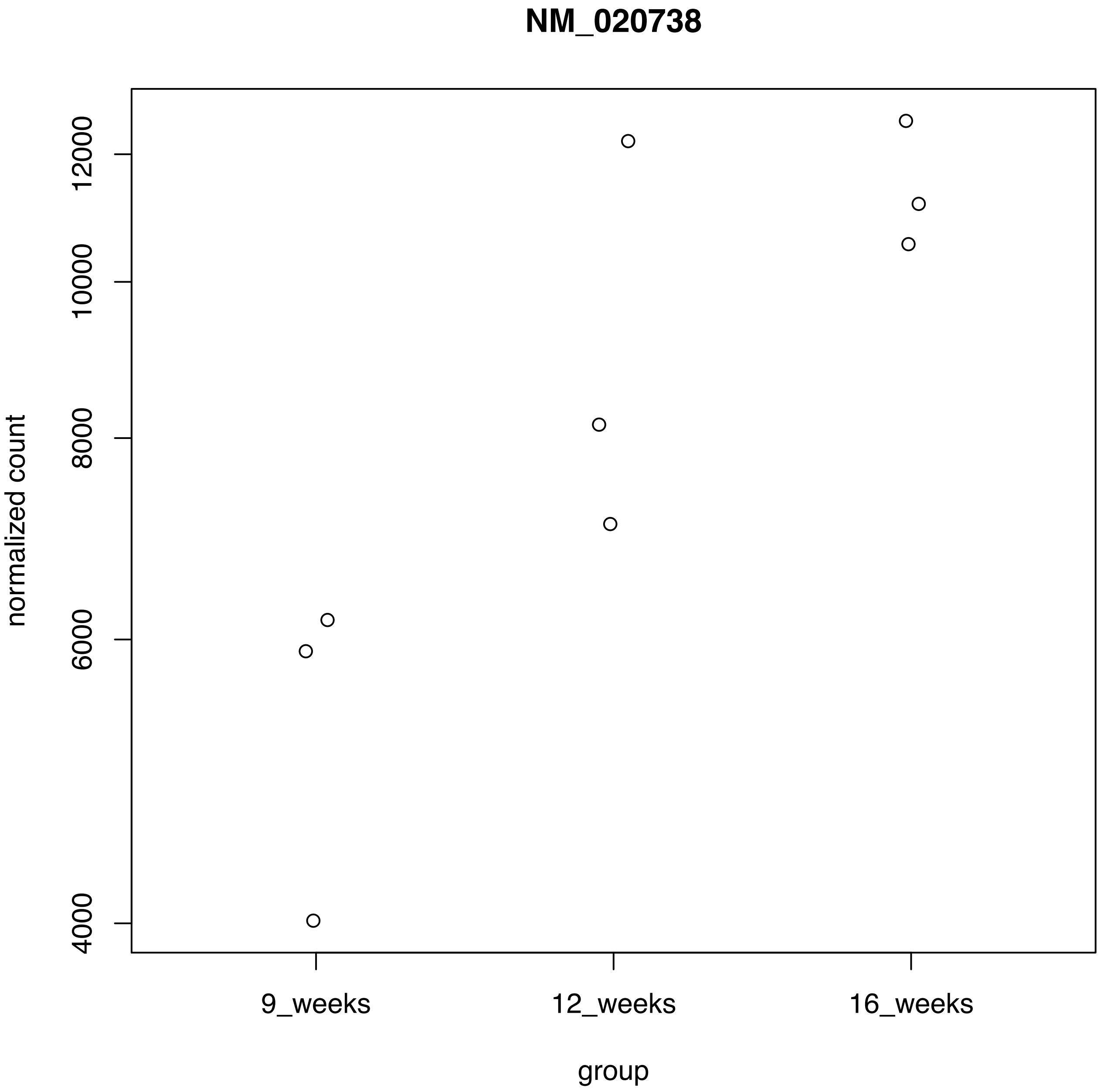

NM_020738
normalized count
4000
6000
8000
10000
12000
9_weeks
12_weeks
16_weeks
group

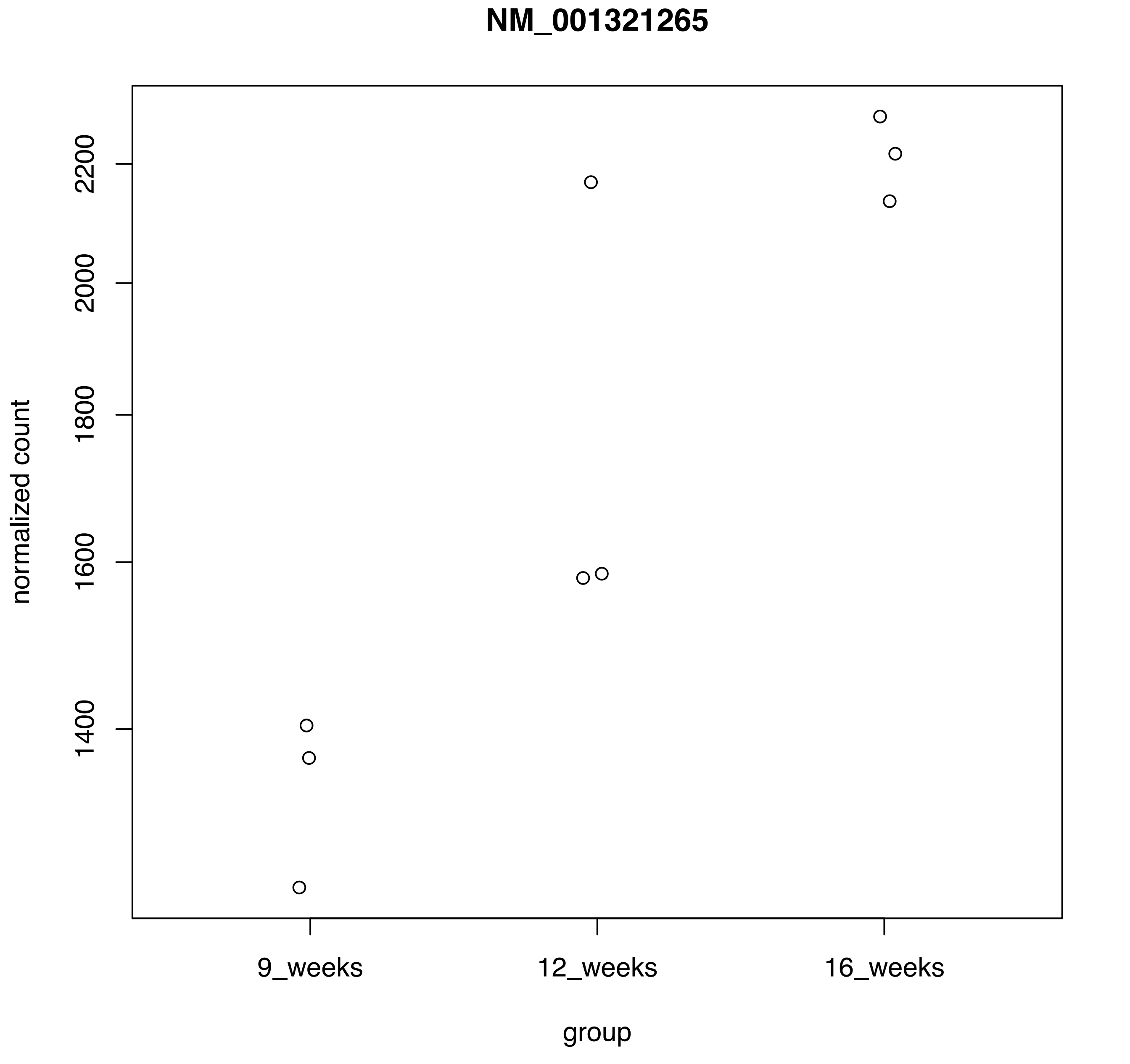

NM_001321265
normalized count
2200
2000
1800
1600
1400
9_weeks
12_weeks
16_weeks
group

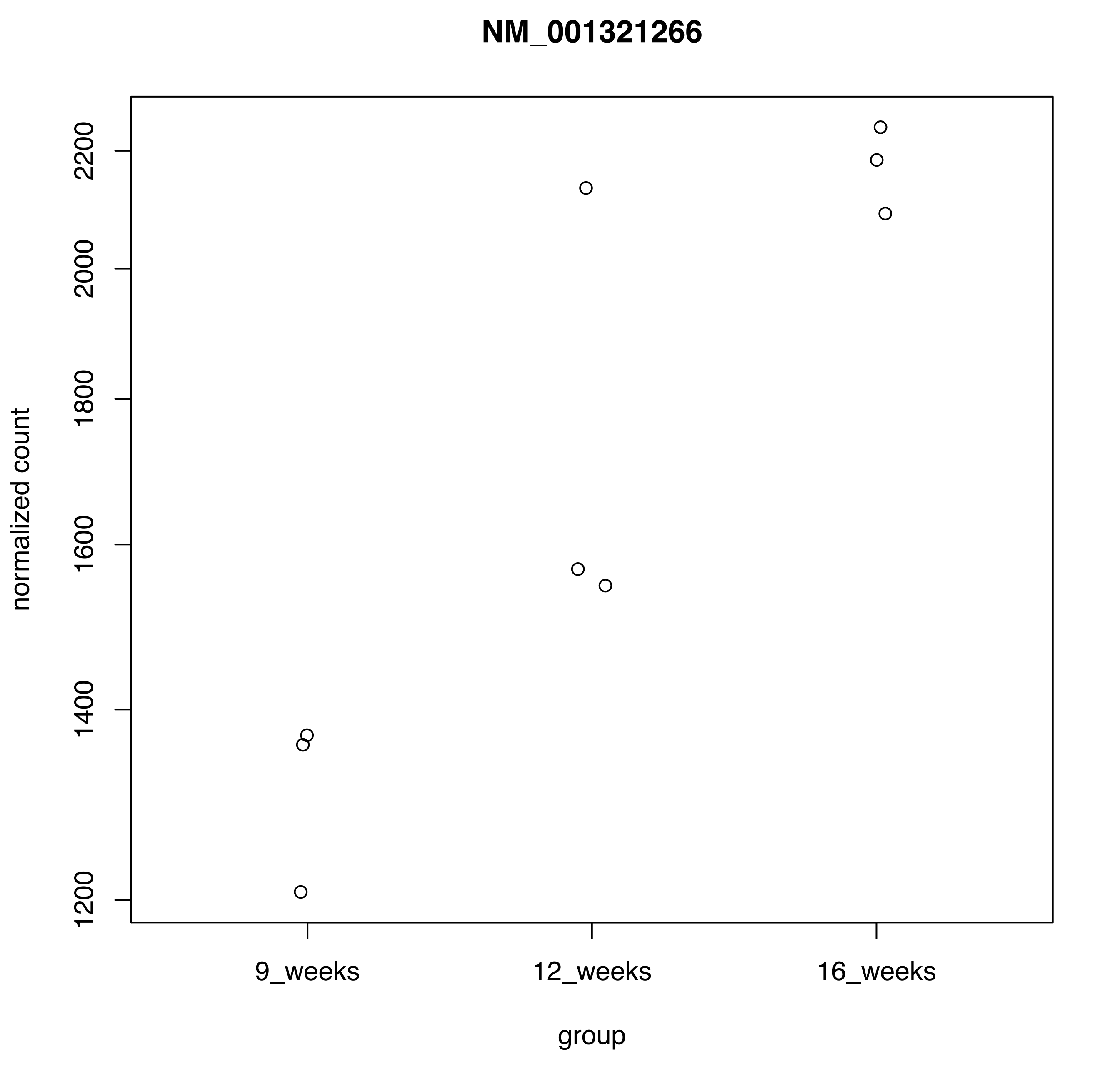

NM_001321266
normalized count
group
1200
1400
1600
1800
2000
2200
9_weeks
12_weeks
16_weeks

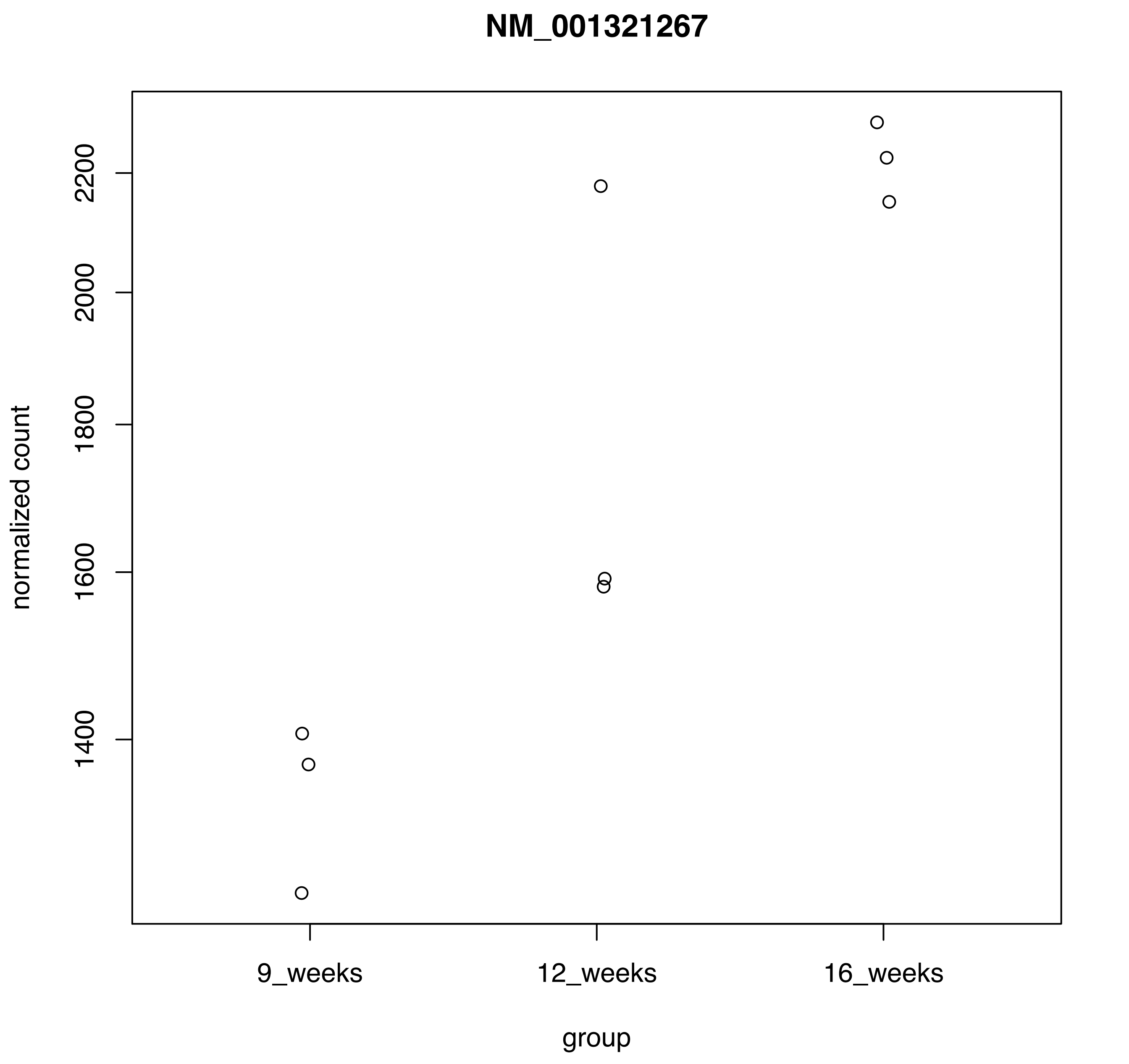
NM_001321267
normalized count
2200
2000
1800
1600
1400
9_weeks
12_weeks
16_weeks
group

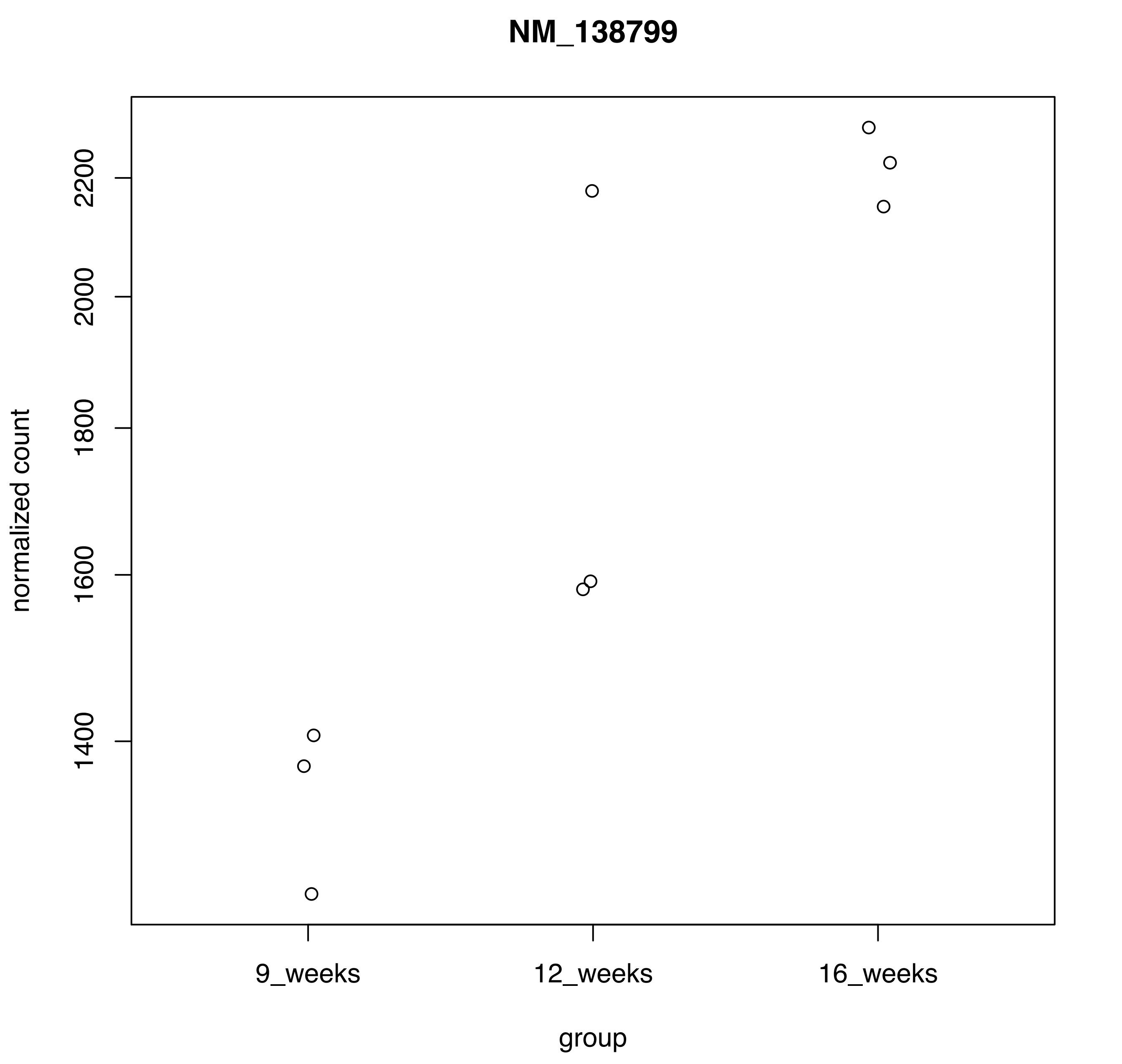
NM_138799
normalized count
2200
2000
1800
1600
1400
9_weeks
12_weeks
16_weeks
group

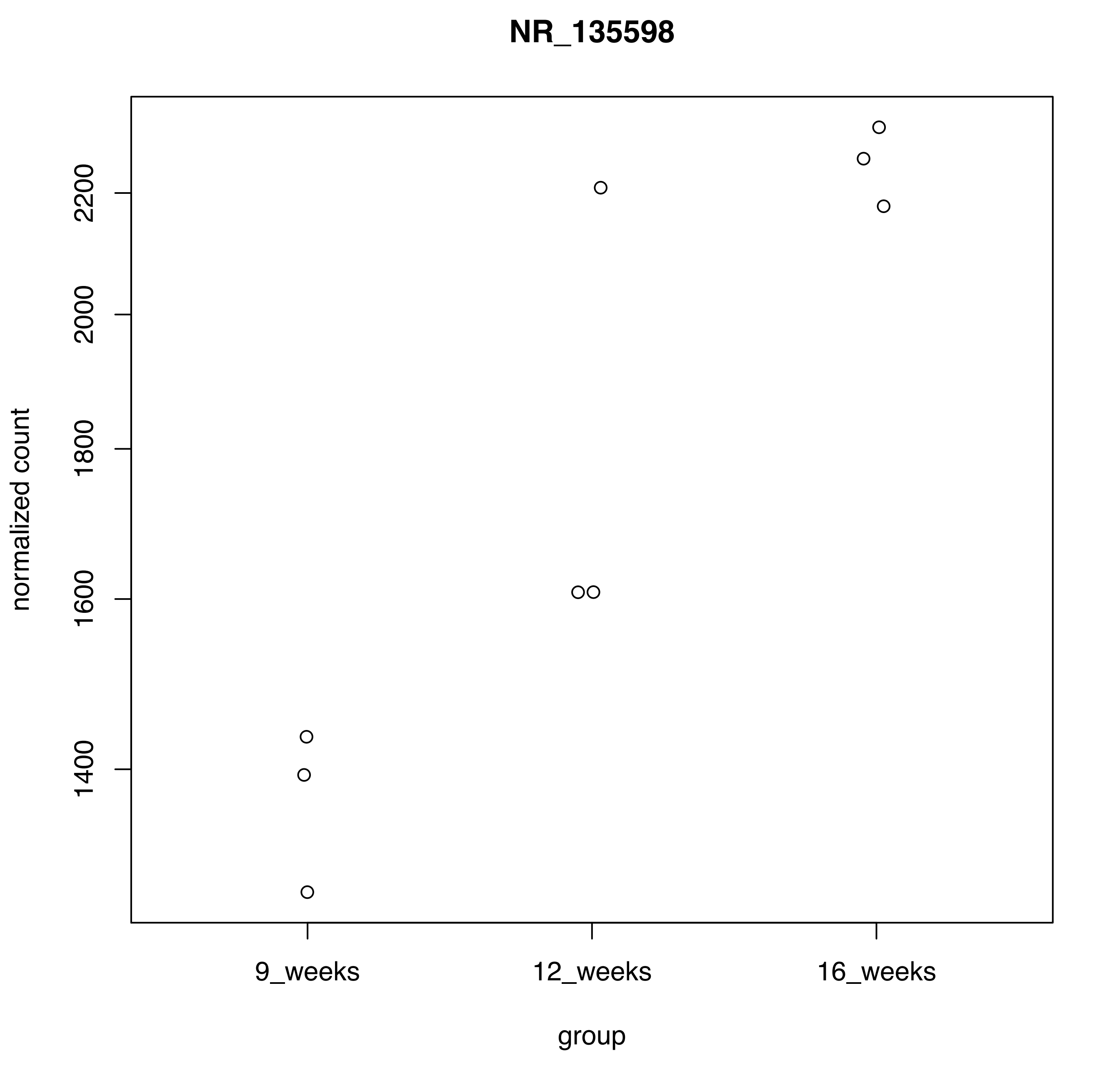
NR_135598
normalized count
2200
2000
1800
1600
1400
9_weeks
12_weeks
16_weeks
group

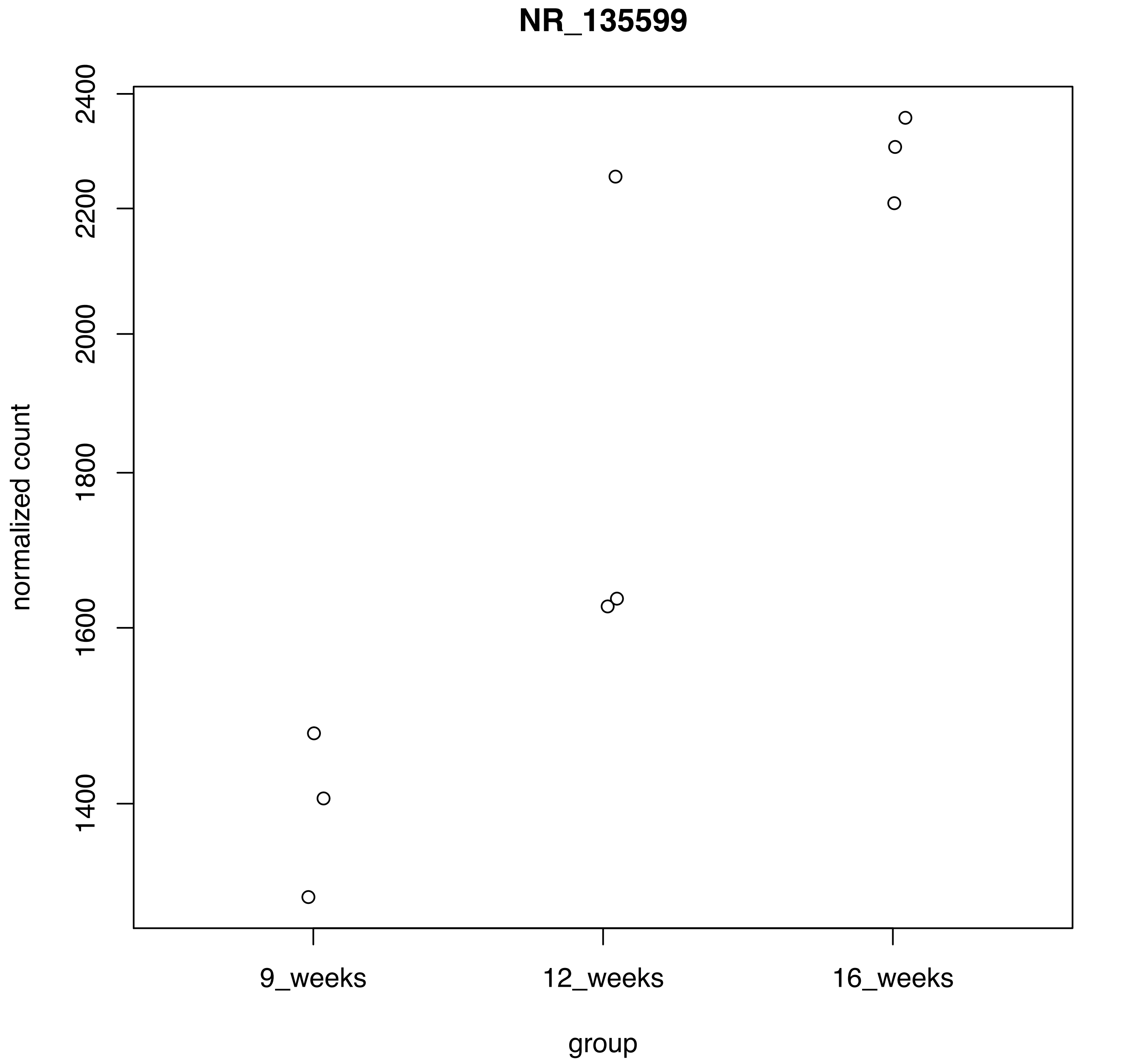

NR_135599
normalized count
2400
2200
2000
1800
1600
1400
9_weeks
12_weeks
16_weeks
group

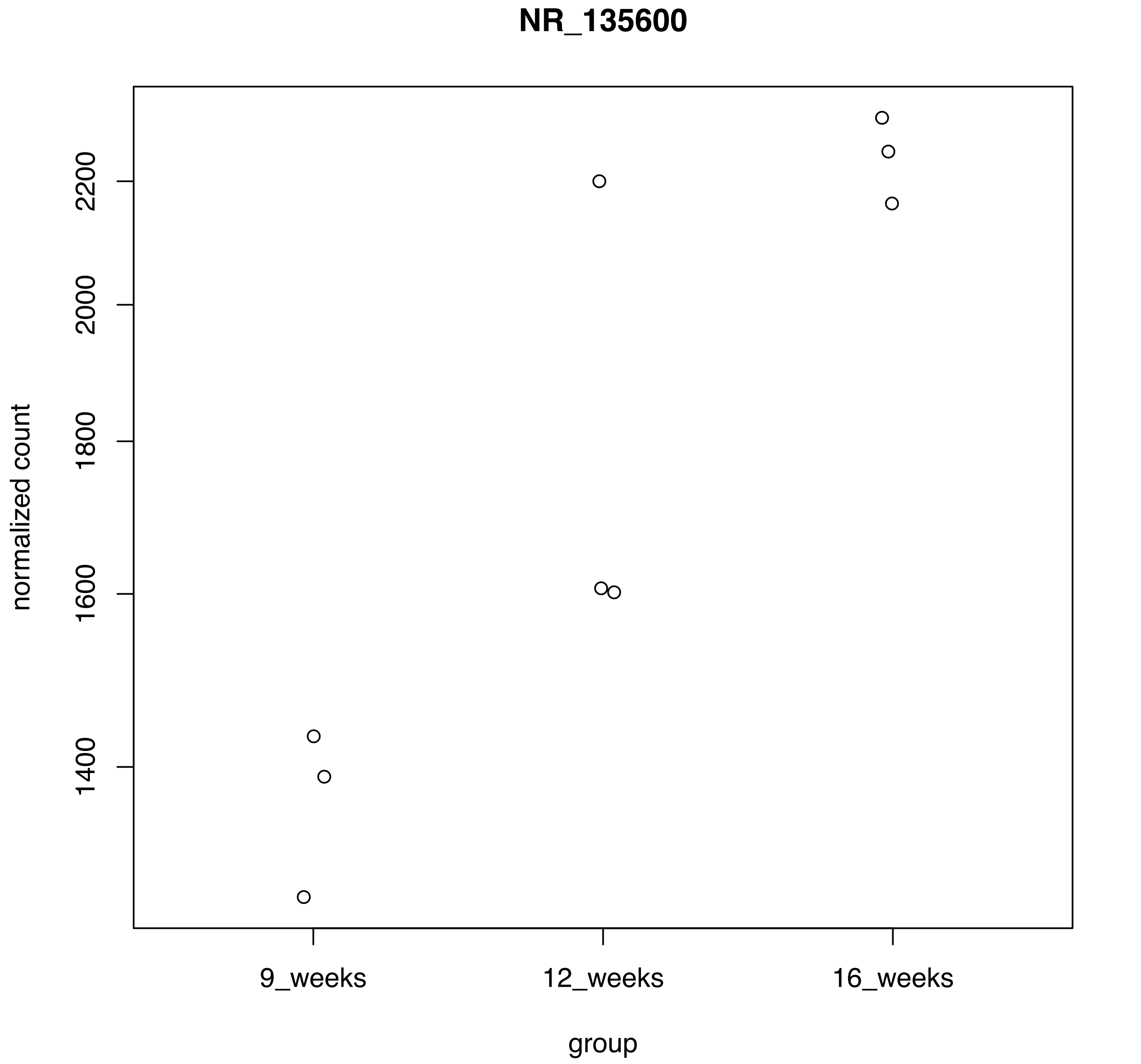

NR_135600
normalized count
2200
2000
1800
1600
1400
9_weeks
12_weeks
16_weeks
group

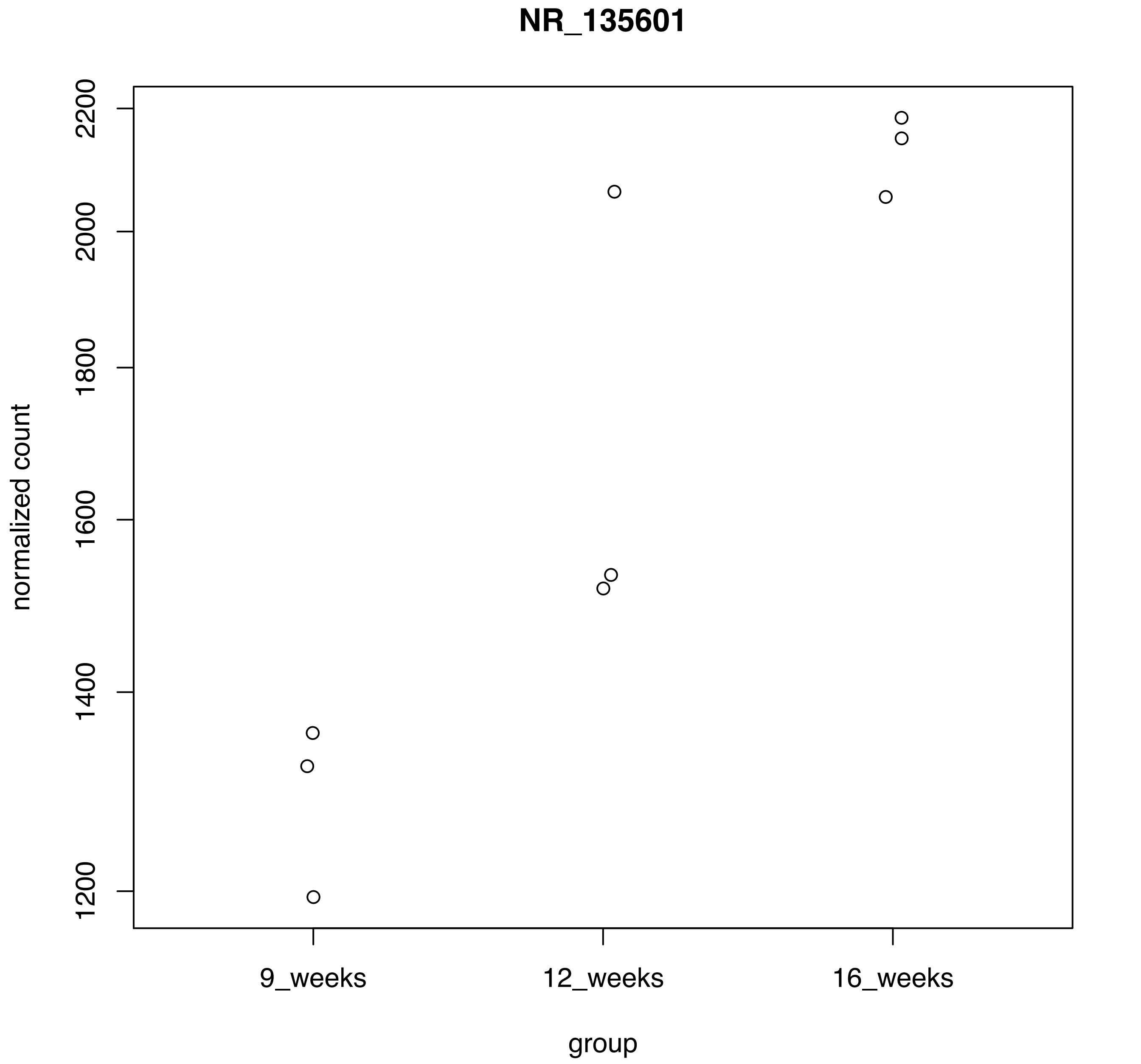
NR_135601
normalized count
2200
2000
1800
1600
1400
1200
9_weeks
12_weeks
16_weeks
group

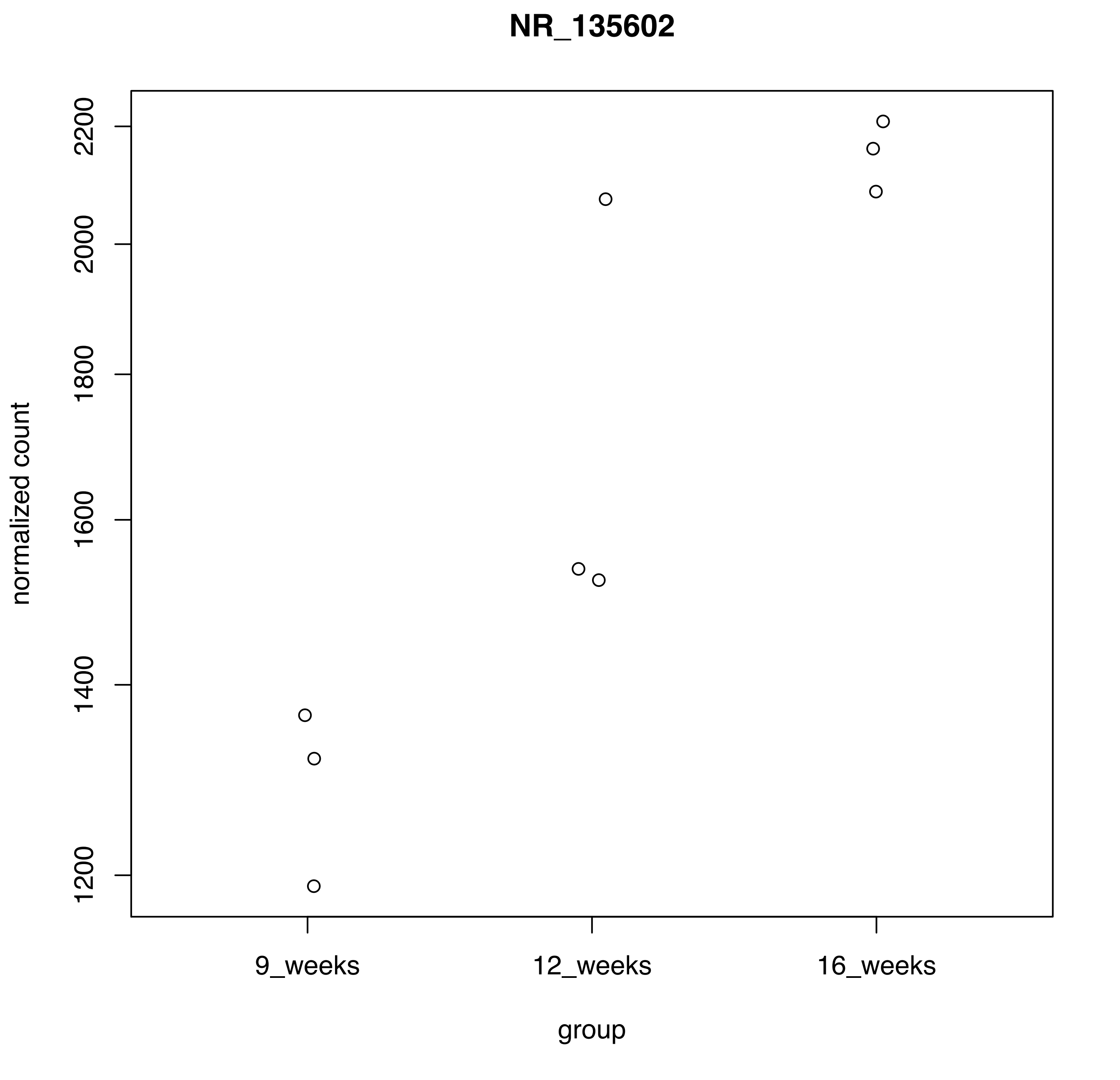
NR_135602
normalized count
2200
2000
1800
1600
1400
1200
9_weeks
12_weeks
16_weeks
group

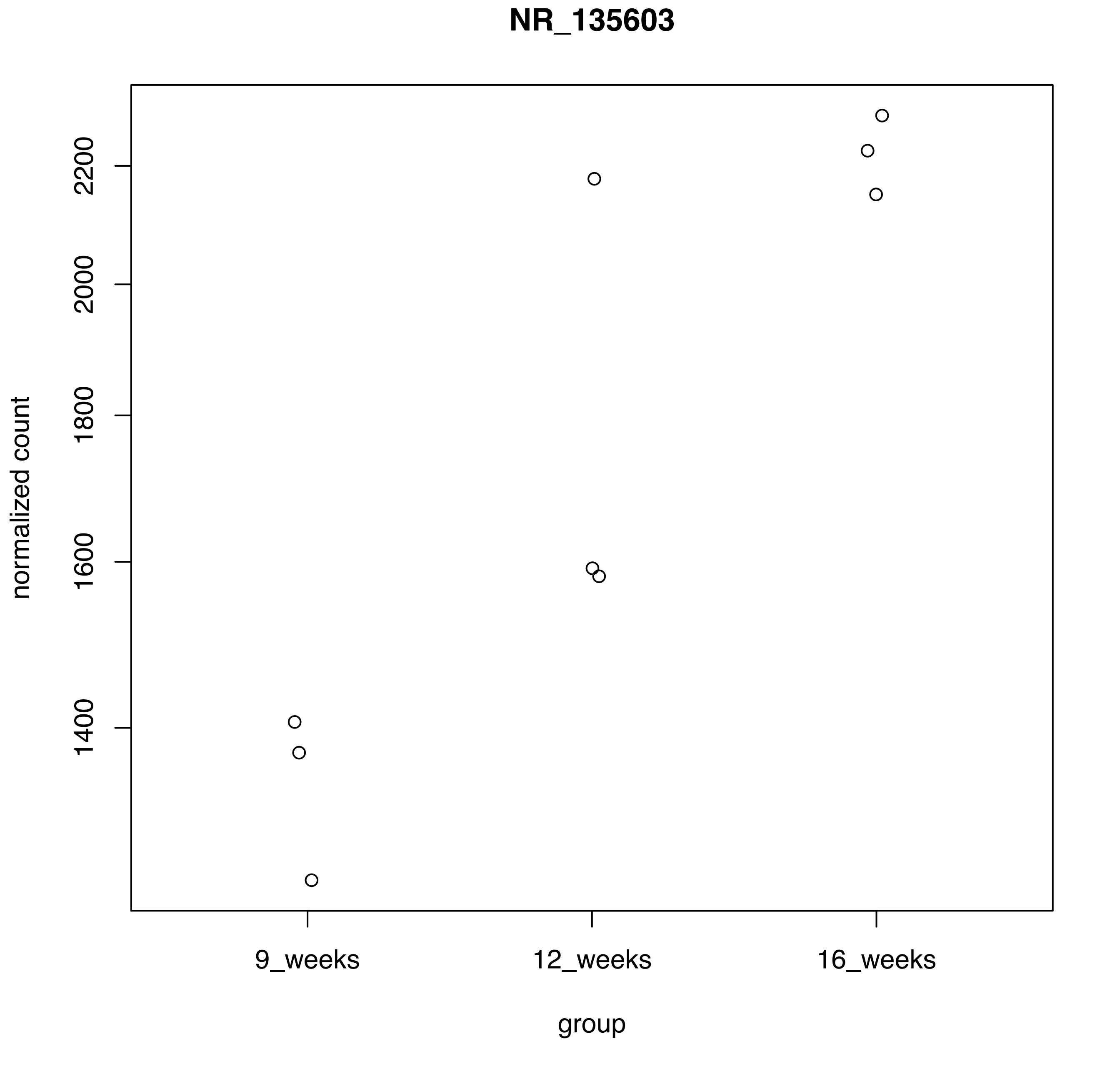

NR_135603
normalized count
2200
2000
1800
1600
1400
9_weeks
12_weeks
16_weeks
group

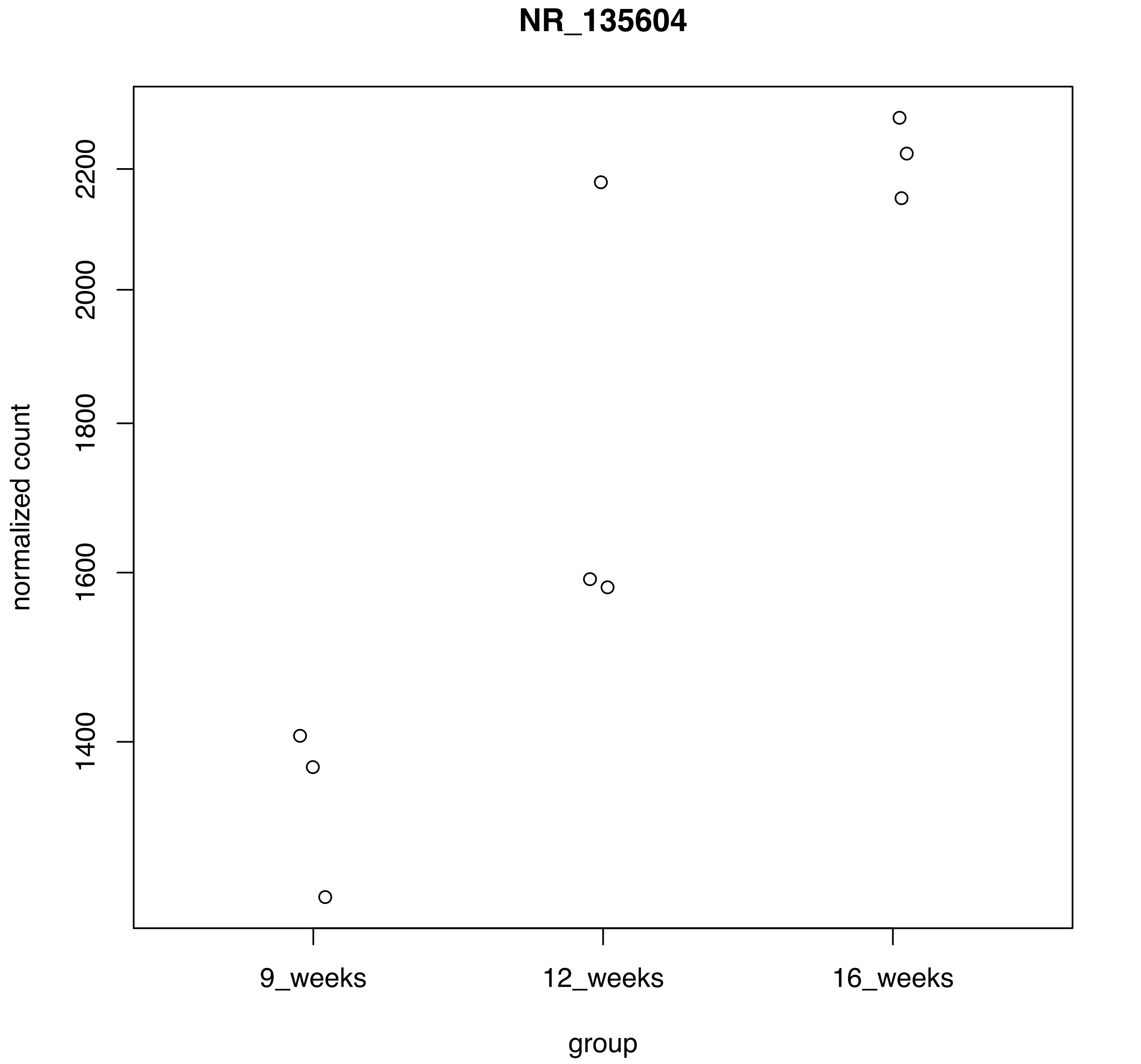

NR_135604
normalized count
1400
1600
1800
2000
2200
9_weeks
12_weeks
16_weeks
group

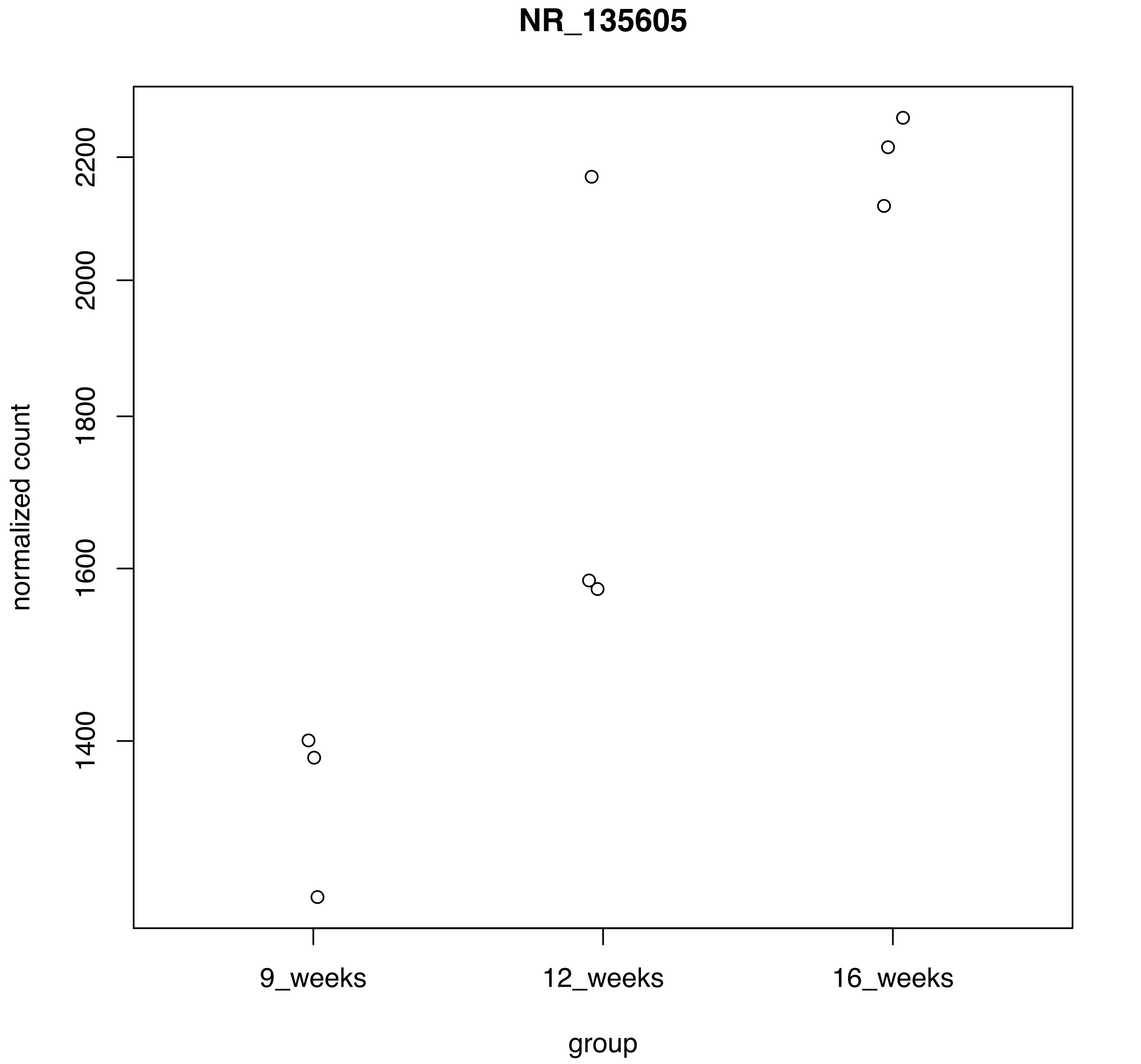

NR_135605
normalized count
2200
2000
1800
1600
1400
9_weeks
12_weeks
16_weeks
group

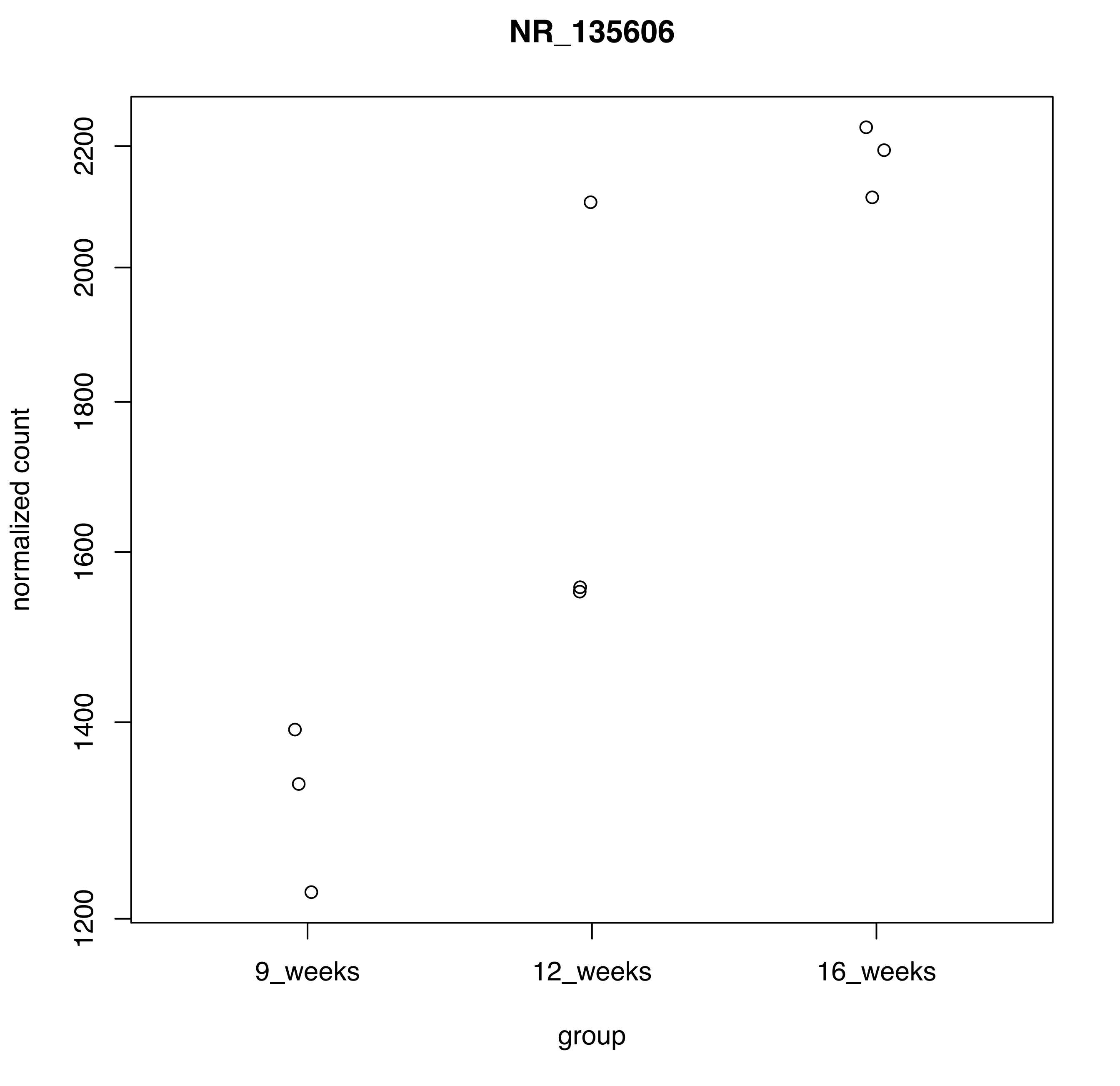

NR_135606
normalized count
2200
2000
1800
1600
1400
1200
9_weeks
12_weeks
16_weeks
group

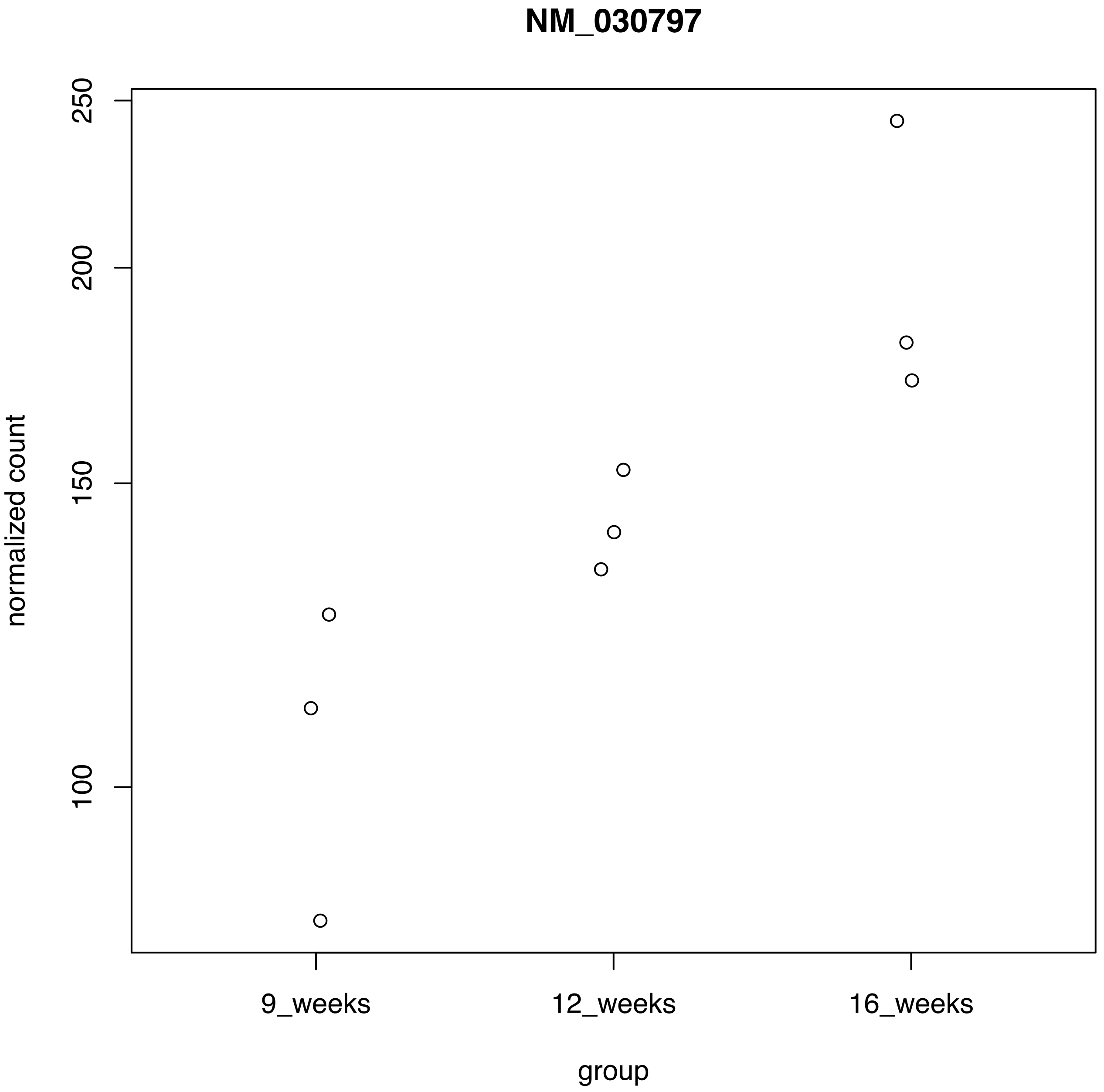
NM_030797
normalized count
250
200
150
100
9_weeks
12_weeks
16_weeks
group

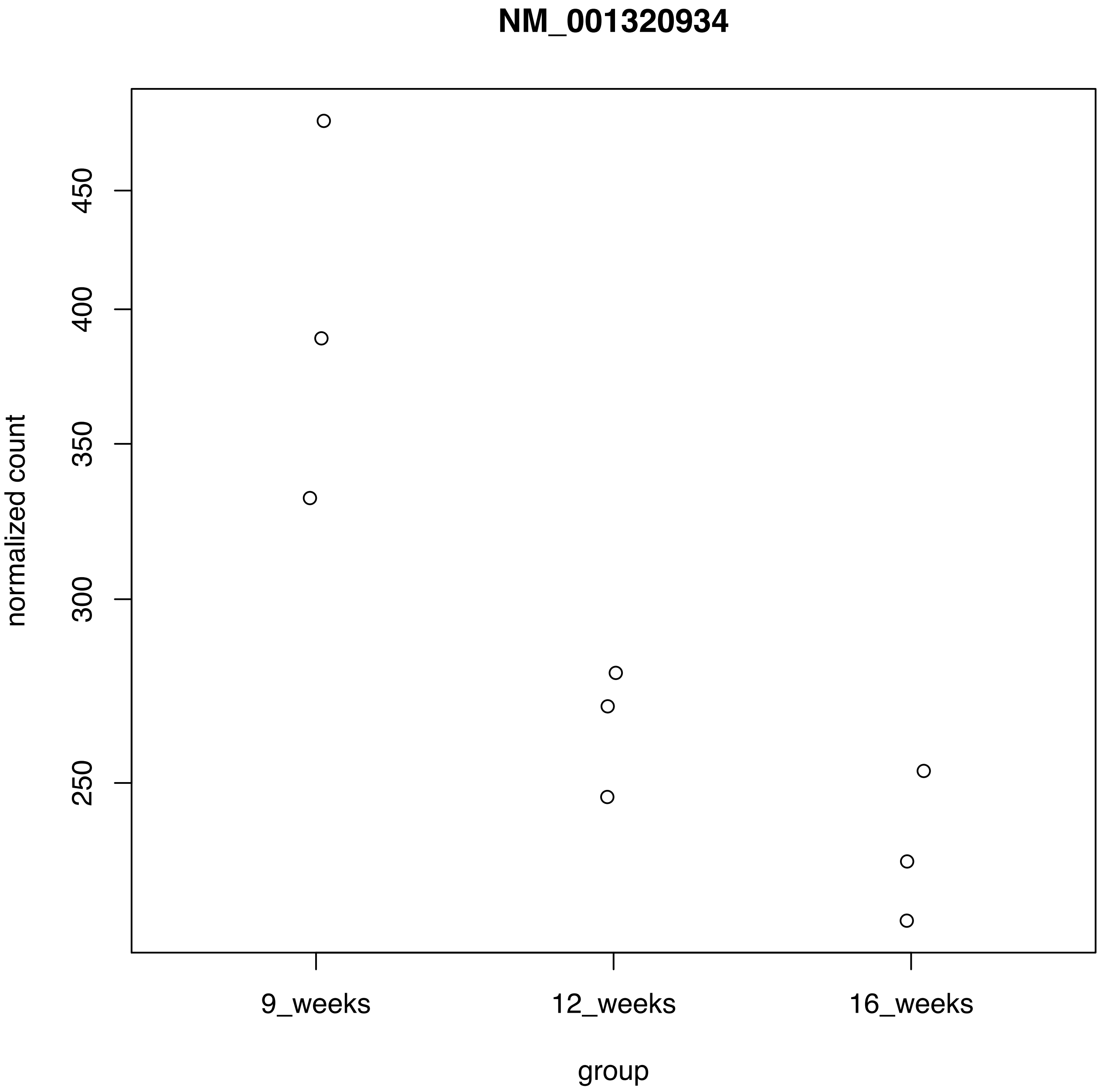
NM_001320934
normalized count
450
400
350
300
250
9_weeks
12_weeks
16_weeks
group

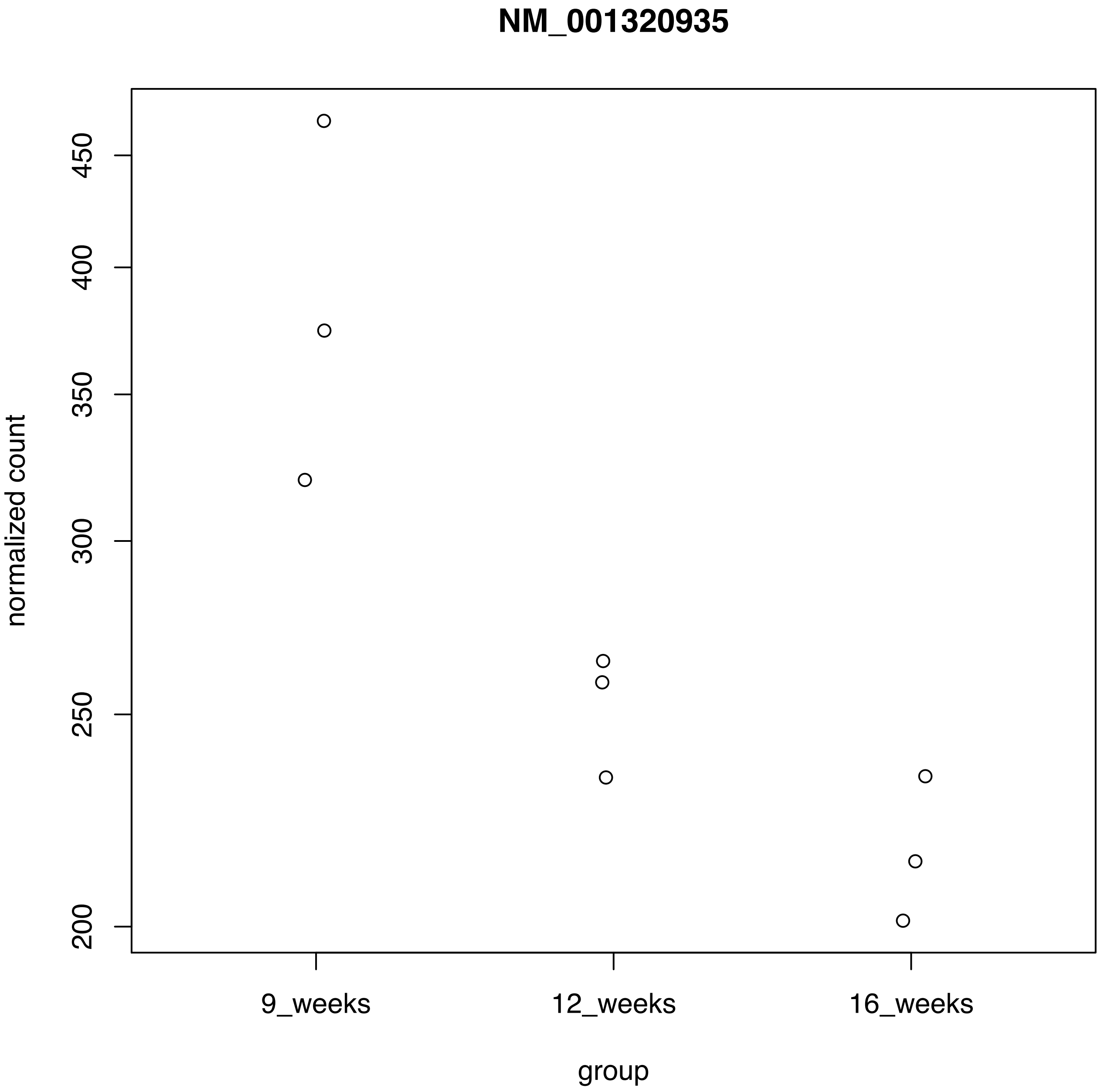
NM_001320935
normalized count
450
400
350
300
250
200
9_weeks
12_weeks
16_weeks
group

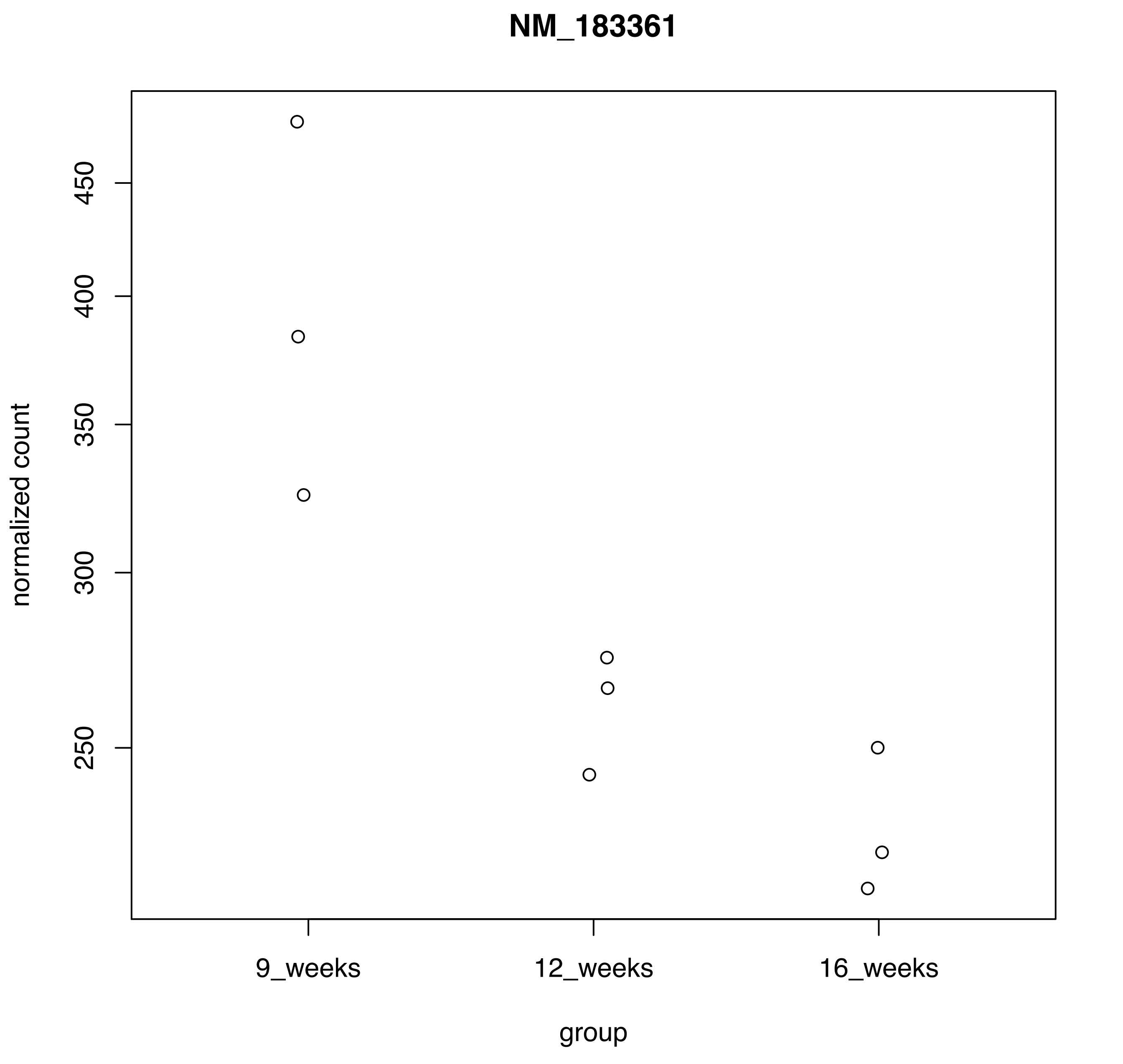
NM_183361
normalized count
450
400
350
300
250
9_weeks
12_weeks
16_weeks
group

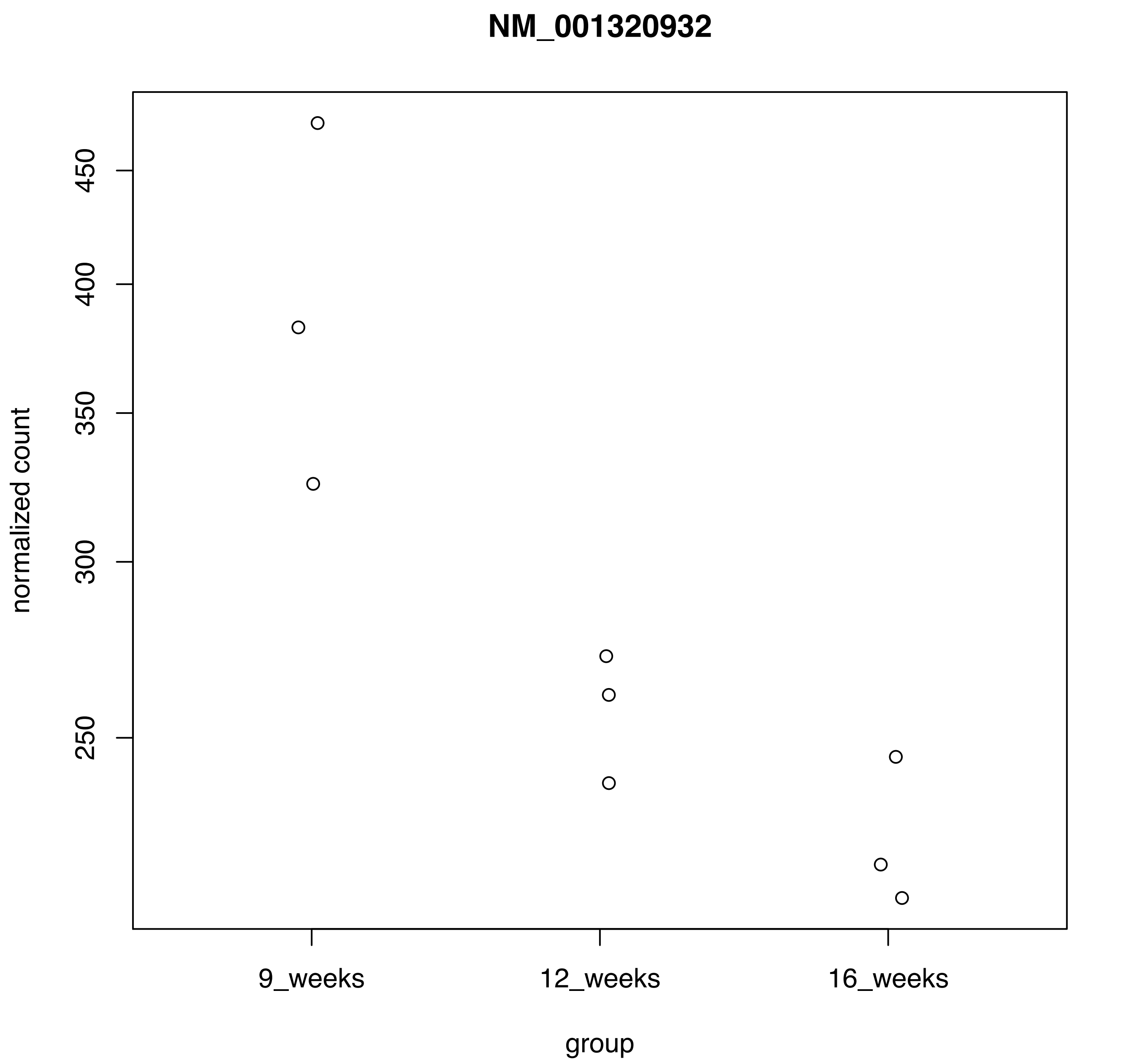
NM_001320932
normalized count
450
400
350
300
250
9_weeks
12_weeks
16_weeks
group

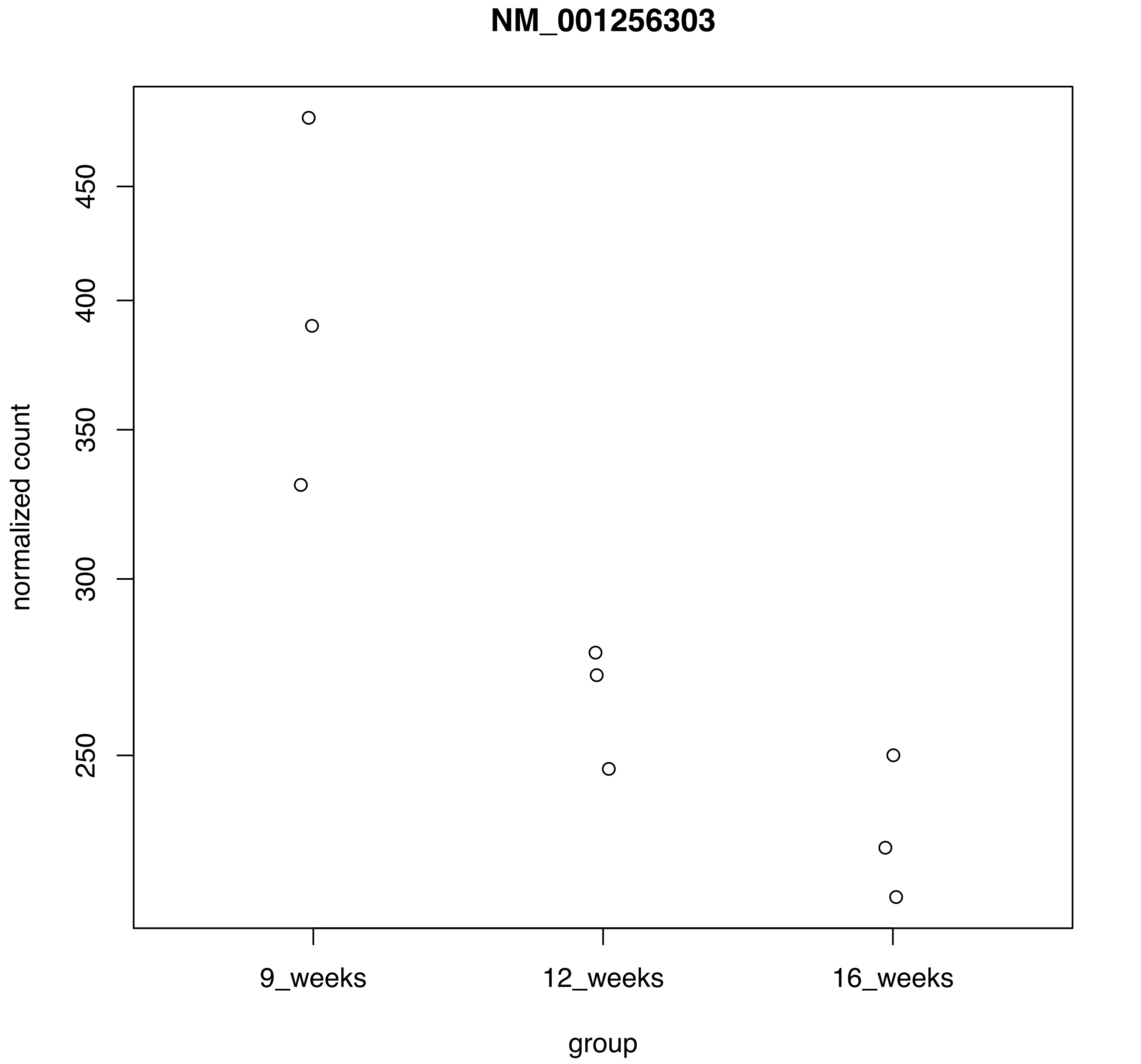
NM_001256303
normalized count
450
400
350
300
250
9_weeks
12_weeks
16_weeks
group

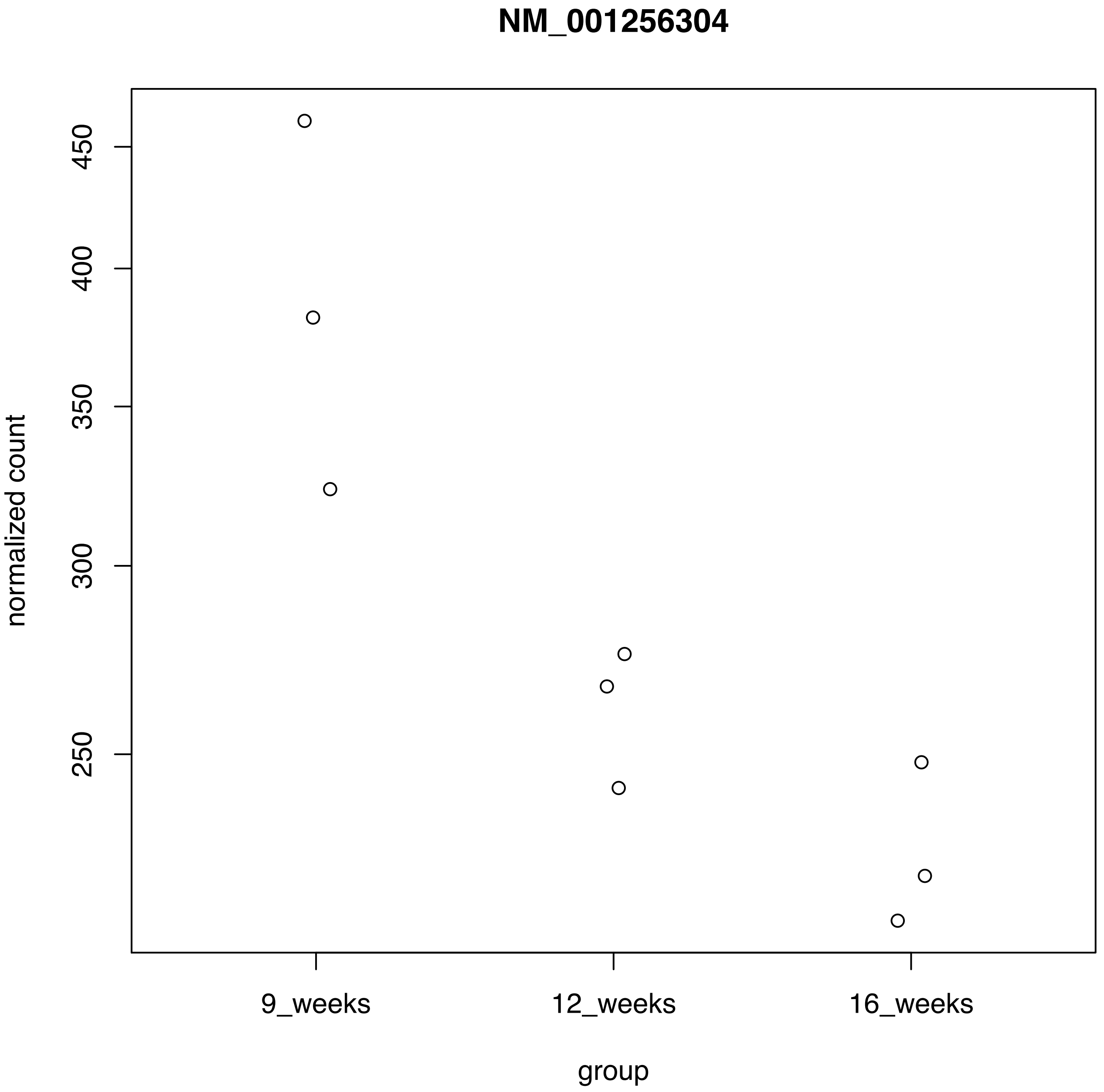

NM_001256304
normalized count
450
400
350
300
250
9_weeks
12_weeks
16_weeks
group

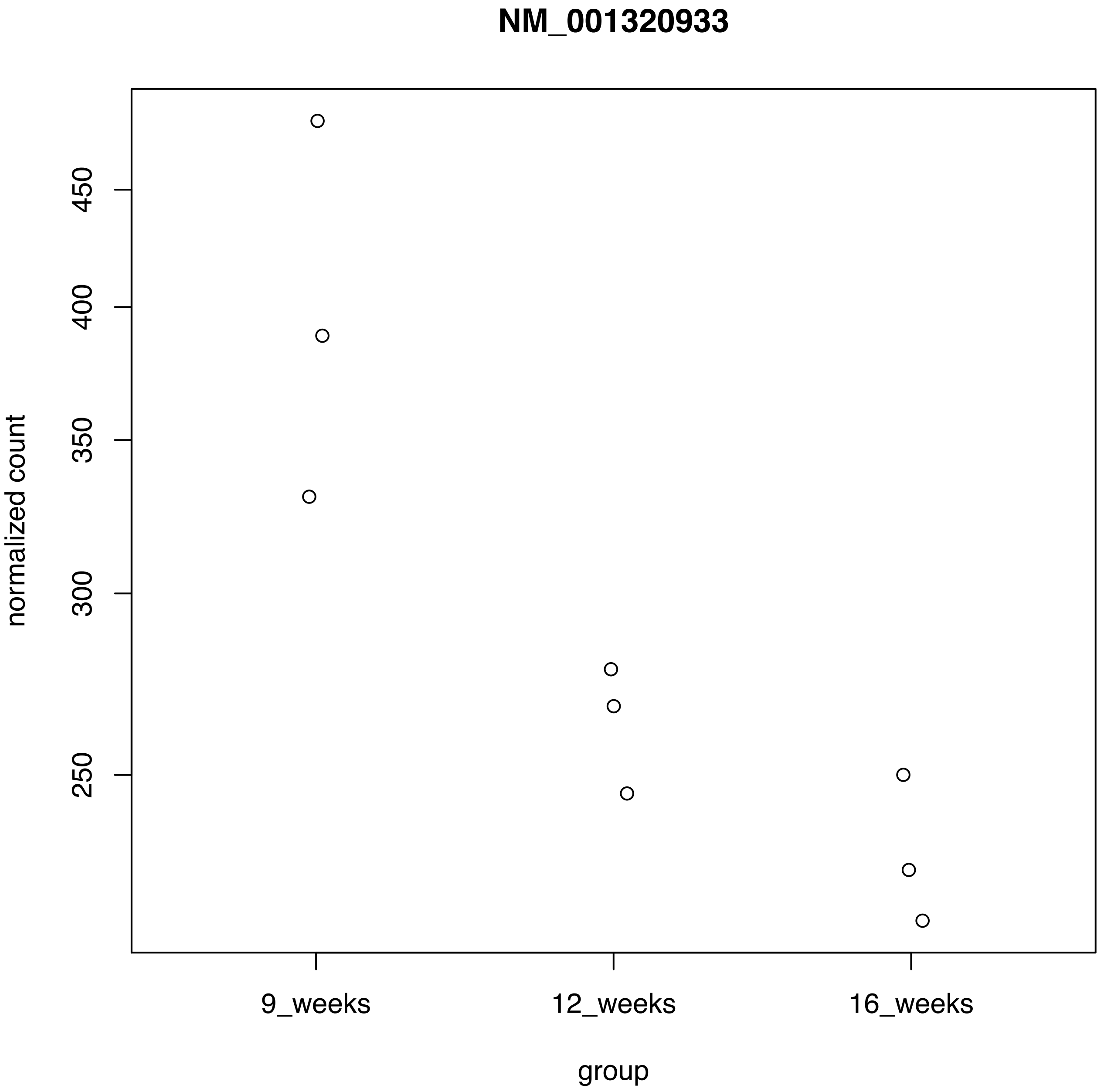
NM_001320933
normalized count
250
300
350
400
450
9_weeks
12_weeks
16_weeks
group

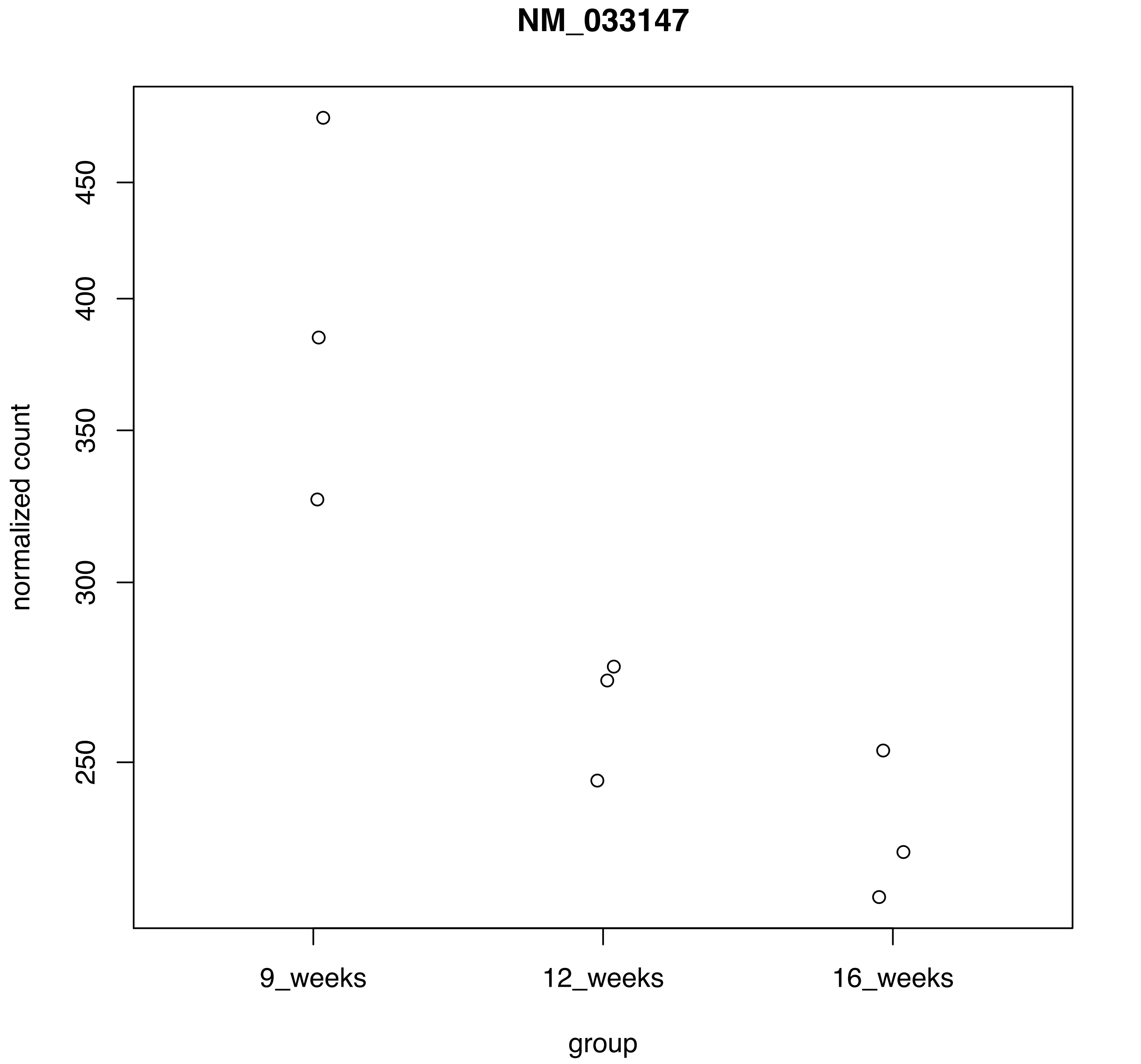

NM_033147
normalized count
450
400
350
300
250
9_weeks
12_weeks
16_weeks
group

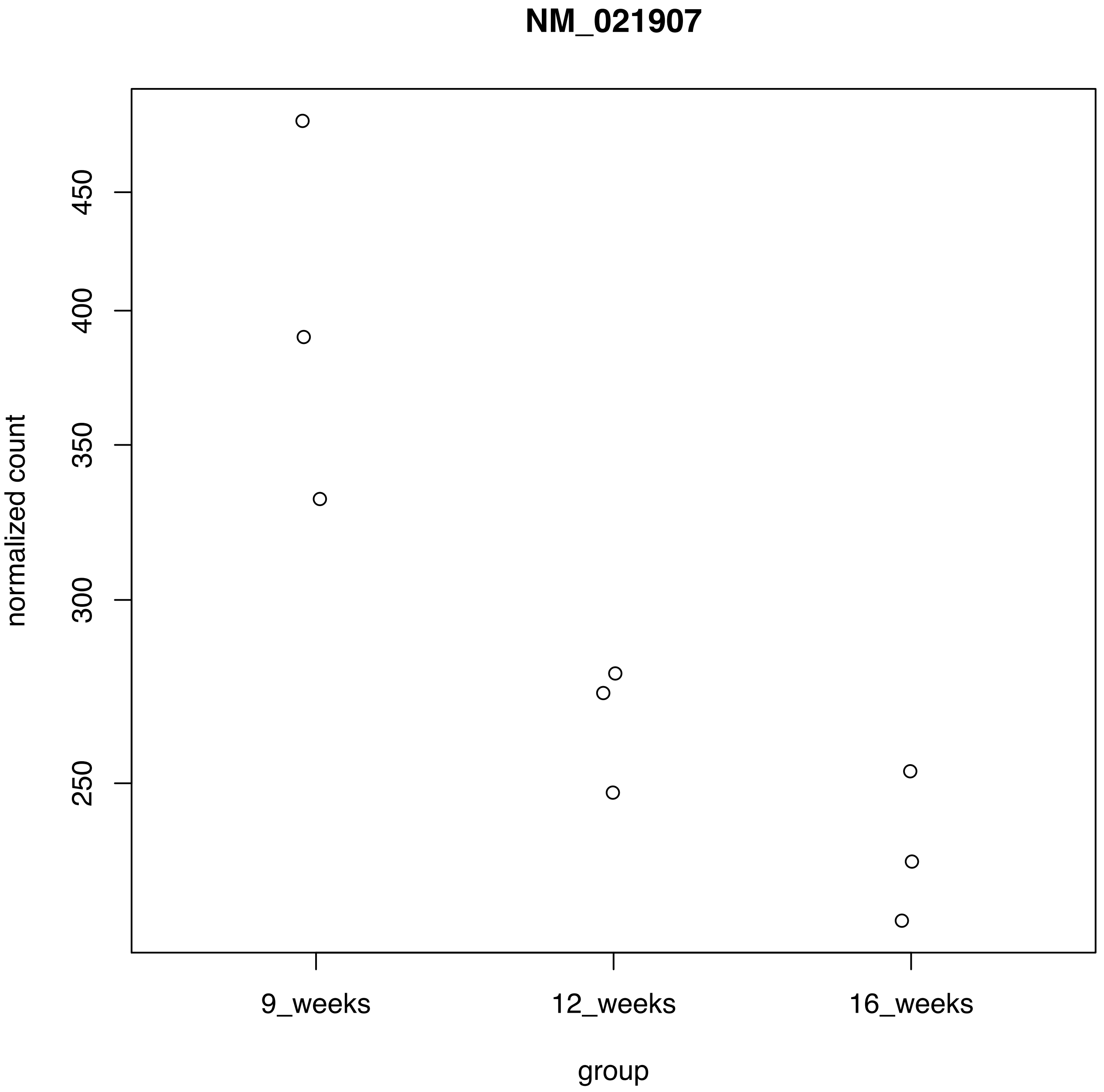
NM_021907
normalized count
250
300
350
400
450
9_weeks
12_weeks
16_weeks
group

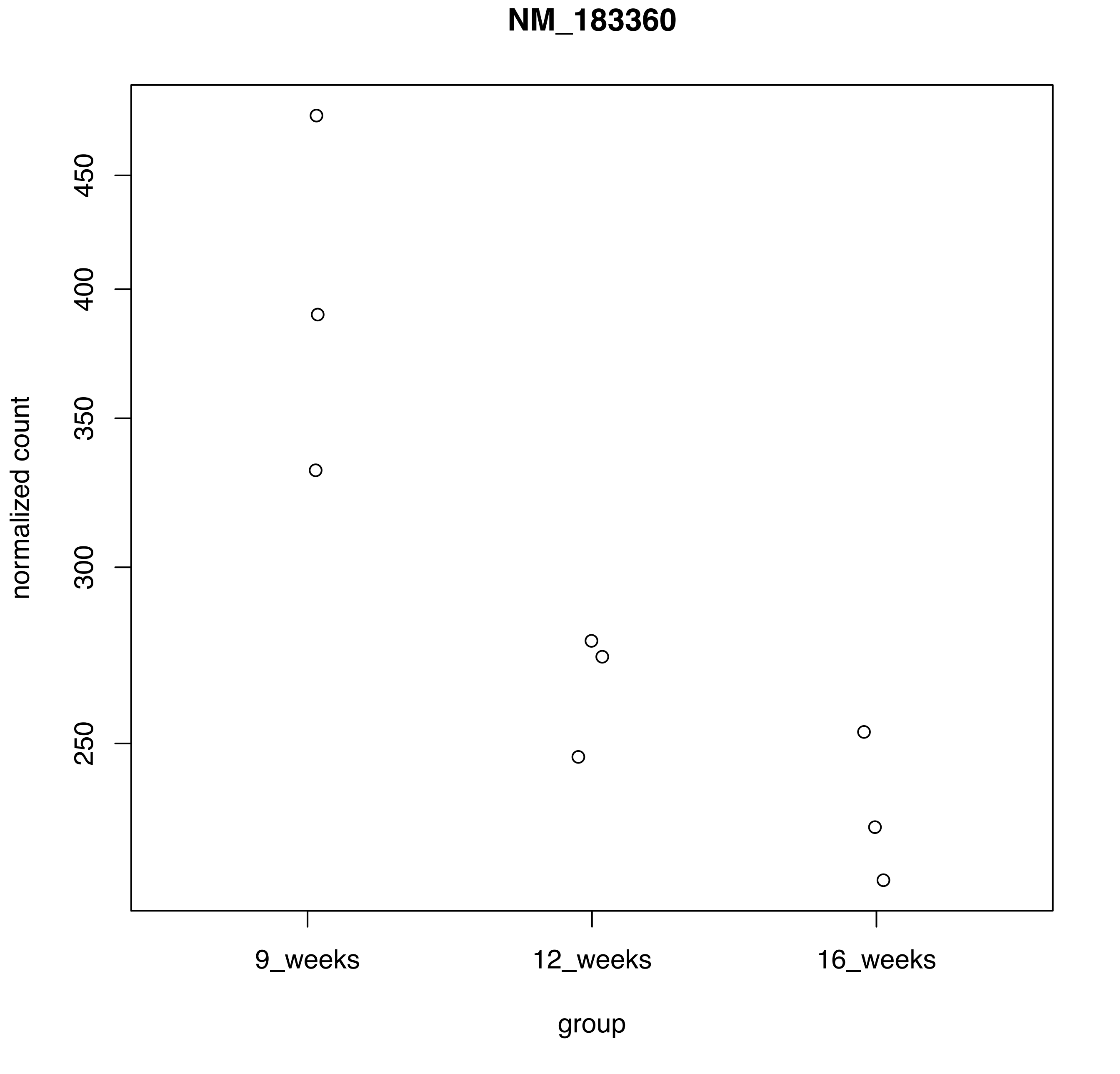

NM_183360
normalized count
450
400
350
300
250
9_weeks
12_weeks
16_weeks
group

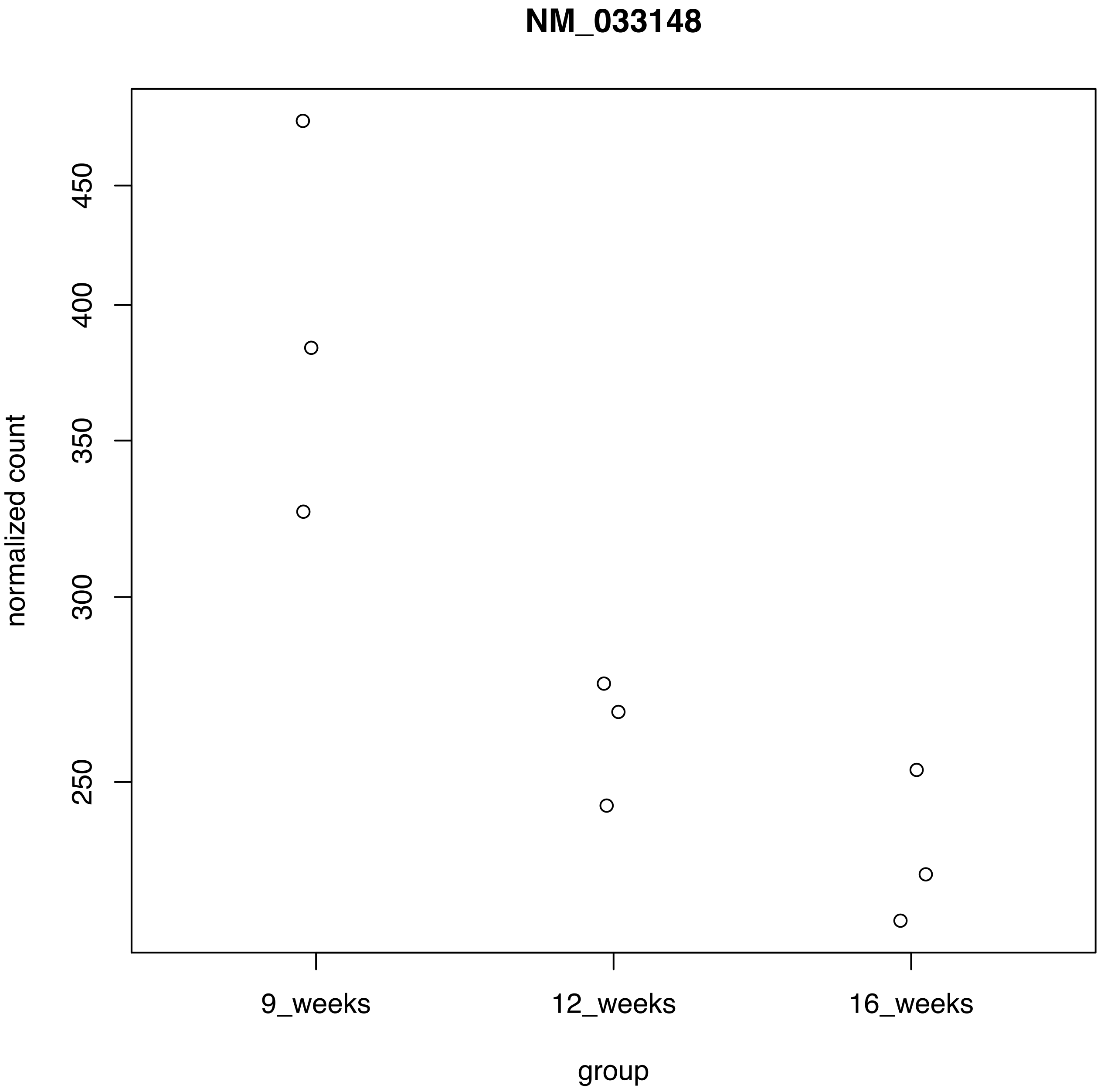

NM_033148
normalized count
250
300
350
400
450
9_weeks
12_weeks
16_weeks
group

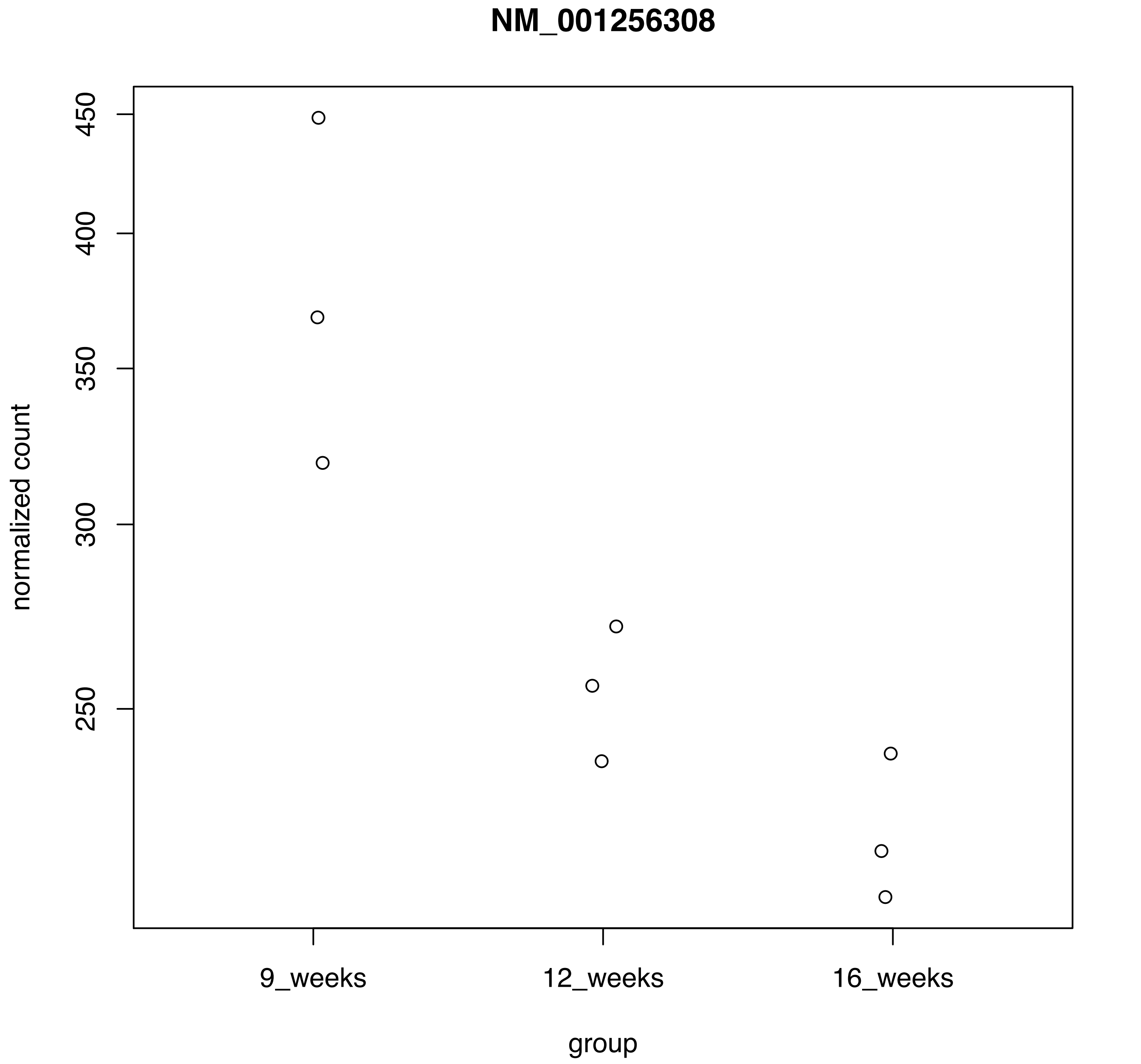
NM_001256308
normalized count
450
400
350
300
250
9_weeks
12_weeks
16_weeks
group

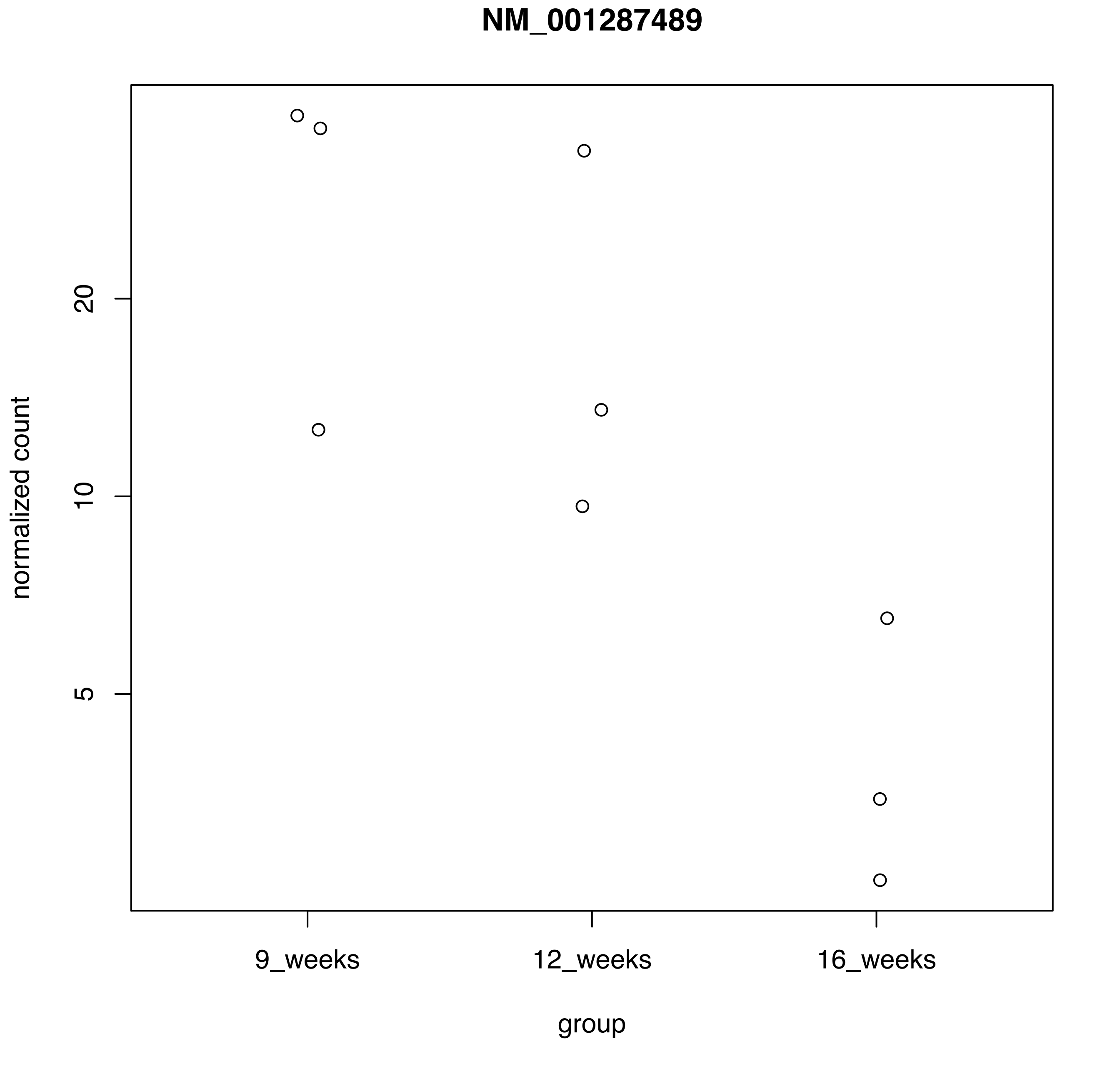
NM_001287489
normalized count
20
10
5
9_weeks
12_weeks
16_weeks
group

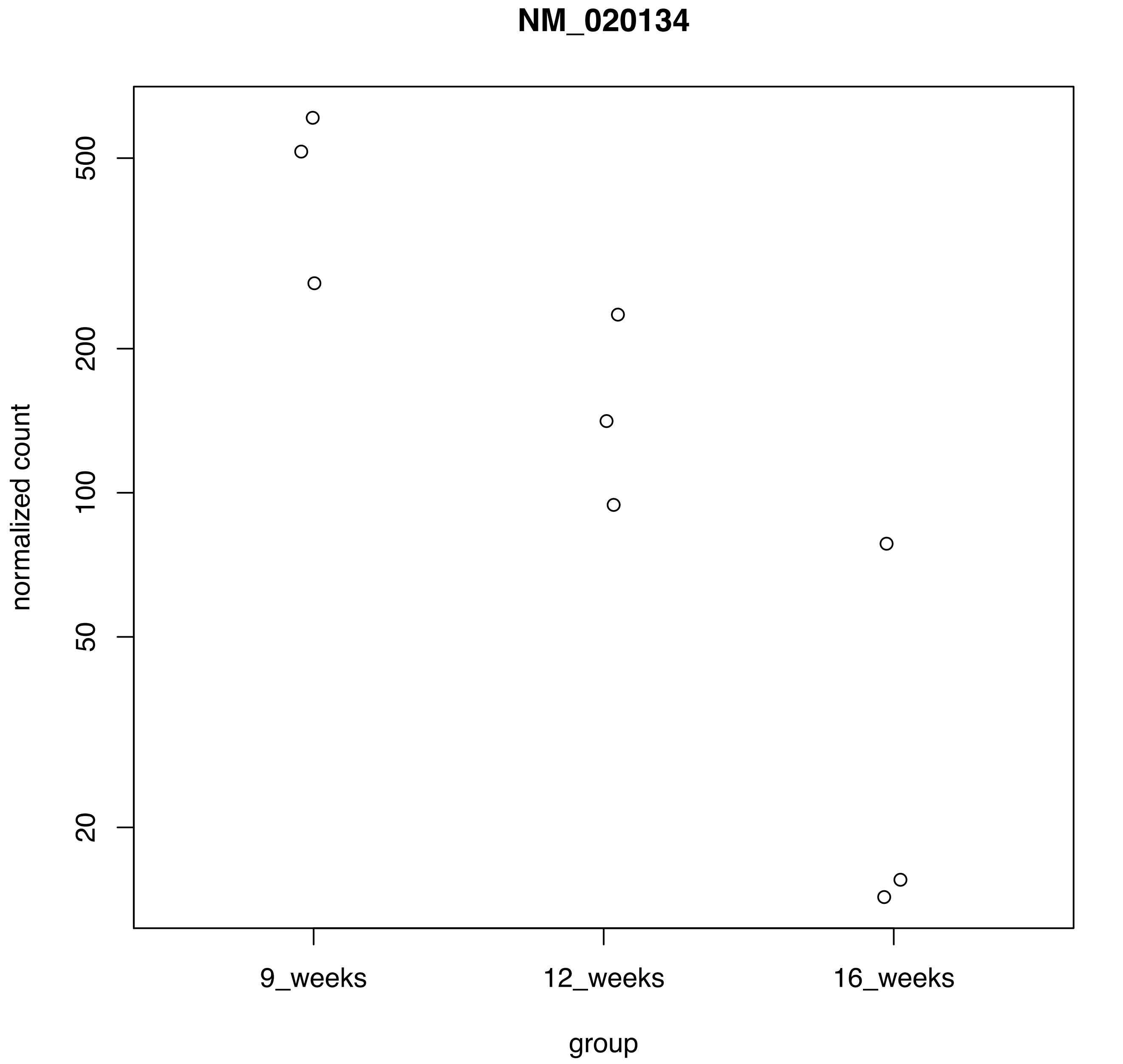

NM_020134
normalized count
500
200
100
50
20
9_weeks
12_weeks
16_weeks
group

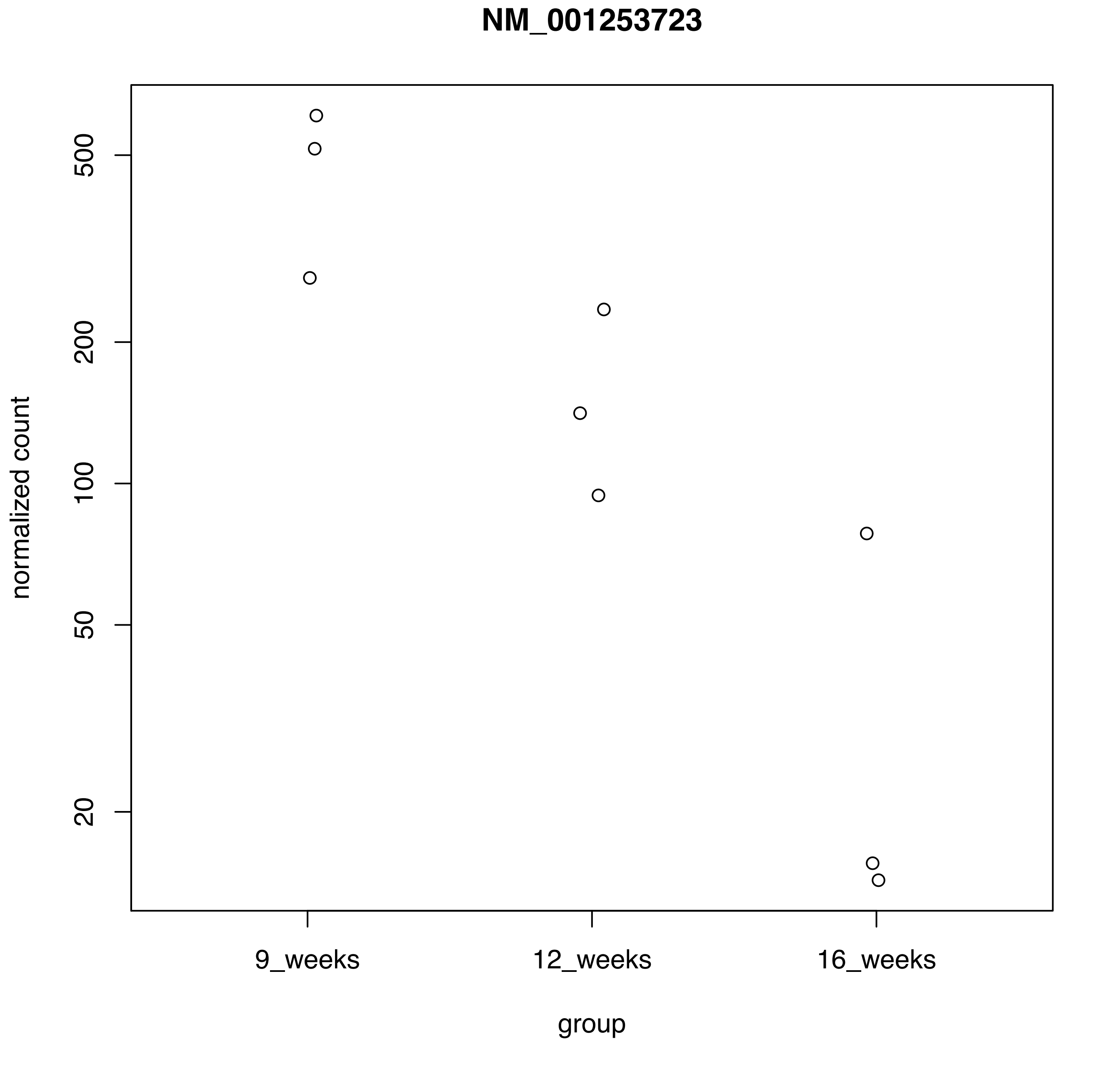

NM_001253723
500
200
100
50
20
normalized count
9_weeks
12_weeks
16_weeks
group

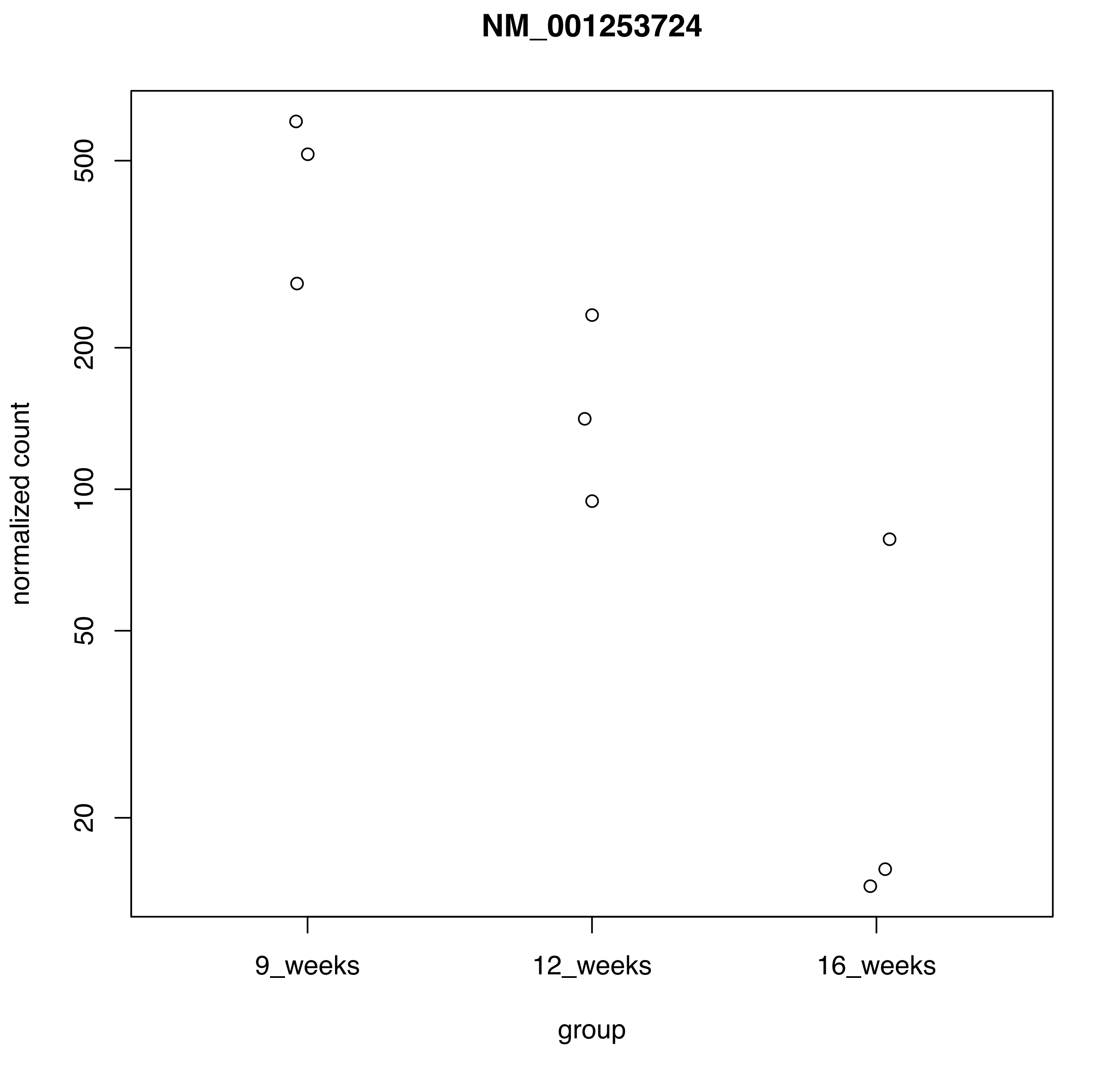

NM_001253724
normalized count
500
200
100
50
20
9_weeks
12_weeks
16_weeks
group

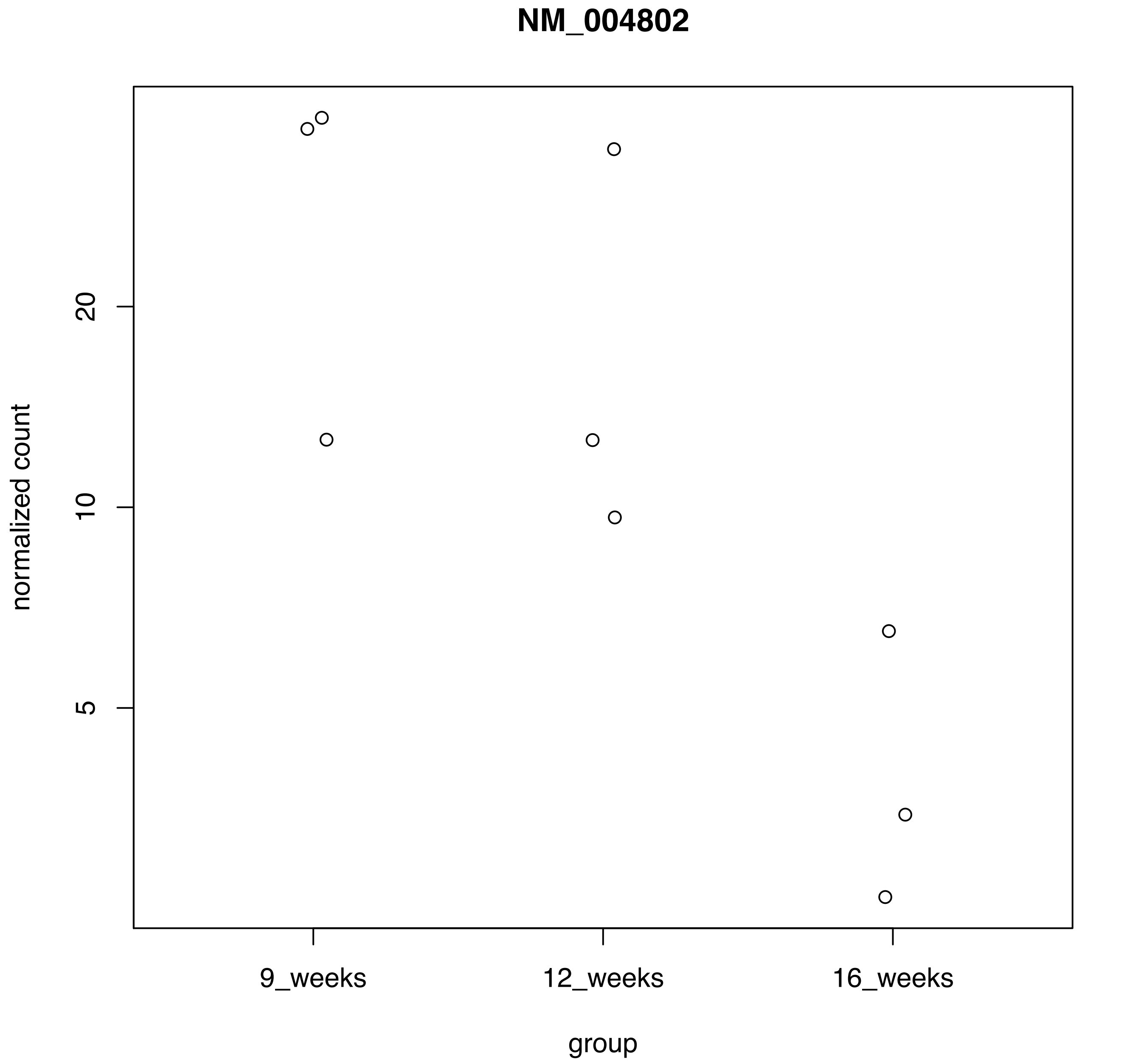
NM_004802
normalized count
20
10
5
9_weeks
12_weeks
16_weeks
group

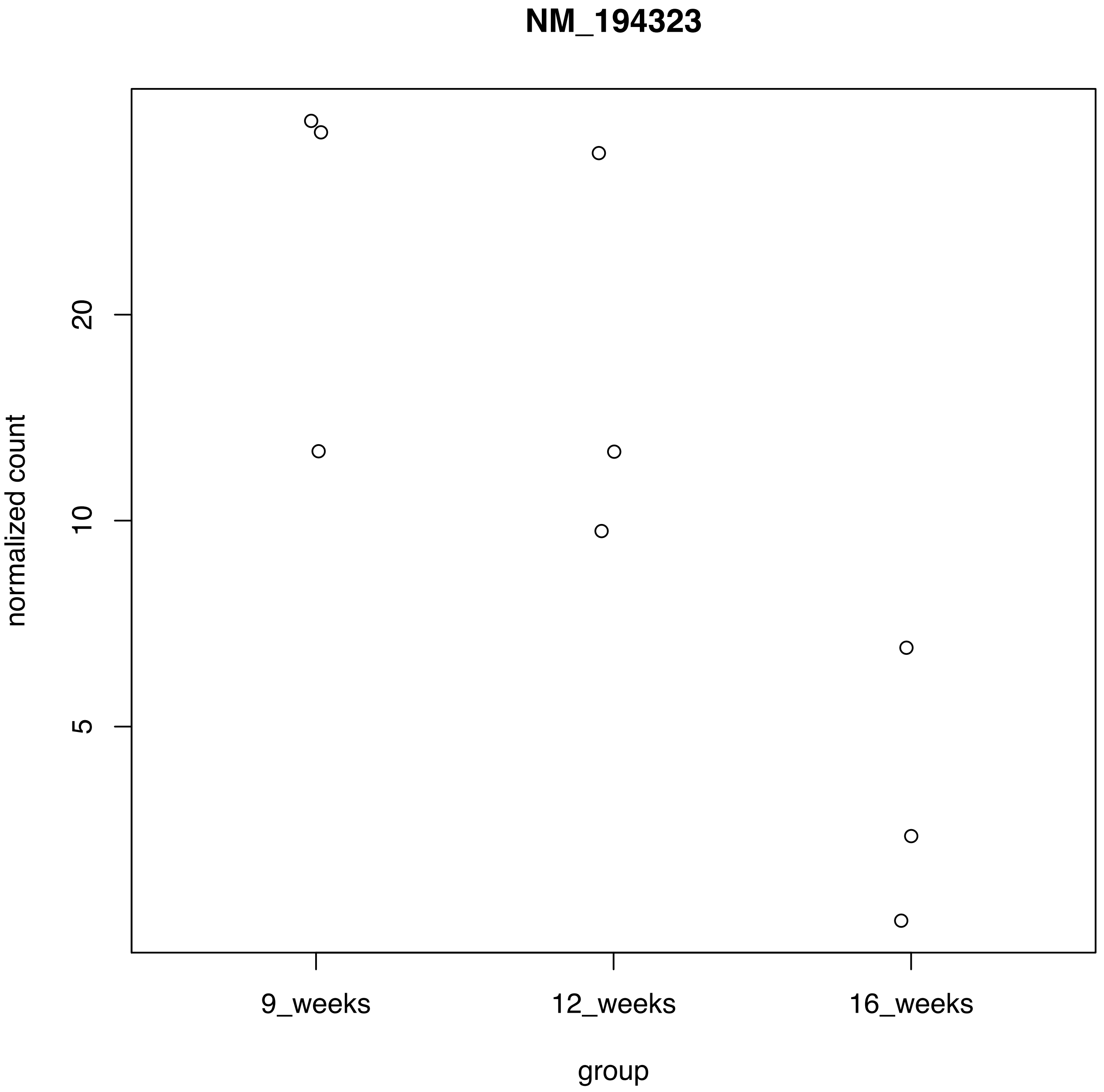
NM_194323
normalized count
20
10
5
9_weeks
12_weeks
16_weeks
group

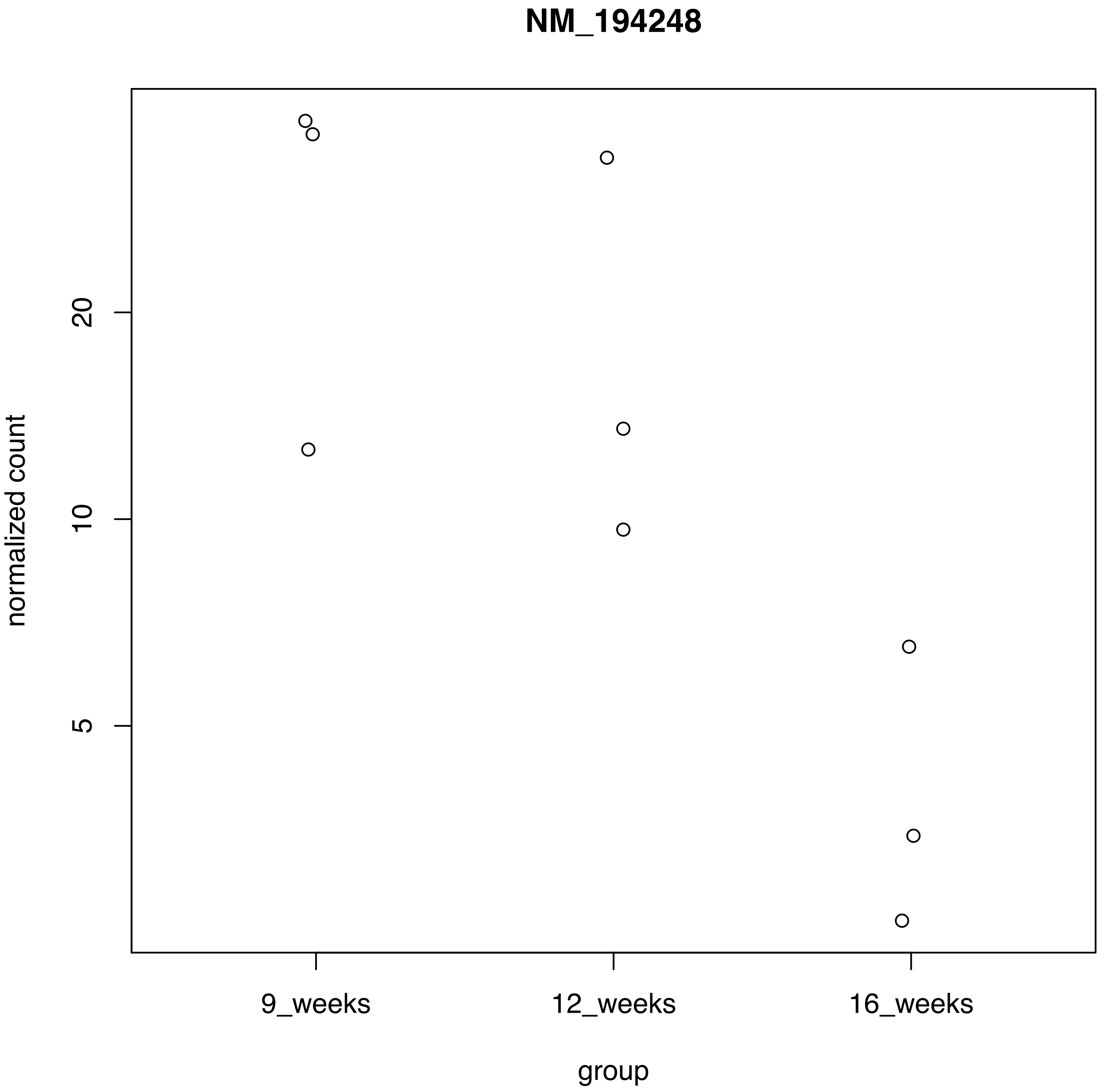

NM_194248
normalized count
20
10
5
9_weeks
12_weeks
16_weeks
group

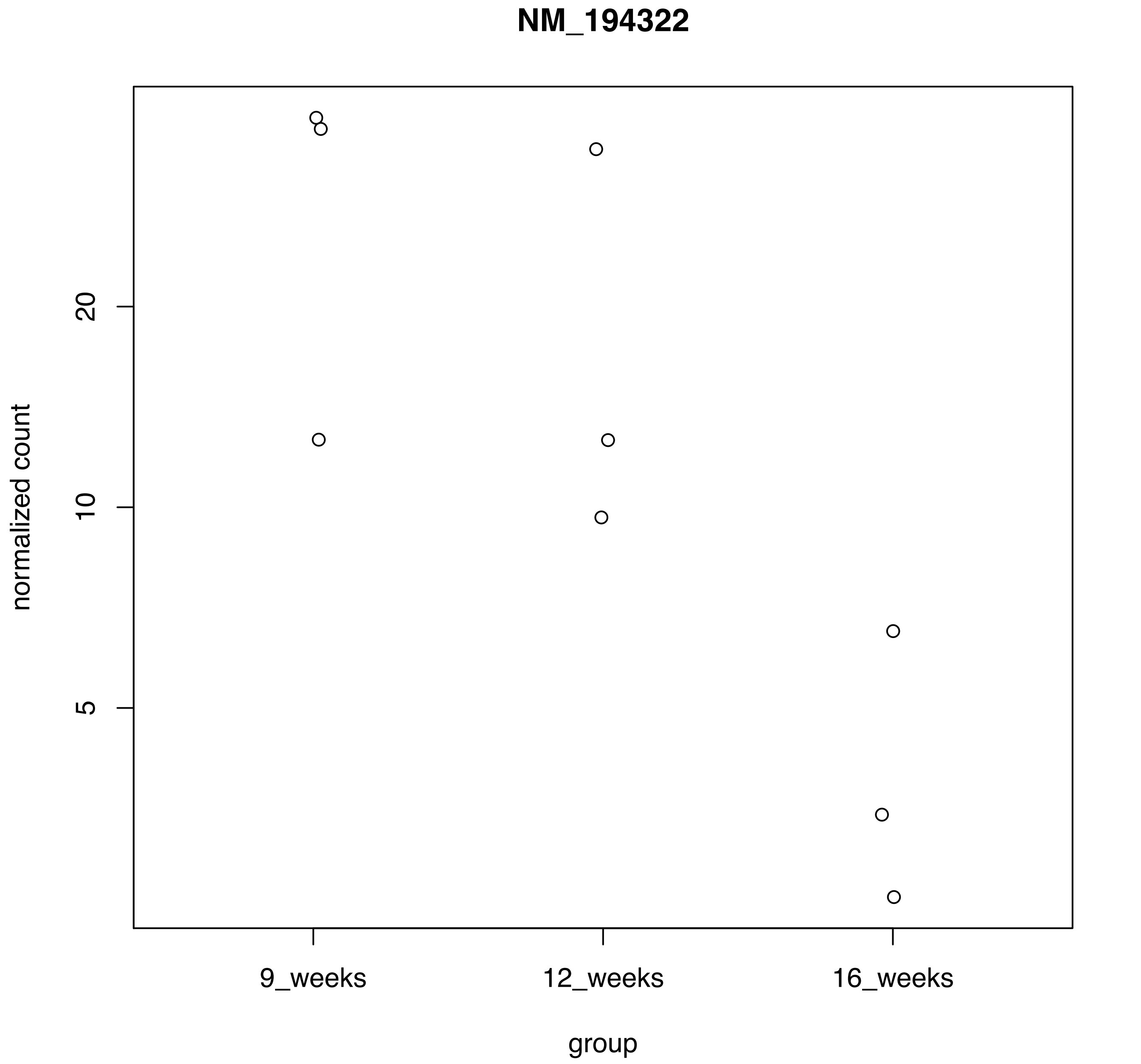

NM_194322
normalized count
20
10
5
9_weeks
12_weeks
16_weeks
group

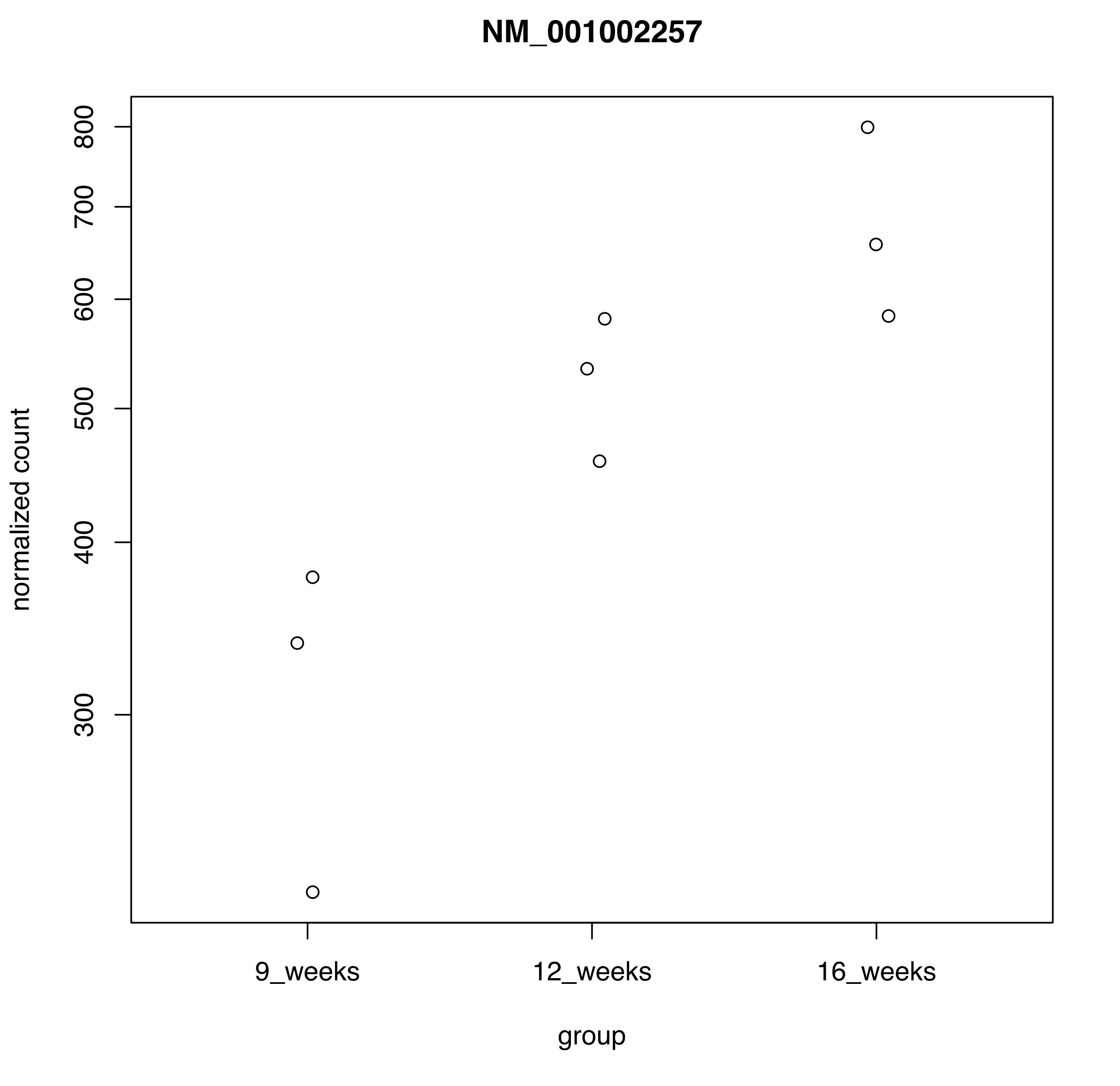
NM_001002257
normalized count
800
700
600
500
400
300
9_weeks
12_weeks
16_weeks
group

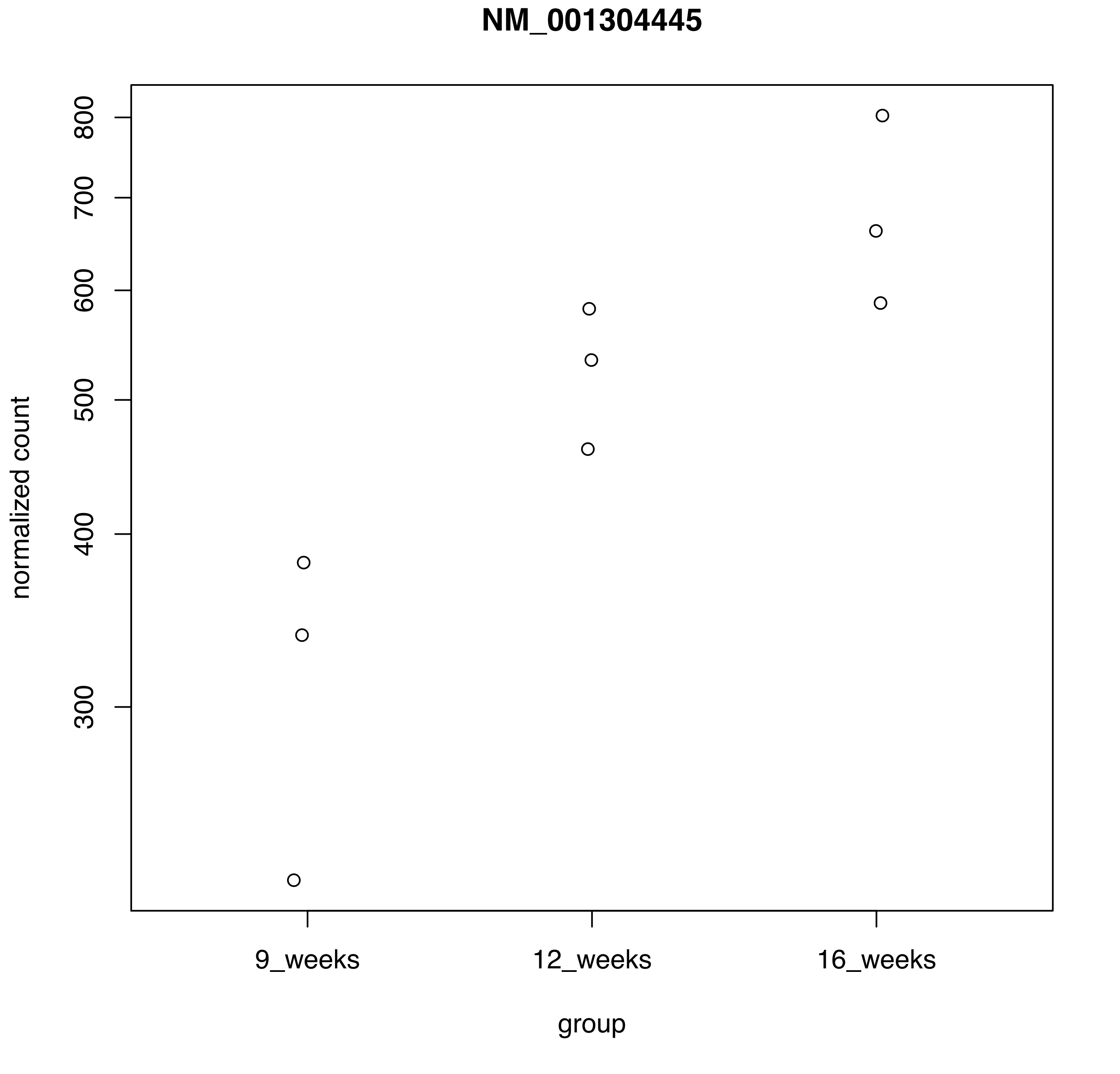
NM_001304445
normalized count
800
700
600
500
400
300
9_weeks
12_weeks
16_weeks
group

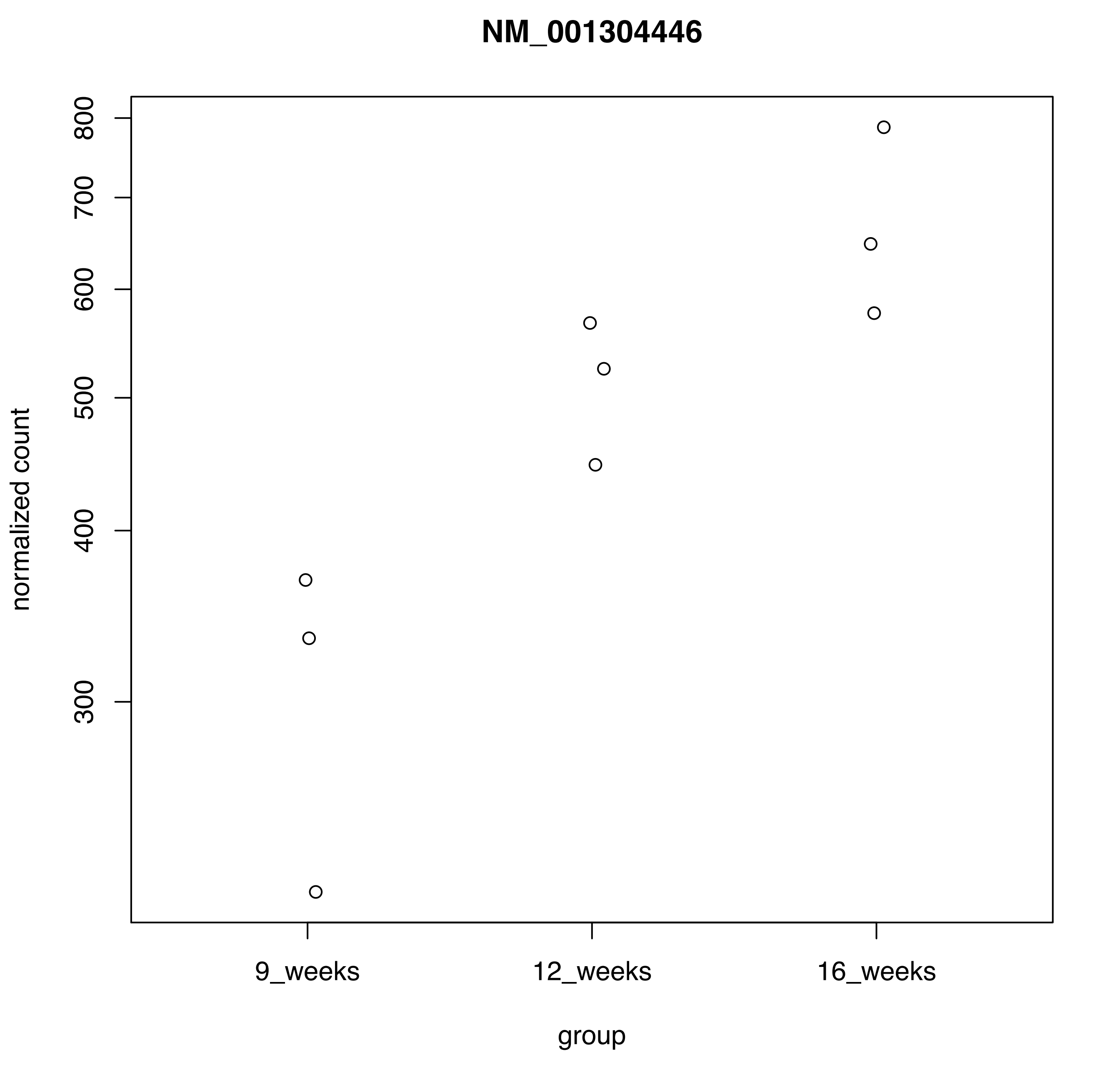
NM_001304446
normalized count
800
700
600
500
400
300
9_weeks
12_weeks
16_weeks
group

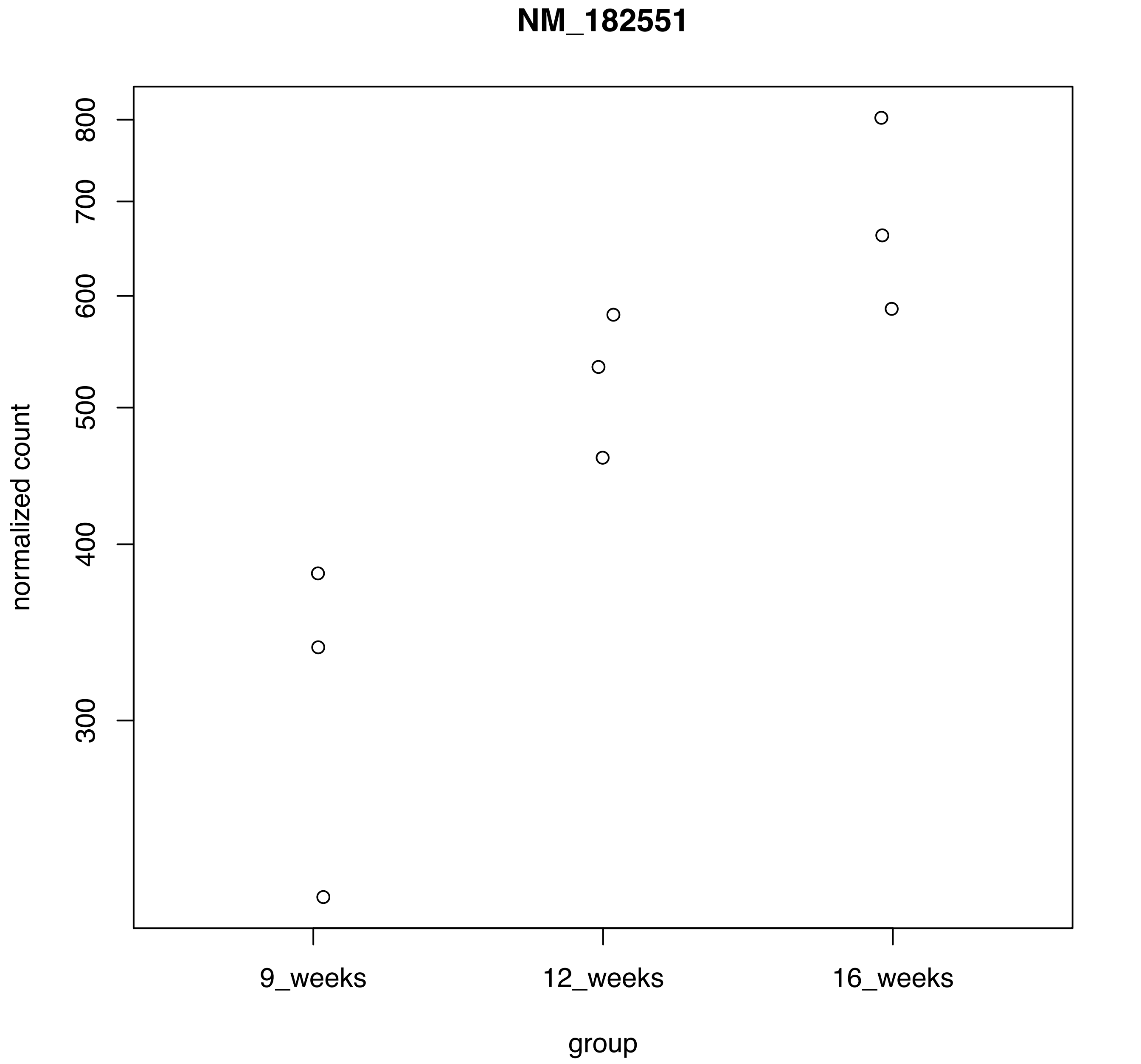

NM_182551
normalized count
800
700
600
500
400
300
9_weeks
12_weeks
16_weeks
group

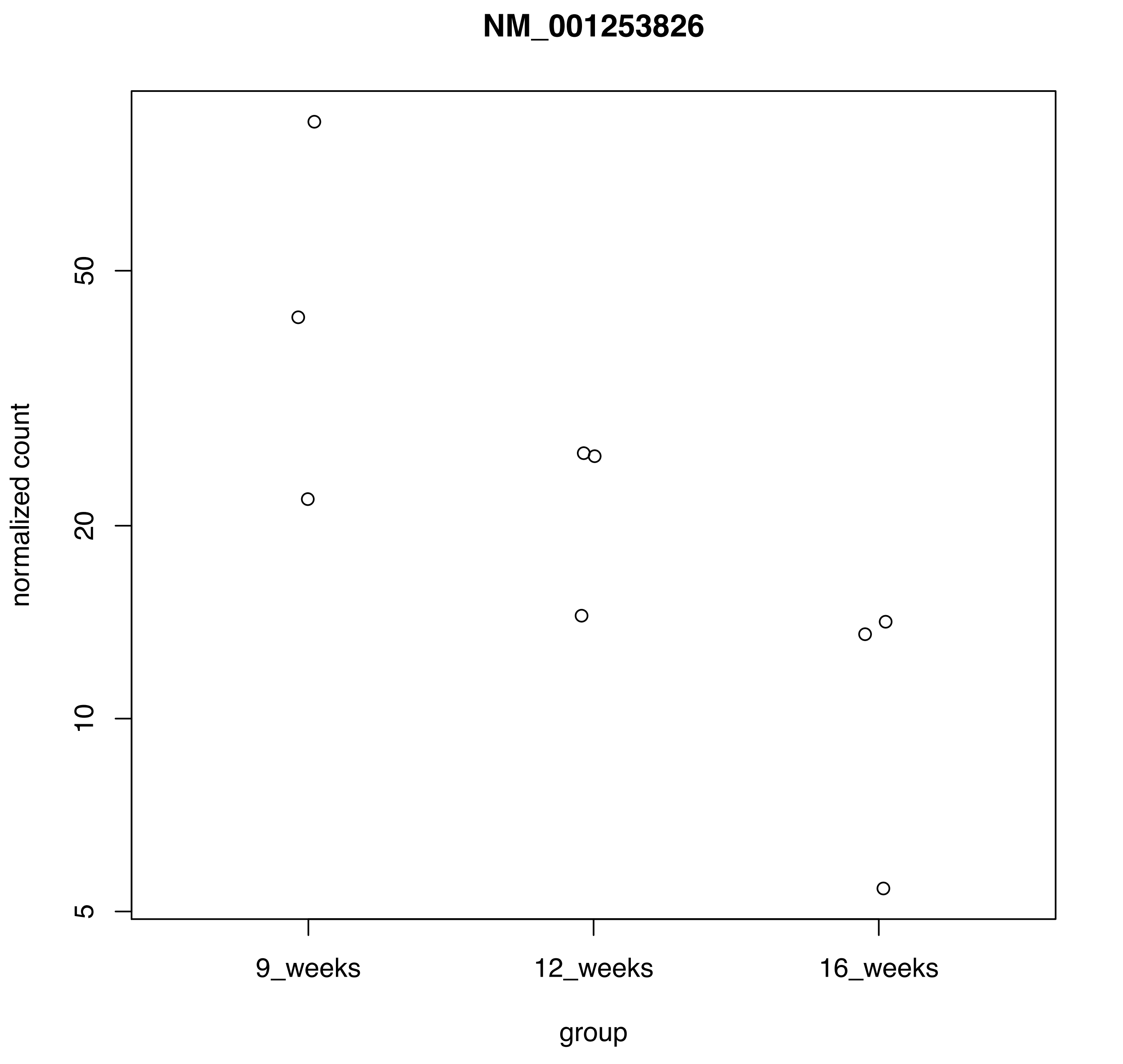
NM_001253826
normalized count
50
20
10
5
9_weeks
12_weeks
16_weeks
group

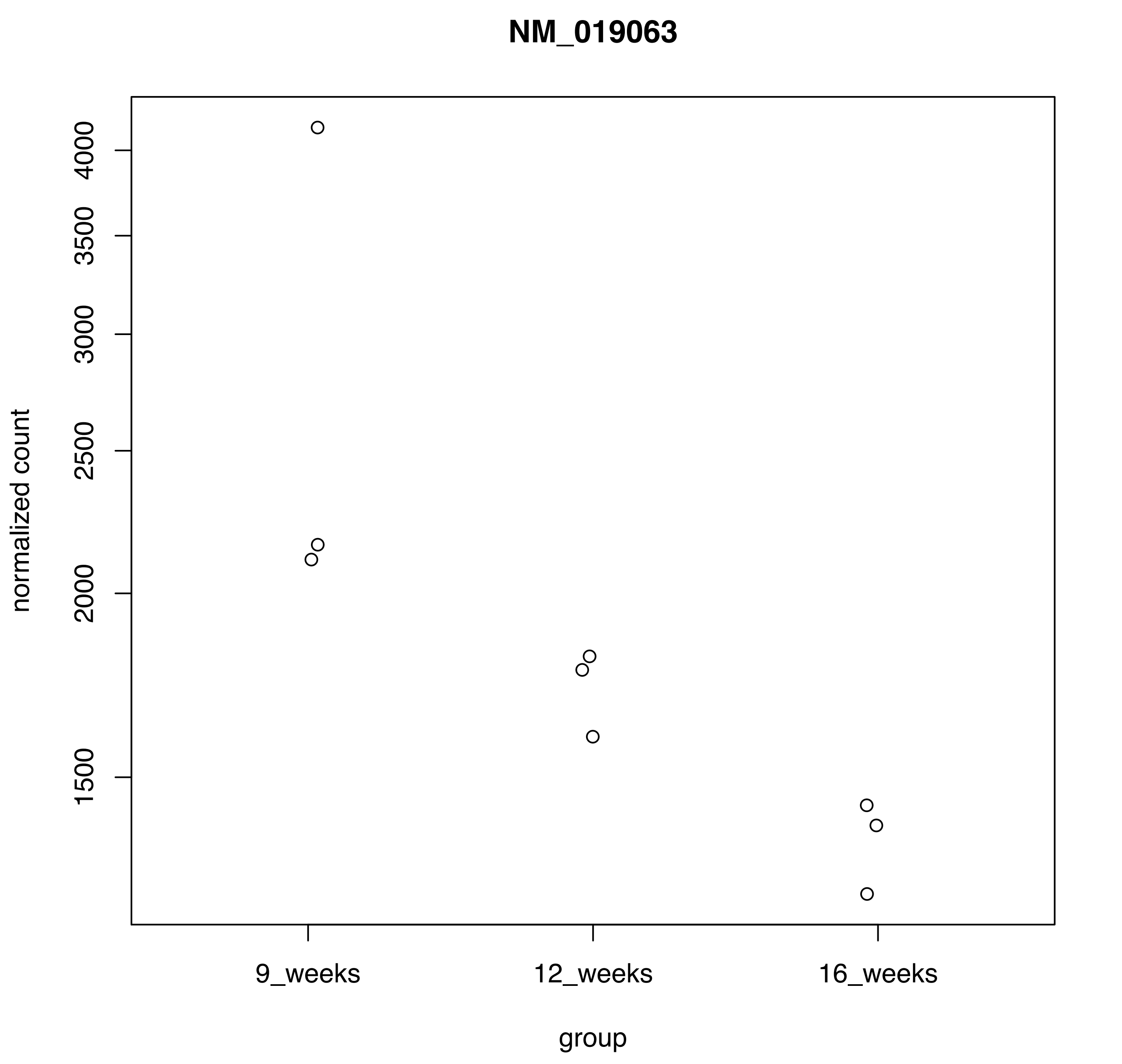

NM_019063
normalized count
4000
3500
3000
2500
2000
1500
9_weeks
12_weeks
16_weeks
group

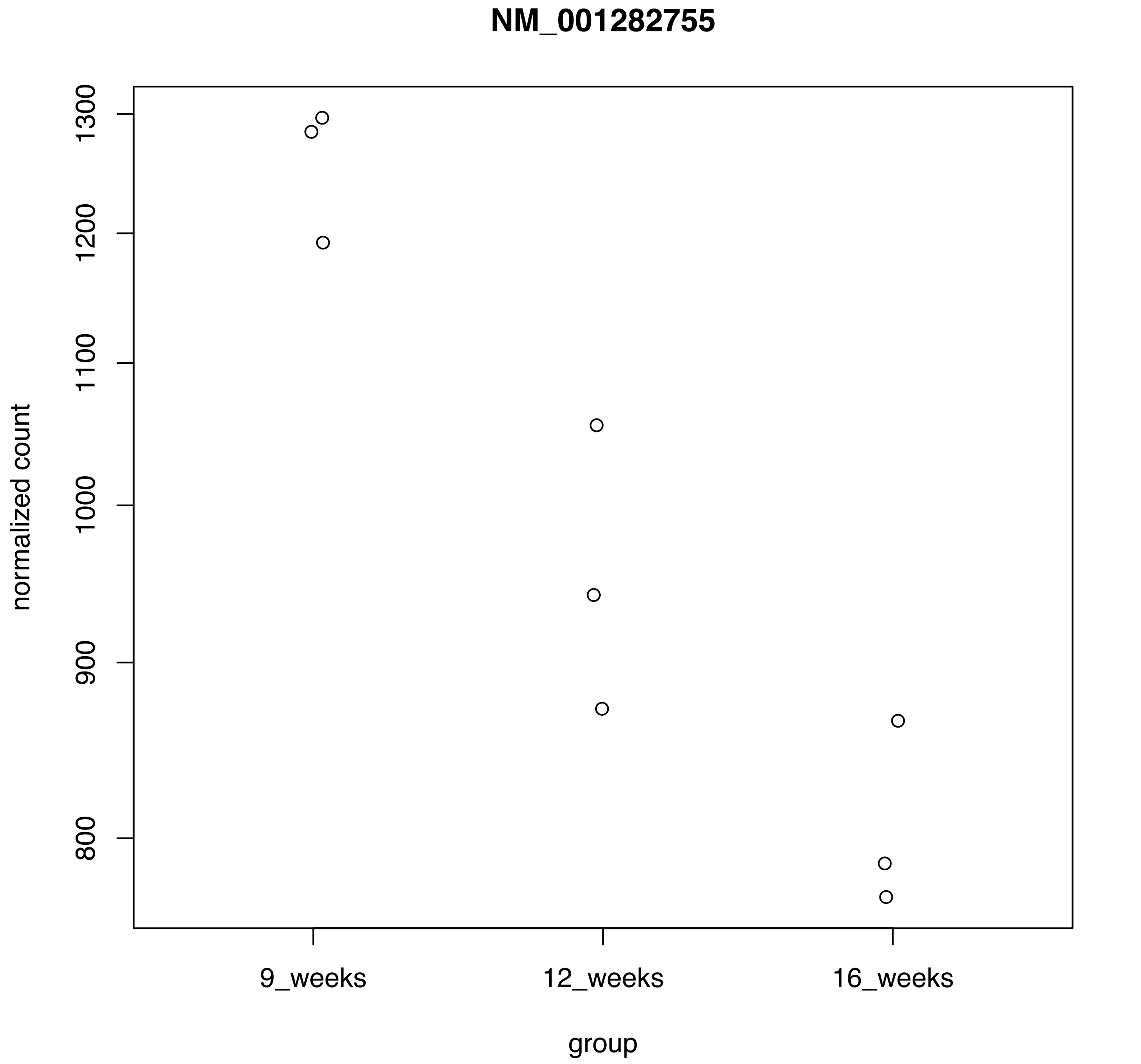
NM_001282755
normalized count
1300
1200
1100
1000
900
800
9_weeks
12_weeks
16_weeks
group

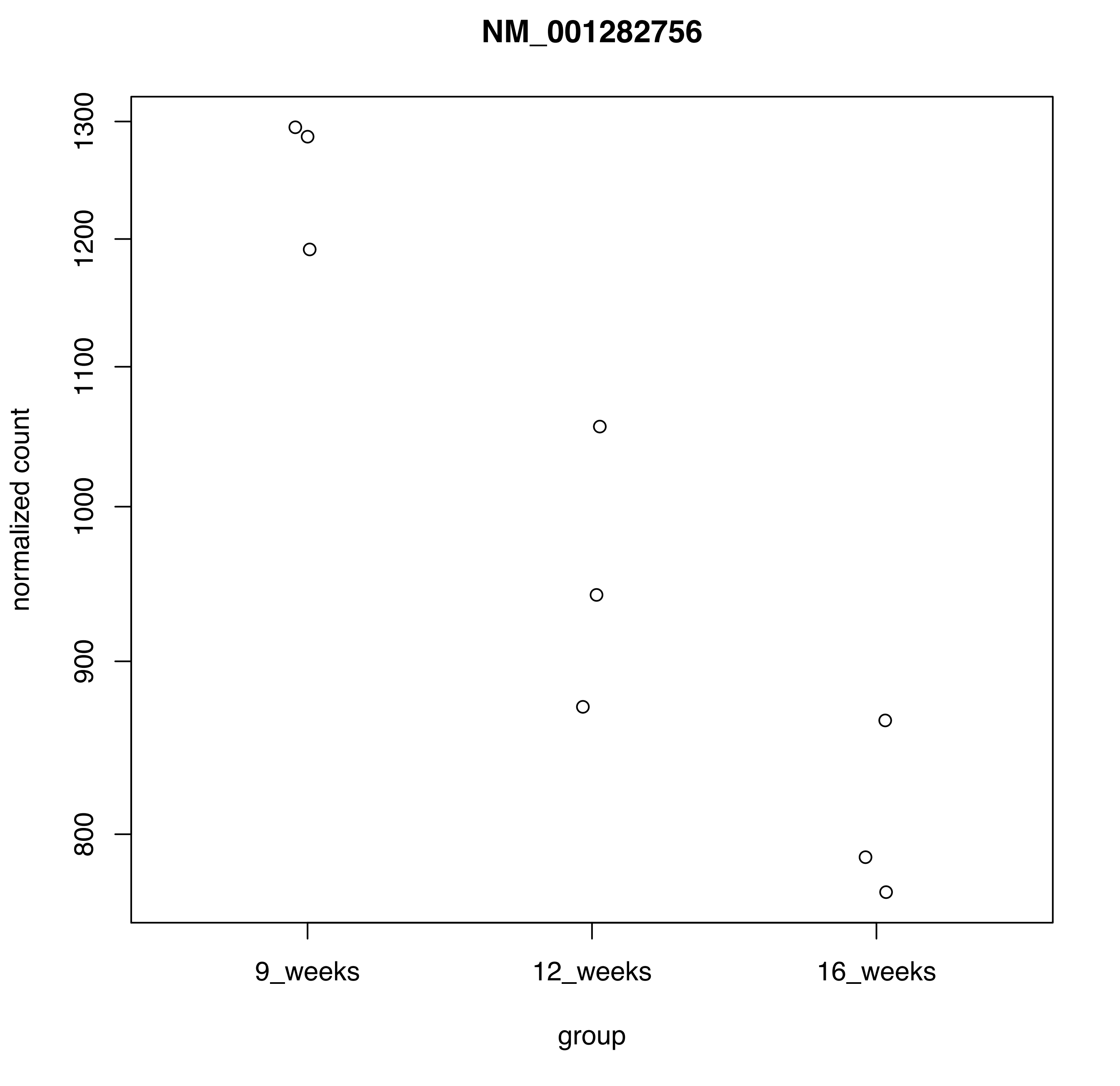
NM_001282756
normalized count
1300
1200
1100
1000
900
800
9_weeks
12_weeks
16_weeks
group

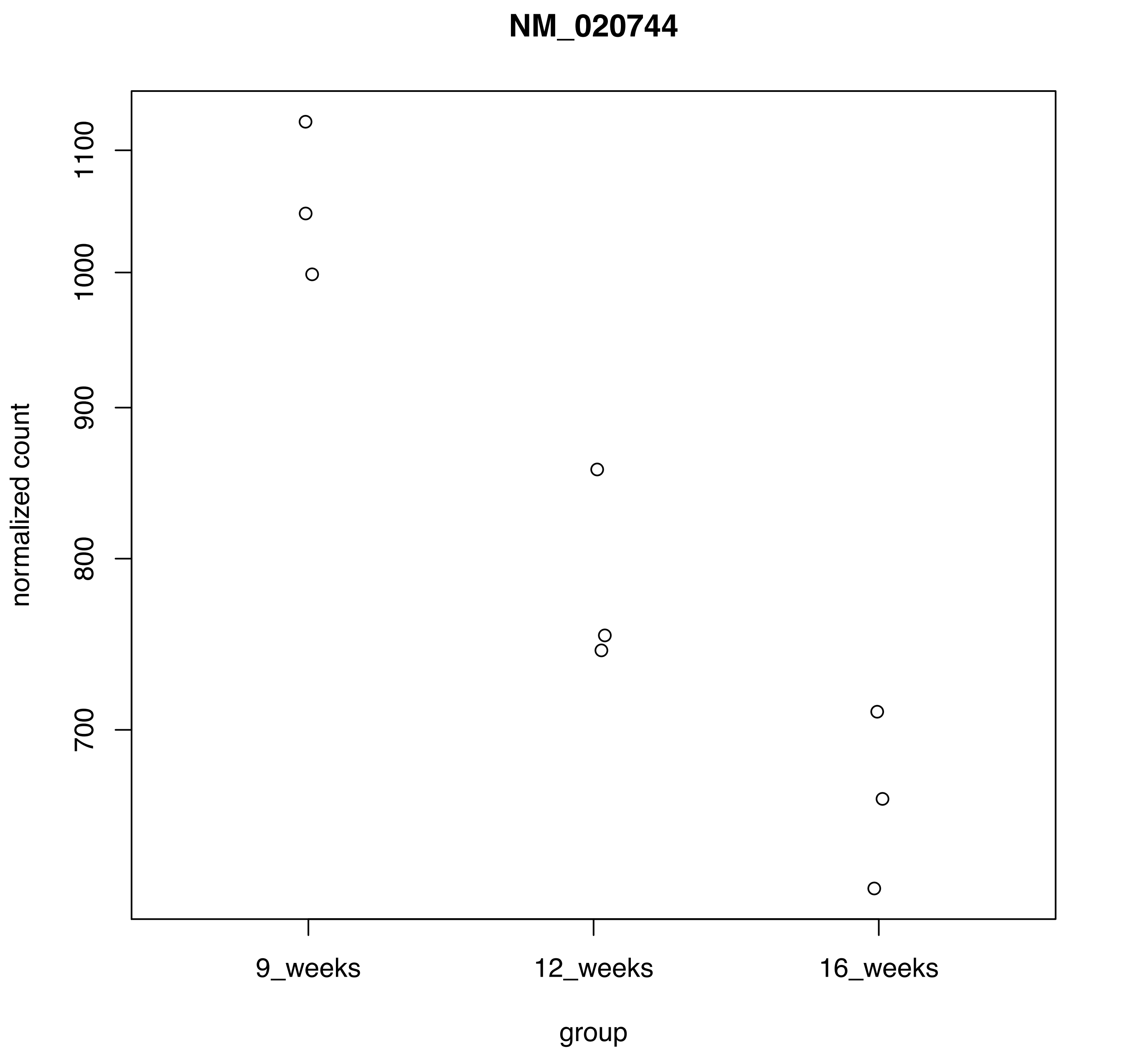

NM_020744
normalized count
1100
1000
900
800
700
9_weeks
12_weeks
16_weeks
group

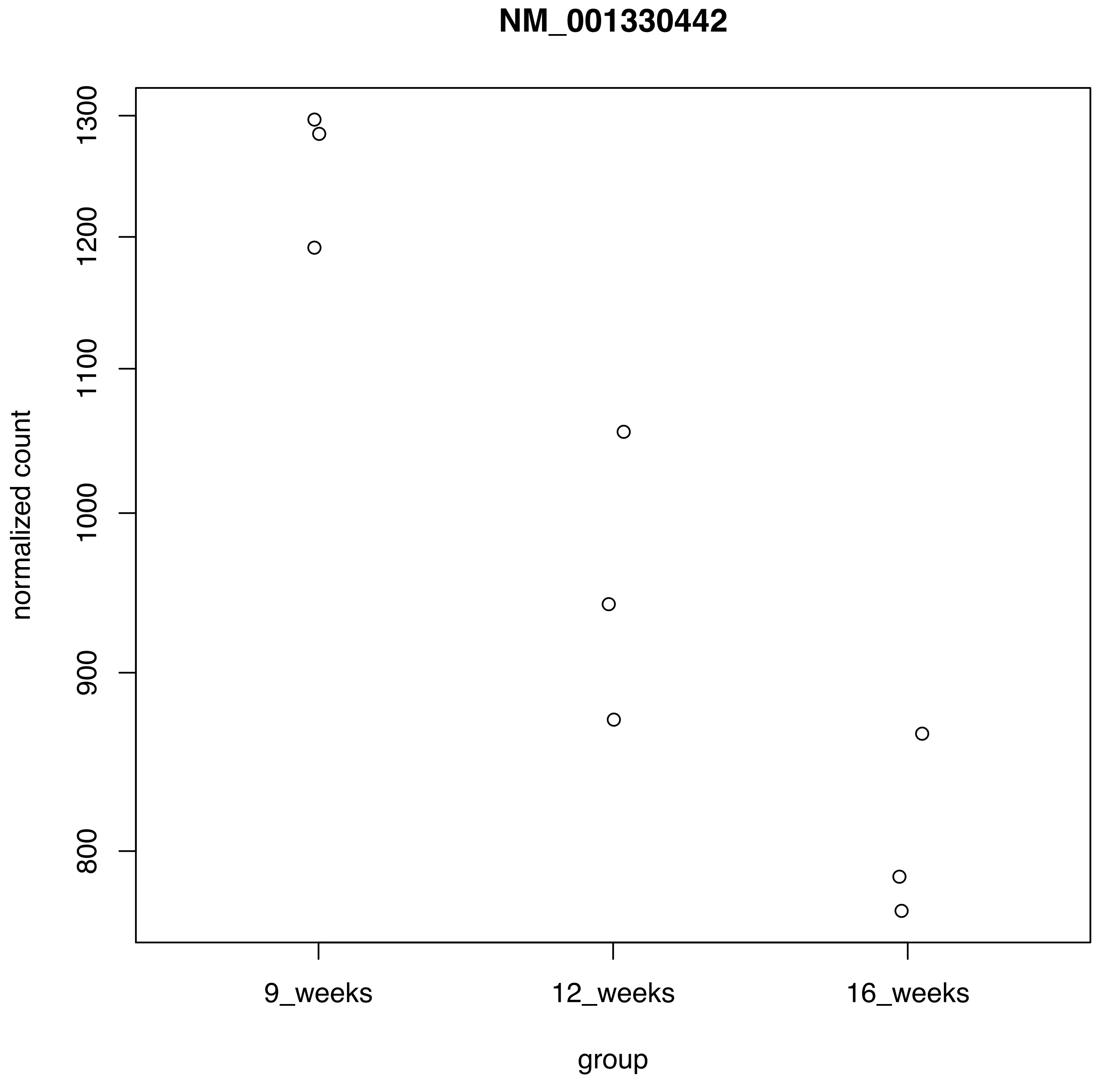
NM_001330442
normalized count
1300
1200
1100
1000
900
800
9_weeks
12_weeks
16_weeks
group

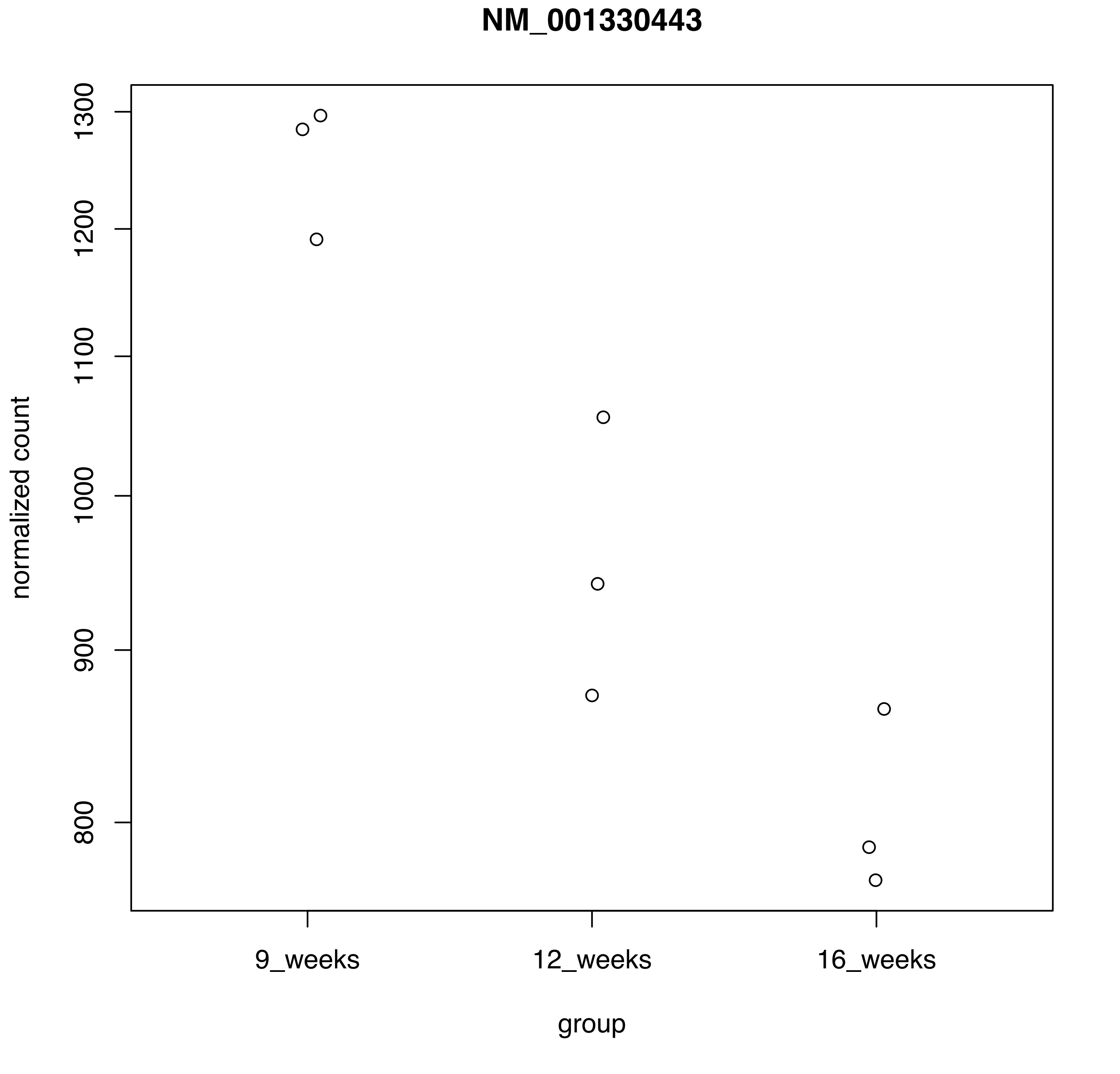

NM_001330443
normalized count
1300
1200
1100
1000
900
800
9_weeks
12_weeks
16_weeks
group

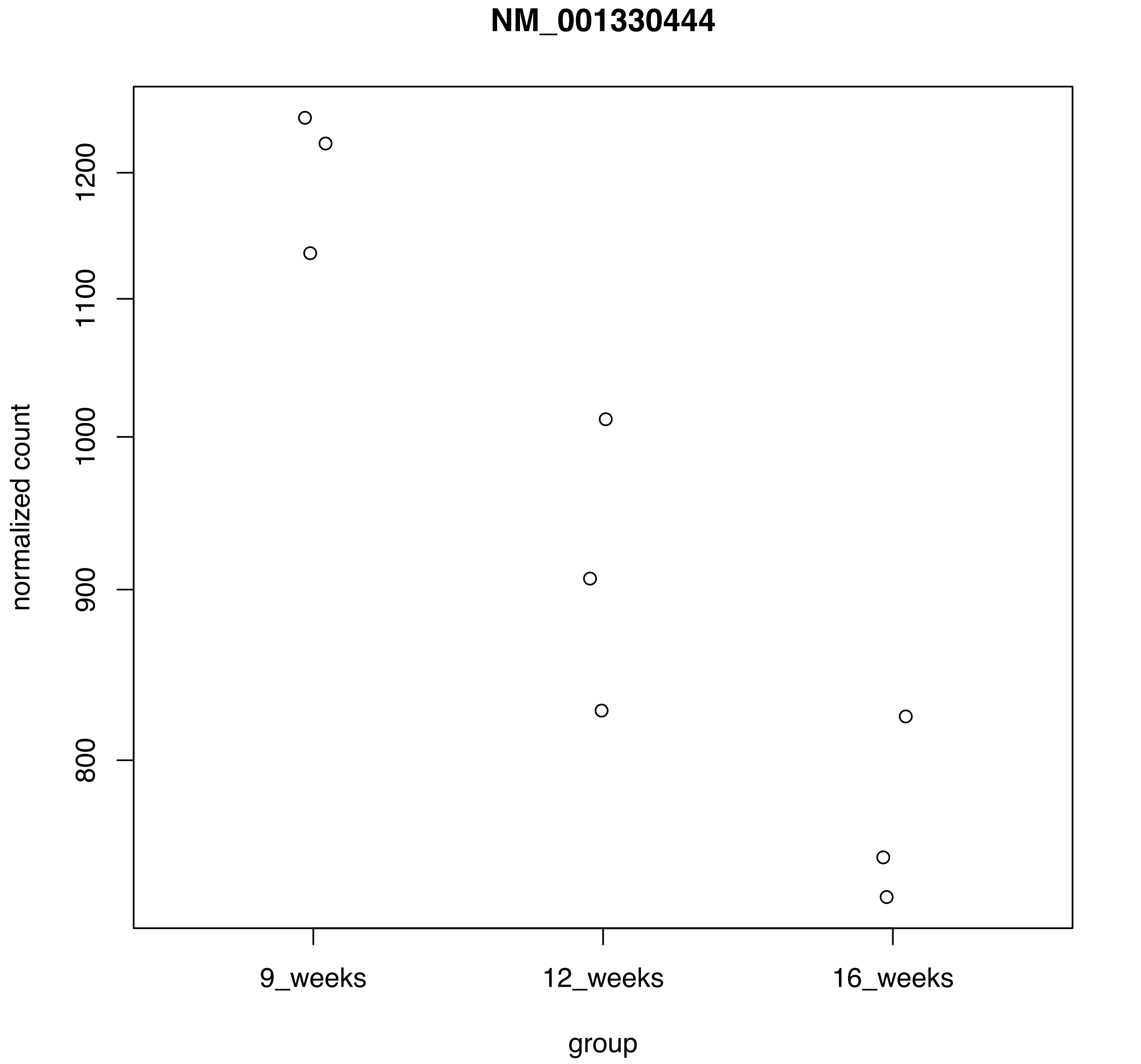
NM_001330444
normalized count
1200
1100
1000
900
800
9_weeks
12_weeks
16_weeks
group

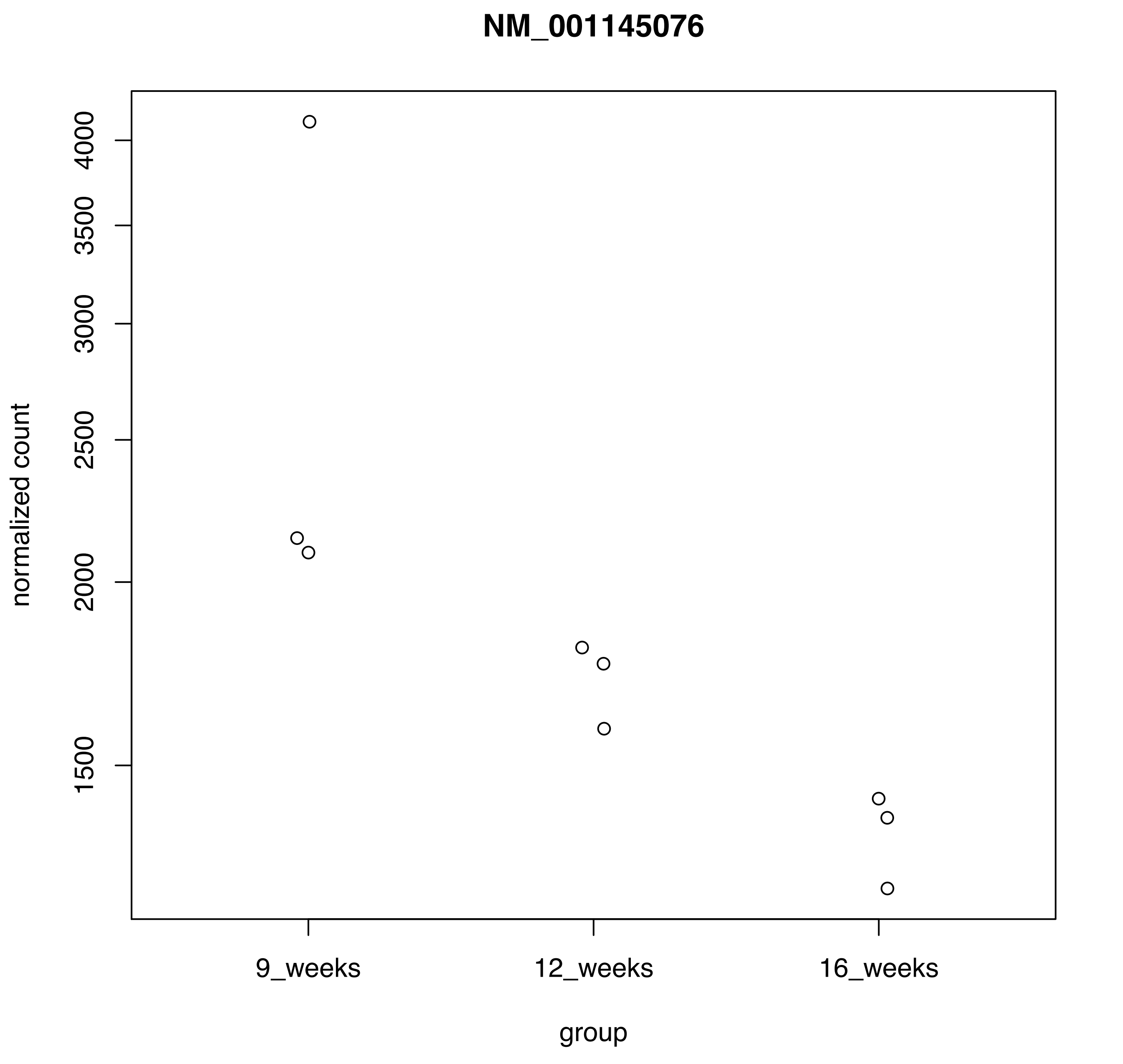

NM_001145076
normalized count
4000
3500
3000
2500
2000
1500
9_weeks
12_weeks
16_weeks
group

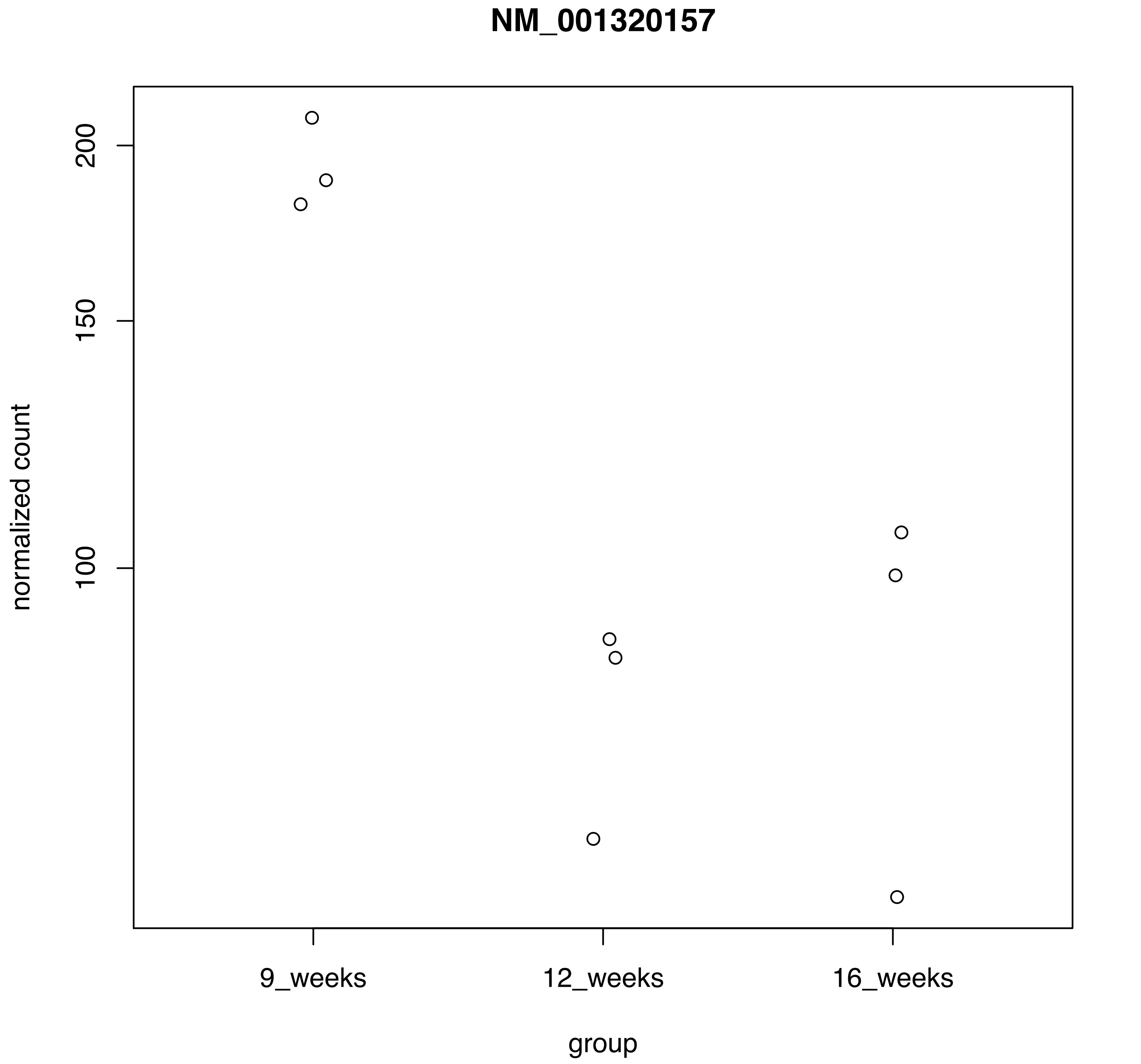
NM_001320157
normalized count
200
150
100
9_weeks
12_weeks
16_weeks
group

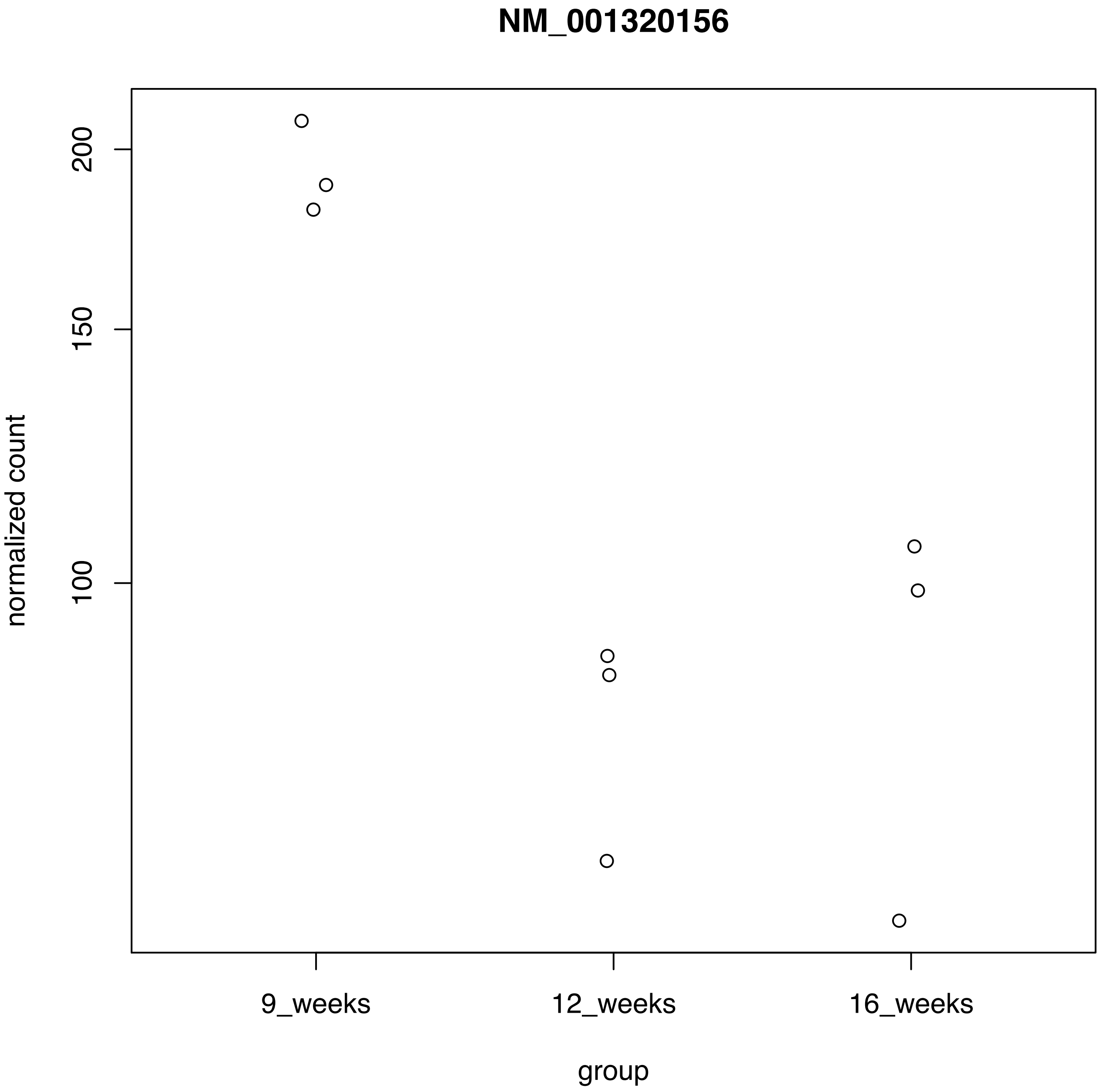
NM_001320156
normalized count
200
150
100
9_weeks
12_weeks
16_weeks
group

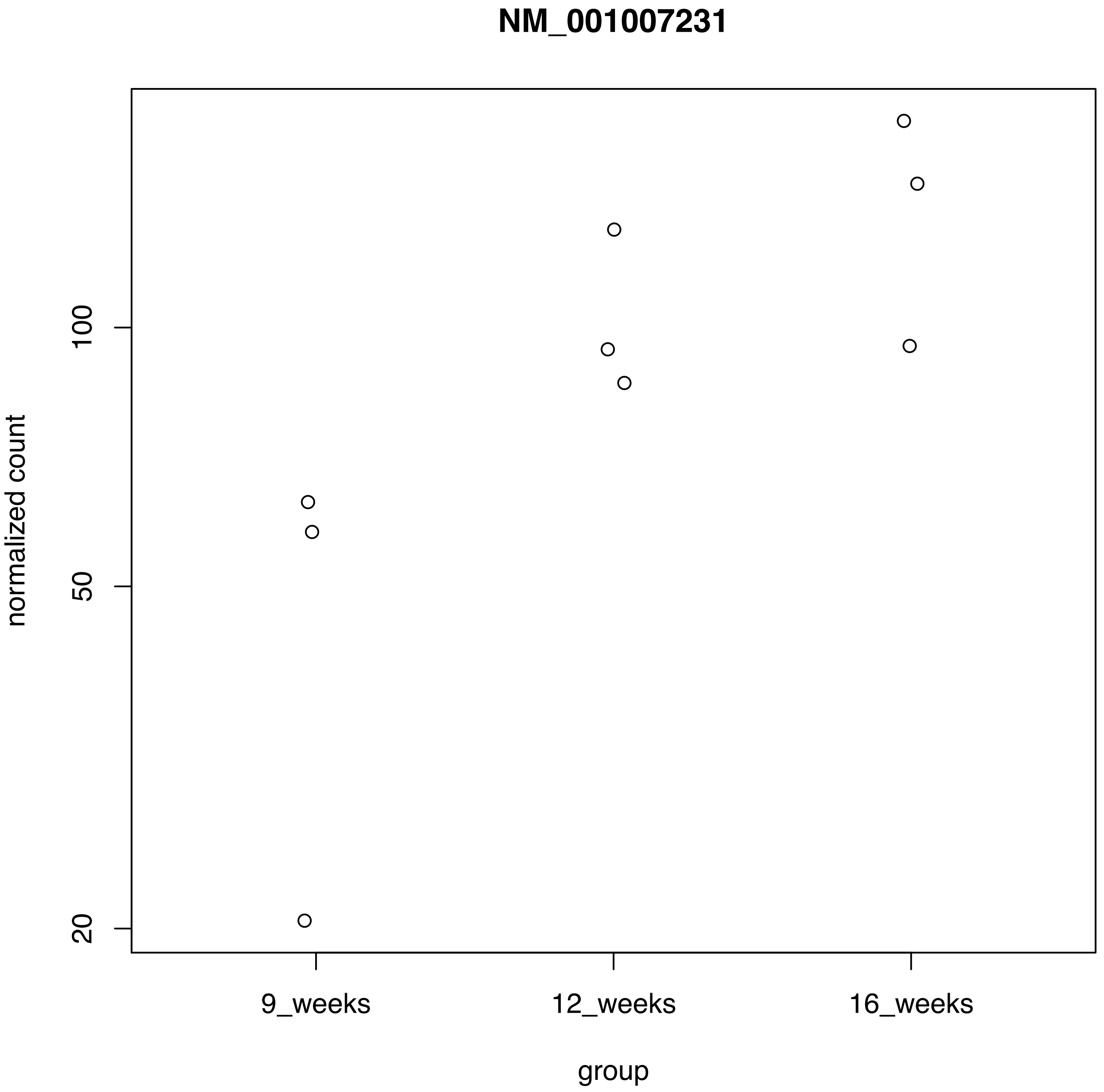
NM_001007231
normalized count
100
50
20
9_weeks
12_weeks
16_weeks
group

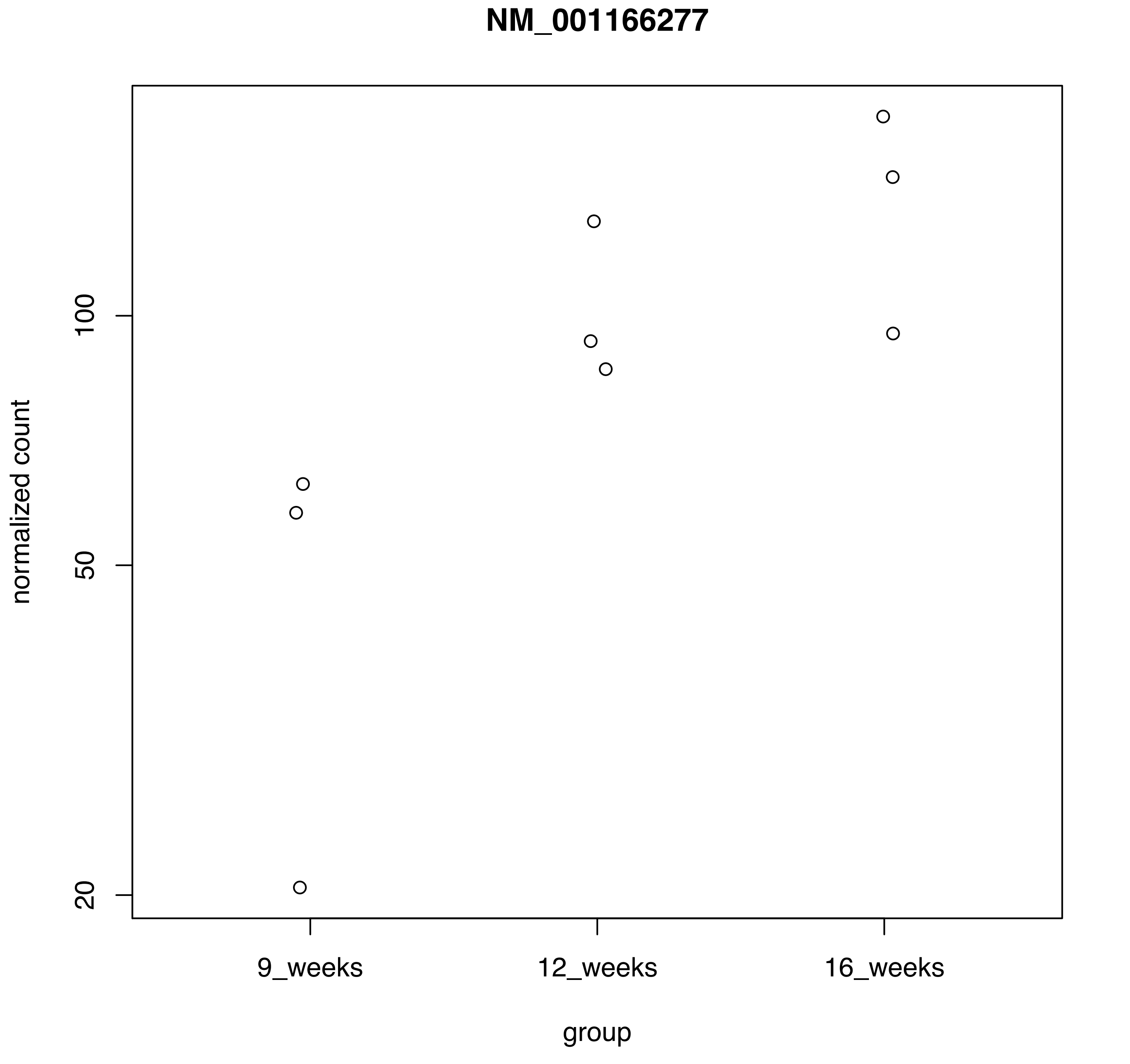

NM_001166277
normalized count
20
50
100
9_weeks
12_weeks
16_weeks
group

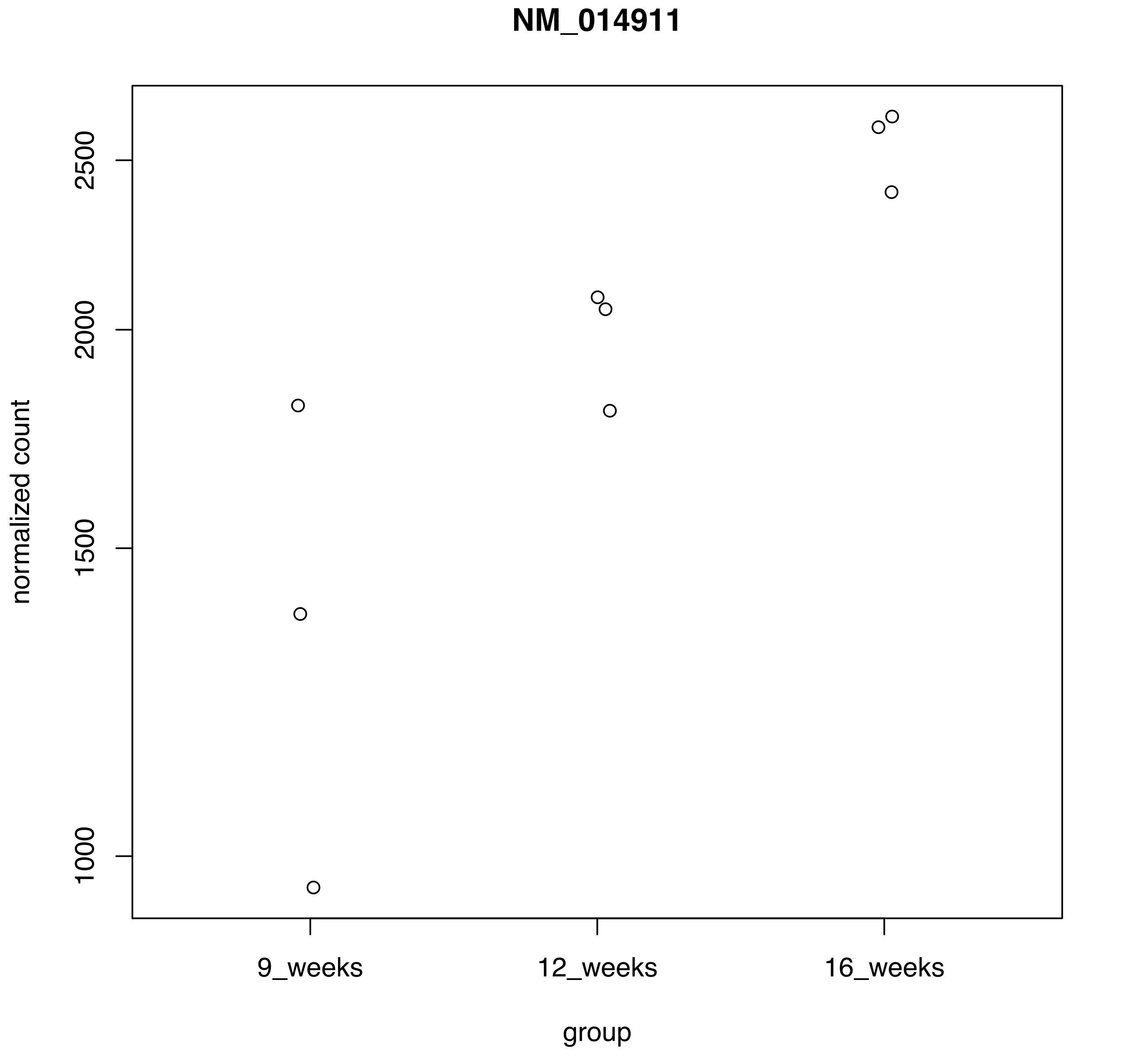

NM_014911
normalized count
1000
1500
2000
2500
9_weeks
12_weeks
16_weeks
group

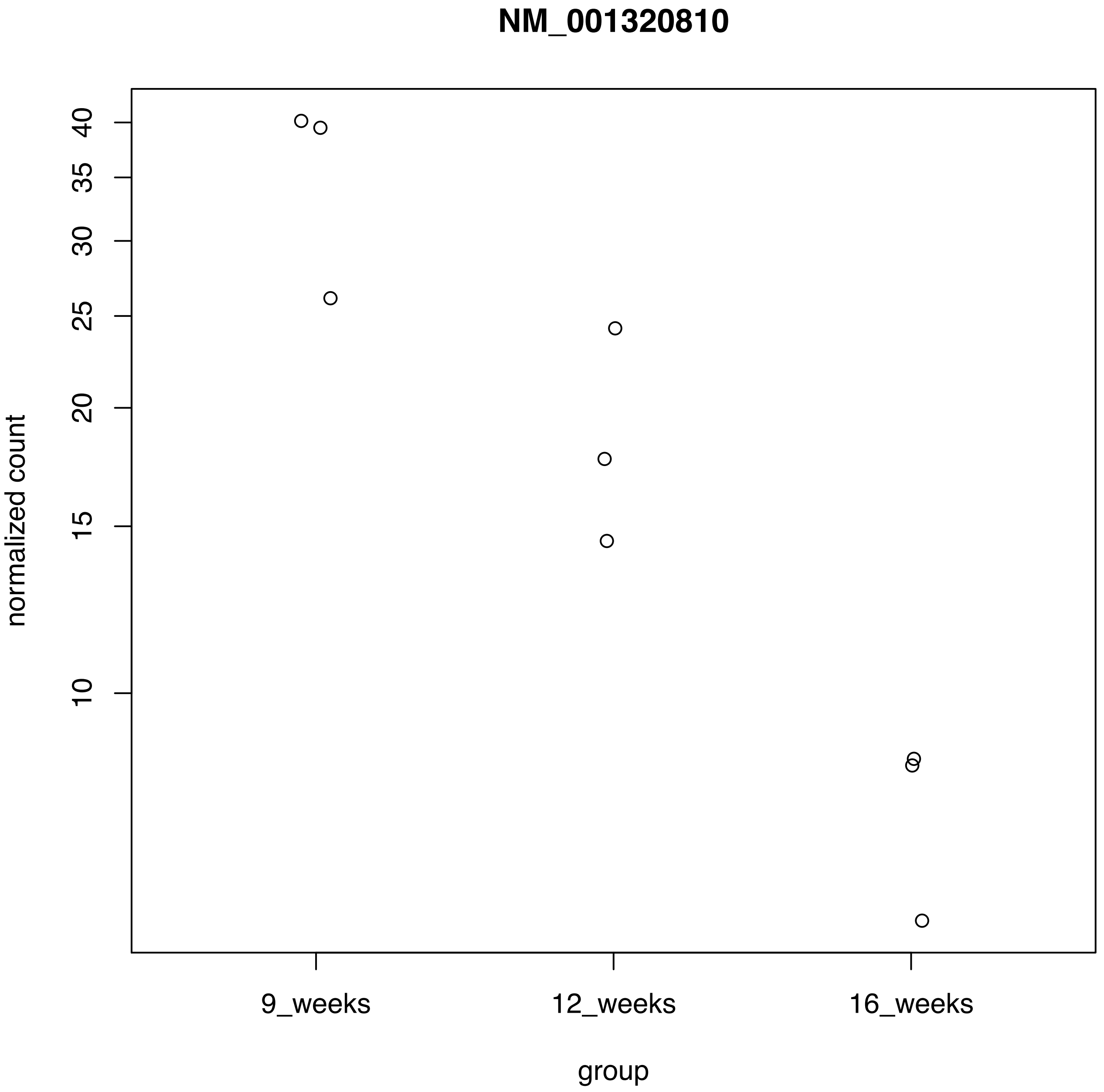
NM_001320810
normalized count
40
35
30
25
20
15
10
9_weeks
12_weeks
16_weeks
group

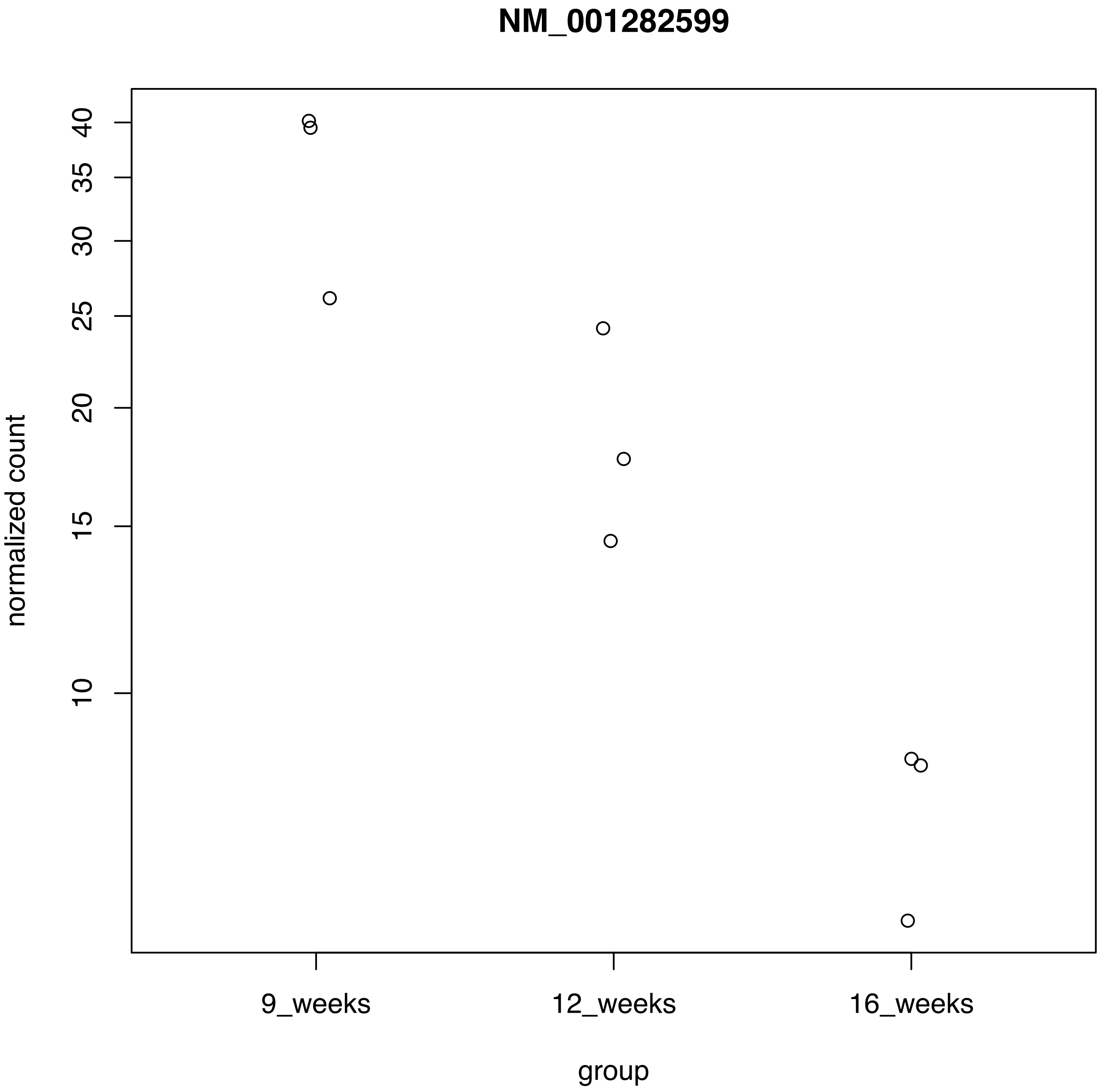

NM_001282599
normalized count
10
15
20
25
30
35
40
9_weeks
12_weeks
16_weeks
group

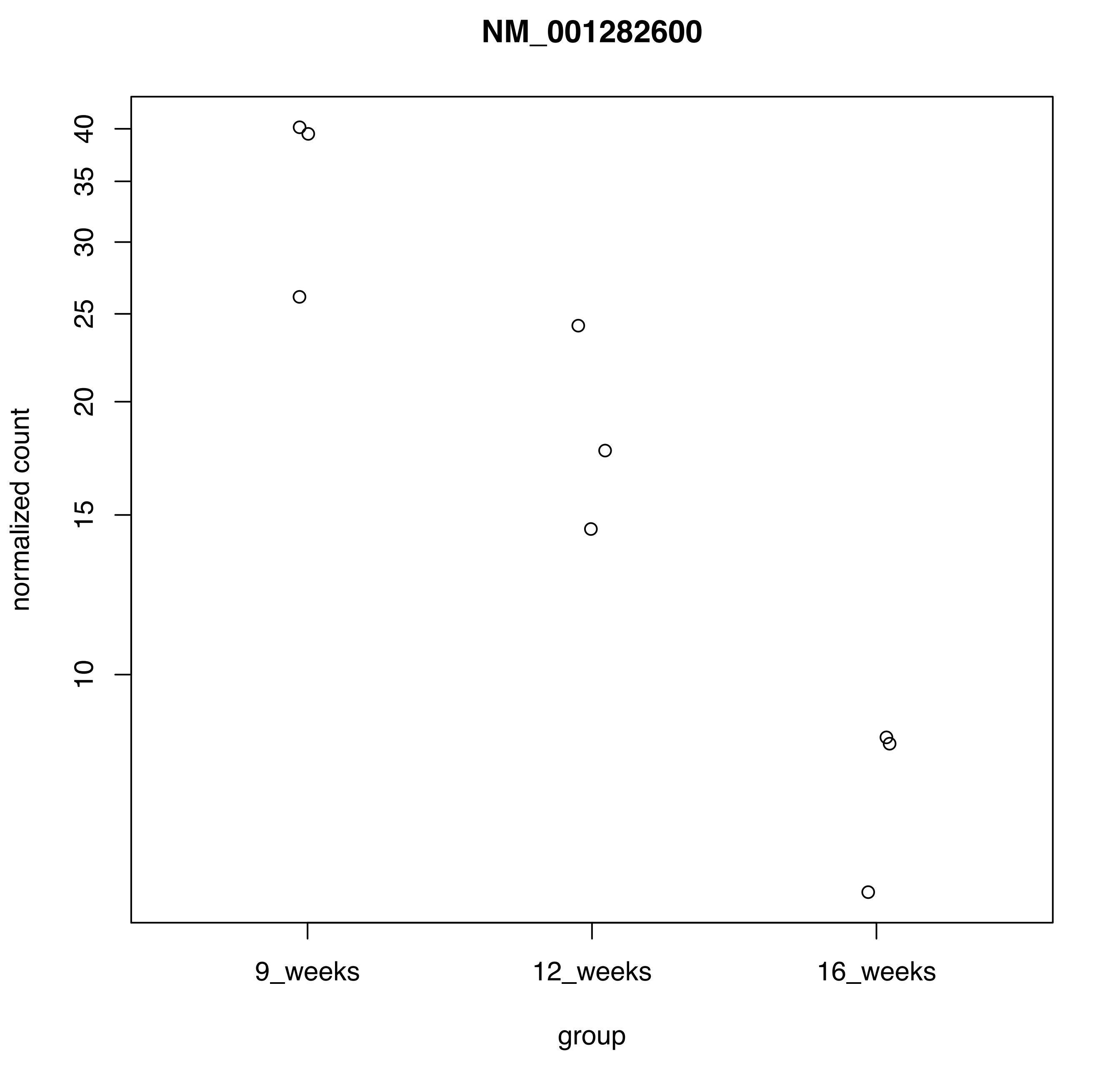

NM_001282600
40
35
30
25
20
15
10
normalized count
9_weeks
12_weeks
16_weeks
group

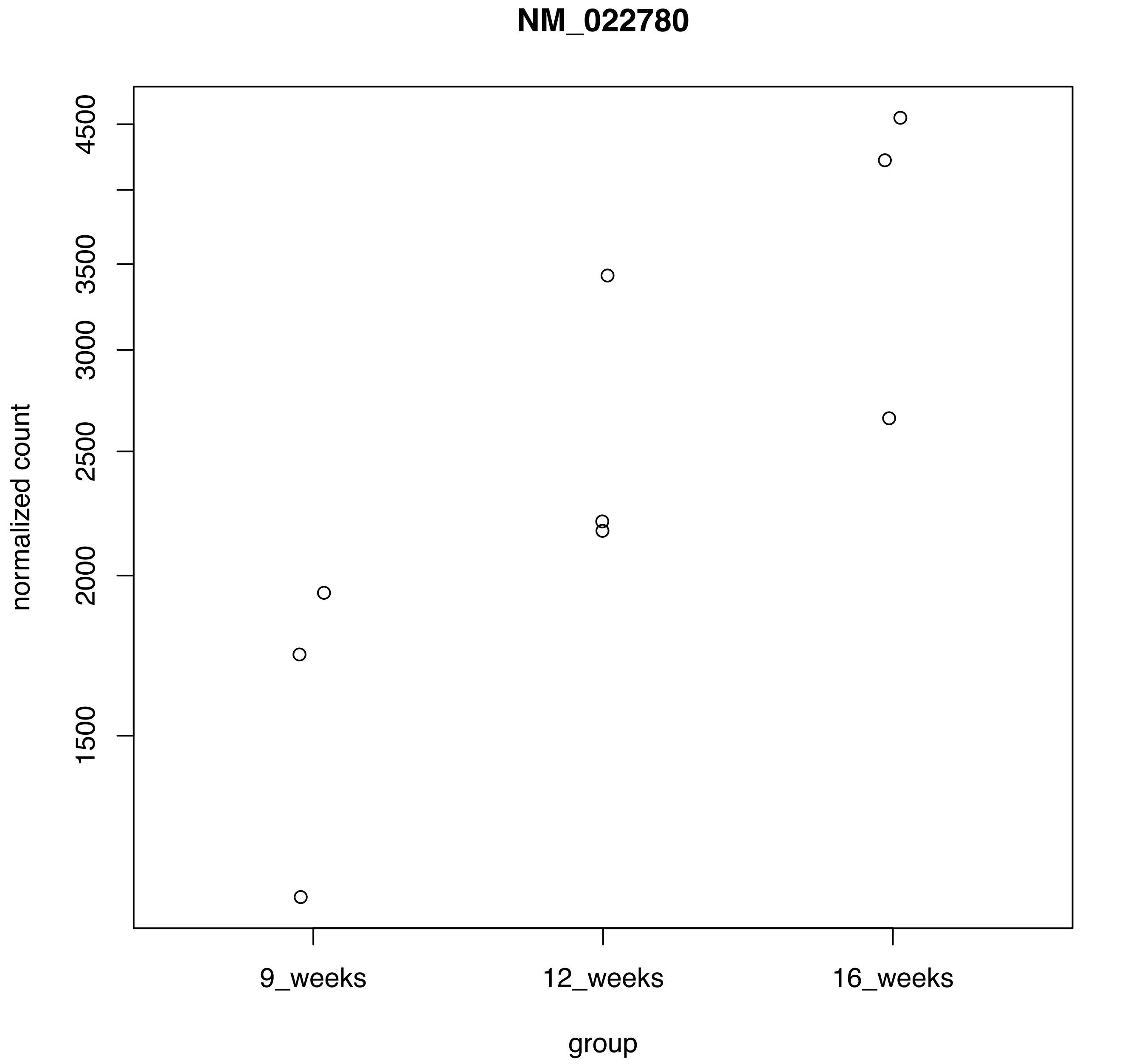
NM_022780
normalized count
1500
2000
2500
3000
3500
4500
9_weeks
12_weeks
16_weeks
group

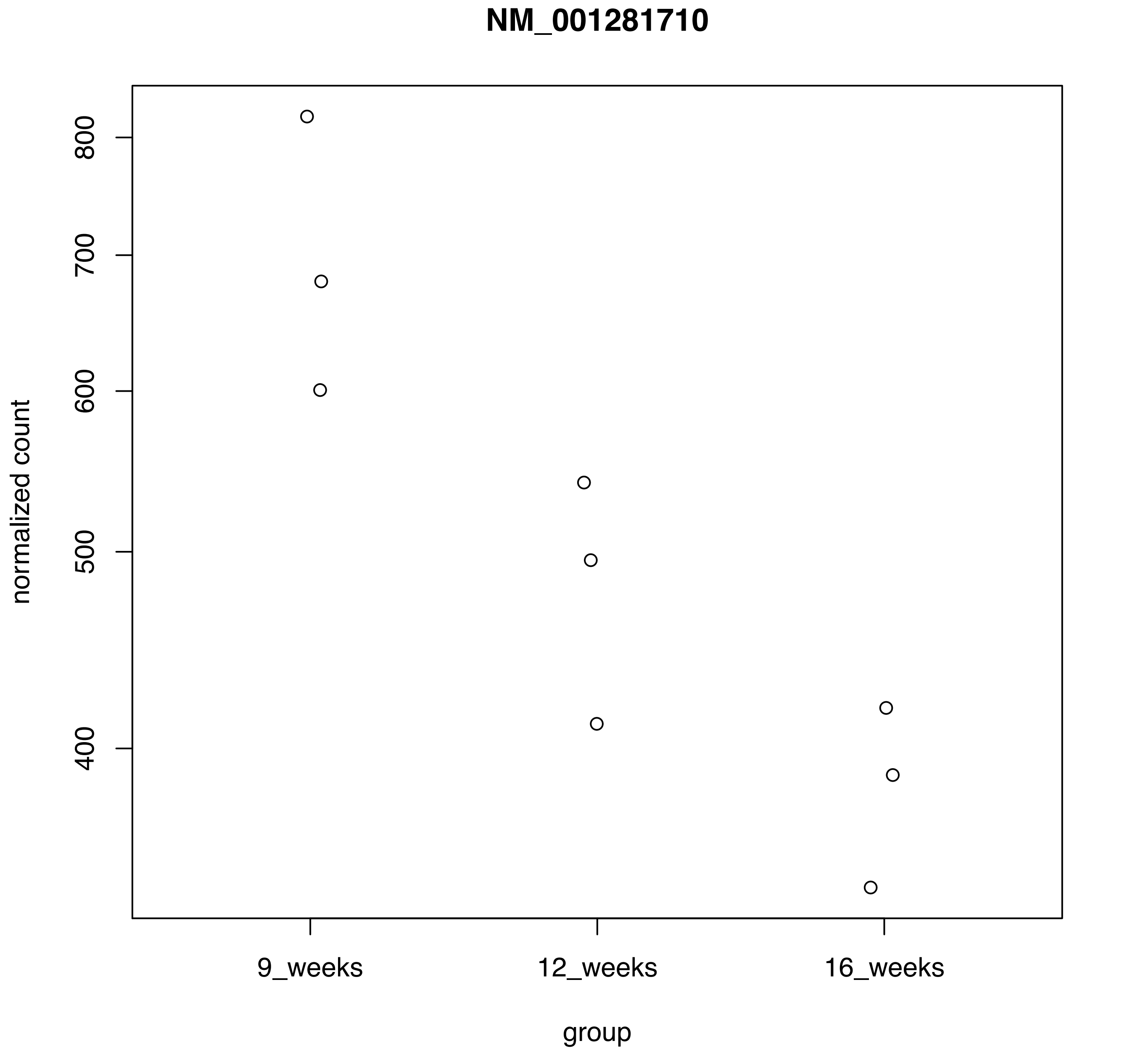
NM_001281710
normalized count
800
700
600
500
400
9_weeks
12_weeks
16_weeks
group

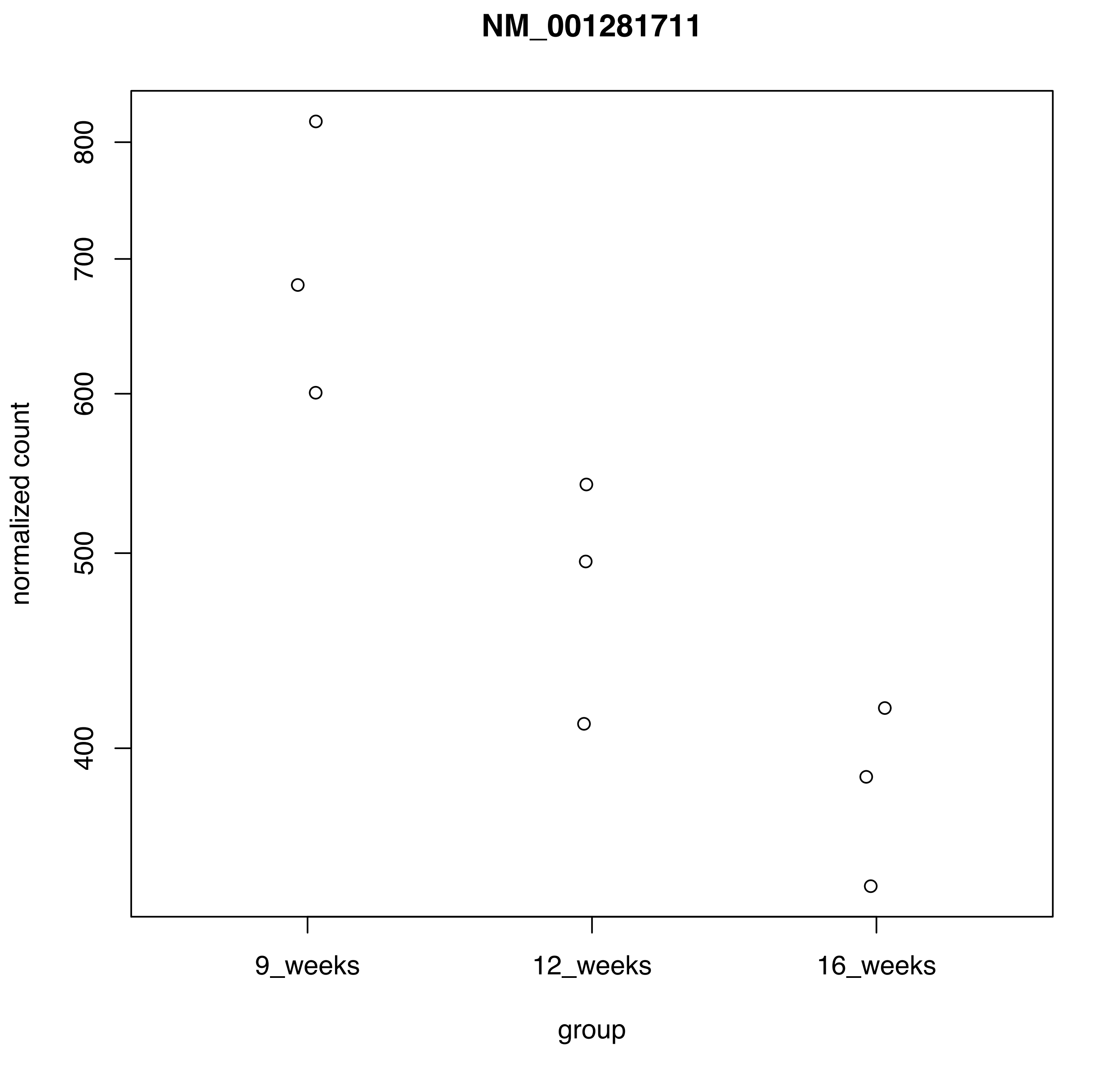
NM_001281711
normalized count
800
700
600
500
400
9_weeks
12_weeks
16_weeks
group

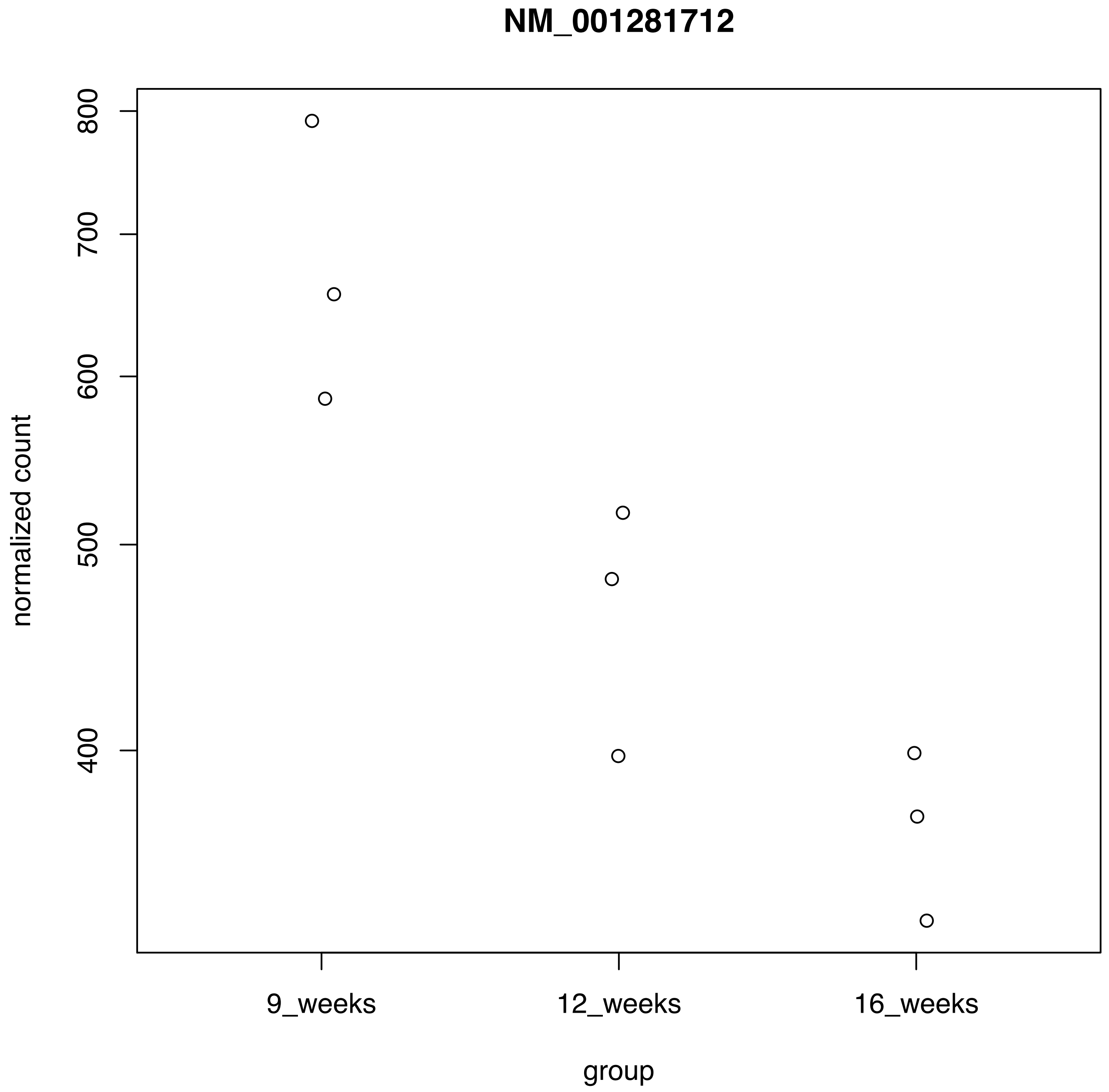
NM_001281712
normalized count
800
700
600
500
400
9_weeks
12_weeks
16_weeks
group

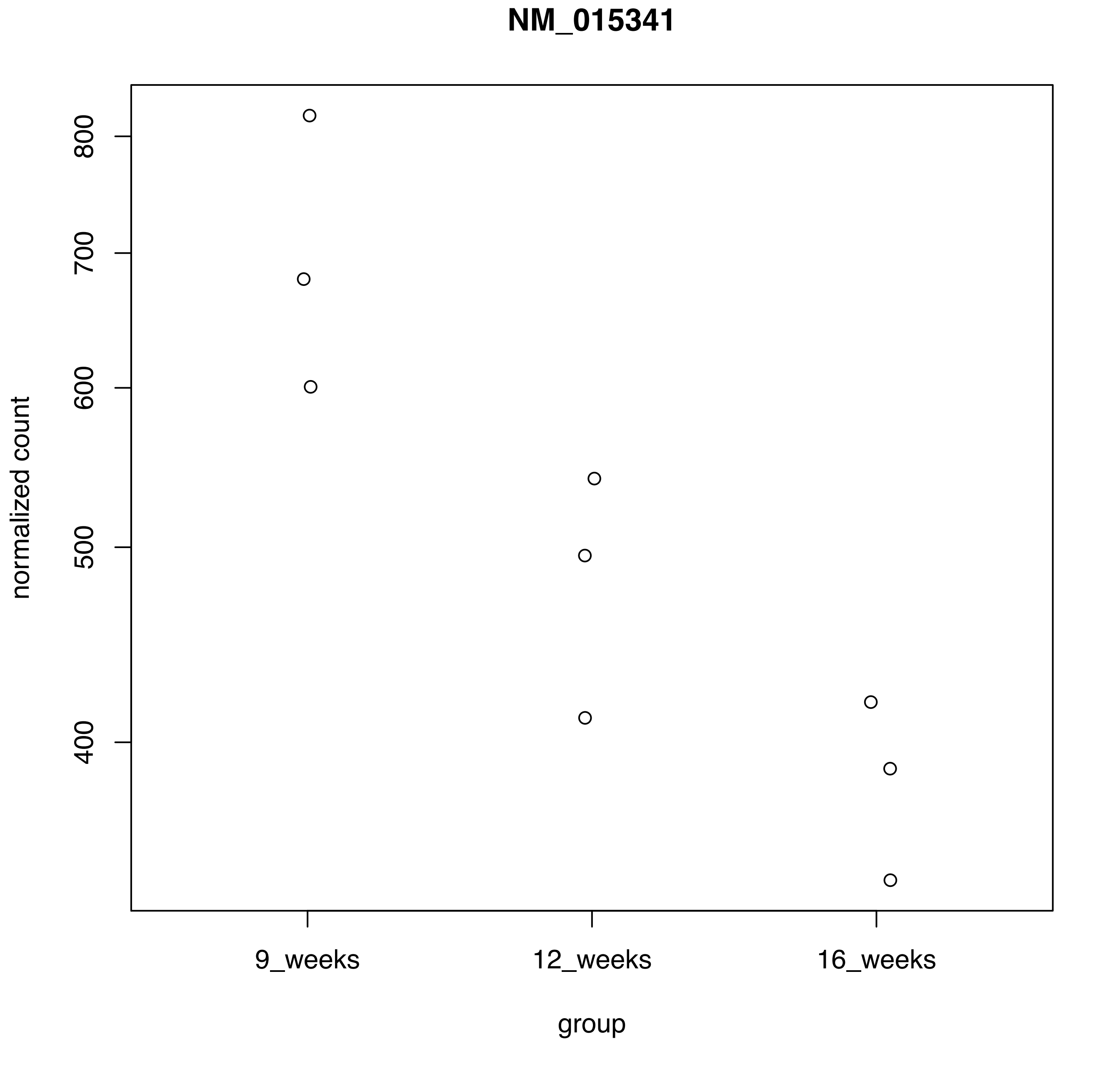
NM_015341
normalized count
800
700
600
500
400
9_weeks
12_weeks
16_weeks
group

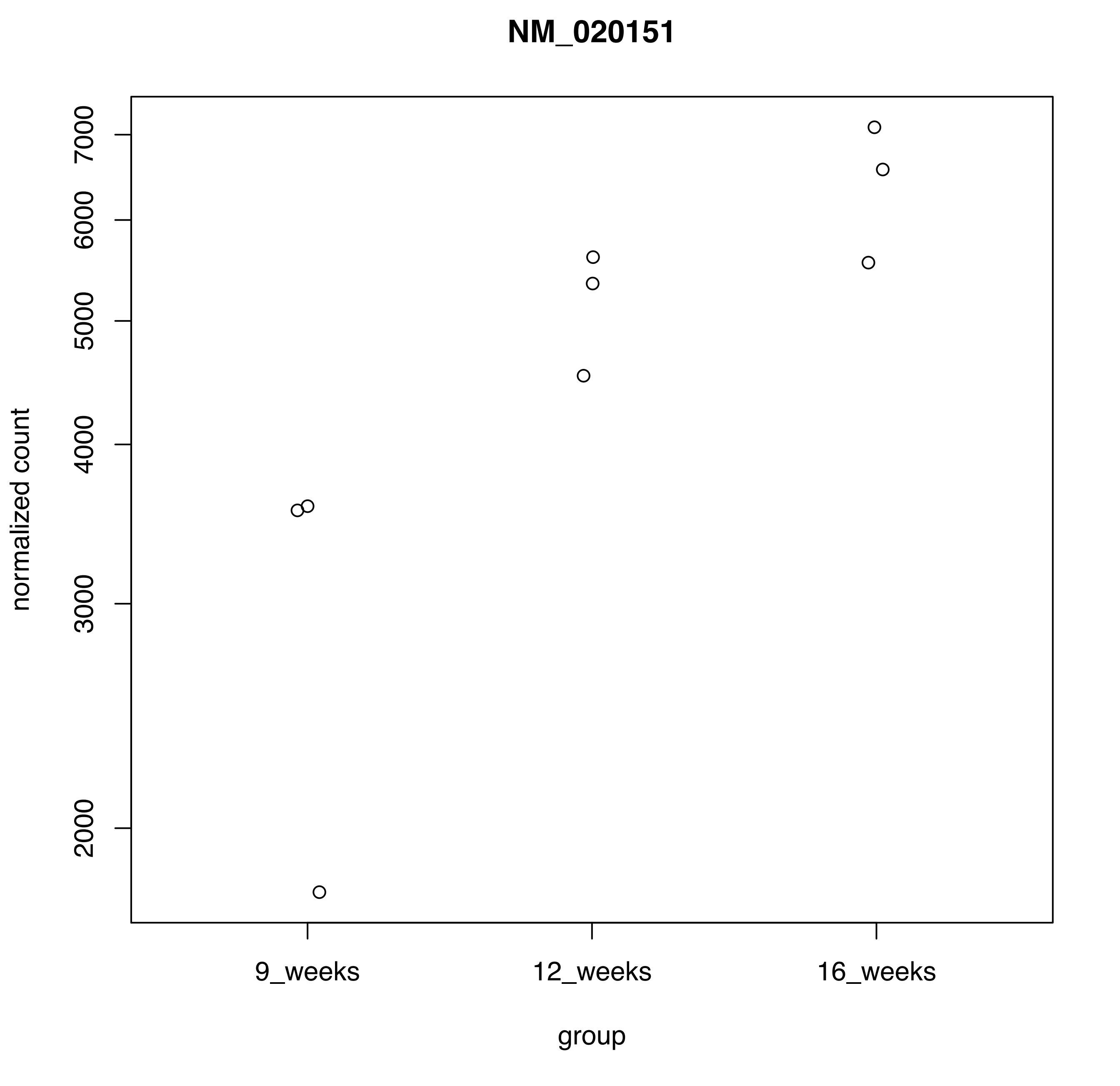

NM_020151
normalized count
7000
6000
5000
4000
3000
2000
9_weeks
12_weeks
16_weeks
group

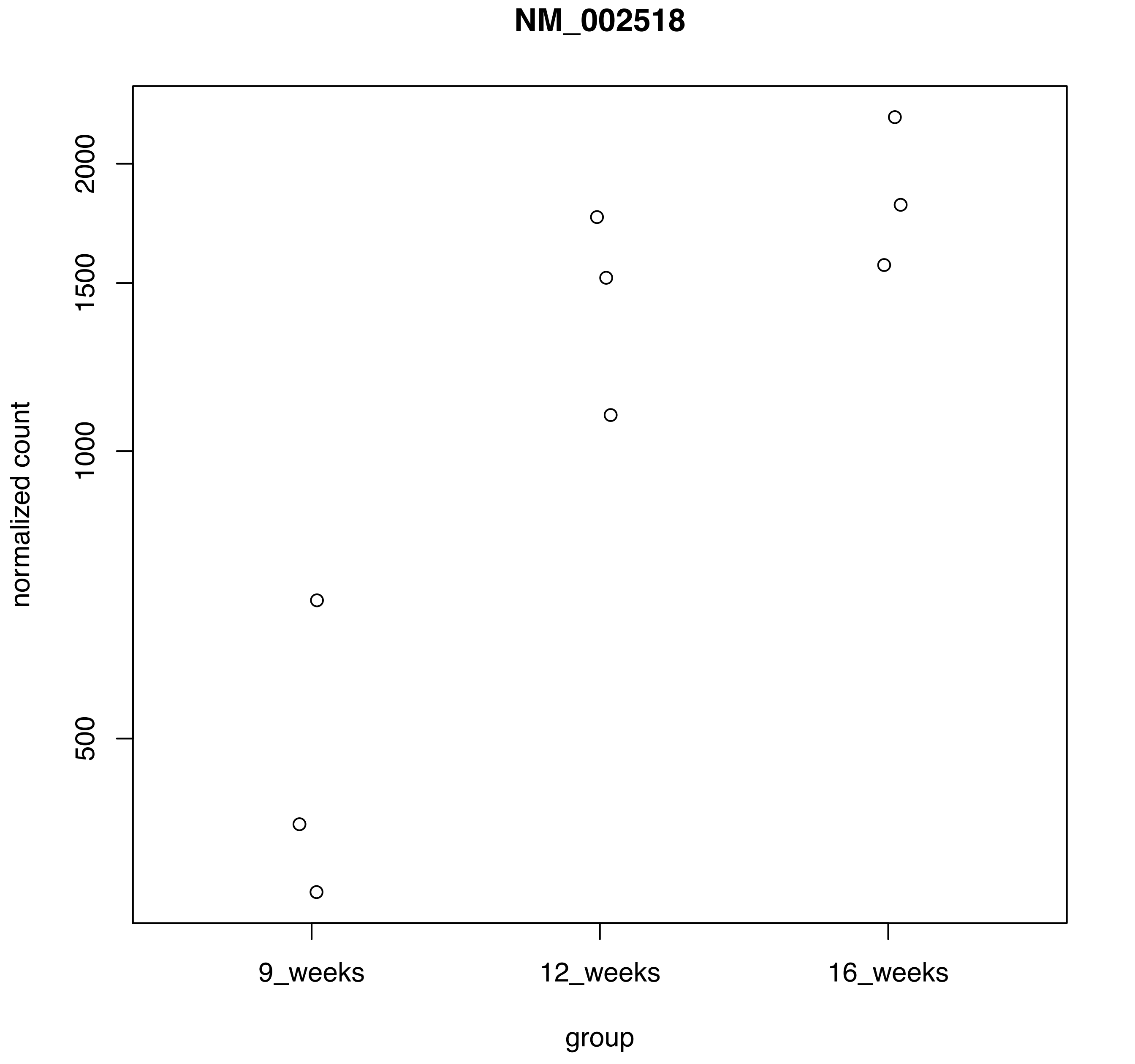

NM_002518
normalized count
2000
1500
1000
500
9_weeks
12_weeks
16_weeks
group

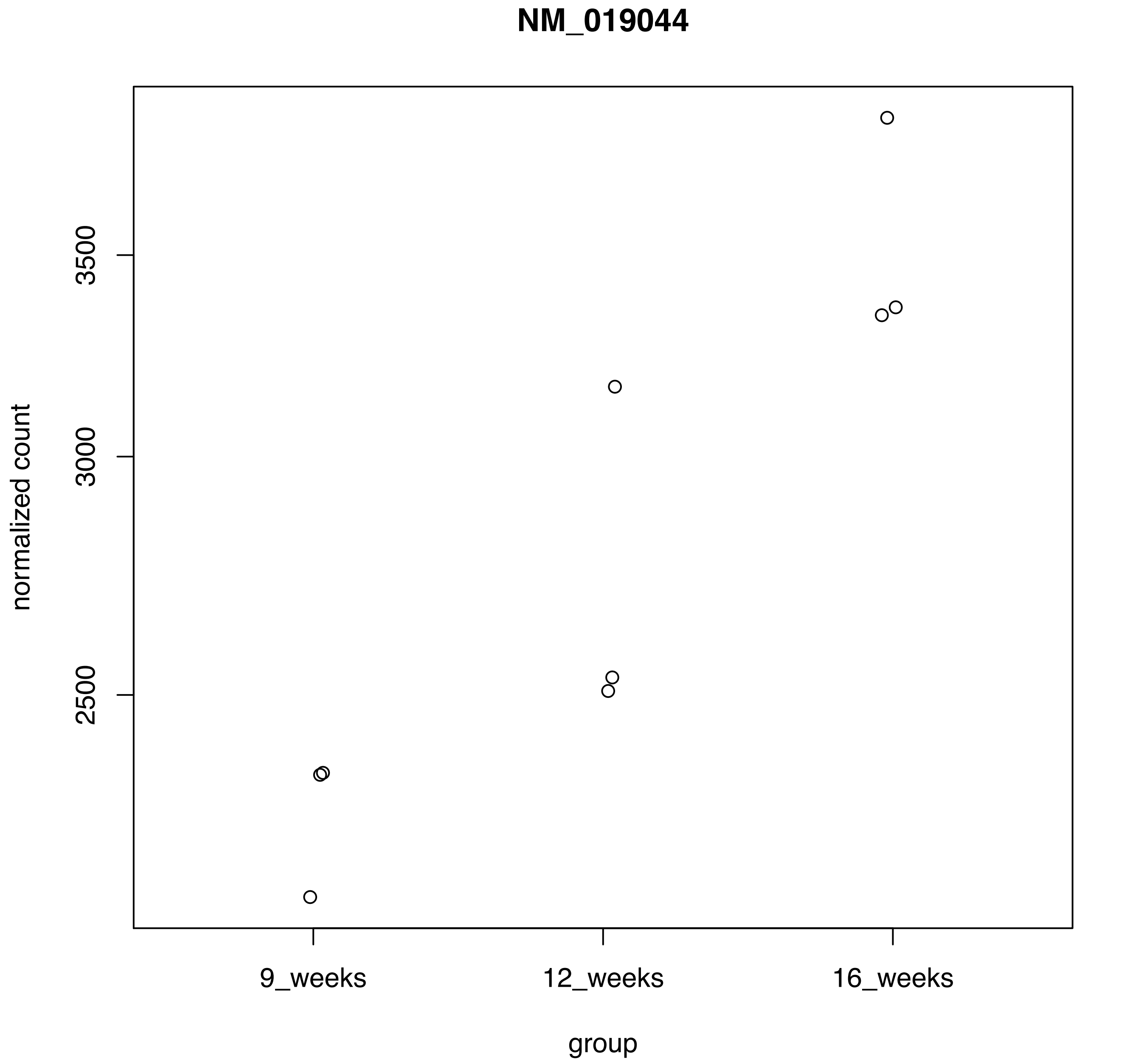

NM_019044
normalized count
3500
3000
2500
9_weeks
12_weeks
16_weeks
group

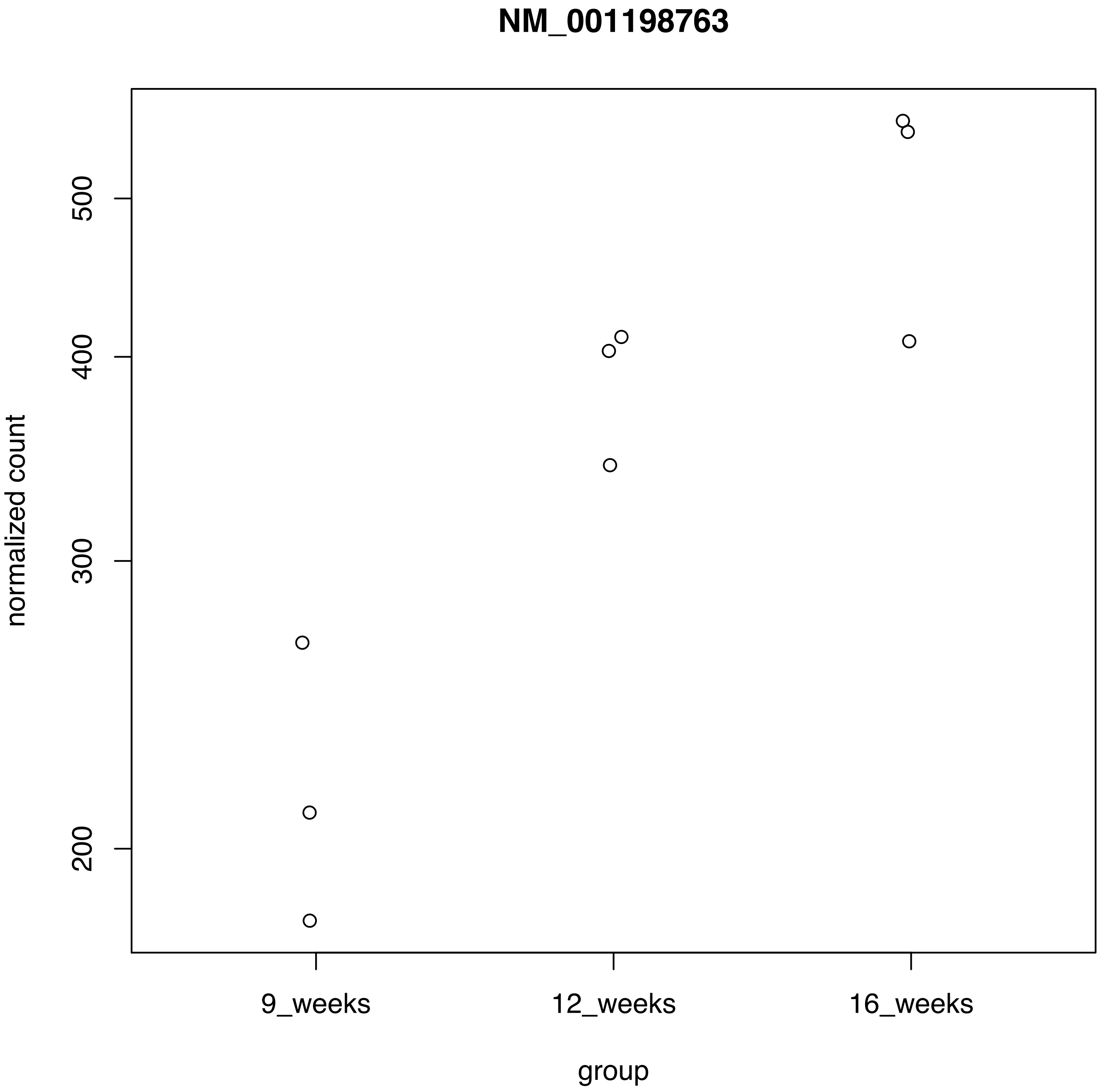

NM_001198763
normalized count
200
300
400
500
9_weeks
12_weeks
16_weeks
group

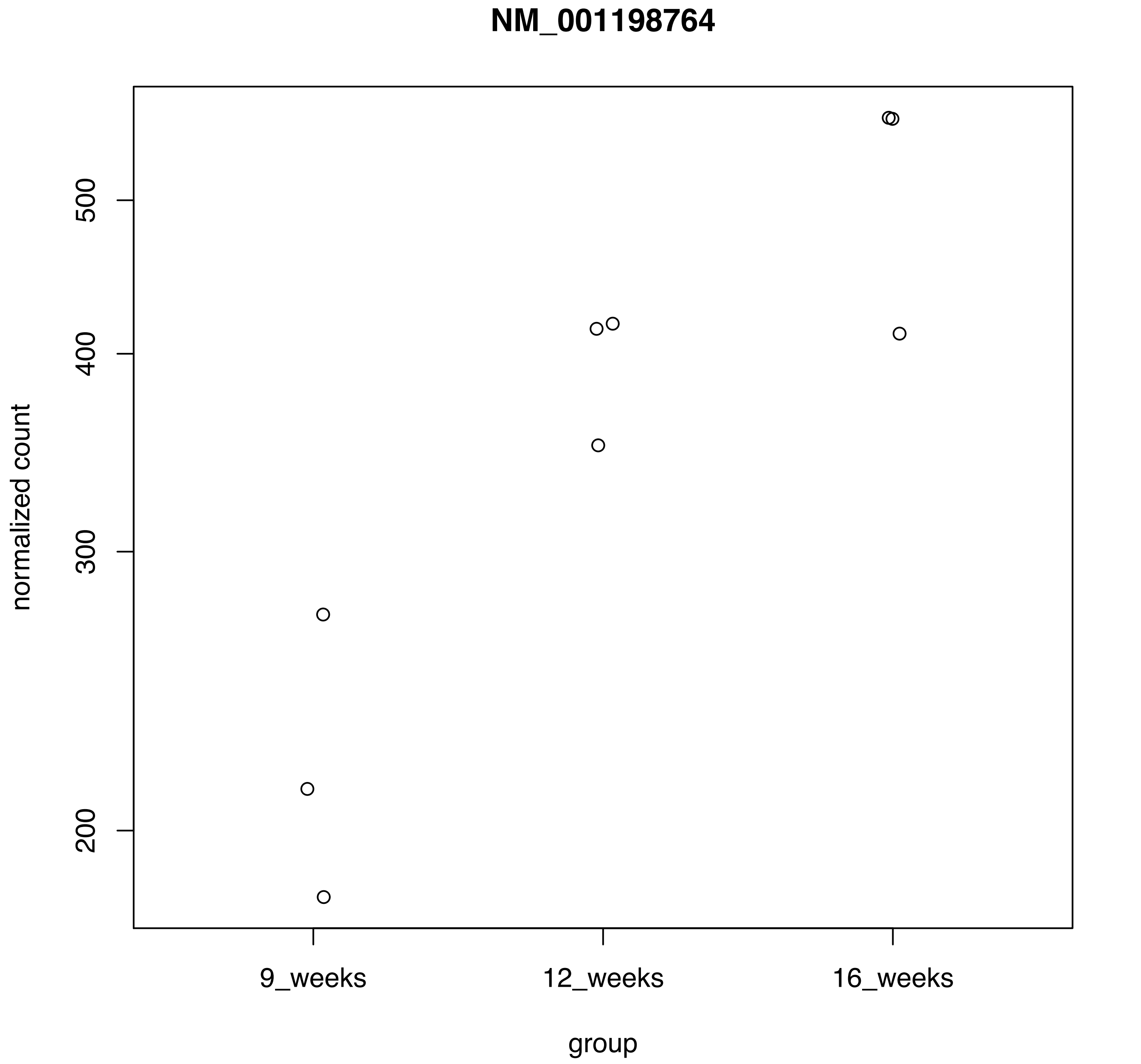

NM_001198764
normalized count
200
300
400
500
9_weeks
12_weeks
16_weeks
group

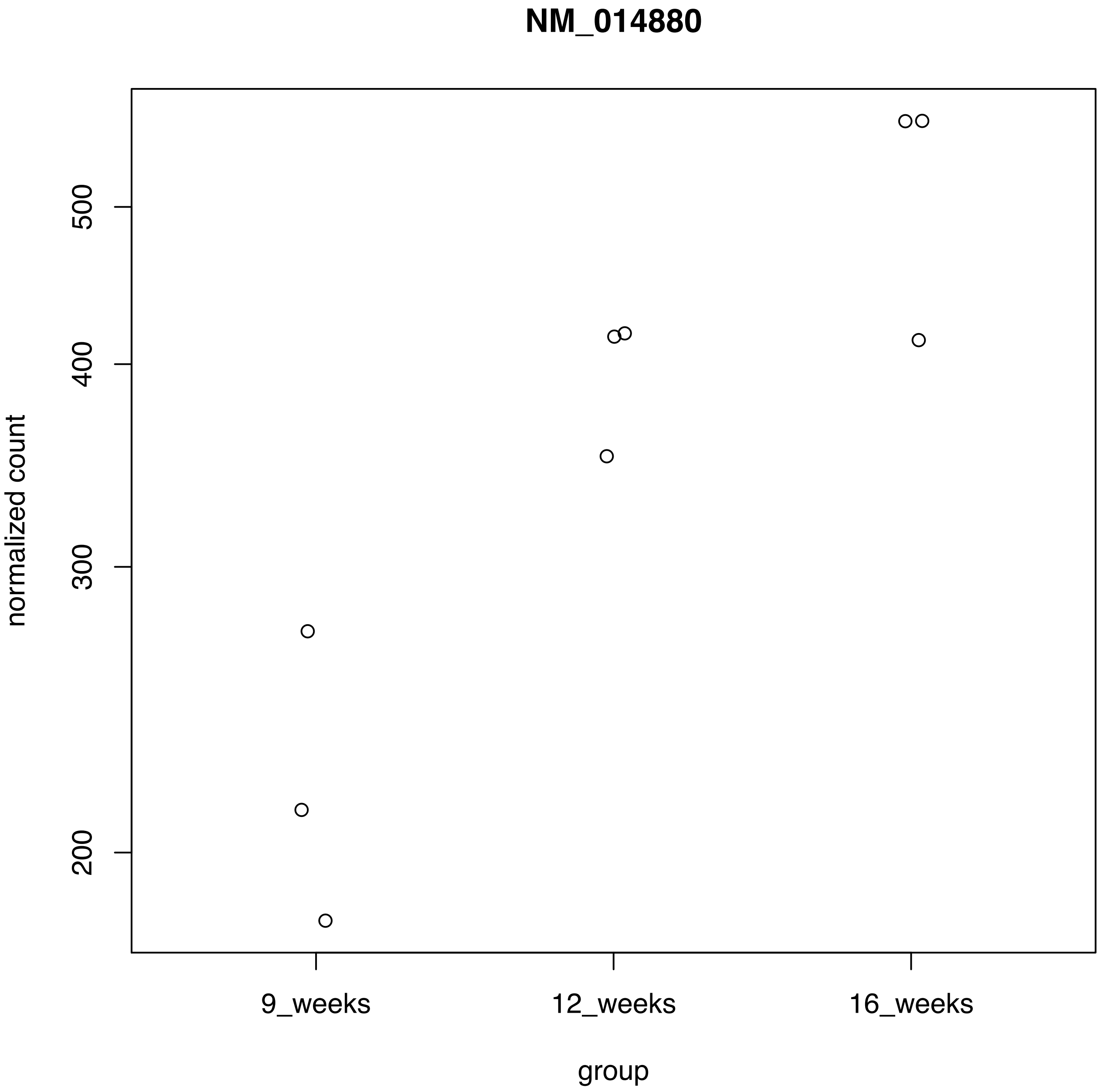
NM_014880
normalized count
500
400
300
200
9_weeks
12_weeks
16_weeks
group

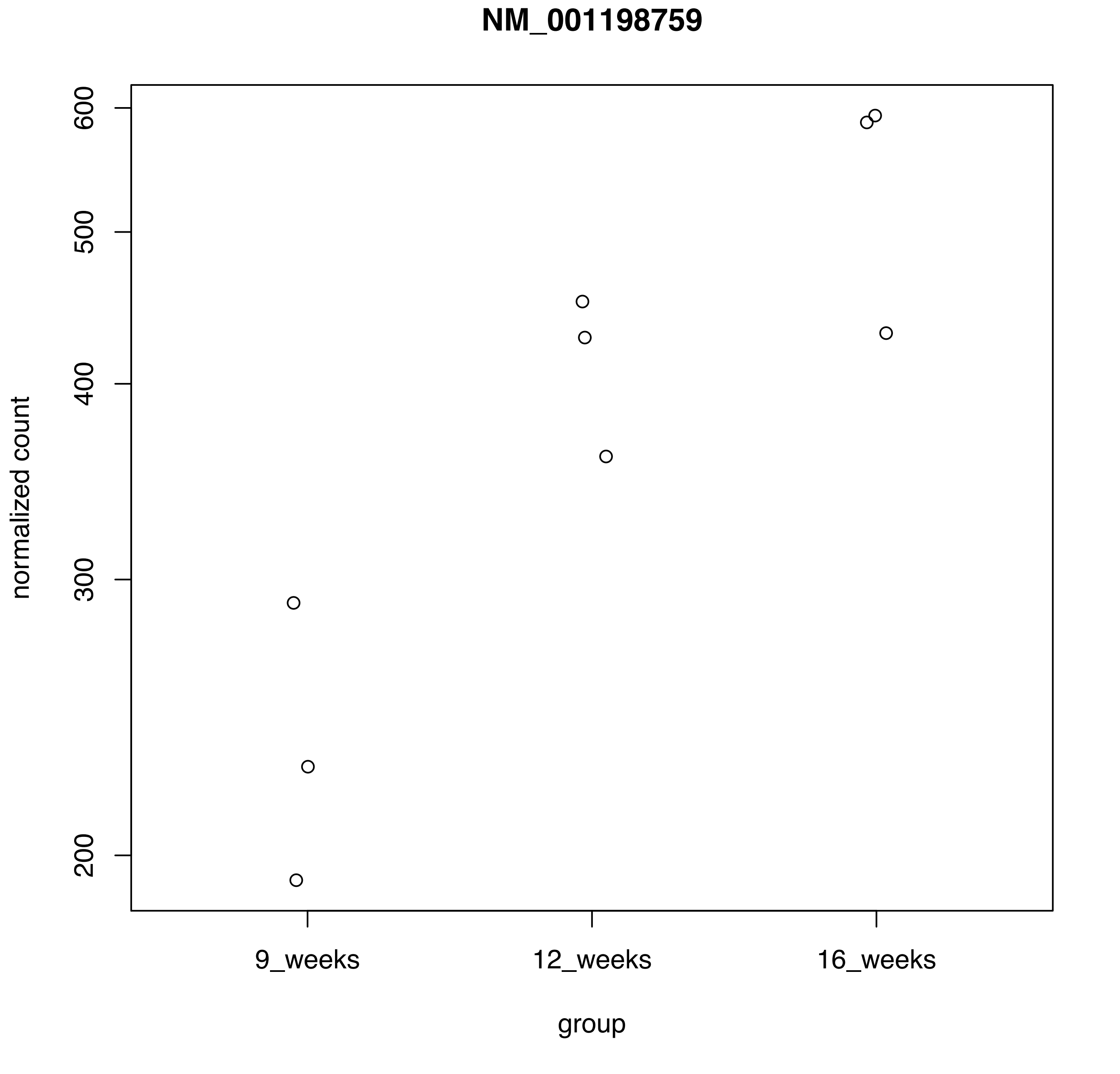

NM_001198759
normalized count
600
500
400
300
200
9_weeks
12_weeks
16_weeks
group

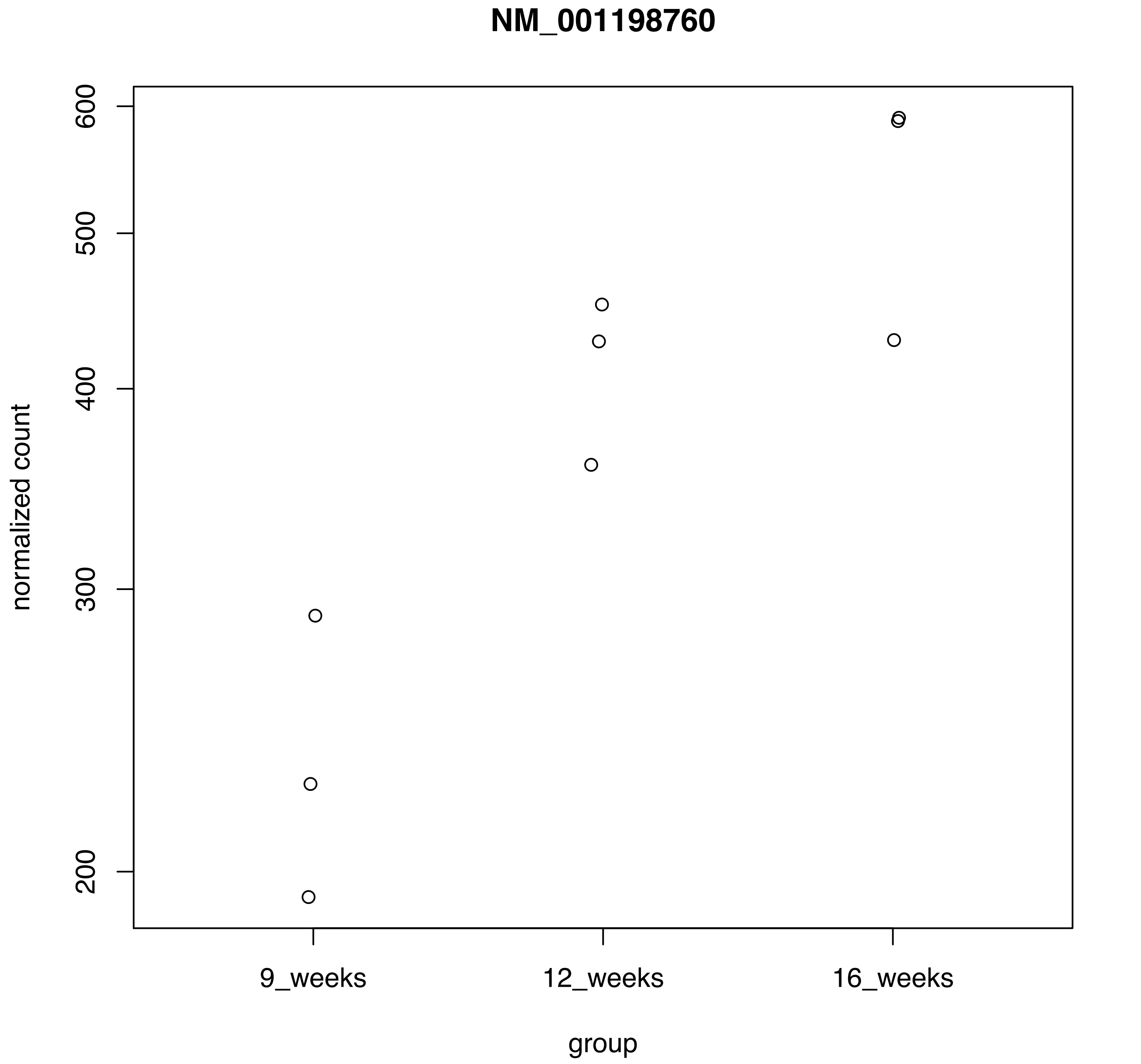
NM_001198760
normalized count
group
9_weeks
12_weeks
16_weeks
200
300
400
500
600

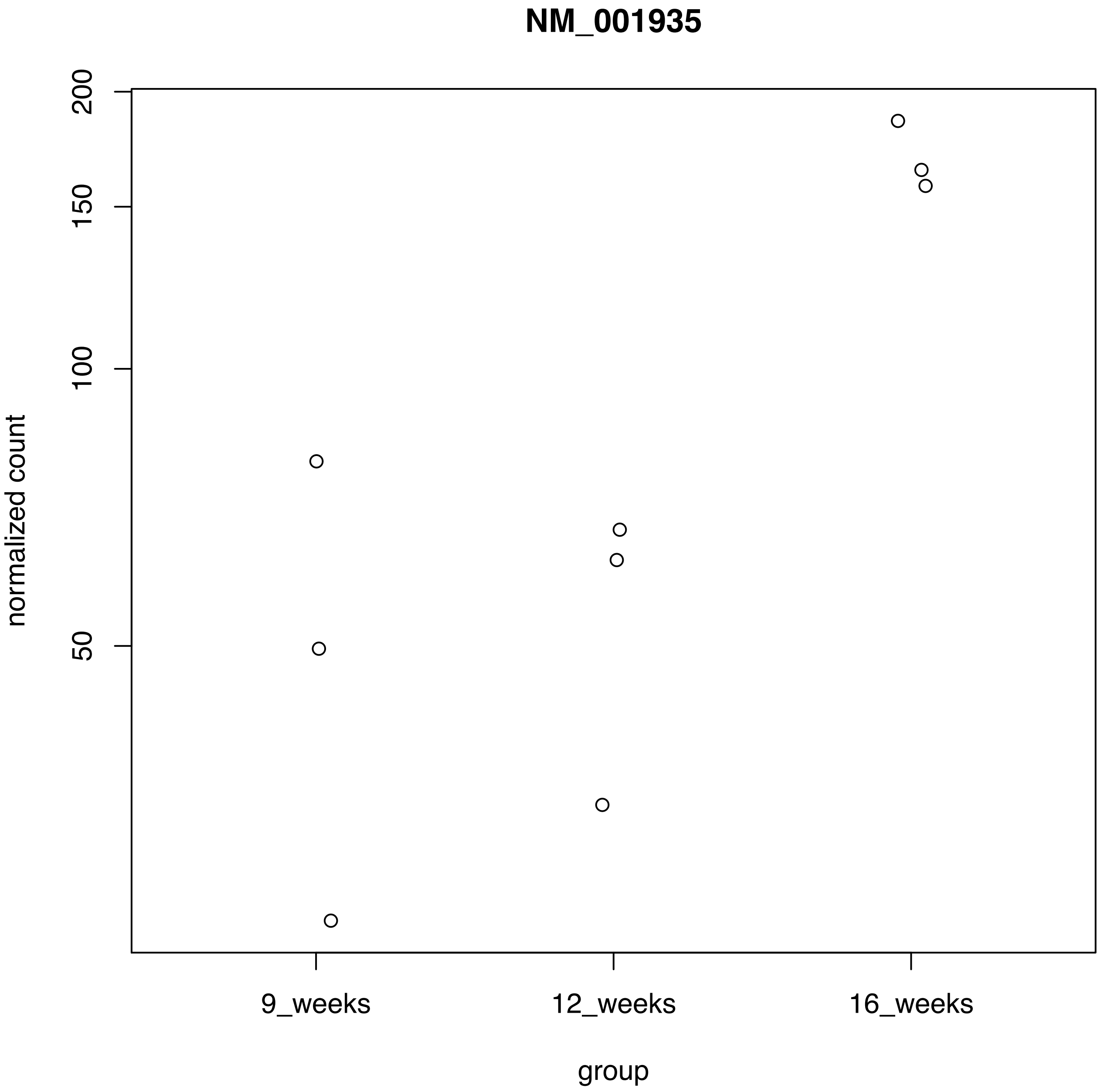

NM_001935
normalized count
200
150
100
50
9_weeks
12_weeks
16_weeks
group

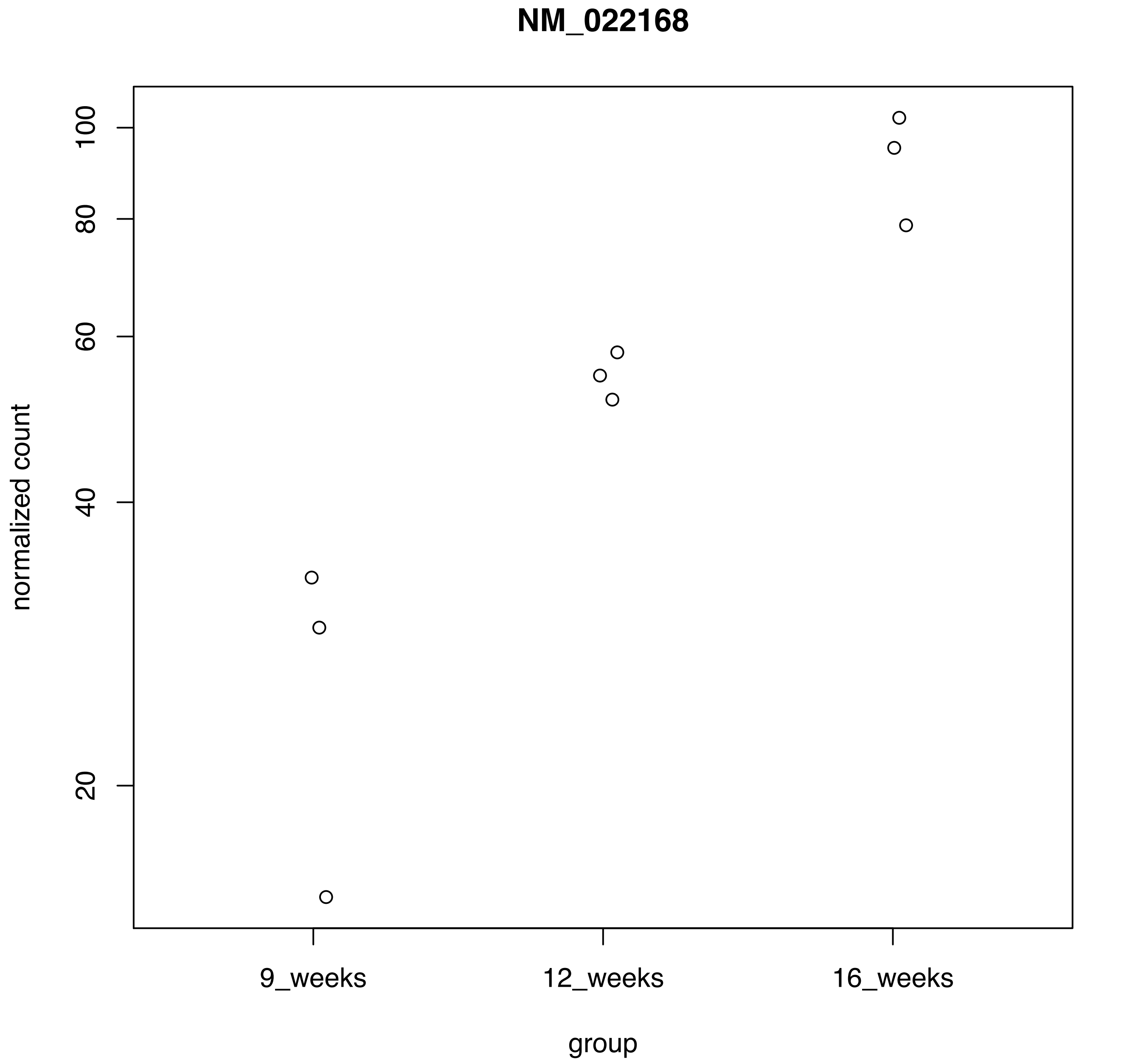

NM_022168
normalized count
100
80
60
40
20
9_weeks
12_weeks
16_weeks
group

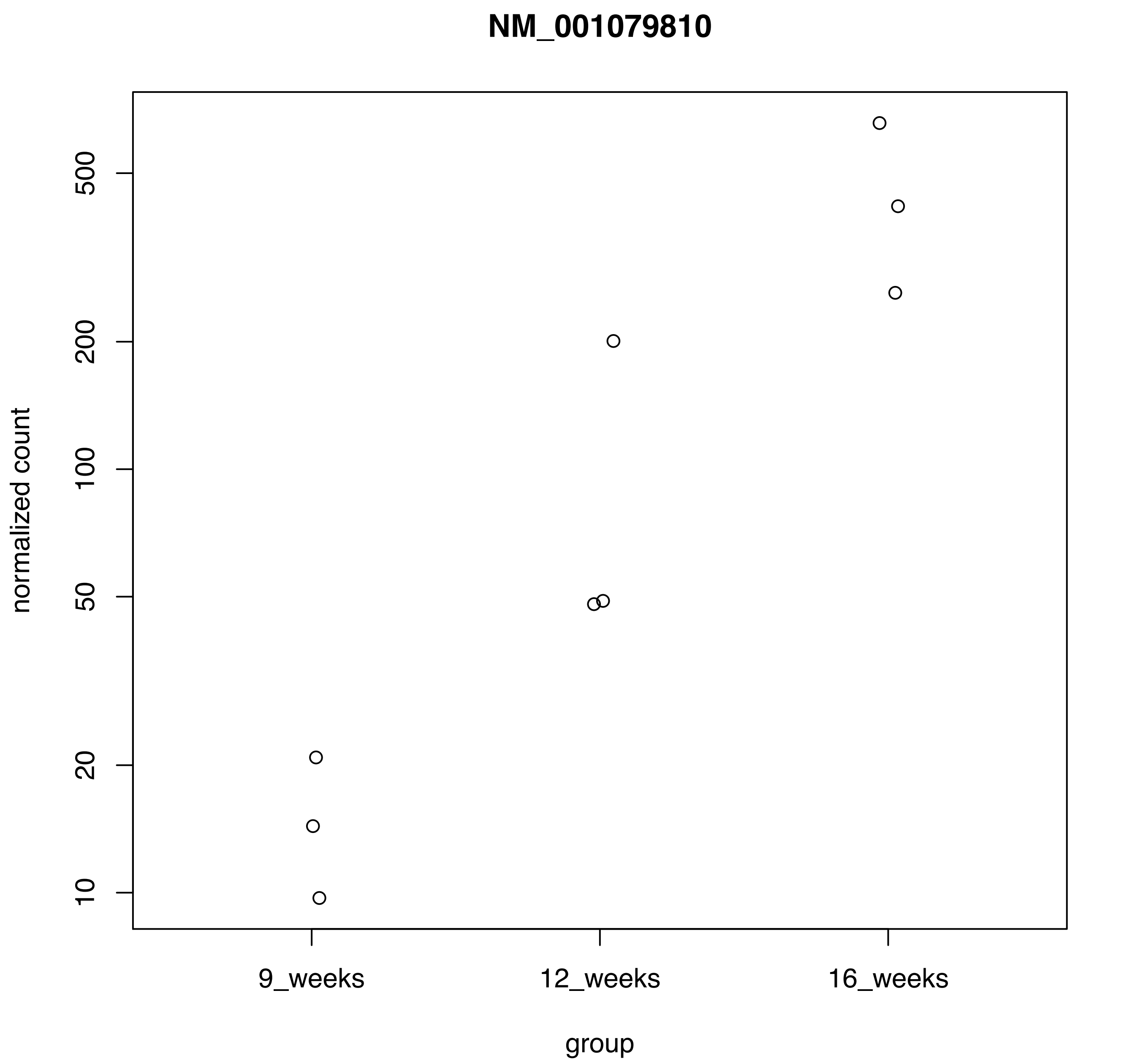
NM_001079810
normalized count
500
200
100
50
20
10
9_weeks
12_weeks
16_weeks
group

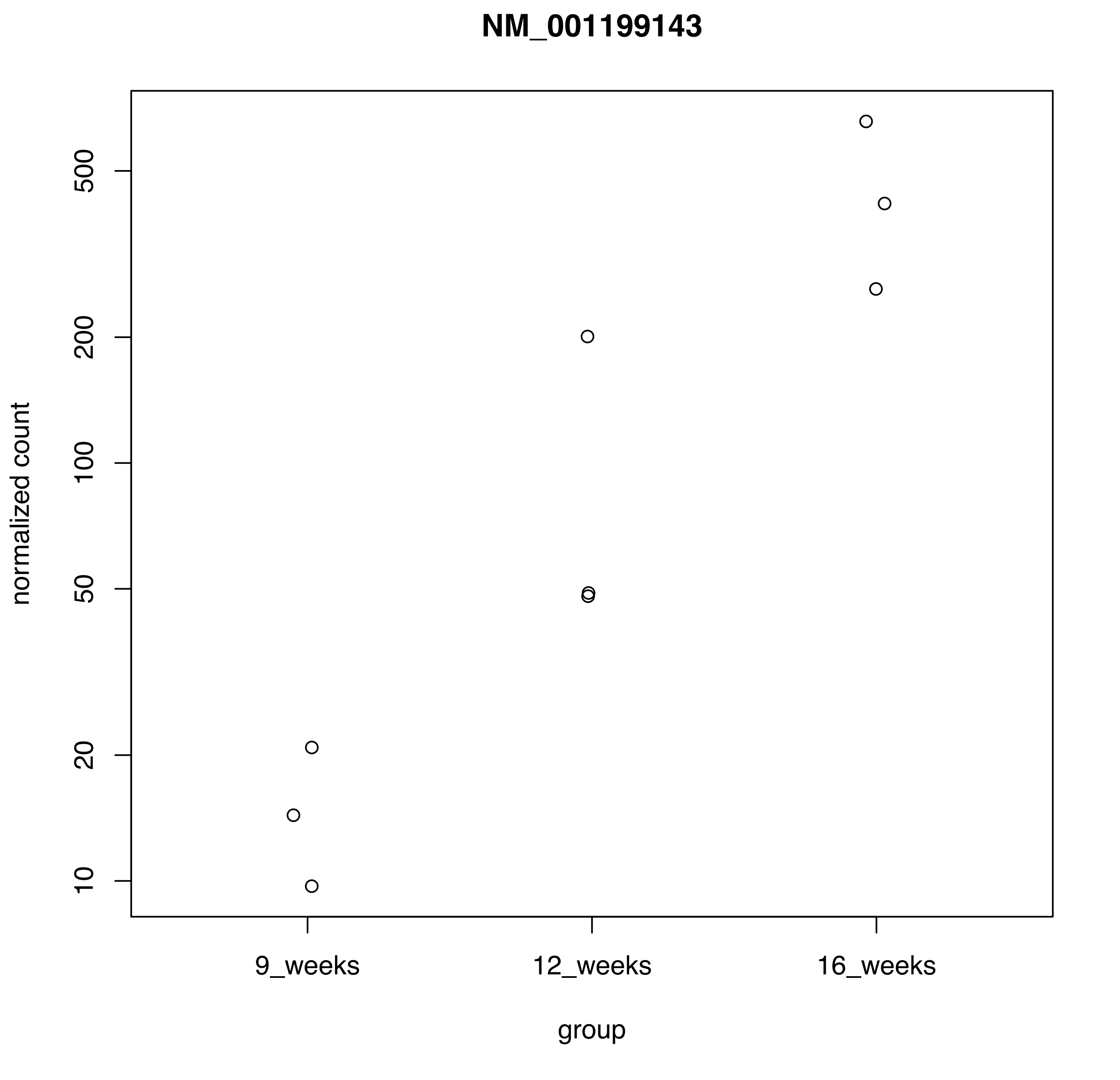
NM_001199143
normalized count
500
200
100
50
20
10
9_weeks
12_weeks
16_weeks
group

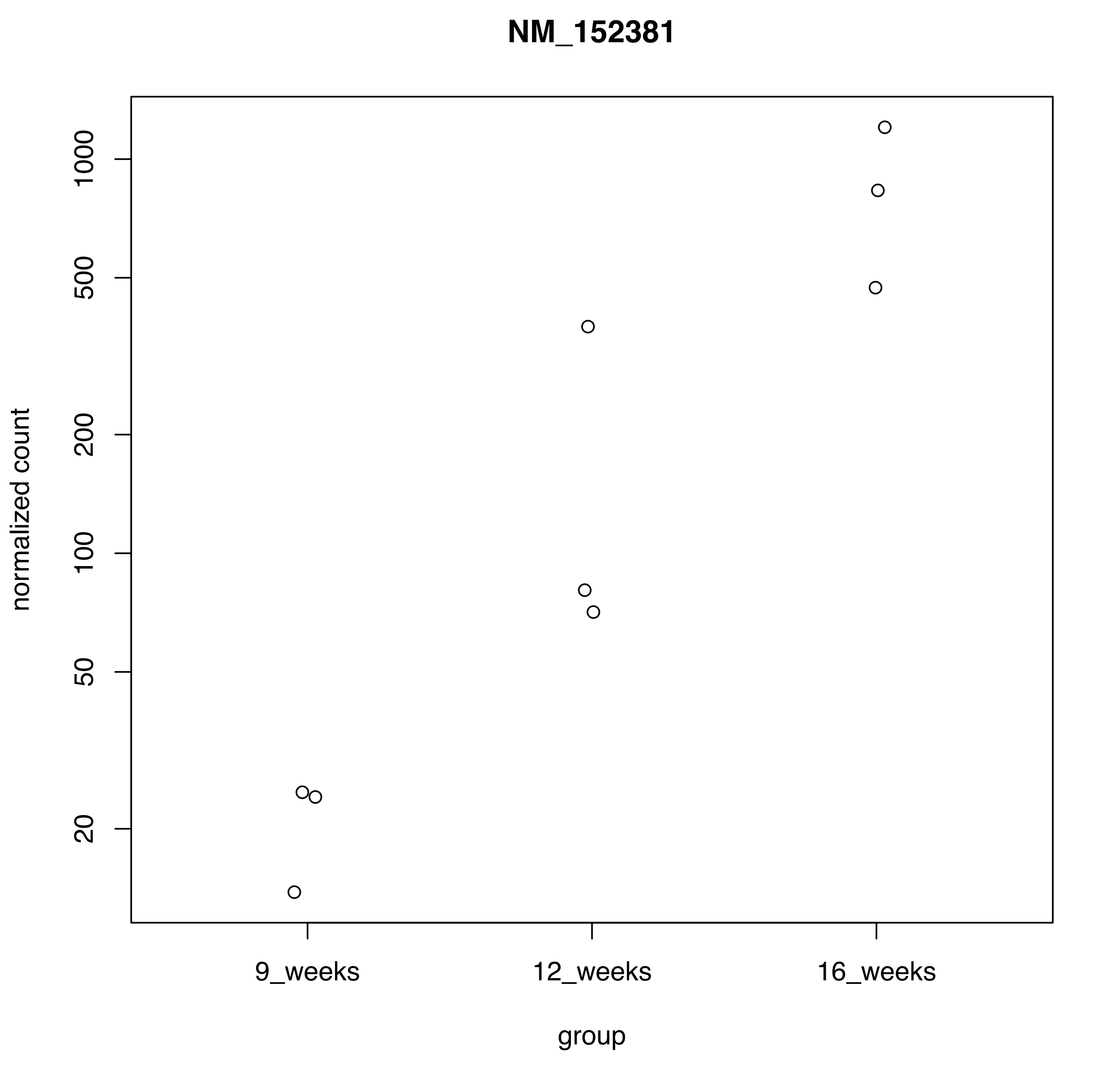
NM_152381
normalized count
1000
500
200
100
50
20
9_weeks
12_weeks
16_weeks
group

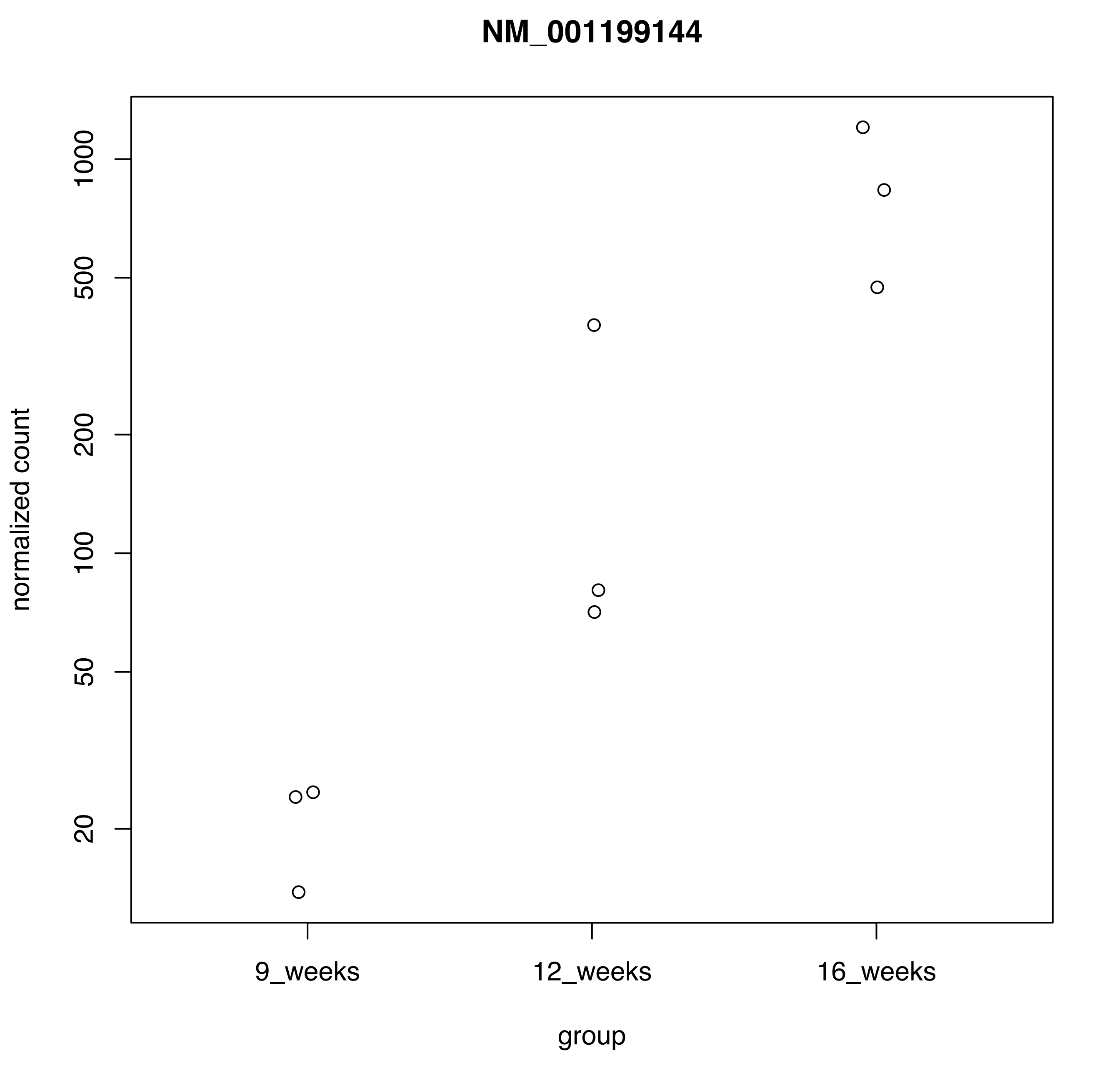

NM_001199144
normalized count
1000
500
200
100
50
20
9_weeks
12_weeks
16_weeks
group

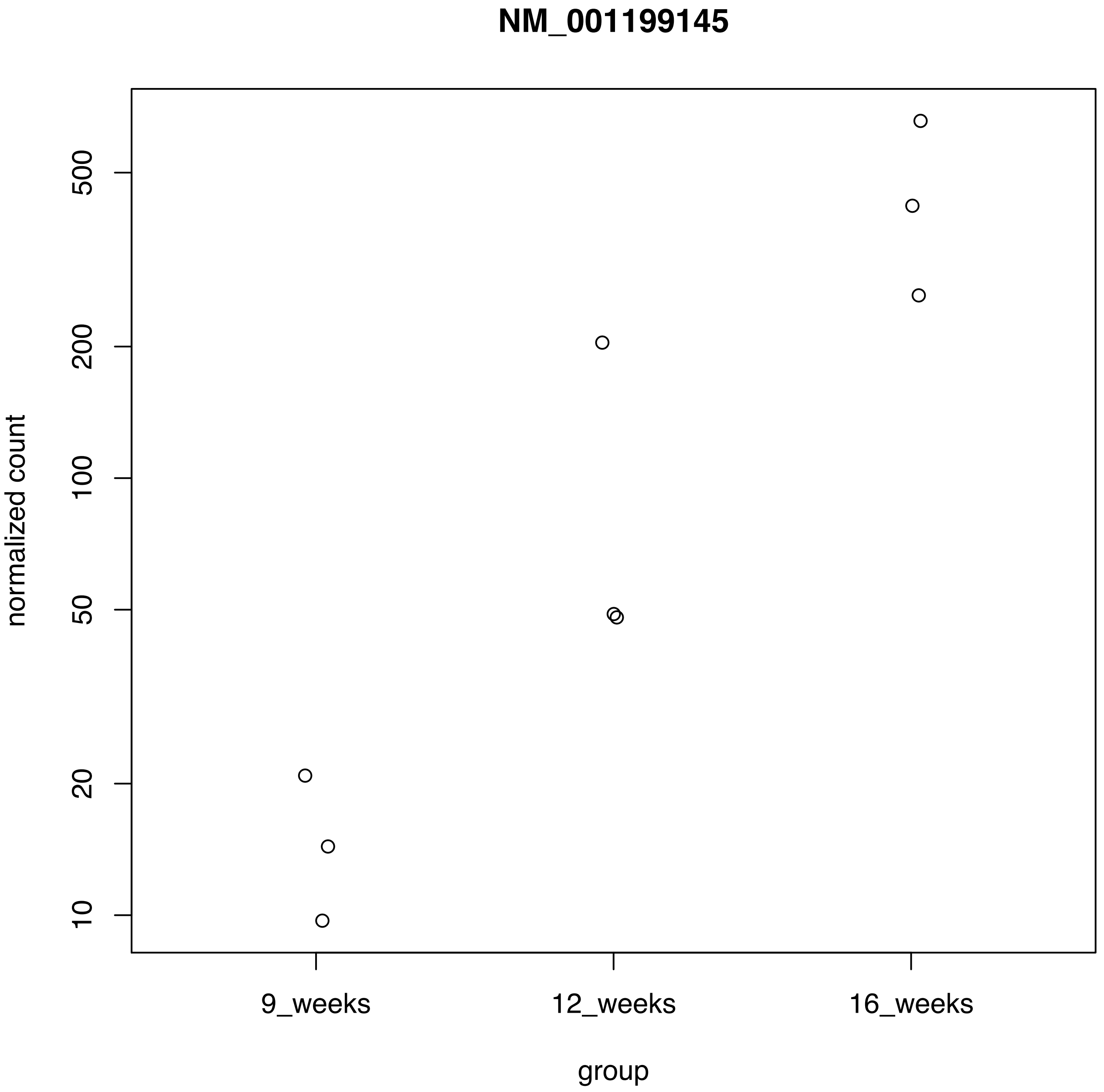
NM_001199145
normalized count
500
200
100
50
20
10
9_weeks
12_weeks
16_weeks
group

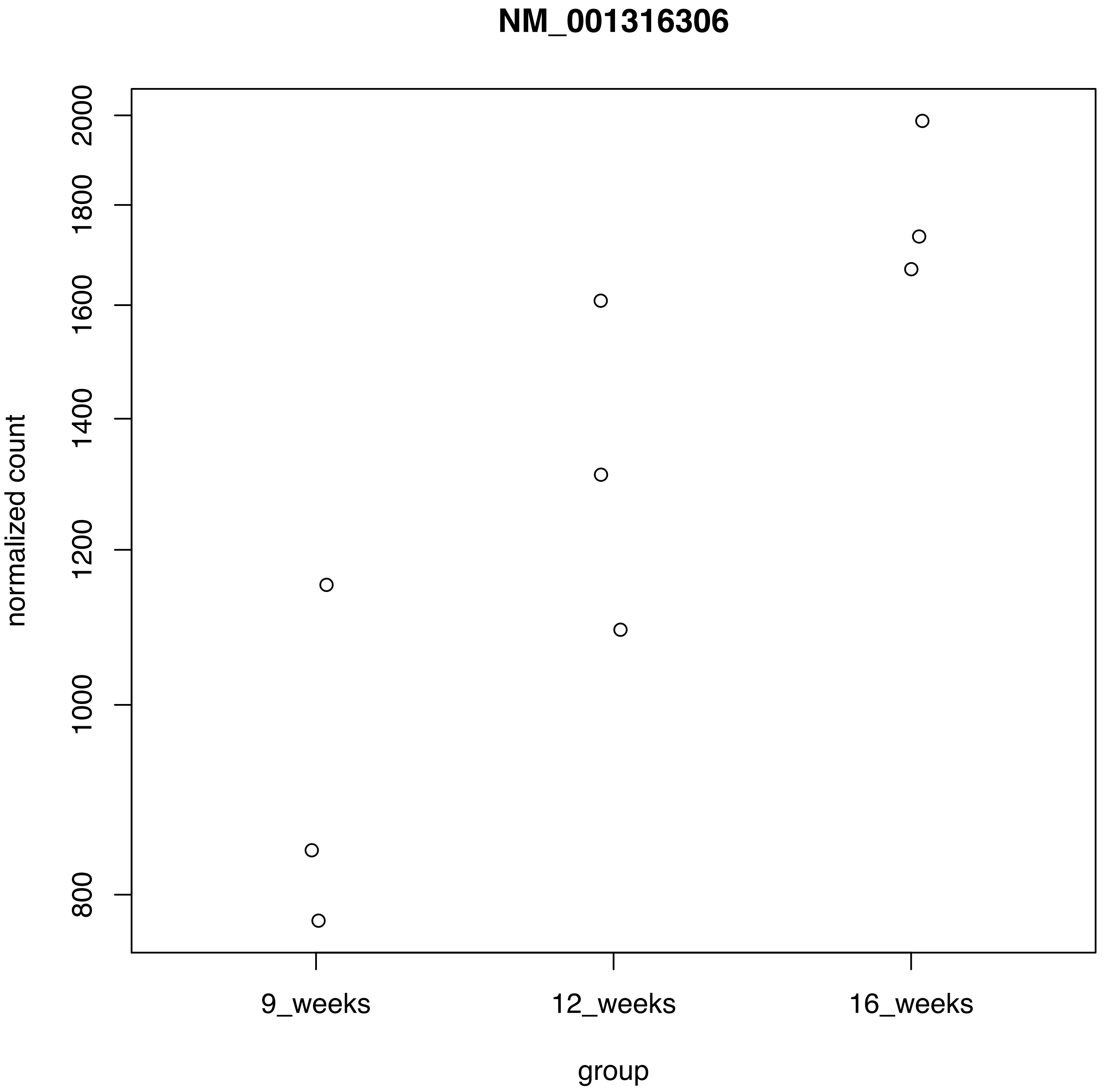
NM_001316306
normalized count
2000
1800
1600
1400
1200
1000
800
9_weeks
12_weeks
16_weeks
group

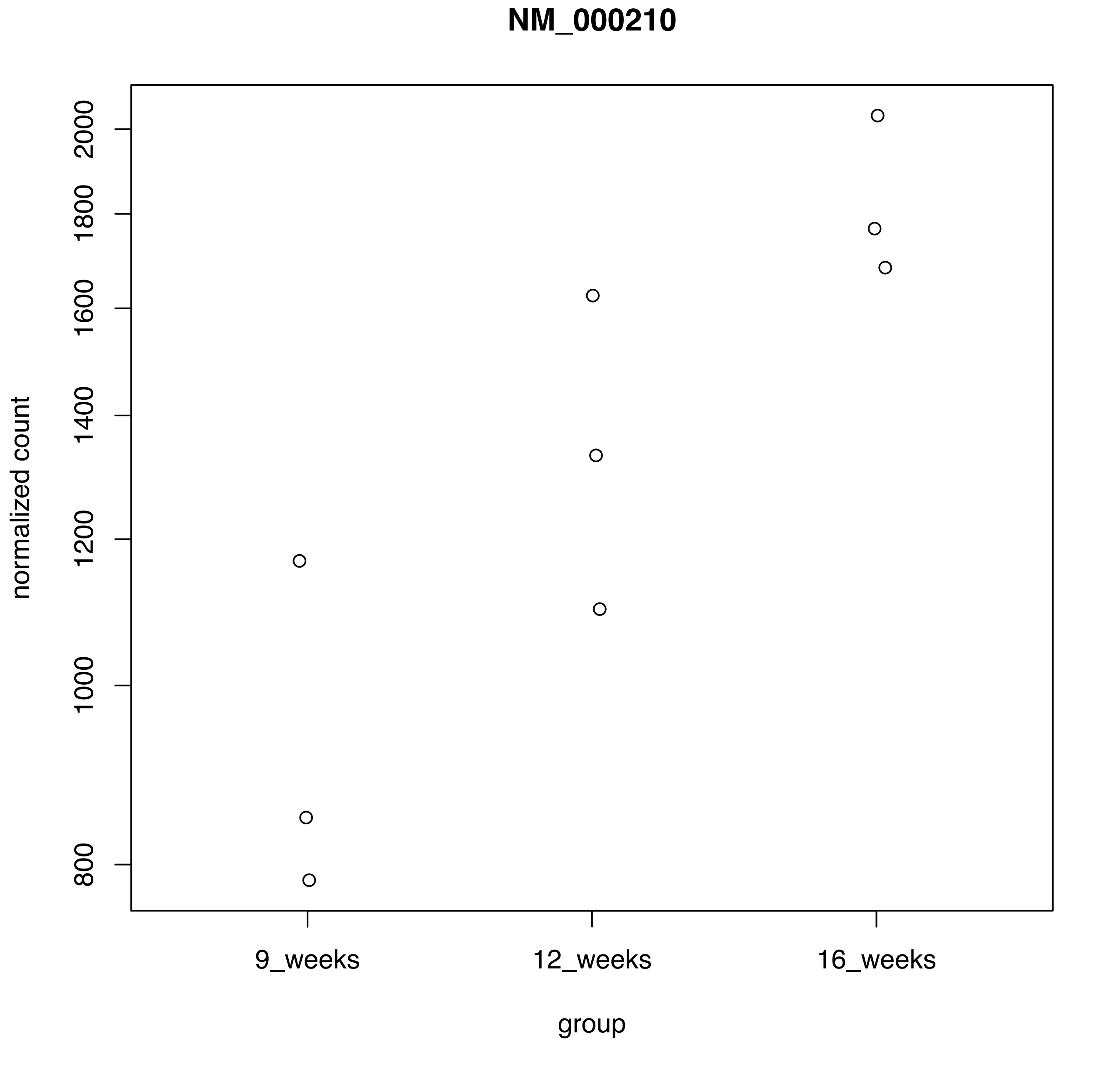

NM_000210
normalized count
800
1000
1200
1400
1600
1800
2000
9_weeks
12_weeks
16_weeks
group

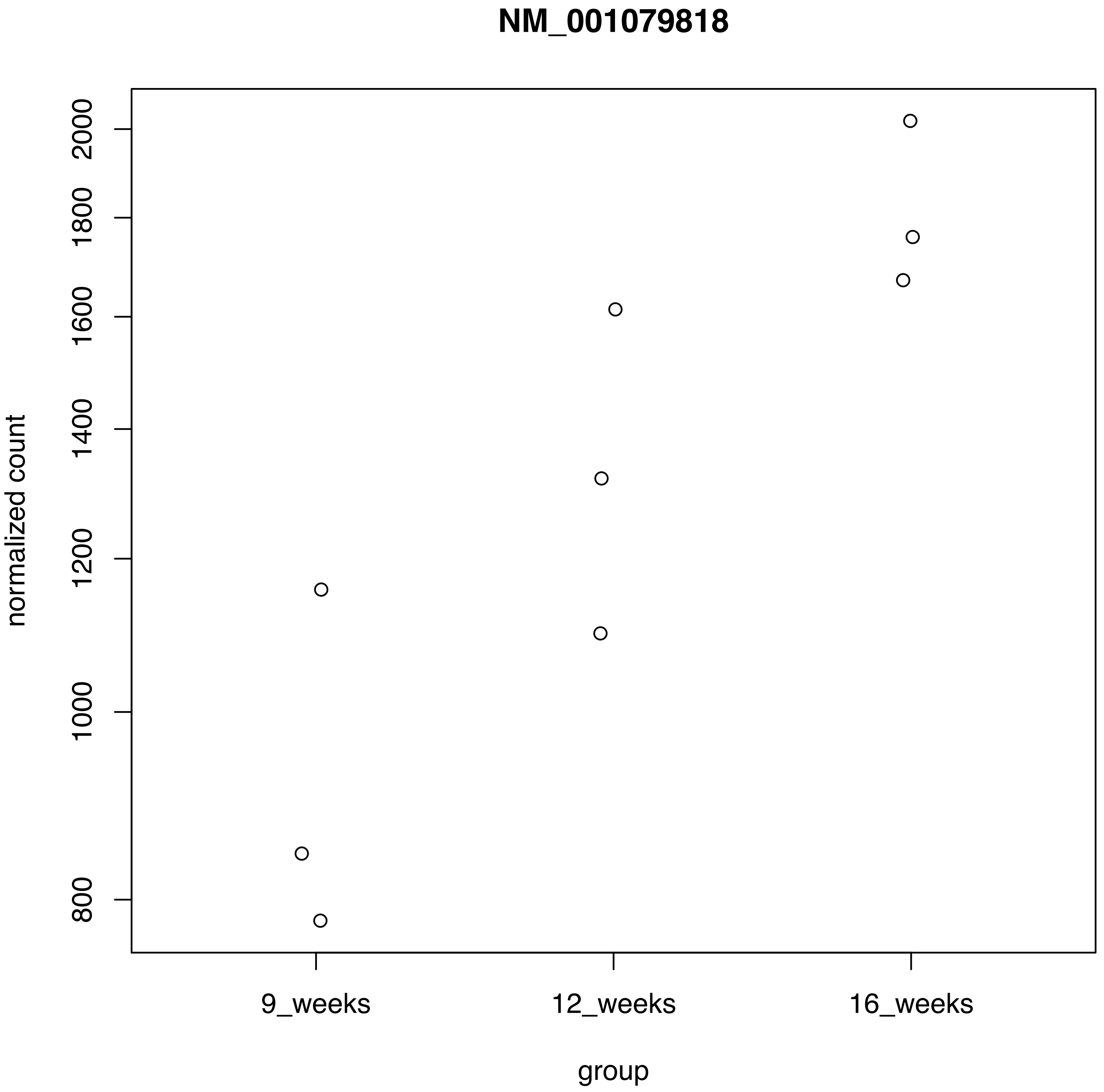
NM_001079818
normalized count
800
1000
1200
1400
1600
1800
2000
9_weeks
12_weeks
16_weeks
group

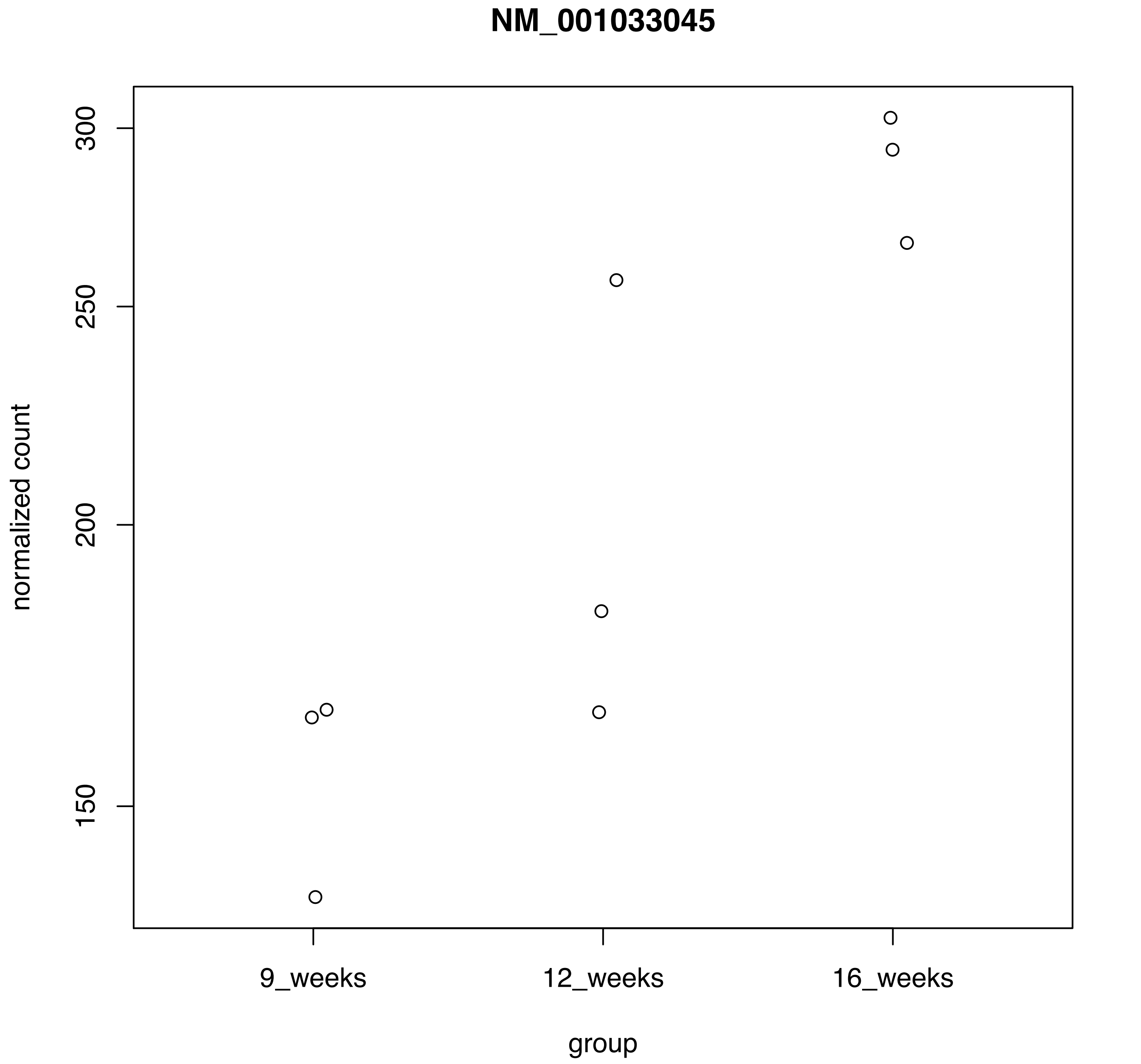
NM_001033045
300
250
200
150
normalized count
9_weeks
12_weeks
16_weeks
group

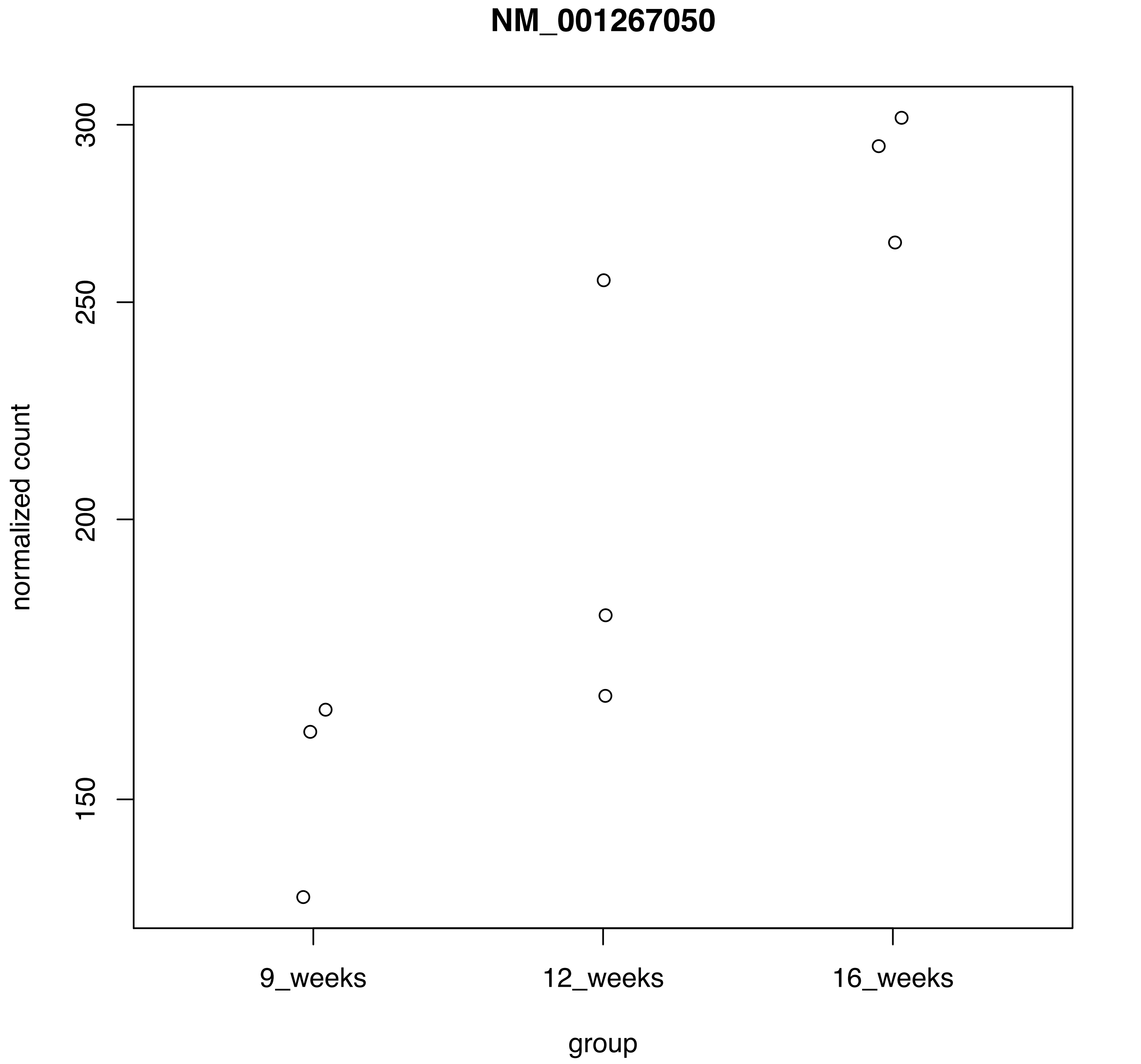
NM_001267050
normalized count
300
250
200
150
9_weeks
12_weeks
16_weeks
group

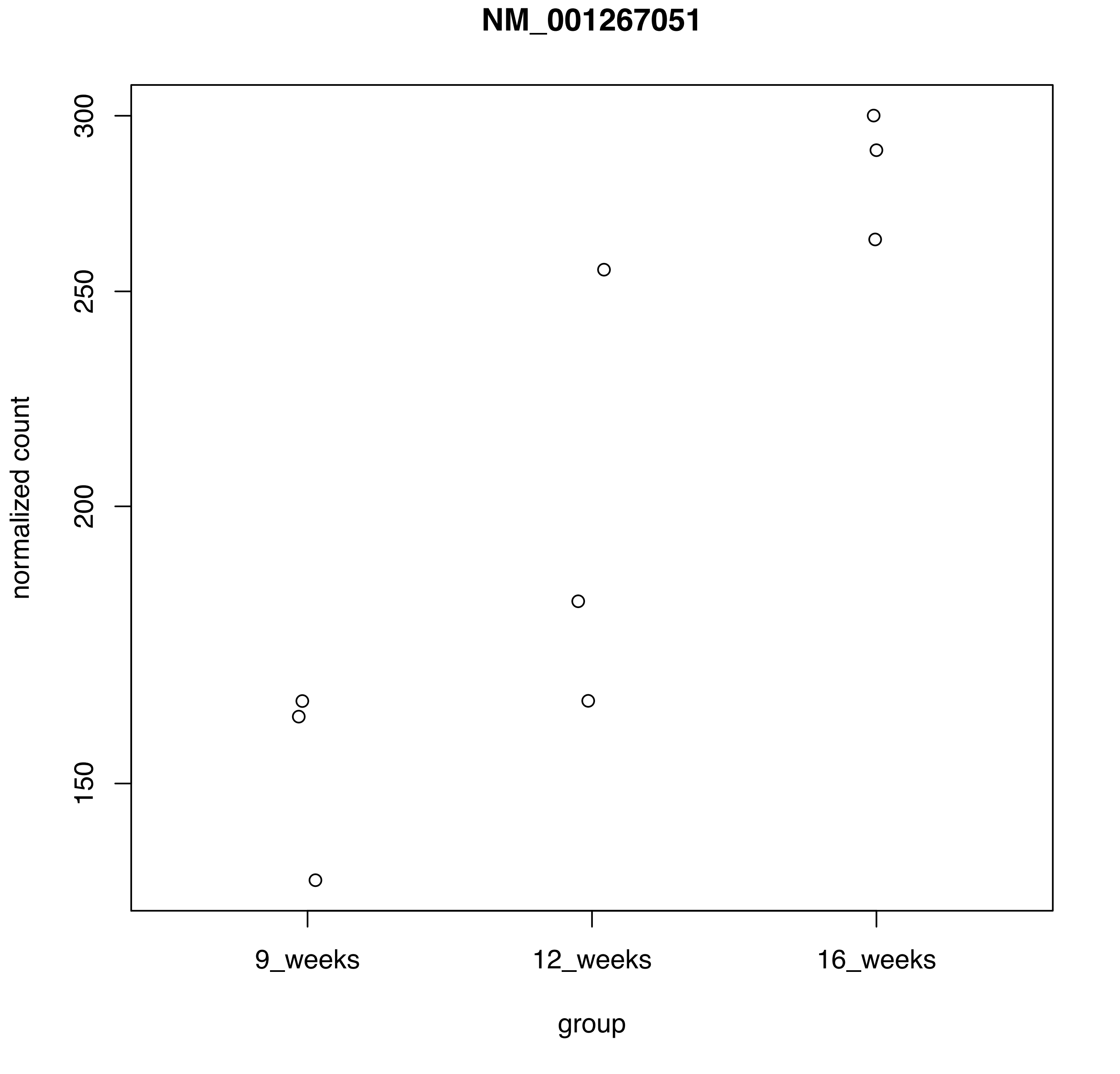
NM_001267051
normalized count
300
250
200
150
9_weeks
12_weeks
16_weeks
group

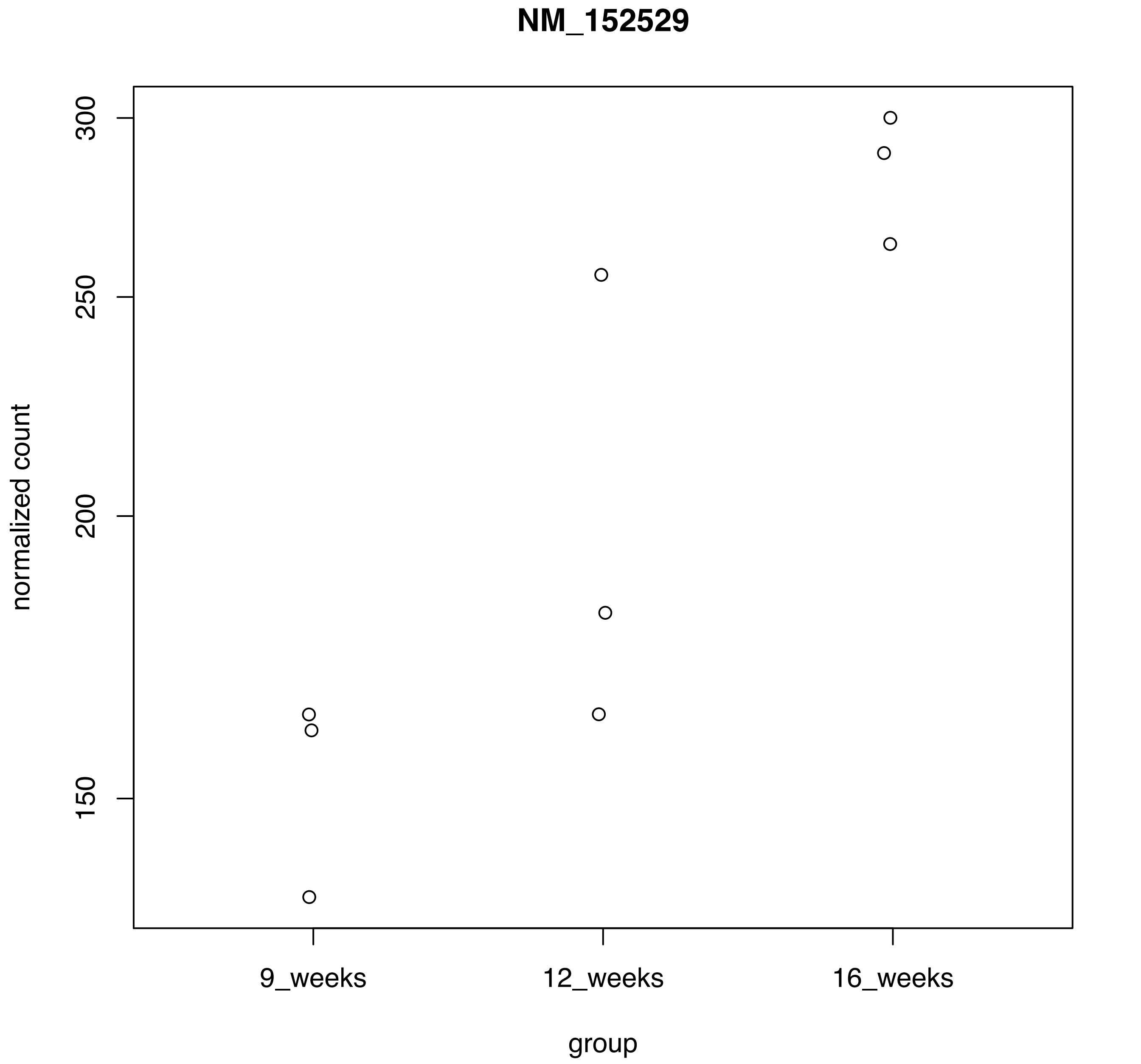

NM_152529
normalized count
300
250
200
150
9_weeks
12_weeks
16_weeks
group

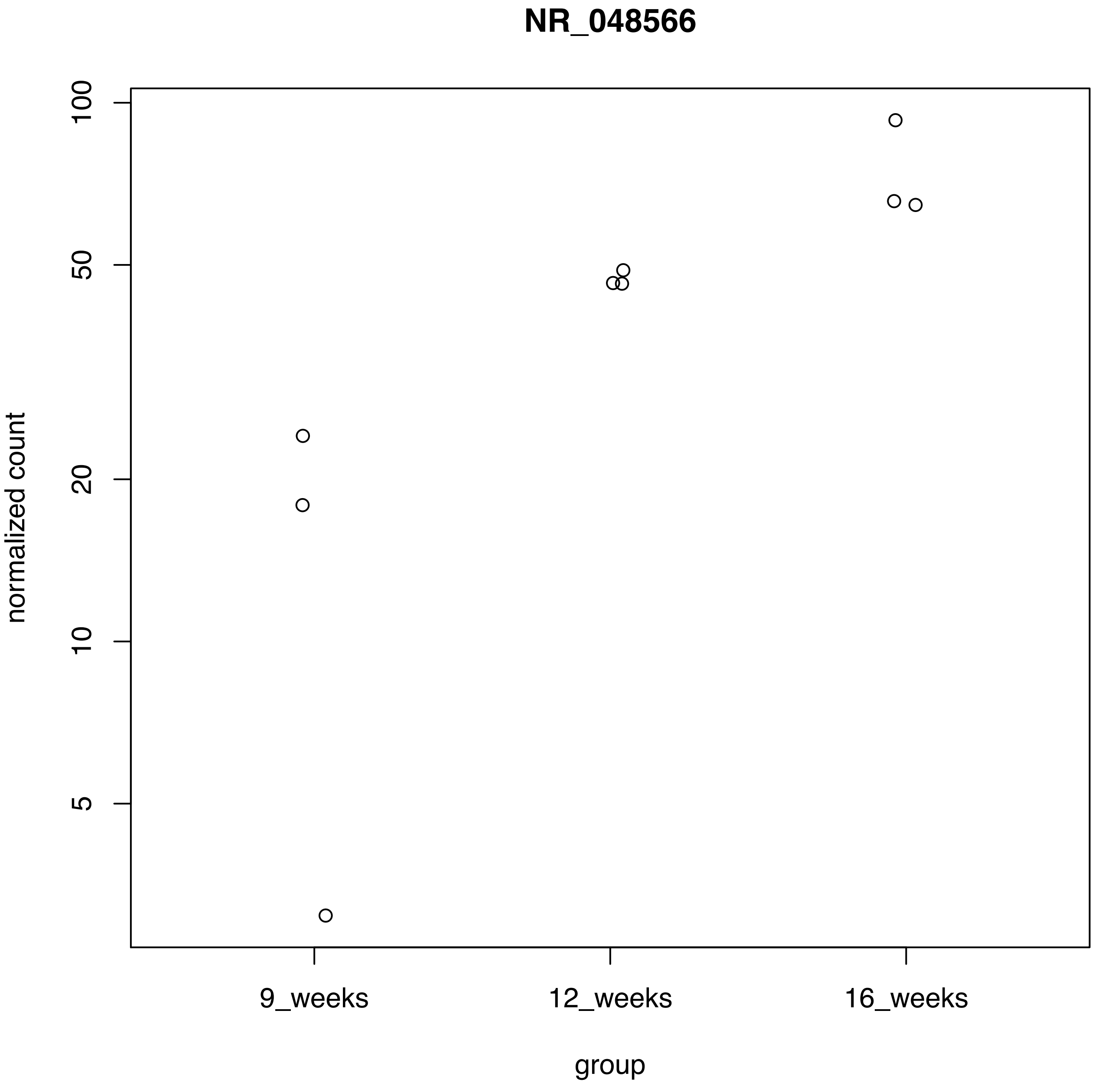

NR_048566
normalized count
100
50
20
10
5
9_weeks
12_weeks
16_weeks
group

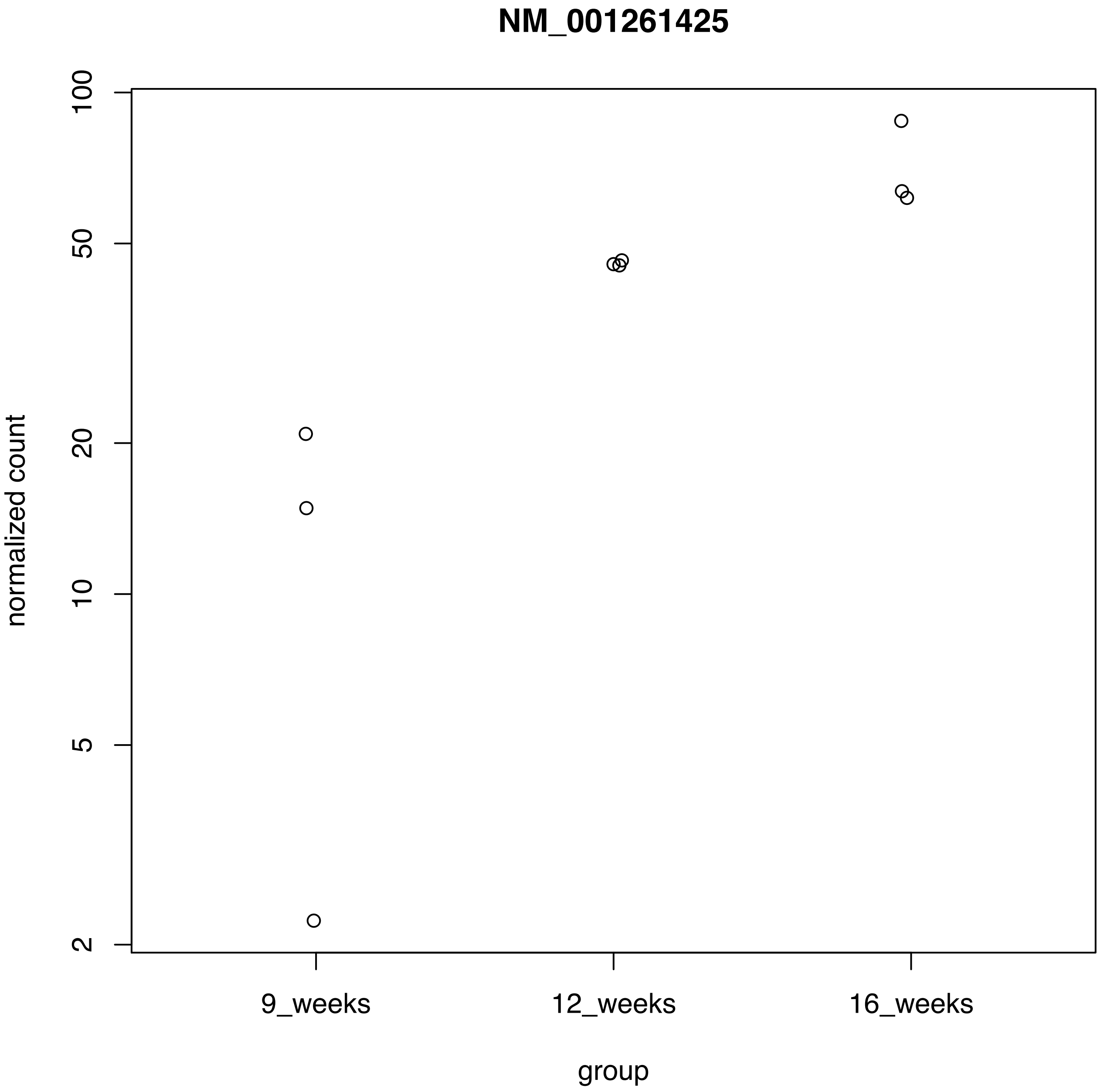
NM_001261425
normalized count
100
50
20
10
5
2
9_weeks
12_weeks
16_weeks
group

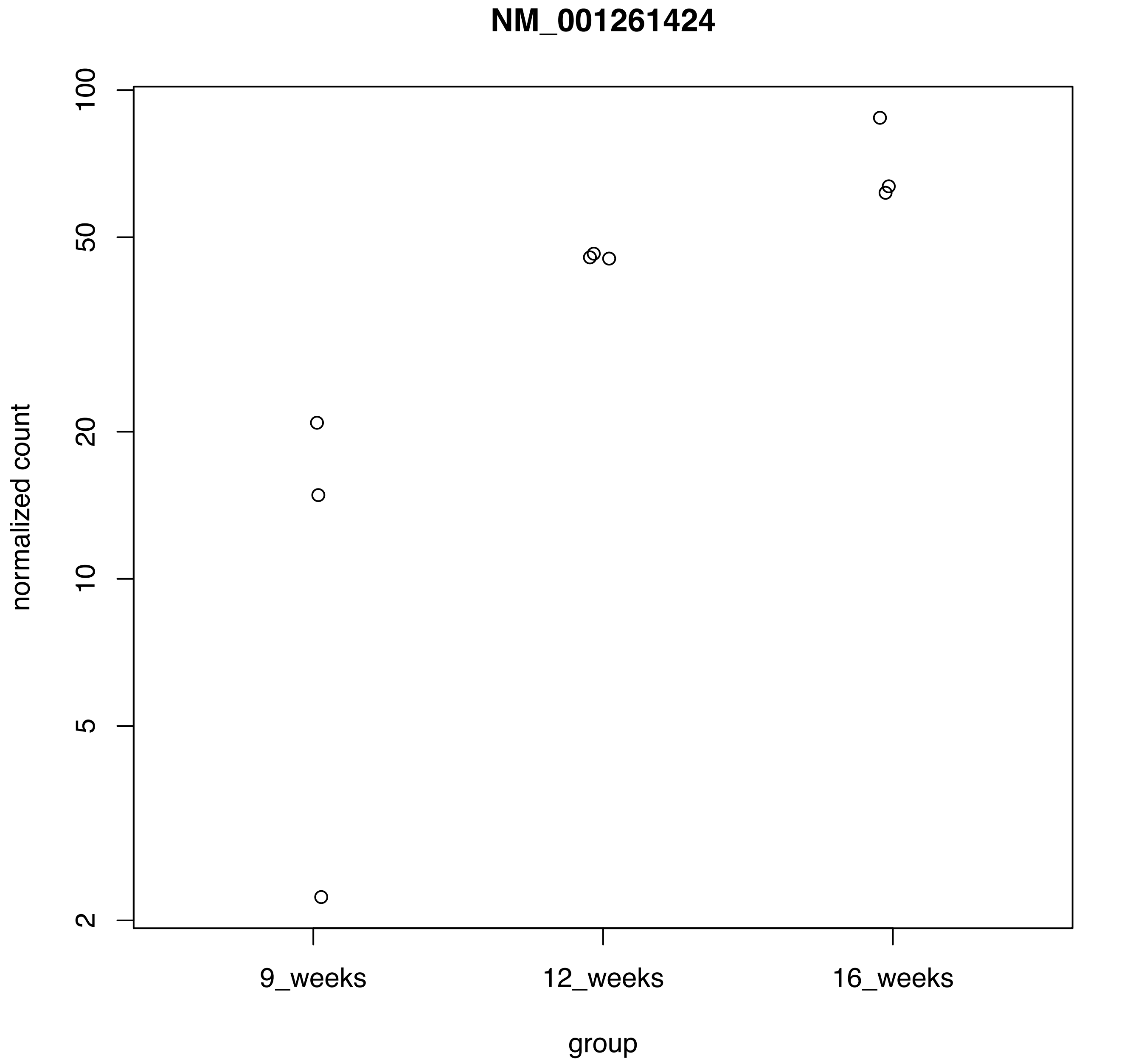

NM_001261424
normalized count
100
50
20
10
5
2
9_weeks
12_weeks
16_weeks
group

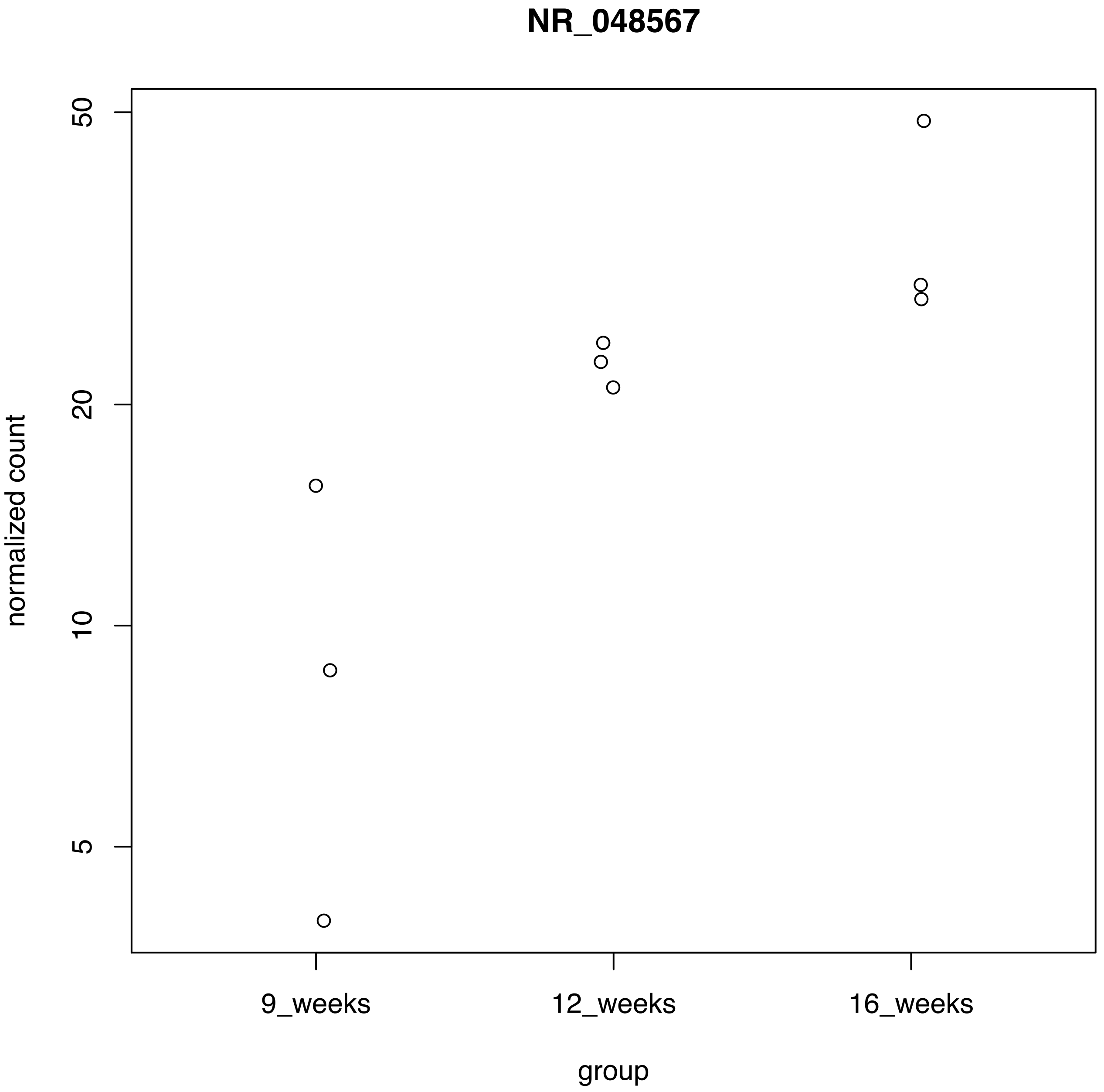
NR_048567
normalized count
50
20
10
5
9_weeks
12_weeks
16_weeks
group

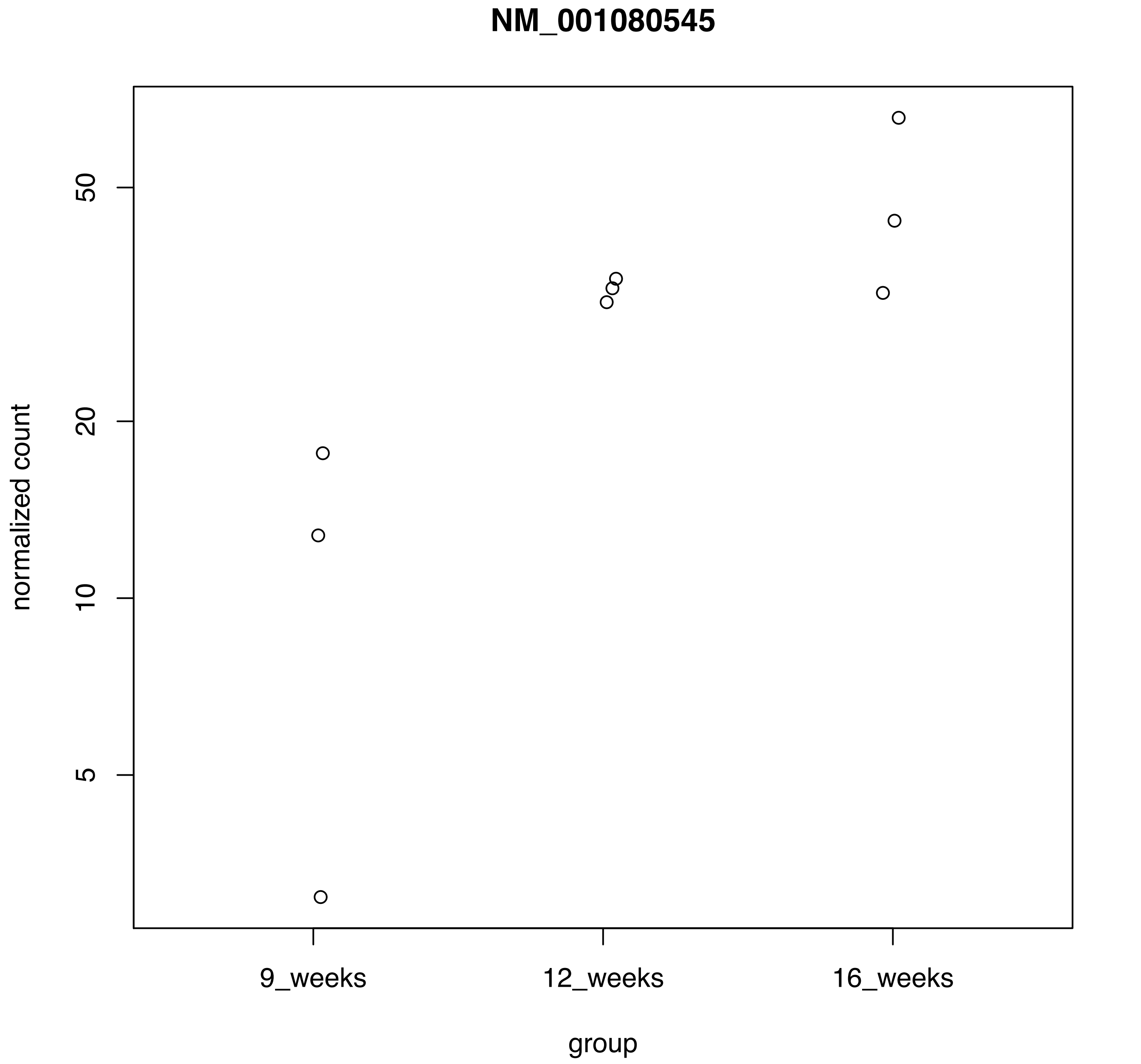
NM_001080545
normalized count
50
20
10
5
9_weeks
12_weeks
16_weeks
group

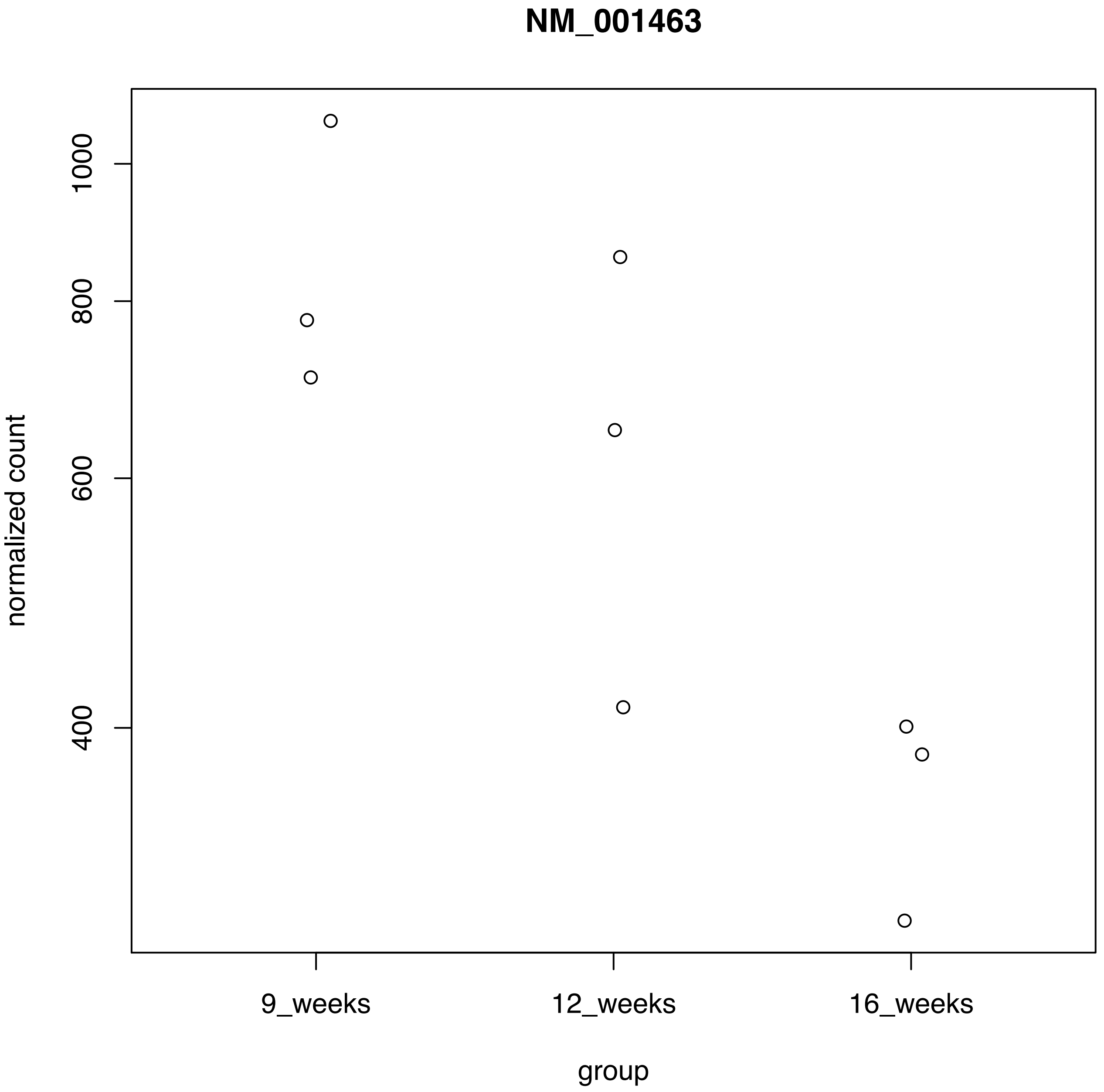
NM_001463
normalized count
1000
800
600
400
9_weeks
12_weeks
16_weeks
group

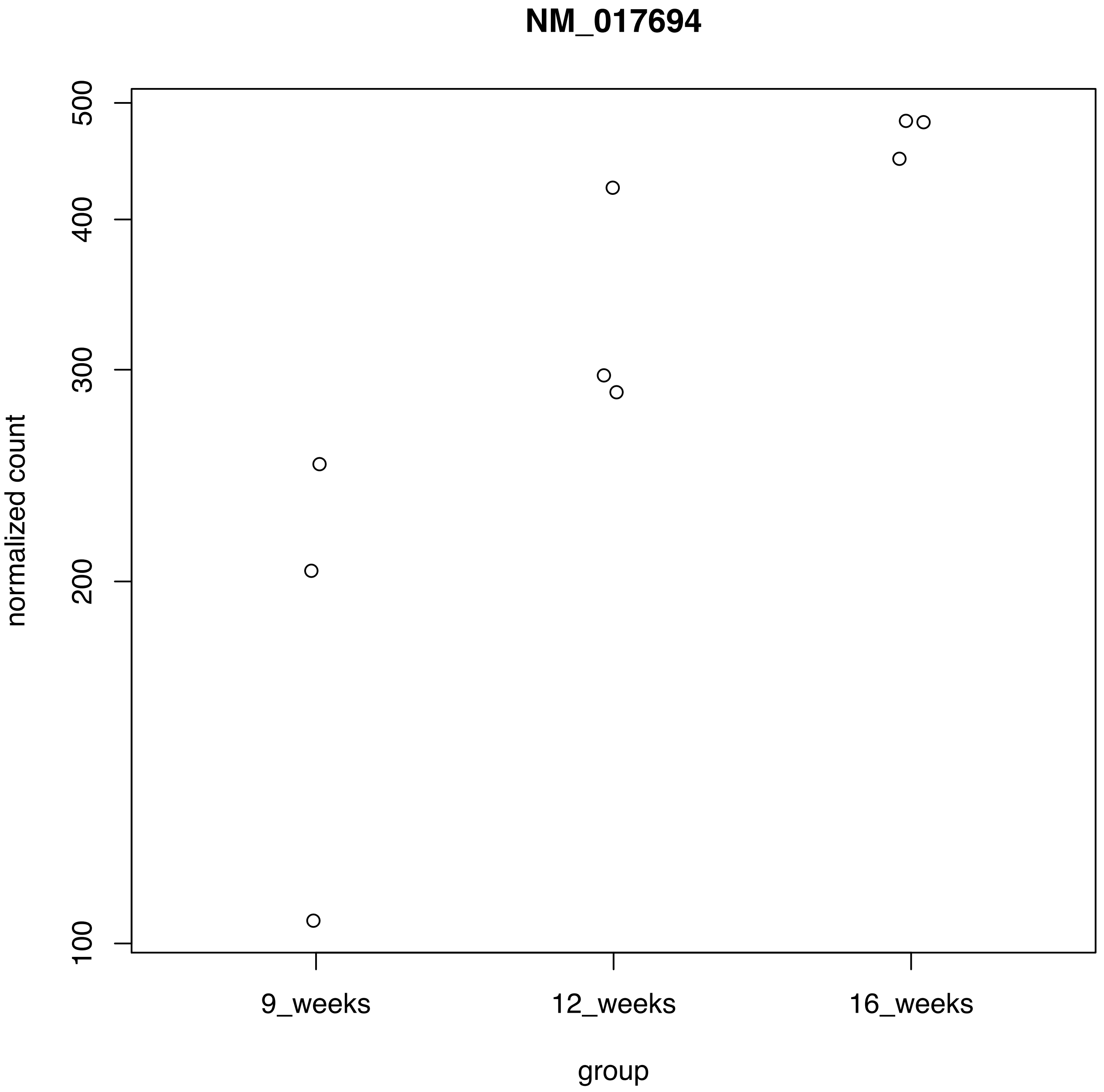
NM_017694
normalized count
500
400
300
200
100
9_weeks
12_weeks
16_weeks
group

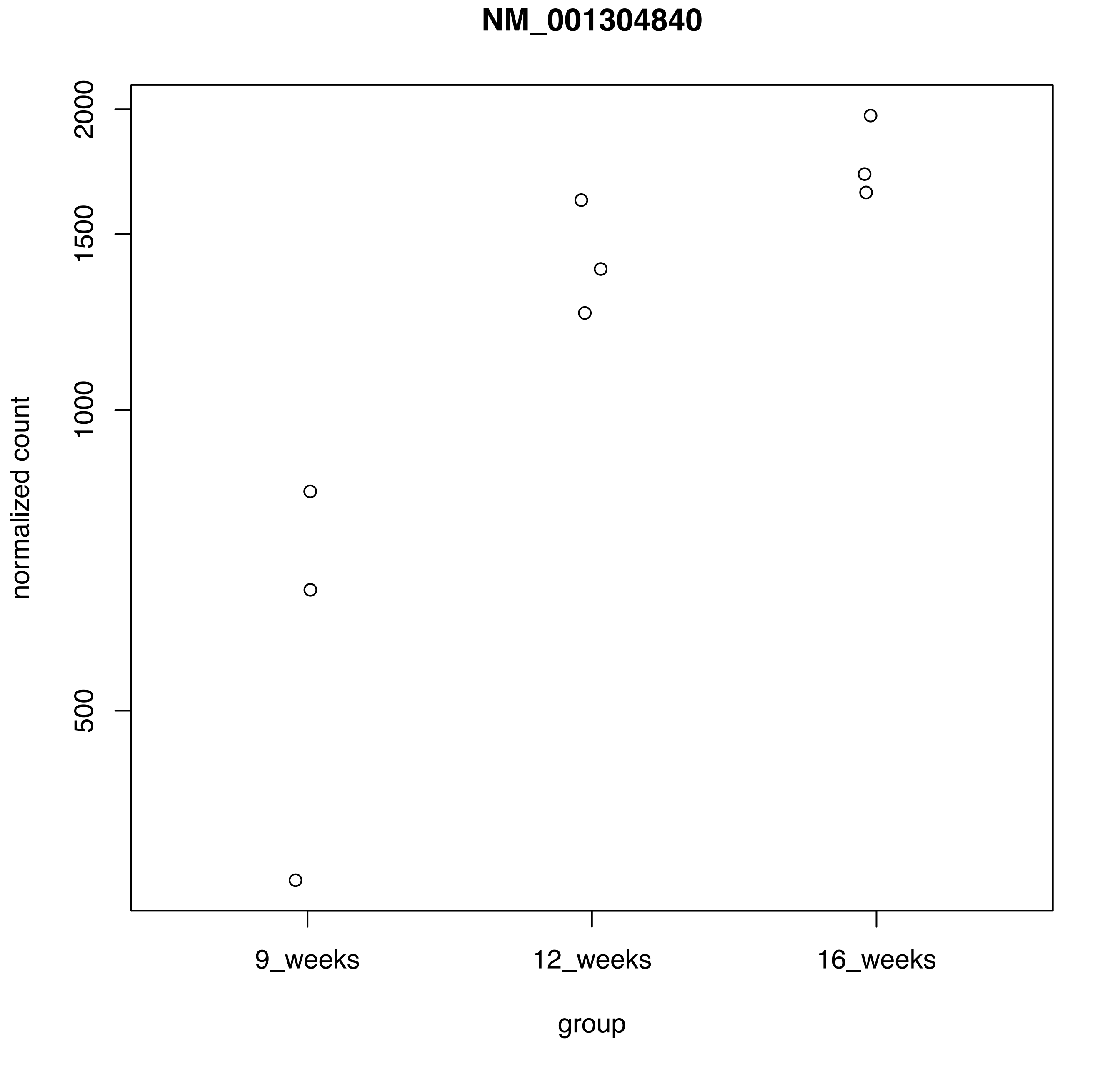

NM_001304840
2000
1500
1000
500
normalized count
9_weeks
12_weeks
16_weeks
group

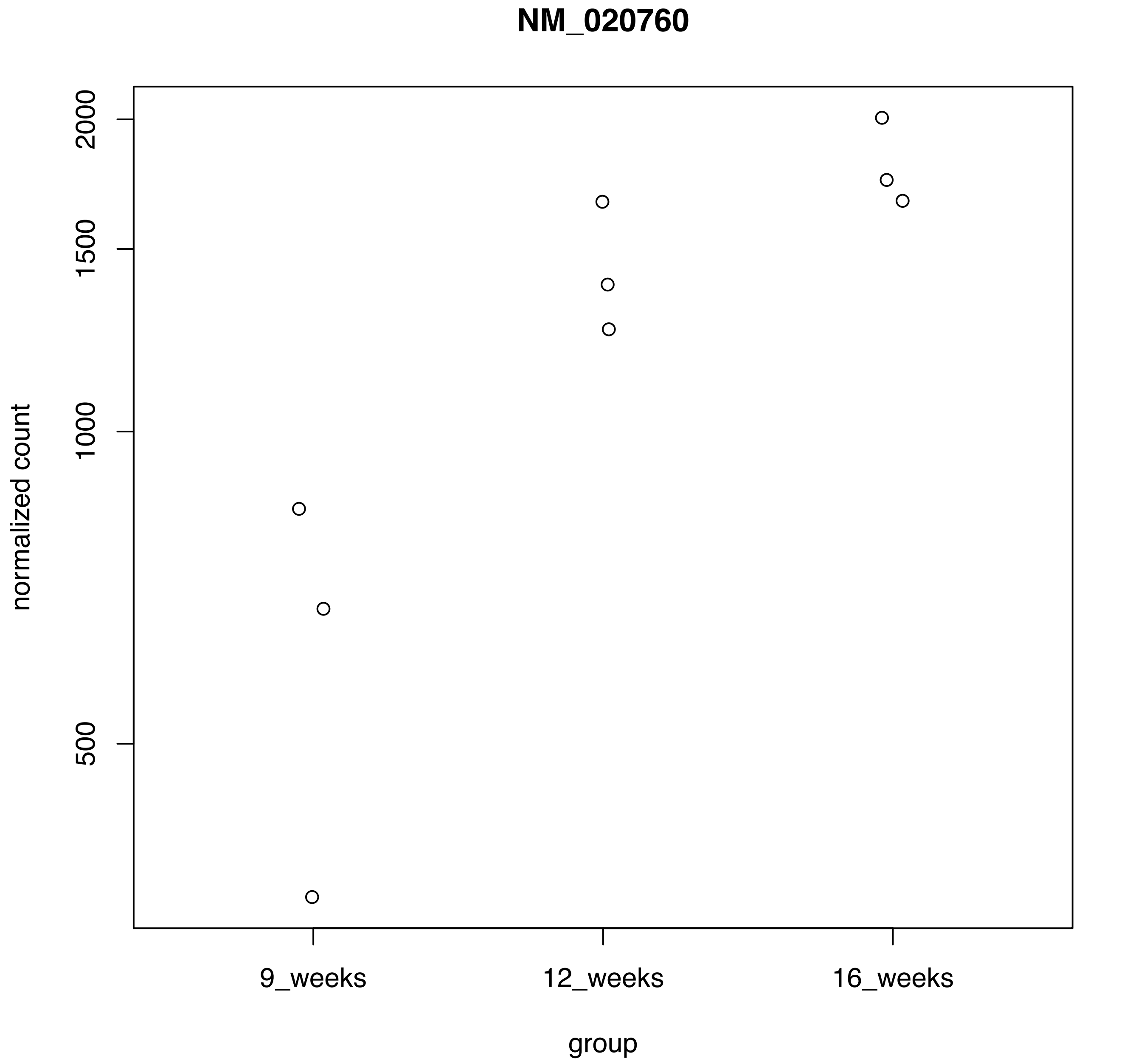
NM_020760
normalized count
2000
1500
1000
500
9_weeks
12_weeks
16_weeks
group

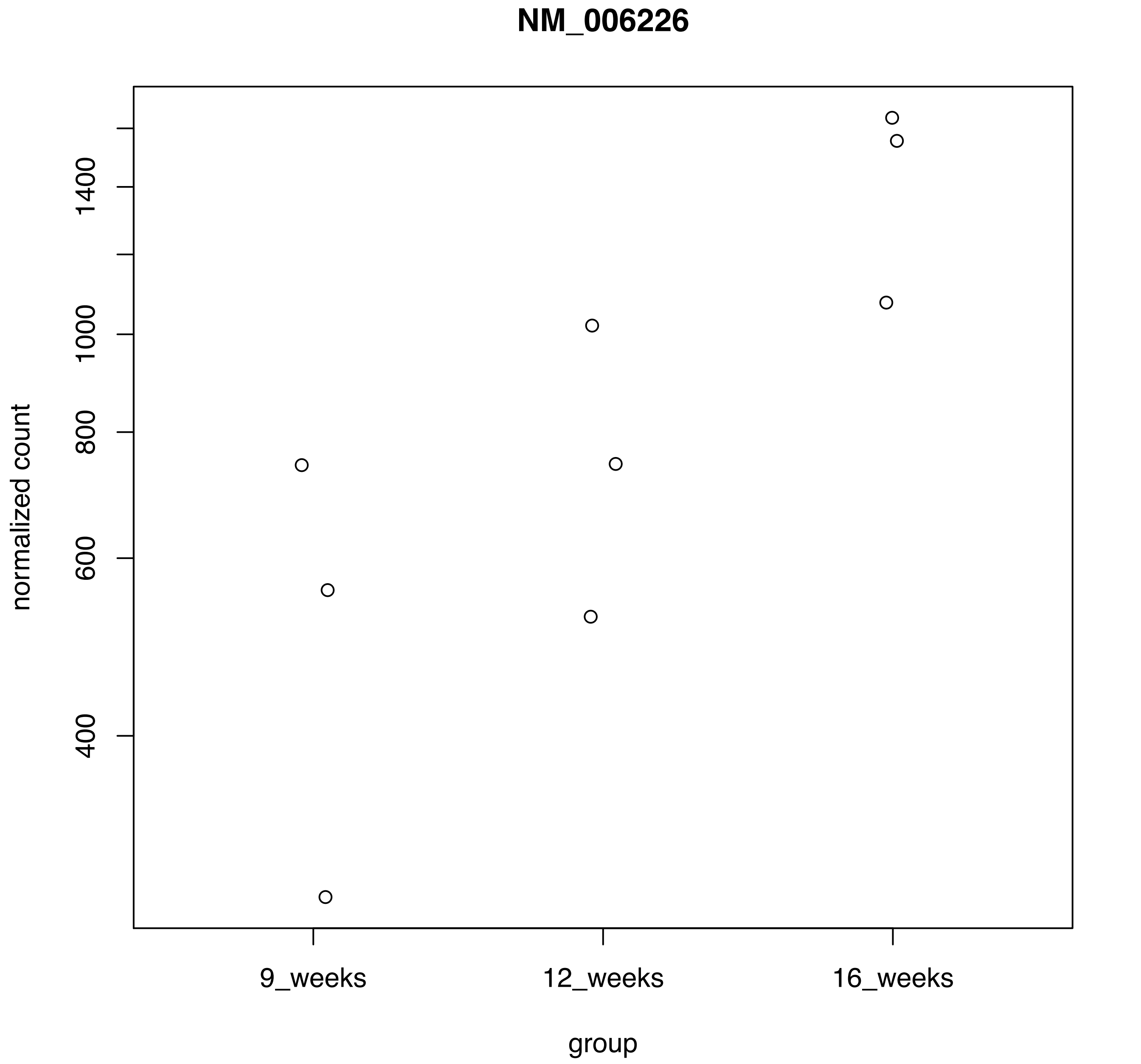
NM_006226
normalized count
400
600
800
1000
1400
9_weeks
12_weeks
16_weeks
group

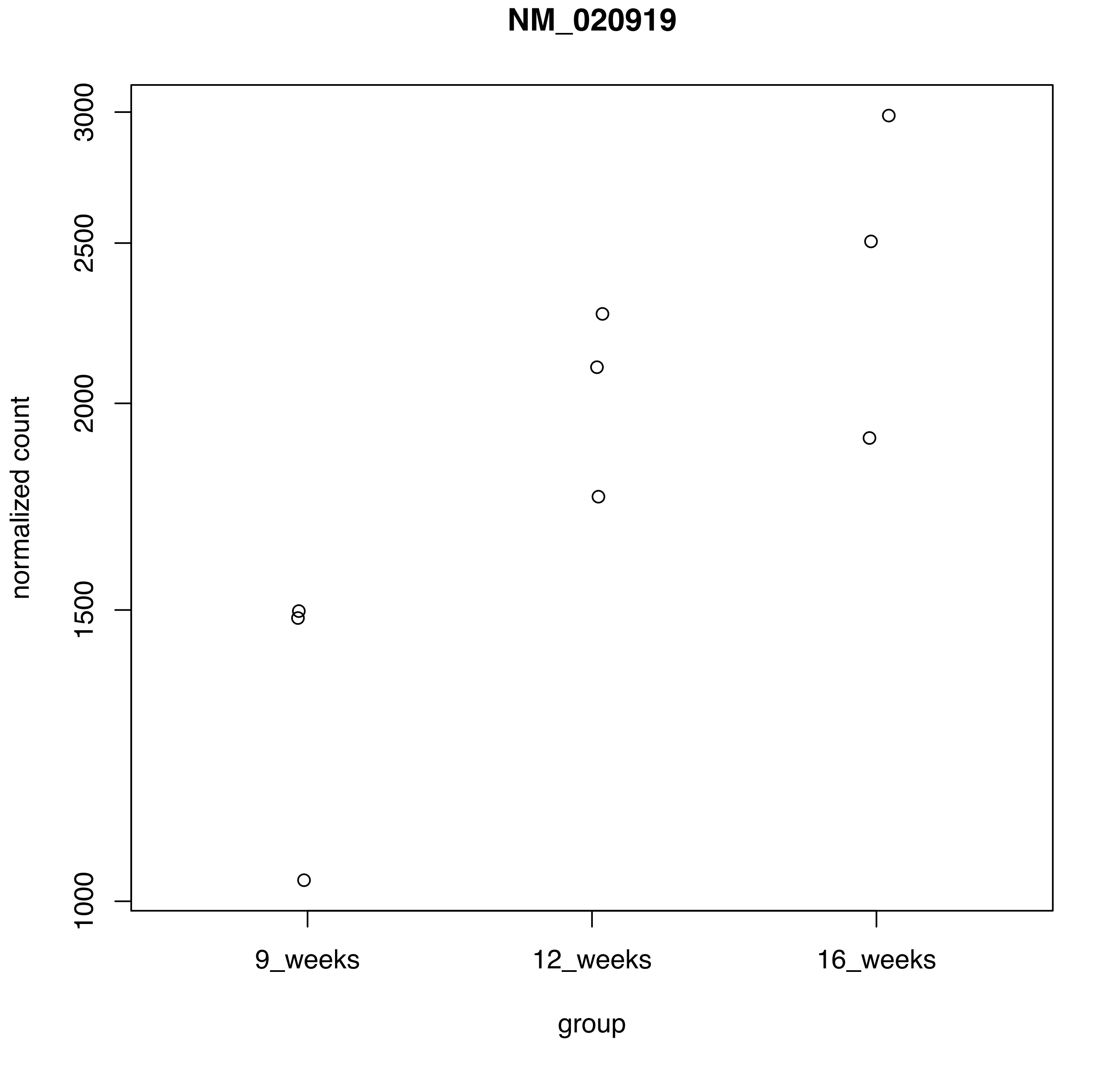

NM_020919
normalized count
3000
2500
2000
1500
1000
9_weeks
12_weeks
16_weeks
group

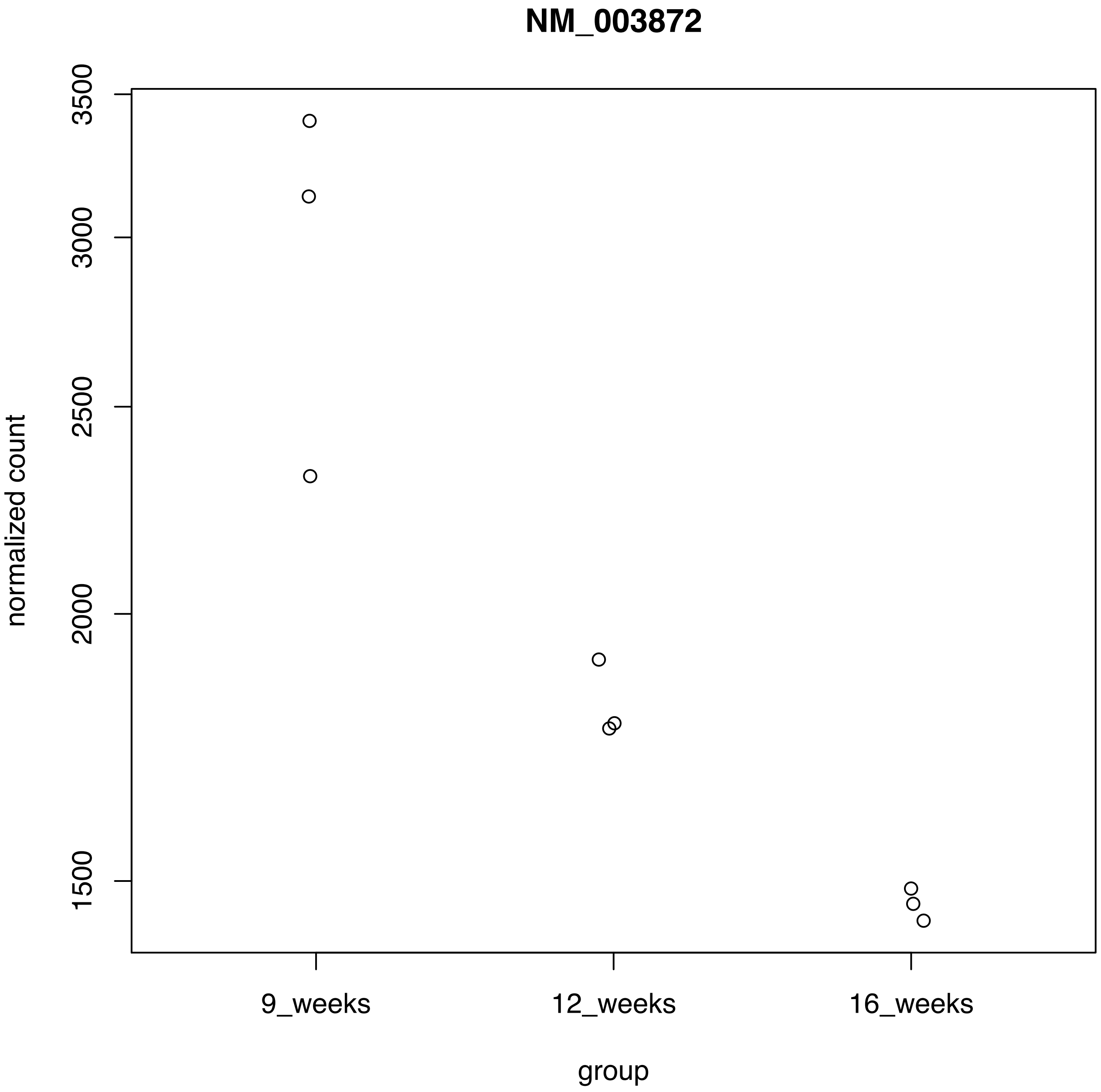
NM_003872
normalized count
3500
3000
2500
2000
1500
9_weeks
12_weeks
16_weeks
group

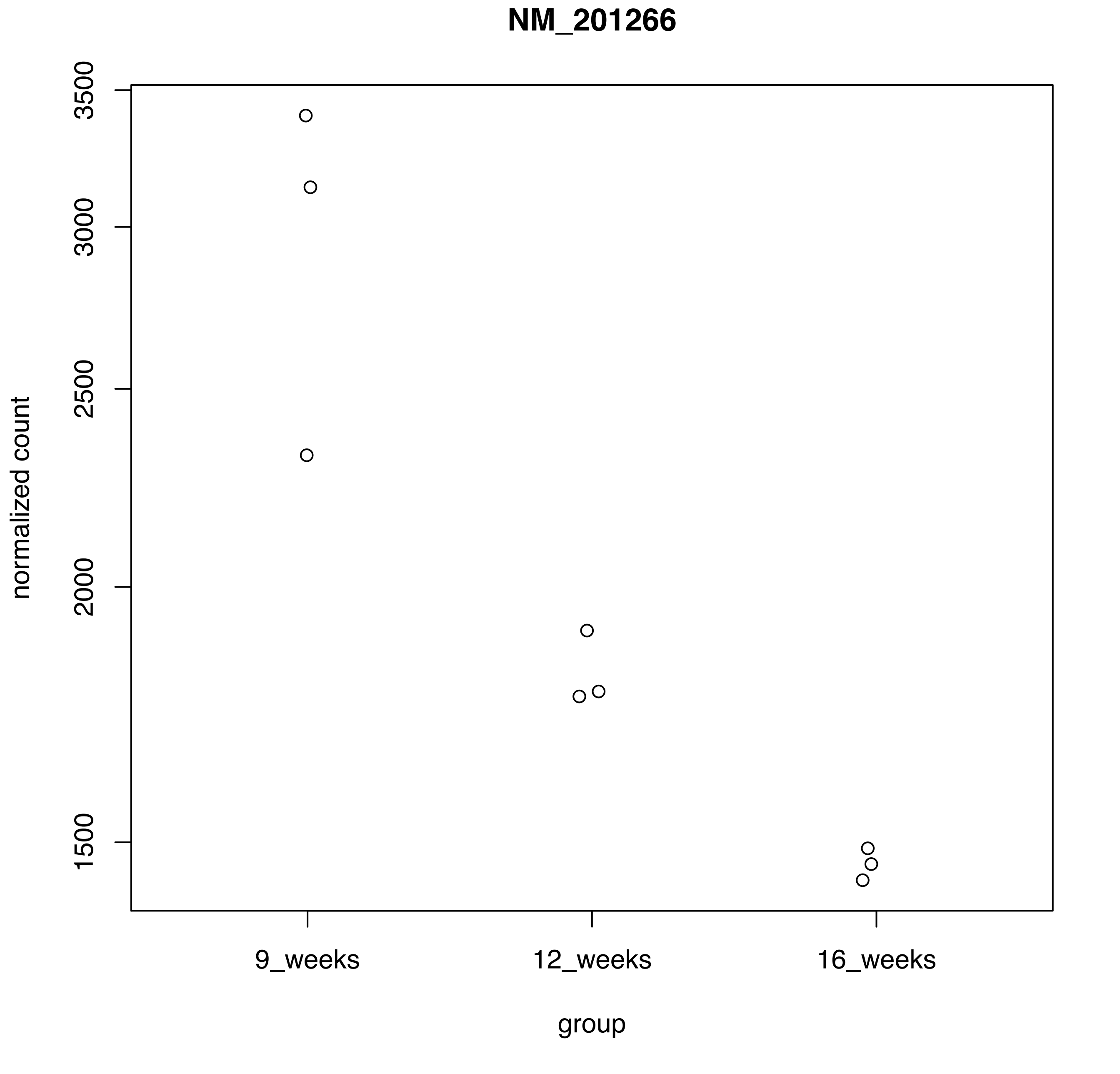

NM_201266
normalized count
3500
3000
2500
2000
1500
9_weeks
12_weeks
16_weeks
group

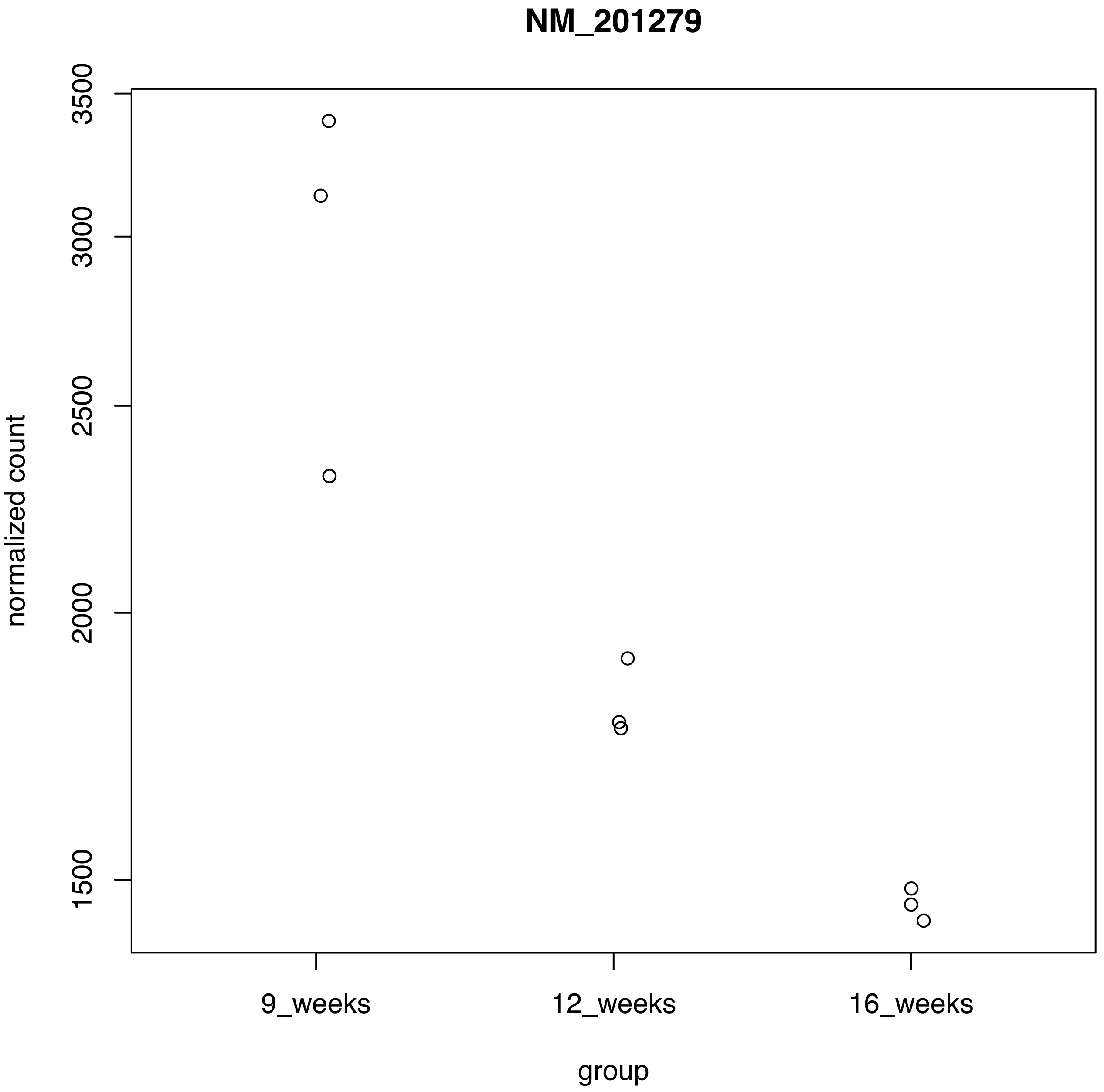

NM_201279
normalized count
3500
3000
2500
2000
1500
9_weeks
12_weeks
16_weeks
group

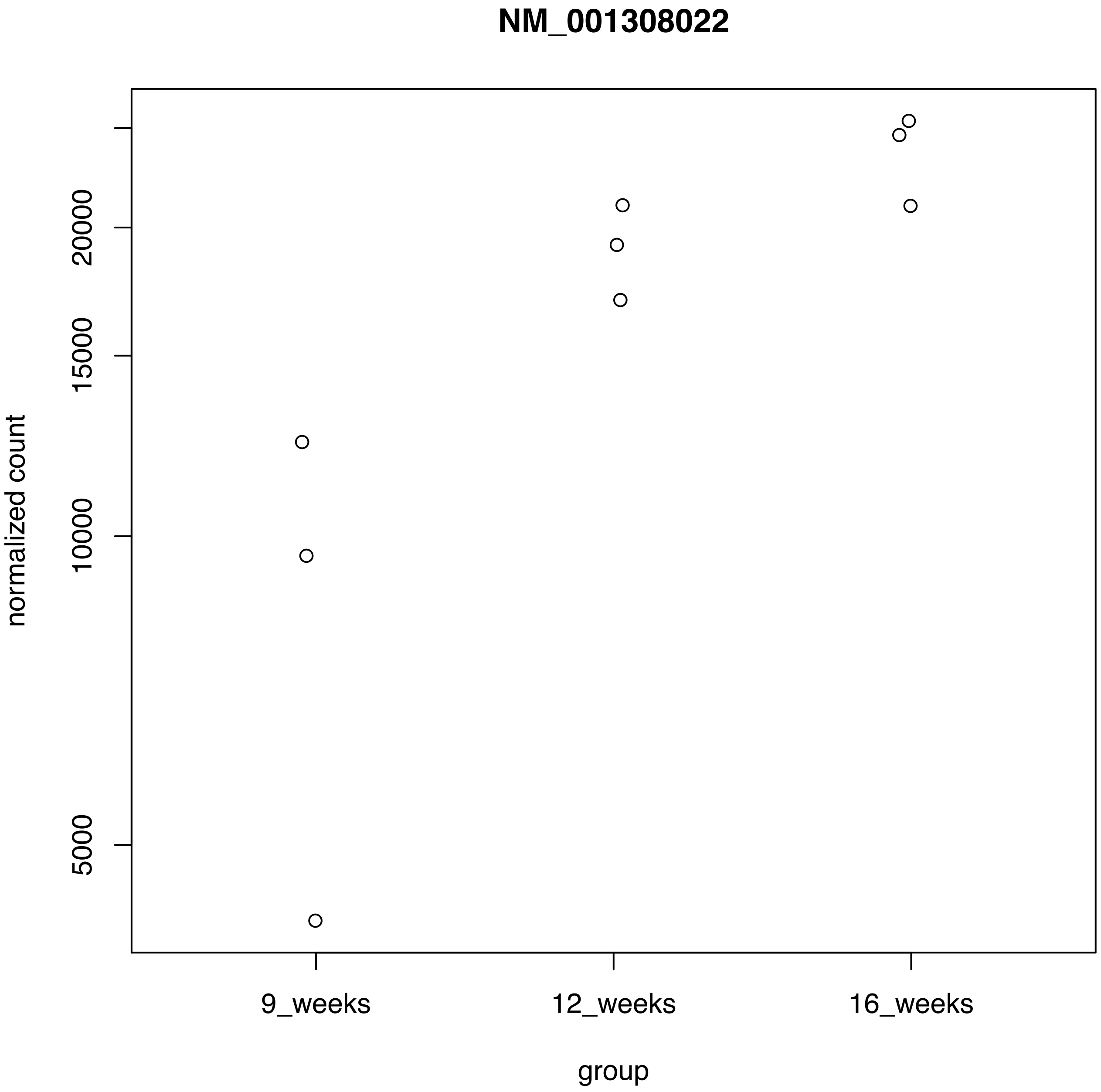
NM_001308022
normalized count
5000
10000
15000
20000
9_weeks
12_weeks
16_weeks
group

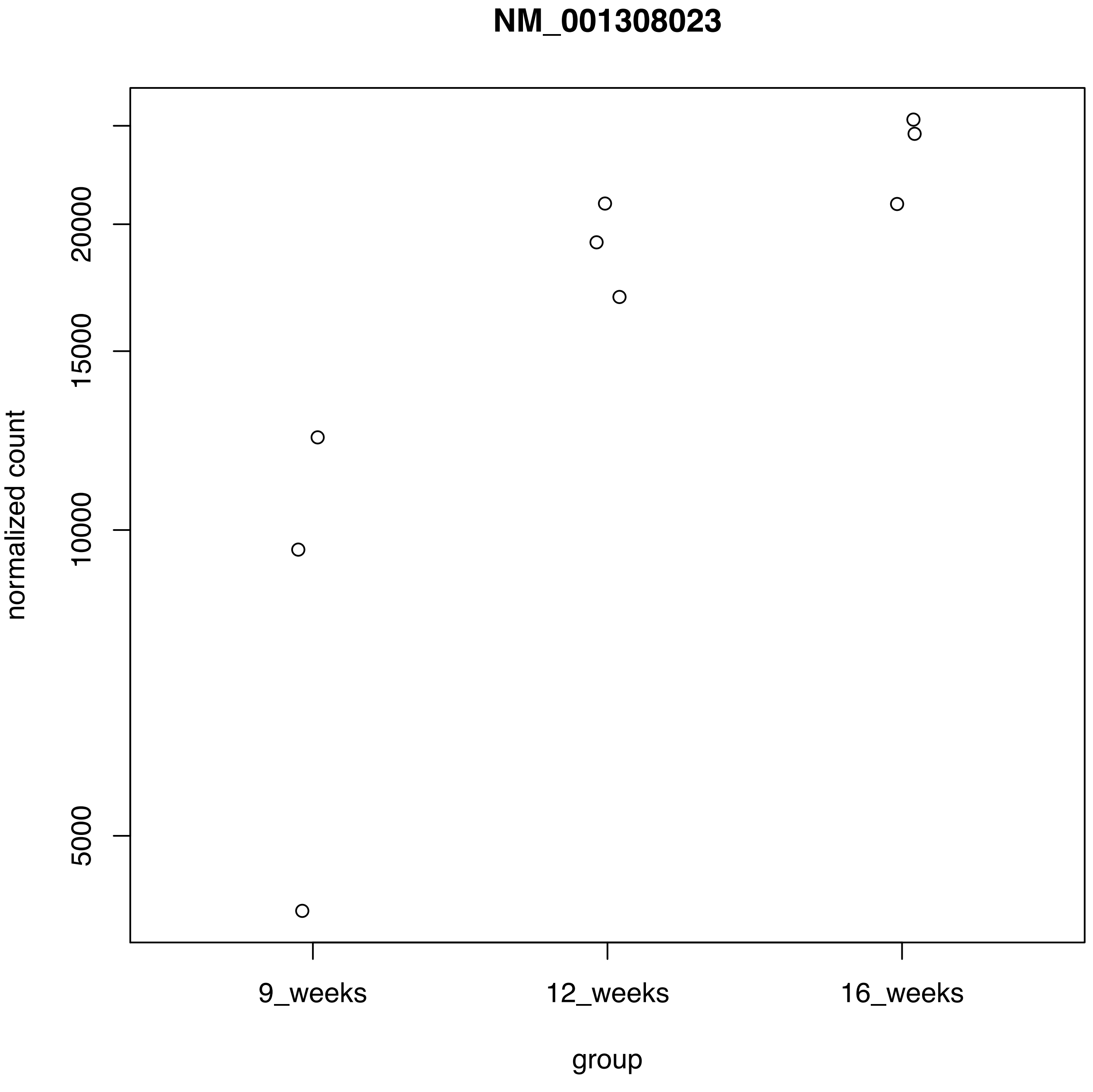

NM_001308023
normalized count
5000
10000
15000
20000
9_weeks
12_weeks
16_weeks
group

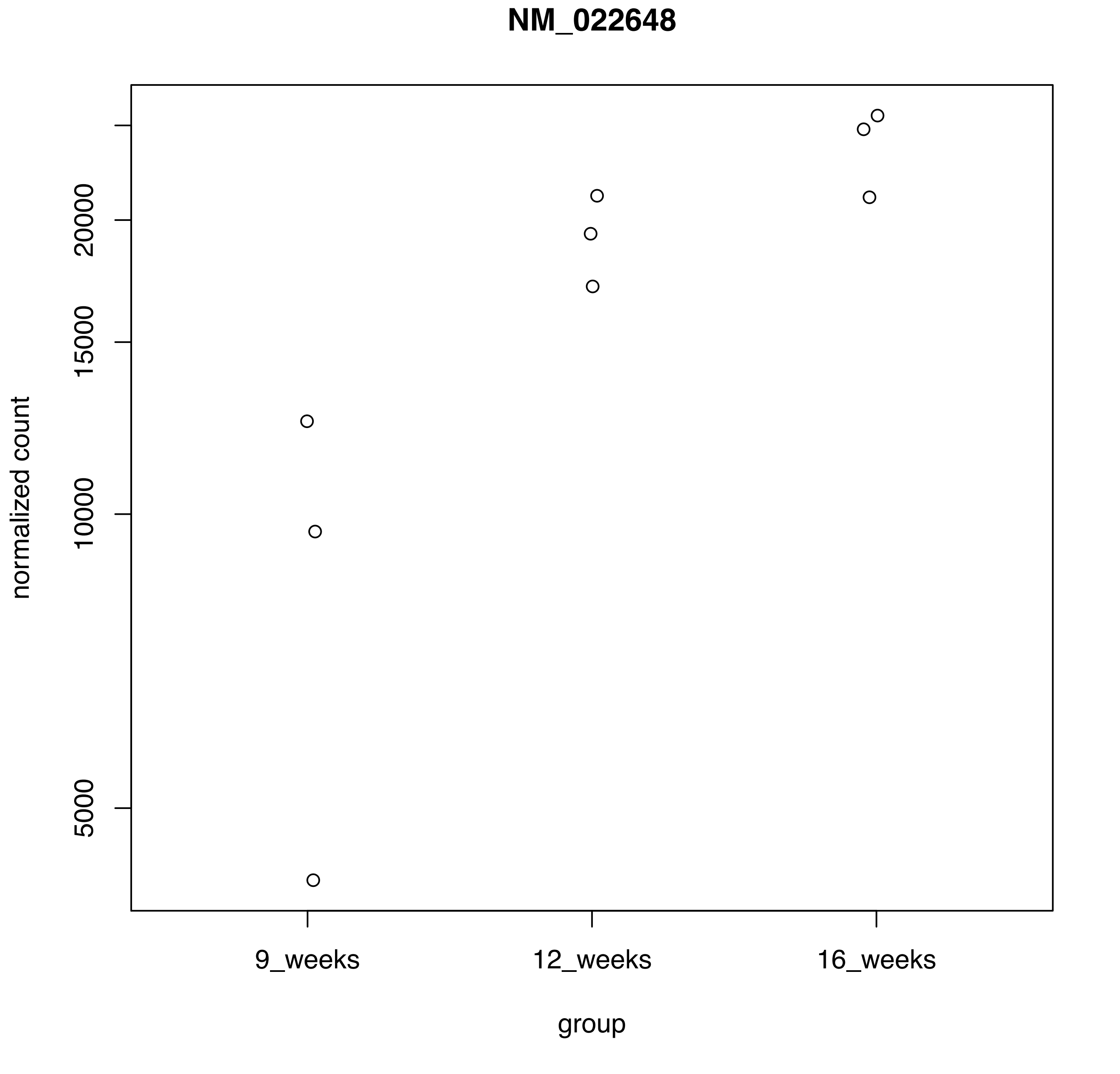

NM_022648
normalized count
5000
10000
15000
20000
9_weeks
12_weeks
16_weeks
group

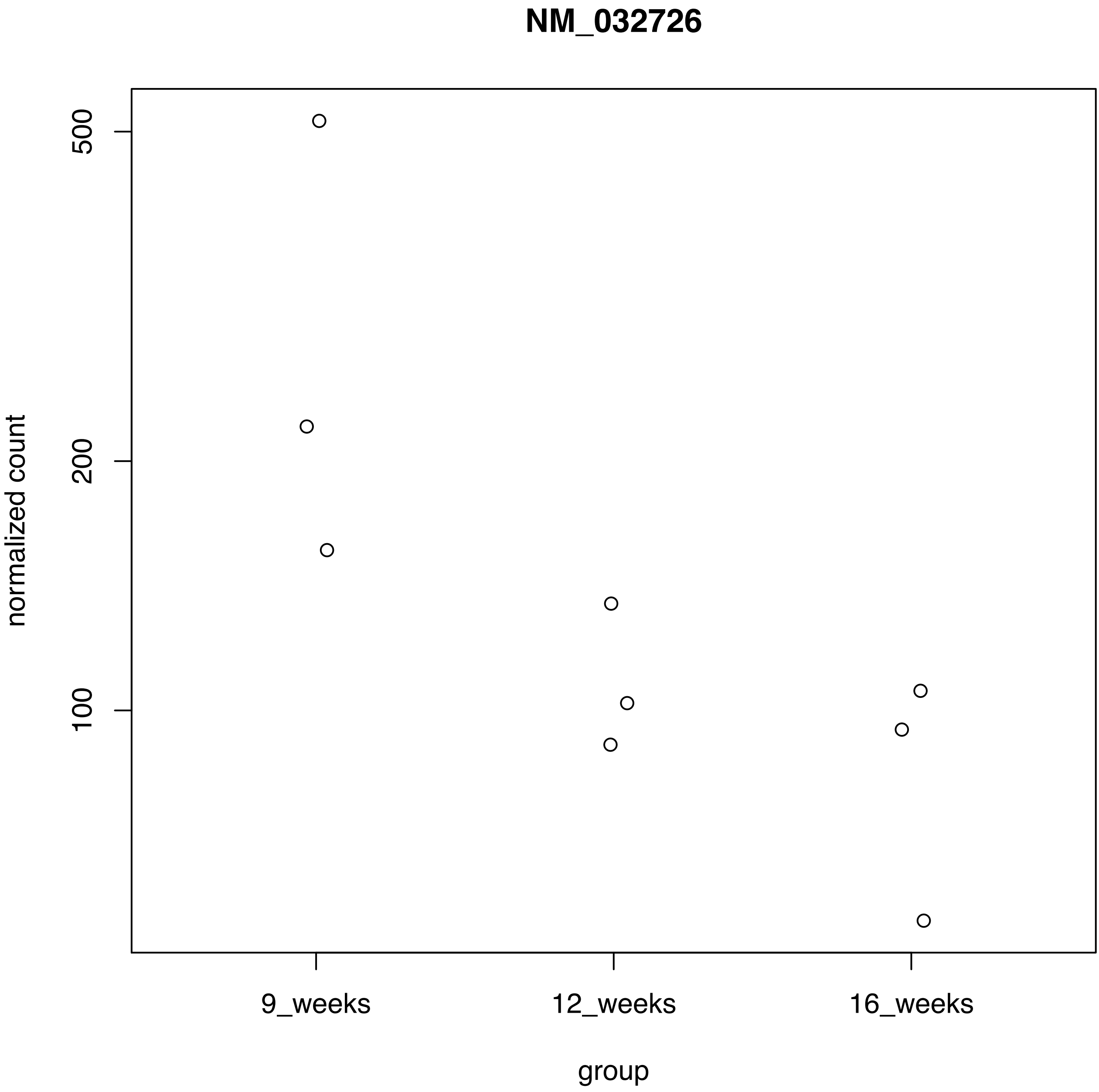

NM_032726
500
200
100
normalized count
9_weeks
12_weeks
16_weeks
group

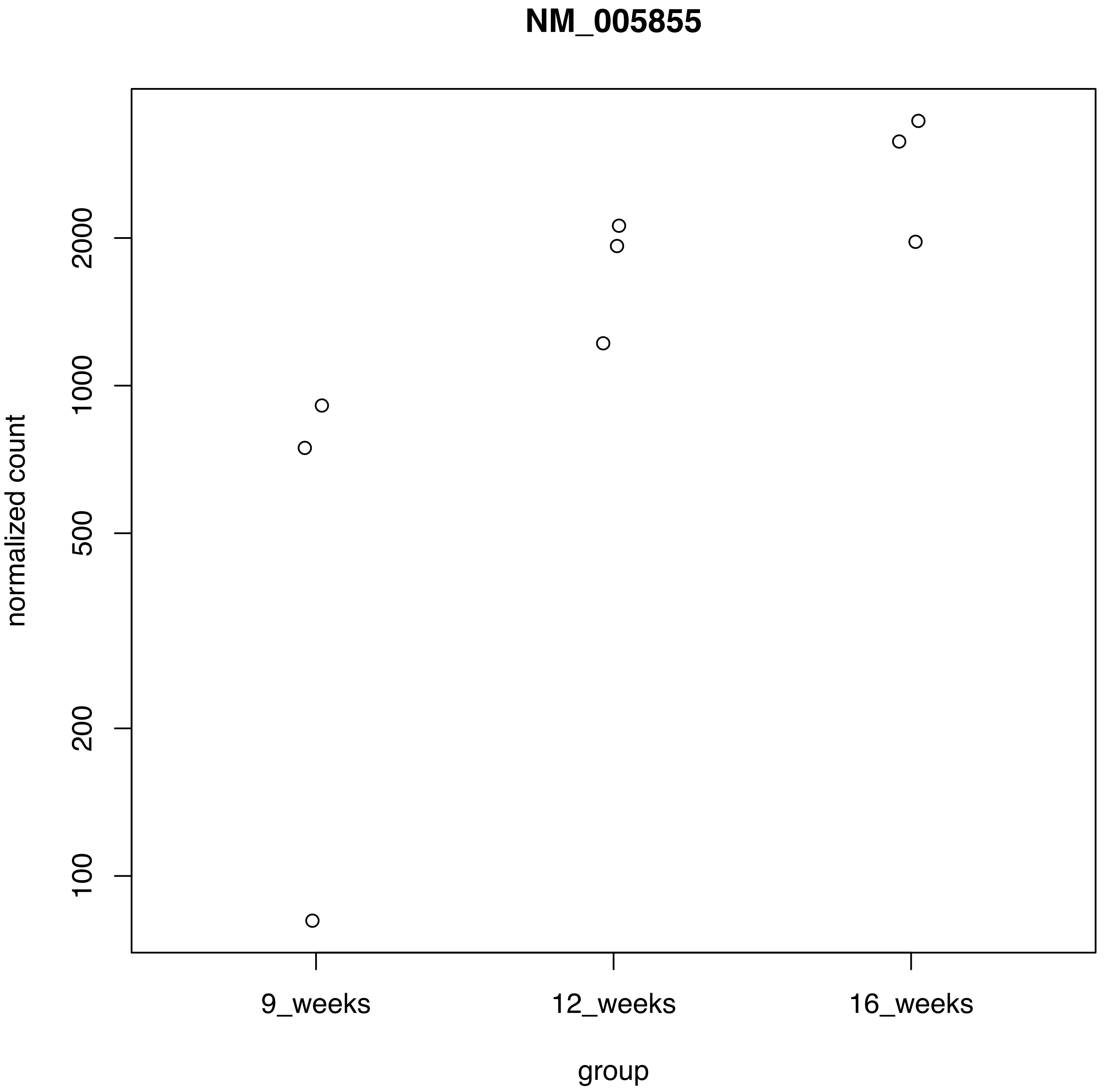
NM_005855
normalized count
2000
1000
500
200
100
9_weeks
12_weeks
16_weeks
group

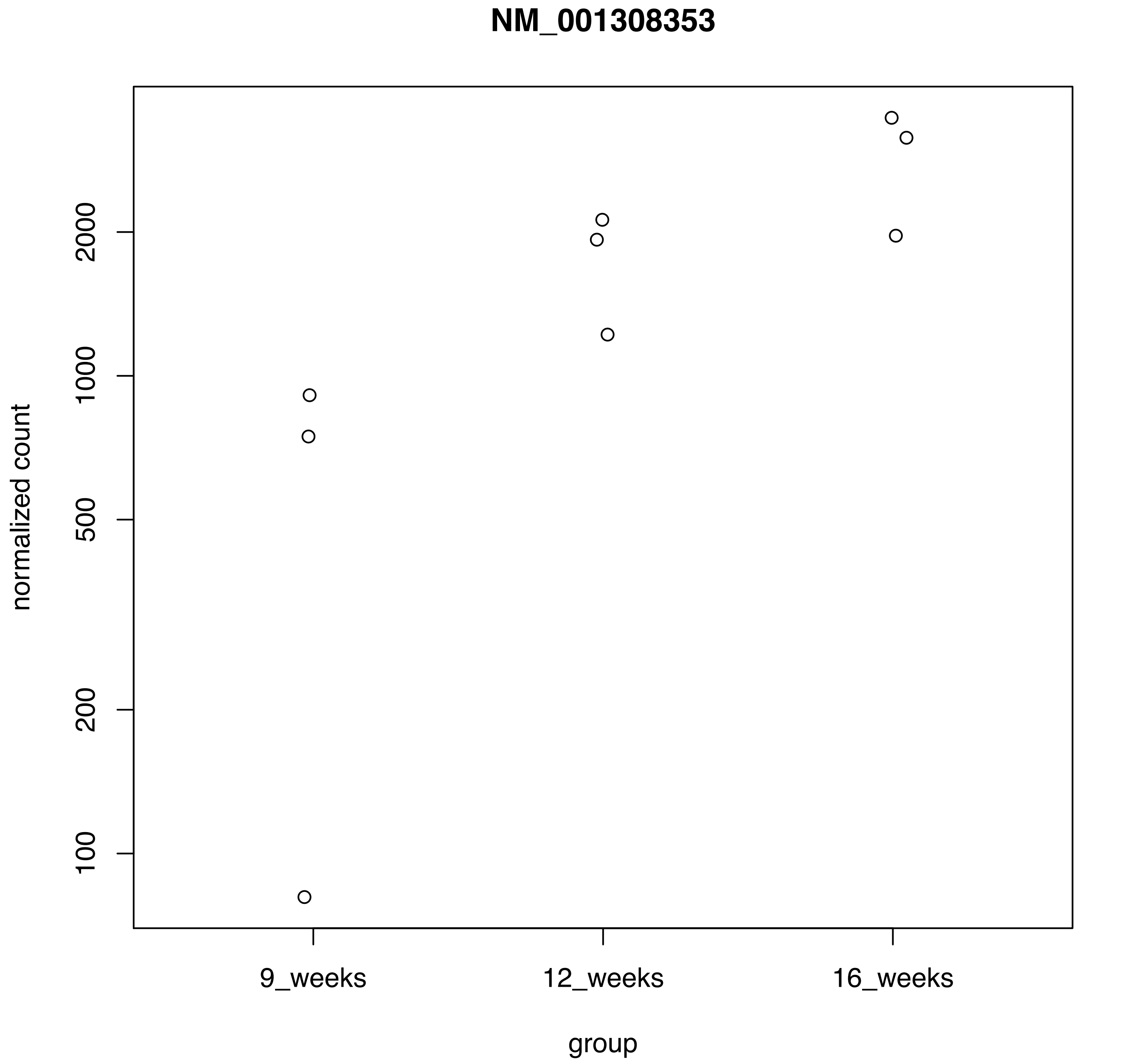

NM_001308353
normalized count
100
200
500
1000
2000
9_weeks
12_weeks
16_weeks
group

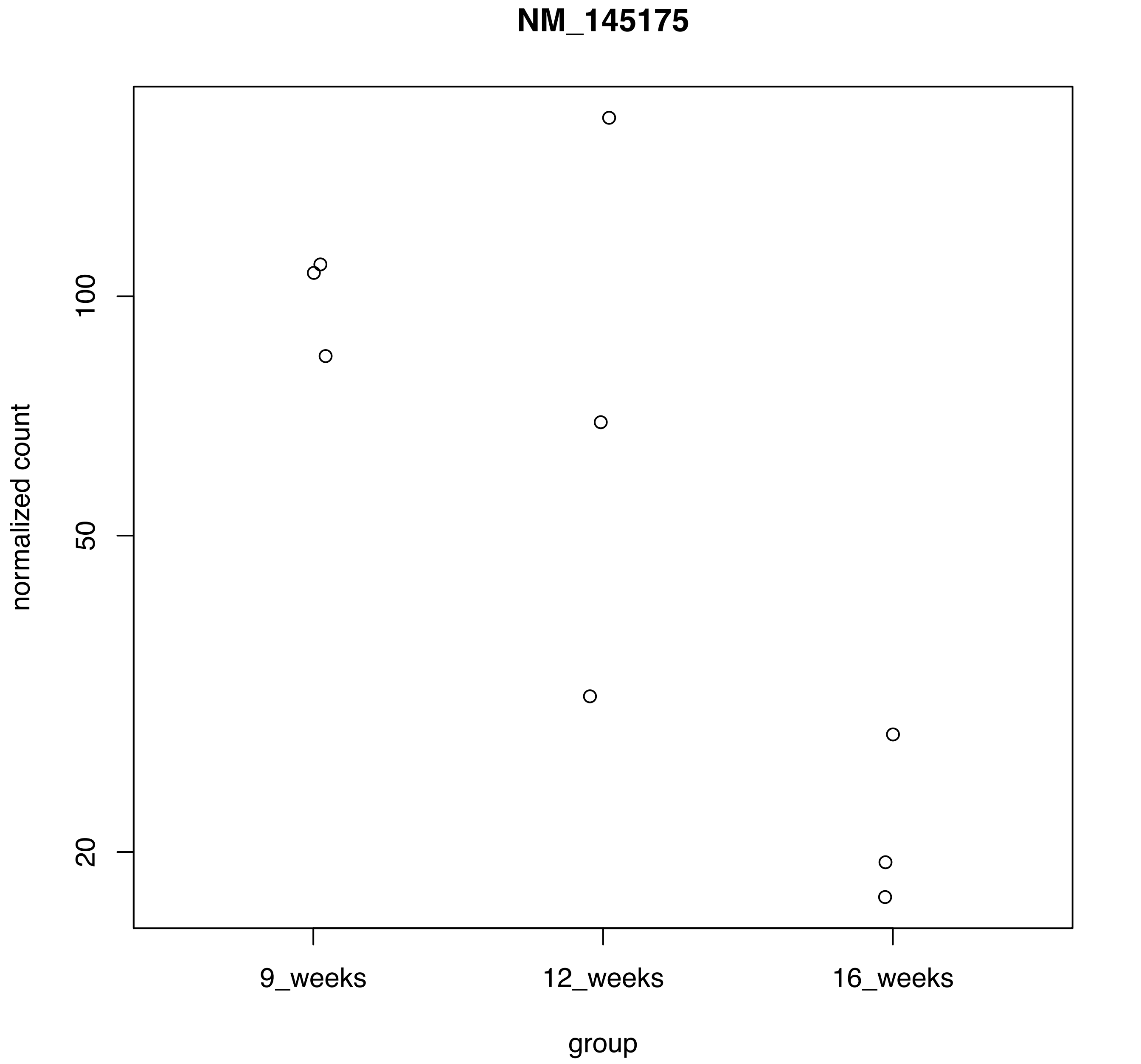
NM_145175
normalized count
100
50
20
9_weeks
12_weeks
16_weeks
group

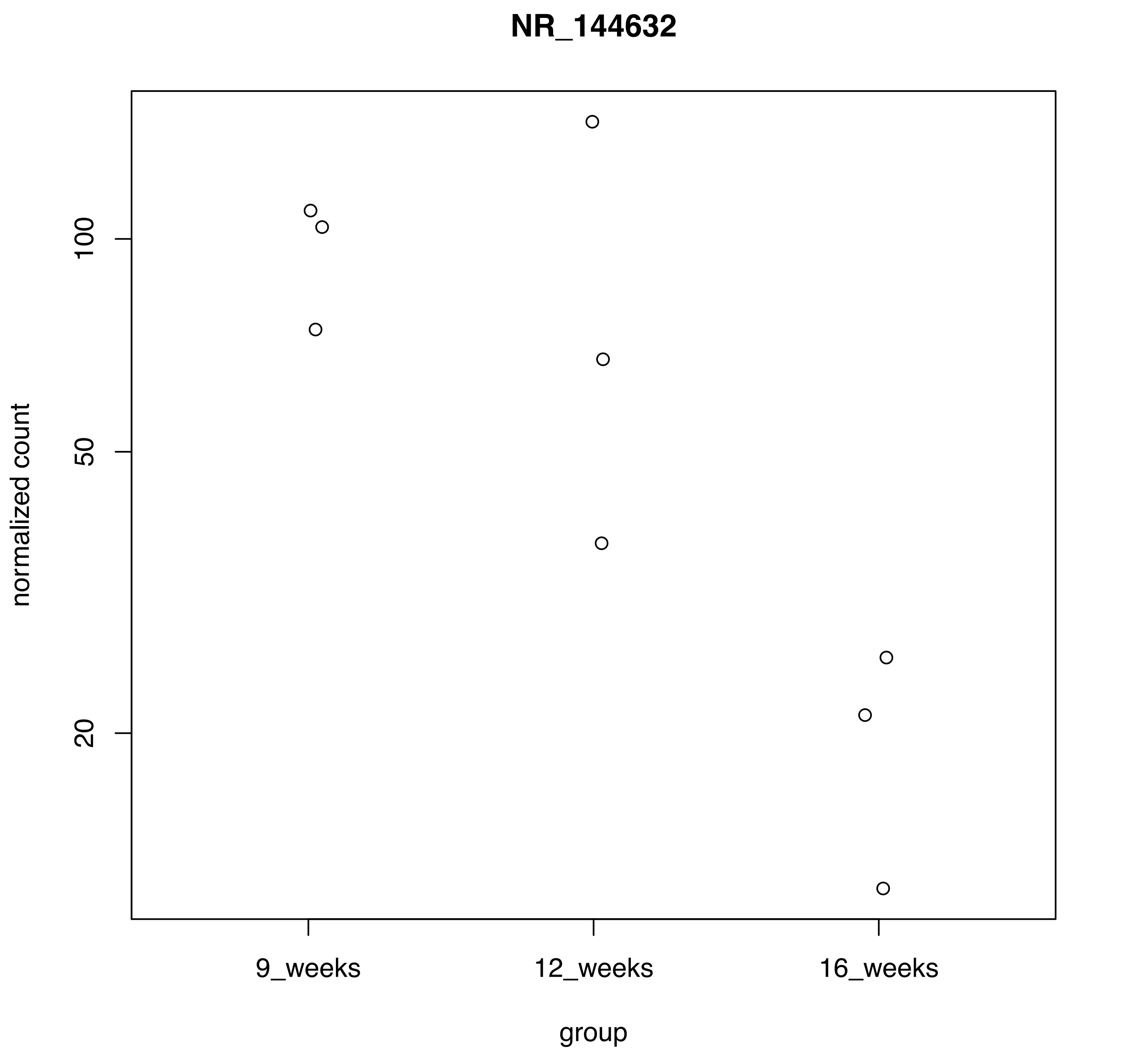
NR_144632
normalized count
100
50
20
9_weeks
12_weeks
16_weeks
group

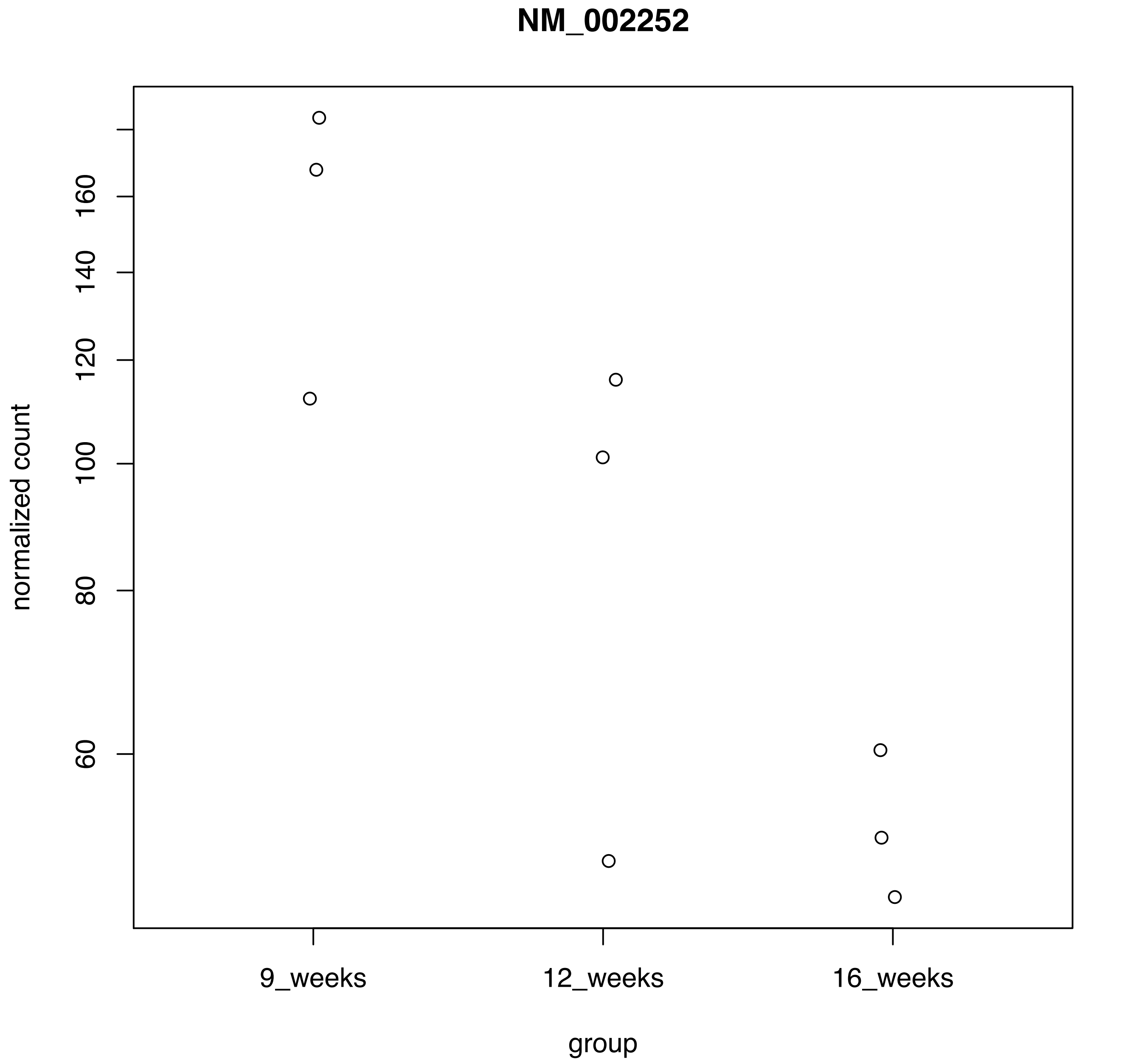

NM_002252
normalized count
160
140
120
100
80
60
9_weeks
12_weeks
16_weeks
group

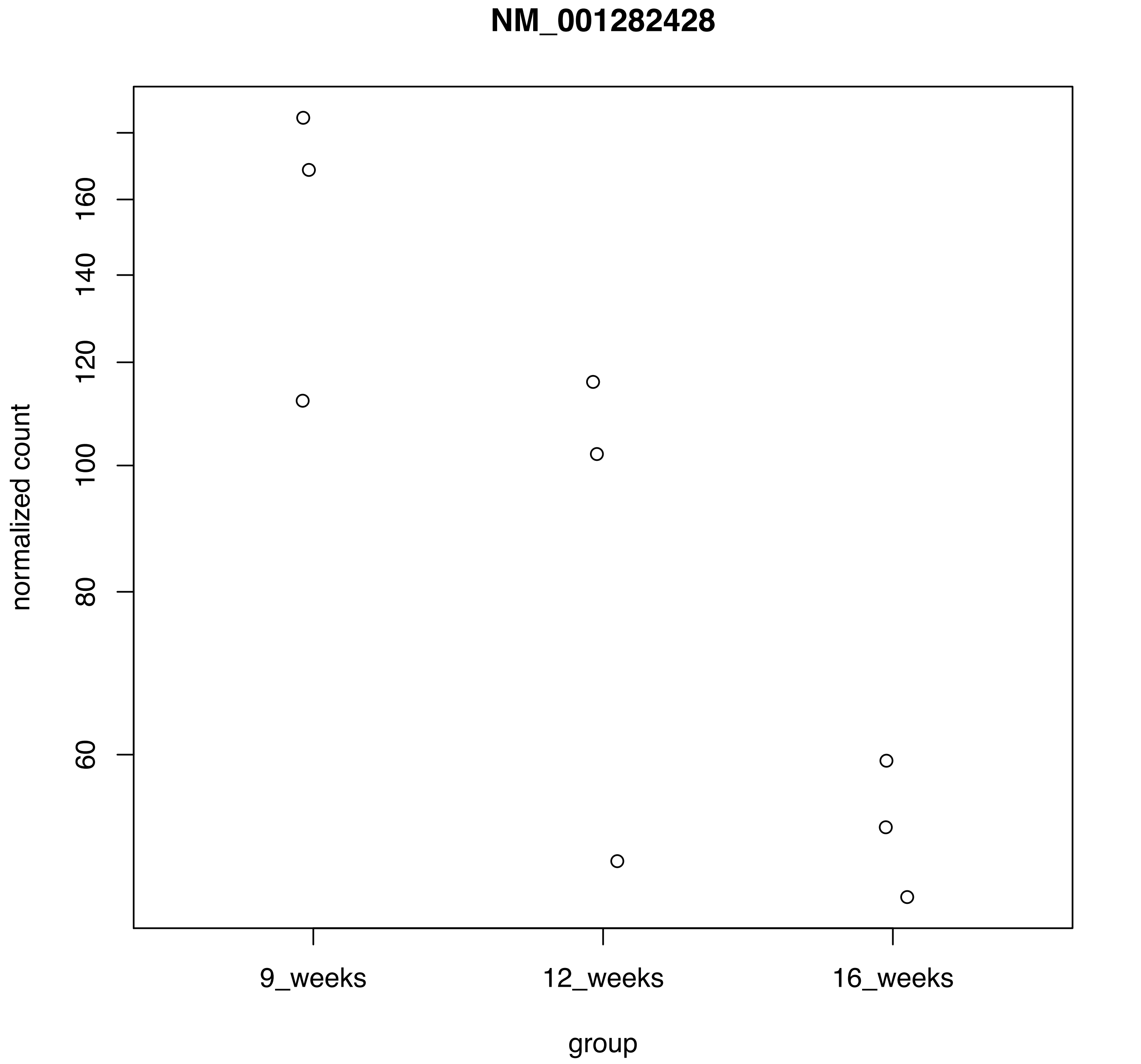

NM_001282428
normalized count
60
80
100
120
140
160
9_weeks
12_weeks
16_weeks
group

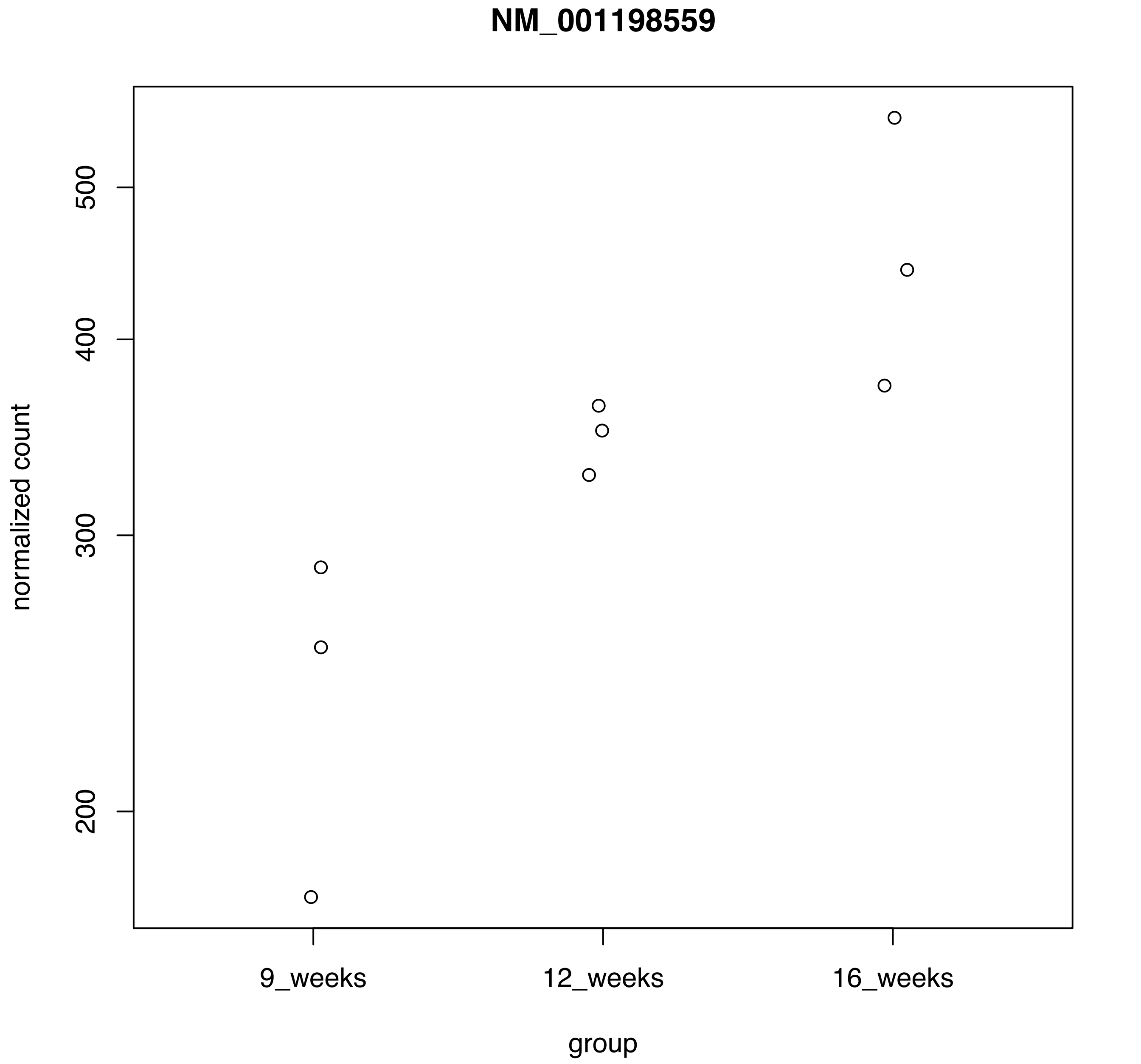
NM_001198559
normalized count
500
400
300
200
9_weeks
12_weeks
16_weeks
group

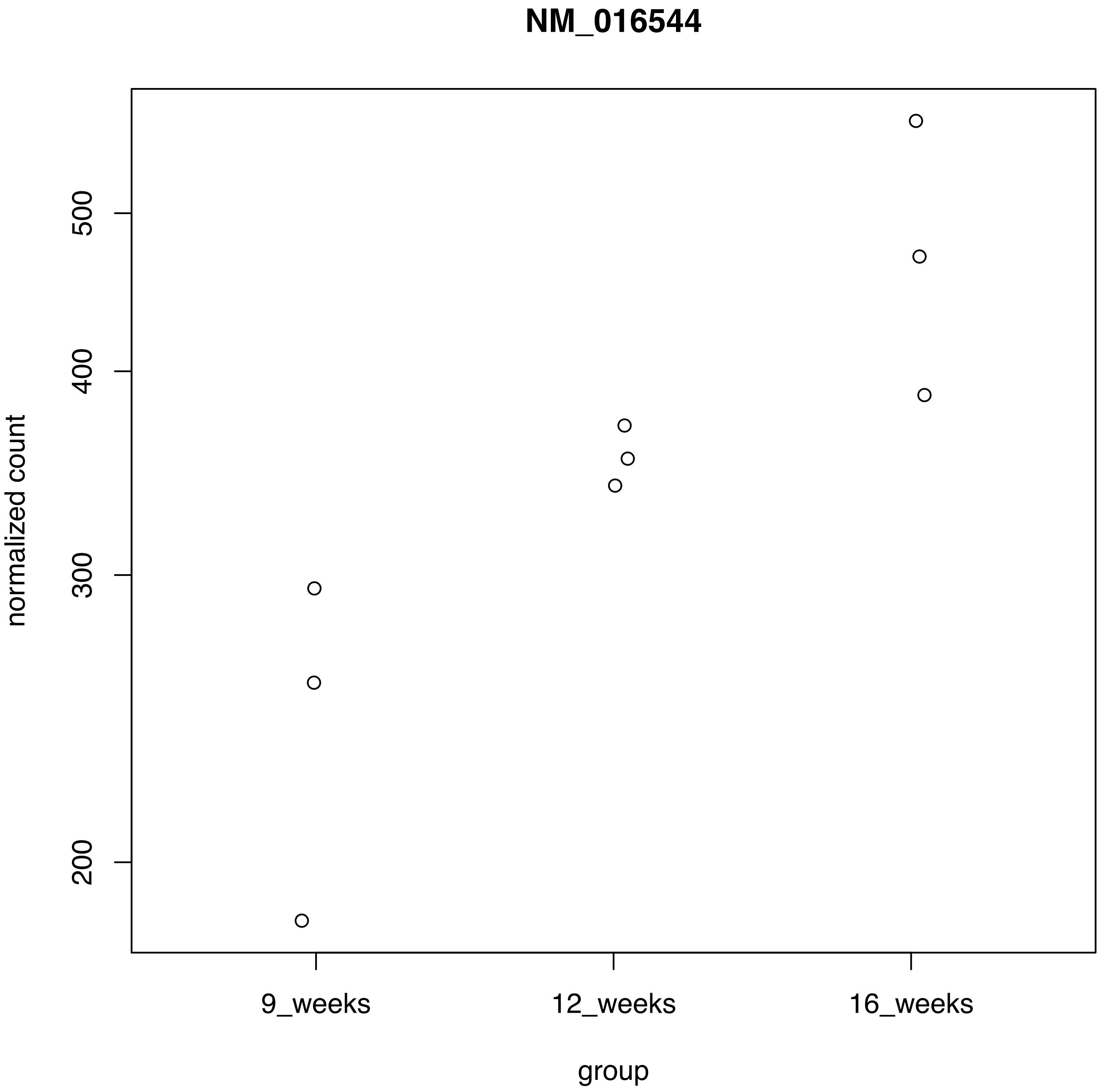
NM_016544
normalized count
200
300
400
500
9_weeks
12_weeks
16_weeks
group

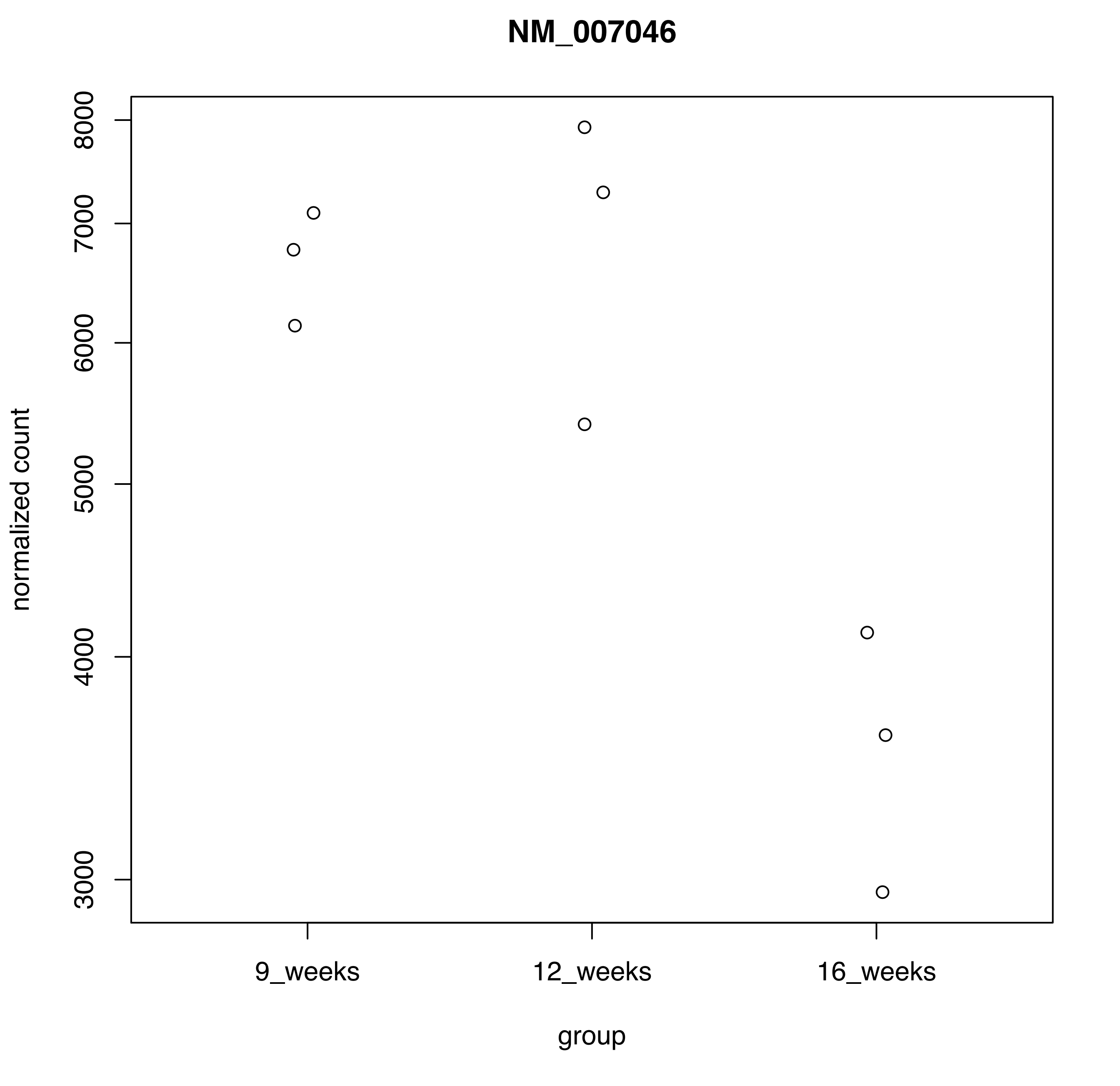
NM_007046
normalized count
8000
7000
6000
5000
4000
3000
9_weeks
12_weeks
16_weeks
group

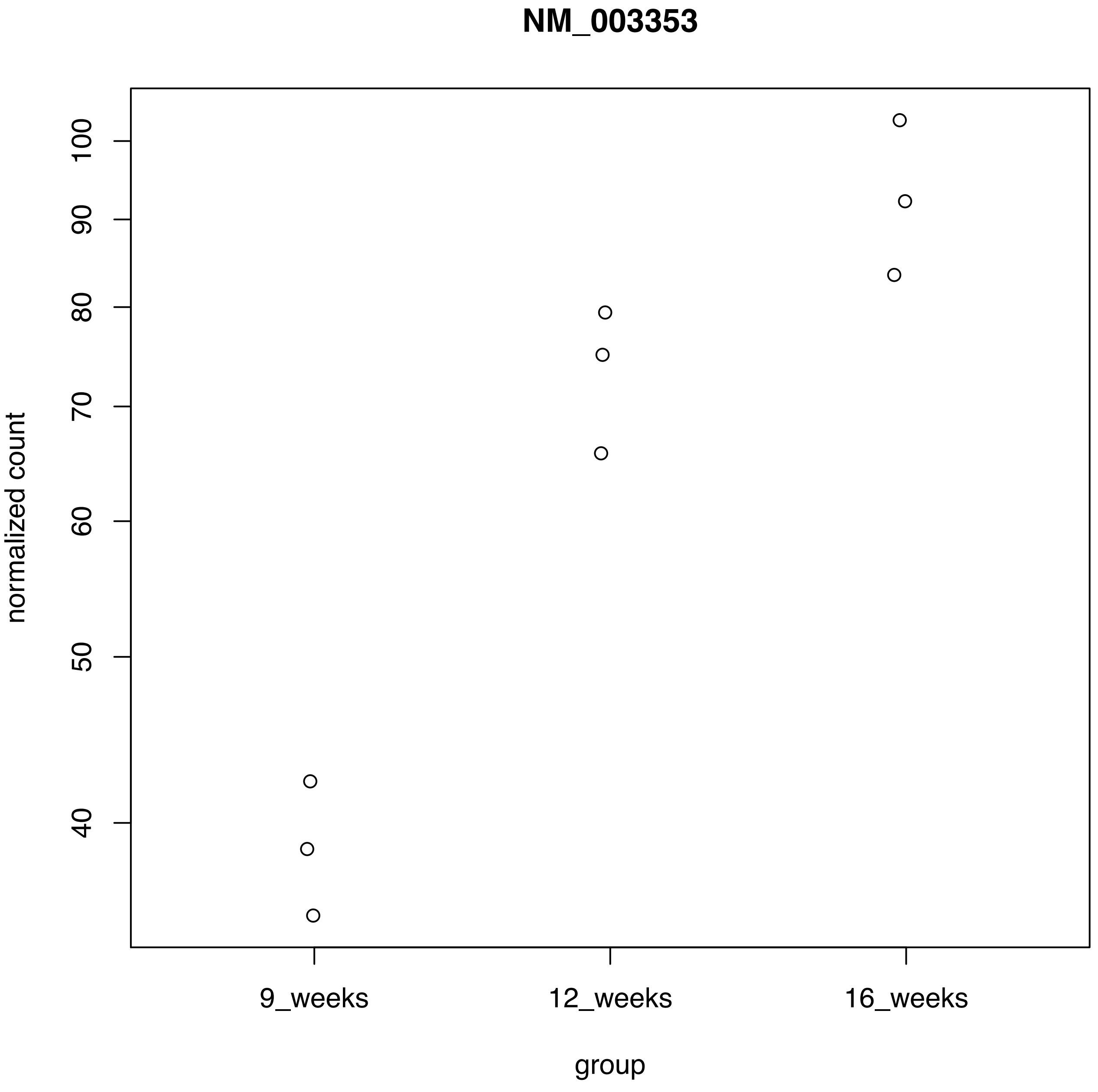
NM_003353
normalized count
100
90
80
70
60
50
40
9_weeks
12_weeks
16_weeks
group

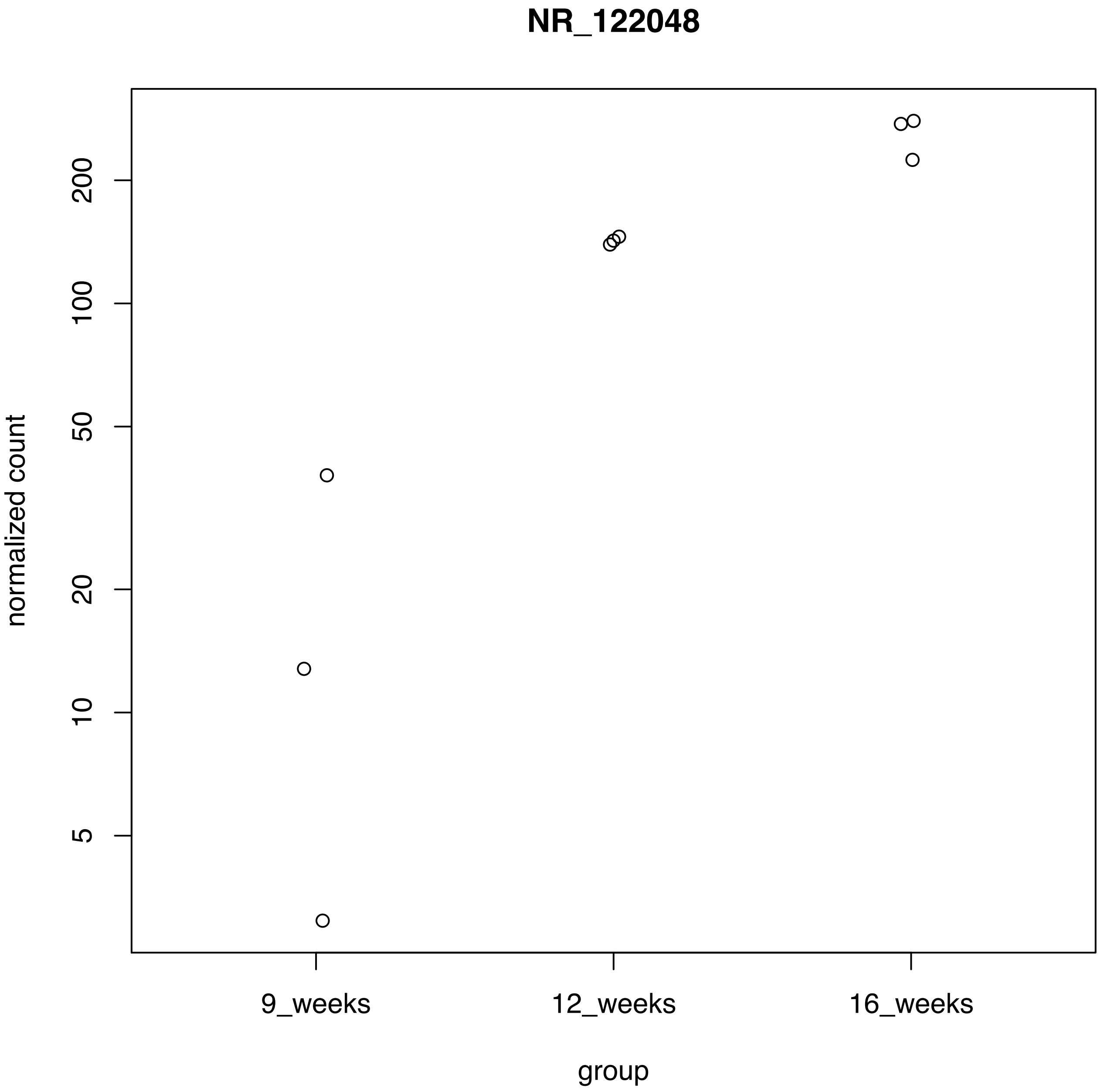

NR_122048
normalized count
5
10
20
50
100
200
9_weeks
12_weeks
16_weeks
group

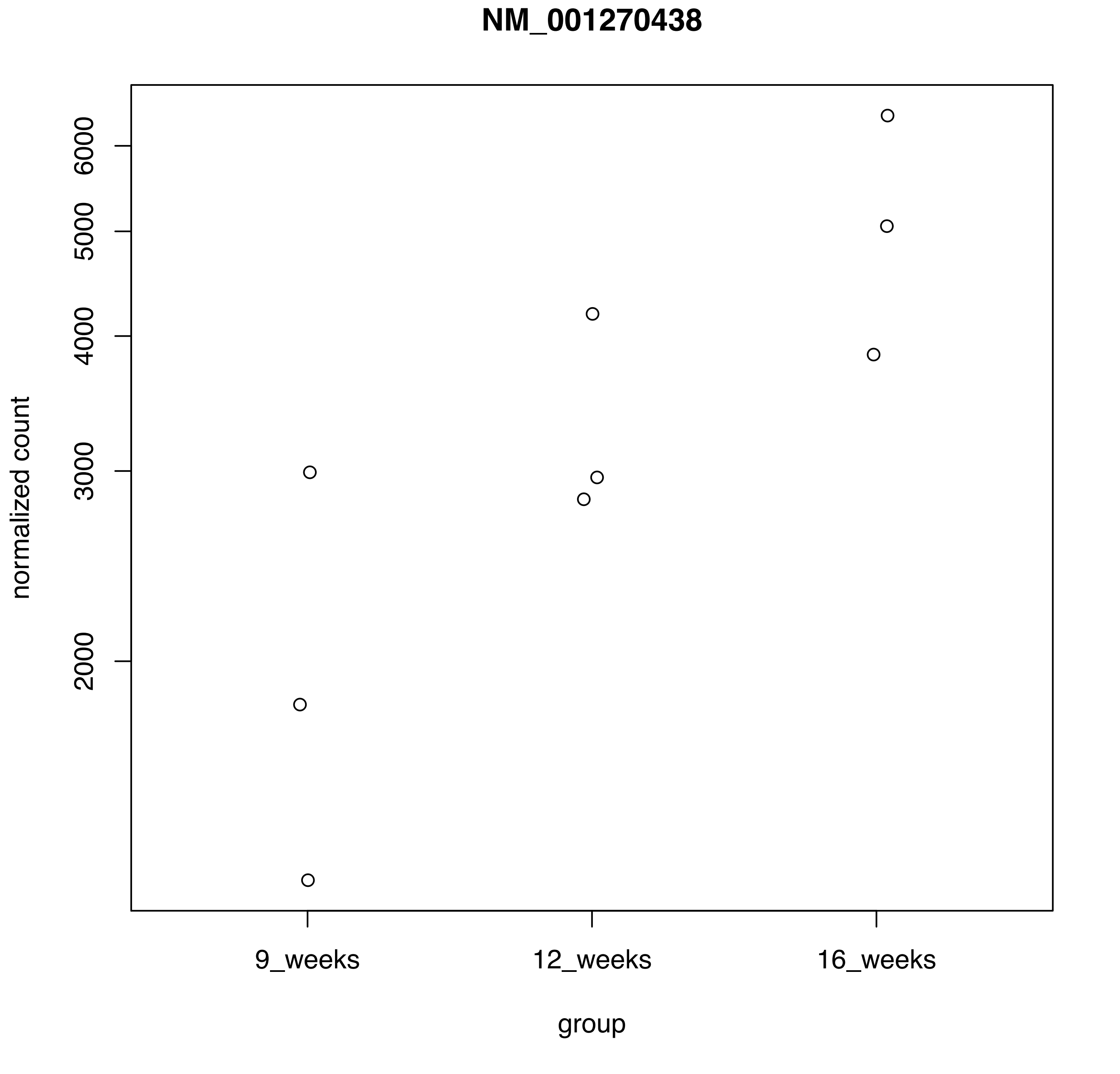

NM_001270438
normalized count
6000
5000
4000
3000
2000
9_weeks
12_weeks
16_weeks
group

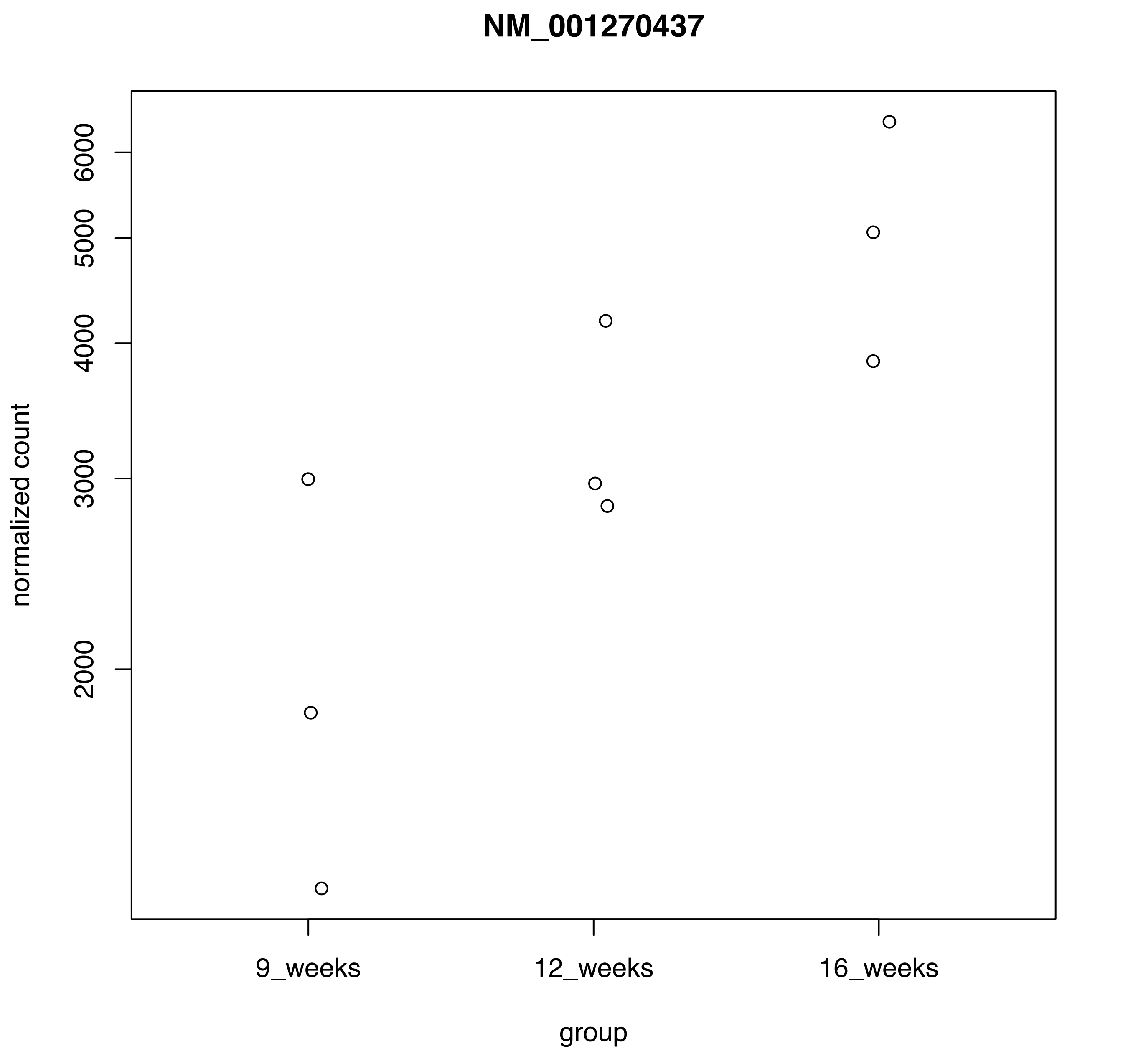

NM_001270437
normalized count
2000
3000
4000
5000
6000
9_weeks
12_weeks
16_weeks
group

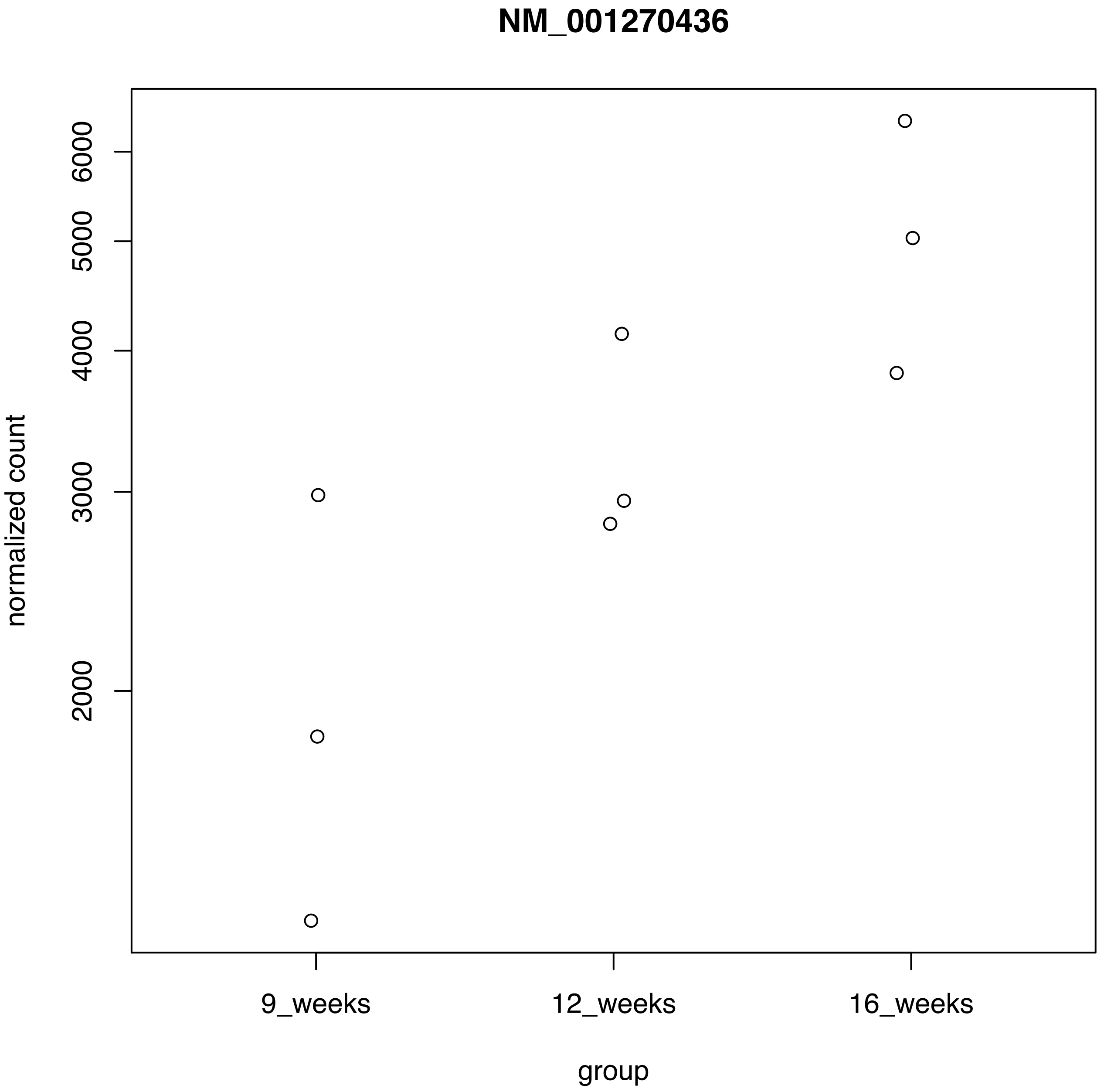
NM_001270436
normalized count
2000
3000
4000
5000
6000
group
9_weeks
12_weeks
16_weeks

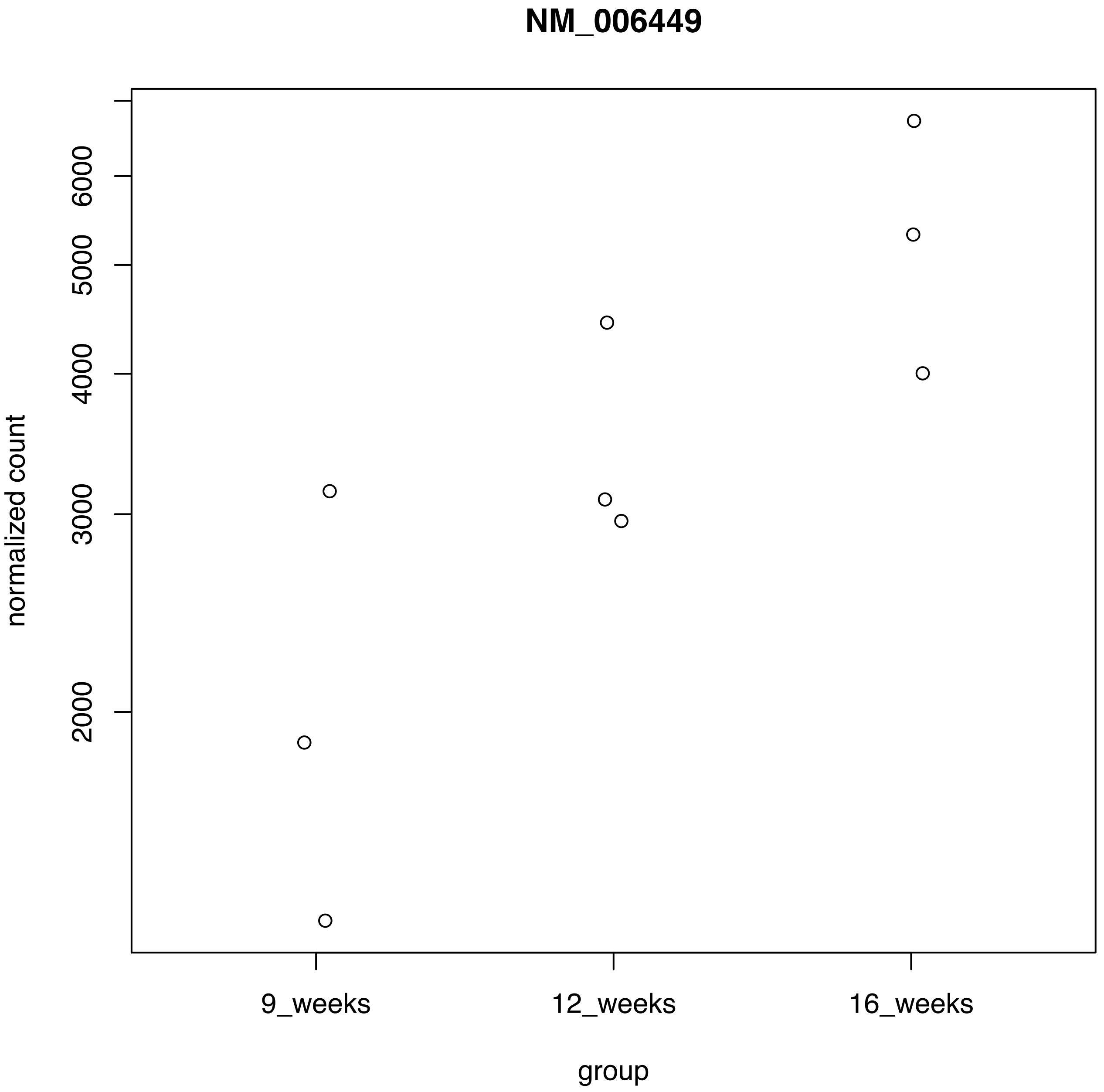
NM_006449
normalized count
2000
3000
4000
5000
6000
9_weeks
12_weeks
16_weeks
group

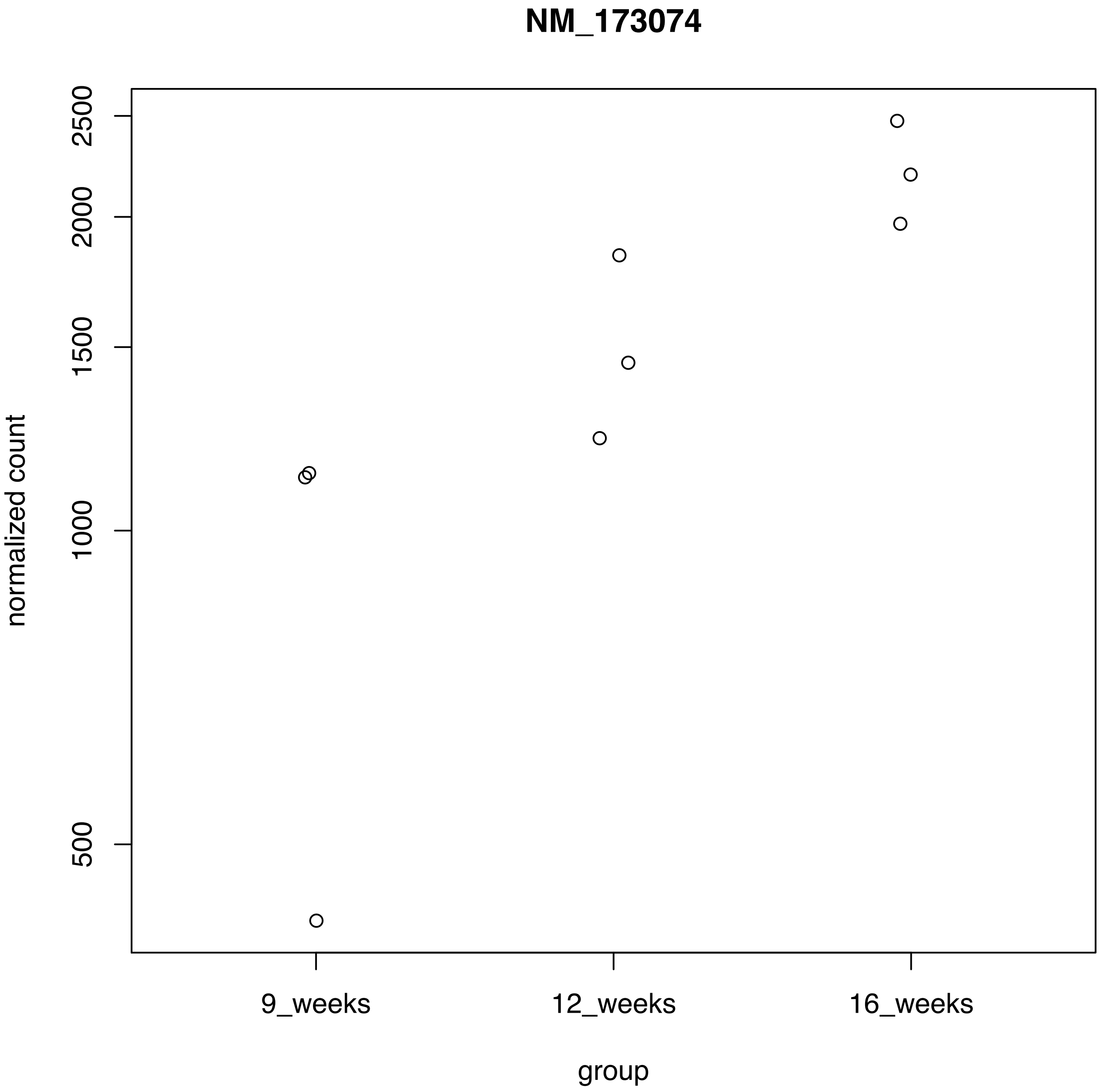
NM_173074
normalized count
2500
2000
1500
1000
500
9_weeks
12_weeks
16_weeks
group

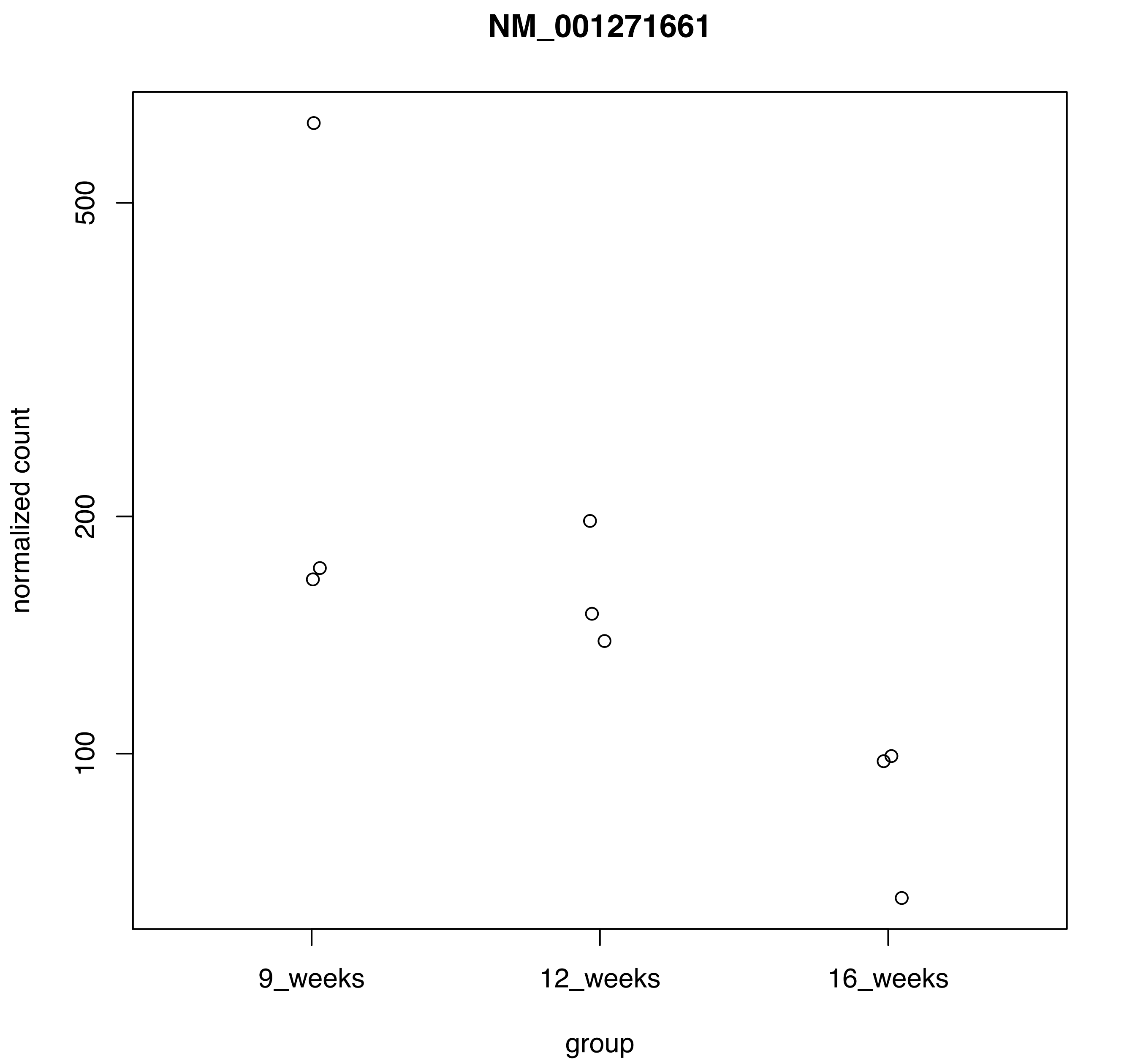
NM_001271661
normalized count
500
200
100
9_weeks
12_weeks
16_weeks
group

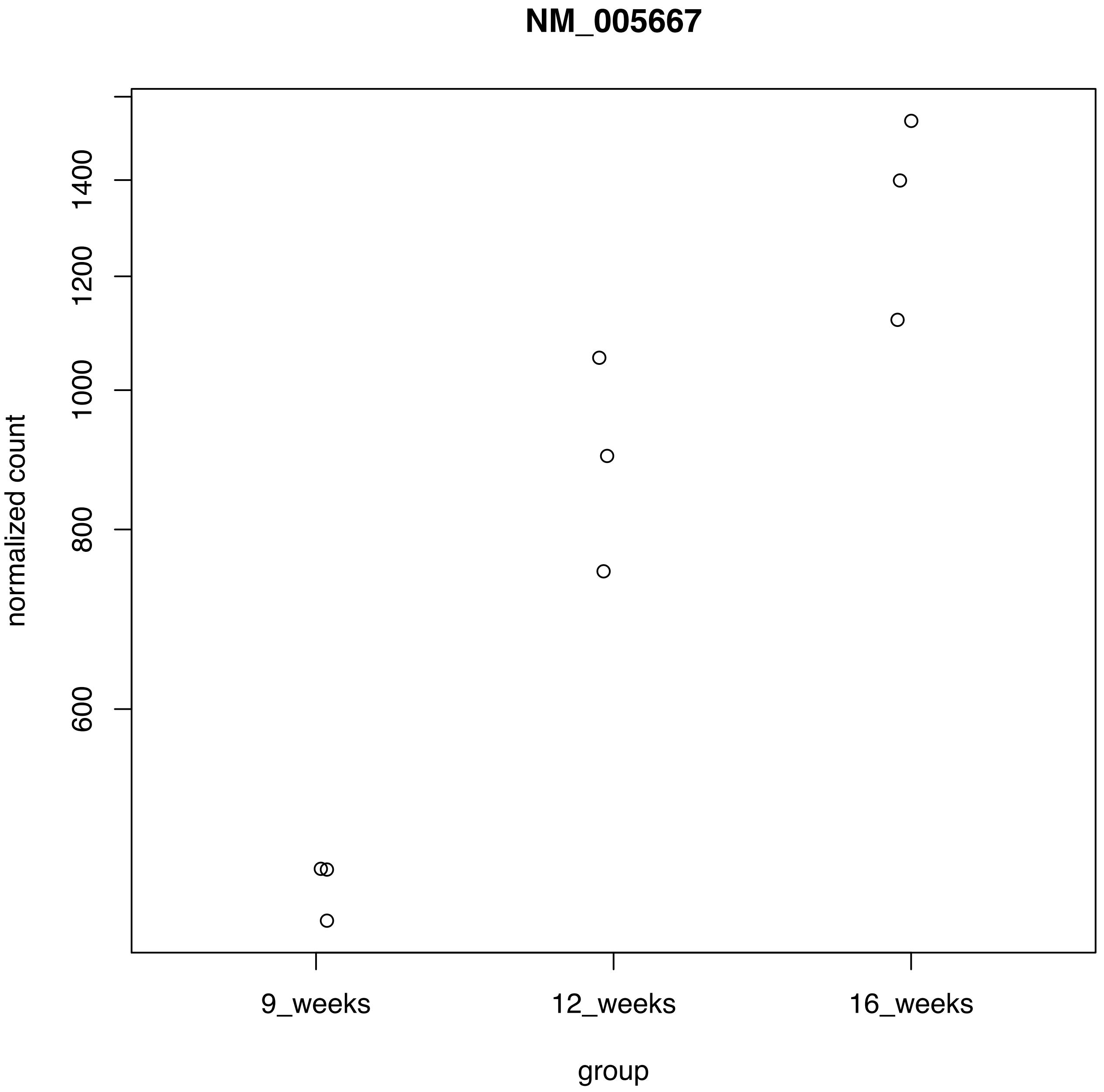
NM_005667
normalized count
600
800
1000
1200
1400
9_weeks
12_weeks
16_weeks
group

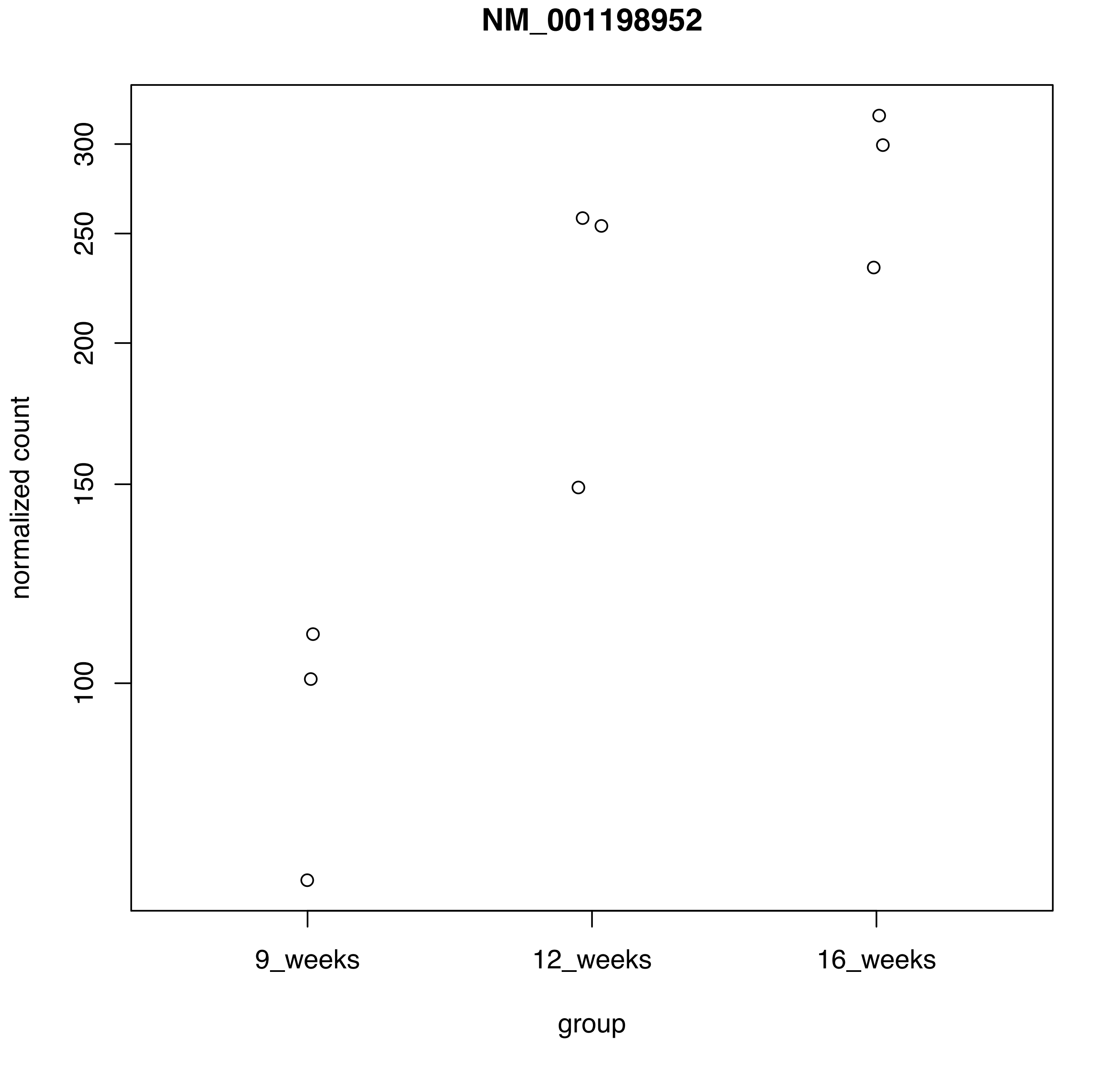

NM_001198952
normalized count
300
250
200
150
100
9_weeks
12_weeks
16_weeks
group

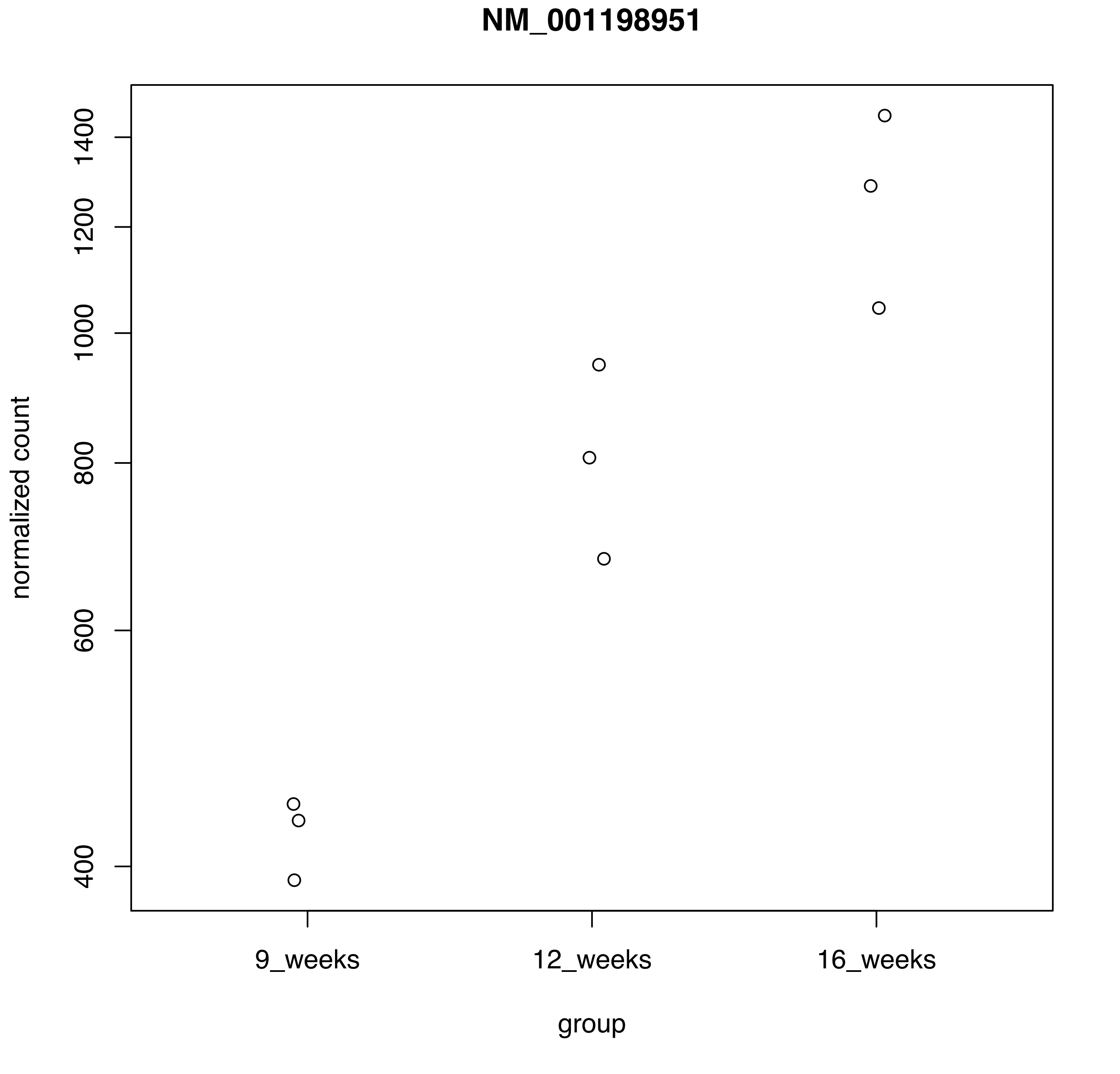

NM_001198951
normalized count
400
600
800
1000
1200
1400
9_weeks
12_weeks
16_weeks
group

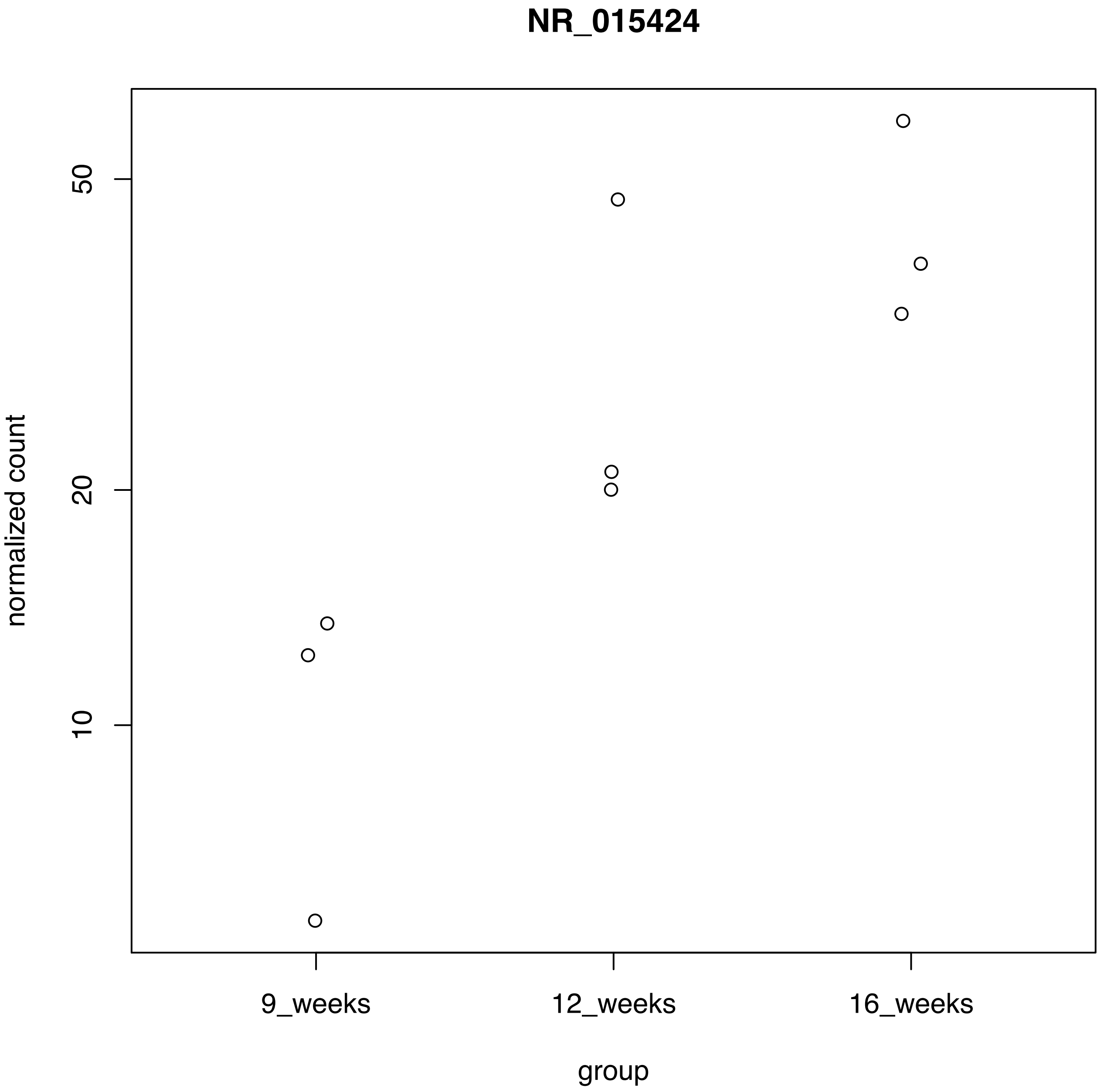
NR_015424
normalized count
50
20
10
9_weeks
12_weeks
16_weeks
group

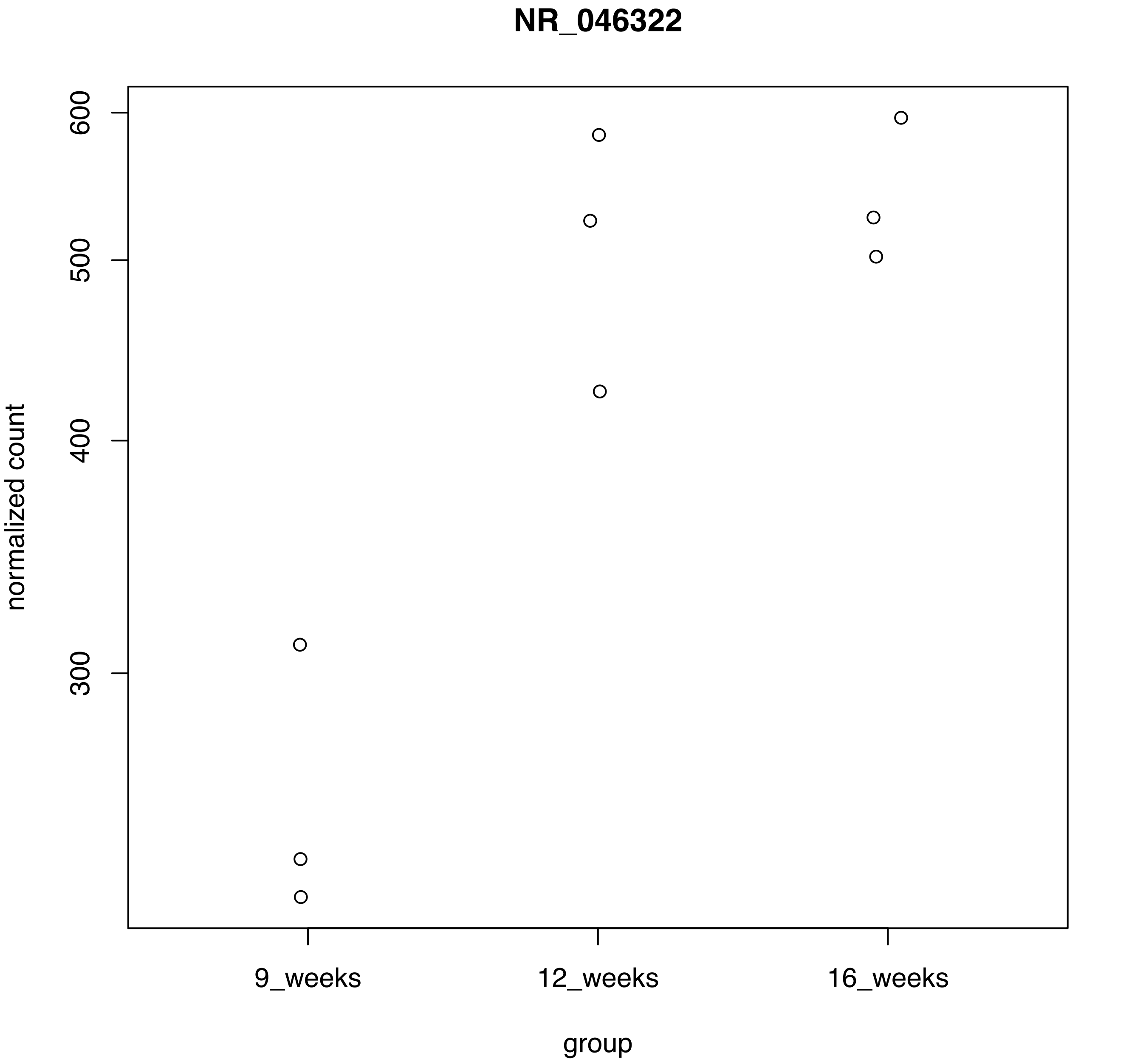
NR_046322
normalized count
600
500
400
300
9_weeks
12_weeks
16_weeks
group

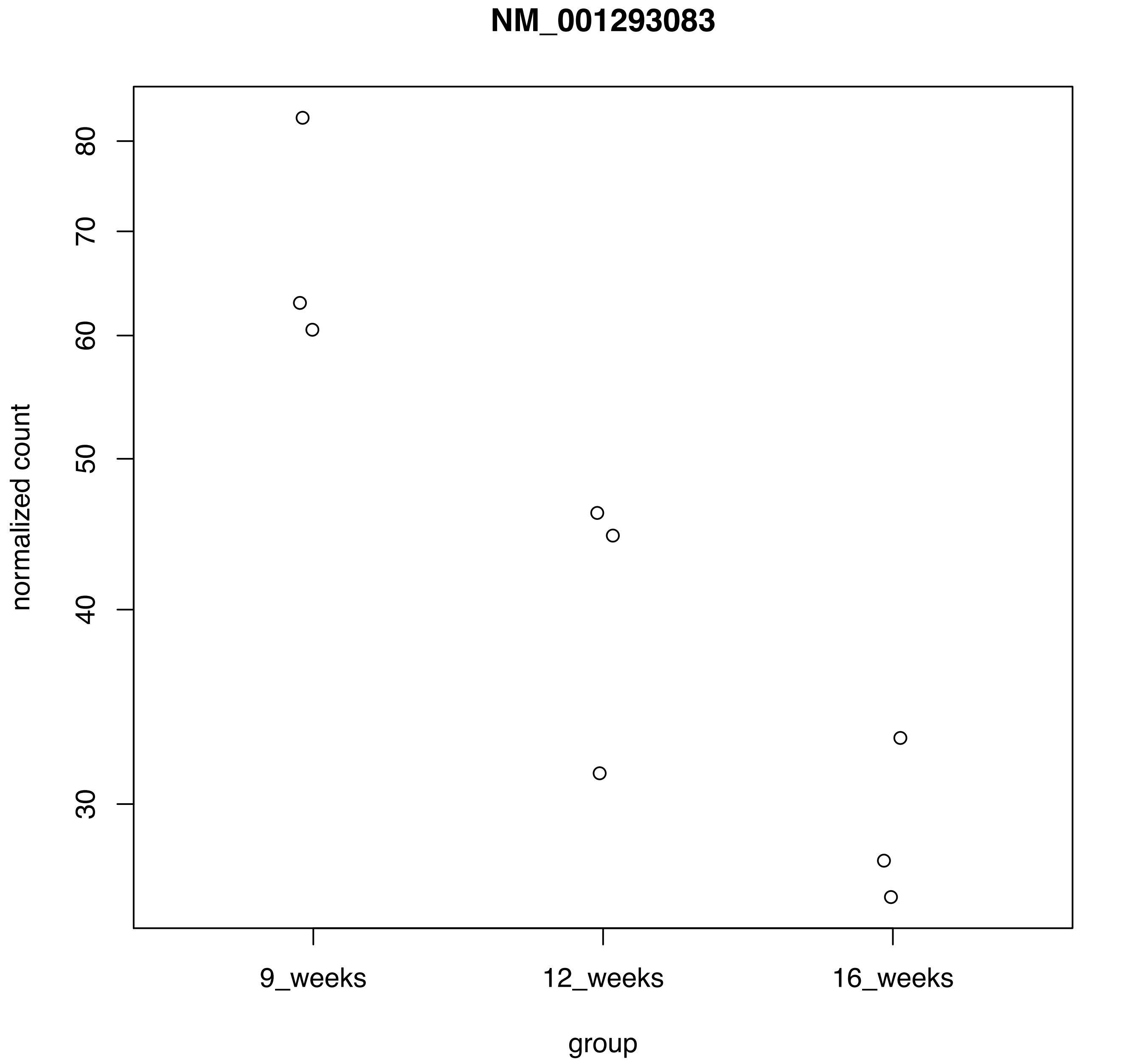

NM_001293083
80
70
60
50
40
30
normalized count
9_weeks
12_weeks
16_weeks
group

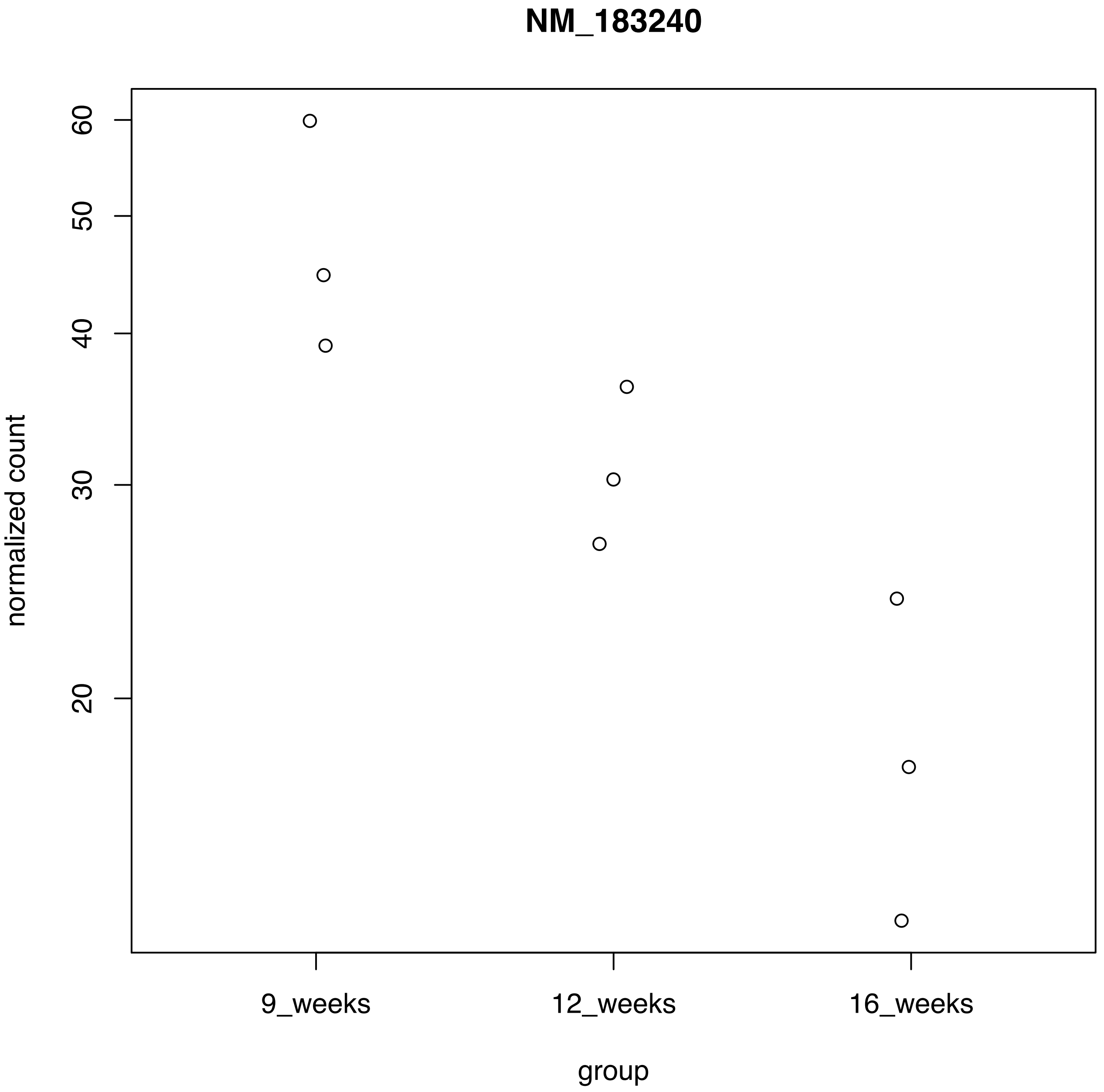
NM_183240
normalized count
60
50
40
30
20
9_weeks
12_weeks
16_weeks
group

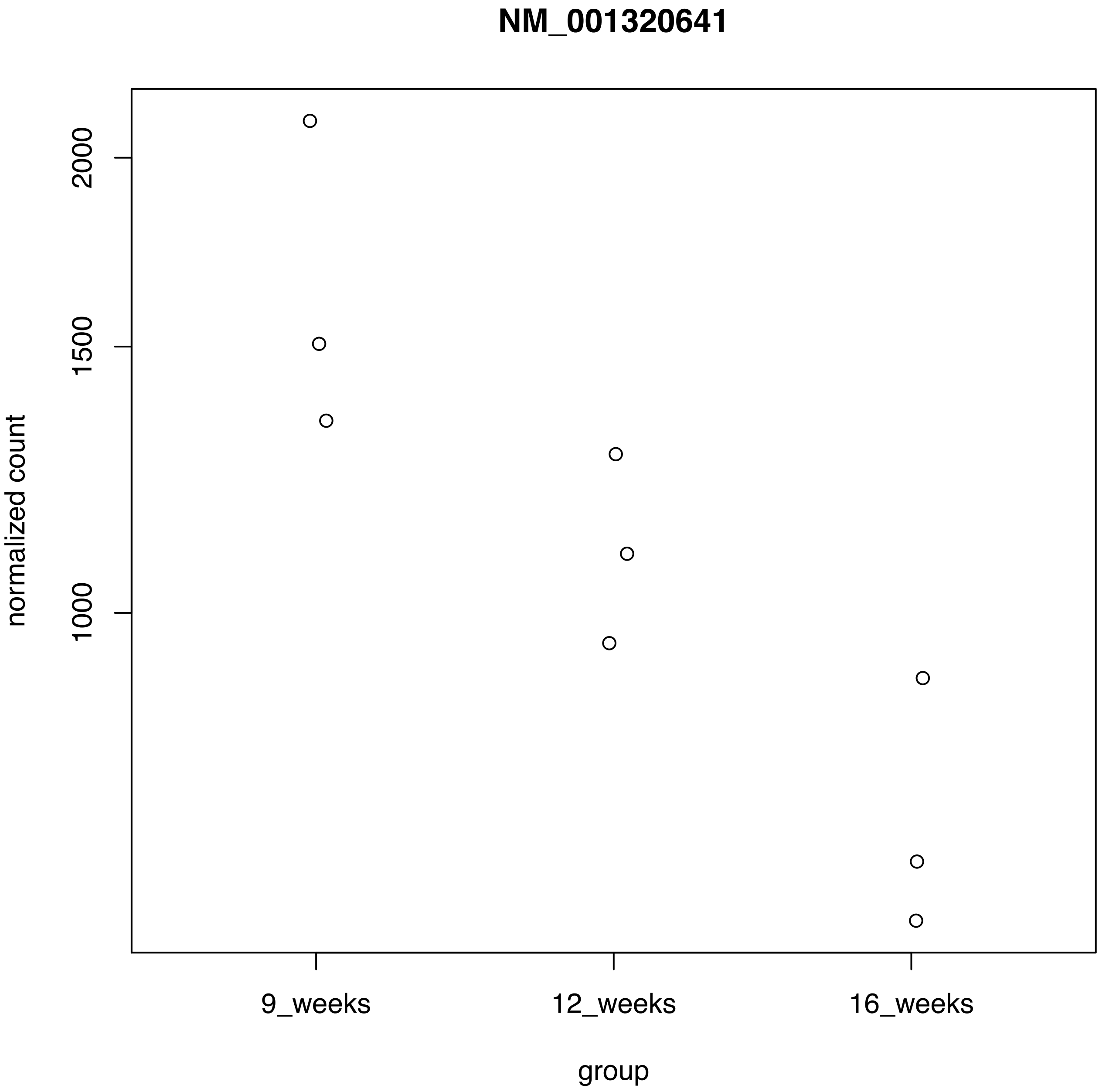
NM_001320641
normalized count
2000
1500
1000
9_weeks
12_weeks
16_weeks
group

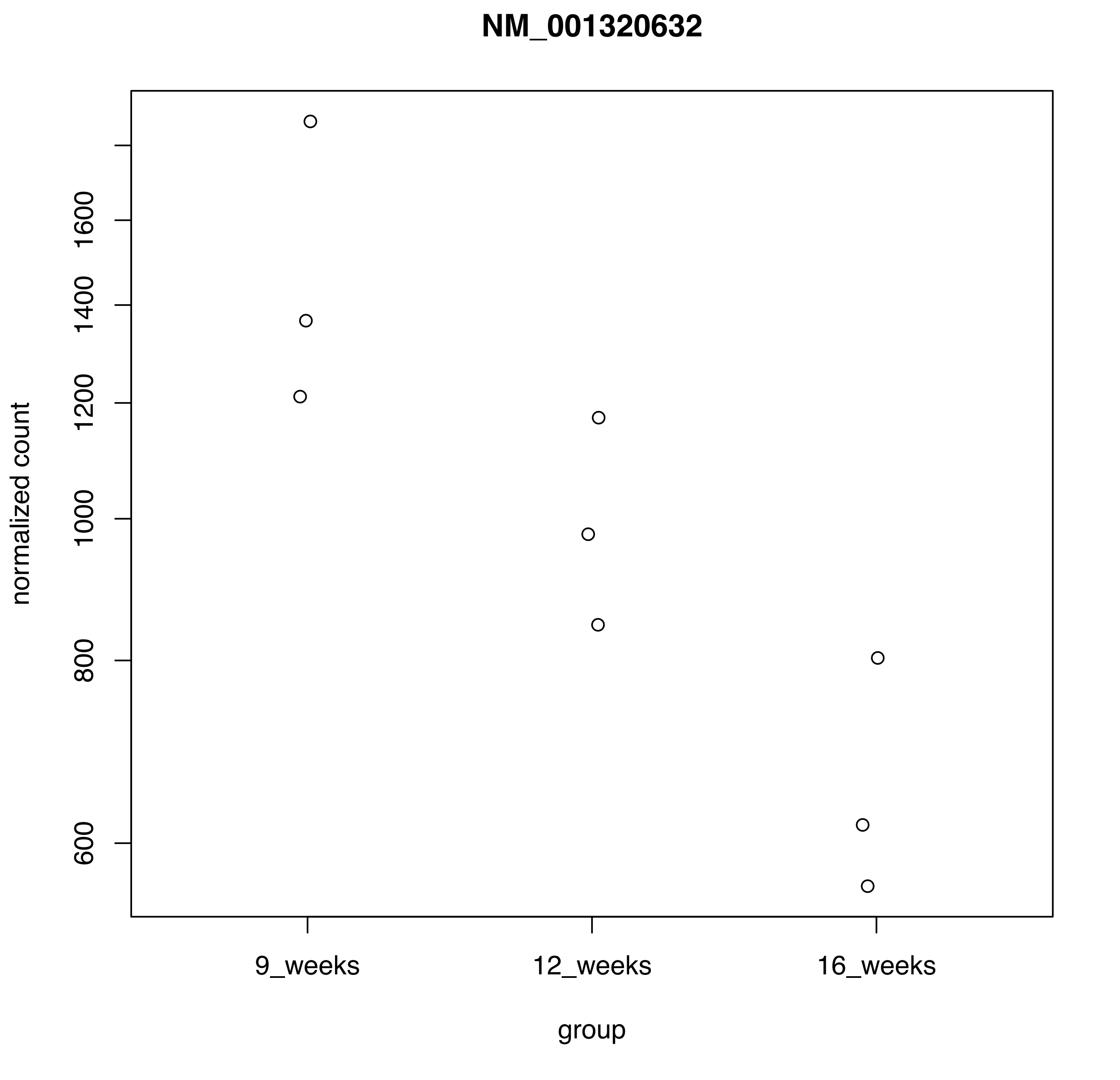
NM_001320632
normalized count
600
800
1000
1200
1400
1600
9_weeks
12_weeks
16_weeks
group

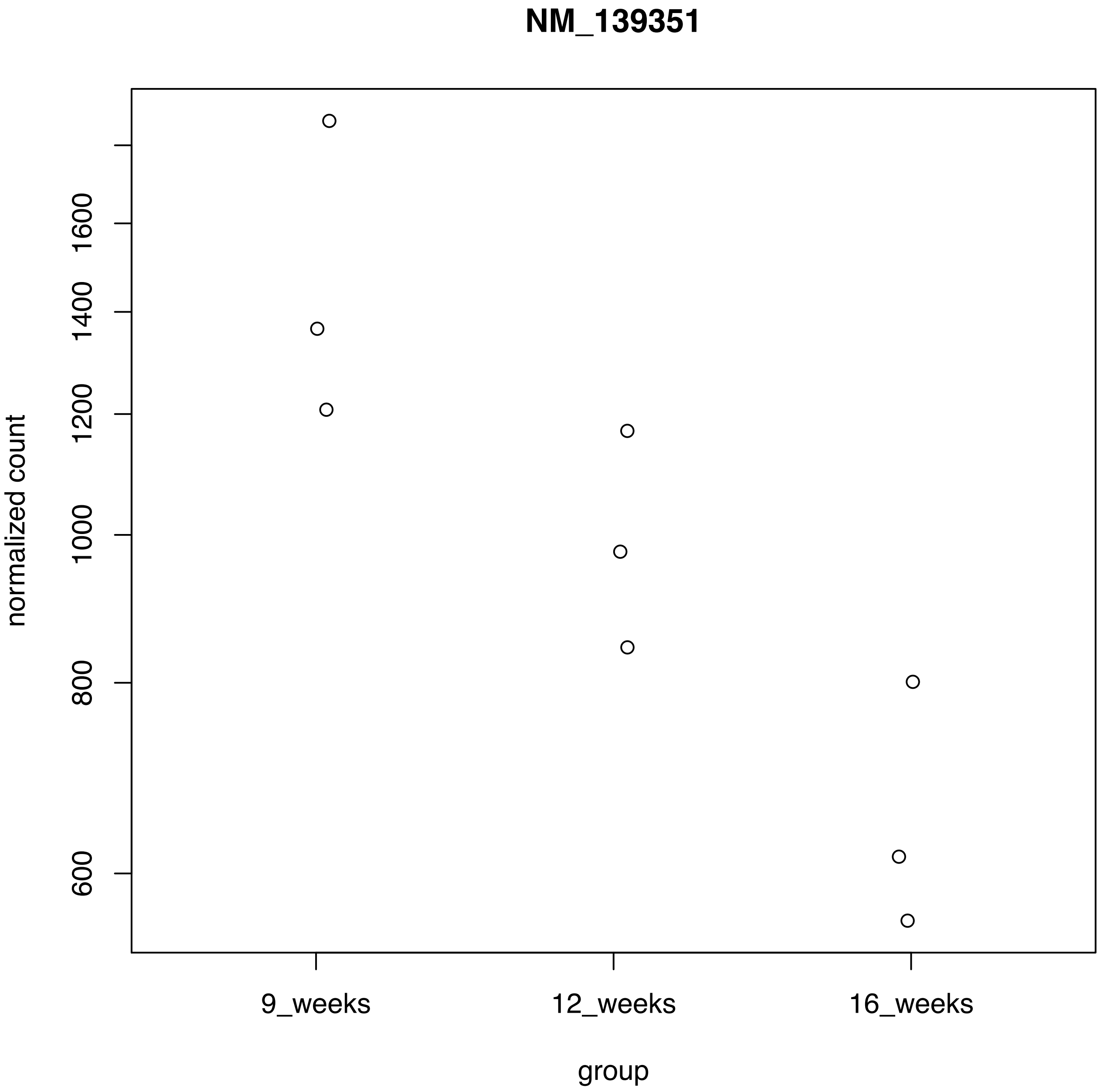
NM_139351
normalized count
1600
1400
1200
1000
800
600
9_weeks
12_weeks
16_weeks
group

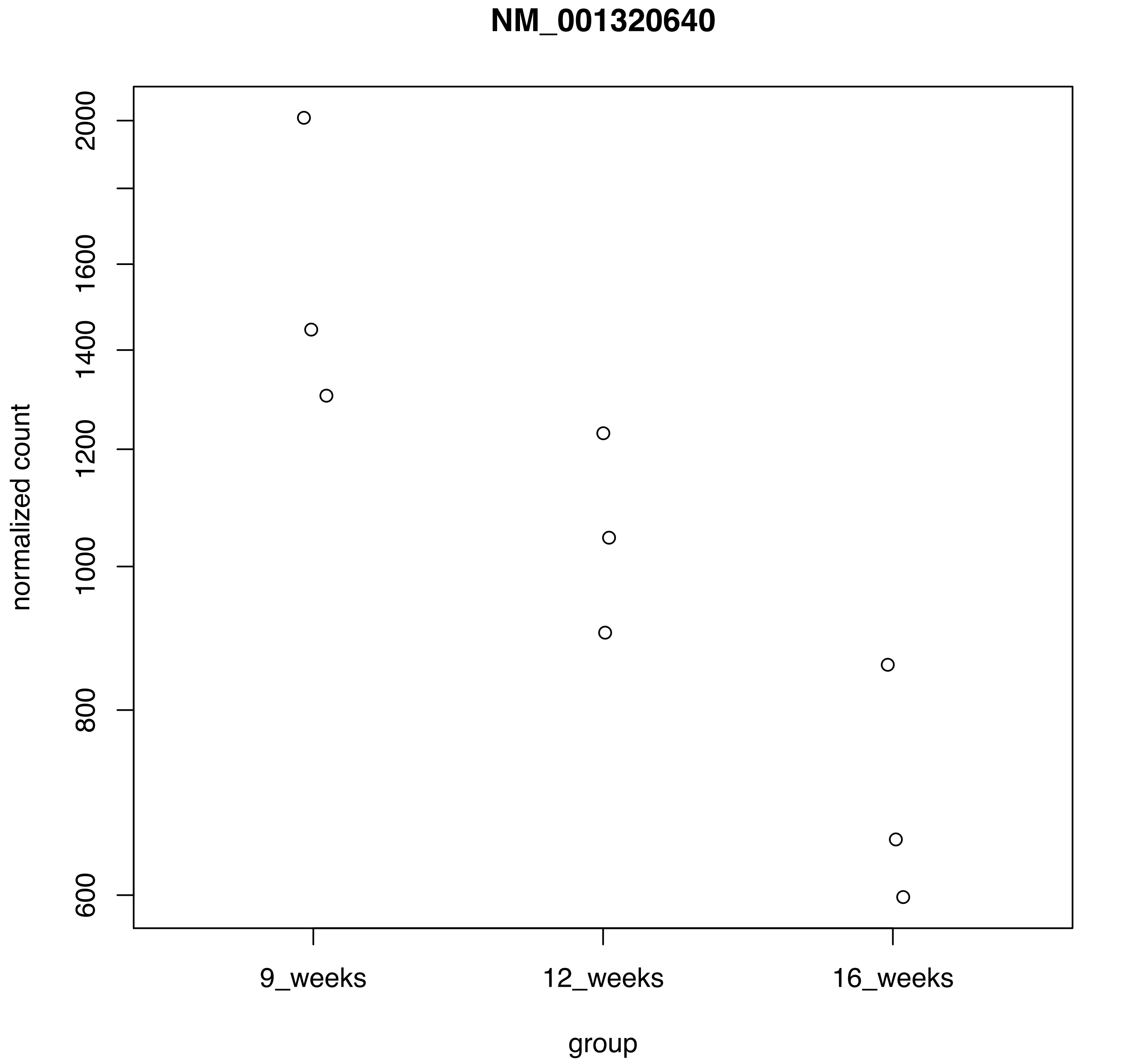
NM_001320640
normalized count
2000
1600
1400
1200
1000
800
600
9_weeks
12_weeks
16_weeks
group

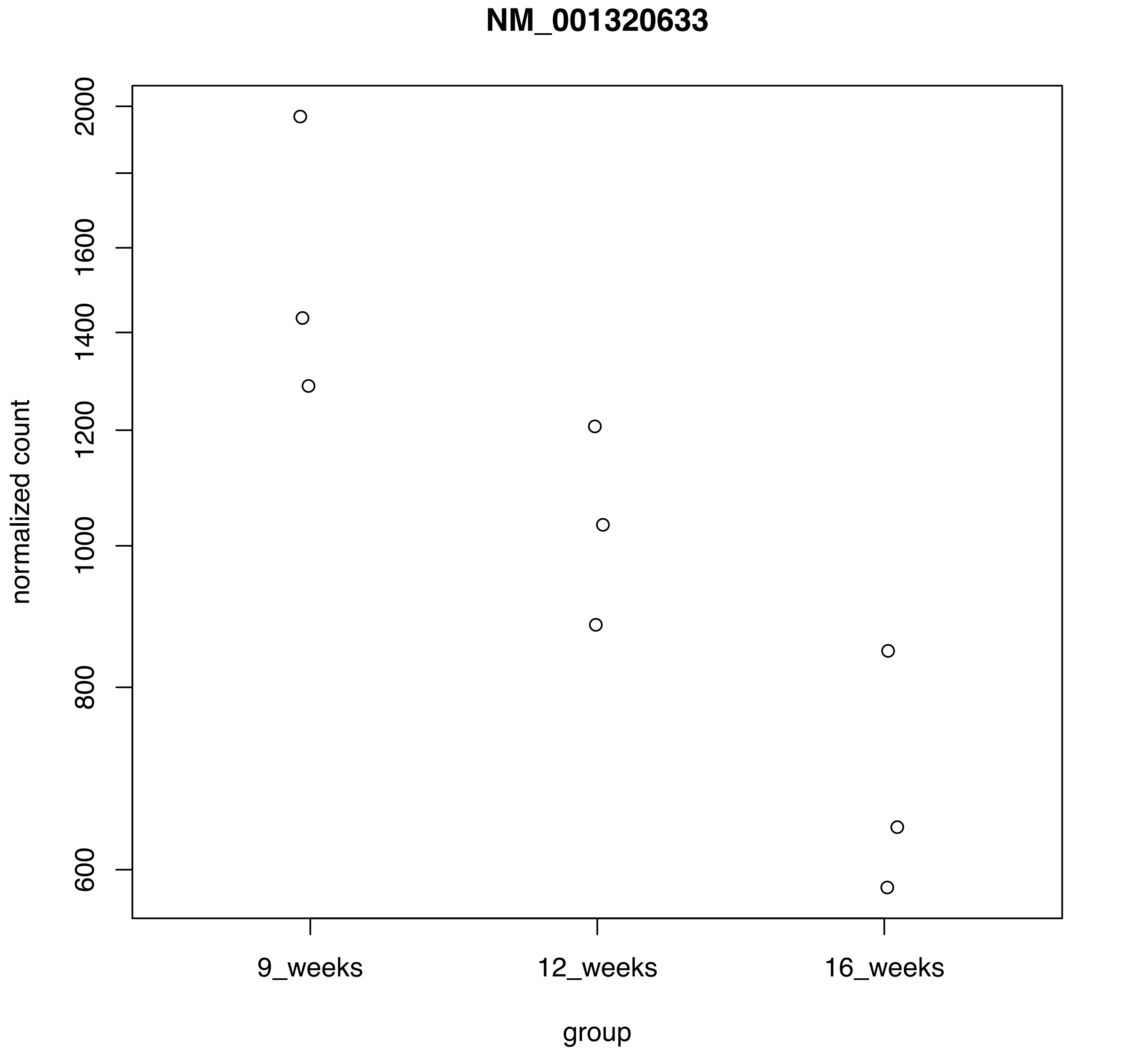
NM_001320633
normalized count
2000
1600
1400
1200
1000
800
600
9_weeks
12_weeks
16_weeks
group

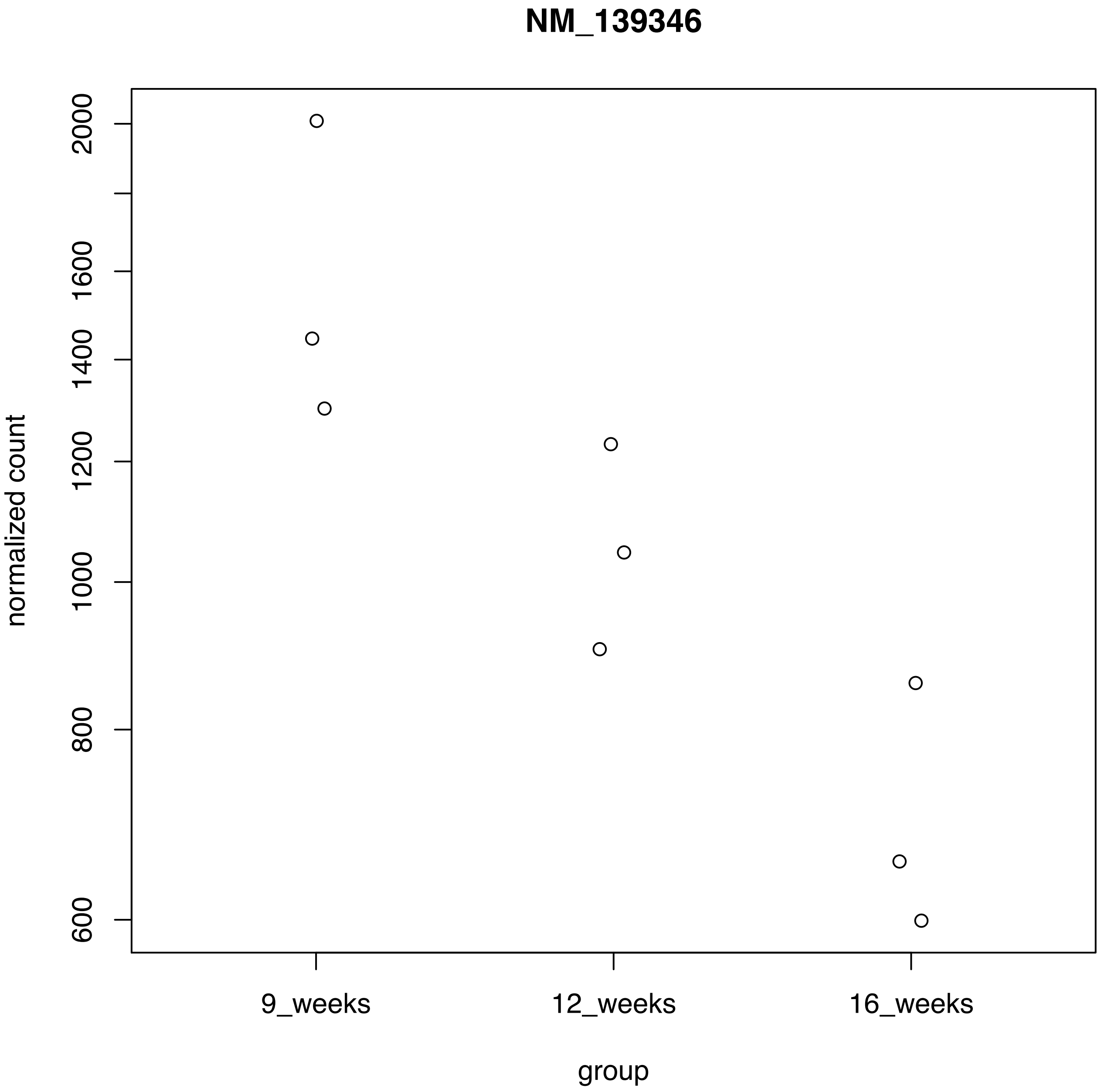
NM_139346
normalized count
2000
1600
1400
1200
1000
800
600
9_weeks
12_weeks
16_weeks
group

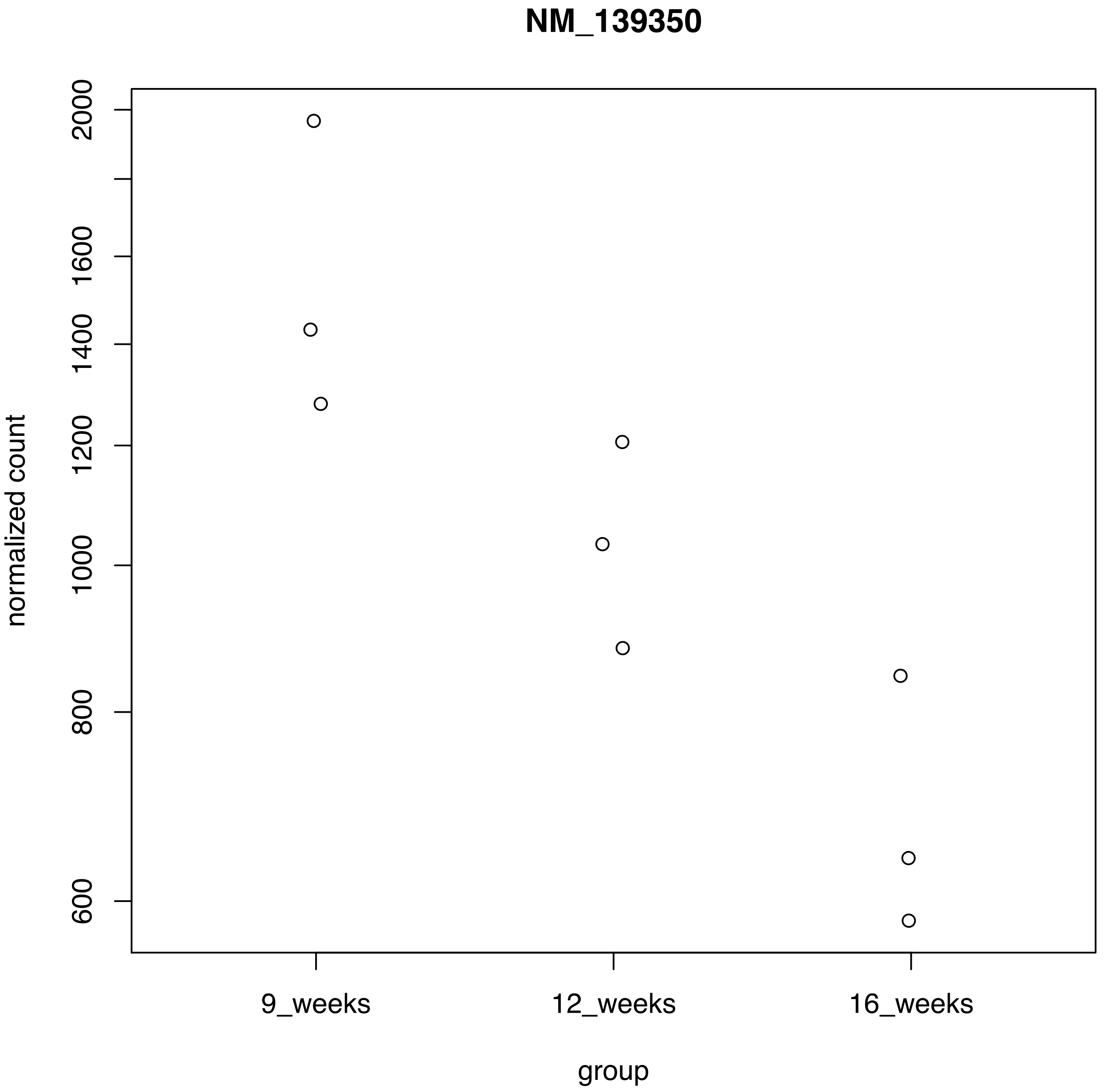
NM_139350
normalized count
2000
1600
1400
1200
1000
800
600
9_weeks
12_weeks
16_weeks
group

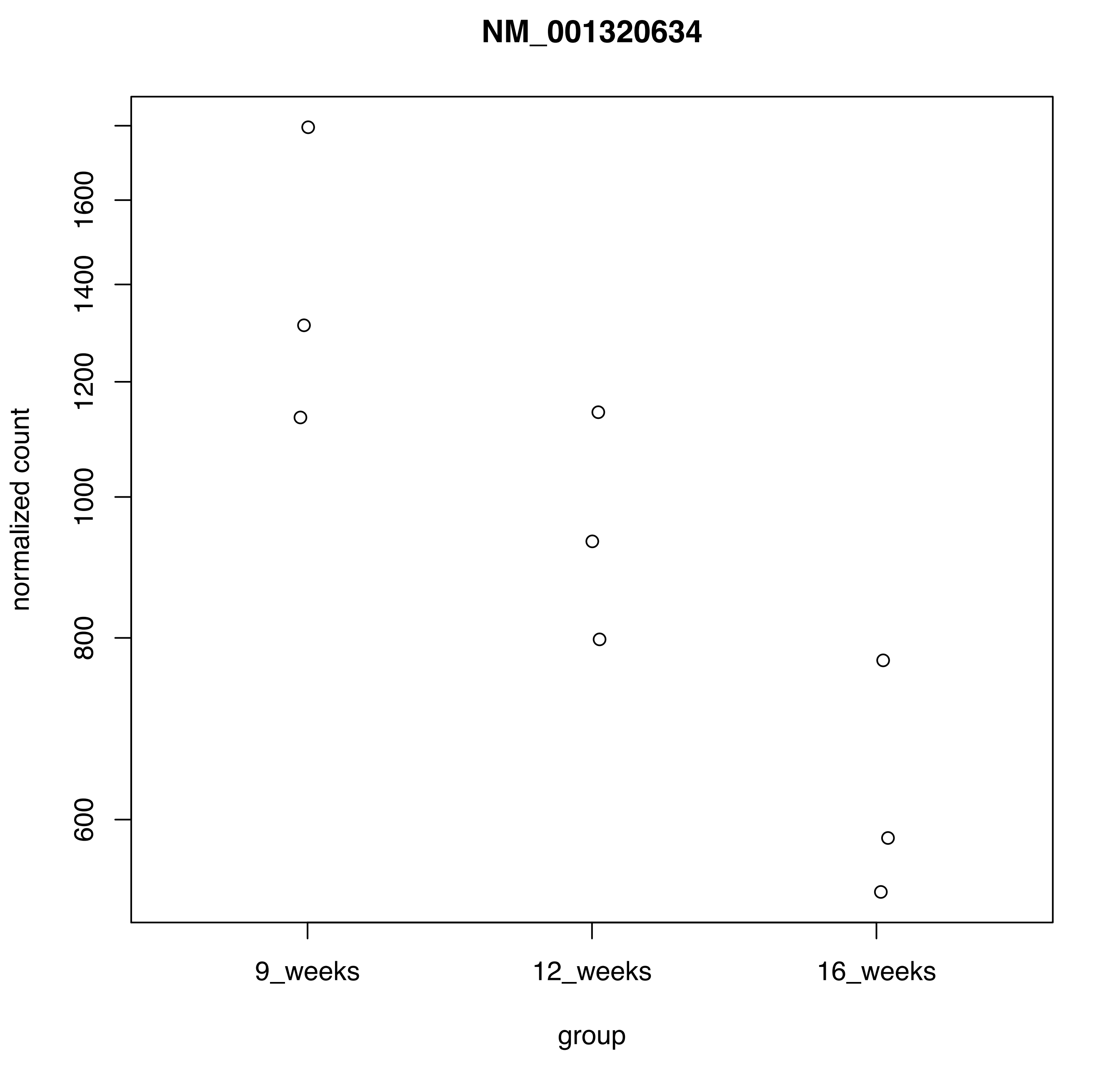

NM_001320634
normalized count
1600
1400
1200
1000
800
600
9_weeks
12_weeks
16_weeks
group

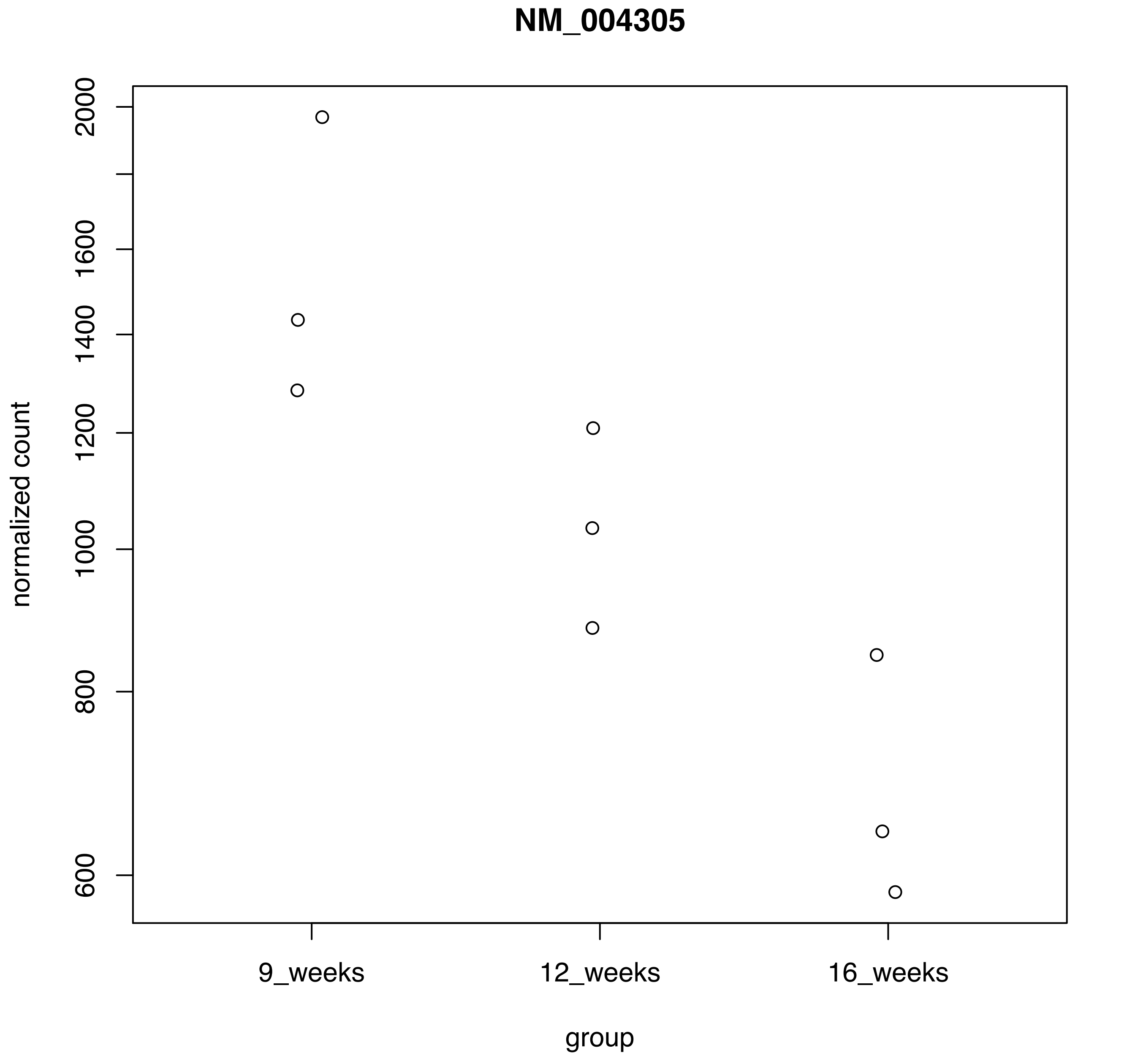

NM_004305
normalized count
2000
1600
1400
1200
1000
800
600
9_weeks
12_weeks
16_weeks
group

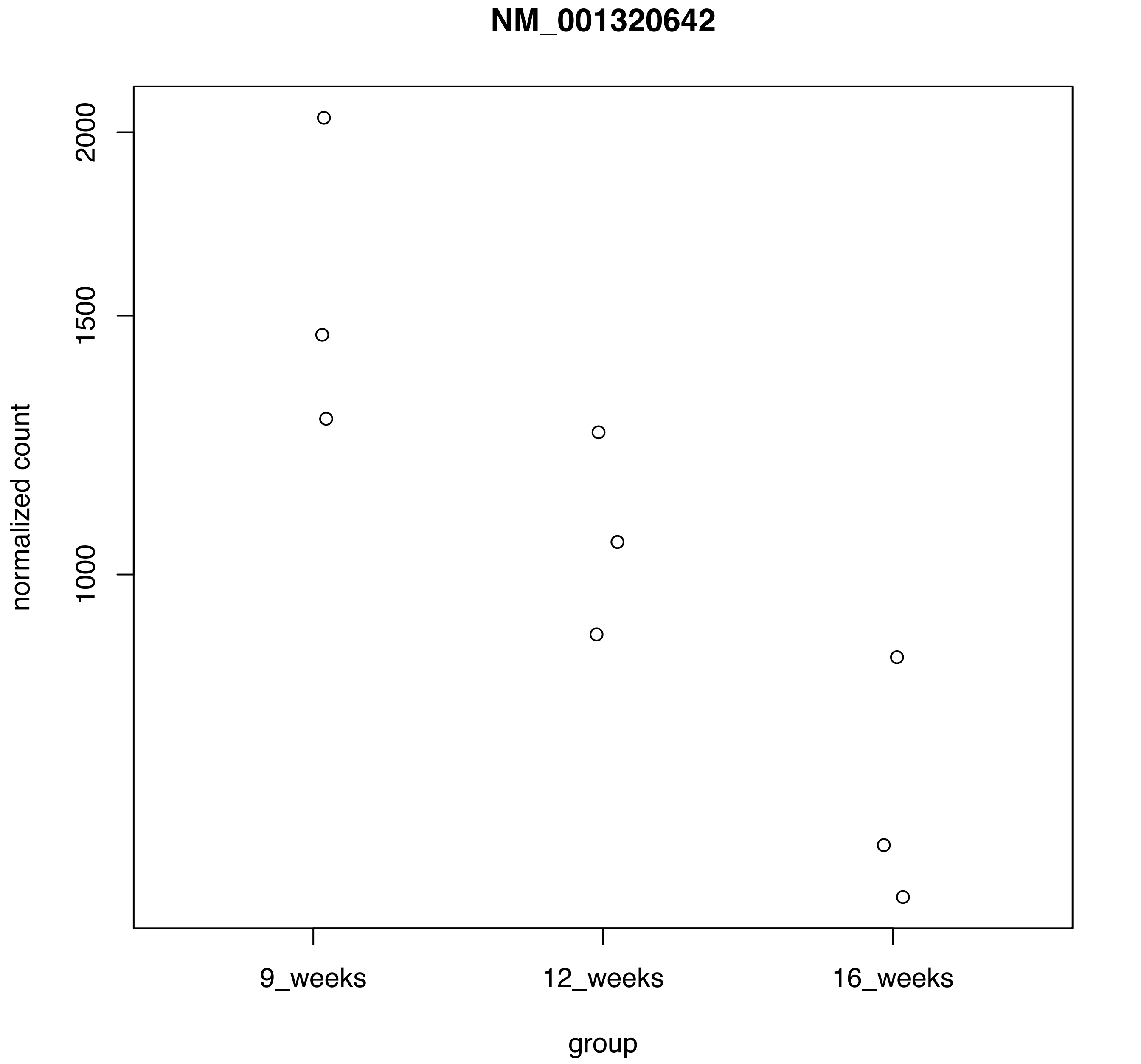
NM_001320642
normalized count
2000
1500
1000
9_weeks
12_weeks
16_weeks
group

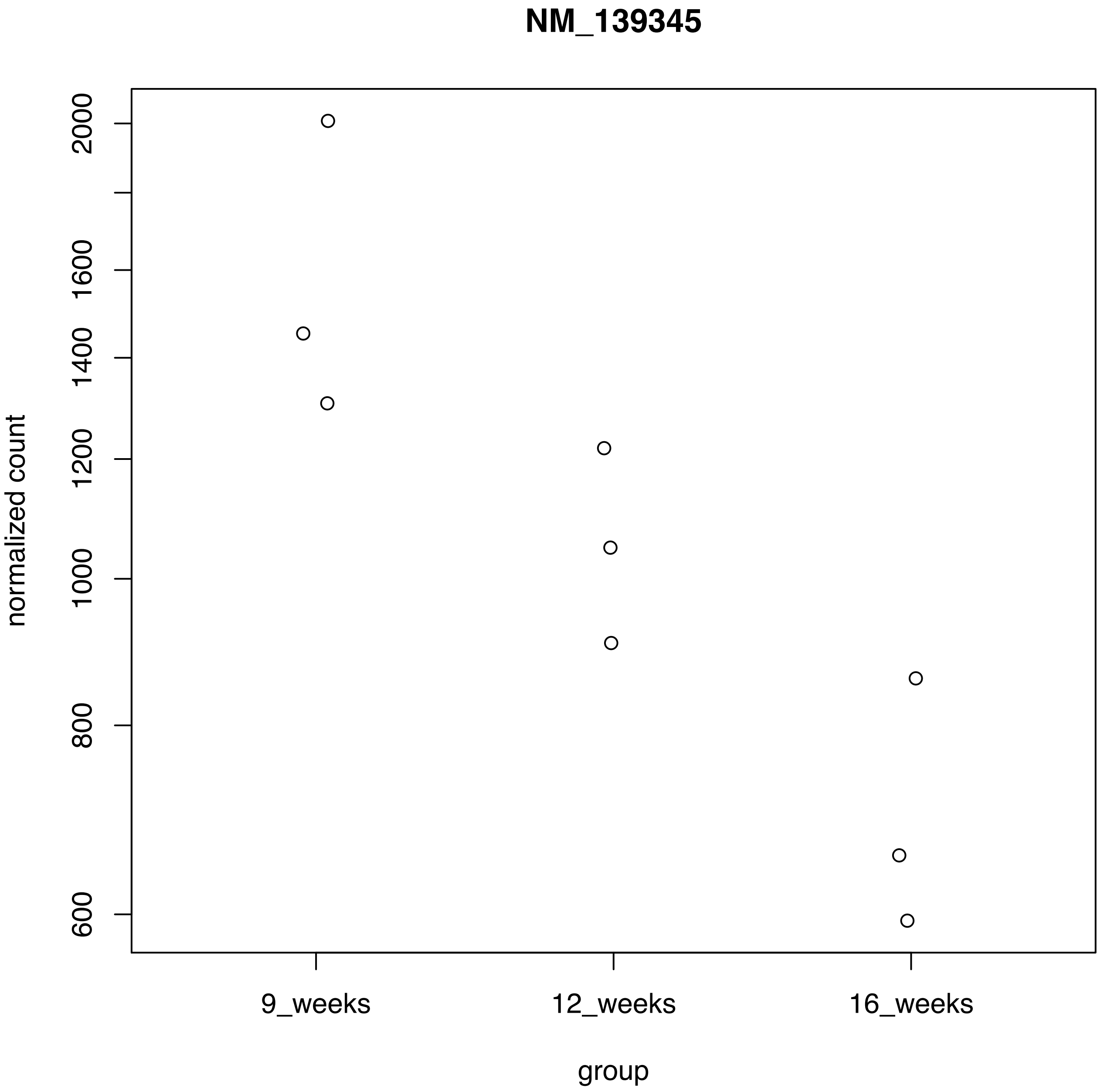

NM_139345
normalized count
2000
1600
1400
1200
1000
800
600
9_weeks
12_weeks
16_weeks
group

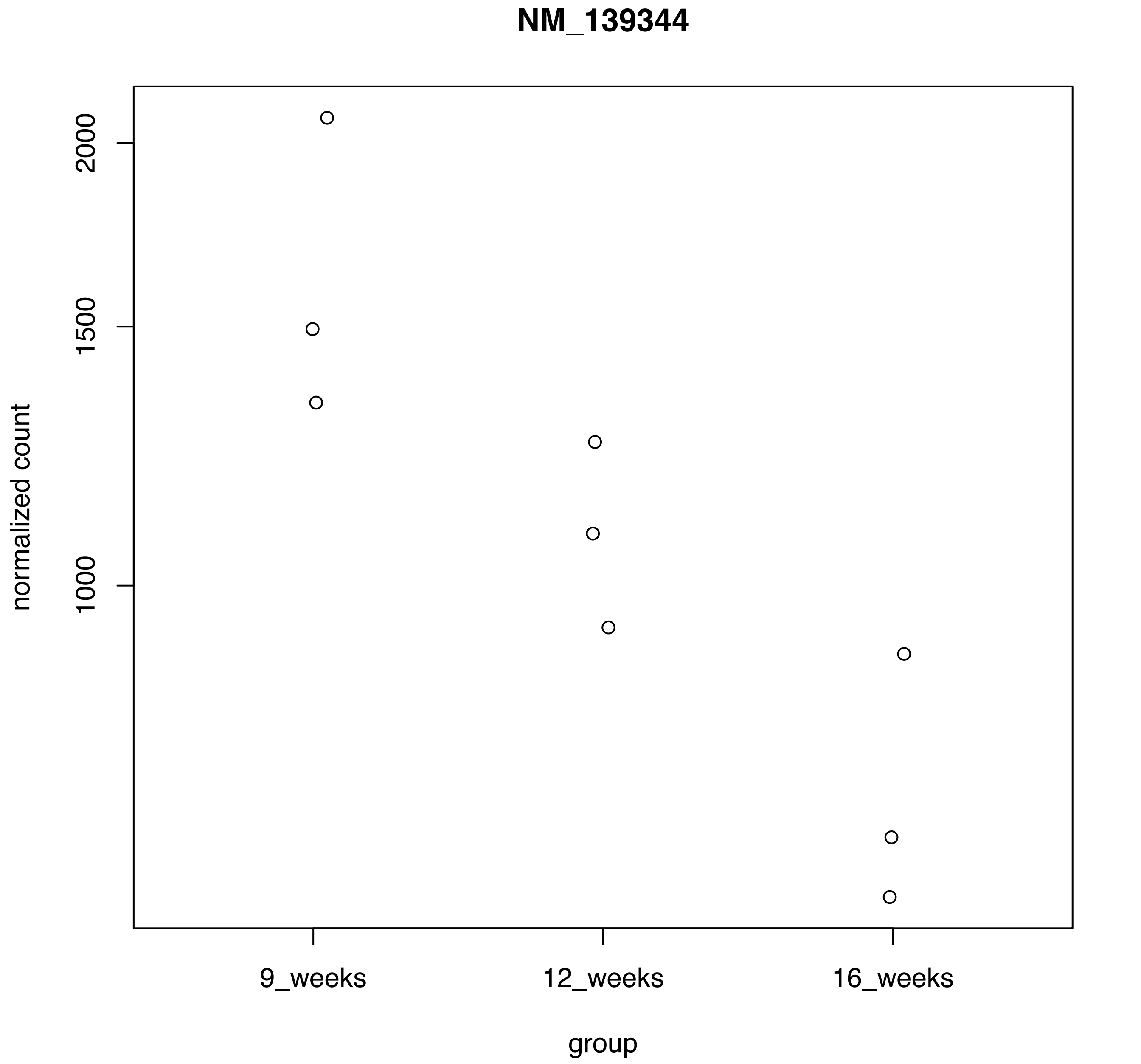

NM_139344
2000
1500
1000
normalized count
9_weeks
12_weeks
16_weeks
group

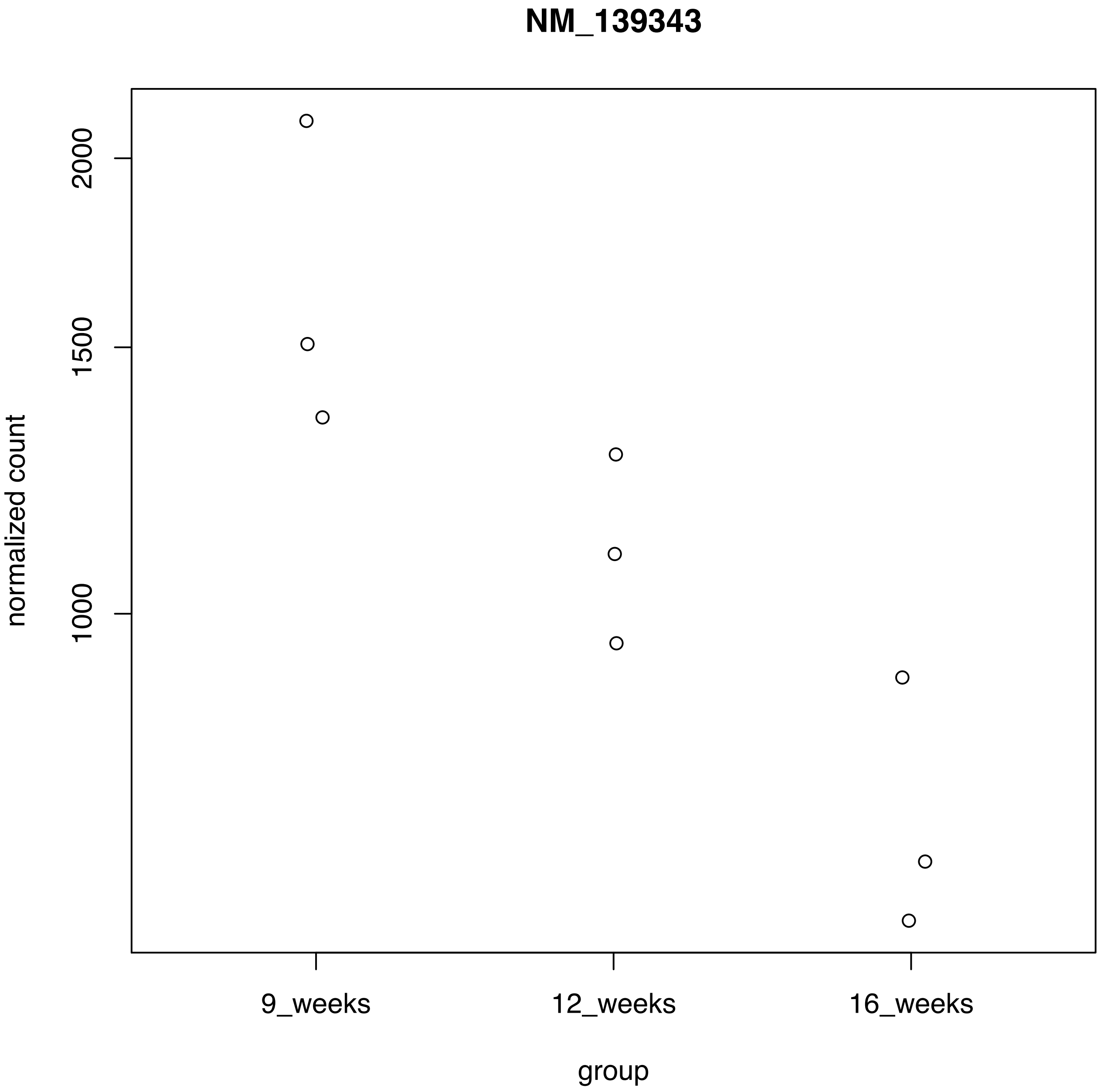
NM_139343
normalized count
2000
1500
1000
9_weeks
12_weeks
16_weeks
group

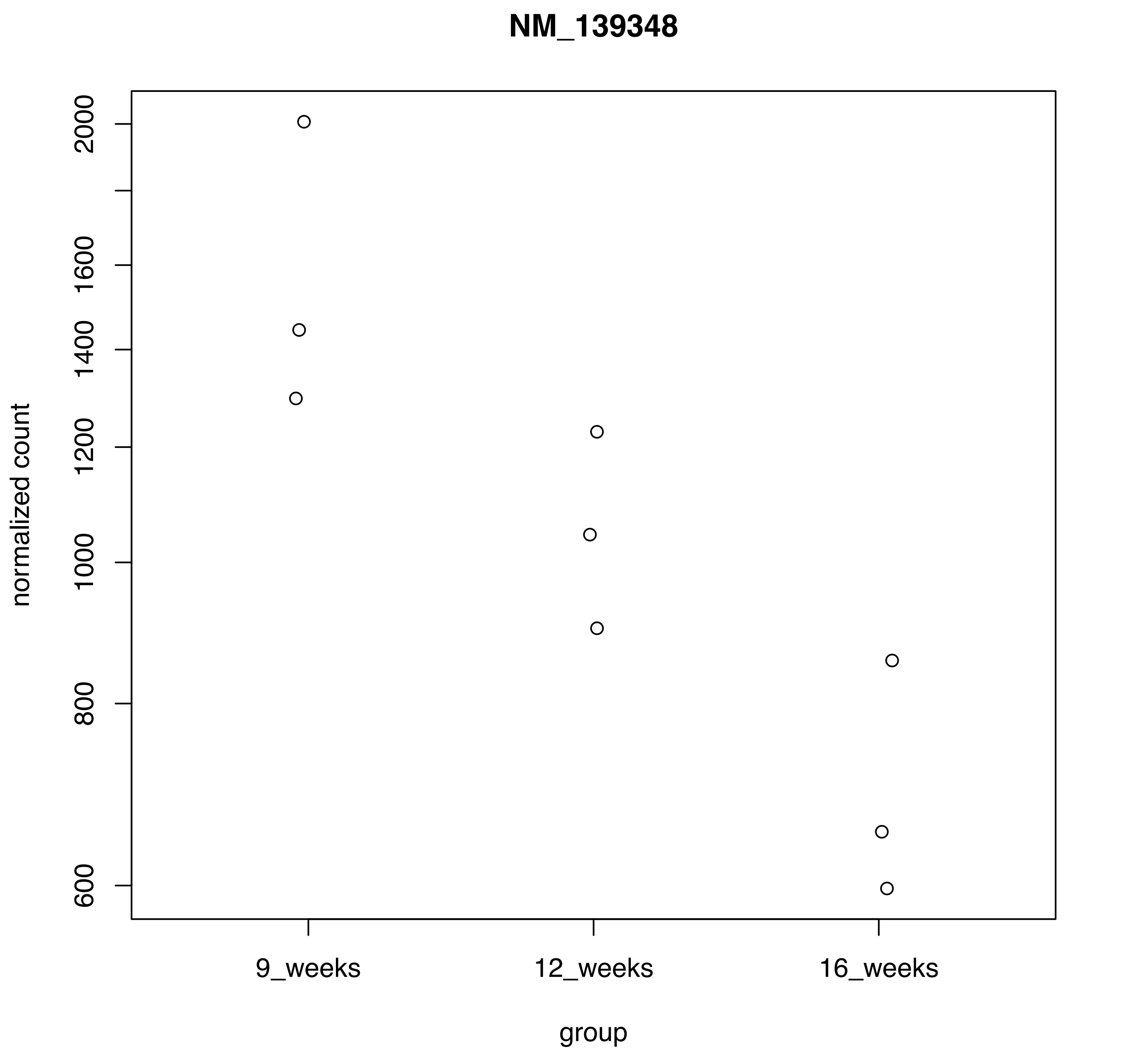
NM_139348
normalized count
600
800
1000
1200
1400
1600
2000
9_weeks
12_weeks
16_weeks
group

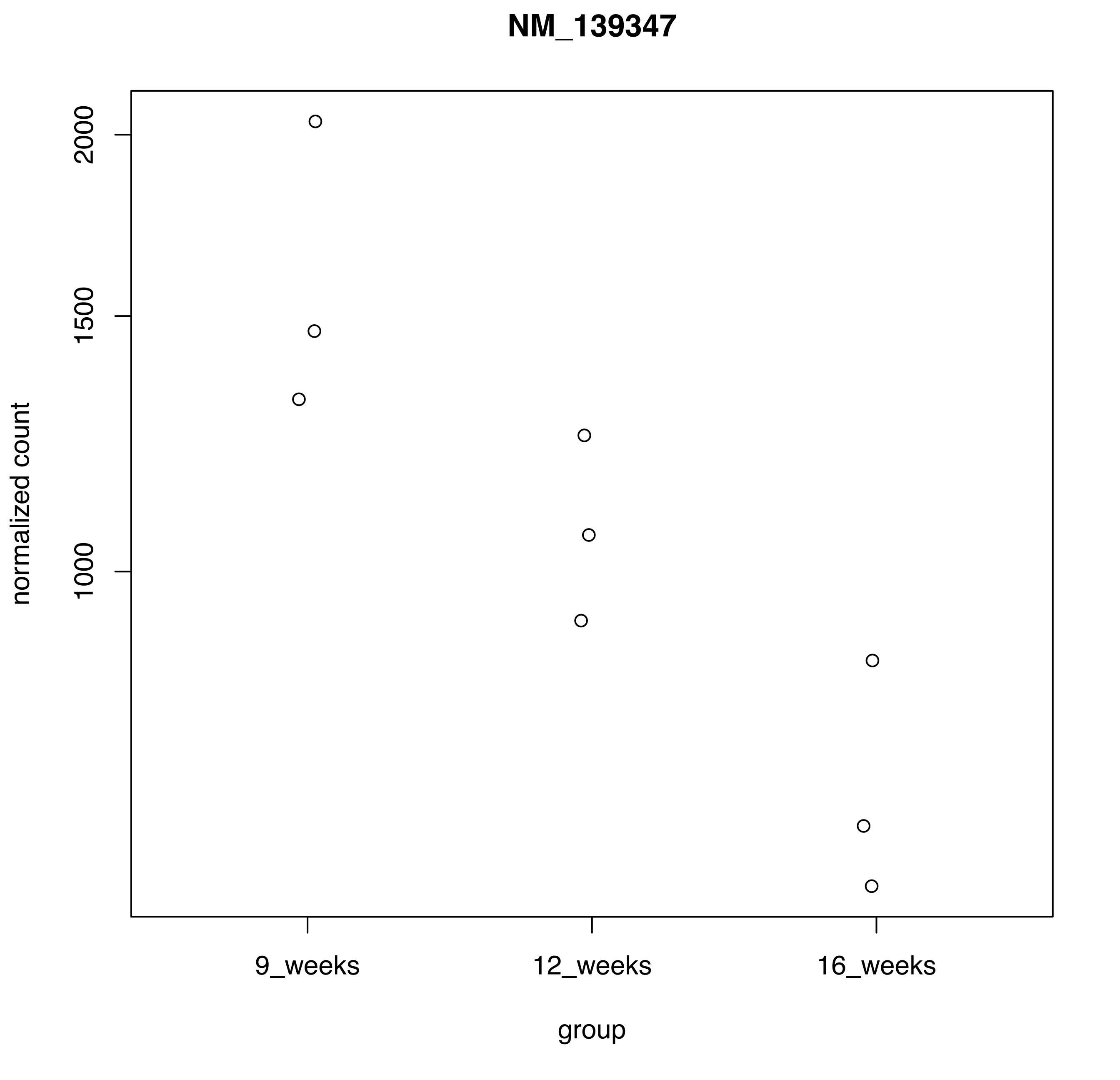
NM_139347
normalized count
2000
1500
1000
9_weeks
12_weeks
16_weeks
group

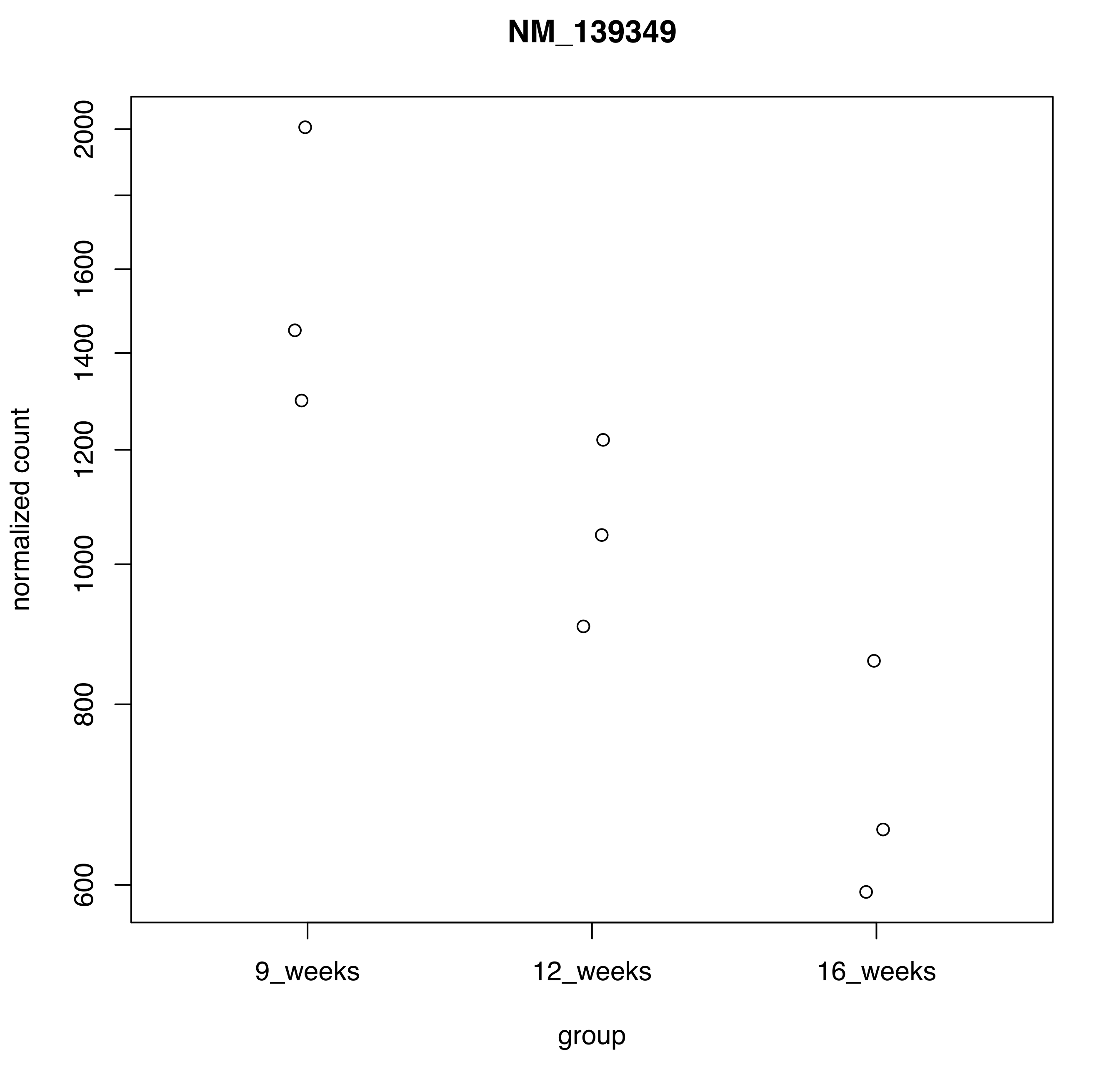

NM_139349
normalized count
2000
1600
1400
1200
1000
800
600
9_weeks
12_weeks
16_weeks
group

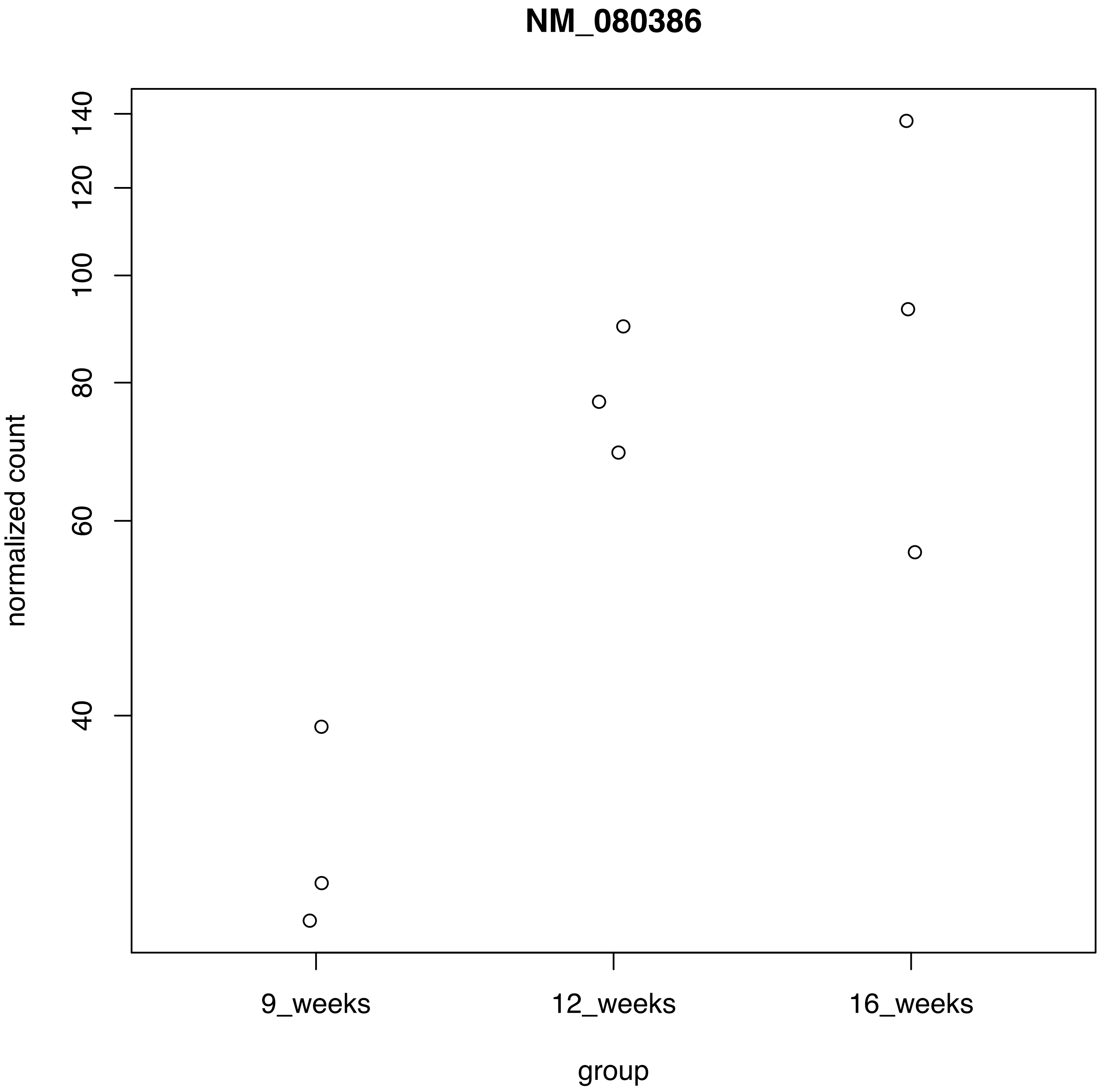
NM_080386
normalized count
140
120
100
80
60
40
9_weeks
12_weeks
16_weeks
group

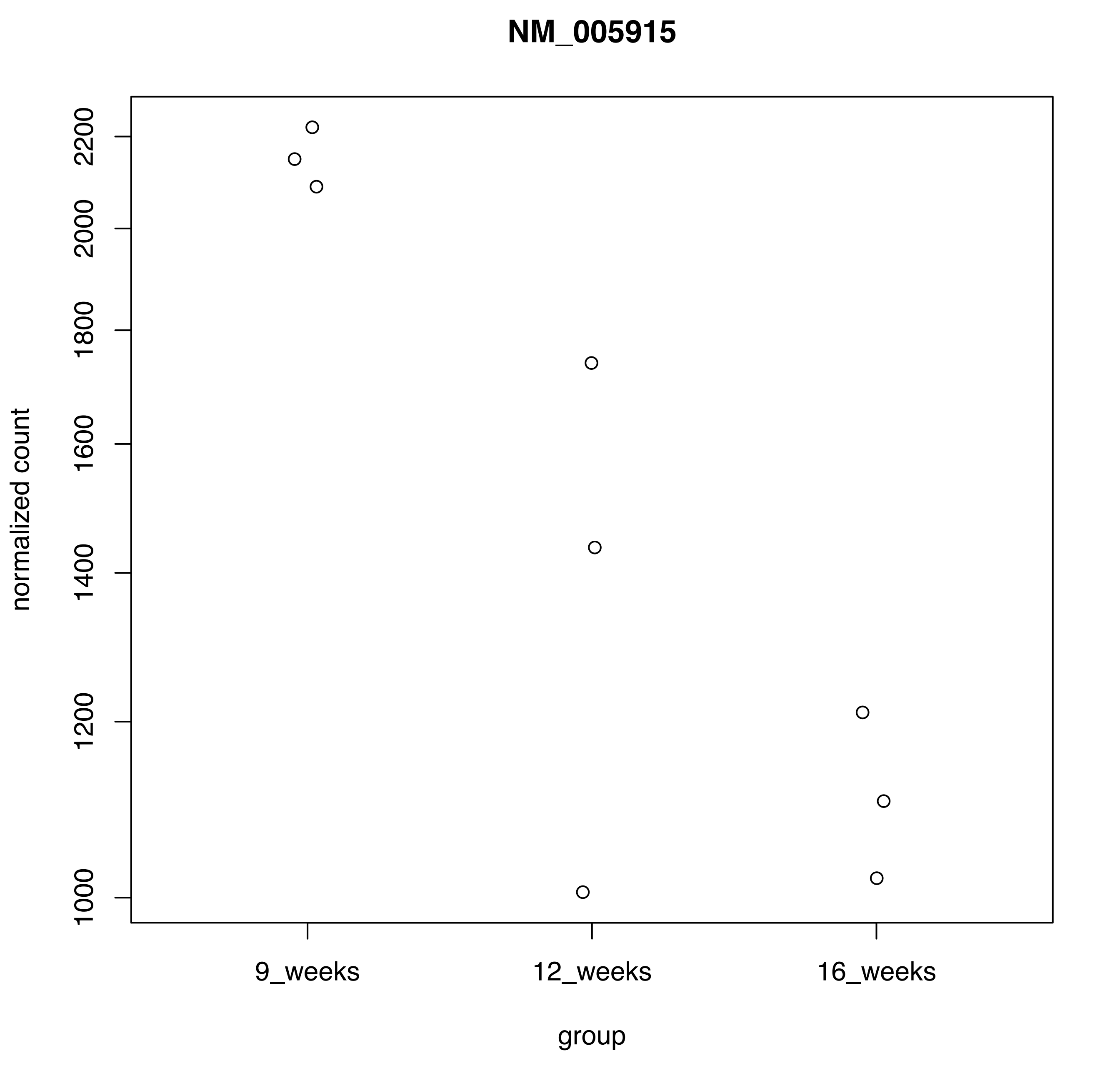
NM_005915
normalized count
2200
2000
1800
1600
1400
1200
1000
9_weeks
12_weeks
16_weeks
group

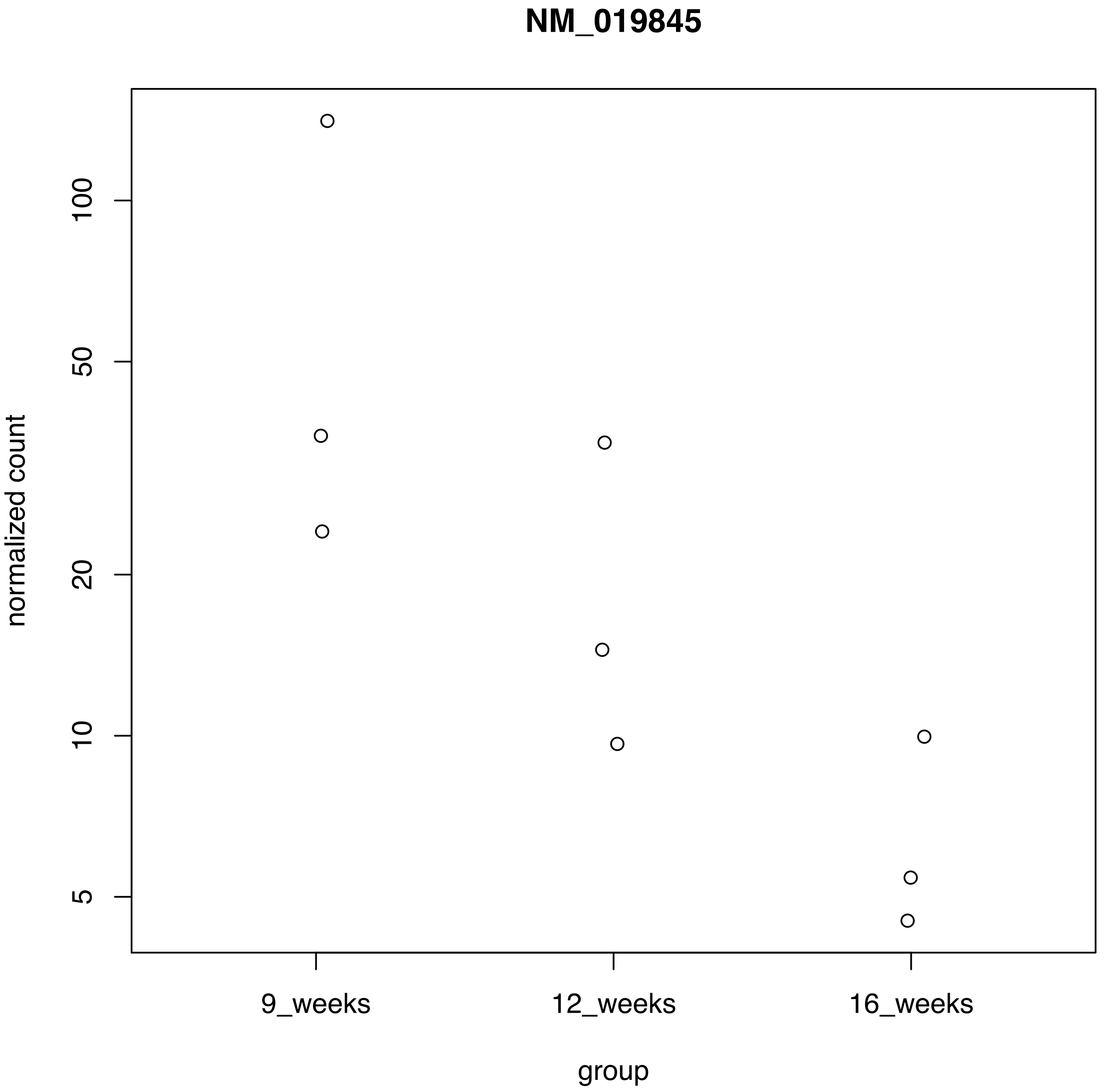

NM_019845
normalized count
100
50
20
10
5
9_weeks
12_weeks
16_weeks
group

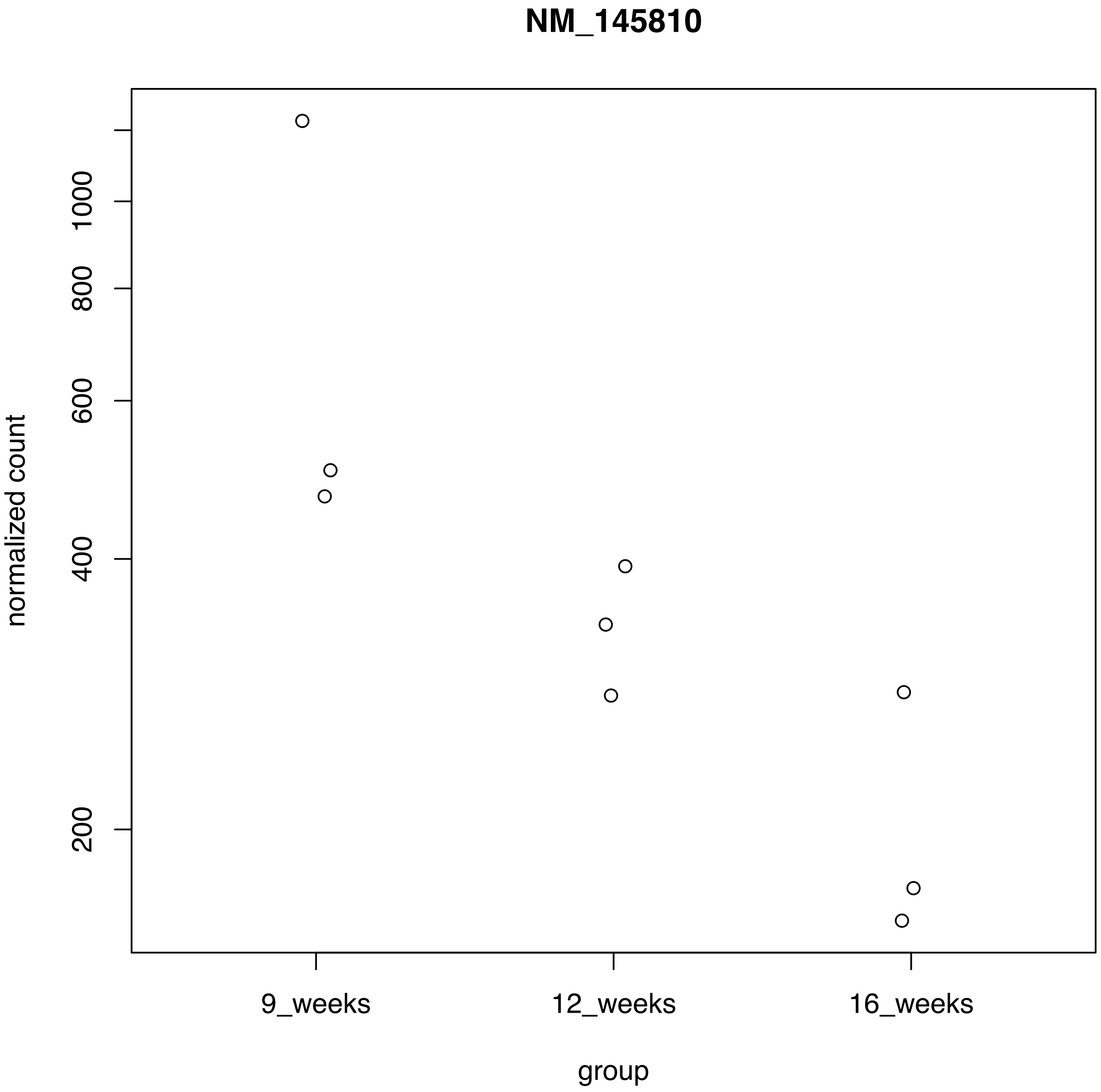
NM_145810
normalized count
1000
800
600
400
200
9_weeks
12_weeks
16_weeks
group

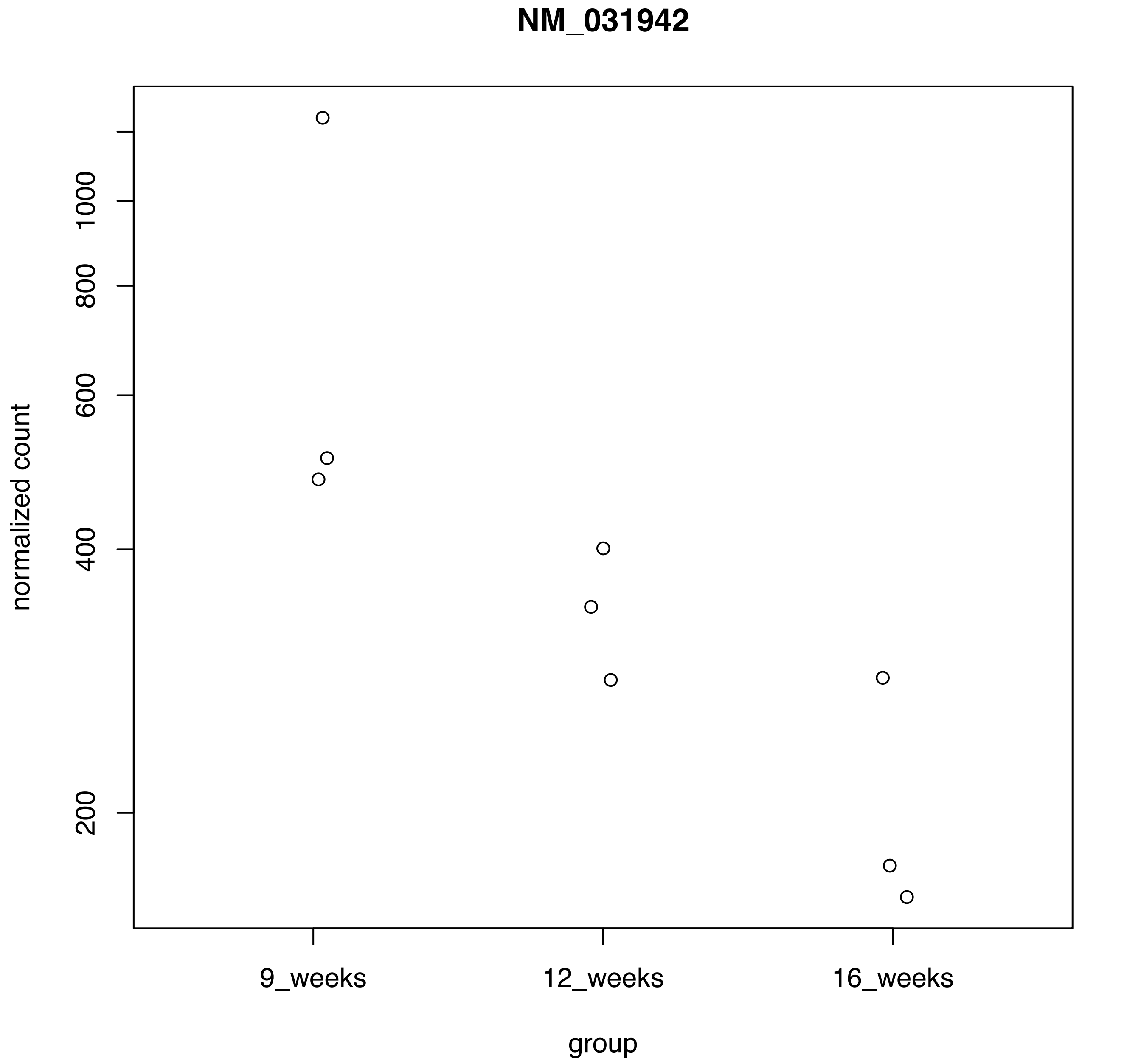
NM_031942
normalized count
200
400
600
800
1000
9_weeks
12_weeks
16_weeks
group

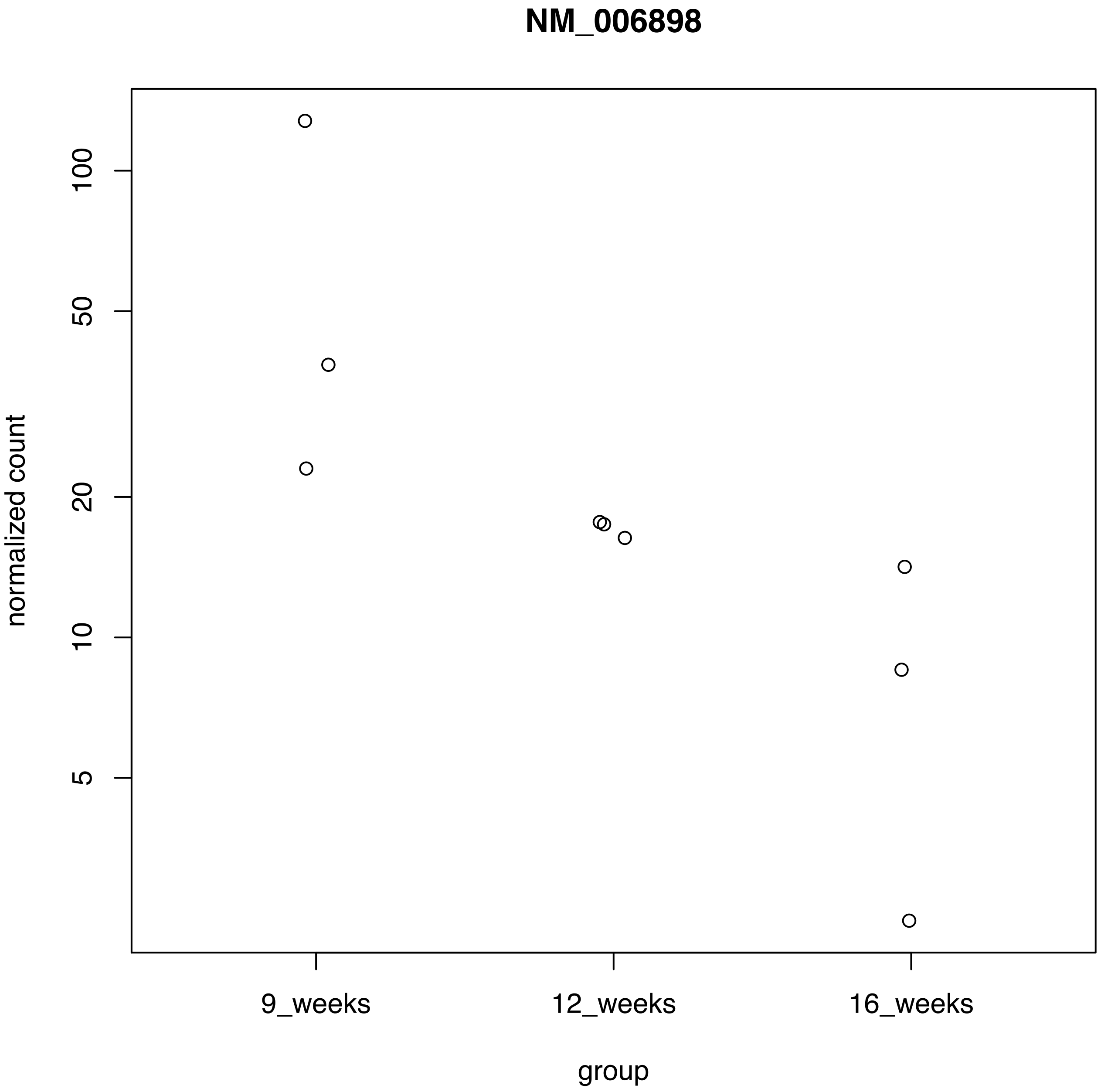
NM_006898
normalized count
100
50
20
10
5
9_weeks
12_weeks
16_weeks
group

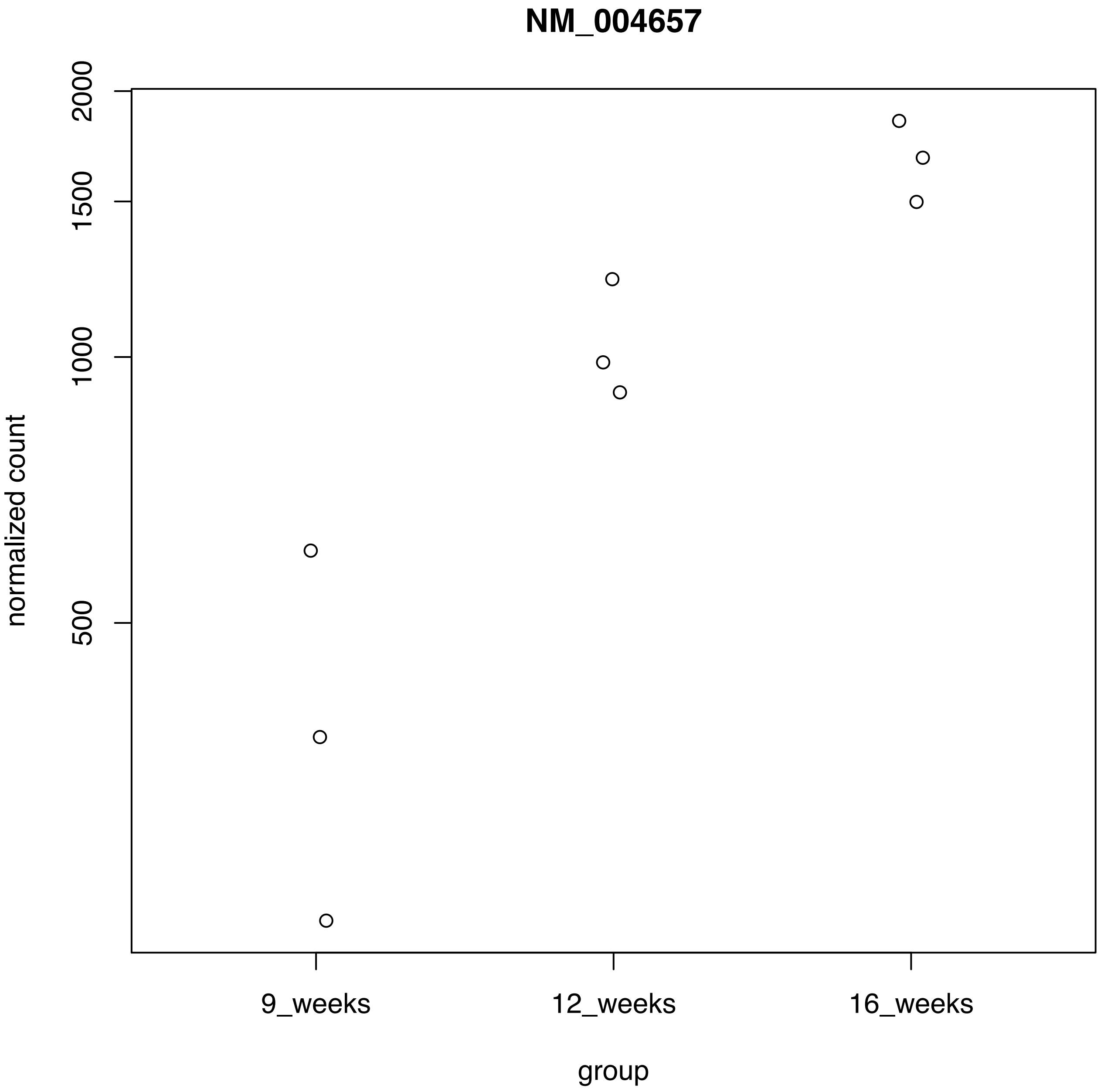
NM_004657
normalized count
2000
1500
1000
500
9_weeks
12_weeks
16_weeks
group

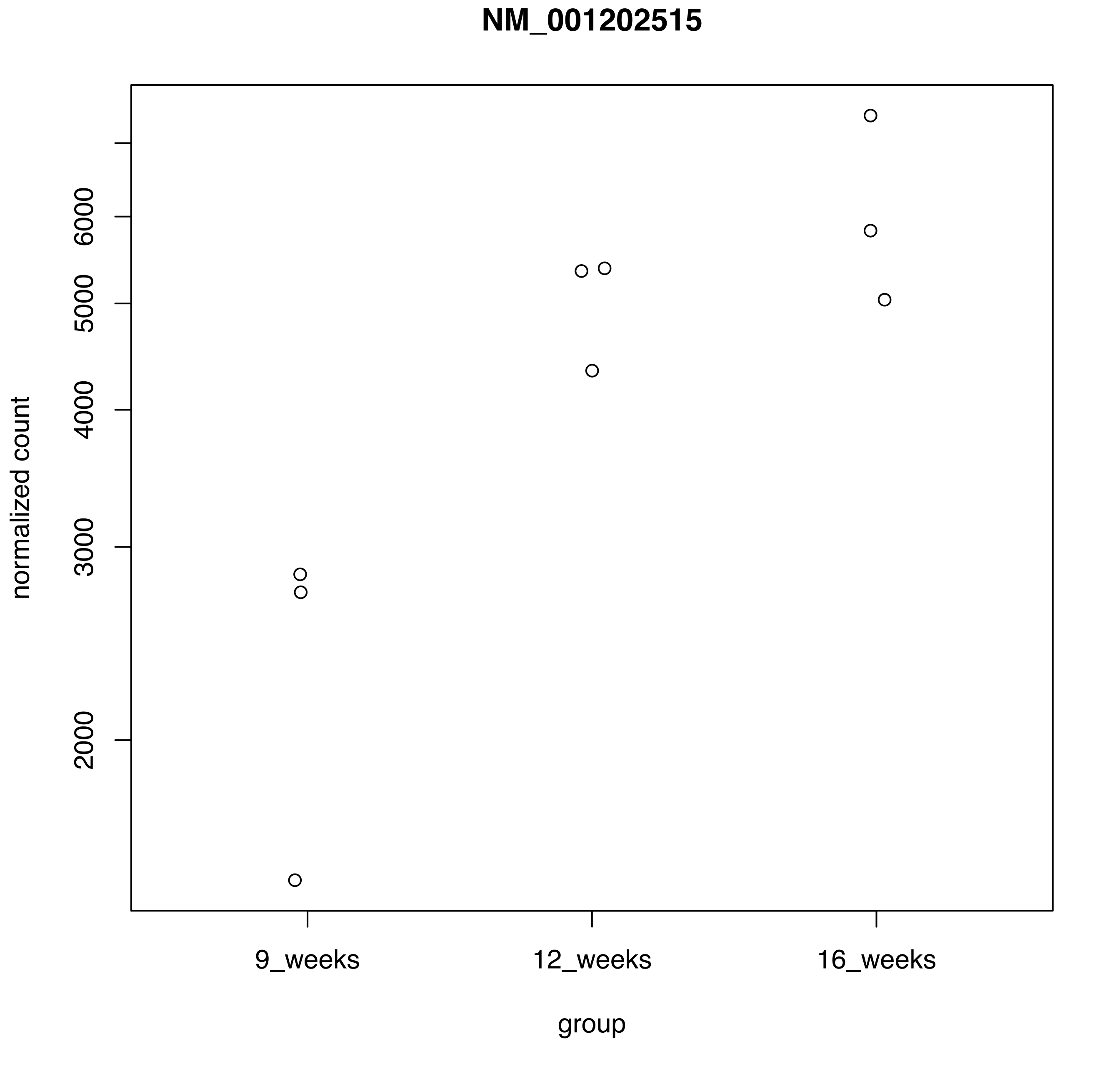
NM_001202515
normalized count
2000
3000
4000
5000
6000
9_weeks
12_weeks
16_weeks
group

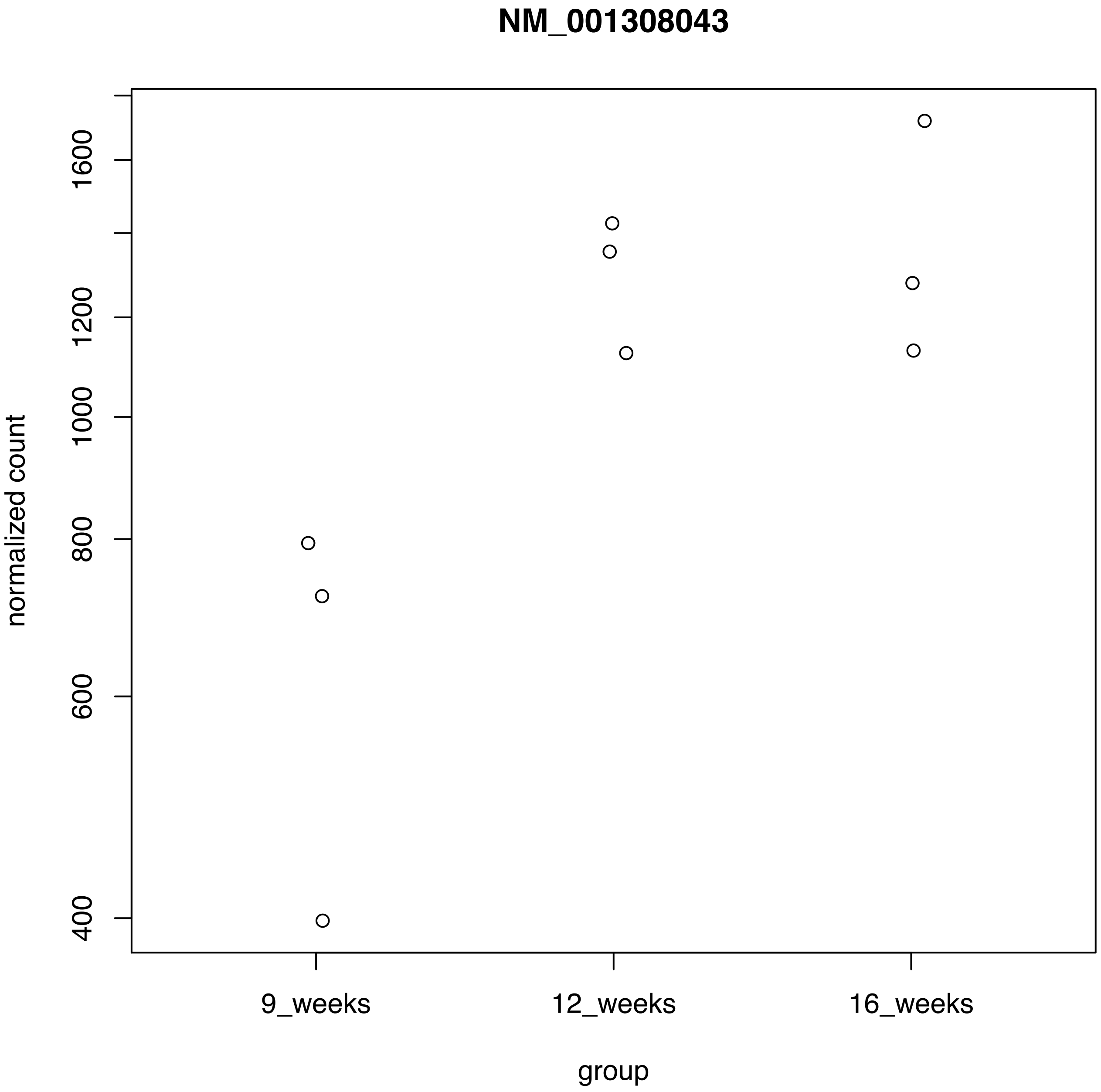

NM_001308043
normalized count
400
600
800
1000
1200
1600
9_weeks
12_weeks
16_weeks
group

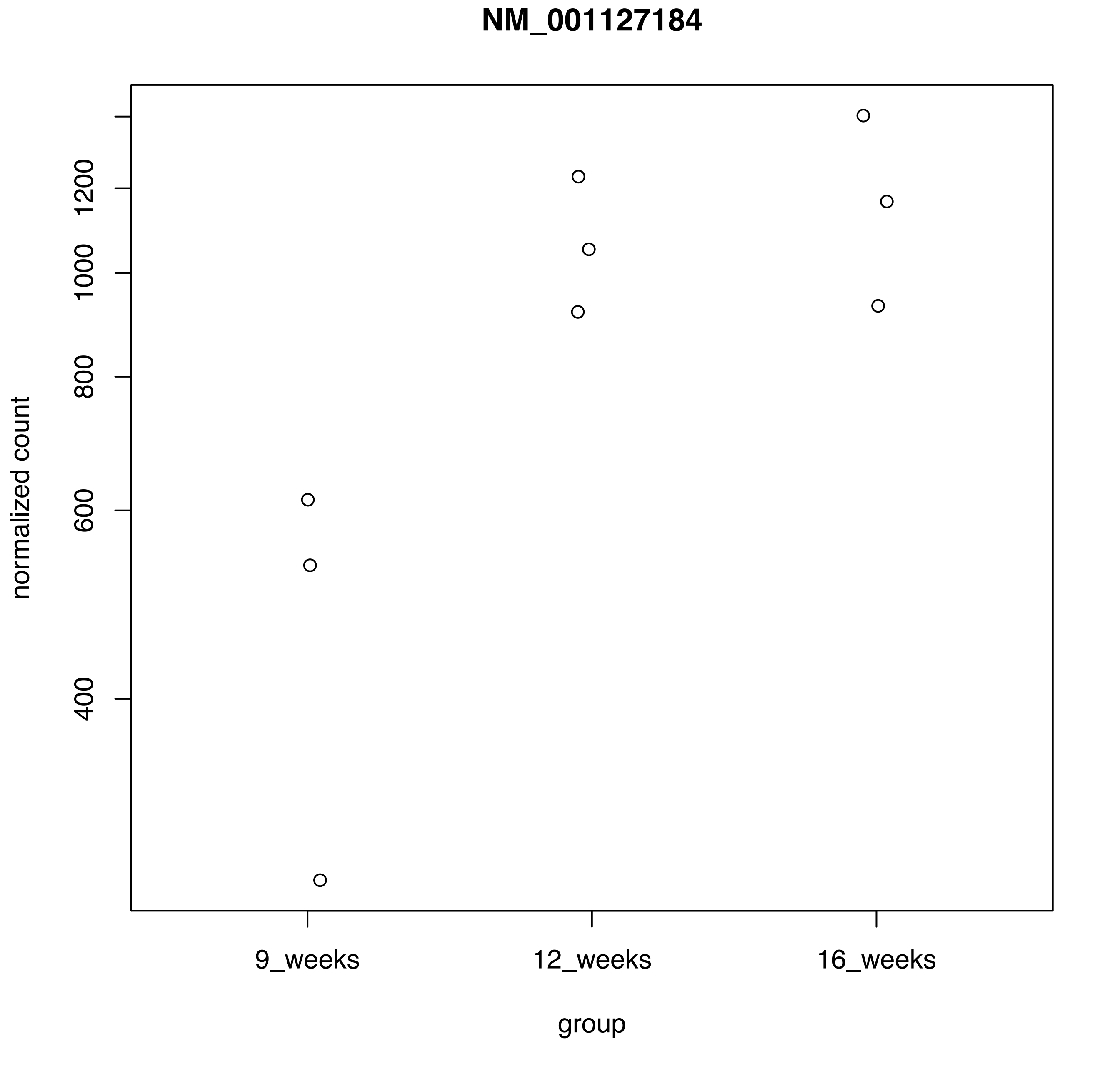

NM_001127184
normalized count
400
600
800
1000
1200
9_weeks
12_weeks
16_weeks
group

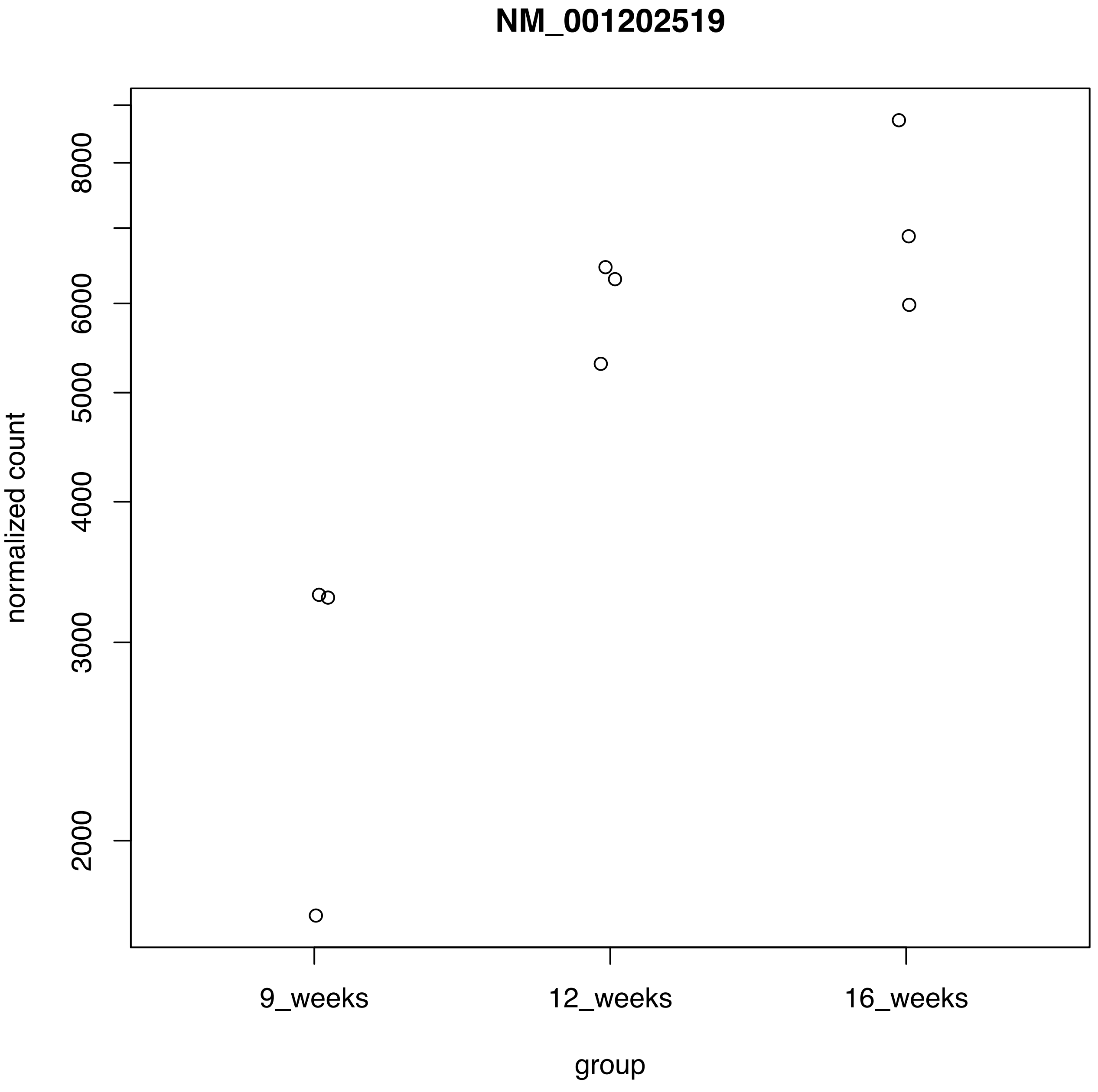
NM_001202519
normalized count
2000
3000
4000
5000
6000
8000
9_weeks
12_weeks
16_weeks
group

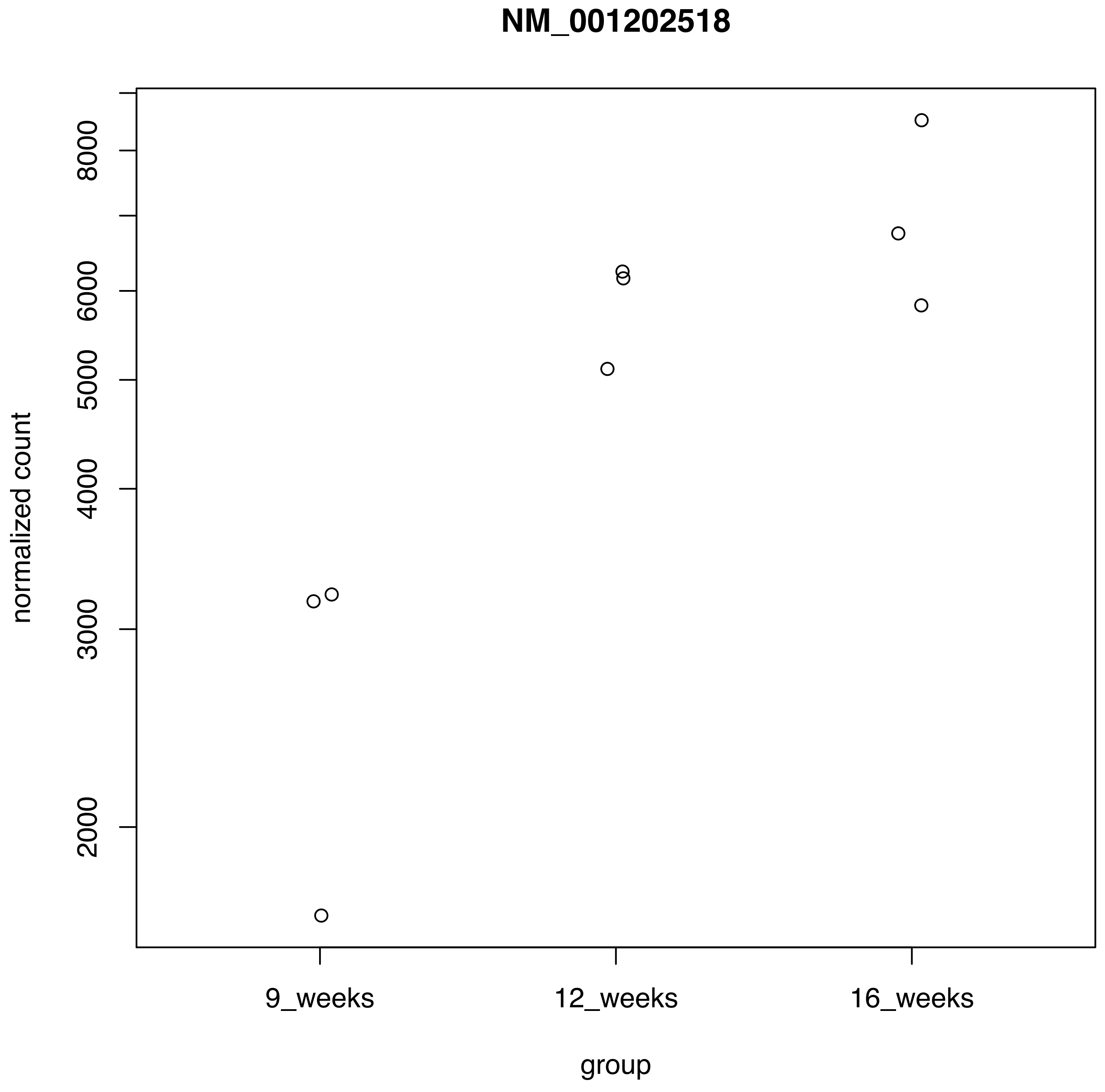
NM_001202518
normalized count
8000
6000
5000
4000
3000
2000
9_weeks
12_weeks
16_weeks
group

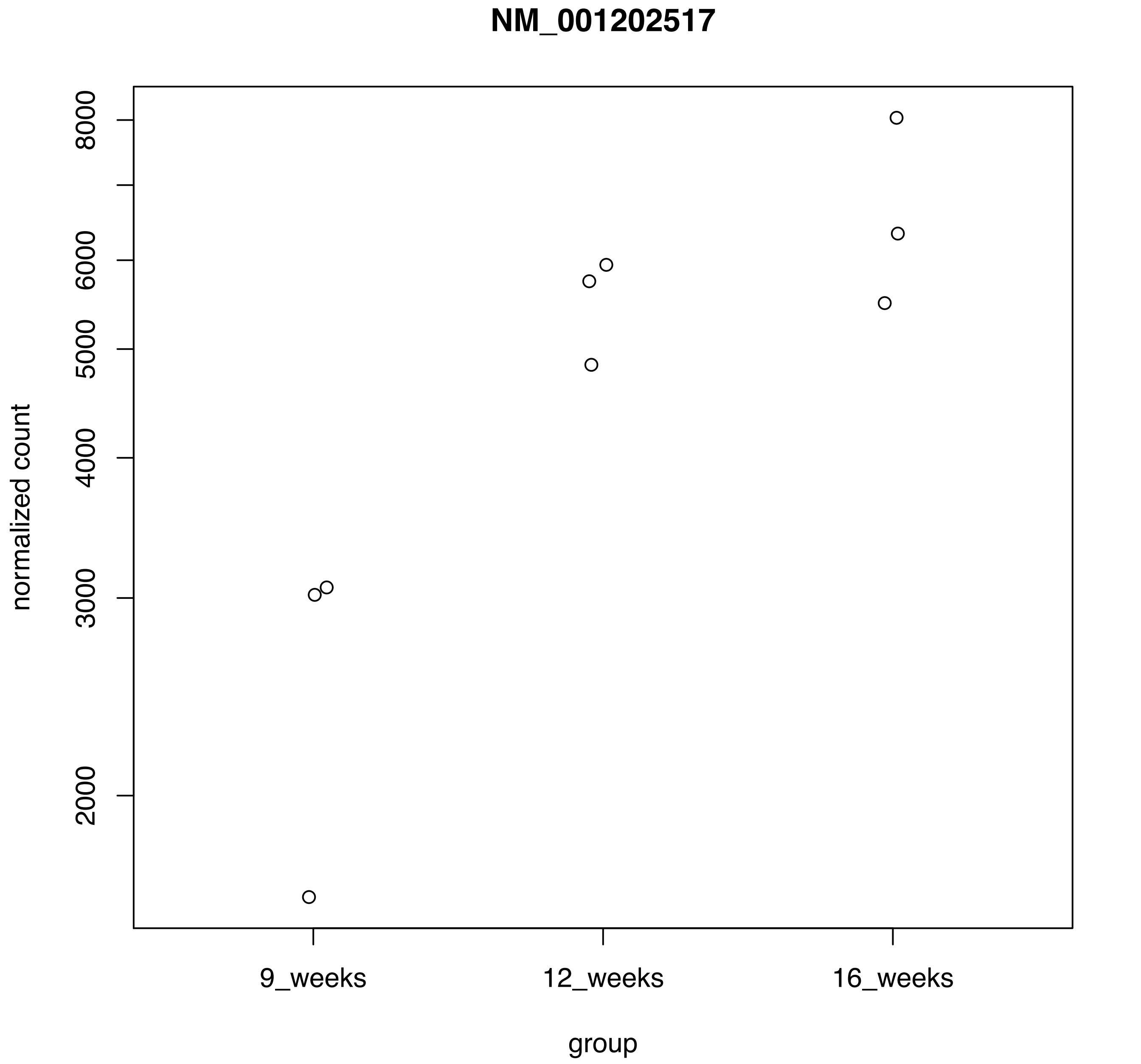

NM_001202517
normalized count
8000
6000
5000
4000
3000
2000
9_weeks
12_weeks
16_weeks
group

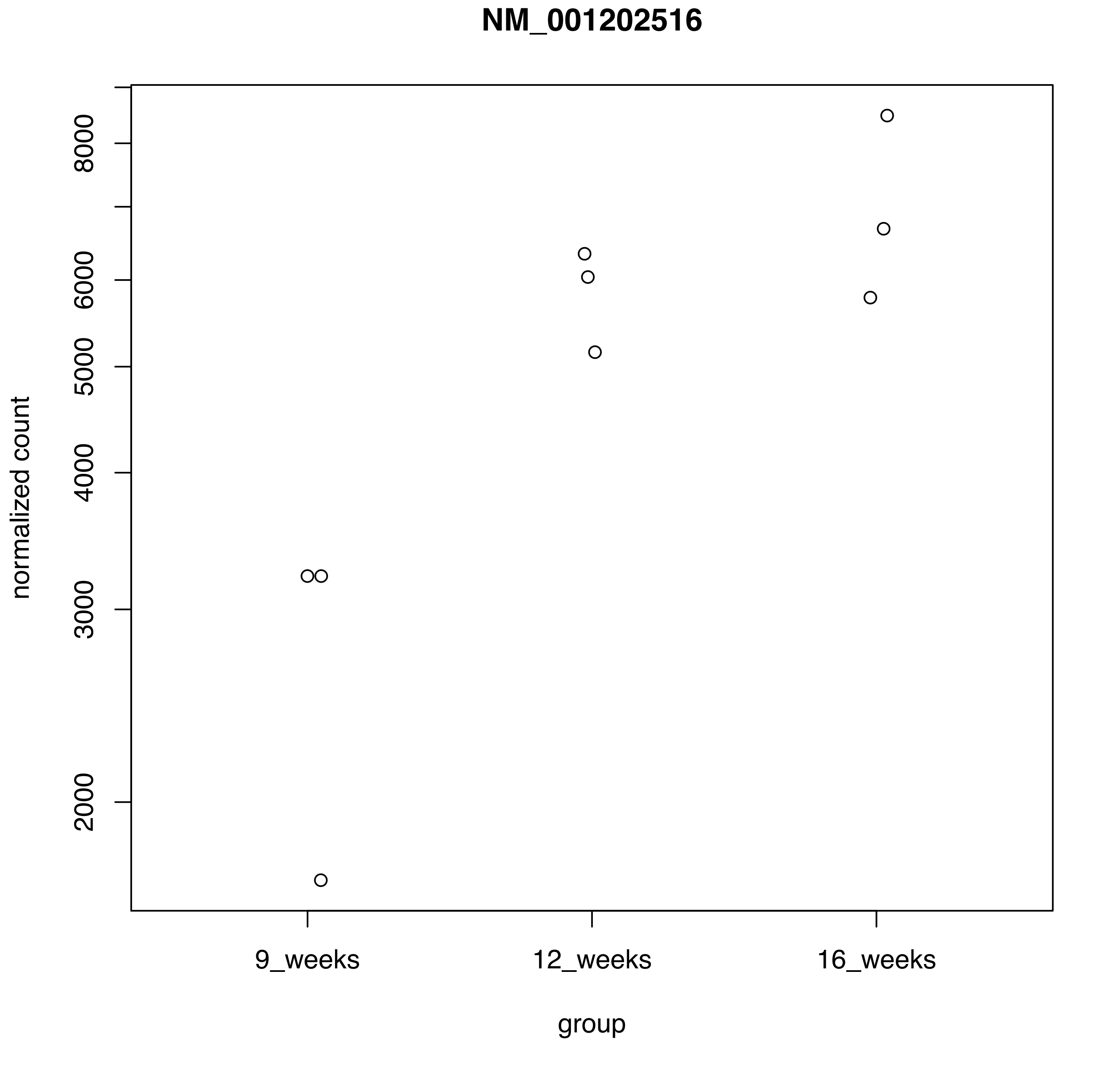

NM_001202516
normalized count
8000
6000
5000
4000
3000
2000
9_weeks
12_weeks
16_weeks
group

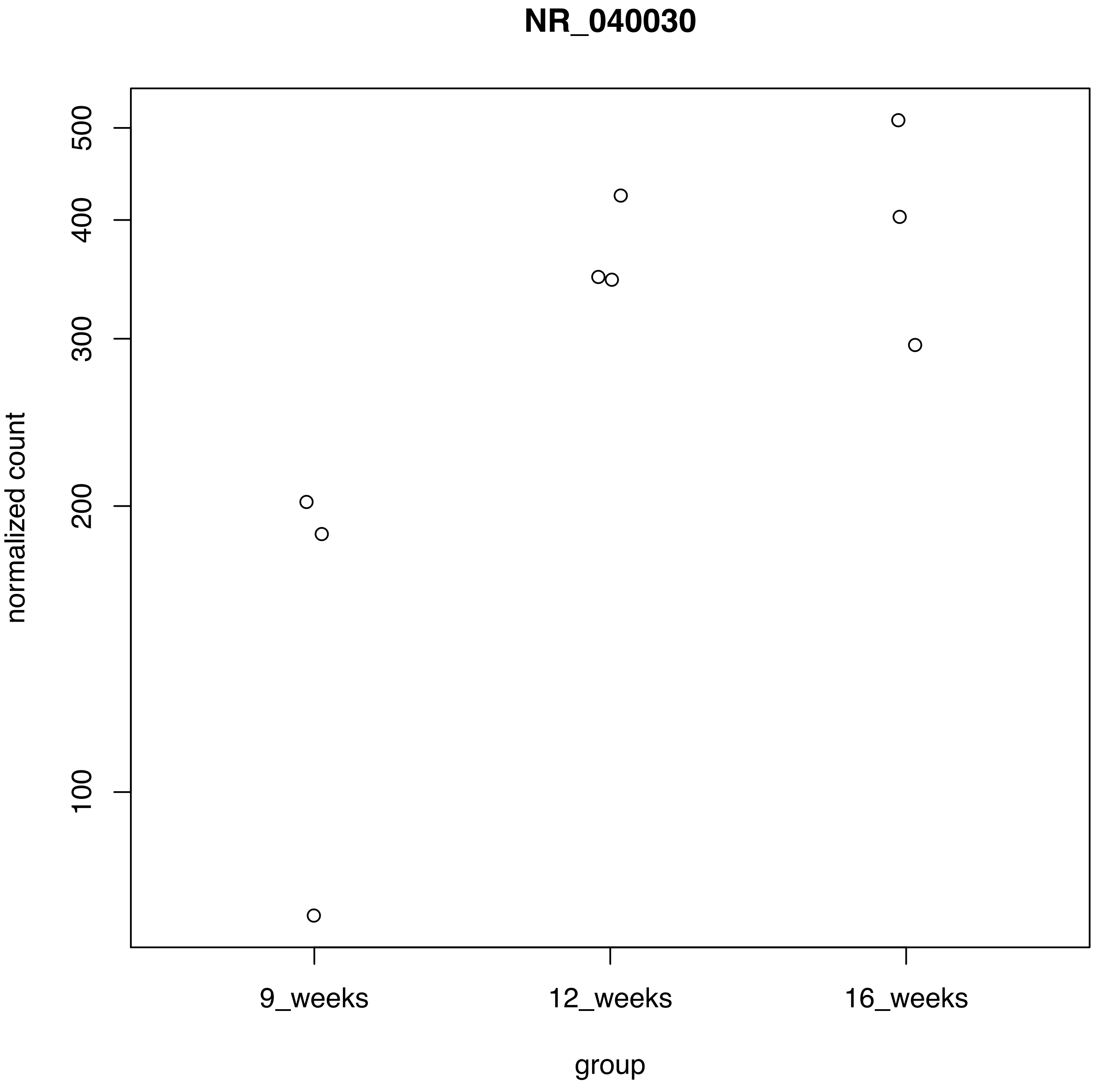

NR_040030
normalized count
100
200
300
400
500
9_weeks
12_weeks
16_weeks
group

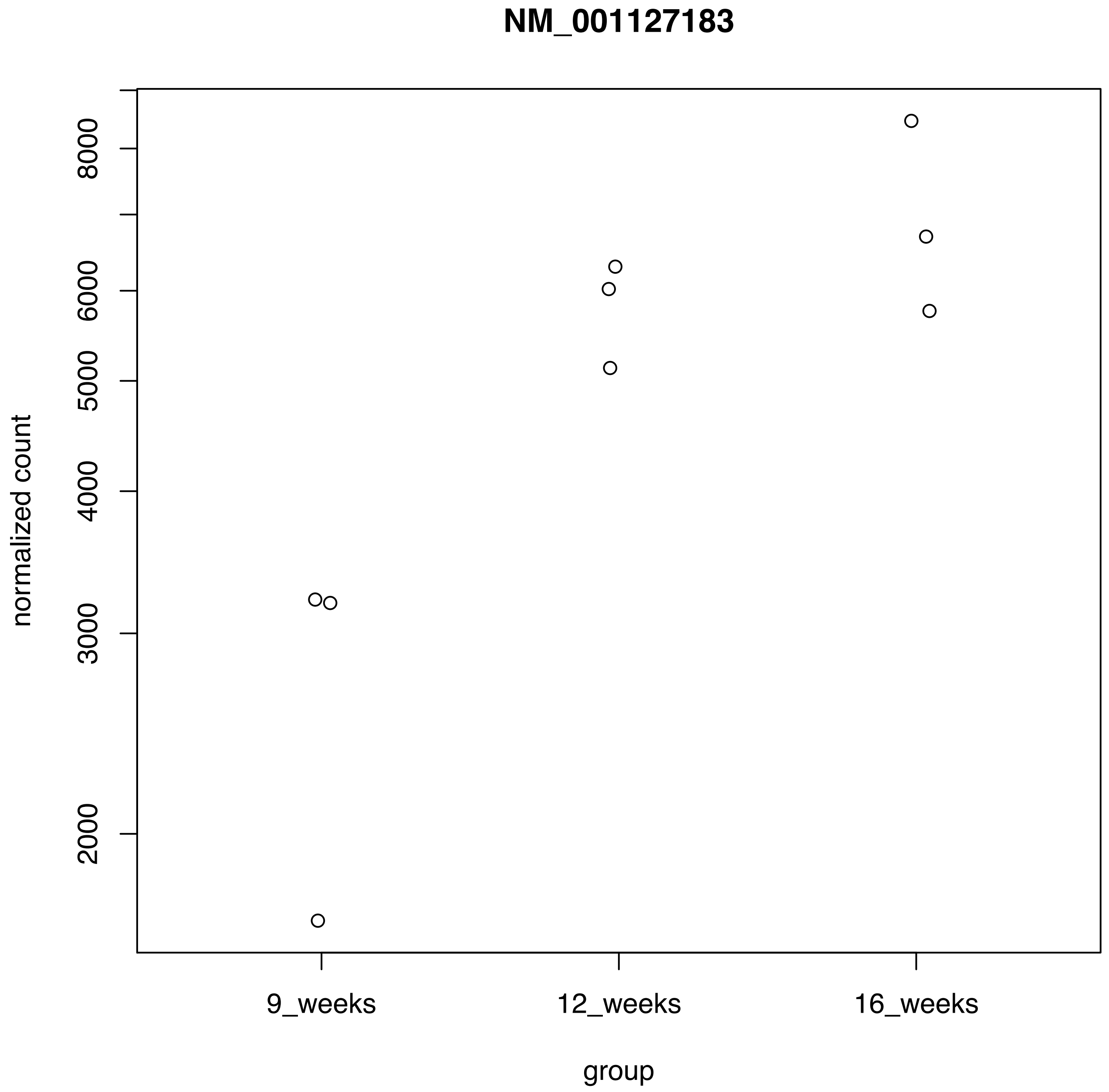
NM_001127183
normalized count
2000
3000
4000
5000
6000
8000
9_weeks
12_weeks
16_weeks
group

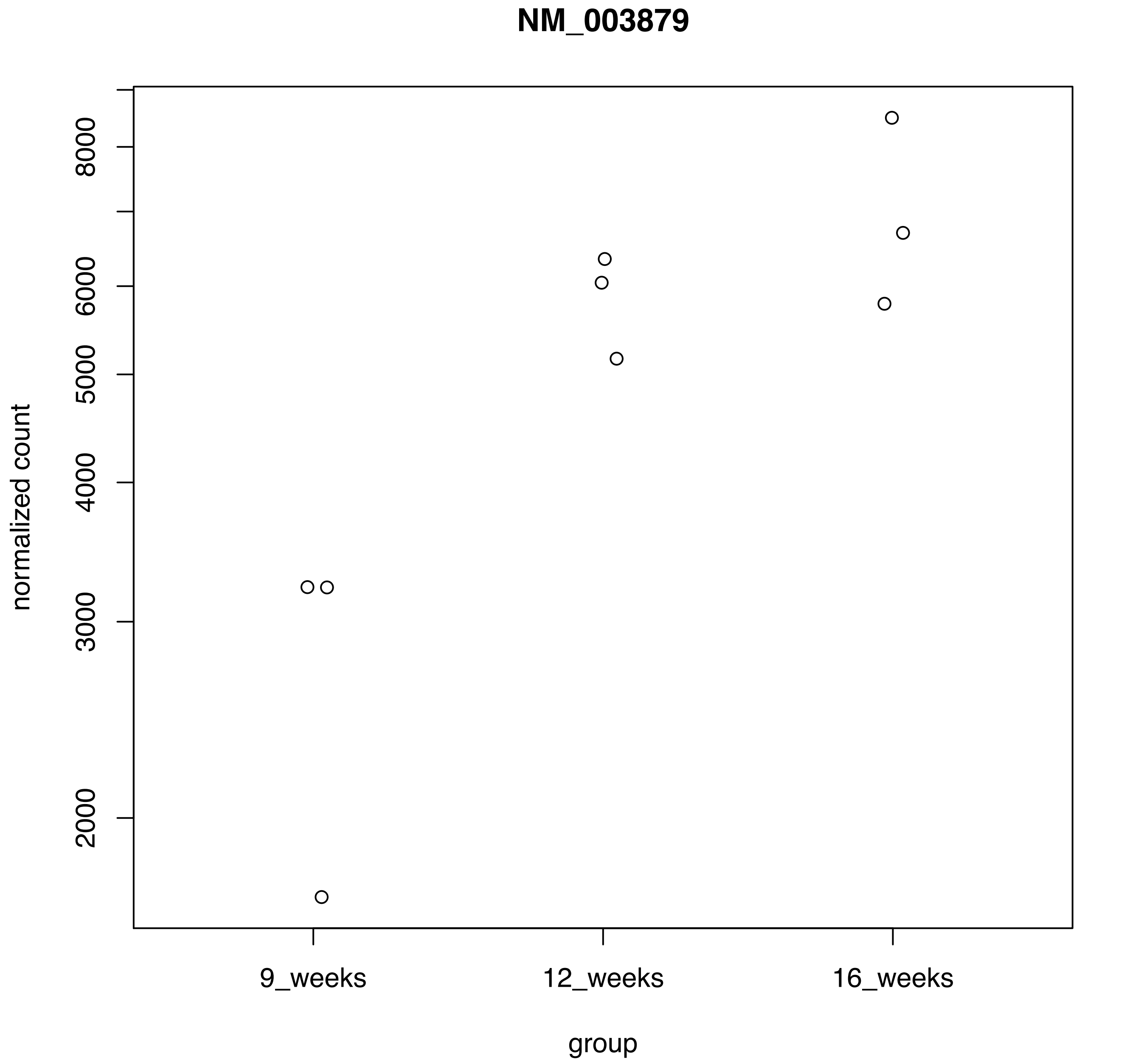

NM_003879
normalized count
8000
6000
5000
4000
3000
2000
9_weeks
12_weeks
16_weeks
group

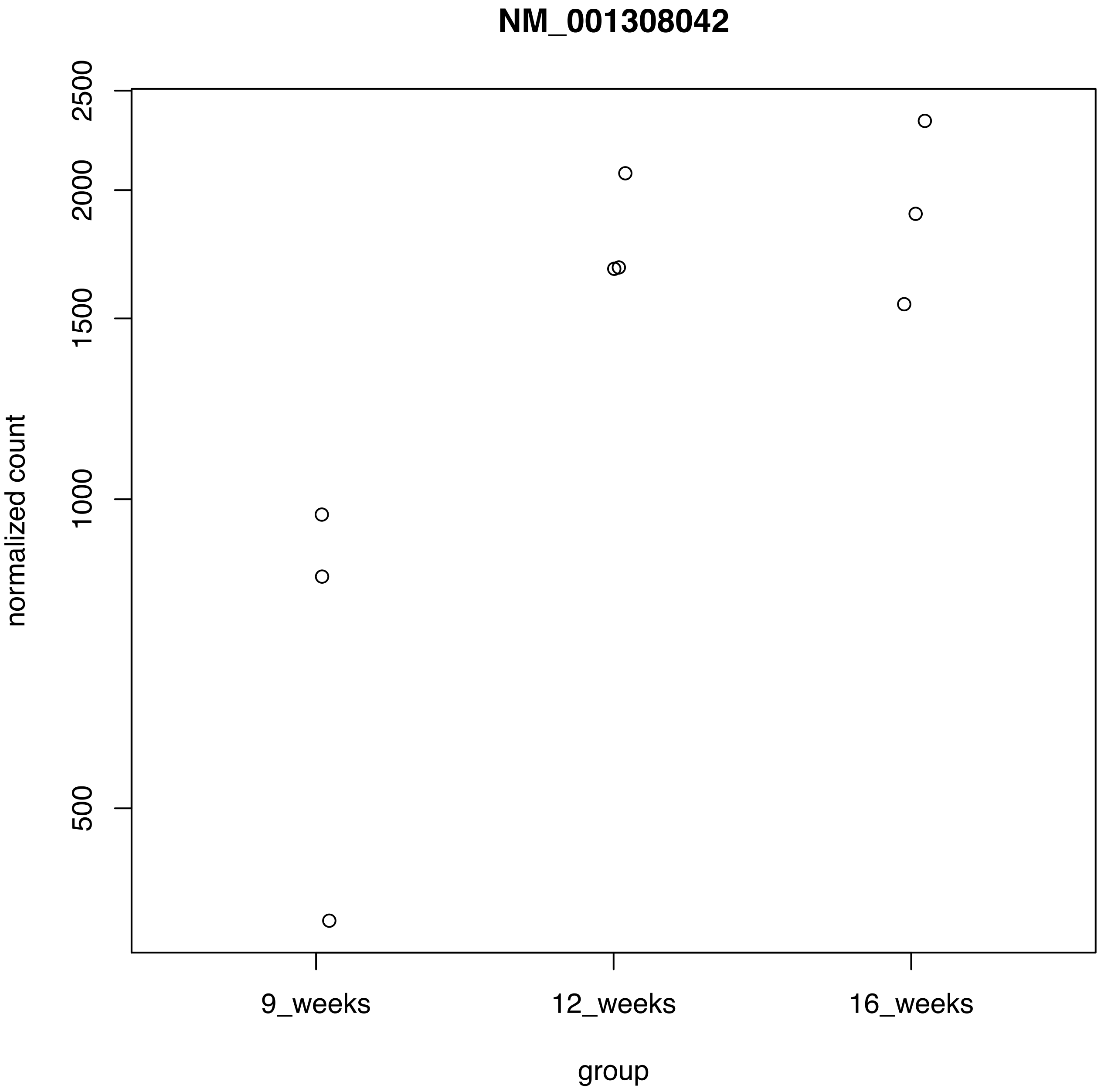
NM_001308042
normalized count
2500
2000
1500
1000
500
9_weeks
12_weeks
16_weeks
group

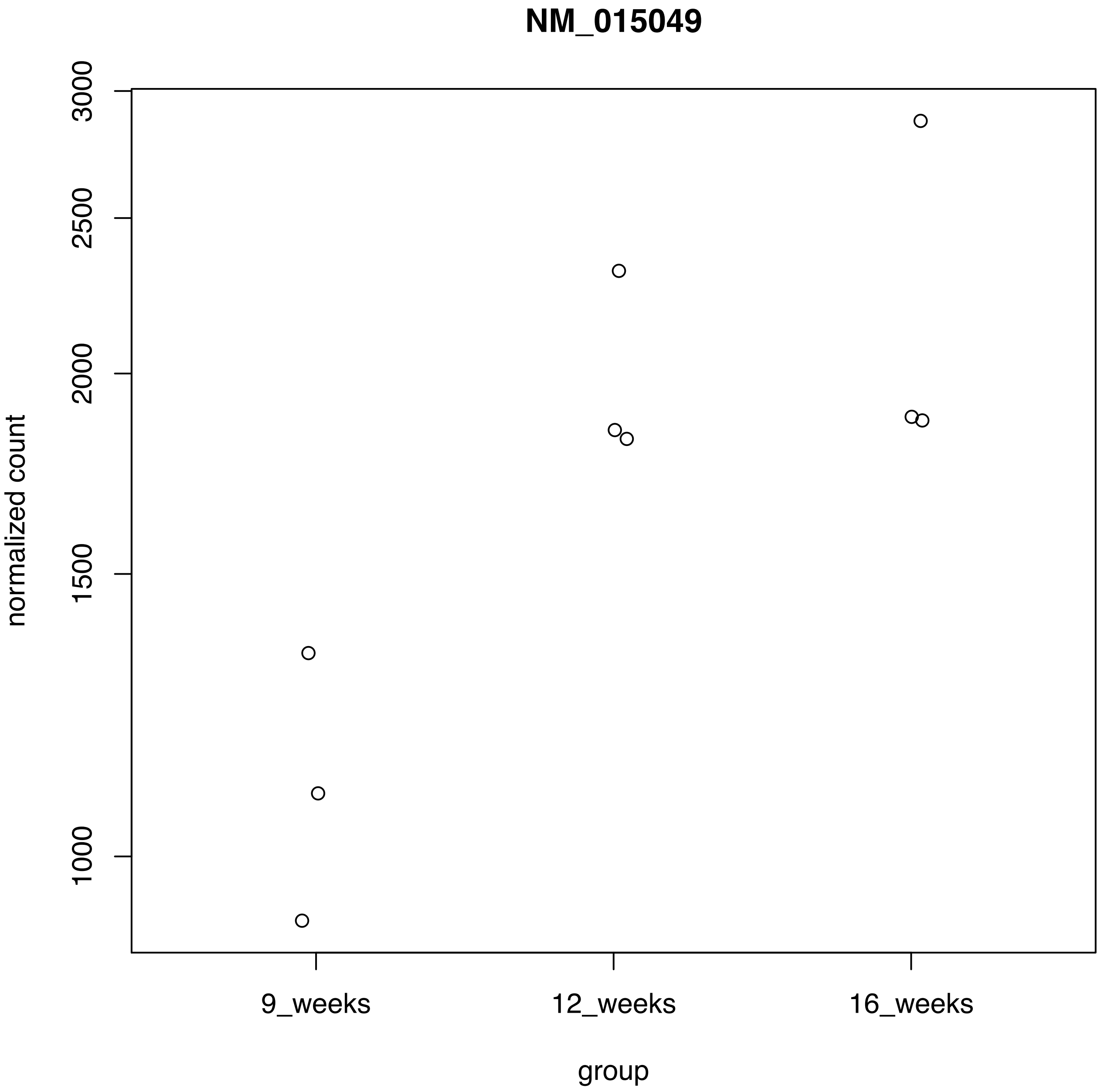
NM_015049
normalized count
3000
2500
2000
1500
1000
9_weeks
12_weeks
16_weeks
group

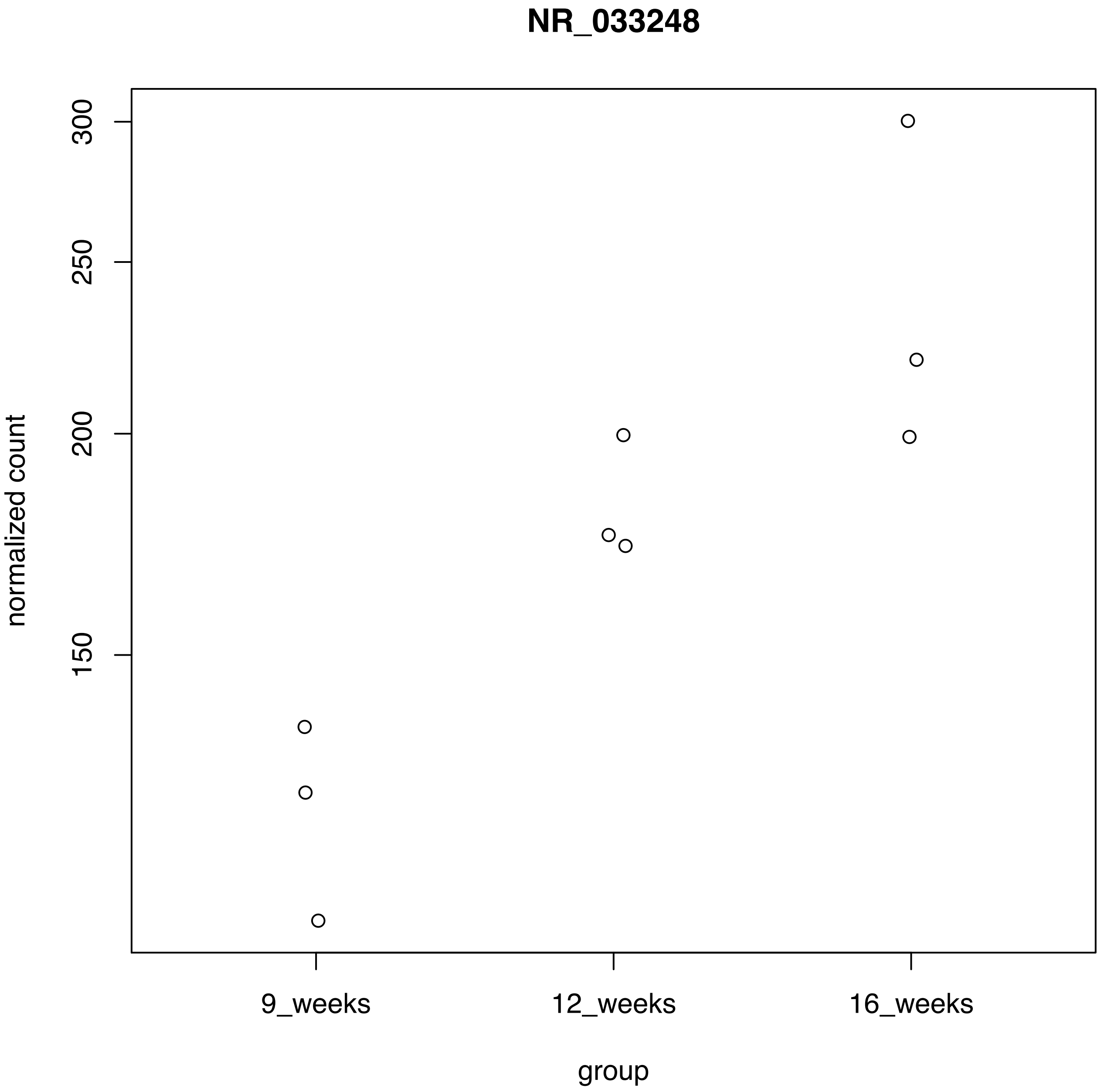

NR_033248
normalized count
300
250
200
150
9_weeks
12_weeks
16_weeks
group

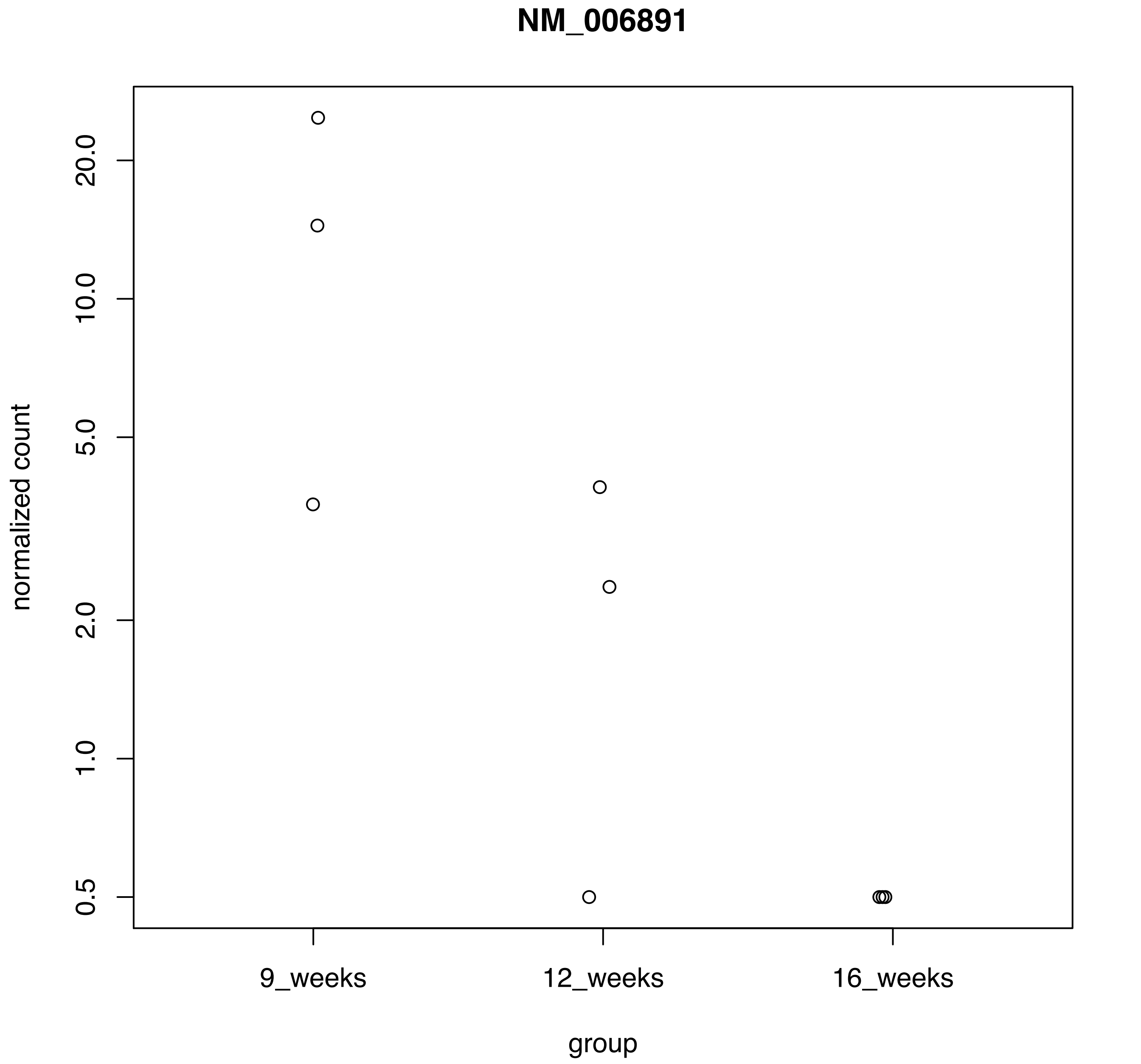
NM_006891
normalized count
20.0
10.0
5.0
2.0
1.0
0.5
9_weeks
12_weeks
16_weeks
group

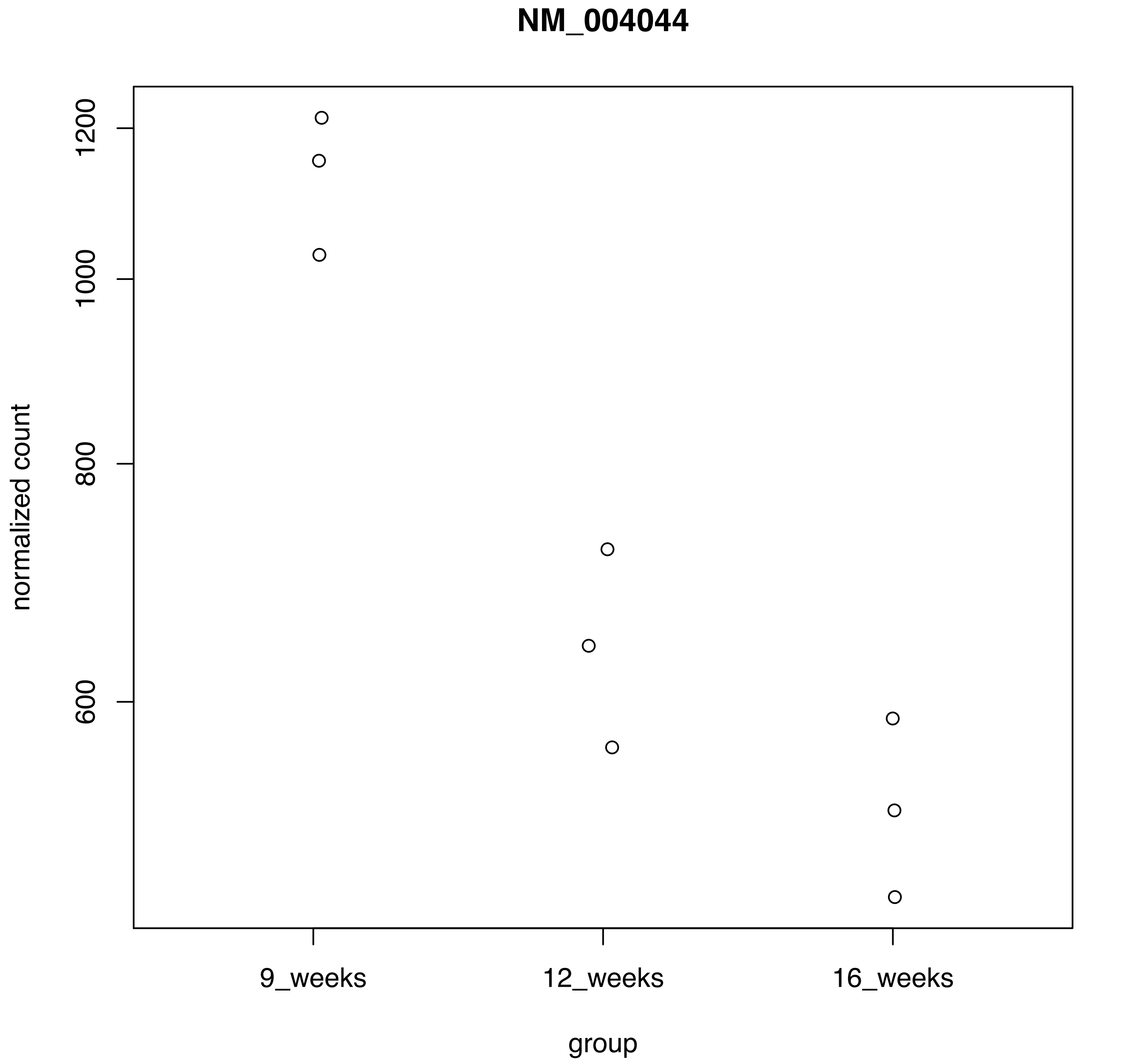

NM_004044
normalized count
1200
1000
800
600
9_weeks
12_weeks
16_weeks
group

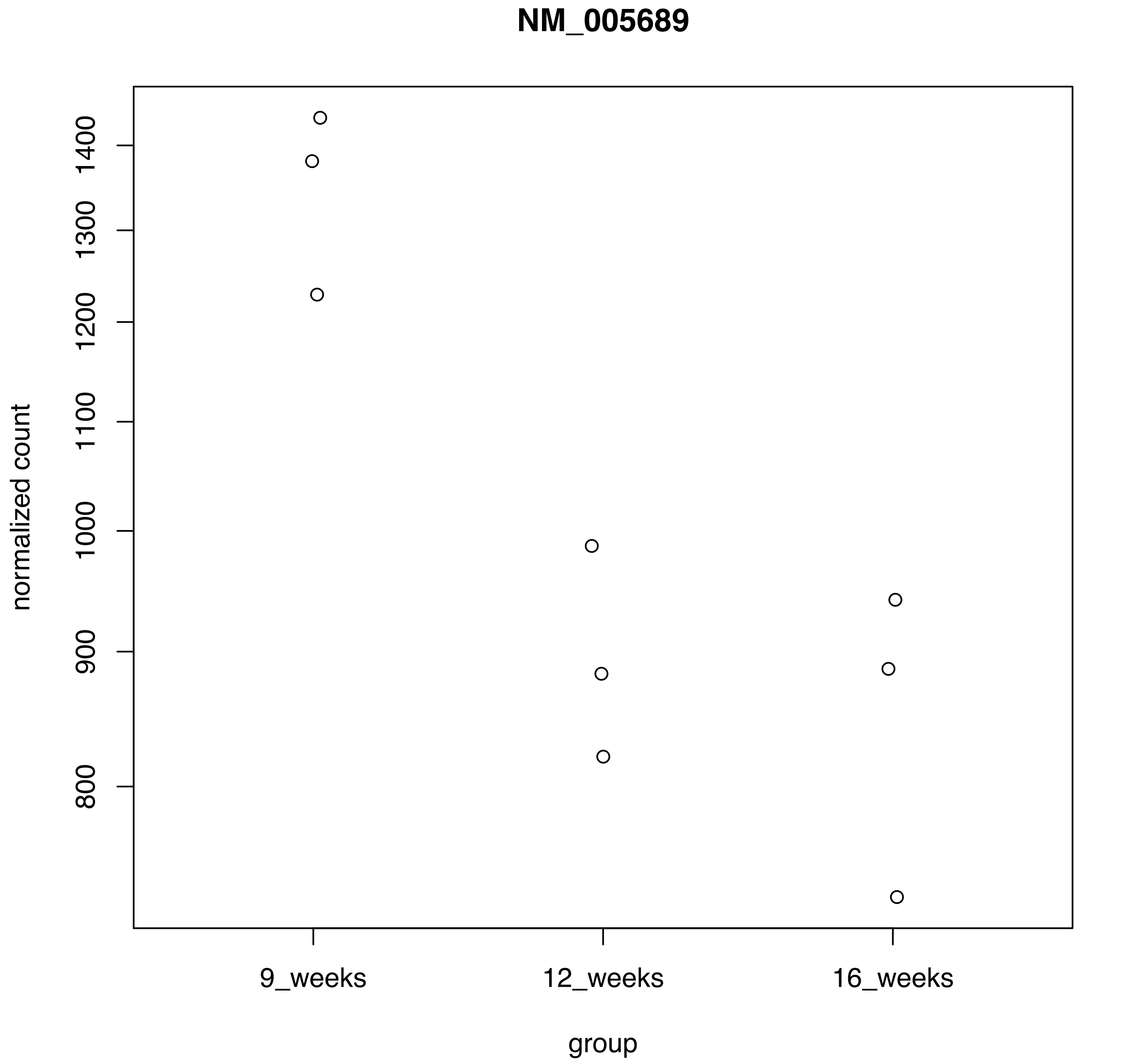
NM_005689
normalized count
800
900
1000
1100
1200
1300
1400
9_weeks
12_weeks
16_weeks
group

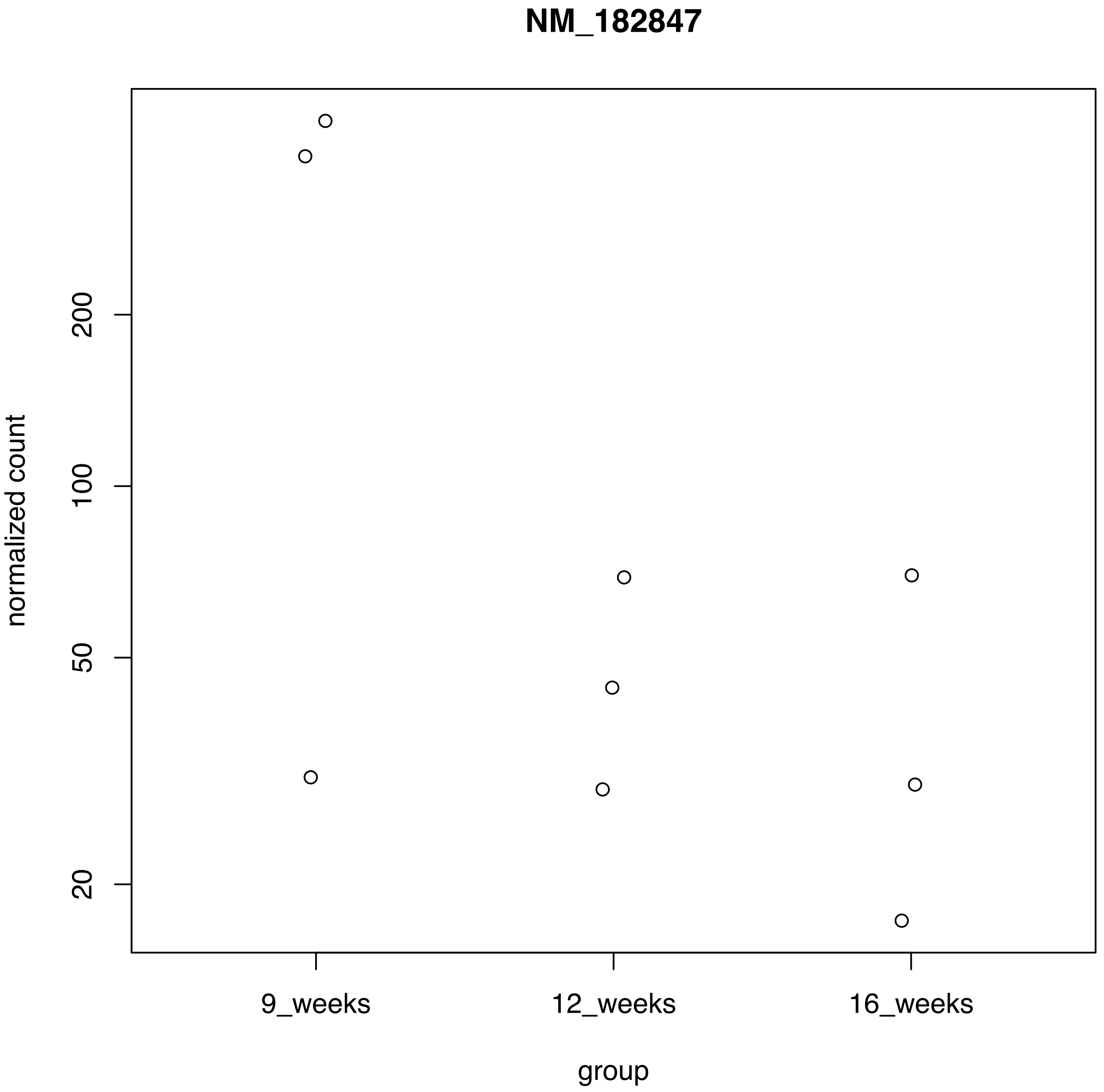
NM_182847
normalized count
200
100
50
20
9_weeks
12_weeks
16_weeks
group

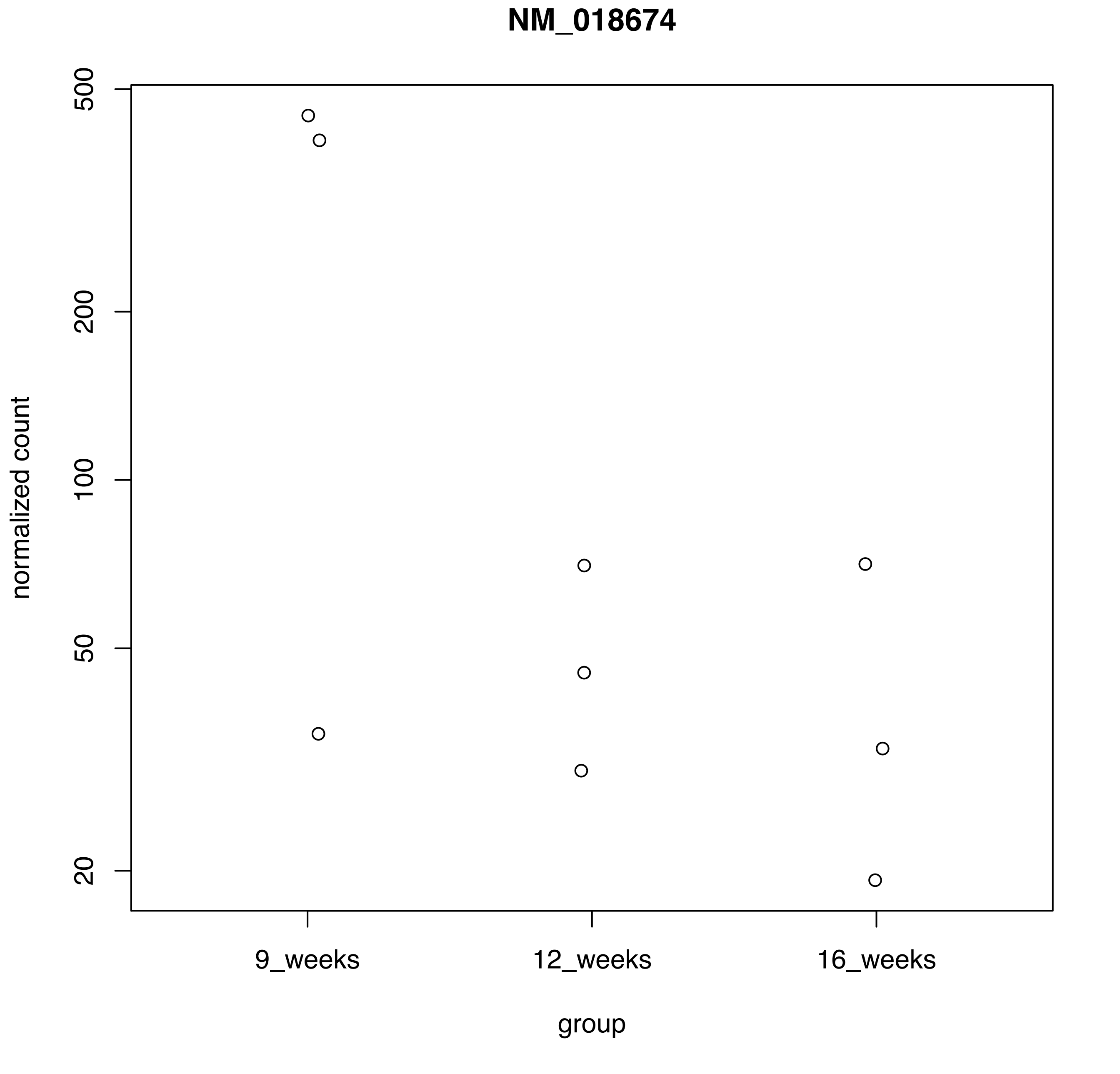
NM_018674
normalized count
500
200
100
50
20
9_weeks
12_weeks
16_weeks
group

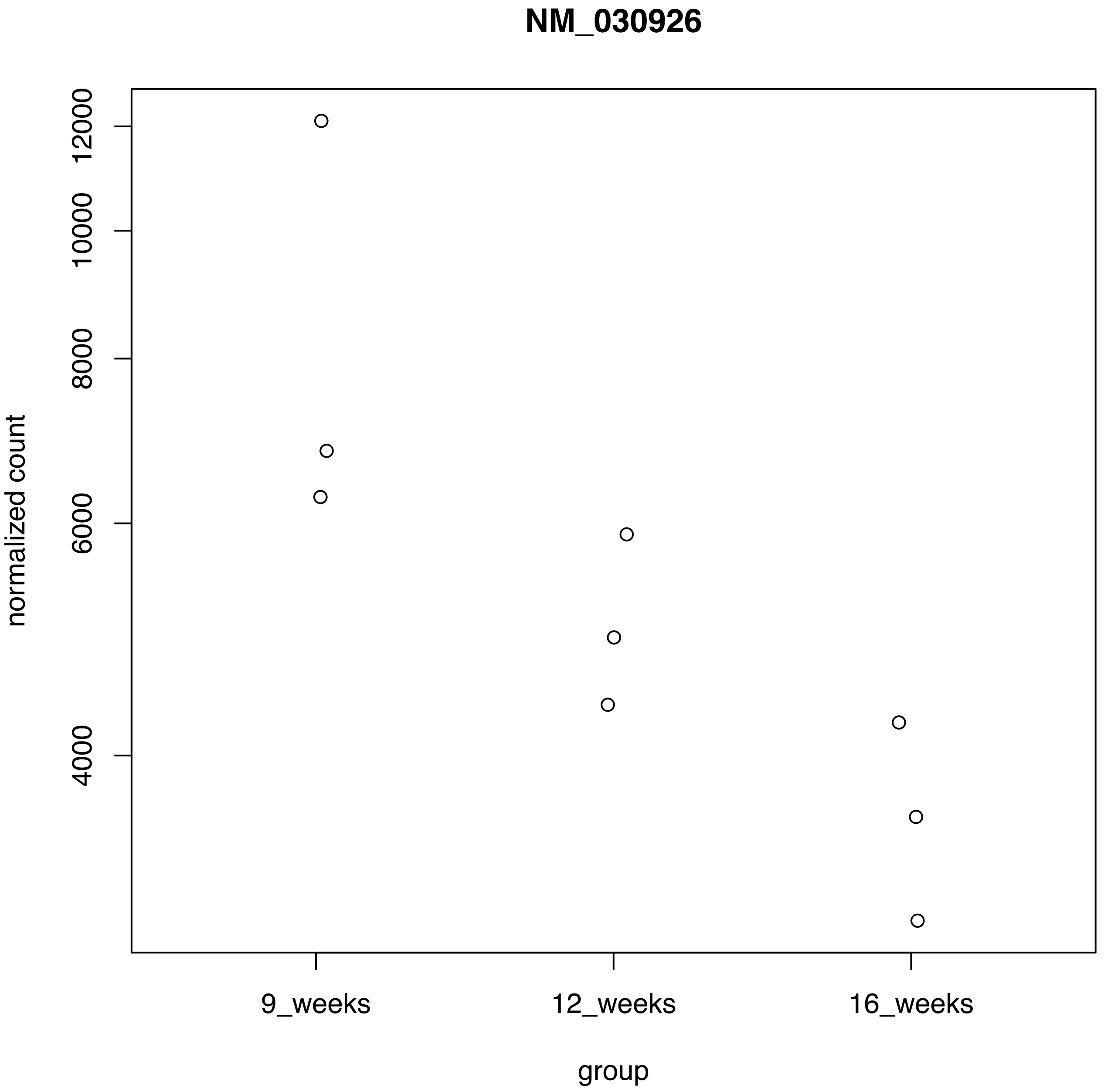

NM_030926
normalized count
12000
10000
8000
6000
4000
9_weeks
12_weeks
16_weeks
group

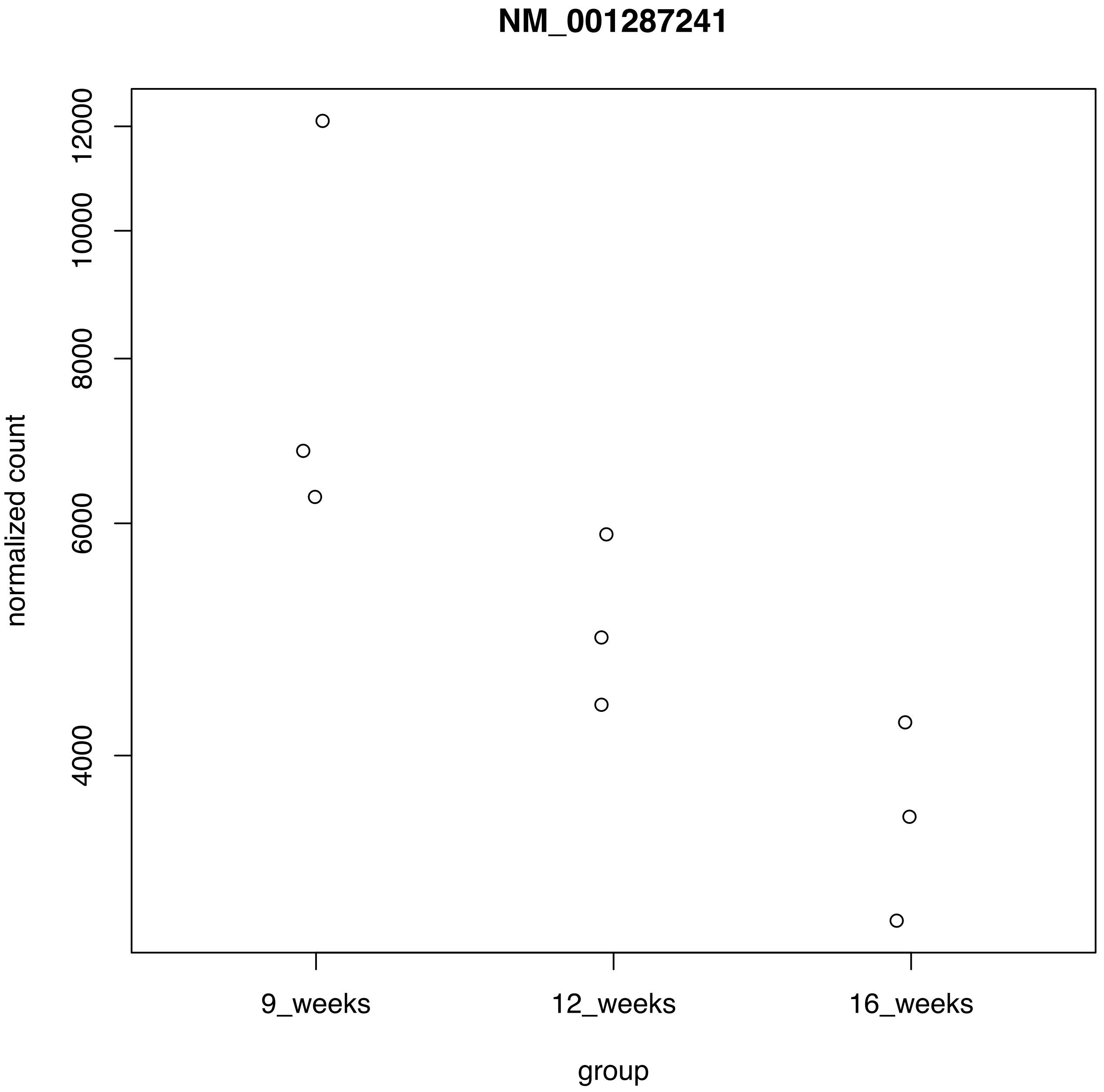

NM_001287241
normalized count
12000
10000
8000
6000
4000
9_weeks
12_weeks
16_weeks
group

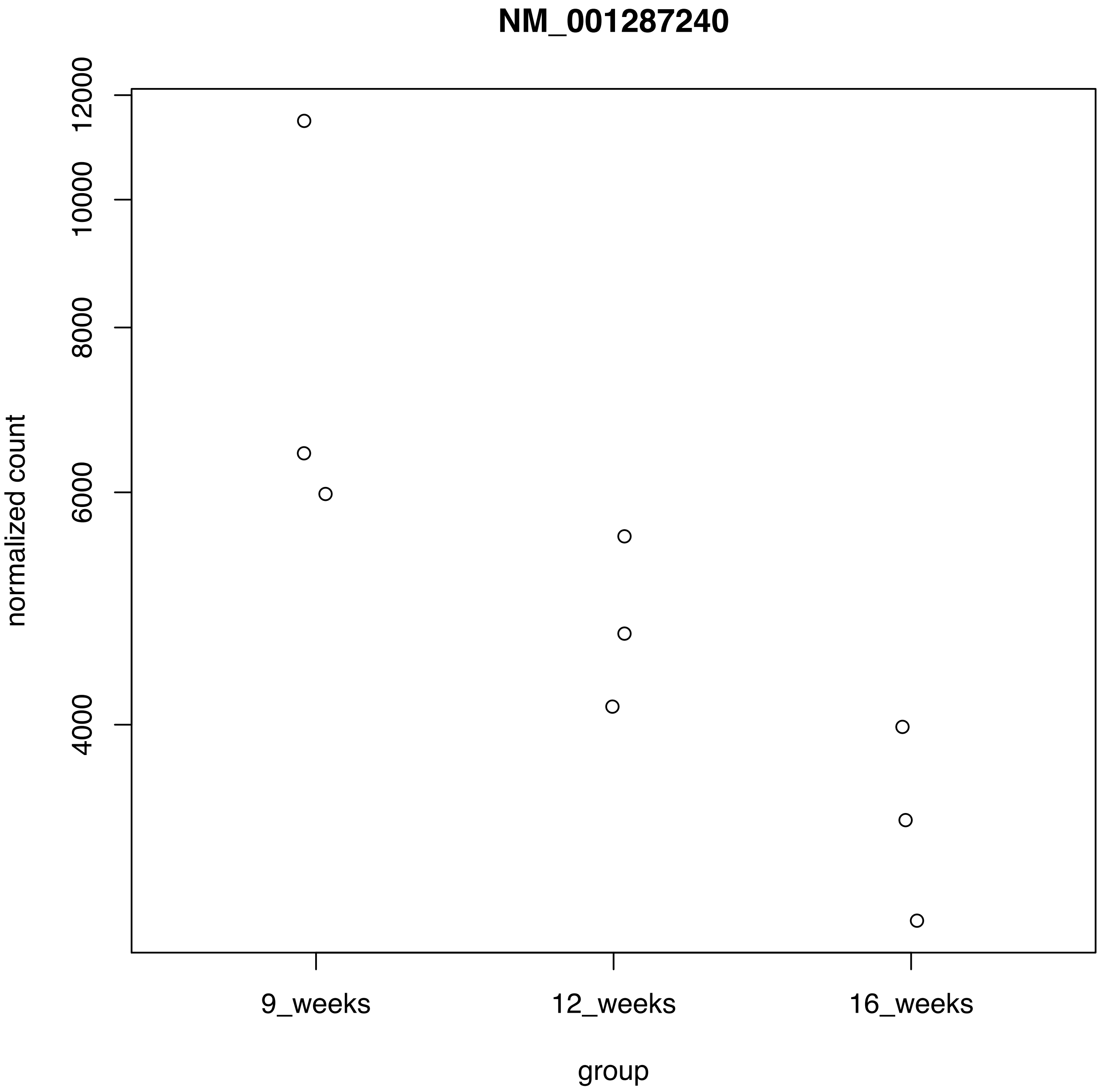
NM_001287240
normalized count
4000
6000
8000
10000
12000
9_weeks
12_weeks
16_weeks
group

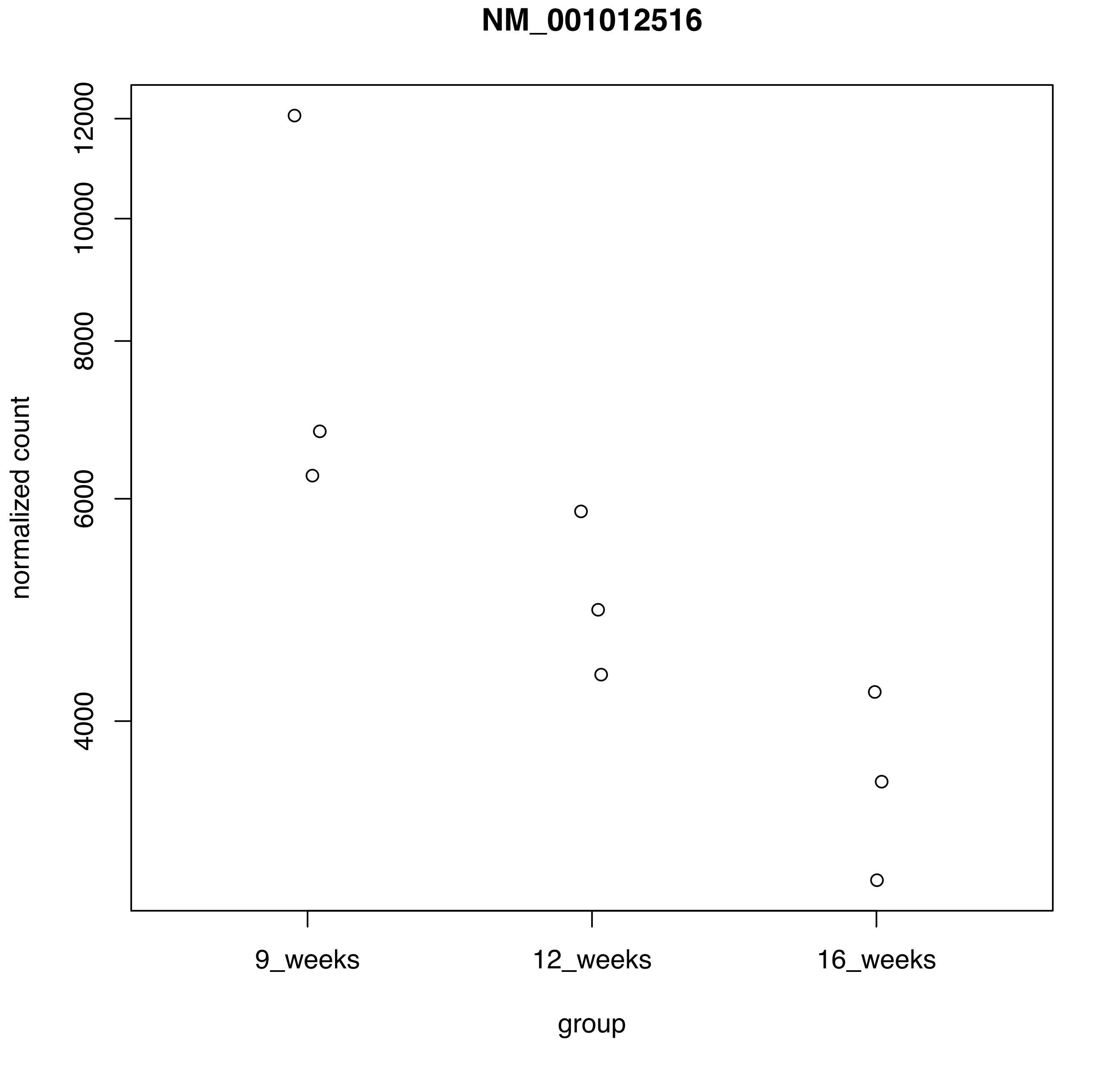
NM_001012516
normalized count
12000
10000
8000
6000
4000
9_weeks
12_weeks
16_weeks
group

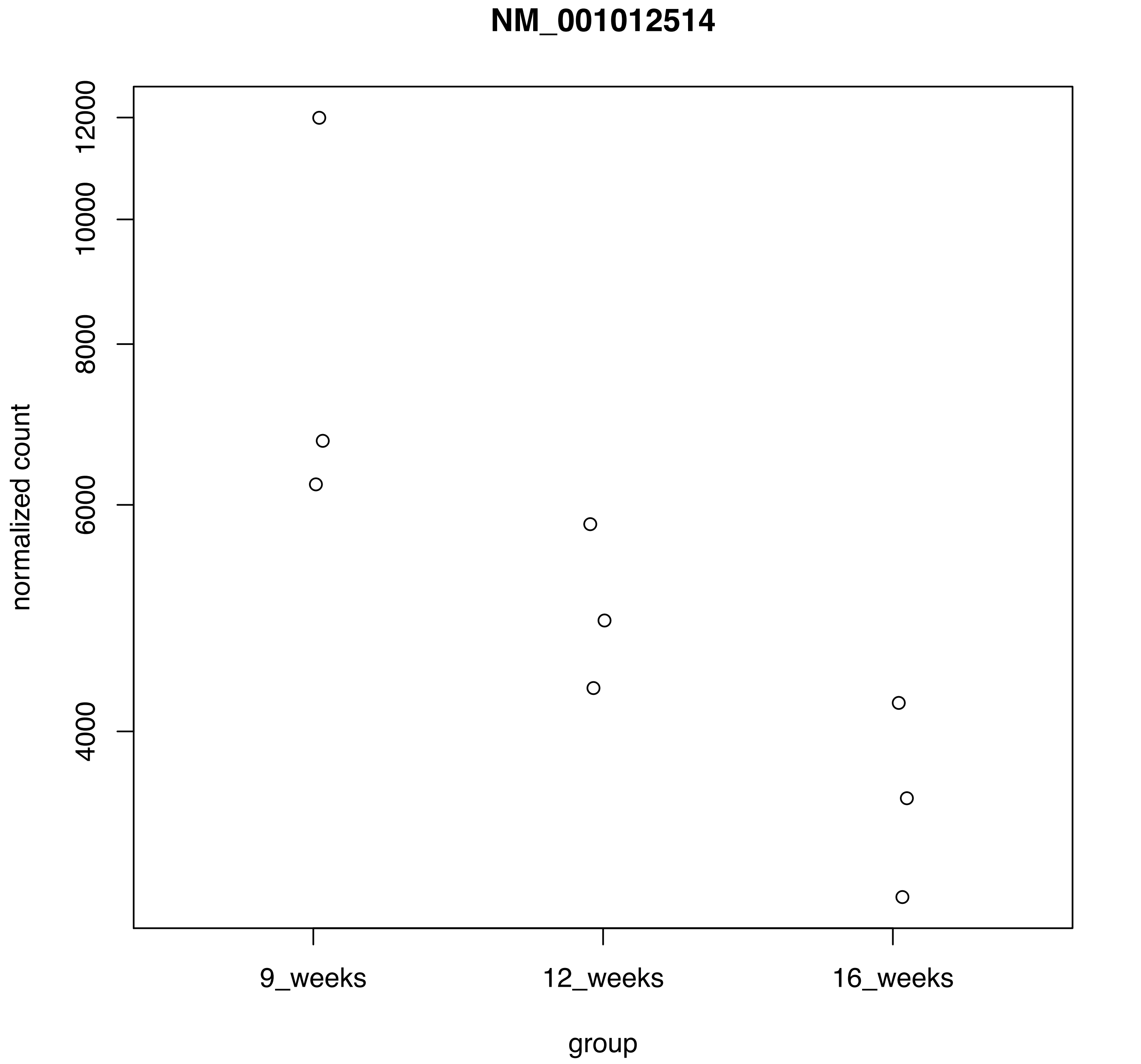

NM_001012514
normalized count
12000
10000
8000
6000
4000
9_weeks
12_weeks
16_weeks
group

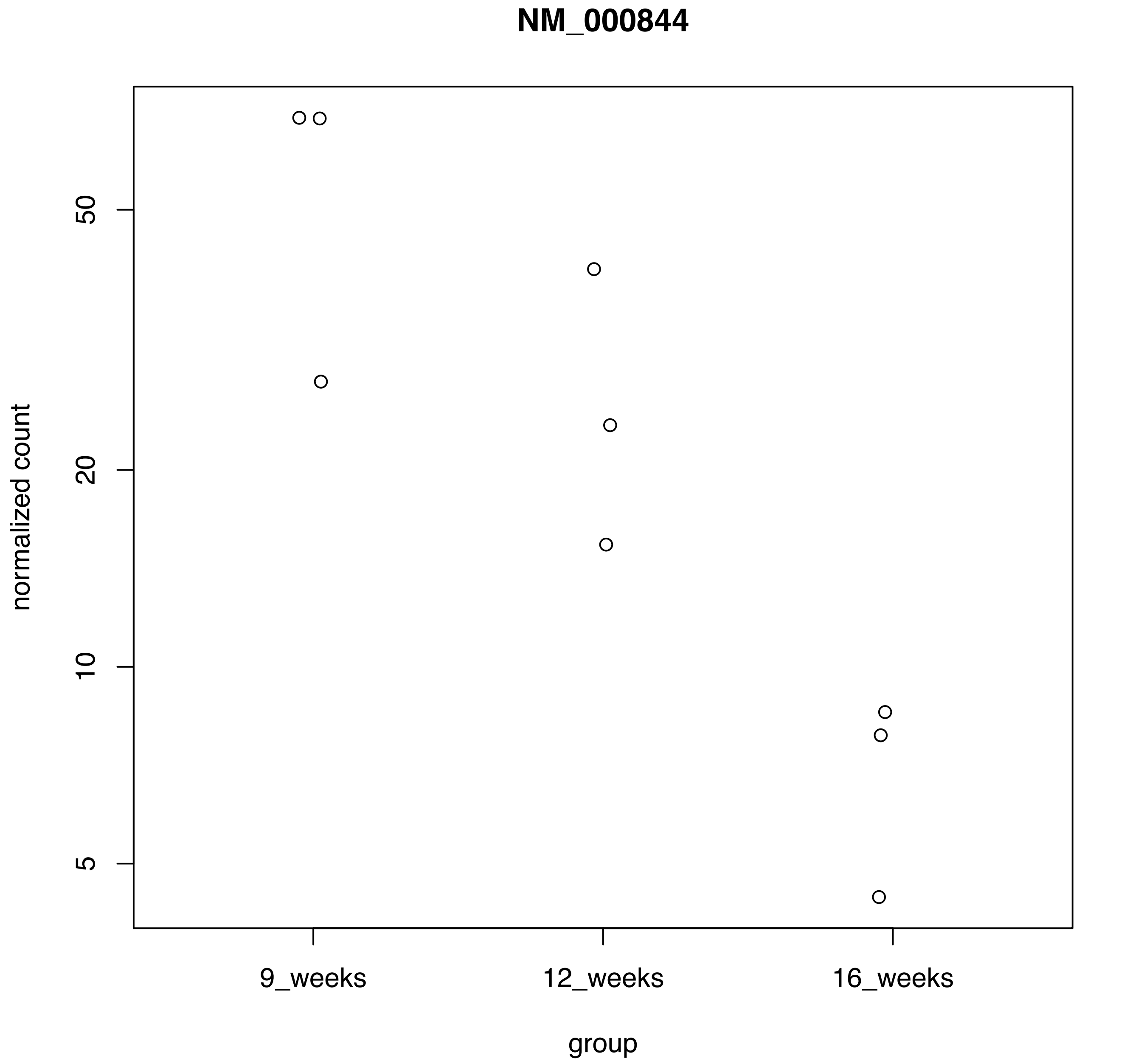
NM_000844
normalized count
50
20
10
5
9_weeks
12_weeks
16_weeks
group

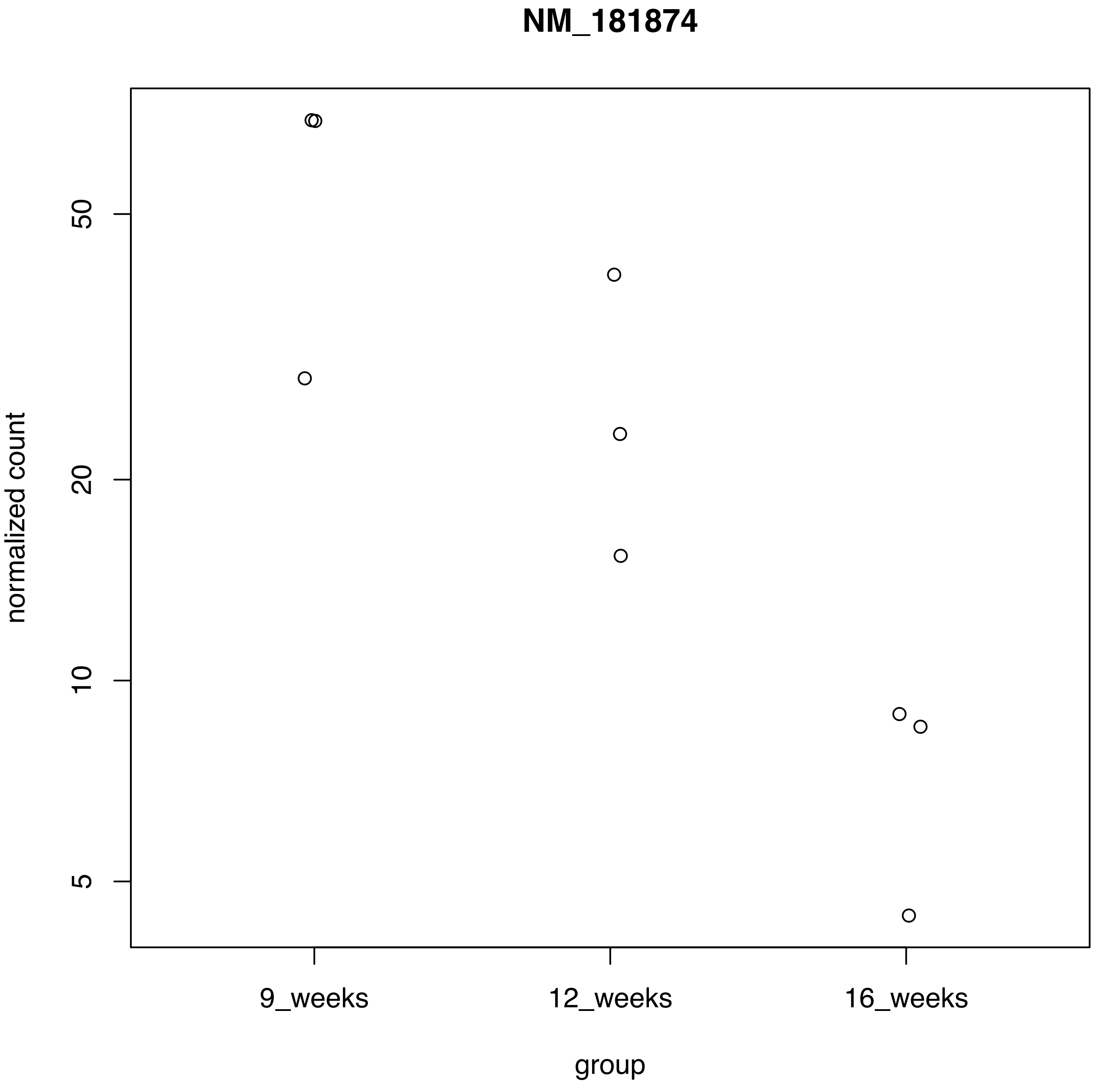

NM_181874
normalized count
50
20
10
5
9_weeks
12_weeks
16_weeks
group

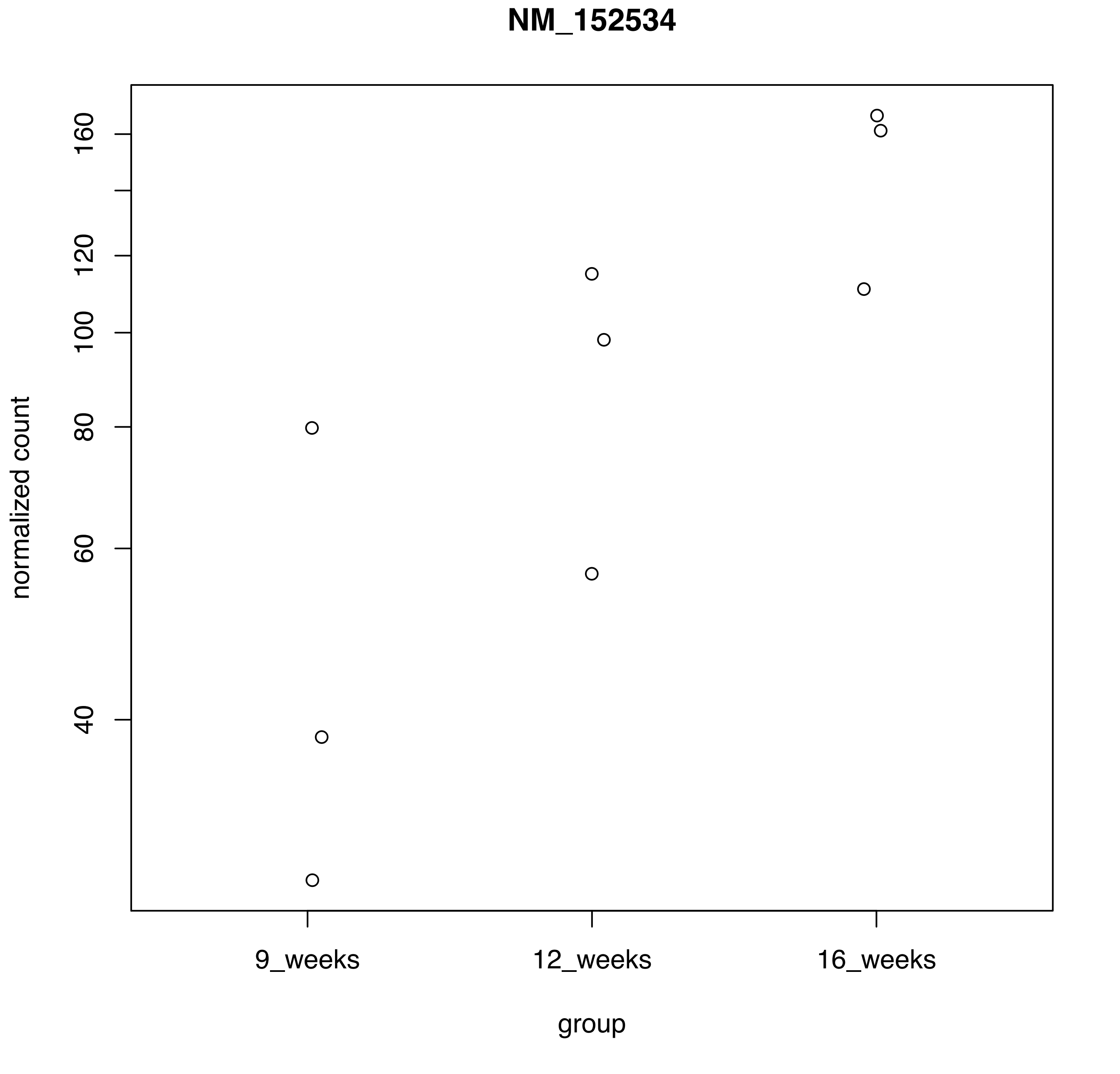

NM_152534
normalized count
40
60
80
100
120
160
9_weeks
12_weeks
16_weeks
group

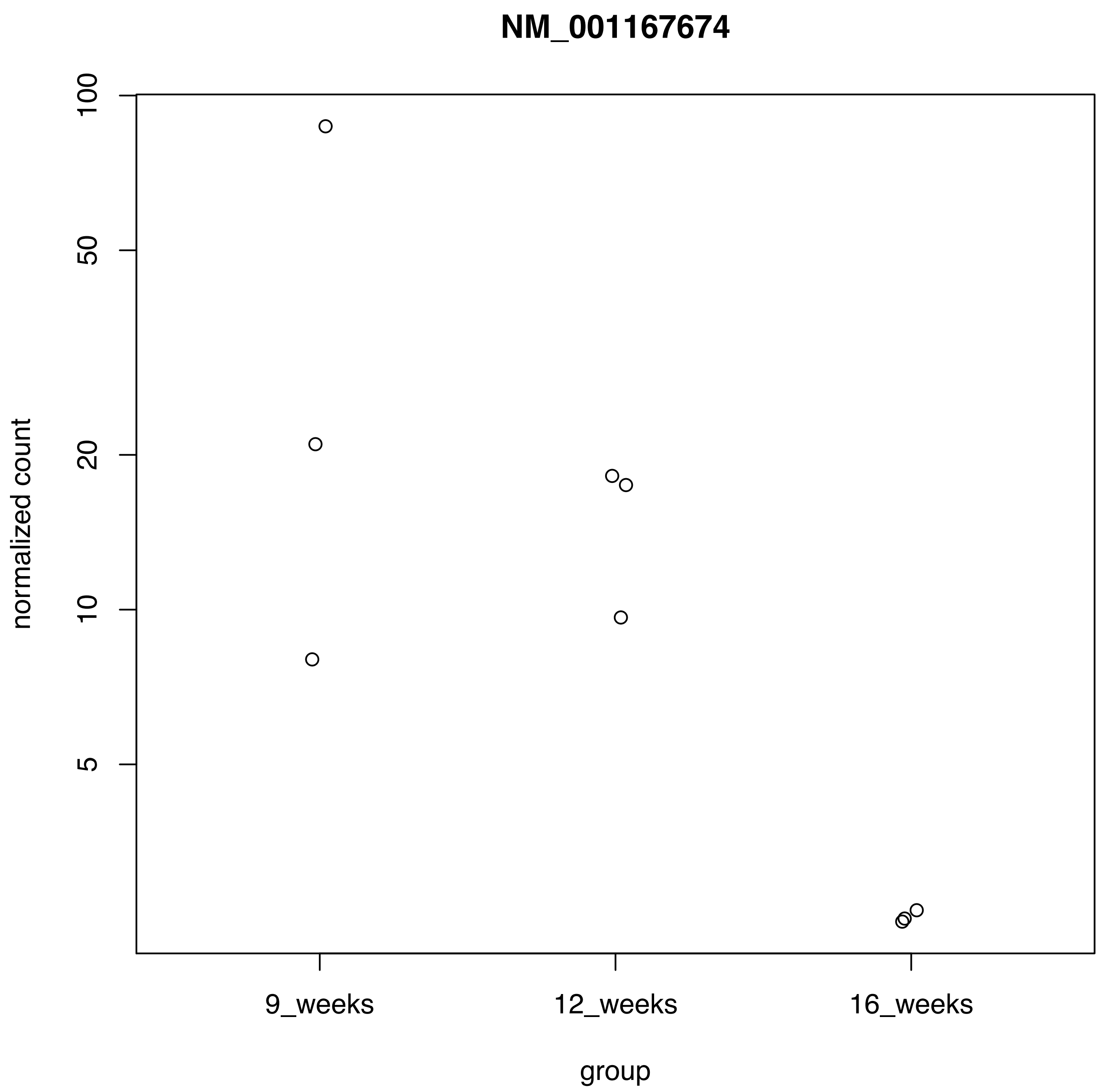

NM_001167674
normalized count
100
50
20
10
5
9_weeks
12_weeks
16_weeks
group

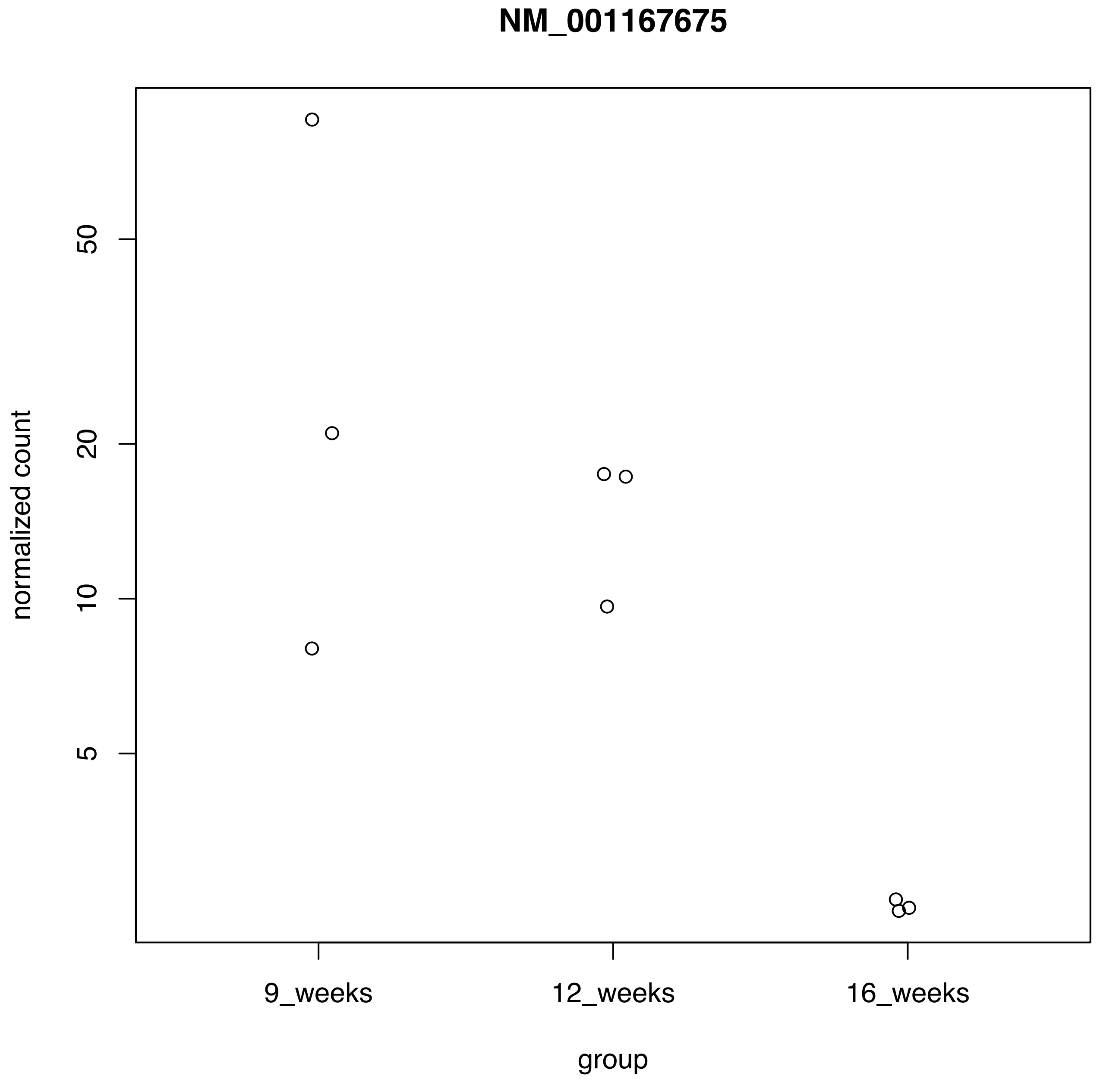

NM_001167675
normalized count
50
20
10
5
9_weeks
12_weeks
16_weeks
group

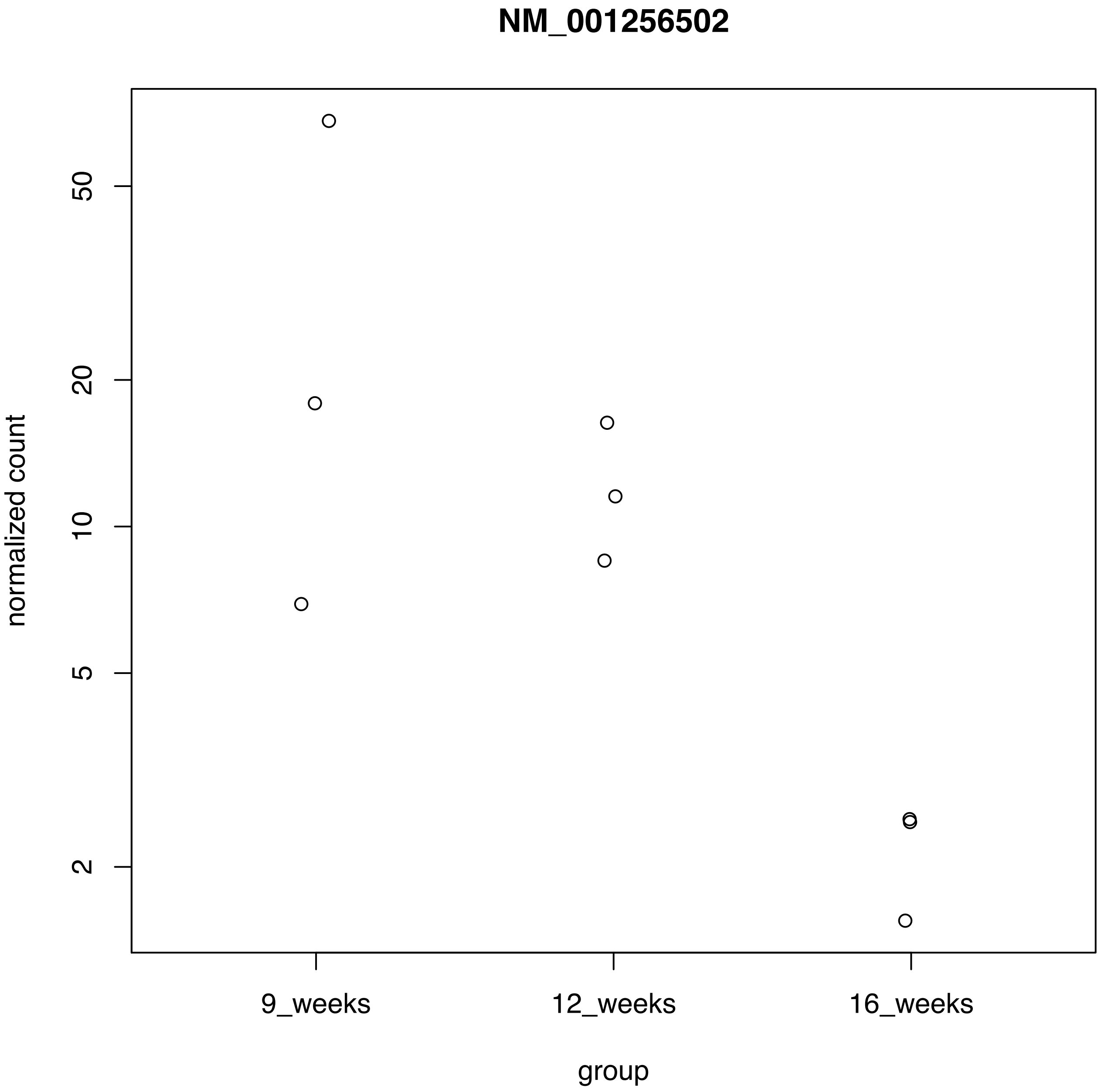
NM_001256502
normalized count
50
20
10
5
2
9_weeks
12_weeks
16_weeks
group

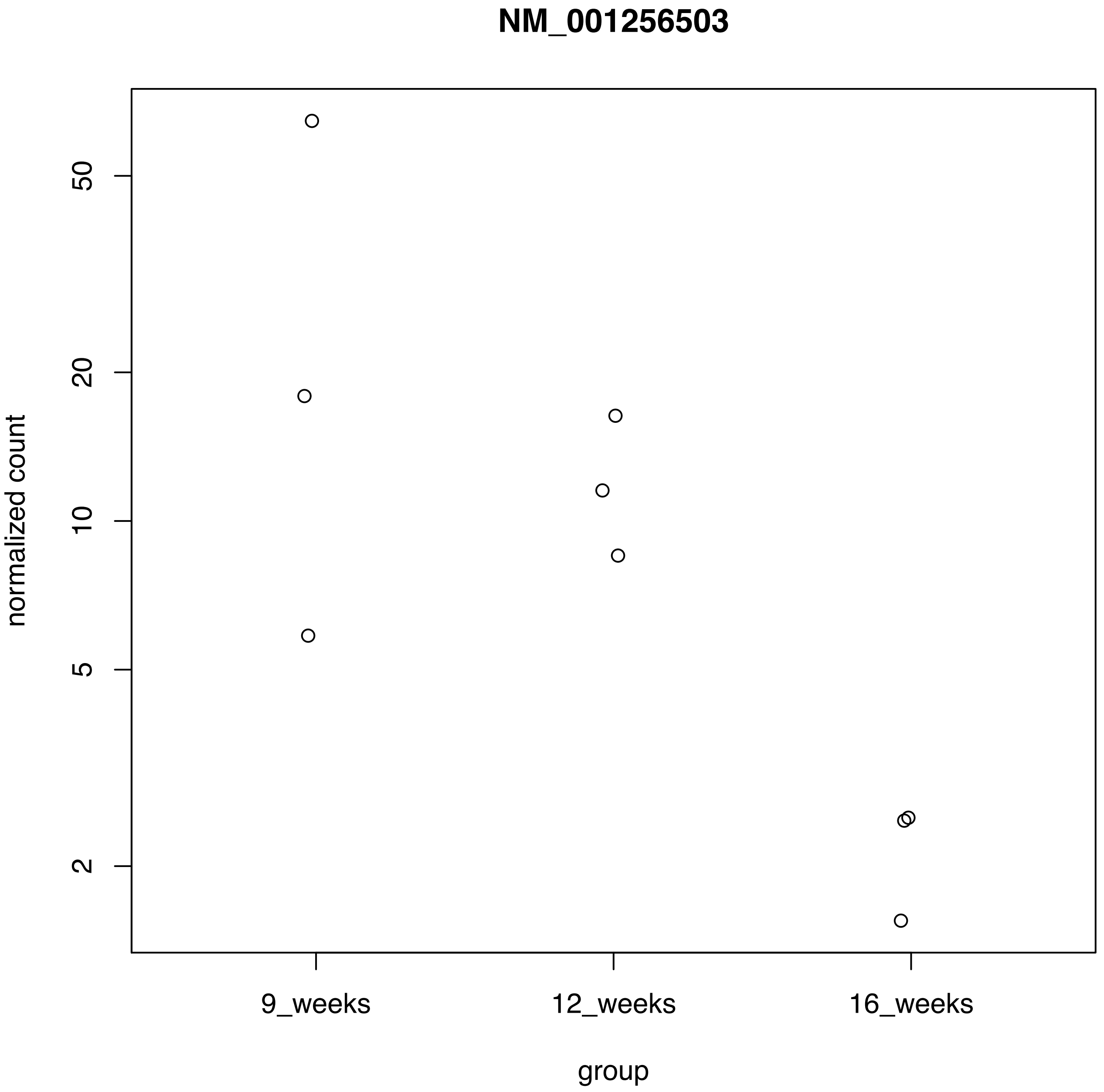
NM_001256503
normalized count
2
5
10
20
50
group
9_weeks
12_weeks
16_weeks

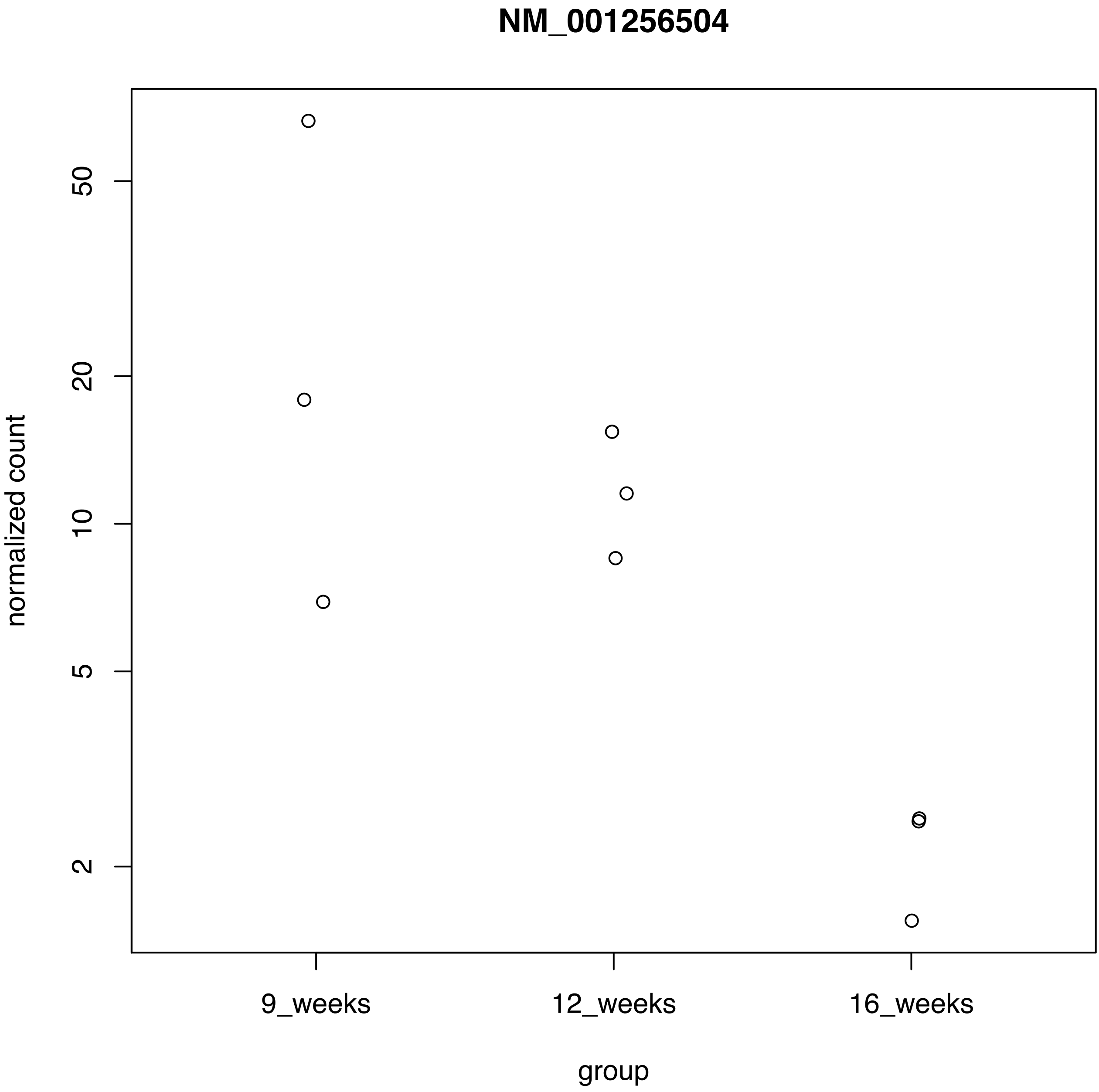
NM_001256504
50
20
10
5
2
normalized count
9_weeks
12_weeks
16_weeks
group

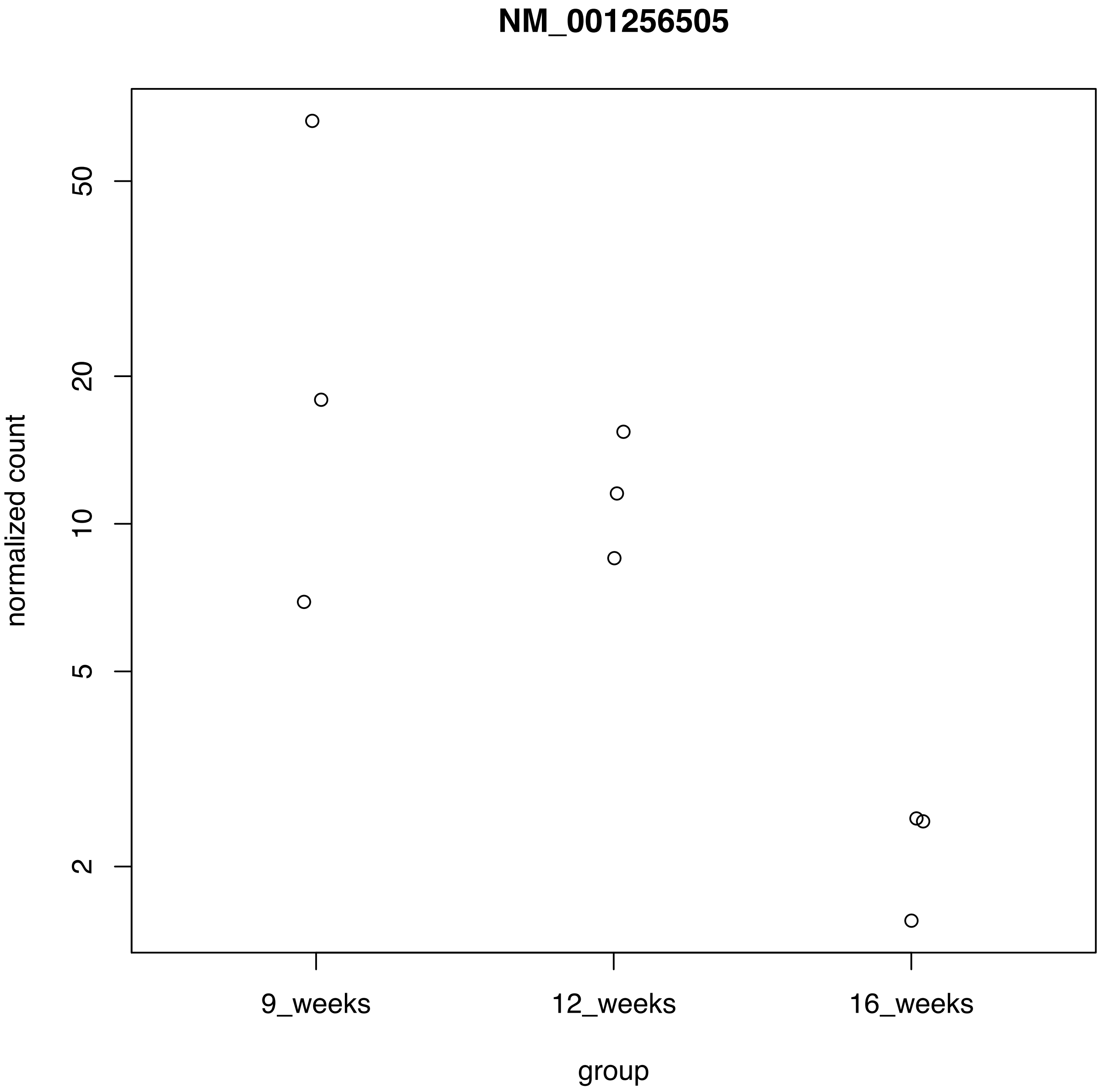
NM_001256505
normalized count
50
20
10
5
2
9_weeks
12_weeks
16_weeks
group

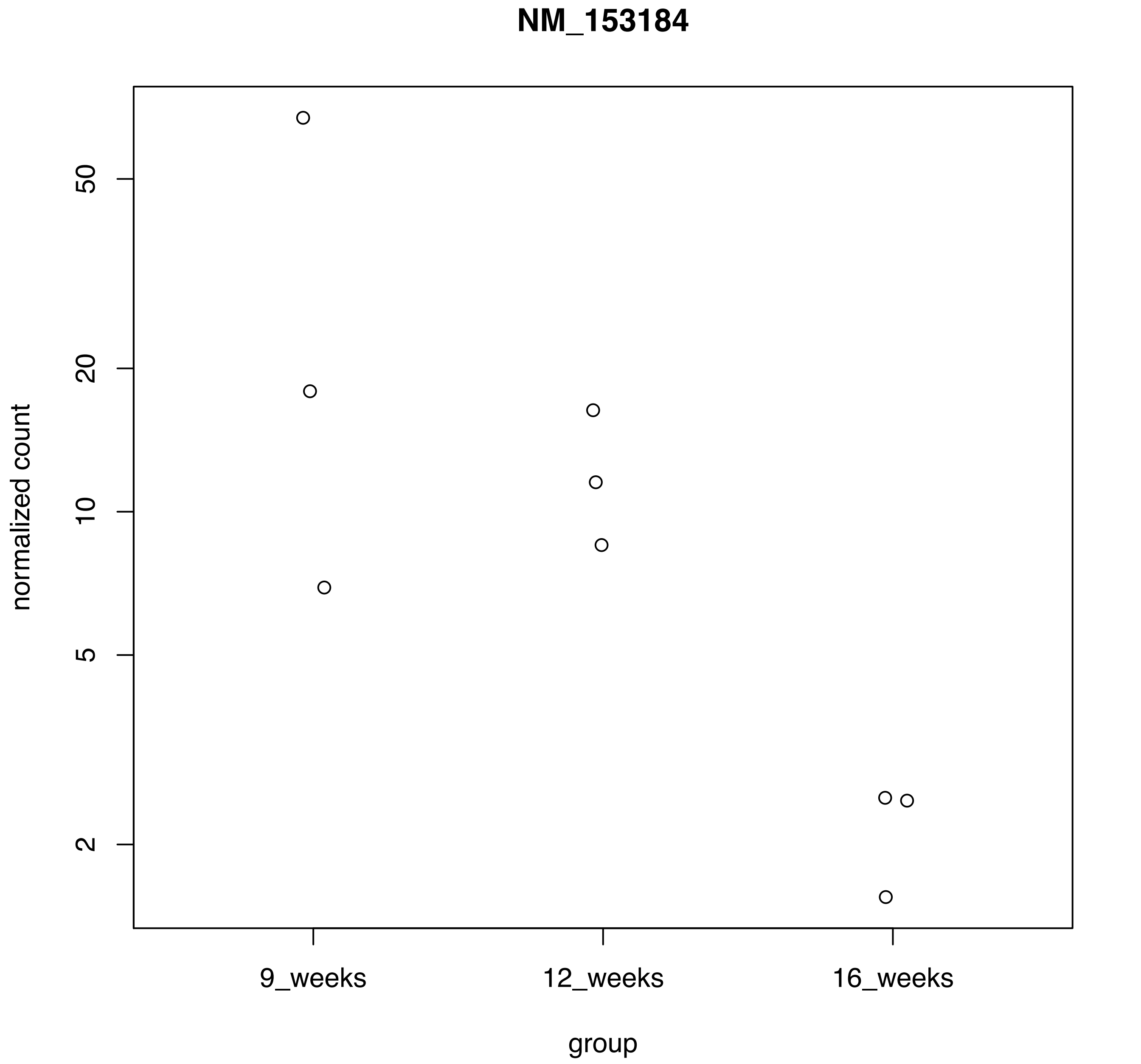

NM_153184
50
20
10
5
2
normalized count
9_weeks
12_weeks
16_weeks
group

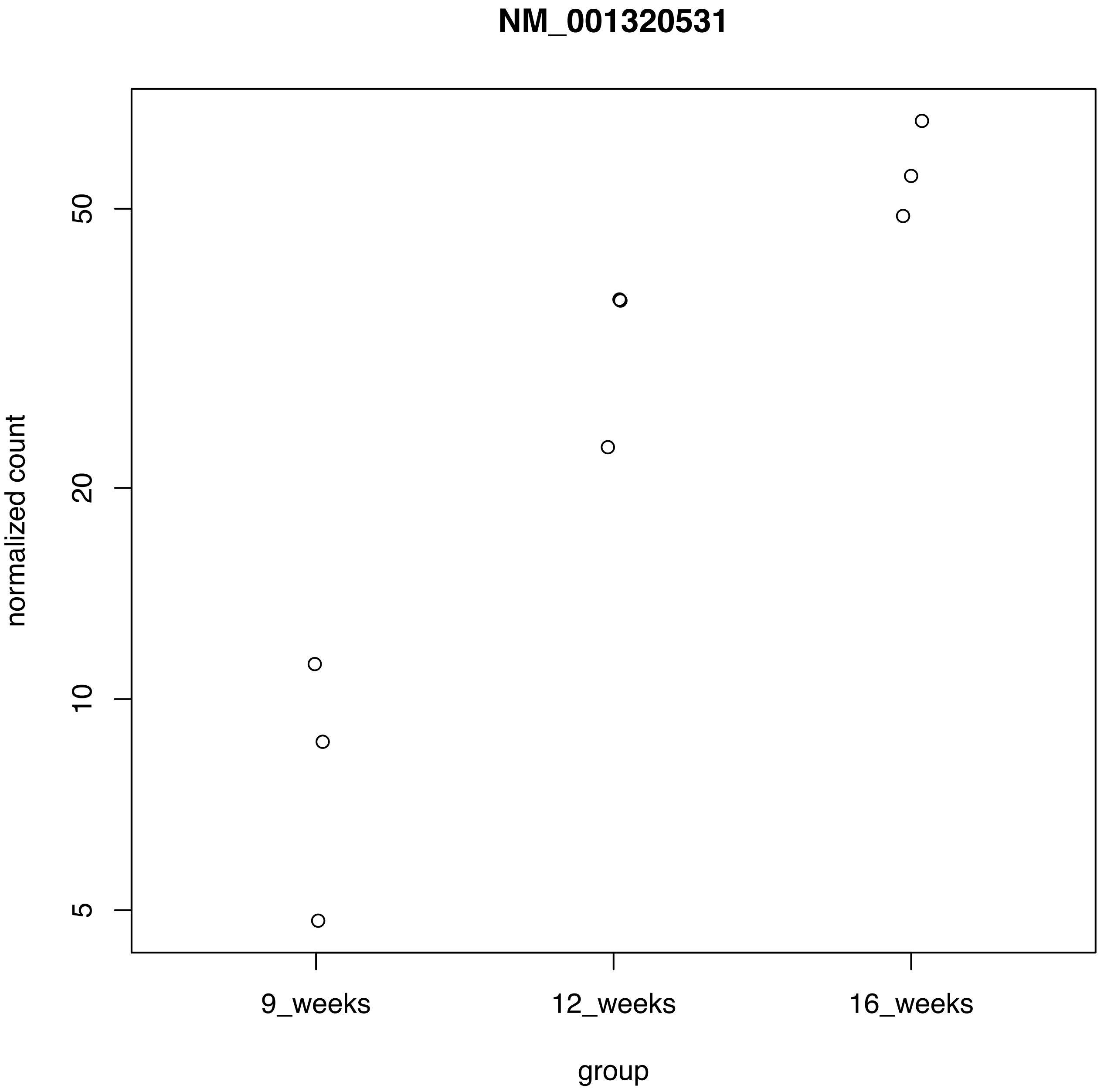
NM_001320531
normalized count
50
20
10
5
9_weeks
12_weeks
16_weeks
group

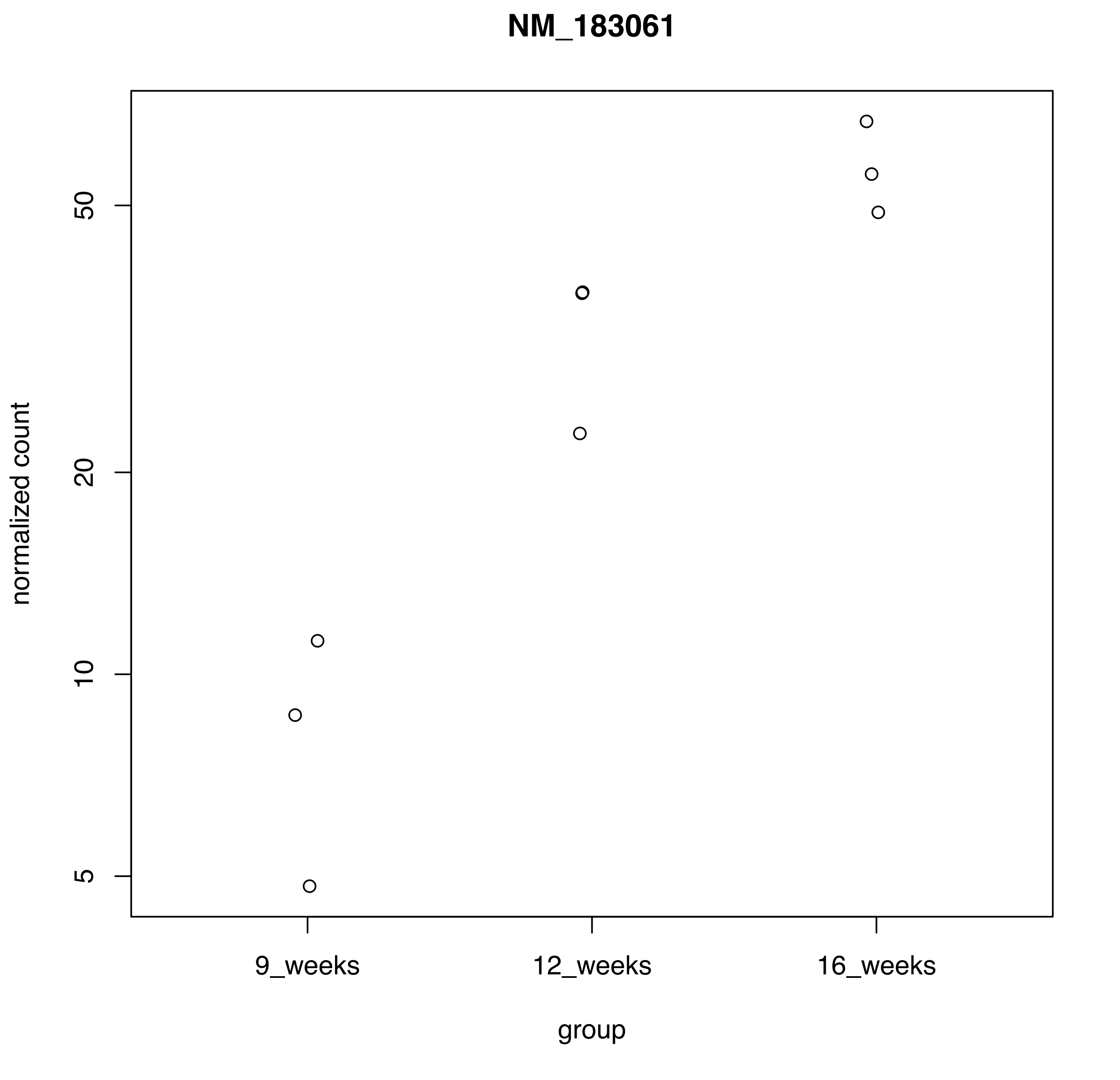
NM_183061
normalized count
50
20
10
5
9_weeks
12_weeks
16_weeks
group

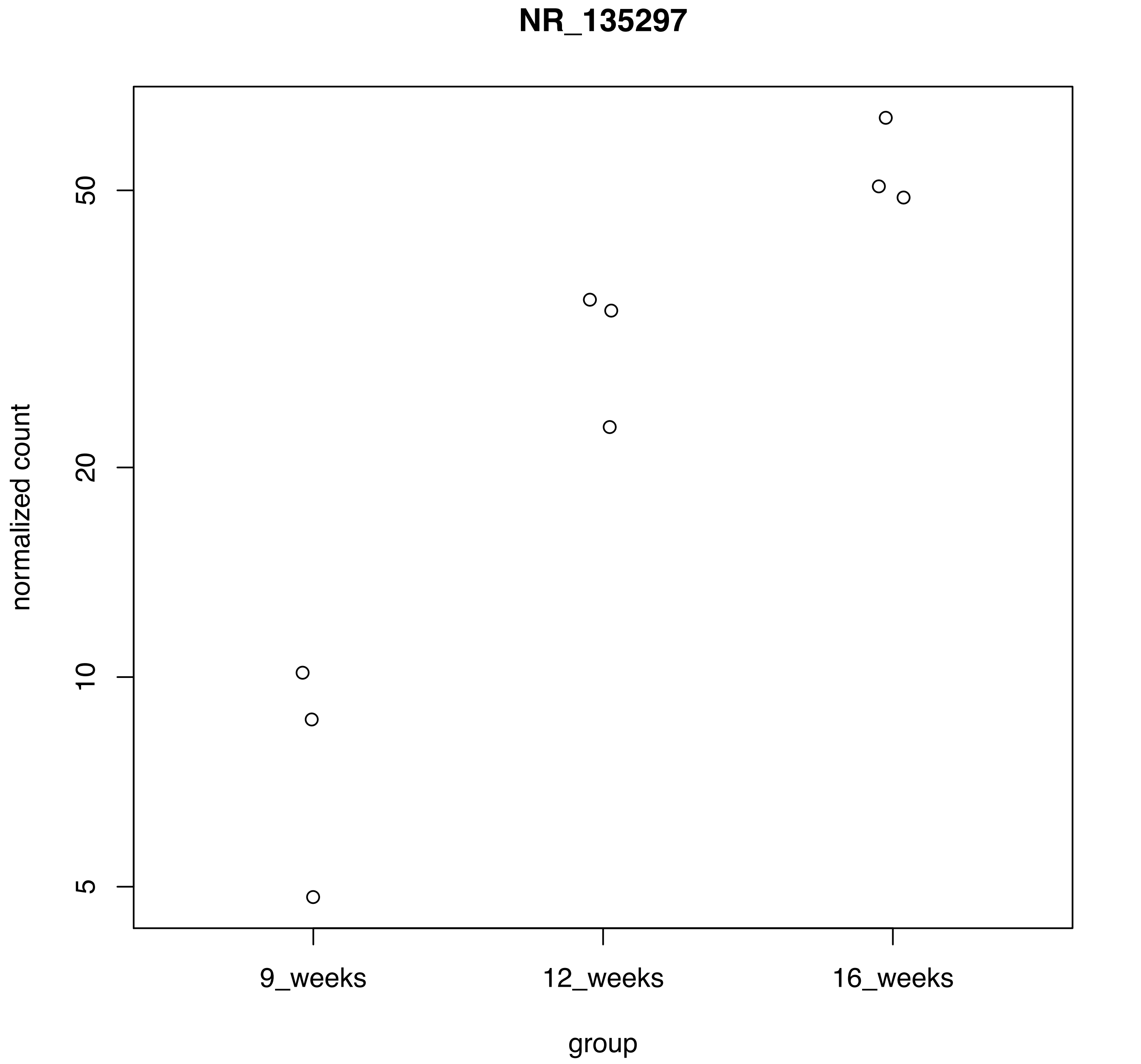

NR_135297
normalized count
50
20
10
5
9_weeks
12_weeks
16_weeks
group

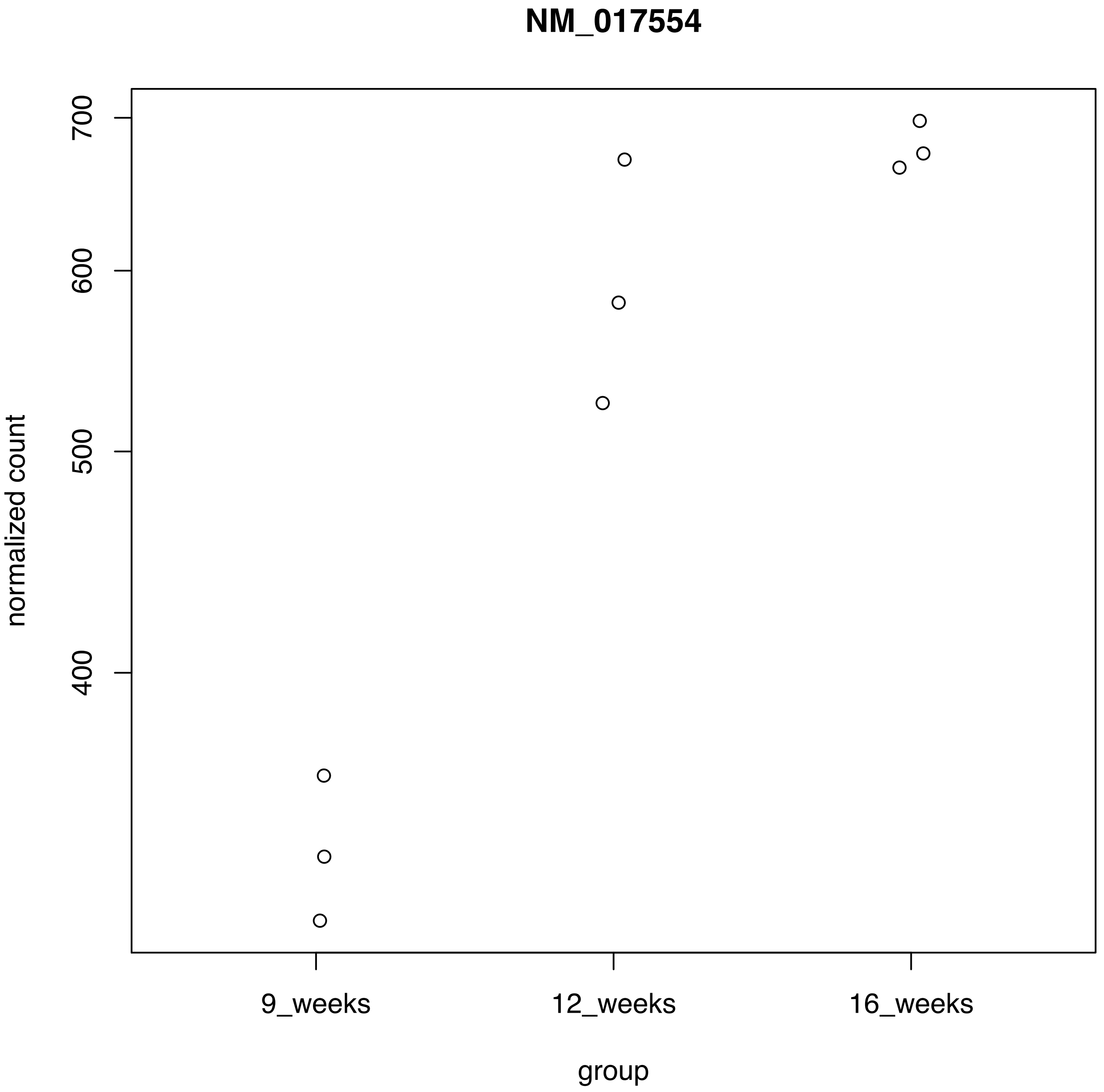
NM_017554
normalized count
700
600
500
400
9_weeks
12_weeks
16_weeks
group

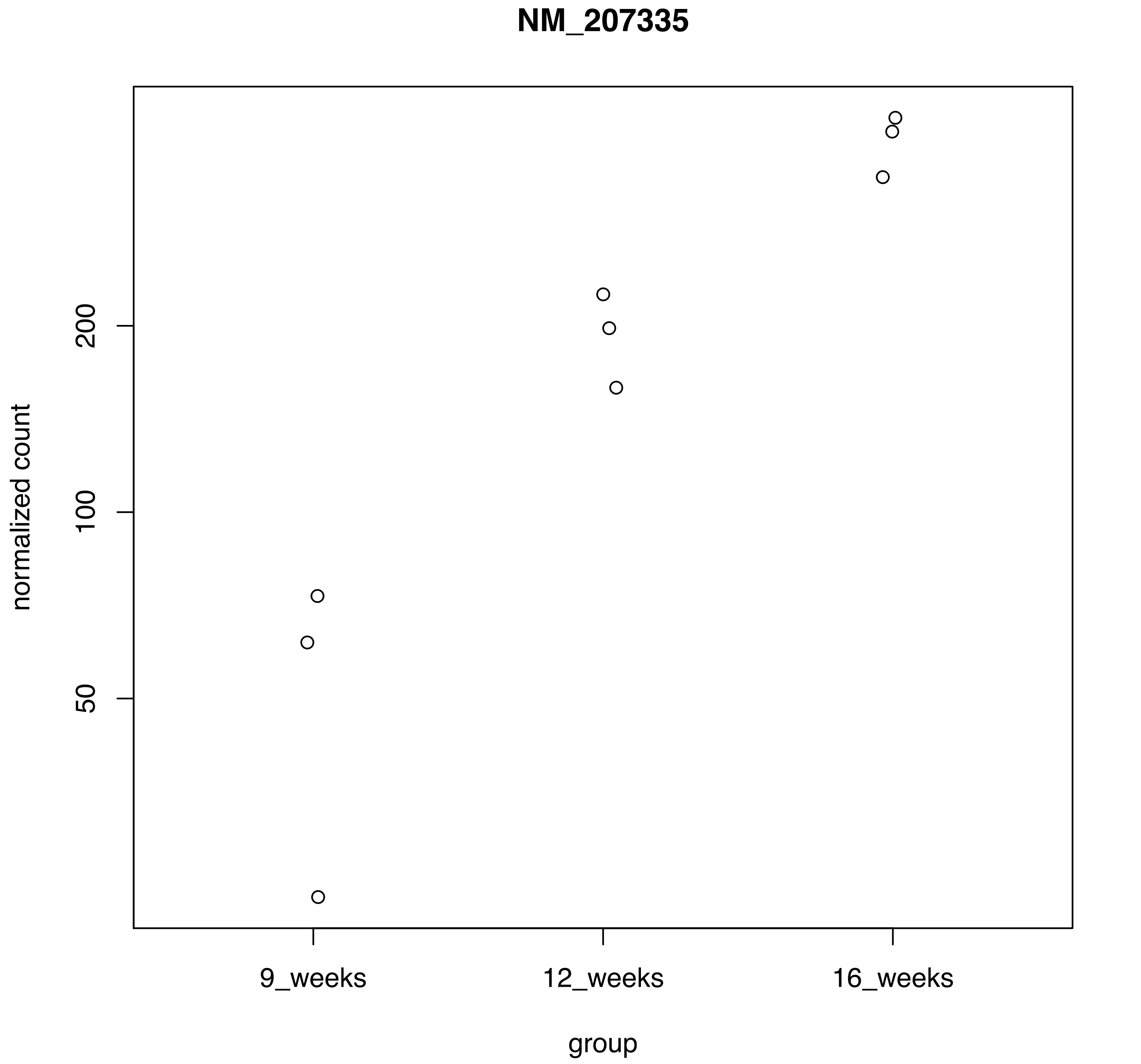
NM_207335
normalized count
200
100
50
9_weeks
12_weeks
16_weeks
group

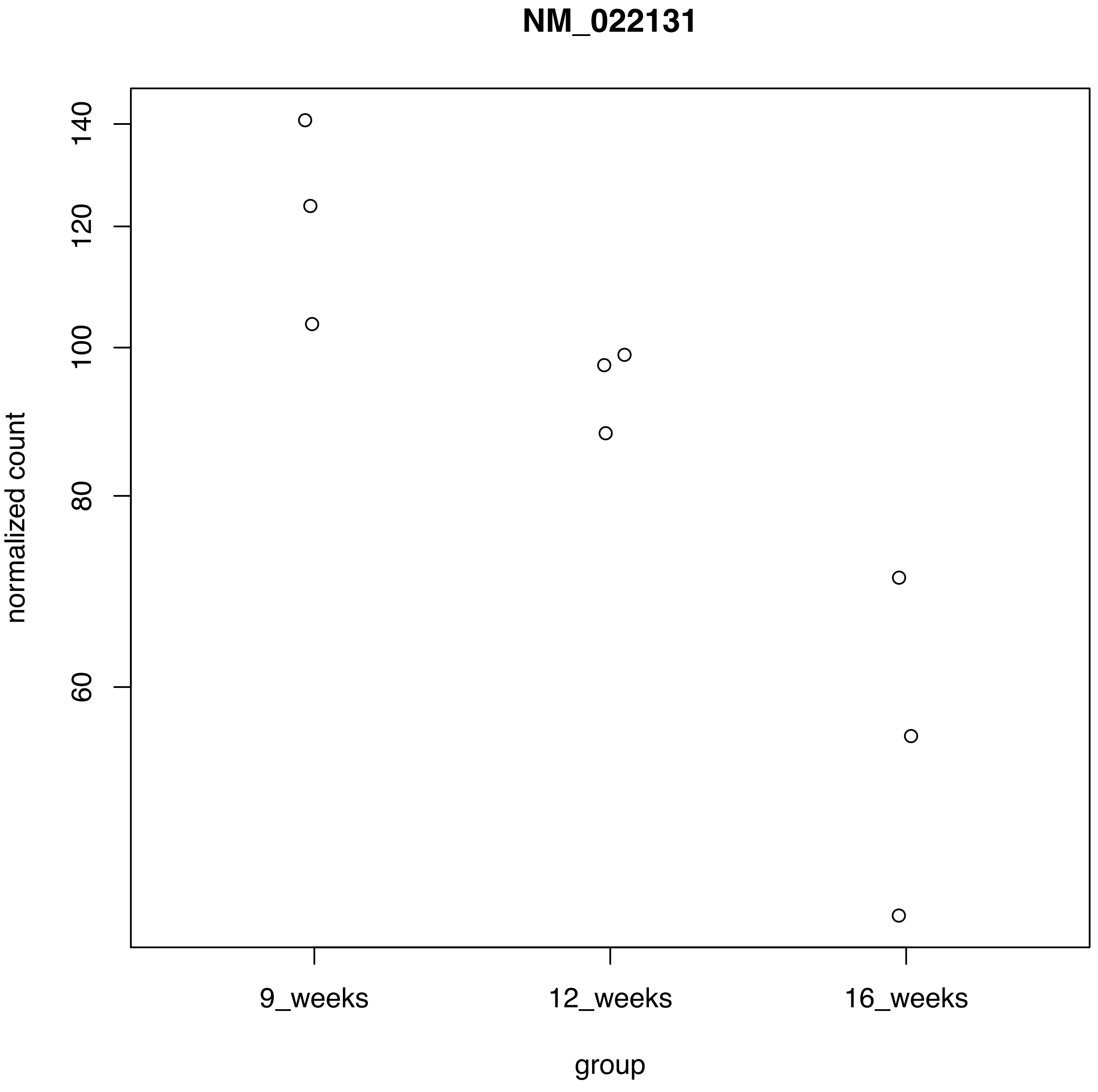
NM_022131
normalized count
140
120
100
80
60
9_weeks
12_weeks
16_weeks
group

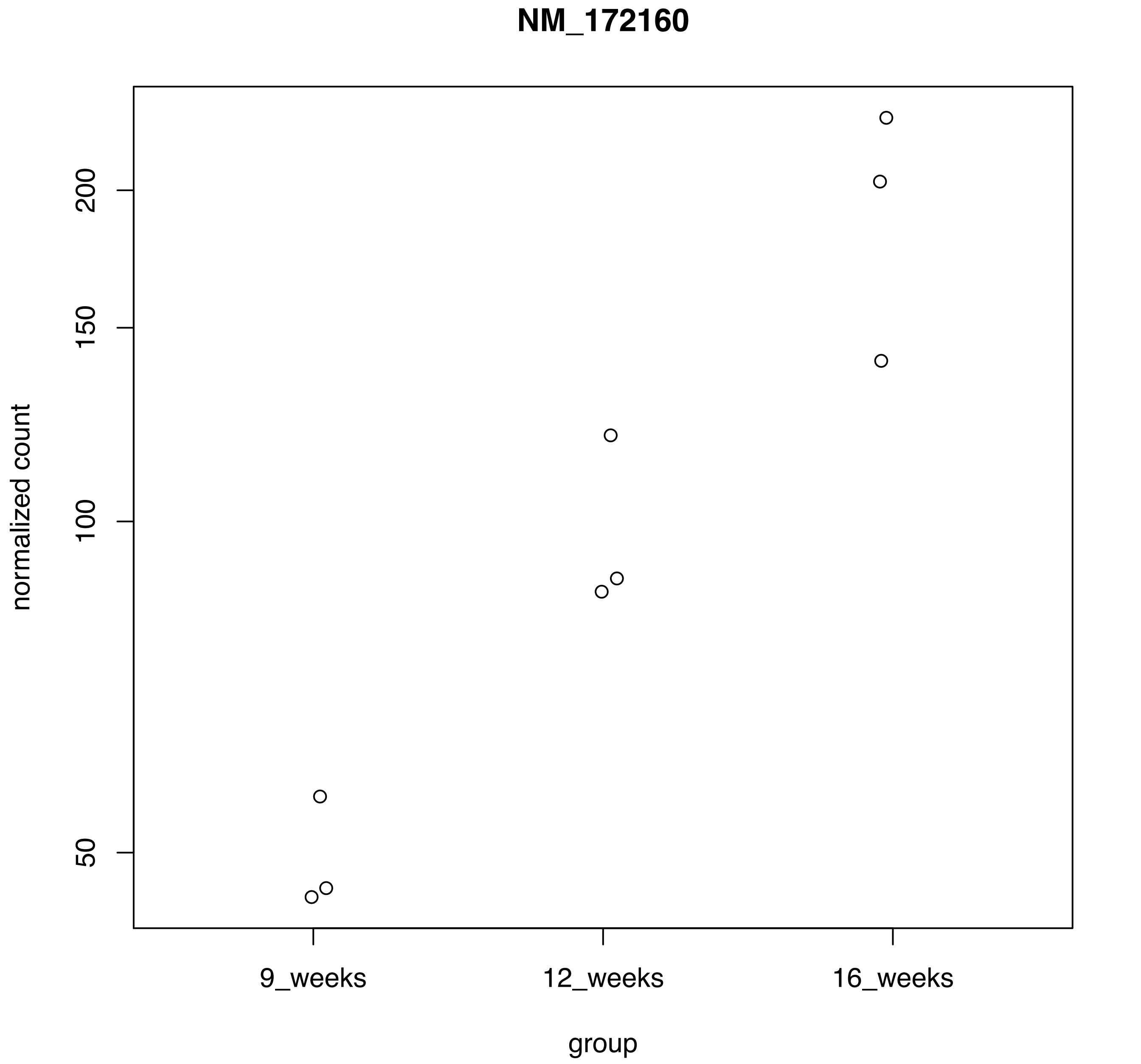
NM_172160
normalized count
200
150
100
50
9_weeks
12_weeks
16_weeks
group

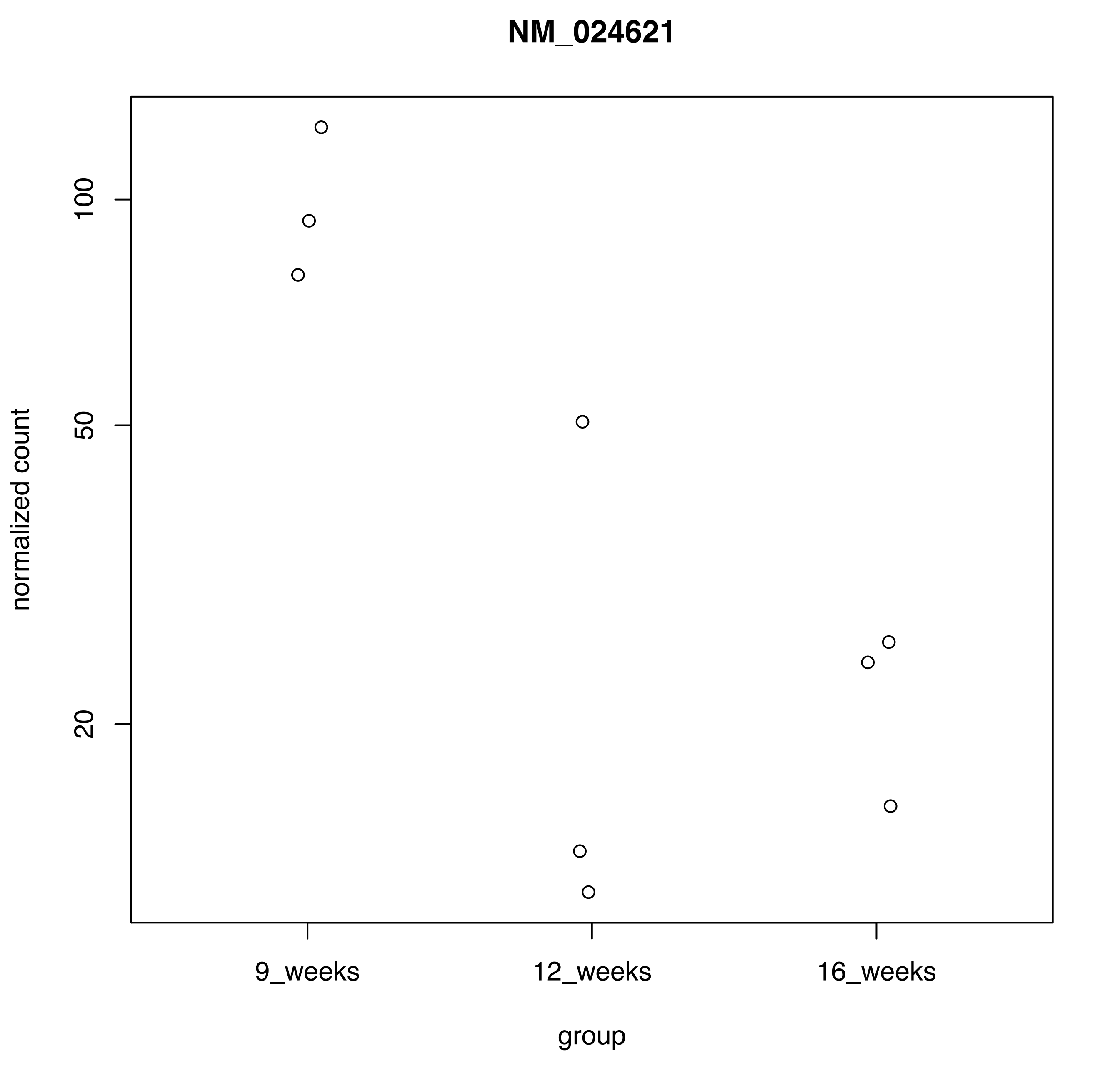

NM_024621
100
50
20
normalized count
9_weeks
12_weeks
16_weeks
group

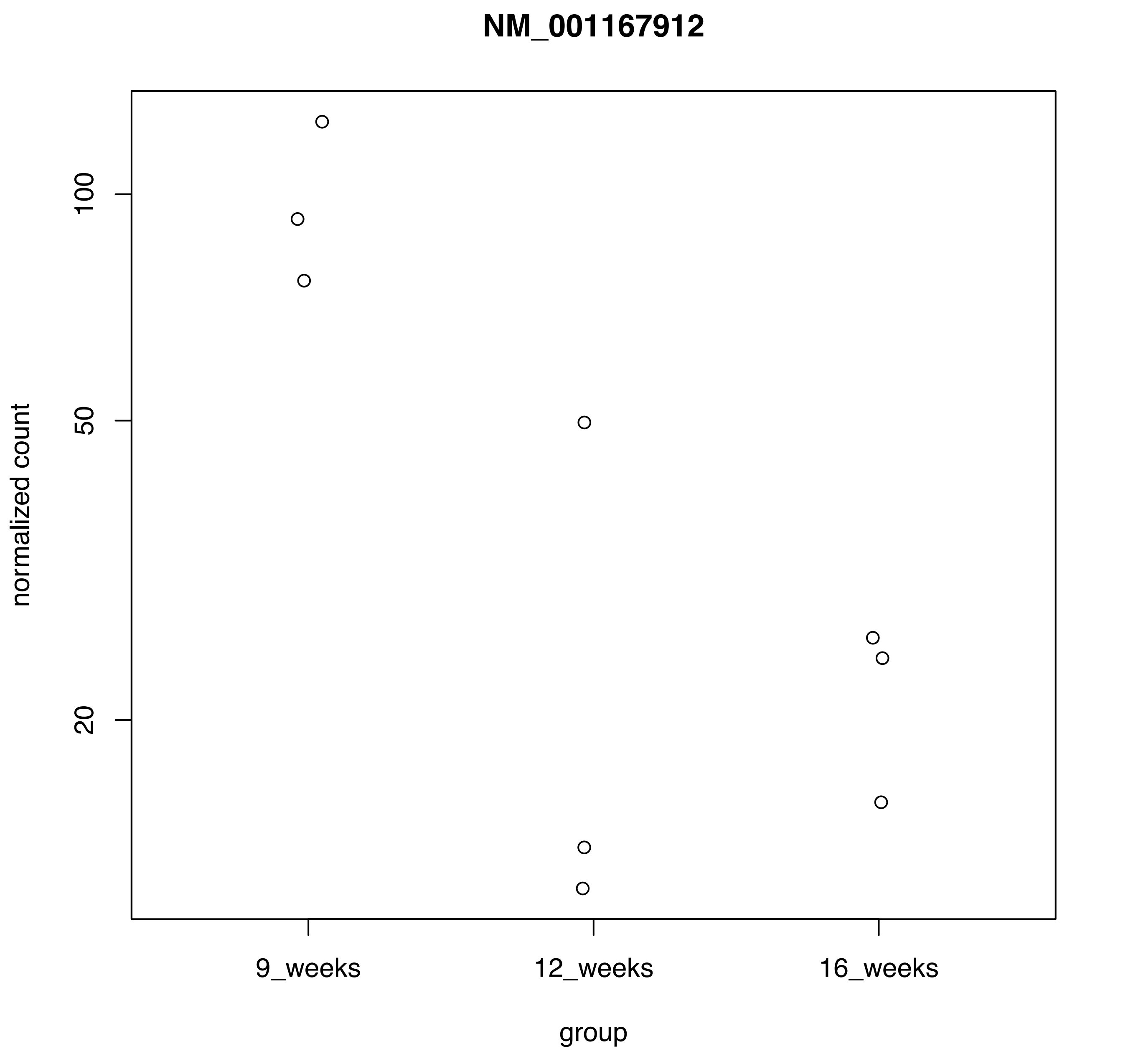
NM_001167912
normalized count
100
50
20
9_weeks
12_weeks
16_weeks
group

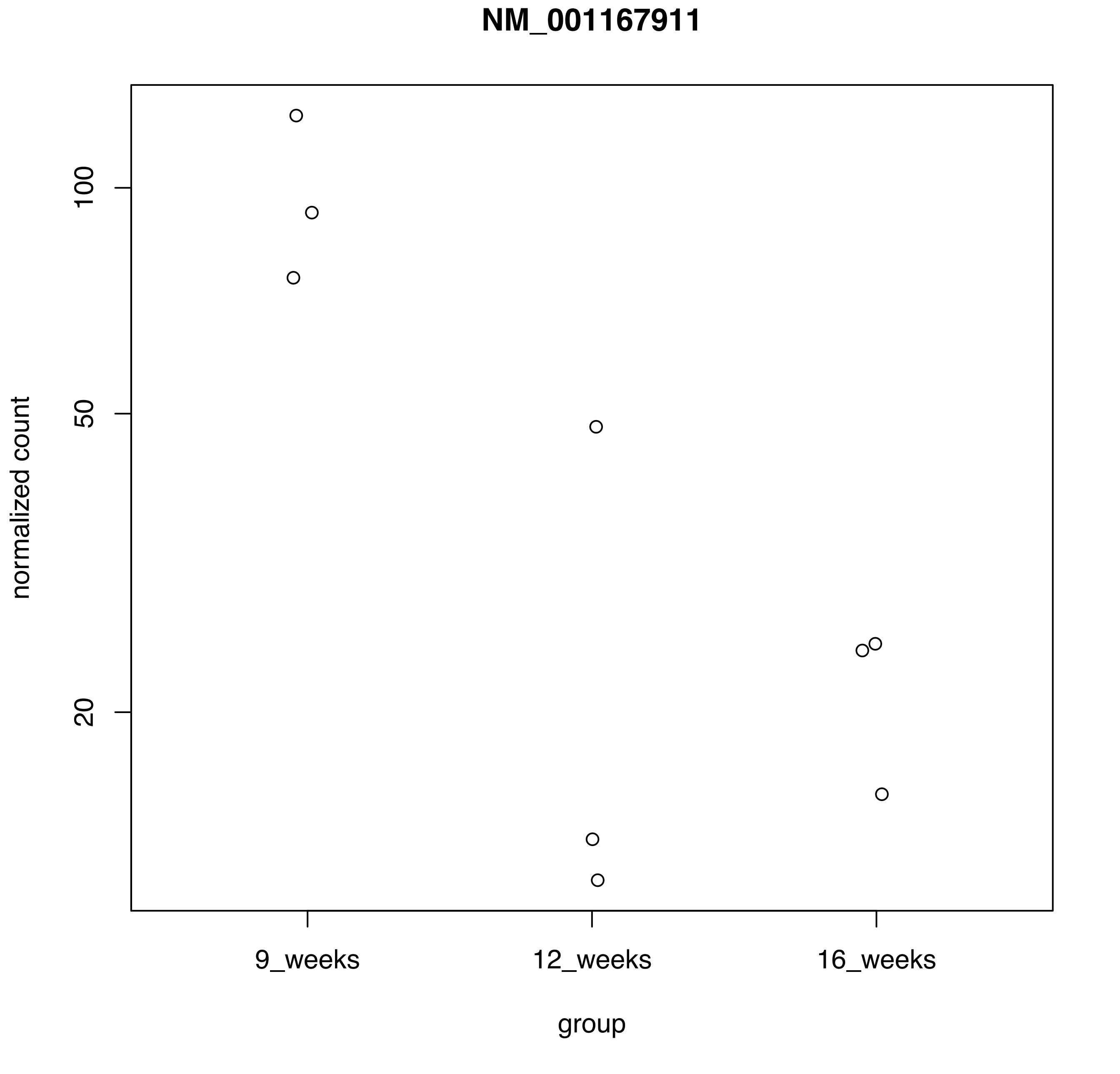
NM_001167911
normalized count
100
50
20
9_weeks
12_weeks
16_weeks
group

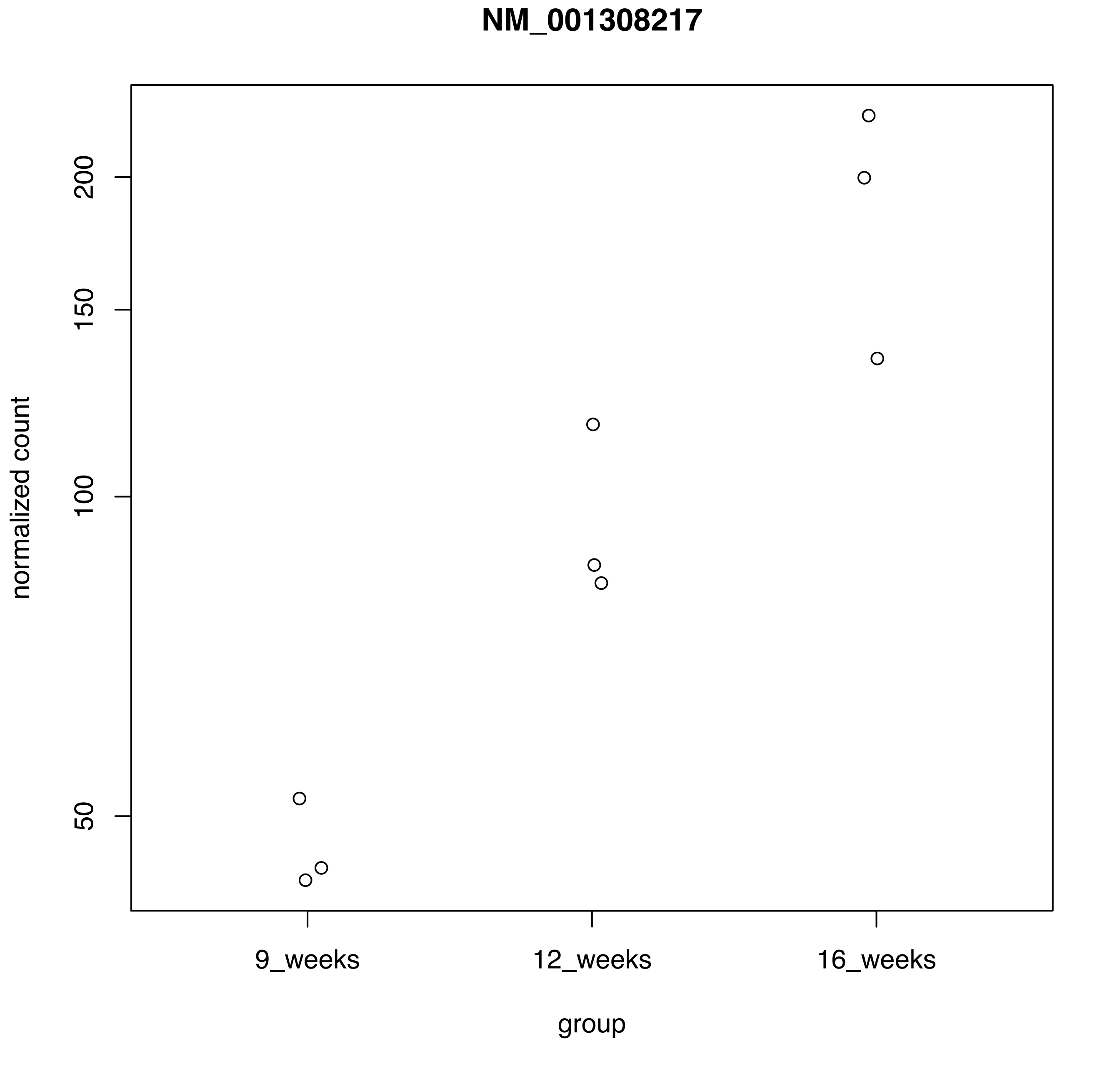
NM_001308217
normalized count
50
100
150
200
9_weeks
12_weeks
16_weeks
group

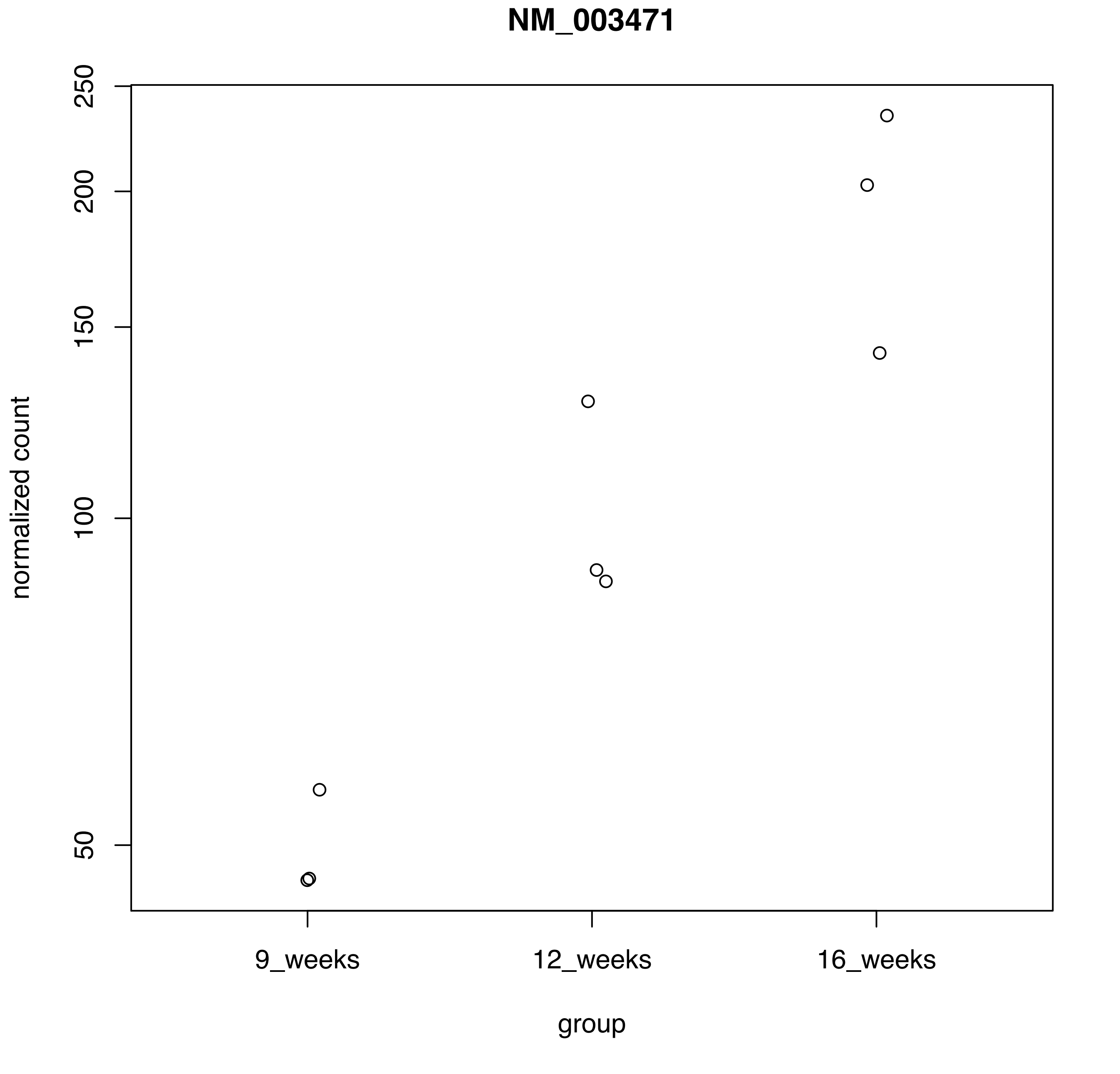

NM_003471
normalized count
50
100
150
200
250
9_weeks
12_weeks
16_weeks
group

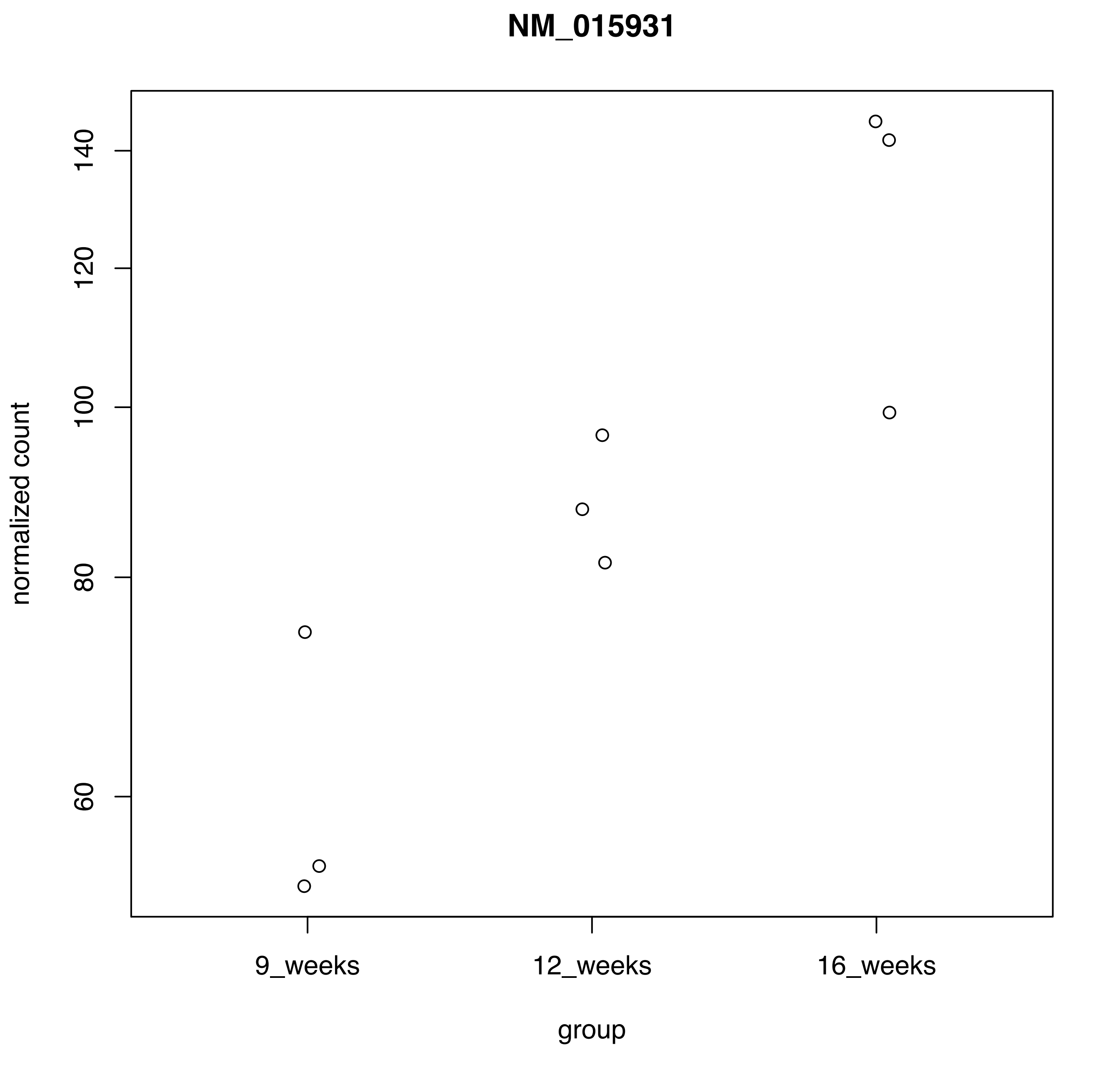
NM_015931
normalized count
60
80
100
120
140
9_weeks
12_weeks
16_weeks
group

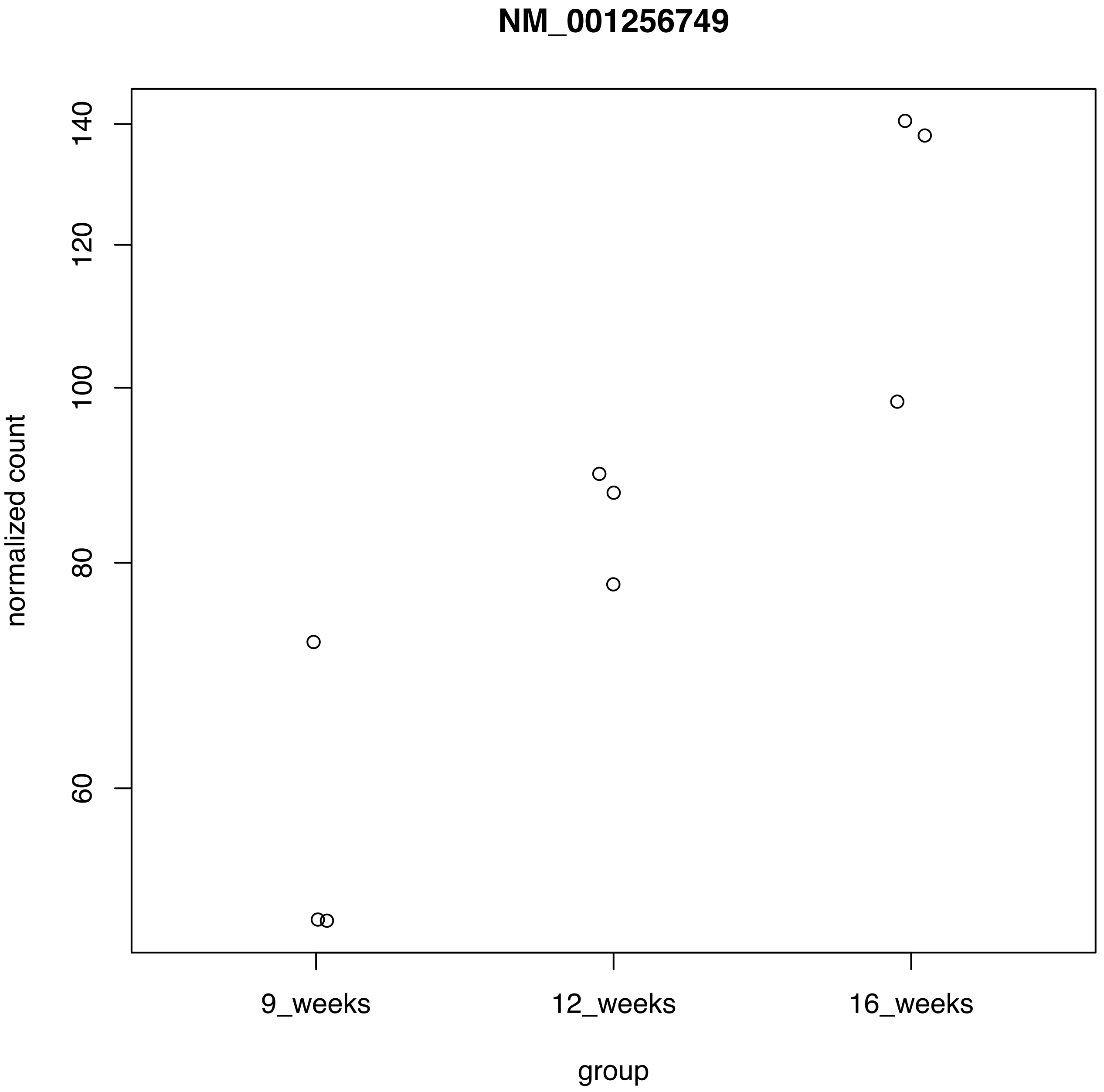
NM_001256749
normalized count
140
120
100
80
60
9_weeks
12_weeks
16_weeks
group

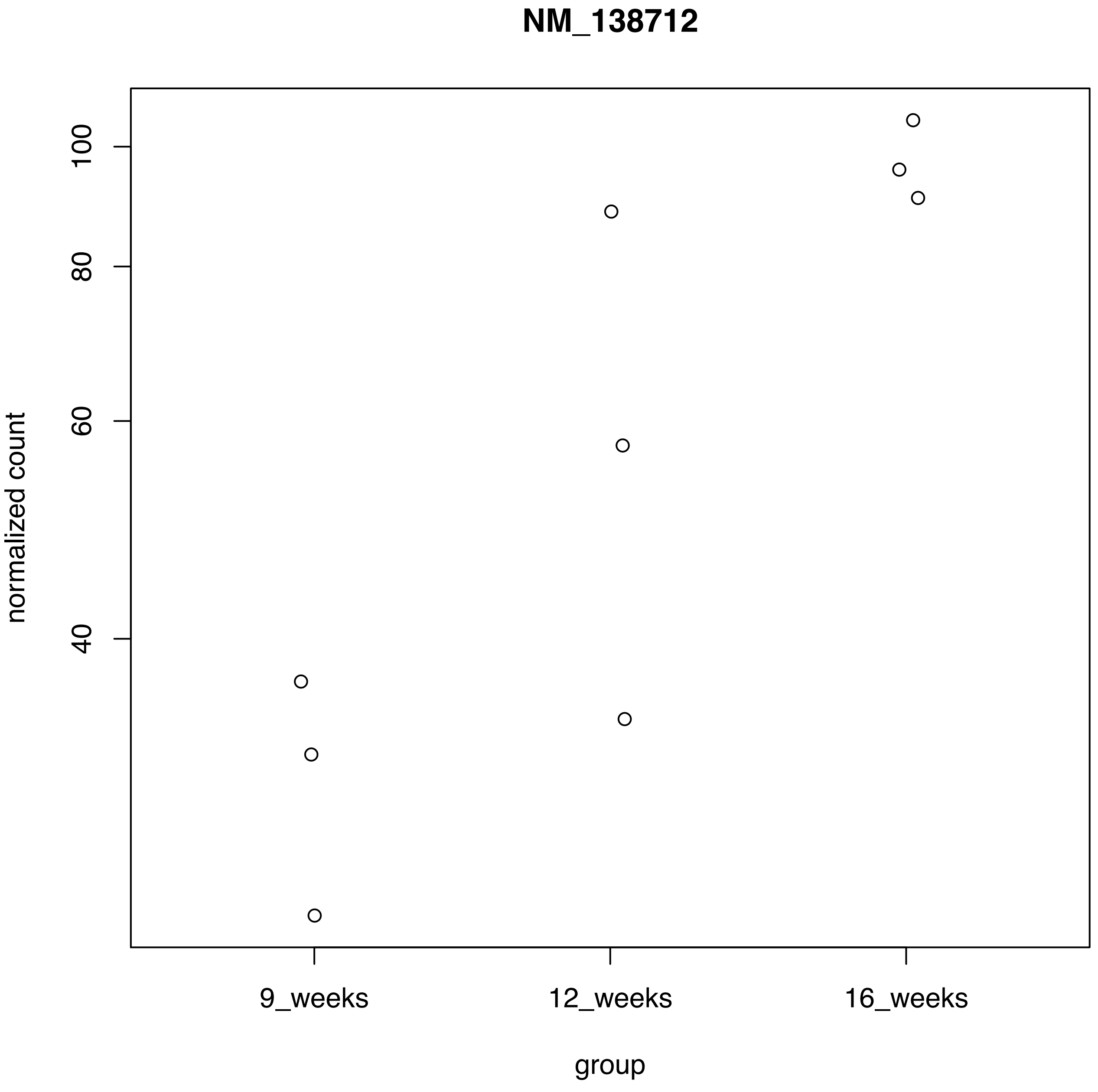
NM_138712
normalized count
100
80
60
40
9_weeks
12_weeks
16_weeks
group

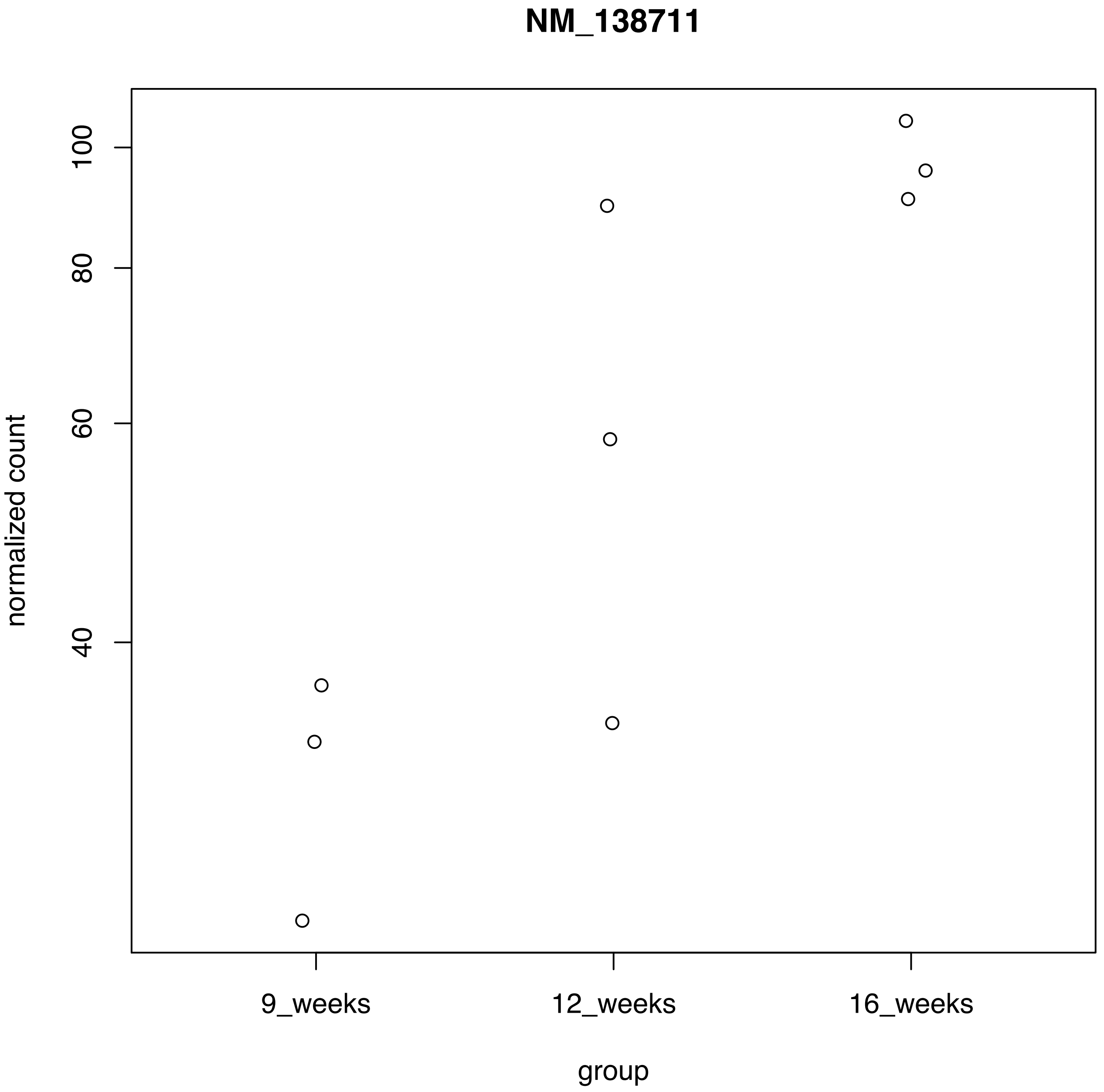
NM_138711
normalized count
100
80
60
40
9_weeks
12_weeks
16_weeks
group

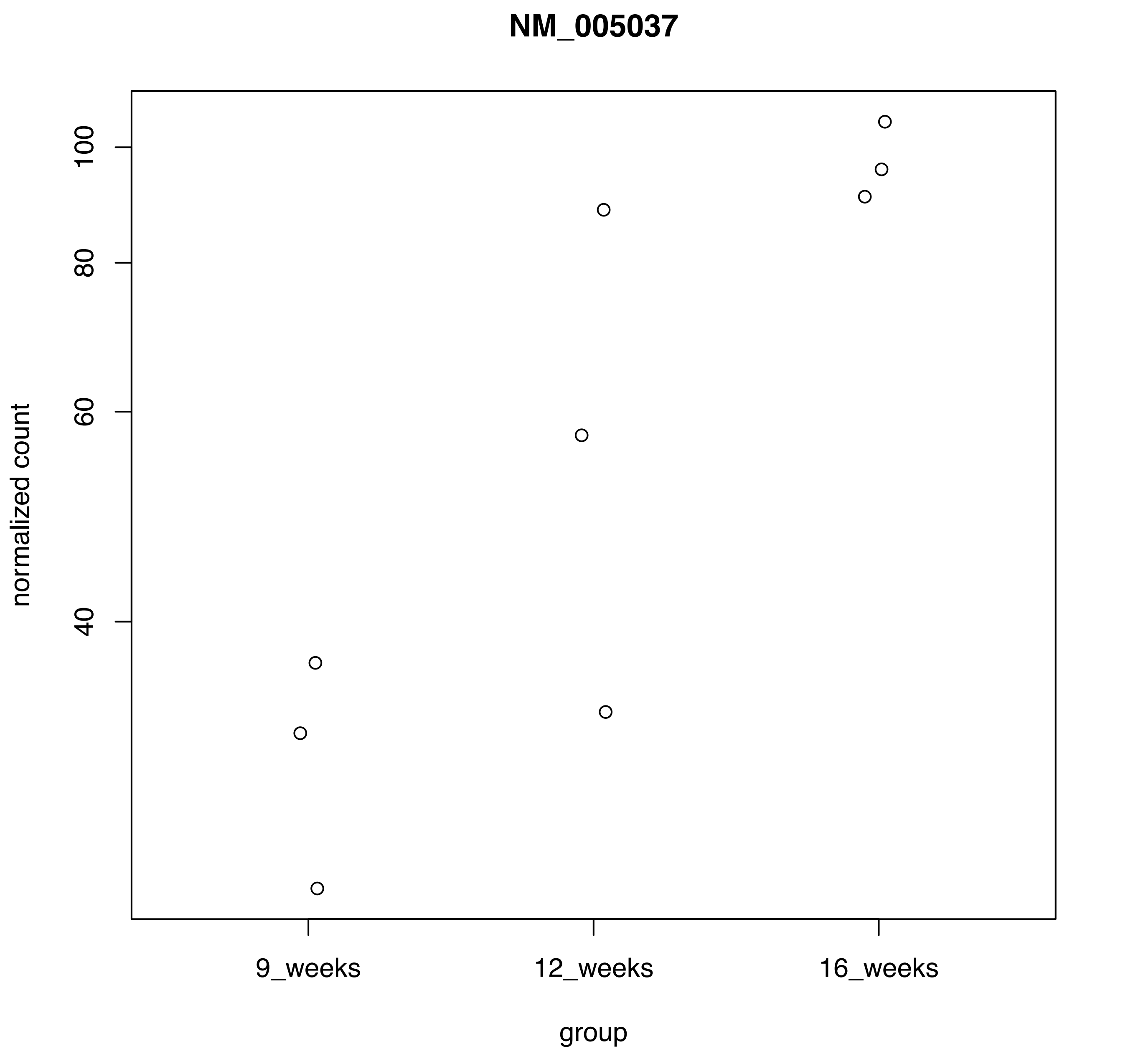
NM_005037
normalized count
100
80
60
40
9_weeks
12_weeks
16_weeks
group

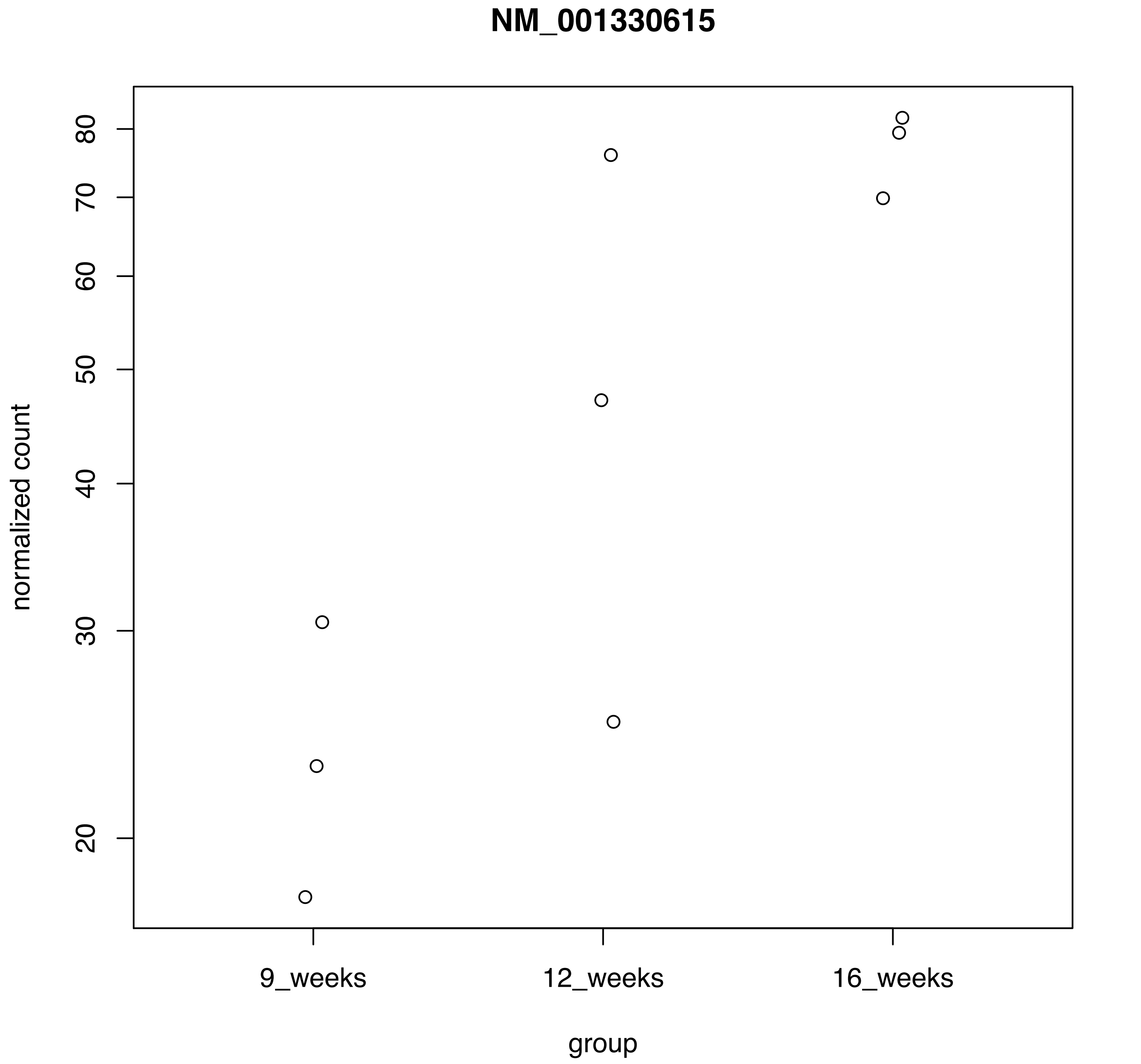
NM_001330615
normalized count
20
30
40
50
60
70
80
9_weeks
12_weeks
16_weeks
group

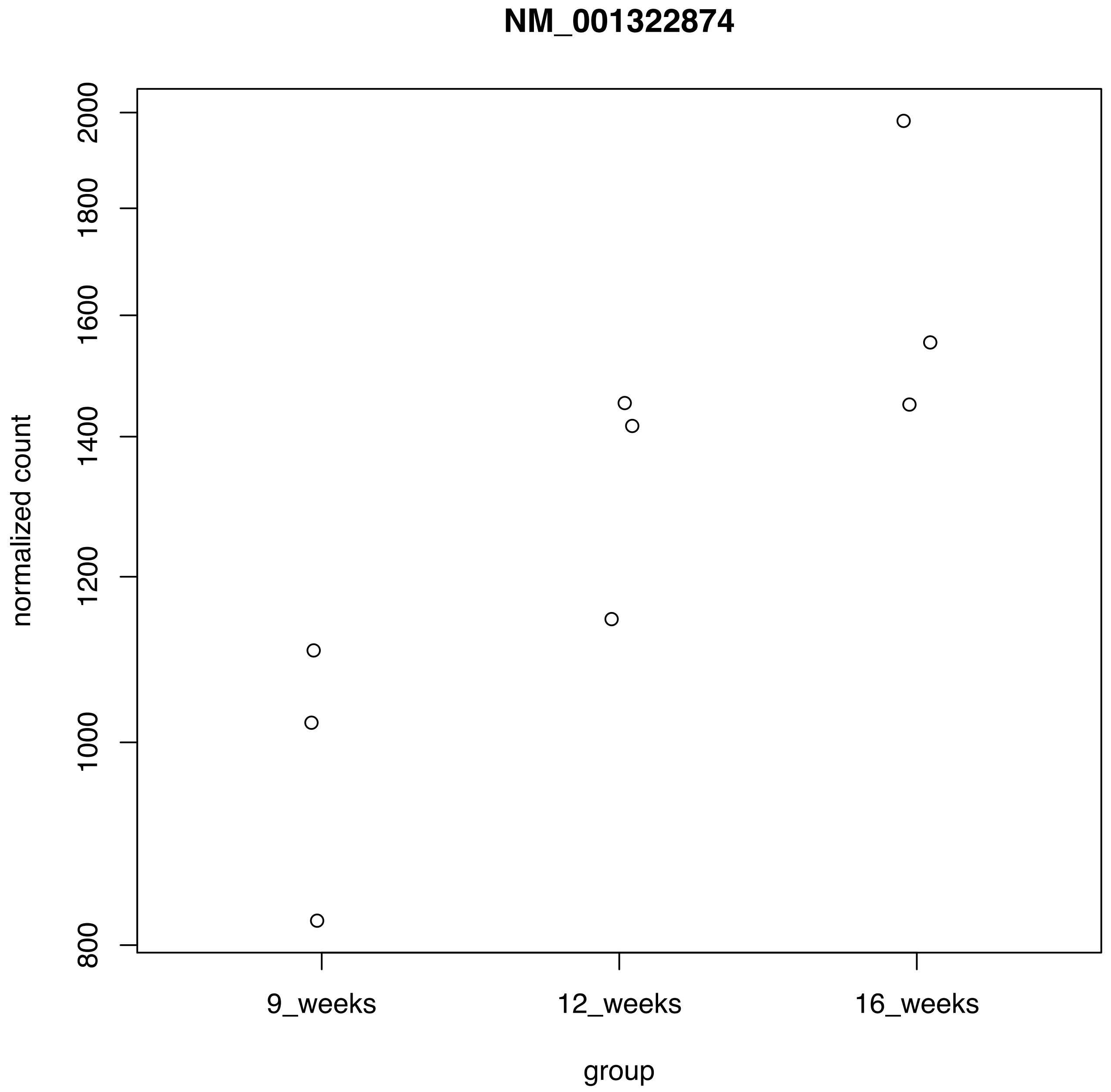

NM_001322874
normalized count
2000
1800
1600
1400
1200
1000
800
9_weeks
12_weeks
16_weeks
group

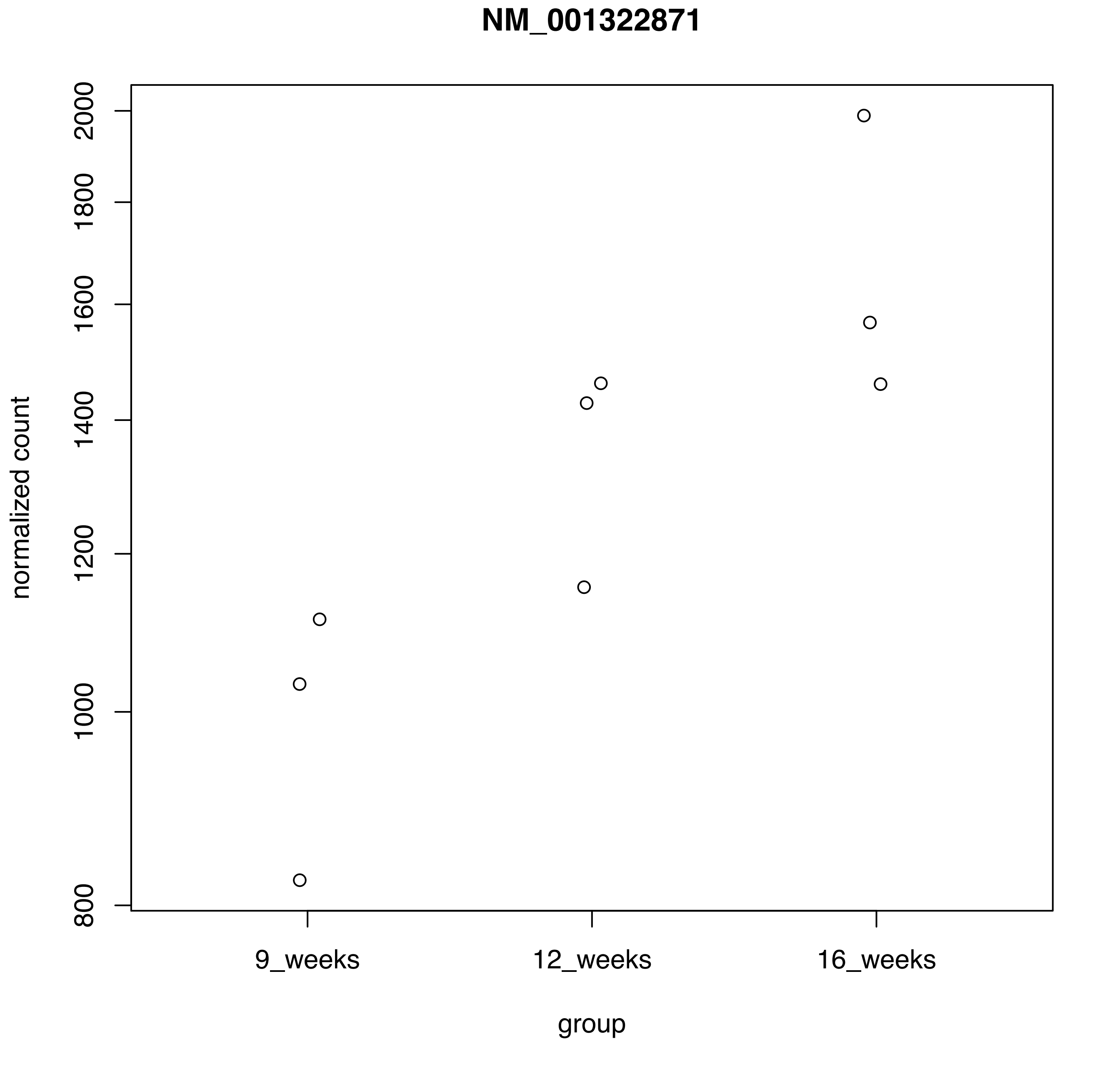
NM_001322871
normalized count
2000
1800
1600
1400
1200
1000
800
9_weeks
12_weeks
16_weeks
group

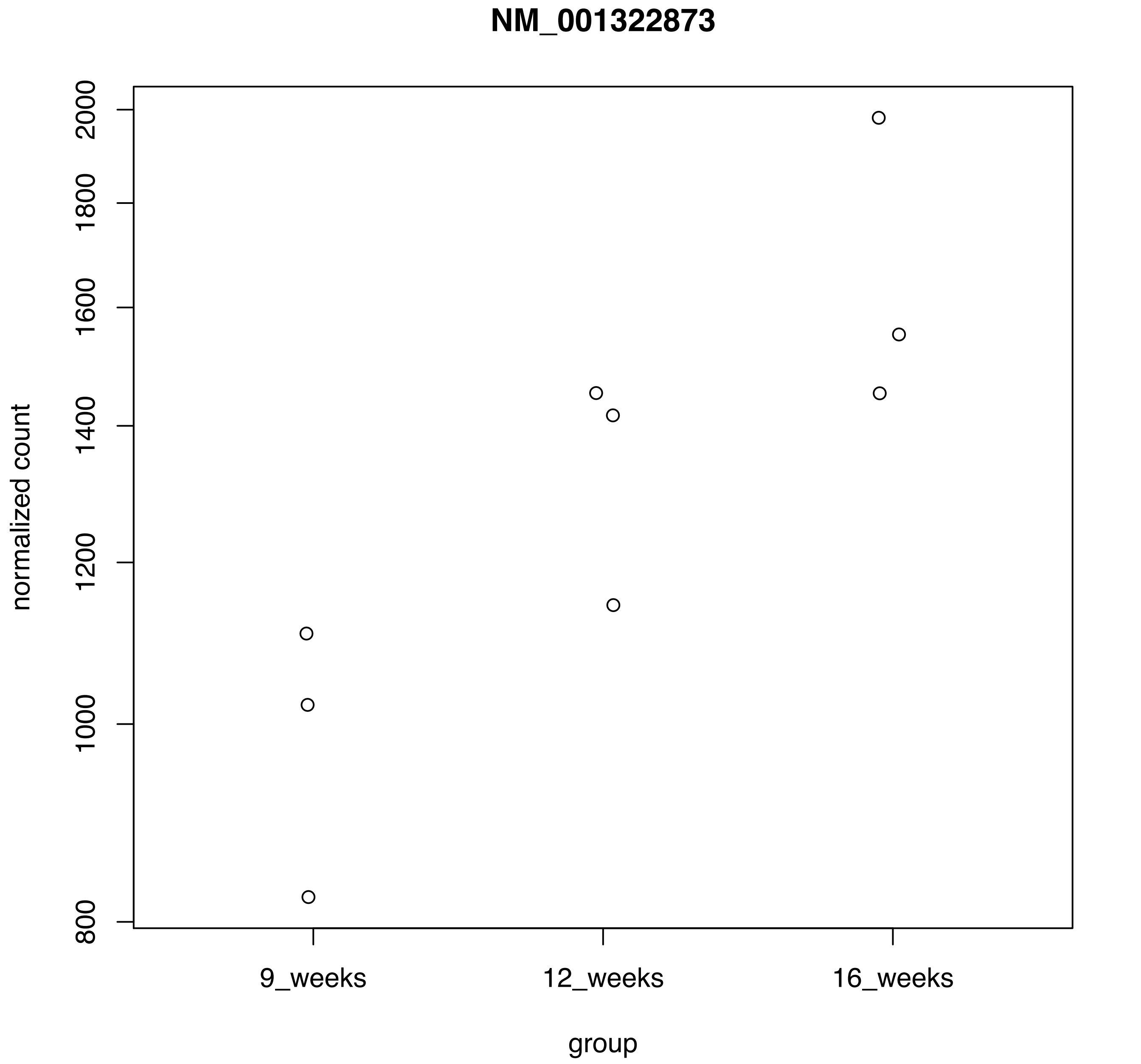
NM_001322873
normalized count
800
1000
1200
1400
1600
1800
2000
9_weeks
12_weeks
16_weeks
group

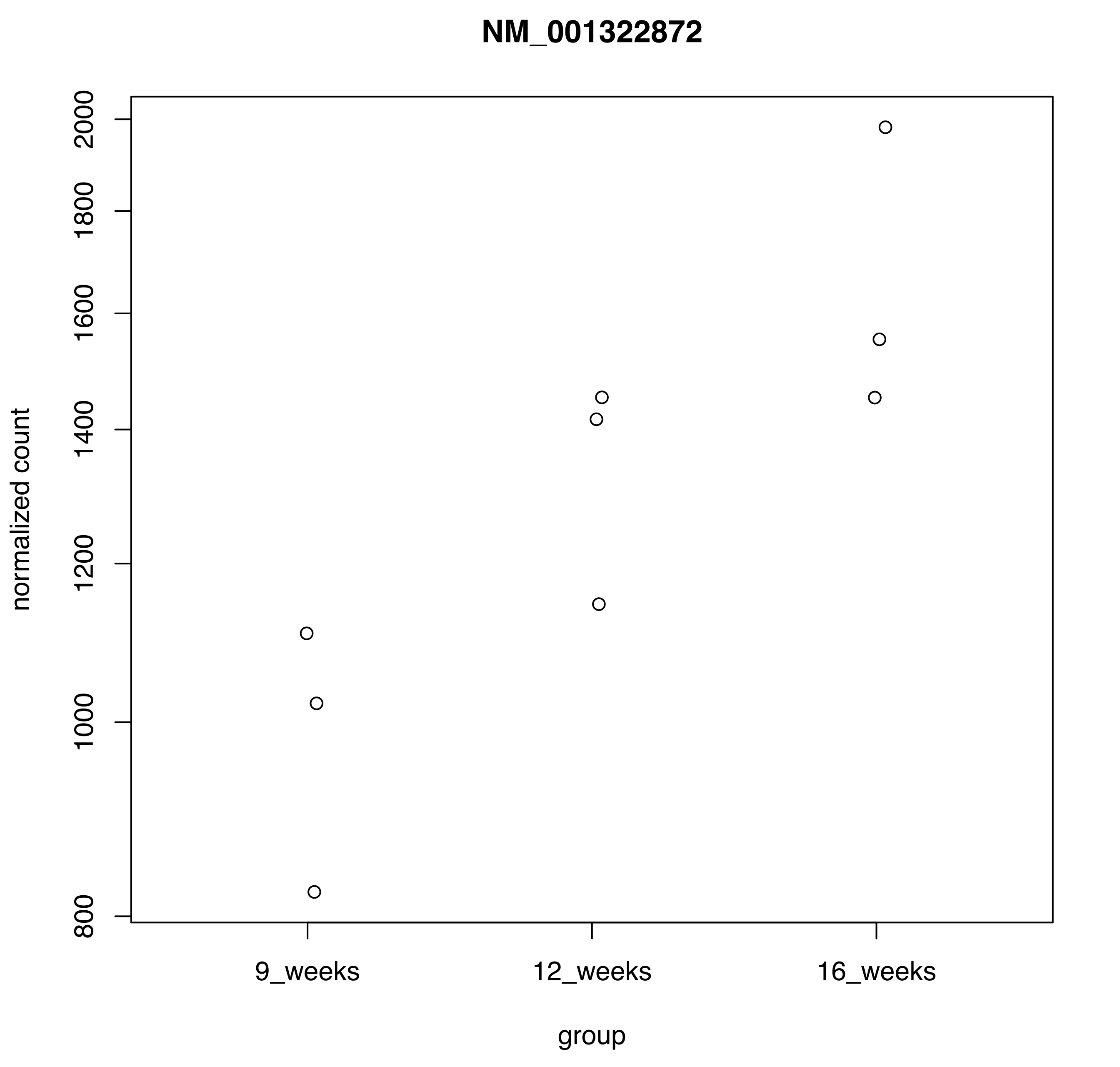

NM_001322872
normalized count
800
1000
1200
1400
1600
1800
2000
9_weeks
12_weeks
16_weeks
group

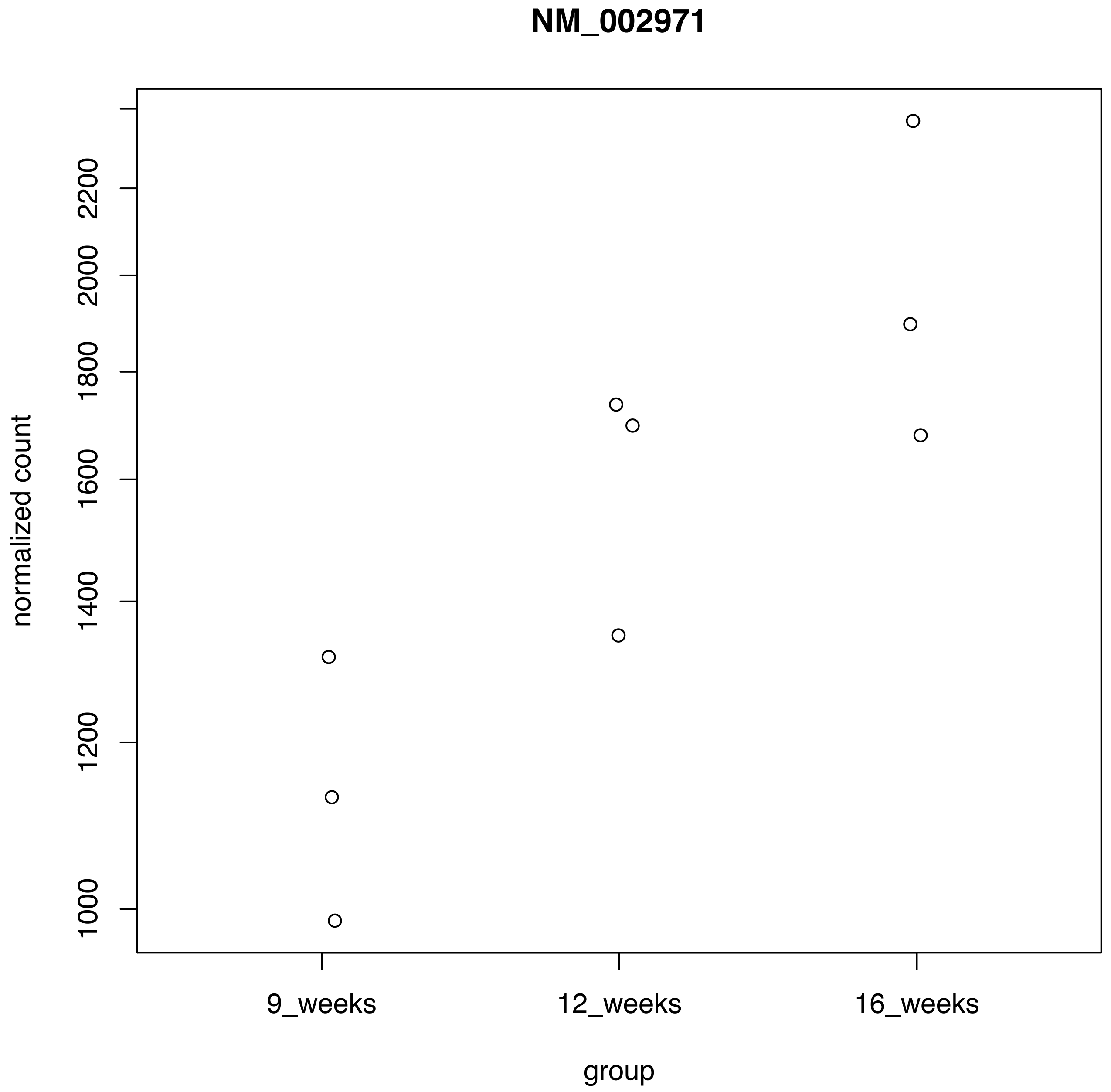
NM_002971
normalized count
1000
1200
1400
1600
1800
2000
2200
9_weeks
12_weeks
16_weeks
group

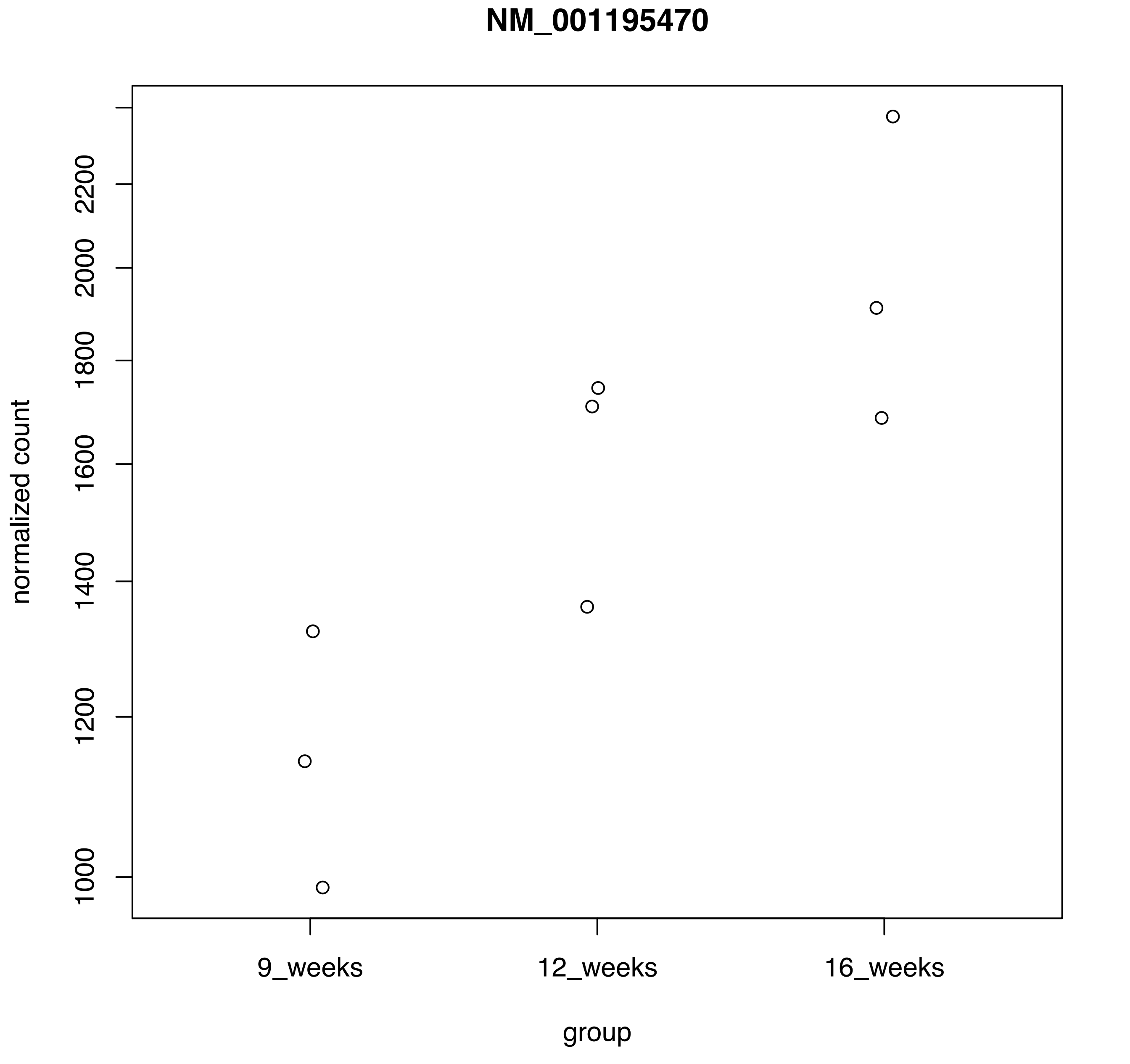
NM_001195470
normalized count
1000
1200
1400
1600
1800
2000
2200
9_weeks
12_weeks
16_weeks
group

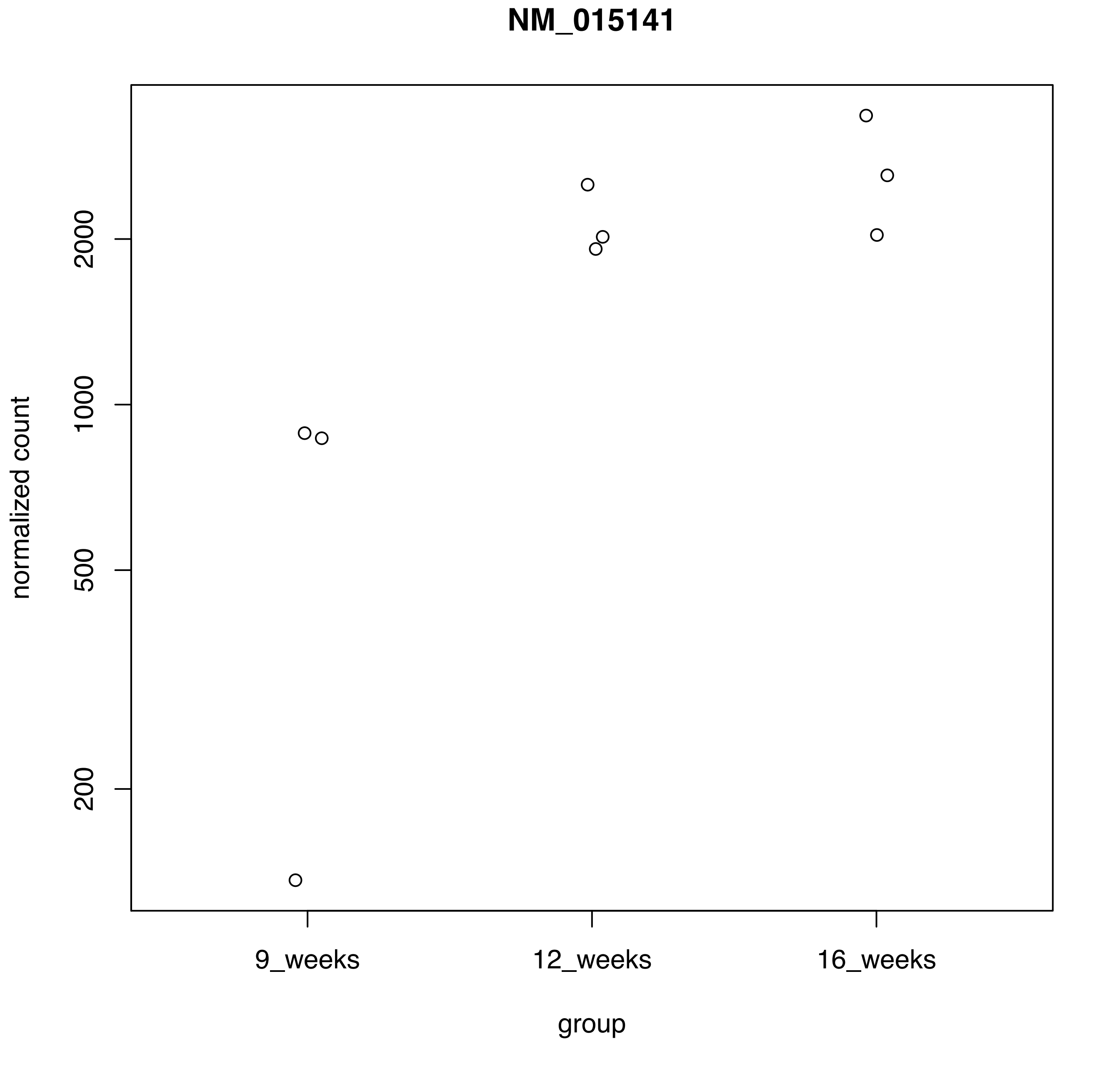

NM_015141
normalized count
2000
1000
500
200
9_weeks
12_weeks
16_weeks
group

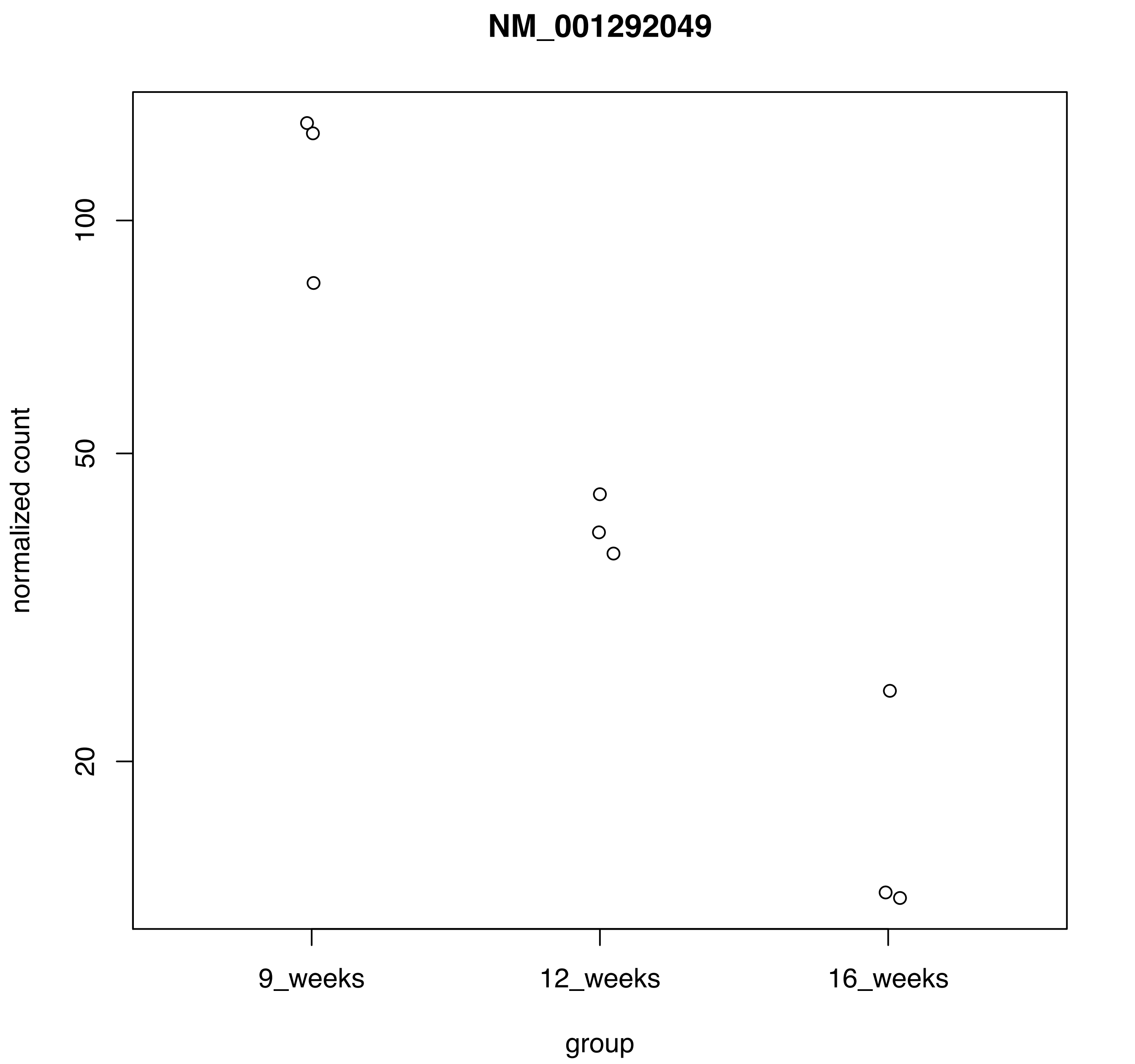
NM_001292049
normalized count
100
50
20
9_weeks
12_weeks
16_weeks
group

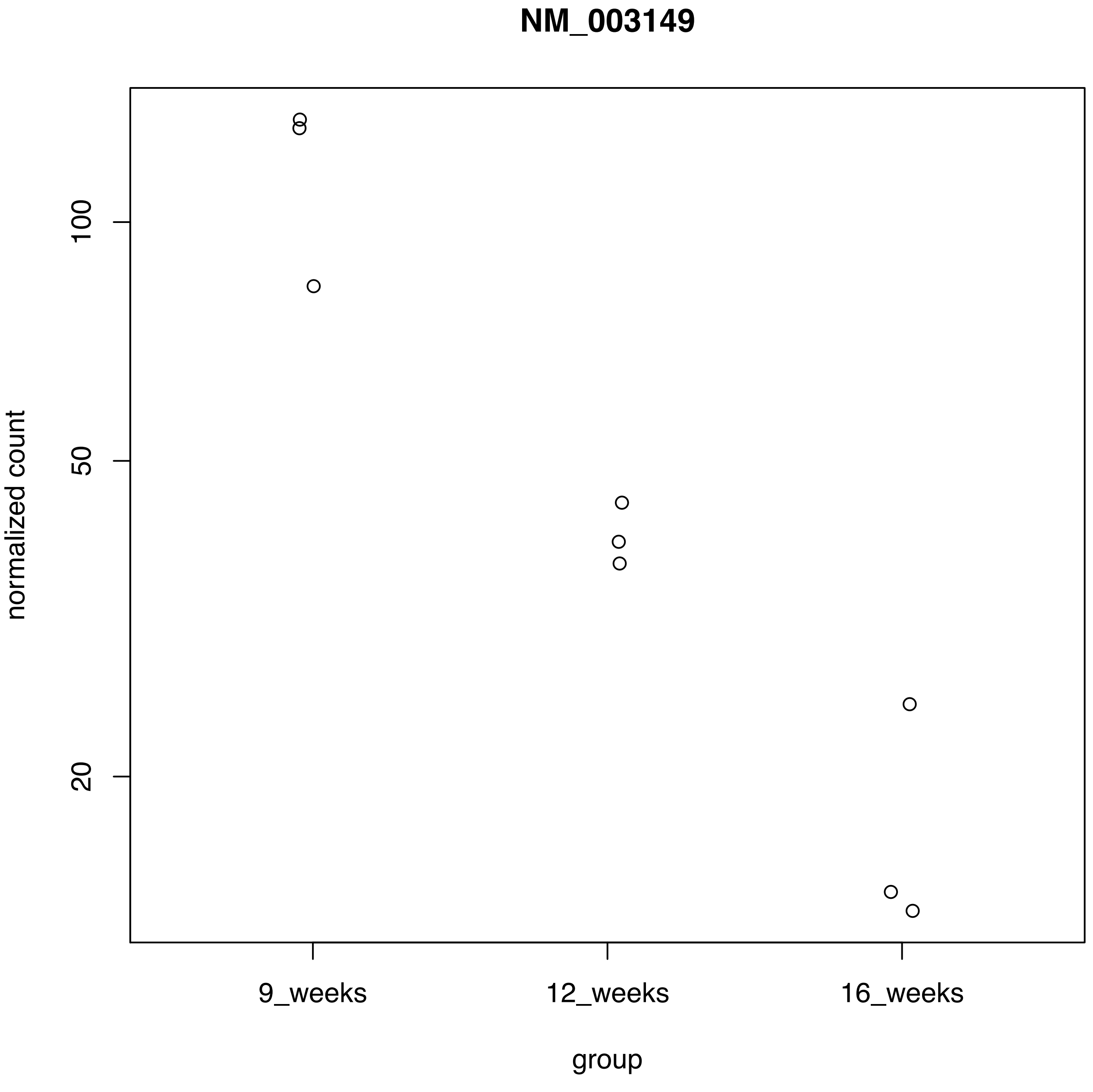
NM_003149
normalized count
100
50
20
9_weeks
12_weeks
16_weeks
group

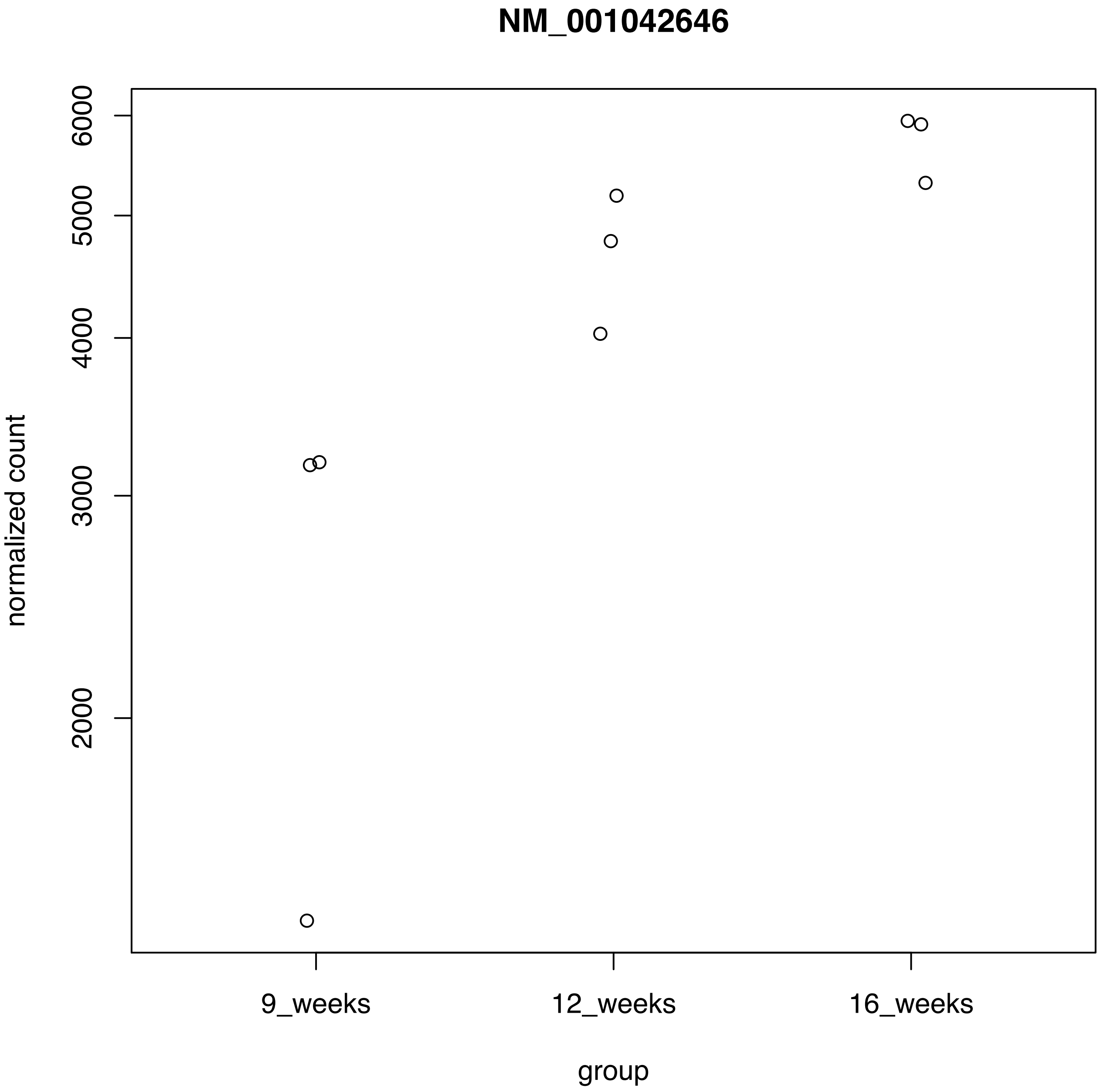
NM_001042646
normalized count
6000
5000
4000
3000
2000
9_weeks
12_weeks
16_weeks
group

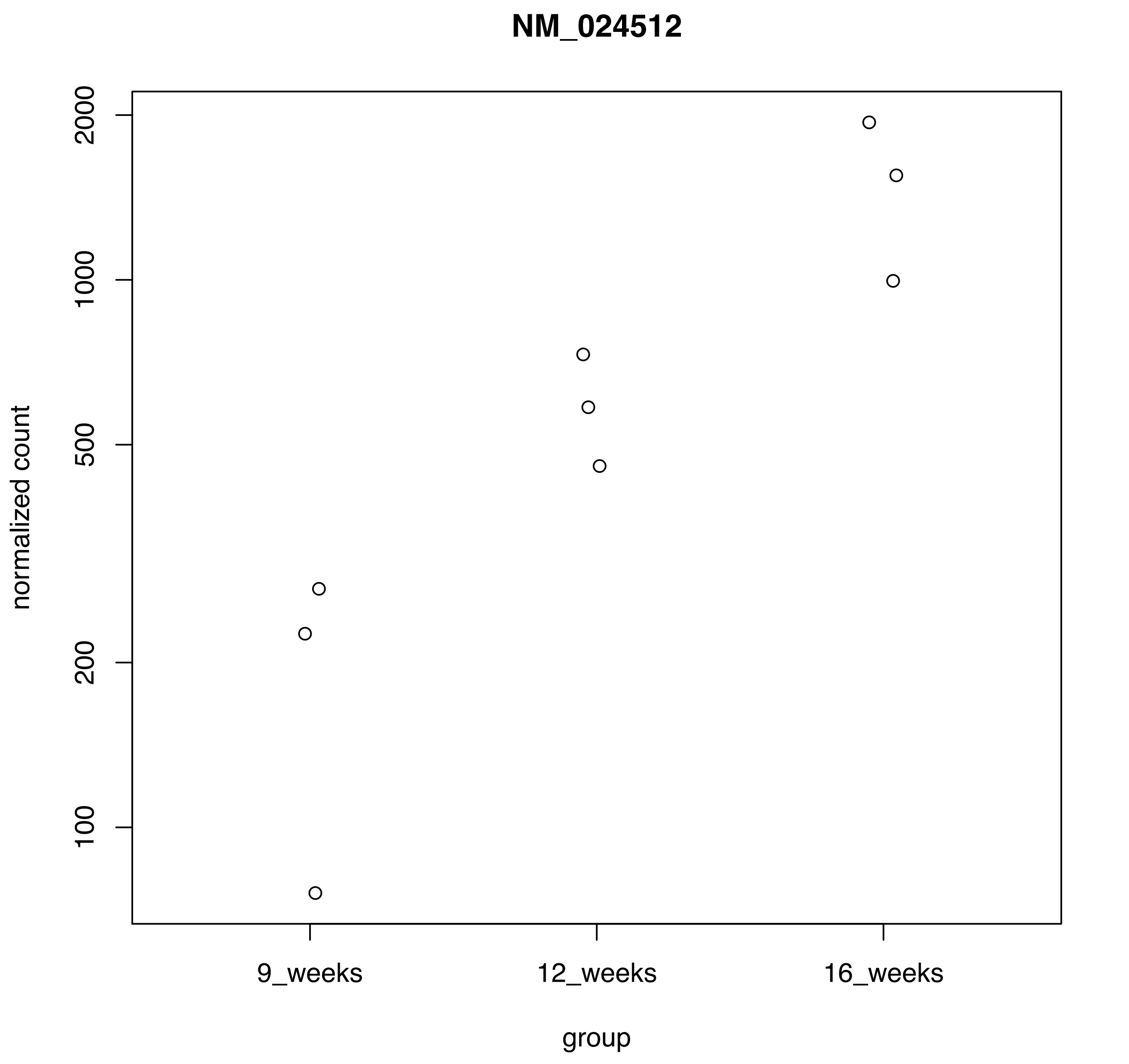
NM_024512
normalized count
2000
1000
500
200
100
9_weeks
12_weeks
16_weeks
group

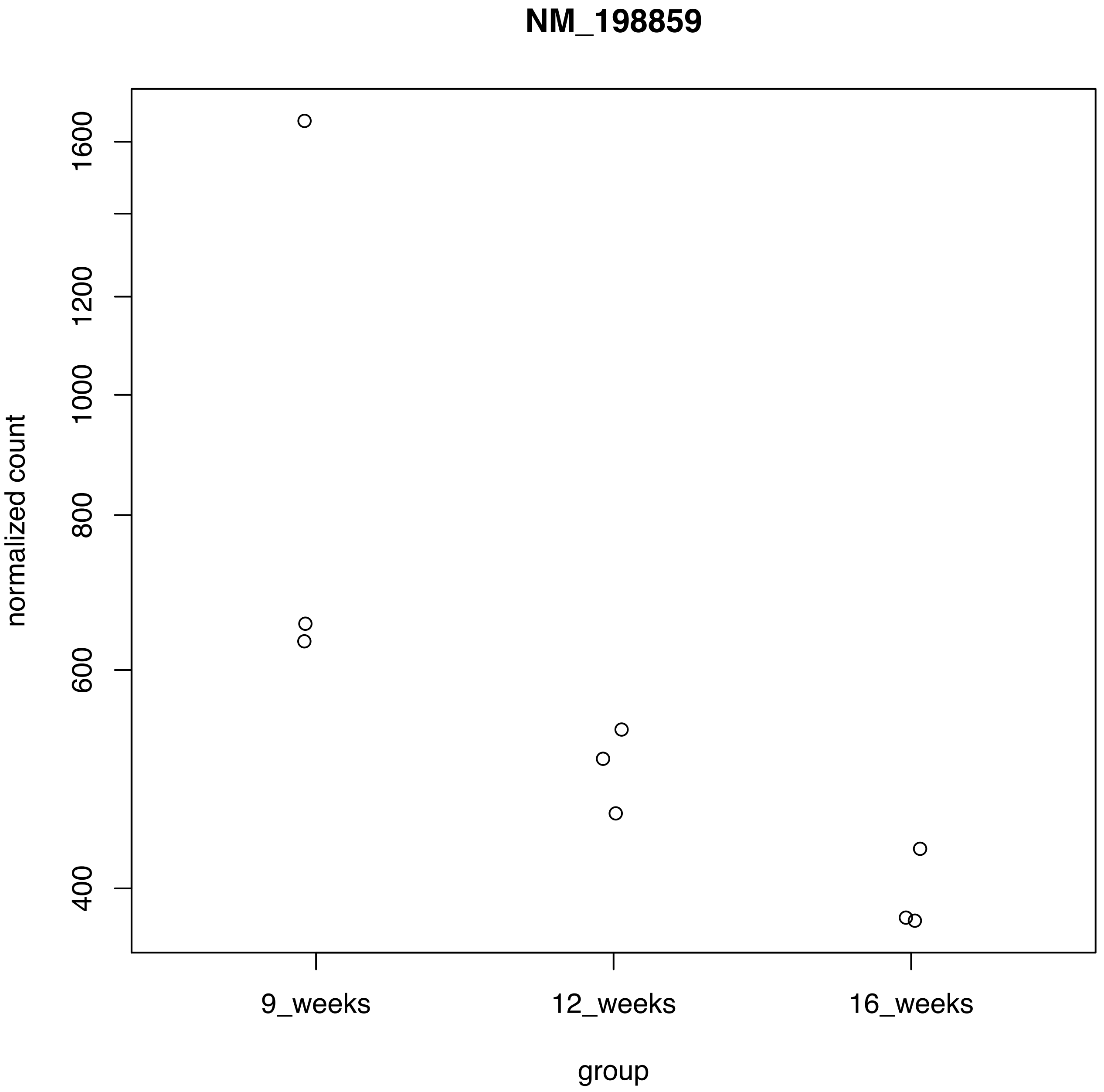
NM_198859
normalized count
1600
1200
1000
800
600
400
9_weeks
12_weeks
16_weeks
group

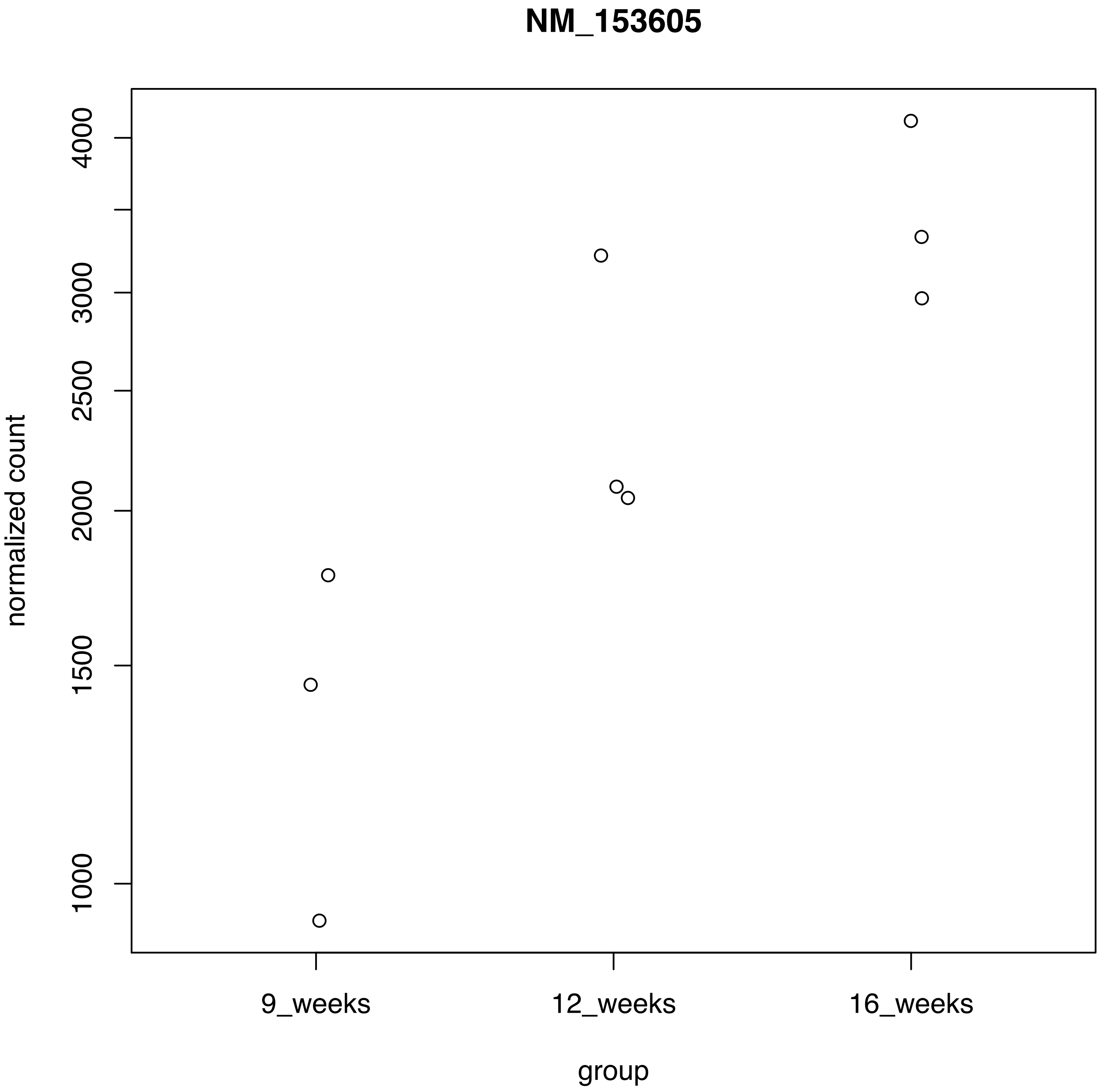

NM_153605
normalized count
4000
3000
2500
2000
1500
1000
9_weeks
12_weeks
16_weeks
group

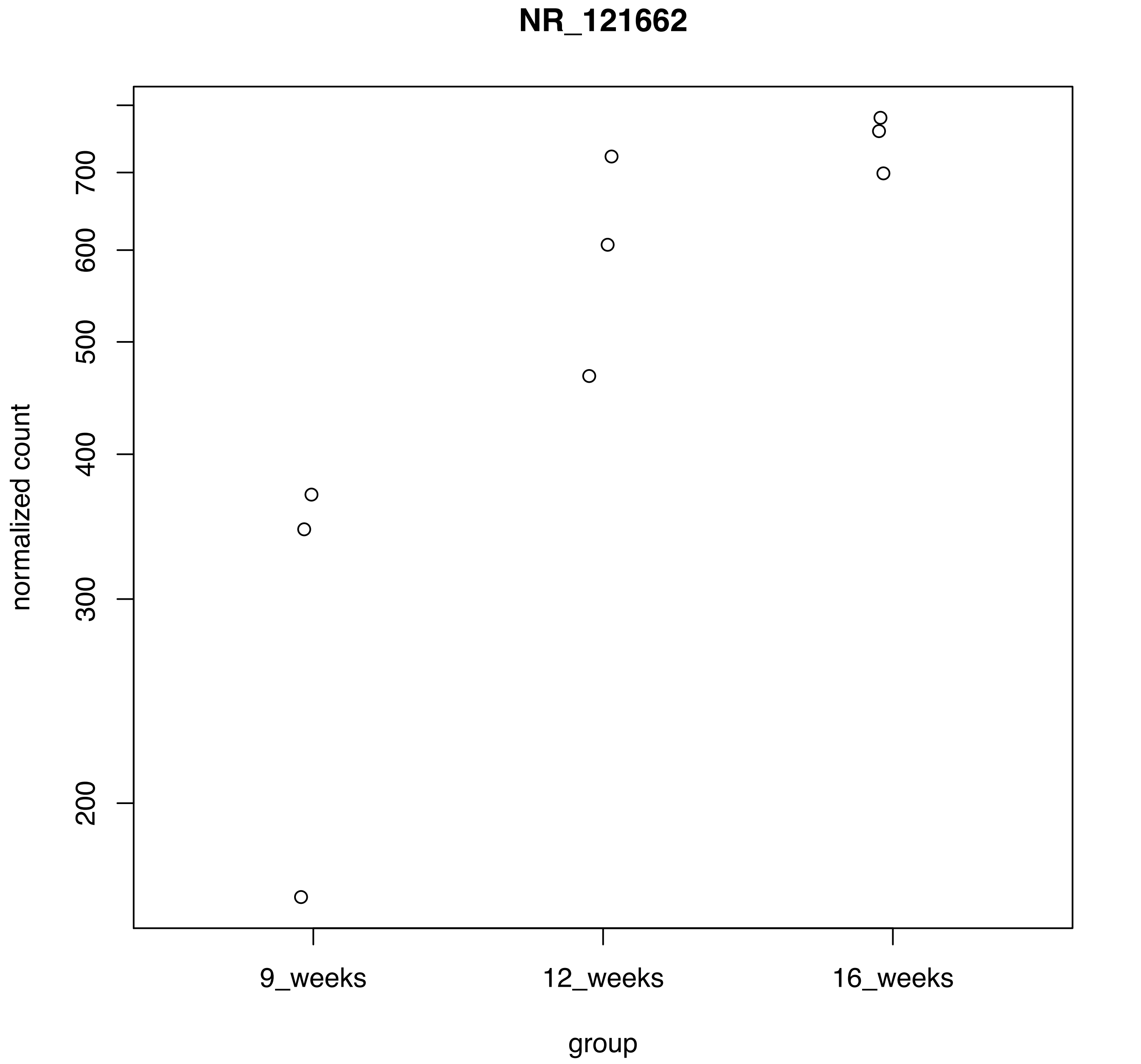
NR_121662
normalized count
700
600
500
400
300
200
9_weeks
12_weeks
16_weeks
group

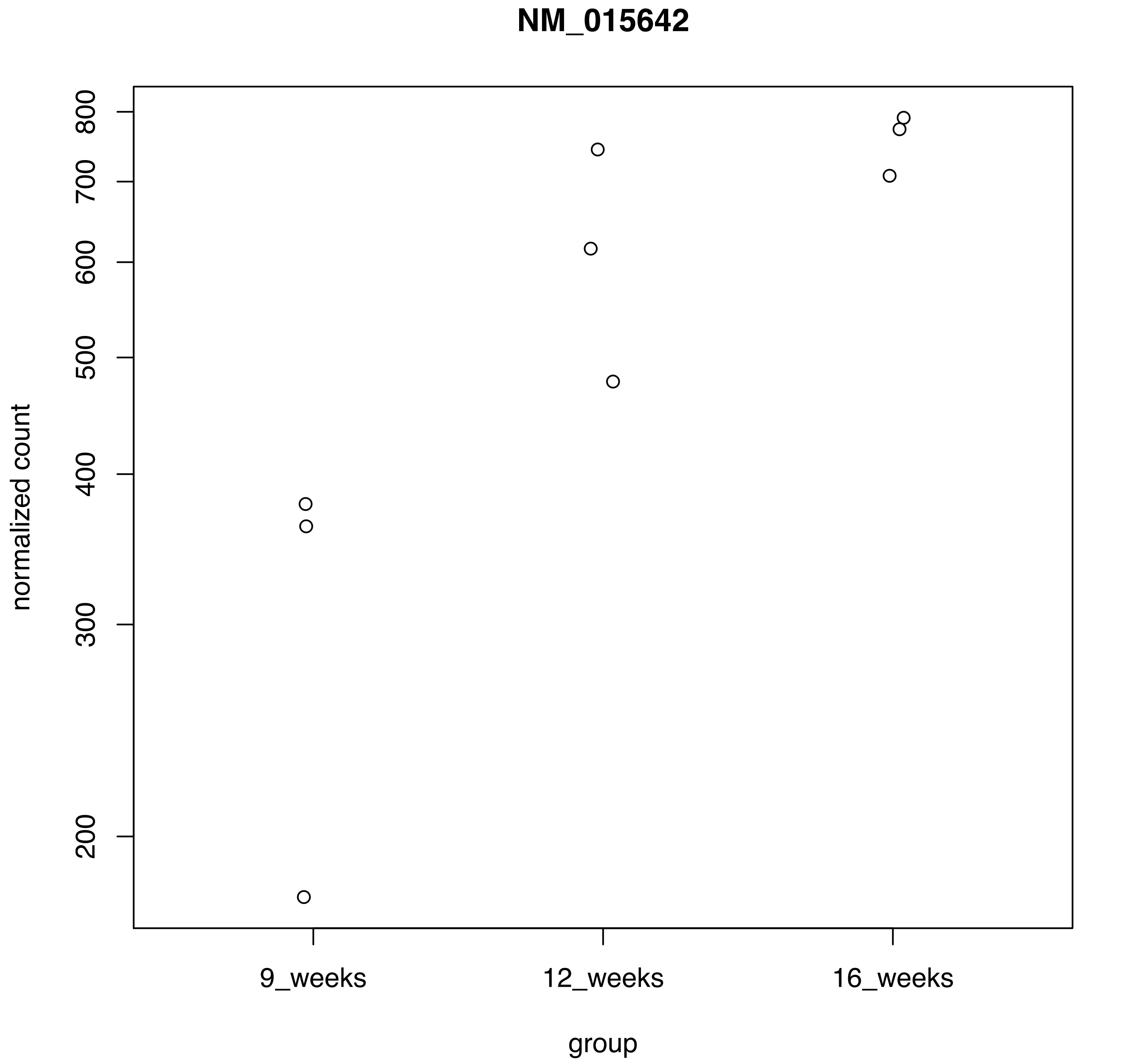
NM_015642
normalized count
800
700
600
500
400
300
200
9_weeks
12_weeks
16_weeks
group

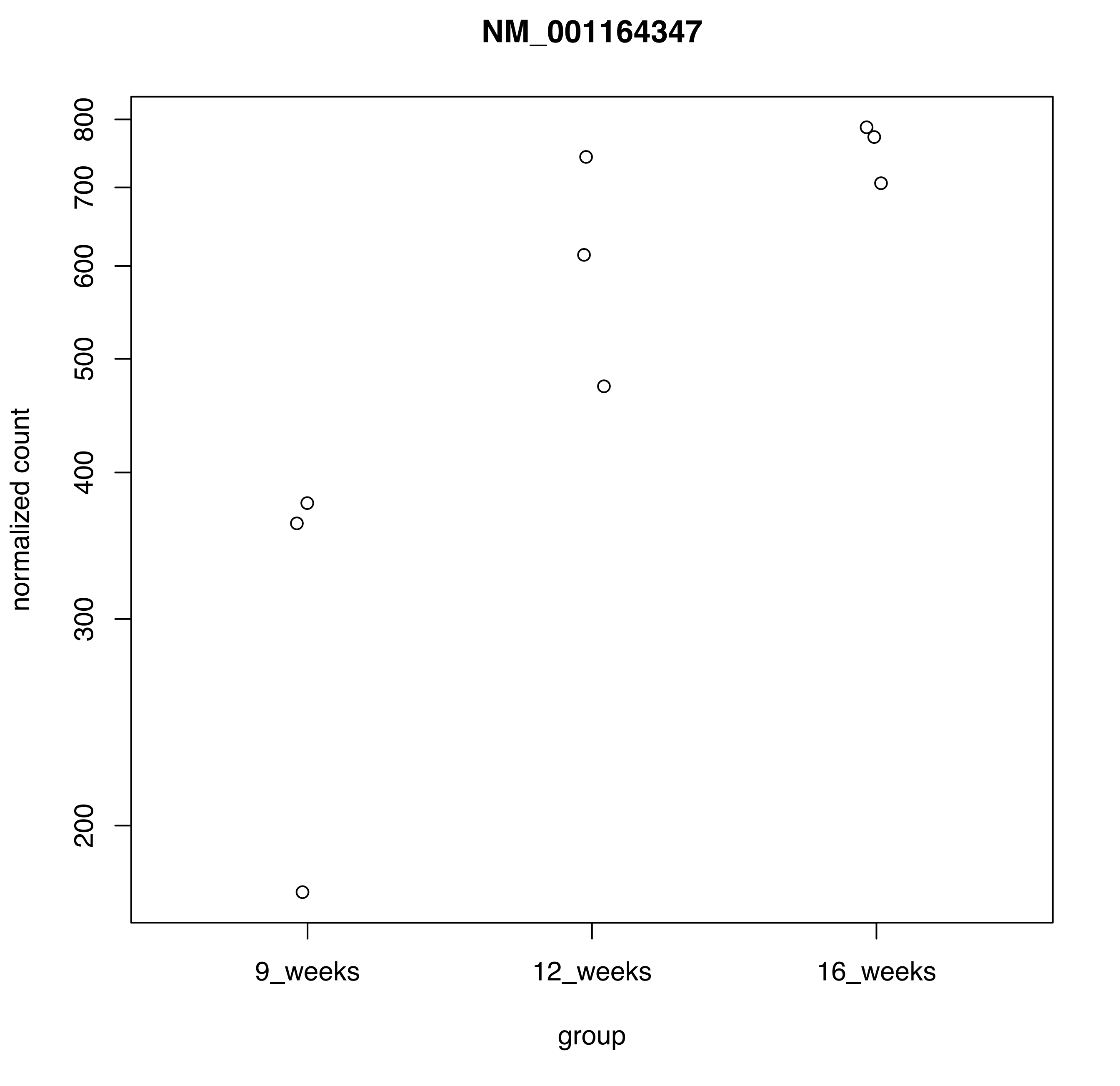
NM_001164347
normalized count
800
700
600
500
400
300
200
9_weeks
12_weeks
16_weeks
group

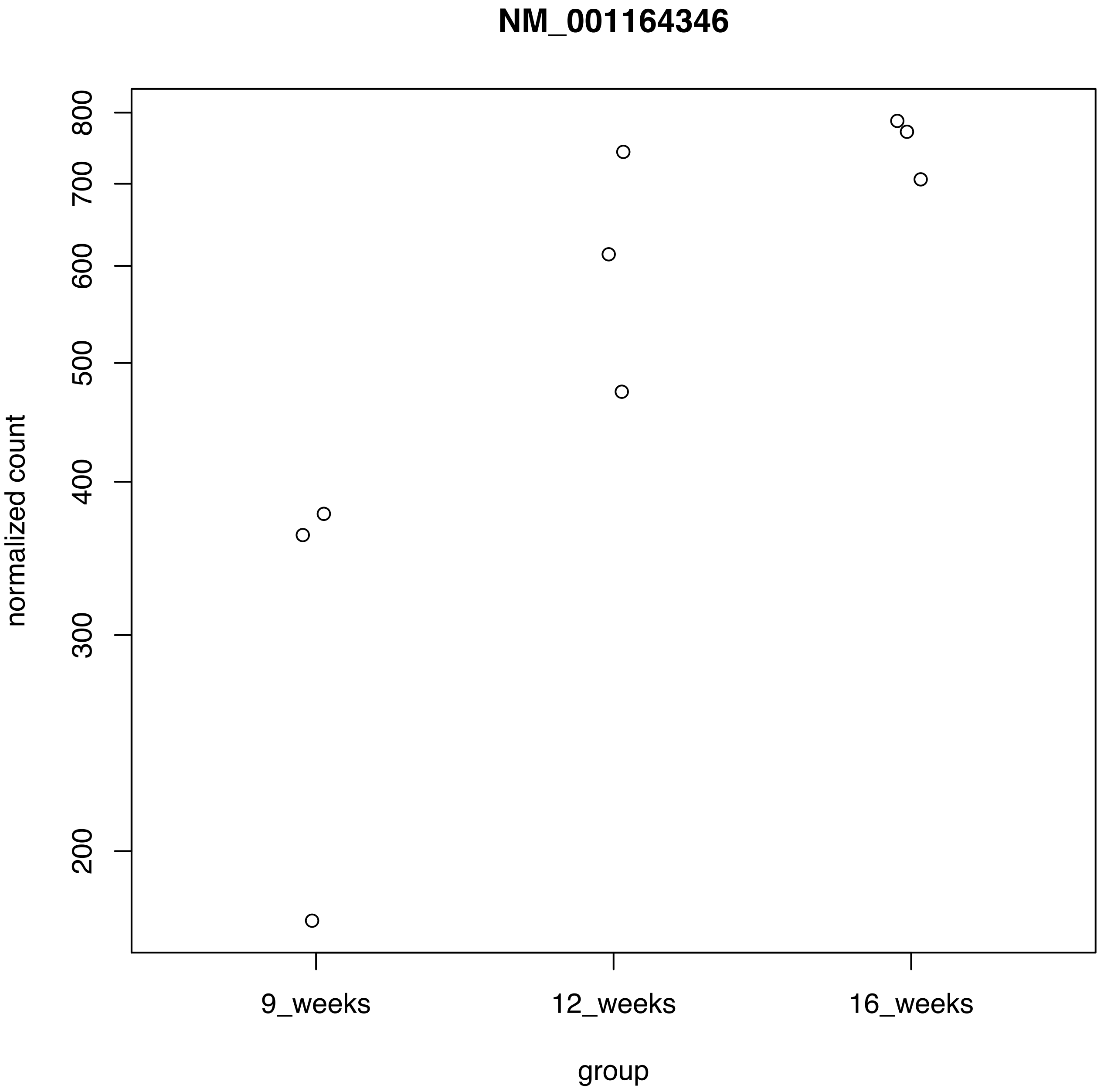
NM_001164346
normalized count
800
700
600
500
400
300
200
9_weeks
12_weeks
16_weeks
group

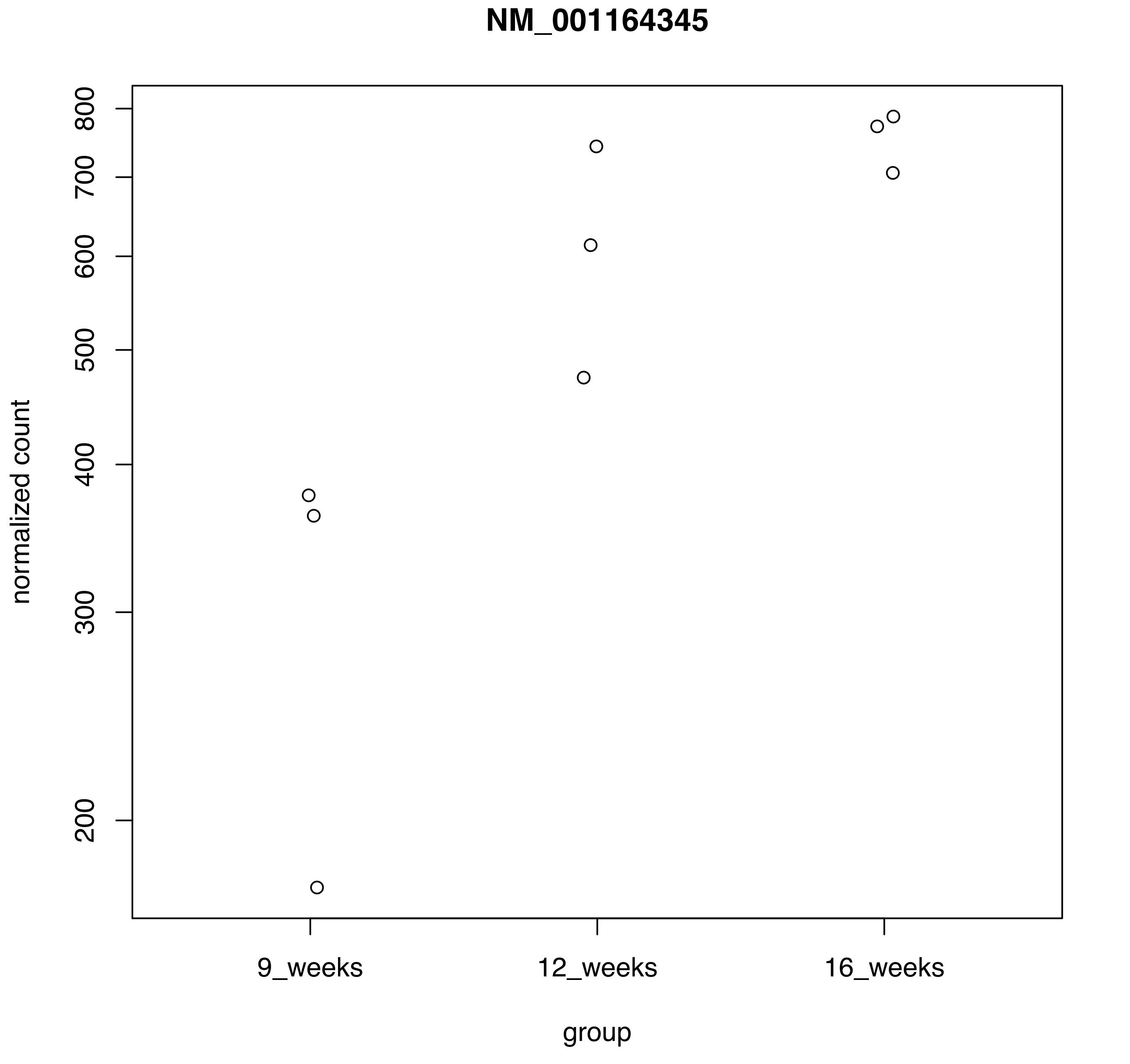
NM_001164345
normalized count
800
700
600
500
400
300
200
9_weeks
12_weeks
16_weeks
group

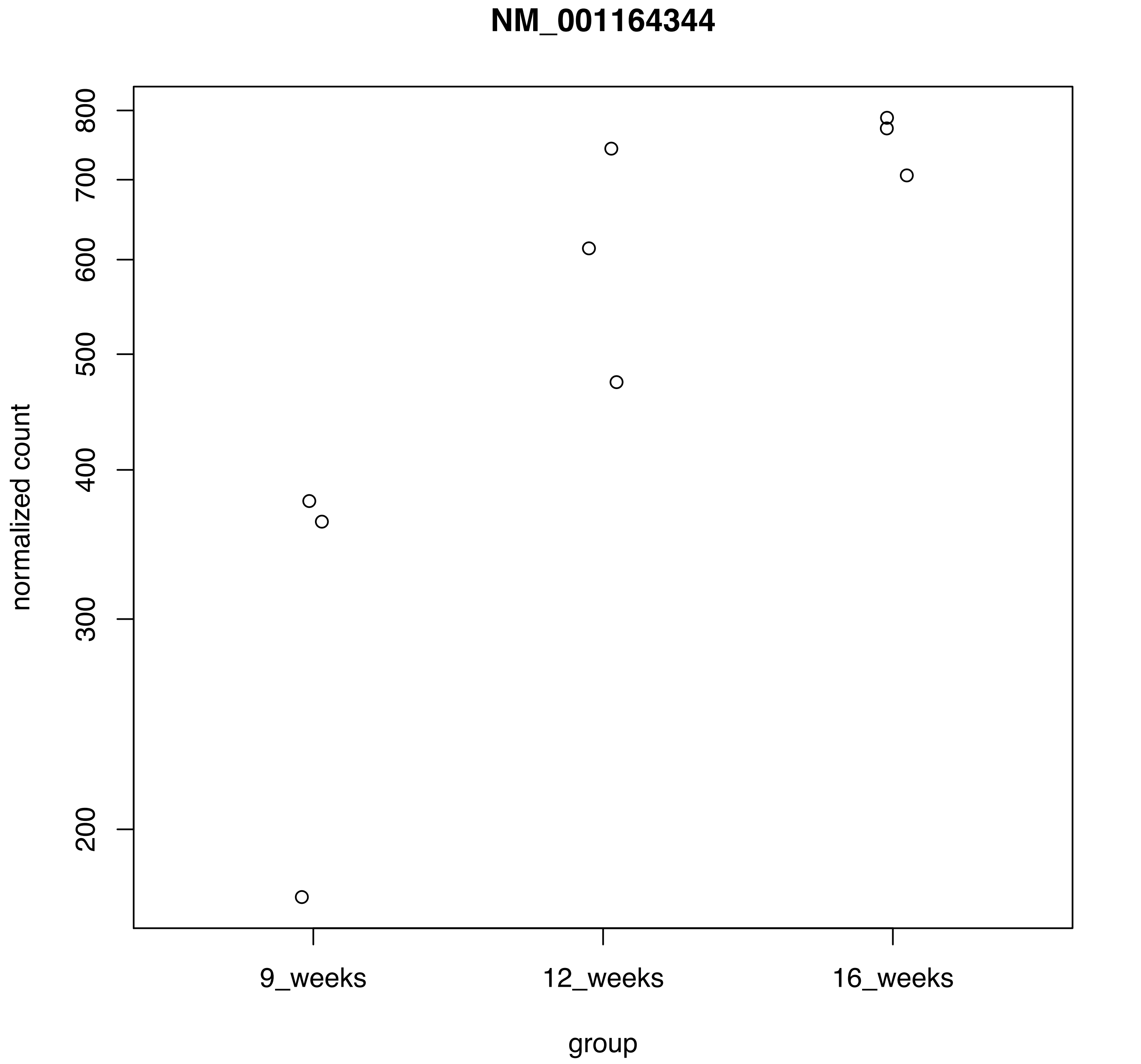

NM_001164344
normalized count
200
300
400
500
600
700
800
9_weeks
12_weeks
16_weeks
group

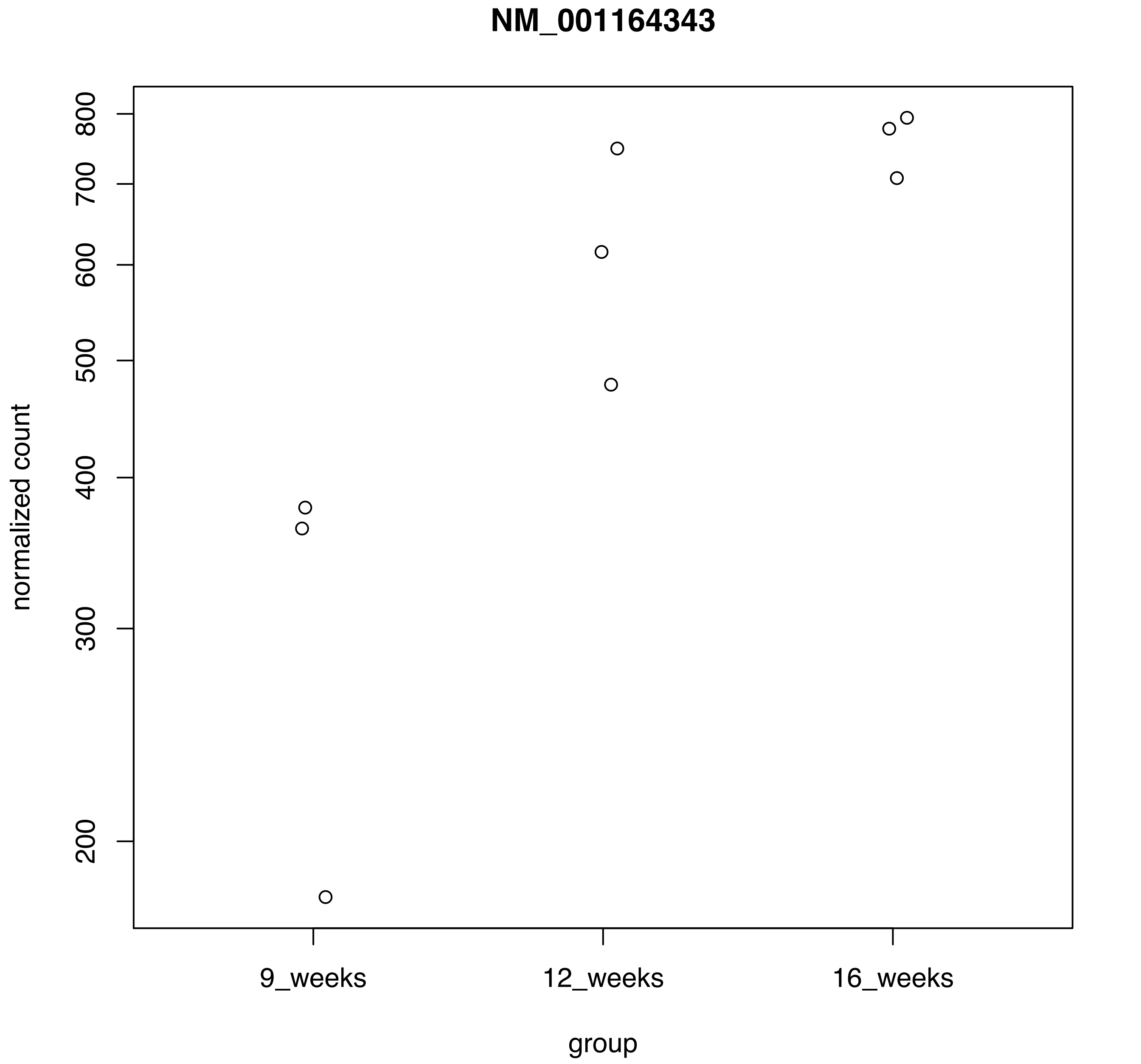
NM_001164343
normalized count
800
700
600
500
400
300
200
9_weeks
12_weeks
16_weeks
group

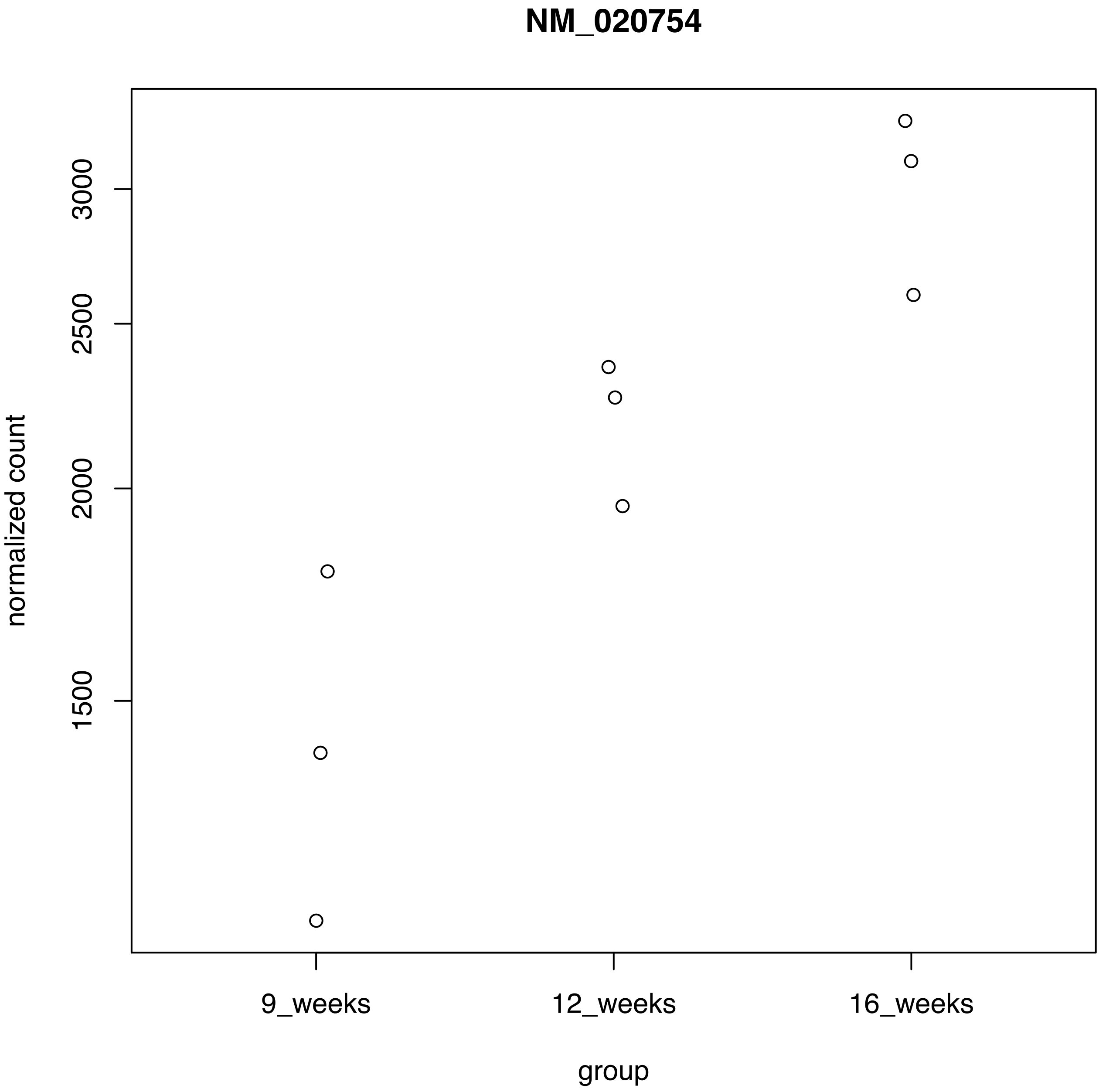
NM_020754
normalized count
3000
2500
2000
1500
9_weeks
12_weeks
16_weeks
group

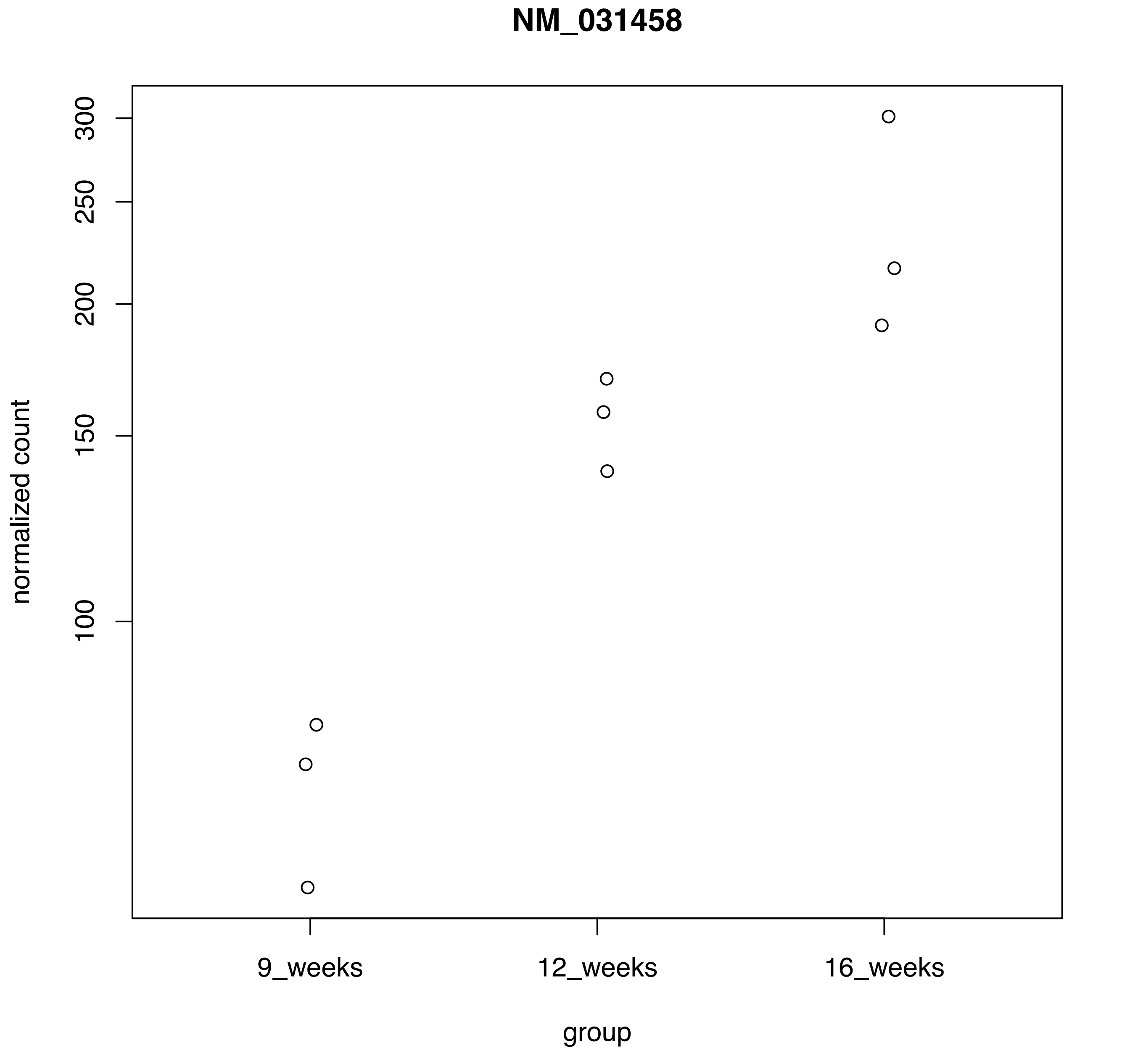

NM_031458
normalized count
300
250
200
150
100
9_weeks
12_weeks
16_weeks
group

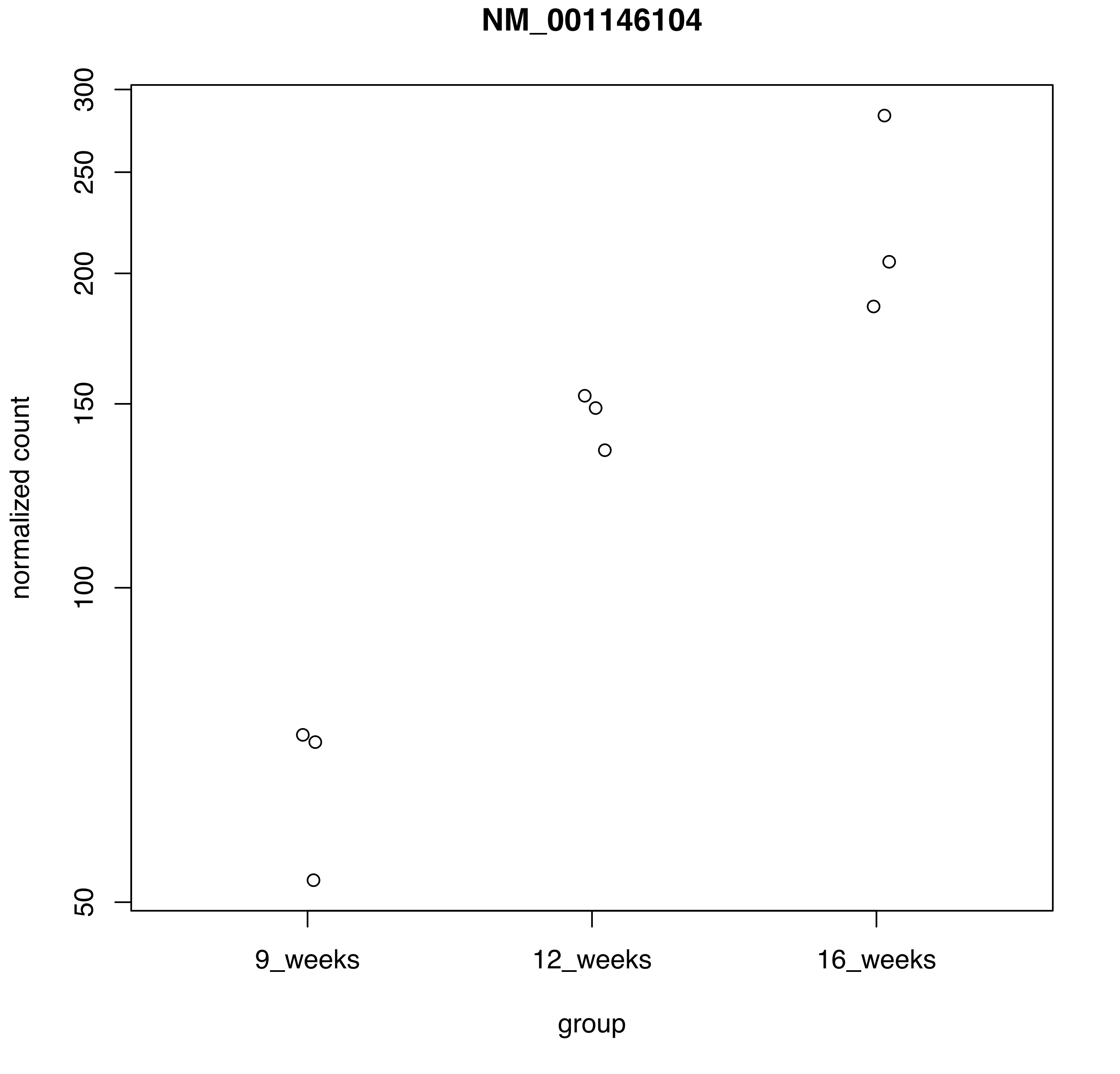

NM_001146104
normalized count
300
250
200
150
100
50
9_weeks
12_weeks
16_weeks
group

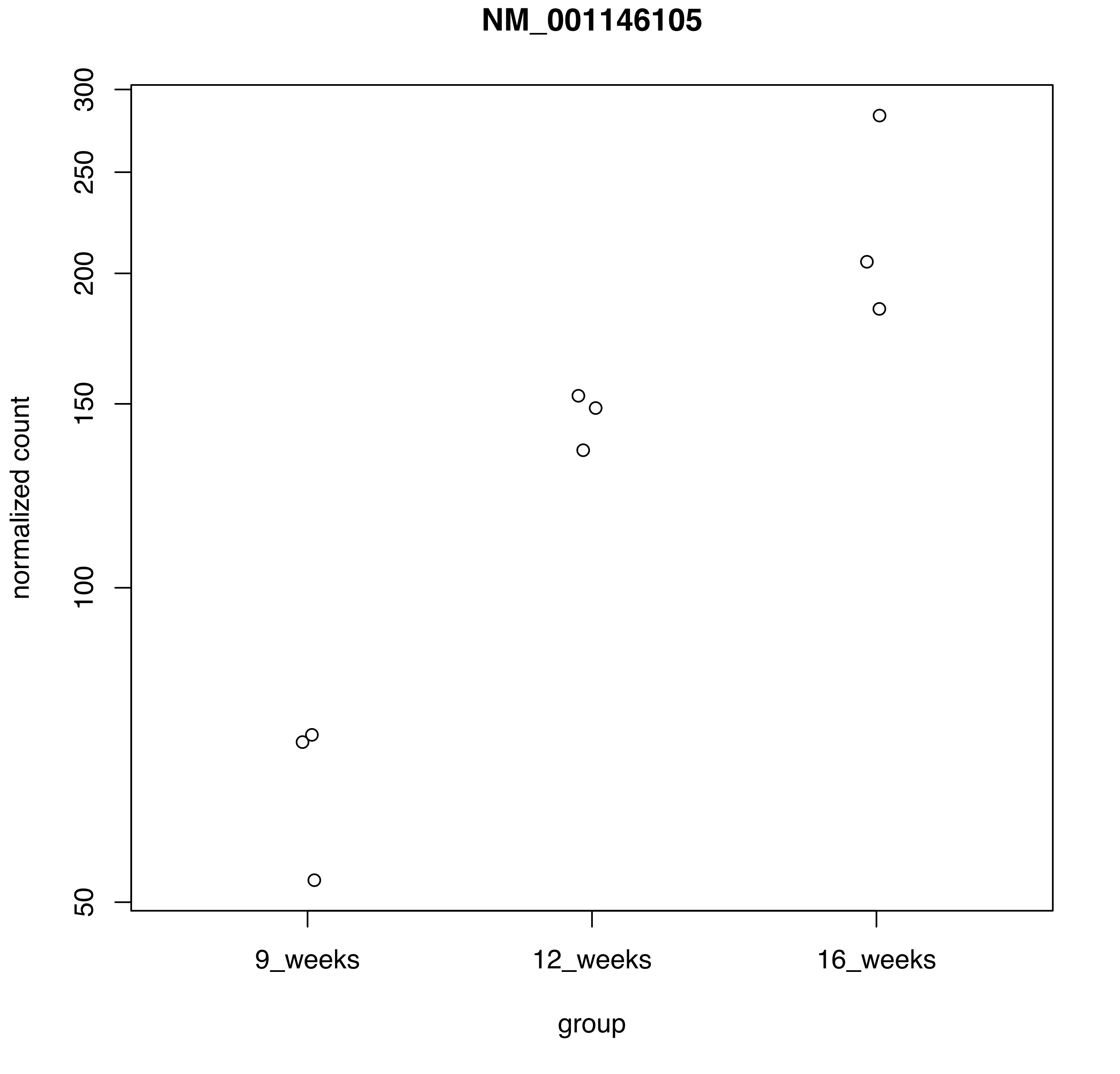
NM_001146105
normalized count
300
250
200
150
100
50
9_weeks
12_weeks
16_weeks
group

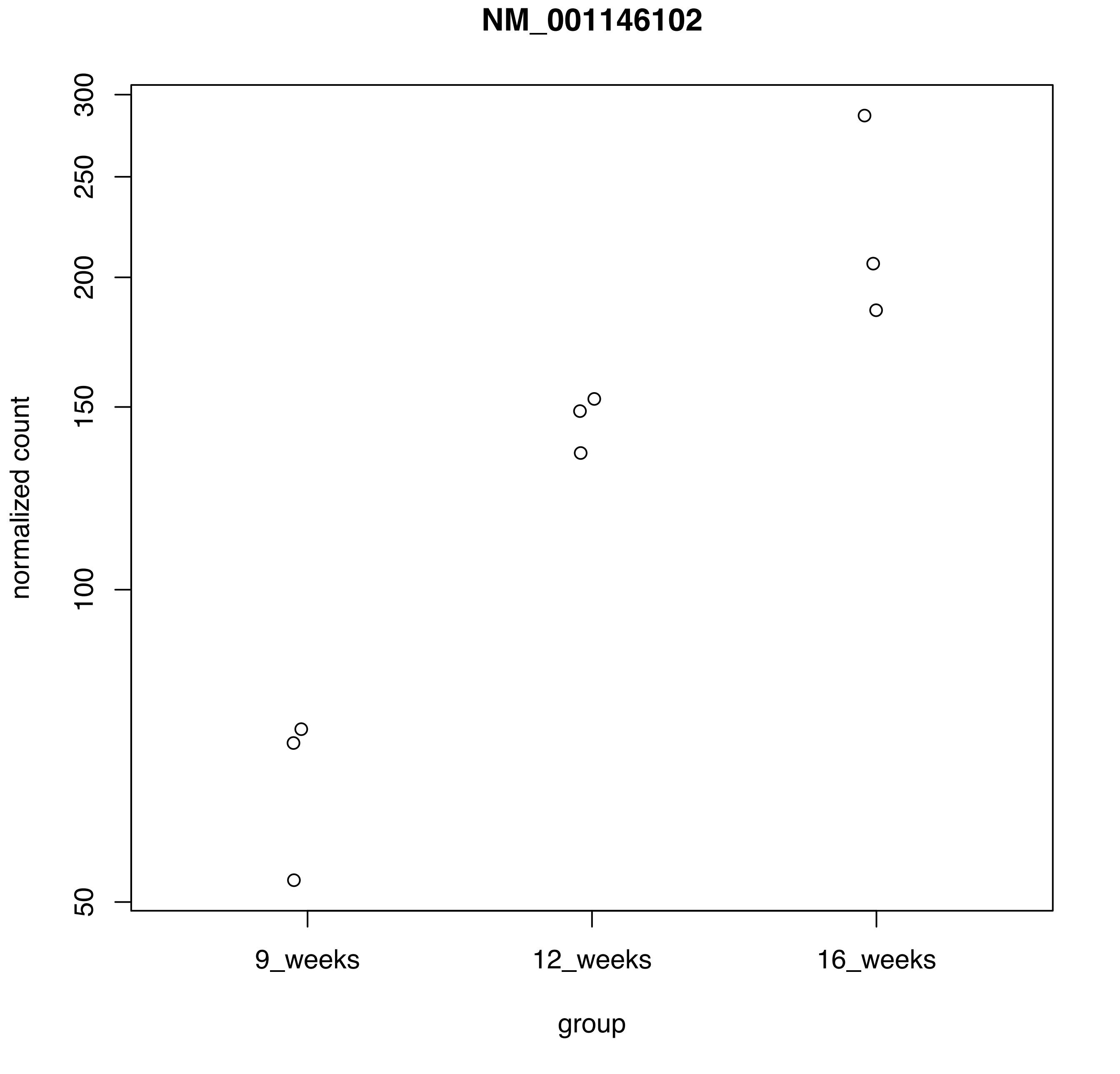
NM_001146102
normalized count
300
250
200
150
100
50
9_weeks
12_weeks
16_weeks
group

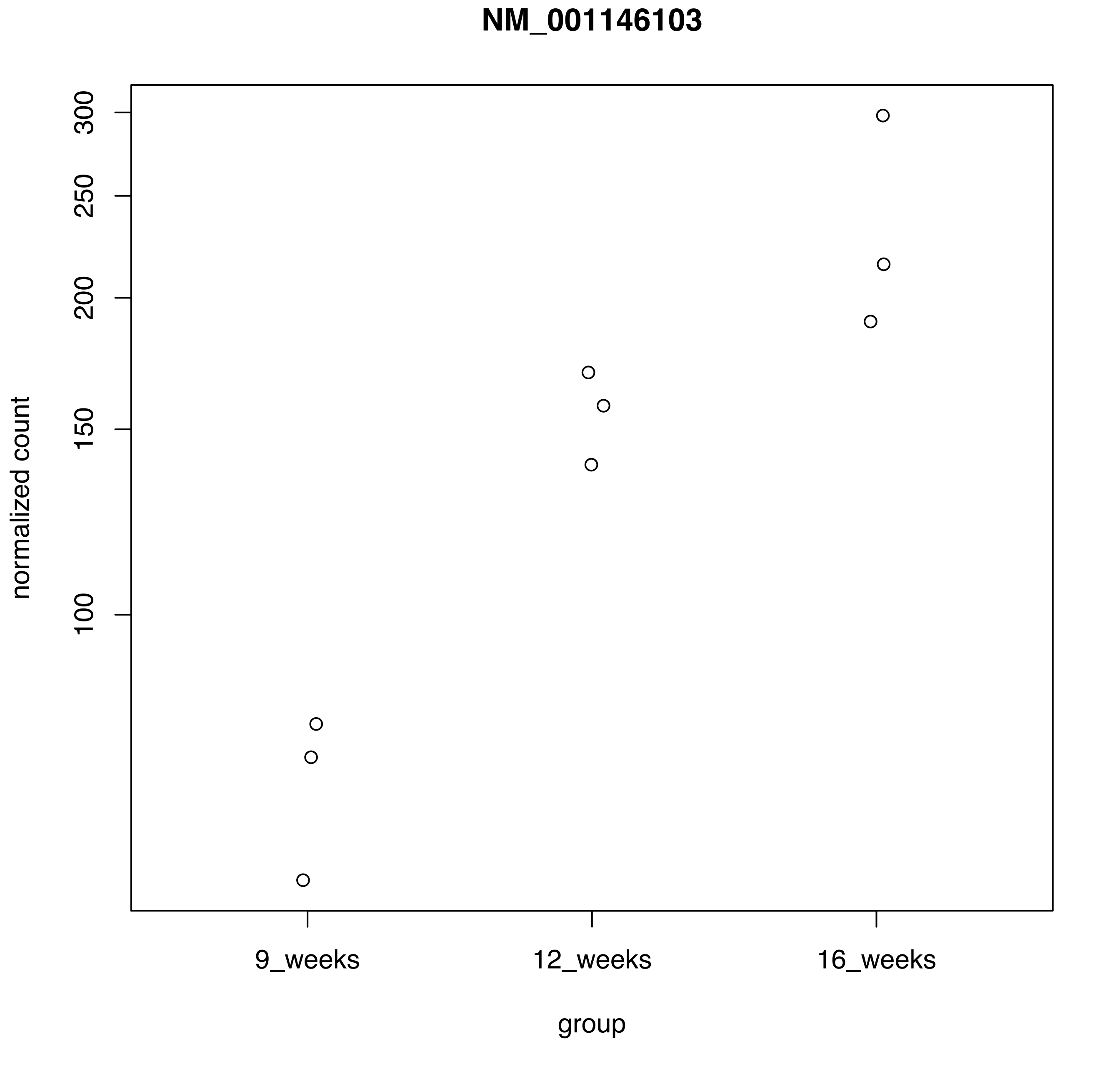
NM_001146103
normalized count
300
250
200
150
100
9_weeks
12_weeks
16_weeks
group

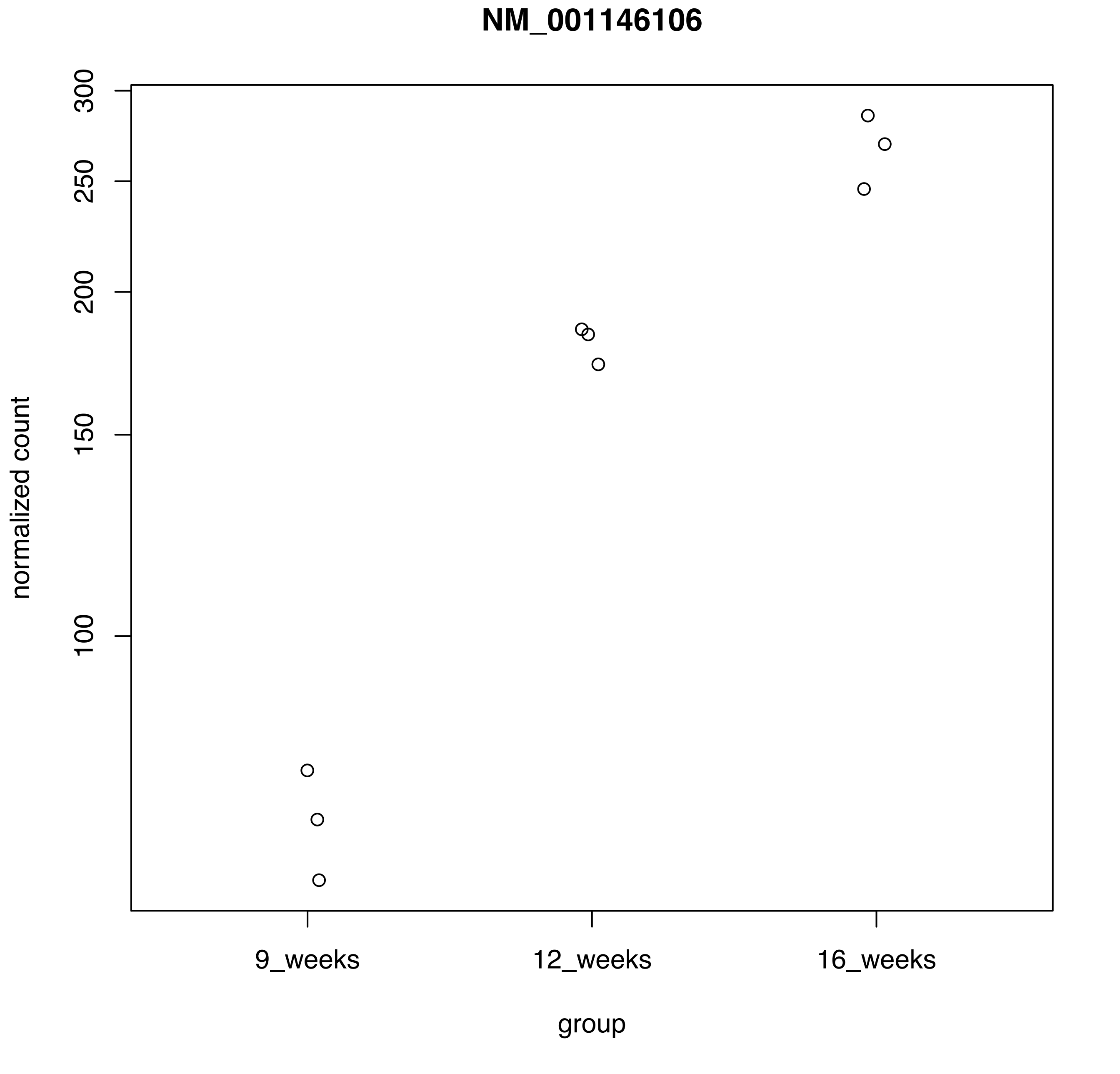
NM_001146106
normalized count
300
250
200
150
100
9_weeks
12_weeks
16_weeks
group

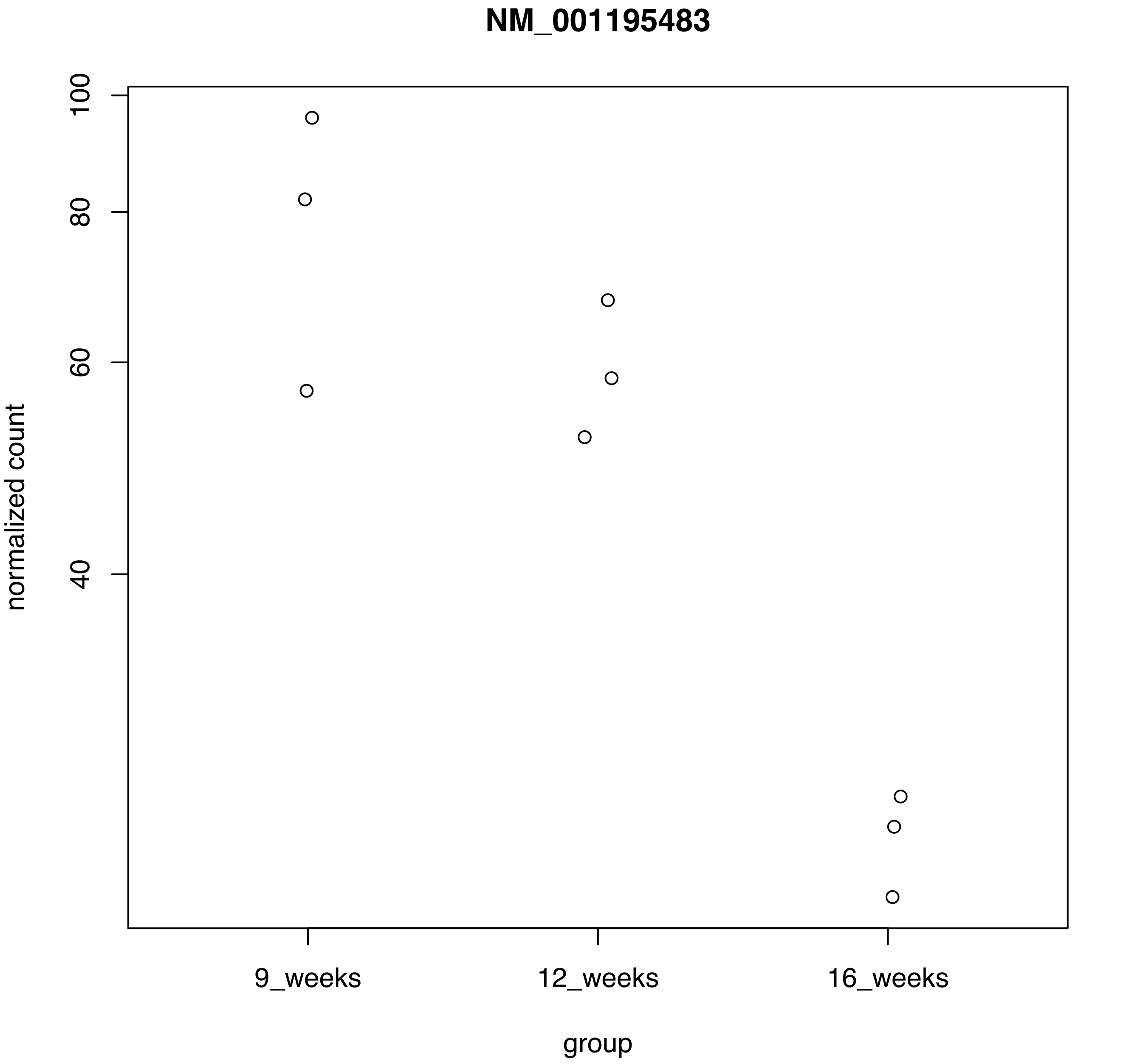
NM_001195483
normalized count
100
80
60
40
9_weeks
12_weeks
16_weeks
group

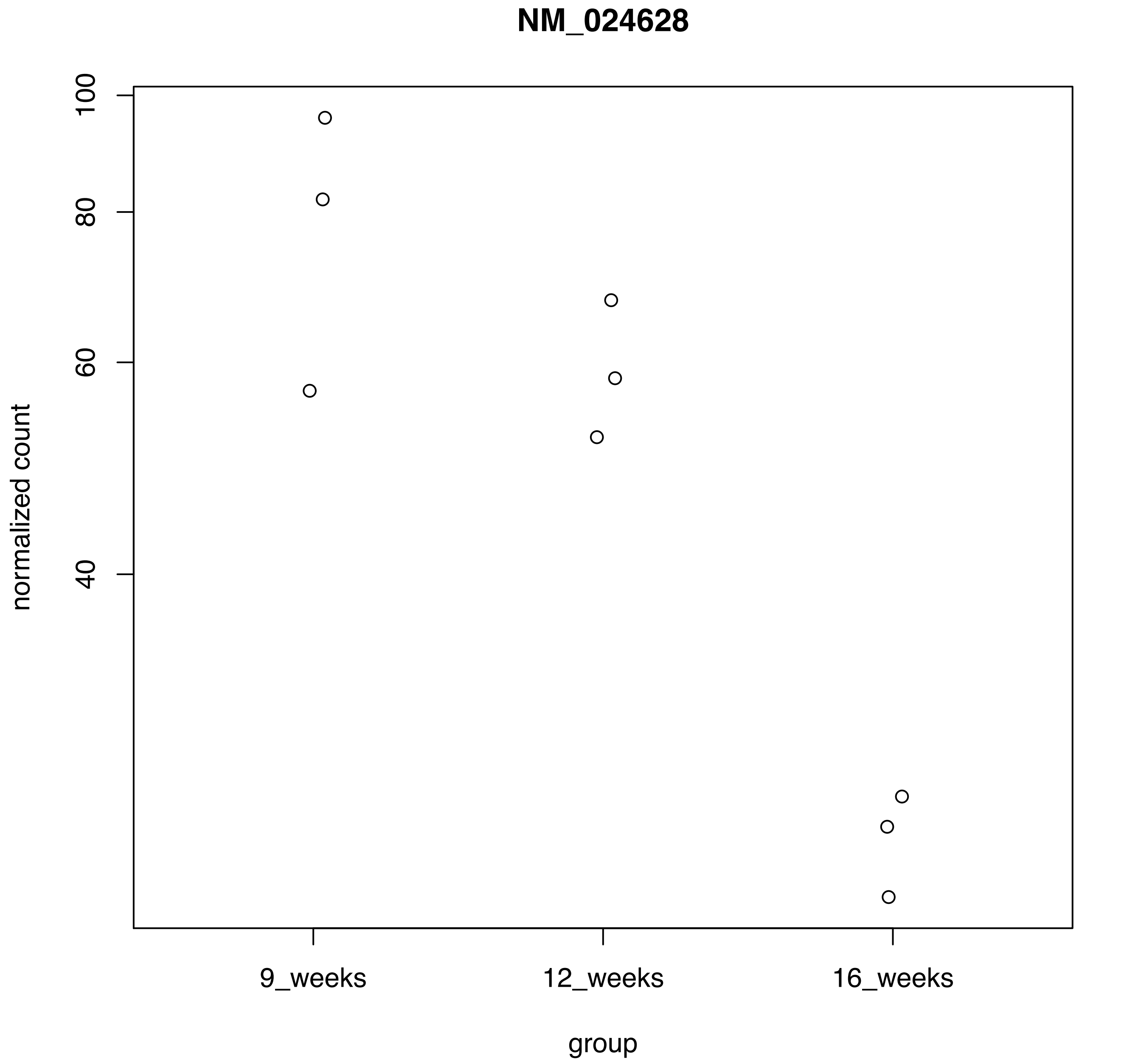

NM_024628
normalized count
100
80
60
40
9_weeks
12_weeks
16_weeks
group

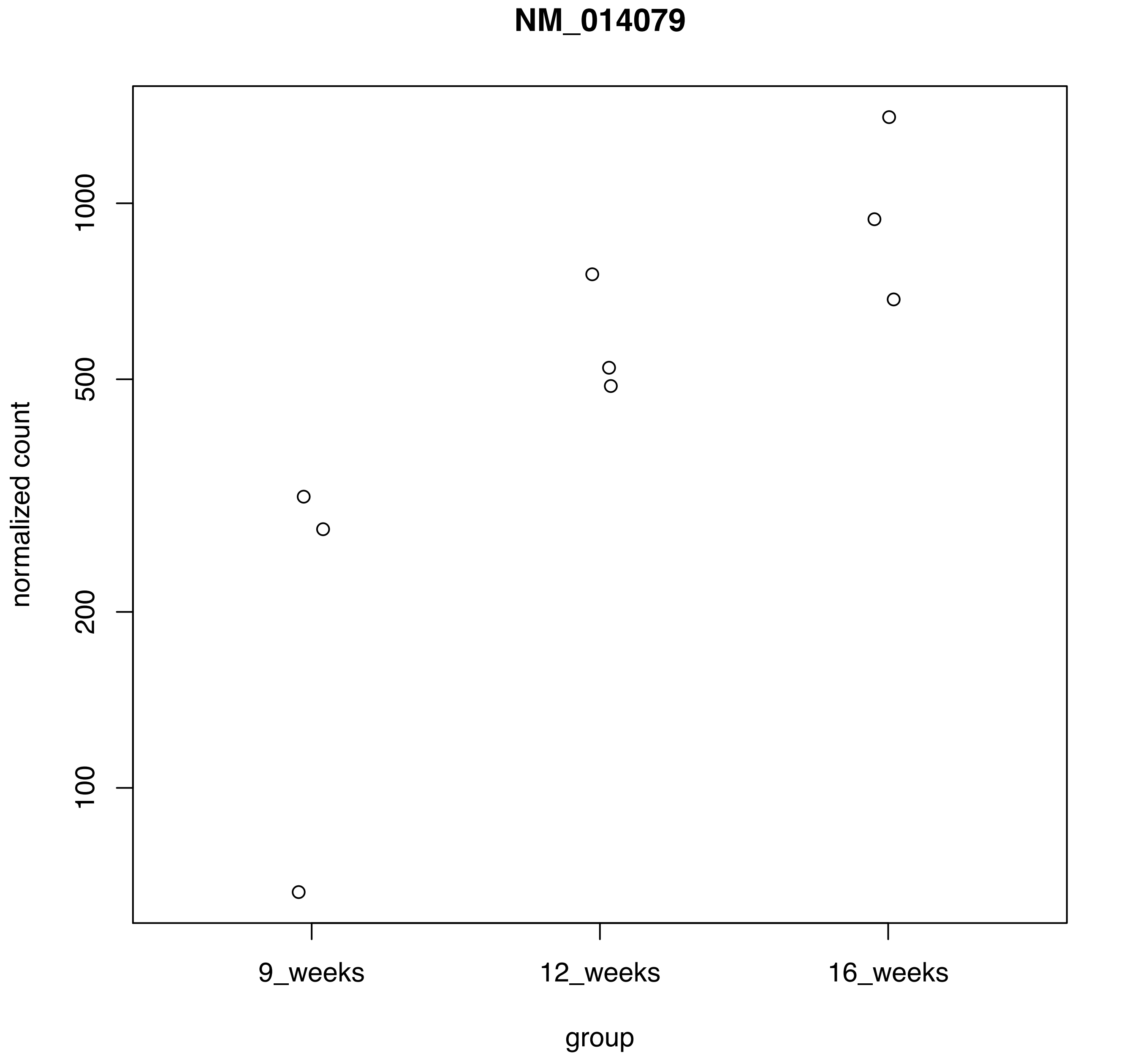

NM_014079
normalized count
1000
500
200
100
9_weeks
12_weeks
16_weeks
group

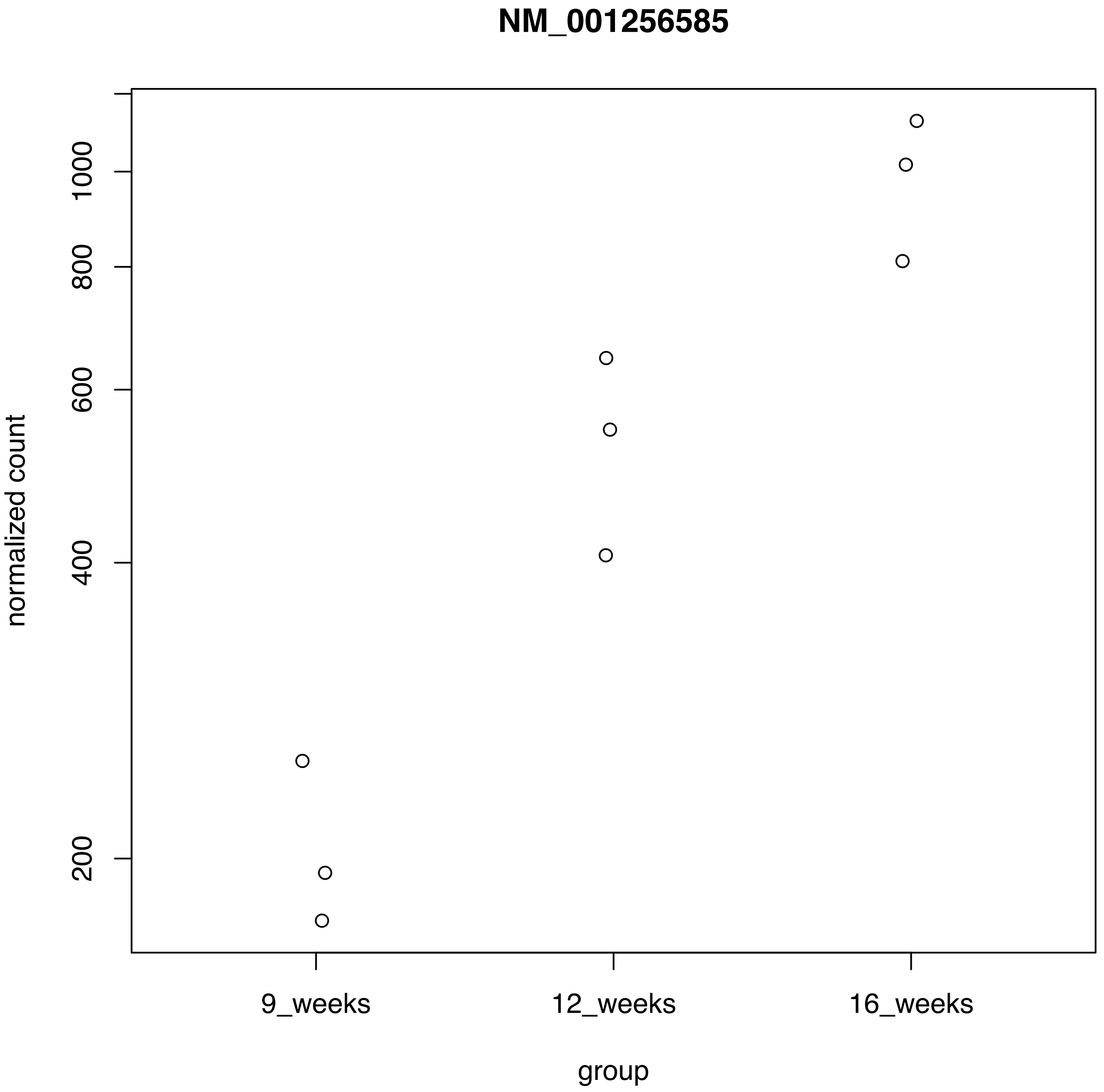
NM_001256585
normalized count
200
400
600
800
1000
9_weeks
12_weeks
16_weeks
group

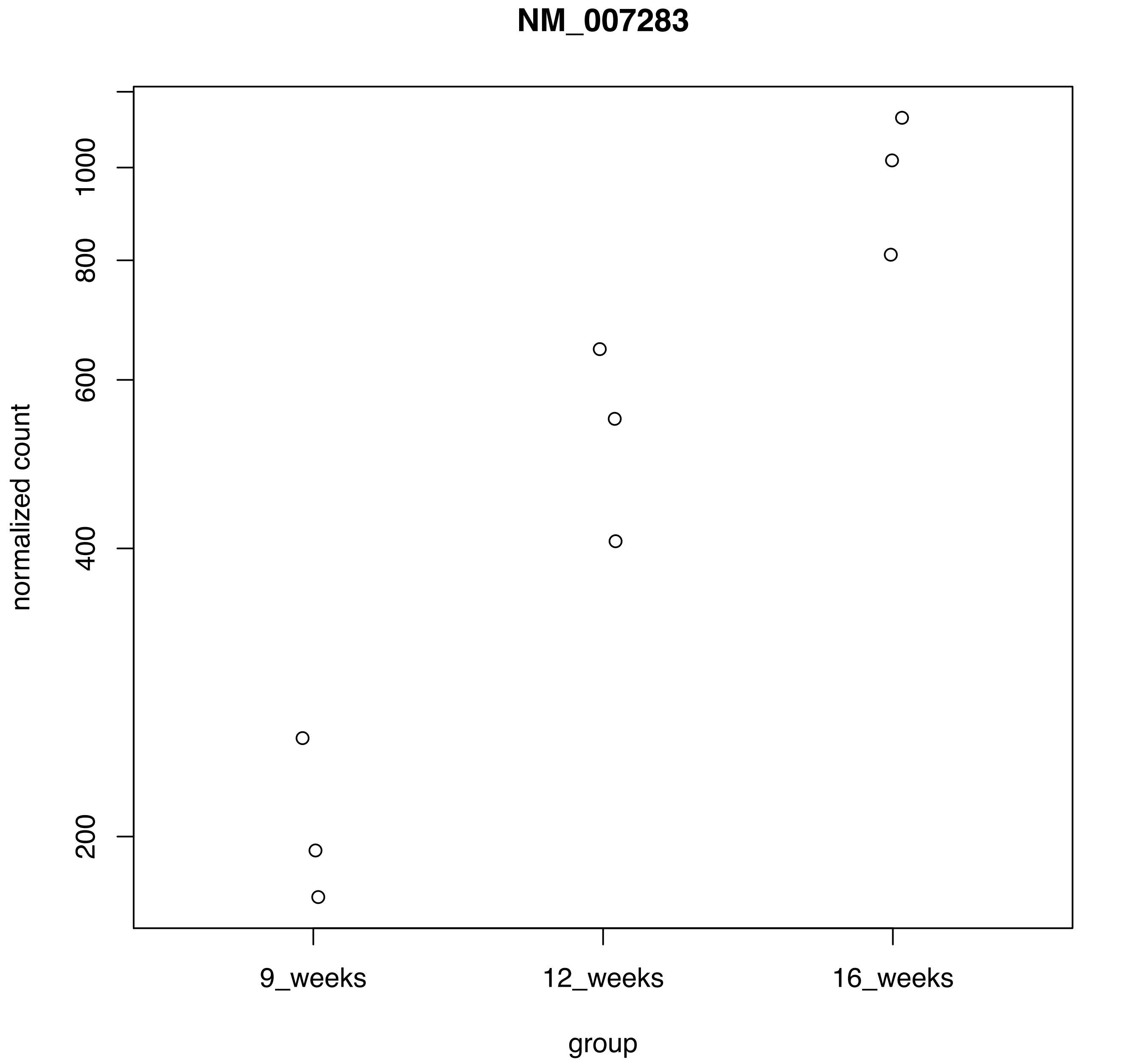

NM_007283
normalized count
200
400
600
800
1000
9_weeks
12_weeks
16_weeks
group

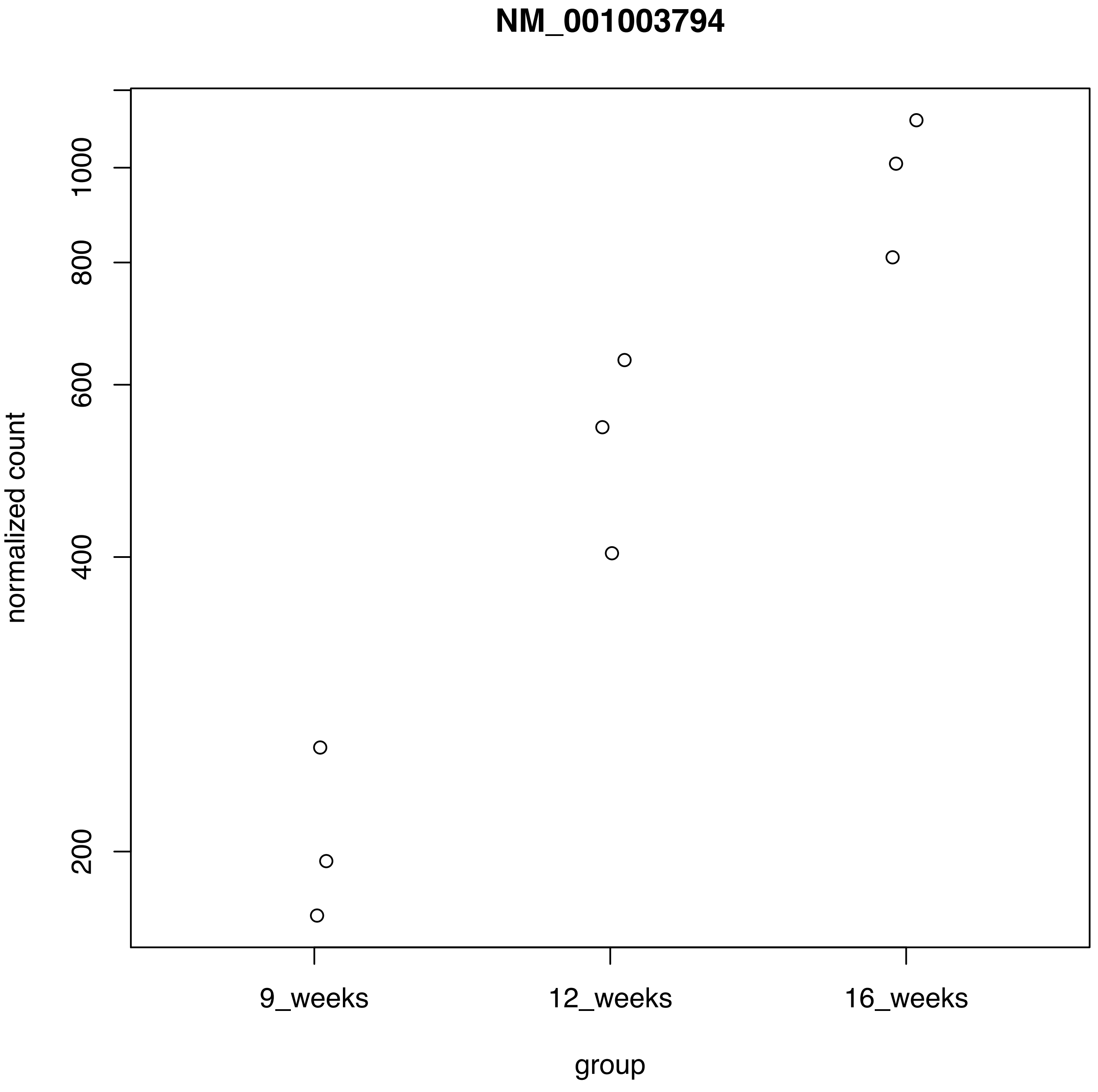
NM_001003794
normalized count
200
400
600
800
1000
9_weeks
12_weeks
16_weeks
group

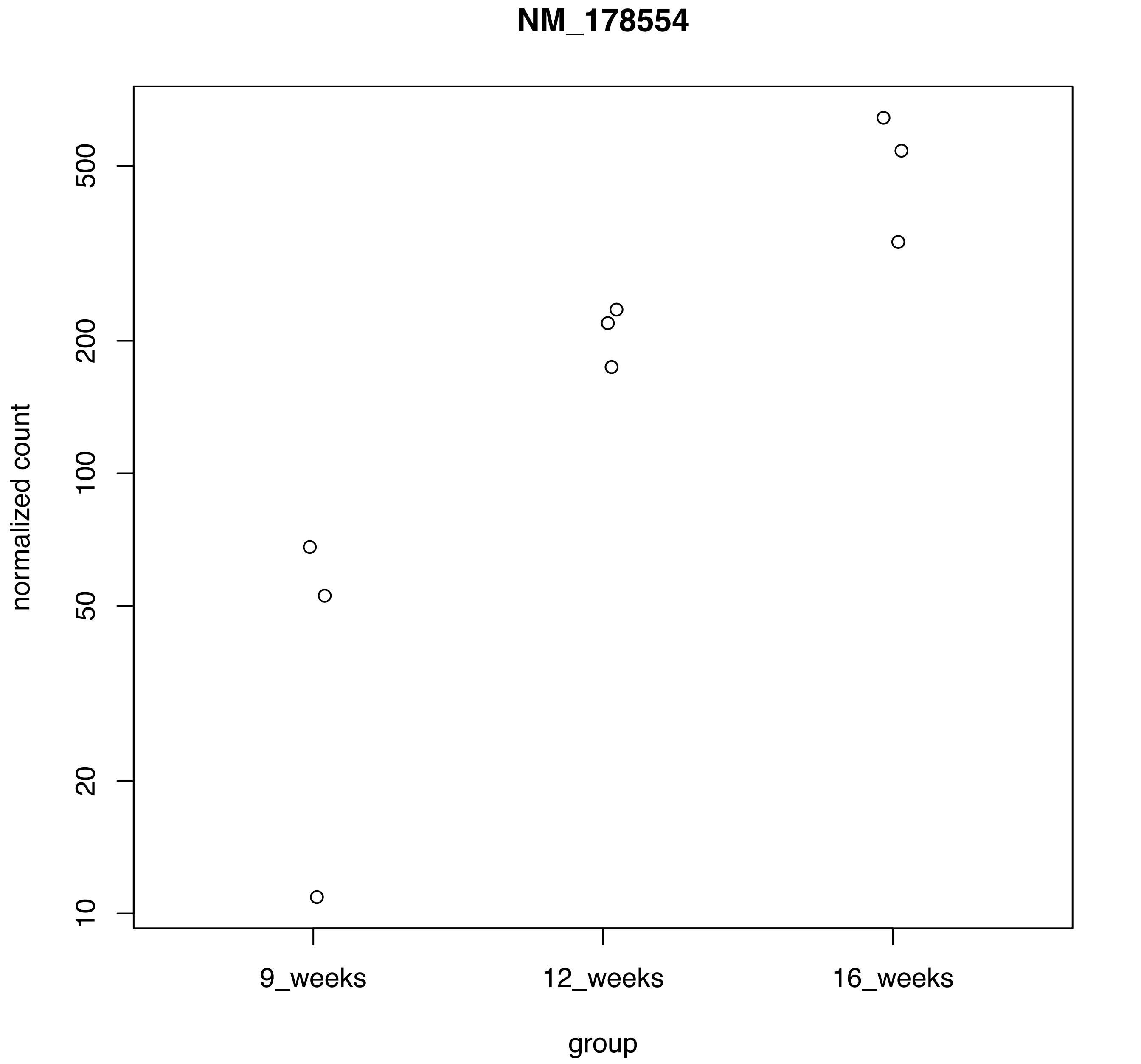

NM_178554
normalized count
500
200
100
50
20
10
9_weeks
12_weeks
16_weeks
group

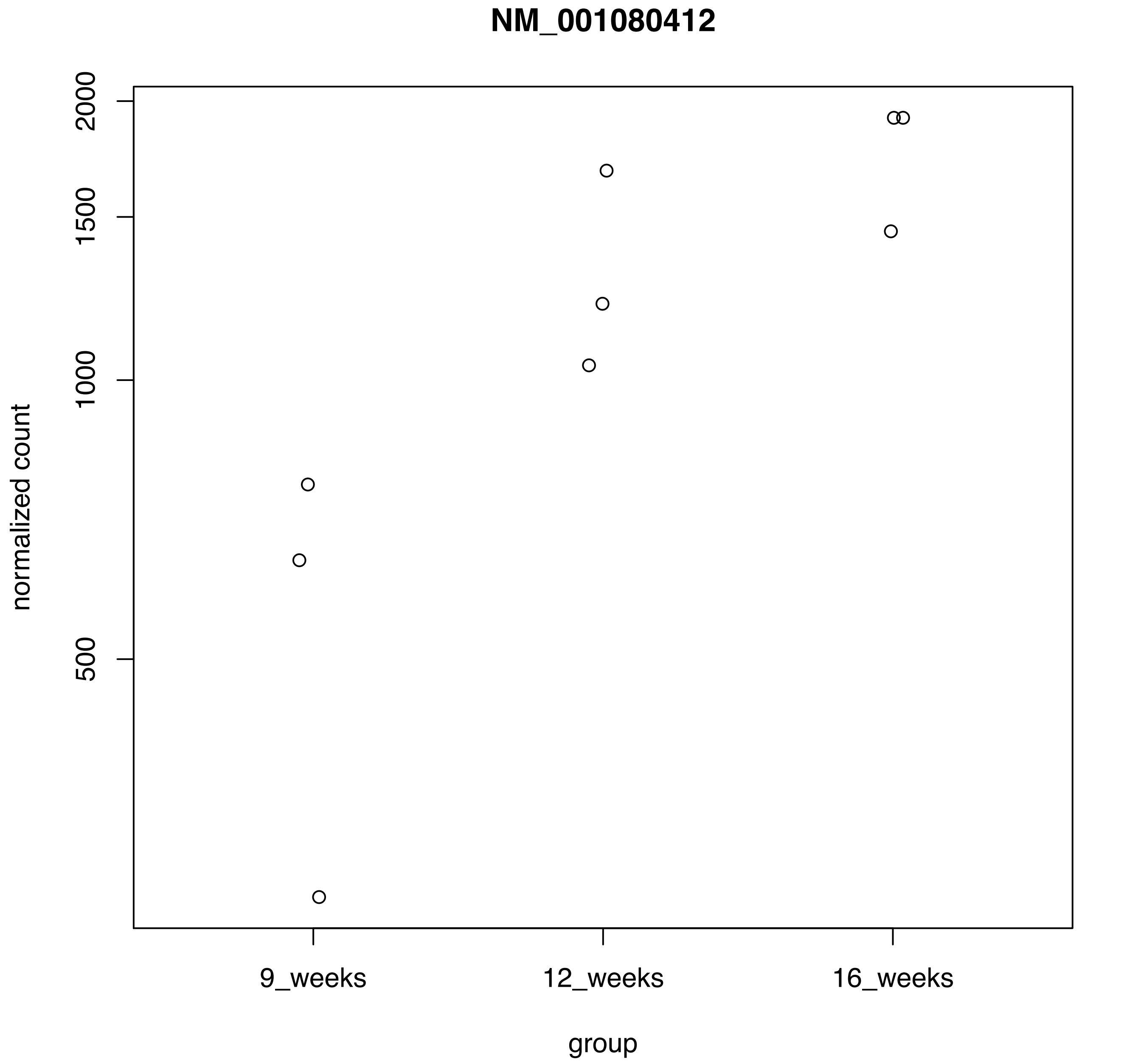
NM_001080412
normalized count
500
1000
1500
2000
9_weeks
12_weeks
16_weeks
group

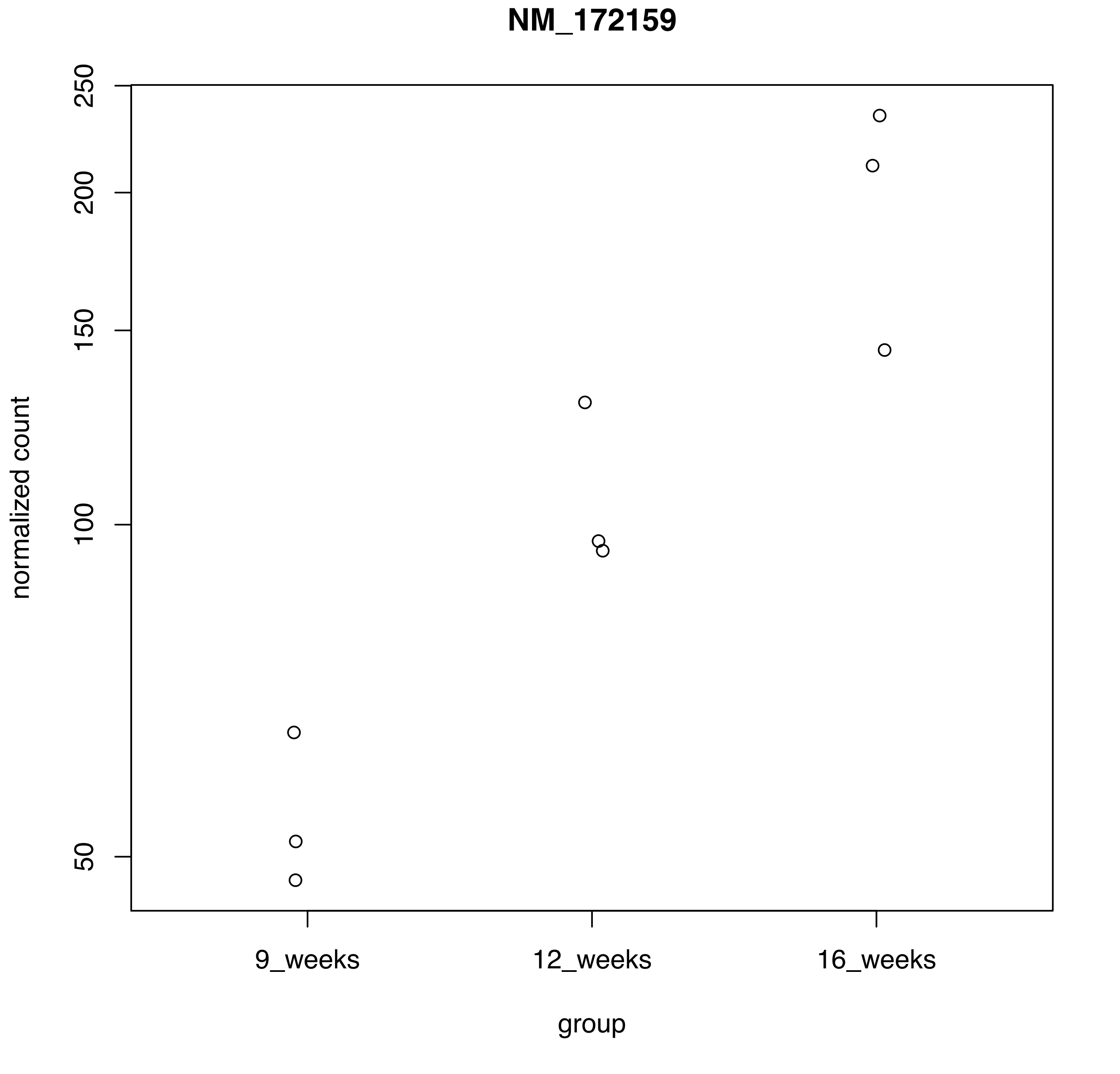
NM_172159
normalized count
250
200
150
100
50
9_weeks
12_weeks
16_weeks
group

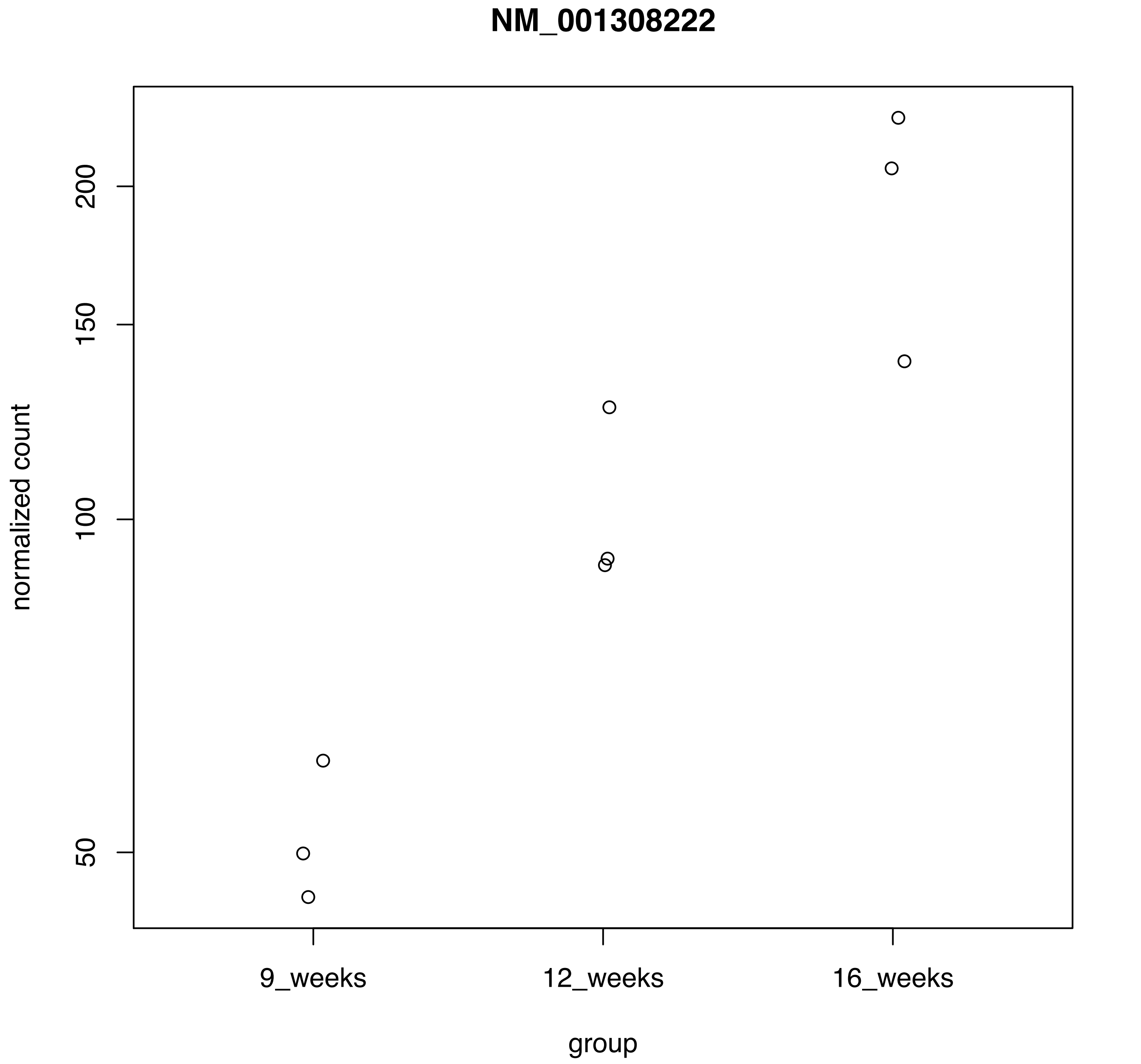

NM_001308222
normalized count
50
100
150
200
9_weeks
12_weeks
16_weeks
group

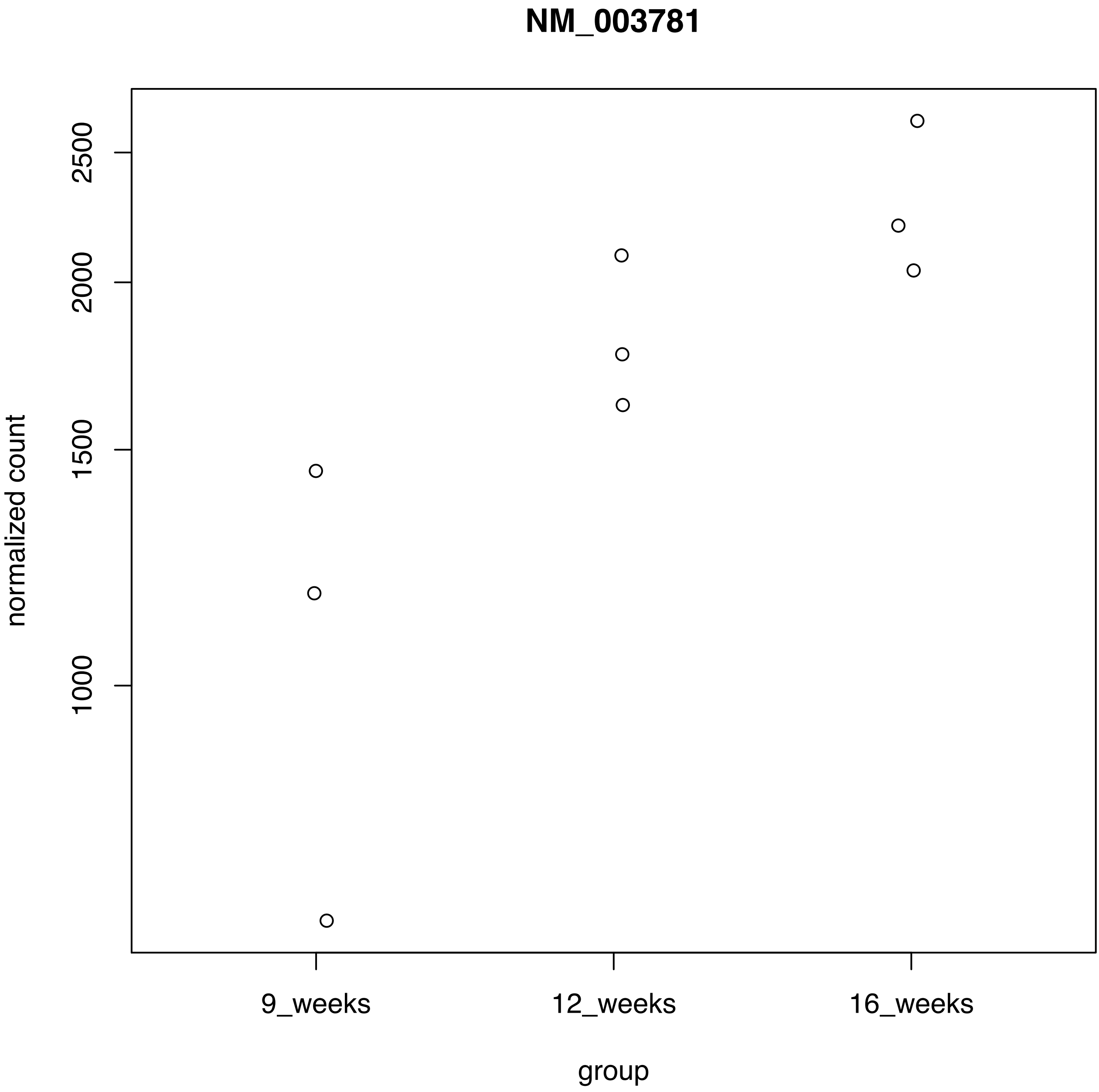
NM_003781
normalized count
2500
2000
1500
1000
9_weeks
12_weeks
16_weeks
group

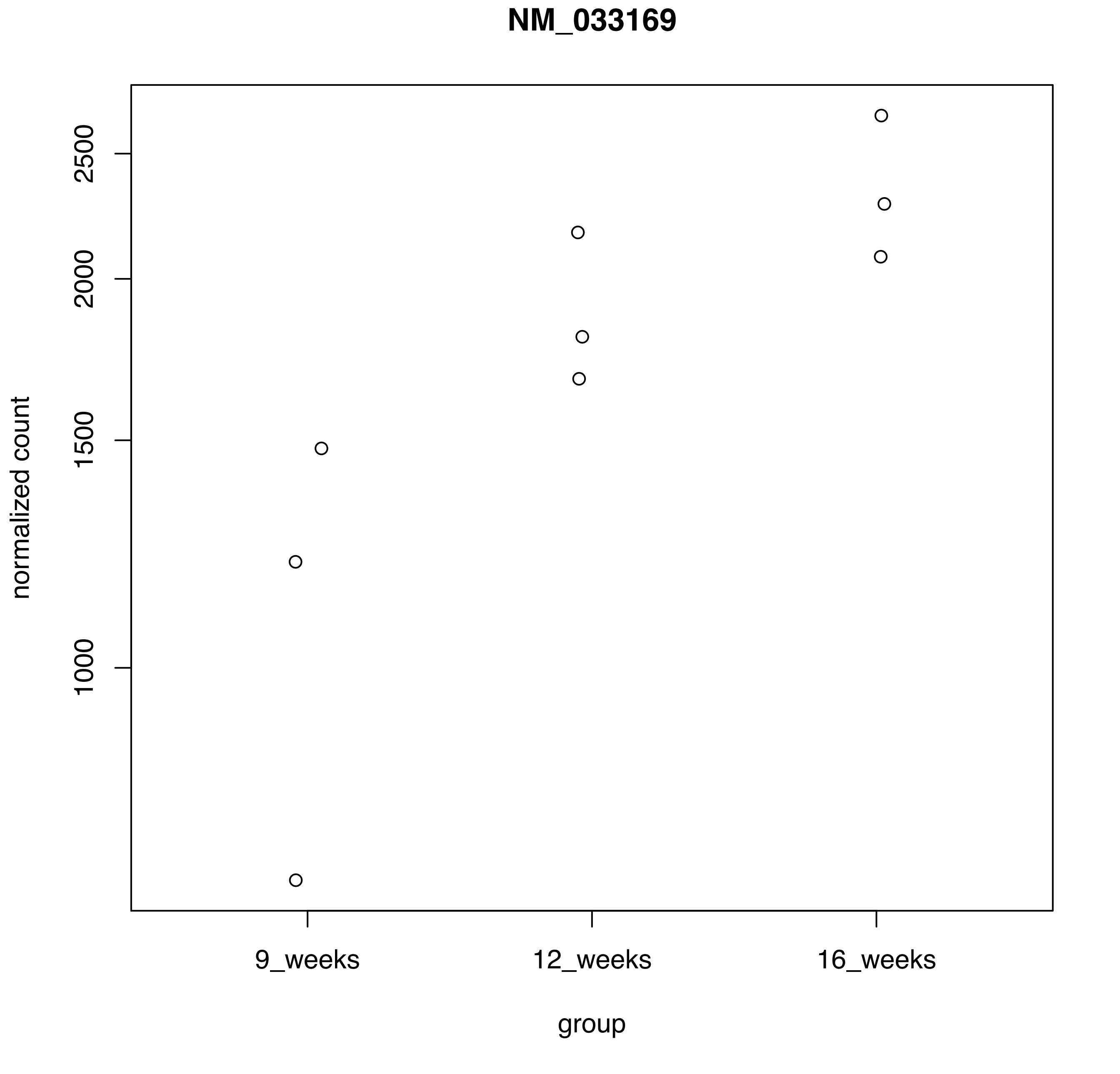
NM_033169
normalized count
2500
2000
1500
1000
9_weeks
12_weeks
16_weeks
group

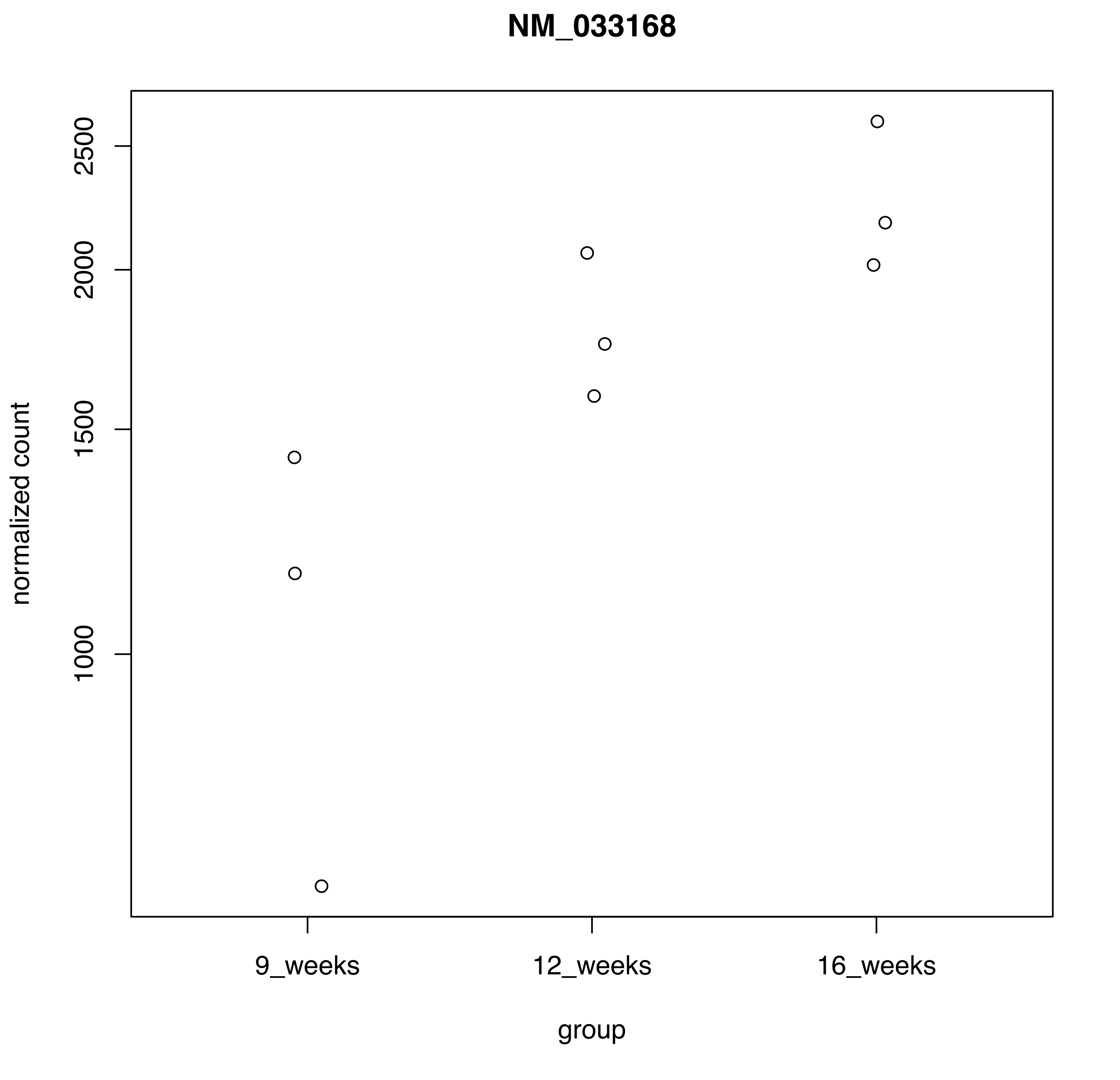

NM_033168
normalized count
2500
2000
1500
1000
9_weeks
12_weeks
16_weeks
group

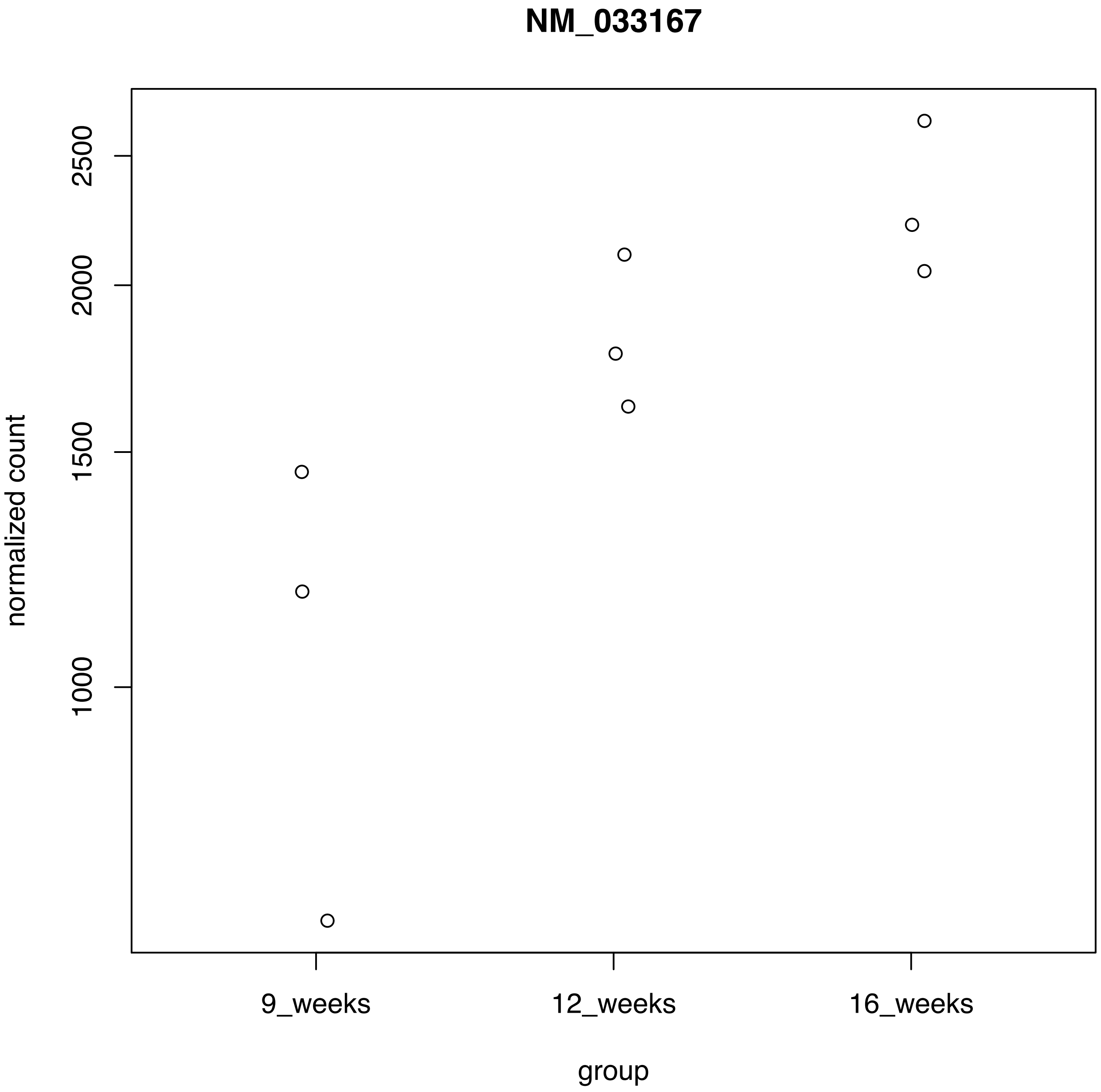
NM_033167
normalized count
2500
2000
1500
1000
9_weeks
12_weeks
16_weeks
group

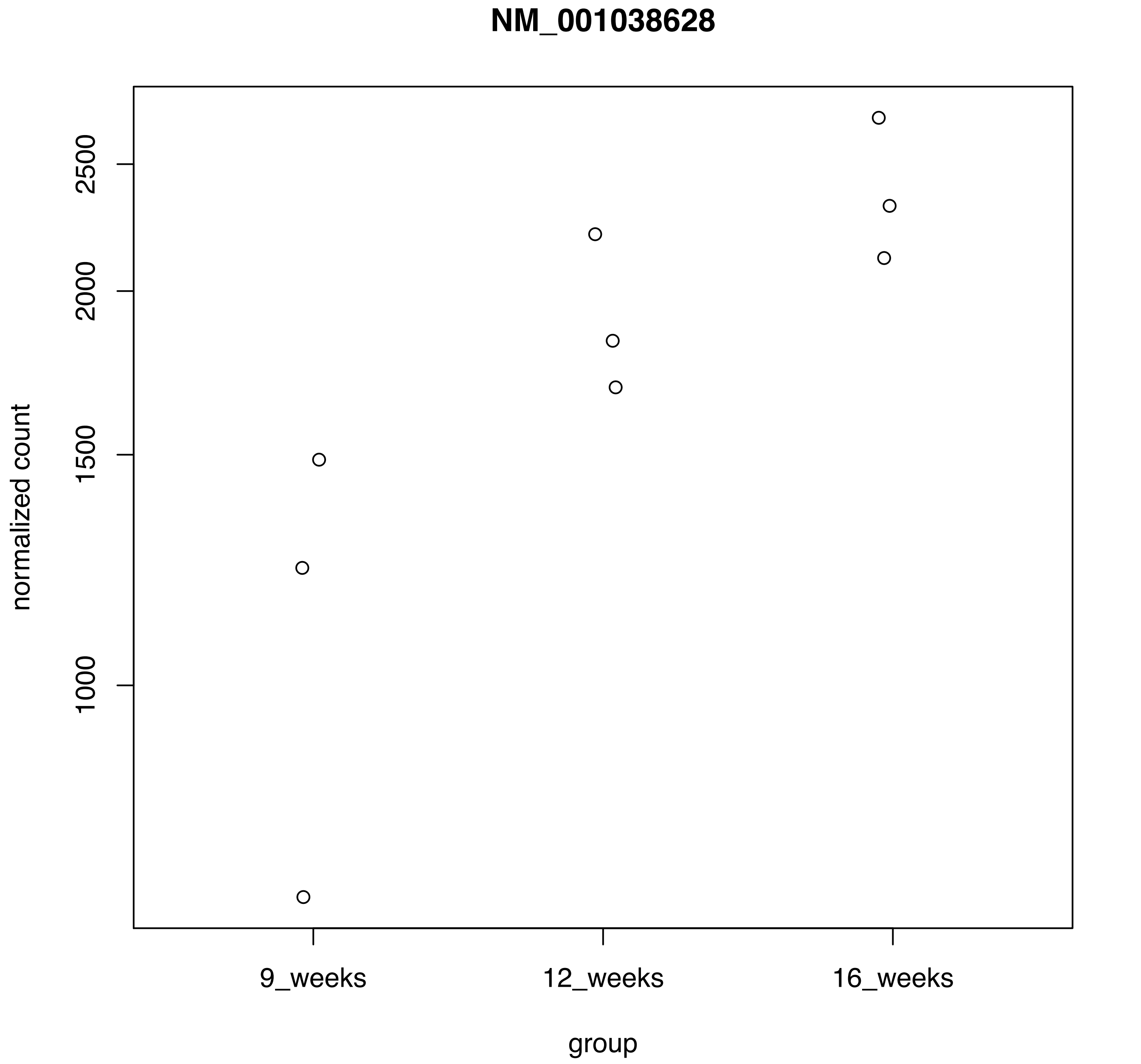

NM_001038628
normalized count
2500
2000
1500
1000
9_weeks
12_weeks
16_weeks
group

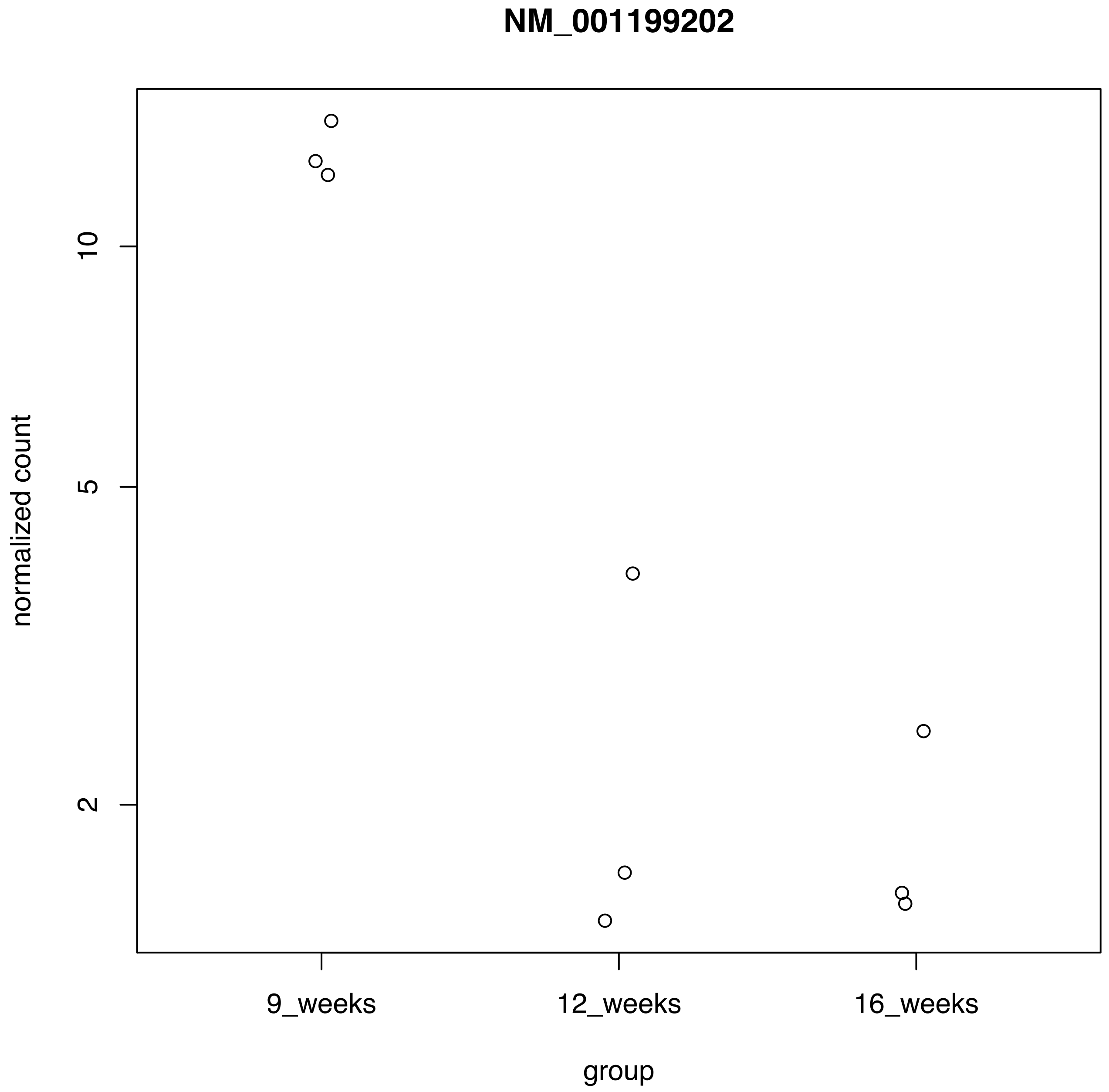
NM_001199202
normalized count
10
5
2
9_weeks
12_weeks
16_weeks
group

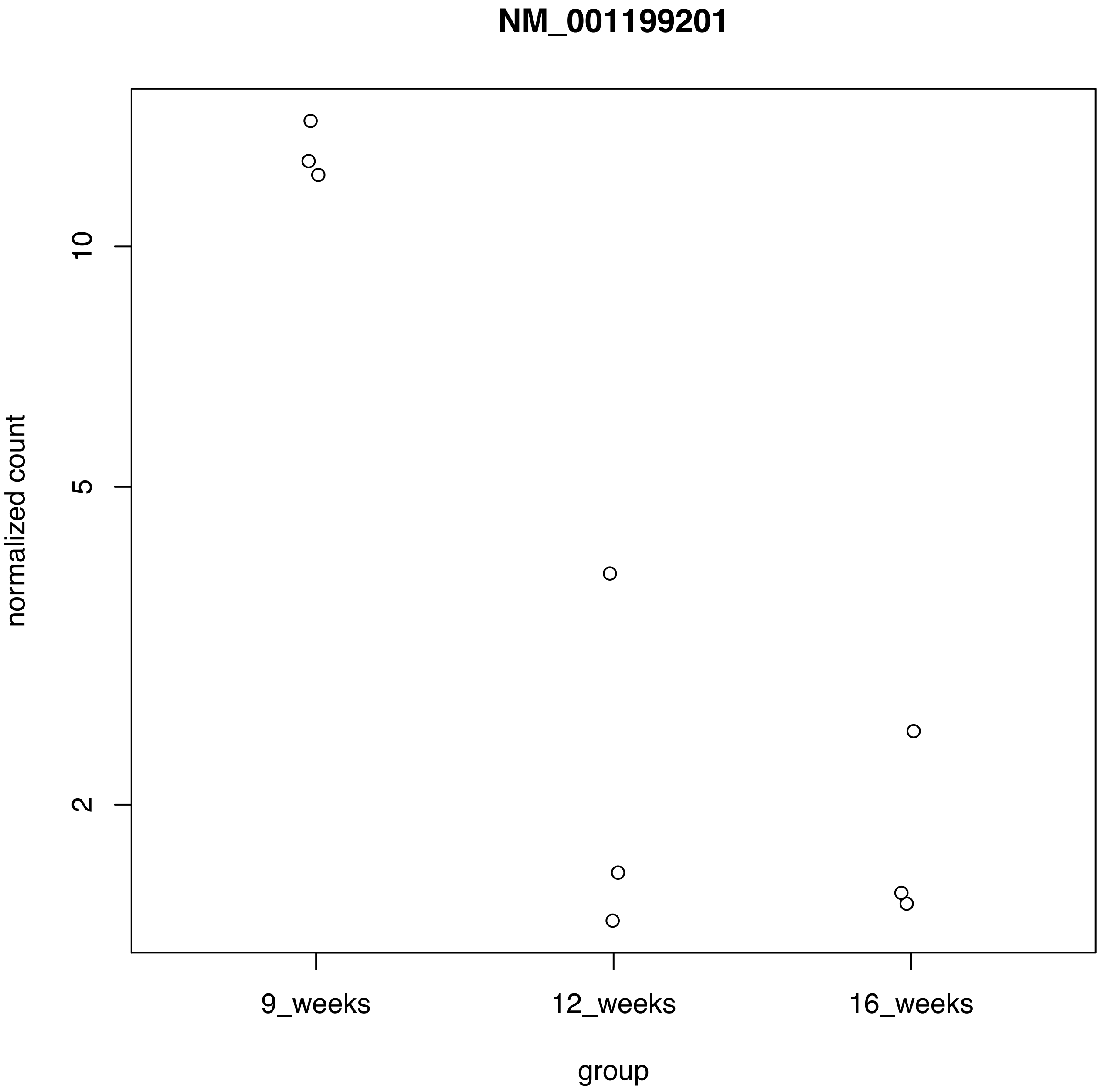

NM_001199201
normalized count
10
5
2
9_weeks
12_weeks
16_weeks
group

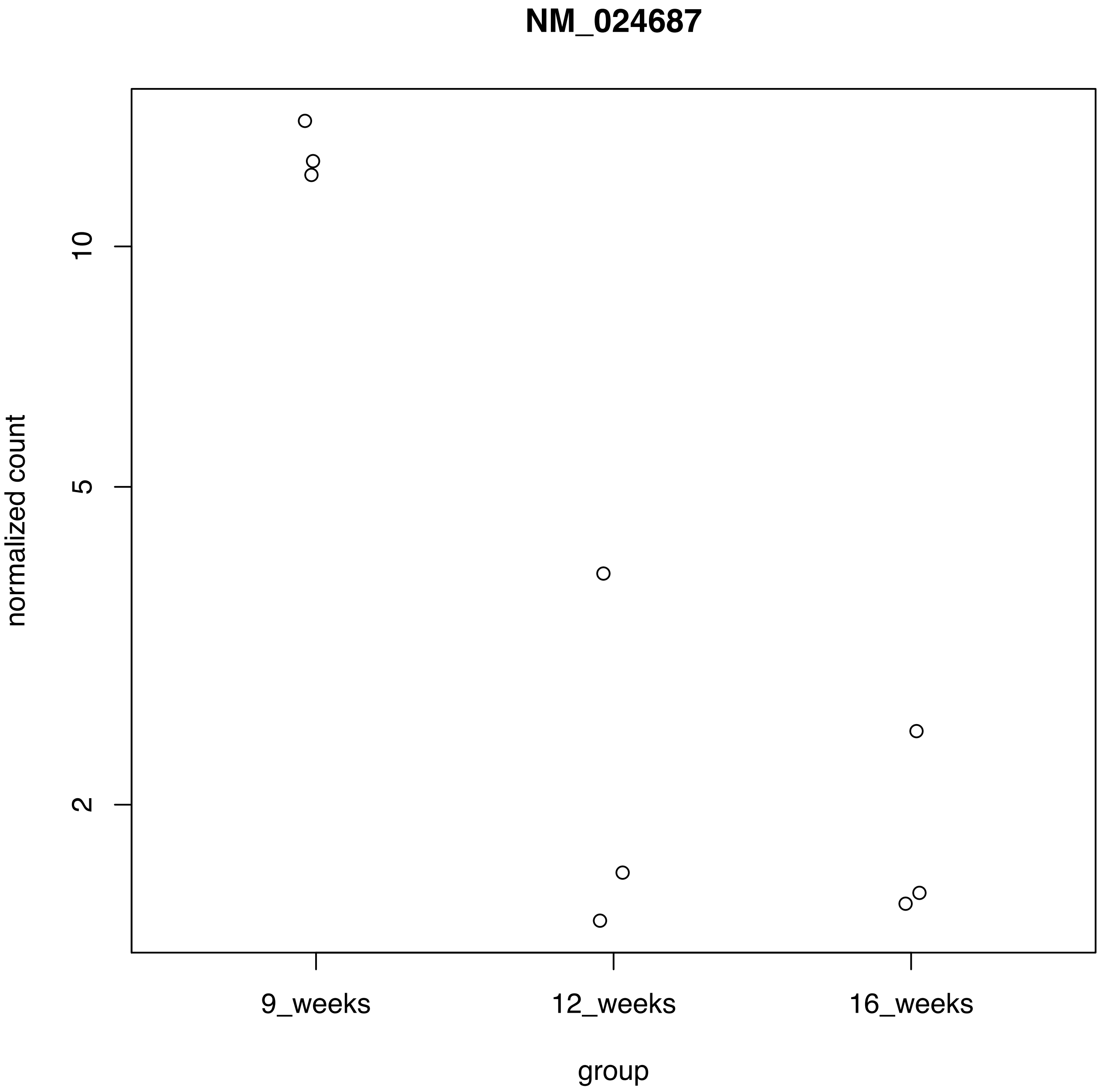

NM_024687
normalized count
10
5
2
9_weeks
12_weeks
16_weeks
group

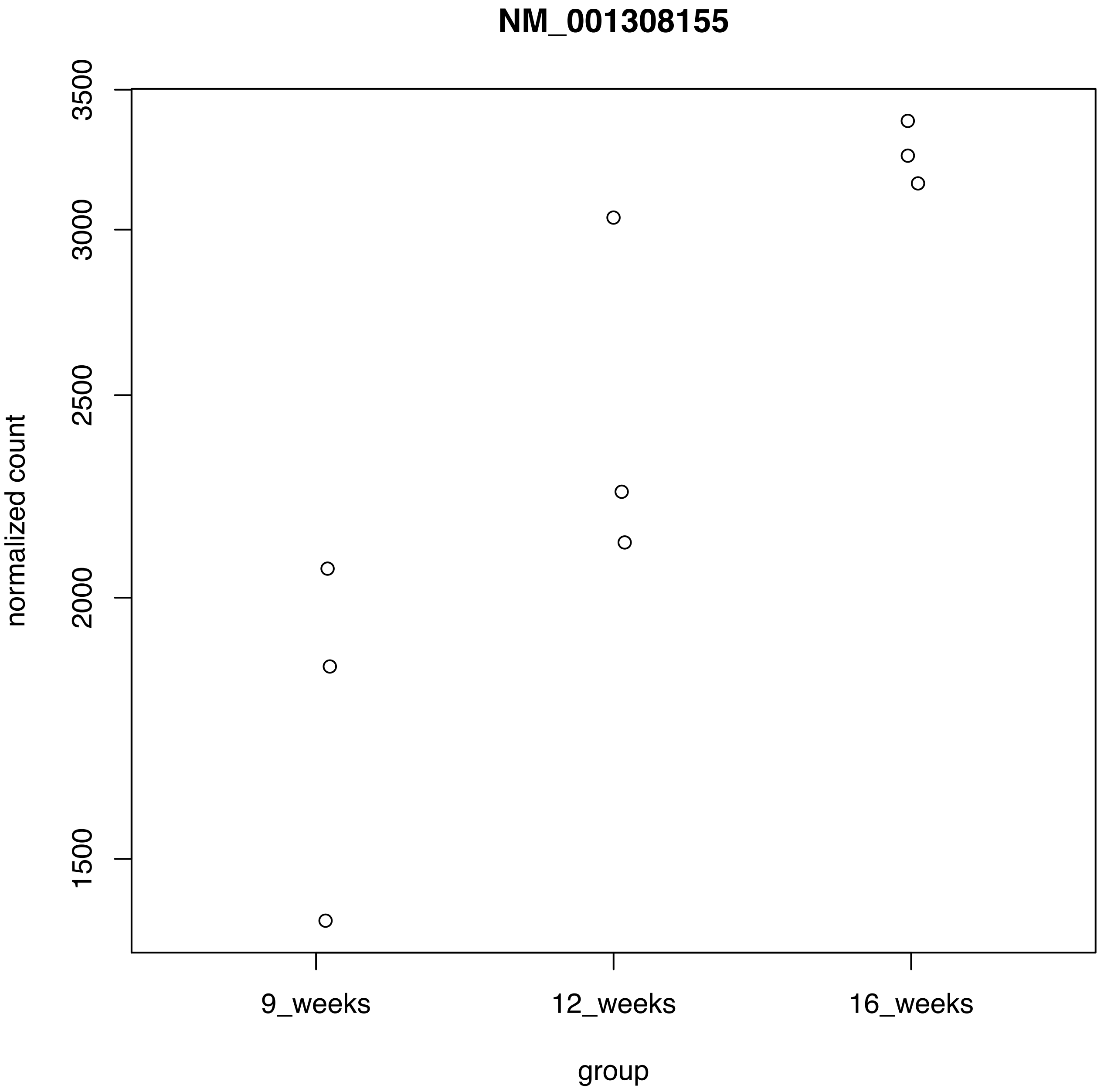

NM_001308155
normalized count
3500
3000
2500
2000
1500
9_weeks
12_weeks
16_weeks
group

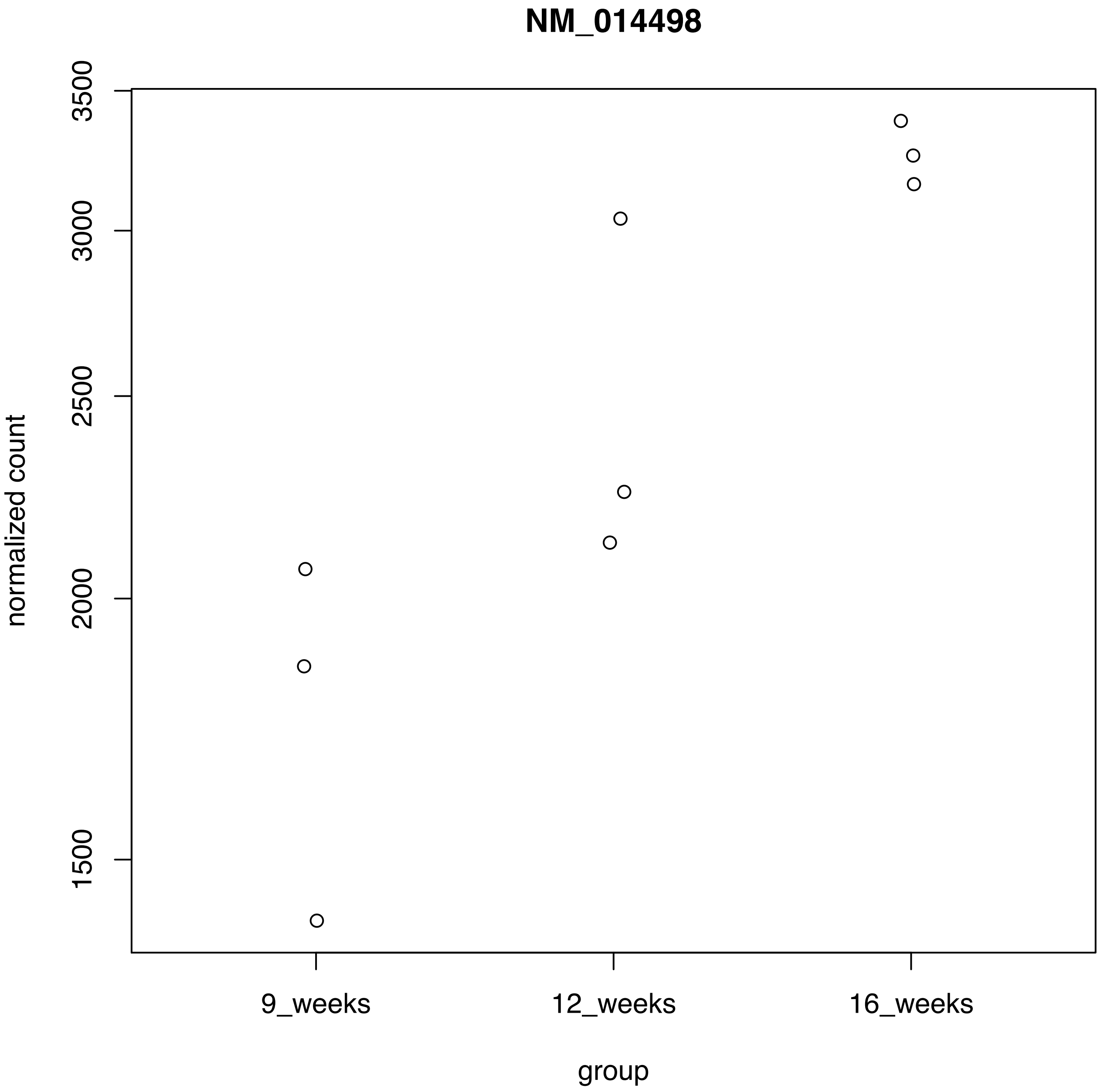

NM_014498
normalized count
3500
3000
2500
2000
1500
9_weeks
12_weeks
16_weeks
group

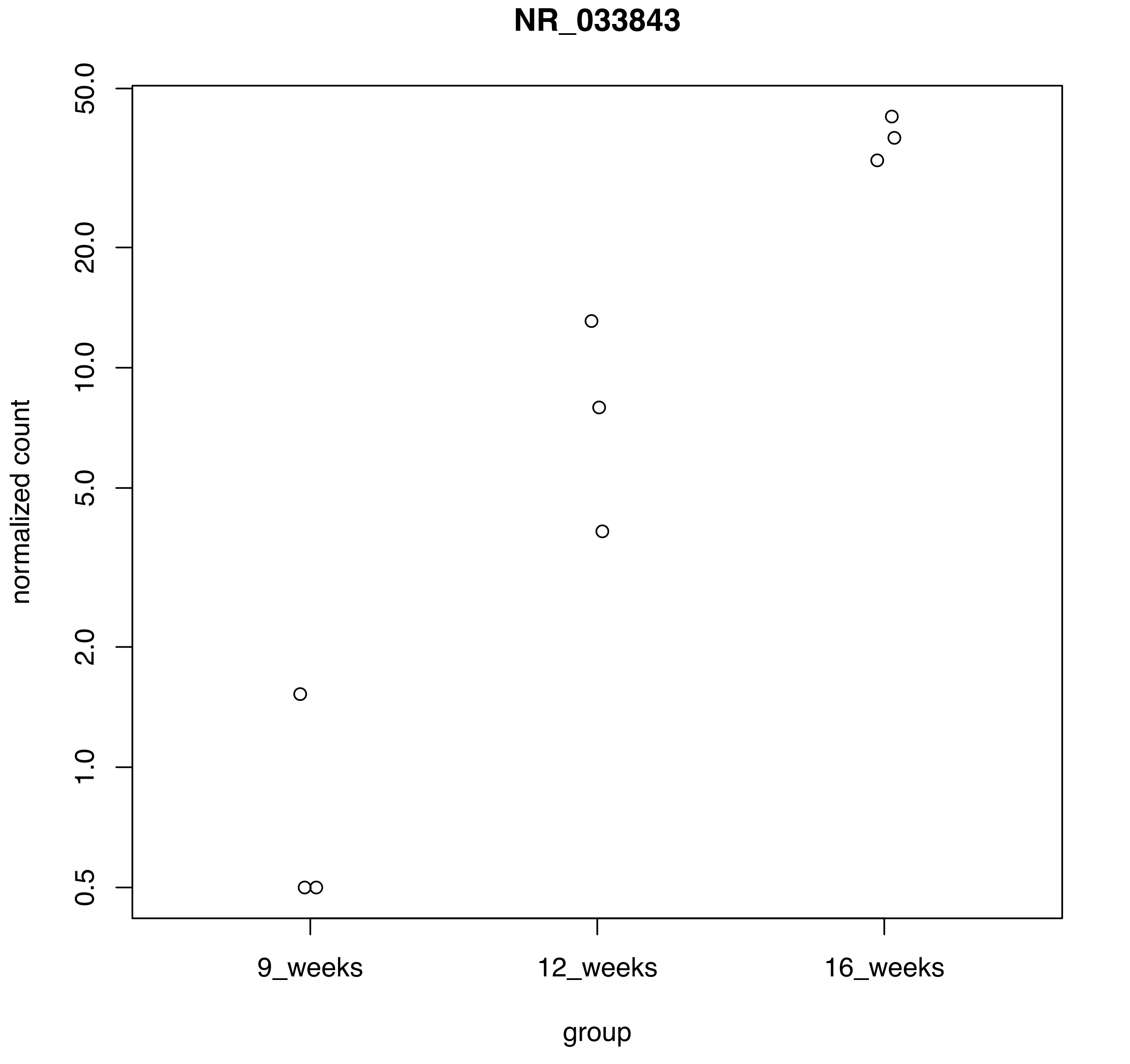
NR_033843
normalized count
50.0
20.0
10.0
5.0
2.0
1.0
0.5
9_weeks
12_weeks
16_weeks
group

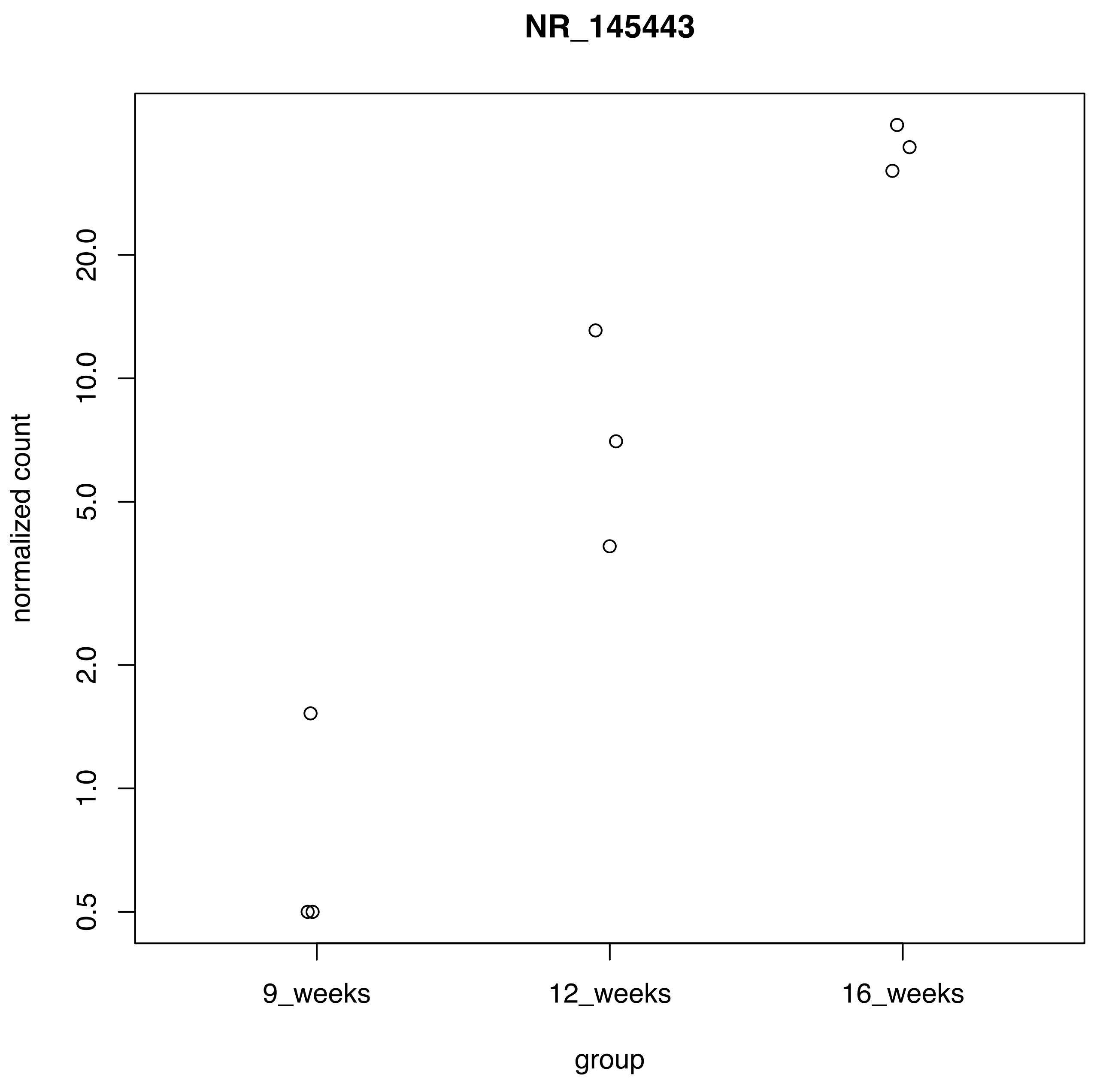
NR_145443
normalized count
0.5
1.0
2.0
5.0
10.0
20.0
9_weeks
12_weeks
16_weeks
group

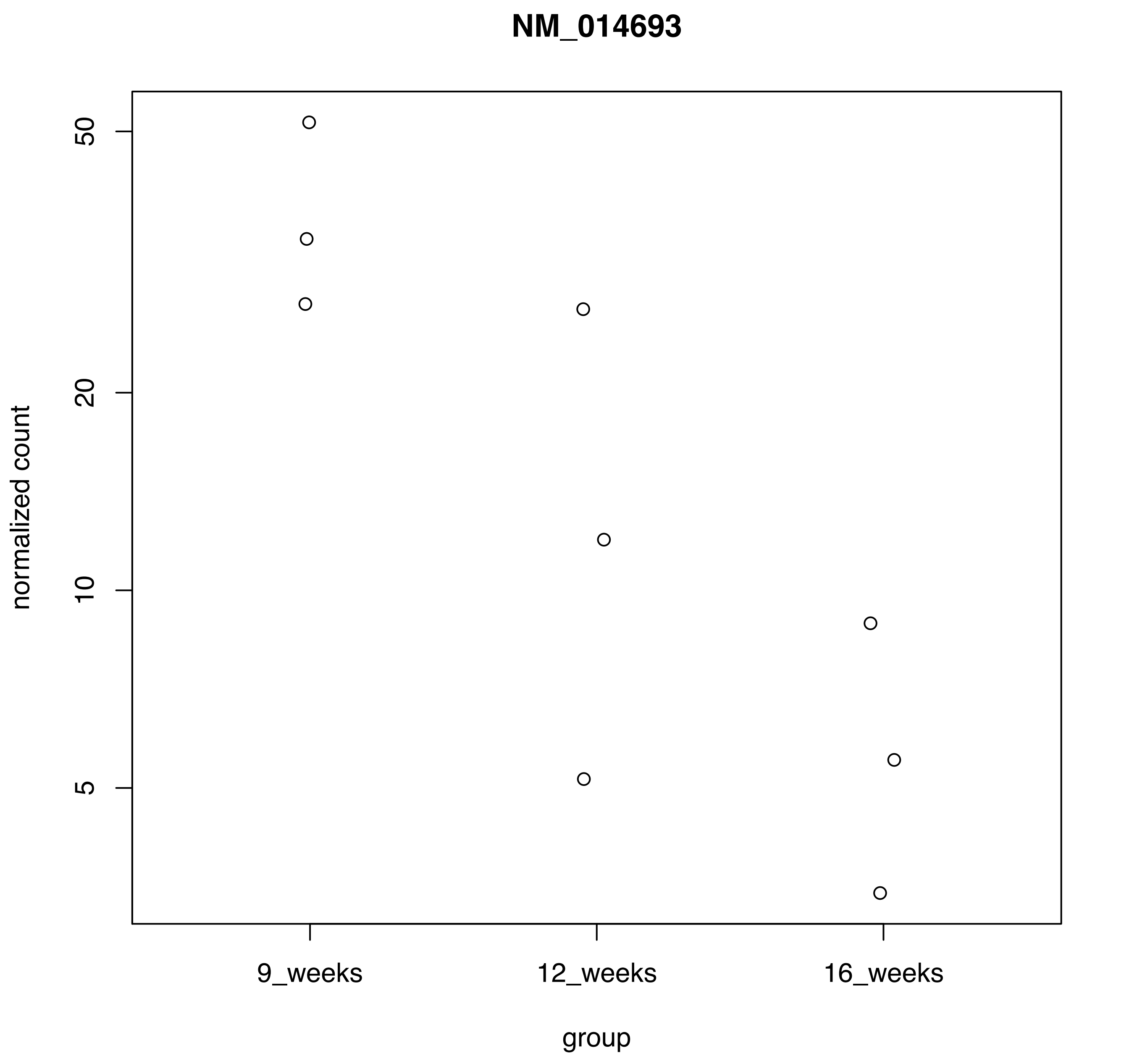
NM_014693
normalized count
50
20
10
5
9_weeks
12_weeks
16_weeks
group

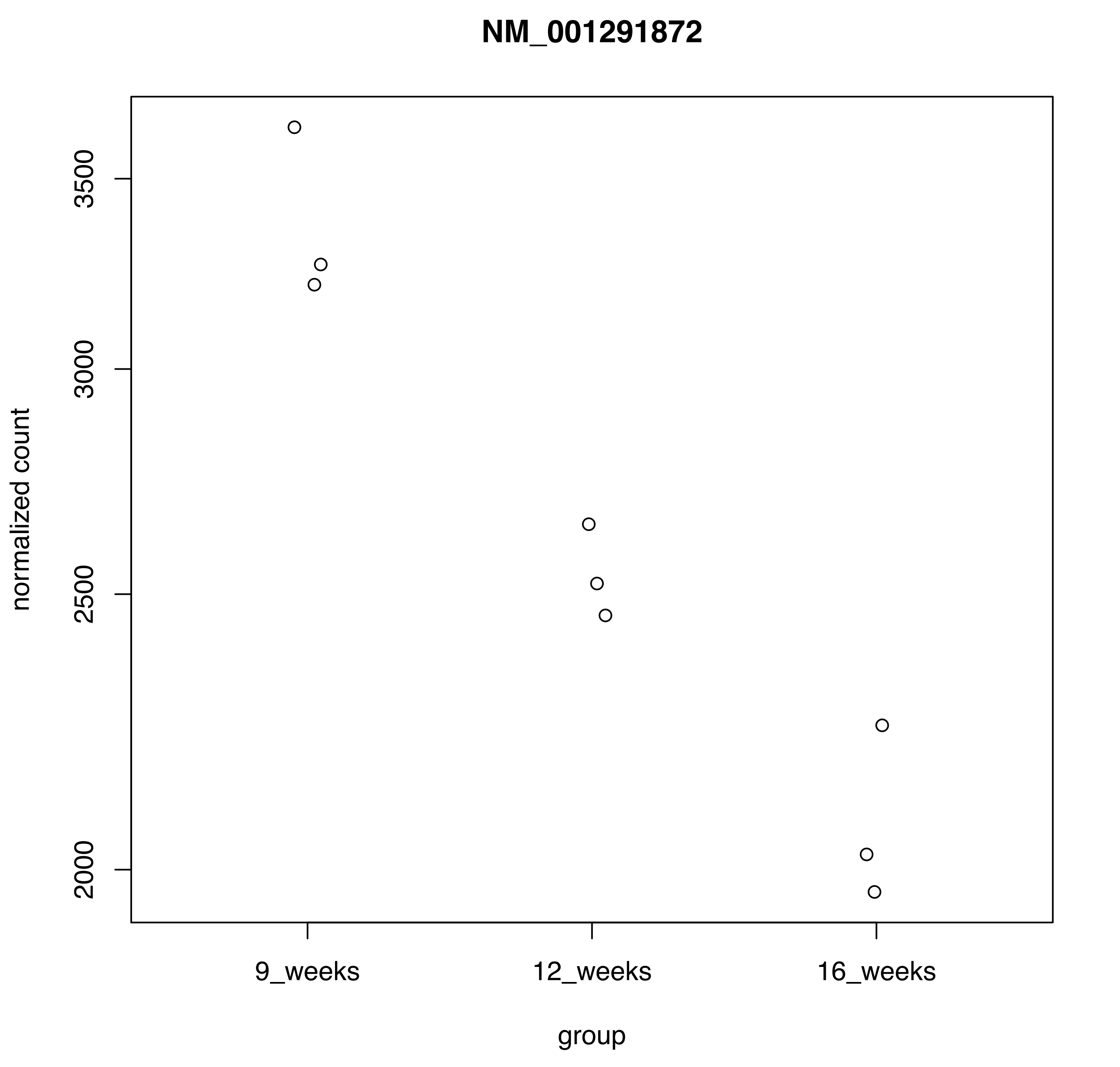
NM_001291872
normalized count
3500
3000
2500
2000
9_weeks
12_weeks
16_weeks
group

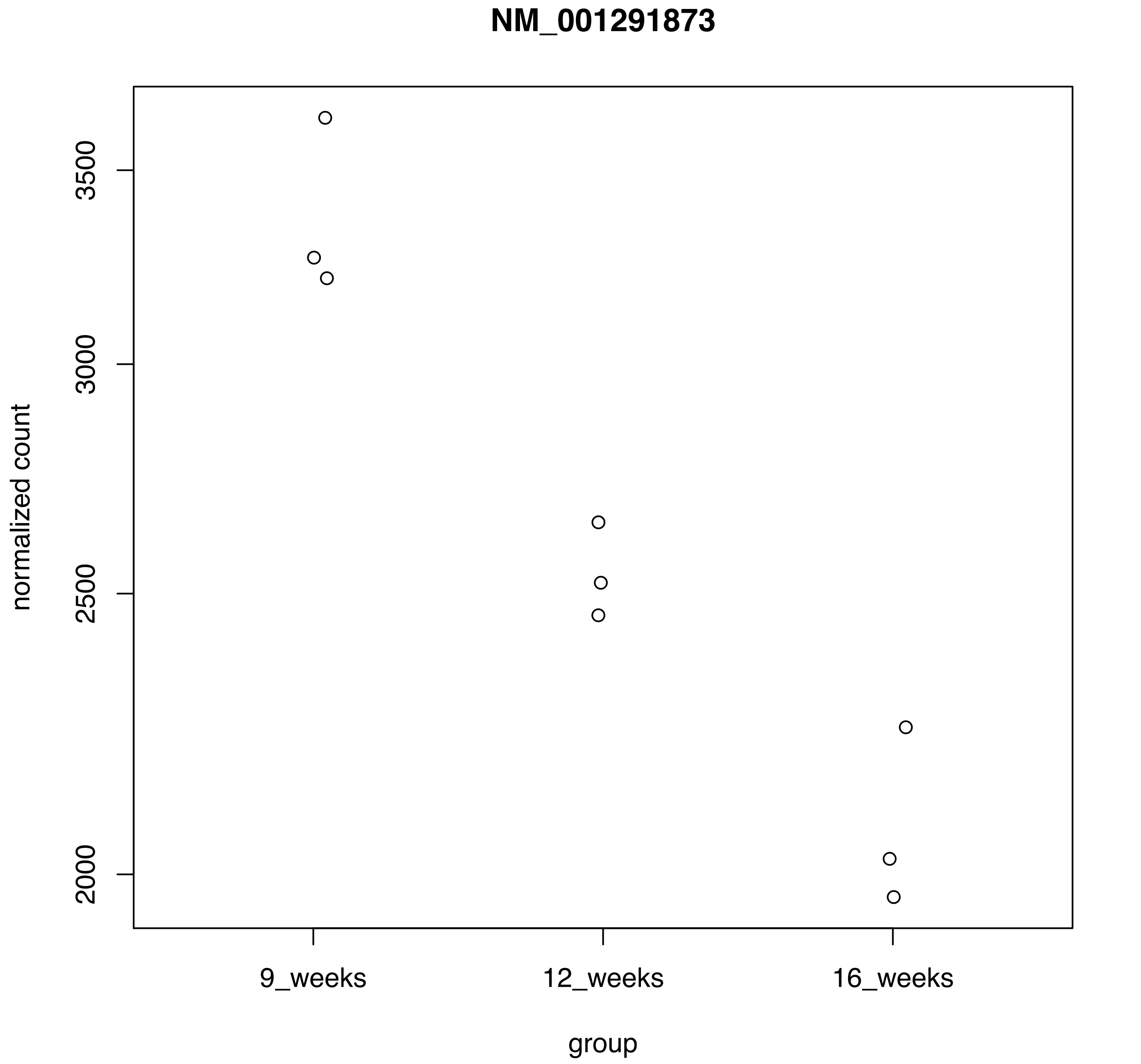

NM_001291873
normalized count
3500
3000
2500
2000
9_weeks
12_weeks
16_weeks
group

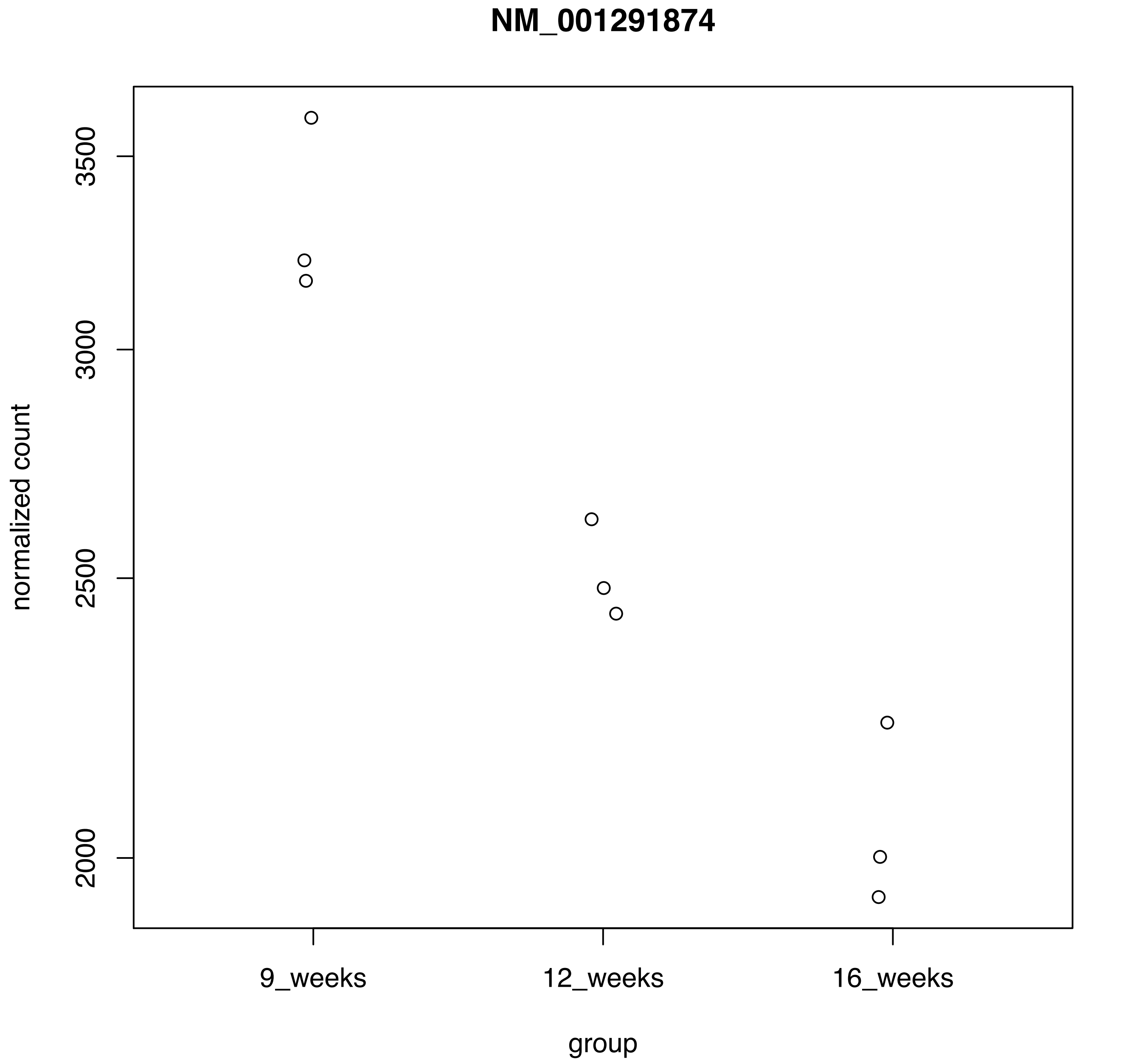
NM_001291874
normalized count
3500
3000
2500
2000
9_weeks
12_weeks
16_weeks
group

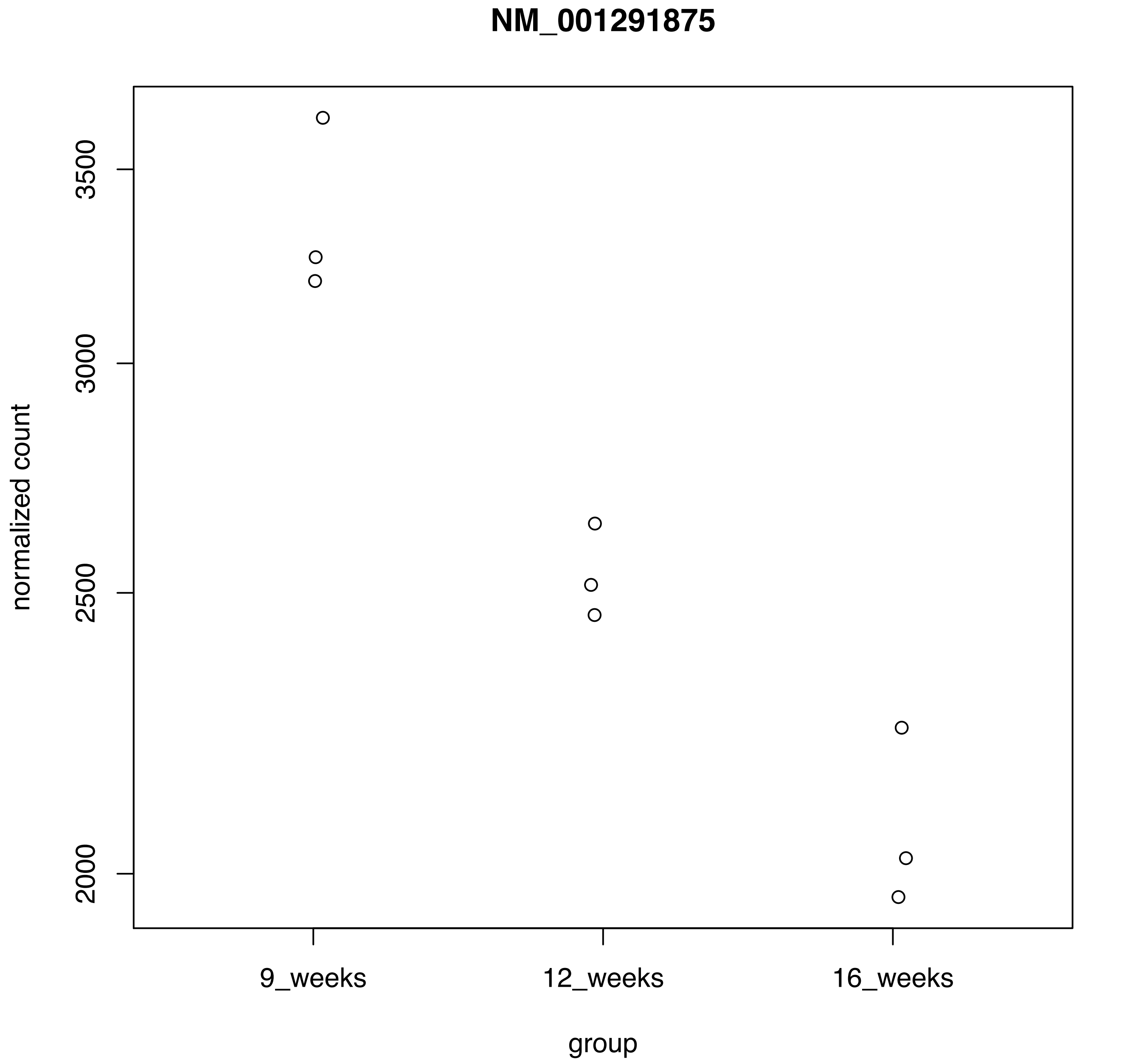
NM_001291875
normalized count
3500
3000
2500
2000
9_weeks
12_weeks
16_weeks
group

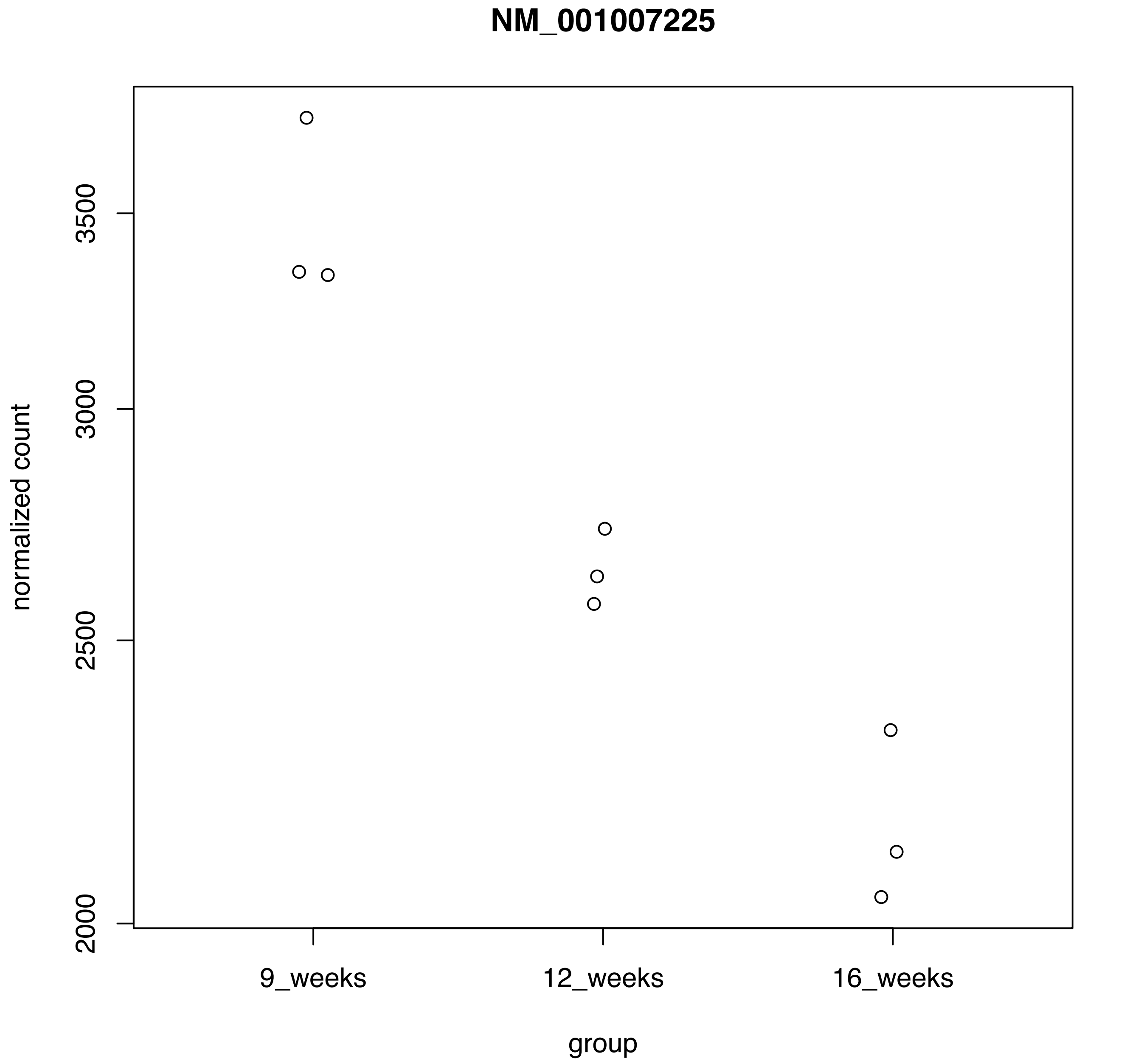
NM_001007225
normalized count
2000
2500
3000
3500
9_weeks
12_weeks
16_weeks
group

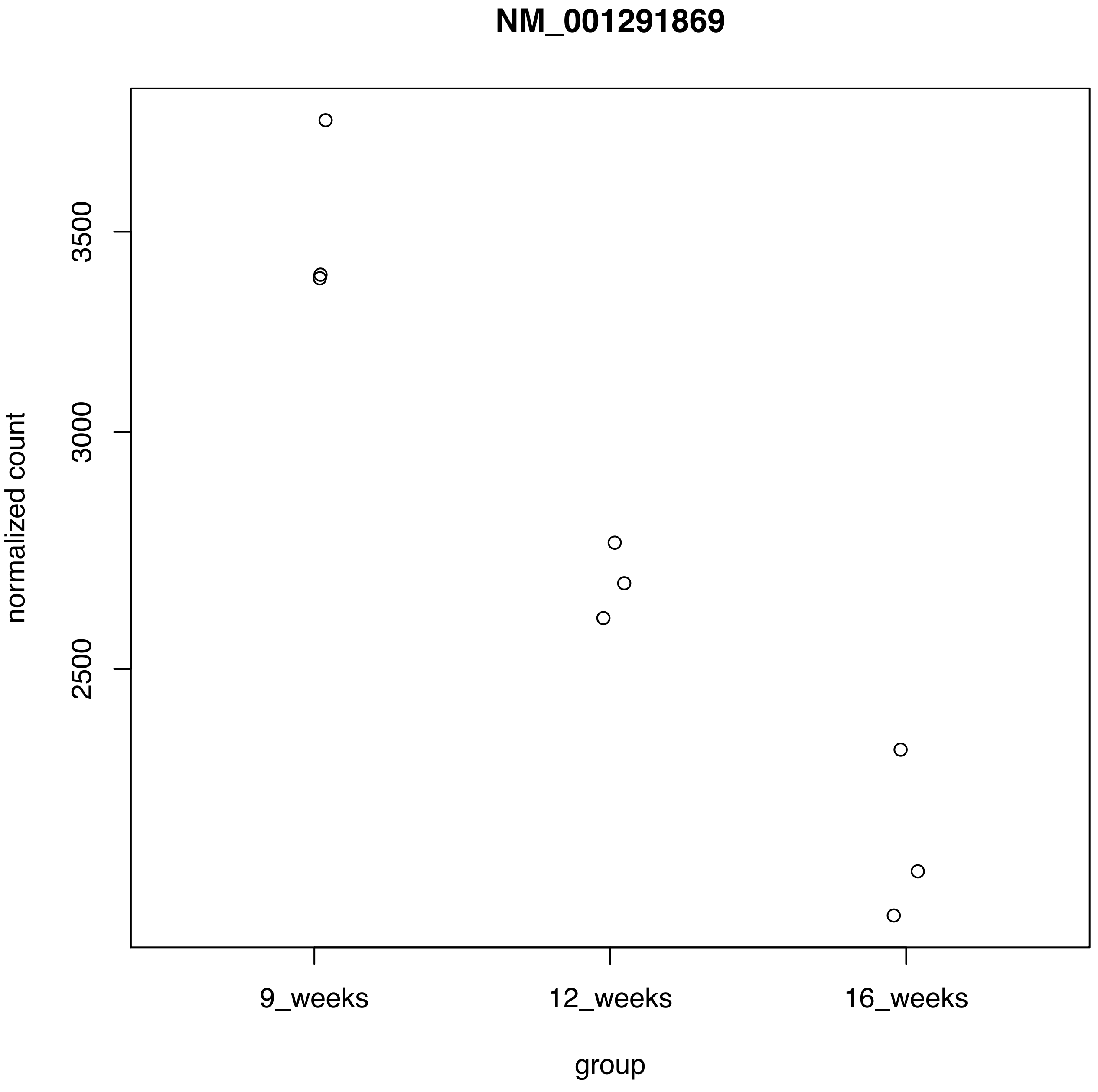
NM_001291869
normalized count
3500
3000
2500
9_weeks
12_weeks
16_weeks
group

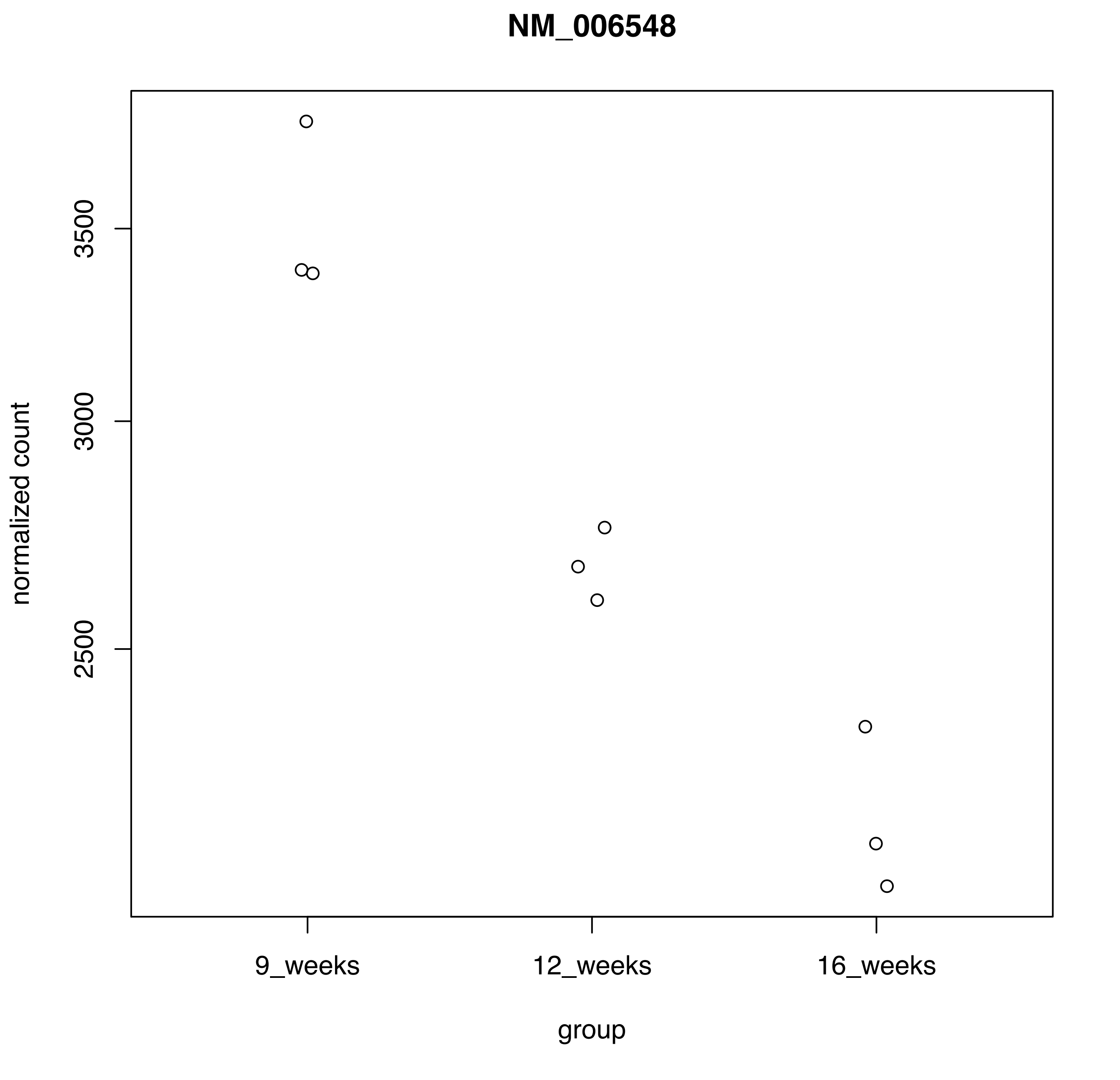

NM_006548
normalized count
2500
3000
3500
9_weeks
12_weeks
16_weeks
group

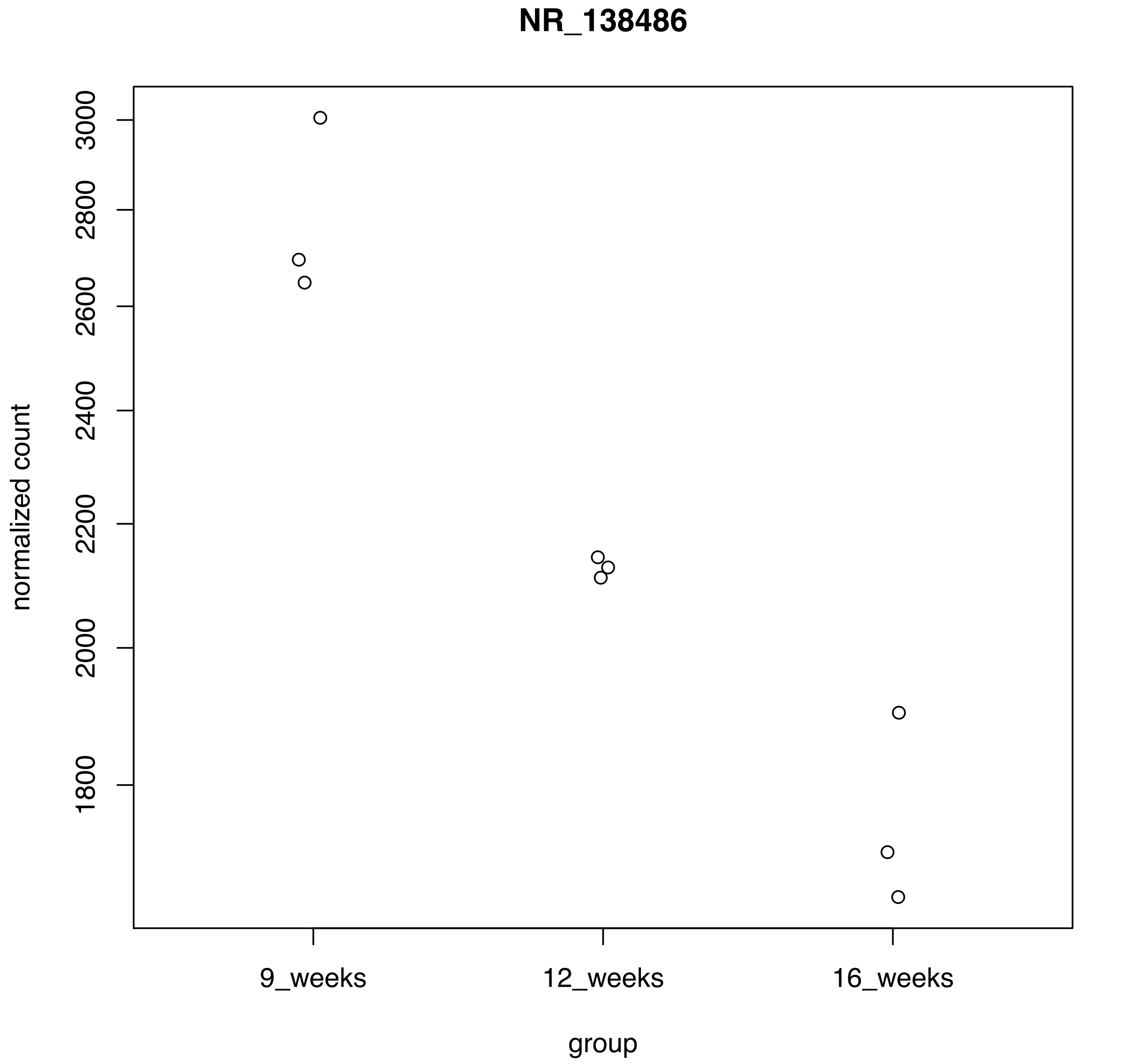
NR_138486
normalized count
3000
2800
2600
2400
2200
2000
1800
9_weeks
12_weeks
16_weeks
group

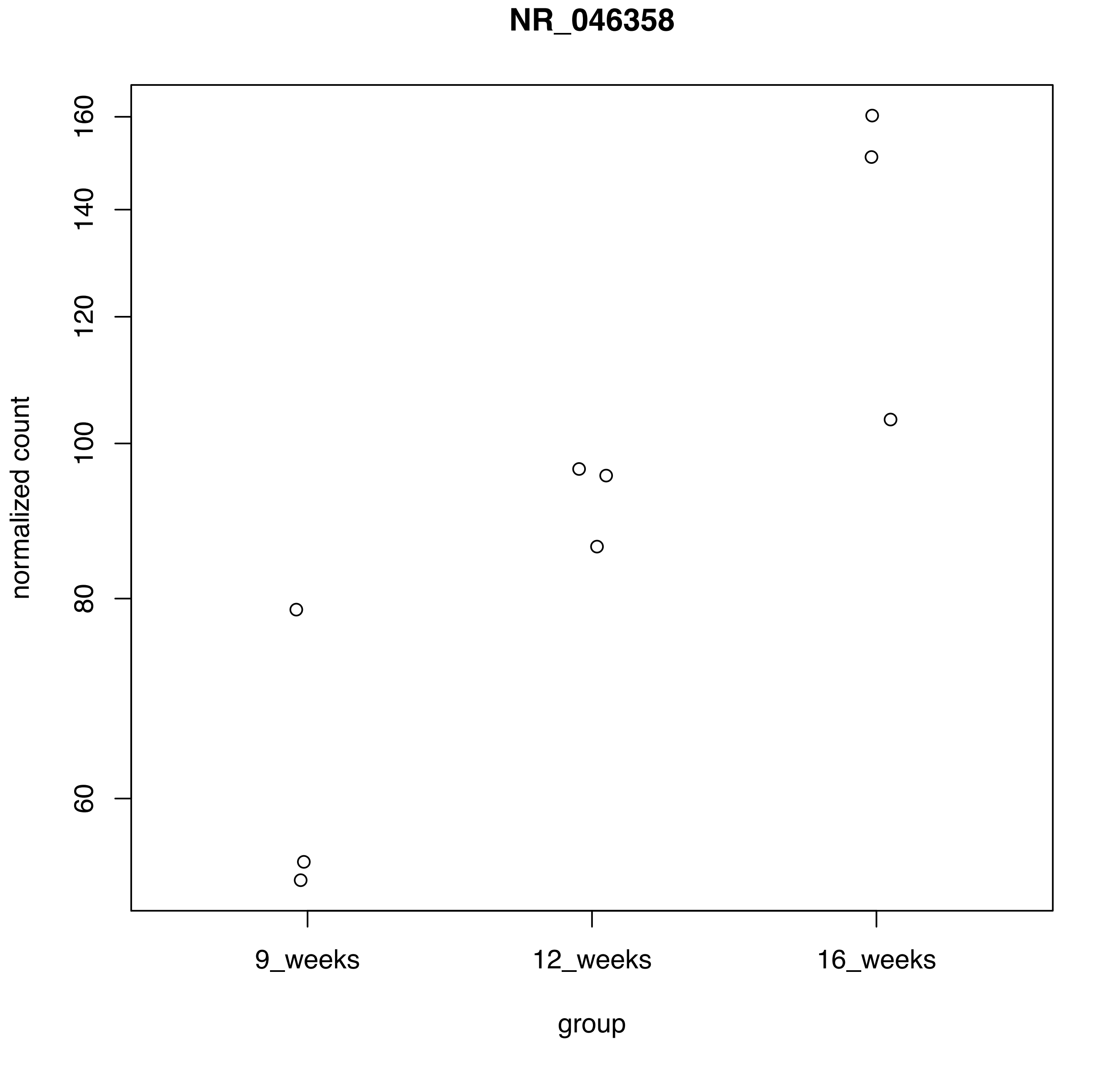
NR_046358
normalized count
60
80
100
120
140
160
9_weeks
12_weeks
16_weeks
group

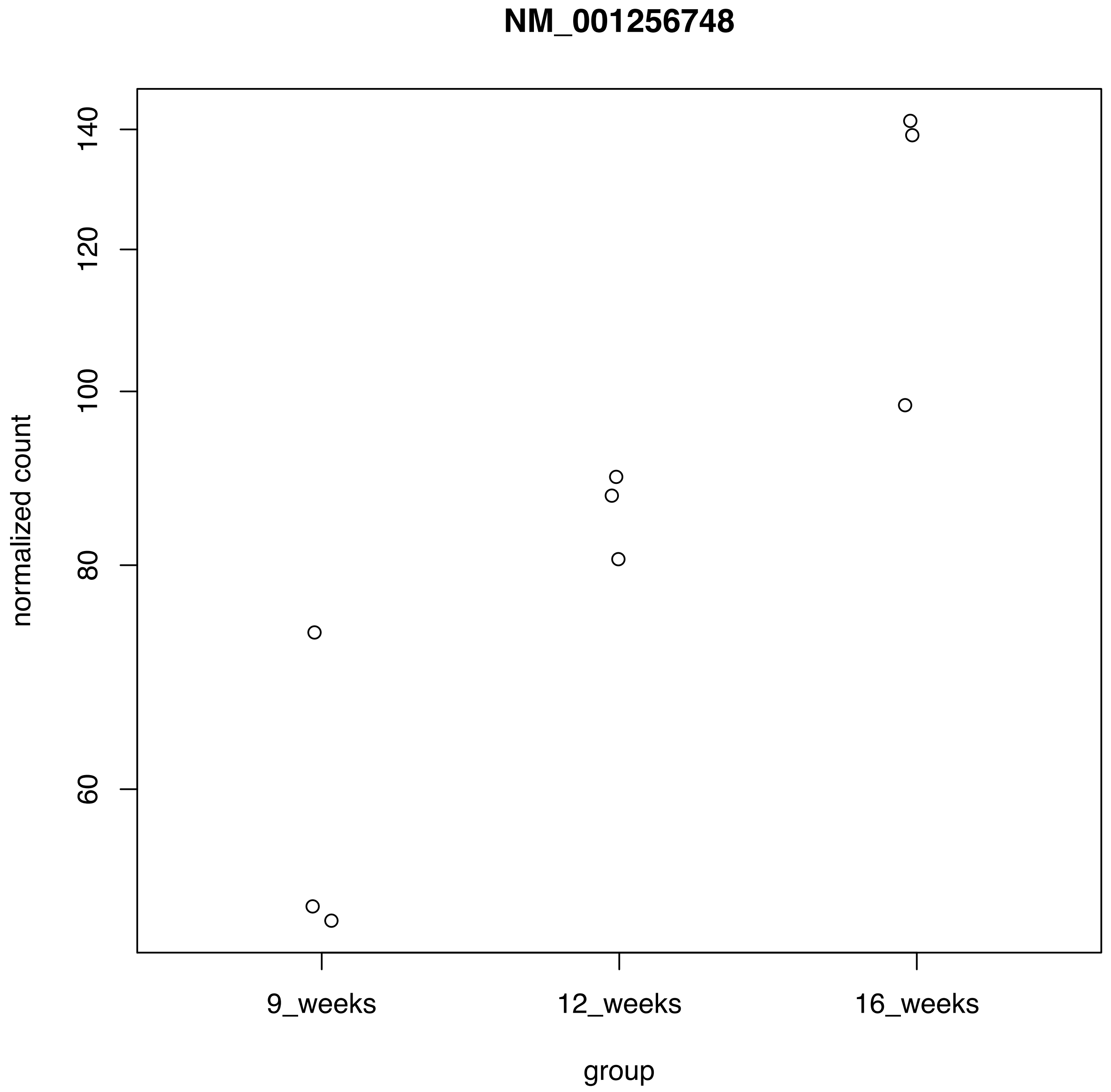
NM_001256748
normalized count
140
120
100
80
60
9_weeks
12_weeks
16_weeks
group

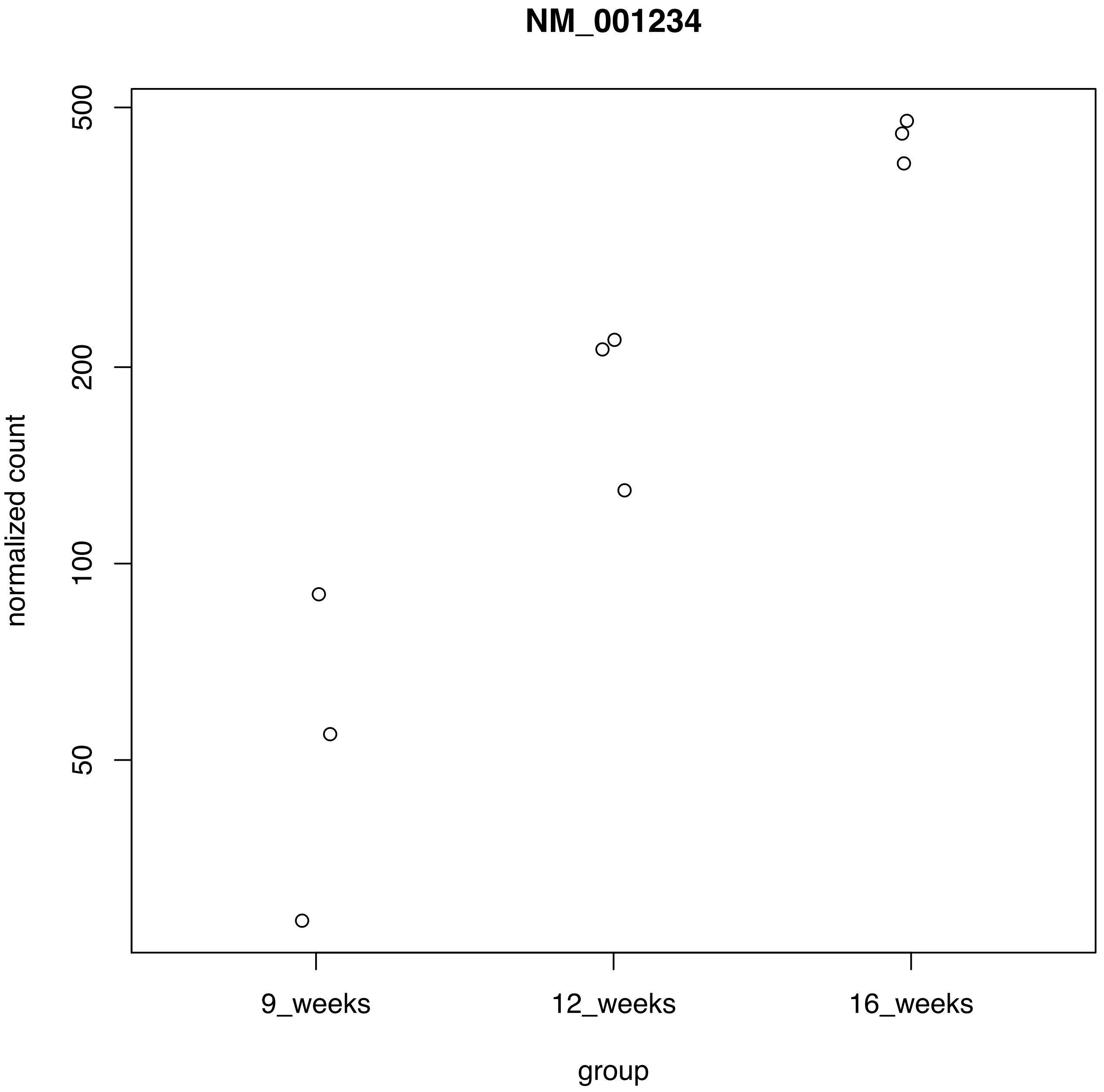
NM_001234
500
200
100
50
normalized count
9_weeks
12_weeks
16_weeks
group

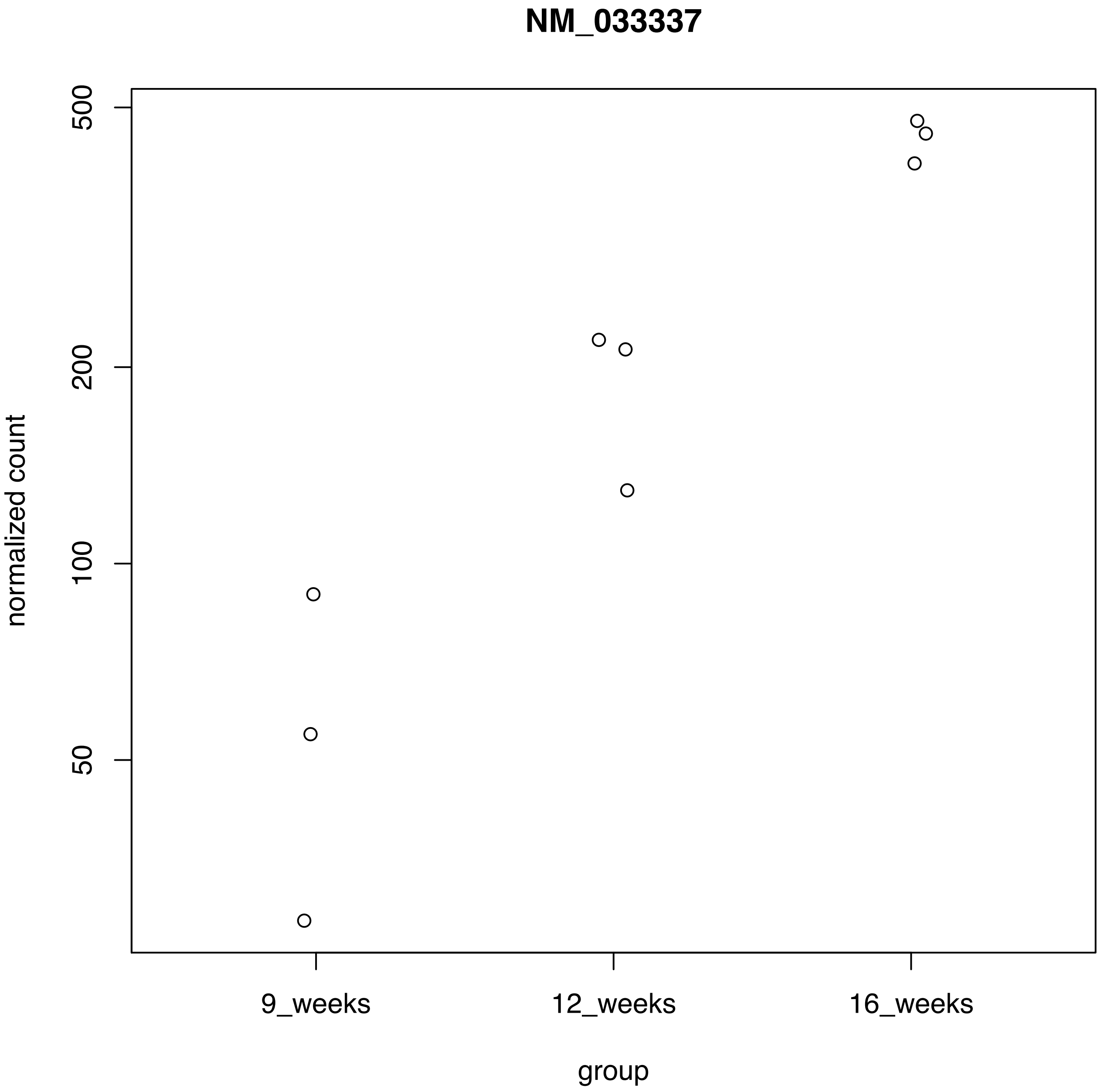
NM_033337
normalized count
500
200
100
50
9_weeks
12_weeks
16_weeks
group

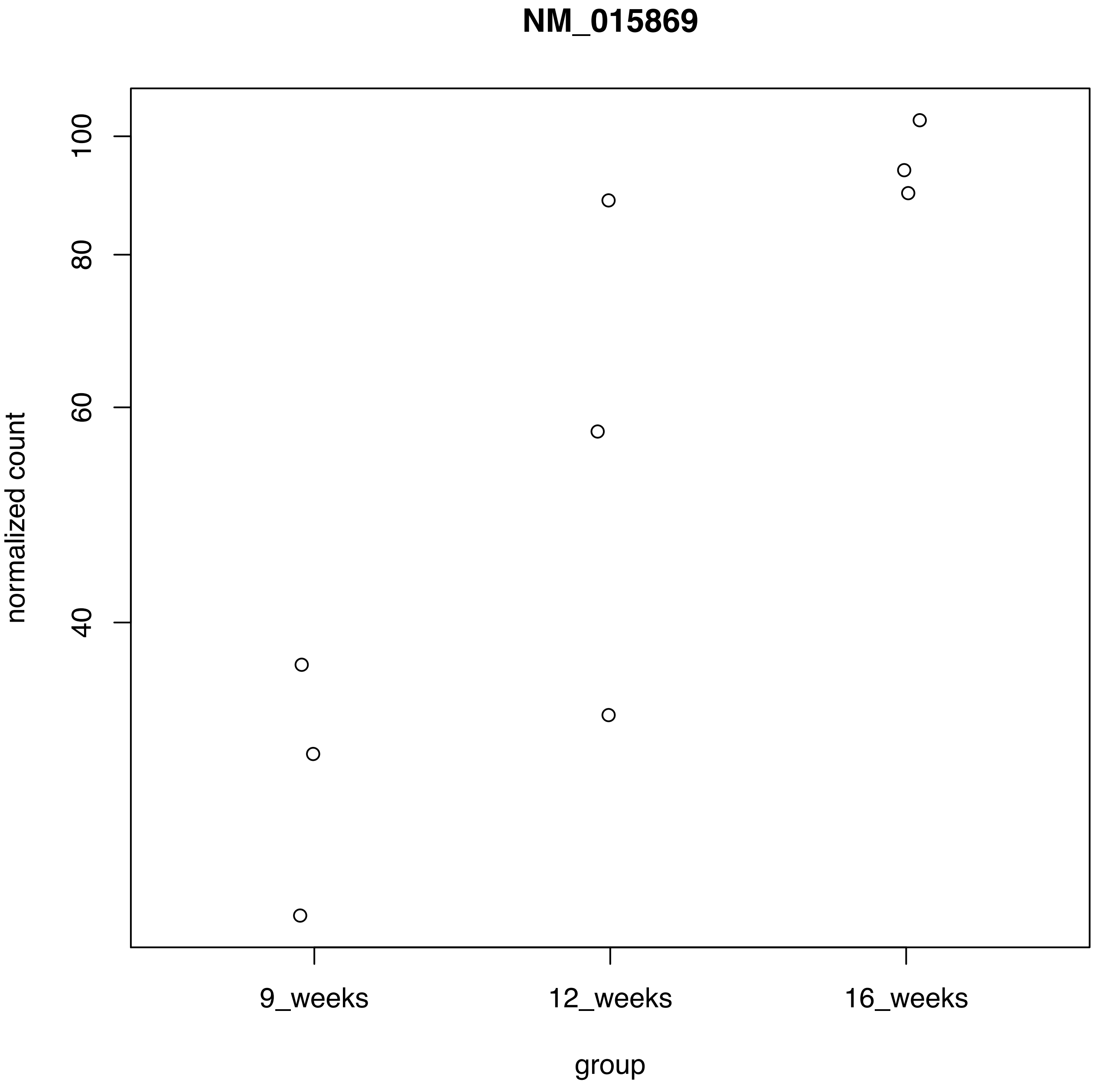
NM_015869
normalized count
100
80
60
40
9_weeks
12_weeks
16_weeks
group

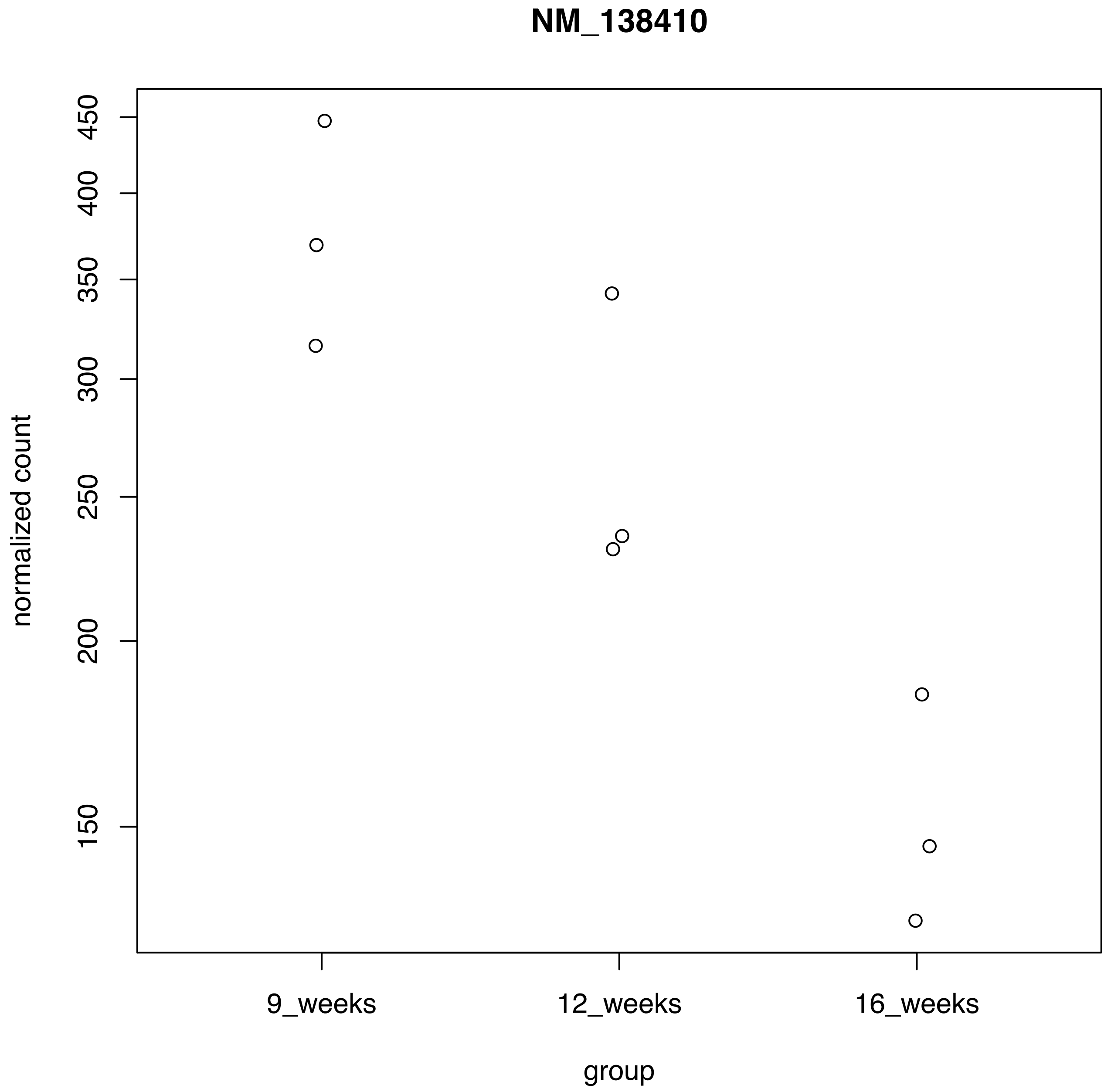

NM_138410
normalized count
450
400
350
300
250
200
150
9_weeks
12_weeks
16_weeks
group

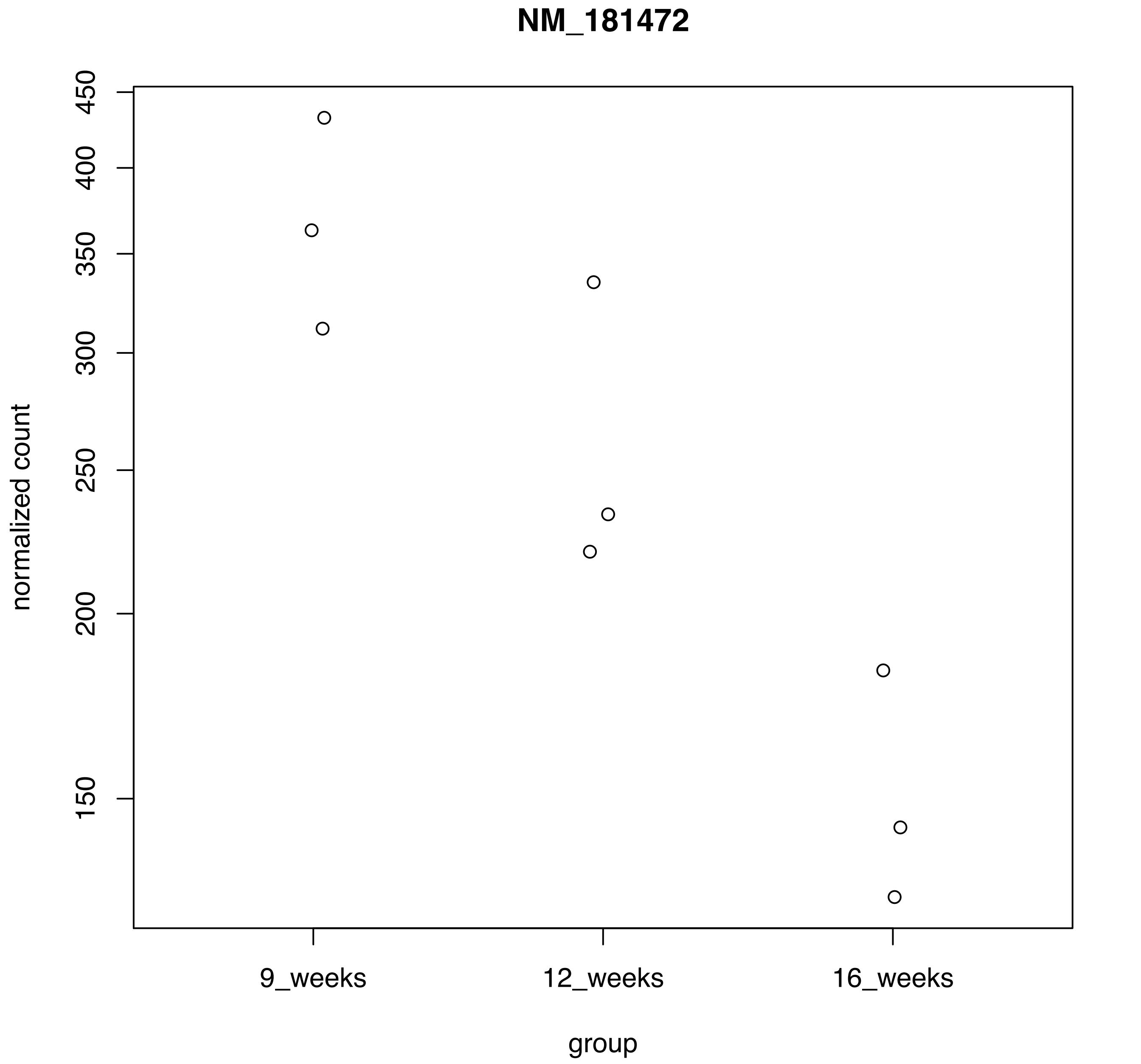
NM_181472
normalized count
450
400
350
300
250
200
150
9_weeks
12_weeks
16_weeks
group

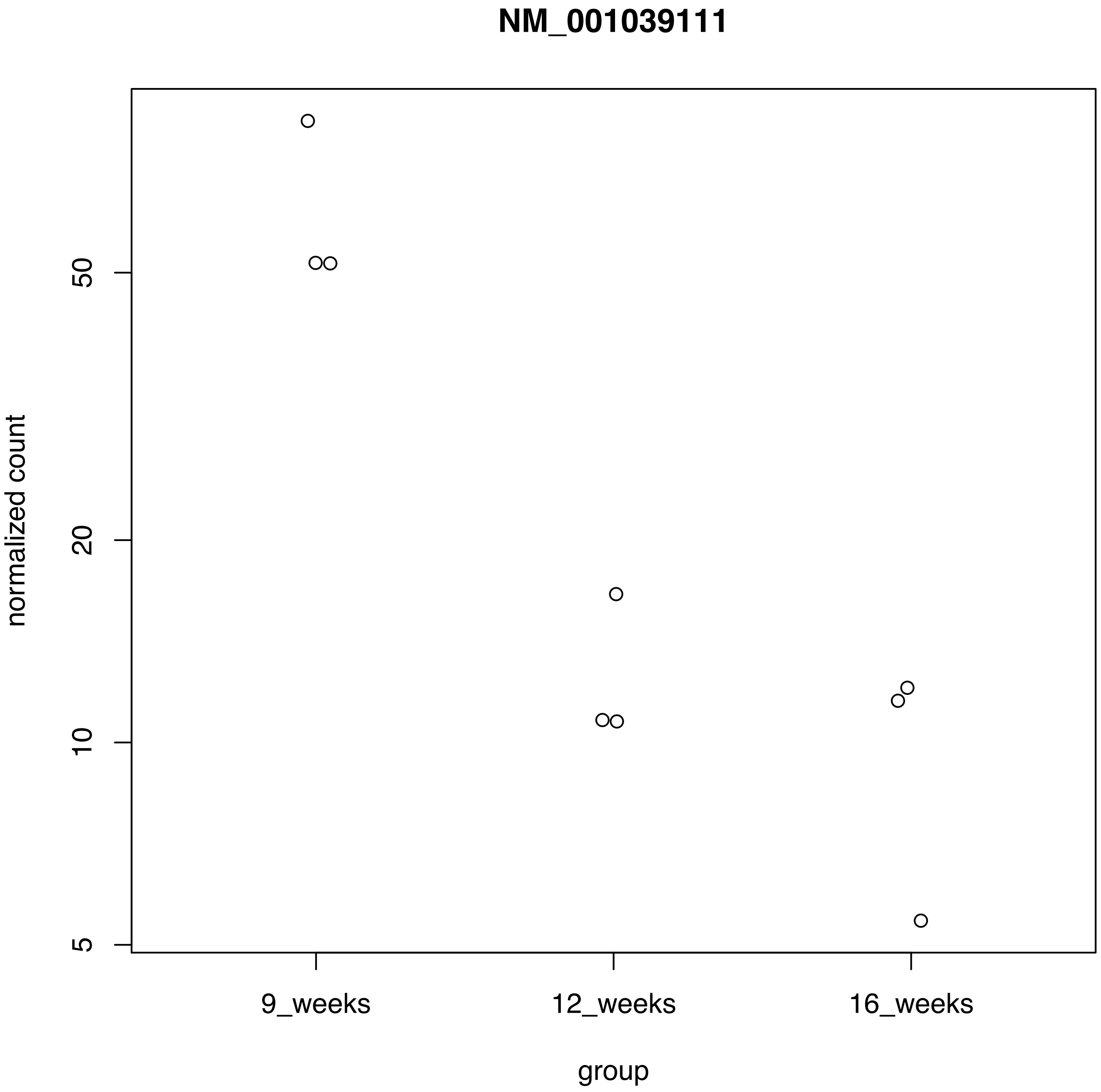
NM_001039111
normalized count
50
20
10
5
9_weeks
12_weeks
16_weeks
group

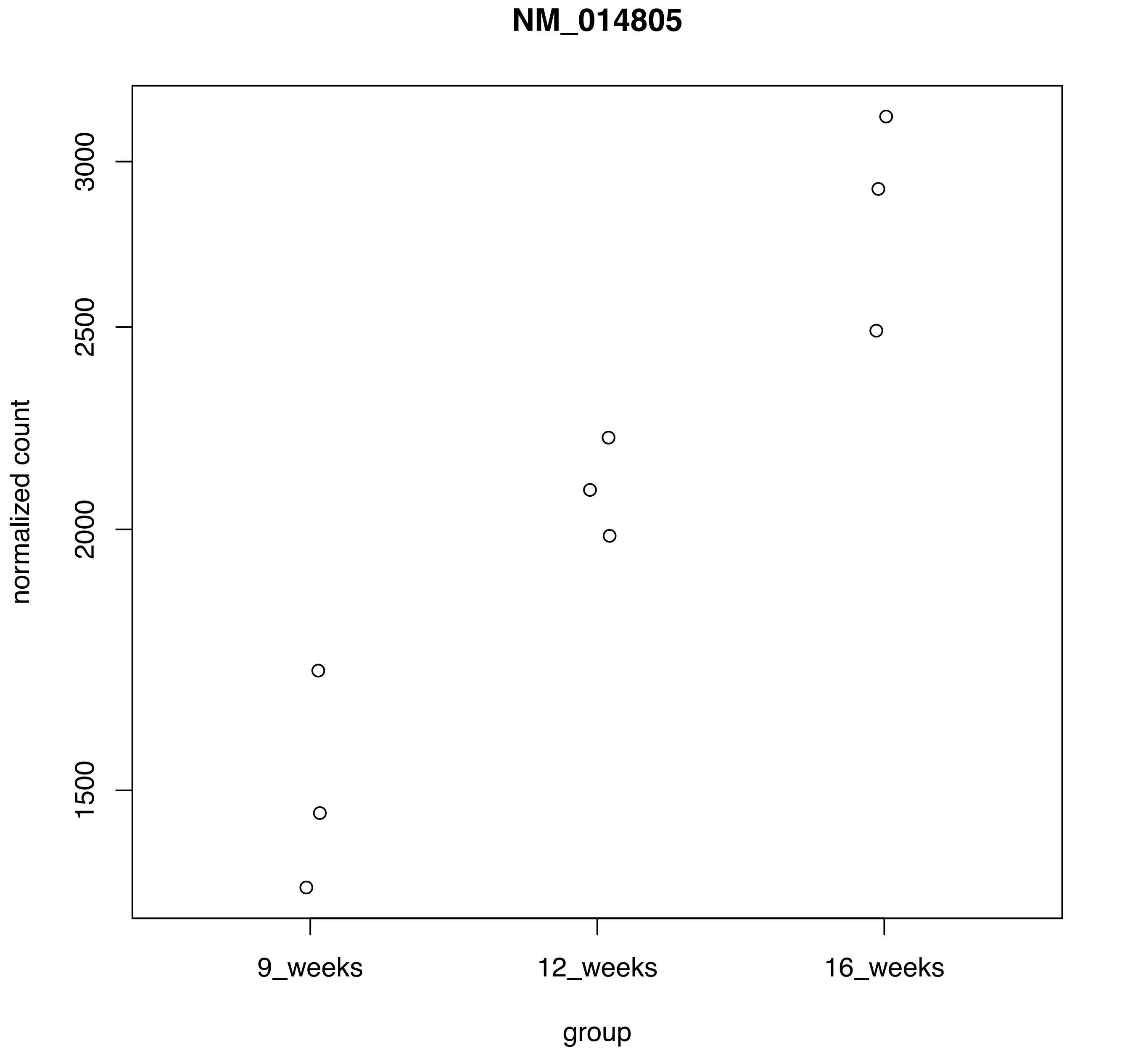

NM_014805
normalized count
3000
2500
2000
1500
9_weeks
12_weeks
16_weeks
group

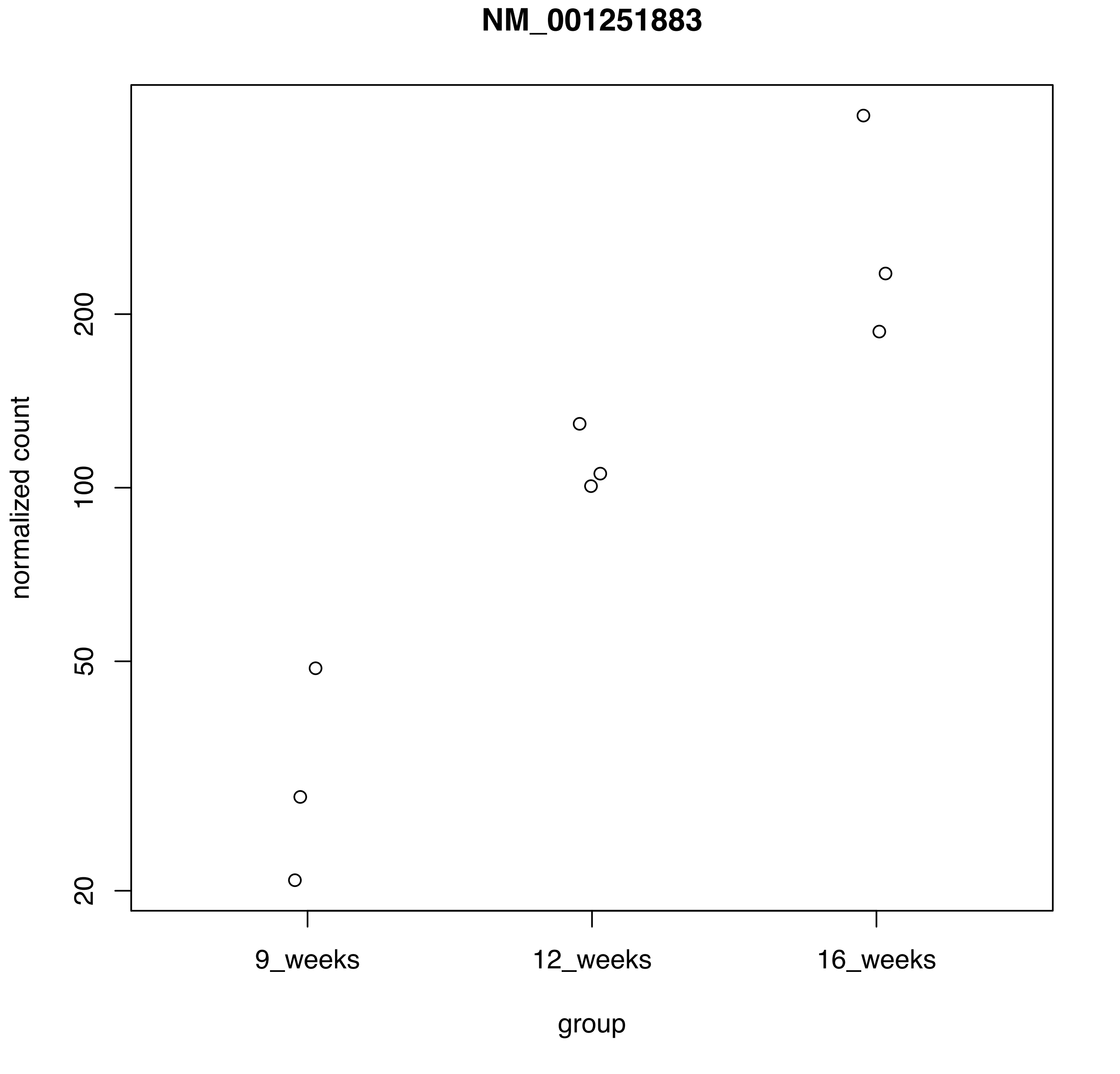
NM_001251883
normalized count
20
50
100
200
9_weeks
12_weeks
16_weeks
group

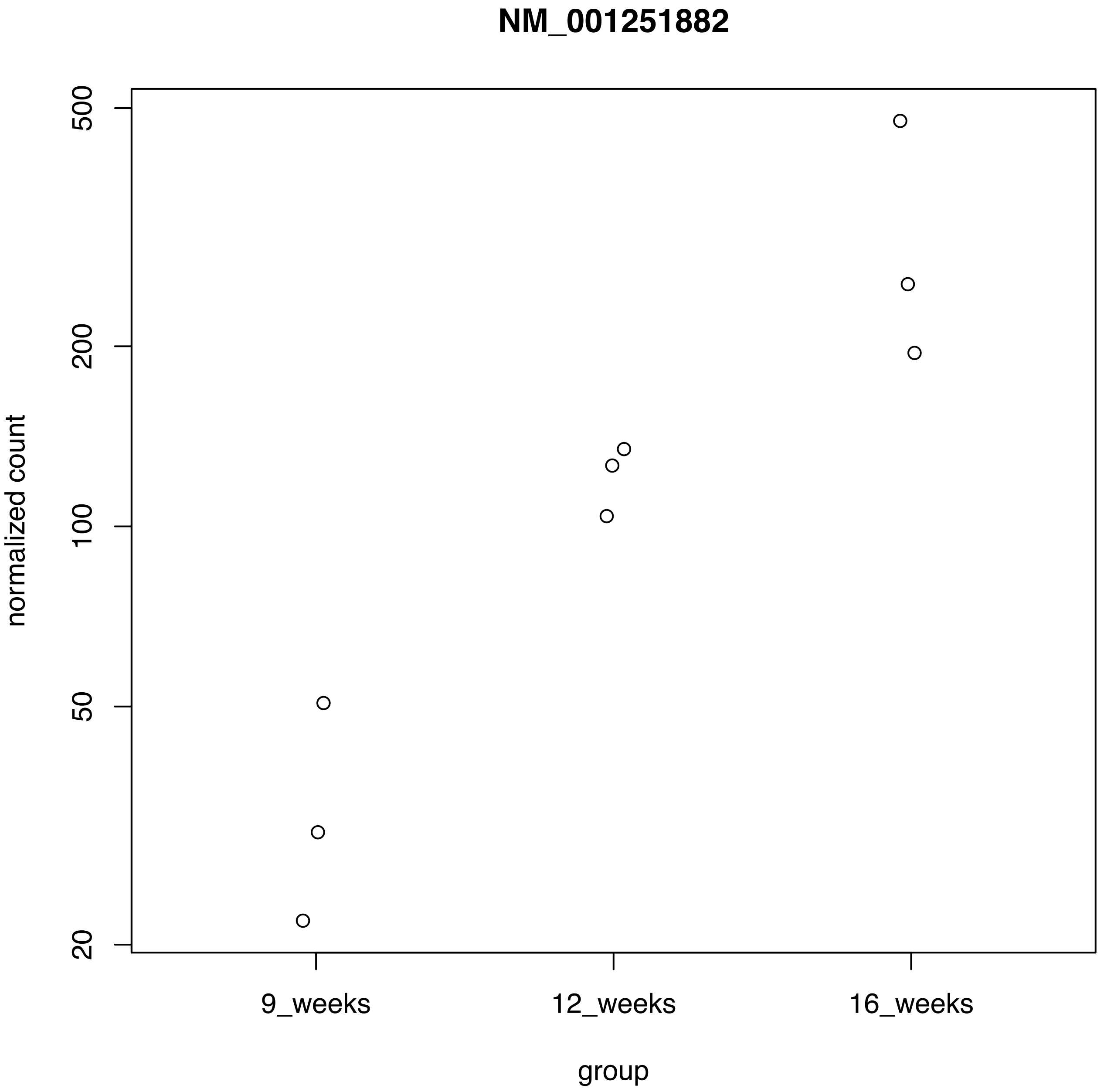
NM_001251882
normalized count
500
200
100
50
20
9_weeks
12_weeks
16_weeks
group

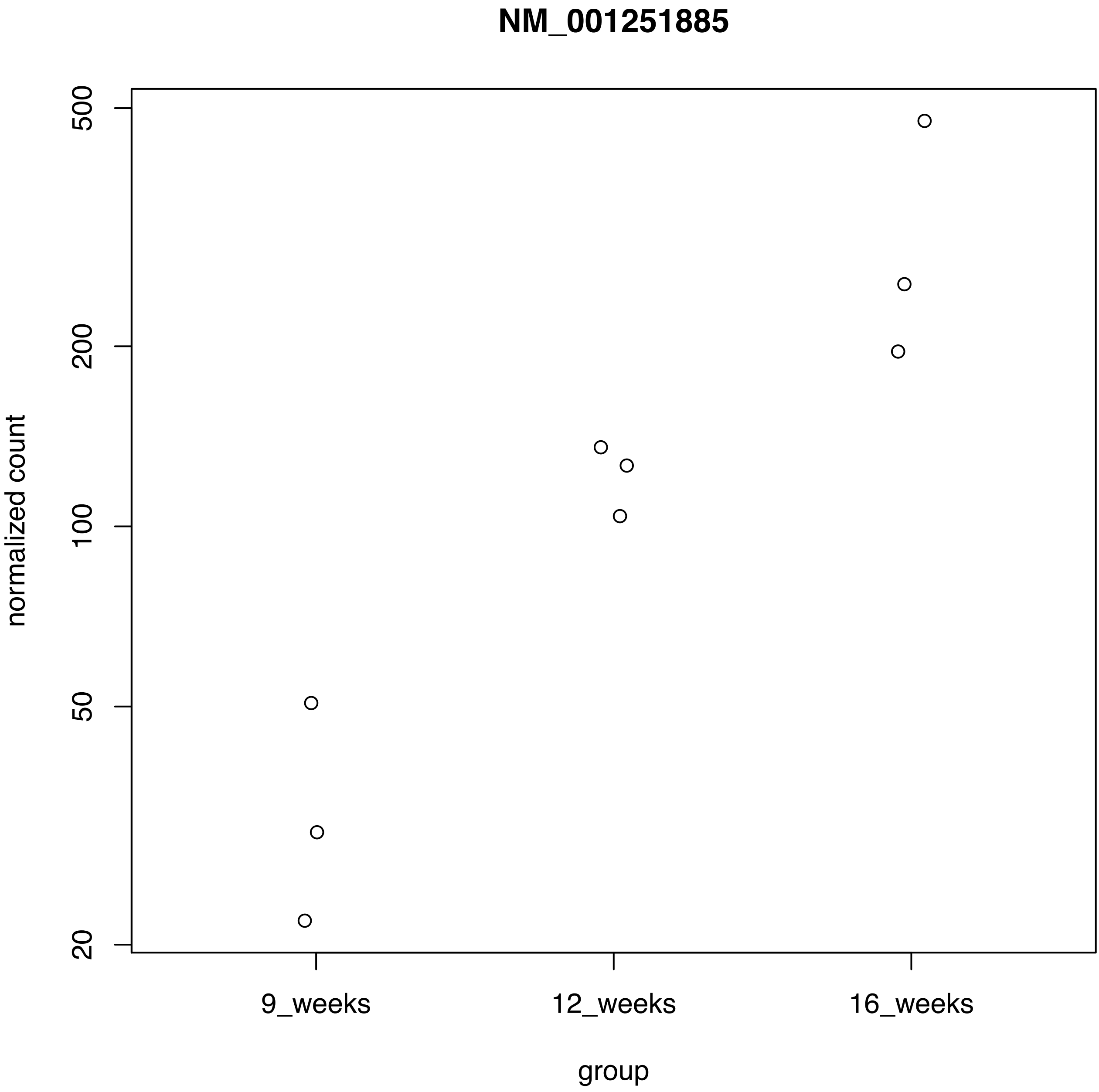
NM_001251885
normalized count
500
200
100
50
20
9_weeks
12_weeks
16_weeks
group

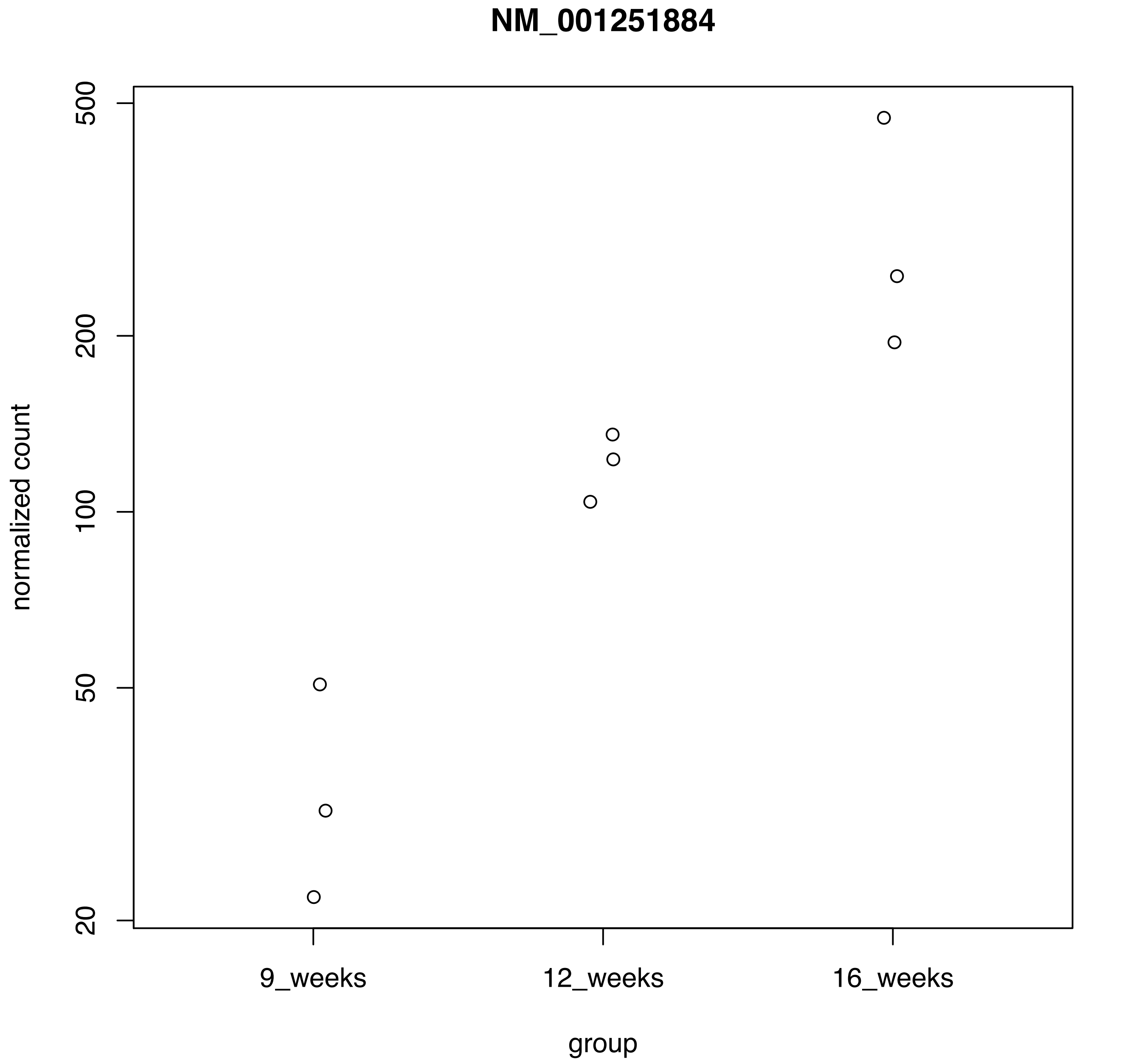

NM_001251884
normalized count
500
200
100
50
20
9_weeks
12_weeks
16_weeks
group

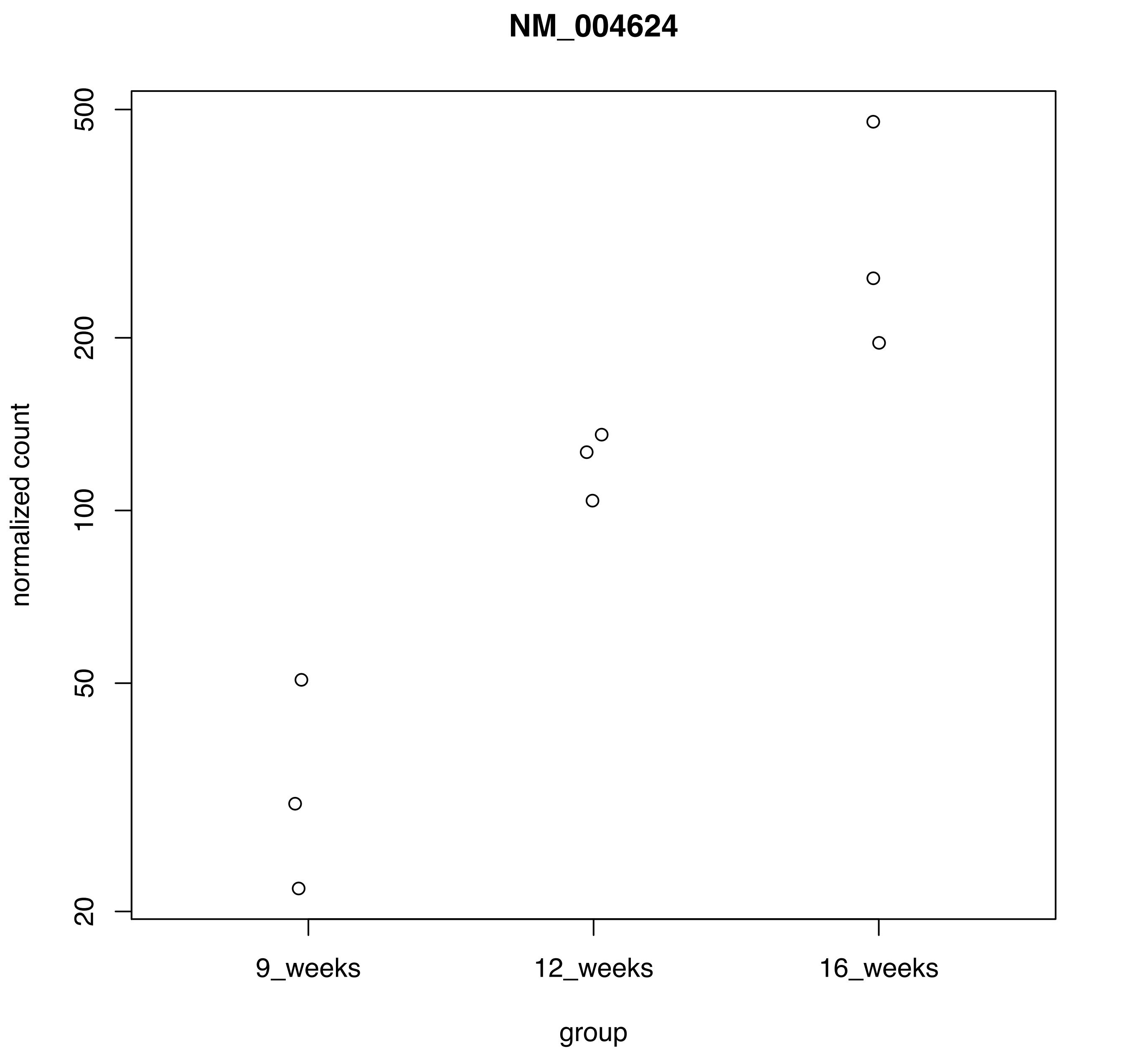

NM_004624
normalized count
500
200
100
50
20
9_weeks
12_weeks
16_weeks
group

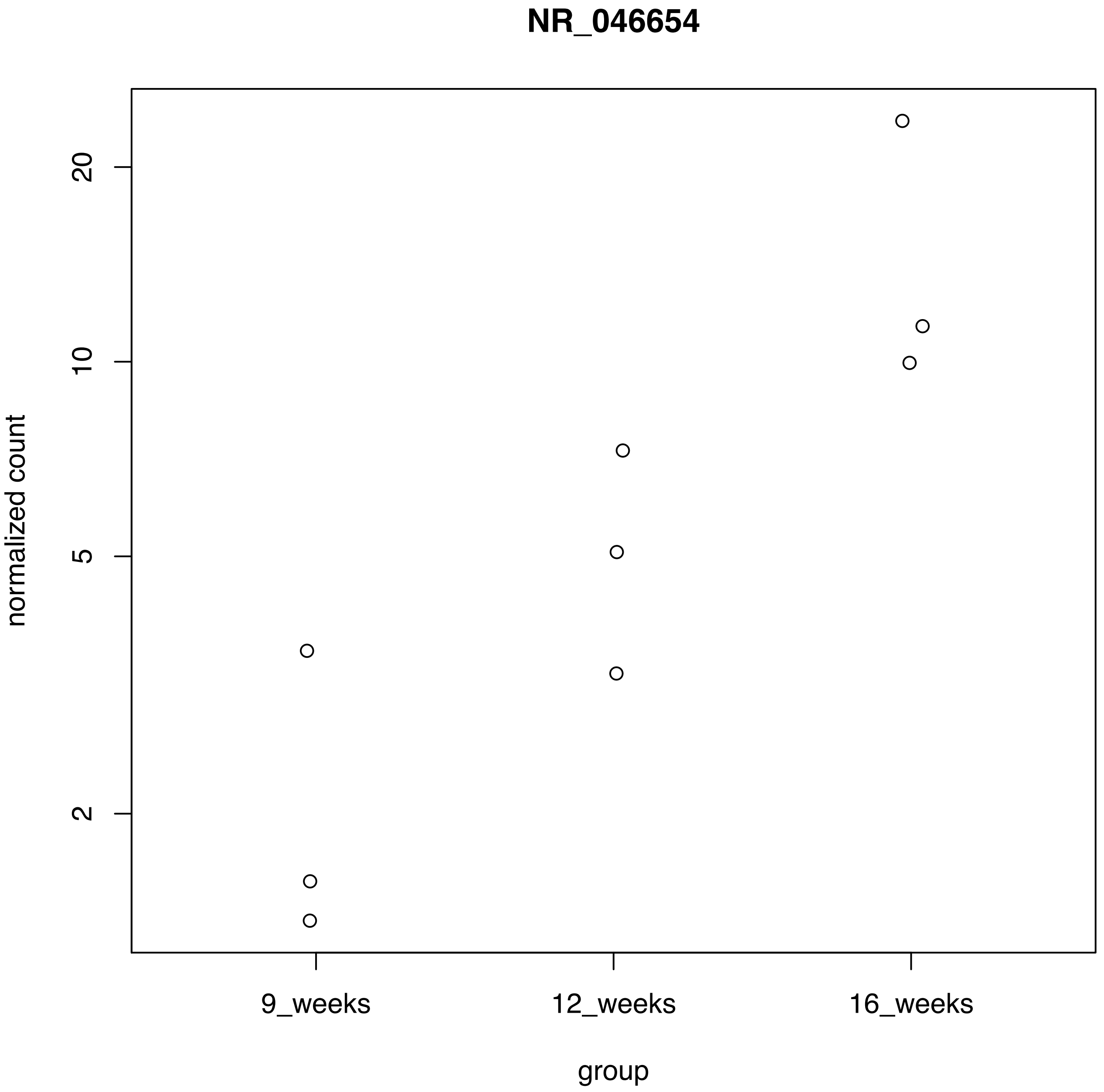

NR_046654
normalized count
20
10
5
2
9_weeks
12_weeks
16_weeks
group

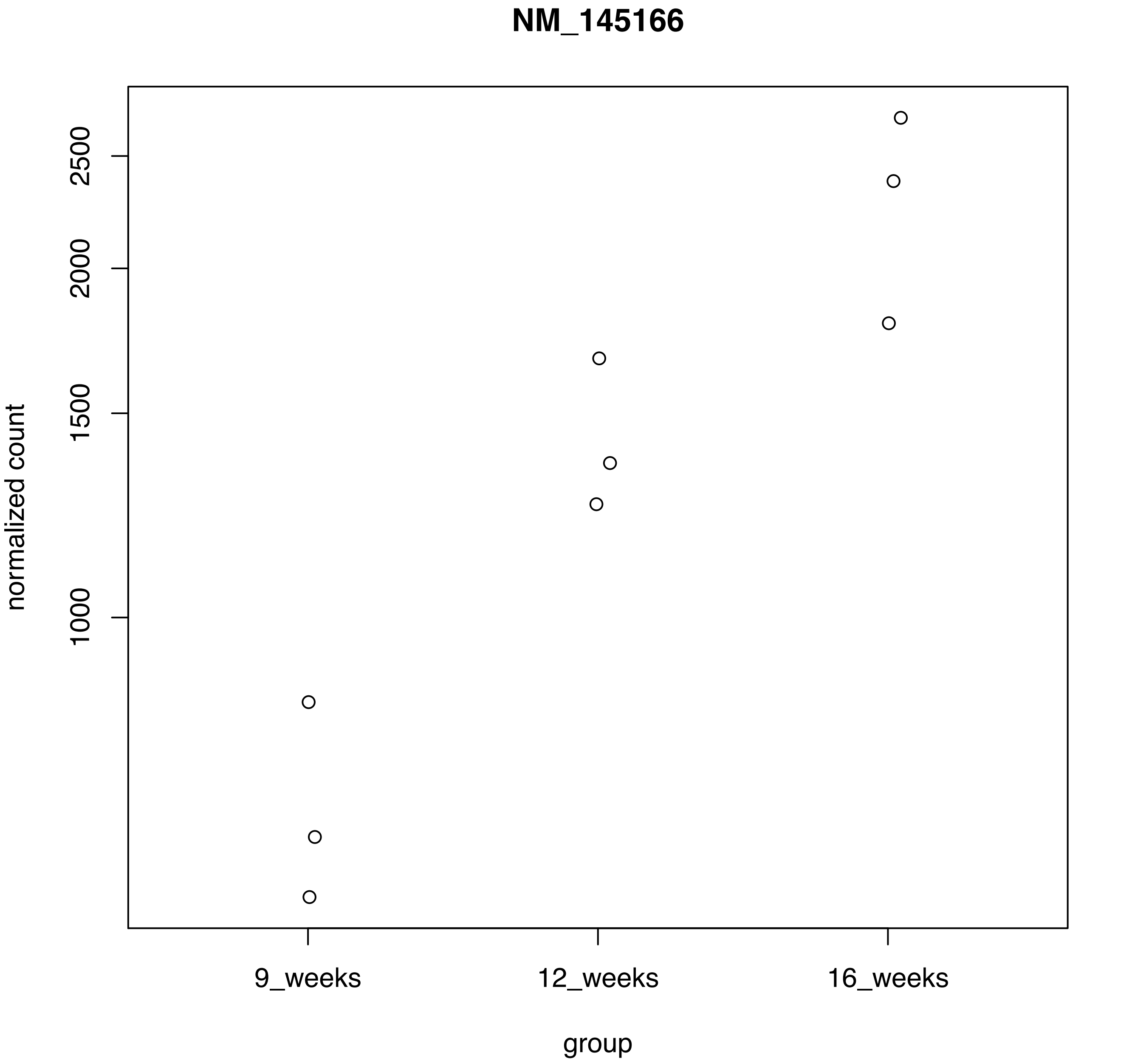

NM_145166
normalized count
1000
1500
2000
2500
9_weeks
12_weeks
16_weeks
group

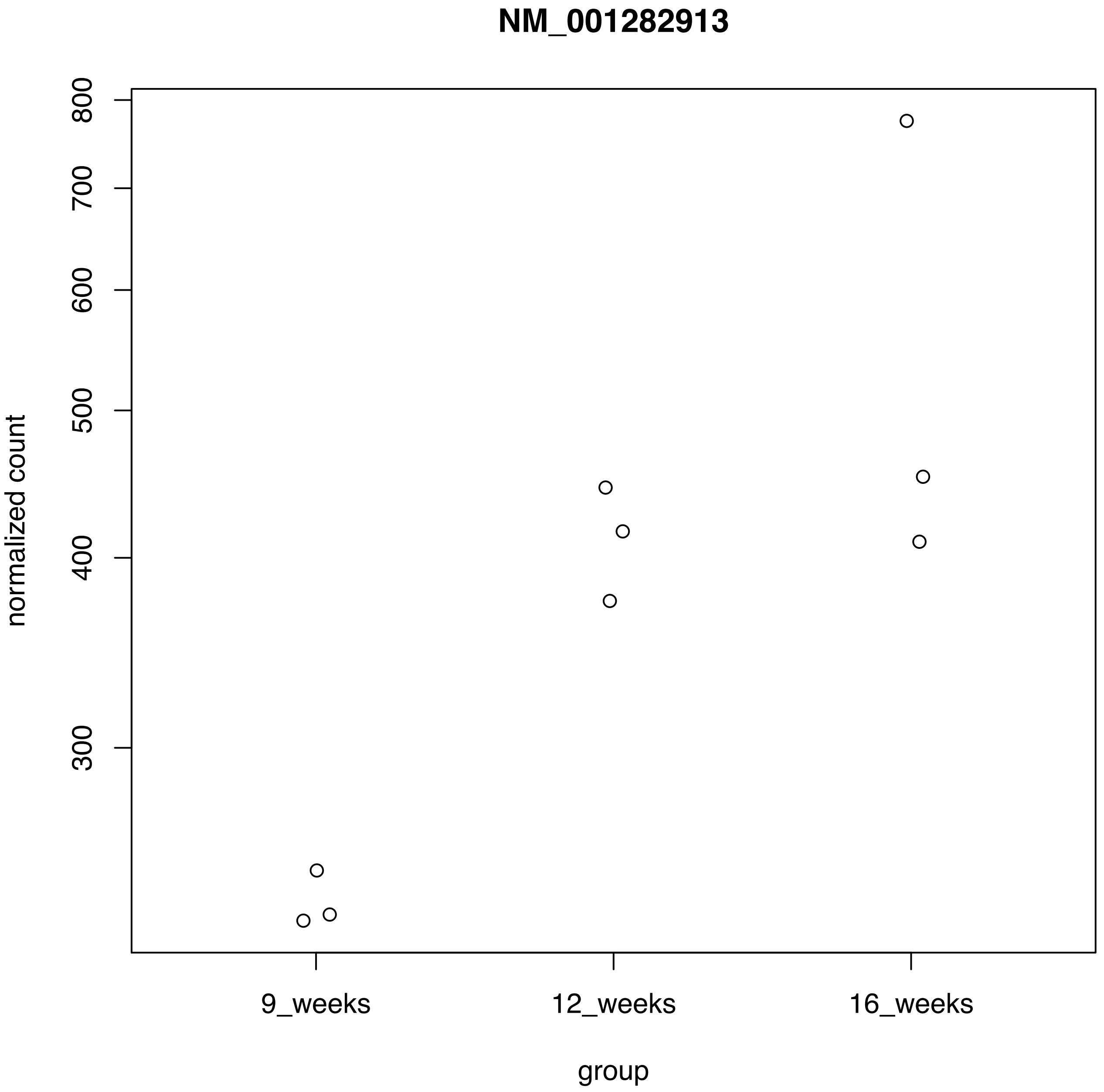

NM_001282913
normalized count
800
700
600
500
400
300
9_weeks
12_weeks
16_weeks
group

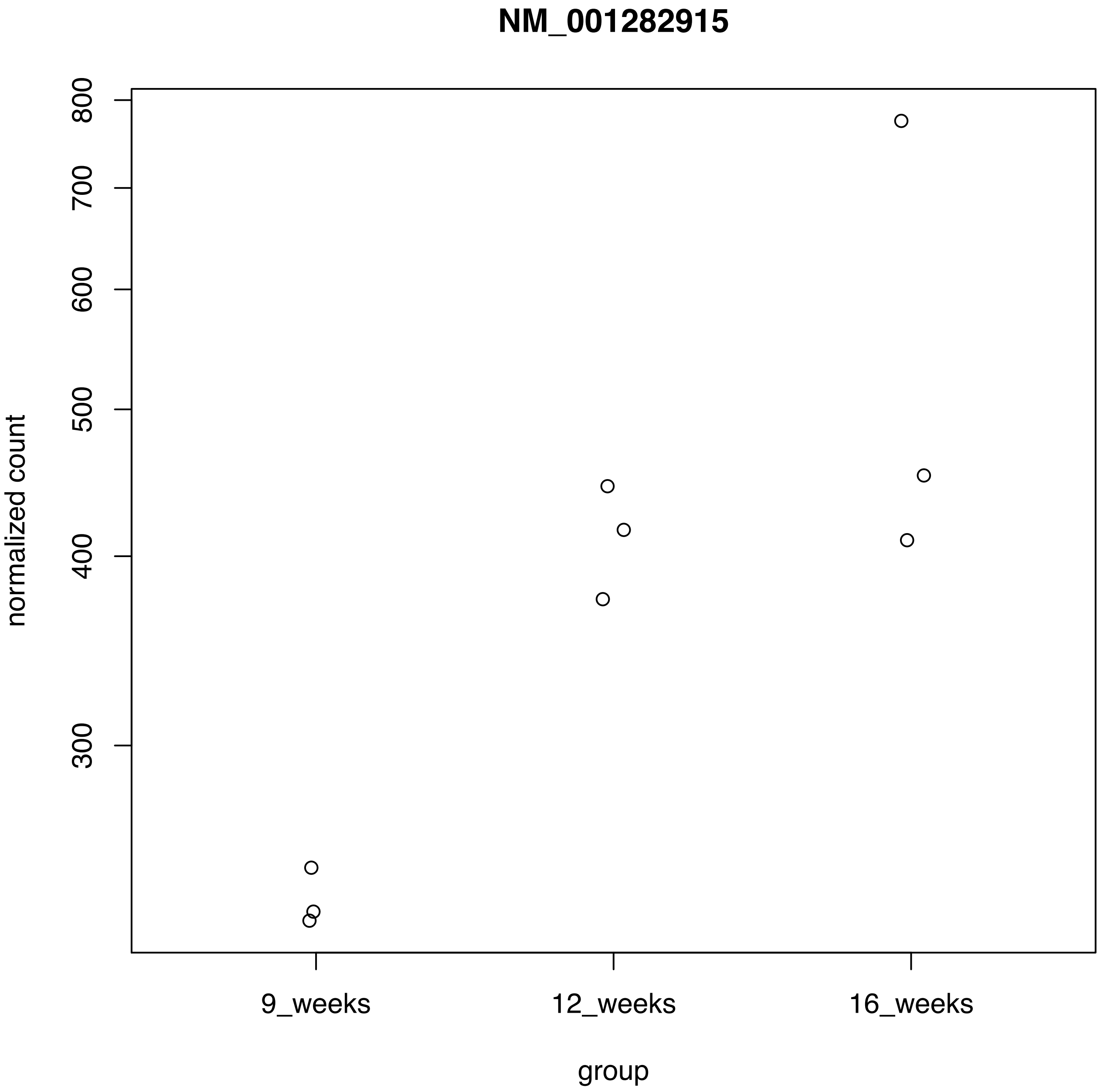
NM_001282915
normalized count
800
700
600
500
400
300
9_weeks
12_weeks
16_weeks
group

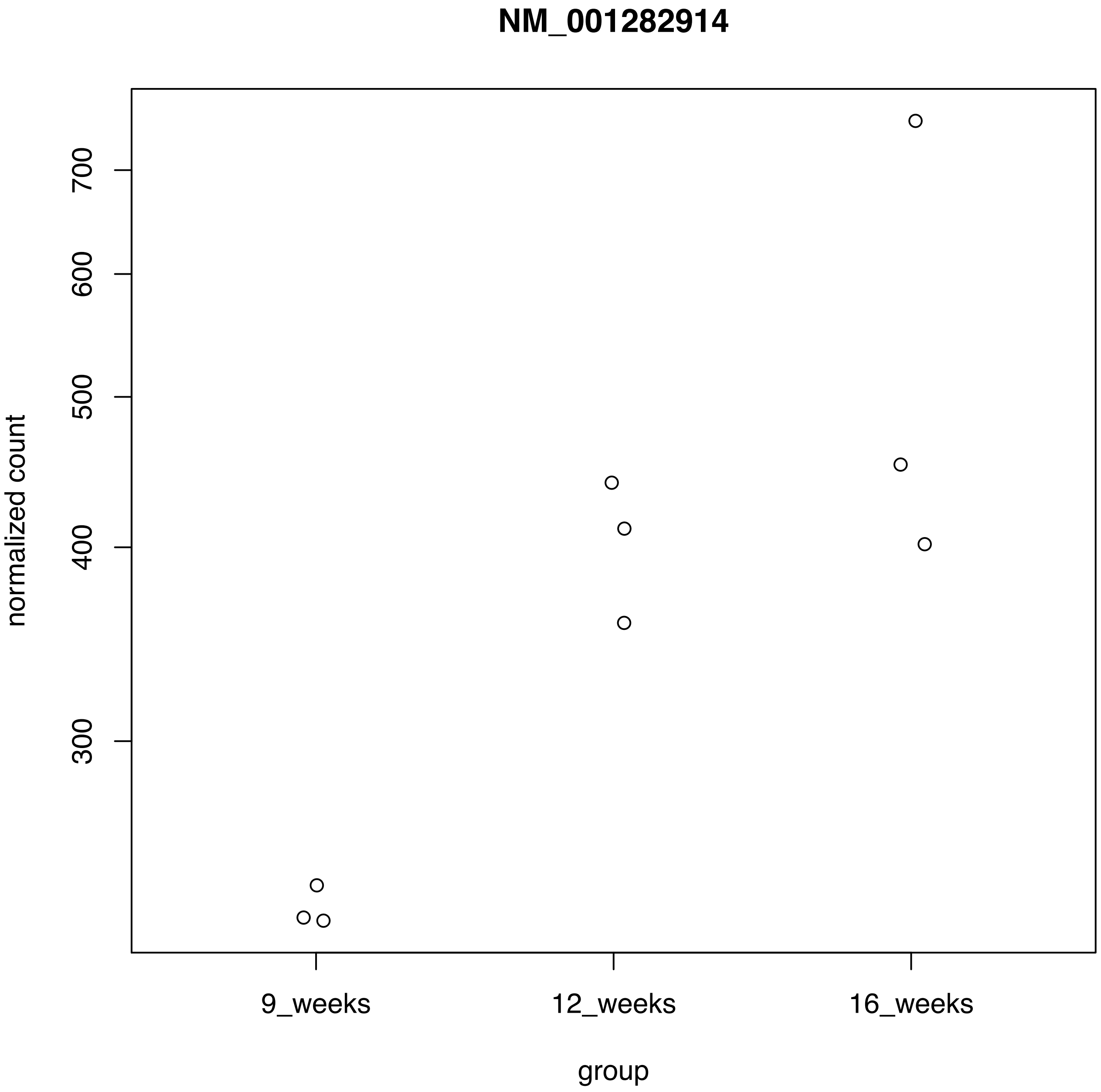
NM_001282914
normalized count
700
600
500
400
300
9_weeks
12_weeks
16_weeks
group

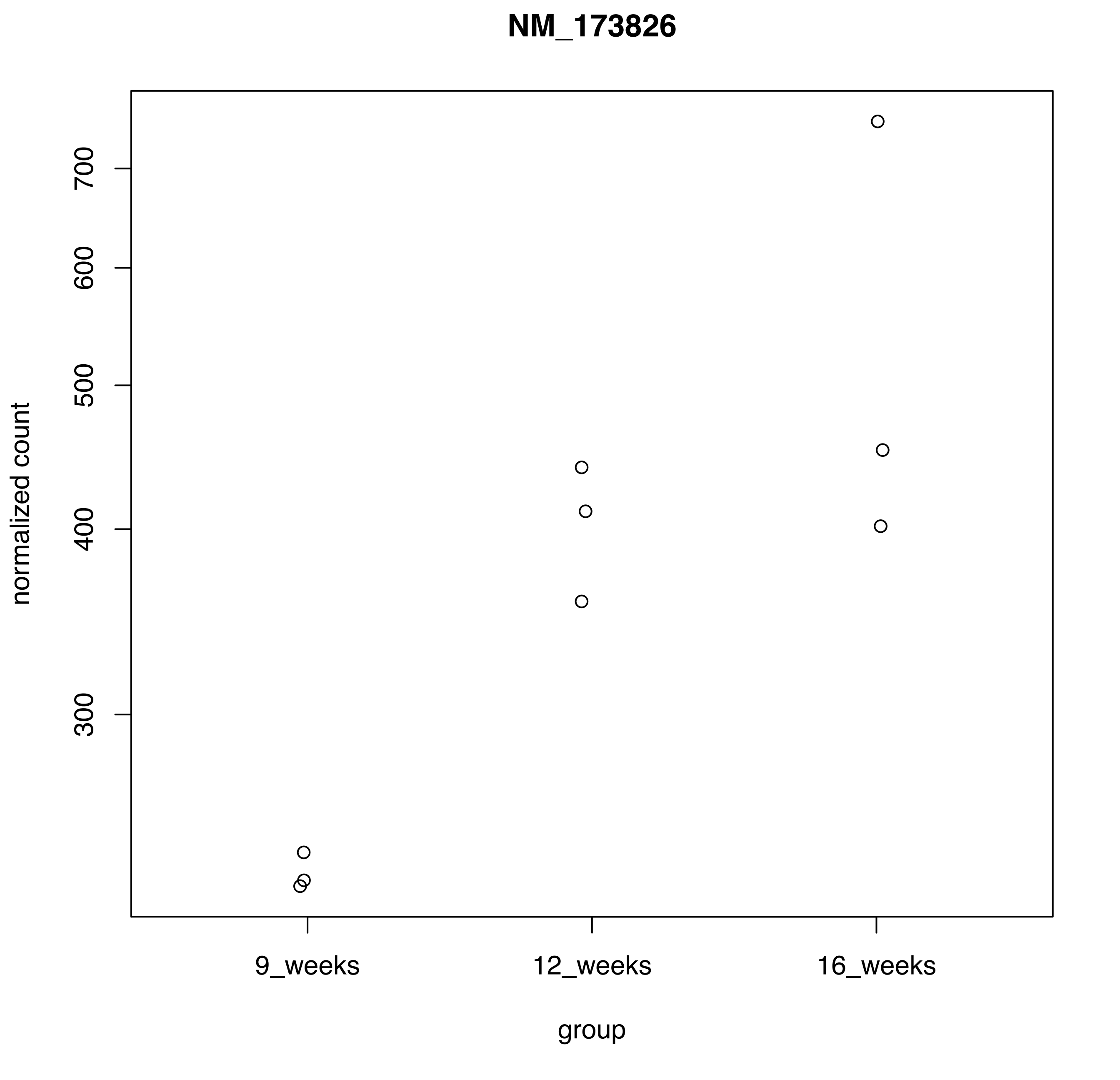

NM_173826
normalized count
300
400
500
600
700
9_weeks
12_weeks
16_weeks
group

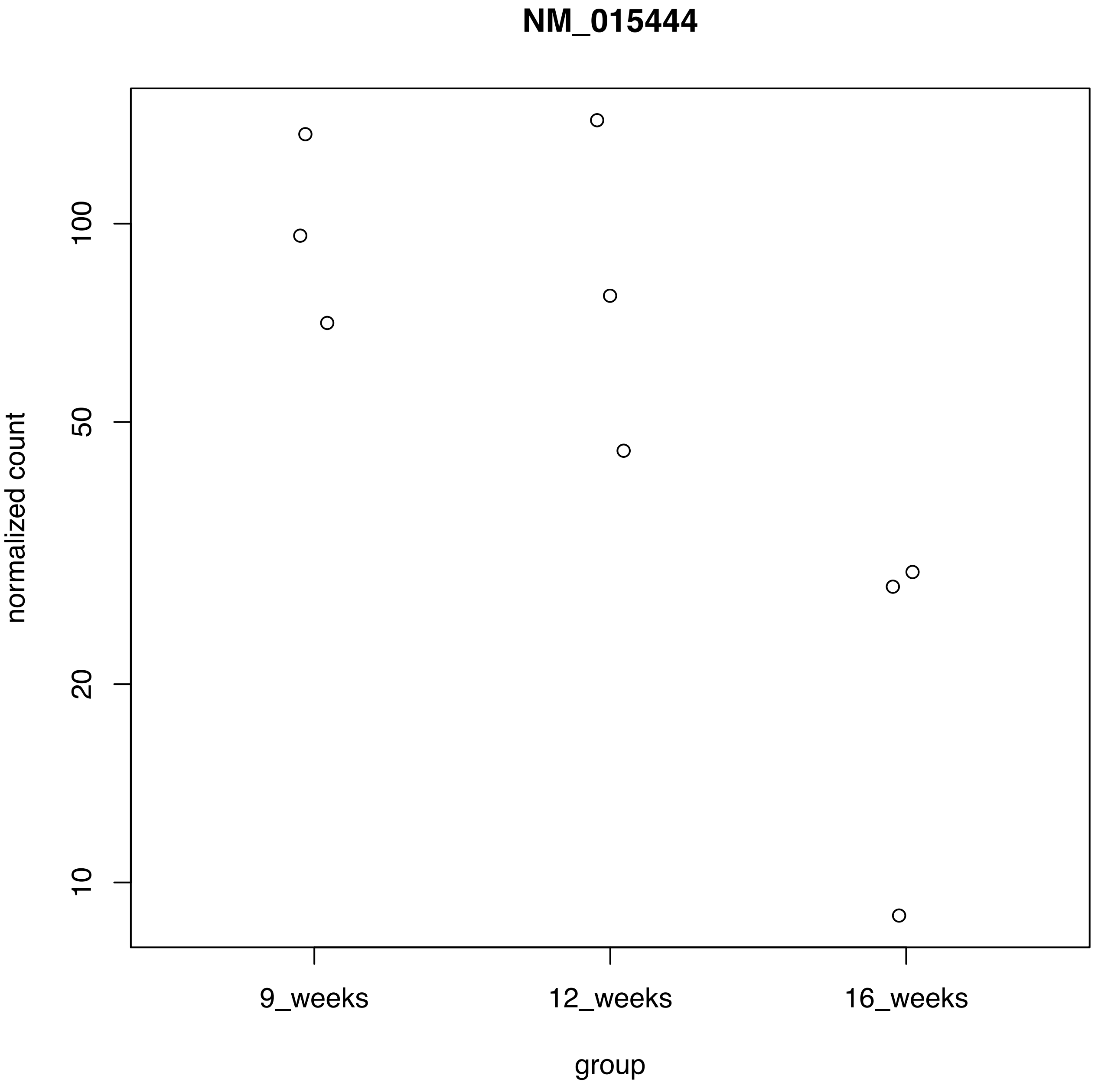

NM_015444
normalized count
100
50
20
10
9_weeks
12_weeks
16_weeks
group

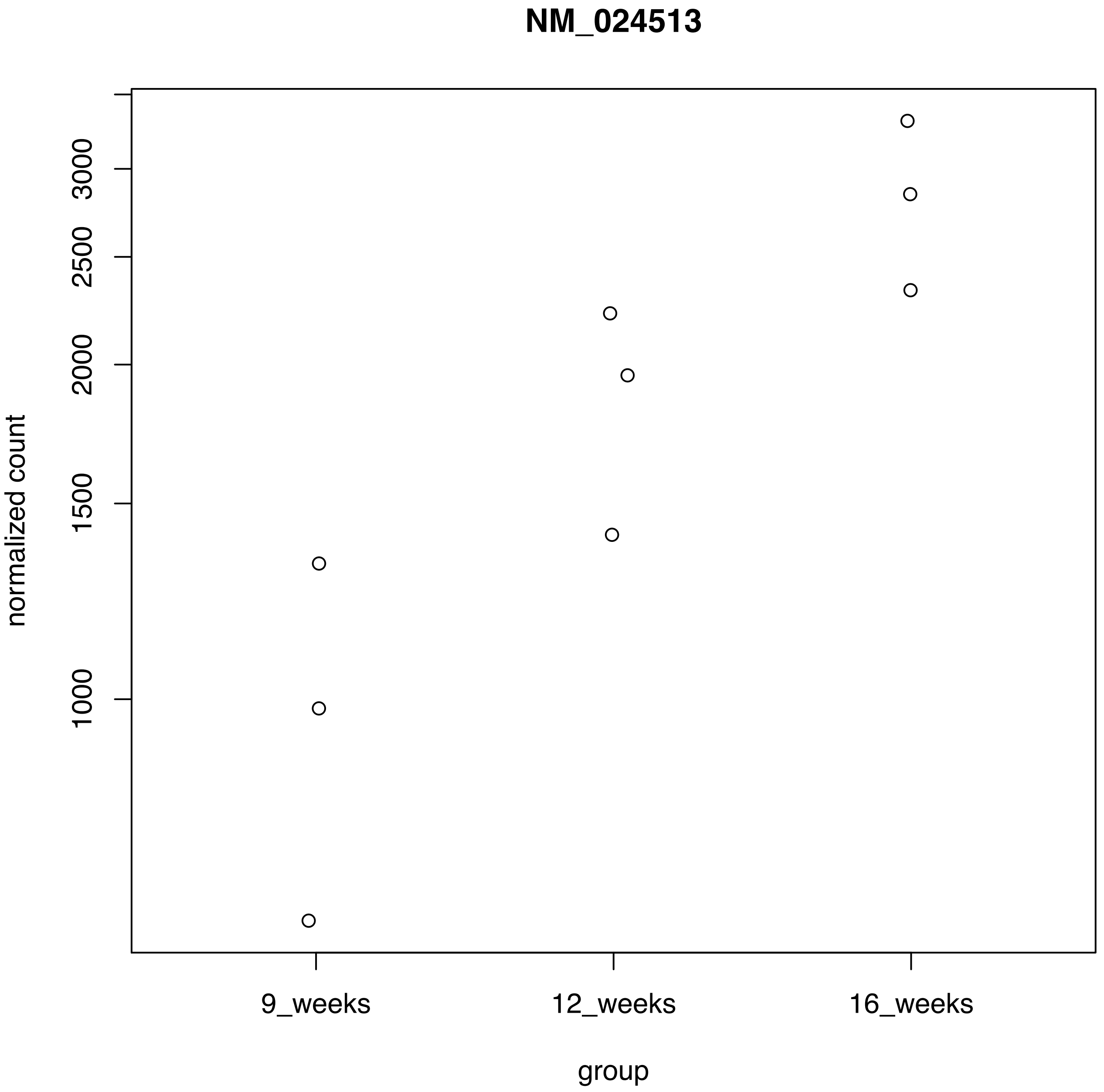
NM_024513
normalized count
1000
1500
2000
2500
3000
9_weeks
12_weeks
16_weeks
group

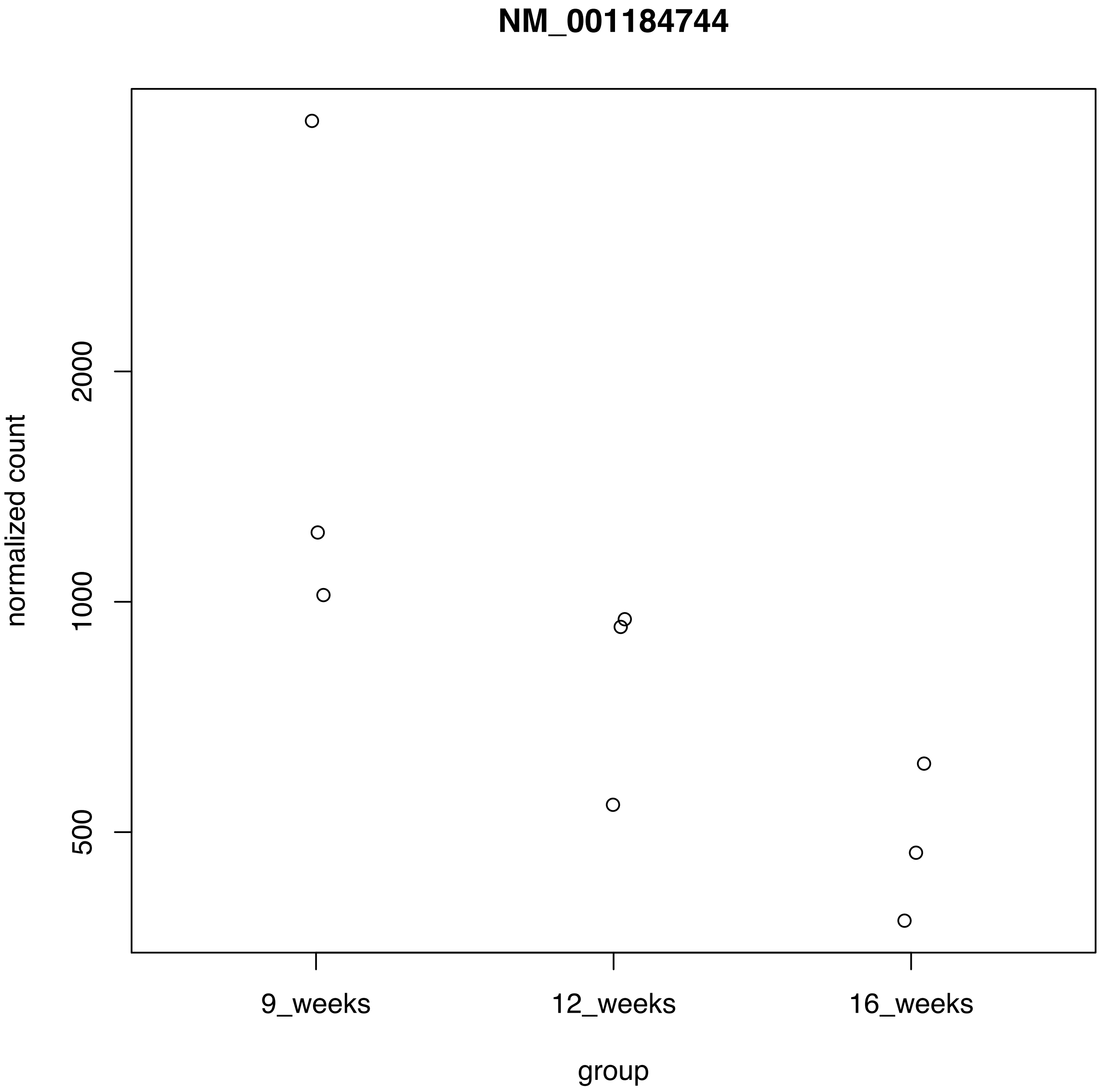
NM_001184744
normalized count
2000
1000
500
9_weeks
12_weeks
16_weeks
group

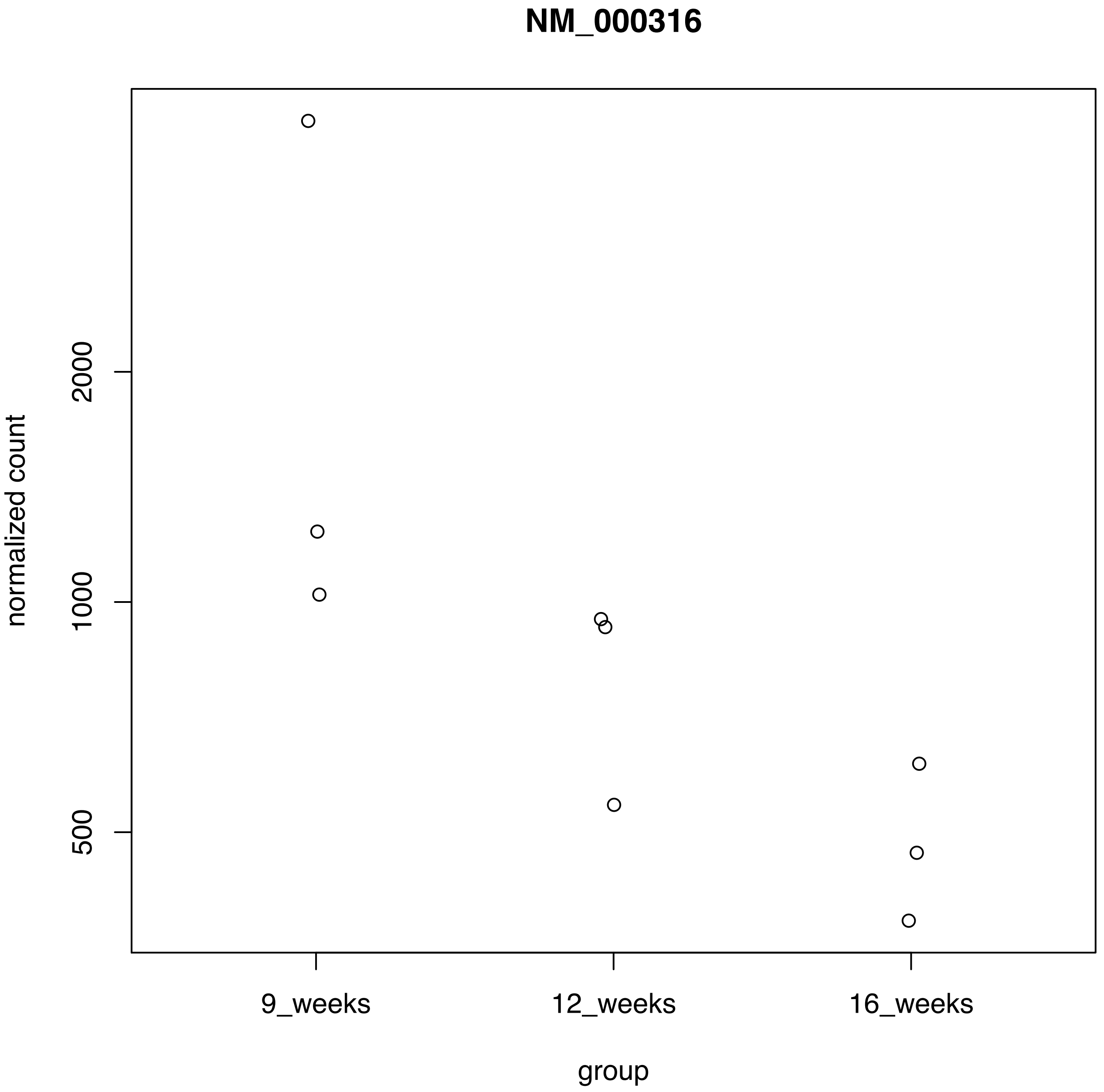

NM_000316
normalized count
500
1000
2000
9_weeks
12_weeks
16_weeks
group

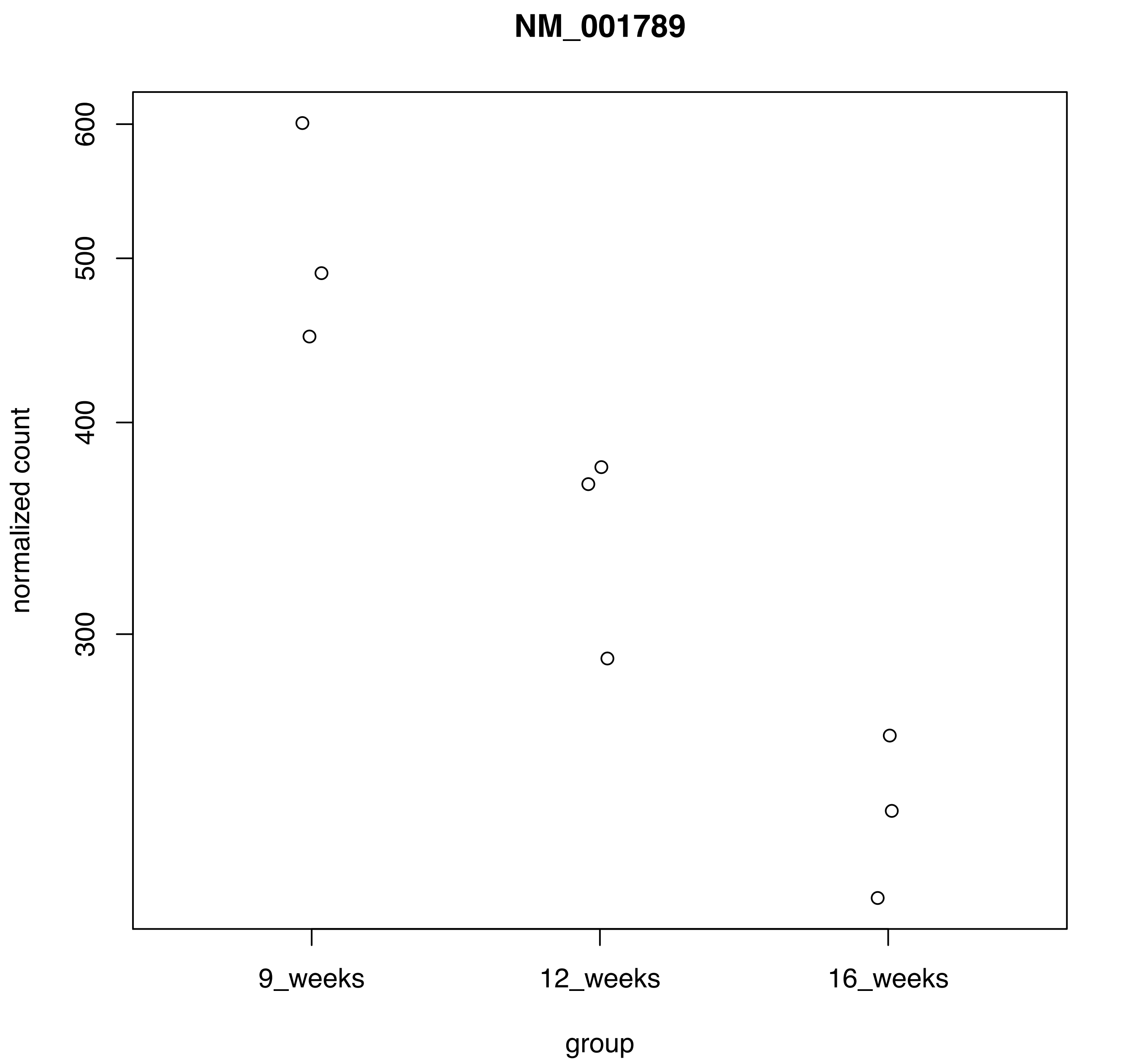

NM_001789
600
500
400
300
normalized count
9_weeks
12_weeks
16_weeks
group

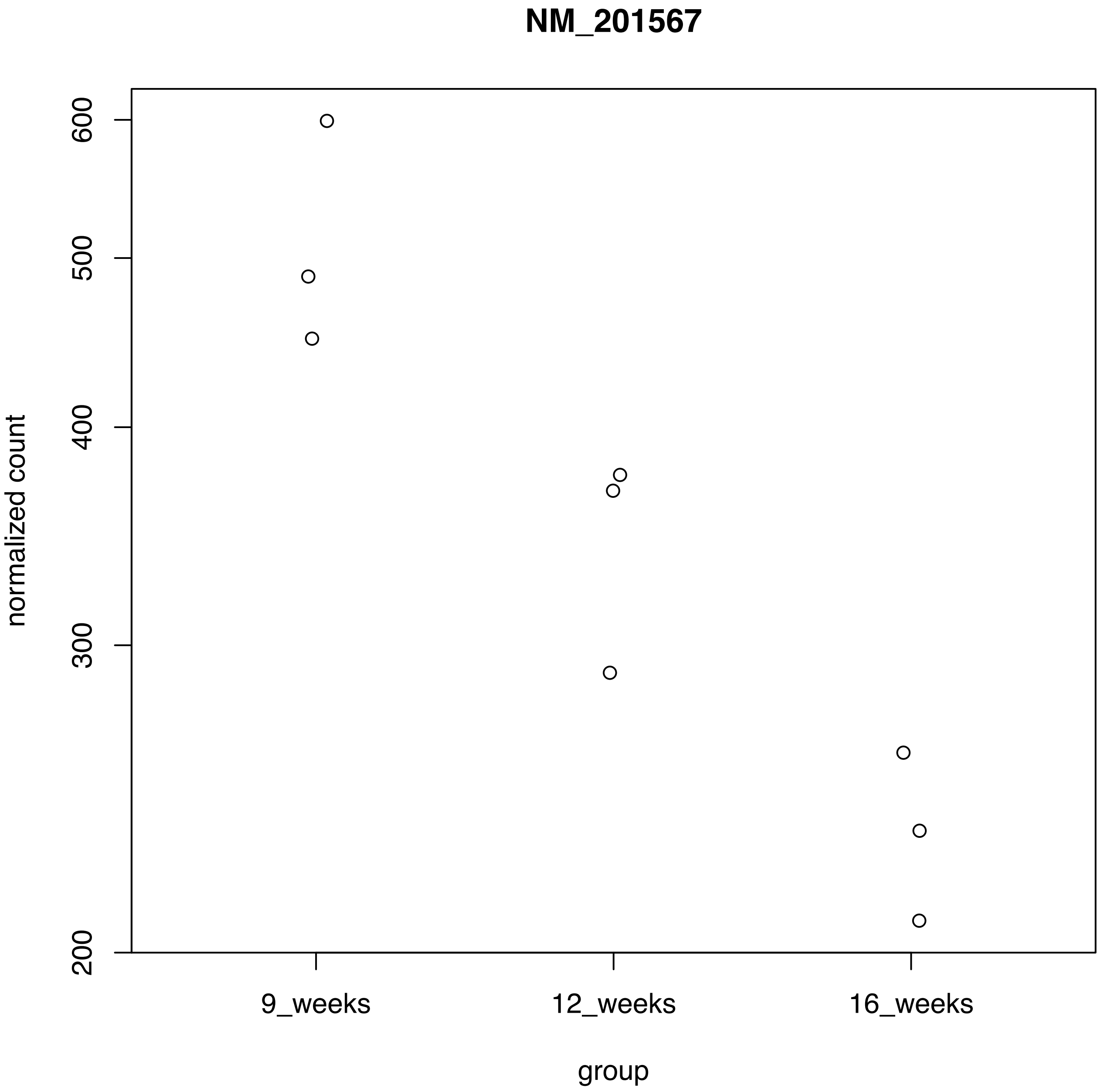
NM_201567
normalized count
600
500
400
300
200
9_weeks
12_weeks
16_weeks
group

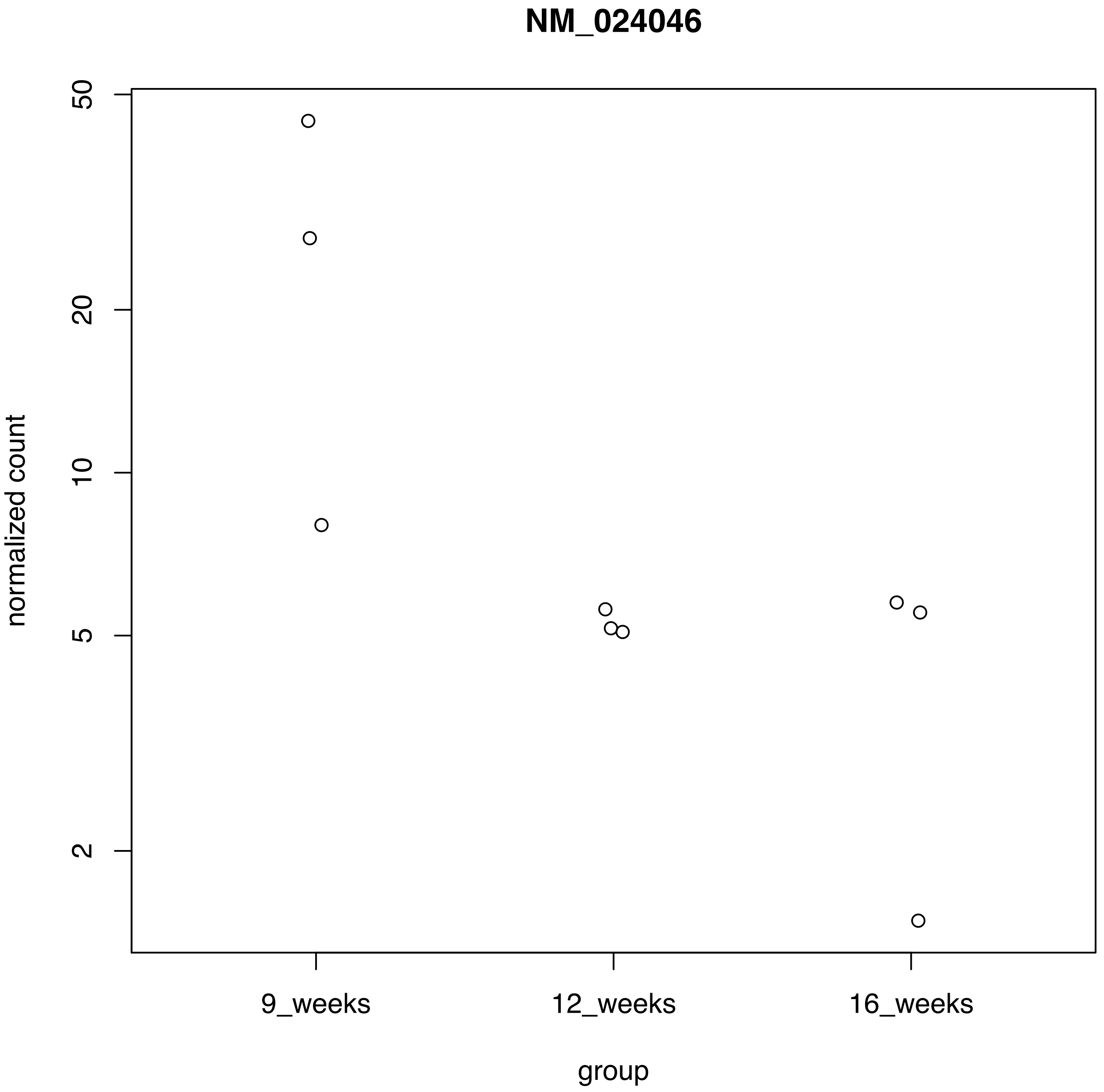

NM_024046
normalized count
50
20
10
5
2
9_weeks
12_weeks
16_weeks
group

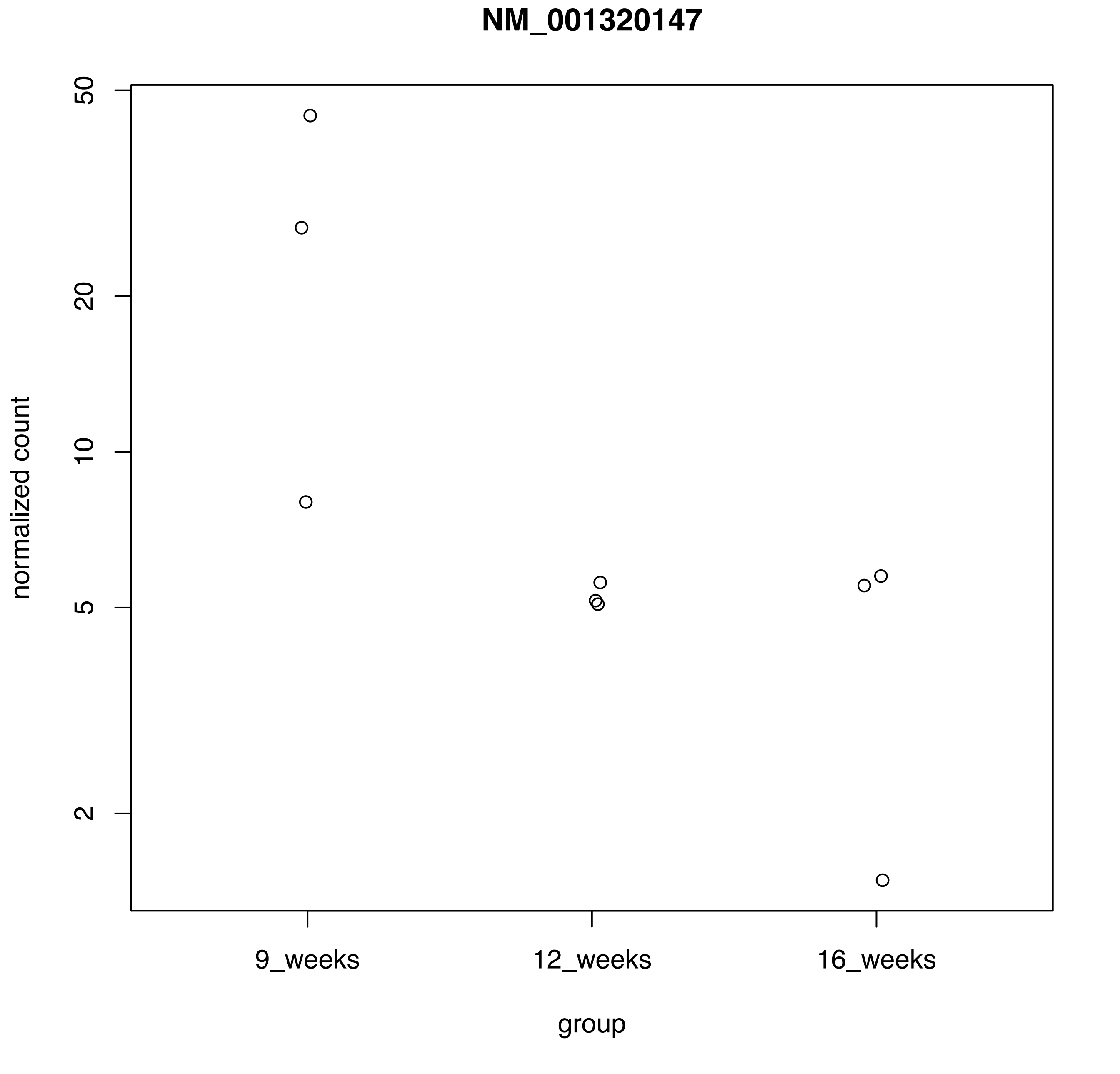

NM_001320147
normalized count
50
20
10
5
2
9_weeks
12_weeks
16_weeks
group

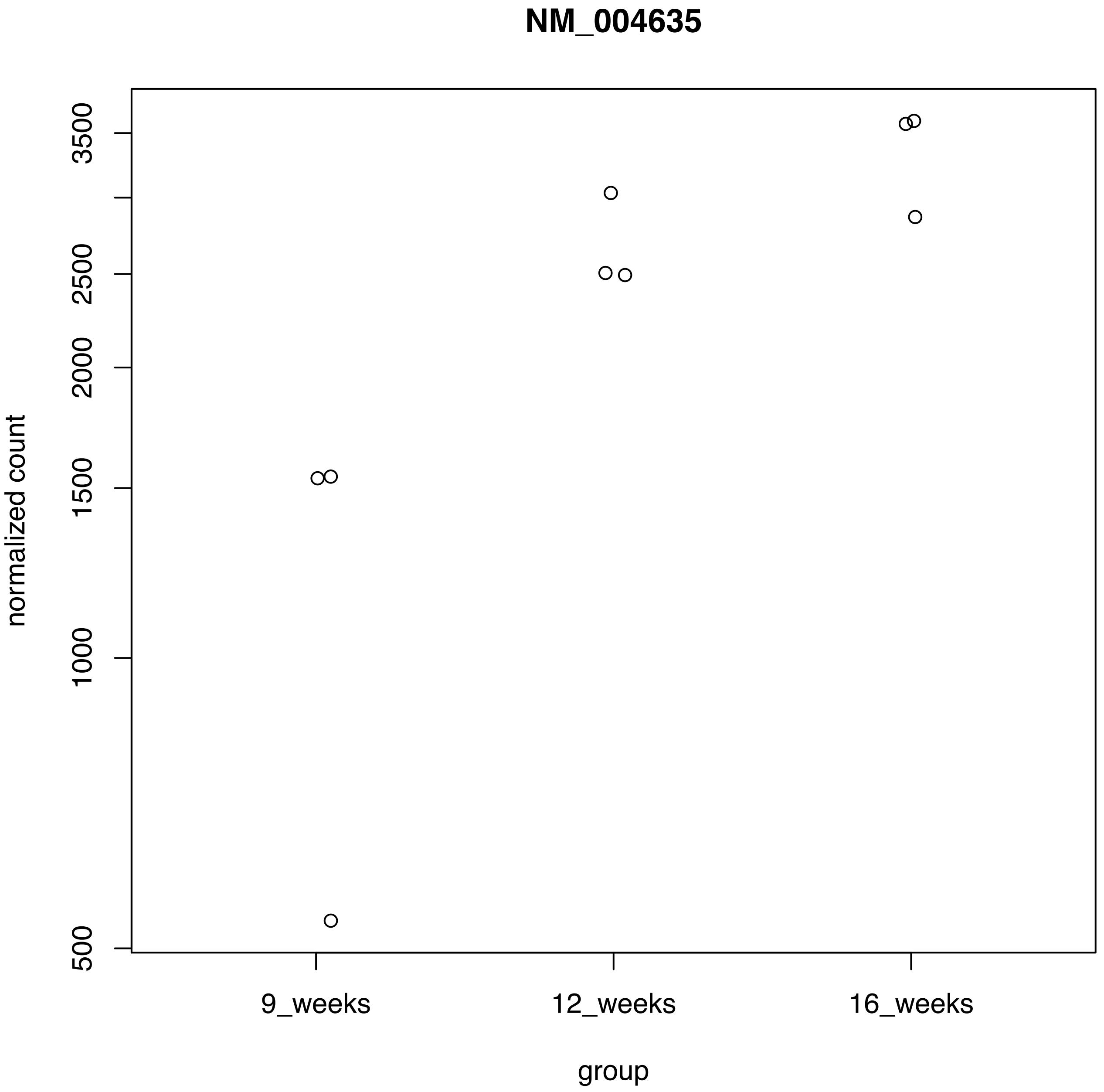

NM_004635
normalized count
3500
2500
2000
1500
1000
500
9_weeks
12_weeks
16_weeks
group

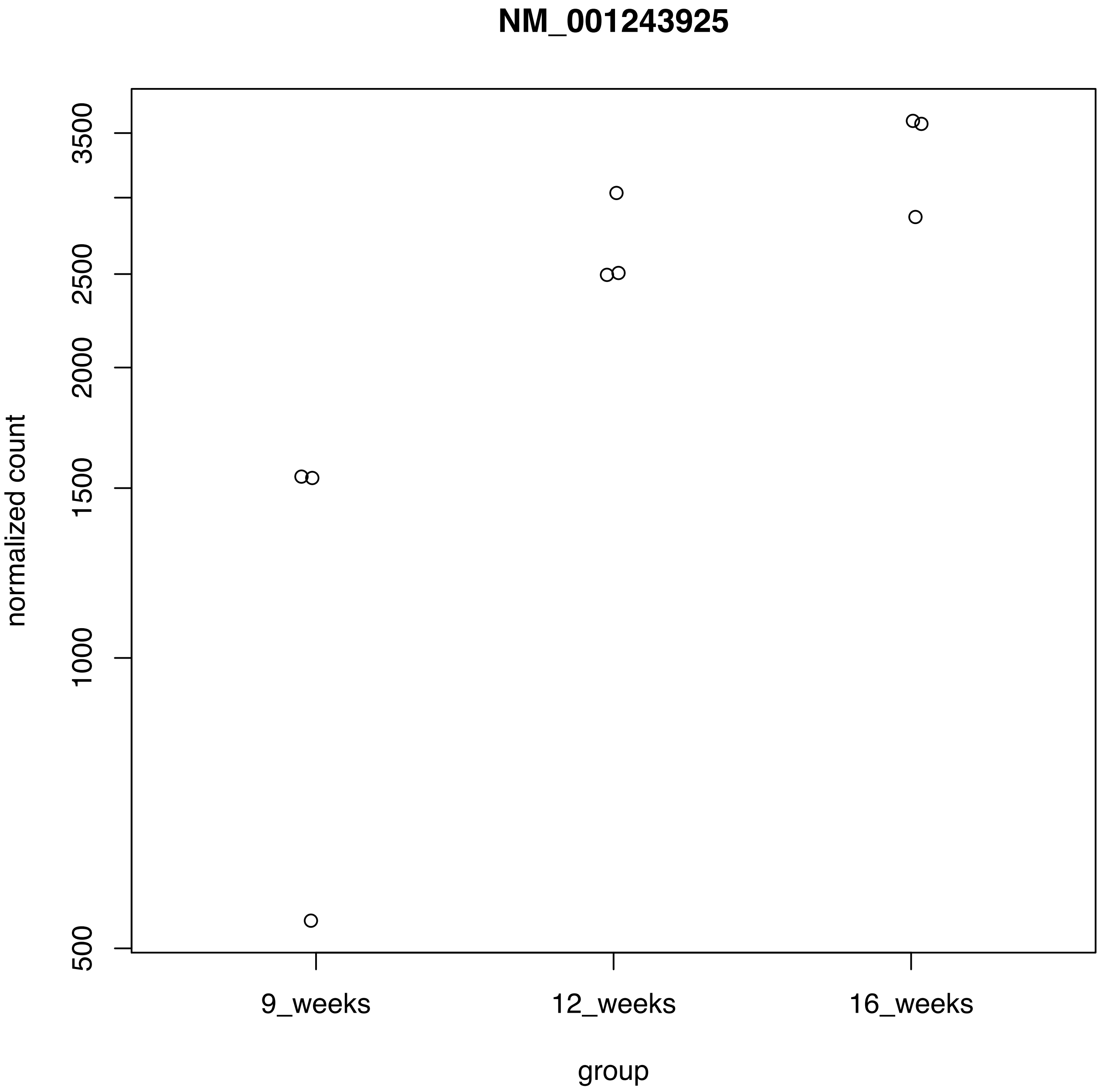
NM_001243925
normalized count
500
1000
1500
2000
2500
3500
9_weeks
12_weeks
16_weeks
group

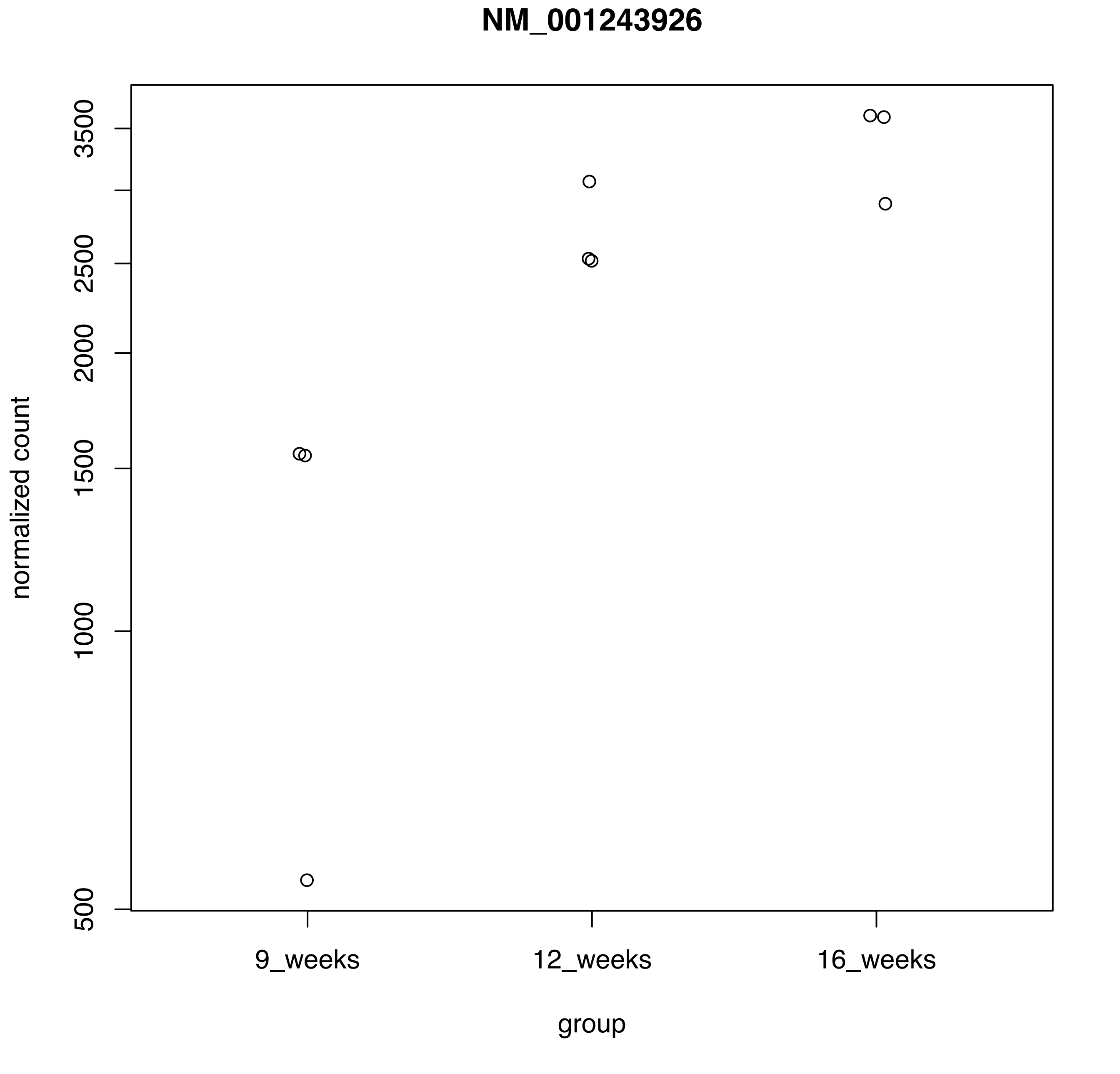
NM_001243926
normalized count
500
1000
1500
2000
2500
3500
9_weeks
12_weeks
16_weeks
group

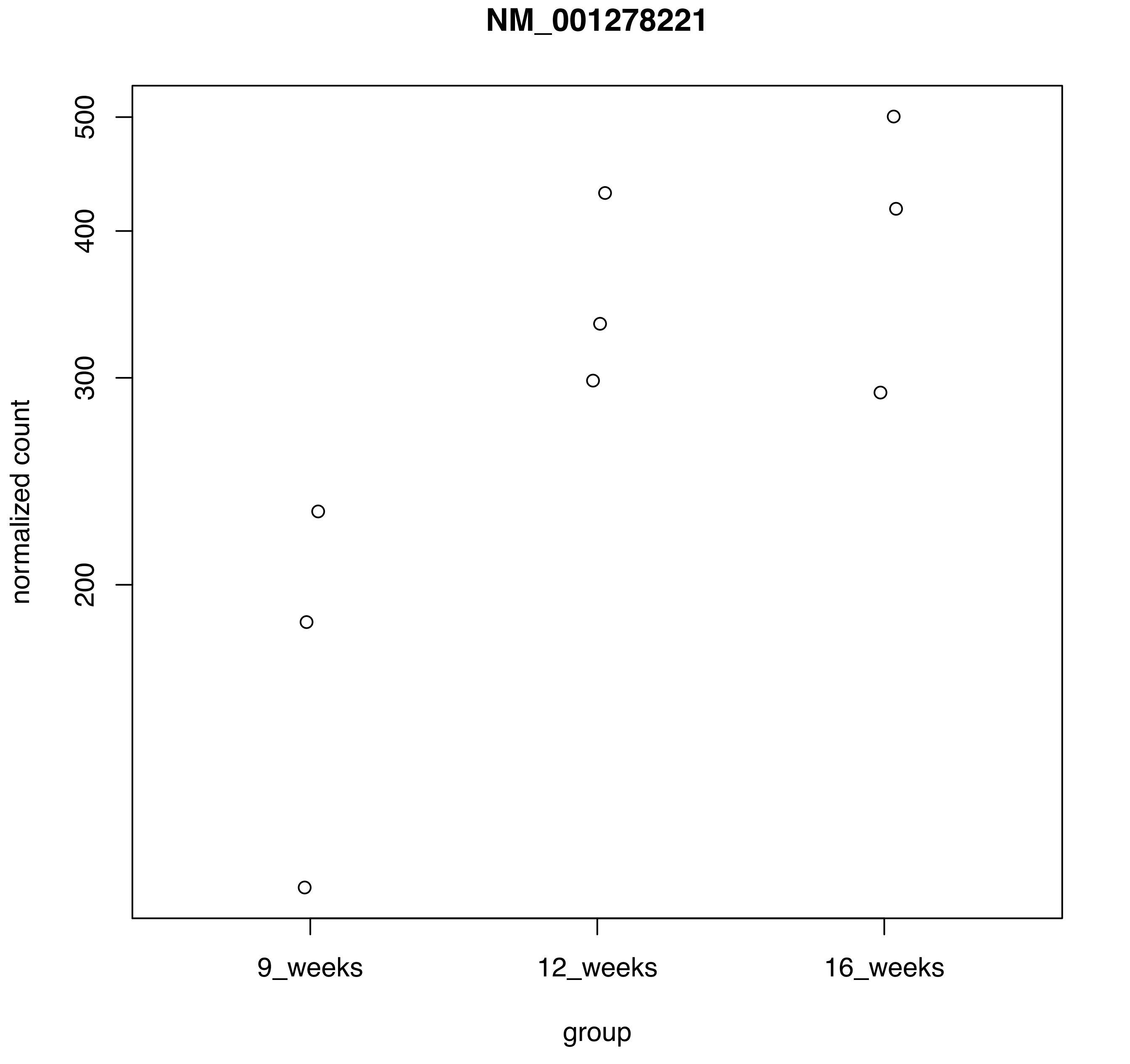

NM_001278221
normalized count
500
400
300
200
9_weeks
12_weeks
16_weeks
group

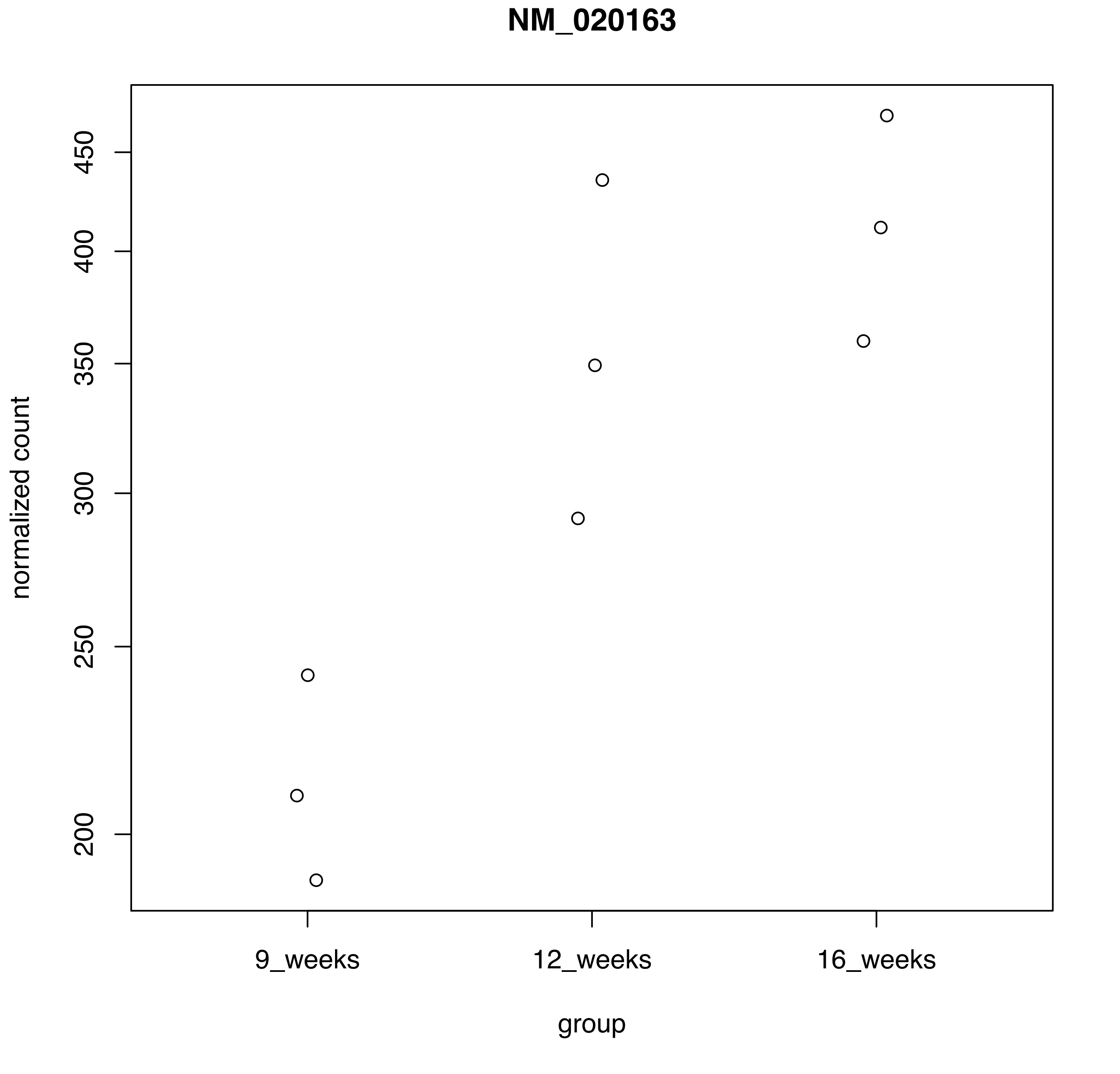
NM_020163
normalized count
450
400
350
300
250
200
9_weeks
12_weeks
16_weeks
group

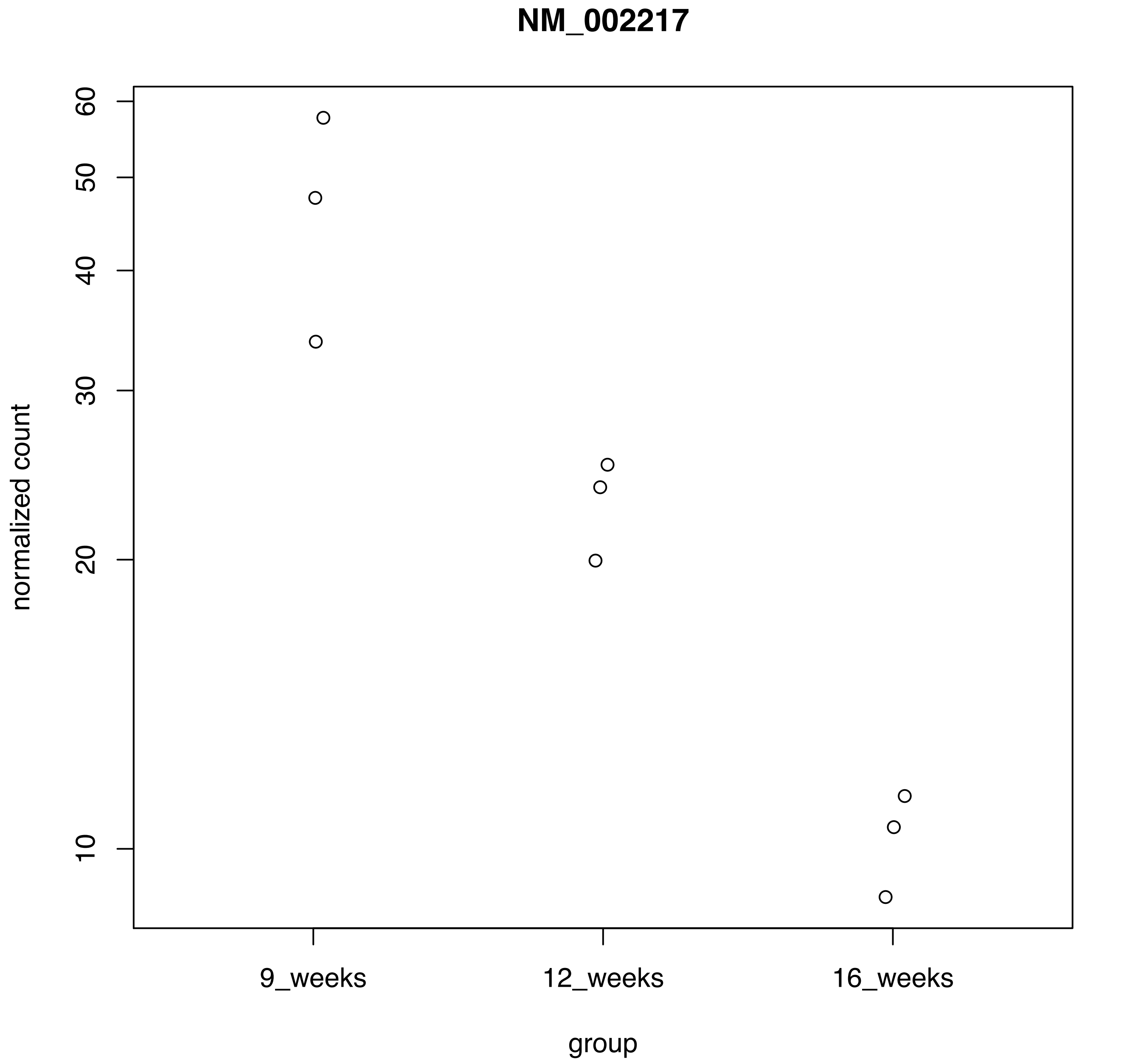
NM_002217
normalized count
60
50
40
30
20
10
9_weeks
12_weeks
16_weeks
group

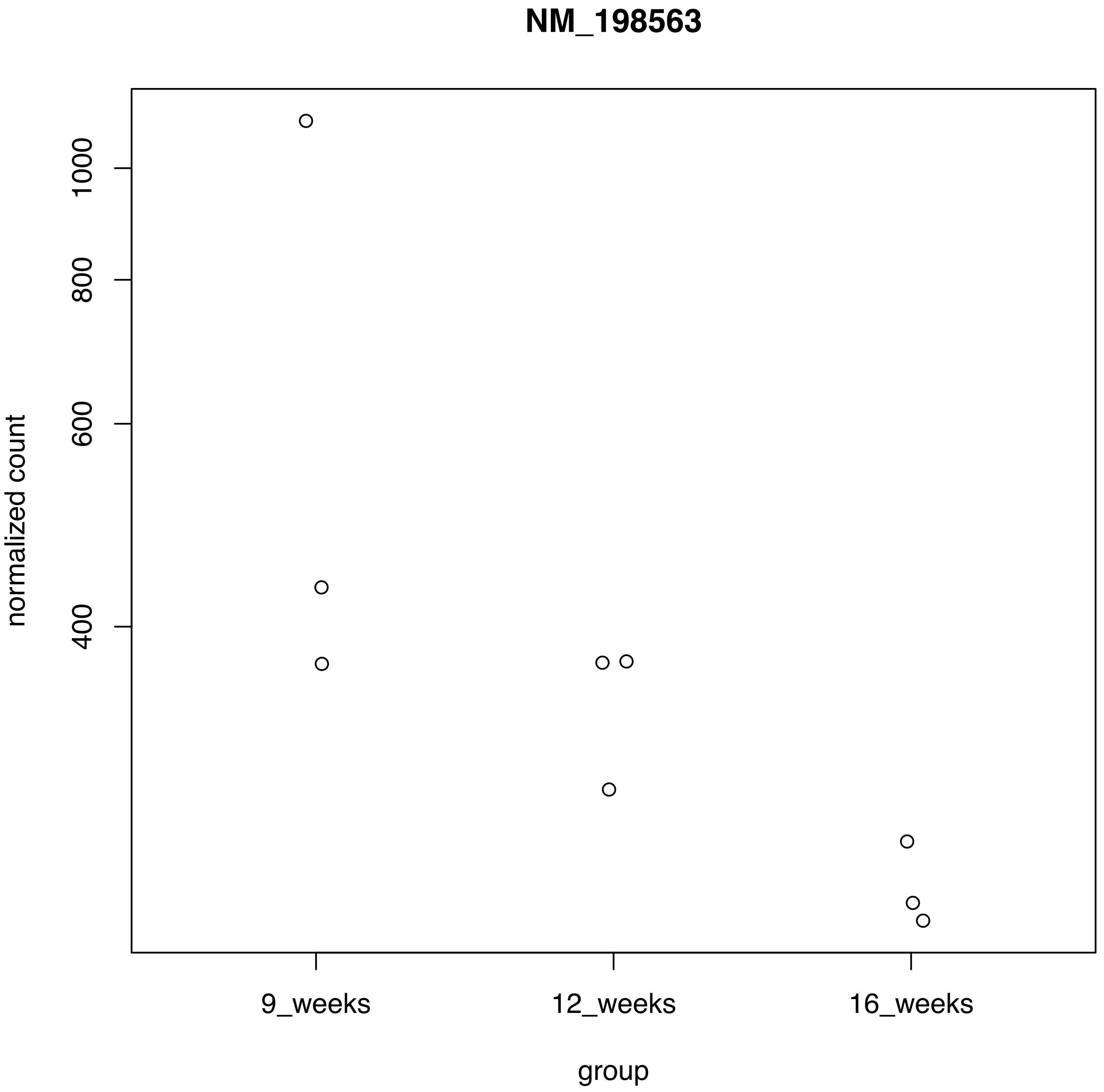
NM_198563
normalized count
1000
800
600
400
9_weeks
12_weeks
16_weeks
group

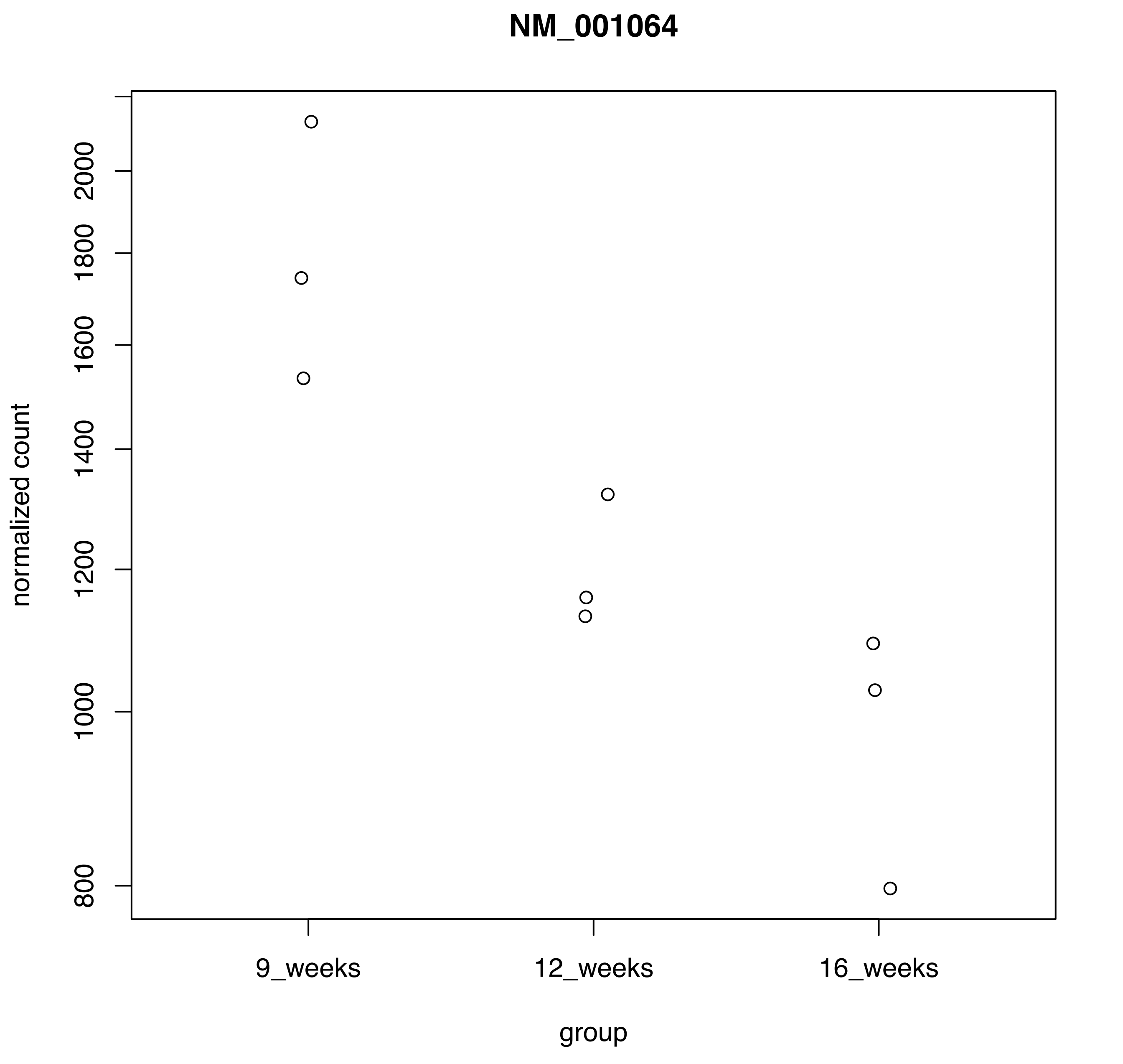
NM_001064
normalized count
800
1000
1200
1400
1600
1800
2000
9_weeks
12_weeks
16_weeks
group

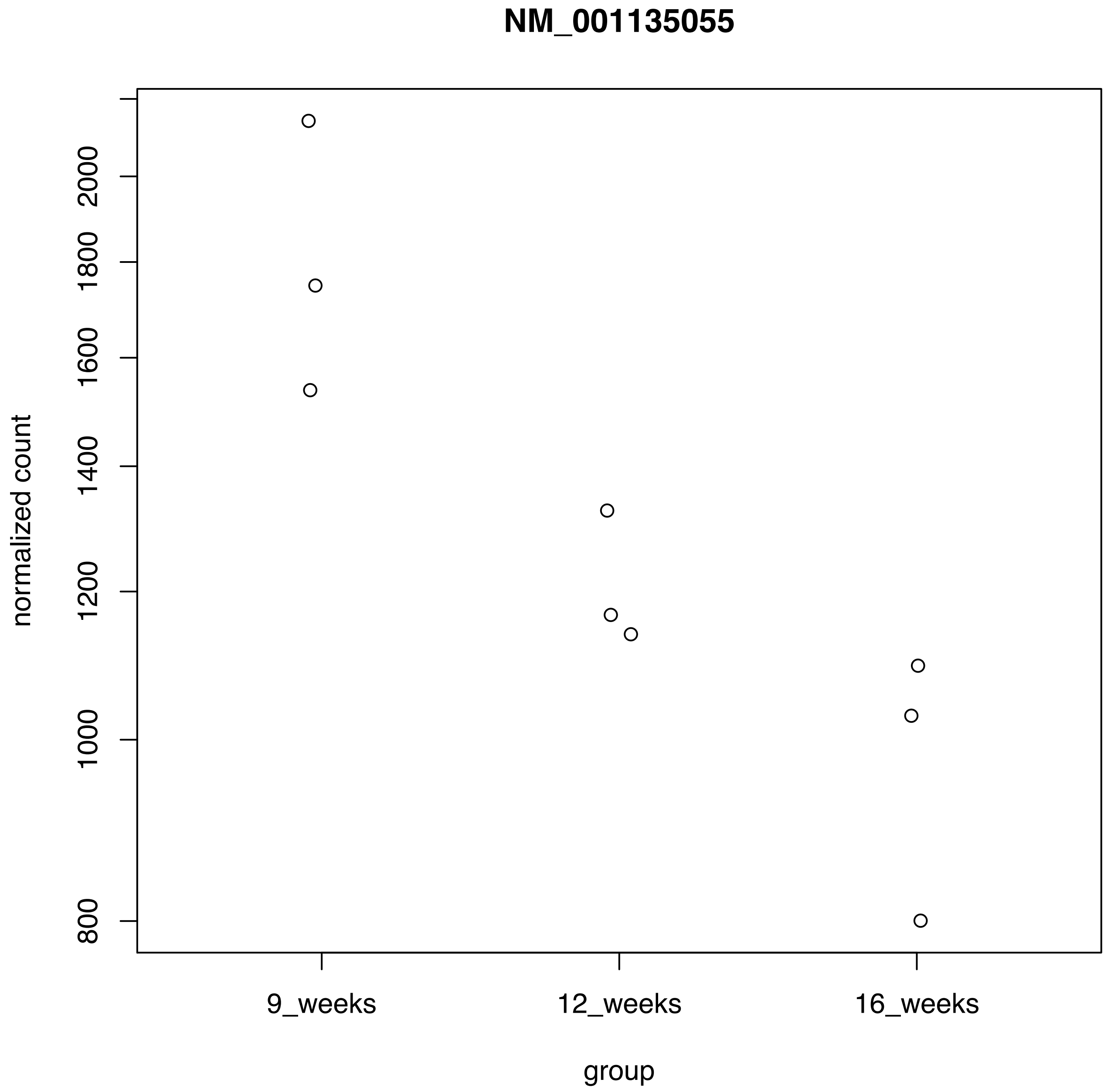
NM_001135055
normalized count
2000
1800
1600
1400
1200
1000
800
9_weeks
12_weeks
16_weeks
group

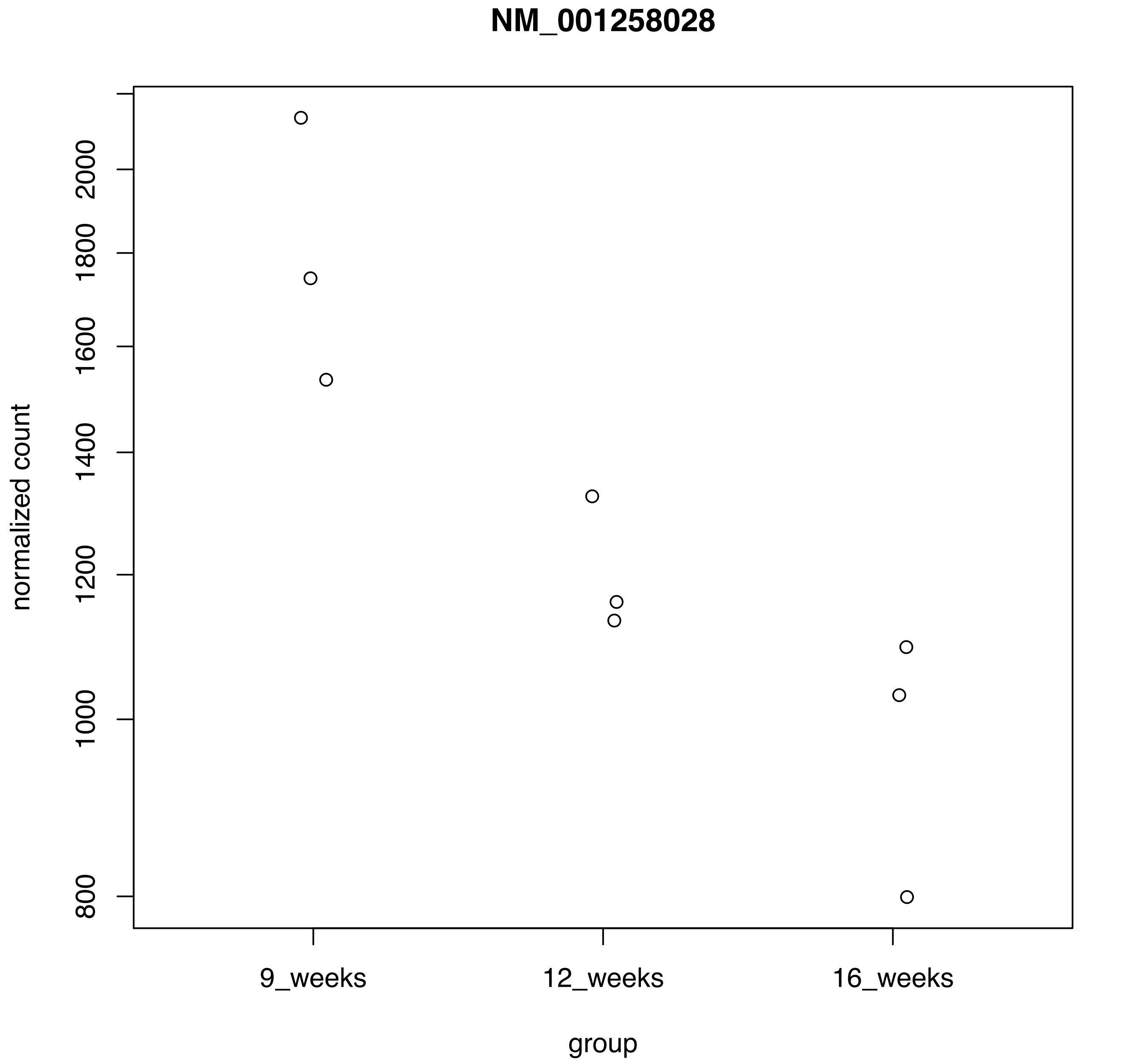
NM_001258028
normalized count
2000
1800
1600
1400
1200
1000
800
9_weeks
12_weeks
16_weeks
group

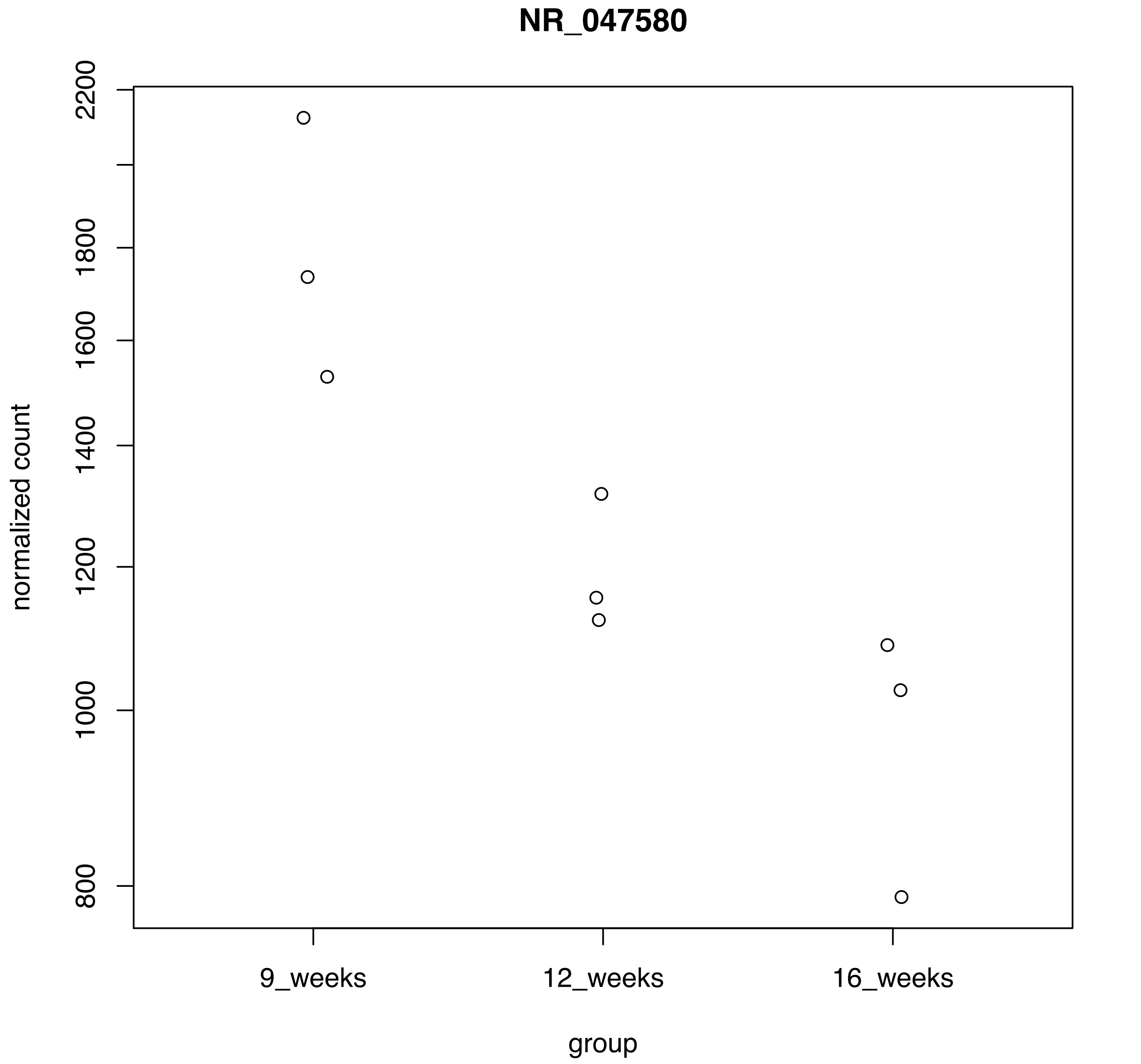

NR_047580
normalized count
2200
1800
1600
1400
1200
1000
800
9_weeks
12_weeks
16_weeks
group

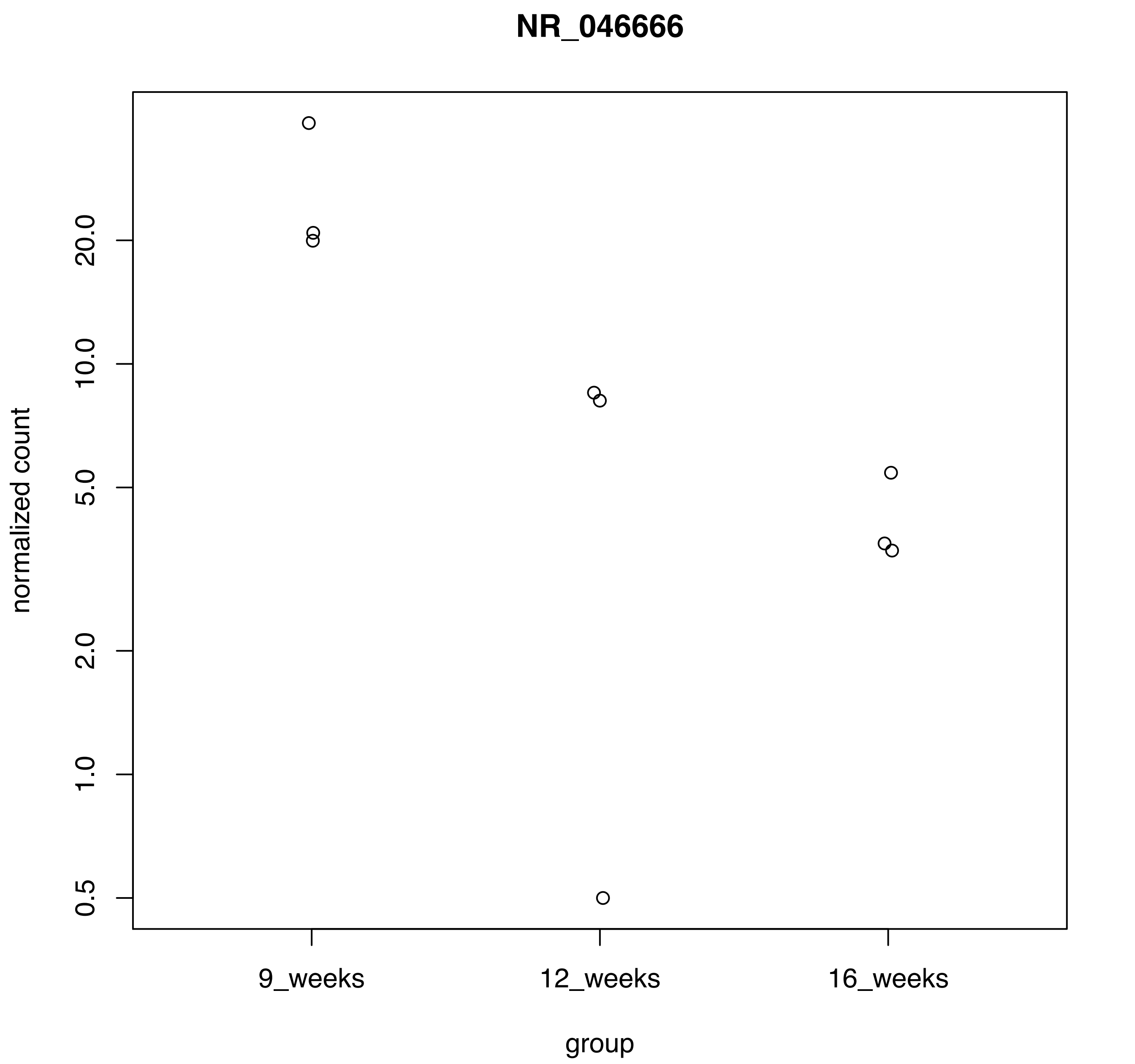
NR_046666
normalized count
0.5
1.0
2.0
5.0
10.0
20.0
9_weeks
12_weeks
16_weeks
group

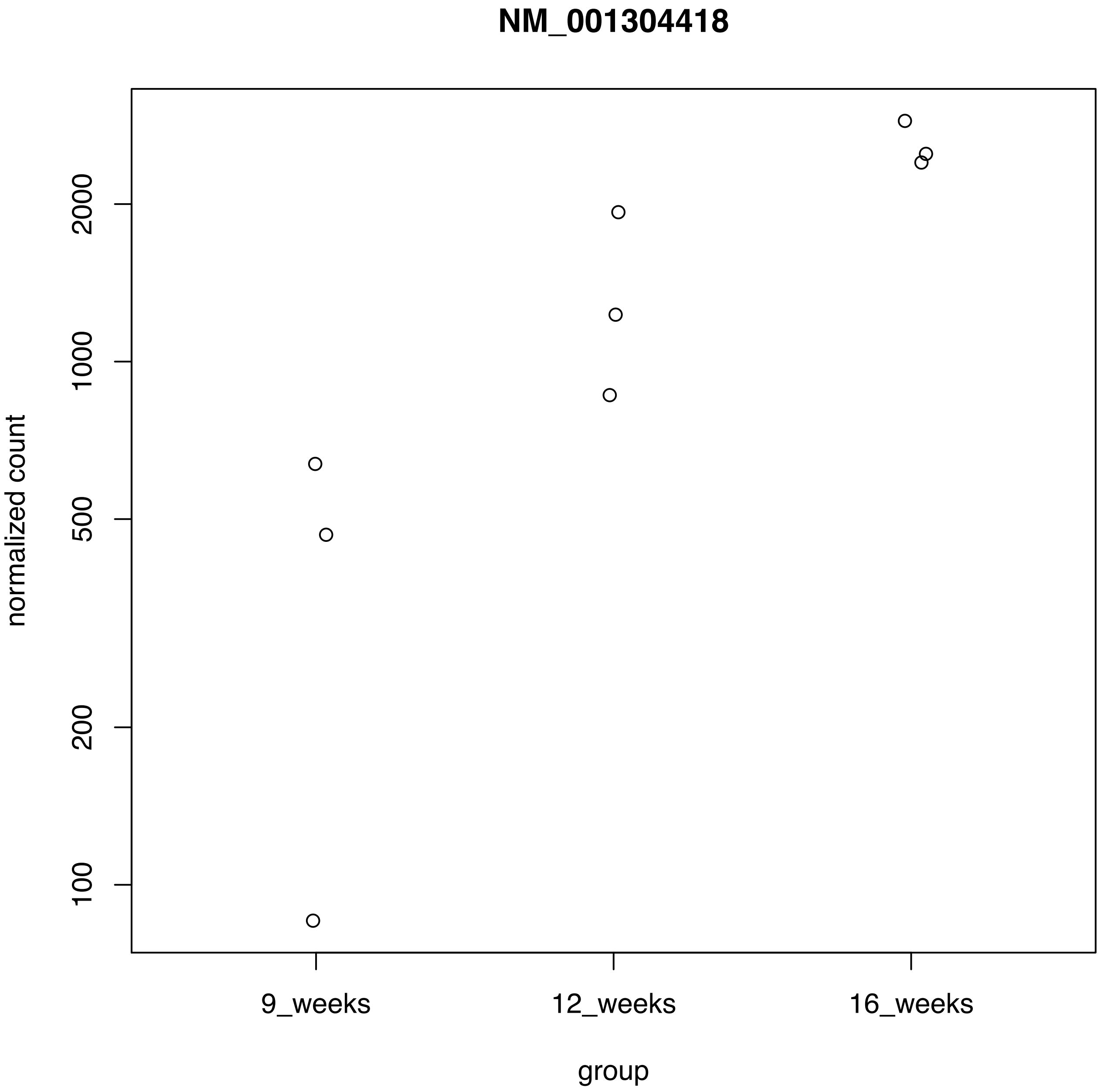

NM_001304418
normalized count
2000
1000
500
200
100
9_weeks
12_weeks
16_weeks
group

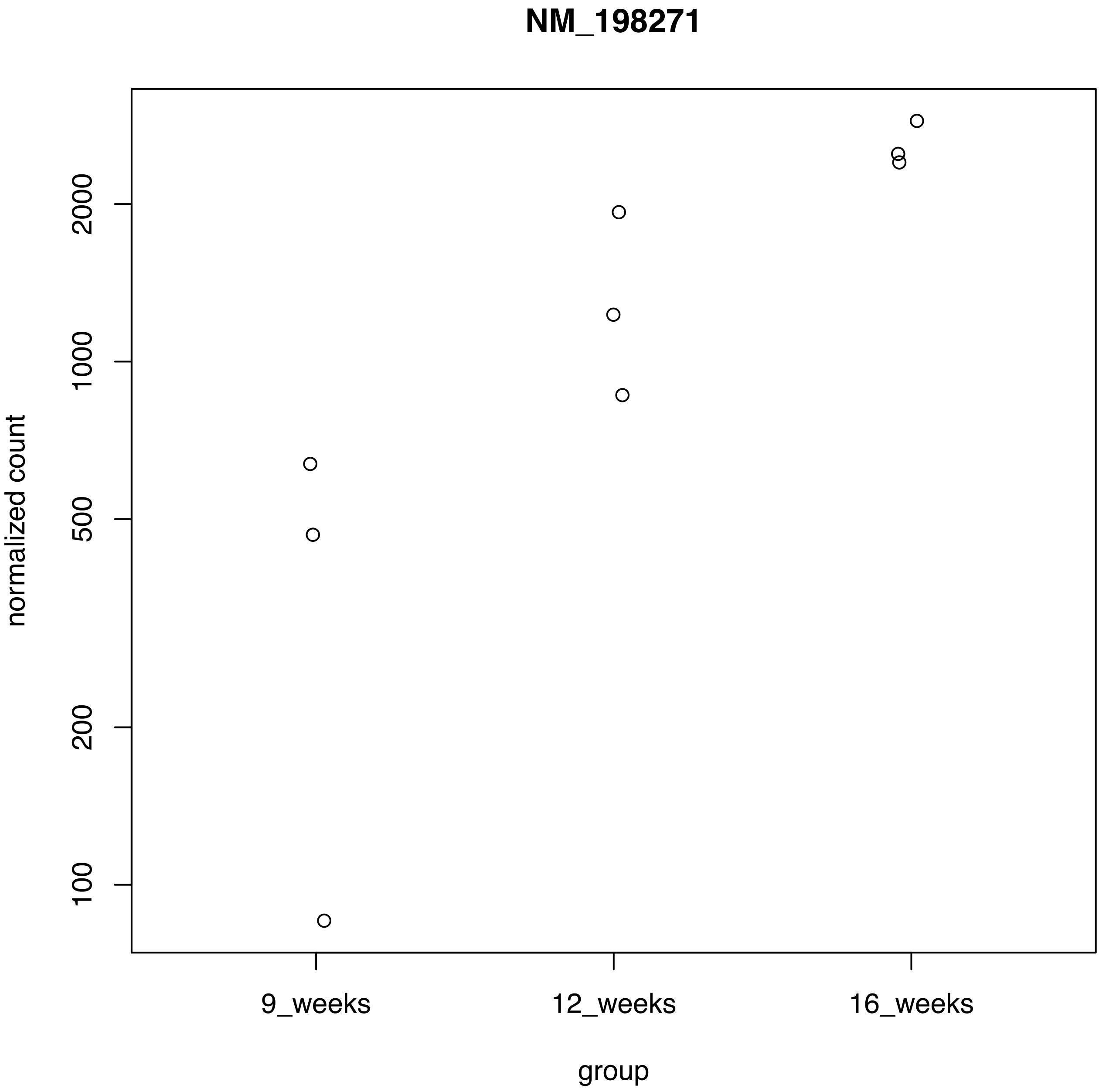
NM_198271
normalized count
2000
1000
500
200
100
9_weeks
12_weeks
16_weeks
group

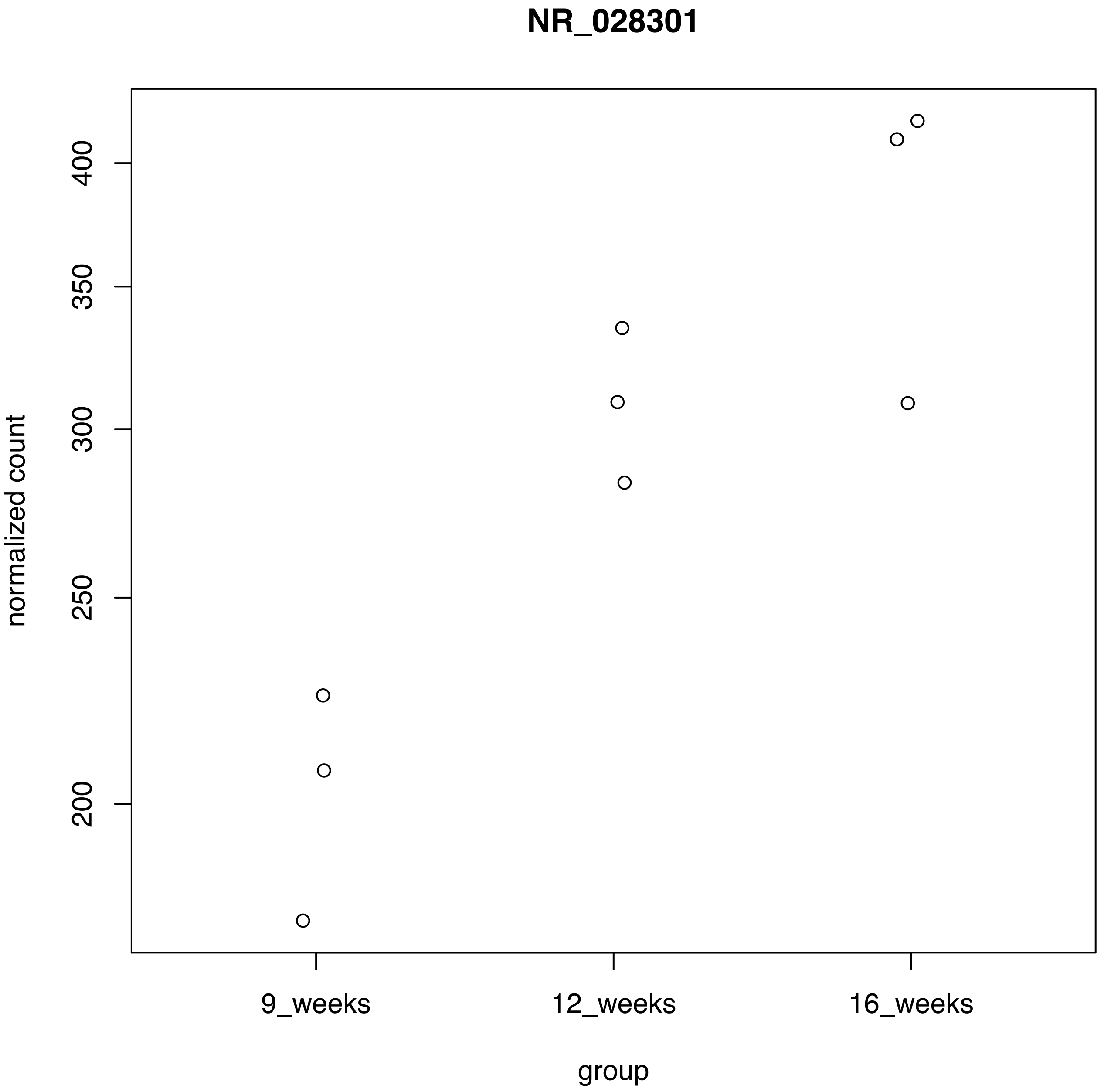

NR_028301
normalized count
200
250
300
350
400
group
9_weeks
12_weeks
16_weeks

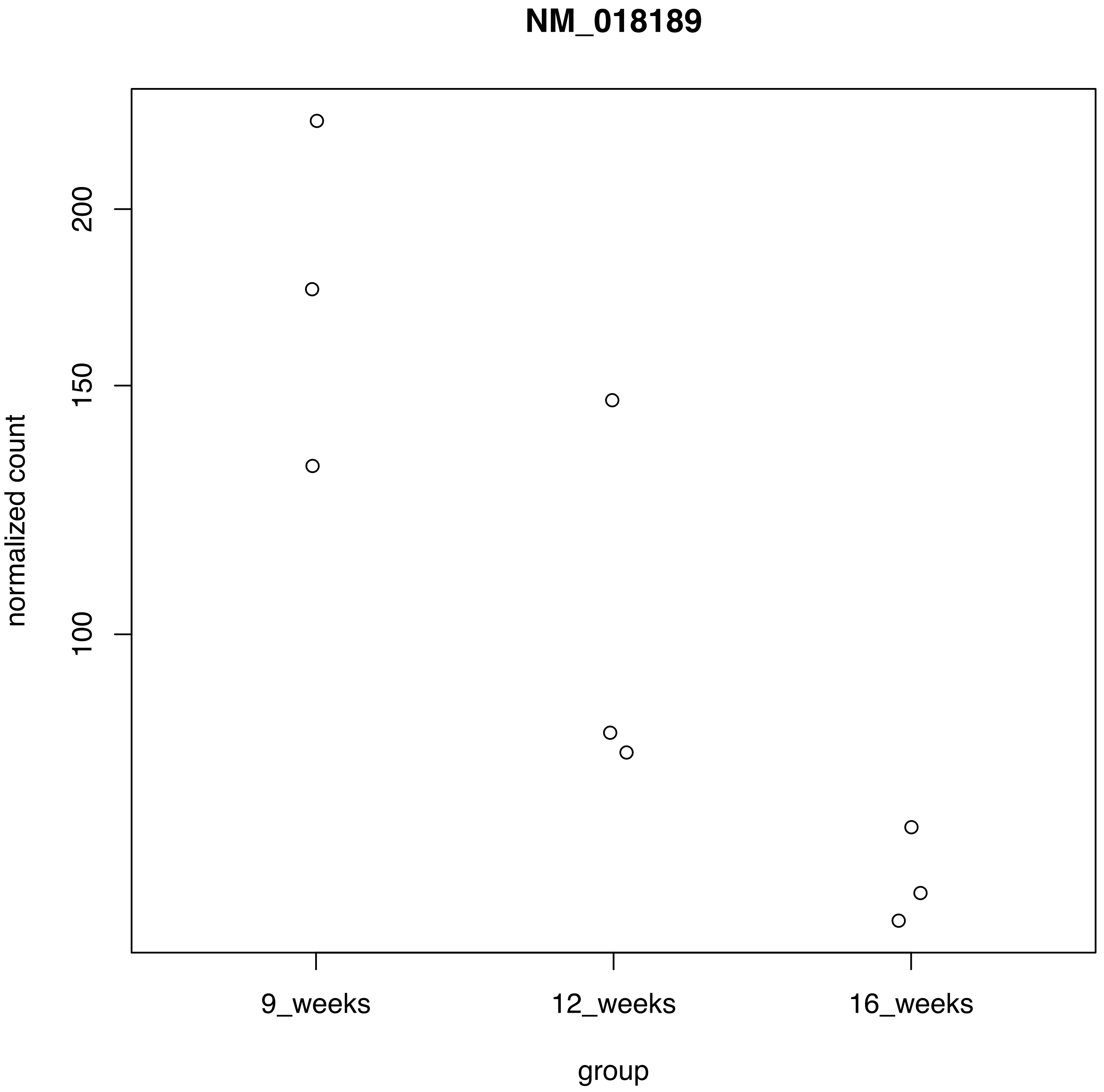
NM_018189
normalized count
200
150
100
9_weeks
12_weeks
16_weeks
group

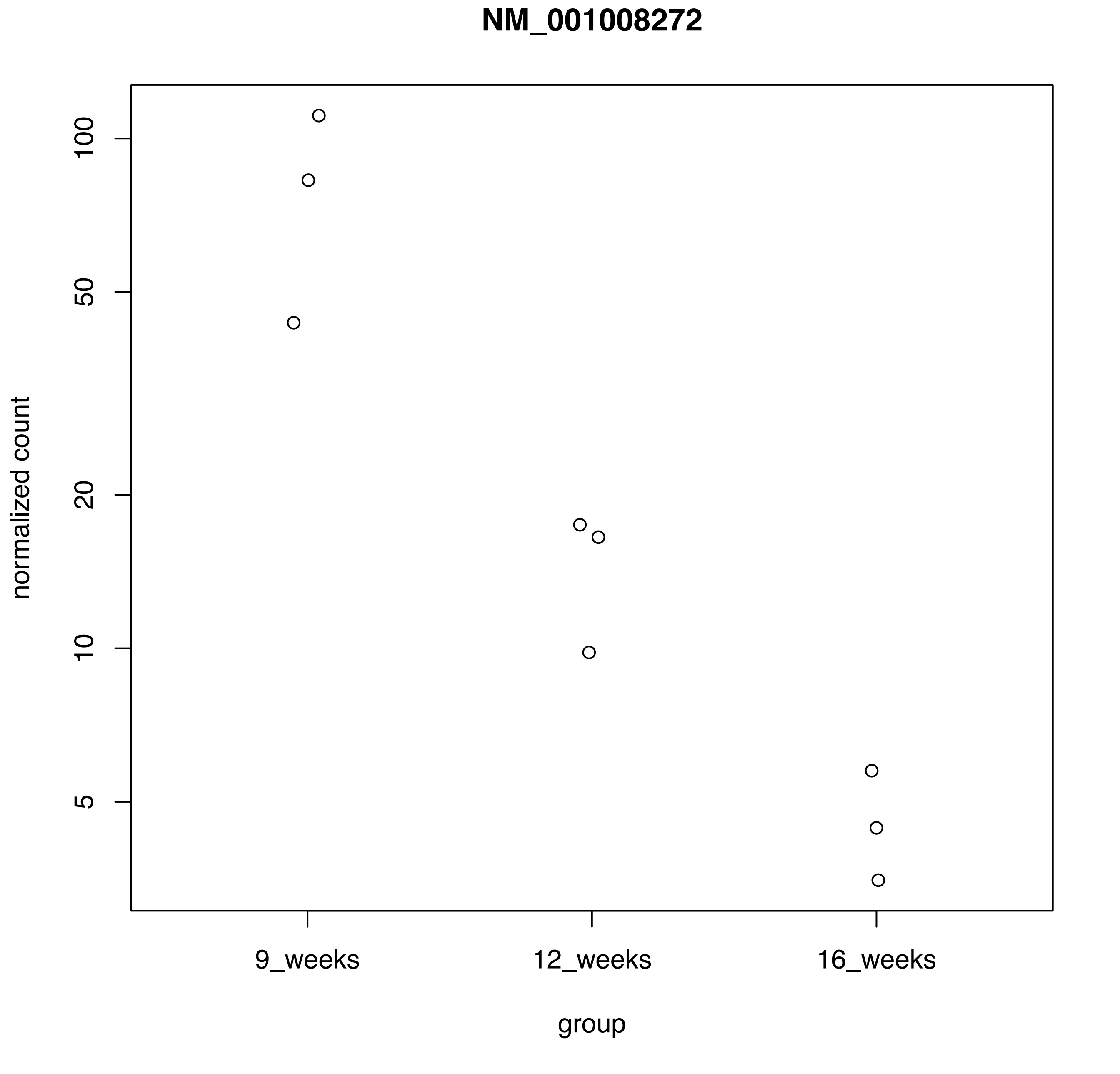

NM_001008272
normalized count
100
50
20
10
5
9_weeks
12_weeks
16_weeks
group

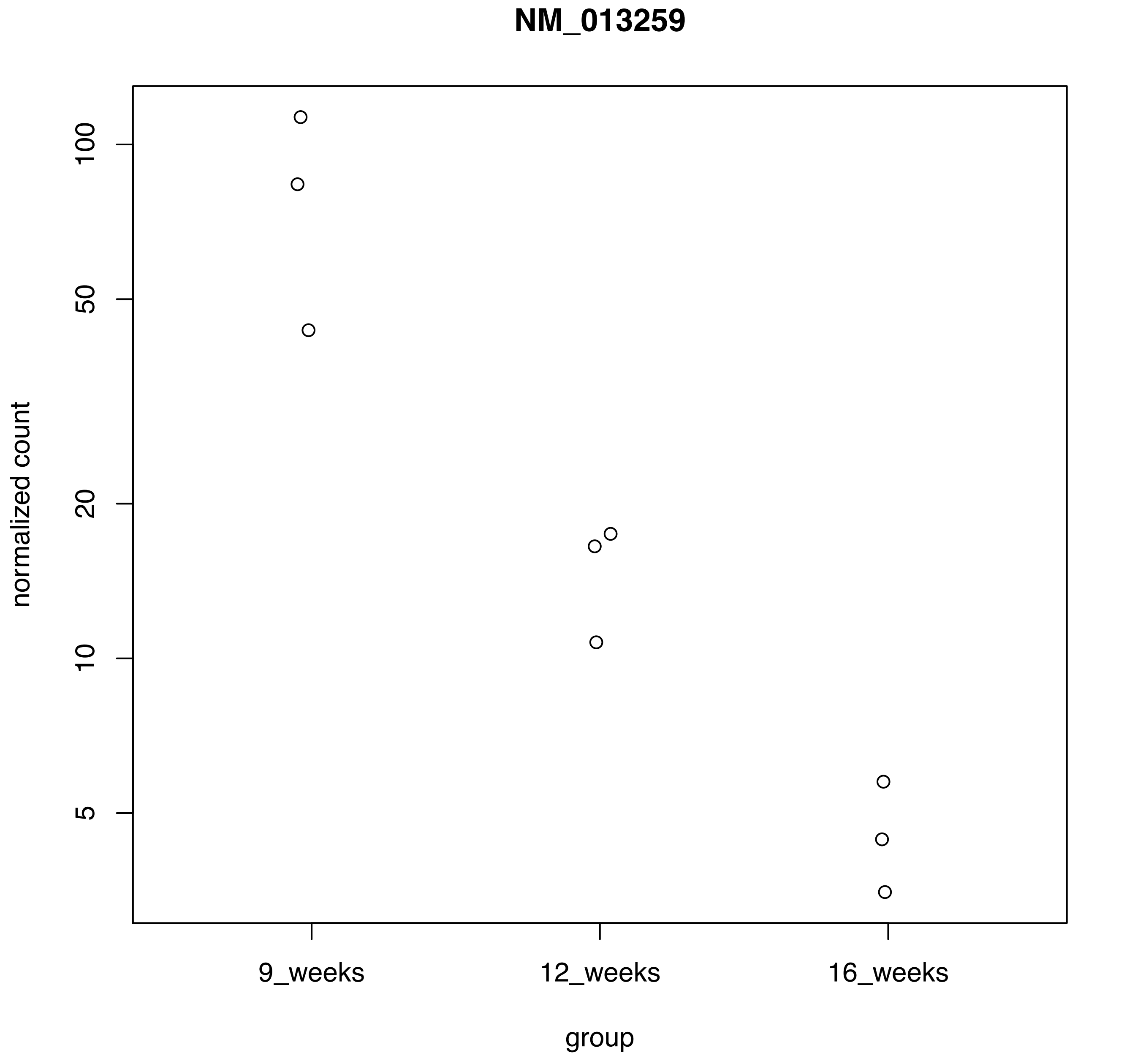
NM_013259
normalized count
100
50
20
10
5
9_weeks
12_weeks
16_weeks
group

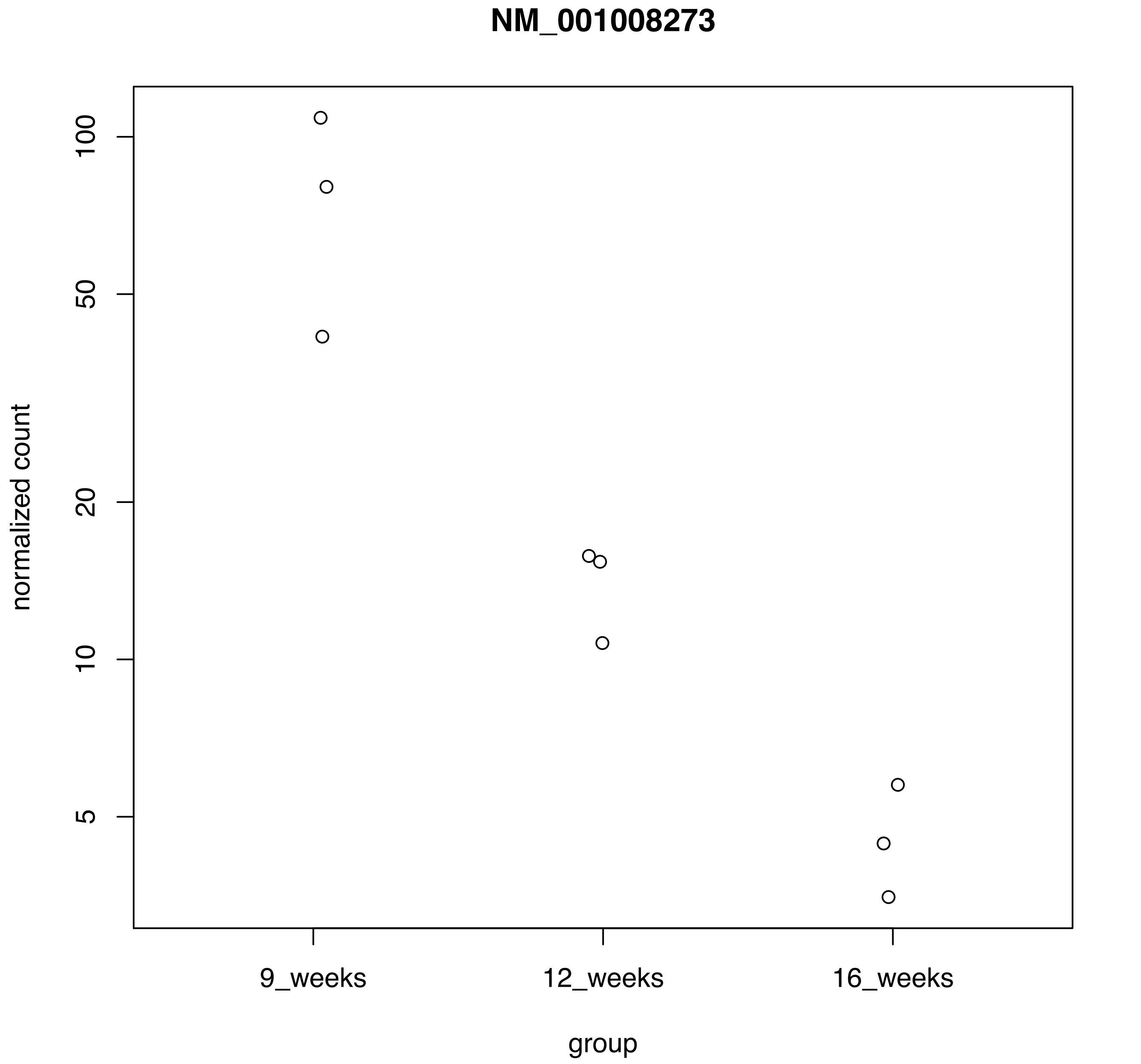

NM_001008273
normalized count
100
50
20
10
5
9_weeks
12_weeks
16_weeks
group

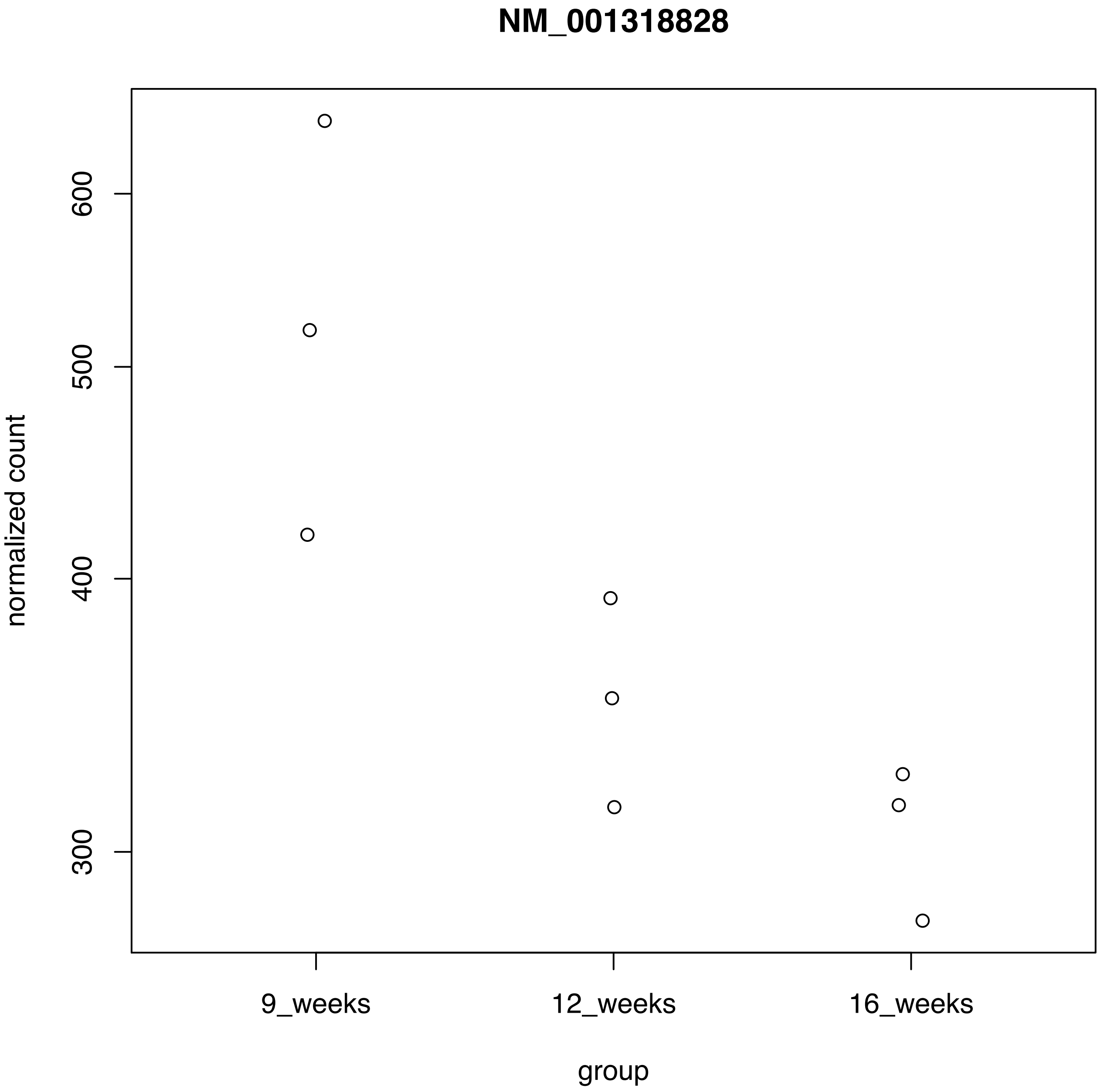
NM_001318828
normalized count
600
500
400
300
9_weeks
12_weeks
16_weeks
group

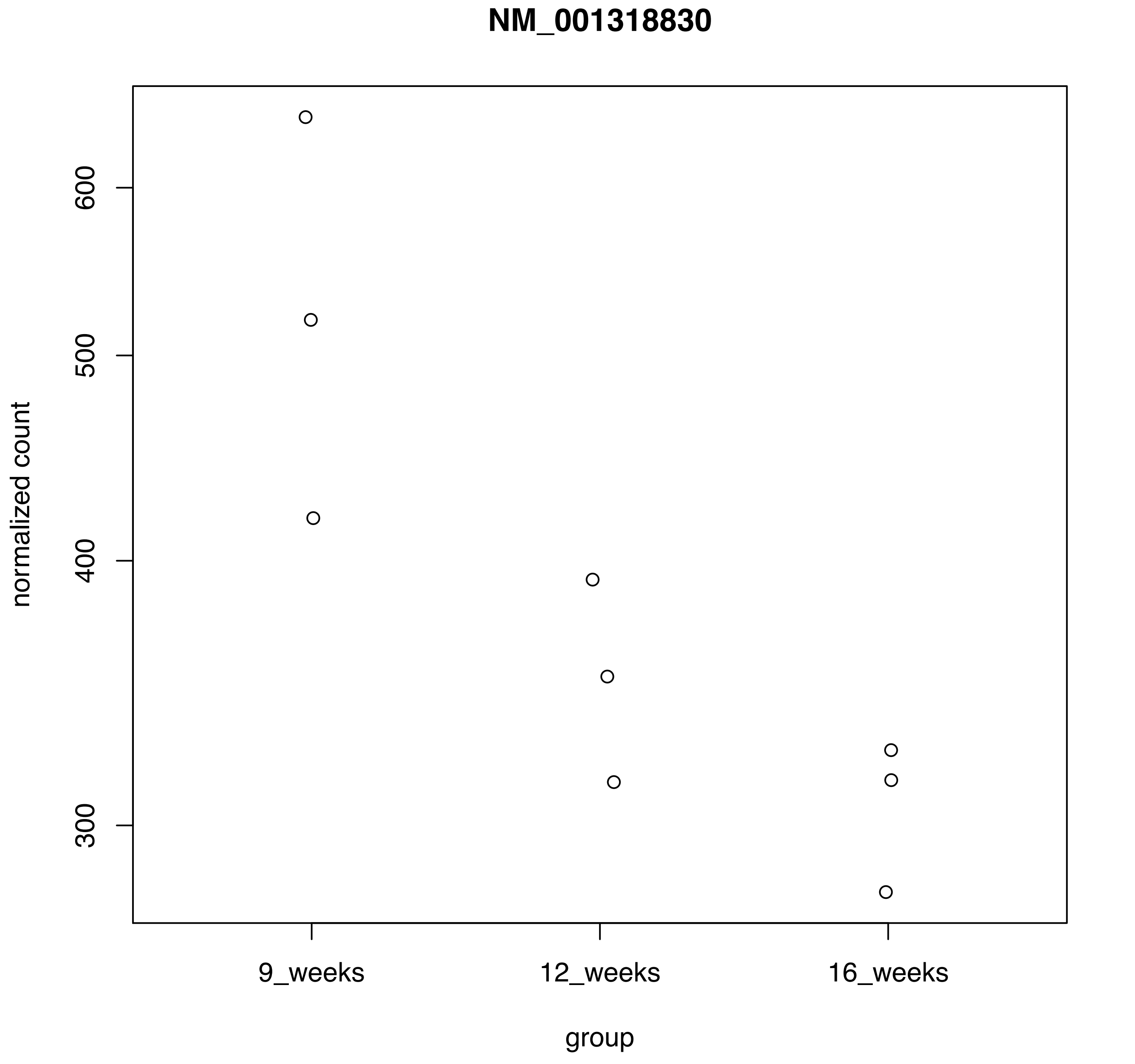
NM_001318830
normalized count
600
500
400
300
9_weeks
12_weeks
16_weeks
group

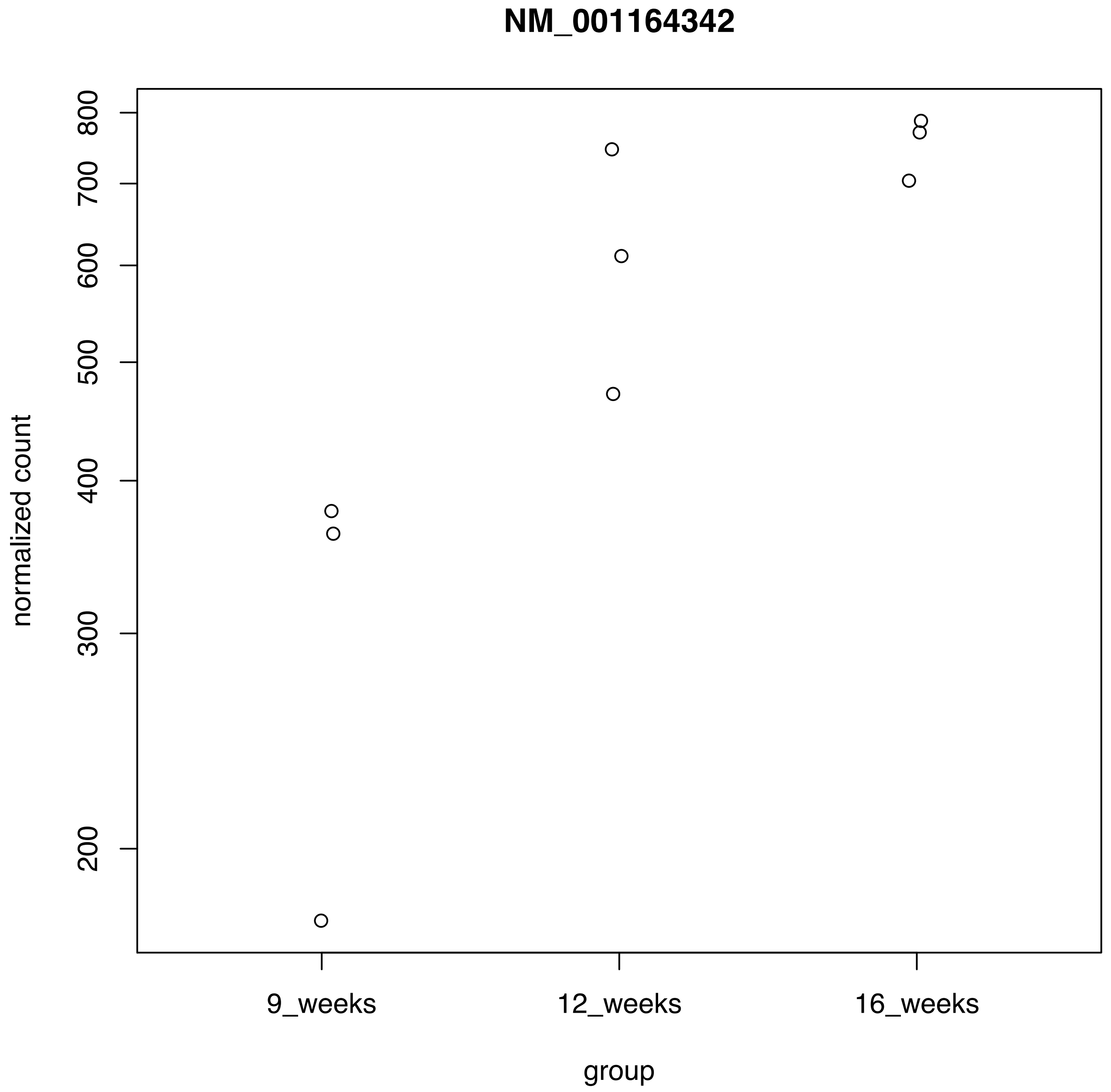

NM_001164342
normalized count
800
700
600
500
400
300
200
9_weeks
12_weeks
16_weeks
group

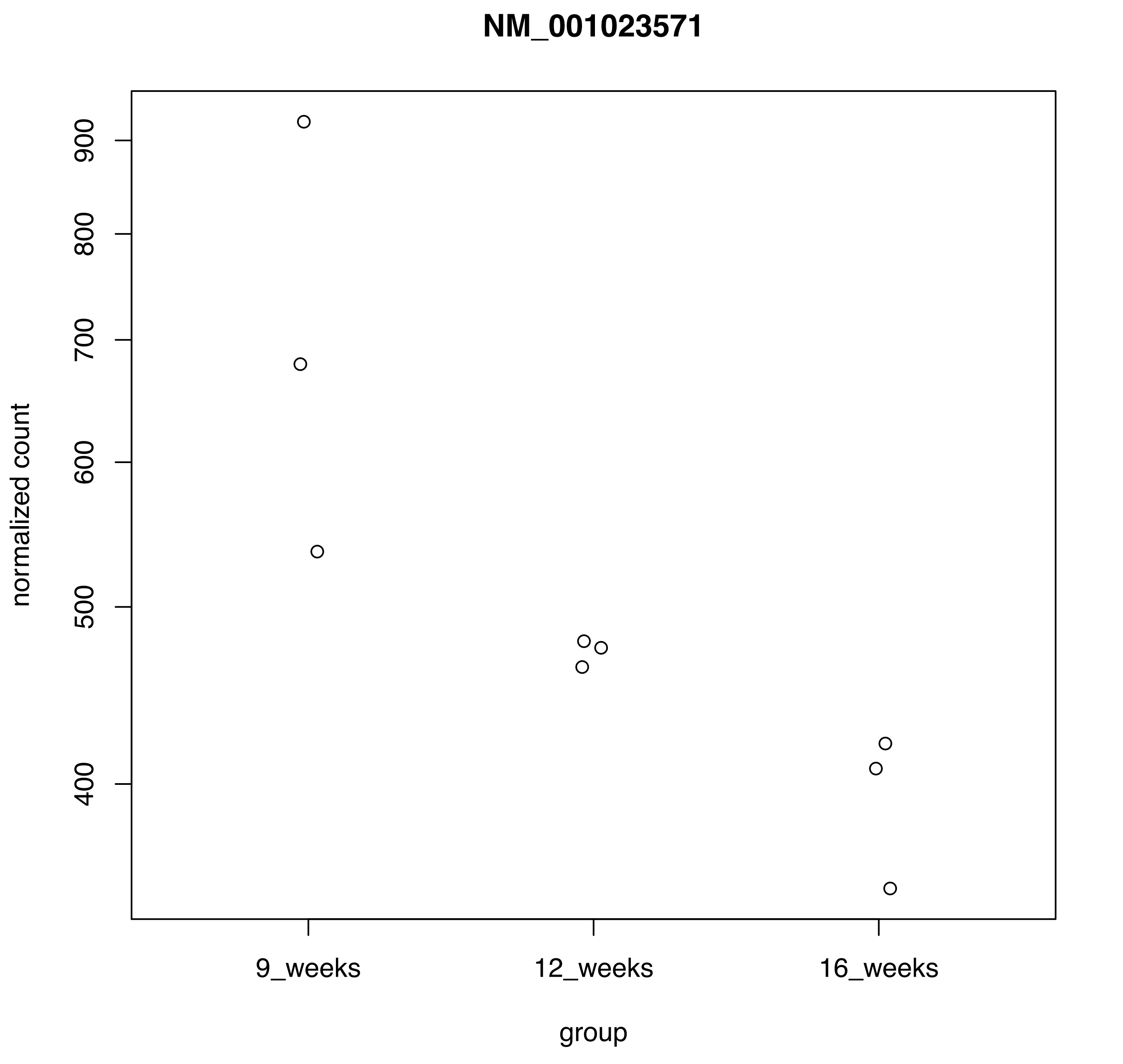

NM_001023571
normalized count
400
500
600
700
800
900
9_weeks
12_weeks
16_weeks
group

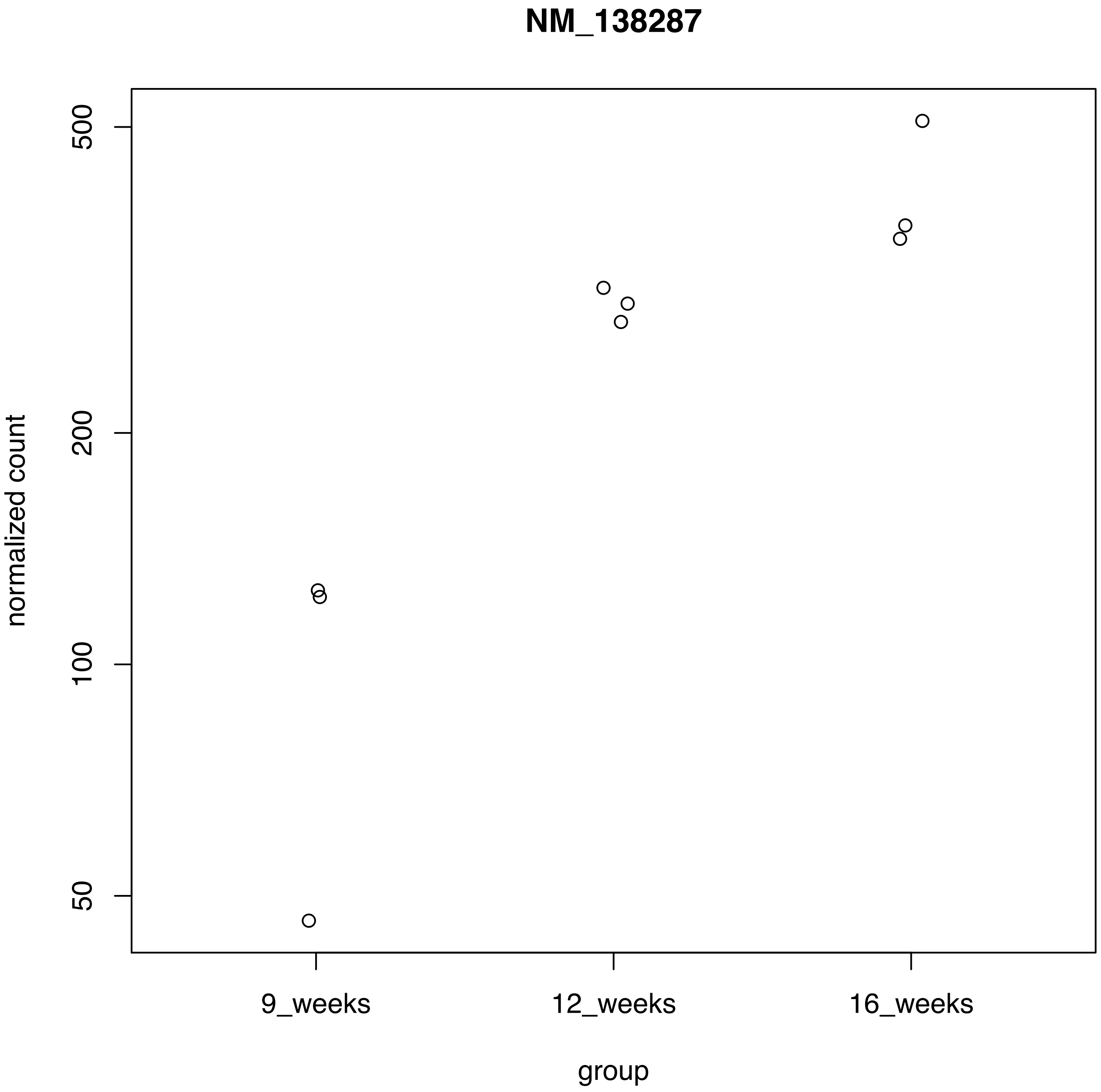
NM_138287
normalized count
500
200
100
50
9_weeks
12_weeks
16_weeks
group

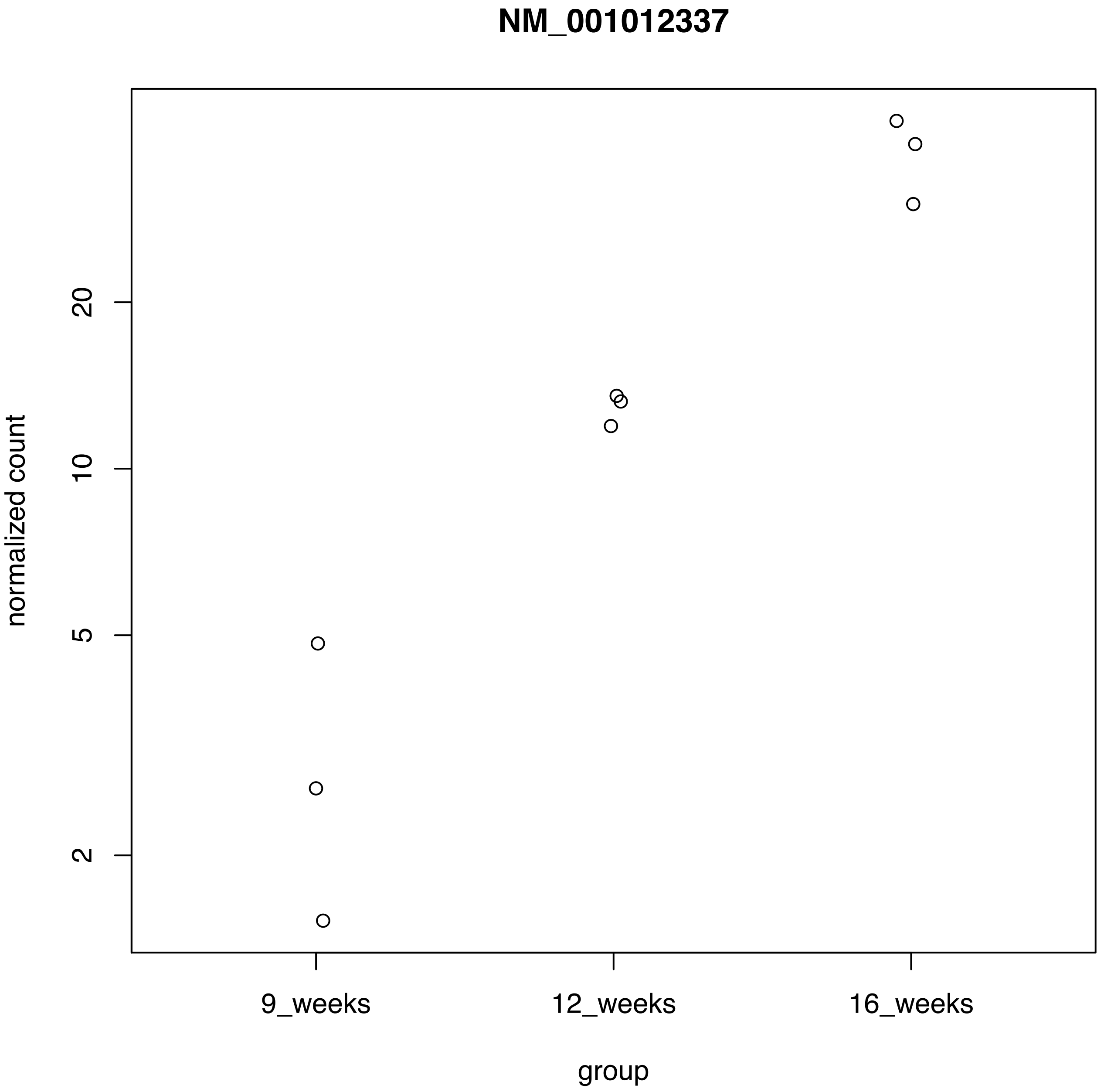
NM_001012337
normalized count
20
10
5
2
9_weeks
12_weeks
16_weeks
group

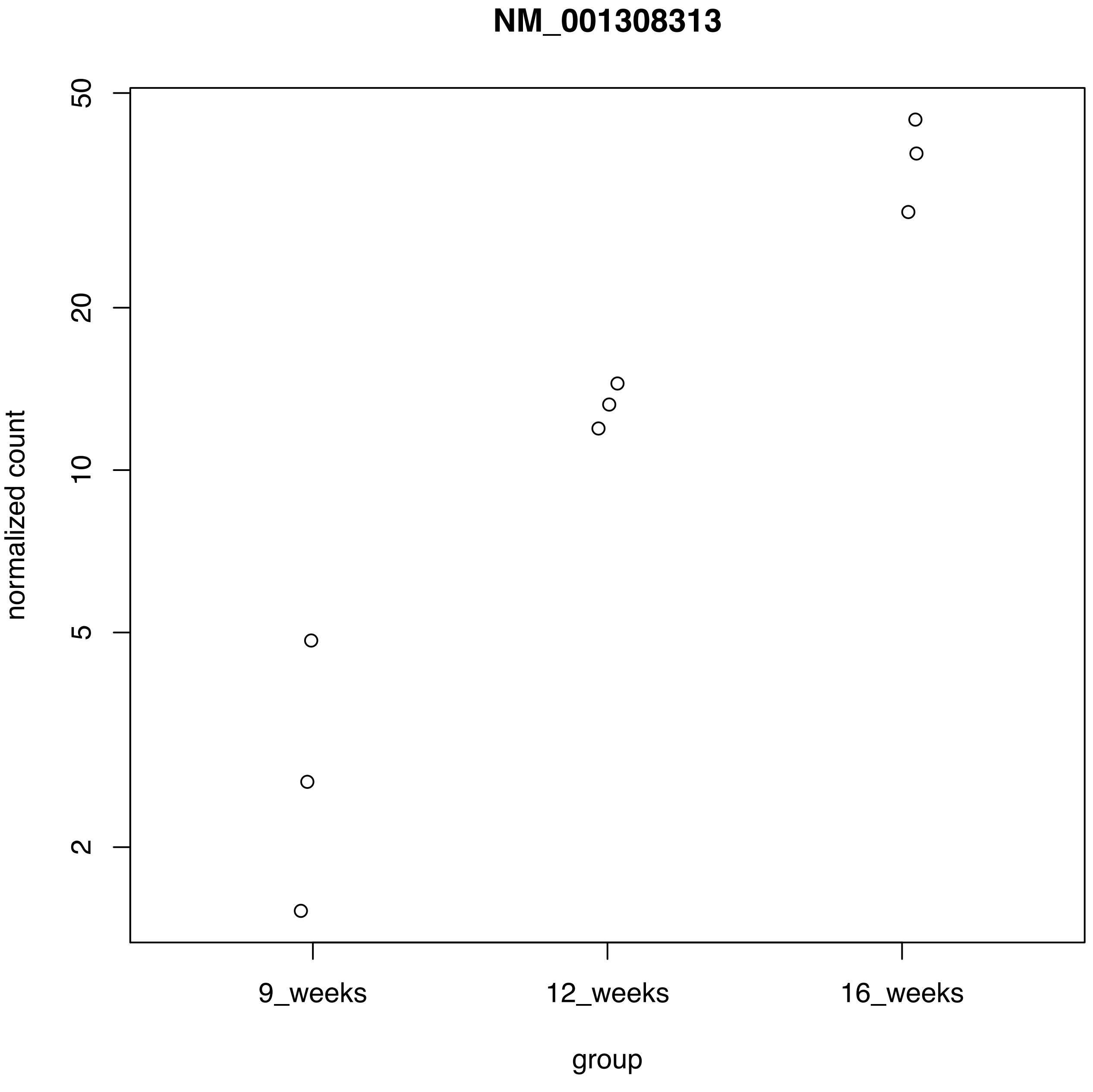
NM_001308313
normalized count
50
20
10
5
2
9_weeks
12_weeks
16_weeks
group

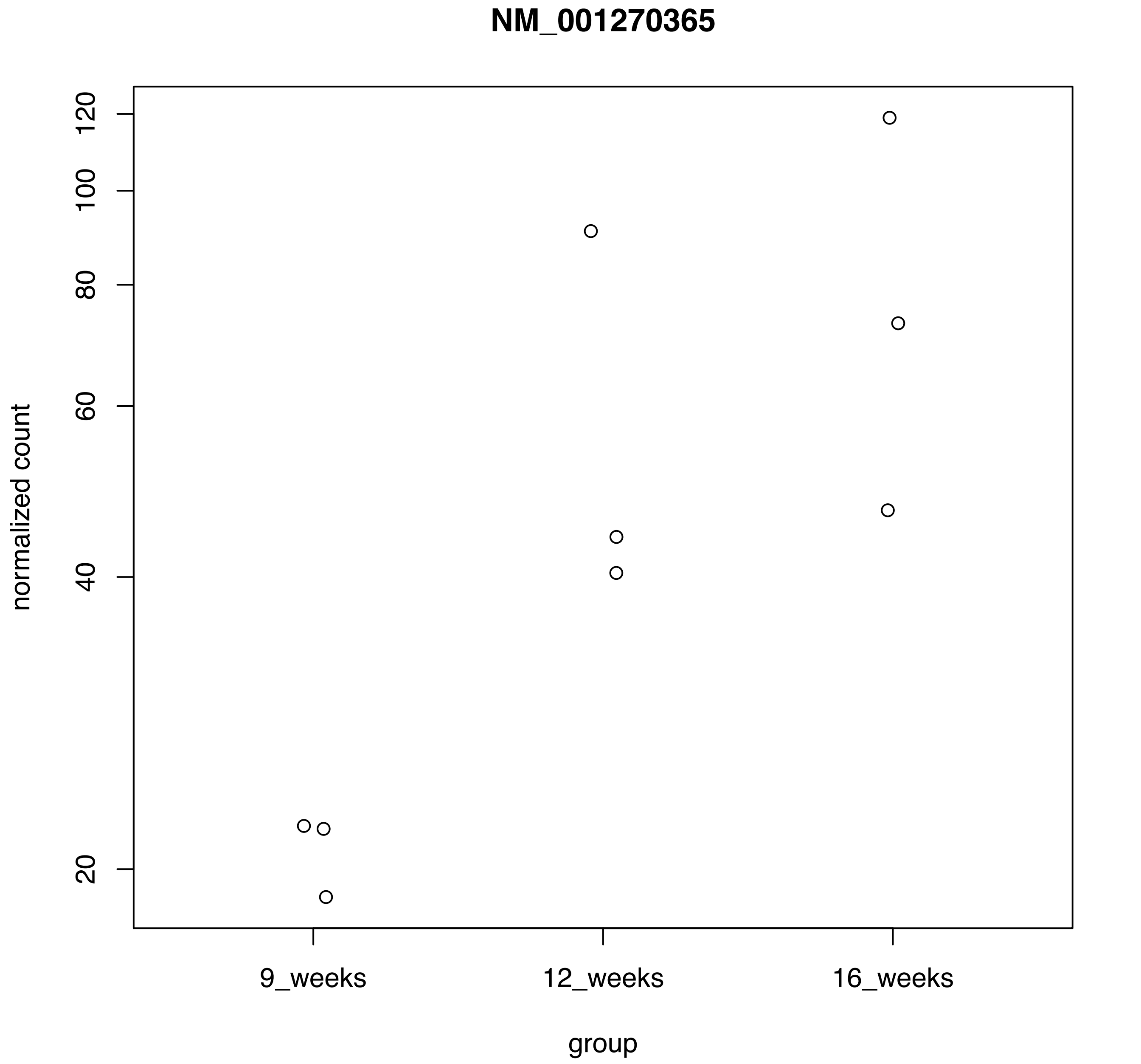

NM_001270365
normalized count
20
40
60
80
100
120
9_weeks
12_weeks
16_weeks
group

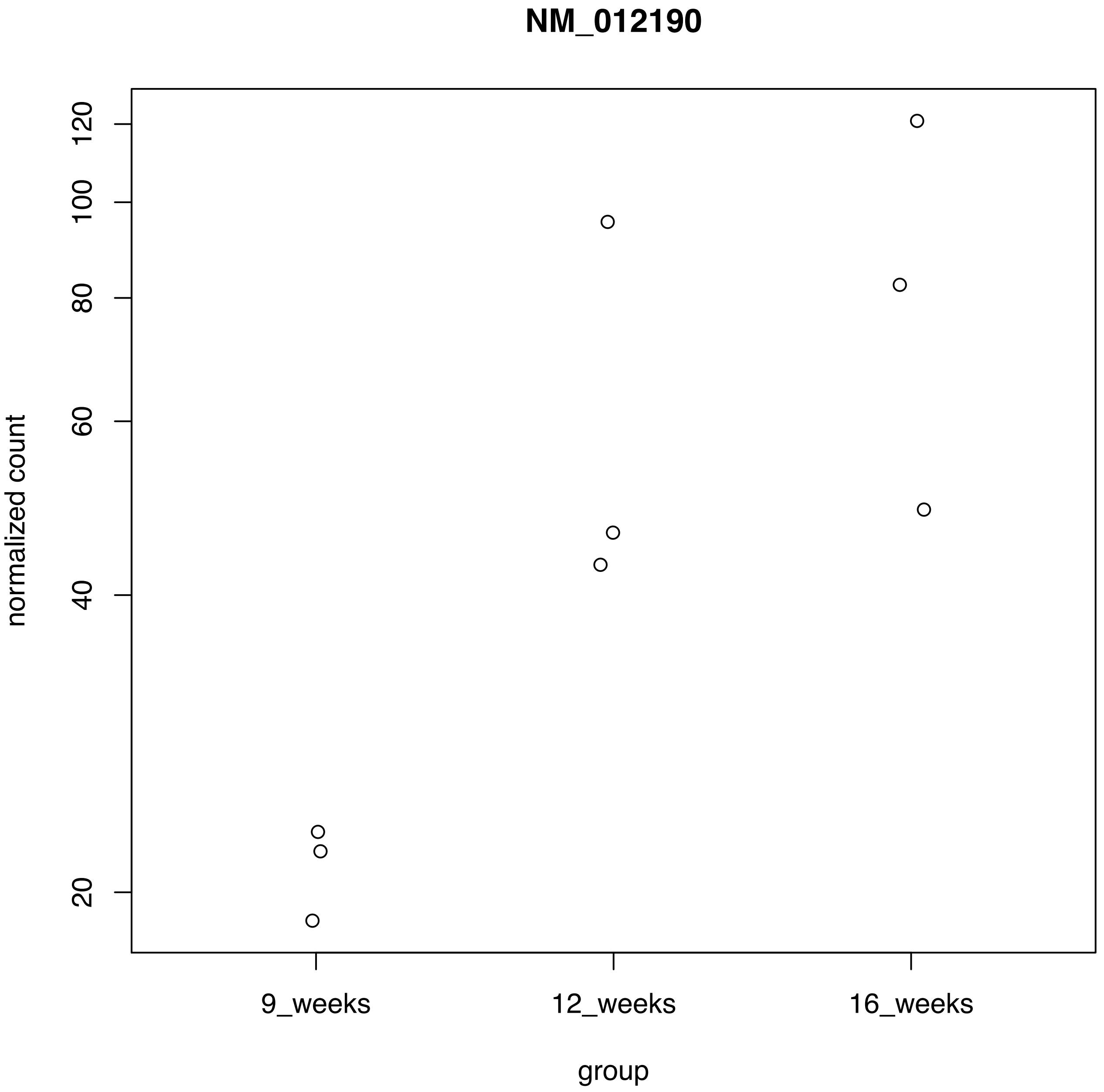

NM_012190
normalized count
120
100
80
60
40
20
9_weeks
12_weeks
16_weeks
group

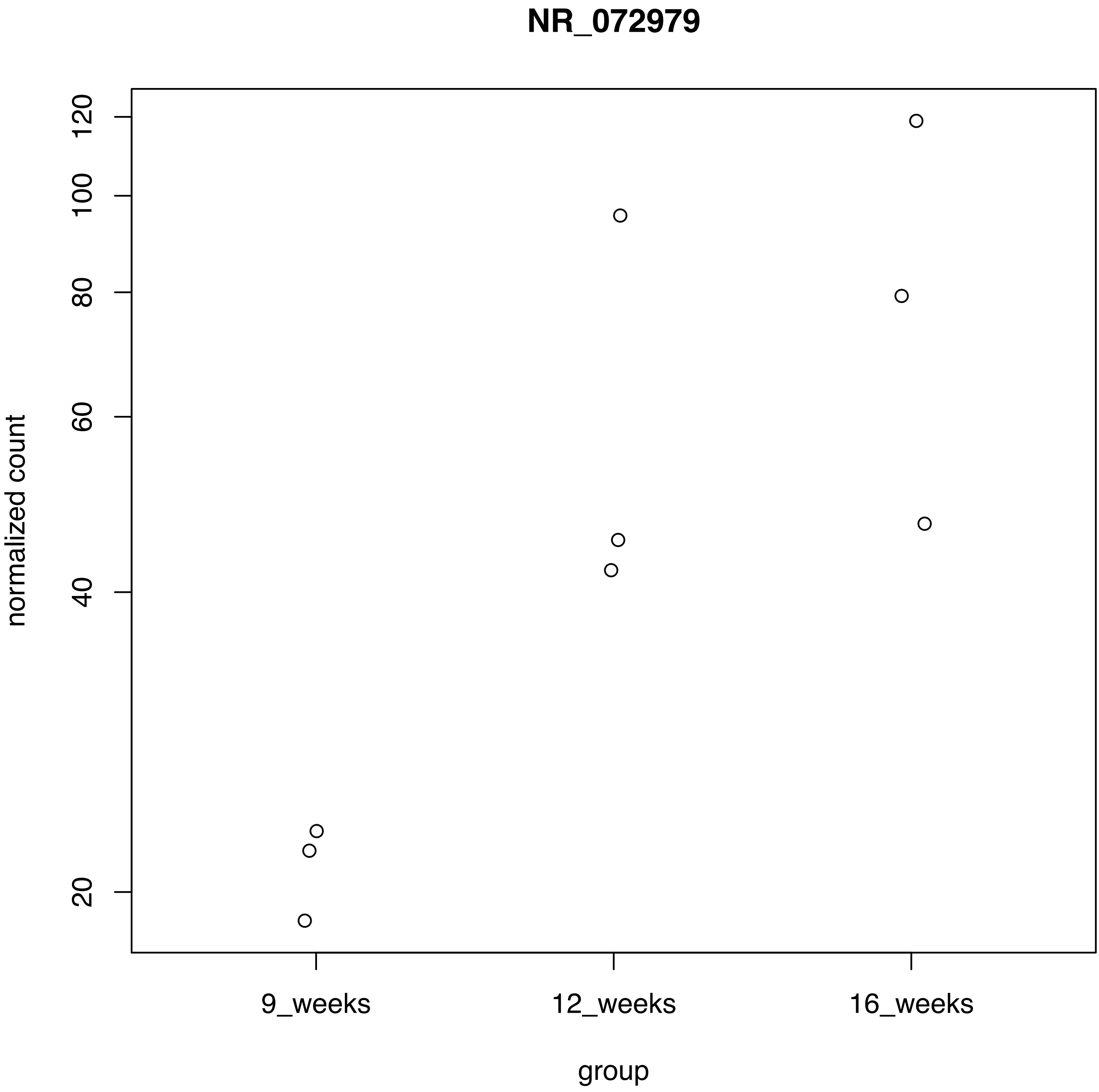

NR_072979
normalized count
120
100
80
60
40
20
9_weeks
12_weeks
16_weeks
group

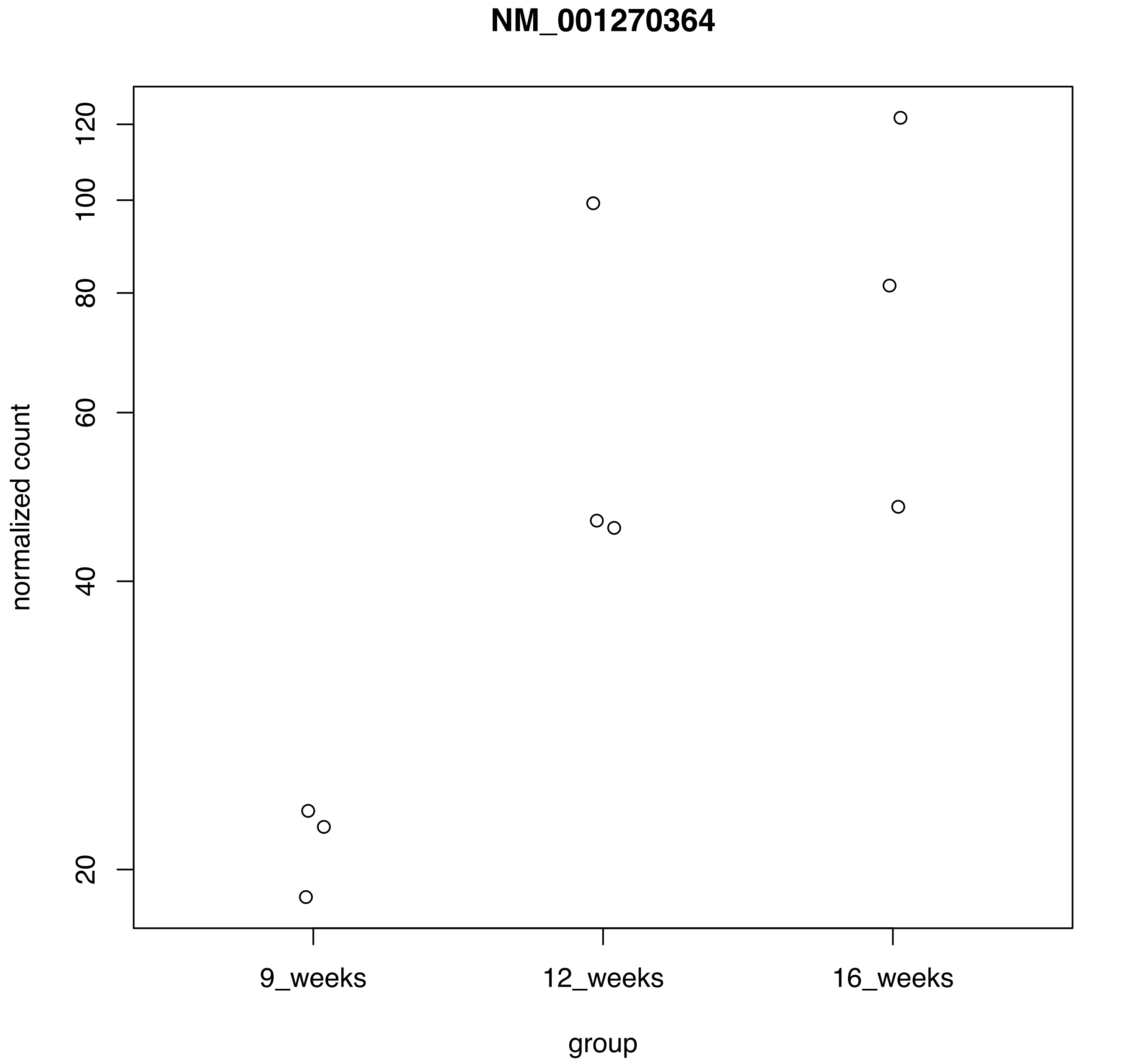

NM_001270364
normalized count
120
100
80
60
40
20
9_weeks
12_weeks
16_weeks
group

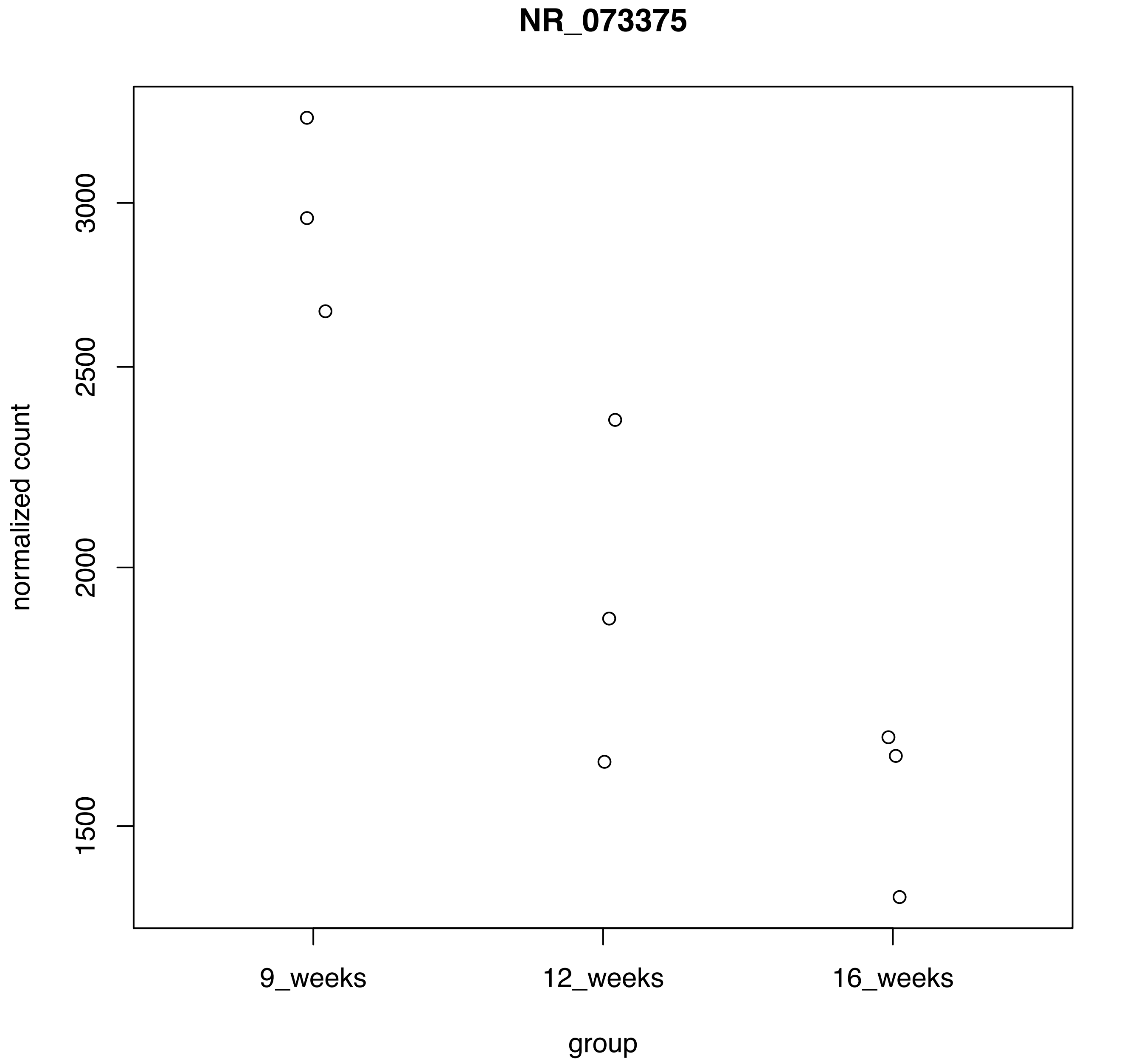
NR_073375
normalized count
3000
2500
2000
1500
9_weeks
12_weeks
16_weeks
group

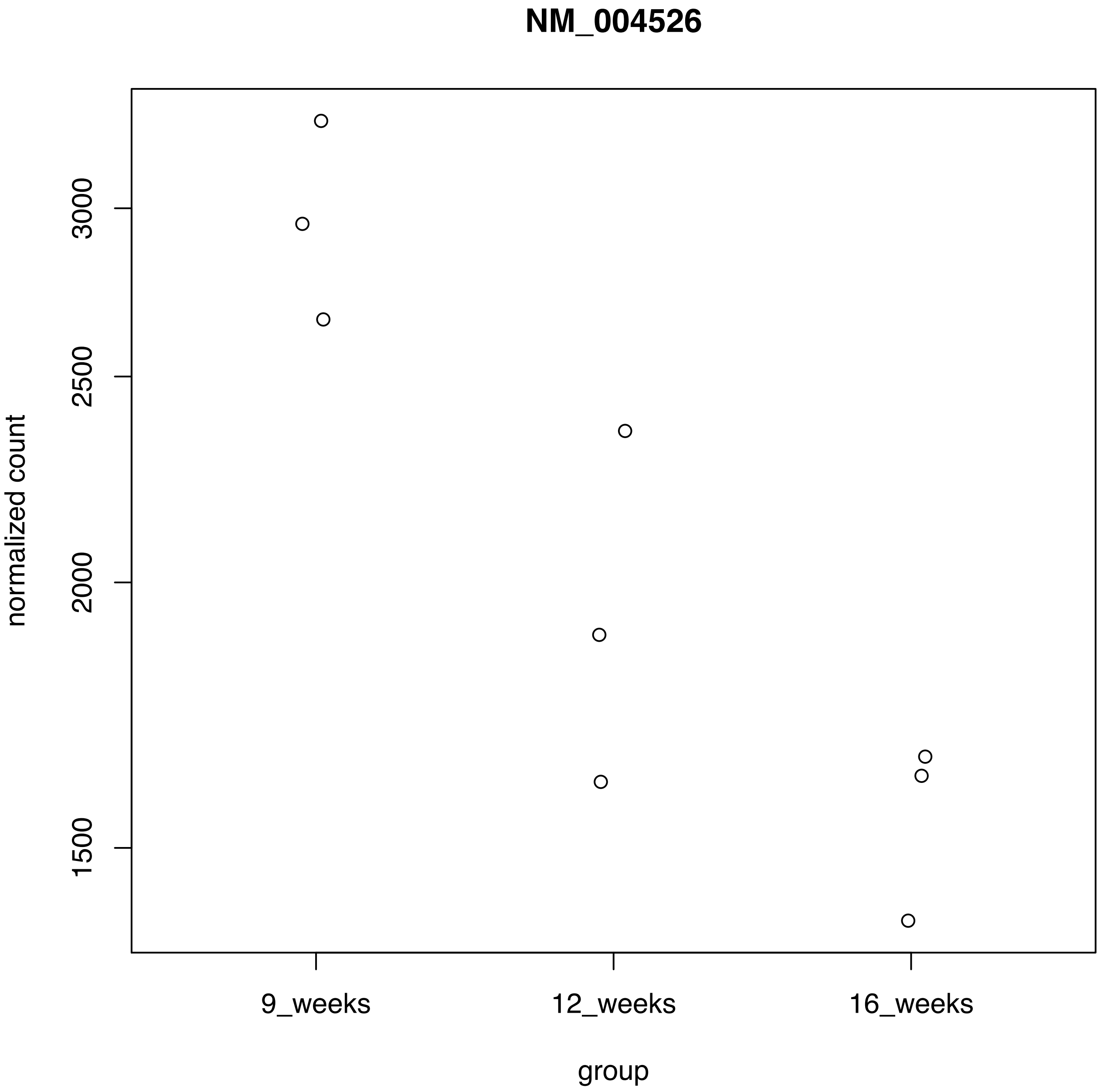
NM_004526
normalized count
3000
2500
2000
1500
9_weeks
12_weeks
16_weeks
group

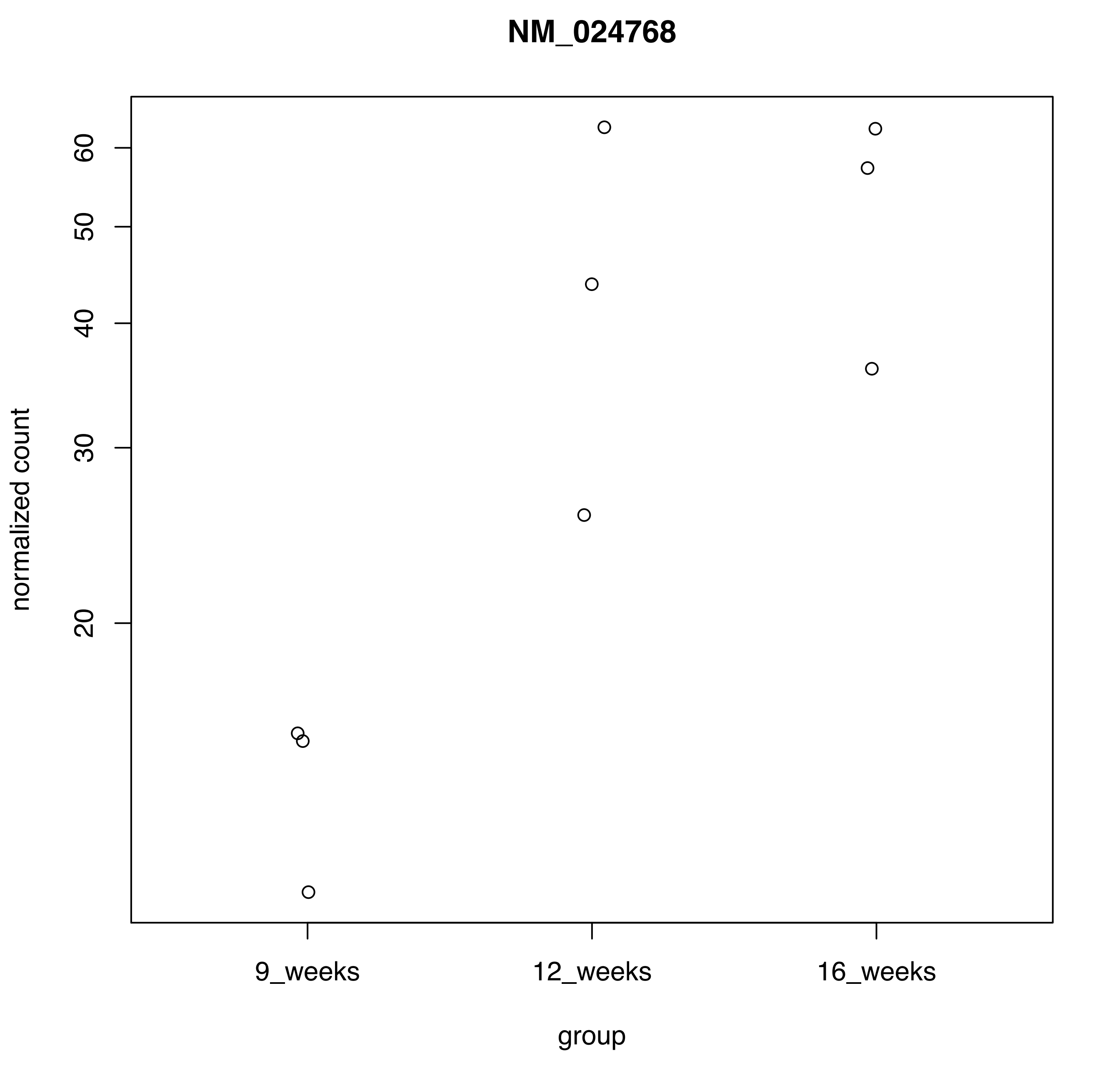
NM_024768
normalized count
20
30
40
50
60
9_weeks
12_weeks
16_weeks
group

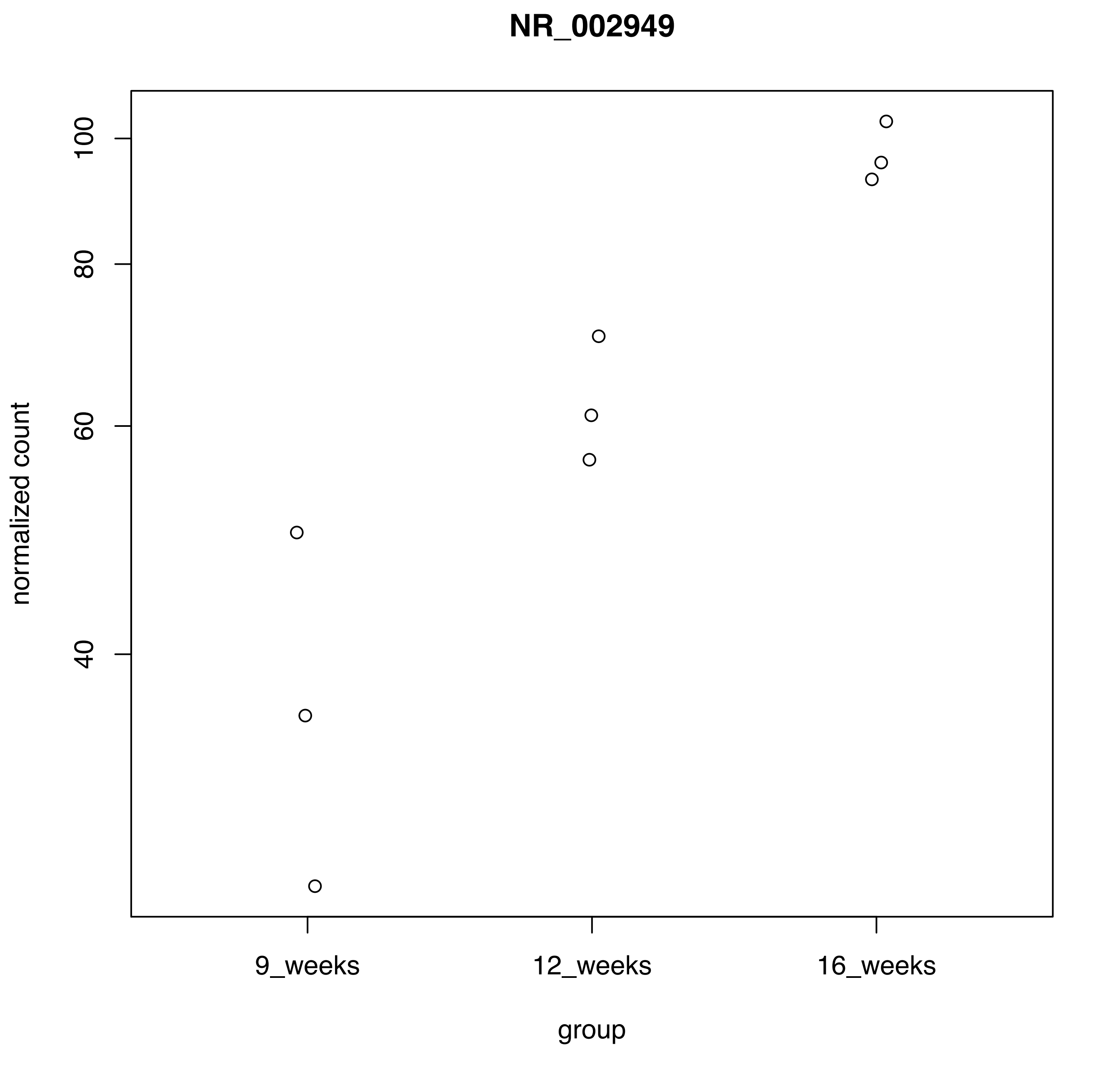

NR_002949
normalized count
100
80
60
40
9_weeks
12_weeks
16_weeks
group

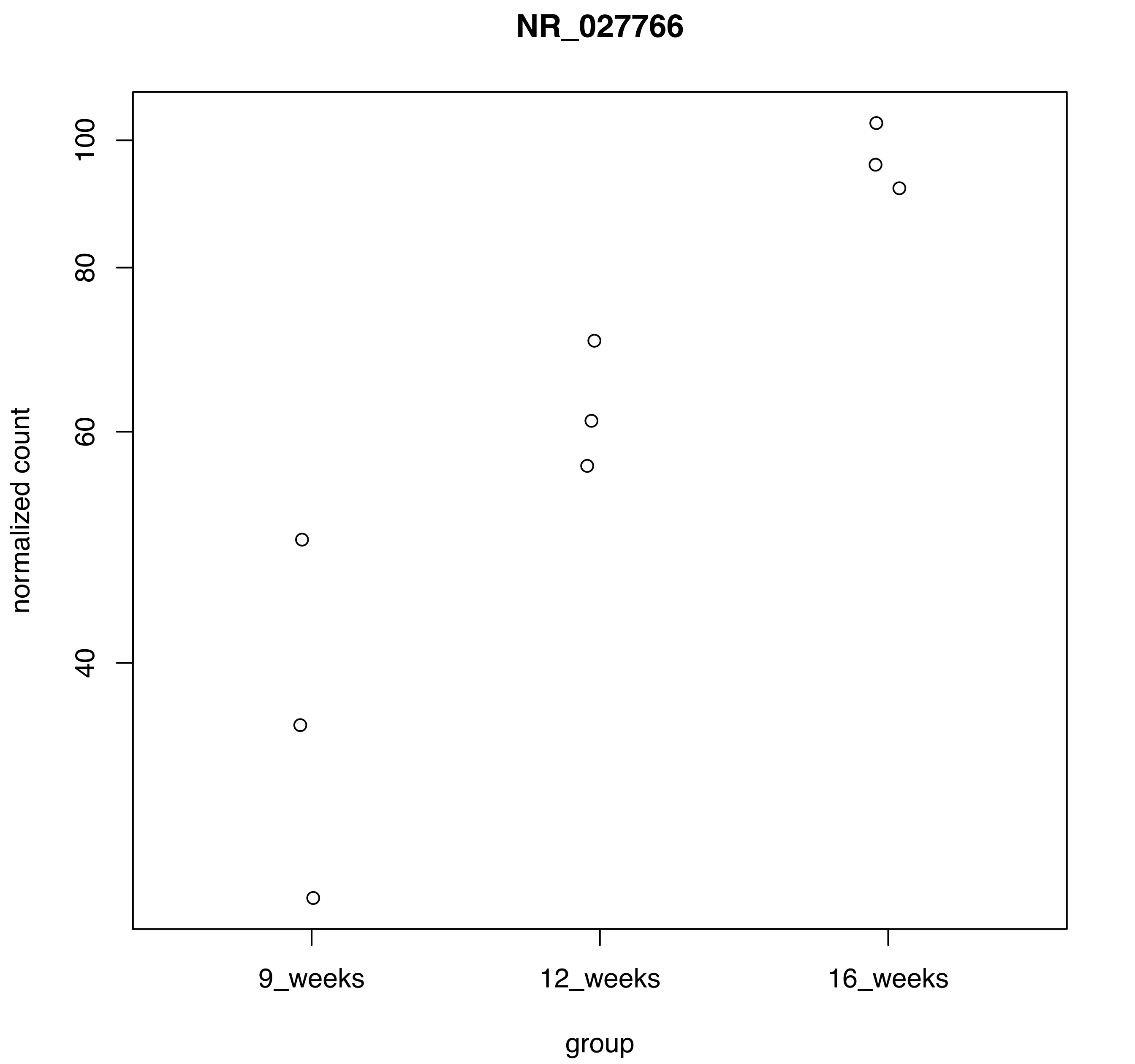
NR_027766
normalized count
100
80
60
40
9_weeks
12_weeks
16_weeks
group

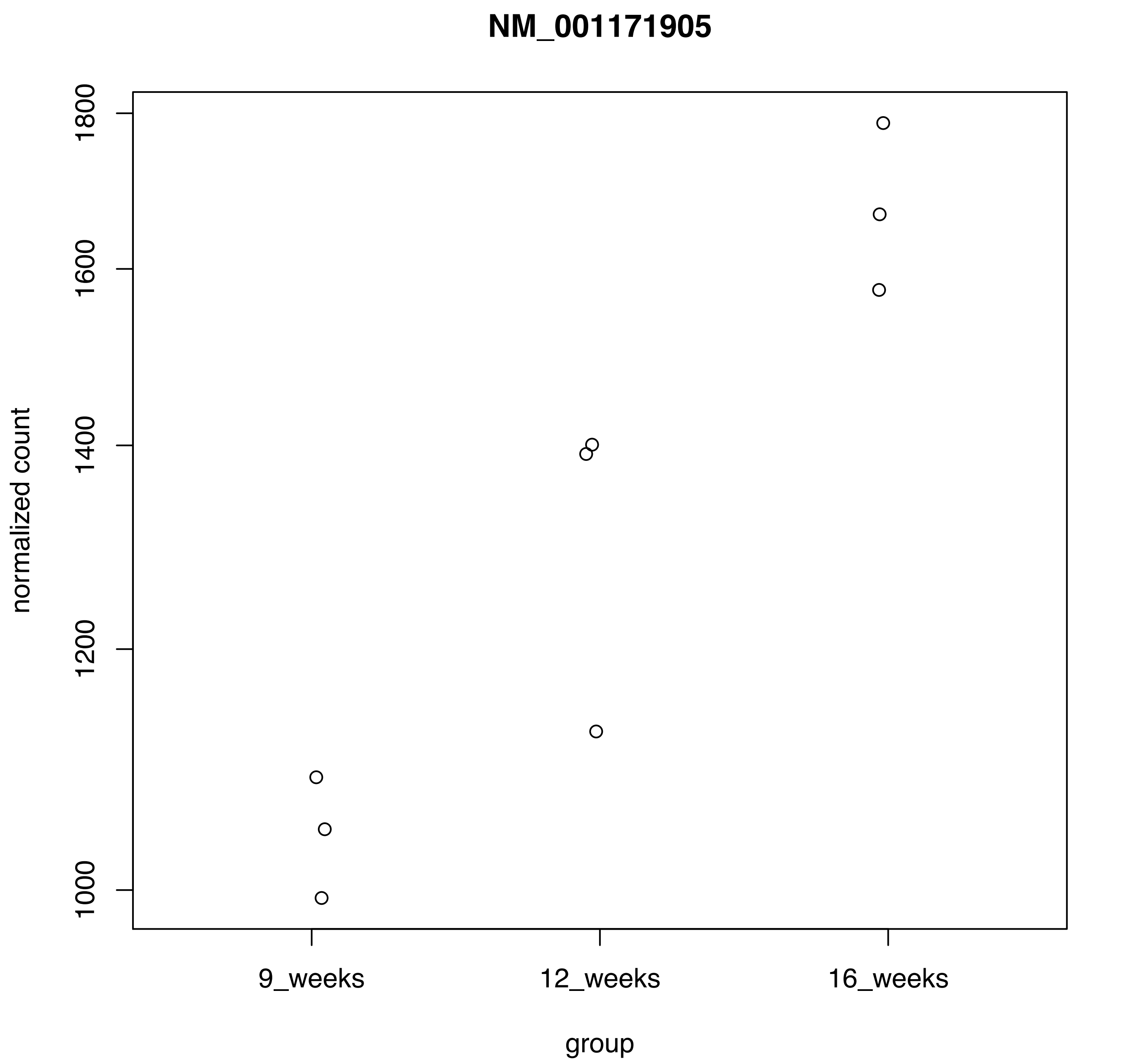

NM_001171905
1800
1600
1400
1200
1000
normalized count
9_weeks
12_weeks
16_weeks
group

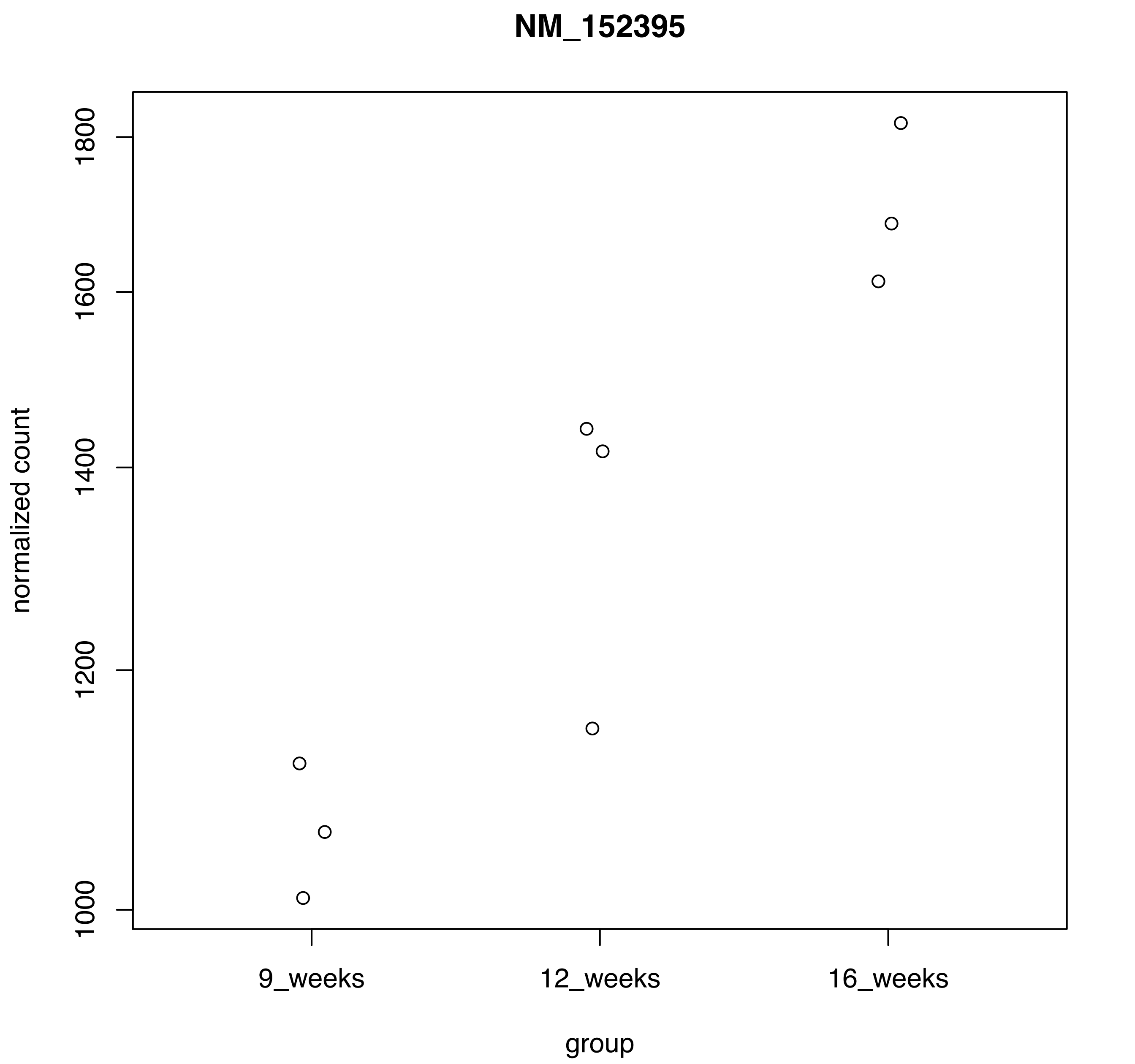
NM_152395
normalized count
1000
1200
1400
1600
1800
9_weeks
12_weeks
16_weeks
group

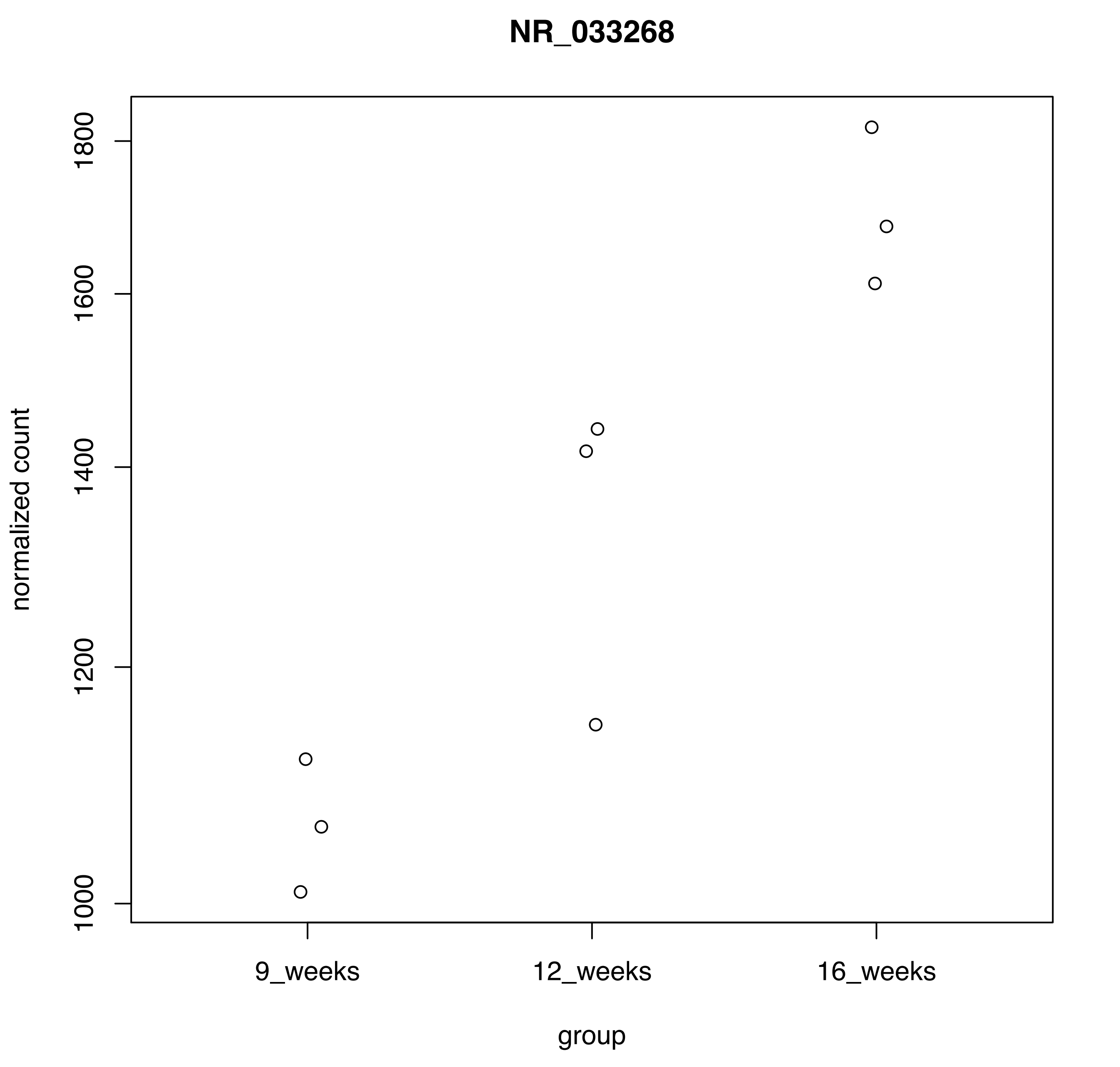
NR_033268
normalized count
1000
1200
1400
1600
1800
9_weeks
12_weeks
16_weeks
group

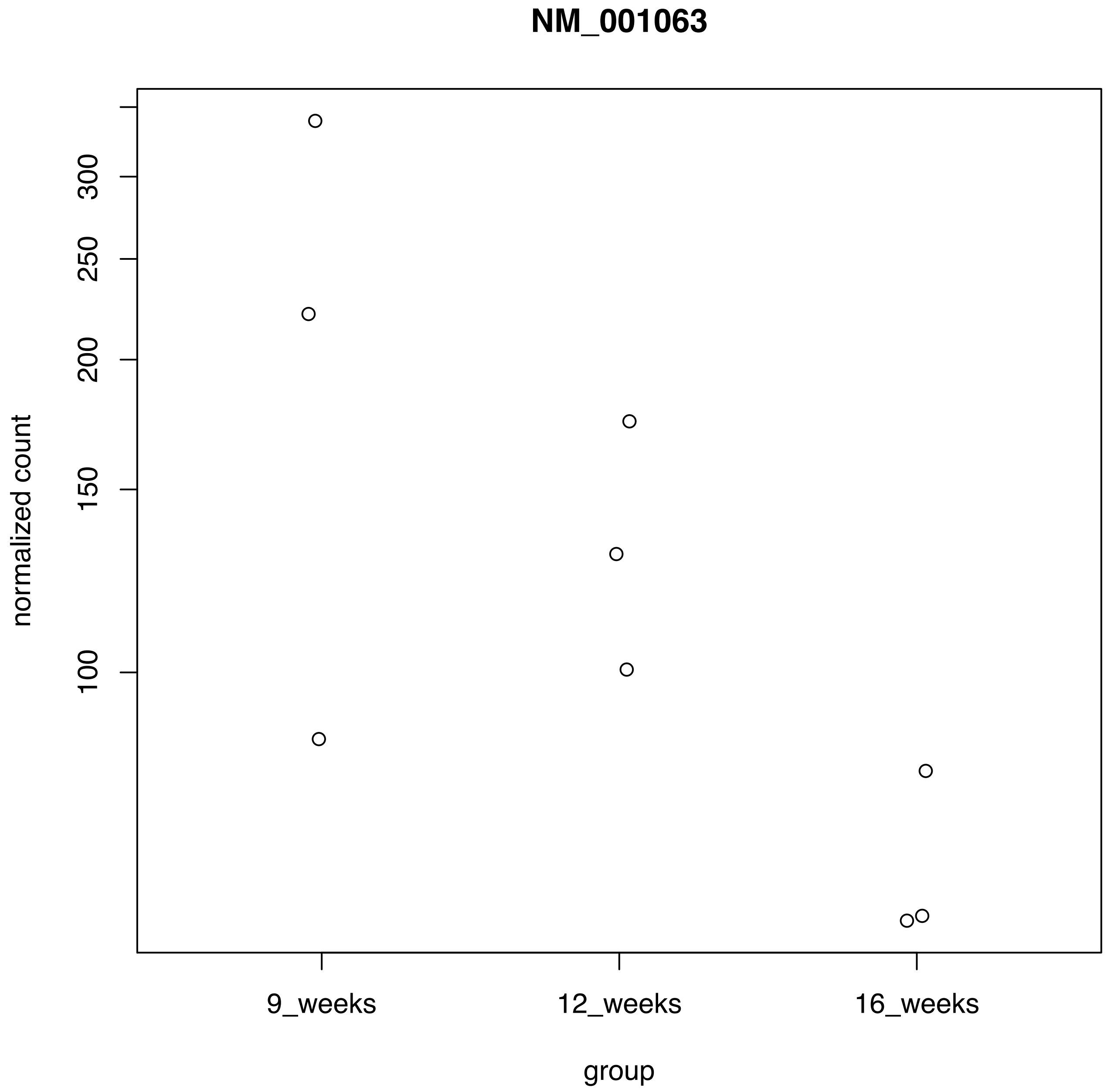
NM_001063
normalized count
300
250
200
150
100
group
9_weeks
12_weeks
16_weeks

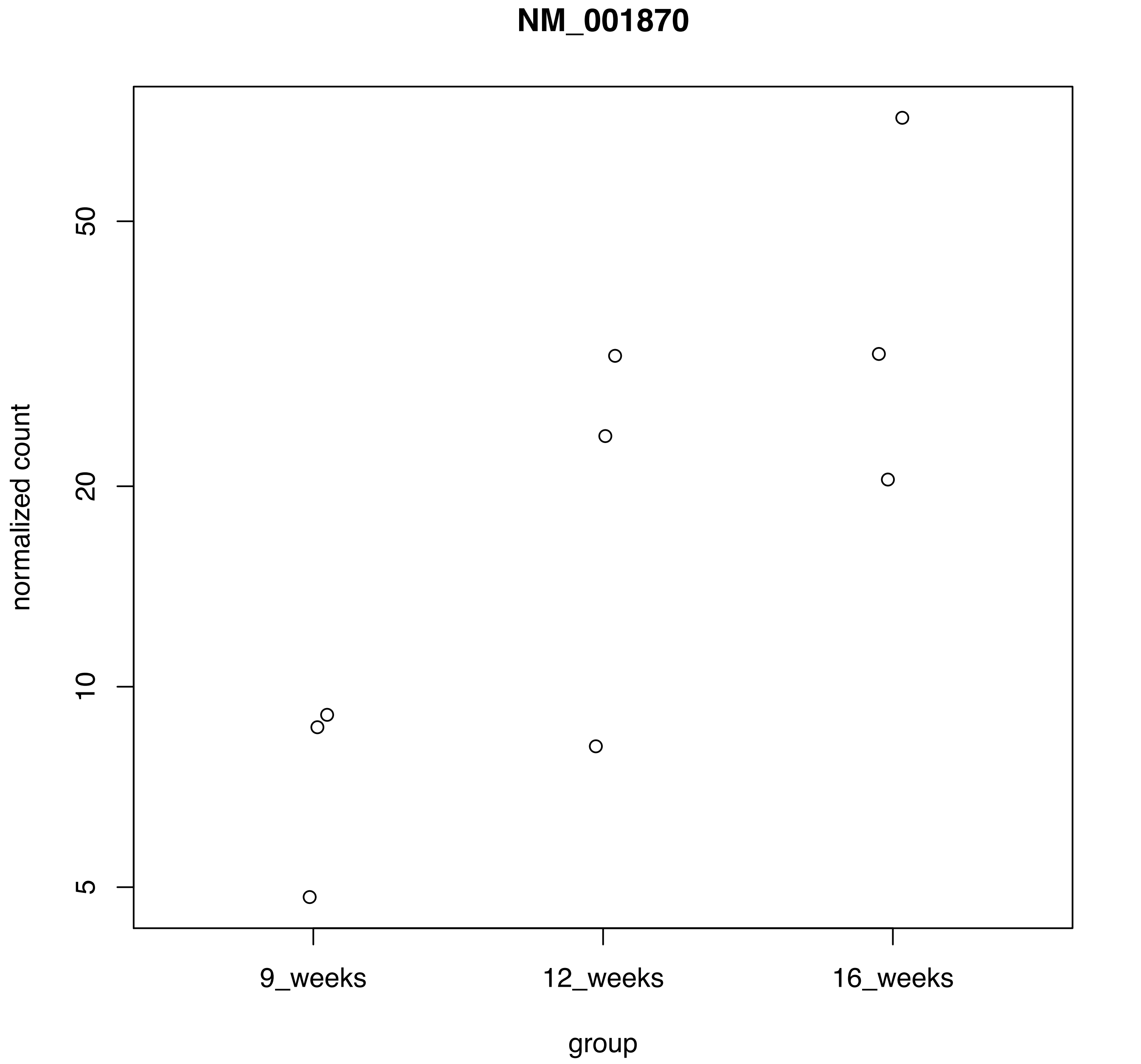
NM_001870
normalized count
50
20
10
5
9_weeks
12_weeks
16_weeks
group

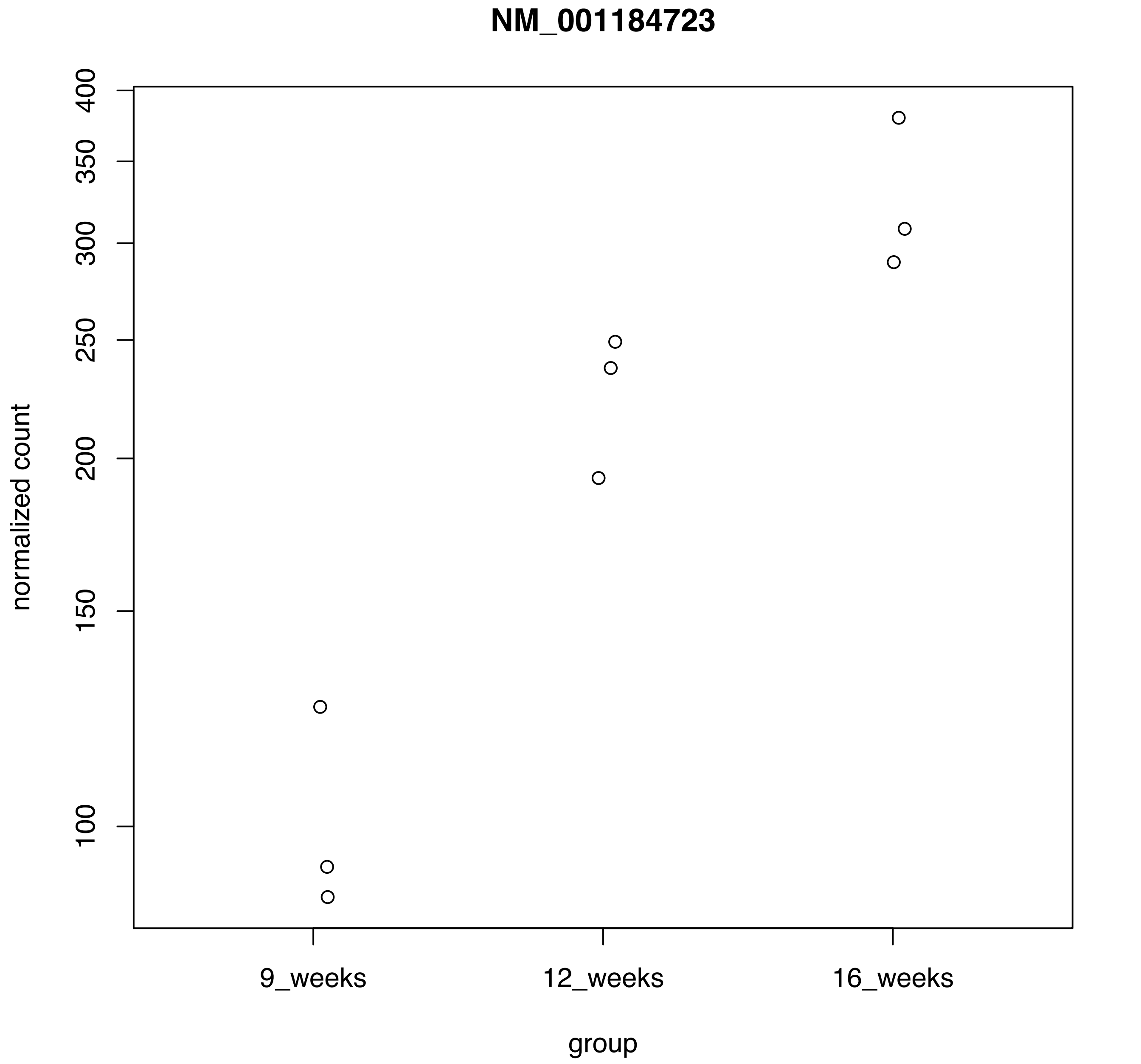
NM_001184723
normalized count
400
350
300
250
200
150
100
9_weeks
12_weeks
16_weeks
group

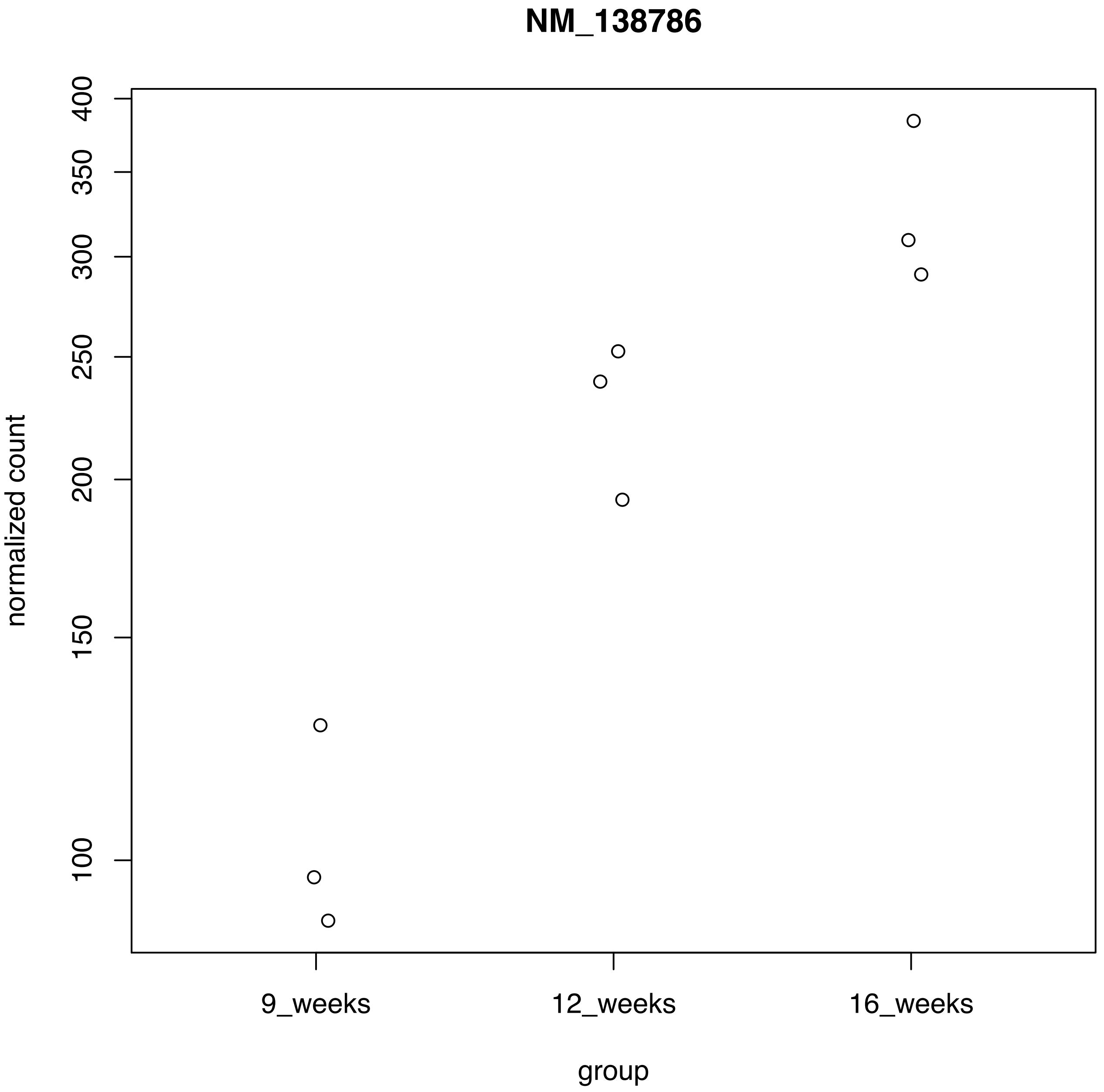
NM_138786
normalized count
400
350
300
250
200
150
100
9_weeks
12_weeks
16_weeks
group

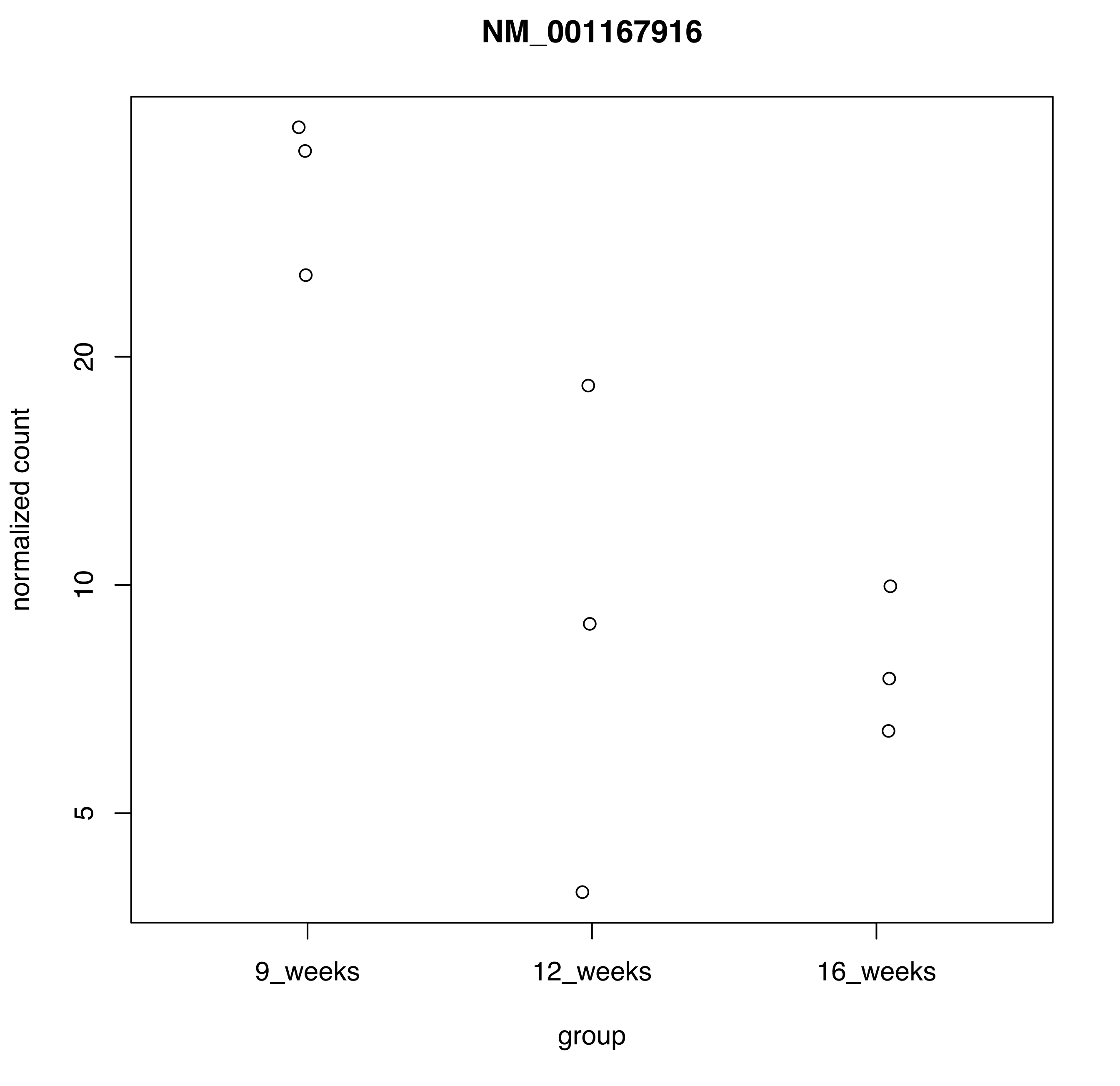
NM_001167916
normalized count
5
10
20
9_weeks
12_weeks
16_weeks
group

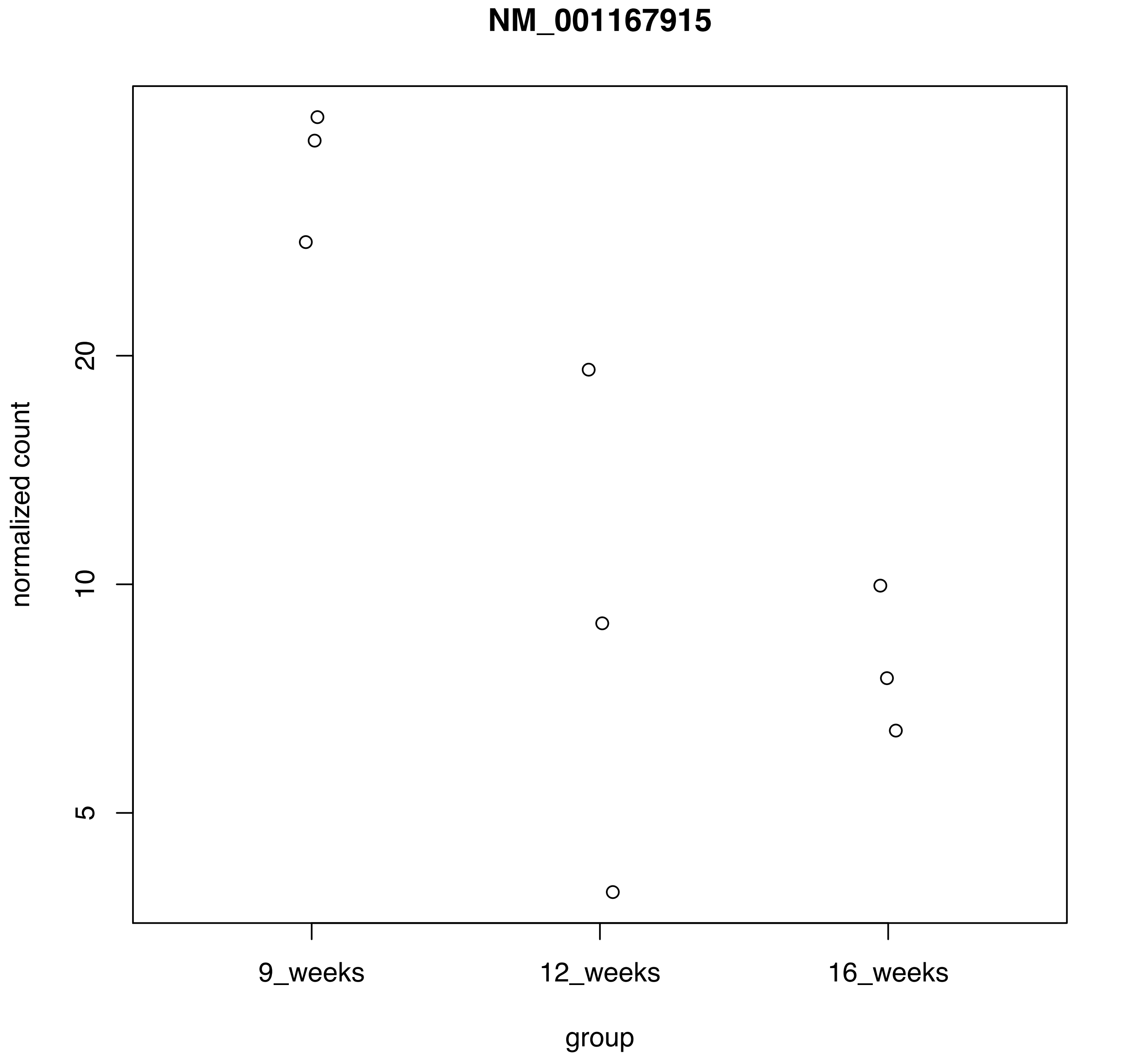

NM_001167915
normalized count
20
10
5
9_weeks
12_weeks
16_weeks
group

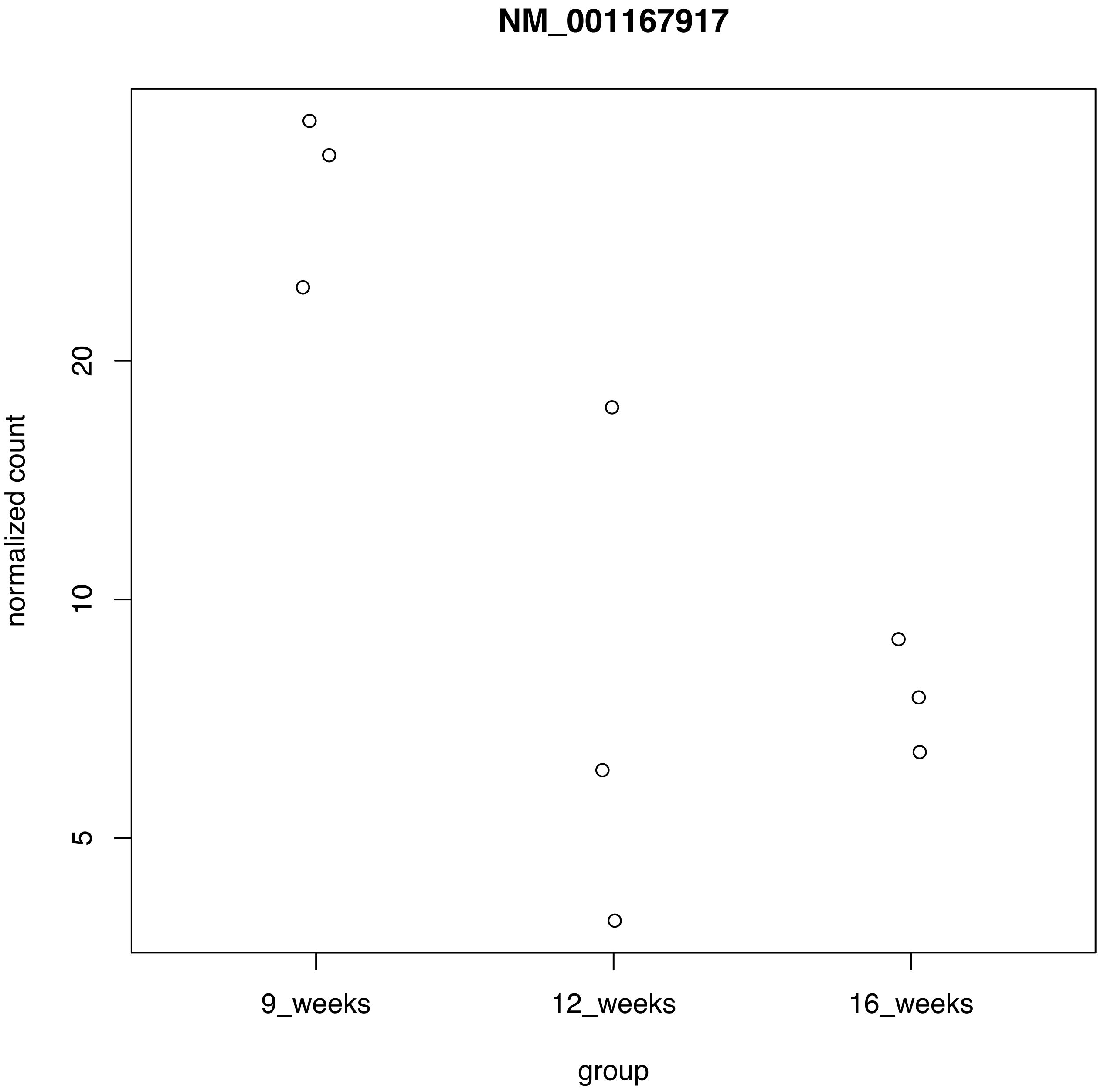

NM_001167917
normalized count
20
10
5
9_weeks
12_weeks
16_weeks
group

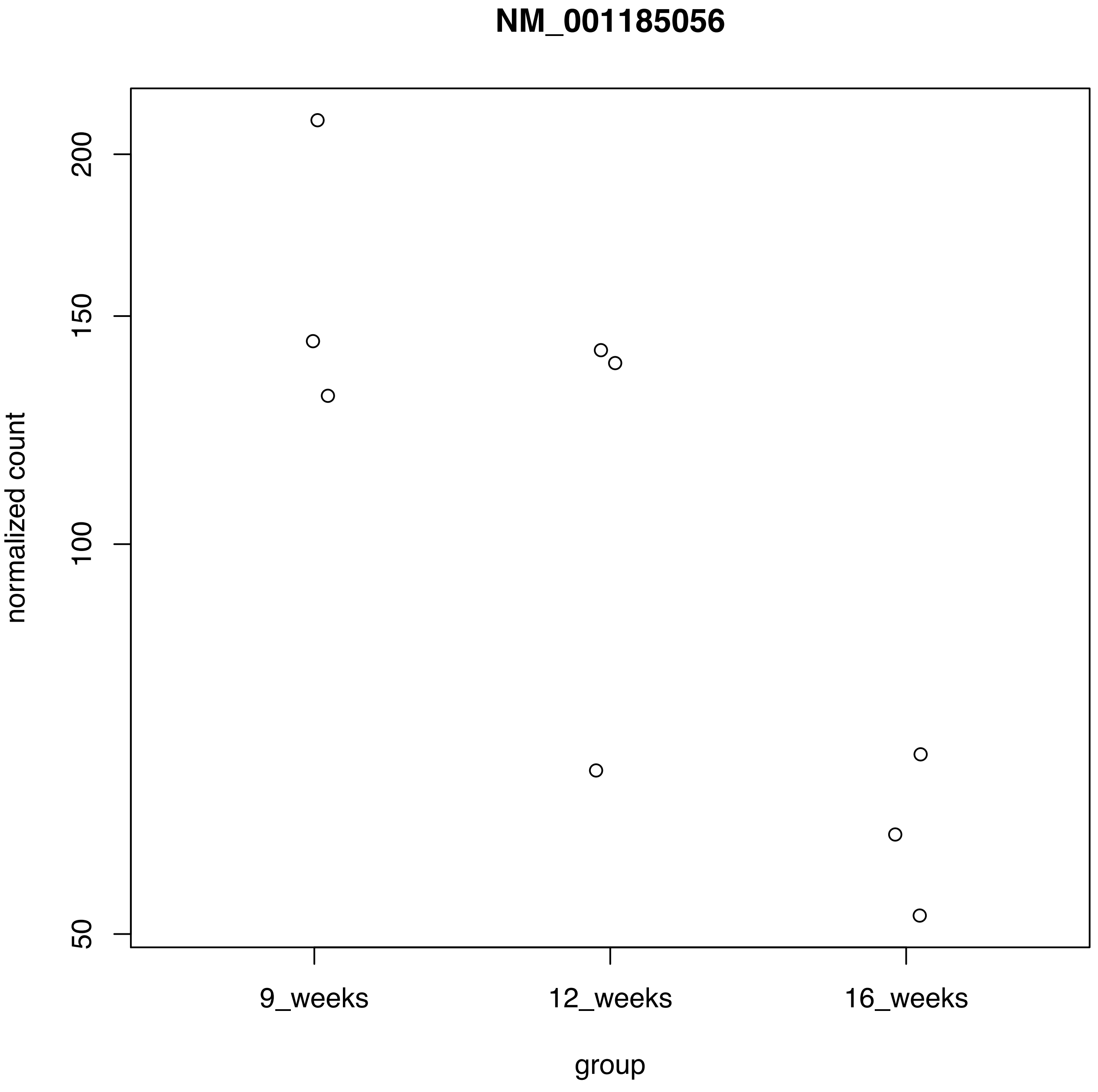

NM_001185056
normalized count
200
150
100
50
9_weeks
12_weeks
16_weeks
group

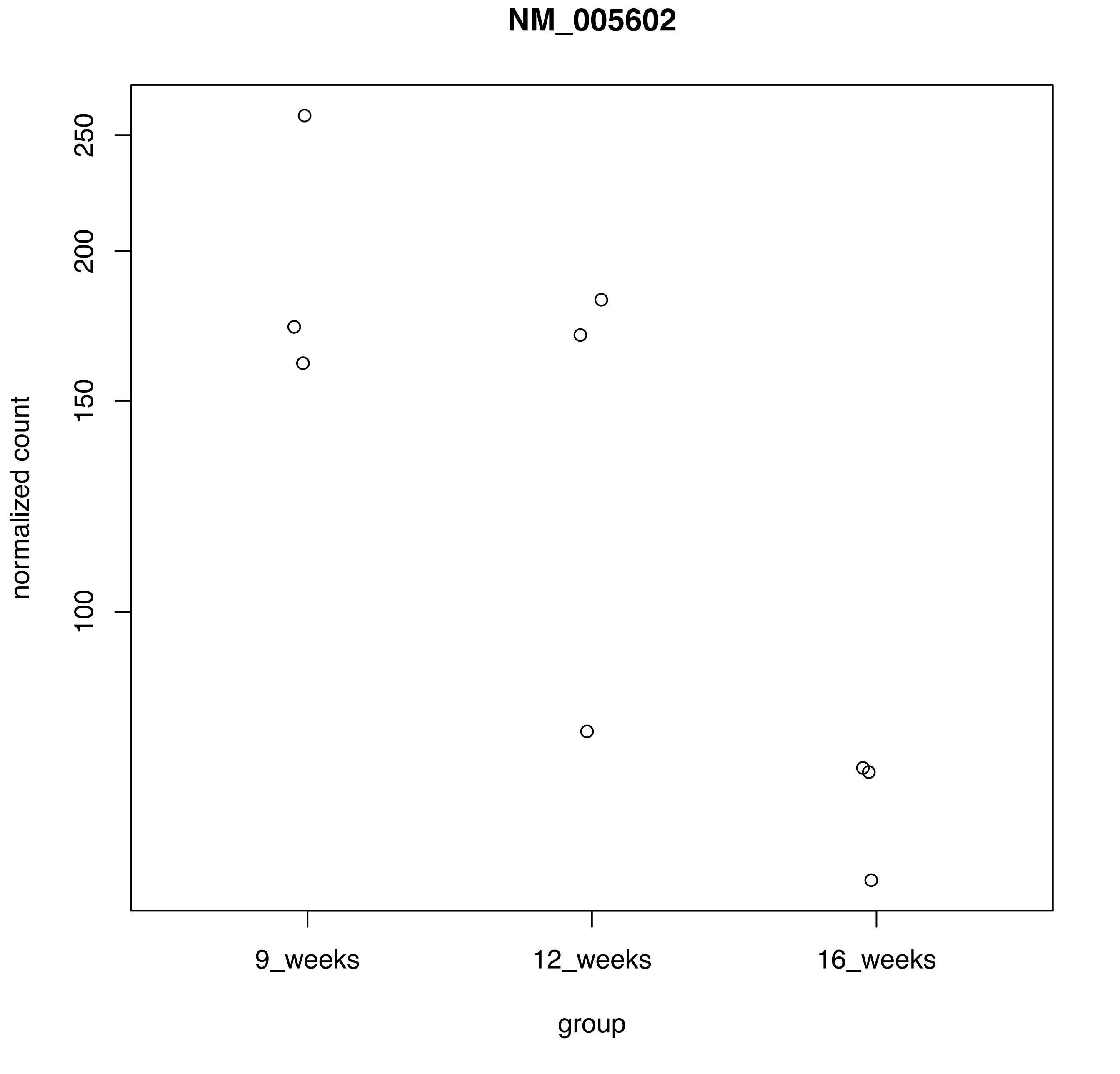

NM_005602
normalized count
100
150
200
250
9_weeks
12_weeks
16_weeks
group

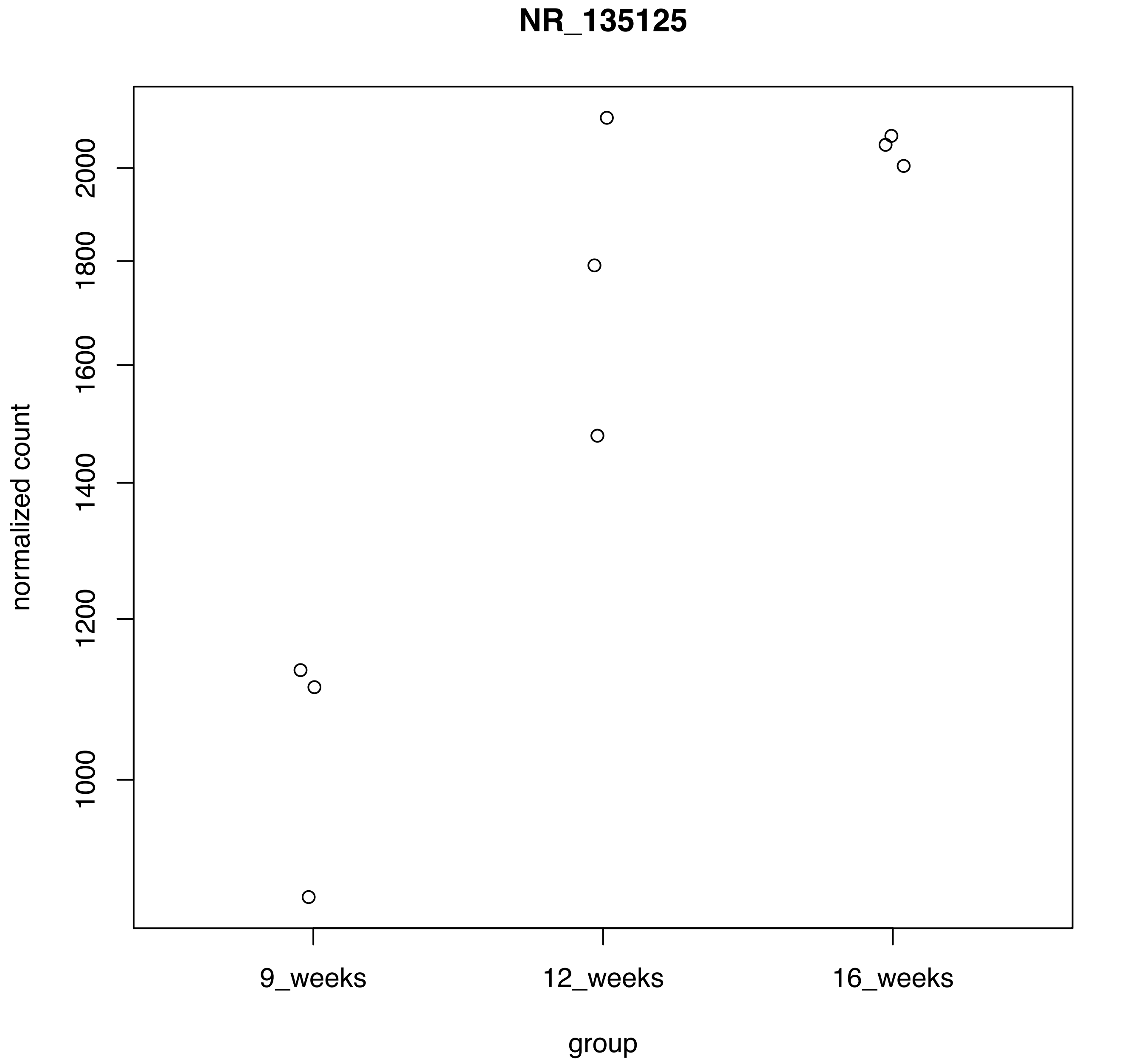

NR_135125
normalized count
1000
1200
1400
1600
1800
2000
9_weeks
12_weeks
16_weeks
group

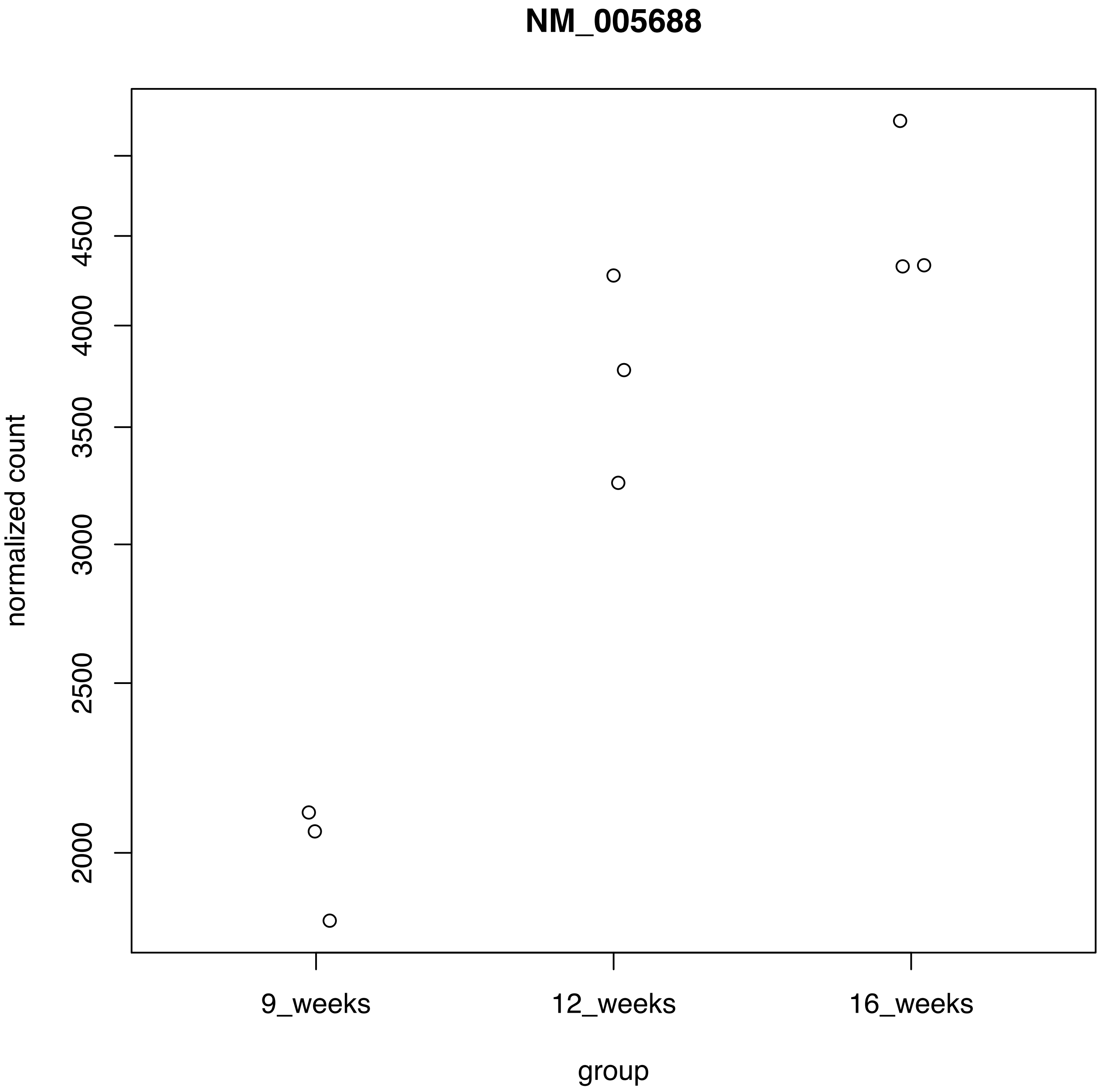
NM_005688
normalized count
2000
2500
3000
3500
4000
4500
9_weeks
12_weeks
16_weeks
group

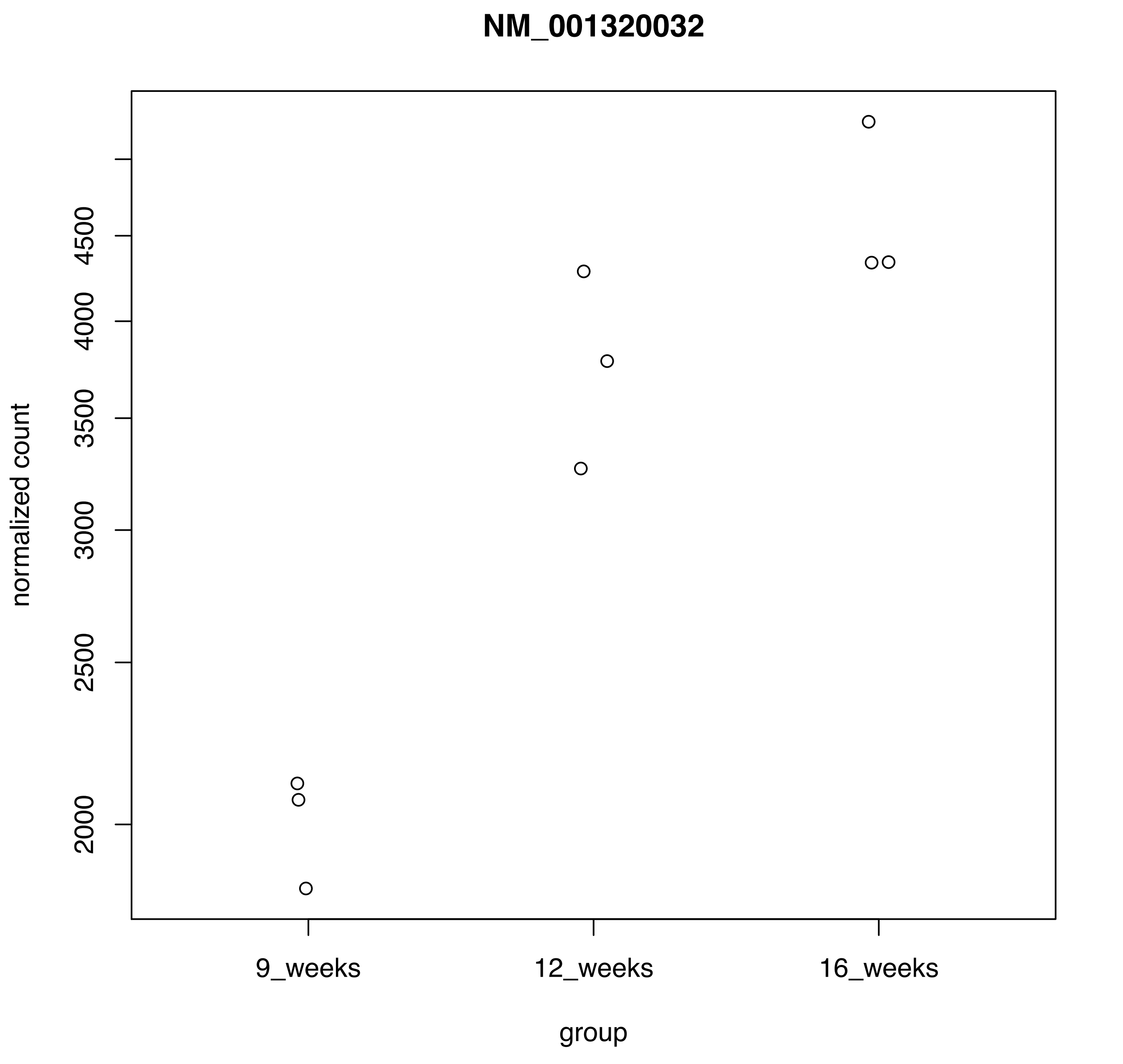
NM_001320032
normalized count
2000
2500
3000
3500
4000
4500
9_weeks
12_weeks
16_weeks
group

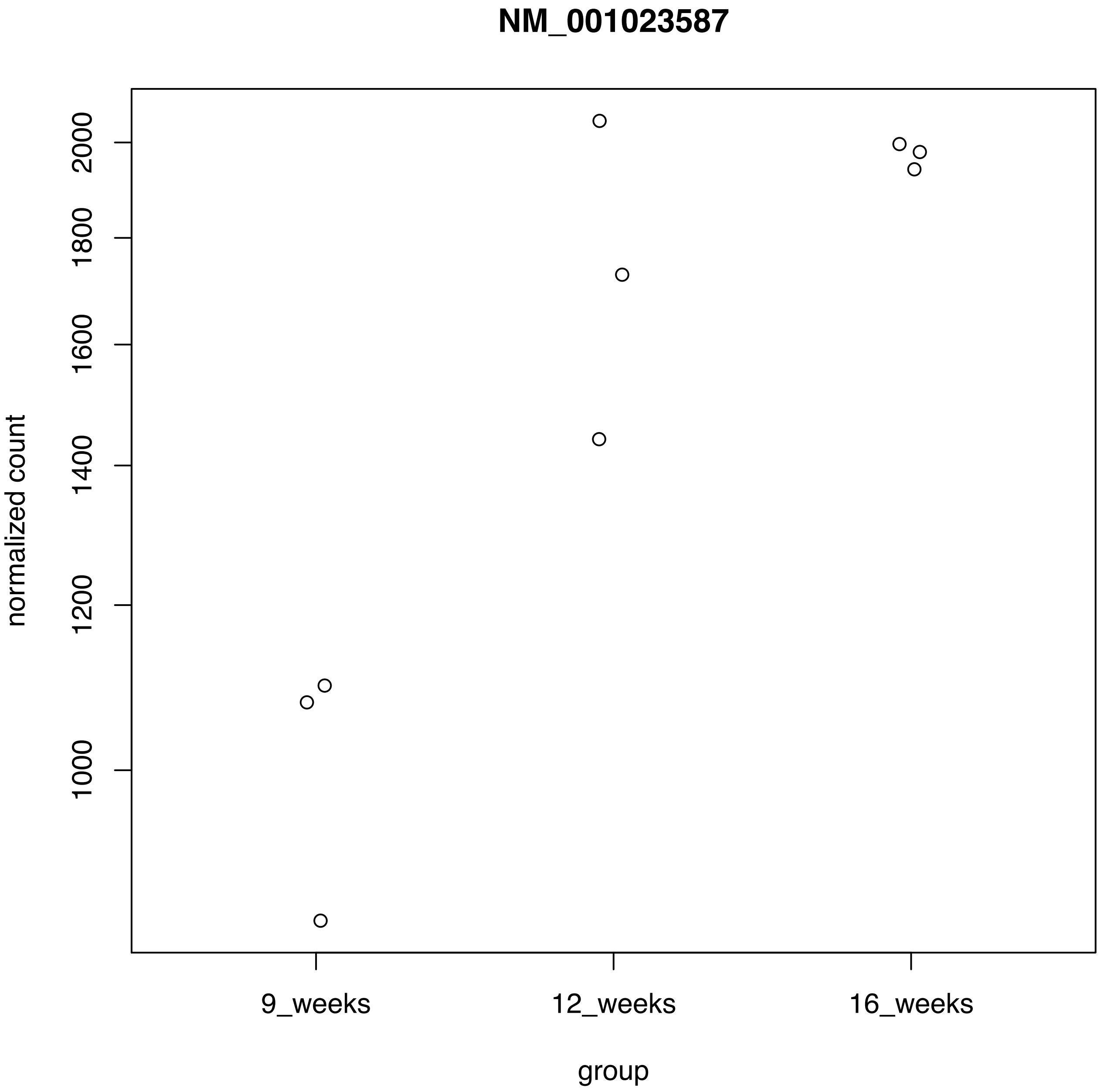
NM_001023587
normalized count
2000
1800
1600
1400
1200
1000
9_weeks
12_weeks
16_weeks
group

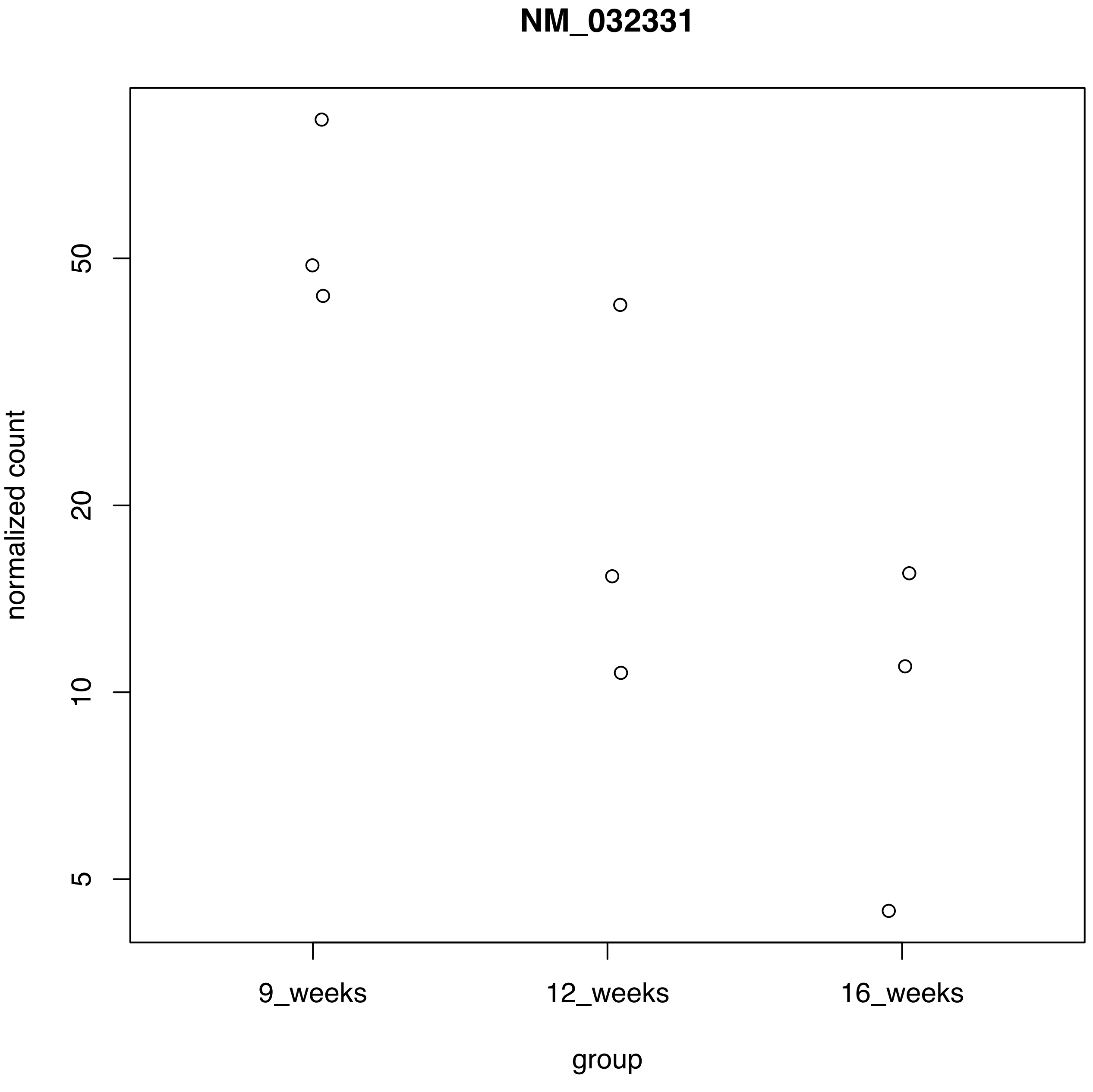
NM_032331
normalized count
50
20
10
5
9_weeks
12_weeks
16_weeks
group

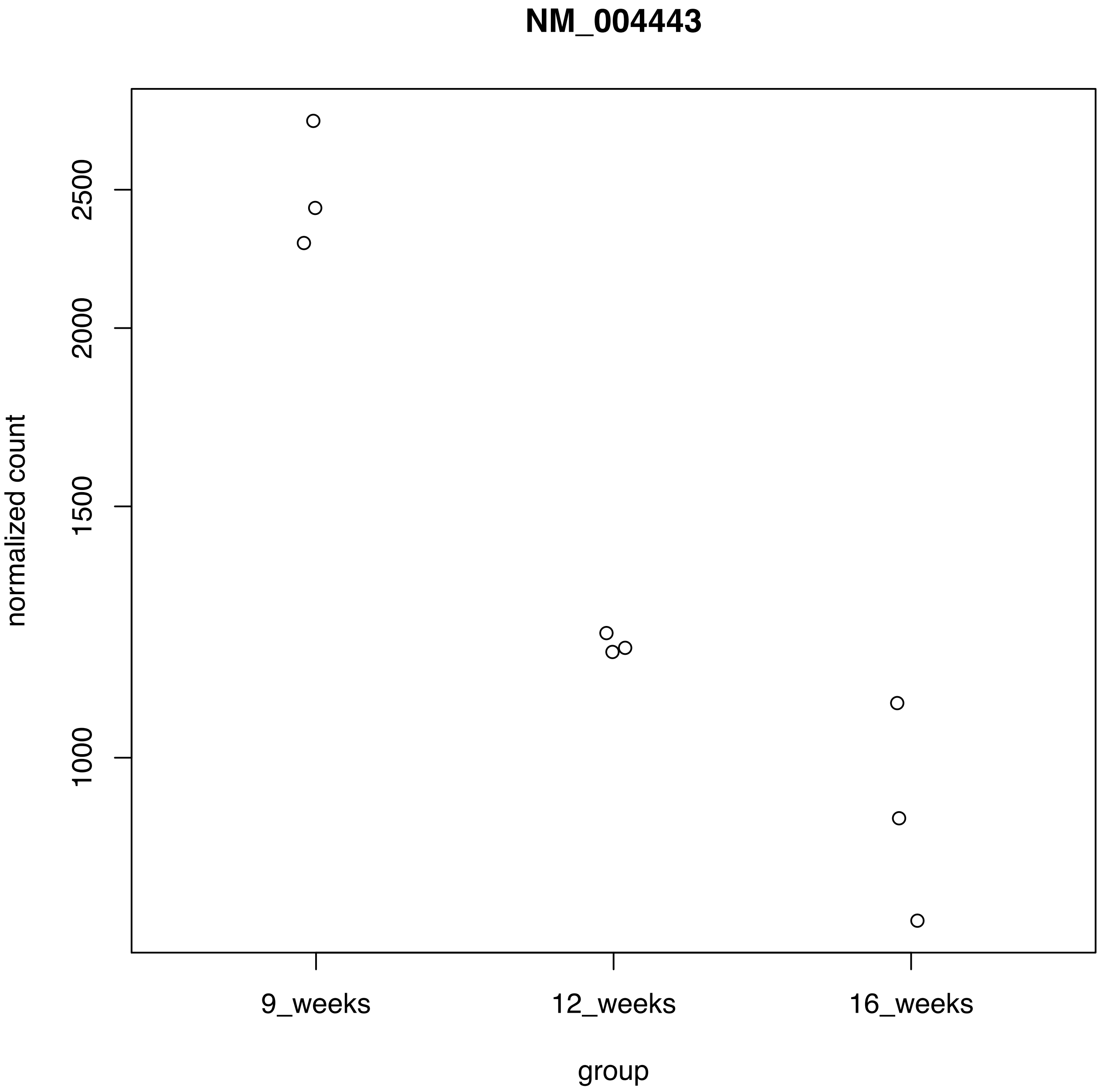
NM_004443
normalized count
2500
2000
1500
1000
9_weeks
12_weeks
16_weeks
group

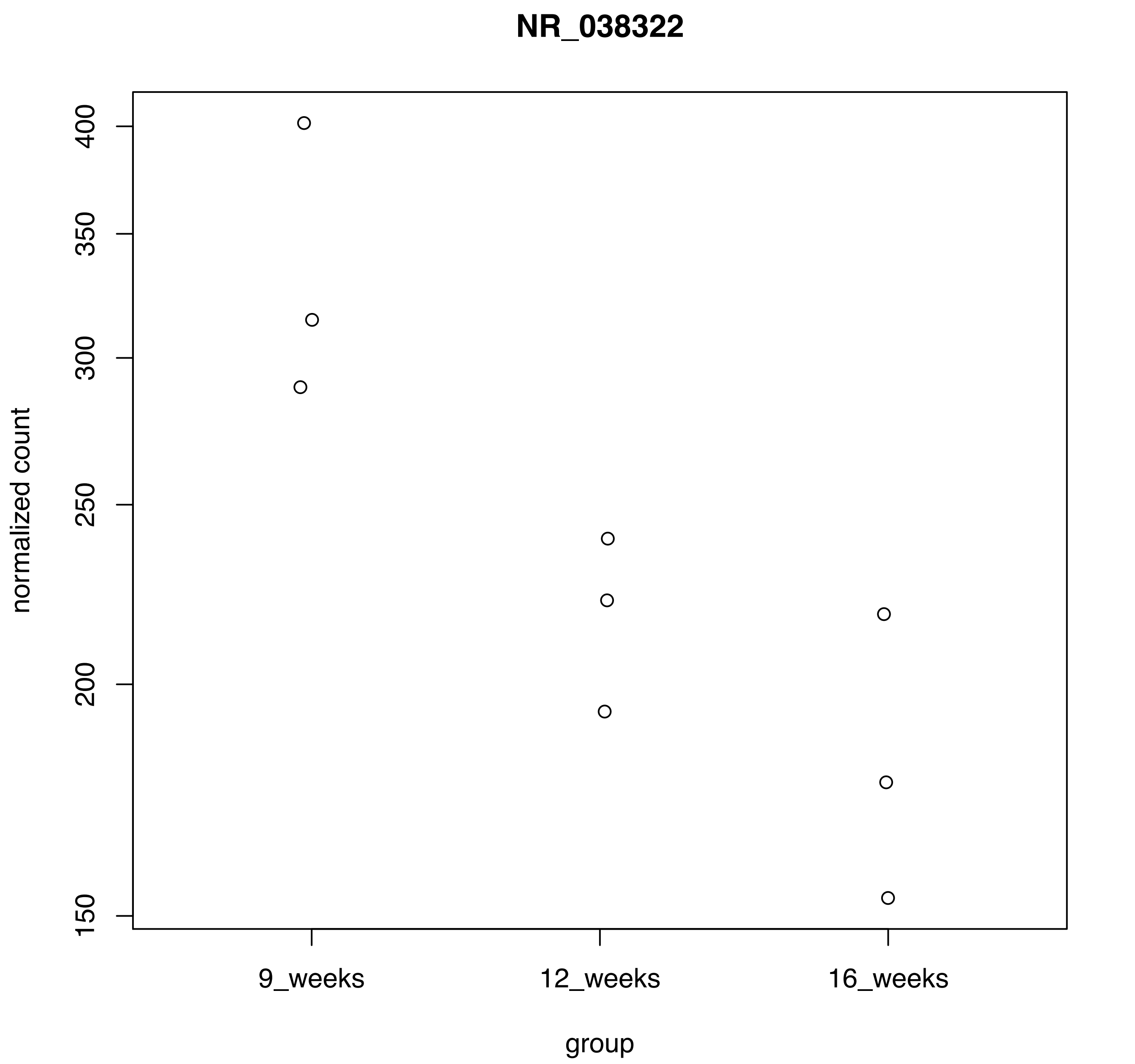

NR_038322
normalized count
400
350
300
250
200
150
9_weeks
12_weeks
16_weeks
group

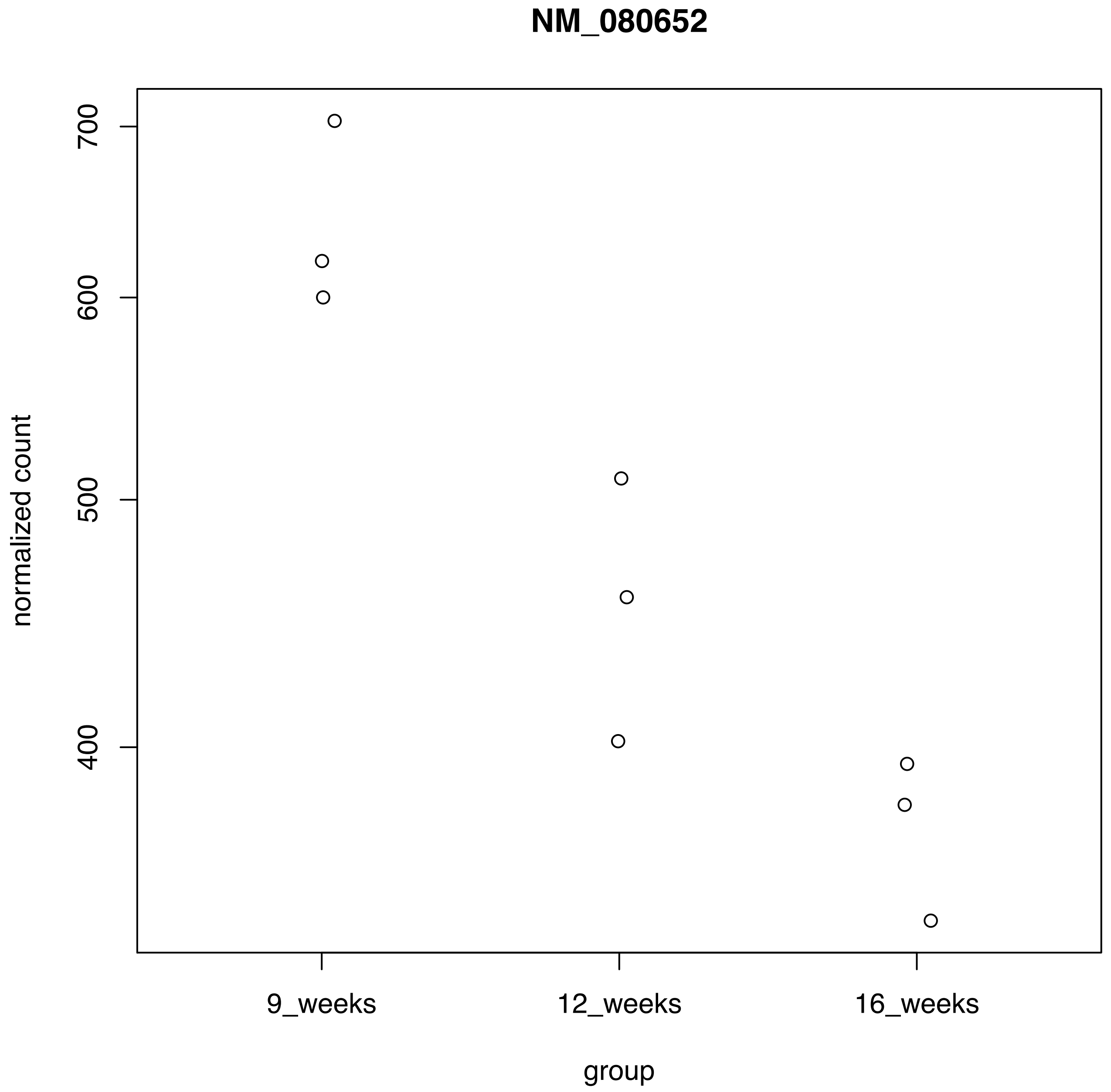
NM_080652
normalized count
700
600
500
400
9_weeks
12_weeks
16_weeks
group

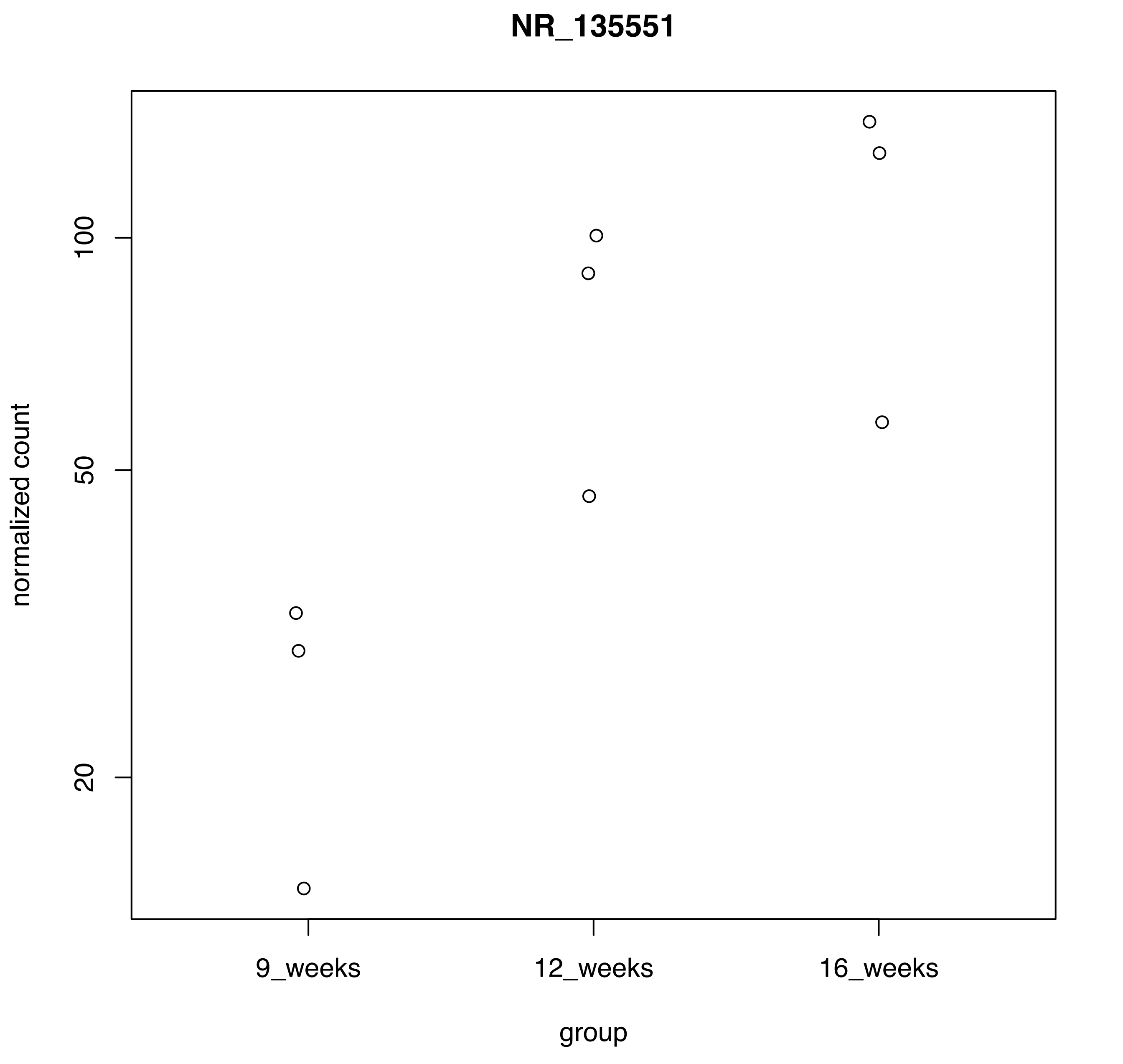

NR_135551
normalized count
100
50
20
9_weeks
12_weeks
16_weeks
group

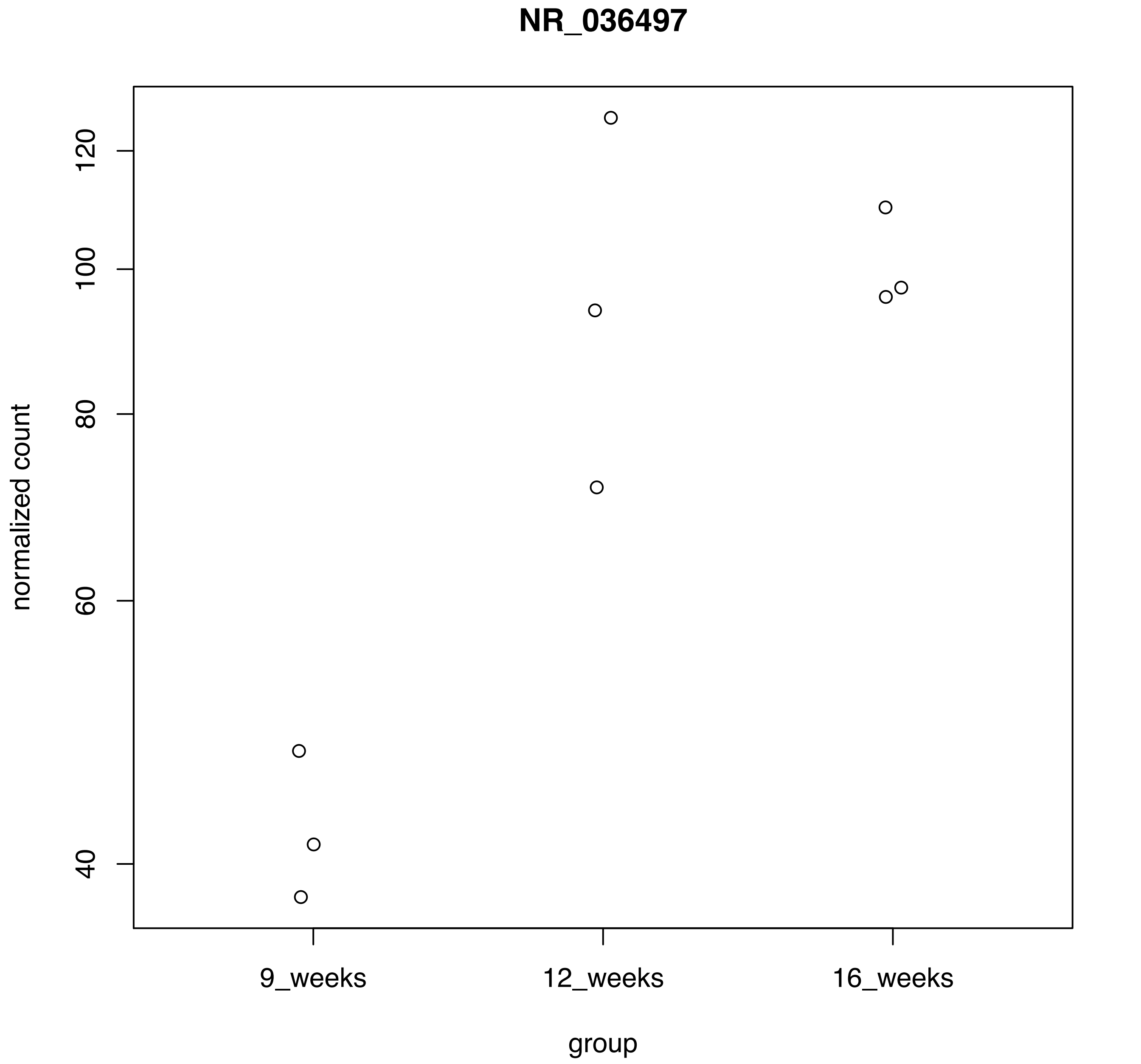

NR_036497
normalized count
120
100
80
60
40
9_weeks
12_weeks
16_weeks
group

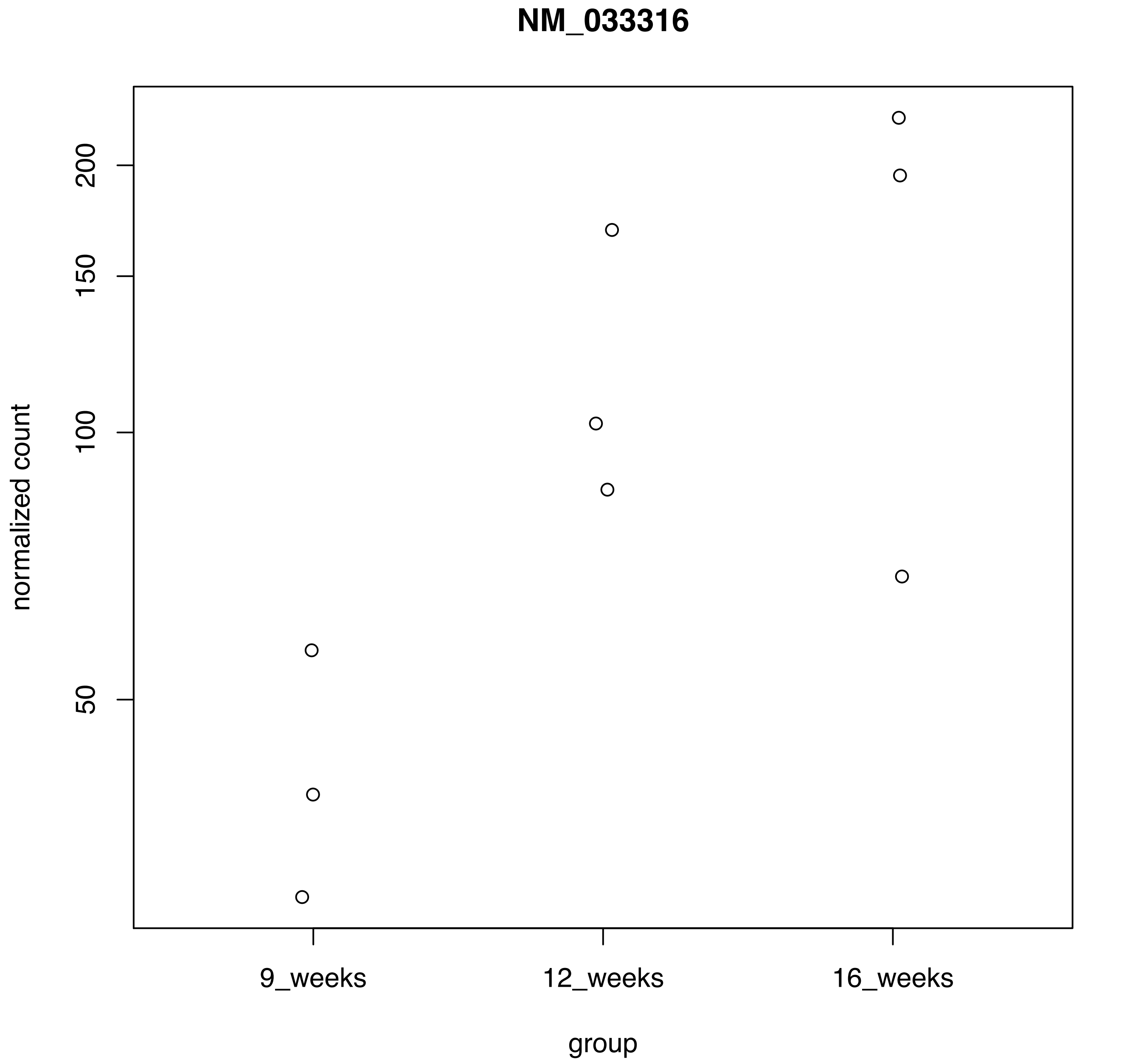
NM_033316
normalized count
200
150
100
50
9_weeks
12_weeks
16_weeks
group

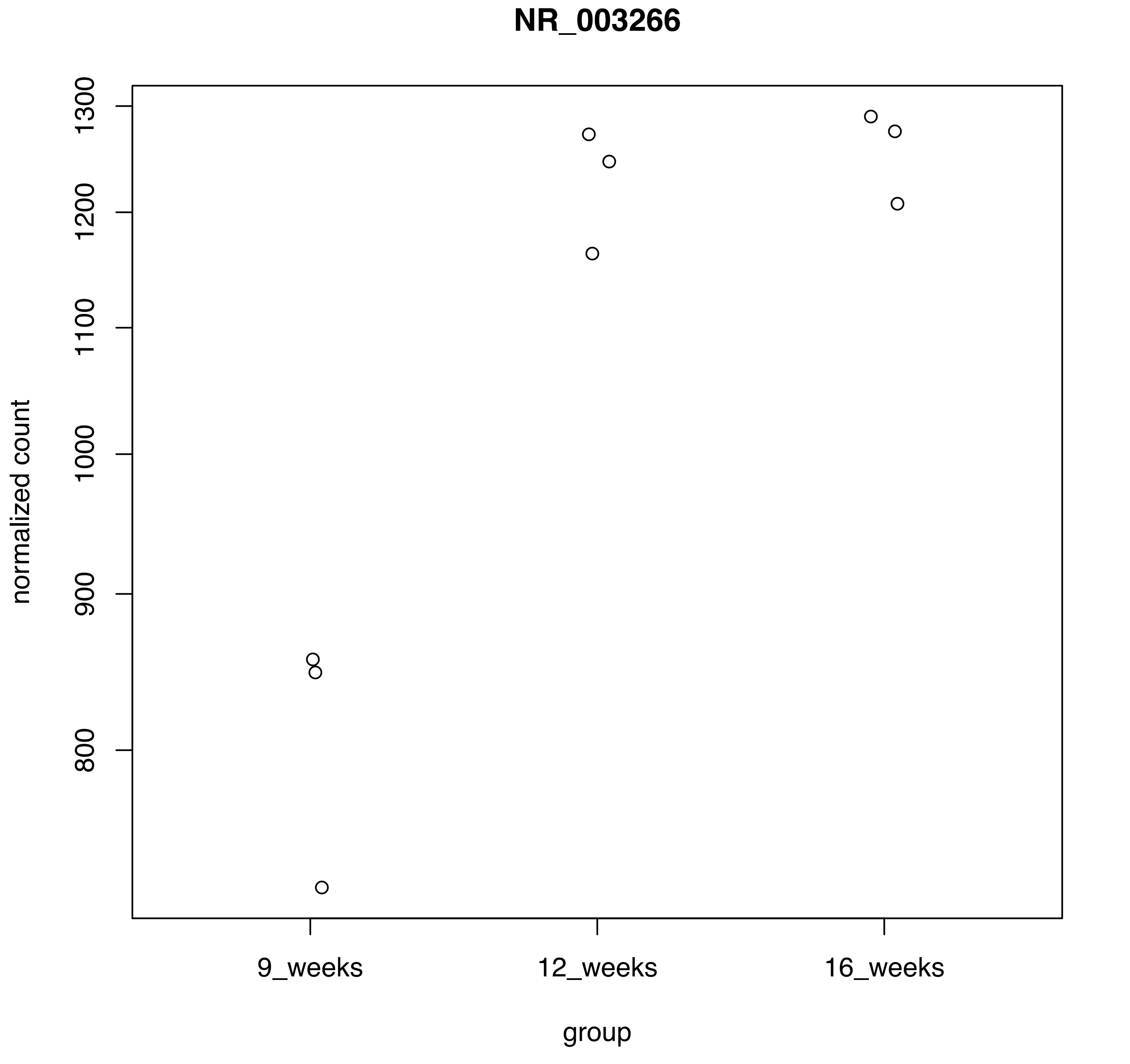

NR_003266
normalized count
1300
1200
1100
1000
900
800
9_weeks
12_weeks
16_weeks
group

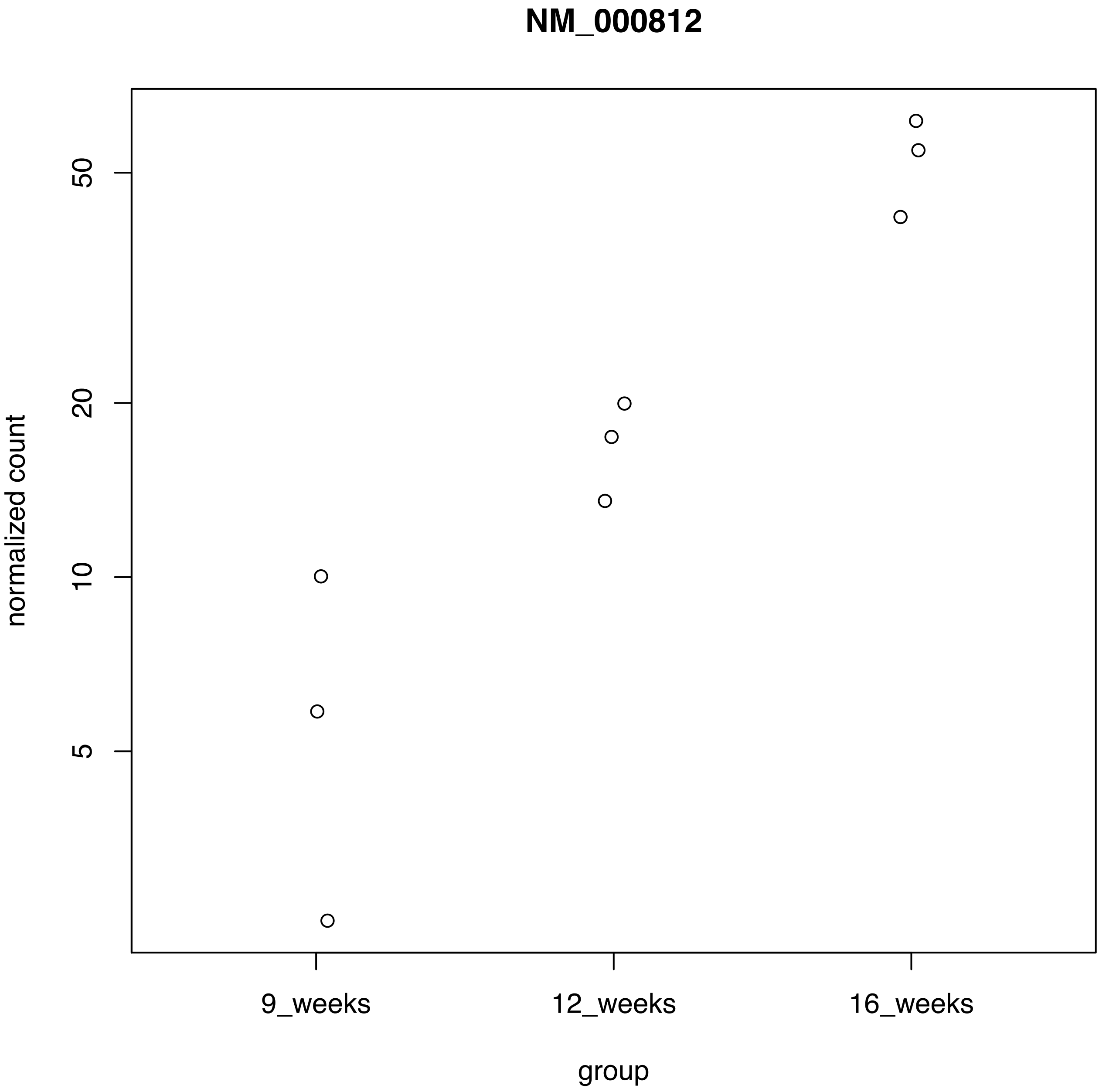
NM_000812
normalized count
group
50
20
10
5
9_weeks
12_weeks
16_weeks

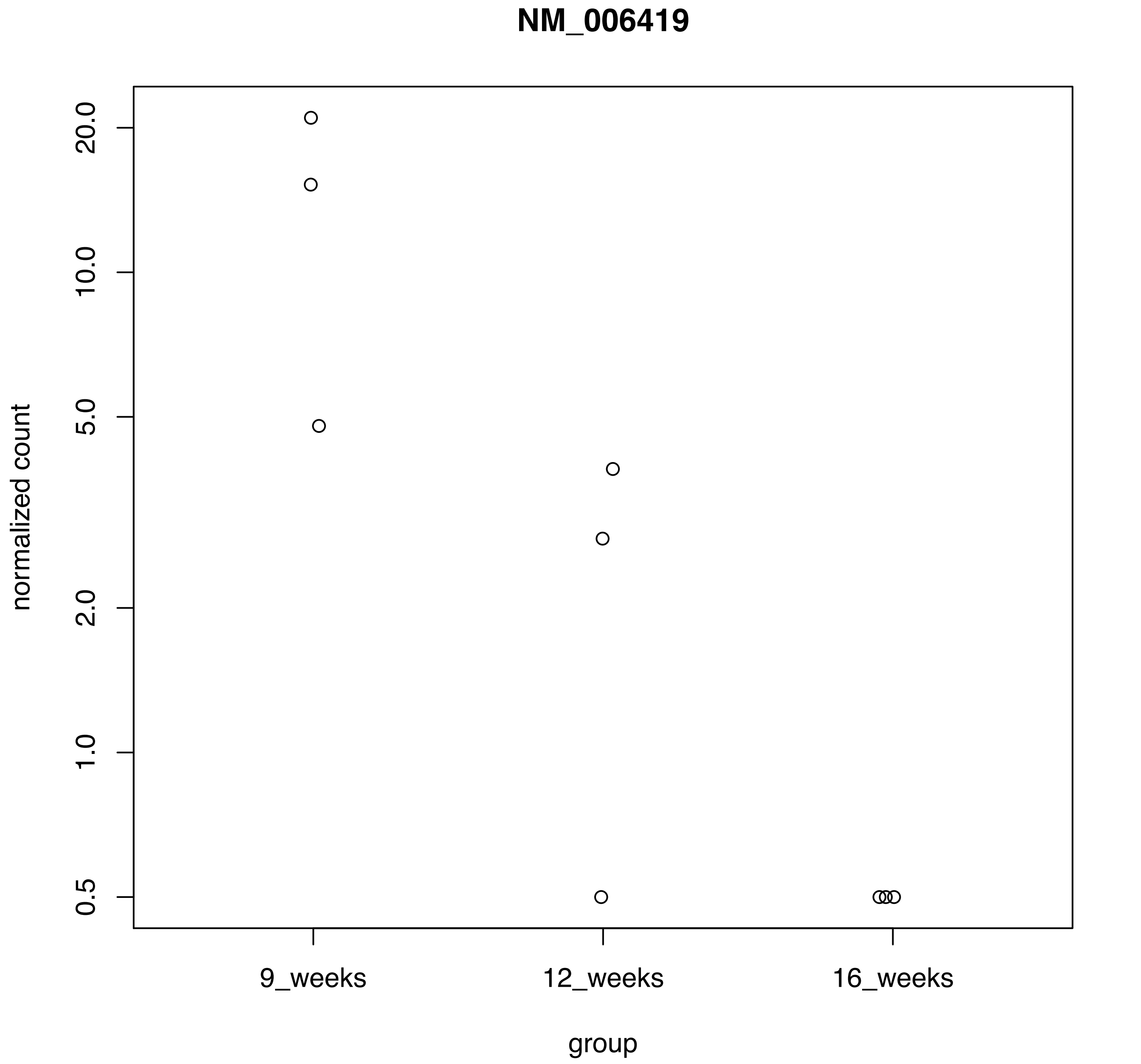

NM_006419
20.0
10.0
5.0
2.0
1.0
0.5
normalized count
9_weeks
12_weeks
16_weeks
group

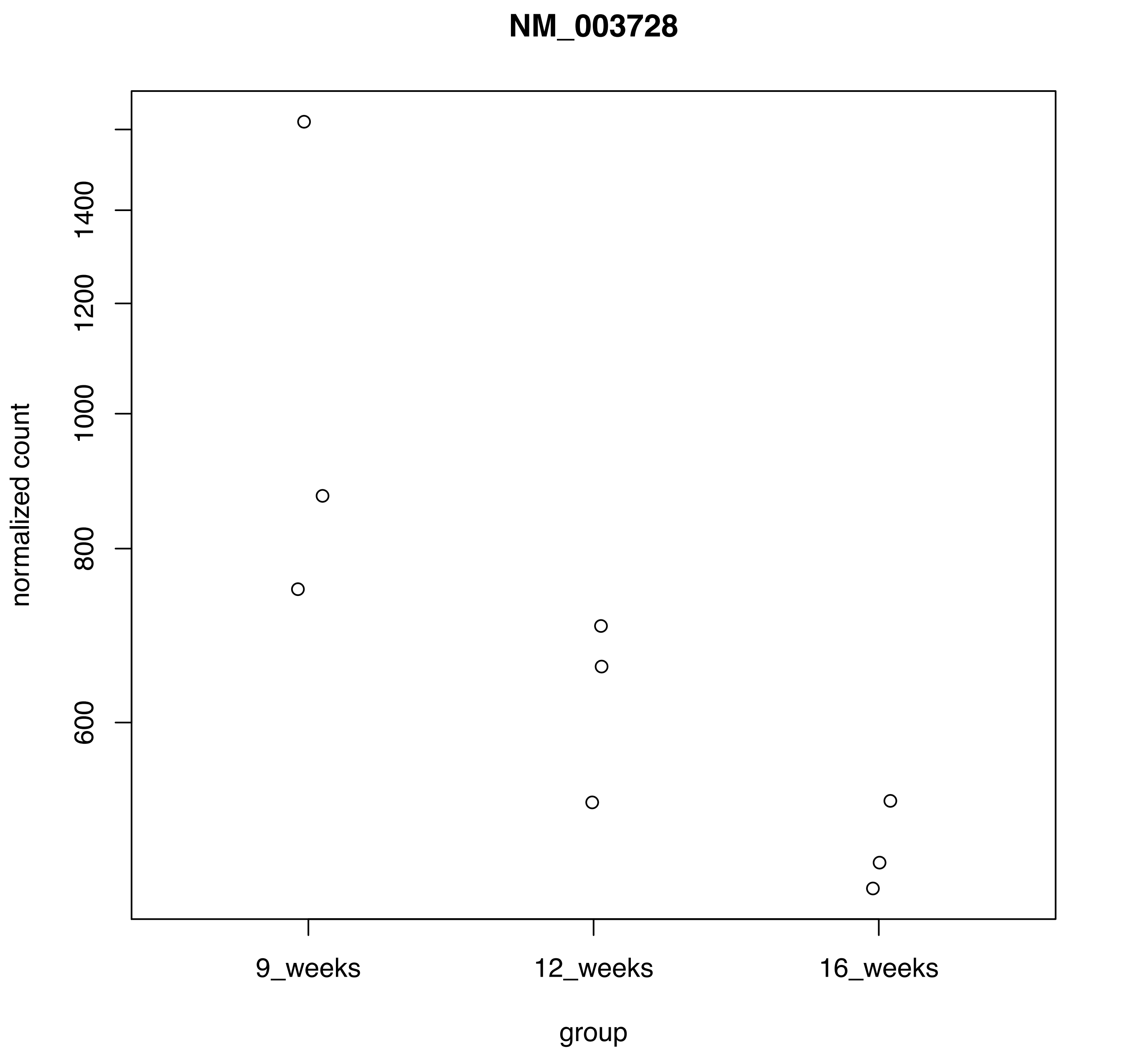

NM_003728
normalized count
600
800
1000
1200
1400
9_weeks
12_weeks
16_weeks
group

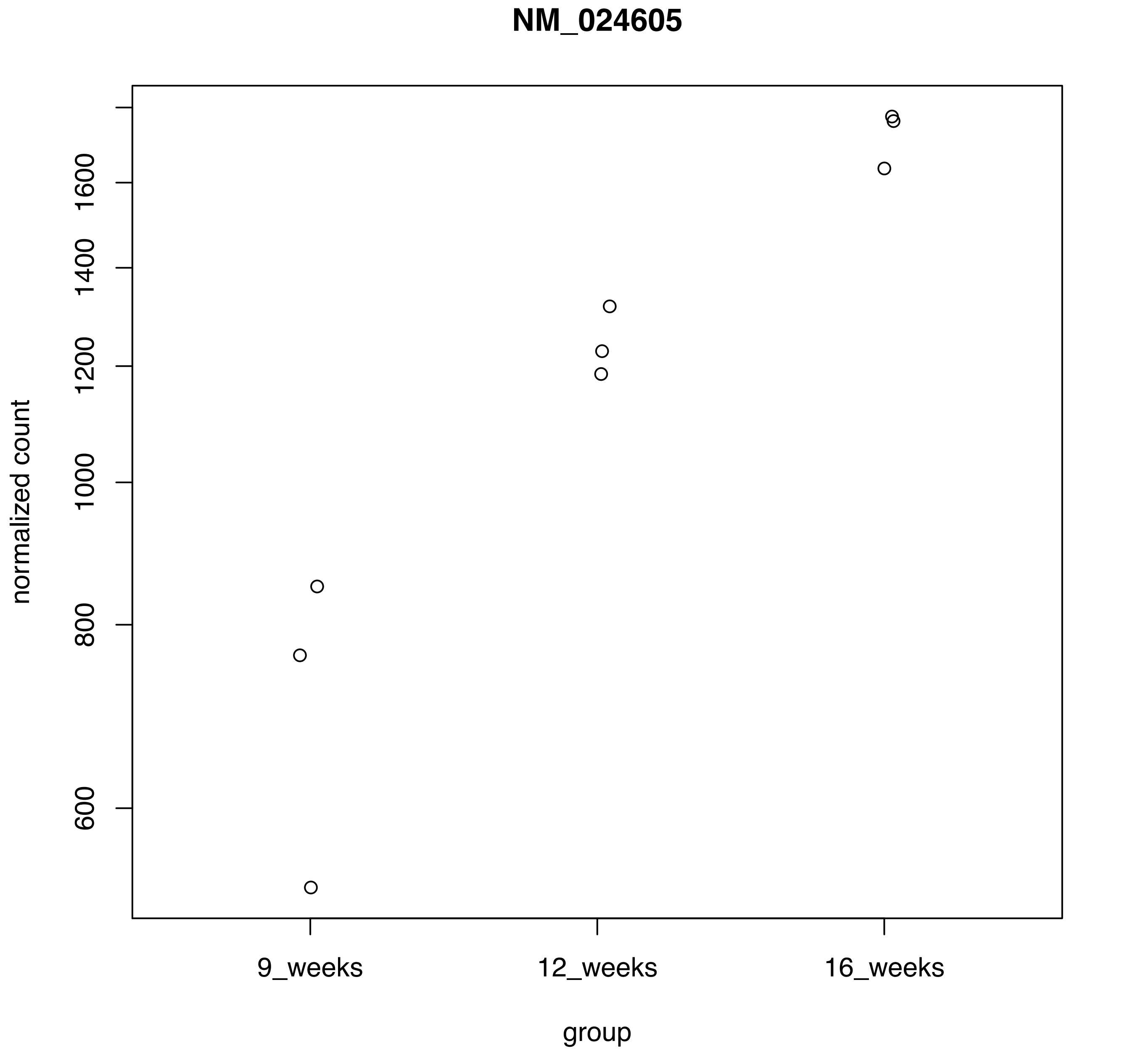

NM_024605
normalized count
600
800
1000
1200
1400
1600
9_weeks
12_weeks
16_weeks
group

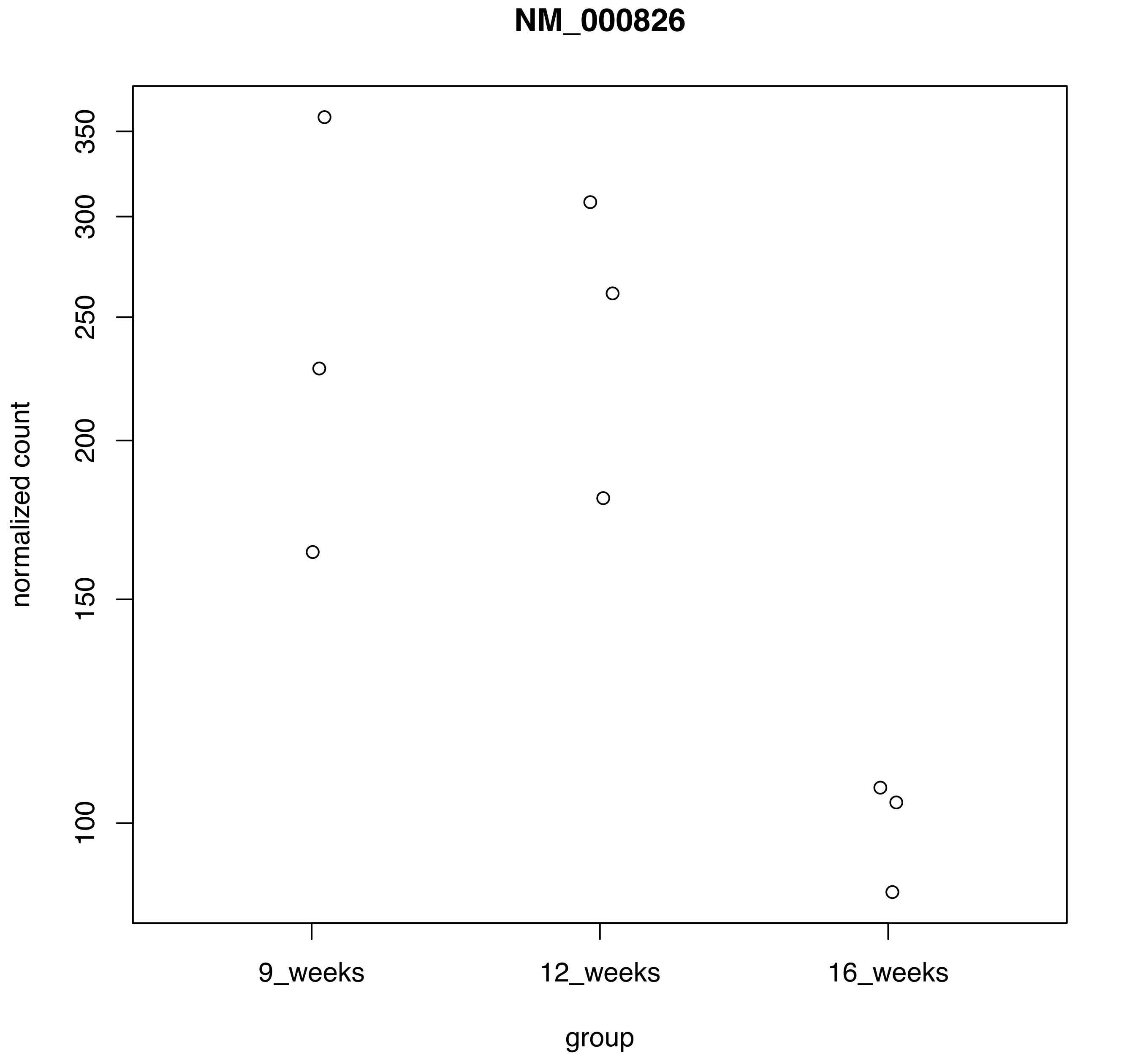

NM_000826
normalized count
100
150
200
250
300
350
9_weeks
12_weeks
16_weeks
group

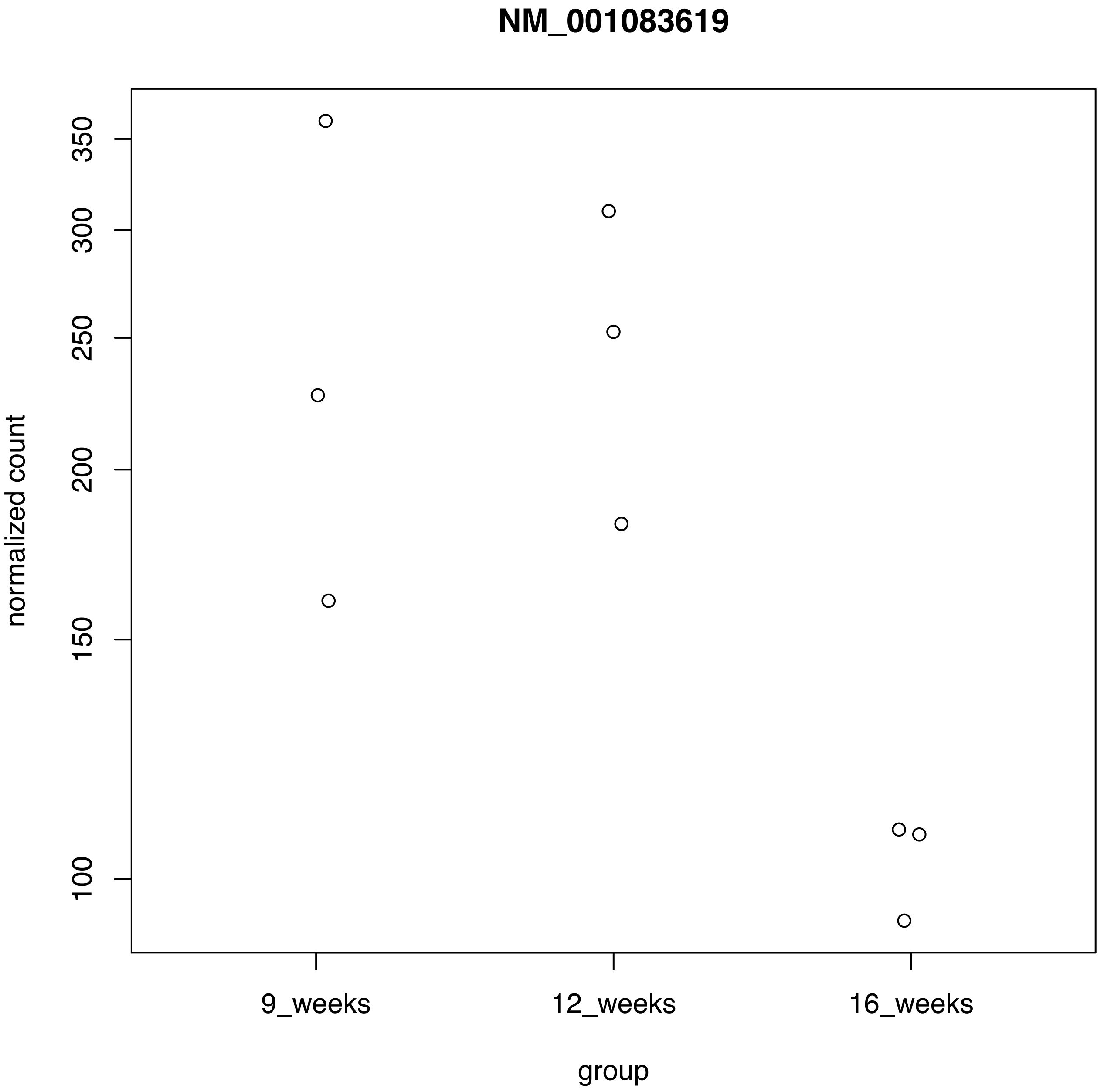
NM_001083619
normalized count
350
300
250
200
150
100
9_weeks
12_weeks
16_weeks
group

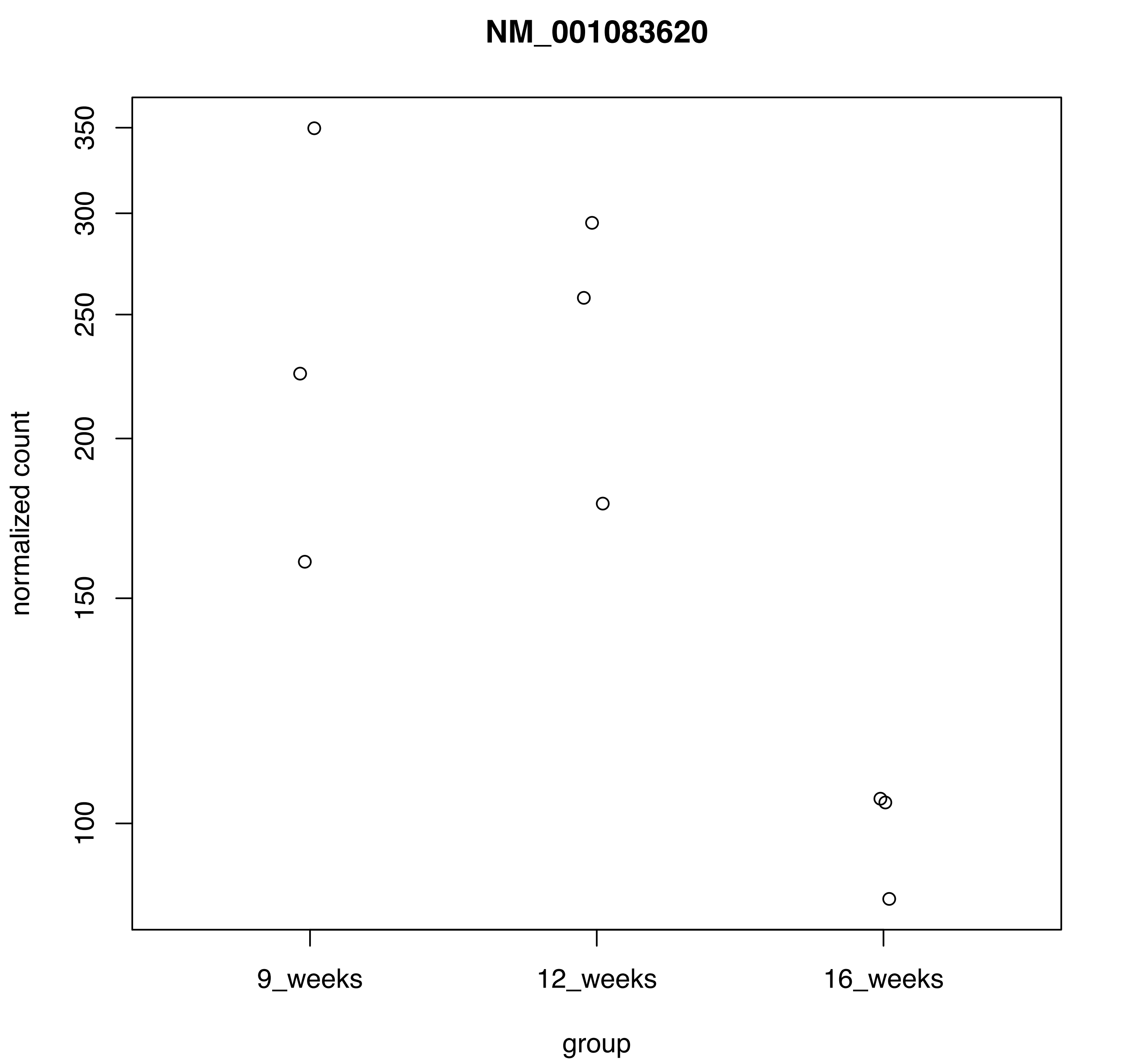
NM_001083620
normalized count
350
300
250
200
150
100
9_weeks
12_weeks
16_weeks
group

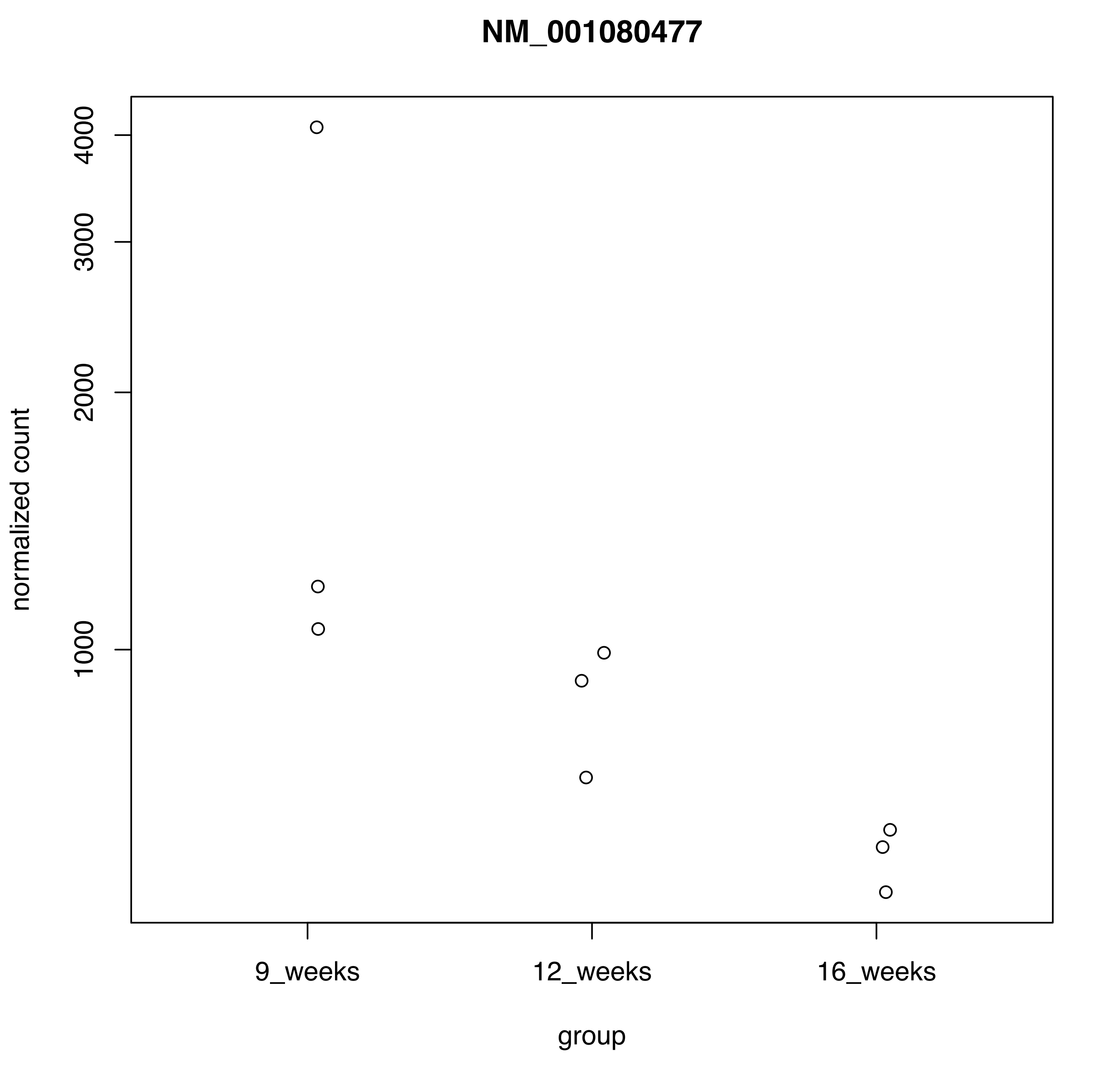
NM_001080477
normalized count
4000
3000
2000
1000
9_weeks
12_weeks
16_weeks
group

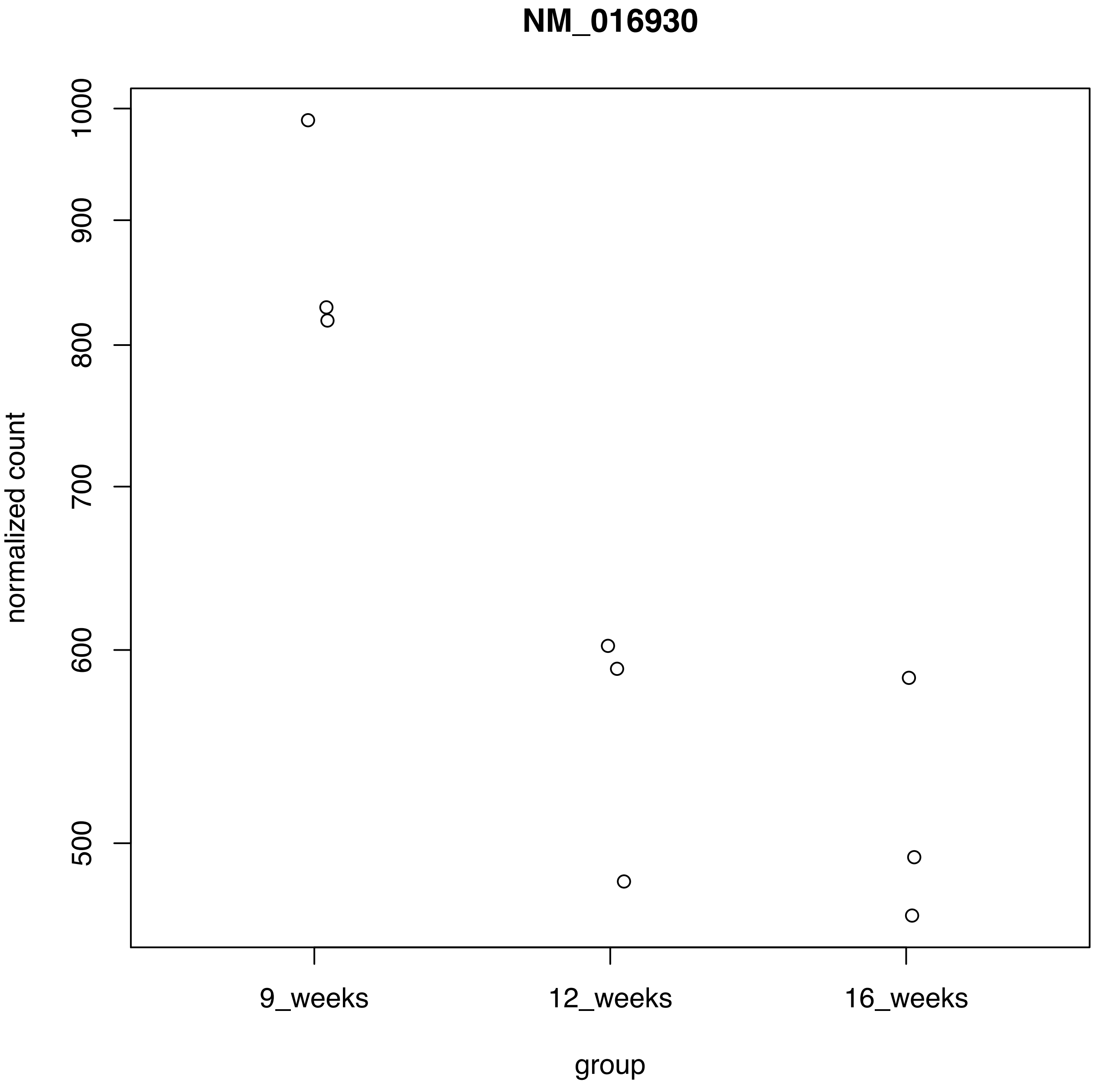

NM_016930
normalized count
1000
900
800
700
600
500
9_weeks
12_weeks
16_weeks
group

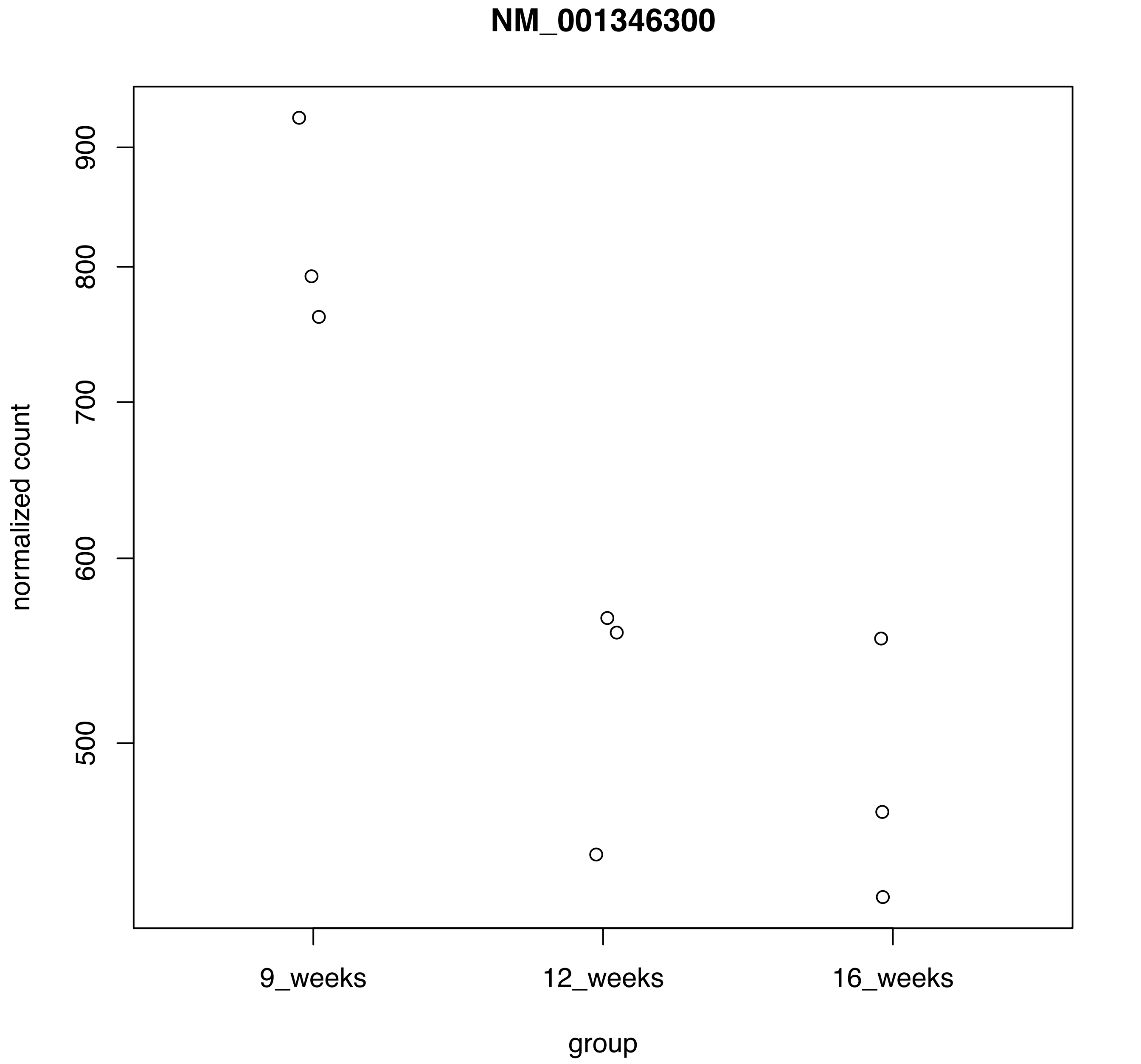
NM_001346300
normalized count
900
800
700
600
500
9_weeks
12_weeks
16_weeks
group

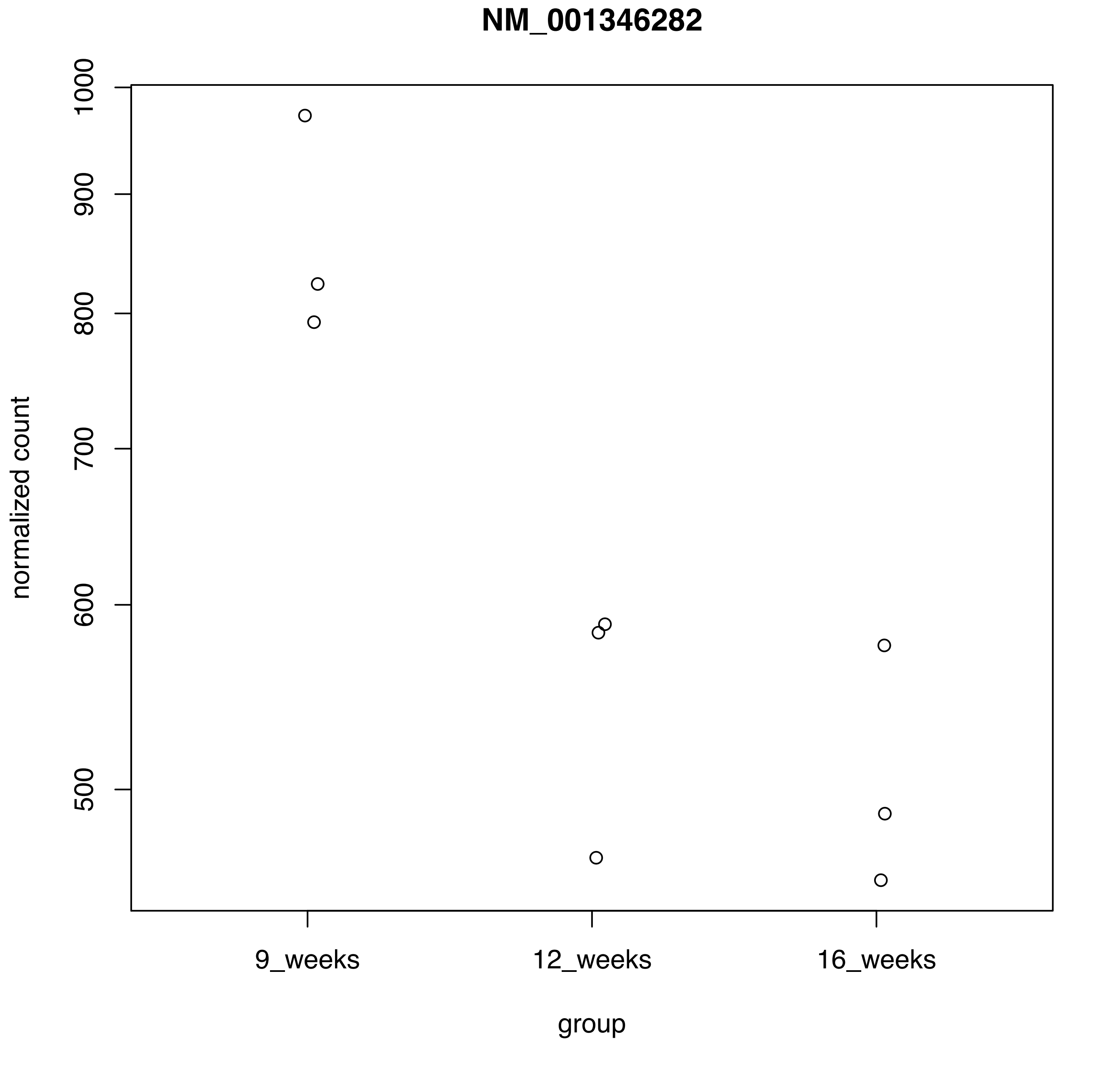
NM_001346282
normalized count
1000
900
800
700
600
500
9_weeks
12_weeks
16_weeks
group

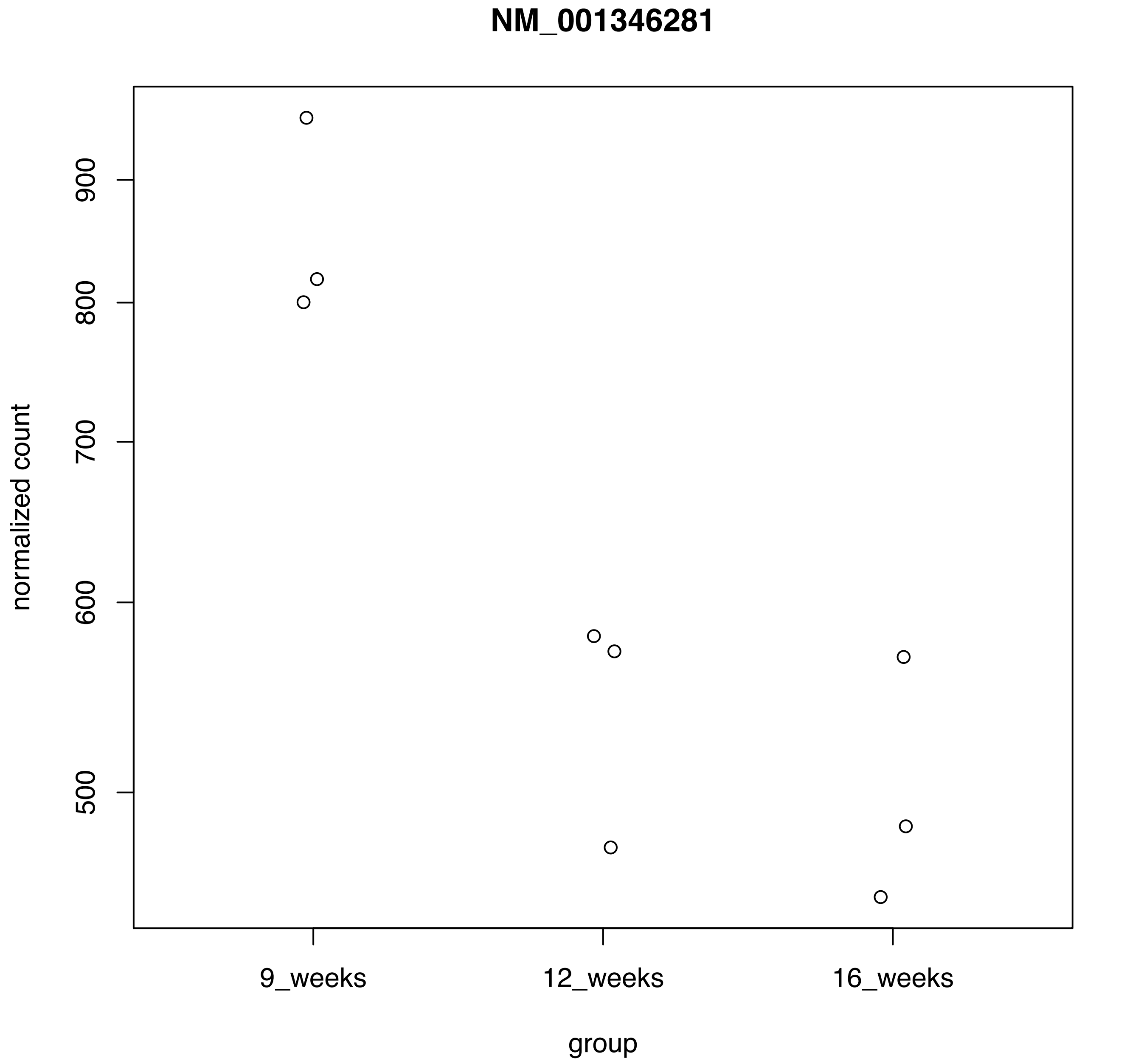

NM_001346281
normalized count
900
800
700
600
500
9_weeks
12_weeks
16_weeks
group

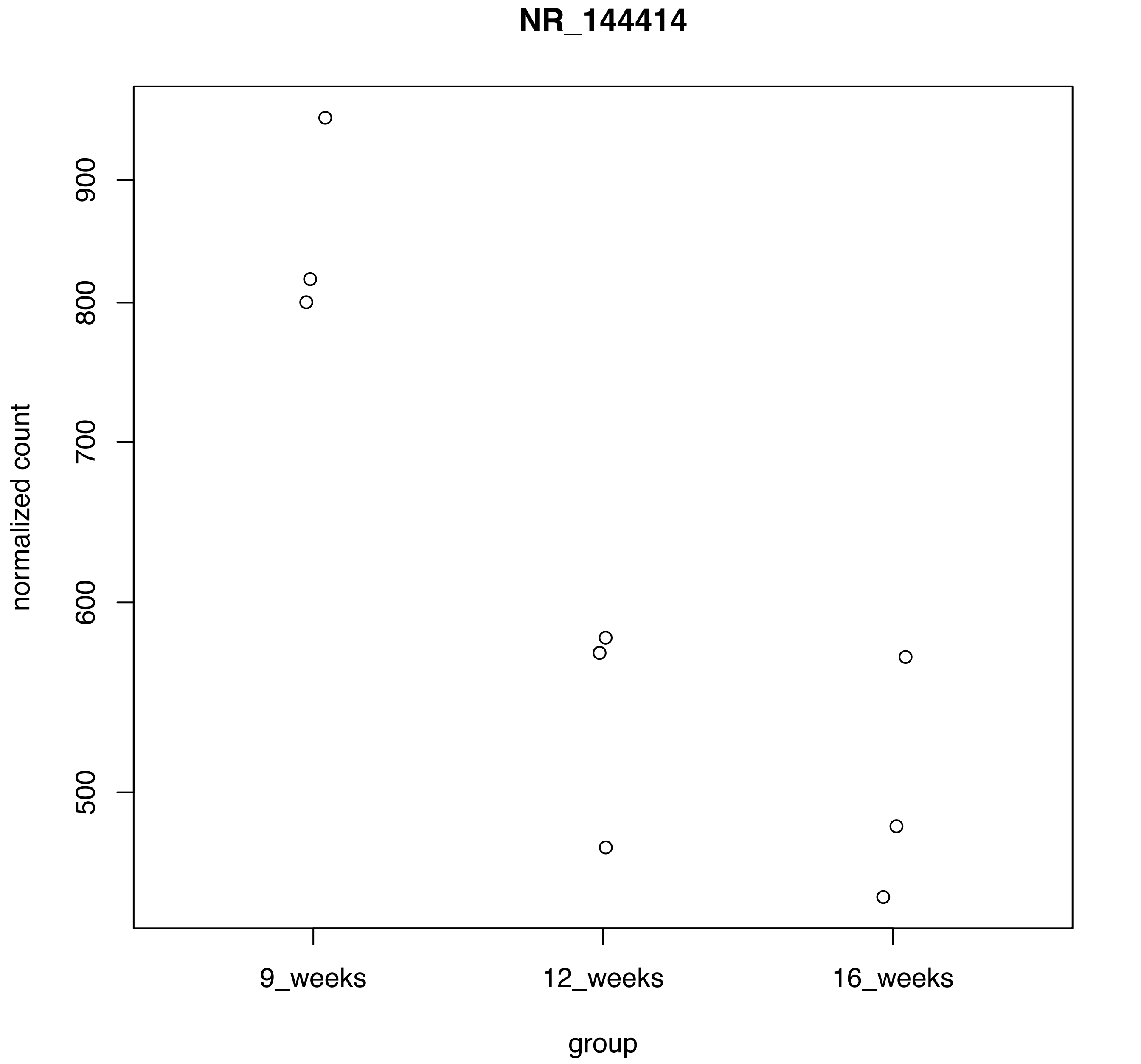
NR_144414
normalized count
500
600
700
800
900
9_weeks
12_weeks
16_weeks
group

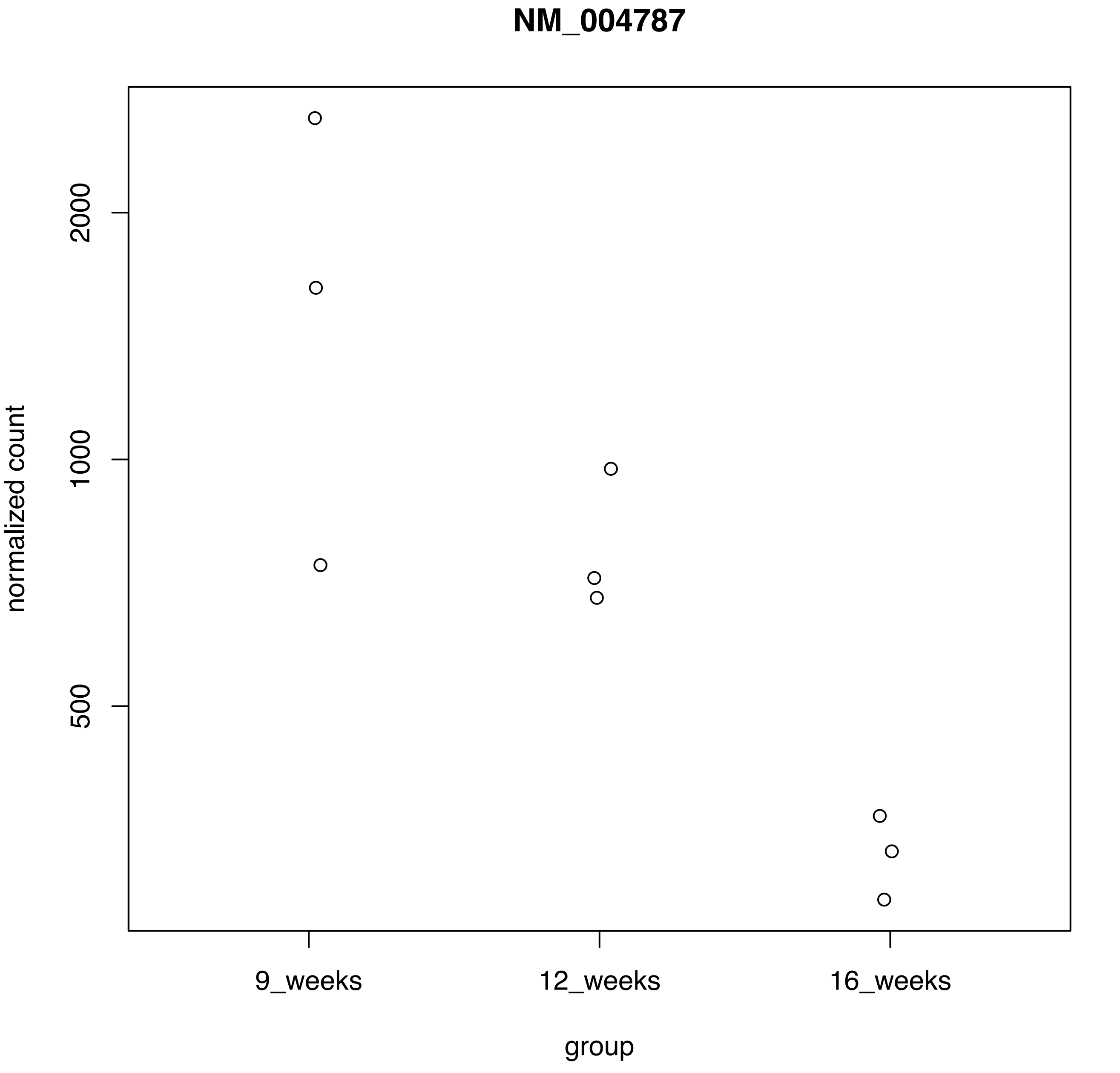

NM_004787
normalized count
2000
1000
500
9_weeks
12_weeks
16_weeks
group

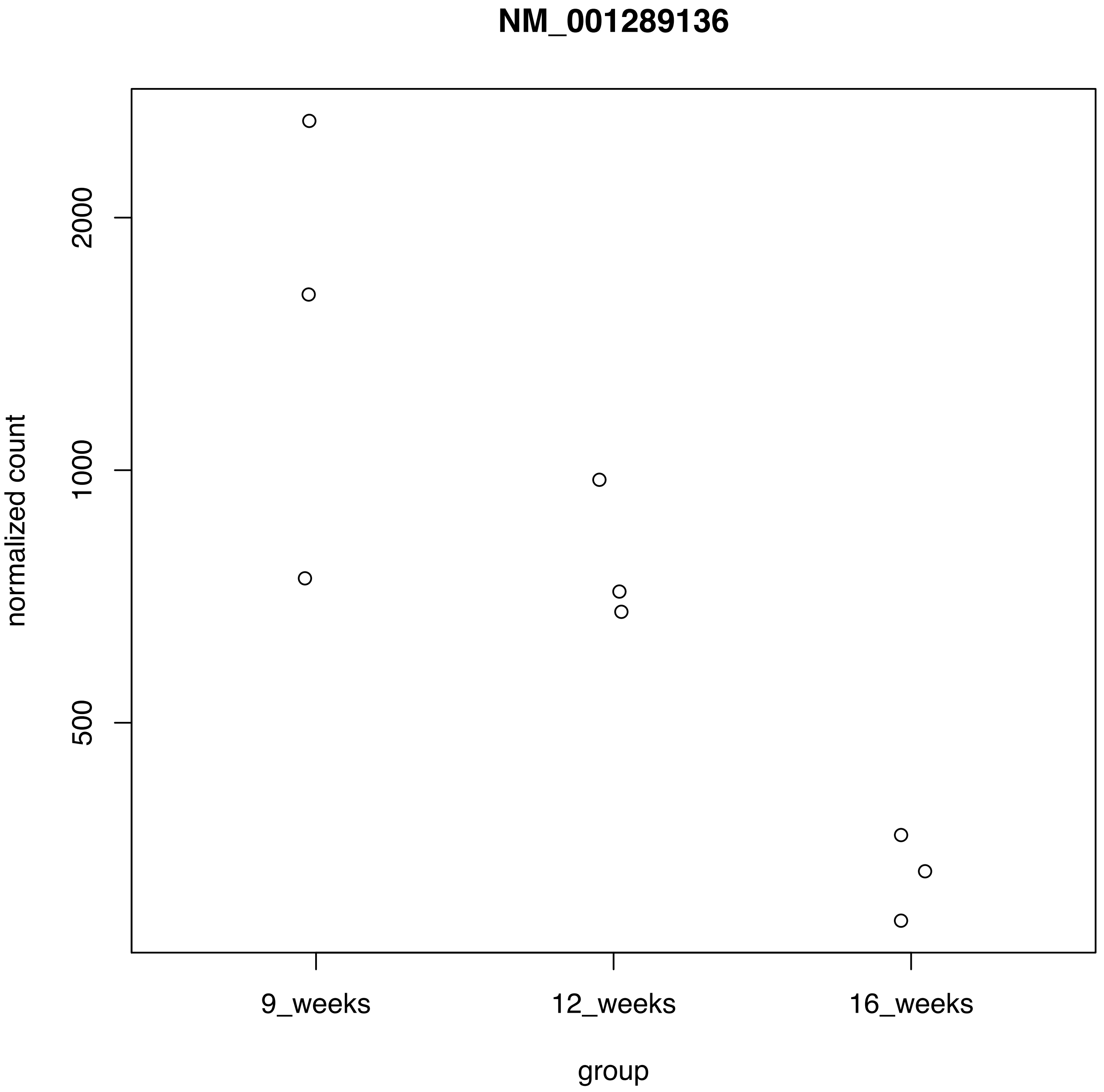
NM_001289136
normalized count
2000
1000
500
9_weeks
12_weeks
16_weeks
group

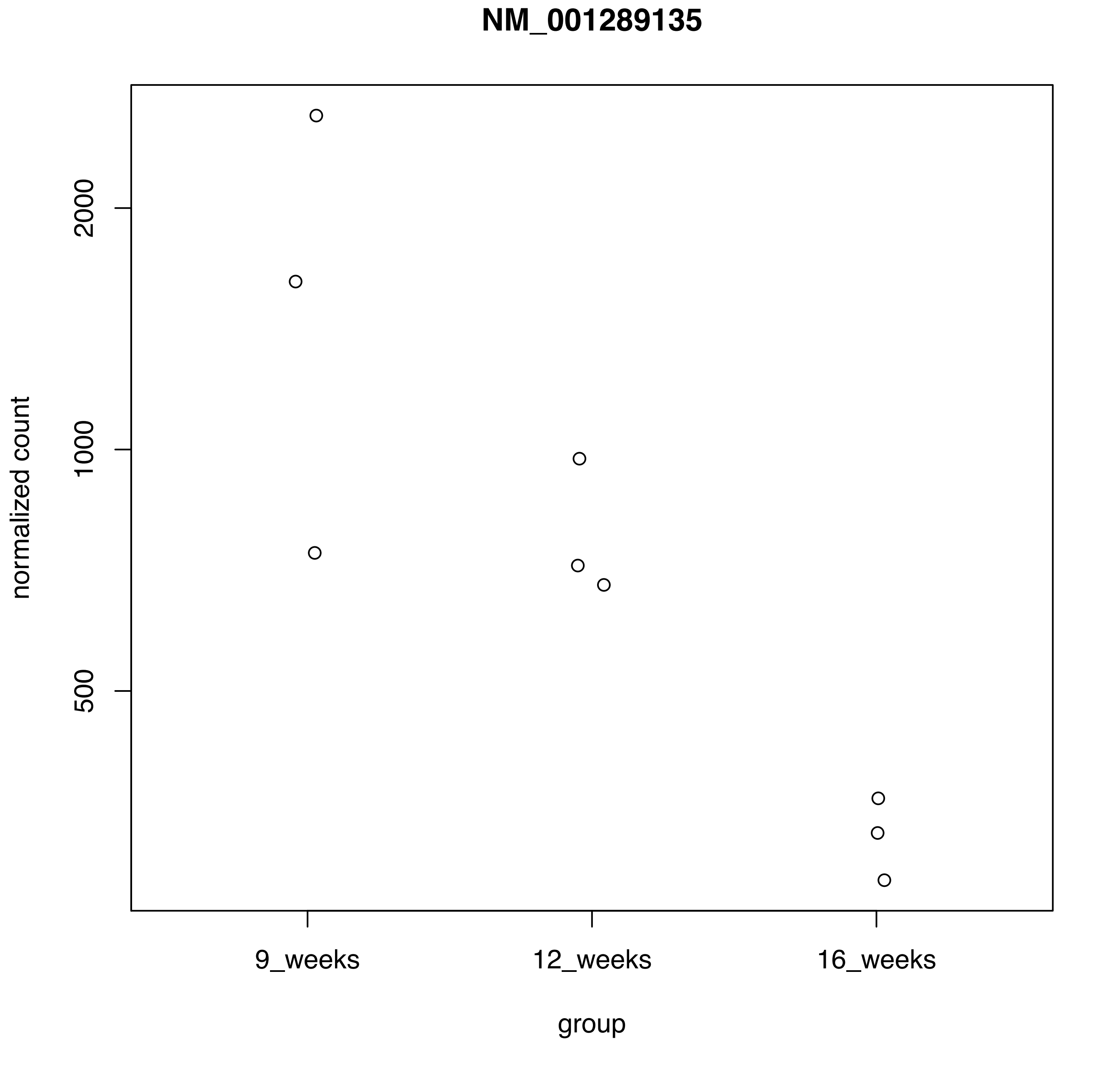
NM_001289135
normalized count
2000
1000
500
9_weeks
12_weeks
16_weeks
group

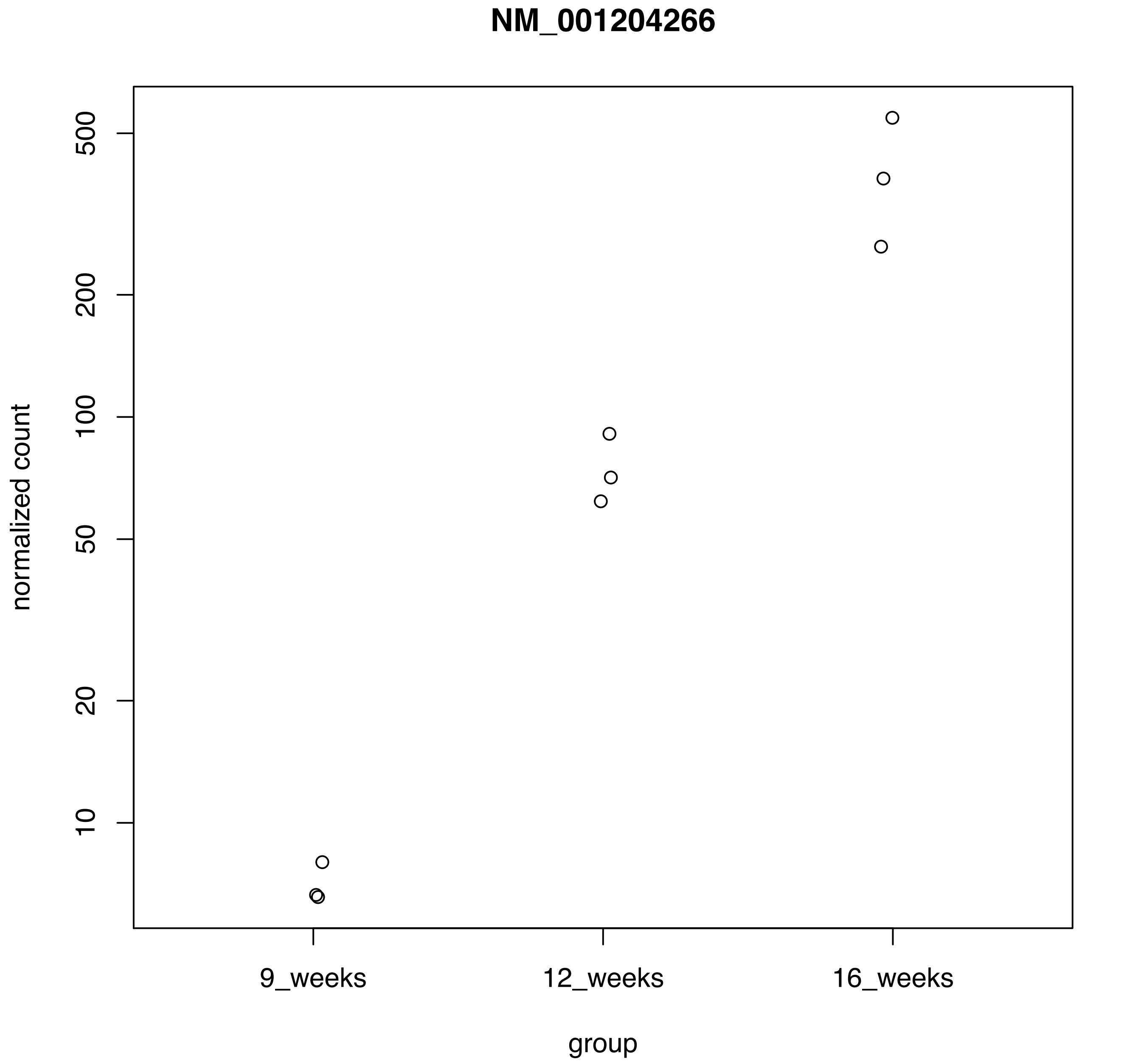
NM_001204266
500
200
100
50
20
10
normalized count
9_weeks
12_weeks
16_weeks
group

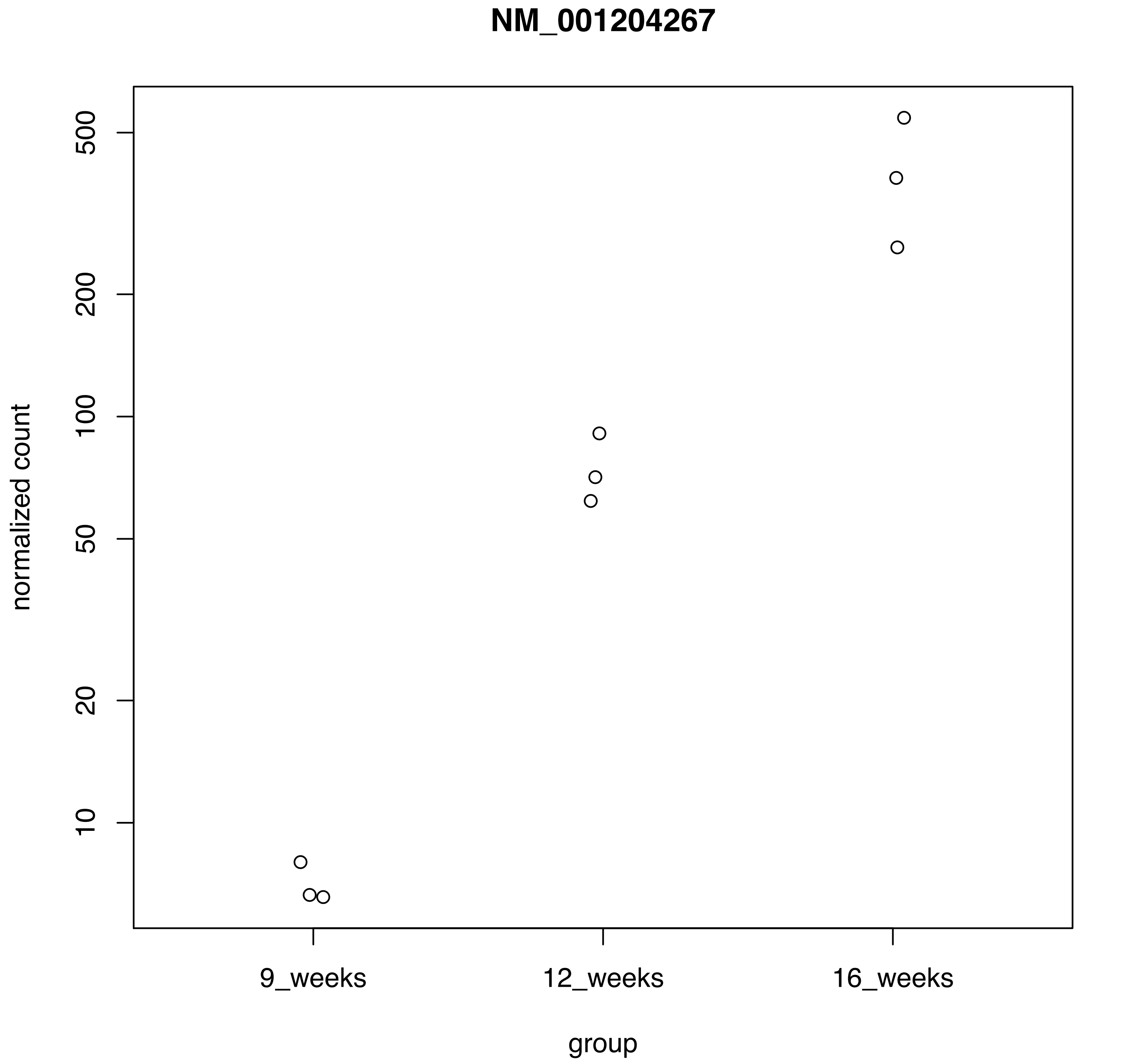
NM_001204267
normalized count
500
200
100
50
20
10
9_weeks
12_weeks
16_weeks
group

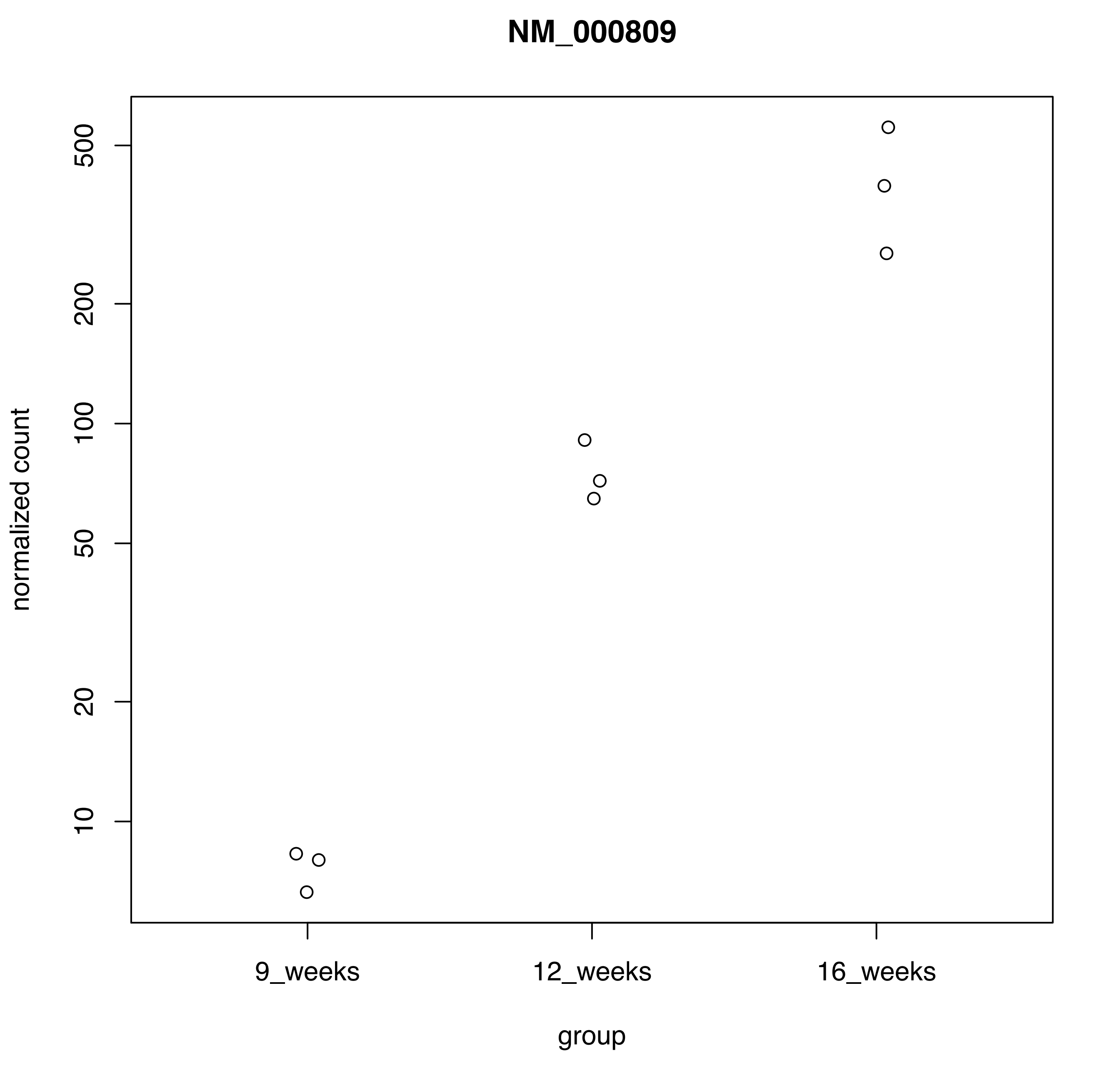

NM_000809
normalized count
500
200
100
50
20
10
9_weeks
12_weeks
16_weeks
group

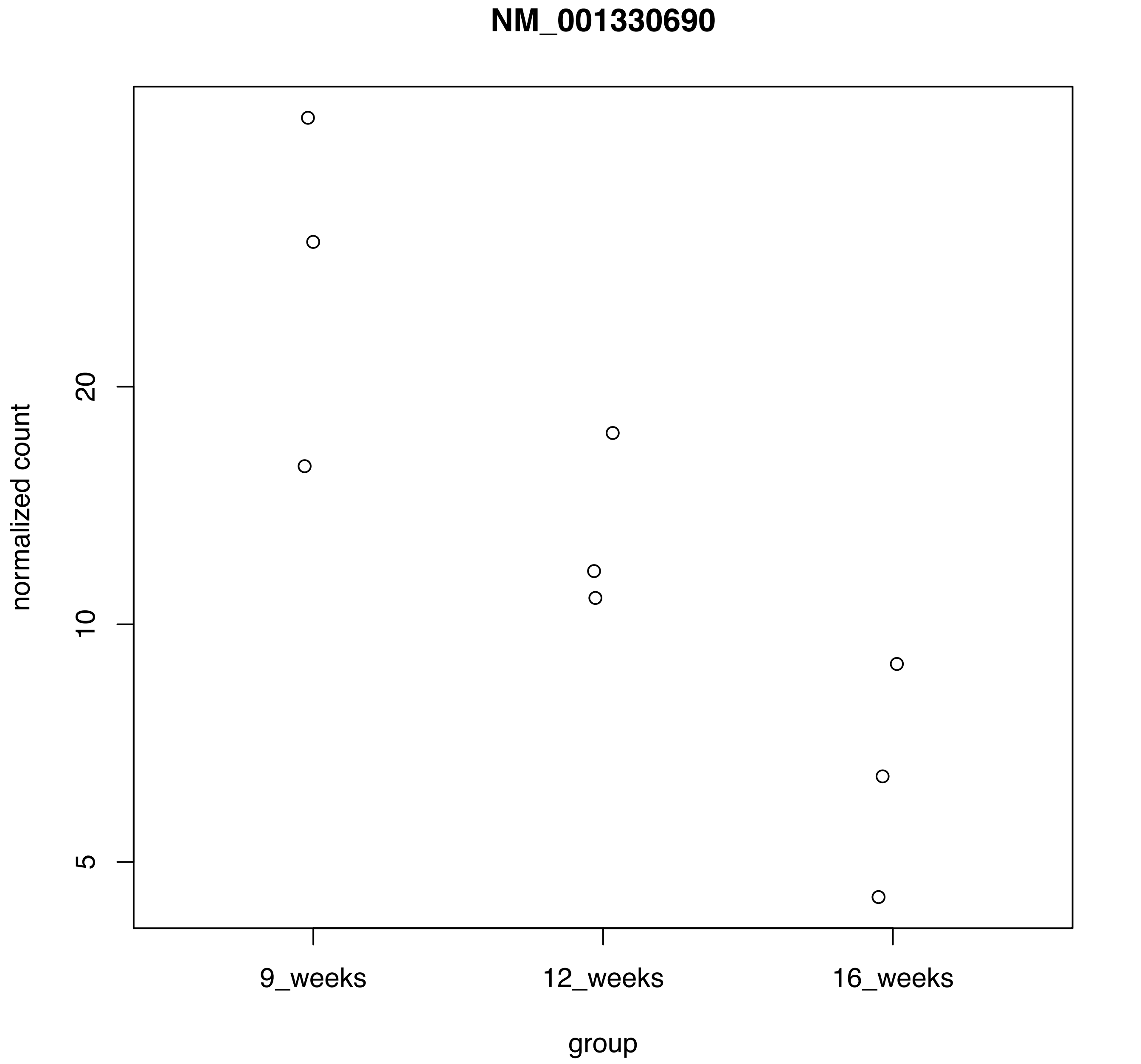
NM_001330690
normalized count
20
10
5
9_weeks
12_weeks
16_weeks
group

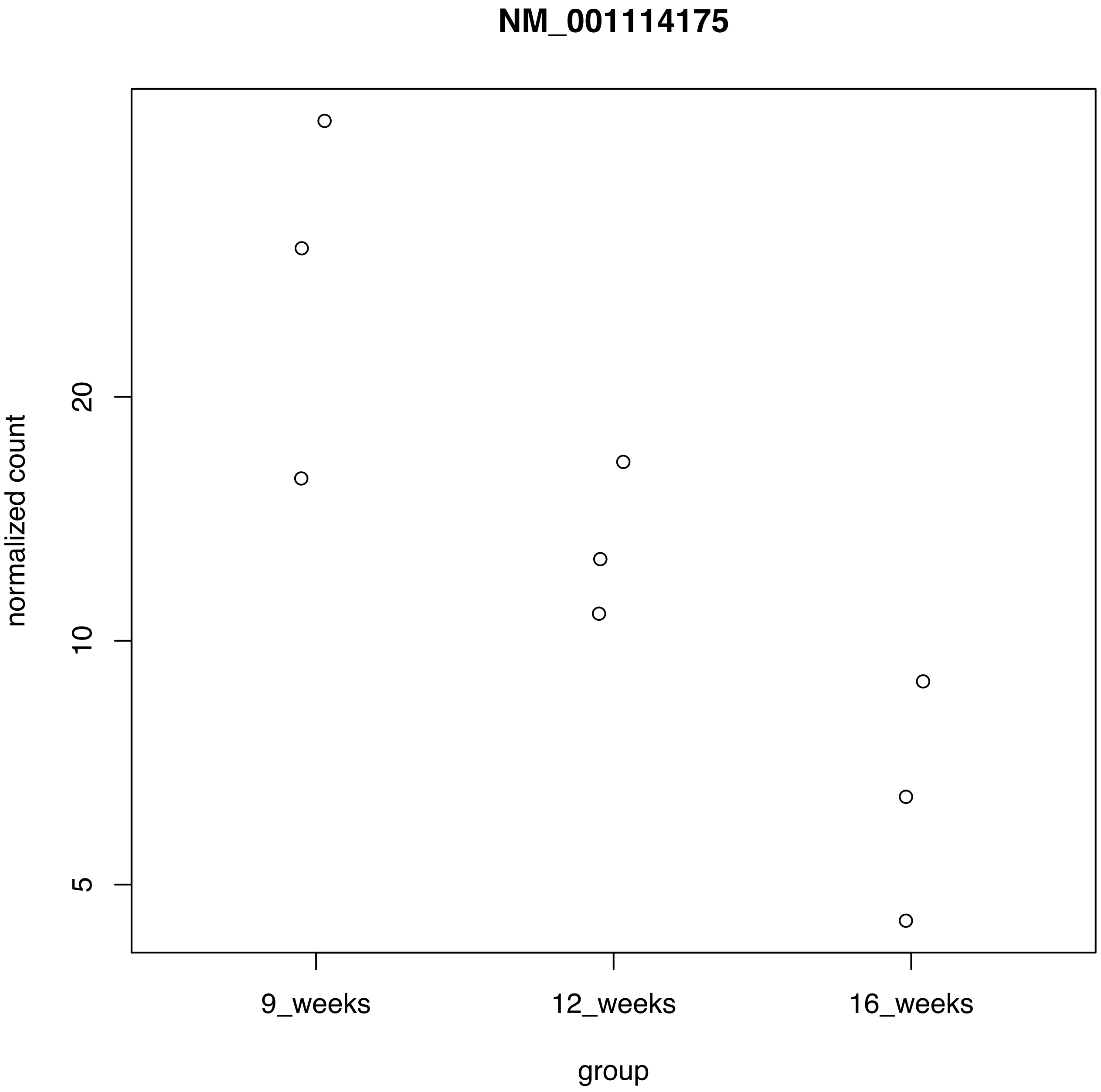

NM_001114175
normalized count
20
10
5
9_weeks
12_weeks
16_weeks
group

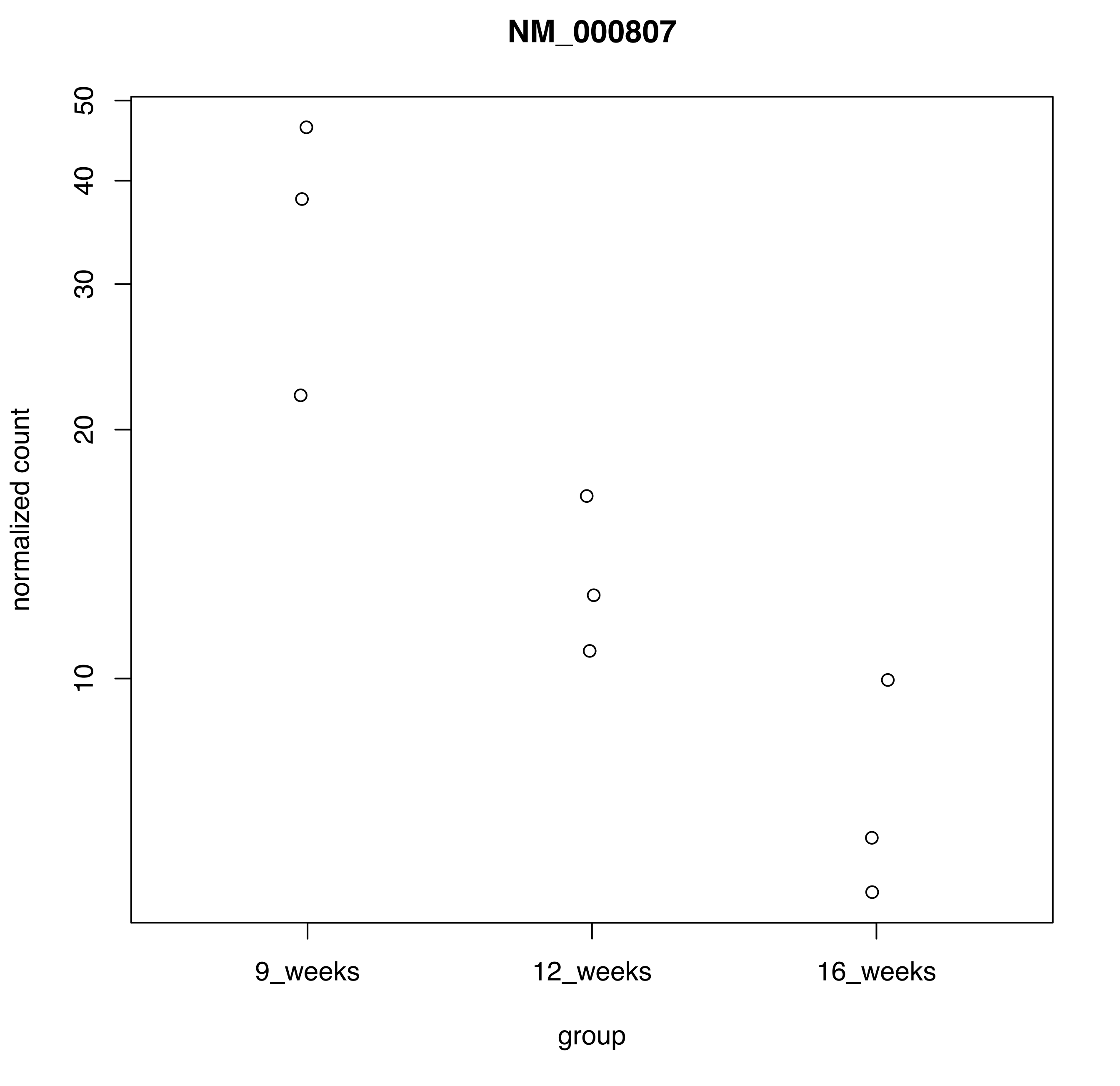

NM_000807
normalized count
50
40
30
20
10
9_weeks
12_weeks
16_weeks
group

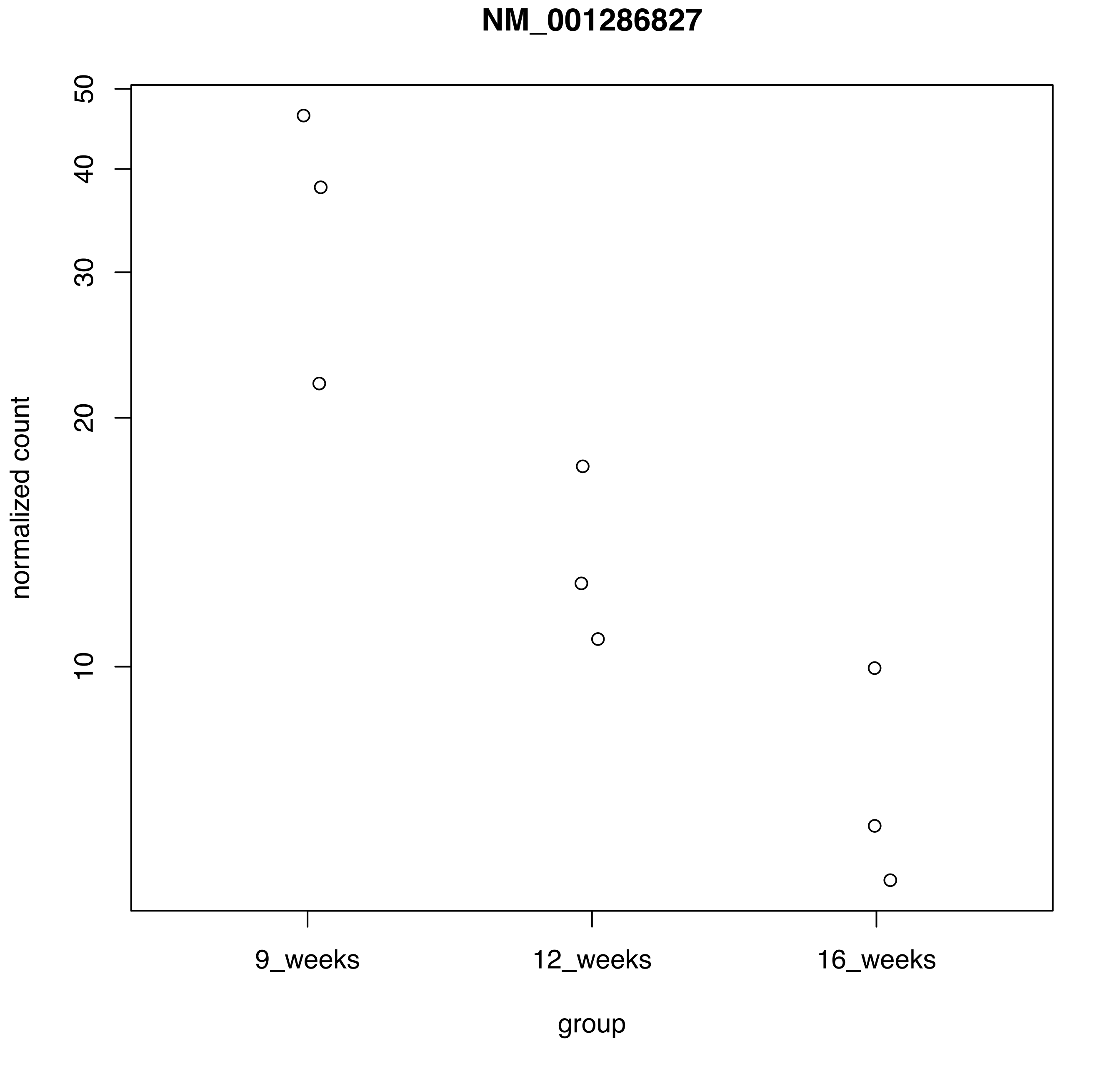

NM_001286827
normalized count
50
40
30
20
10
9_weeks
12_weeks
16_weeks
group

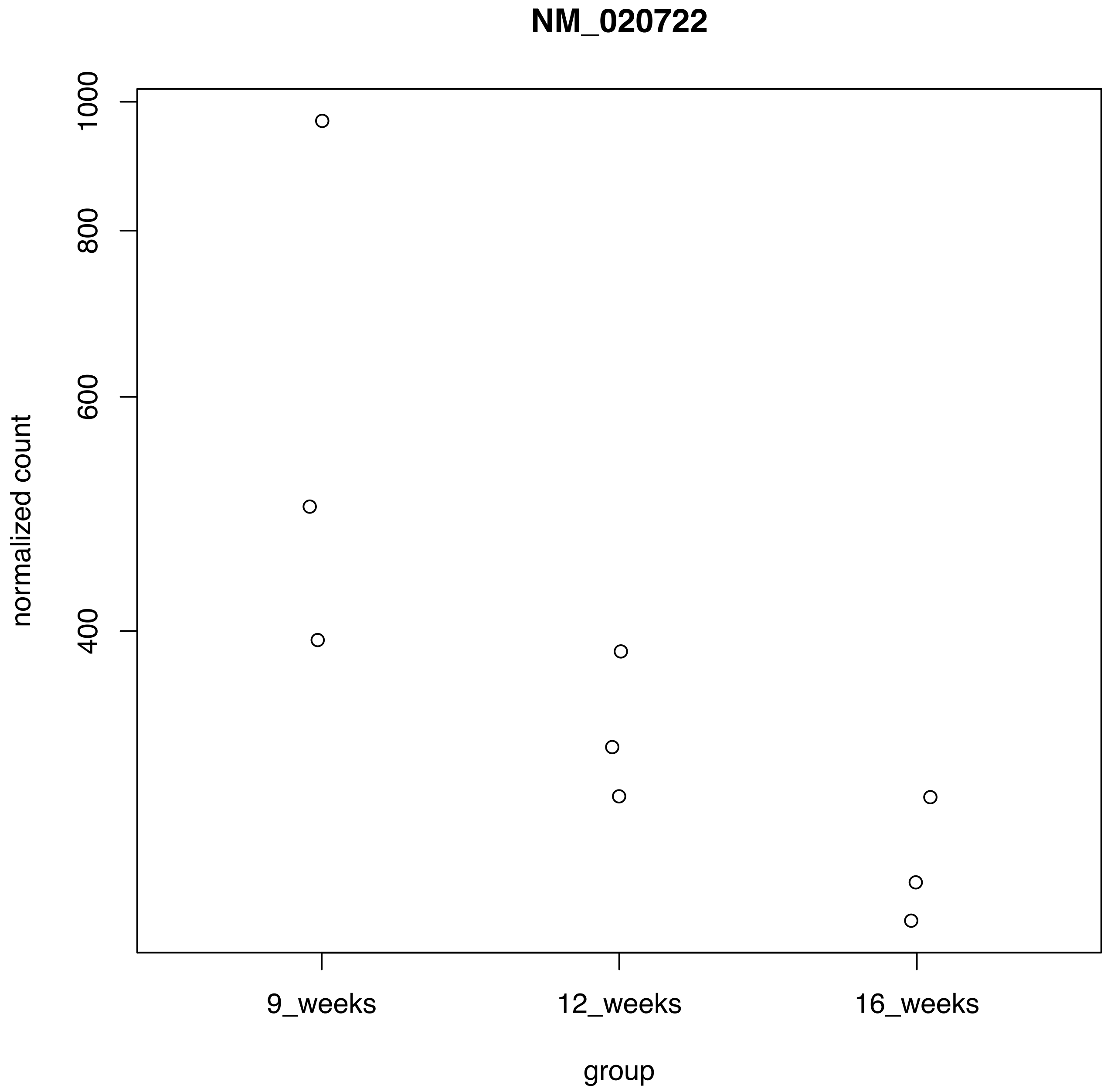
NM_020722
normalized count
1000
800
600
400
9_weeks
12_weeks
16_weeks
group

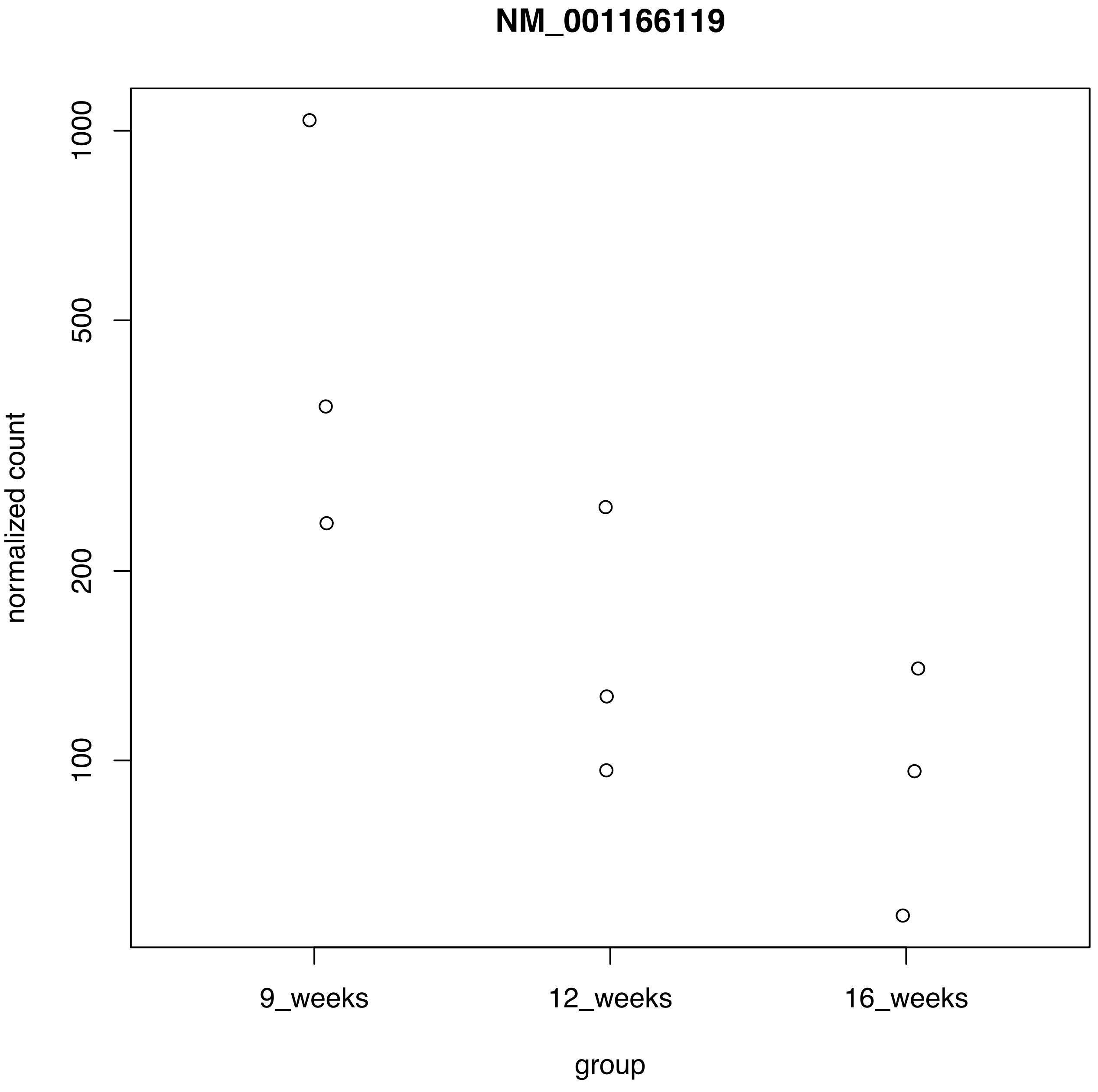
NM_001166119
normalized count
1000
500
200
100
9_weeks
12_weeks
16_weeks
group

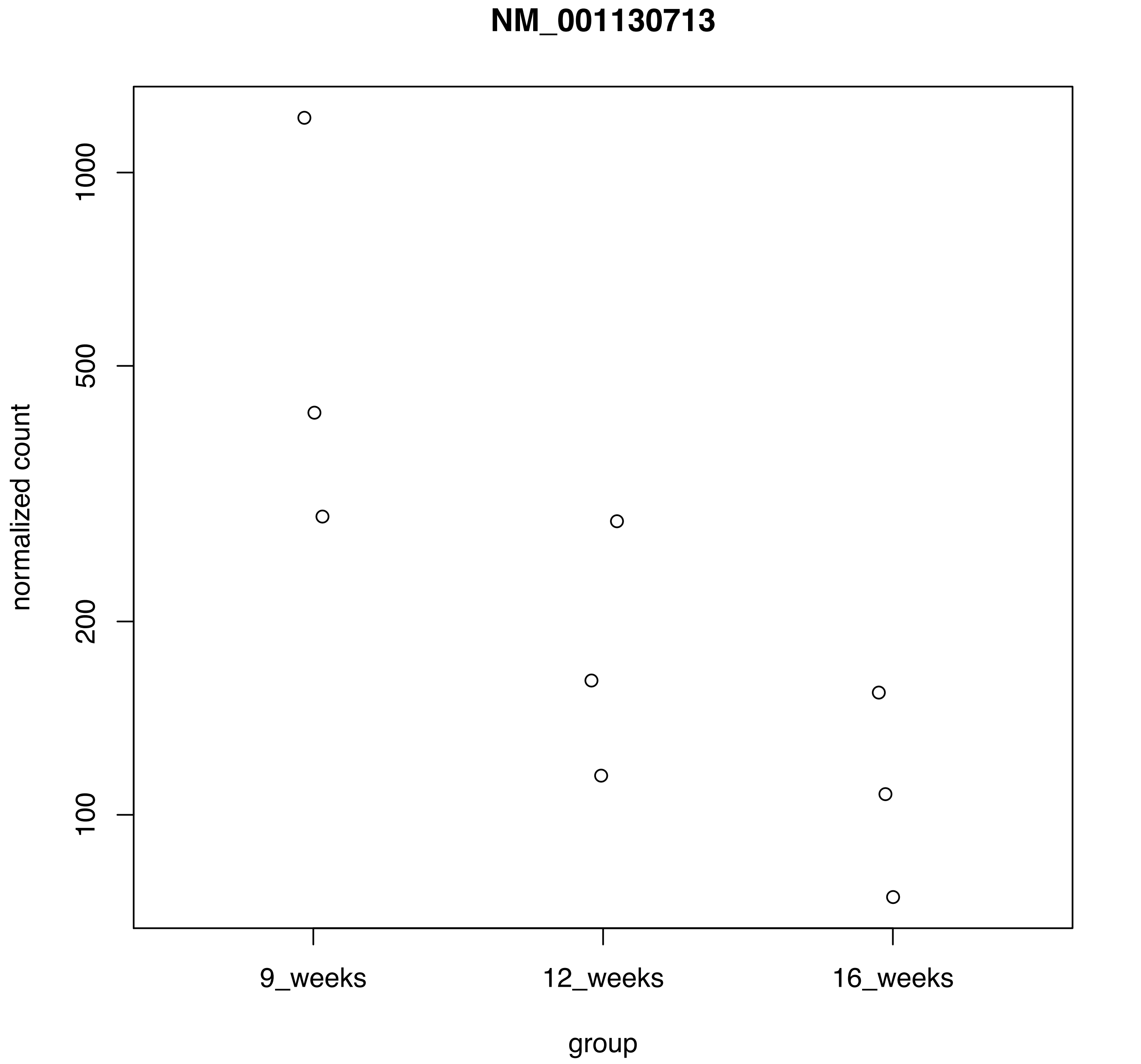

NM_001130713
normalized count
1000
500
200
100
9_weeks
12_weeks
16_weeks
group

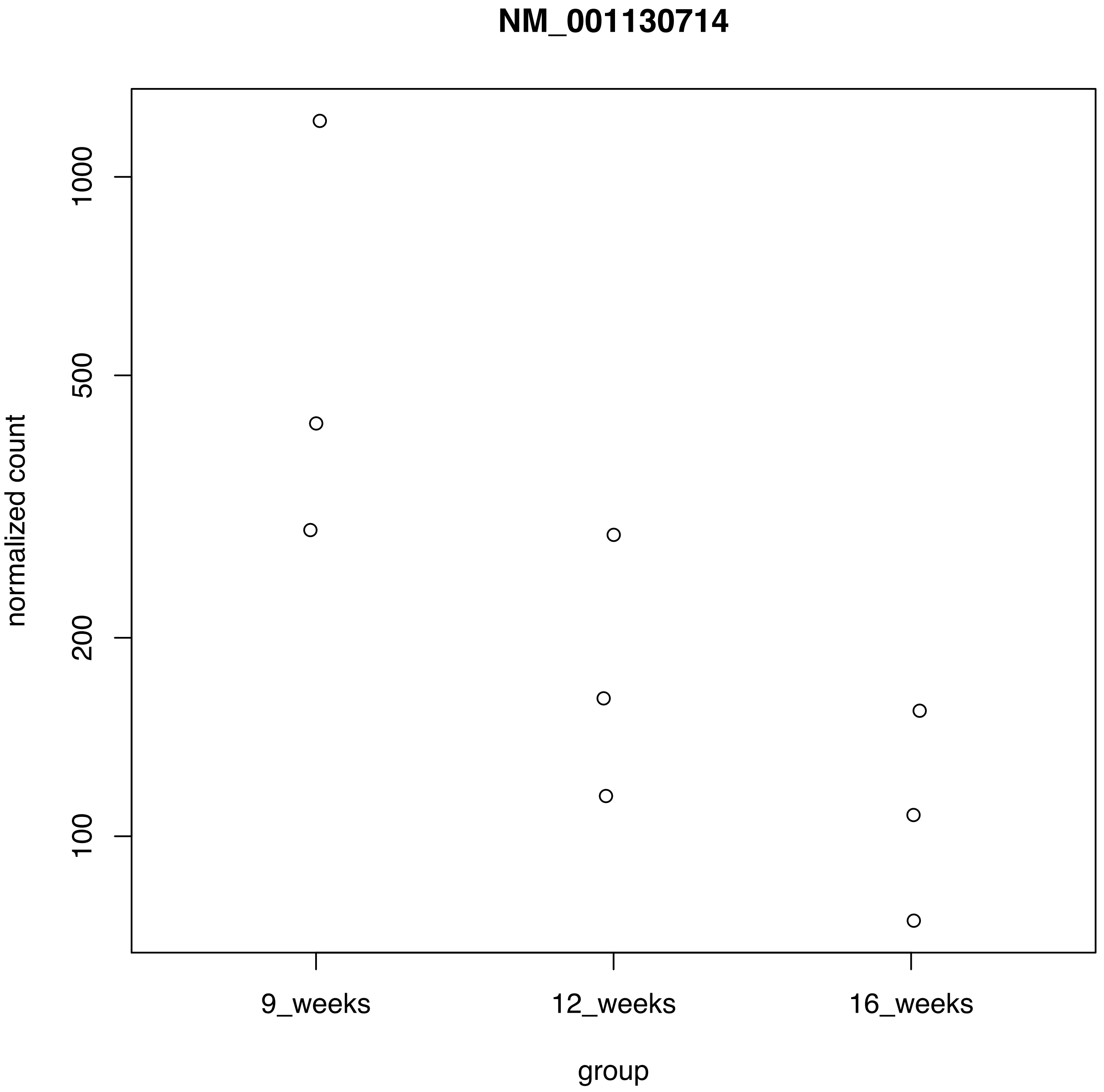
NM_001130714
normalized count
1000
500
200
100
9_weeks
12_weeks
16_weeks
group

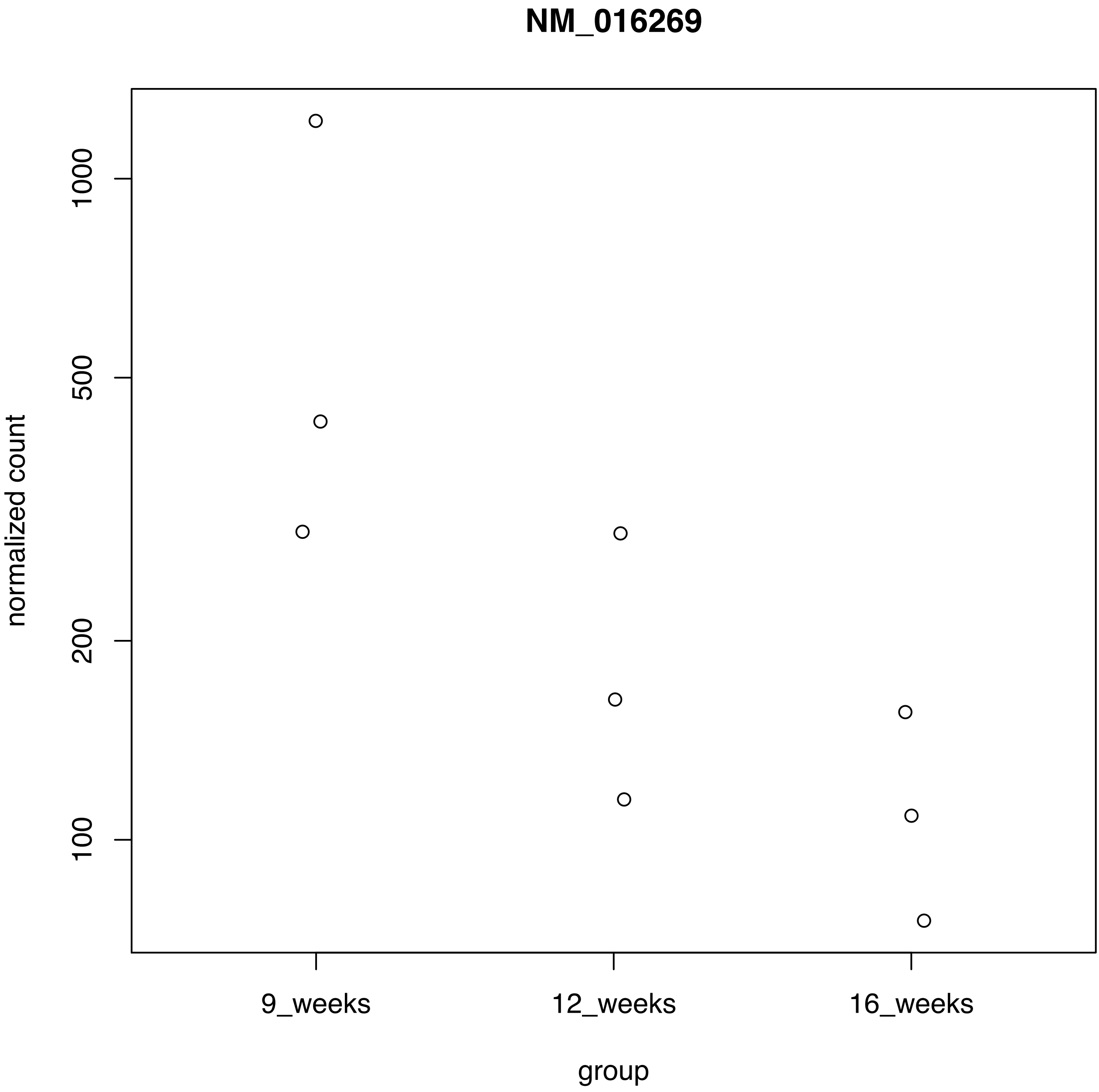

NM_016269
1000
500
200
100
normalized count
9_weeks
12_weeks
16_weeks
group

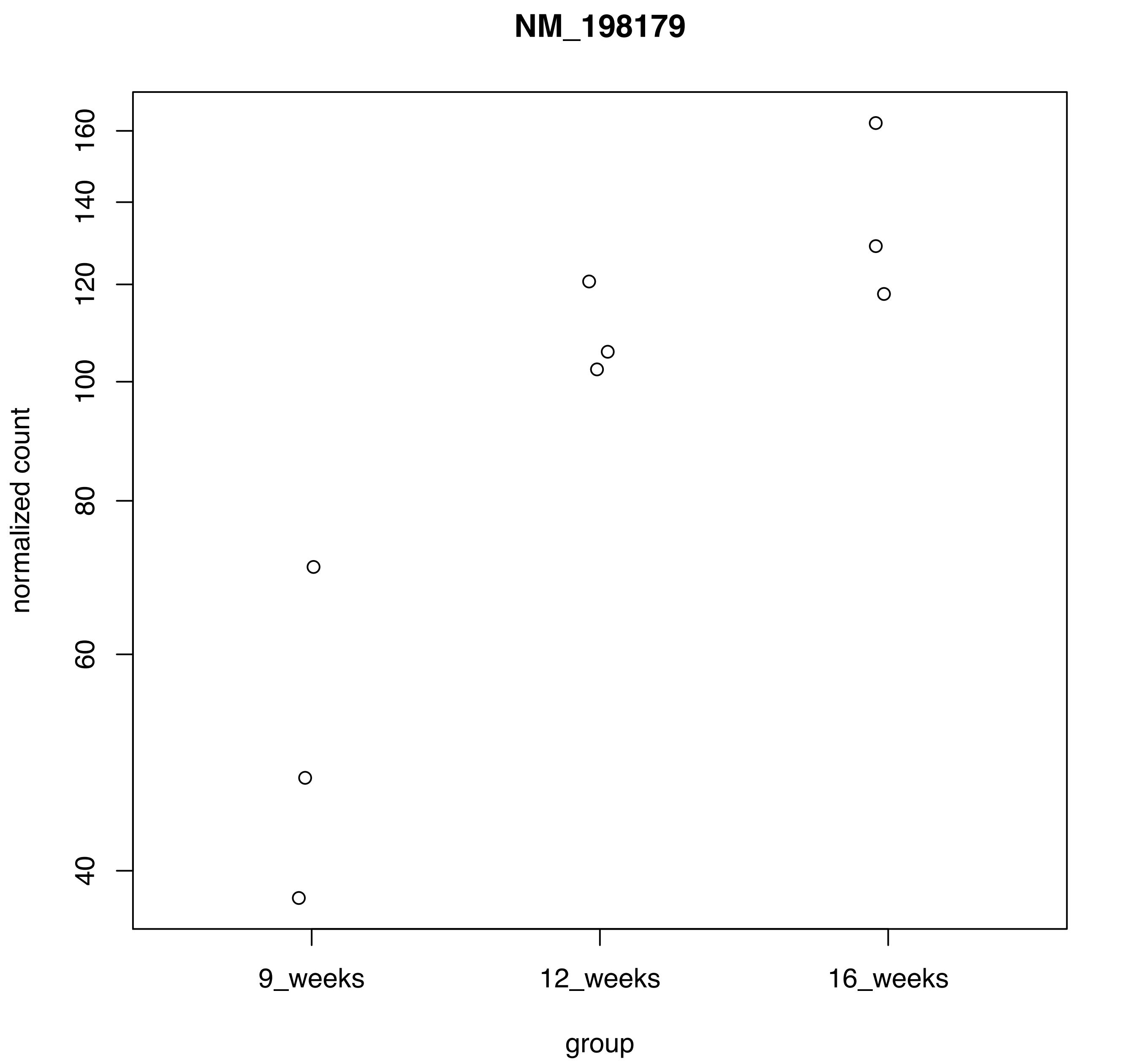
NM_198179
normalized count
160
140
120
100
80
60
40
9_weeks
12_weeks
16_weeks
group

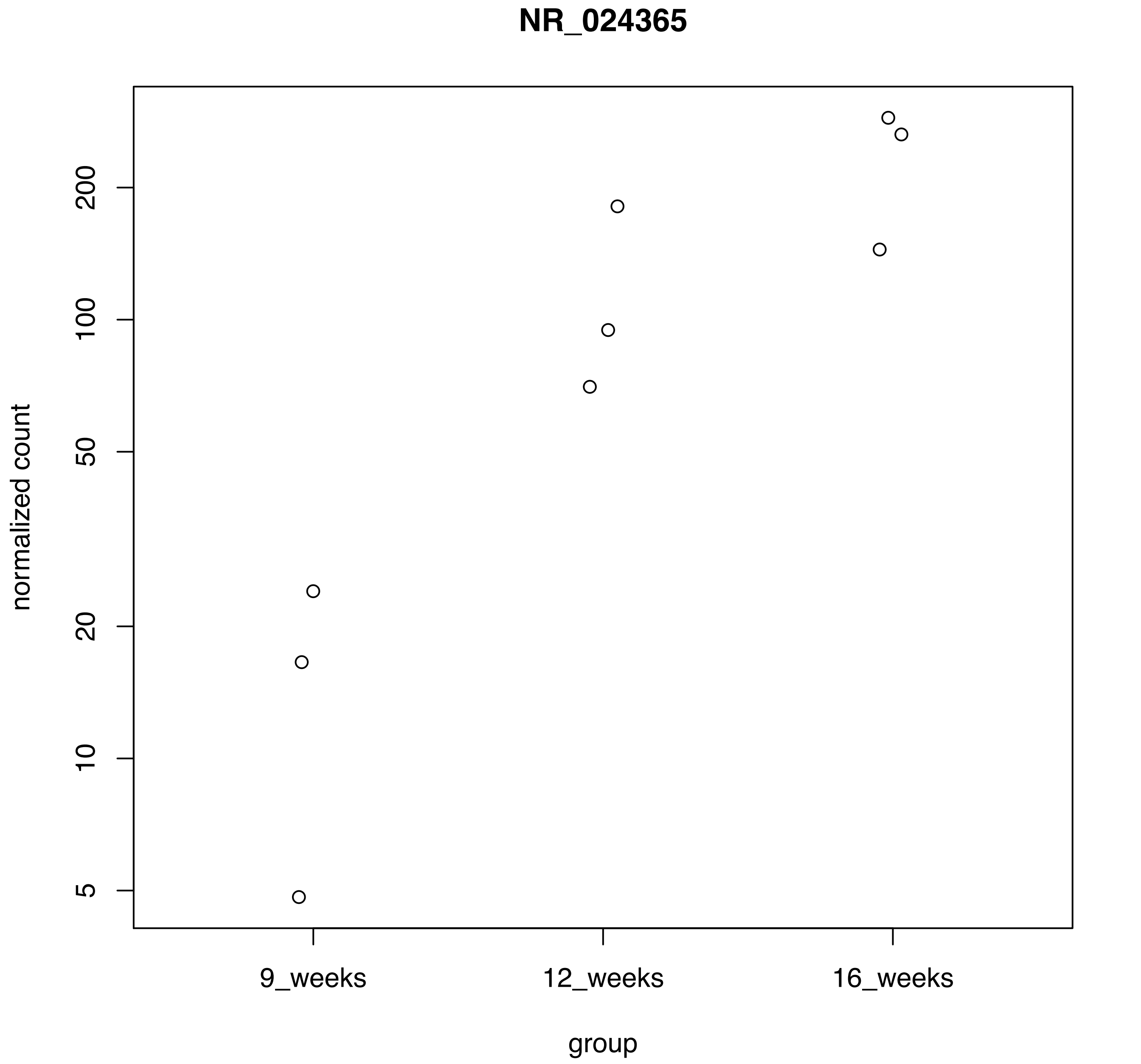

NR_024365
normalized count
200
100
50
20
10
5
9_weeks
12_weeks
16_weeks
group

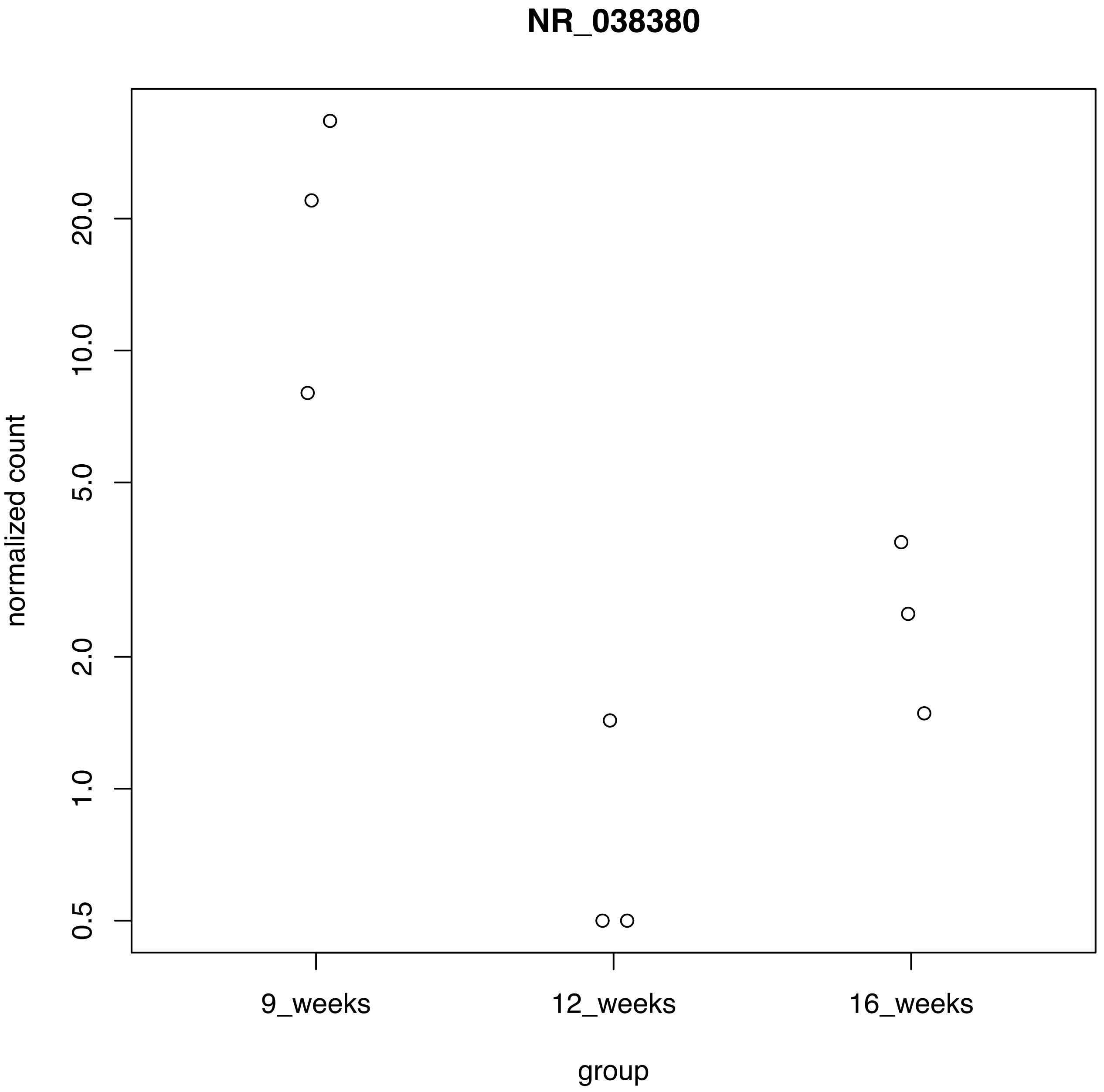
NR_038380
normalized count
0.5
1.0
2.0
5.0
10.0
20.0
9_weeks
12_weeks
16_weeks
group

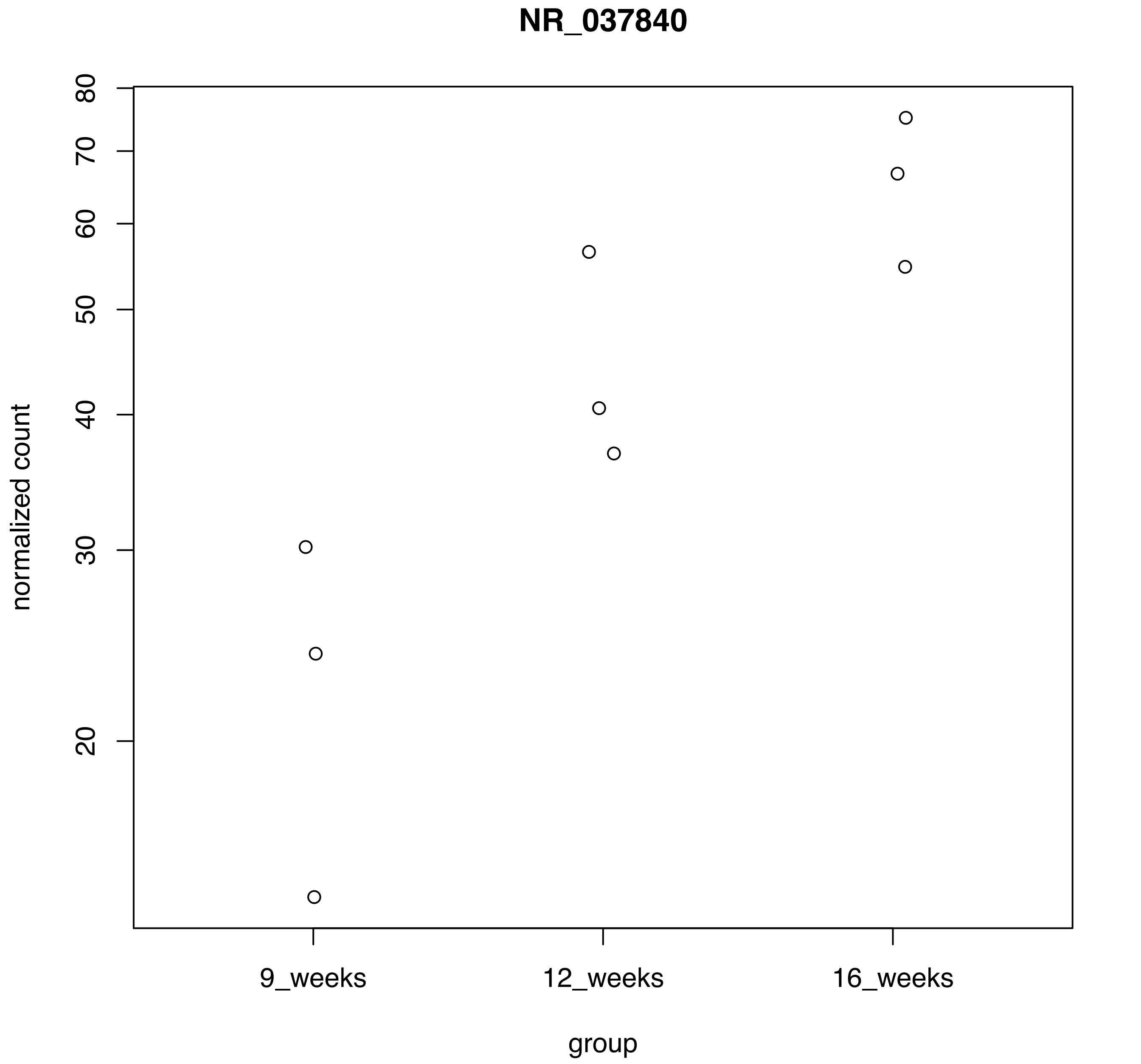
NR_037840
normalized count
80
70
60
50
40
30
20
9_weeks
12_weeks
16_weeks
group

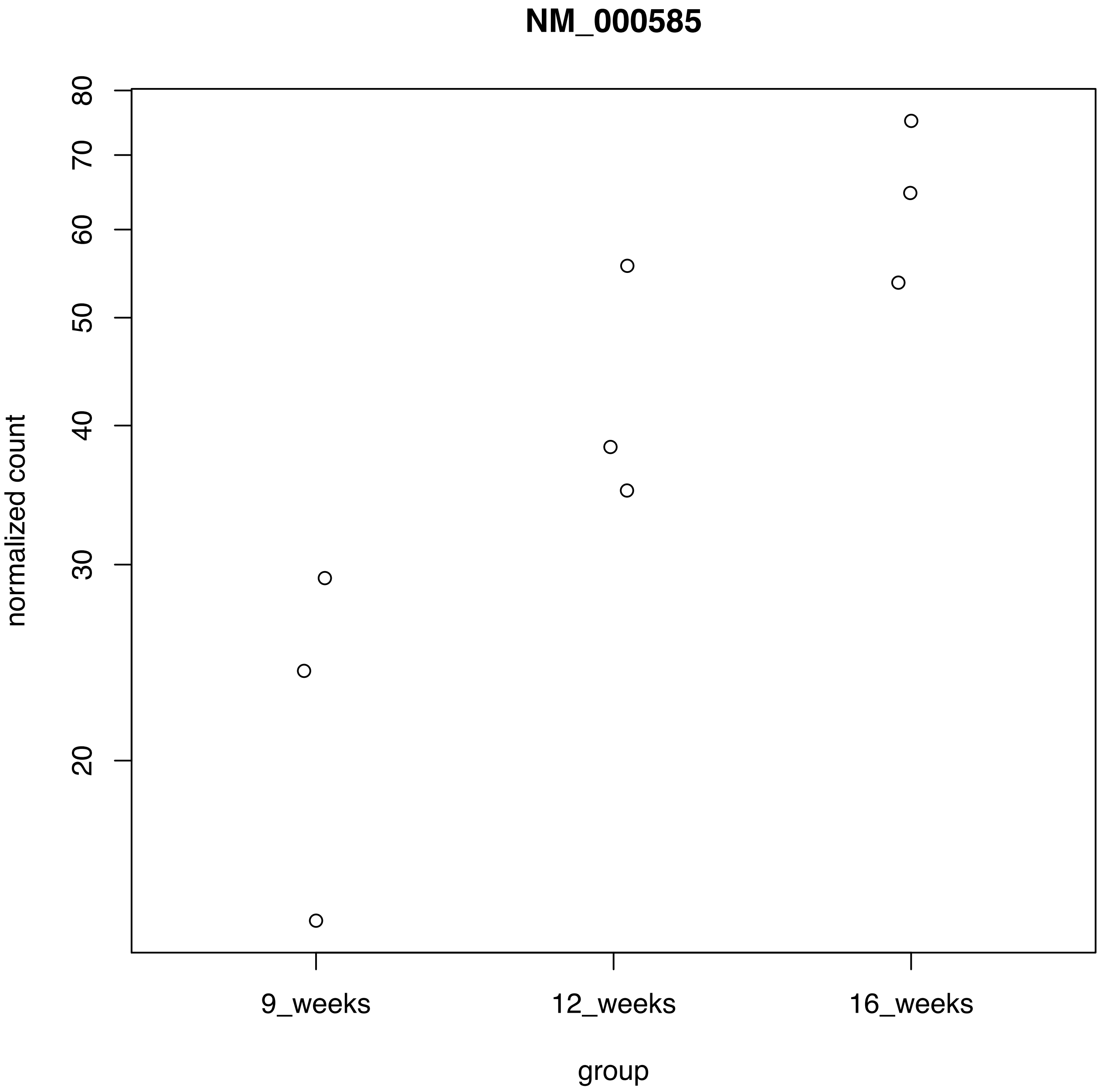

NM_000585
normalized count
80
70
60
50
40
30
20
9_weeks
12_weeks
16_weeks
group

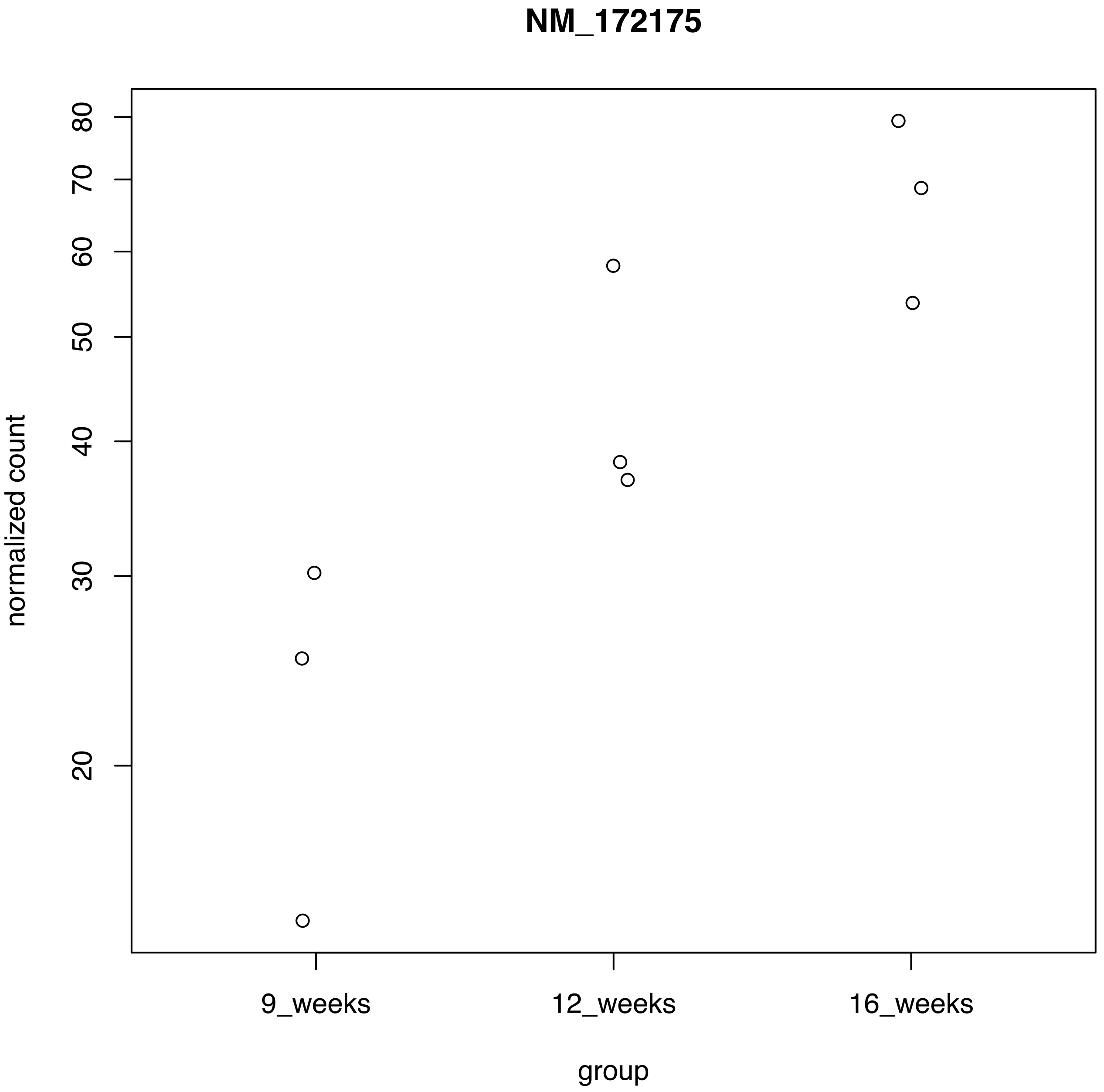

NM_172175
normalized count
80
70
60
50
40
30
20
9_weeks
12_weeks
16_weeks
group

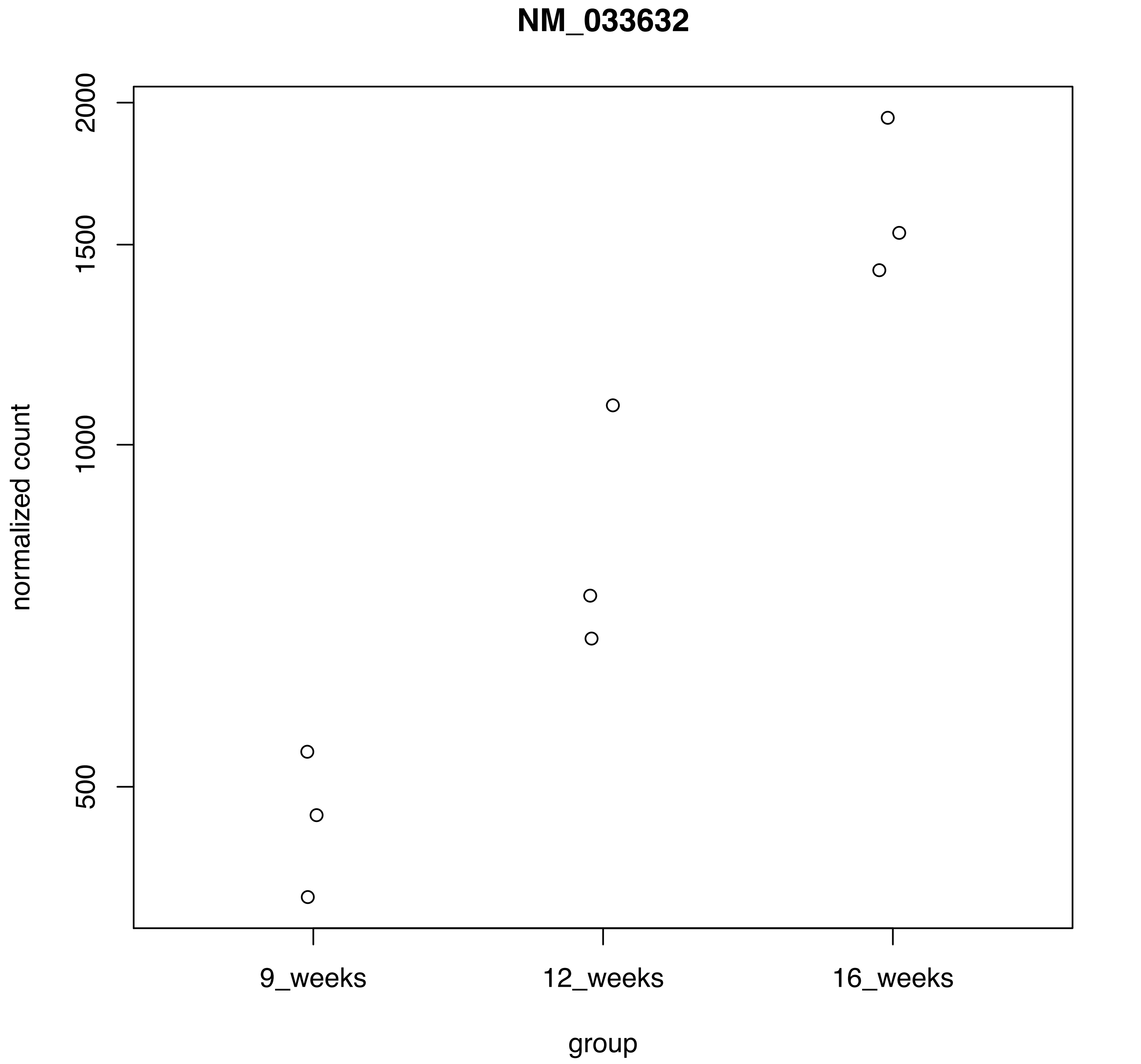
NM_033632
normalized count
2000
1500
1000
500
9_weeks
12_weeks
16_weeks
group

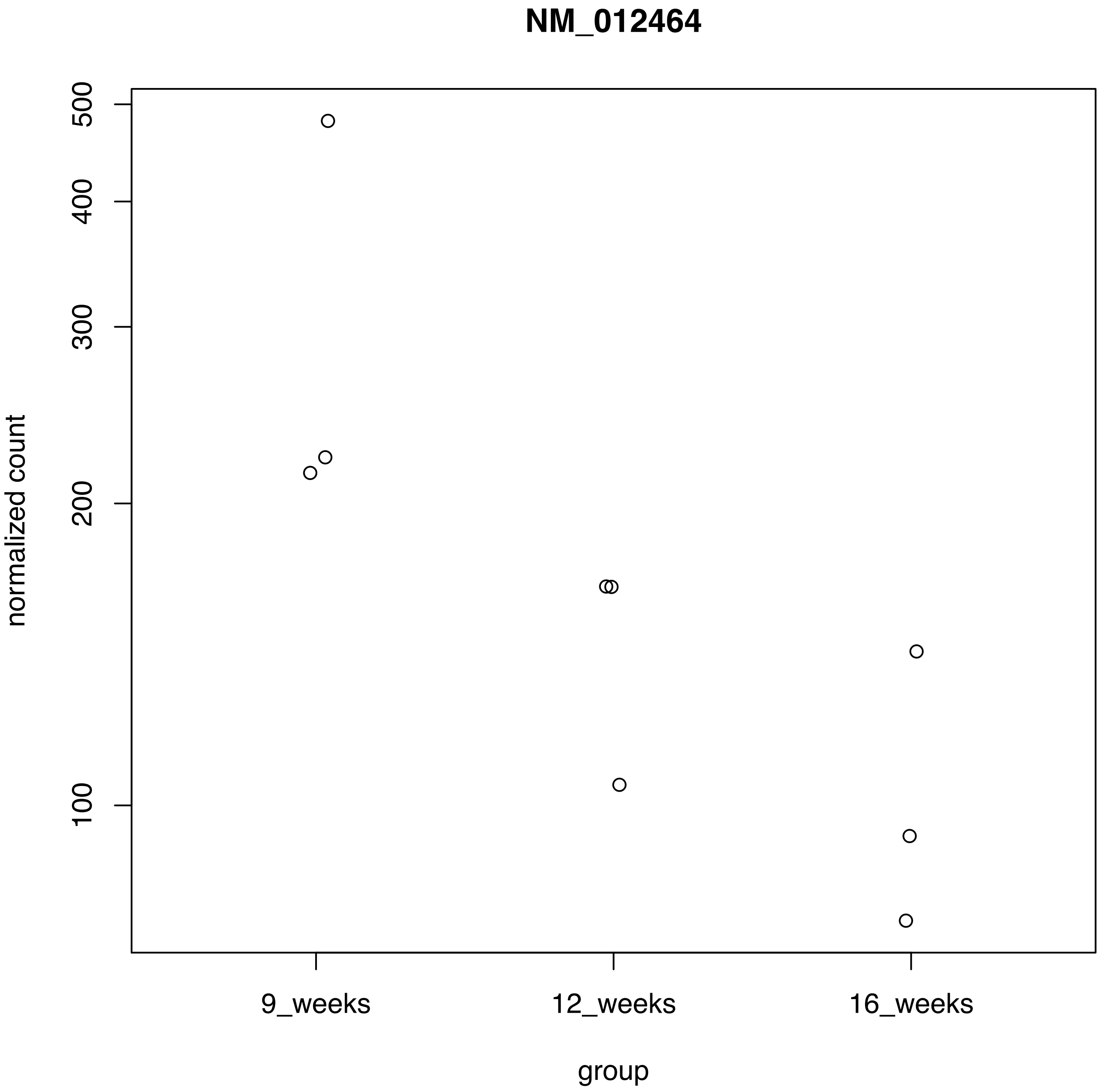
NM_012464
normalized count
500
400
300
200
100
9_weeks
12_weeks
16_weeks
group

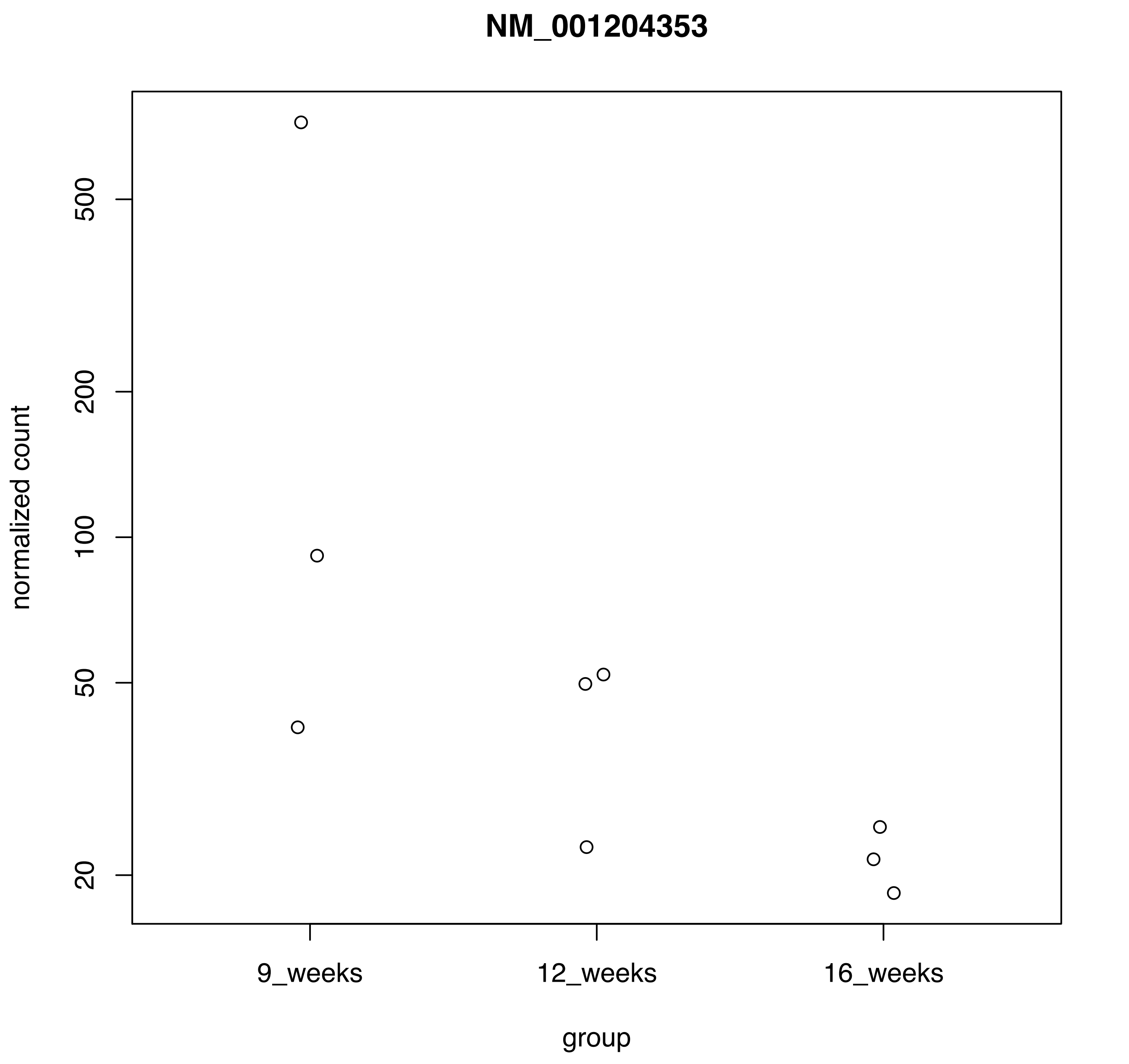
NM_001204353
normalized count
500
200
100
50
20
9_weeks
12_weeks
16_weeks
group

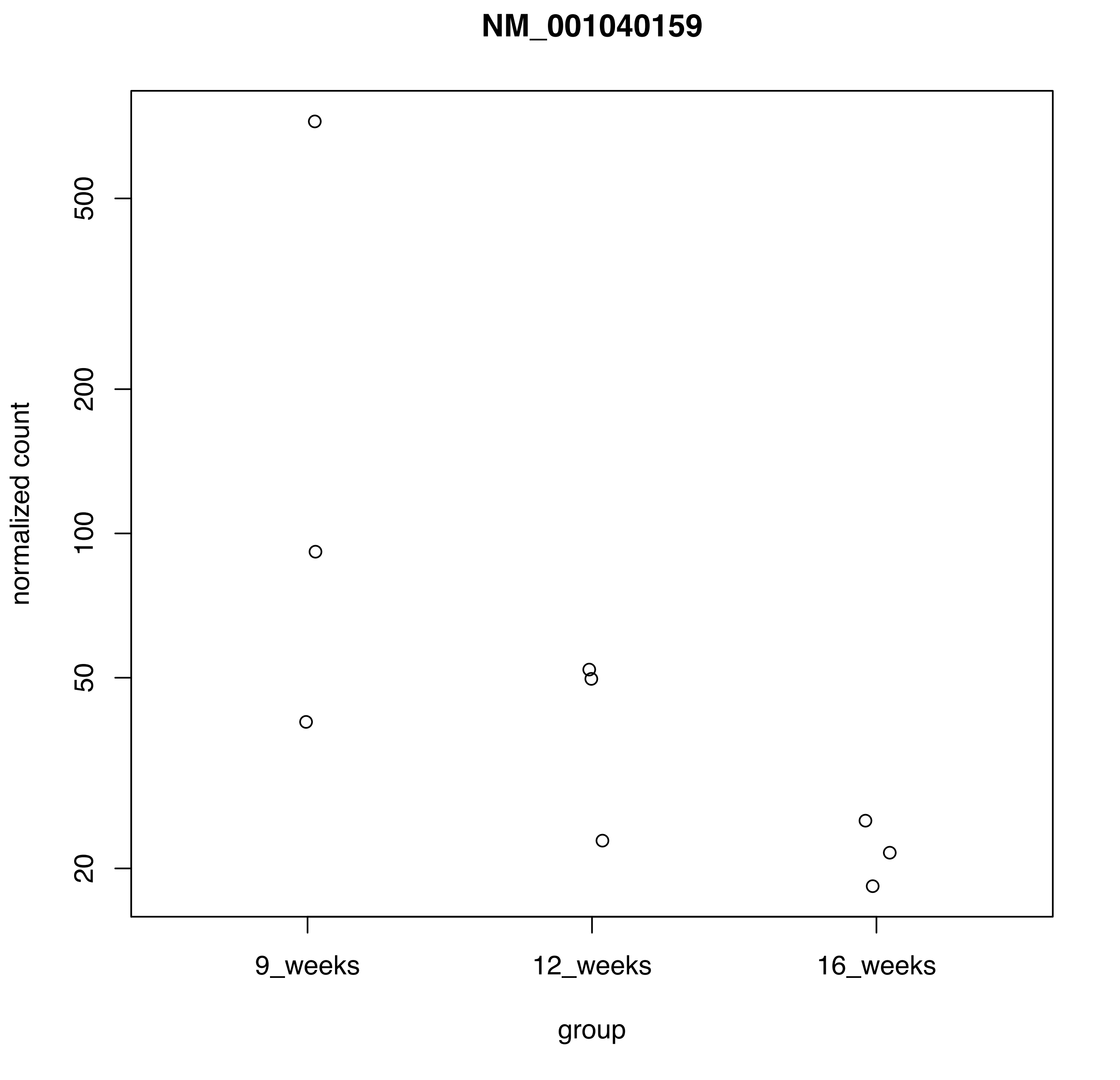
NM_001040159
normalized count
500
200
100
50
20
9_weeks
12_weeks
16_weeks
group

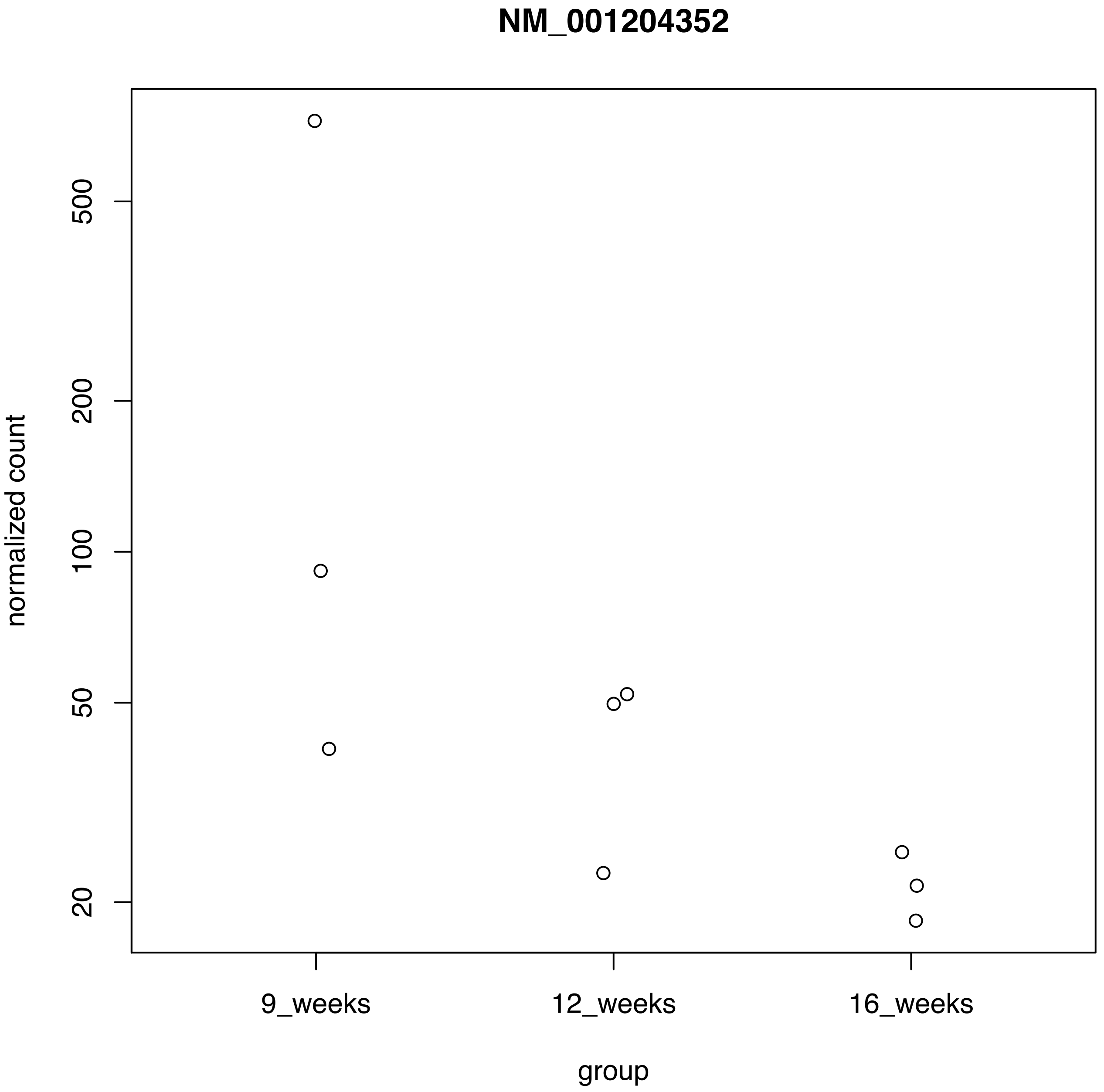
NM_001204352
normalized count
500
200
100
50
20
9_weeks
12_weeks
16_weeks
group

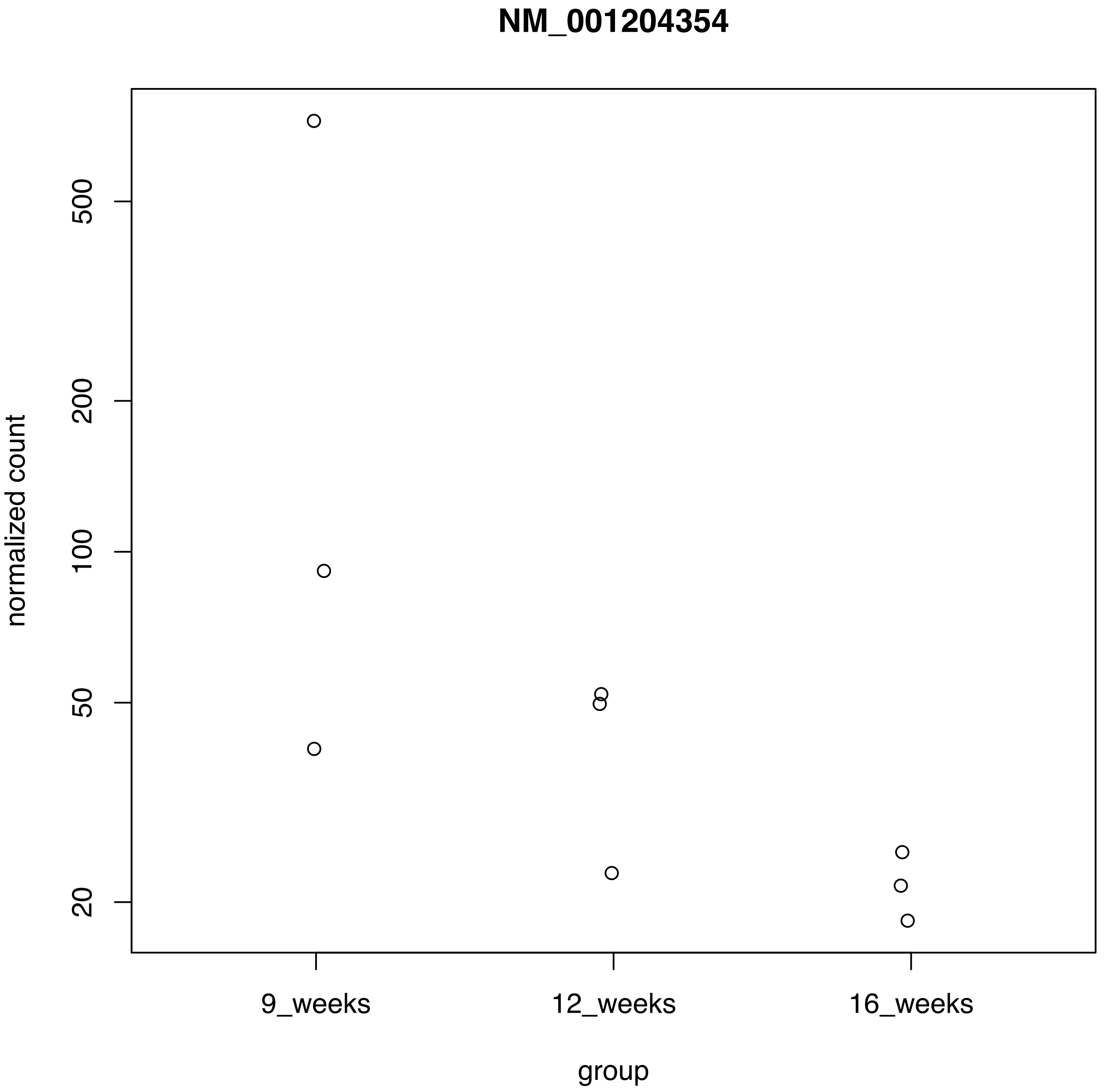
NM_001204354
normalized count
500
200
100
50
20
9_weeks
12_weeks
16_weeks
group

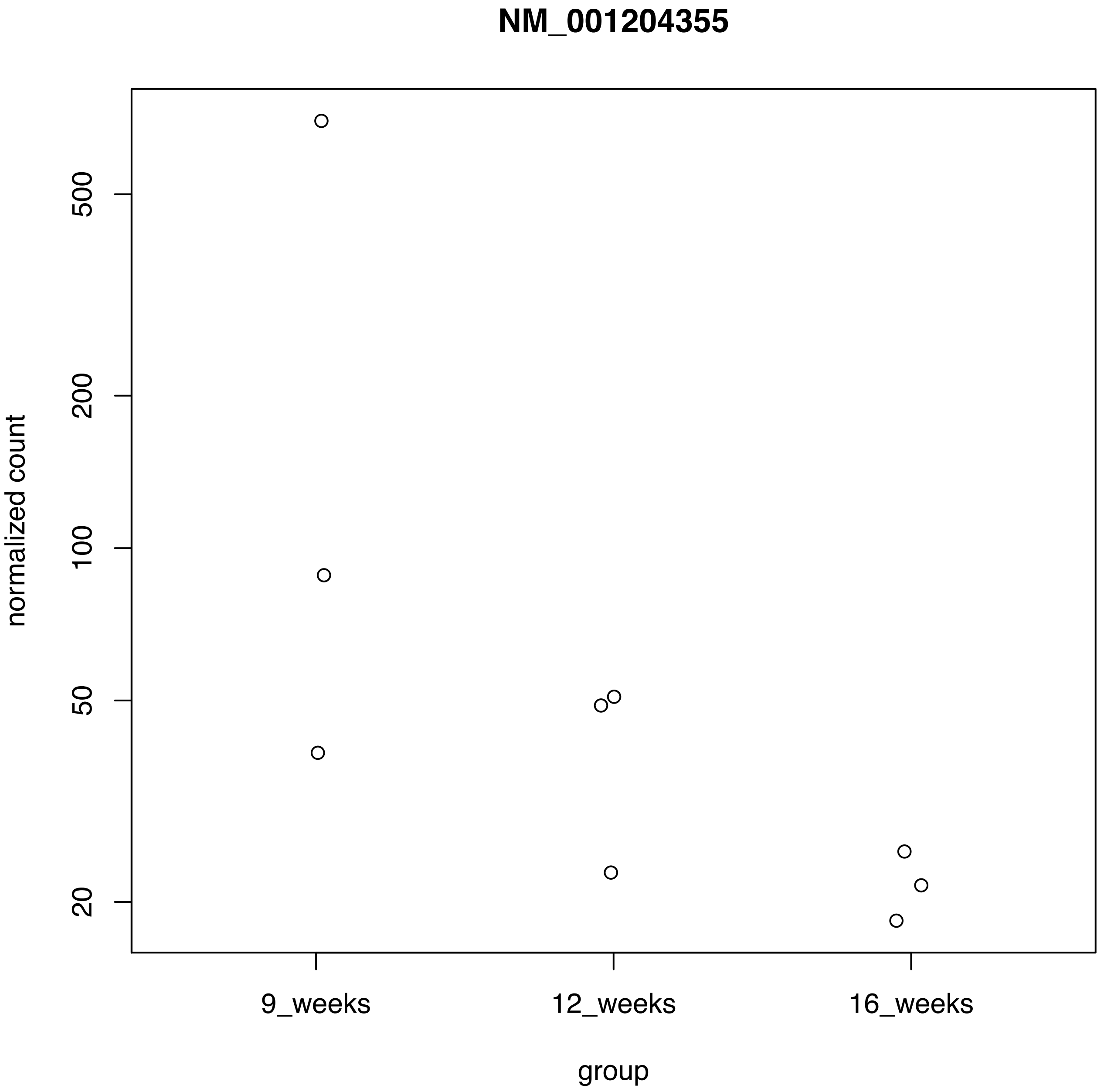

NM_001204355
normalized count
500
200
100
50
20
9_weeks
12_weeks
16_weeks
group

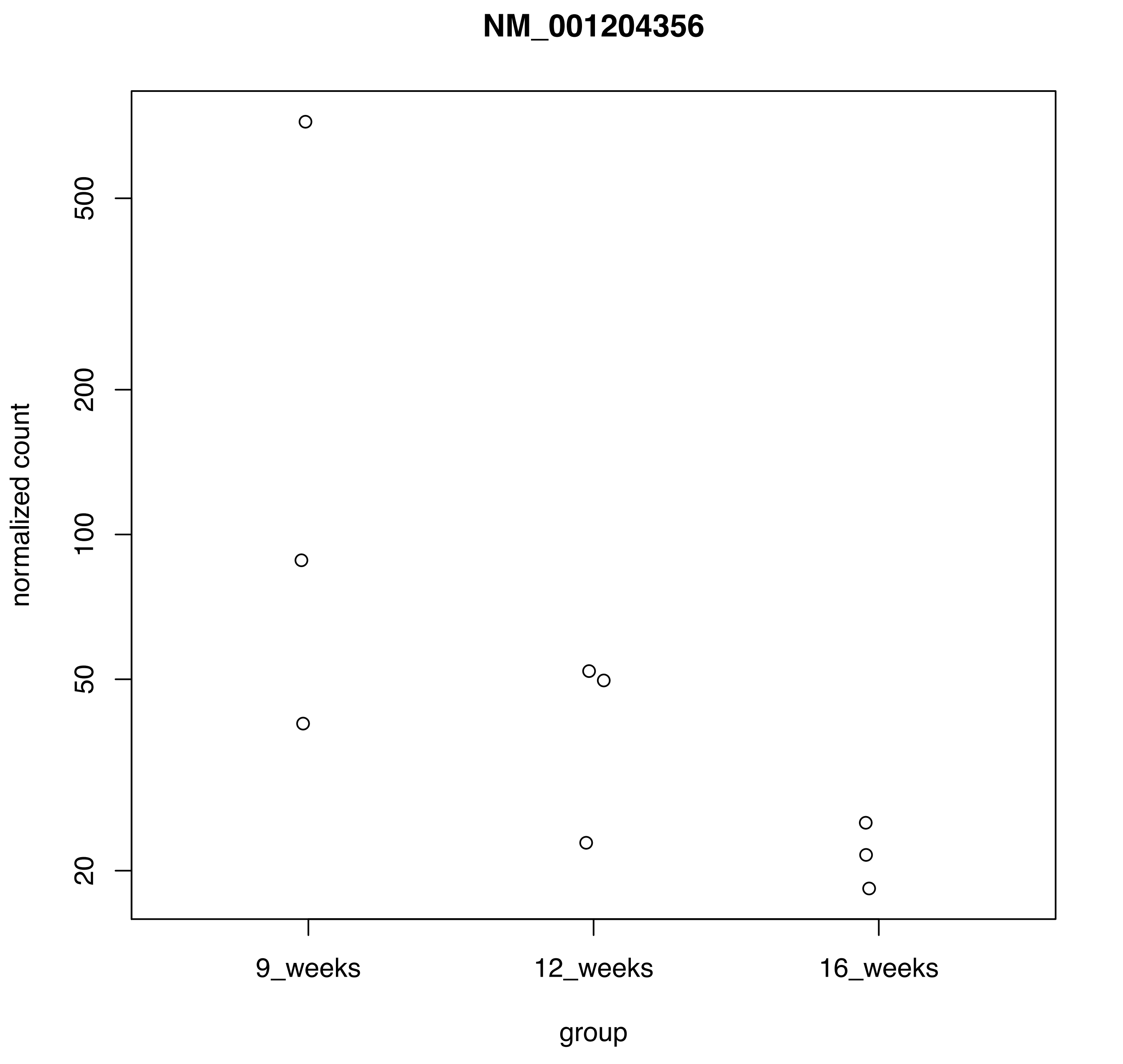
NM_001204356
normalized count
500
200
100
50
20
9_weeks
12_weeks
16_weeks
group

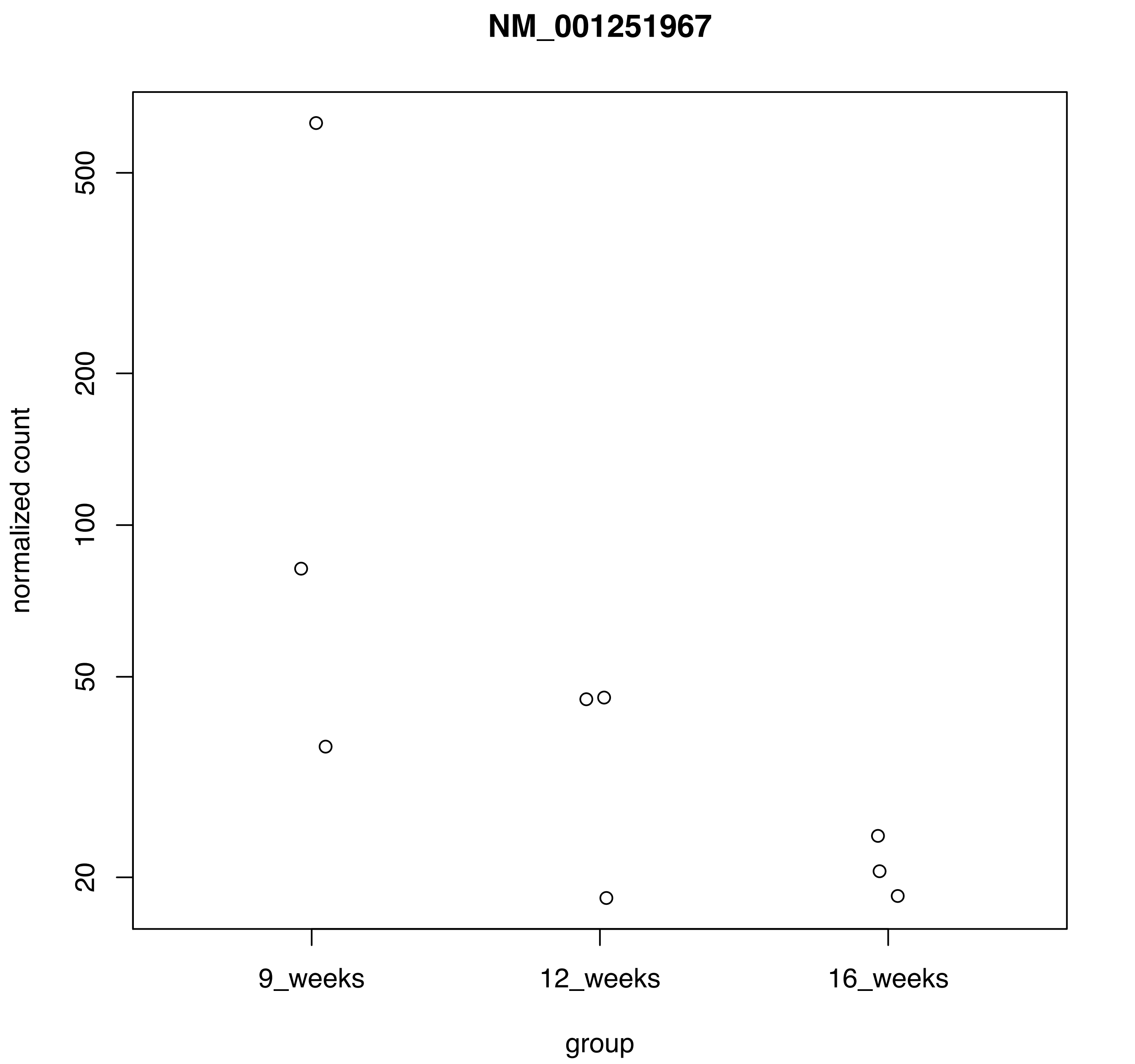
NM_001251967
normalized count
500
200
100
50
20
9_weeks
12_weeks
16_weeks
group

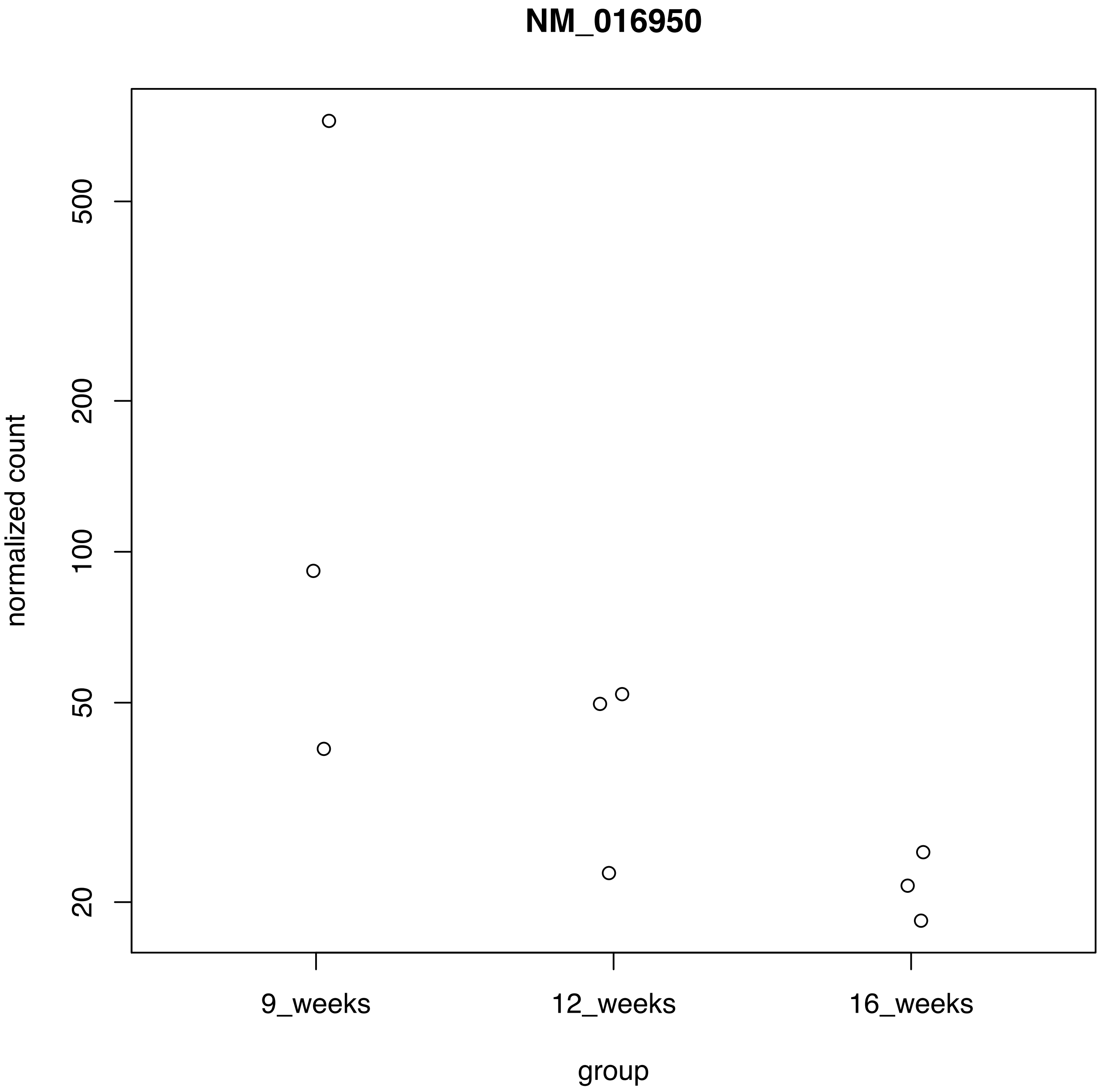
NM_016950
normalized count
500
200
100
50
20
9_weeks
12_weeks
16_weeks
group

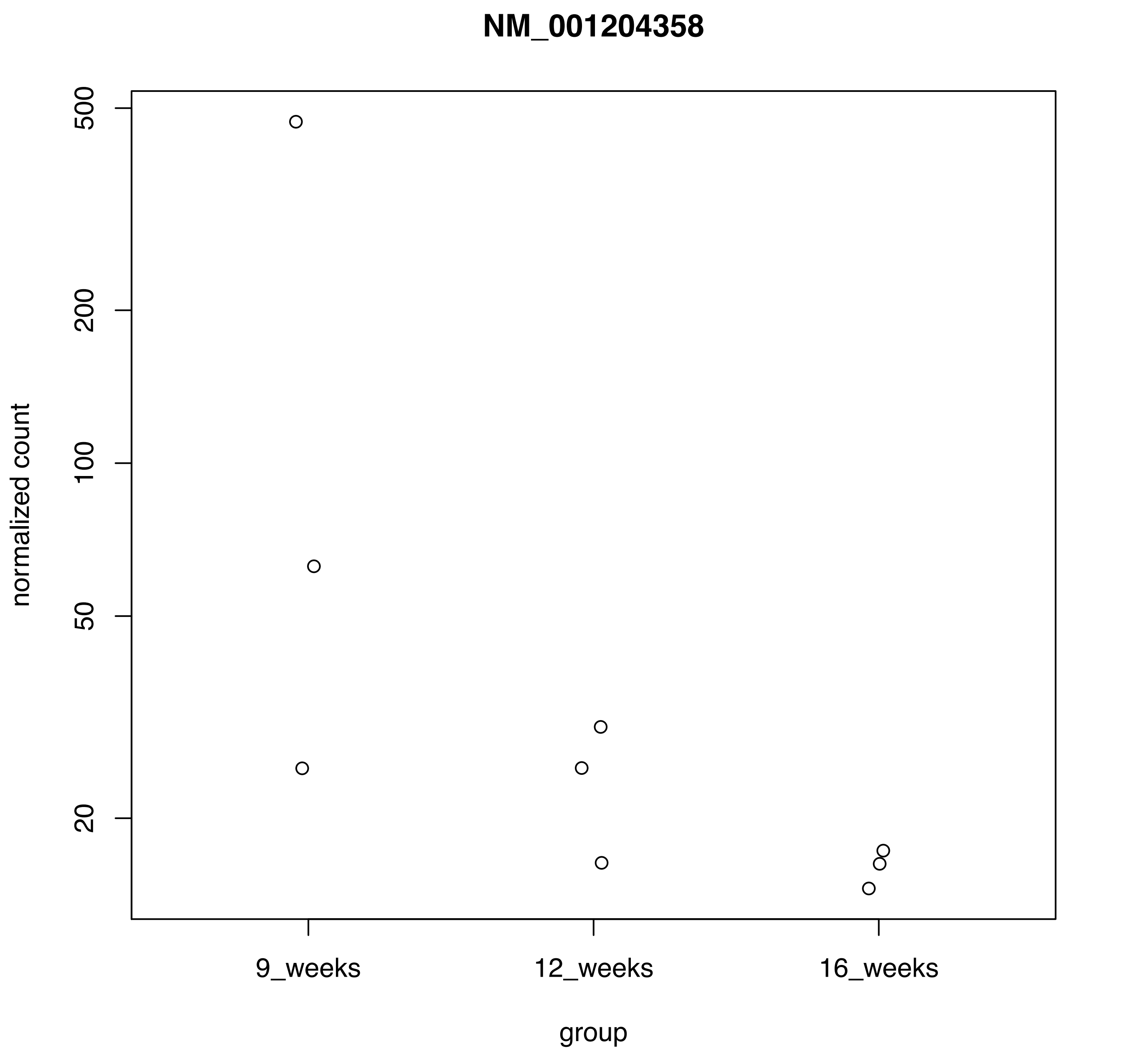

NM_001204358
500
200
100
50
20
normalized count
9_weeks
12_weeks
16_weeks
group

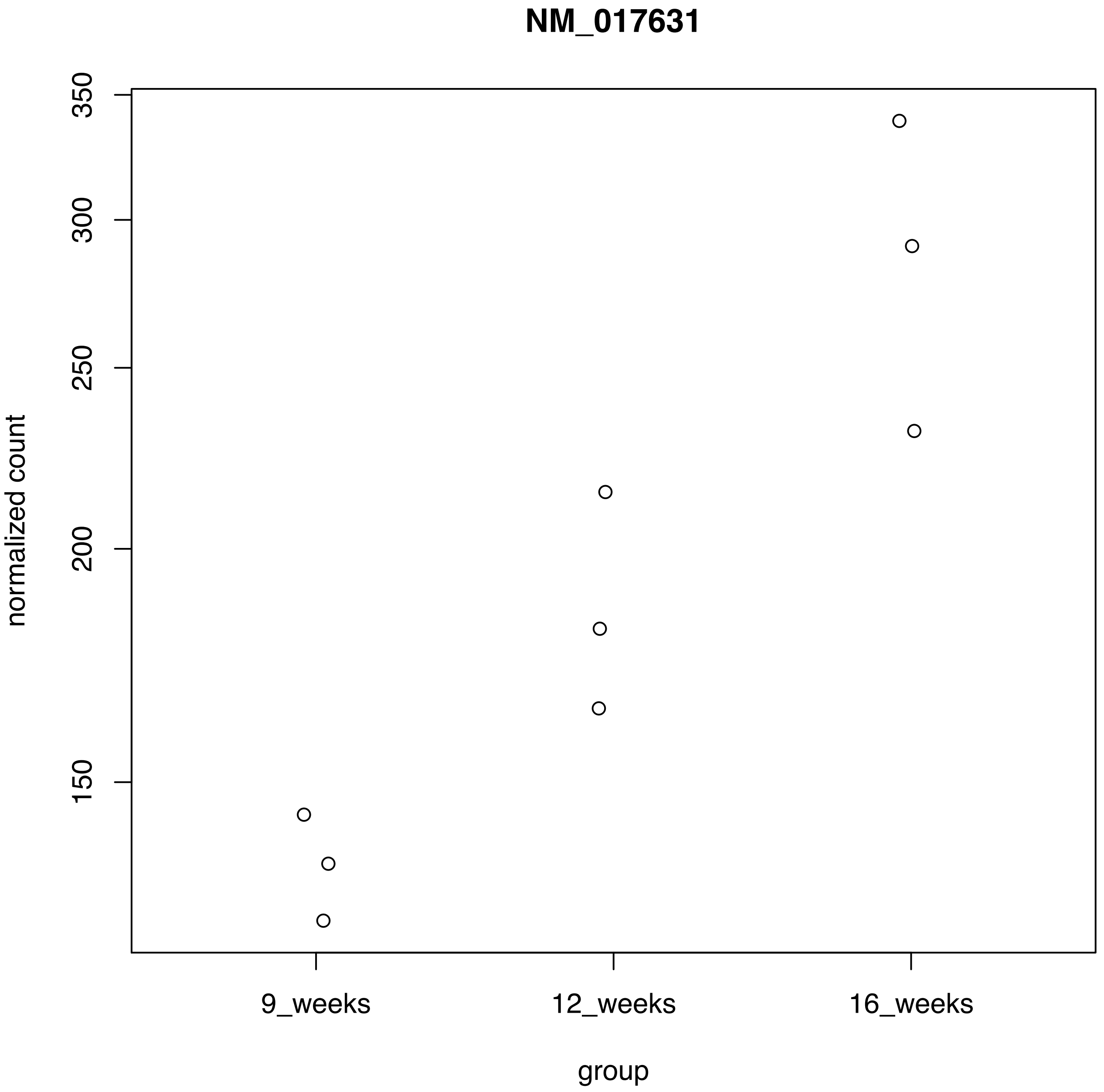

NM_017631
normalized count
350
300
250
200
150
9_weeks
12_weeks
16_weeks
group

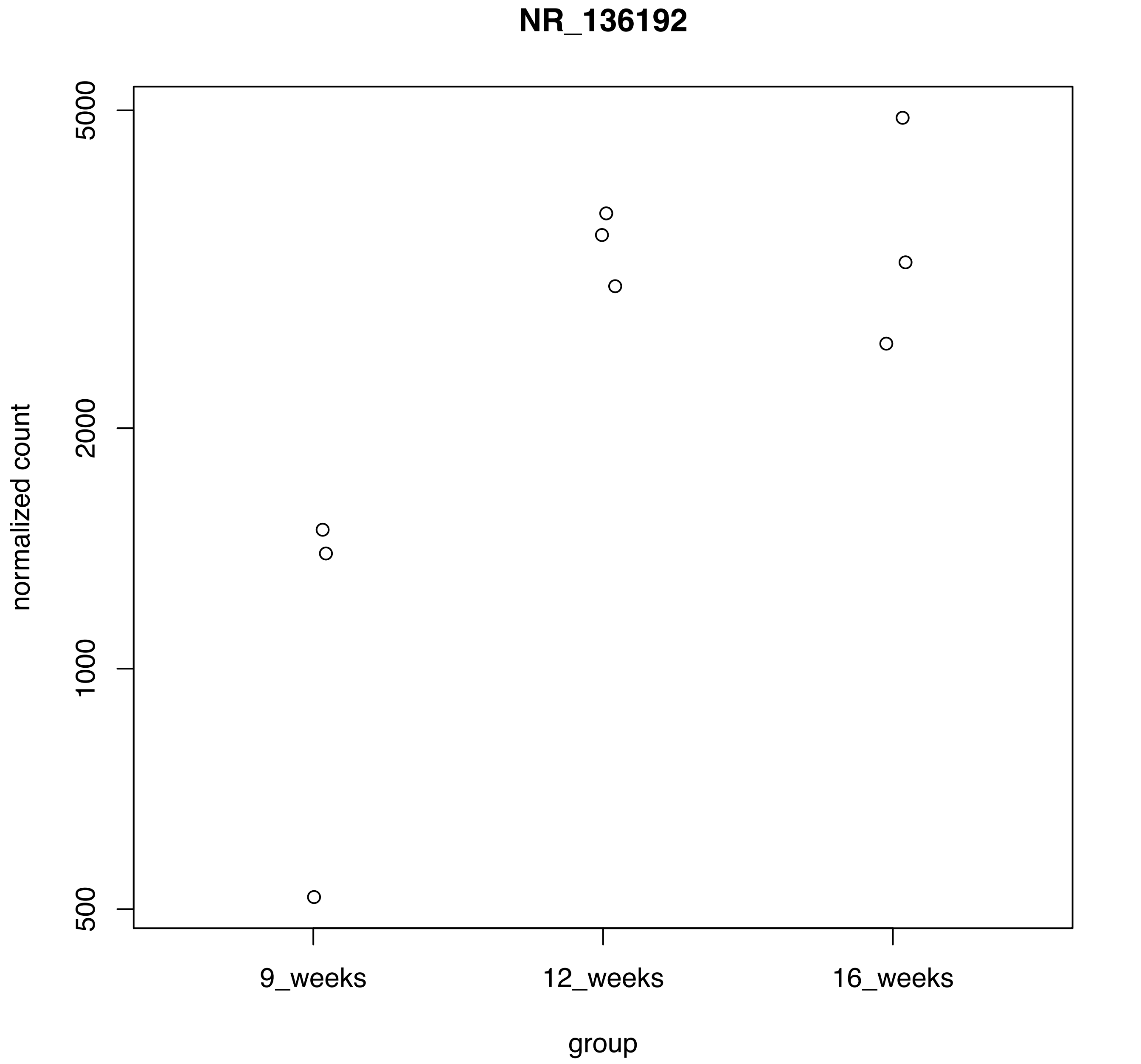
NR_136192
normalized count
5000
2000
1000
500
9_weeks
12_weeks
16_weeks
group

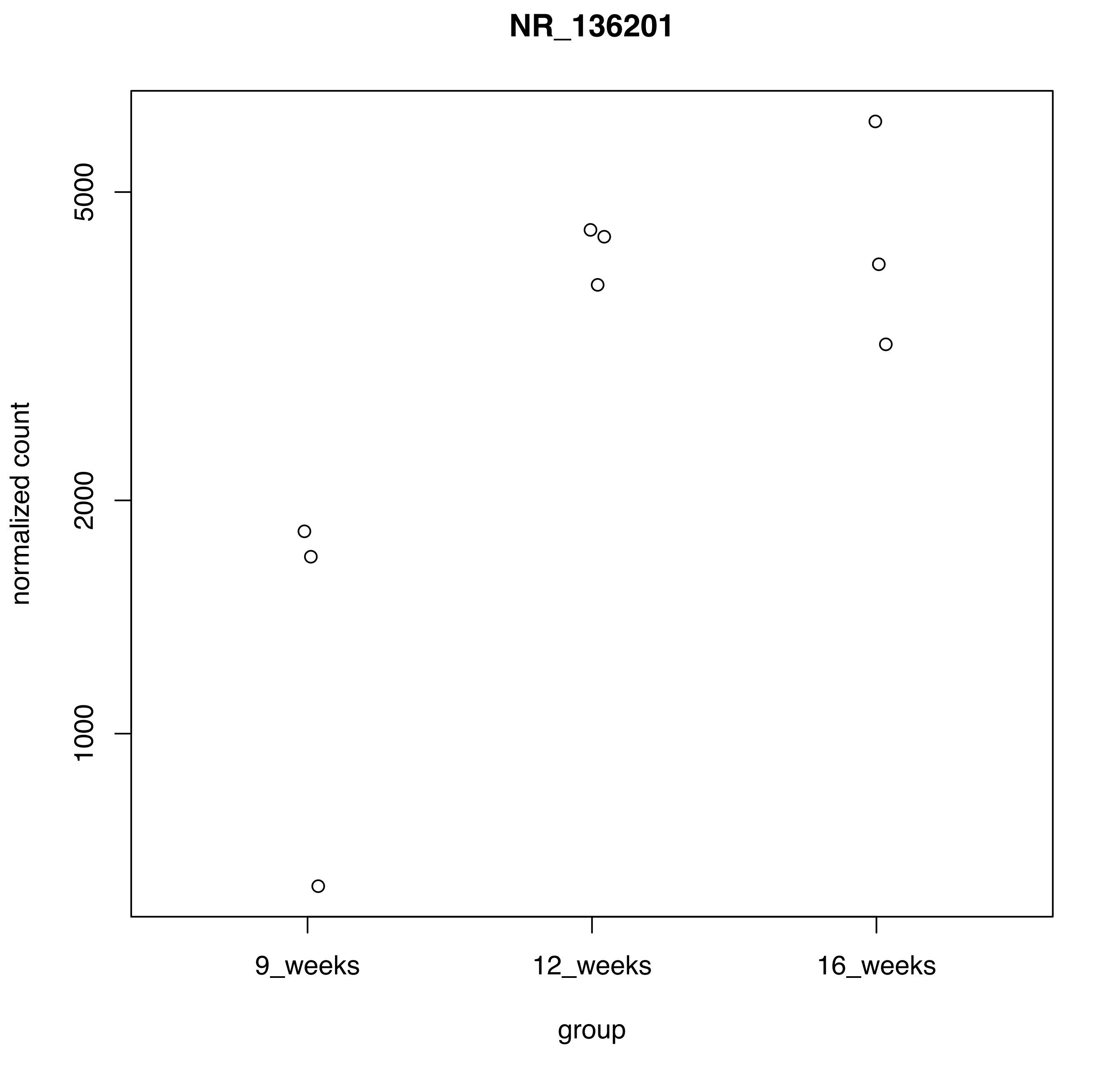

NR_136201
normalized count
5000
2000
1000
9_weeks
12_weeks
16_weeks
group

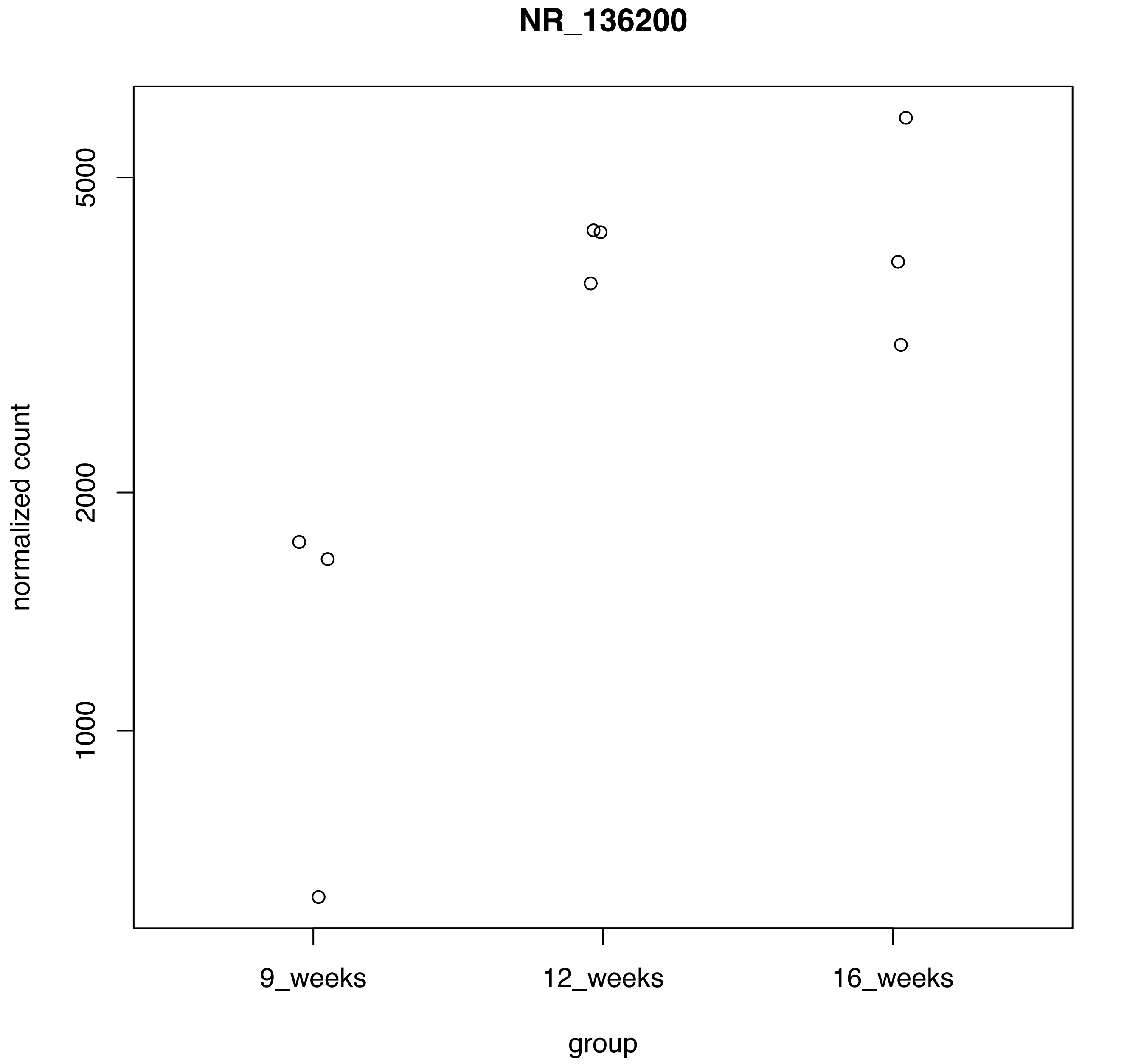
NR_136200
normalized count
5000
2000
1000
9_weeks
12_weeks
16_weeks
group

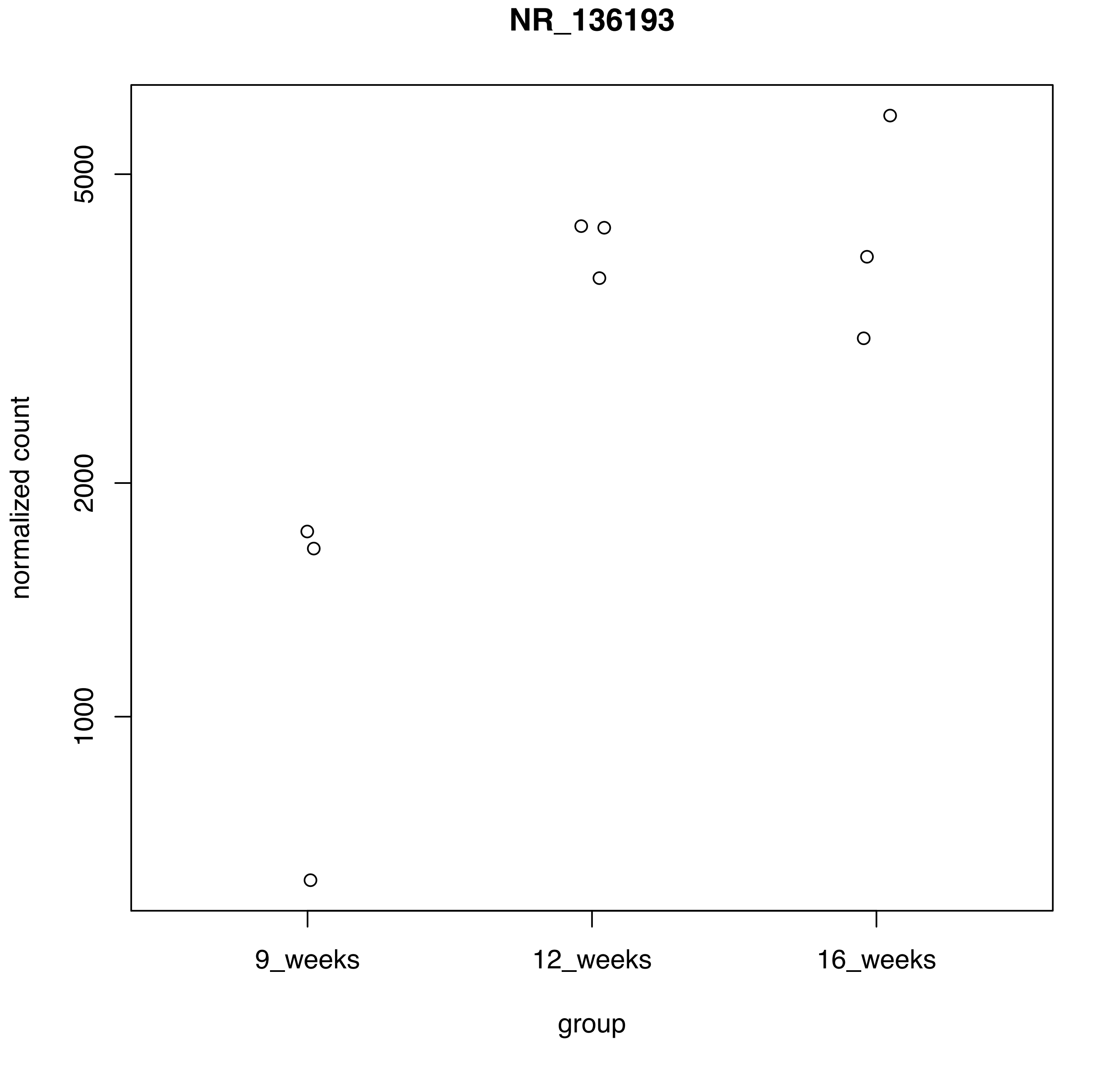

NR_136193
normalized count
5000
2000
1000
9_weeks
12_weeks
16_weeks
group

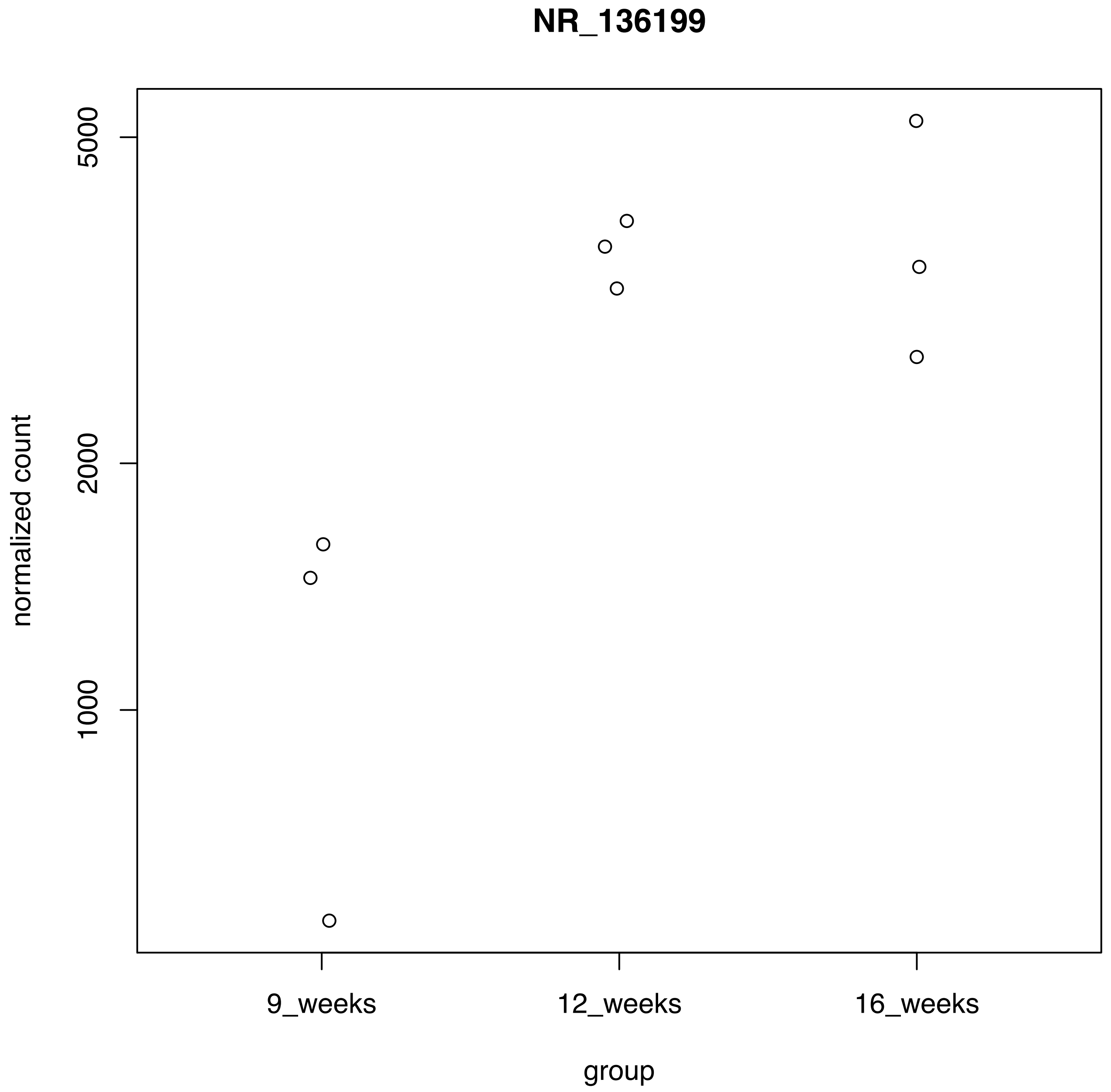
NR_136199
normalized count
5000
2000
1000
9_weeks
12_weeks
16_weeks
group

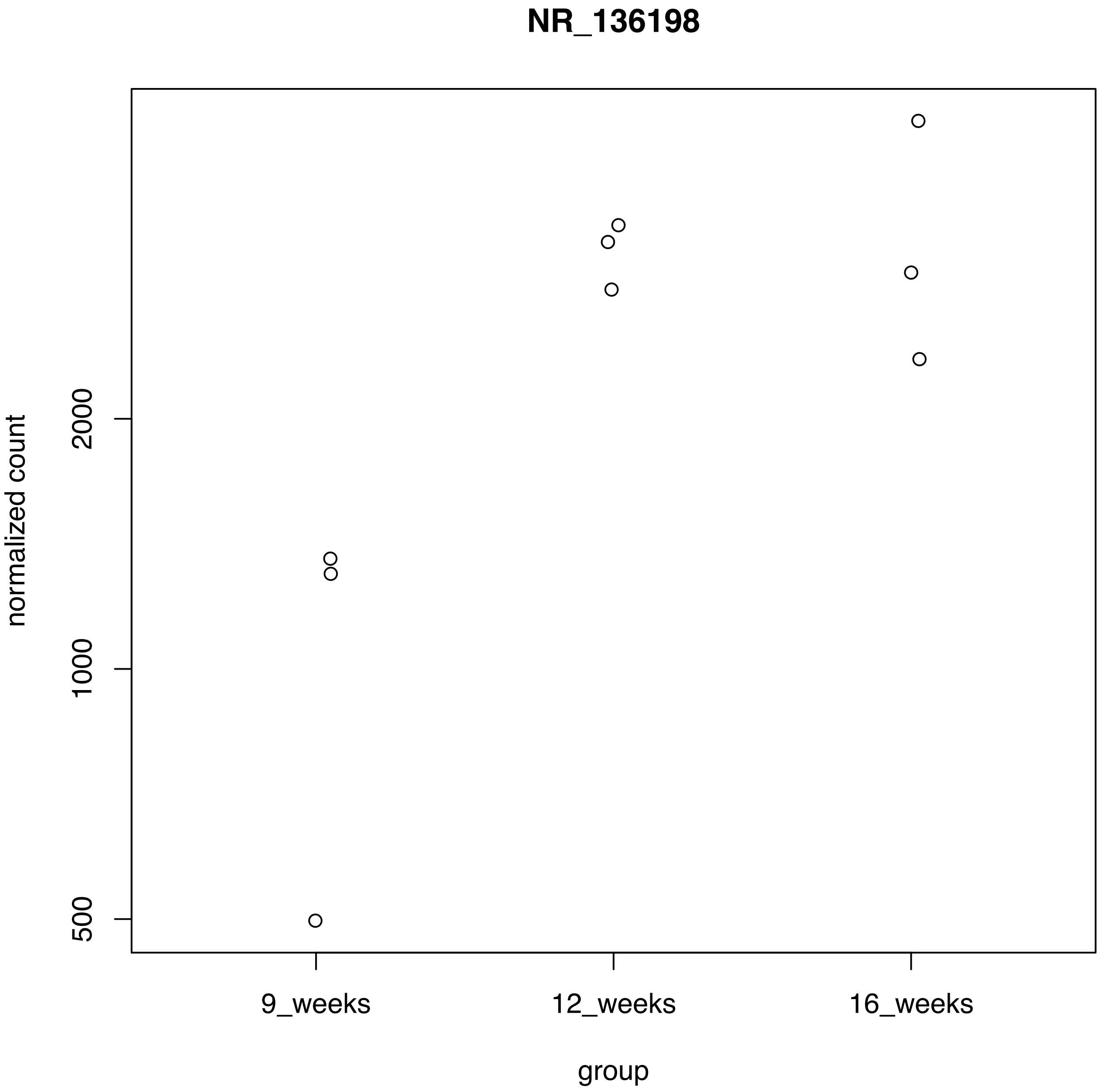
NR_136198
normalized count
2000
1000
500
9_weeks
12_weeks
16_weeks
group

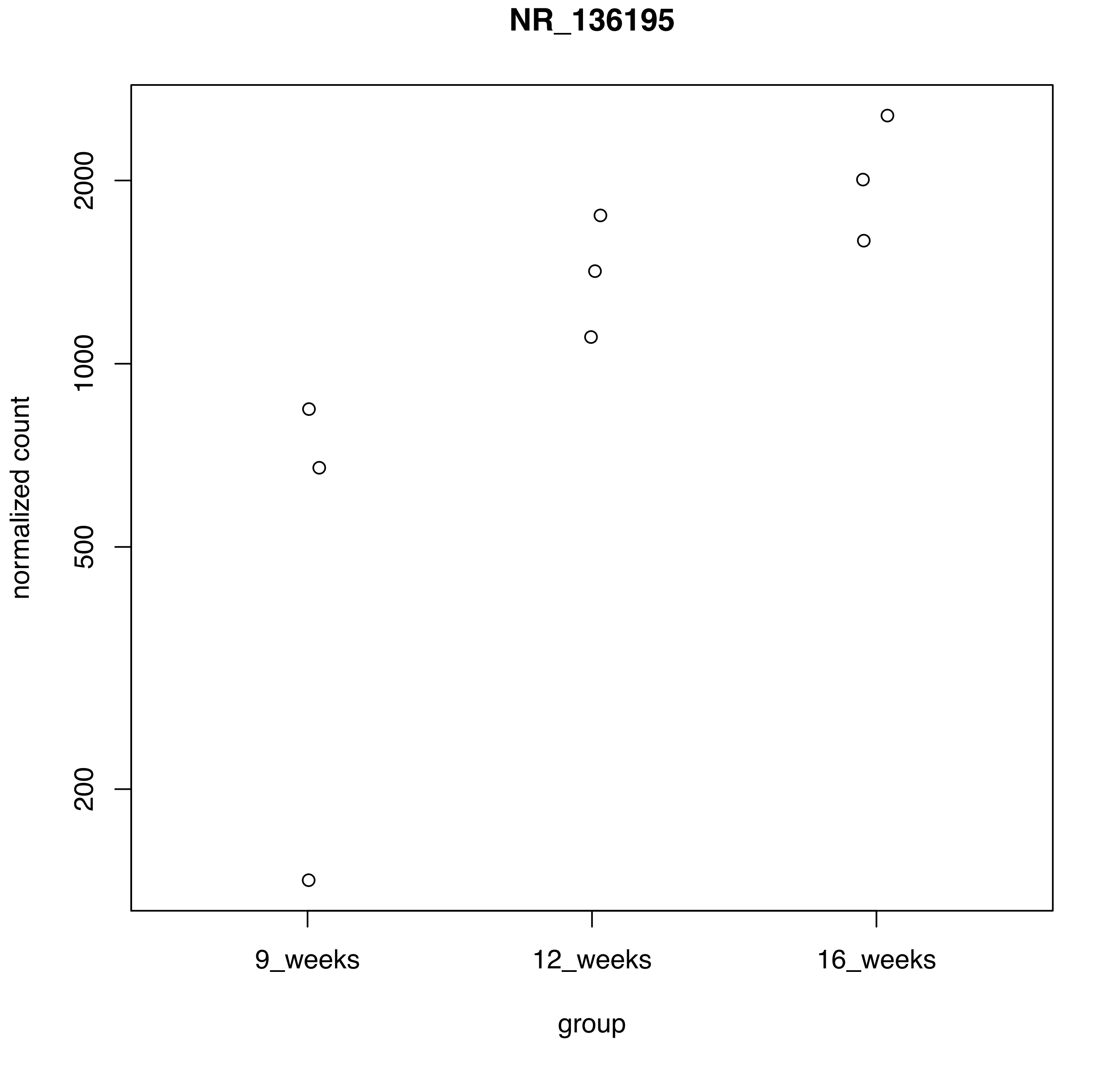

NR_136195
normalized count
2000
1000
500
200
9_weeks
12_weeks
16_weeks
group

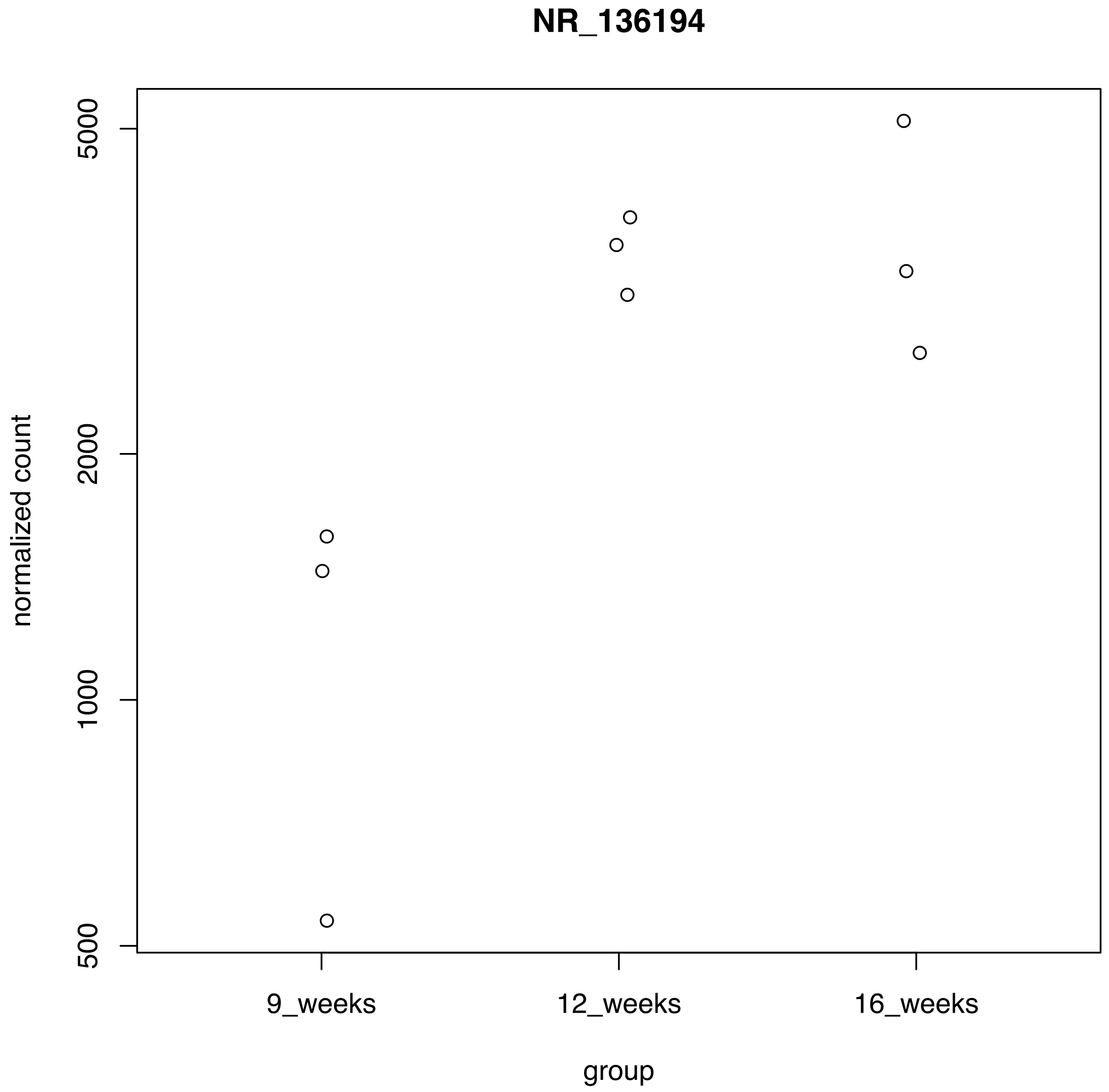
NR_136194
normalized count
5000
2000
1000
500
9_weeks
12_weeks
16_weeks
group

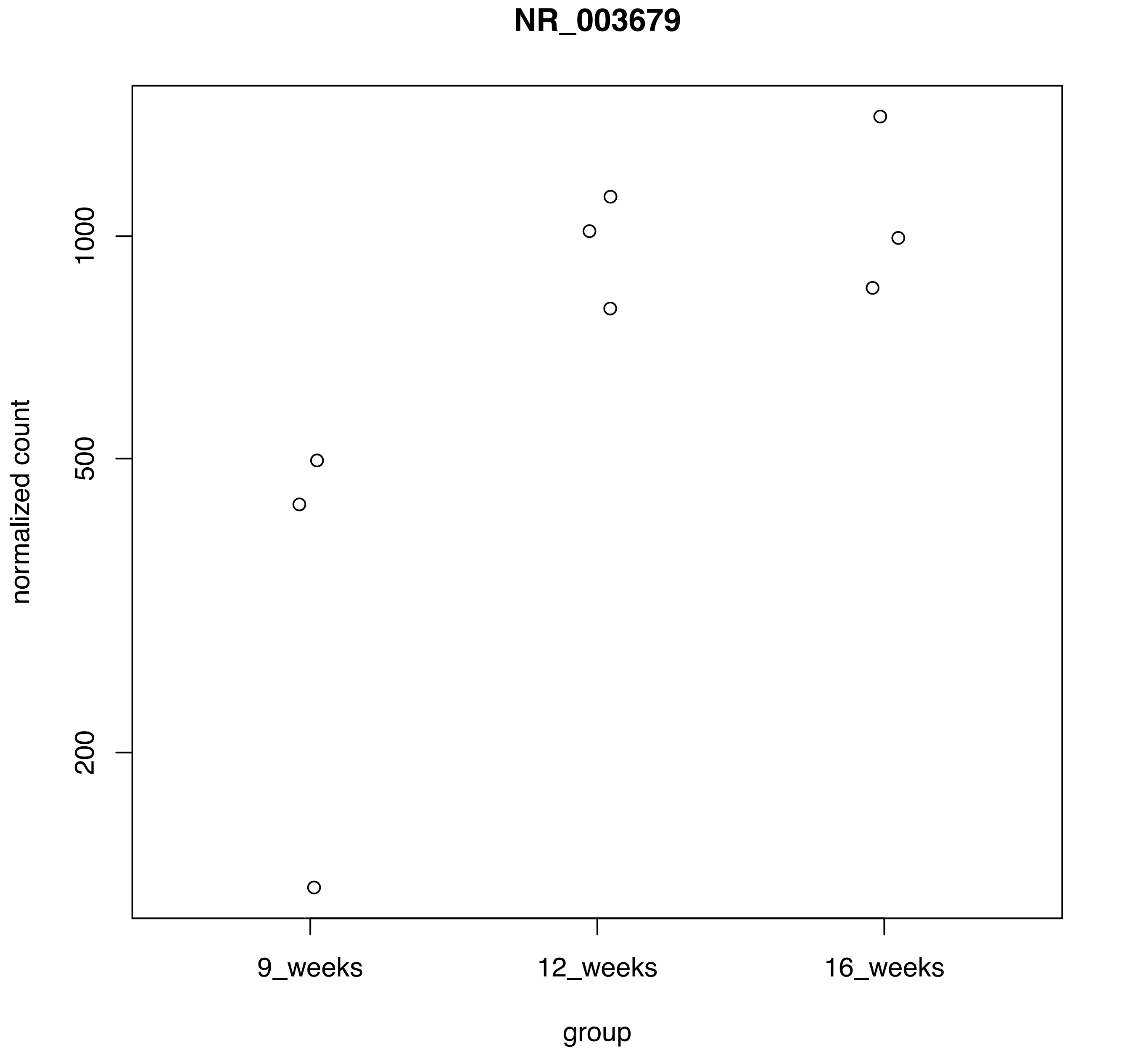

NR_003679
normalized count
1000
500
200
9_weeks
12_weeks
16_weeks
group

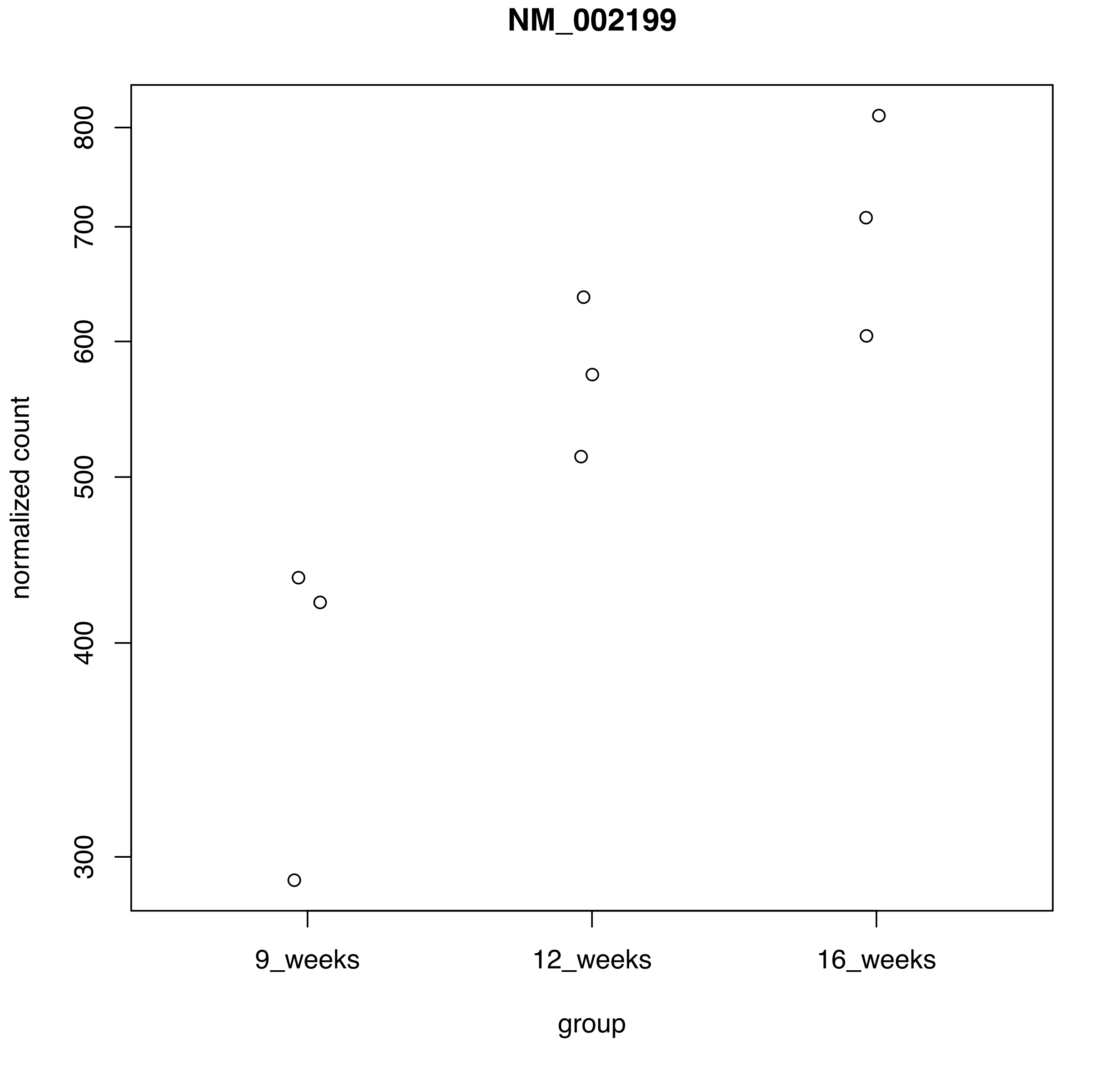
NM_002199
normalized count
300
400
500
600
700
800
9_weeks
12_weeks
16_weeks
group

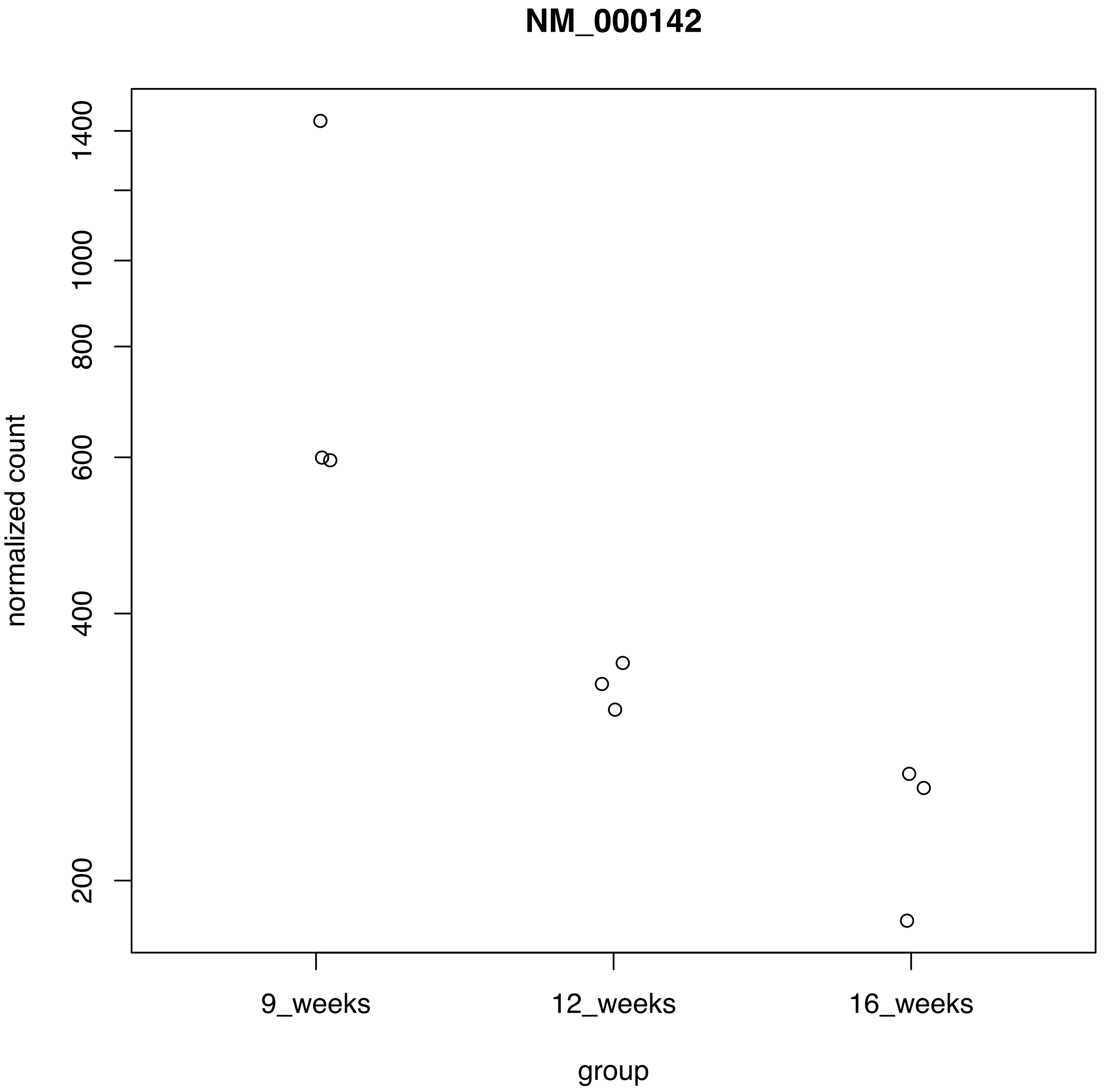

NM_000142
normalized count
1400
1000
800
600
400
200
9_weeks
12_weeks
16_weeks
group

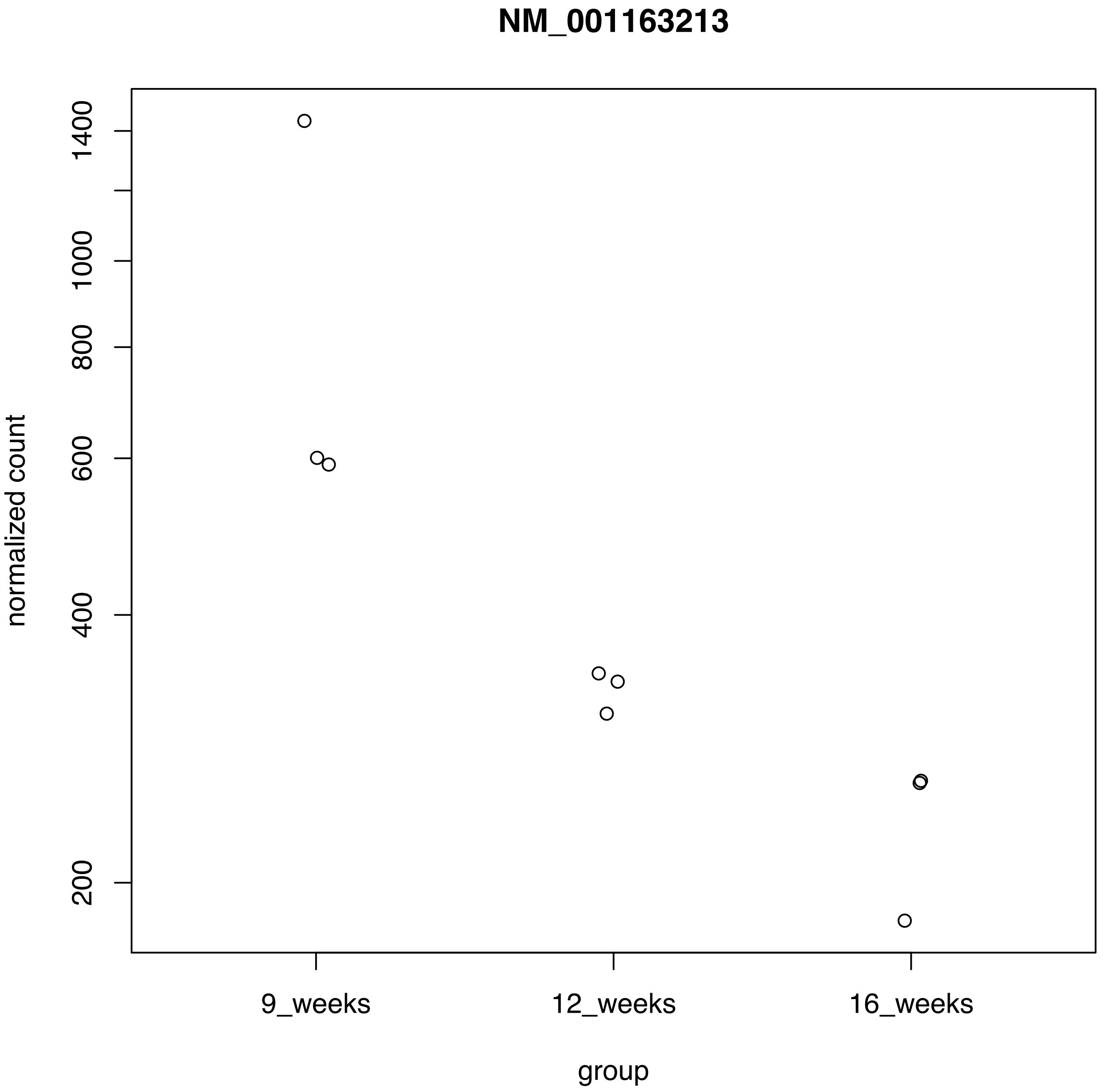
NM_001163213
normalized count
1400
1000
800
600
400
200
9_weeks
12_weeks
16_weeks
group

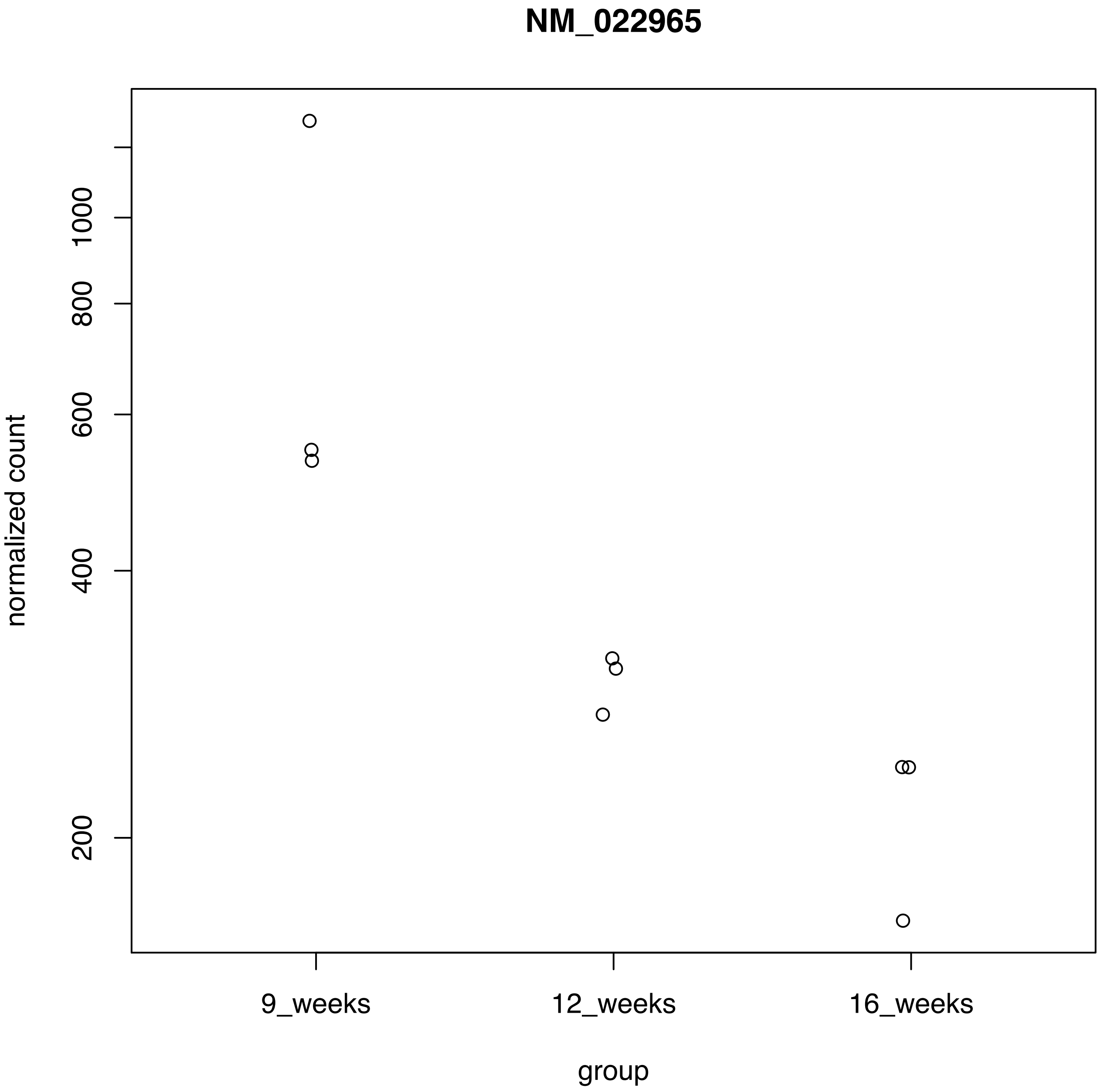
NM_022965
normalized count
200
400
600
800
1000
9_weeks
12_weeks
16_weeks
group

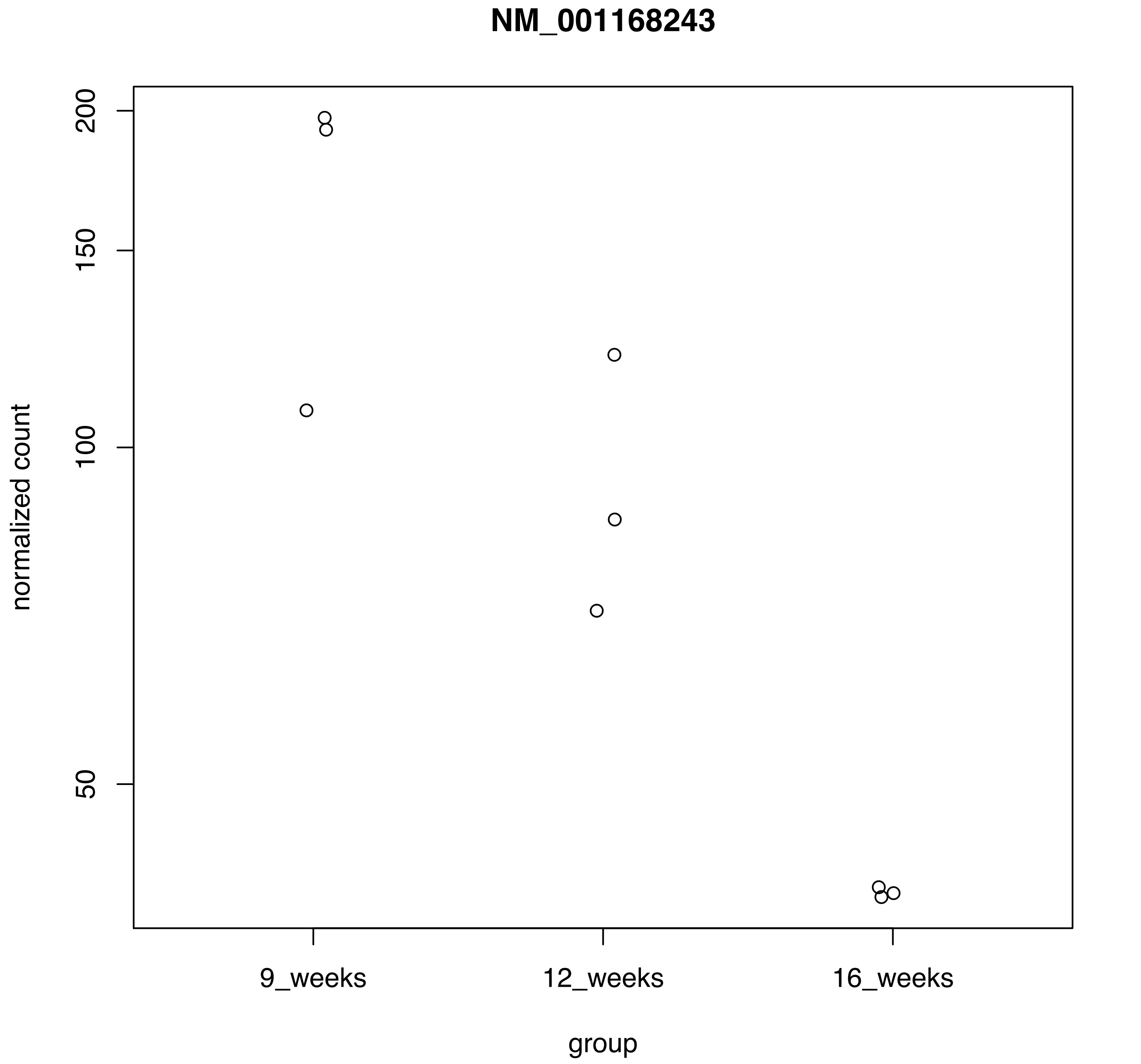

NM_001168243
normalized count
200
150
100
50
9_weeks
12_weeks
16_weeks
group

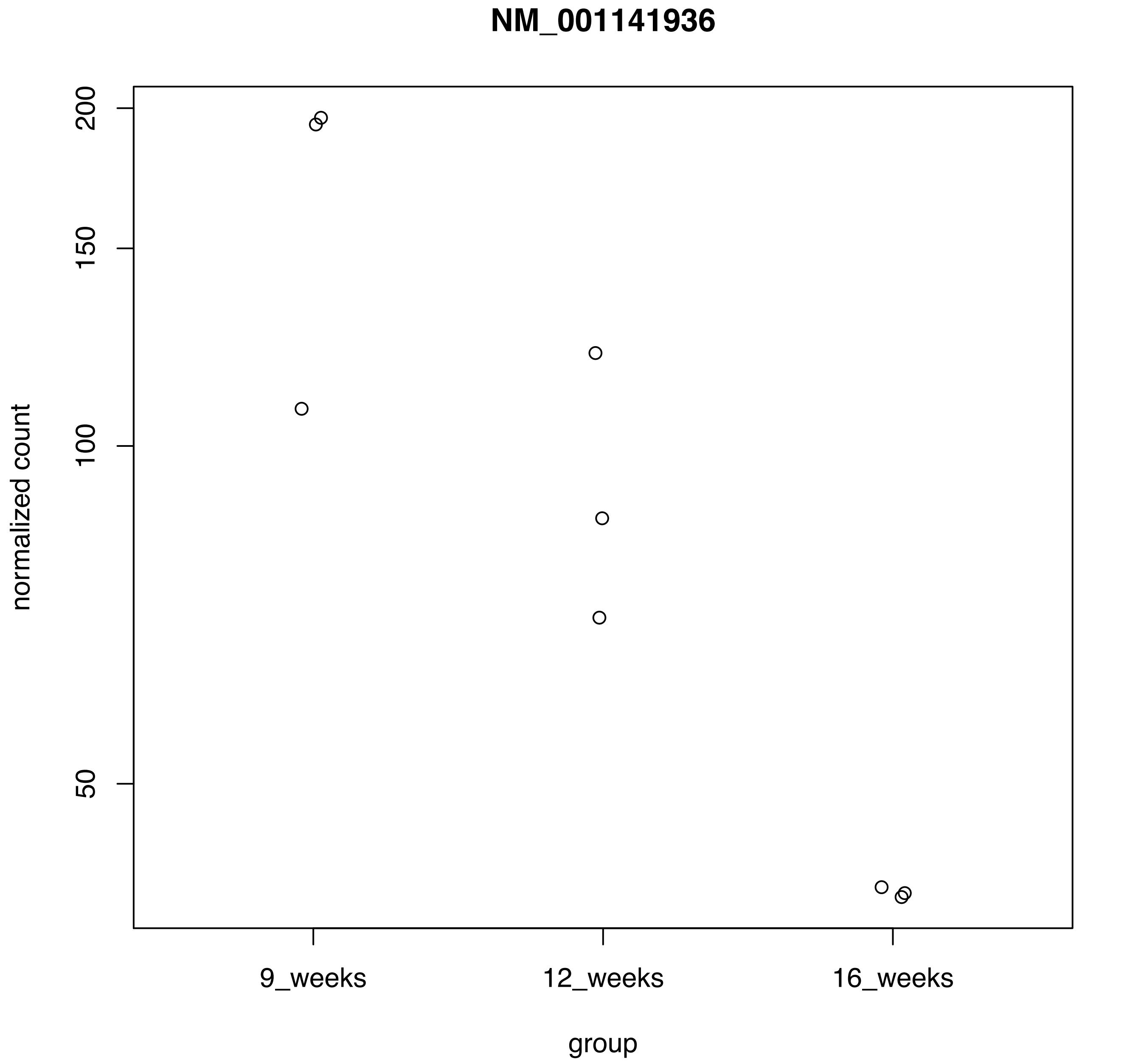
NM_001141936
normalized count
200
150
100
50
9_weeks
12_weeks
16_weeks
group

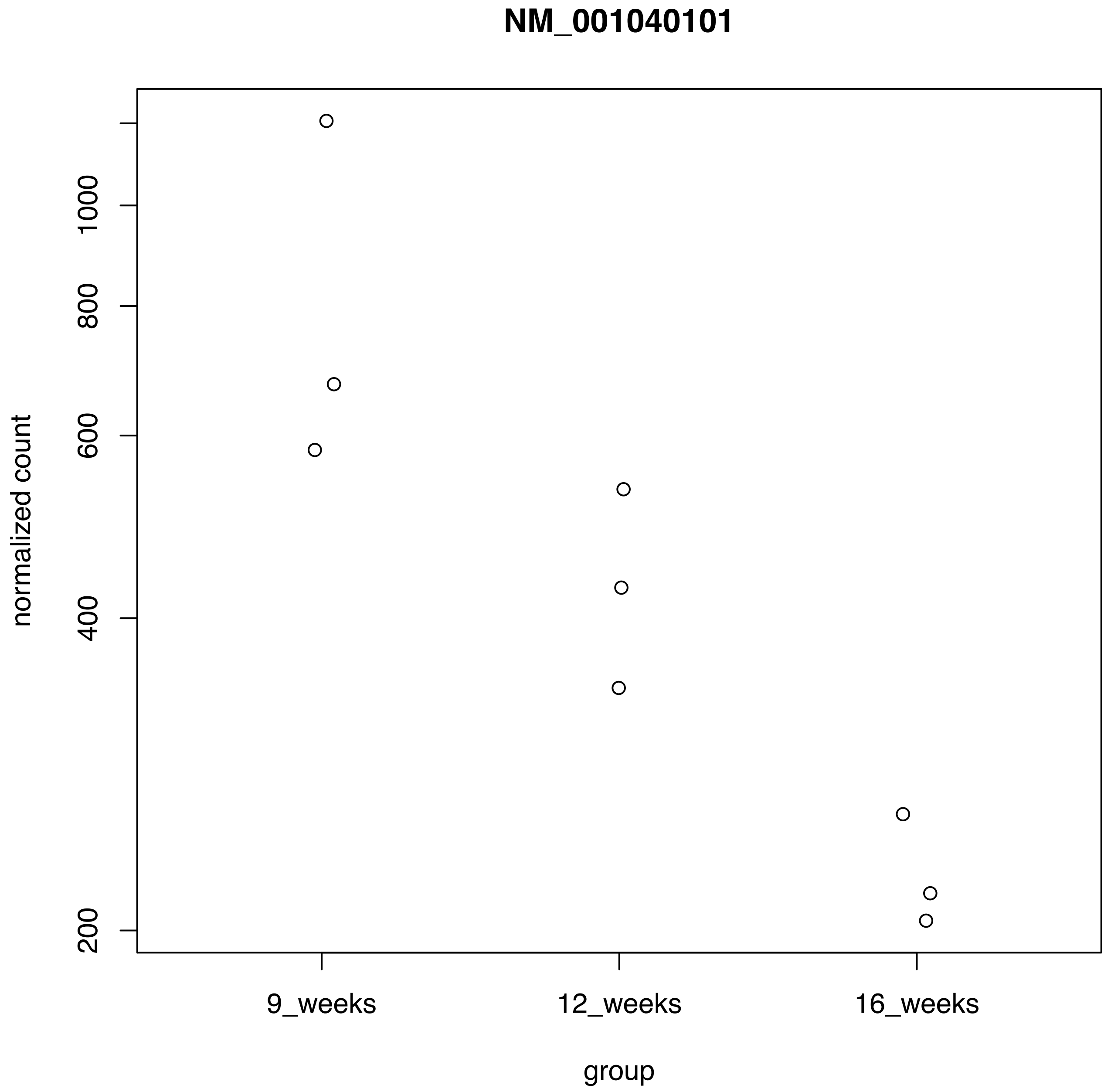

NM_001040101
normalized count
200
400
600
800
1000
9_weeks
12_weeks
16_weeks
group

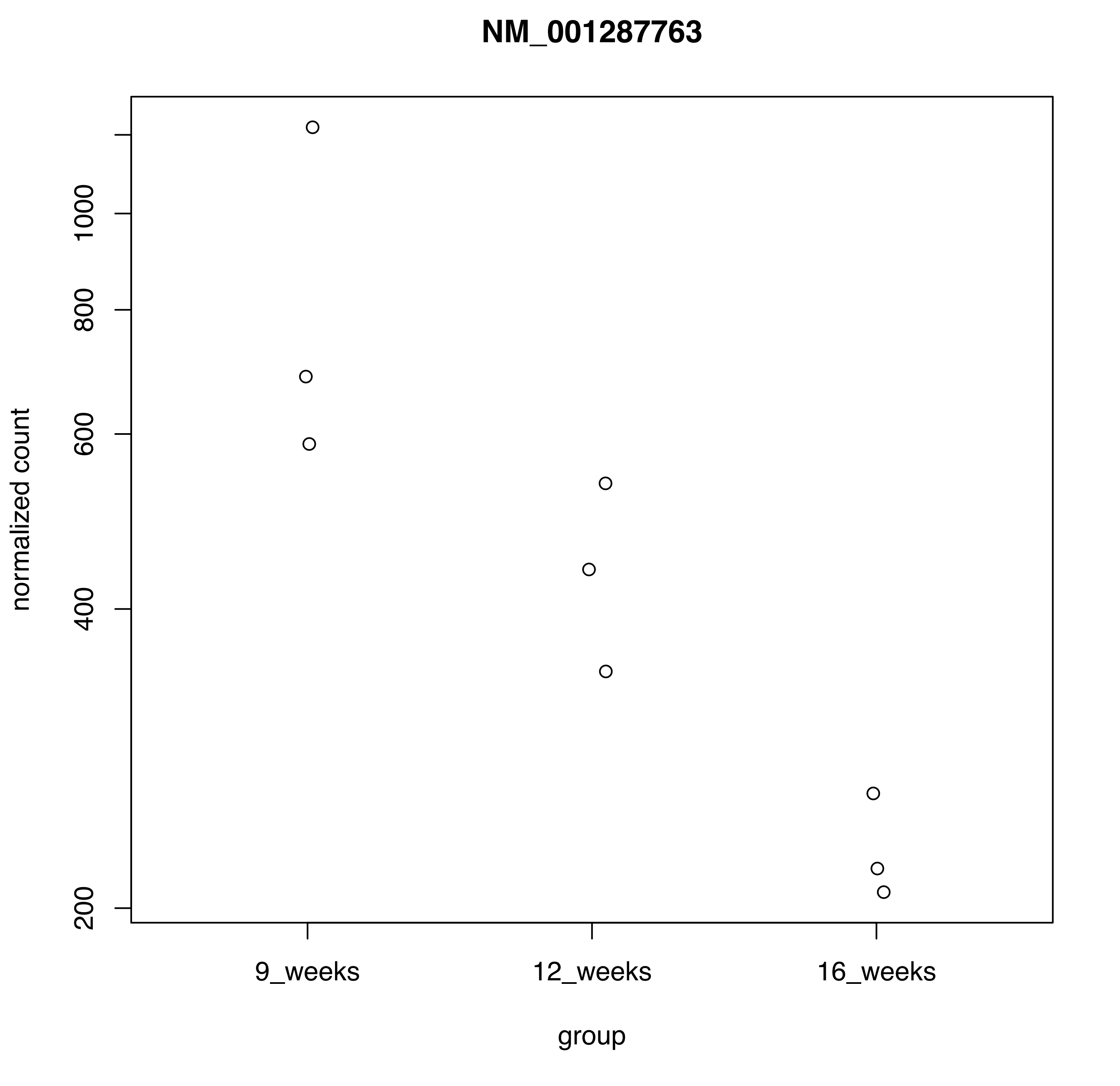

NM_001287763
normalized count
1000
800
600
400
200
9_weeks
12_weeks
16_weeks
group

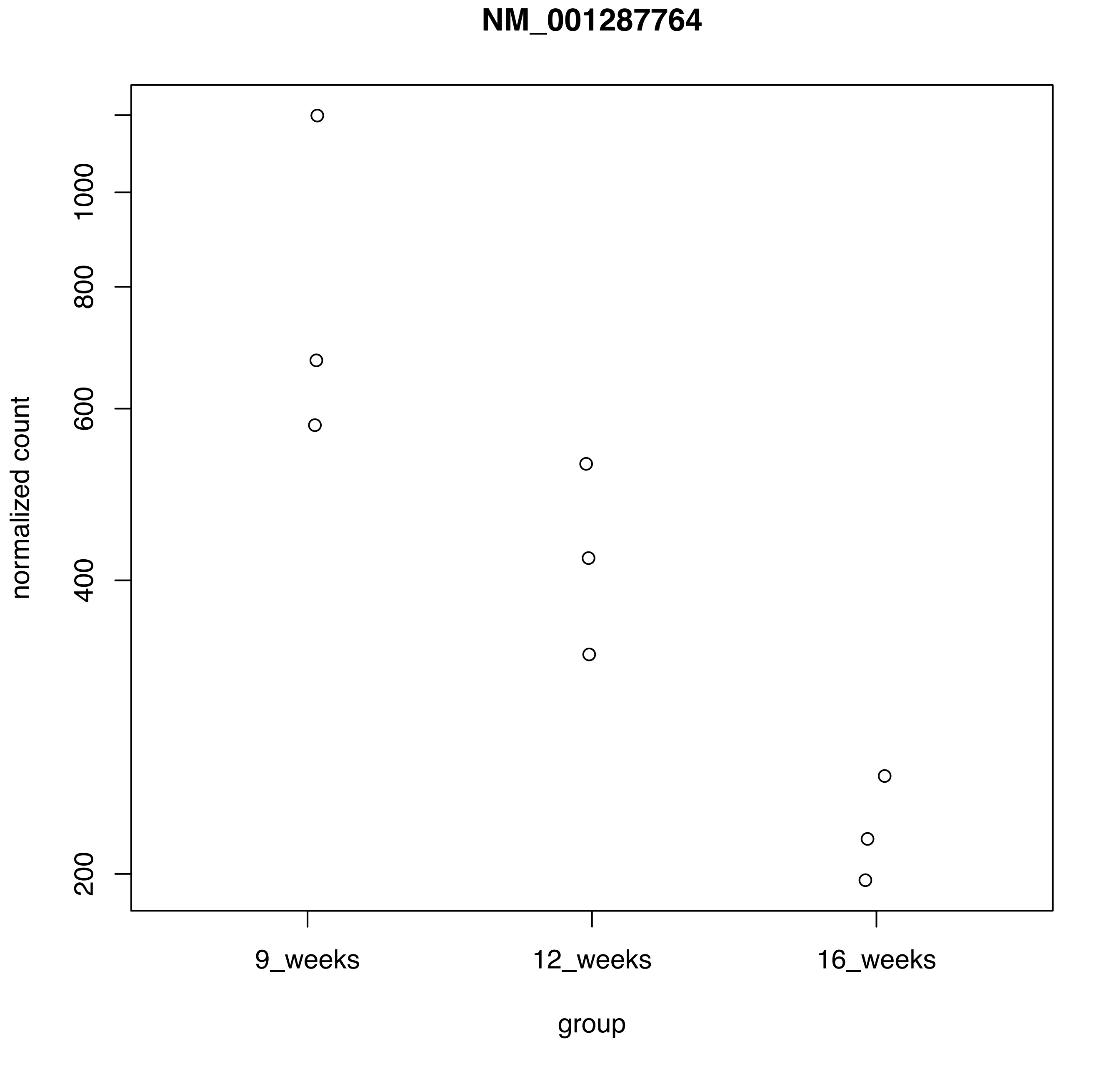
NM_001287764
normalized count
1000
800
600
400
200
9_weeks
12_weeks
16_weeks
group

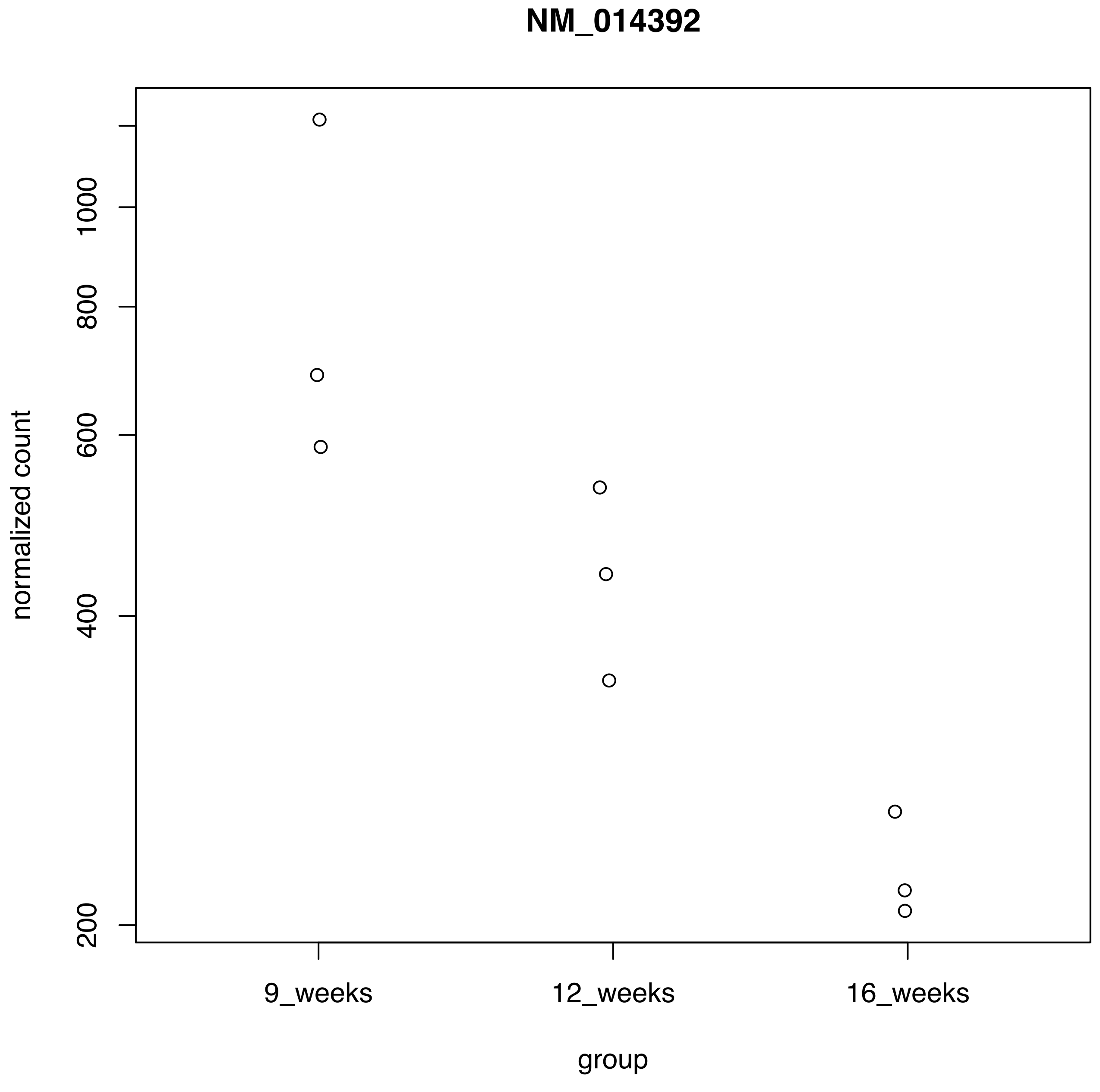
NM_014392
normalized count
200
400
600
800
1000
9_weeks
12_weeks
16_weeks
group

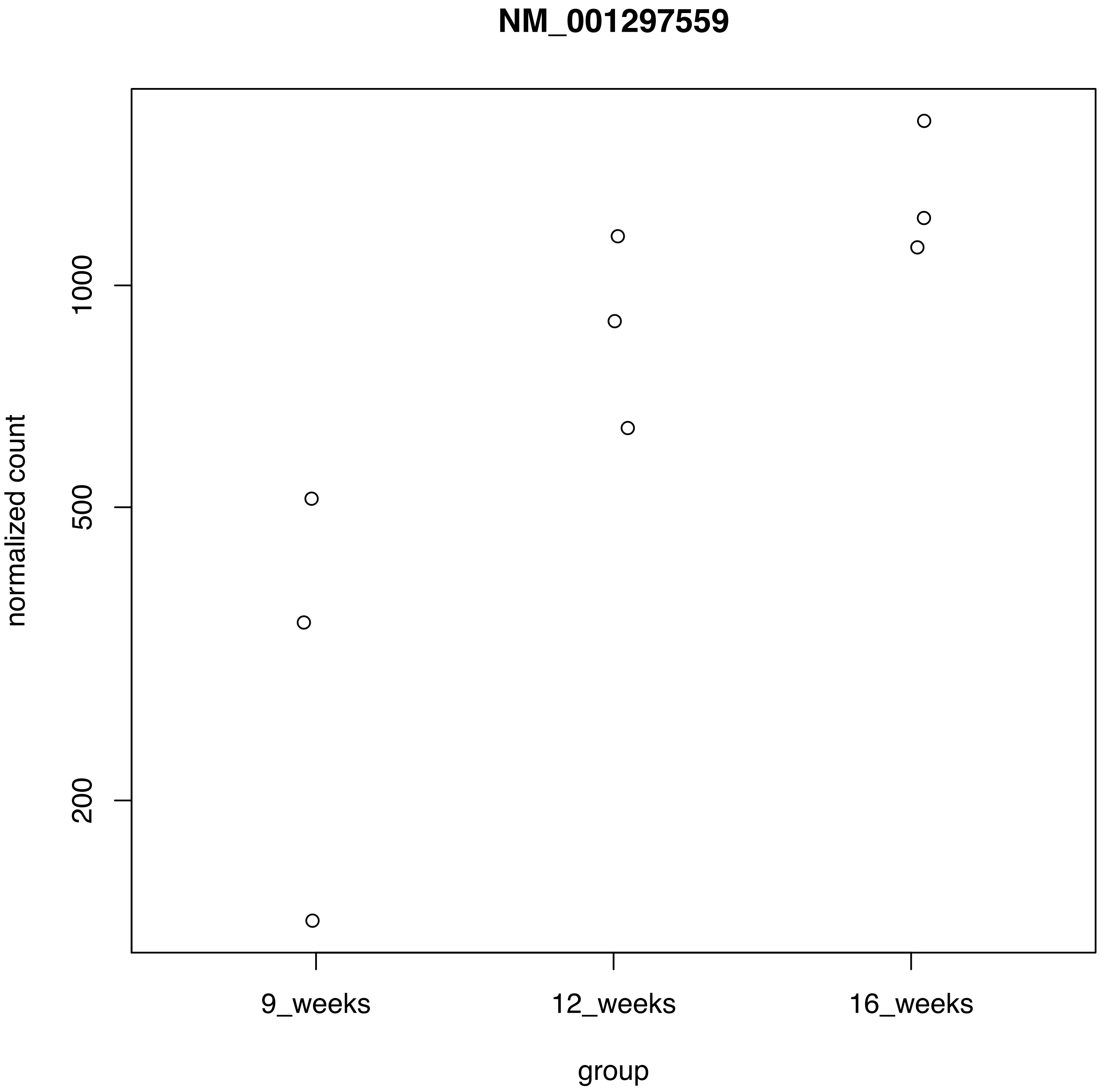

NM_001297559
normalized count
1000
500
200
9_weeks
12_weeks
16_weeks
group

NM_053044

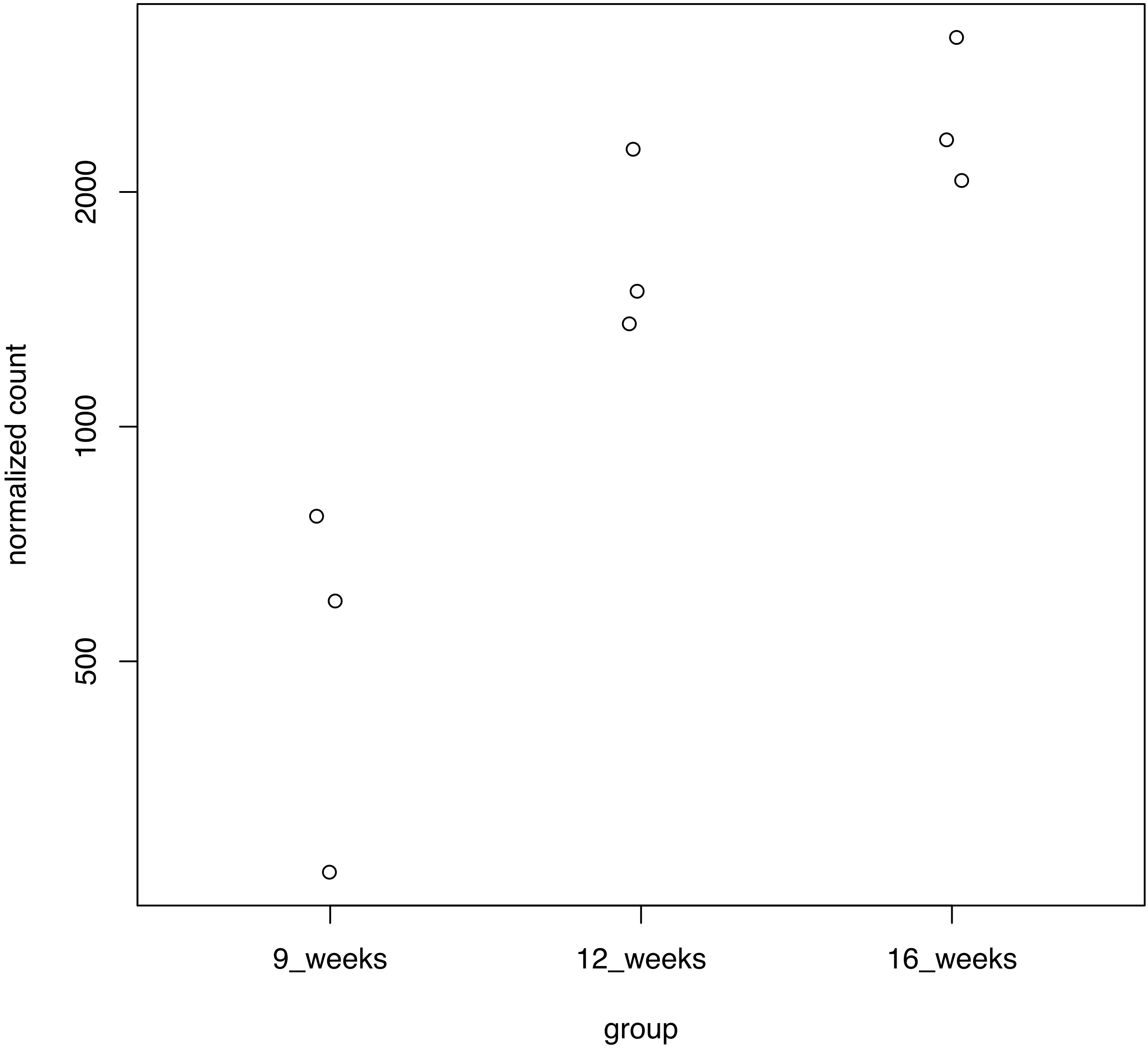

normalized count
2000
1000
500
9_weeks
12_weeks
16_weeks
group

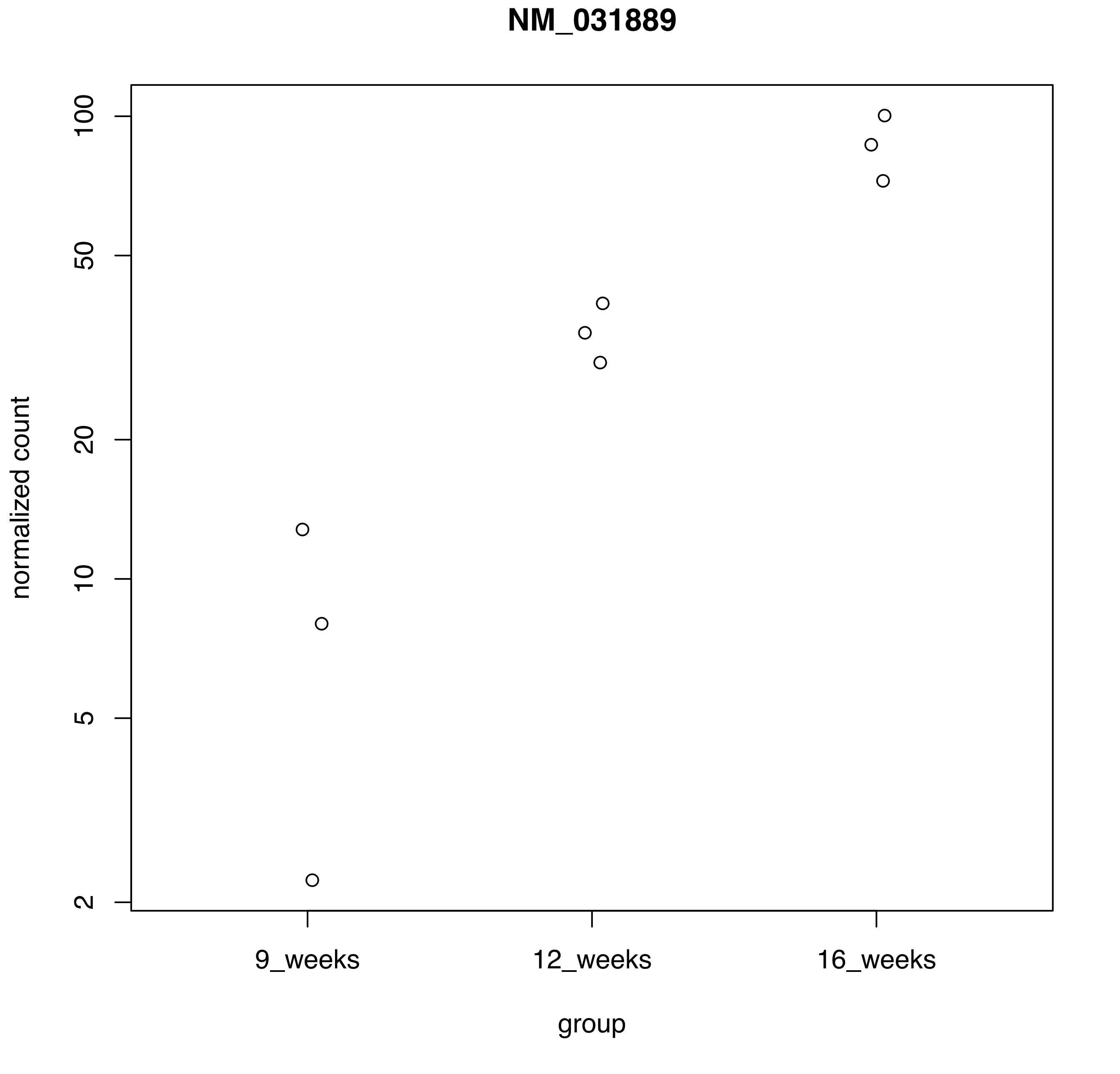

NM_031889
normalized count
100
50
20
10
5
2
9_weeks
12_weeks
16_weeks
group

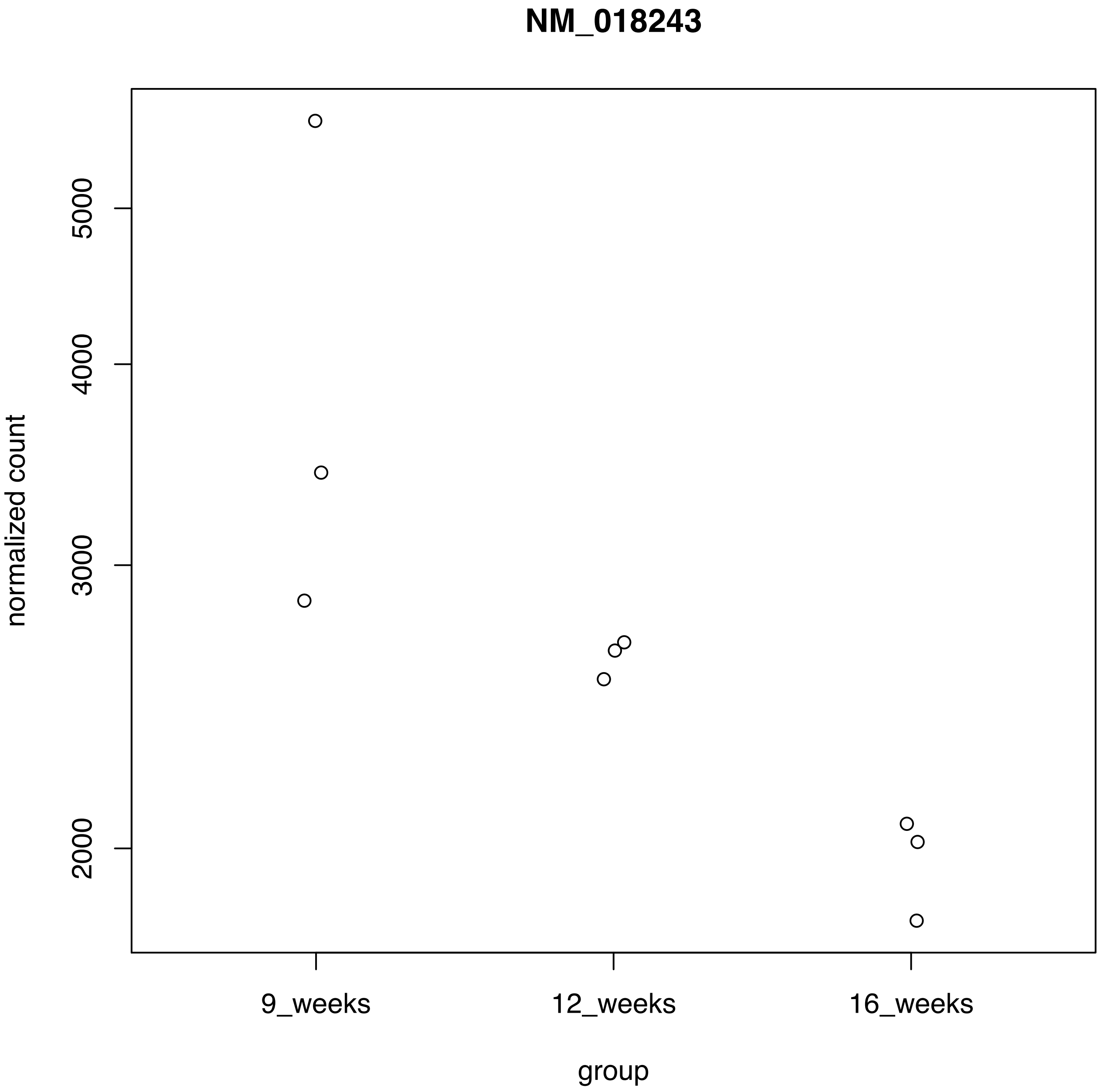

NM_018243
normalized count
5000
4000
3000
2000
9_weeks
12_weeks
16_weeks
group

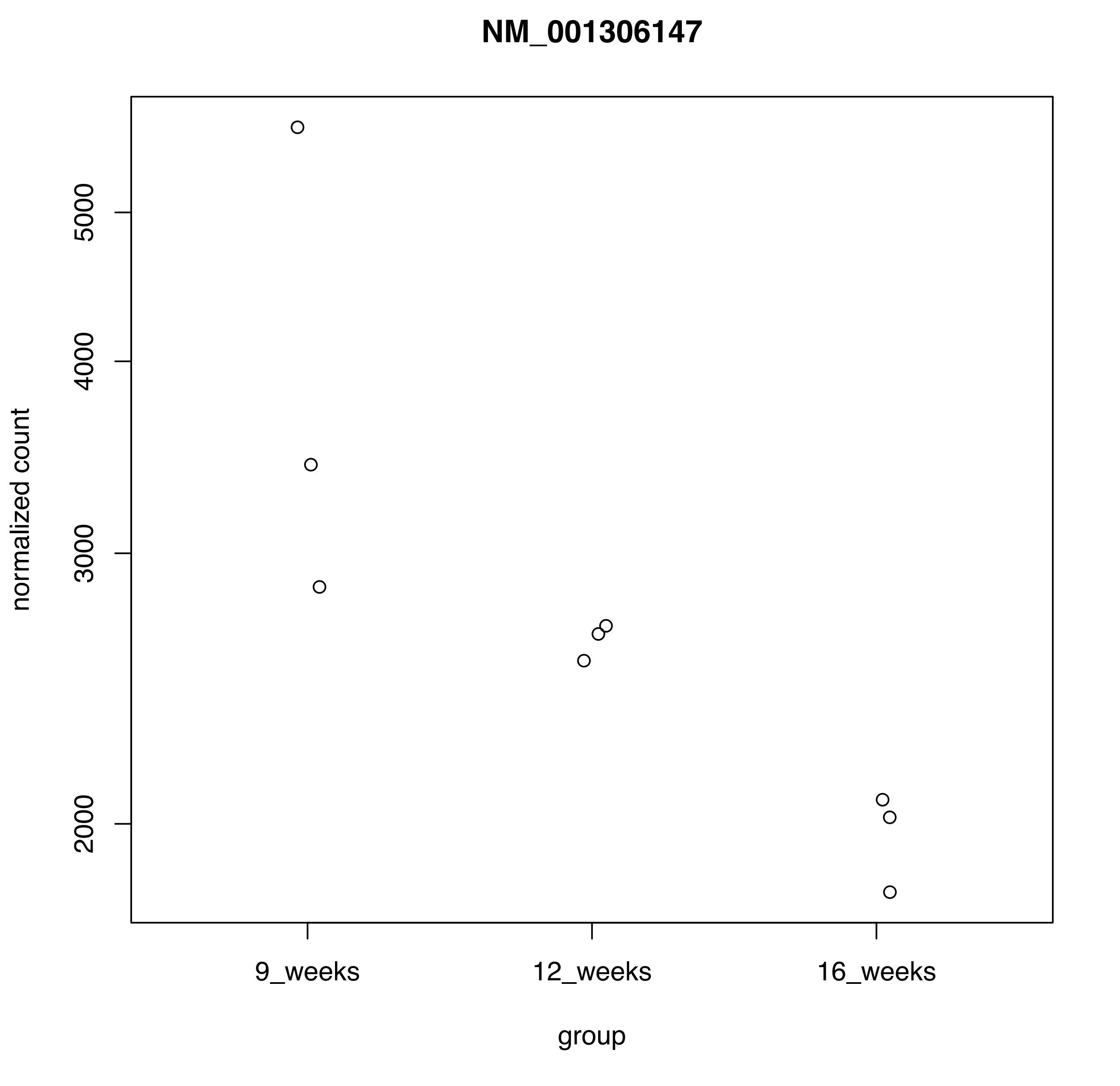
NM_001306147
normalized count
5000
4000
3000
2000
9_weeks
12_weeks
16_weeks
group

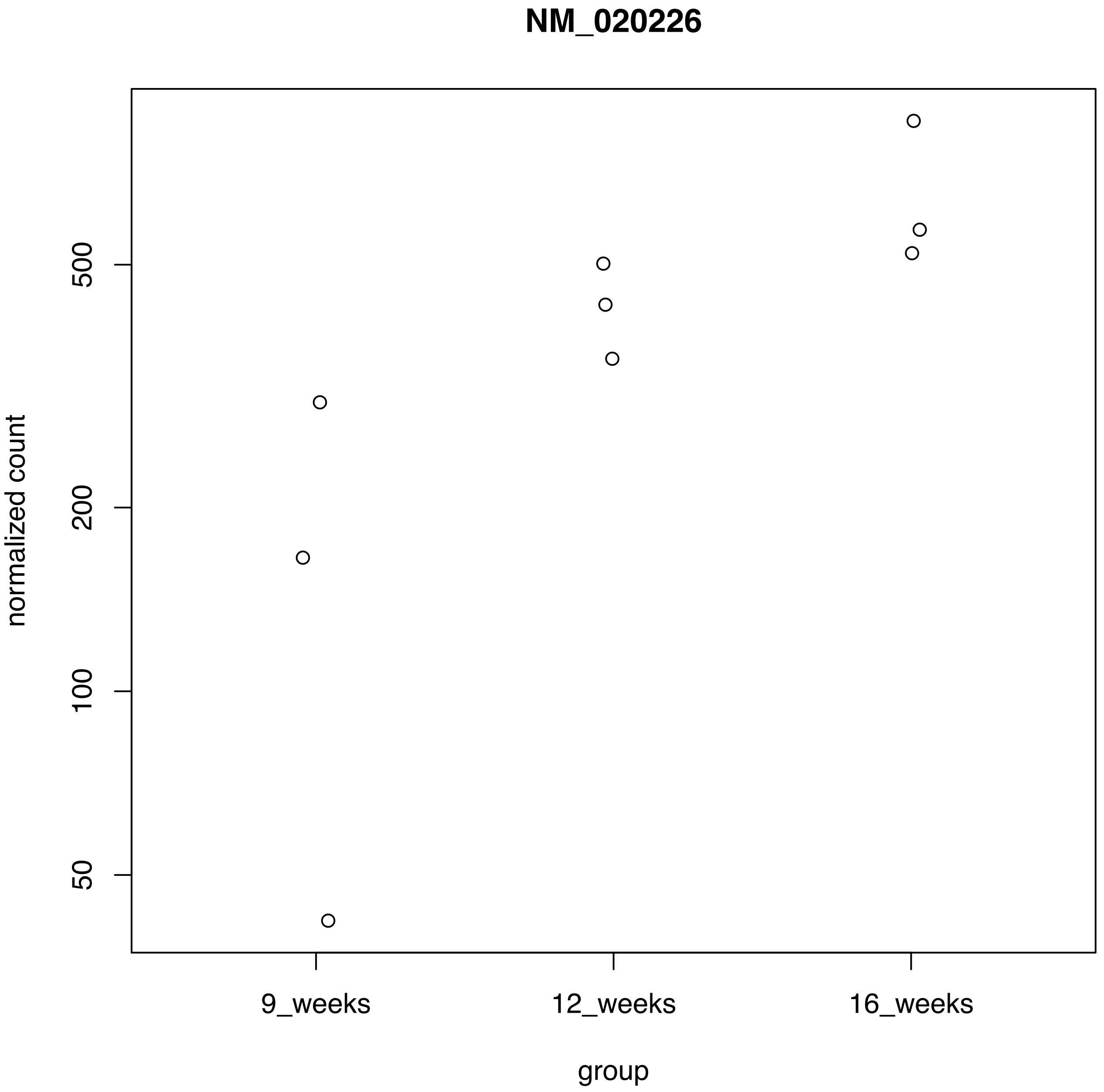
NM_020226
normalized count
500
200
100
50
9_weeks
12_weeks
16_weeks
group

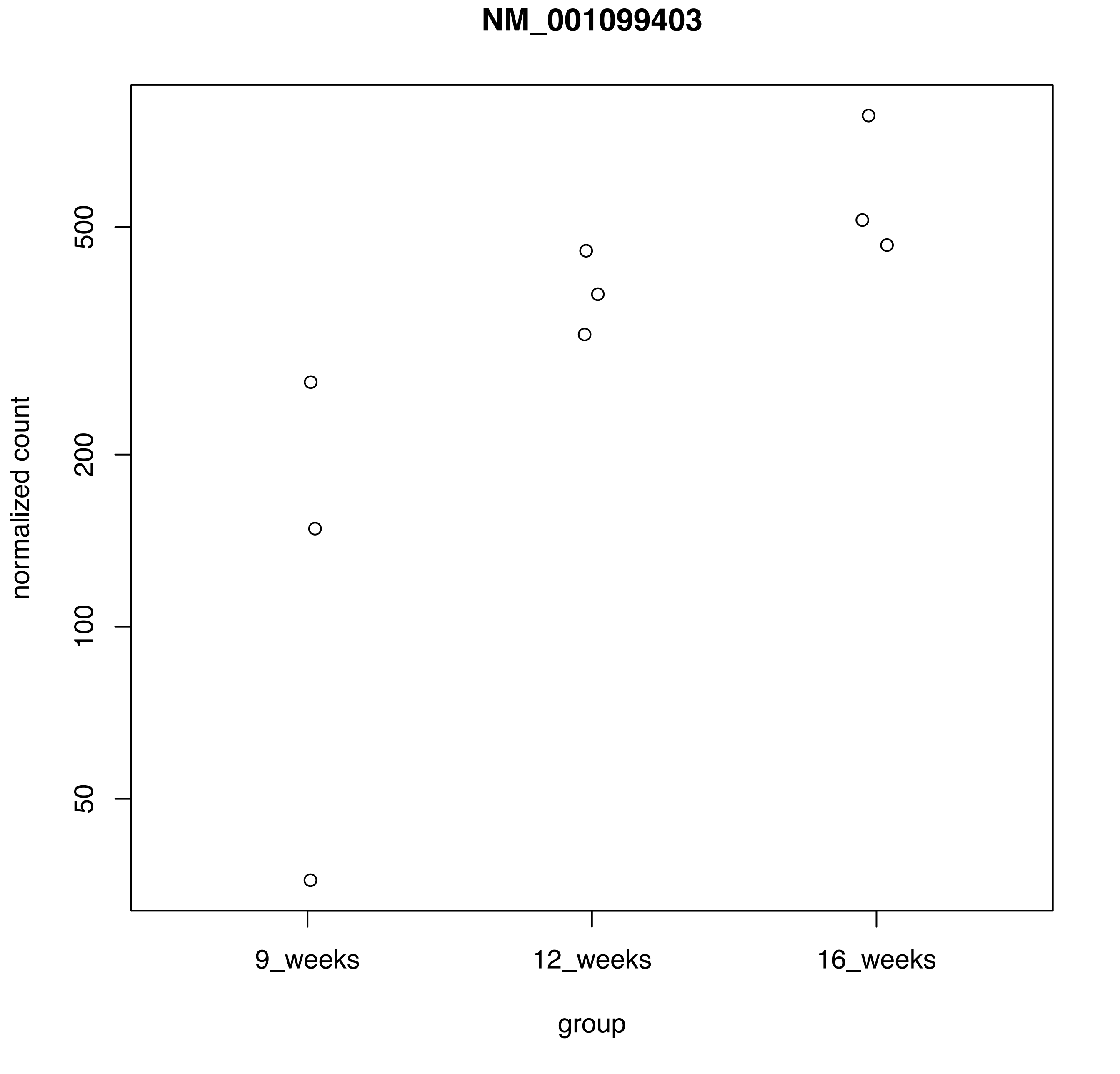
NM_001099403
normalized count
500
200
100
50
9_weeks
12_weeks
16_weeks
group

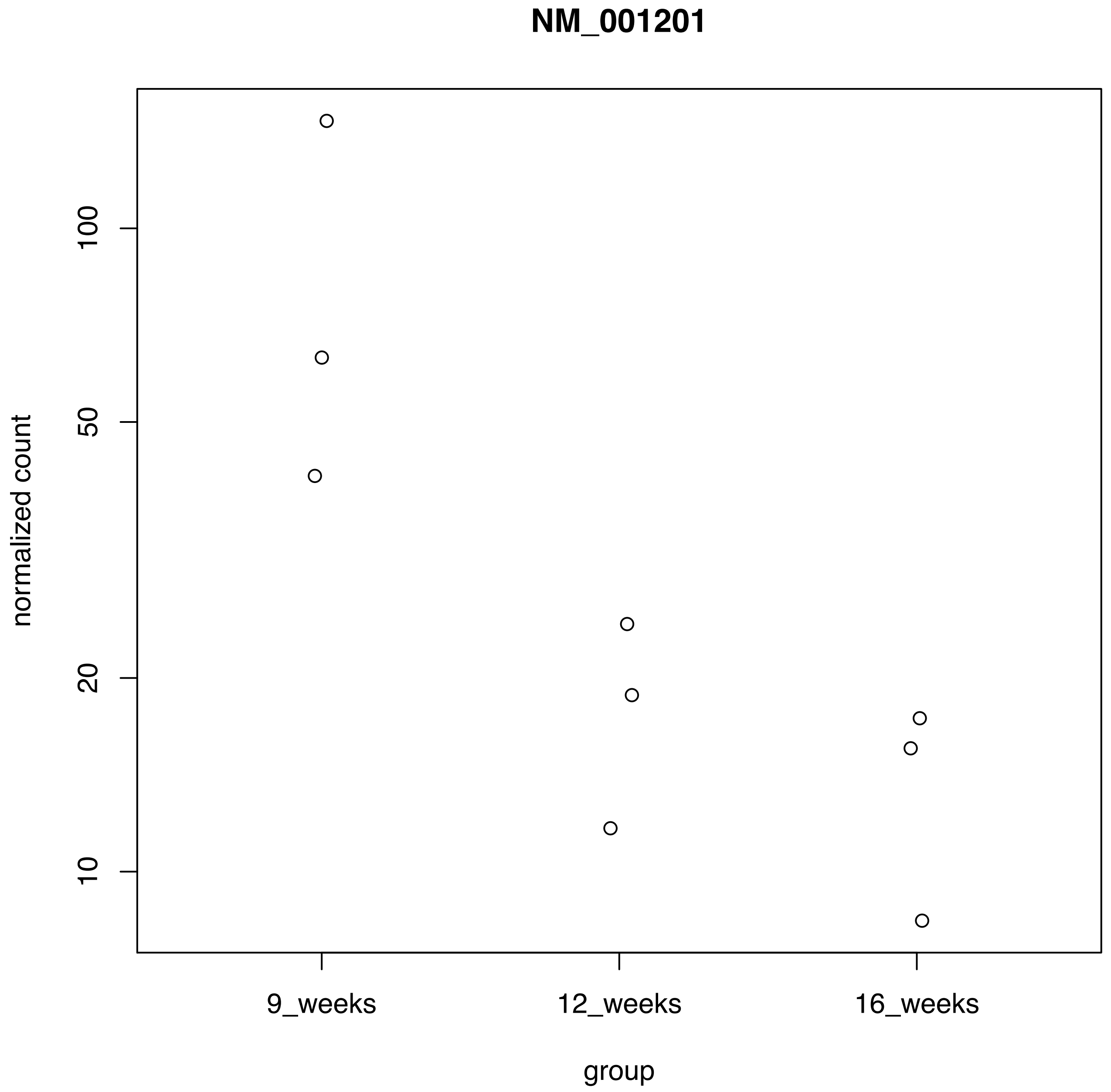
NM_001201
normalized count
100
50
20
10
9_weeks
12_weeks
16_weeks
group

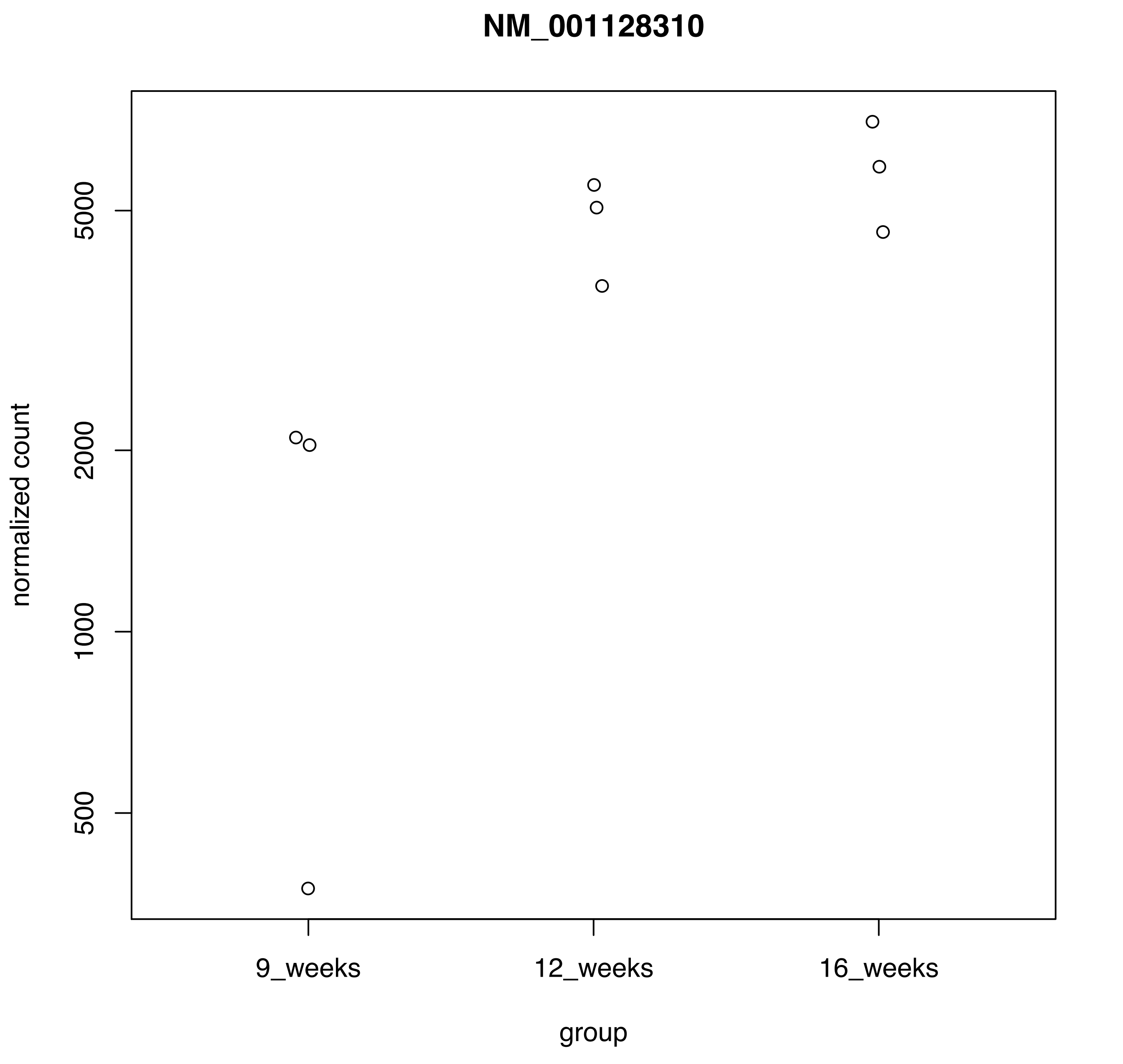
NM_001128310
normalized count
5000
2000
1000
500
9_weeks
12_weeks
16_weeks
group

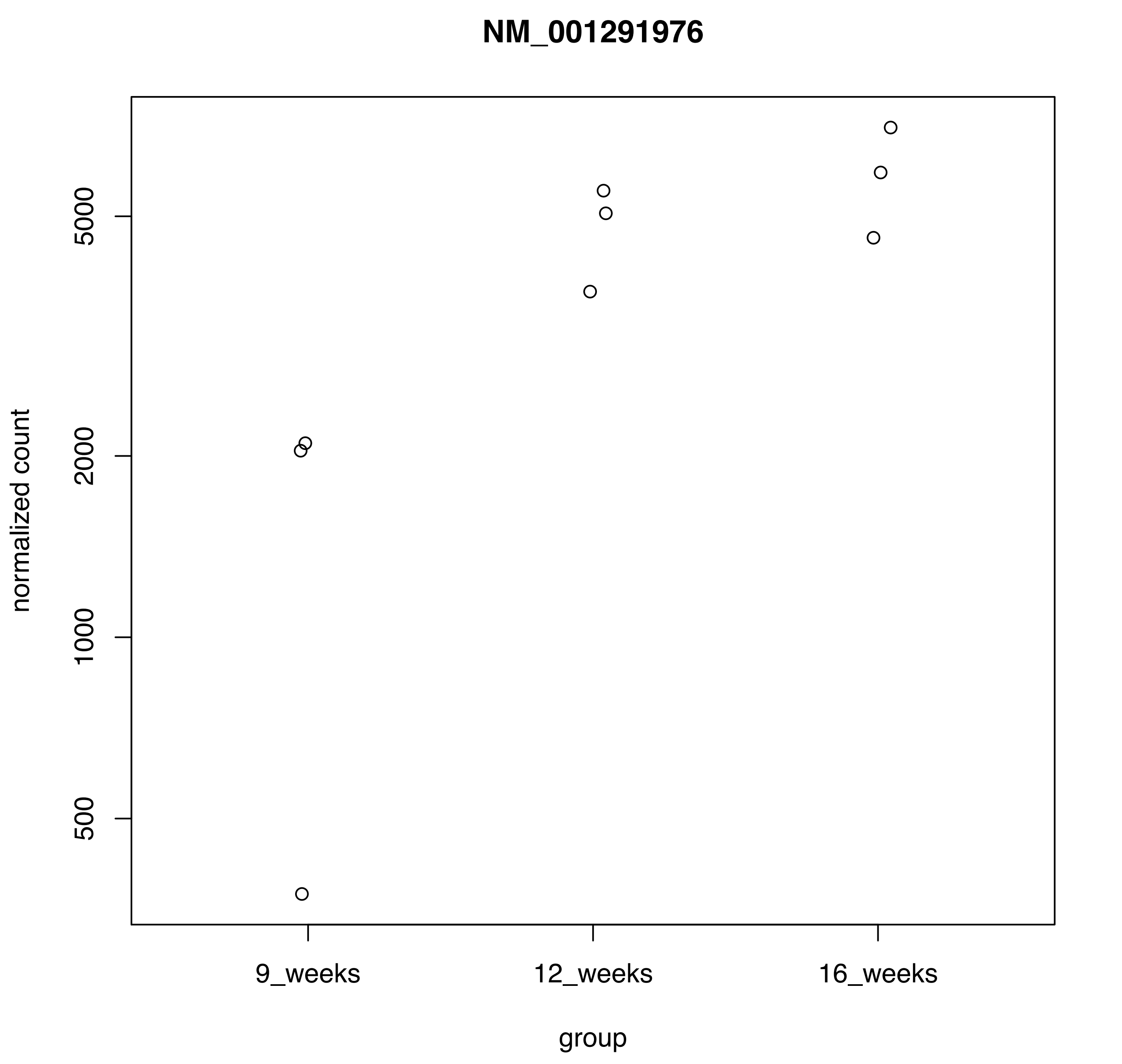

NM_001291976
normalized count
5000
2000
1000
500
9_weeks
12_weeks
16_weeks
group

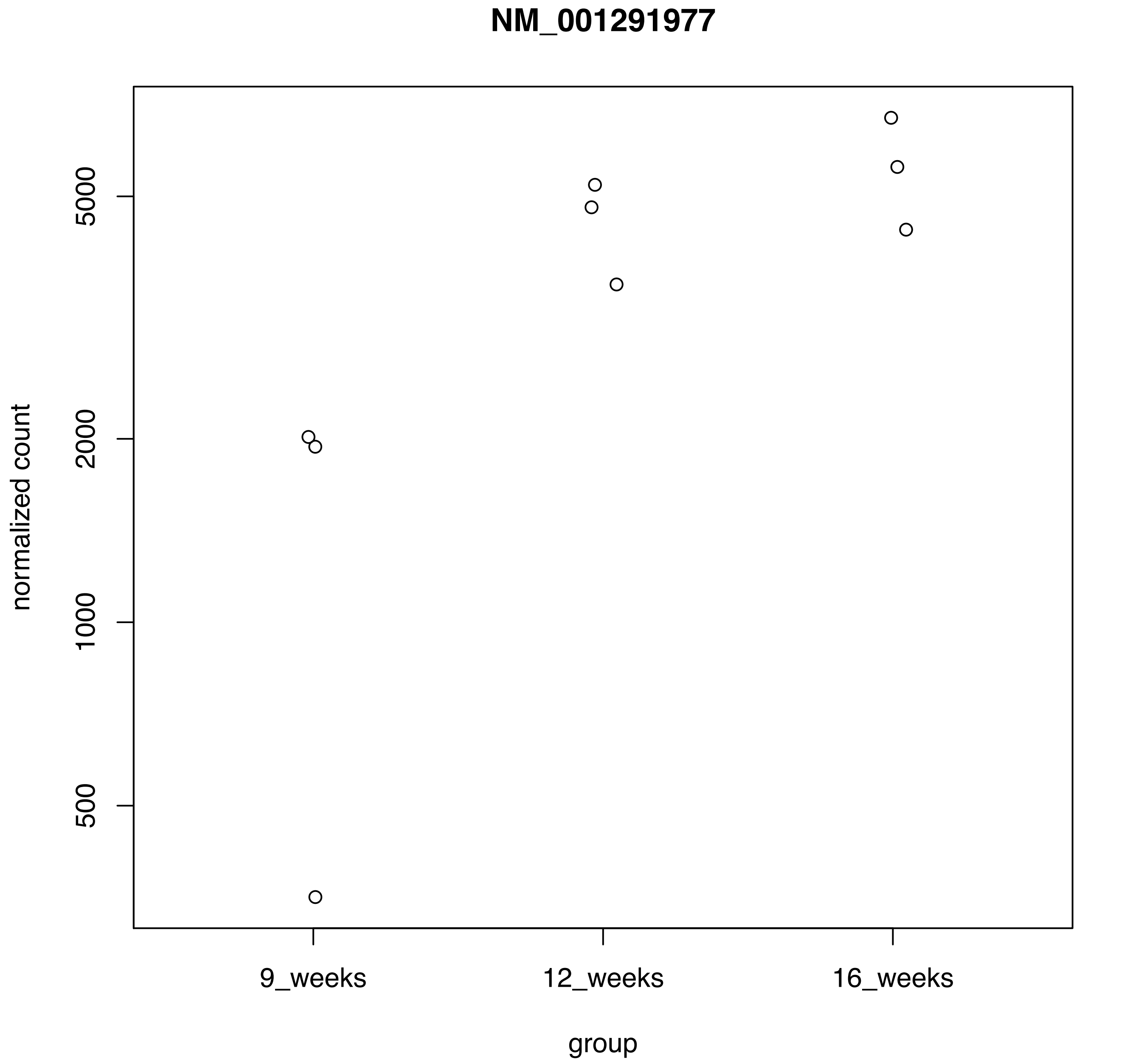

NM_001291977
normalized count
5000
2000
1000
500
9_weeks
12_weeks
16_weeks
group

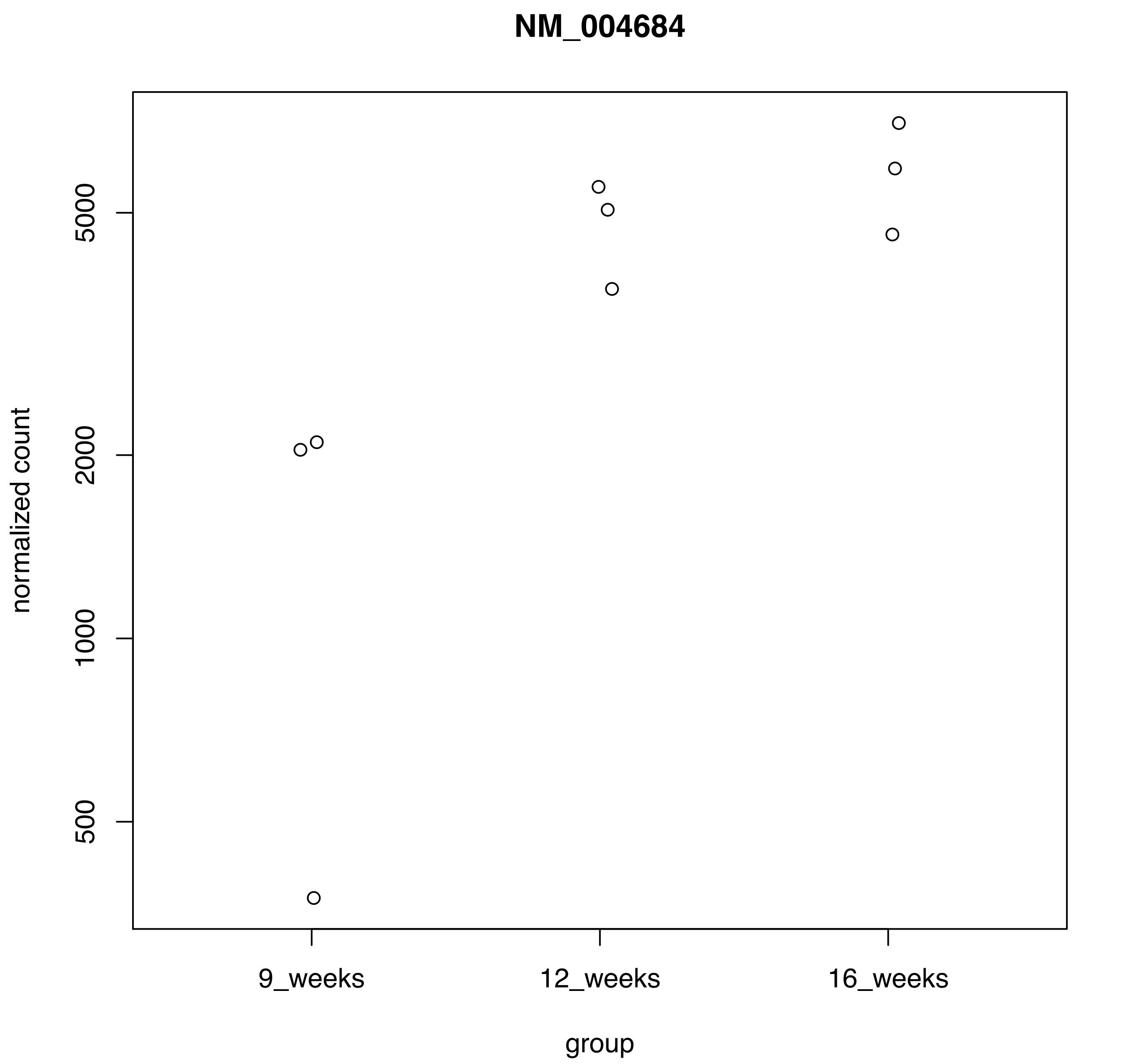
NM_004684
normalized count
5000
2000
1000
500
9_weeks
12_weeks
16_weeks
group

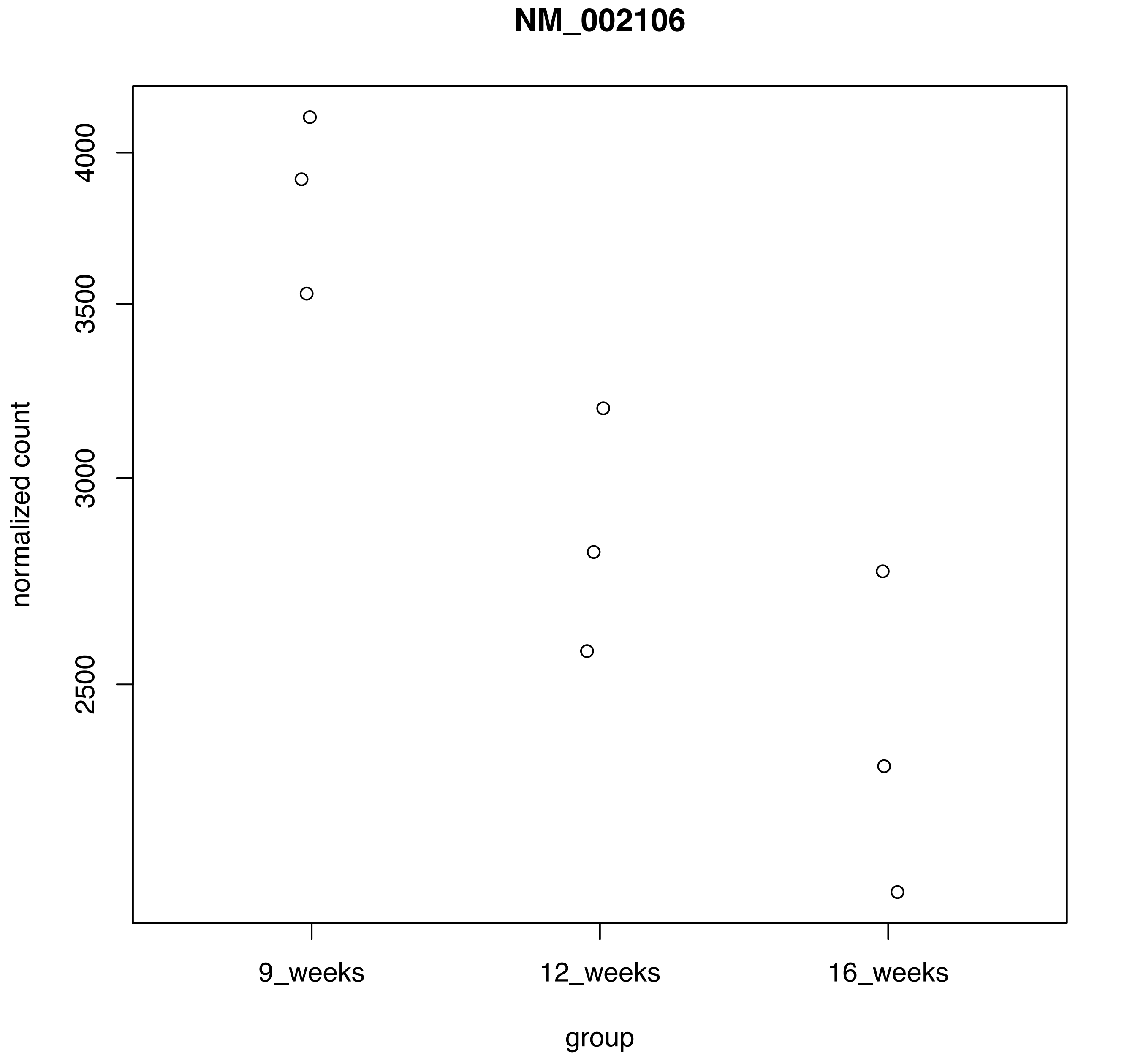

NM_002106
normalized count
4000
3500
3000
2500
9_weeks
12_weeks
16_weeks
group

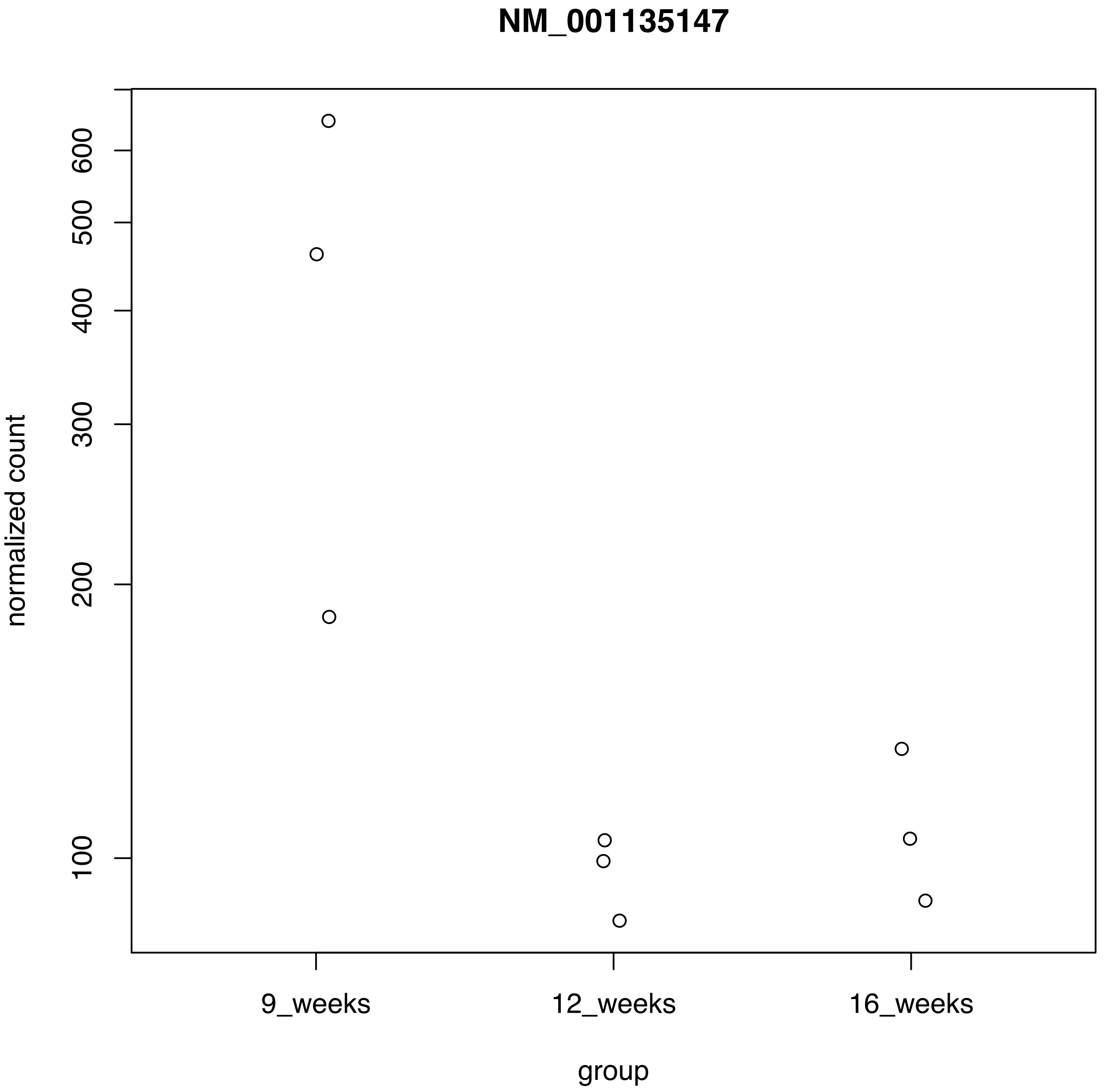

NM_001135147
normalized count
100
200
300
400
500
600
9_weeks
12_weeks
16_weeks
group

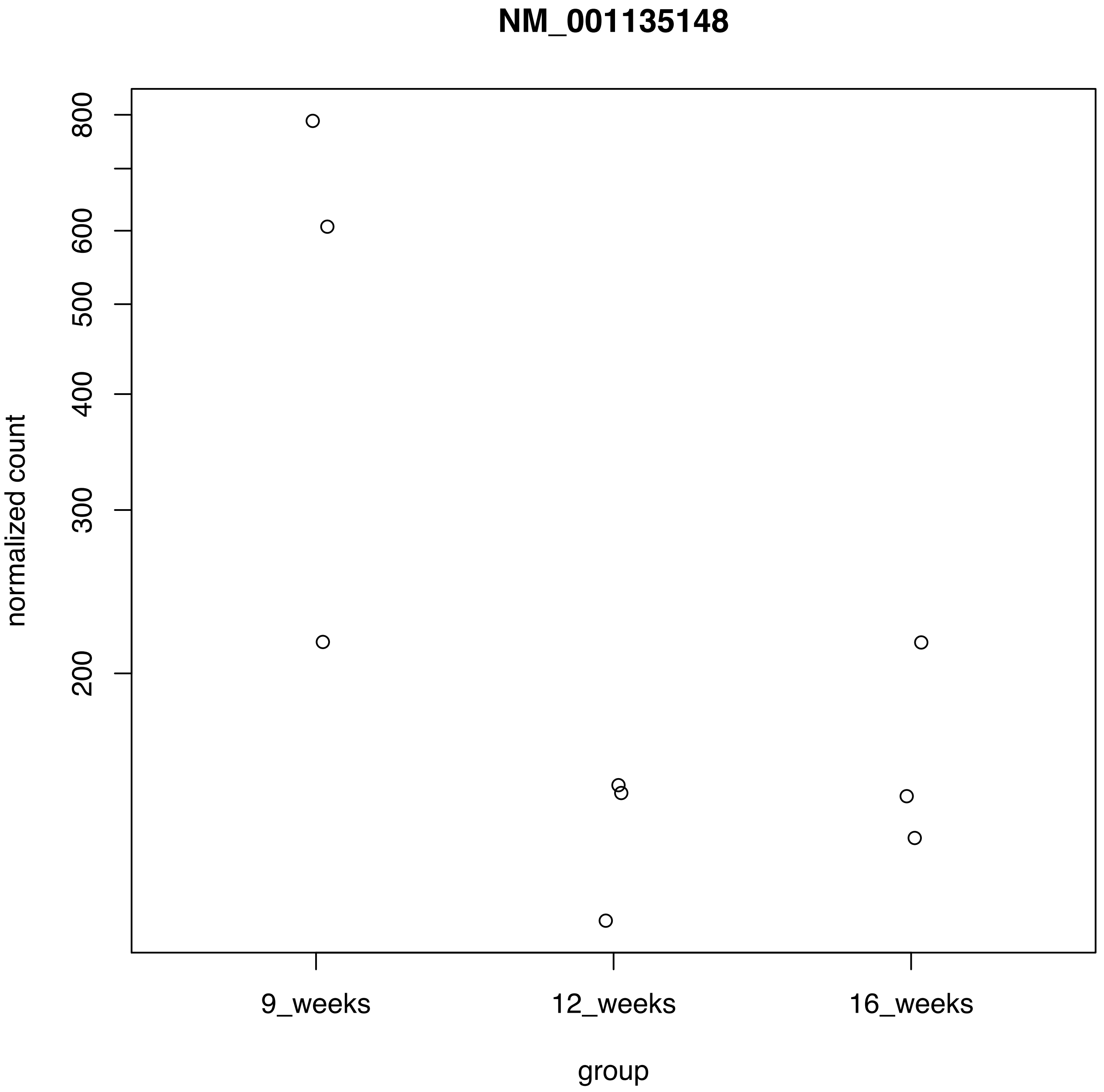
NM_001135148
normalized count
800
600
500
400
300
200
9_weeks
12_weeks
16_weeks
group

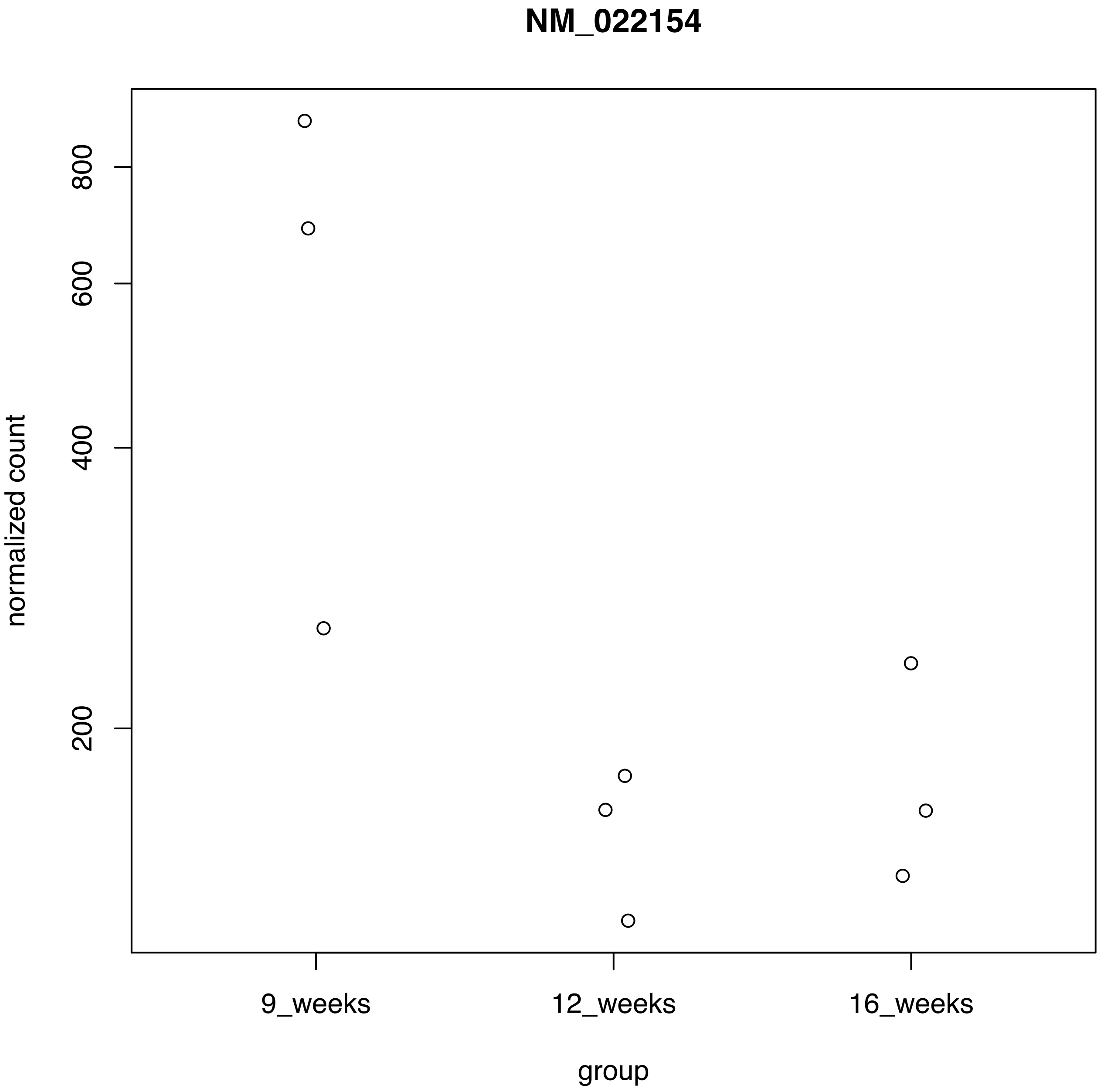

NM_022154
800
600
400
200
normalized count
9_weeks
12_weeks
16_weeks
group

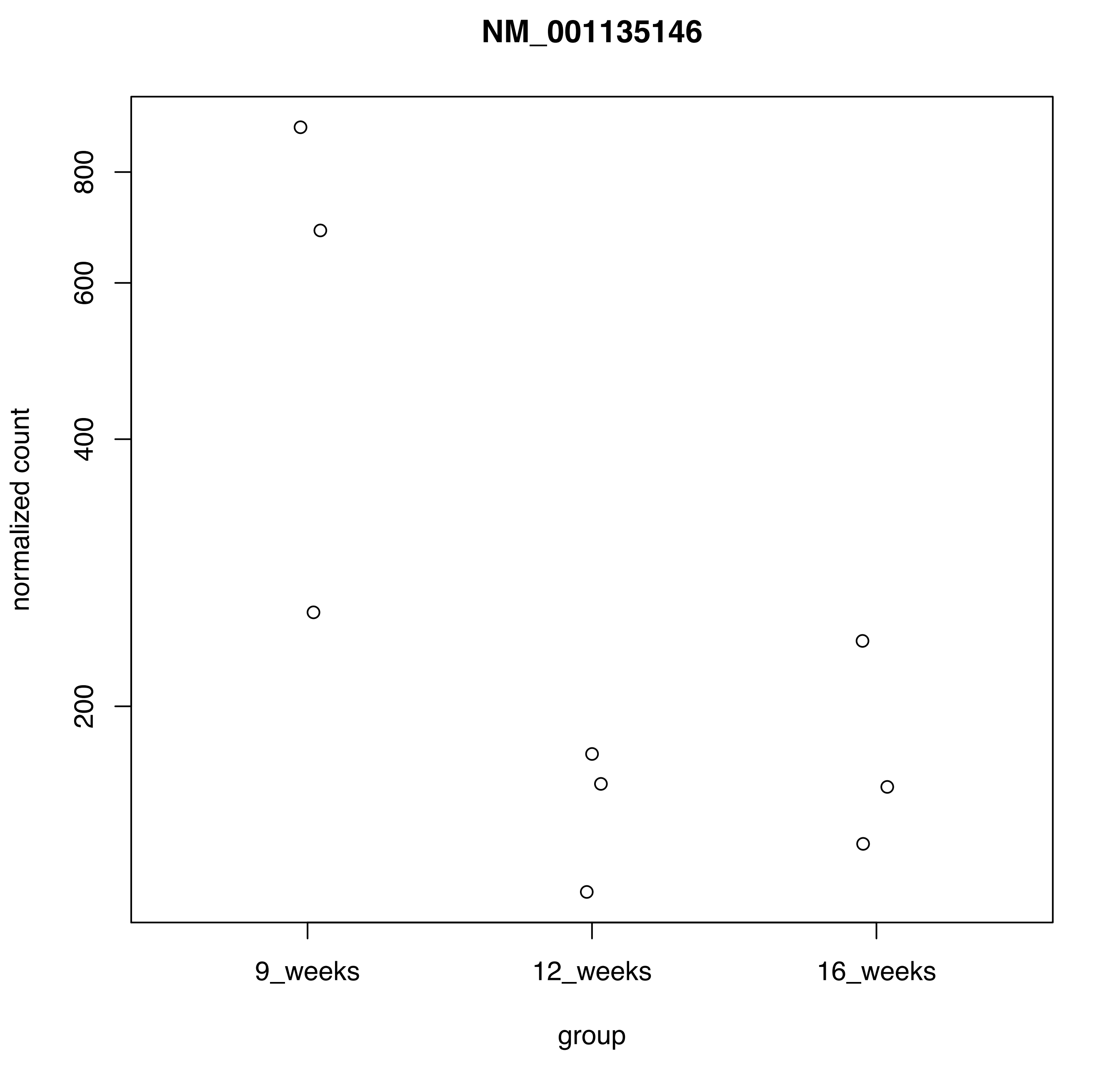
NM_001135146
normalized count
800
600
400
200
9_weeks
12_weeks
16_weeks
group

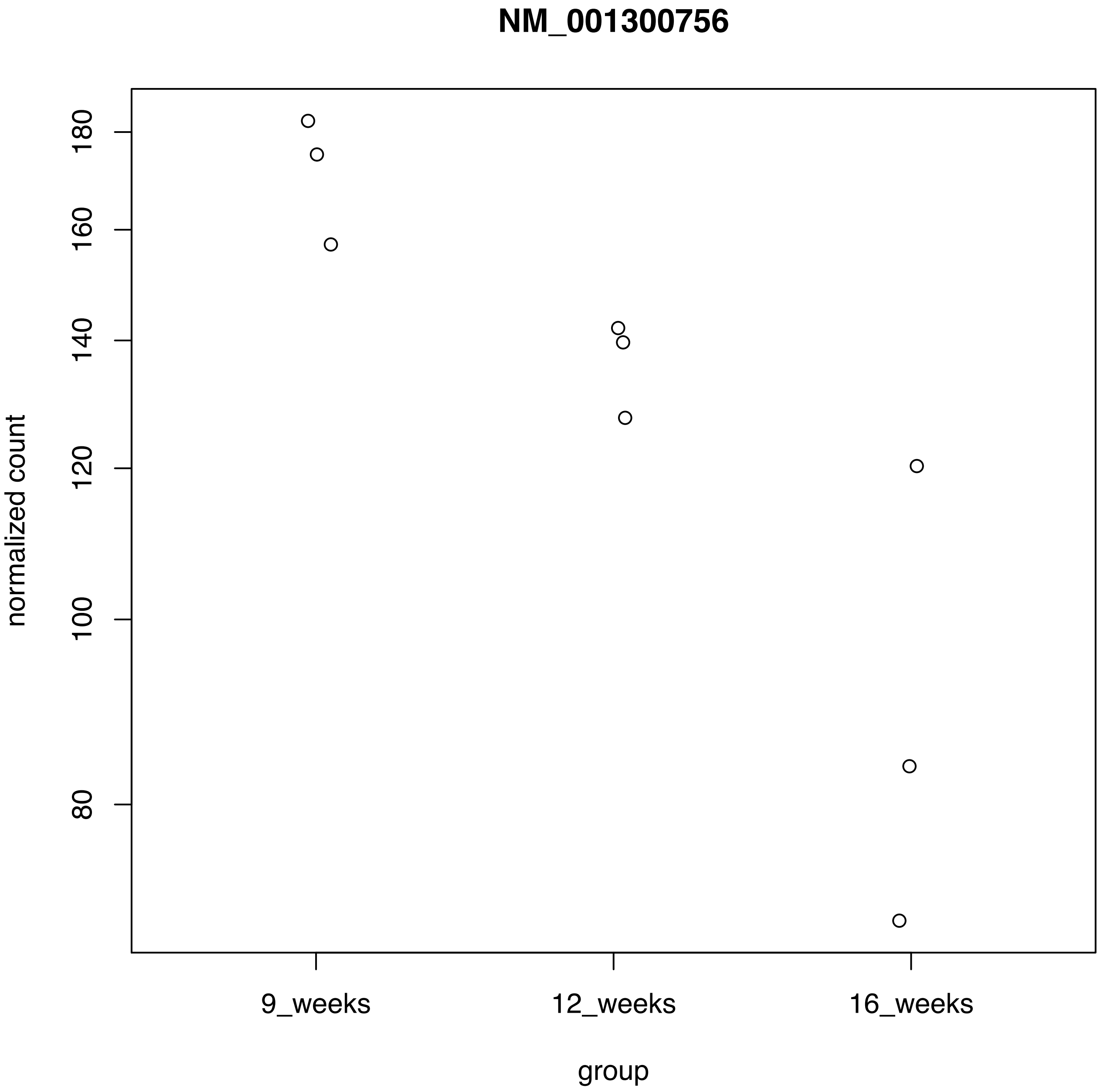
NM_001300756
normalized count
80
100
120
140
160
180
9_weeks
12_weeks
16_weeks
group

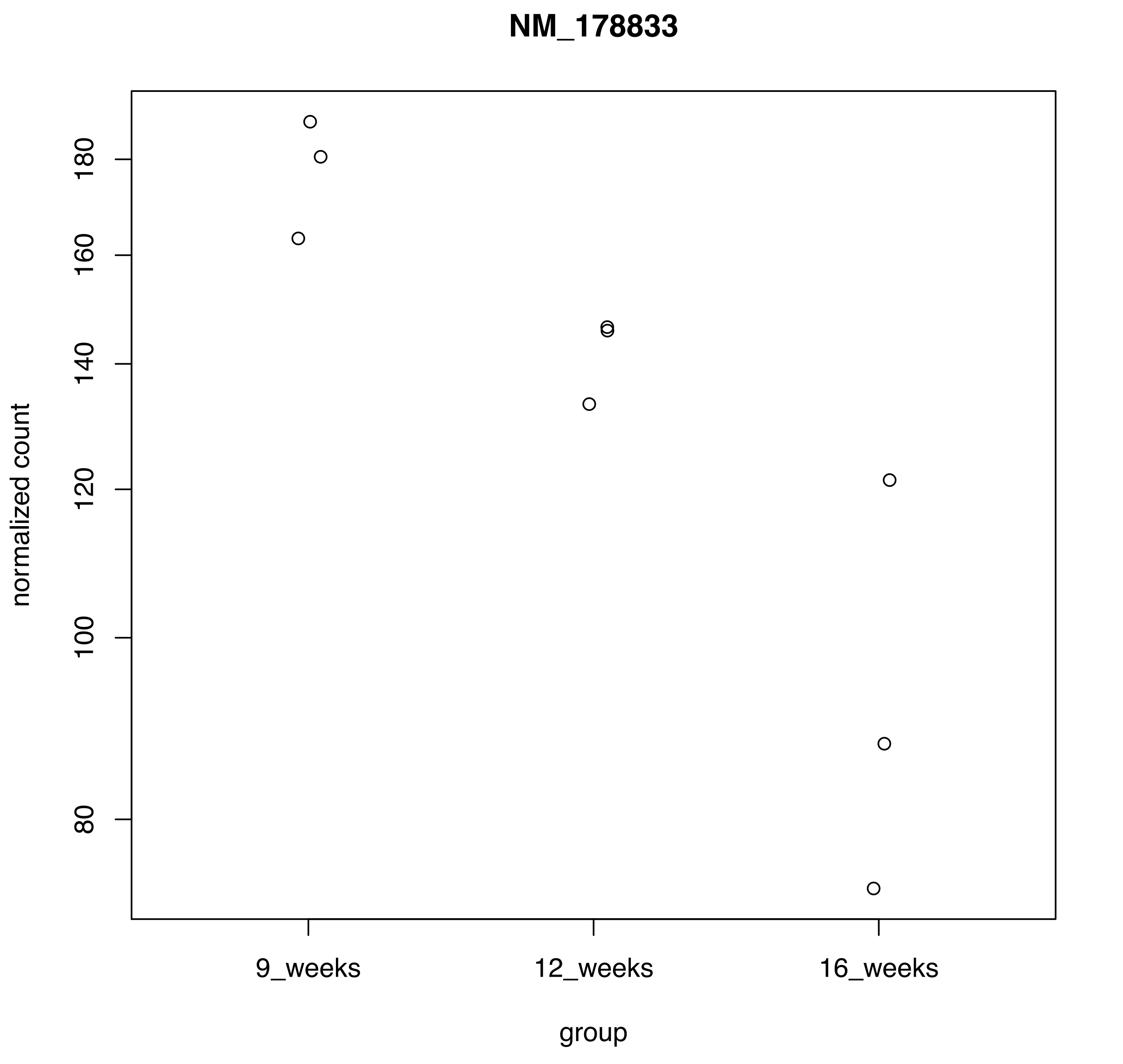

NM_178833
normalized count
80
100
120
140
160
180
9_weeks
12_weeks
16_weeks
group

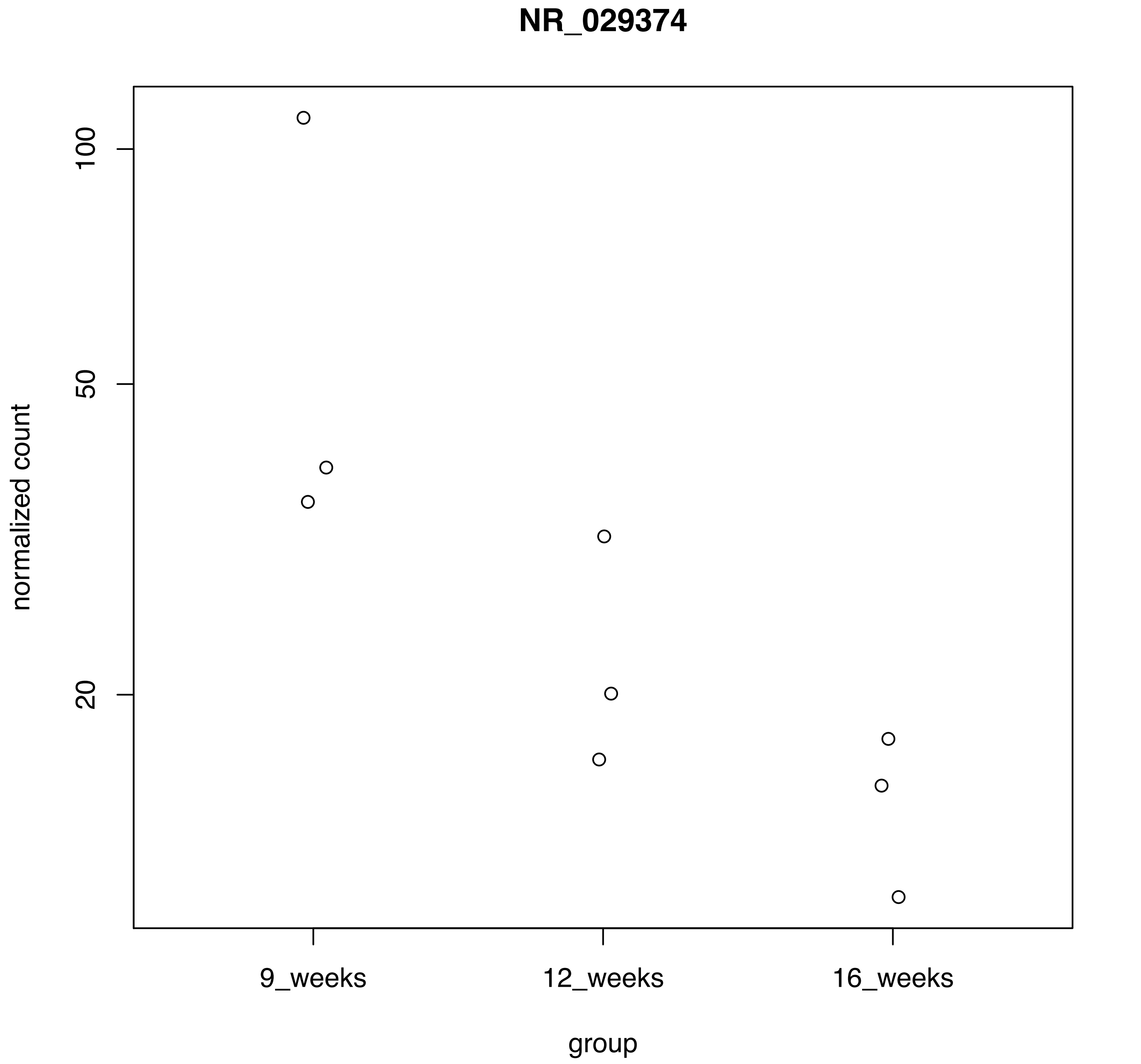
NR_029374
normalized count
100
50
20
9_weeks
12_weeks
16_weeks
group

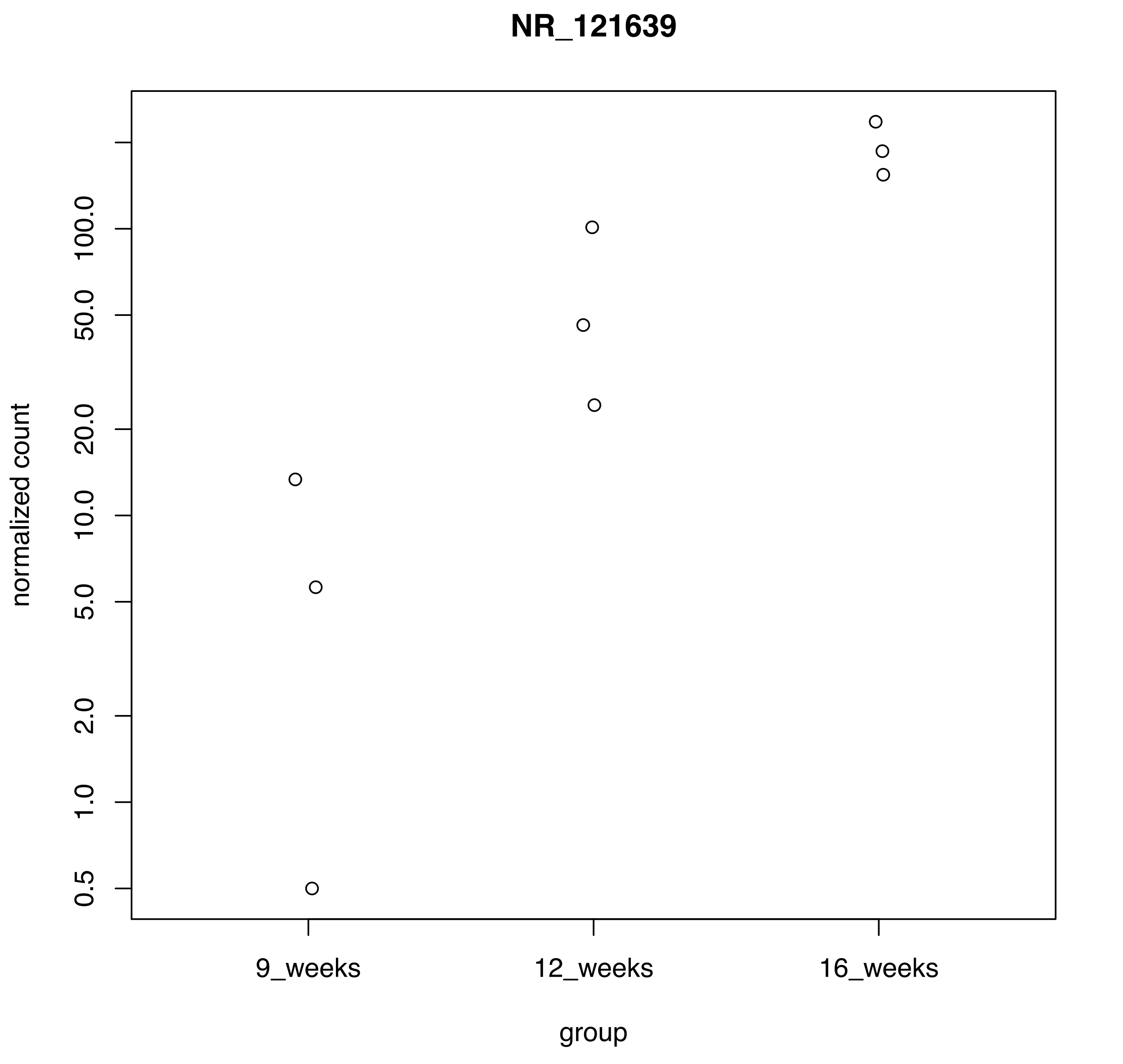
NR_121639
normalized count
0.5
1.0
2.0
5.0
10.0
20.0
50.0
100.0
9_weeks
12_weeks
16_weeks
group

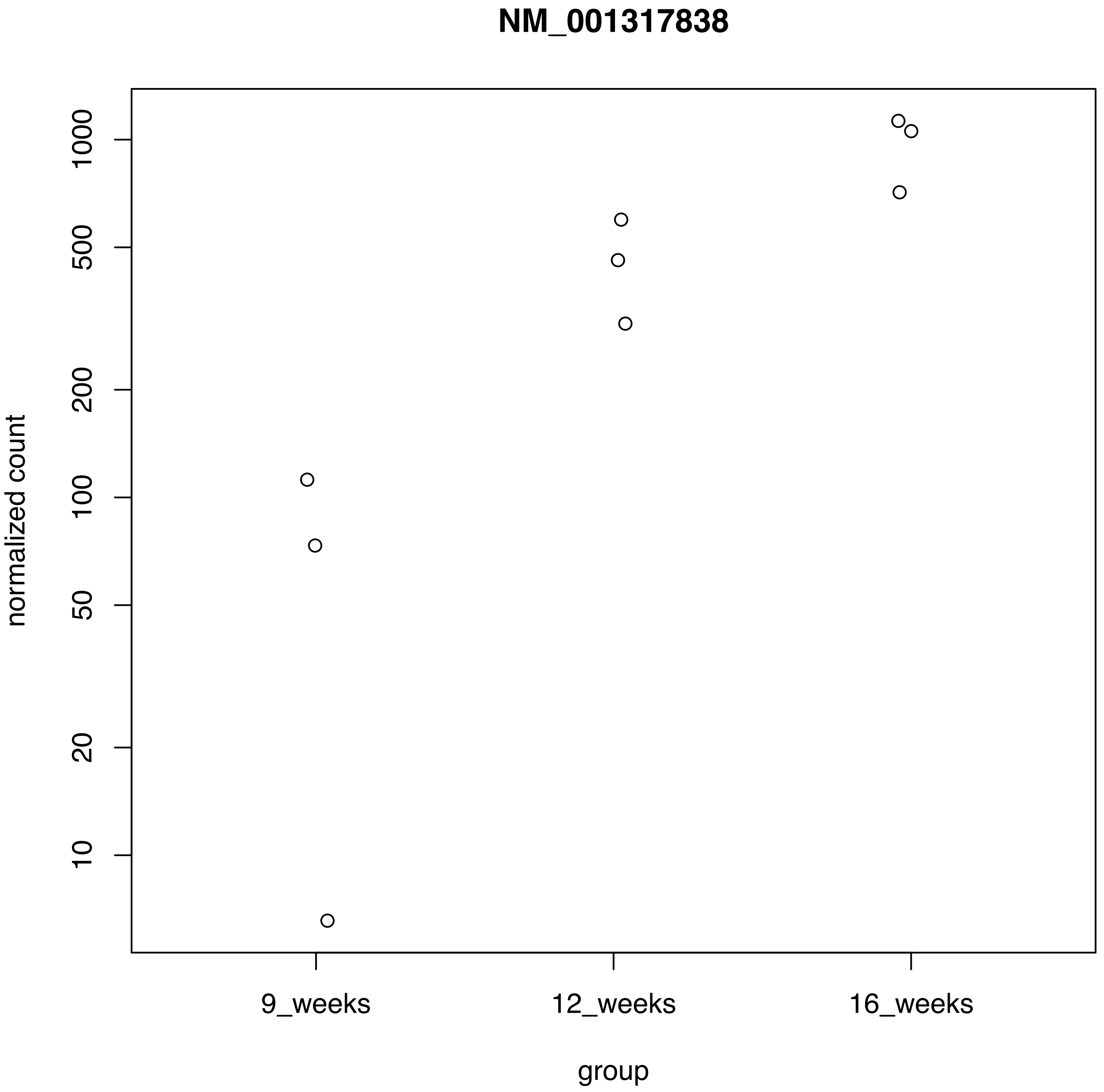
NM_001317838
normalized count
1000
500
200
100
50
20
10
9_weeks
12_weeks
16_weeks
group

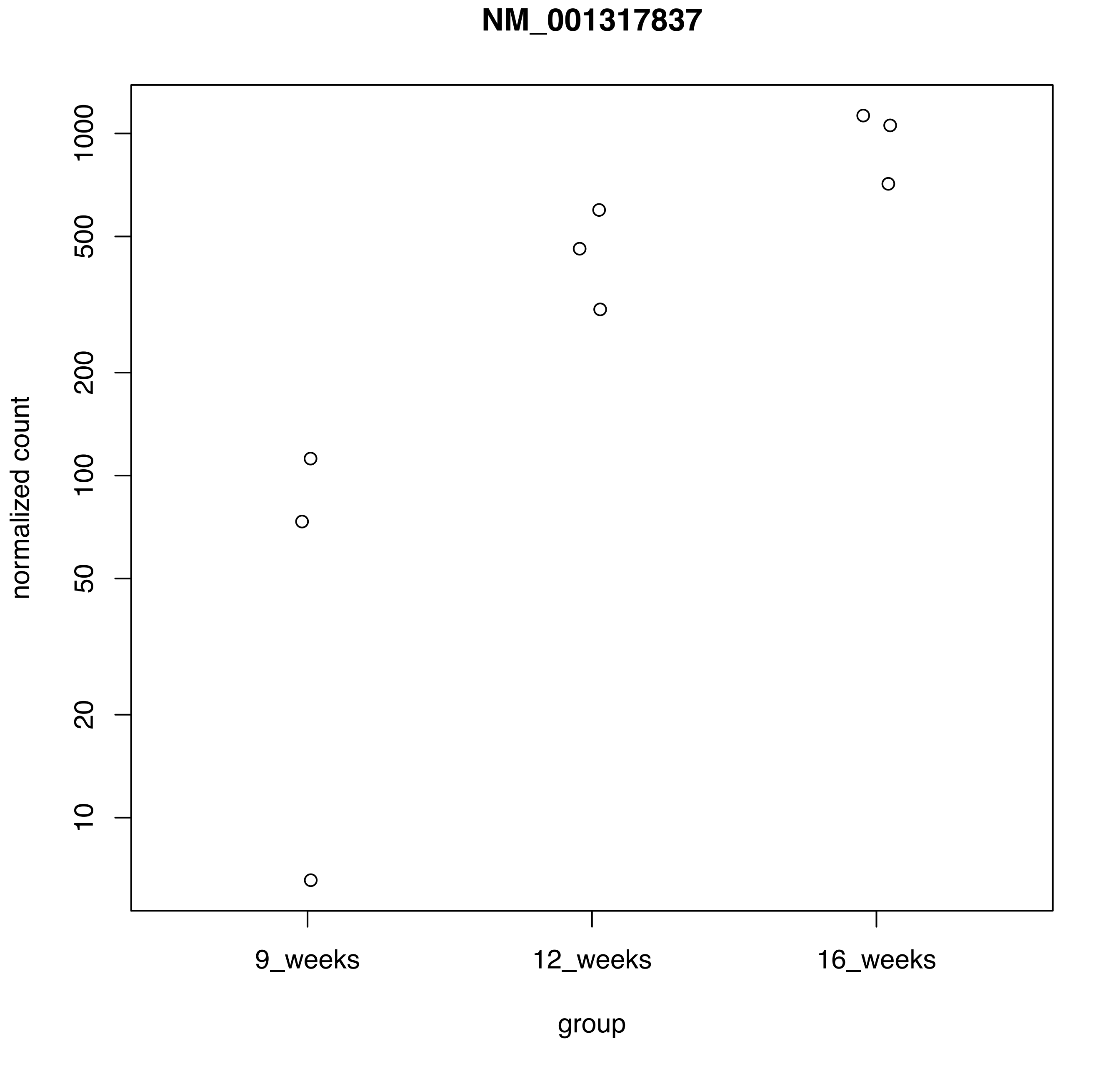
NM_001317837
normalized count
1000
500
200
100
50
20
10
9_weeks
12_weeks
16_weeks
group

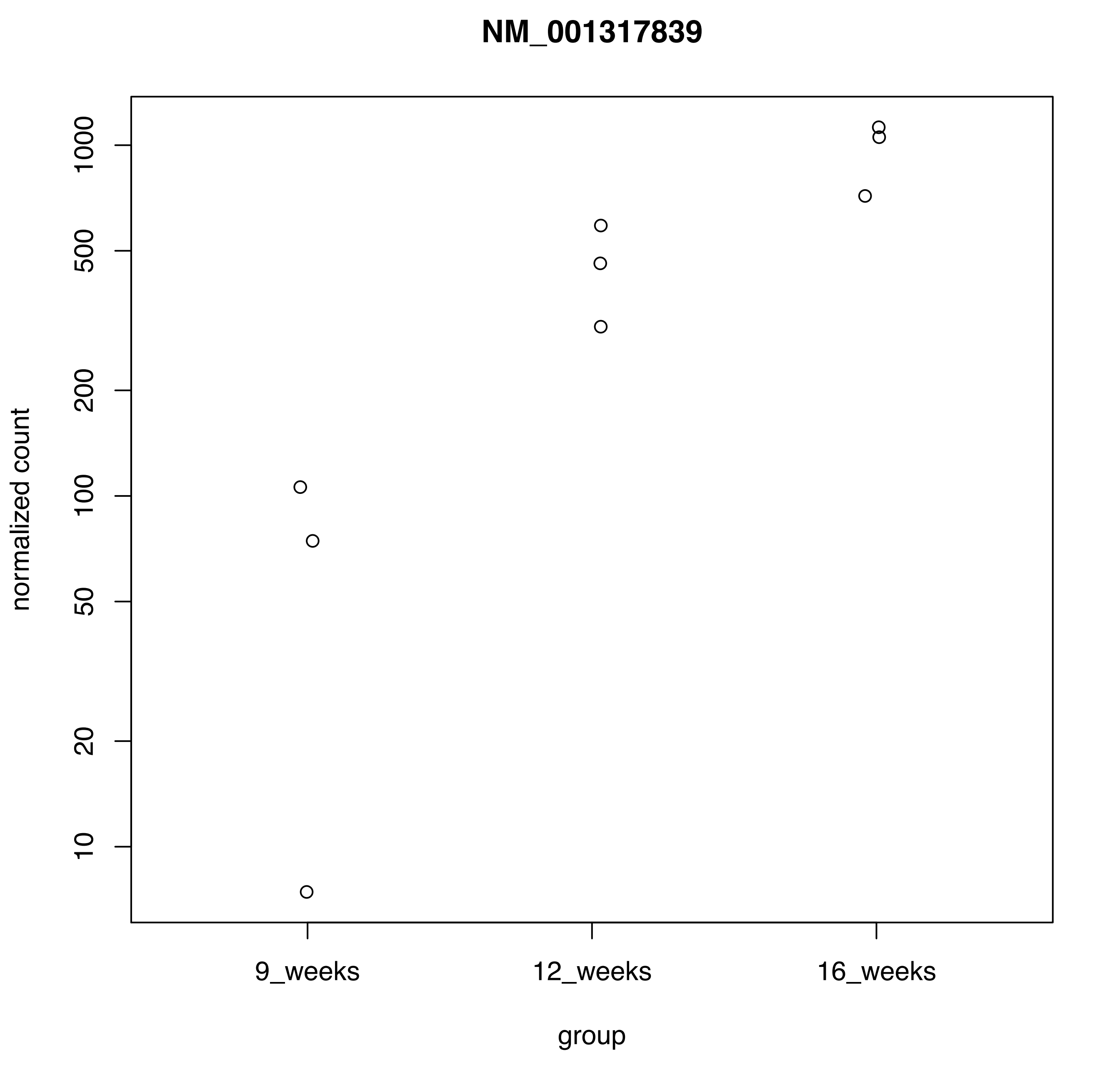

NM_001317839
normalized count
1000
500
200
100
50
20
10
9_weeks
12_weeks
16_weeks
group

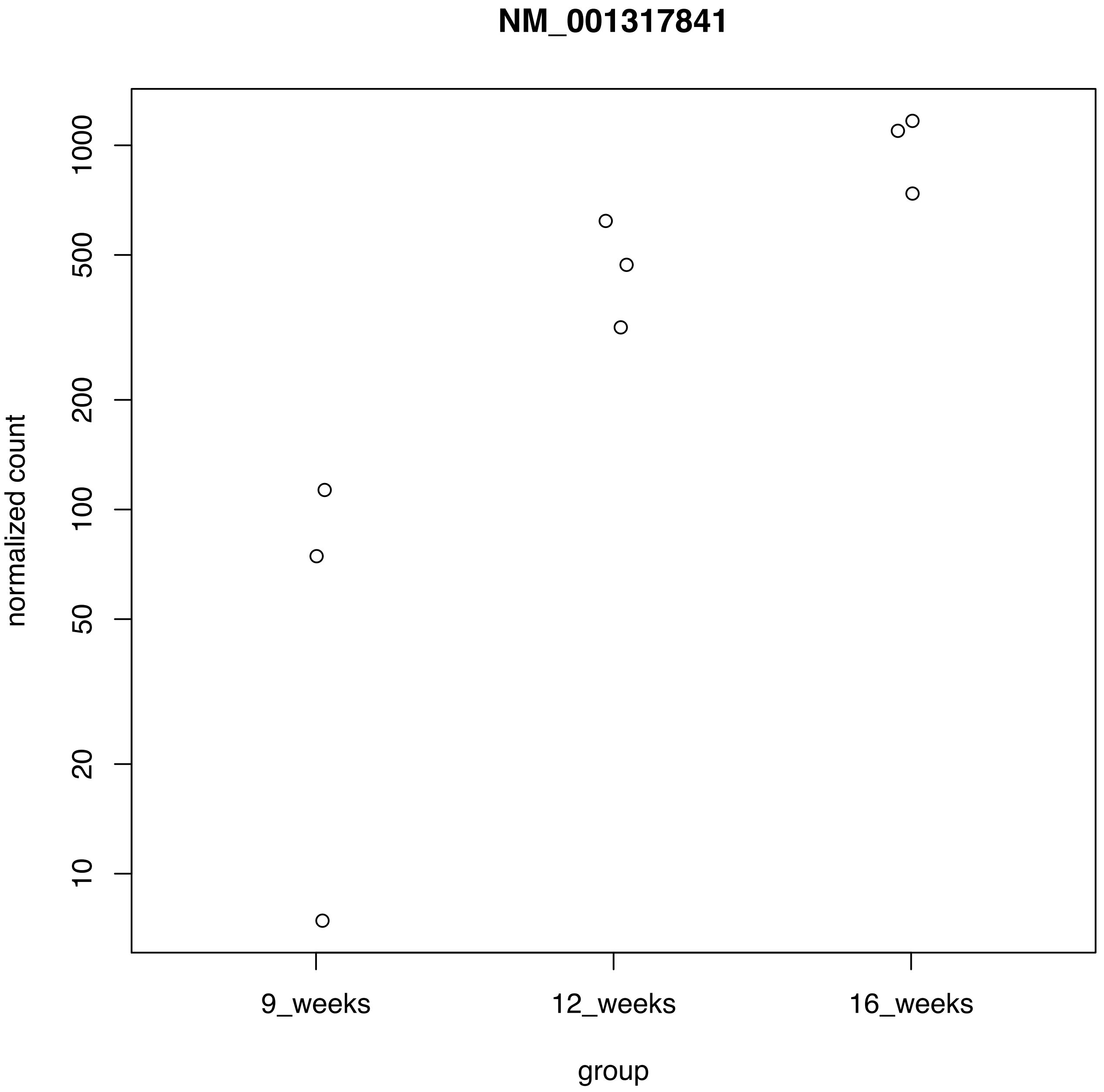
NM_001317841
normalized count
1000
500
200
100
50
20
10
9_weeks
12_weeks
16_weeks
group

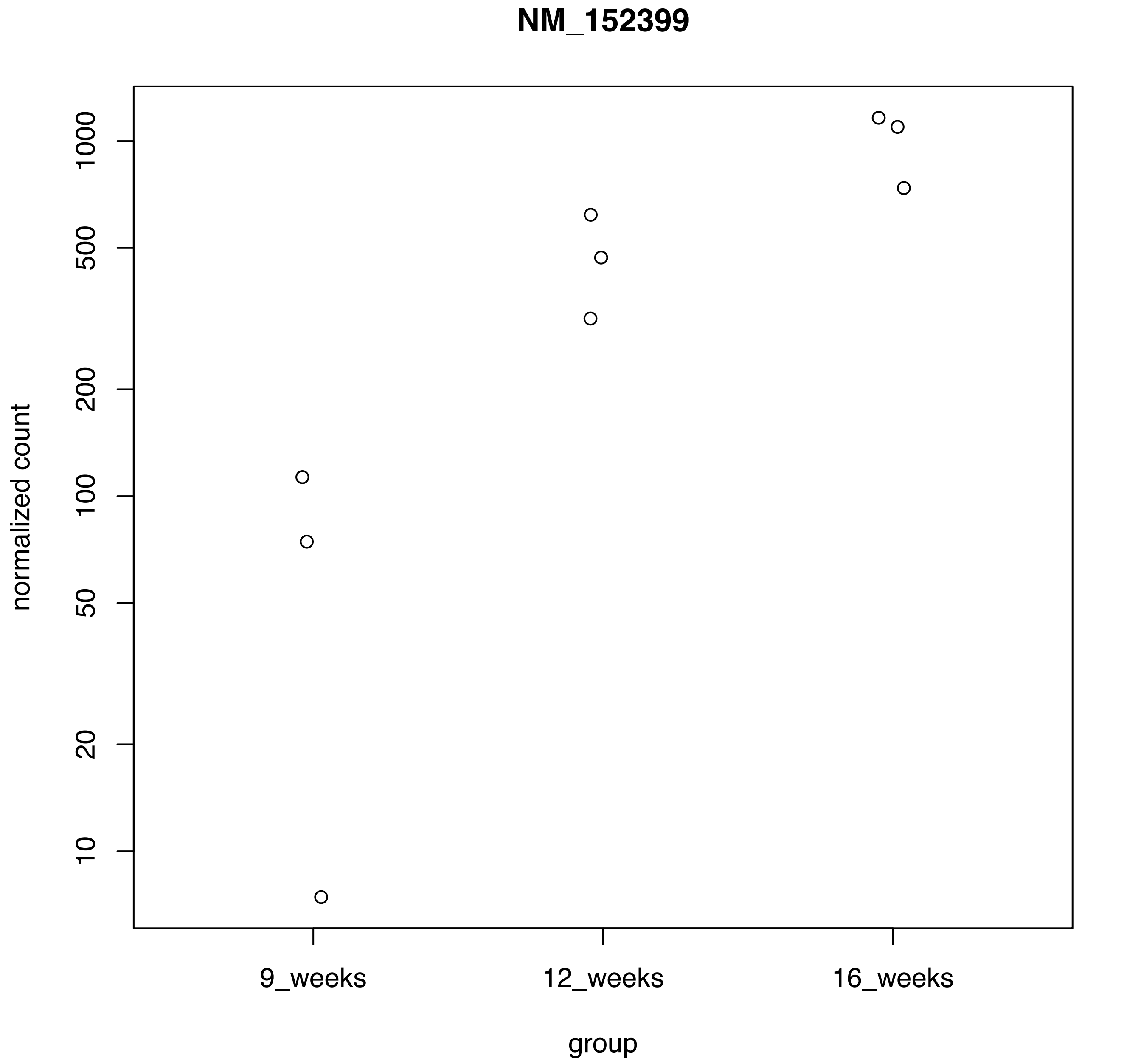
NM_152399
normalized count
1000
500
200
100
50
20
10
9_weeks
12_weeks
16_weeks
group

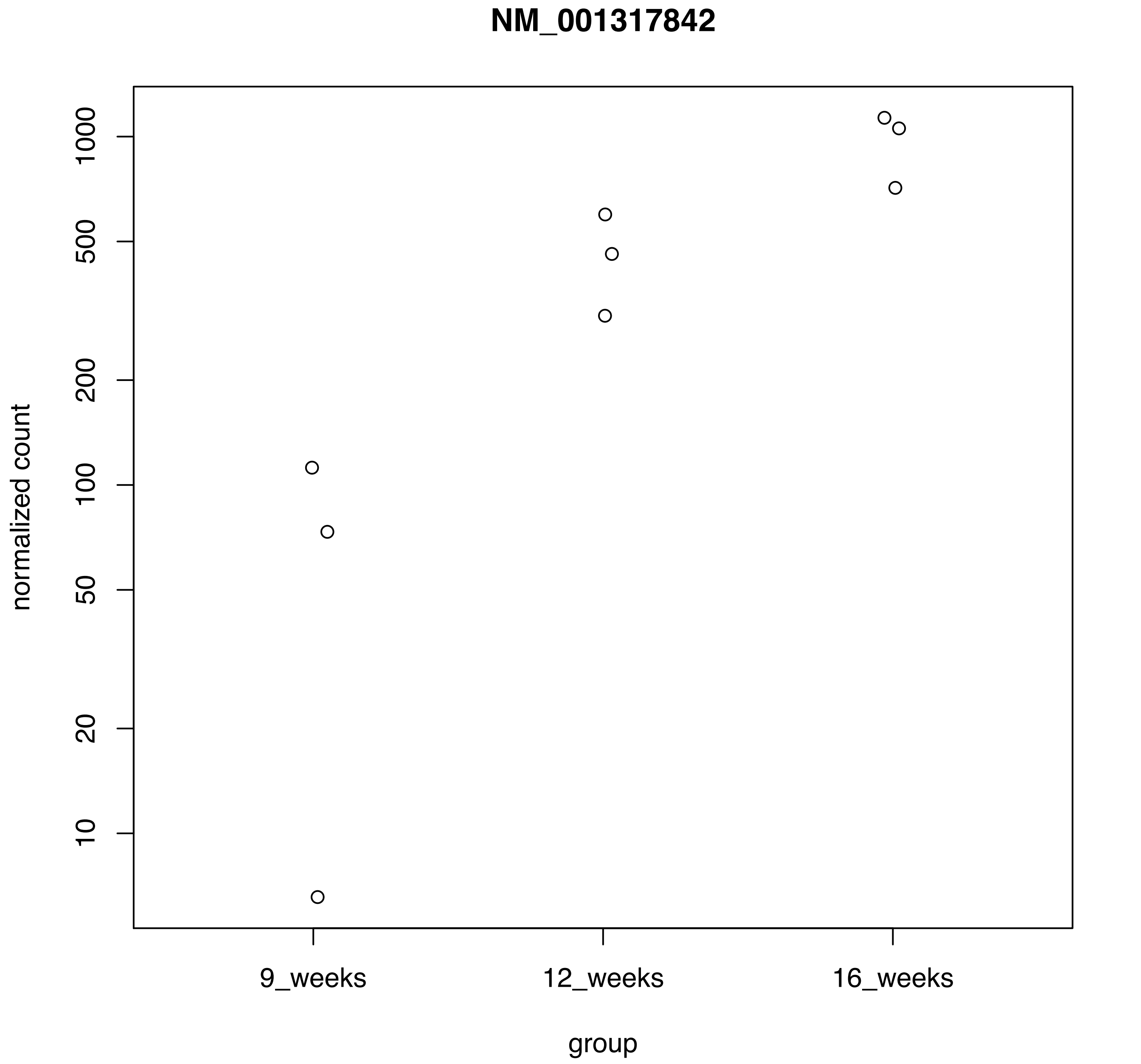

NM_001317842
normalized count
1000
500
200
100
50
20
10
9_weeks
12_weeks
16_weeks
group

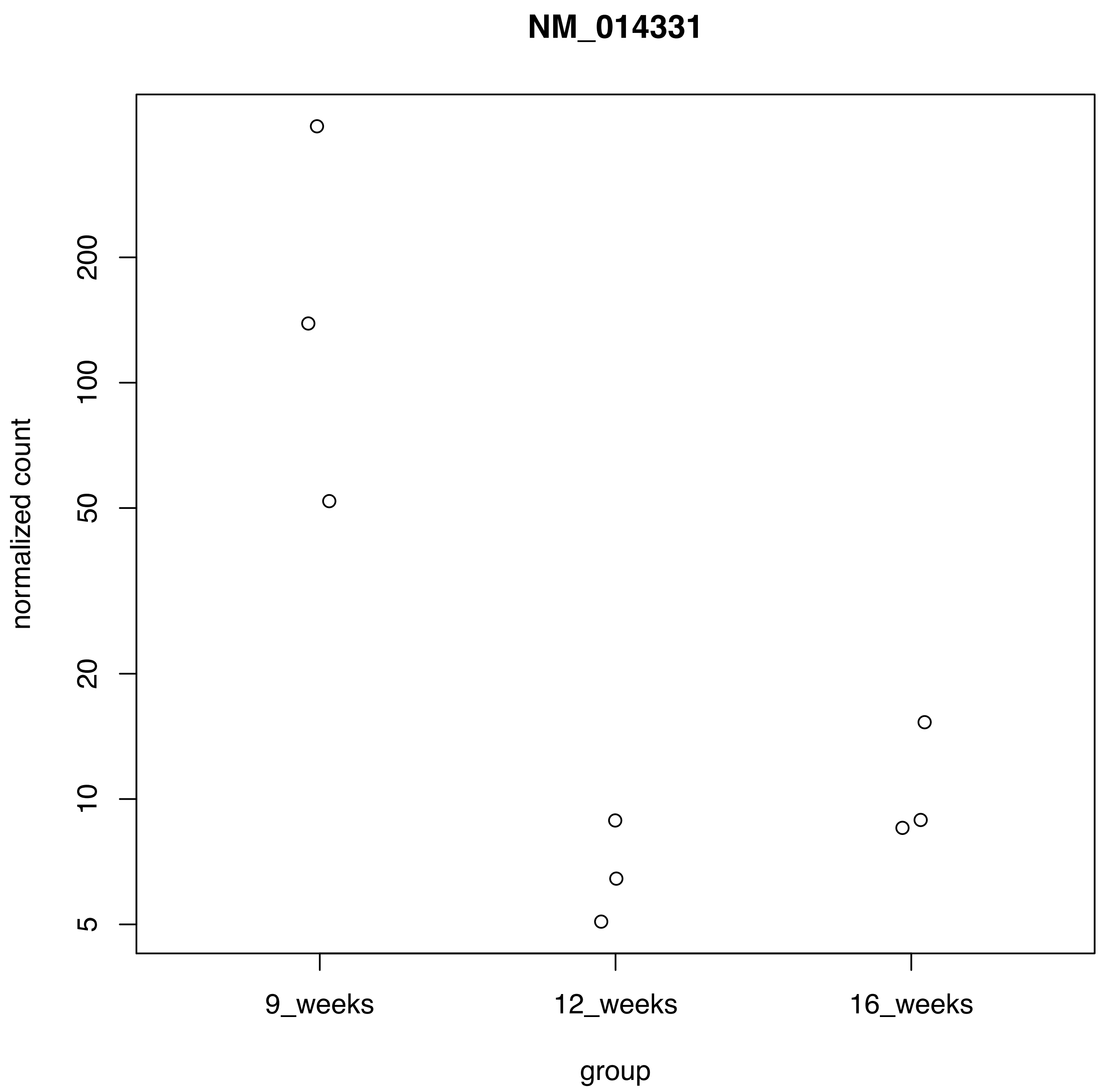

NM_014331
normalized count
200
100
50
20
10
5
9_weeks
12_weeks
16_weeks
group

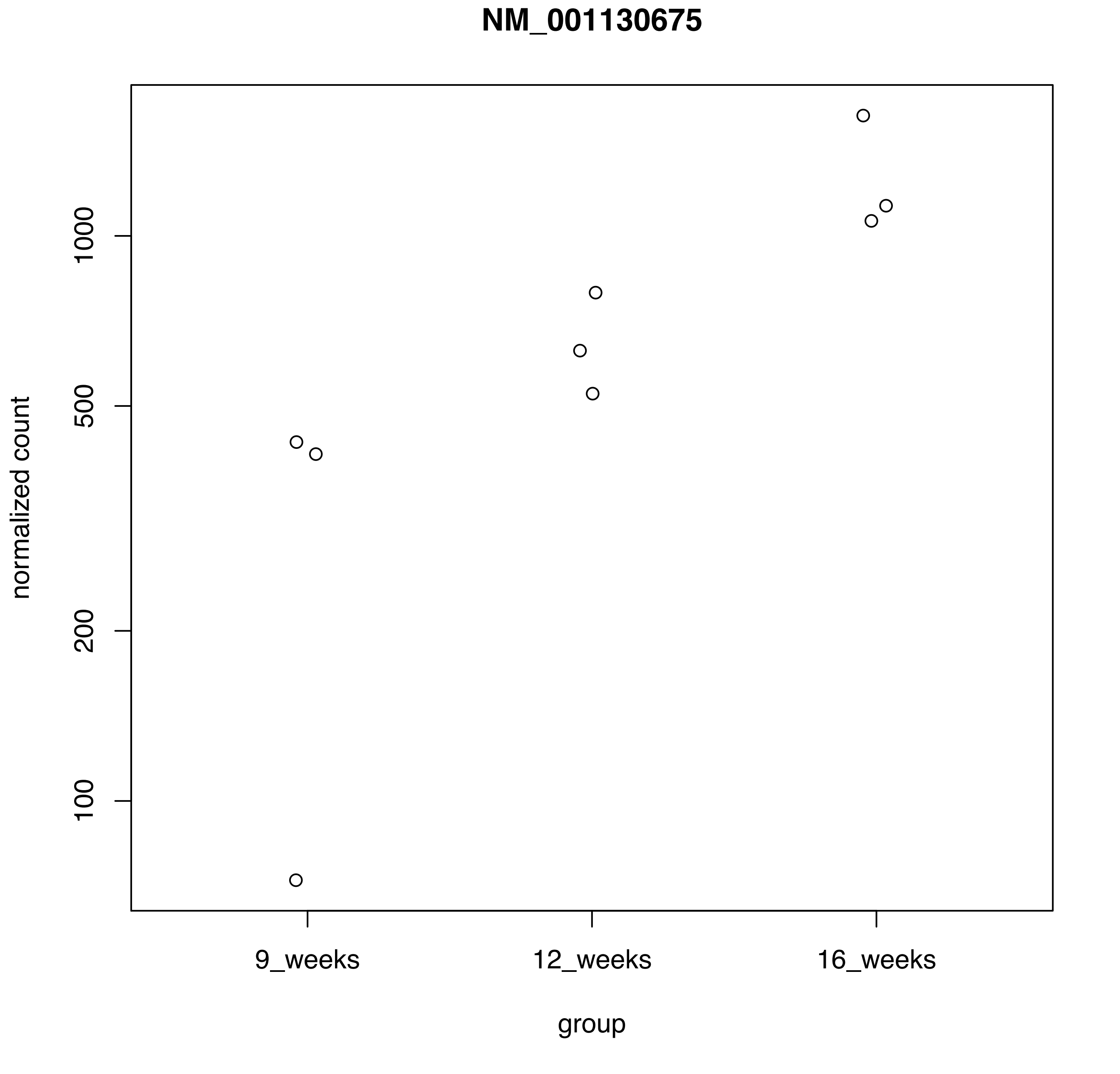
NM_001130675
normalized count
1000
500
200
100
9_weeks
12_weeks
16_weeks
group

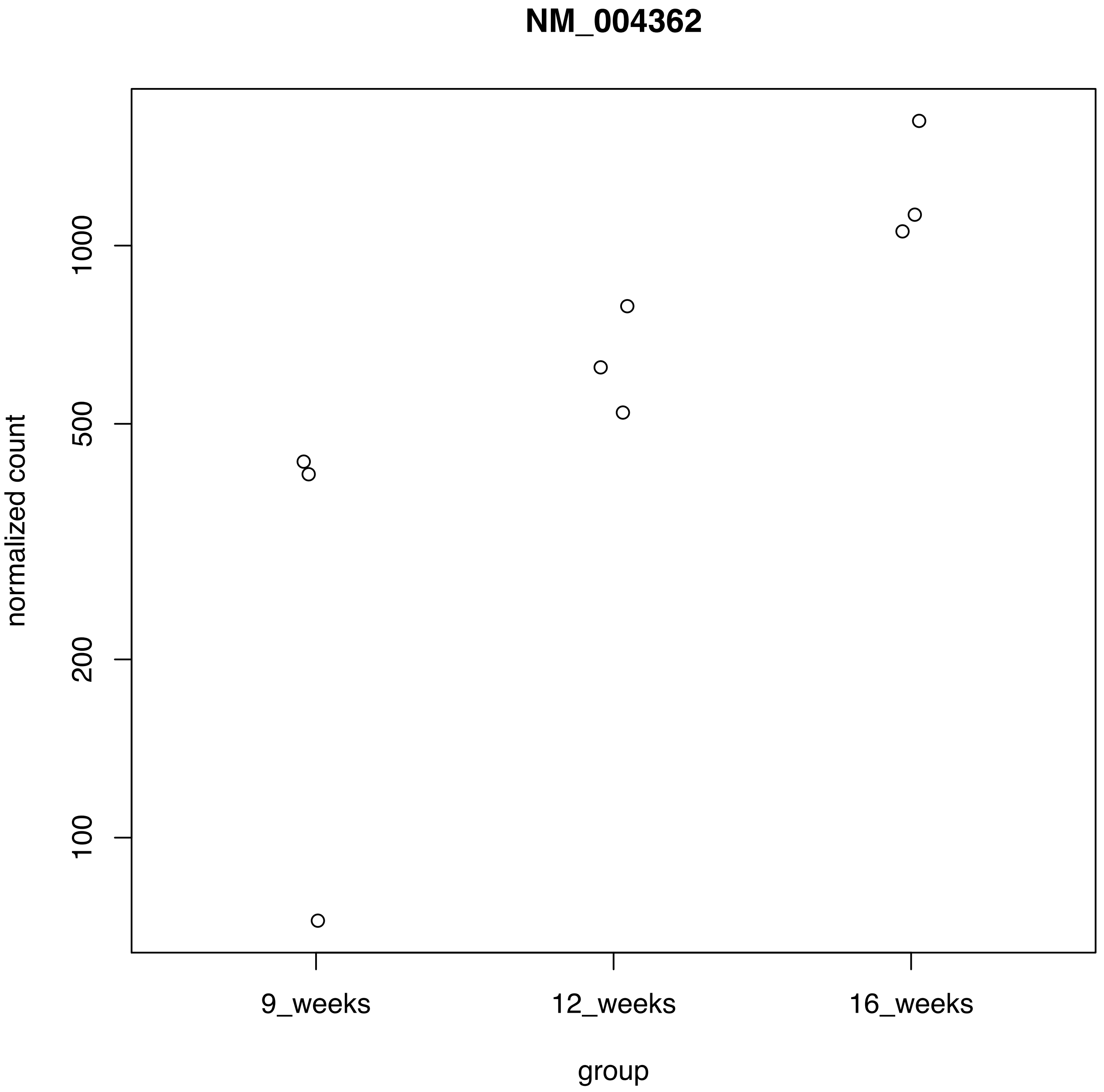

NM_004362
normalized count
100
200
500
1000
9_weeks
12_weeks
16_weeks
group

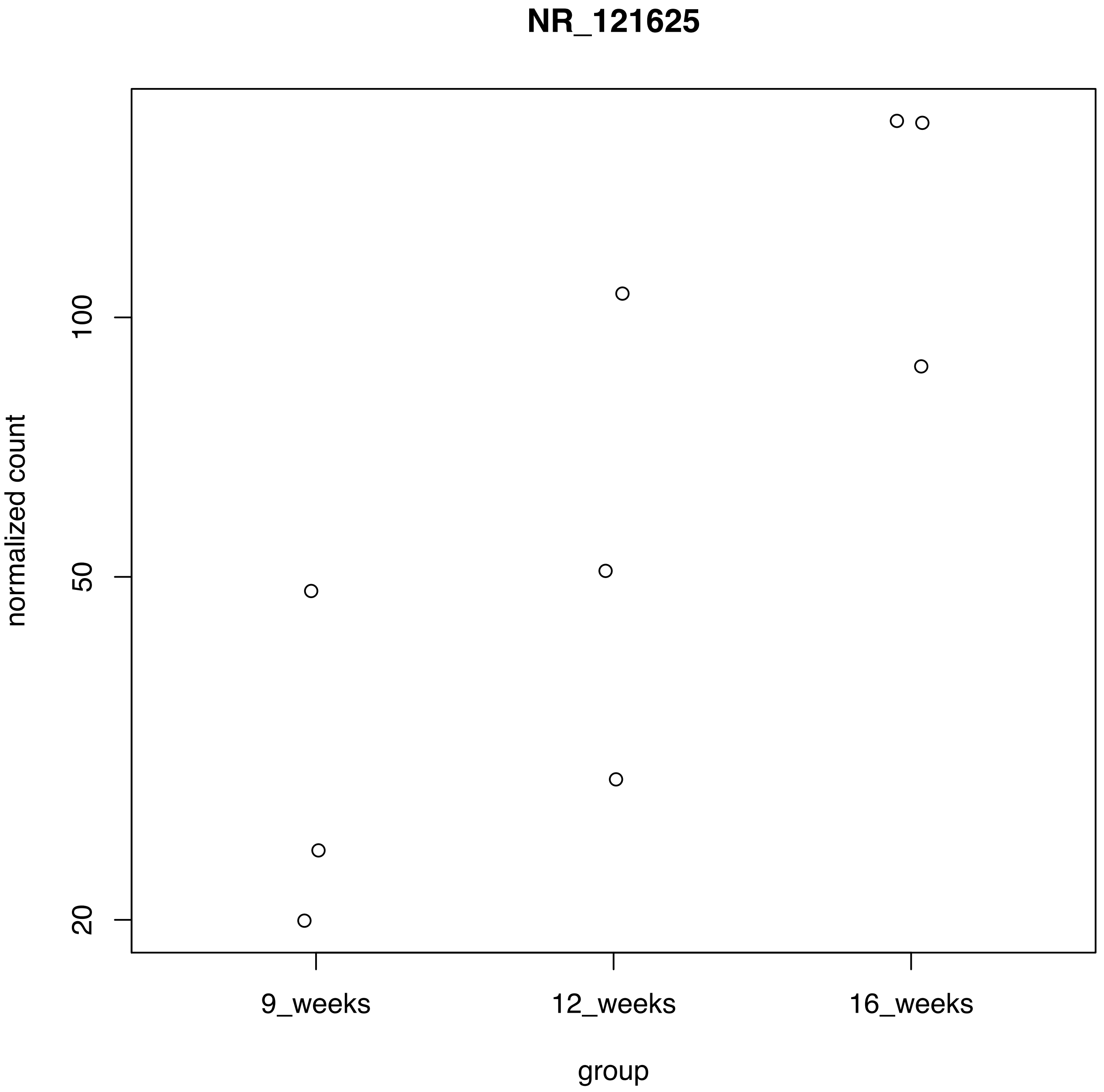
NR_121625
normalized count
100
50
20
9_weeks
12_weeks
16_weeks
group

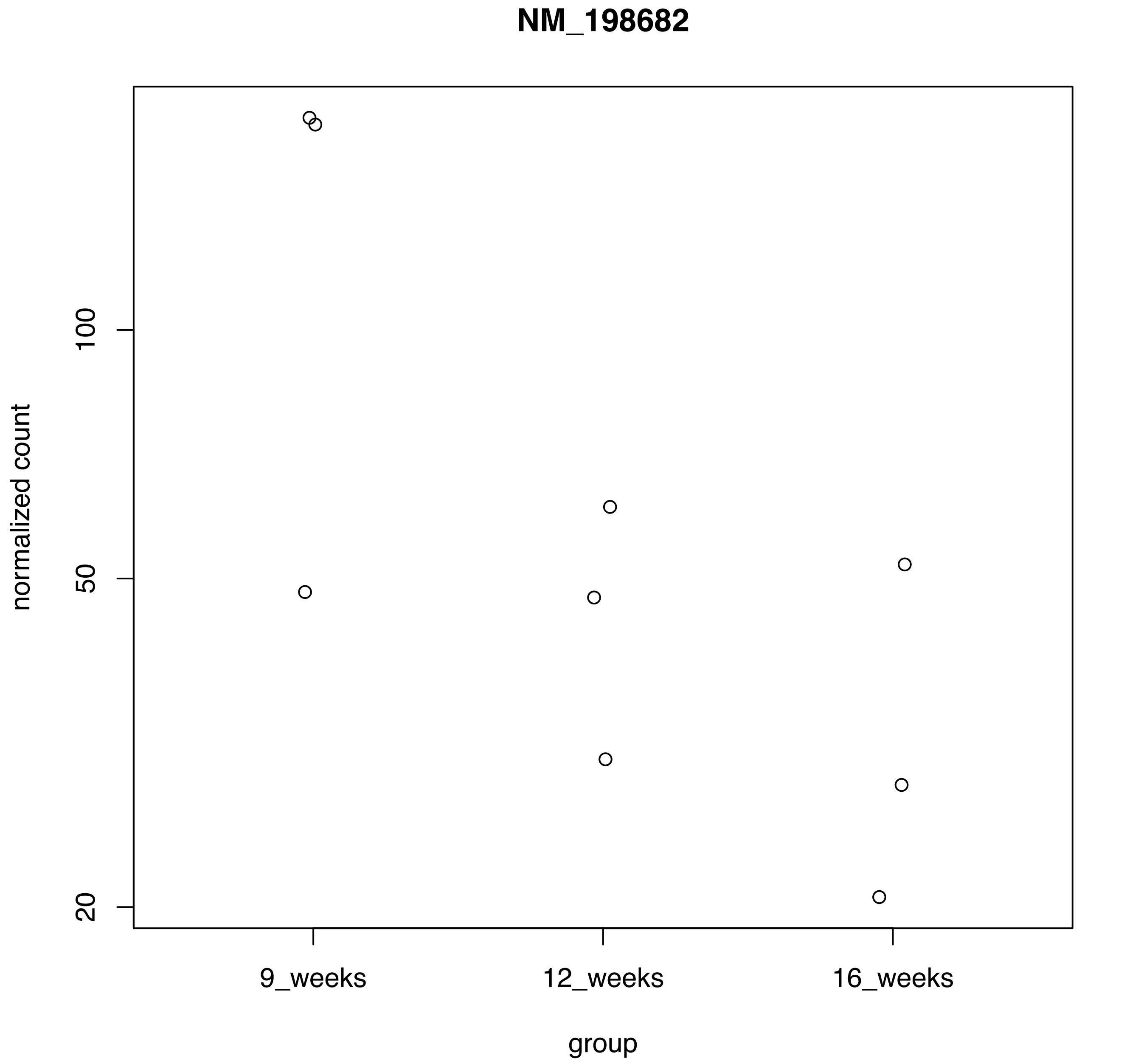

NM_198682
normalized count
100
50
20
9_weeks
12_weeks
16_weeks
group

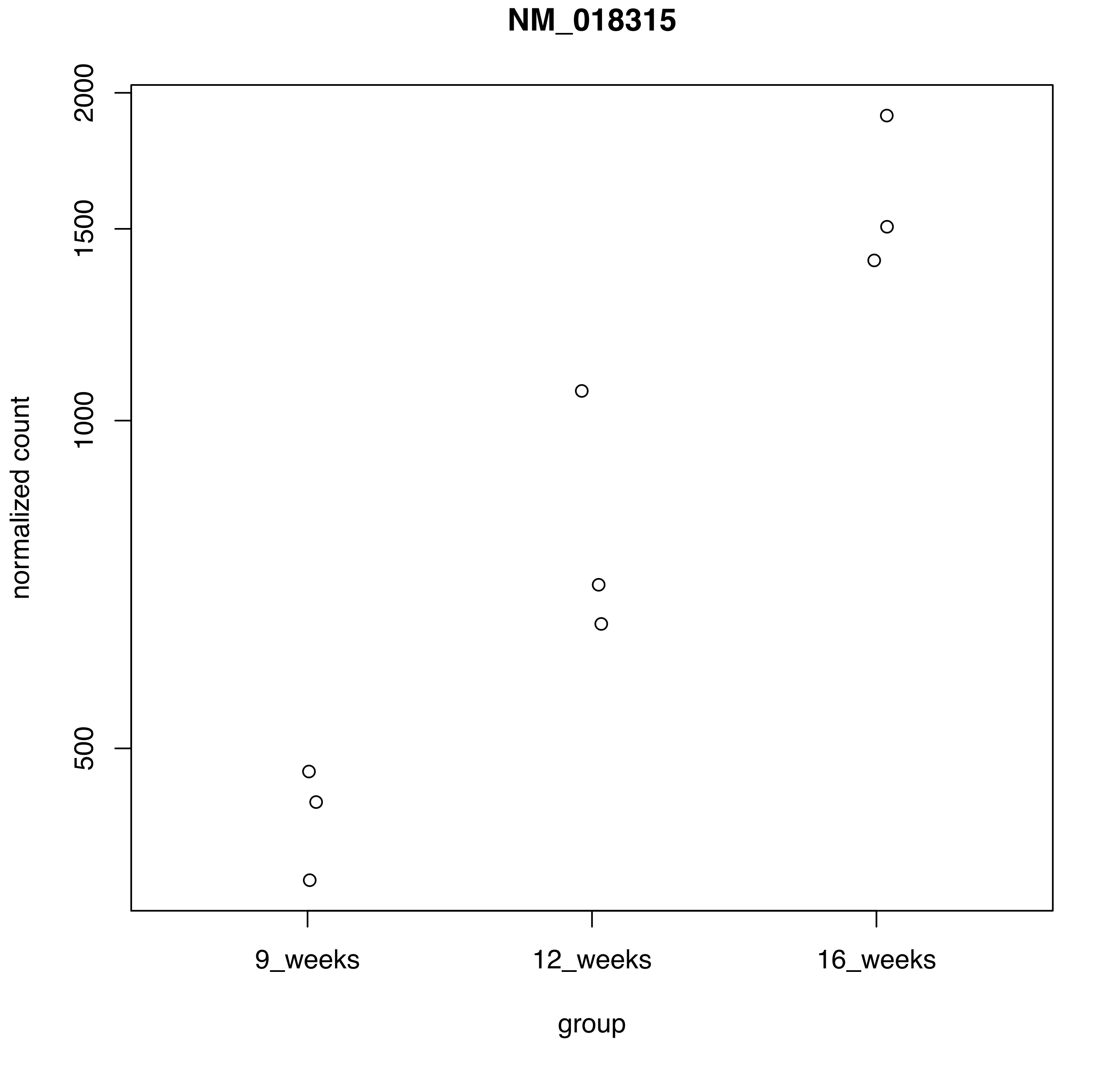

NM_018315
2000
1500
1000
500
normalized count
9_weeks
12_weeks
16_weeks
group

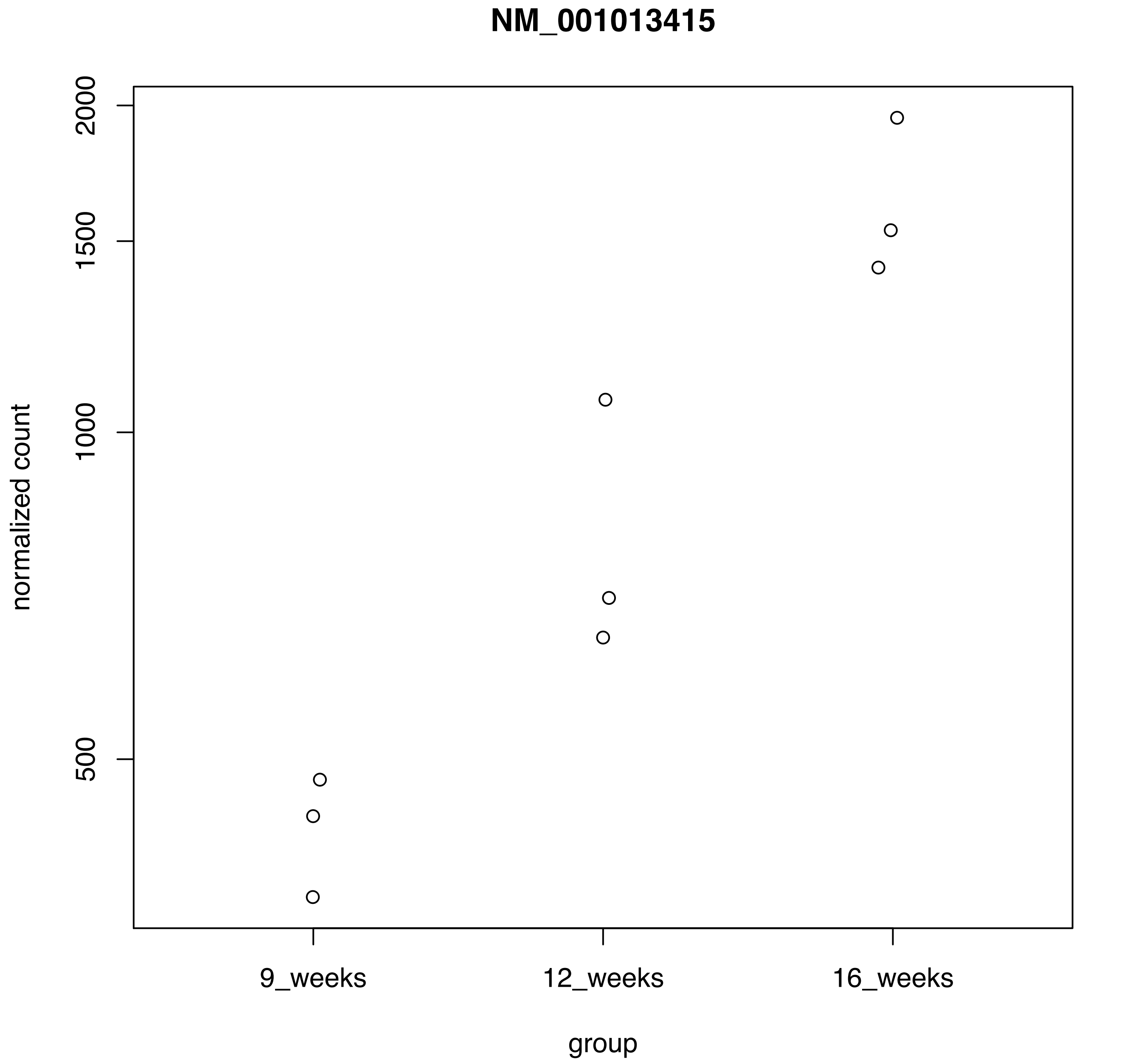
NM_001013415
normalized count
500
1000
1500
2000
9_weeks
12_weeks
16_weeks
group

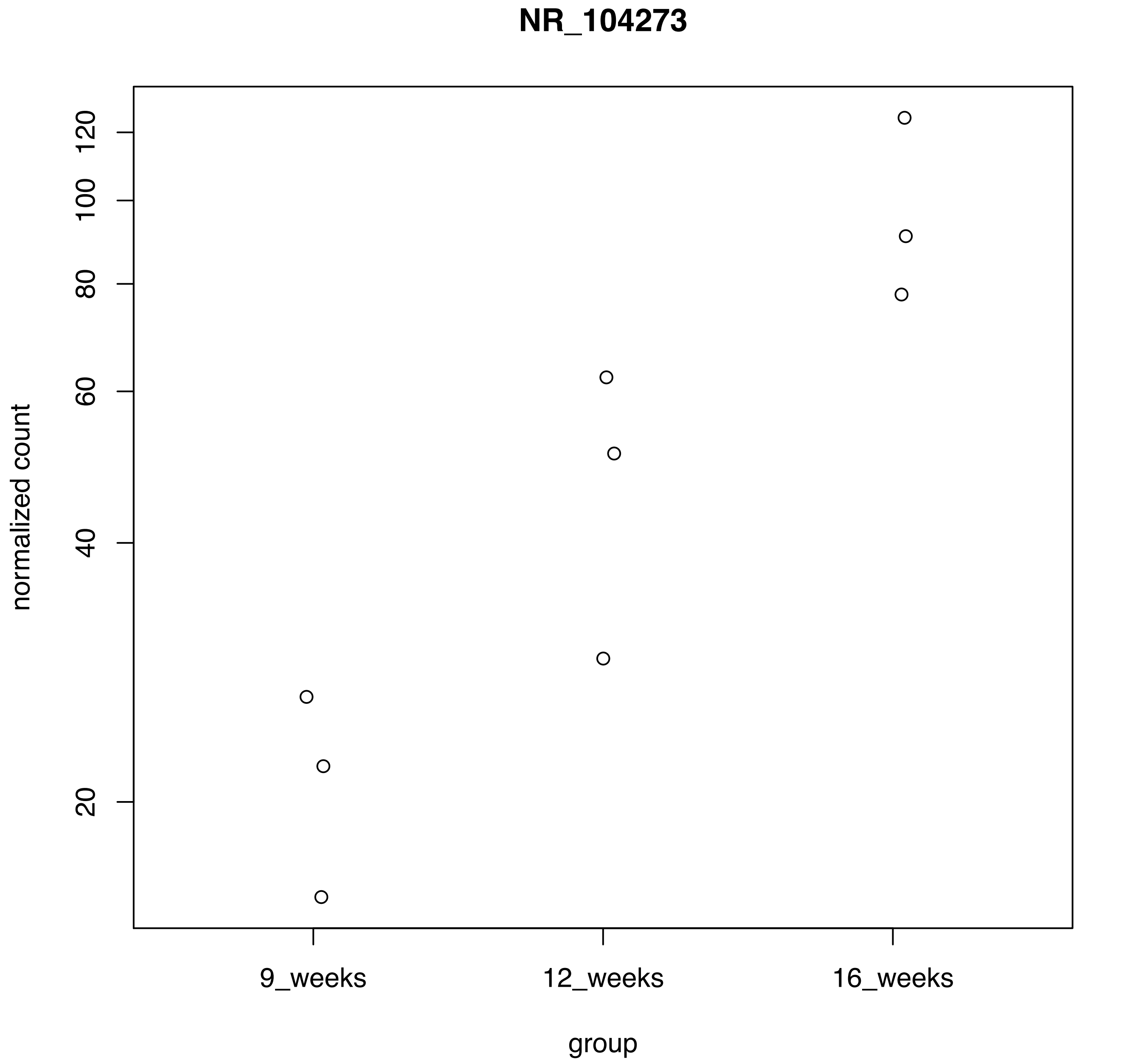

NR_104273
normalized count
120
100
80
60
40
20
9_weeks
12_weeks
16_weeks
group

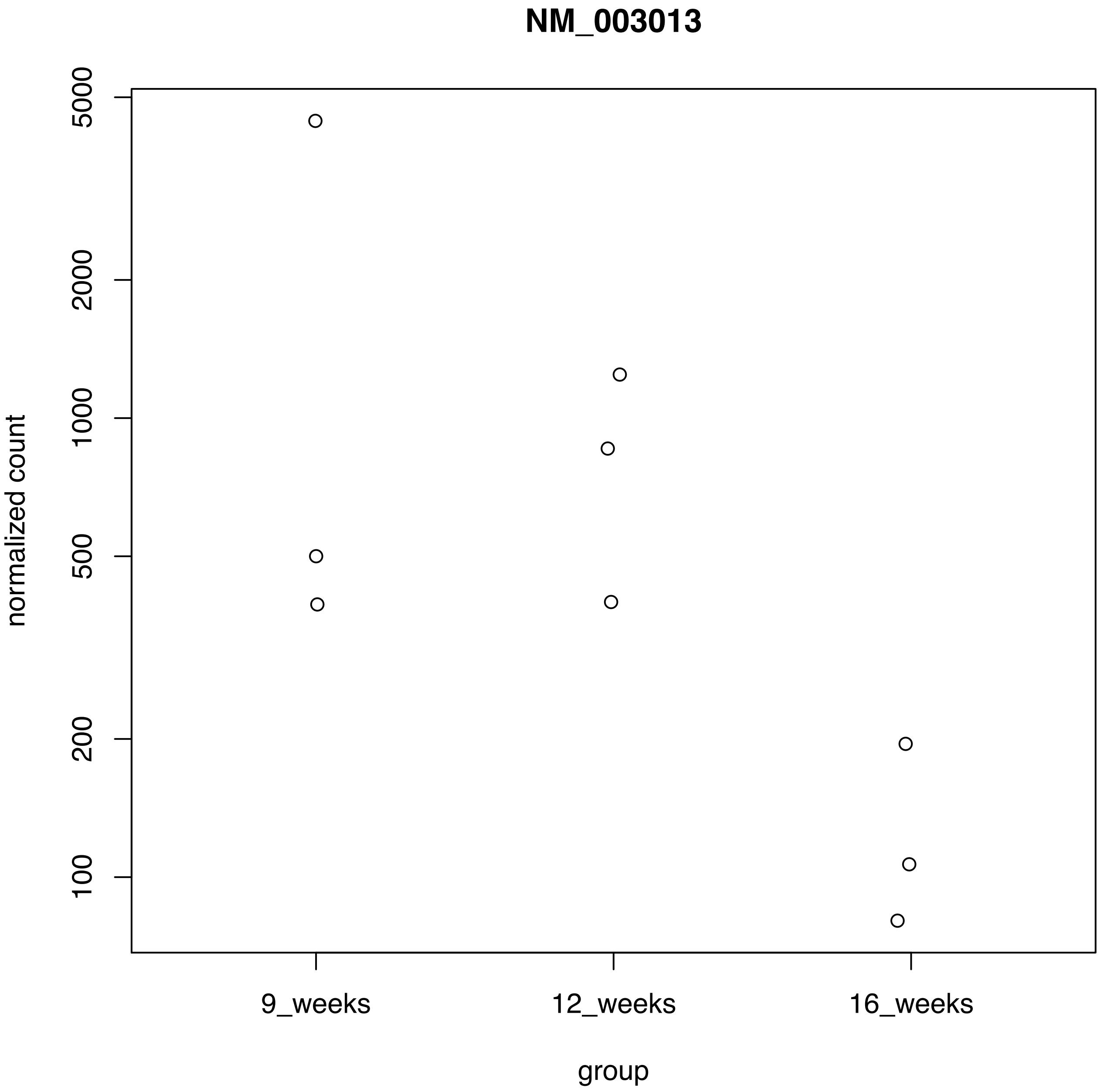
NM_003013
normalized count
5000
2000
1000
500
200
100
9_weeks
12_weeks
16_weeks
group

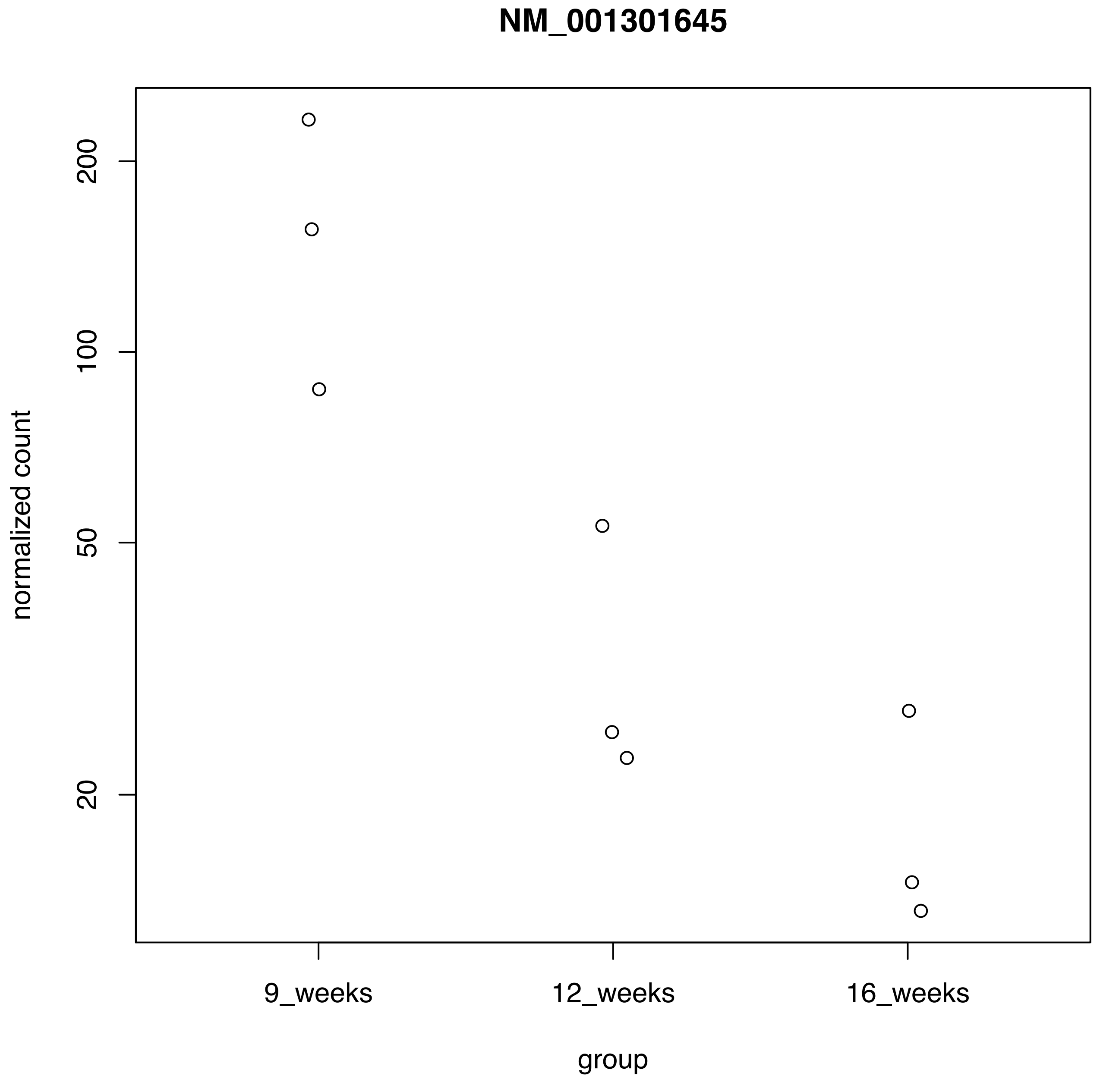

NM_001301645
normalized count
200
100
50
20
9_weeks
12_weeks
16_weeks
group

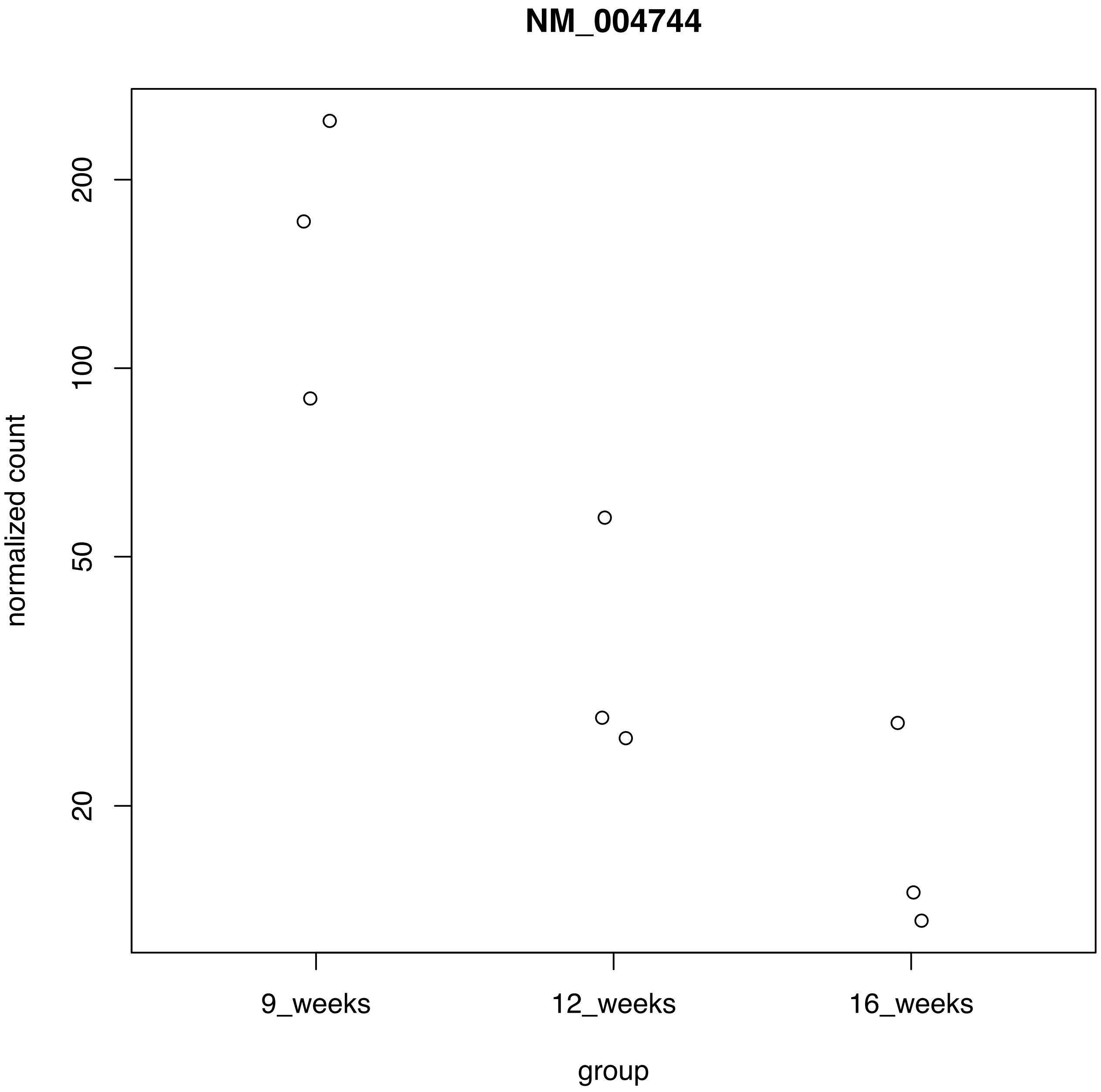

NM_004744
normalized count
200
100
50
20
9_weeks
12_weeks
16_weeks
group

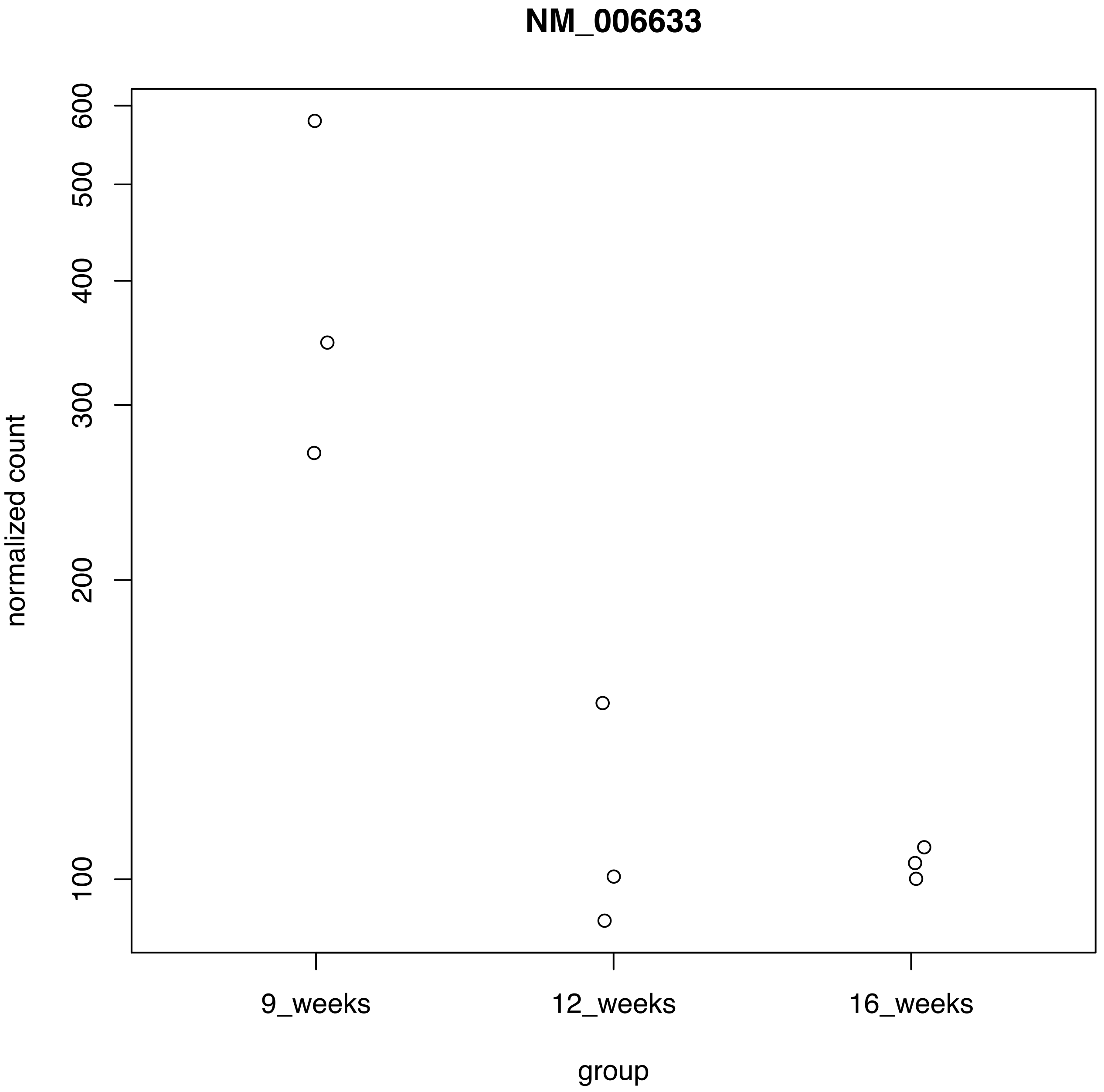
NM_006633
normalized count
600
500
400
300
200
100
9_weeks
12_weeks
16_weeks
group

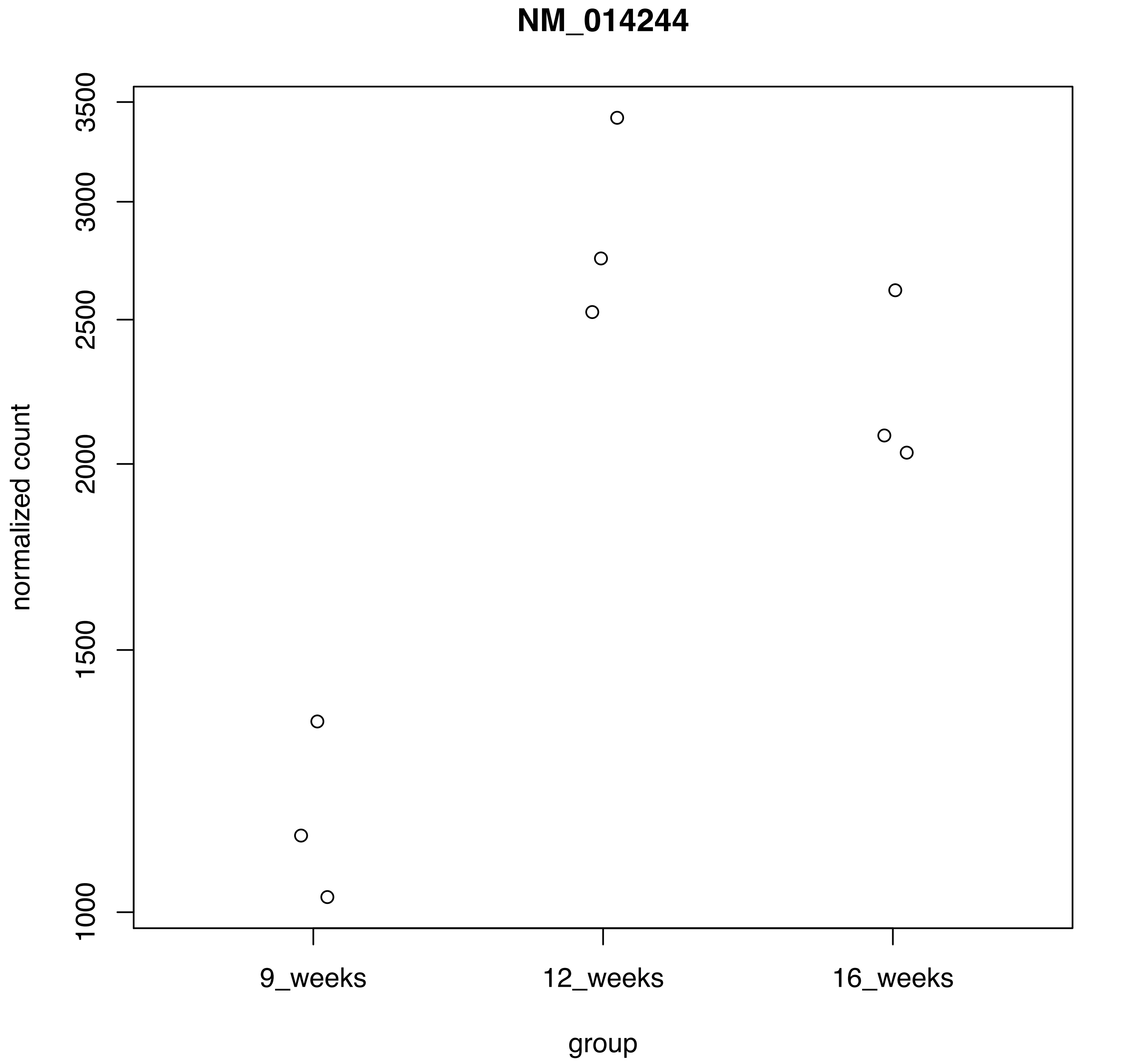
NM_014244
normalized count
3500
3000
2500
2000
1500
1000
9_weeks
12_weeks
16_weeks
group

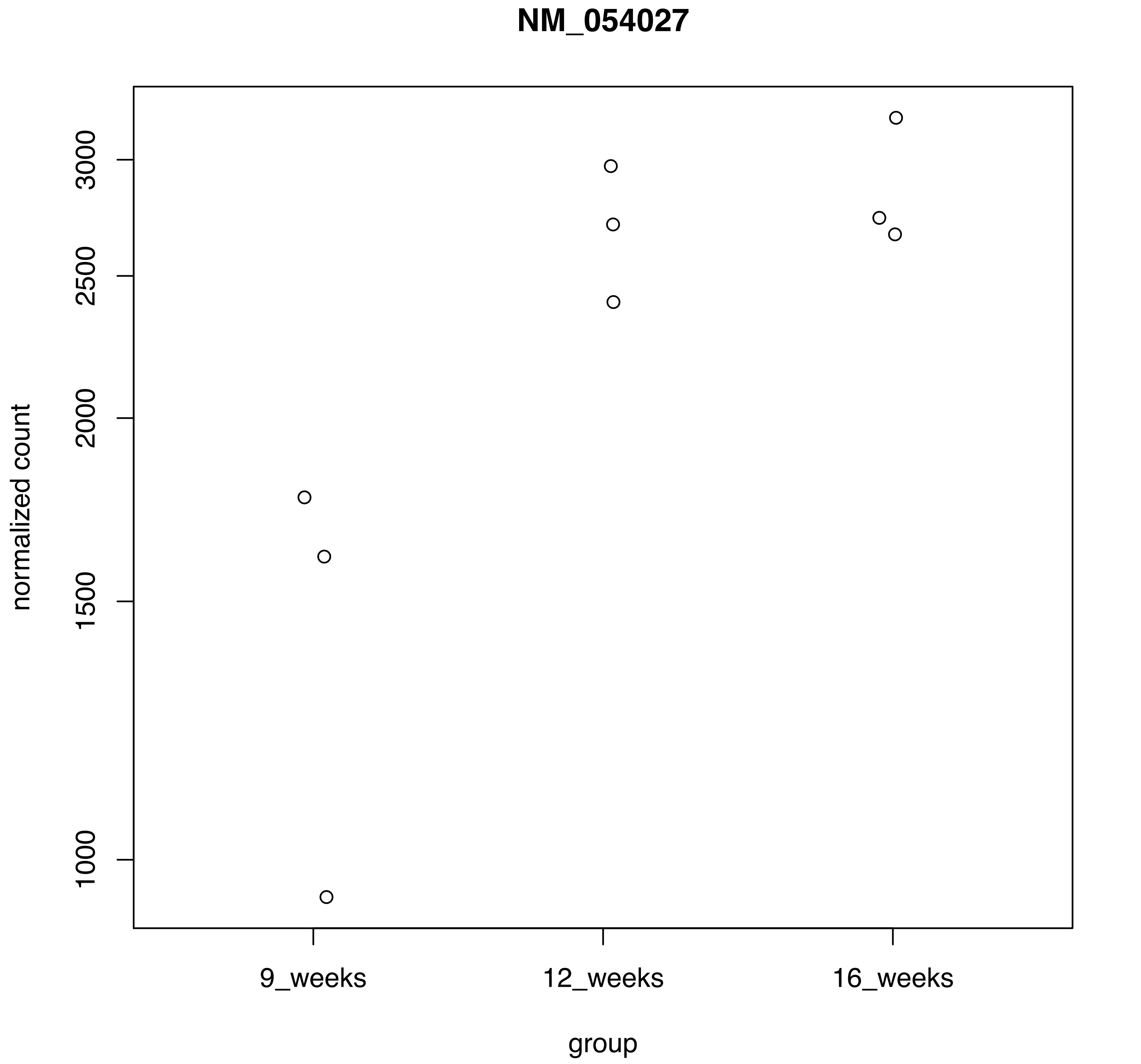

NM_054027
normalized count
1000
1500
2000
2500
3000
9_weeks
12_weeks
16_weeks
group

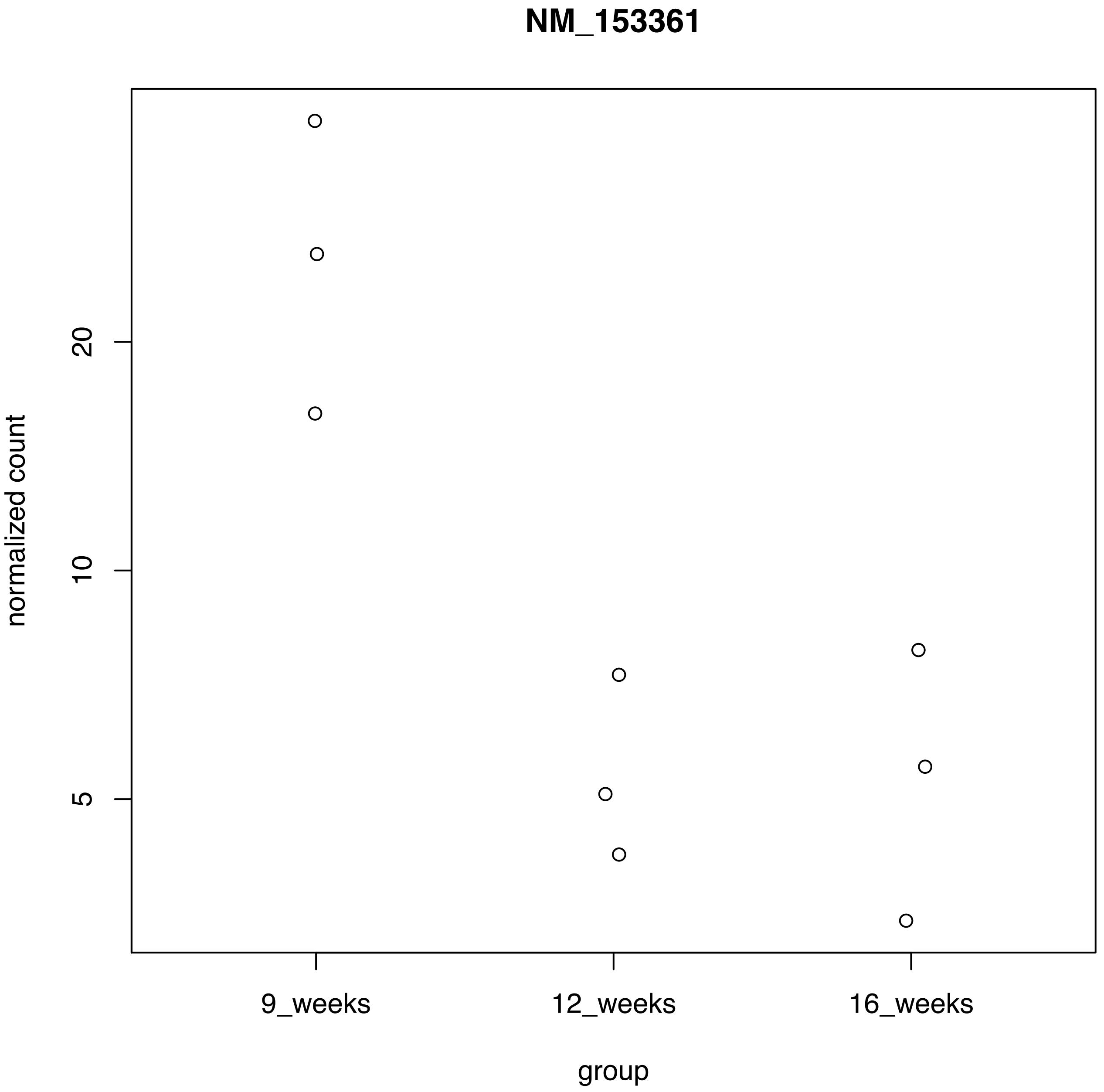
NM_153361
normalized count
20
10
5
9_weeks
12_weeks
16_weeks
group

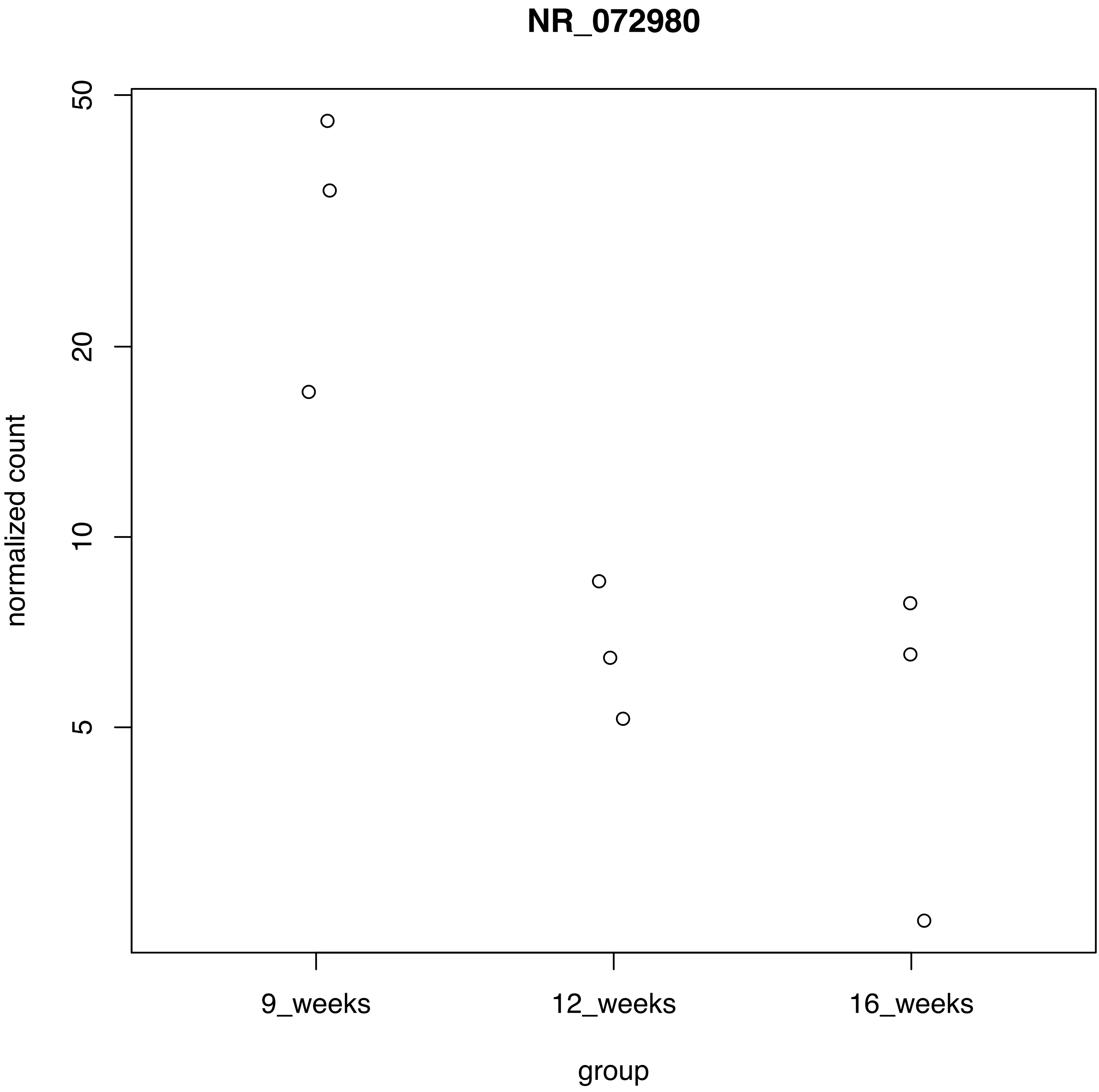
NR_072980
normalized count
50
20
10
5
9_weeks
12_weeks
16_weeks
group

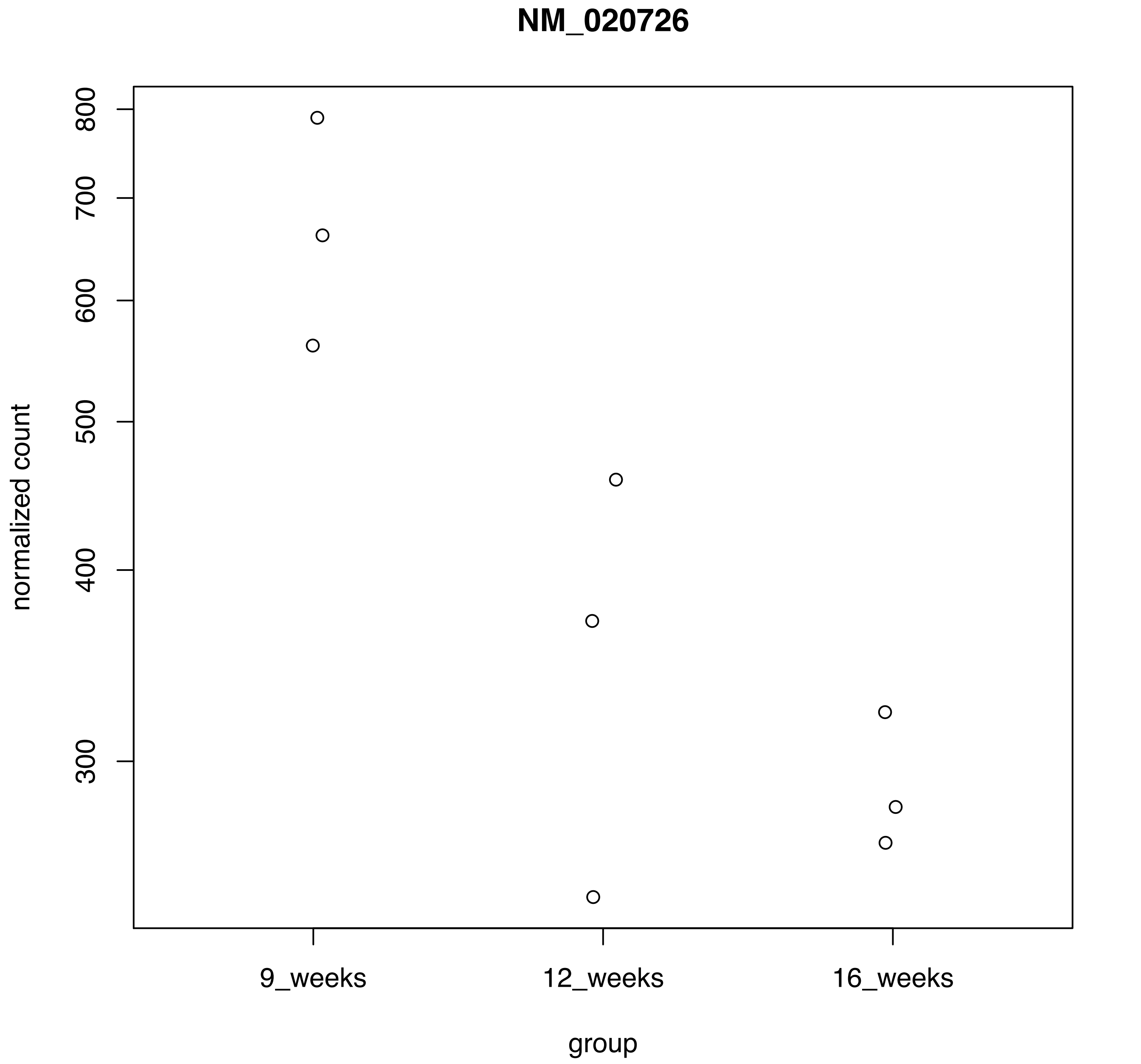

NM_020726
normalized count
800
700
600
500
400
300
9_weeks
12_weeks
16_weeks
group

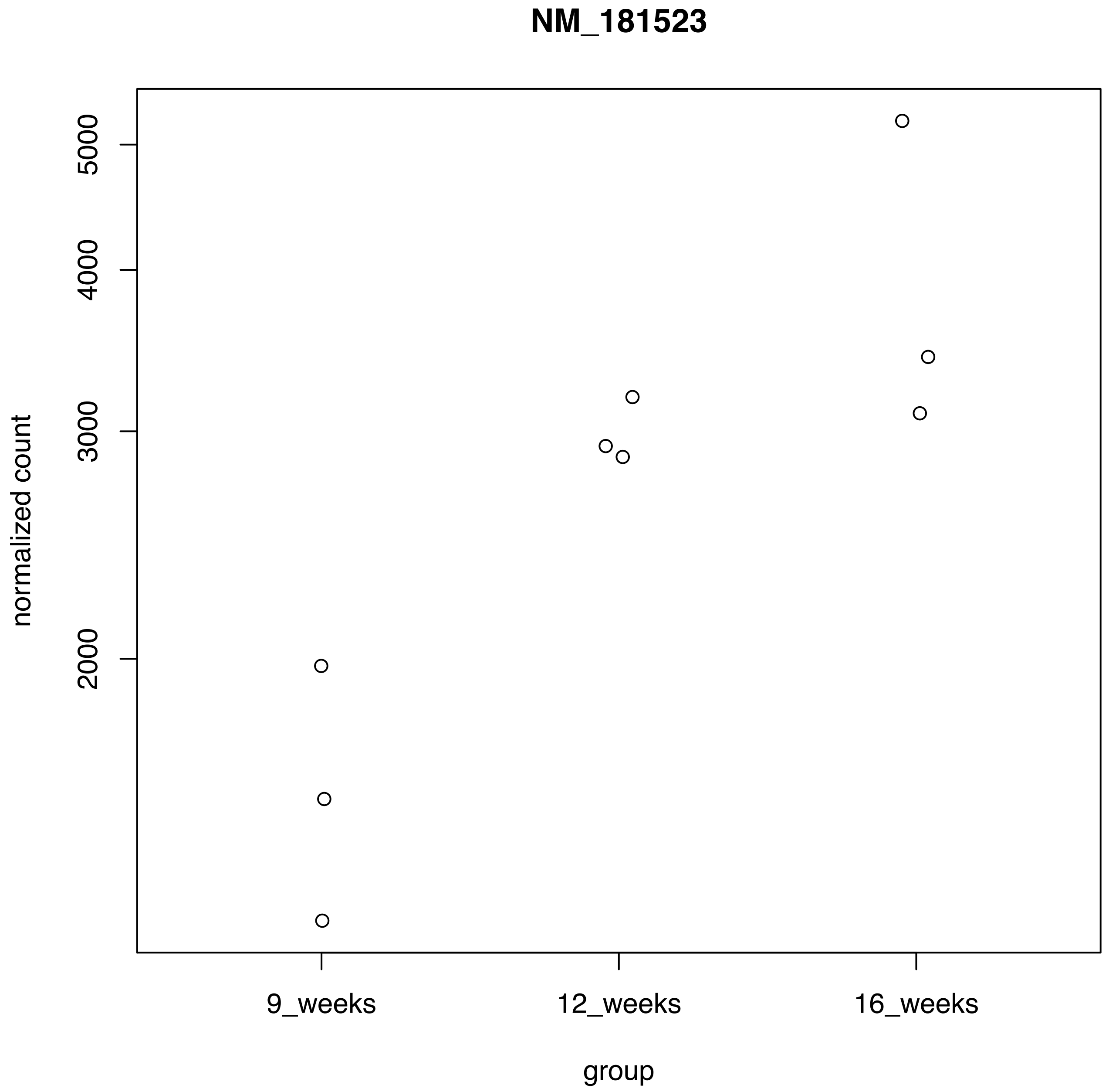

NM_181523
normalized count
5000
4000
3000
2000
9_weeks
12_weeks
16_weeks
group

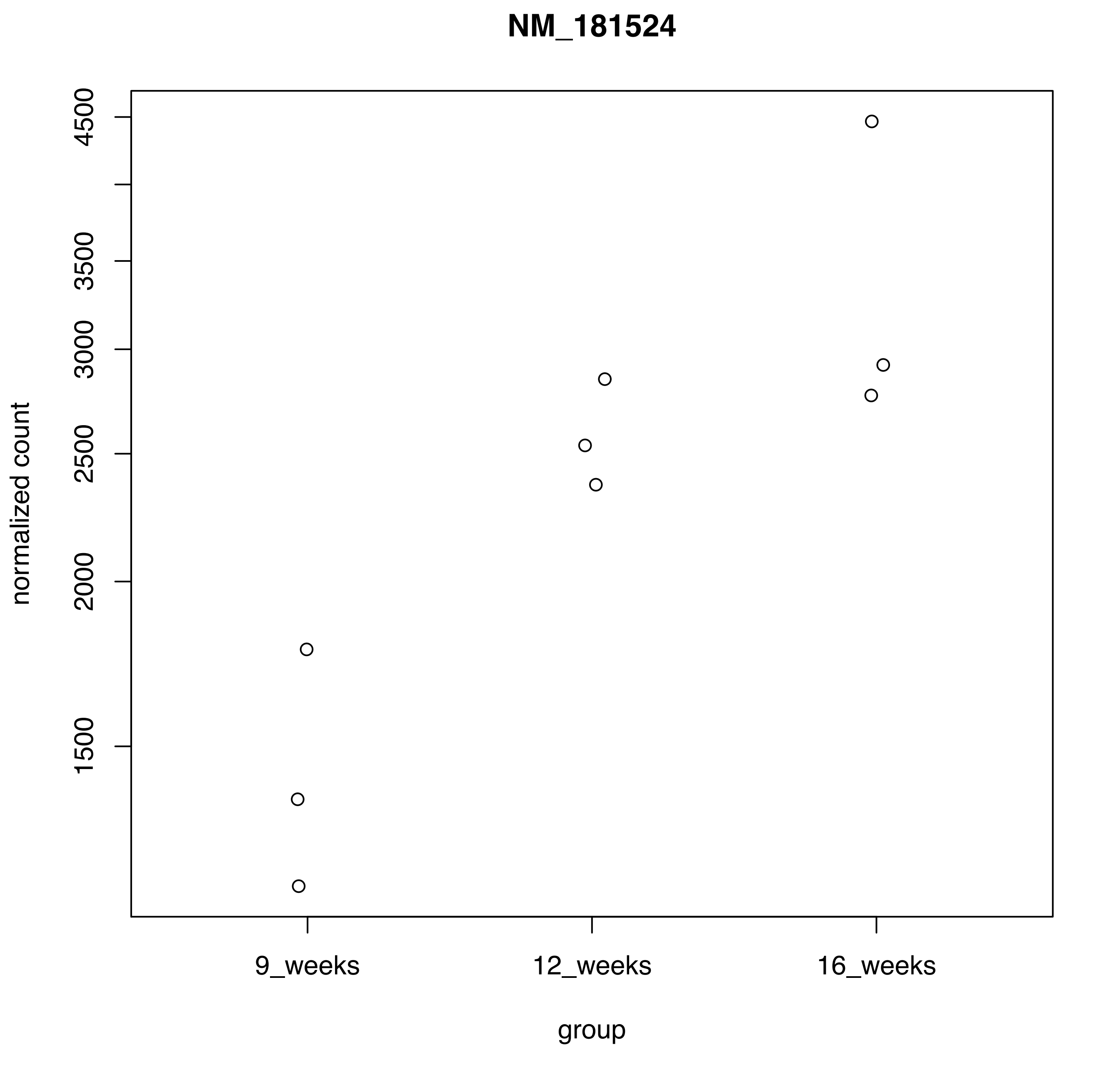

NM_181524
normalized count
4500
3500
3000
2500
2000
1500
9_weeks
12_weeks
16_weeks
group

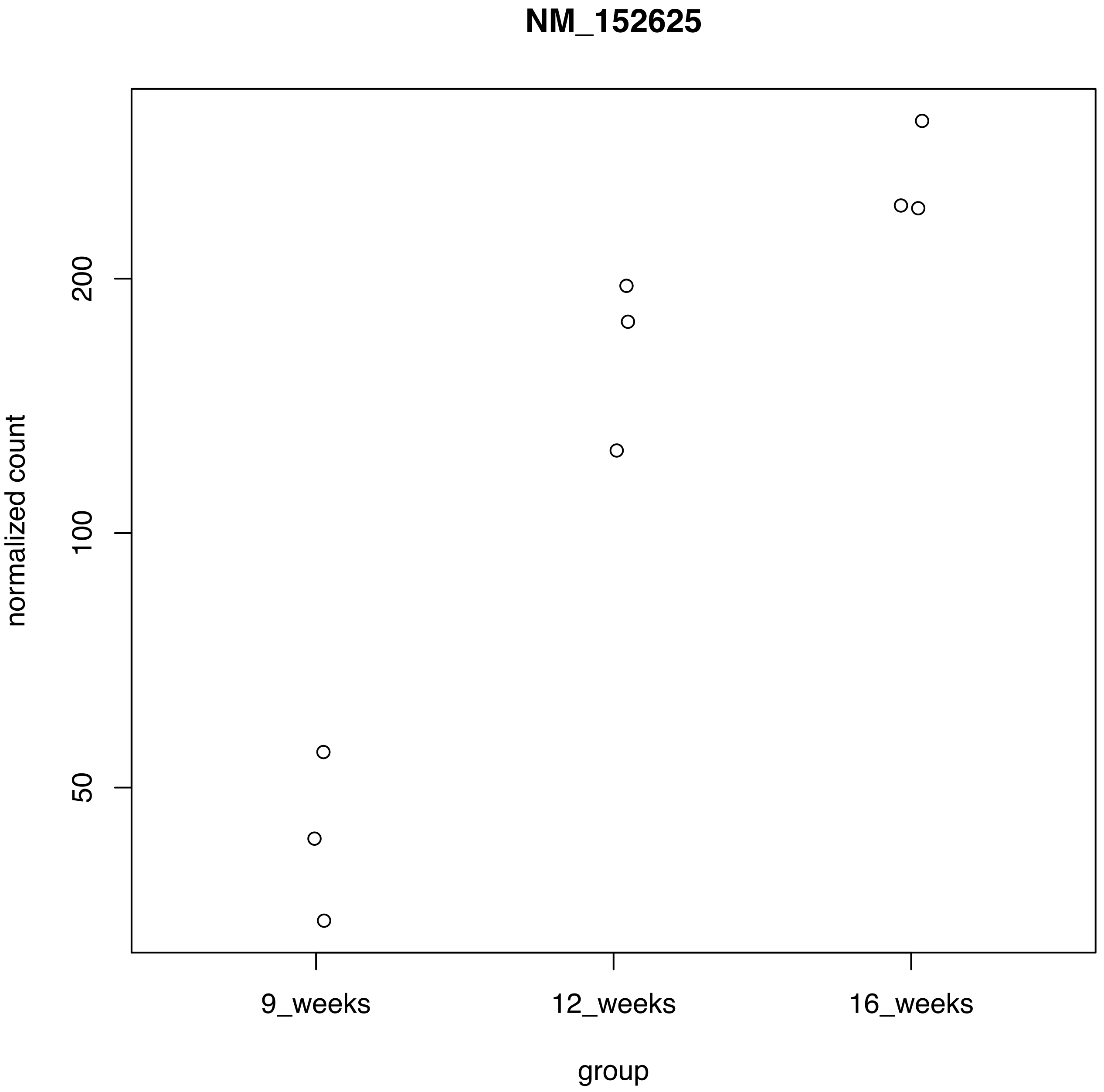

NM_152625
normalized count
200
100
50
9_weeks
12_weeks
16_weeks
group

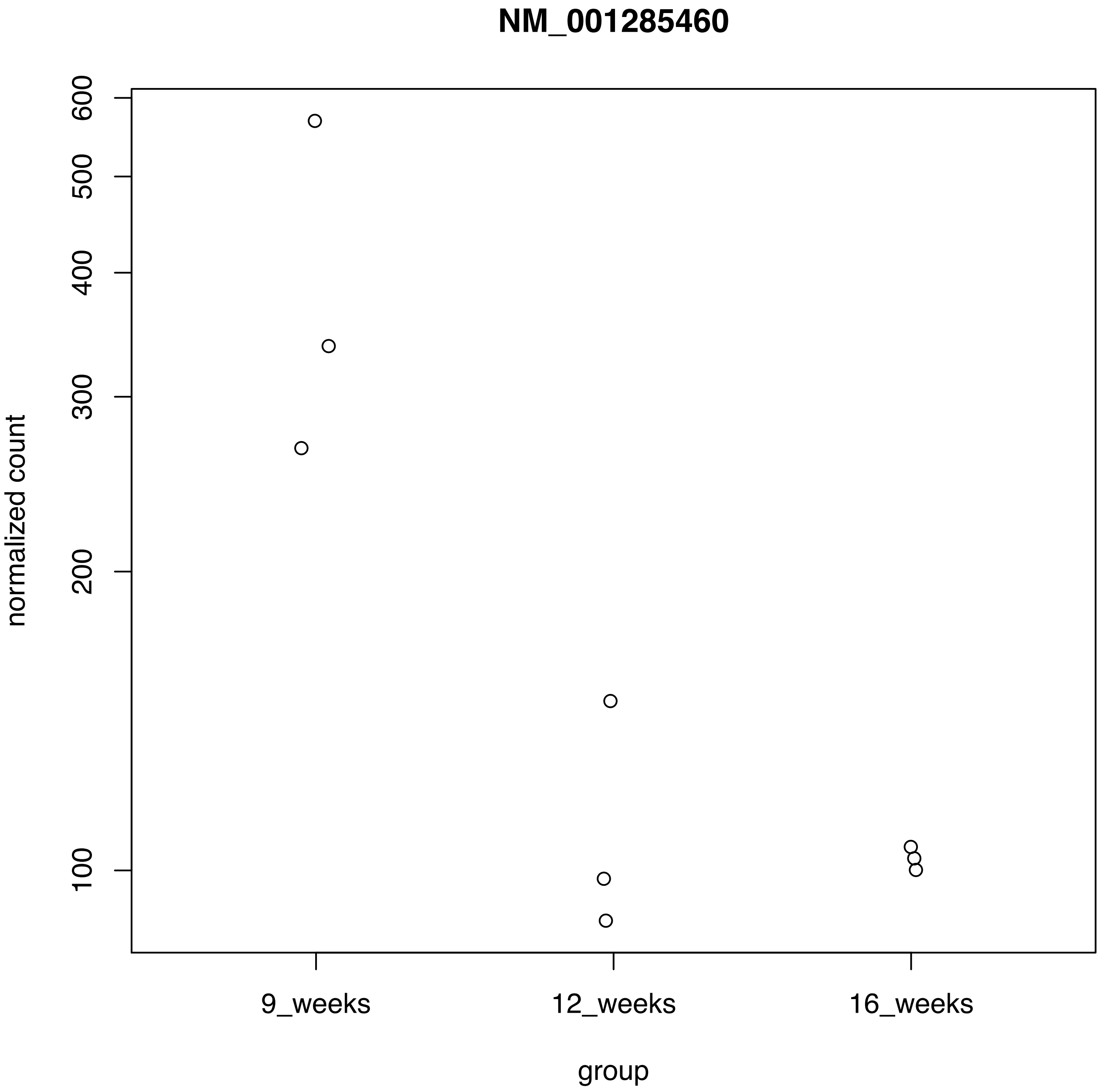
NM_001285460
normalized count
600
500
400
300
200
100
9_weeks
12_weeks
16_weeks
group

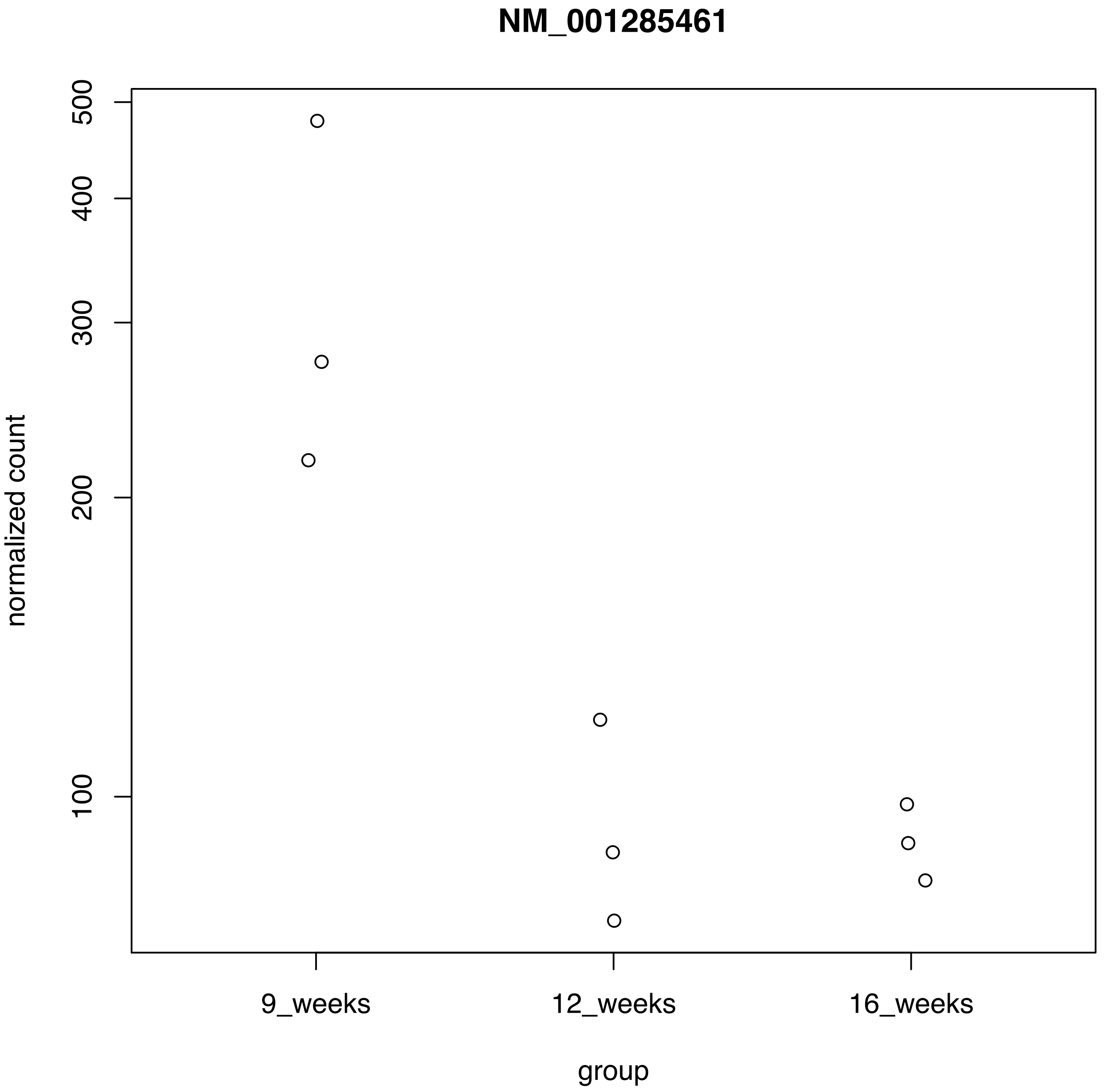

NM_001285461
normalized count
500
400
300
200
100
9_weeks
12_weeks
16_weeks
group

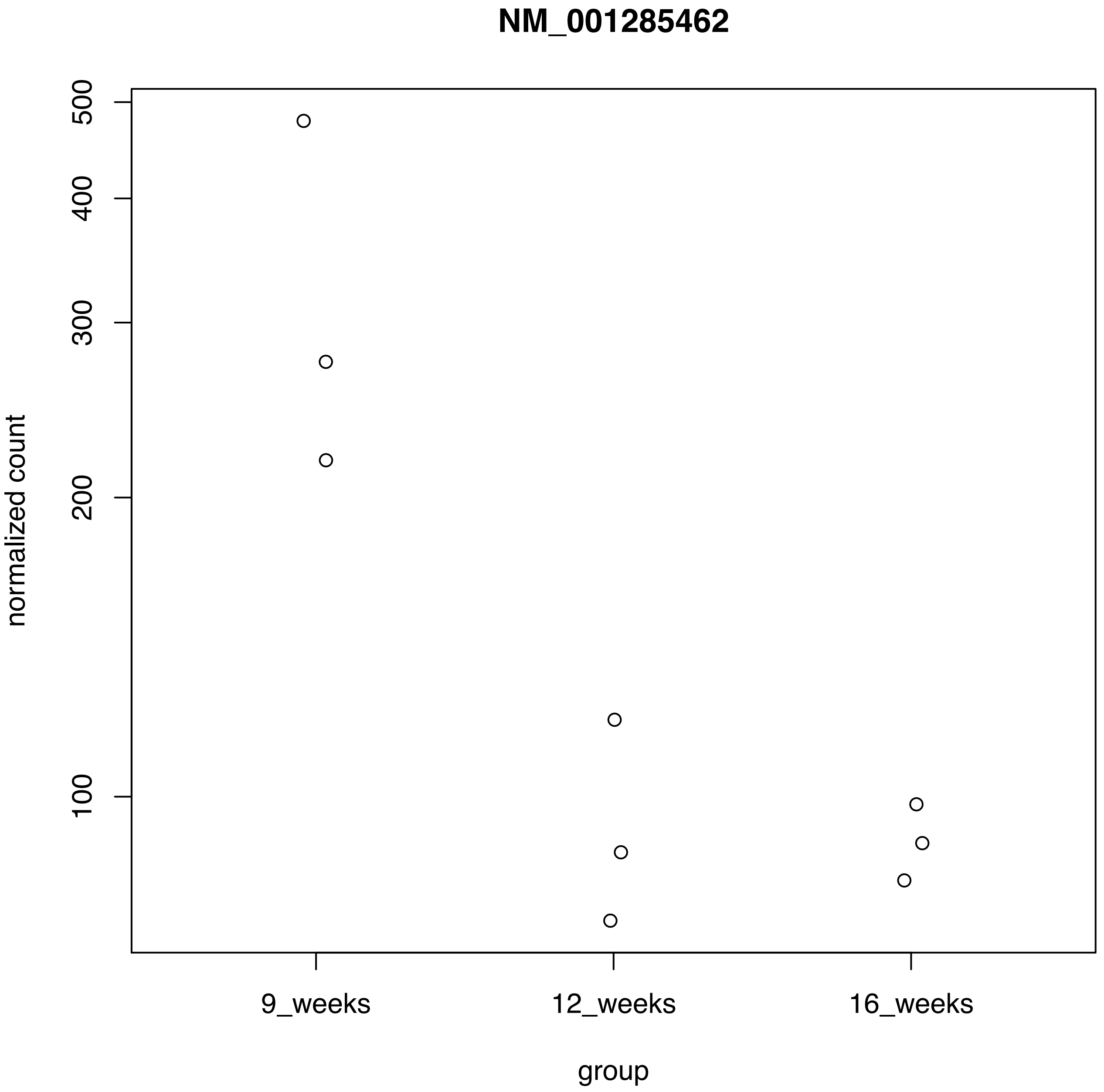
NM_001285462
normalized count
500
400
300
200
100
9_weeks
12_weeks
16_weeks
group

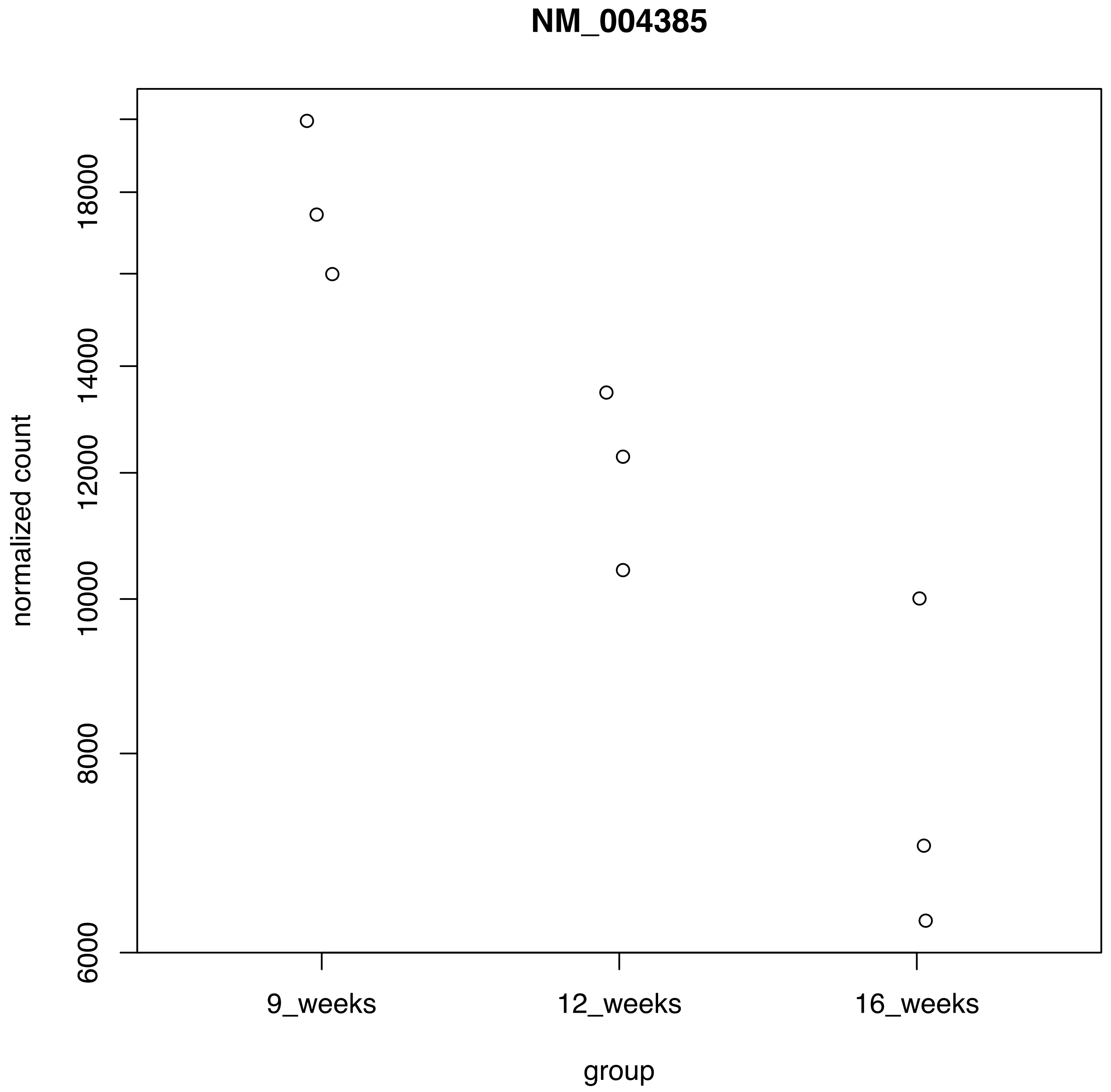NM_004385
normalized count
6000
8000
10000
12000
14000
18000
9_weeks
12_weeks
16_weeks
group

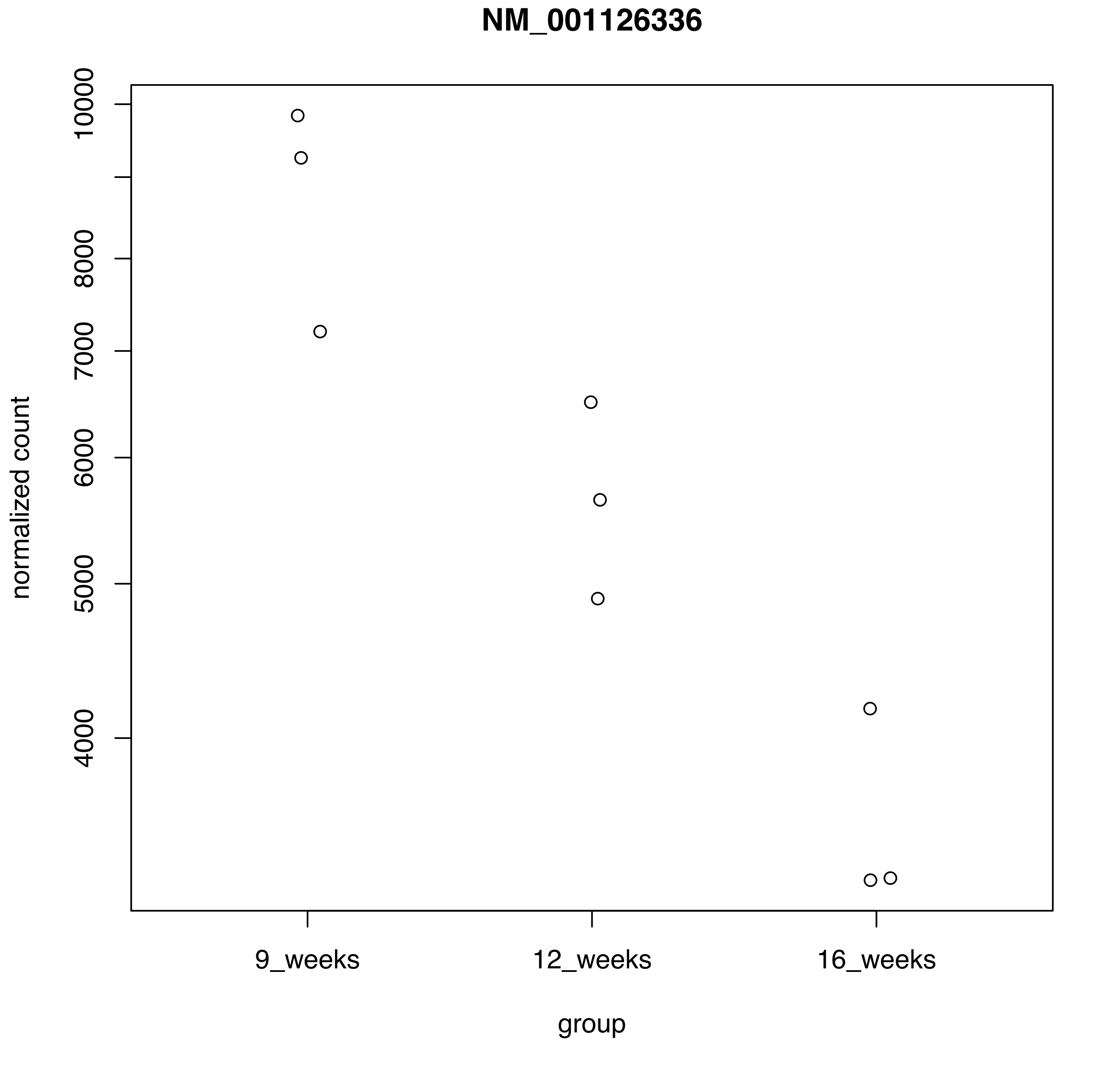

NM_001126336
normalized count
10000
8000
7000
6000
5000
4000
9_weeks
12_weeks
16_weeks
group

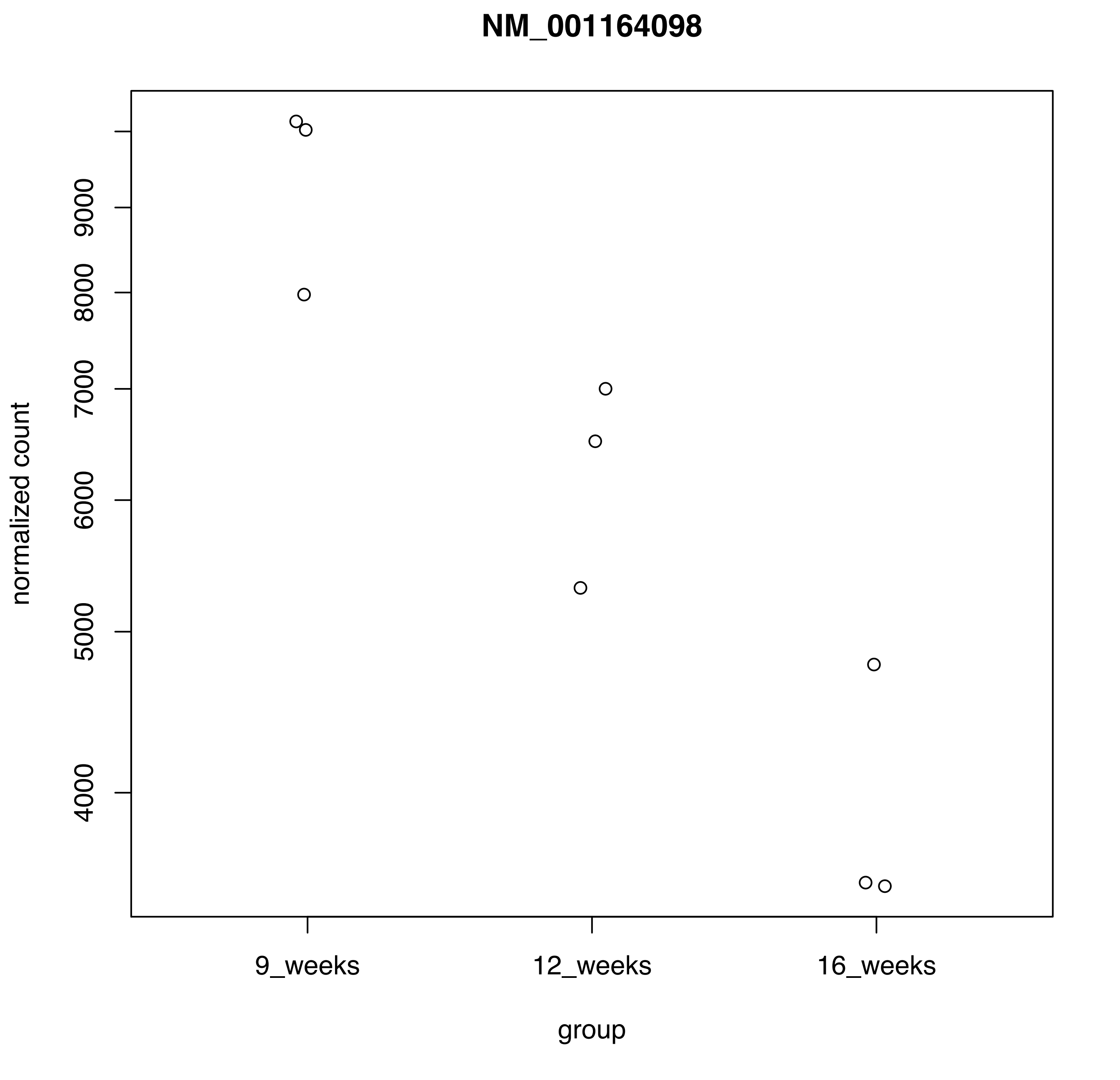
NM_001164098
normalized count
4000
5000
6000
7000
8000
9000
9_weeks
12_weeks
16_weeks
group

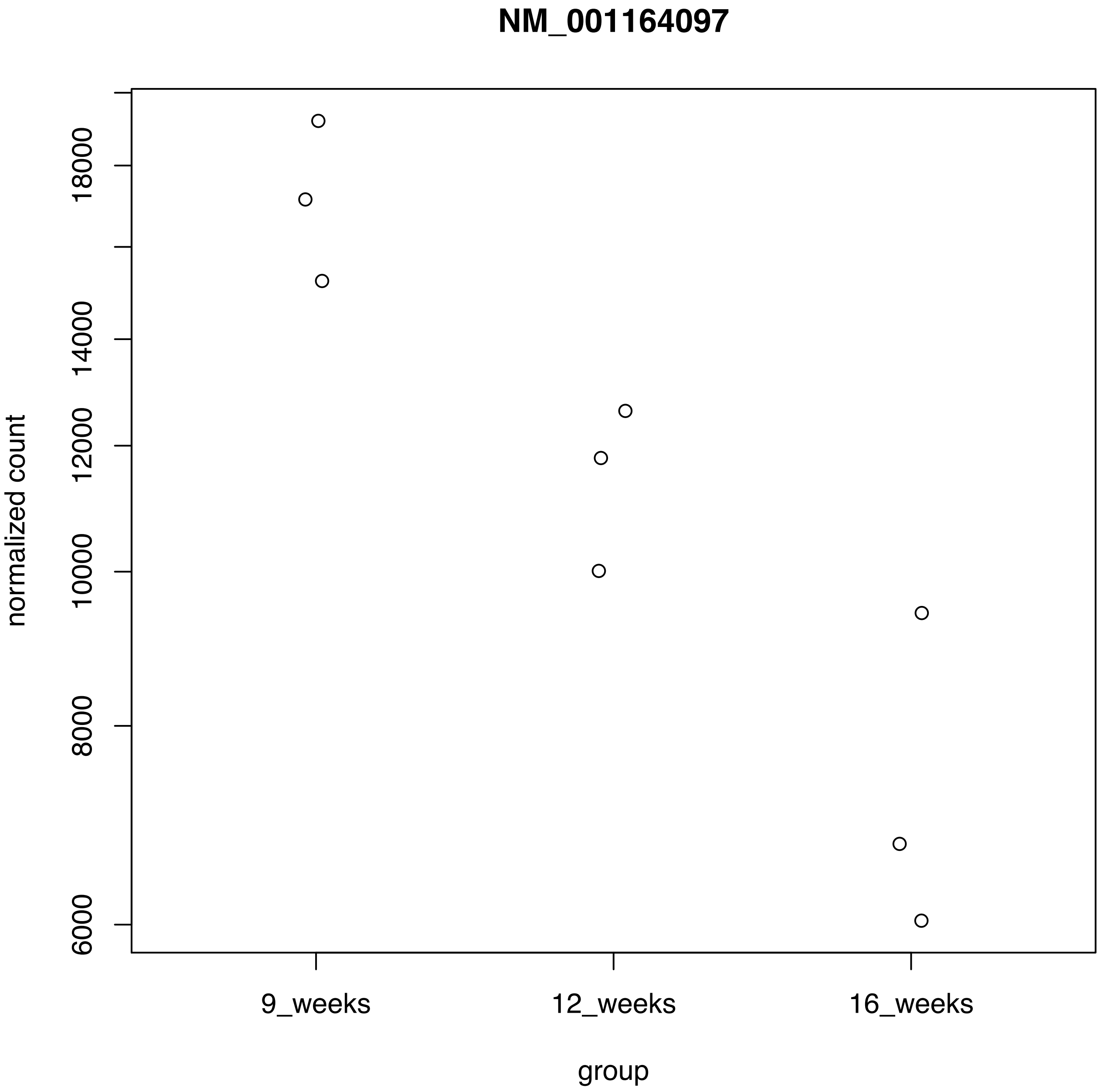
NM_001164097
normalized count
18000
14000
12000
10000
8000
6000
9_weeks
12_weeks
16_weeks
group

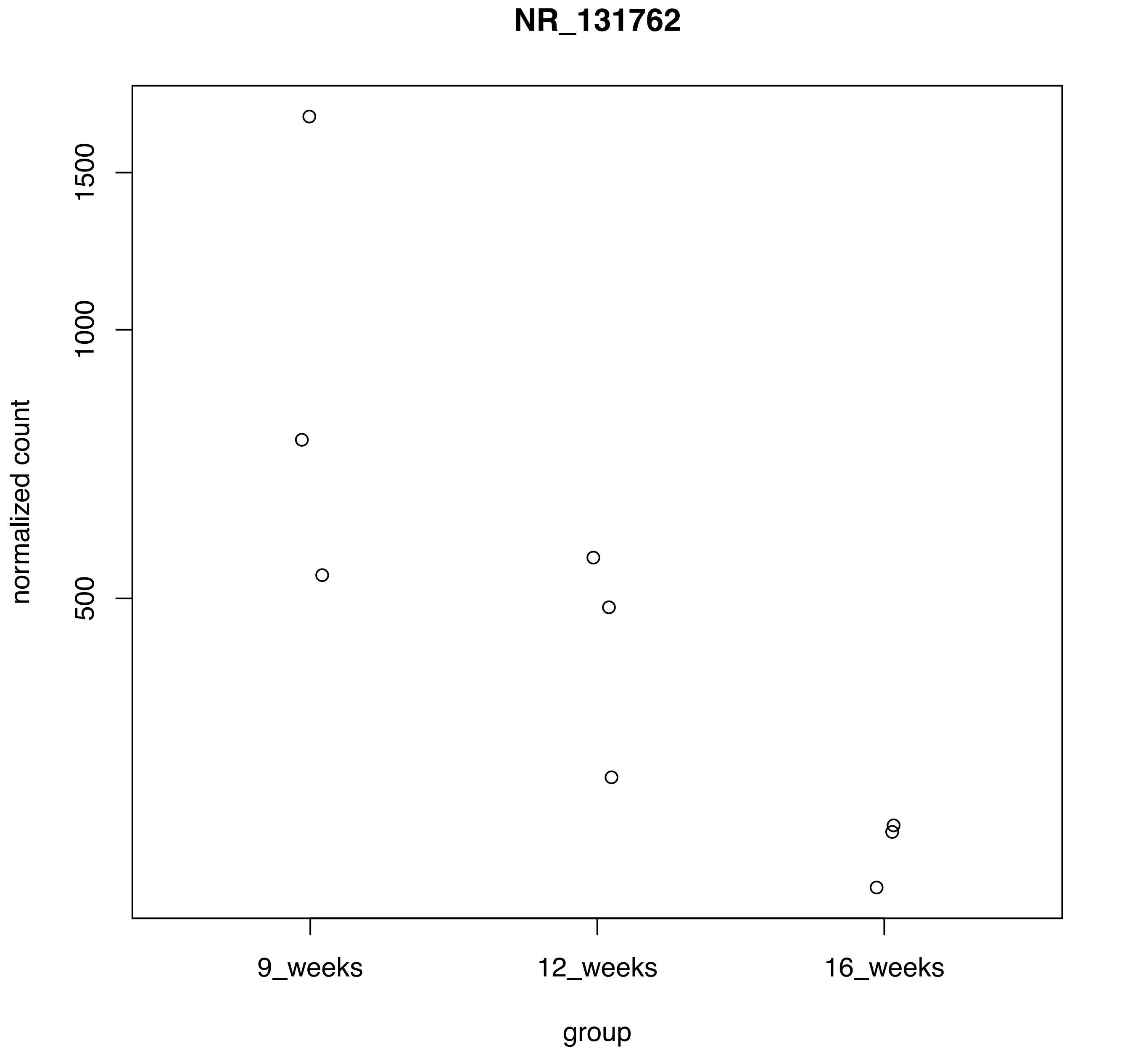

NR_131762
1500
1000
500
normalized count
9_weeks
12_weeks
16_weeks
group

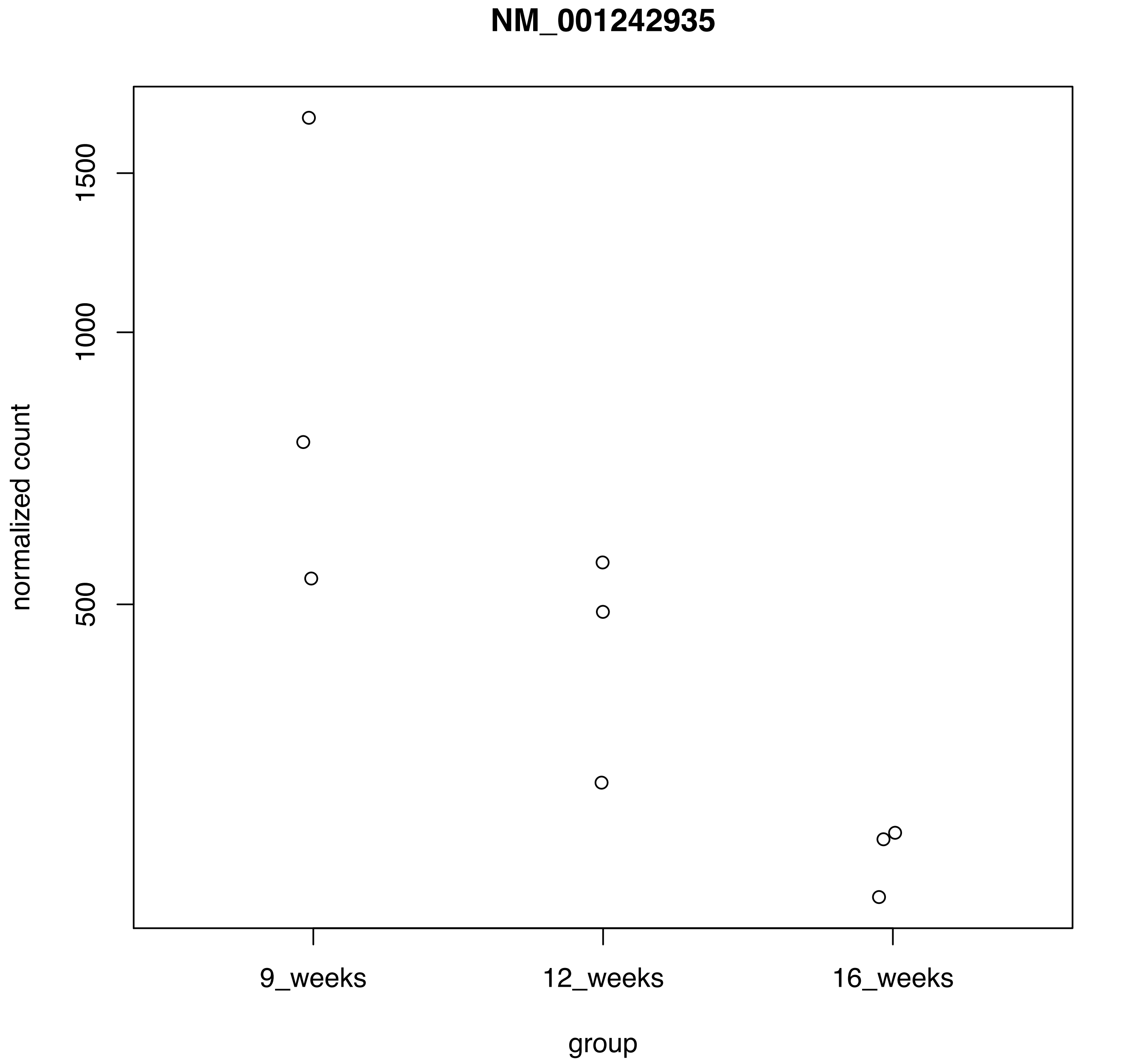
NM_001242935
normalized count
1500
1000
500
9_weeks
12_weeks
16_weeks
group

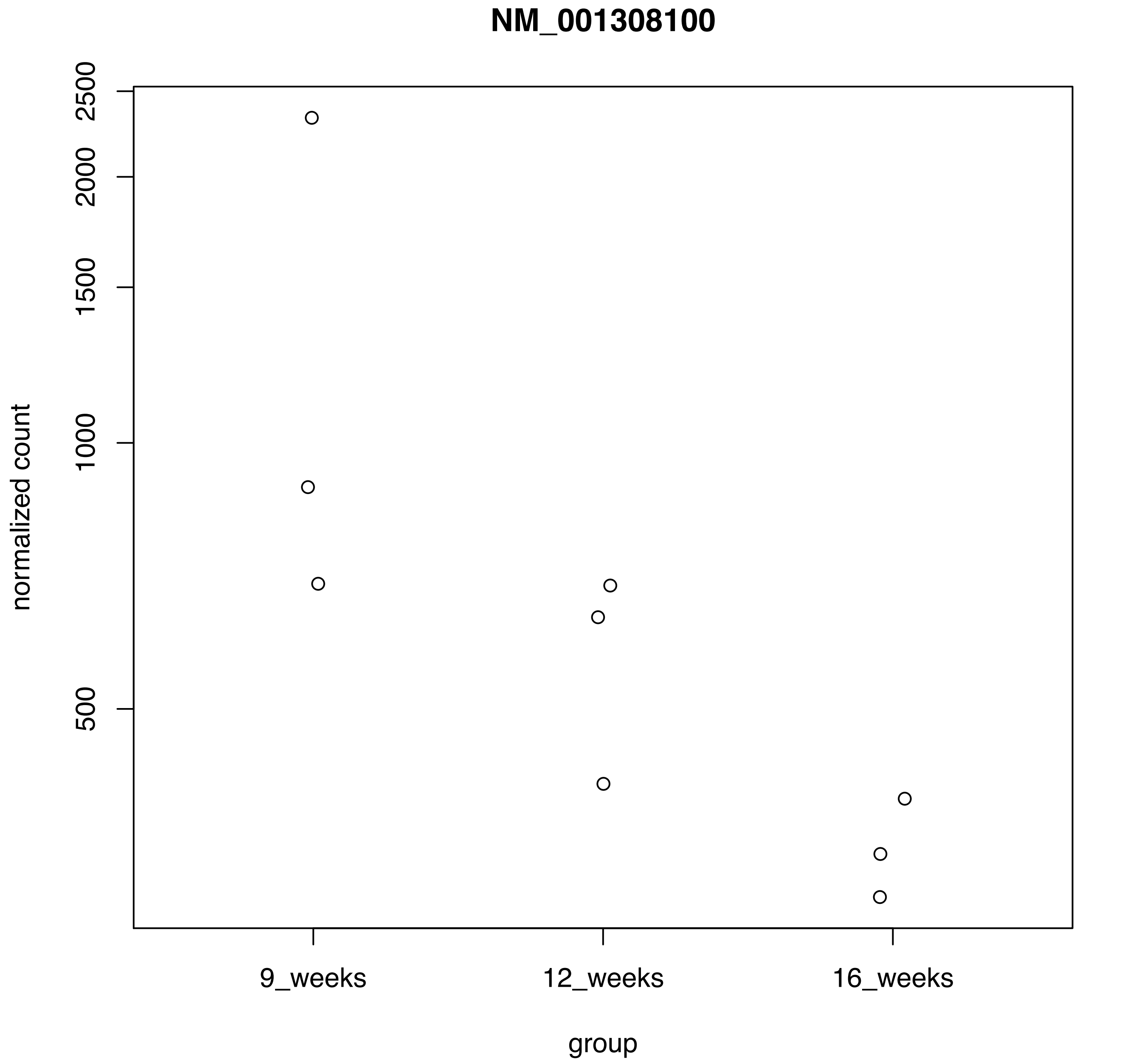

NM_001308100
normalized count
2500
2000
1500
1000
500
9_weeks
12_weeks
16_weeks
group

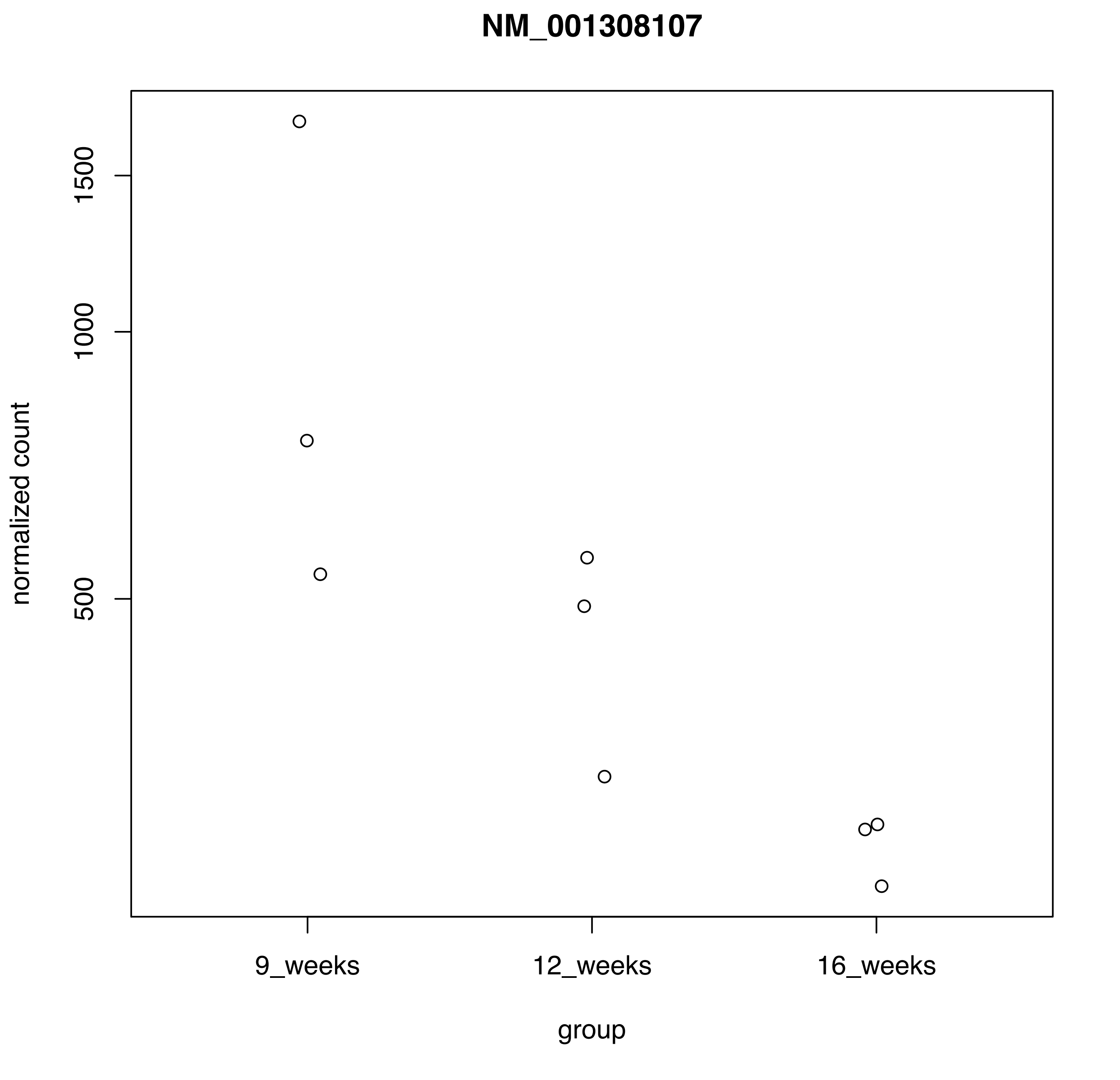
NM_001308107
normalized count
500
1000
1500
9_weeks
12_weeks
16_weeks
group

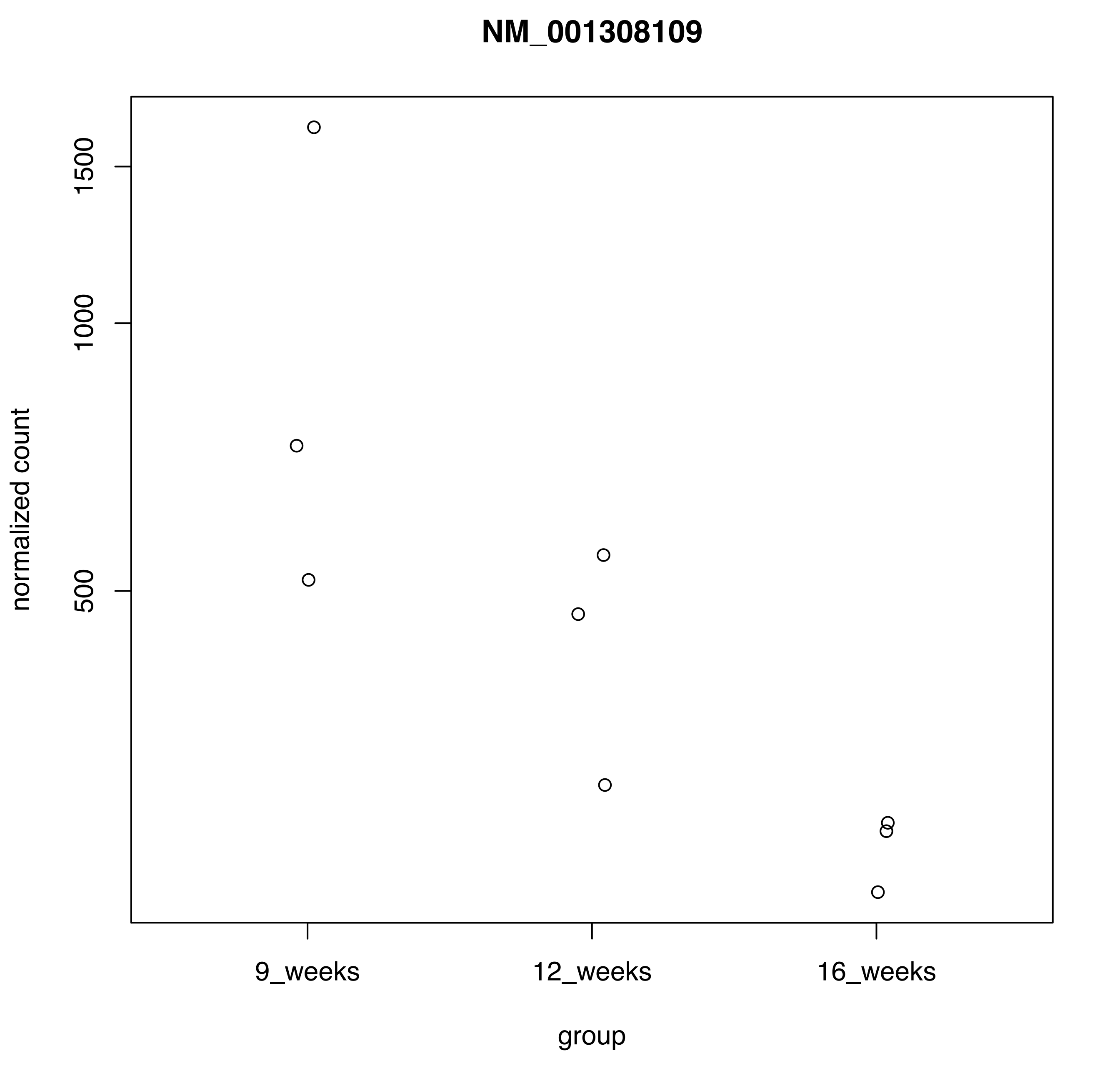

NM_001308109
normalized count
1500
1000
500
group
9_weeks
12_weeks
16_weeks

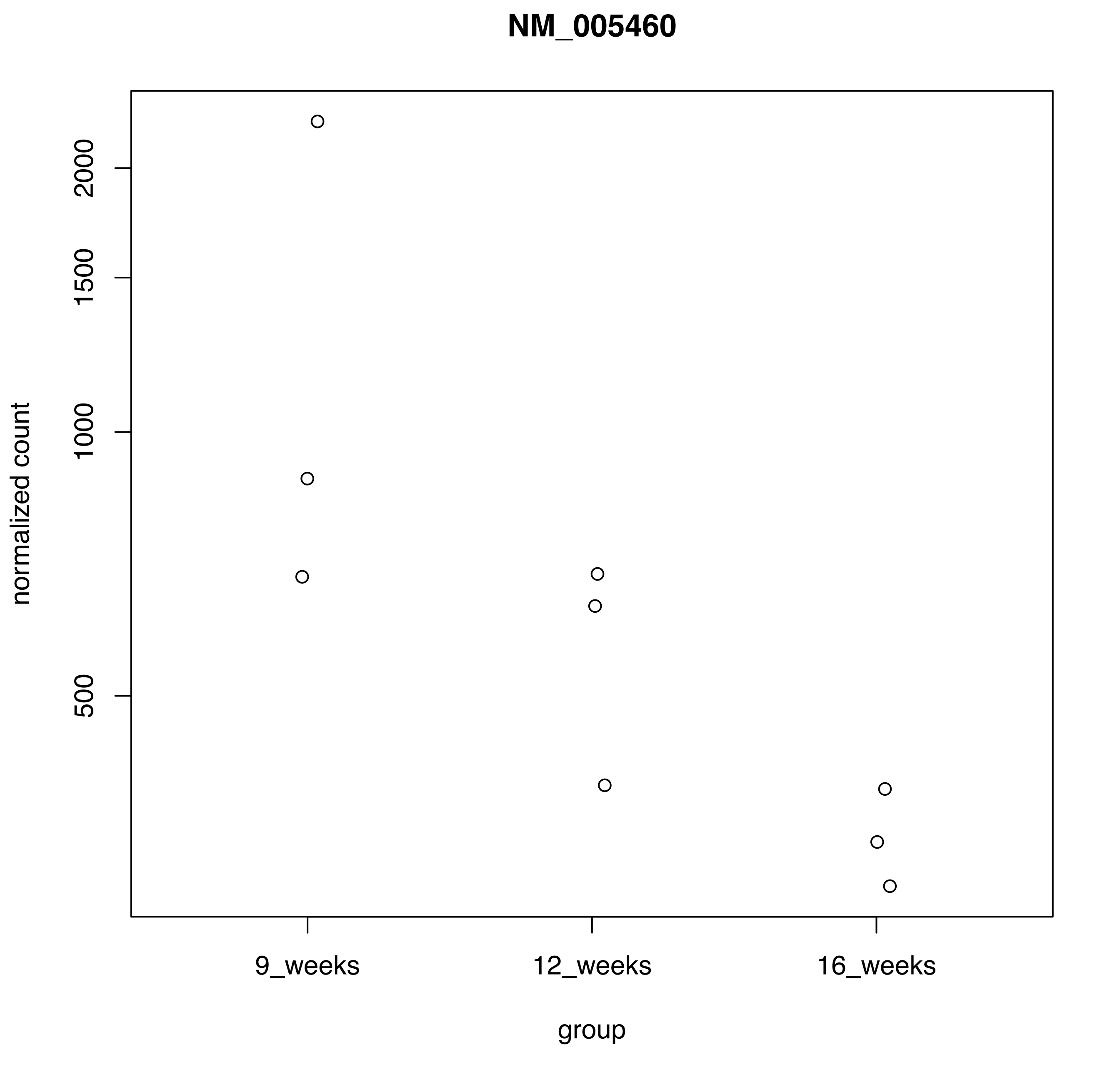
NM_005460
normalized count
2000
1500
1000
500
9_weeks
12_weeks
16_weeks
group

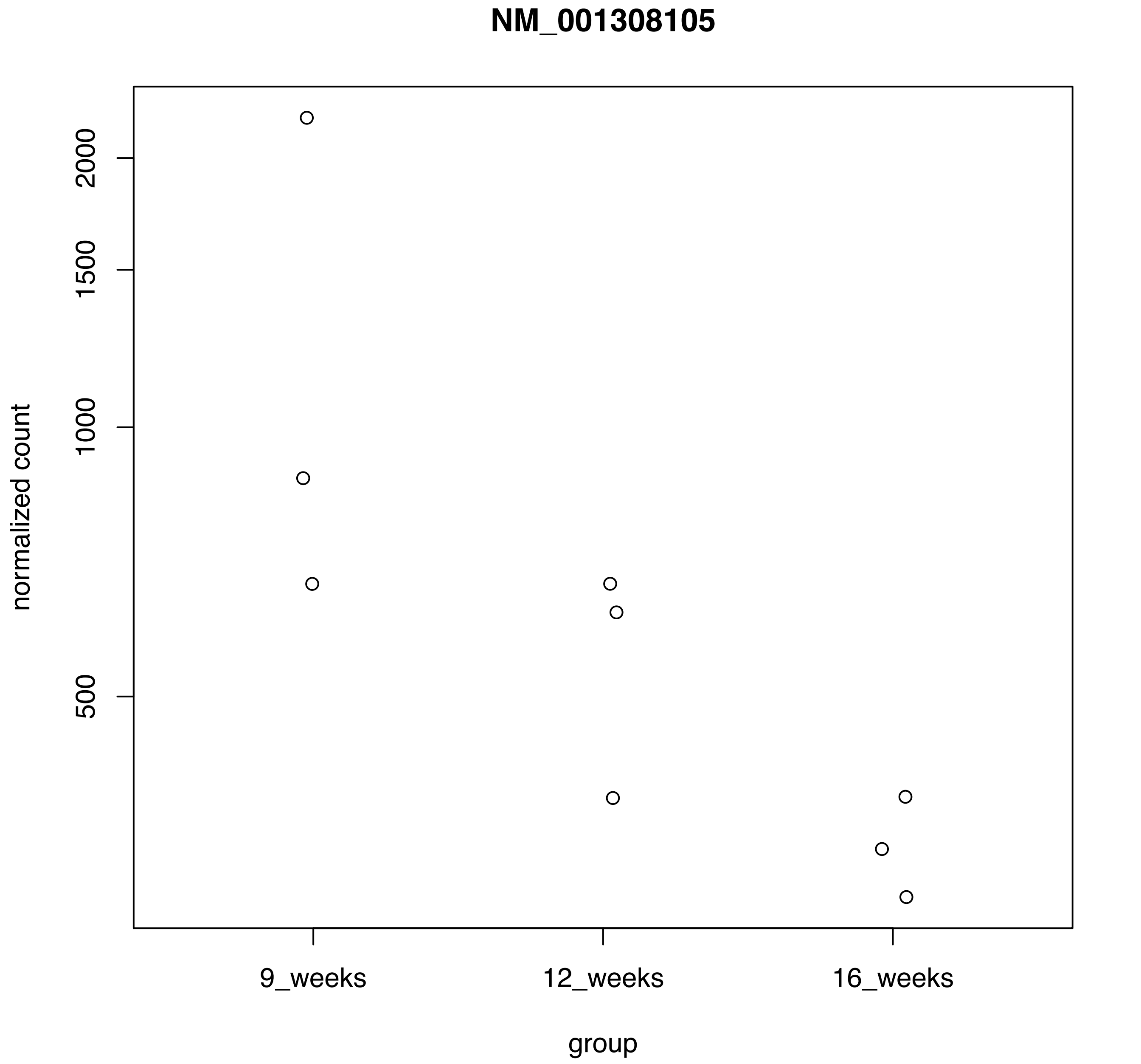

NM_001308105
normalized count
2000
1500
1000
500
9_weeks
12_weeks
16_weeks
group

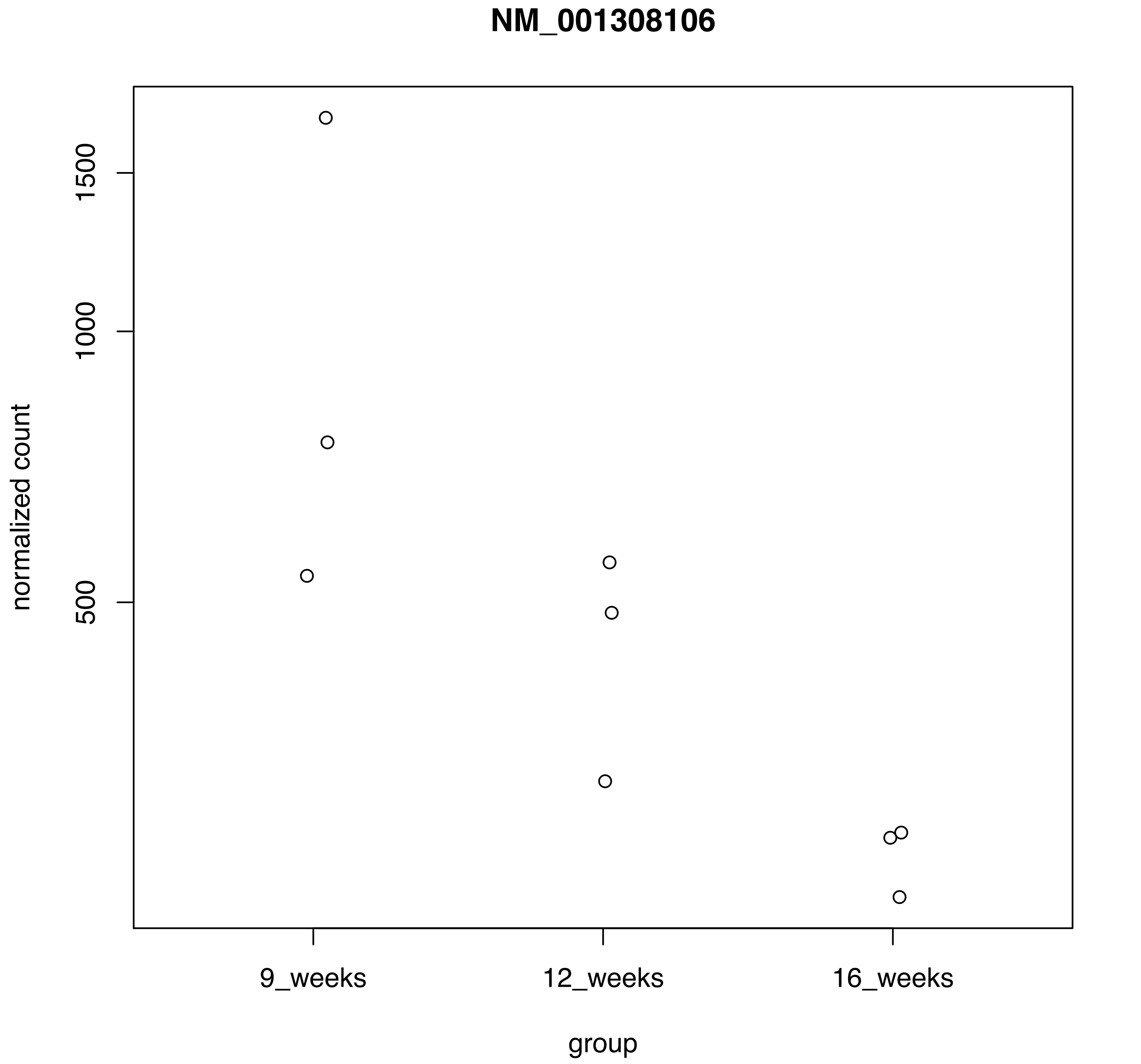
NM_001308106
normalized count
1500
1000
500
9_weeks
12_weeks
16_weeks
group

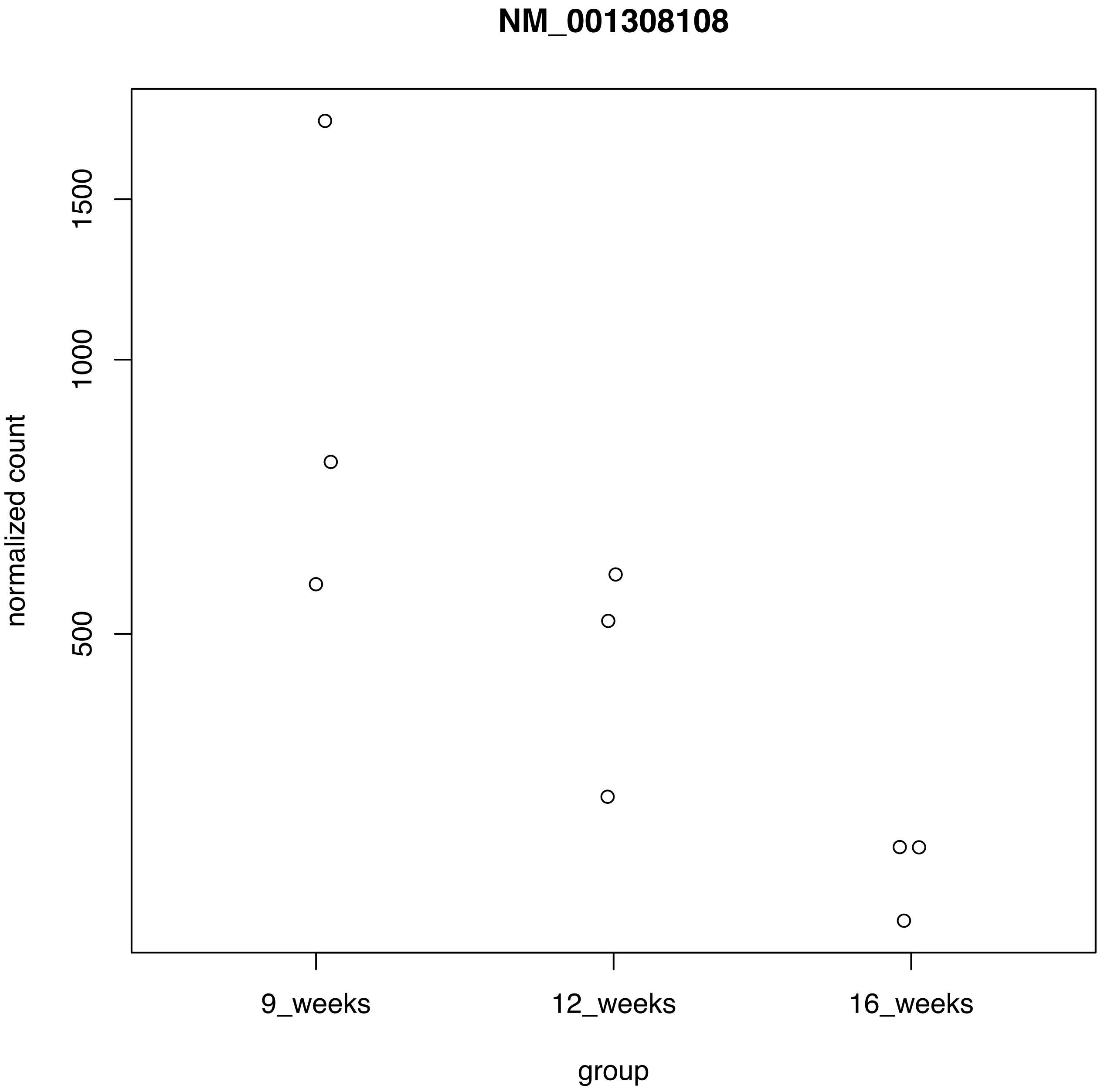

NM_001308108
normalized count
500
1000
1500
9_weeks
12_weeks
16_weeks
group

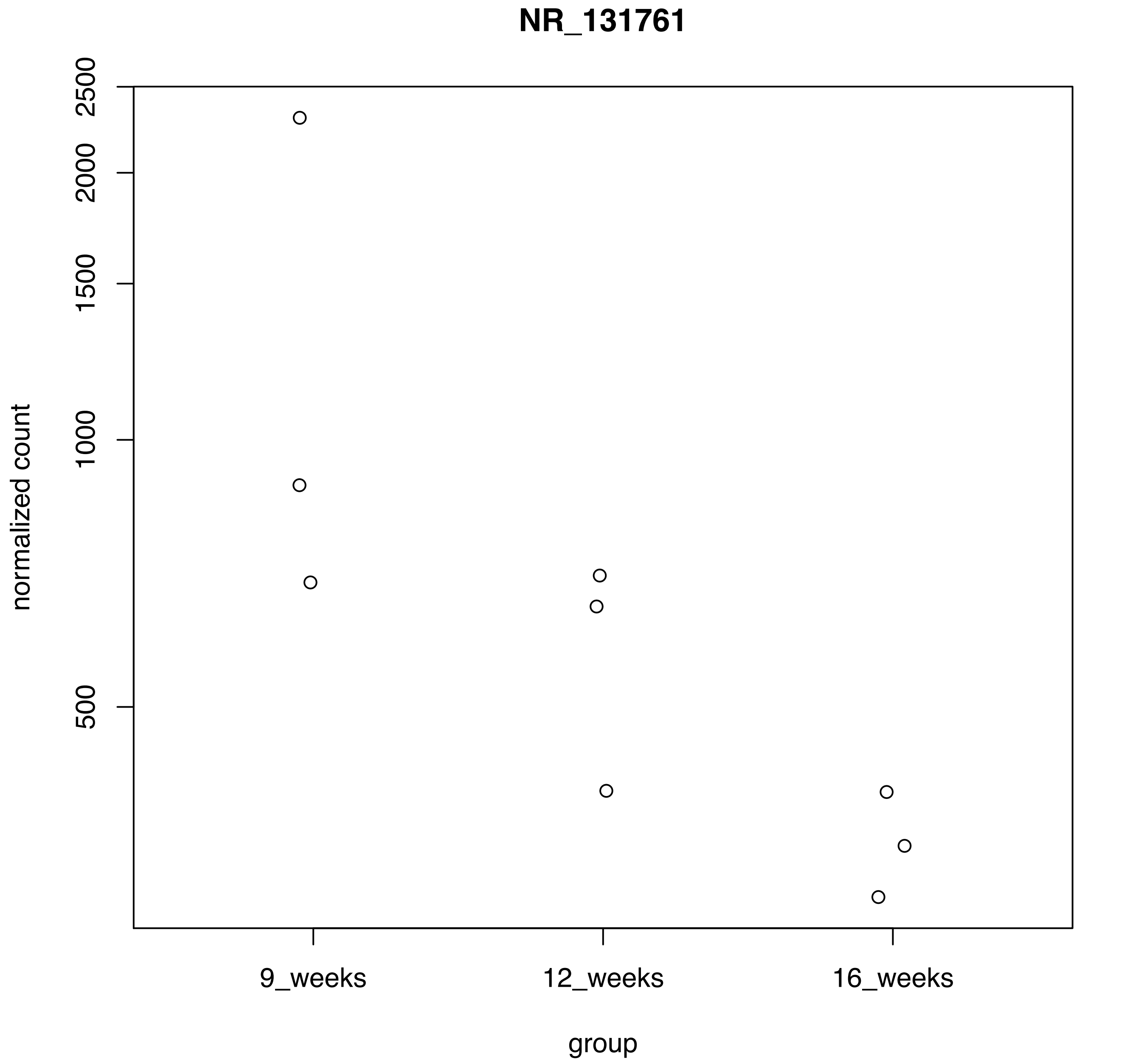

NR_131761
normalized count
2500
2000
1500
1000
500
9_weeks
12_weeks
16_weeks
group

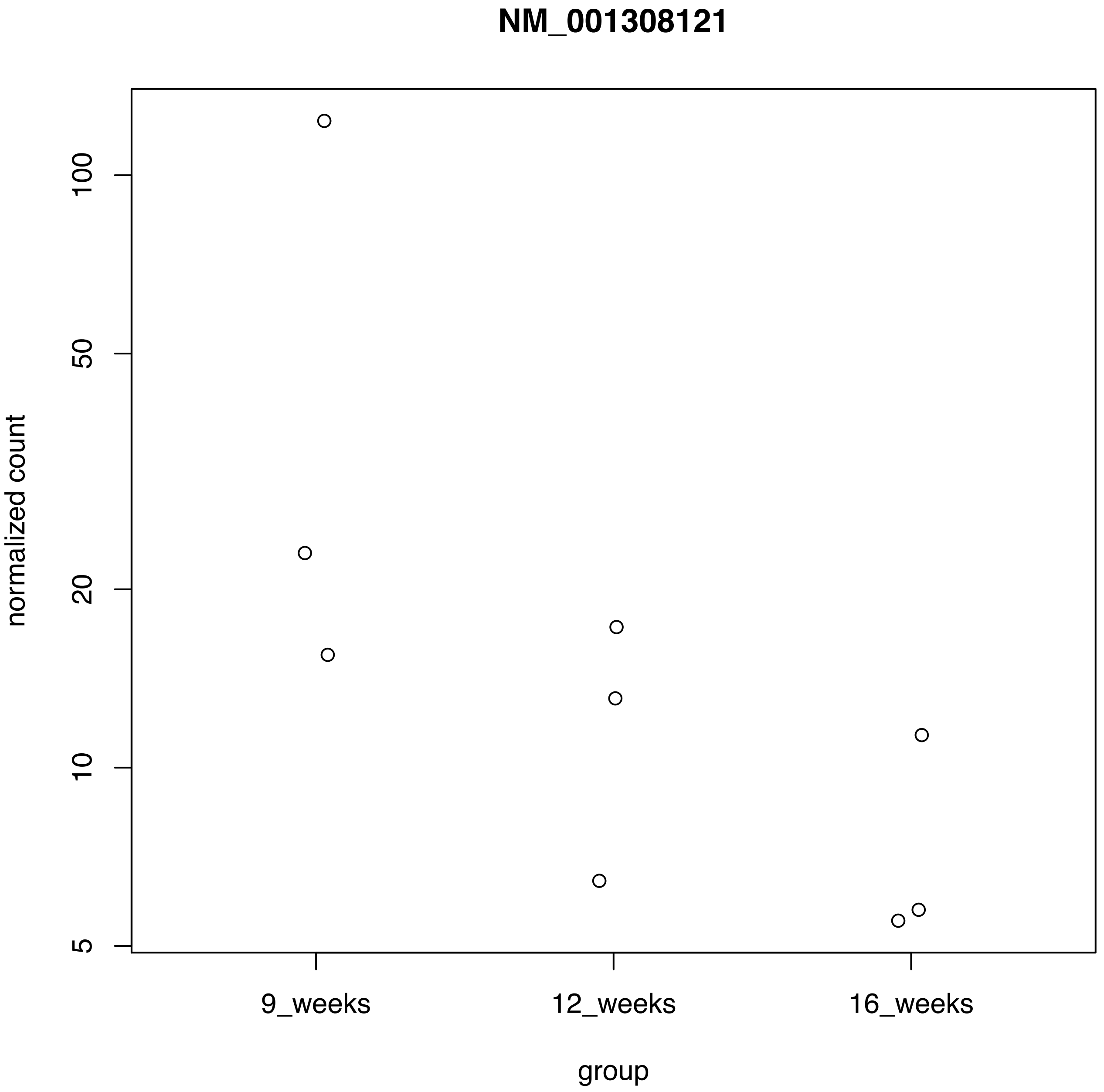
NM_001308121
normalized count
100
50
20
10
5
9_weeks
12_weeks
16_weeks
group

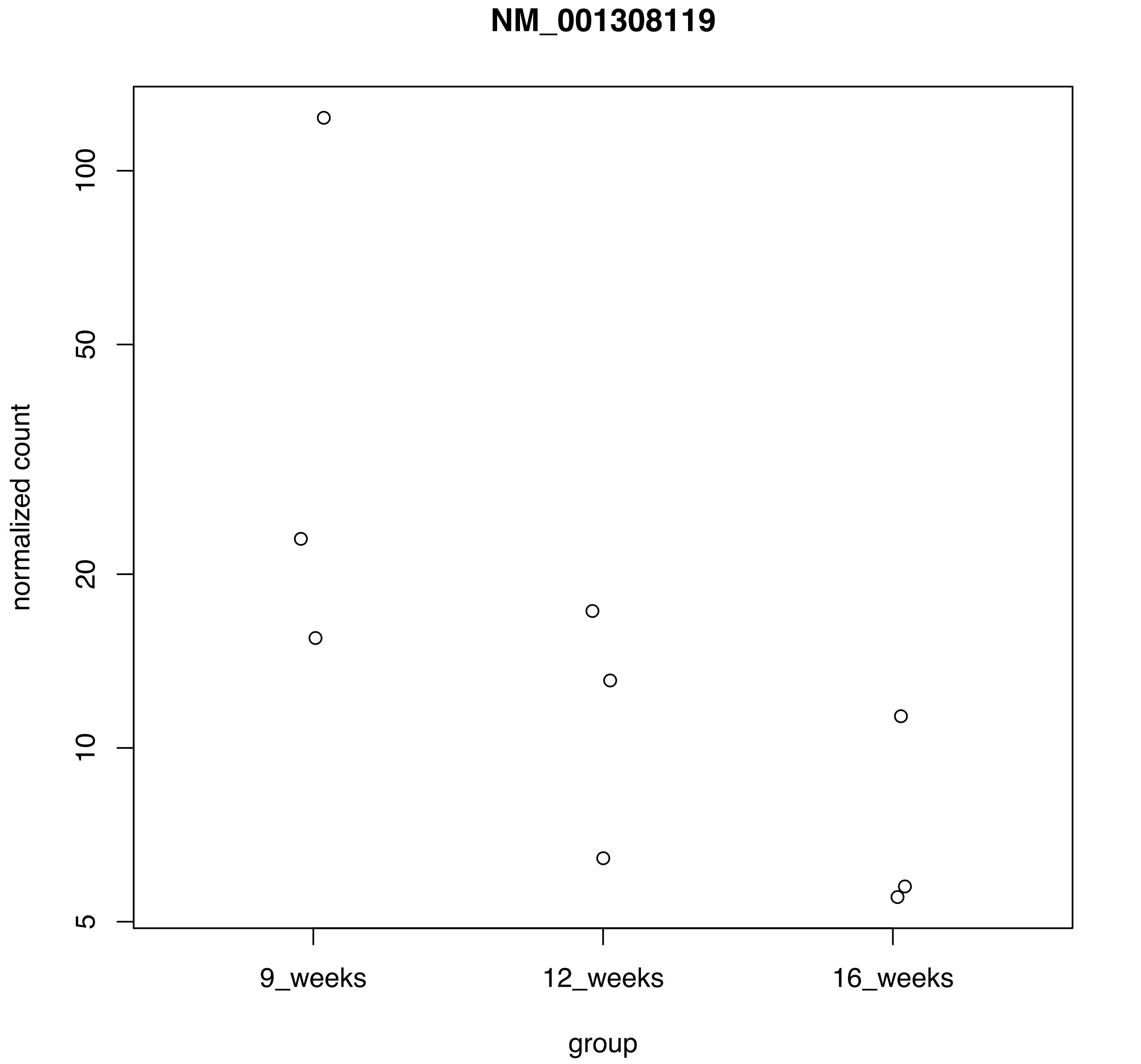

NM_001308119
normalized count
100
50
20
10
5
9_weeks
12_weeks
16_weeks
group

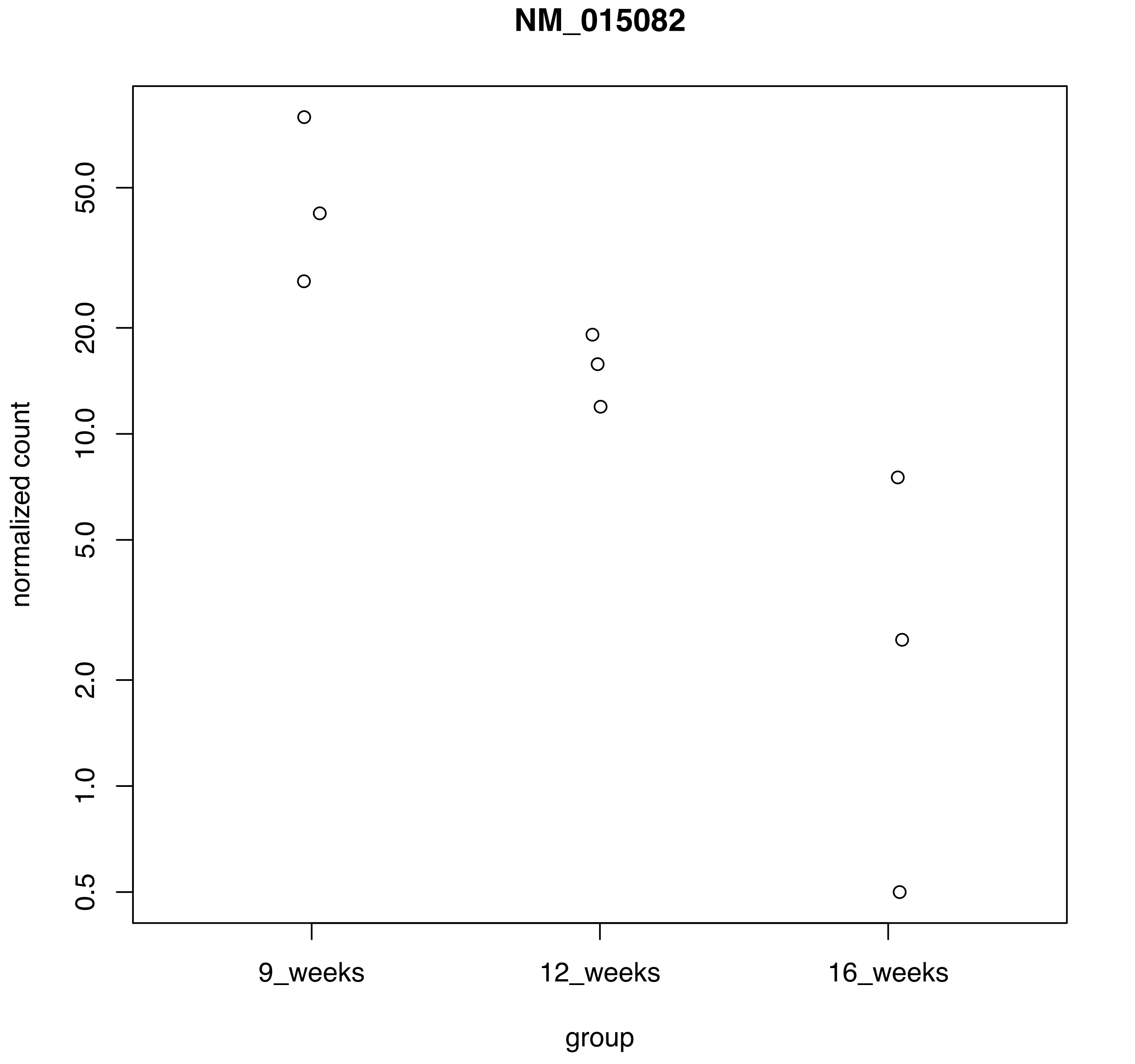

NM_015082
normalized count
50.0
20.0
10.0
5.0
2.0
1.0
0.5
9_weeks
12_weeks
16_weeks
group

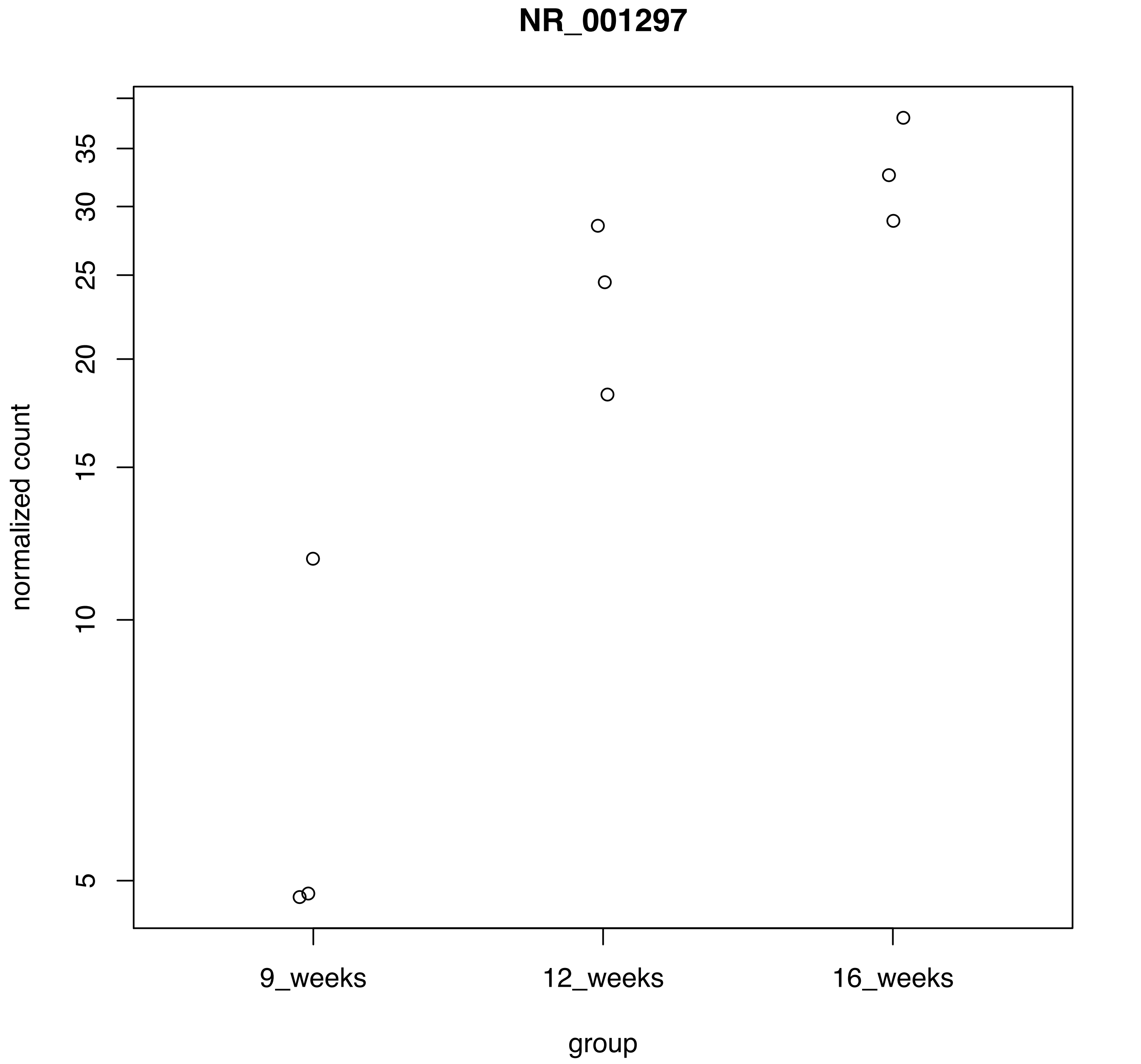
NR_001297
normalized count
35
30
25
20
15
10
5
9_weeks
12_weeks
16_weeks
group

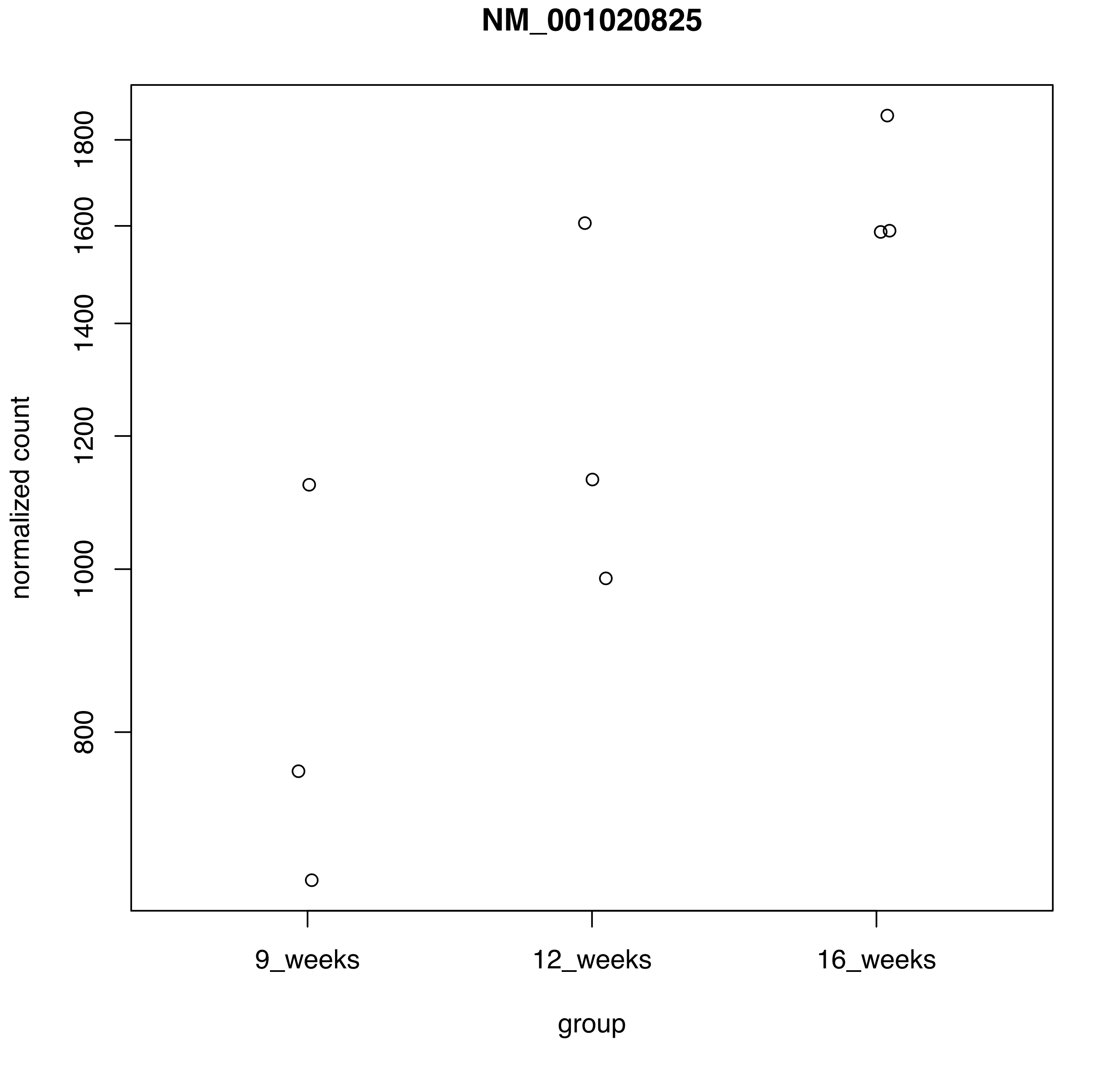
NM_001020825
normalized count
1800
1600
1400
1200
1000
800
9_weeks
12_weeks
16_weeks
group

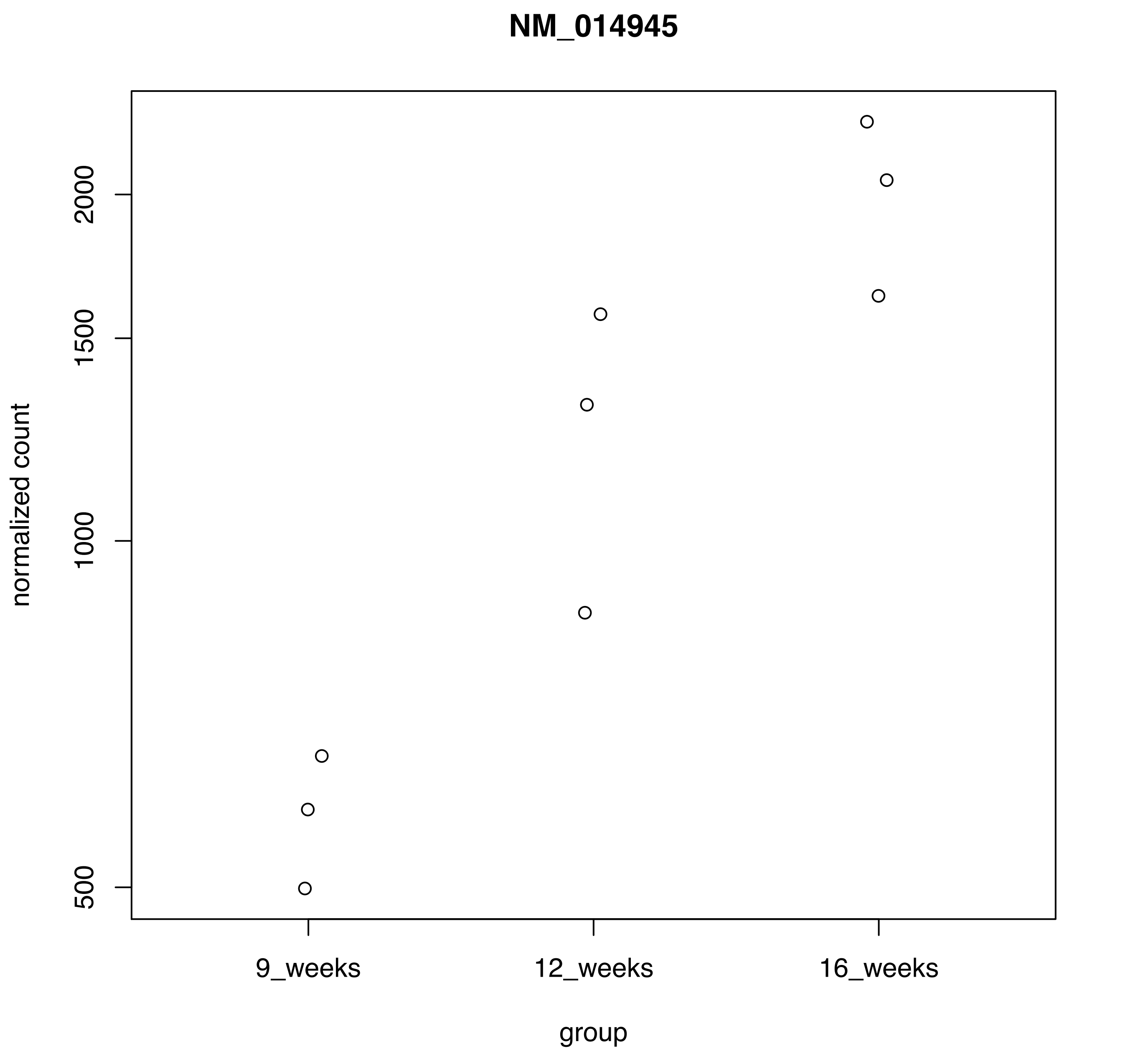
NM_014945
normalized count
500
1000
1500
2000
9_weeks
12_weeks
16_weeks
group

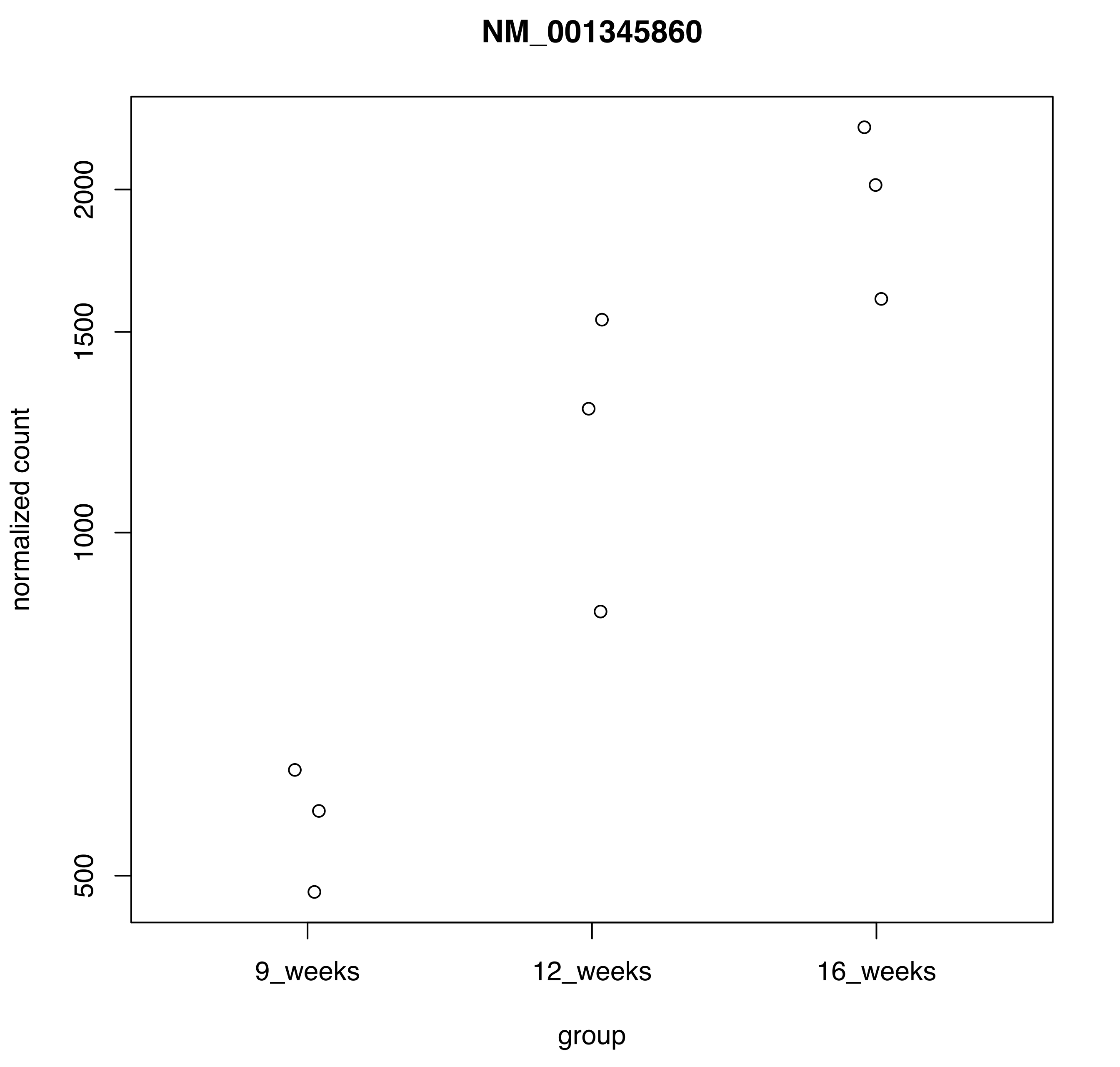
NM_001345860
normalized count
500
1000
1500
2000
9_weeks
12_weeks
16_weeks
group

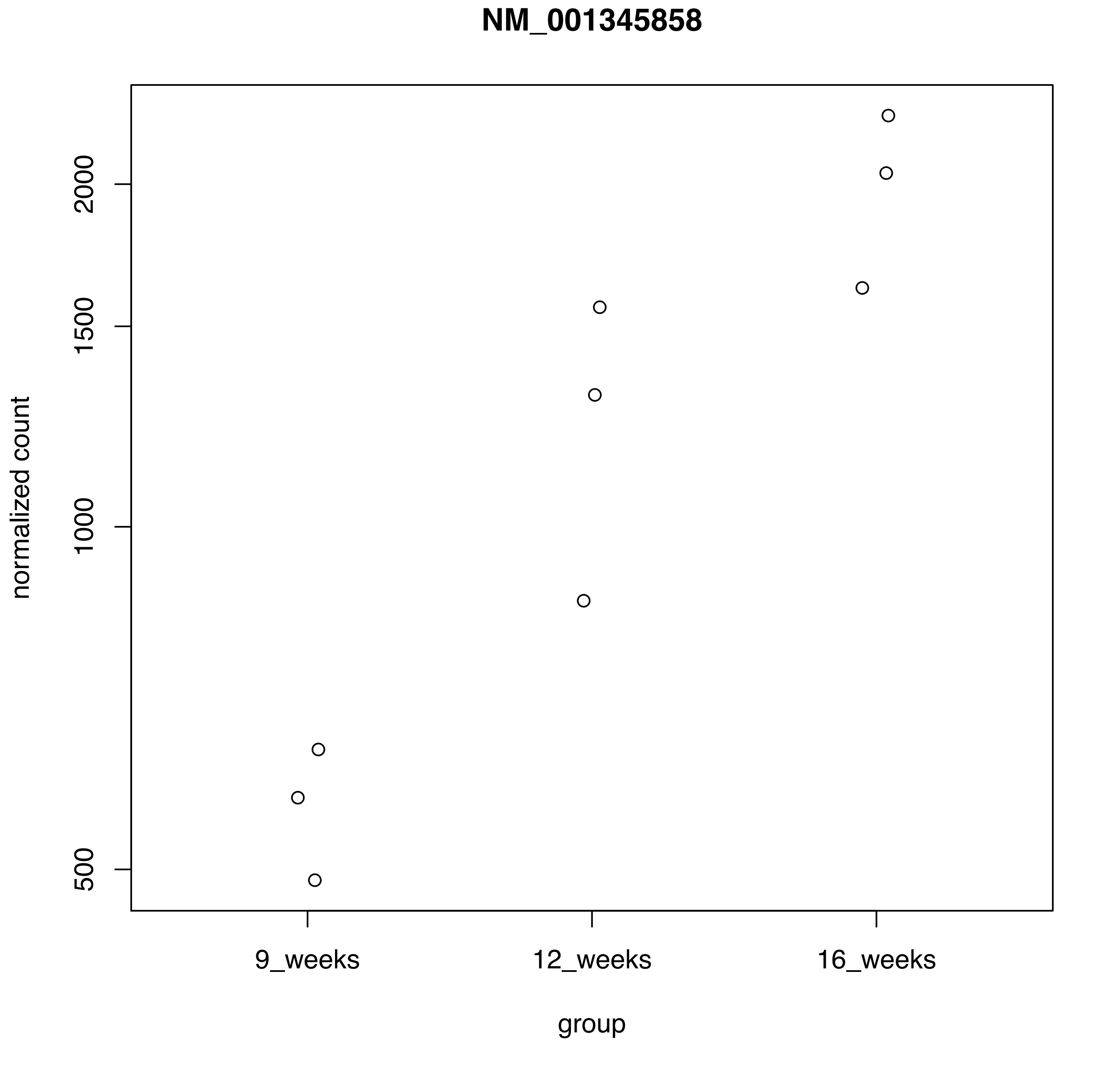

NM_001345858
normalized count
500
1000
1500
2000
9_weeks
12_weeks
16_weeks
group

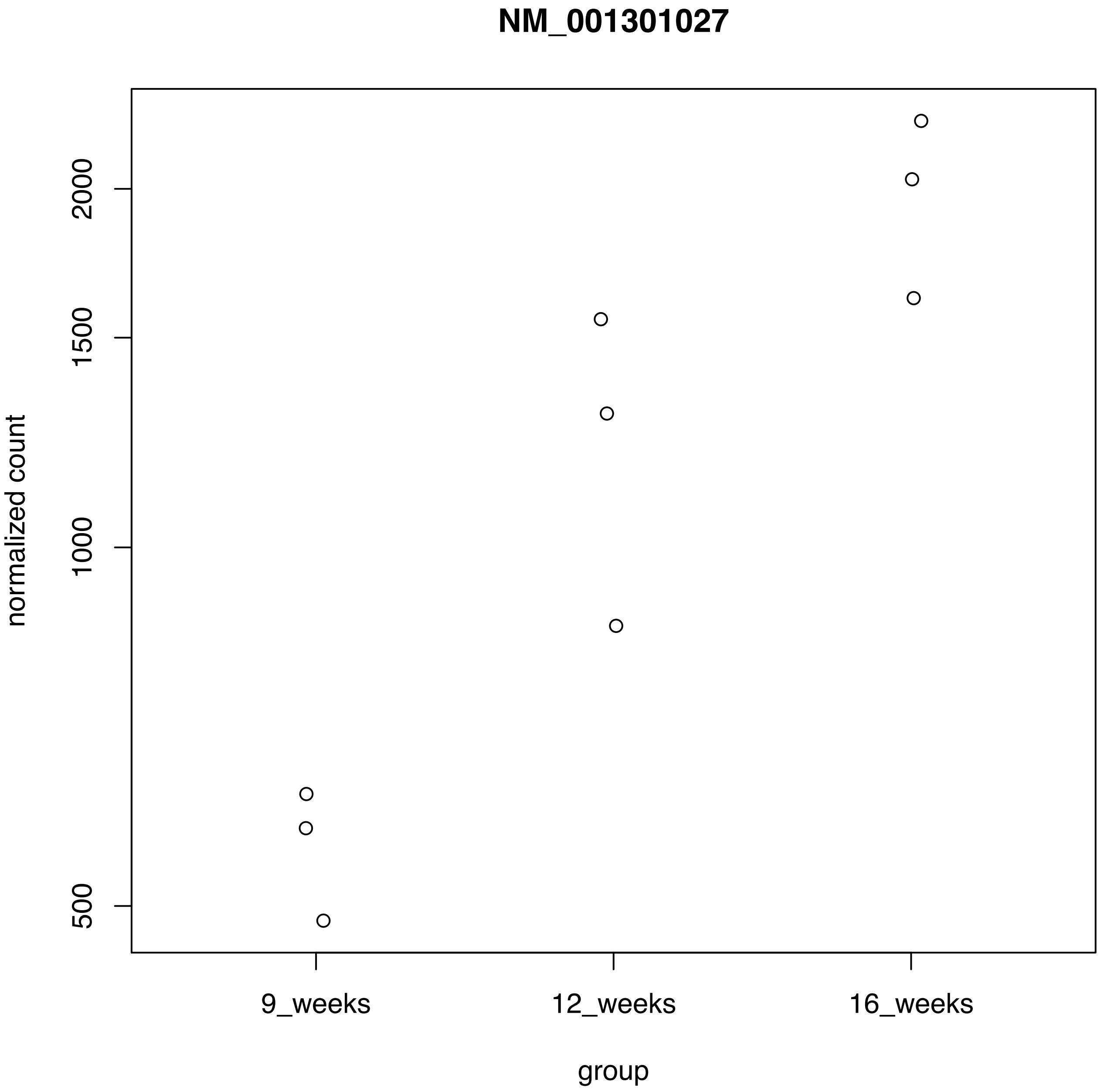

NM_001301027
normalized count
500
1000
1500
2000
9_weeks
12_weeks
16_weeks
group

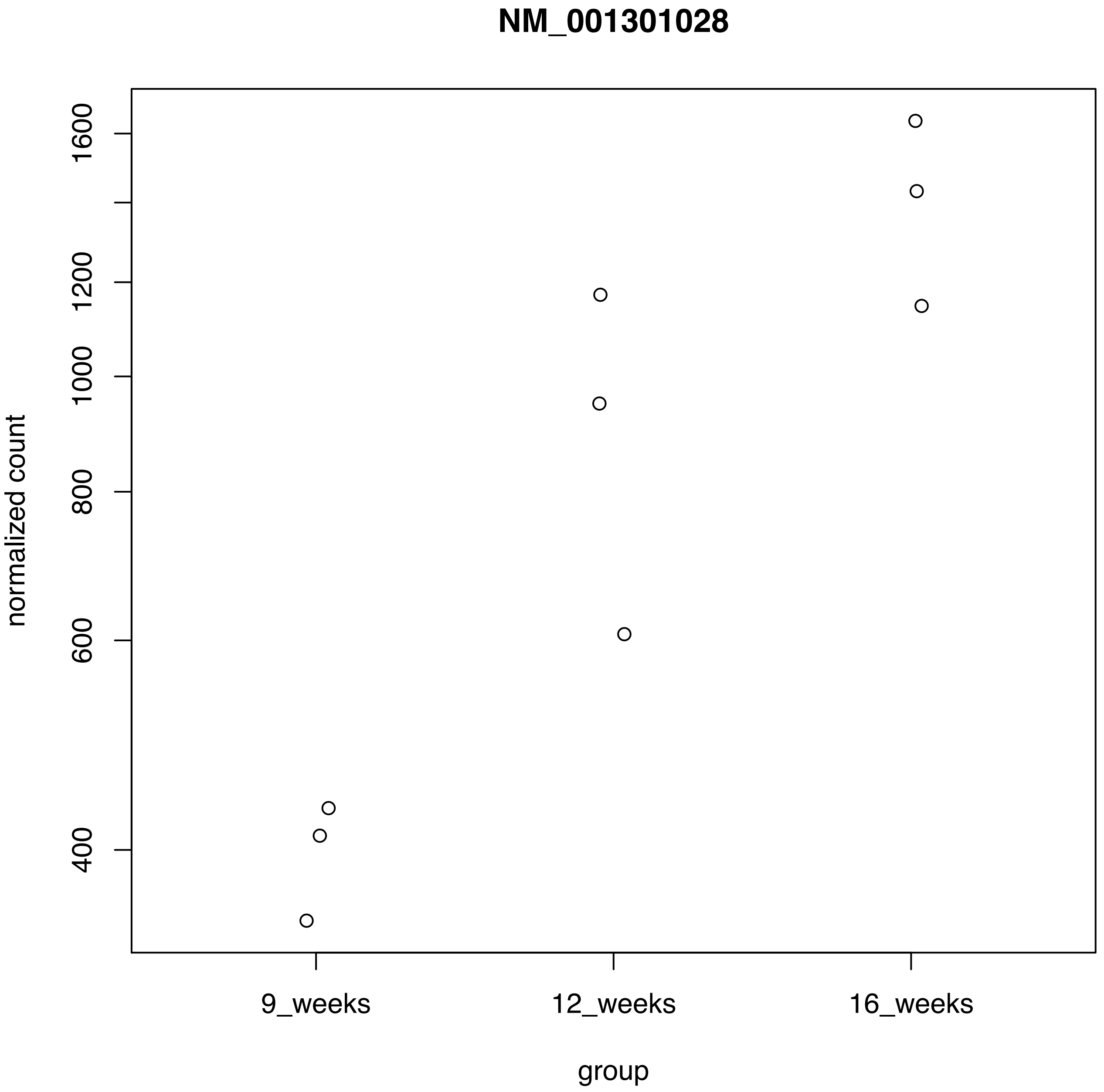
NM_001301028
normalized count
400
600
800
1000
1200
1600
9_weeks
12_weeks
16_weeks
group

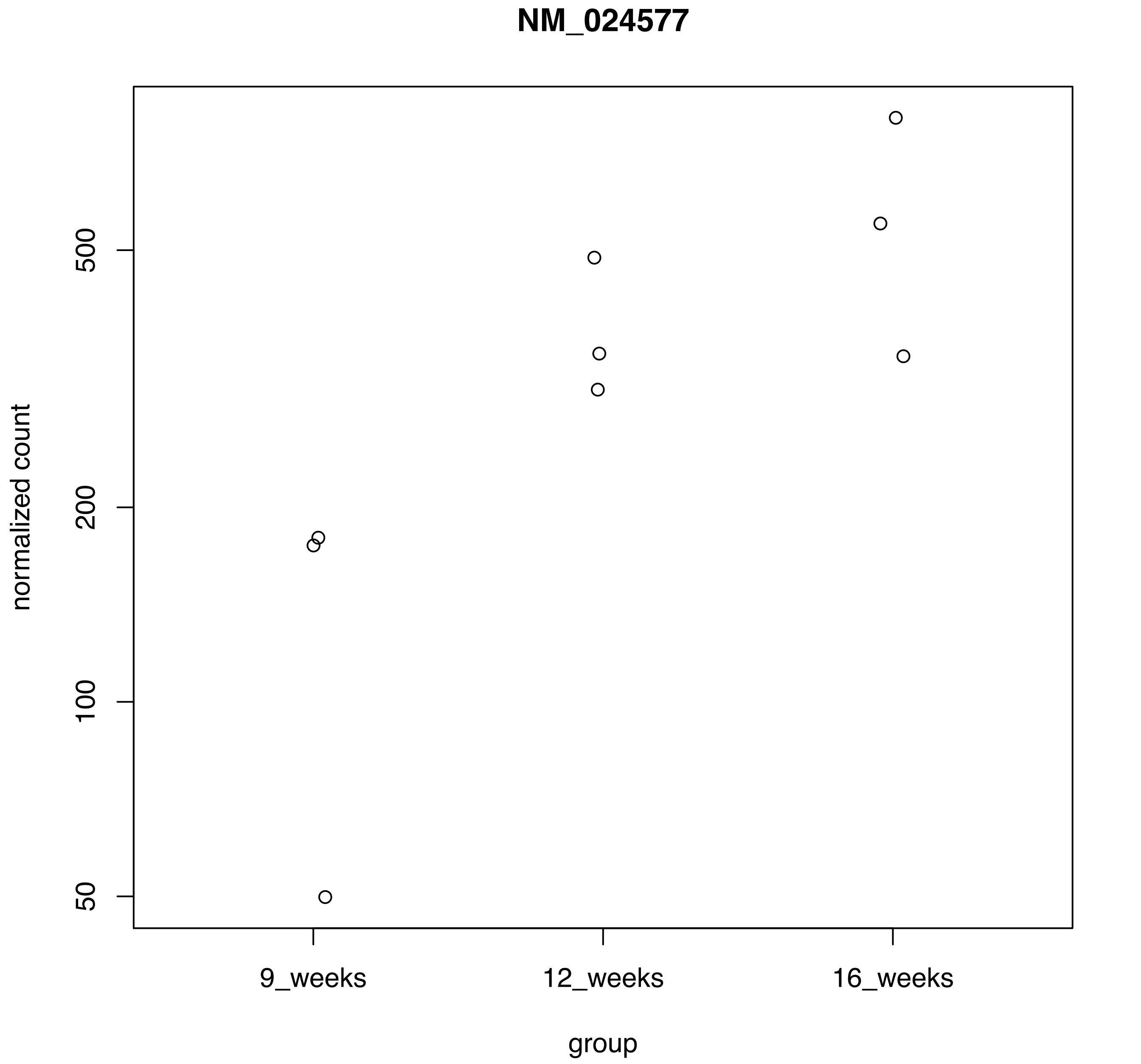
NM_024577
normalized count
500
200
100
50
9_weeks
12_weeks
16_weeks
group

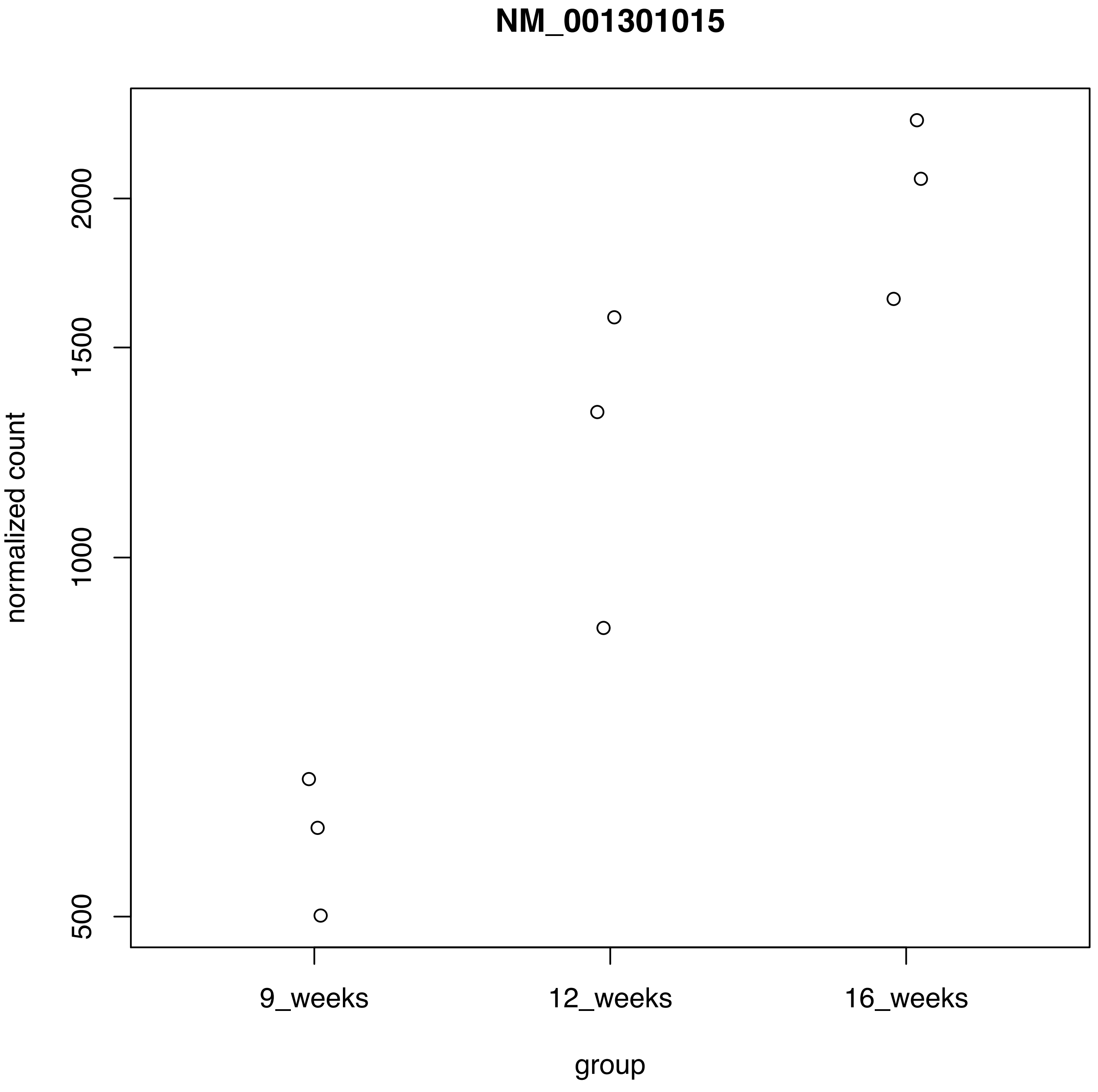
NM_001301015
normalized count
2000
1500
1000
500
9_weeks
12_weeks
16_weeks
group

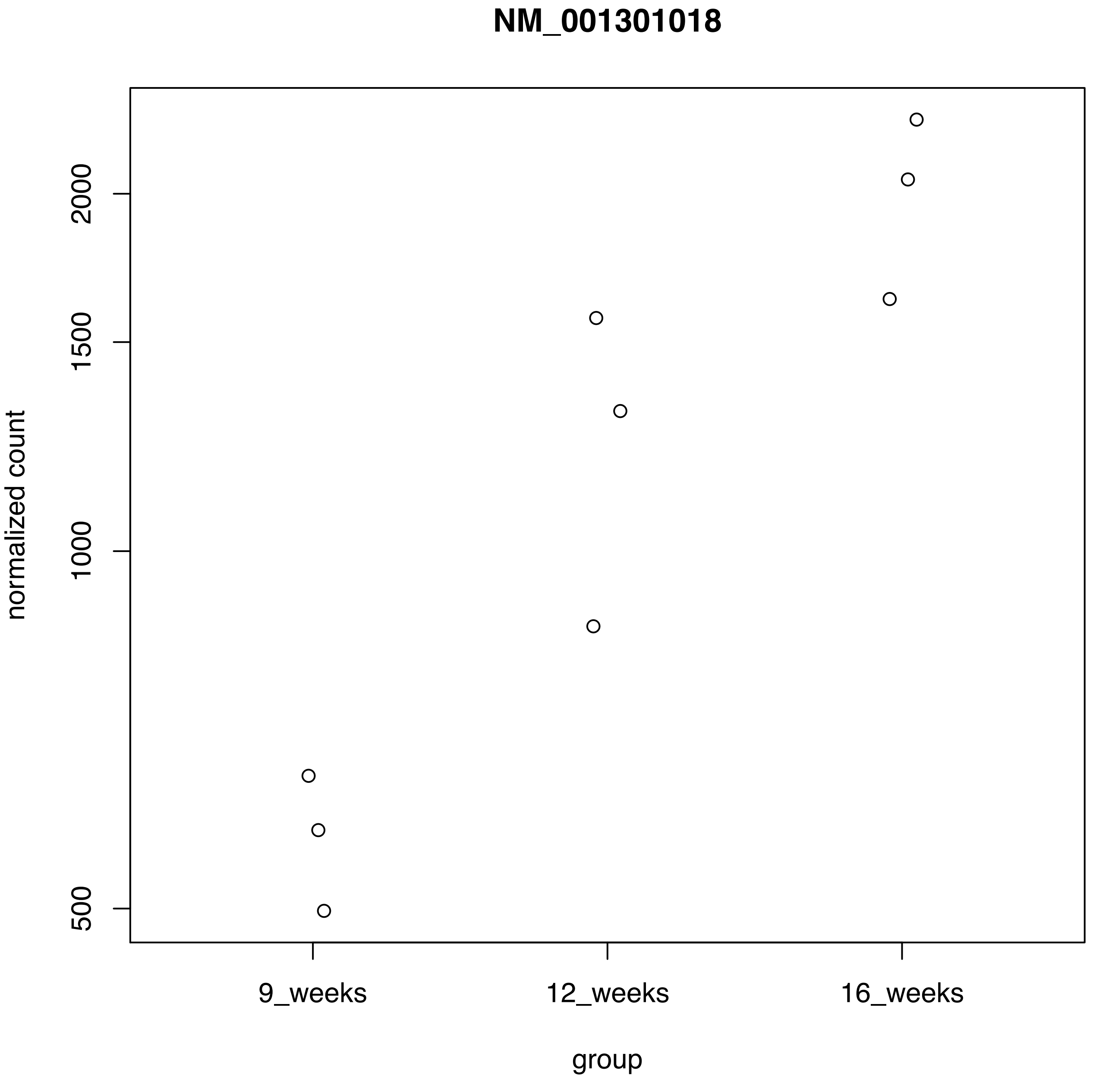

NM_001301018
normalized count
500
1000
1500
2000
9_weeks
12_weeks
16_weeks
group

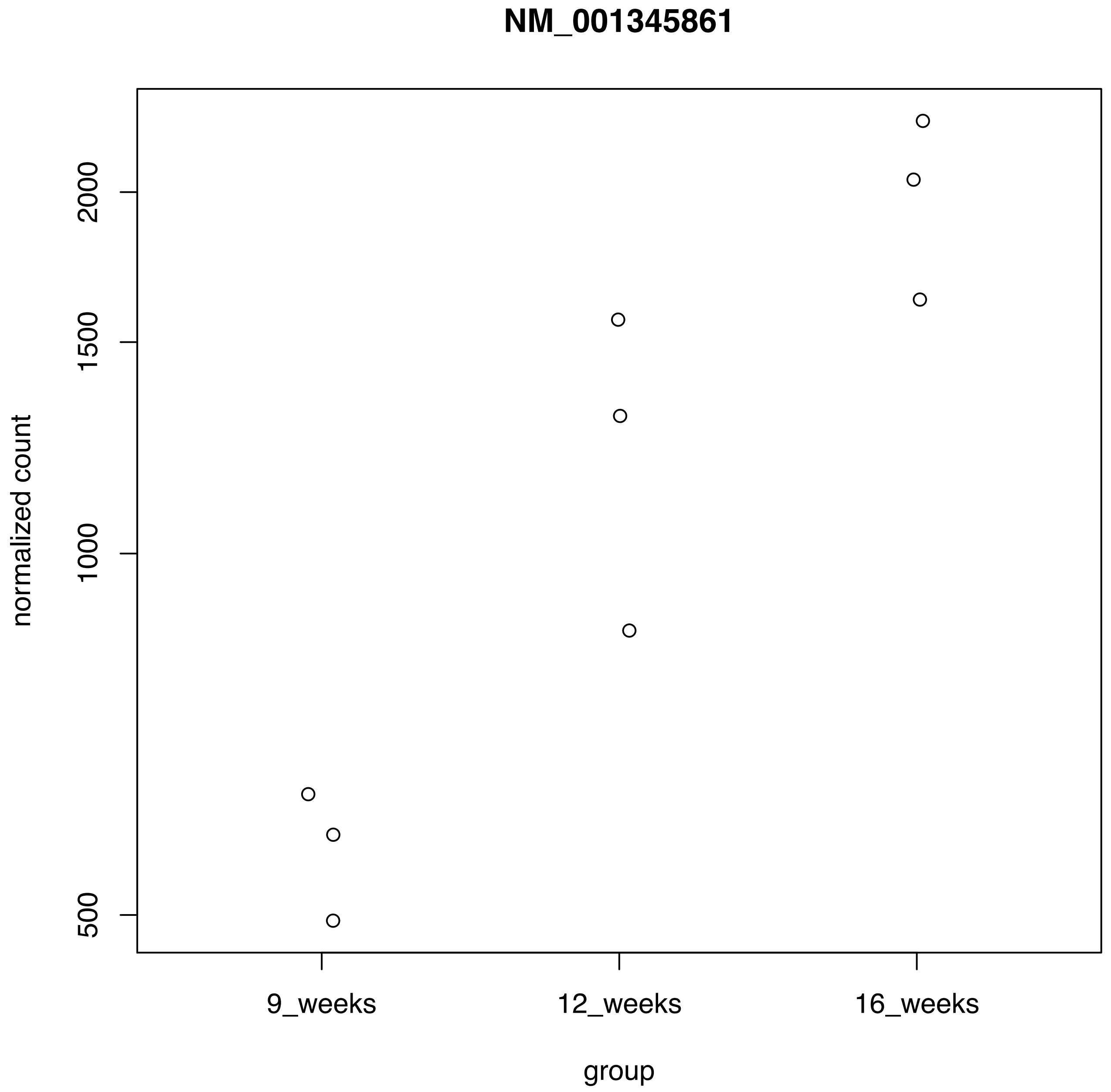

NM_001345861
normalized count
500
1000
1500
2000
9_weeks
12_weeks
16_weeks
group

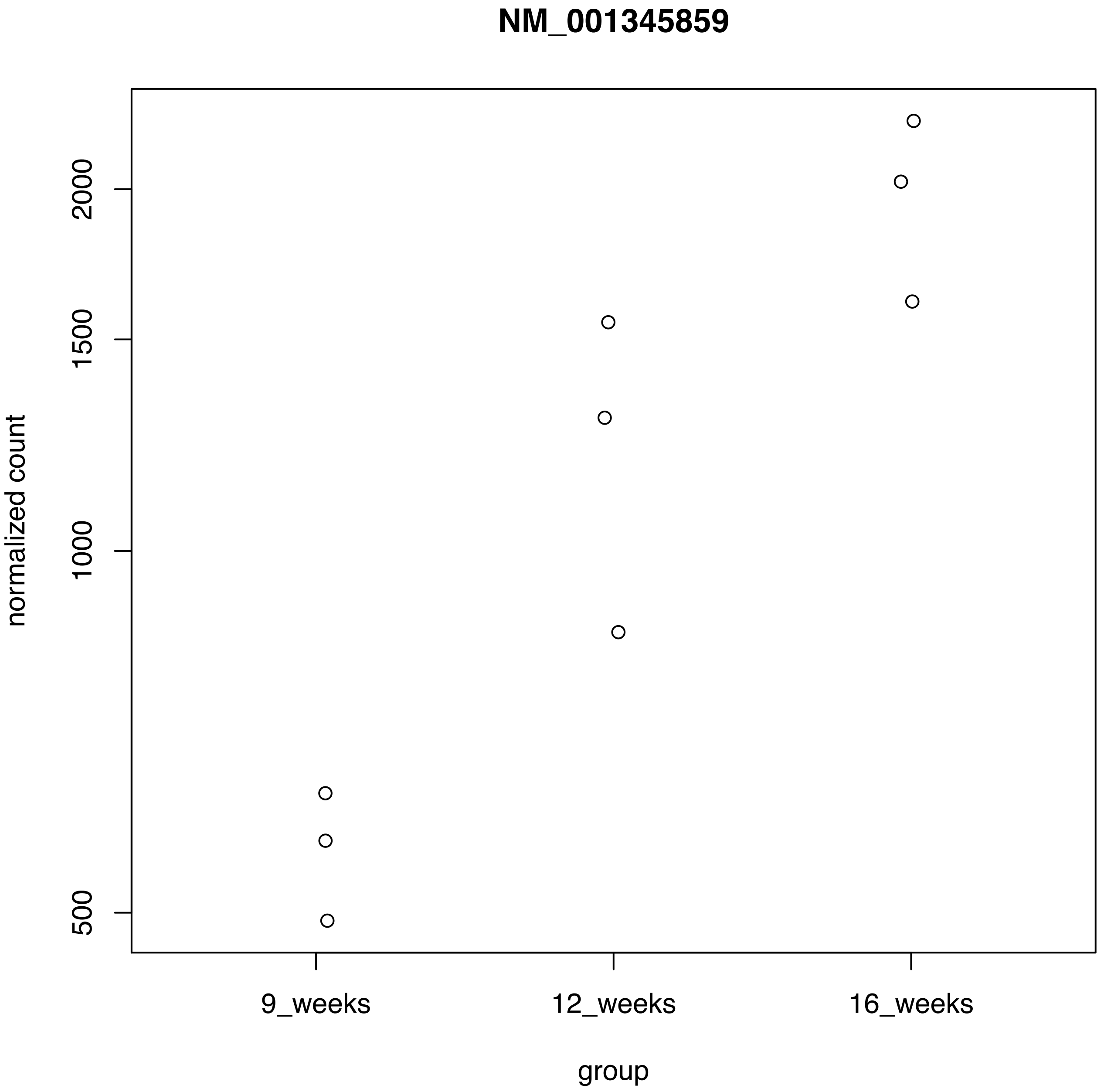
NM_001345859
normalized count
500
1000
1500
2000
9_weeks
12_weeks
16_weeks
group

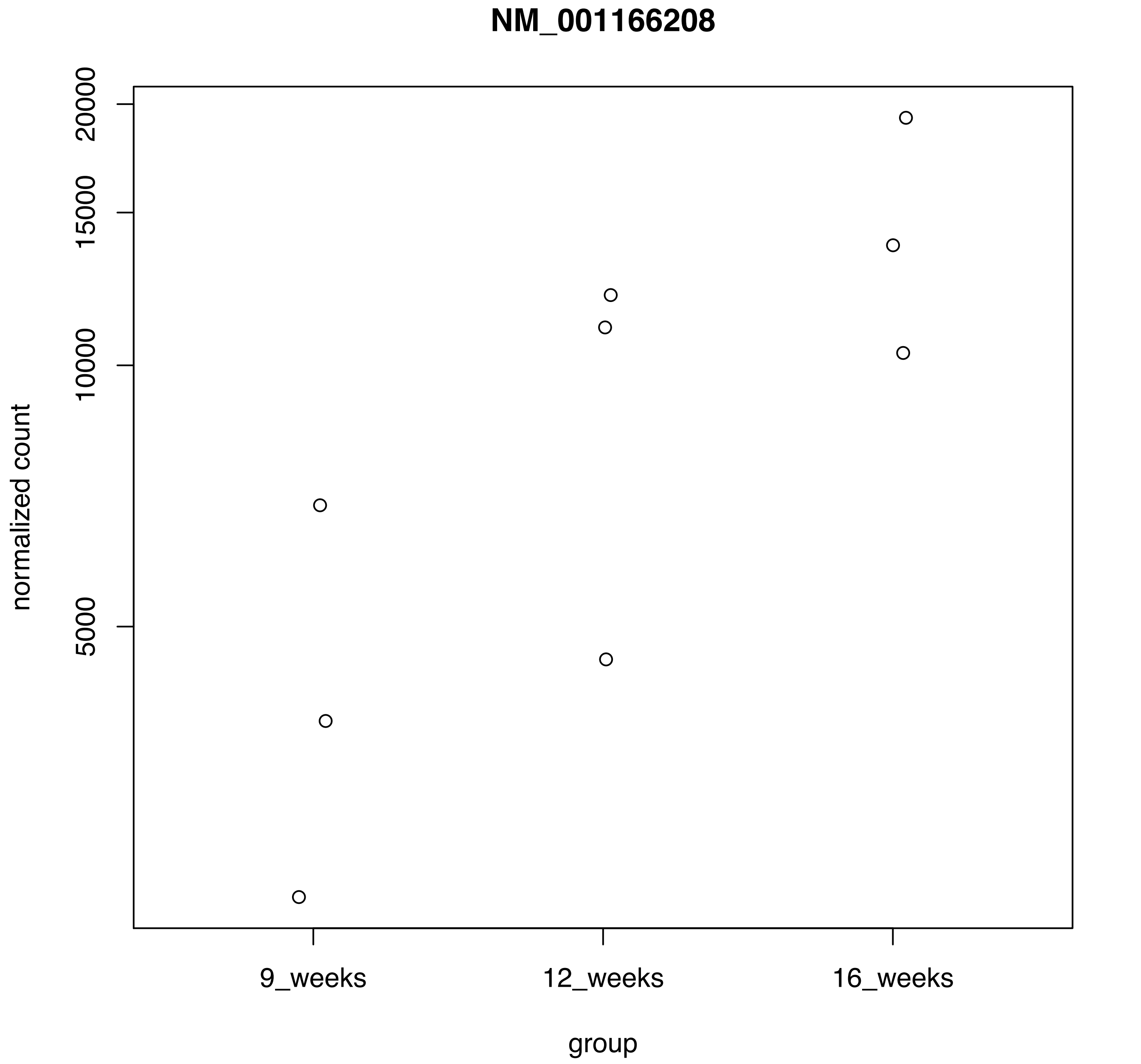
NM_001166208
normalized count
20000
15000
10000
5000
9_weeks
12_weeks
16_weeks
group

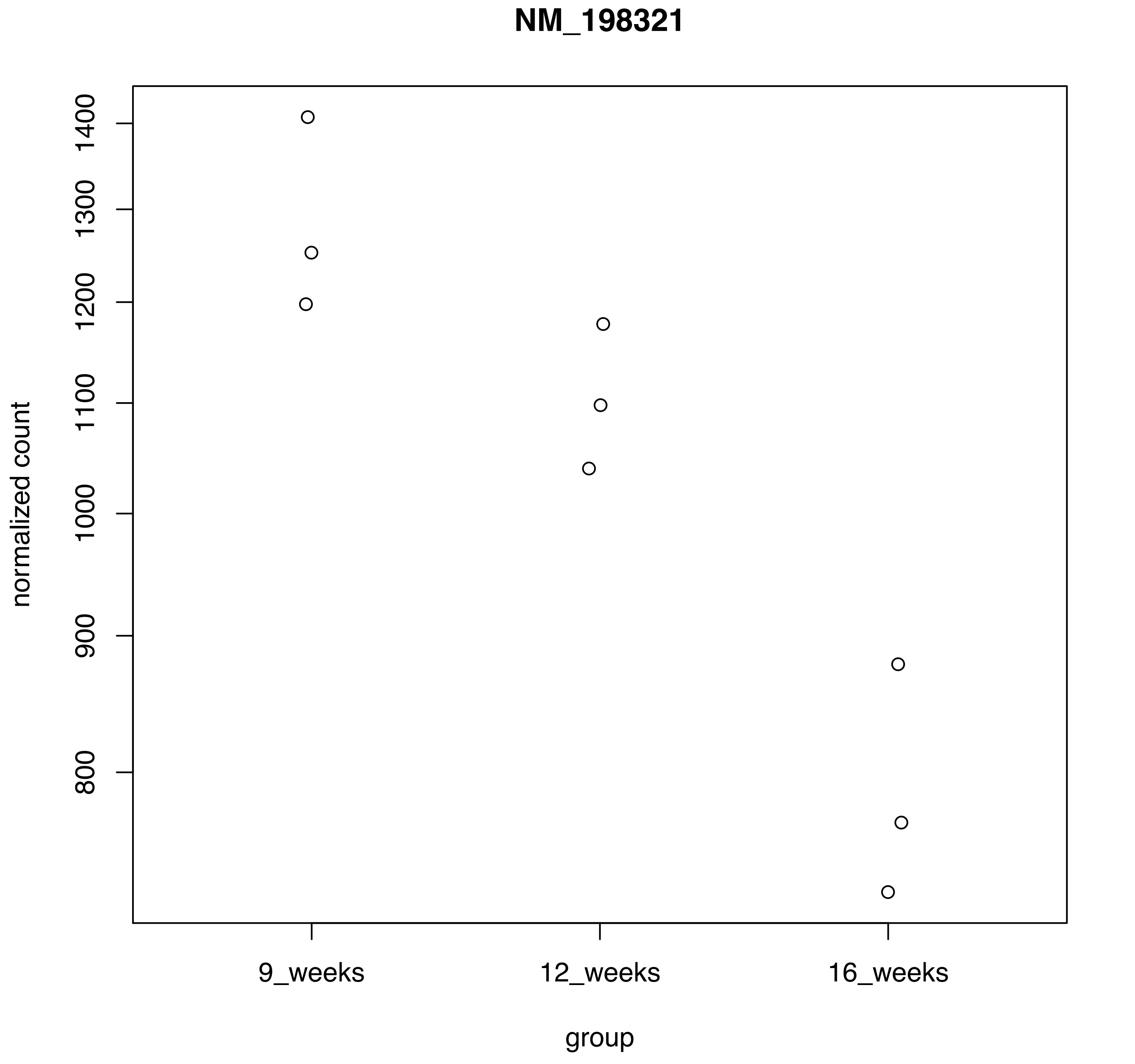

NM_198321
normalized count
1400
1300
1200
1100
1000
900
800
9_weeks
12_weeks
16_weeks
group

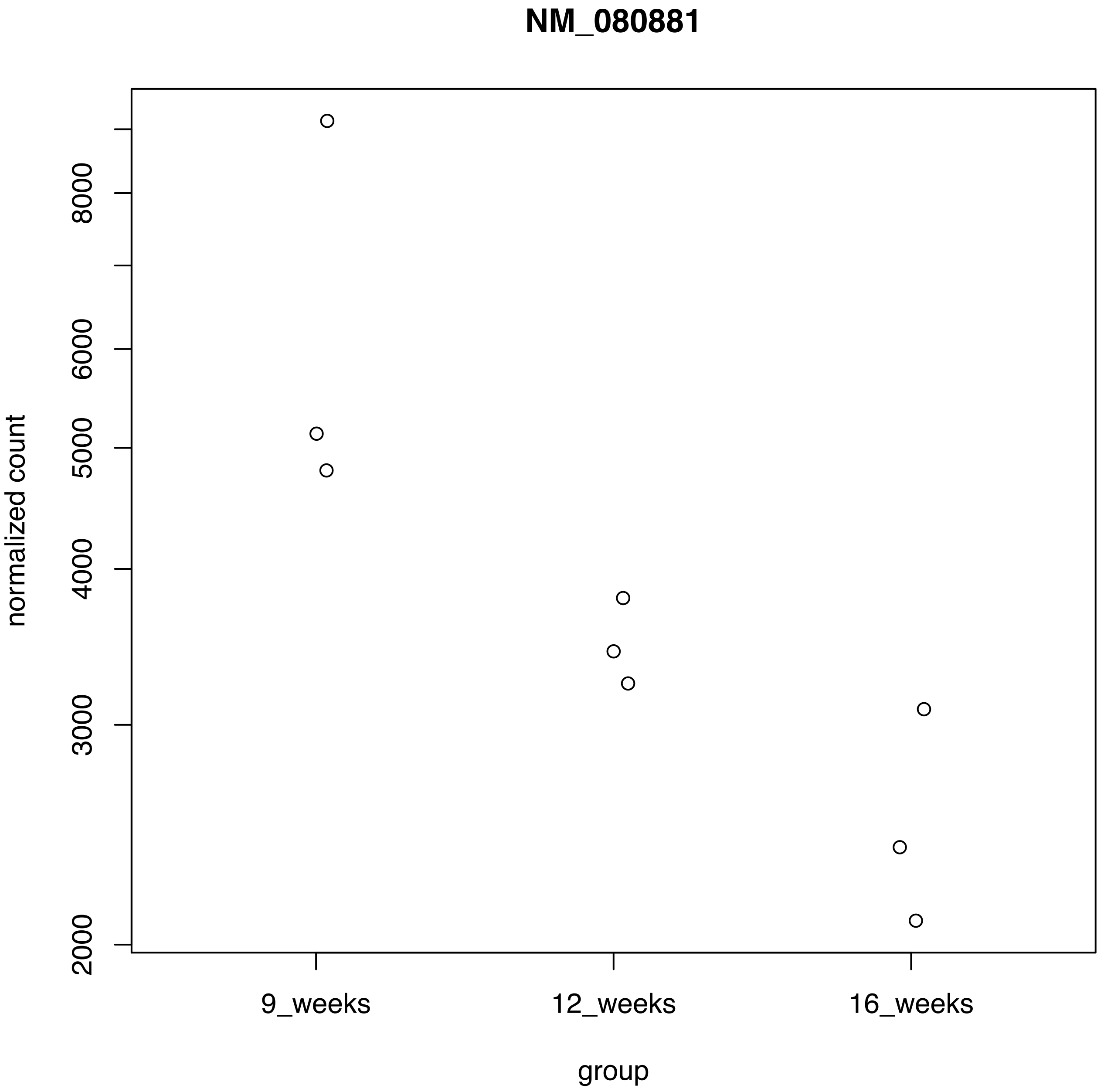
NM_080881
normalized count
2000
3000
4000
5000
6000
8000
9_weeks
12_weeks
16_weeks
group

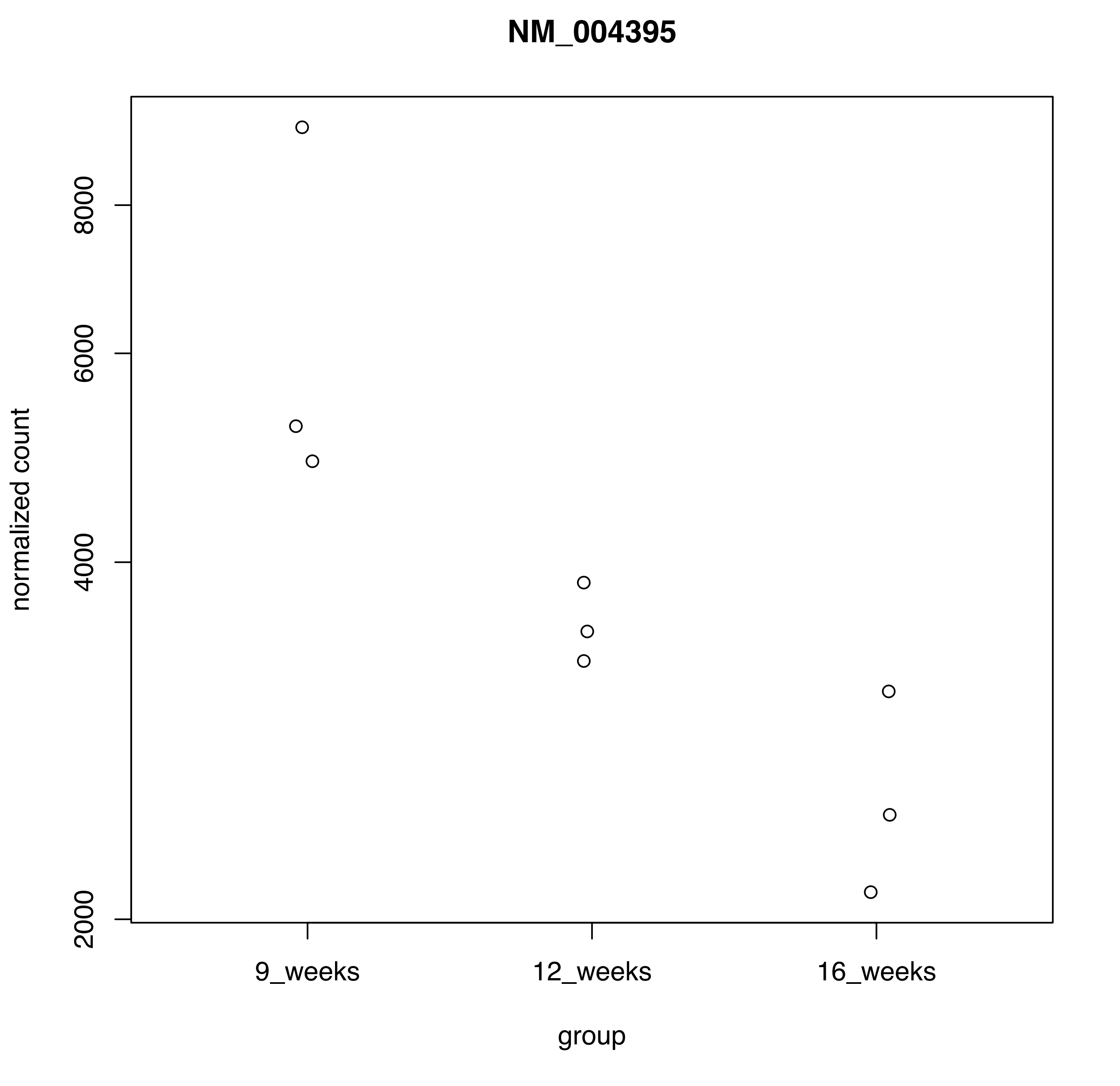
NM_004395
normalized count
8000
6000
4000
2000
9_weeks
12_weeks
16_weeks
group

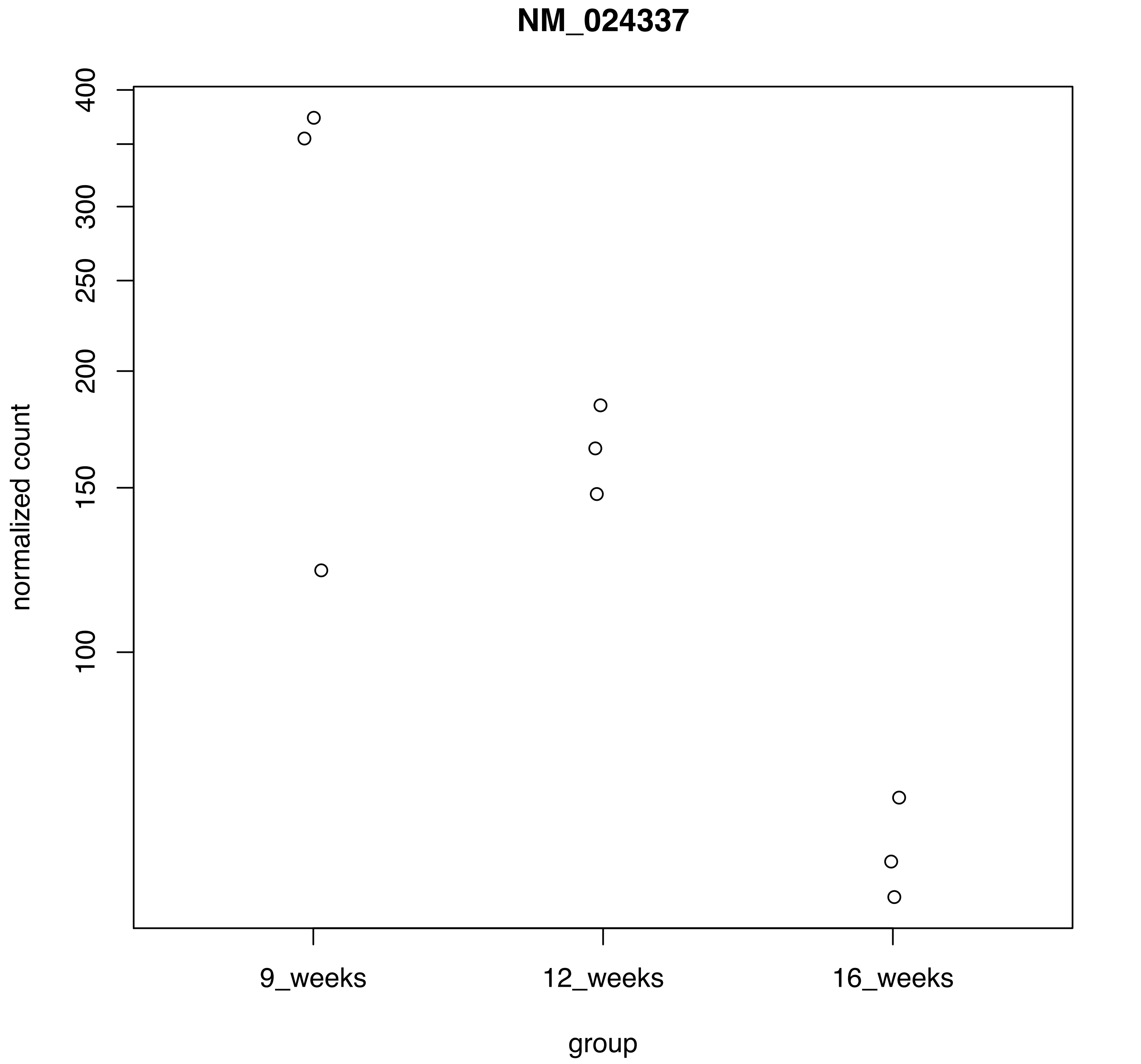

NM_024337
normalized count
400
300
250
200
150
100
9_weeks
12_weeks
16_weeks
group

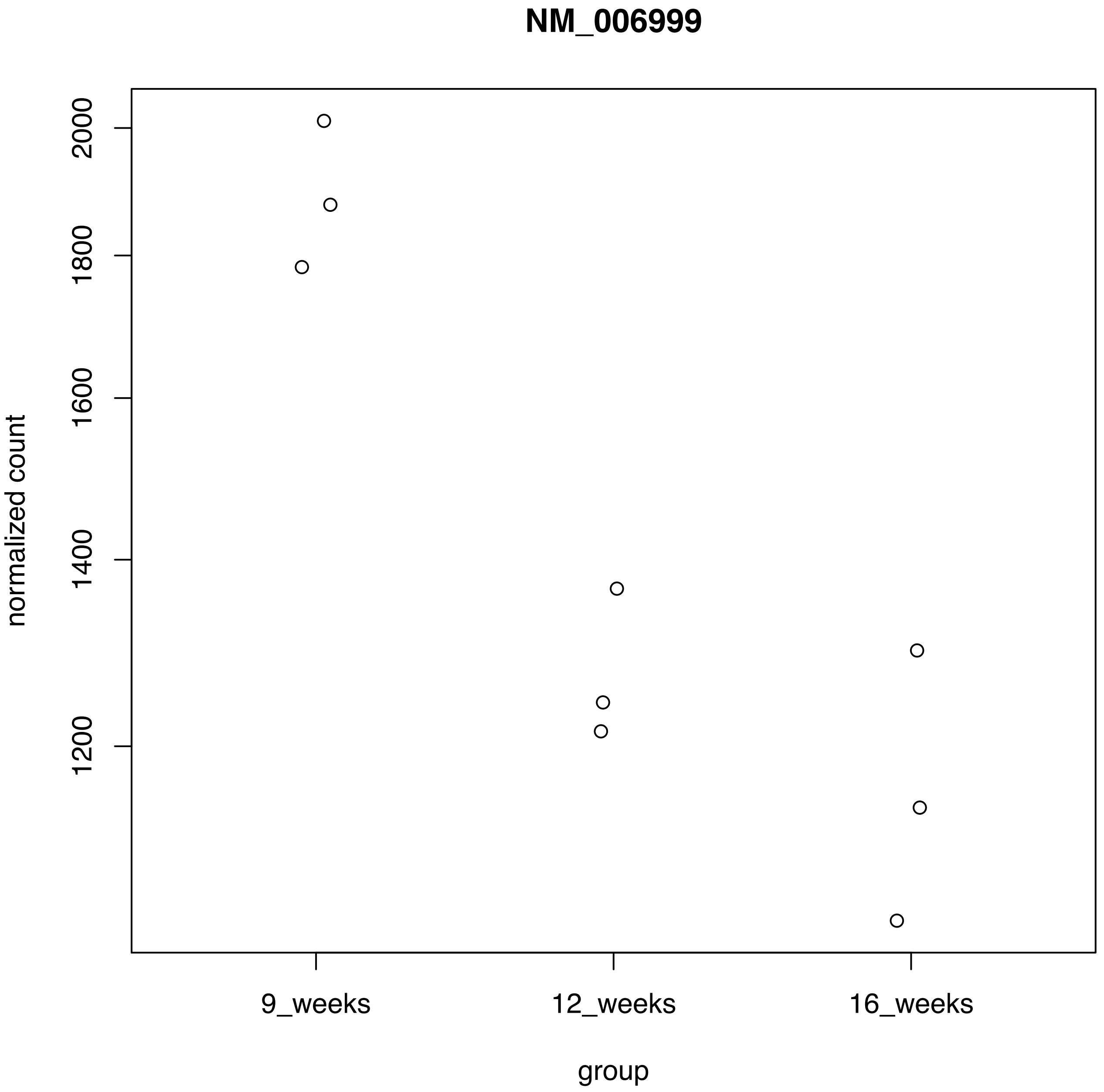
NM_006999
normalized count
2000
1800
1600
1400
1200
9_weeks
12_weeks
16_weeks
group

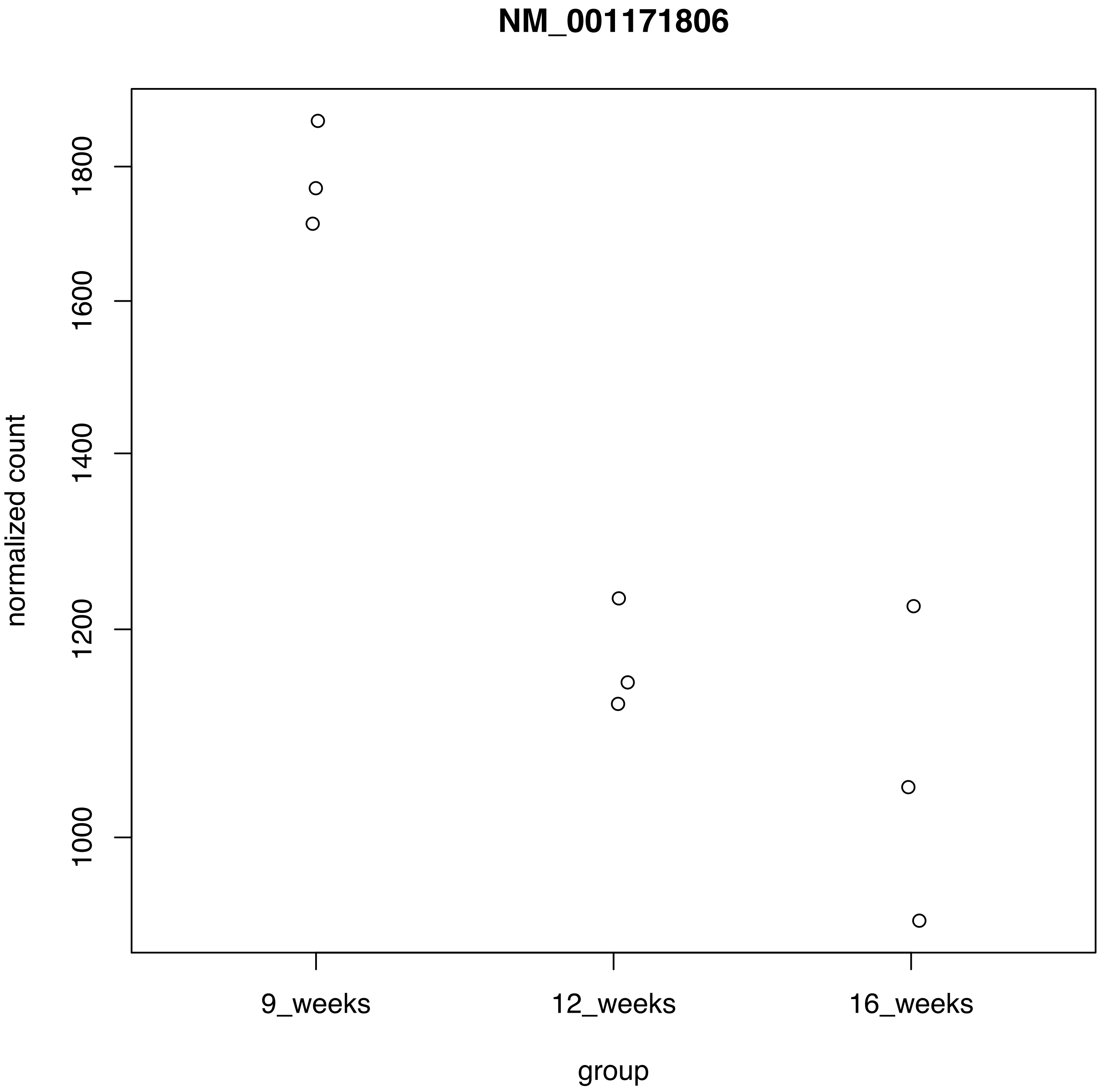
NM_001171806
normalized count
1000
1200
1400
1600
1800
9_weeks
12_weeks
16_weeks
group

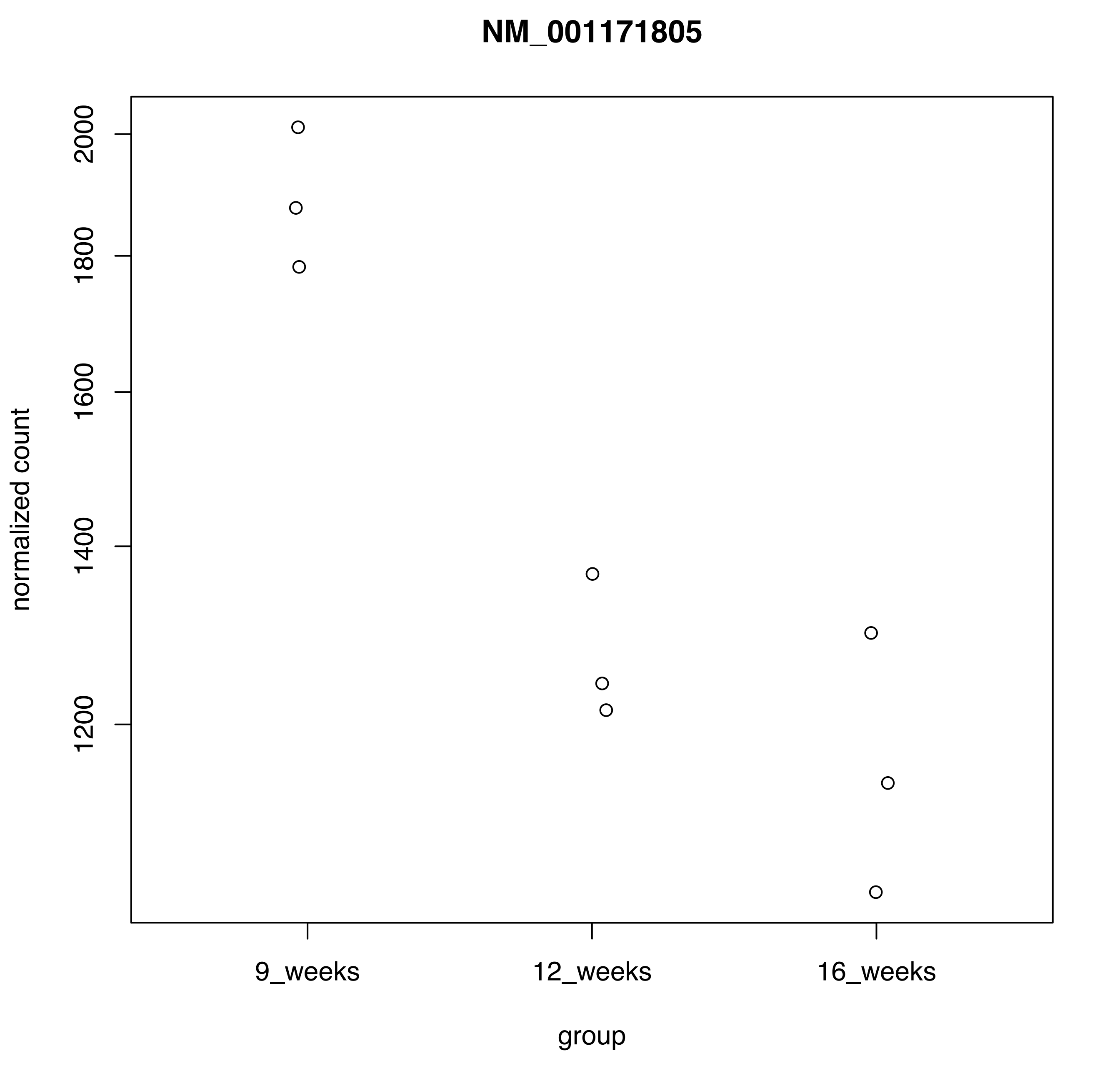
NM_001171805
normalized count
2000
1800
1600
1400
1200
9_weeks
12_weeks
16_weeks
group

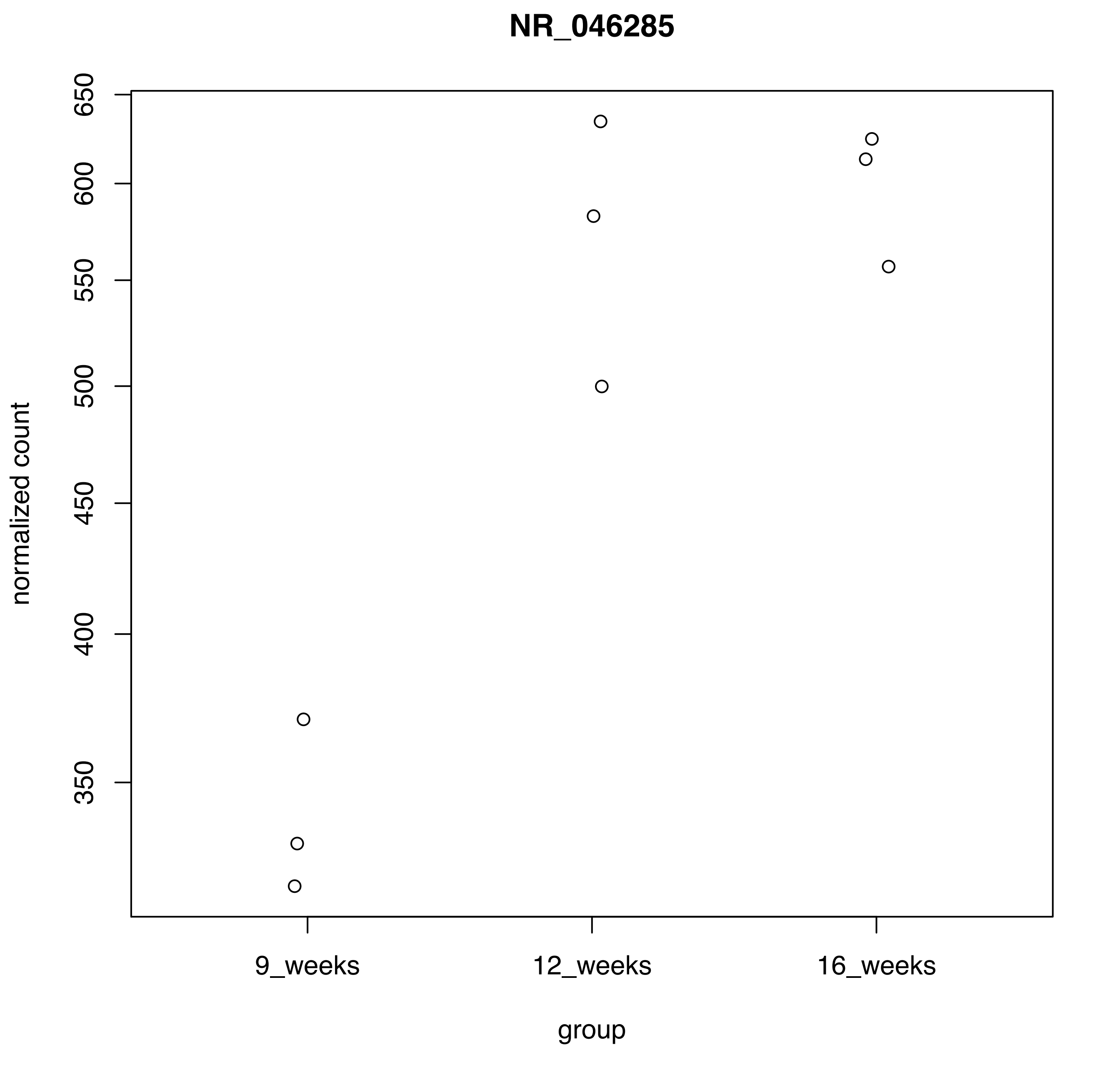
NR_046285
normalized count
650
600
550
500
450
400
350
9_weeks
12_weeks
16_weeks
group

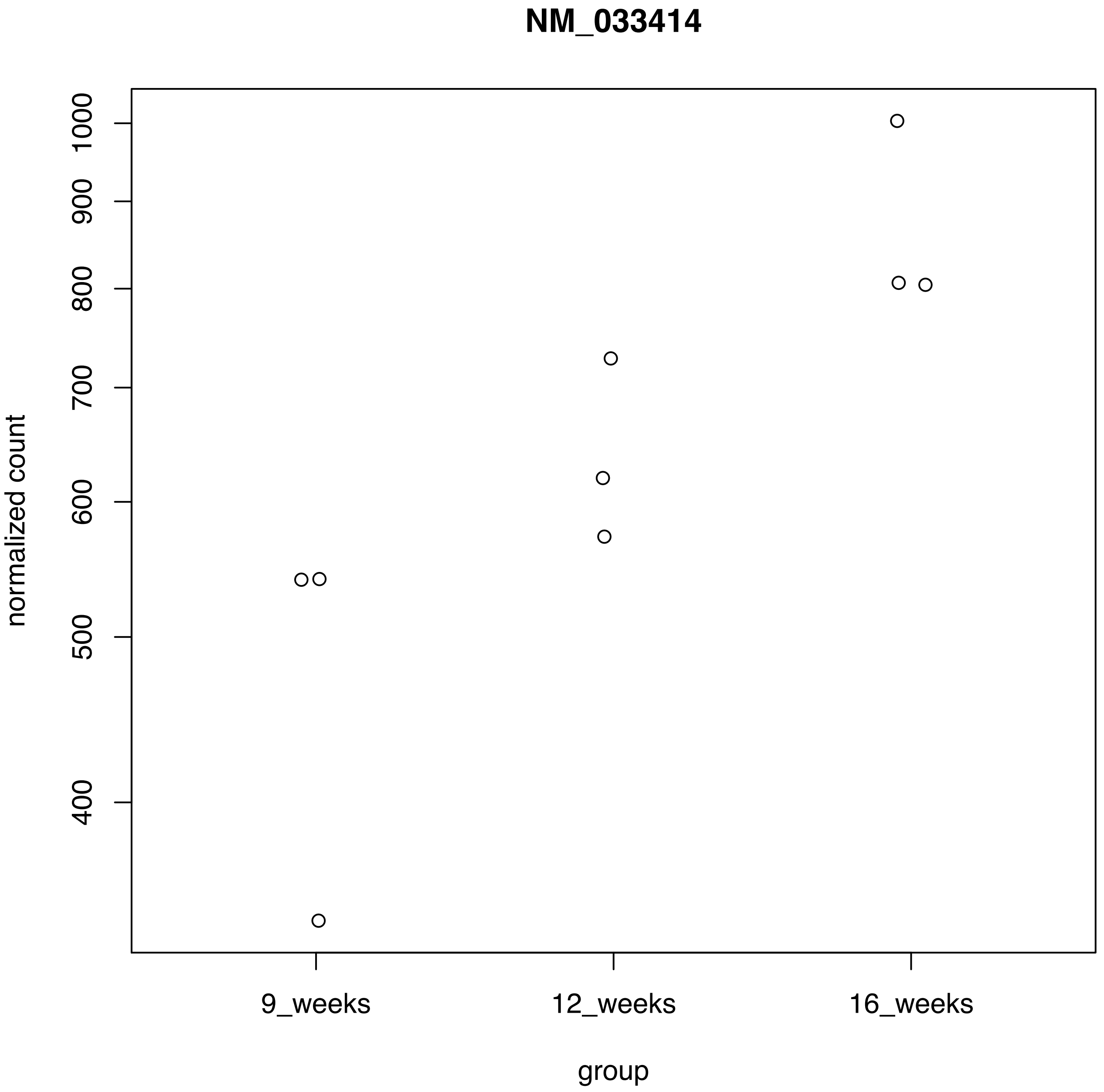
NM_033414
normalized count
1000
900
800
700
600
500
400
9_weeks
12_weeks
16_weeks
group

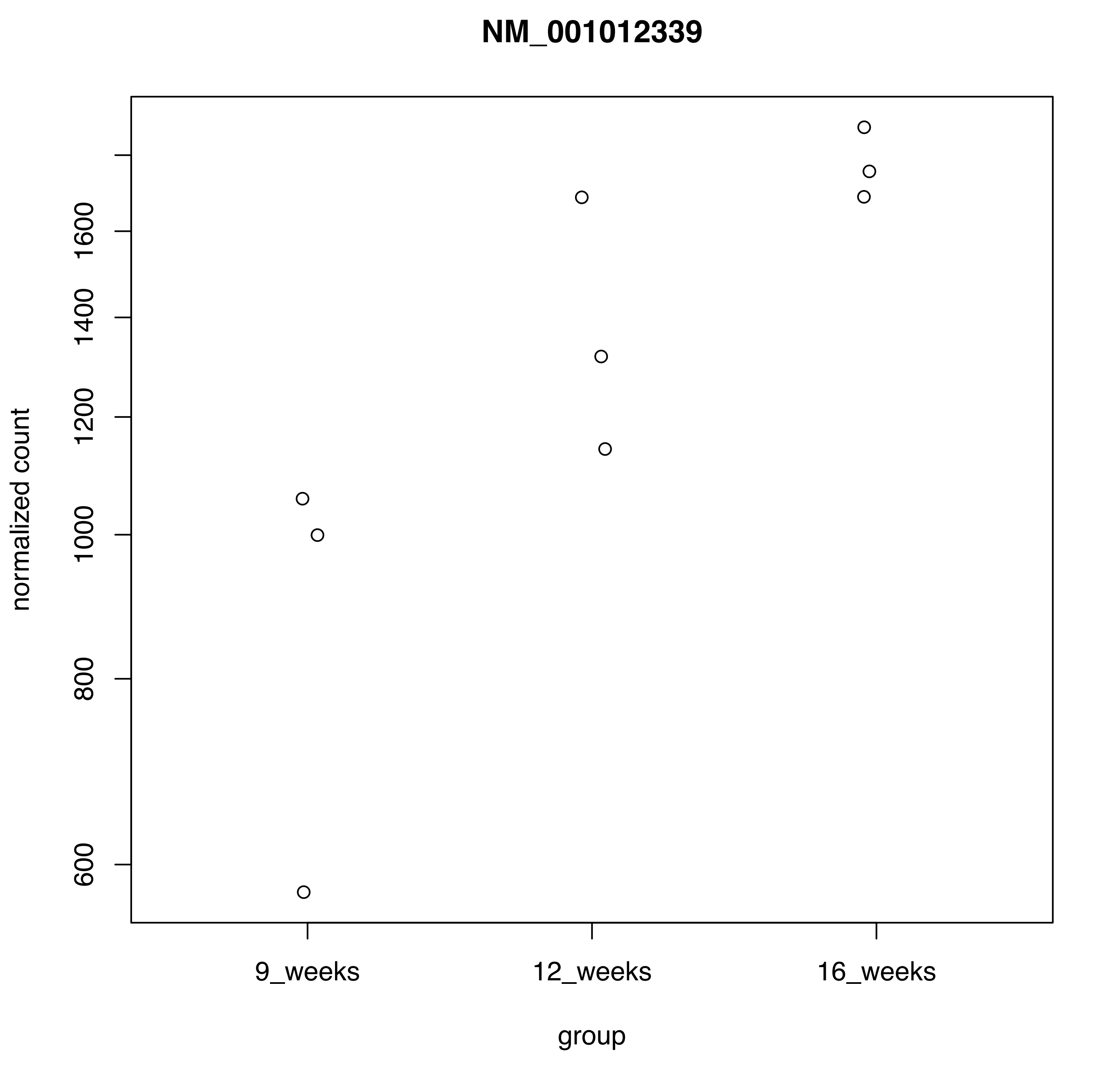

NM_001012339
normalized count
600
800
1000
1200
1400
1600
9_weeks
12_weeks
16_weeks
group

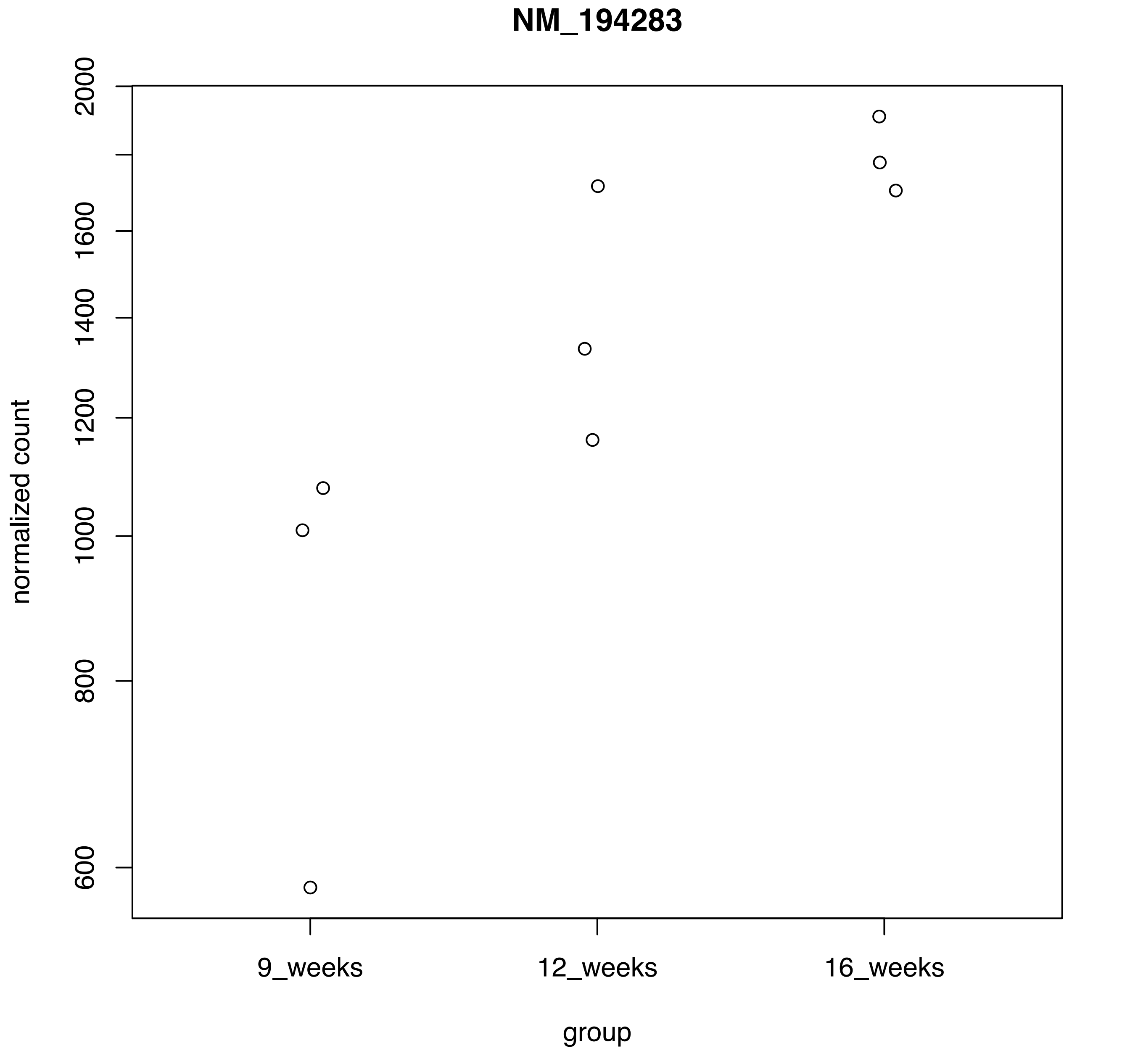

NM_194283
normalized count
2000
1600
1400
1200
1000
800
600
9_weeks
12_weeks
16_weeks
group

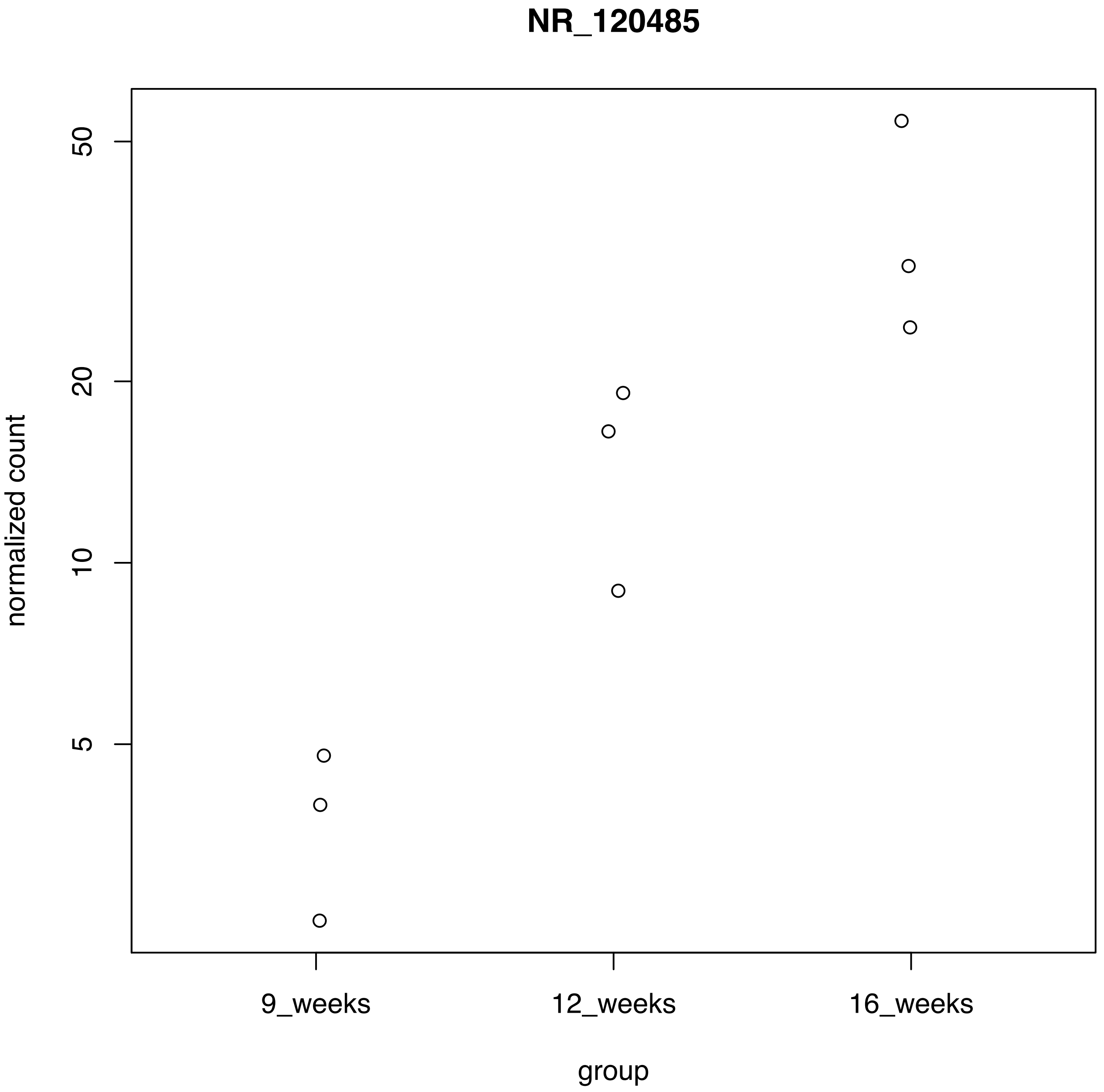

NR_120485
normalized count
50
20
10
5
9_weeks
12_weeks
16_weeks
group

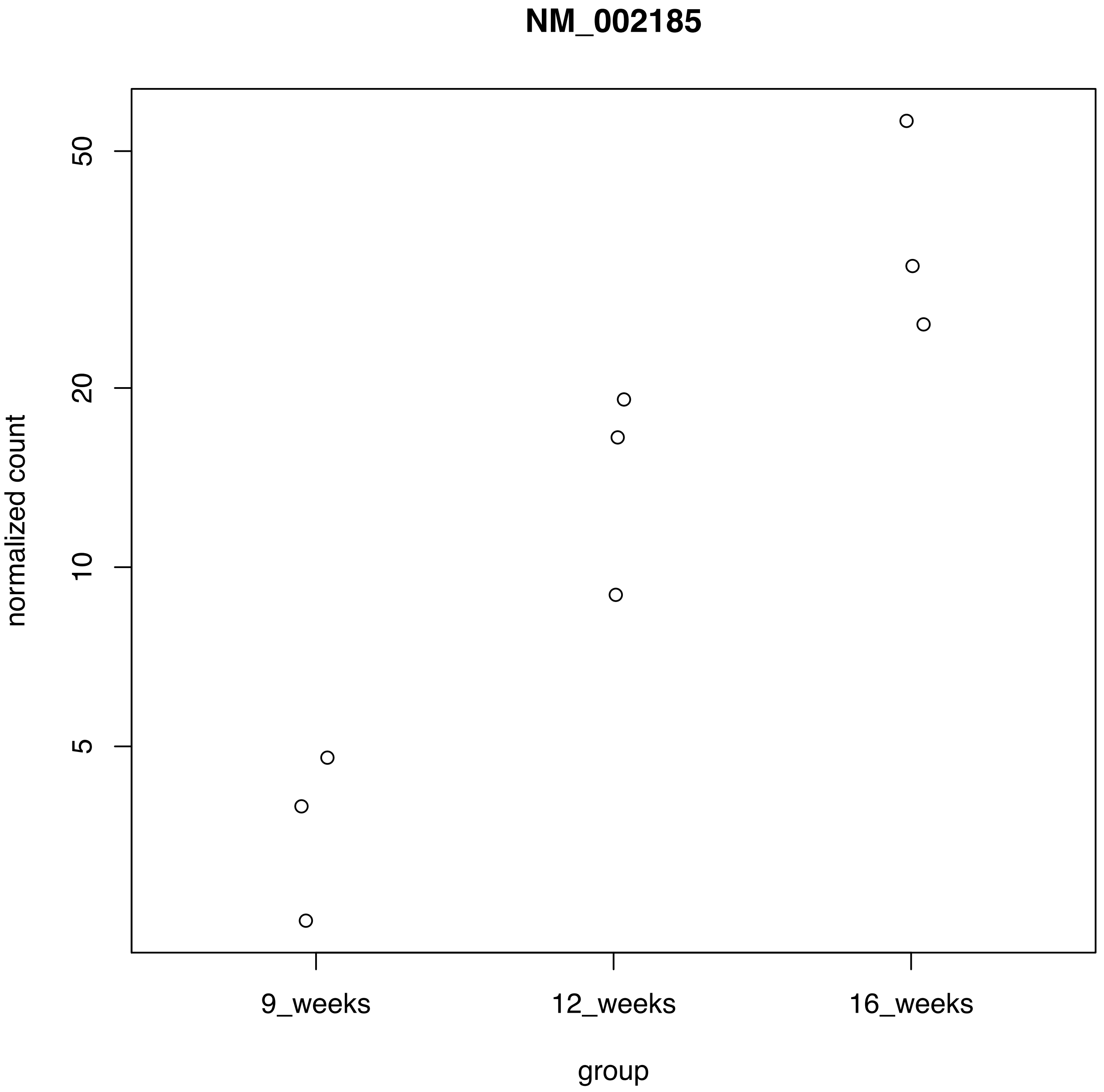
NM_002185
normalized count
50
20
10
5
9_weeks
12_weeks
16_weeks
group

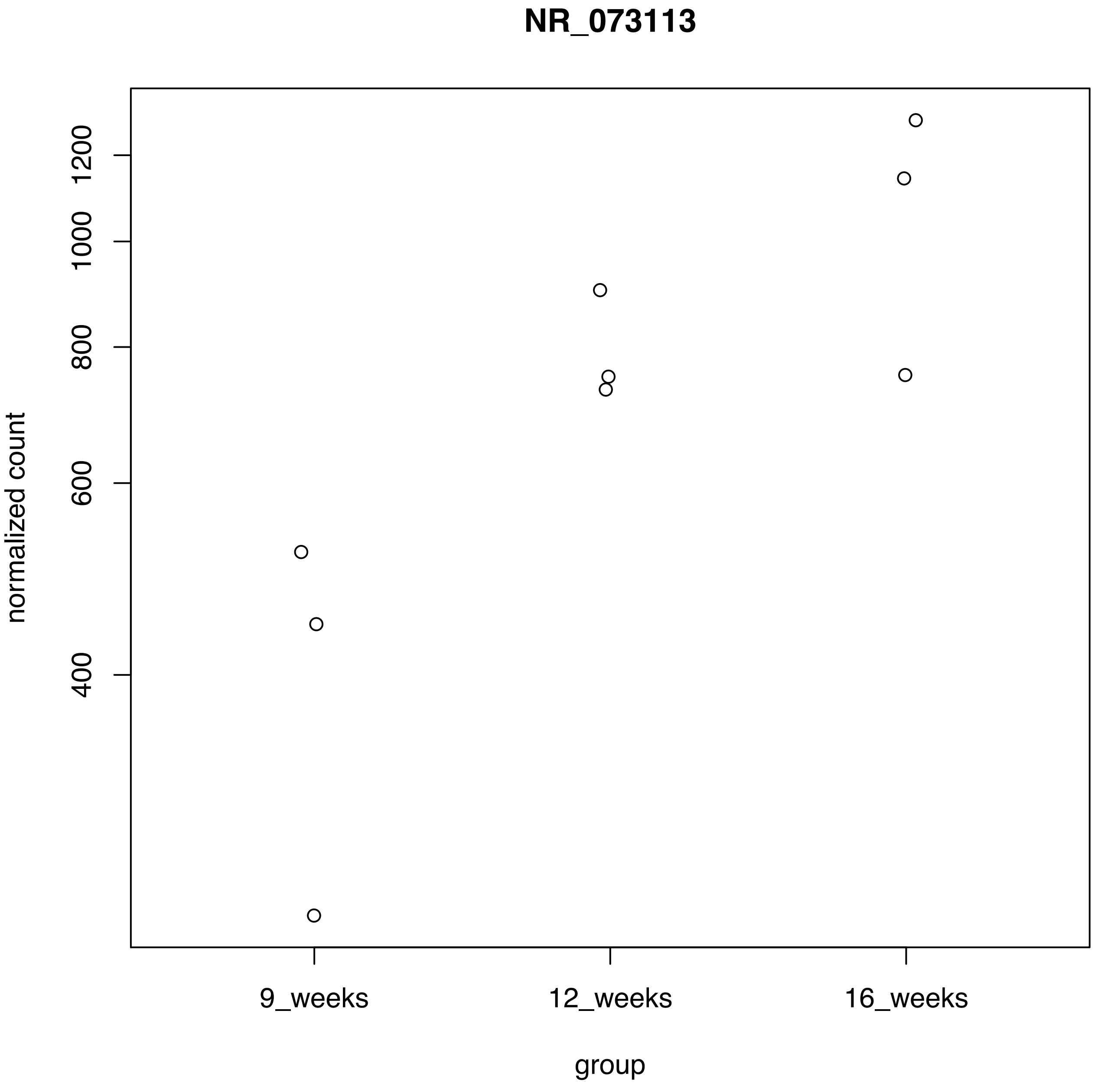
NR_073113
normalized count
400
600
800
1000
1200
9_weeks
12_weeks
16_weeks
group

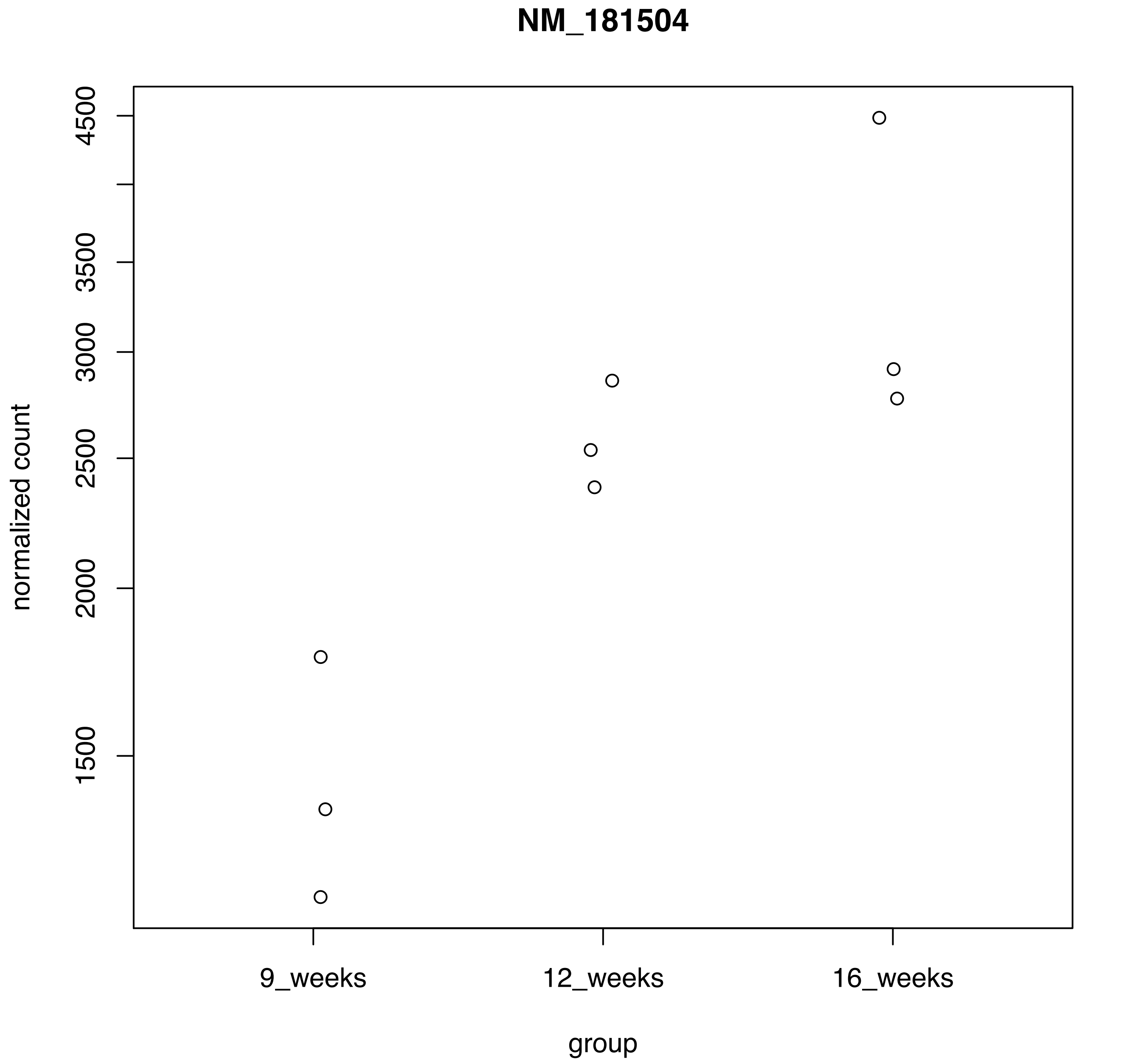

NM_181504
normalized count
4500
3500
3000
2500
2000
1500
9_weeks
12_weeks
16_weeks
group

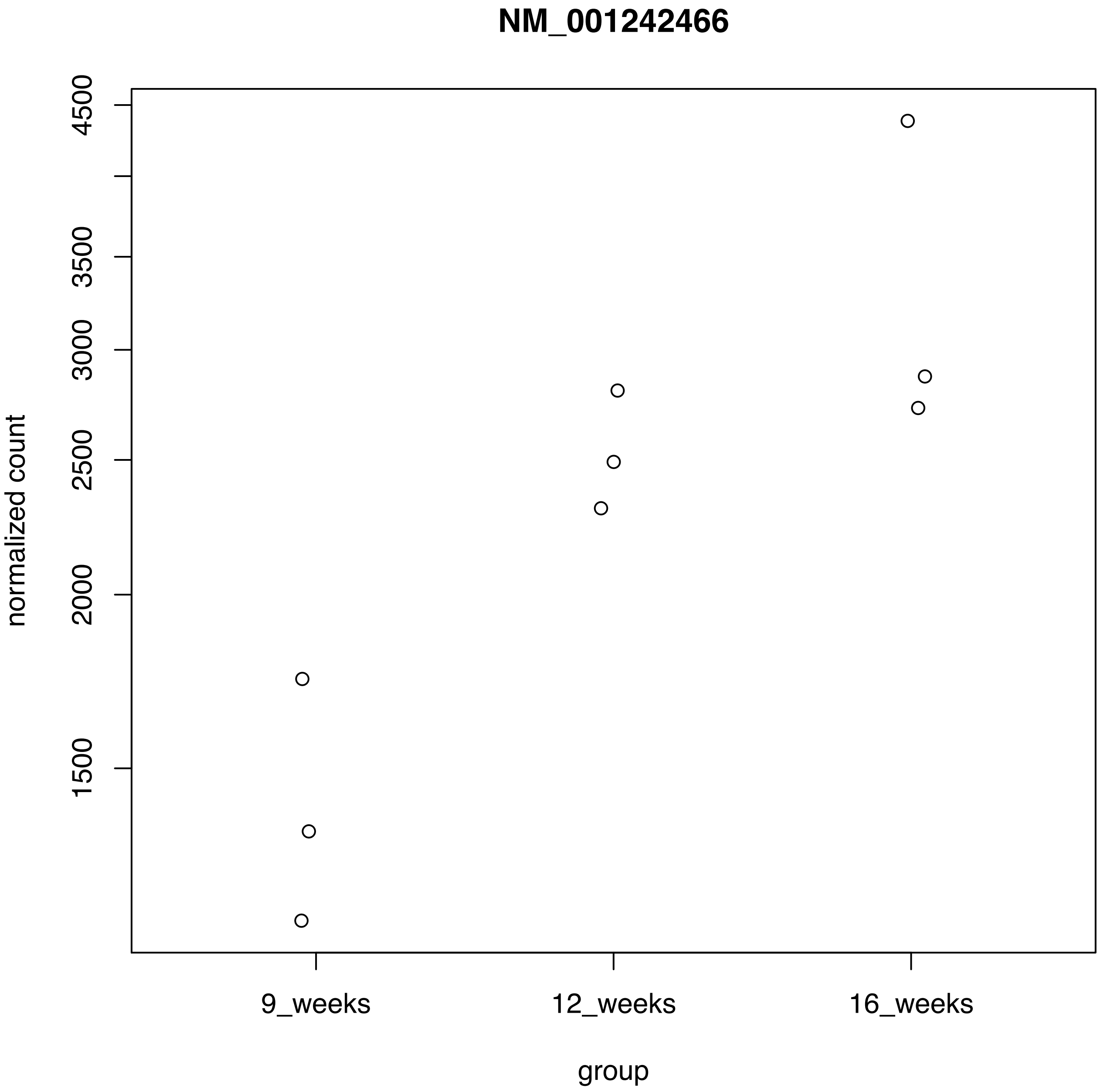
NM_001242466
normalized count
1500
2000
2500
3000
3500
4500
9_weeks
12_weeks
16_weeks
group

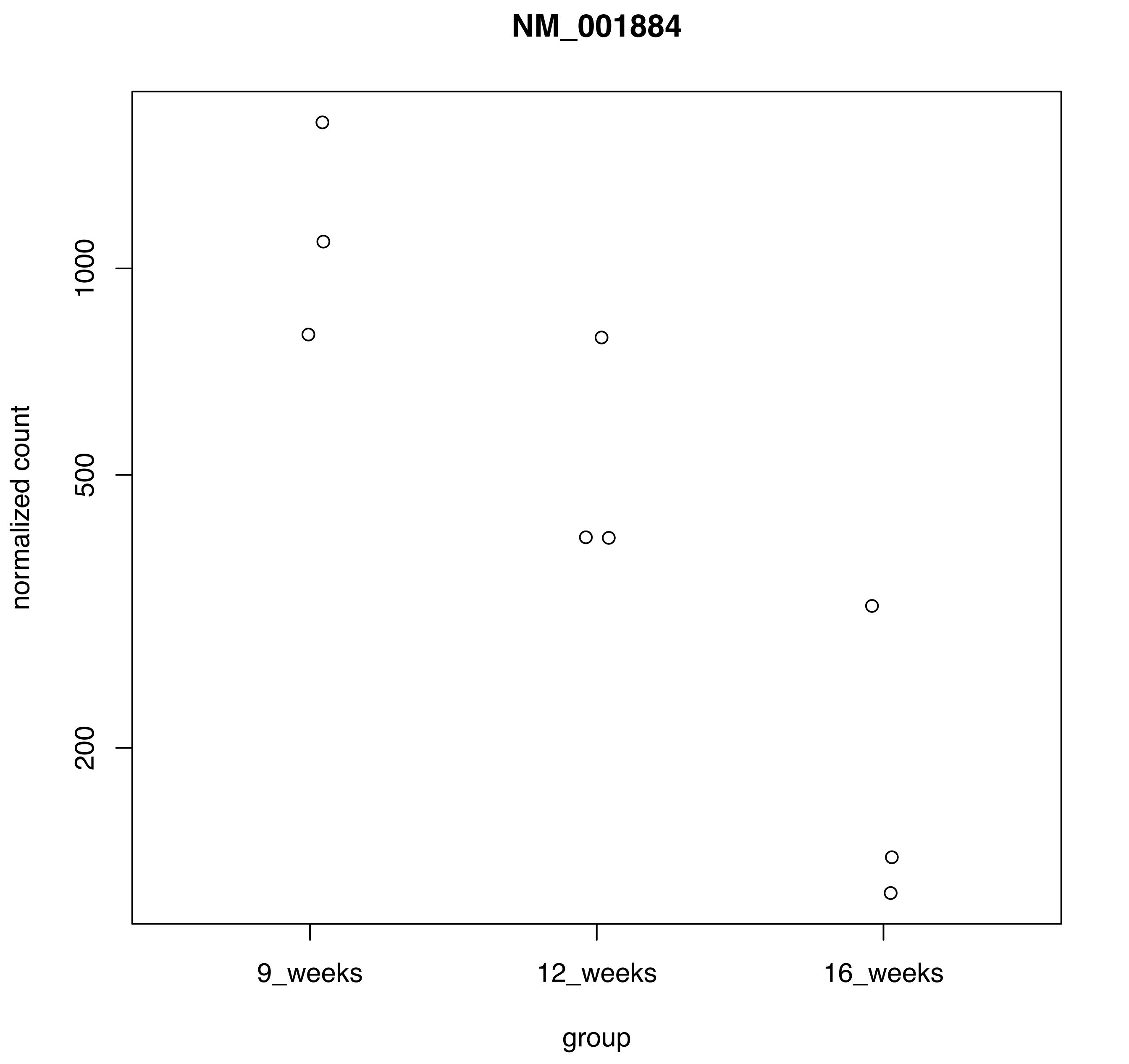

NM_001884
normalized count
1000
500
200
9_weeks
12_weeks
16_weeks
group

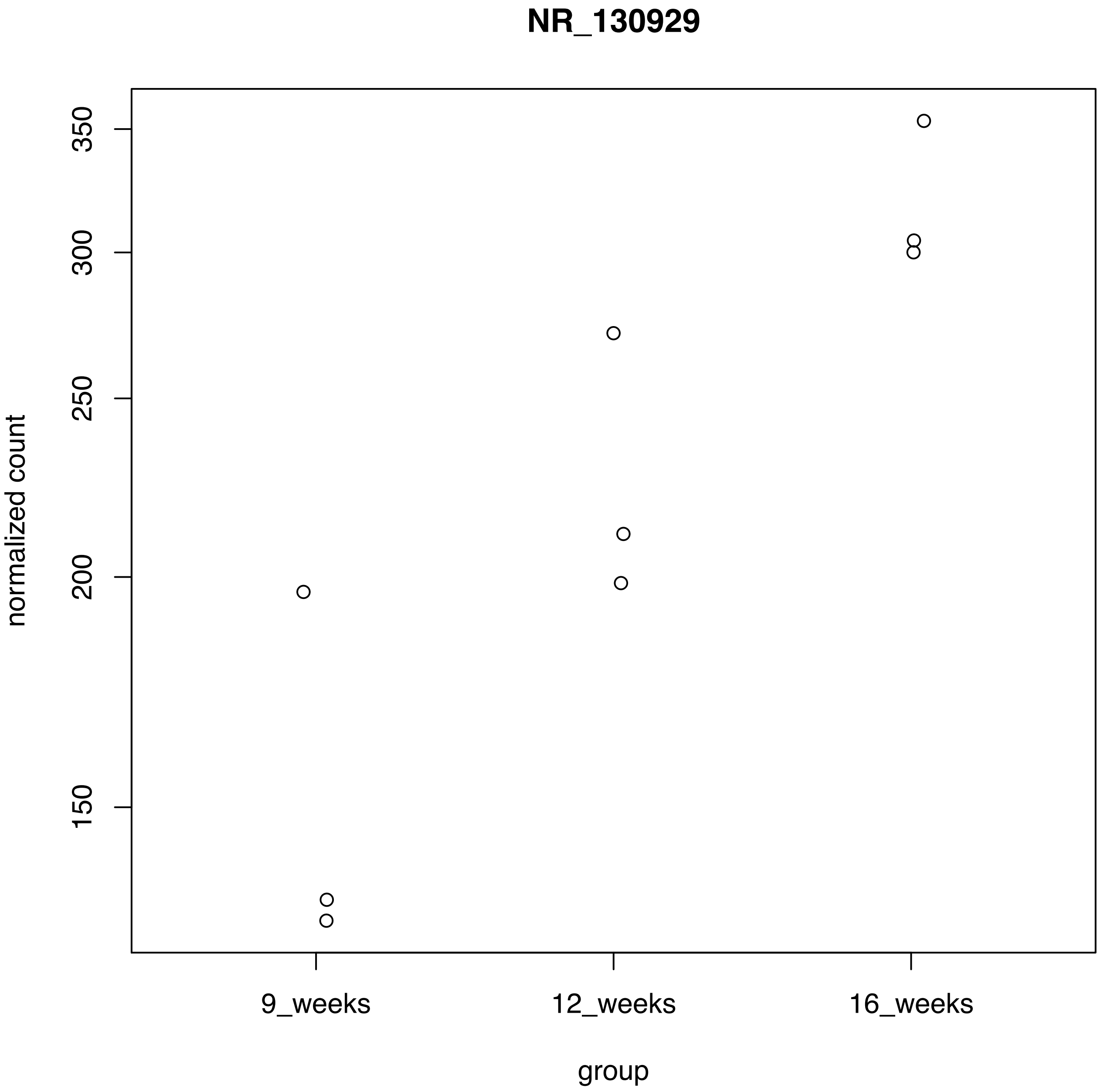
NR_130929
normalized count
150
200
250
300
350
9_weeks
12_weeks
16_weeks
group

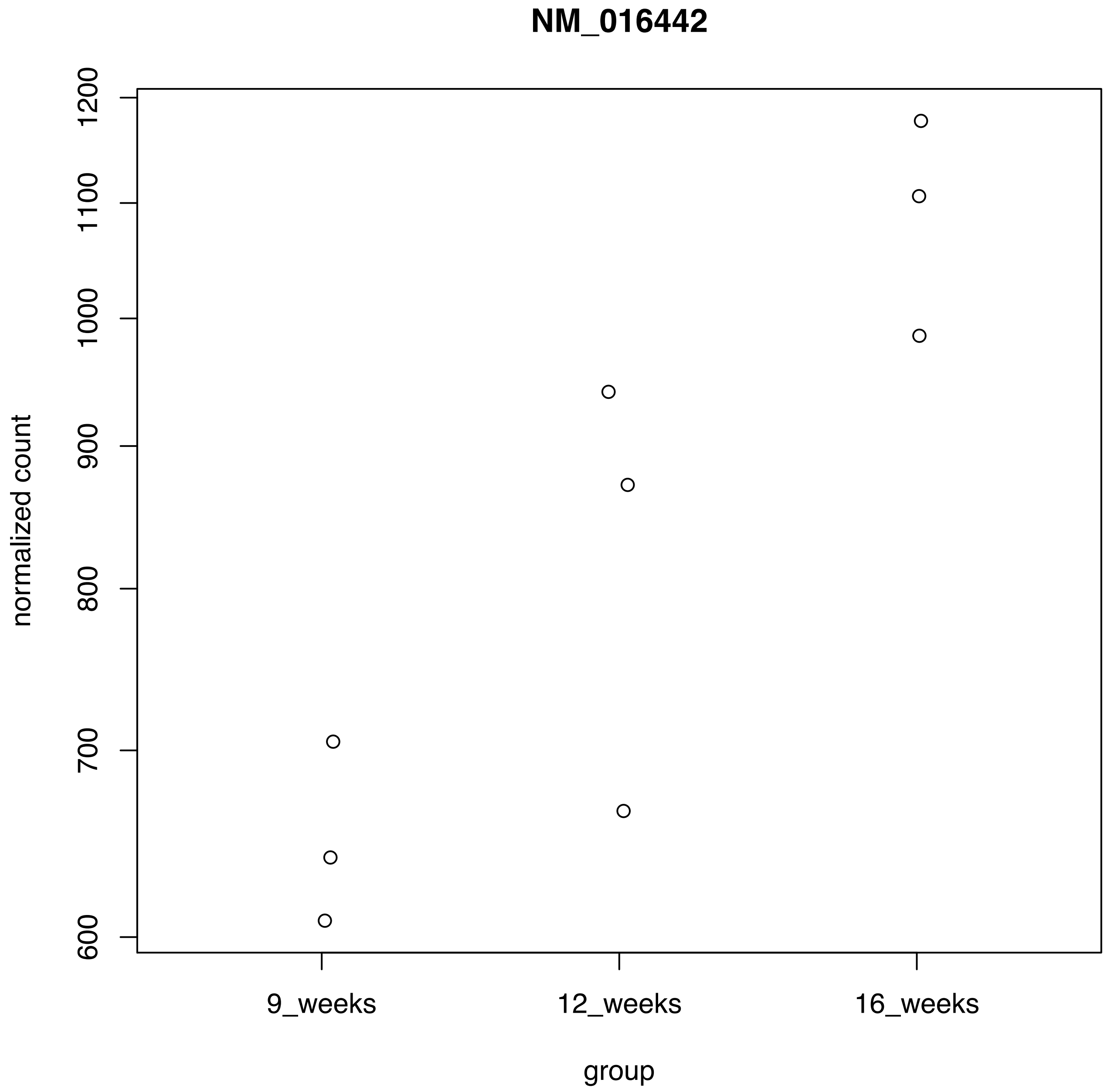

NM_016442
normalized count
1200
1100
1000
900
800
700
600
9_weeks
12_weeks
16_weeks
group

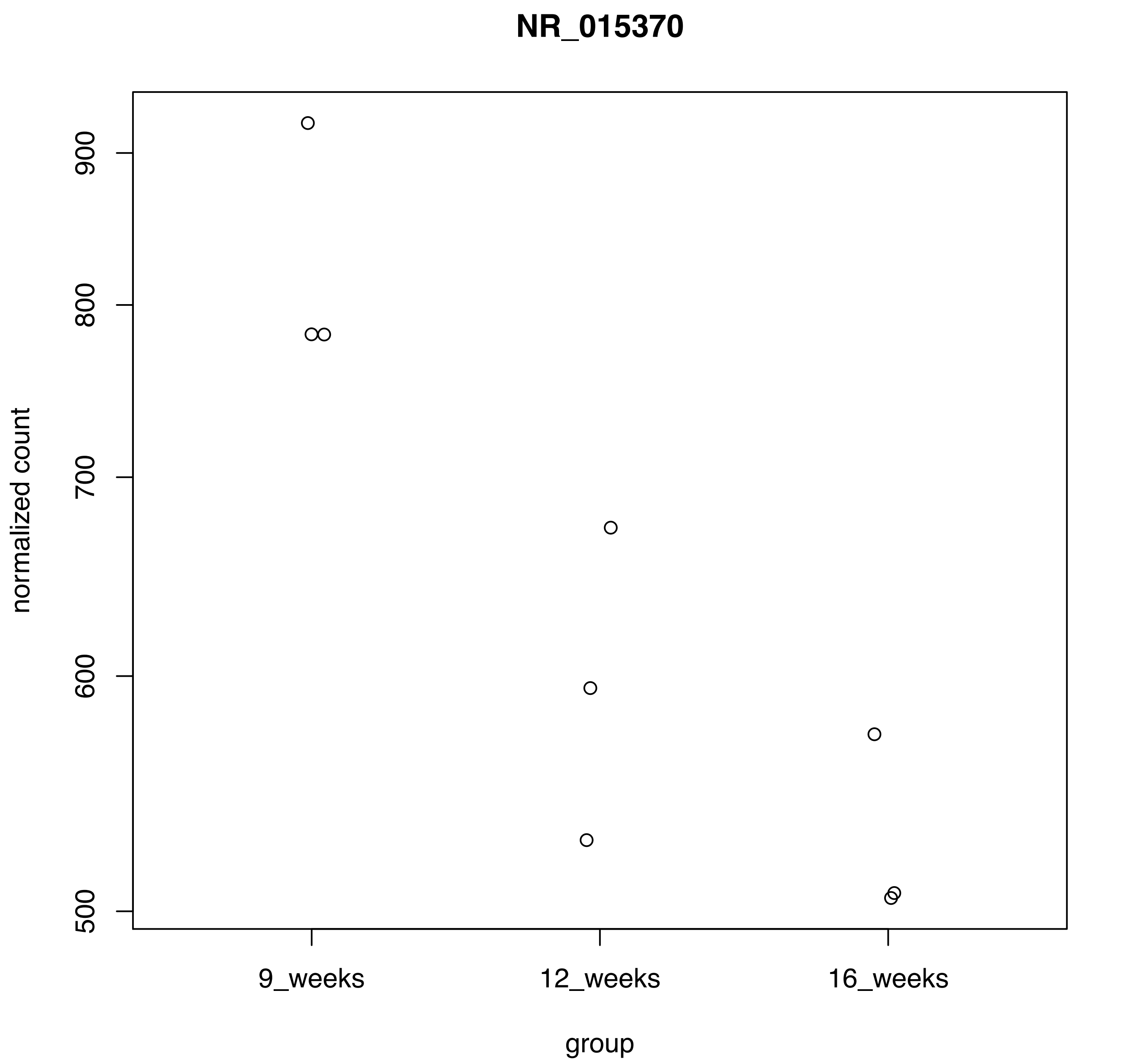

NR_015370
normalized count
900
800
700
600
500
9_weeks
12_weeks
16_weeks
group

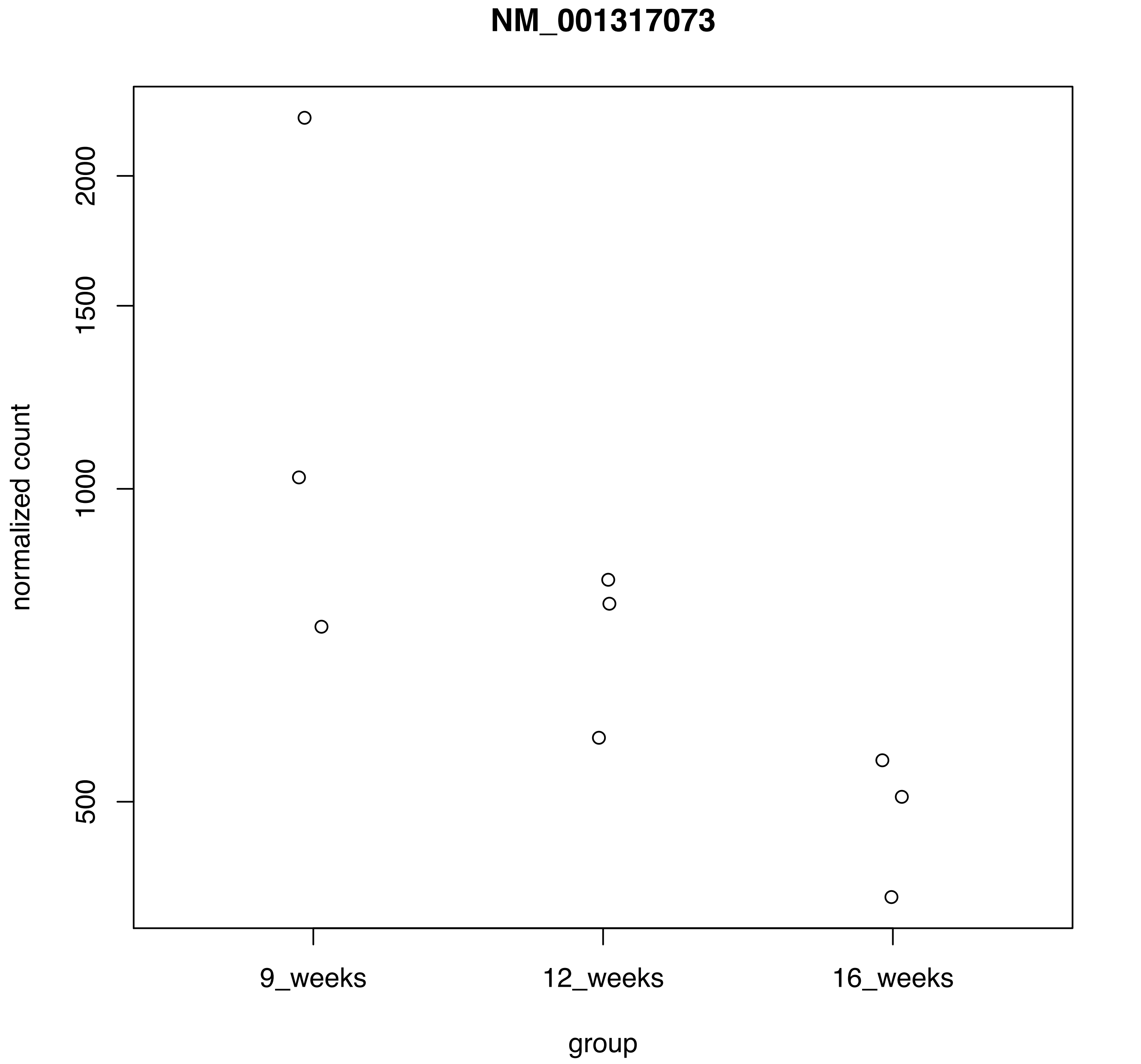
NM_001317073
normalized count
2000
1500
1000
500
9_weeks
12_weeks
16_weeks
group

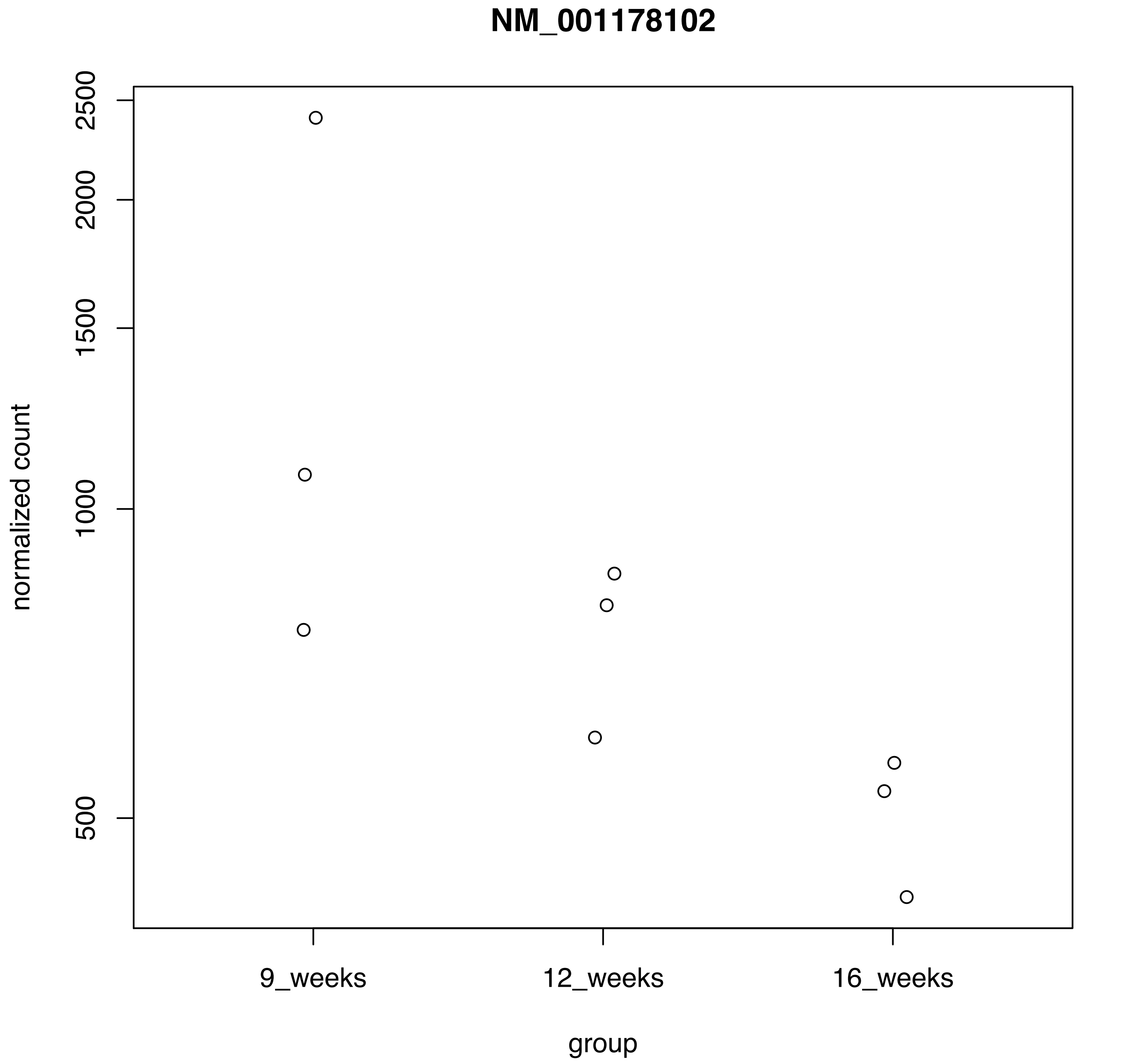

NM_001178102
normalized count
2500
2000
1500
1000
500
9_weeks
12_weeks
16_weeks
group

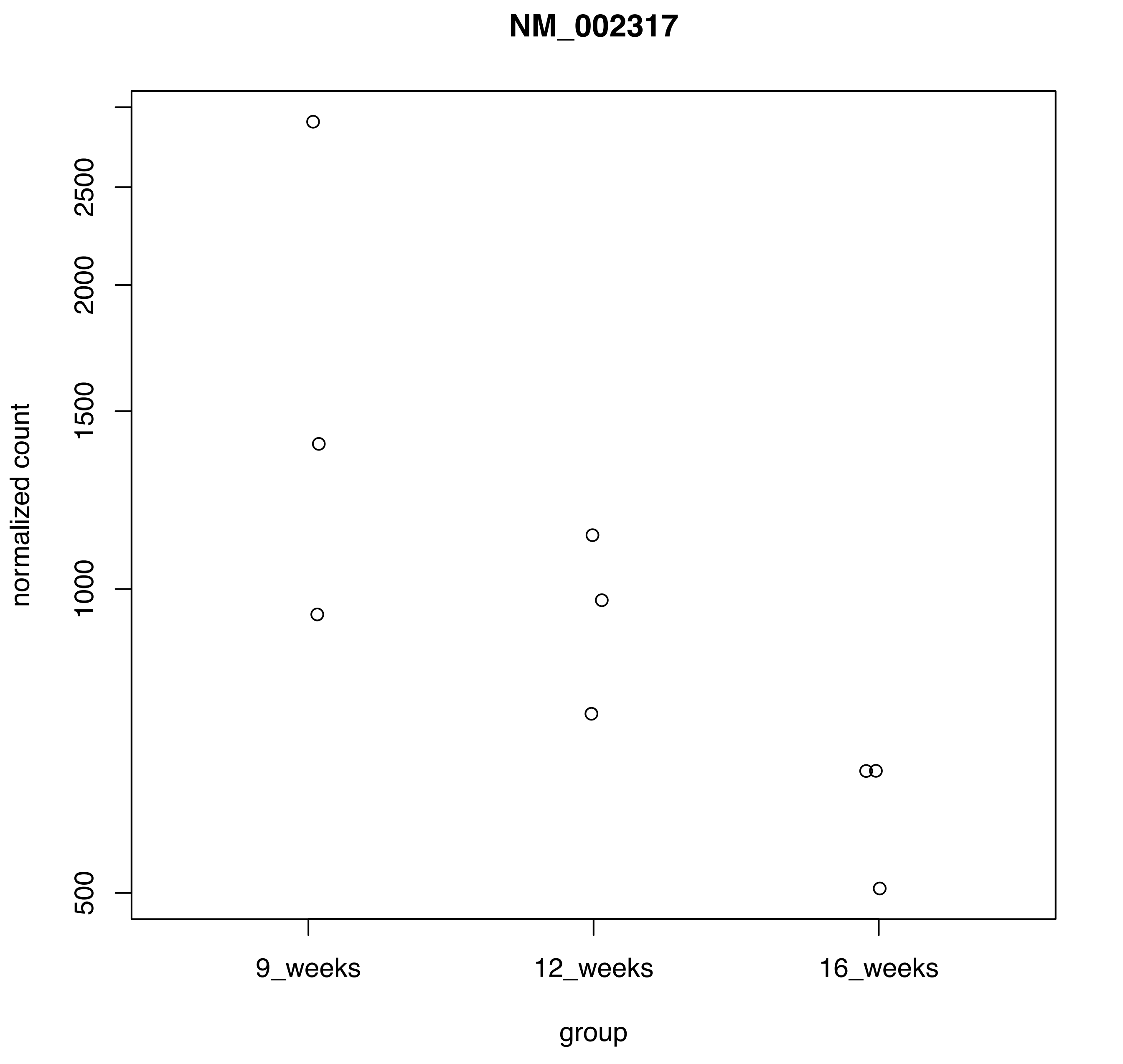

NM_002317
normalized count
500
1000
1500
2000
2500
9_weeks
12_weeks
16_weeks
group

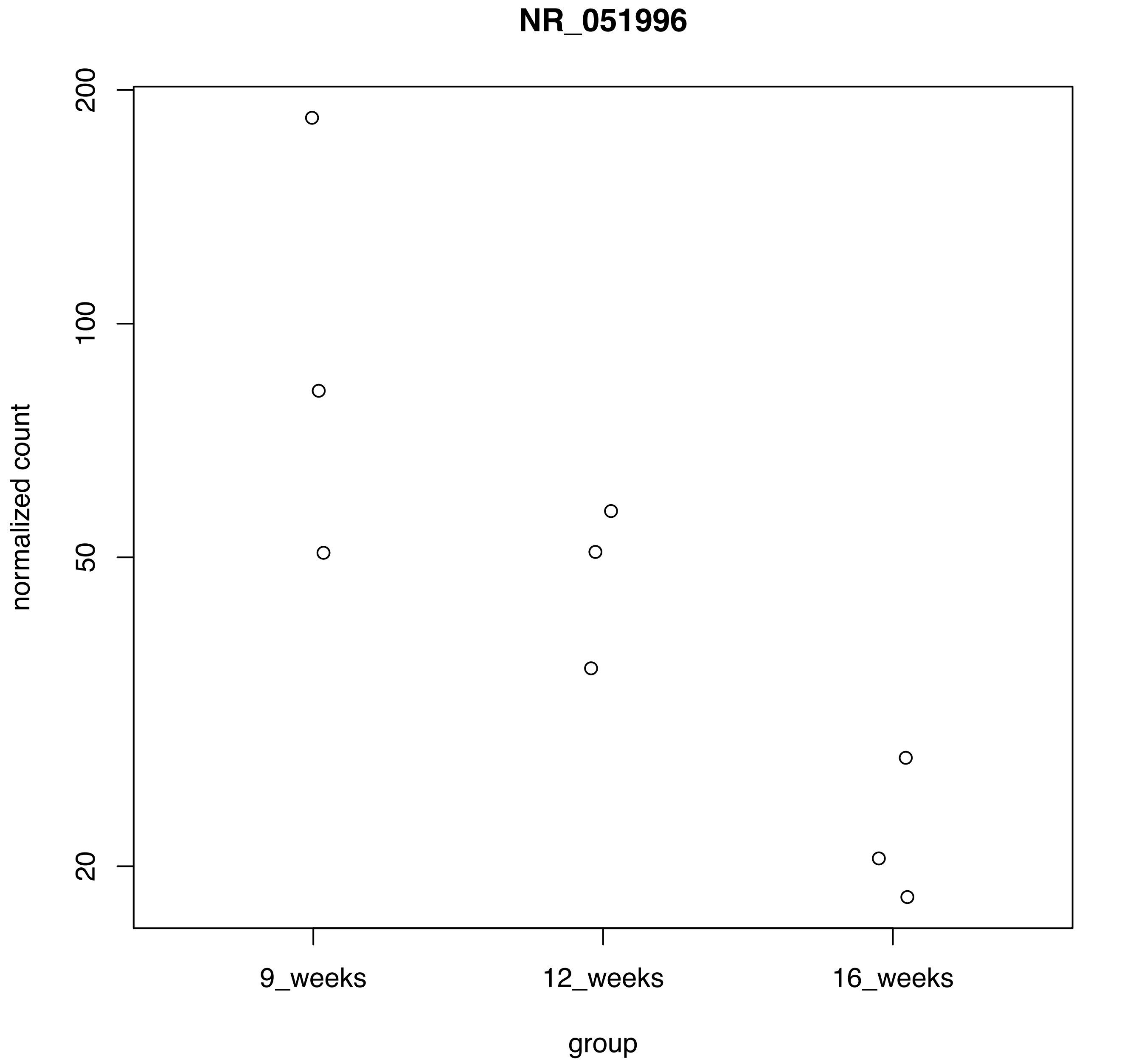

NR_051996
normalized count
200
100
50
20
9_weeks
12_weeks
16_weeks
group

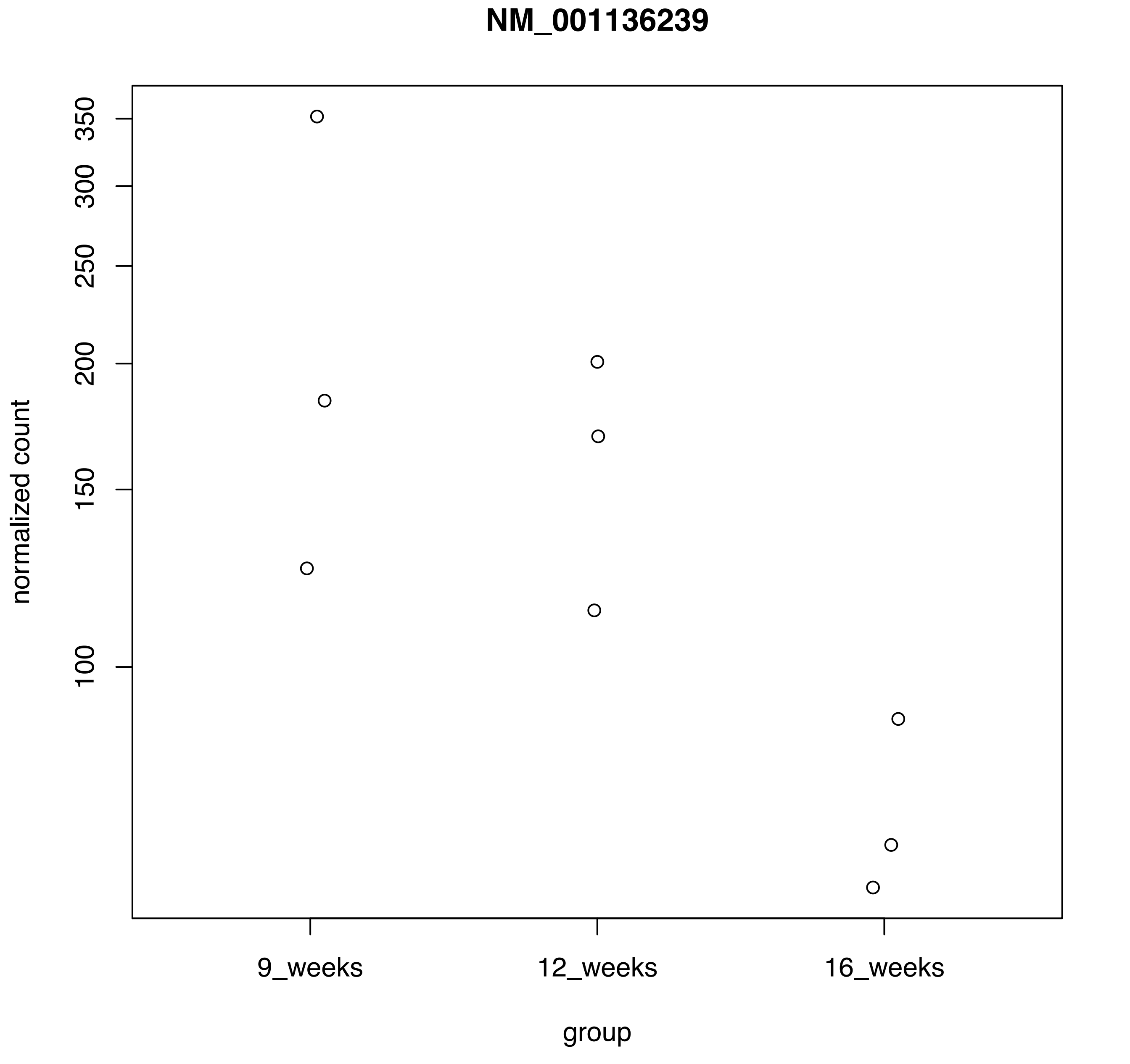
NM_001136239
normalized count
350
300
250
200
150
100
9_weeks
12_weeks
16_weeks
group

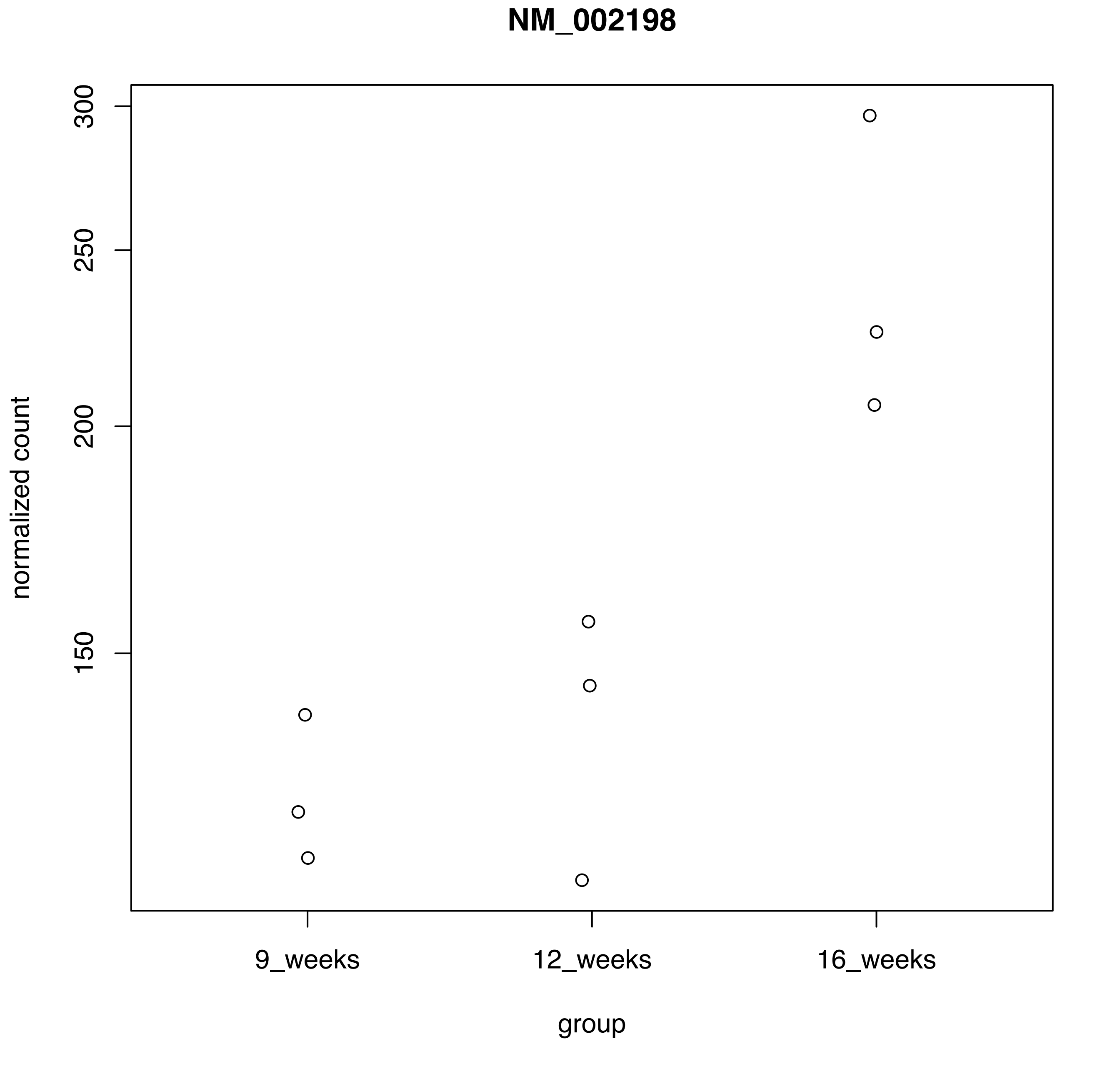

NM_002198
300
250
200
150
normalized count
9_weeks
12_weeks
16_weeks
group

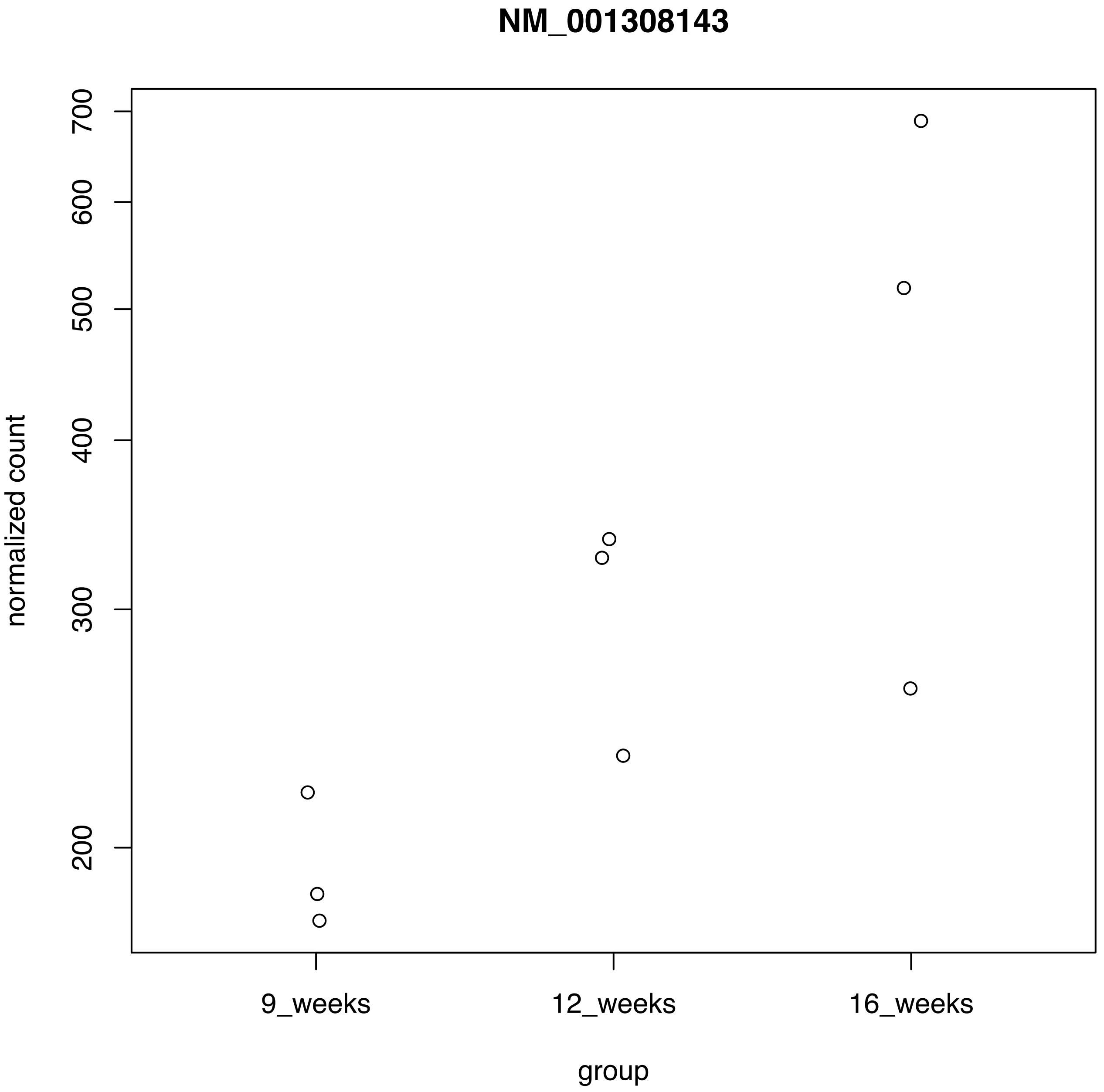
NM_001308143
normalized count
700
600
500
400
300
200
9_weeks
12_weeks
16_weeks
group

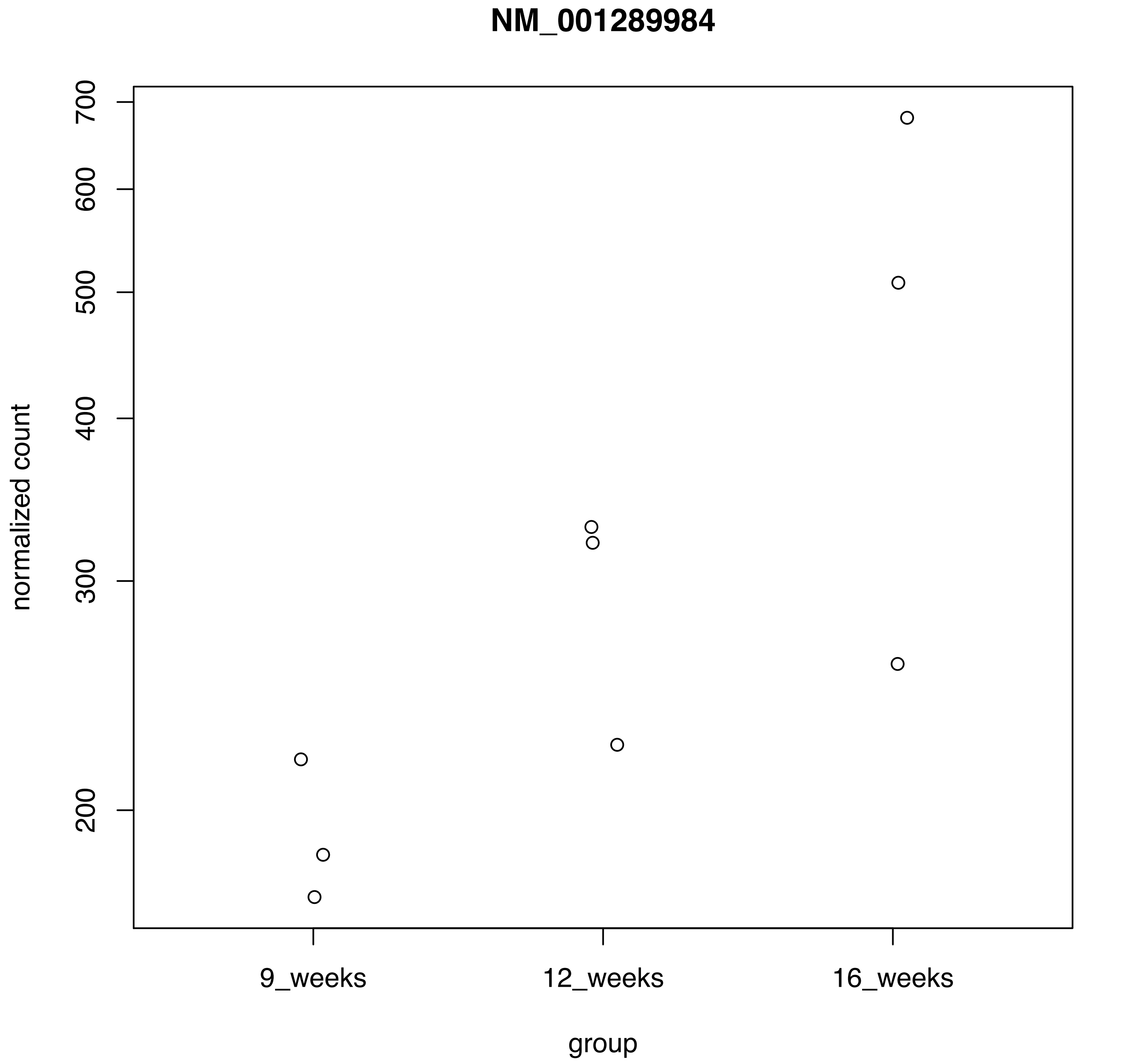
NM_001289984
normalized count
700
600
500
400
300
200
9_weeks
12_weeks
16_weeks
group

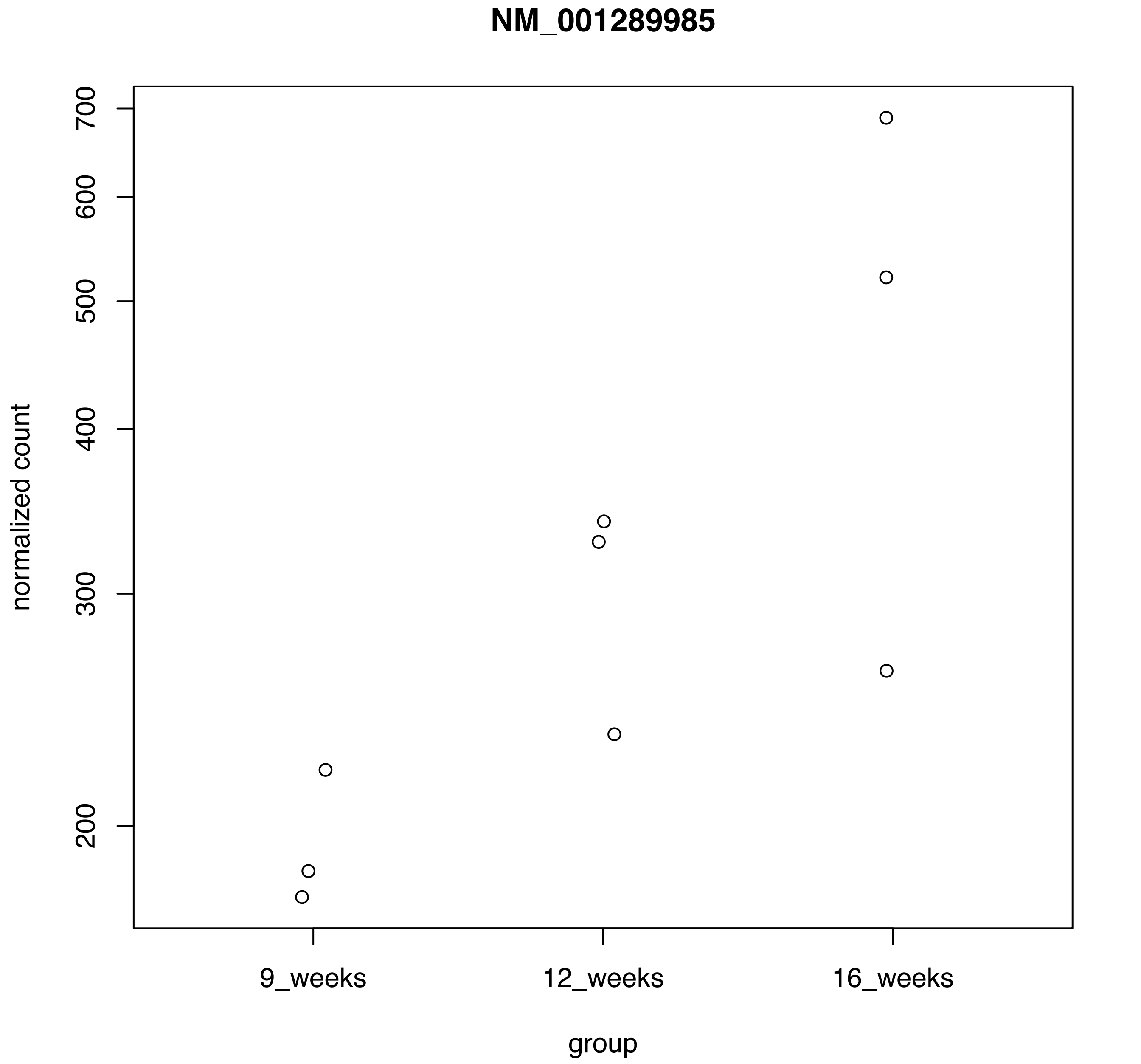
NM_001289985
normalized count
700
600
500
400
300
200
9_weeks
12_weeks
16_weeks
group

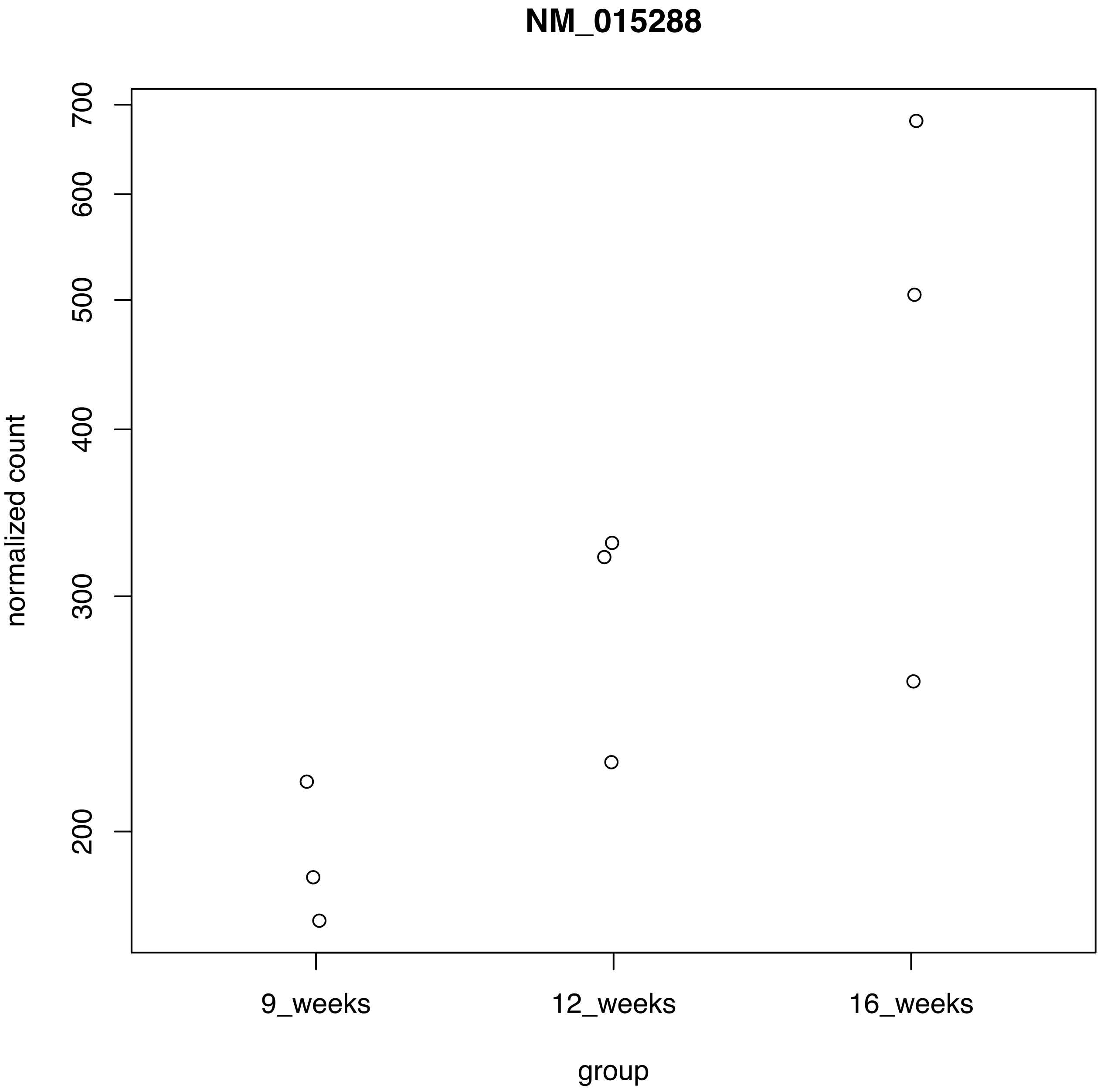
NM_015288
normalized count
700
600
500
400
300
200
9_weeks
12_weeks
16_weeks
group

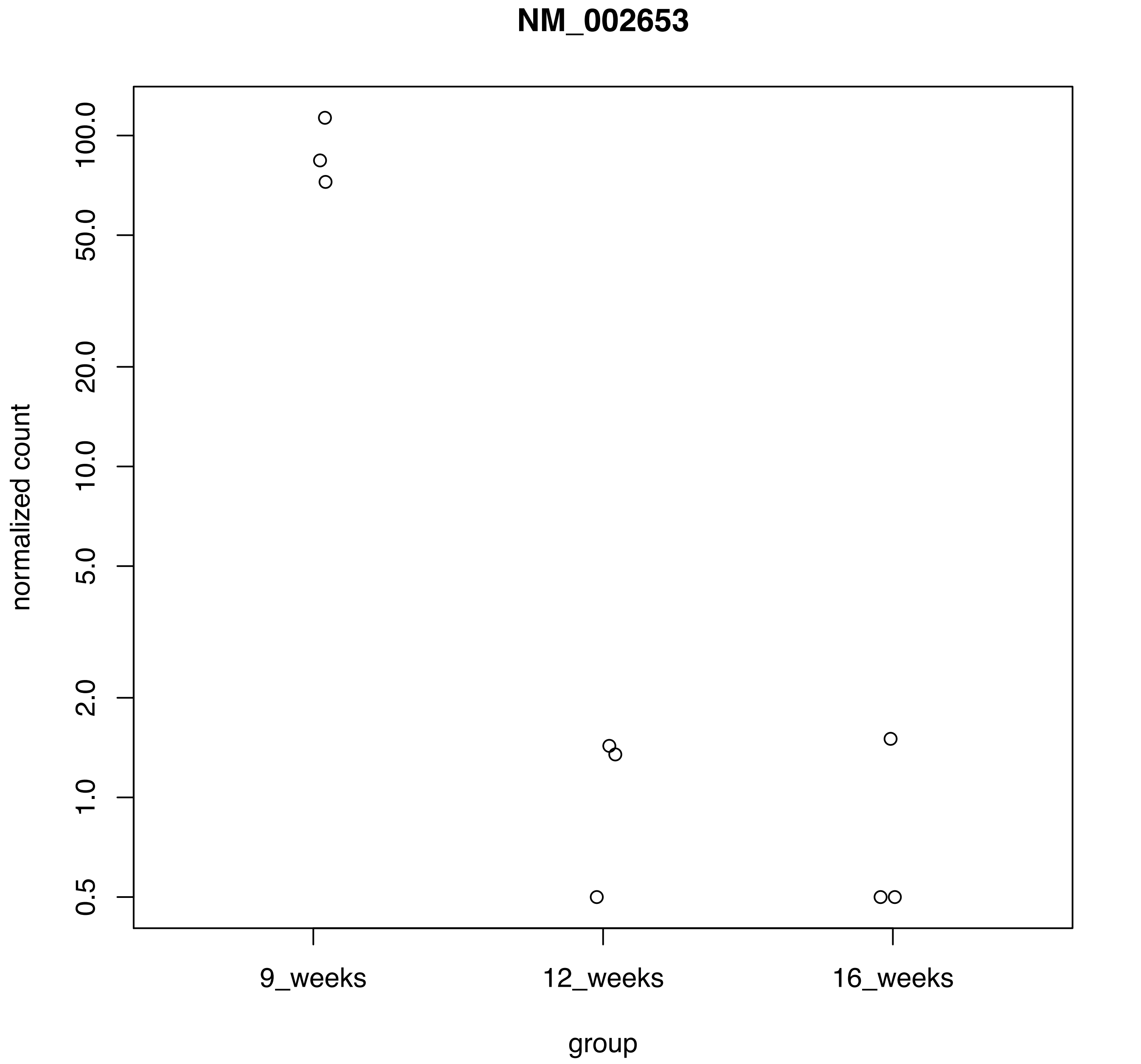
NM_002653
normalized count
100.0
50.0
20.0
10.0
5.0
2.0
1.0
0.5
9_weeks
12_weeks
16_weeks
group

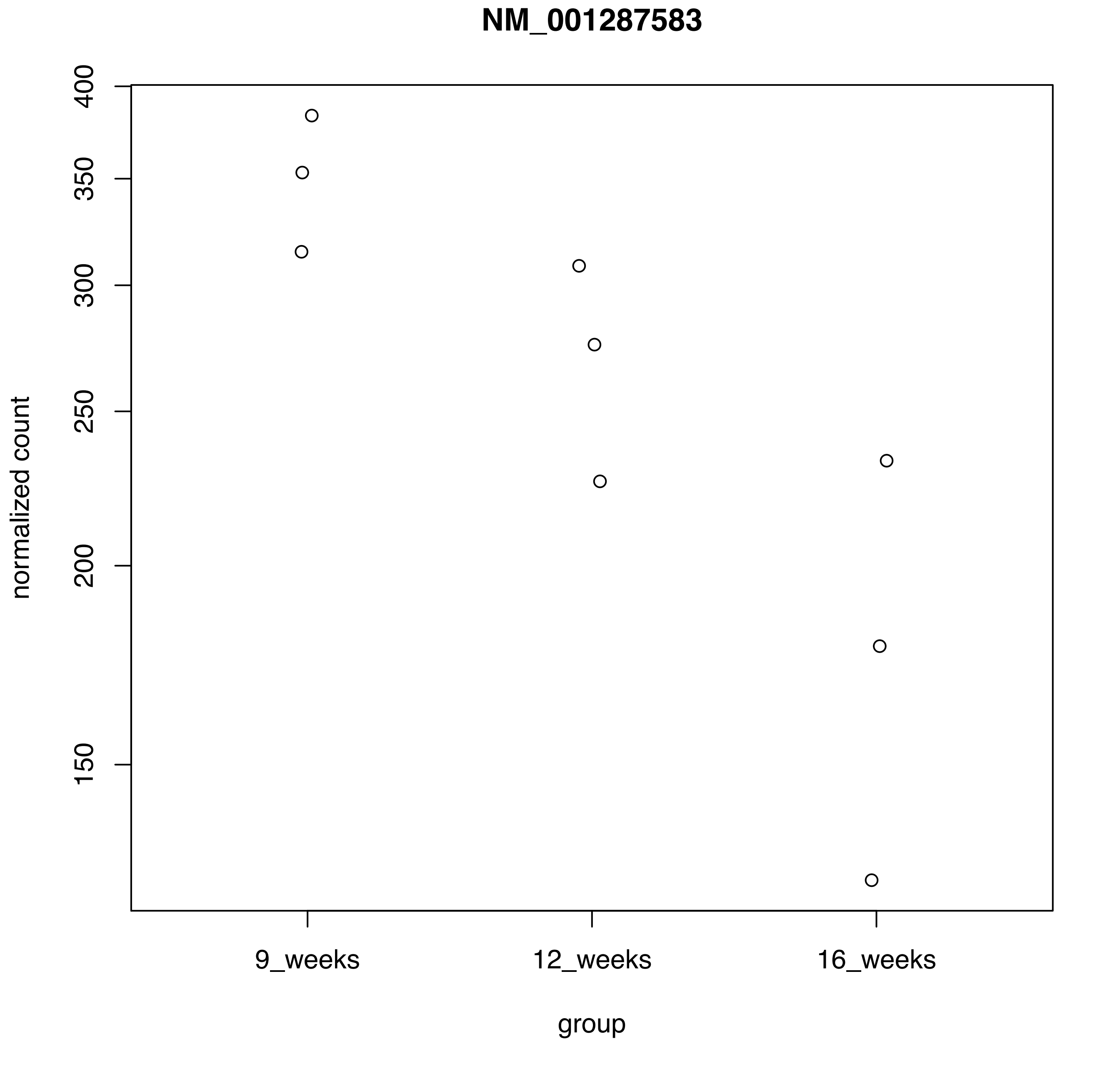
NM_001287583
normalized count
400
350
300
250
200
150
9_weeks
12_weeks
16_weeks
group

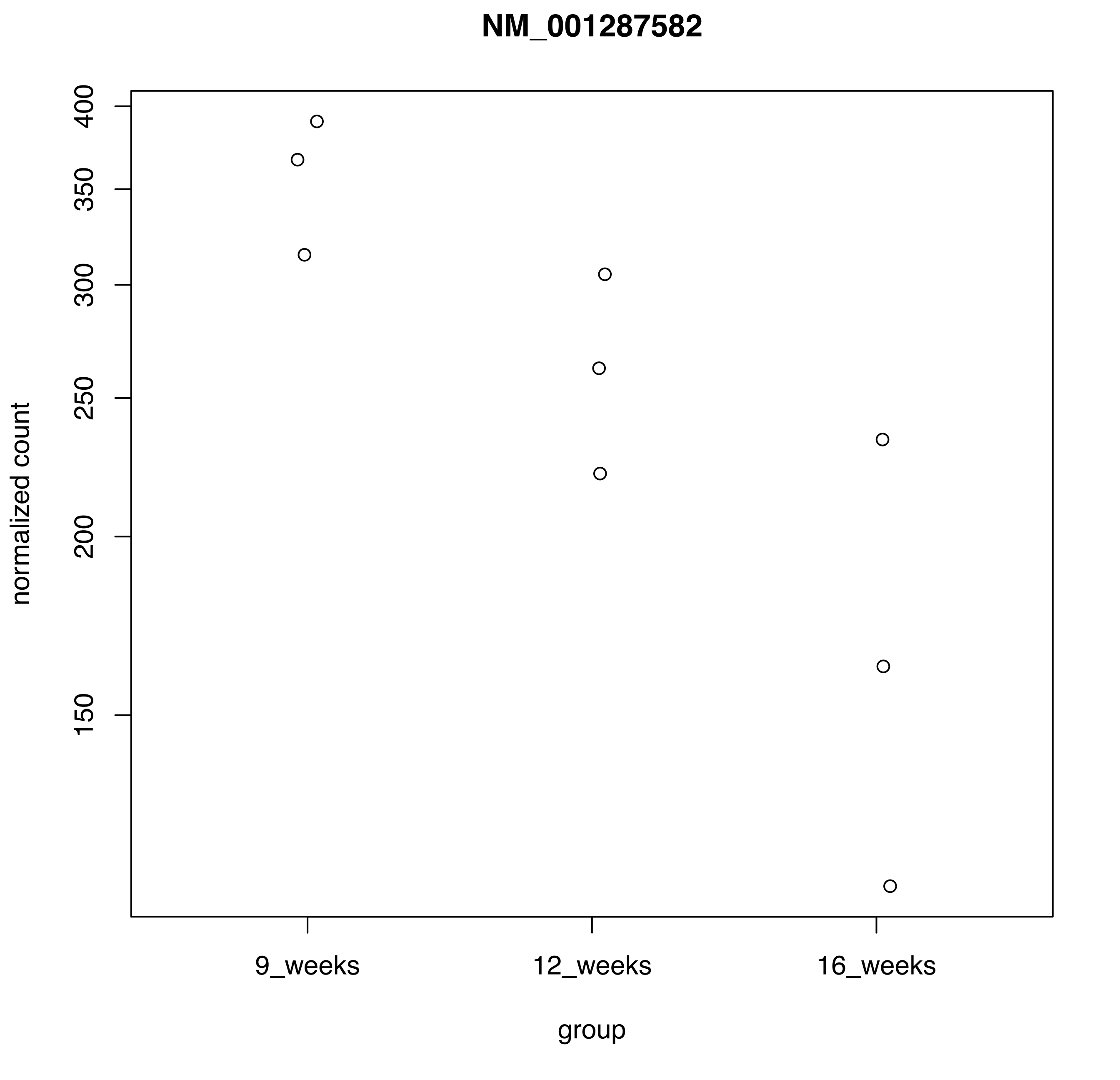

NM_001287582
normalized count
400
350
300
250
200
150
9_weeks
12_weeks
16_weeks
group

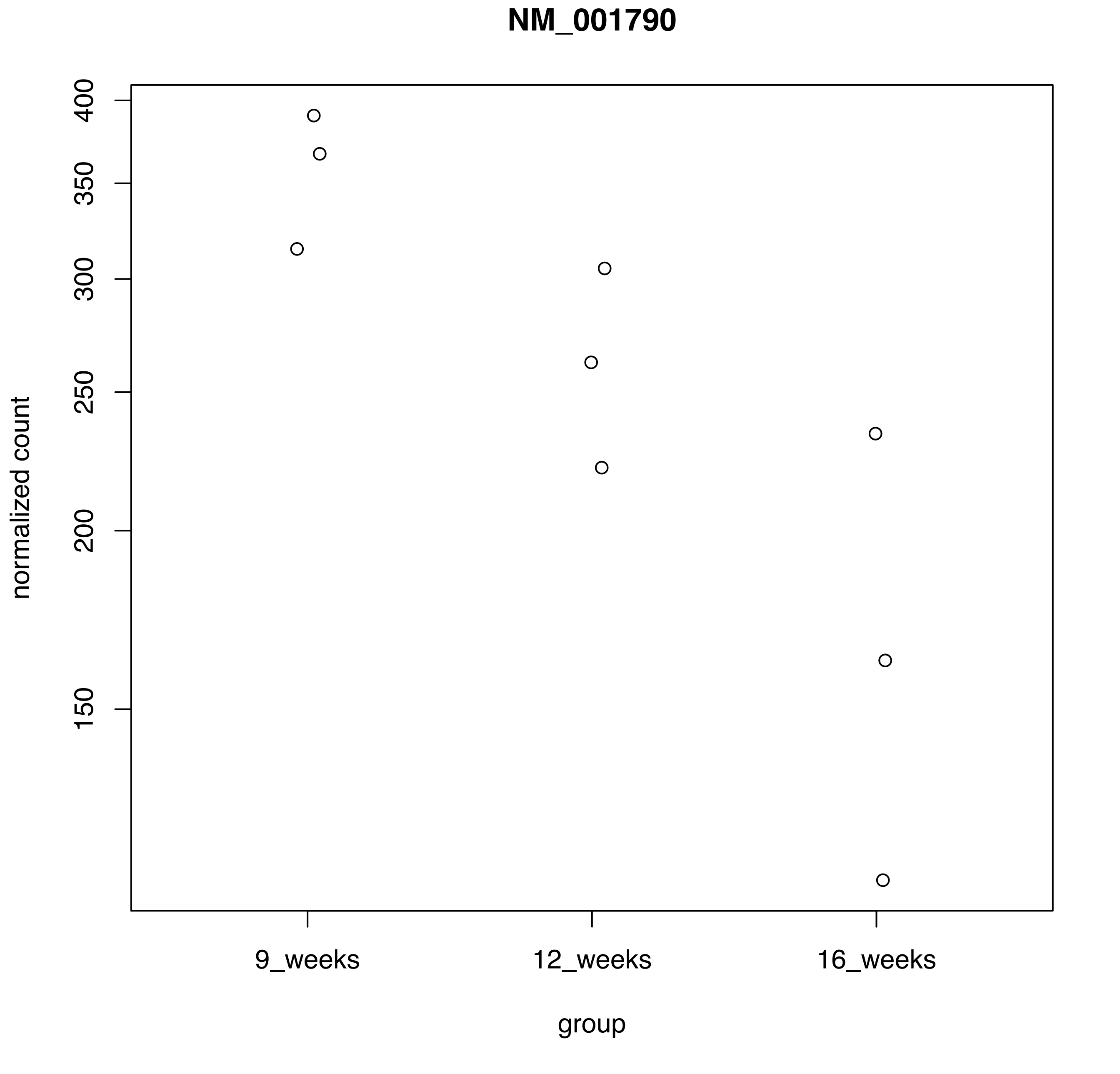
NM_001790
normalized count
400
350
300
250
200
150
9_weeks
12_weeks
16_weeks
group

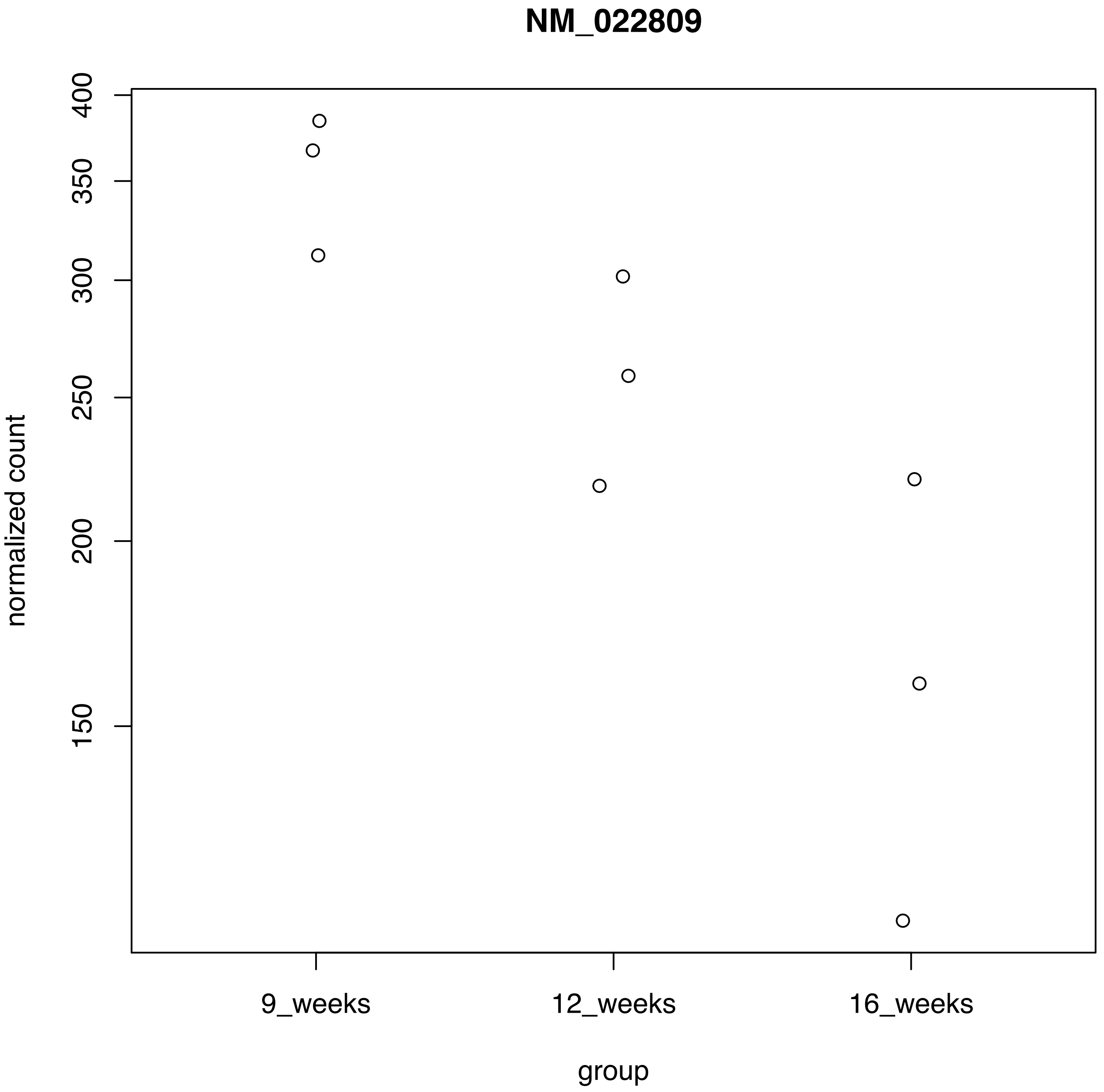

NM_022809
normalized count
400
350
300
250
200
150
9_weeks
12_weeks
16_weeks
group

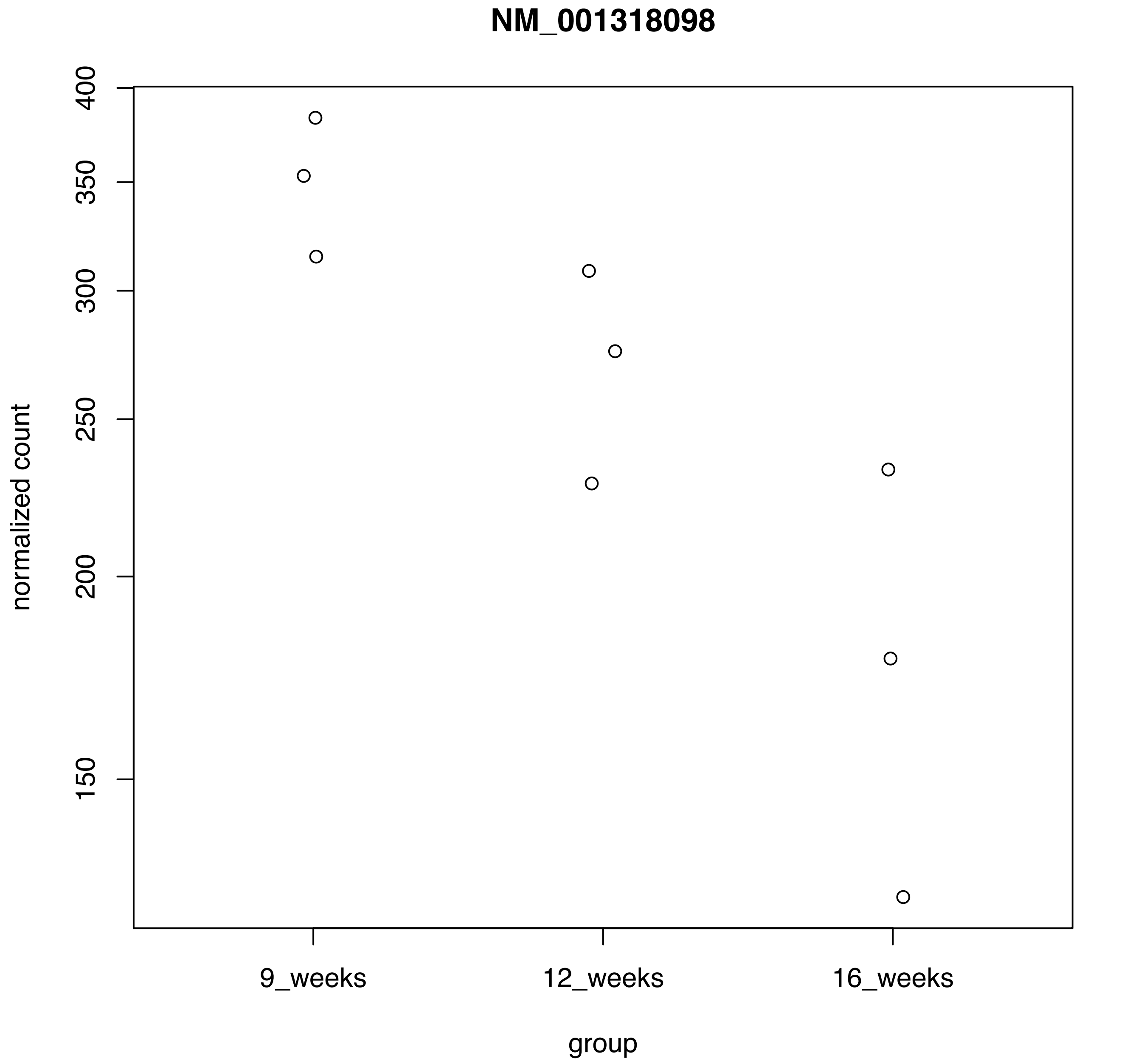
NM_001318098
normalized count
400
350
300
250
200
150
9_weeks
12_weeks
16_weeks
group

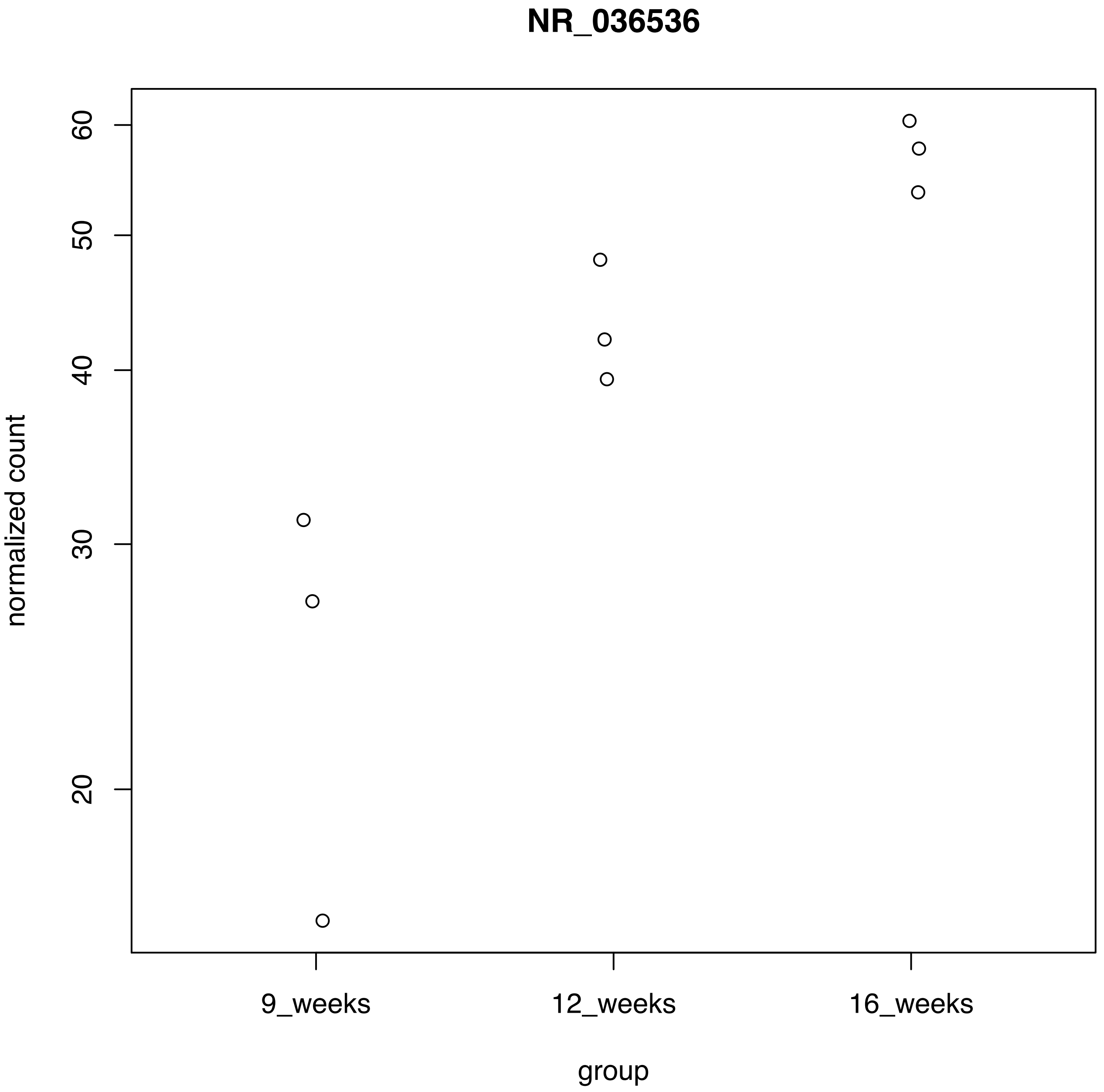
NR_036536
normalized count
20
30
40
50
60
9_weeks
12_weeks
16_weeks
group

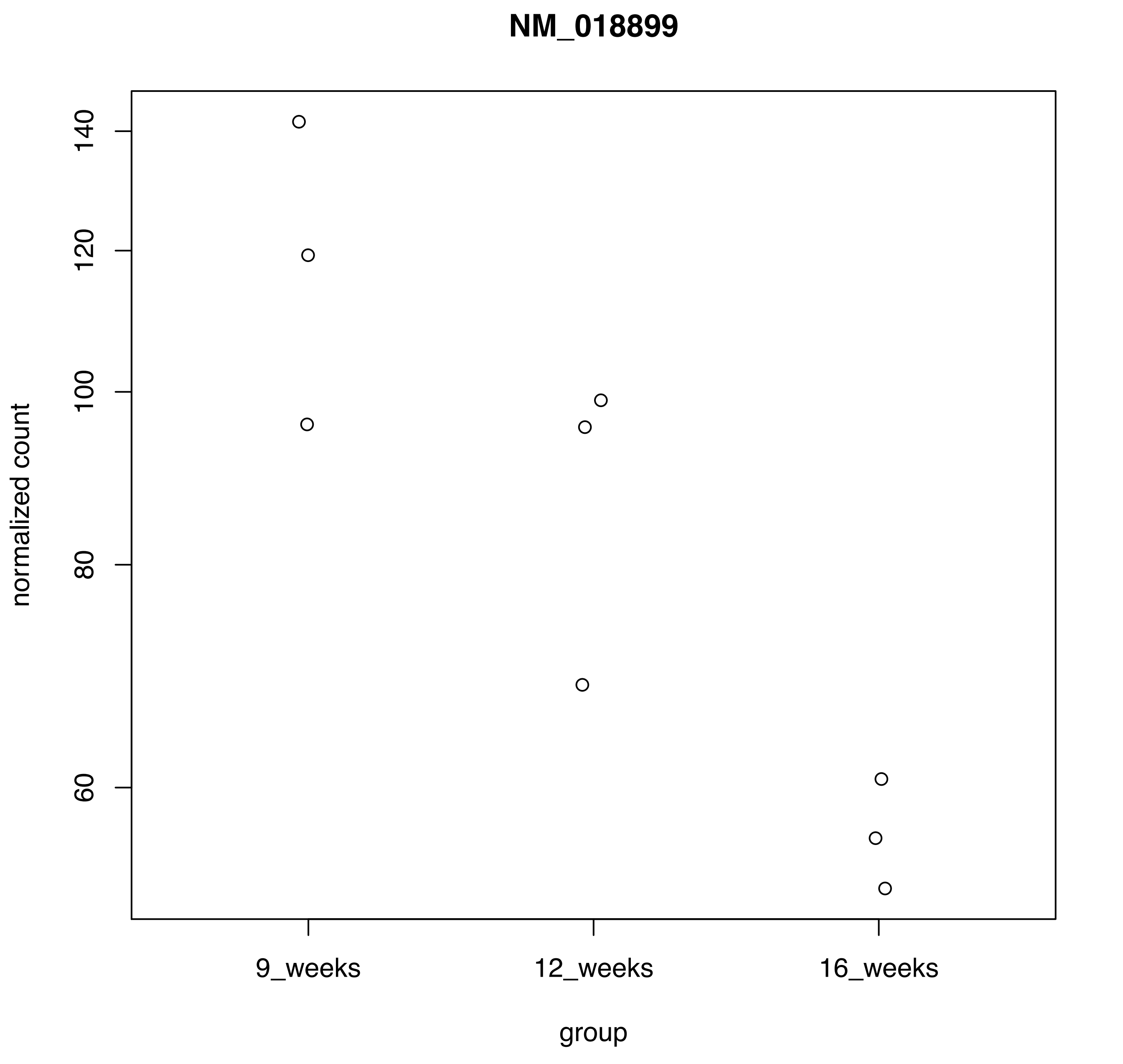
NM_018899
normalized count
140
120
100
80
60
9_weeks
12_weeks
16_weeks
group

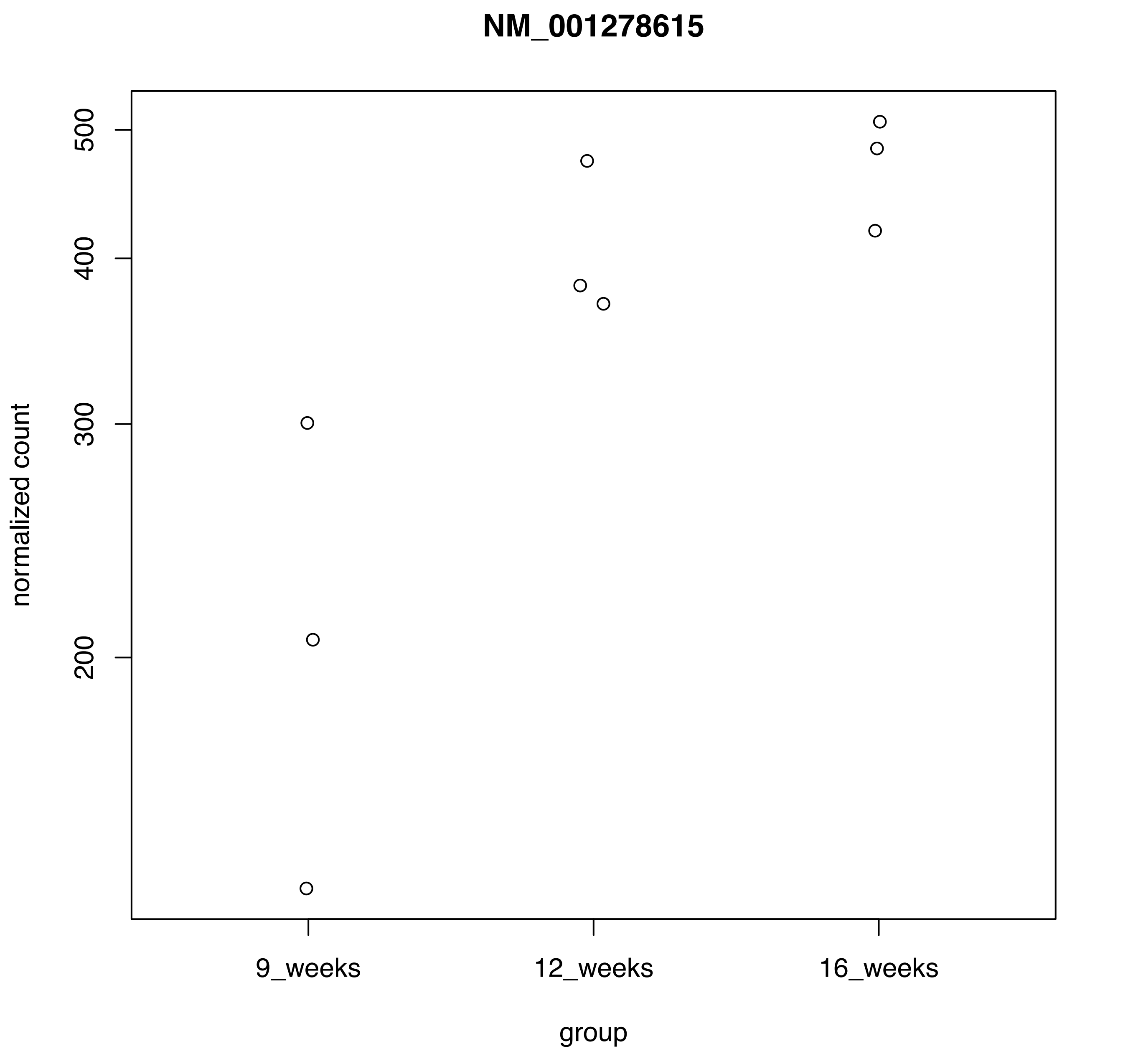

NM_001278615
normalized count
500
400
300
200
9_weeks
12_weeks
16_weeks
group

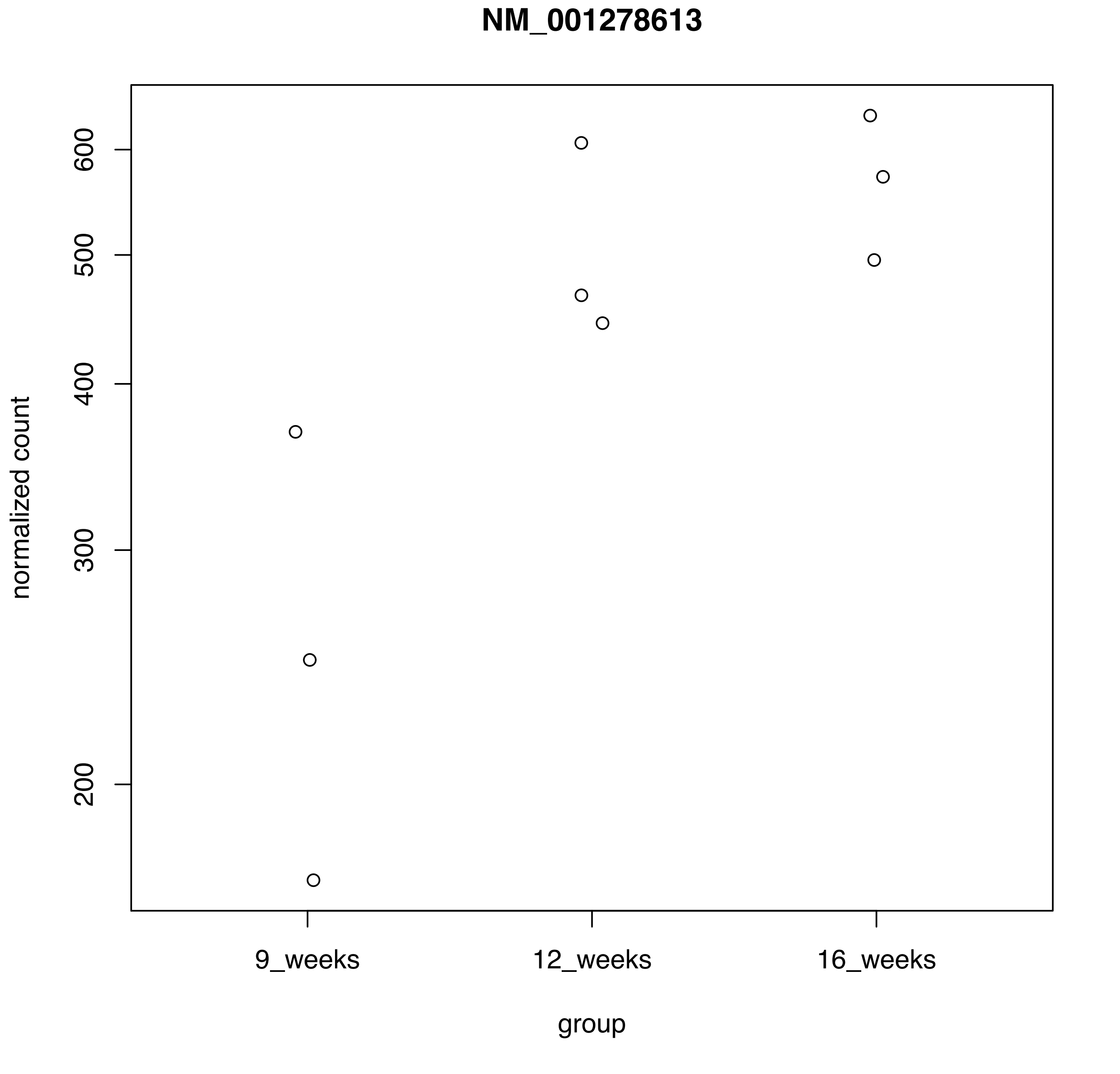
NM_001278613
normalized count
200
300
400
500
600
9_weeks
12_weeks
16_weeks
group

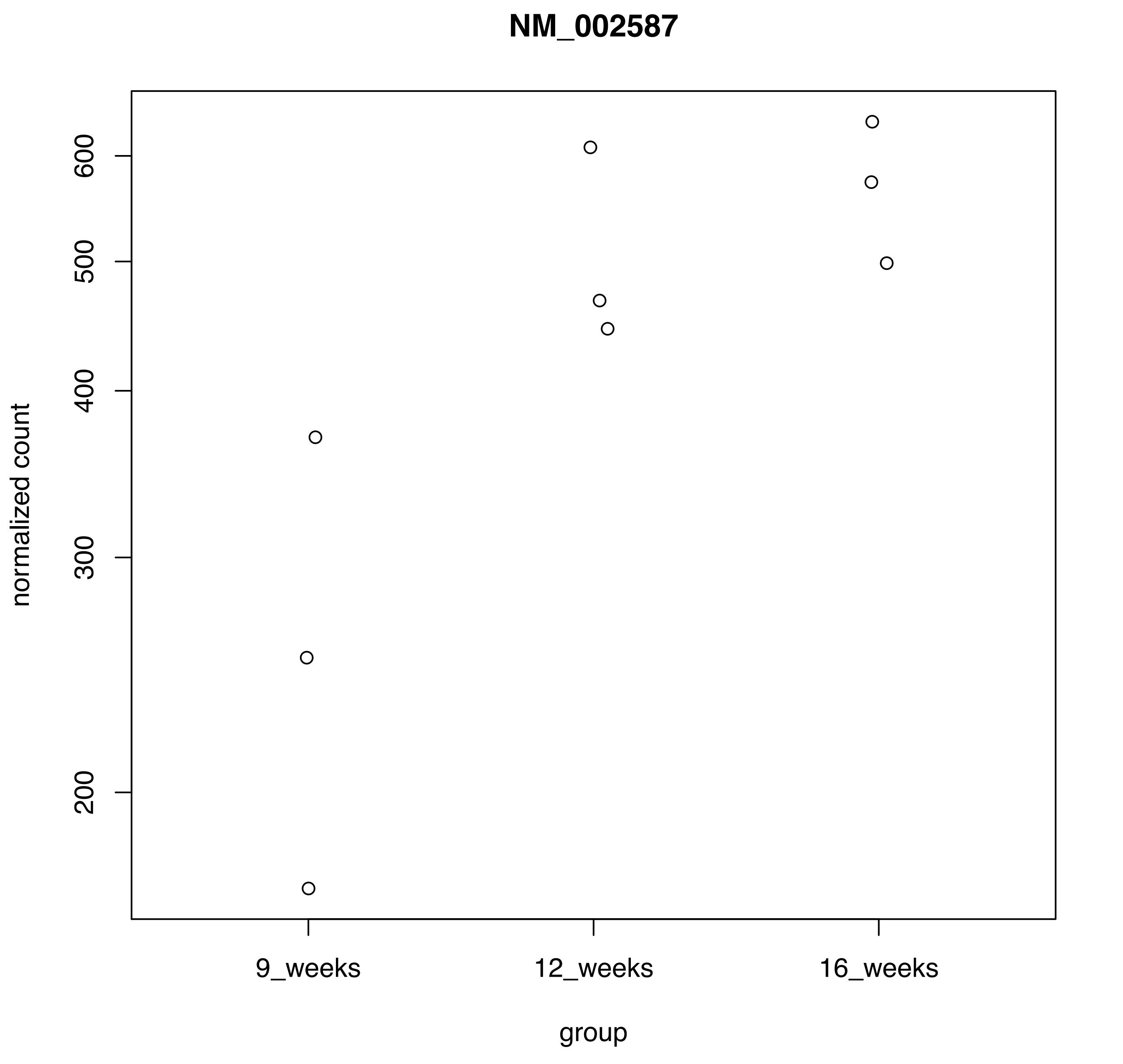
NM_002587
normalized count
600
500
400
300
200
9_weeks
12_weeks
16_weeks
group

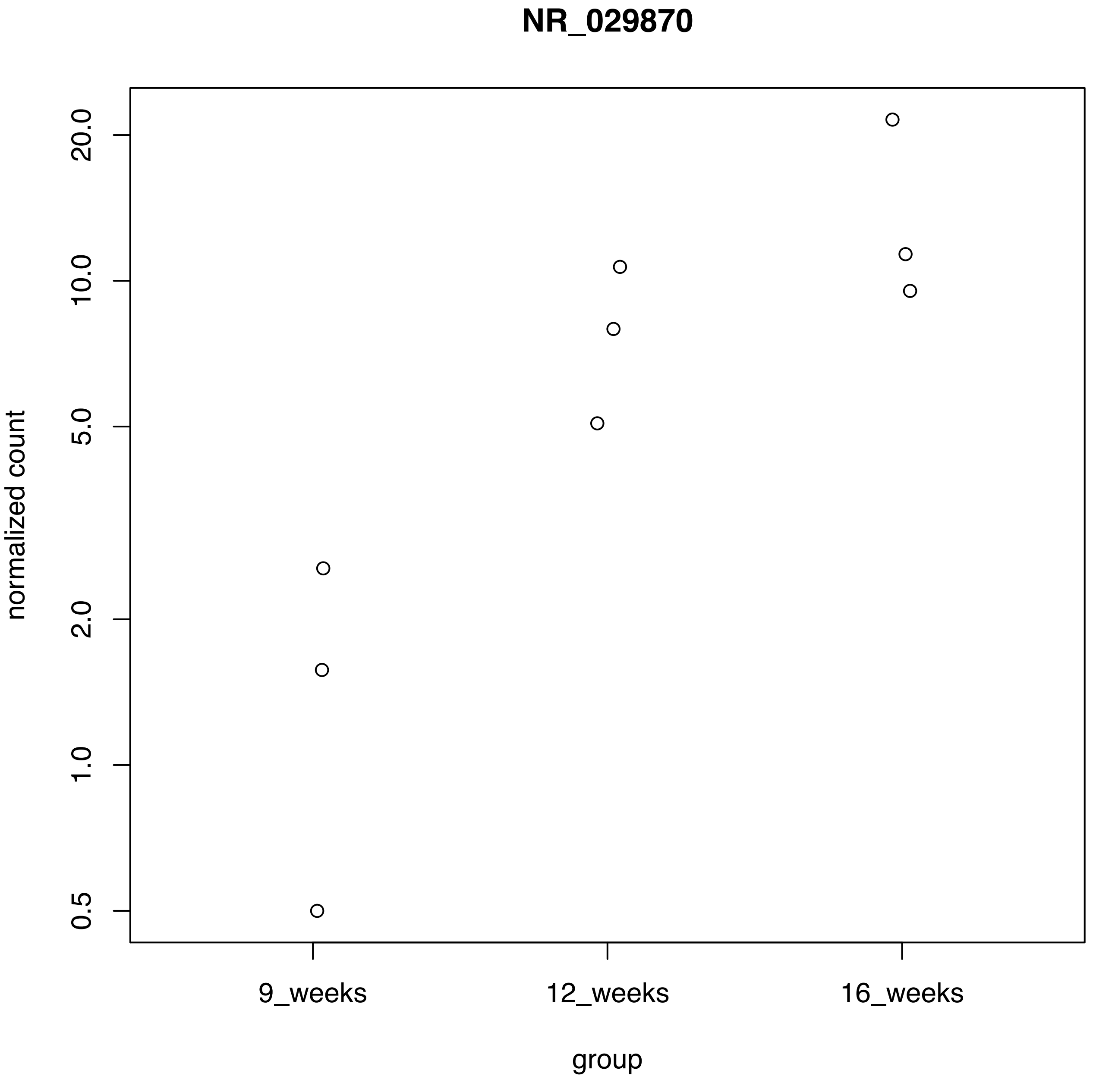

NR_029870
normalized count
20.0
10.0
5.0
2.0
1.0
0.5
9_weeks
12_weeks
16_weeks
group

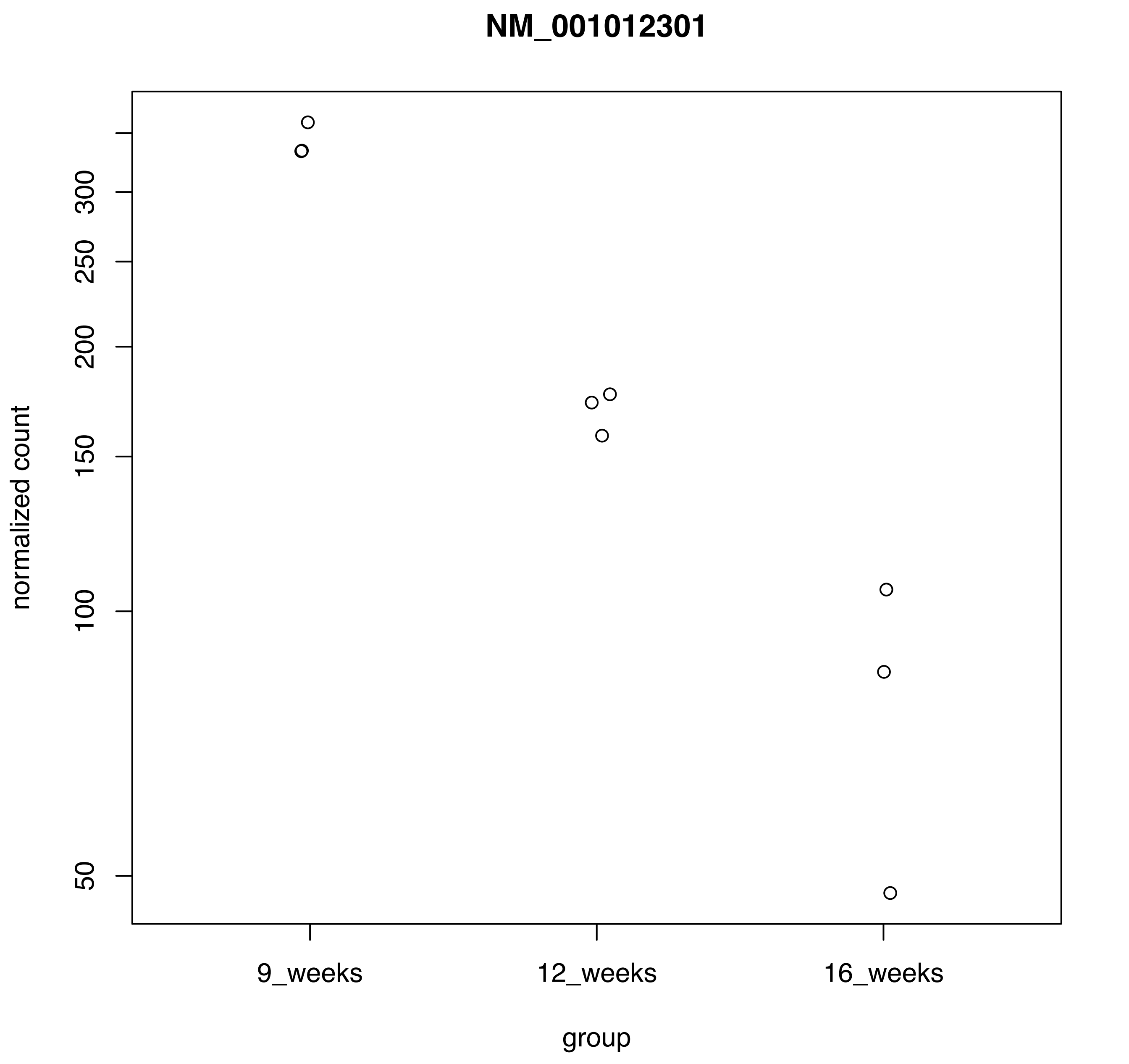
NM_001012301
normalized count
300
250
200
150
100
50
9_weeks
12_weeks
16_weeks
group

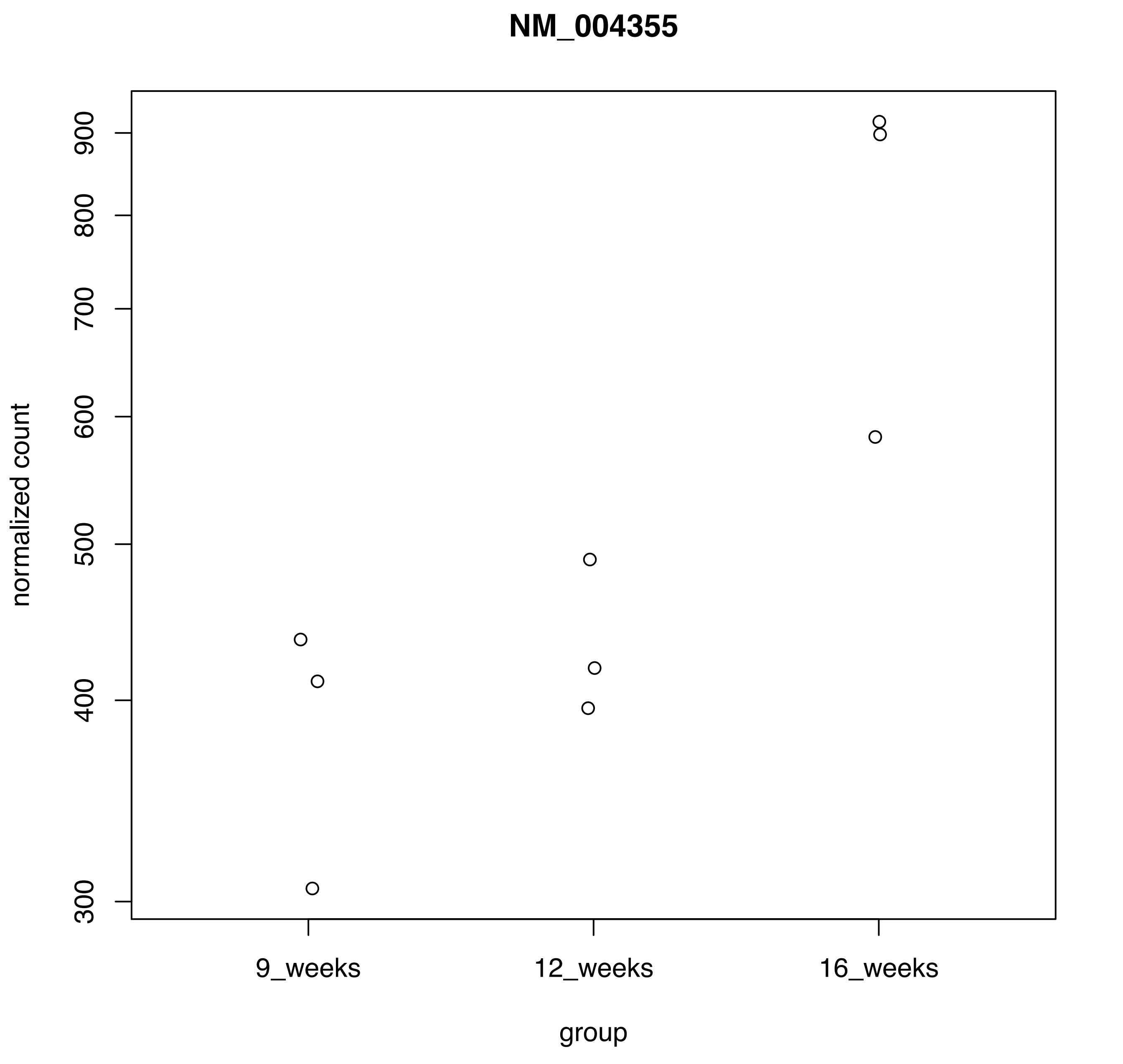
NM_004355
normalized count
300
400
500
600
700
800
900
9_weeks
12_weeks
16_weeks
group

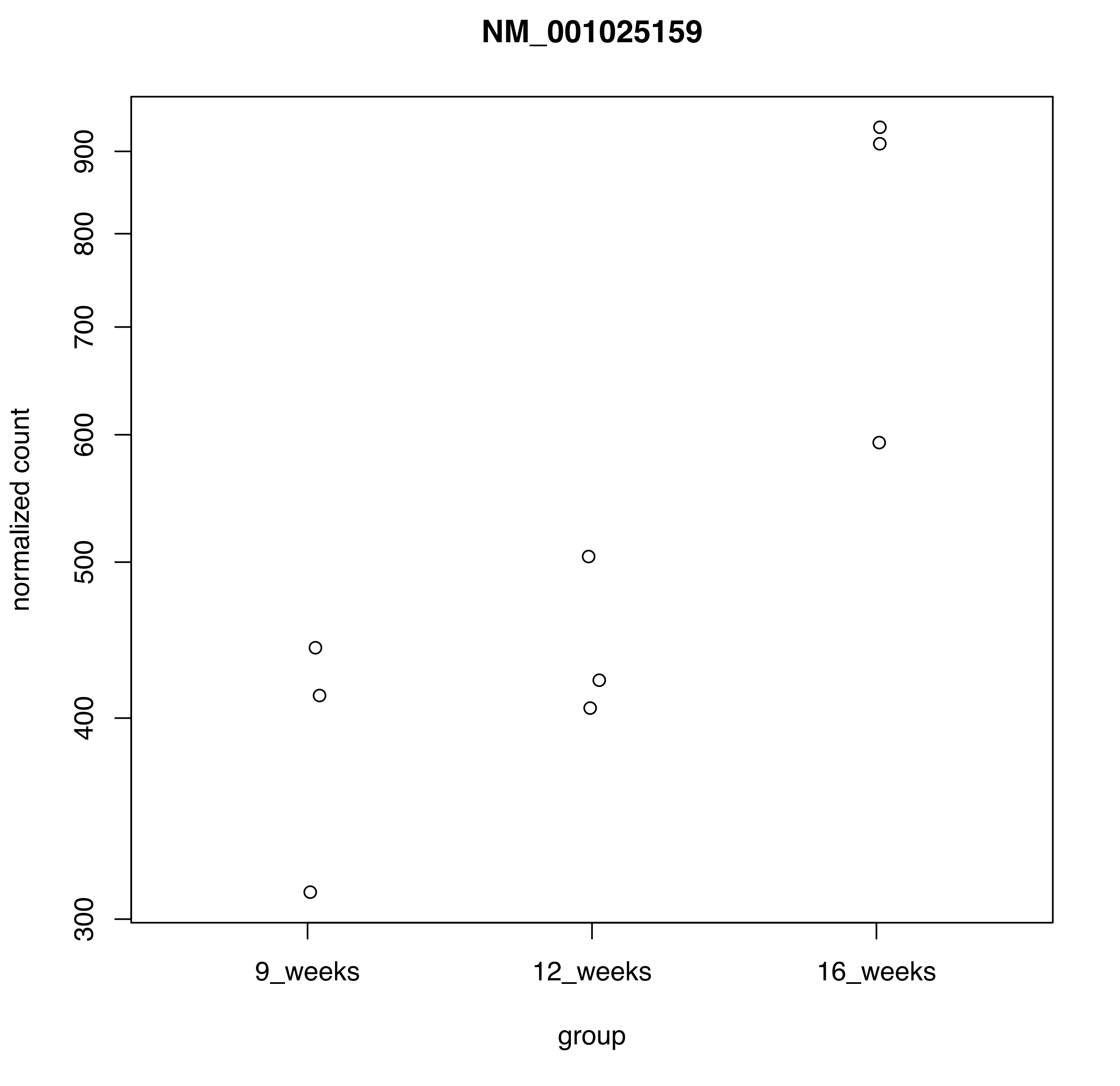
NM_001025159
normalized count
300
400
500
600
700
800
900
9_weeks
12_weeks
16_weeks
group

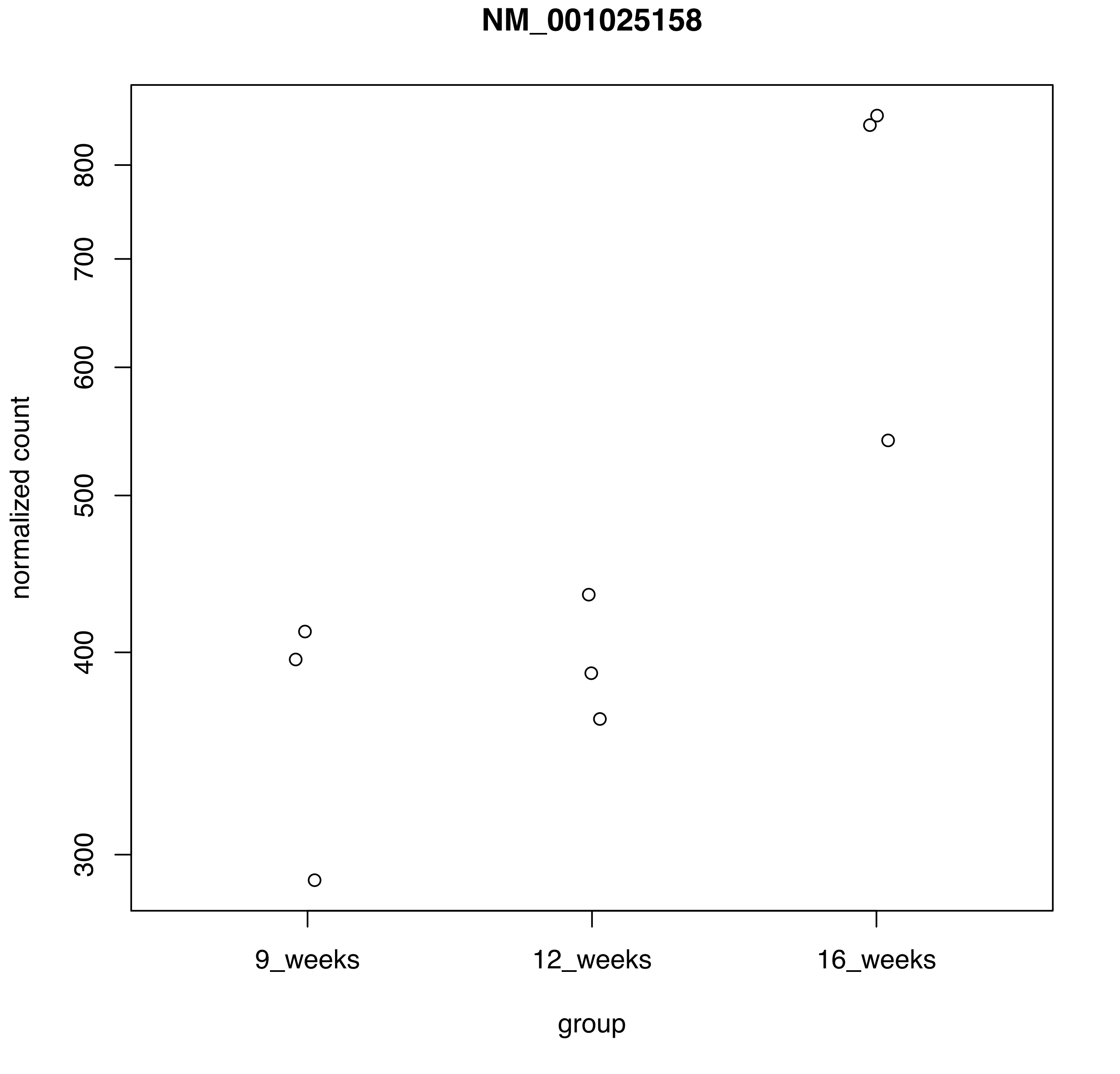

NM_001025158
normalized count
800
700
600
500
400
300
9_weeks
12_weeks
16_weeks
group

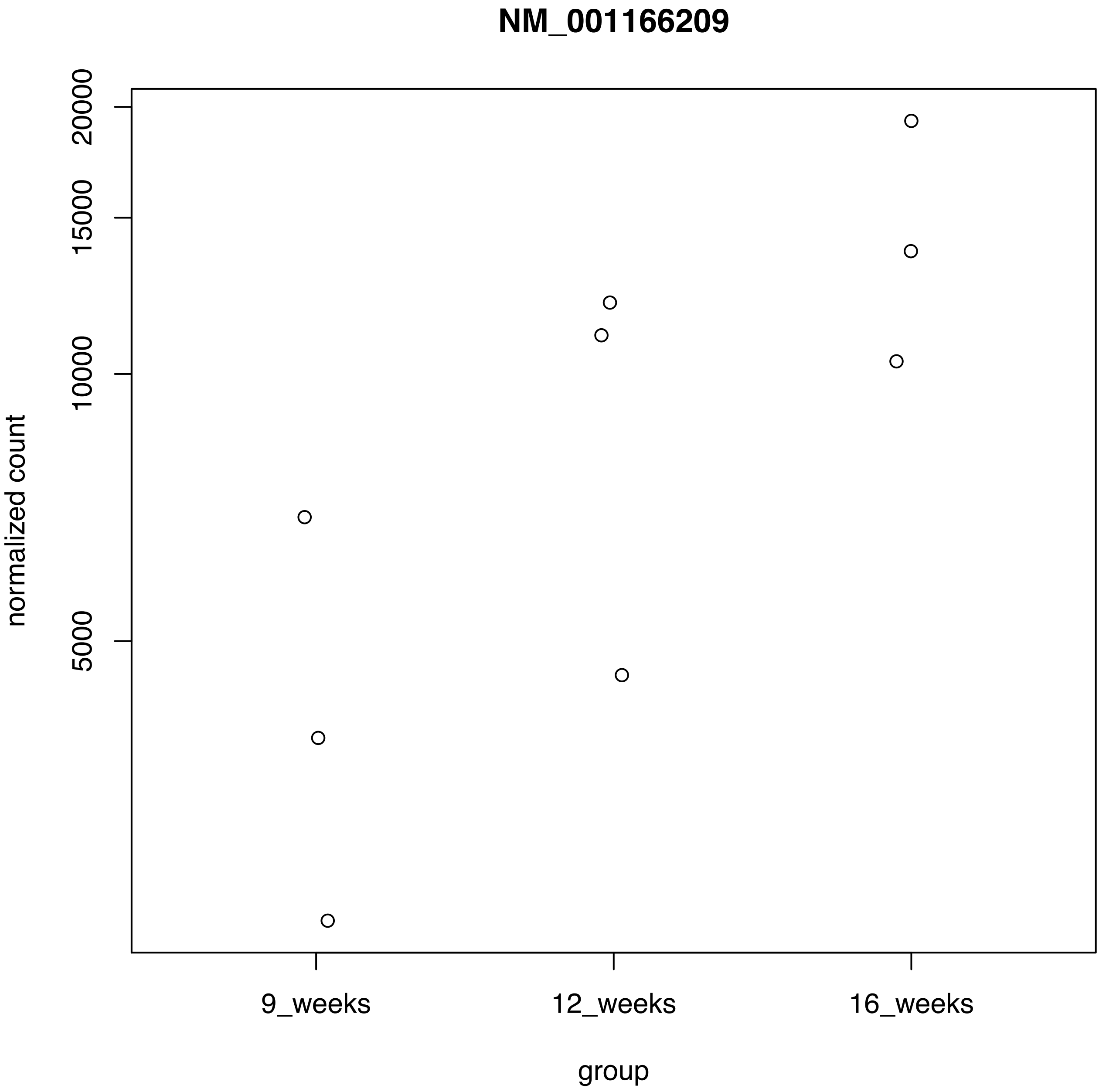

NM_001166209
normalized count
20000
15000
10000
5000
9_weeks
12_weeks
16_weeks
group

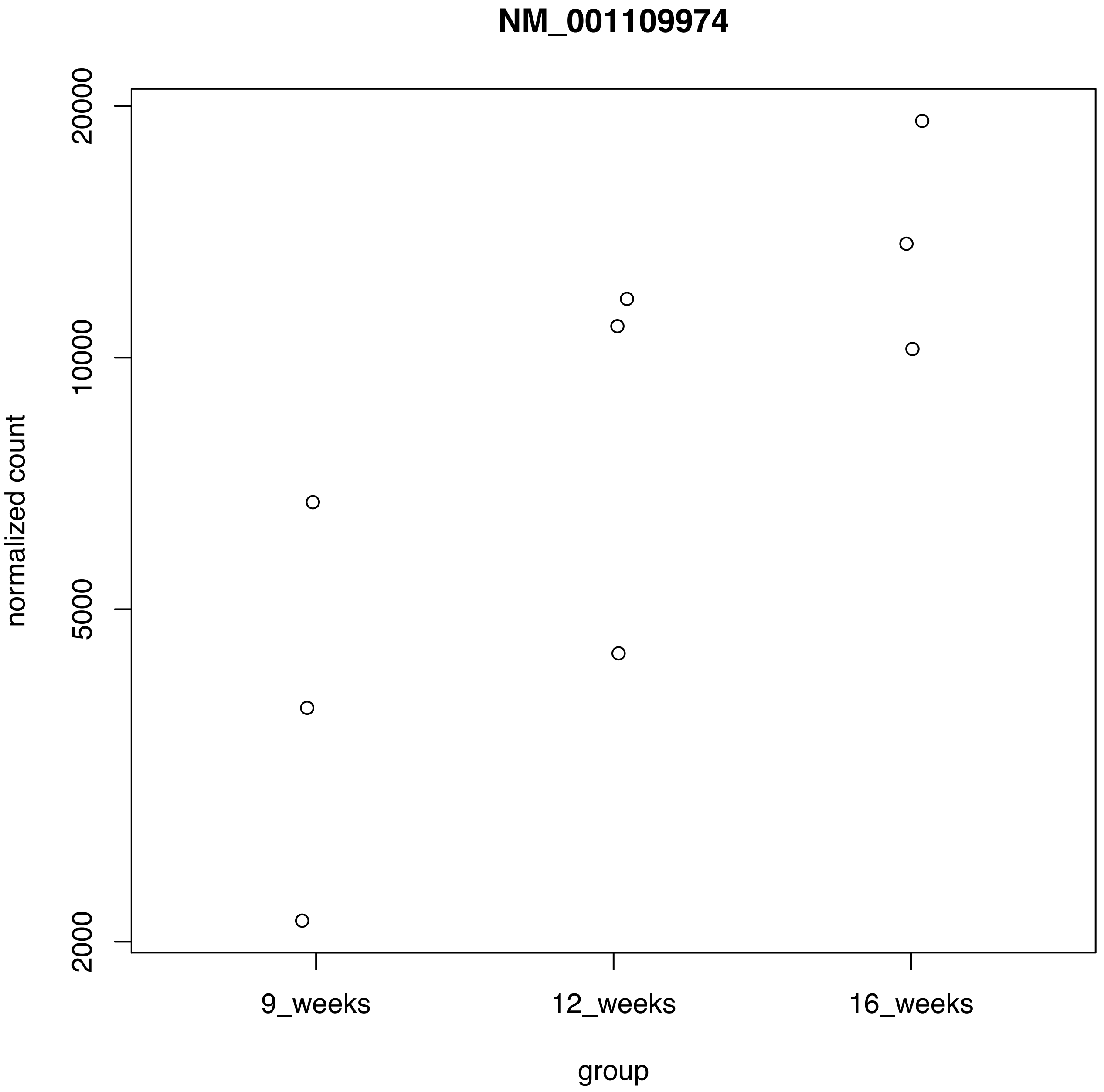
NM_001109974
normalized count
20000
10000
5000
2000
9_weeks
12_weeks
16_weeks
group

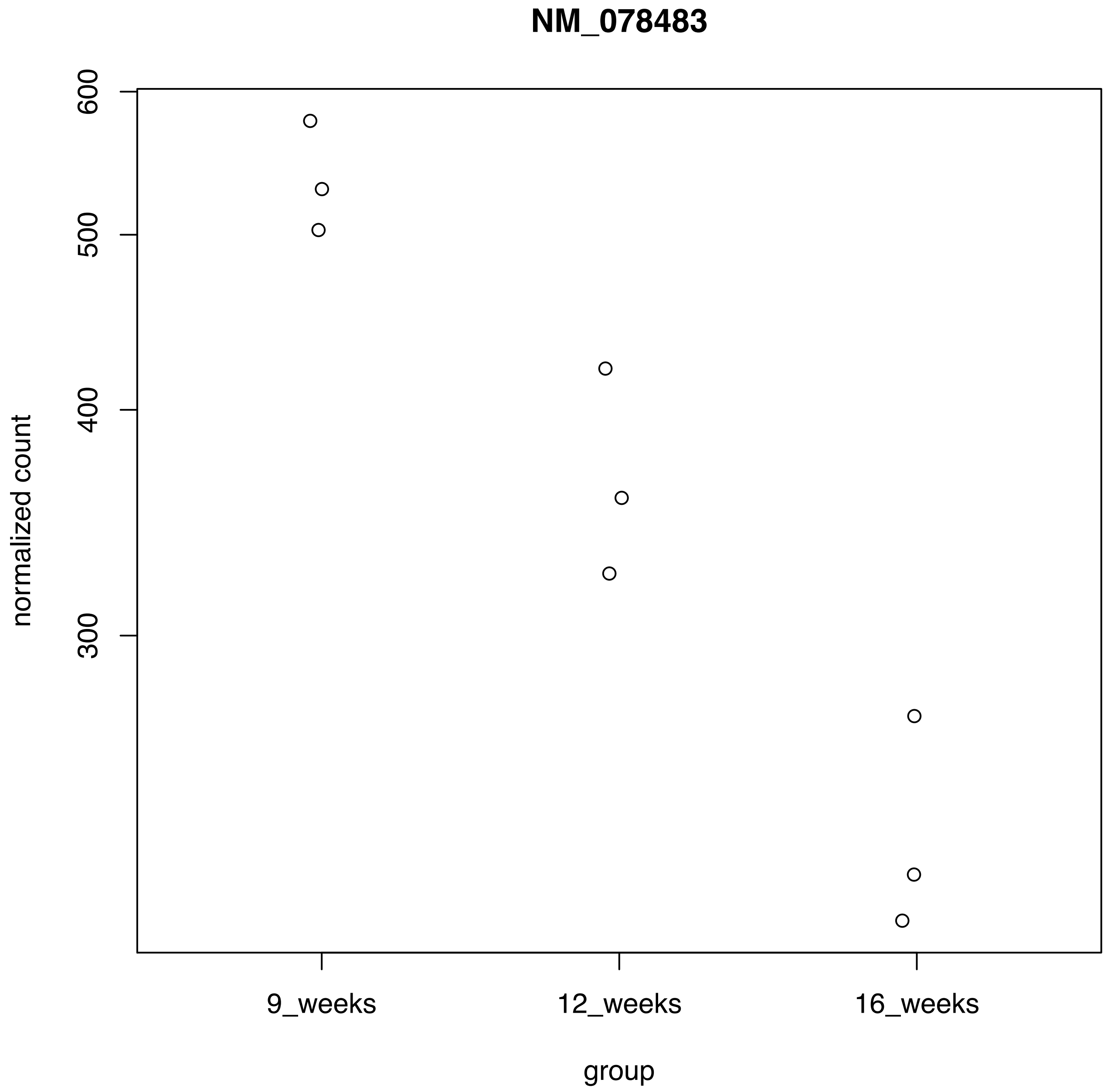
NM_078483
normalized count
600
500
400
300
9_weeks
12_weeks
16_weeks
group

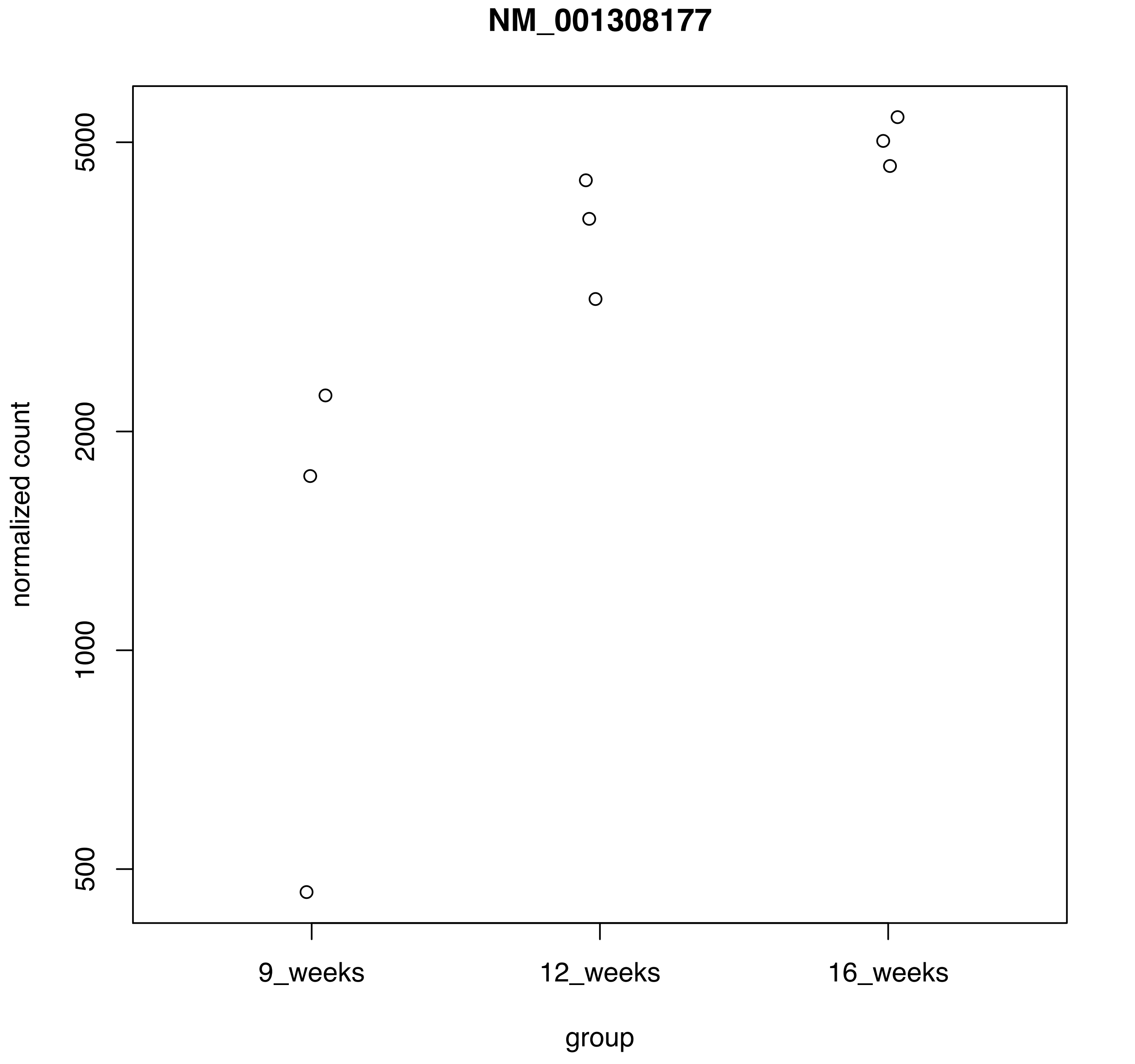

NM_001308177
normalized count
5000
2000
1000
500
9_weeks
12_weeks
16_weeks
group

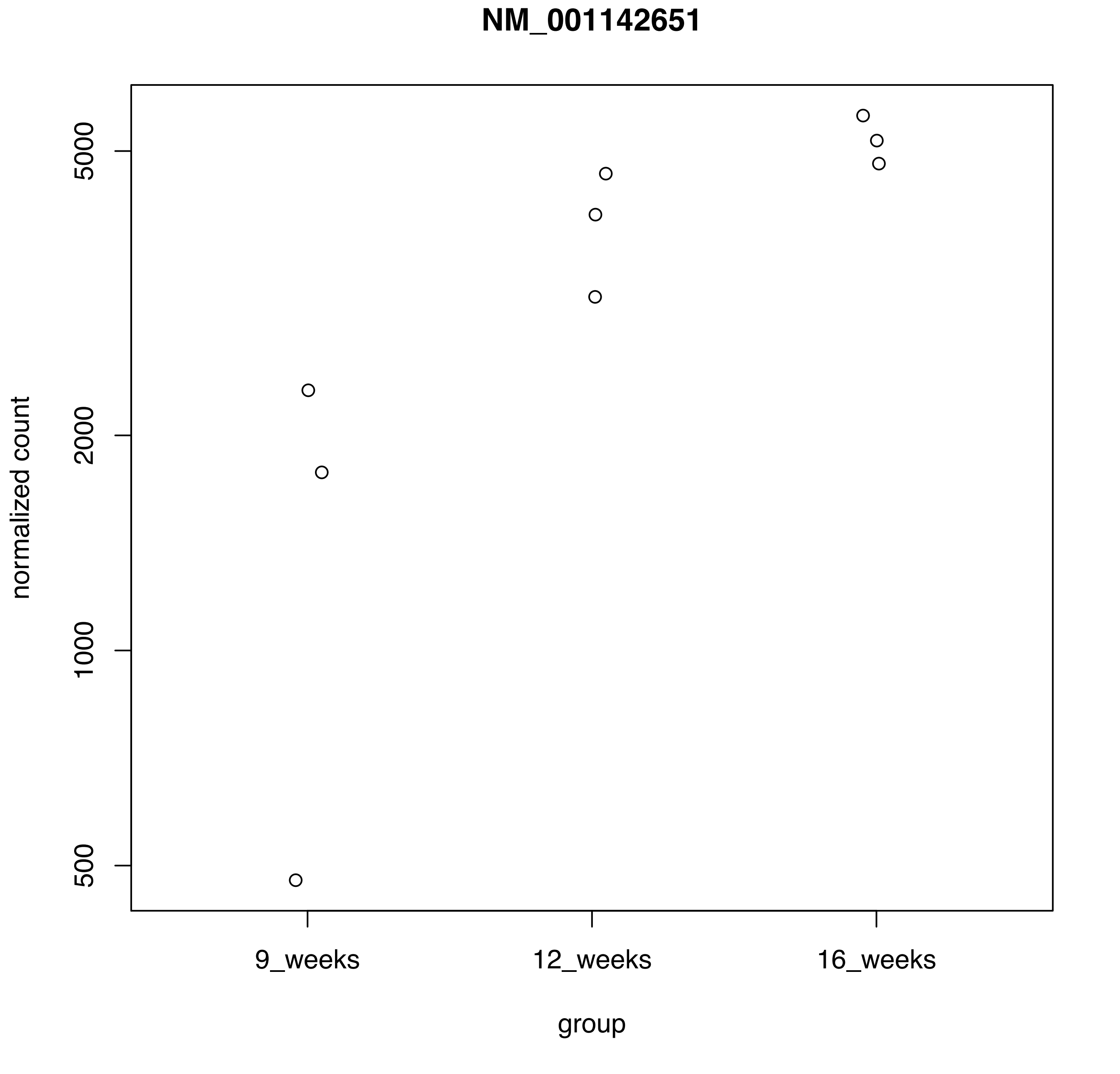

NM_001142651
normalized count
5000
2000
1000
500
9_weeks
12_weeks
16_weeks
group

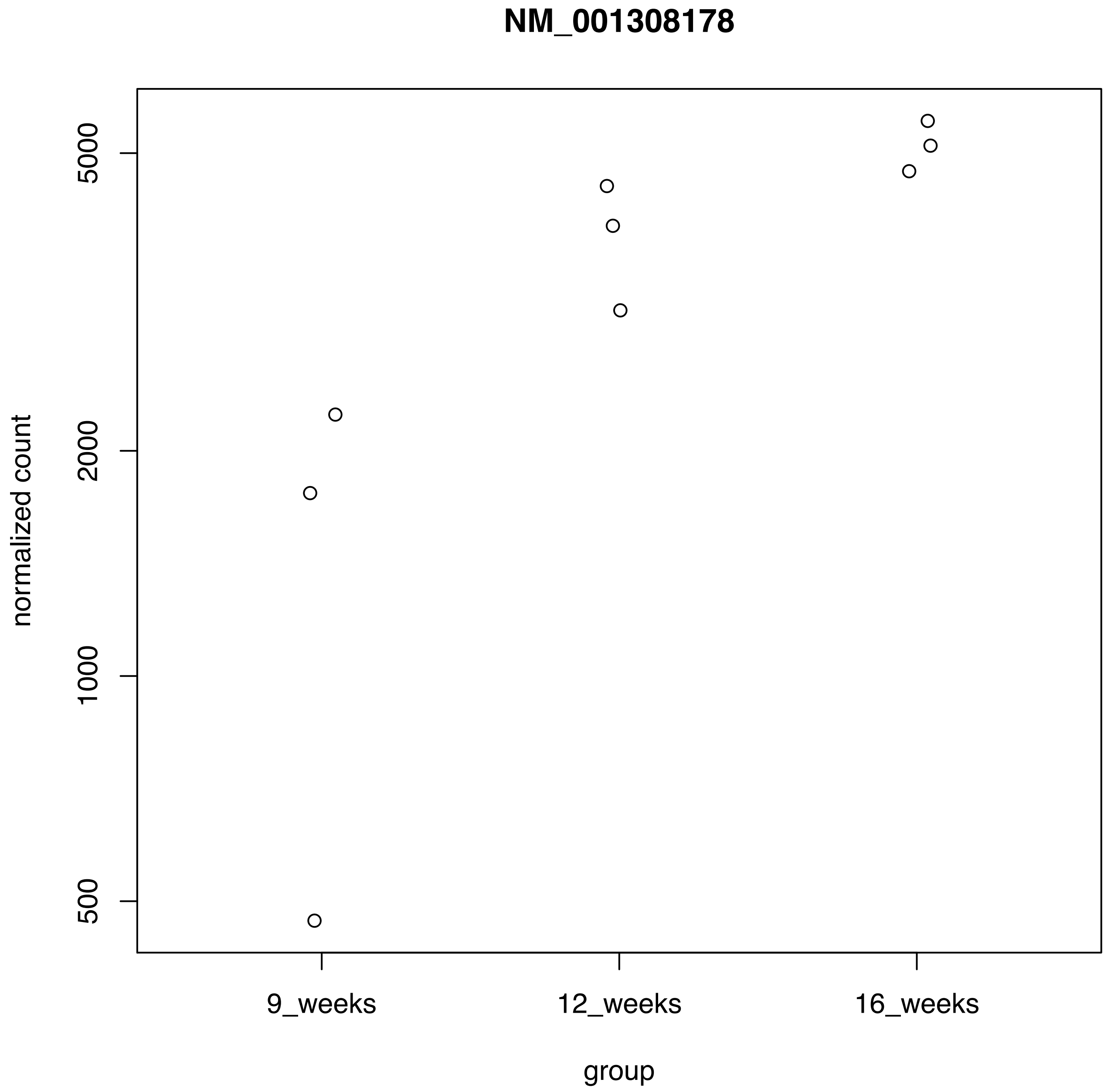

NM_001308178
normalized count
5000
2000
1000
500
9_weeks
12_weeks
16_weeks
group

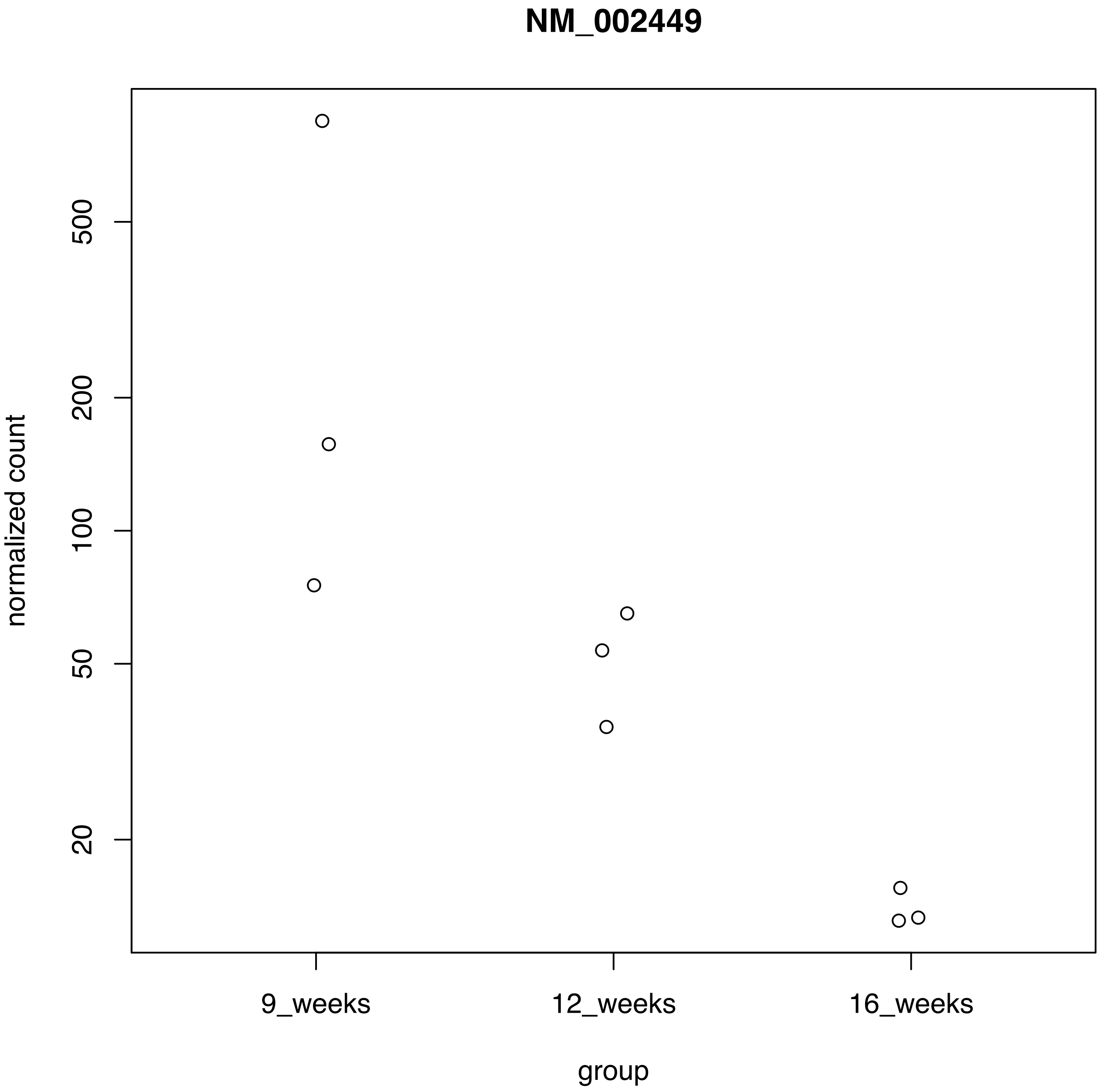
NM_002449
normalized count
500
200
100
50
20
9_weeks
12_weeks
16_weeks
group

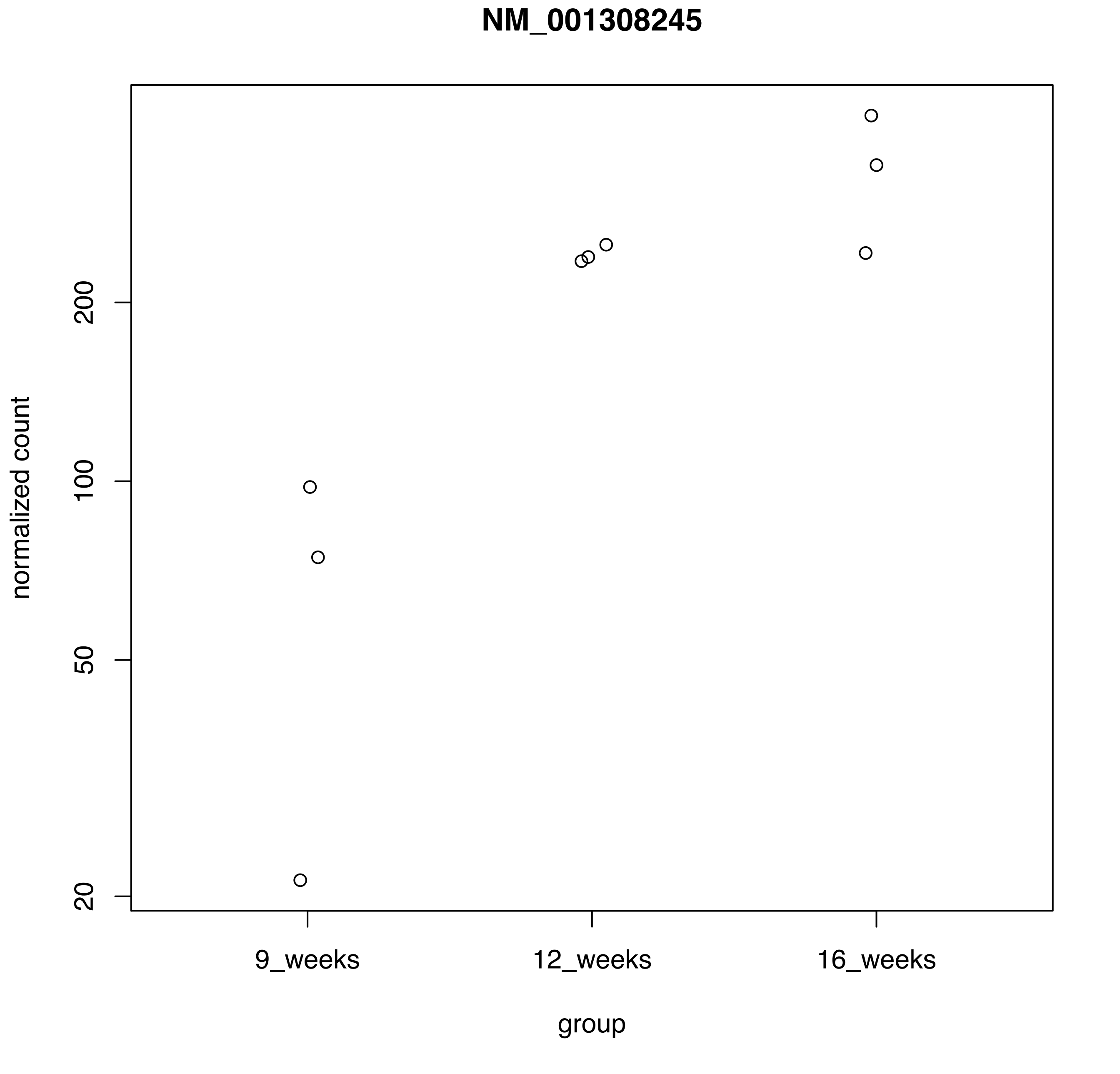

NM_001308245
normalized count
20
50
100
200
9_weeks
12_weeks
16_weeks
group

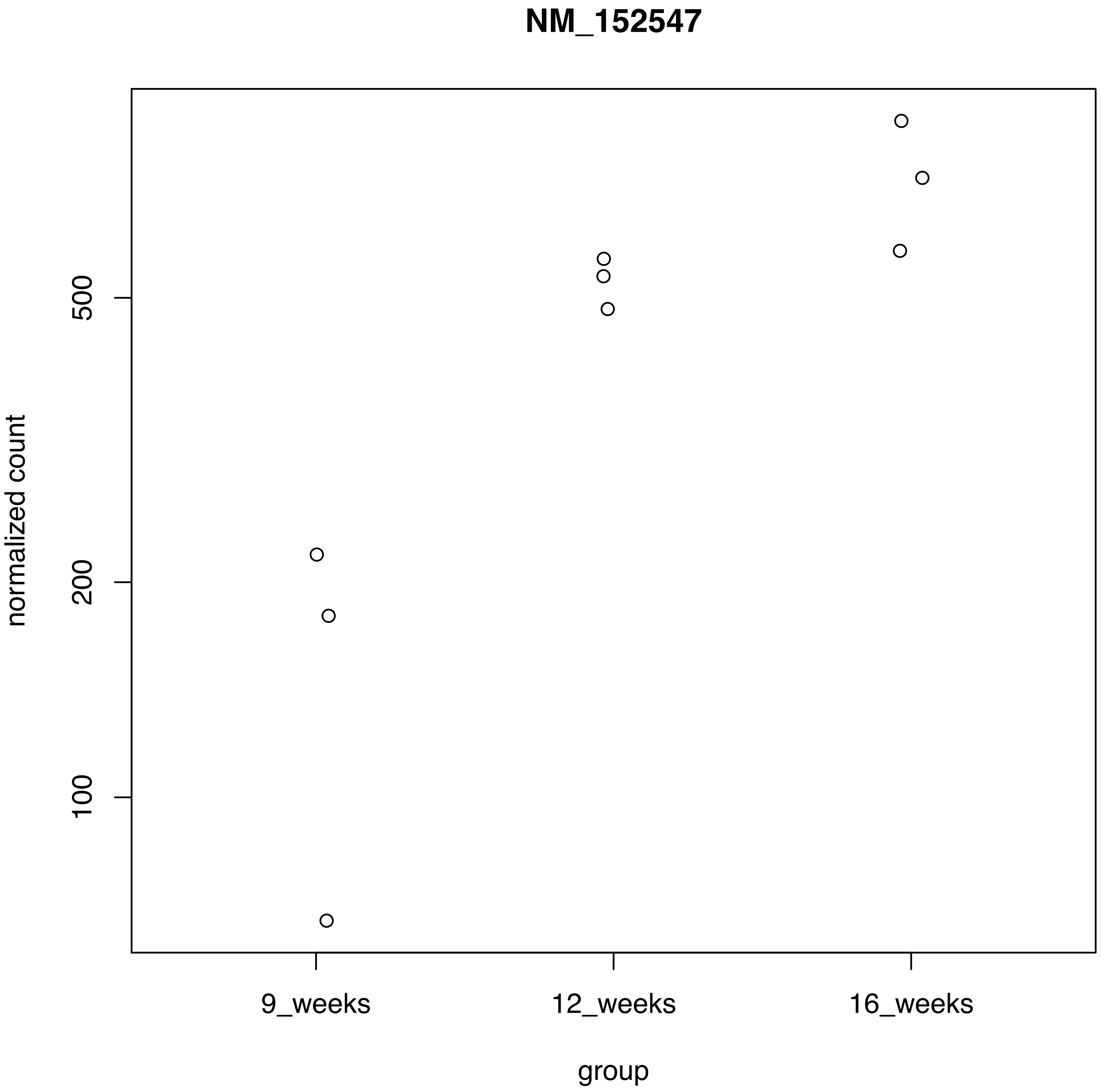
NM_152547
normalized count
500
200
100
9_weeks
12_weeks
16_weeks
group

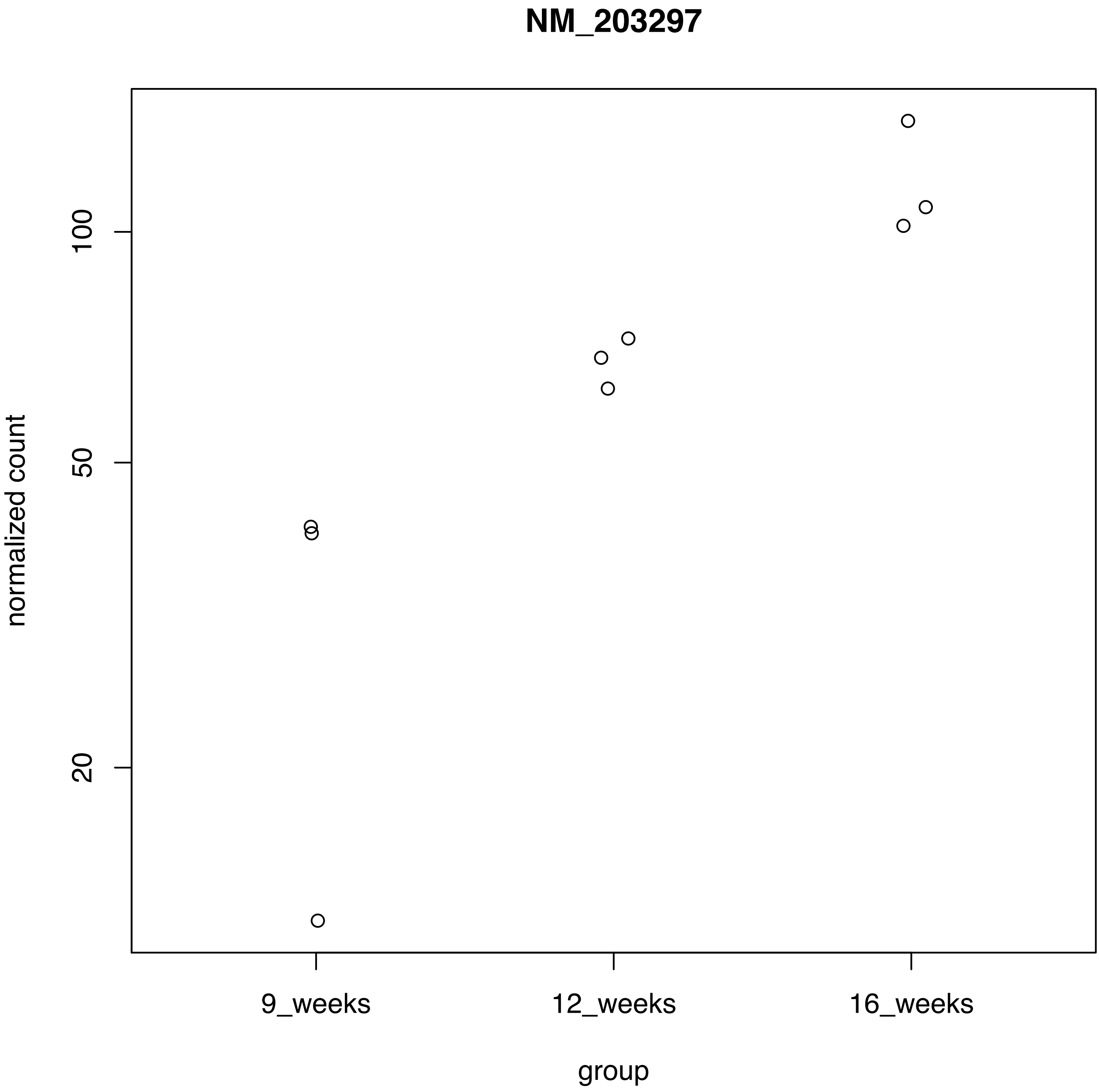
NM_203297
normalized count
100
50
20
9_weeks
12_weeks
16_weeks
group

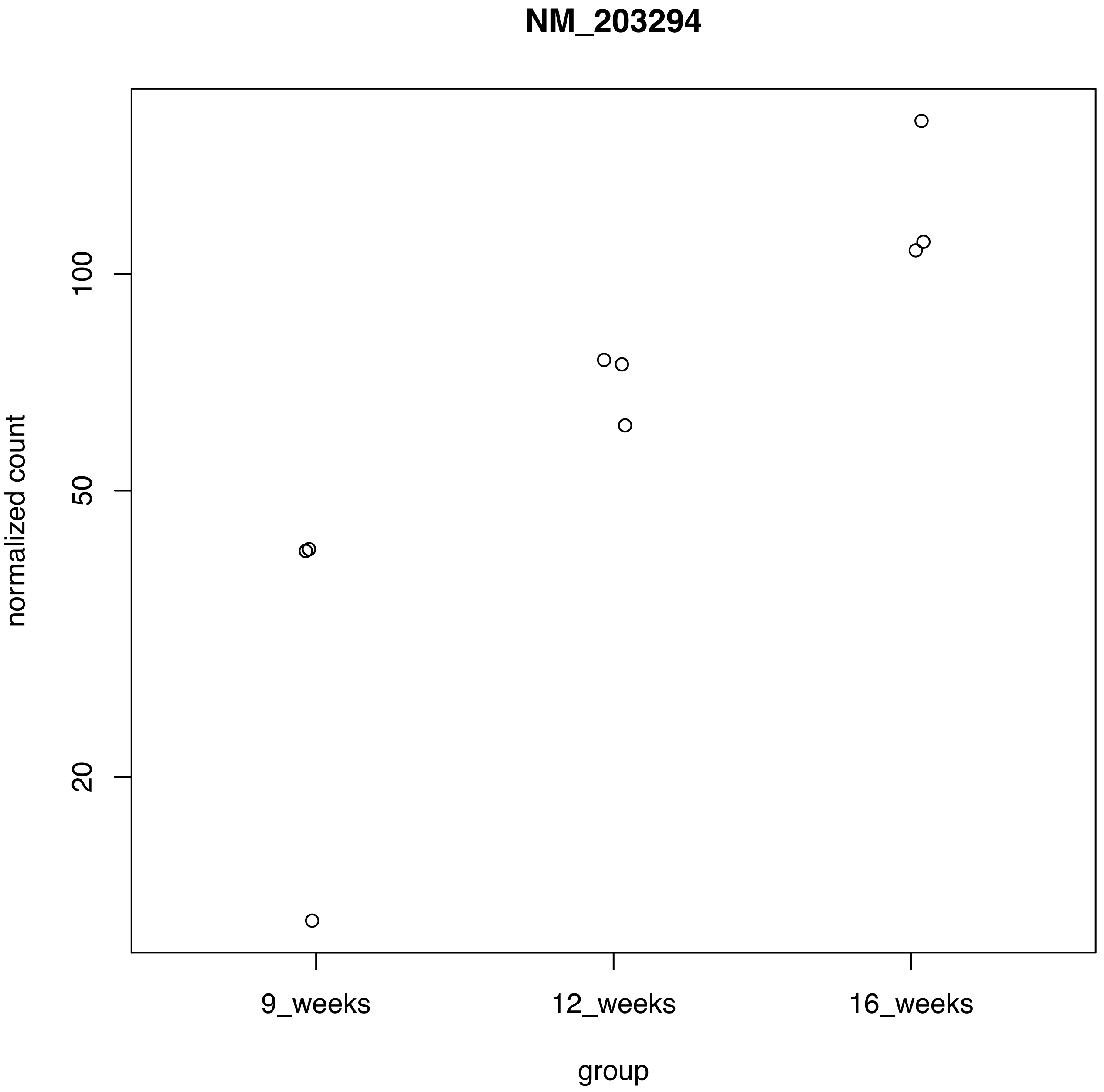
NM_203294
normalized count
100
50
20
9_weeks
12_weeks
16_weeks
group

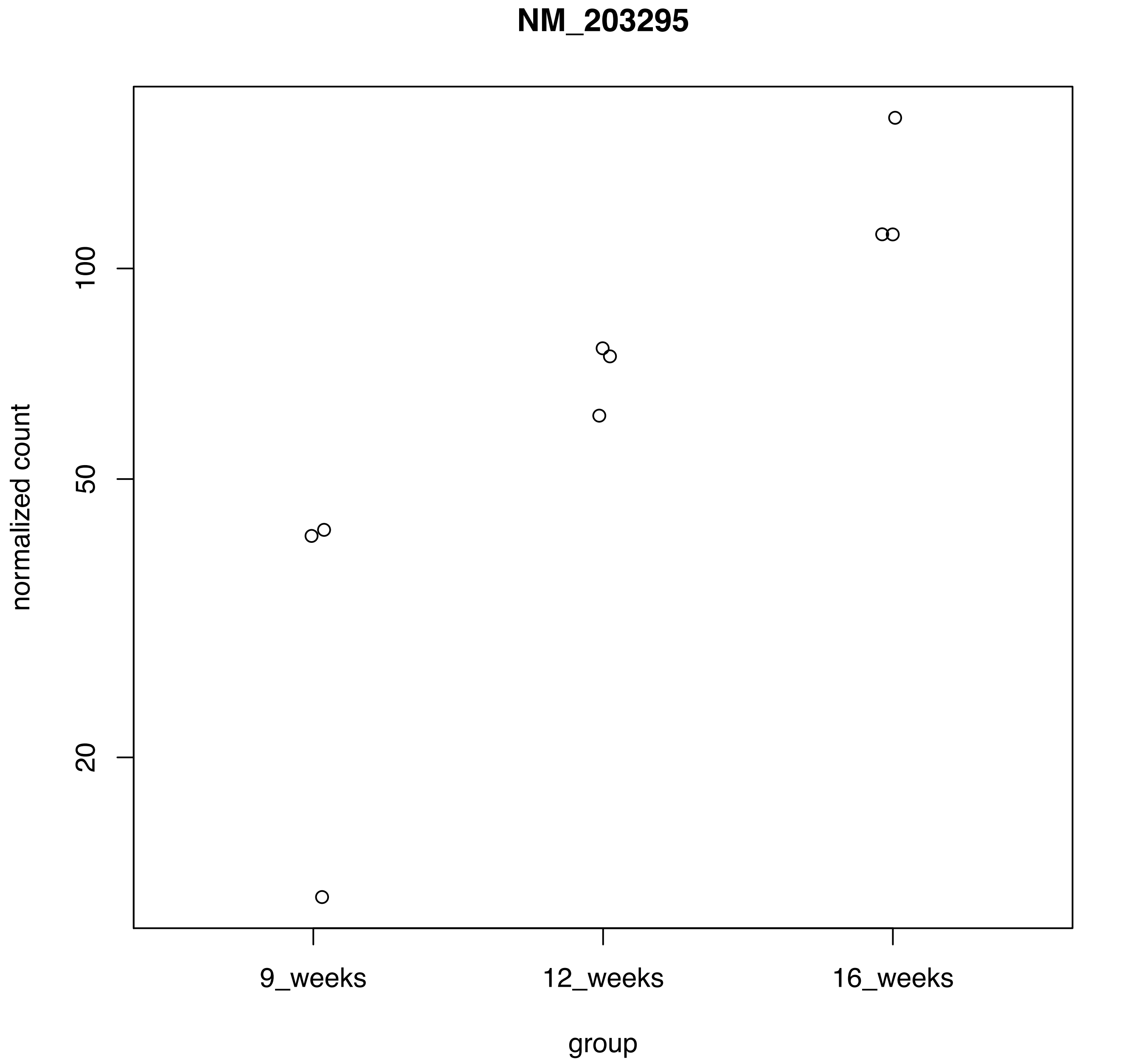

NM_203295
normalized count
100
50
20
9_weeks
12_weeks
16_weeks
group

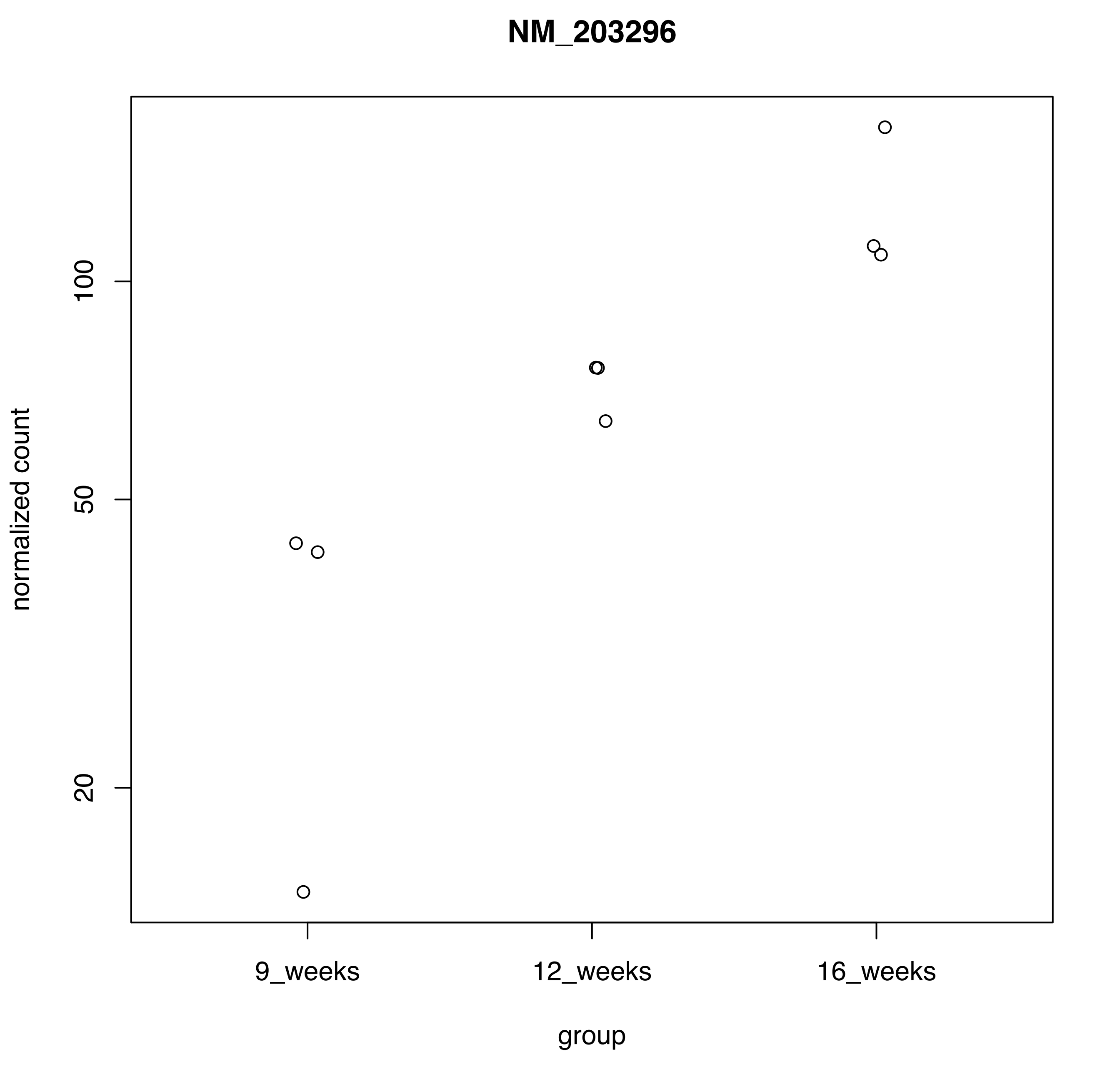
NM_203296
normalized count
100
50
20
9_weeks
12_weeks
16_weeks
group

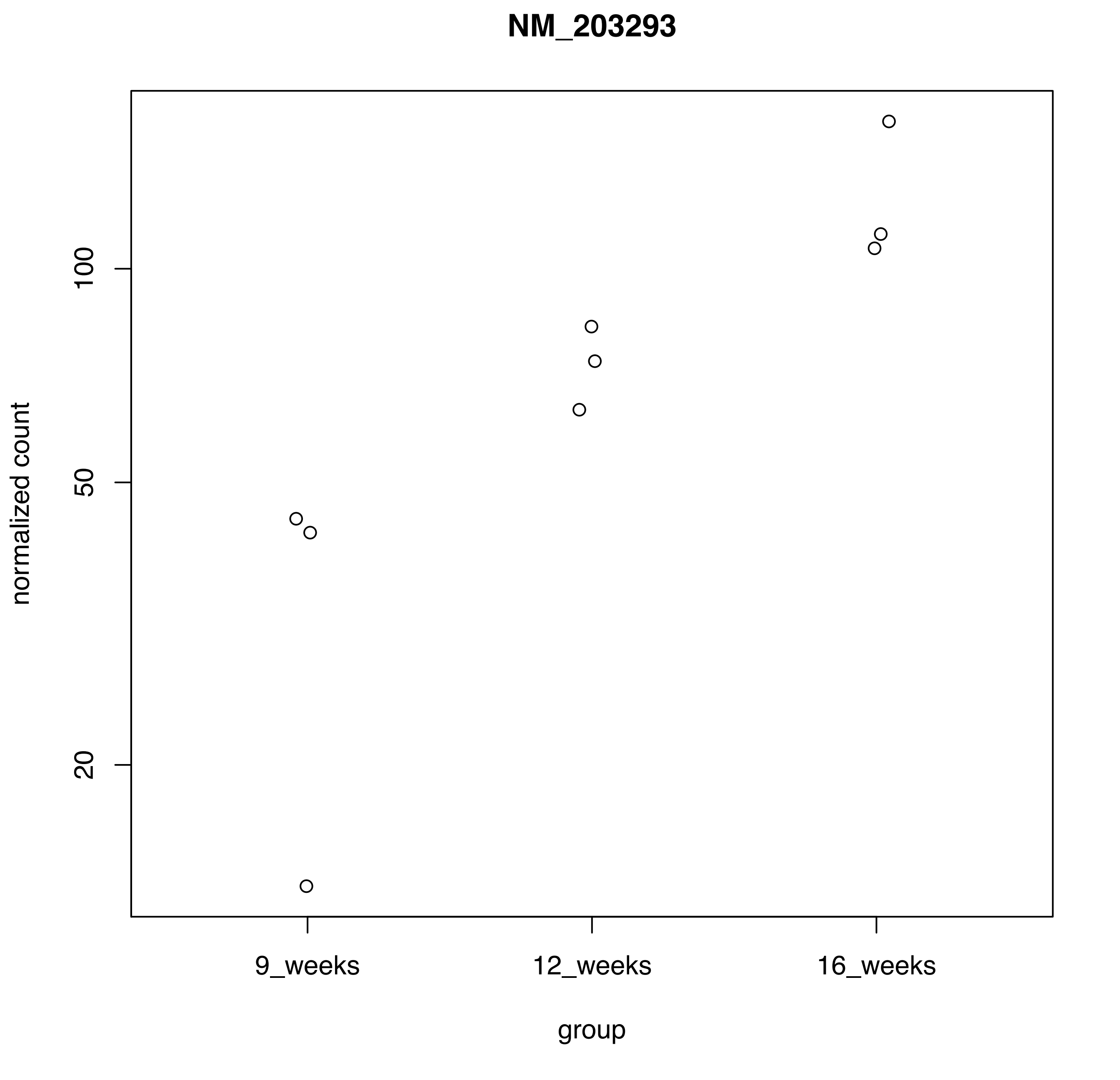

NM_203293
normalized count
100
50
20
9_weeks
12_weeks
16_weeks
group

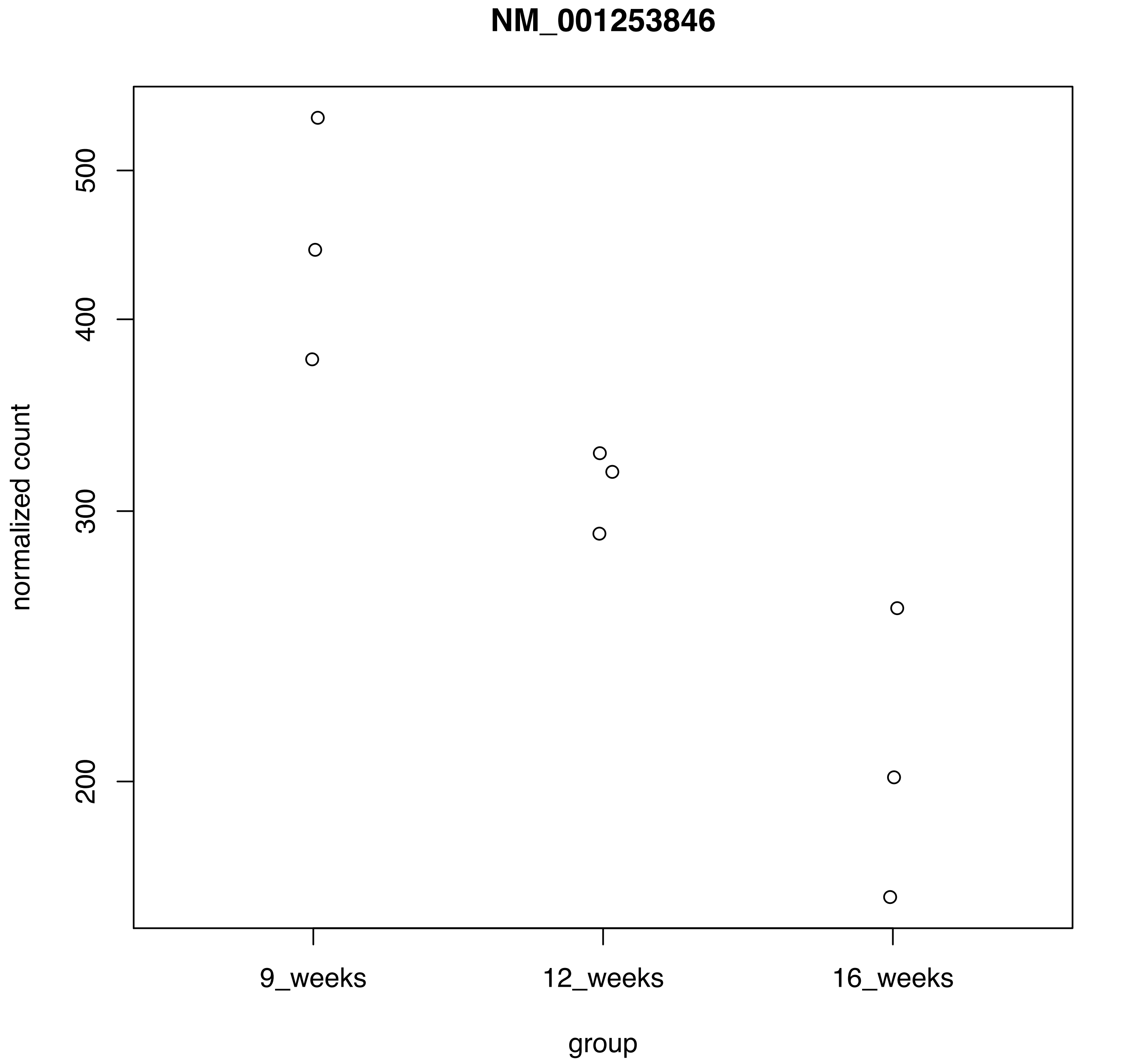

NM_001253846
normalized count
200
300
400
500
9_weeks
12_weeks
16_weeks
group

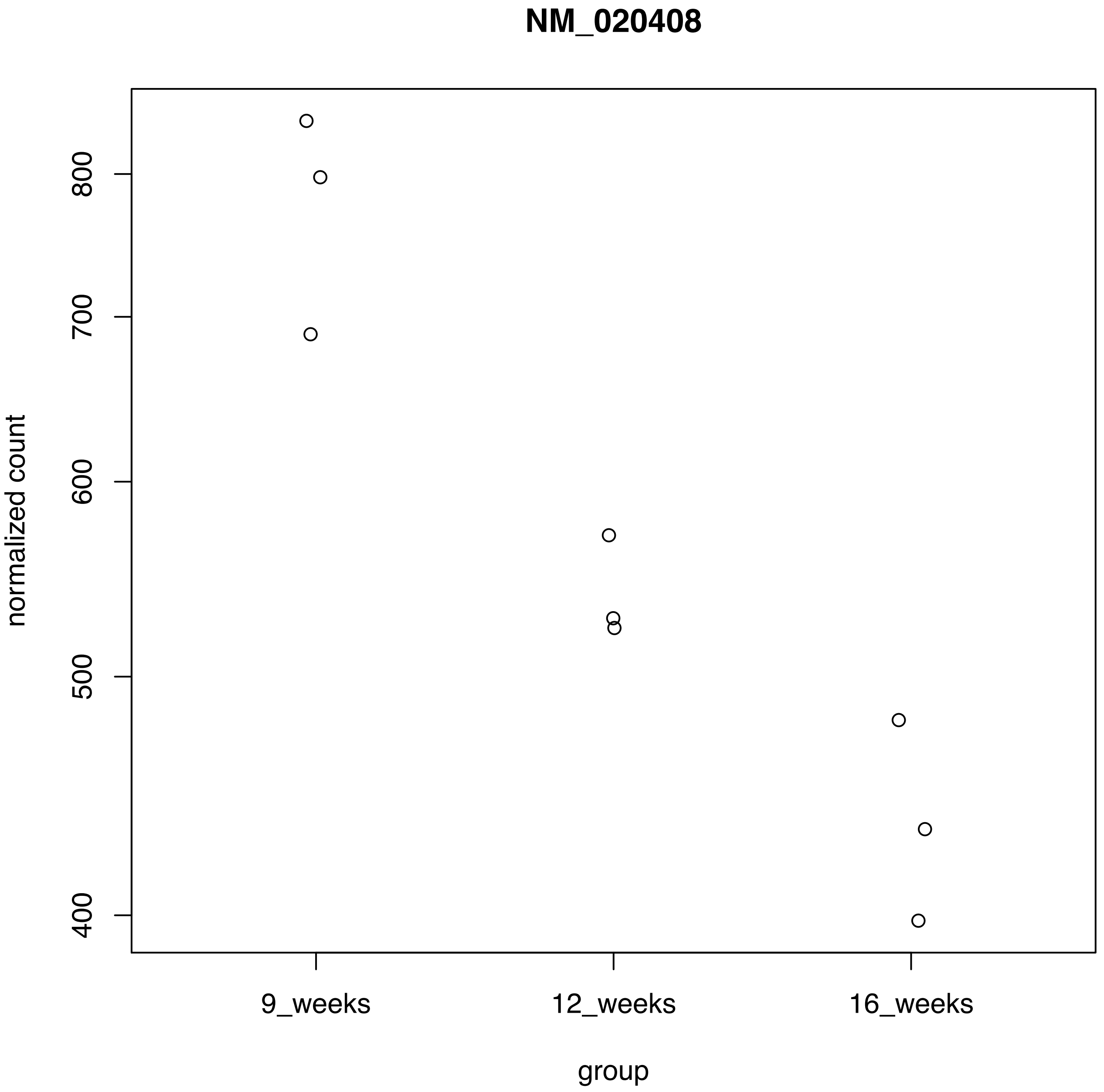
NM_020408
normalized count
800
700
600
500
400
9_weeks
12_weeks
16_weeks
group

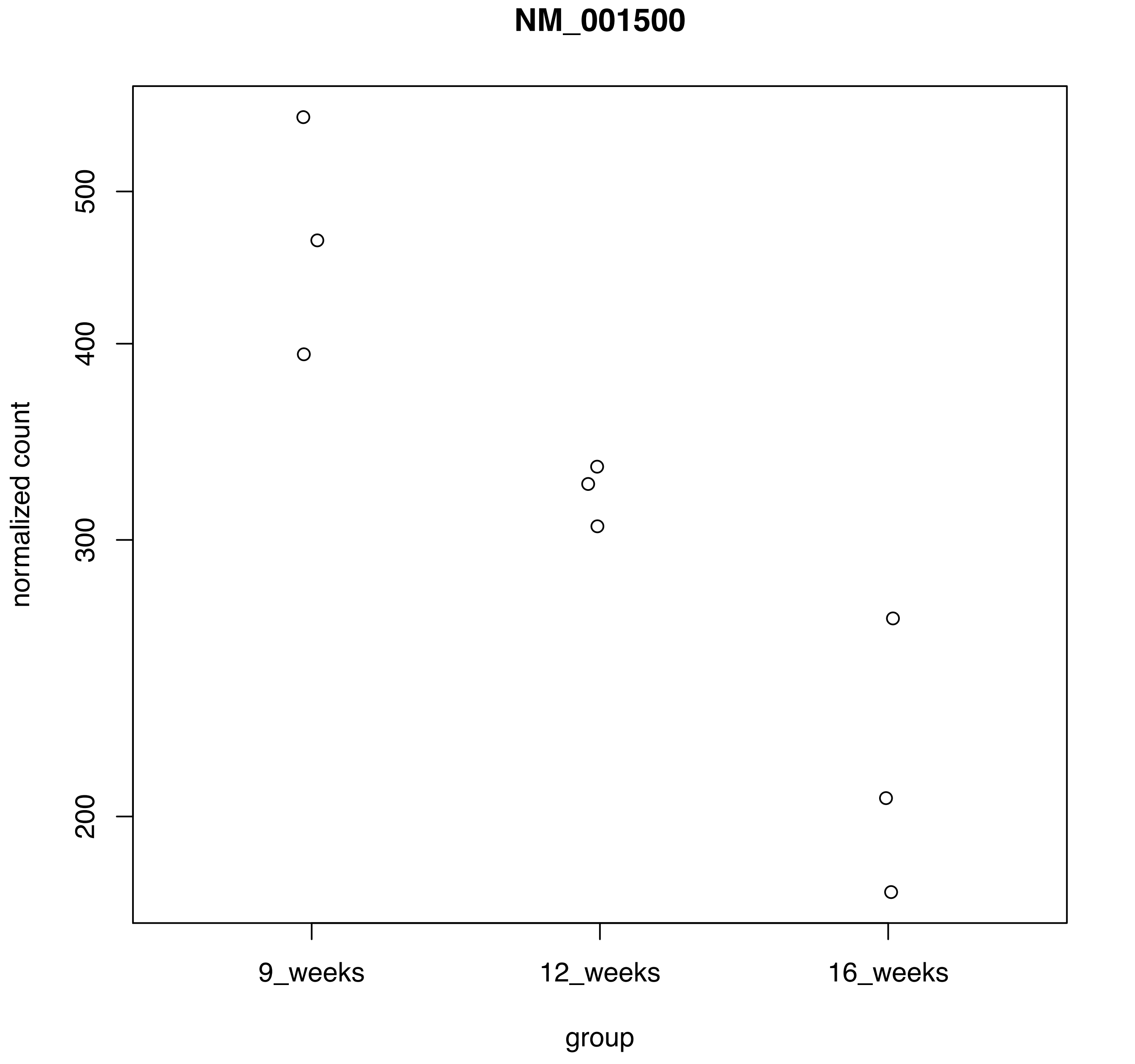
NM_001500
normalized count
500
400
300
200
9_weeks
12_weeks
16_weeks
group

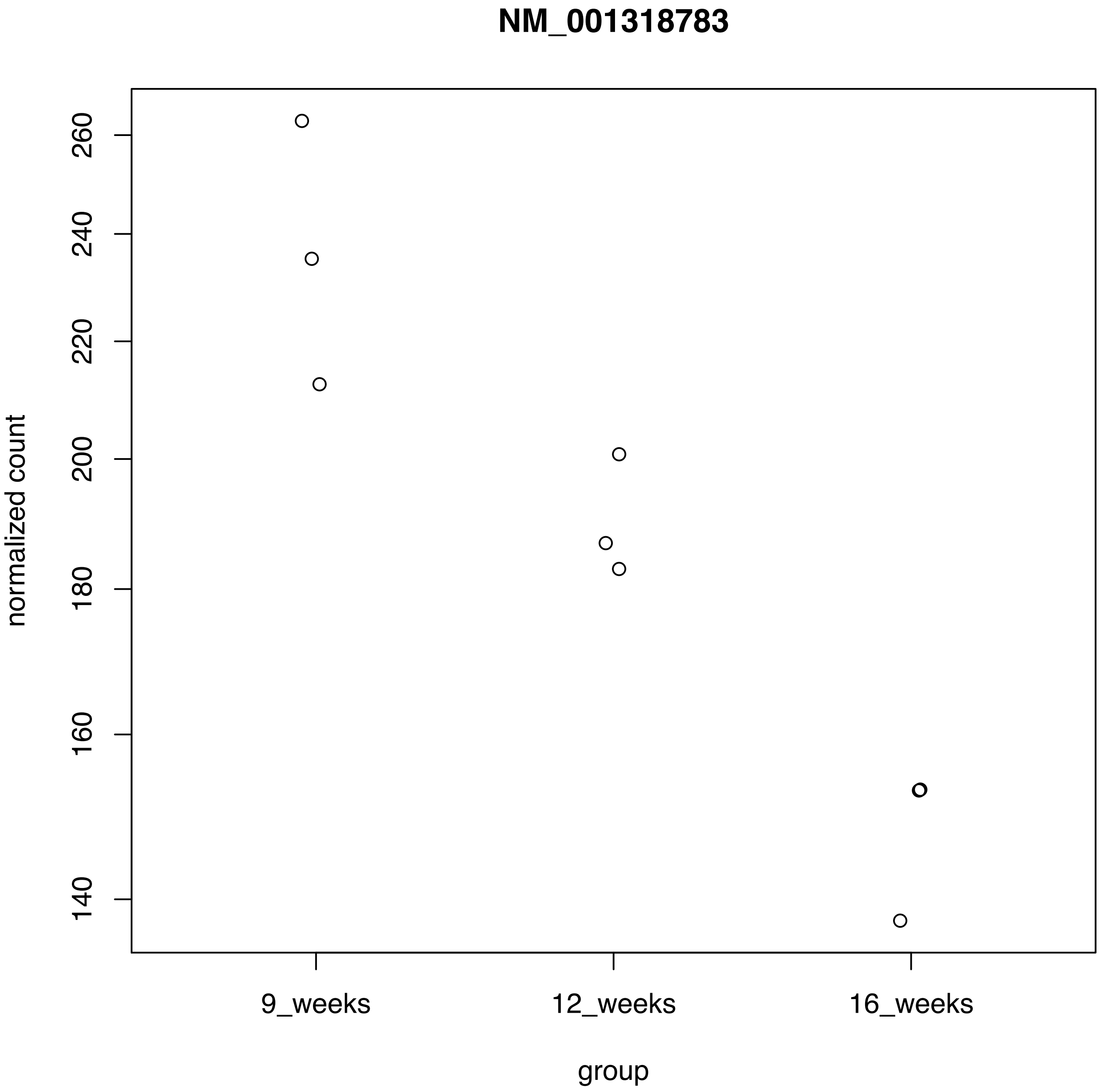

NM_001318783
normalized count
260
240
220
200
180
160
140
9_weeks
12_weeks
16_weeks
group

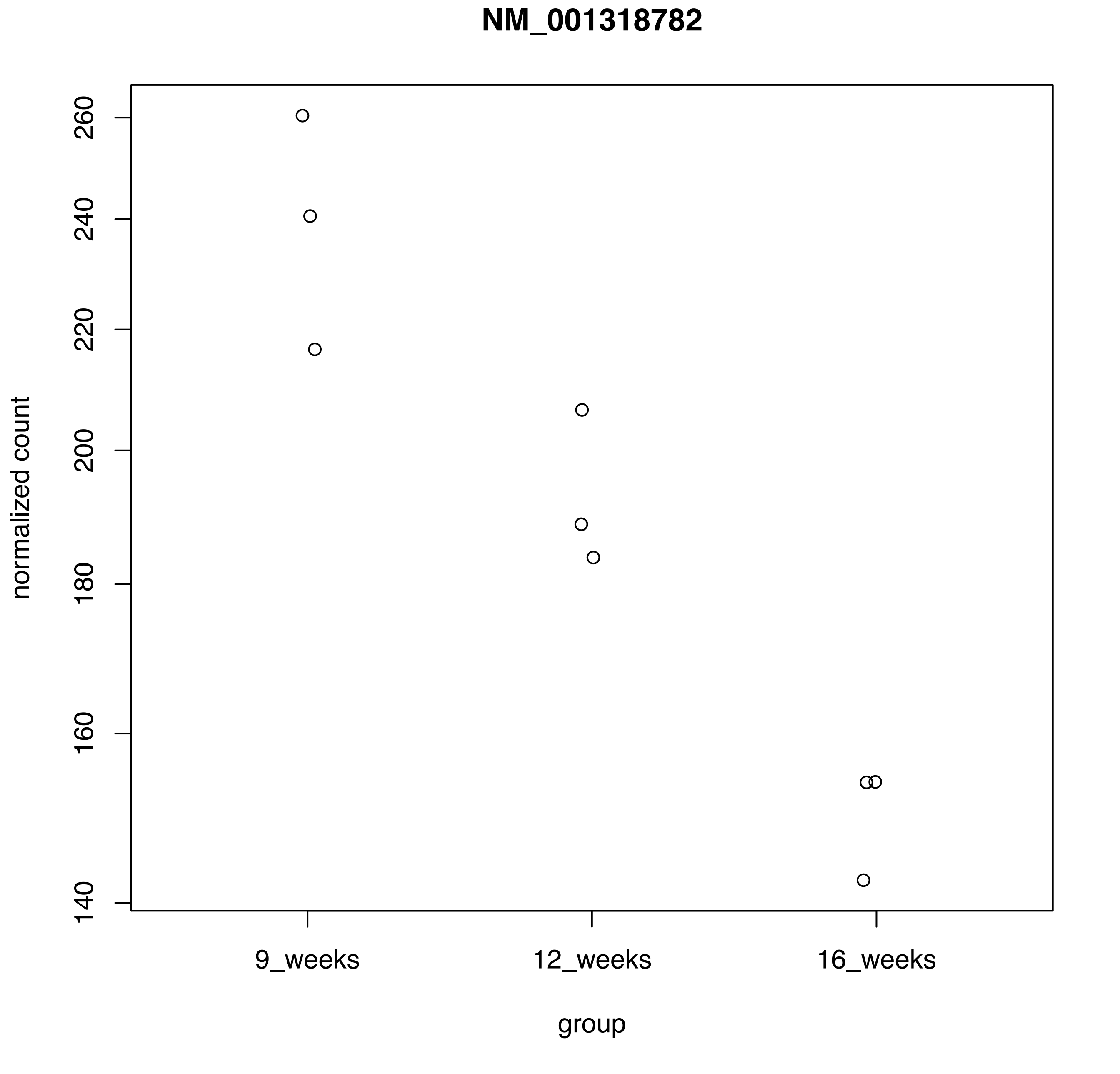

NM_001318782
normalized count
260
240
220
200
180
160
140
9_weeks
12_weeks
16_weeks
group

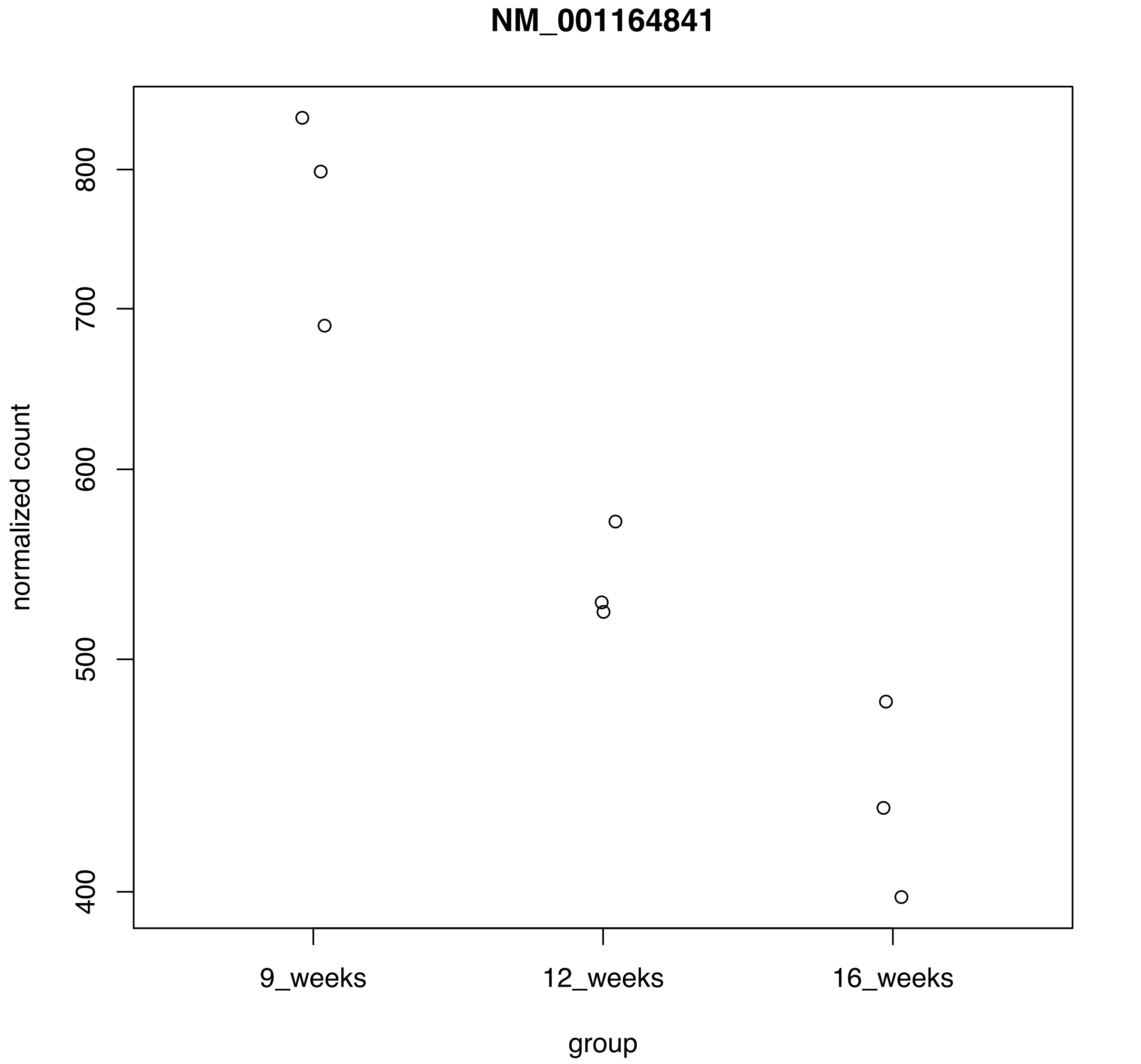
NM_001164841
normalized count
800
700
600
500
400
9_weeks
12_weeks
16_weeks
group

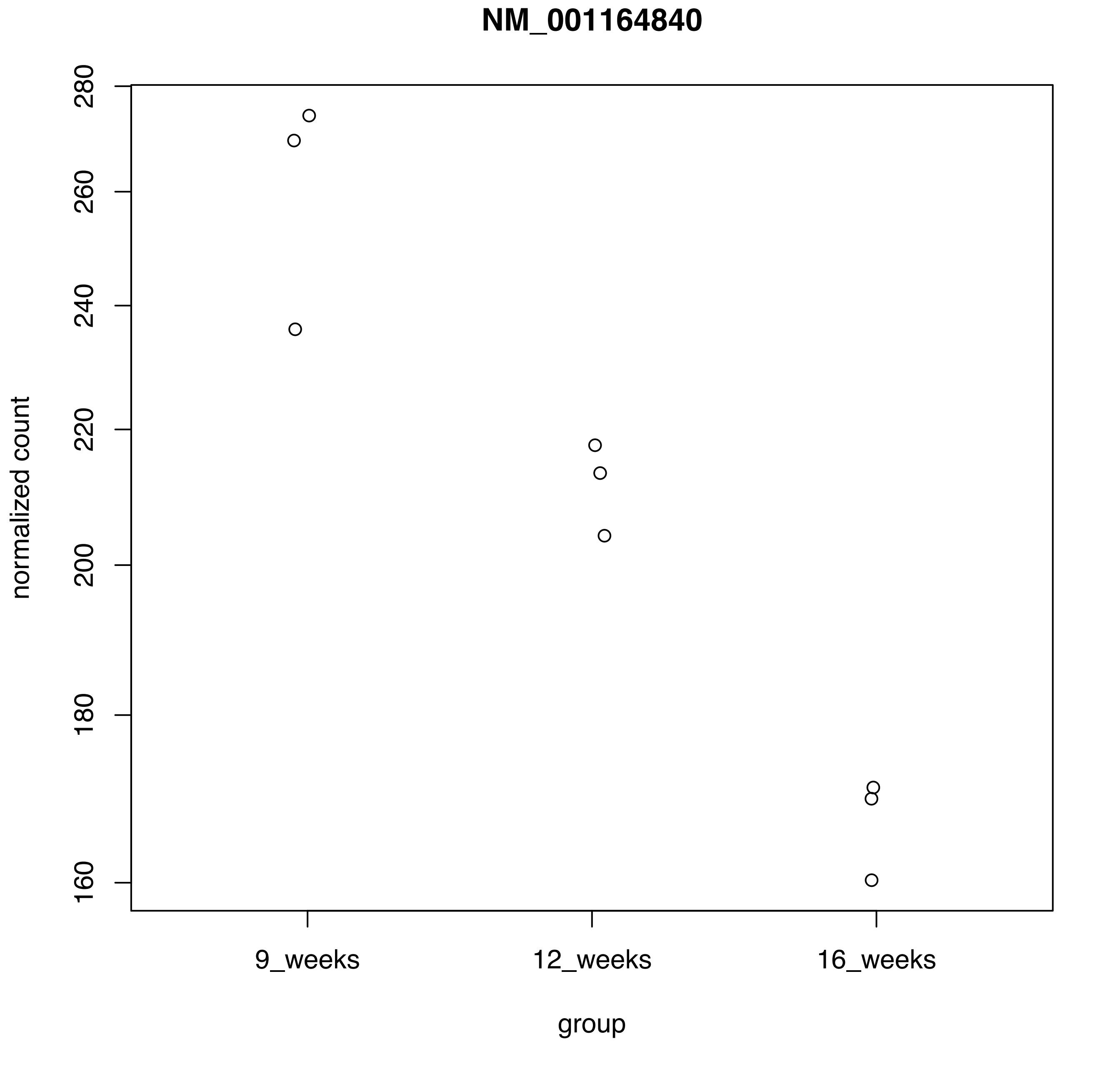
NM_001164840
normalized count
280
260
240
220
200
180
160
9_weeks
12_weeks
16_weeks
group

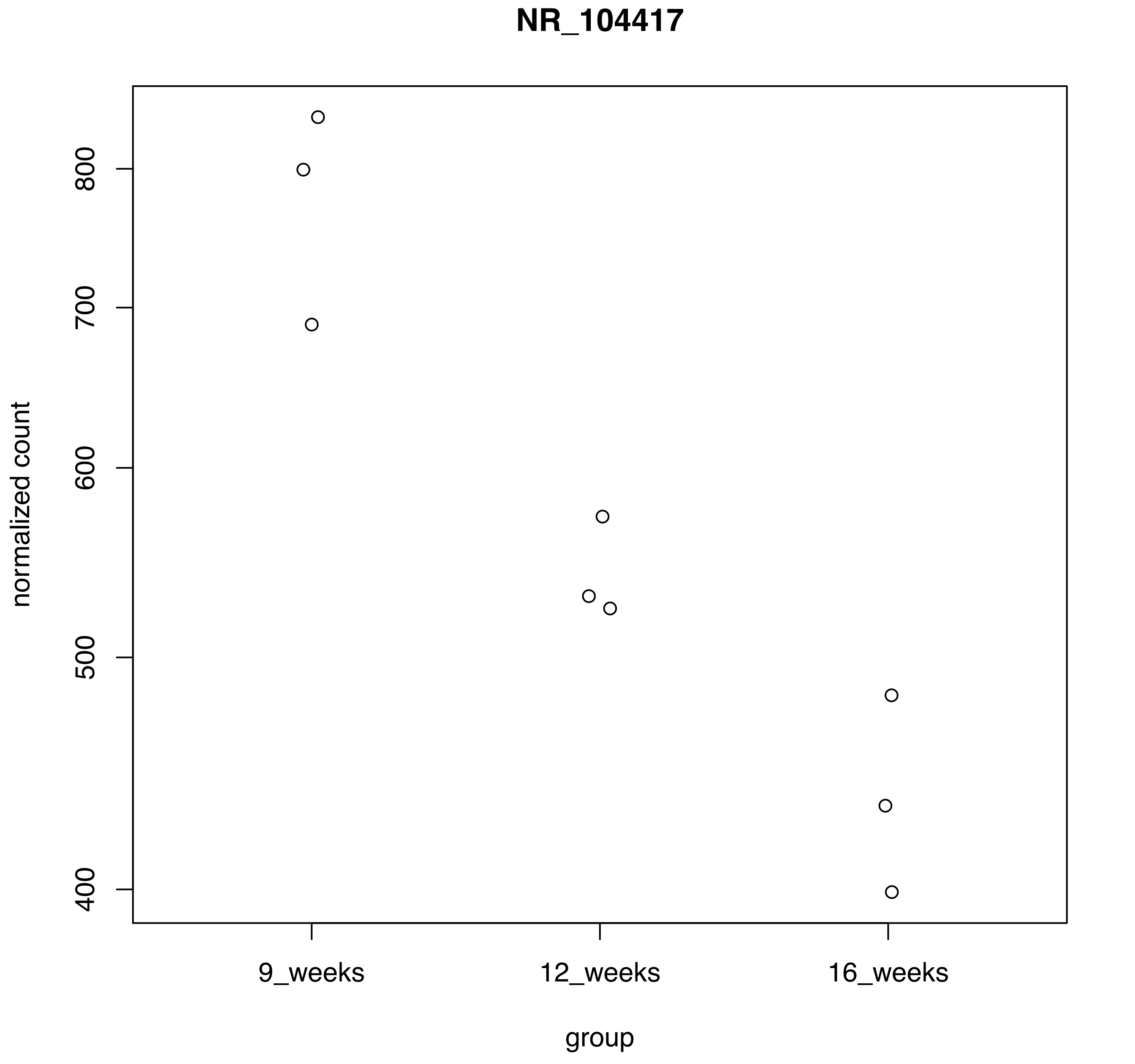

NR_104417
normalized count
800
700
600
500
400
9_weeks
12_weeks
16_weeks
group

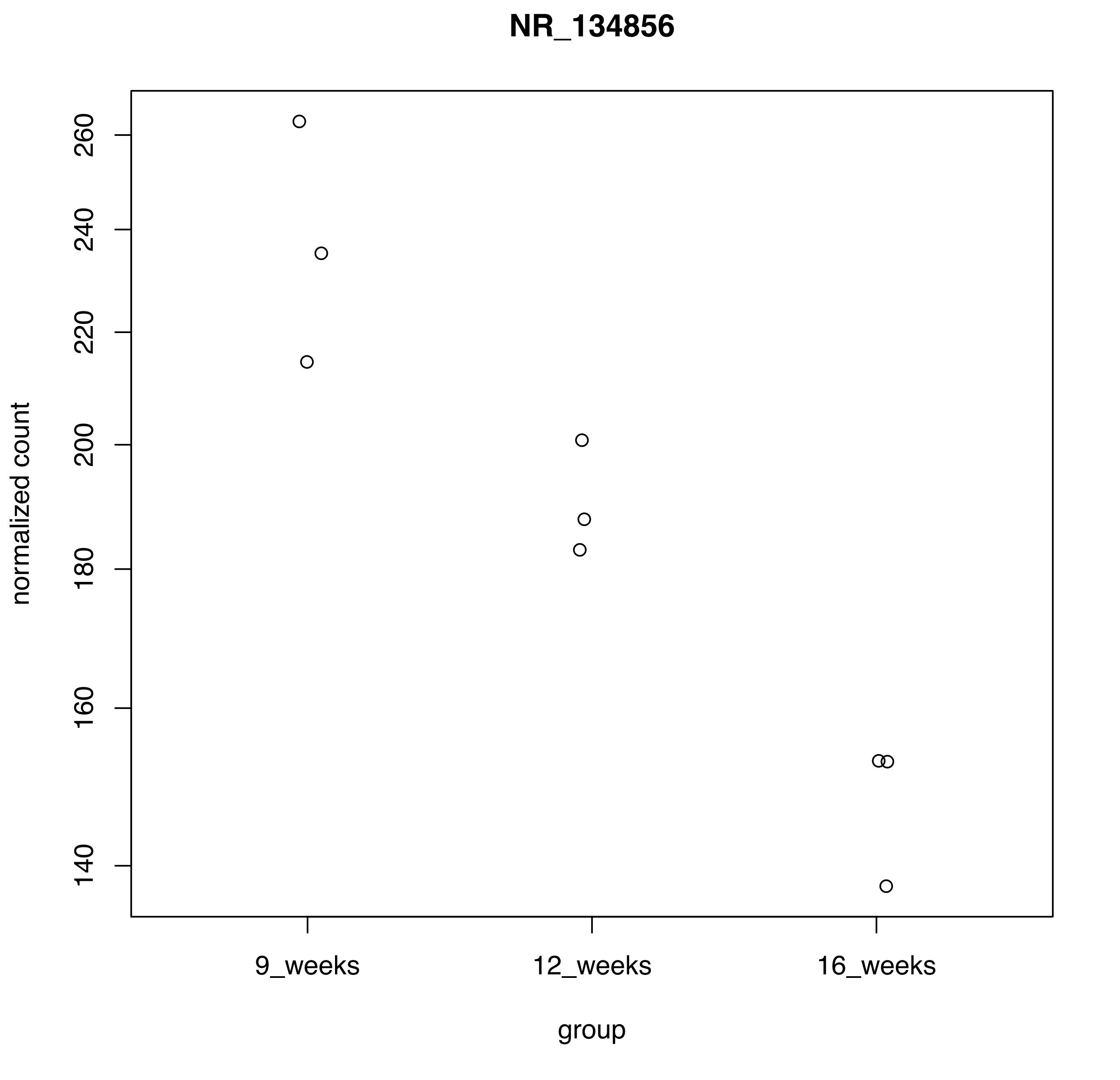
NR_134856
normalized count
260
240
220
200
180
160
140
9_weeks
12_weeks
16_weeks
group

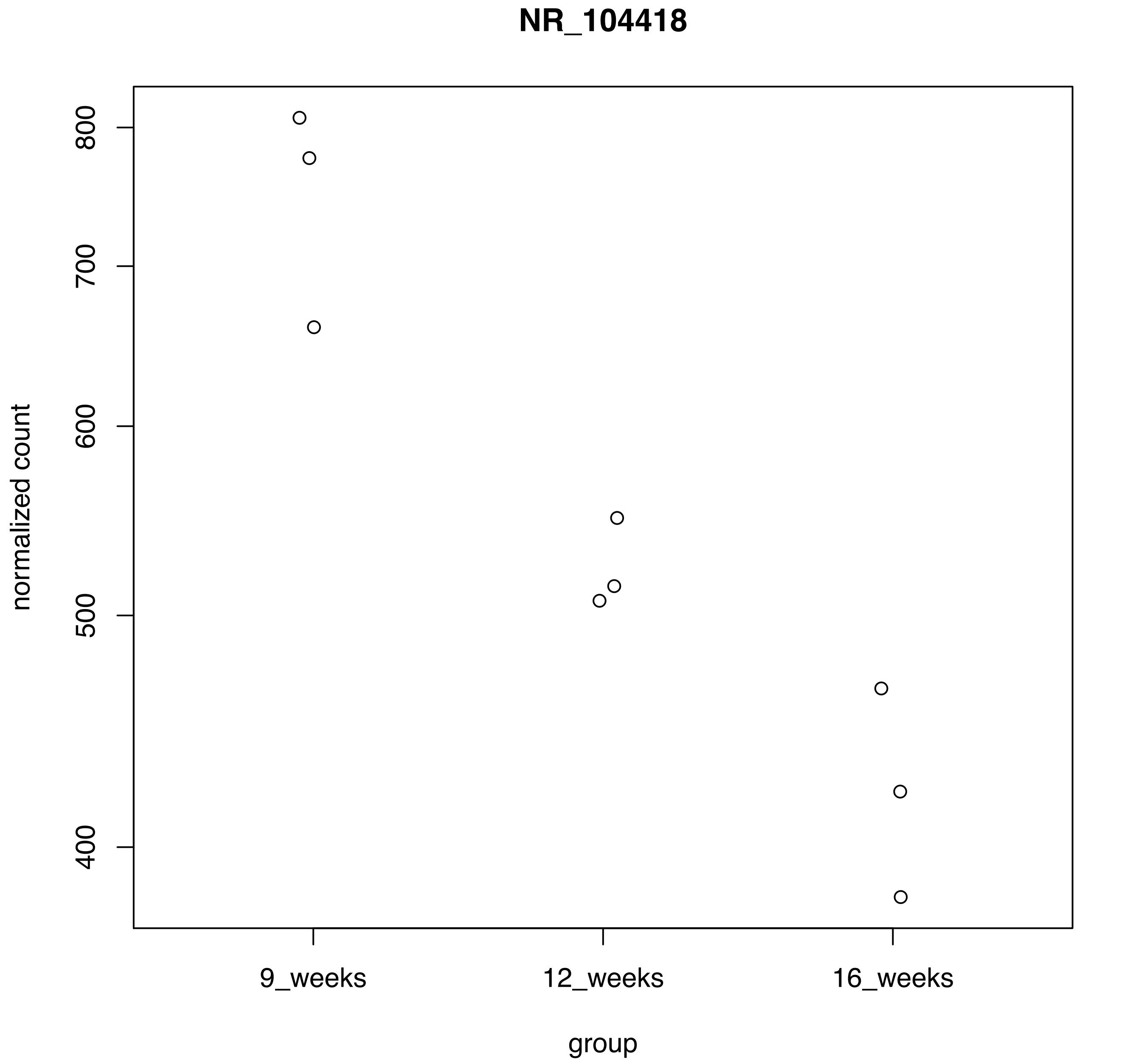

NR_104418
normalized count
800
700
600
500
400
9_weeks
12_weeks
16_weeks
group

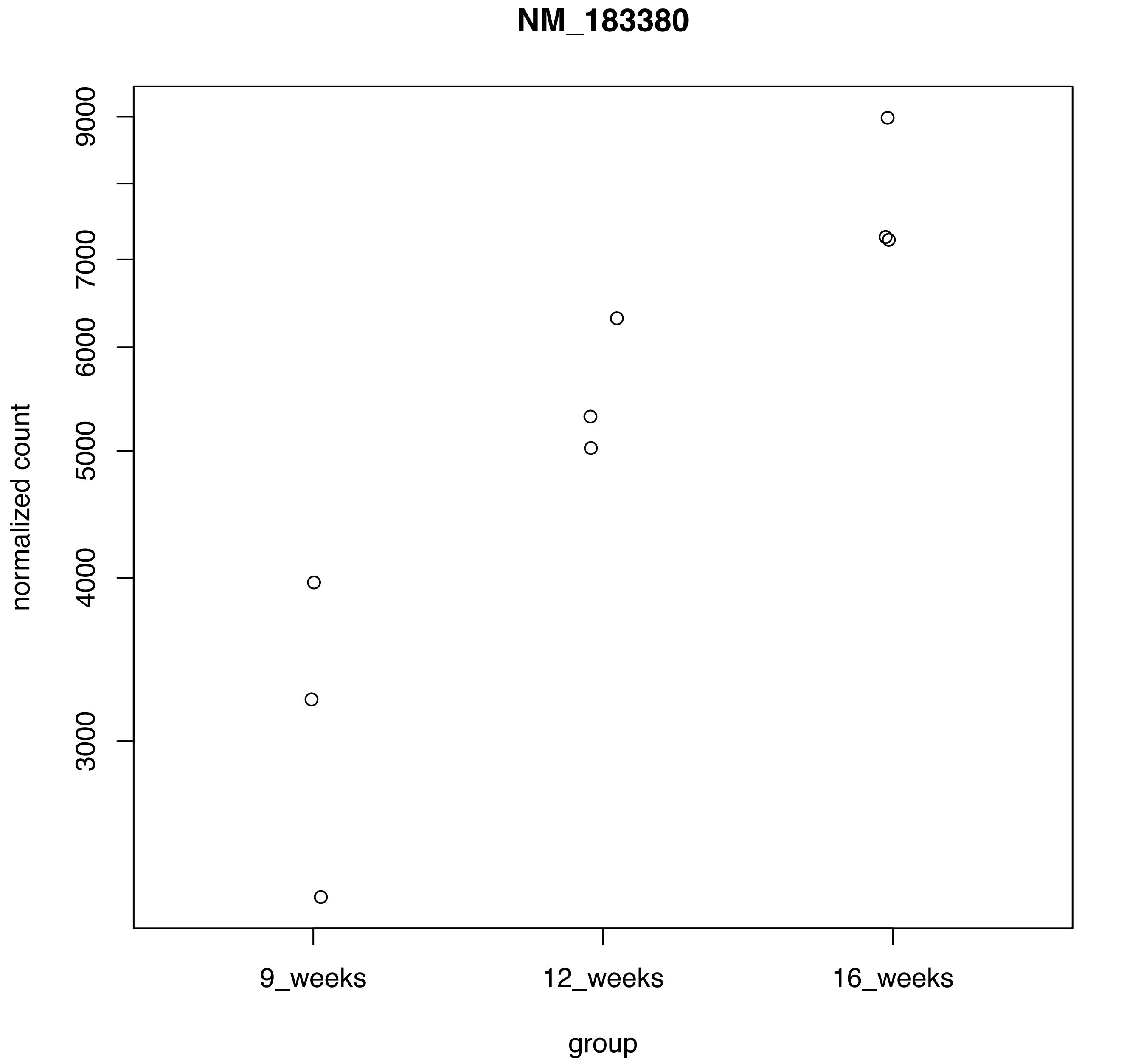

NM_183380
normalized count
9000
7000
6000
5000
4000
3000
9_weeks
12_weeks
16_weeks
group

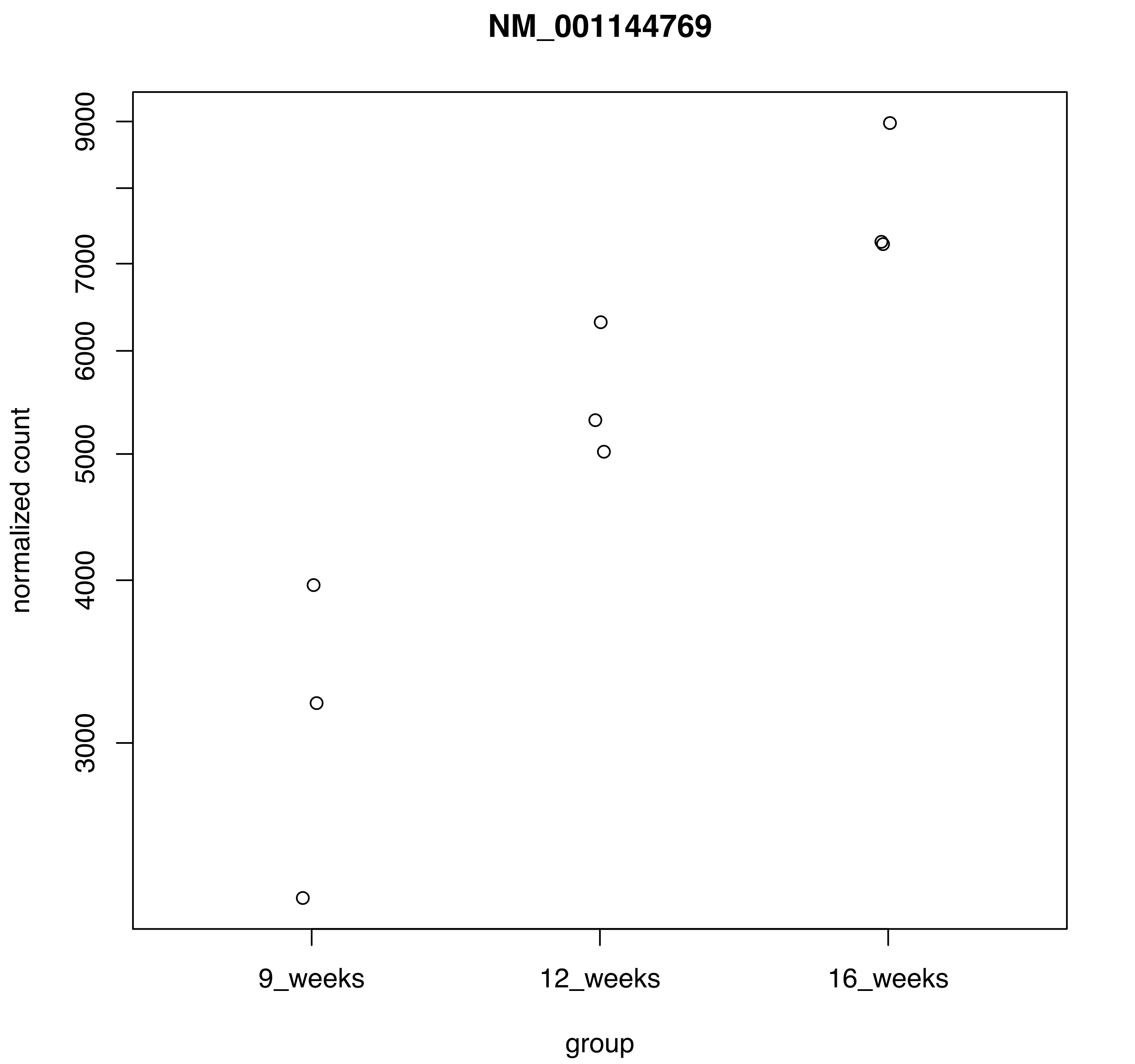
NM_001144769
normalized count
9000
7000
6000
5000
4000
3000
9_weeks
12_weeks
16_weeks
group

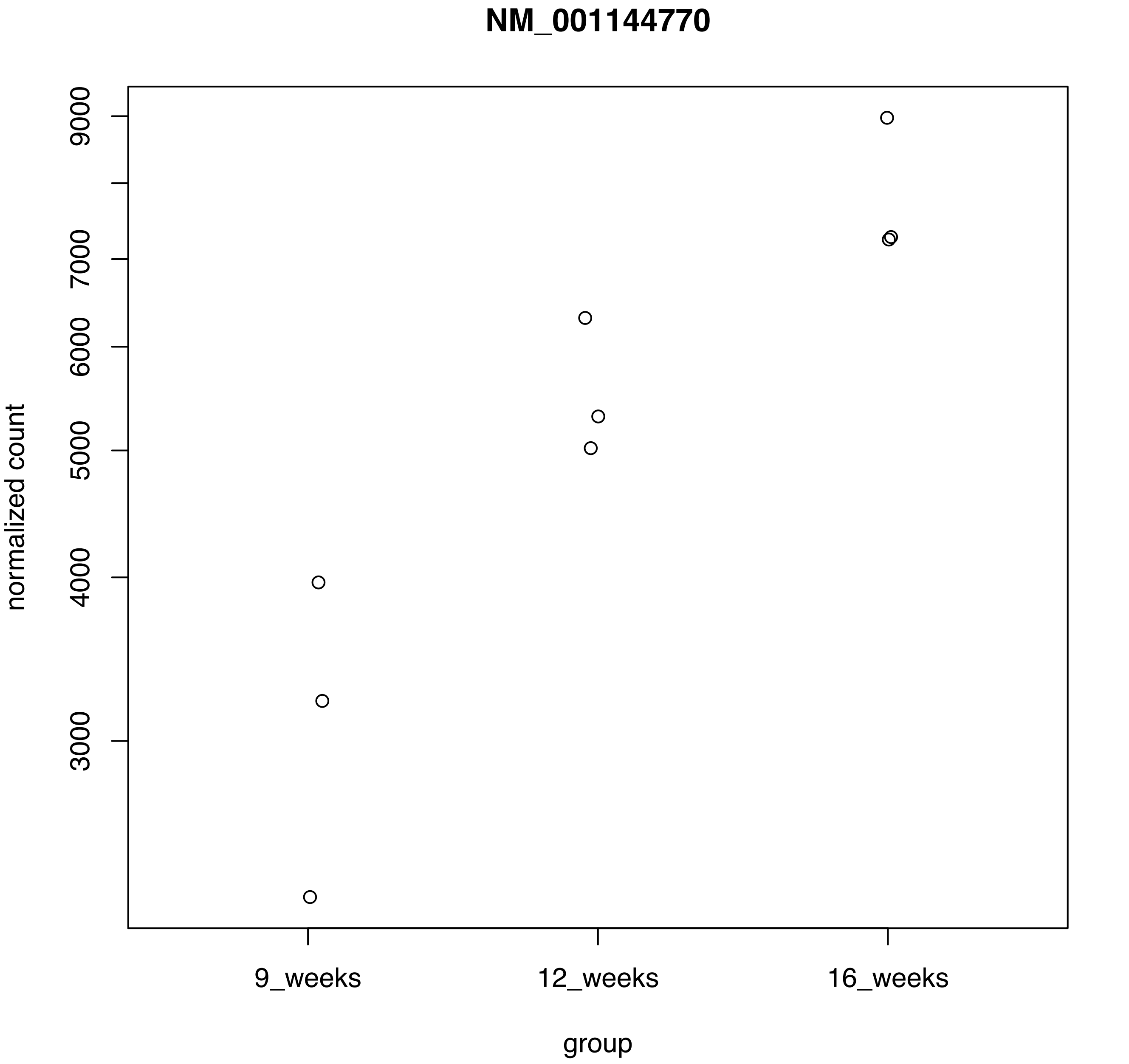
NM_001144770
normalized count
9000
7000
6000
5000
4000
3000
9_weeks
12_weeks
16_weeks
group

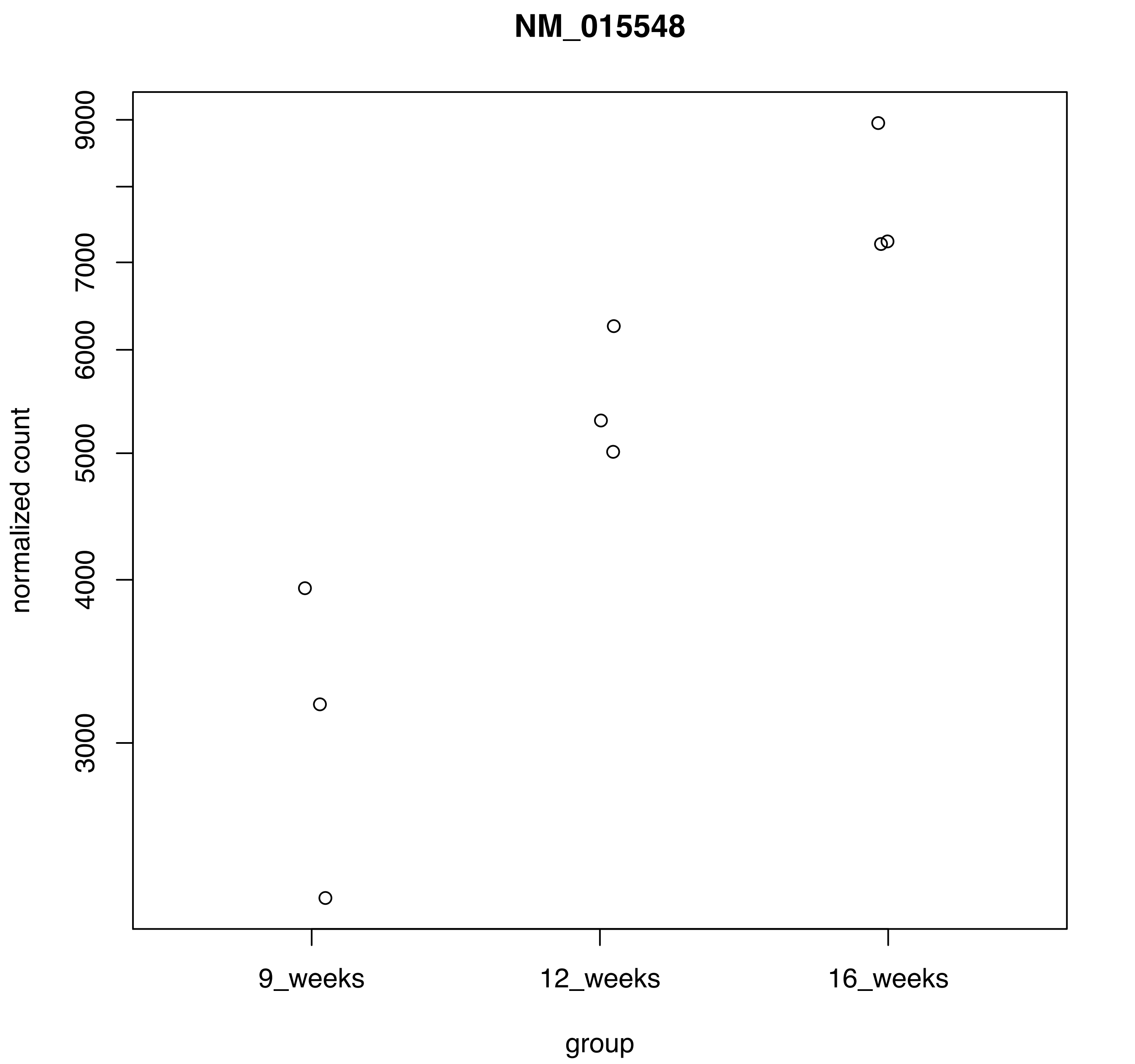

NM_015548
normalized count
9000
7000
6000
5000
4000
3000
9_weeks
12_weeks
16_weeks
group

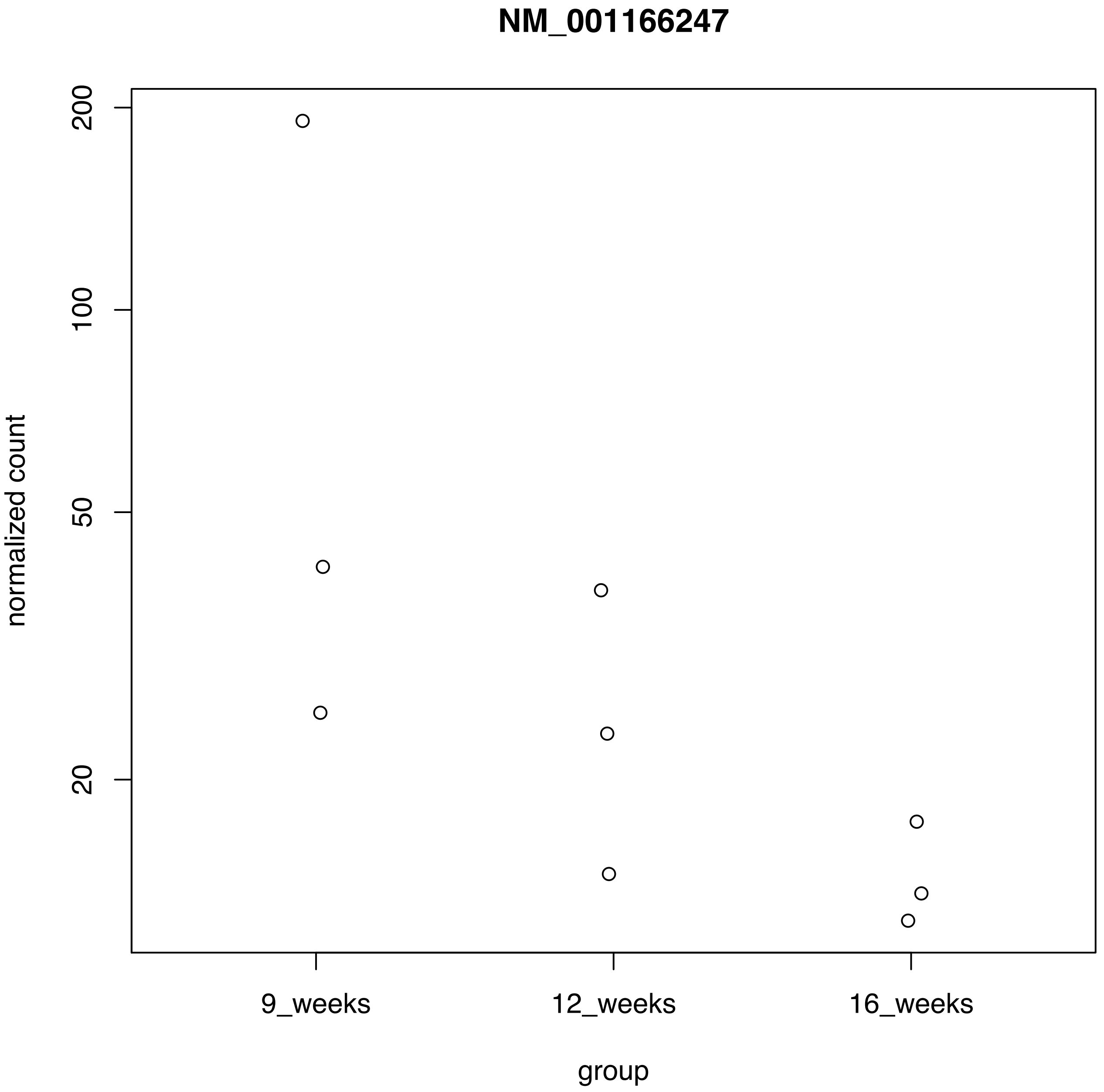
NM_001166247
200
100
50
20
normalized count
9_weeks
12_weeks
16_weeks
group

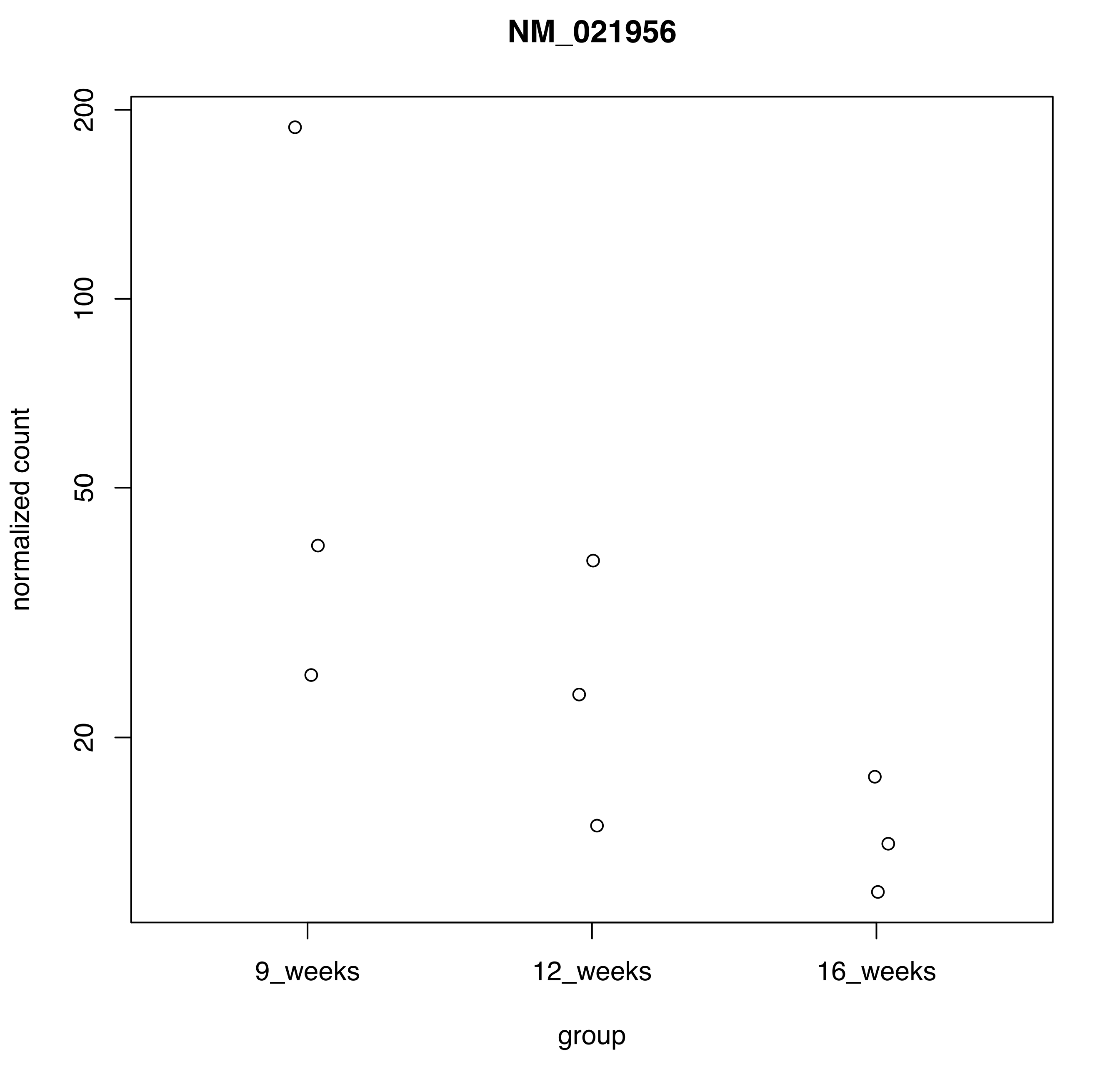
NM_021956
normalized count
200
100
50
20
9_weeks
12_weeks
16_weeks
group

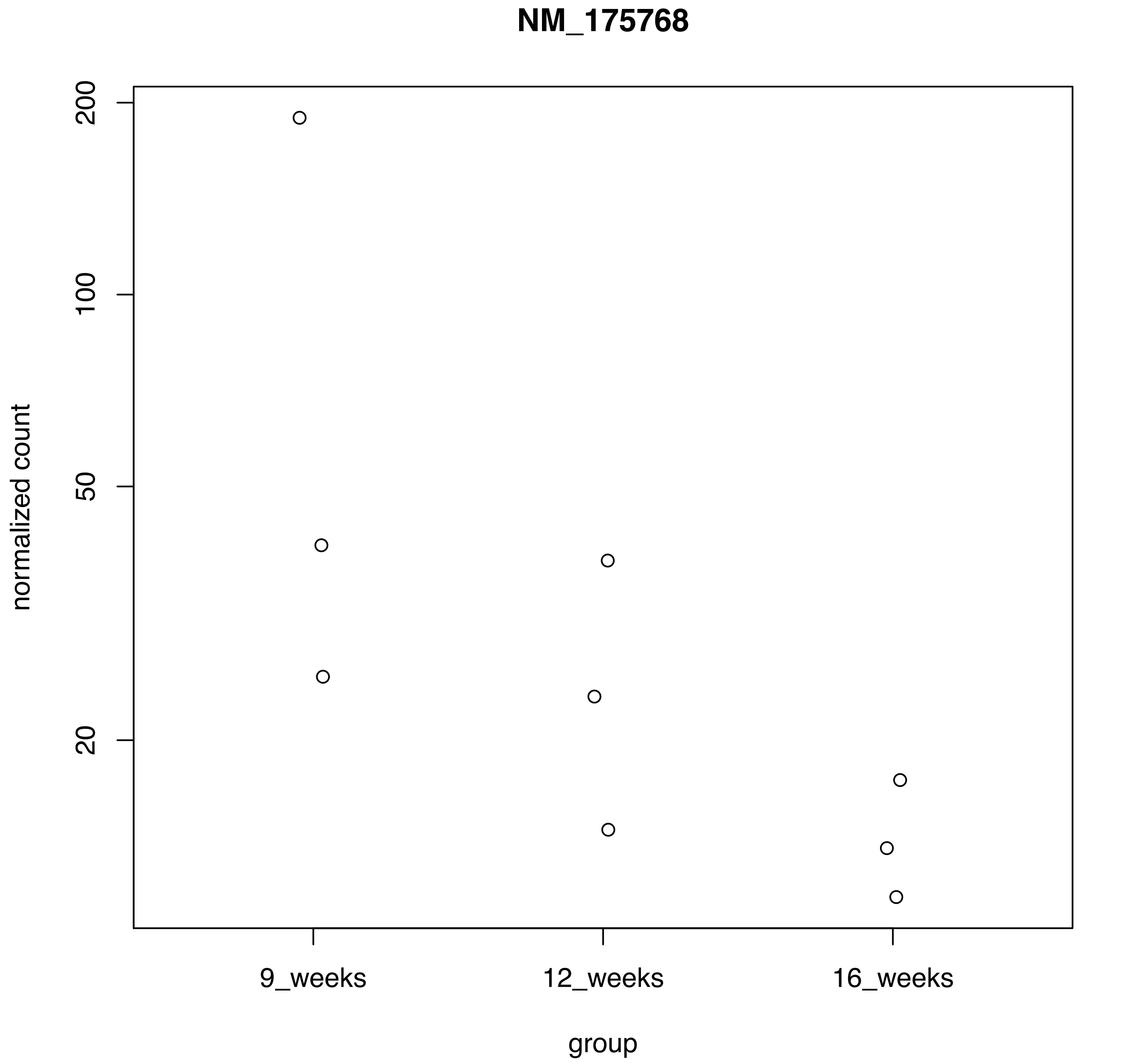

NM_175768
200
100
50
20
normalized count
9_weeks
12_weeks
16_weeks
group

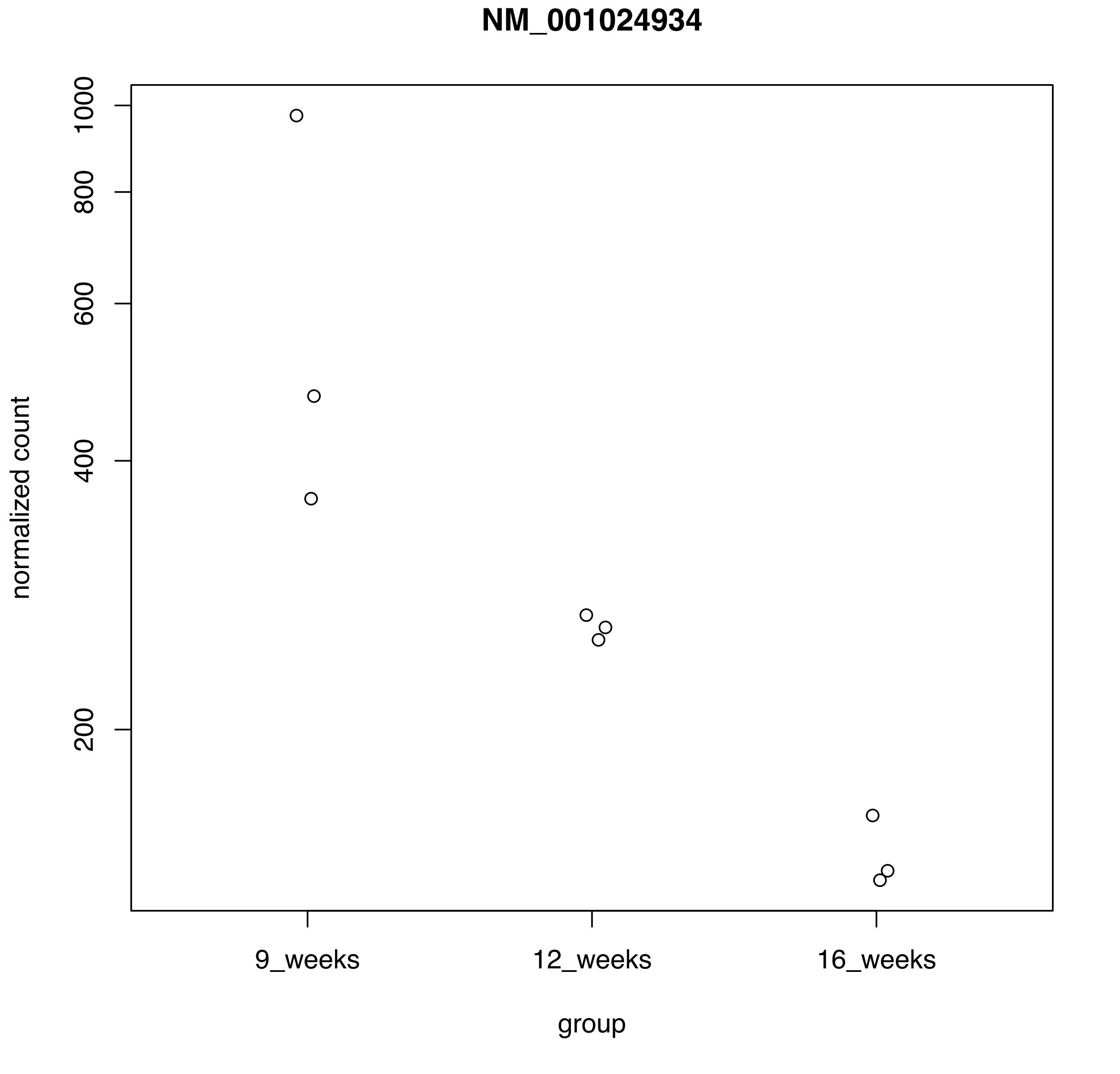
NM_001024934
1000
800
600
400
200
normalized count
9_weeks
12_weeks
16_weeks
group

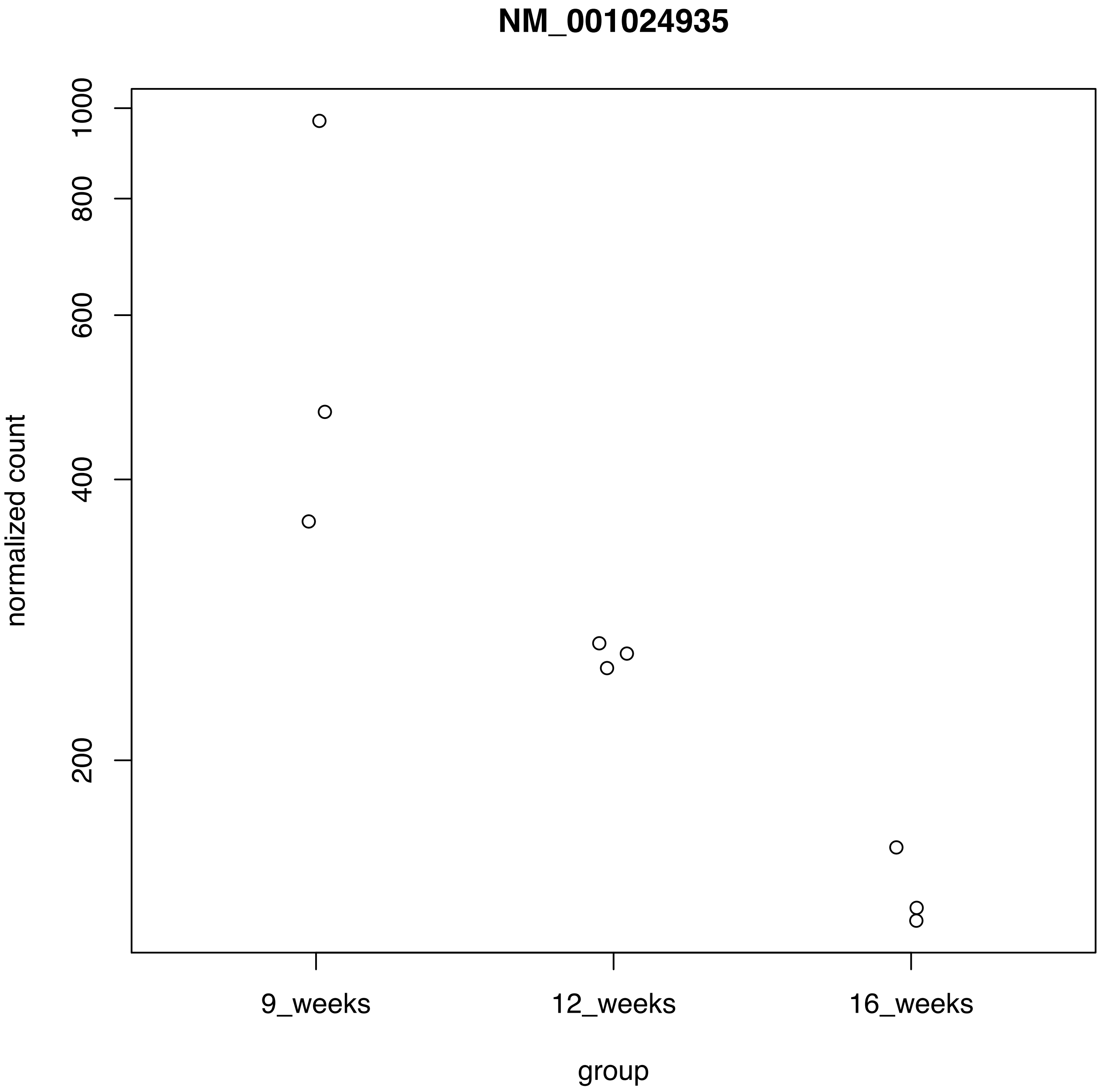
NM_001024935
normalized count
1000
800
600
400
200
9_weeks
12_weeks
16_weeks
group

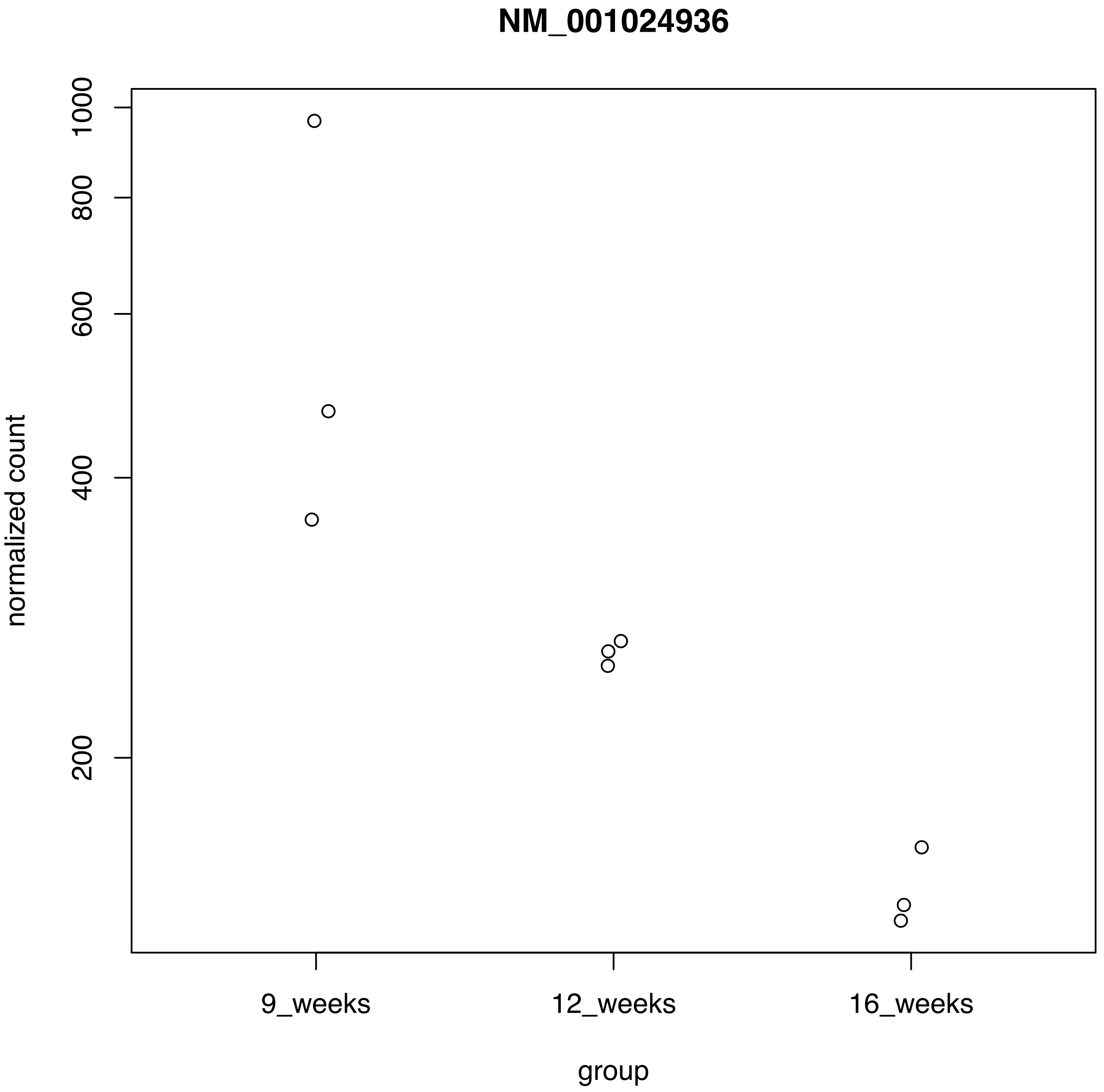
NM_001024936
1000
800
600
400
200
normalized count
9_weeks
12_weeks
16_weeks
group

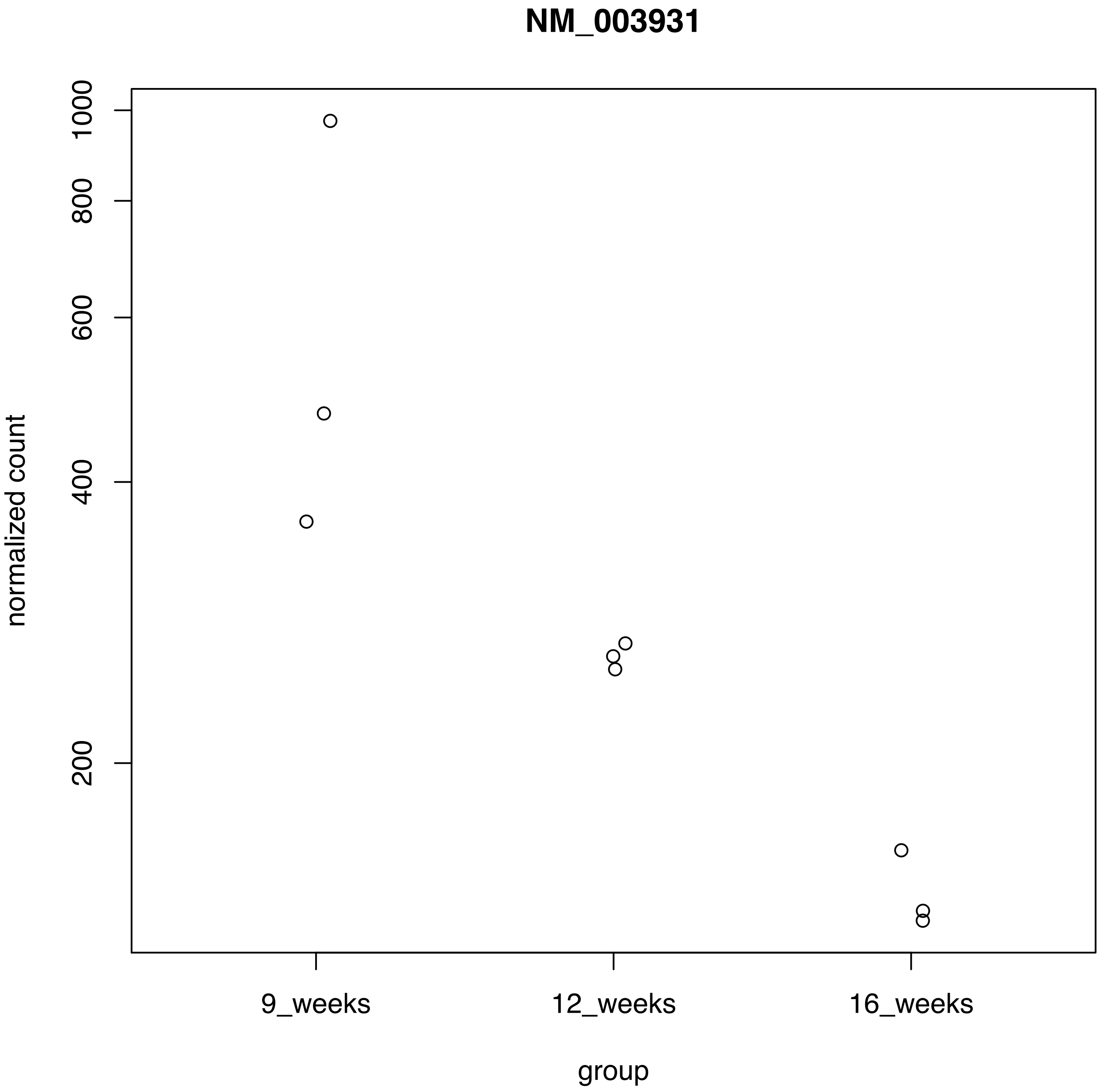

NM_003931
normalized count
1000
800
600
400
200
9_weeks
12_weeks
16_weeks
group

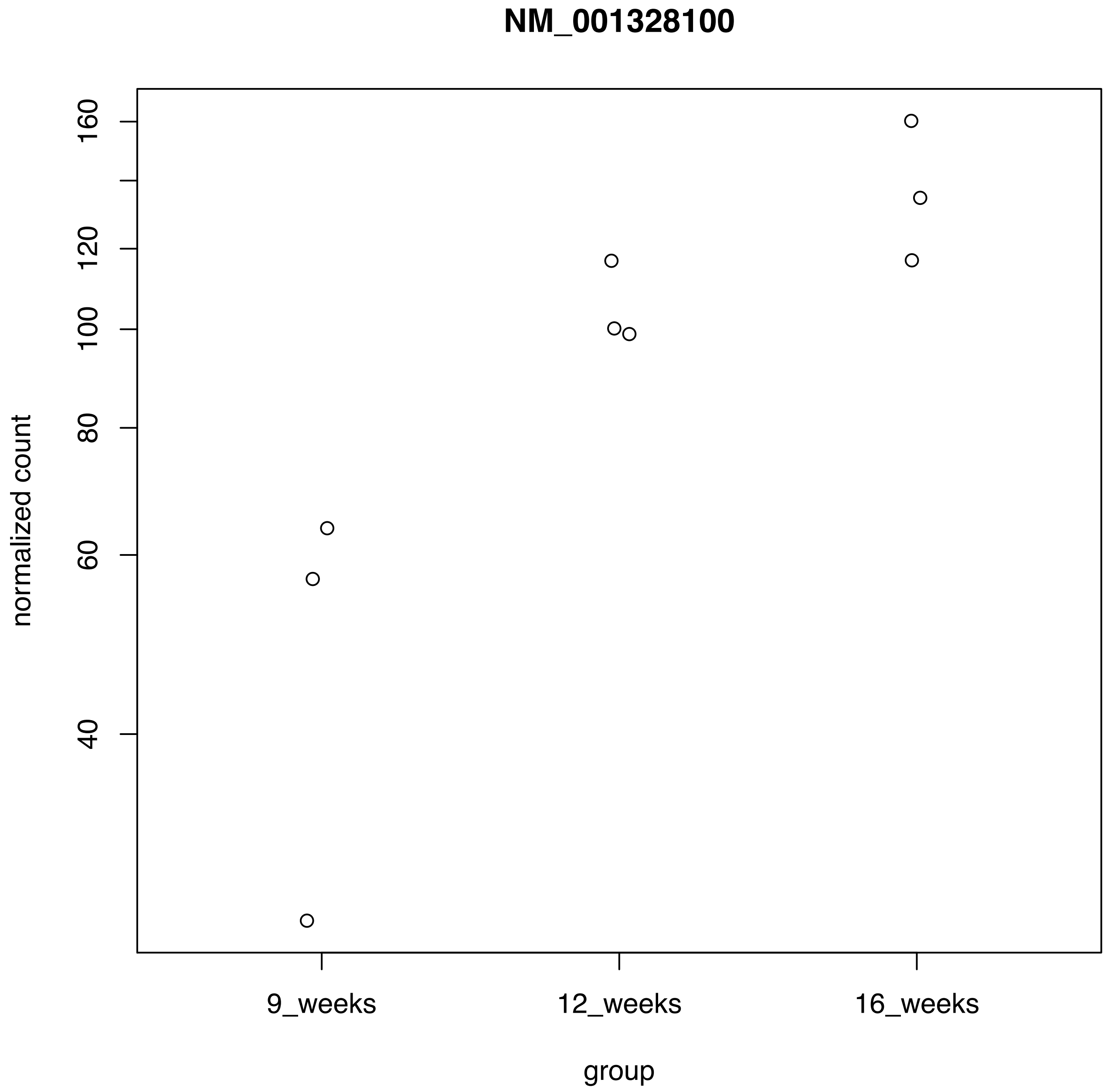
NM_001328100
normalized count
160
120
100
80
60
40
9_weeks
12_weeks
16_weeks
group

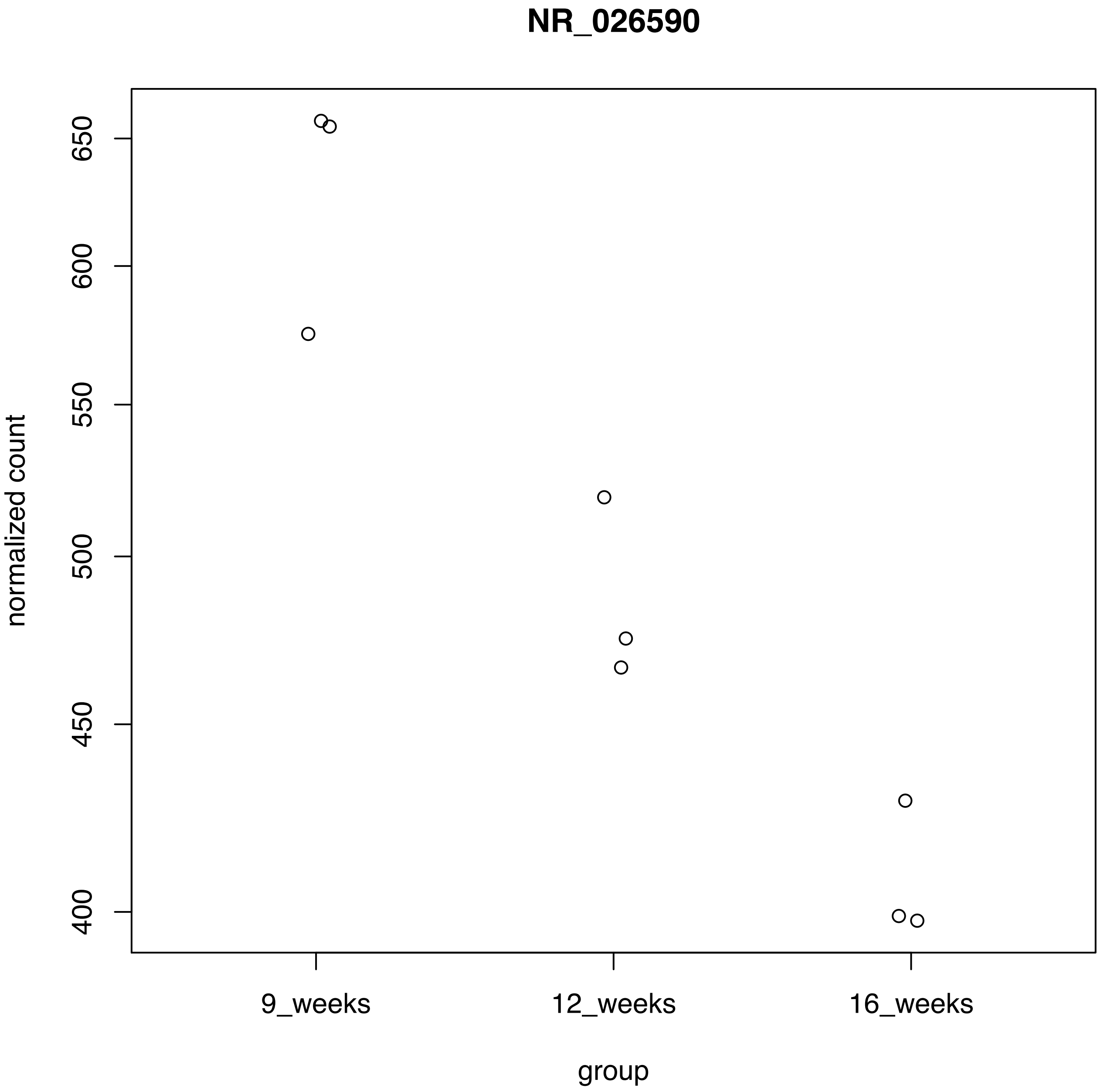
NR_026590
normalized count
650
600
550
500
450
400
9_weeks
12_weeks
16_weeks
group

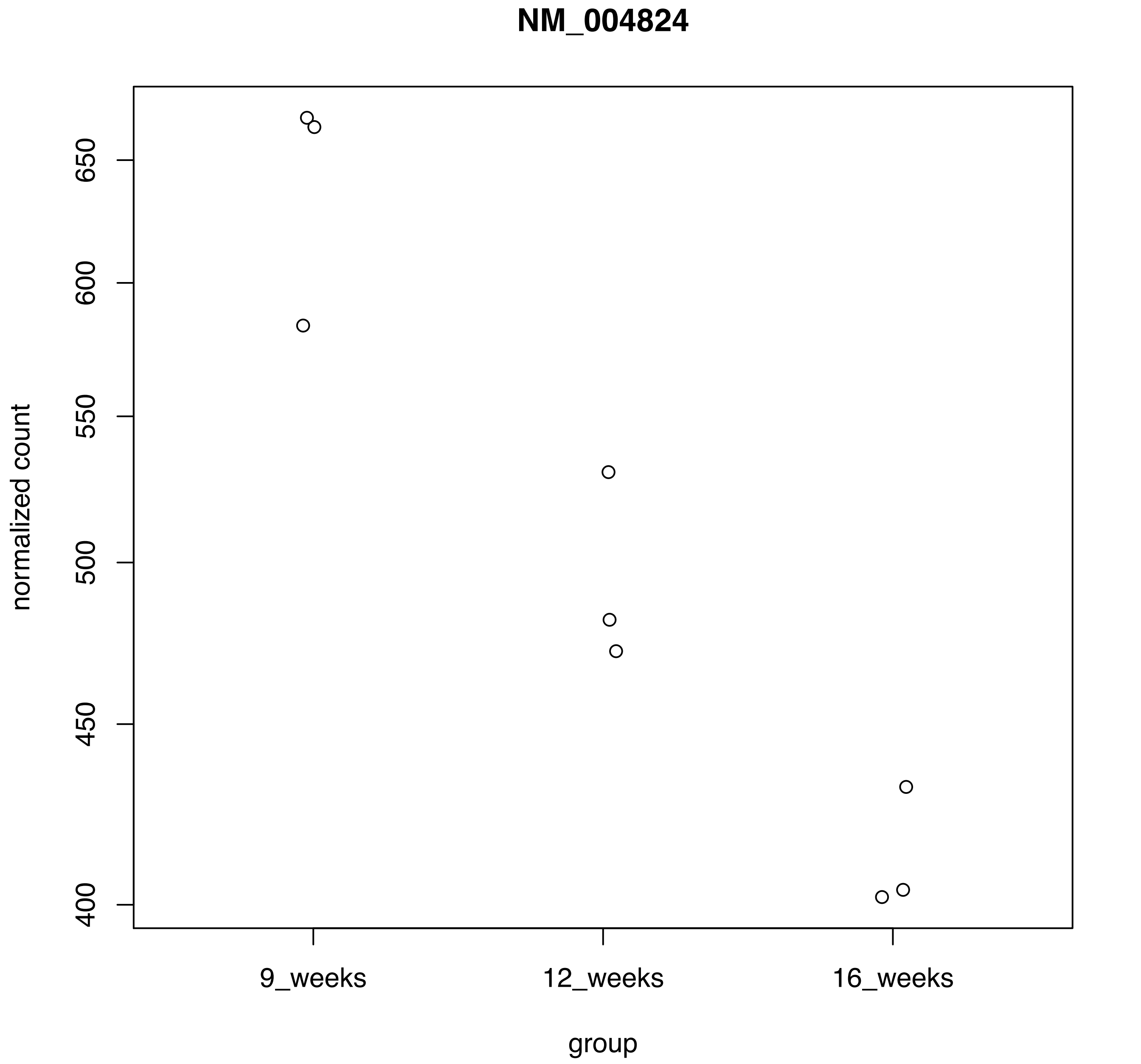
NM_004824
normalized count
650
600
550
500
450
400
9_weeks
12_weeks
16_weeks
group

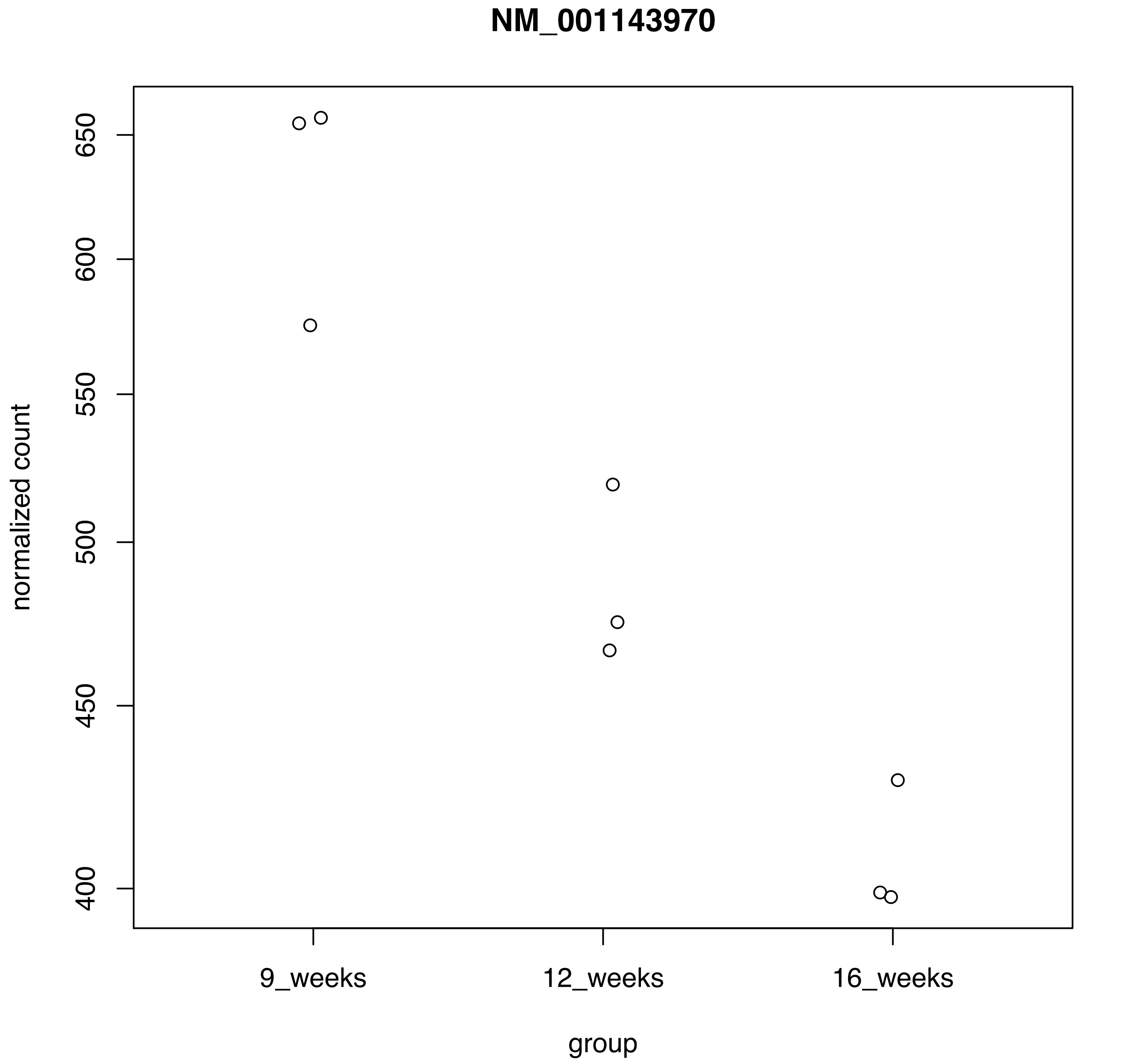
NM_001143970
normalized count
650
600
550
500
450
400
9_weeks
12_weeks
16_weeks
group

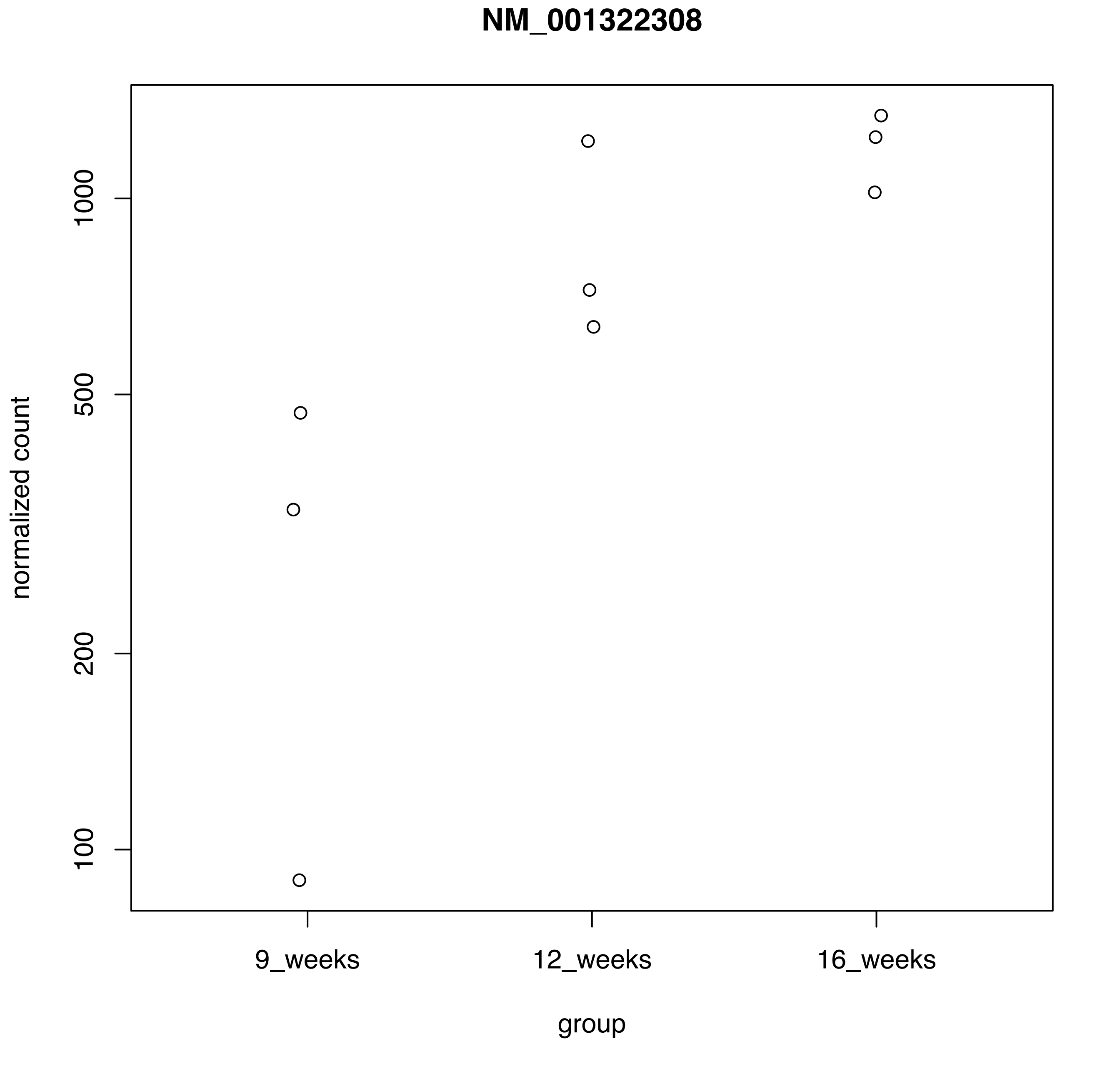

NM_001322308
normalized count
1000
500
200
100
9_weeks
12_weeks
16_weeks
group

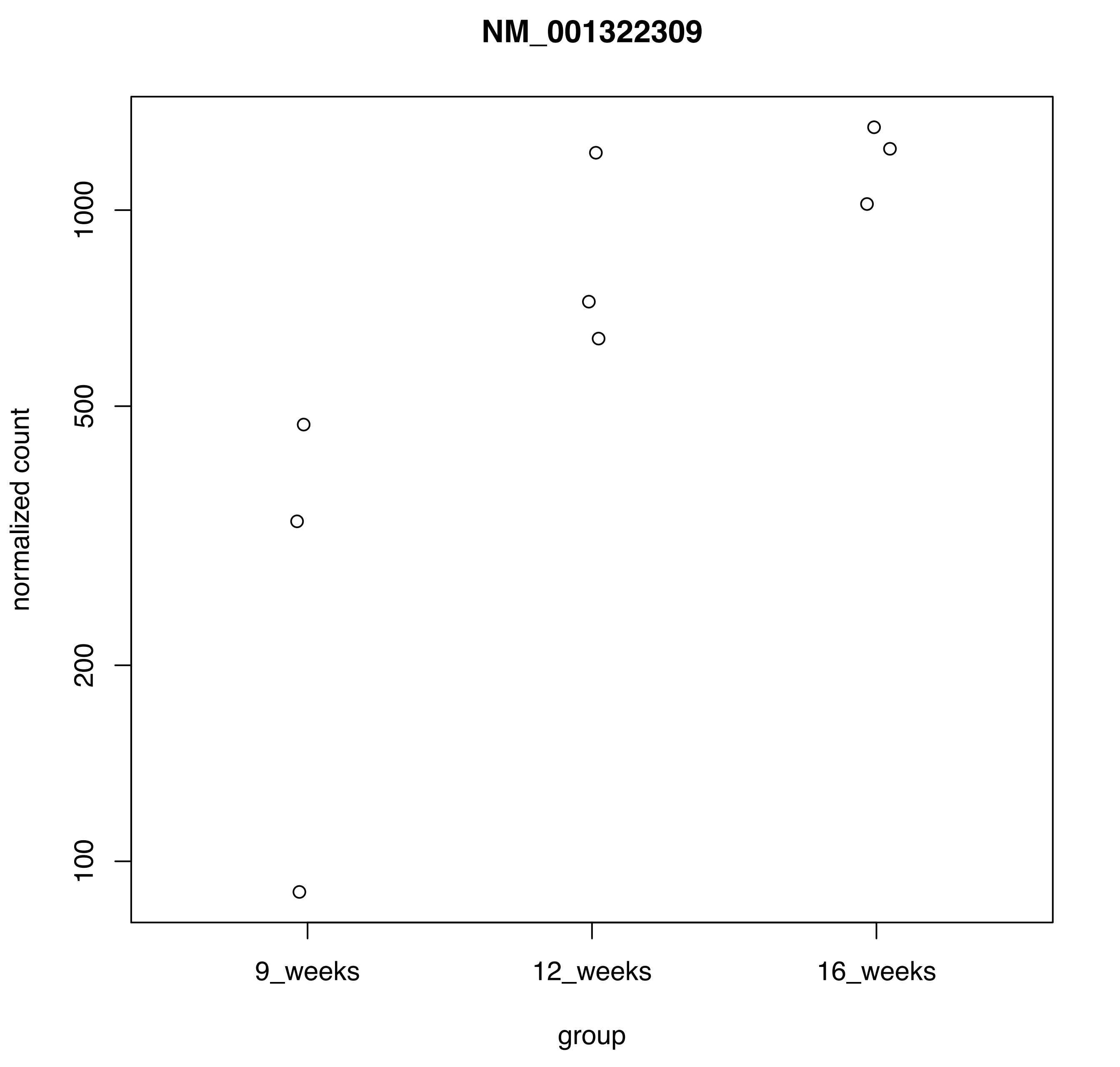
NM_001322309
normalized count
100
200
500
1000
9_weeks
12_weeks
16_weeks
group

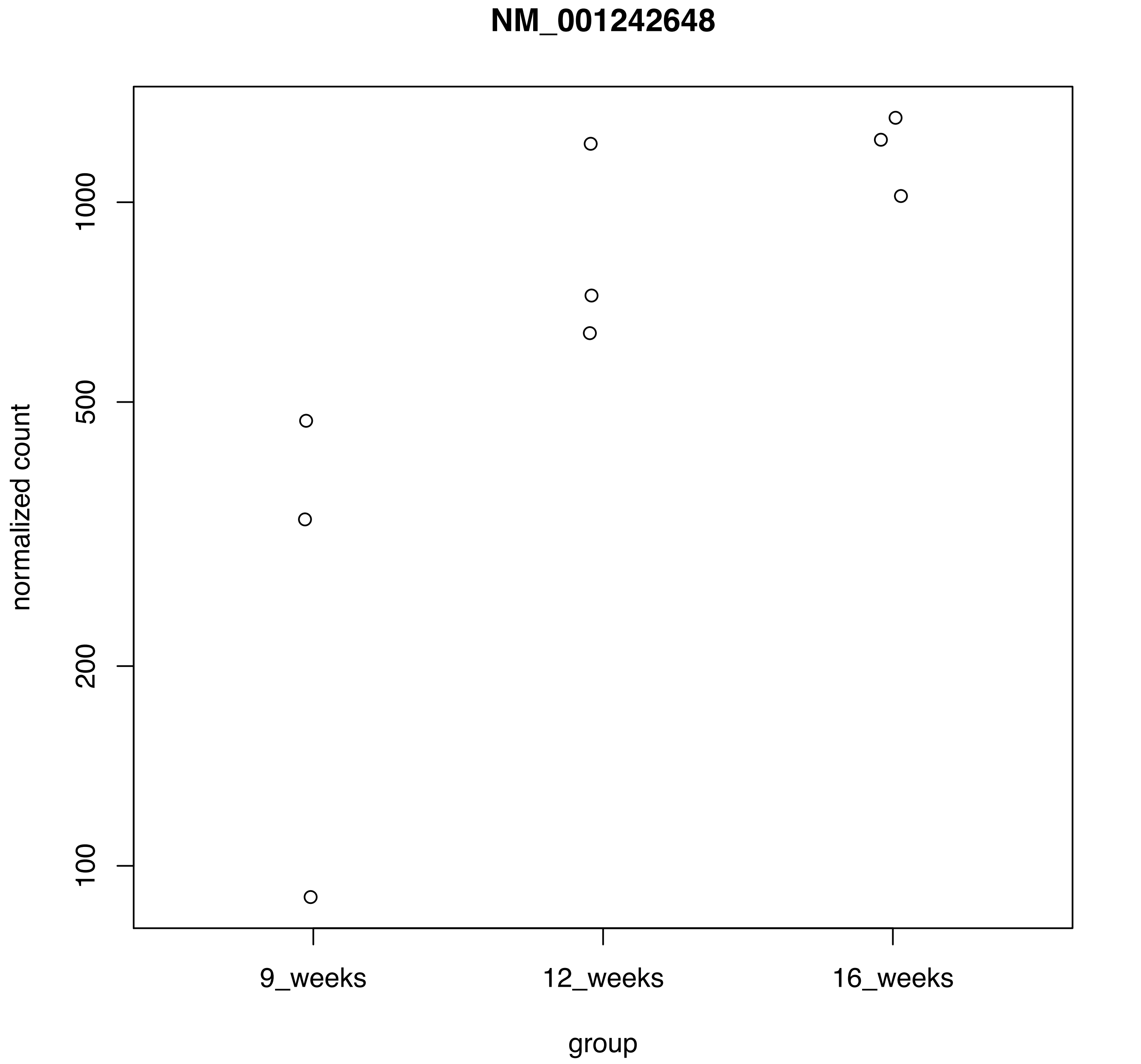
NM_001242648
normalized count
1000
500
200
100
9_weeks
12_weeks
16_weeks
group

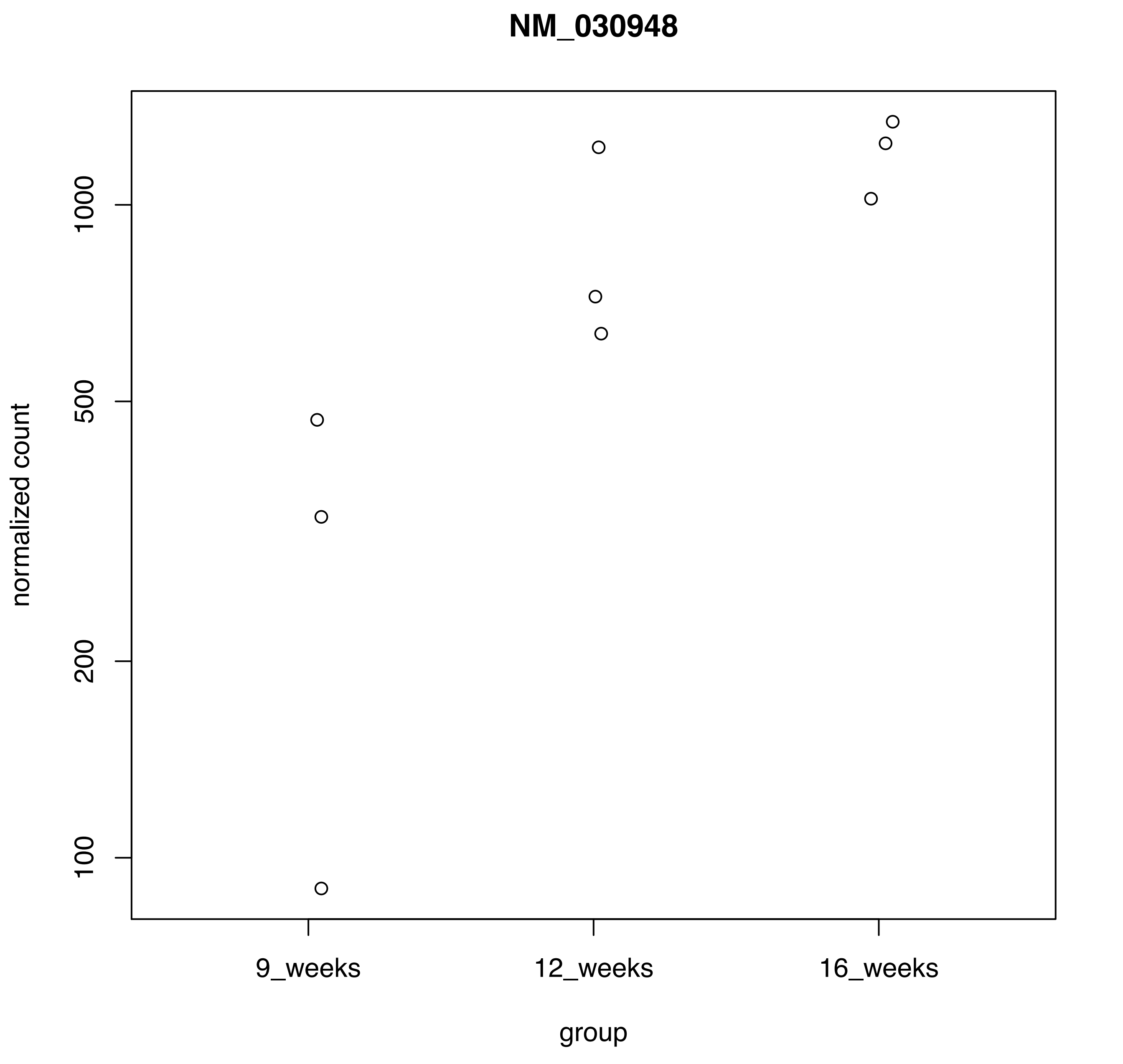

NM_030948
normalized count
1000
500
200
100
9_weeks
12_weeks
16_weeks
group

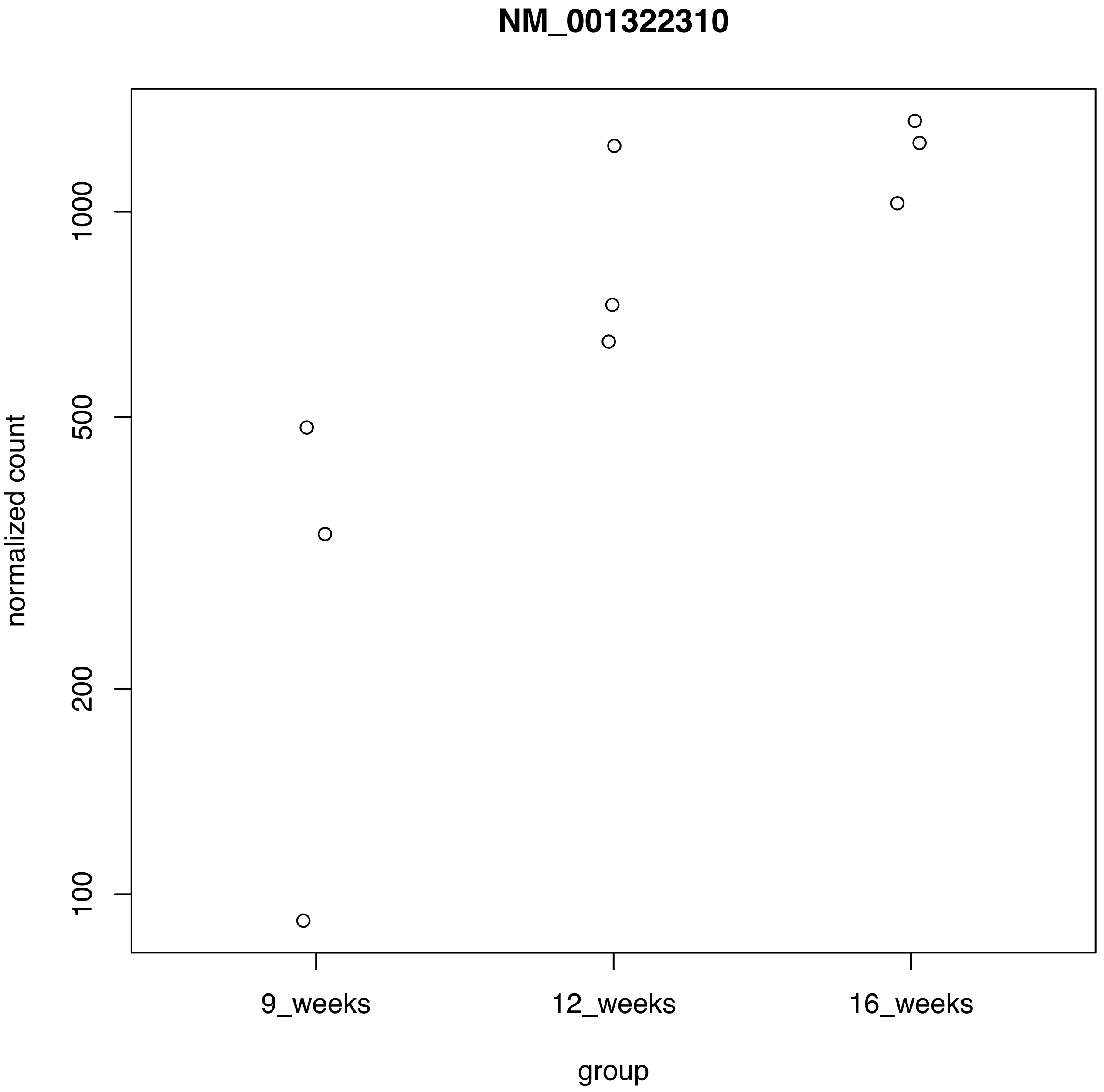
NM_001322310
normalized count
1000
500
200
100
9_weeks
12_weeks
16_weeks
group

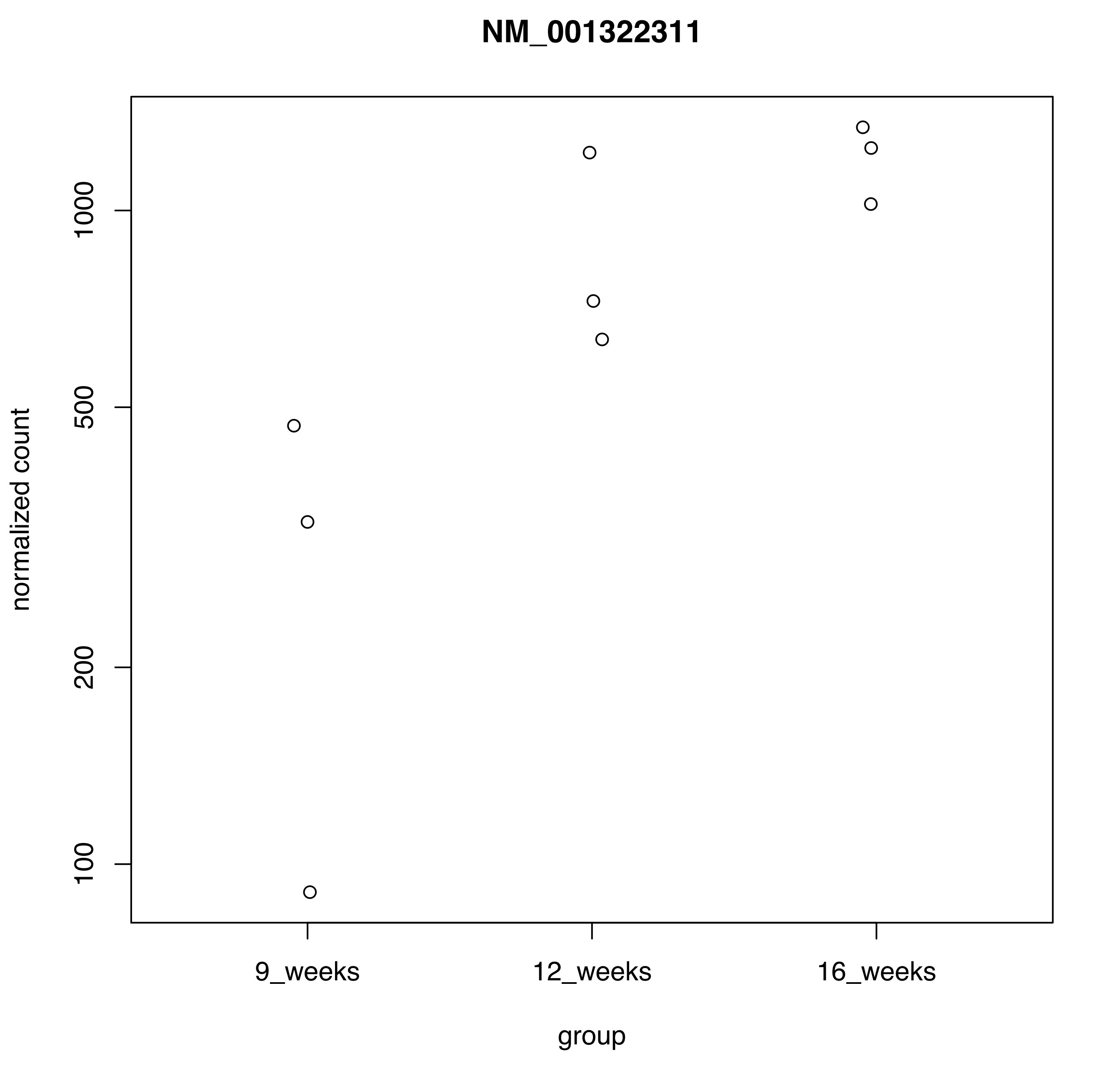

NM_001322311
normalized count
1000
500
200
100
9_weeks
12_weeks
16_weeks
group

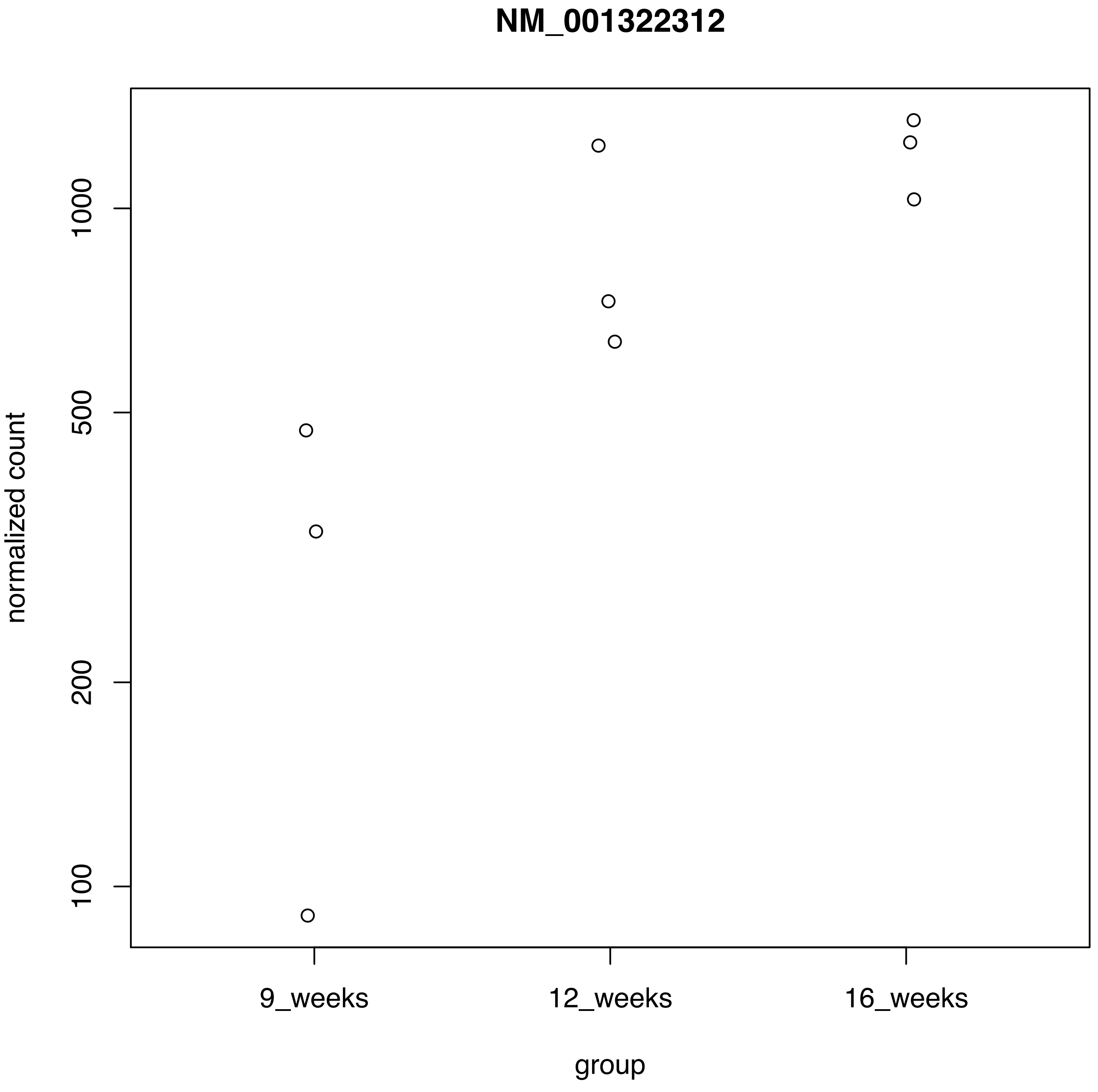
NM_001322312
normalized count
1000
500
200
100
9_weeks
12_weeks
16_weeks
group

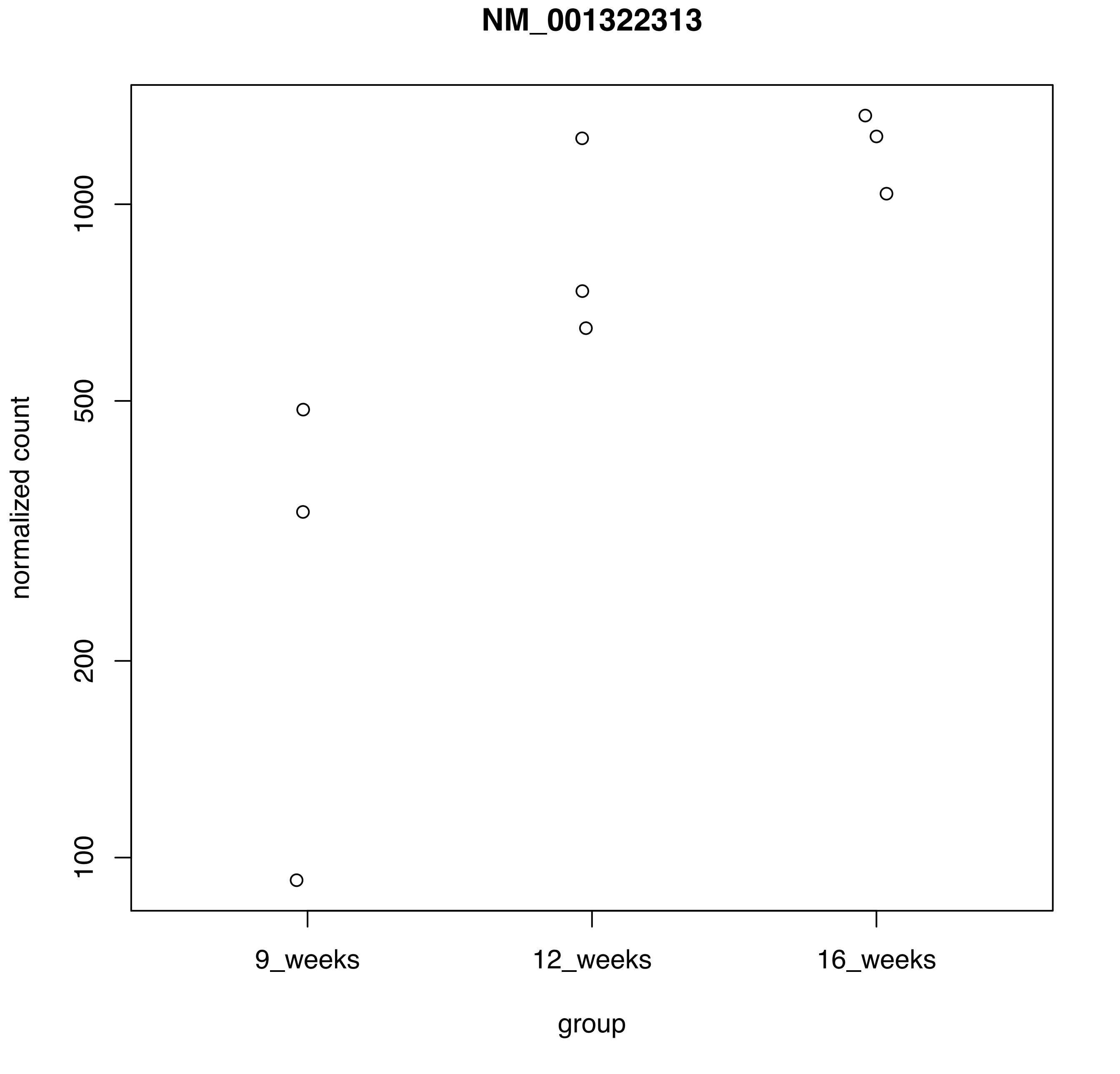
NM_001322313
normalized count
1000
500
200
100
9_weeks
12_weeks
16_weeks
group

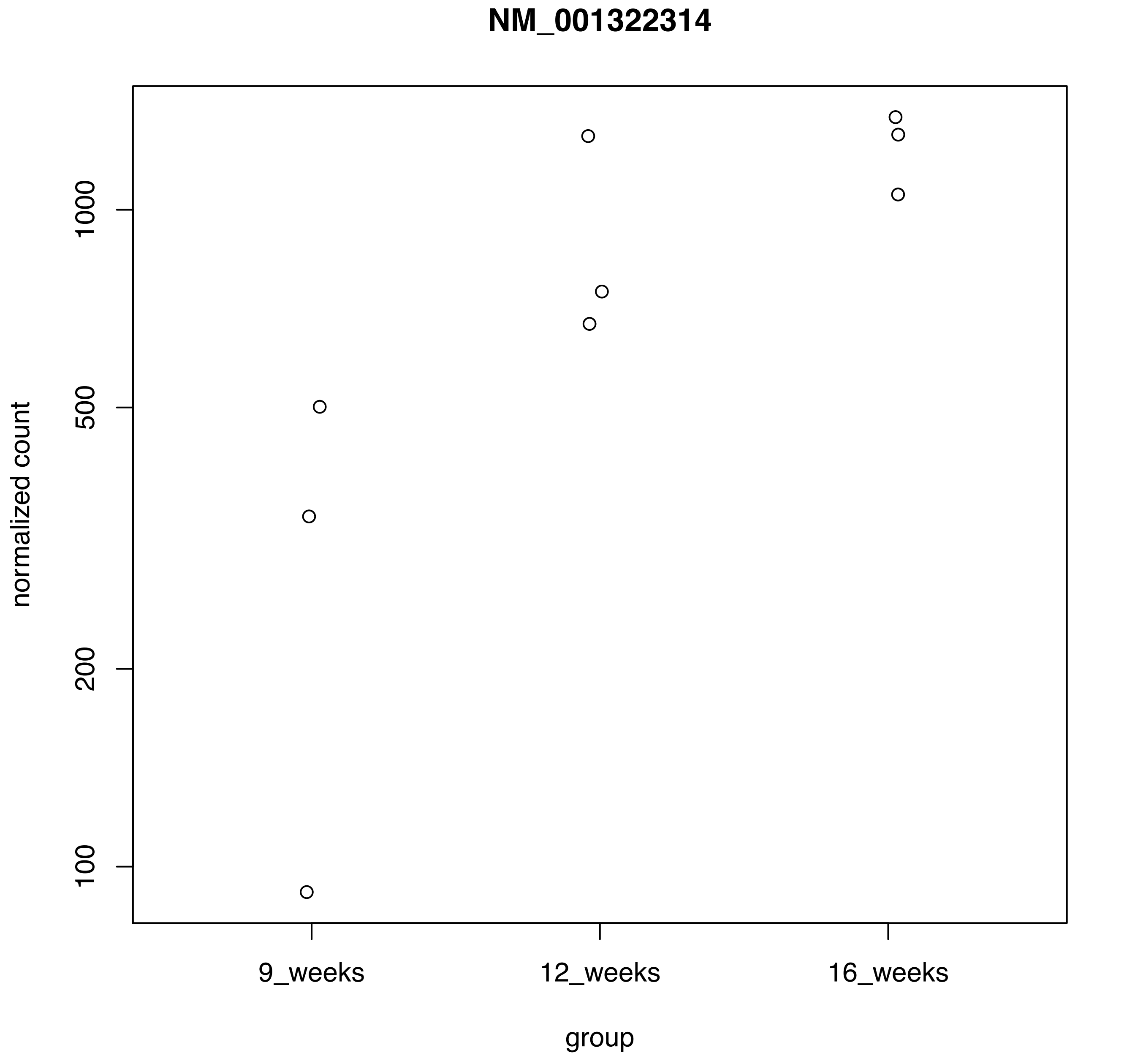
NM_001322314
normalized count
1000
500
200
100
9_weeks
12_weeks
16_weeks
group

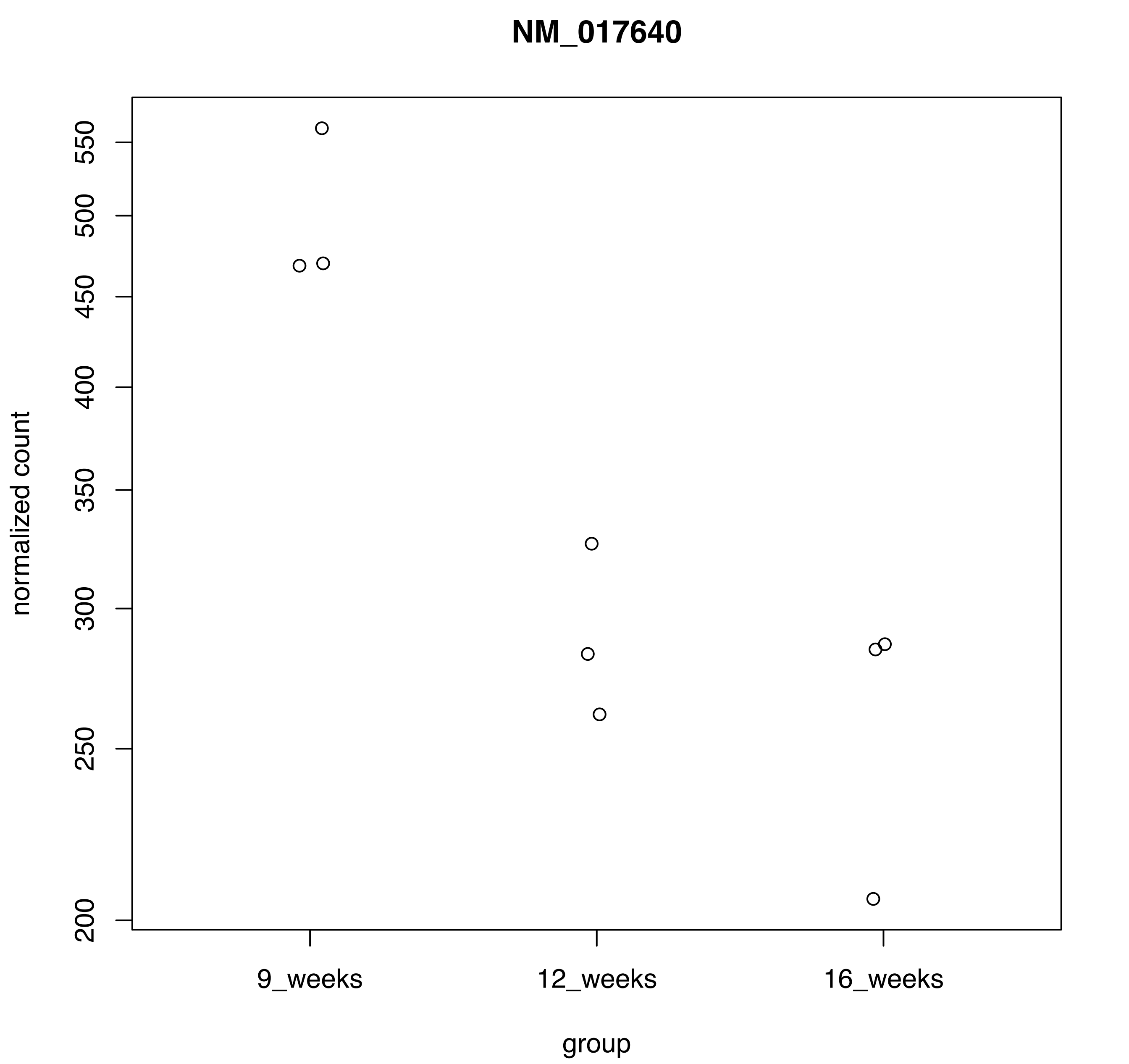

NM_017640
normalized count
550
500
450
400
350
300
250
200
9_weeks
12_weeks
16_weeks
group

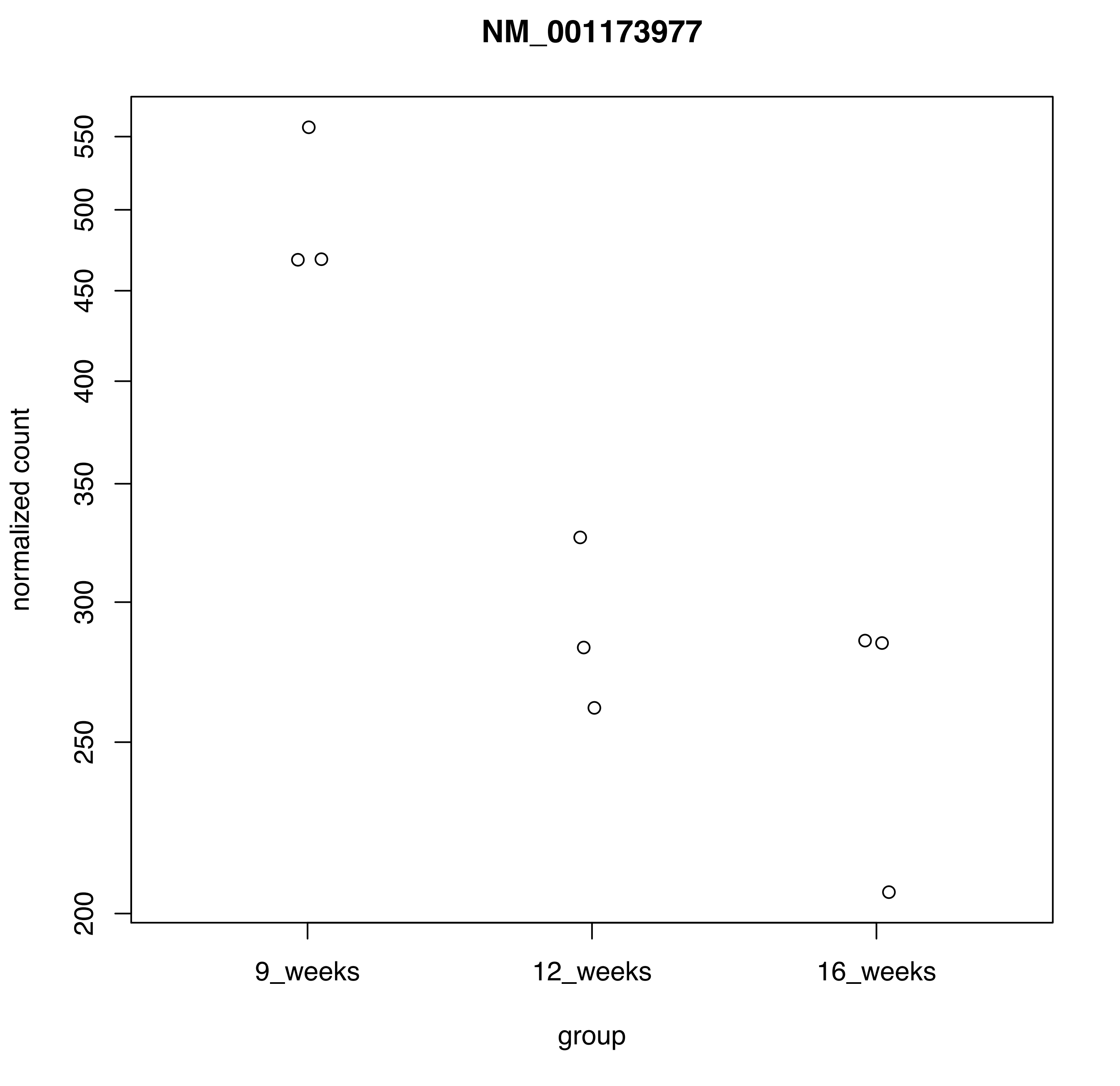

NM_001173977
normalized count
550
500
450
400
350
300
250
200
9_weeks
12_weeks
16_weeks
group

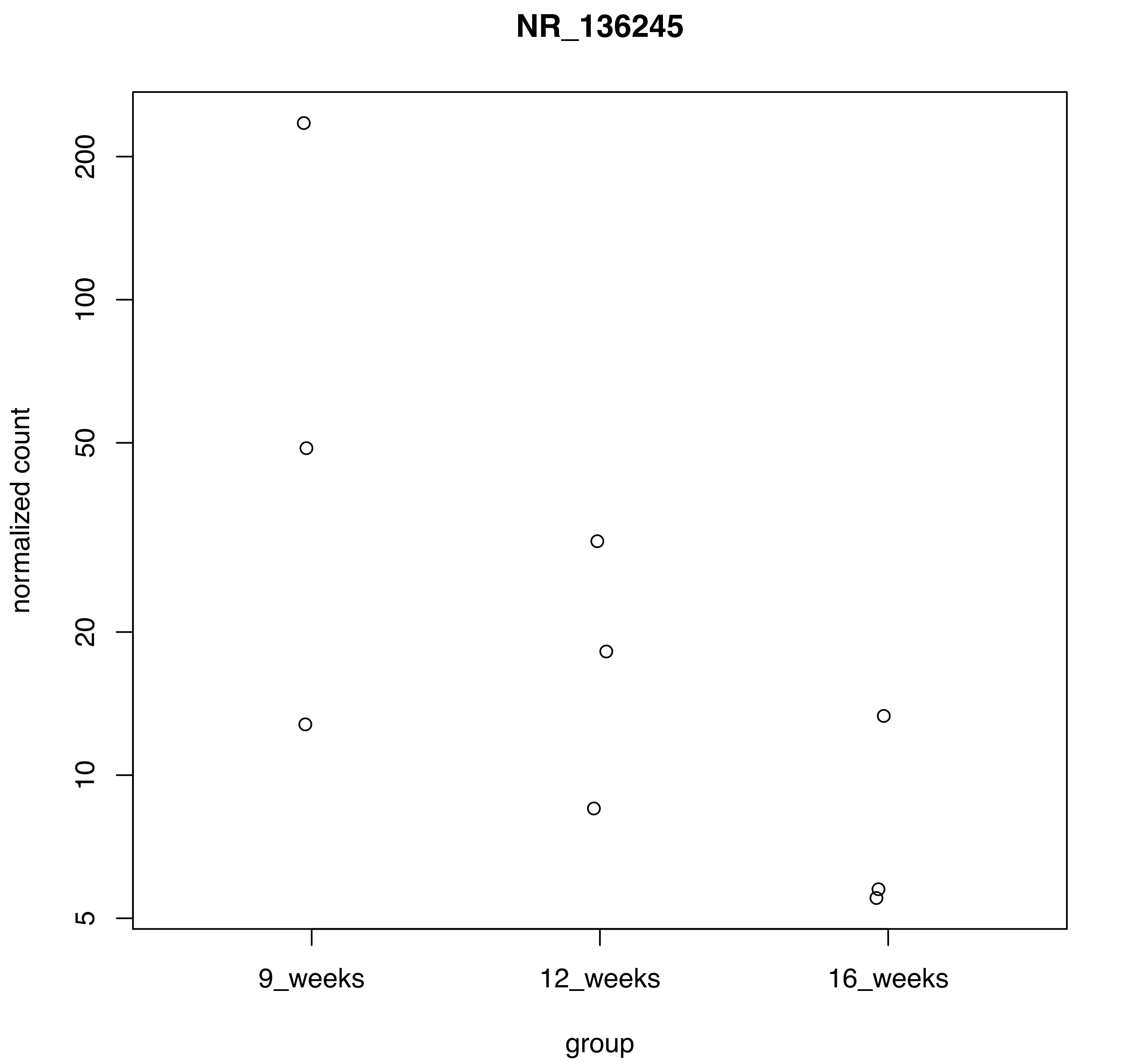

NR_136245
normalized count
200
100
50
20
10
5
9_weeks
12_weeks
16_weeks
group

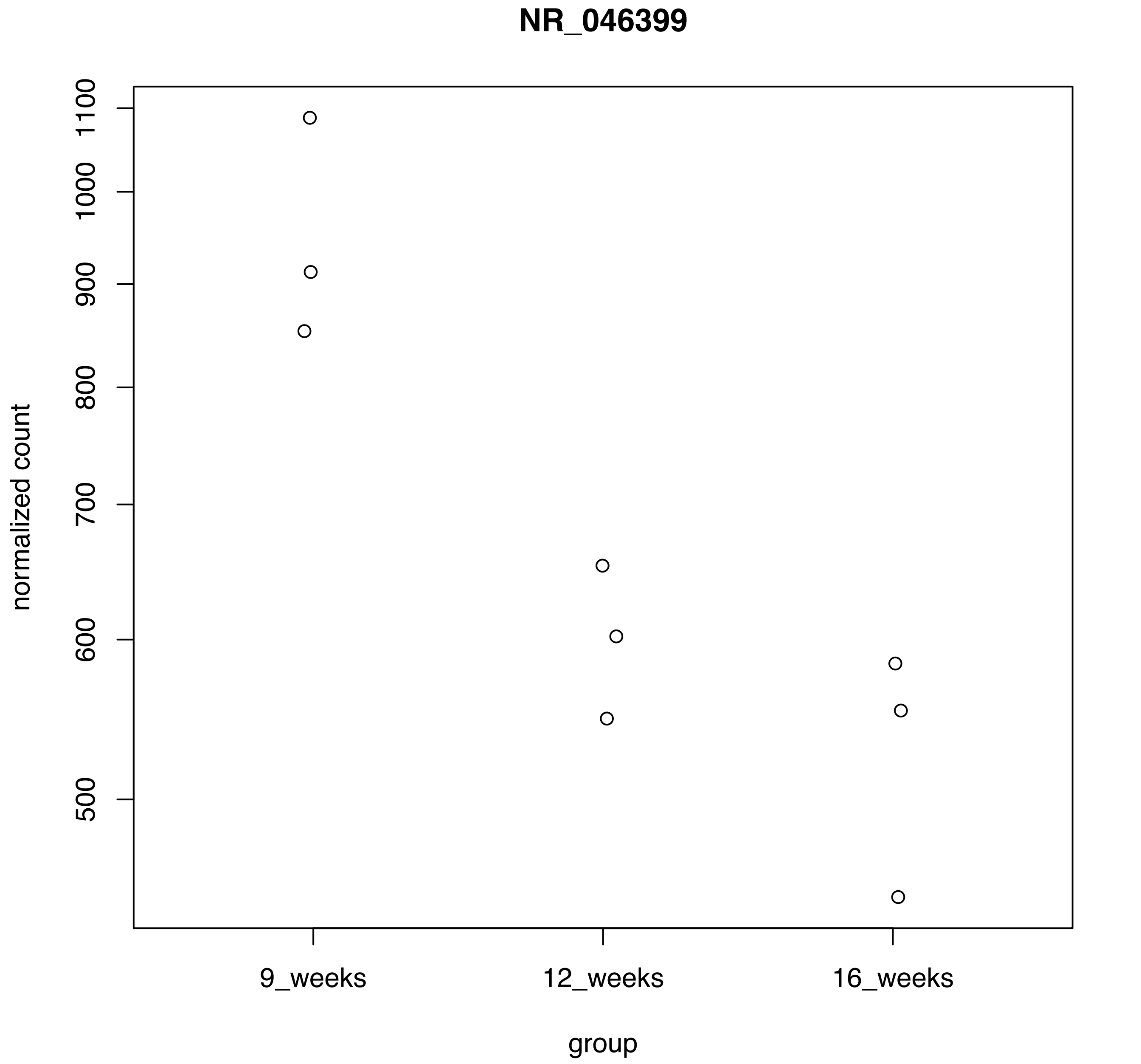

NR_046399
normalized count
1100
1000
900
800
700
600
500
9_weeks
12_weeks
16_weeks
group

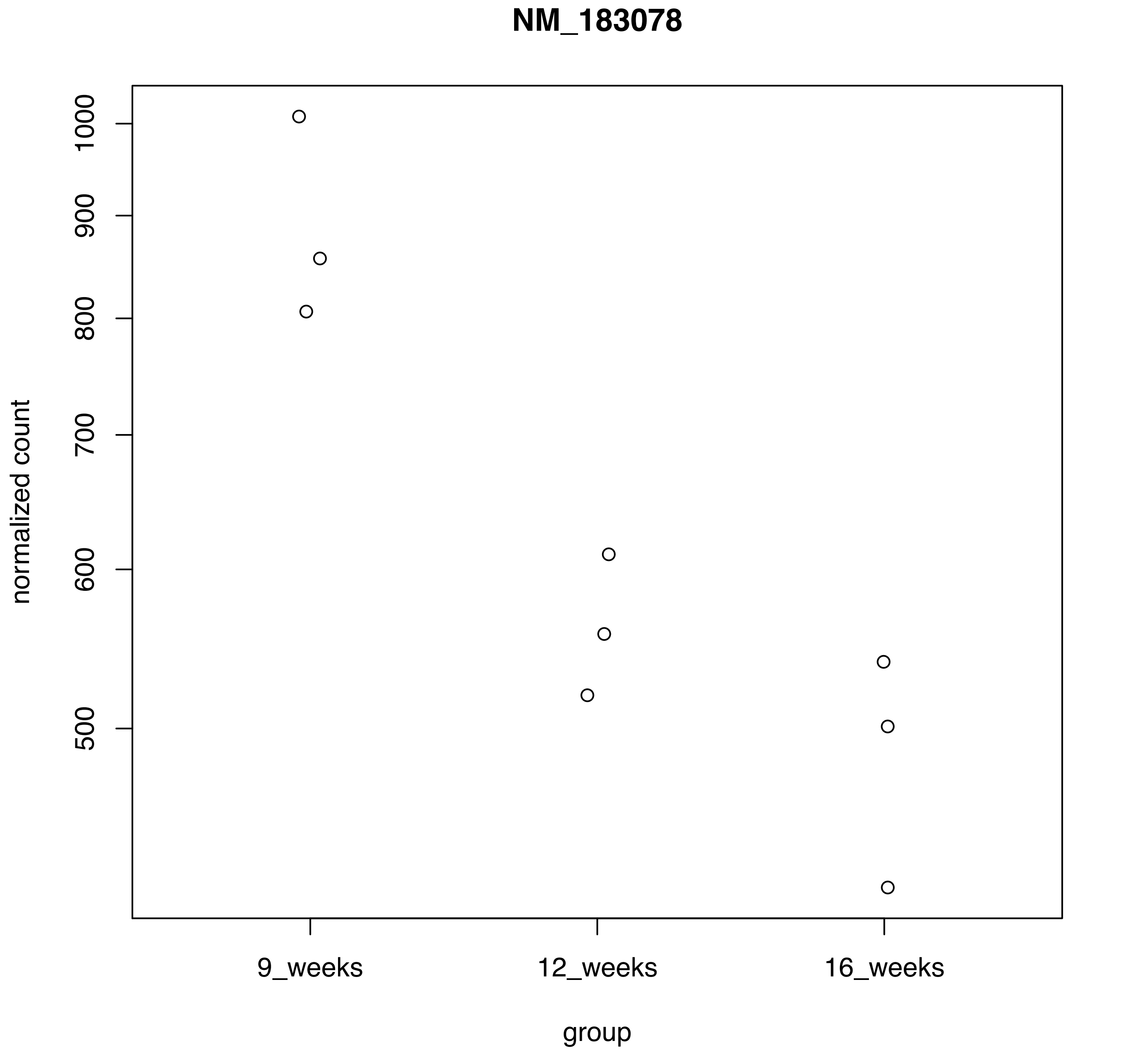
NM_183078
normalized count
1000
900
800
700
600
500
group
9_weeks
12_weeks
16_weeks

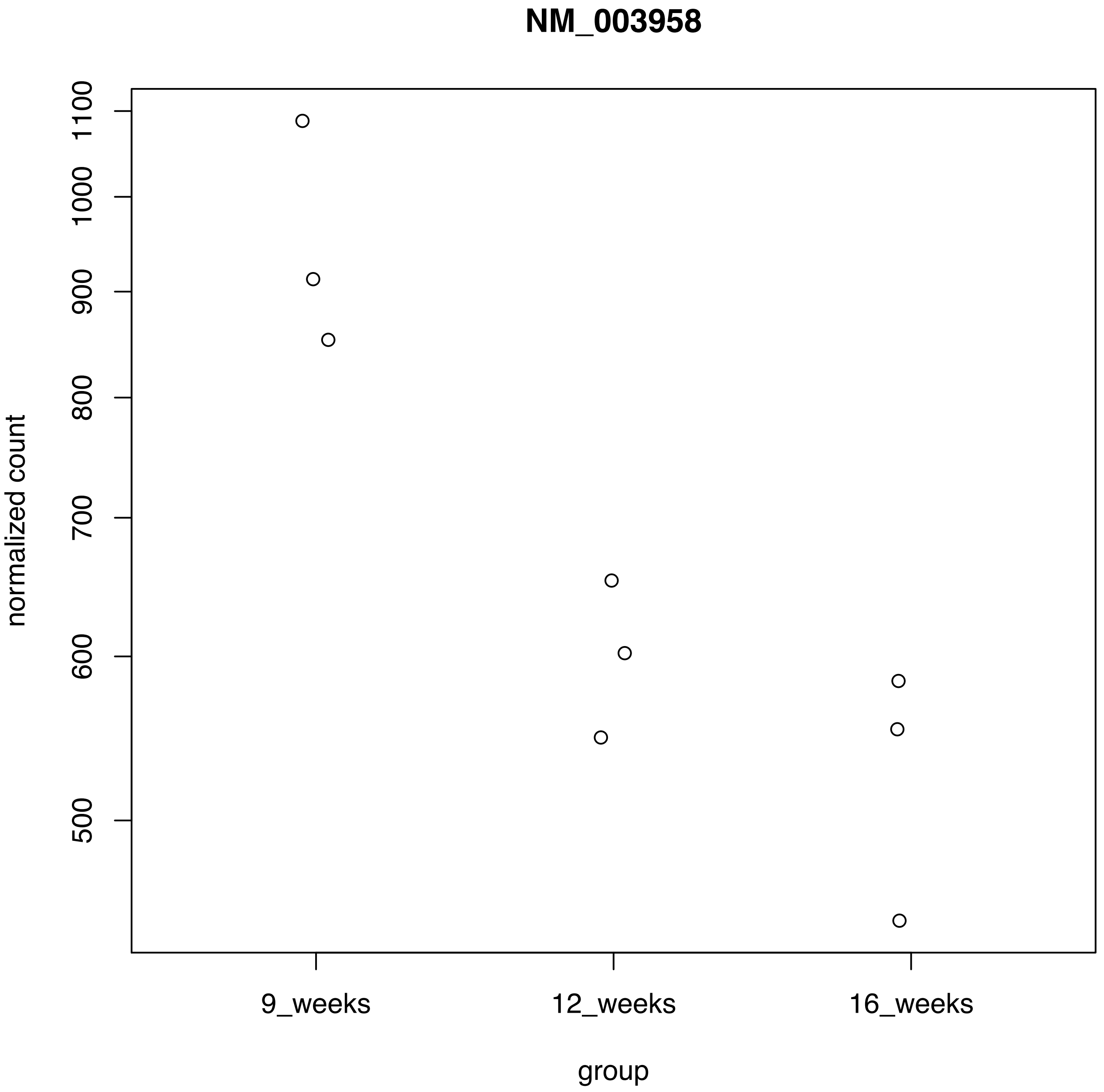

NM_003958
normalized count
1100
1000
900
800
700
600
500
9_weeks
12_weeks
16_weeks
group

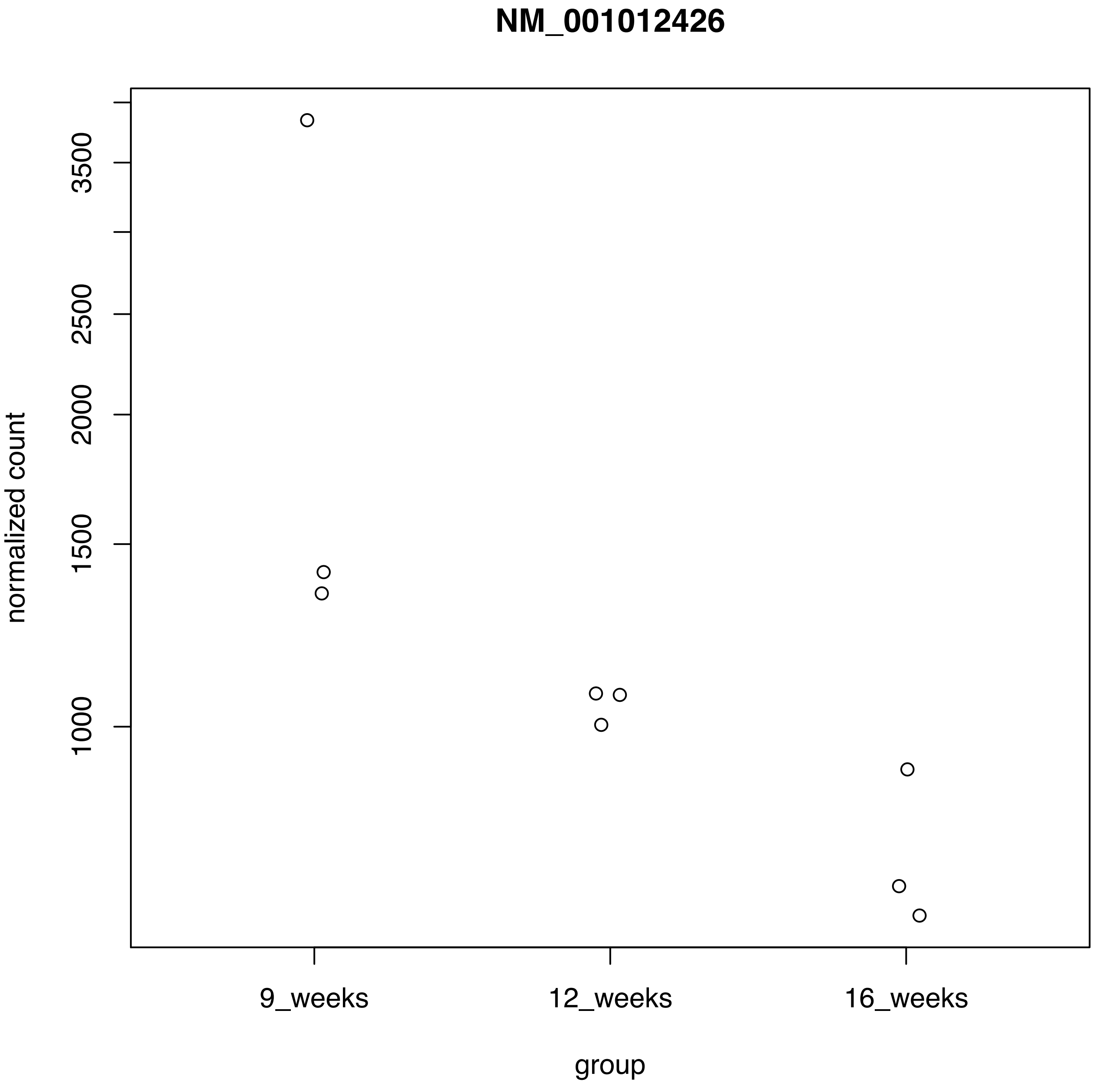

NM_001012426
normalized count
3500
2500
2000
1500
1000
9_weeks
12_weeks
16_weeks
group

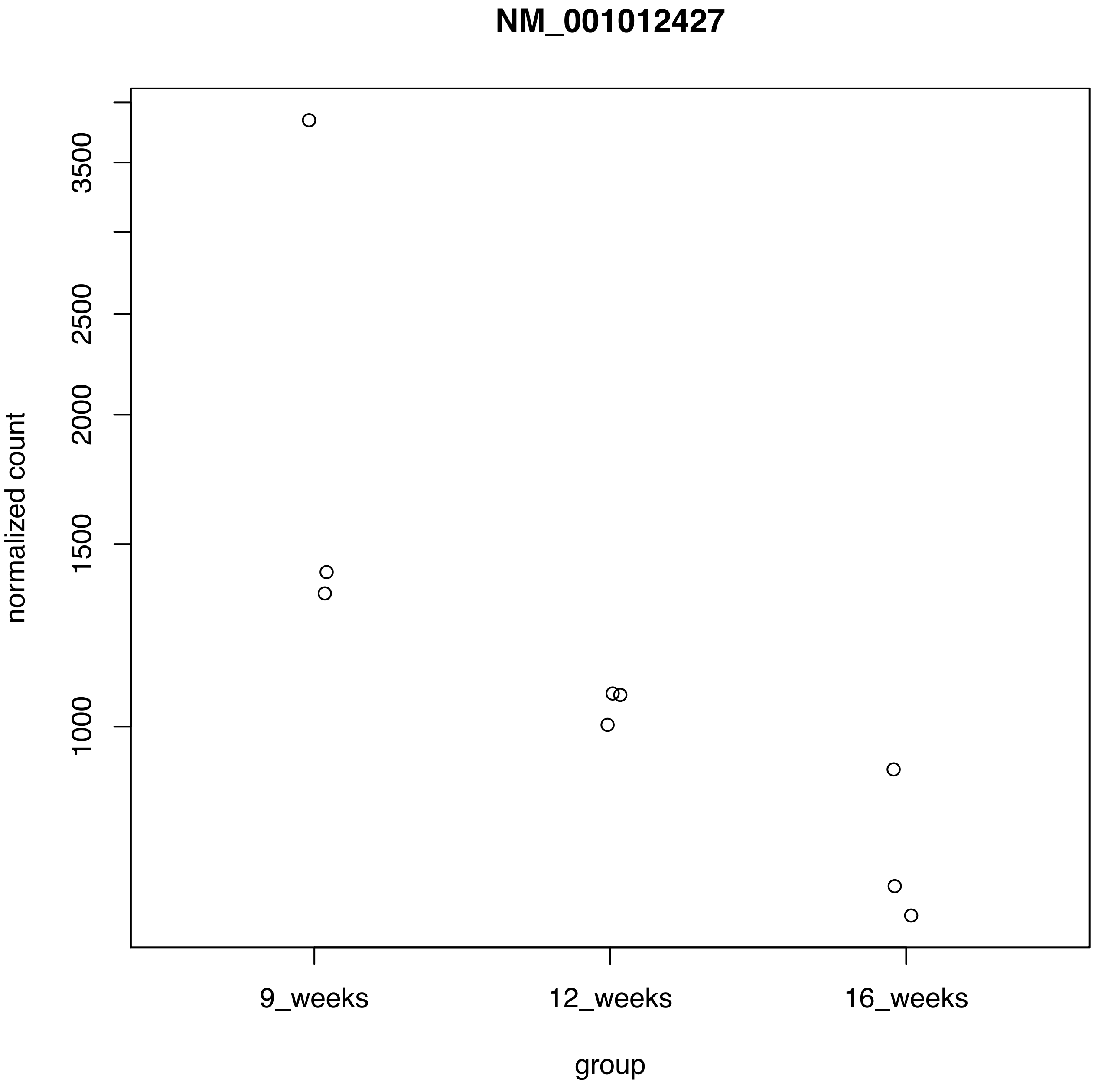

NM_001012427
normalized count
1000
1500
2000
2500
3500
9_weeks
12_weeks
16_weeks
group

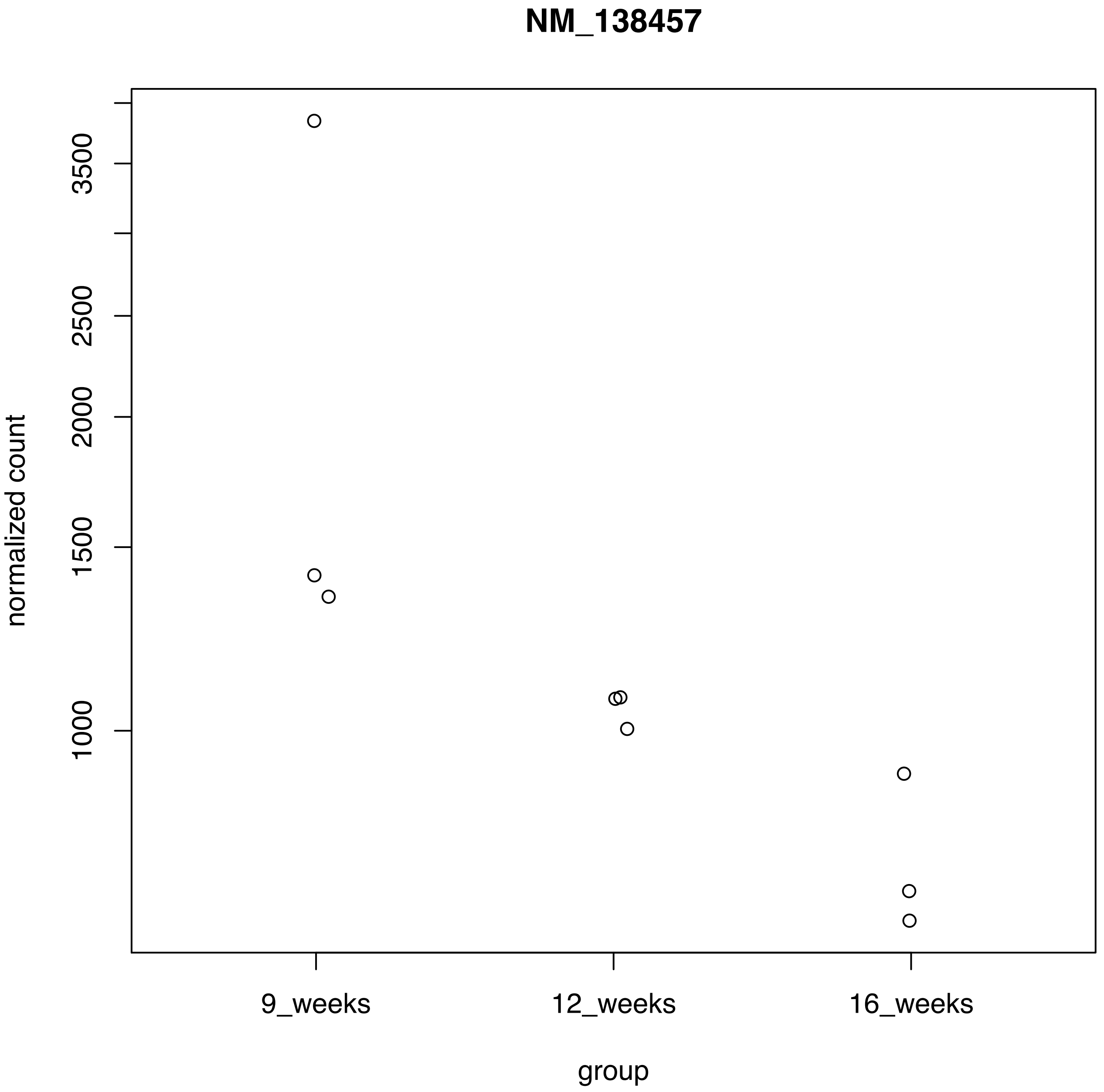
NM_138457
normalized count
1000
1500
2000
2500
3500
9_weeks
12_weeks
16_weeks
group

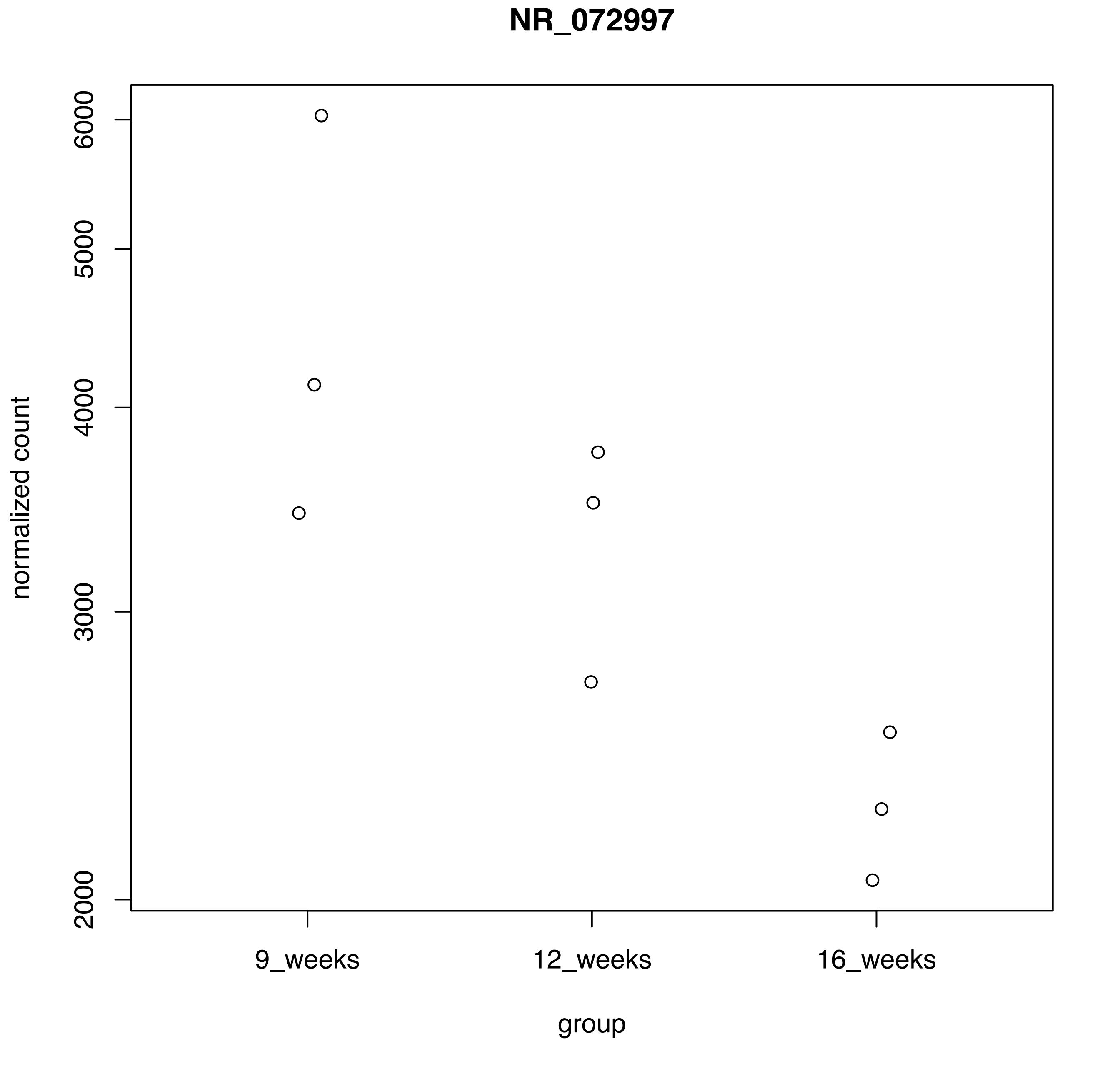

NR_072997
normalized count
6000
5000
4000
3000
2000
9_weeks
12_weeks
16_weeks
group

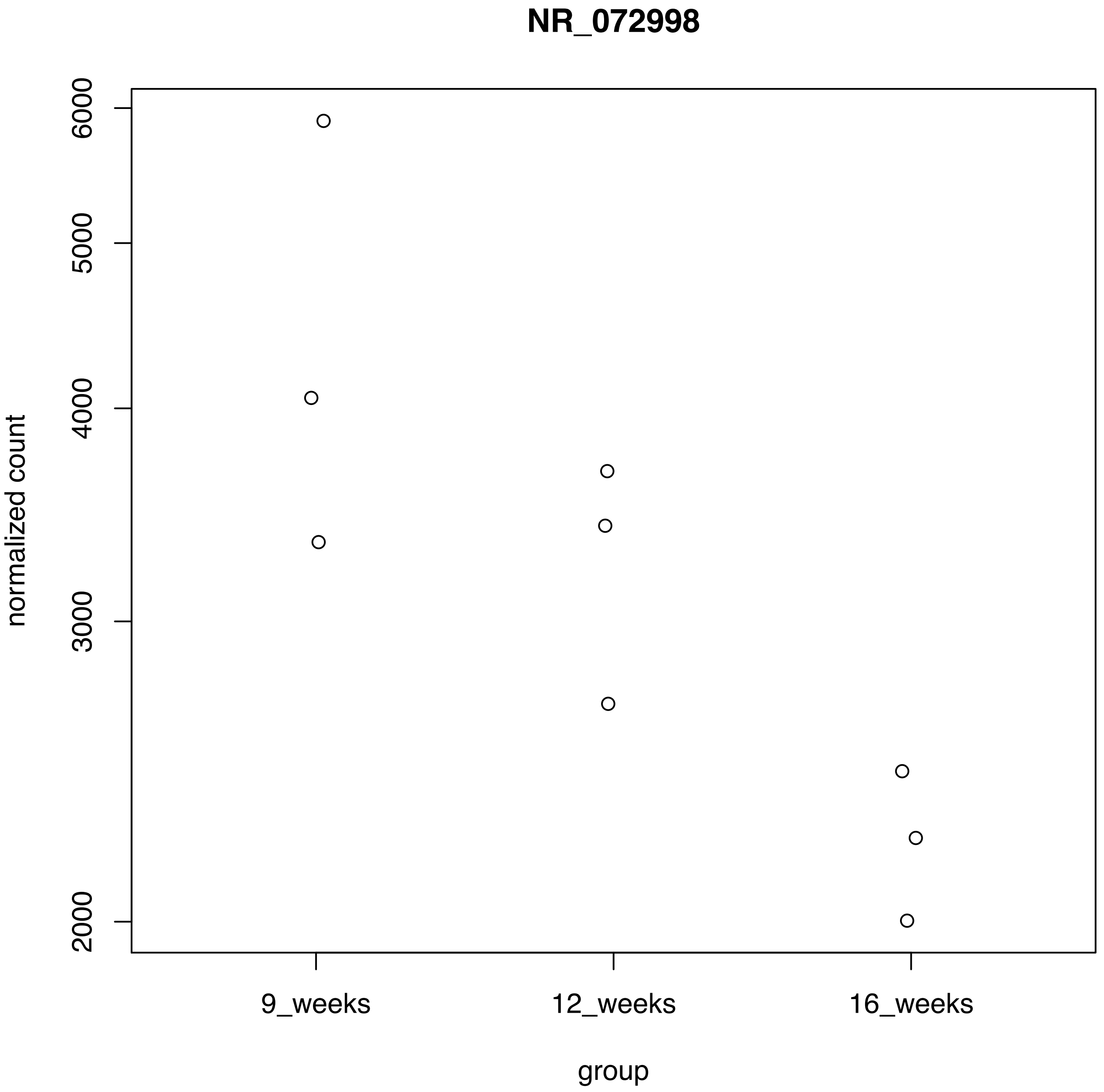
NR_072998
normalized count
6000
5000
4000
3000
2000
9_weeks
12_weeks
16_weeks
group

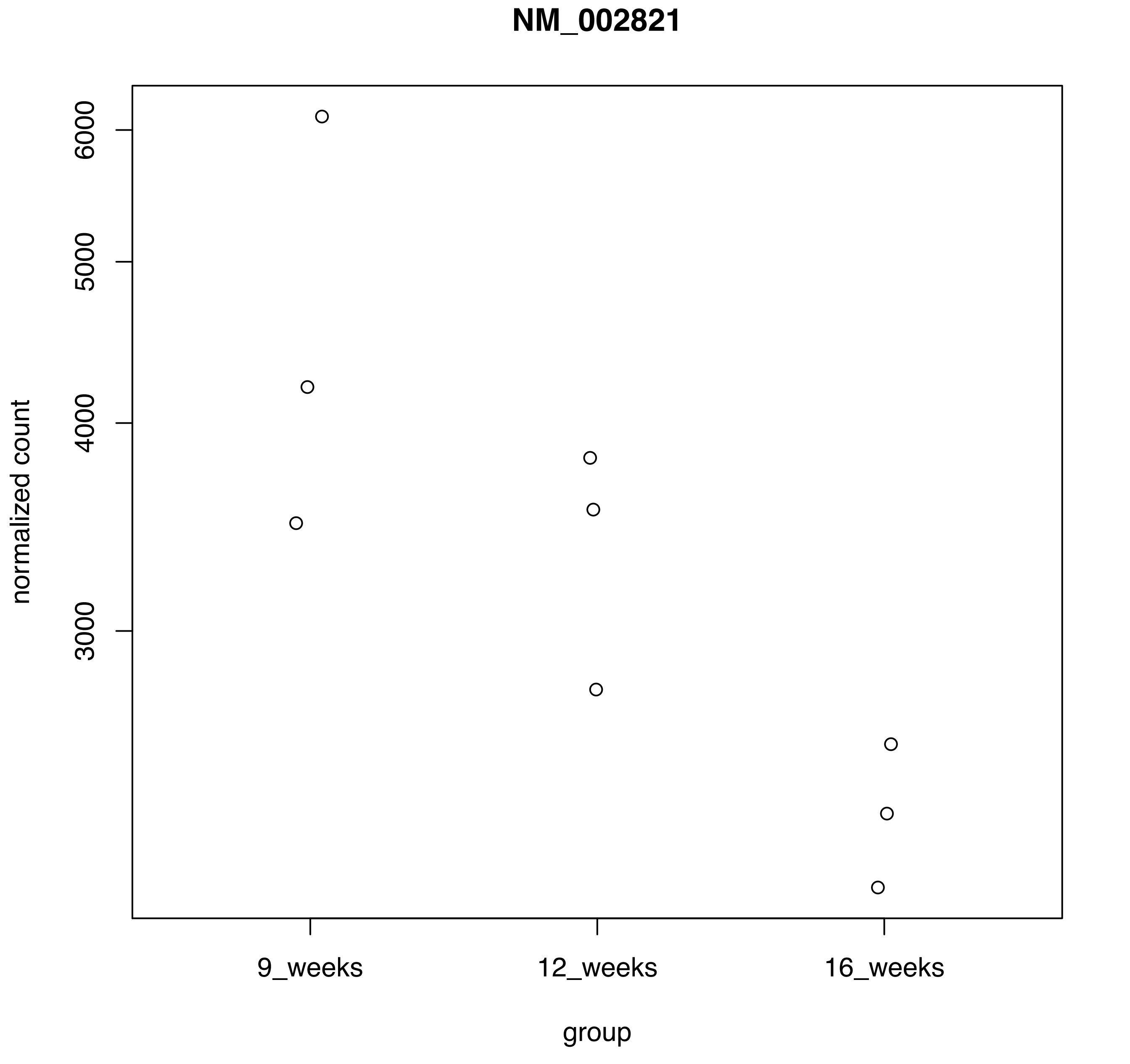

NM_002821
normalized count
6000
5000
4000
3000
9_weeks
12_weeks
16_weeks
group

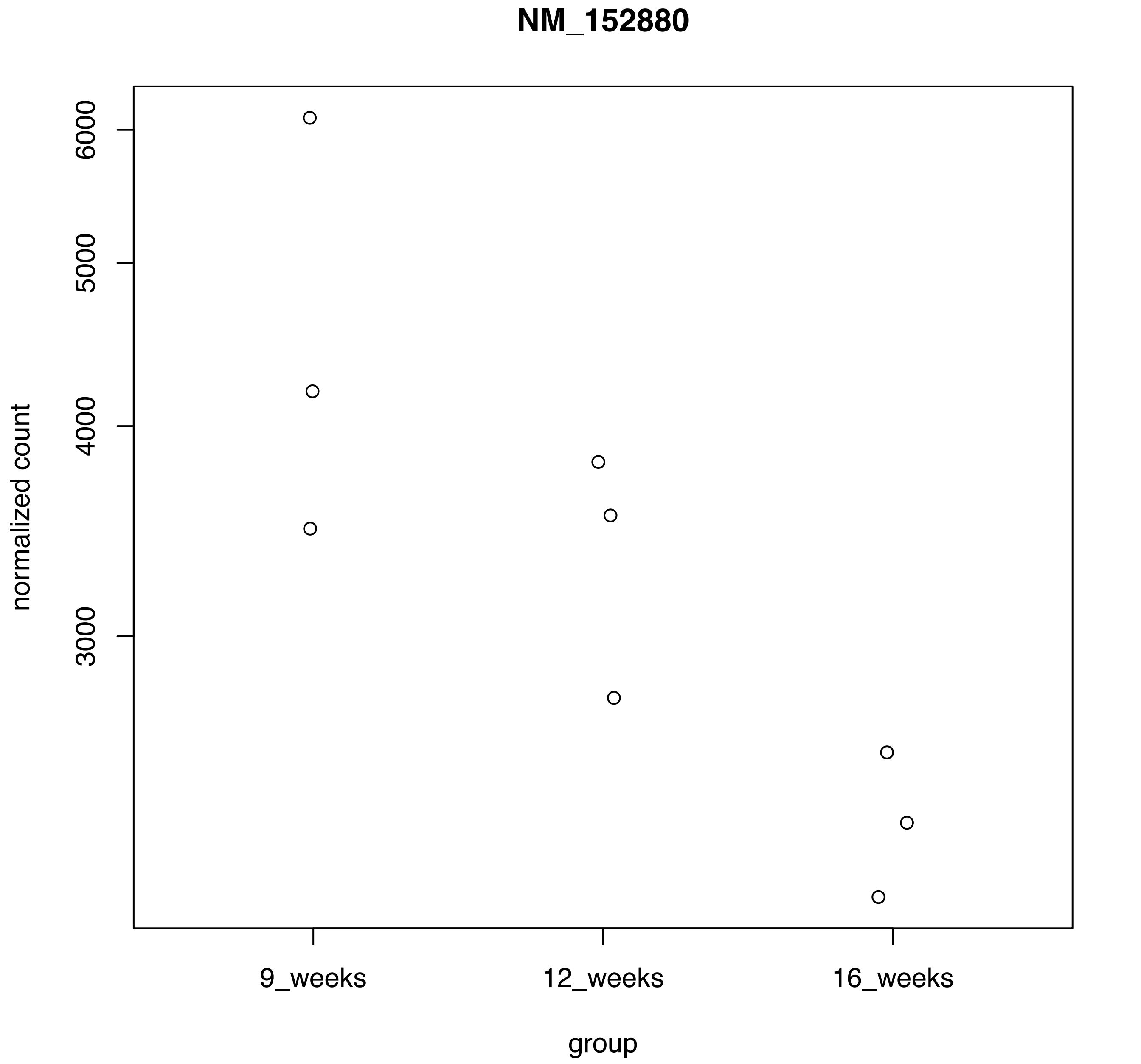
NM_152880
normalized count
6000
5000
4000
3000
9_weeks
12_weeks
16_weeks
group

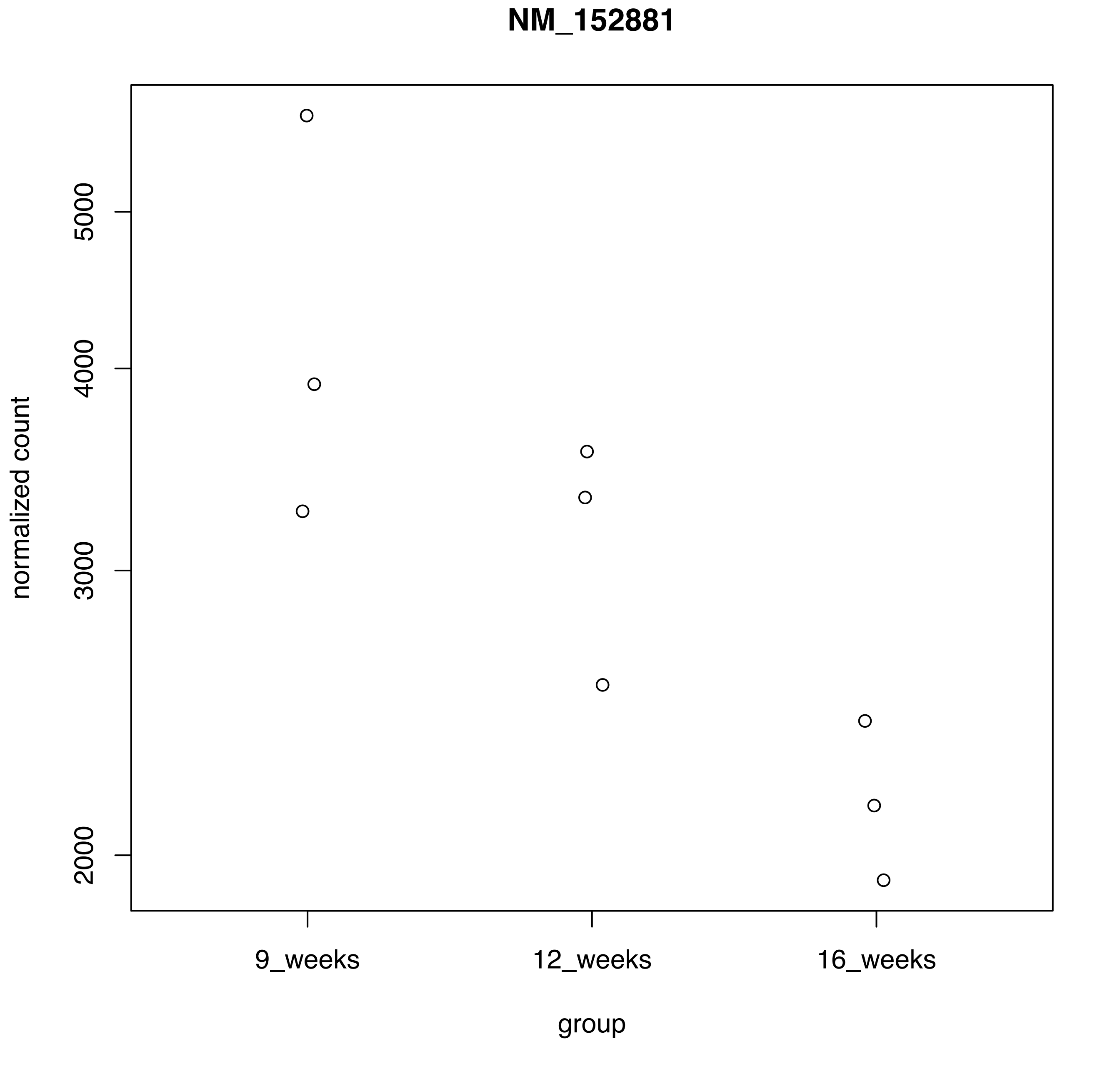

NM_152881
normalized count
5000
4000
3000
2000
9_weeks
12_weeks
16_weeks
group

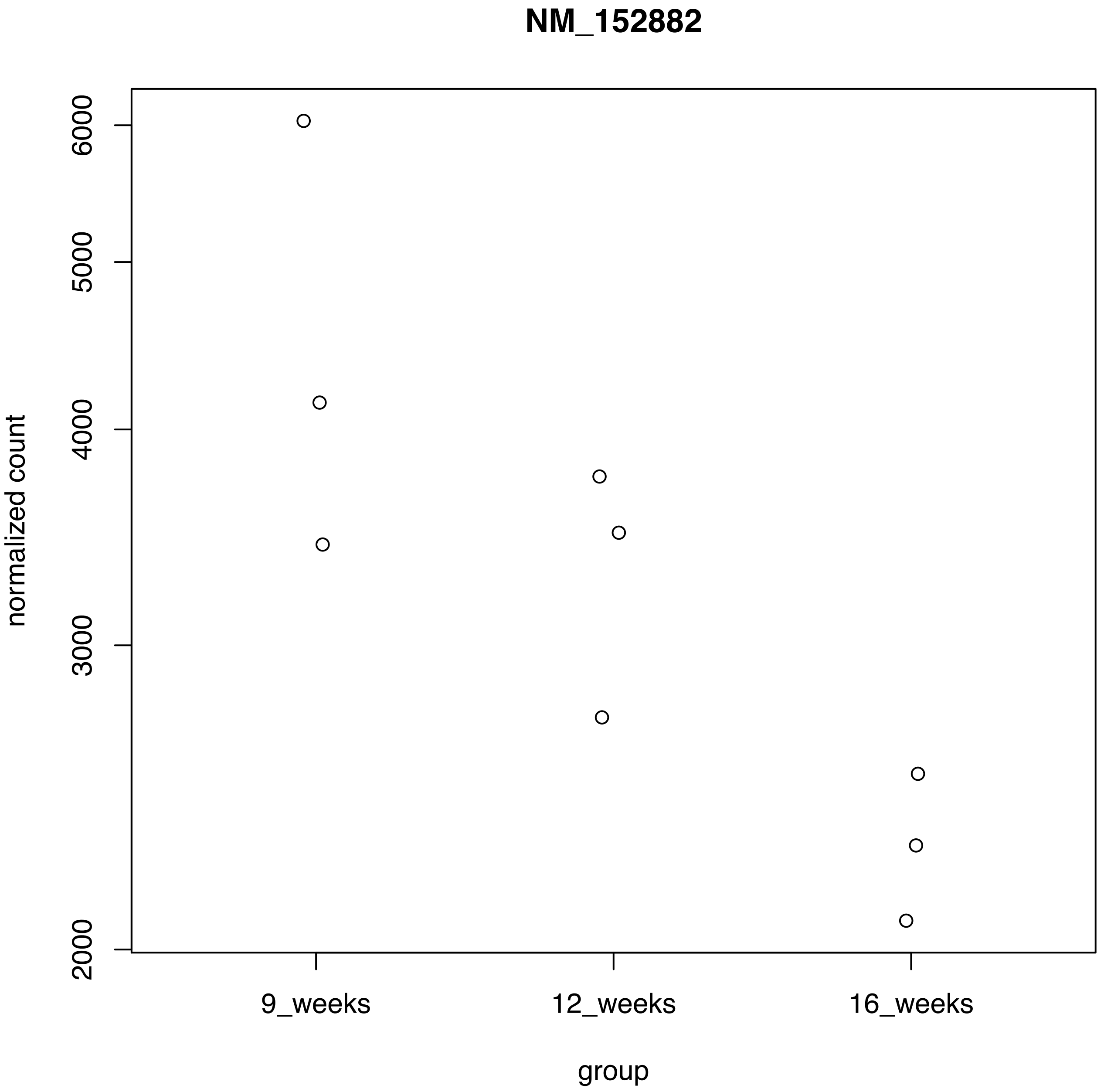
NM_152882
normalized count
6000
5000
4000
3000
2000
9_weeks
12_weeks
16_weeks
group

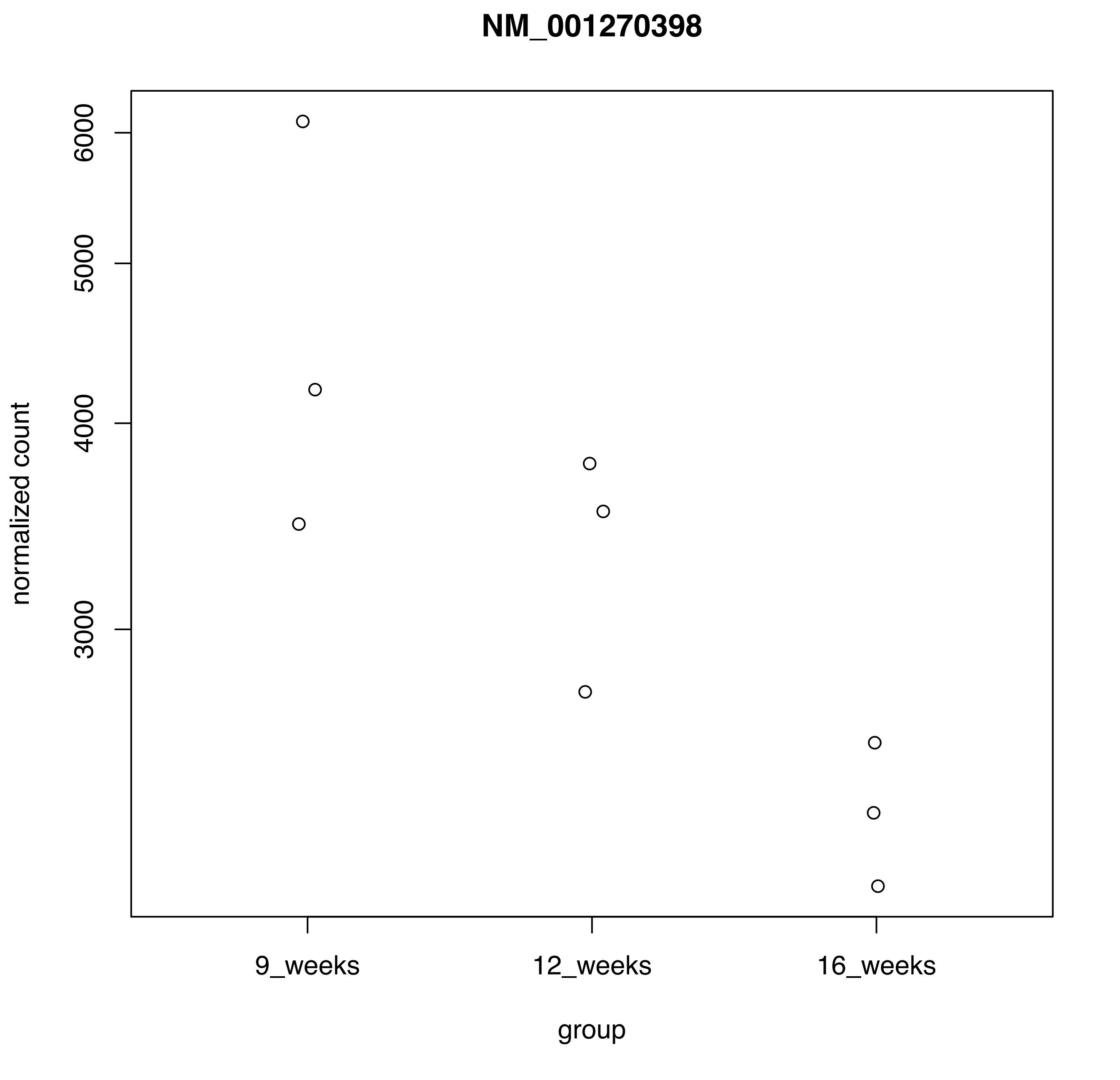

NM_001270398
normalized count
6000
5000
4000
3000
9_weeks
12_weeks
16_weeks
group

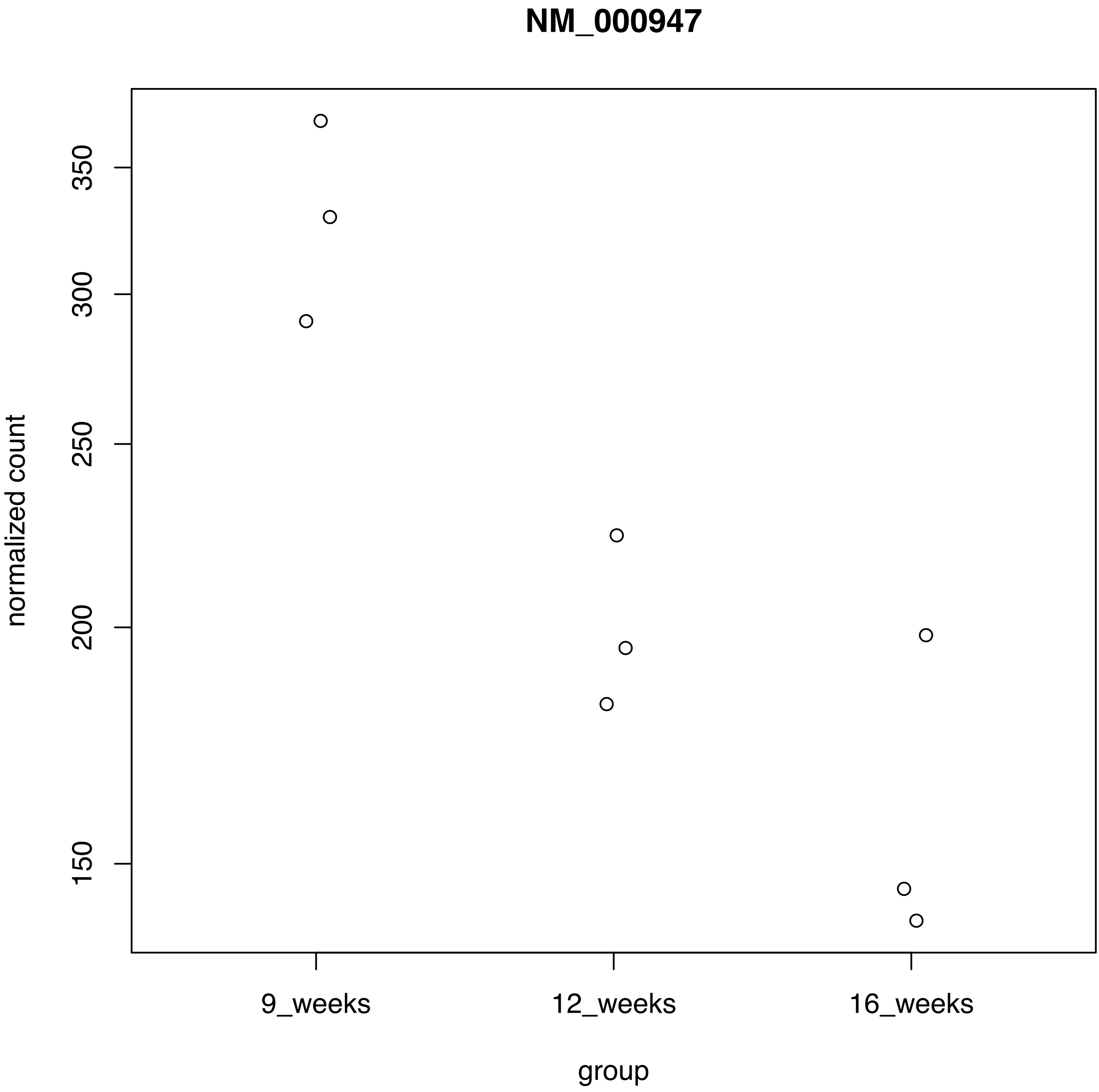
NM_000947
normalized count
350
300
250
200
150
9_weeks
12_weeks
16_weeks
group

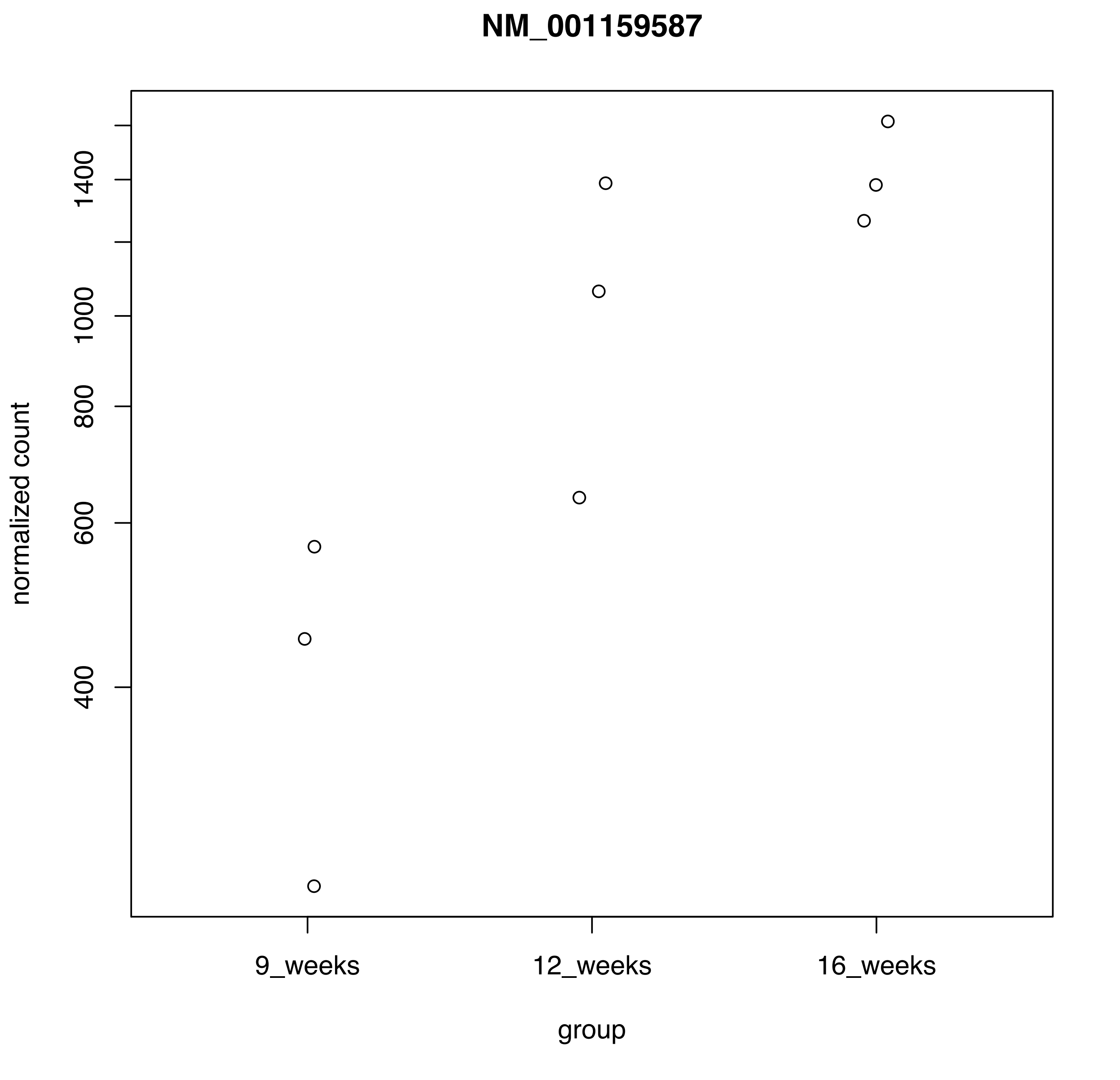

NM_001159587
normalized count
400
600
800
1000
1400
9_weeks
12_weeks
16_weeks
group

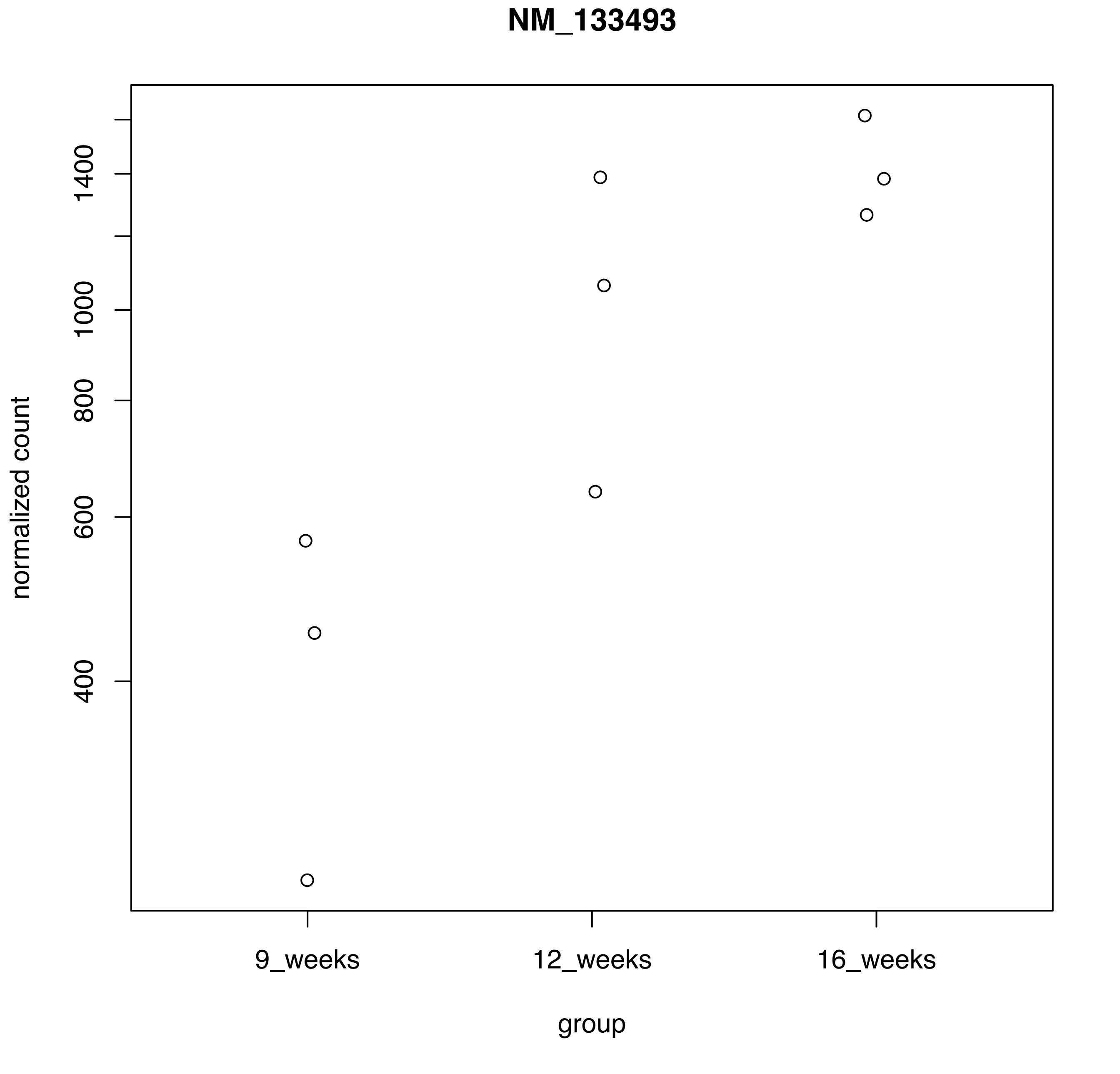
NM_133493
normalized count
1400
1000
800
600
400
9_weeks
12_weeks
16_weeks
group

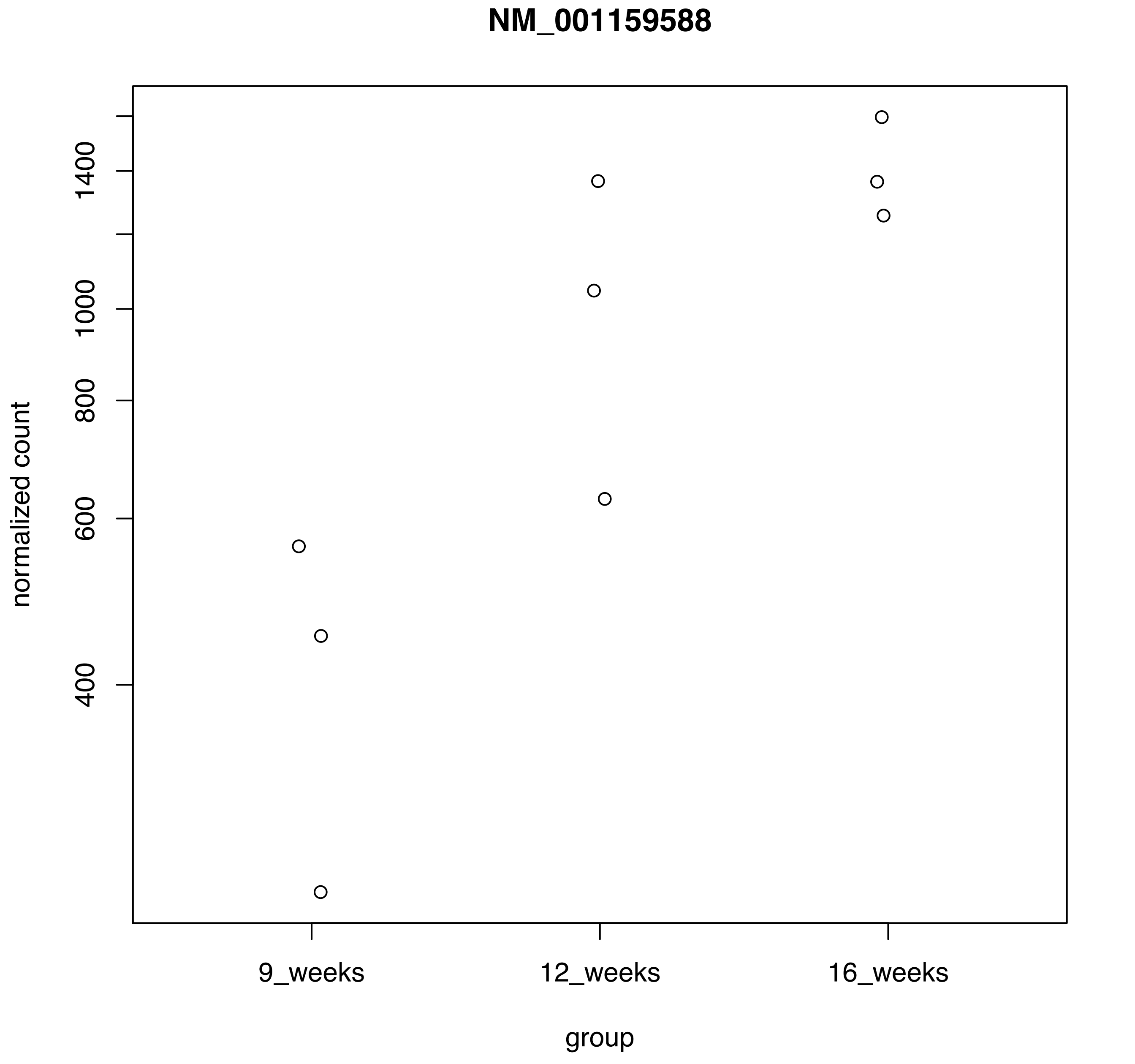

NM_001159588
normalized count
400
600
800
1000
1400
9_weeks
12_weeks
16_weeks
group

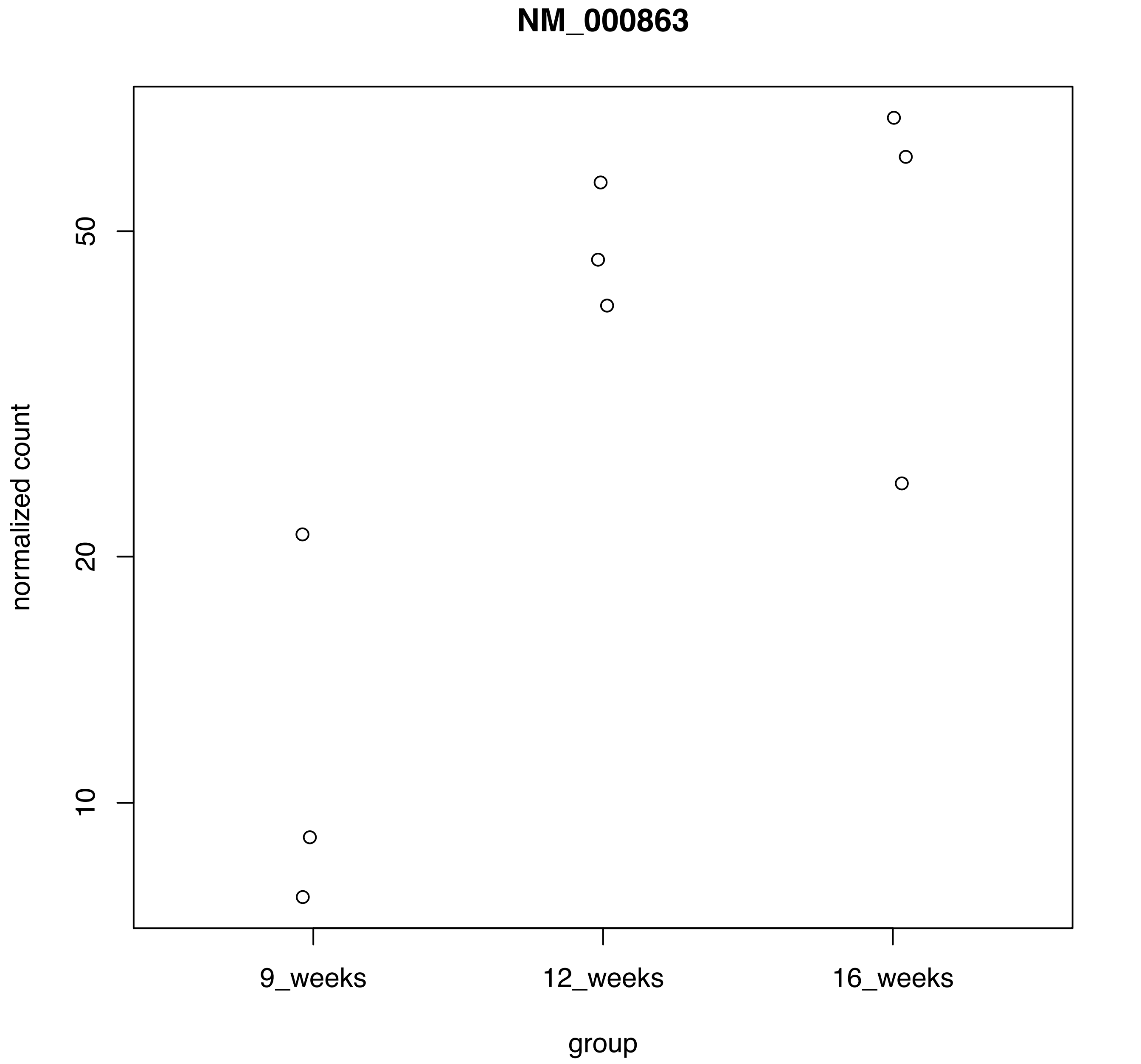
NM_000863
normalized count
50
20
10
9_weeks
12_weeks
16_weeks
group

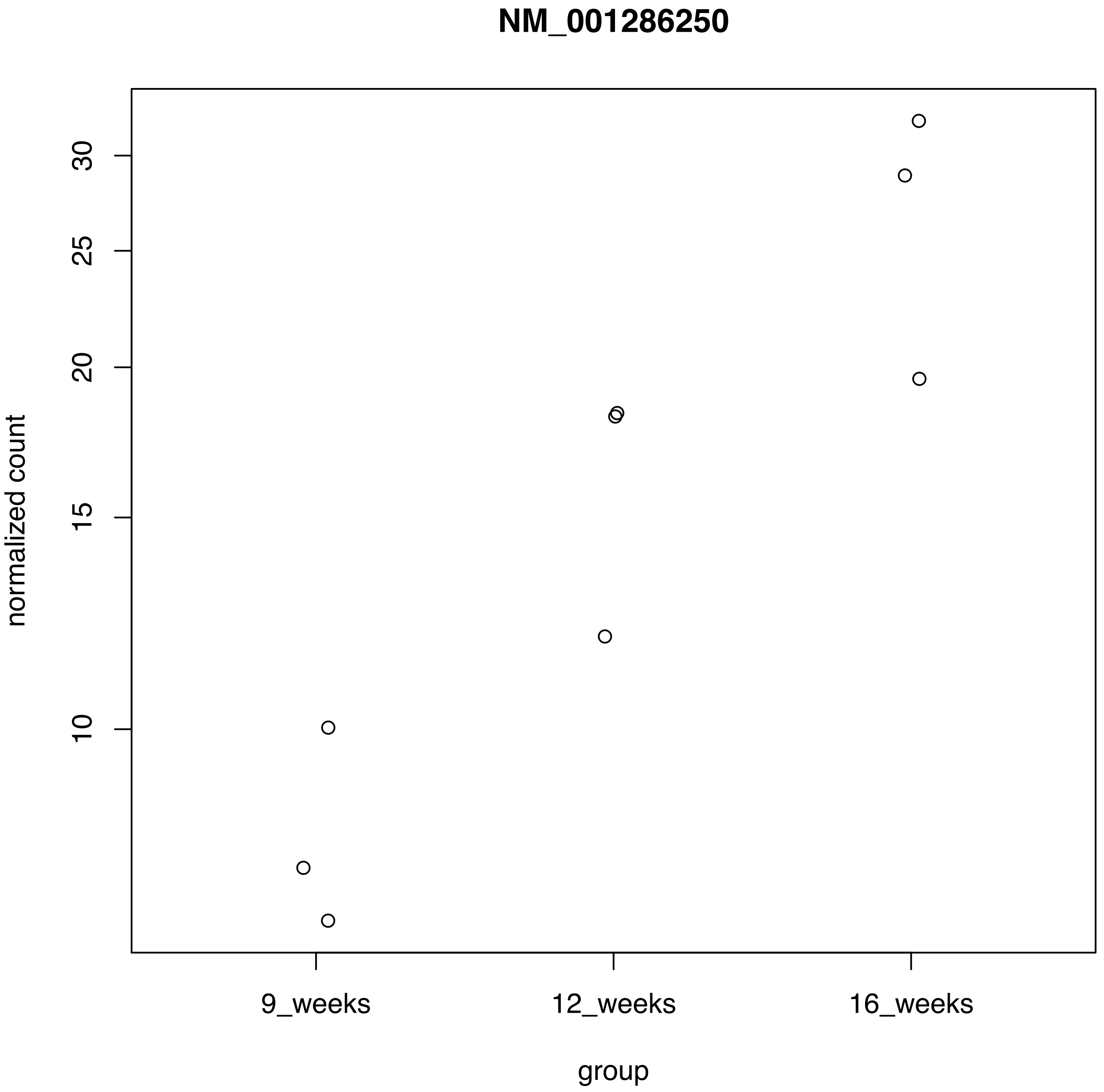

NM_001286250
normalized count
10
15
20
25
30
9_weeks
12_weeks
16_weeks
group

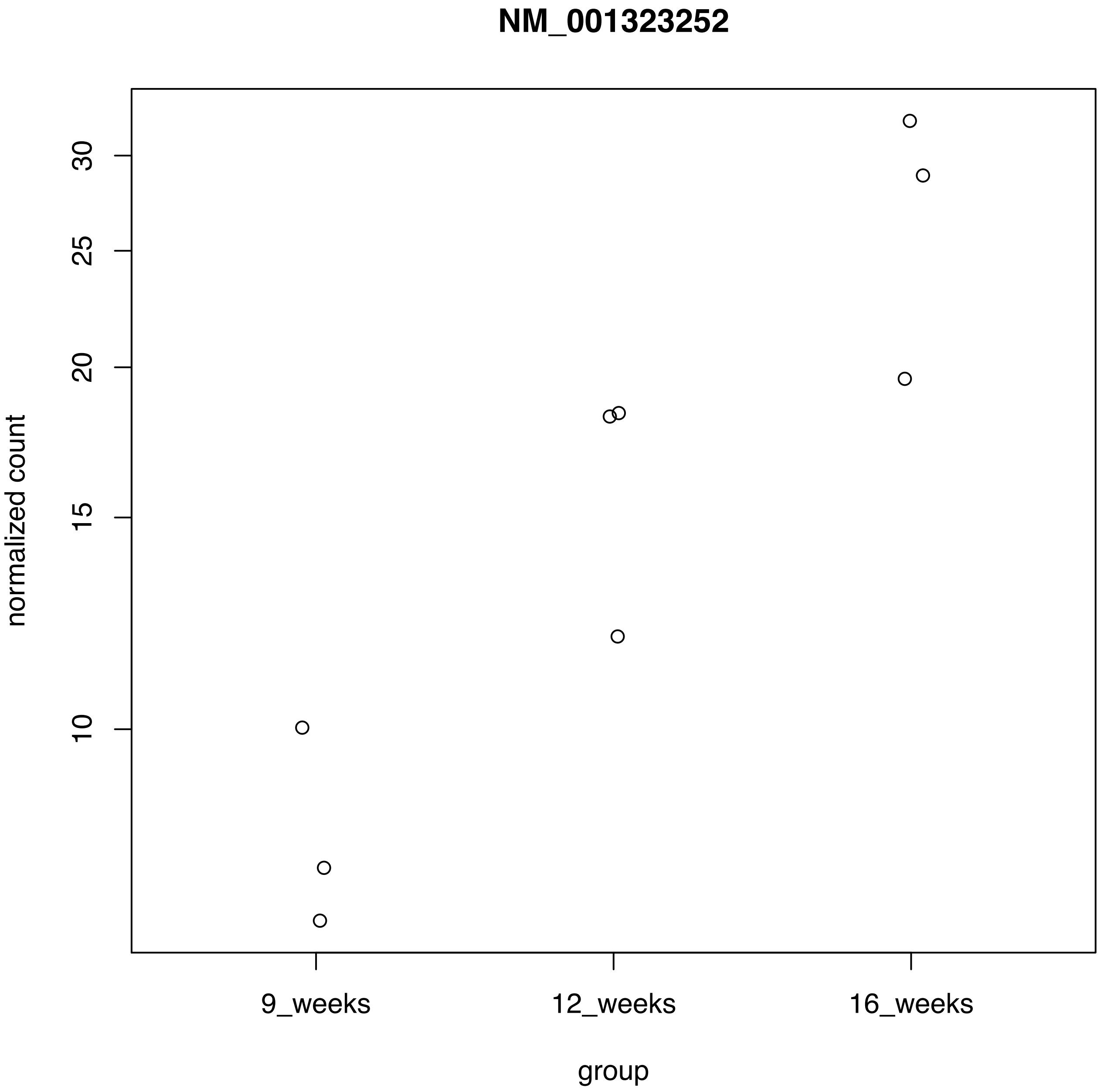
NM_001323252
normalized count
30
25
20
15
10
9_weeks
12_weeks
16_weeks
group

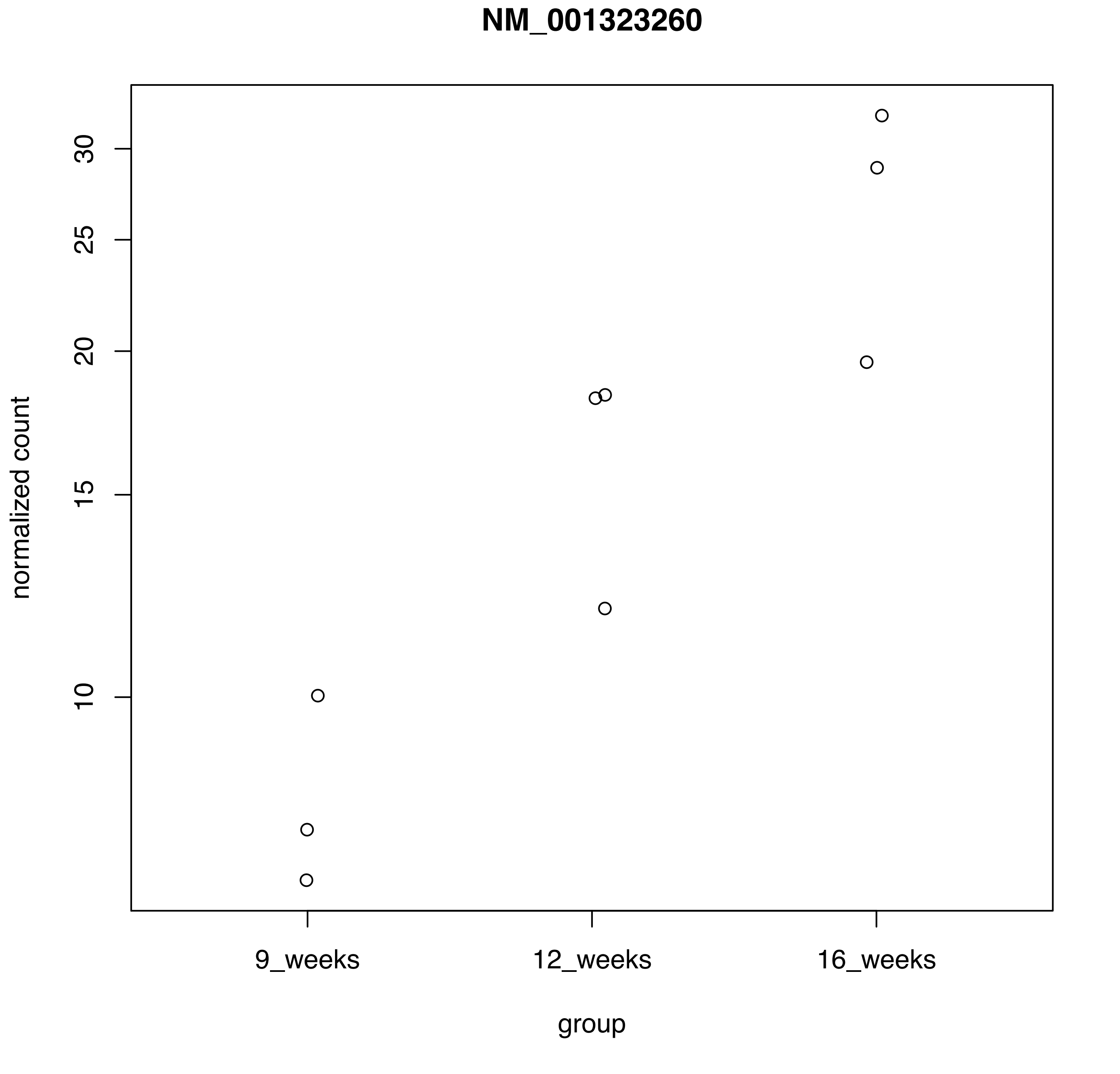
NM_001323260
normalized count
10
15
20
25
30
9_weeks
12_weeks
16_weeks
group

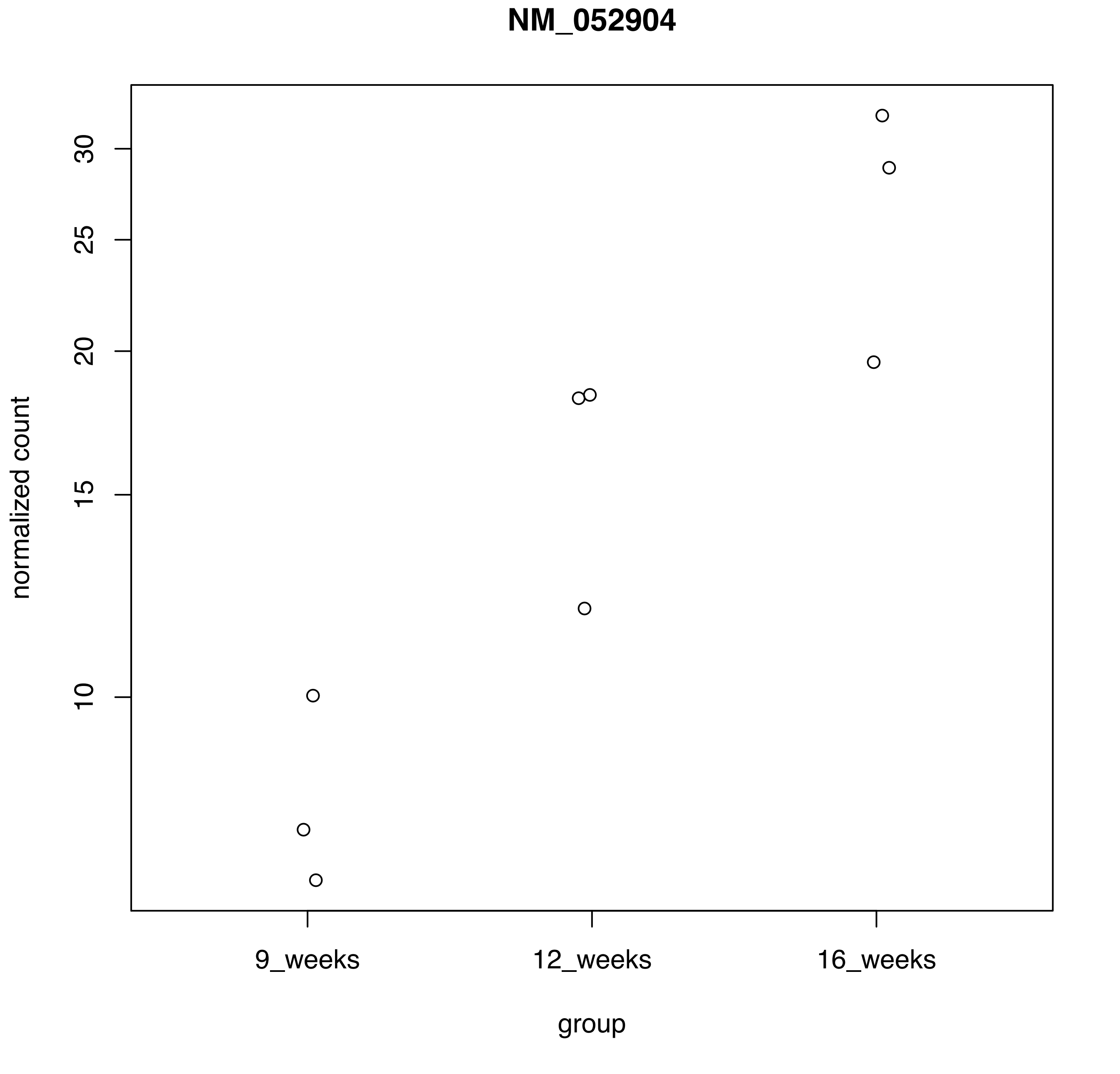
NM_052904
normalized count
30
25
20
15
10
9_weeks
12_weeks
16_weeks
group

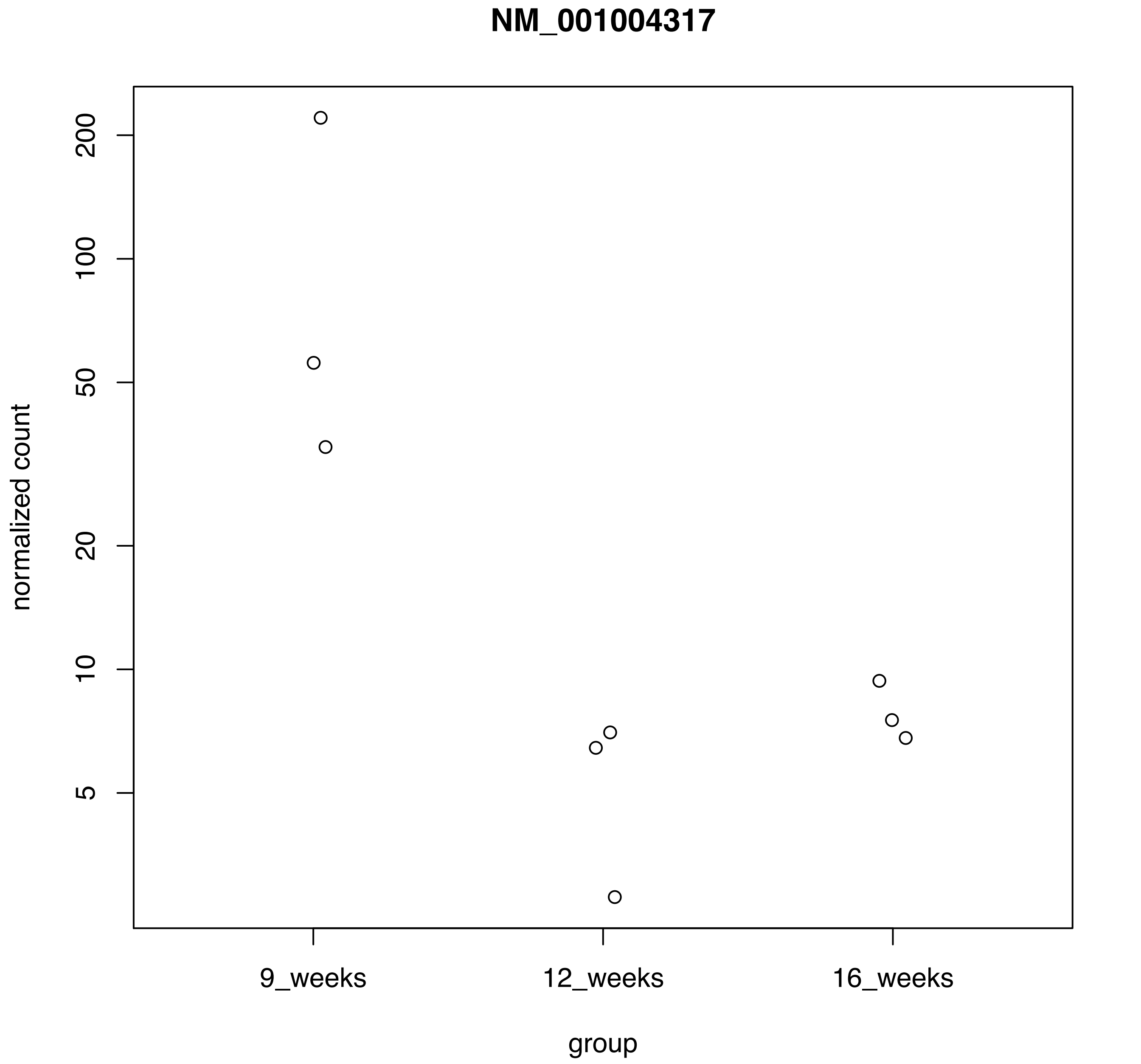

NM_001004317
normalized count
200
100
50
20
10
5
9_weeks
12_weeks
16_weeks
group

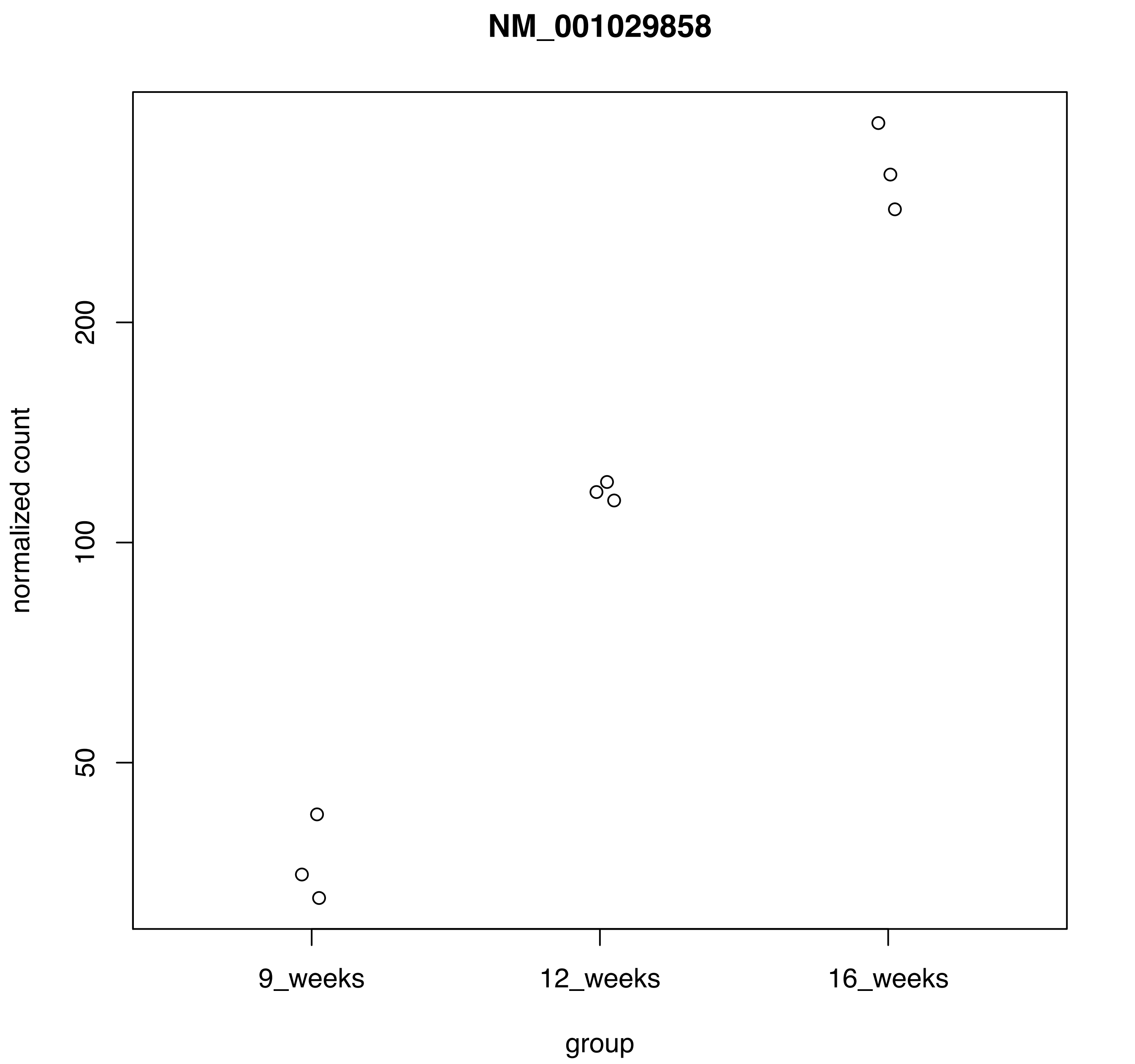
NM_001029858
normalized count
200
100
50
9_weeks
12_weeks
16_weeks
group

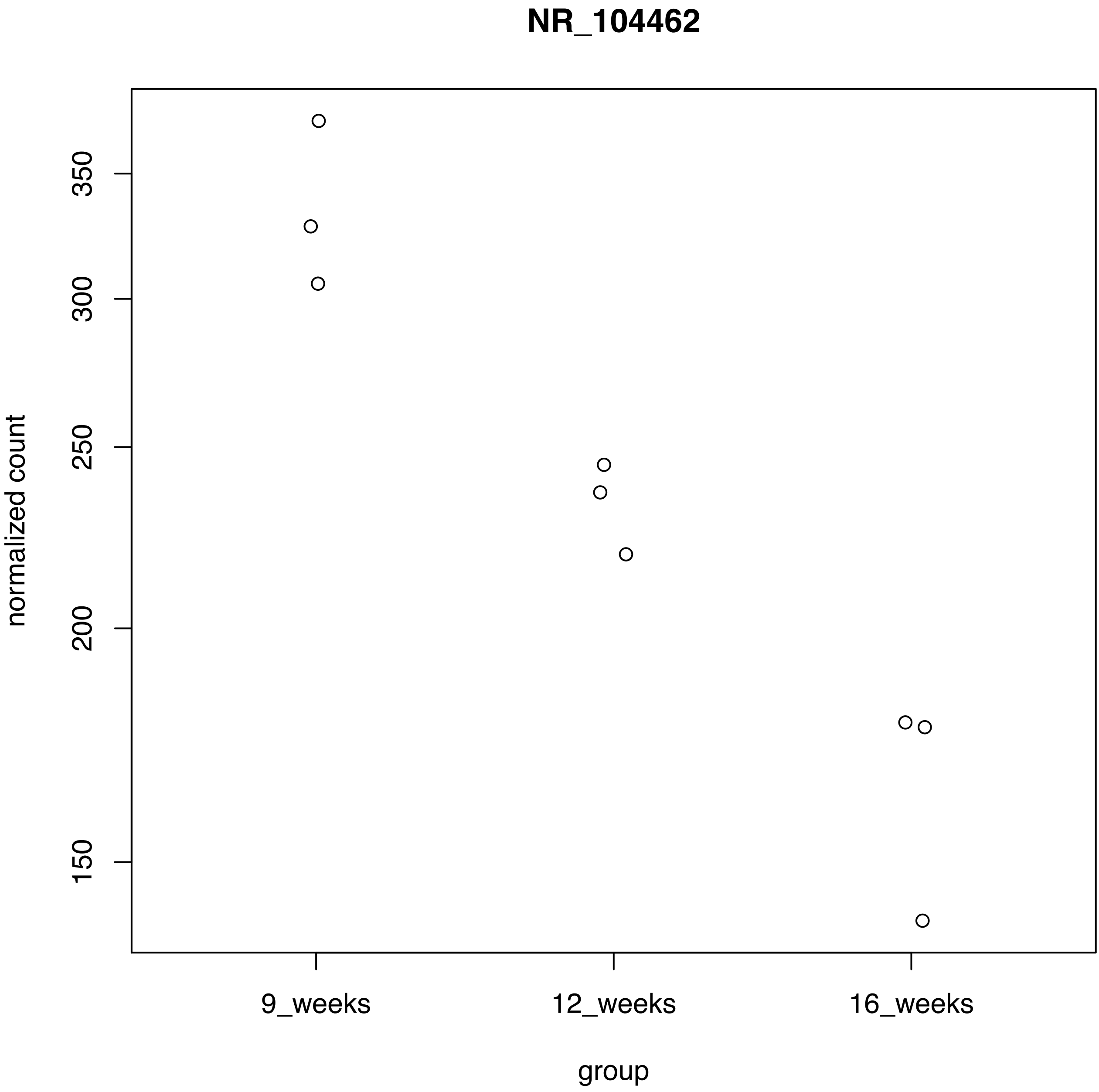
NR_104462
normalized count
350
300
250
200
150
9_weeks
12_weeks
16_weeks
group

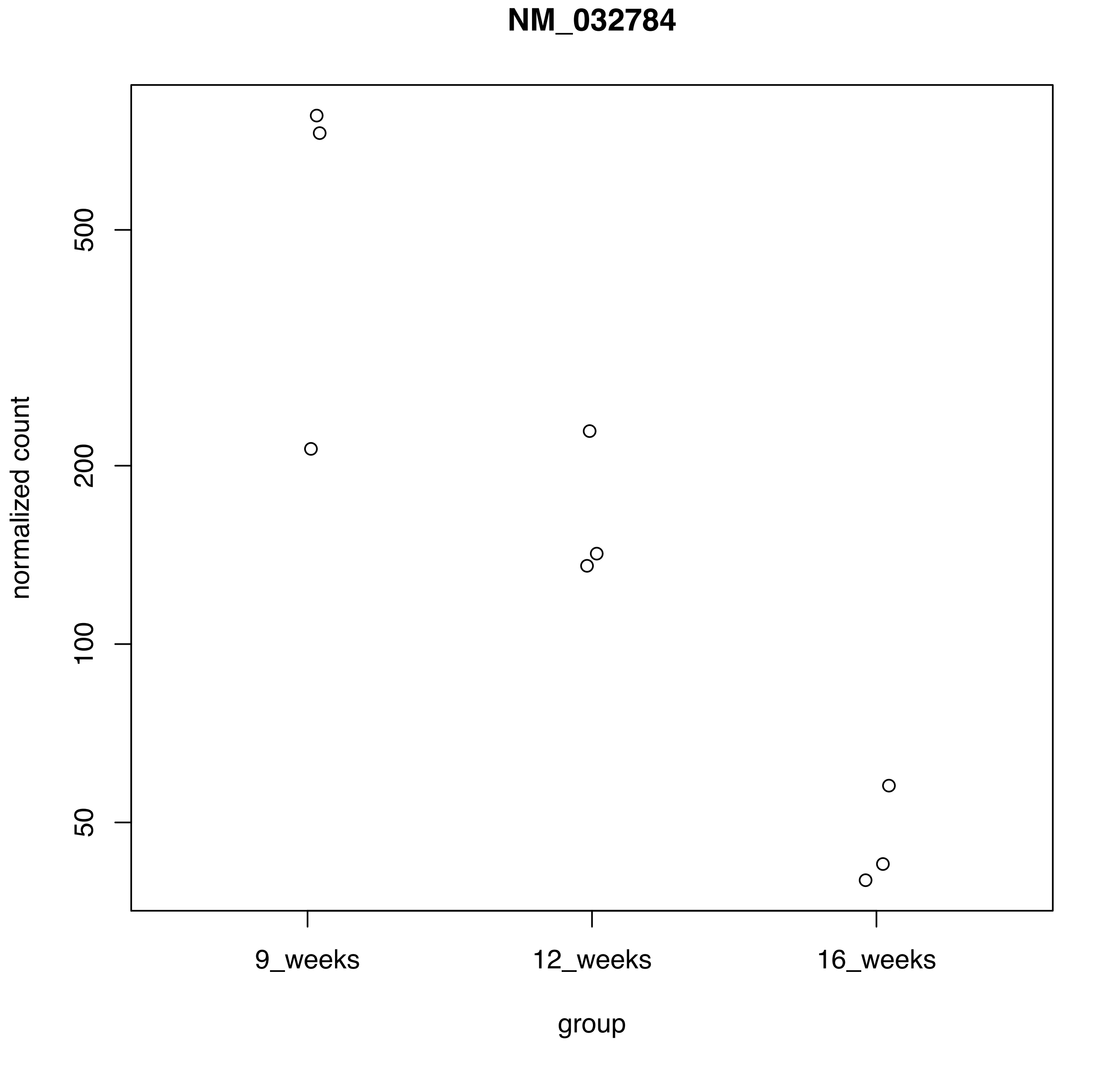
NM_032784
normalized count
500
200
100
50
9_weeks
12_weeks
16_weeks
group

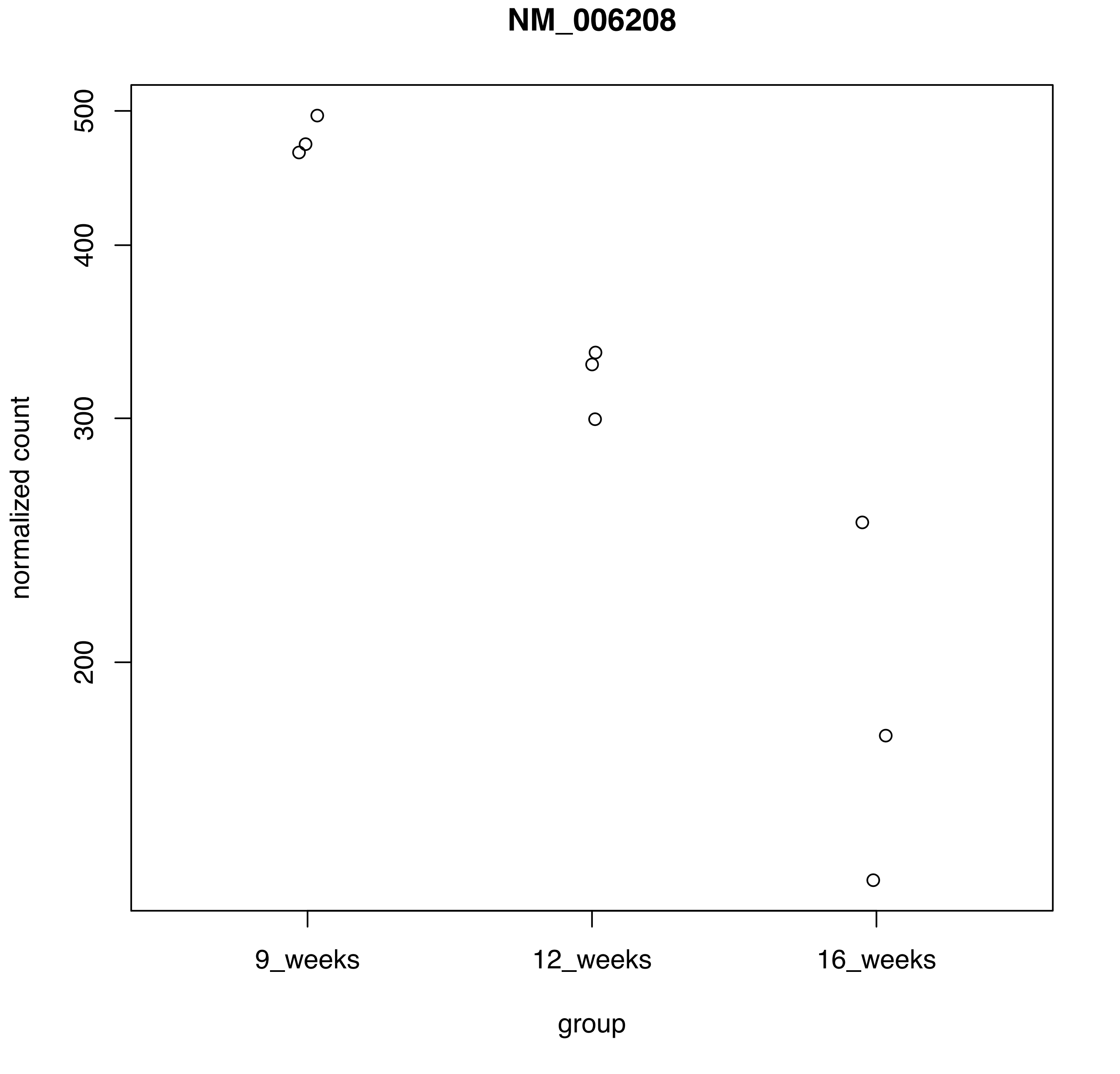

NM_006208
normalized count
500
400
300
200
9_weeks
12_weeks
16_weeks
group

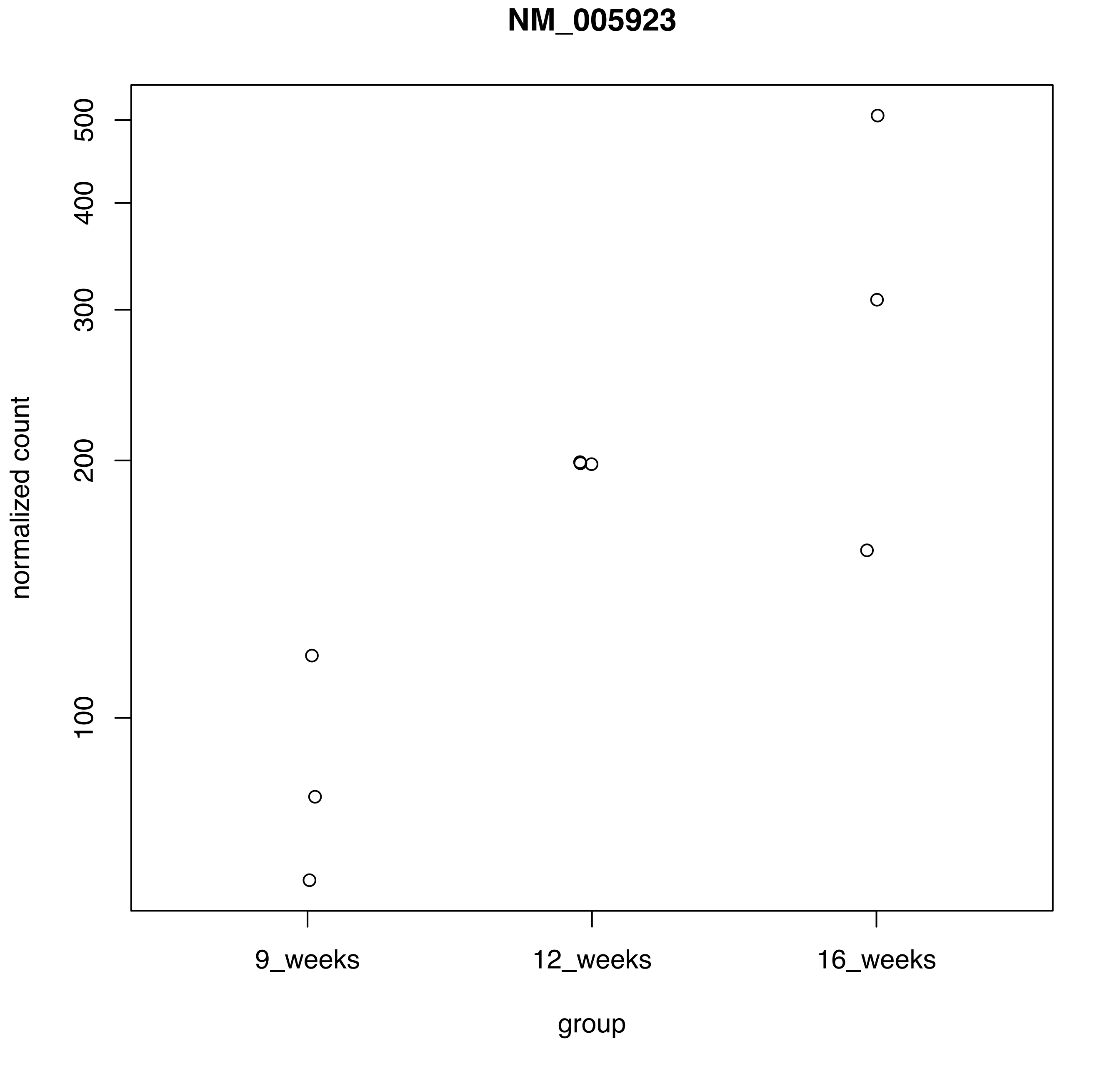

NM_005923
normalized count
500
400
300
200
100
9_weeks
12_weeks
16_weeks
group

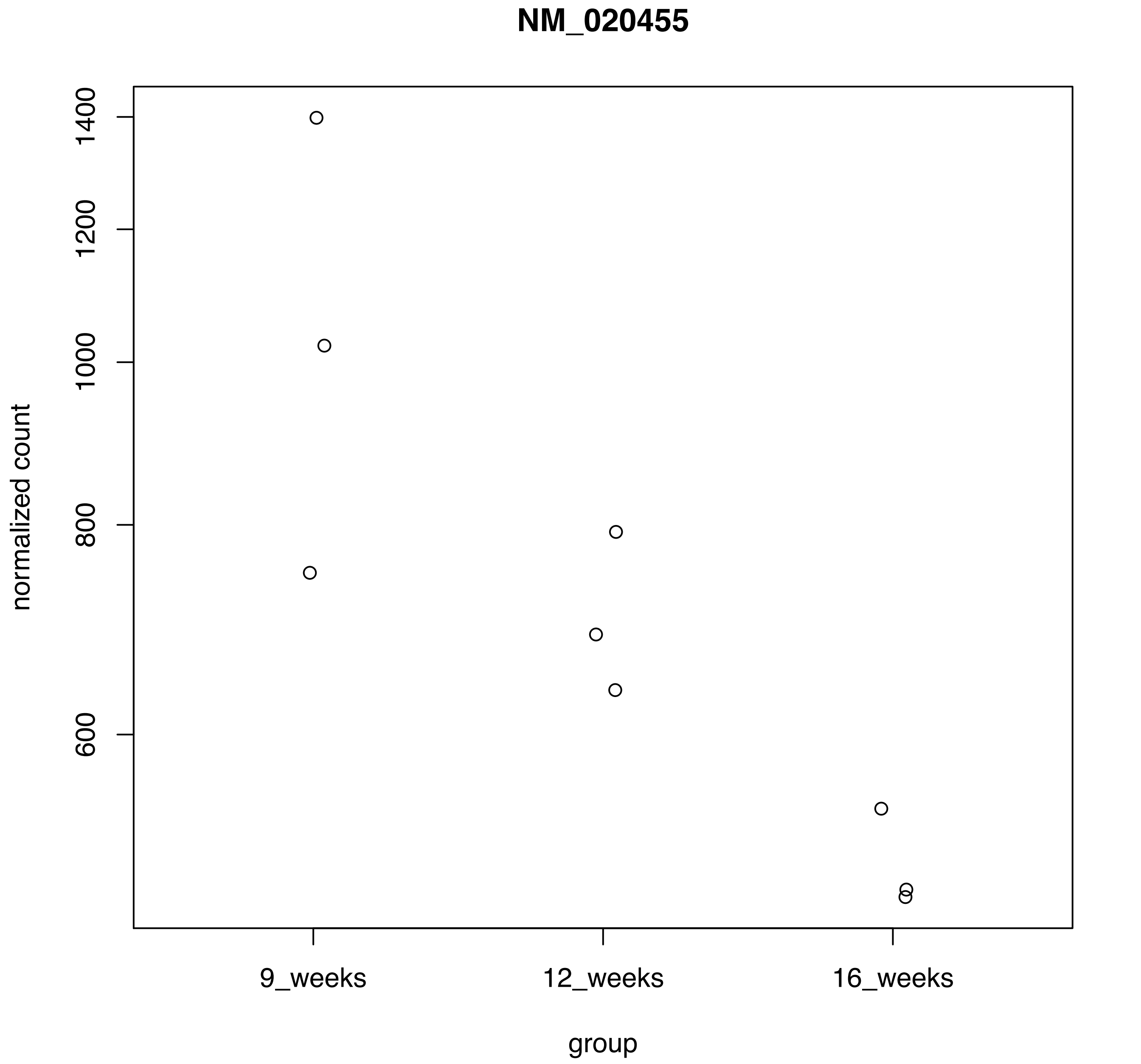

NM_020455
normalized count
1400
1200
1000
800
600
9_weeks
12_weeks
16_weeks
group

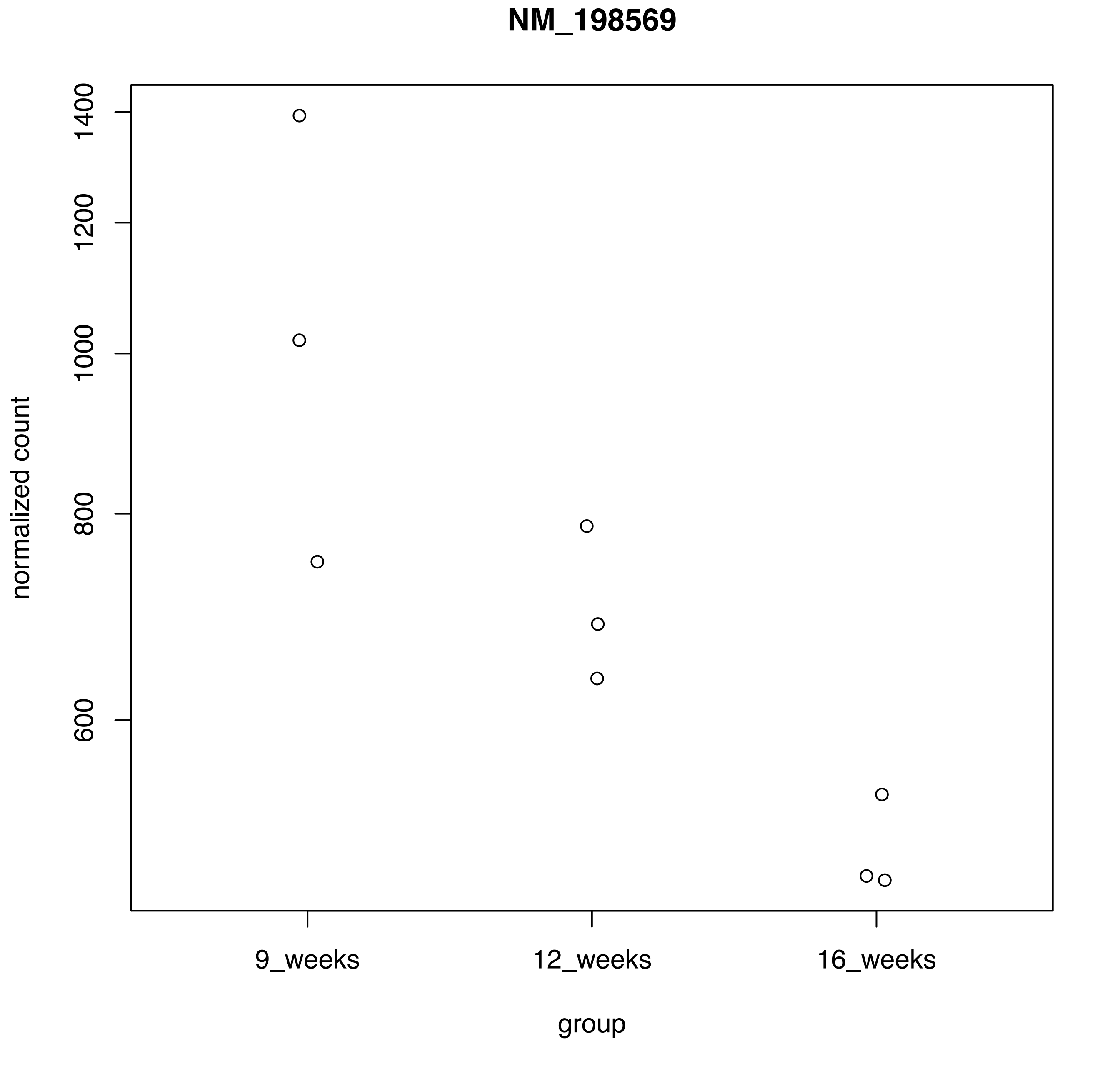

NM_198569
normalized count
1400
1200
1000
800
600
9_weeks
12_weeks
16_weeks
group

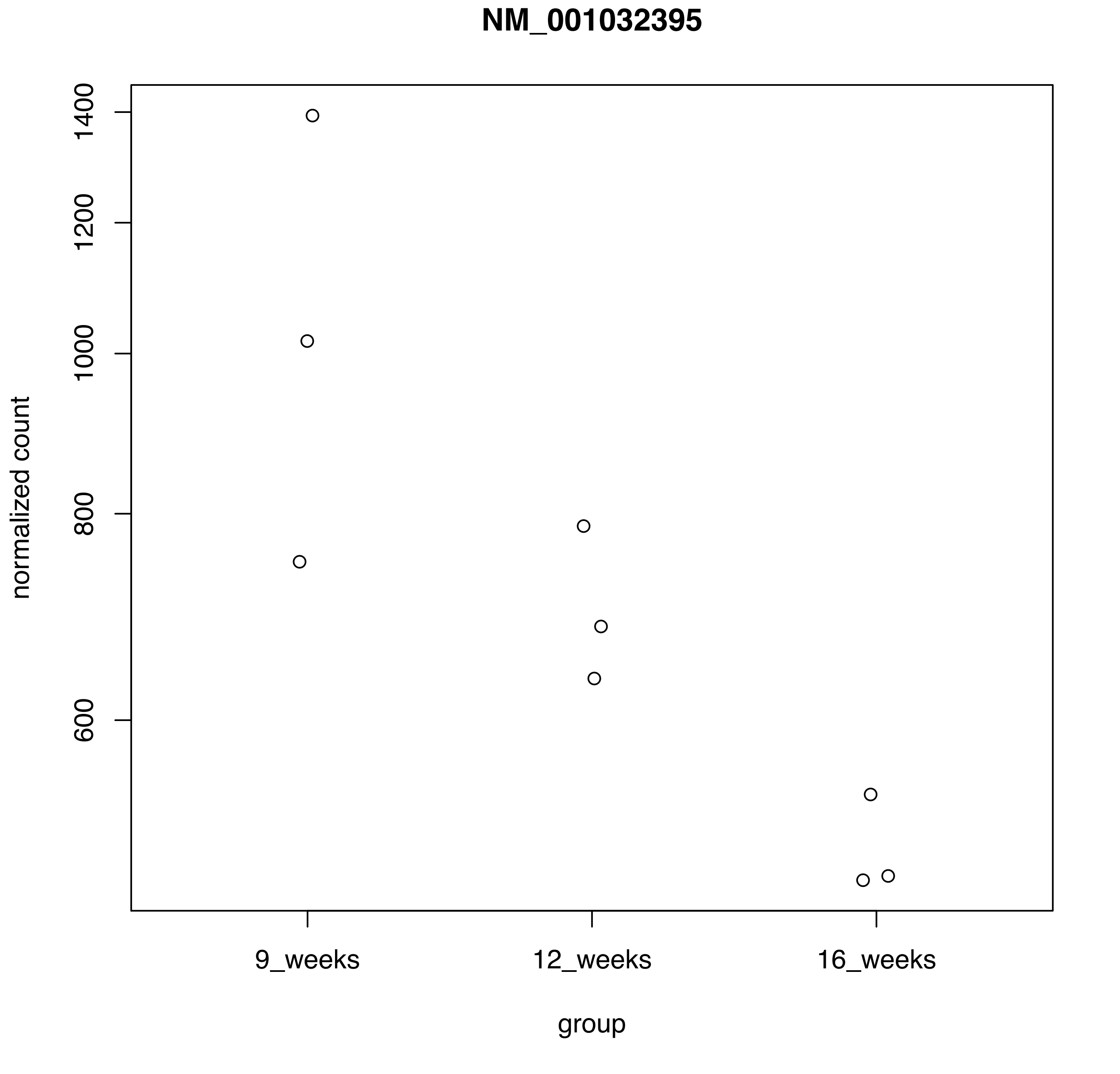
NM_001032395
normalized count
1400
1200
1000
800
600
9_weeks
12_weeks
16_weeks
group

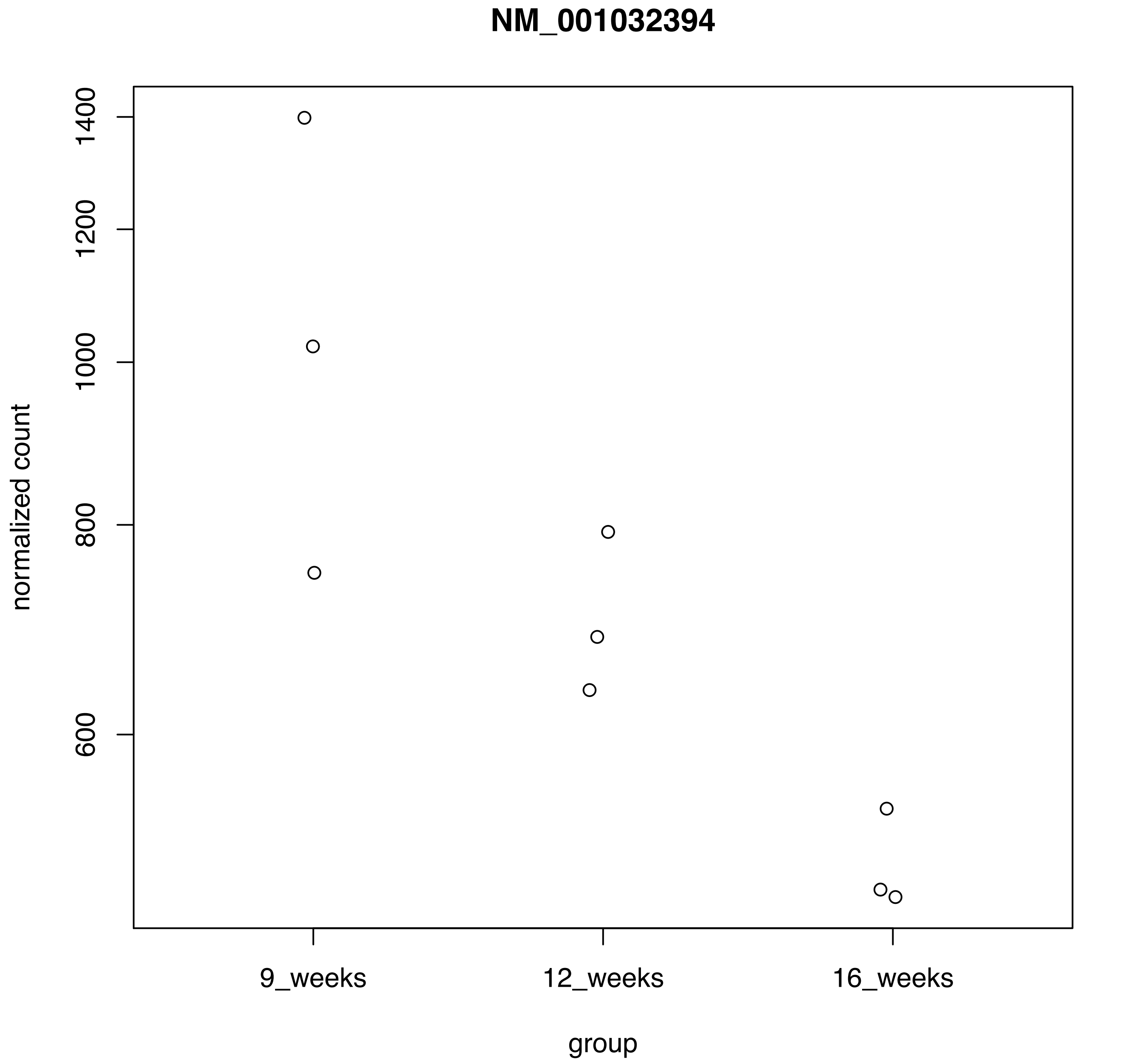

NM_001032394
normalized count
1400
1200
1000
800
600
9_weeks
12_weeks
16_weeks
group

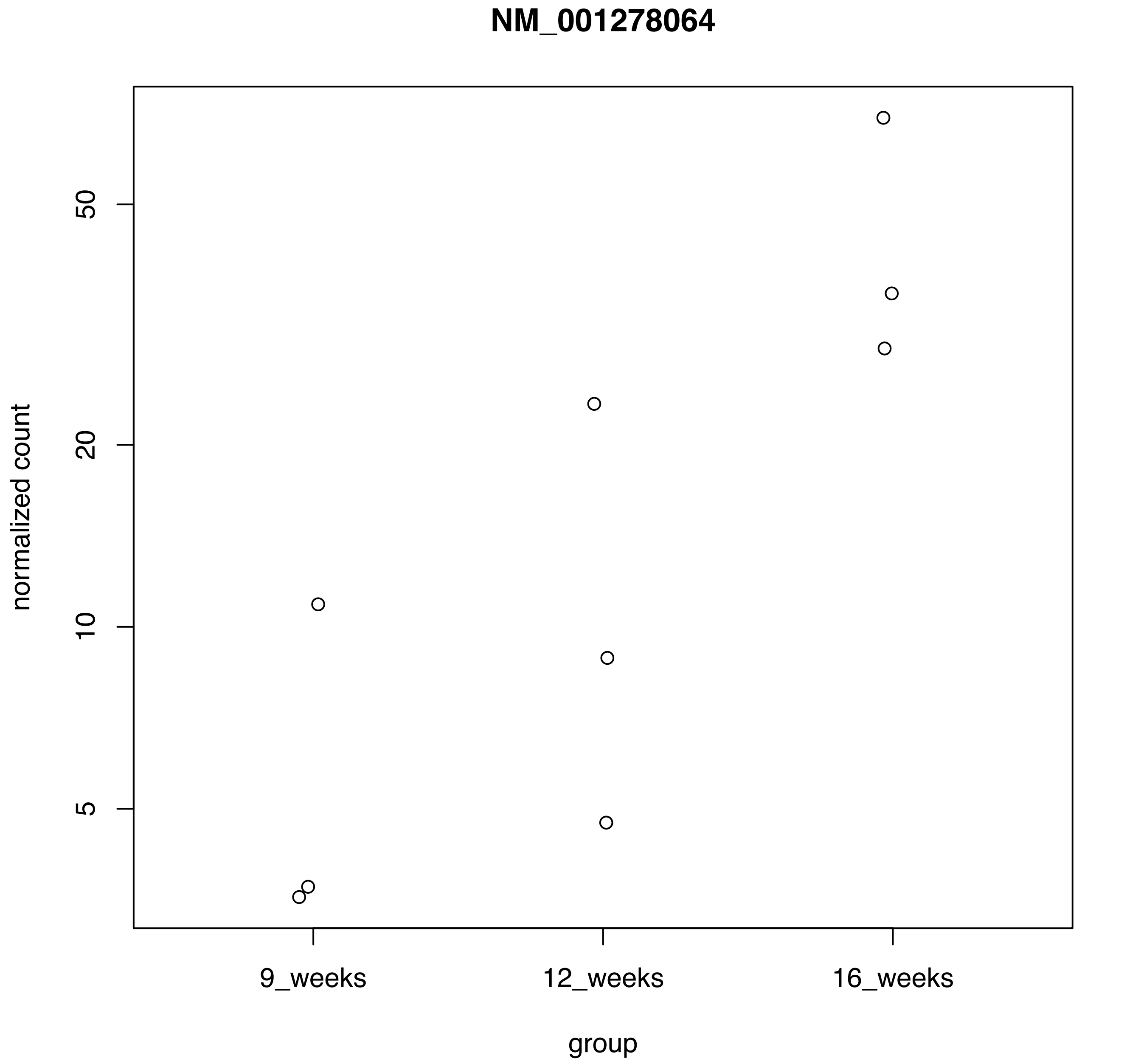
NM_001278064
normalized count
50
20
10
5
9_weeks
12_weeks
16_weeks
group

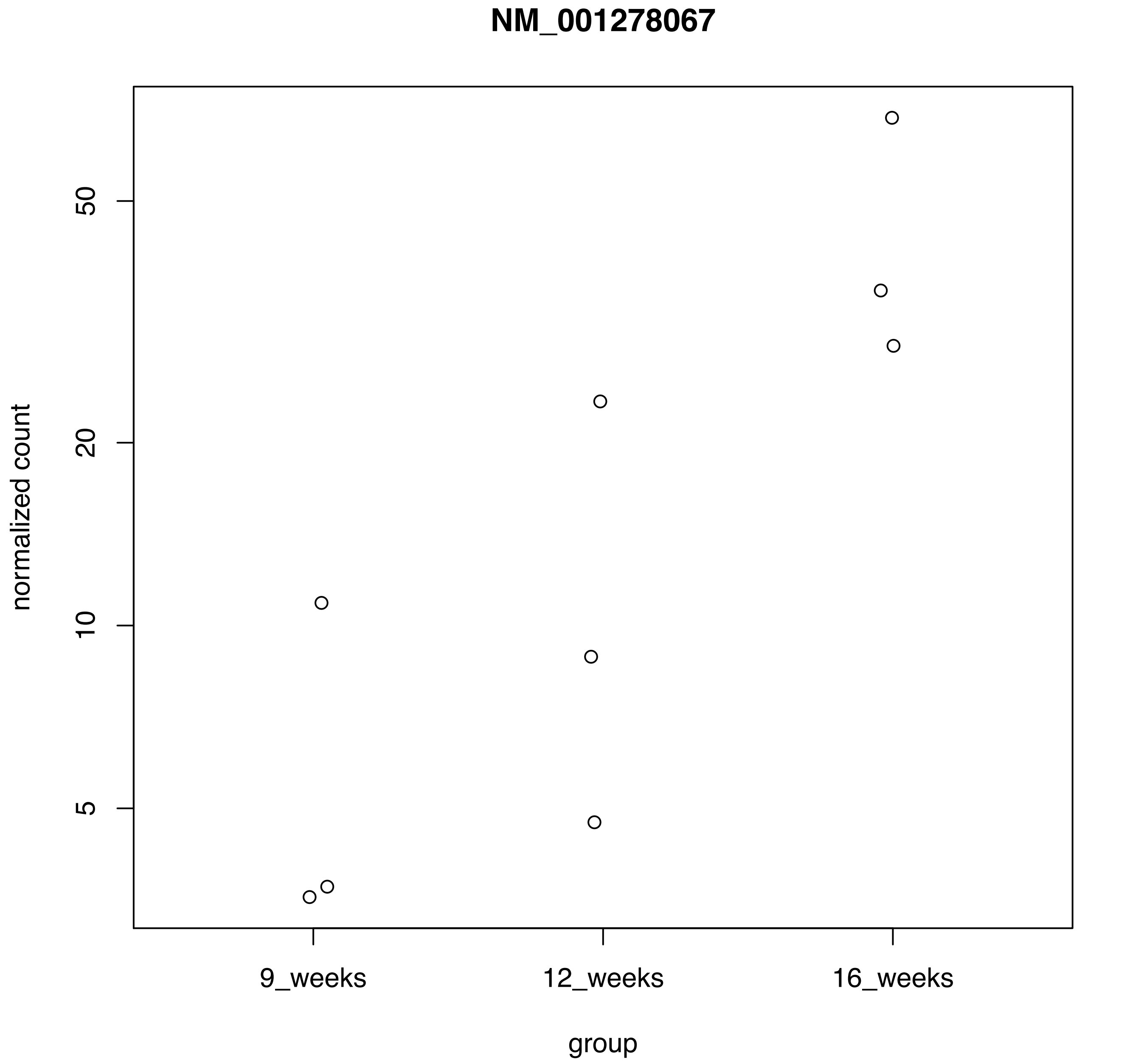

NM_001278067
normalized count
50
20
10
5
9_weeks
12_weeks
16_weeks
group

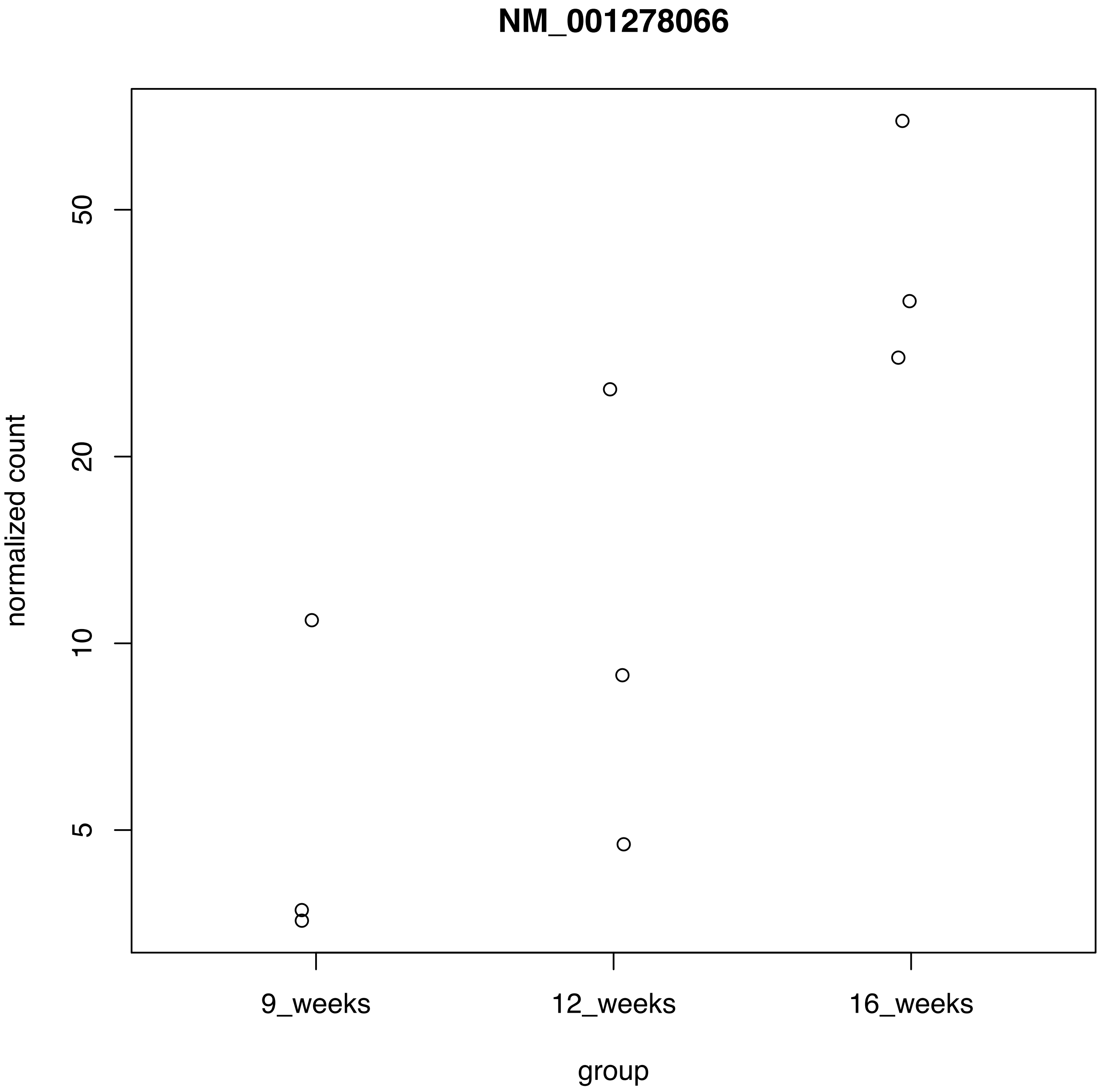
NM_001278066
normalized count
50
20
10
5
9_weeks
12_weeks
16_weeks
group

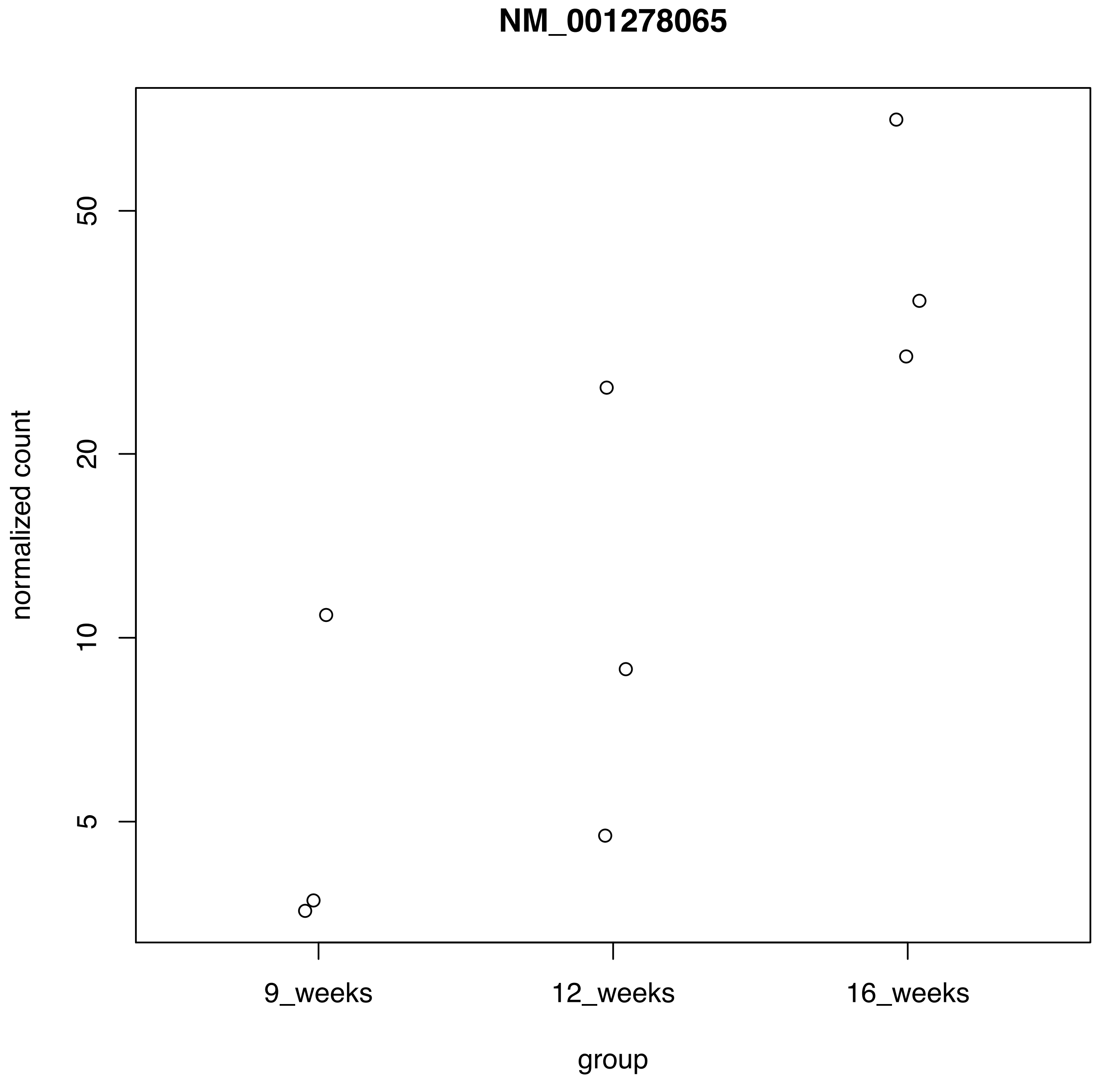

NM_001278065
normalized count
50
20
10
5
9_weeks
12_weeks
16_weeks
group

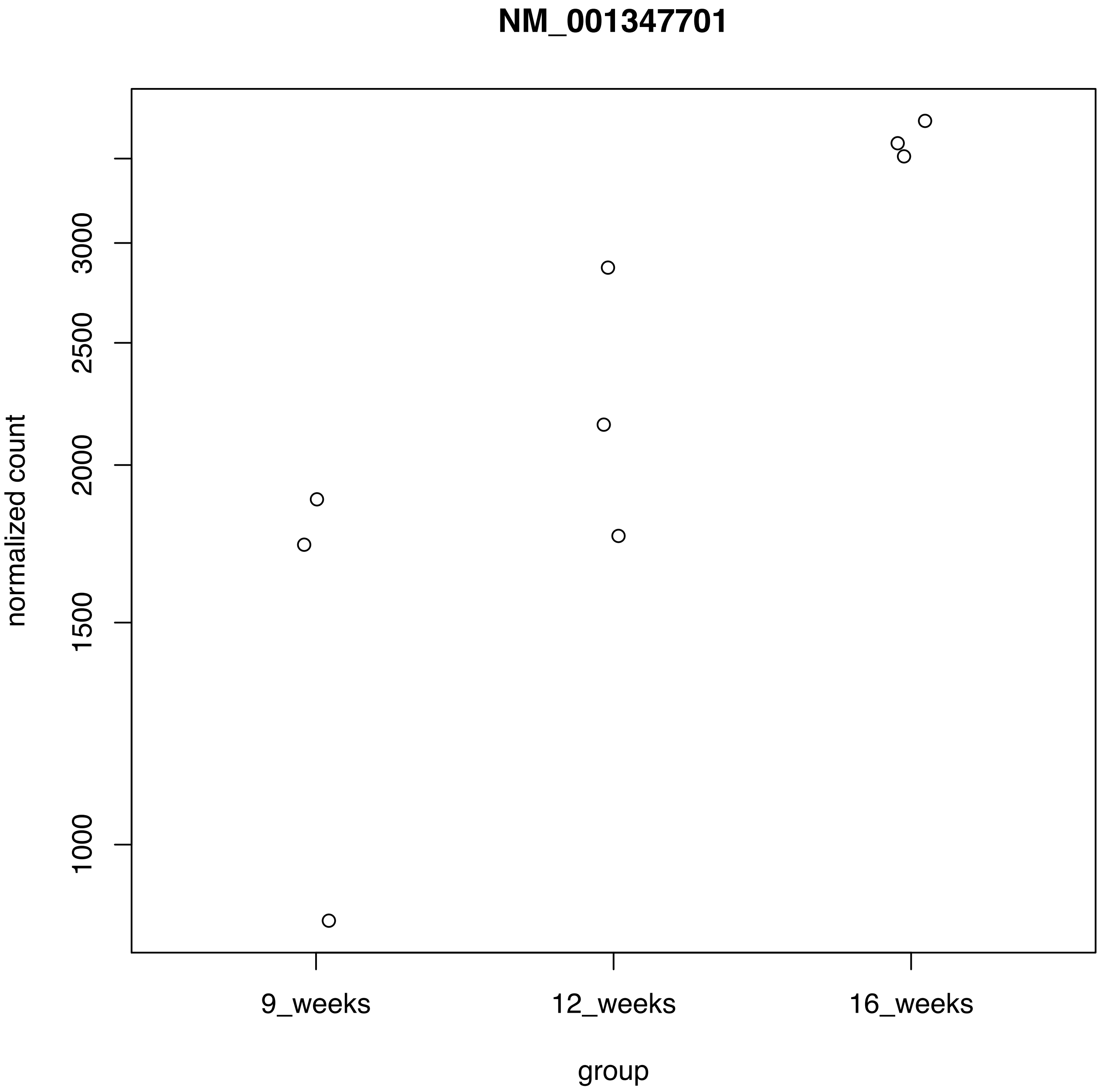

NM_001347701
normalized count
1000
1500
2000
2500
3000
9_weeks
12_weeks
16_weeks
group

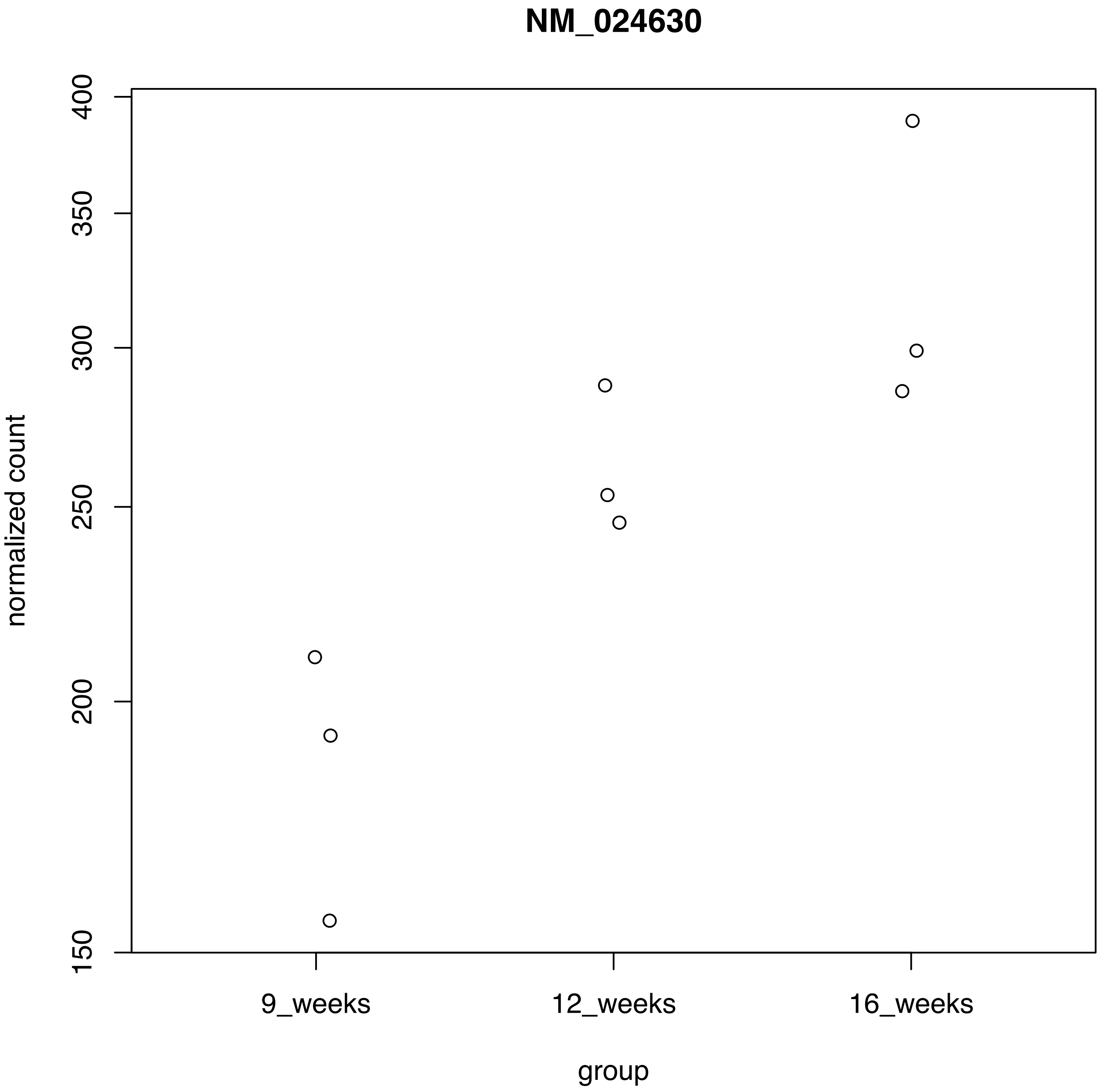
NM_024630
normalized count
400
350
300
250
200
150
9_weeks
12_weeks
16_weeks
group

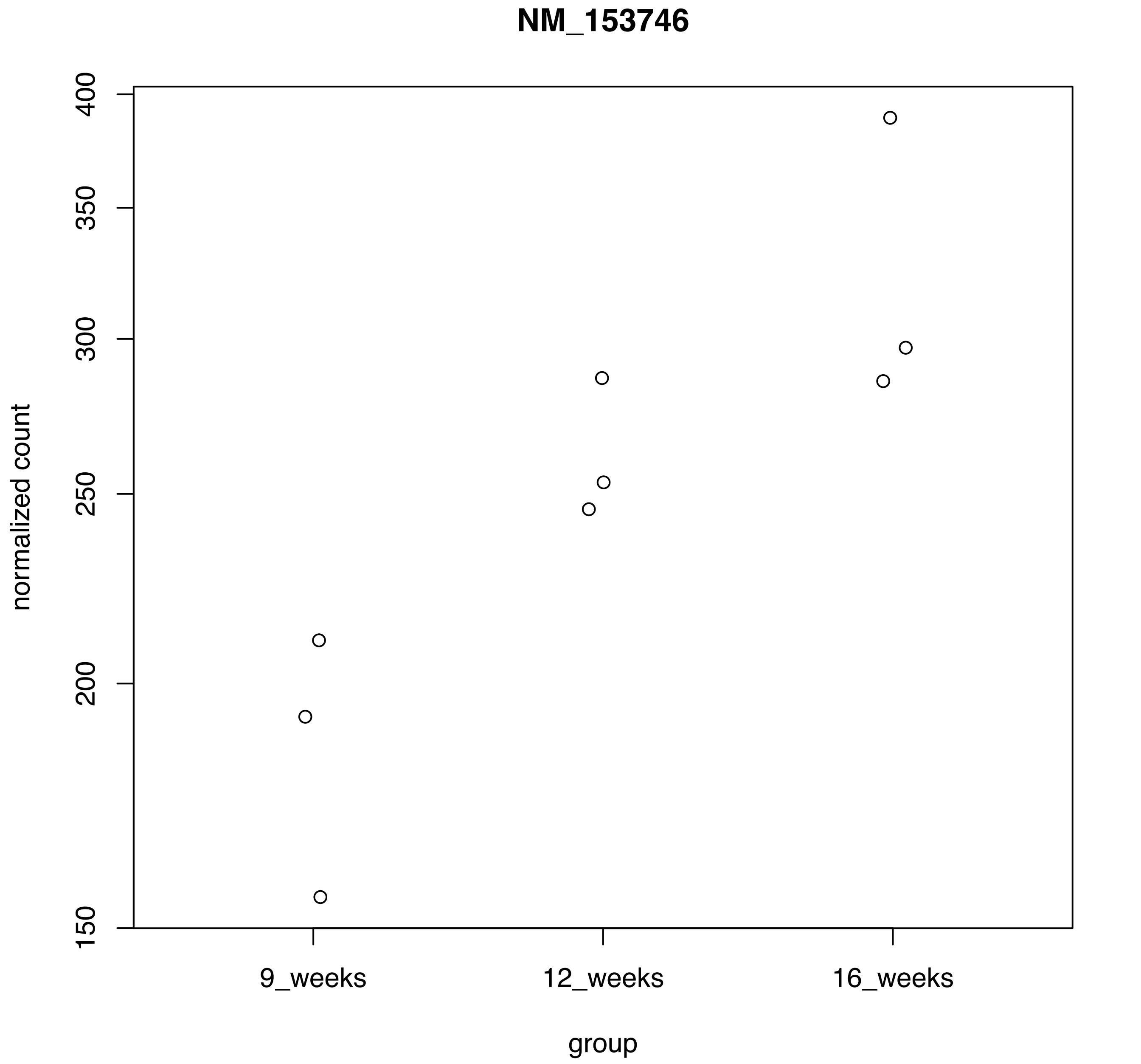
NM_153746
normalized count
400
350
300
250
200
150
9_weeks
12_weeks
16_weeks
group

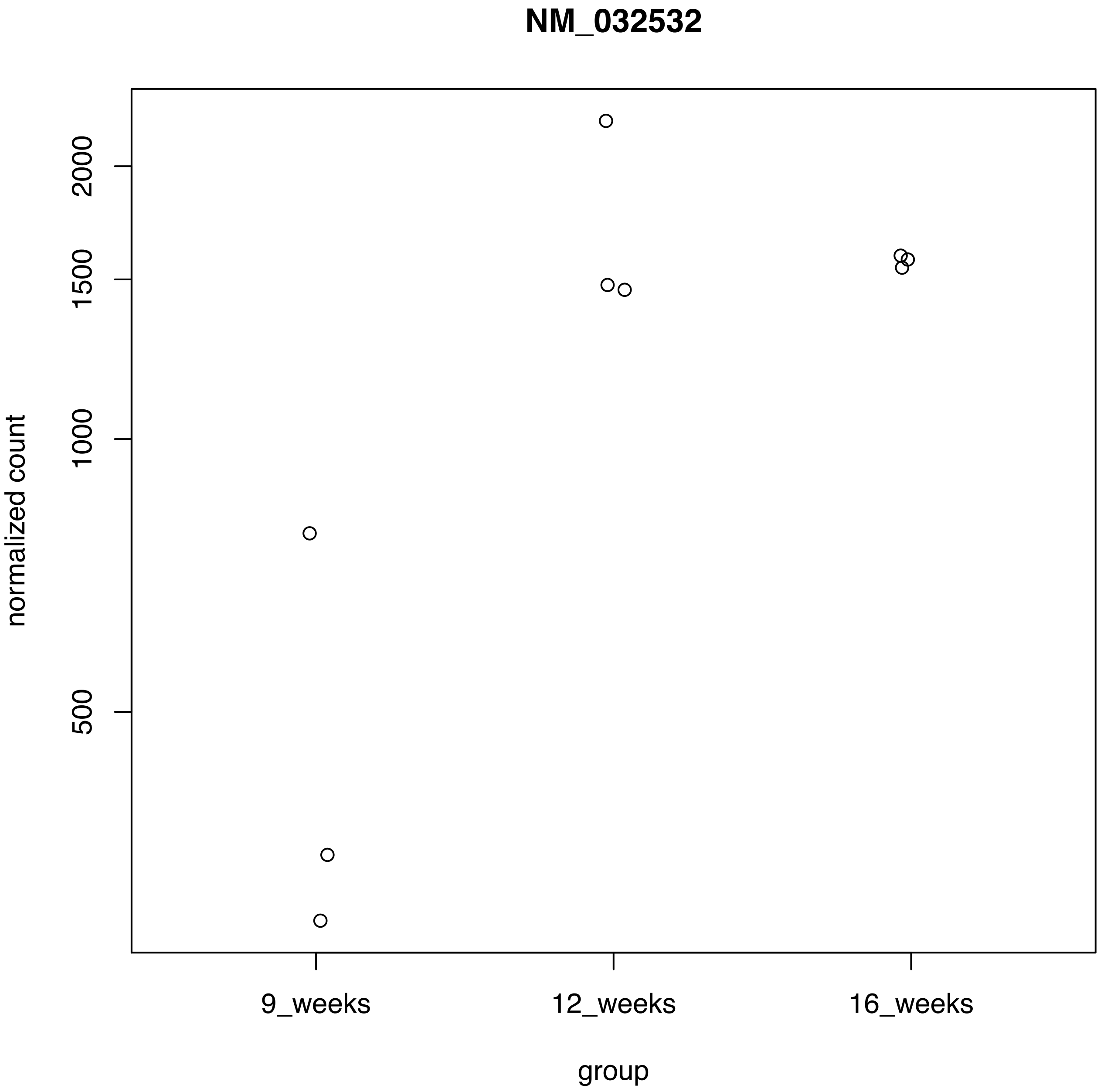
NM_032532
normalized count
500
1000
1500
2000
9_weeks
12_weeks
16_weeks
group

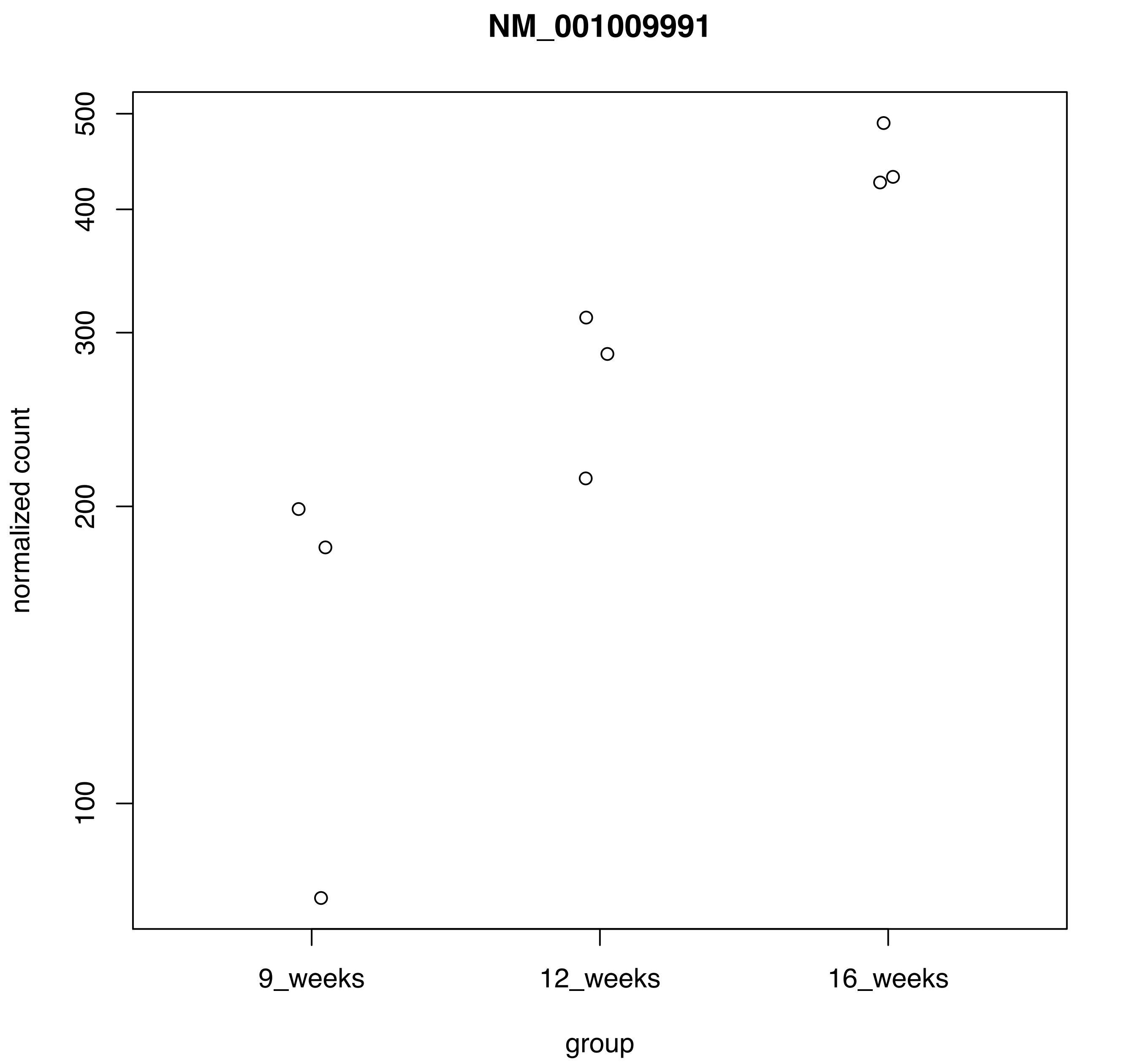

NM_001009991
normalized count
500
400
300
200
100
9_weeks
12_weeks
16_weeks
group

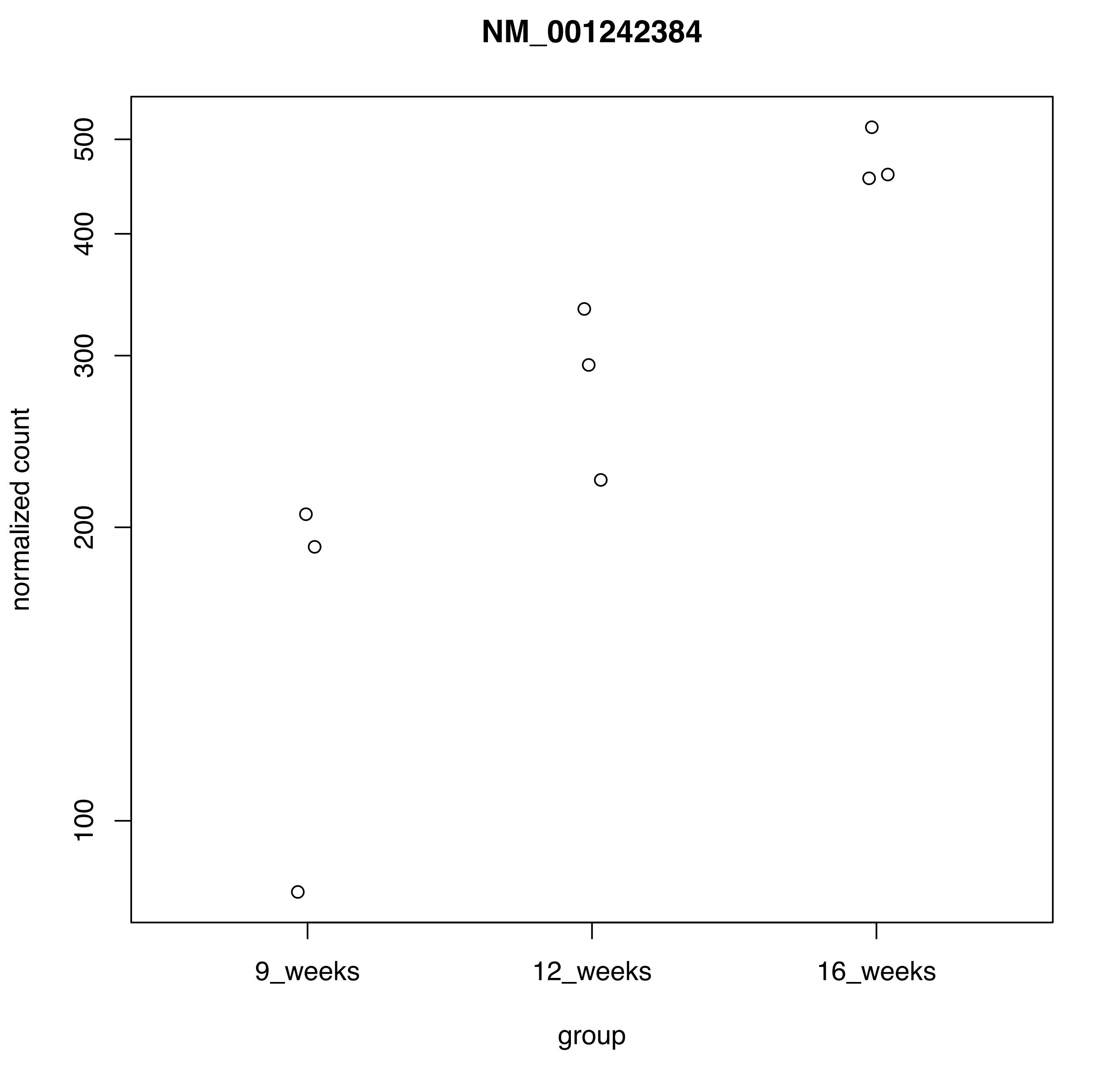
NM_001242384
normalized count
100
200
300
400
500
9_weeks
12_weeks
16_weeks
group

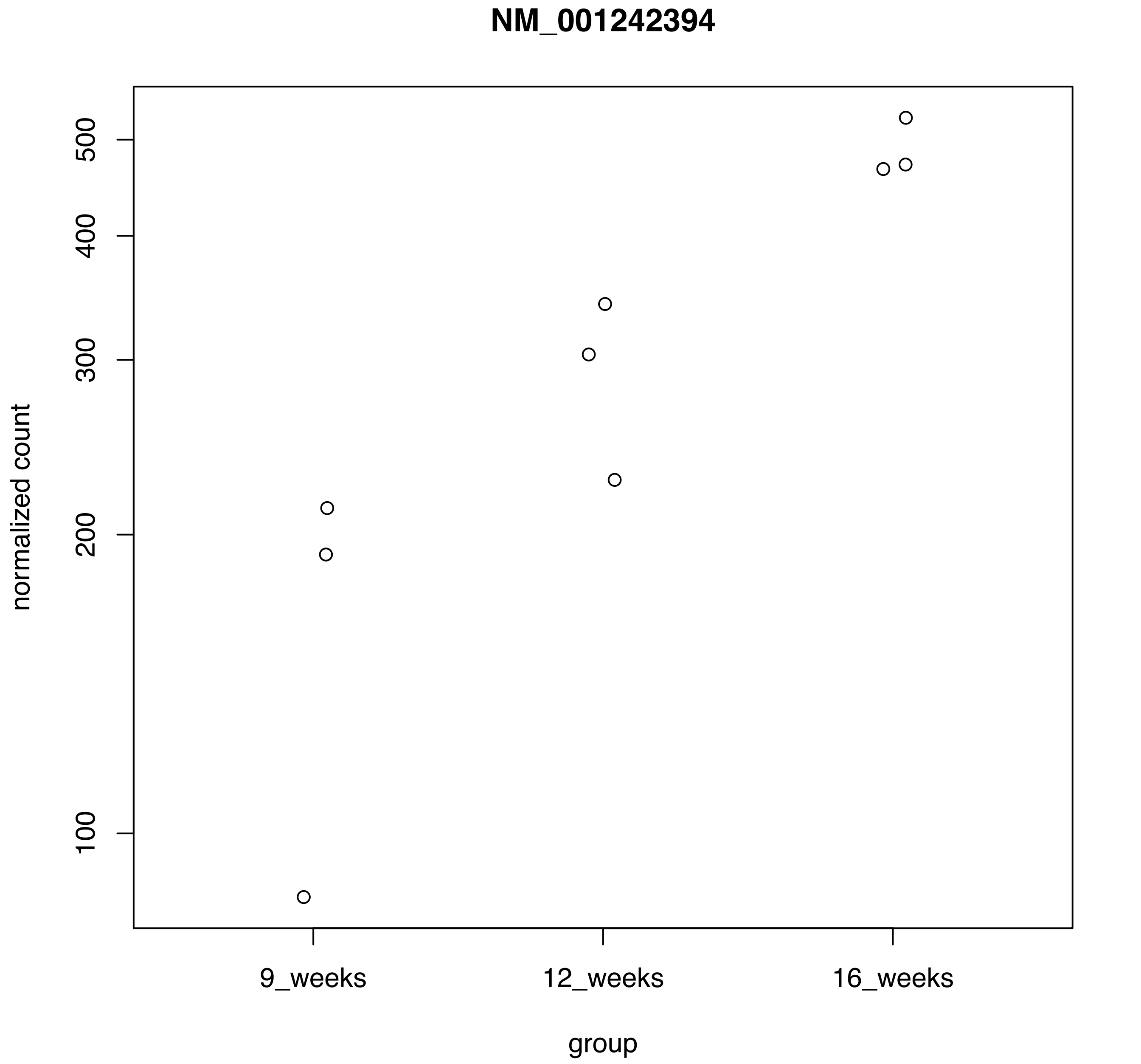

NM_001242394
normalized count
500
400
300
200
100
9_weeks
12_weeks
16_weeks
group

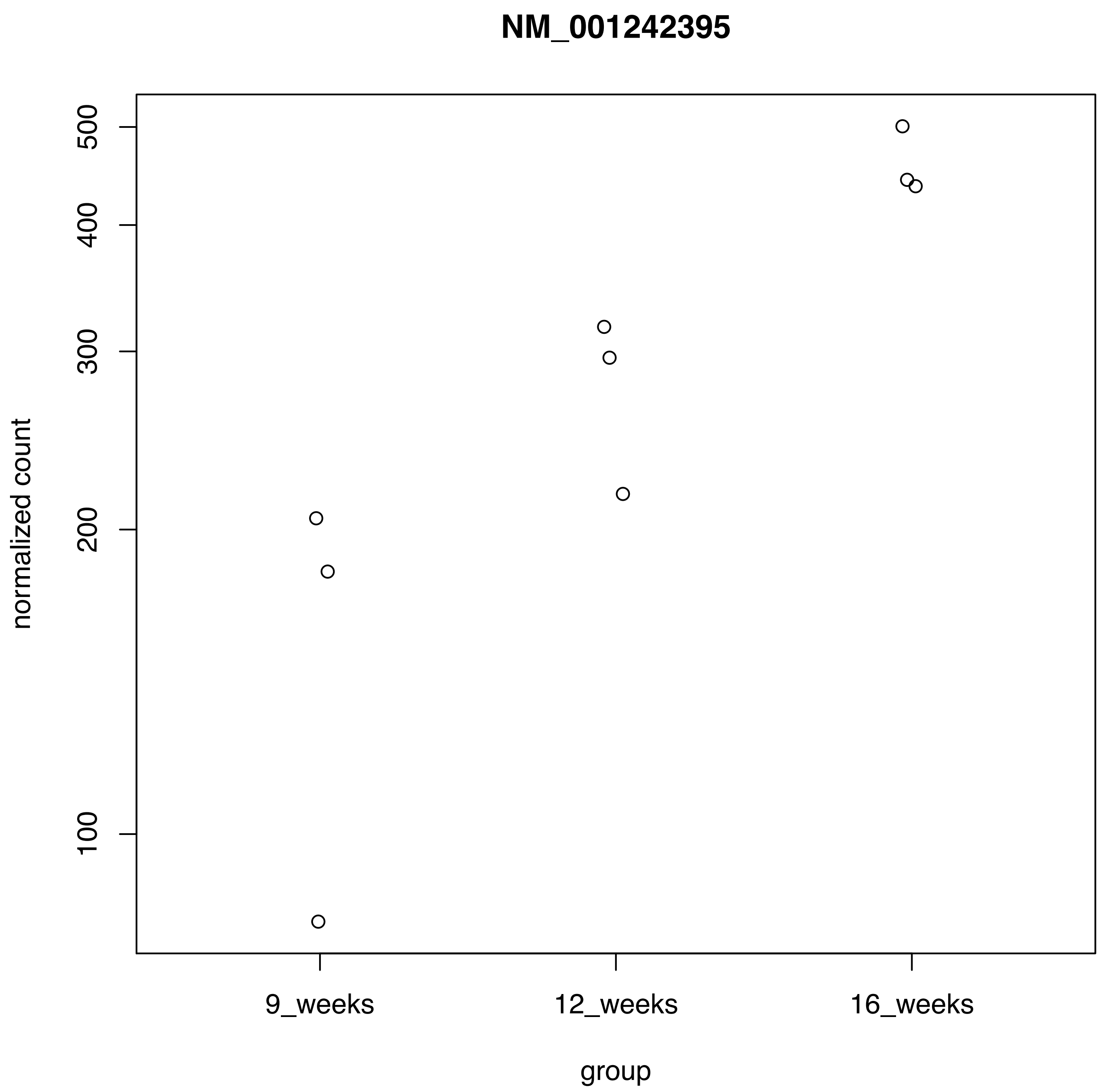

NM_001242395
normalized count
500
400
300
200
100
9_weeks
12_weeks
16_weeks
group

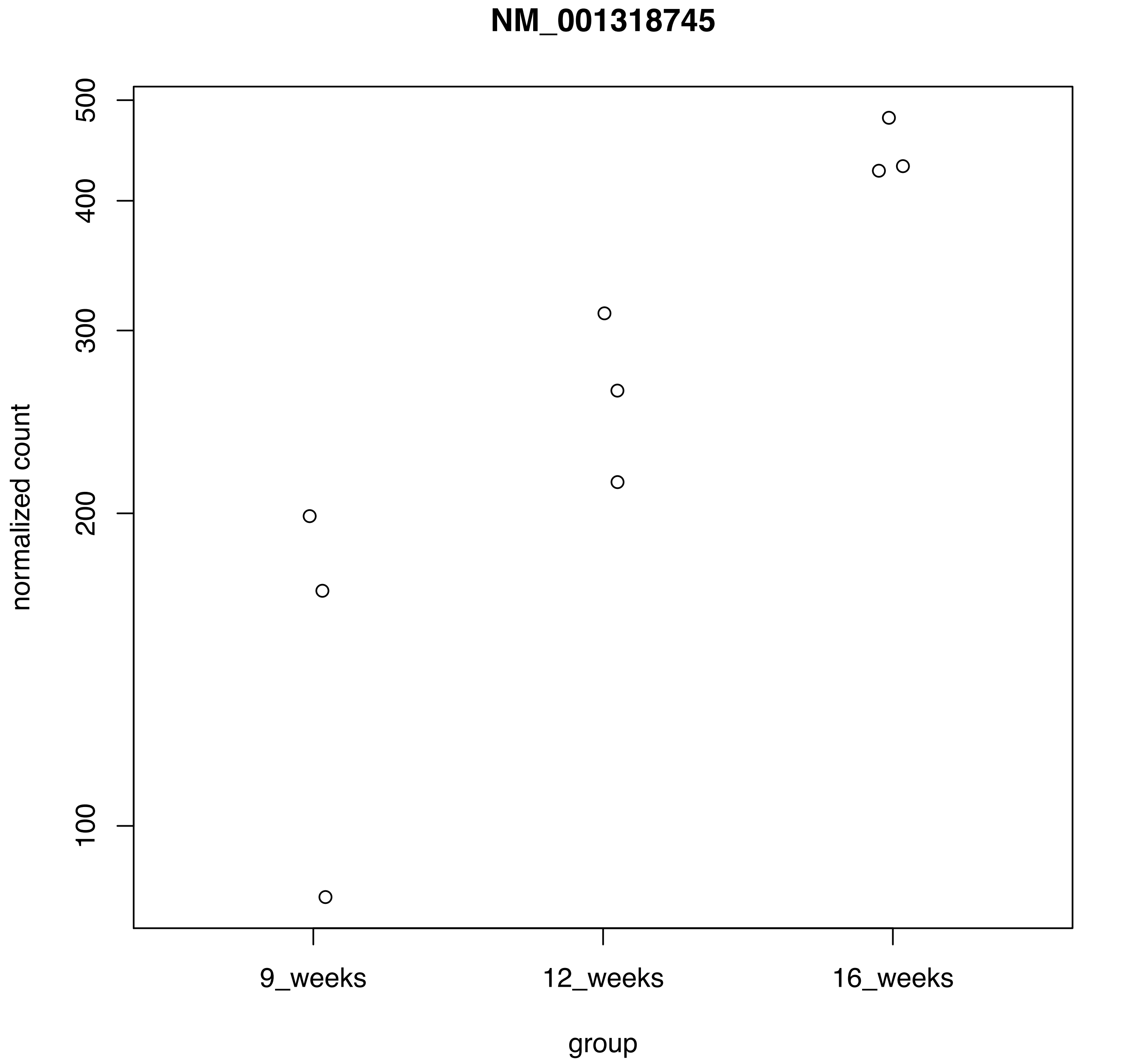
NM_001318745
normalized count
500
400
300
200
100
9_weeks
12_weeks
16_weeks
group

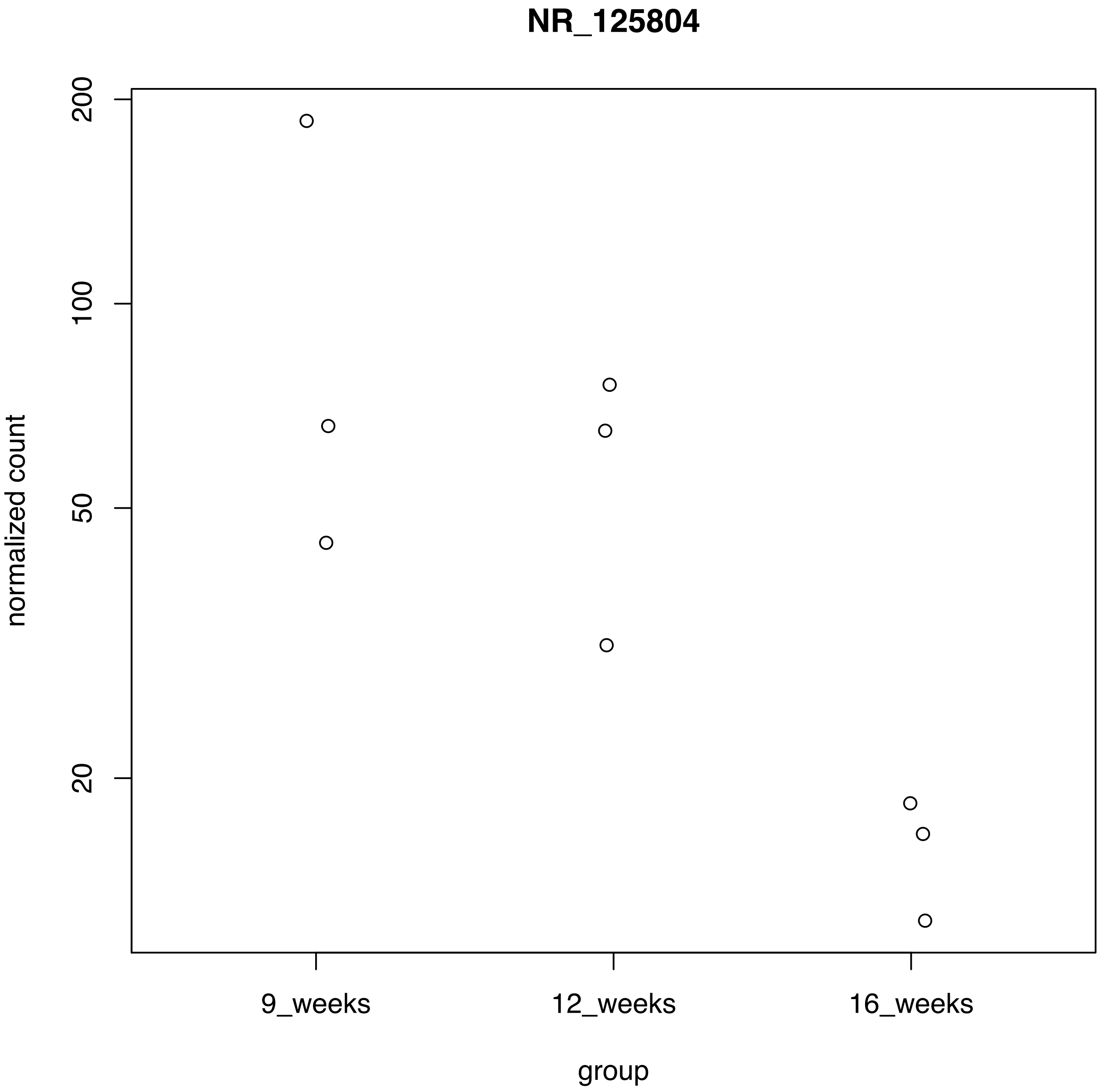

NR_125804
normalized count
200
100
50
20
9_weeks
12_weeks
16_weeks
group

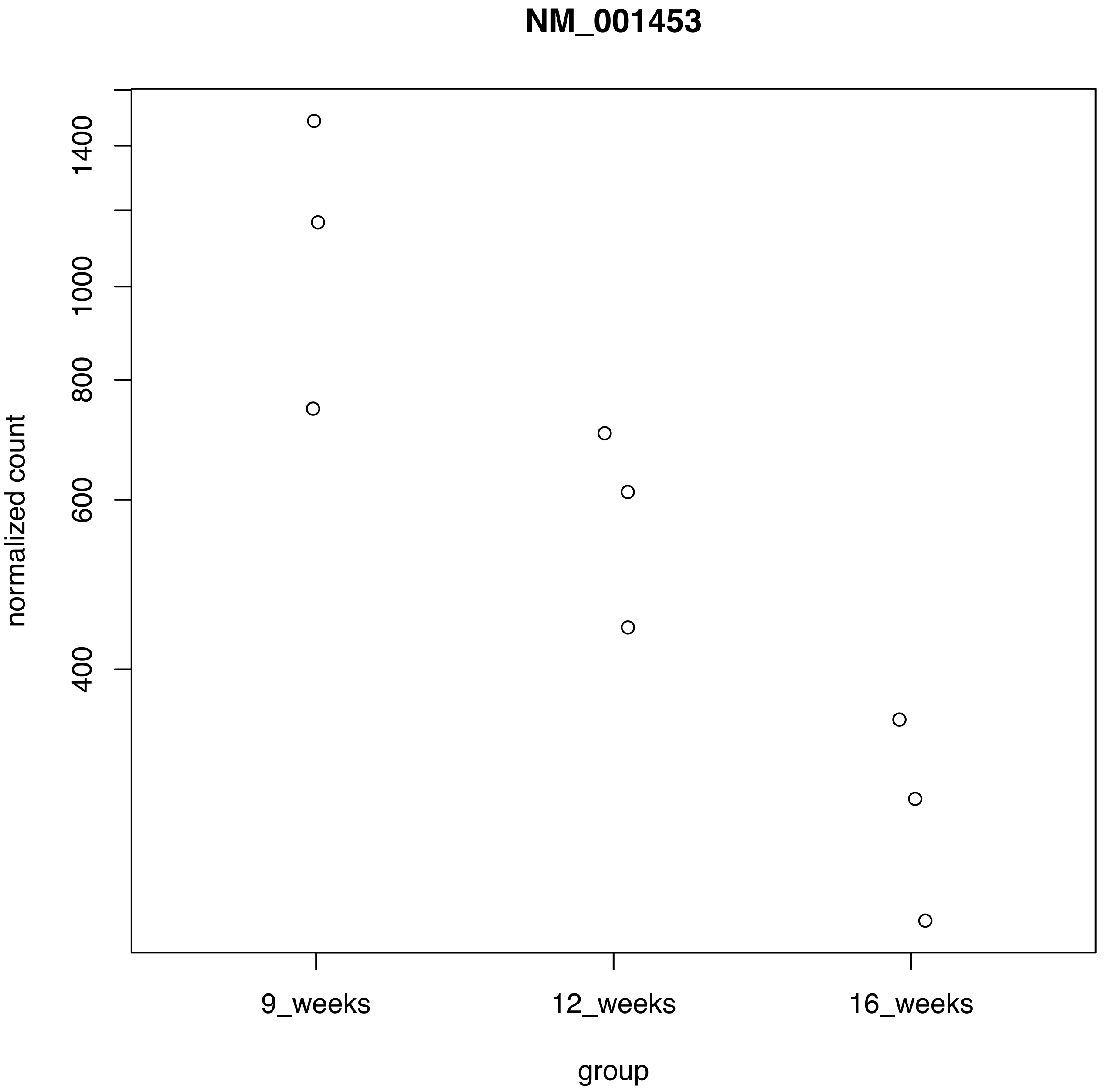
NM_001453
normalized count
1400
1000
800
600
400
9_weeks
12_weeks
16_weeks
group

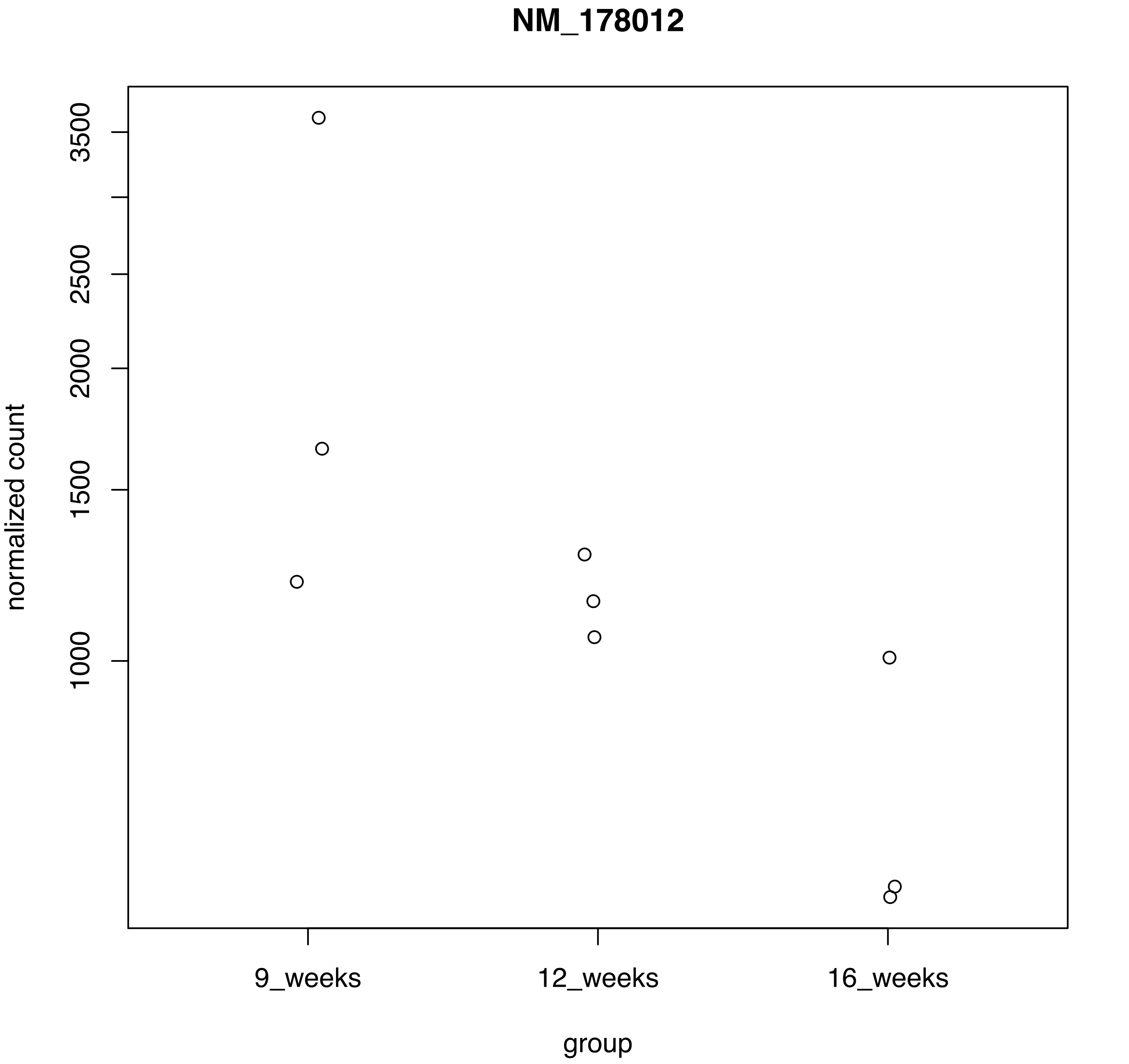
NM_178012
normalized count
3500
2500
2000
1500
1000
9_weeks
12_weeks
16_weeks
group

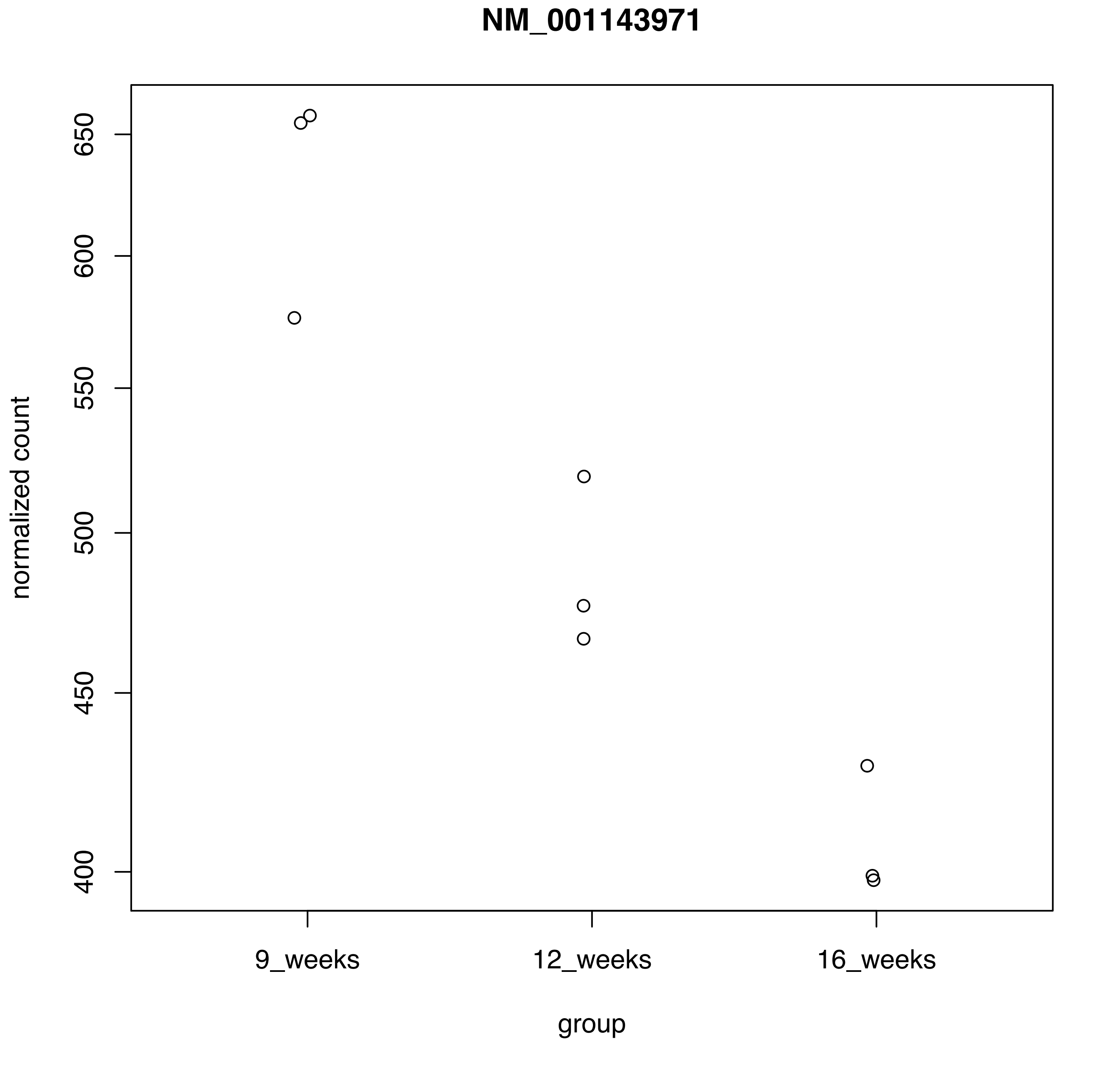

NM_001143971
normalized count
650
600
550
500
450
400
9_weeks
12_weeks
16_weeks
group

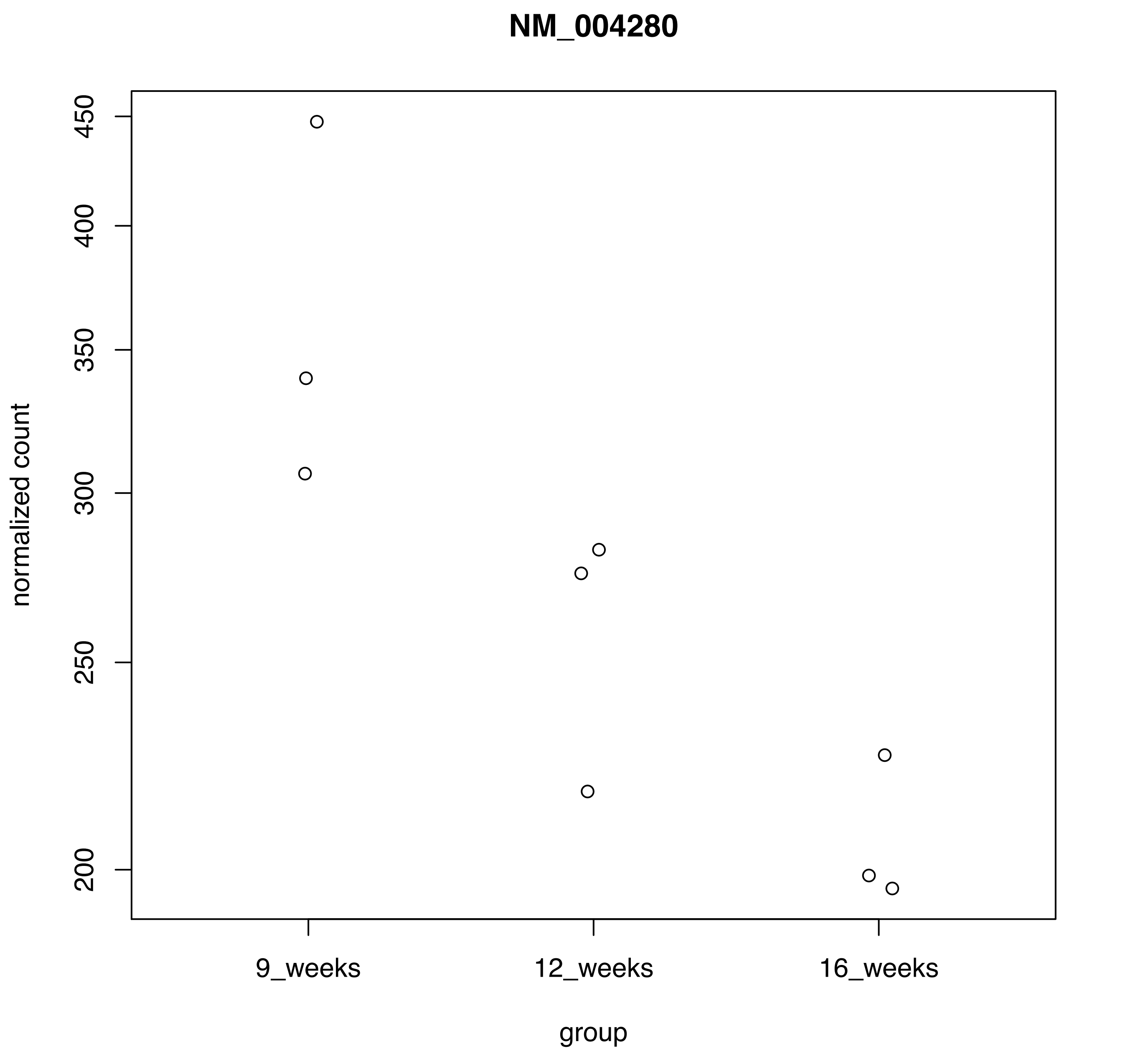
NM_004280
normalized count
450
400
350
300
250
200
9_weeks
12_weeks
16_weeks
group

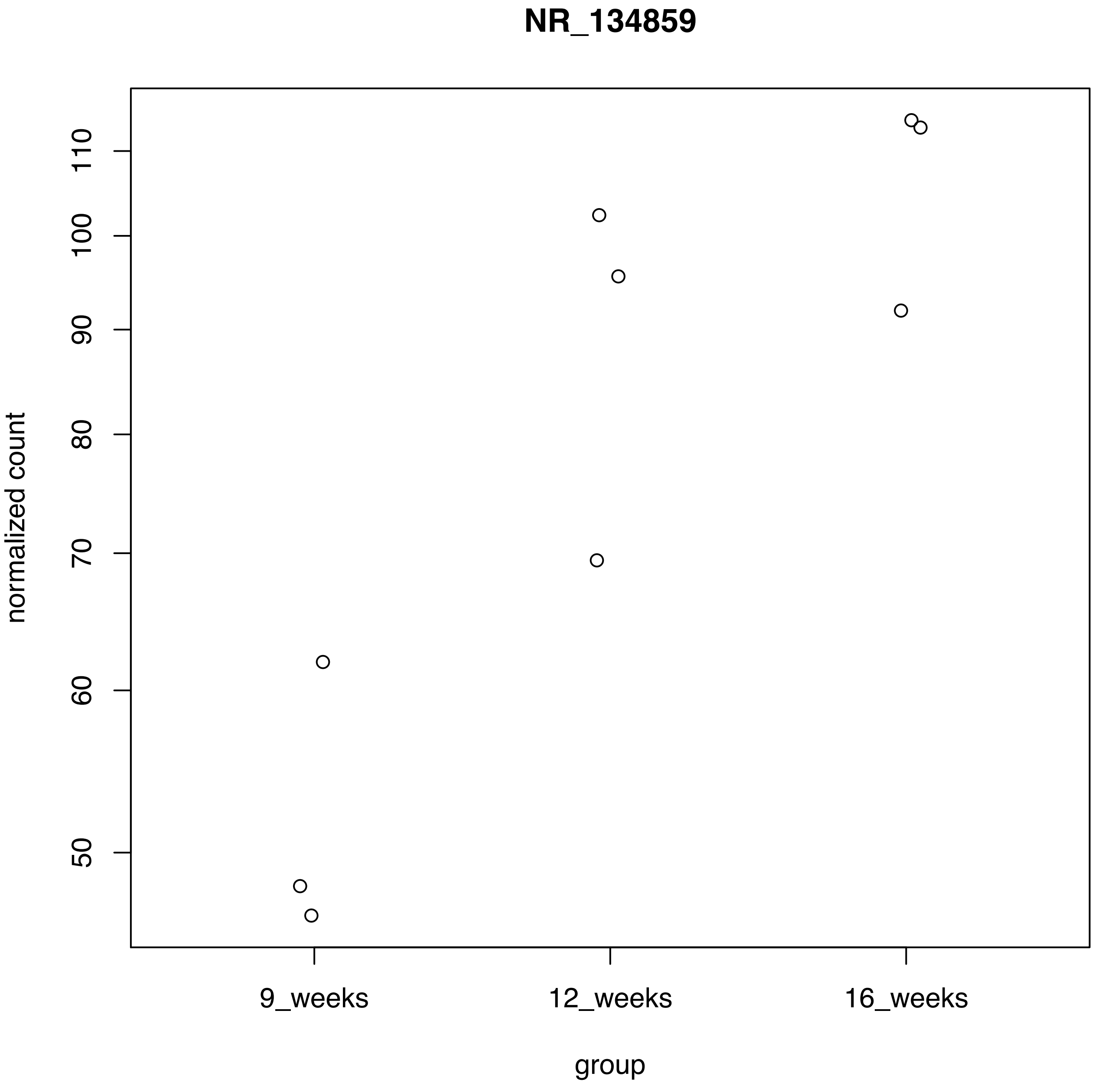
NR_134859
normalized count
50
60
70
80
90
100
110
9_weeks
12_weeks
16_weeks
group

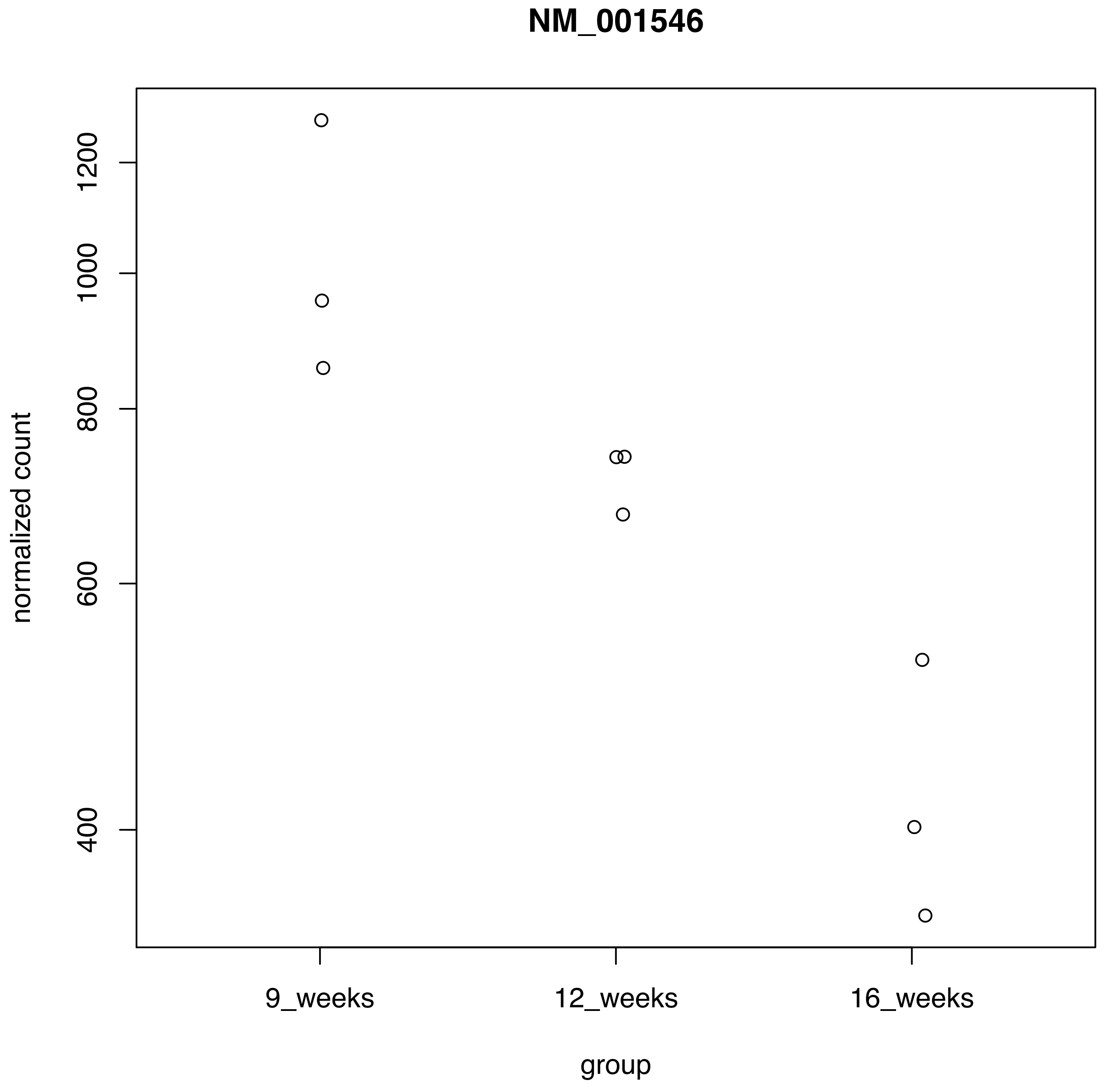

NM_001546
normalized count
400
600
800
1000
1200
9_weeks
12_weeks
16_weeks
group

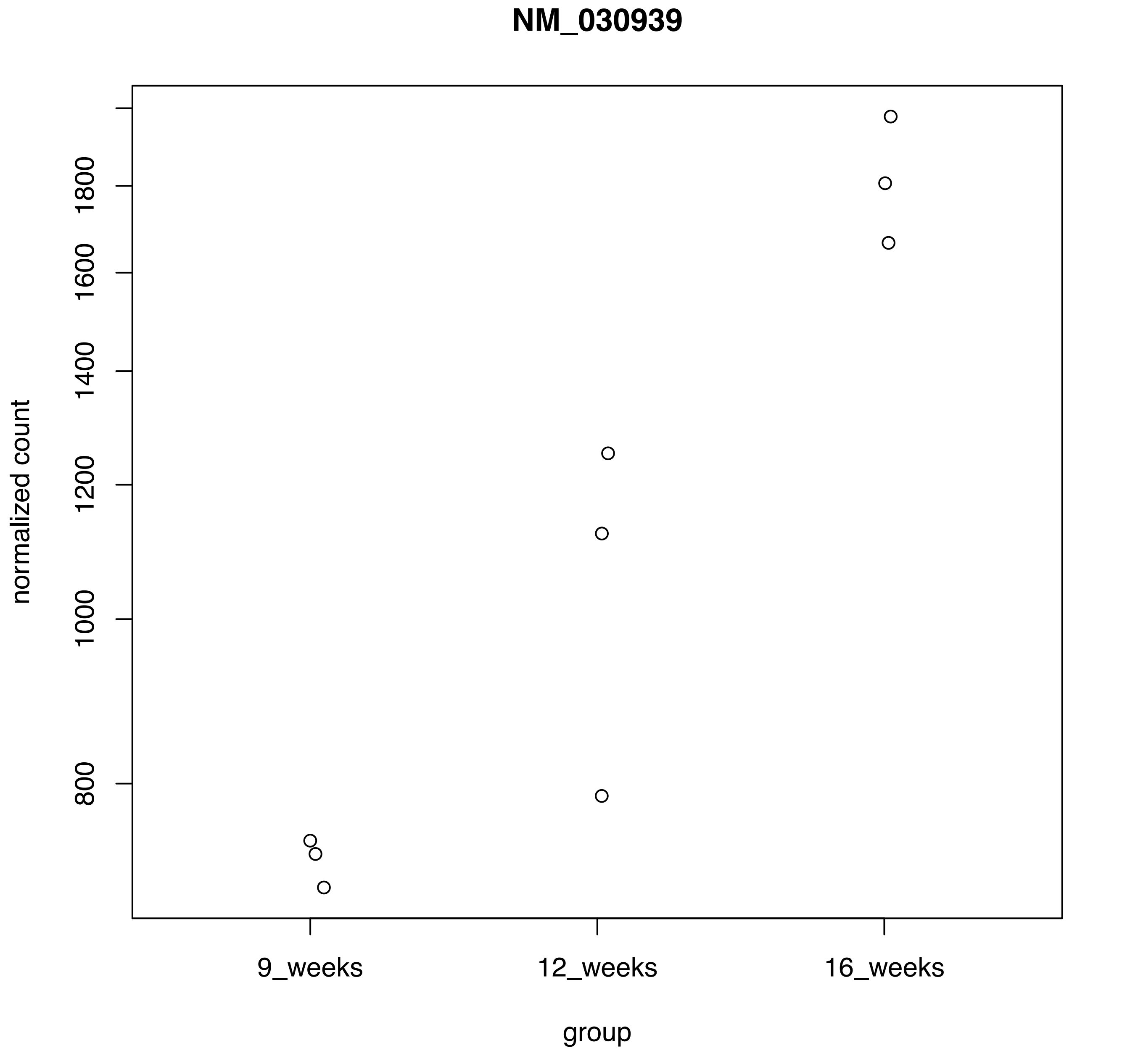
NM_030939
normalized count
800
1000
1200
1400
1600
1800
9_weeks
12_weeks
16_weeks
group

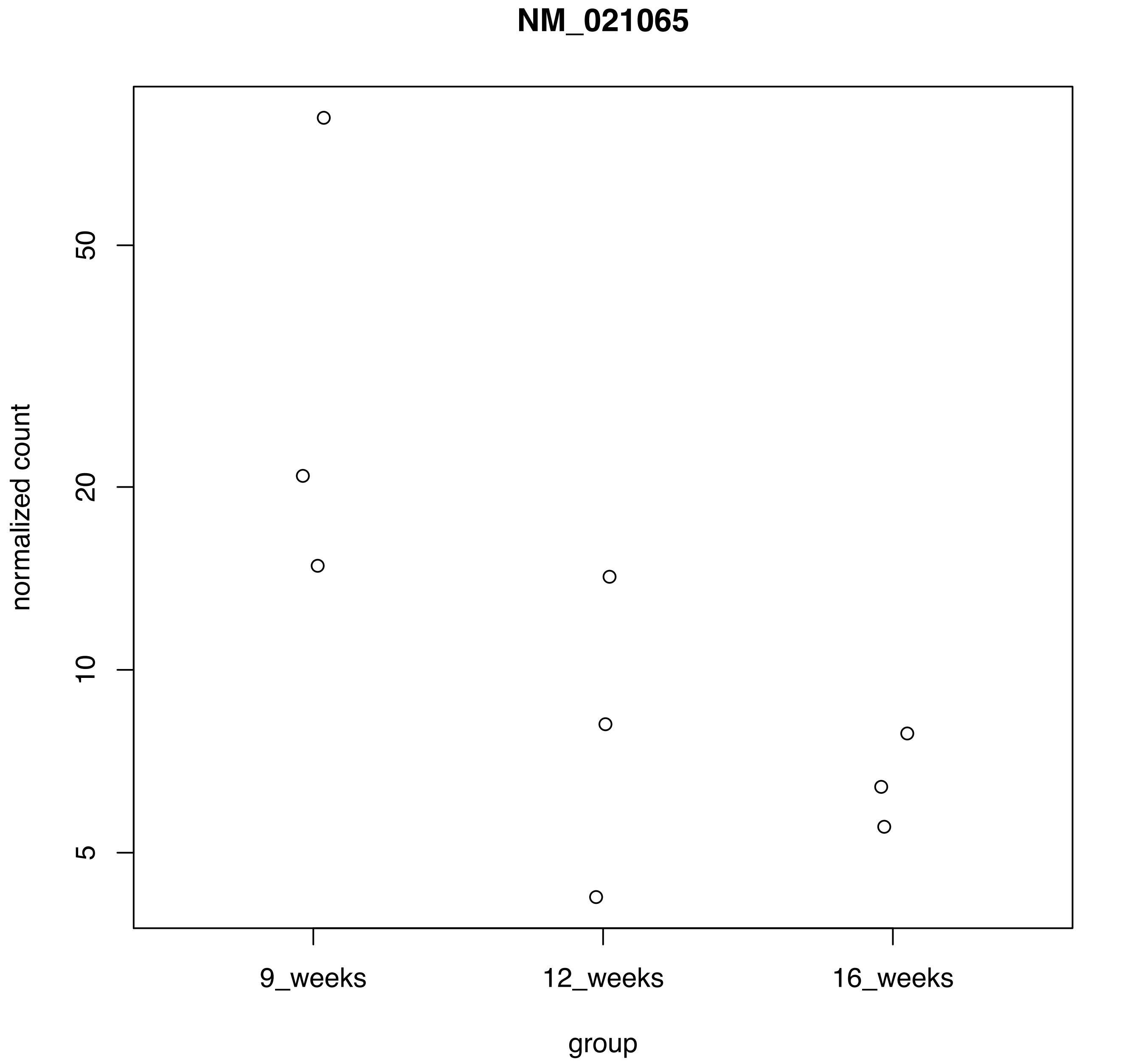
NM_021065
normalized count
50
20
10
5
9_weeks
12_weeks
16_weeks
group

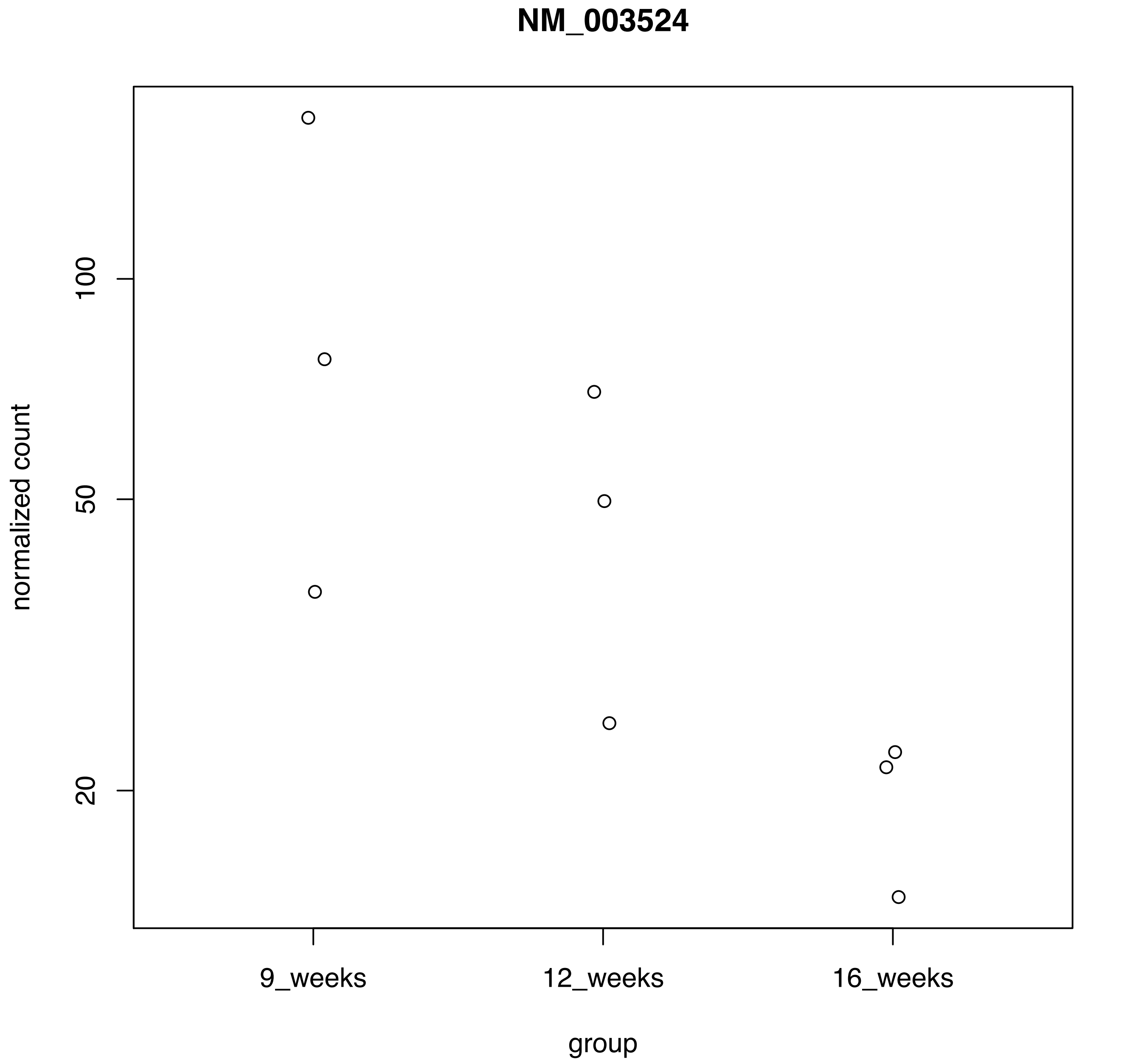
NM_003524
normalized count
100
50
20
9_weeks
12_weeks
16_weeks
group

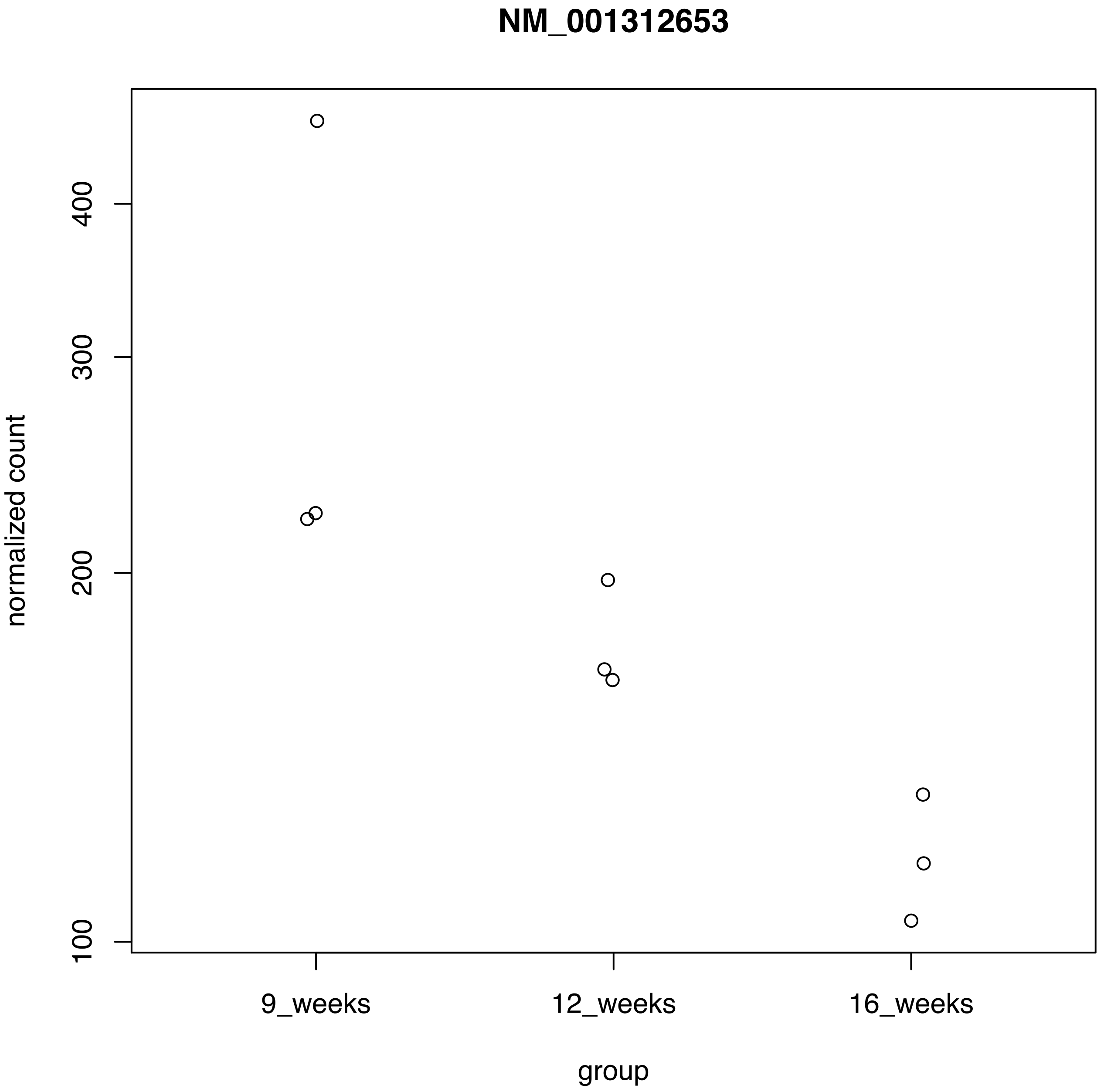
NM_001312653
normalized count
100
200
300
400
group
9_weeks
12_weeks
16_weeks

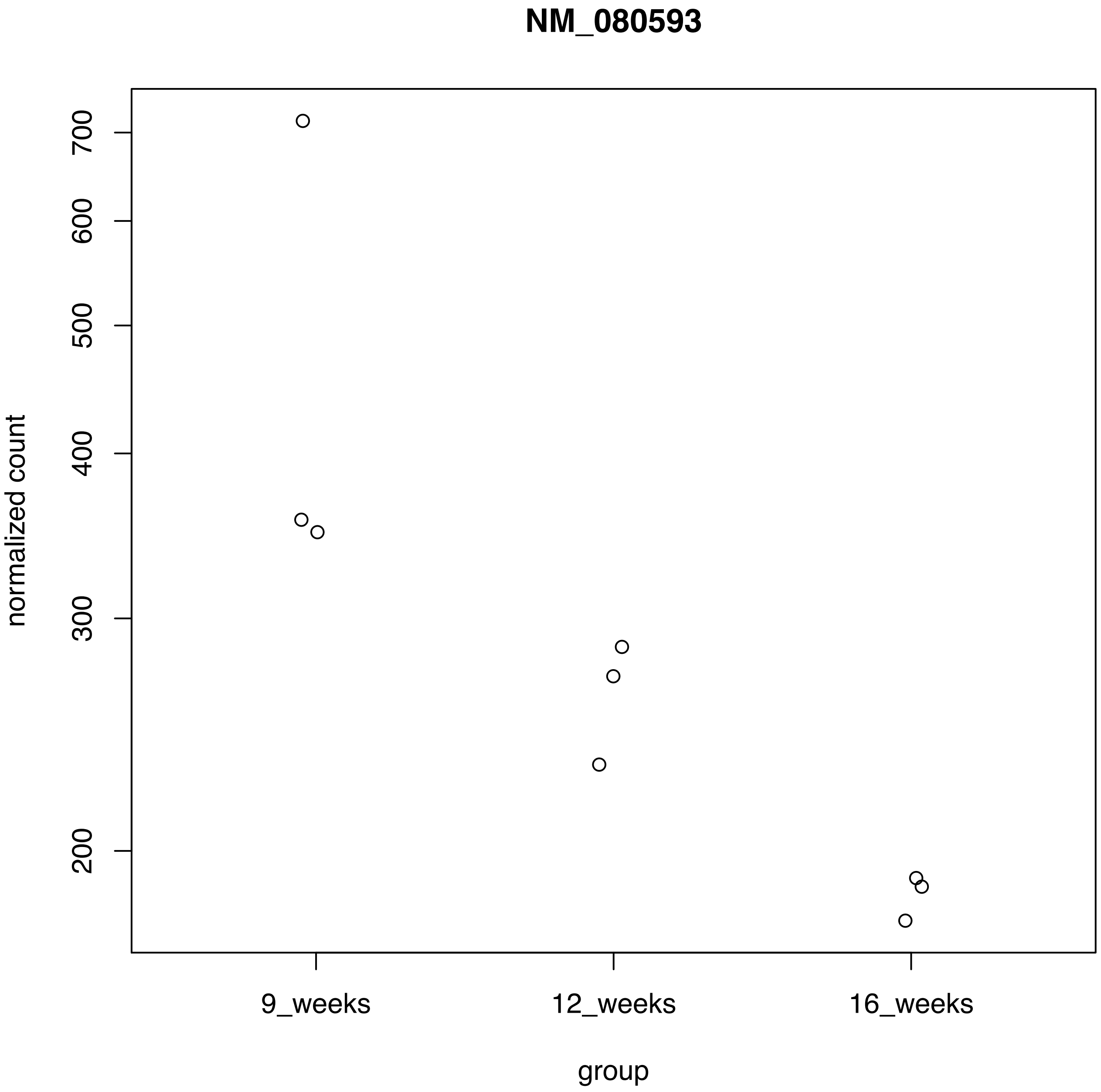
NM_080593
normalized count
700
600
500
400
300
200
9_weeks
12_weeks
16_weeks
group

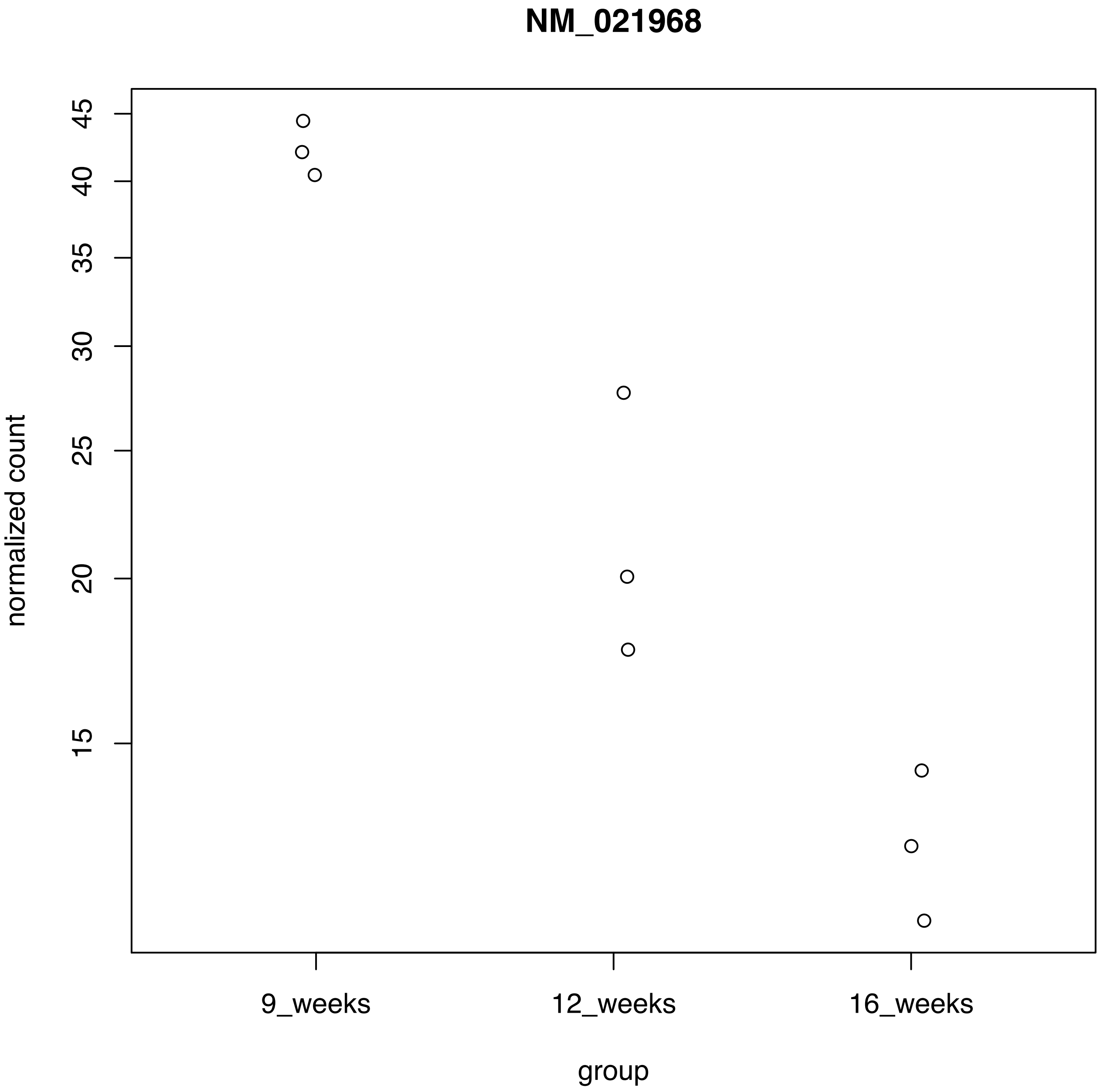
NM_021968
normalized count
45
40
35
30
25
20
15
9_weeks
12_weeks
16_weeks
group

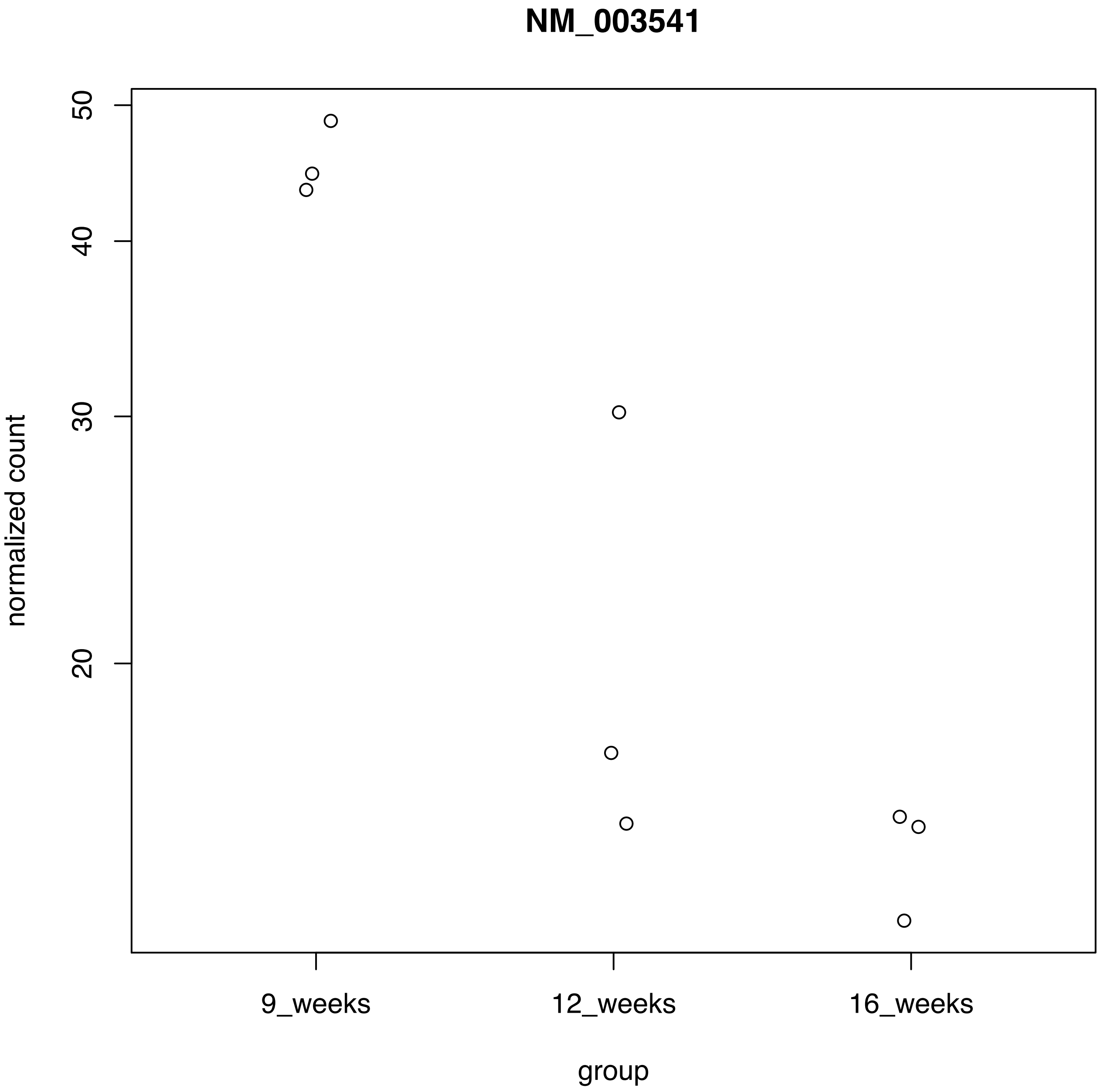
NM_003541
normalized count
50
40
30
20
9_weeks
12_weeks
16_weeks
group

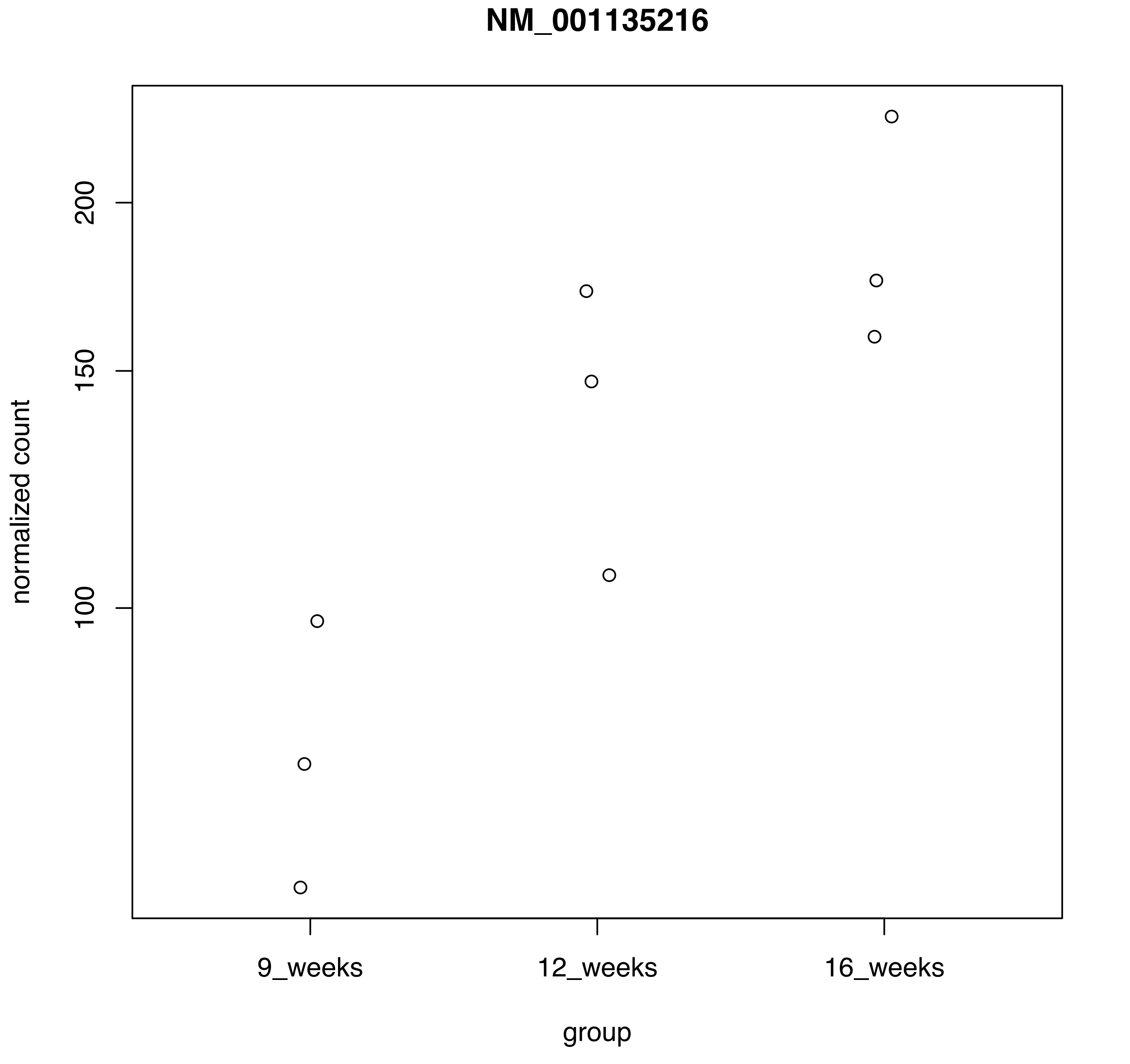
NM_001135216
normalized count
200
150
100
9_weeks
12_weeks
16_weeks
group

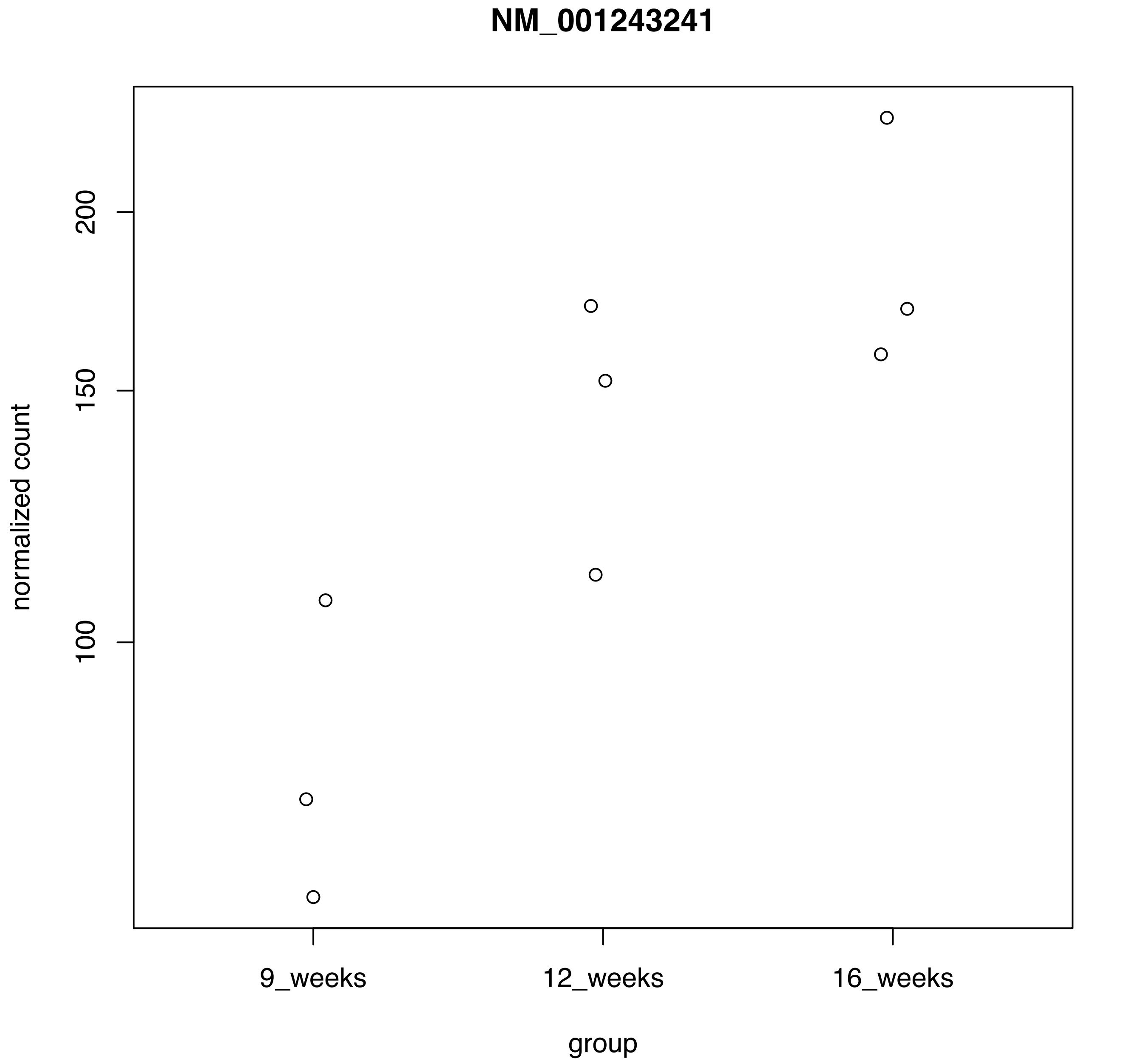
NM_001243241
normalized count
200
150
100
9_weeks
12_weeks
16_weeks
group

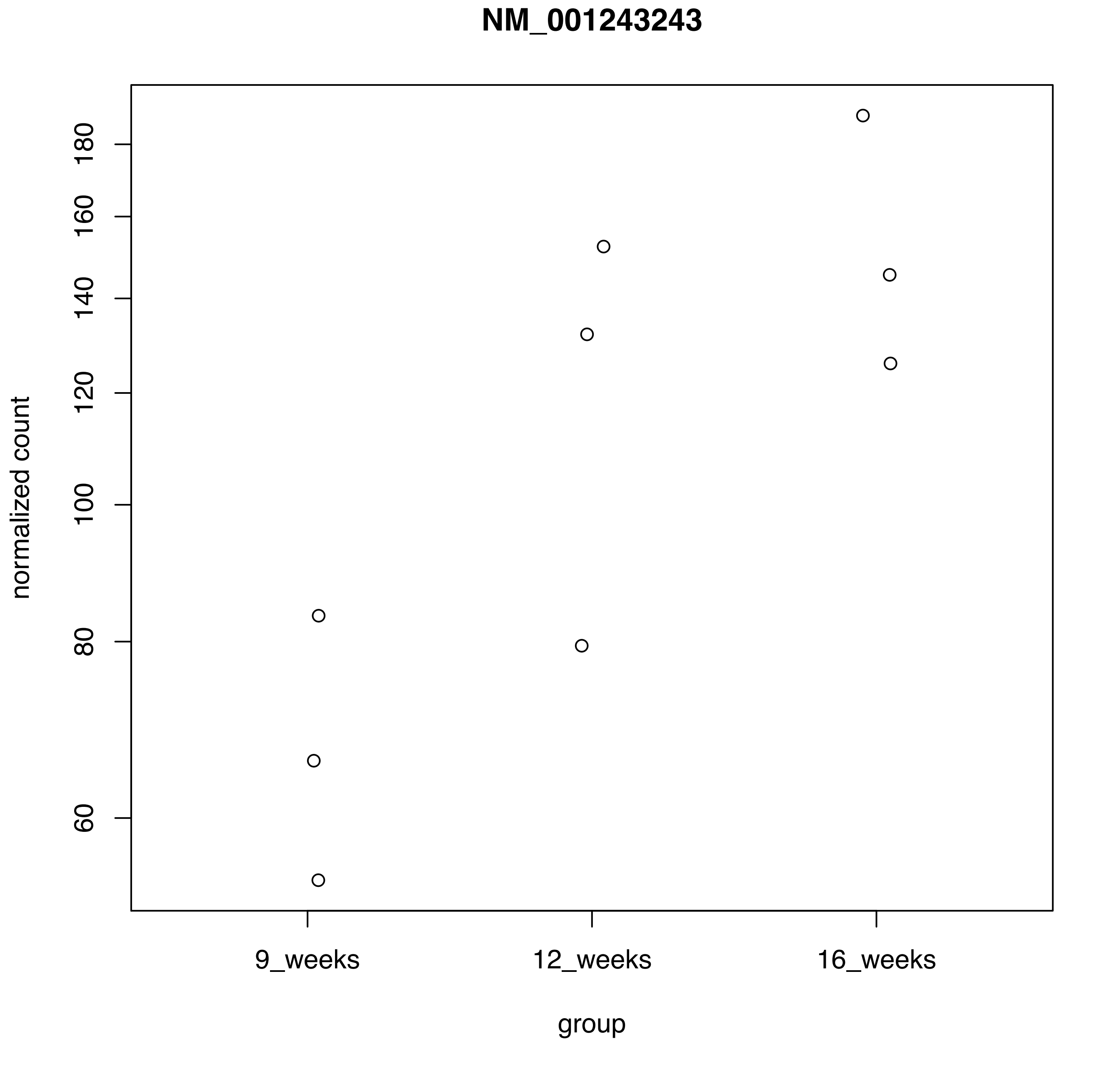
NM_001243243
normalized count
60
80
100
120
140
160
180
9_weeks
12_weeks
16_weeks
group

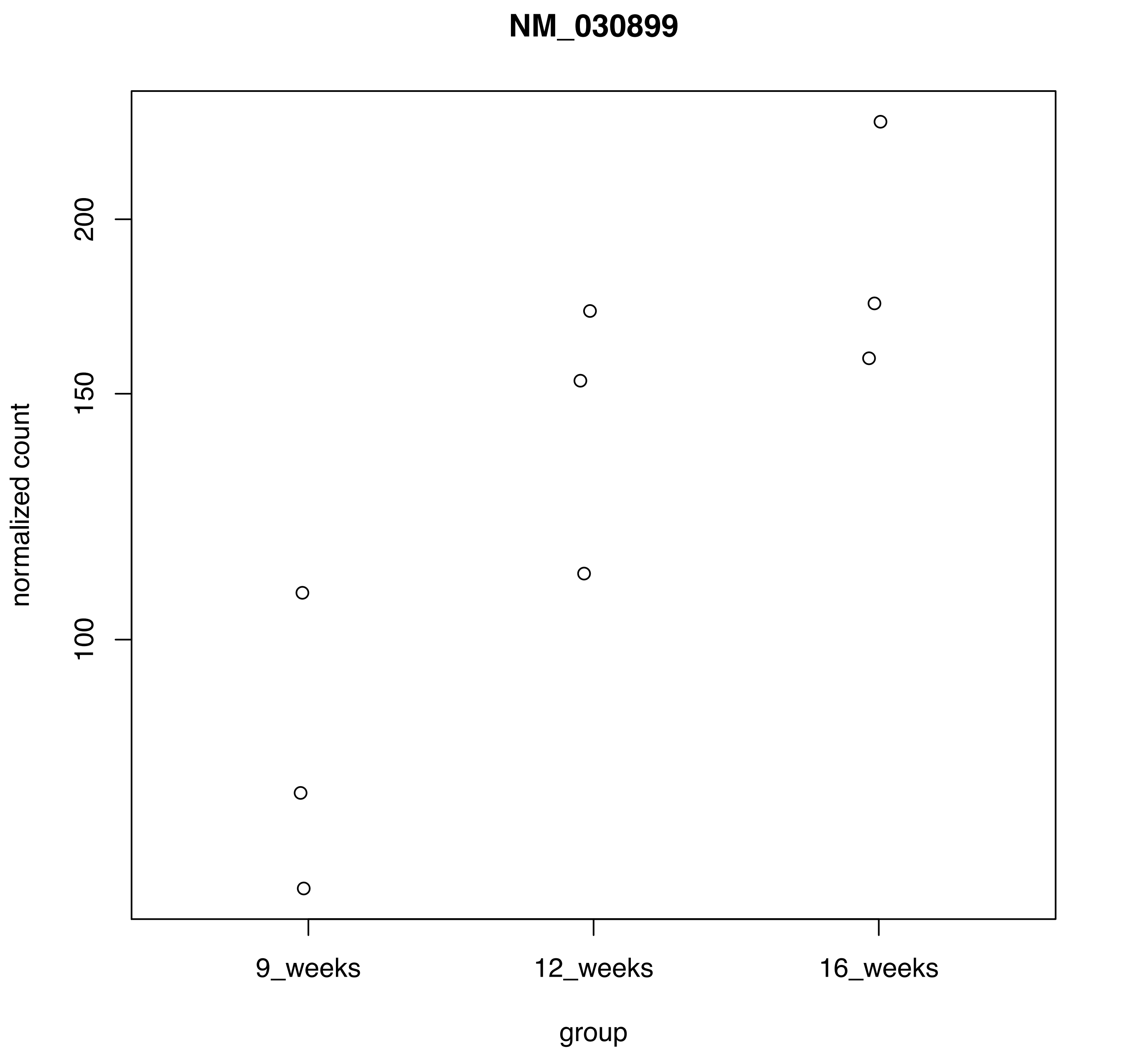
NM_030899
normalized count
200
150
100
9_weeks
12_weeks
16_weeks
group

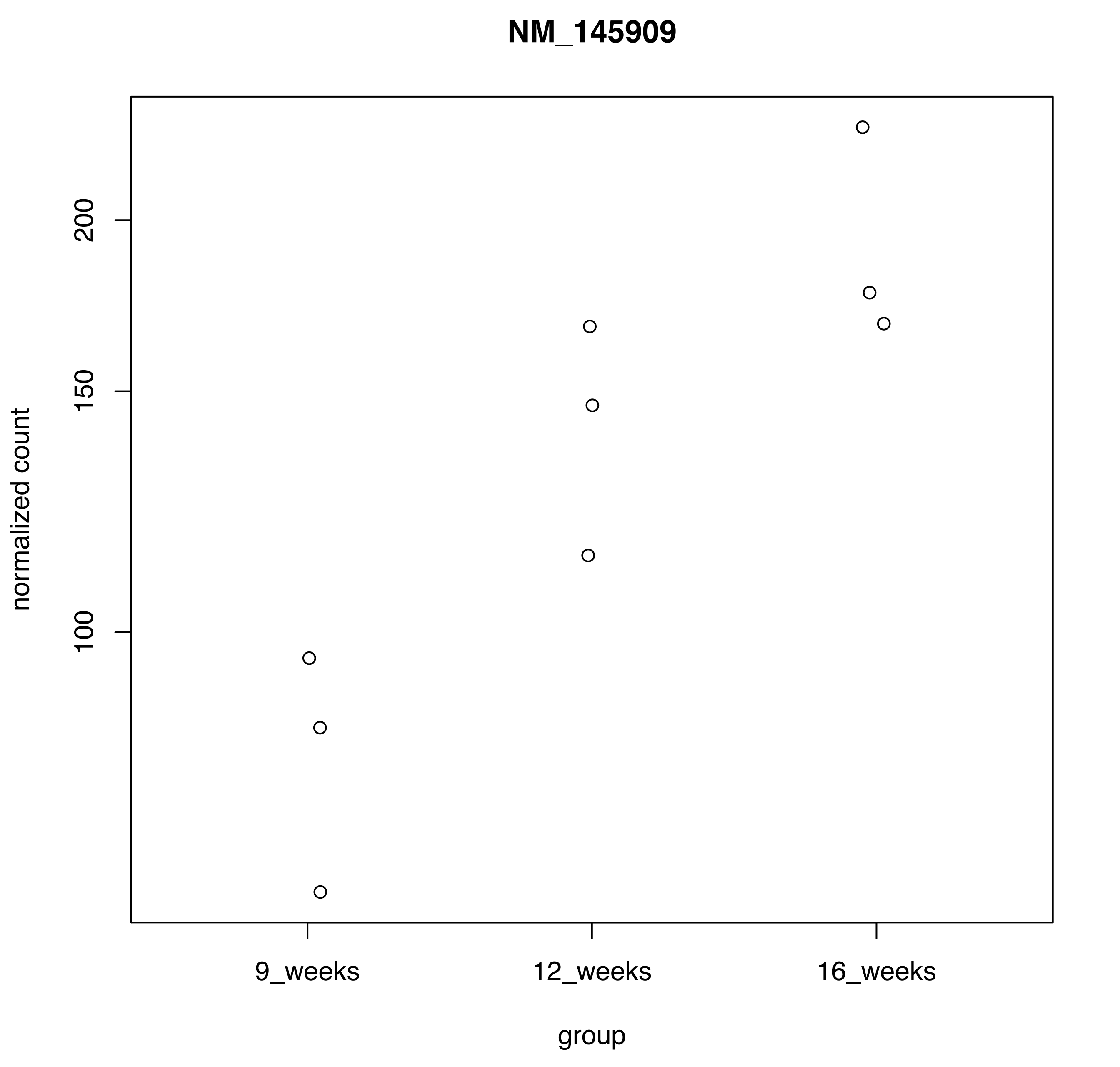
NM_145909
normalized count
200
150
100
9_weeks
12_weeks
16_weeks
group

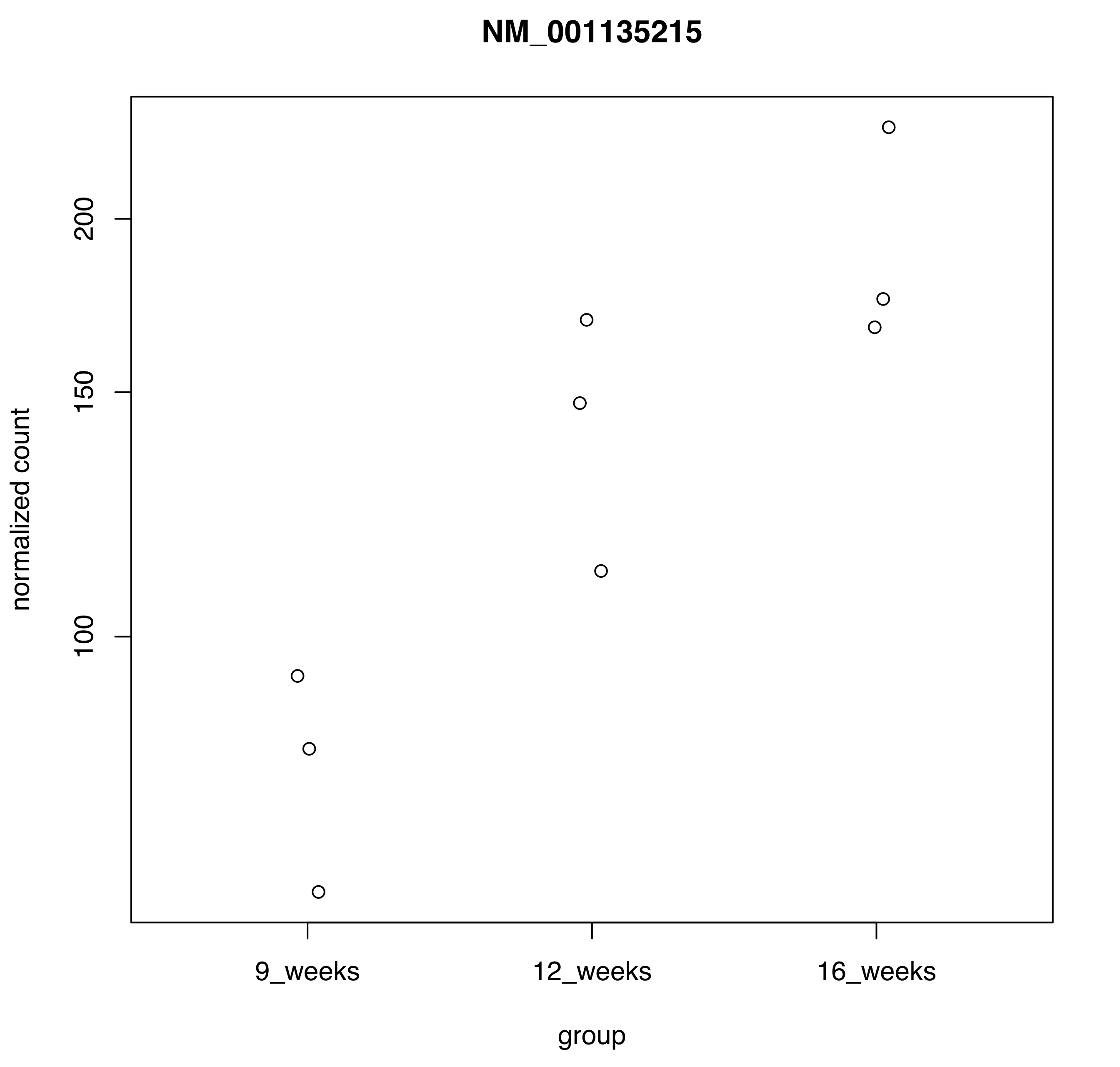
NM_001135215
normalized count
200
150
100
9_weeks
12_weeks
16_weeks
group

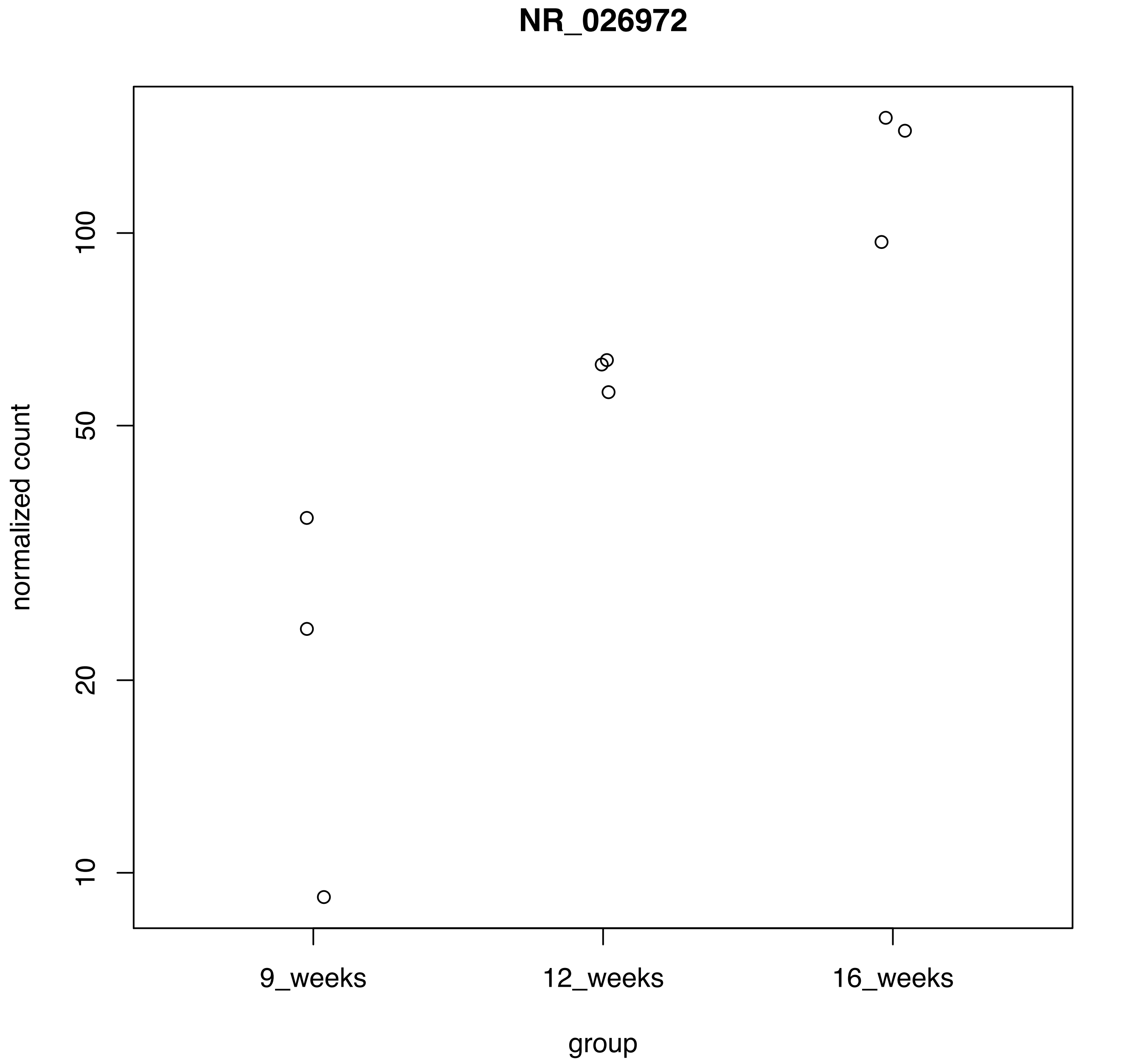
NR_026972
normalized count
100
50
20
10
9_weeks
12_weeks
16_weeks
group

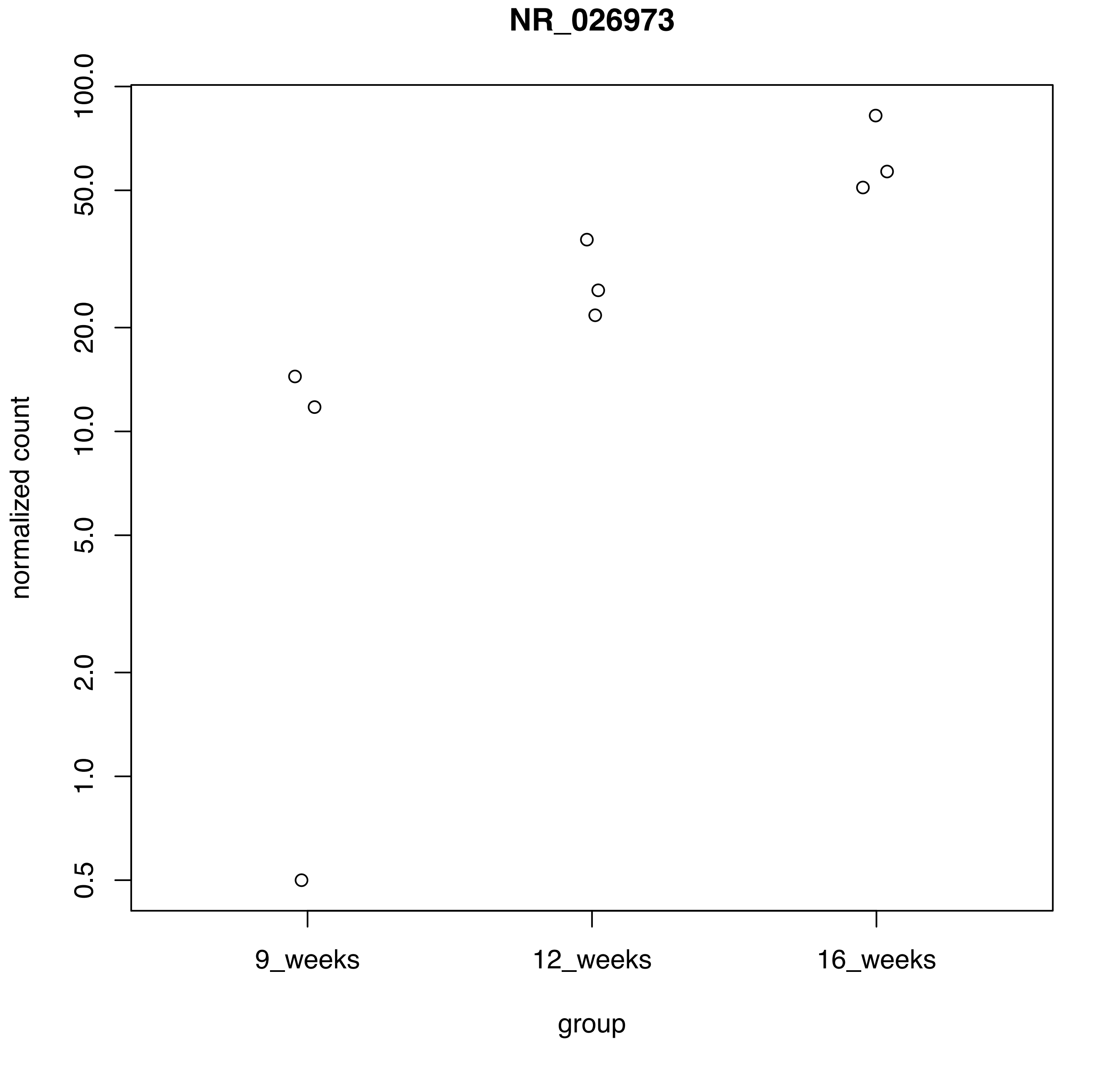

NR_026973
100.0
50.0
20.0
10.0
5.0
2.0
1.0
0.5
normalized count
9_weeks
12_weeks
16_weeks
group

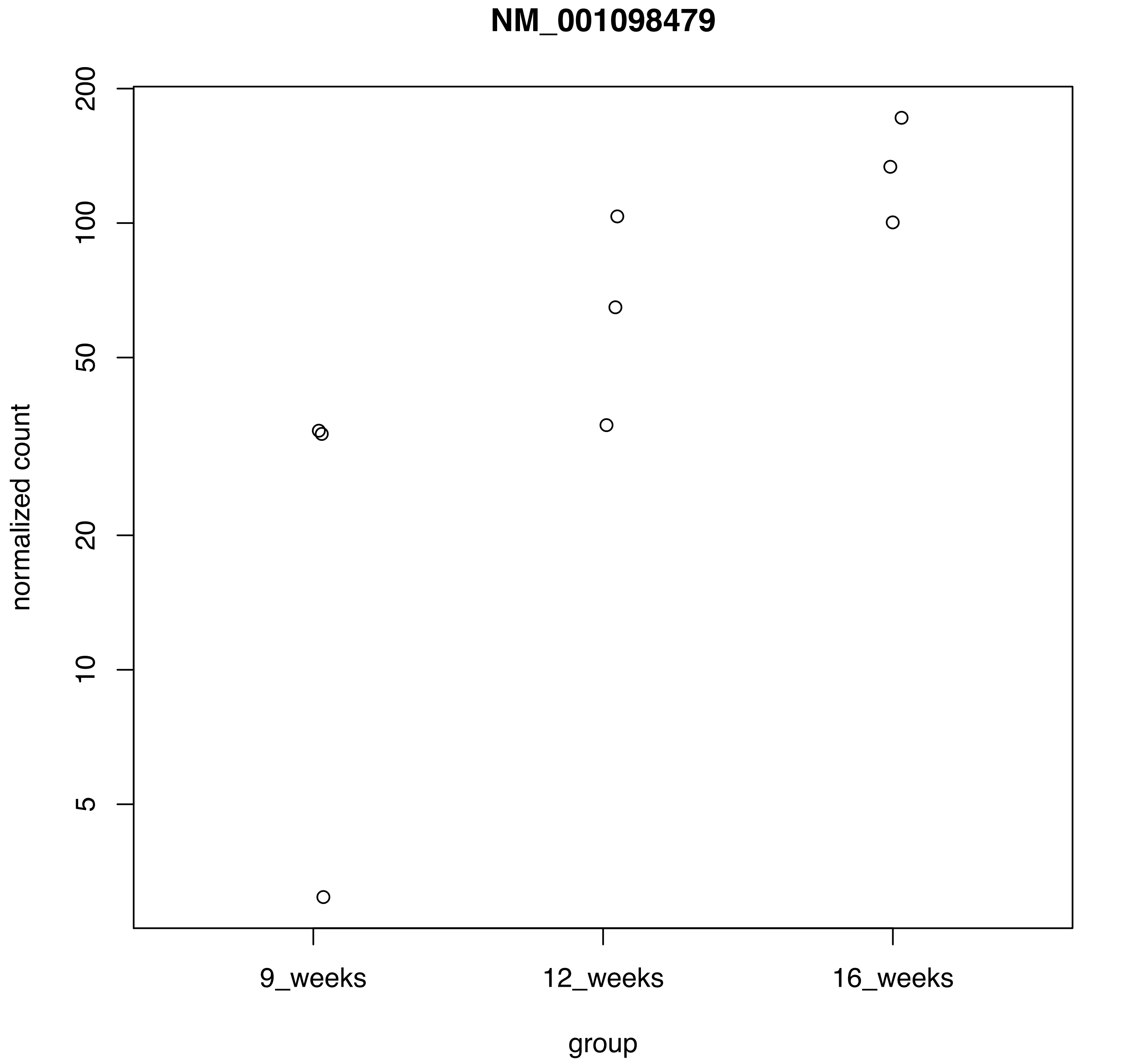
NM_001098479
normalized count
200
100
50
20
10
5
9_weeks
12_weeks
16_weeks
group

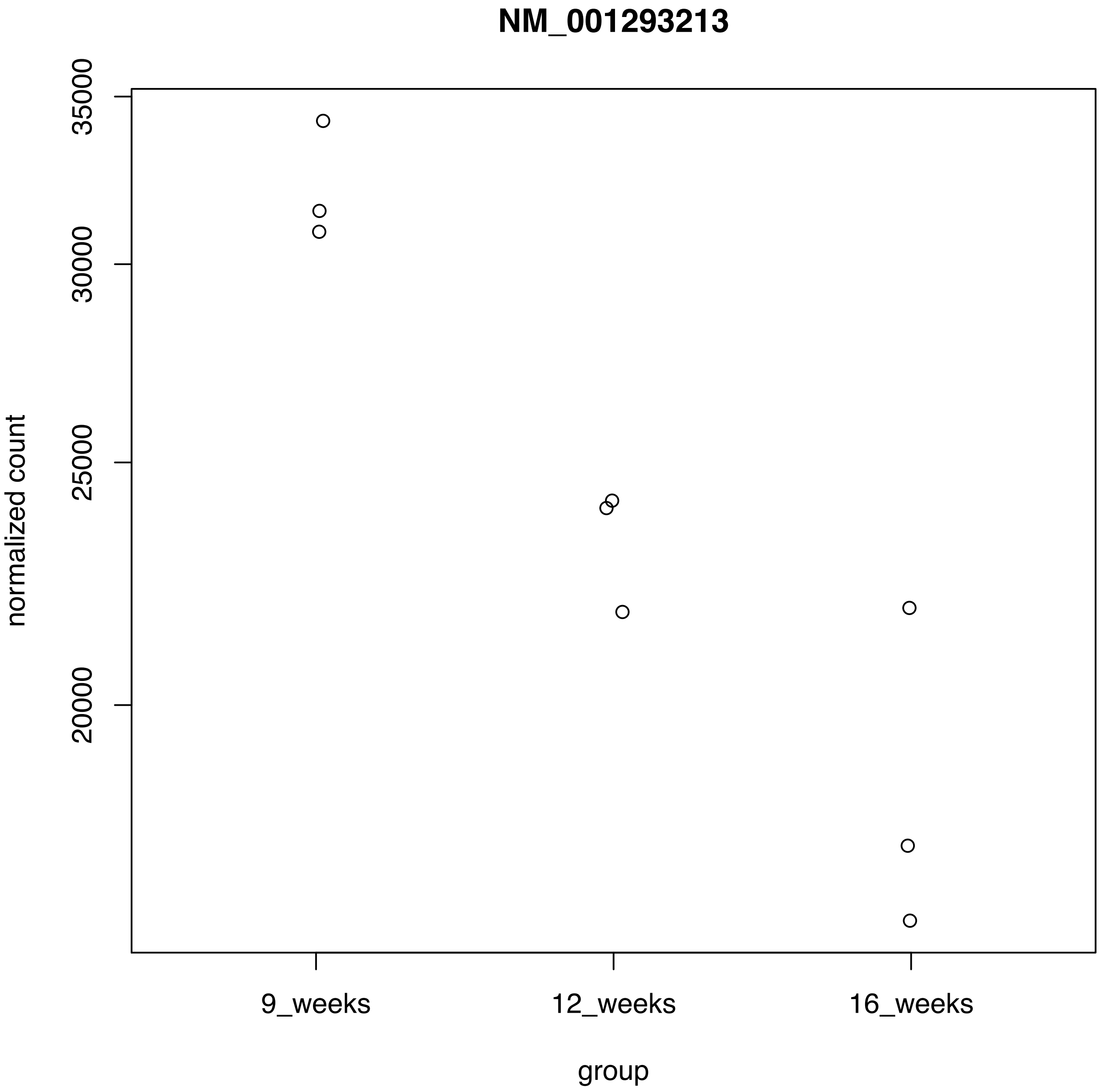
NM_001293213
35000
30000
25000
20000
normalized count
9_weeks
12_weeks
16_weeks
group

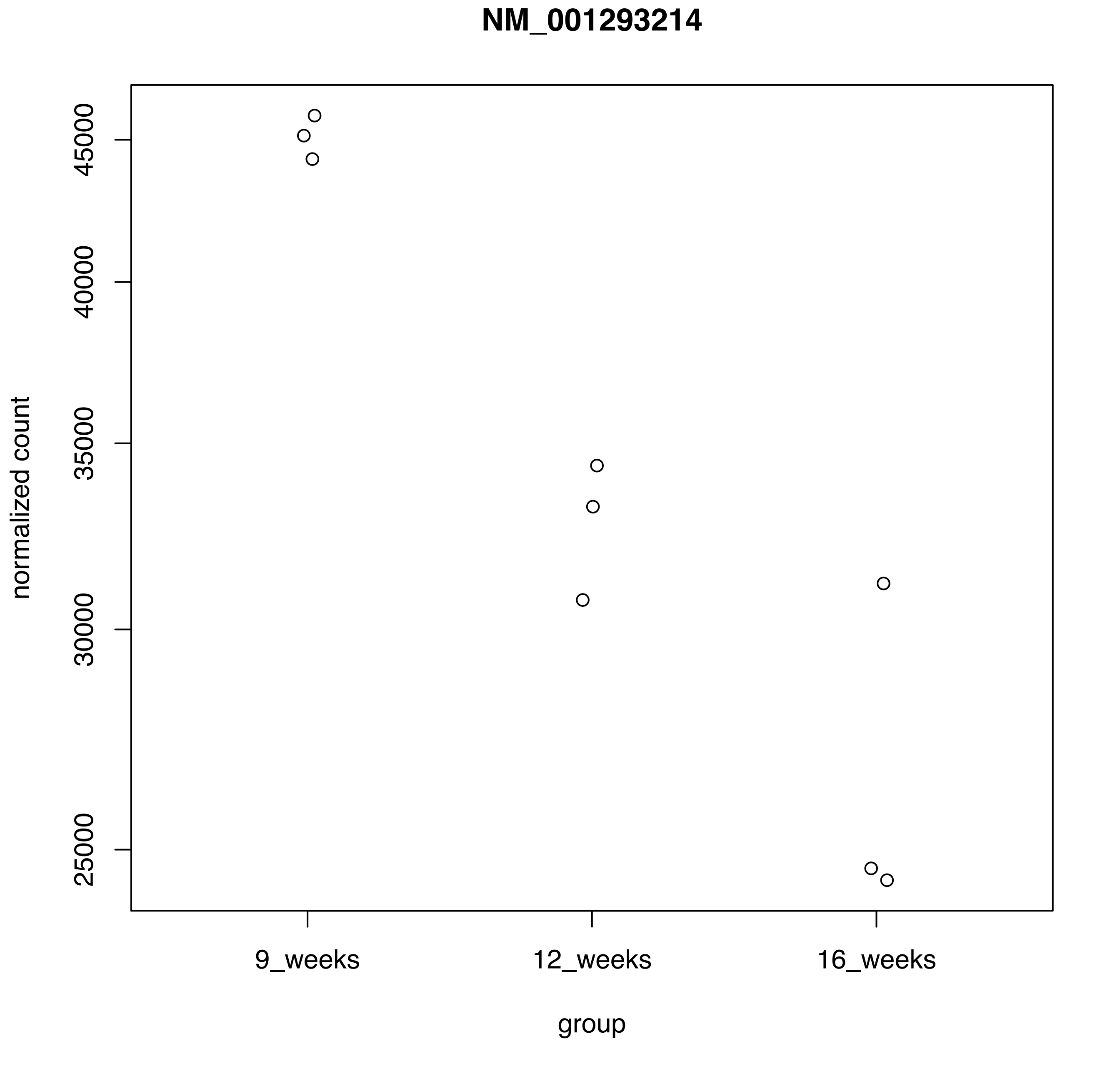
NM_001293214
normalized count
45000
40000
35000
30000
25000
9_weeks
12_weeks
16_weeks
group

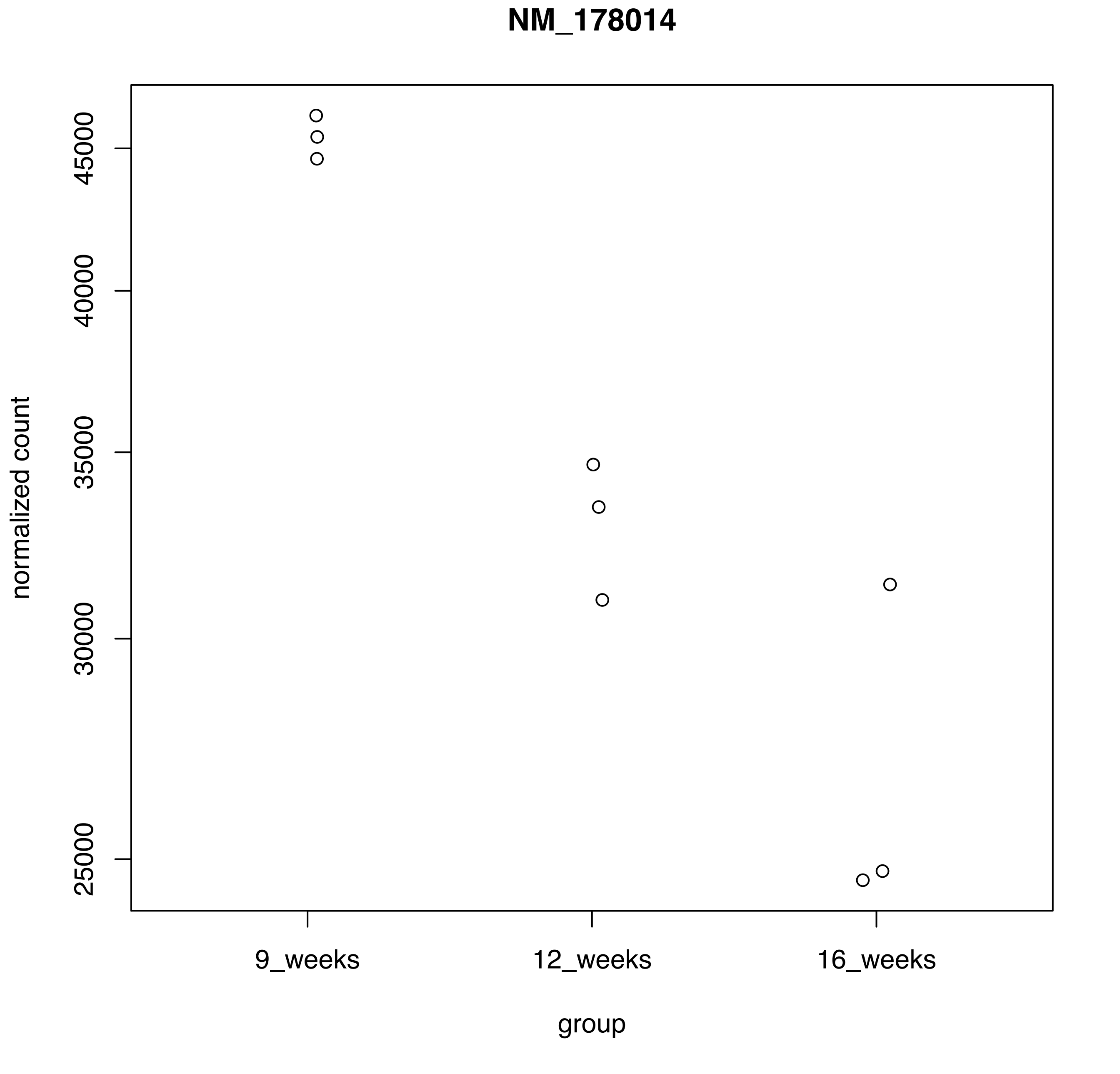
NM_178014
normalized count
45000
40000
35000
30000
25000
9_weeks
12_weeks
16_weeks
group

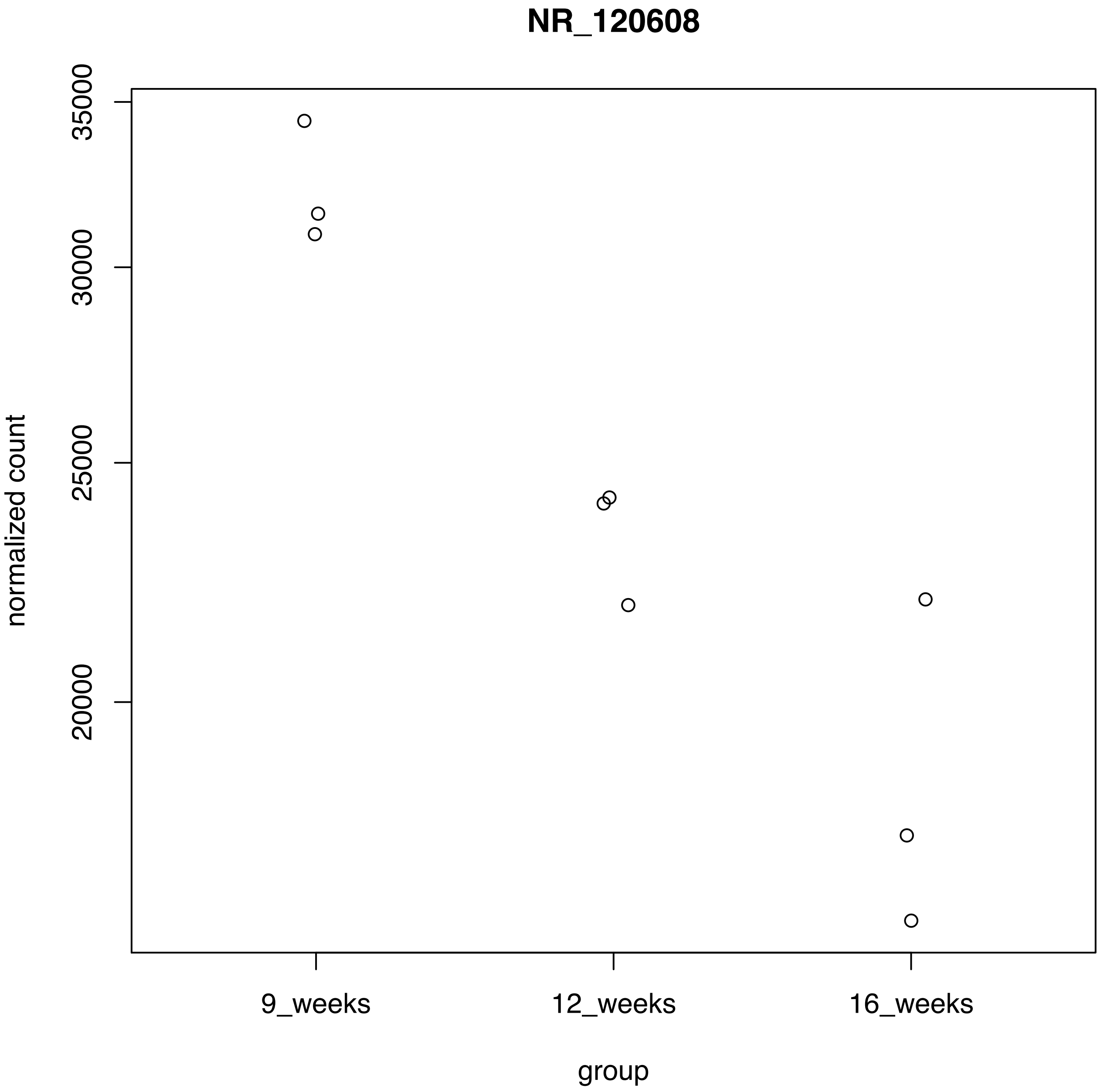
NR_120608
normalized count
35000
30000
25000
20000
9_weeks
12_weeks
16_weeks
group

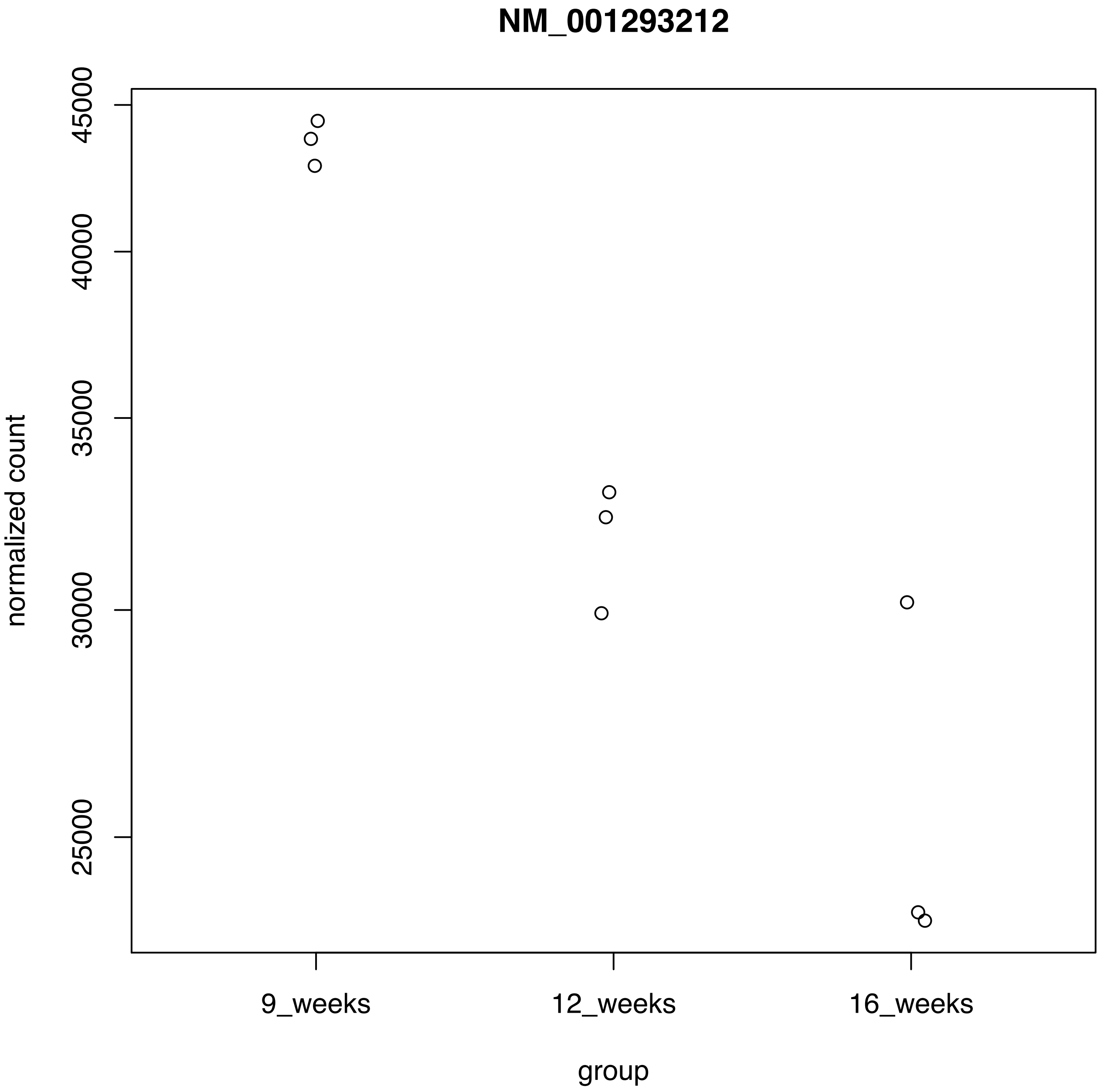
NM_001293212
normalized count
45000
40000
35000
30000
25000
9_weeks
12_weeks
16_weeks
group

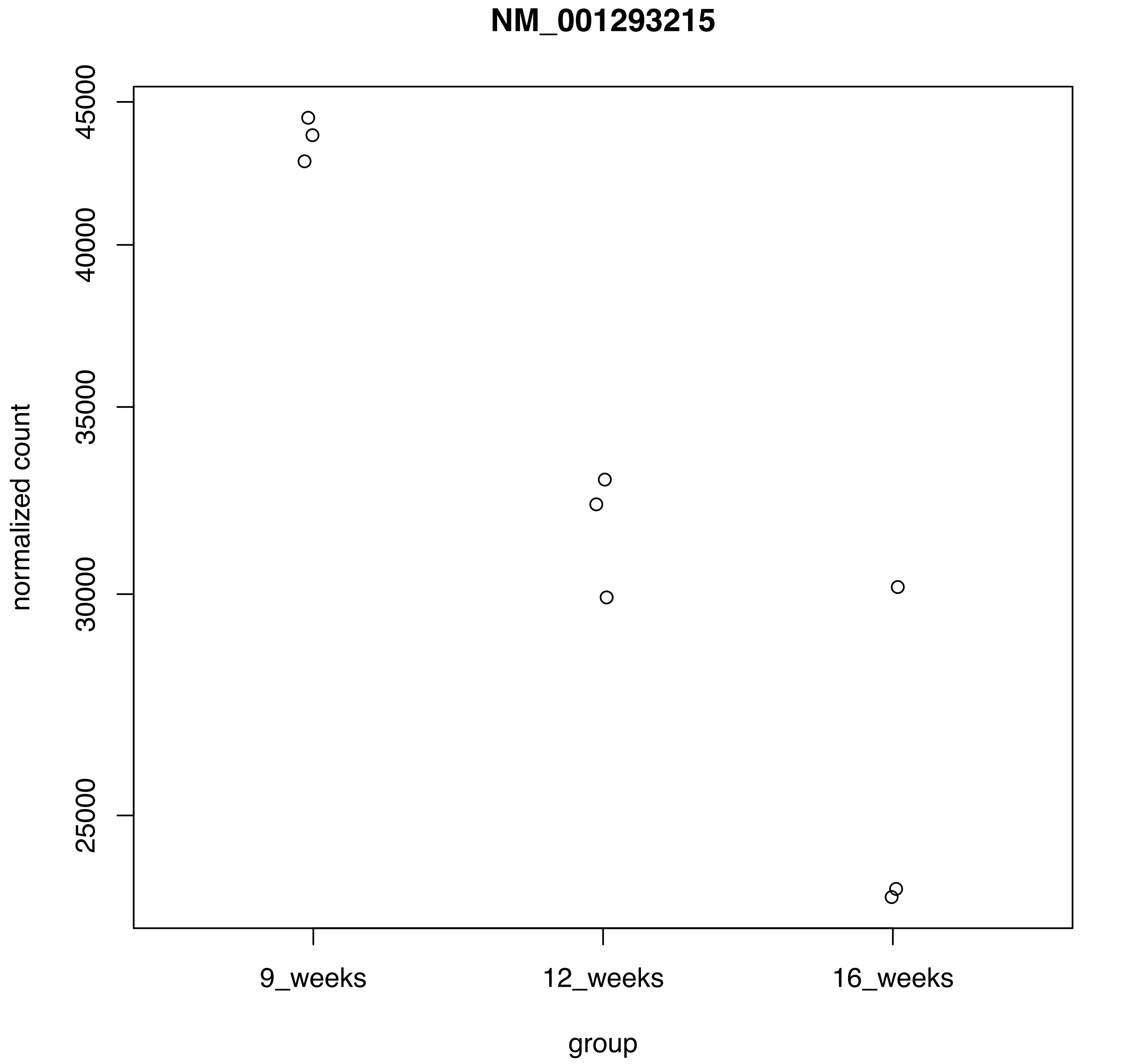

NM_001293215
normalized count
45000
40000
35000
30000
25000
9_weeks
12_weeks
16_weeks
group

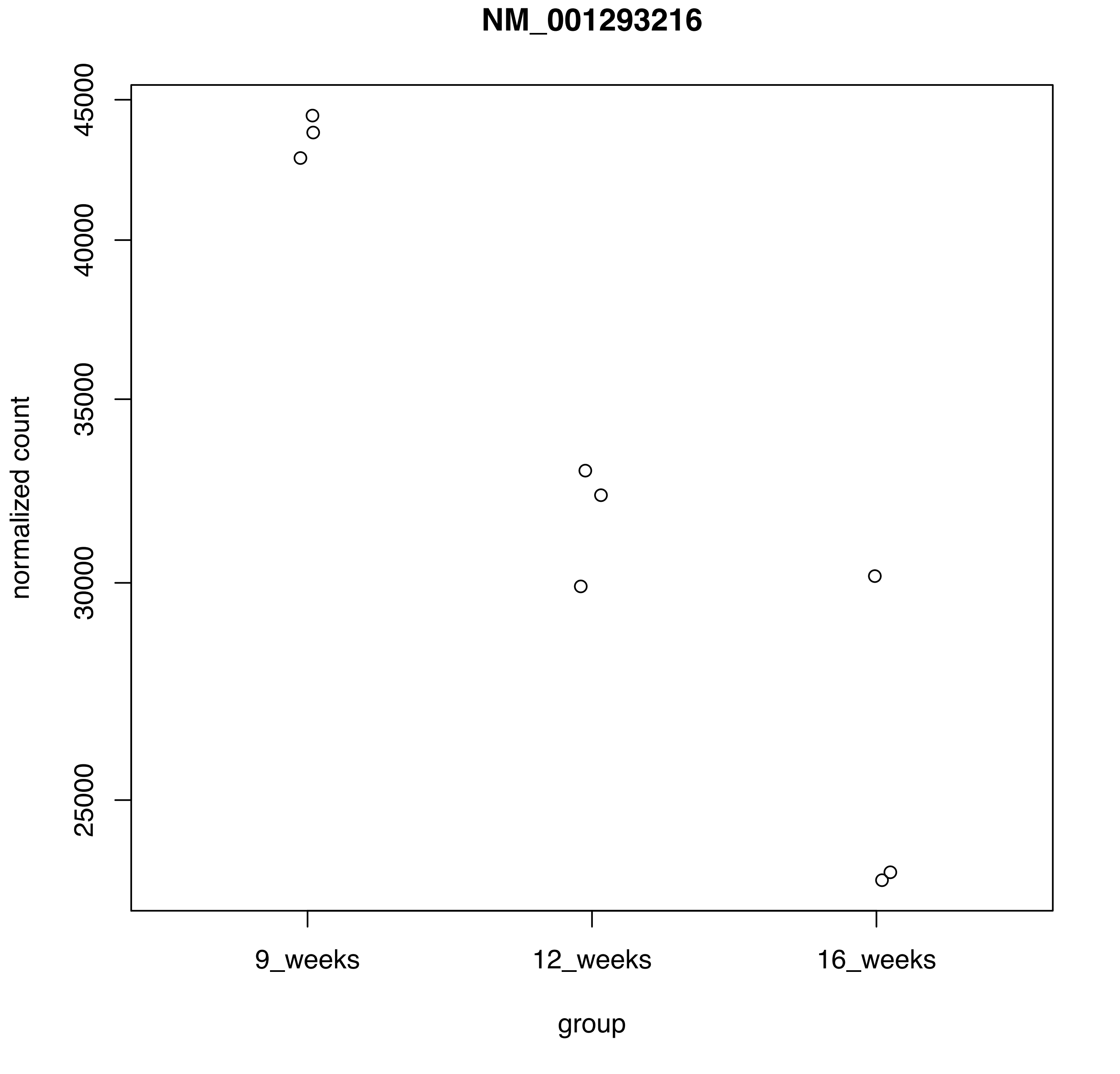

NM_001293216
normalized count
45000
40000
35000
30000
25000
9_weeks
12_weeks
16_weeks
group

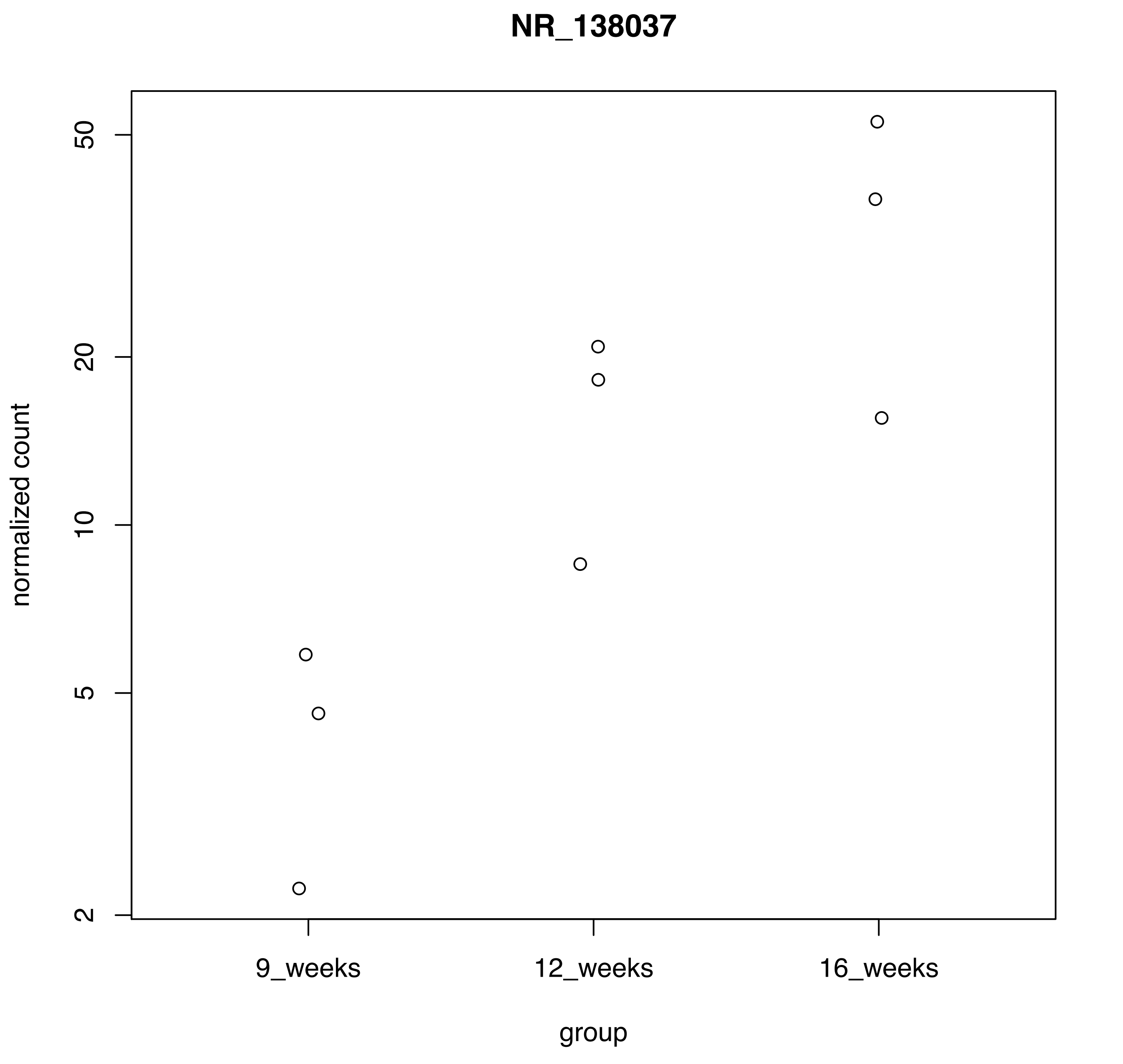

NR_138037
normalized count
50
20
10
5
2
9_weeks
12_weeks
16_weeks
group

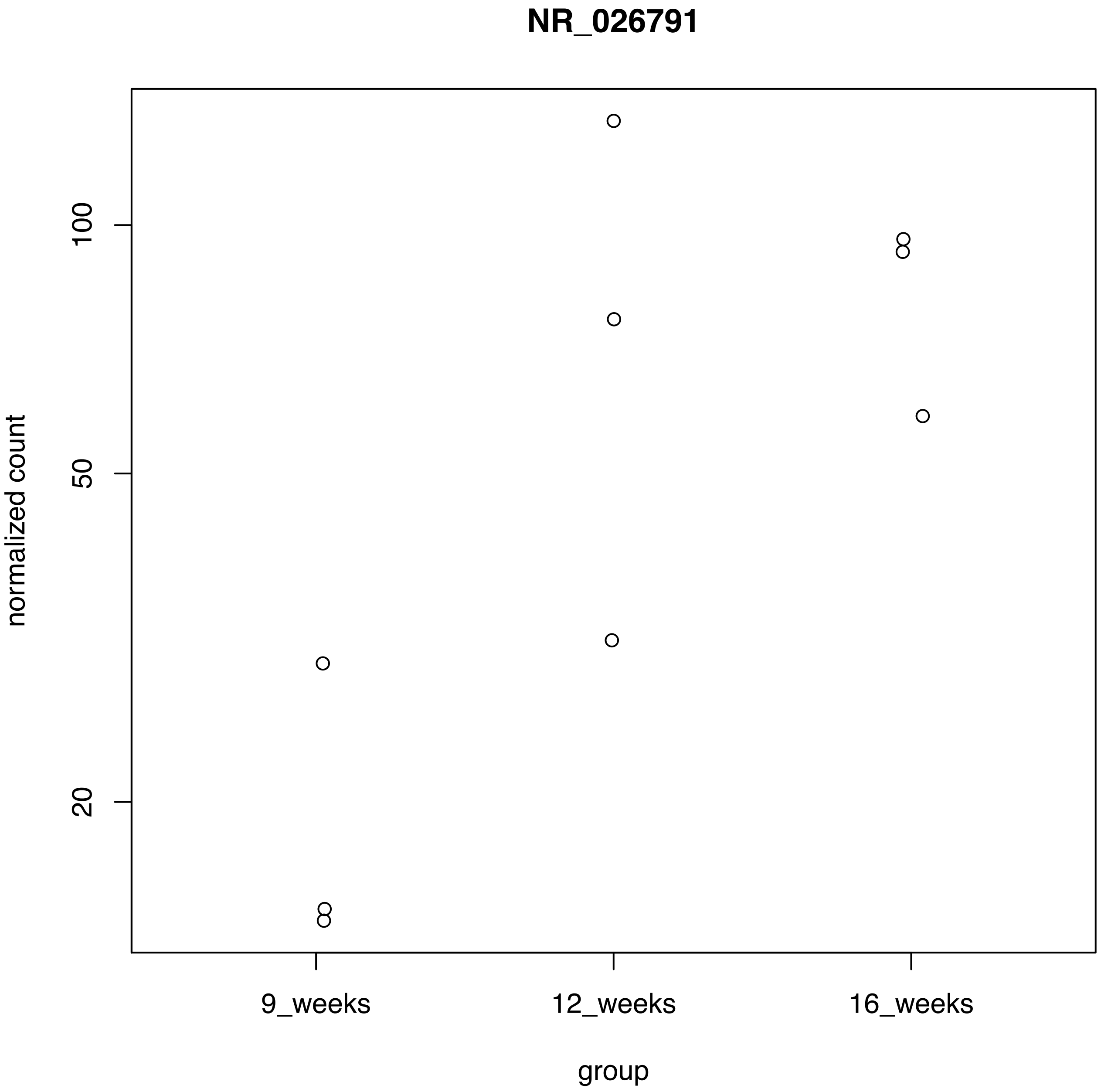

NR_026791
100
50
20
normalized count
9_weeks
12_weeks
16_weeks
group

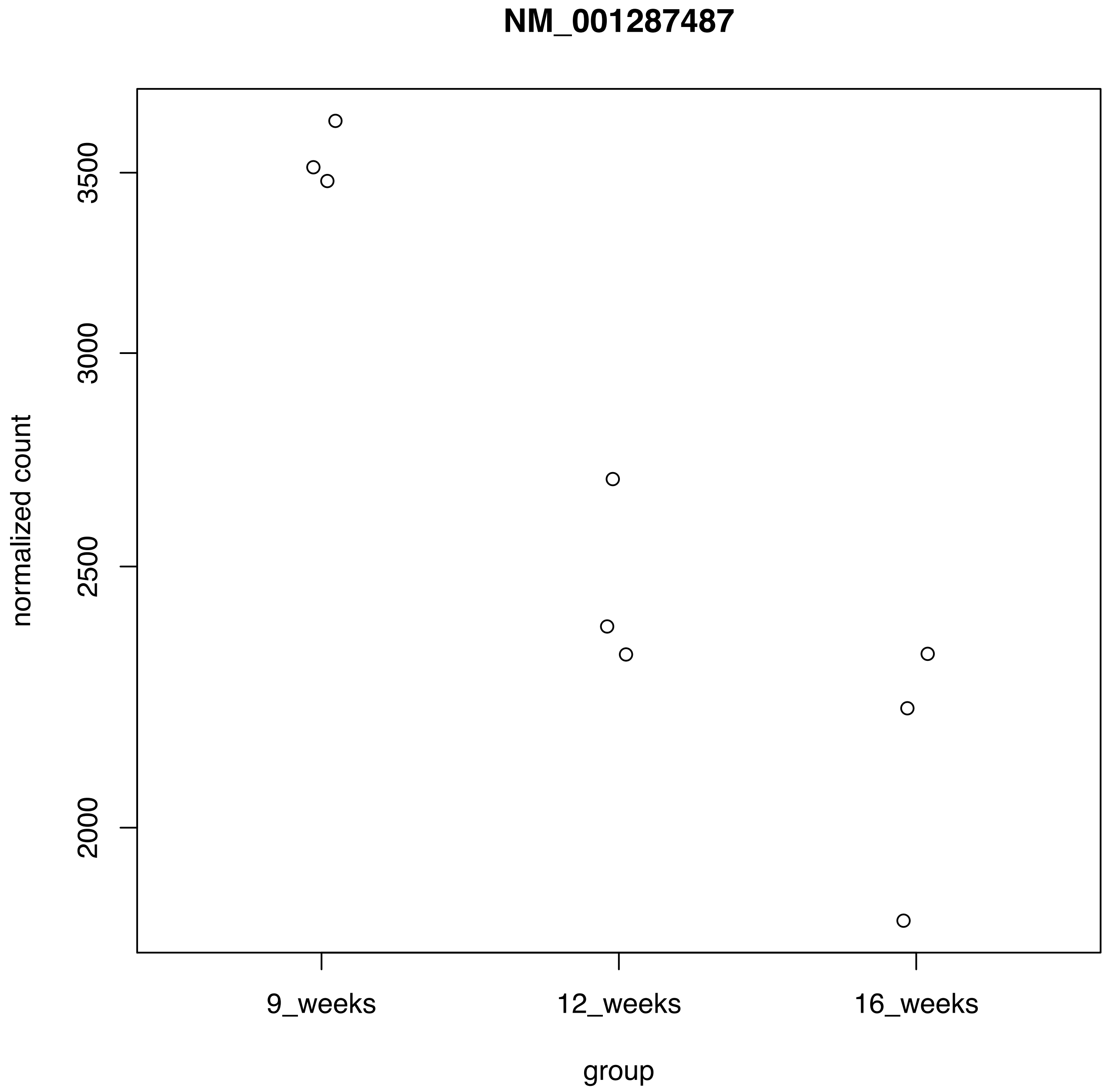
NM_001287487
normalized count
3500
3000
2500
2000
9_weeks
12_weeks
16_weeks
group

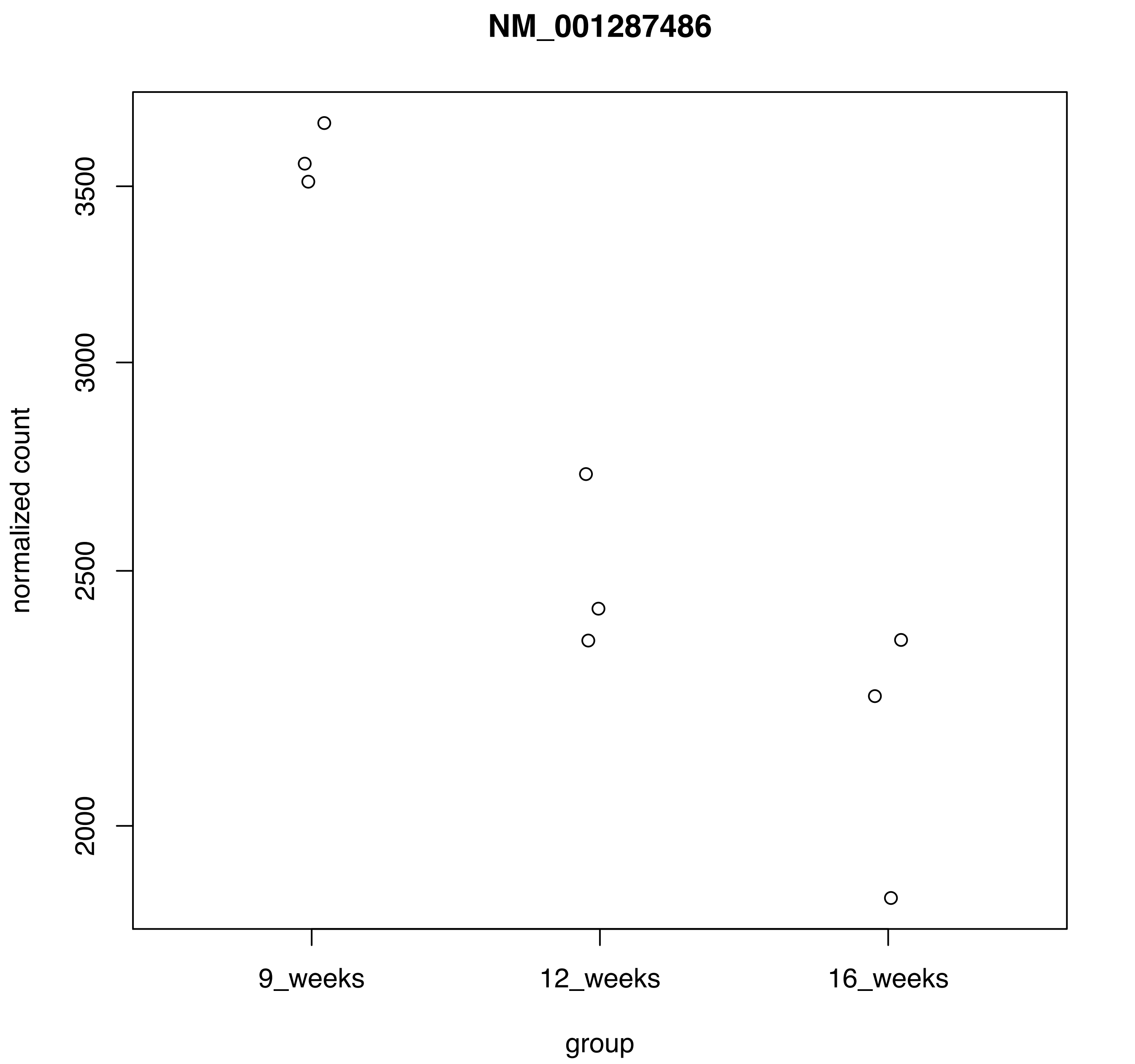
NM_001287486
normalized count
3500
3000
2500
2000
9_weeks
12_weeks
16_weeks
group

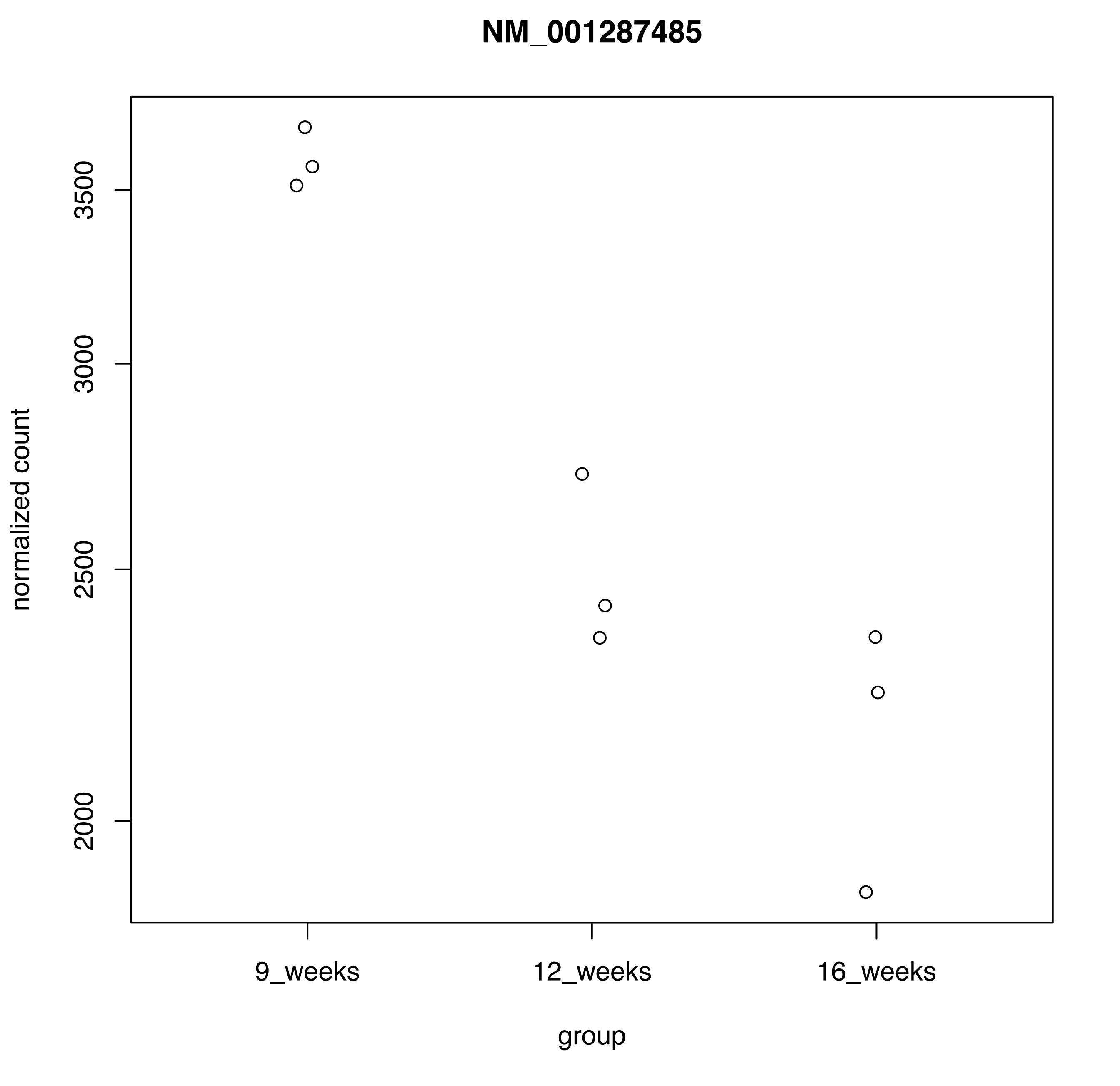
NM_001287485
normalized count
3500
3000
2500
2000
9_weeks
12_weeks
16_weeks
group

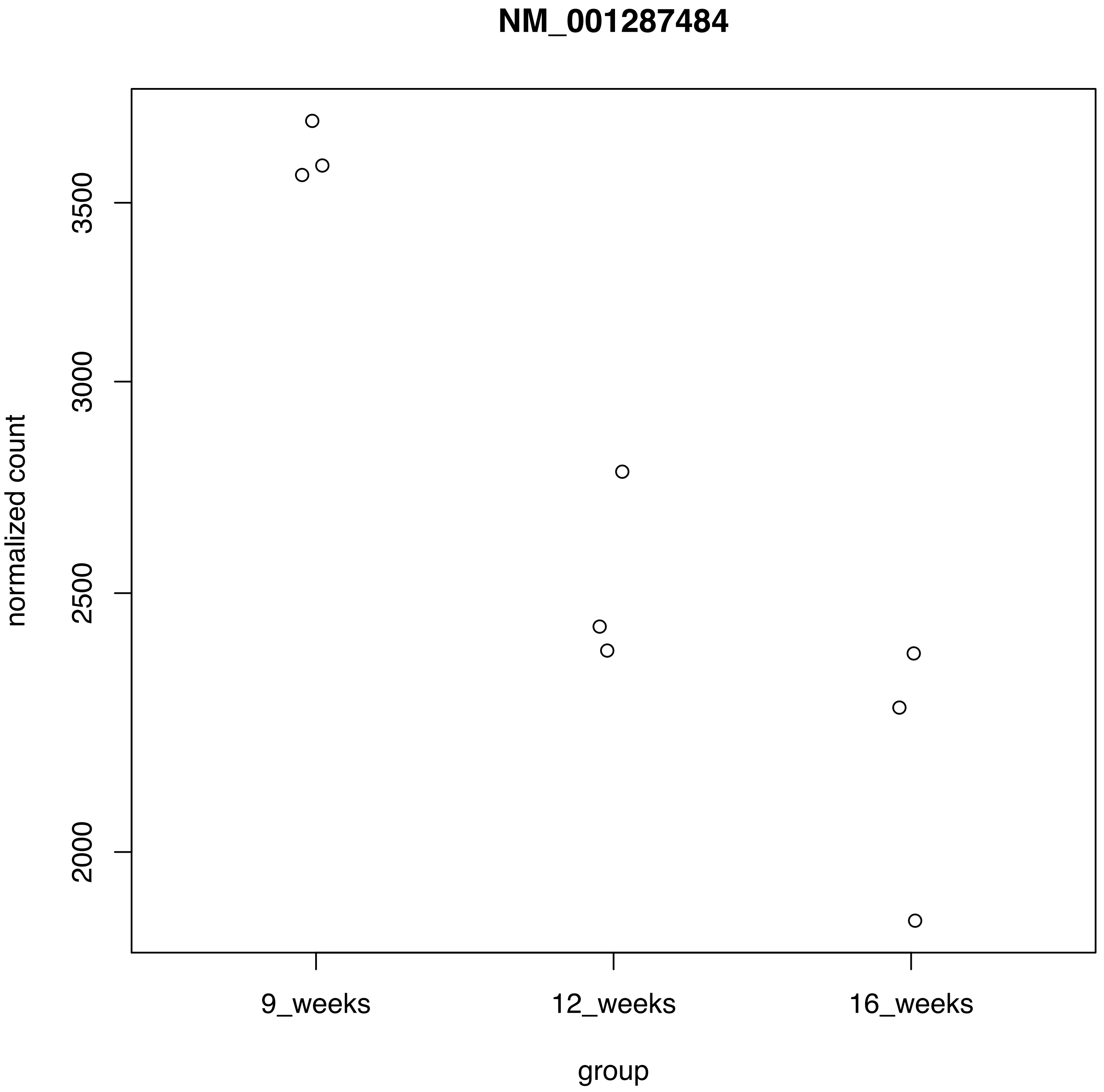

NM_001287484
normalized count
2000
2500
3000
3500
9_weeks
12_weeks
16_weeks
group

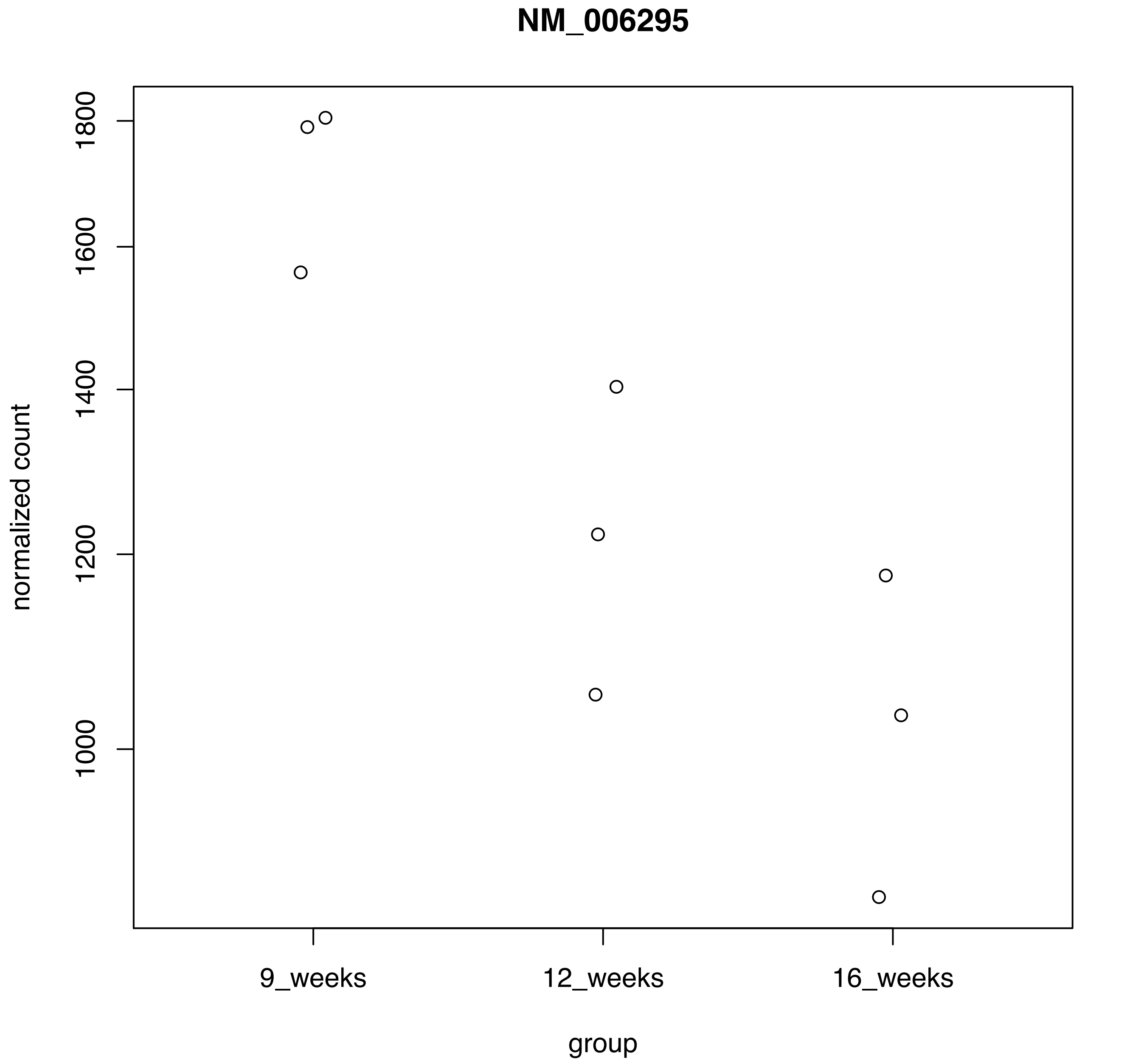
NM_006295
normalized count
1800
1600
1400
1200
1000
9_weeks
12_weeks
16_weeks
group

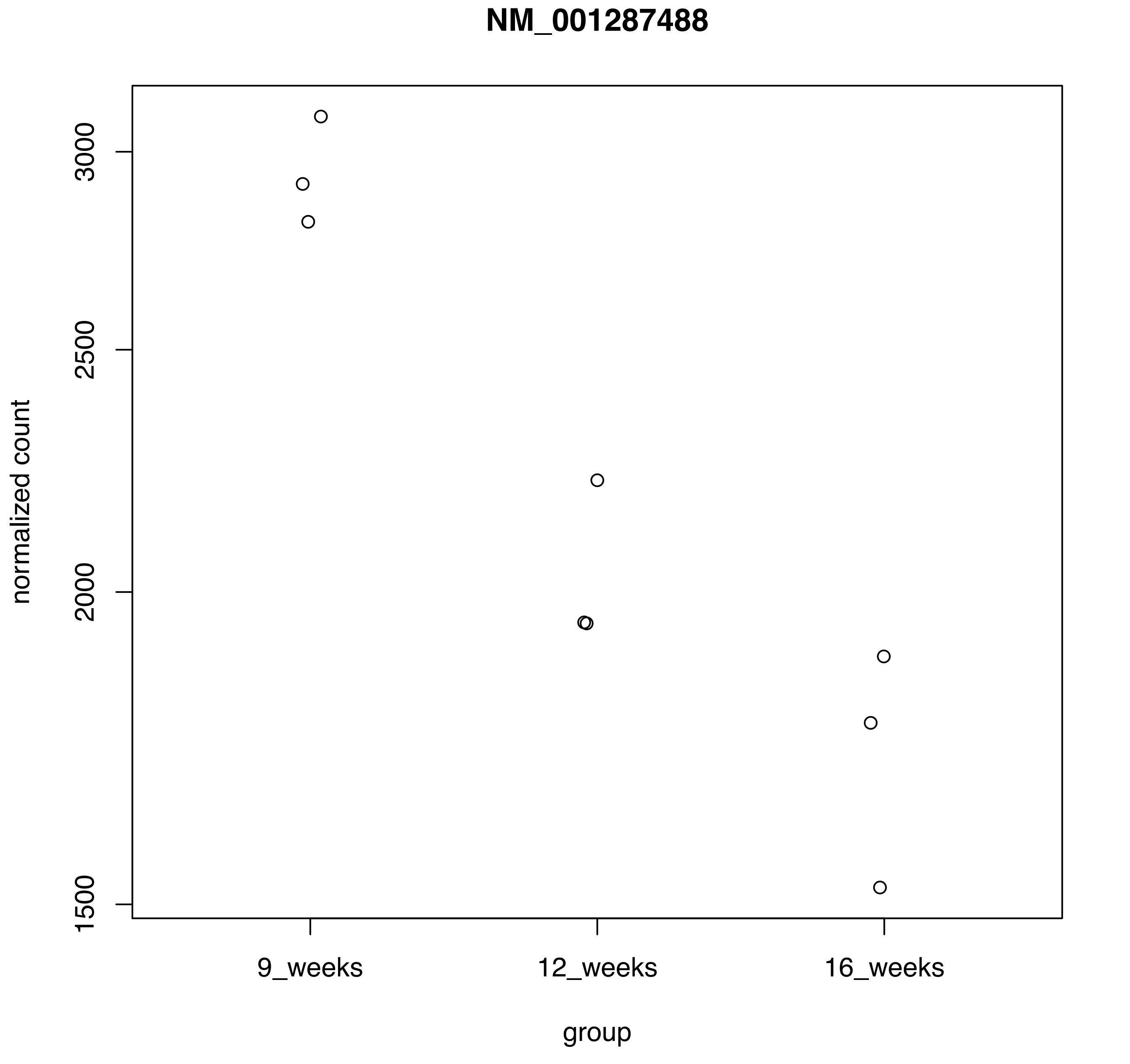
NM_001287488
normalized count
3000
2500
2000
1500
9_weeks
12_weeks
16_weeks
group

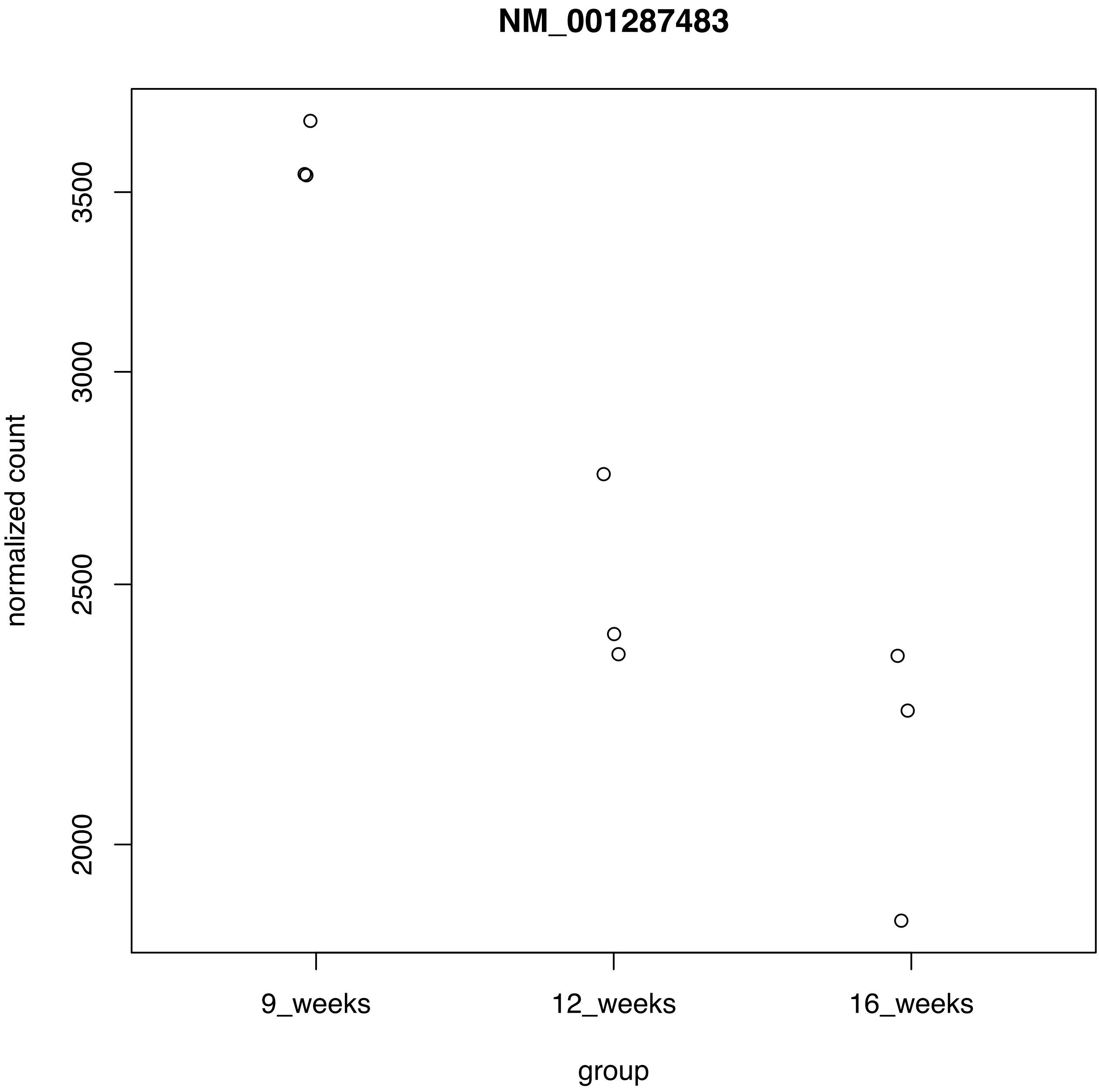
NM_001287483
normalized count
2000
2500
3000
3500
9_weeks
12_weeks
16_weeks
group

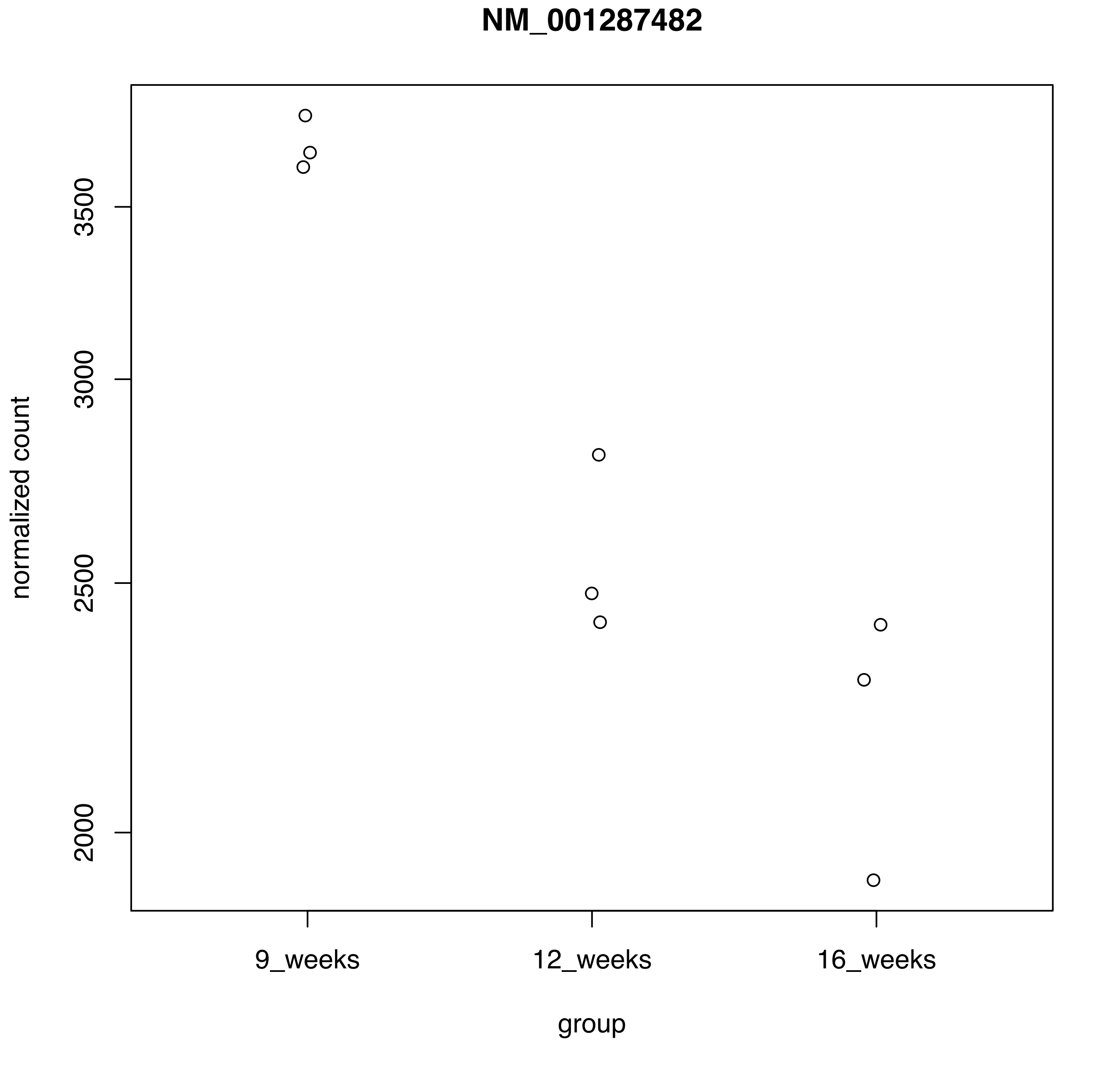

NM_001287482
normalized count
3500
3000
2500
2000
9_weeks
12_weeks
16_weeks
group

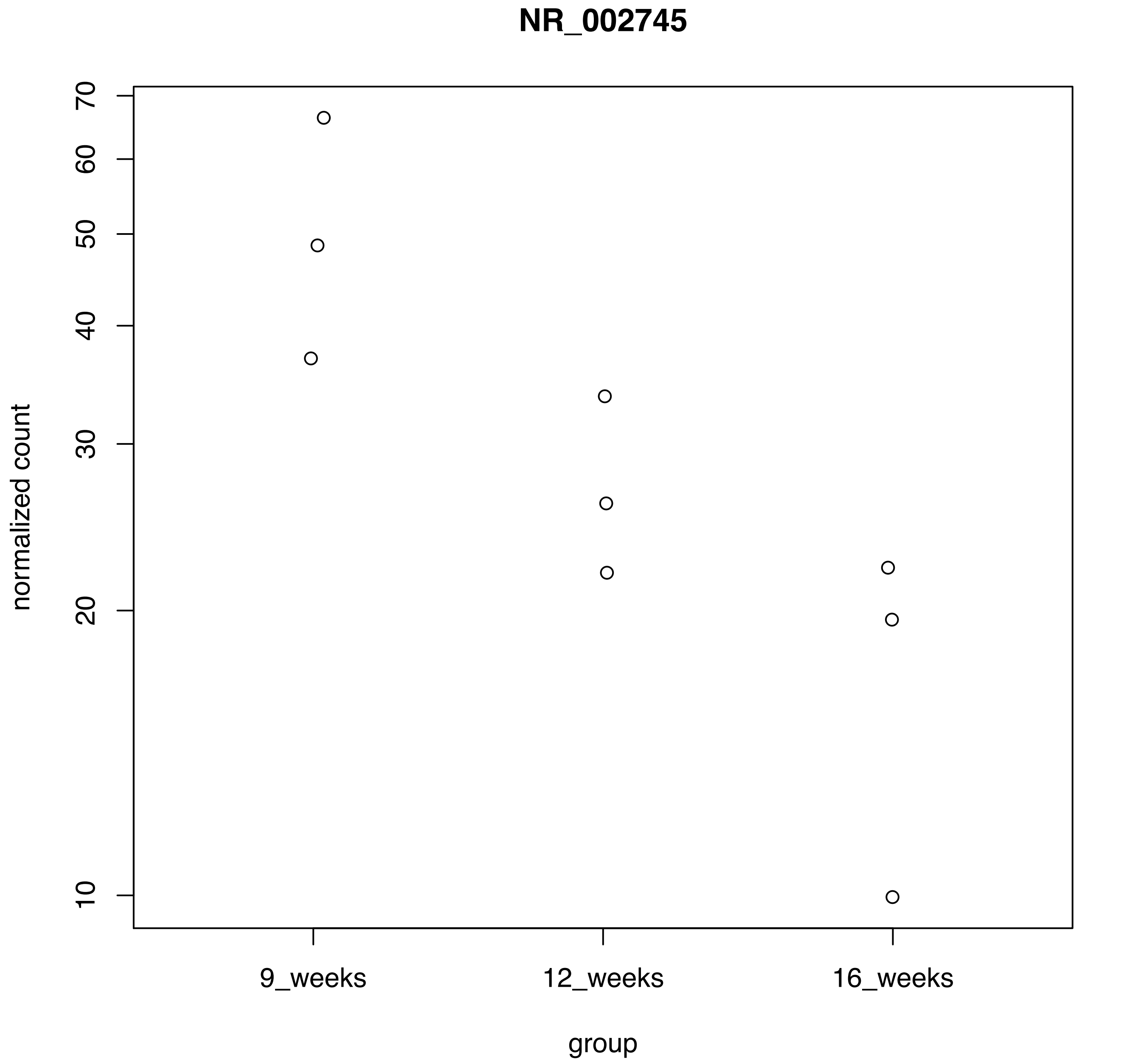
NR_002745
normalized count
70
60
50
40
30
20
10
9_weeks
12_weeks
16_weeks
group

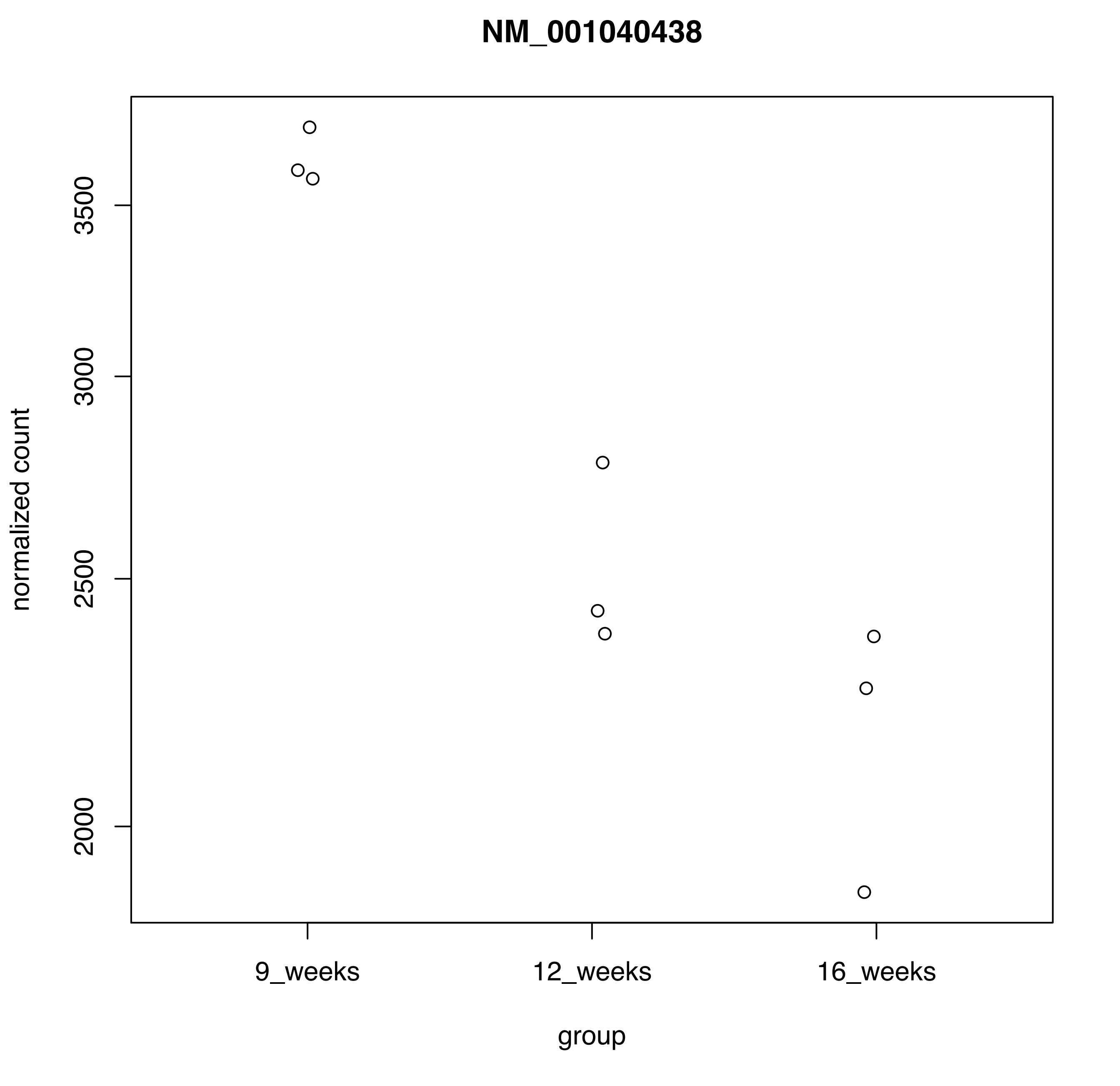
NM_001040438
normalized count
3500
3000
2500
2000
9_weeks
12_weeks
16_weeks
group

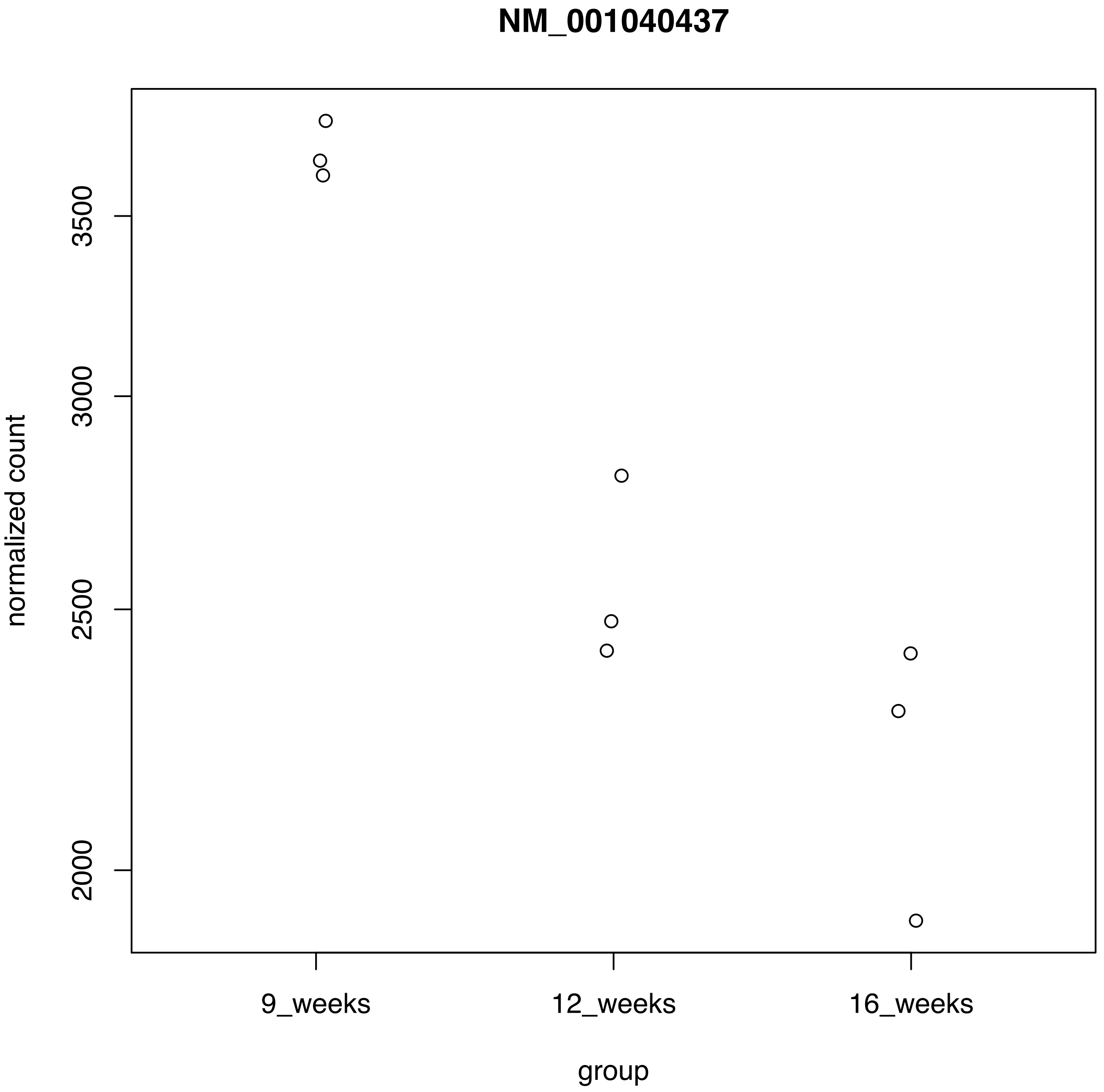
NM_001040437
normalized count
3500
3000
2500
2000
9_weeks
12_weeks
16_weeks
group

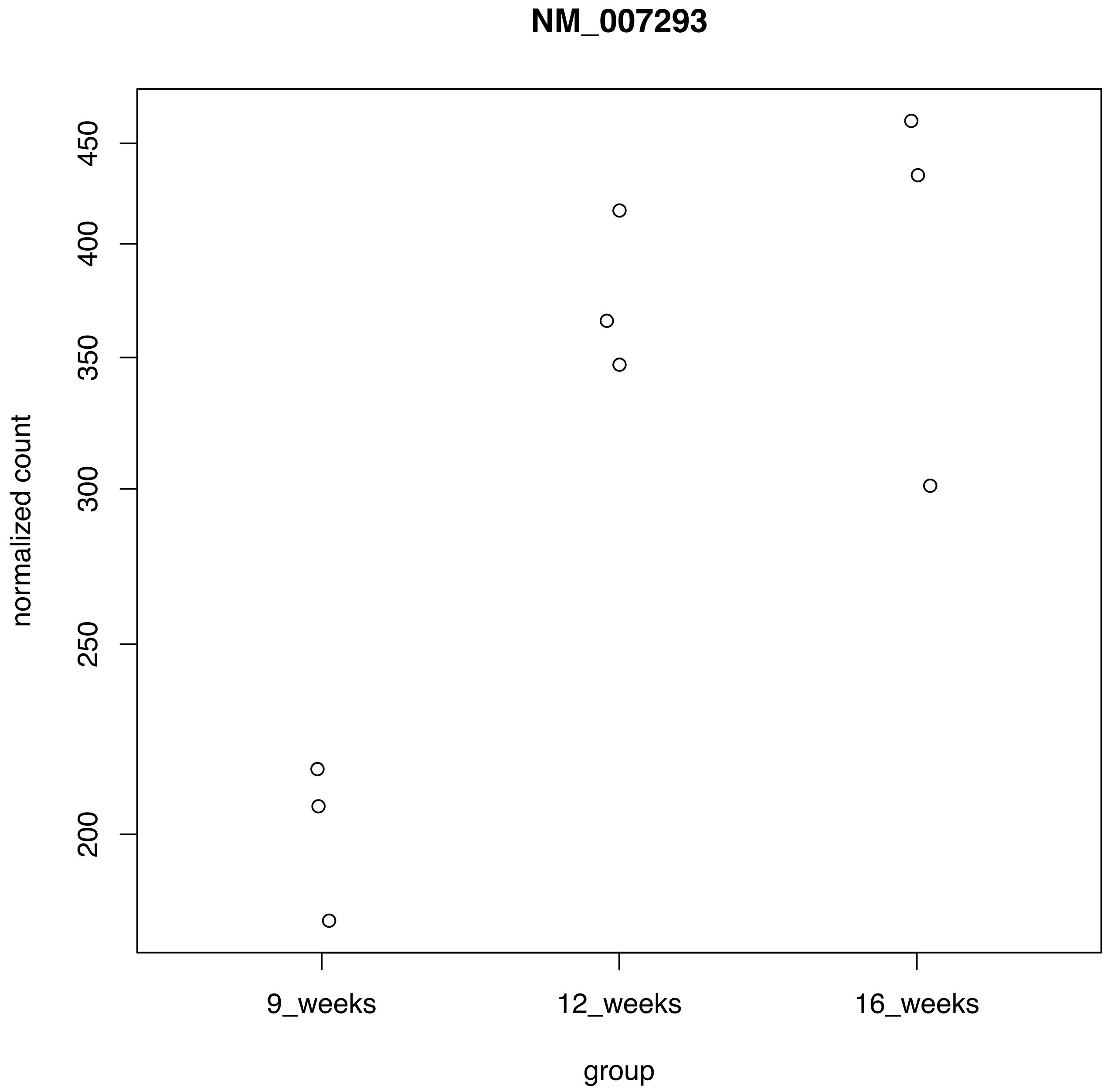
NM_007293
normalized count
450
400
350
300
250
200
9_weeks
12_weeks
16_weeks
group

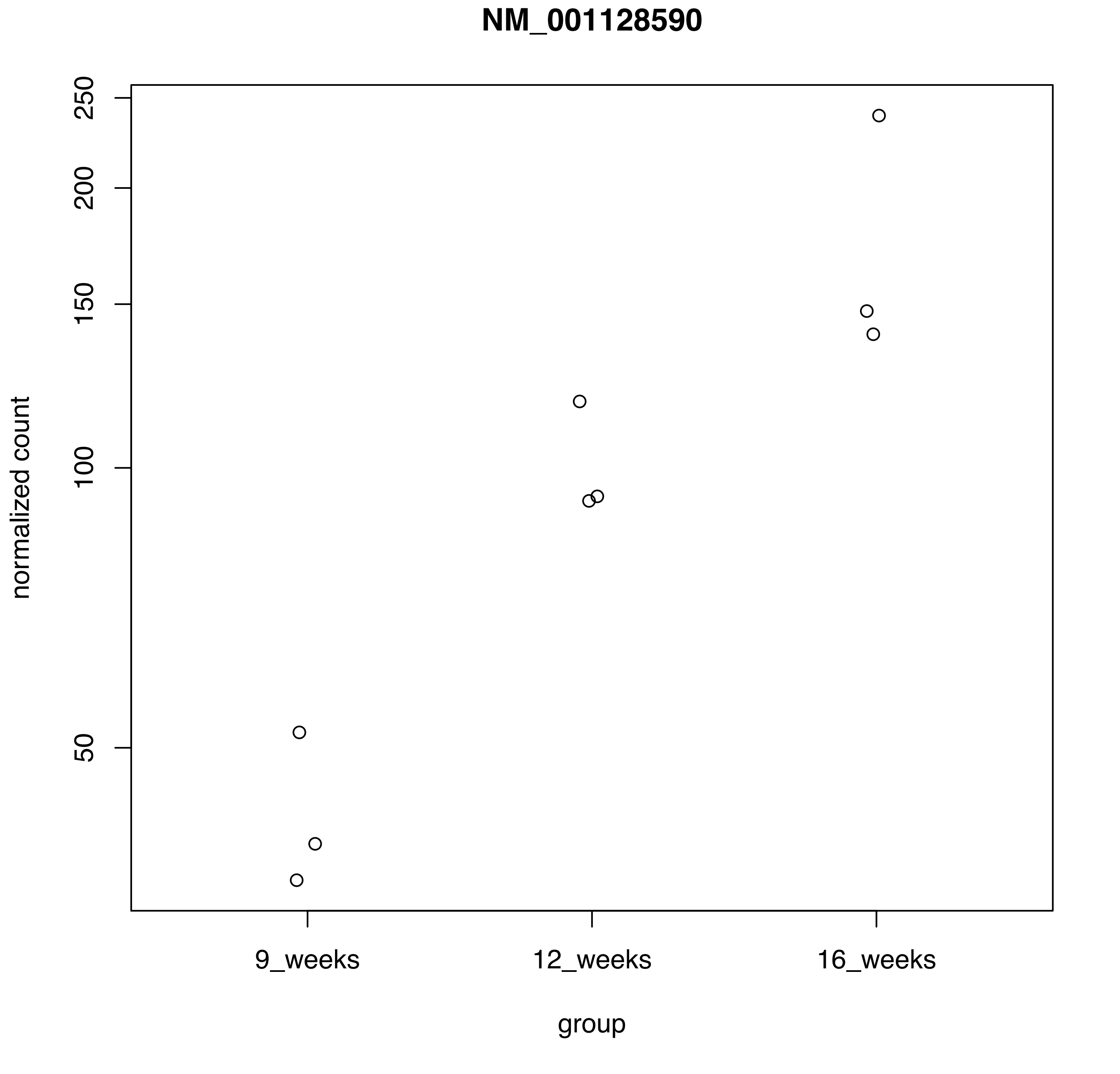
NM_001128590
normalized count
250
200
150
100
50
9_weeks
12_weeks
16_weeks
group

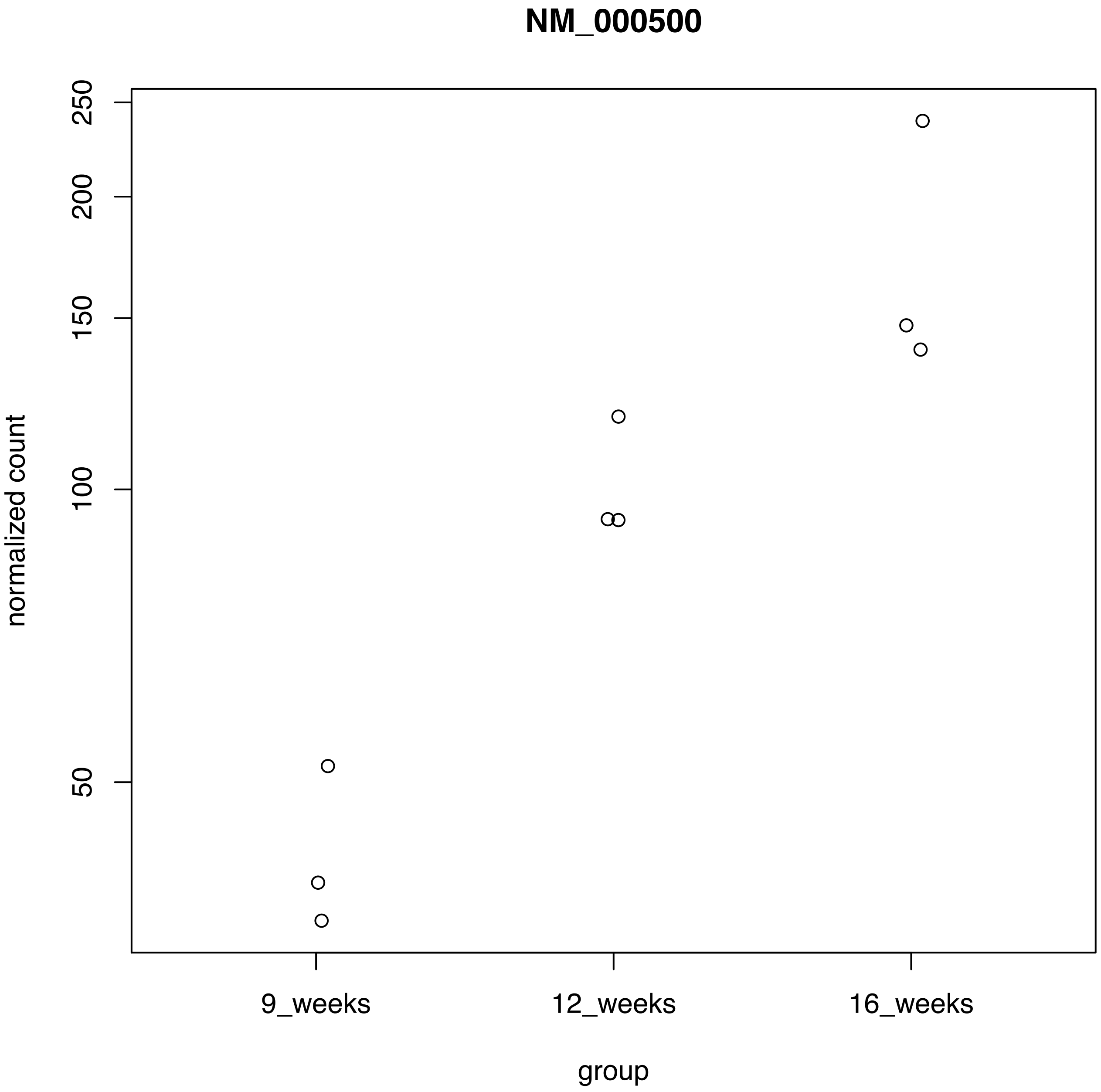
NM_000500
normalized count
250
200
150
100
50
9_weeks
12_weeks
16_weeks
group

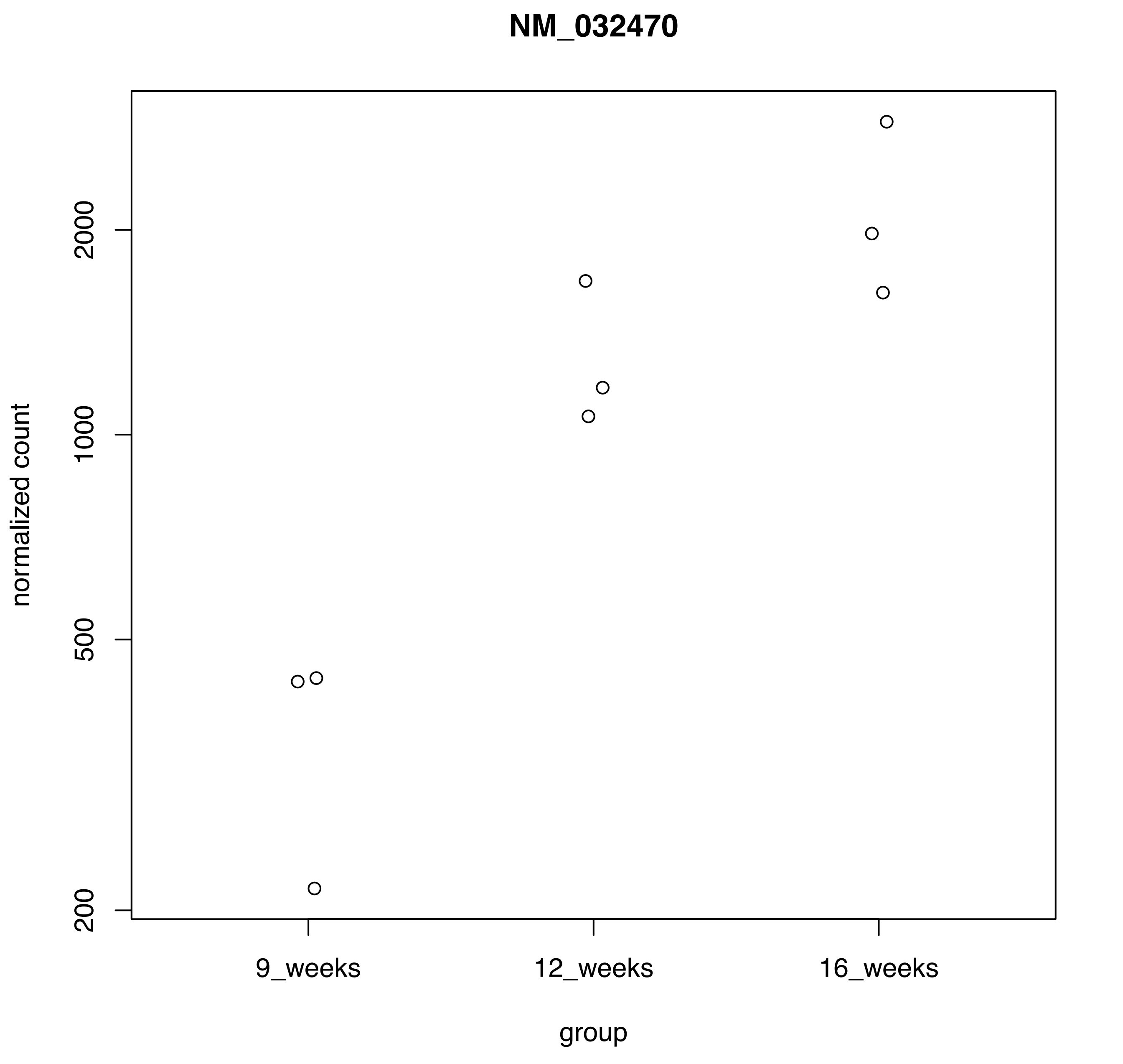
NM_032470
normalized count
2000
1000
500
200
9_weeks
12_weeks
16_weeks
group

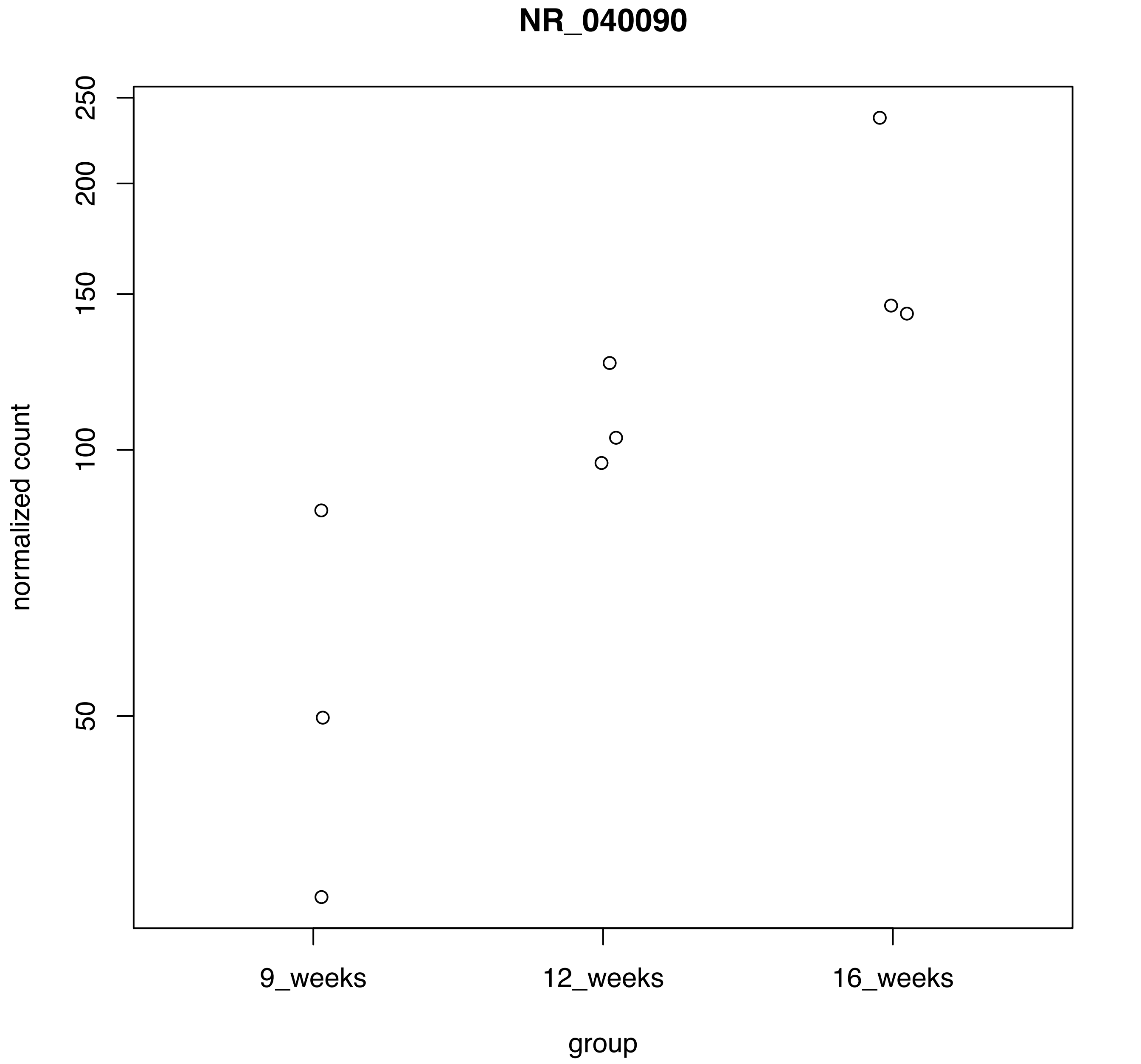

NR_040090
normalized count
250
200
150
100
50
9_weeks
12_weeks
16_weeks
group

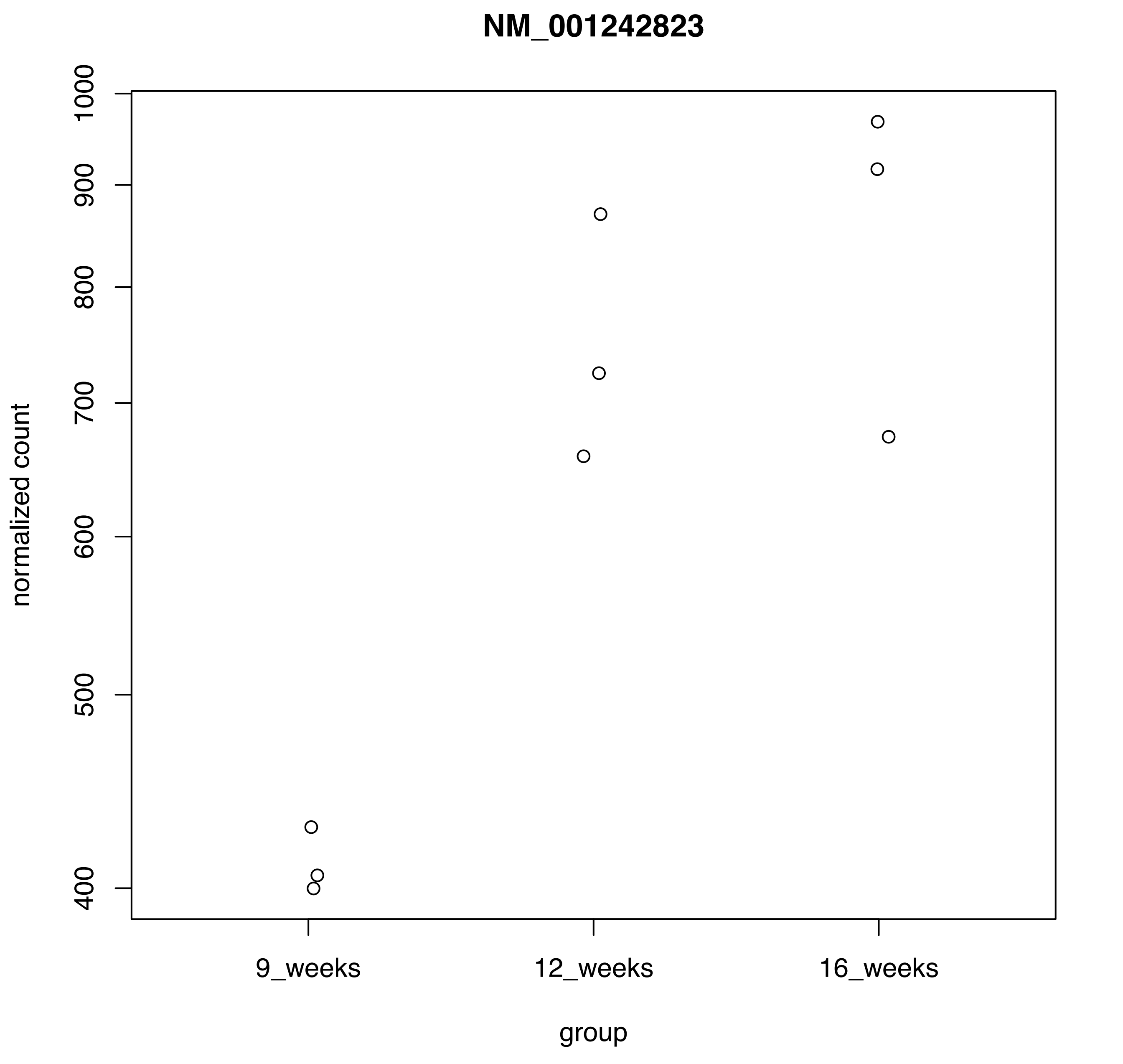

NM_001242823
normalized count
1000
900
800
700
600
500
400
9_weeks
12_weeks
16_weeks
group

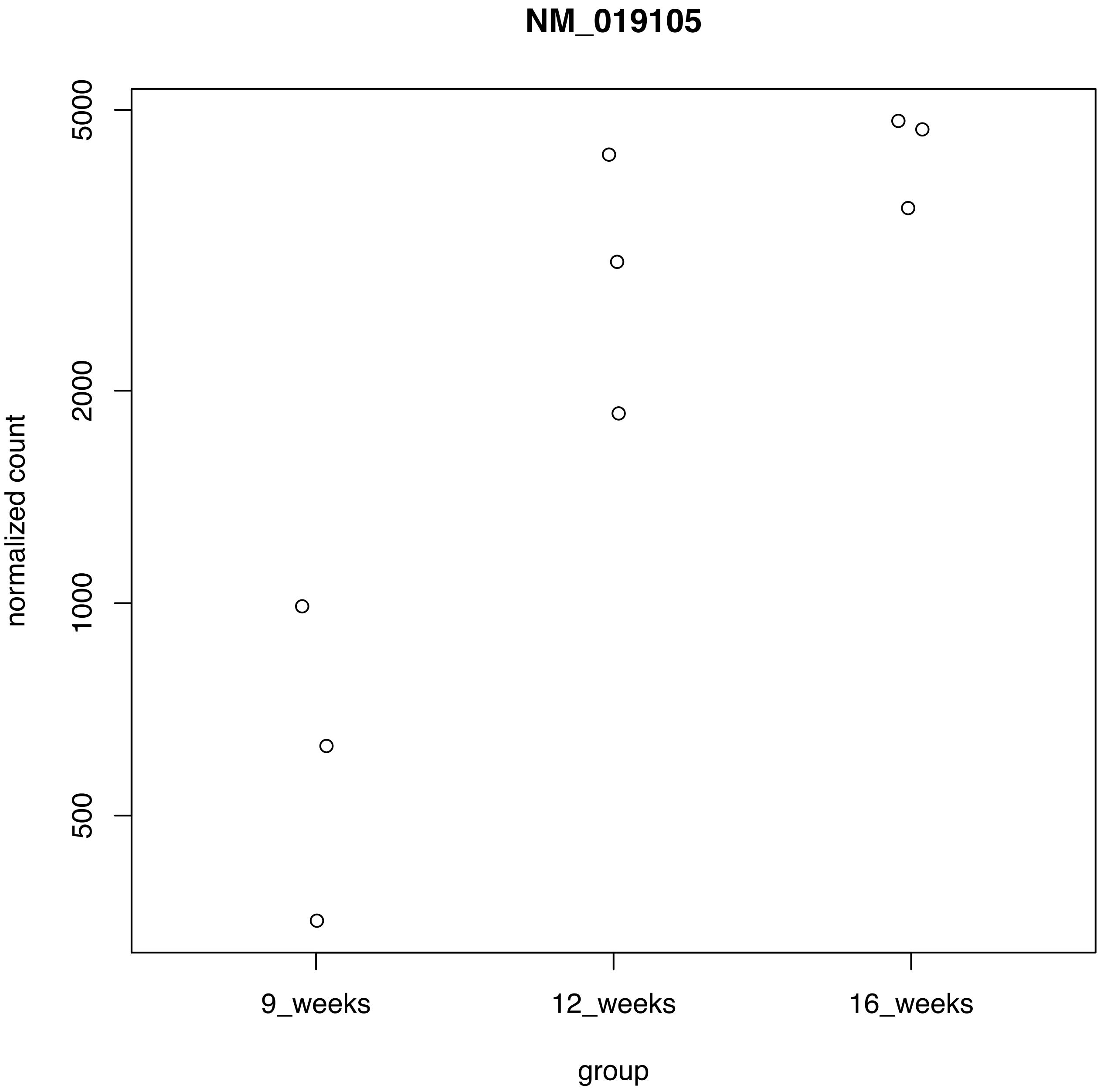
NM_019105
normalized count
5000
2000
1000
500
9_weeks
12_weeks
16_weeks
group

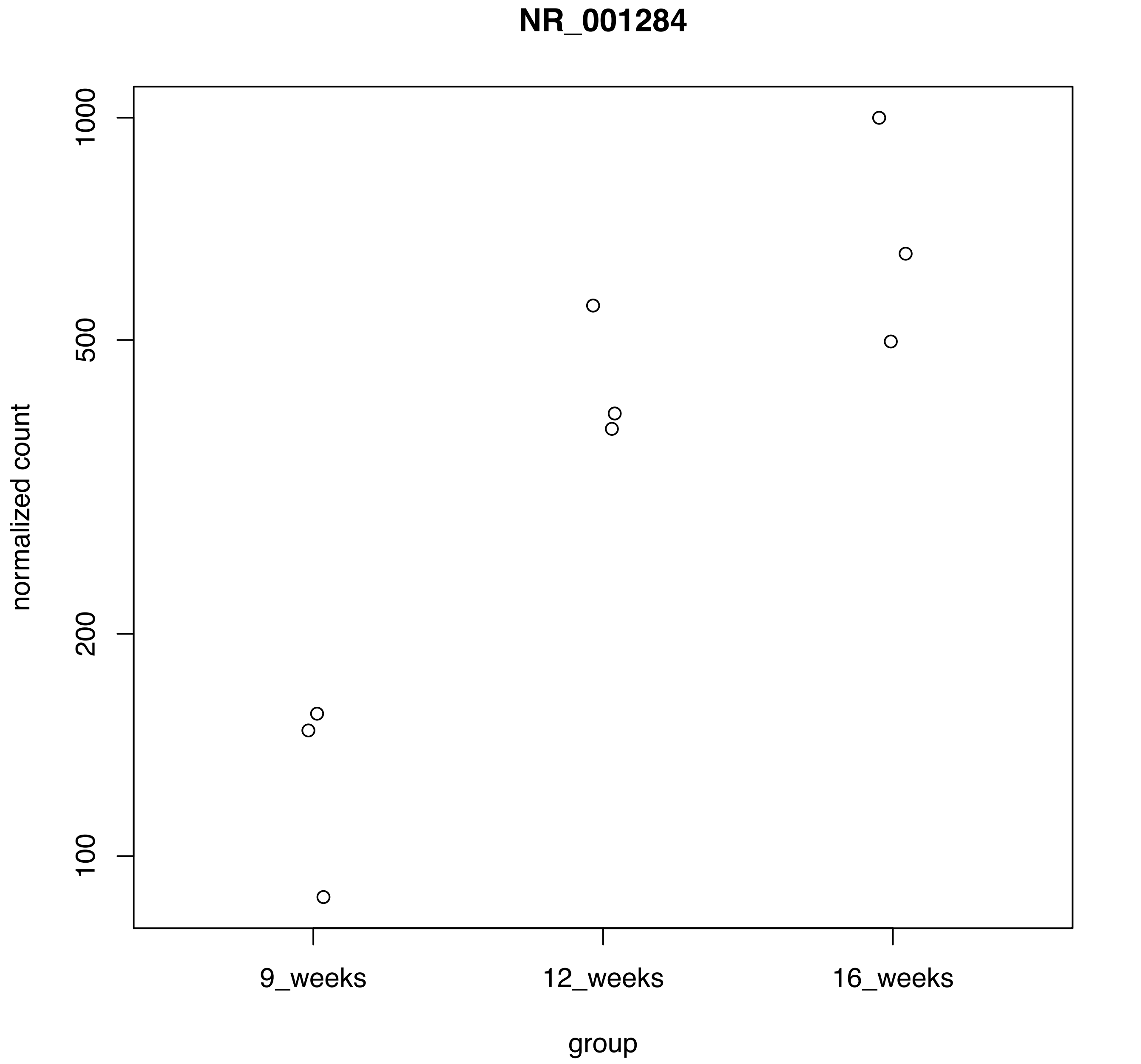
NR_001284
normalized count
1000
500
200
100
9_weeks
12_weeks
16_weeks
group

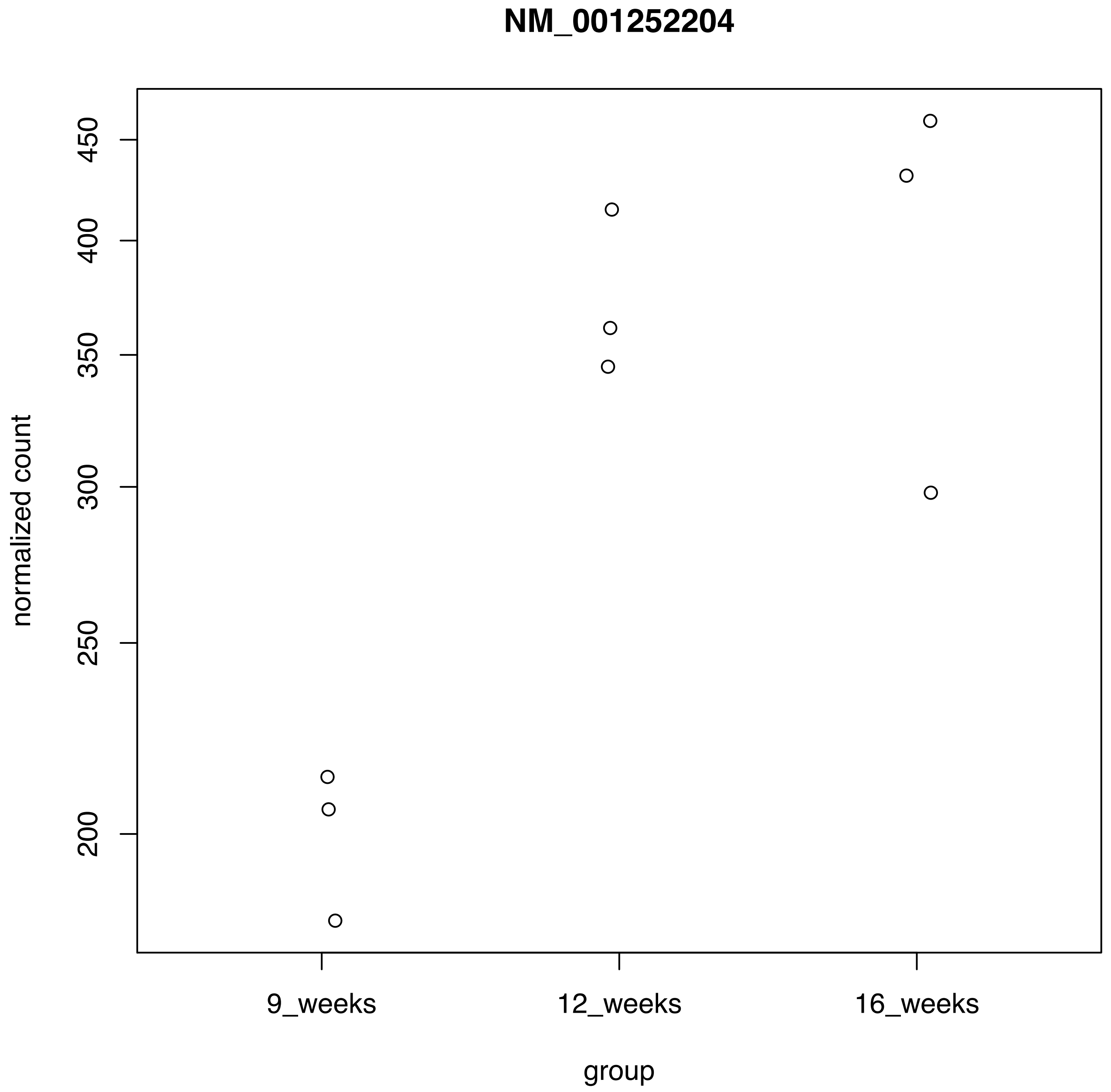
NM_001252204
normalized count
200
250
300
350
400
450
9_weeks
12_weeks
16_weeks
group

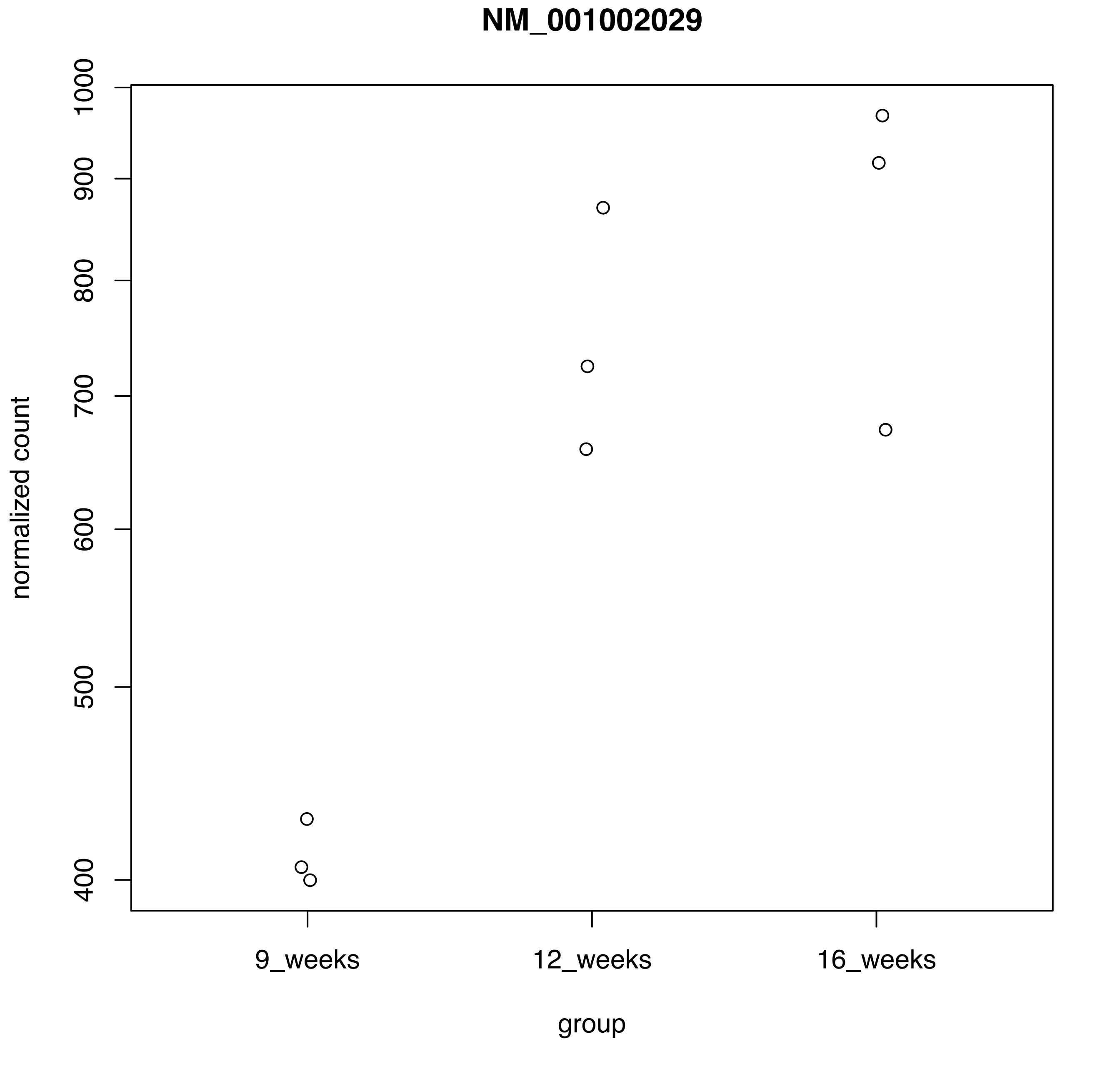
NM_001002029
normalized count
1000
900
800
700
600
500
400
9_weeks
12_weeks
16_weeks
group

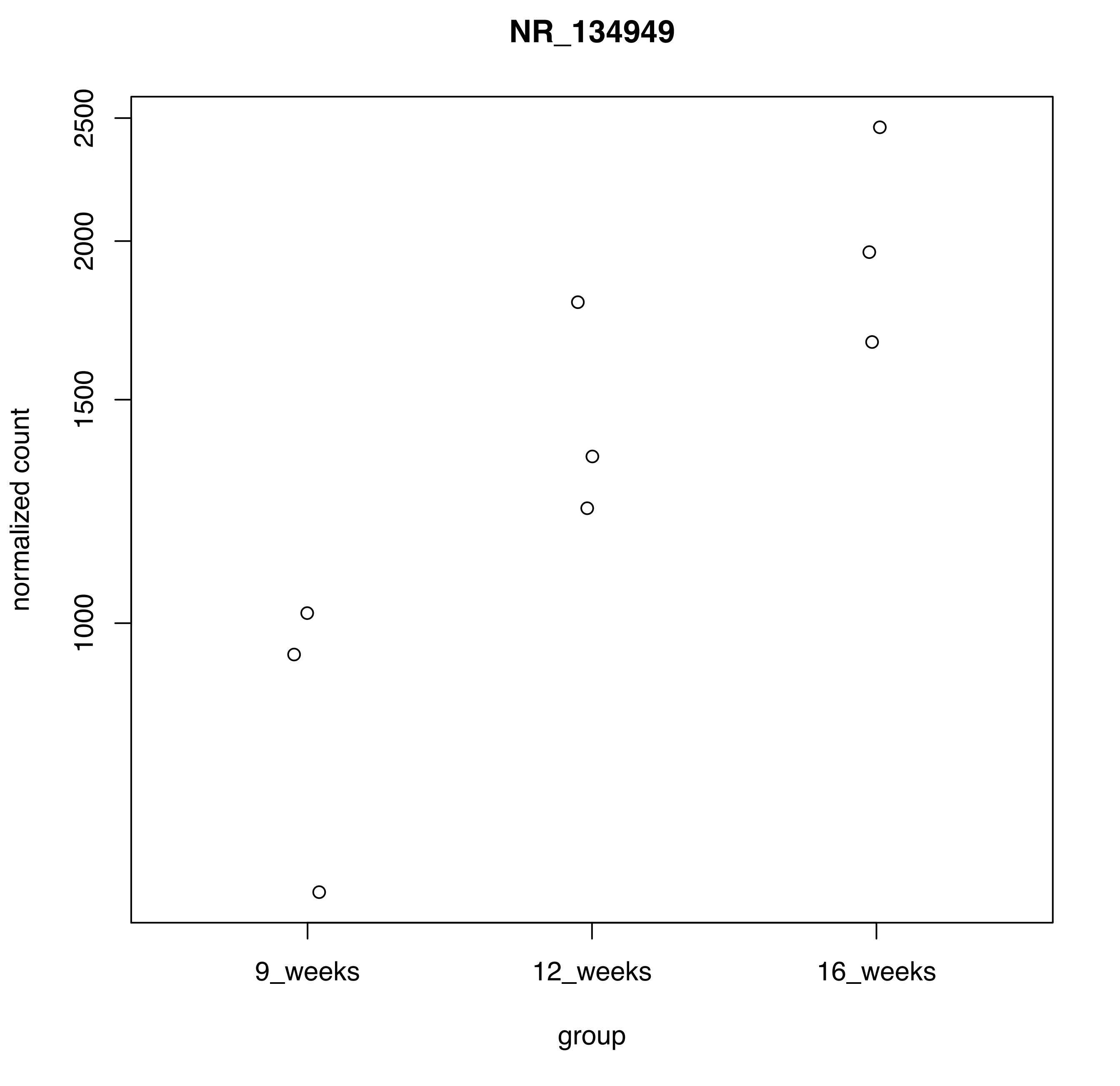
NR_134949
normalized count
2500
2000
1500
1000
9_weeks
12_weeks
16_weeks
group

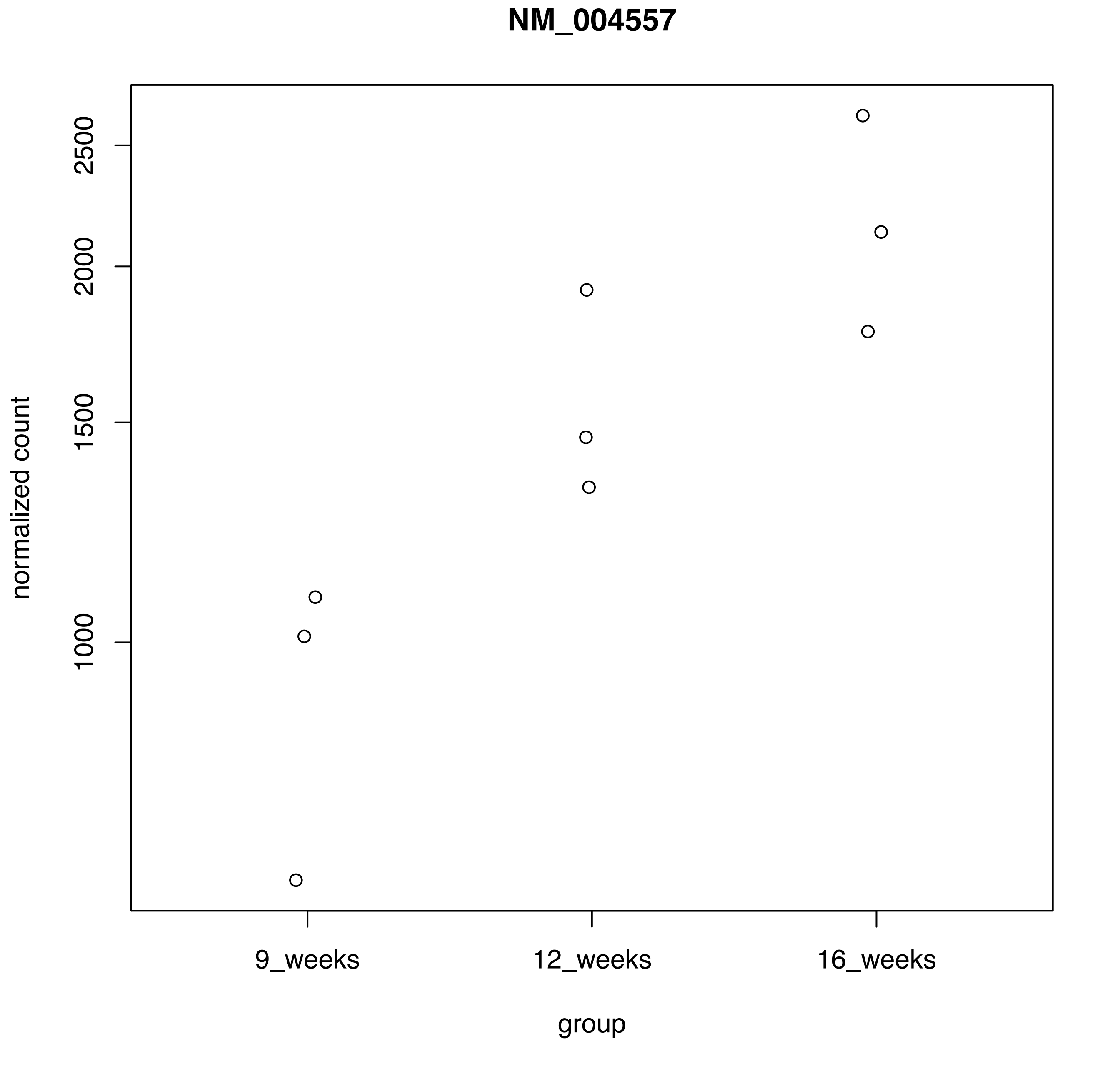
NM_004557
normalized count
2500
2000
1500
1000
9_weeks
12_weeks
16_weeks
group

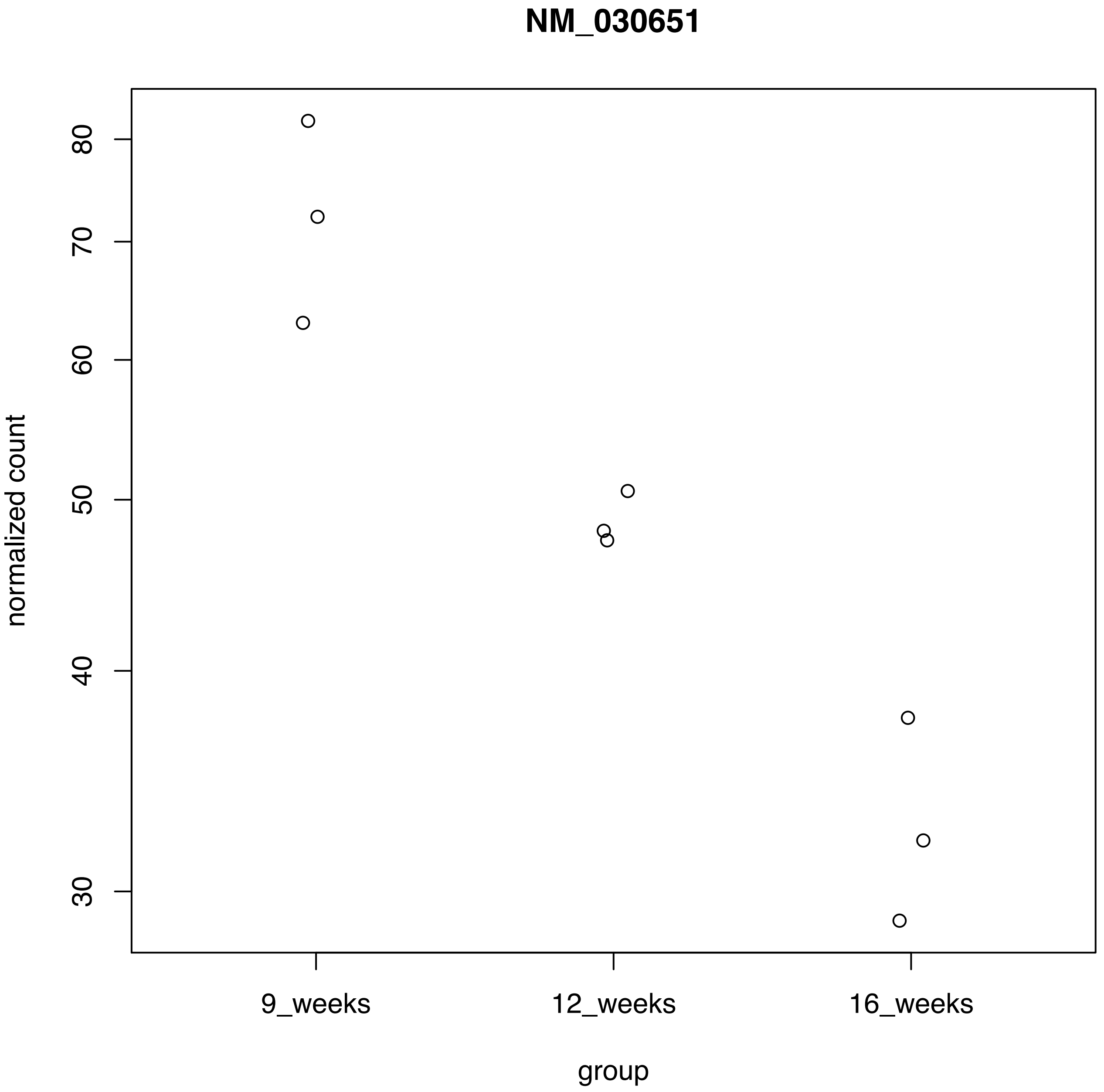
NM_030651
normalized count
30
40
50
60
70
80
9_weeks
12_weeks
16_weeks
group

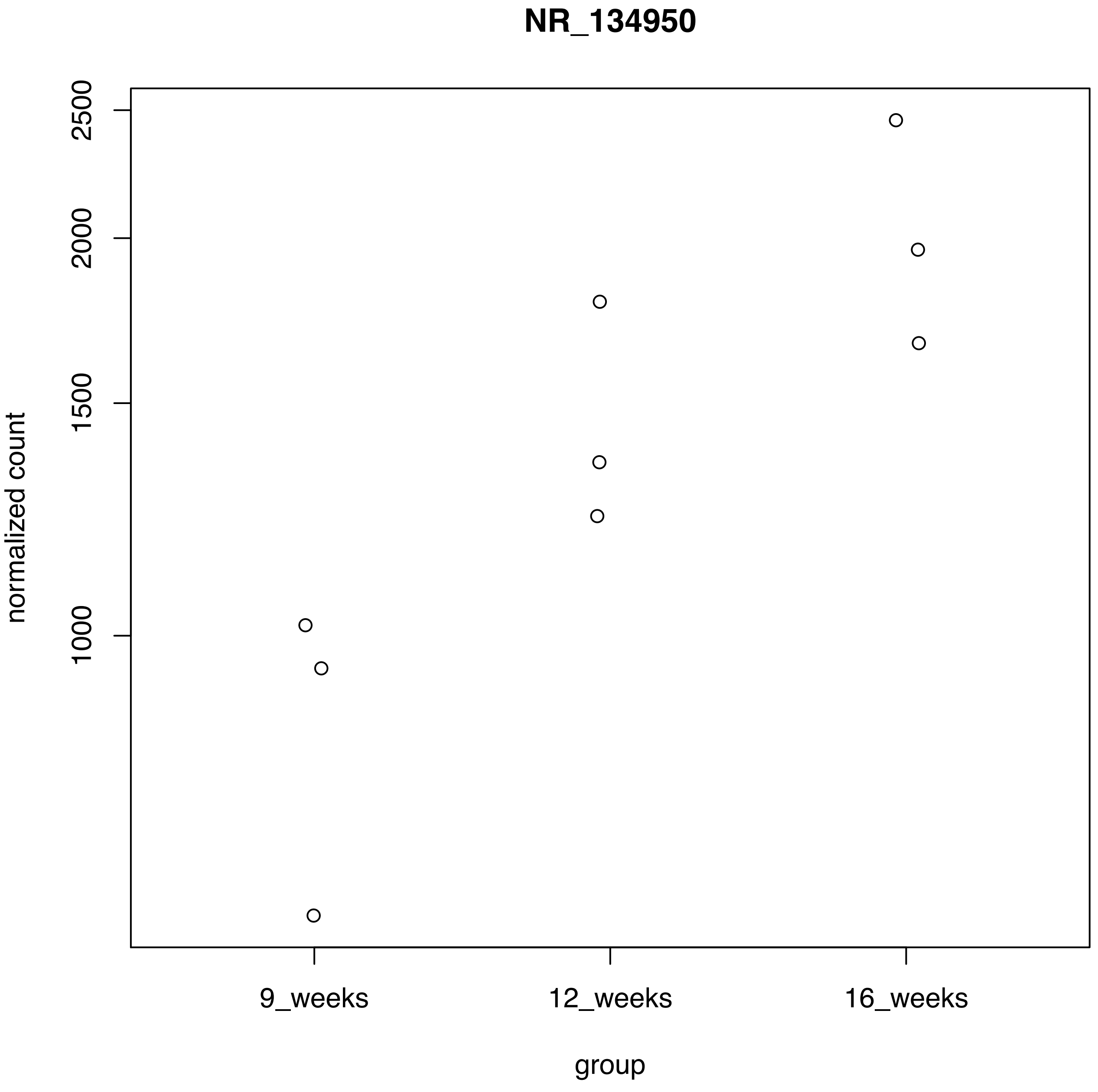
NR_134950
2500
2000
1500
1000
normalized count
9_weeks
12_weeks
16_weeks
group

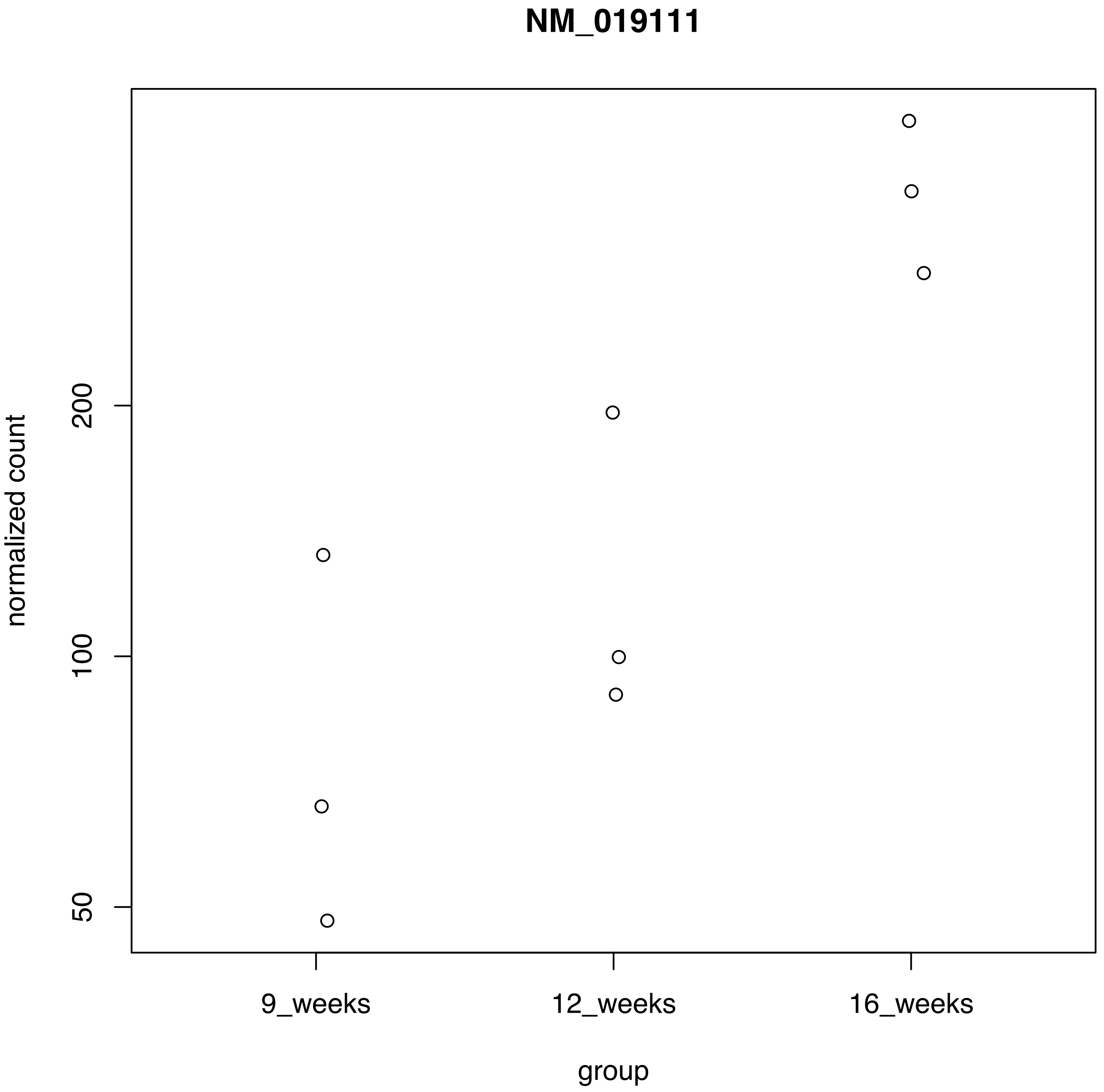
NM_019111
normalized count
200
100
50
9_weeks
12_weeks
16_weeks
group

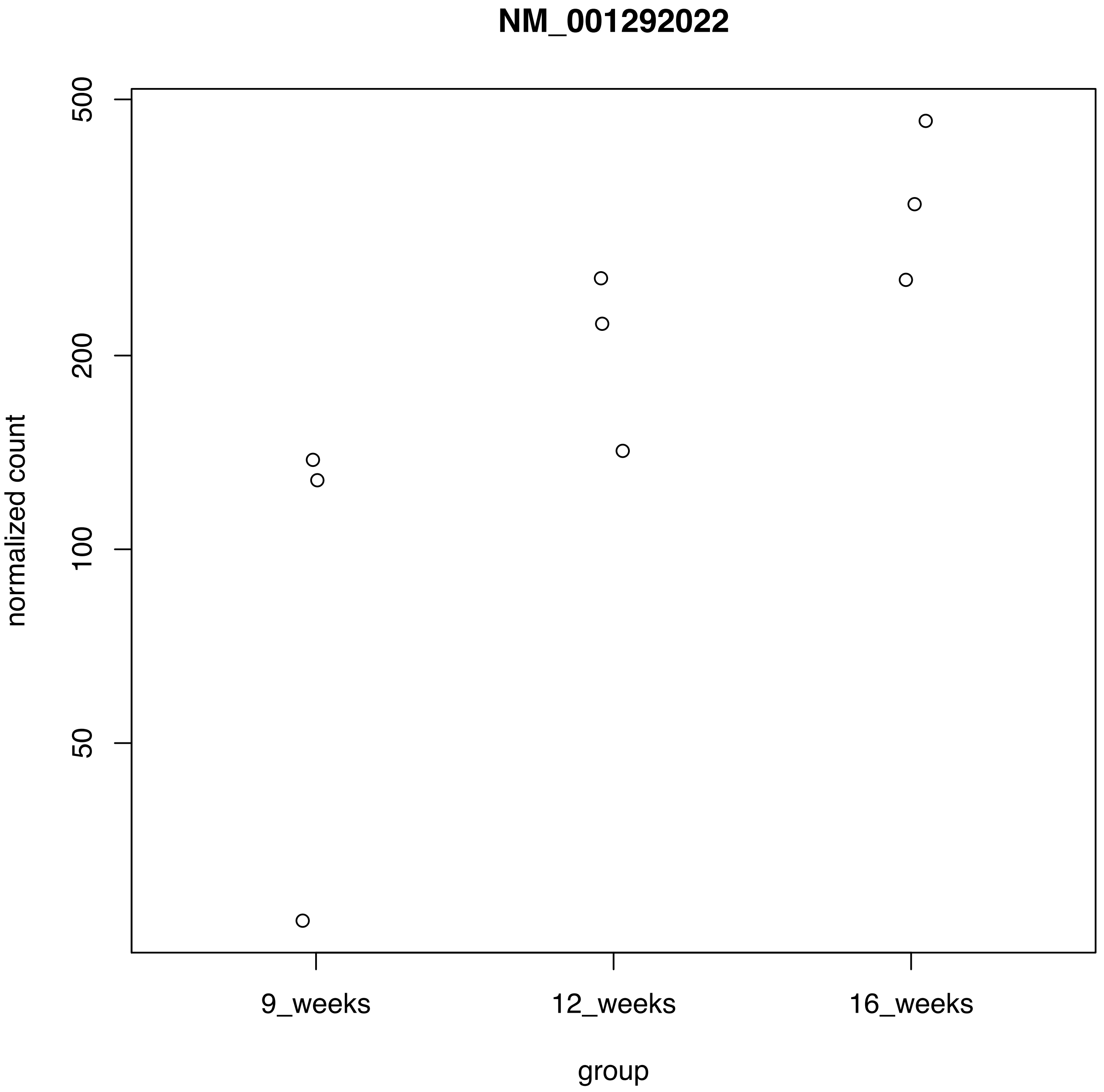

NM_001292022
500
200
100
50
normalized count
9_weeks
12_weeks
16_weeks
group

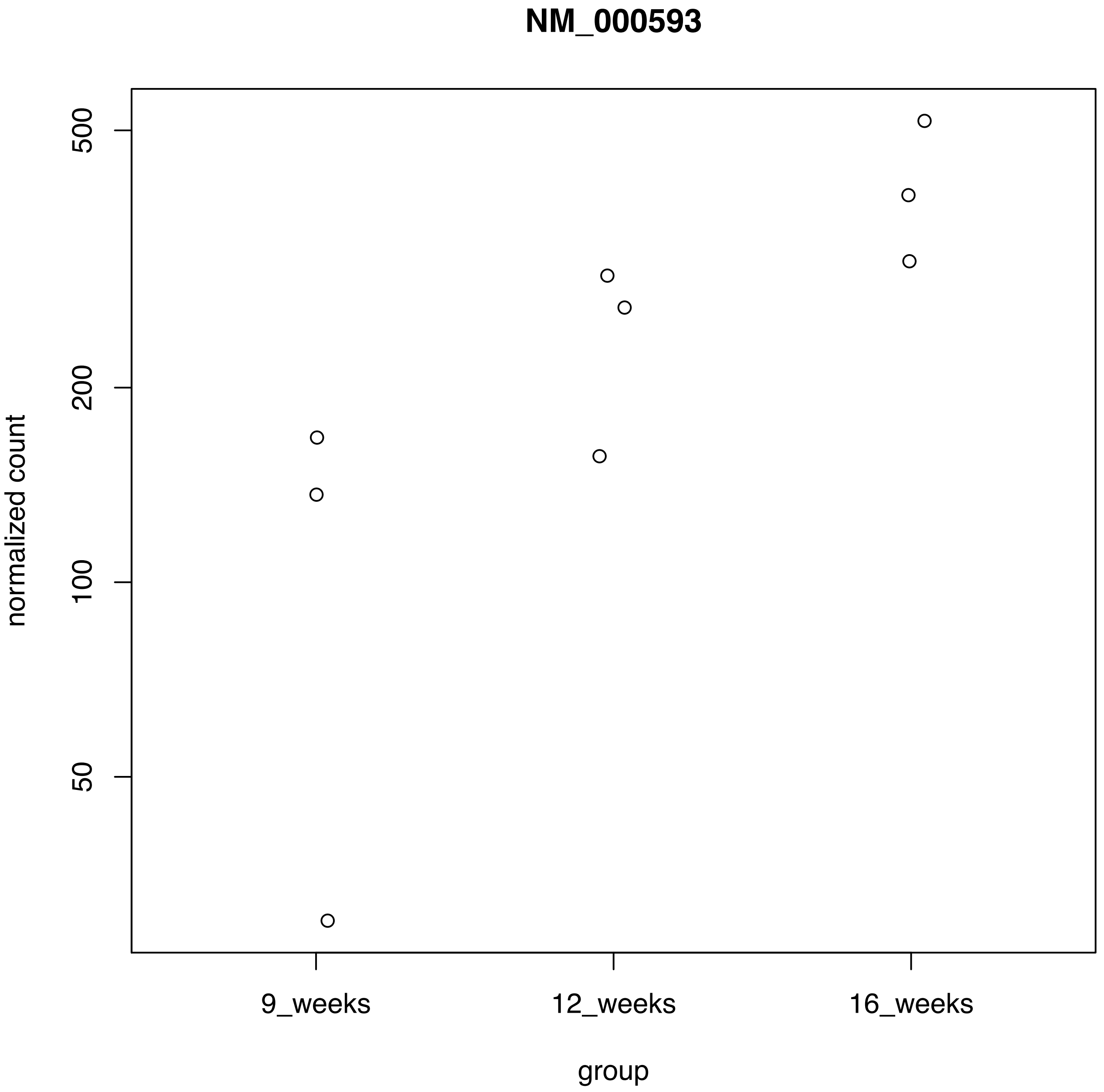
NM_000593
normalized count
500
200
100
50
9_weeks
12_weeks
16_weeks
group

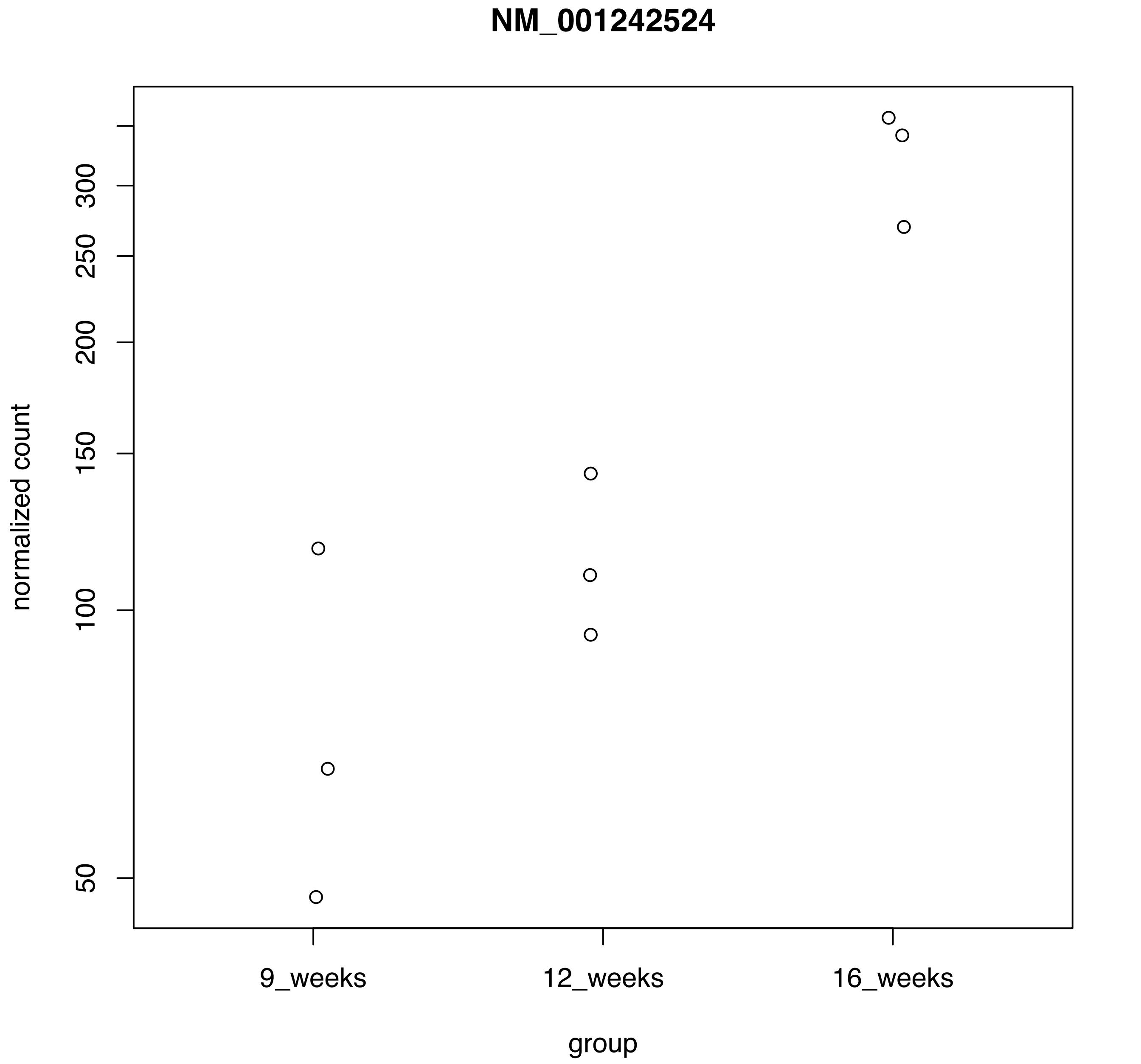

NM_001242524
normalized count
50
100
150
200
250
300
9_weeks
12_weeks
16_weeks
group

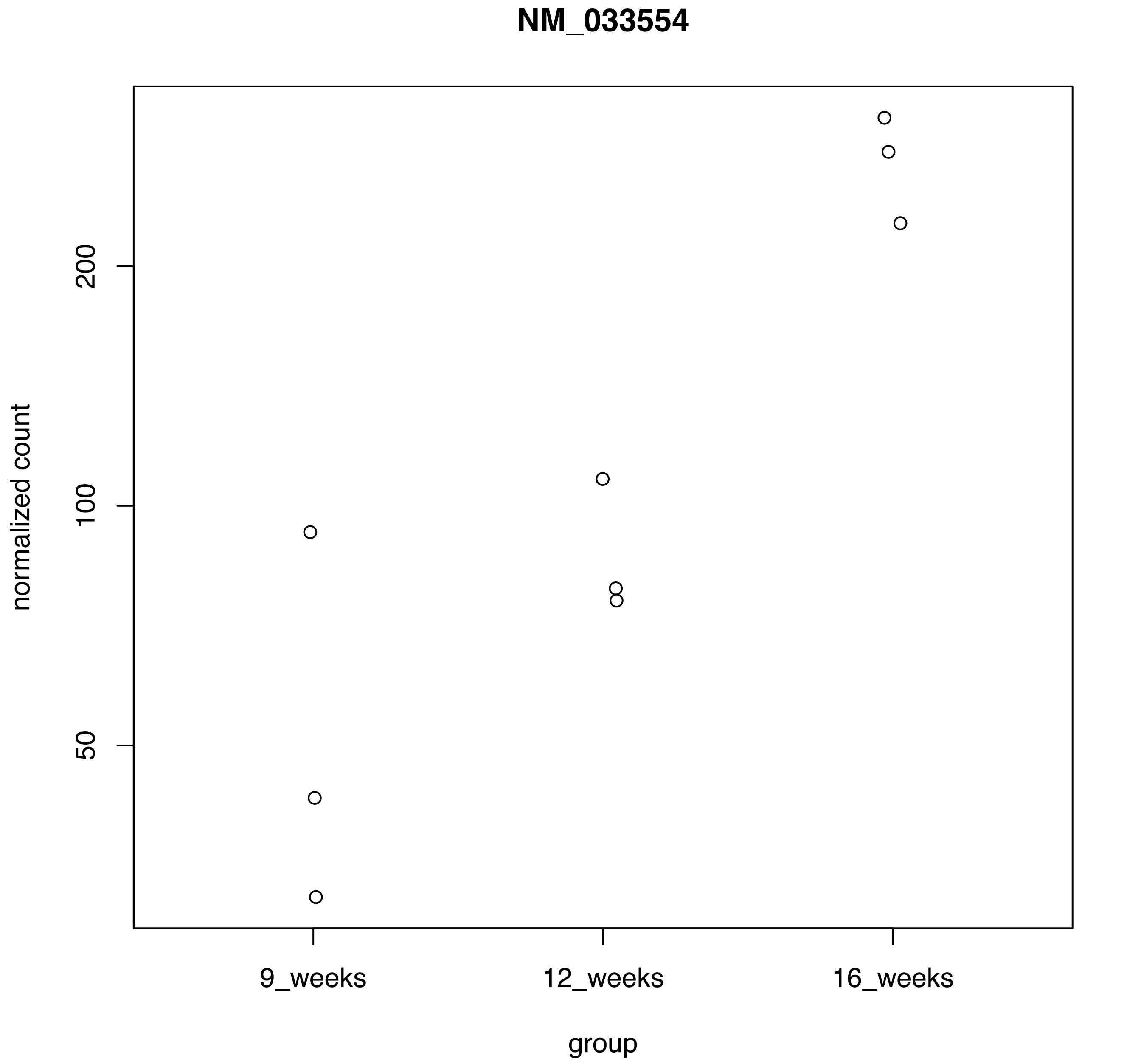
NM_033554
normalized count
200
100
50
9_weeks
12_weeks
16_weeks
group

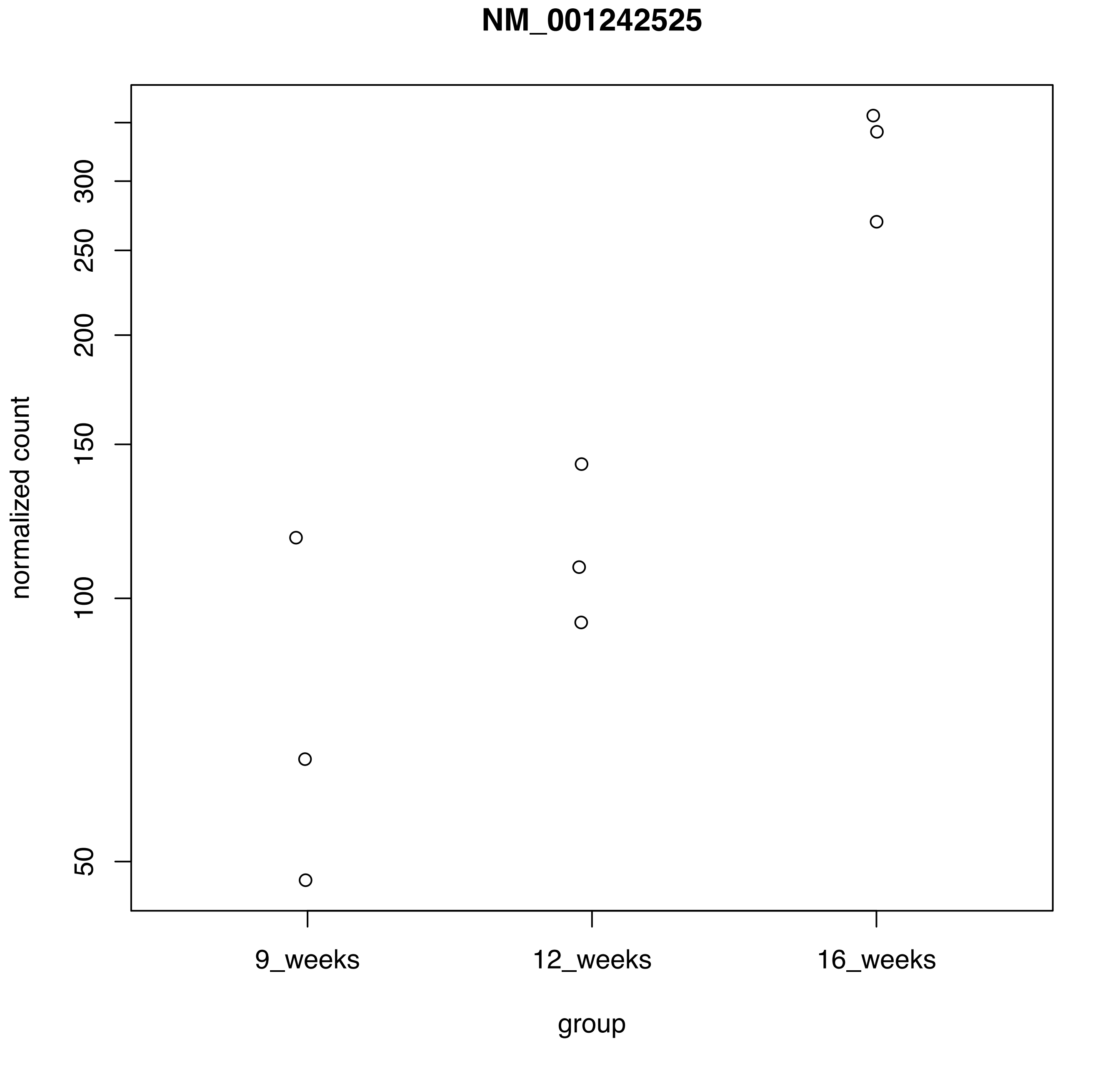

NM_001242525
normalized count
50
100
150
200
250
300
9_weeks
12_weeks
16_weeks
group

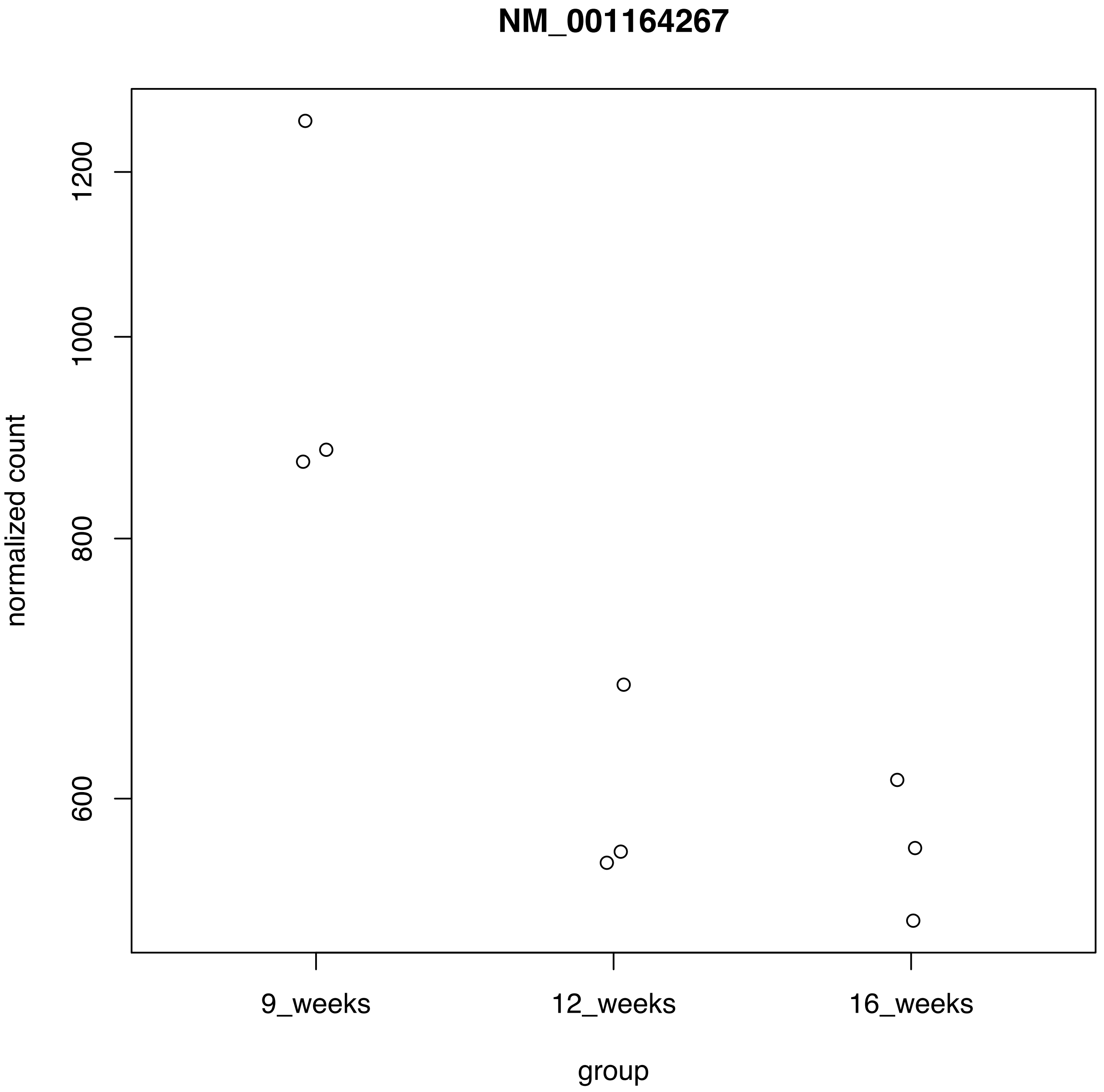

NM_001164267
normalized count
1200
1000
800
600
9_weeks
12_weeks
16_weeks
group

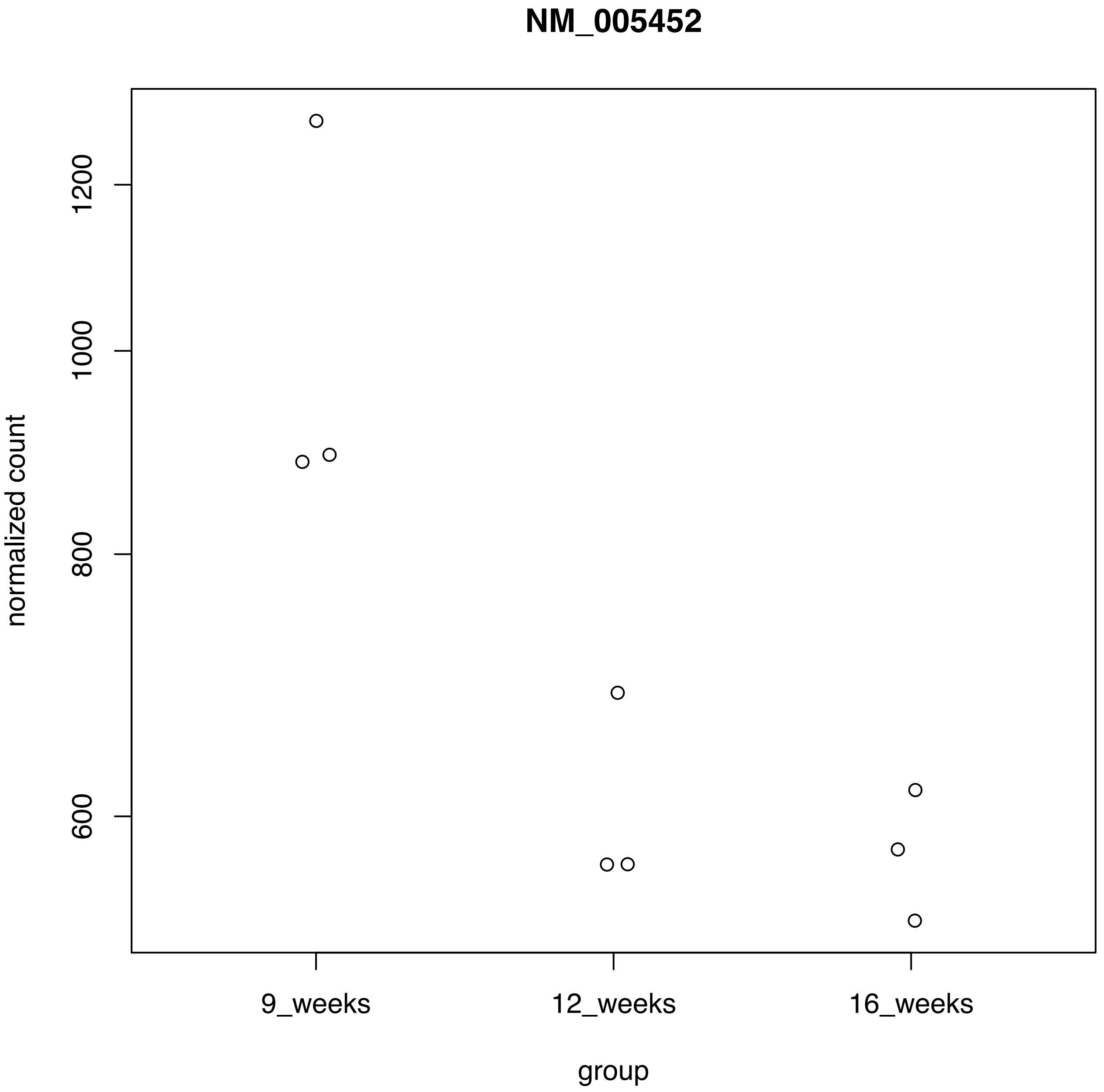
NM_005452
normalized count
1200
1000
800
600
9_weeks
12_weeks
16_weeks
group

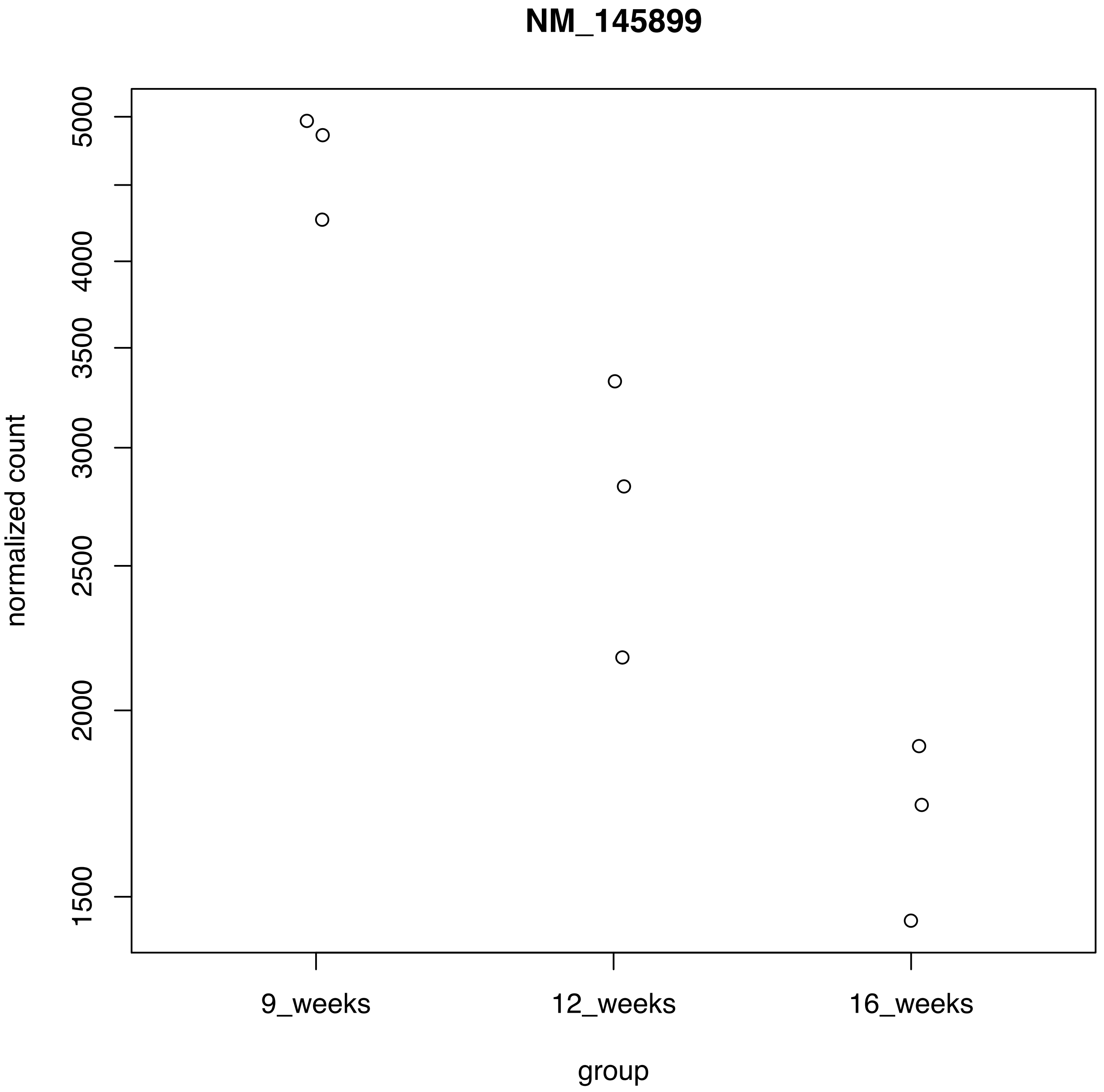
NM_145899
normalized count
5000
4000
3500
3000
2500
2000
1500
9_weeks
12_weeks
16_weeks
group

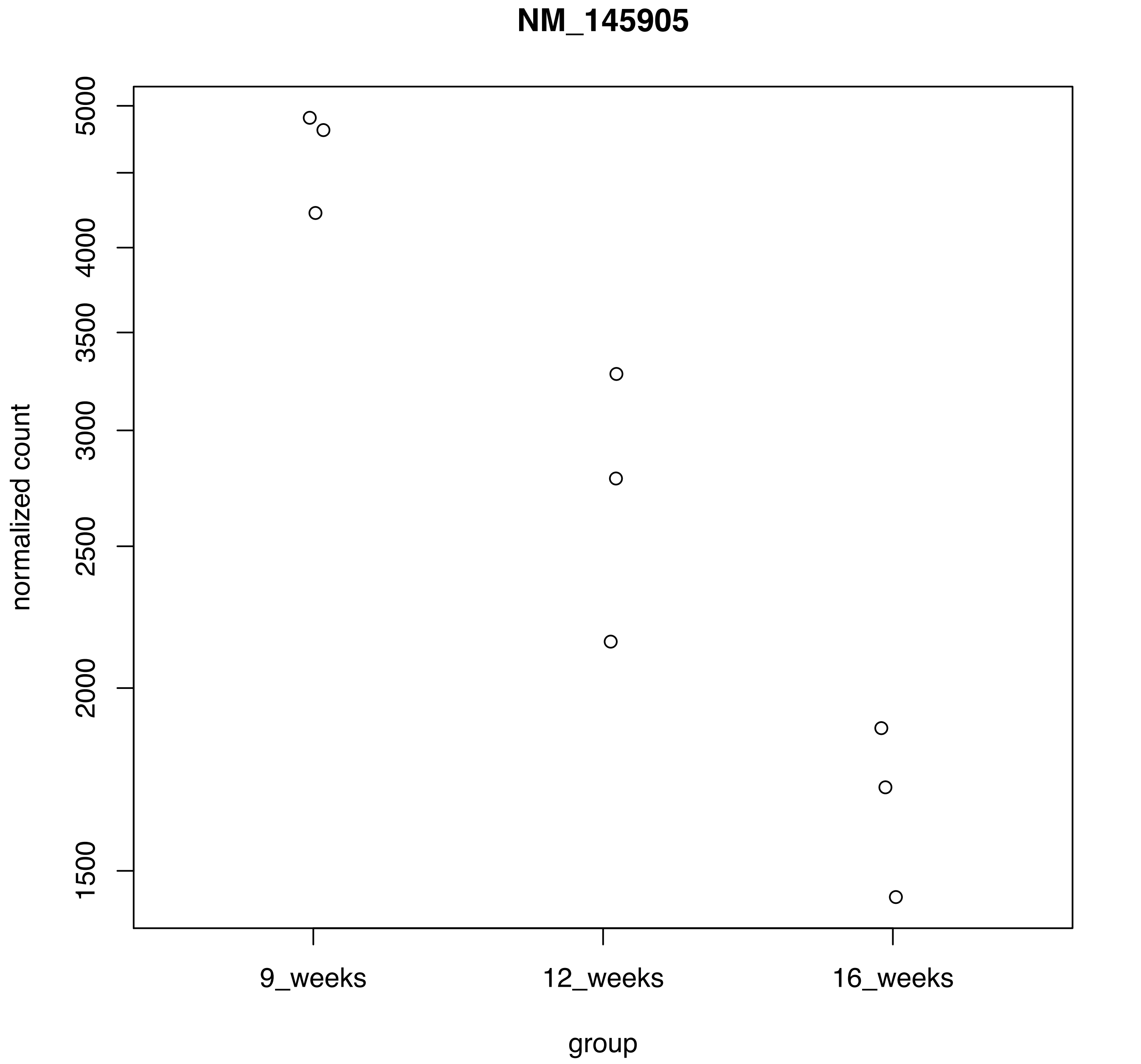
NM_145905
normalized count
1500
2000
2500
3000
3500
4000
5000
9_weeks
12_weeks
16_weeks
group

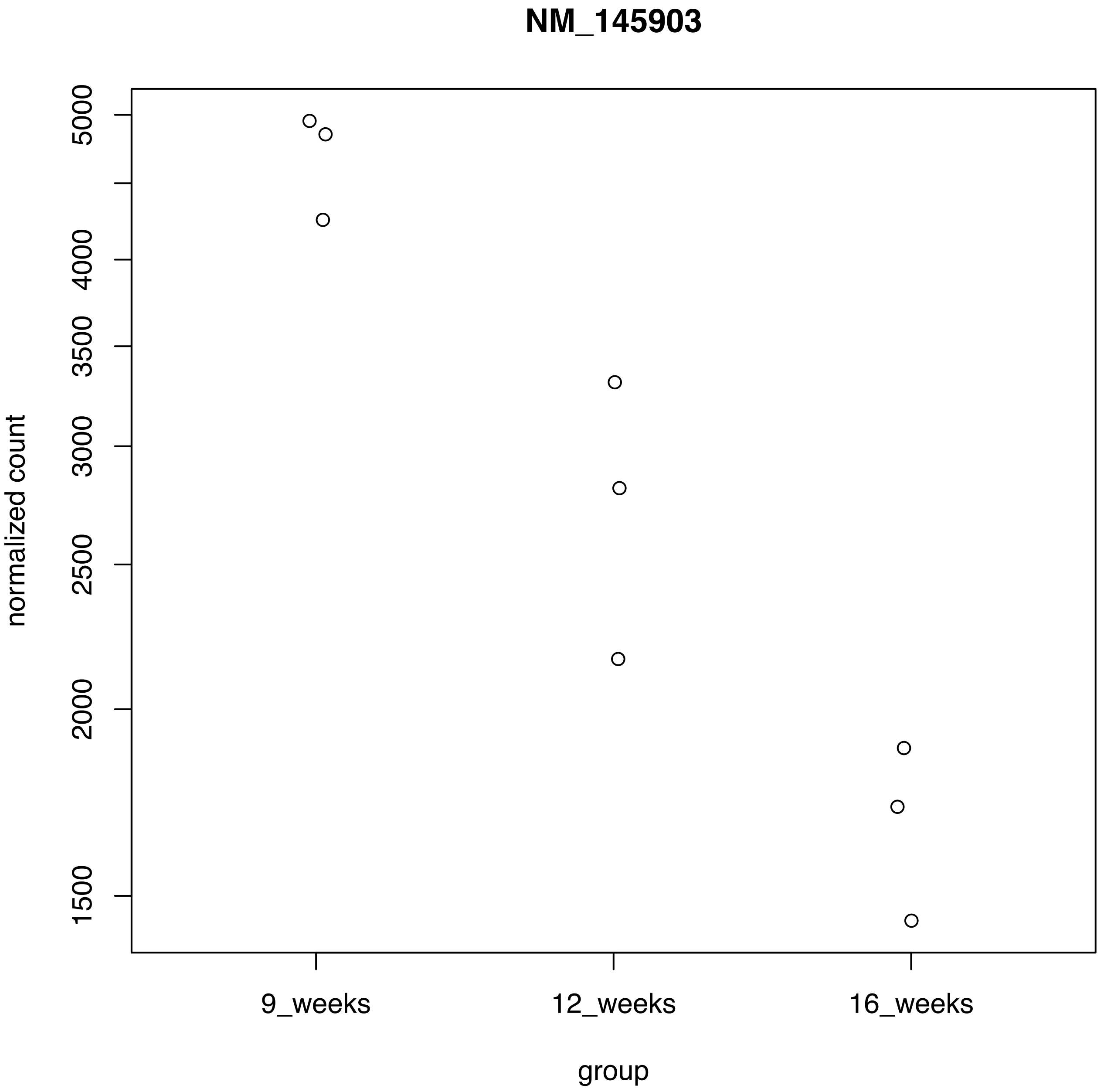
NM_145903
normalized count
5000
4000
3500
3000
2500
2000
1500
9_weeks
12_weeks
16_weeks
group

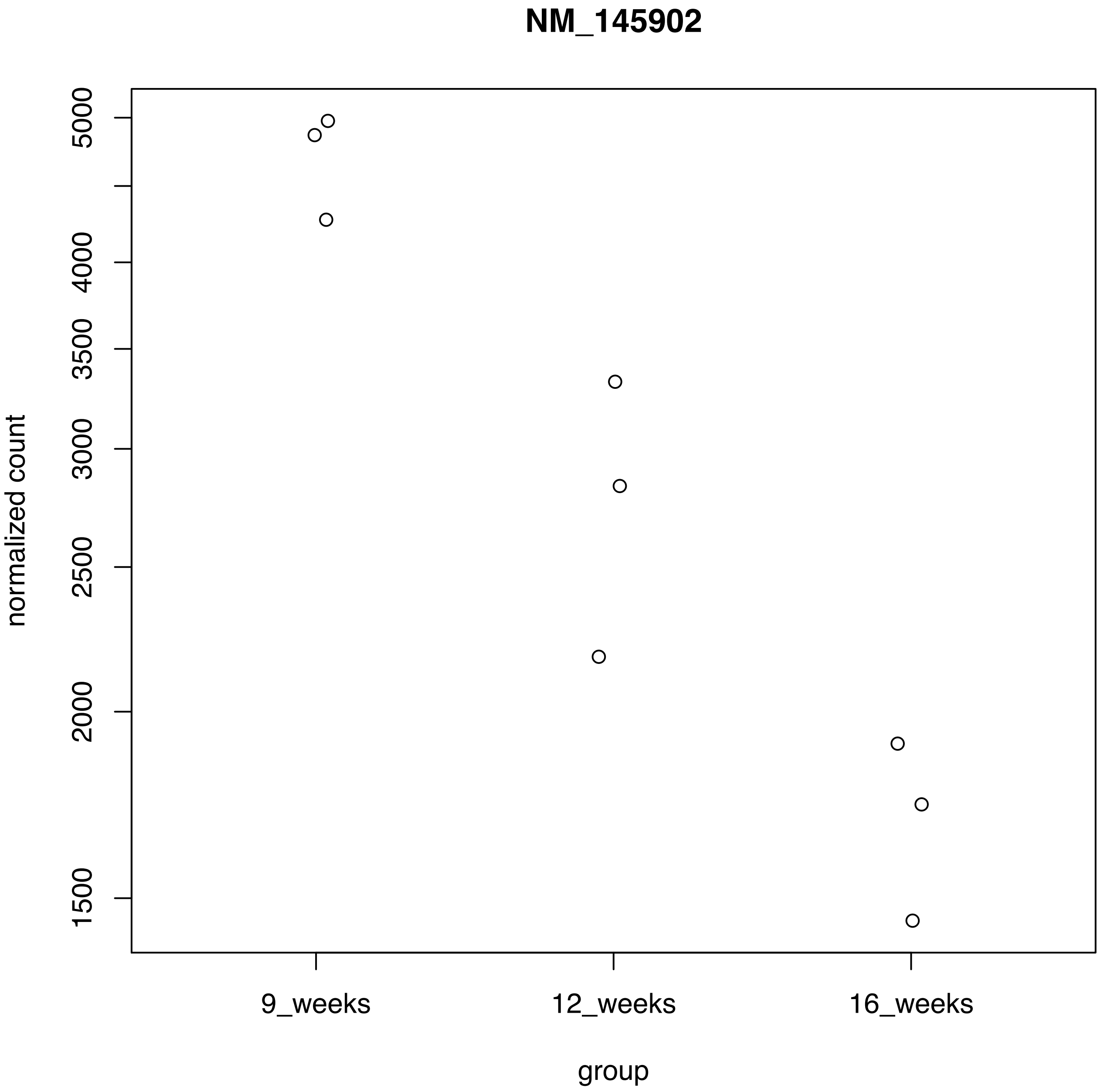
NM_145902
normalized count
5000
4000
3500
3000
2500
2000
1500
9_weeks
12_weeks
16_weeks
group

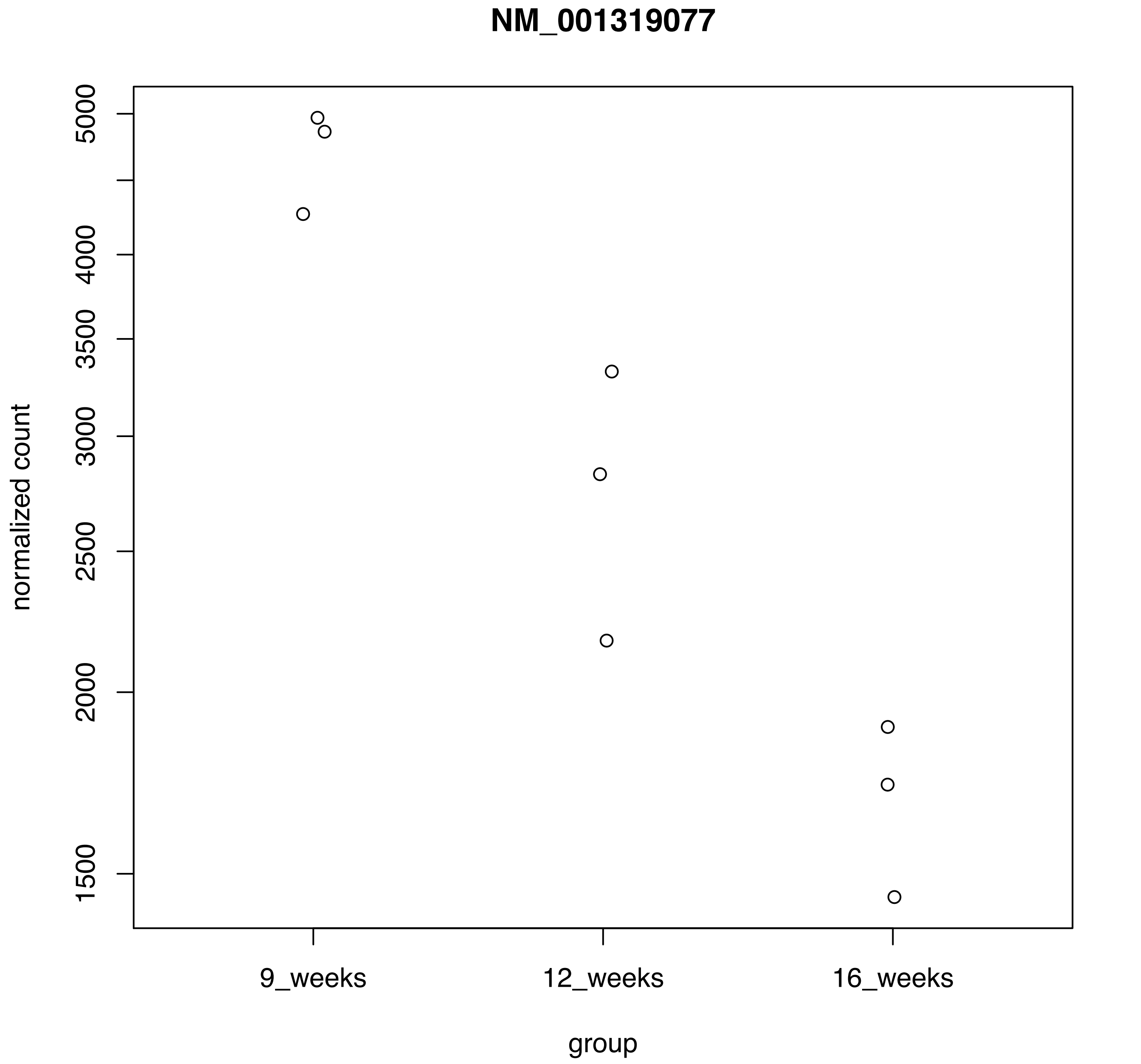

NM_001319077
normalized count
5000
4000
3500
3000
2500
2000
1500
9_weeks
12_weeks
16_weeks
group

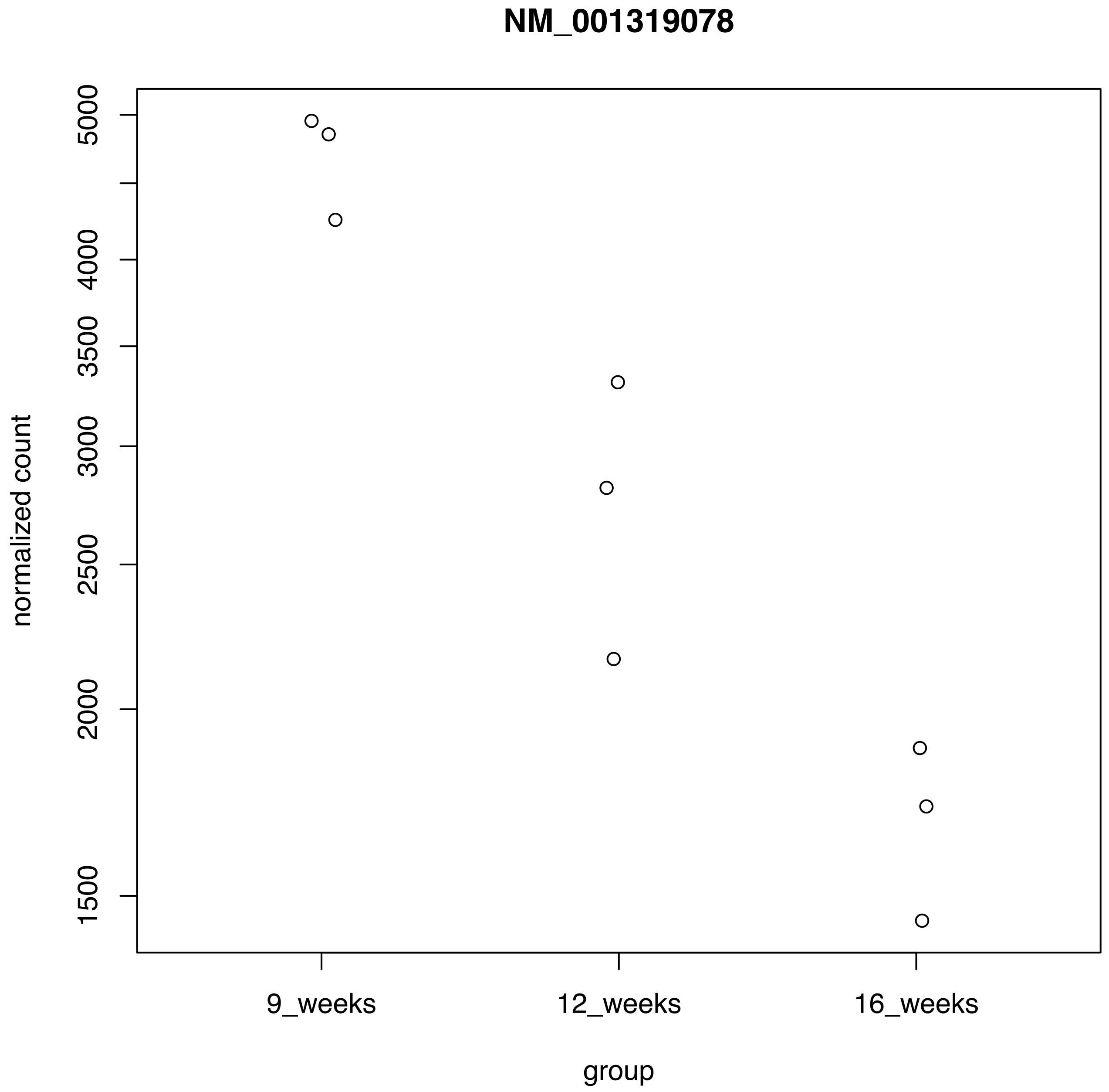
NM_001319078
normalized count
5000
4000
3500
3000
2500
2000
1500
9_weeks
12_weeks
16_weeks
group

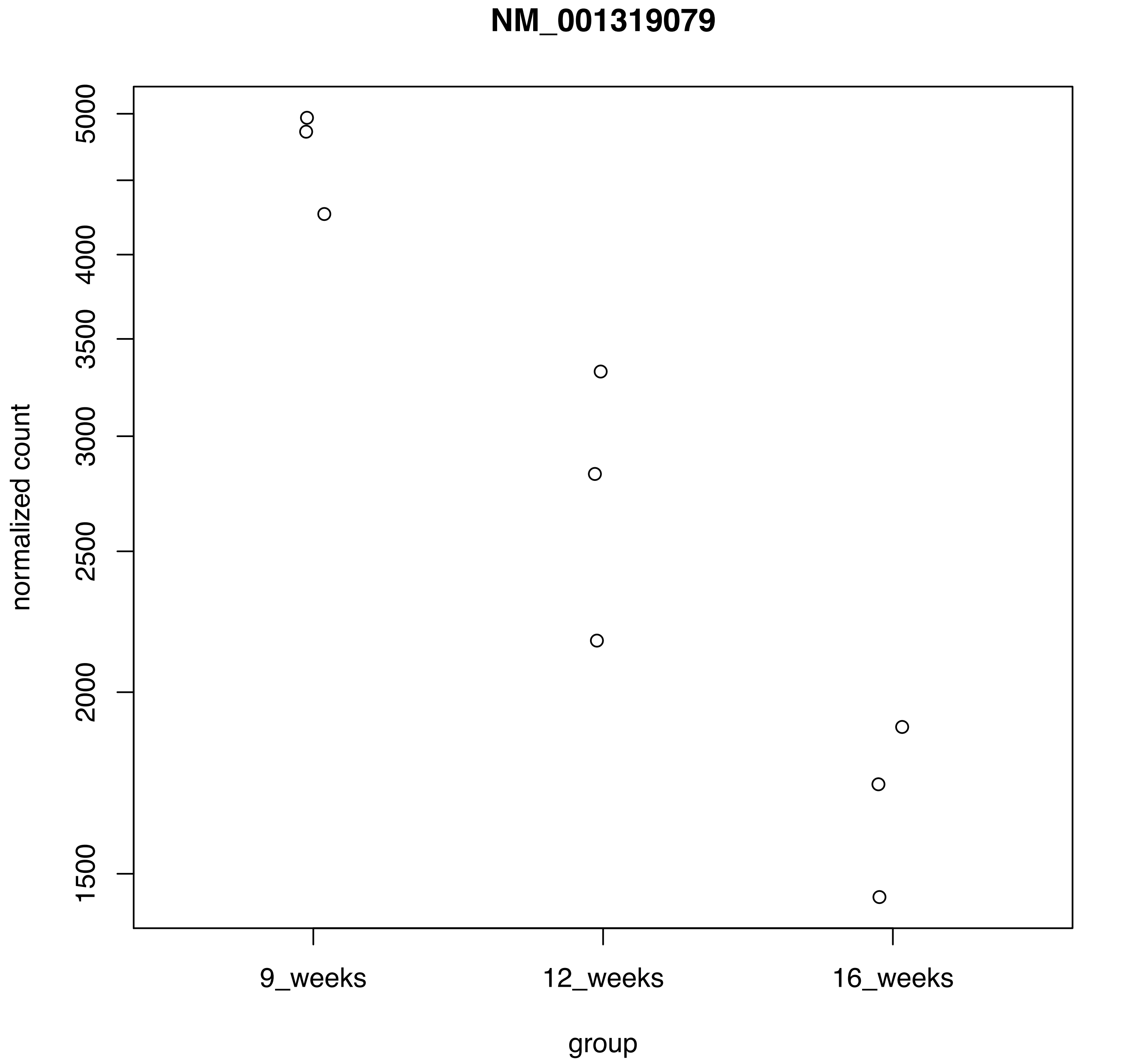
NM_001319079
normalized count
5000
4000
3500
3000
2500
2000
1500
9_weeks
12_weeks
16_weeks
group

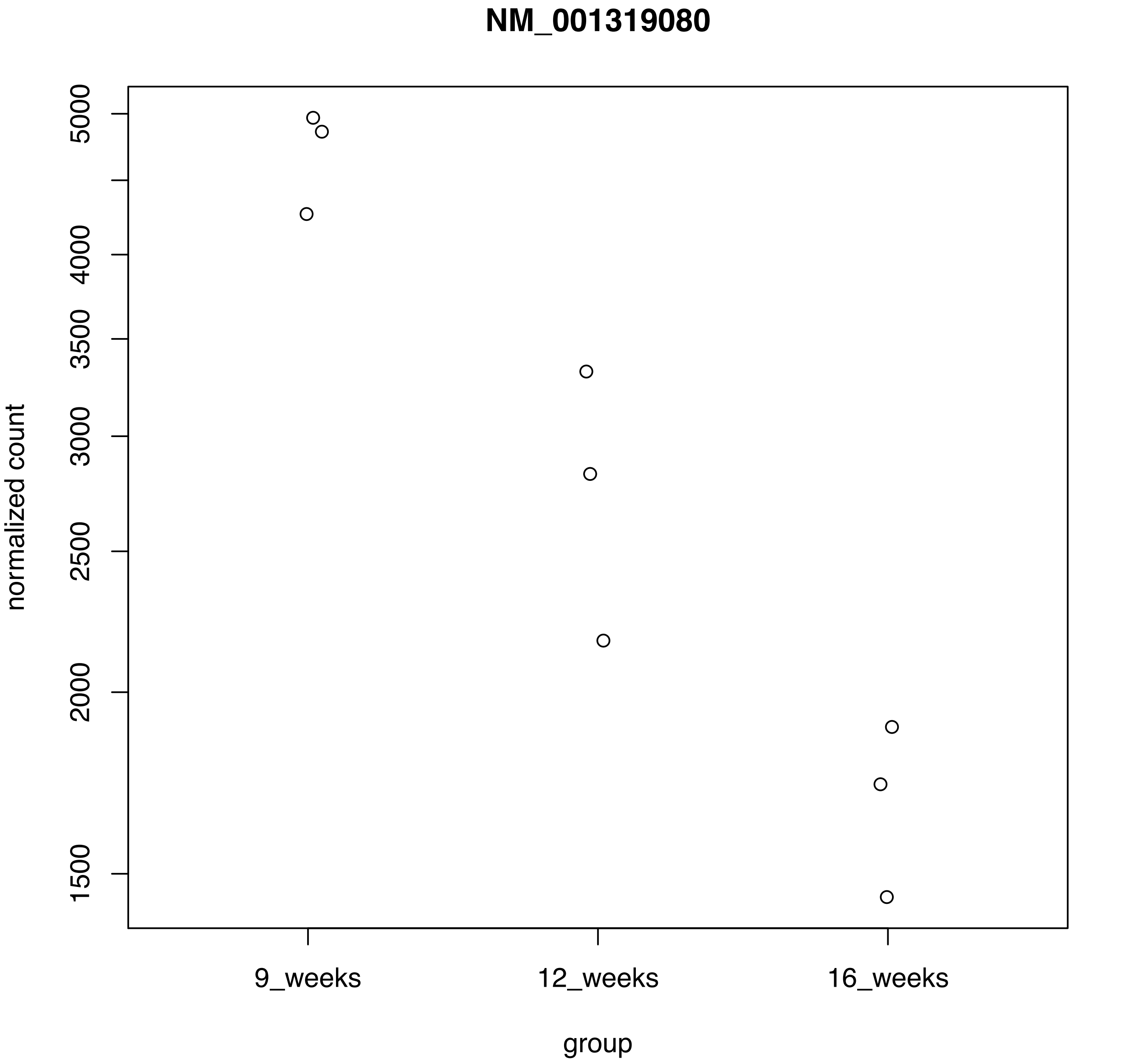

NM_001319080
normalized count
5000
4000
3500
3000
2500
2000
1500
9_weeks
12_weeks
16_weeks
group

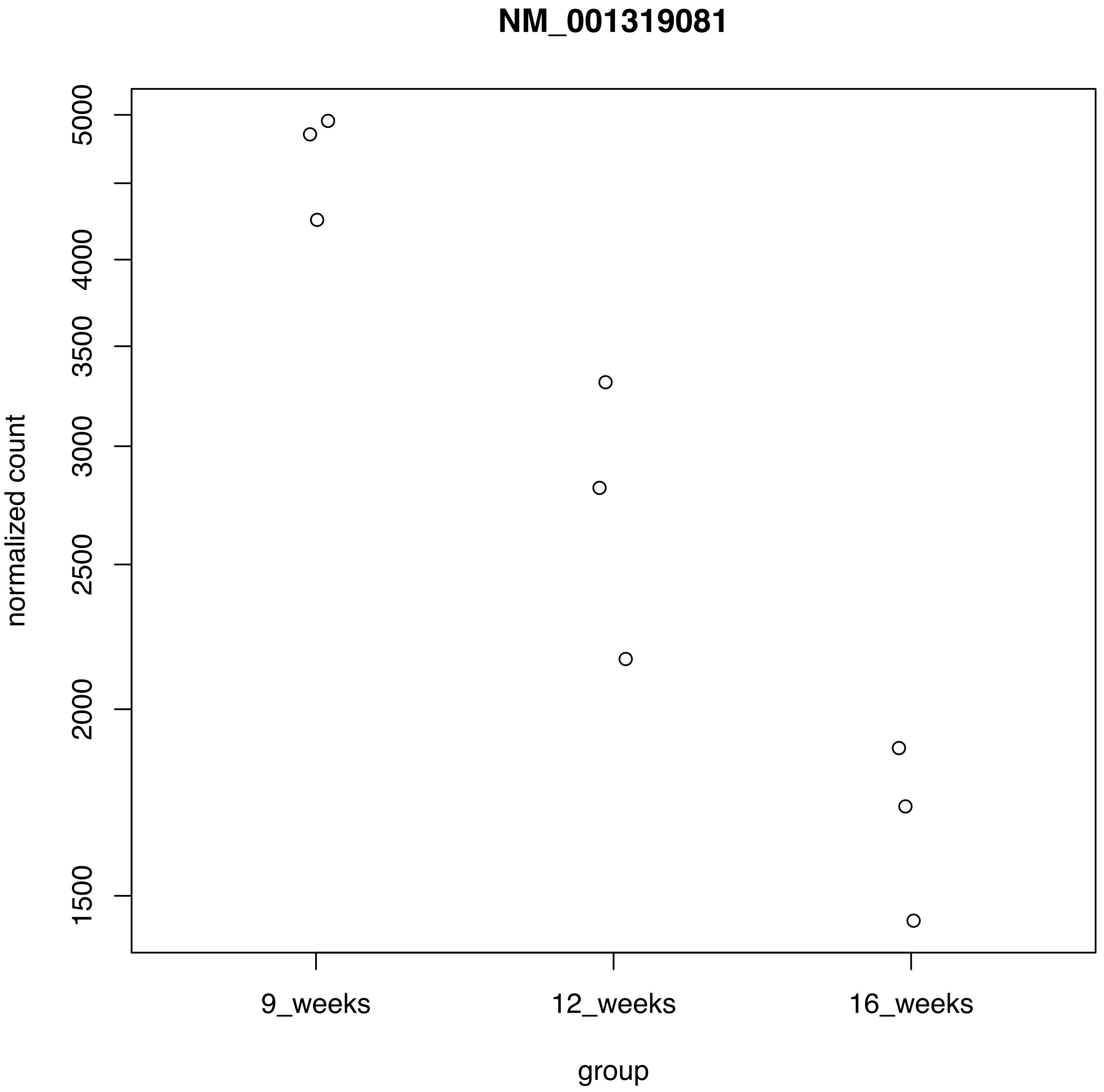
NM_001319081
normalized count
1500
2000
2500
3000
3500
4000
5000
9_weeks
12_weeks
16_weeks
group

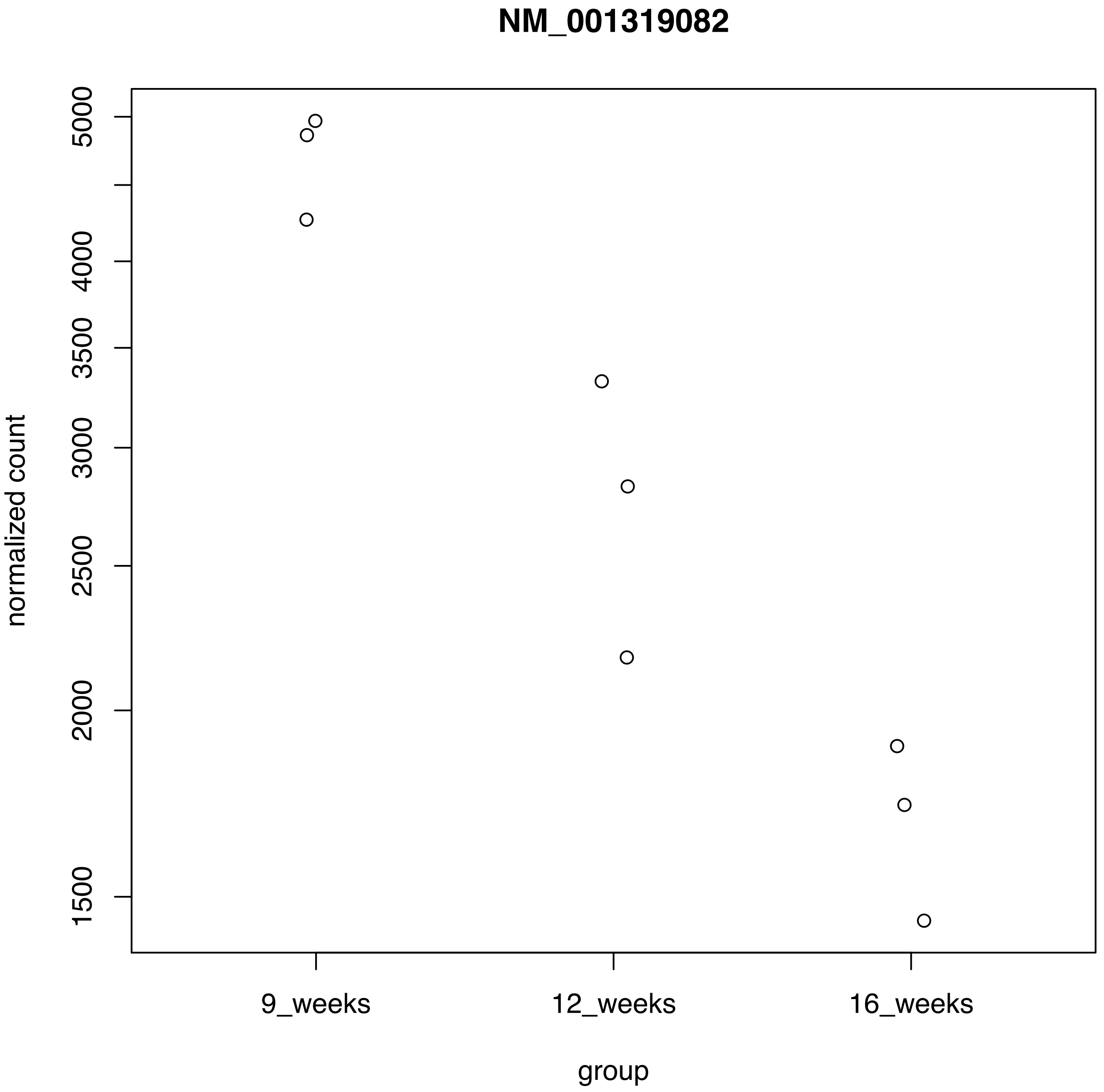
NM_001319082
normalized count
5000
4000
3500
3000
2500
2000
1500
9_weeks
12_weeks
16_weeks
group

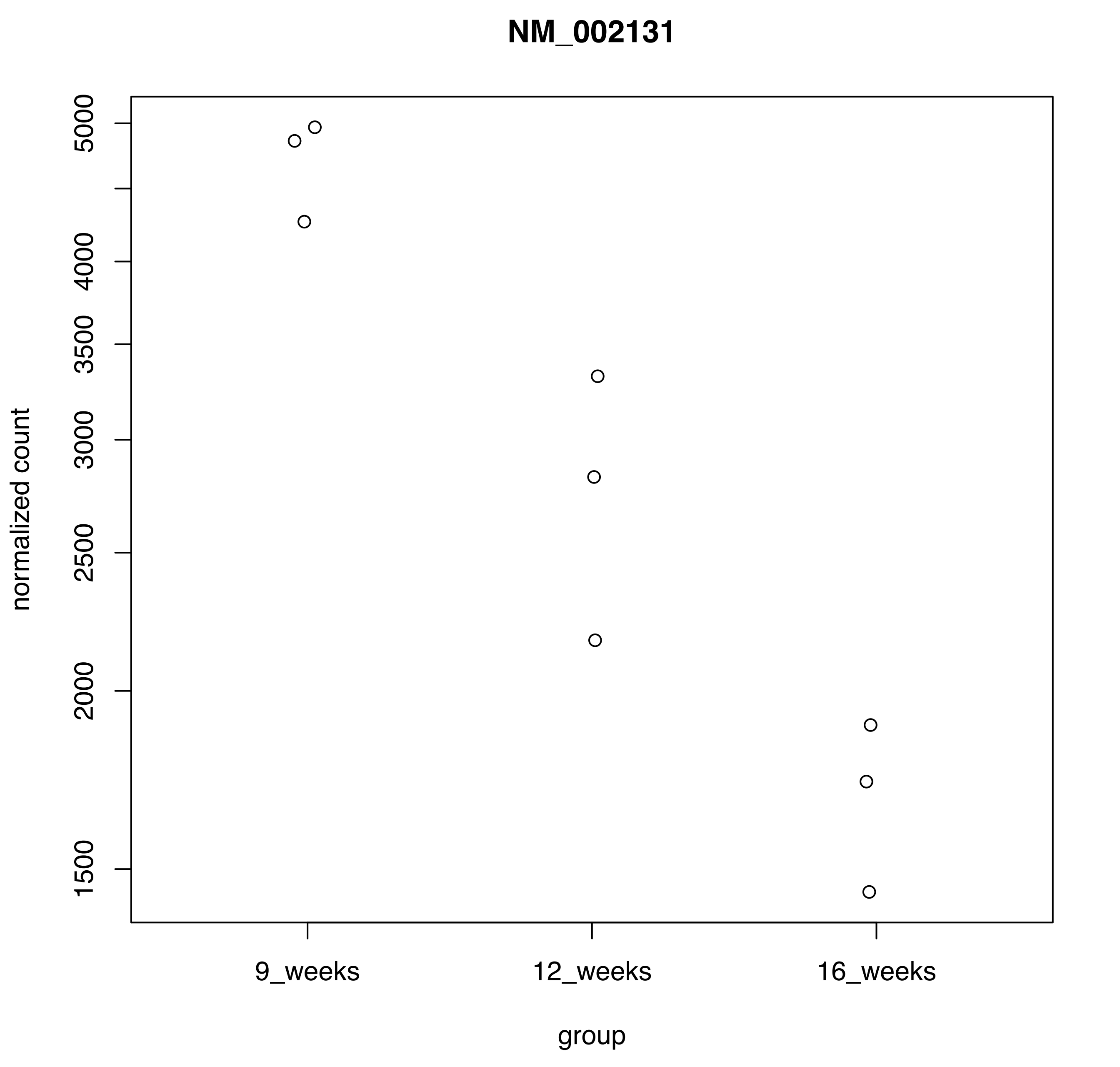
NM_002131
normalized count
5000
4000
3500
3000
2500
2000
1500
9_weeks
12_weeks
16_weeks
group

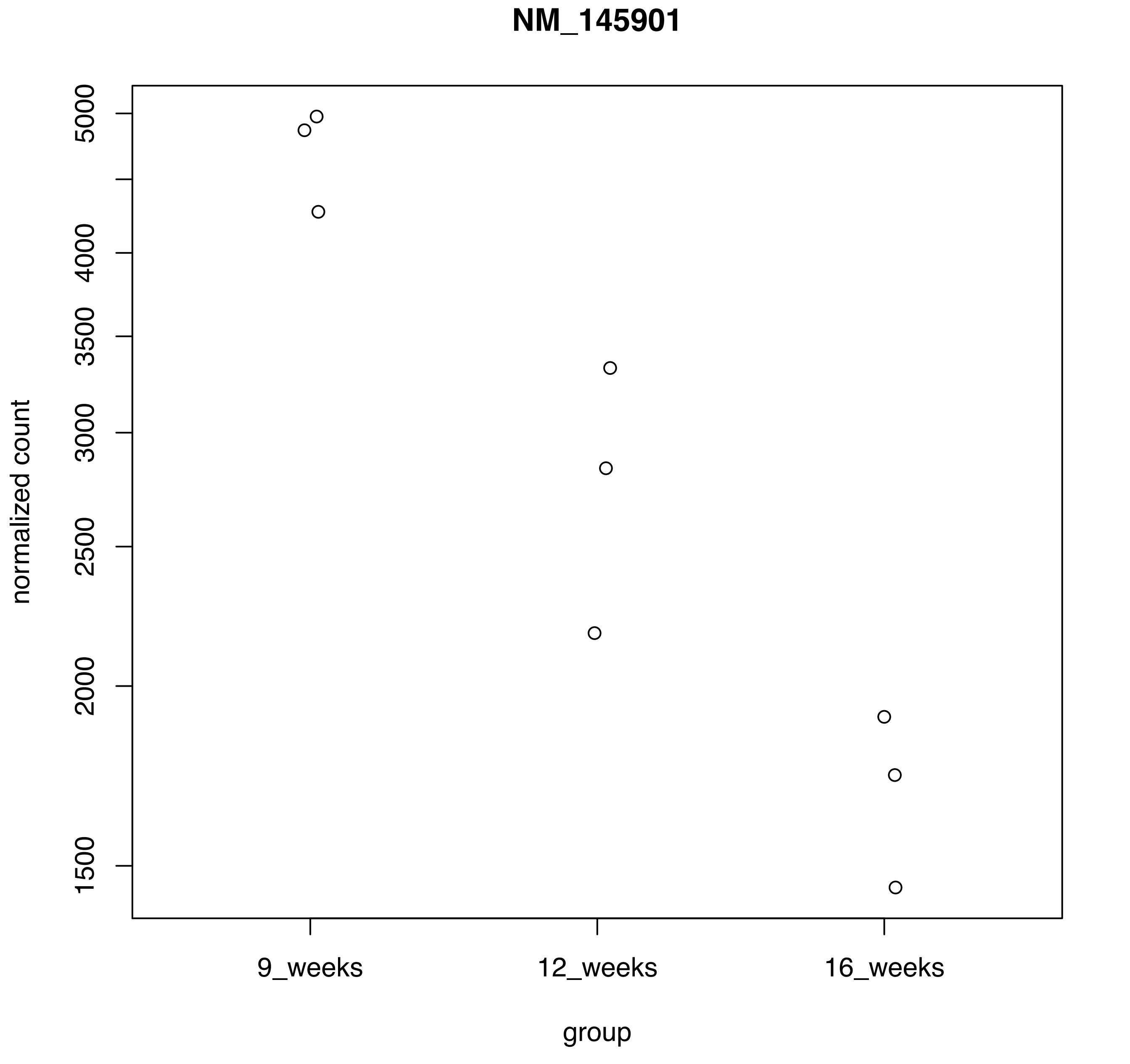

NM_145901
normalized count
5000
4000
3500
3000
2500
2000
1500
9_weeks
12_weeks
16_weeks
group

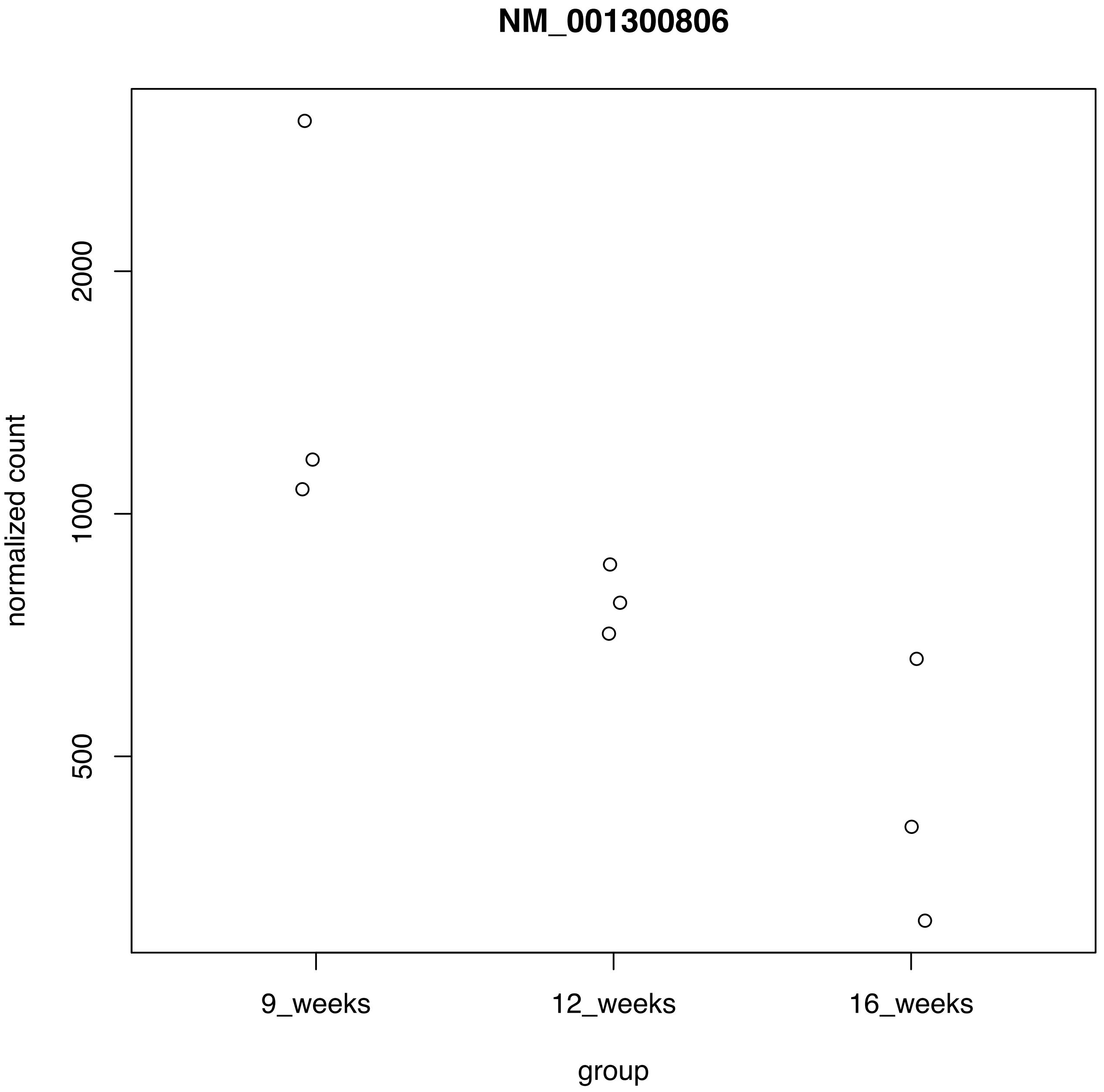
NM_001300806
normalized count
2000
1000
500
9_weeks
12_weeks
16_weeks
group

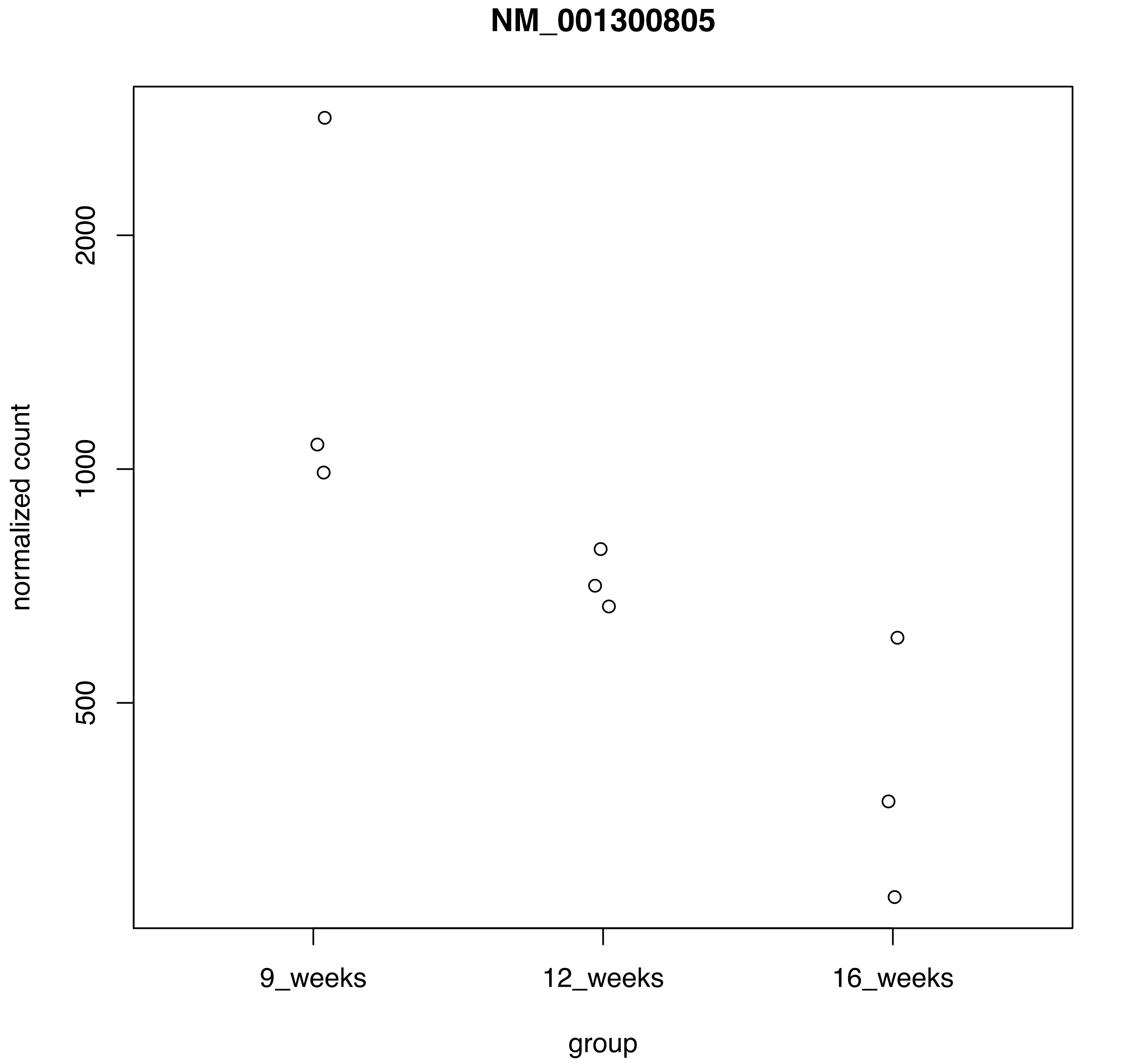
NM_001300805
normalized count
2000
1000
500
9_weeks
12_weeks
16_weeks
group

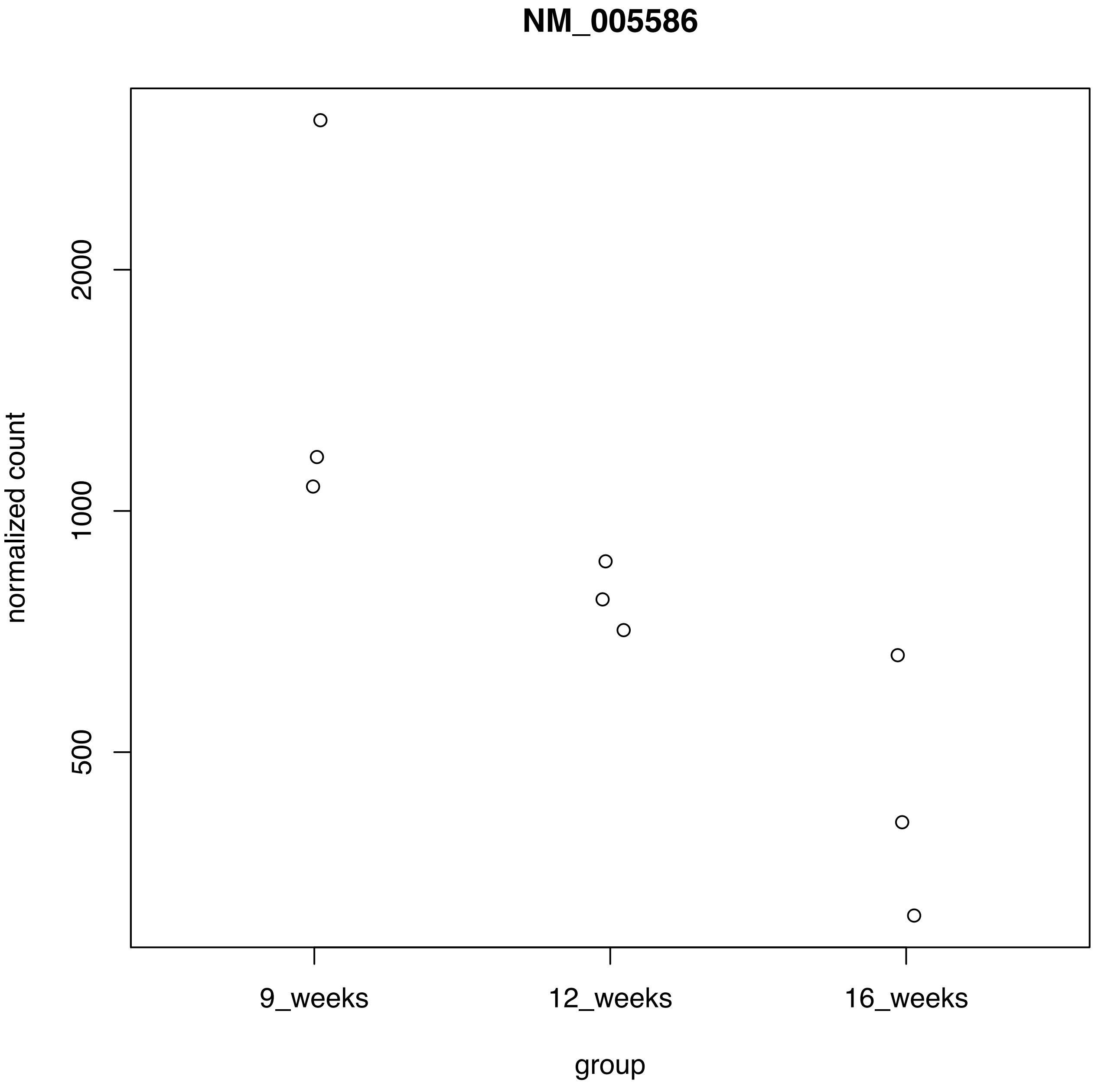
NM_005586
normalized count
2000
1000
500
9_weeks
12_weeks
16_weeks
group

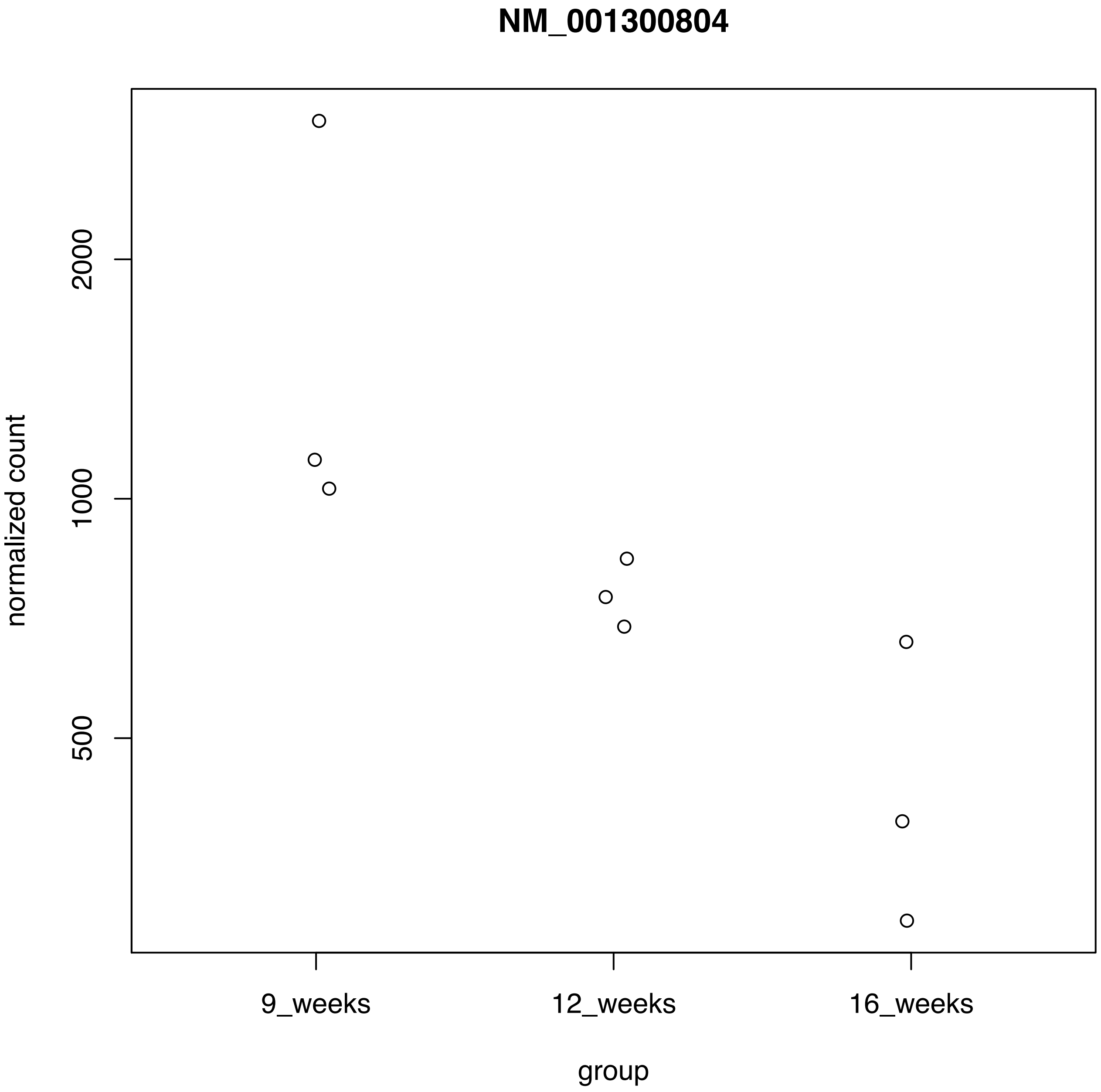
NM_001300804
normalized count
2000
1000
500
9_weeks
12_weeks
16_weeks
group

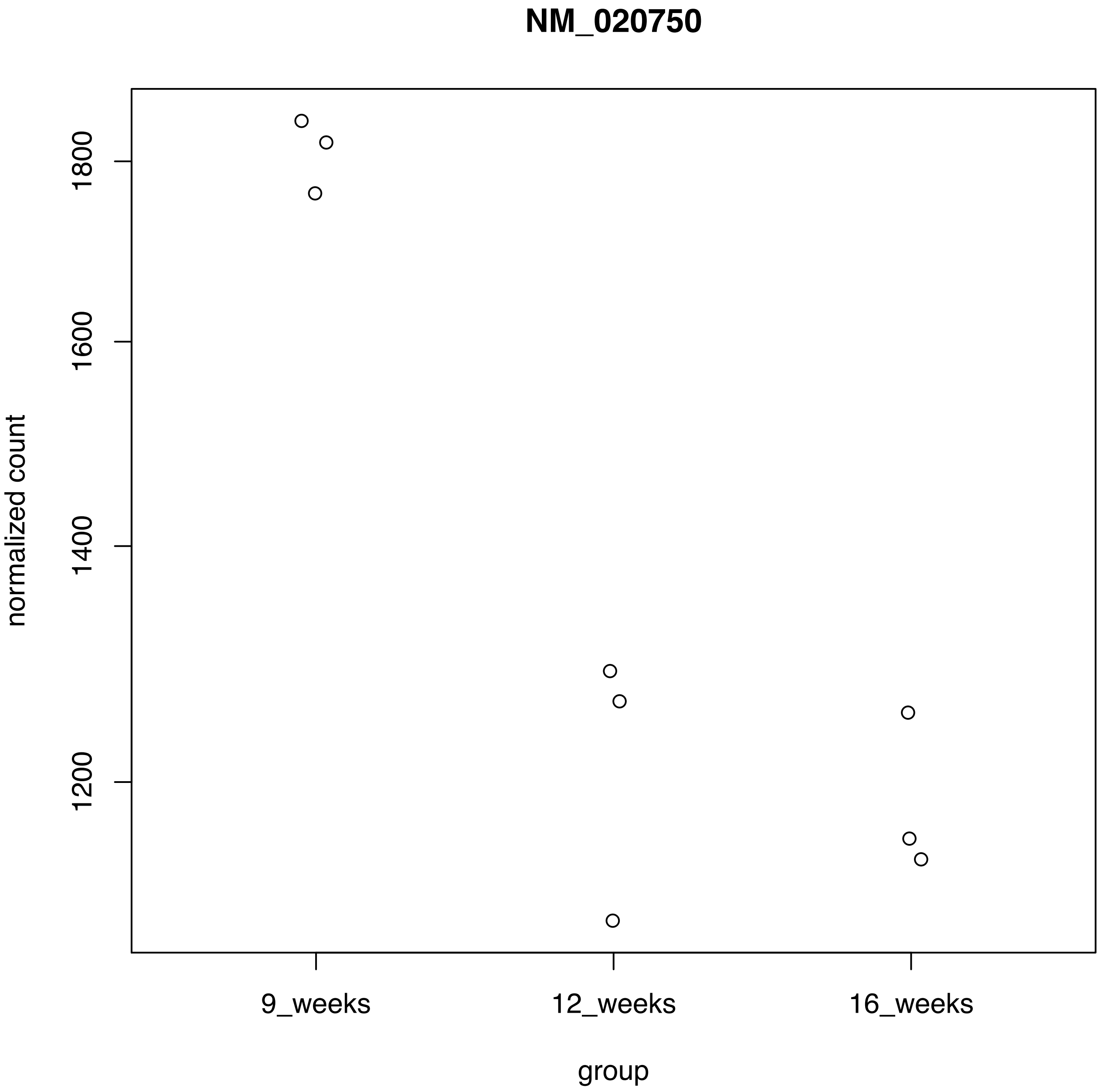
NM_020750
normalized count
1200
1400
1600
1800
9_weeks
12_weeks
16_weeks
group

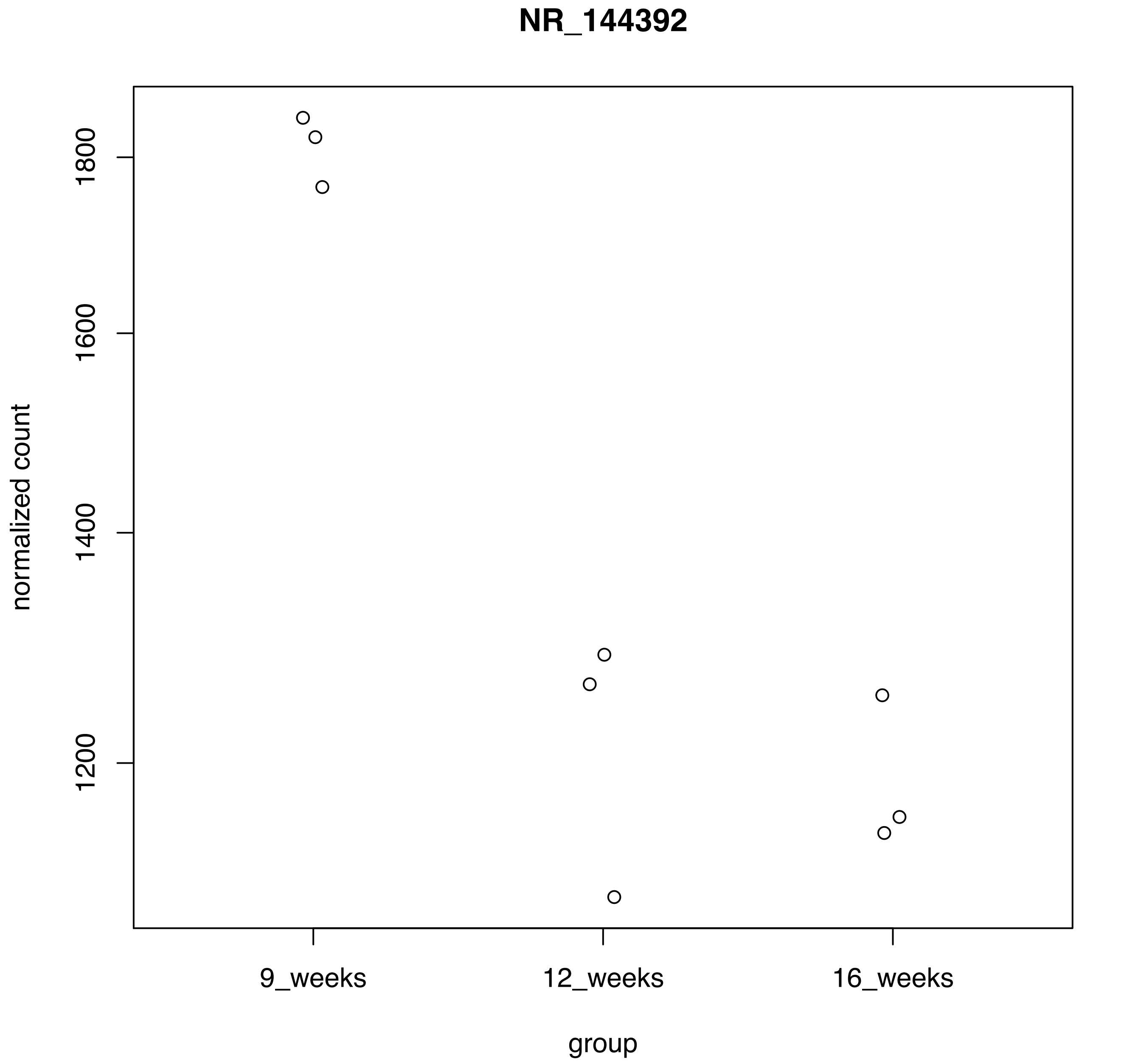
NR_144392
normalized count
1200
1400
1600
1800
9_weeks
12_weeks
16_weeks
group

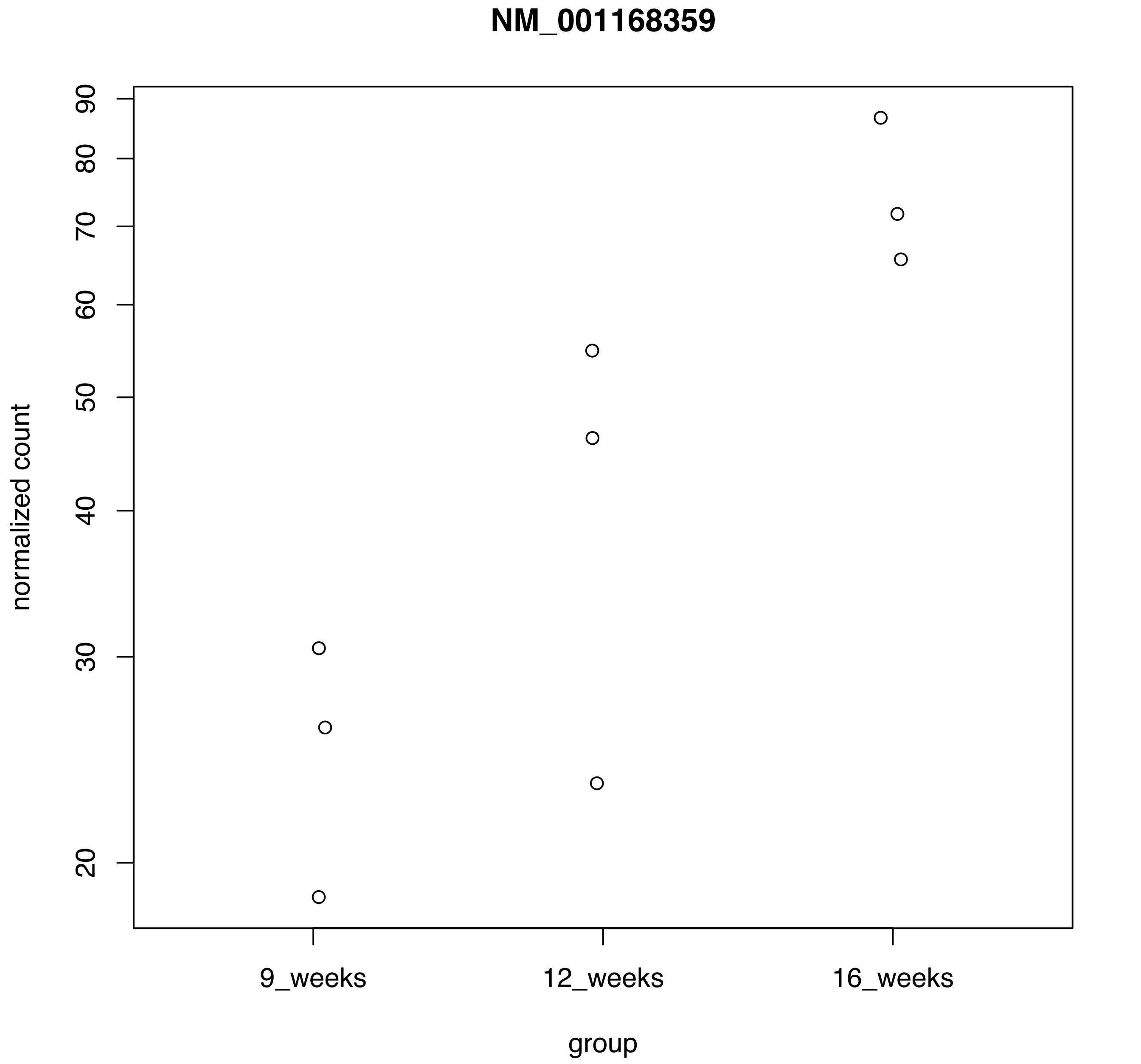
NM_001168359
normalized count
90
80
70
60
50
40
30
20
9_weeks
12_weeks
16_weeks
group

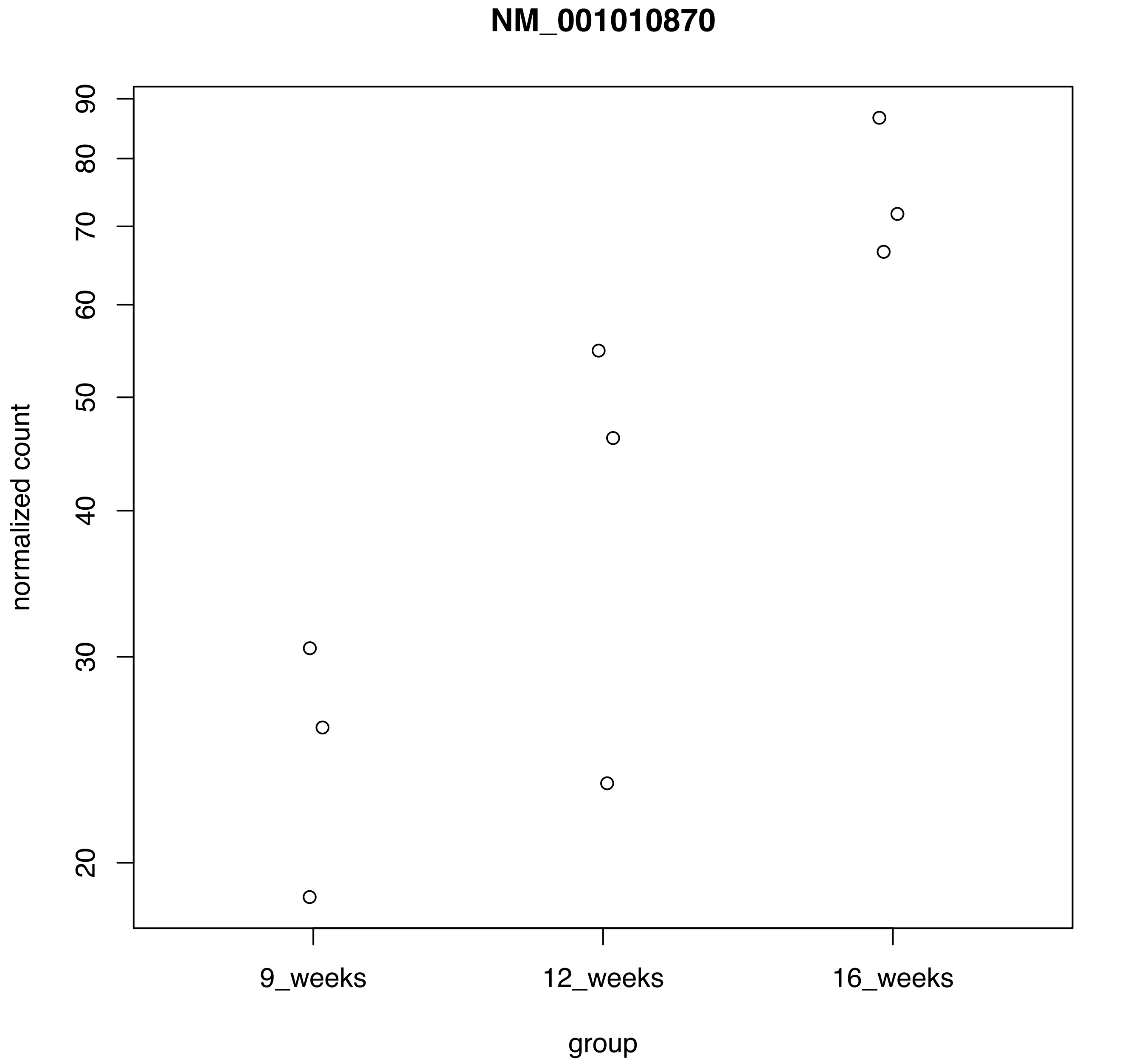

NM_001010870
normalized count
90
80
70
60
50
40
30
20
9_weeks
12_weeks
16_weeks
group

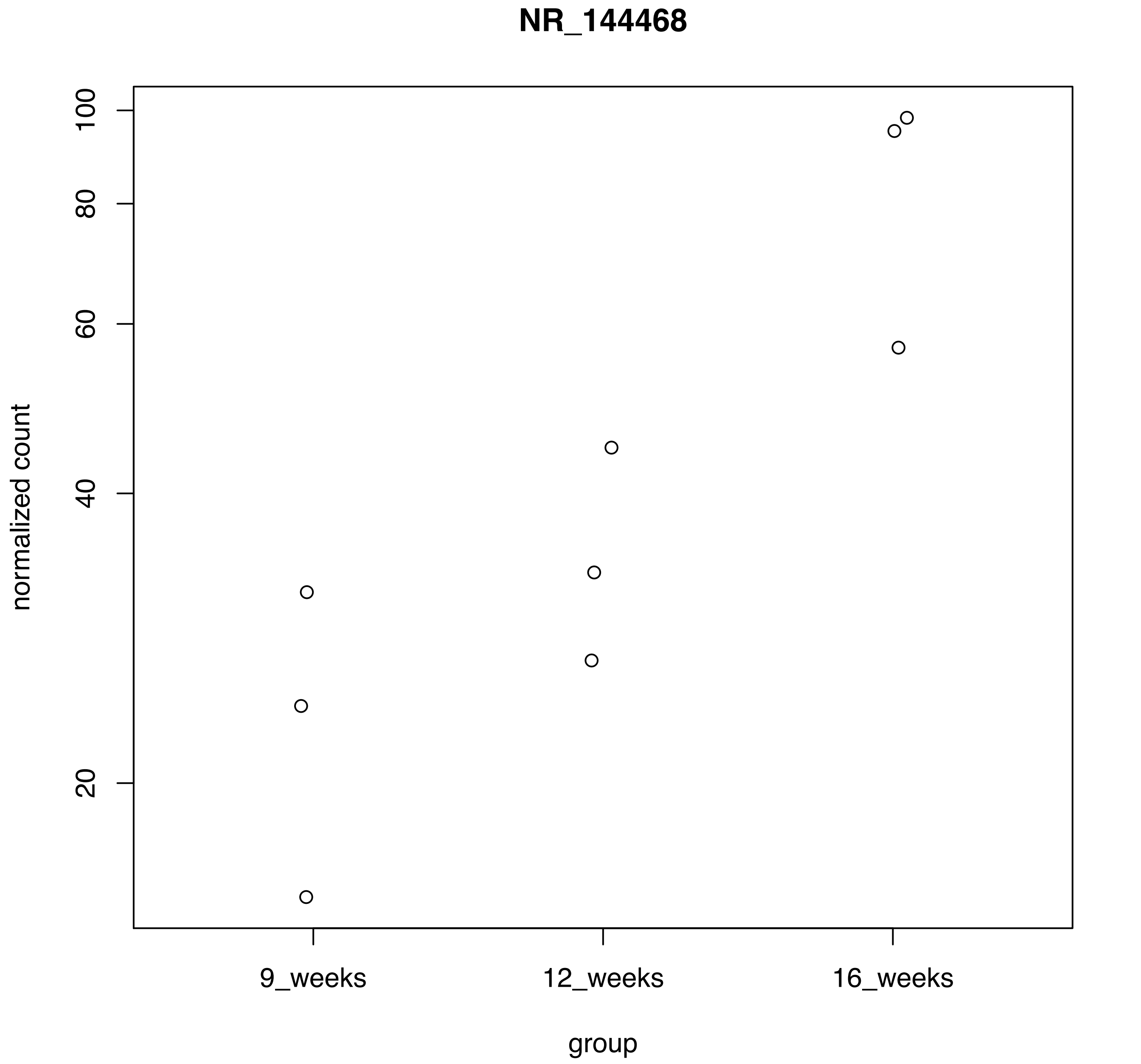

NR_144468
100
80
60
40
20
normalized count
9_weeks
12_weeks
16_weeks
group

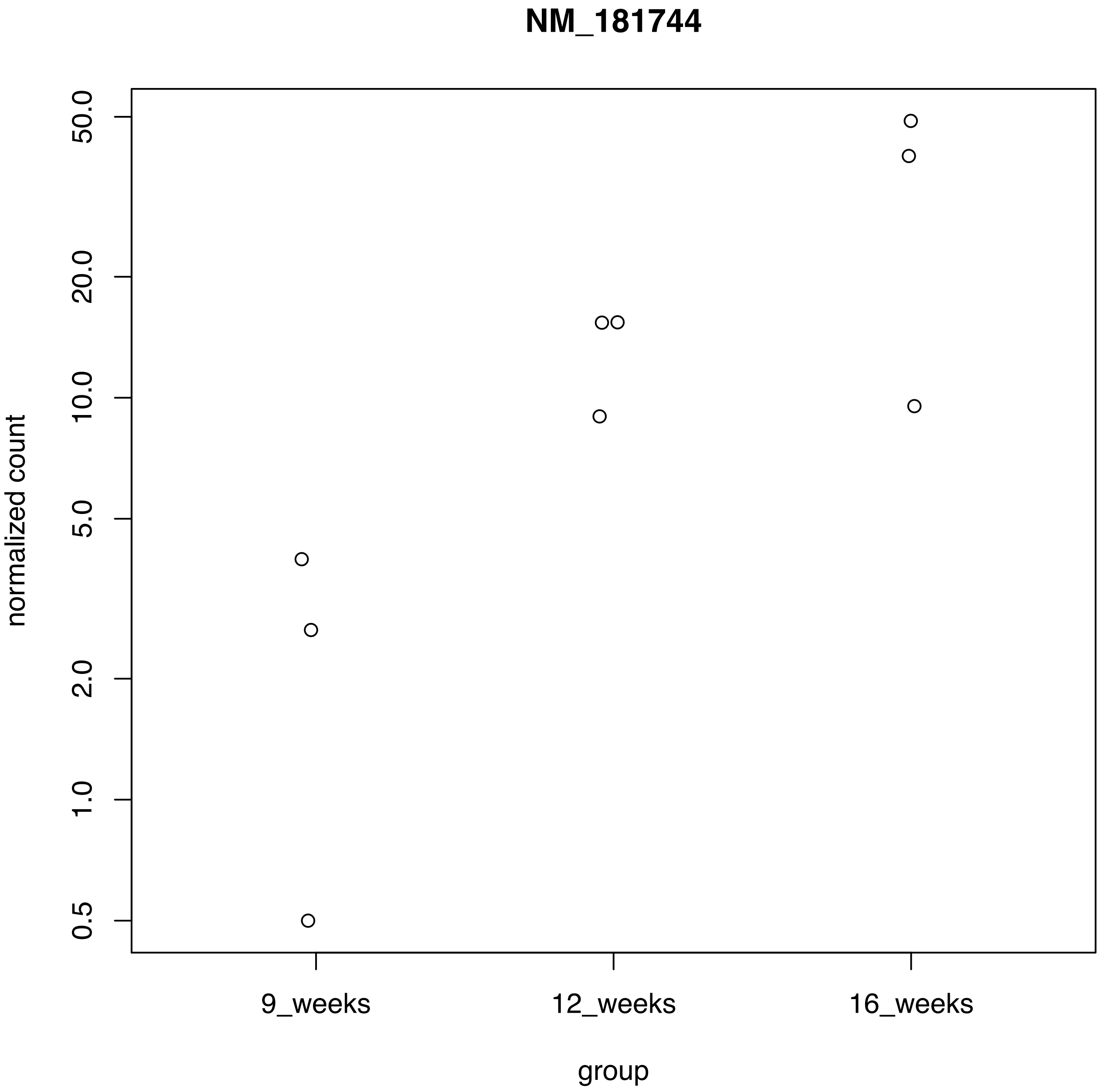

NM_181744
normalized count
50.0
20.0
10.0
5.0
2.0
1.0
0.5
9_weeks
12_weeks
16_weeks
group

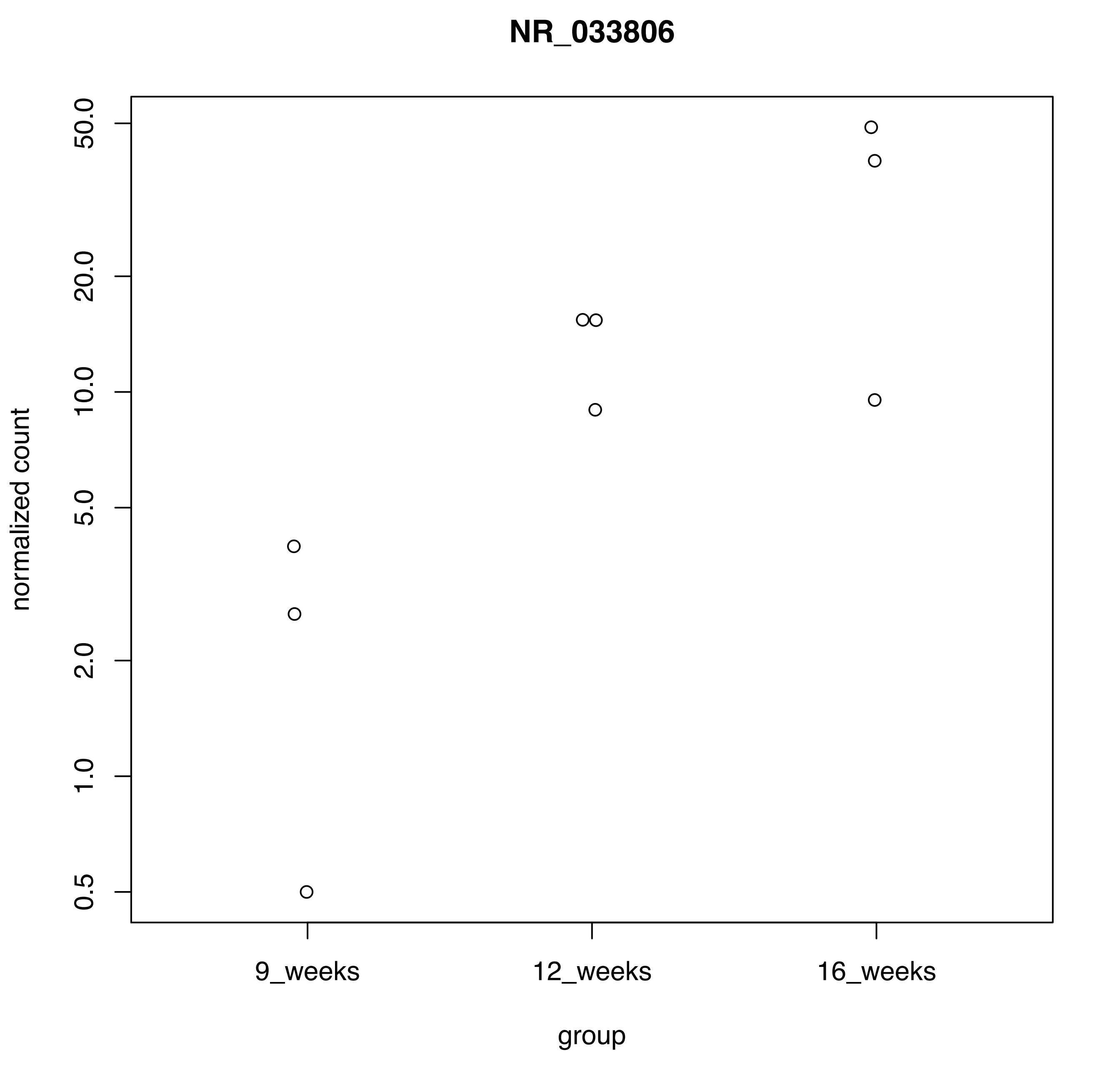

NR_033806
50.0
20.0
10.0
5.0
2.0
1.0
0.5
normalized count
9_weeks
12_weeks
16_weeks
group

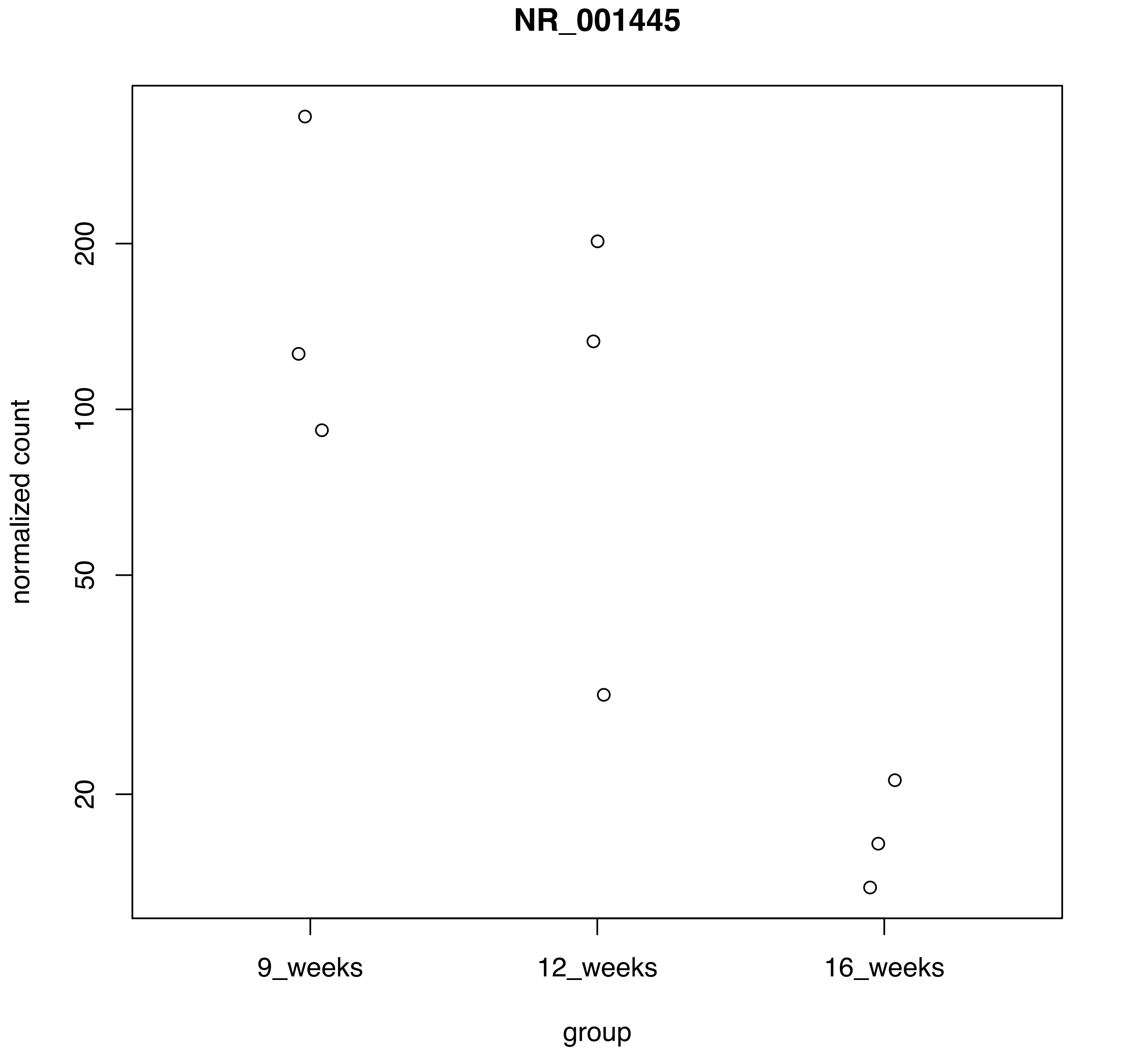
NR_001445
normalized count
200
100
50
20
9_weeks
12_weeks
16_weeks
group

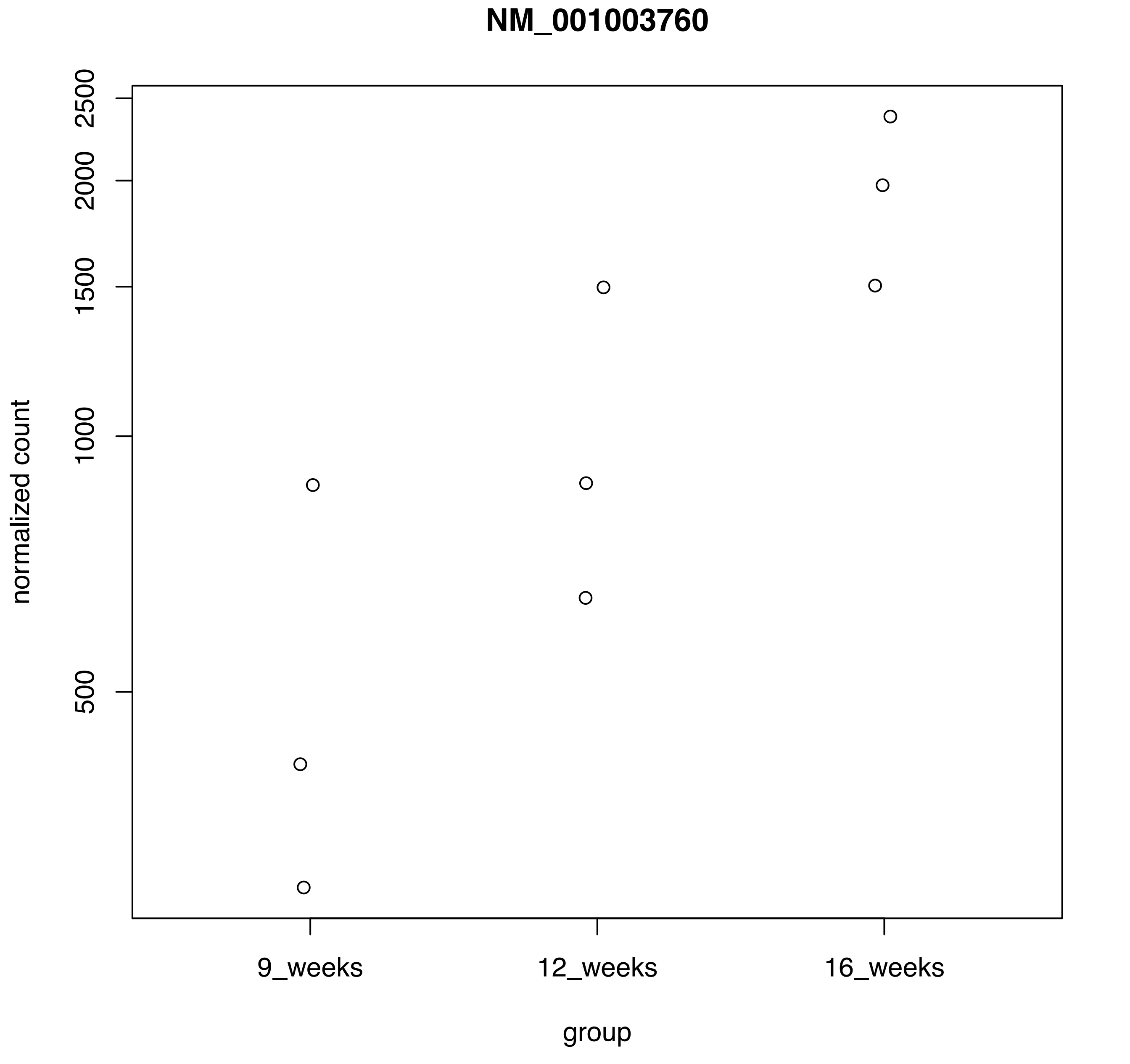
NM_001003760
normalized count
2500
2000
1500
1000
500
9_weeks
12_weeks
16_weeks
group

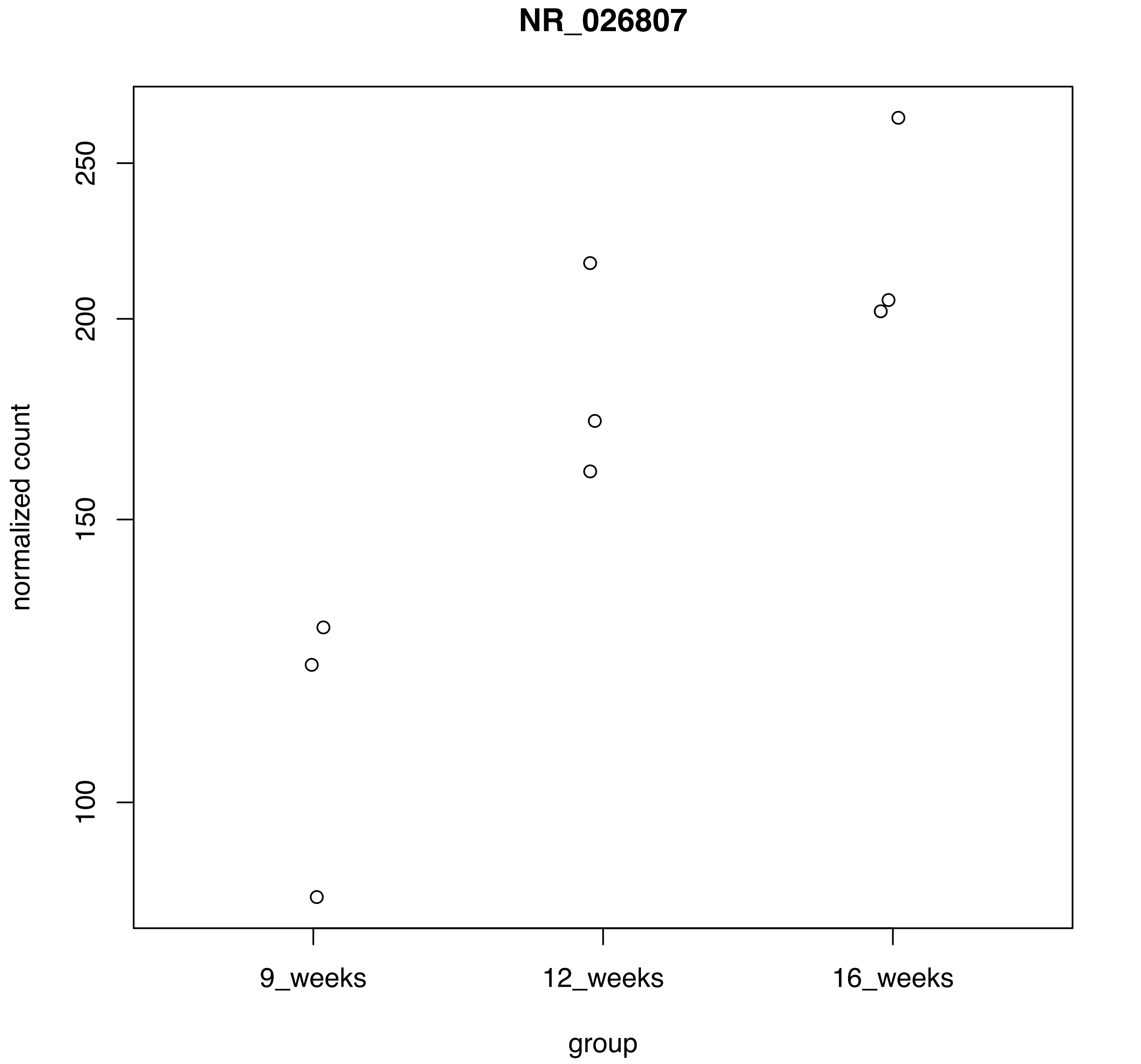

NR_026807
normalized count
250
200
150
100
9_weeks
12_weeks
16_weeks
group

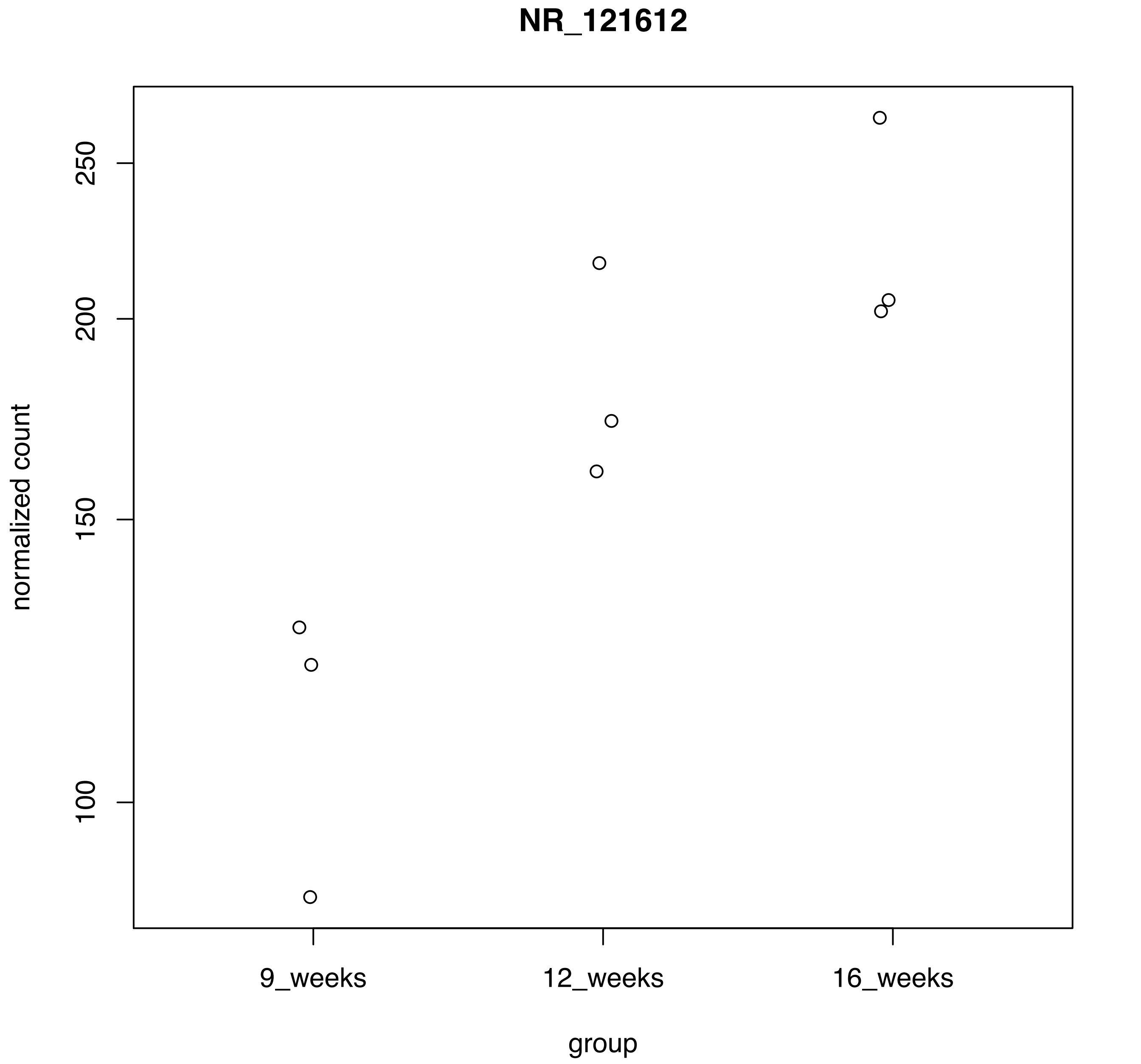

NR_121612
normalized count
250
200
150
100
9_weeks
12_weeks
16_weeks
group

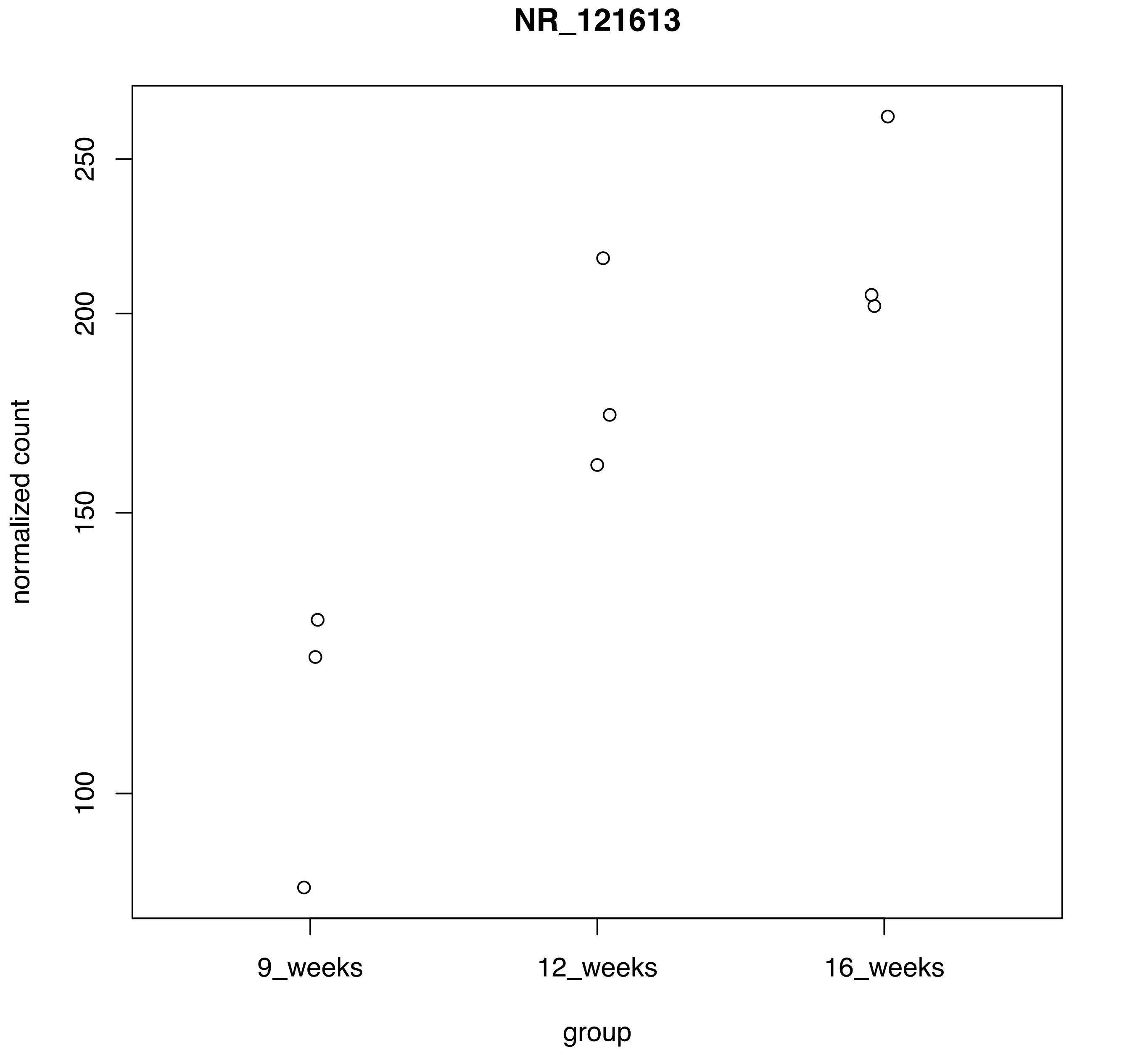

NR_121613
normalized count
250
200
150
100
9_weeks
12_weeks
16_weeks
group

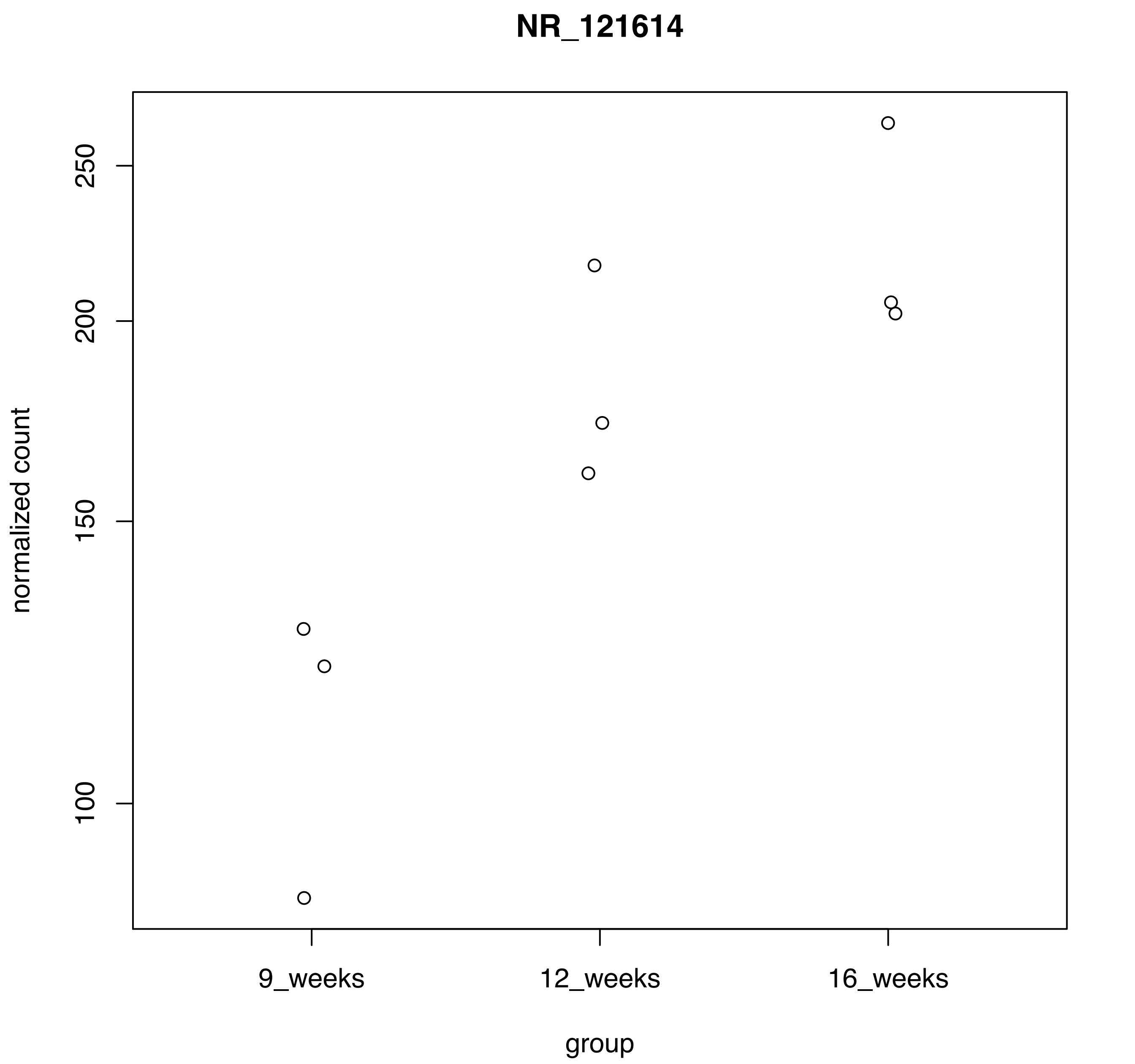
NR_121614
normalized count
250
200
150
100
9_weeks
12_weeks
16_weeks
group

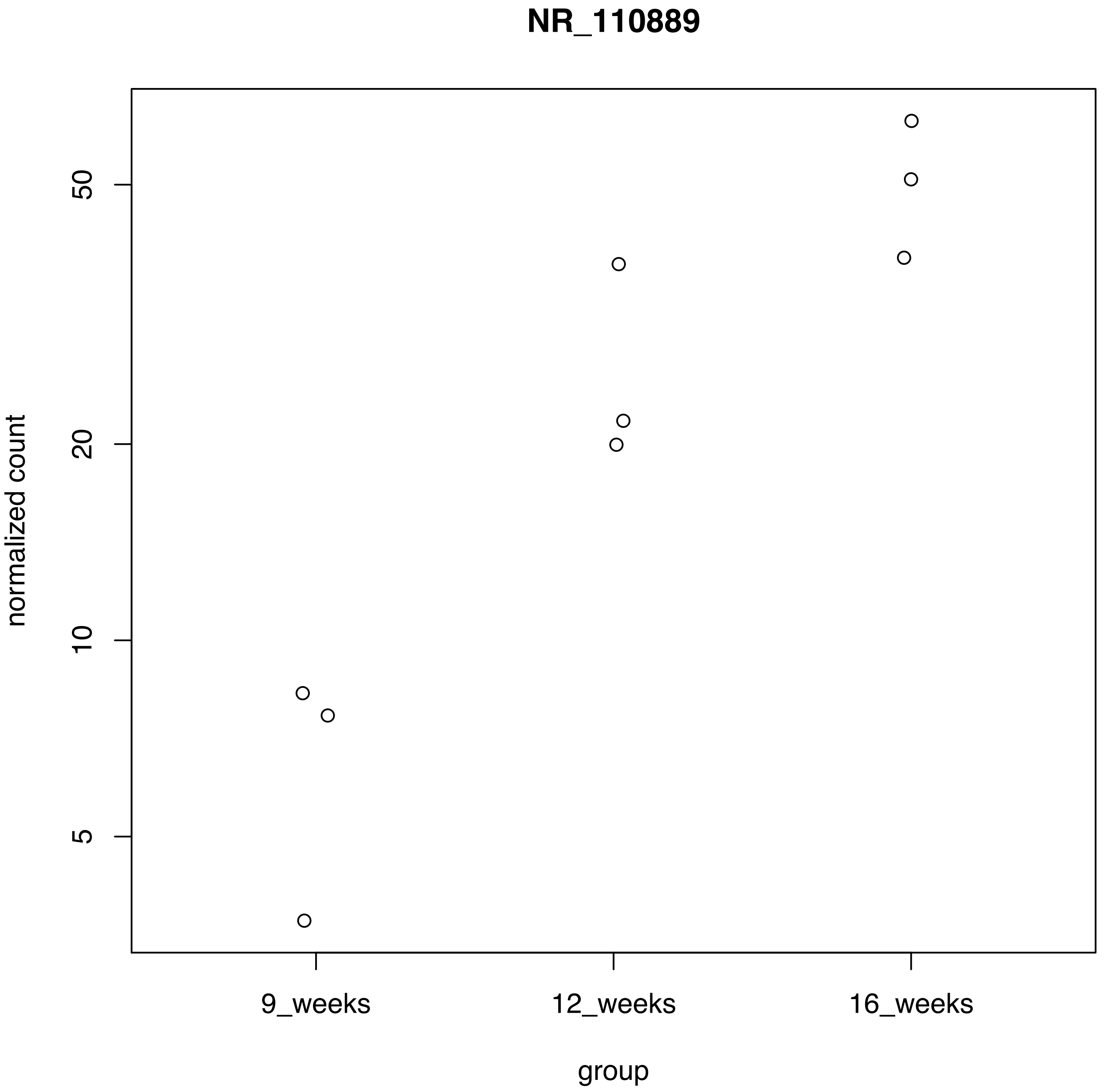
NR_110889
normalized count
5
10
20
50
9_weeks
12_weeks
16_weeks
group

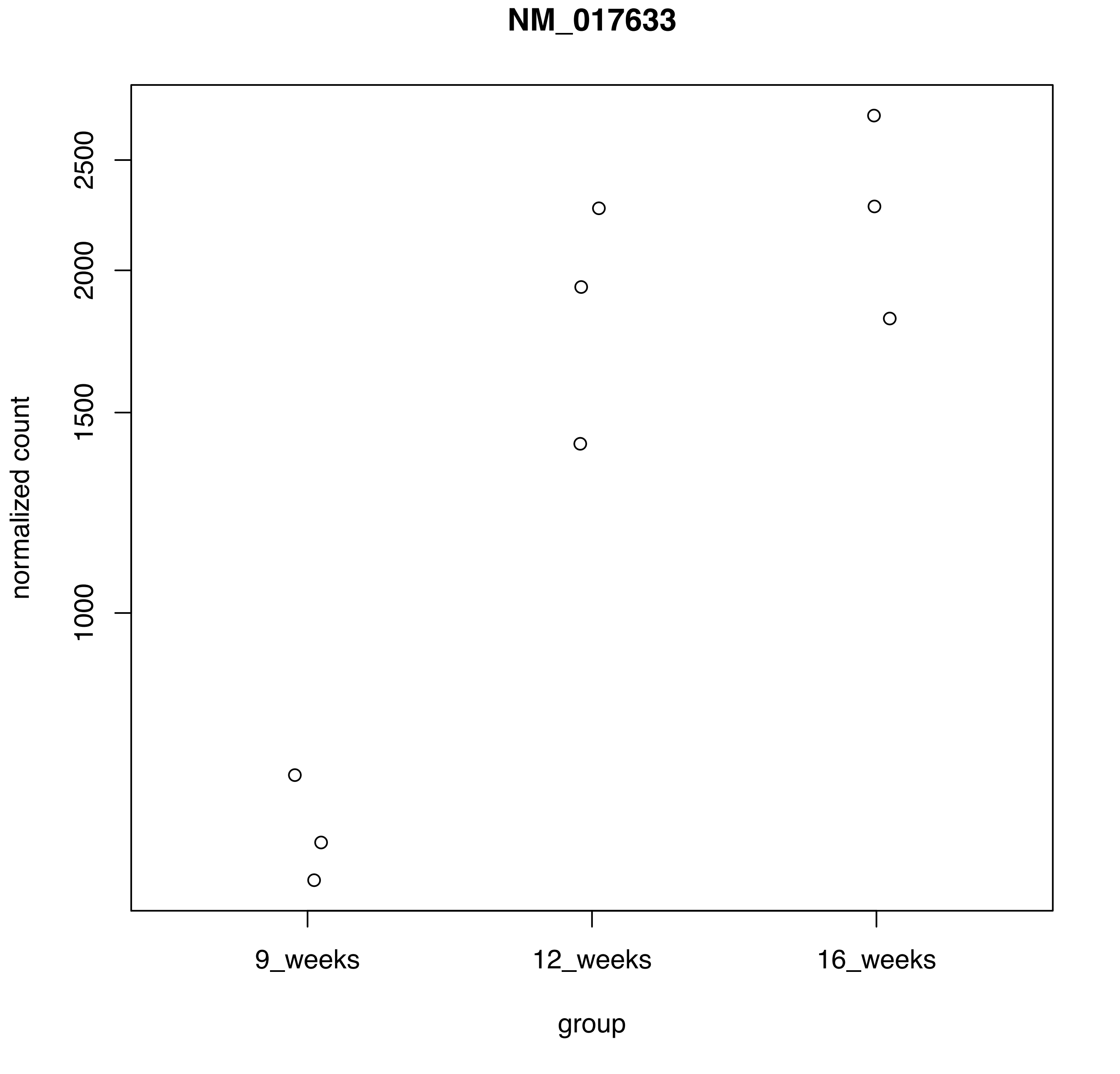
NM_017633
normalized count
2500
2000
1500
1000
9_weeks
12_weeks
16_weeks
group

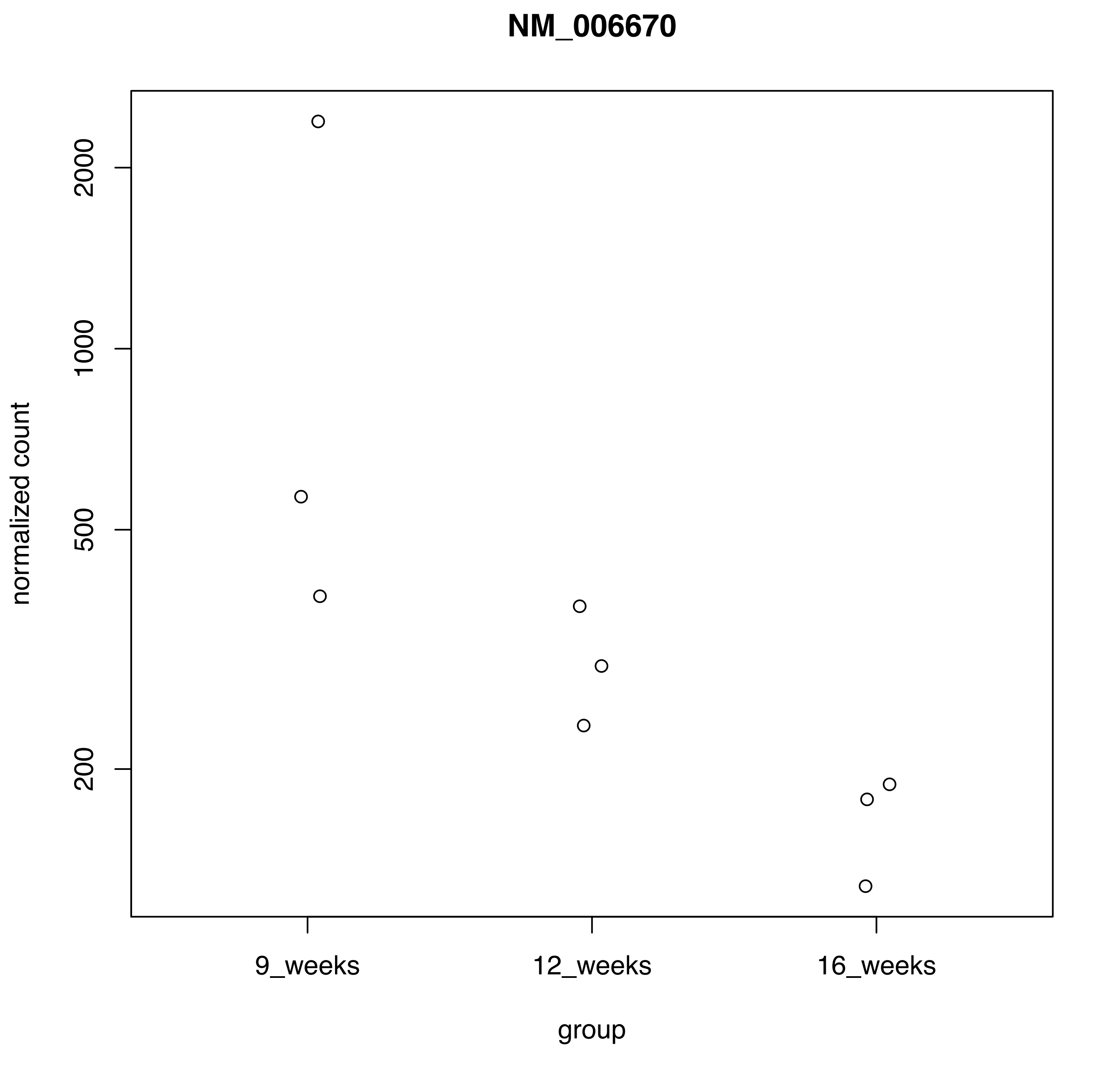
NM_006670
normalized count
2000
1000
500
200
9_weeks
12_weeks
16_weeks
group

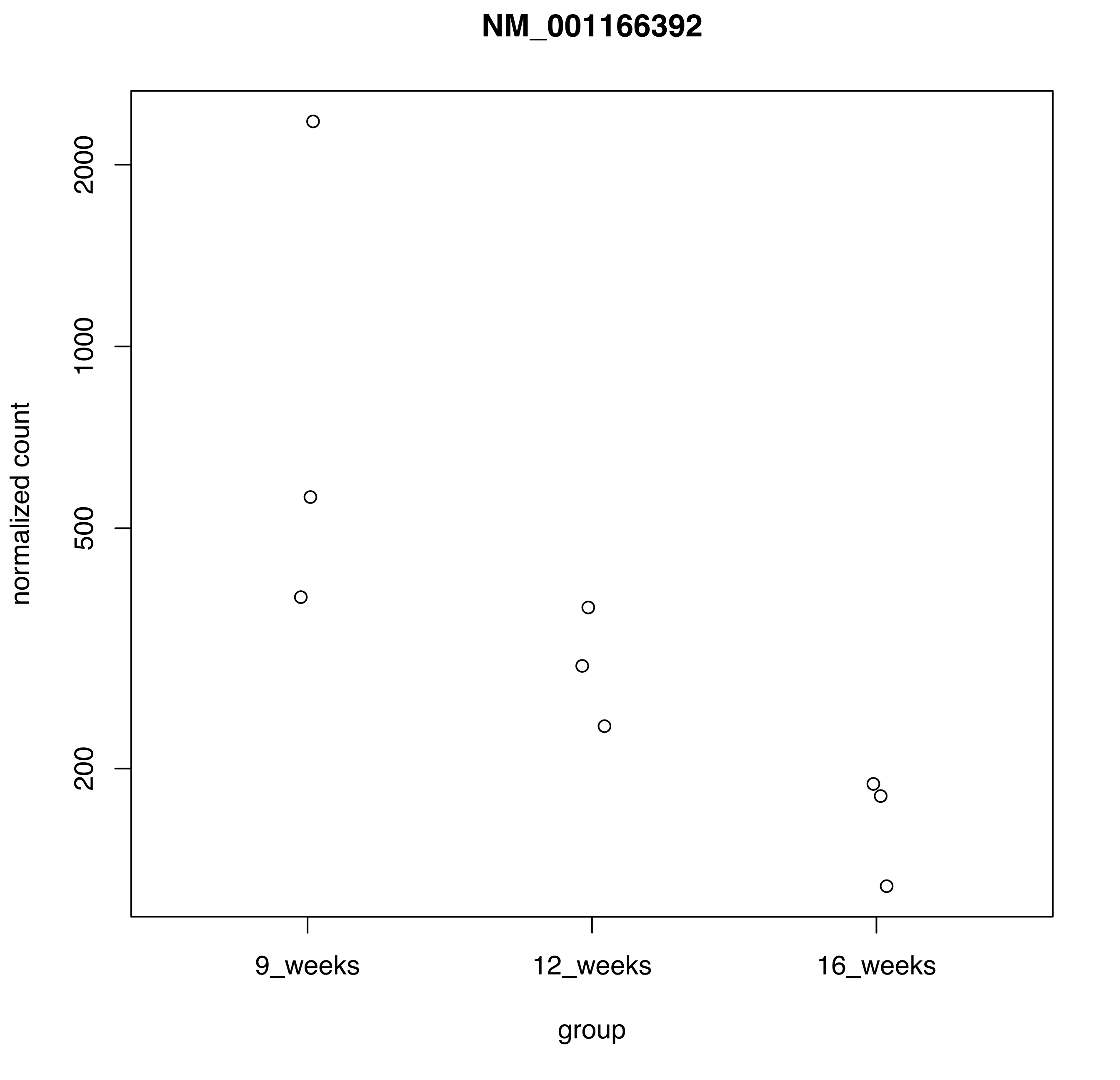
NM_001166392
normalized count
2000
1000
500
200
9_weeks
12_weeks
16_weeks
group

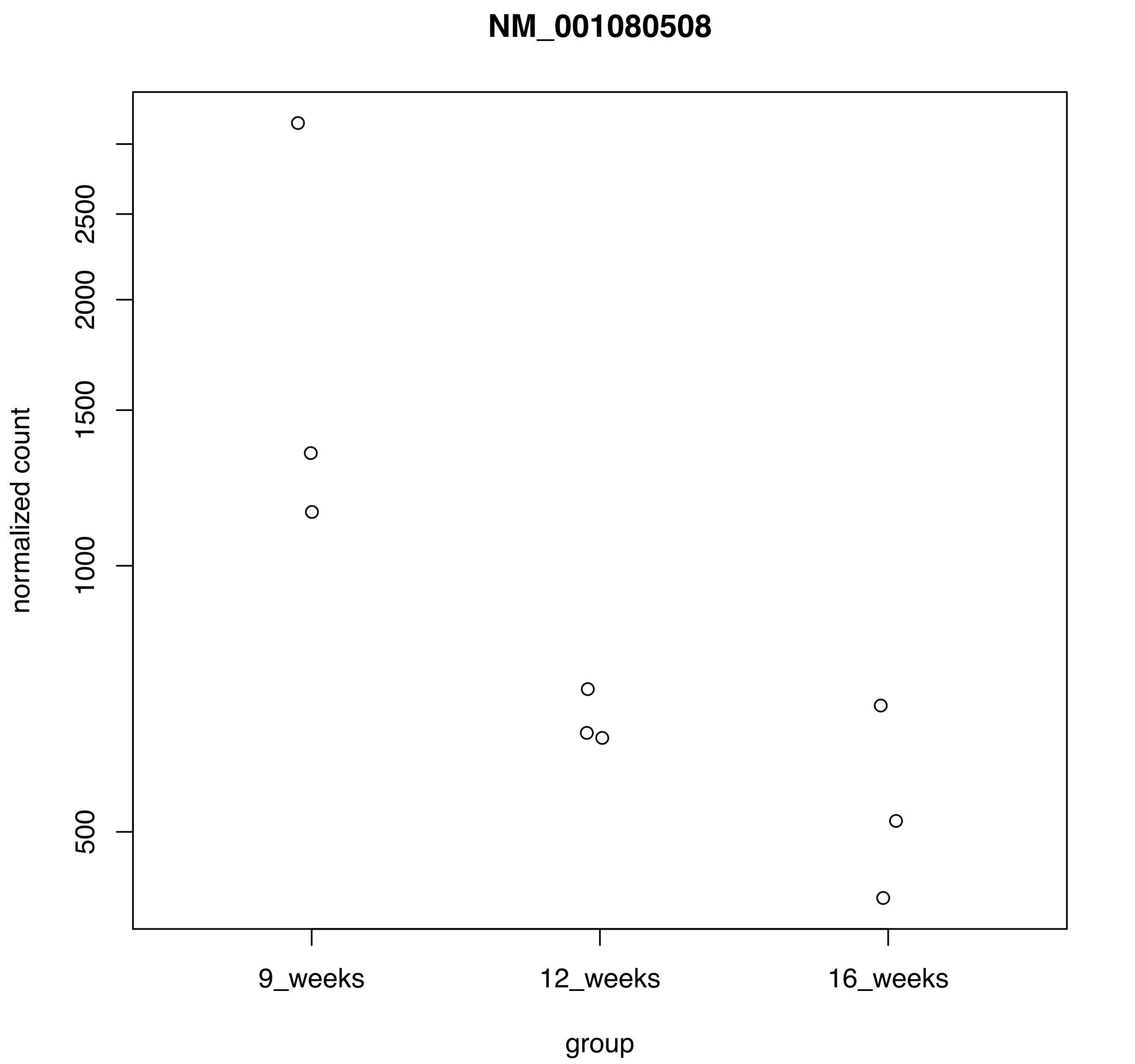
NM_001080508
normalized count
500
1000
1500
2000
2500
9_weeks
12_weeks
16_weeks
group

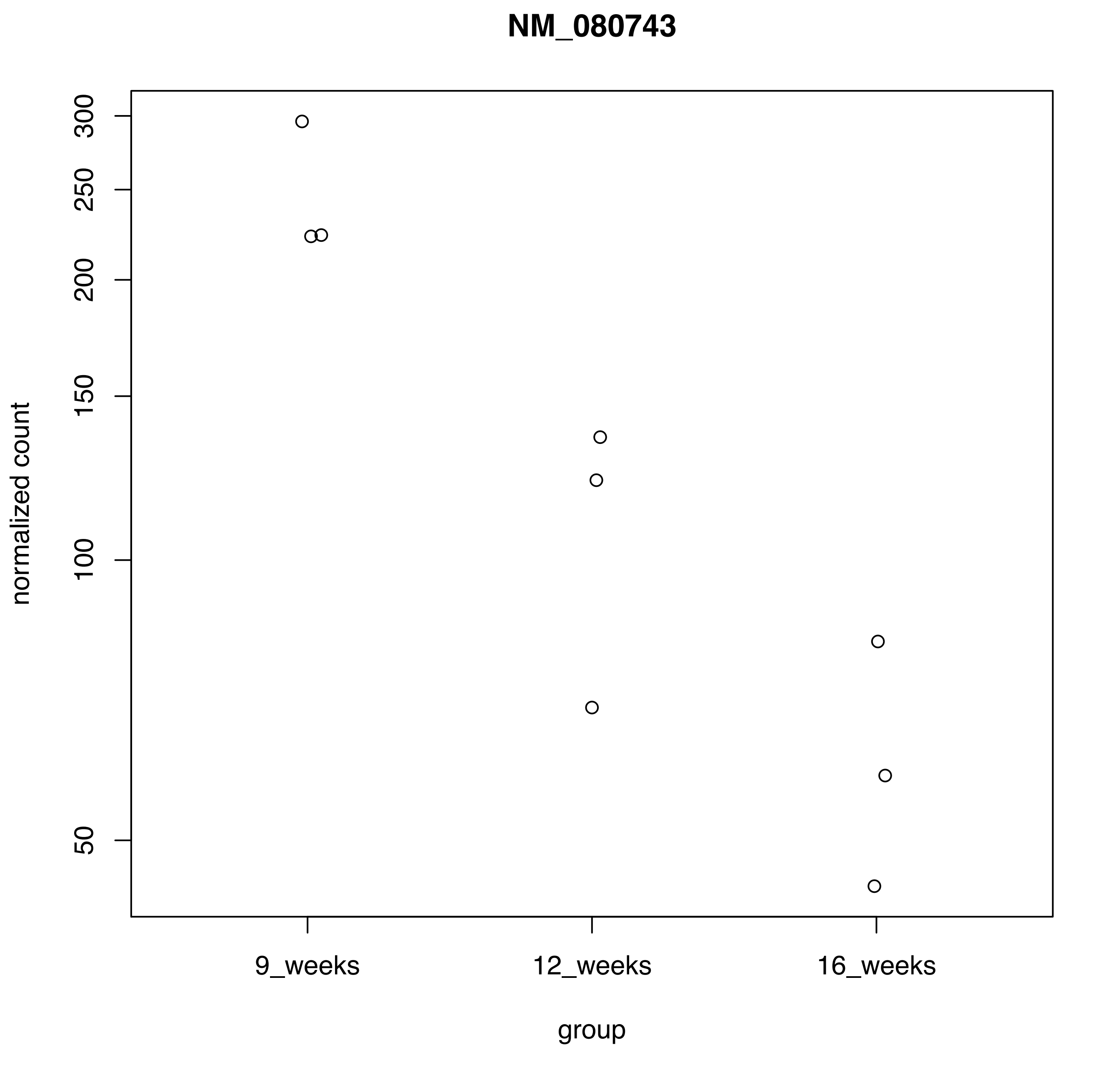
NM_080743
normalized count
300
250
200
150
100
50
9_weeks
12_weeks
16_weeks
group

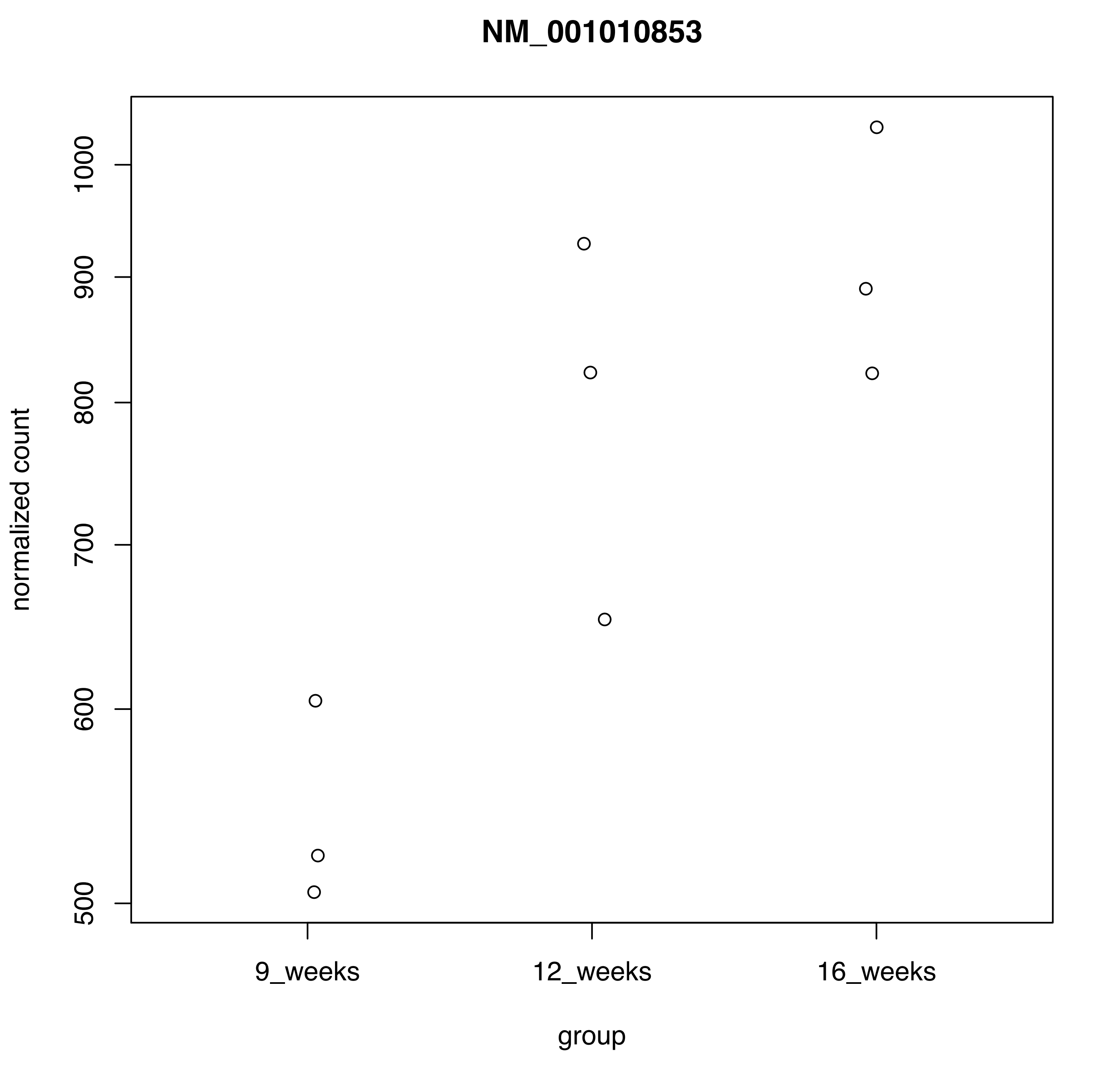
NM_001010853
normalized count
500
600
700
800
900
1000
9_weeks
12_weeks
16_weeks
group

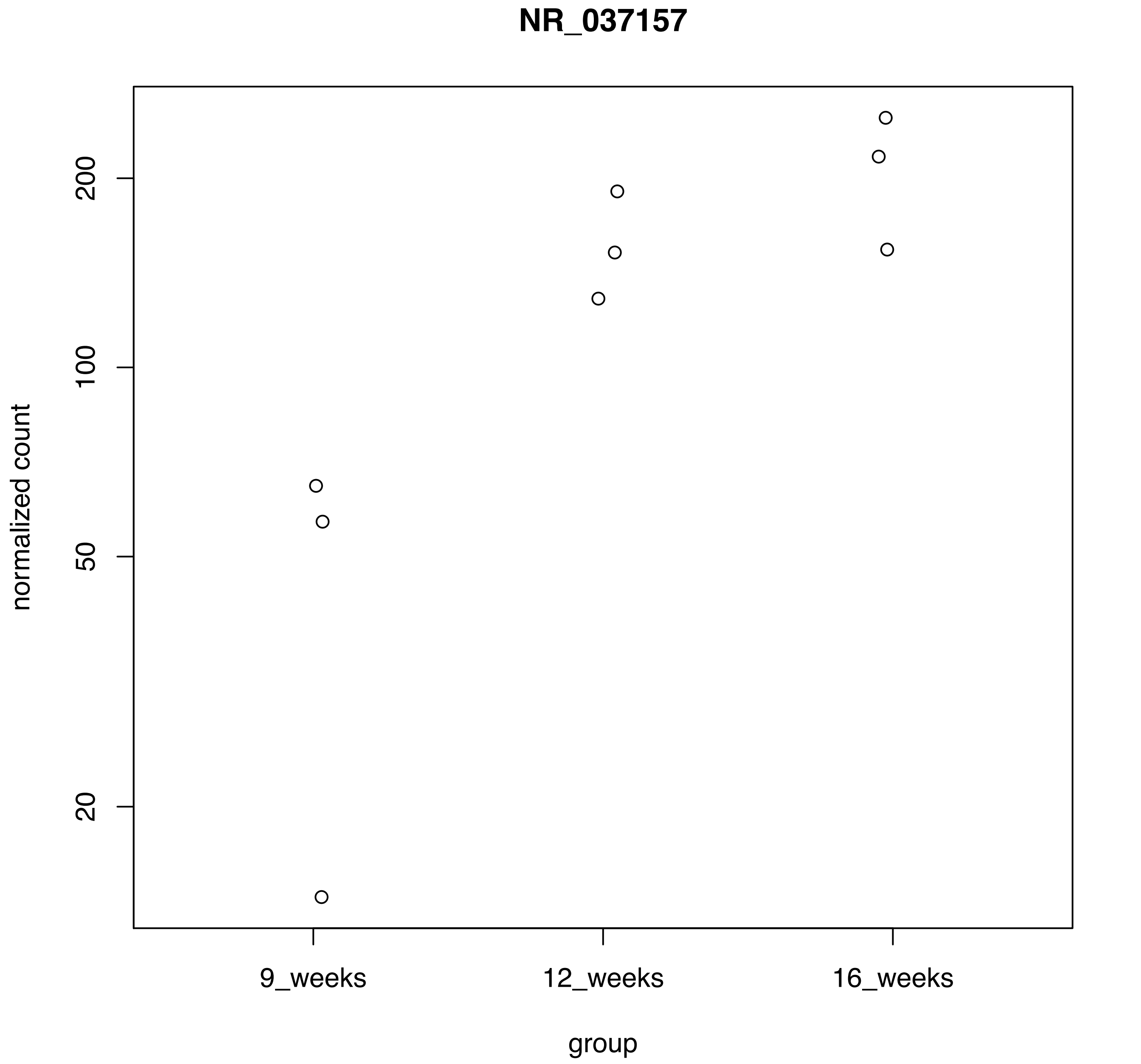
NR_037157
normalized count
200
100
50
20
9_weeks
12_weeks
16_weeks
group

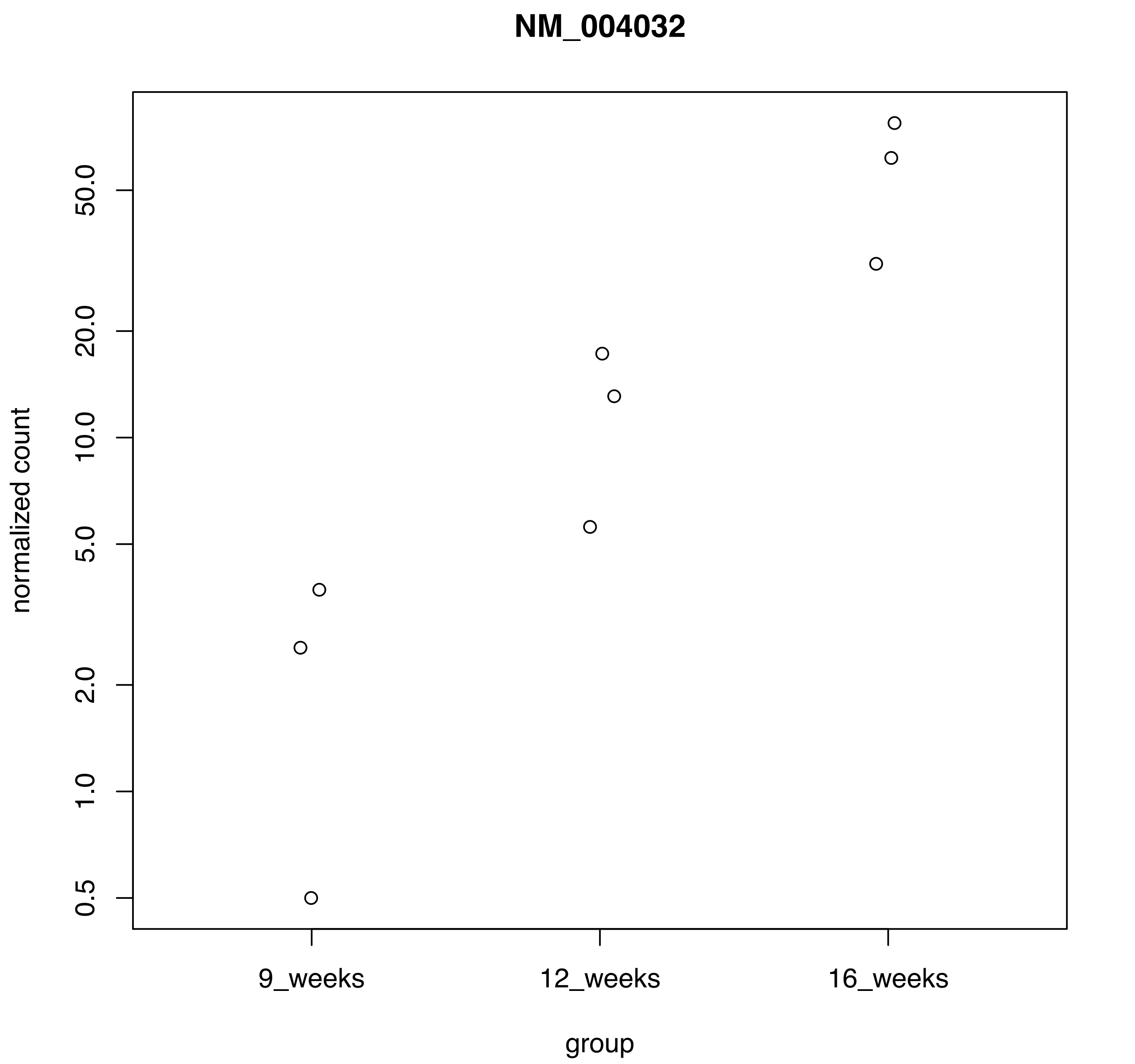
NM_004032
normalized count
0.5
1.0
2.0
5.0
10.0
20.0
50.0
9_weeks
12_weeks
16_weeks
group

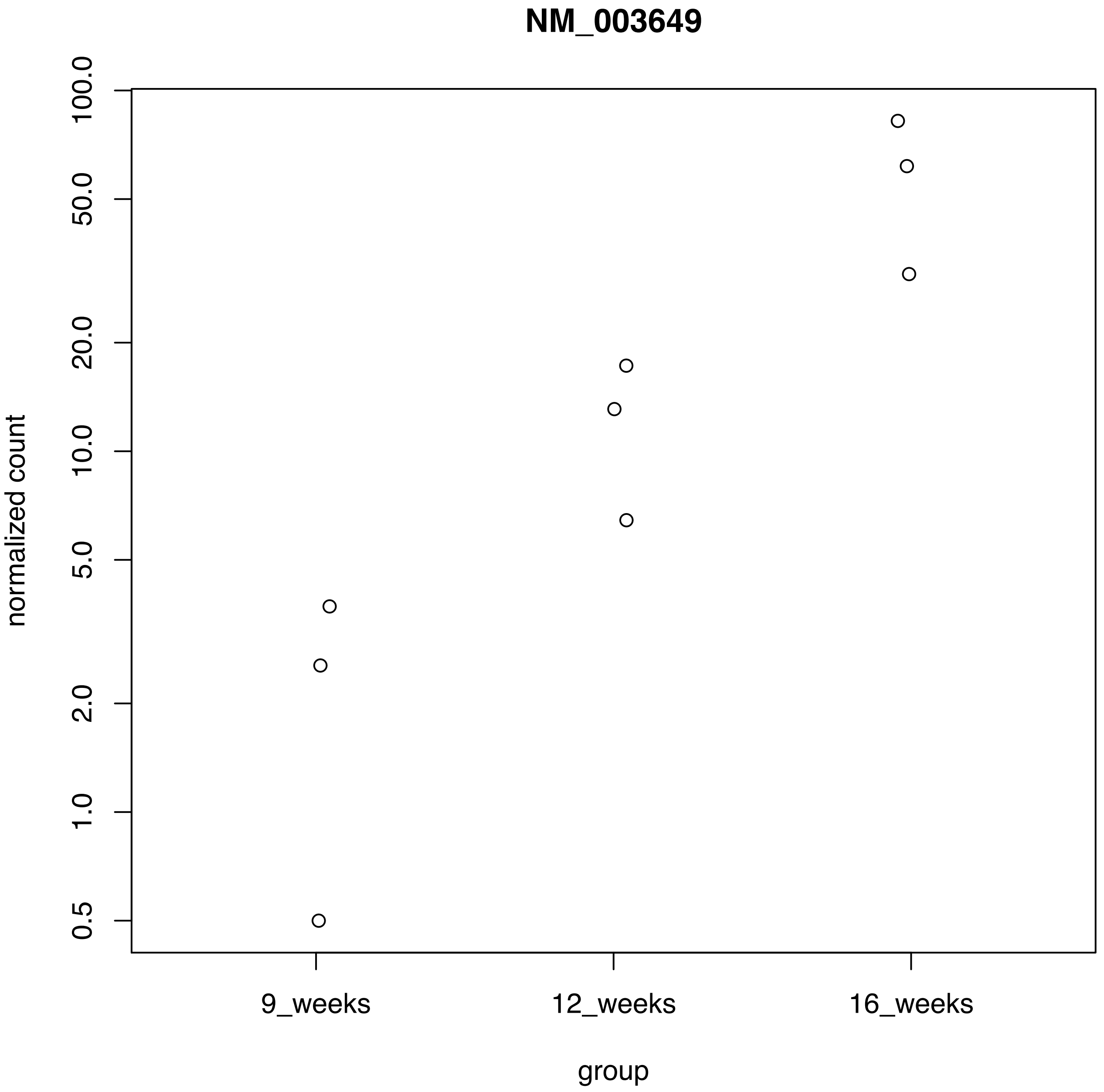
NM_003649
normalized count
100.0
50.0
20.0
10.0
5.0
2.0
1.0
0.5
9_weeks
12_weeks
16_weeks
group

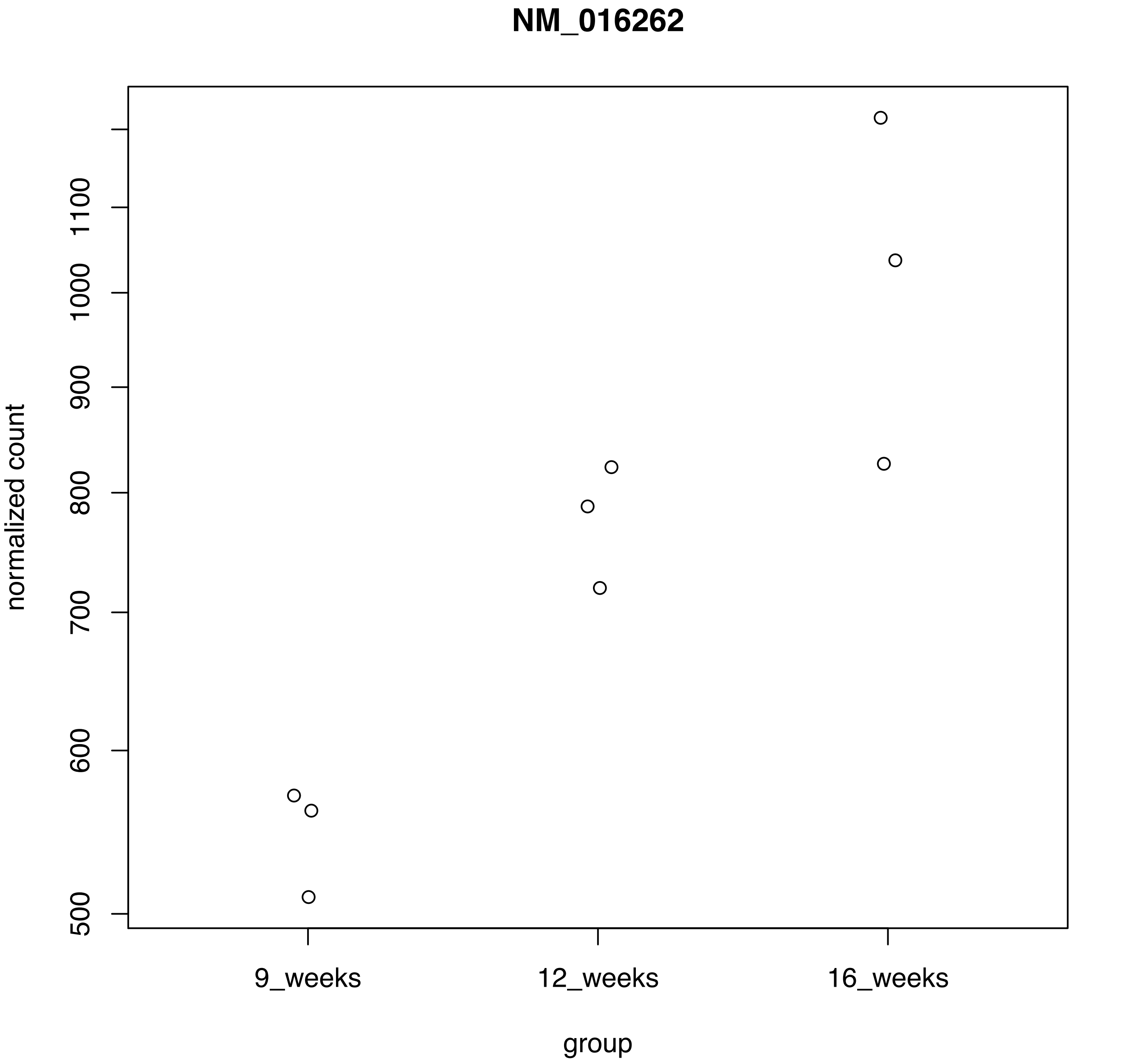
NM_016262
normalized count
500
600
700
800
900
1000
1100
9_weeks
12_weeks
16_weeks
group

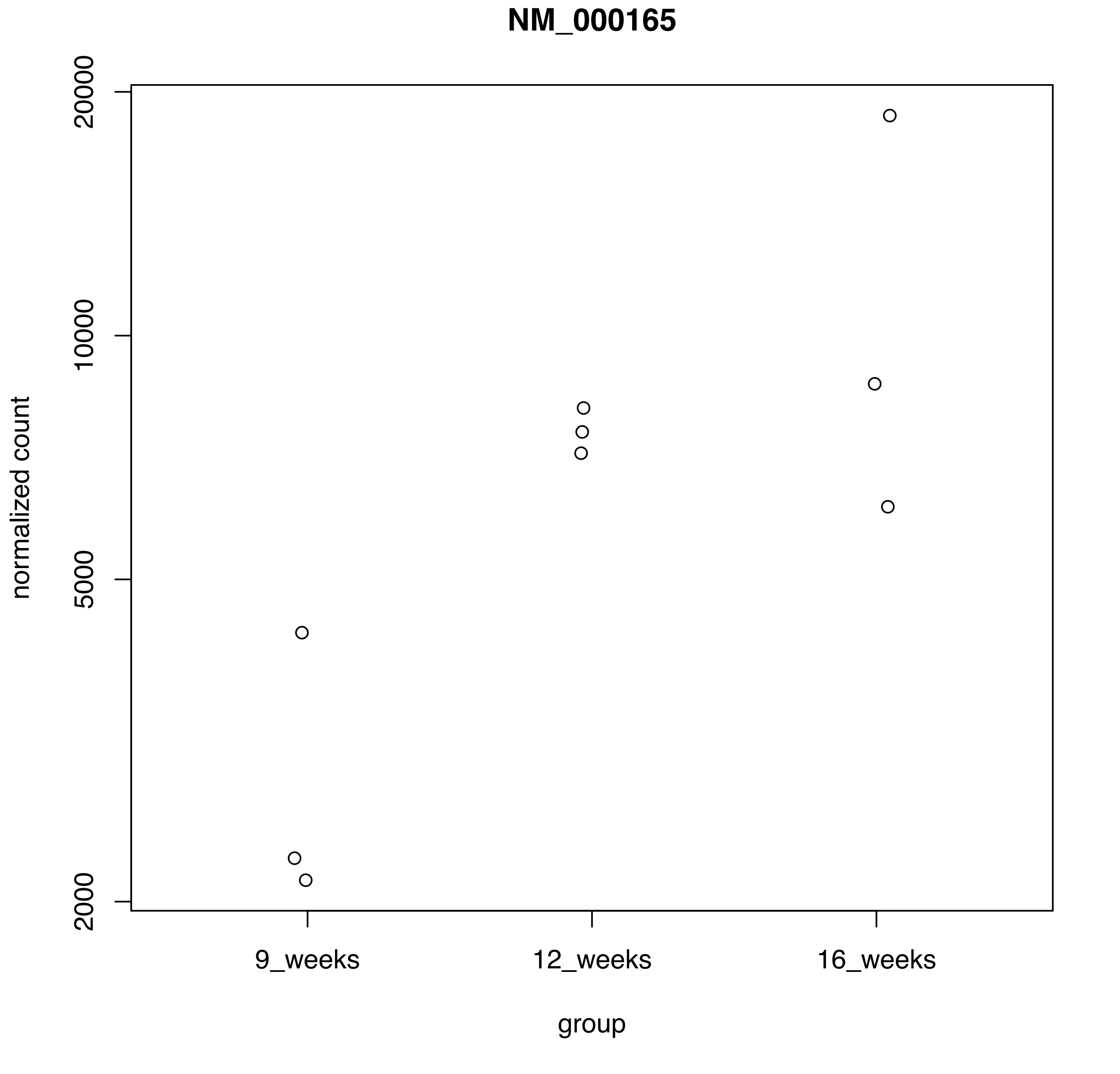
NM_000165
normalized count
20000
10000
5000
2000
9_weeks
12_weeks
16_weeks
group

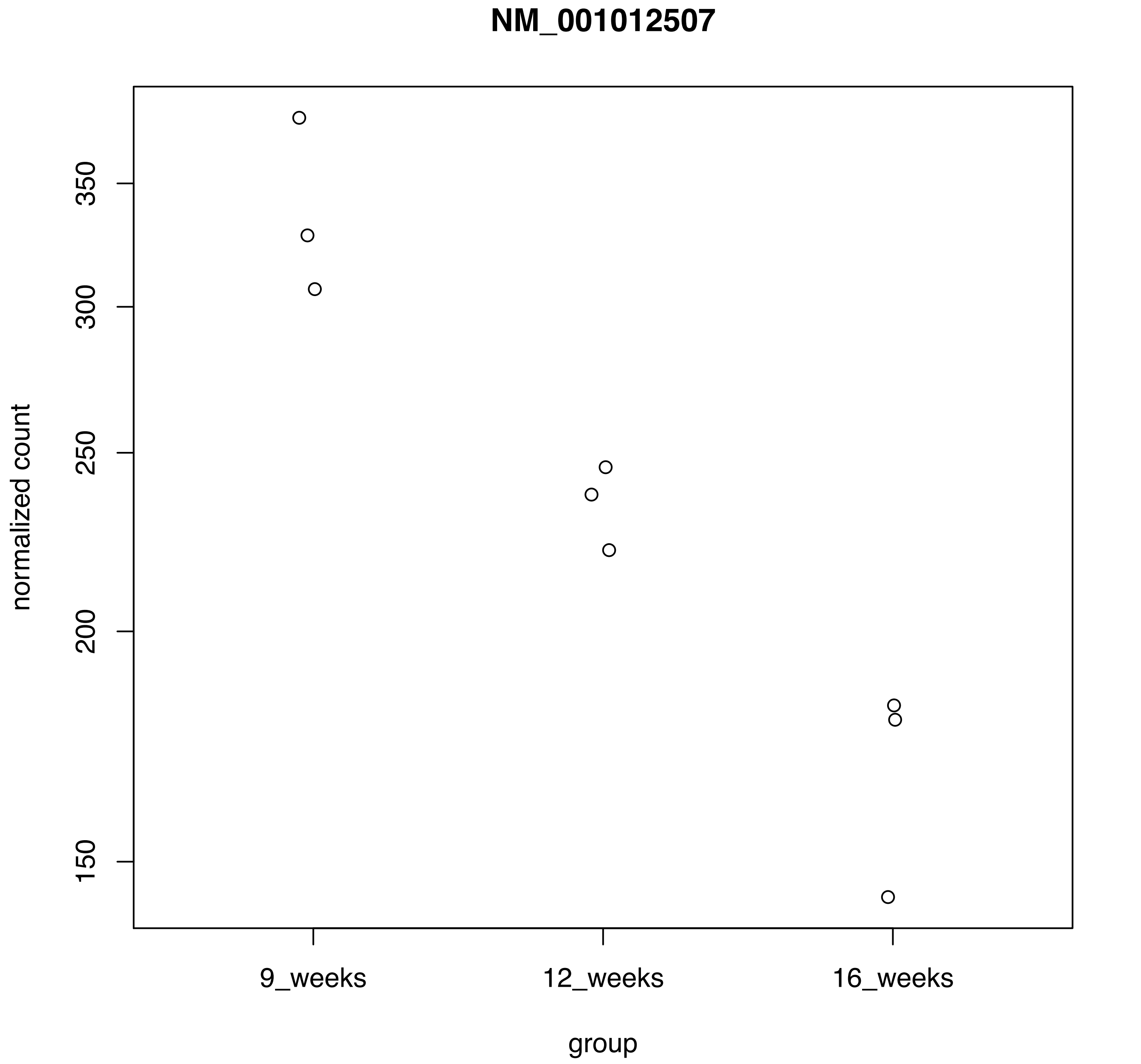
NM_001012507
normalized count
350
300
250
200
150
9_weeks
12_weeks
16_weeks
group

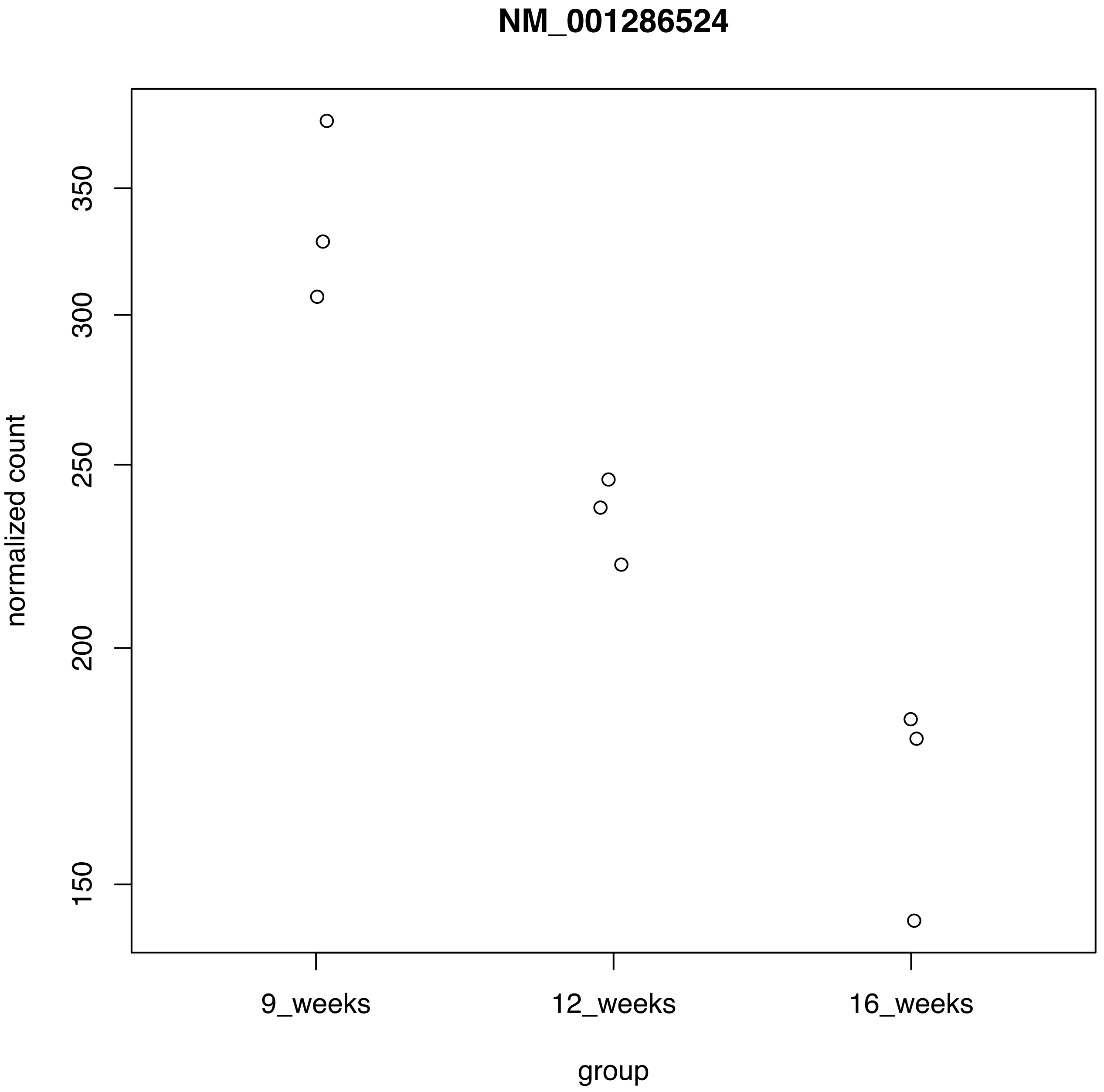
NM_001286524
normalized count
350
300
250
200
150
9_weeks
12_weeks
16_weeks
group

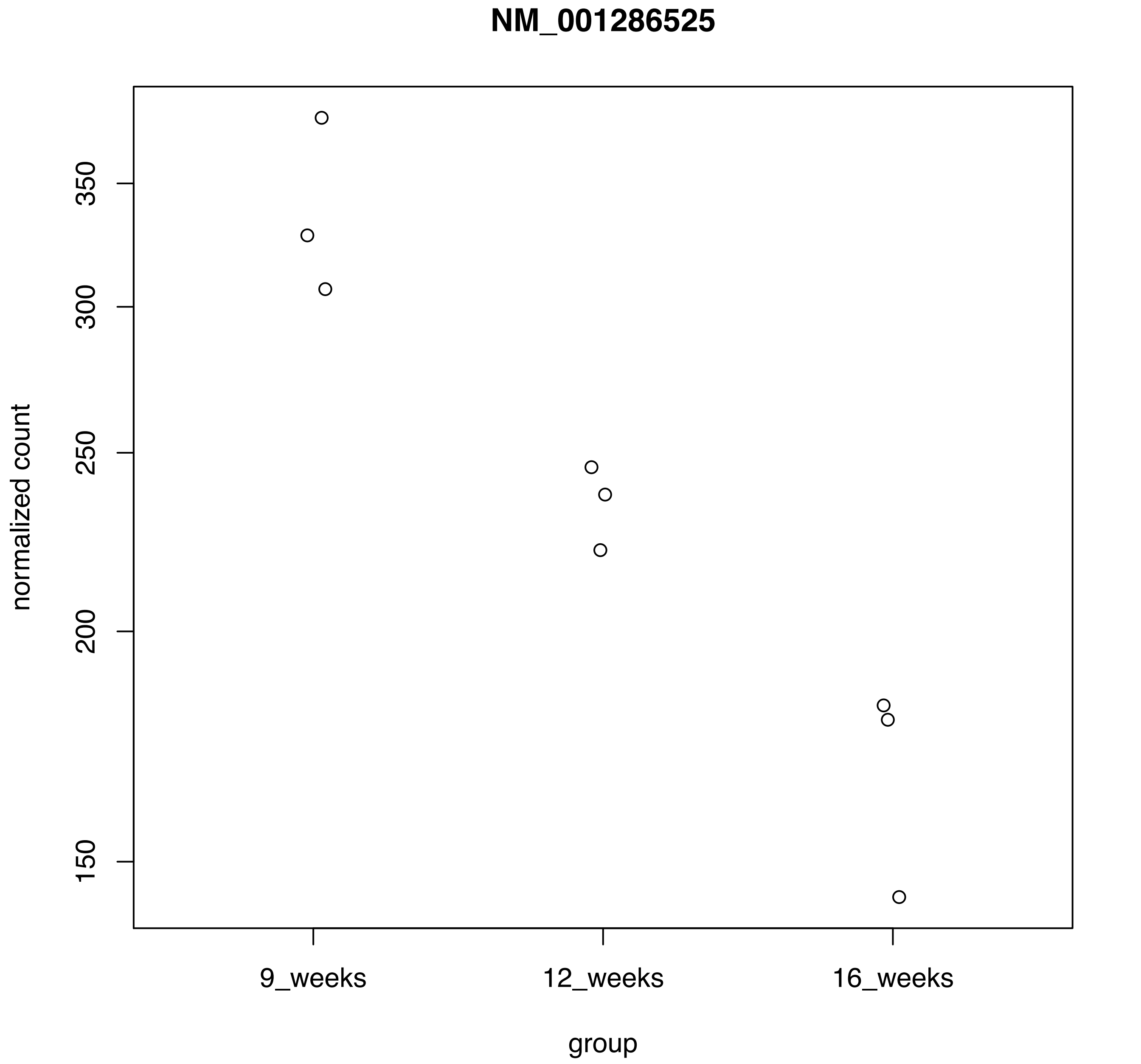
NM_001286525
normalized count
350
300
250
200
150
9_weeks
12_weeks
16_weeks
group

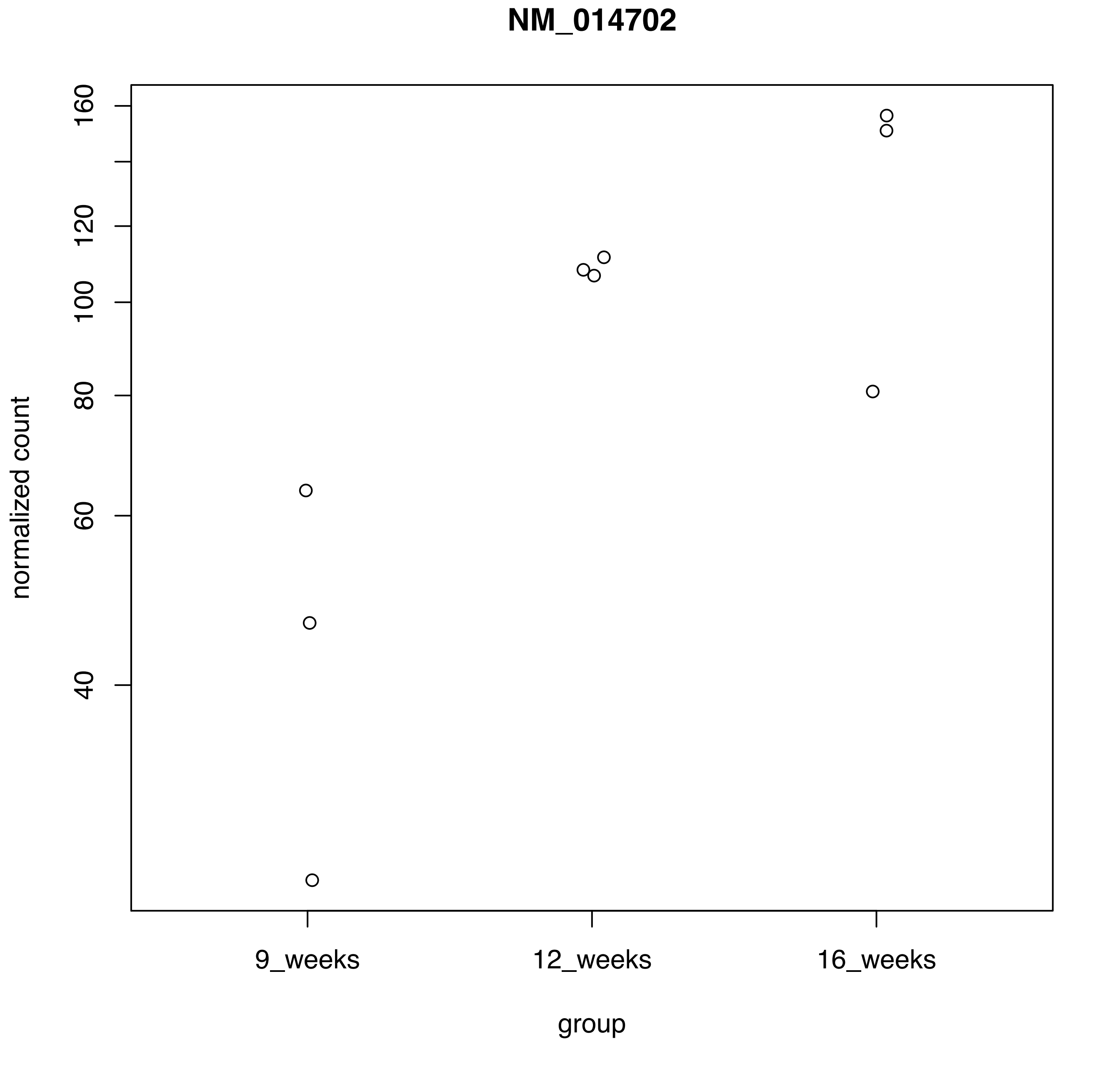
NM_014702
normalized count
160
120
100
80
60
40
9_weeks
12_weeks
16_weeks
group

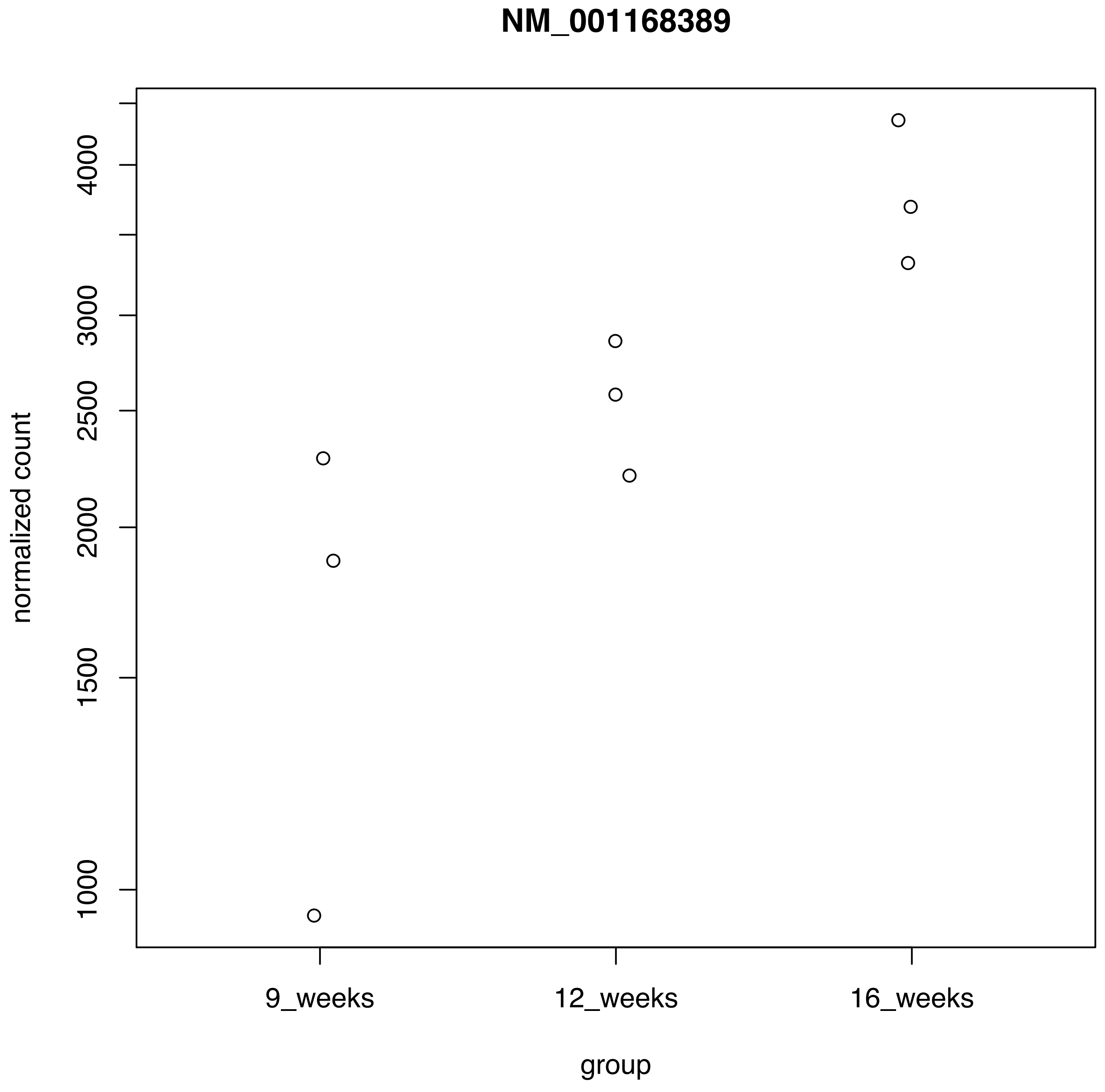
NM_001168389
normalized count
1000
1500
2000
2500
3000
4000
9_weeks
12_weeks
16_weeks
group

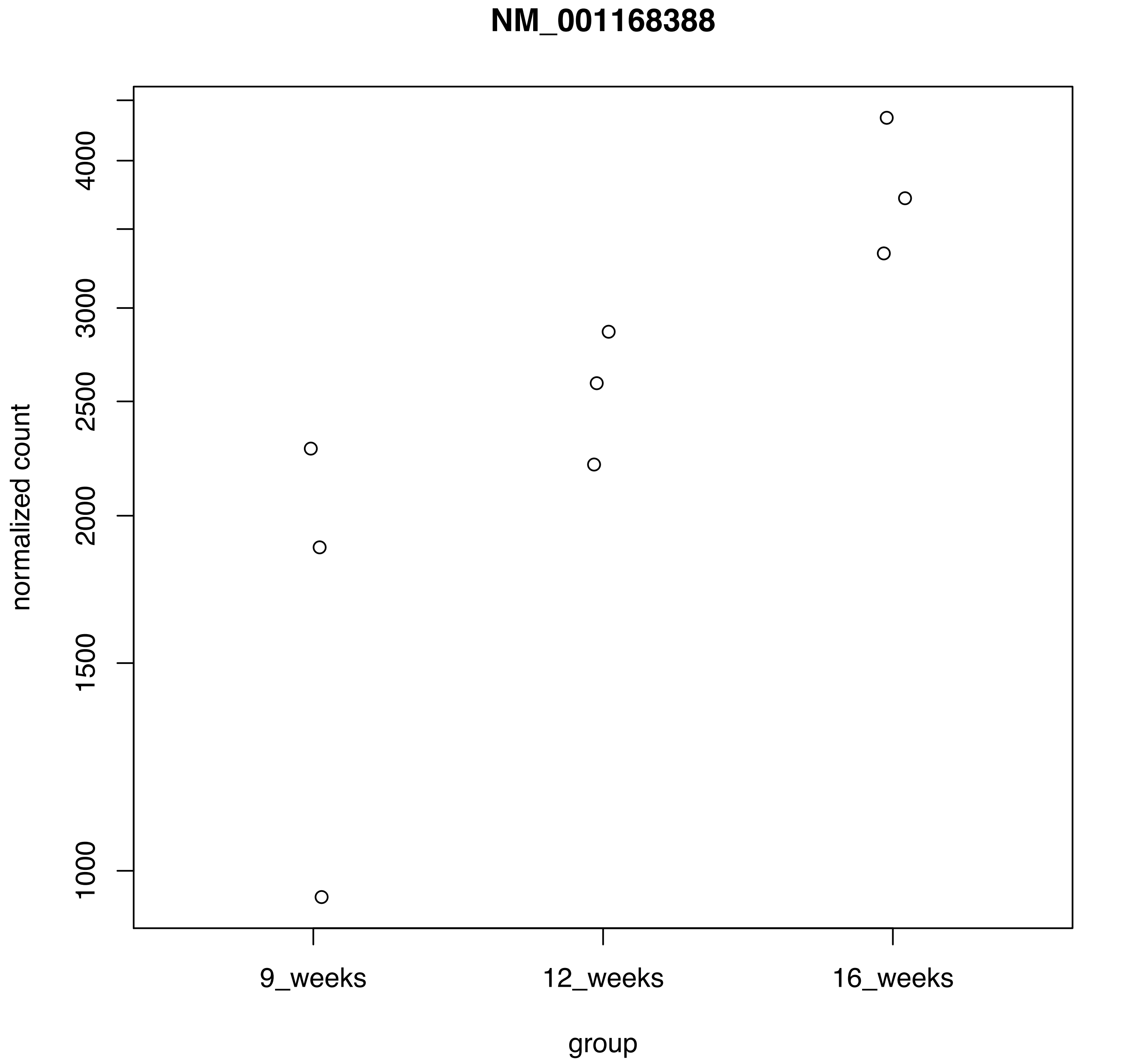

NM_001168388
normalized count
4000
3000
2500
2000
1500
1000
9_weeks
12_weeks
16_weeks
group

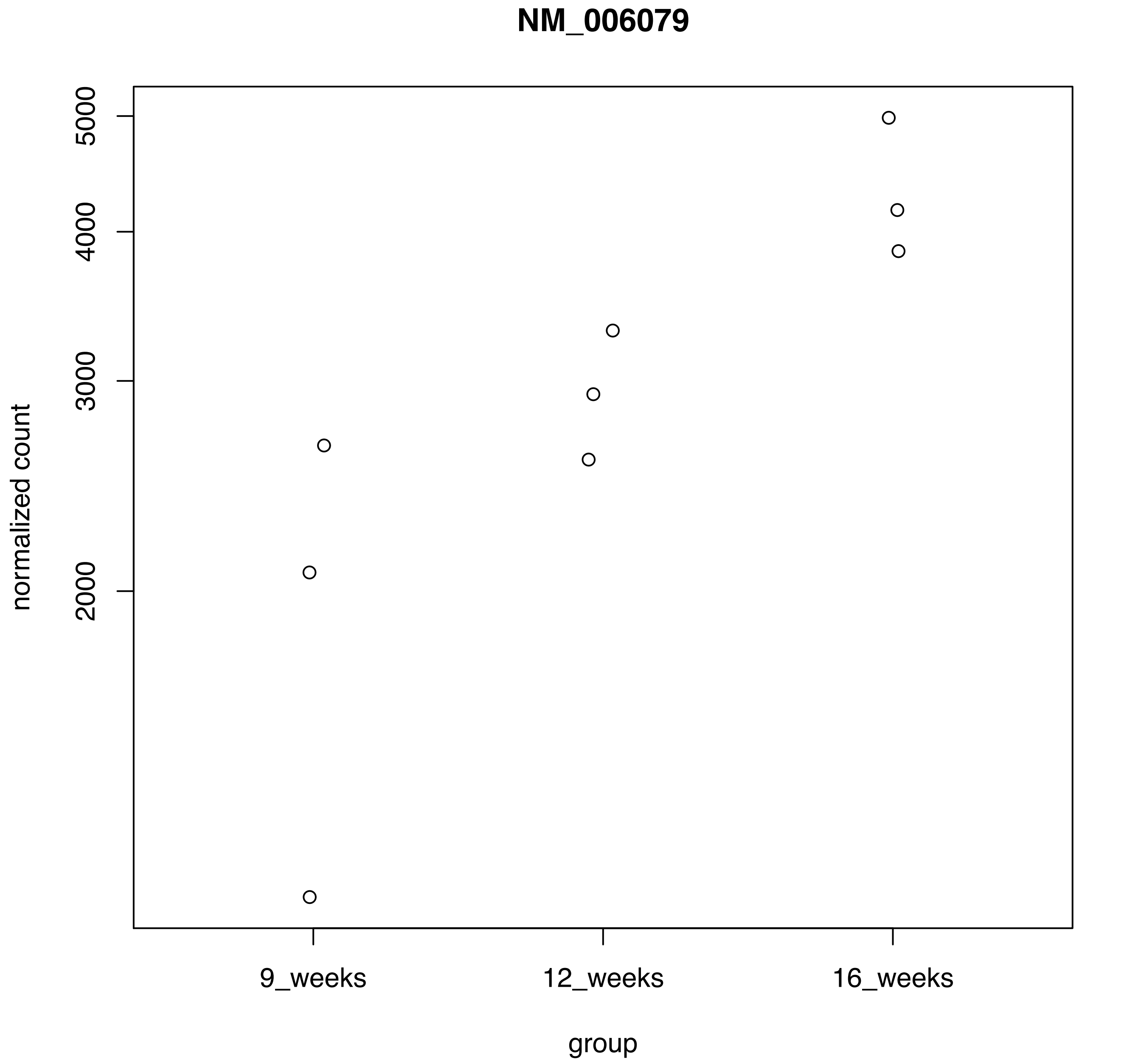

NM_006079
normalized count
5000
4000
3000
2000
9_weeks
12_weeks
16_weeks
group

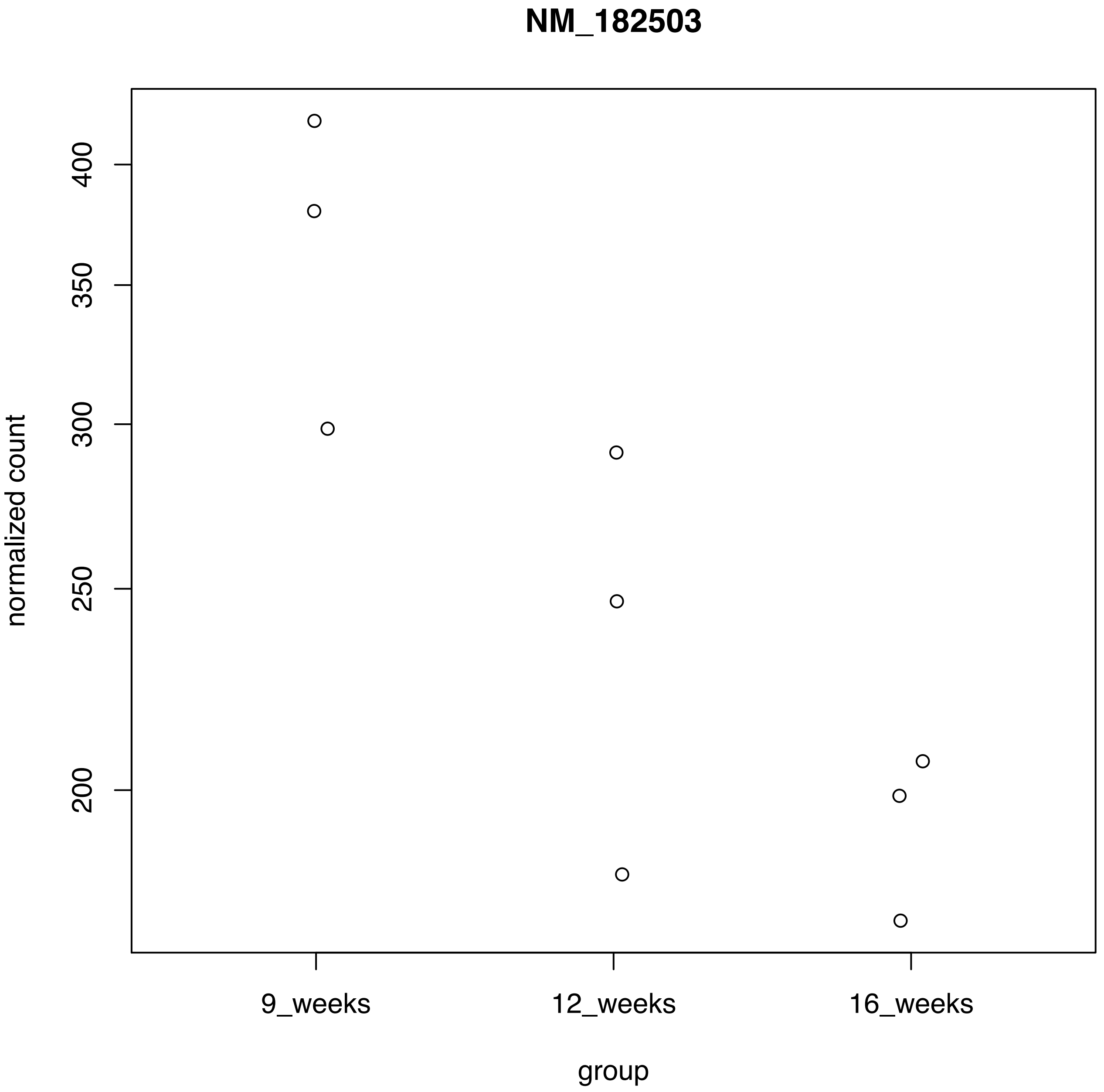
NM_182503
normalized count
200
250
300
350
400
9_weeks
12_weeks
16_weeks
group

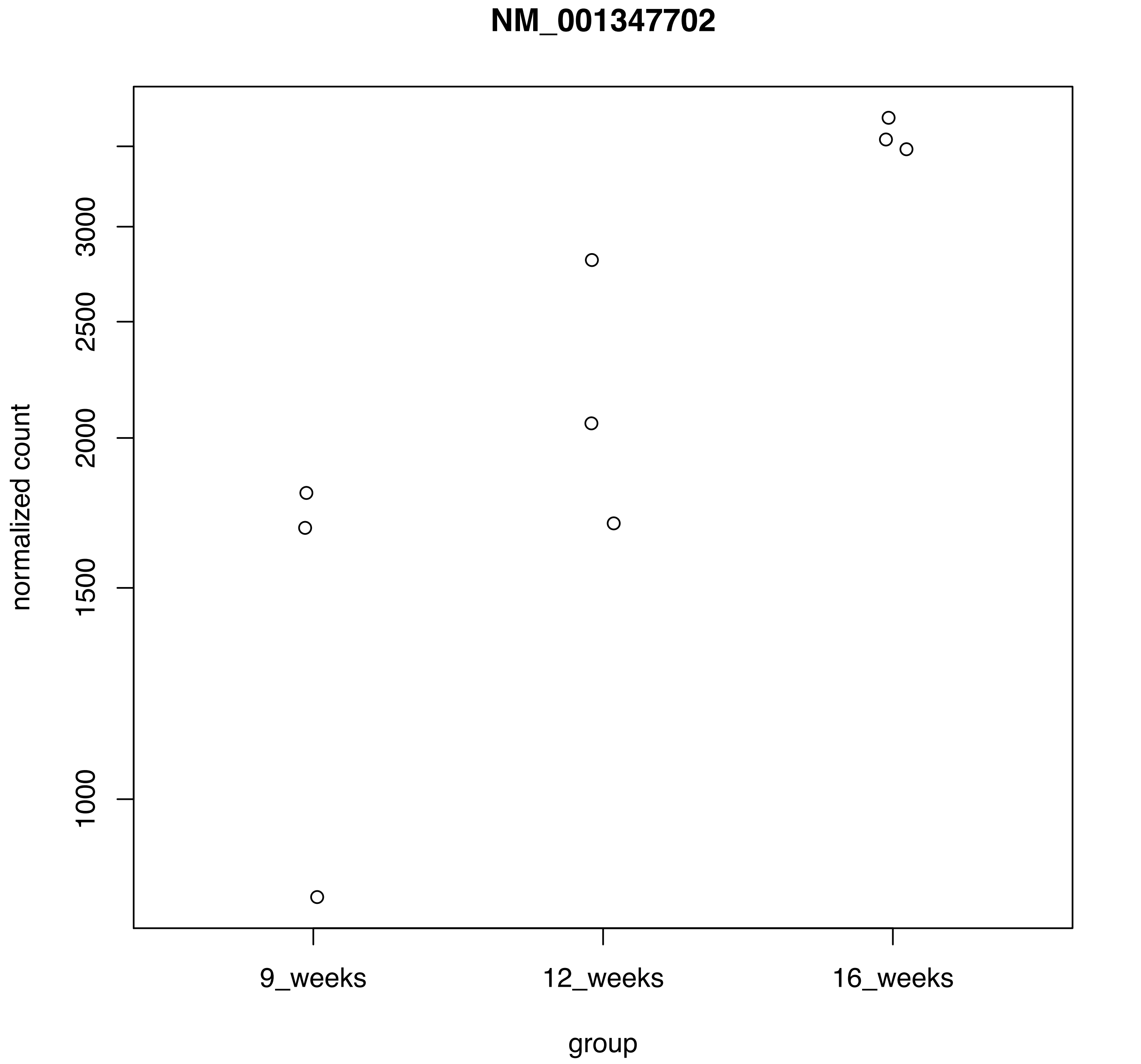

NM_001347702
normalized count
1000
1500
2000
2500
3000
9_weeks
12_weeks
16_weeks
group

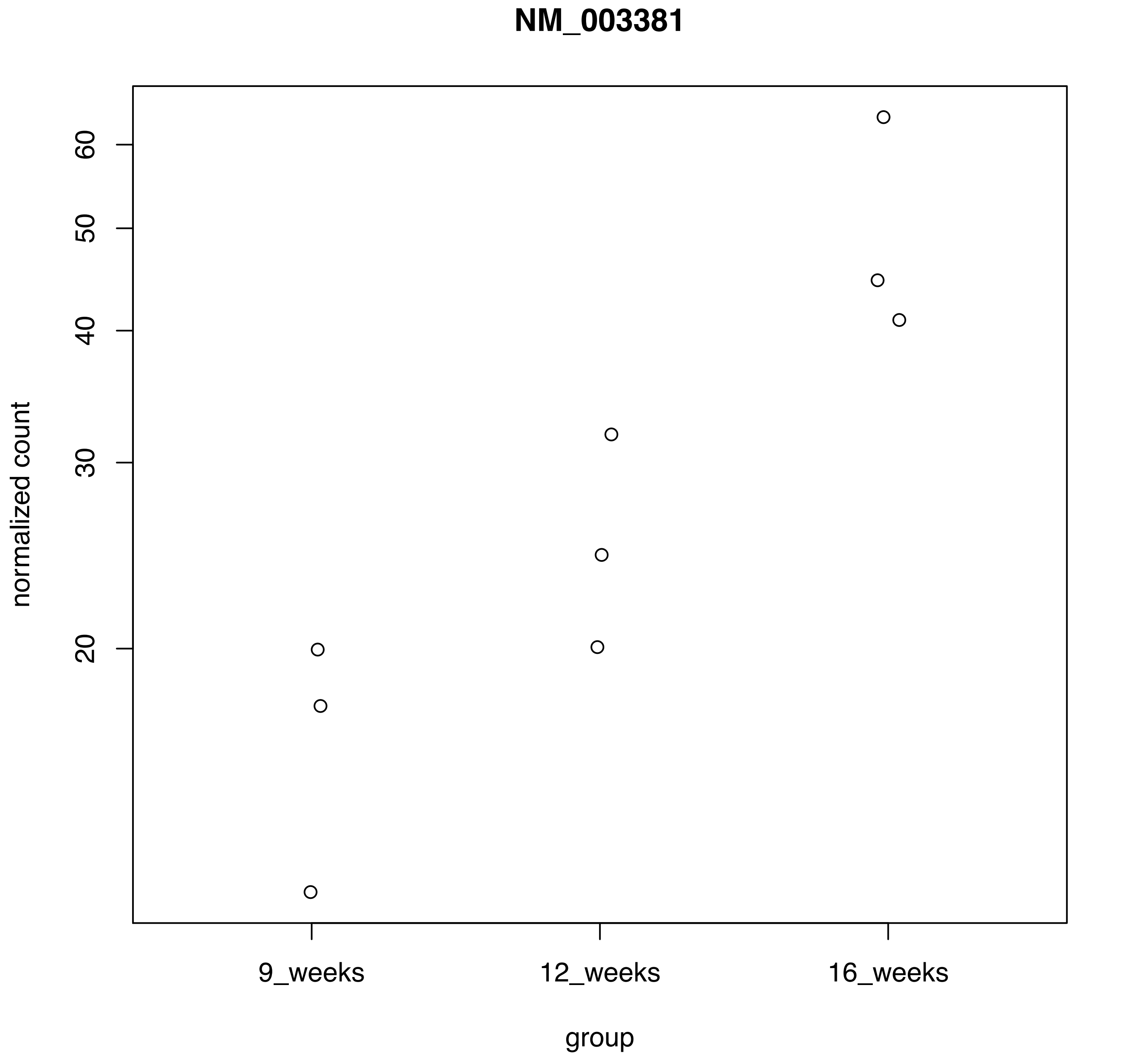
NM_003381
normalized count
60
50
40
30
20
9_weeks
12_weeks
16_weeks
group

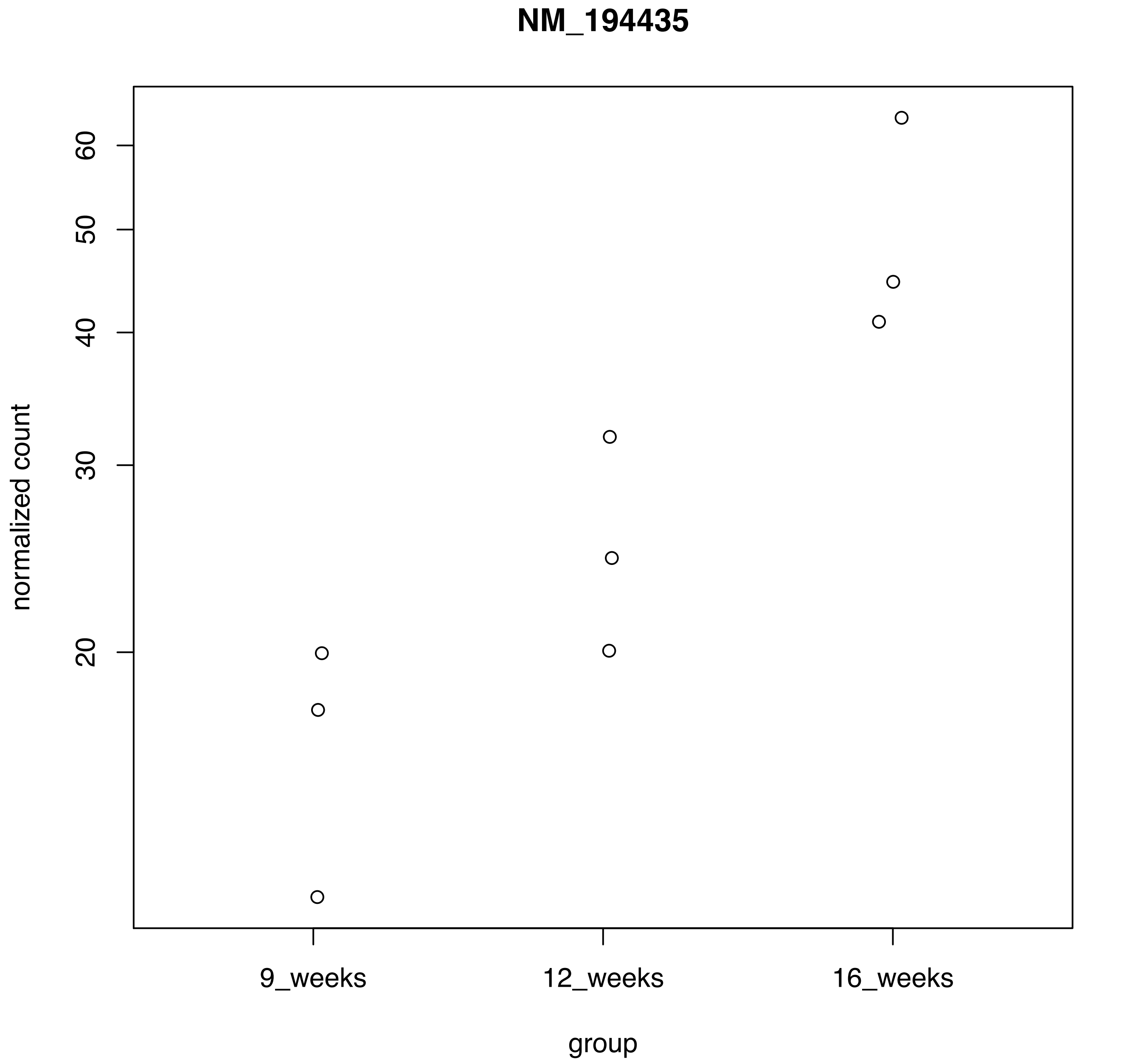
NM_194435
normalized count
20
30
40
50
60
9_weeks
12_weeks
16_weeks
group

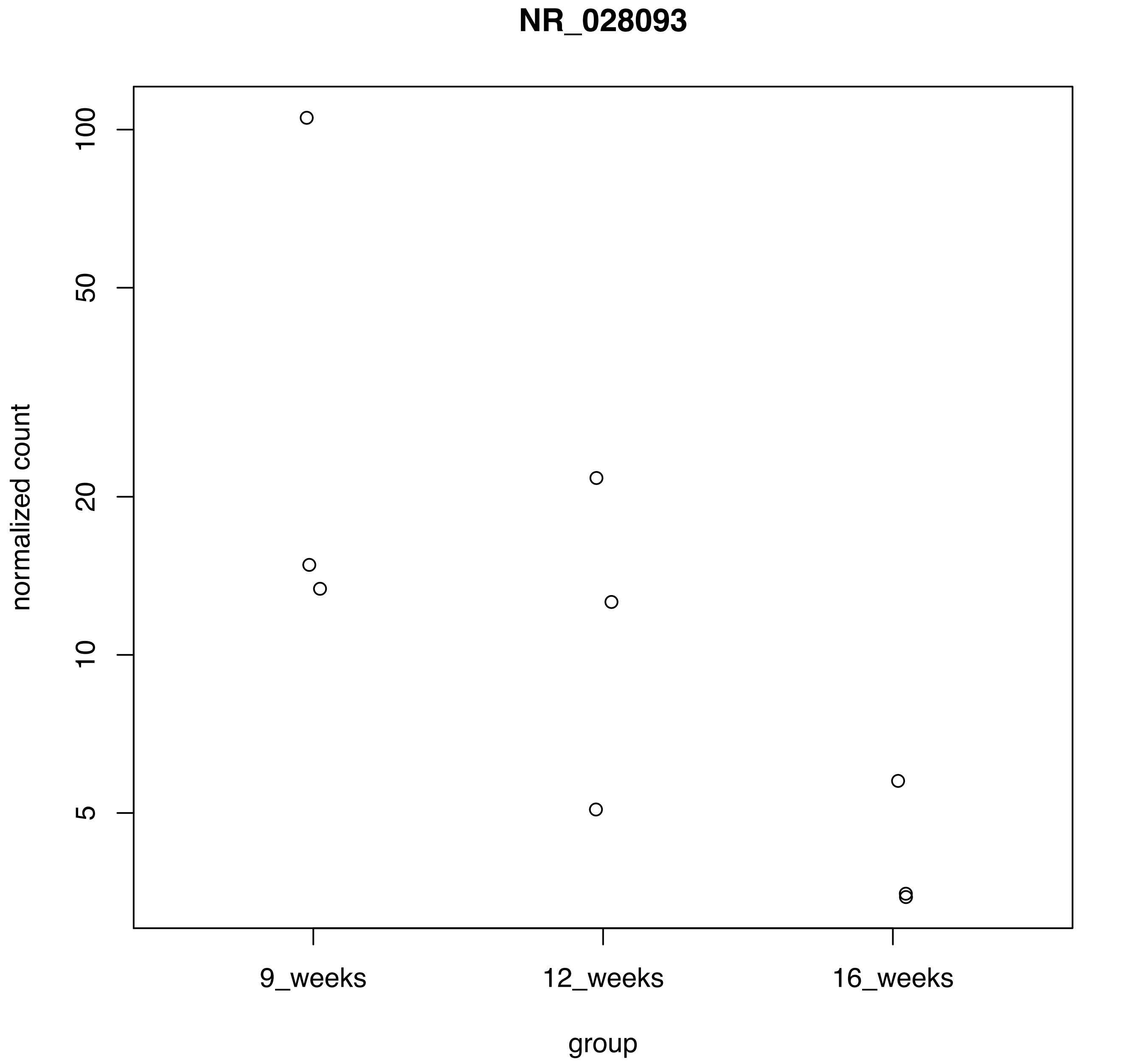
NR_028093
normalized count
100
50
20
10
5
9_weeks
12_weeks
16_weeks
group

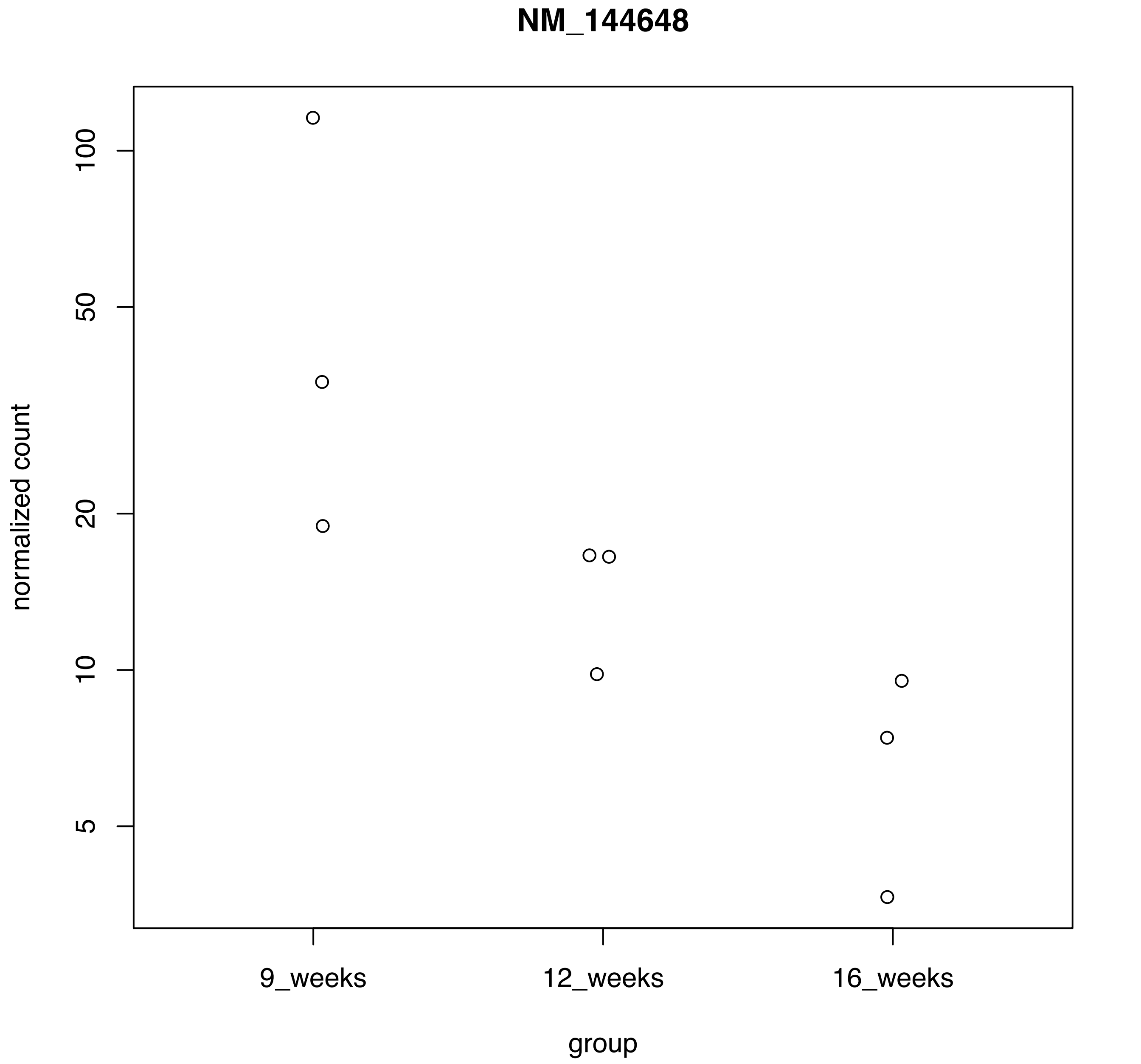
NM_144648
normalized count
100
50
20
10
5
9_weeks
12_weeks
16_weeks
group

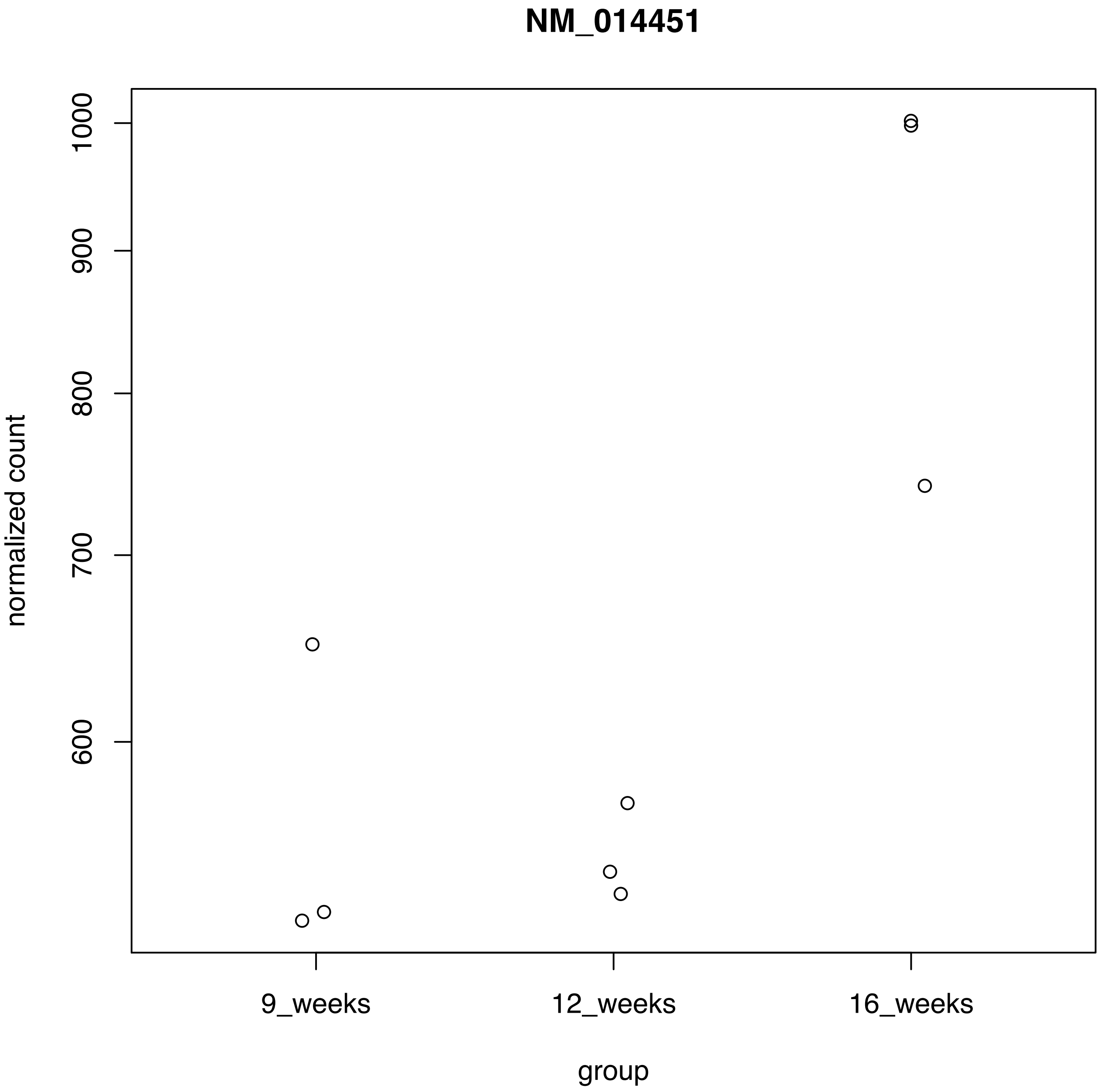

NM_014451
normalized count
1000
900
800
700
600
9_weeks
12_weeks
16_weeks
group

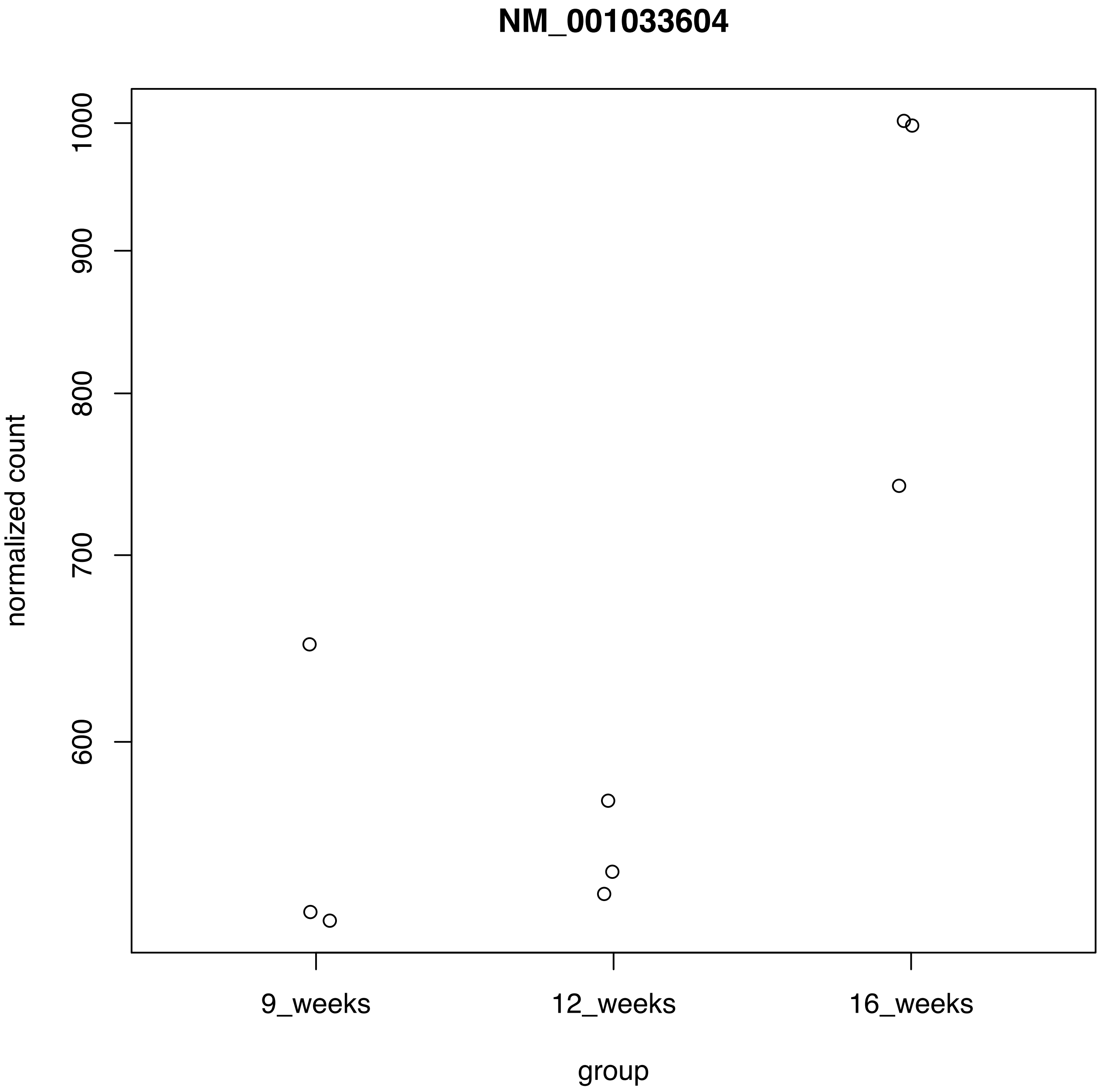

NM_001033604
normalized count
1000
900
800
700
600
9_weeks
12_weeks
16_weeks
group

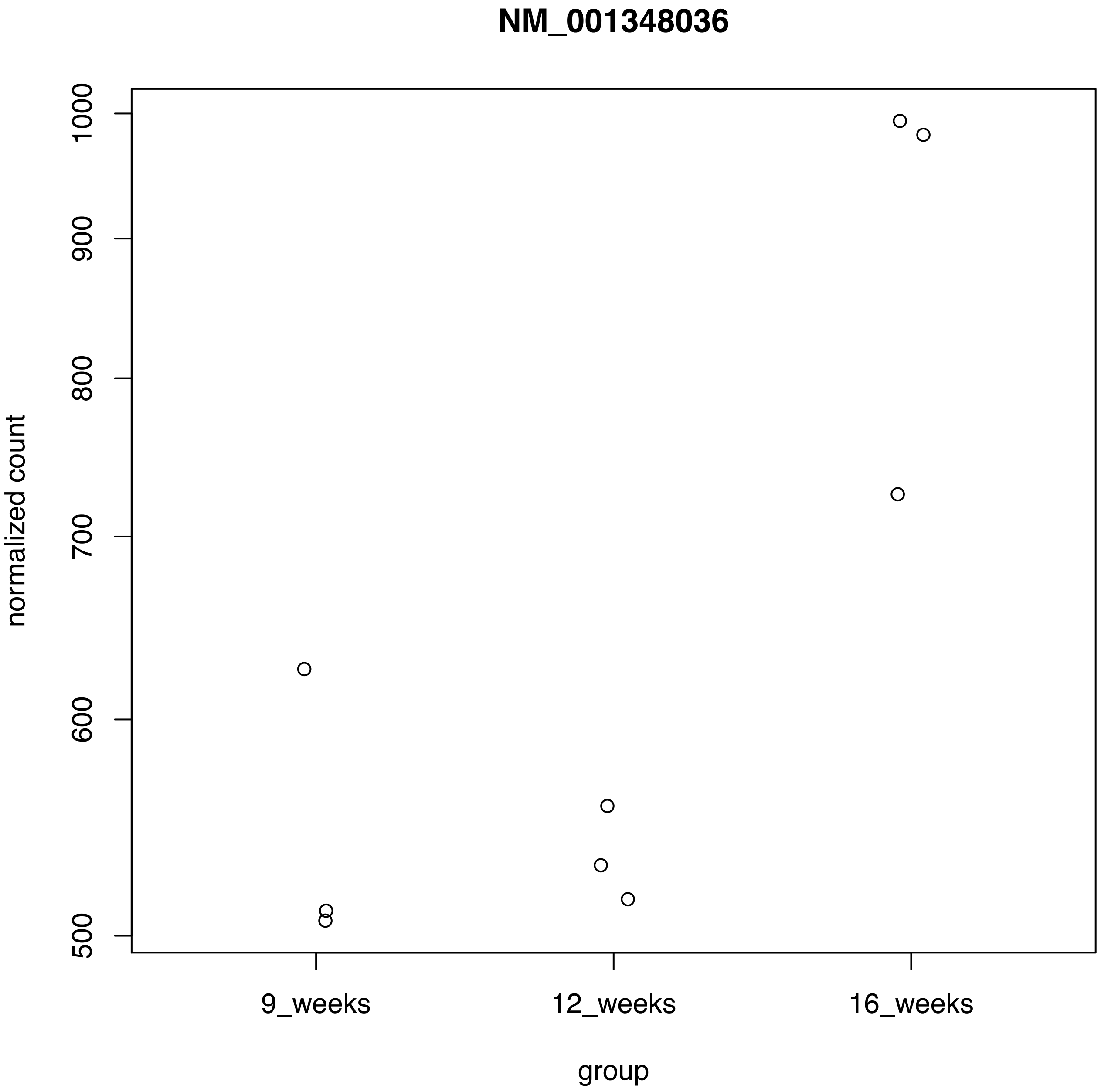
NM_001348036
normalized count
500
600
700
800
900
1000
9_weeks
12_weeks
16_weeks
group

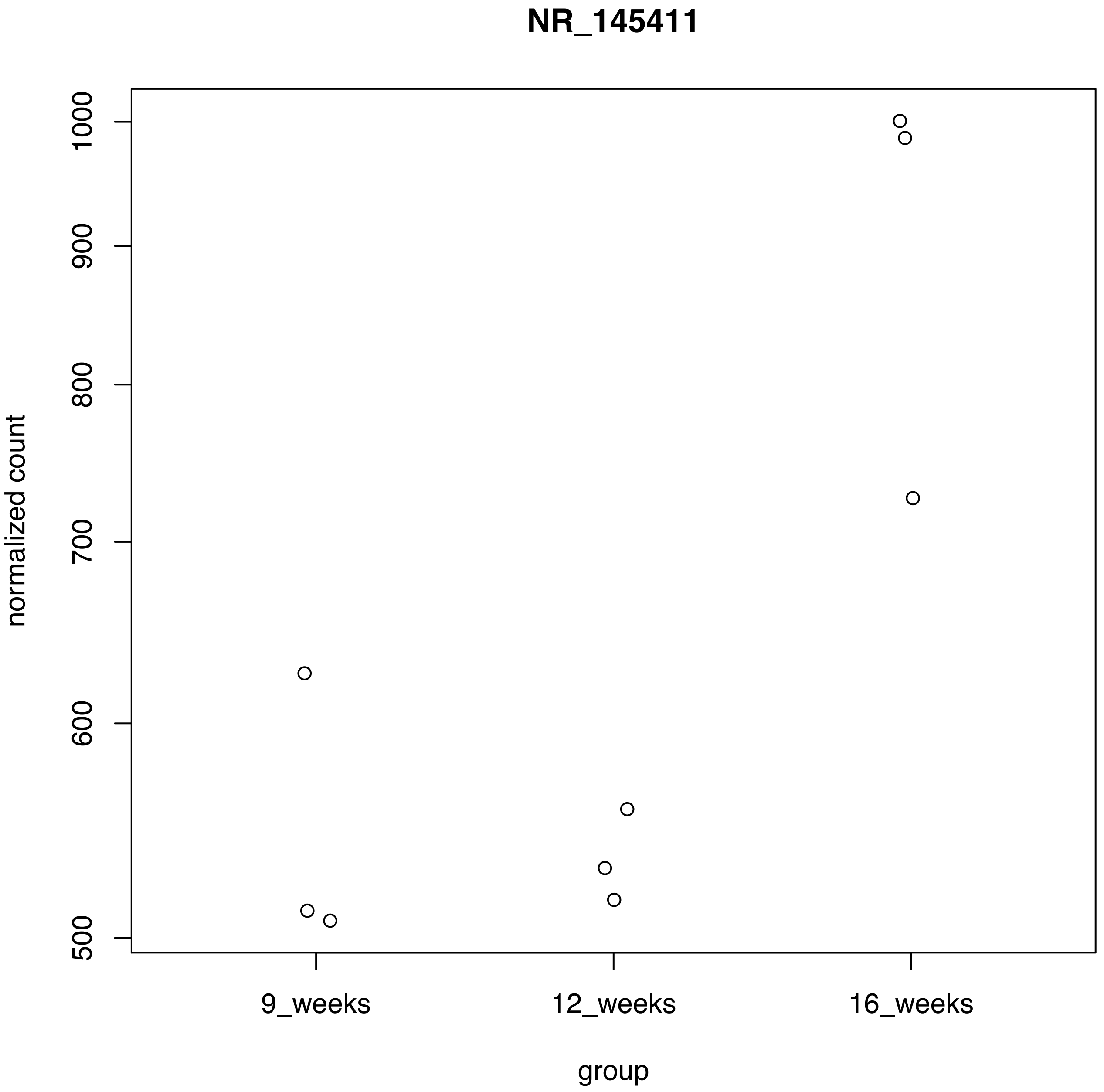
NR_145411
normalized count
1000
900
800
700
600
500
9_weeks
12_weeks
16_weeks
group

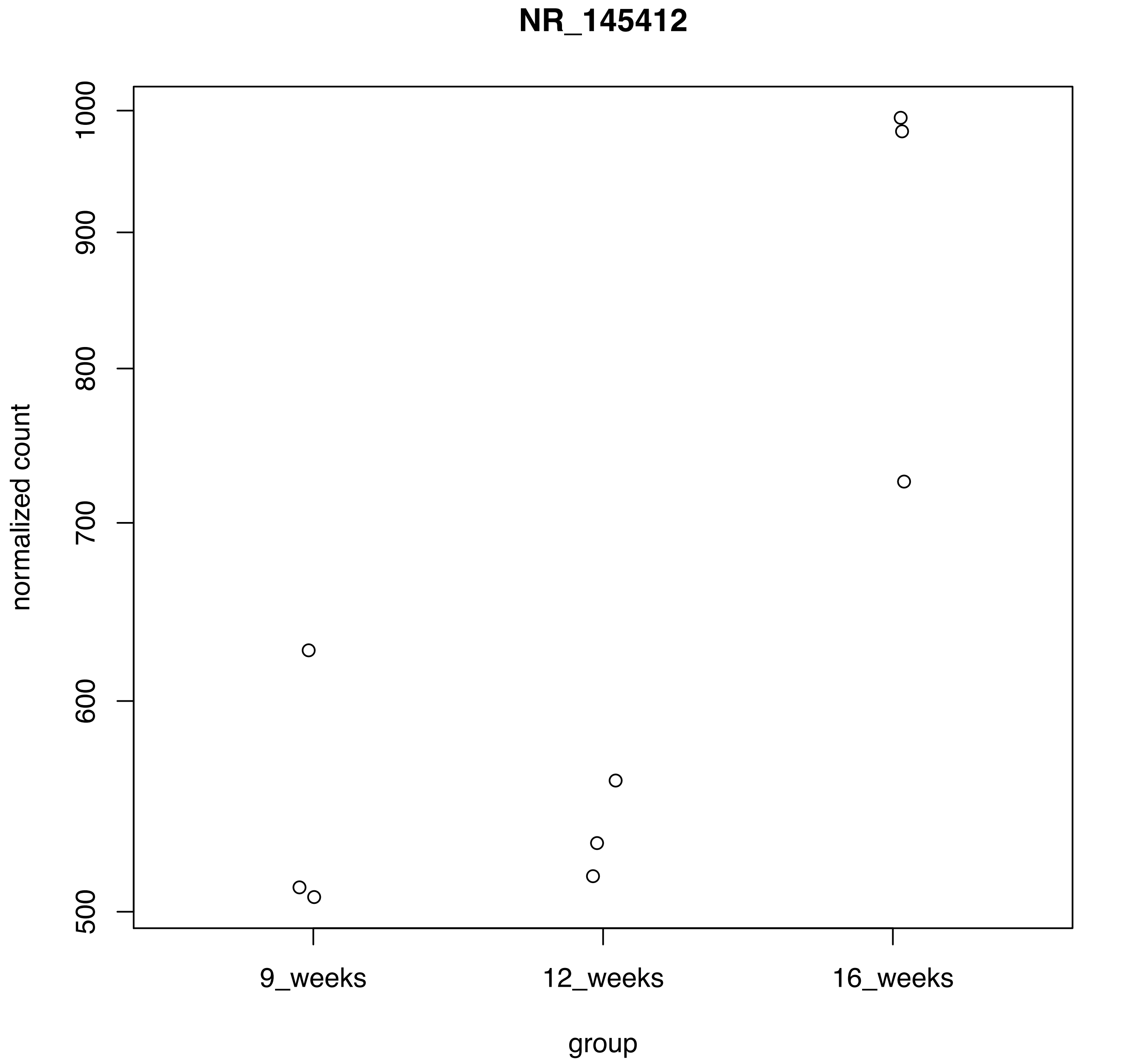
NR_145412
normalized count
1000
900
800
700
600
500
9_weeks
12_weeks
16_weeks
group

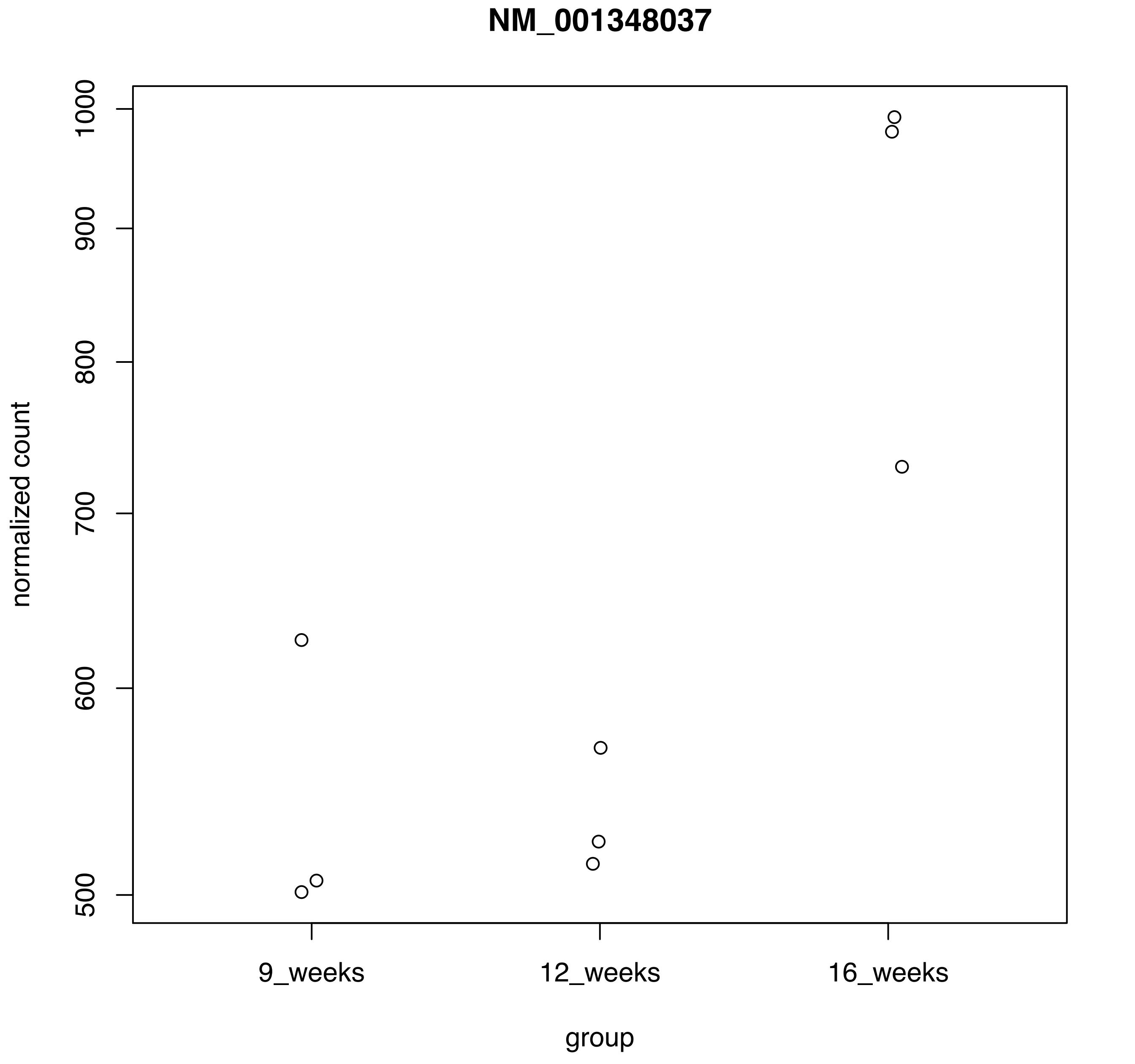
NM_001348037
normalized count
1000
900
800
700
600
500
9_weeks
12_weeks
16_weeks
group

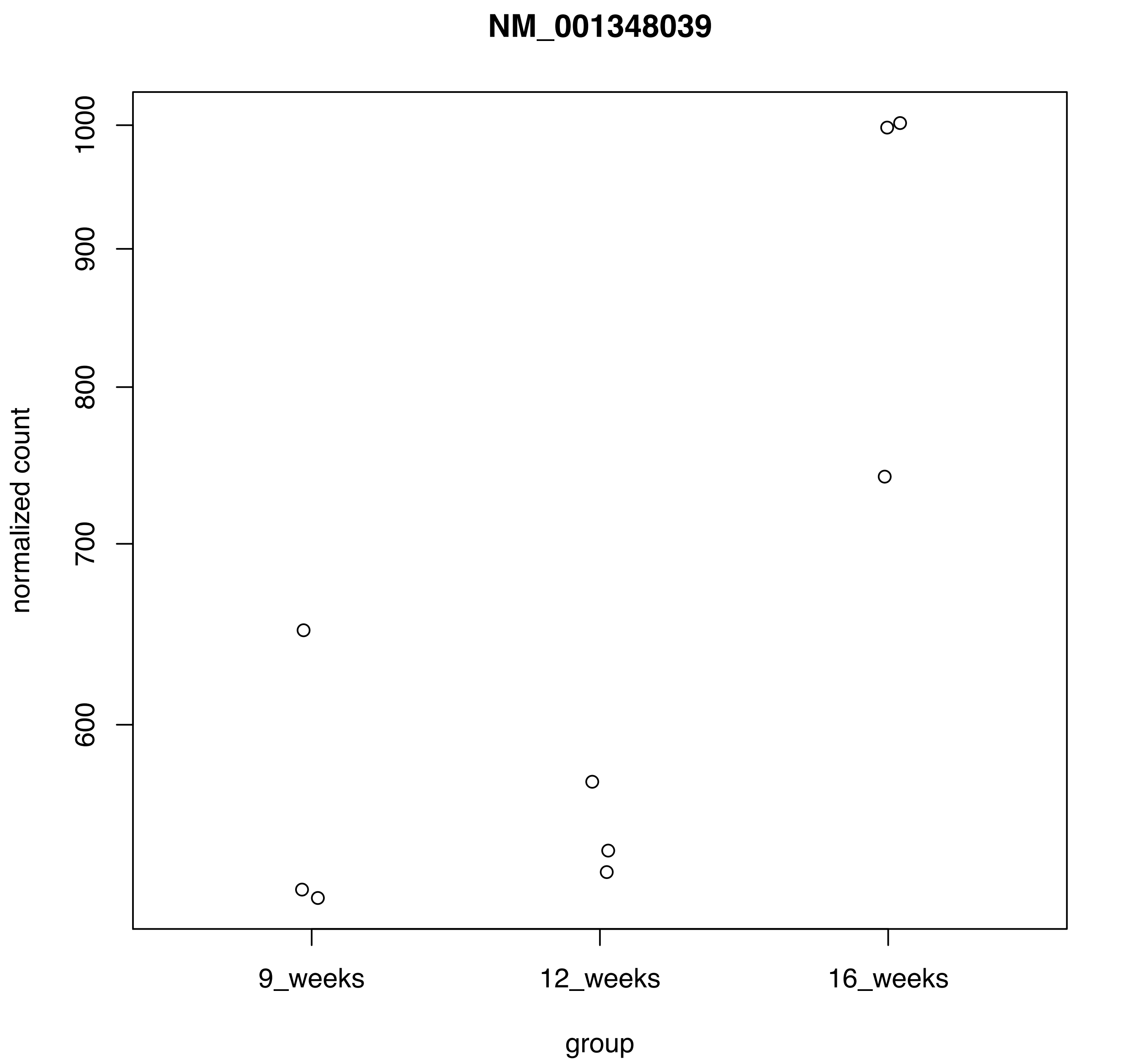
NM_001348039
normalized count
600
700
800
900
1000
9_weeks
12_weeks
16_weeks
group

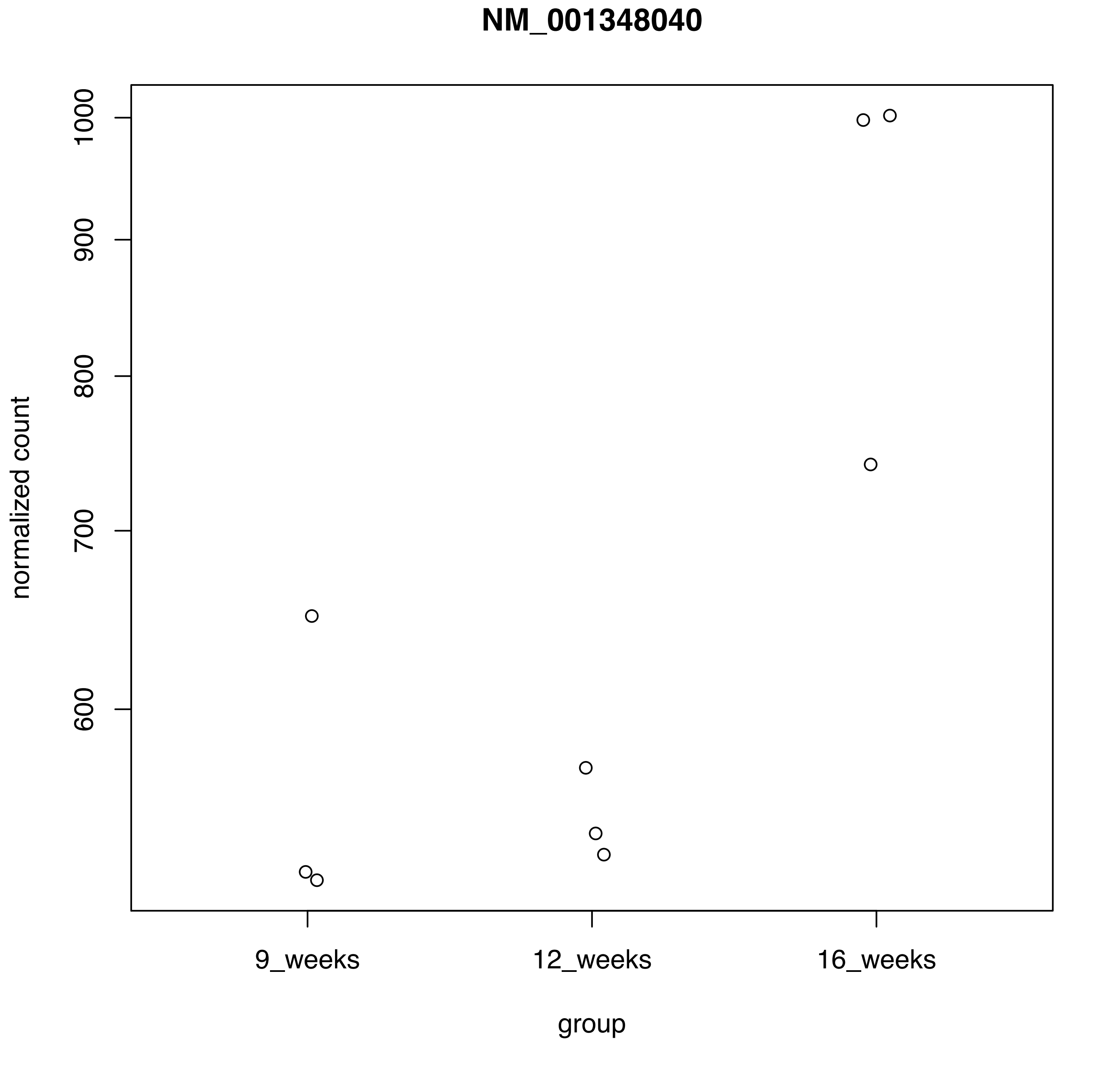
NM_001348040
normalized count
1000
900
800
700
600
9_weeks
12_weeks
16_weeks
group

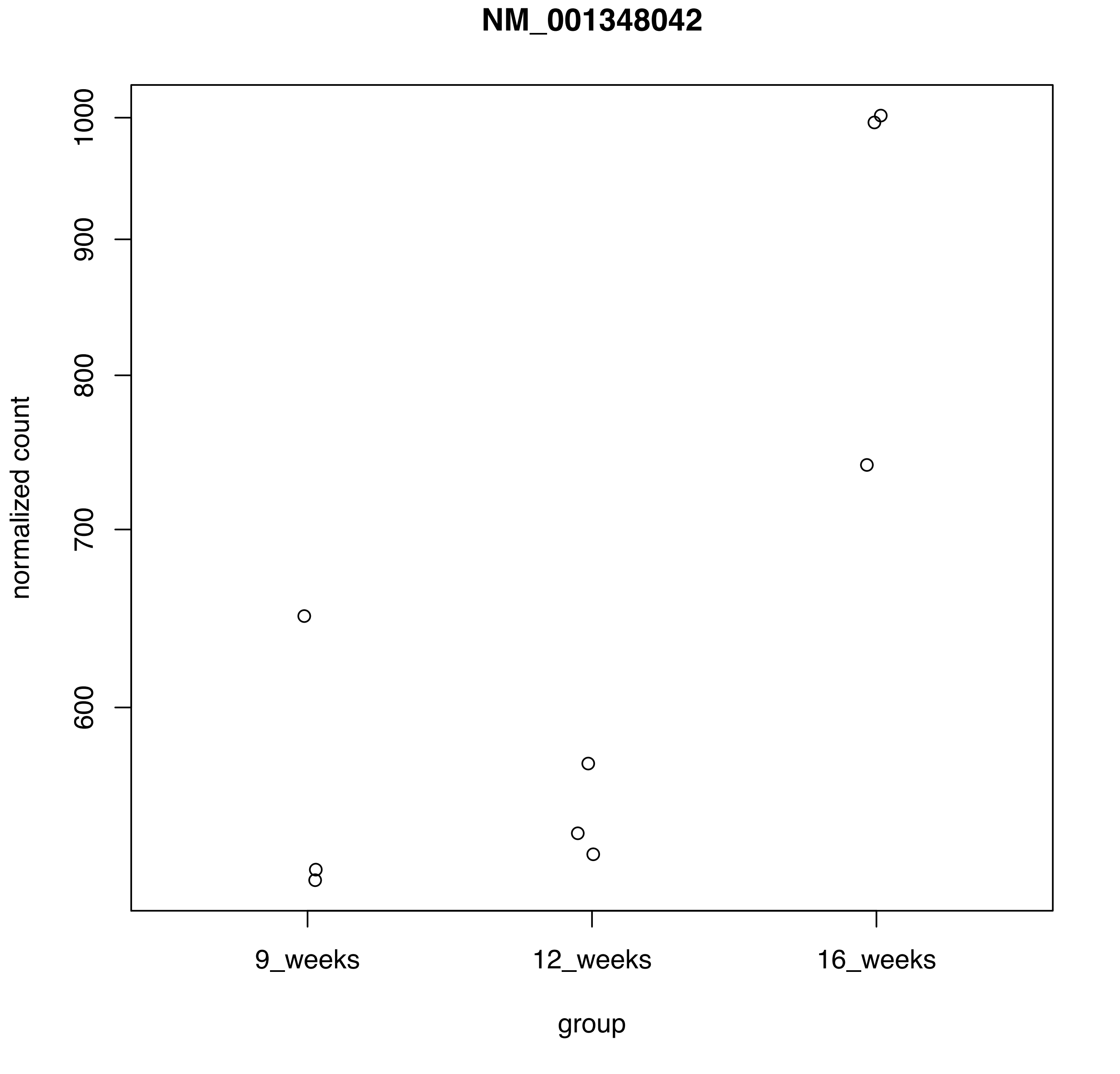
NM_001348042
normalized count
1000
900
800
700
600
9_weeks
12_weeks
16_weeks
group

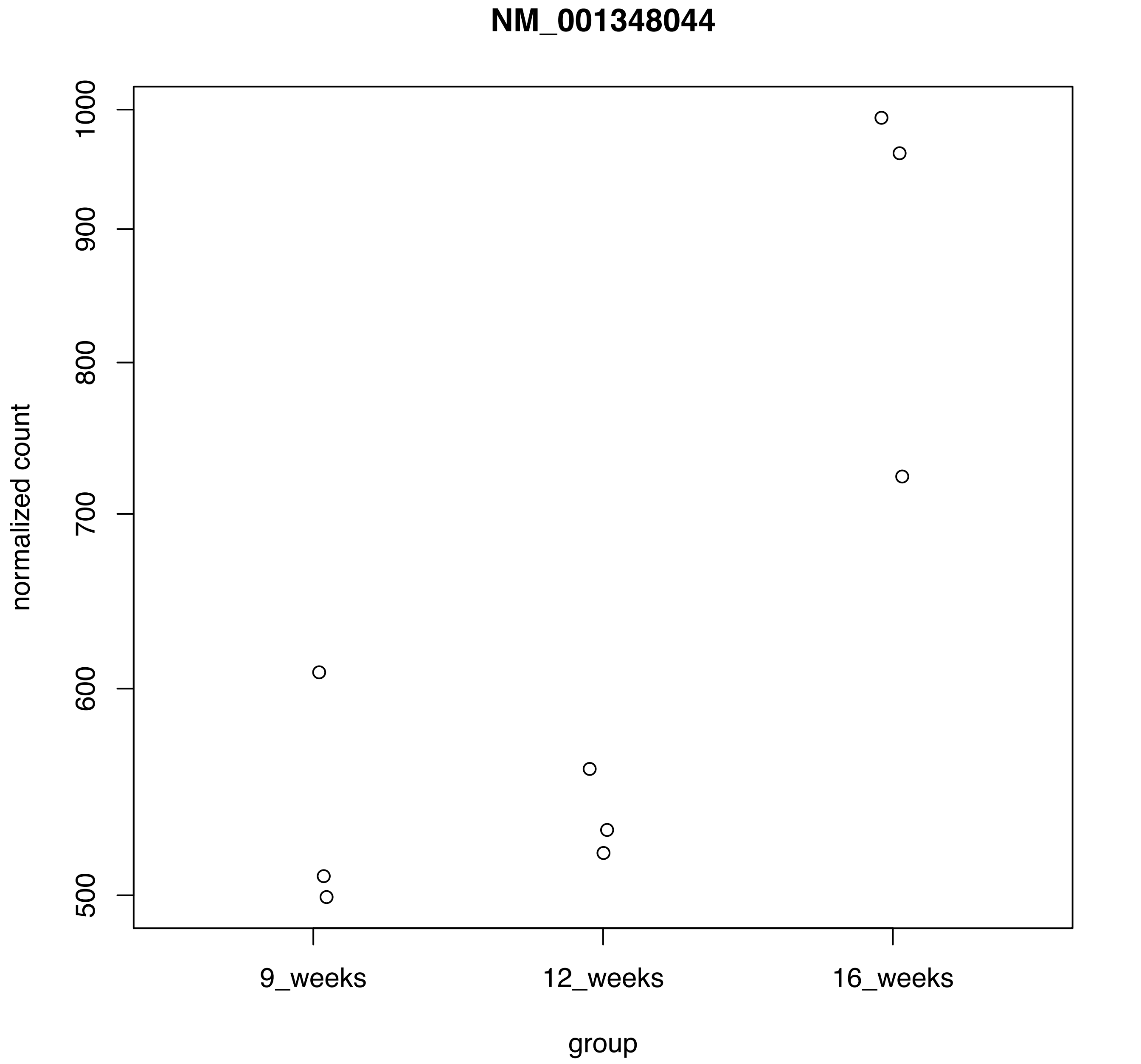
NM_001348044
normalized count
1000
900
800
700
600
500
9_weeks
12_weeks
16_weeks
group

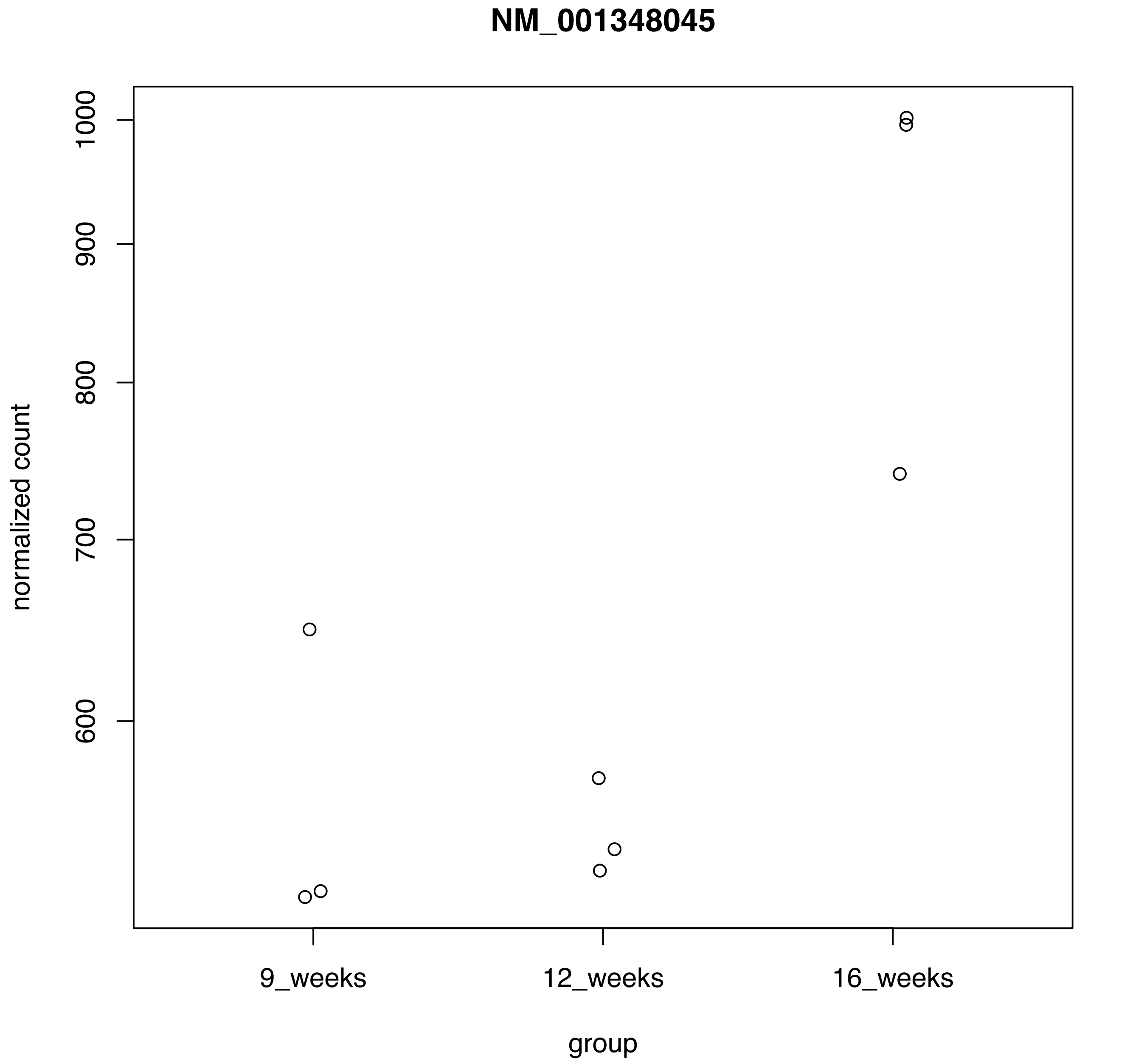
NM_001348045
normalized count
1000
900
800
700
600
9_weeks
12_weeks
16_weeks
group

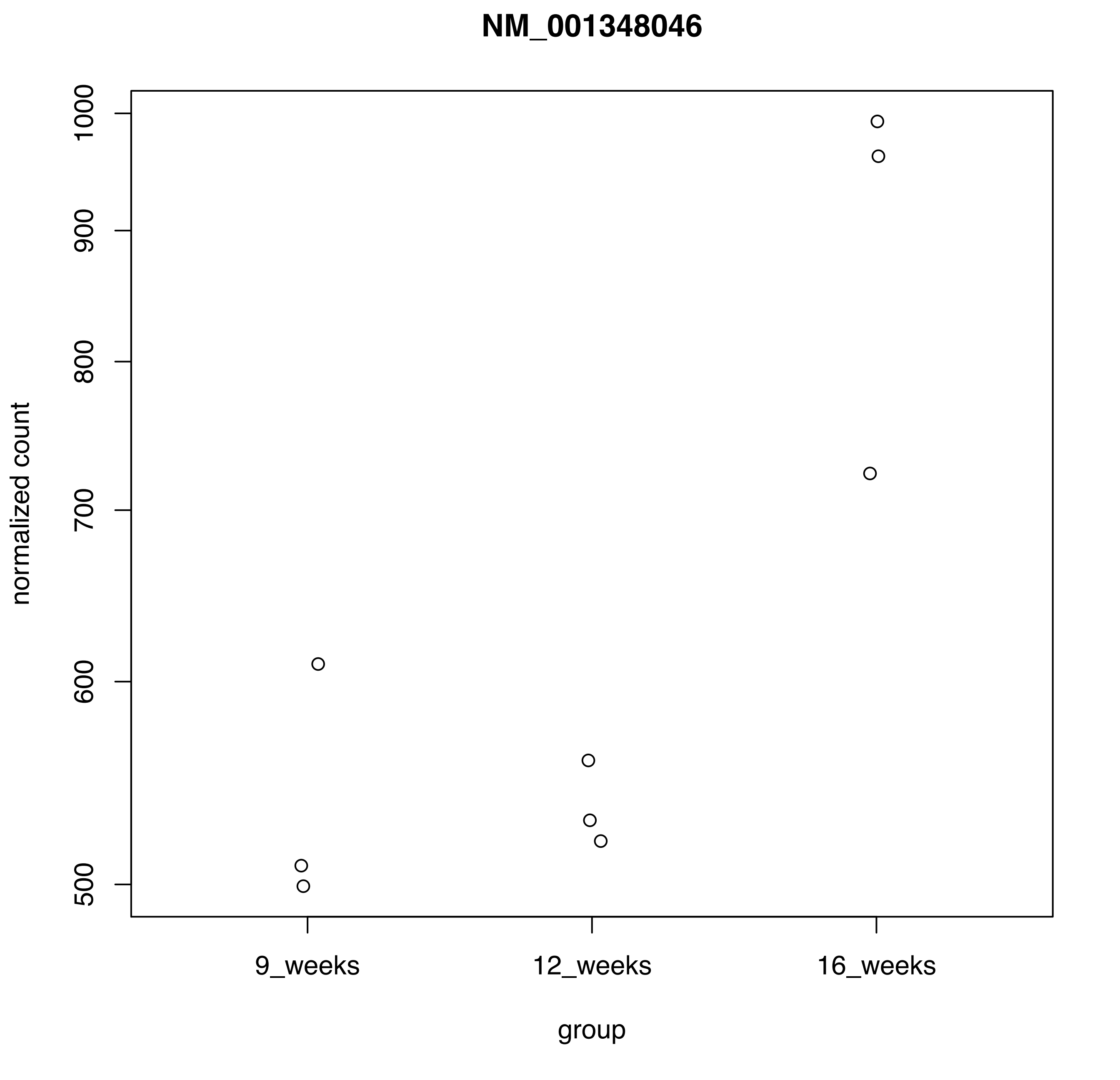

NM_001348046
normalized count
1000
900
800
700
600
500
9_weeks
12_weeks
16_weeks
group

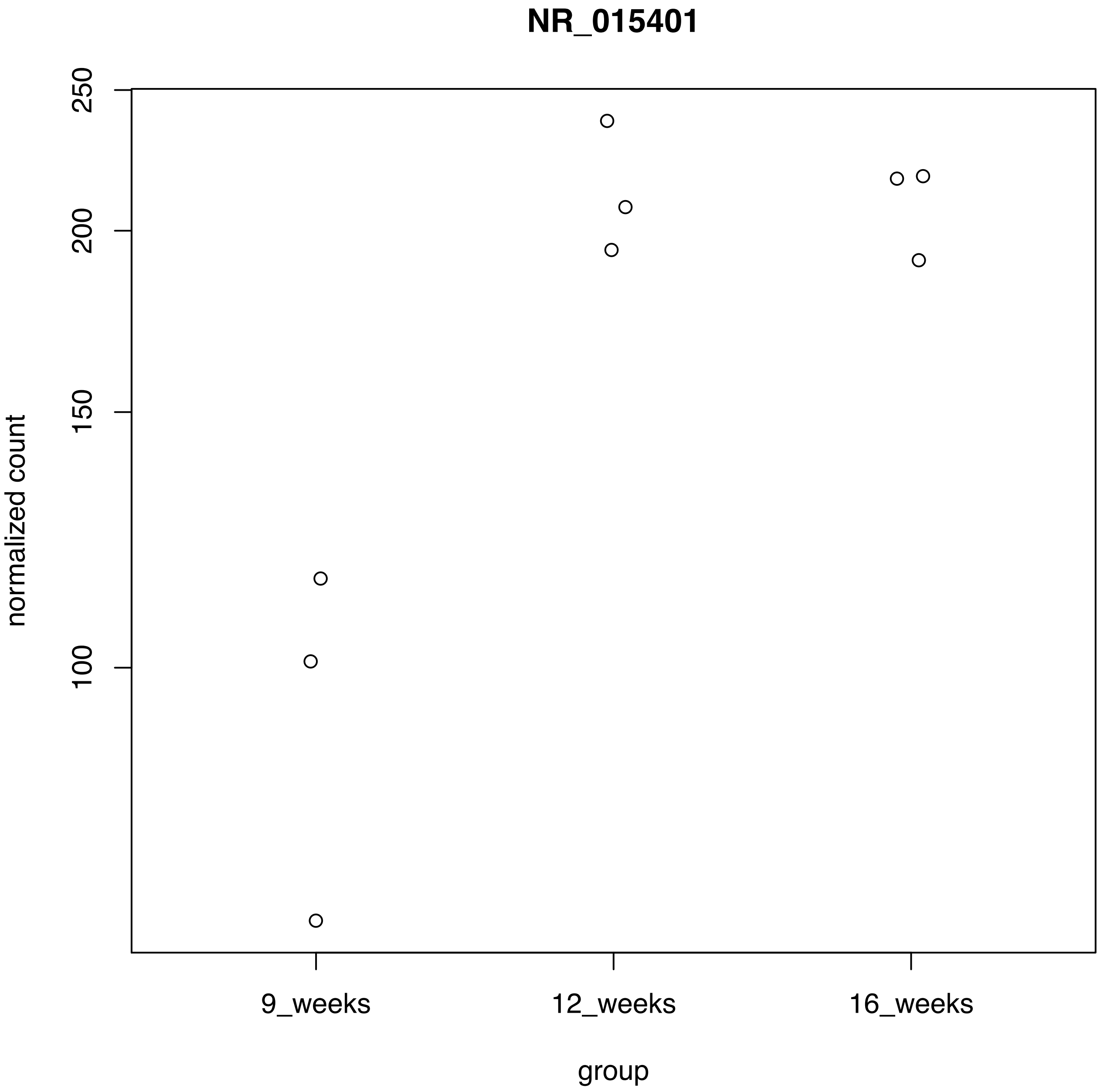
NR_015401
normalized count
250
200
150
100
9_weeks
12_weeks
16_weeks
group

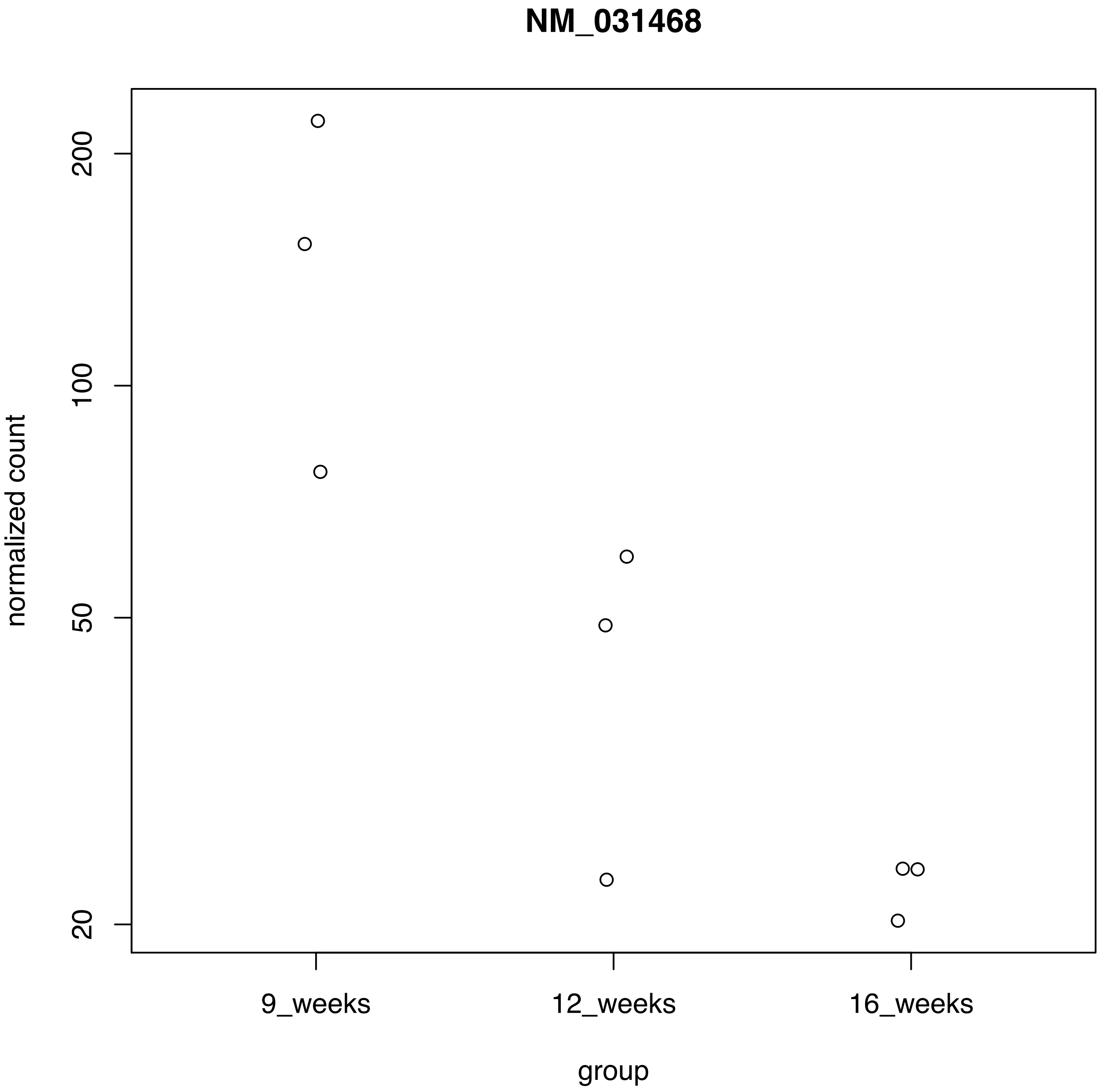
NM_031468
normalized count
200
100
50
20
9_weeks
12_weeks
16_weeks
group

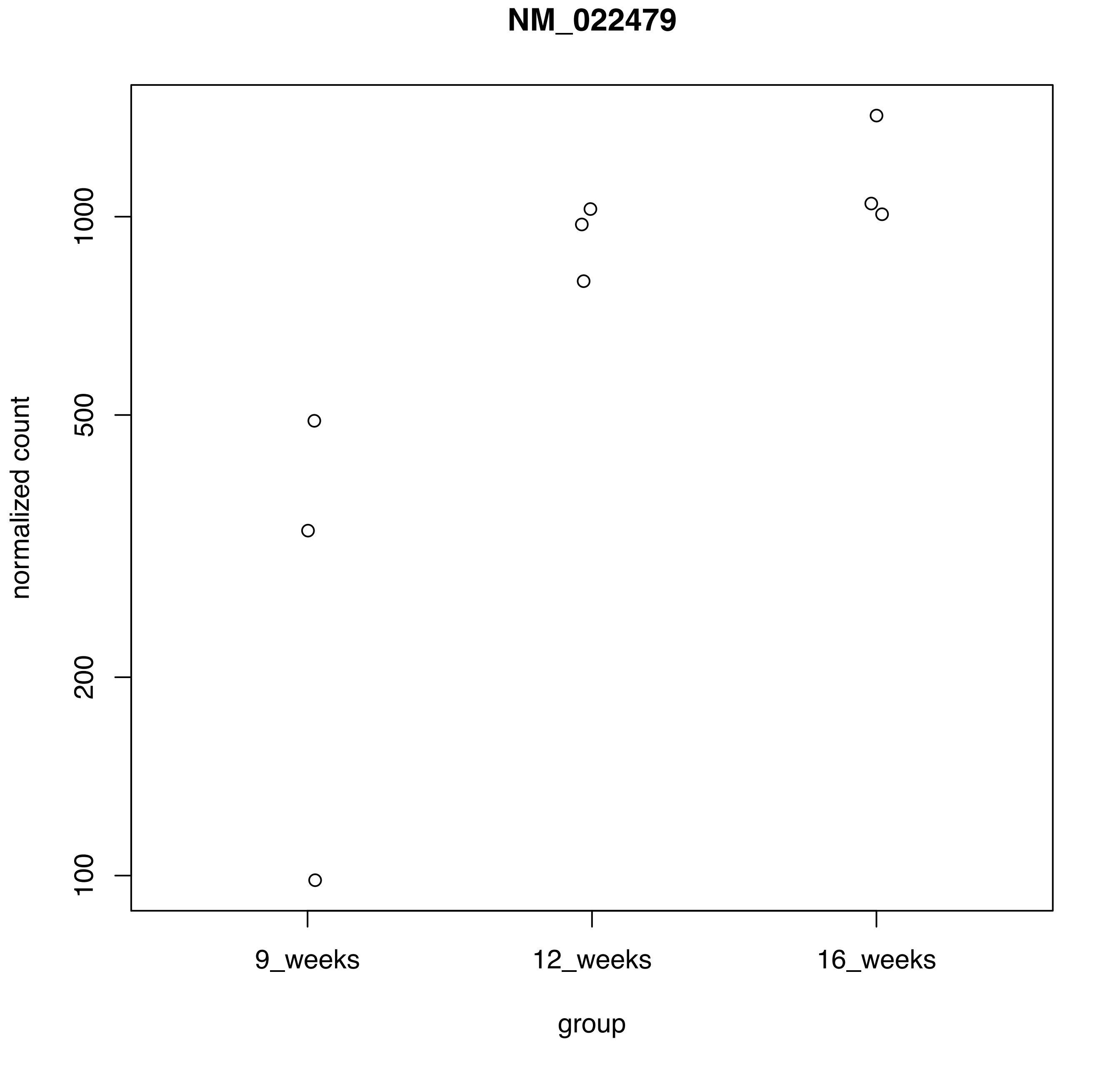
NM_022479
normalized count
1000
500
200
100
9_weeks
12_weeks
16_weeks
group

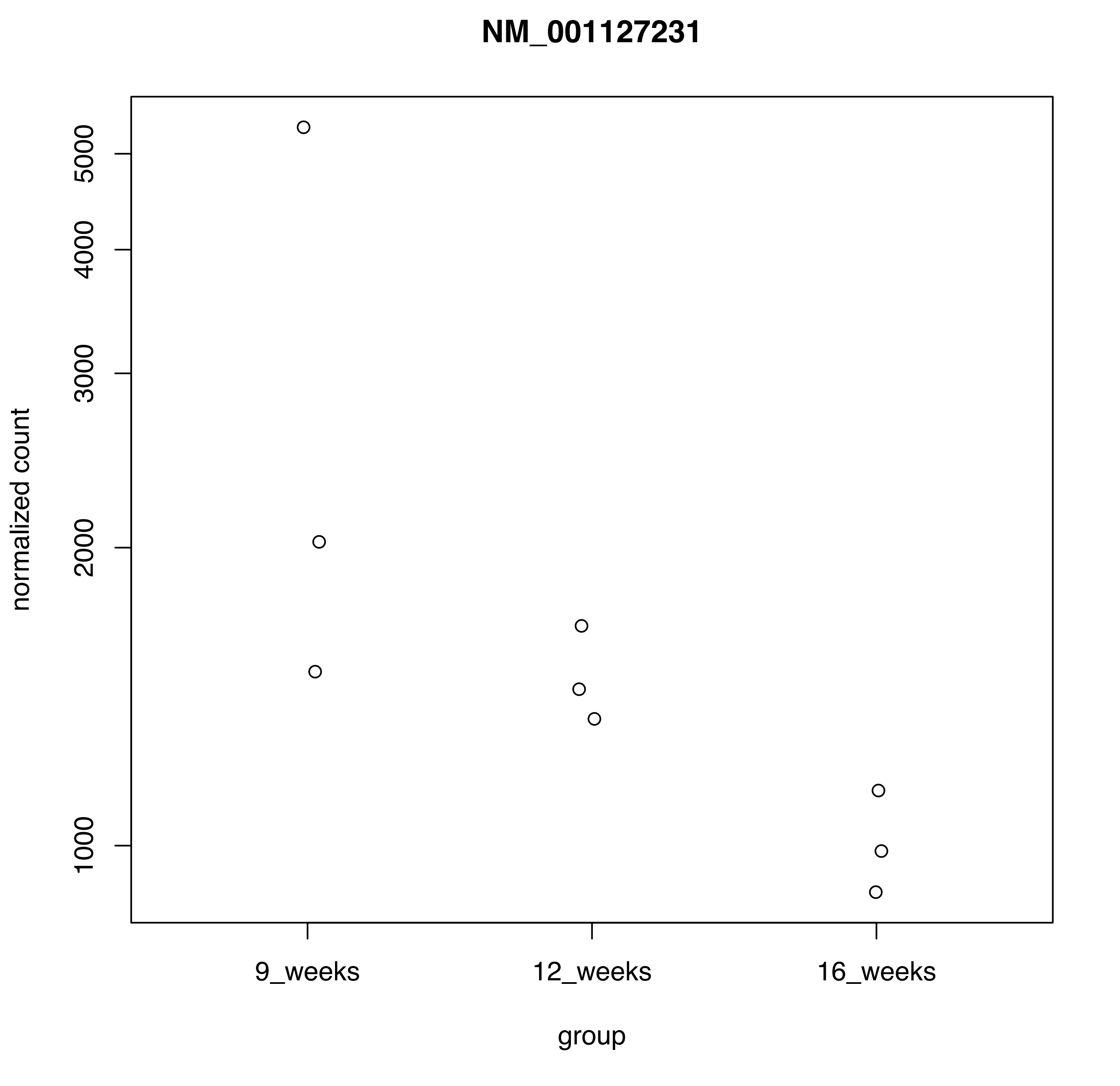
NM_001127231
normalized count
5000
4000
3000
2000
1000
9_weeks
12_weeks
16_weeks
group

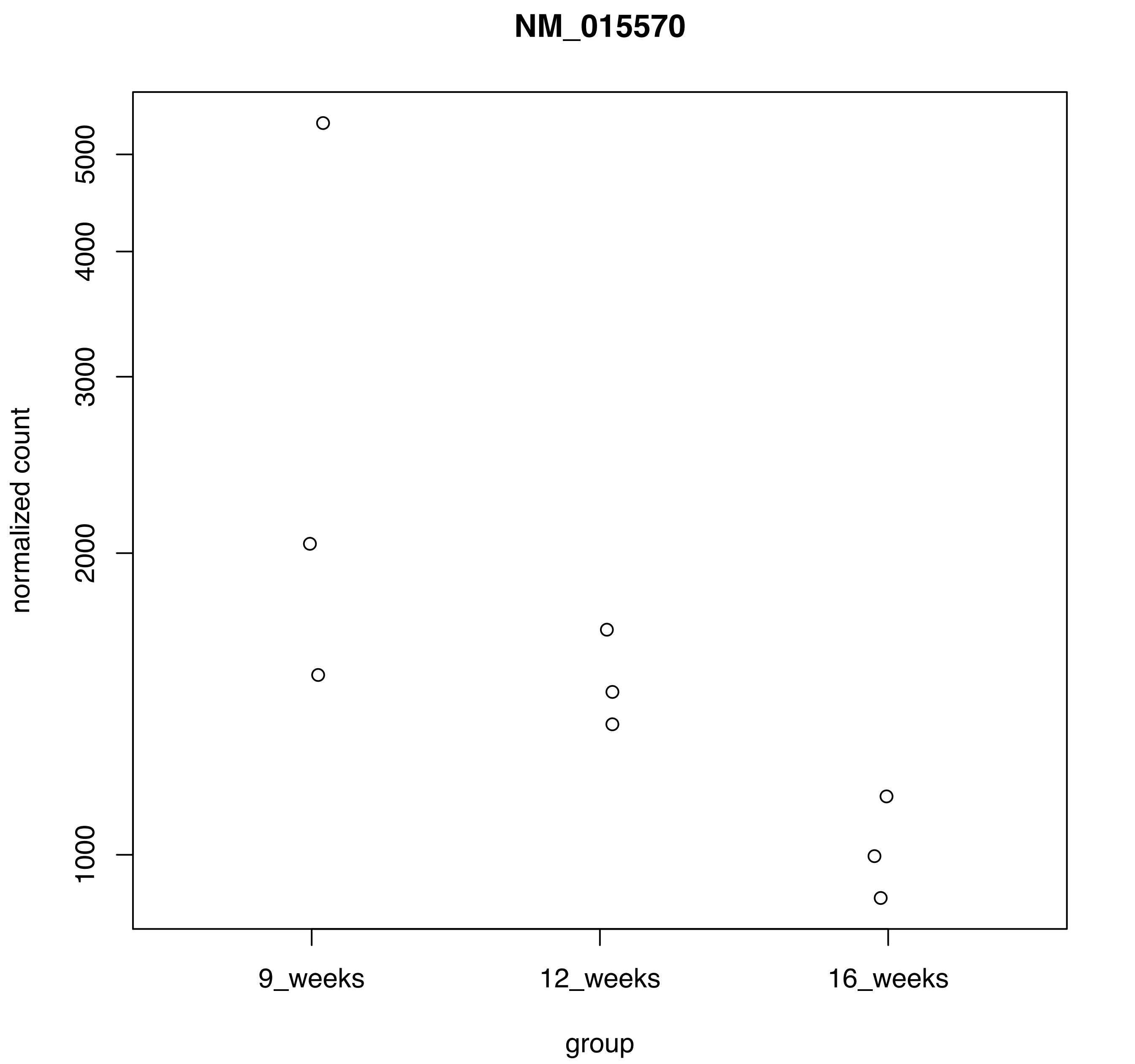
NM_015570
normalized count
5000
4000
3000
2000
1000
9_weeks
12_weeks
16_weeks
group

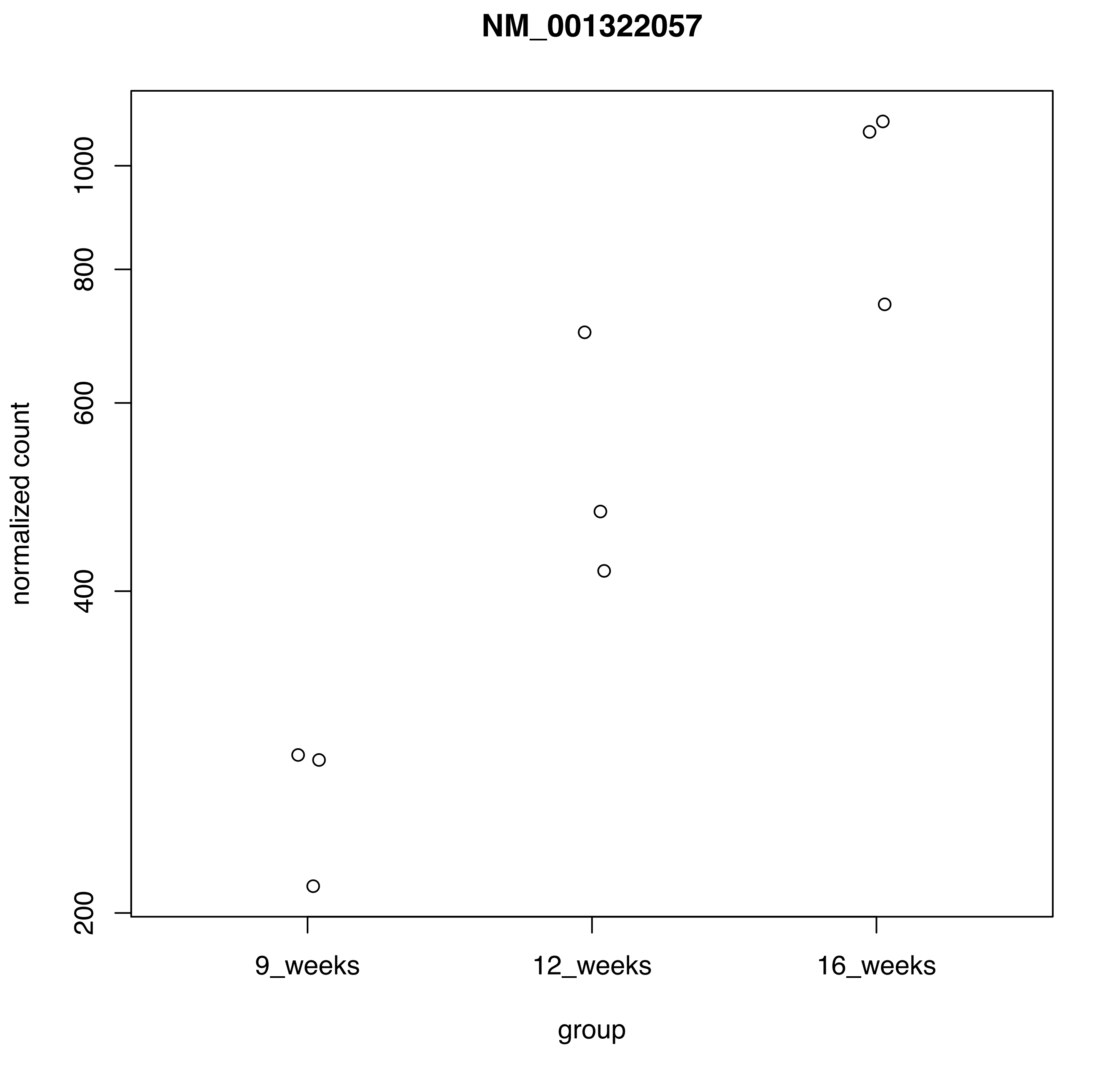

NM_001322057
normalized count
1000
800
600
400
200
9_weeks
12_weeks
16_weeks
group

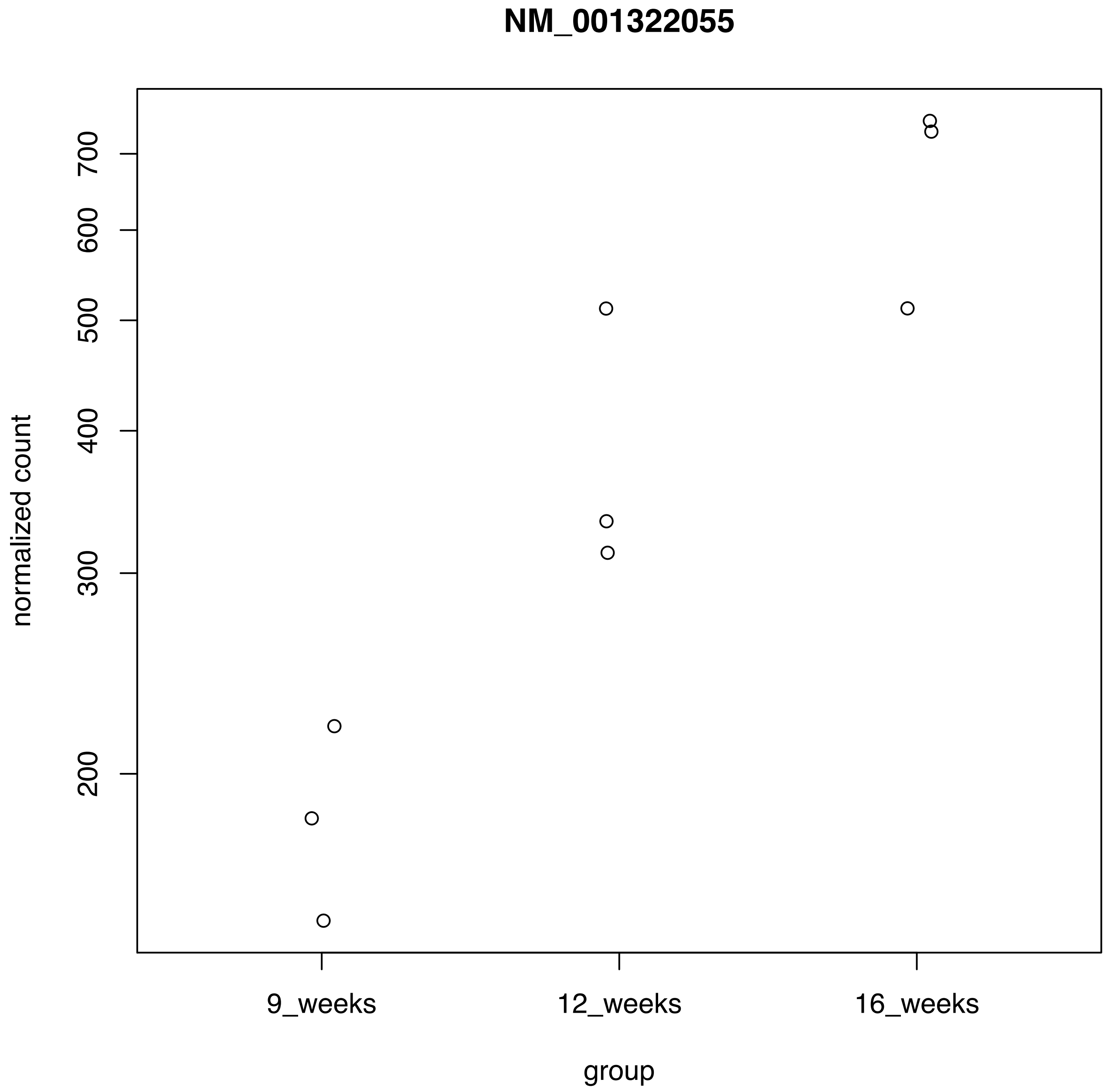
NM_001322055
normalized count
200
300
400
500
600
700
9_weeks
12_weeks
16_weeks
group

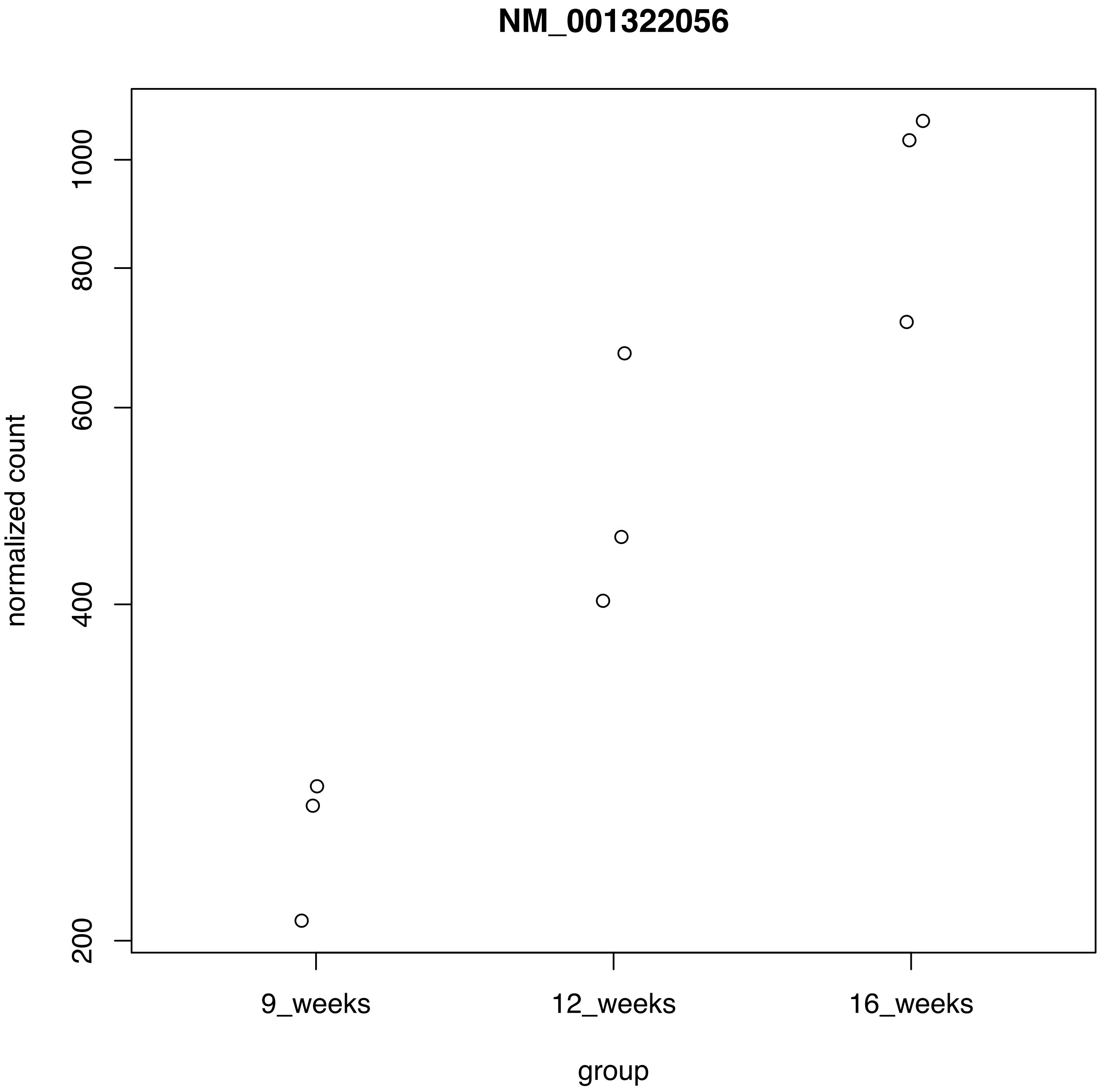
NM_001322056
normalized count
200
400
600
800
1000
9_weeks
12_weeks
16_weeks
group

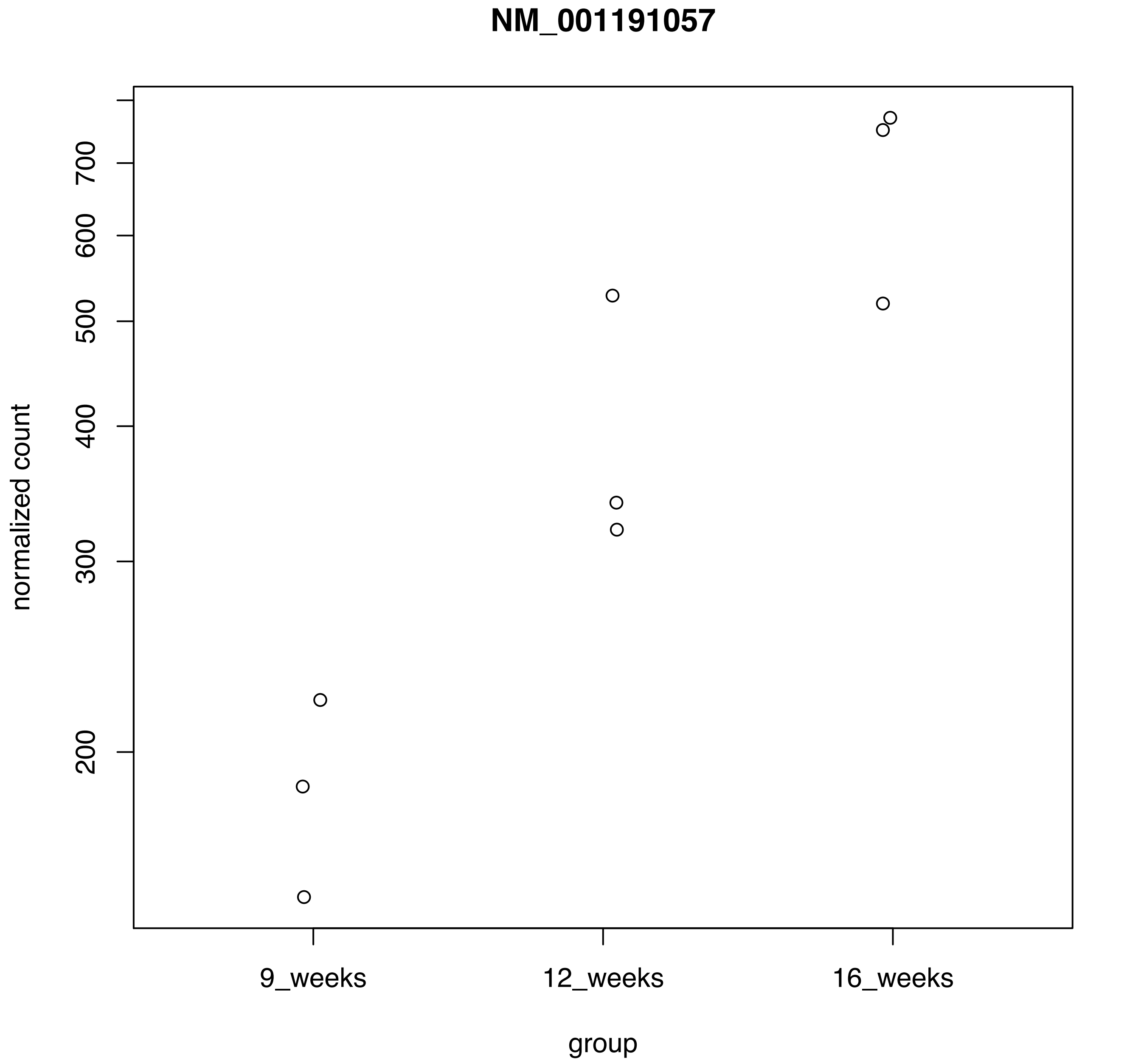
NM_001191057
normalized count
700
600
500
400
300
200
9_weeks
12_weeks
16_weeks
group

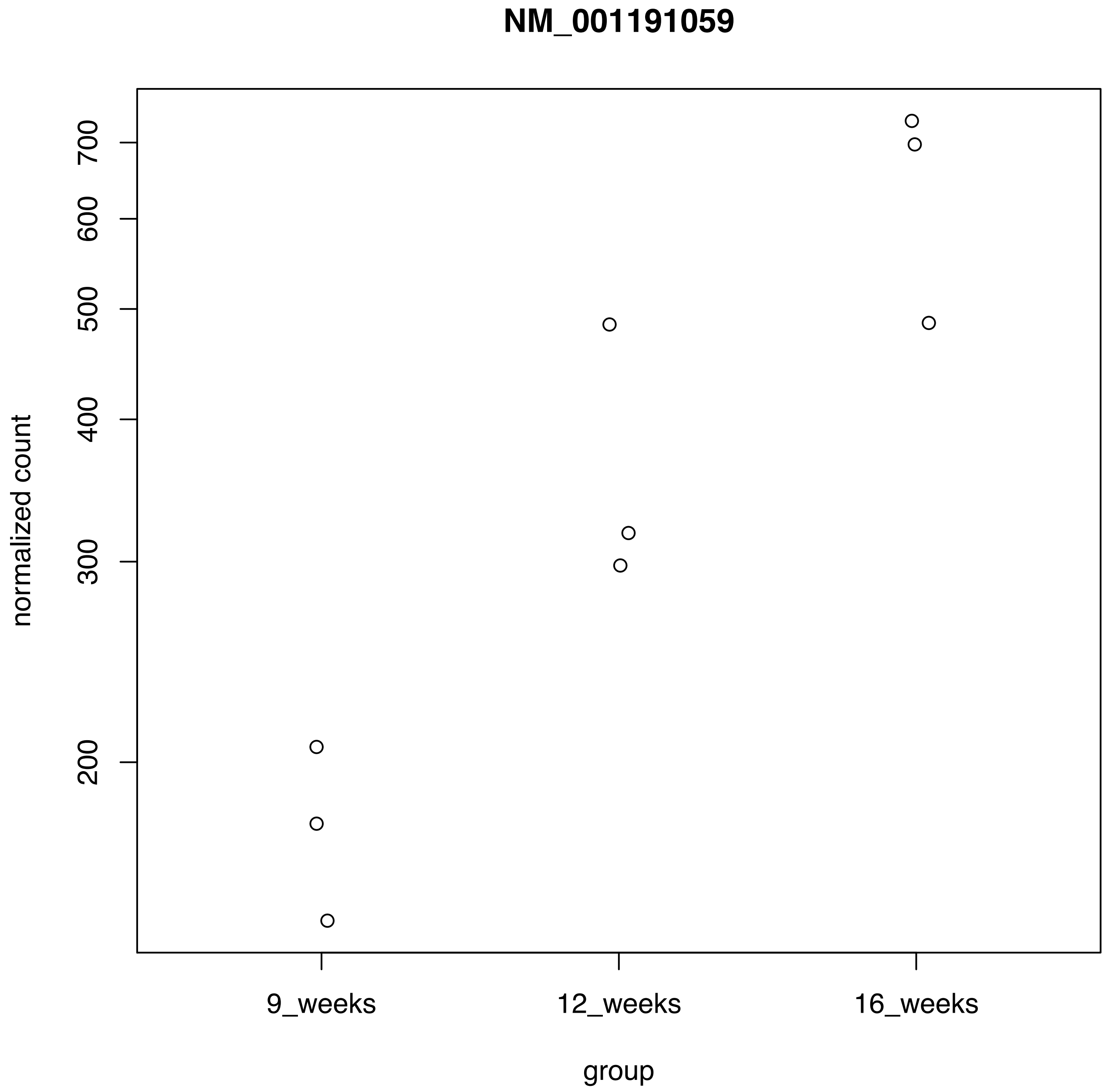
NM_001191059
normalized count
700
600
500
400
300
200
9_weeks
12_weeks
16_weeks
group

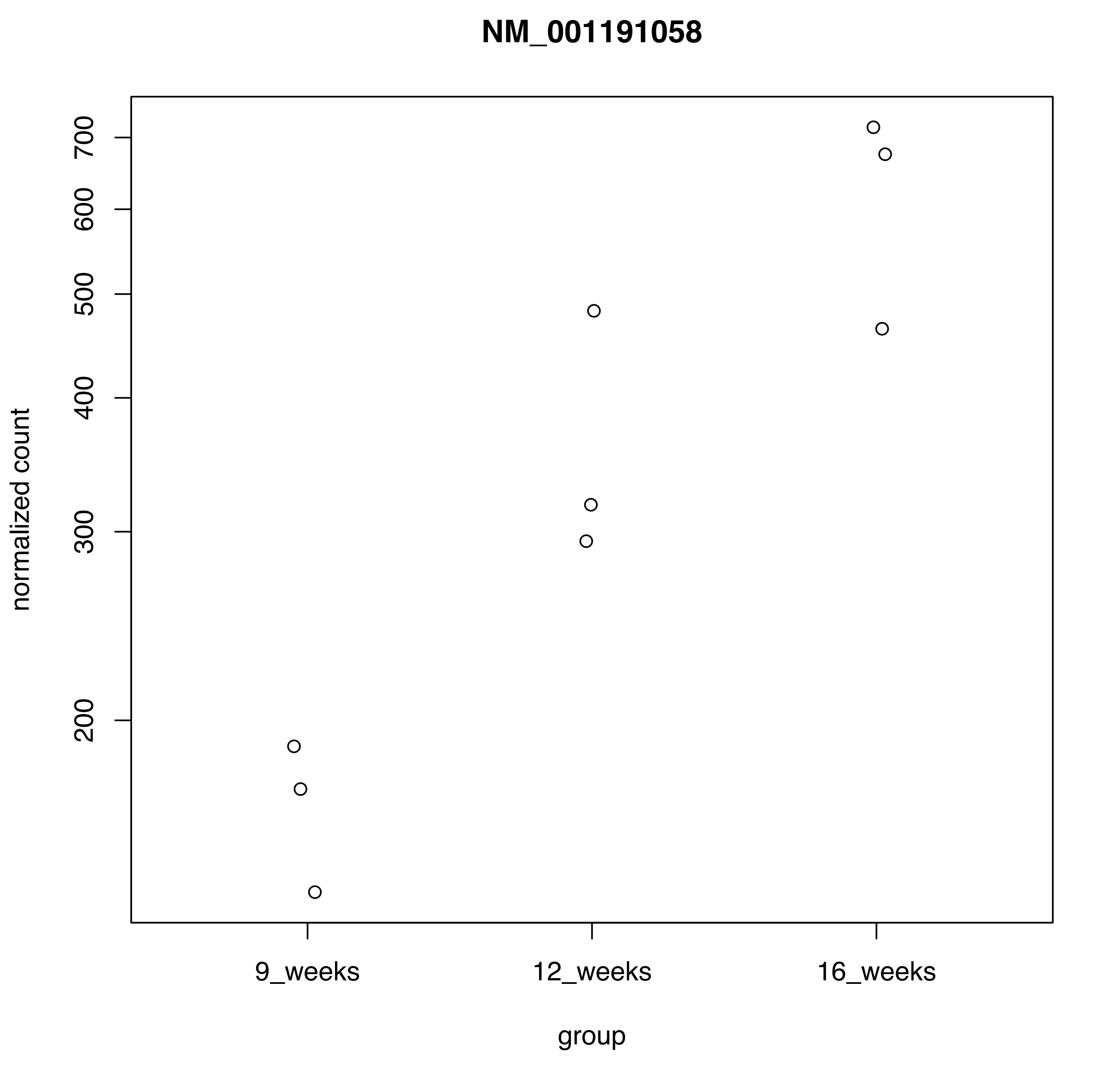
NM_001191058
normalized count
700
600
500
400
300
200
9_weeks
12_weeks
16_weeks
group

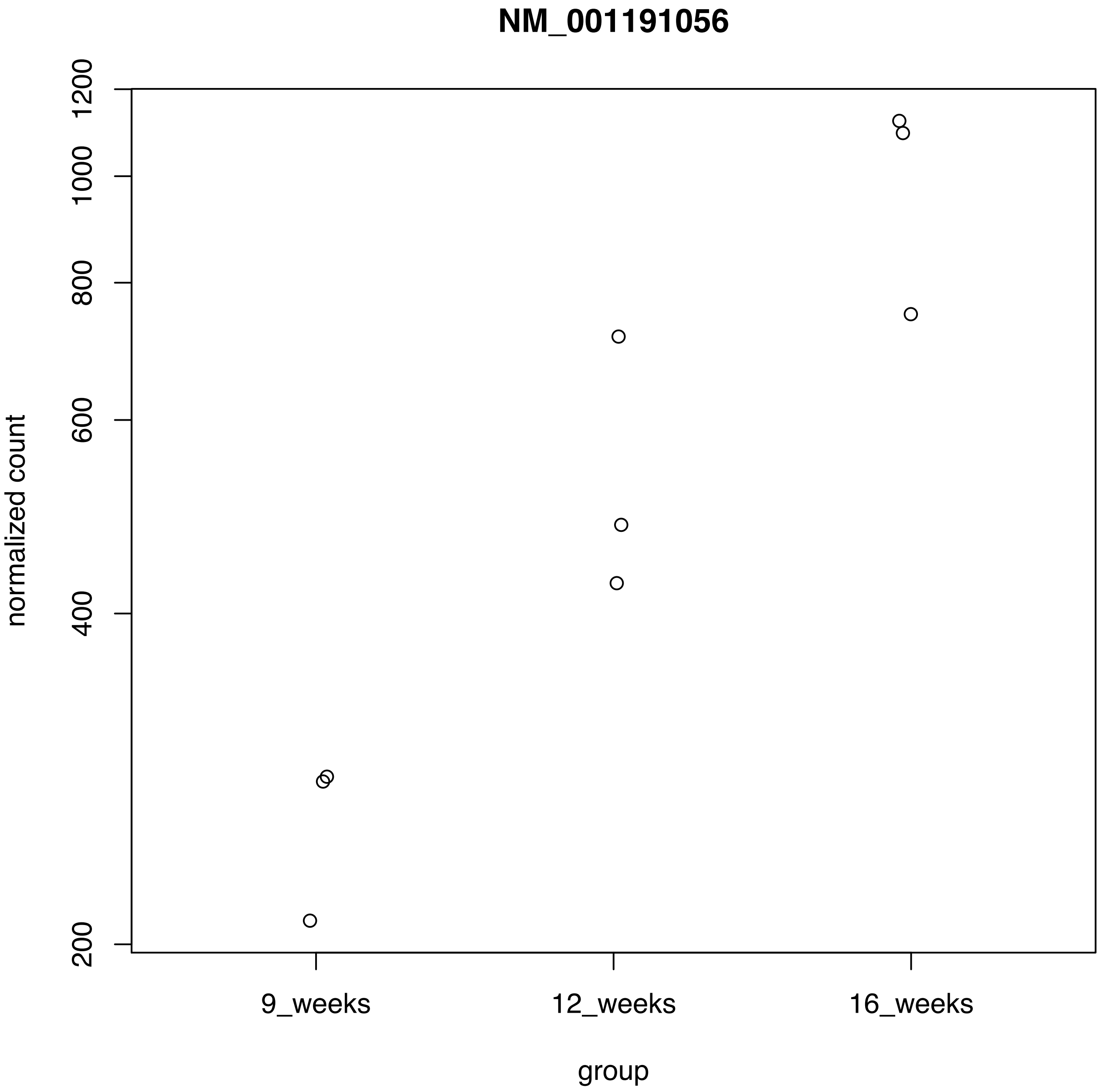
NM_001191056
normalized count
200
400
600
800
1000
1200
9_weeks
12_weeks
16_weeks
group

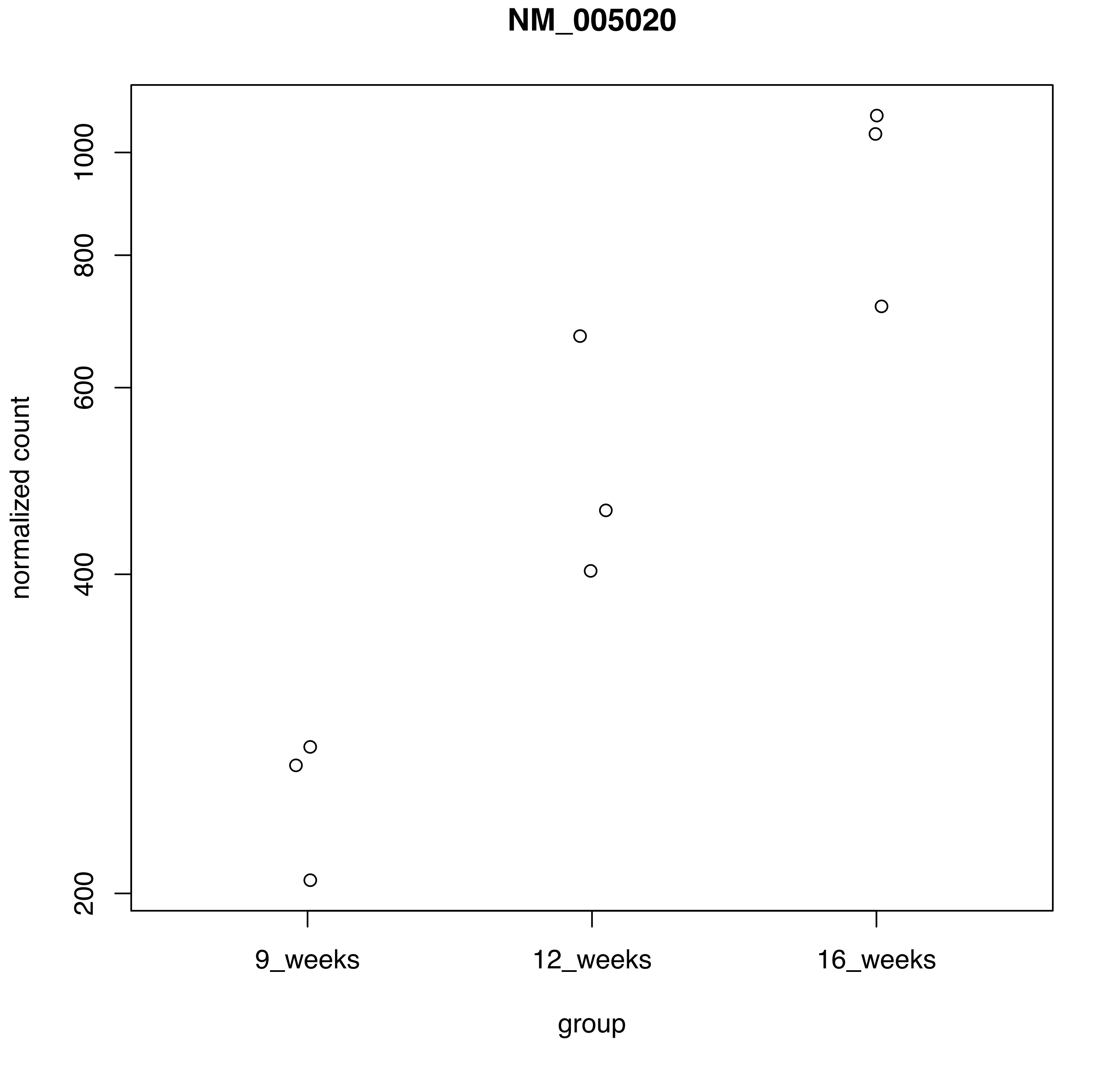
NM_005020
normalized count
200
400
600
800
1000
9_weeks
12_weeks
16_weeks
group

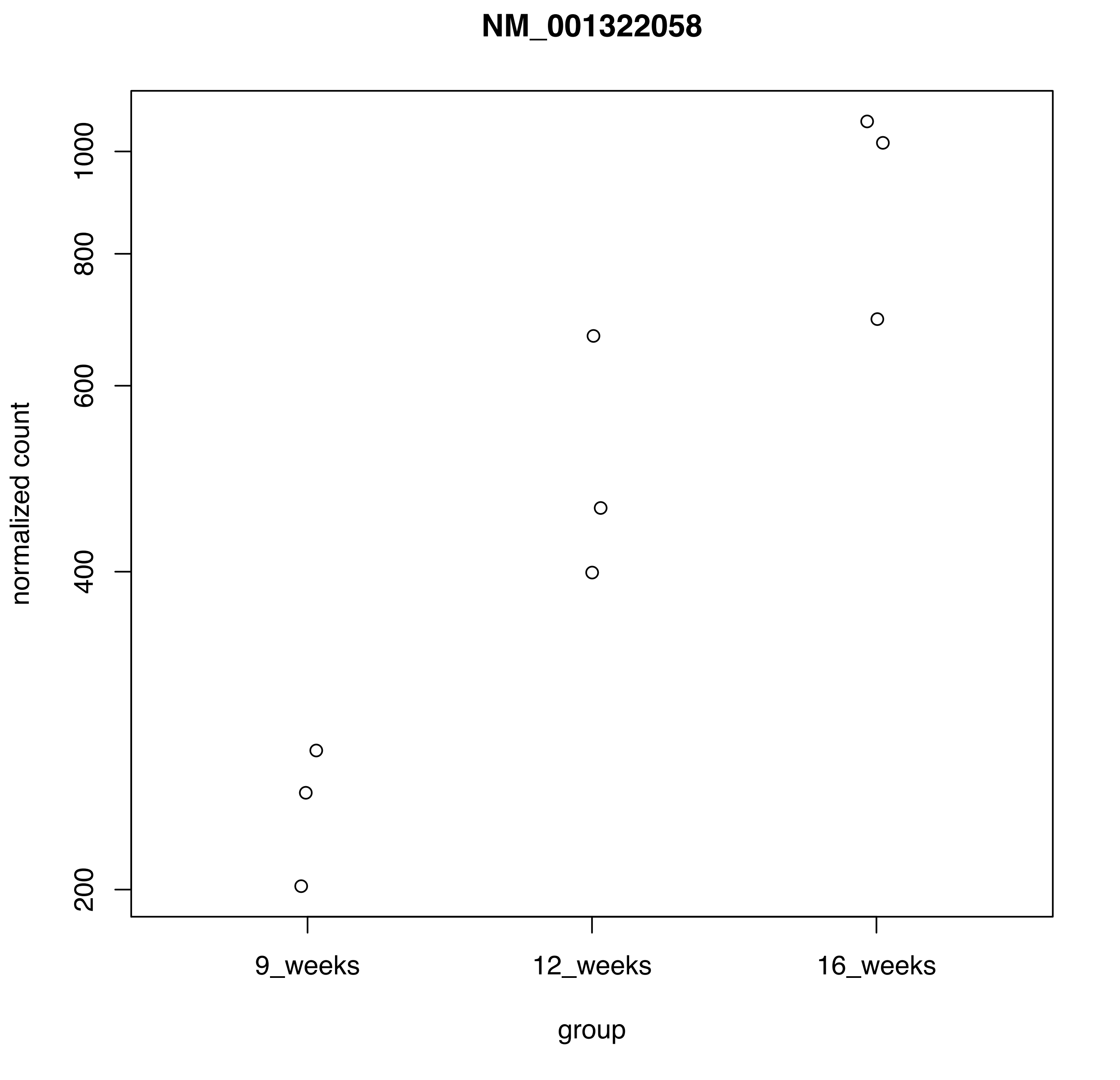
NM_001322058
normalized count
1000
800
600
400
200
9_weeks
12_weeks
16_weeks
group

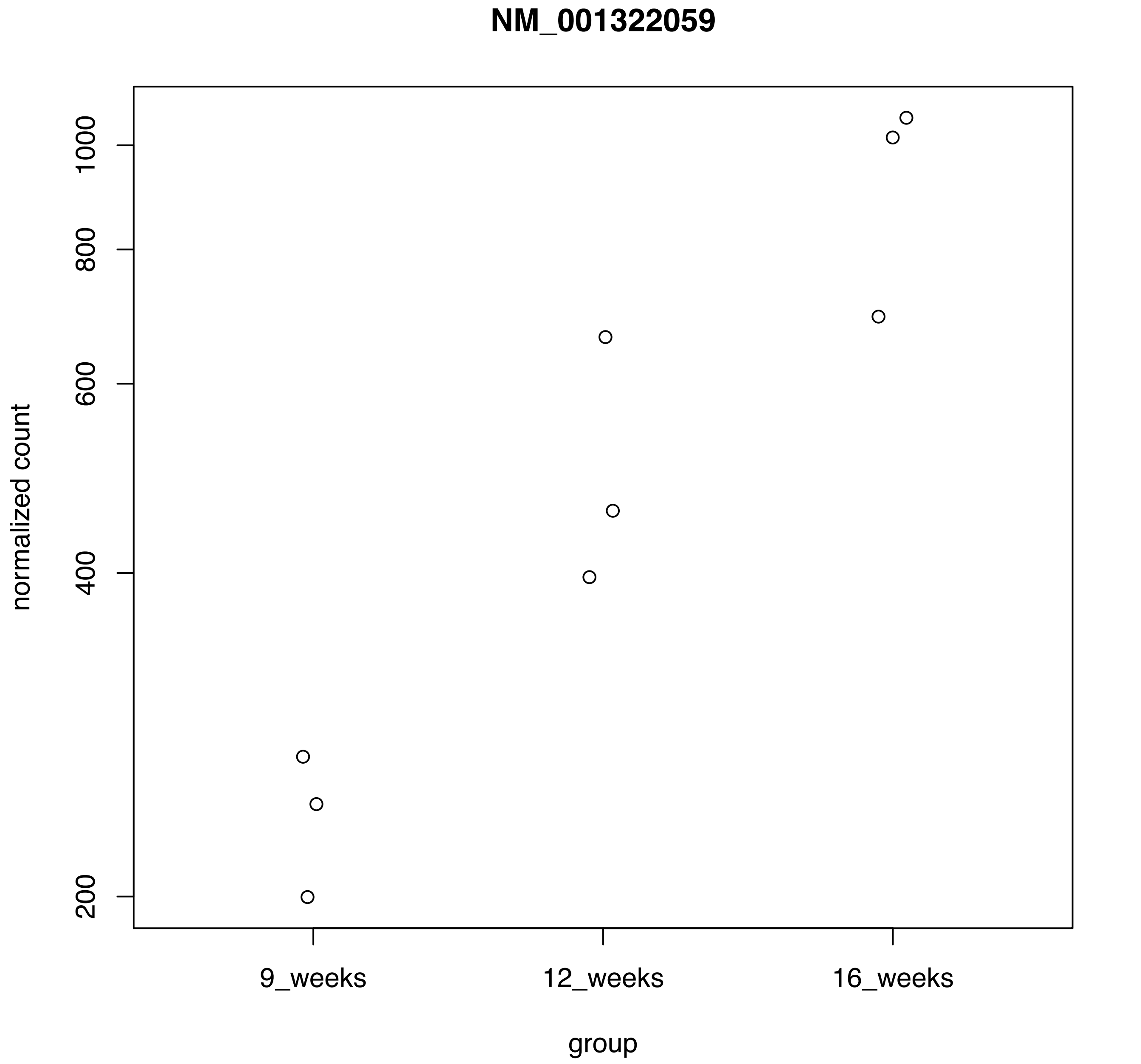
NM_001322059
normalized count
1000
800
600
400
200
9_weeks
12_weeks
16_weeks
group

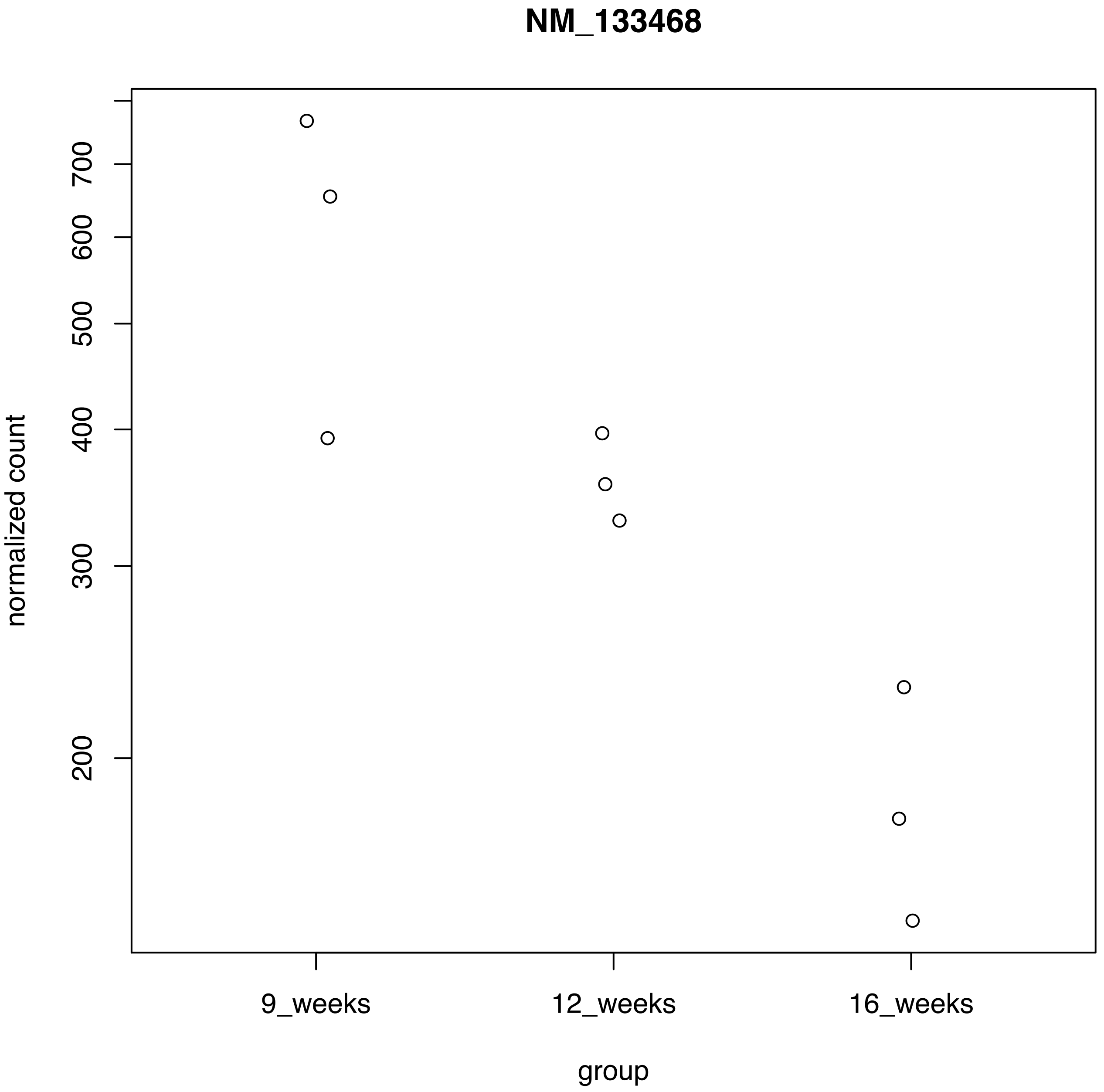
NM_133468
normalized count
700
600
500
400
300
200
9_weeks
12_weeks
16_weeks
group

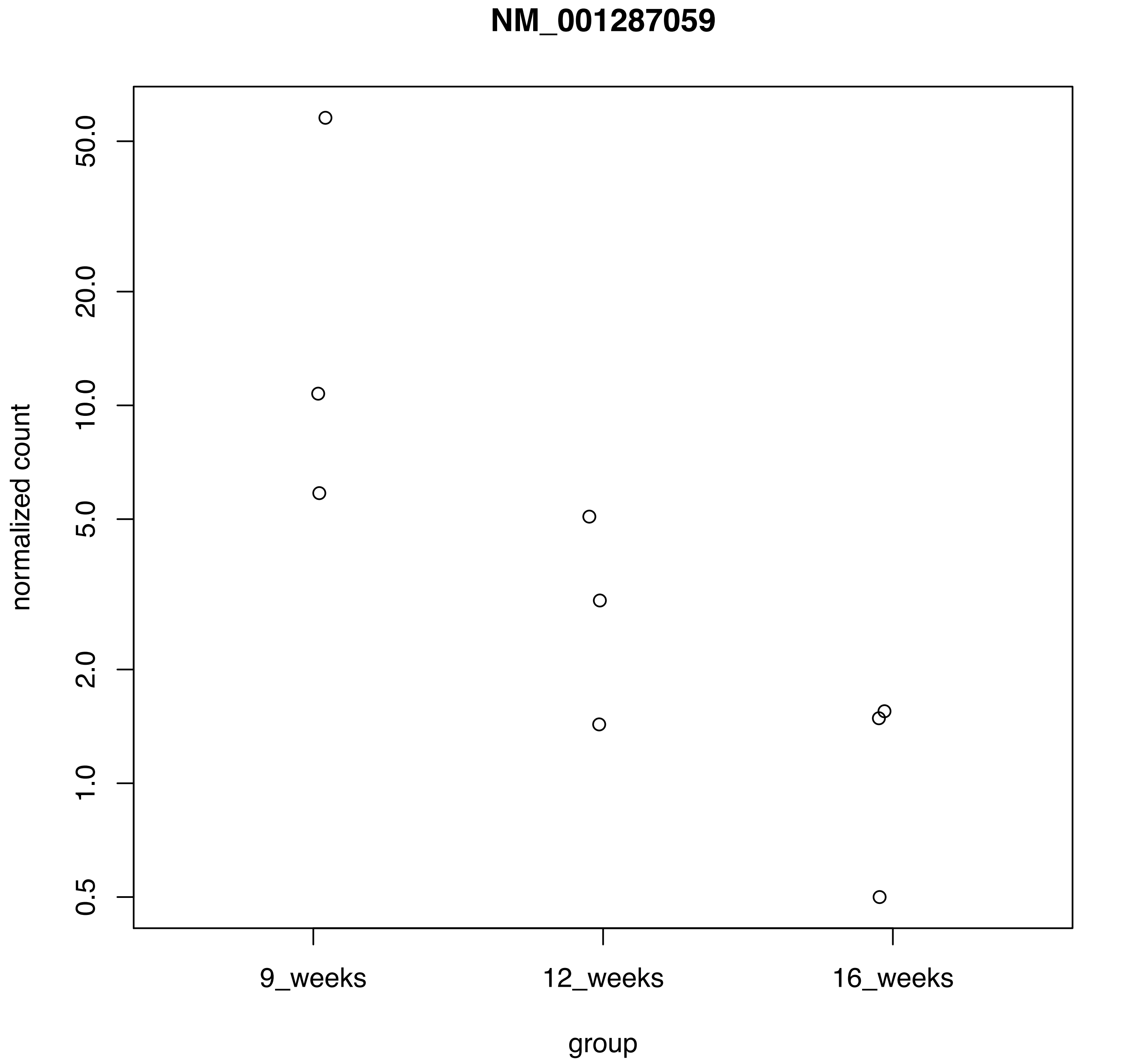
NM_001287059
normalized count
50.0
20.0
10.0
5.0
2.0
1.0
0.5
9_weeks
12_weeks
16_weeks
group

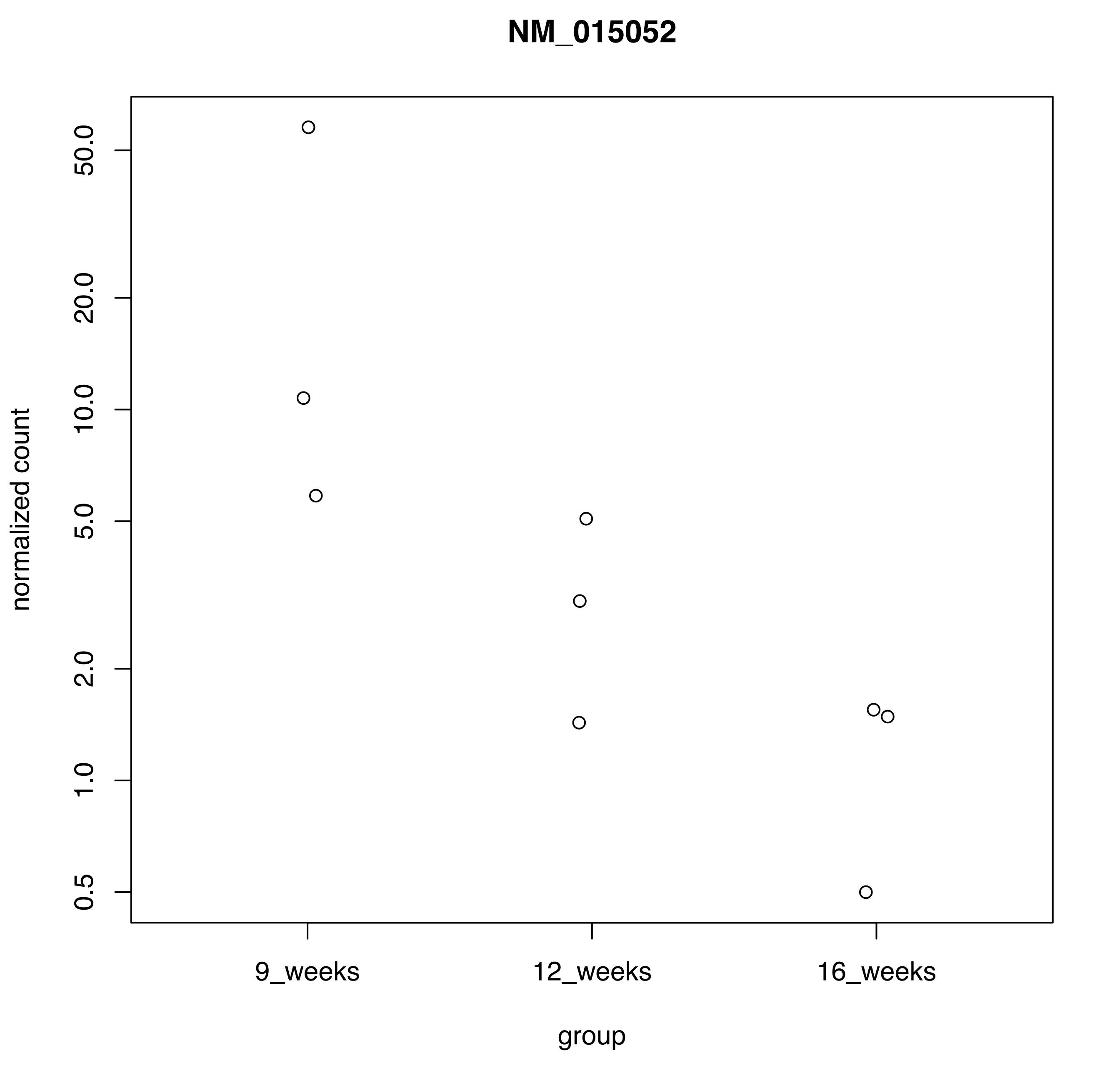
NM_015052
normalized count
50.0
20.0
10.0
5.0
2.0
1.0
0.5
9_weeks
12_weeks
16_weeks
group

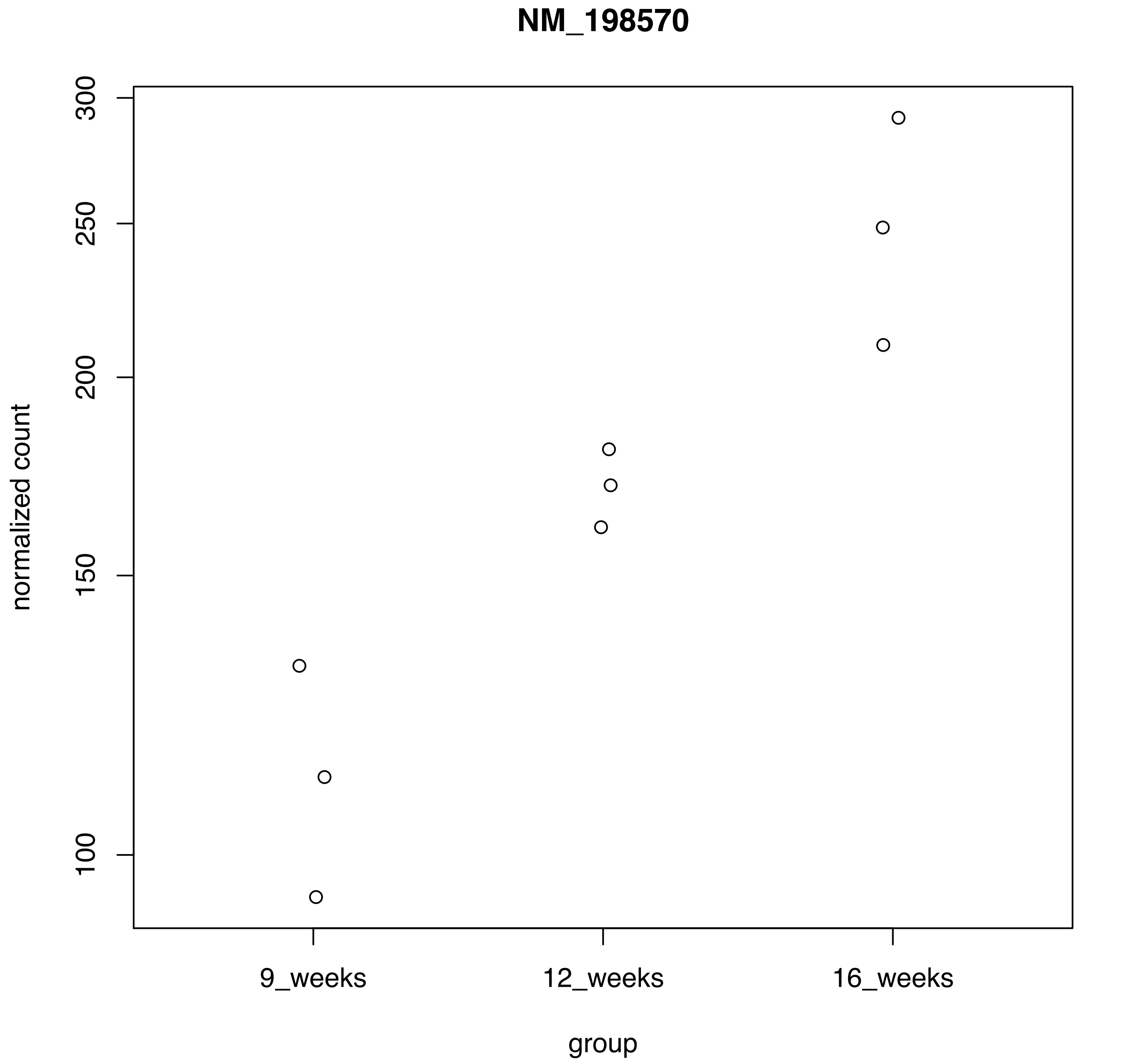
NM_198570
normalized count
300
250
200
150
100
9_weeks
12_weeks
16_weeks
group

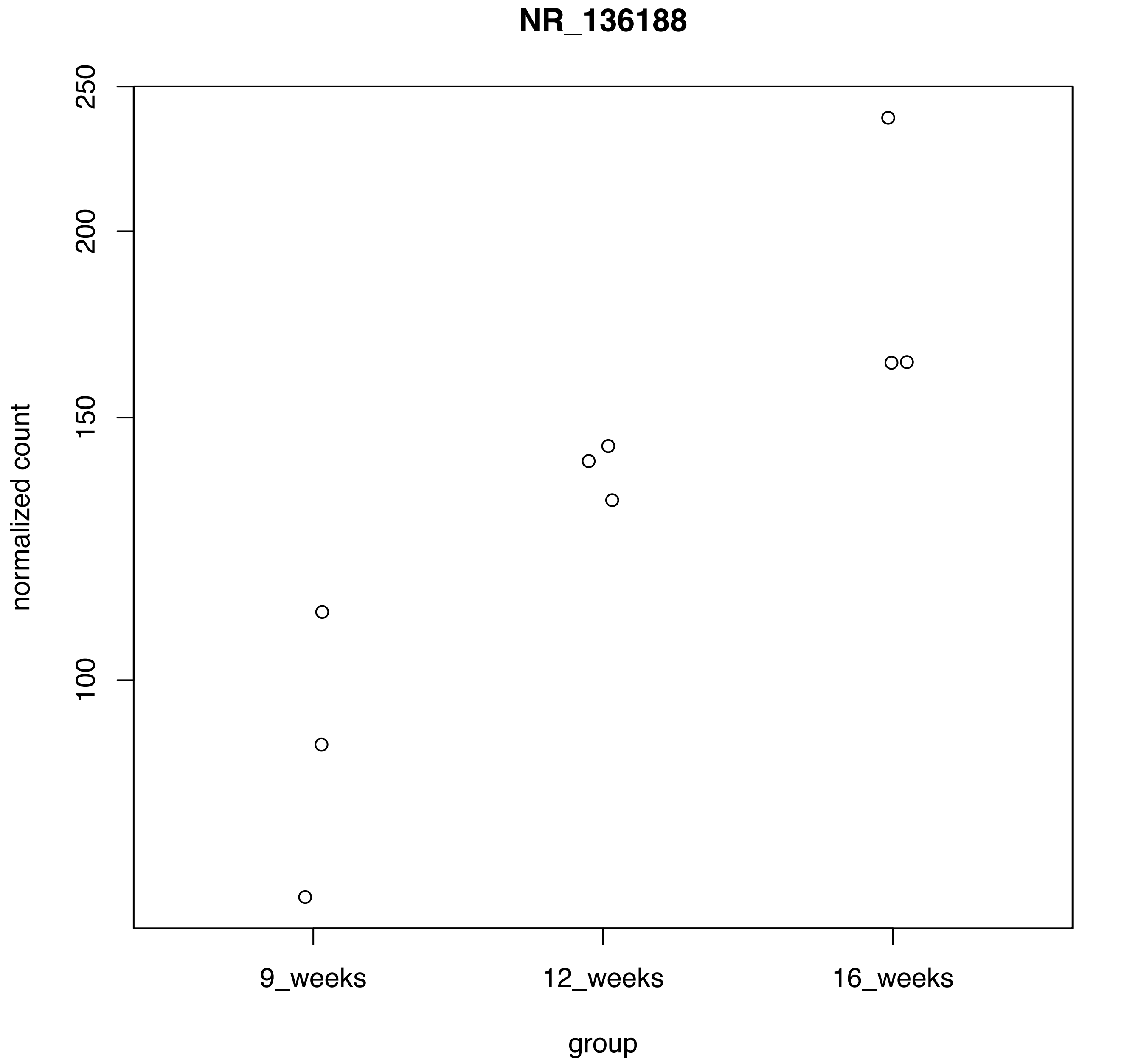

NR_136188
250
200
150
100
normalized count
9_weeks
12_weeks
16_weeks
group

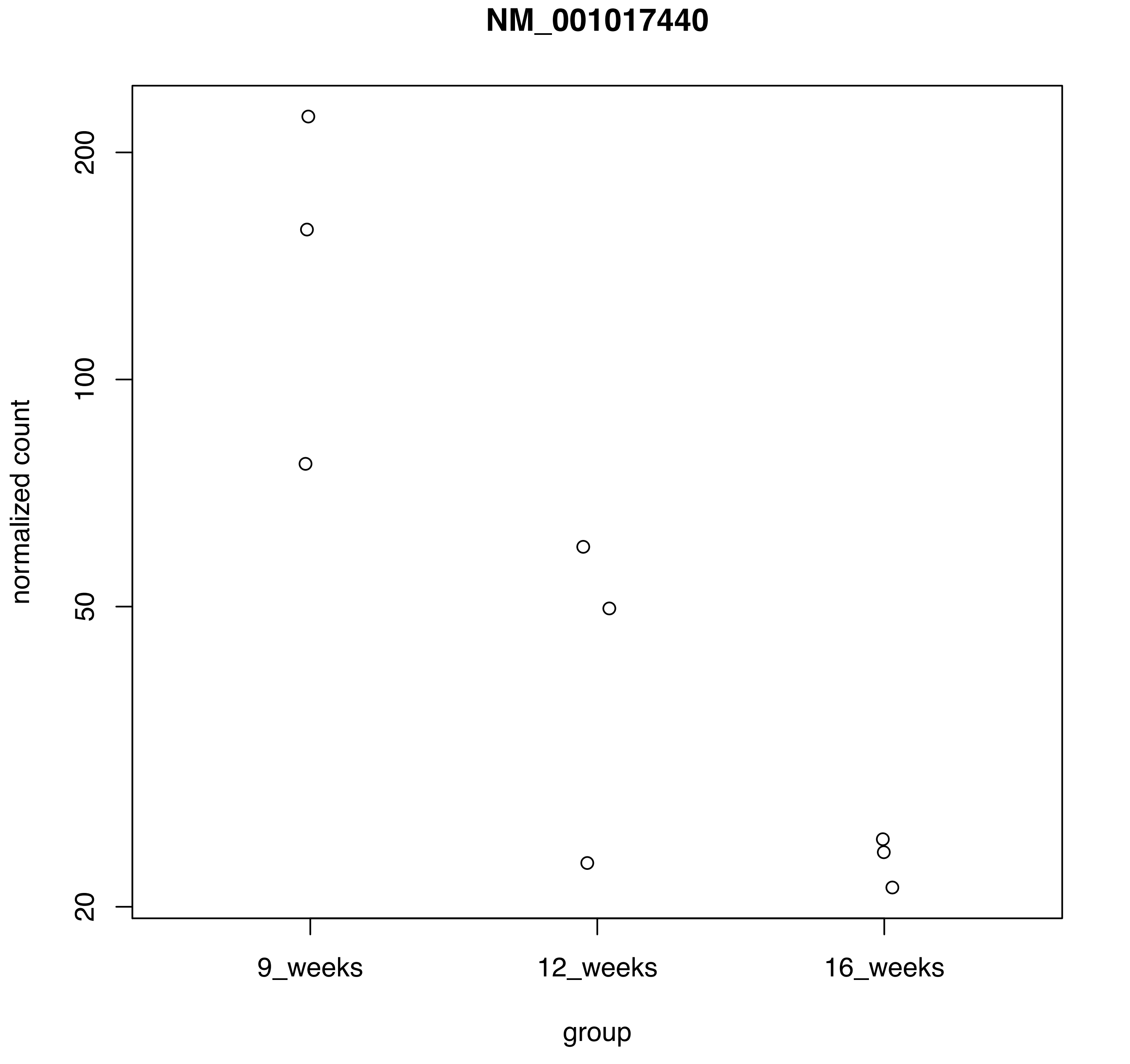
NM_001017440
normalized count
200
100
50
20
9_weeks
12_weeks
16_weeks
group

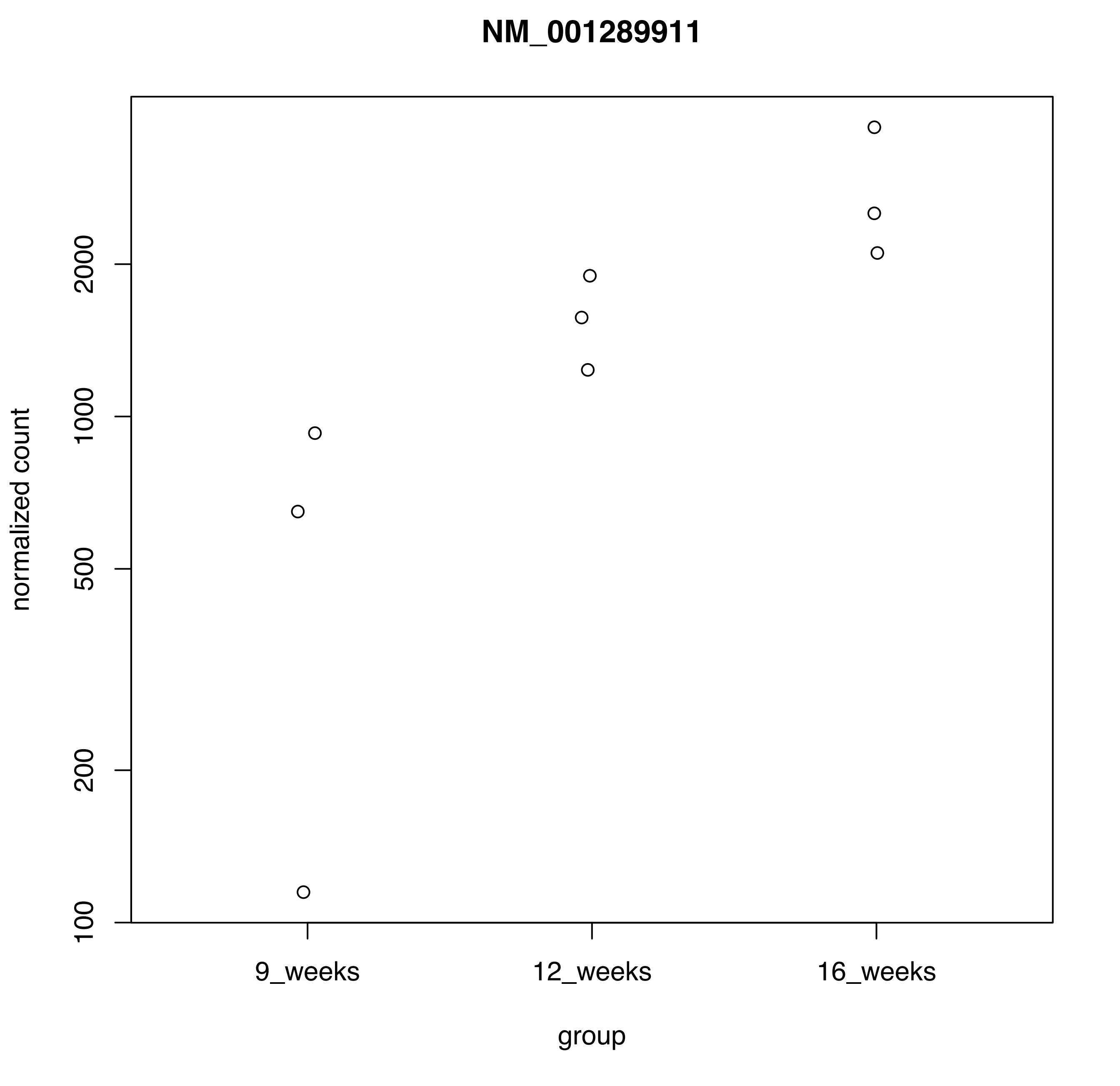
NM_001289911
normalized count
100
200
500
1000
2000
9_weeks
12_weeks
16_weeks
group

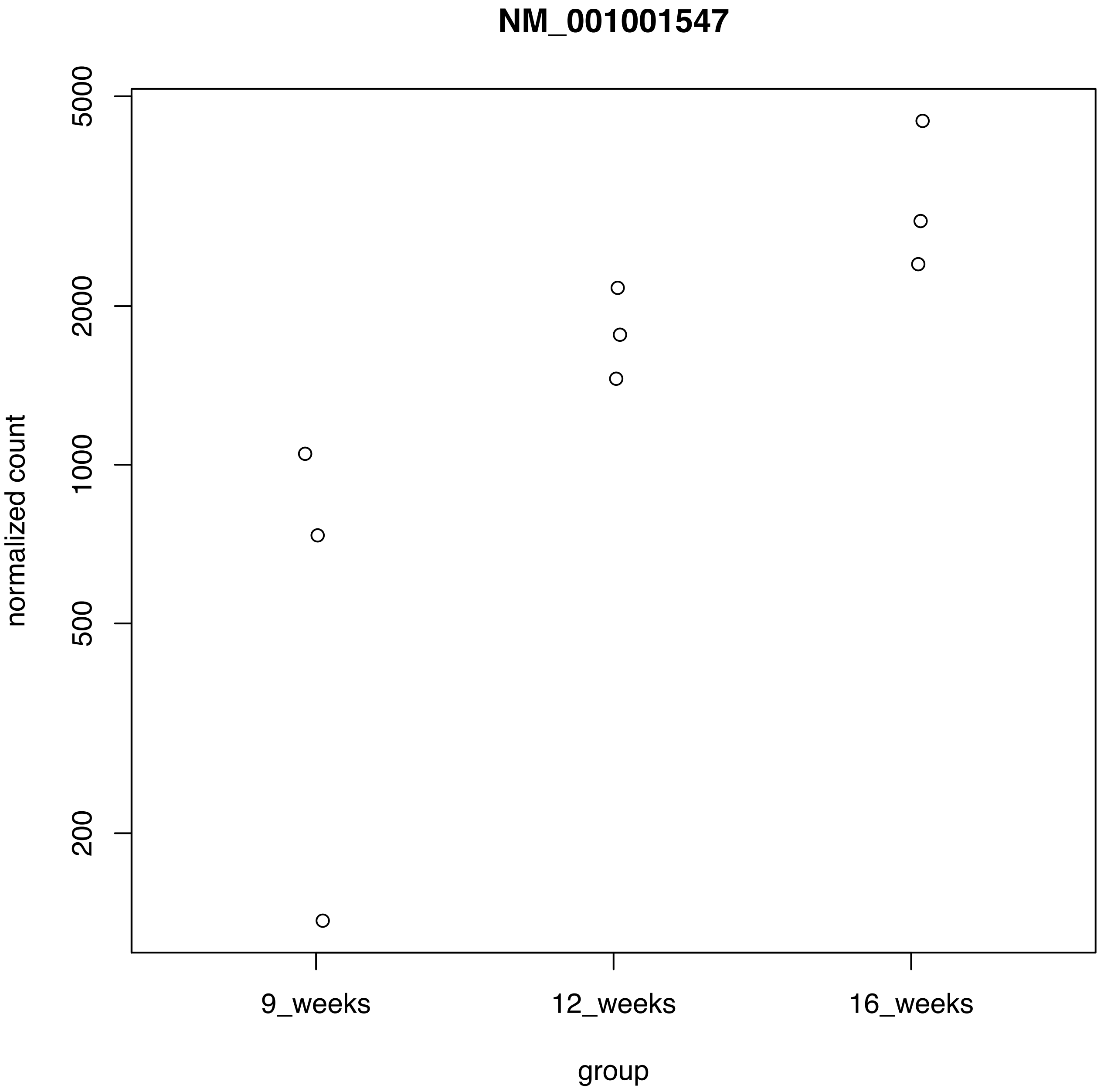
NM_001001547
normalized count
5000
2000
1000
500
200
9_weeks
12_weeks
16_weeks
group

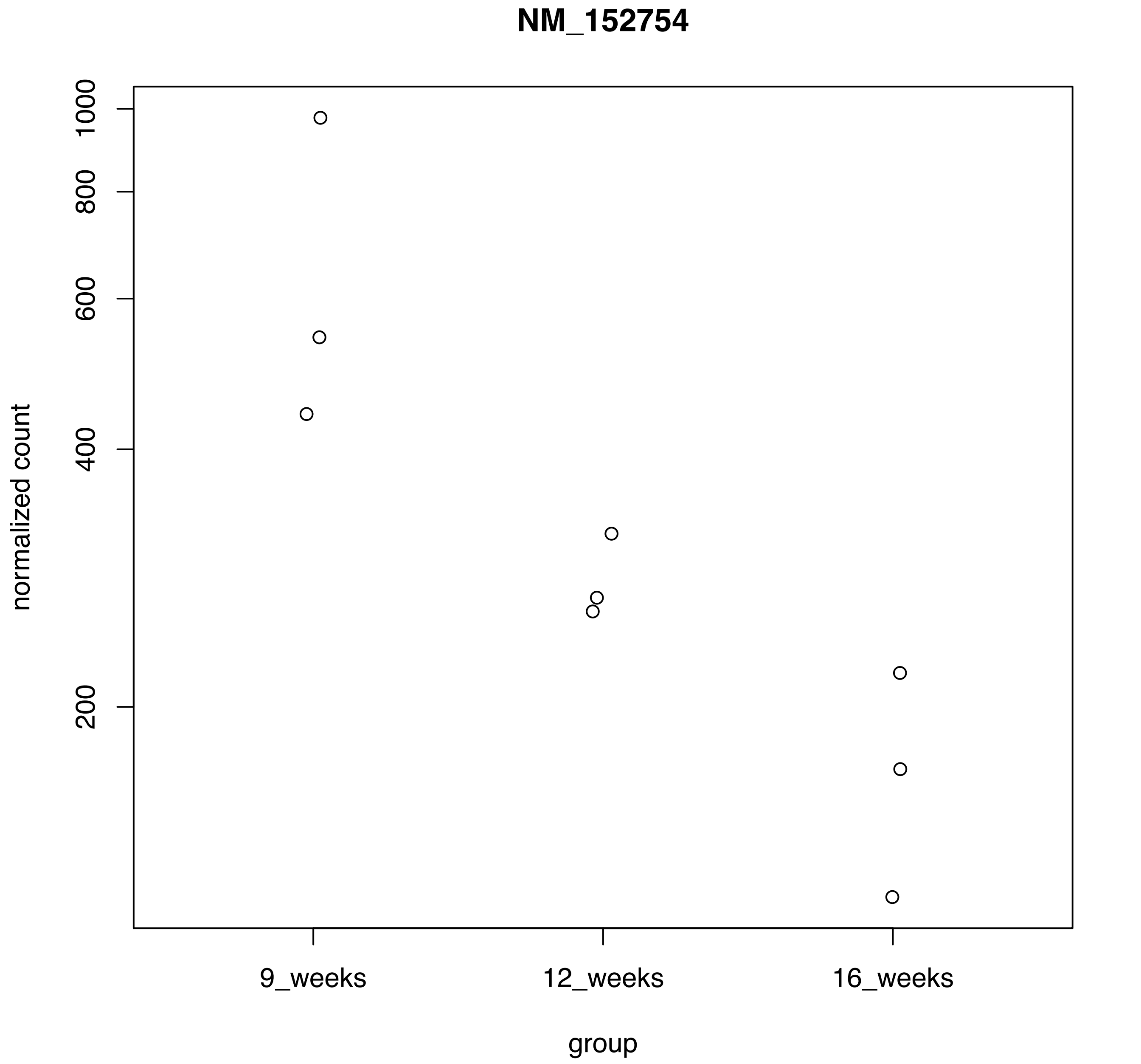
NM_152754
normalized count
1000
800
600
400
200
9_weeks
12_weeks
16_weeks
group

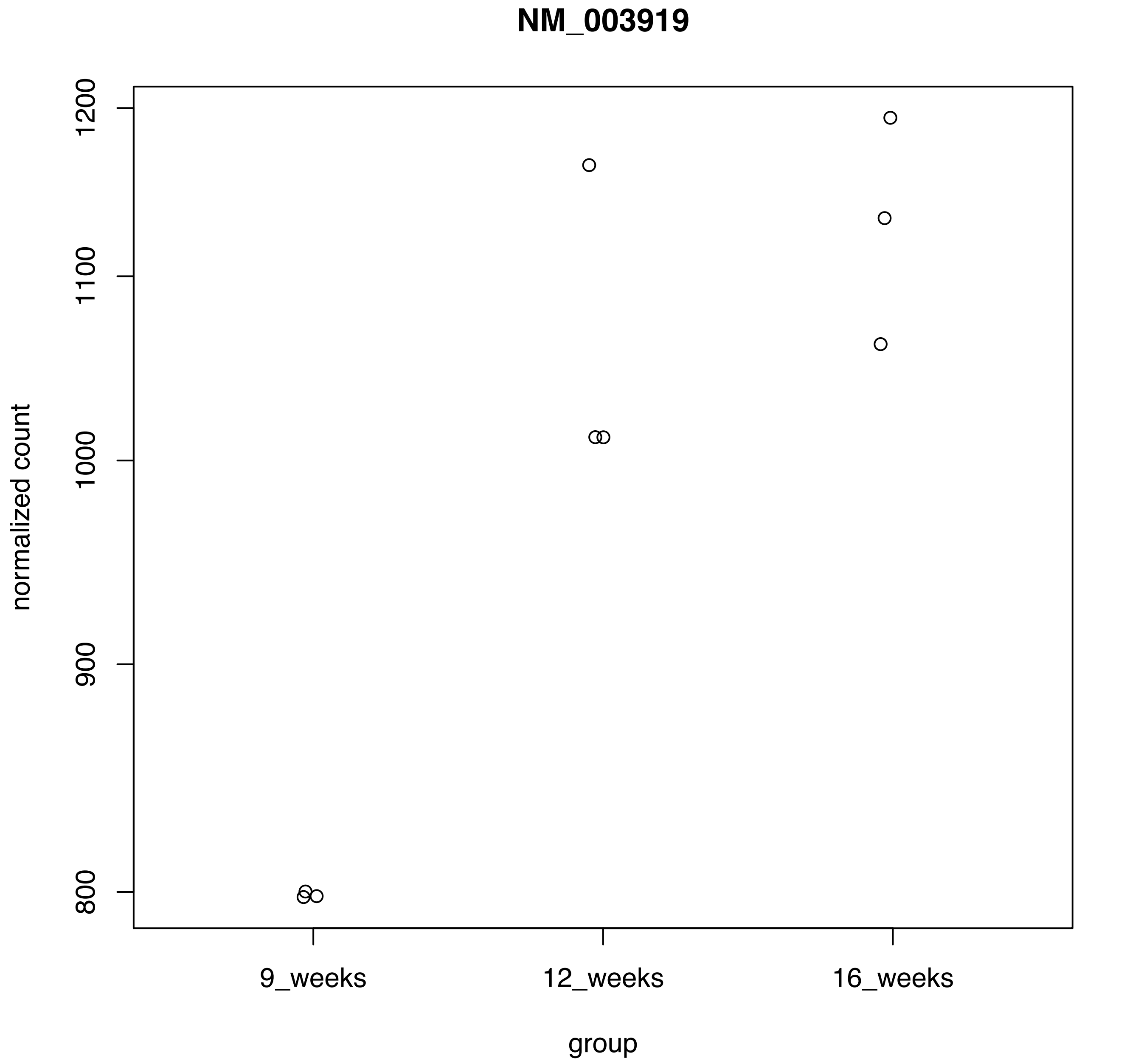
NM_003919
normalized count
1200
1100
1000
900
800
9_weeks
12_weeks
16_weeks
group

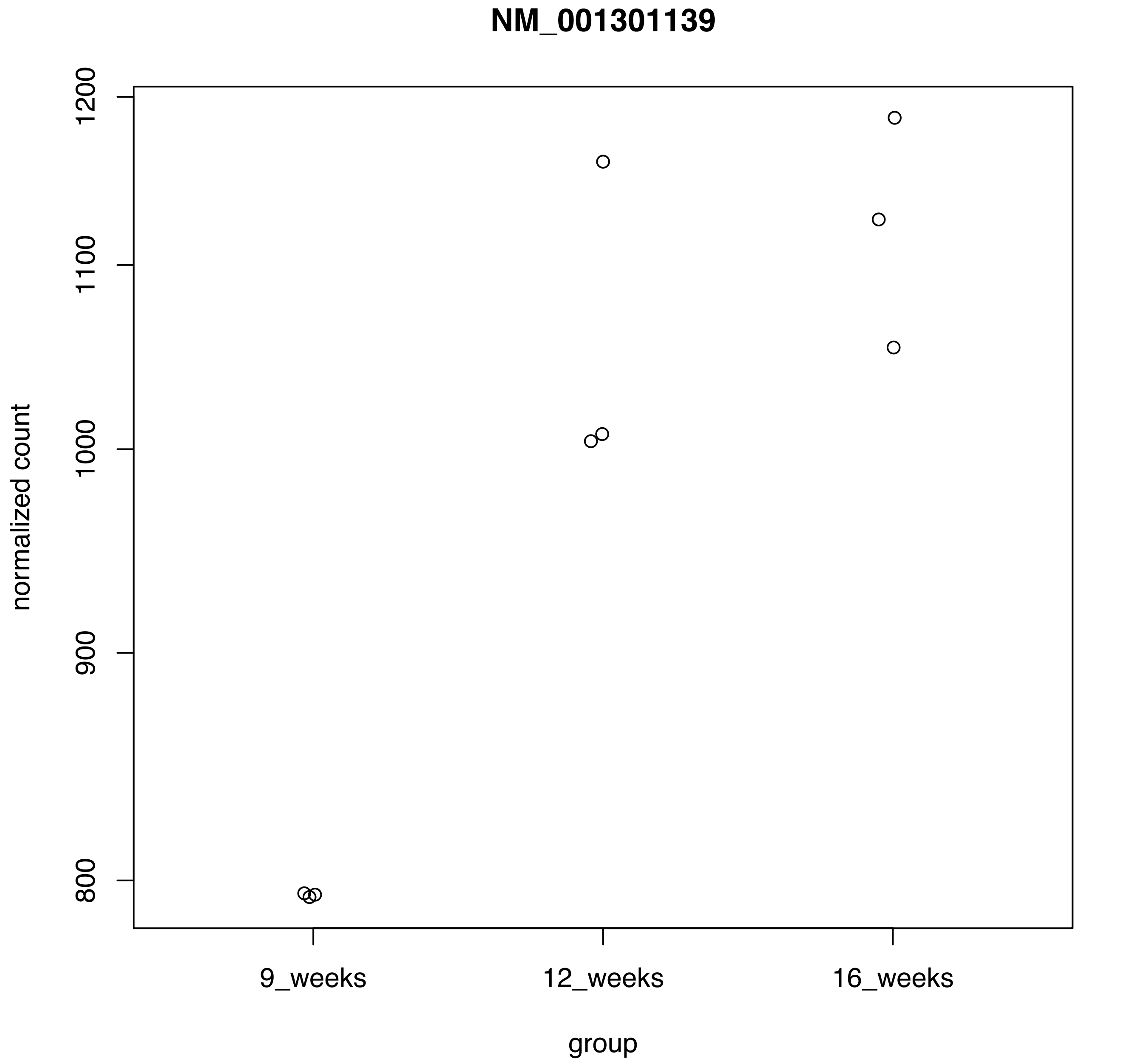
NM_001301139
normalized count
1200
1100
1000
900
800
9_weeks
12_weeks
16_weeks
group

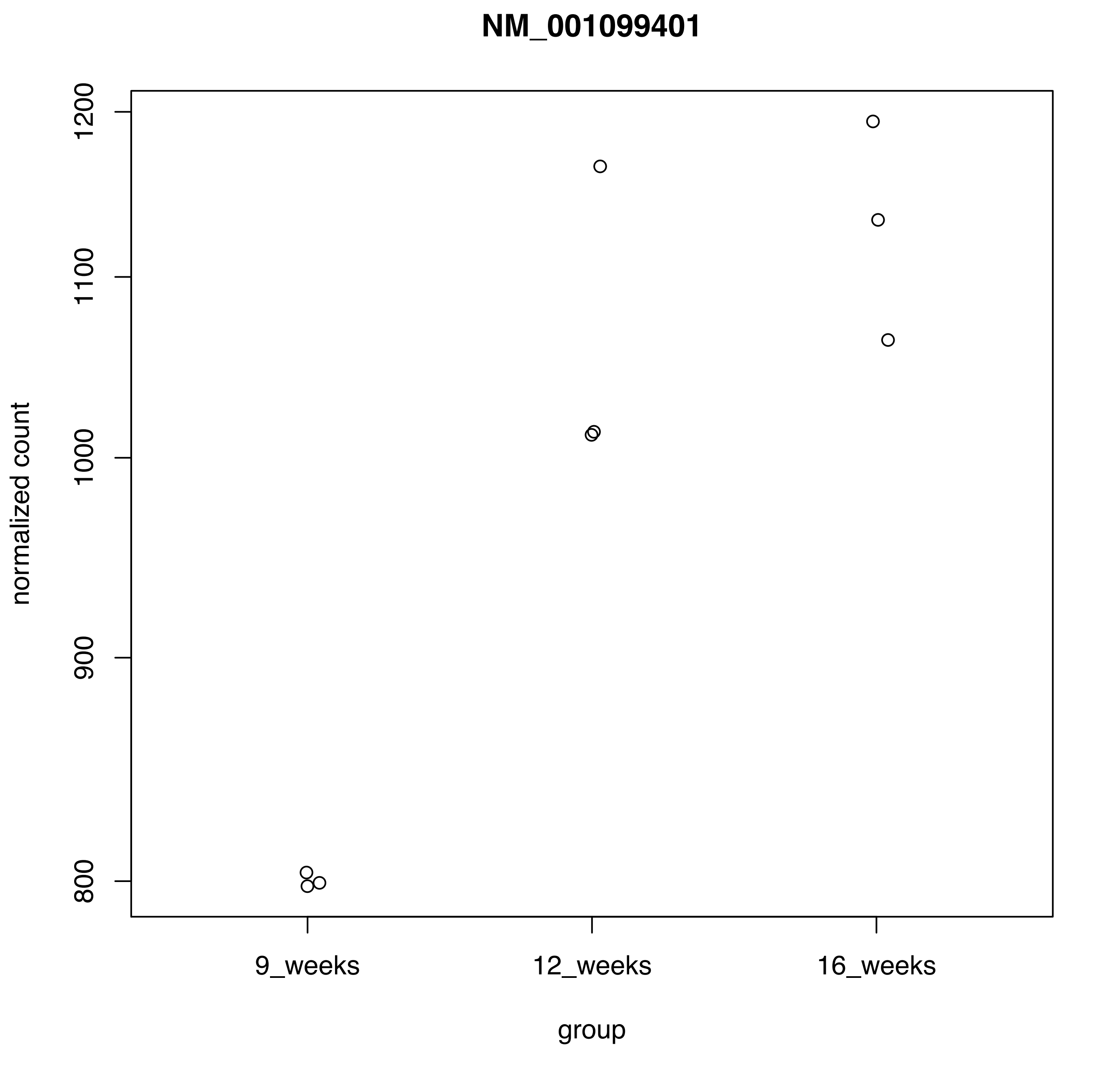
NM_001099401
normalized count
1200
1100
1000
900
800
9_weeks
12_weeks
16_weeks
group

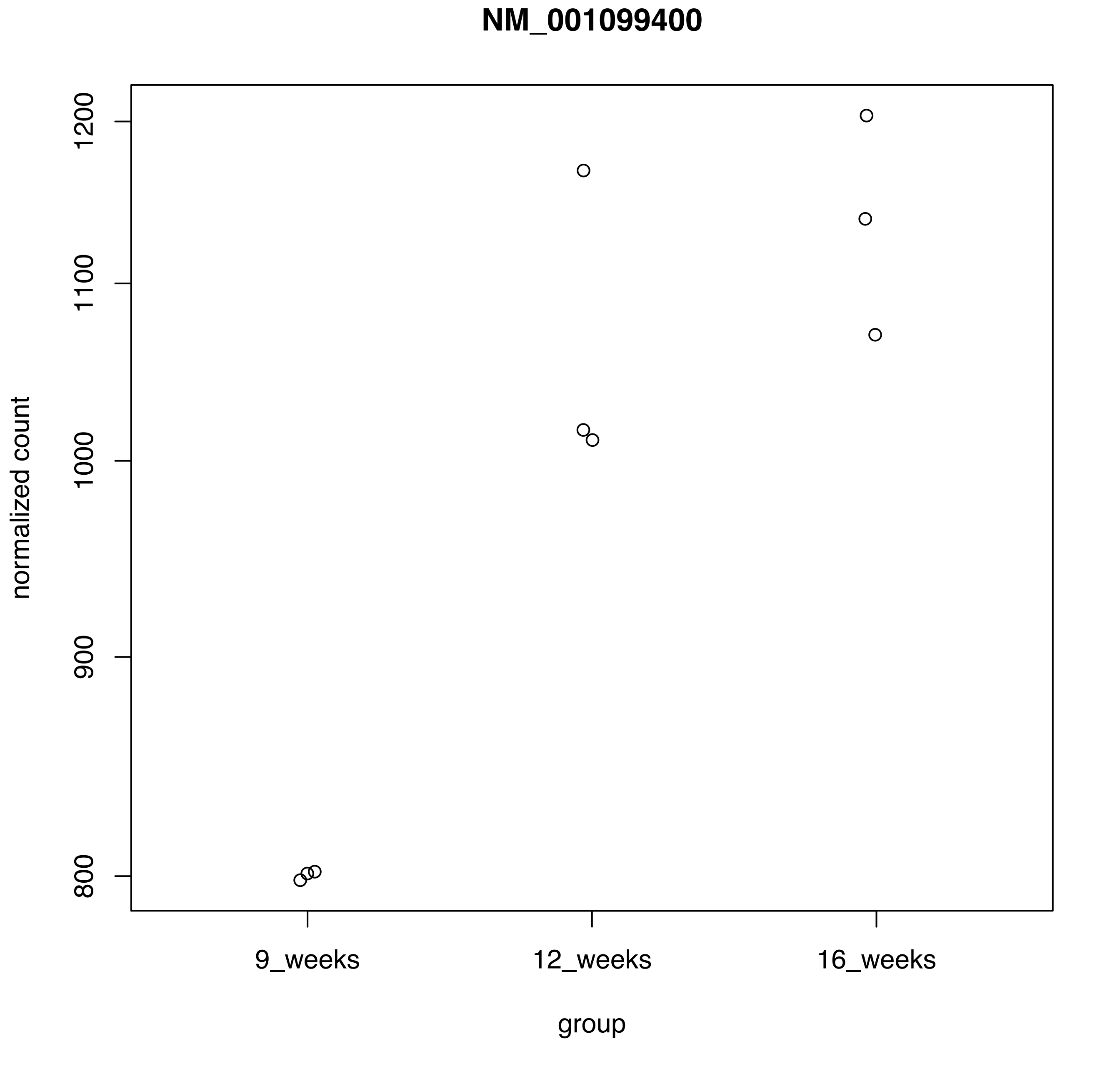

NM_001099400
normalized count
1200
1100
1000
900
800
9_weeks
12_weeks
16_weeks
group

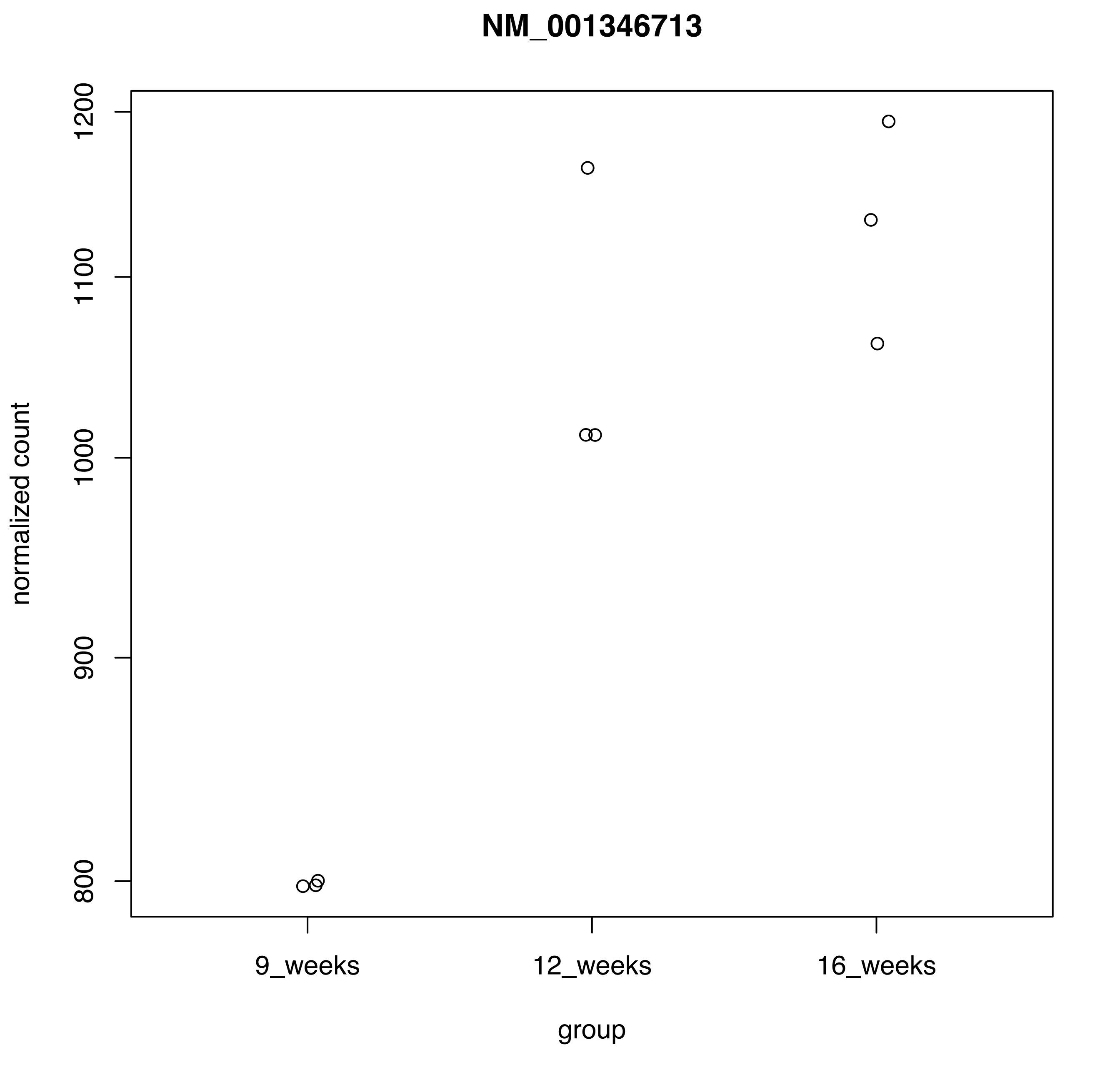
NM_001346713
normalized count
1200
1100
1000
900
800
9_weeks
12_weeks
16_weeks
group

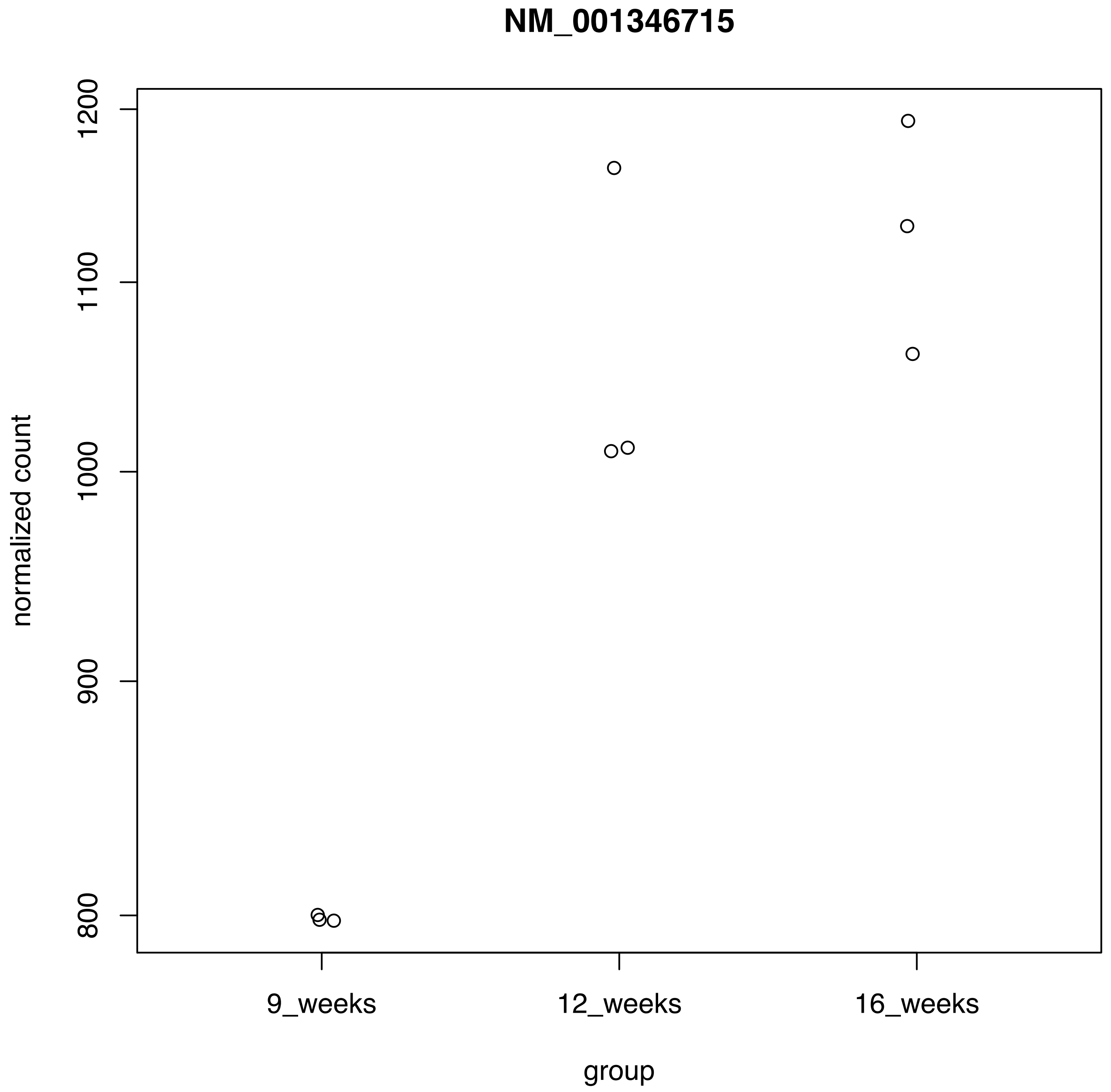
NM_001346715
normalized count
1200
1100
1000
900
800
9_weeks
12_weeks
16_weeks
group

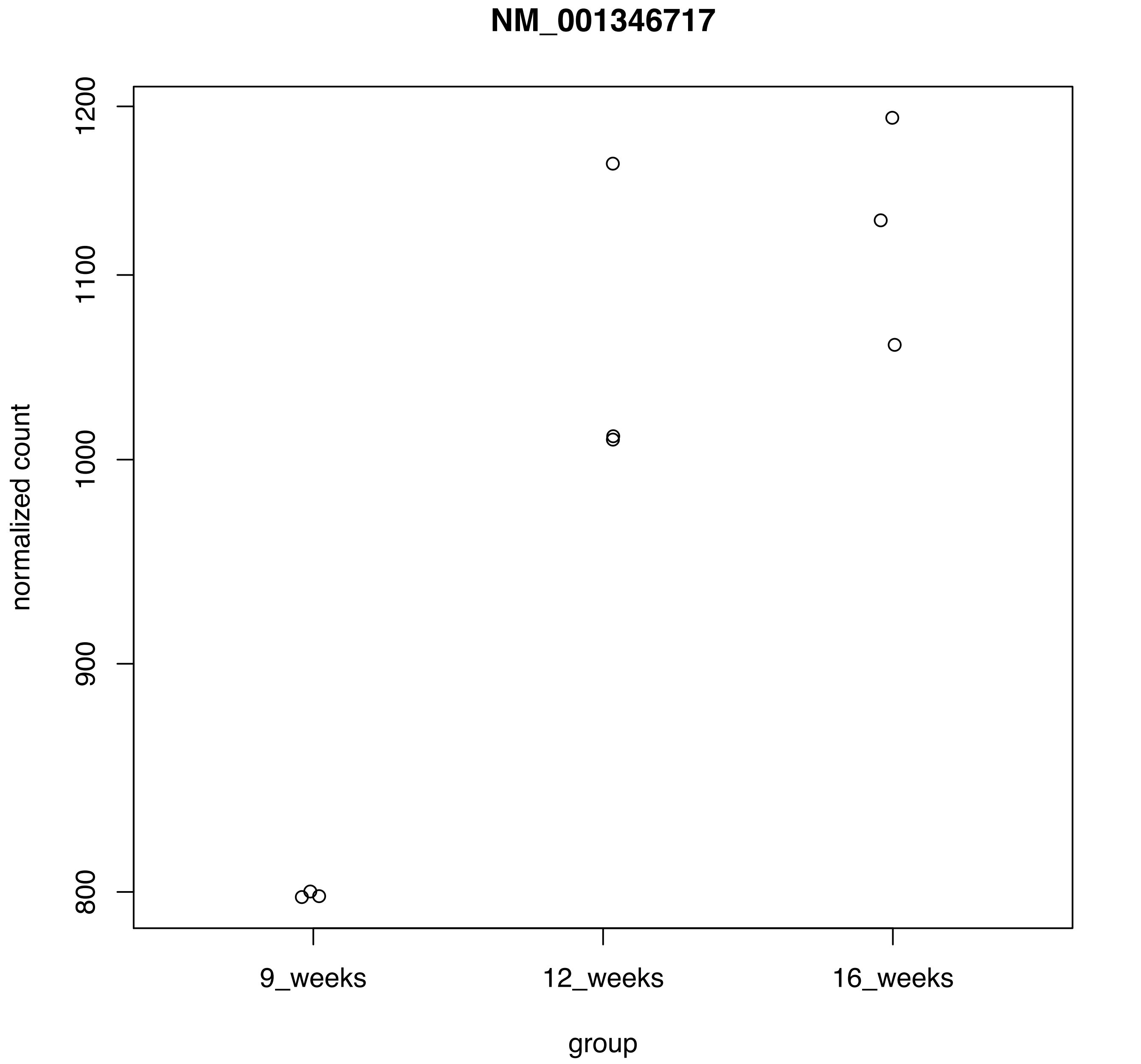

NM_001346717
normalized count
1200
1100
1000
900
800
9_weeks
12_weeks
16_weeks
group

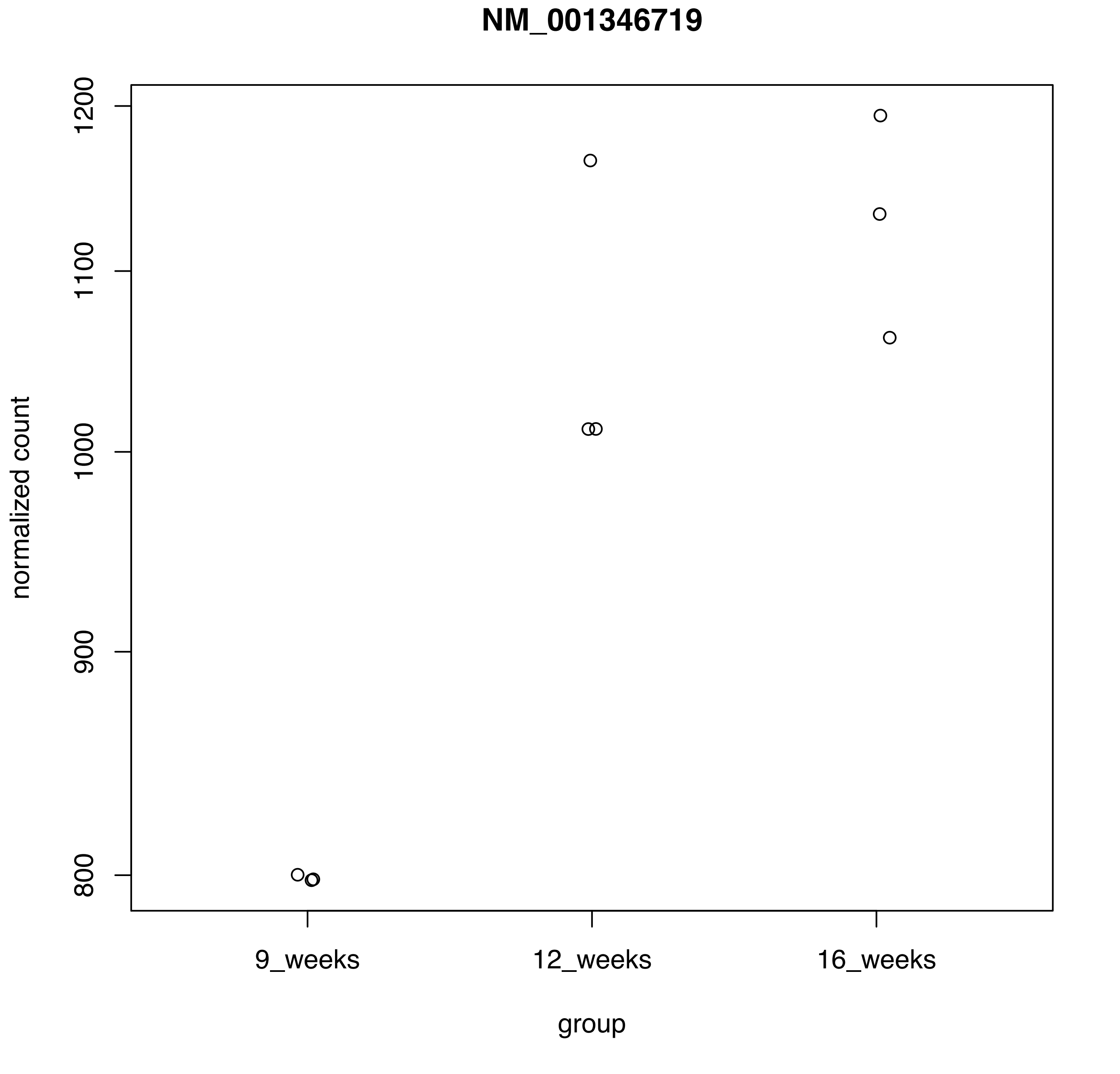
NM_001346719
normalized count
1200
1100
1000
900
800
9_weeks
12_weeks
16_weeks
group

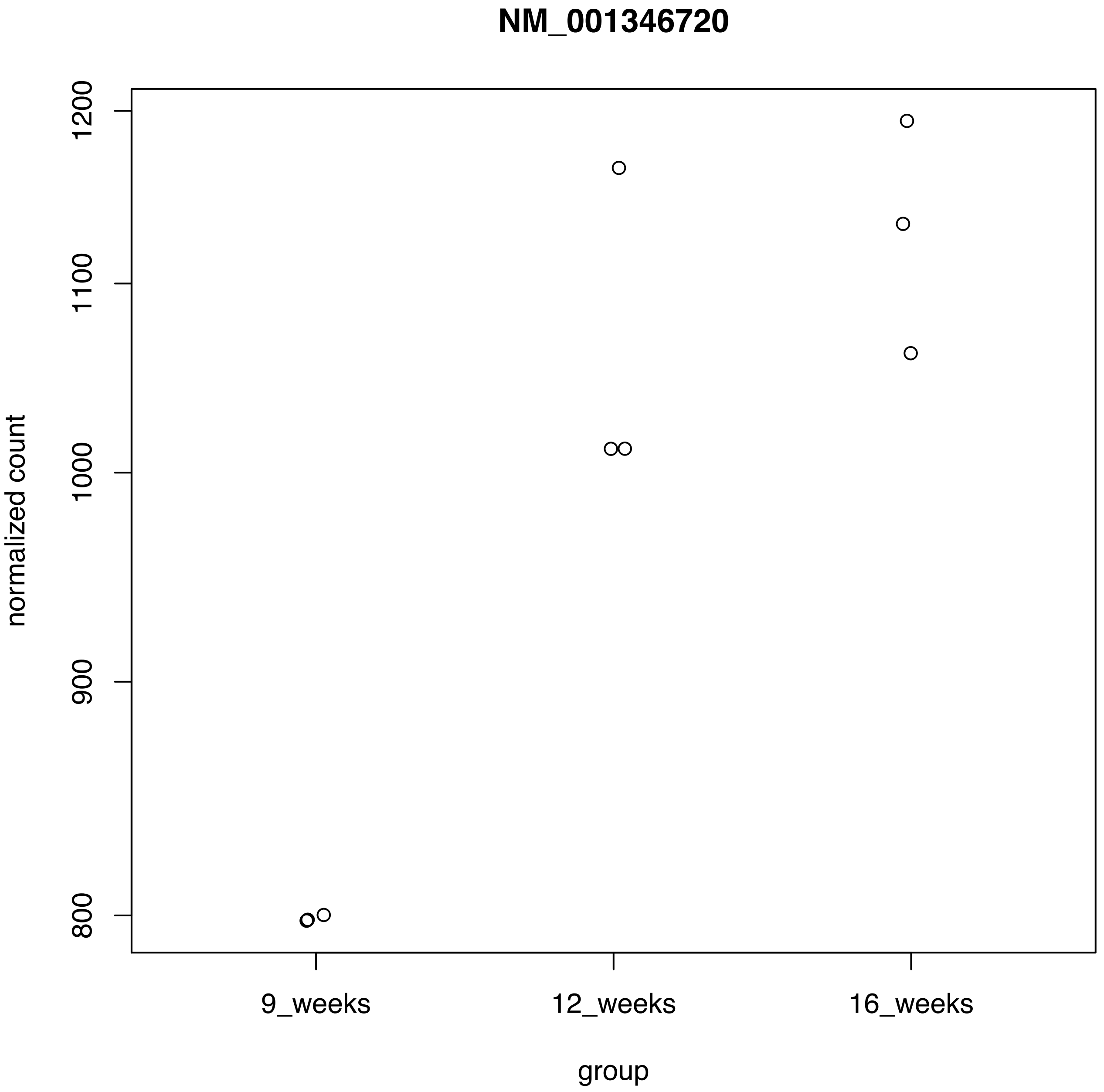

NM_001346720
normalized count
1200
1100
1000
900
800
9_weeks
12_weeks
16_weeks
group

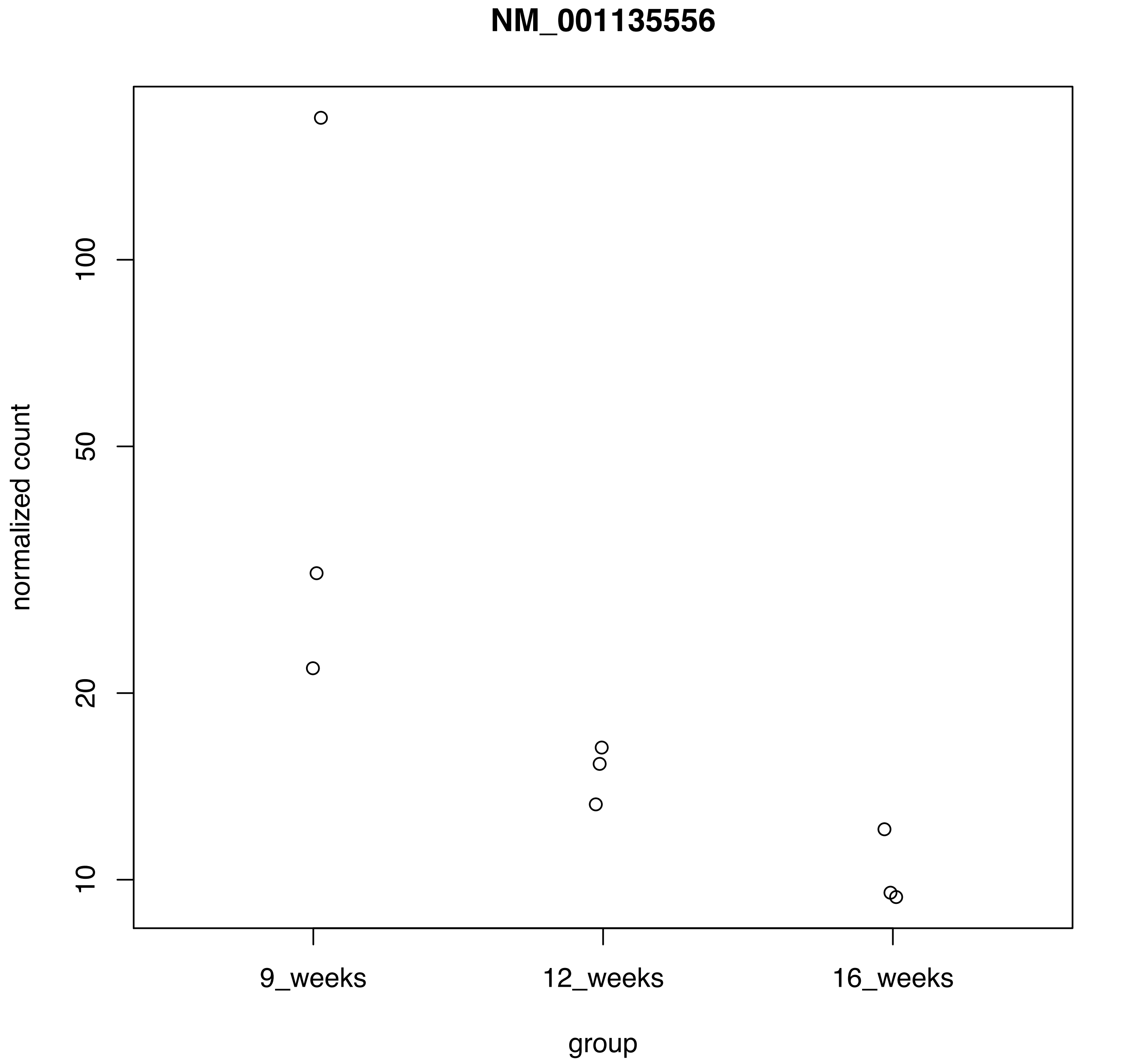

NM_001135556
normalized count
100
50
20
10
9_weeks
12_weeks
16_weeks
group

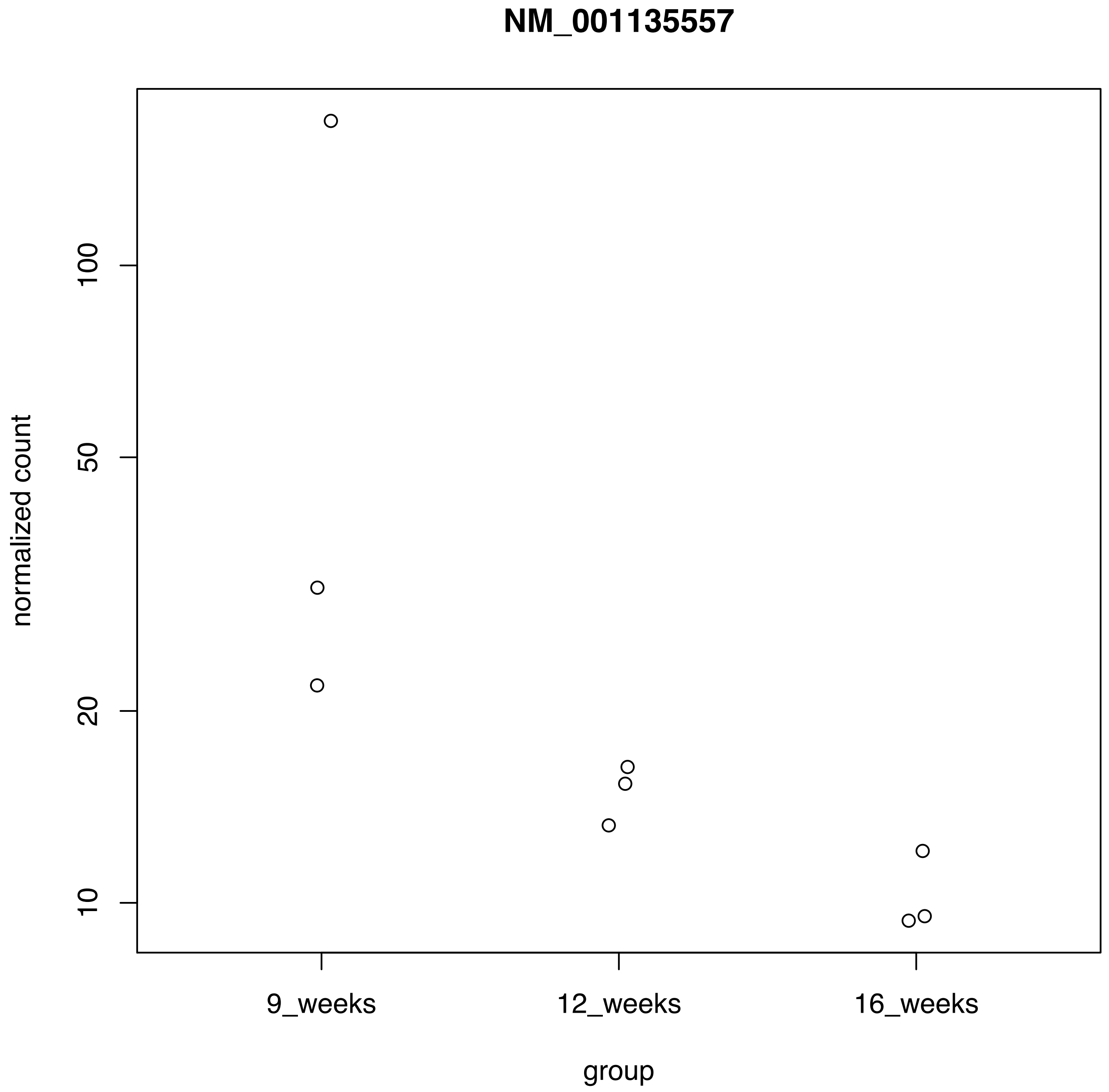
NM_001135557
normalized count
100
50
20
10
9_weeks
12_weeks
16_weeks
group

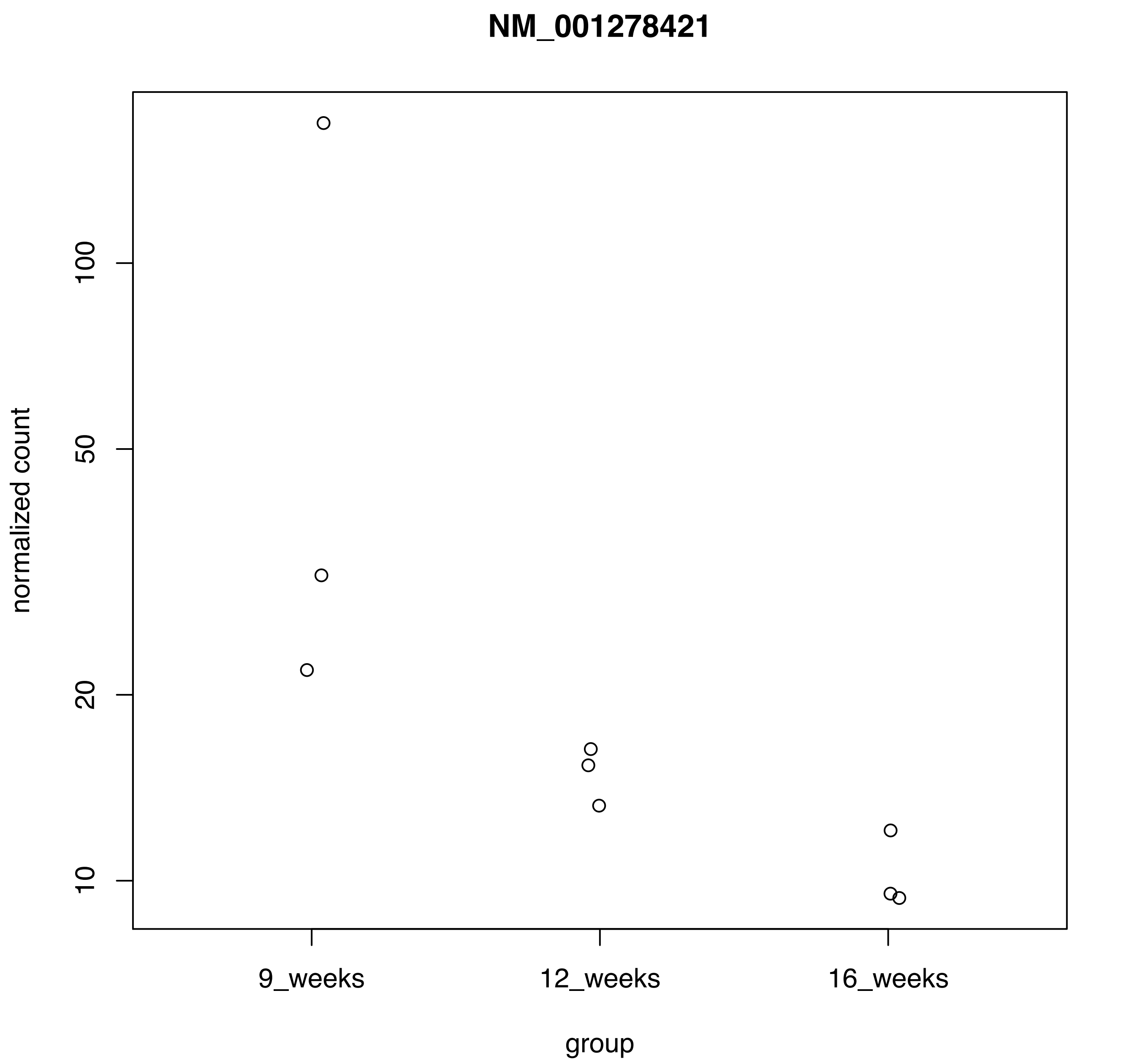
NM_001278421
normalized count
100
50
20
10
9_weeks
12_weeks
16_weeks
group

NM_004411

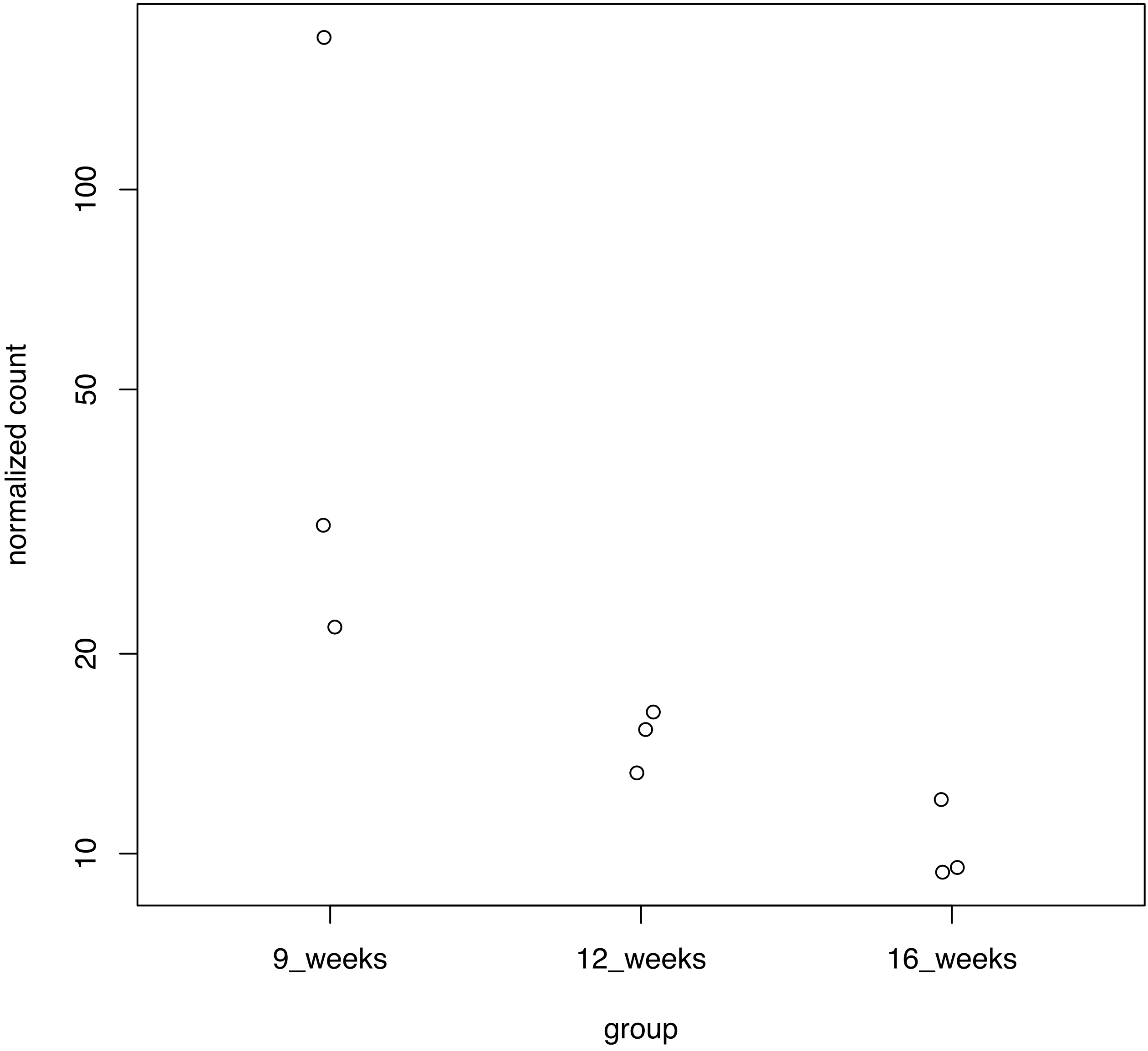

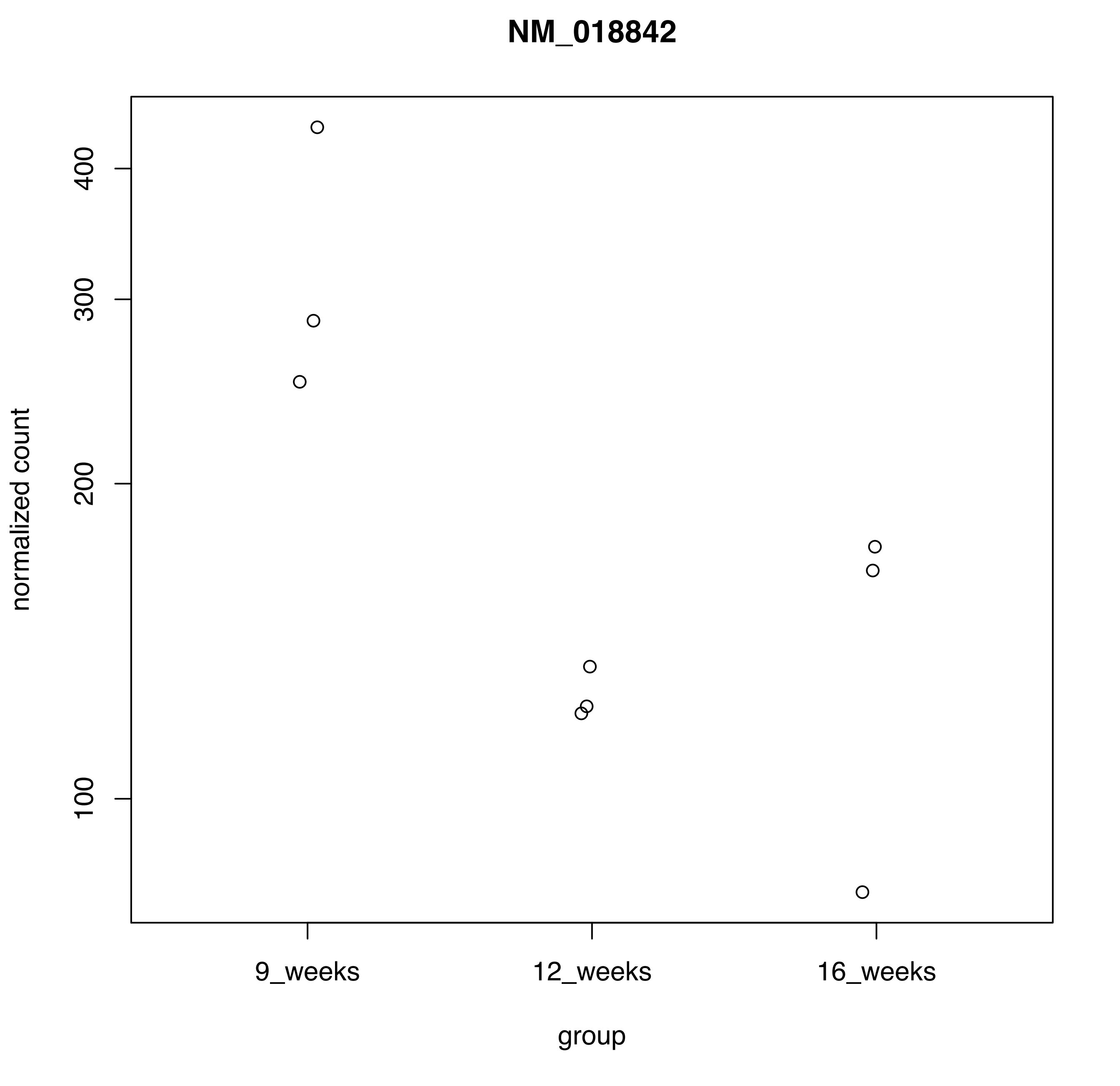
NM_018842
normalized count
100
200
300
400
9_weeks
12_weeks
16_weeks
group

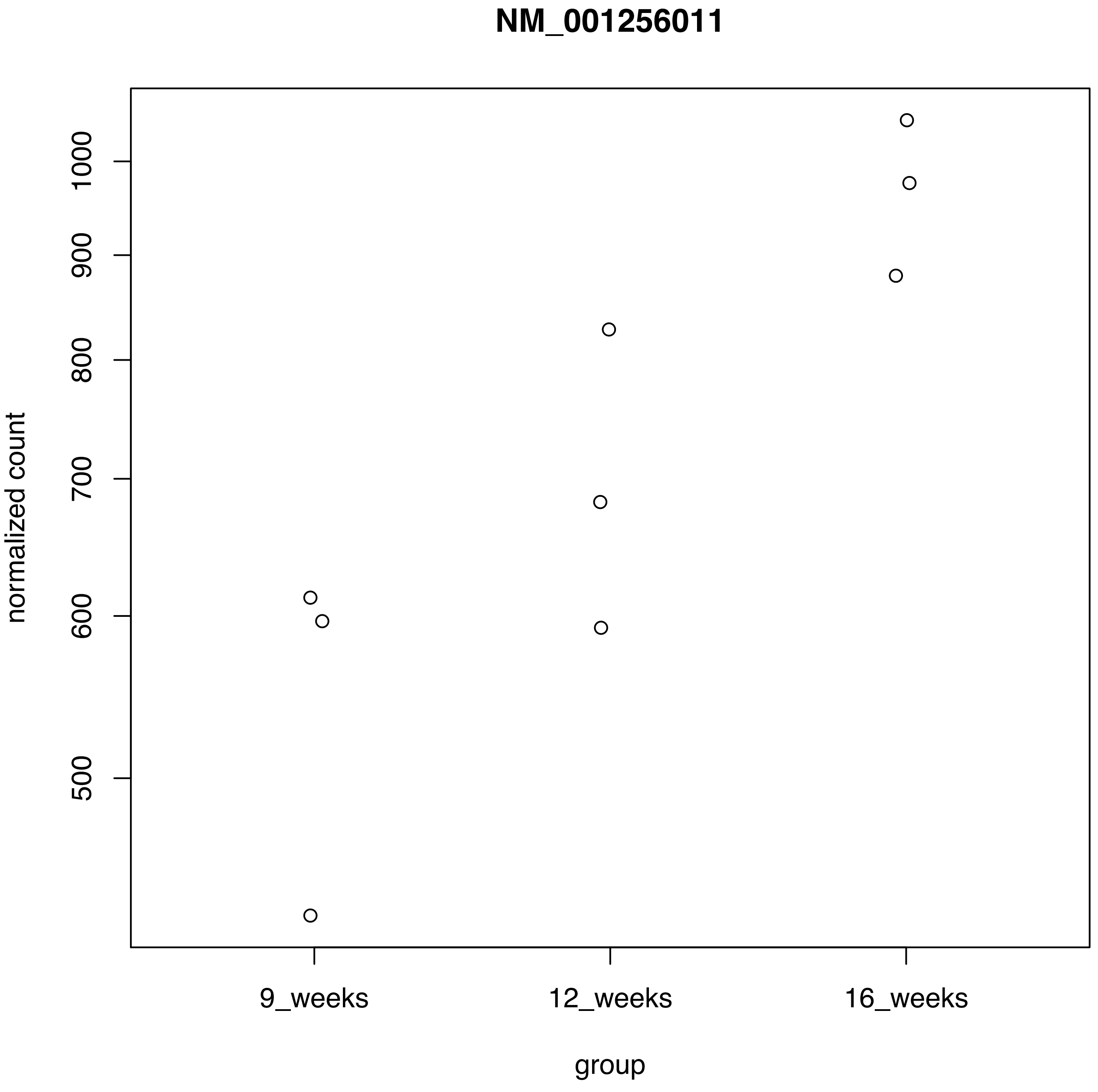

NM_001256011
normalized count
500
600
700
800
900
1000
9_weeks
12_weeks
16_weeks
group

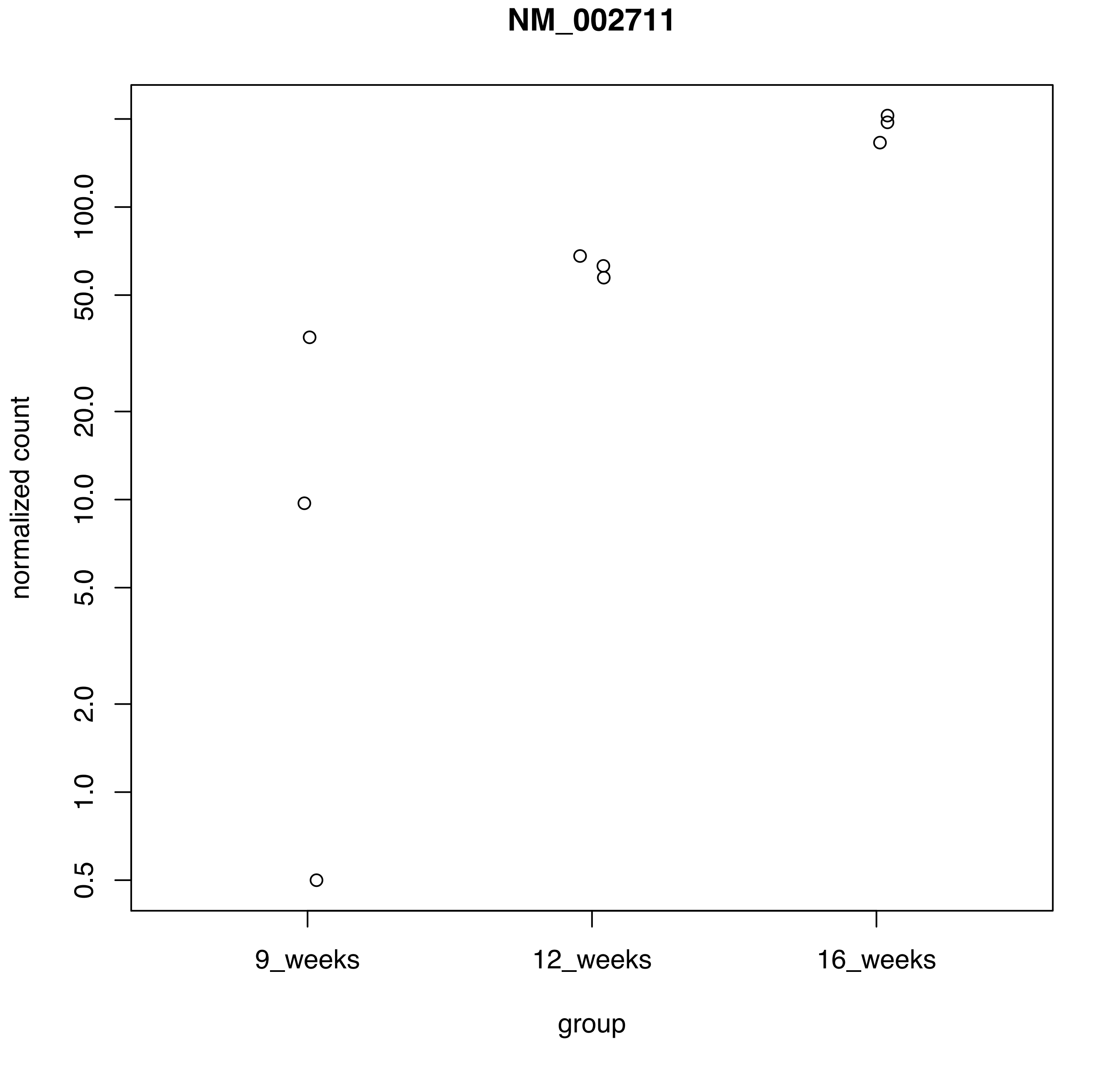

NM_002711
normalized count
100.0
50.0
20.0
10.0
5.0
2.0
1.0
0.5
9_weeks
12_weeks
16_weeks
group

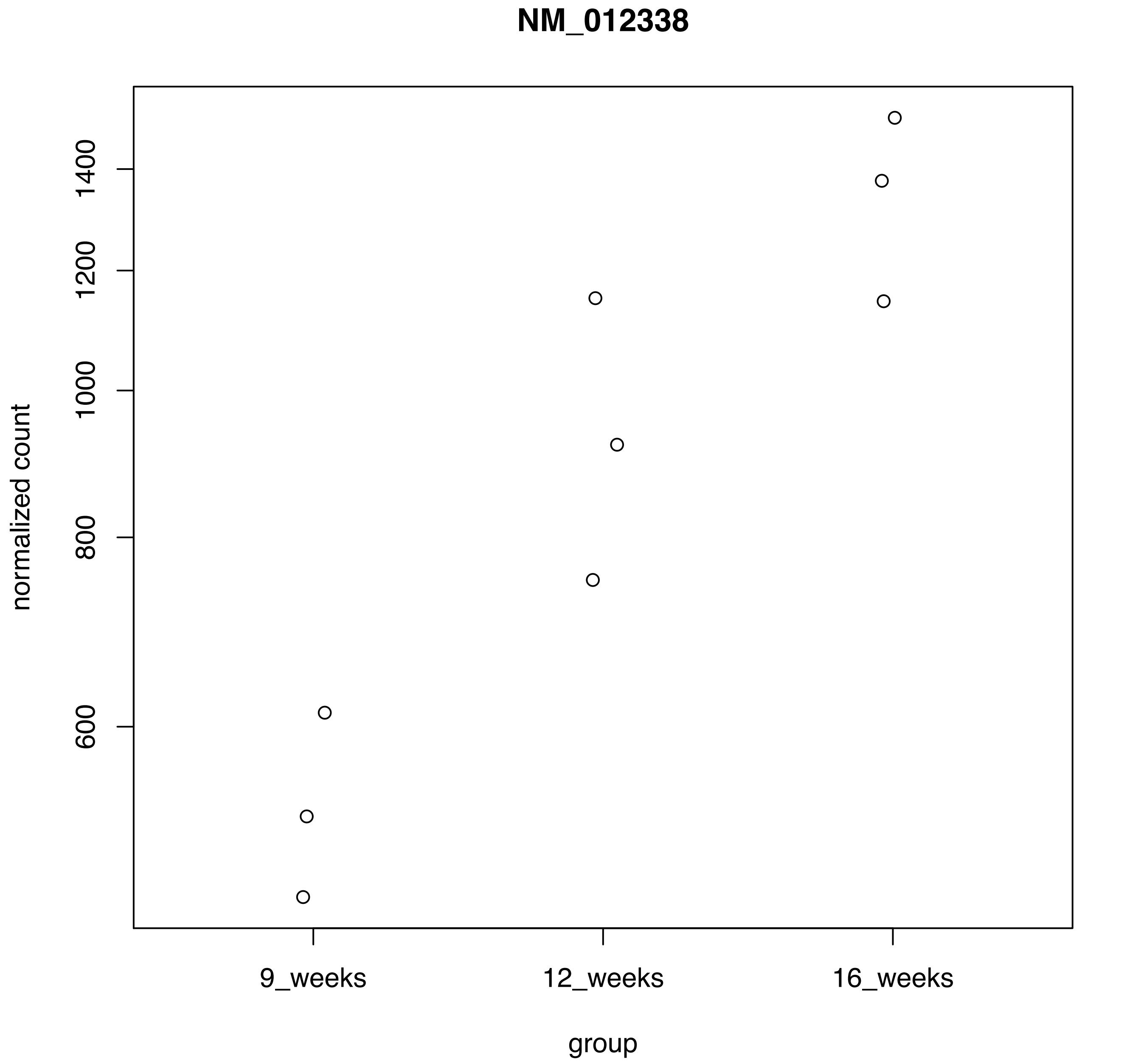
NM_012338
normalized count
1400
1200
1000
800
600
9_weeks
12_weeks
16_weeks
group

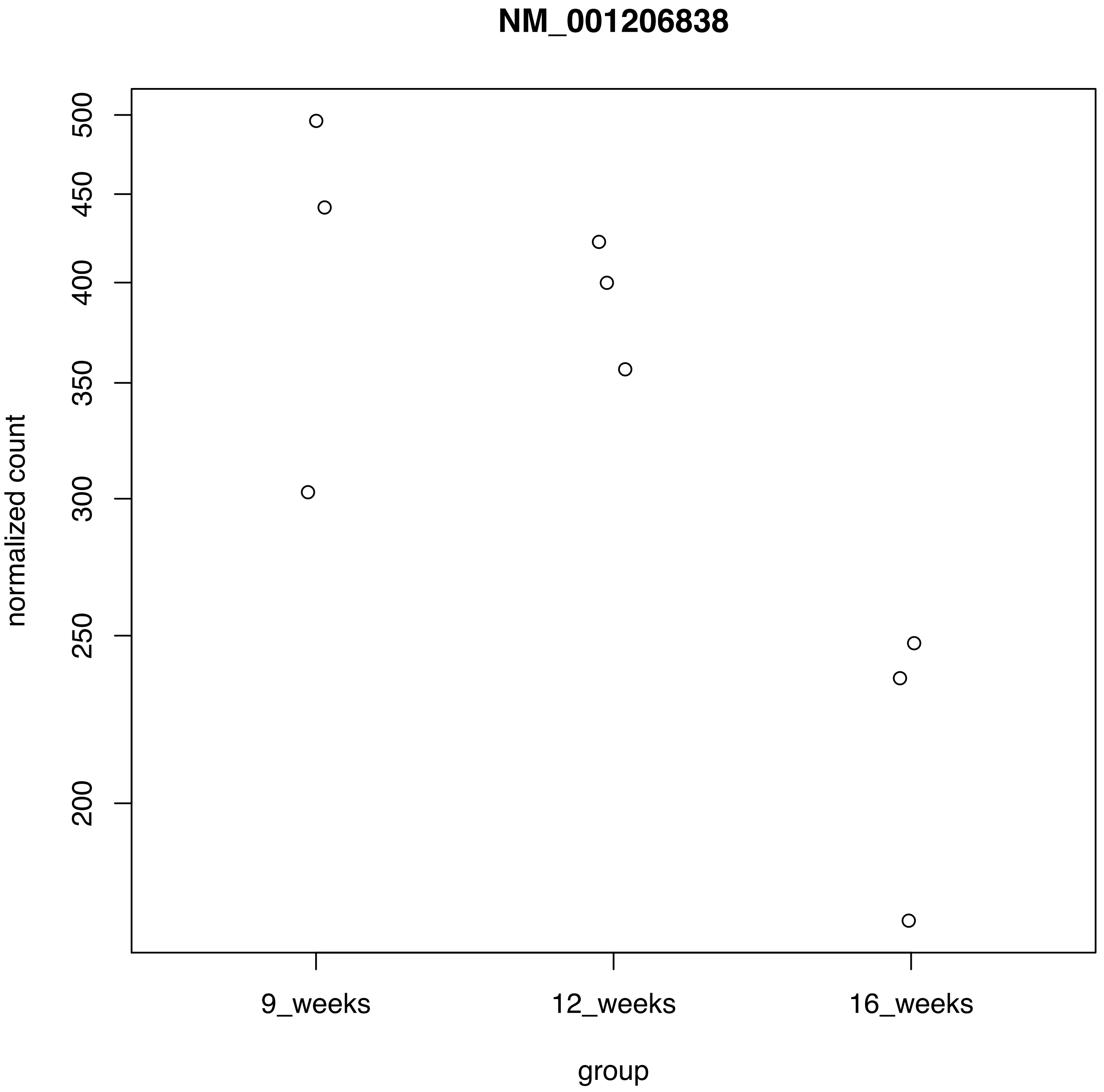
NM_001206838
normalized count
500
450
400
350
300
250
200
9_weeks
12_weeks
16_weeks
group

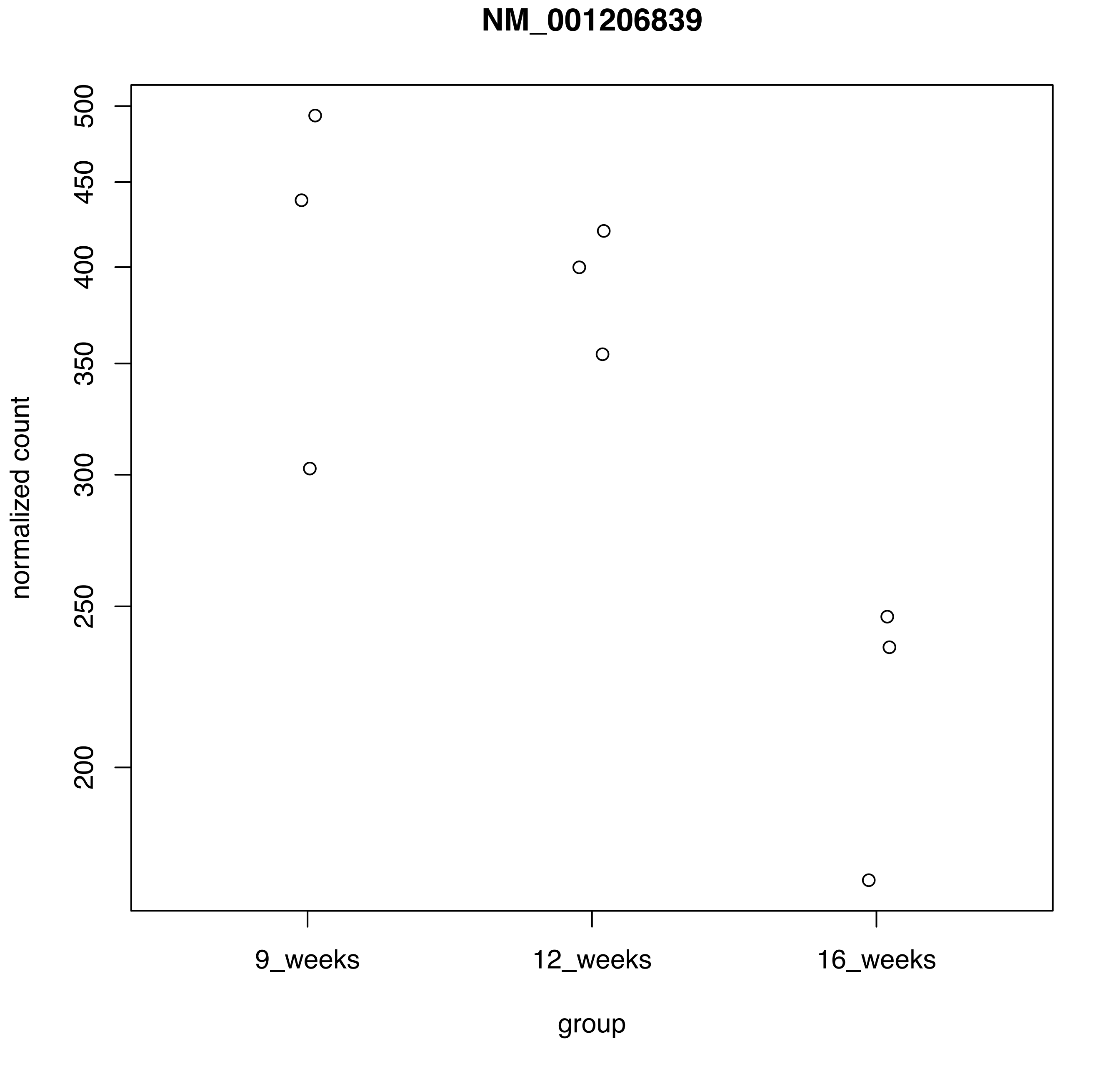
NM_001206839
normalized count
500
450
400
350
300
250
200
9_weeks
12_weeks
16_weeks
group

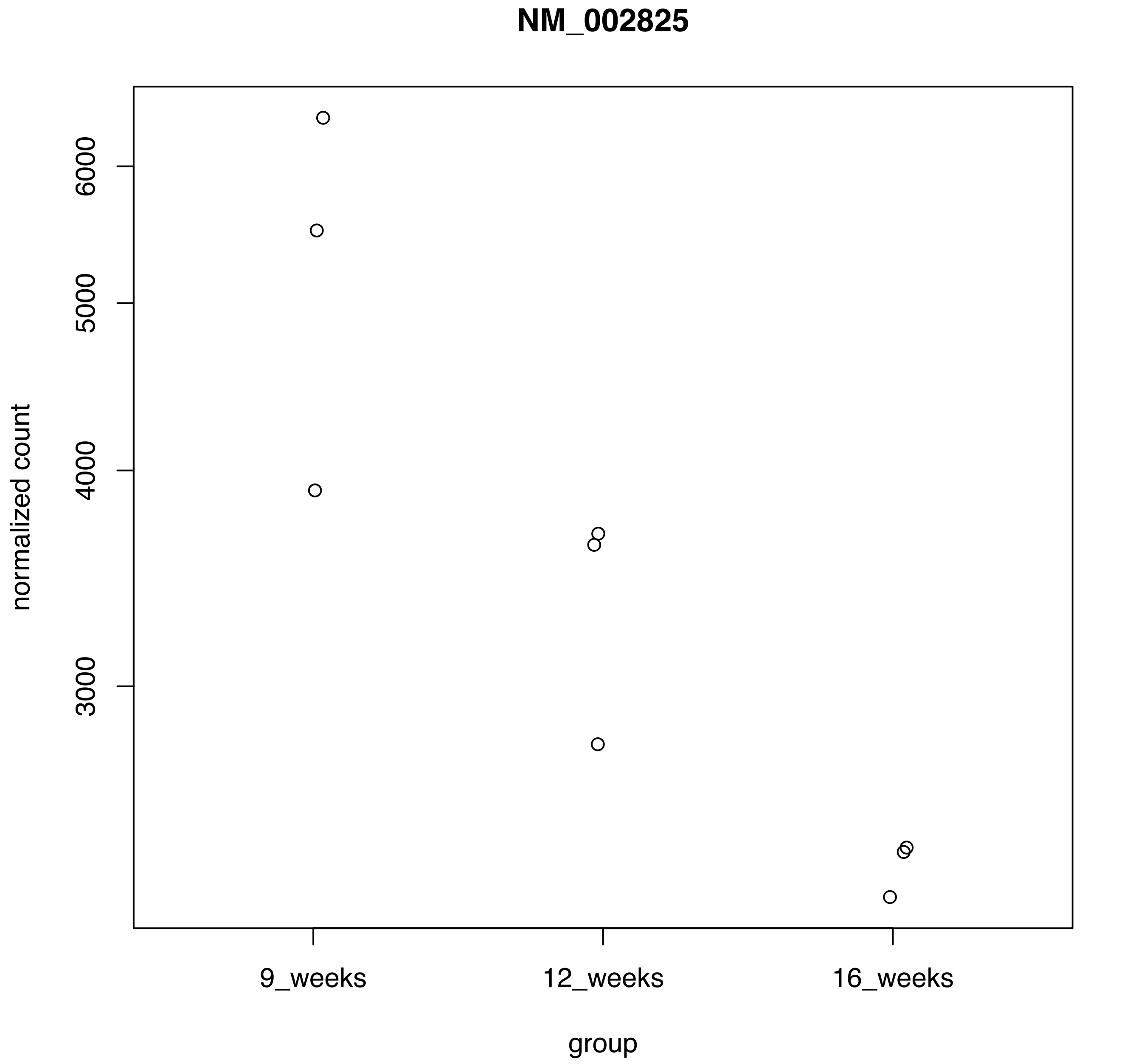
NM_002825
normalized count
6000
5000
4000
3000
9_weeks
12_weeks
16_weeks
group

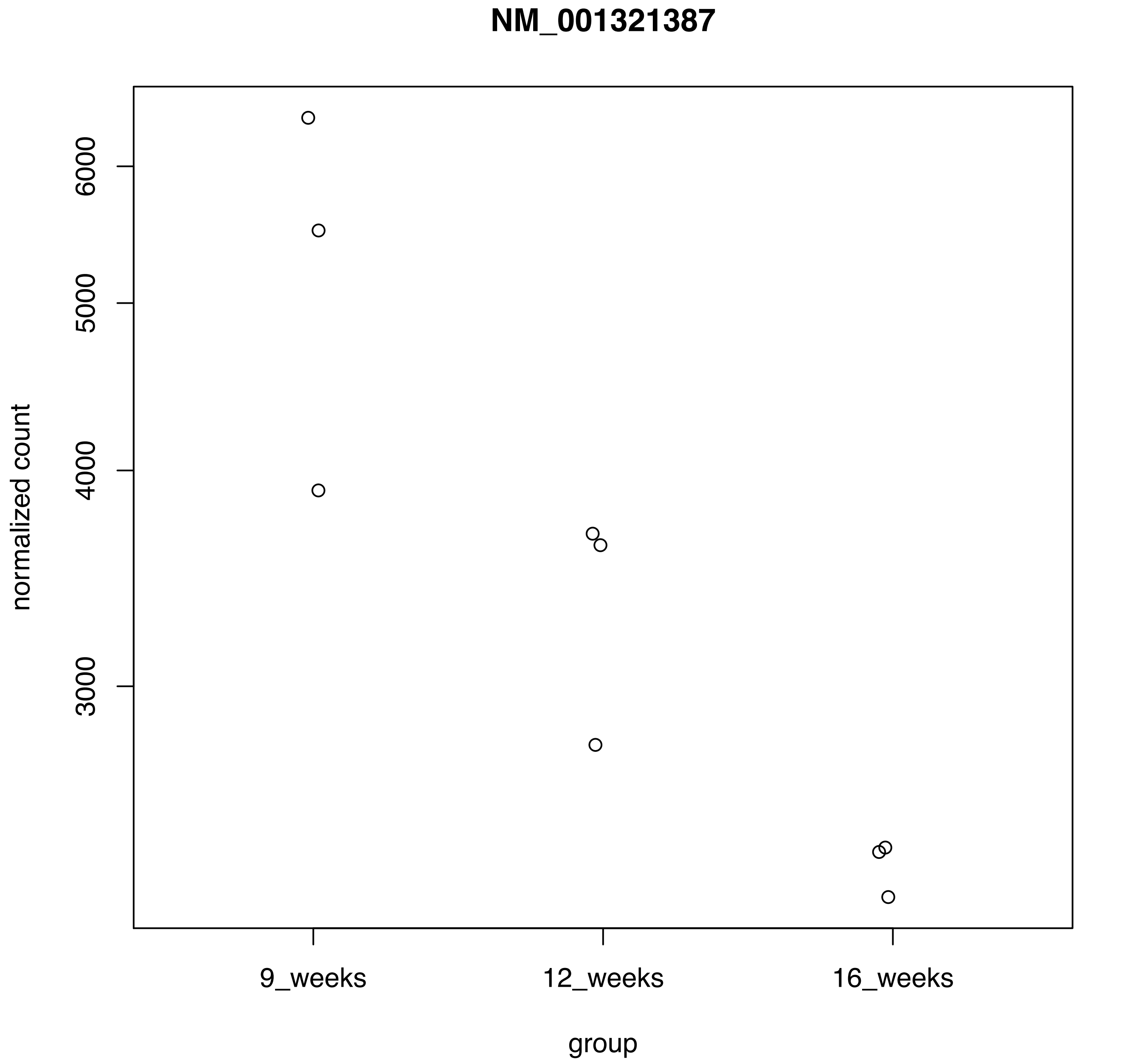

NM_001321387
6000
5000
4000
3000
normalized count
9_weeks
12_weeks
16_weeks
group

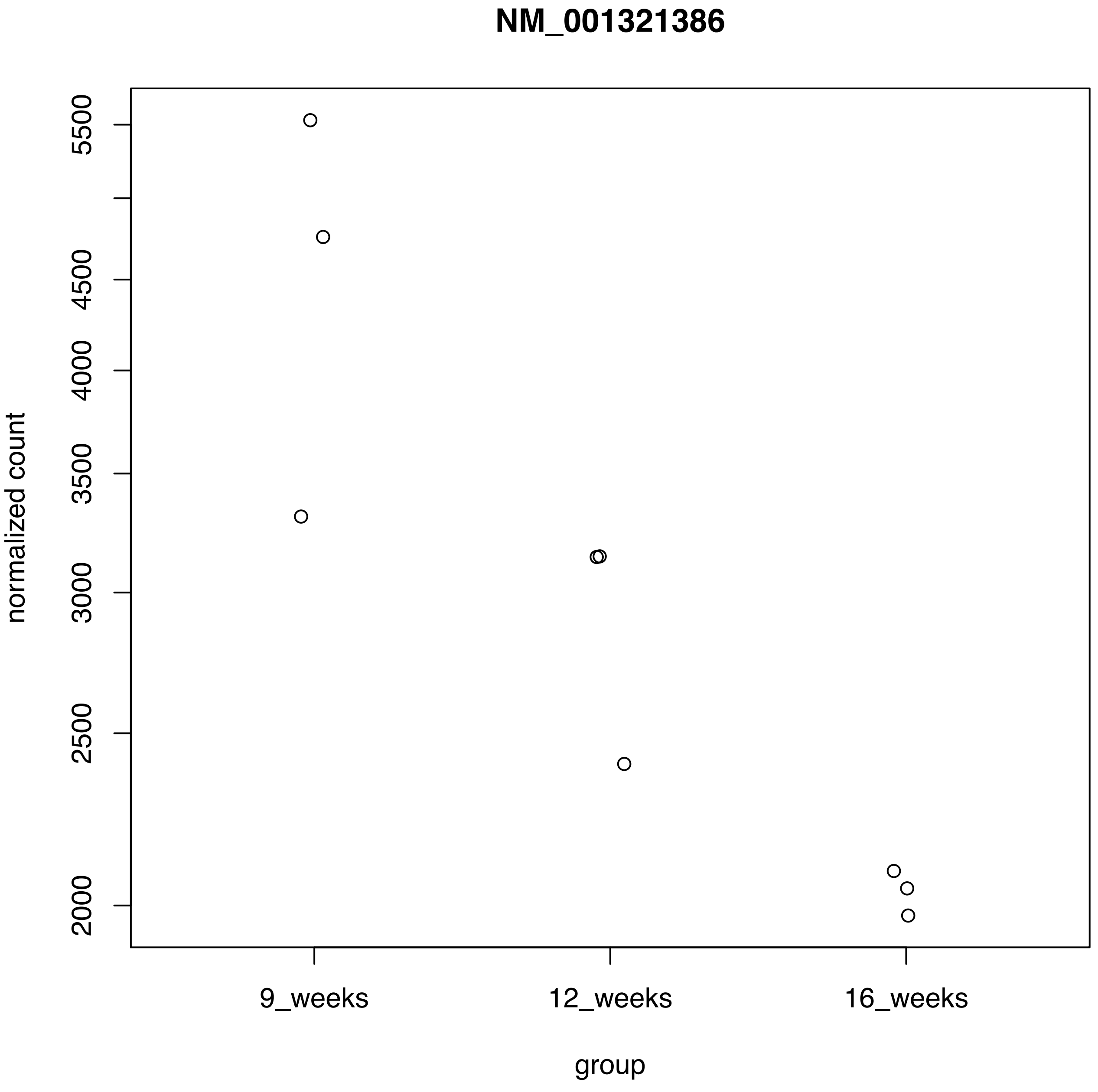
NM_001321386
normalized count
5500
4500
4000
3500
3000
2500
2000
9_weeks
12_weeks
16_weeks
group

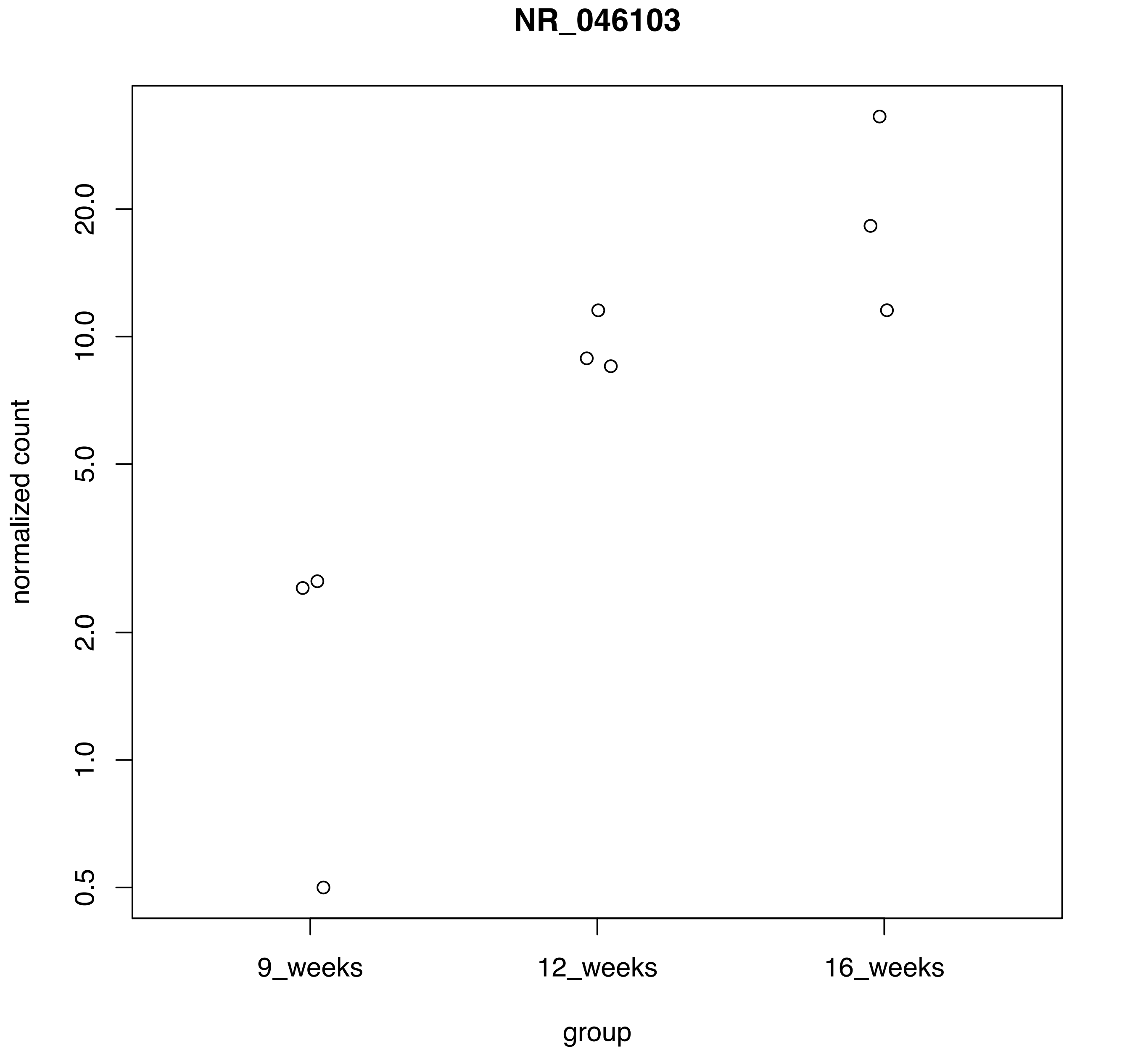
NR_046103
normalized count
0.5
1.0
2.0
5.0
10.0
20.0
9_weeks
12_weeks
16_weeks
group

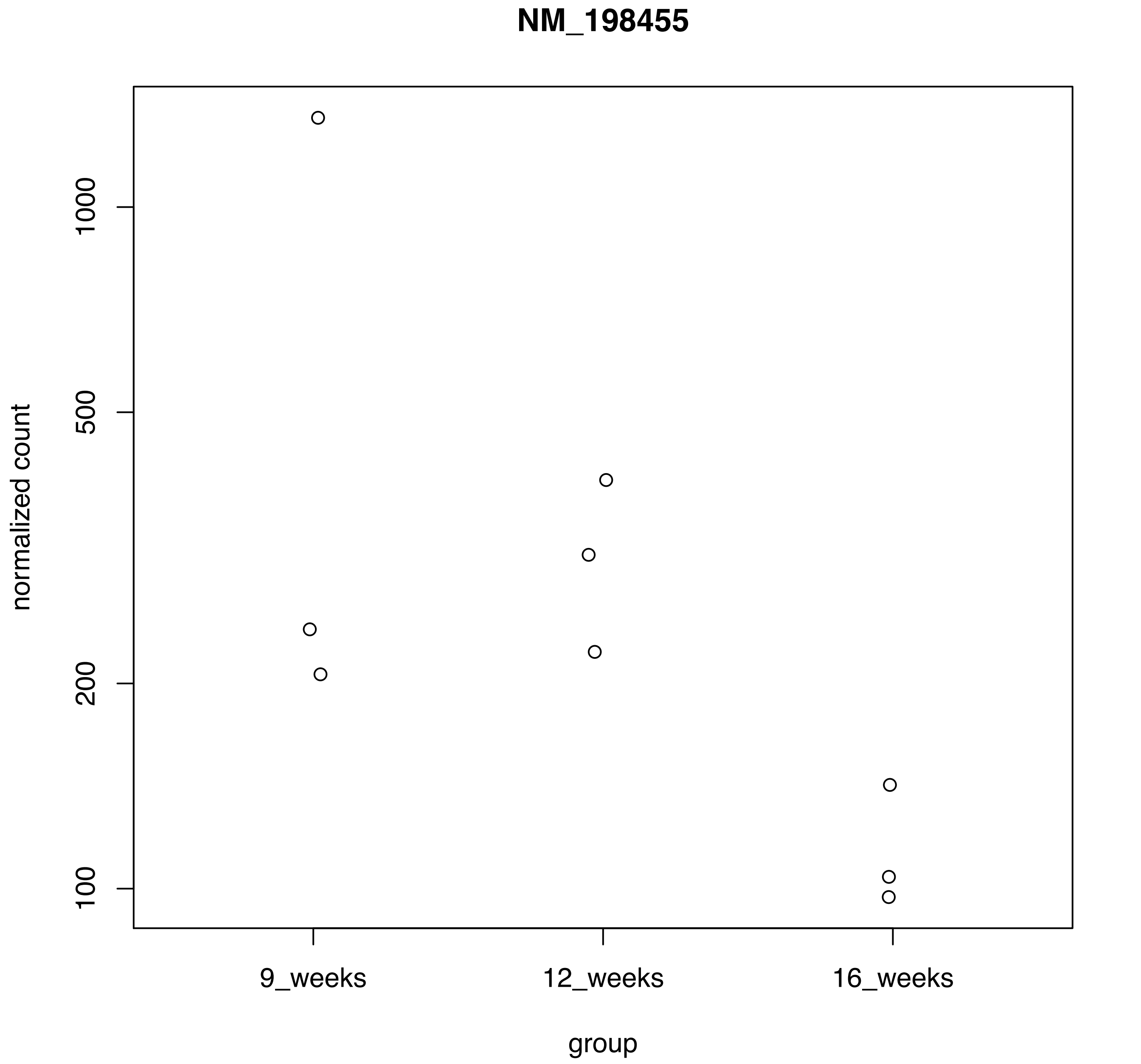
NM_198455
normalized count
1000
500
200
100
9_weeks
12_weeks
16_weeks
group

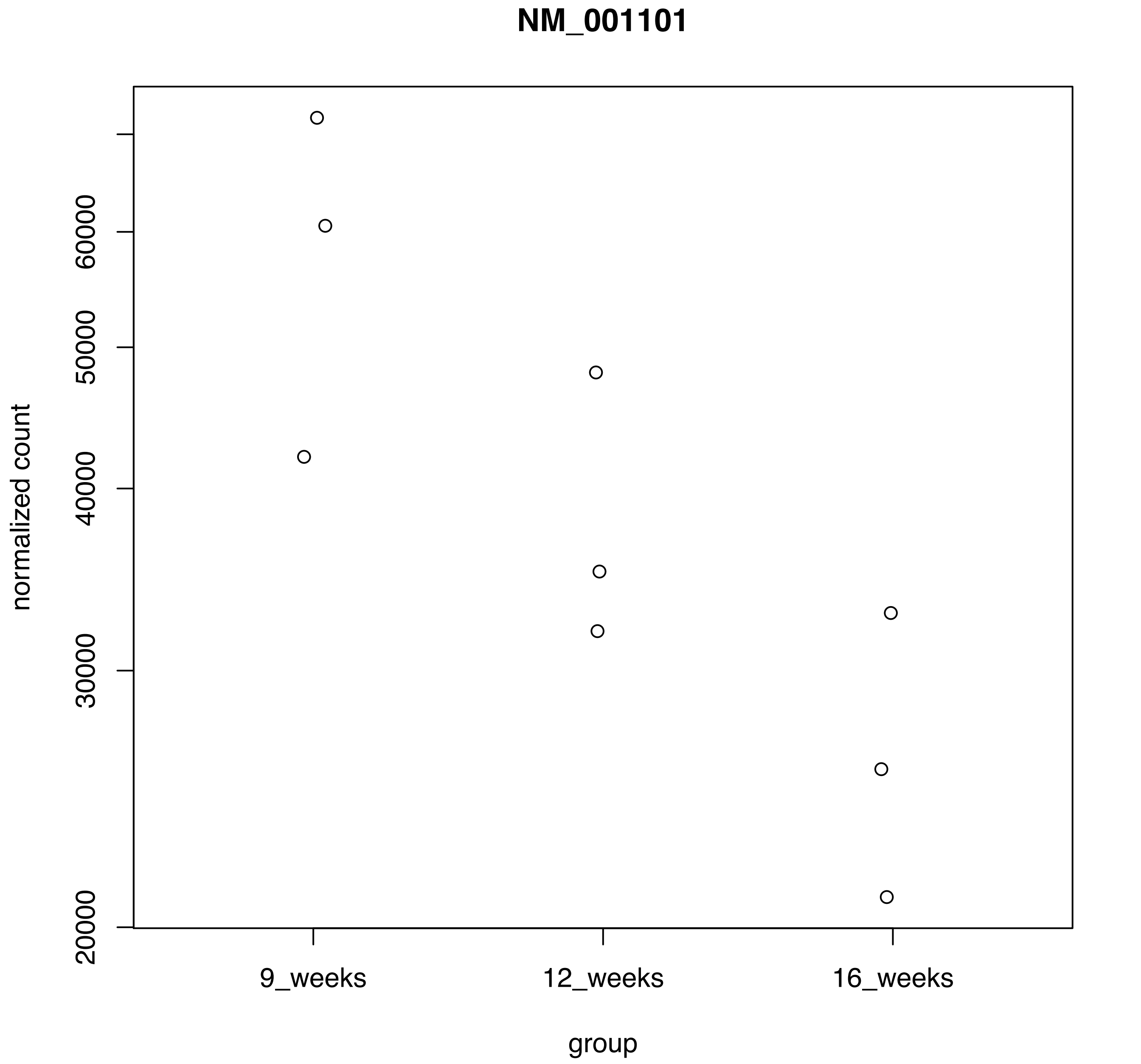

NM_001101
normalized count
20000
30000
40000
50000
60000
9_weeks
12_weeks
16_weeks
group

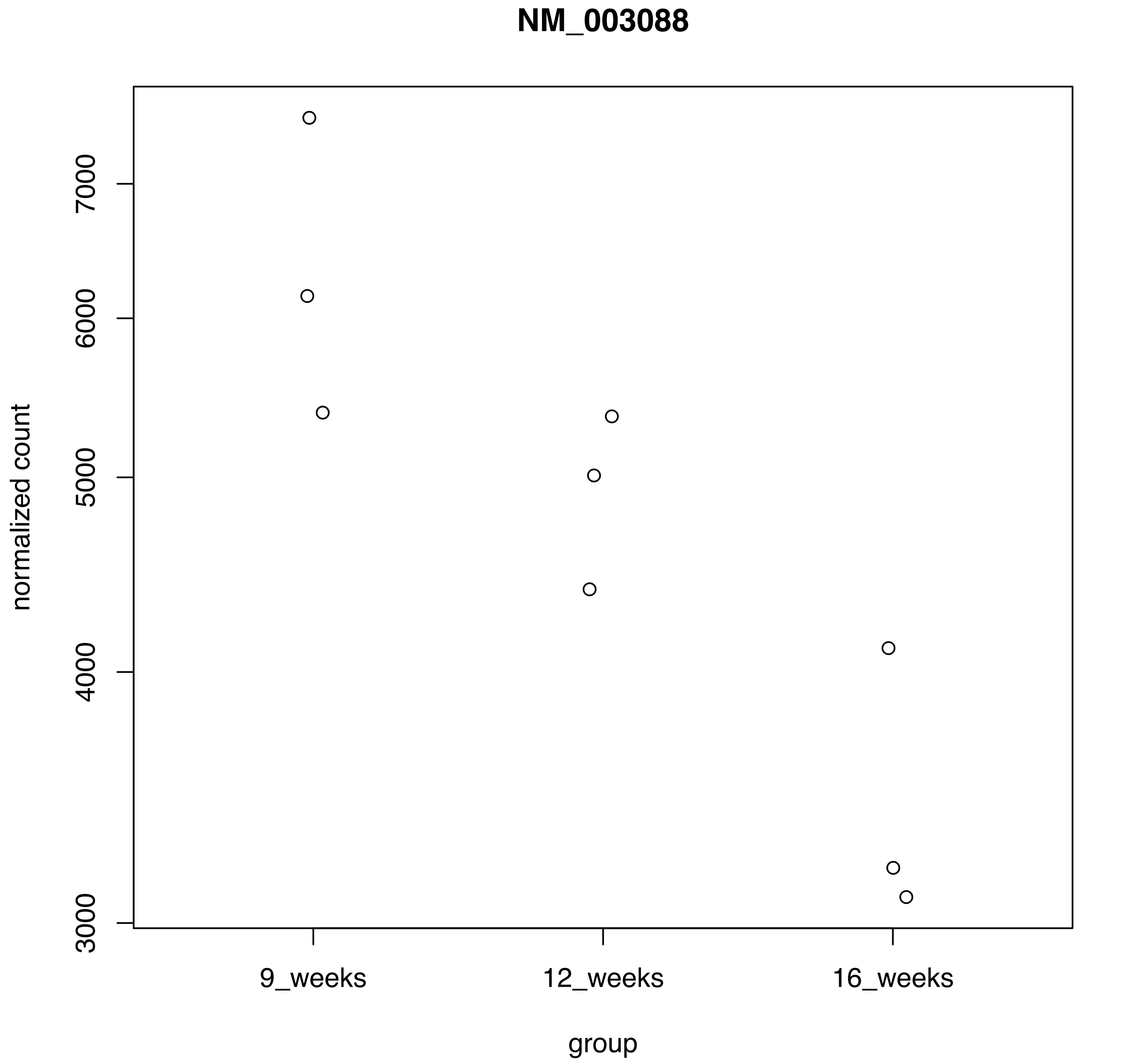
NM_003088
normalized count
7000
6000
5000
4000
3000
9_weeks
12_weeks
16_weeks
group

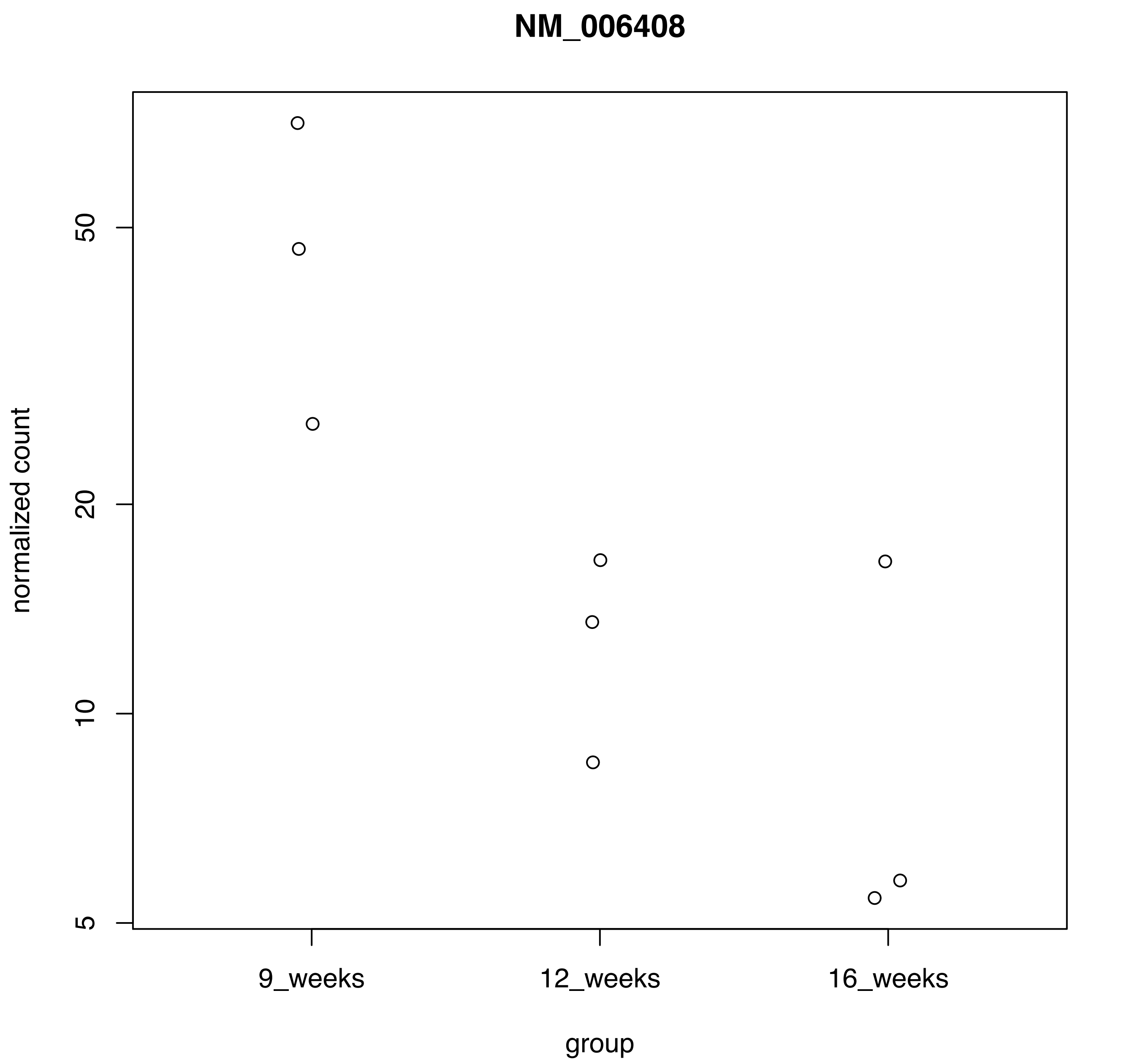
NM_006408
normalized count
50
20
10
5
9_weeks
12_weeks
16_weeks
group

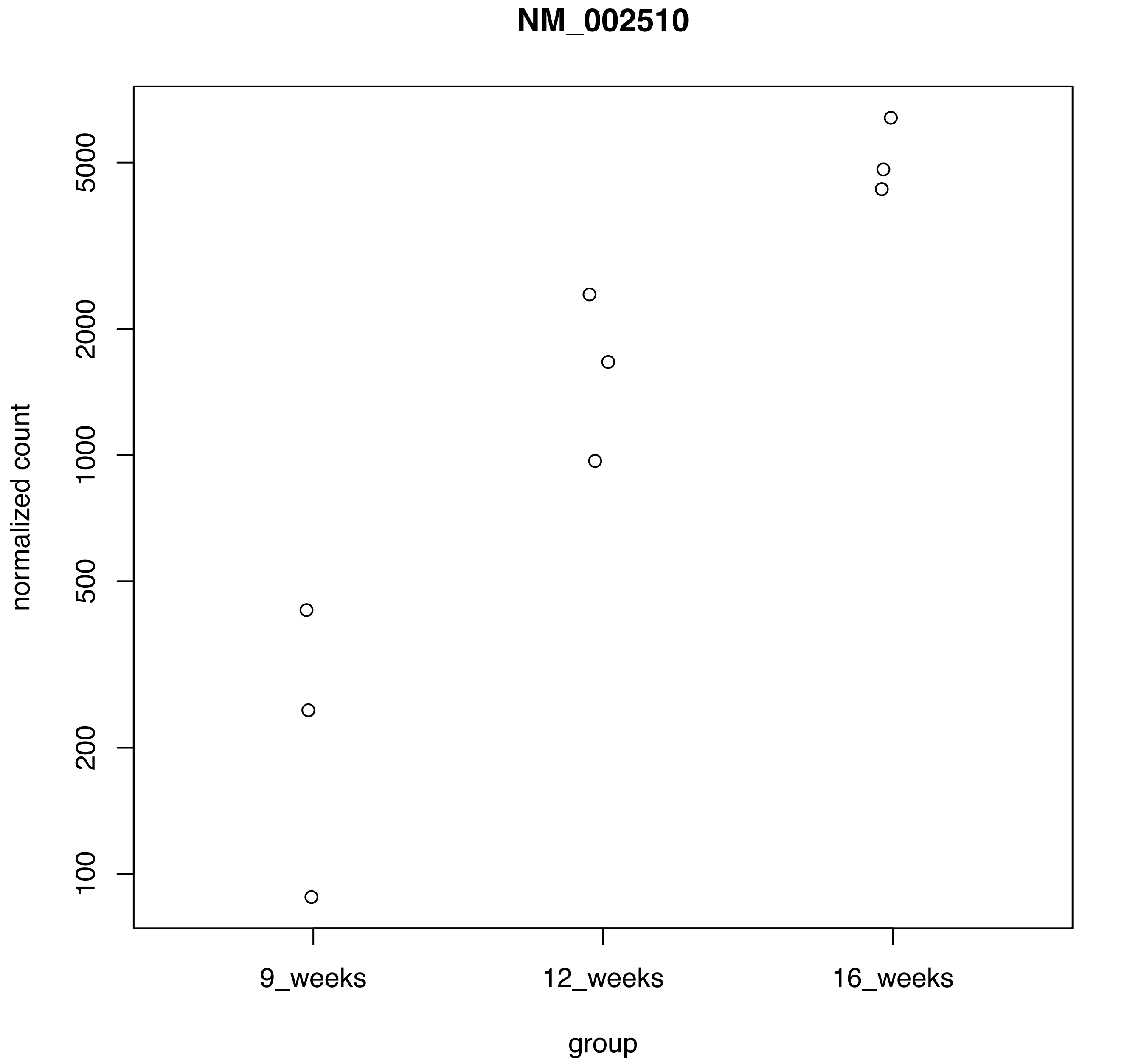

NM_002510
normalized count
5000
2000
1000
500
200
100
9_weeks
12_weeks
16_weeks
group

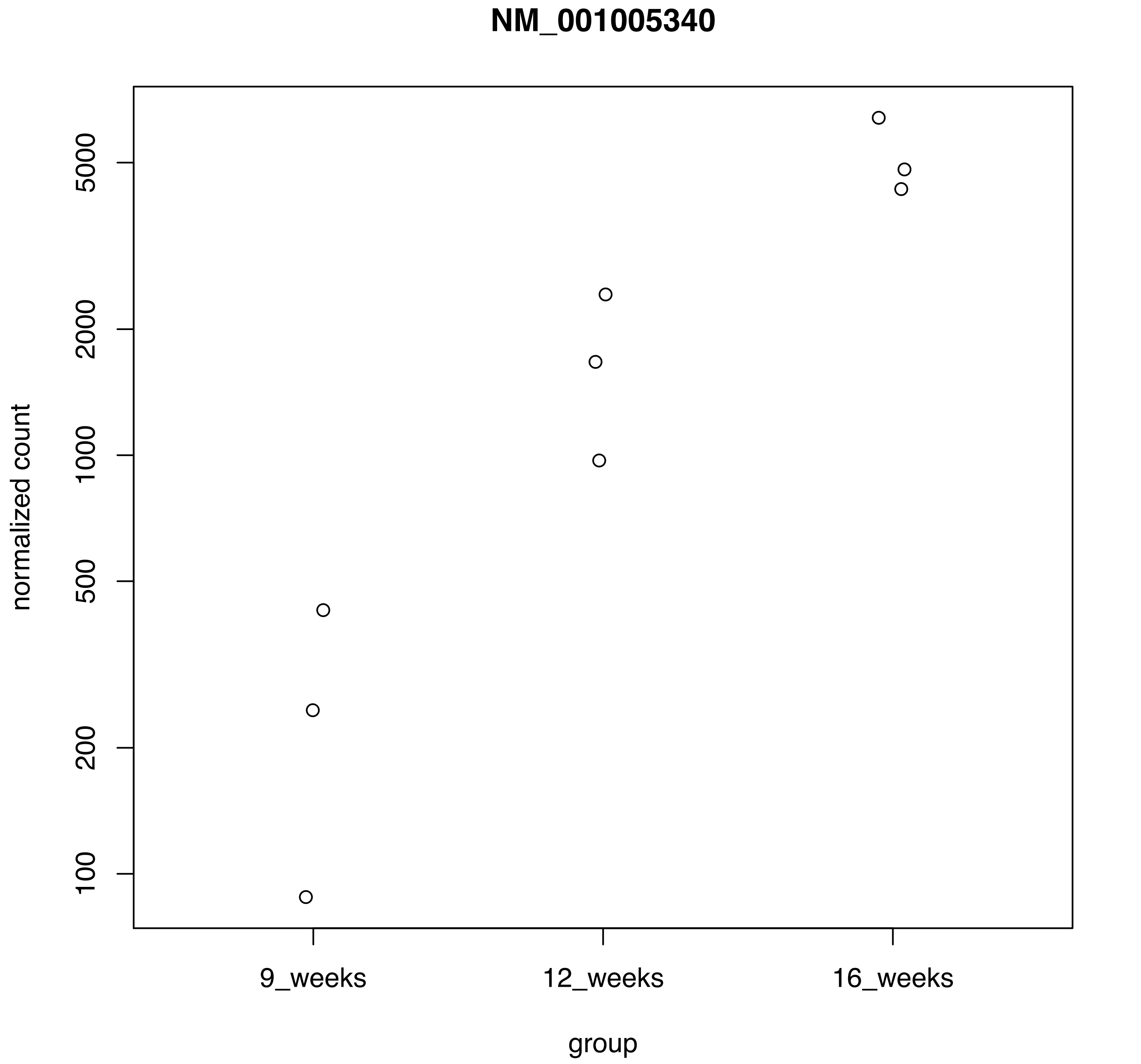
NM_001005340
normalized count
5000
2000
1000
500
200
100
9_weeks
12_weeks
16_weeks
group

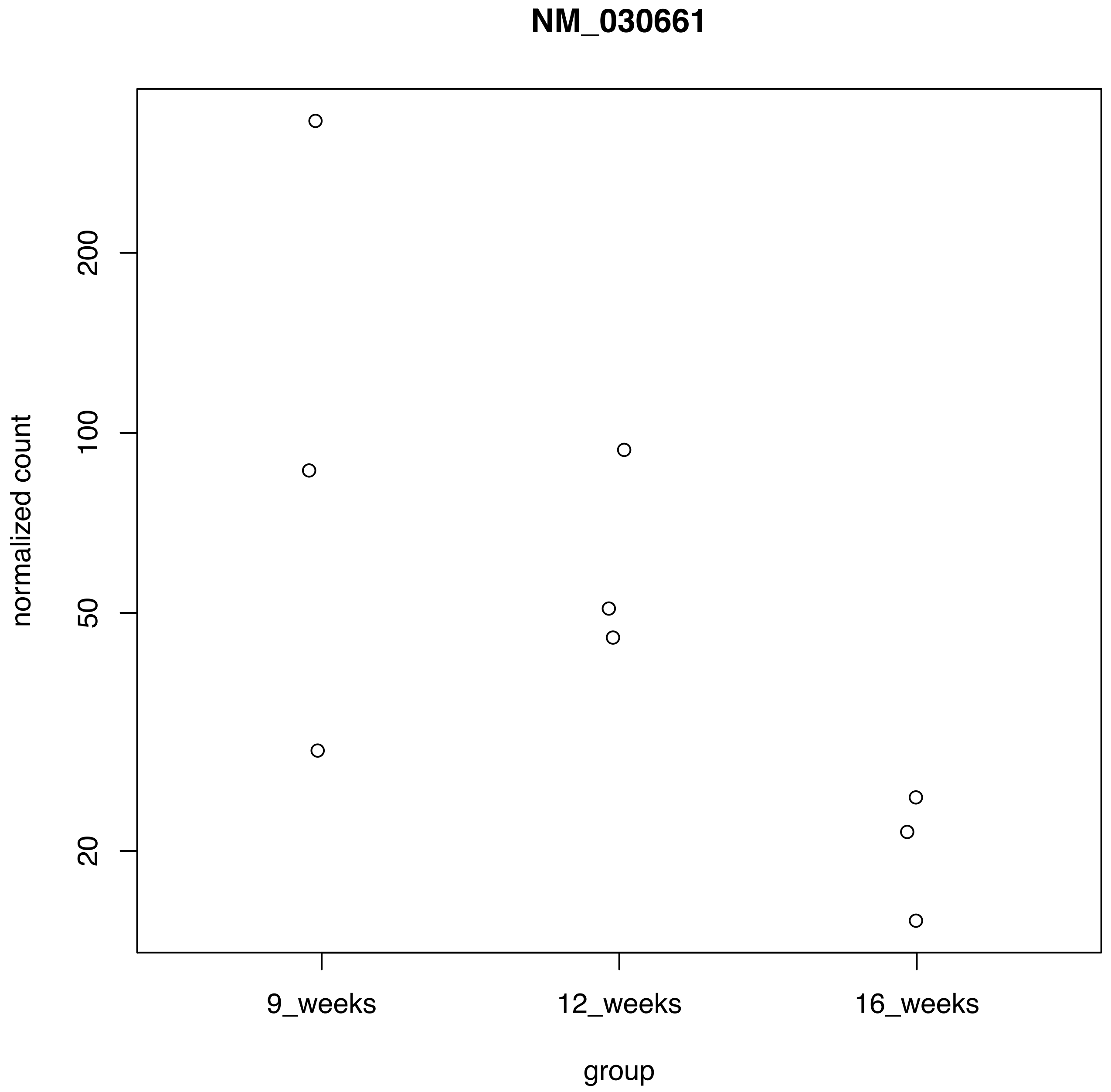
NM_030661
normalized count
200
100
50
20
9_weeks
12_weeks
16_weeks
group

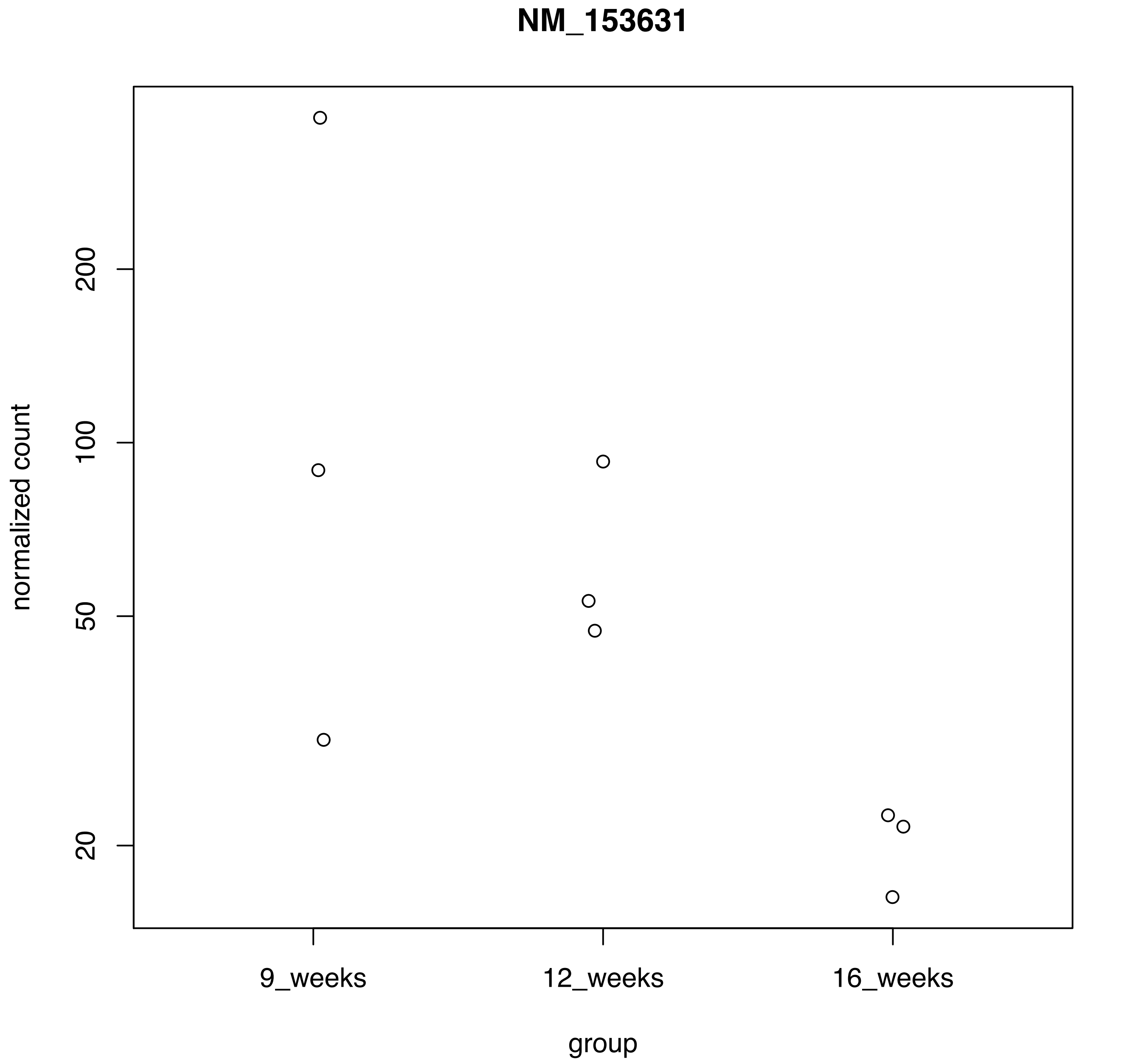

NM_153631
normalized count
200
100
50
20
9_weeks
12_weeks
16_weeks
group

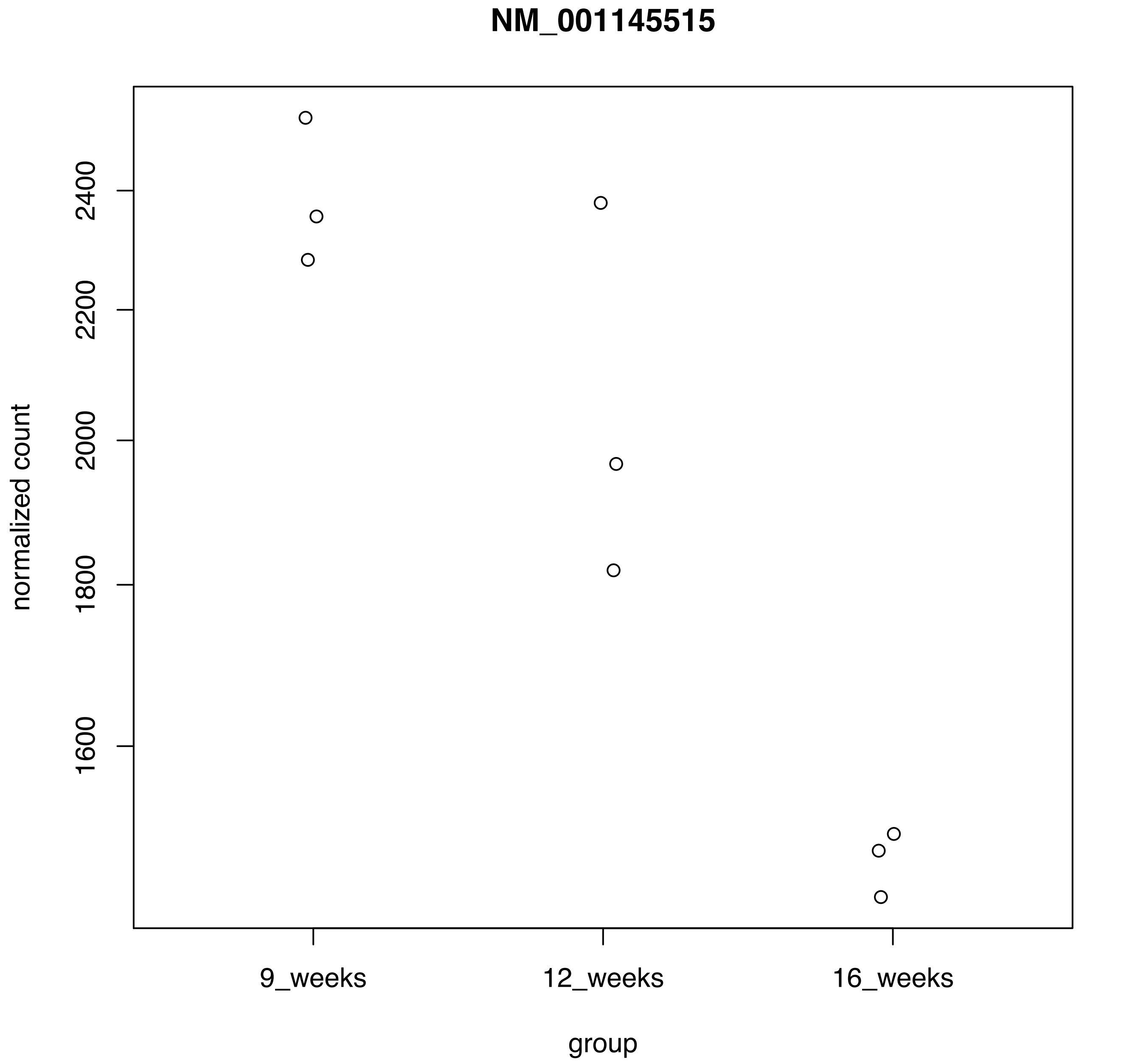

NM_001145515
normalized count
2400
2200
2000
1800
1600
9_weeks
12_weeks
16_weeks
group

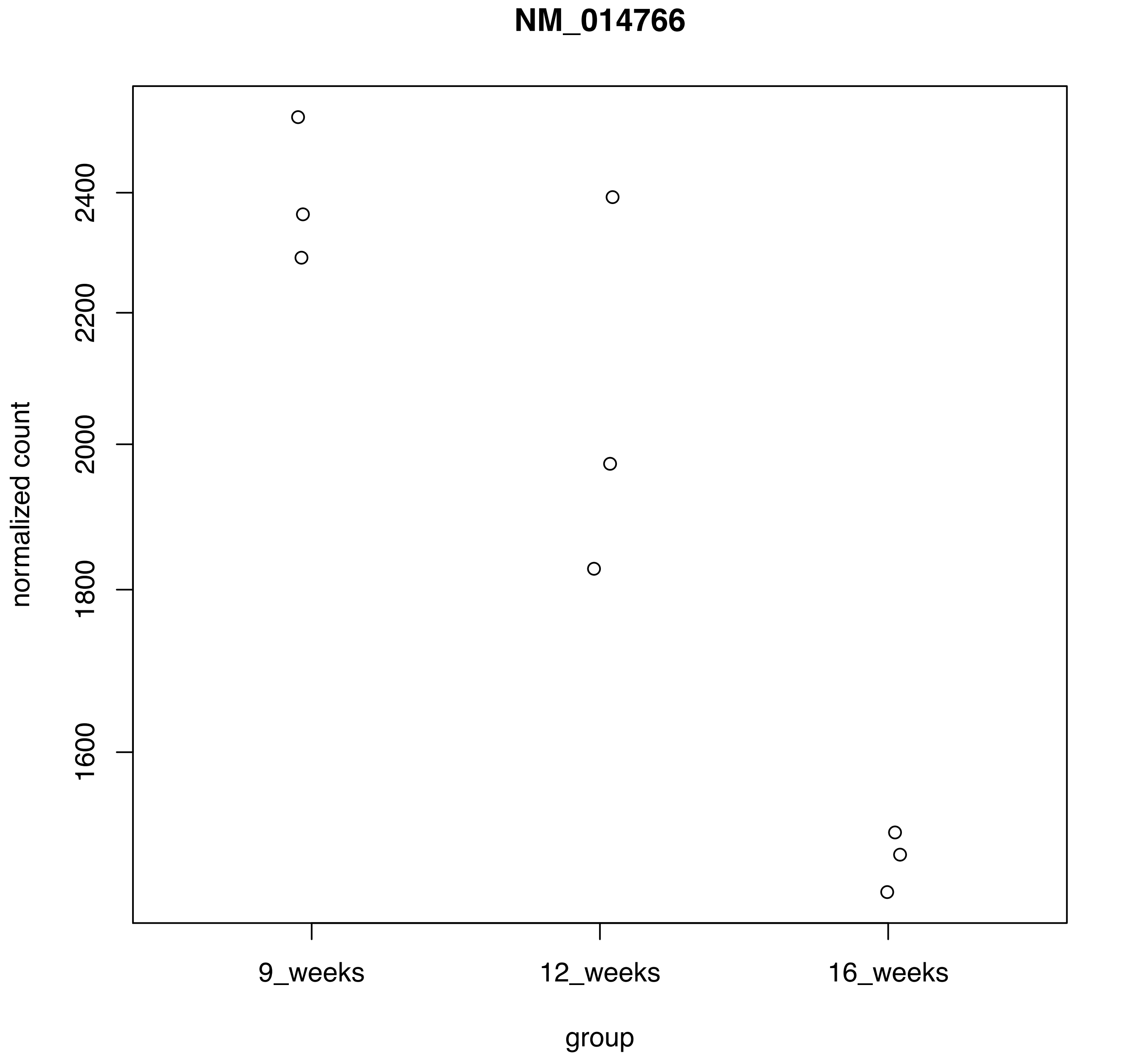

NM_014766
normalized count
2400
2200
2000
1800
1600
9_weeks
12_weeks
16_weeks
group

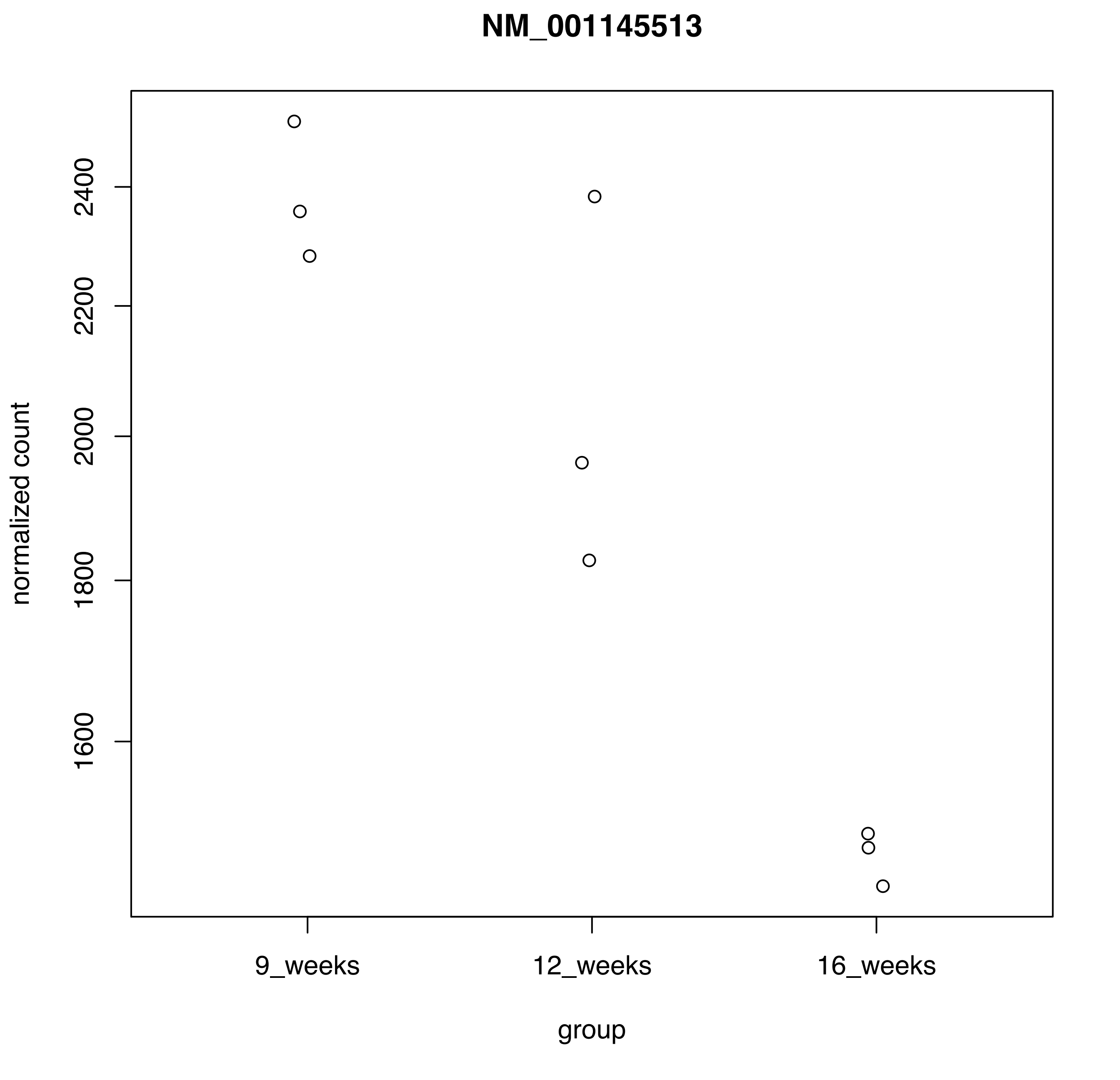

NM_001145513
normalized count
2400
2200
2000
1800
1600
9_weeks
12_weeks
16_weeks
group

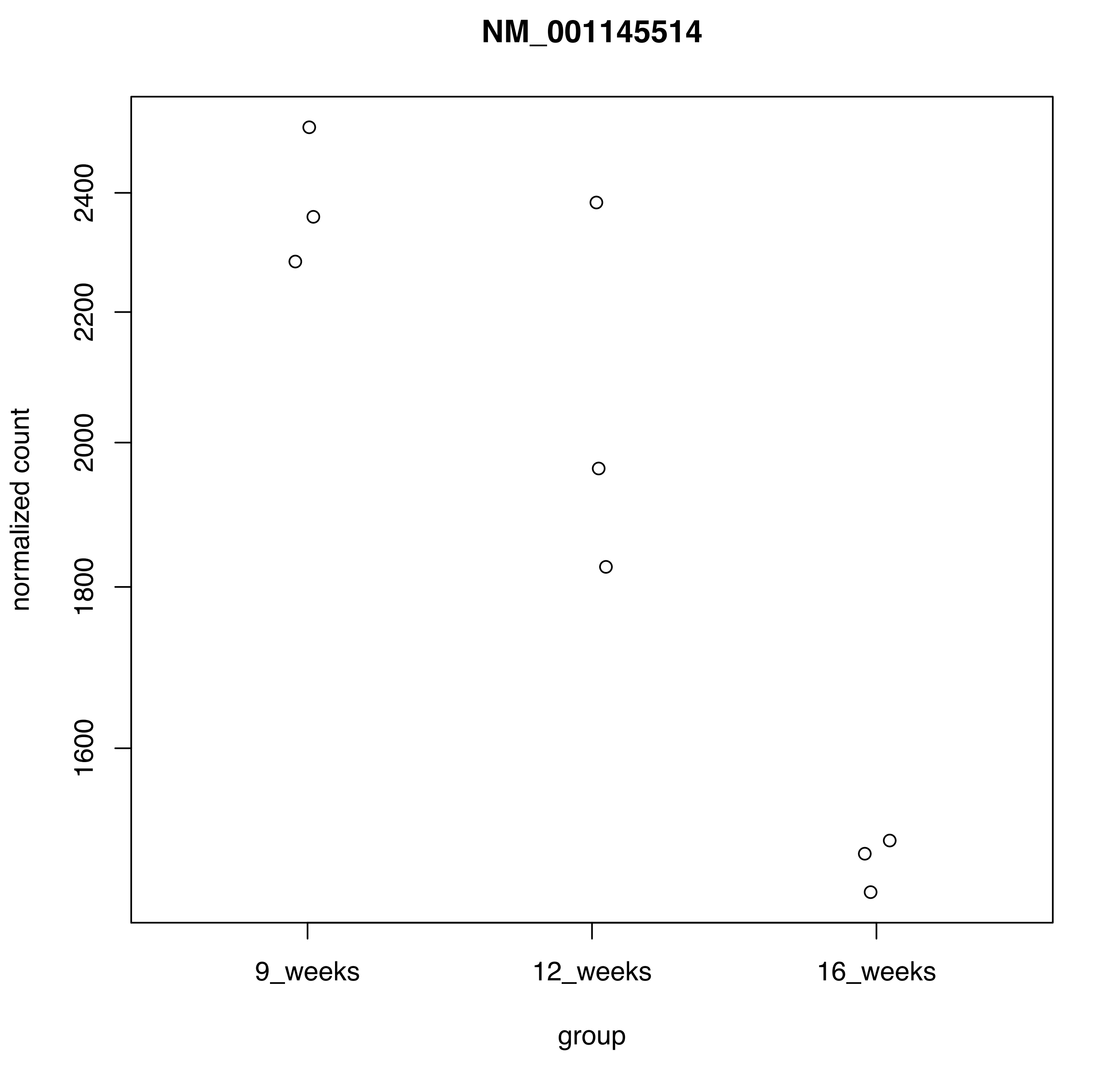
NM_001145514
normalized count
2400
2200
2000
1800
1600
9_weeks
12_weeks
16_weeks
group

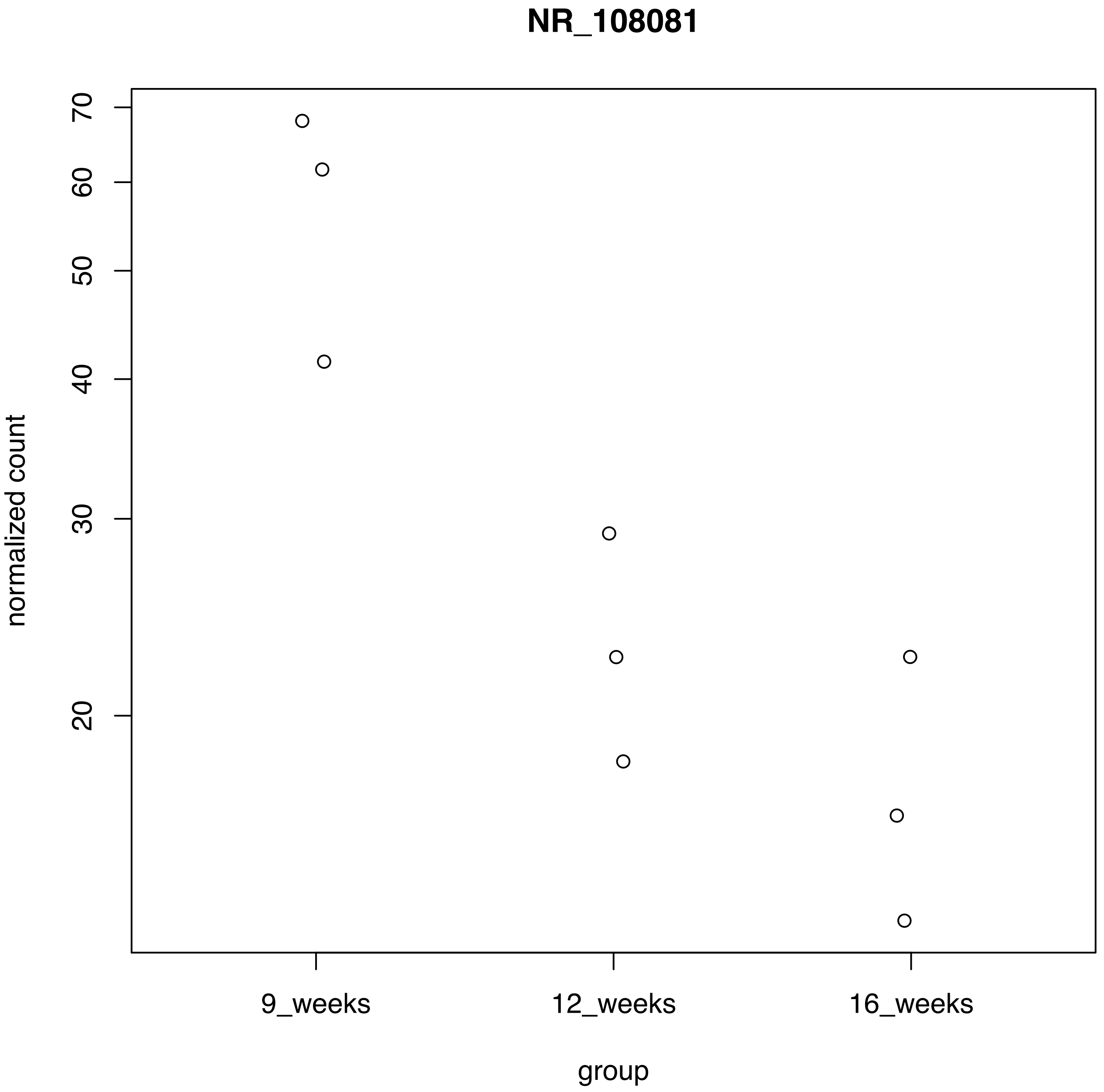
NR_108081
normalized count
70
60
50
40
30
20
9_weeks
12_weeks
16_weeks
group

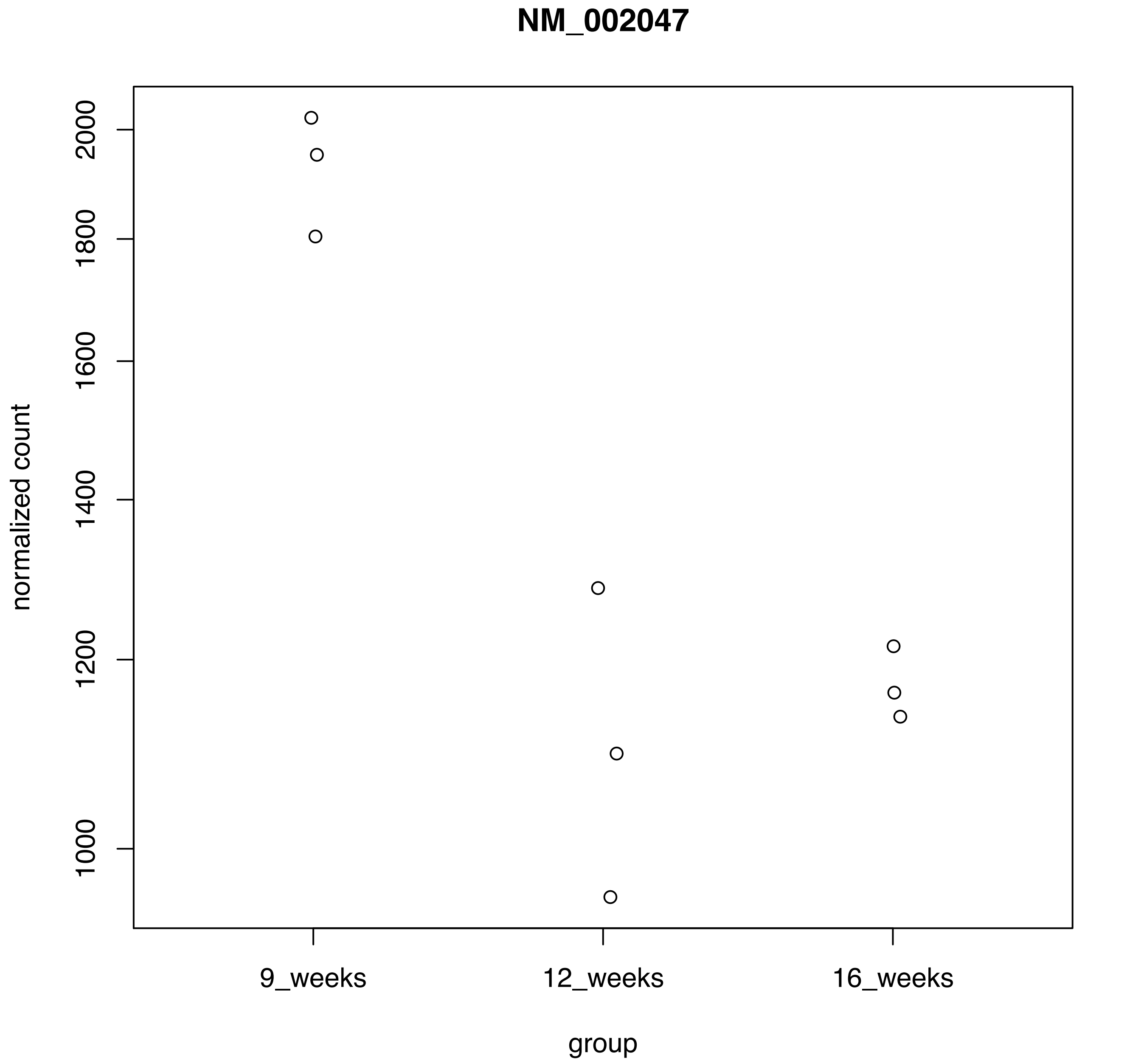
NM_002047
normalized count
1000
1200
1400
1600
1800
2000
9_weeks
12_weeks
16_weeks
group

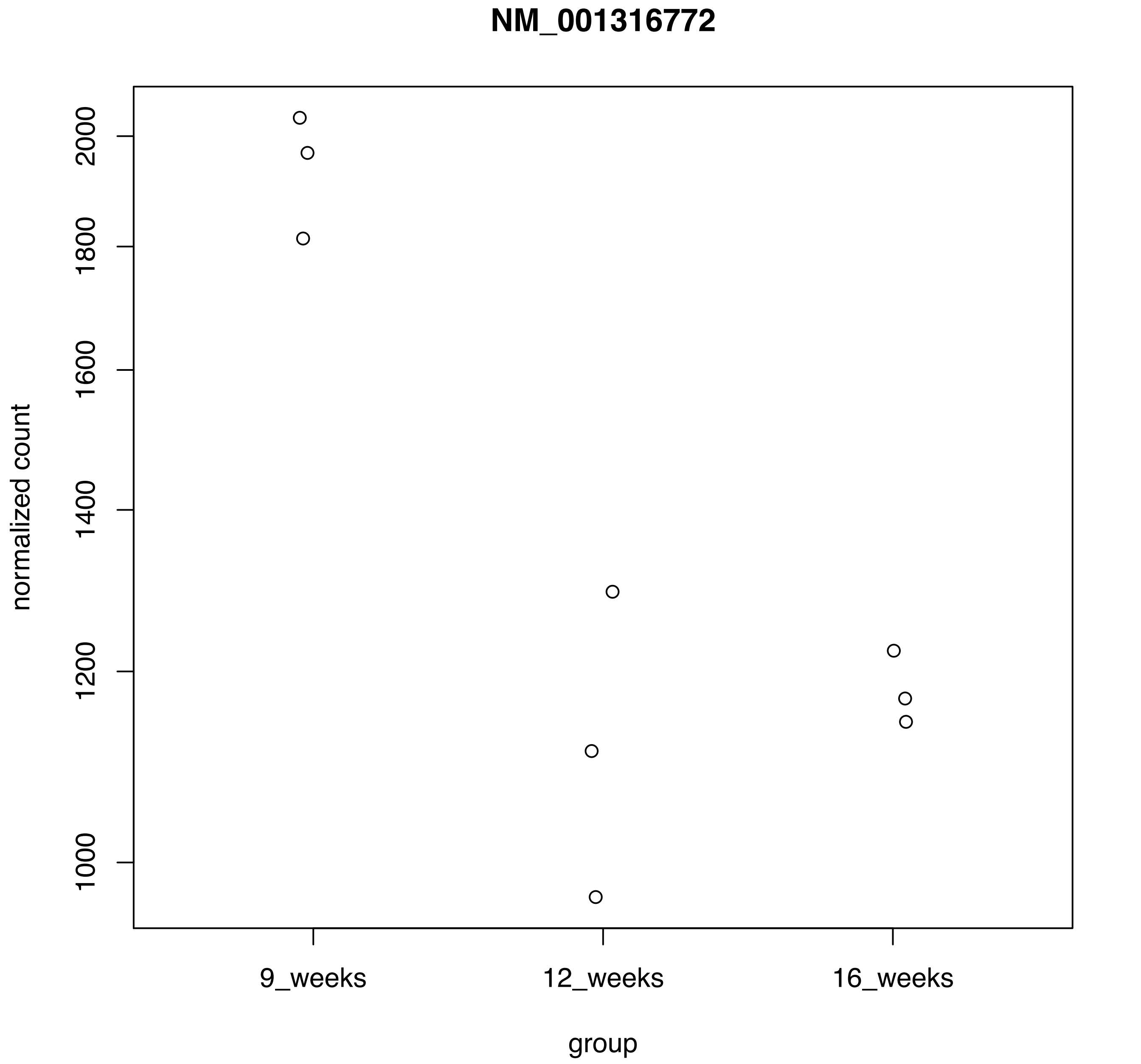
NM_001316772
normalized count
2000
1800
1600
1400
1200
1000
9_weeks
12_weeks
16_weeks
group

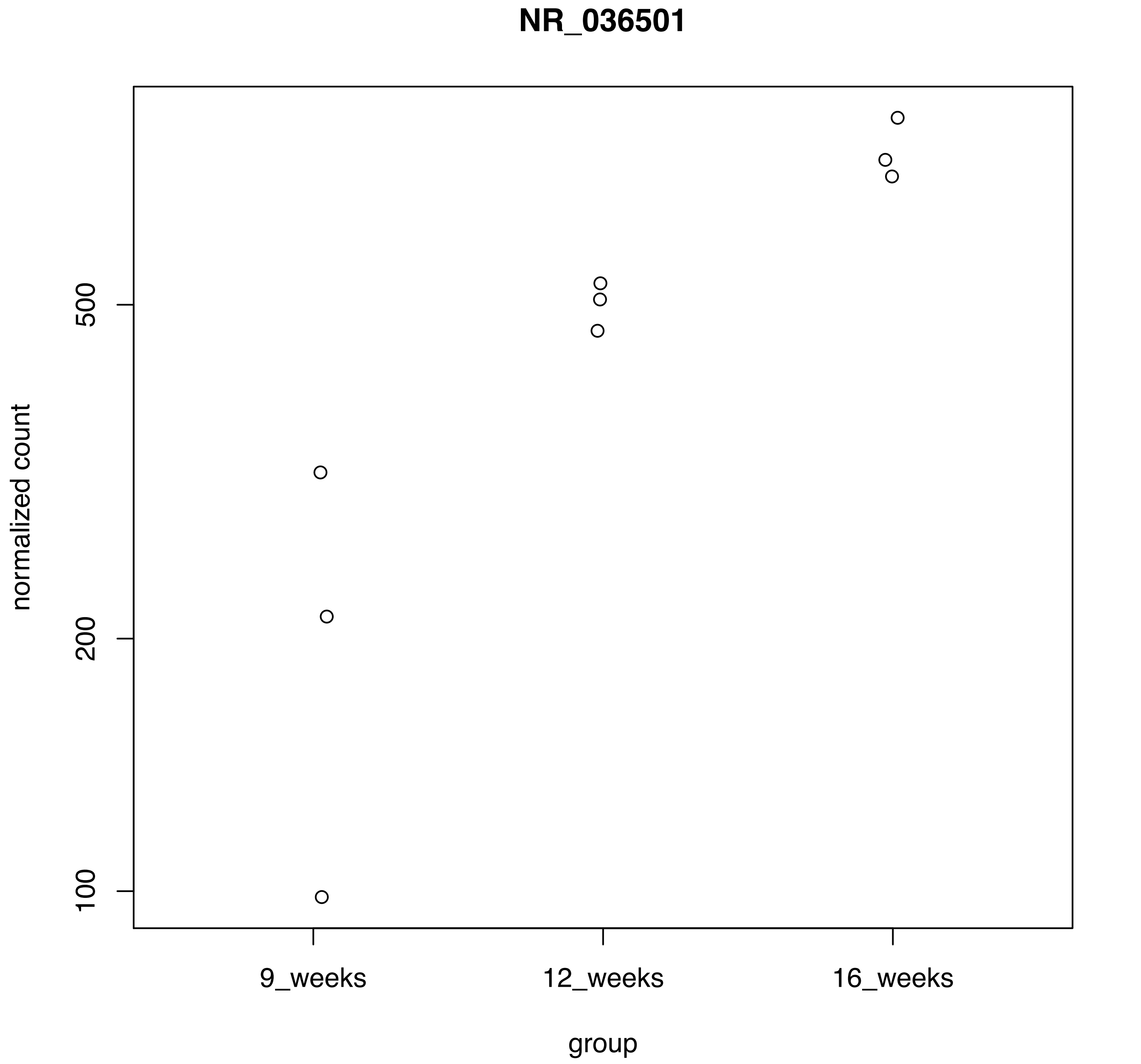
NR_036501
normalized count
100
200
500
9_weeks
12_weeks
16_weeks
group

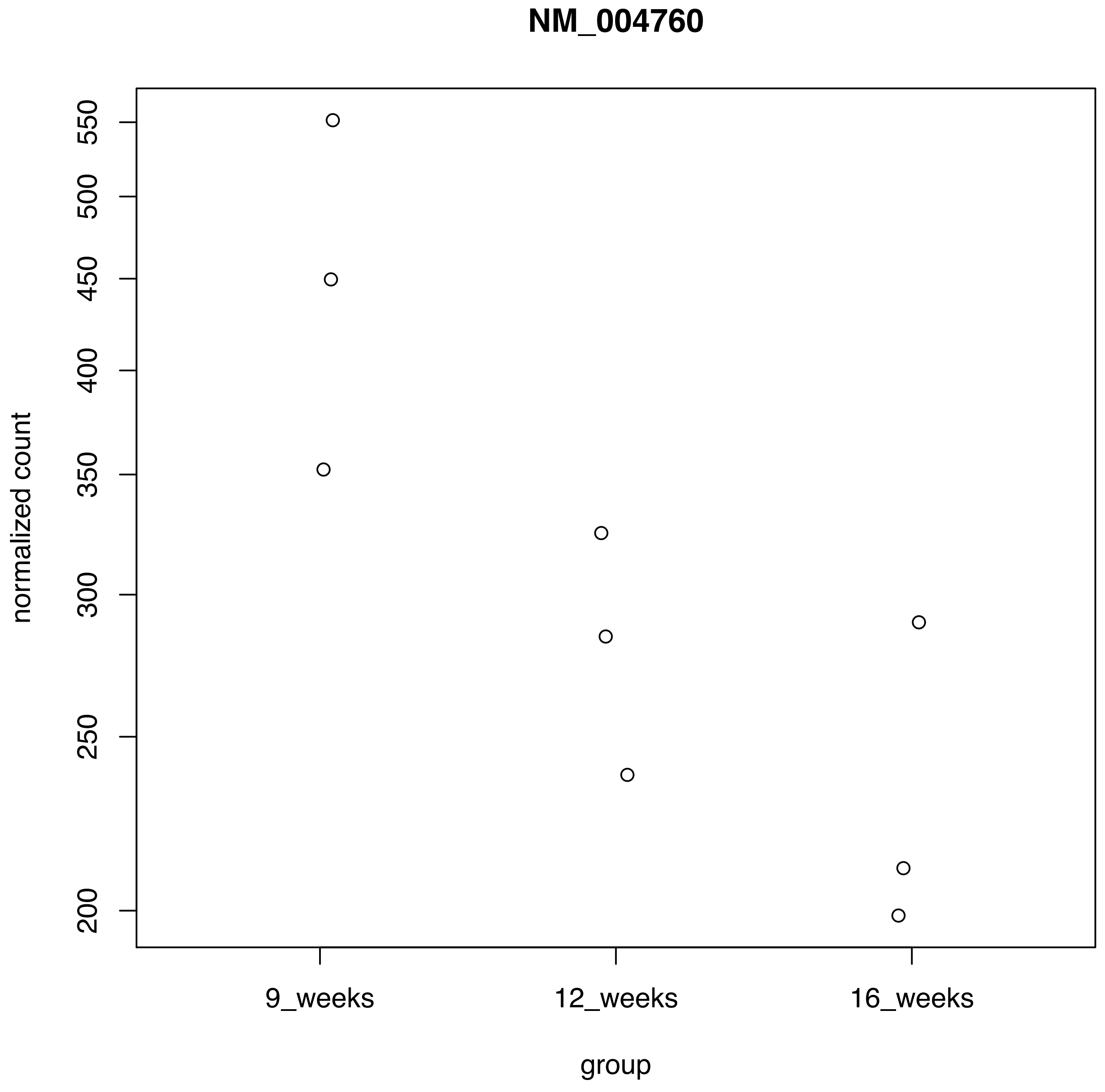

NM_004760
normalized count
550
500
450
400
350
300
250
200
9_weeks
12_weeks
16_weeks
group

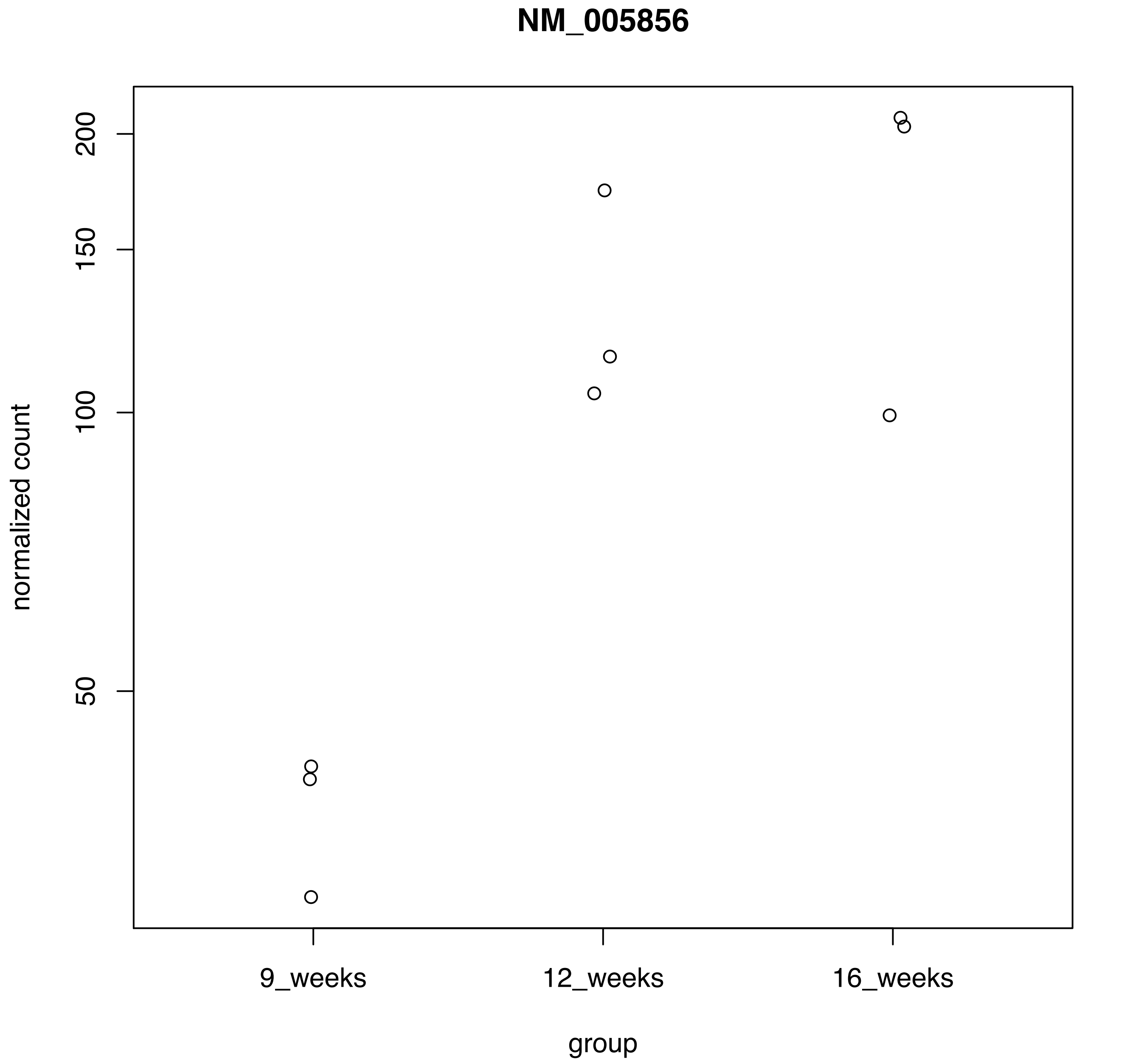
NM_005856
normalized count
200
150
100
50
9_weeks
12_weeks
16_weeks
group

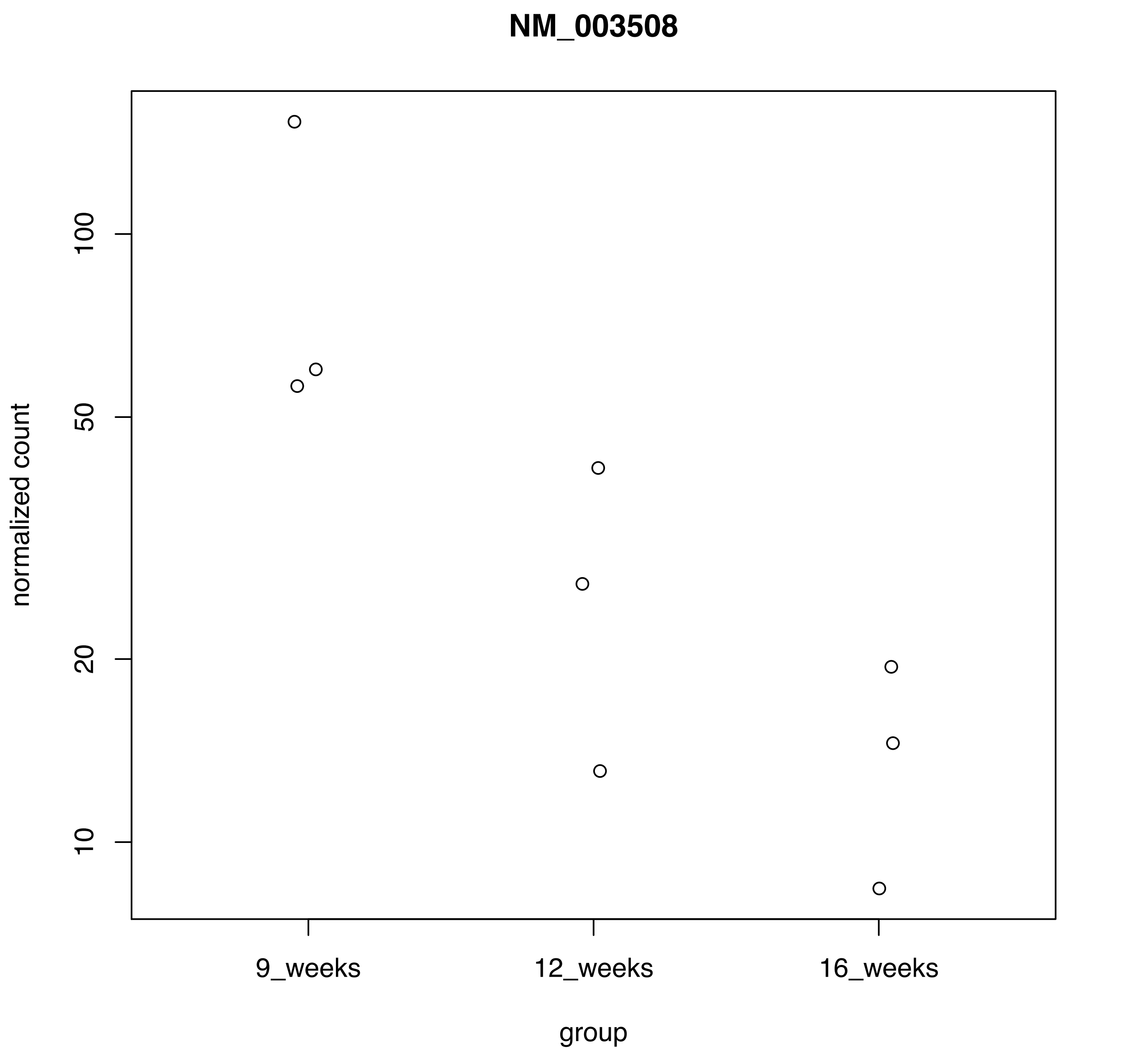
NM_003508
normalized count
100
50
20
10
9_weeks
12_weeks
16_weeks
group

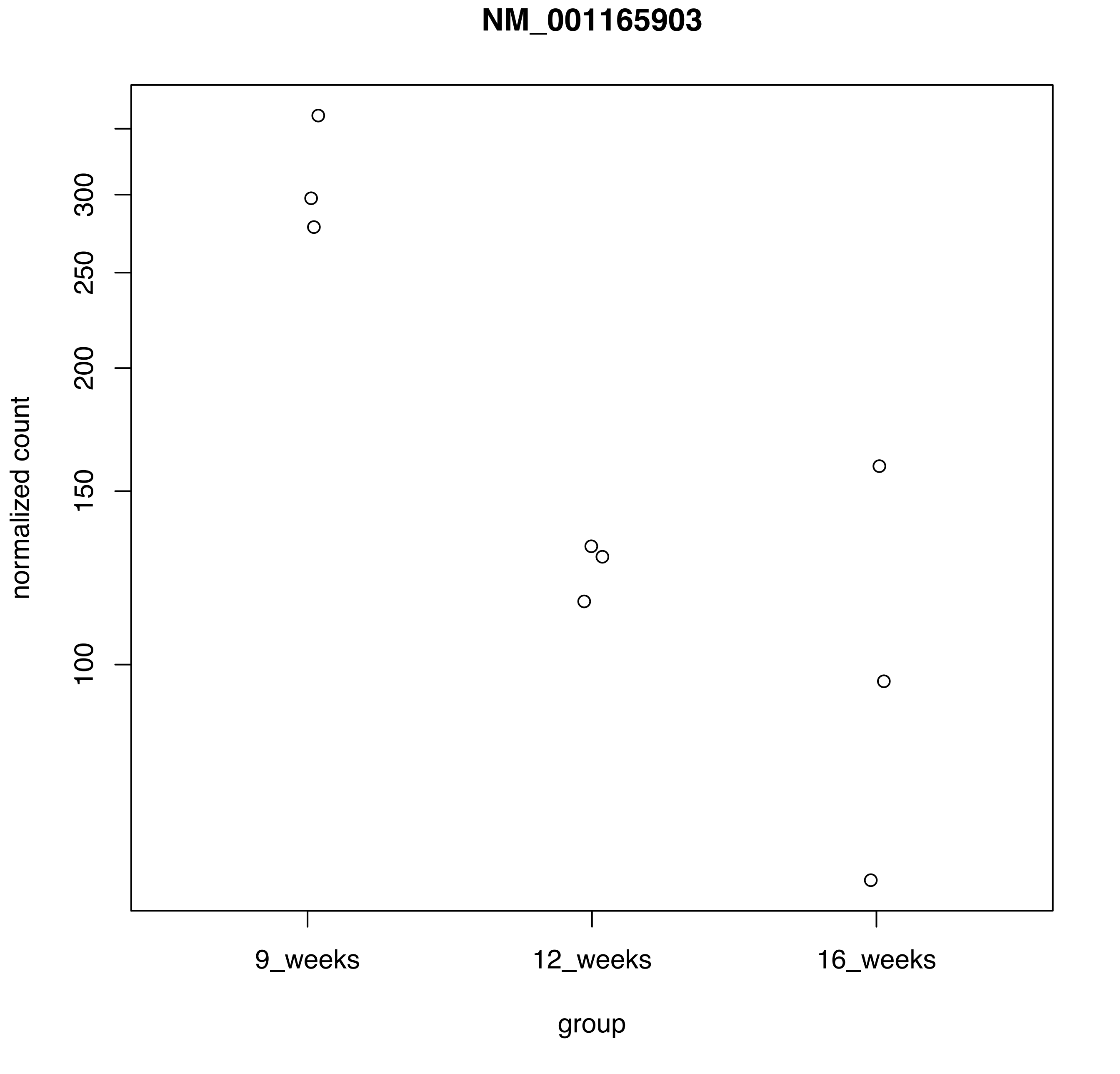
NM_001165903
normalized count
100
150
200
250
300
group
9_weeks
12_weeks
16_weeks

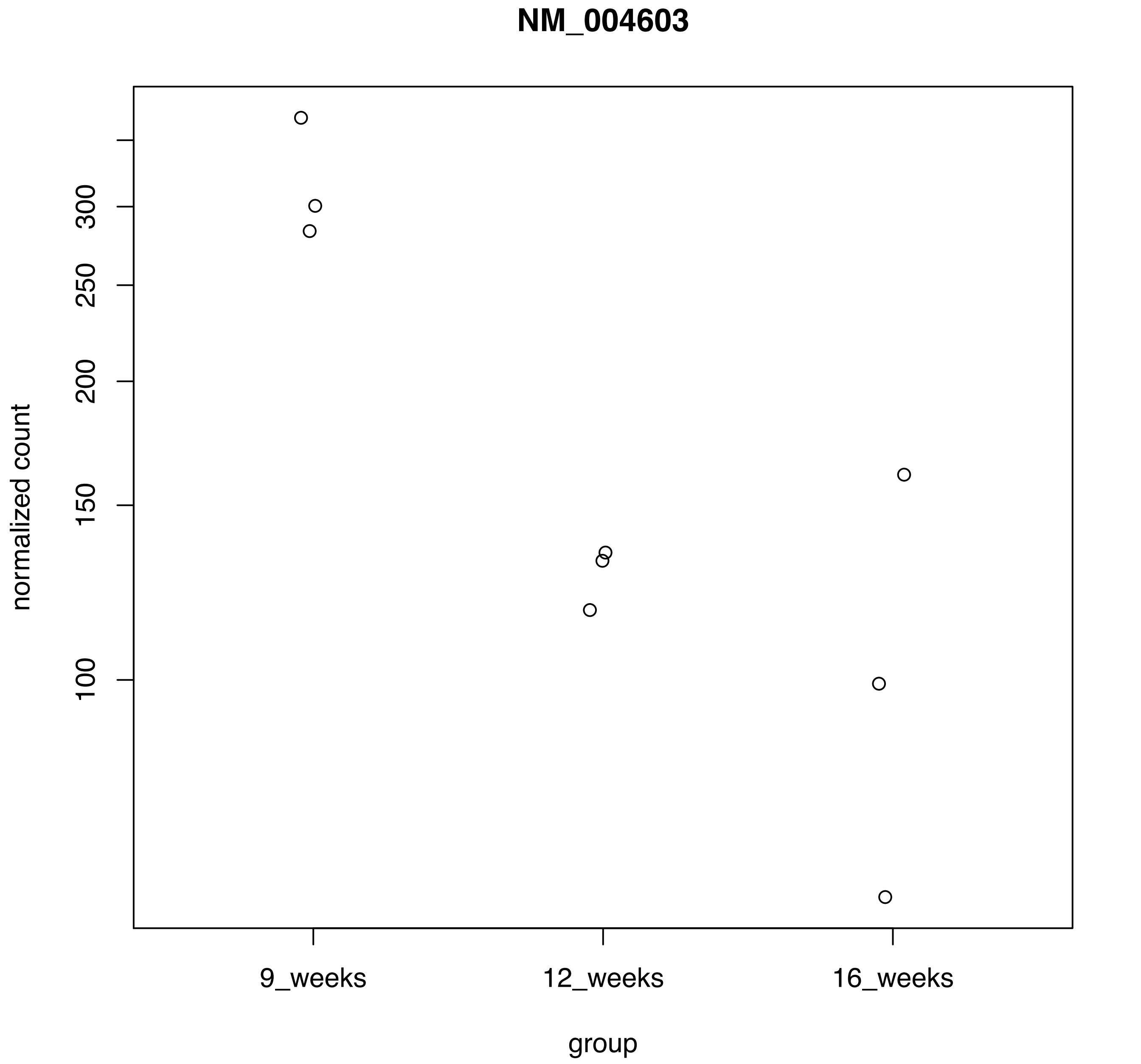
NM_004603
normalized count
100
150
200
250
300
9_weeks
12_weeks
16_weeks
group

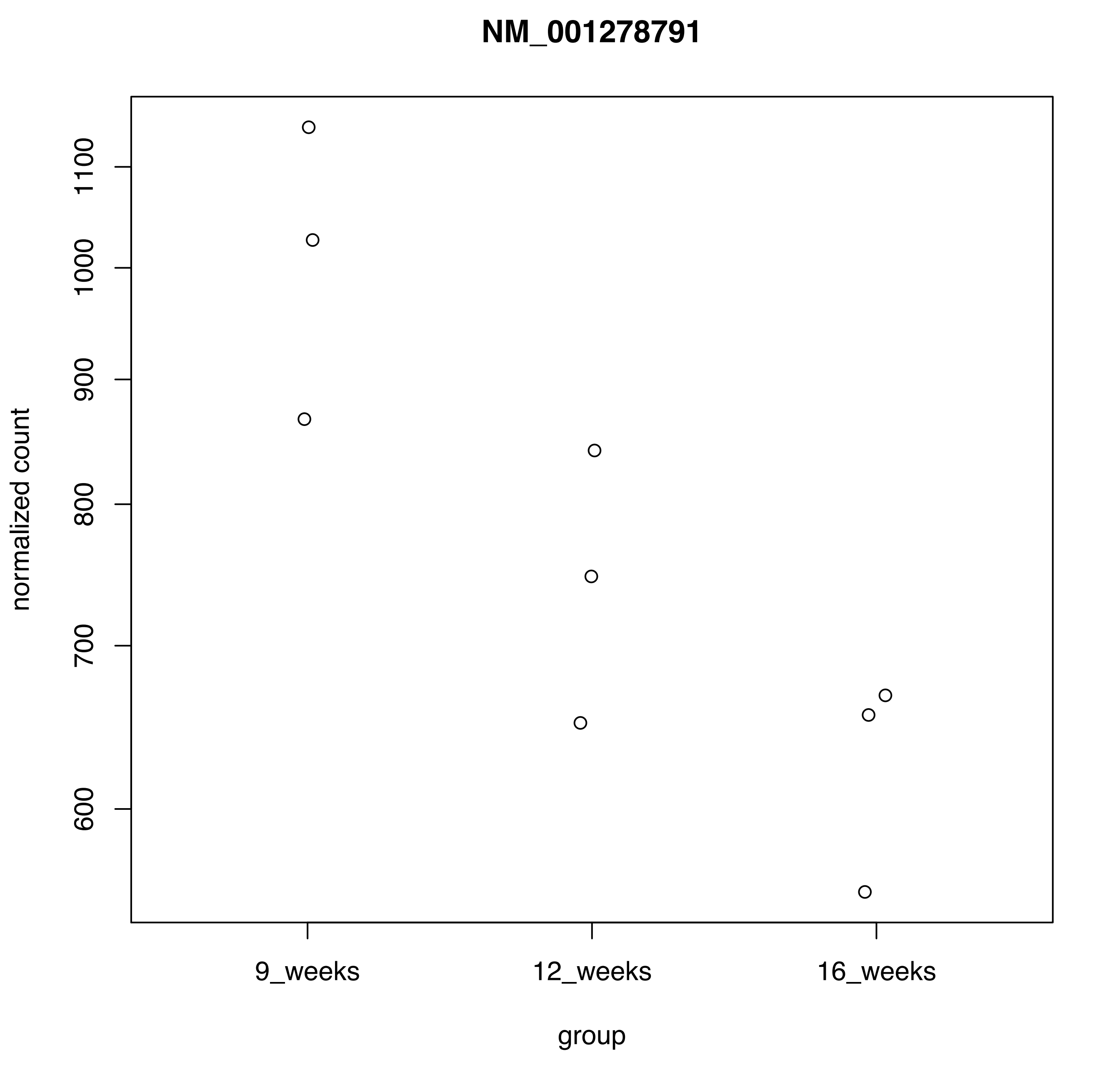
NM_001278791
normalized count
1100
1000
900
800
700
600
9_weeks
12_weeks
16_weeks
group

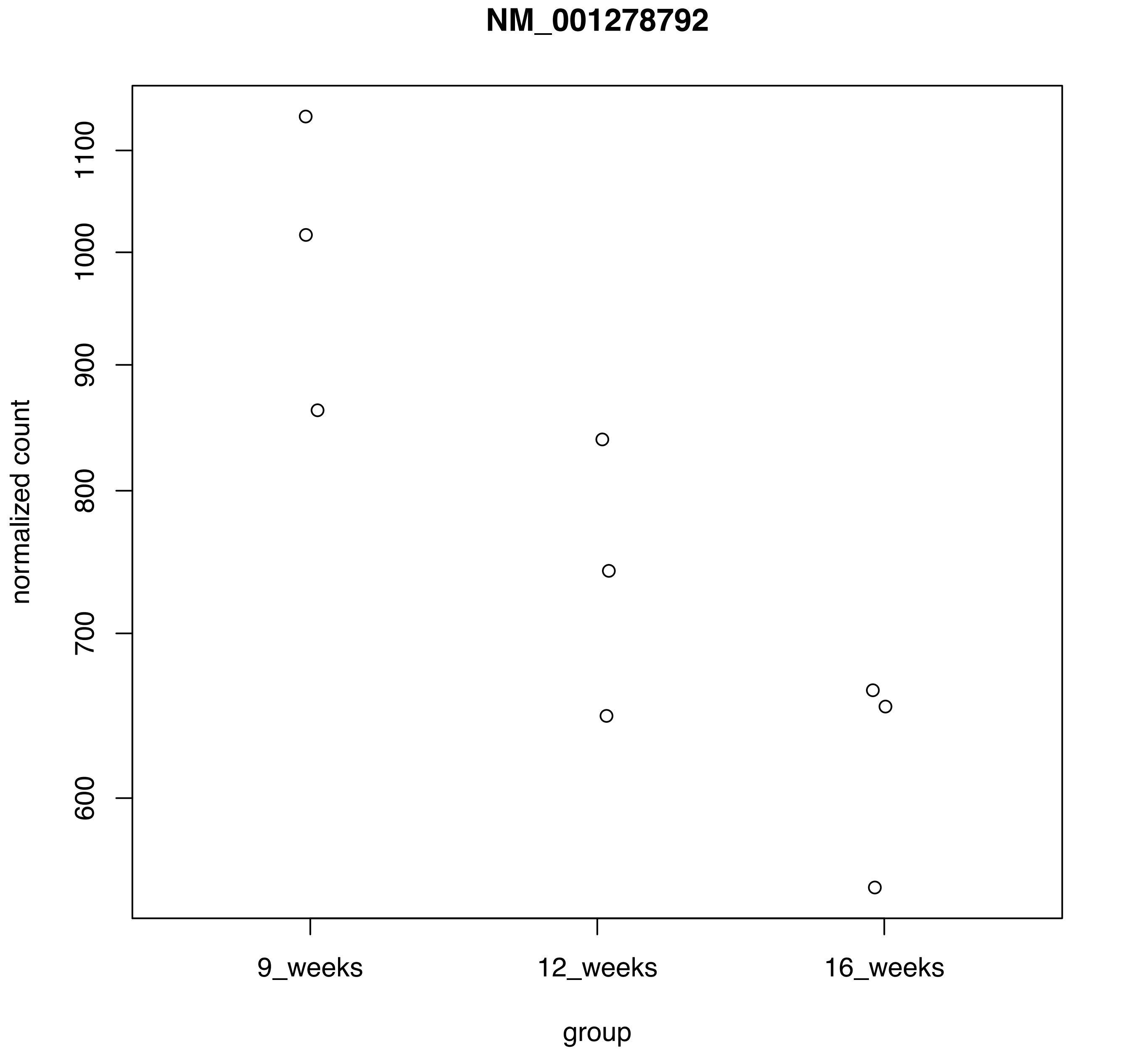

NM_001278792
normalized count
1100
1000
900
800
700
600
9_weeks
12_weeks
16_weeks
group

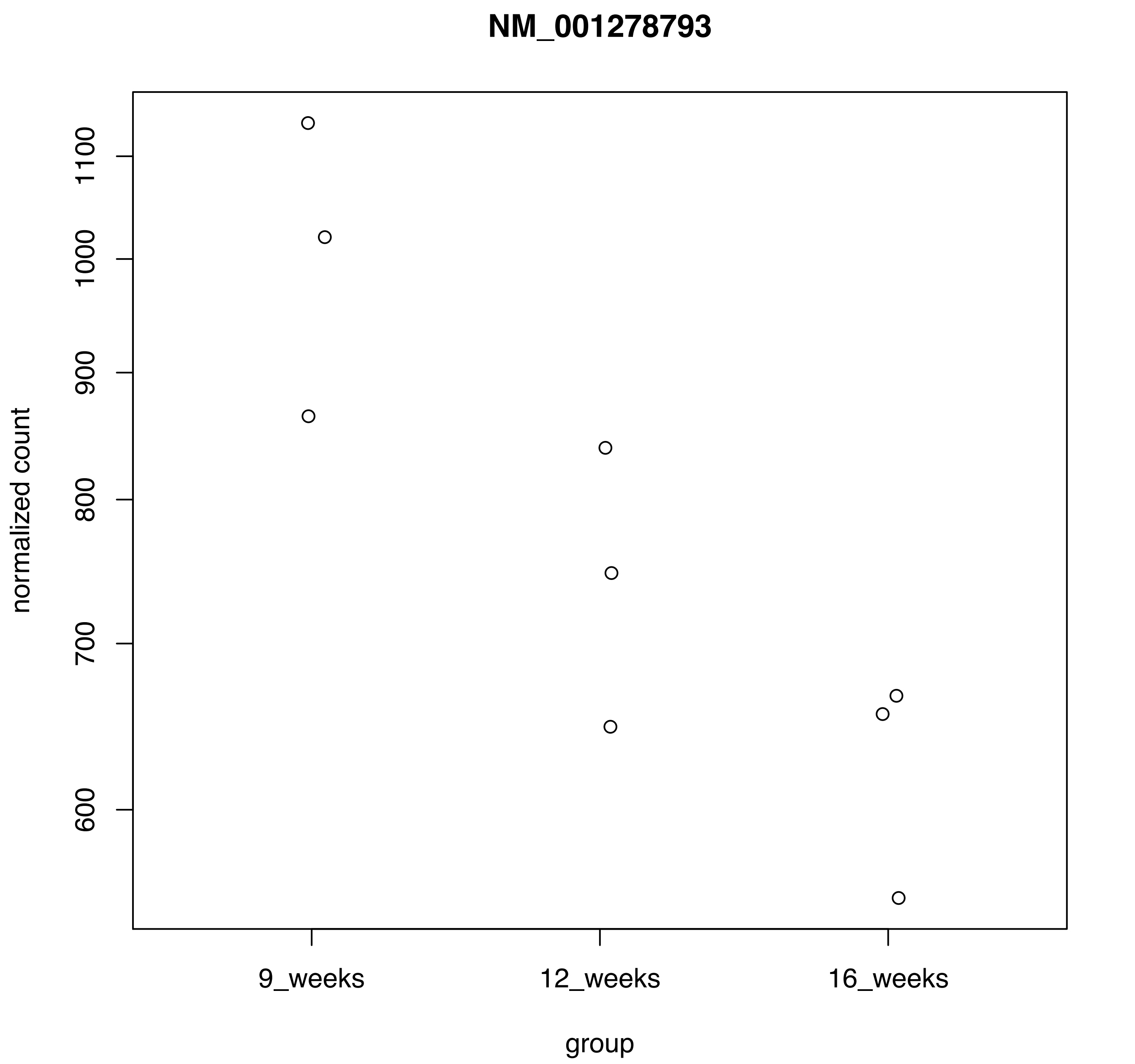

NM_001278793
normalized count
600
700
800
900
1000
1100
9_weeks
12_weeks
16_weeks
group

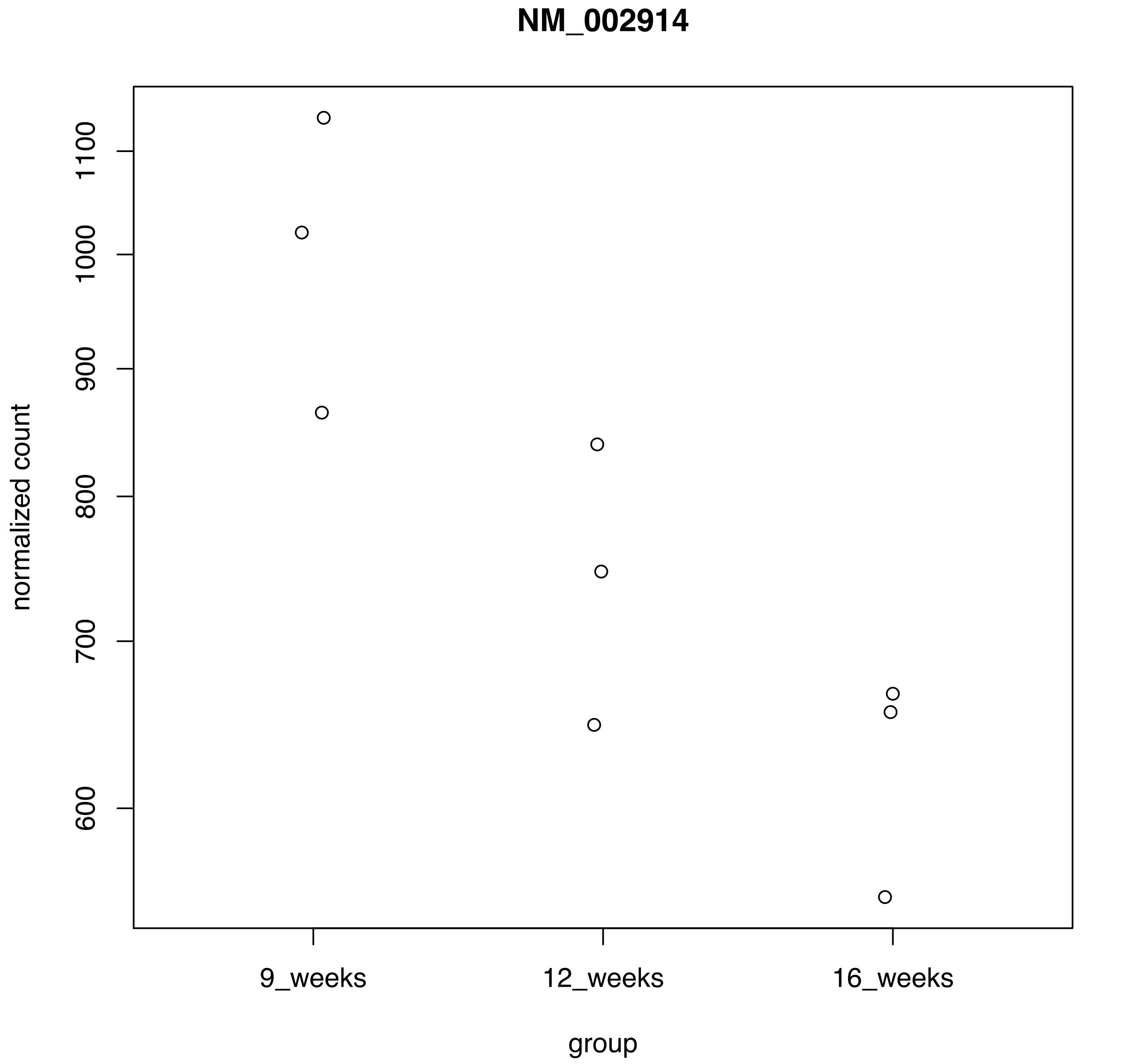

NM_002914
normalized count
1100
1000
900
800
700
600
9_weeks
12_weeks
16_weeks
group

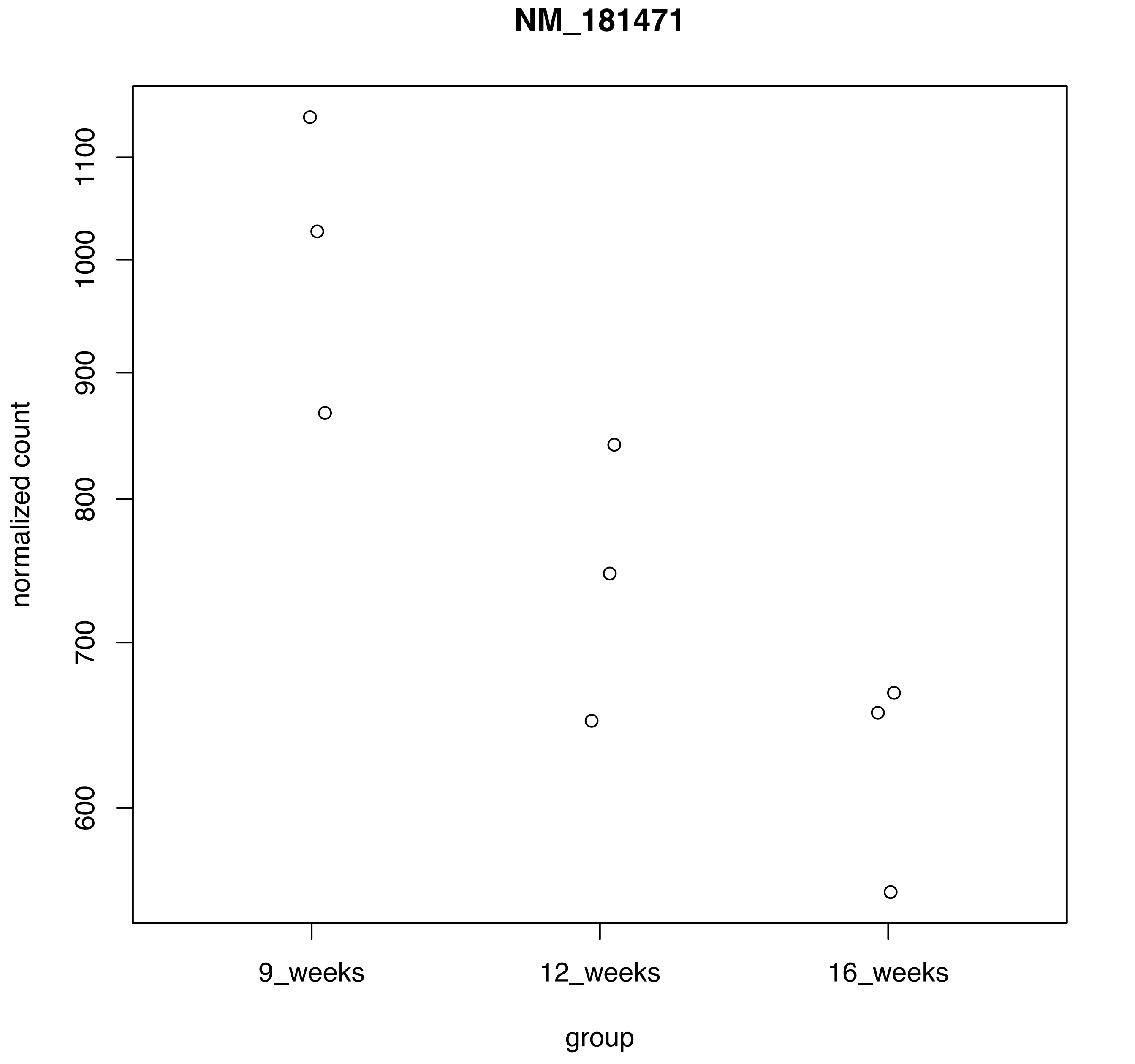
NM_181471
normalized count
1100
1000
900
800
700
600
9_weeks
12_weeks
16_weeks
group

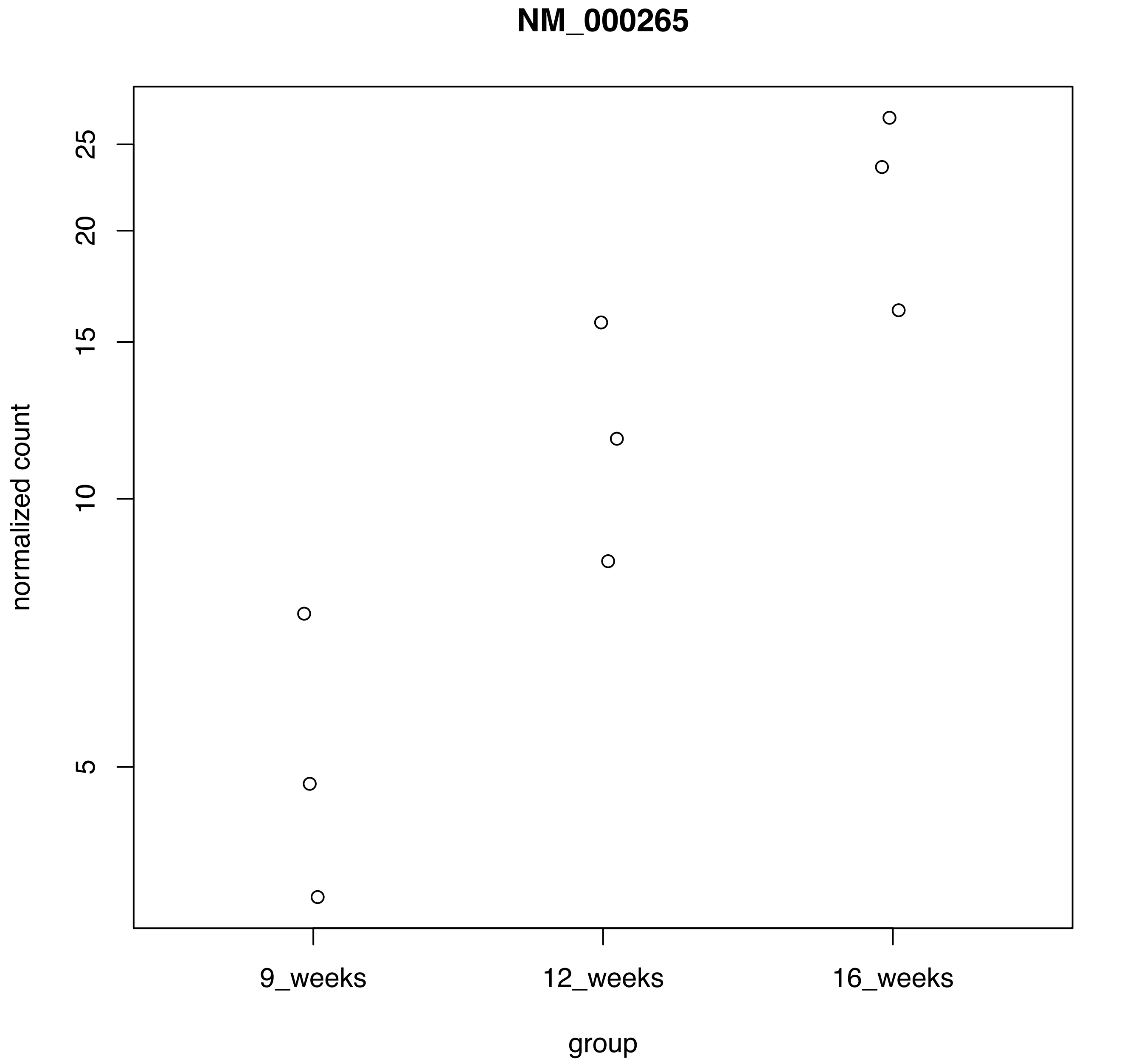
NM_000265
normalized count
5
10
15
20
25
9_weeks
12_weeks
16_weeks
group

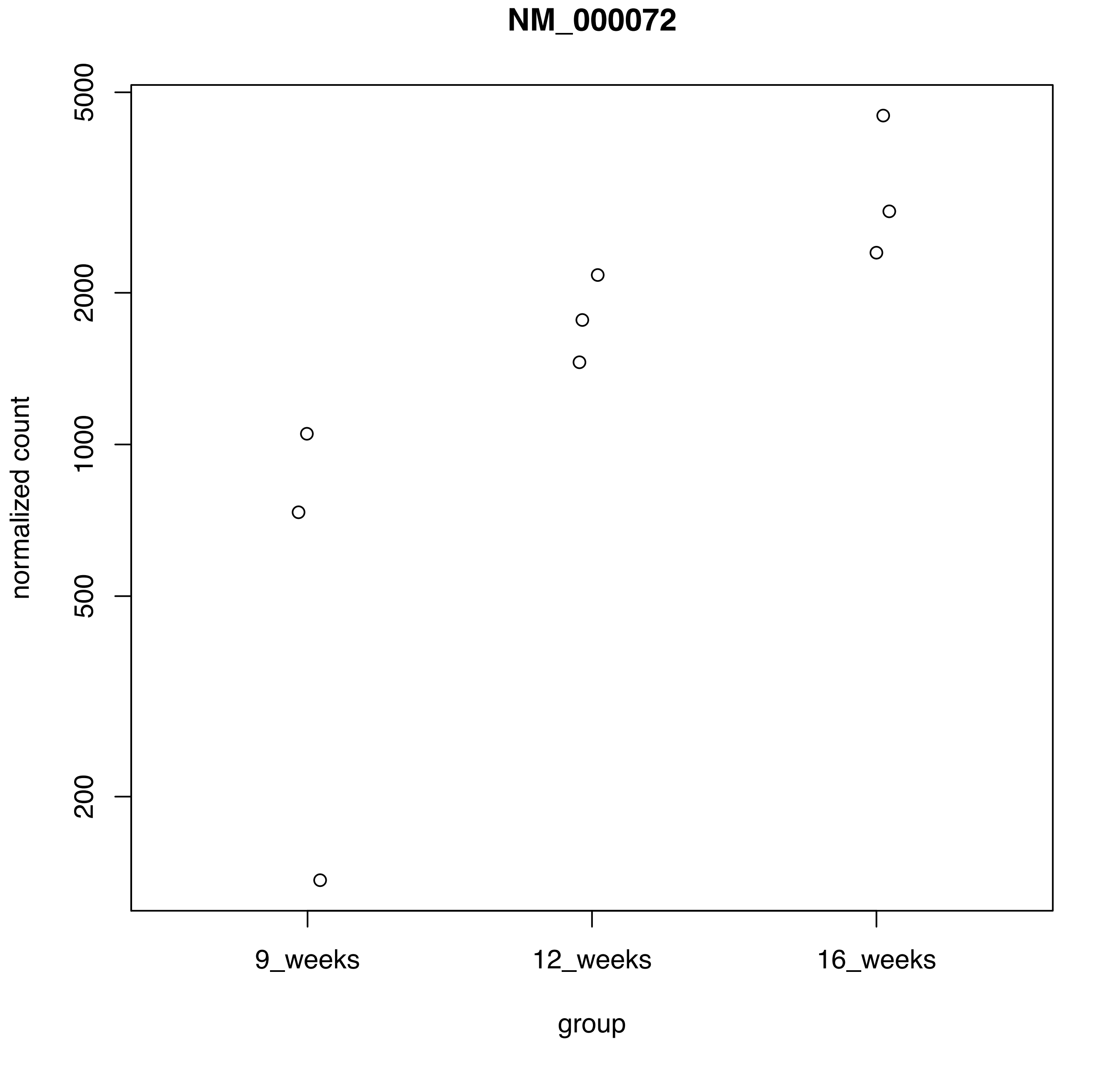

NM_000072
normalized count
5000
2000
1000
500
200
9_weeks
12_weeks
16_weeks
group

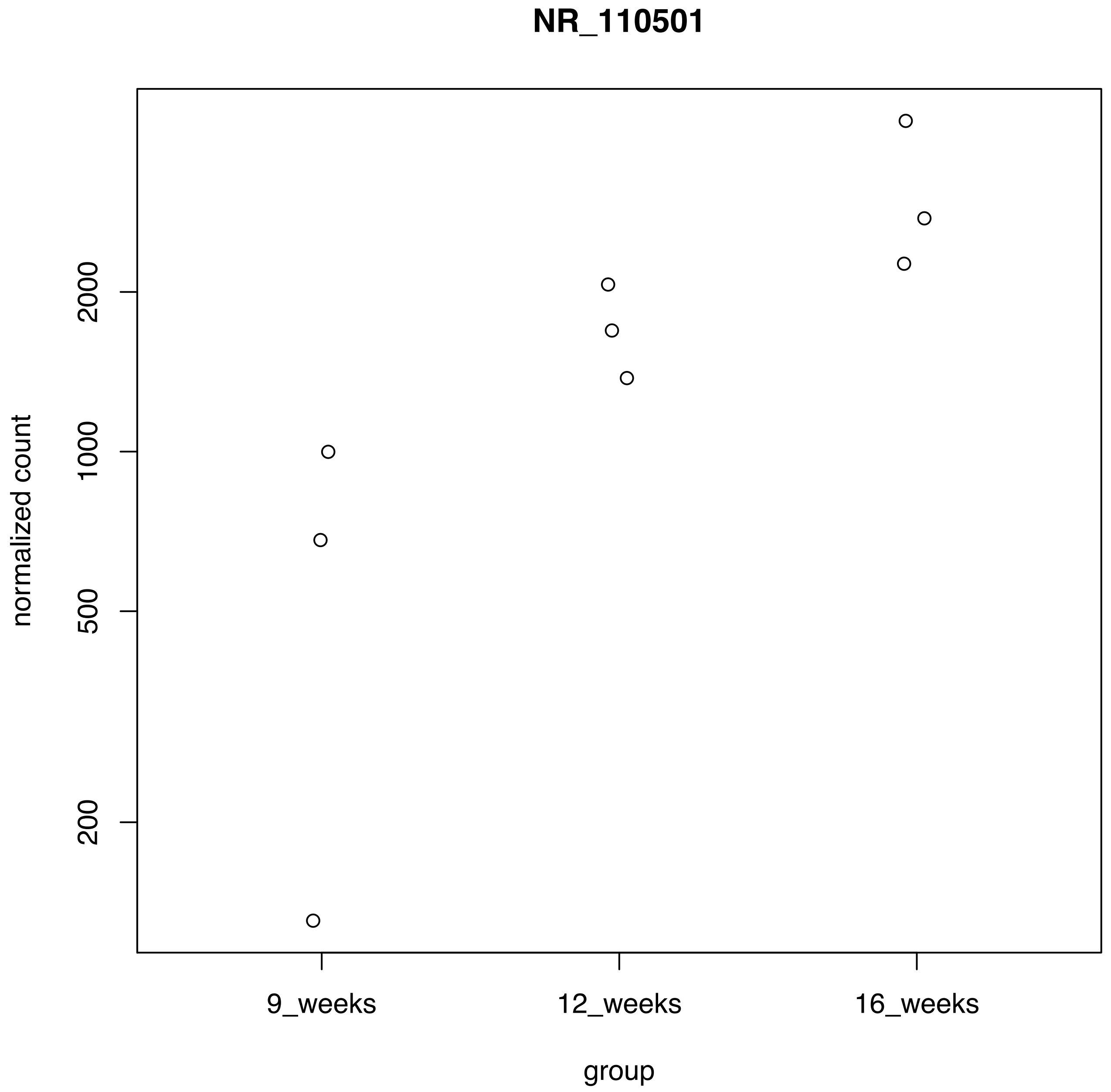
NR_110501
normalized count
2000
1000
500
200
9_weeks
12_weeks
16_weeks
group

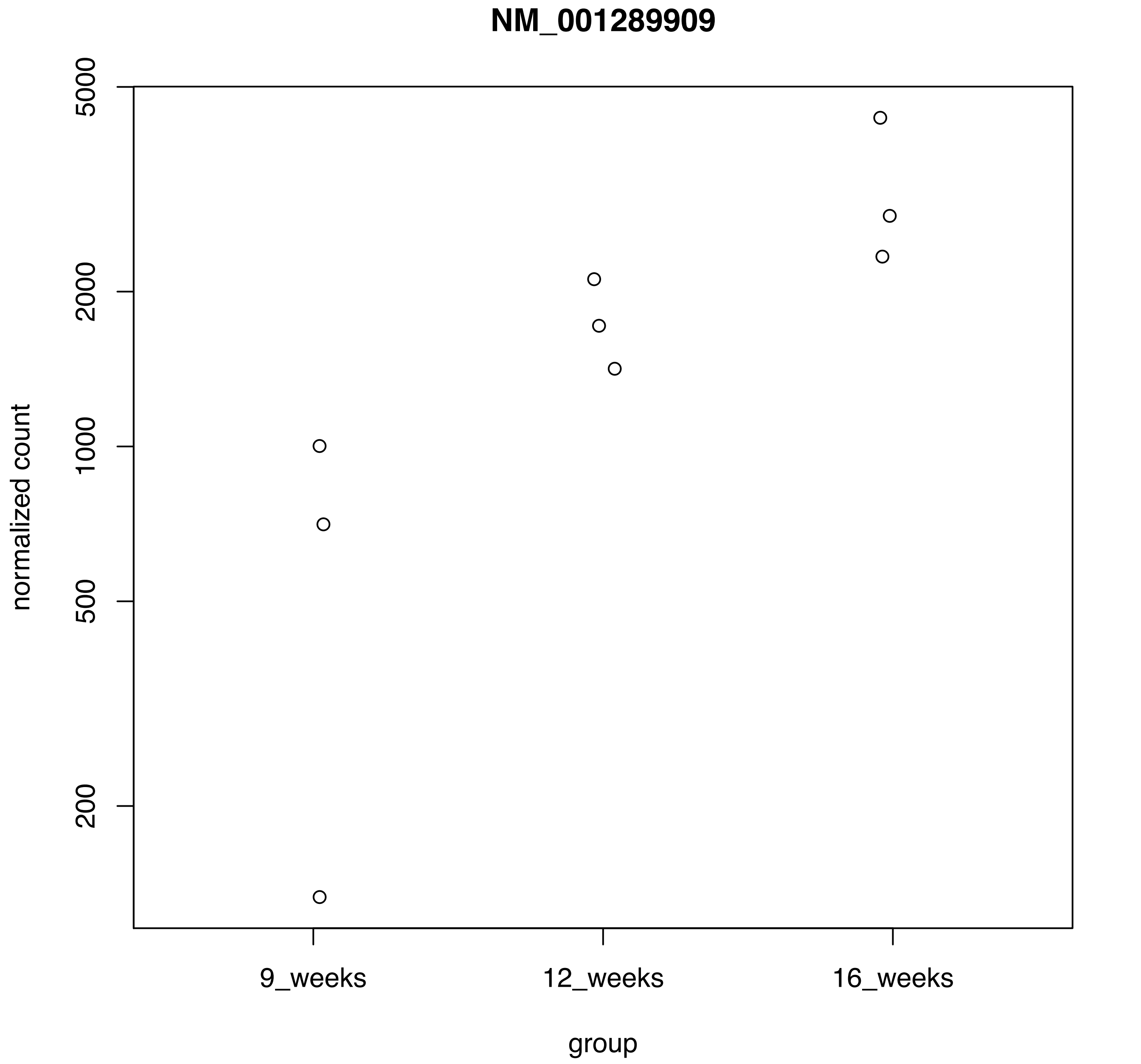
NM_001289909
normalized count
5000
2000
1000
500
200
9_weeks
12_weeks
16_weeks
group

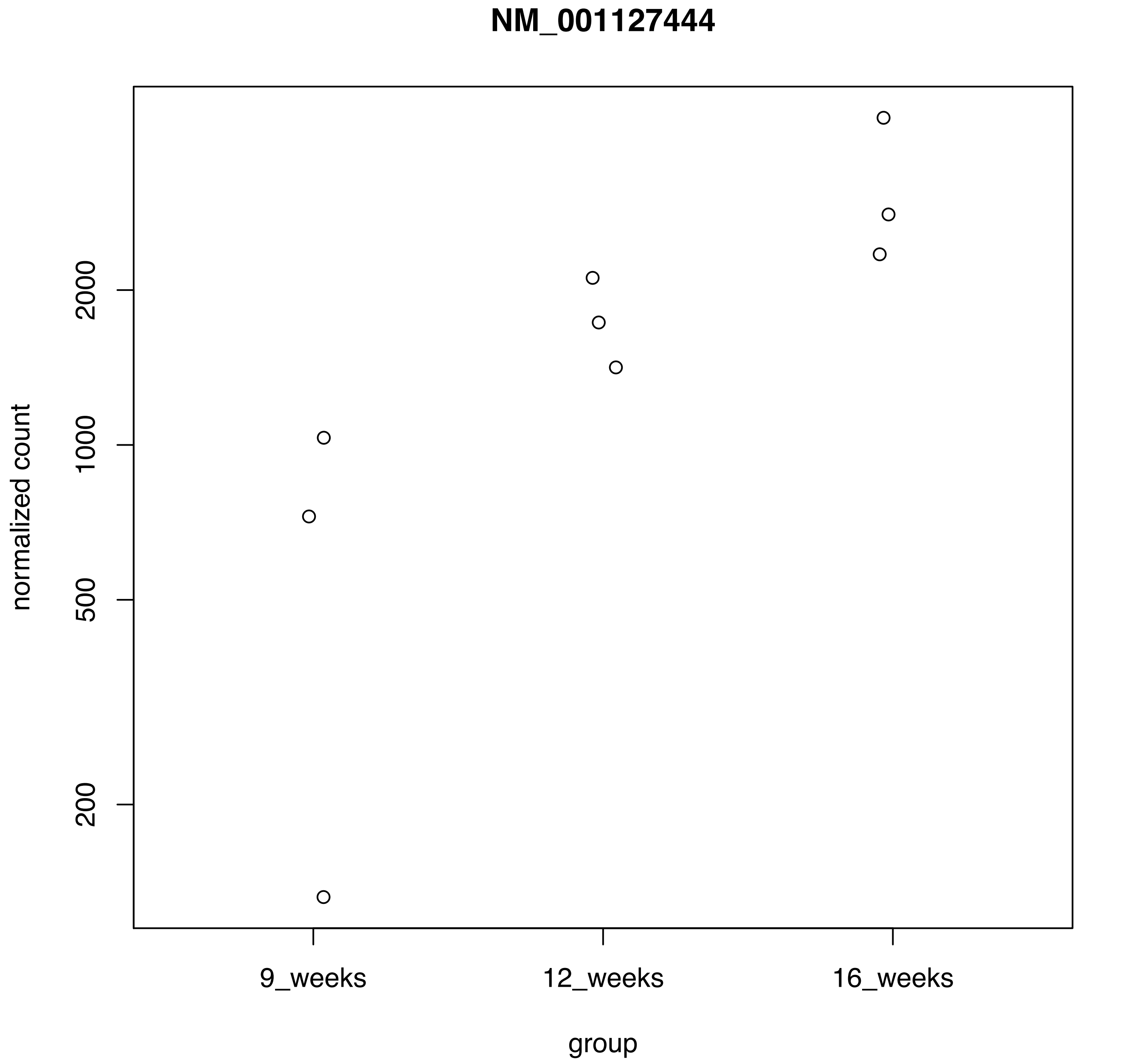

NM_001127444
normalized count
2000
1000
500
200
9_weeks
12_weeks
16_weeks
group

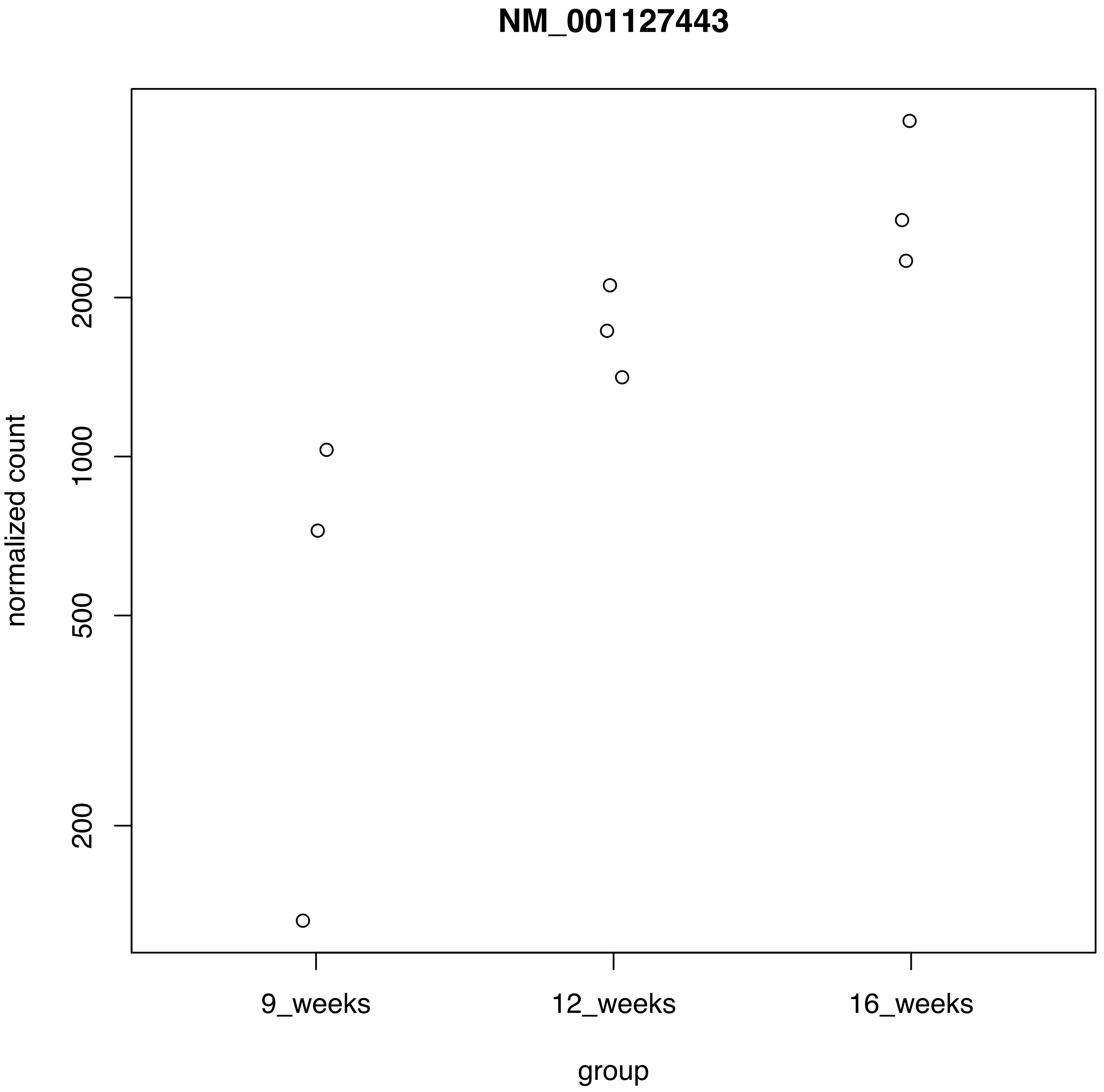
NM_001127443
normalized count
2000
1000
500
200
9_weeks
12_weeks
16_weeks
group

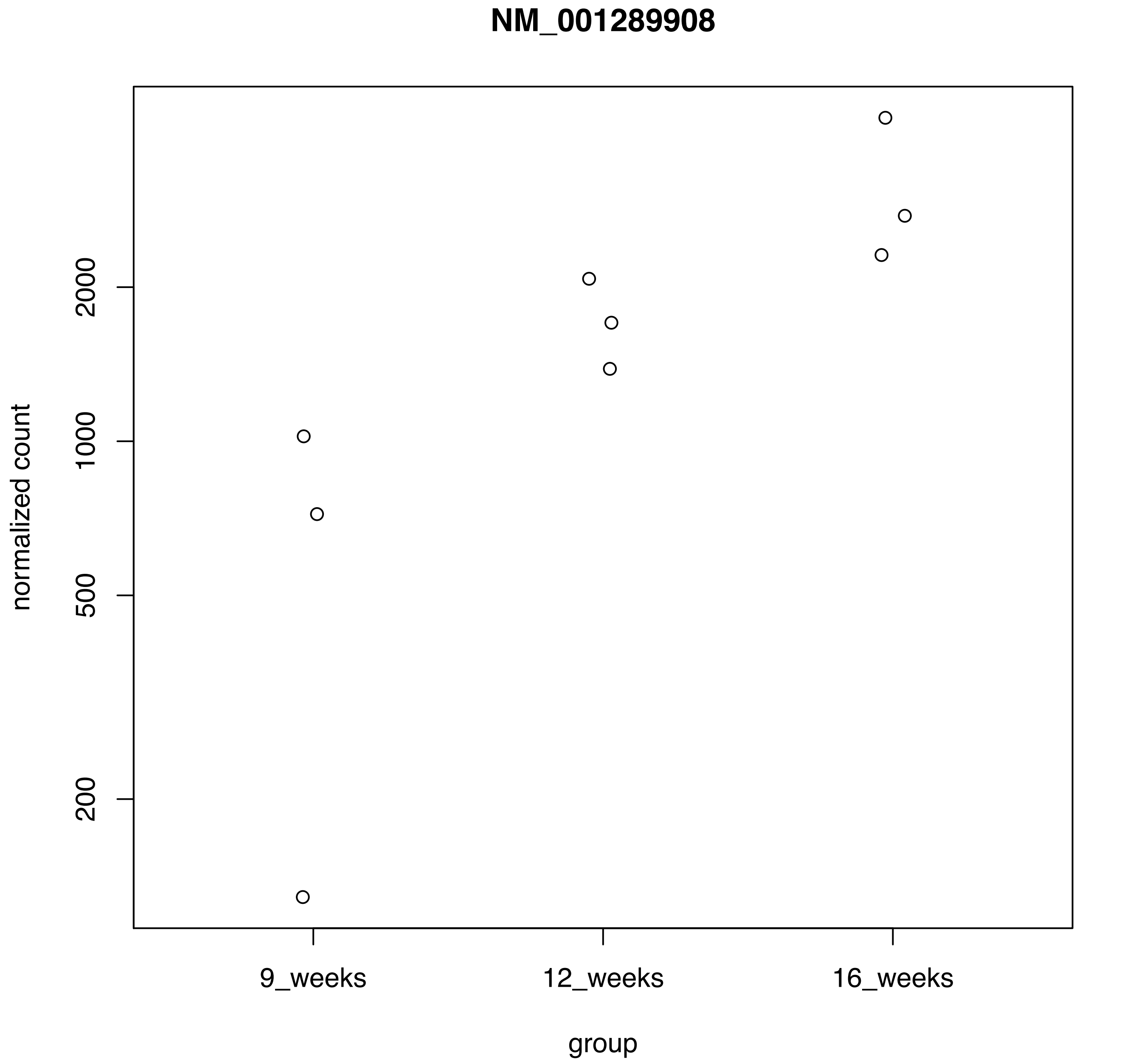
NM_001289908
normalized count
2000
1000
500
200
9_weeks
12_weeks
16_weeks
group

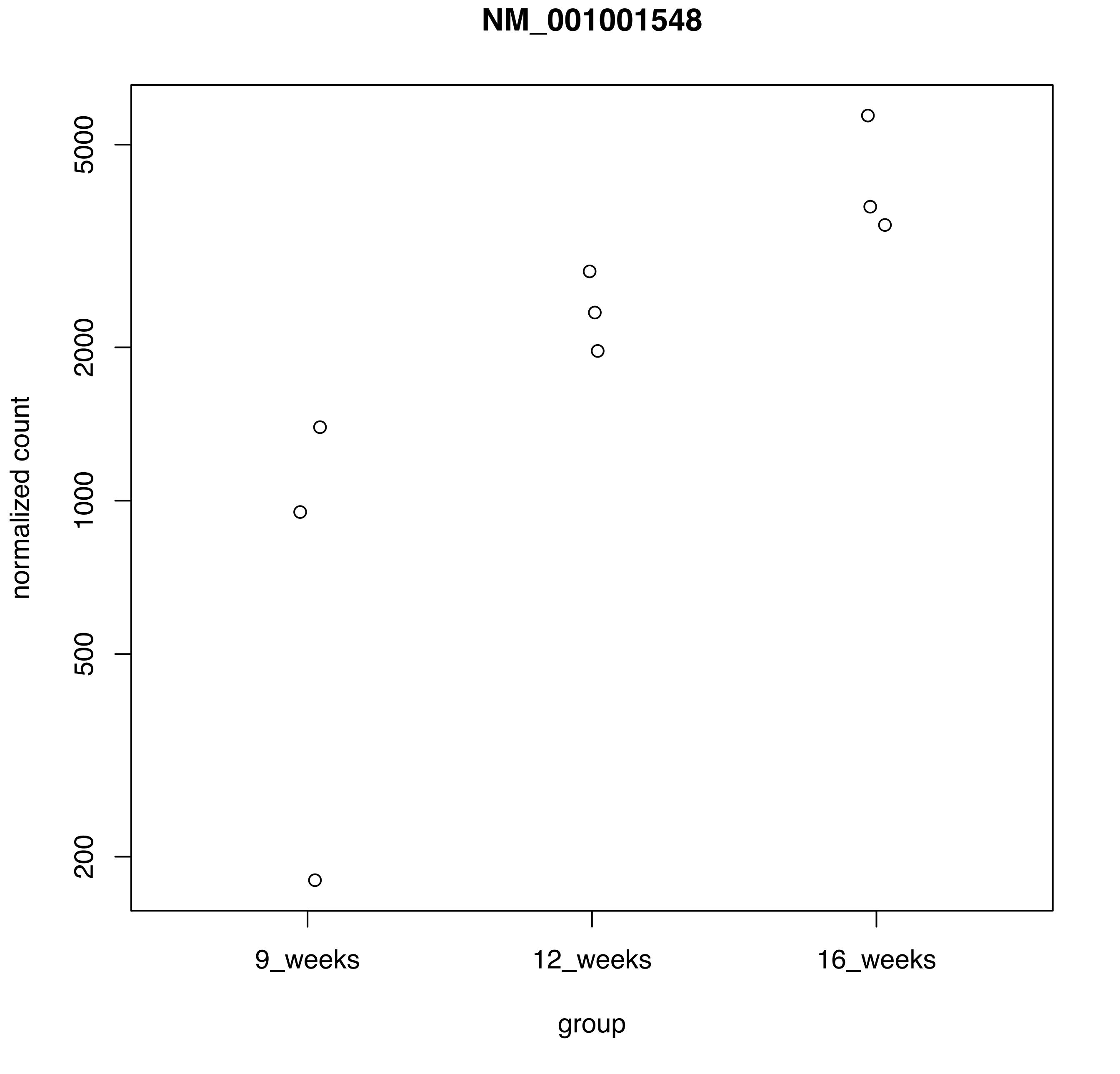
NM_001001548
normalized count
5000
2000
1000
500
200
9_weeks
12_weeks
16_weeks
group

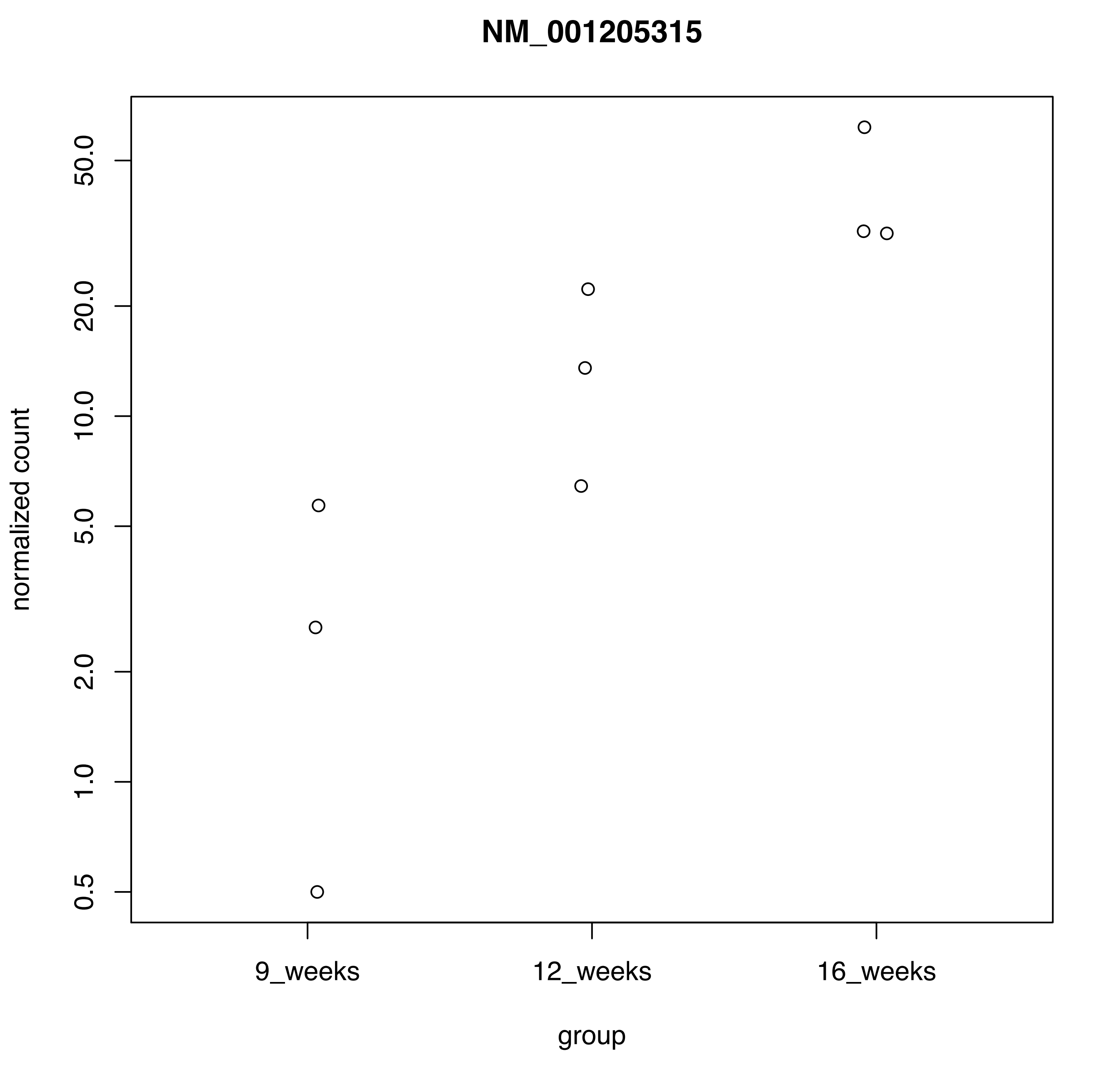
NM_001205315
normalized count
50.0
20.0
10.0
5.0
2.0
1.0
0.5
9_weeks
12_weeks
16_weeks
group

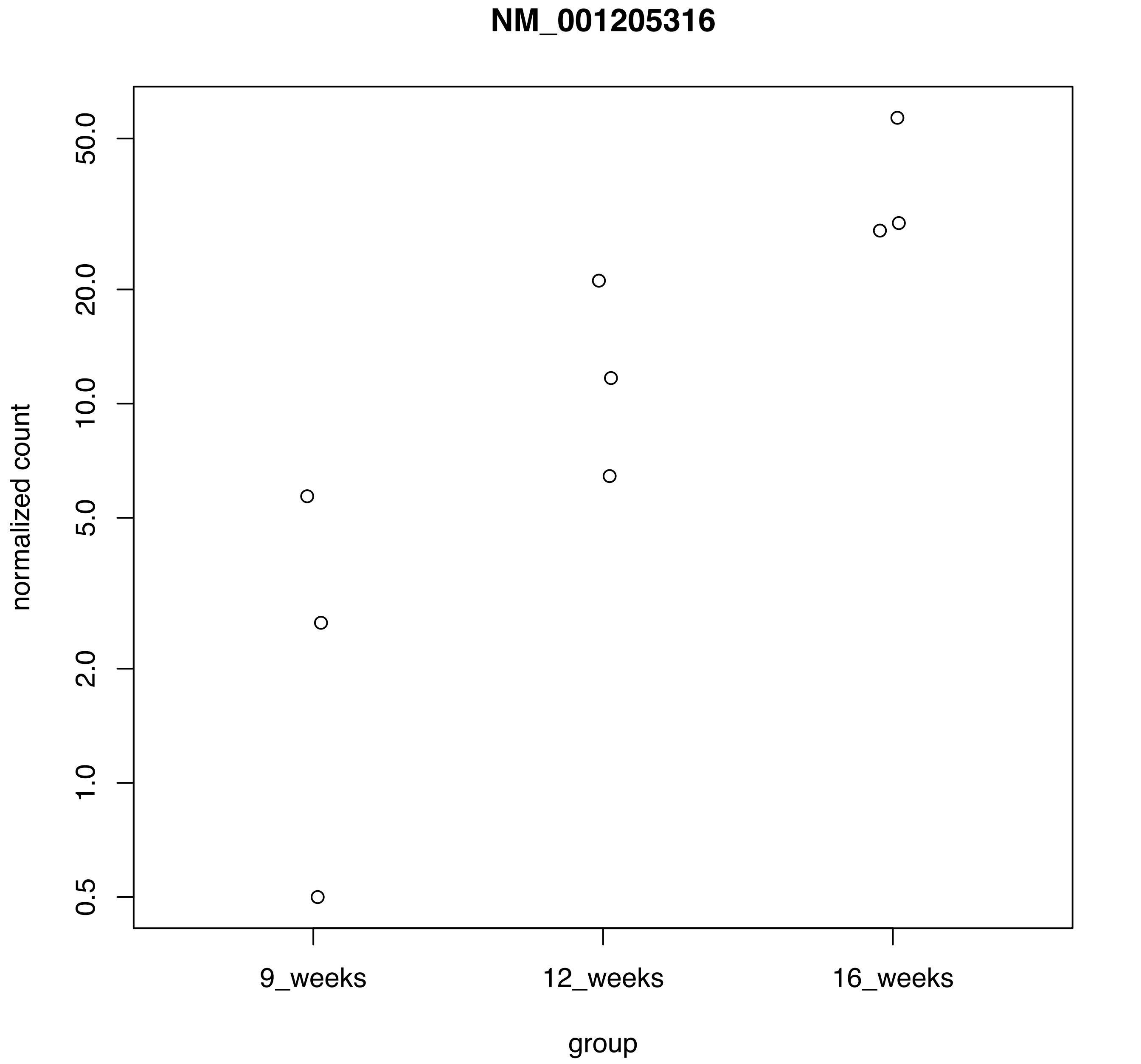

NM_001205316
normalized count
50.0
20.0
10.0
5.0
2.0
1.0
0.5
9_weeks
12_weeks
16_weeks
group

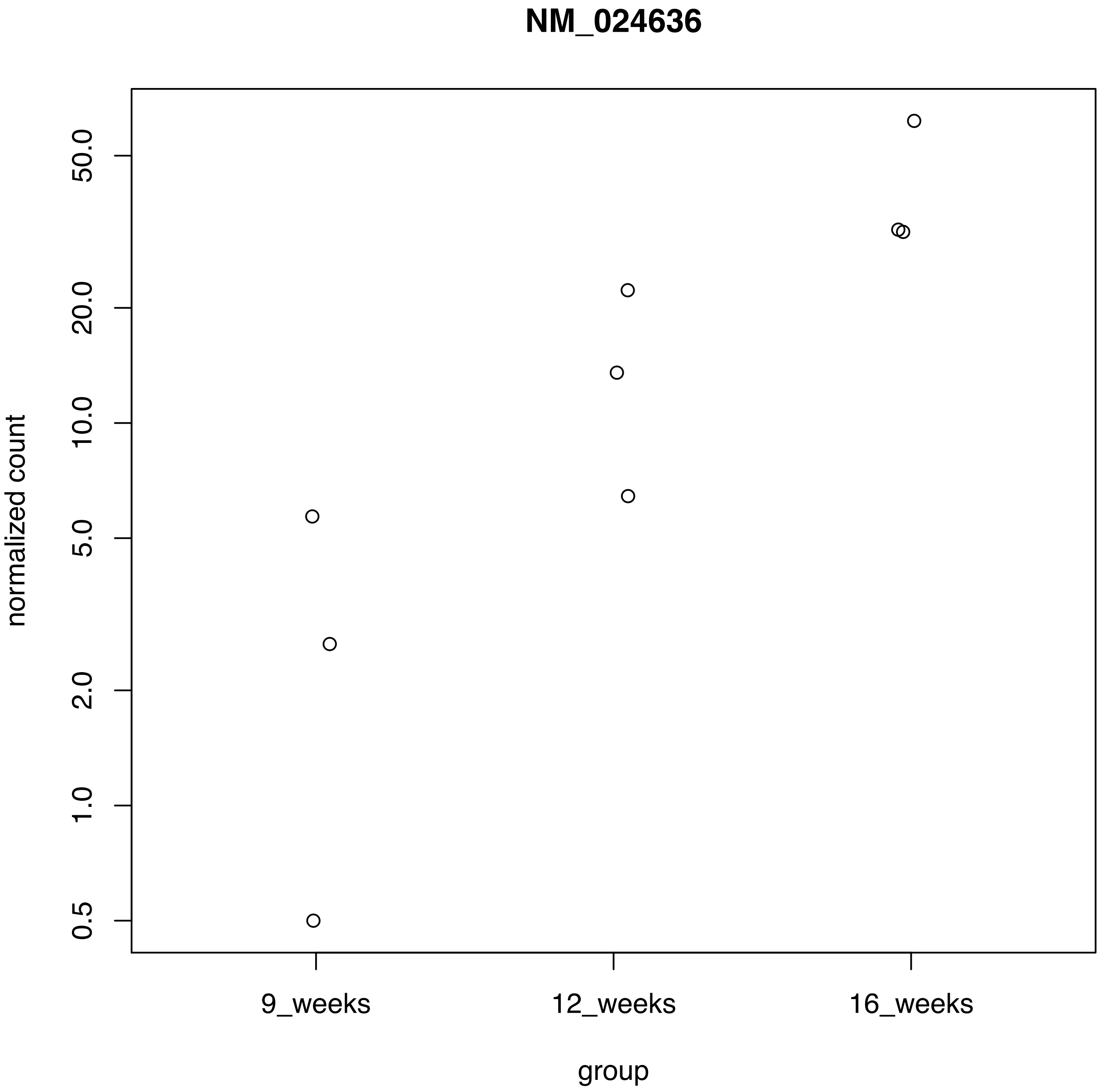
NM_024636
normalized count
50.0
20.0
10.0
5.0
2.0
1.0
0.5
9_weeks
12_weeks
16_weeks
group

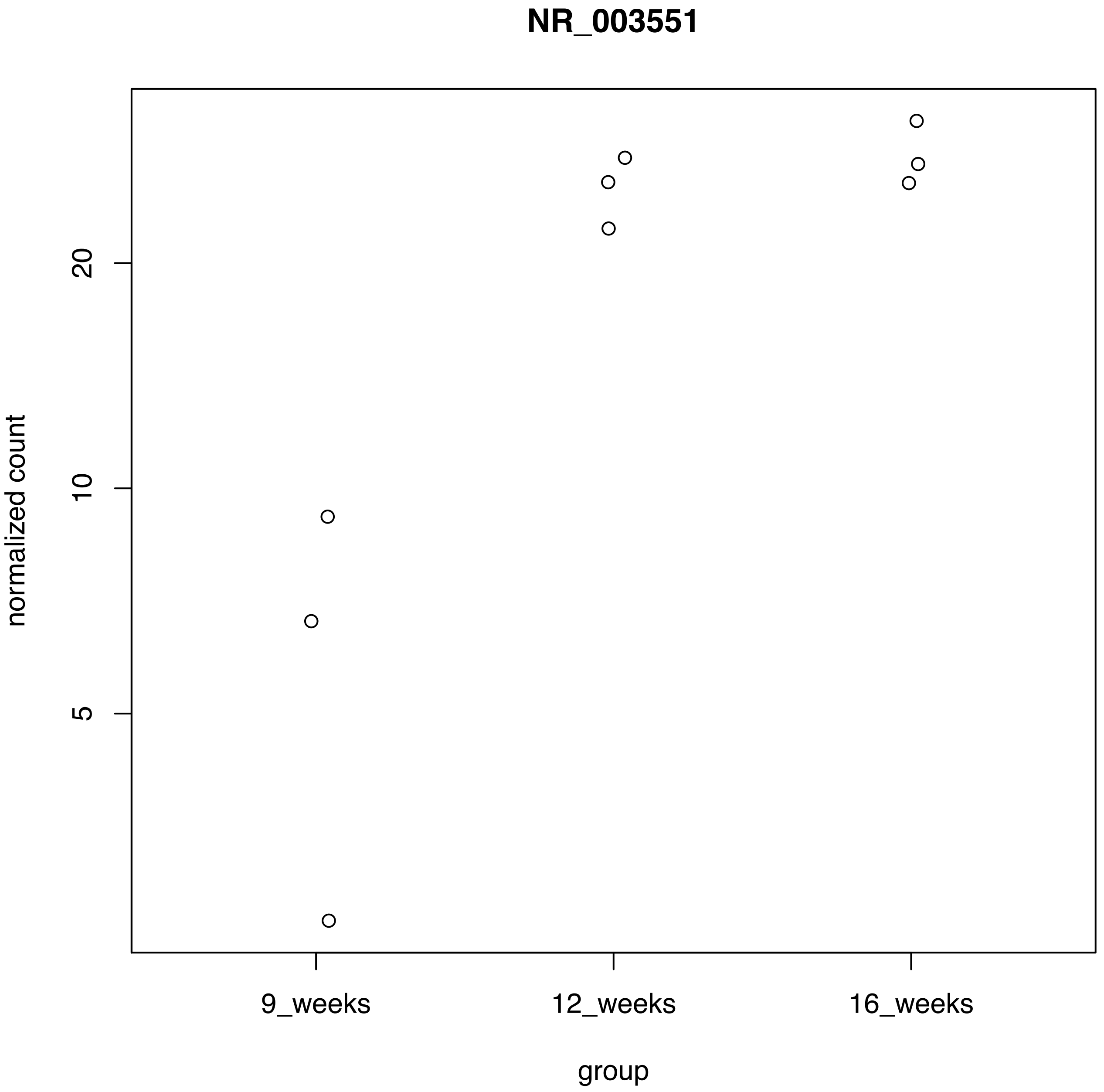

NR_003551
normalized count
20
10
5
9_weeks
12_weeks
16_weeks
group

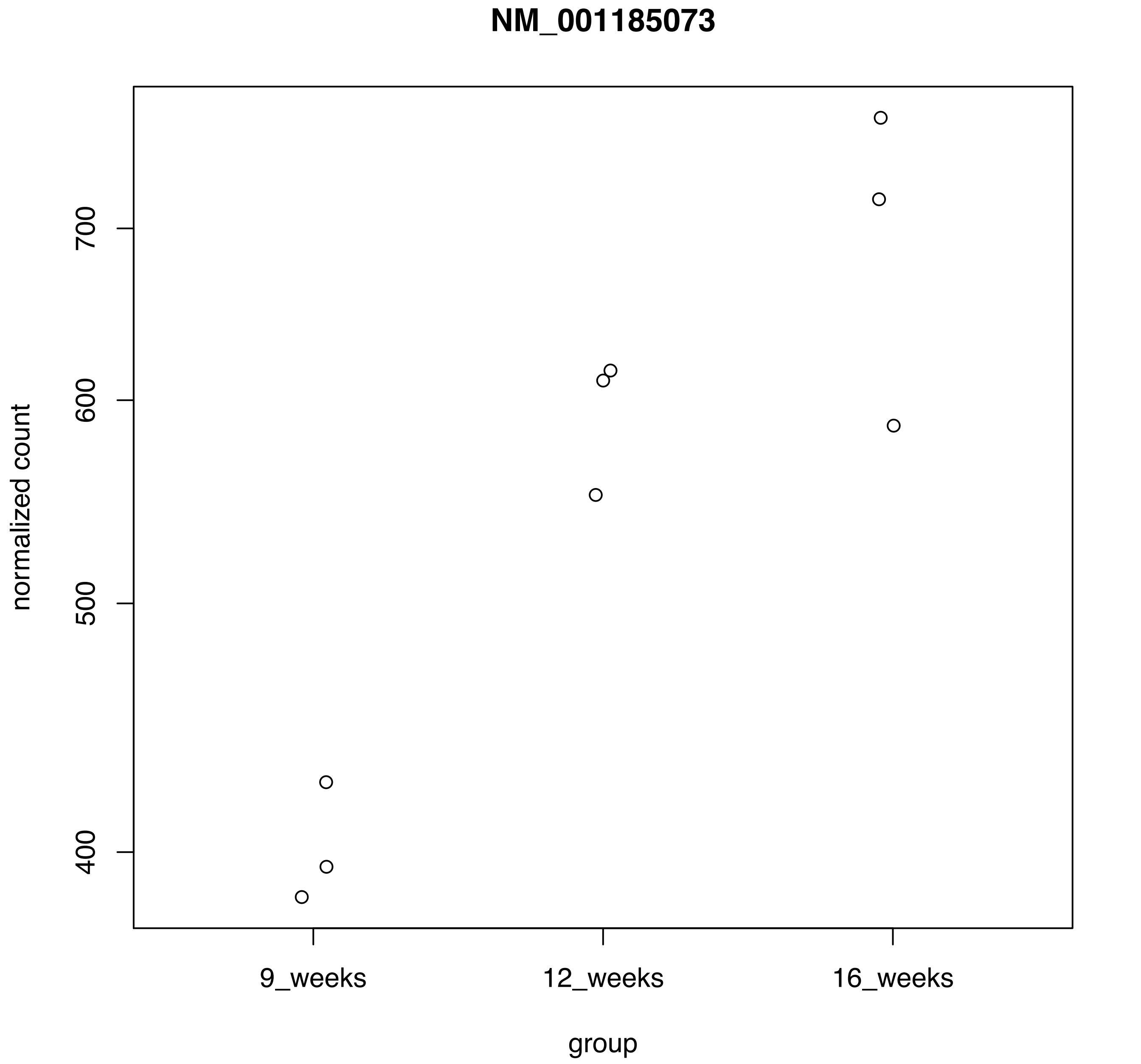
NM_001185073
normalized count
400
500
600
700
9_weeks
12_weeks
16_weeks
group

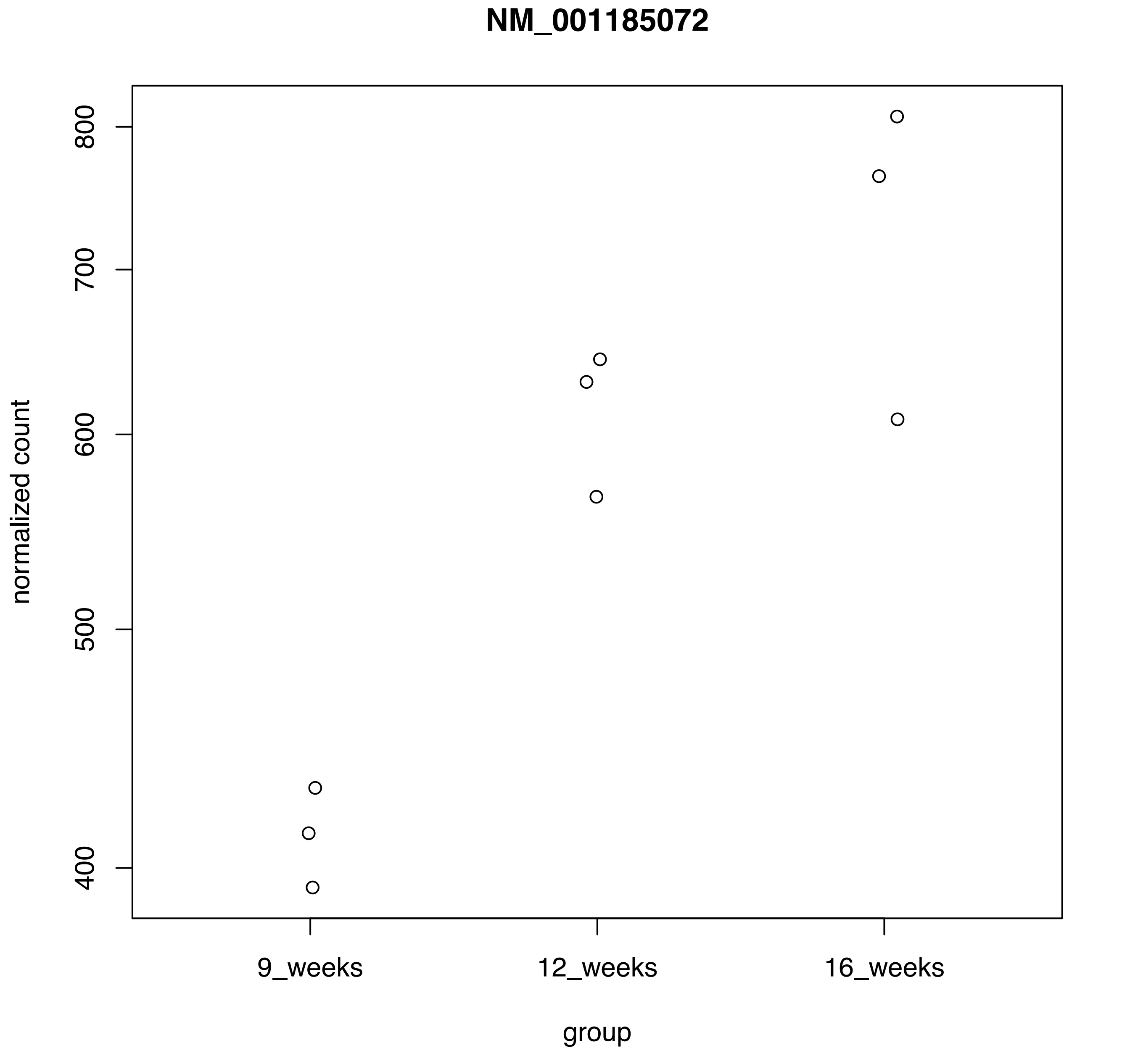
NM_001185072
normalized count
800
700
600
500
400
9_weeks
12_weeks
16_weeks
group

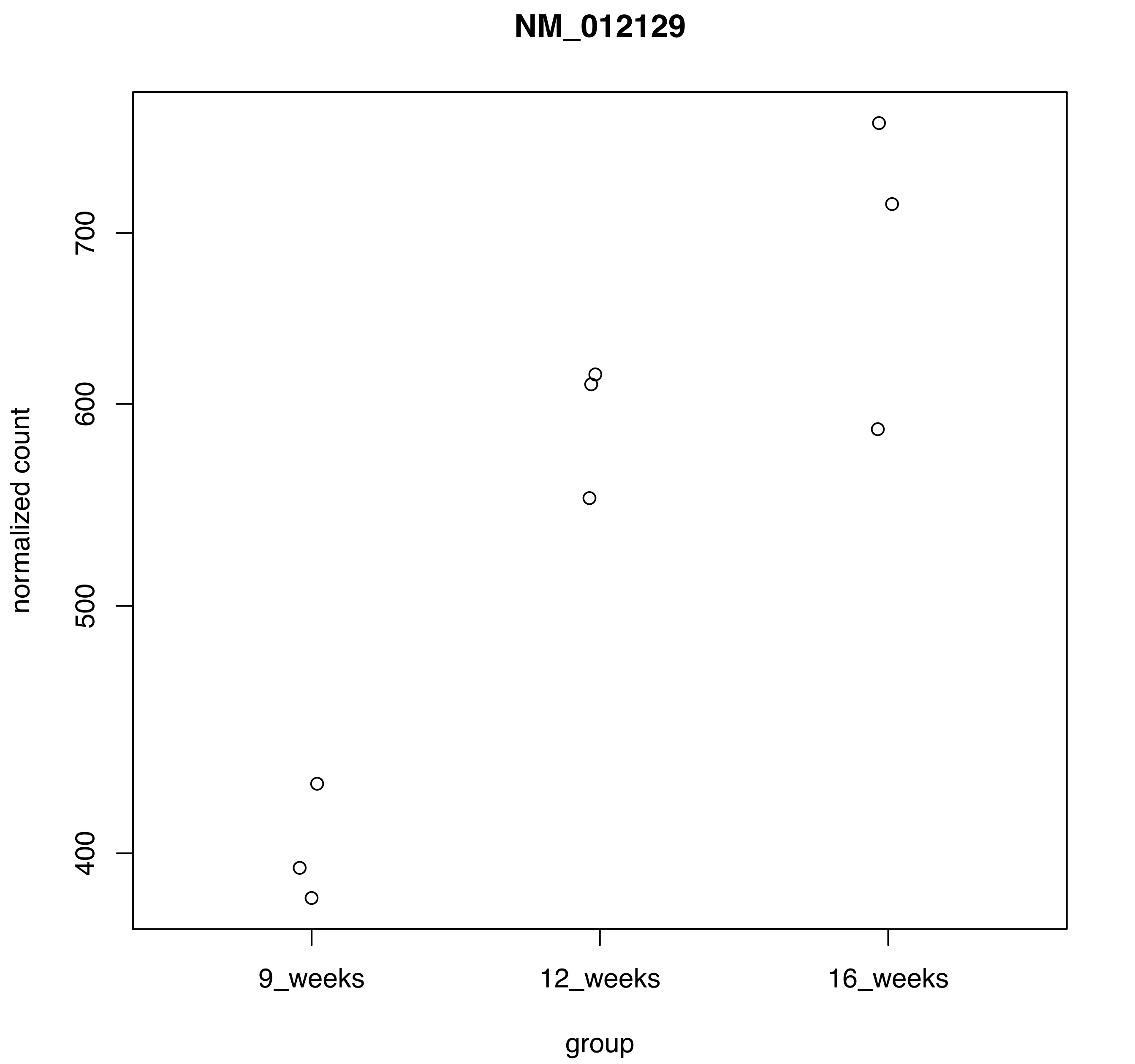
NM_012129
normalized count
700
600
500
400
9_weeks
12_weeks
16_weeks
group

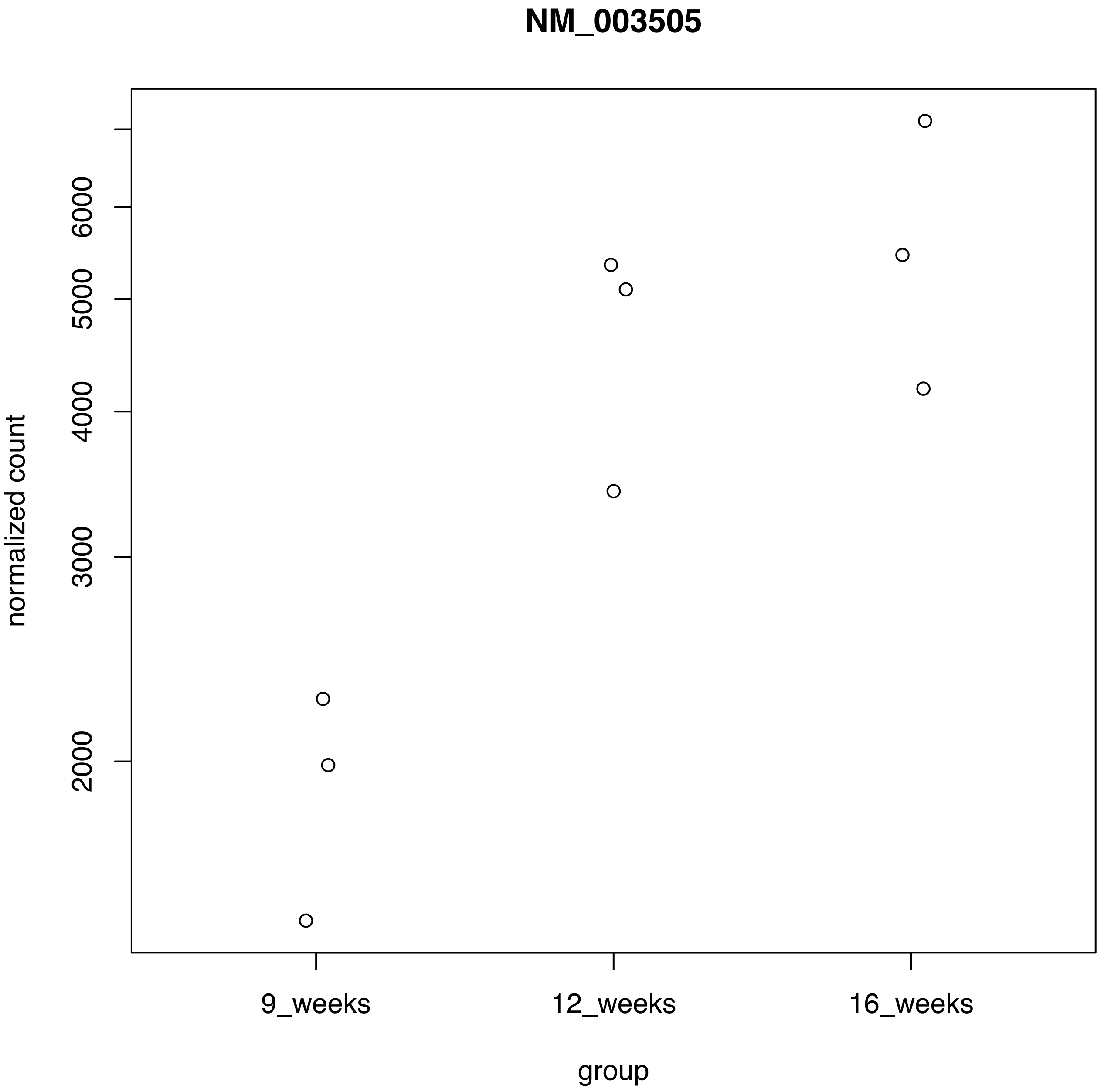

NM_003505
normalized count
6000
5000
4000
3000
2000
9_weeks
12_weeks
16_weeks
group

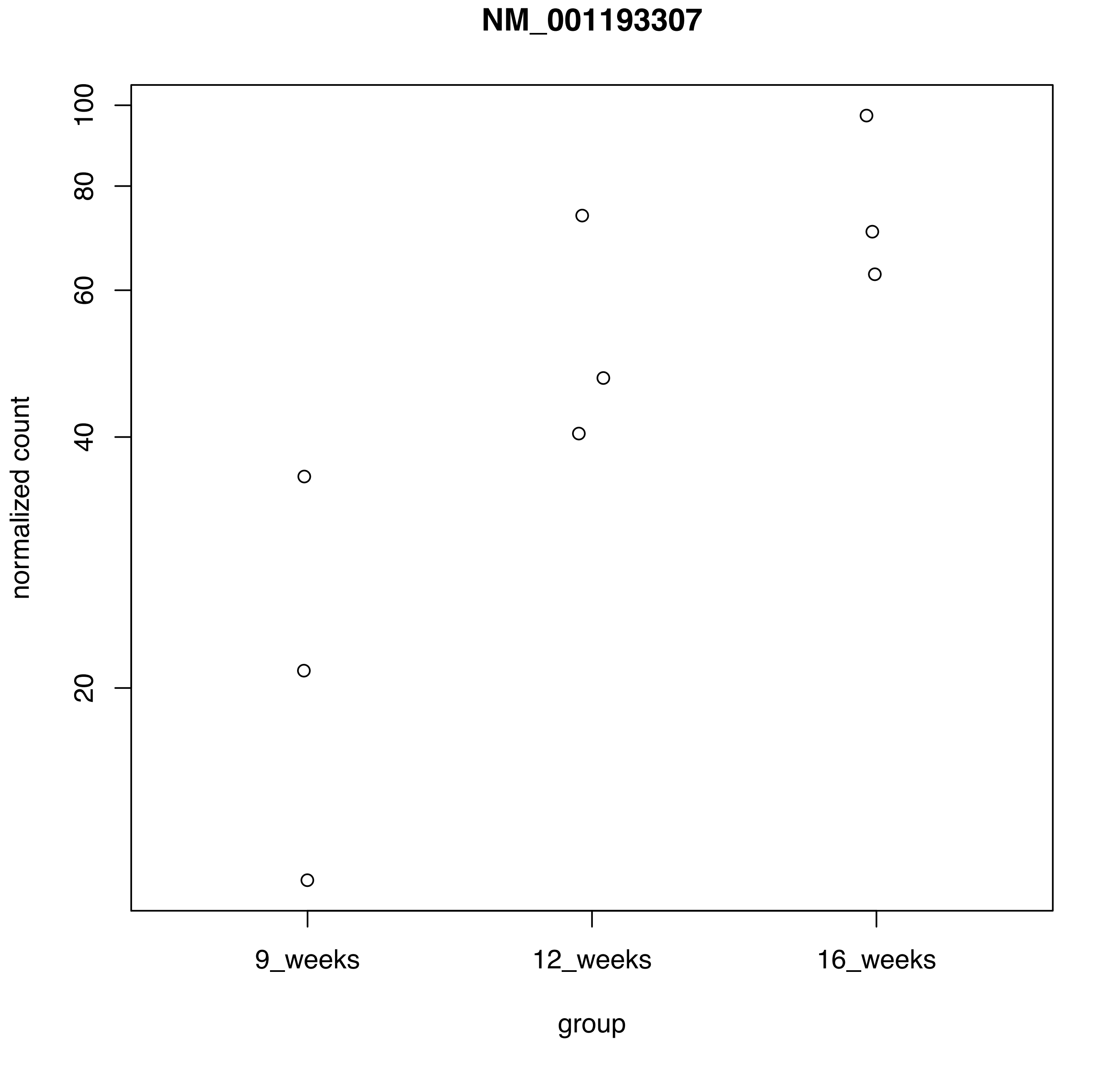
NM_001193307
normalized count
100
80
60
40
20
9_weeks
12_weeks
16_weeks
group

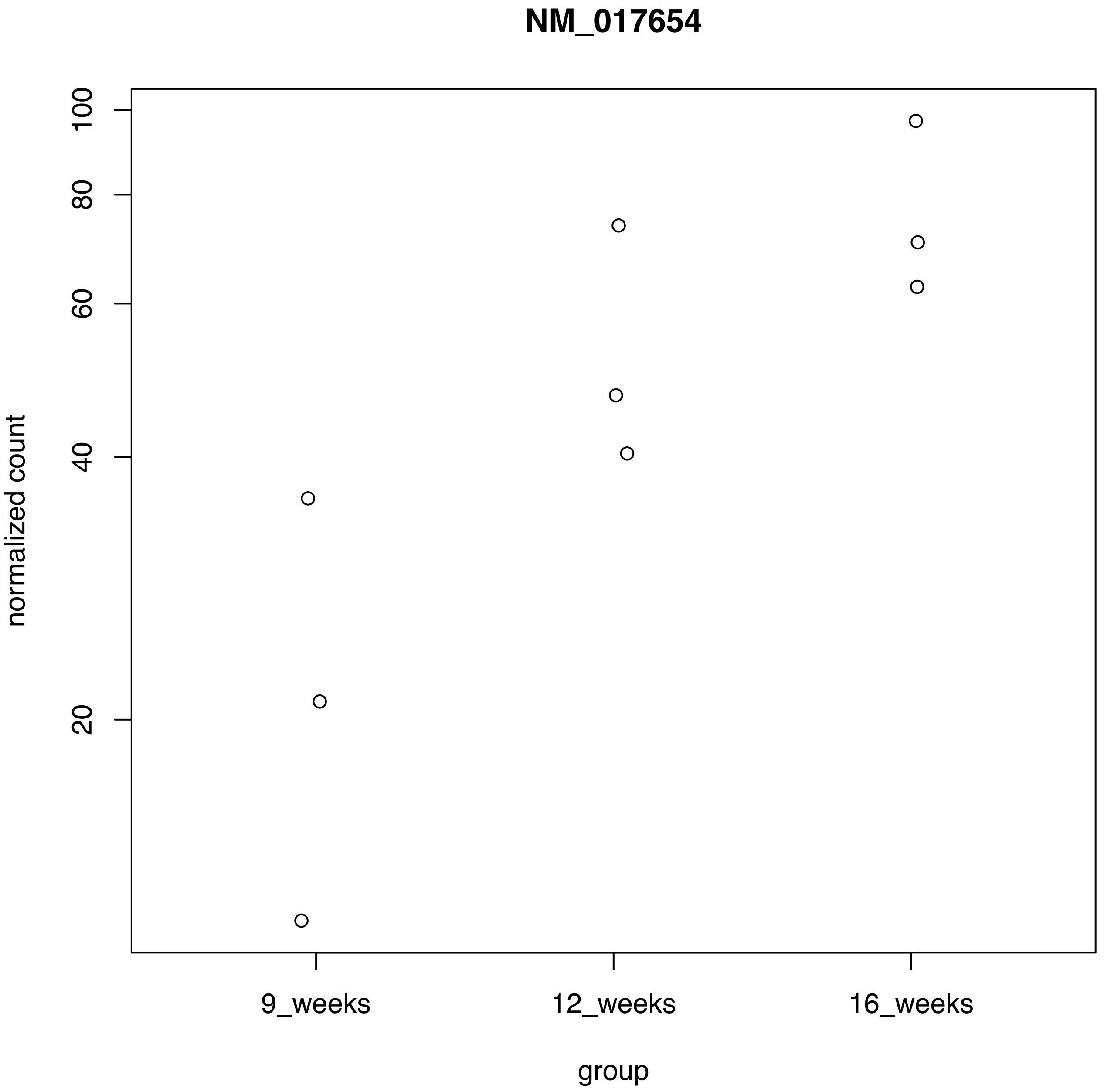
NM_017654
normalized count
100
80
60
40
20
9_weeks
12_weeks
16_weeks
group

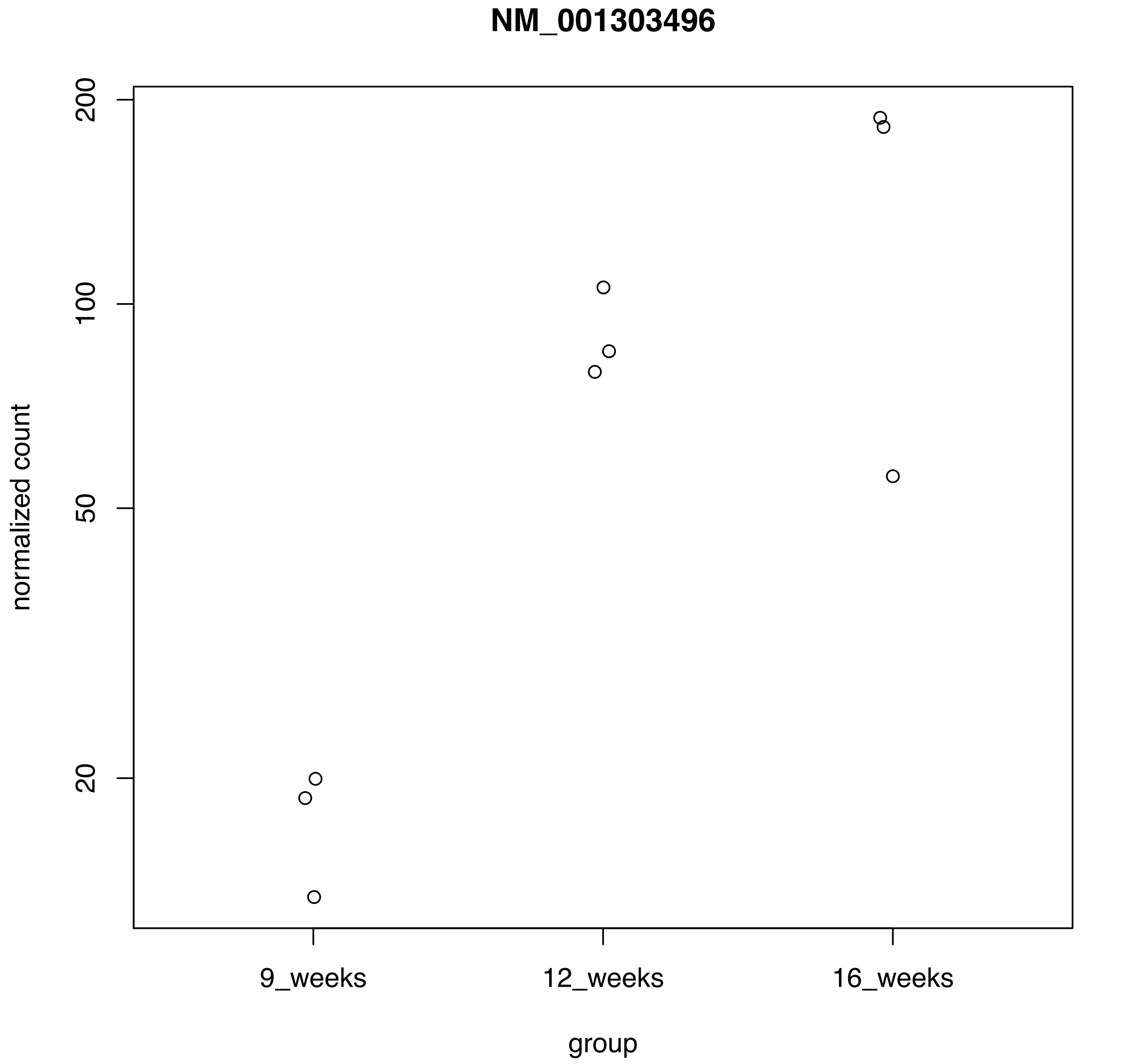

NM_001303496
normalized count
200
100
50
20
9_weeks
12_weeks
16_weeks
group

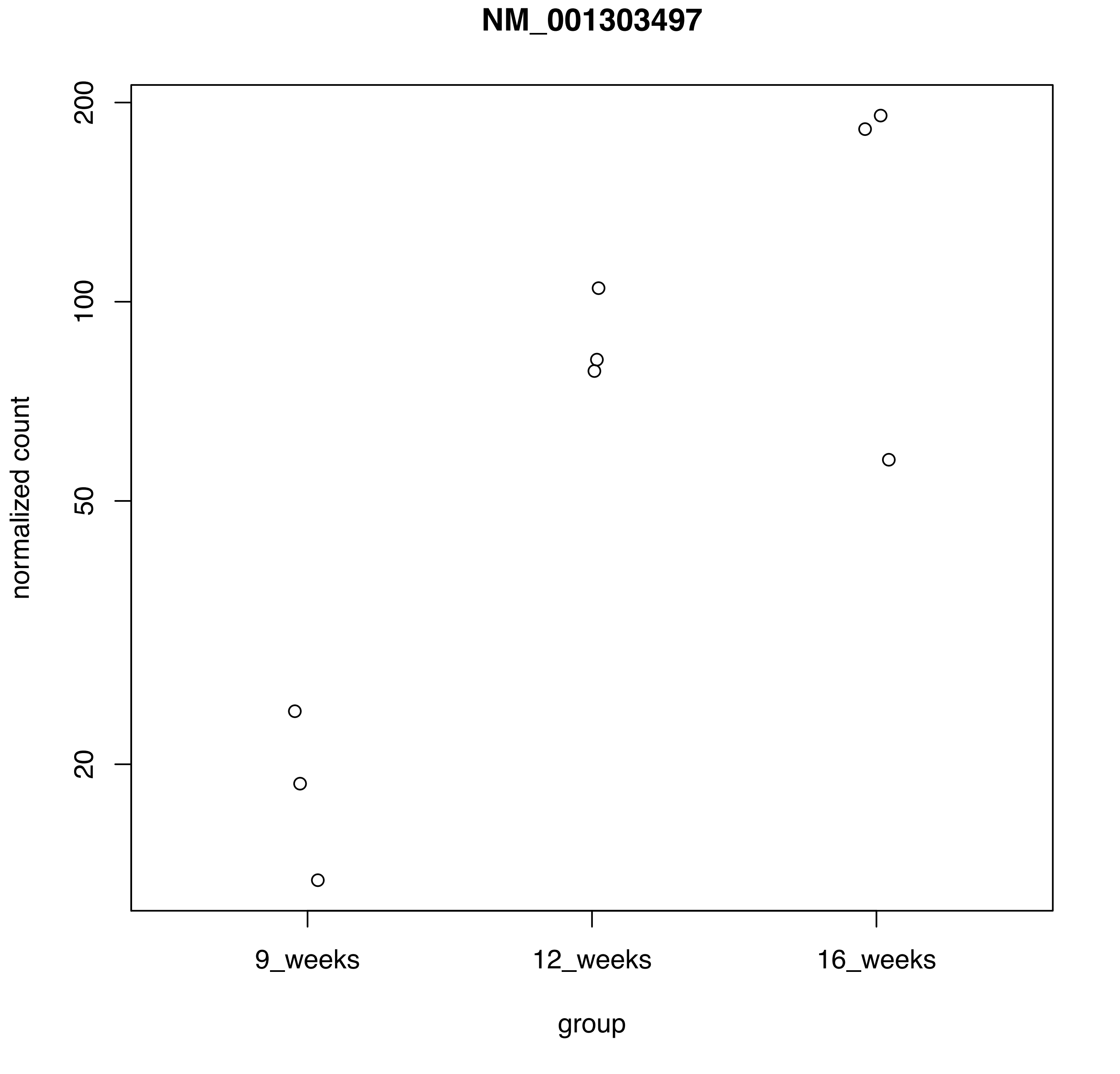

NM_001303497
normalized count
200
100
50
20
9_weeks
12_weeks
16_weeks
group

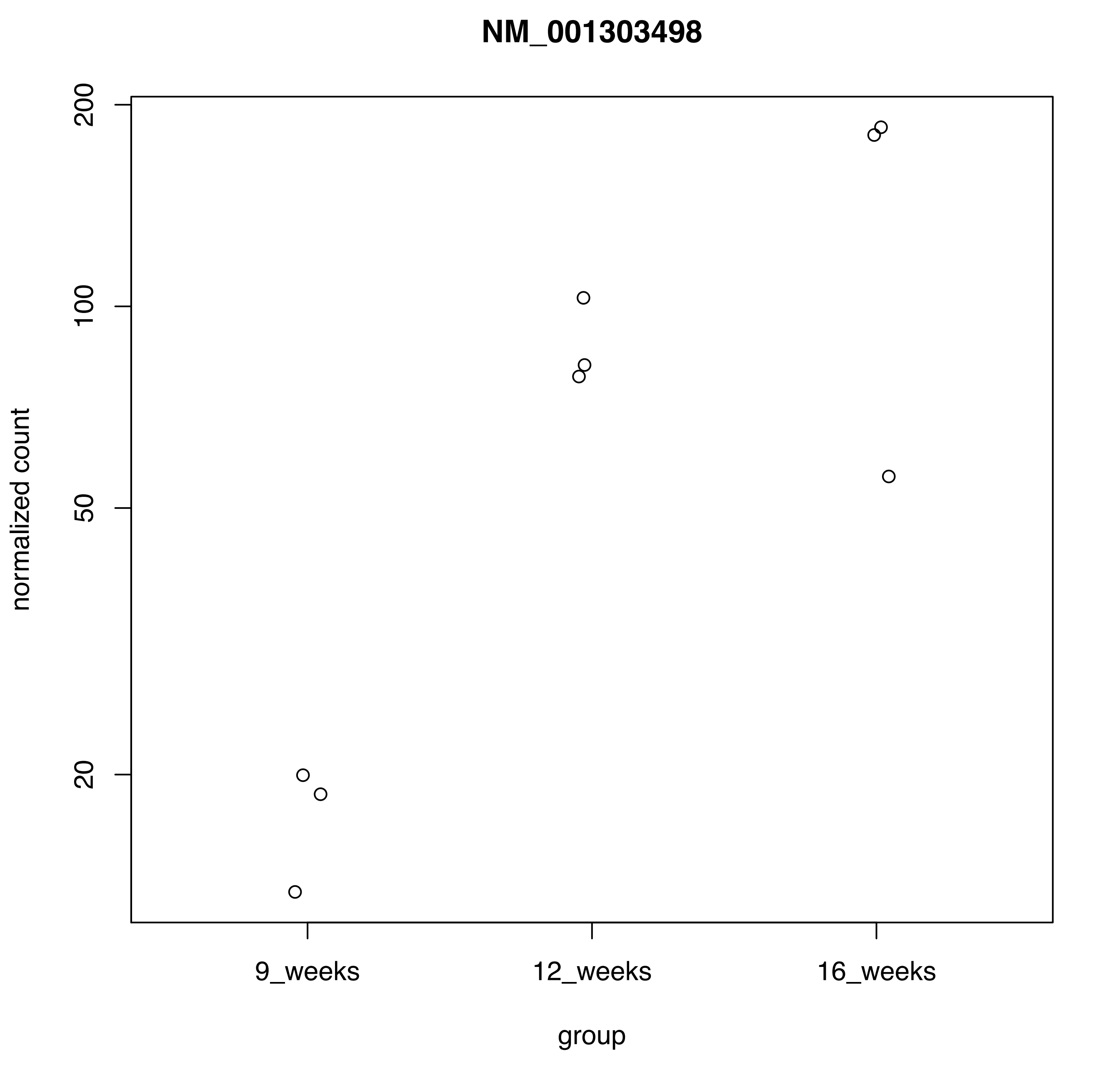
NM_001303498
normalized count
200
100
50
20
9_weeks
12_weeks
16_weeks
group

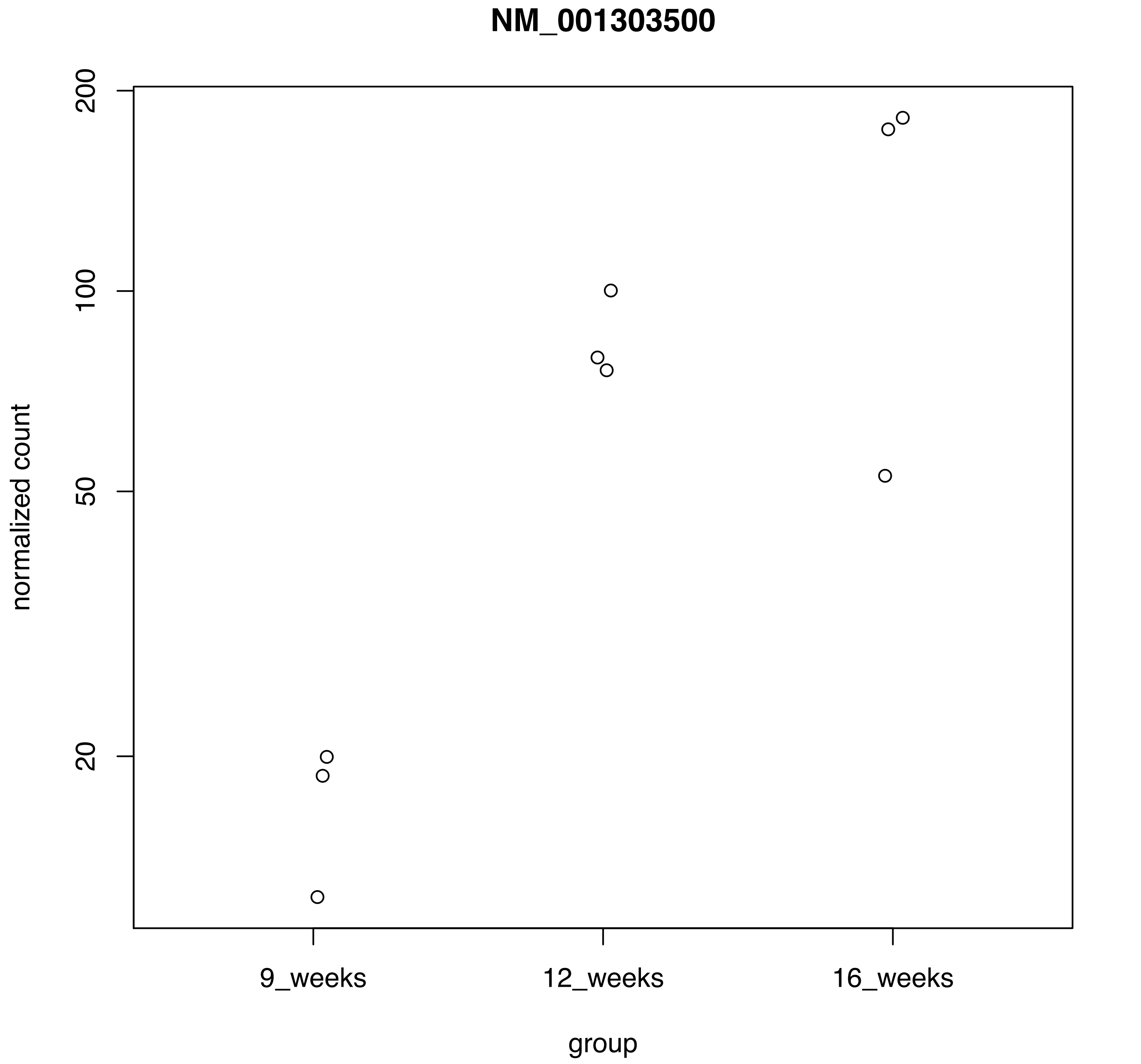
NM_001303500
normalized count
200
100
50
20
9_weeks
12_weeks
16_weeks
group

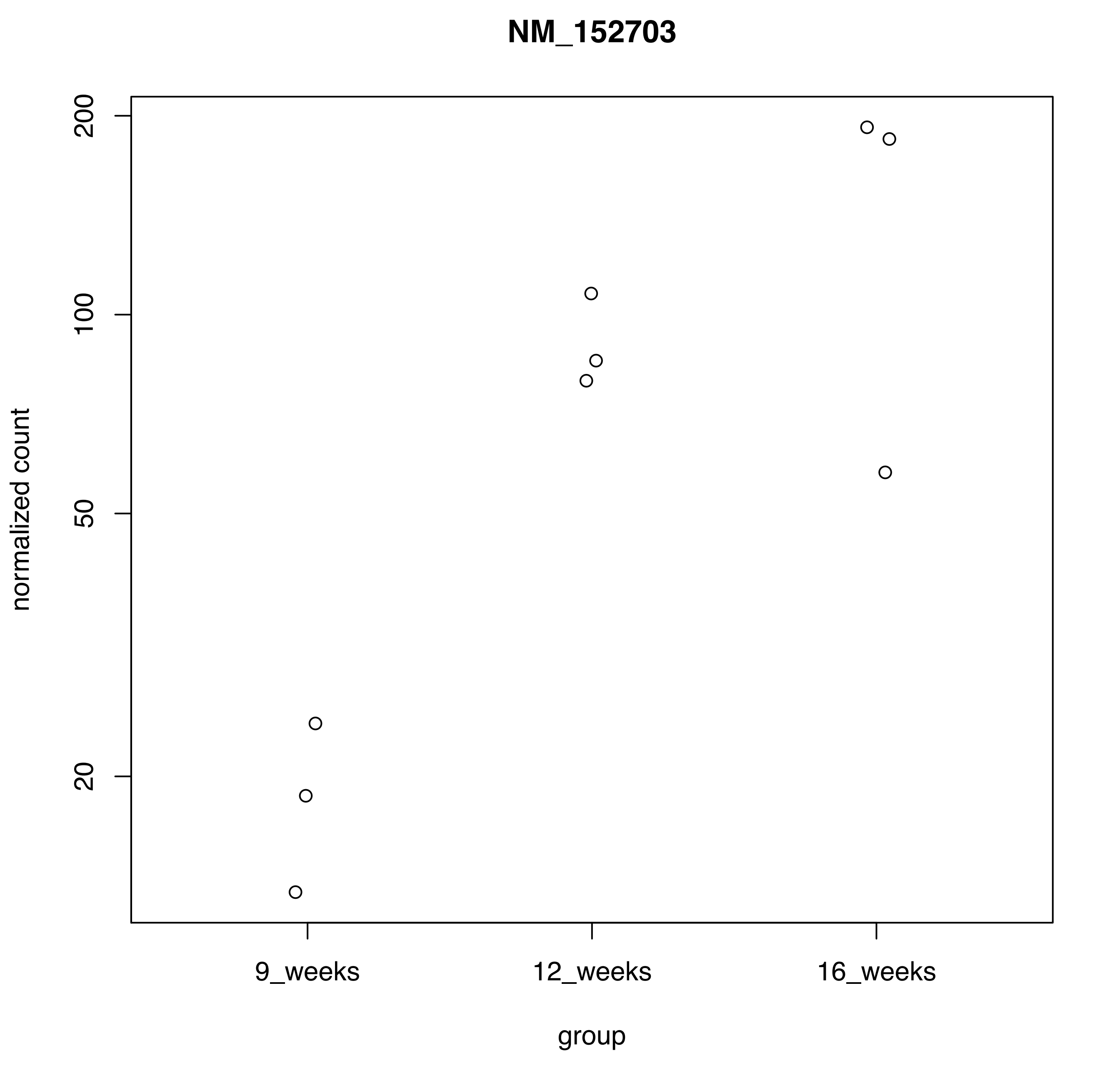
NM_152703
normalized count
200
100
50
20
9_weeks
12_weeks
16_weeks
group

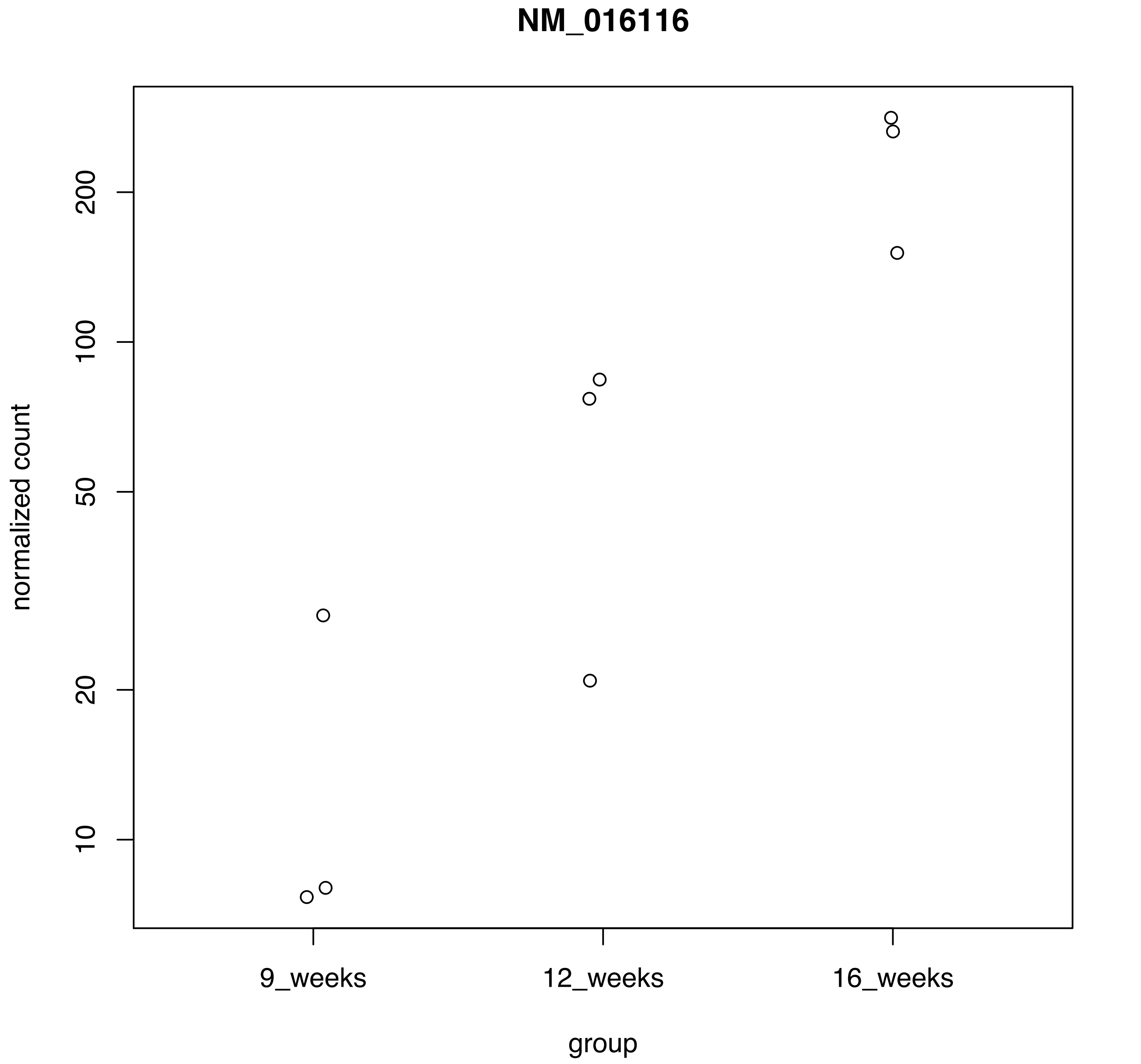

NM_016116
normalized count
10
20
50
100
200
9_weeks
12_weeks
16_weeks
group

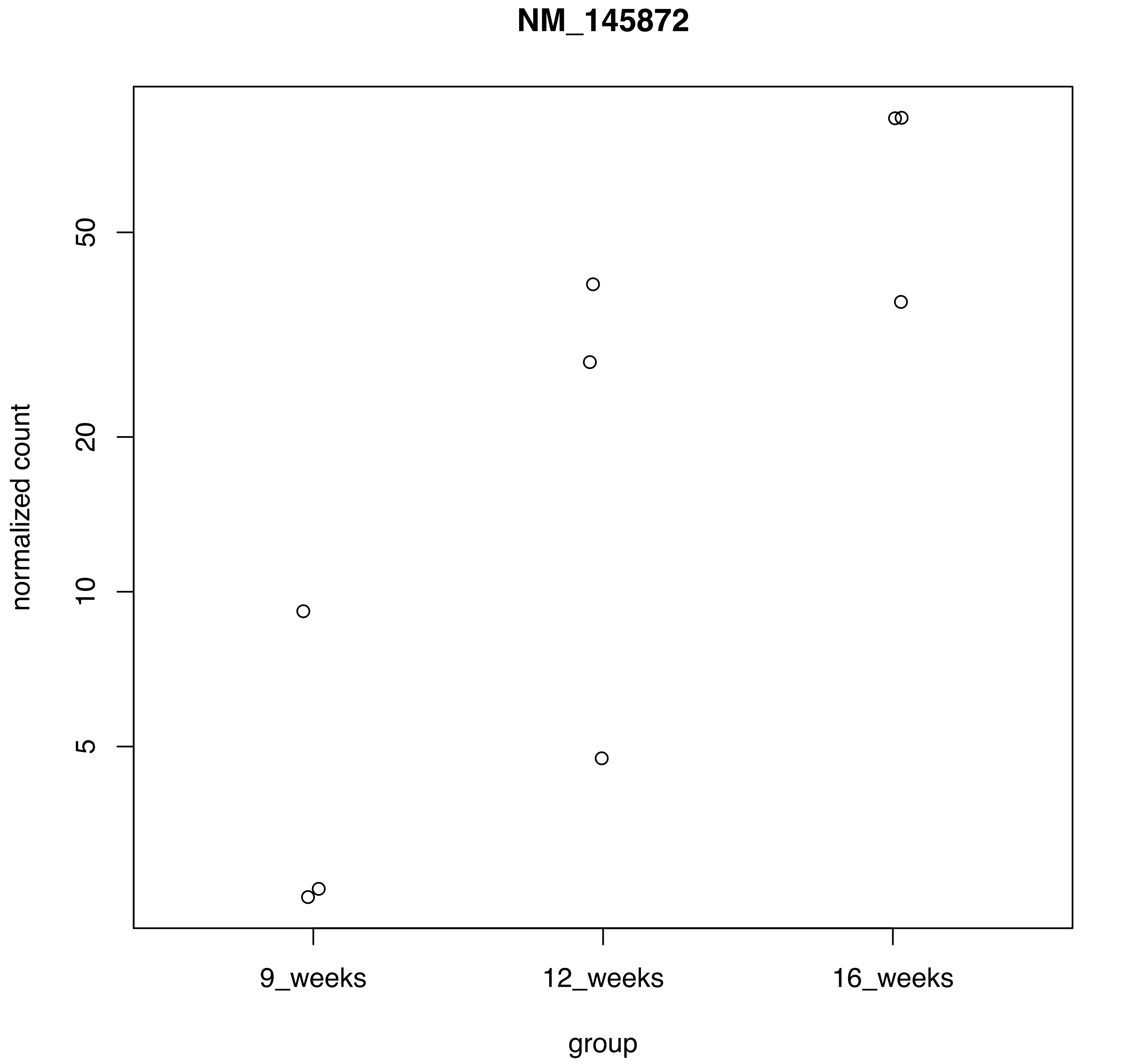
NM_145872
normalized count
50
20
10
5
9_weeks
12_weeks
16_weeks
group

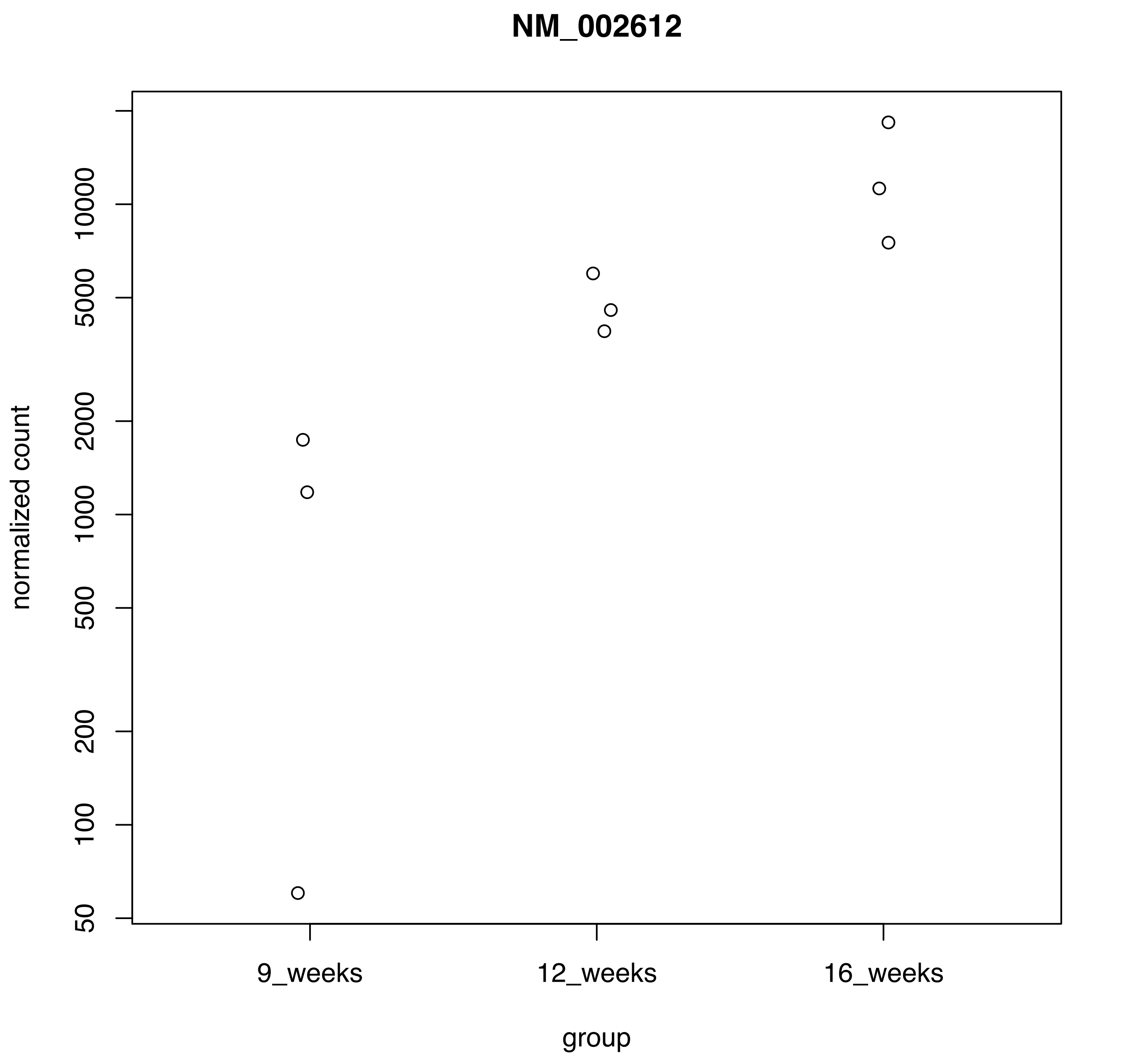
NM_002612
normalized count
10000
5000
2000
1000
500
200
100
50
9_weeks
12_weeks
16_weeks
group

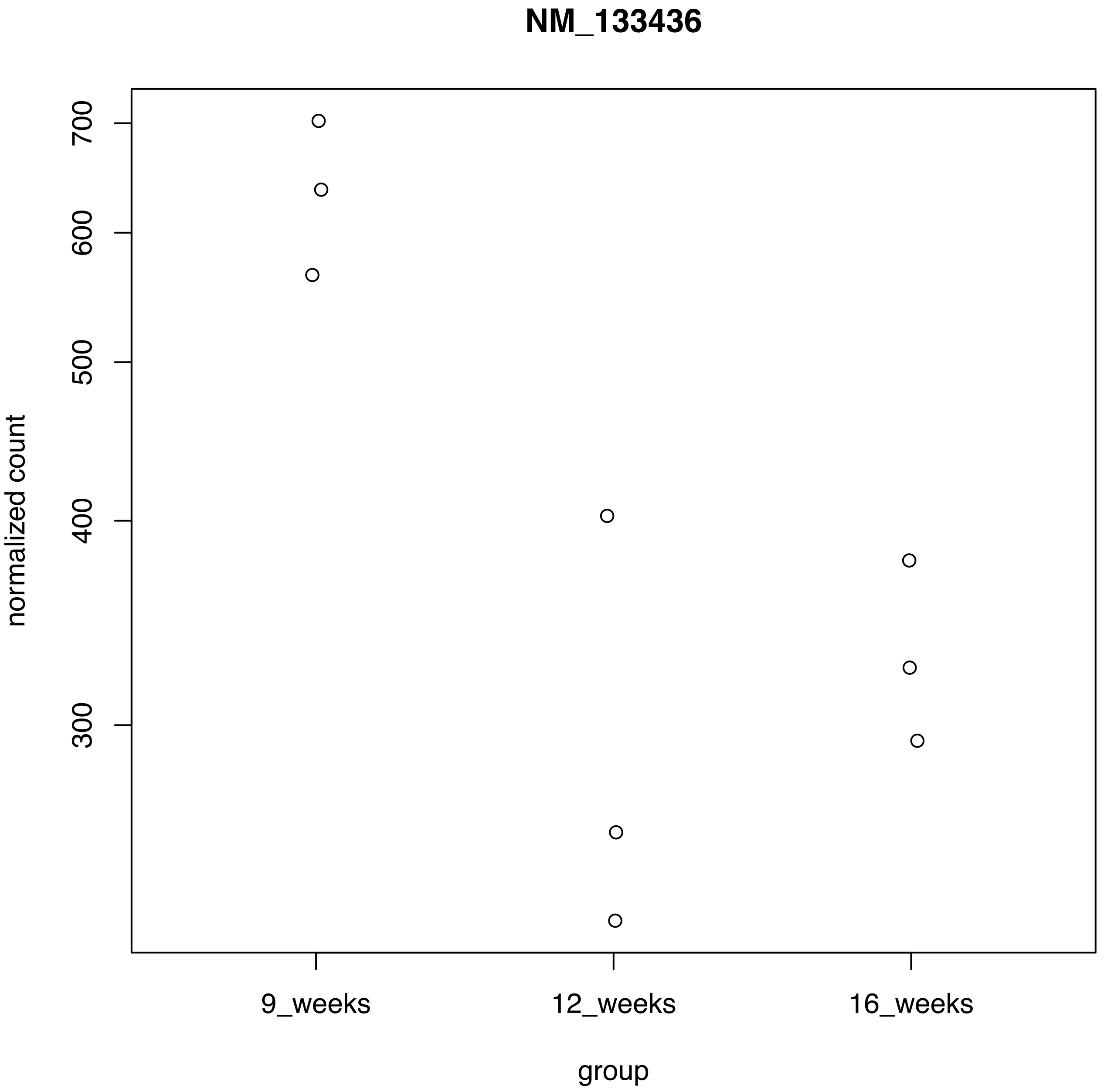
NM_133436
normalized count
700
600
500
400
300
9_weeks
12_weeks
16_weeks
group

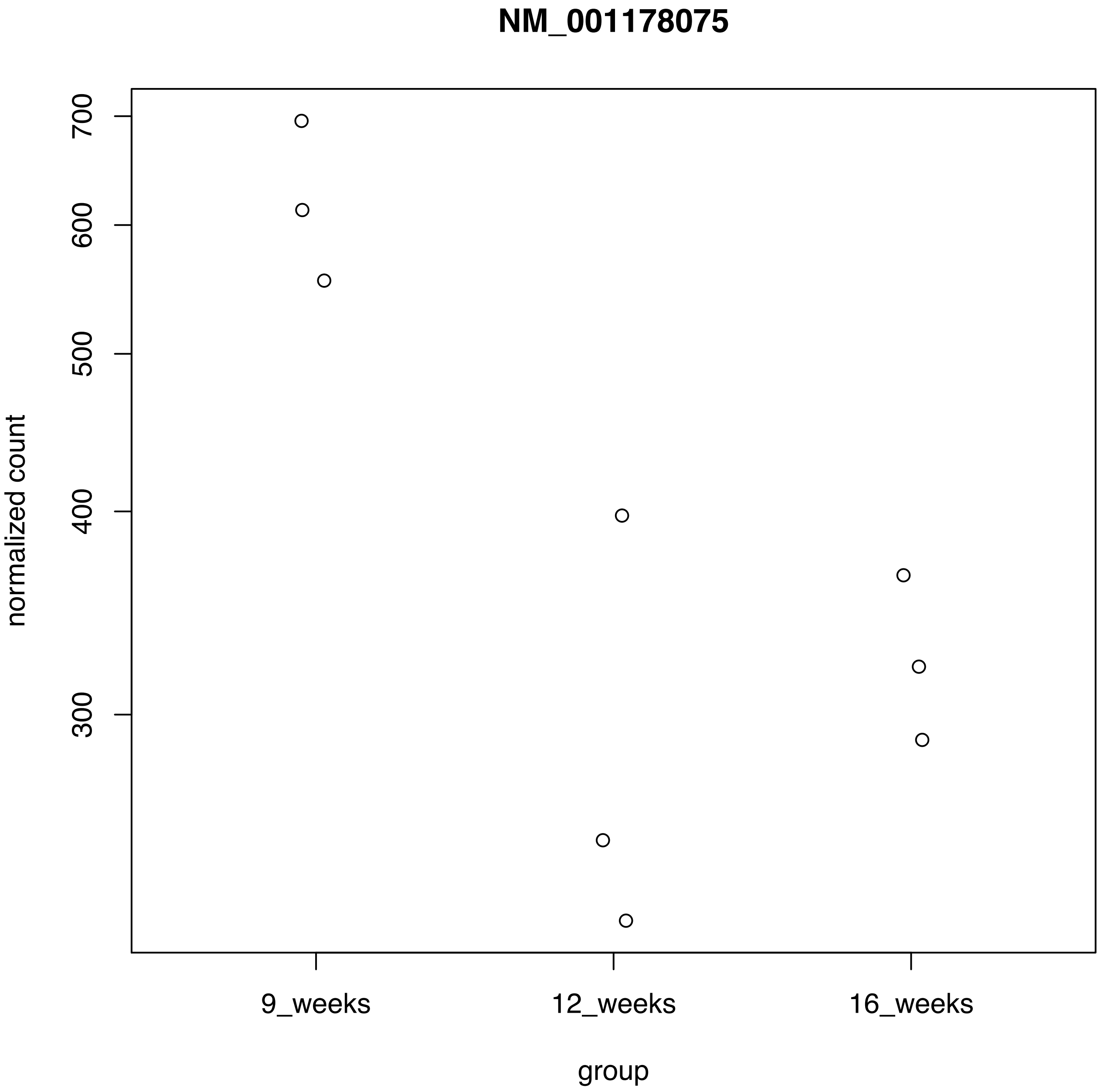
NM_001178075
normalized count
700
600
500
400
300
9_weeks
12_weeks
16_weeks
group

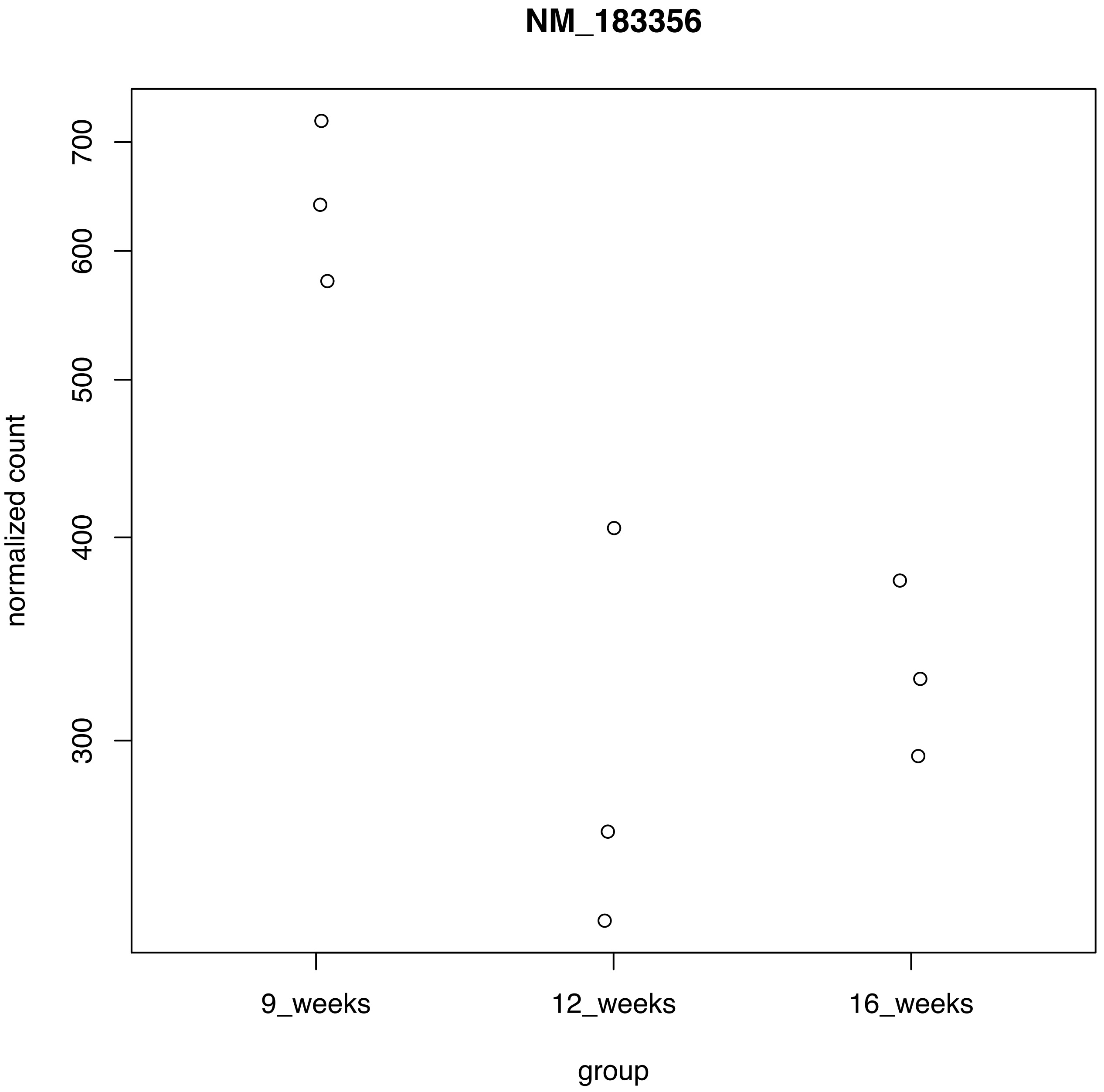
NM_183356
normalized count
700
600
500
400
300
9_weeks
12_weeks
16_weeks
group

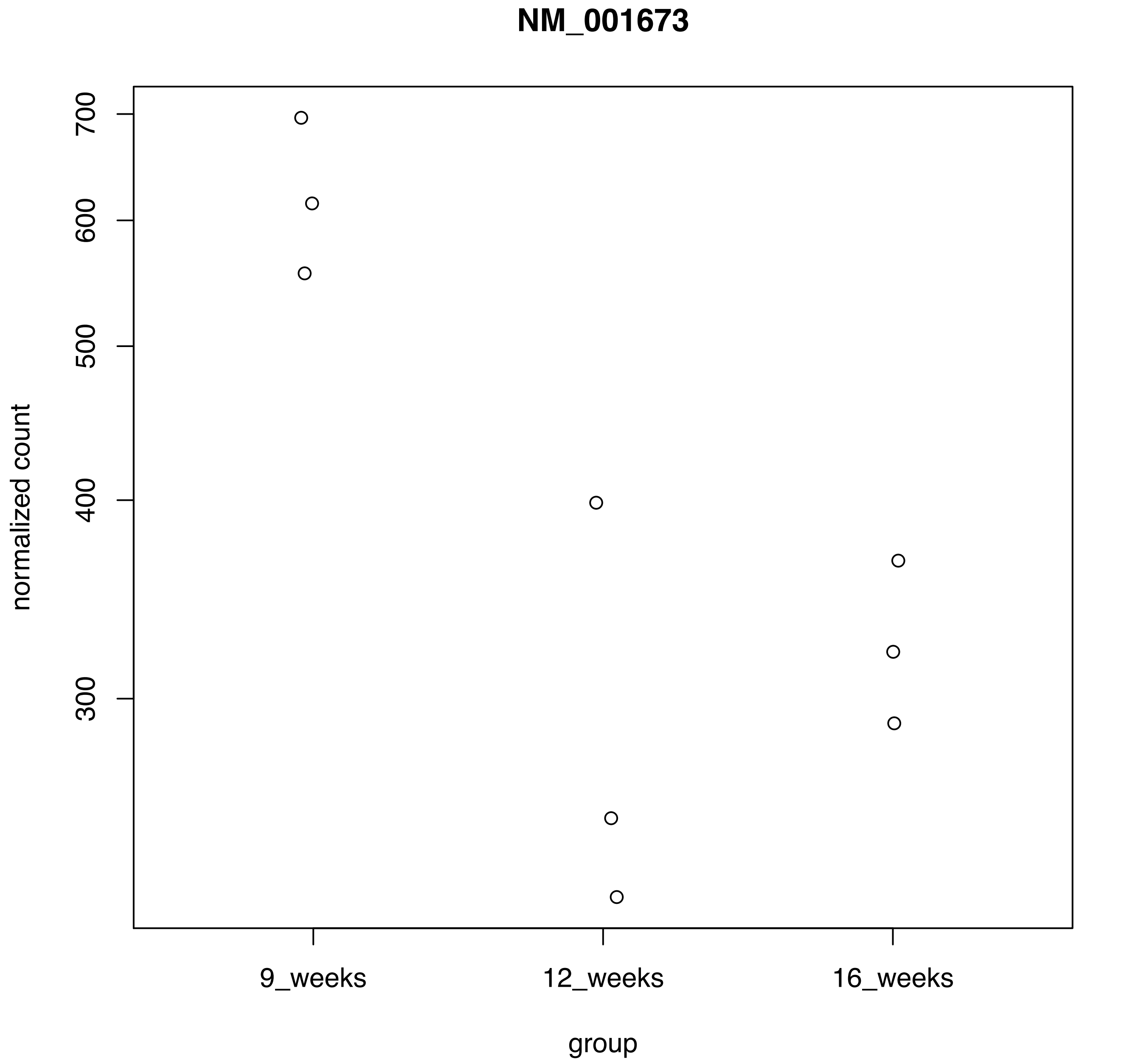
NM_001673
normalized count
700
600
500
400
300
9_weeks
12_weeks
16_weeks
group

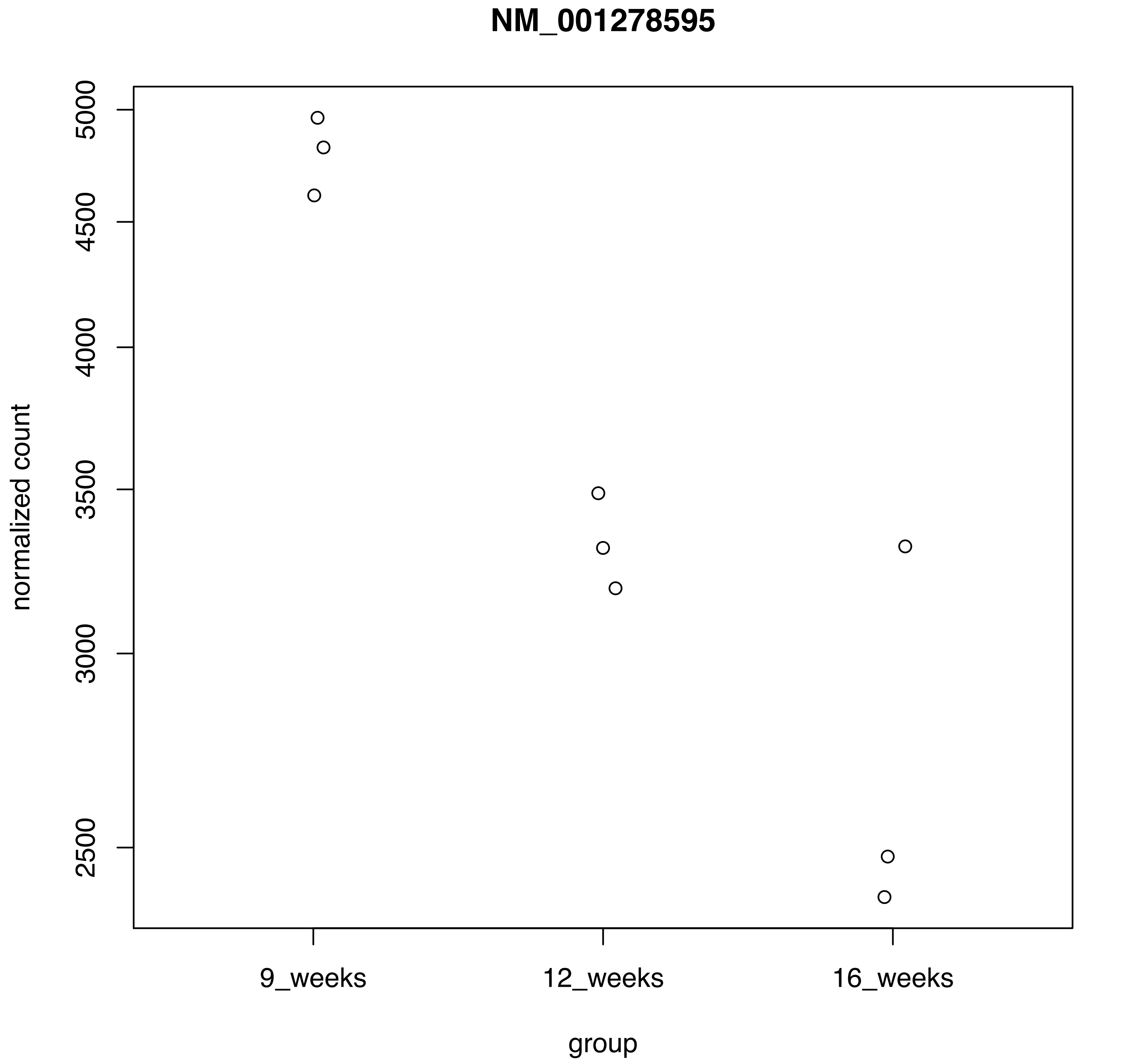
NM_001278595
normalized count
5000
4500
4000
3500
3000
2500
9_weeks
12_weeks
16_weeks
group

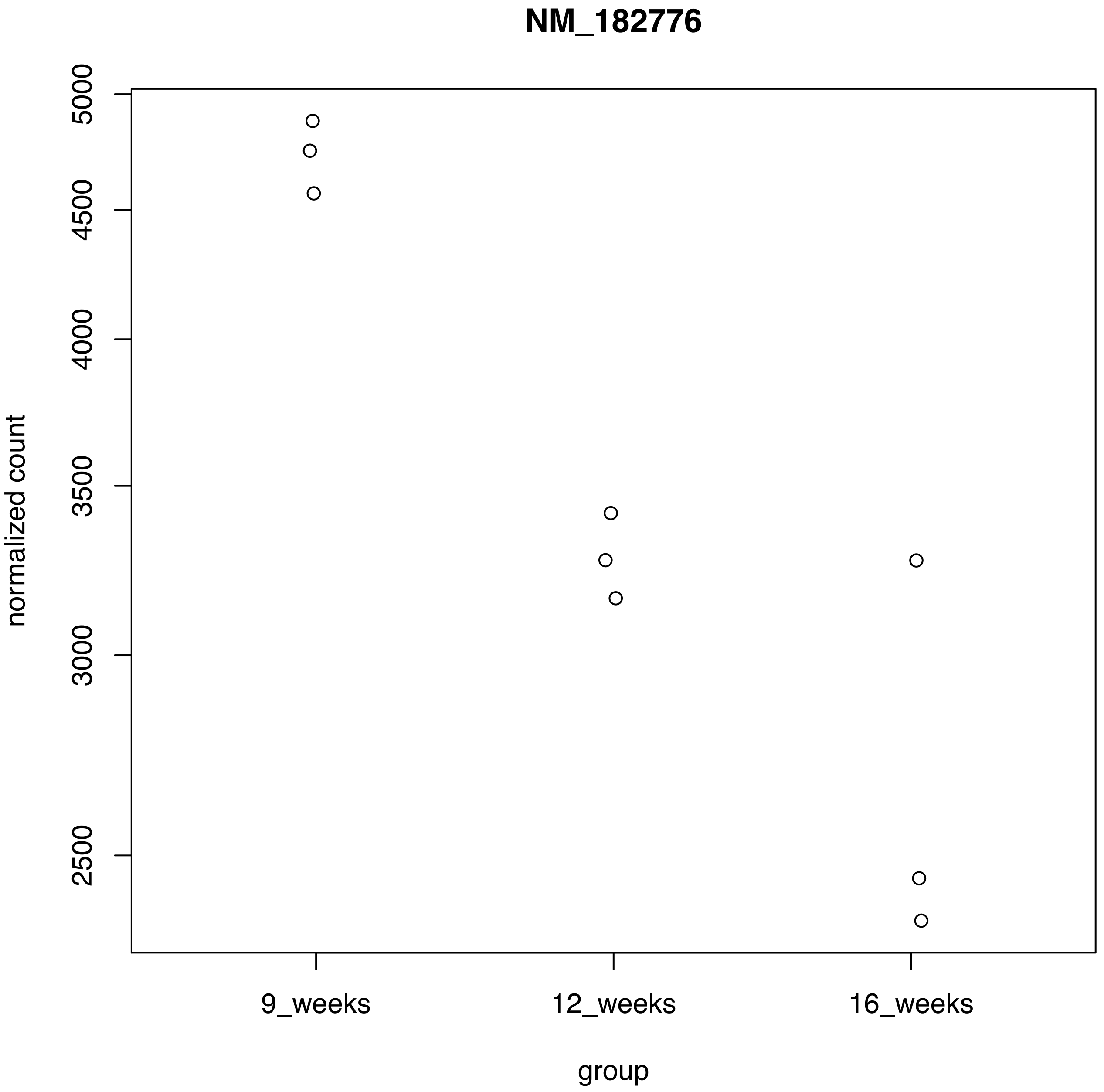
NM_182776
normalized count
5000
4500
4000
3500
3000
2500
9_weeks
12_weeks
16_weeks
group

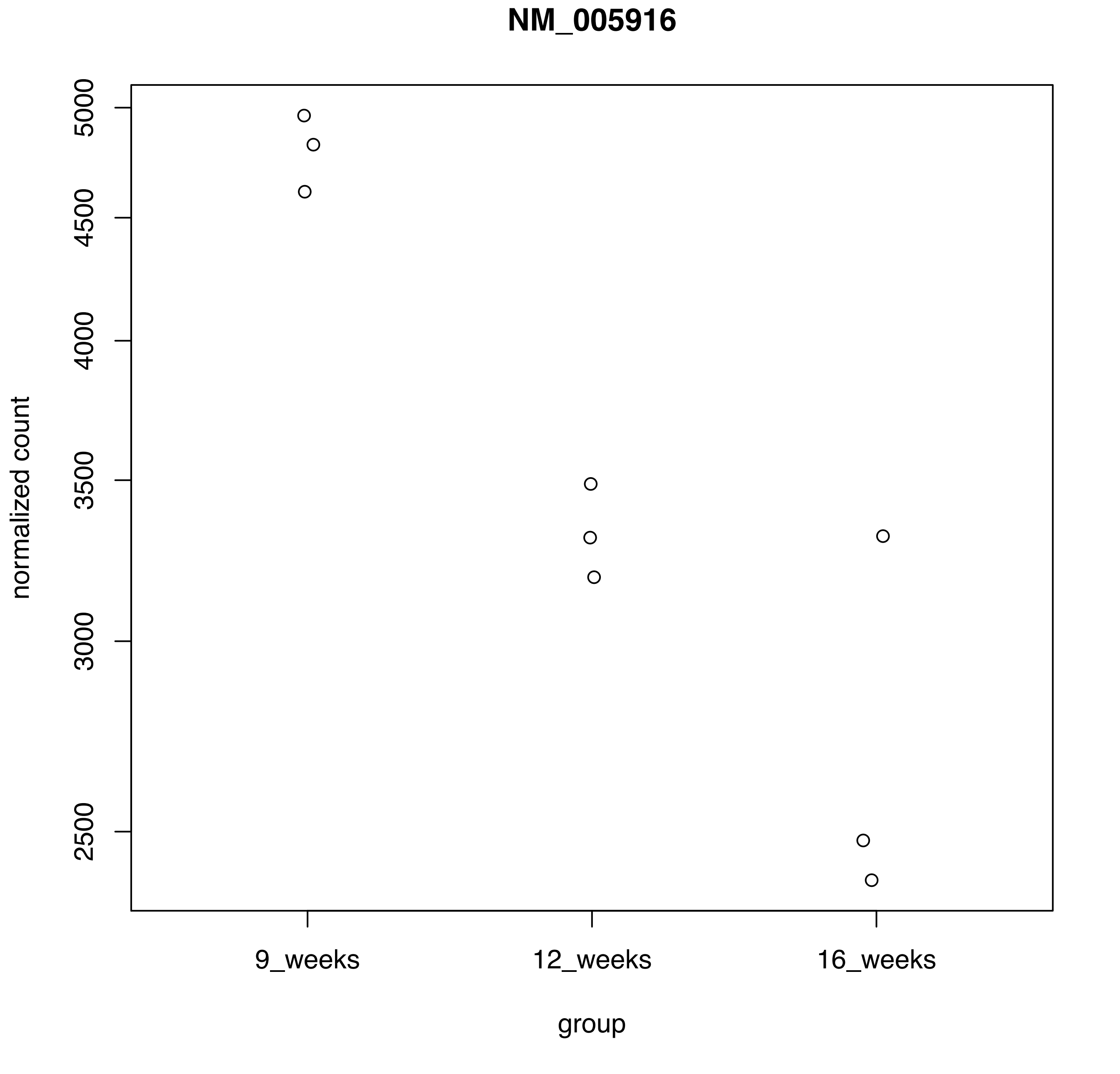

NM_005916
normalized count
5000
4500
4000
3500
3000
2500
9_weeks
12_weeks
16_weeks
group

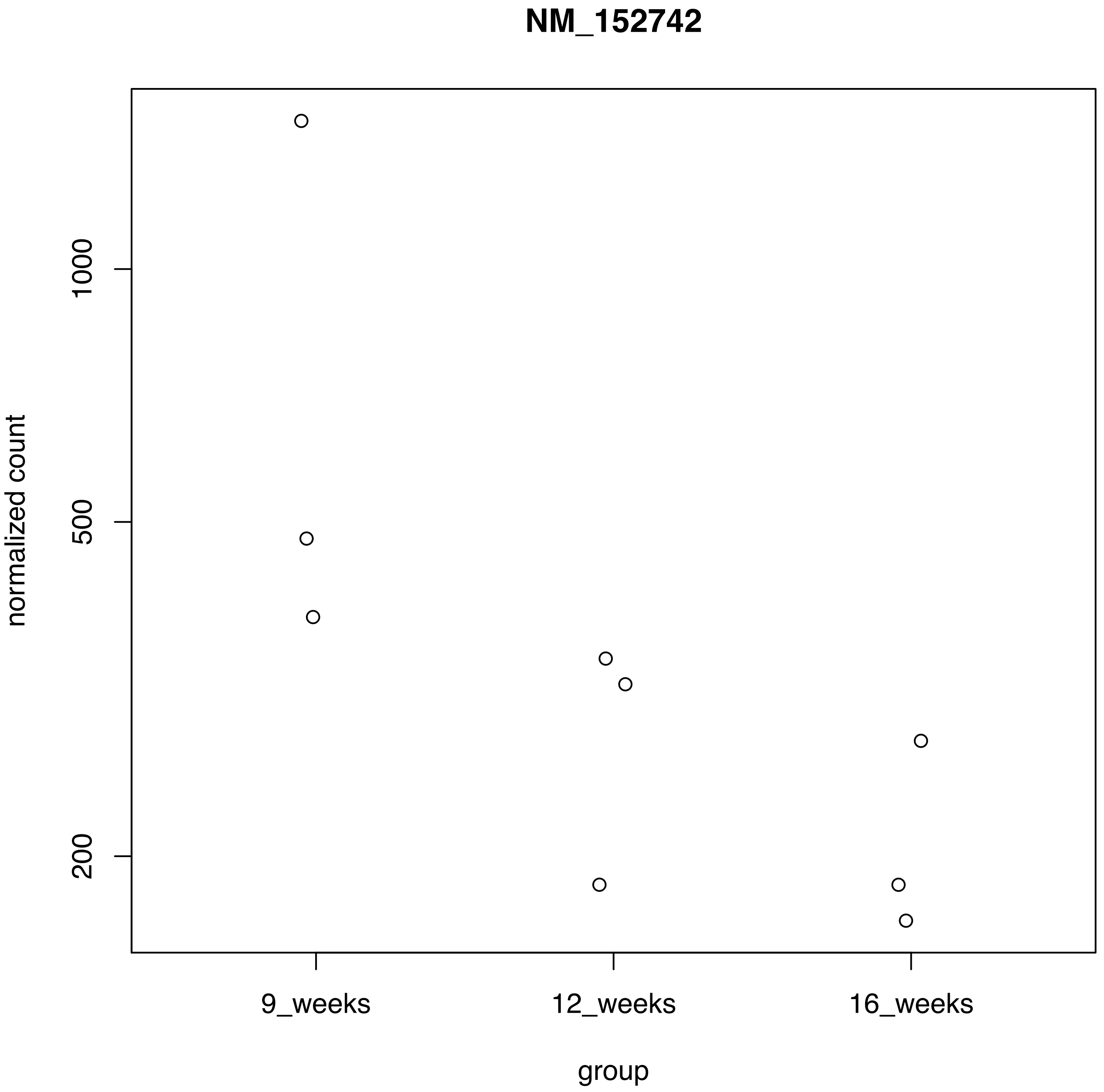
NM_152742
normalized count
1000
500
200
9_weeks
12_weeks
16_weeks
group

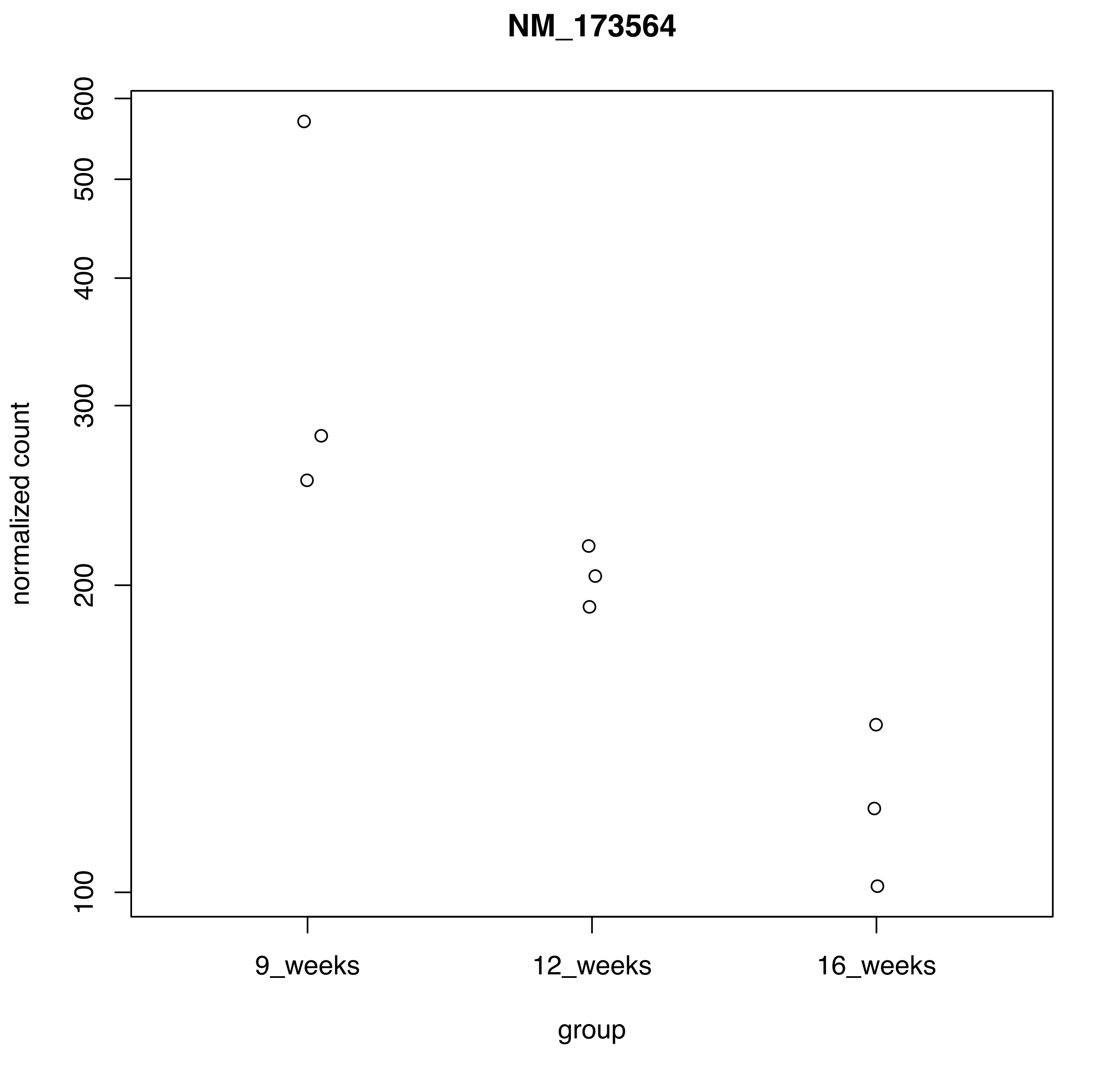

NM_173564
normalized count
600
500
400
300
200
100
9_weeks
12_weeks
16_weeks
group

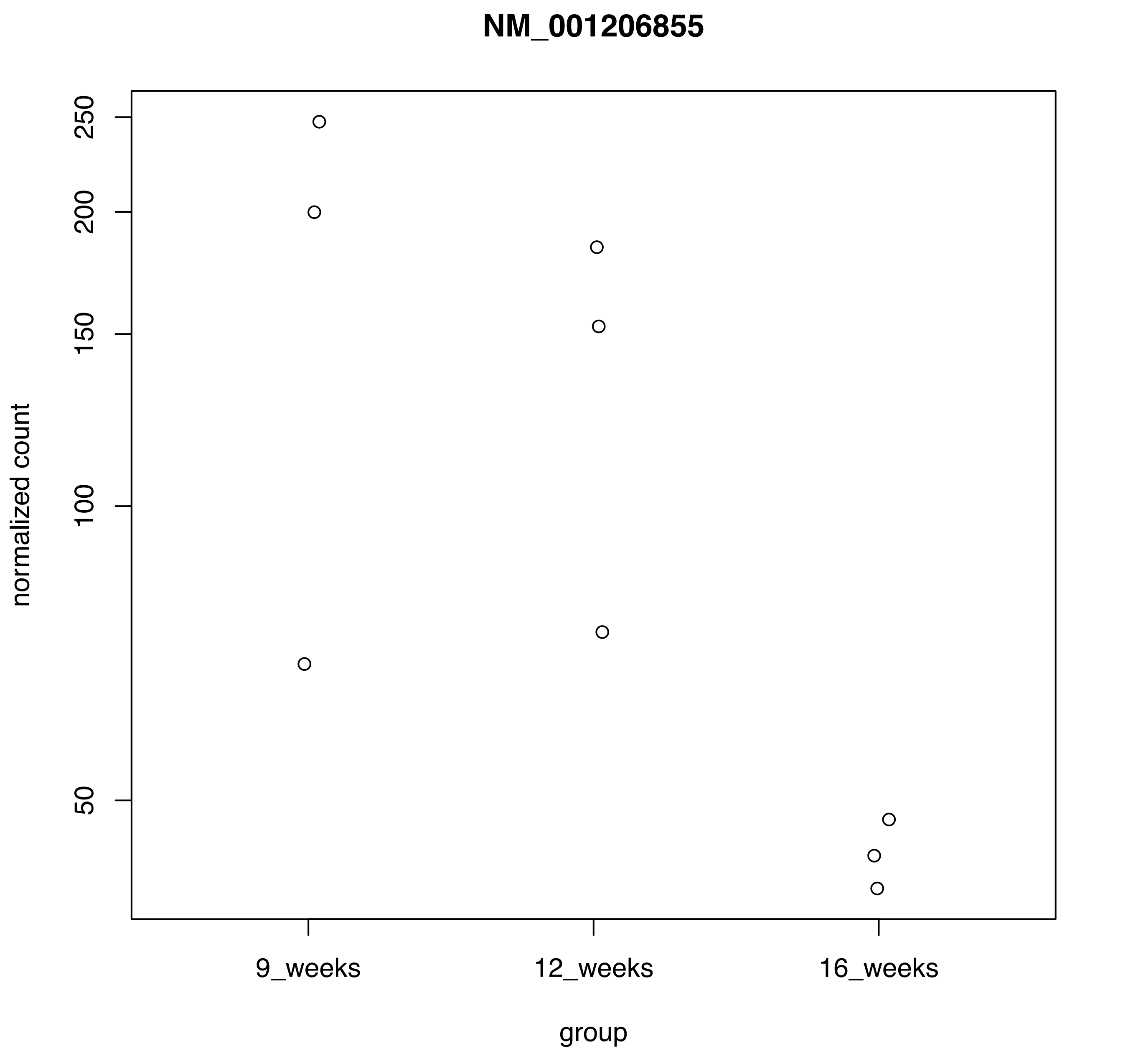

NM_001206855
normalized count
250
200
150
100
50
9_weeks
12_weeks
16_weeks
group

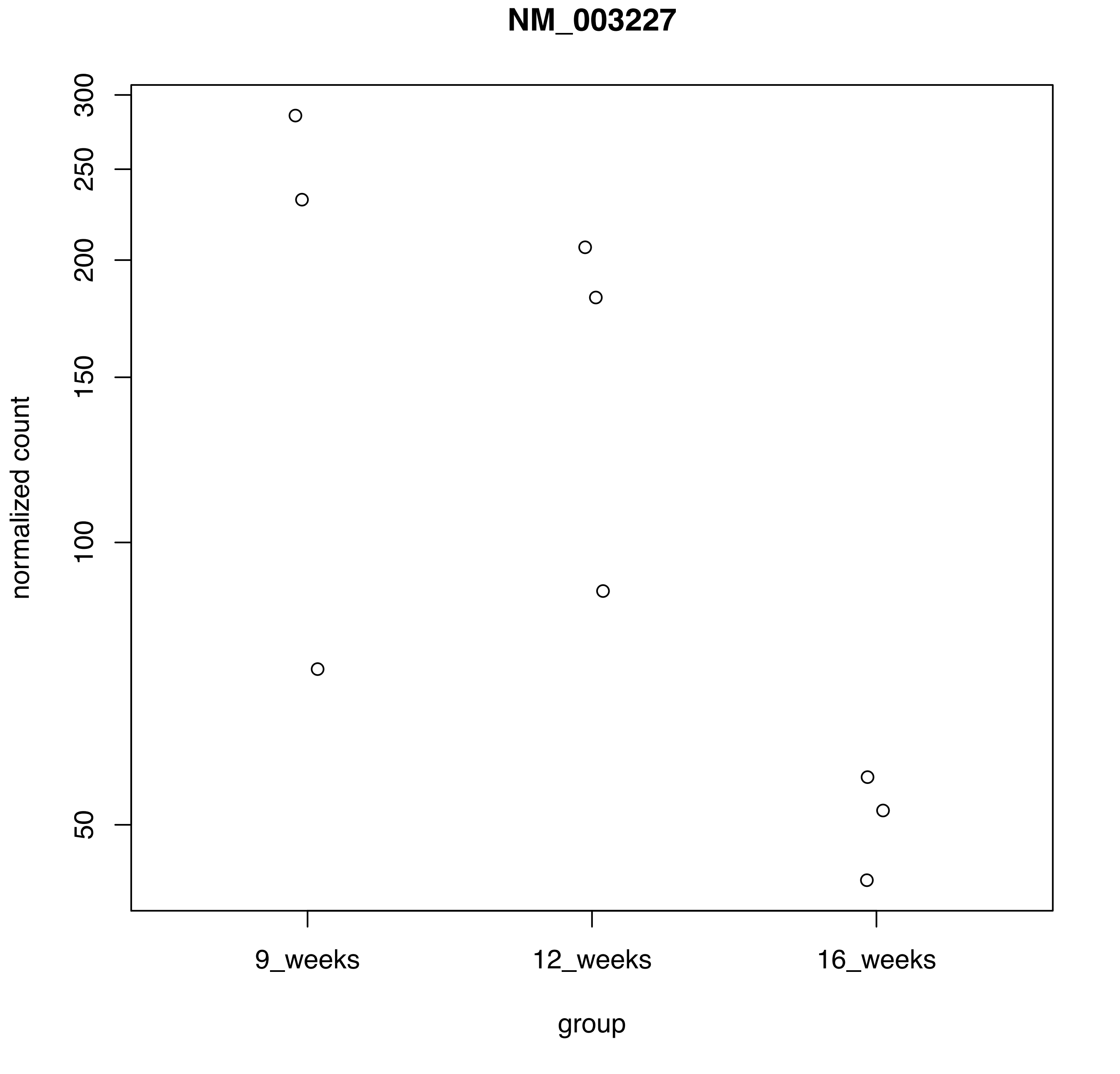
NM_003227
normalized count
300
250
200
150
100
50
9_weeks
12_weeks
16_weeks
group

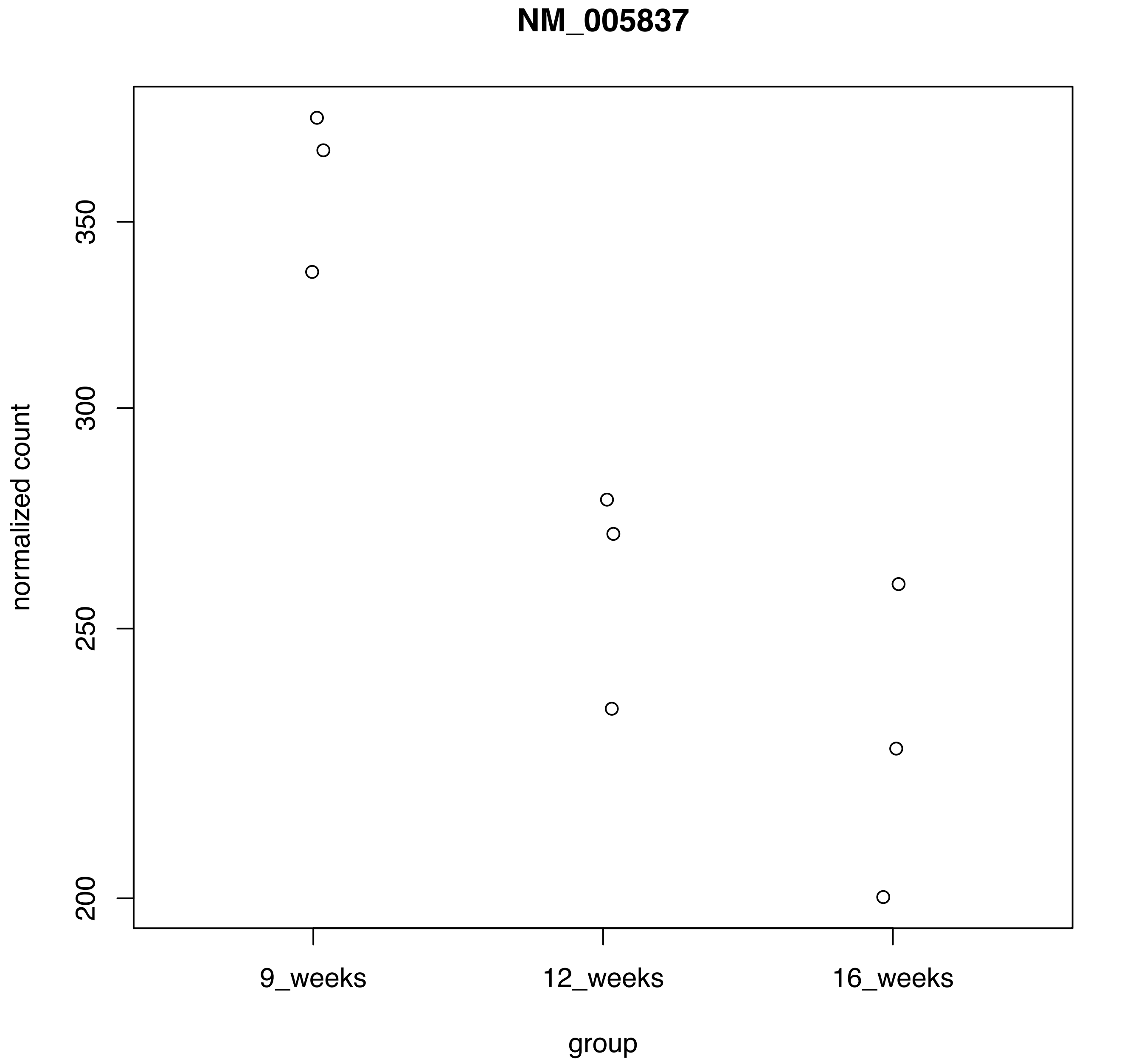
NM_005837
normalized count
350
300
250
200
9_weeks
12_weeks
16_weeks
group

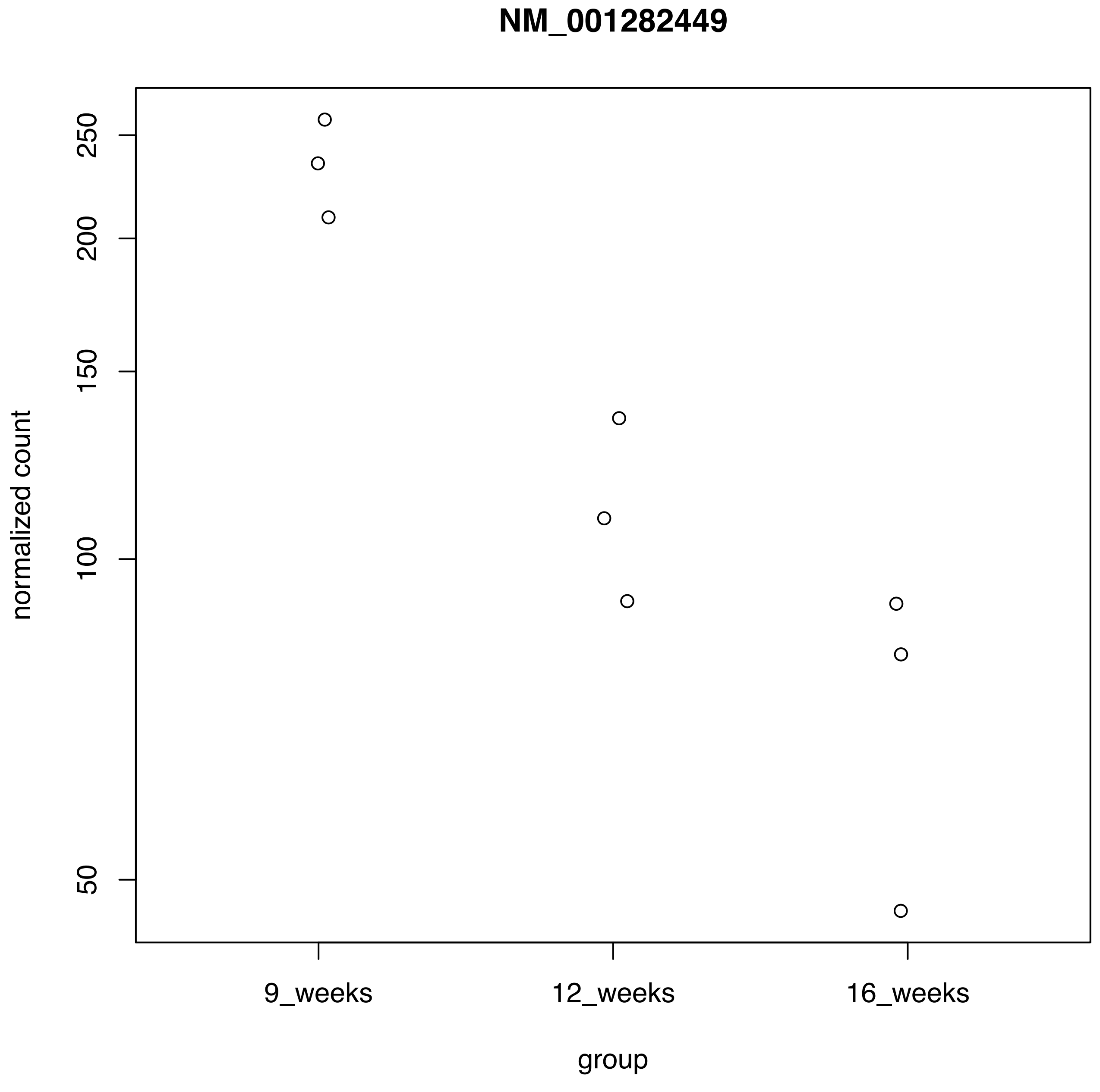
NM_001282449
normalized count
250
200
150
100
50
9_weeks
12_weeks
16_weeks
group

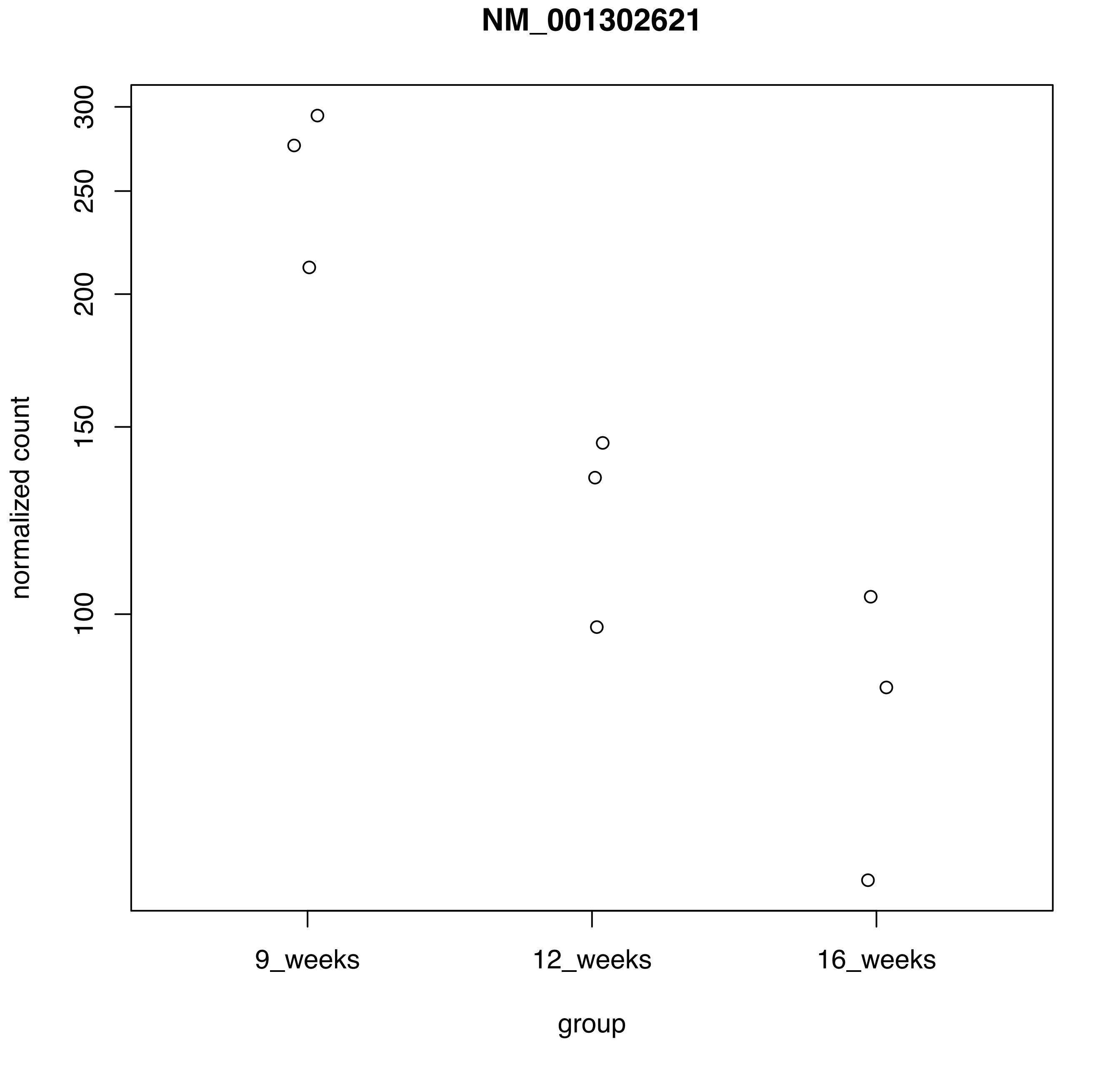
NM_001302621
normalized count
300
250
200
150
100
9_weeks
12_weeks
16_weeks
group

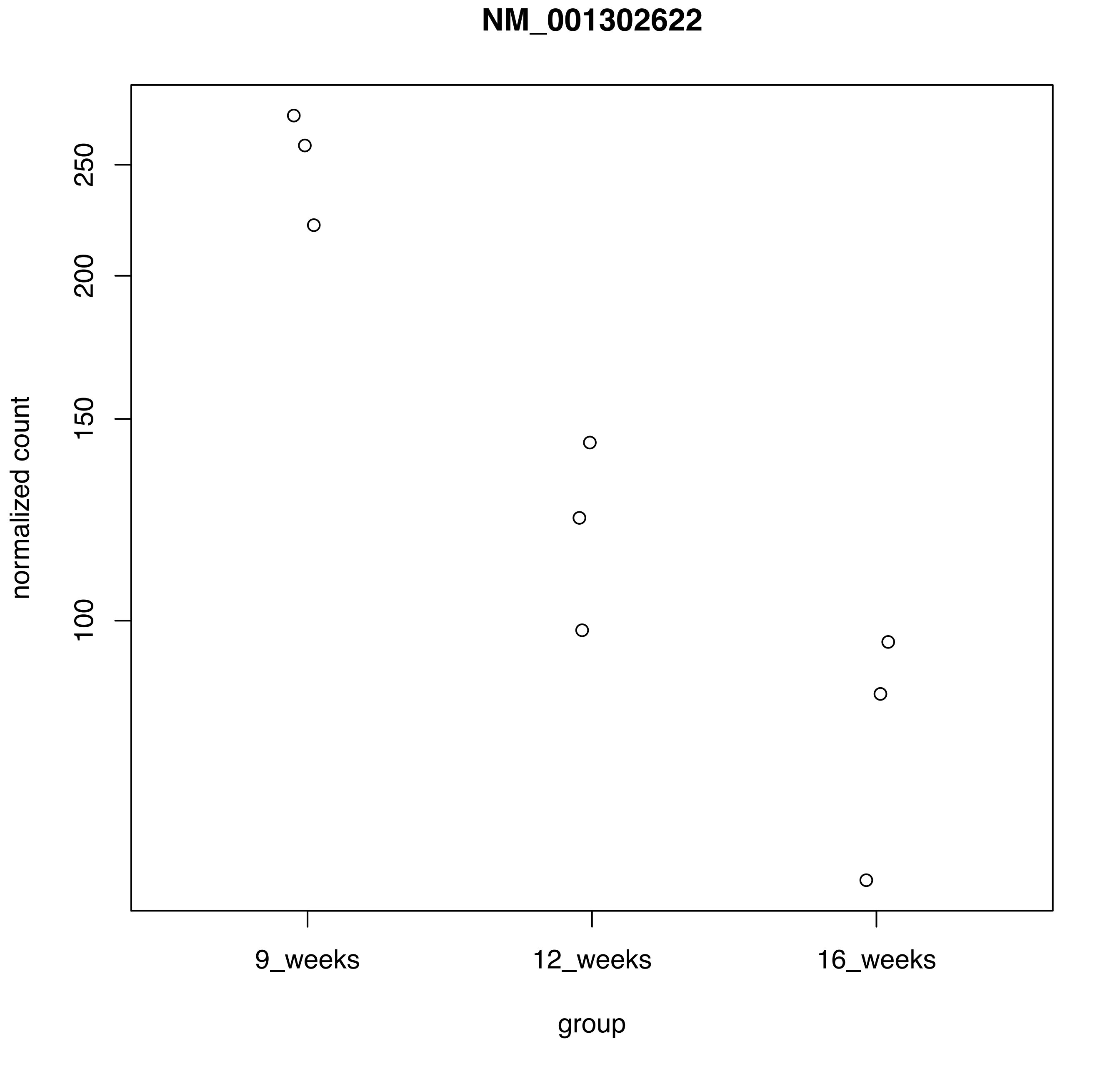
NM_001302622
normalized count
250
200
150
100
9_weeks
12_weeks
16_weeks
group

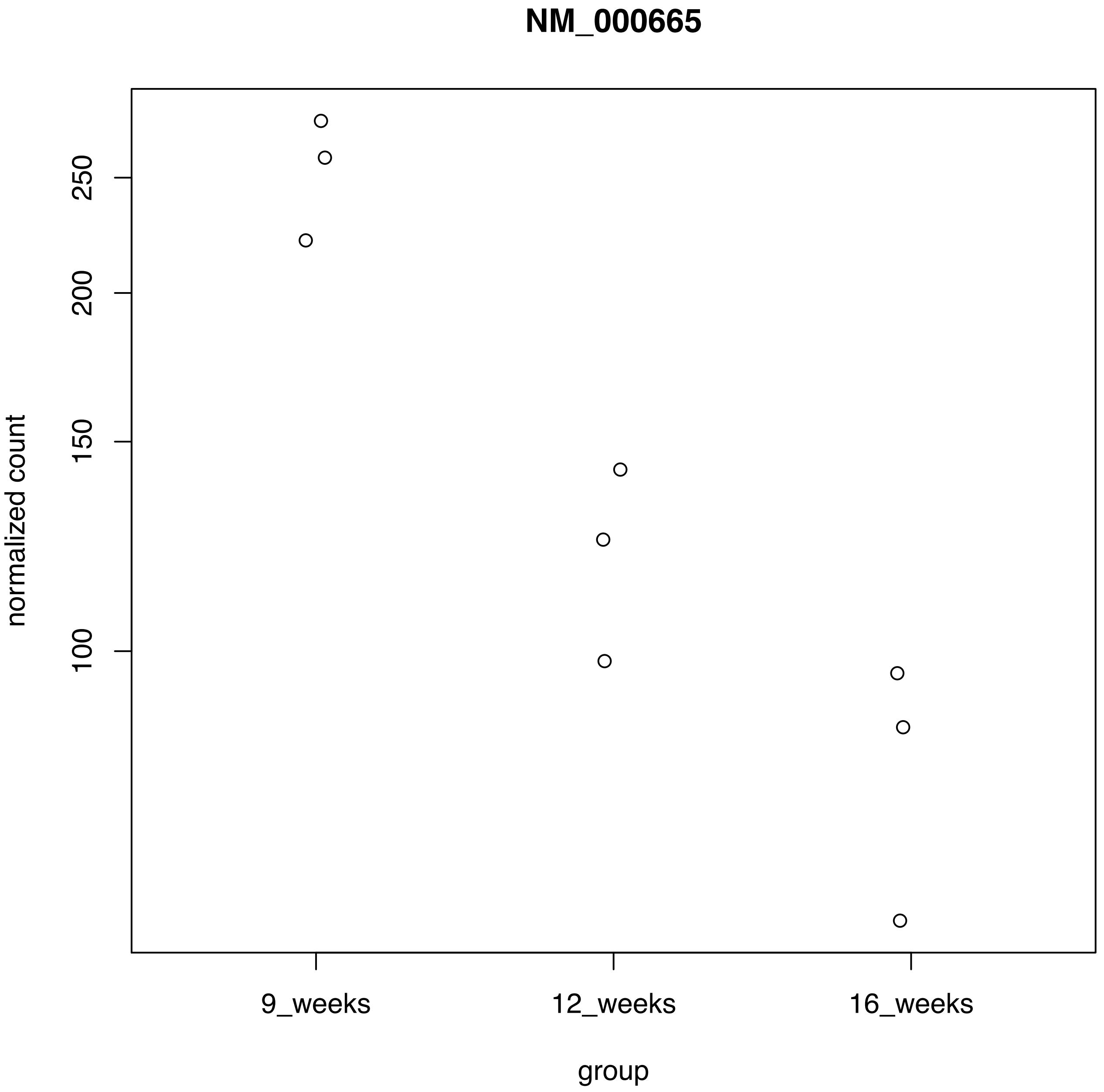
NM_000665
normalized count
250
200
150
100
9_weeks
12_weeks
16_weeks
group

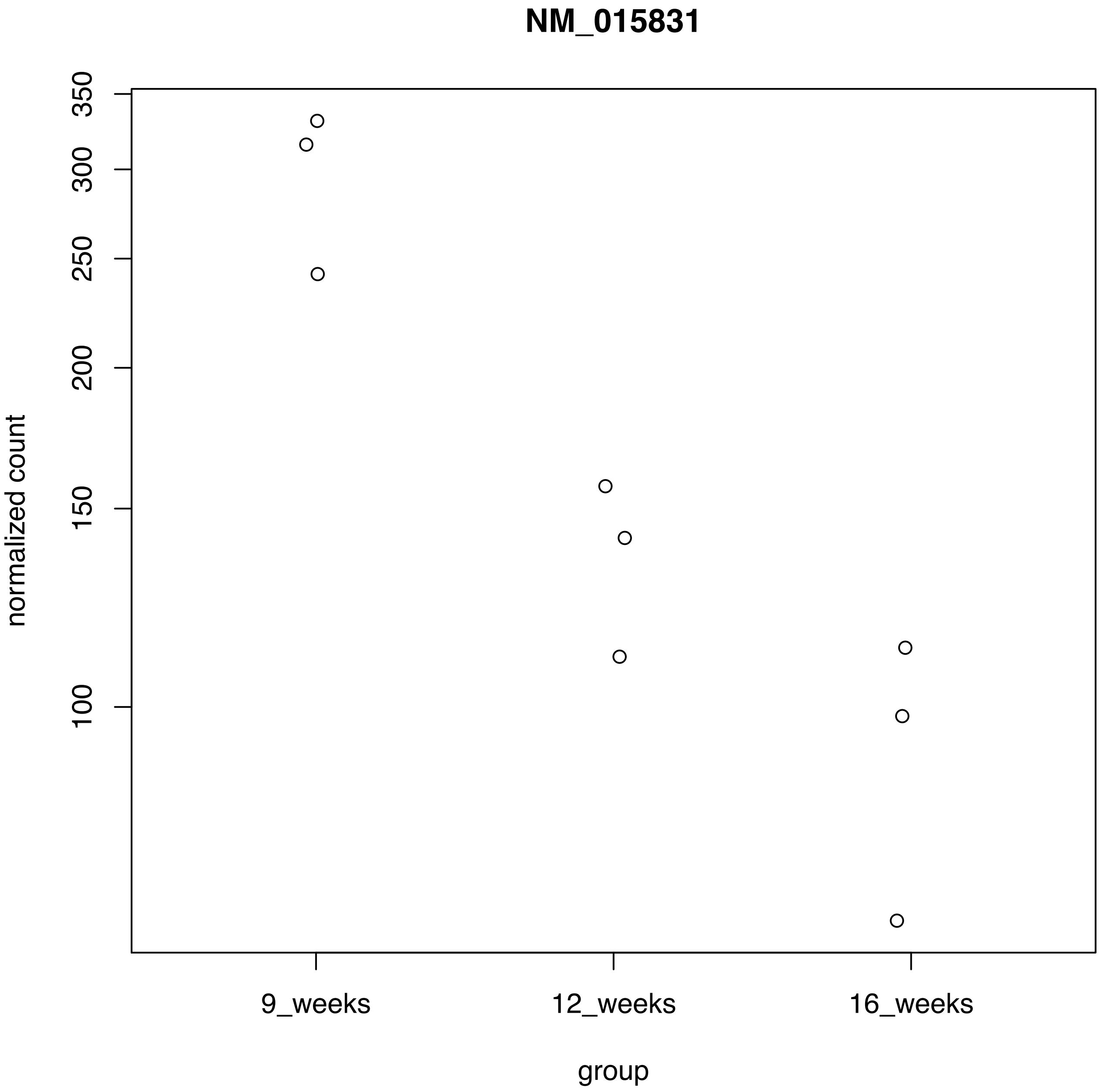
NM_015831
normalized count
350
300
250
200
150
100
9_weeks
12_weeks
16_weeks
group

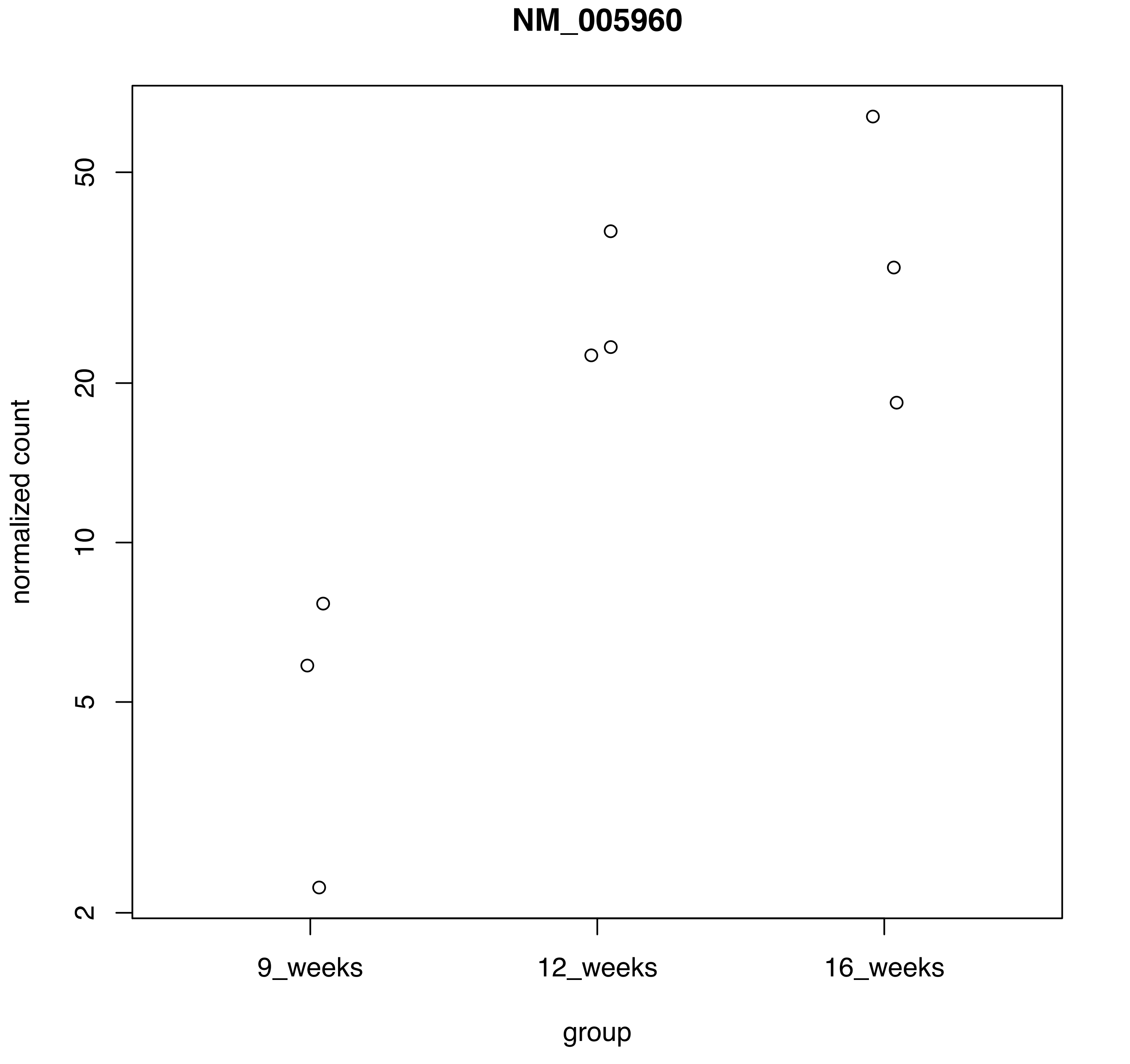
NM_005960
normalized count
50
20
10
5
2
9_weeks
12_weeks
16_weeks
group

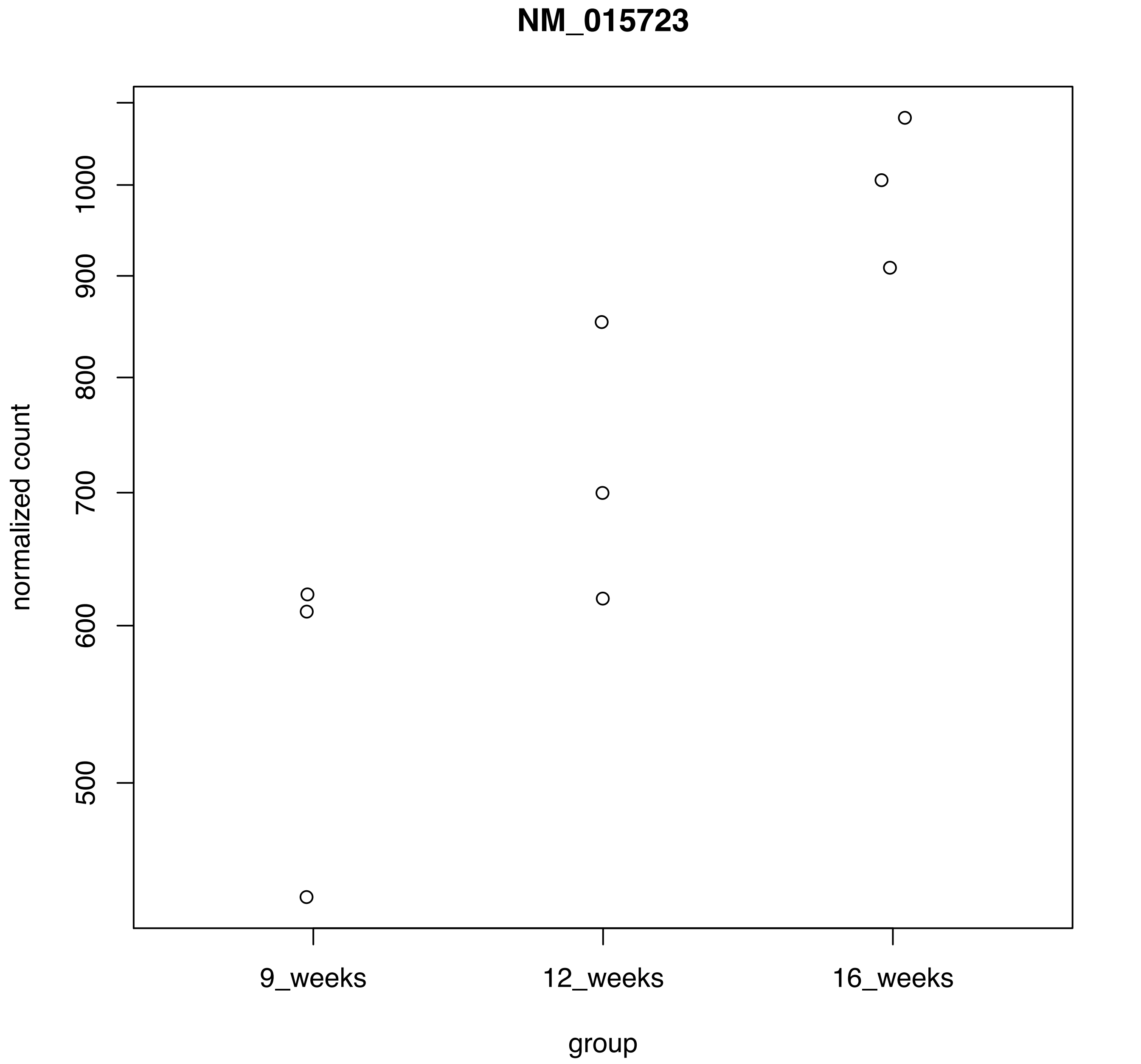
NM_015723
normalized count
1000
900
800
700
600
500
9_weeks
12_weeks
16_weeks
group

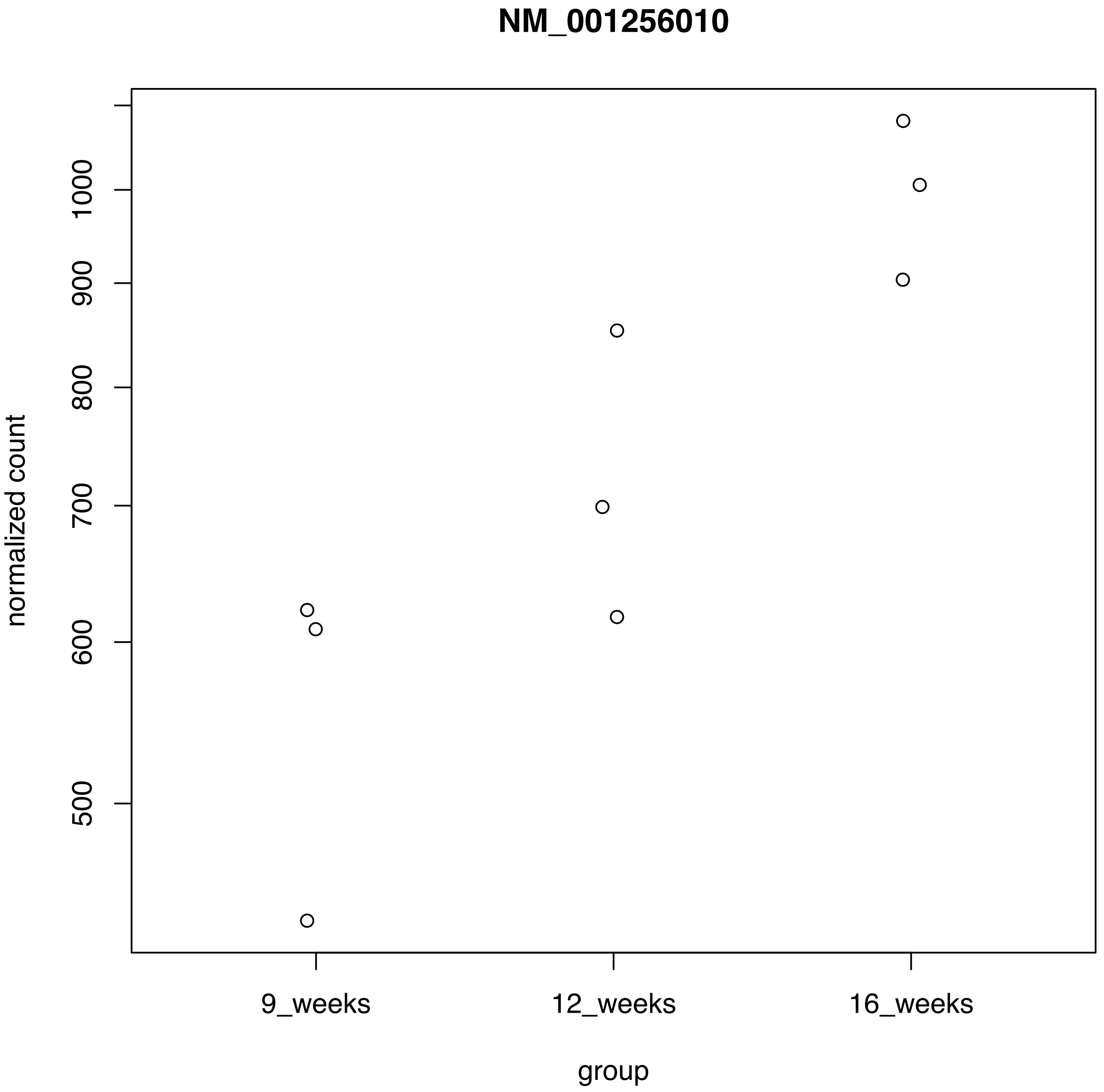

NM_001256010
normalized count
500
600
700
800
900
1000
9_weeks
12_weeks
16_weeks
group

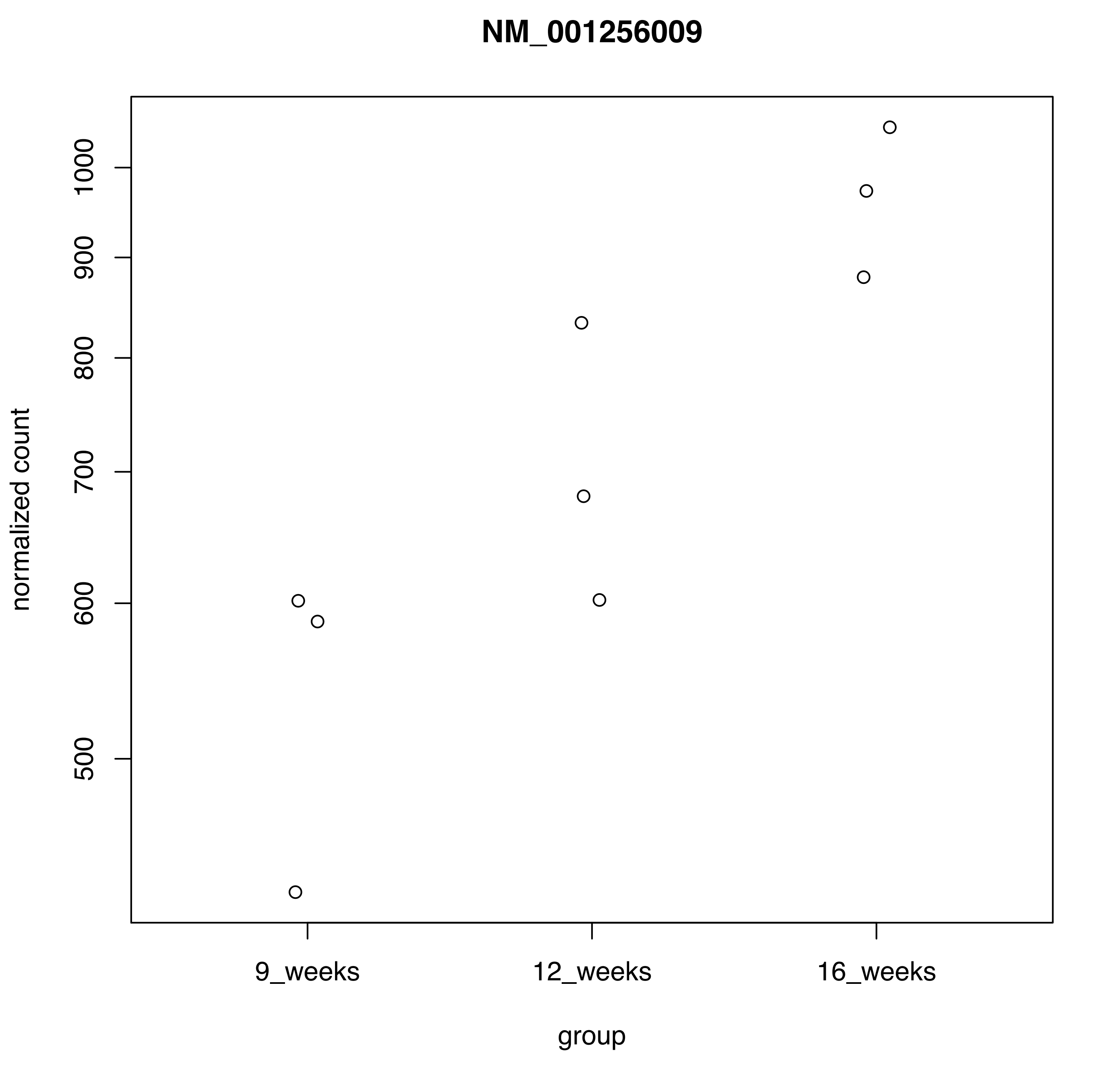
NM_001256009
normalized count
500
600
700
800
900
1000
9_weeks
12_weeks
16_weeks
group

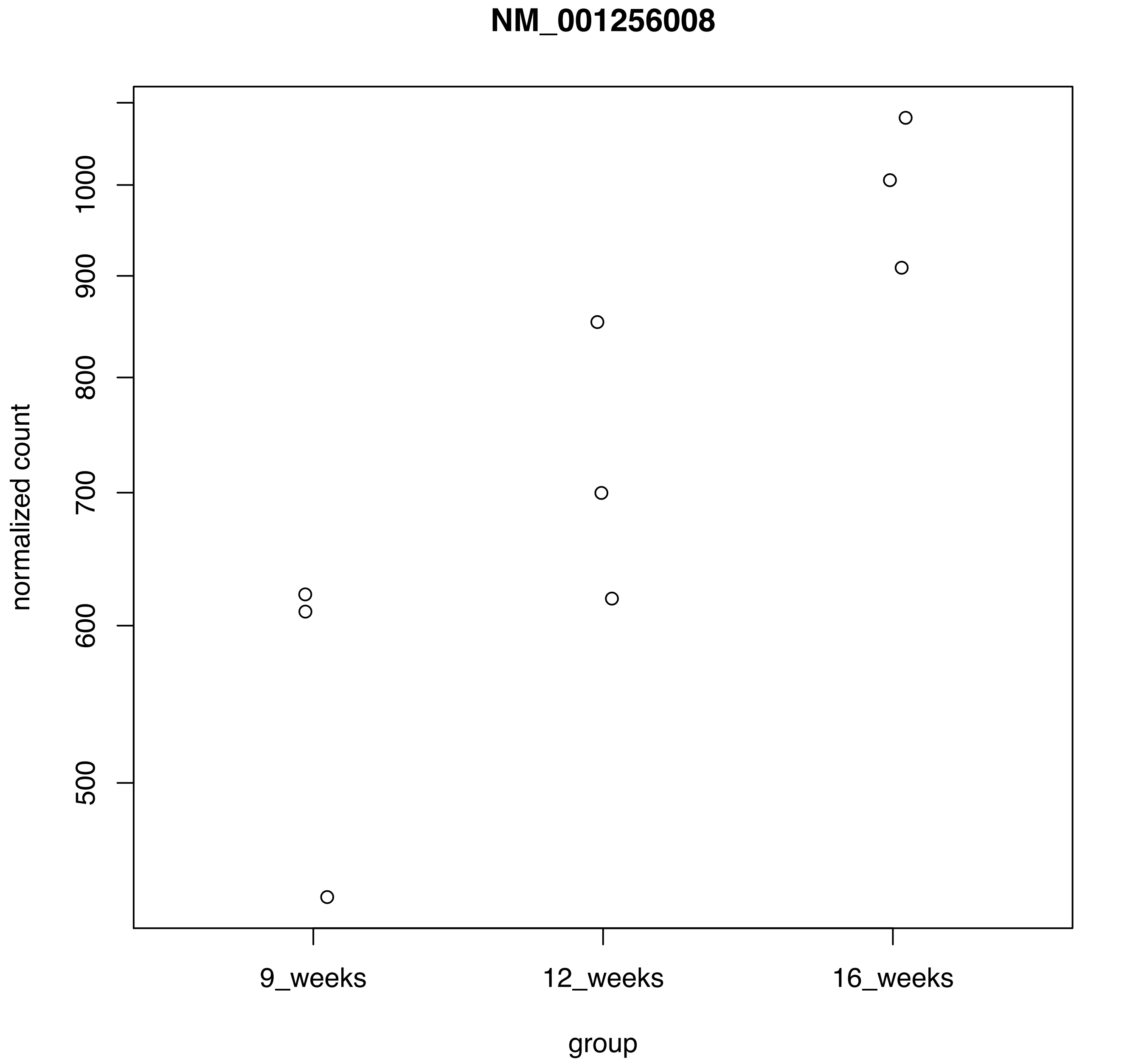
NM_001256008
normalized count
1000
900
800
700
600
500
9_weeks
12_weeks
16_weeks
group

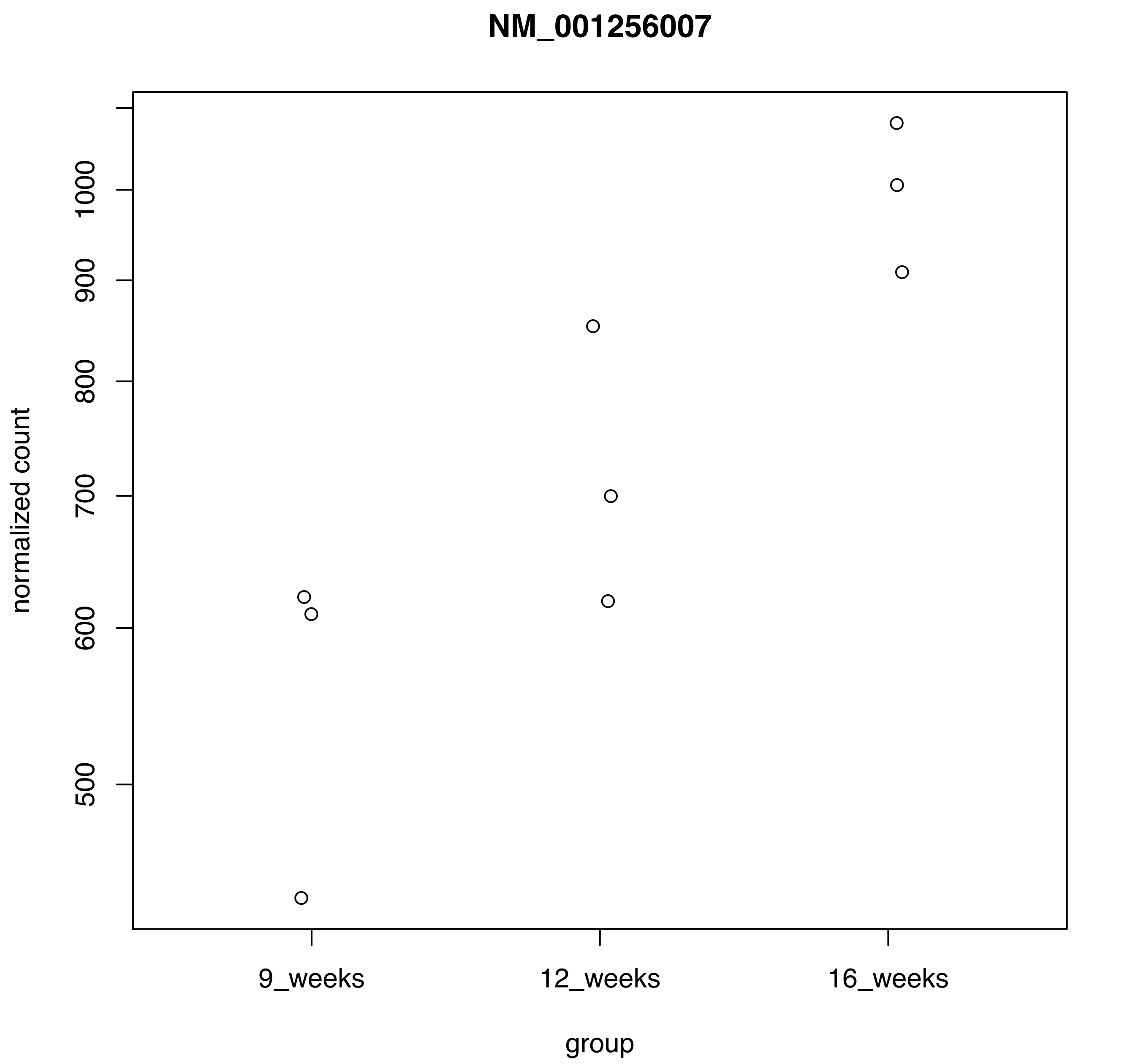
NM_001256007
normalized count
1000
900
800
700
600
500
9_weeks
12_weeks
16_weeks
group

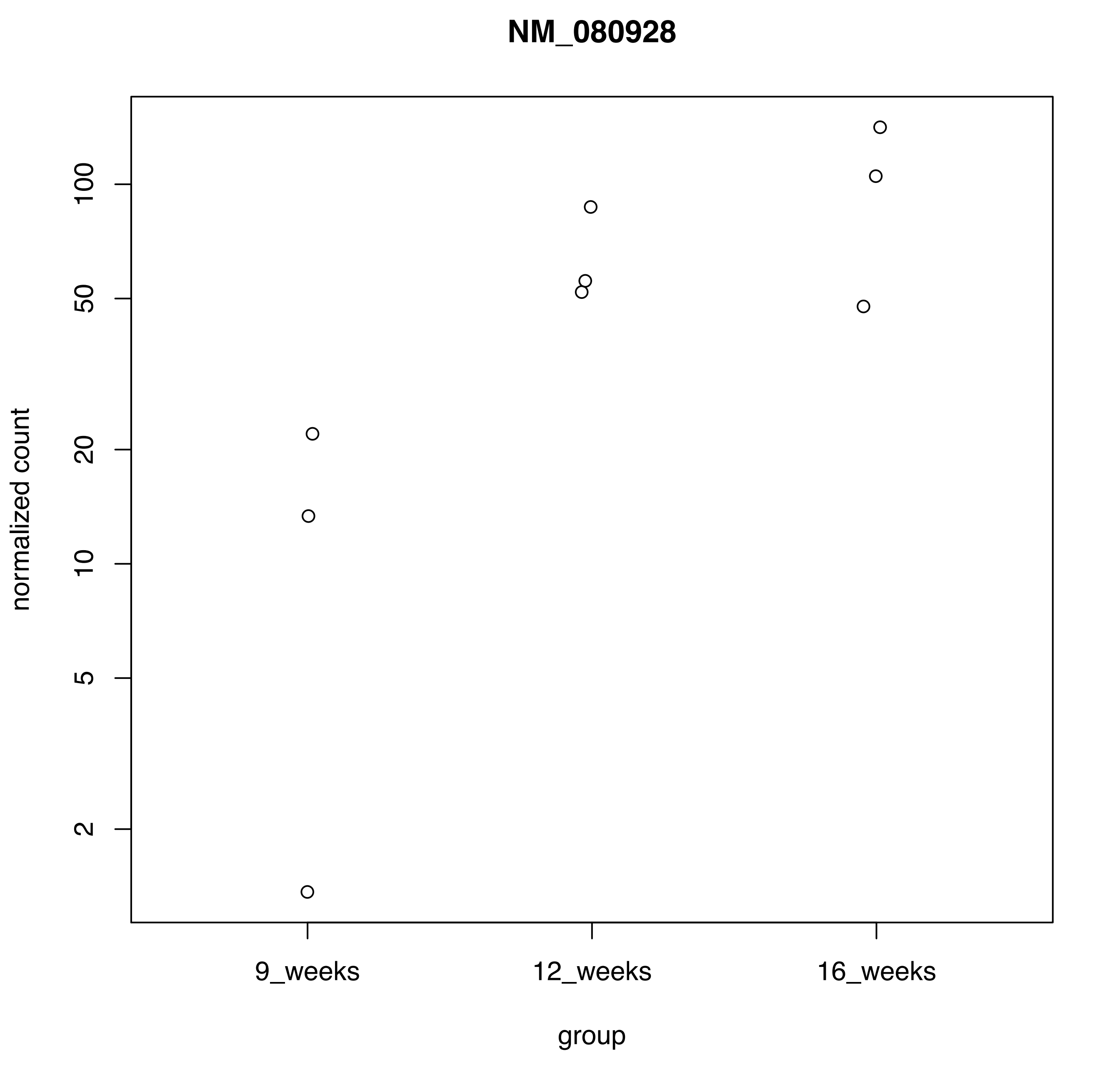

NM_080928
normalized count
100
50
20
10
5
2
9_weeks
12_weeks
16_weeks
group

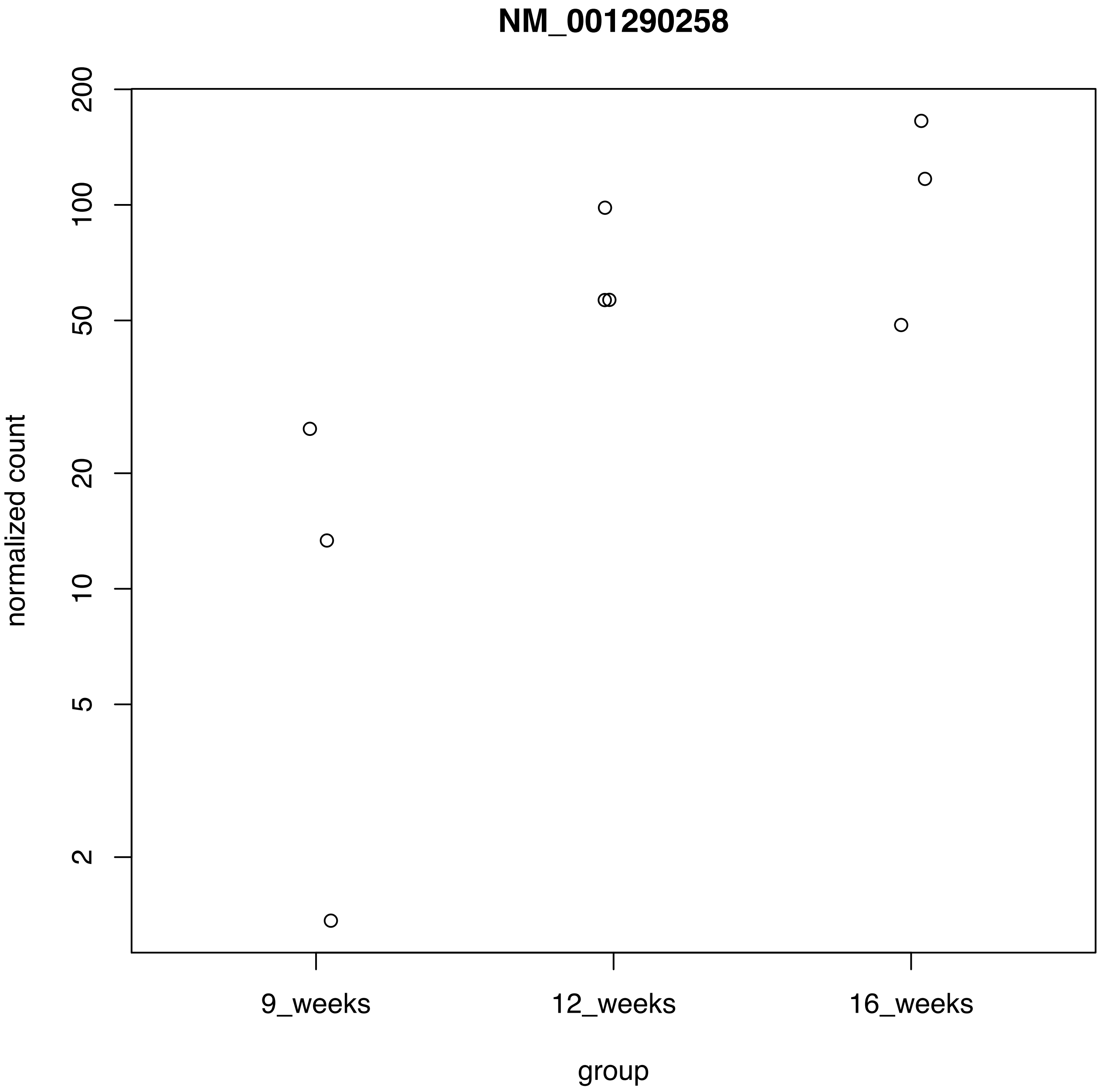
NM_001290258
normalized count
200
100
50
20
10
5
2
9_weeks
12_weeks
16_weeks
group

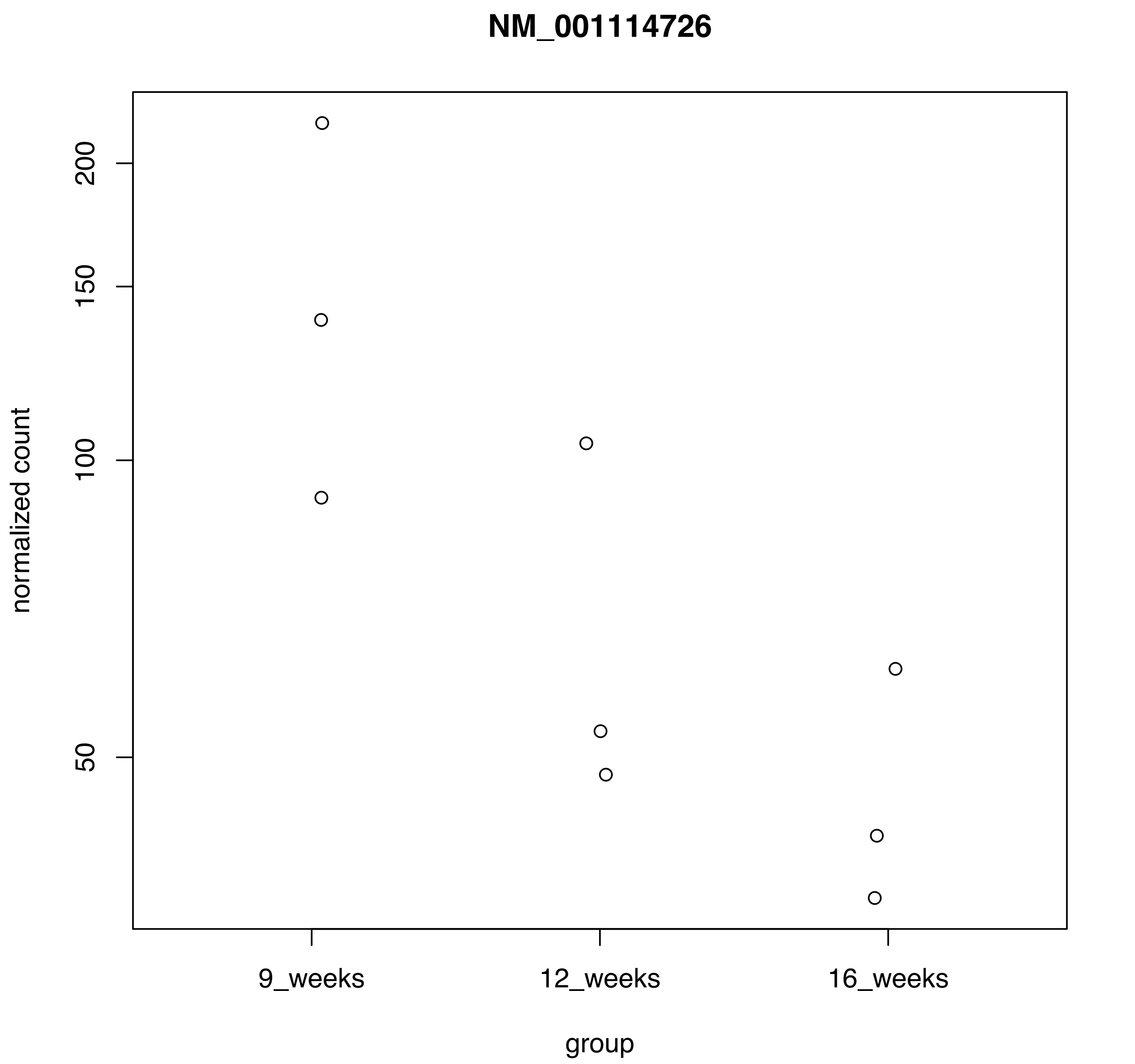
NM_001114726
normalized count
200
150
100
50
9_weeks
12_weeks
16_weeks
group

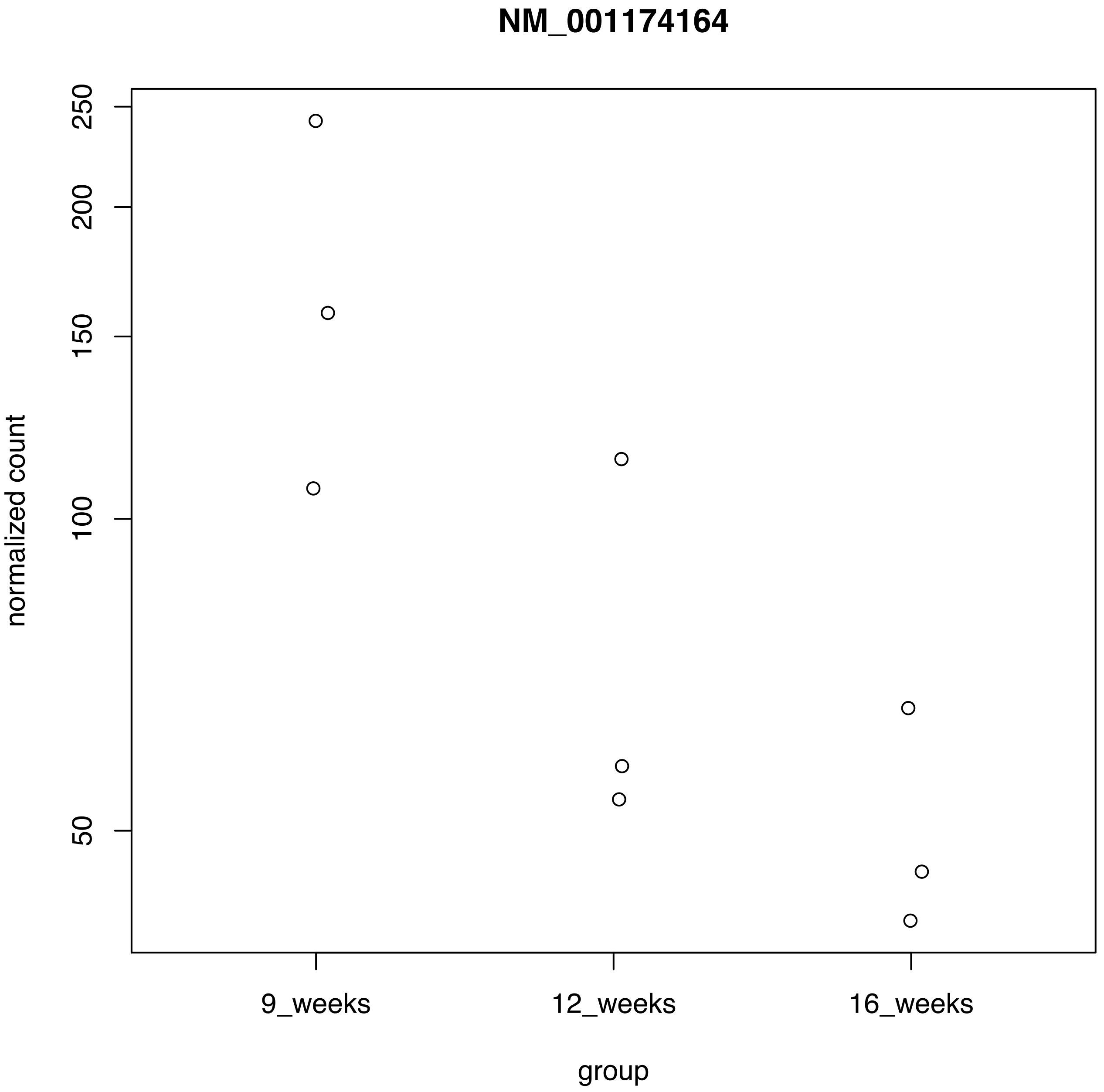
NM_001174164
normalized count
250
200
150
100
50
9_weeks
12_weeks
16_weeks
group

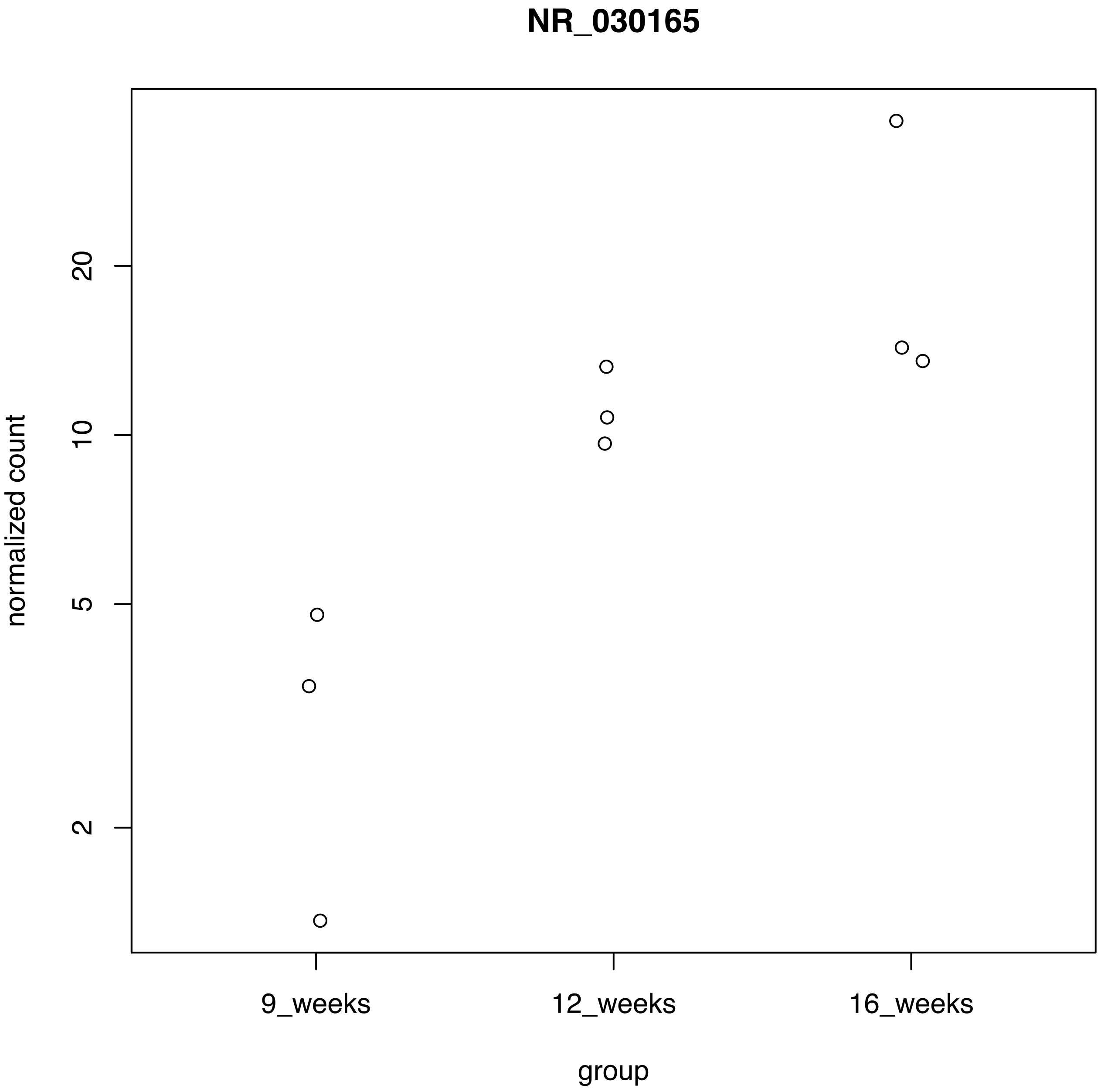

NR_030165
normalized count
20
10
5
2
9_weeks
12_weeks
16_weeks
group

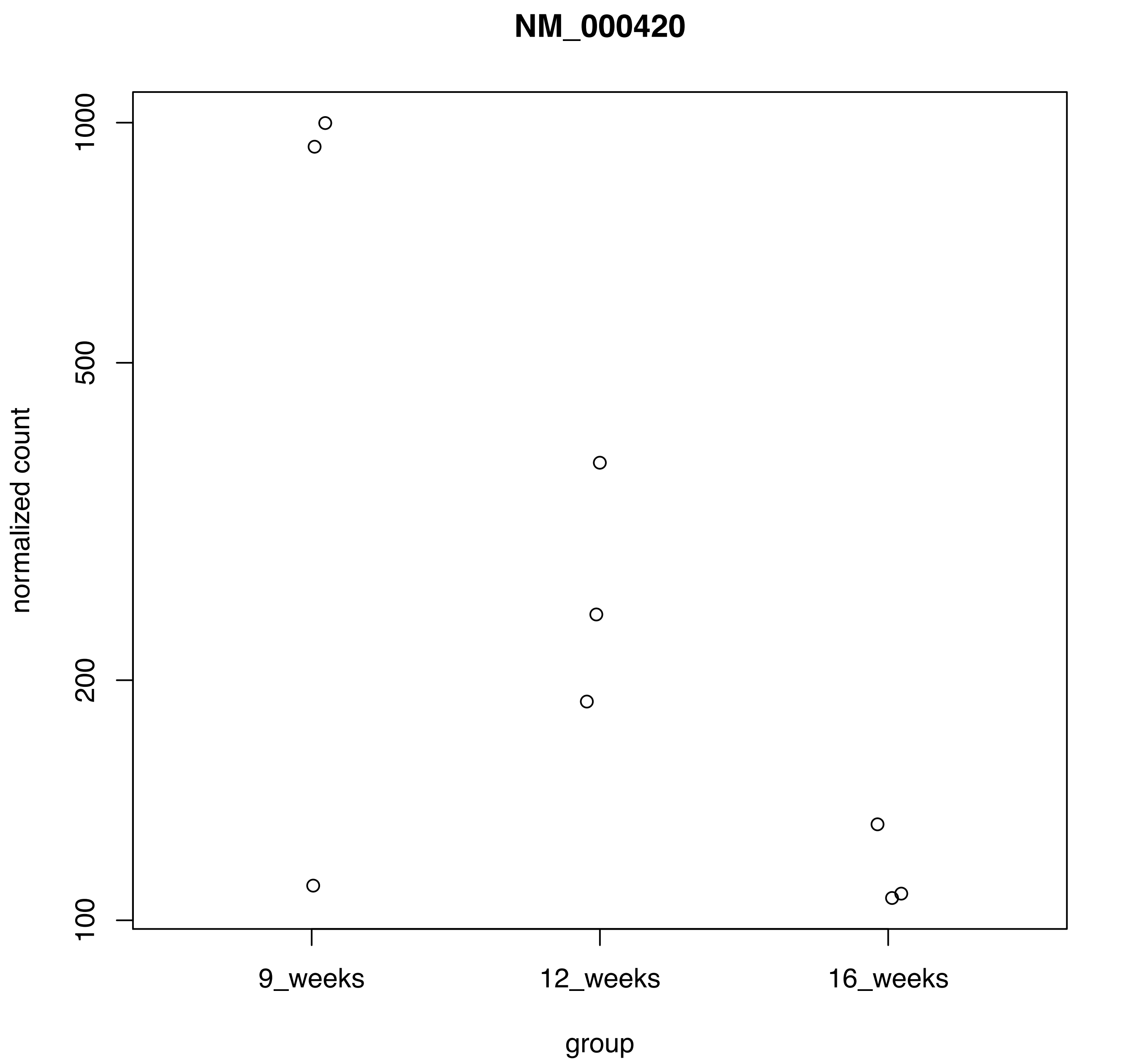
NM_000420
normalized count
1000
500
200
100
9_weeks
12_weeks
16_weeks
group

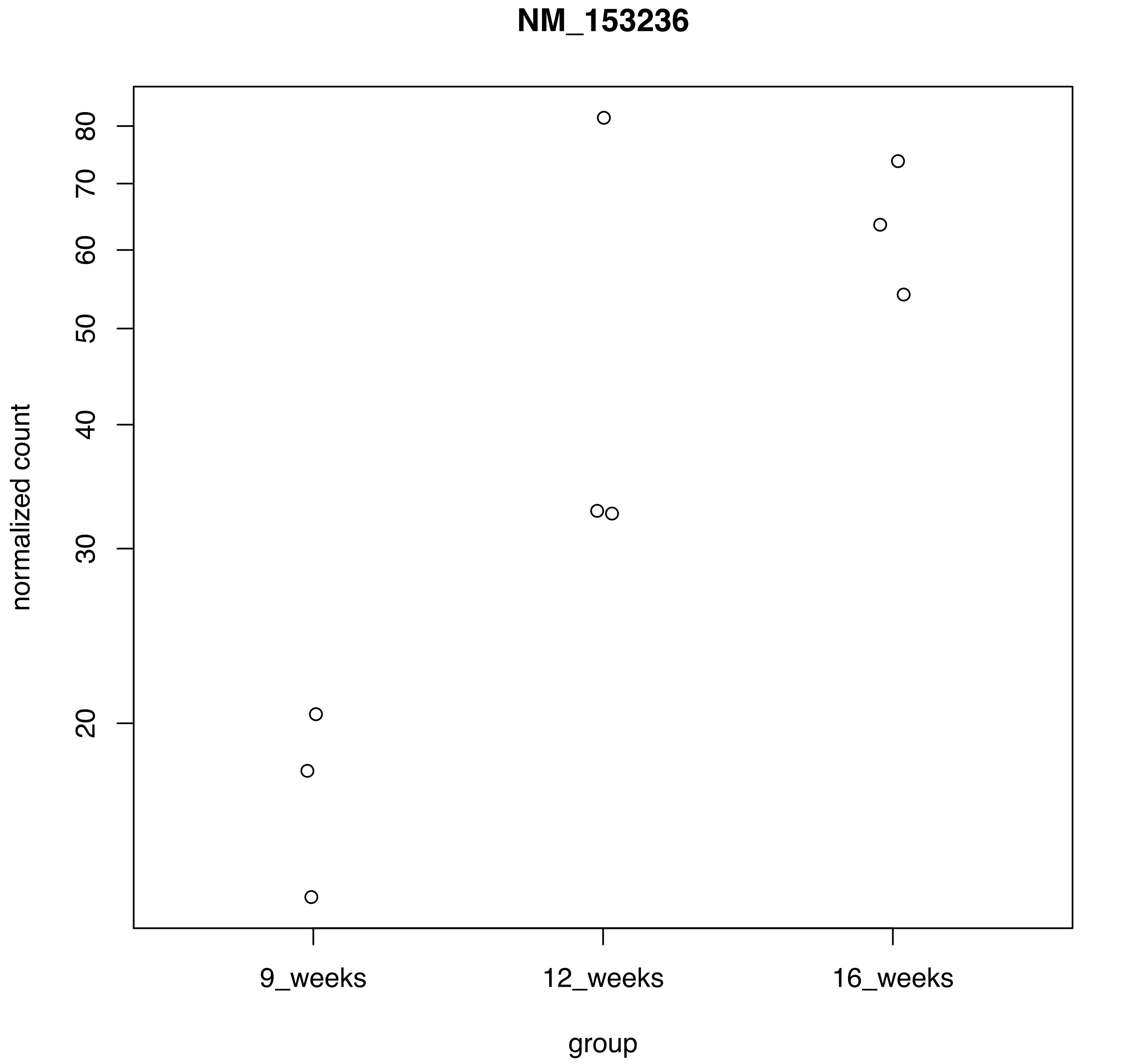
NM_153236
normalized count
80
70
60
50
40
30
20
9_weeks
12_weeks
16_weeks
group

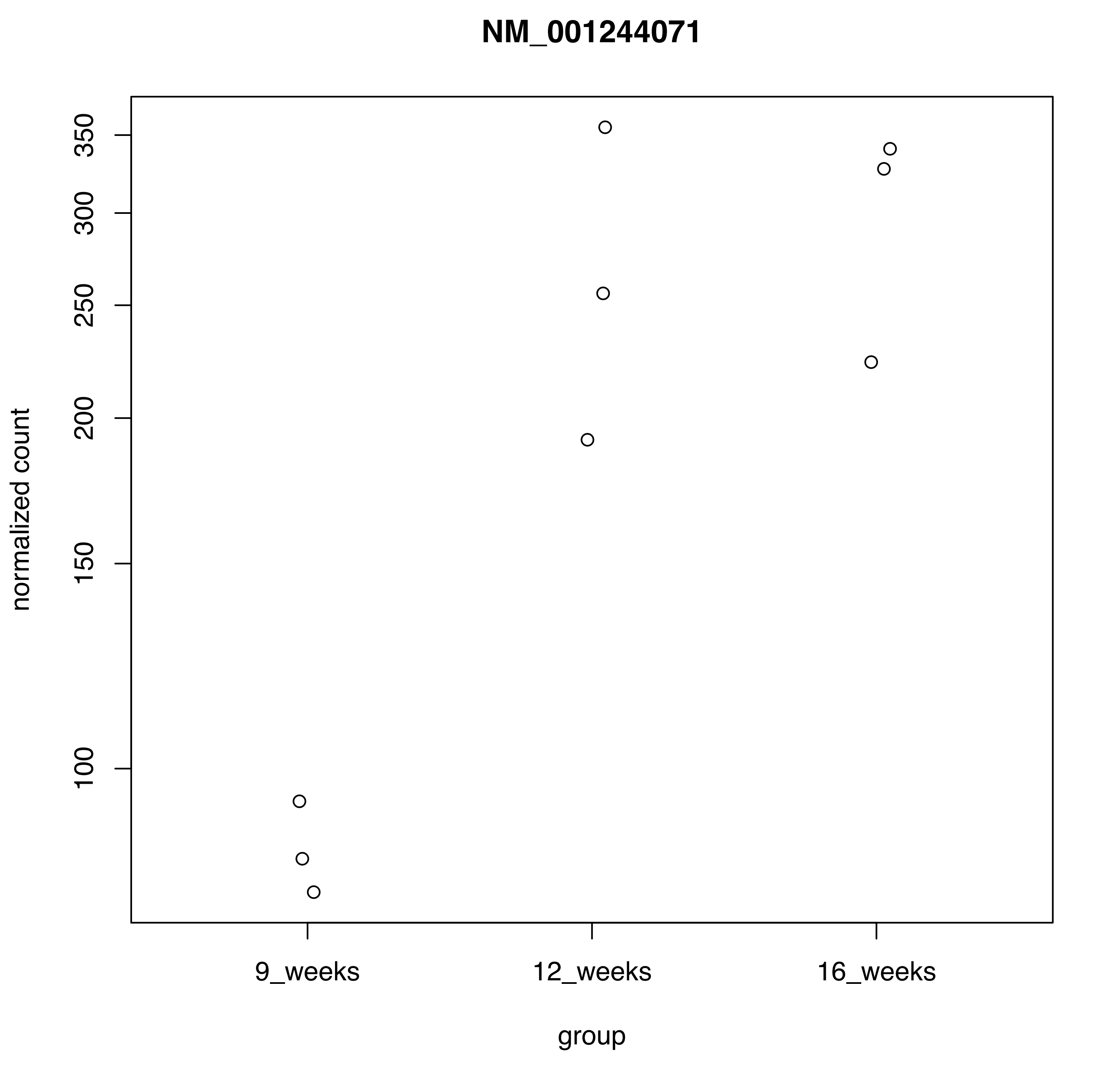

NM_001244071
normalized count
350
300
250
200
150
100
9_weeks
12_weeks
16_weeks
group

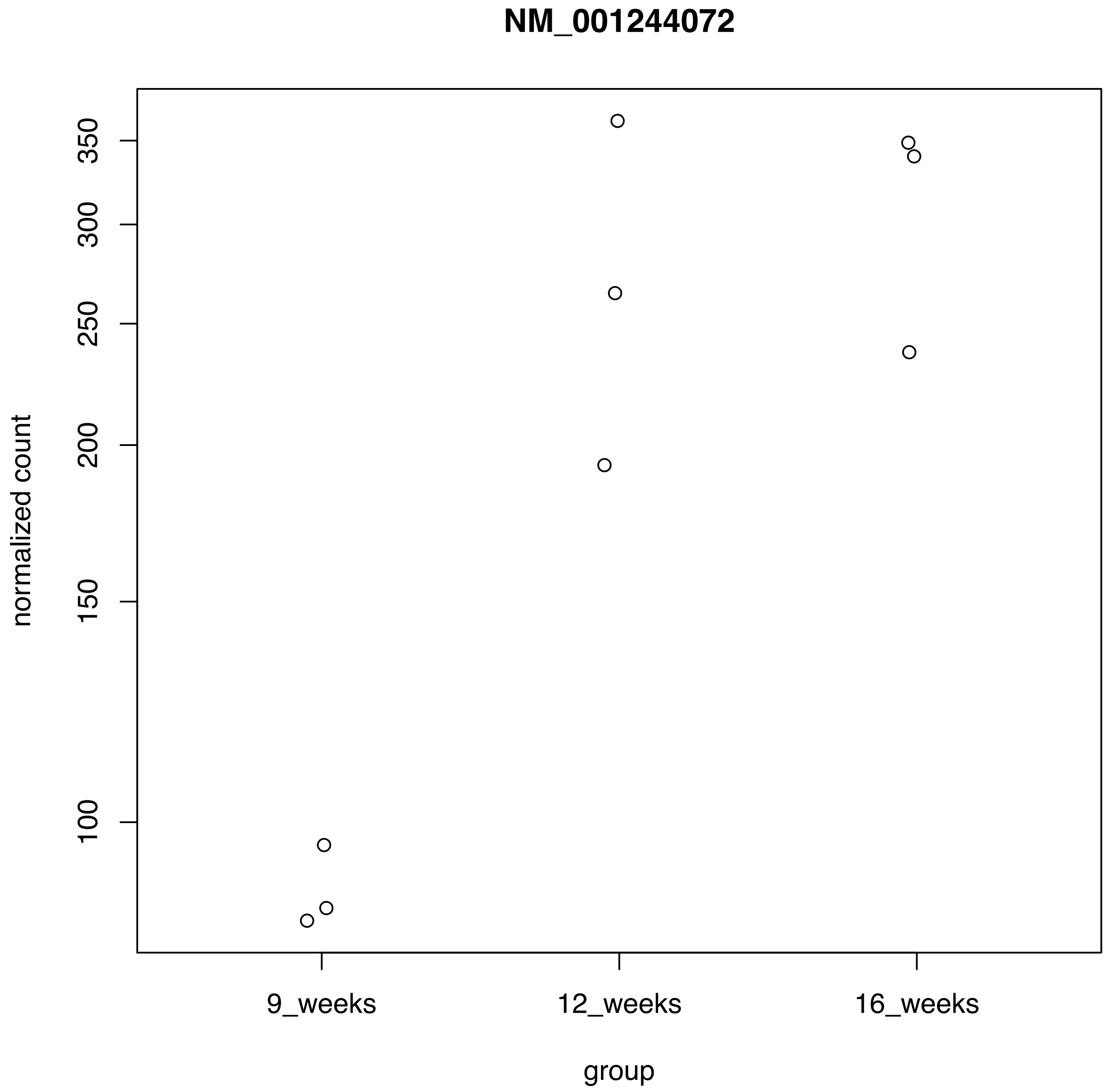

NM_001244072
normalized count
100
150
200
250
300
350
9_weeks
12_weeks
16_weeks
group

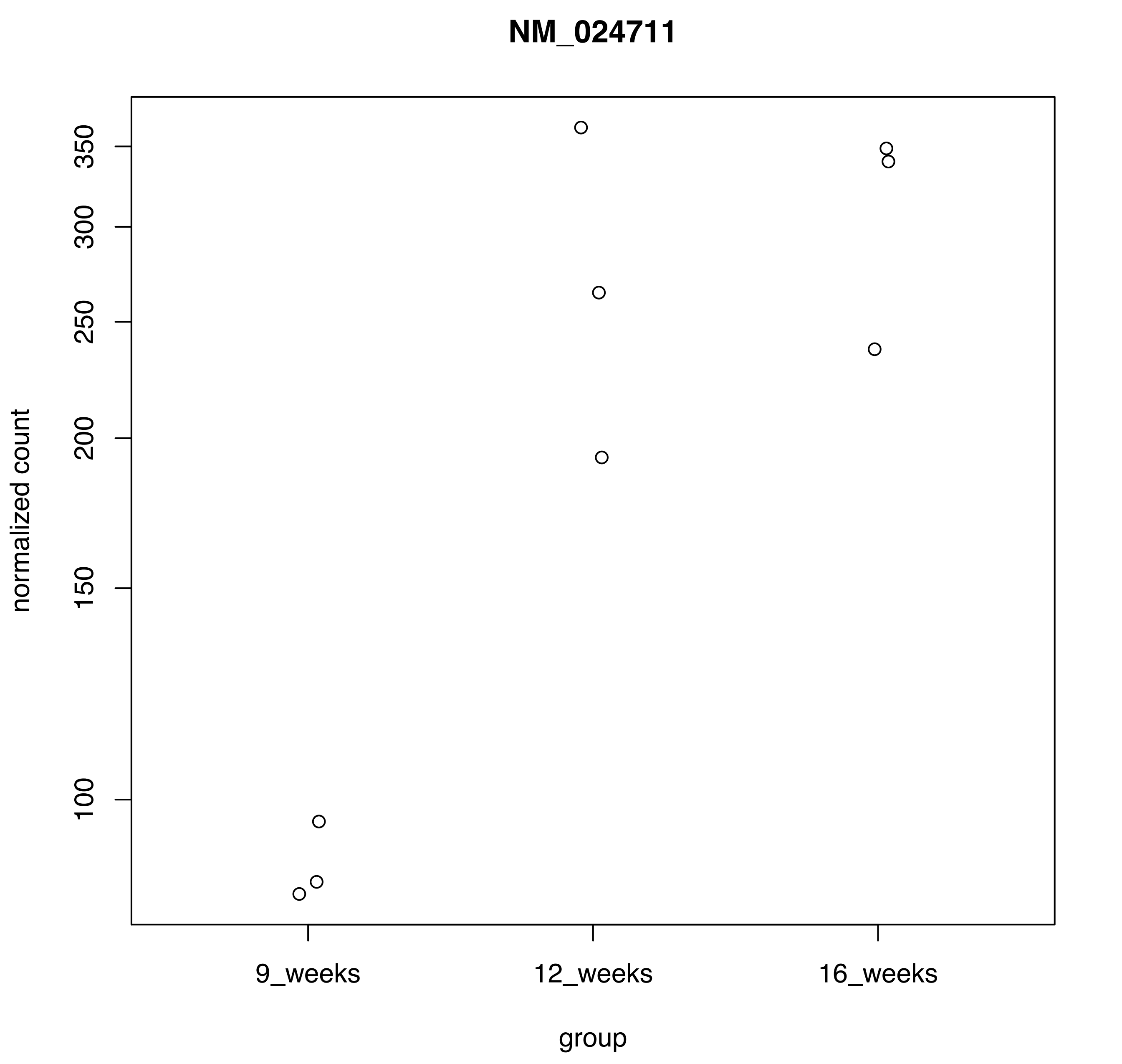

NM_024711
normalized count
100
150
200
250
300
350
9_weeks
12_weeks
16_weeks
group

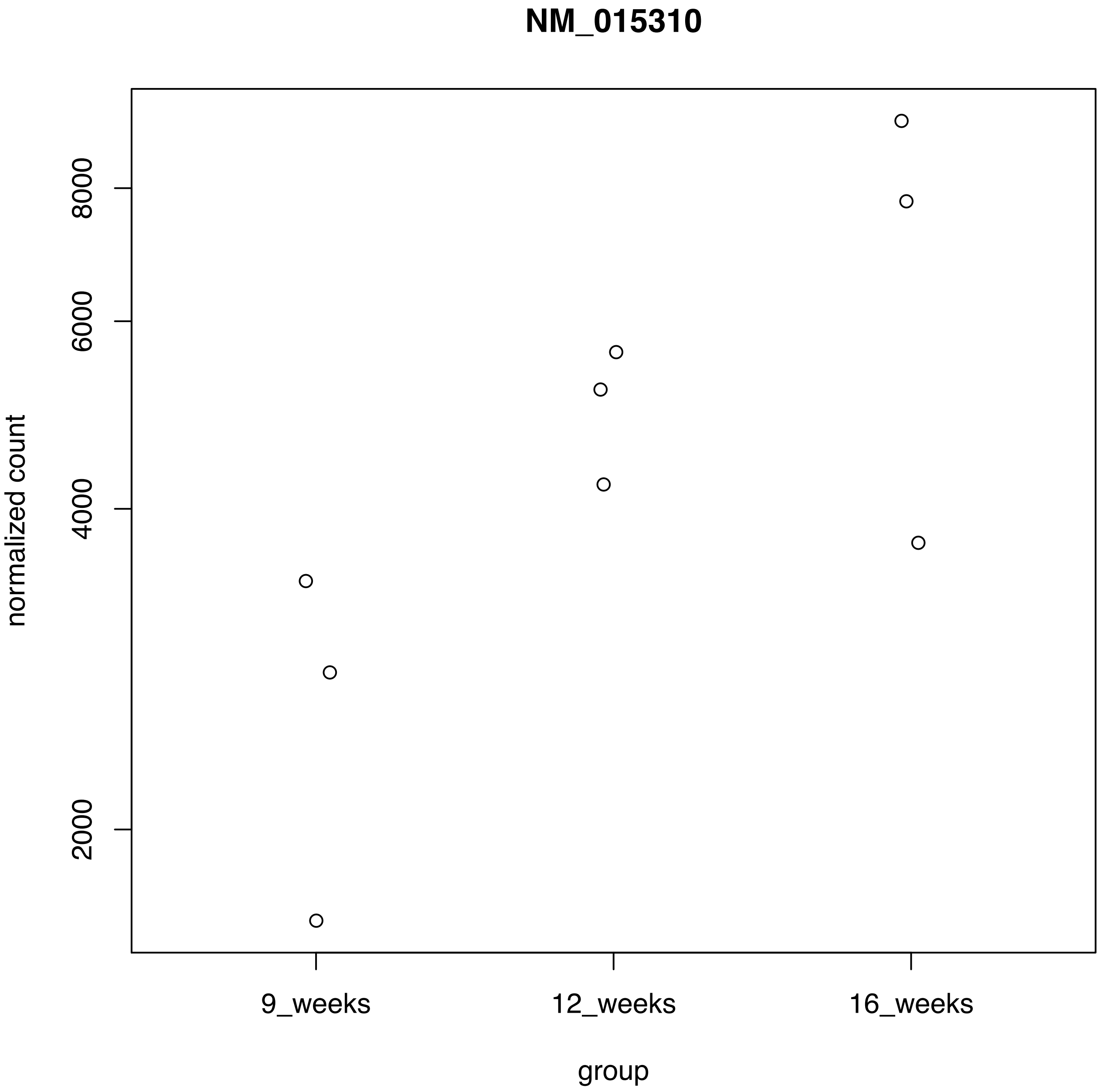
NM_015310
normalized count
8000
6000
4000
2000
9_weeks
12_weeks
16_weeks
group

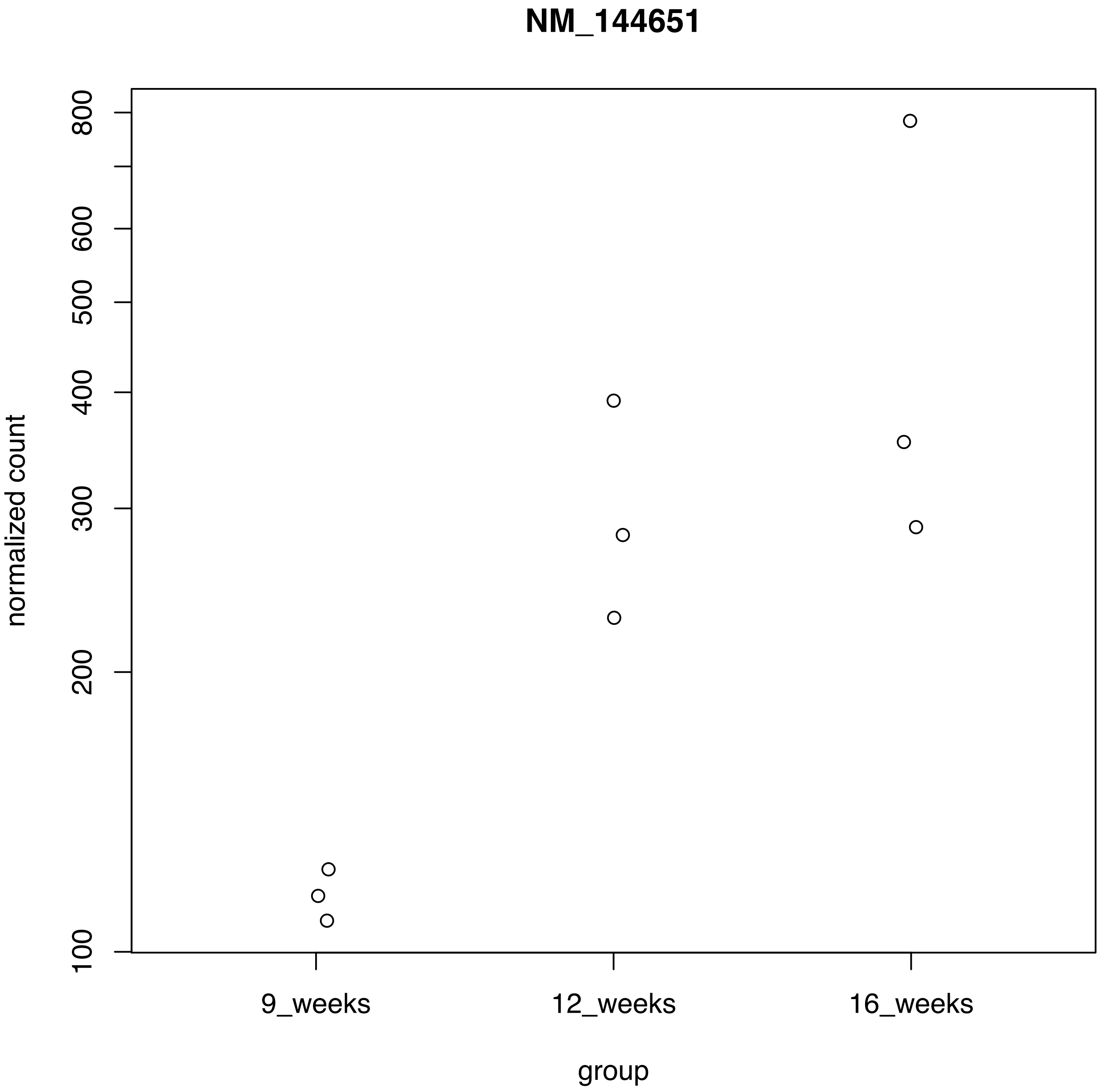
NM_144651
normalized count
800
600
500
400
300
200
100
9_weeks
12_weeks
16_weeks
group

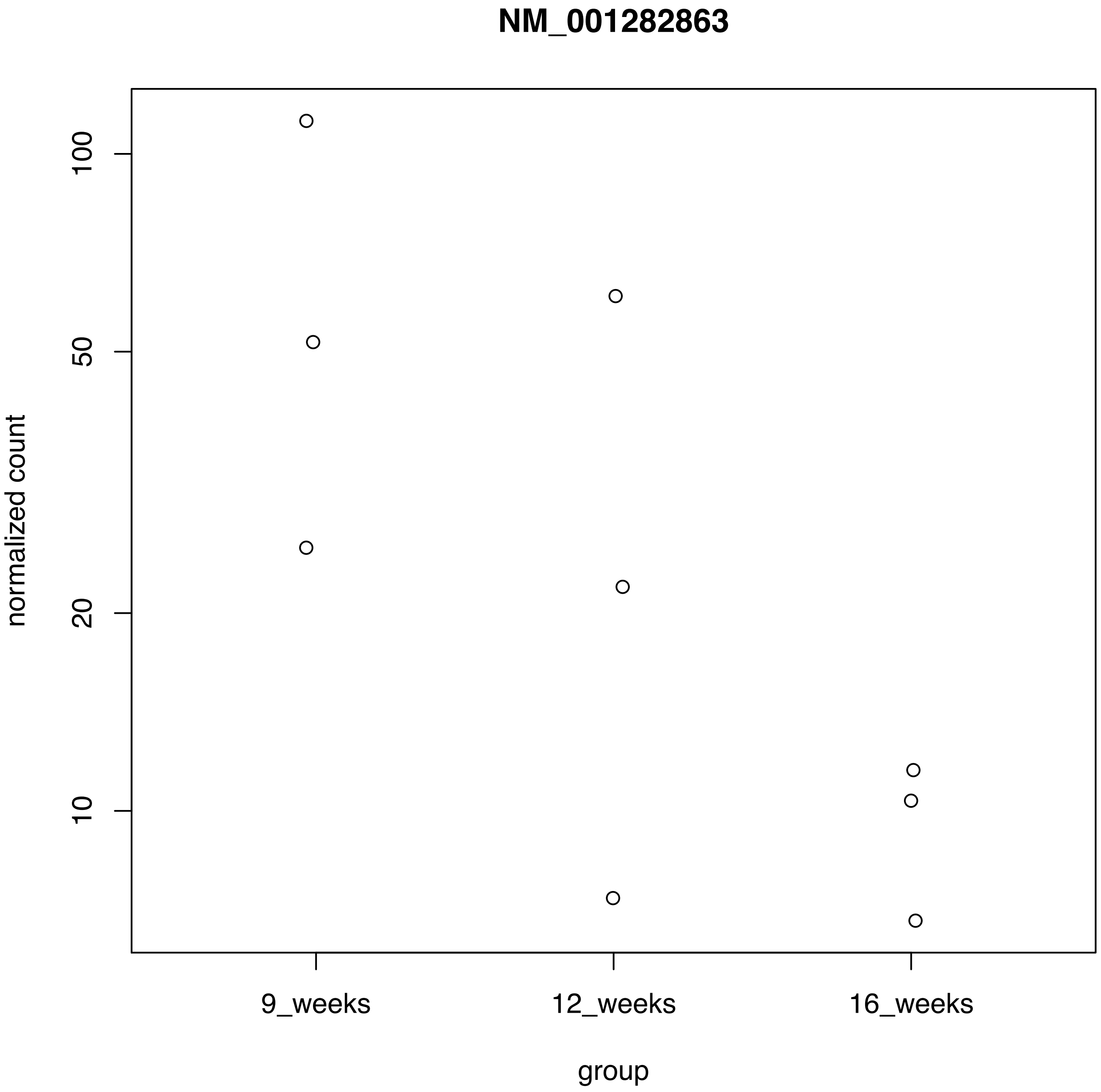
NM_001282863
normalized count
100
50
20
10
9_weeks
12_weeks
16_weeks
group

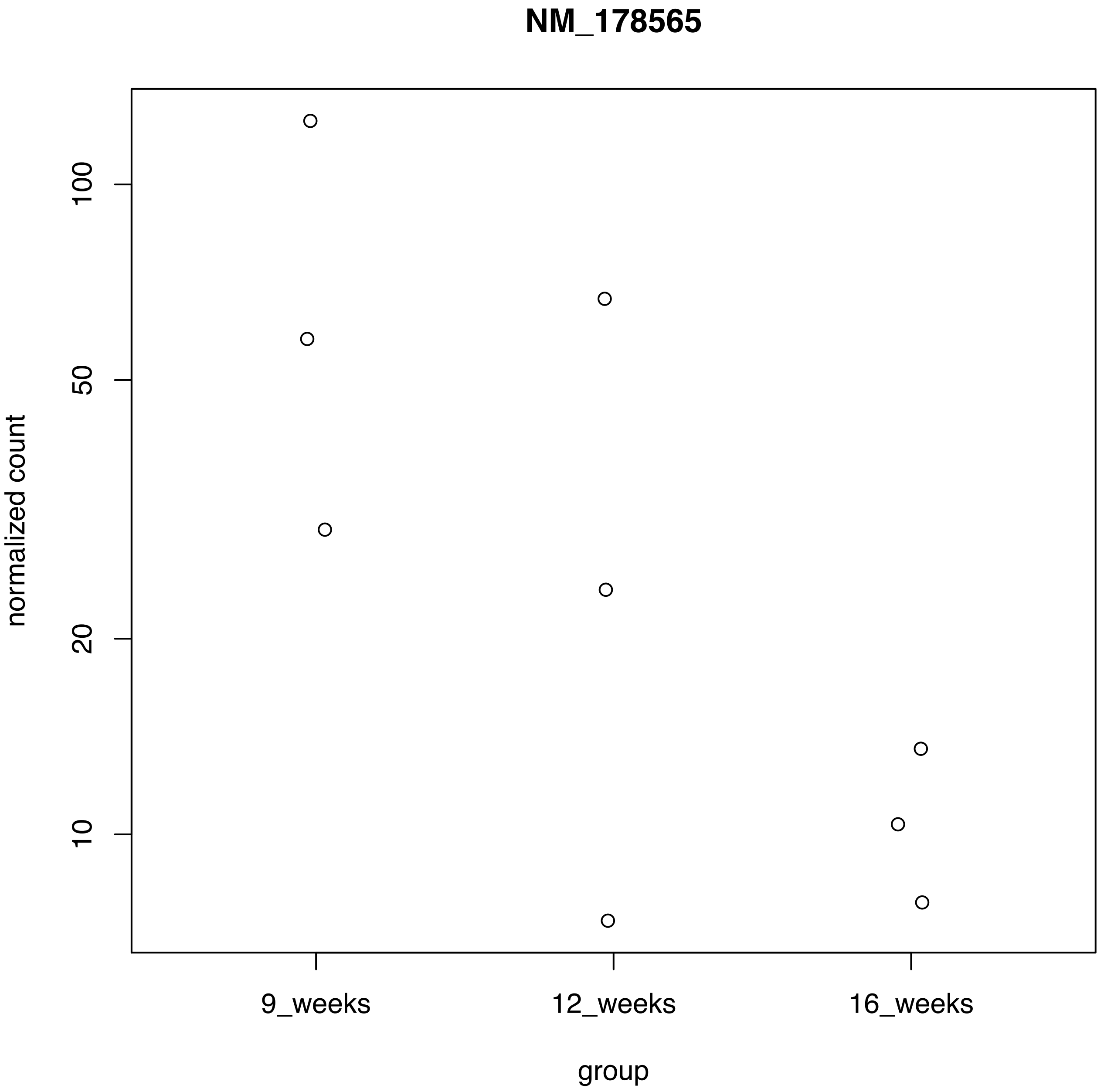
NM_178565
normalized count
100
50
20
10
9_weeks
12_weeks
16_weeks
group

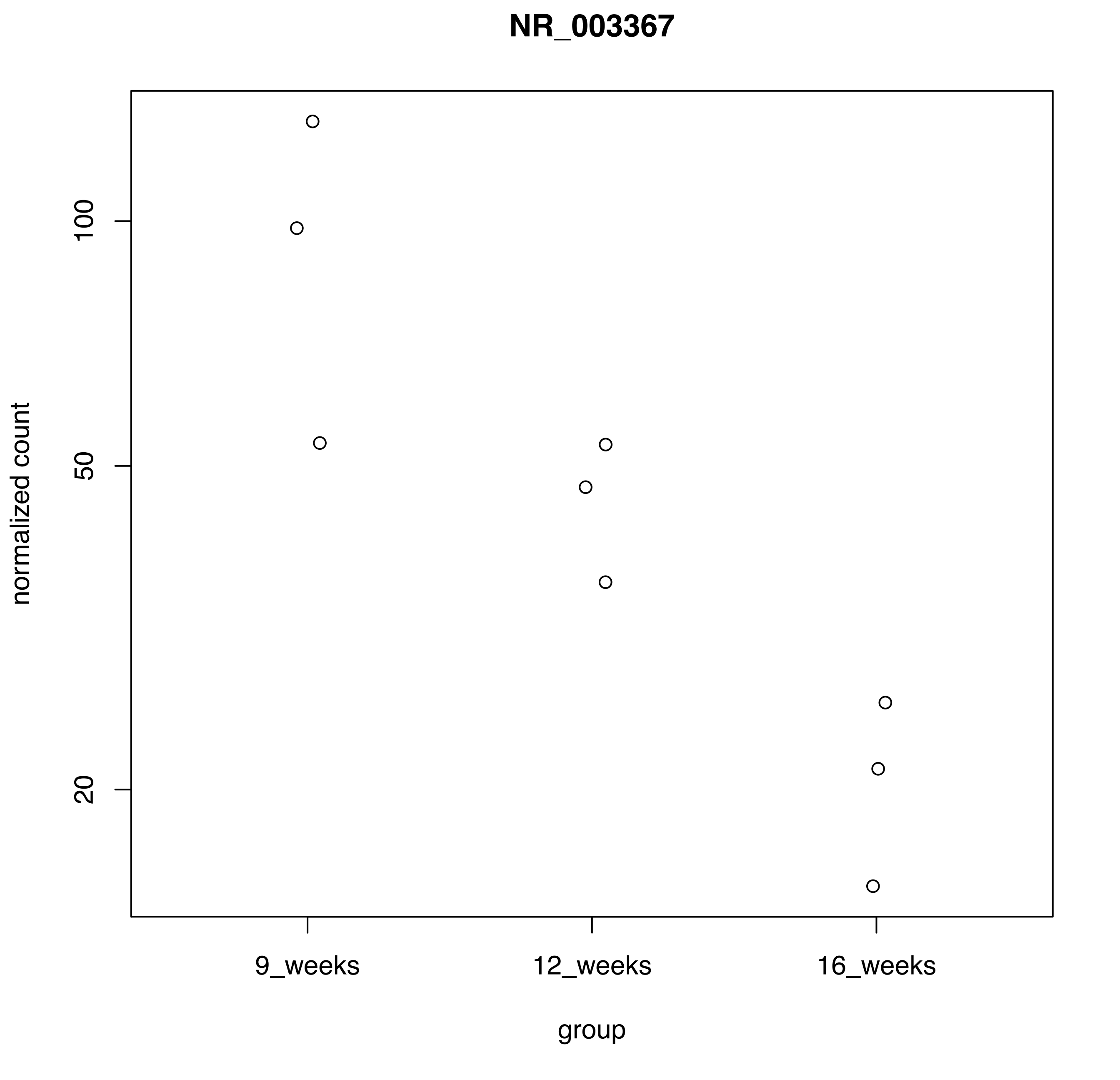
NR_003367
normalized count
100
50
20
9_weeks
12_weeks
16_weeks
group

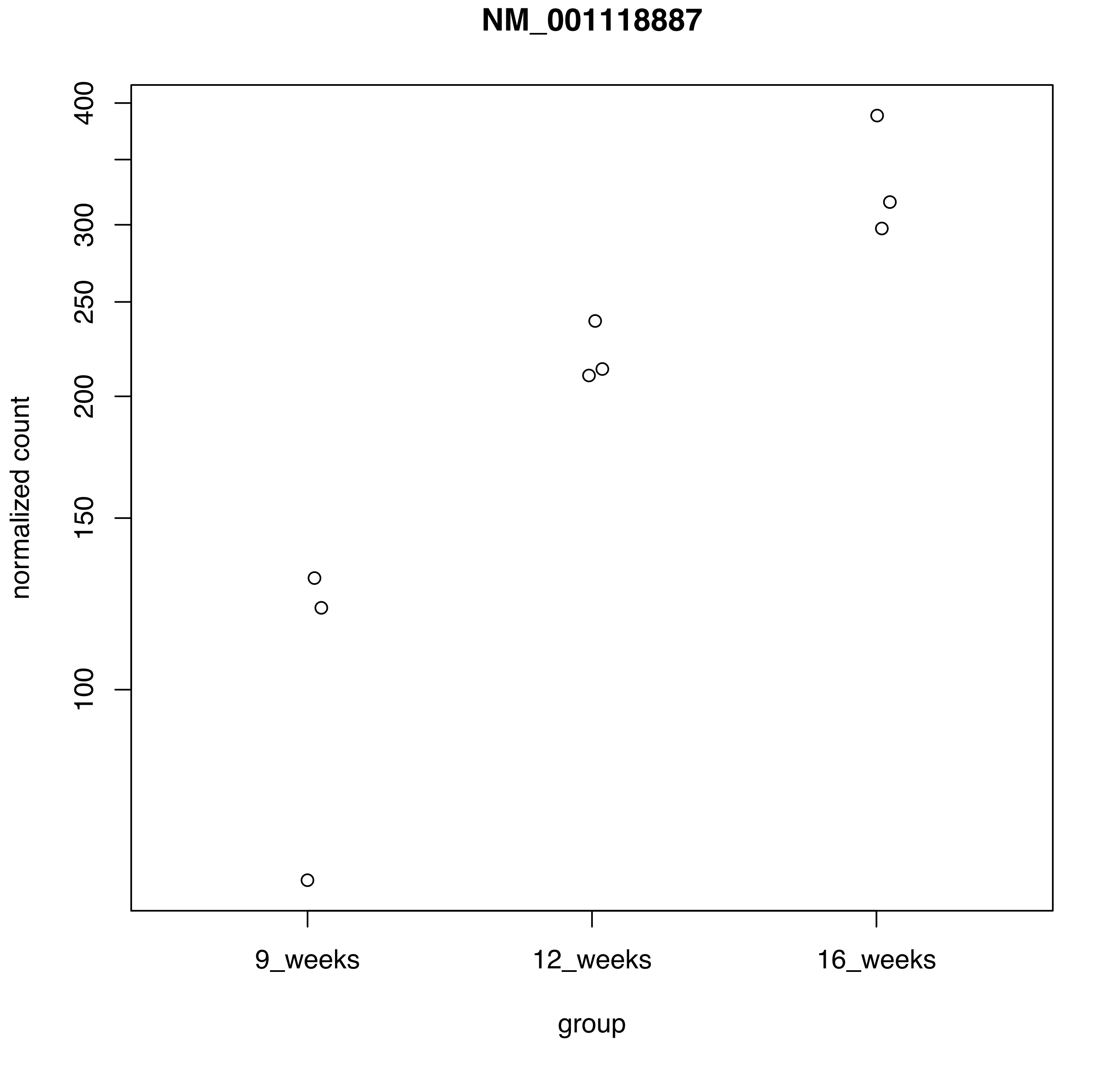
NM_001118887
normalized count
400
300
250
200
150
100
9_weeks
12_weeks
16_weeks
group

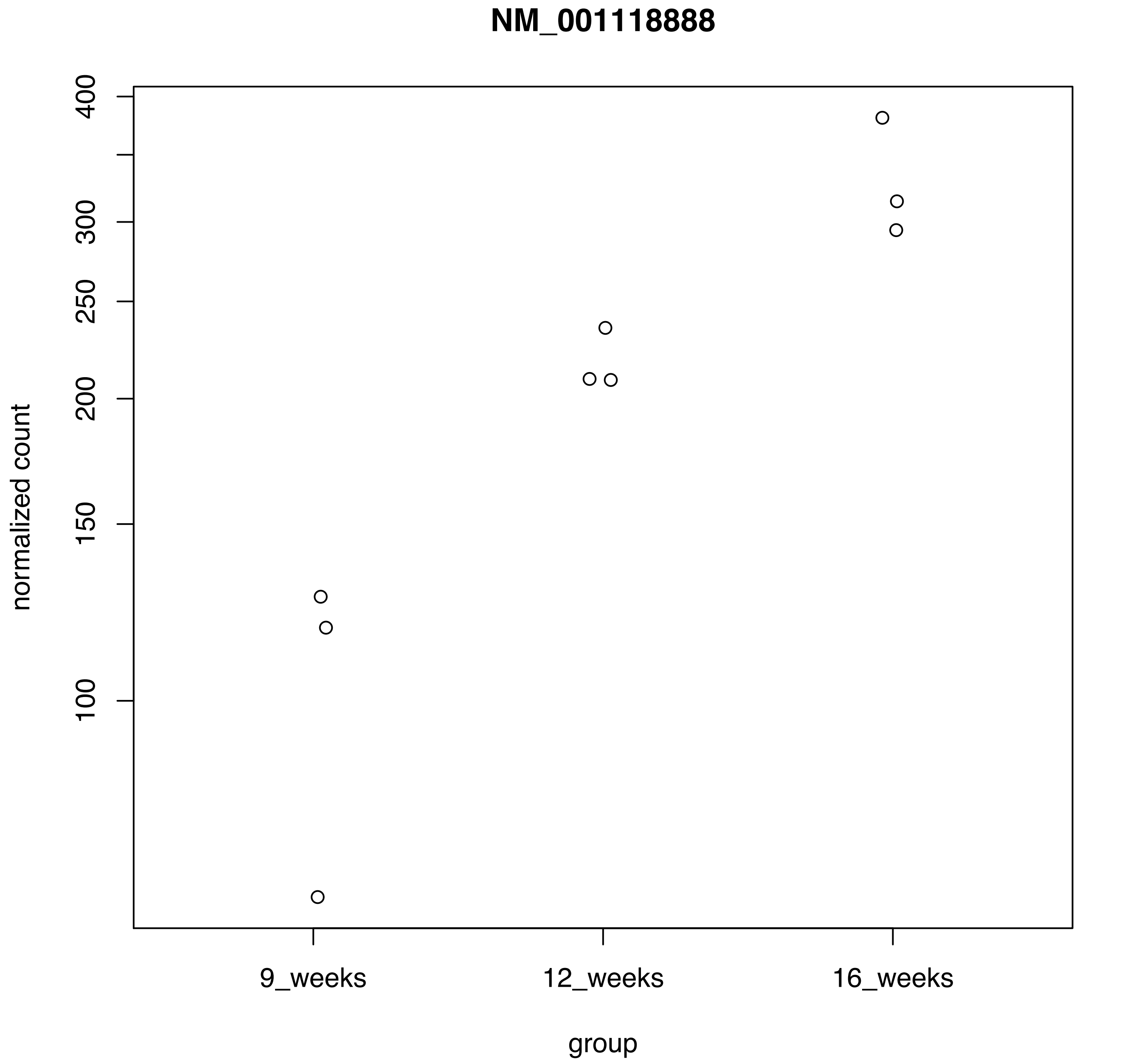
NM_001118888
normalized count
400
300
250
200
150
100
9_weeks
12_weeks
16_weeks
group

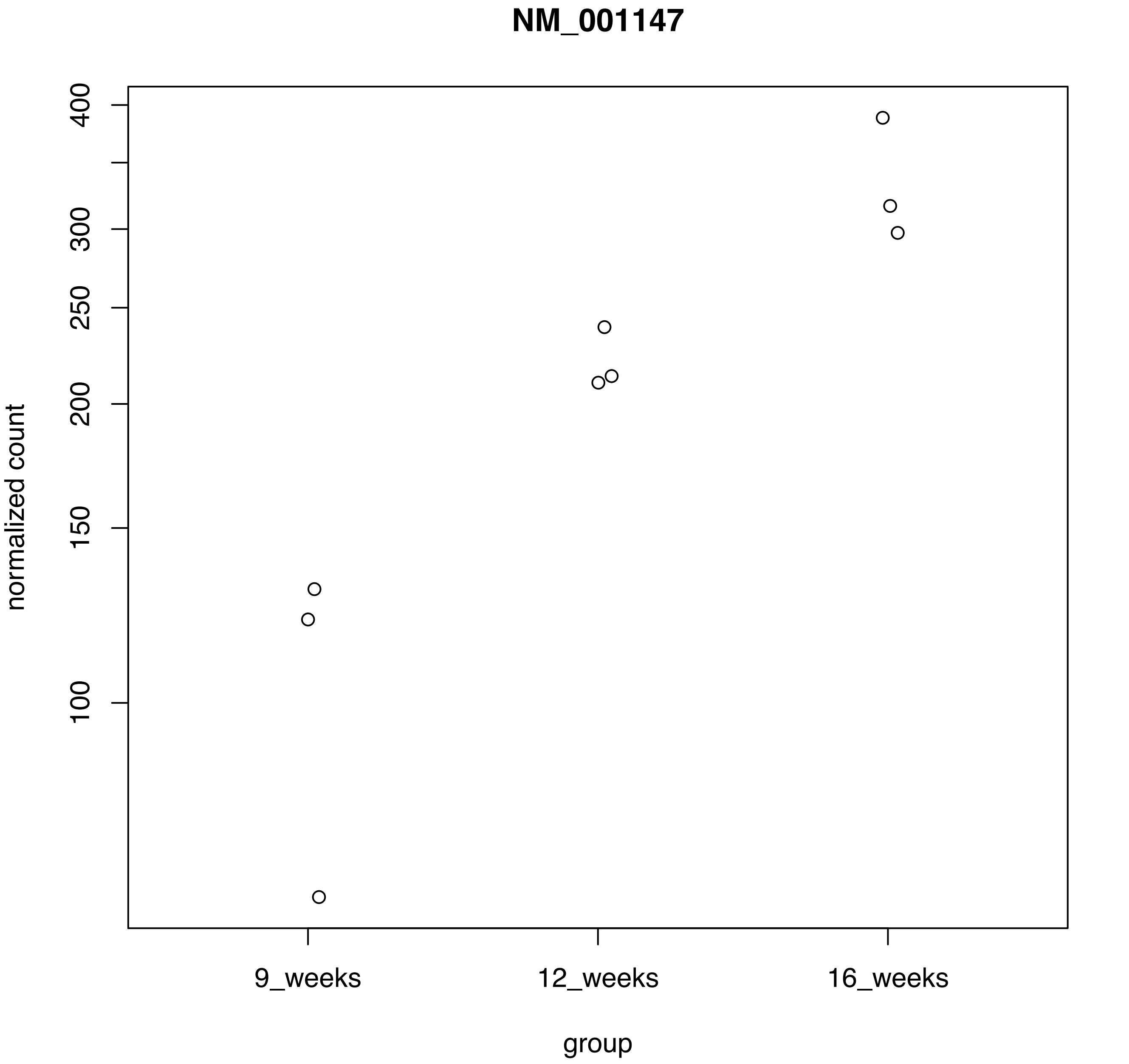

NM_001147
normalized count
400
300
250
200
150
100
9_weeks
12_weeks
16_weeks
group

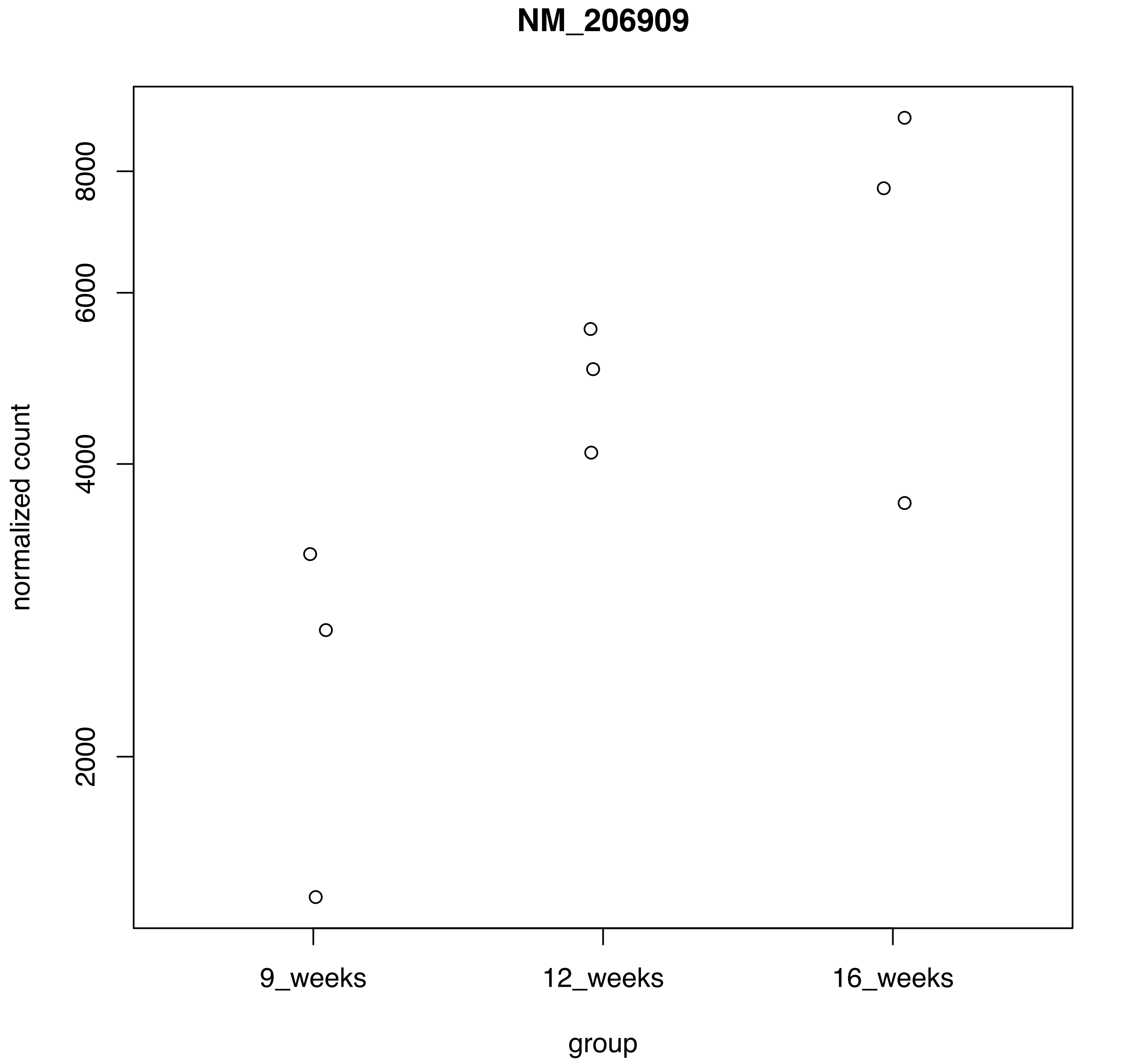

NM_206909
normalized count
8000
6000
4000
2000
9_weeks
12_weeks
16_weeks
group

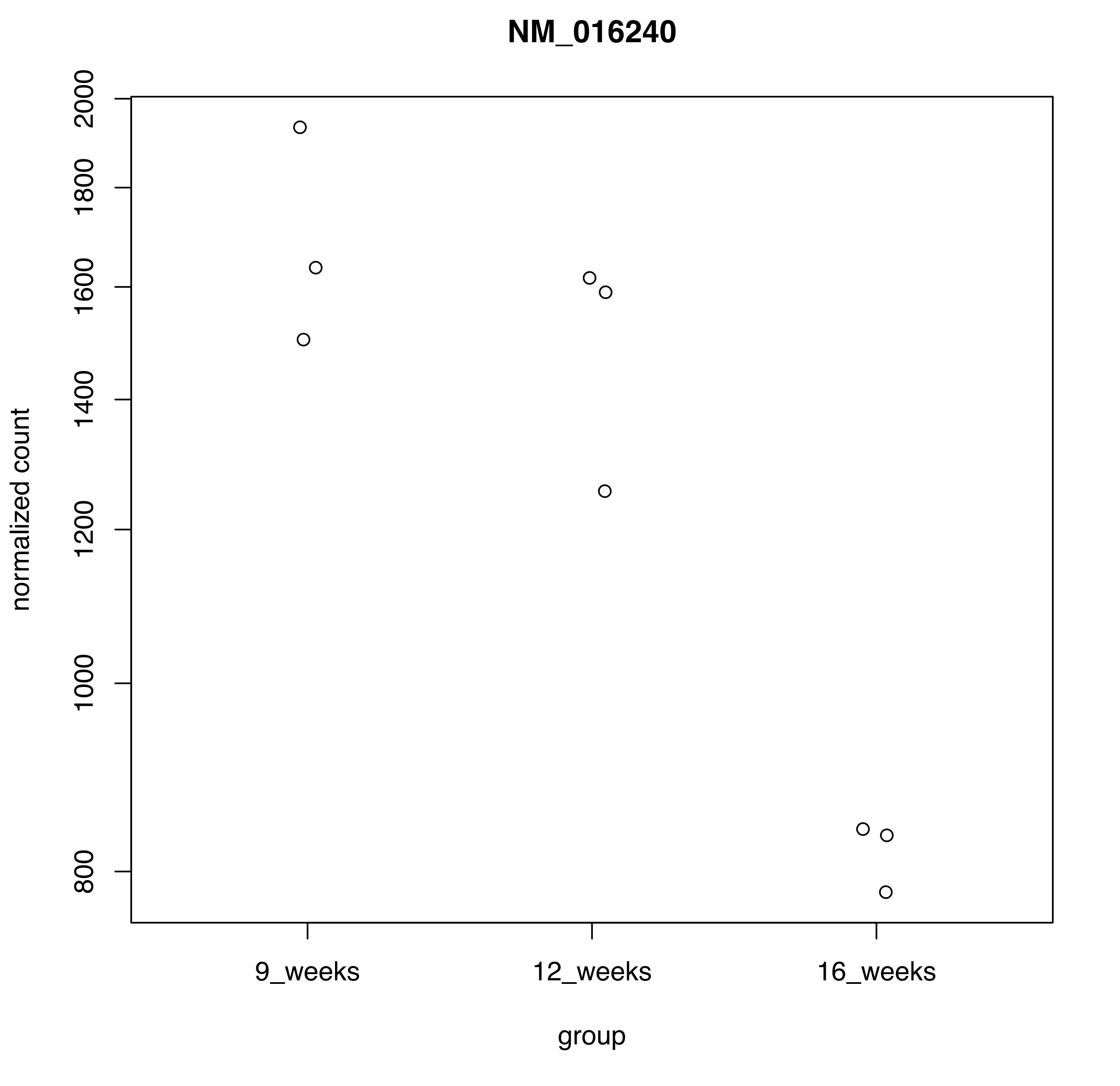
NM_016240
normalized count
2000
1800
1600
1400
1200
1000
800
9_weeks
12_weeks
16_weeks
group

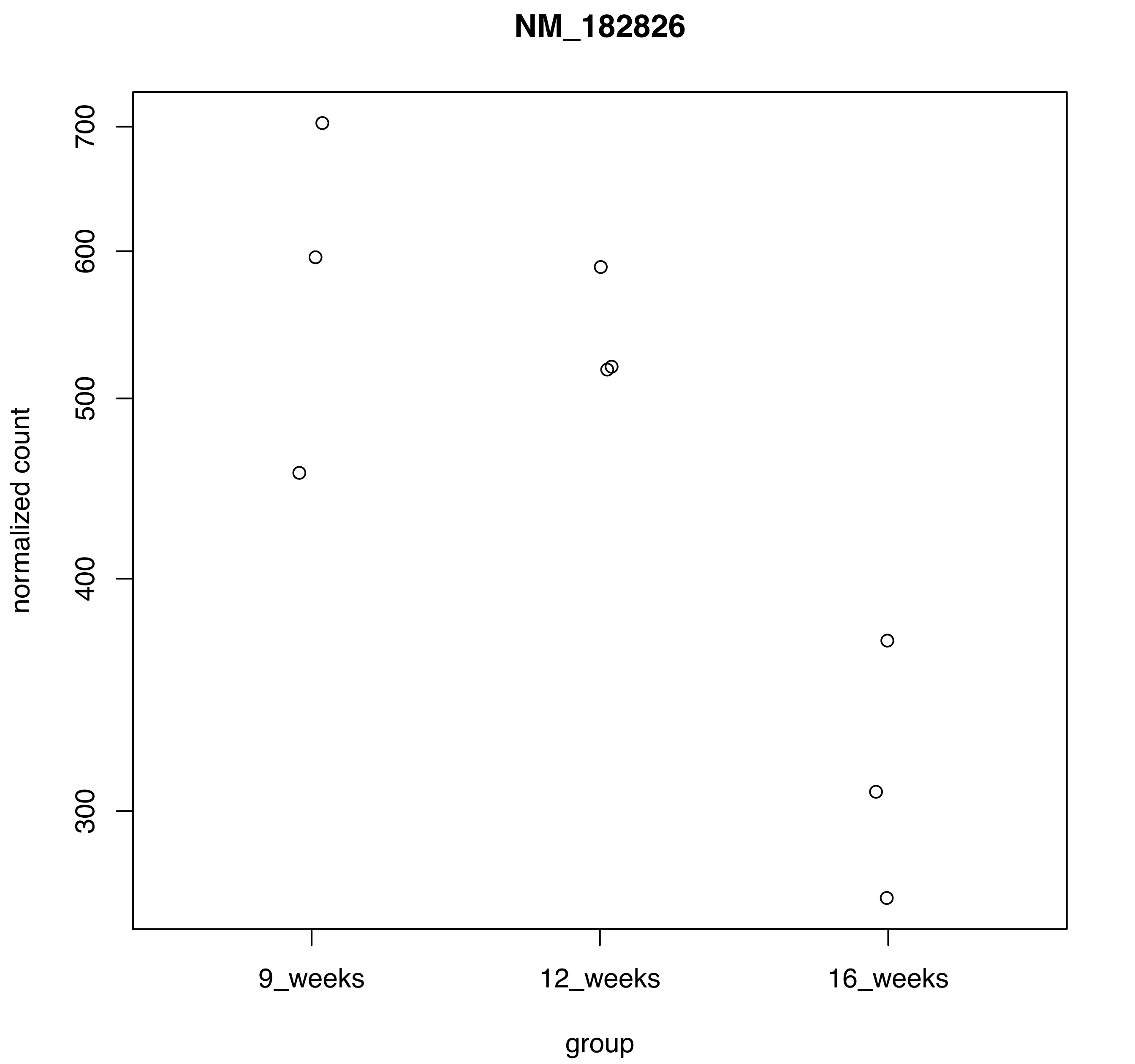

NM_182826
normalized count
700
600
500
400
300
9_weeks
12_weeks
16_weeks
group

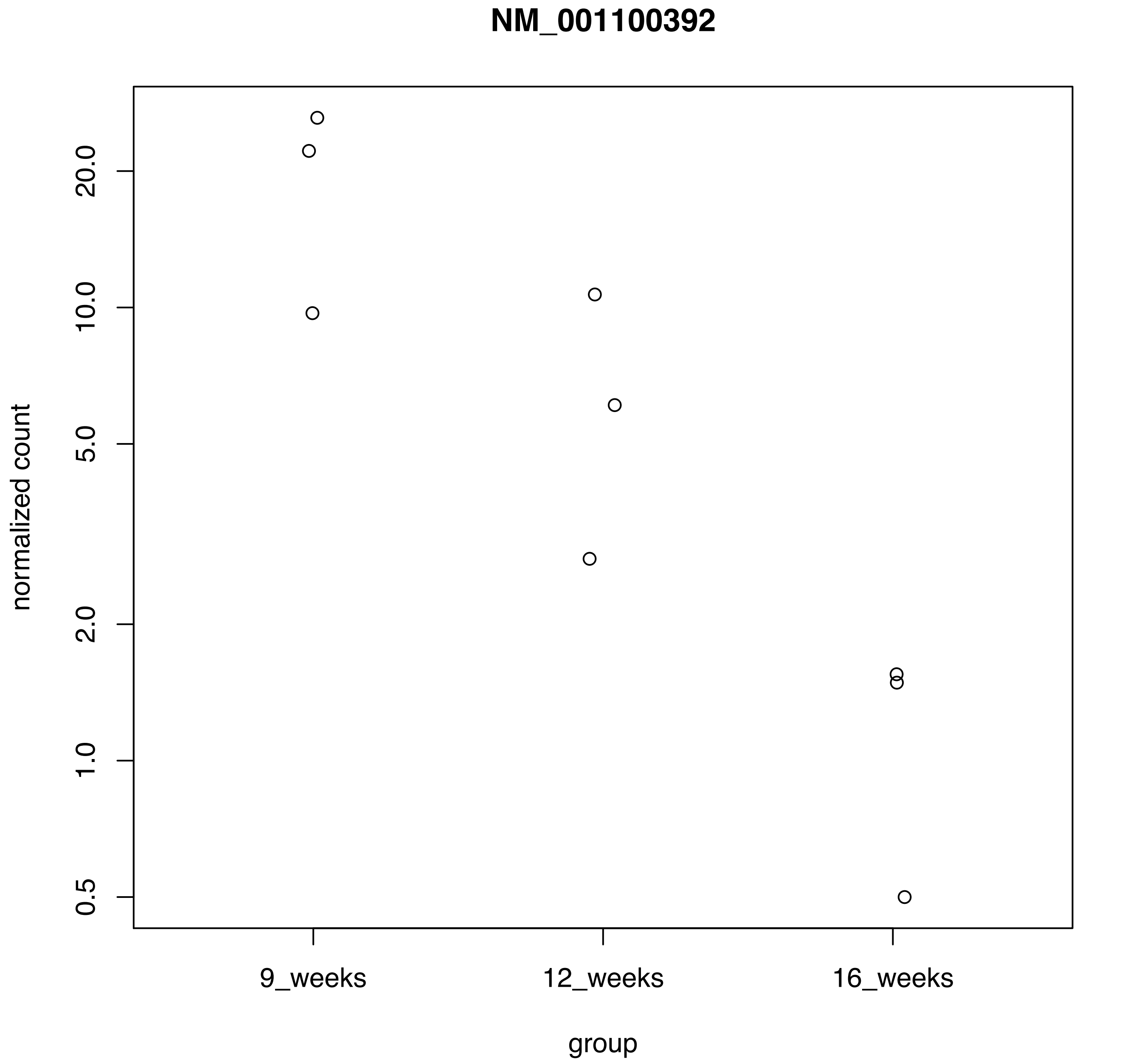
NM_001100392
normalized count
20.0
10.0
5.0
2.0
1.0
0.5
9_weeks
12_weeks
16_weeks
group

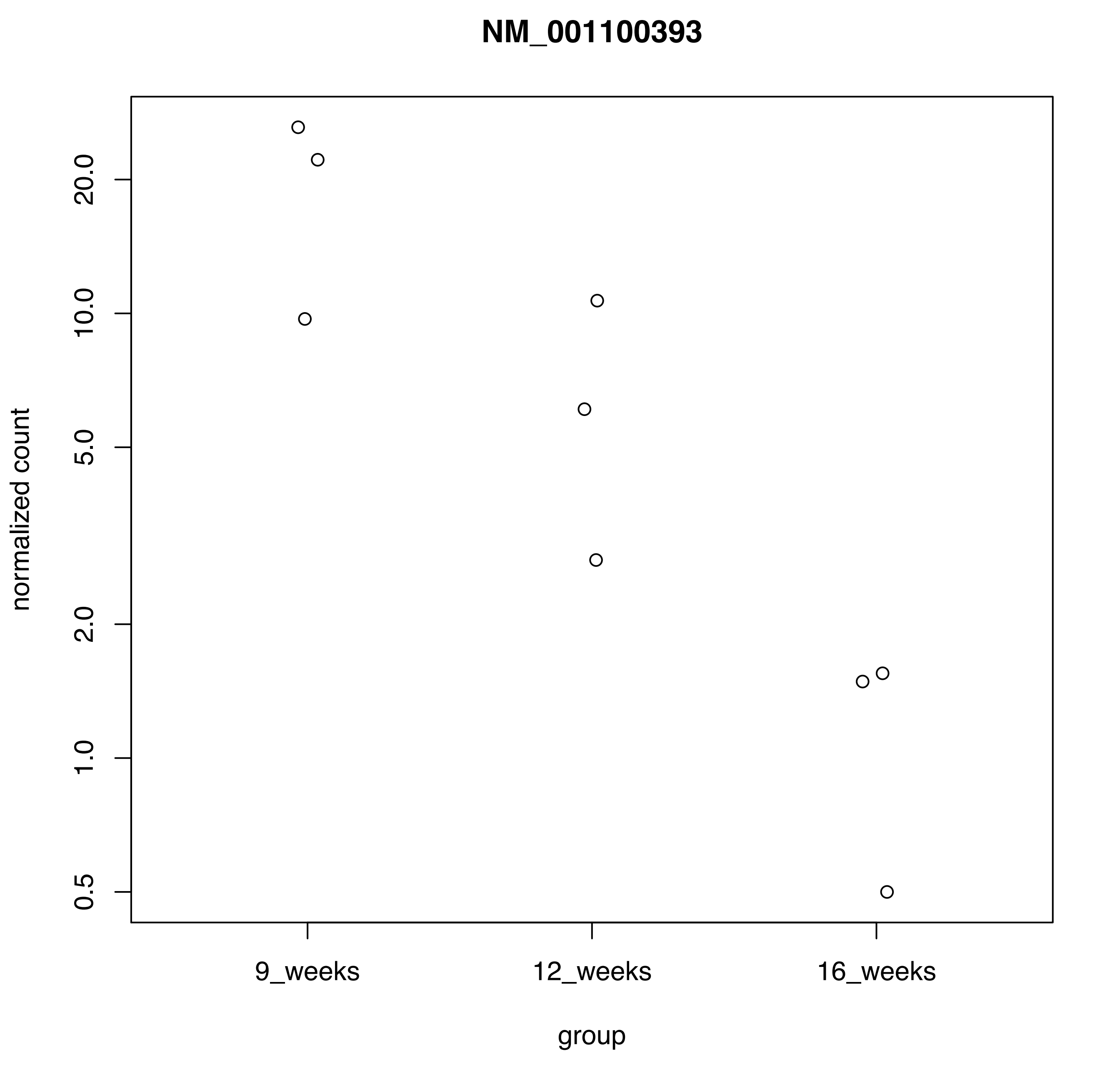
NM_001100393
normalized count
20.0
10.0
5.0
2.0
1.0
0.5
9_weeks
12_weeks
16_weeks
group

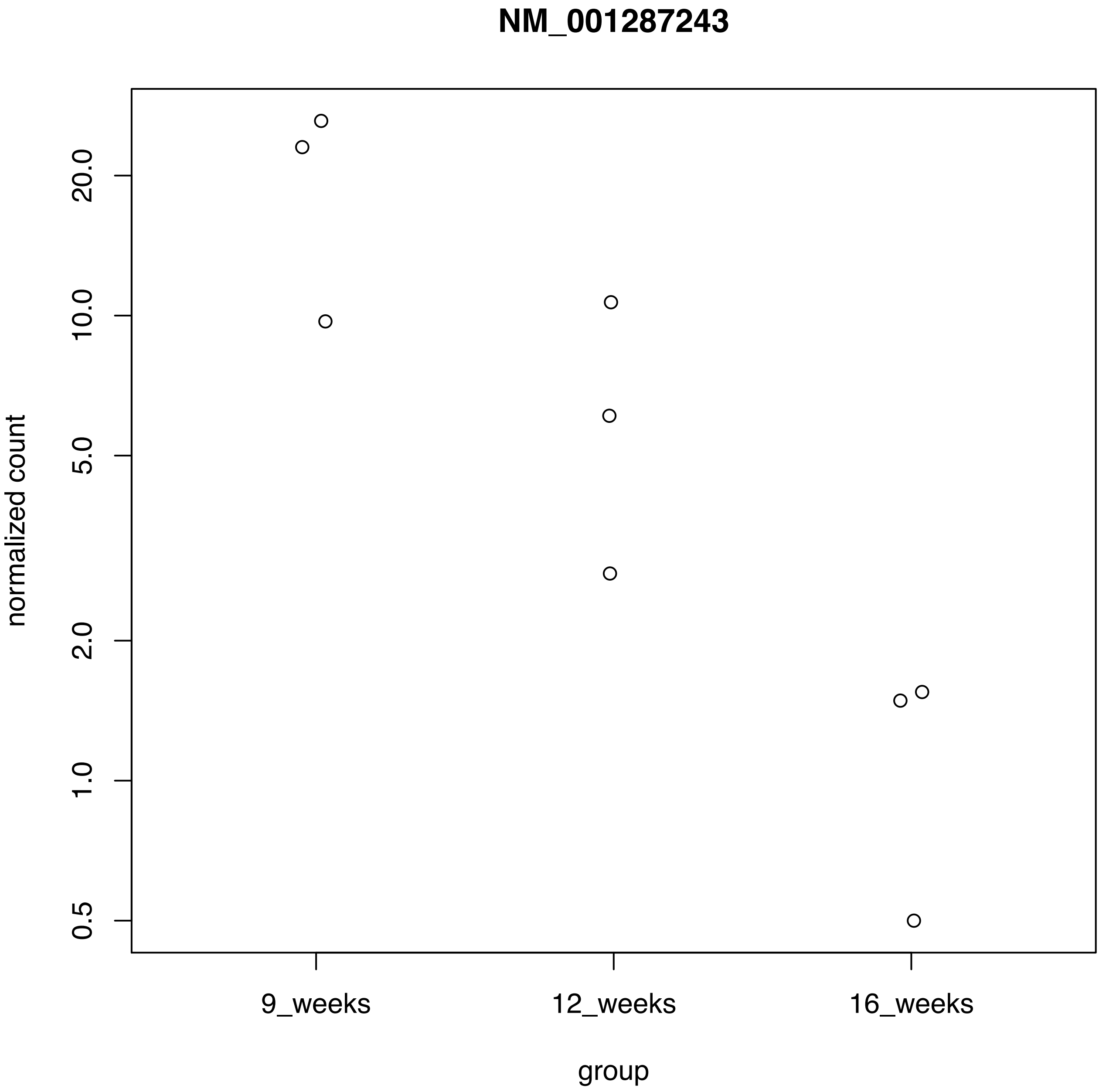
NM_001287243
normalized count
20.0
10.0
5.0
2.0
1.0
0.5
9_weeks
12_weeks
16_weeks
group

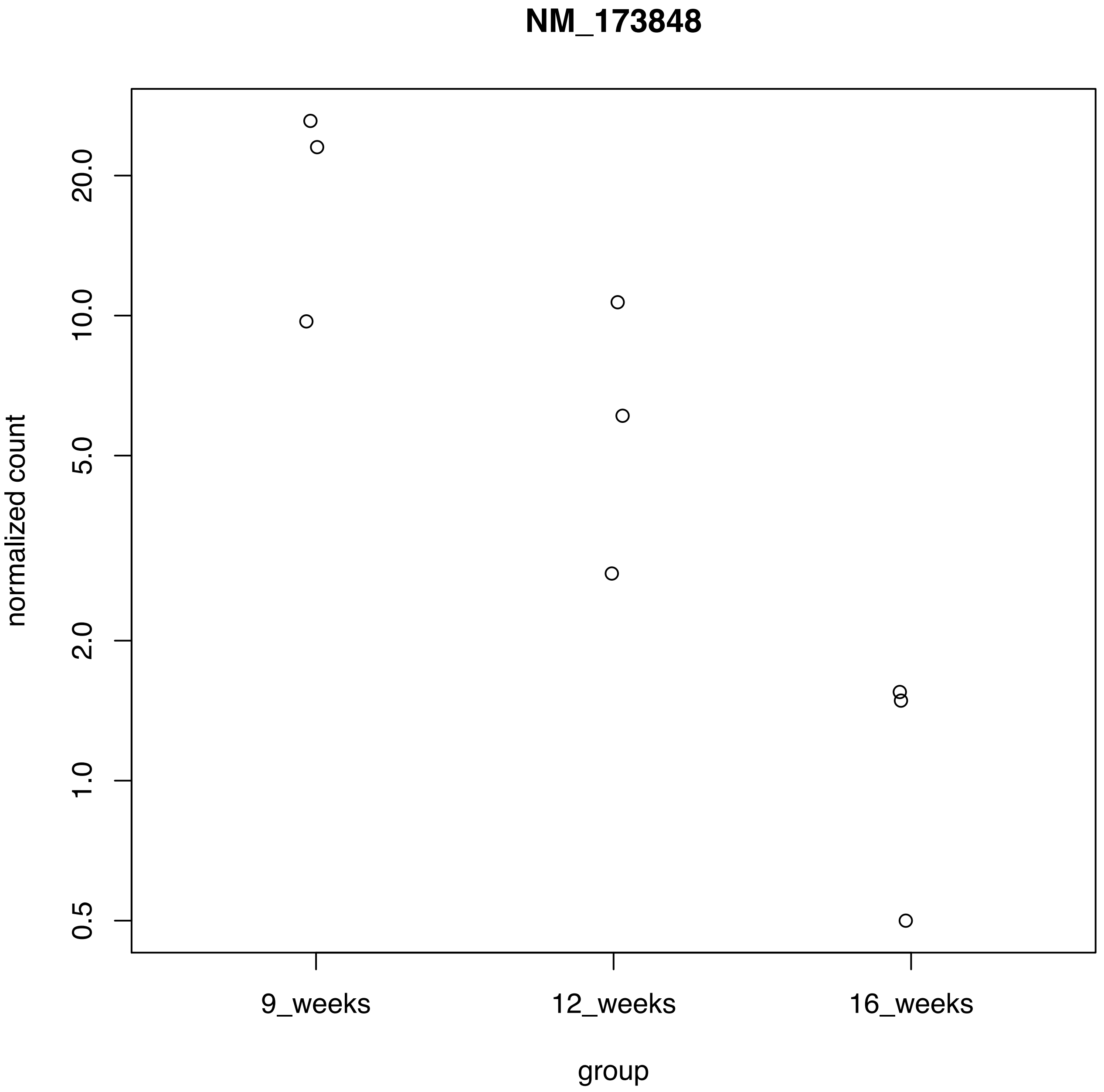
NM_173848
normalized count
20.0
10.0
5.0
2.0
1.0
0.5
9_weeks
12_weeks
16_weeks
group

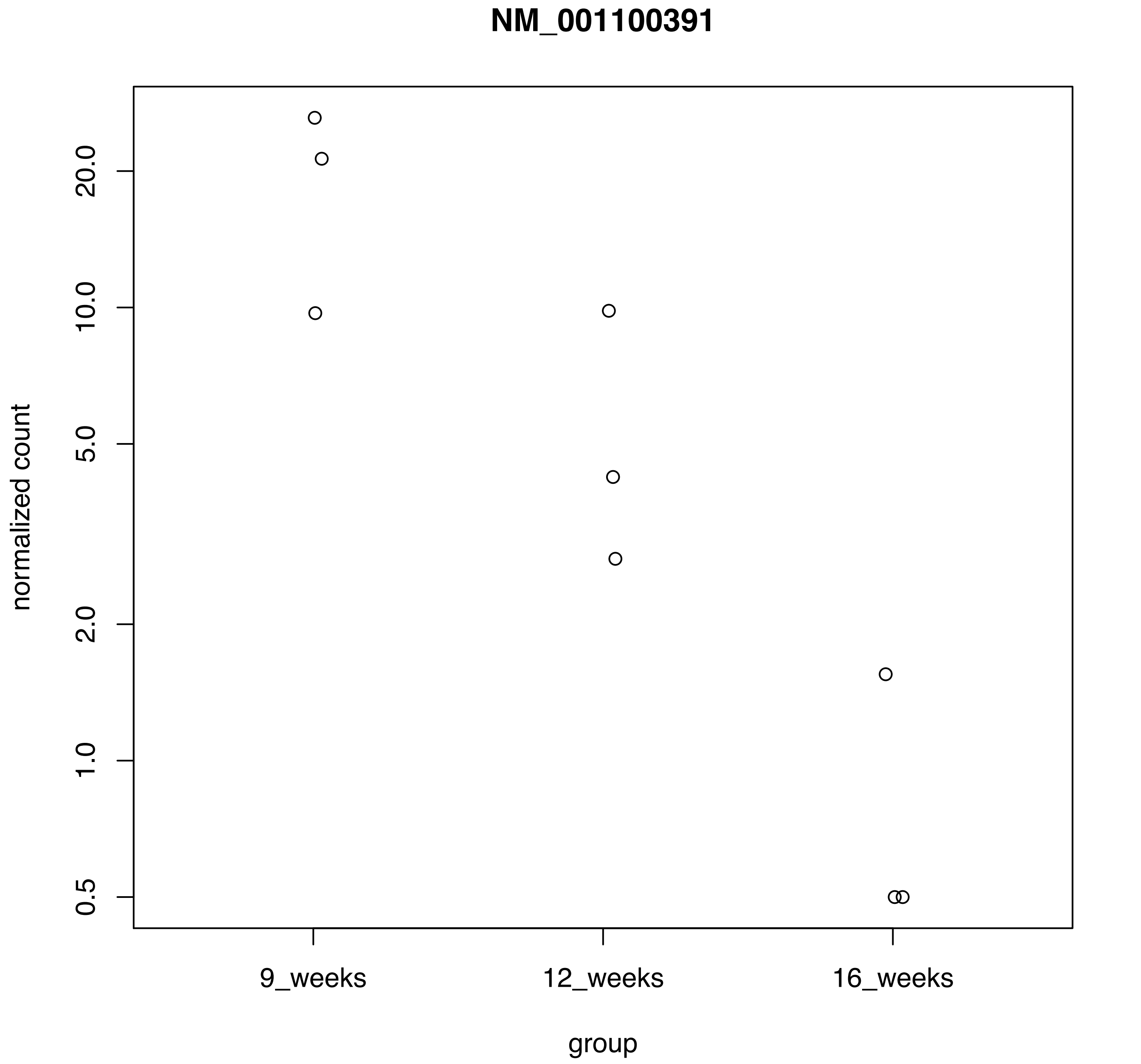

NM_001100391
normalized count
20.0
10.0
5.0
2.0
1.0
0.5
9_weeks
12_weeks
16_weeks
group

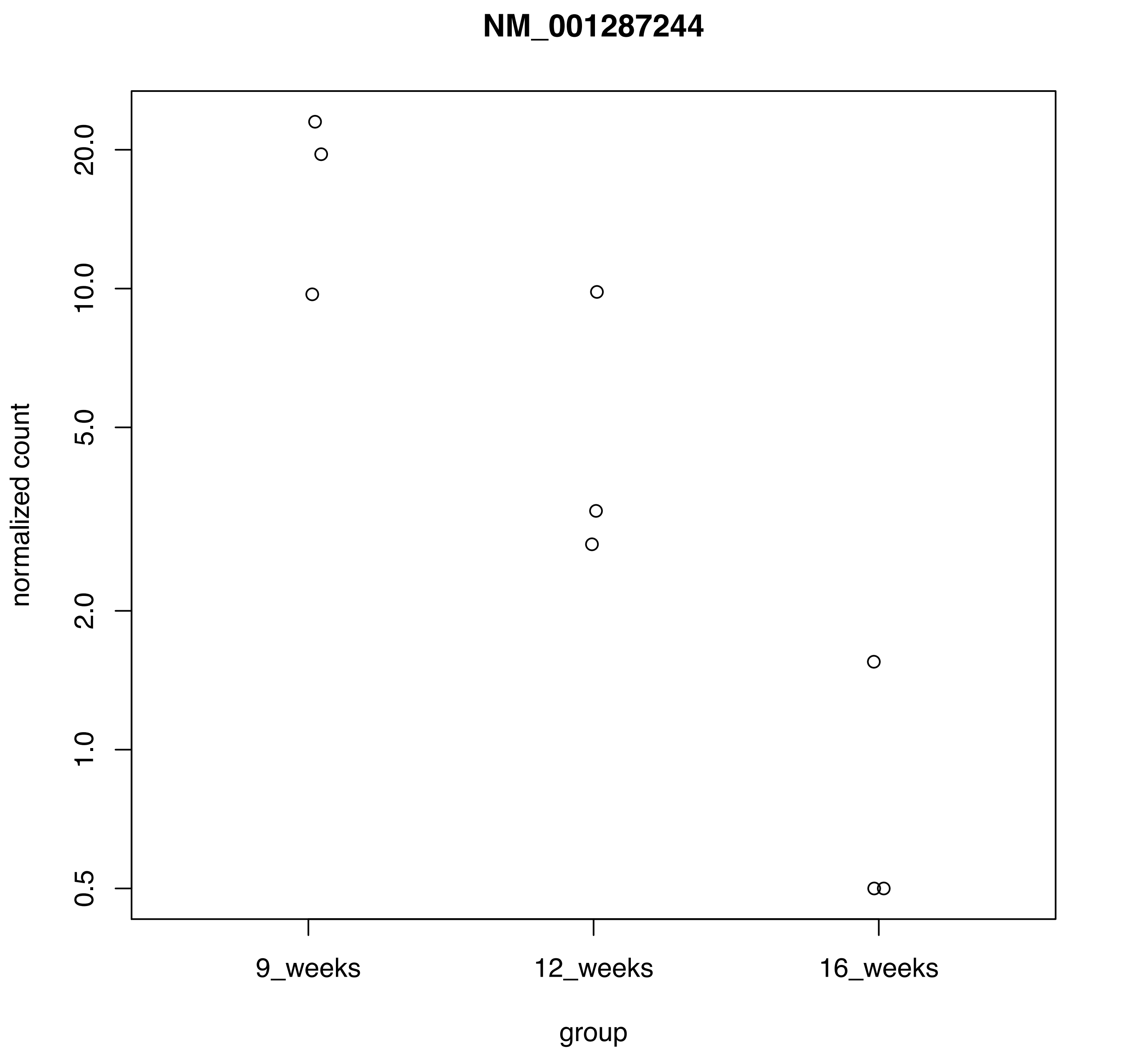
NM_001287244
normalized count
20.0
10.0
5.0
2.0
1.0
0.5
9_weeks
12_weeks
16_weeks
group

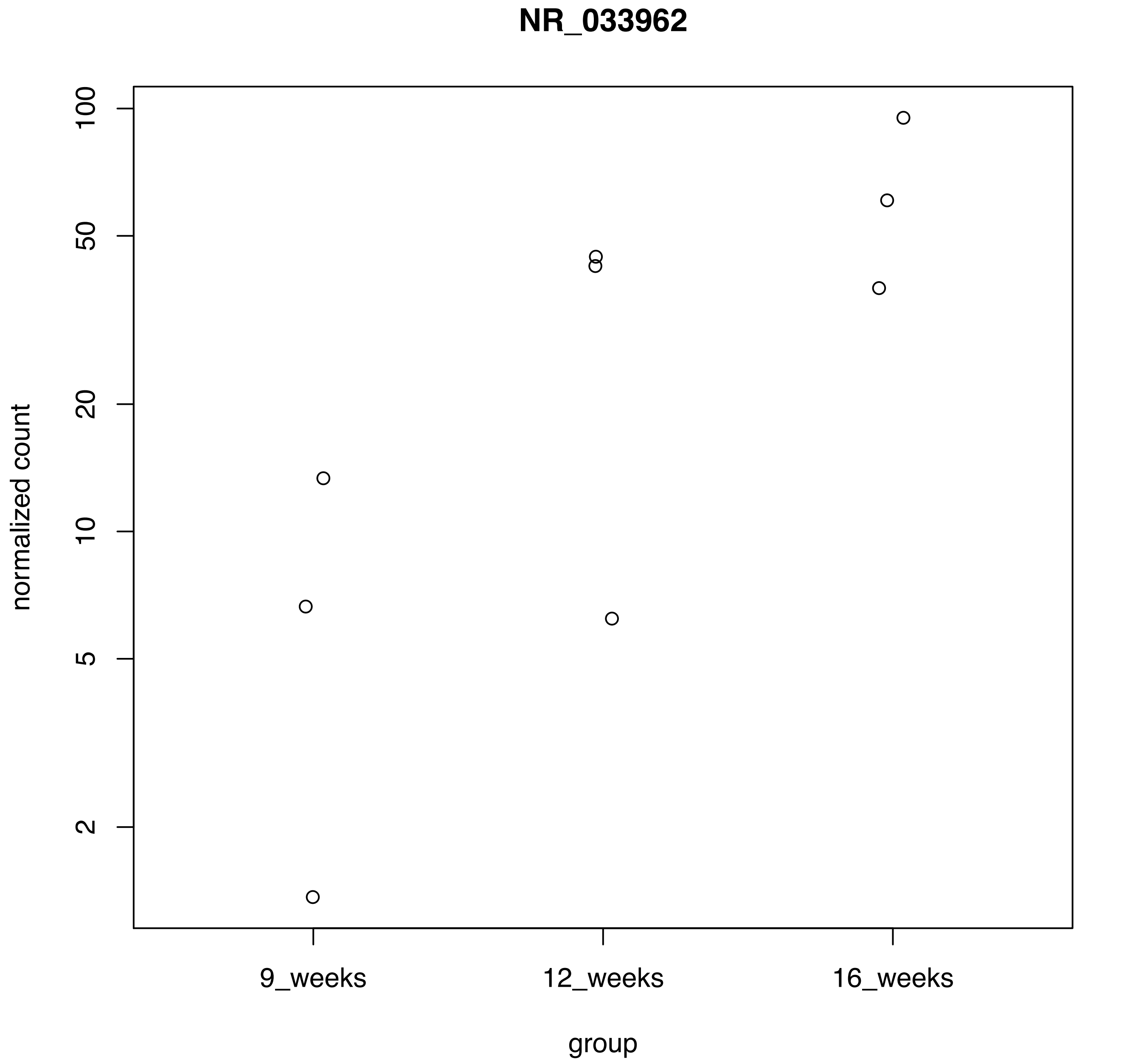
NR_033962
normalized count
100
50
20
10
5
2
9_weeks
12_weeks
16_weeks
group

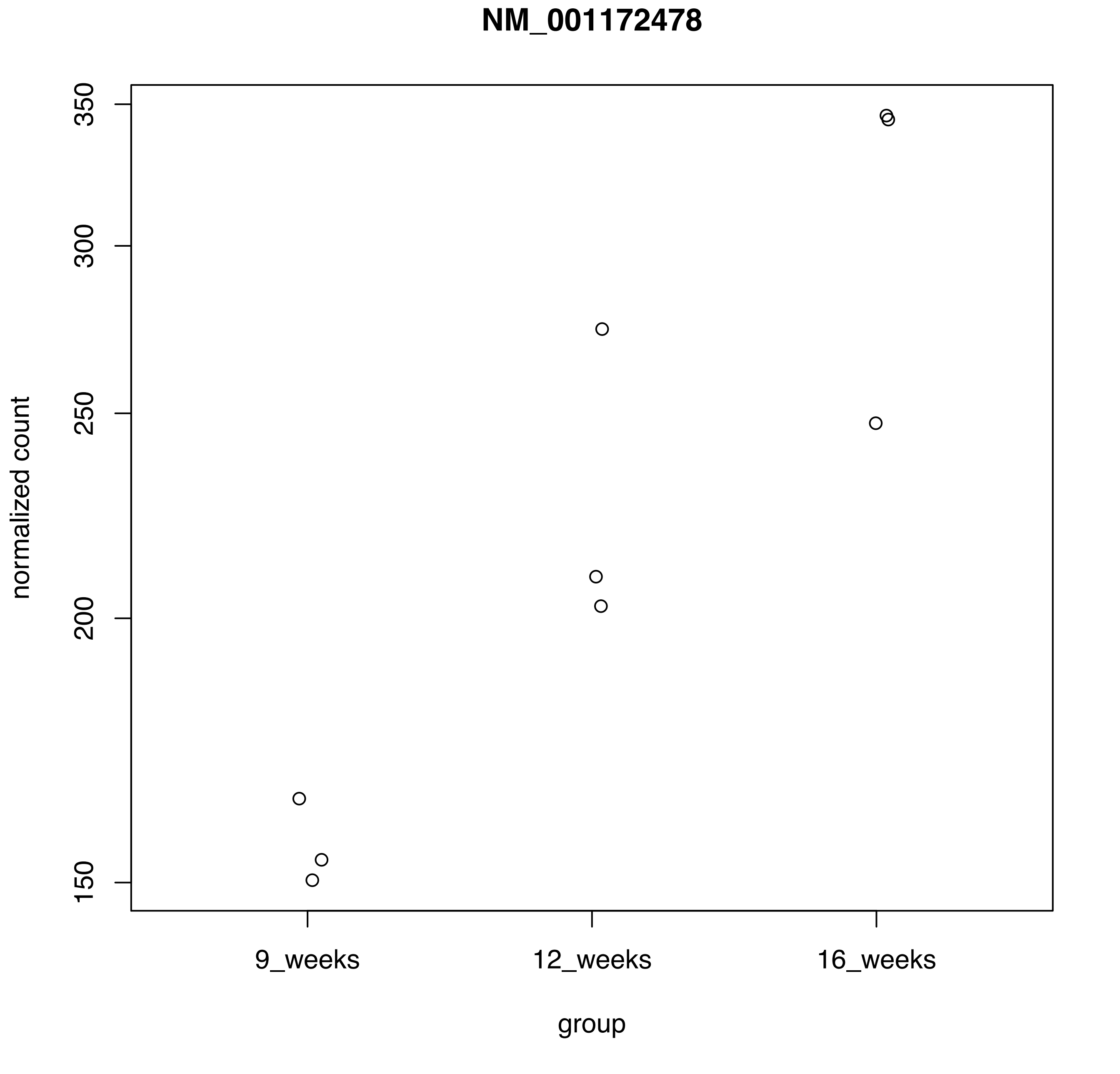
NM_001172478
normalized count
150
200
250
300
350
9_weeks
12_weeks
16_weeks
group

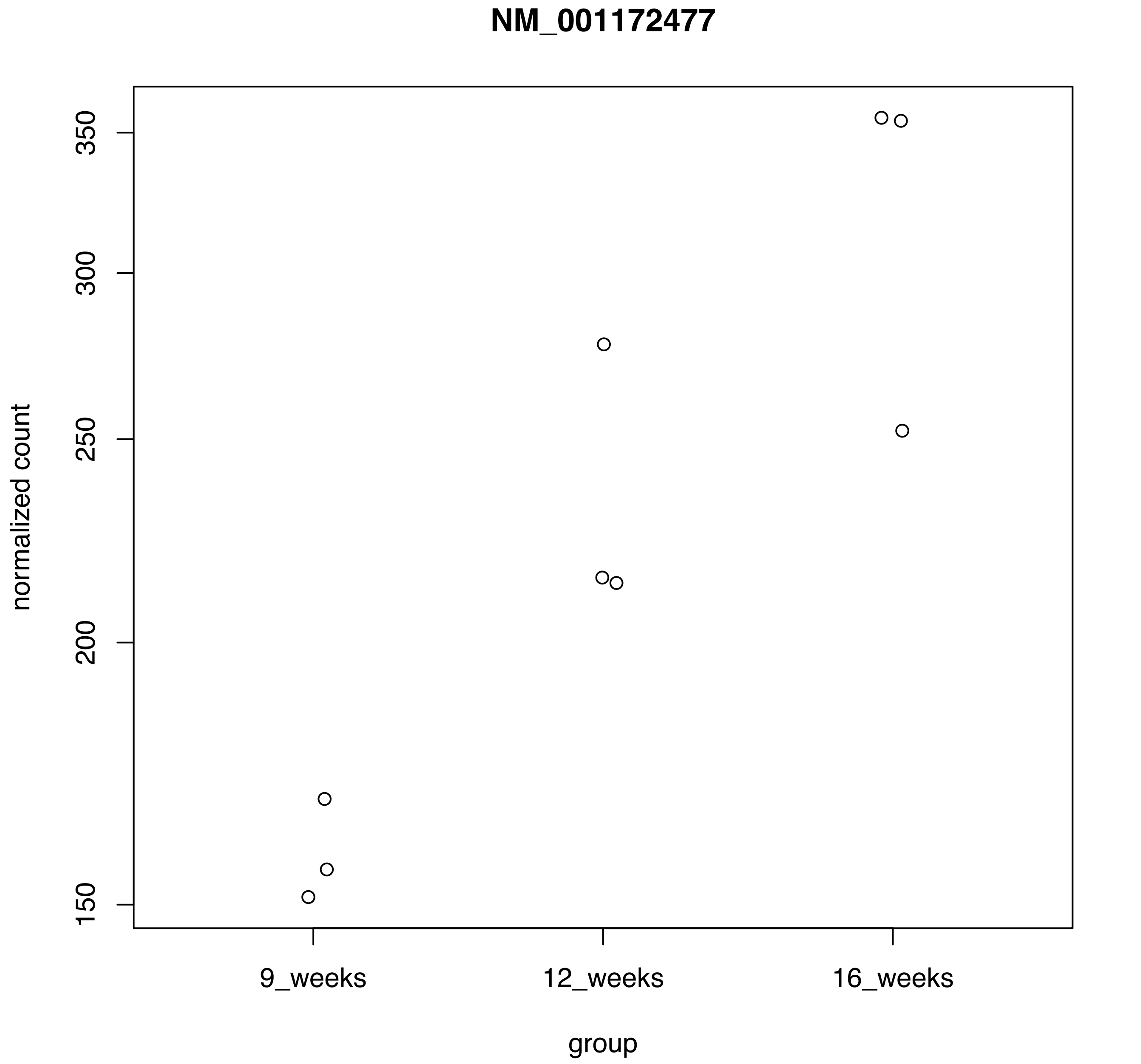
NM_001172477
normalized count
150
200
250
300
350
9_weeks
12_weeks
16_weeks
group

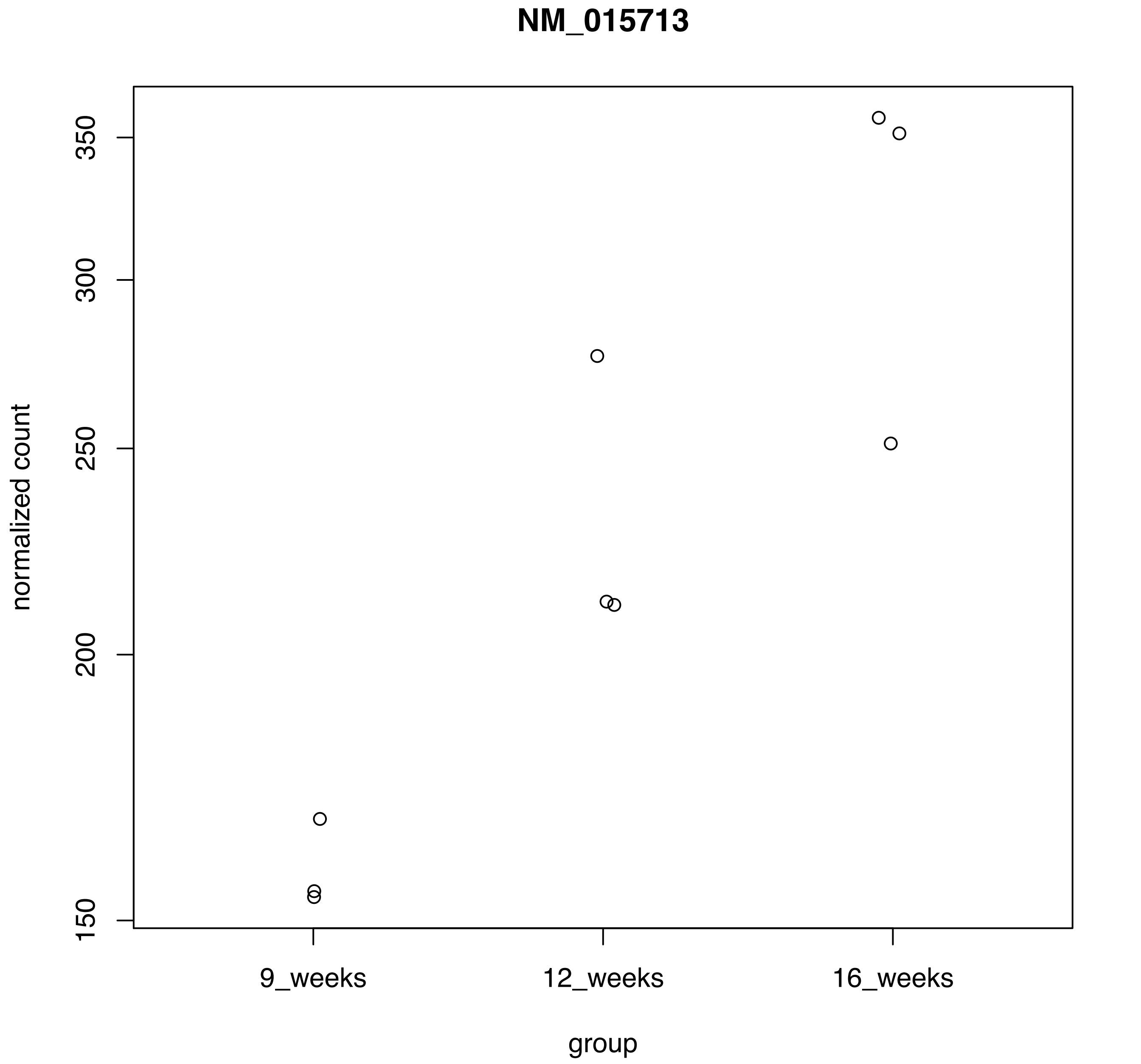
NM_015713
normalized count
350
300
250
200
150
9_weeks
12_weeks
16_weeks
group

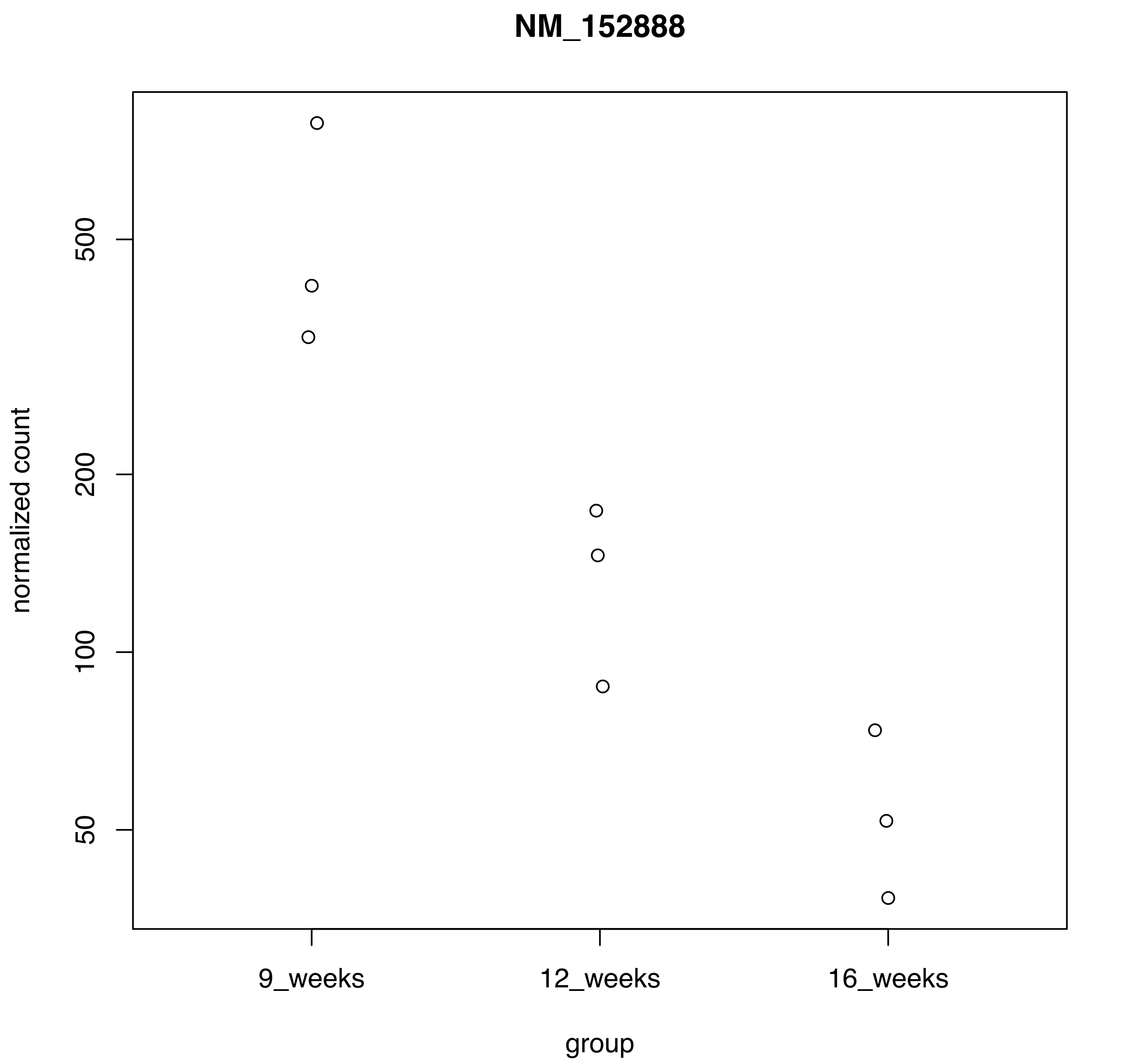
NM_152888
normalized count
500
200
100
50
9_weeks
12_weeks
16_weeks
group

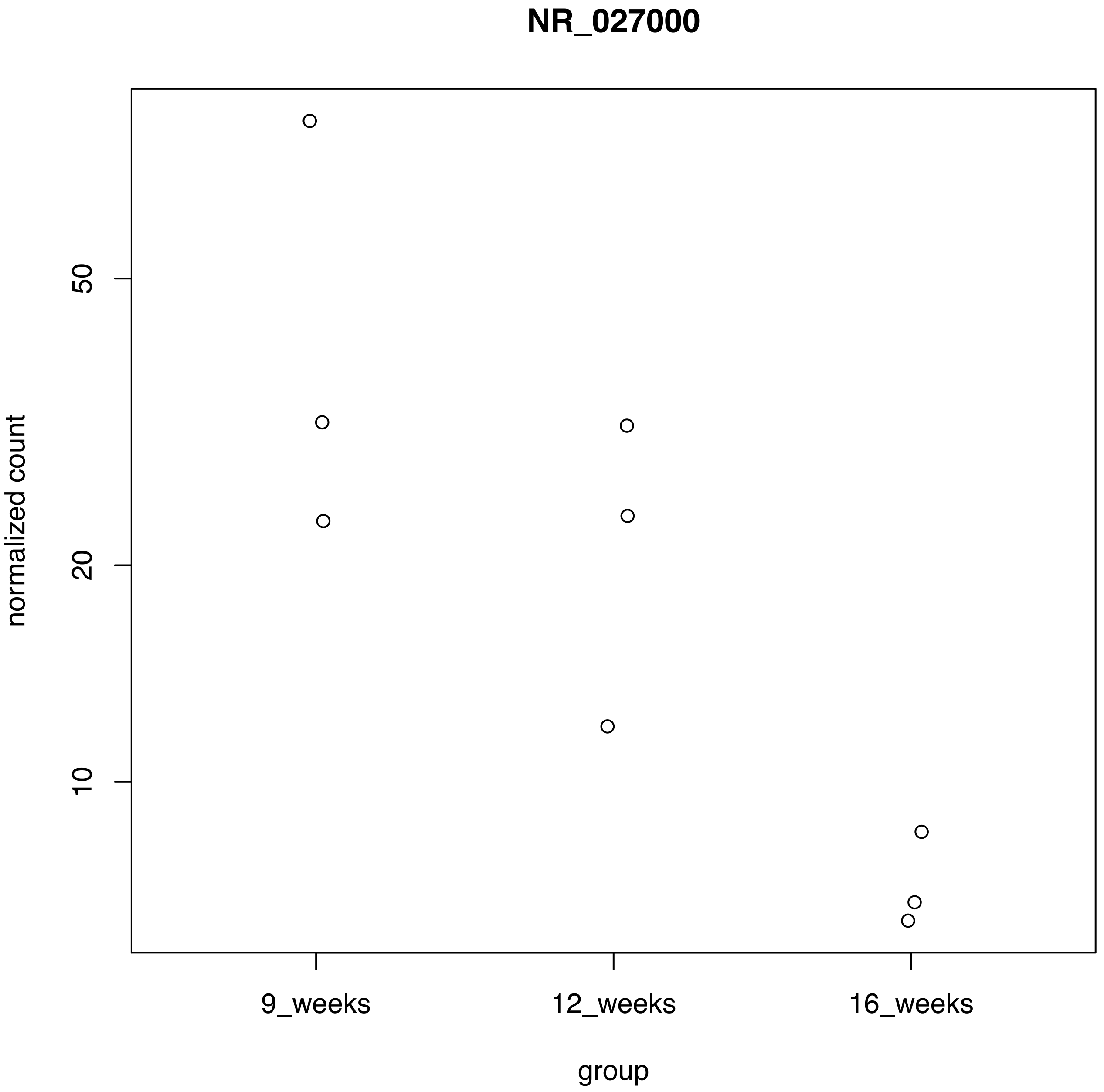
NR_027000
normalized count
50
20
10
9_weeks
12_weeks
16_weeks
group

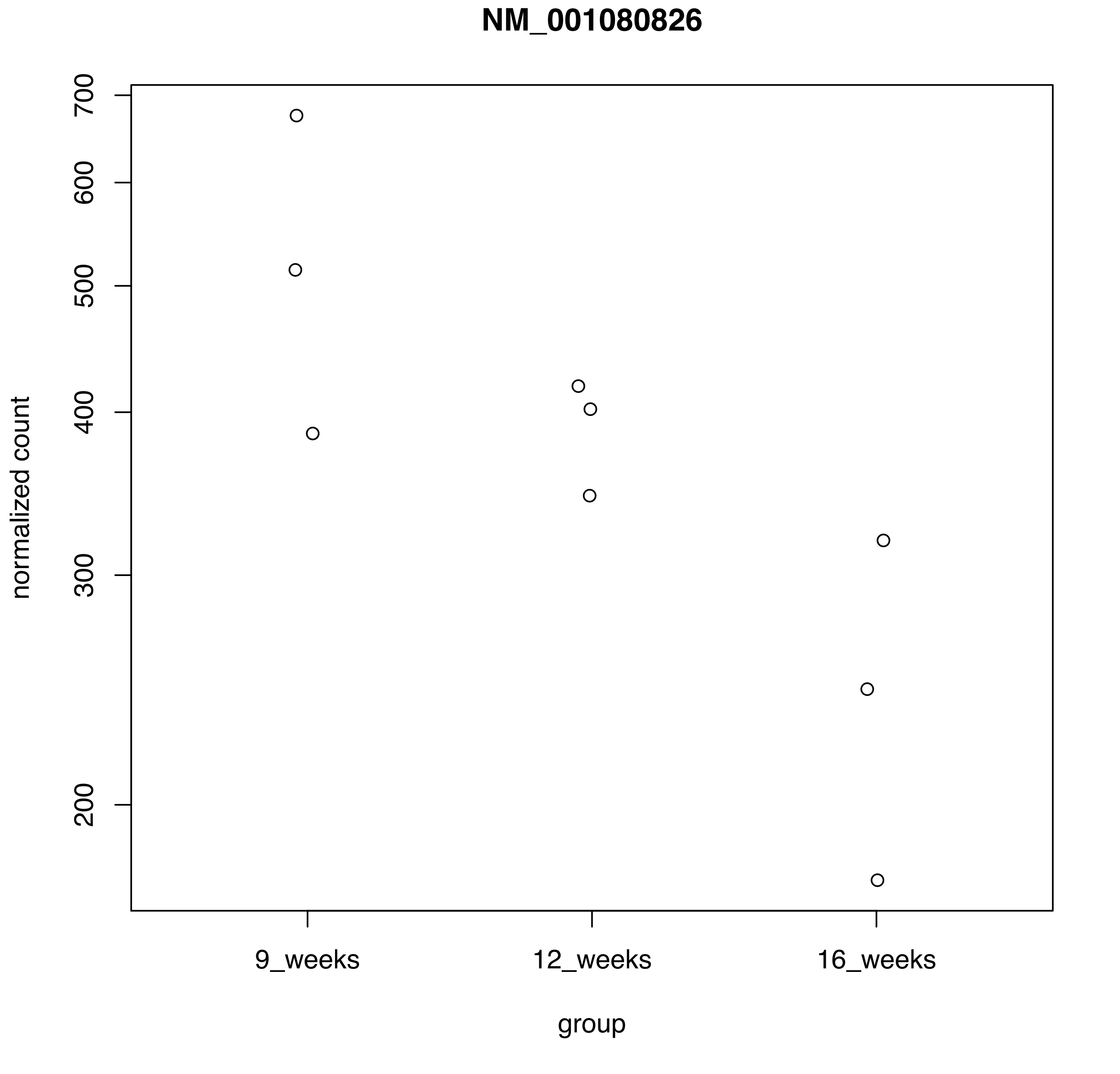
NM_001080826
normalized count
700
600
500
400
300
200
9_weeks
12_weeks
16_weeks
group

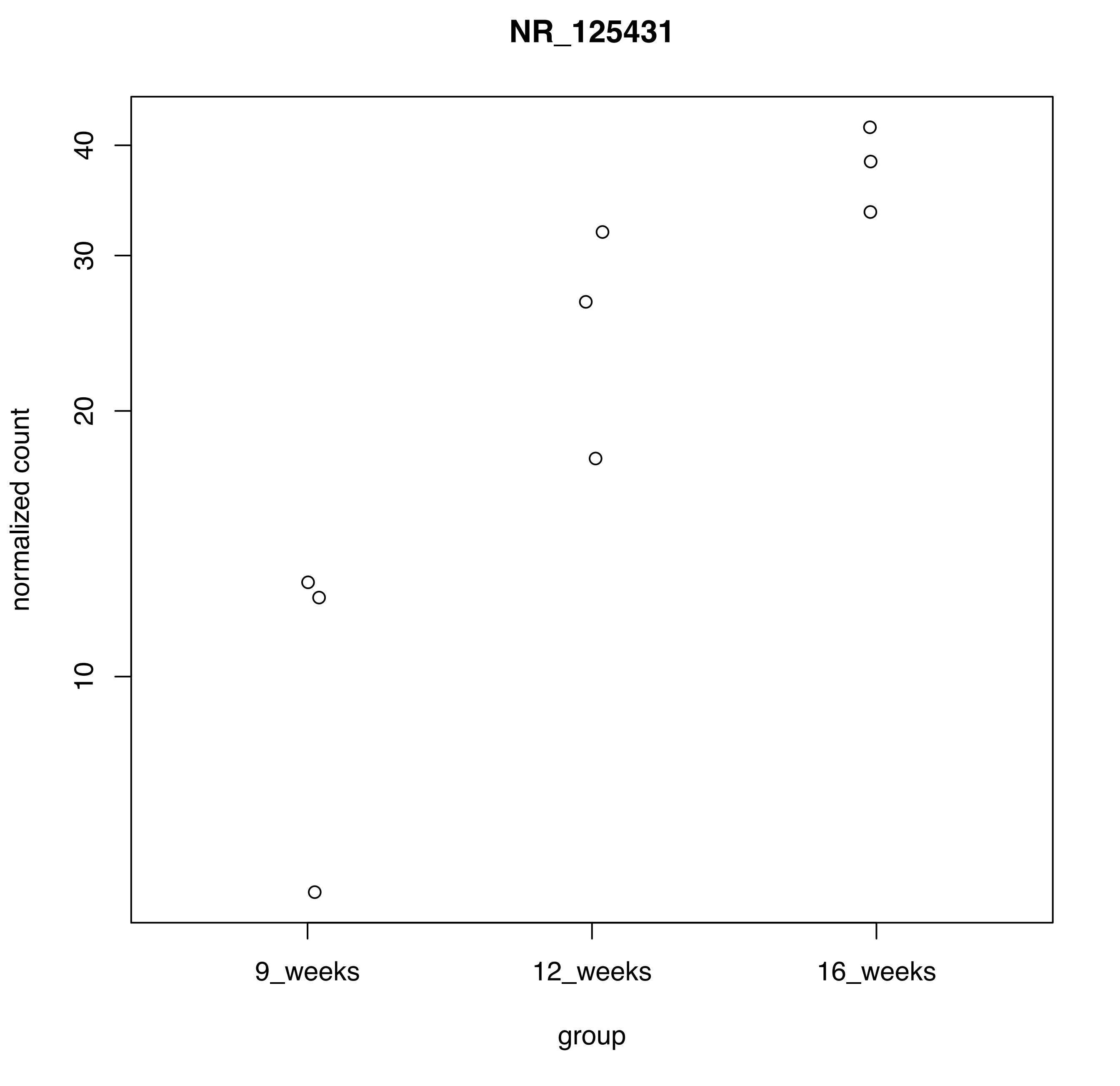
NR_125431
normalized count
10
20
30
40
9_weeks
12_weeks
16_weeks
group

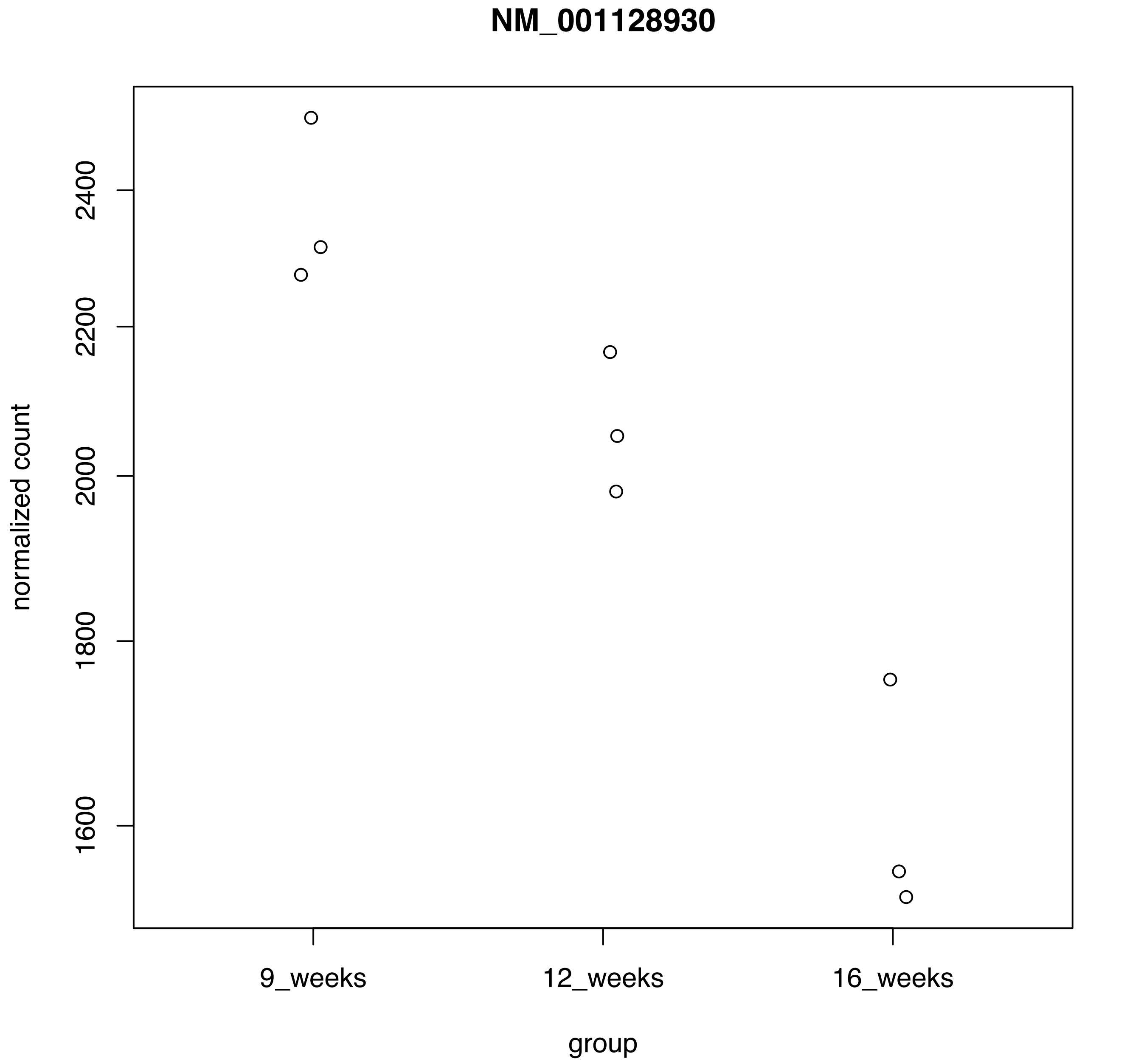
NM_001128930
2400
2200
2000
1800
1600
normalized count
9_weeks
12_weeks
16_weeks
group

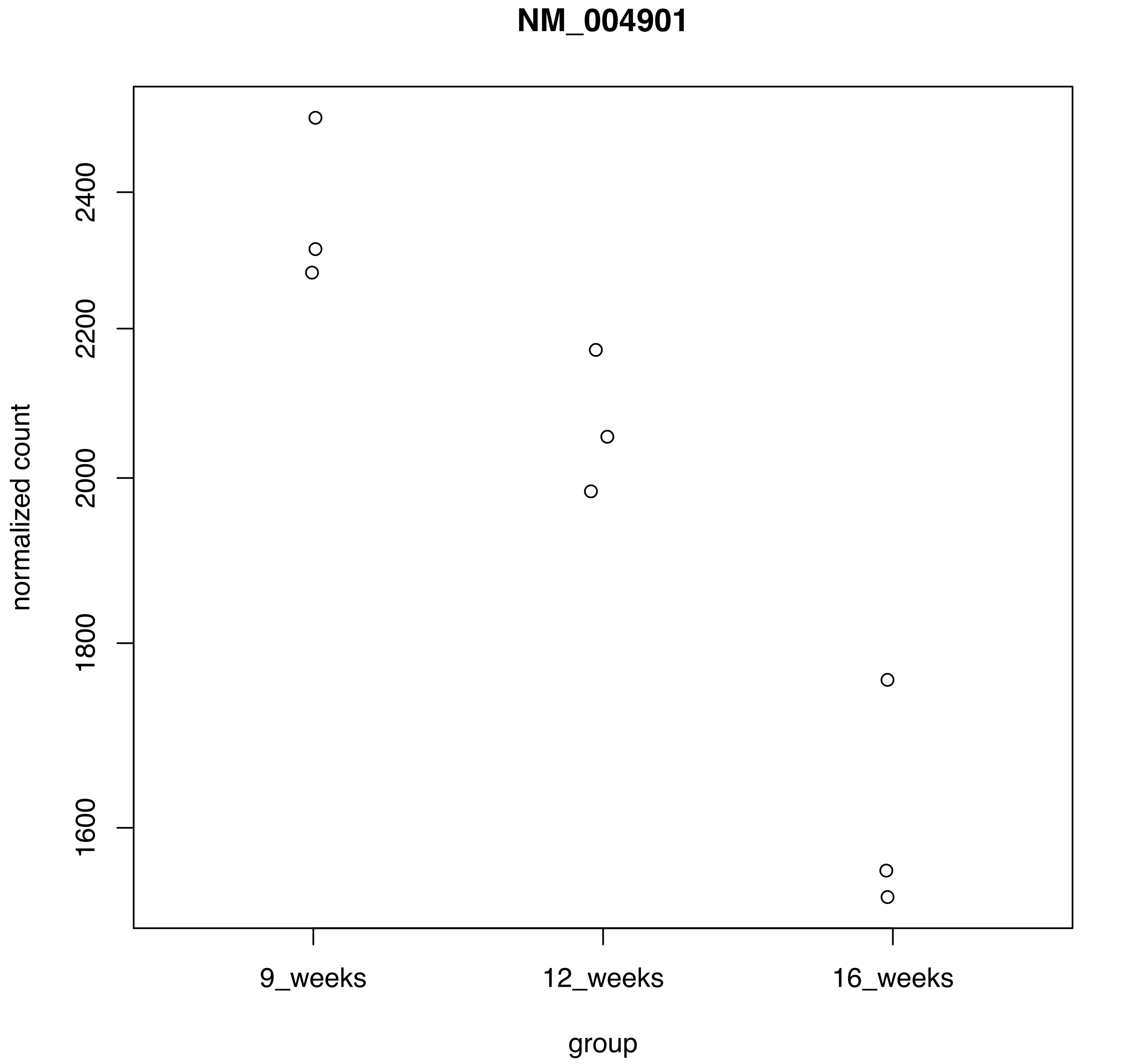
NM_004901
normalized count
2400
2200
2000
1800
1600
9_weeks
12_weeks
16_weeks
group

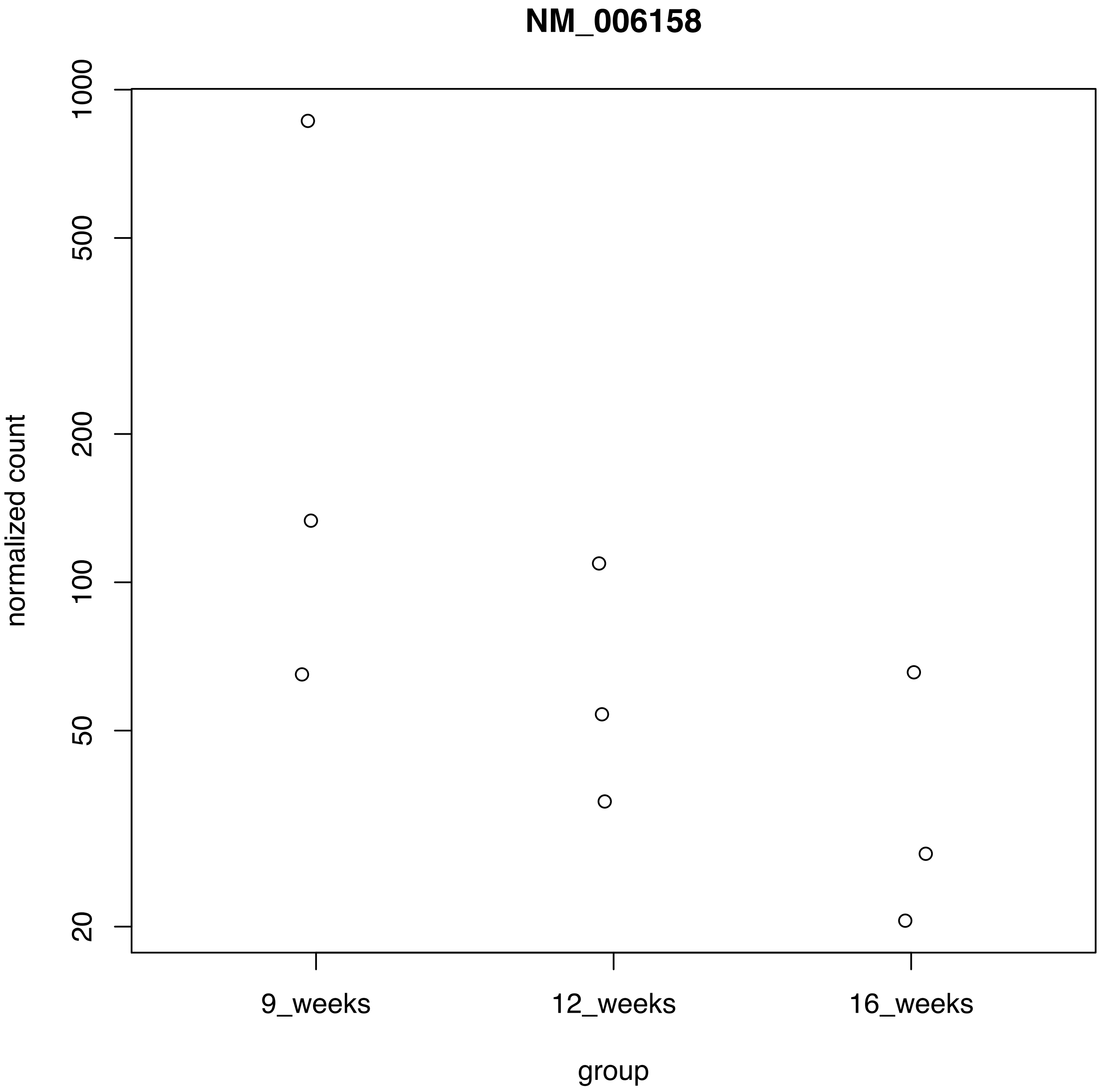

NM_006158
normalized count
1000
500
200
100
50
20
9_weeks
12_weeks
16_weeks
group

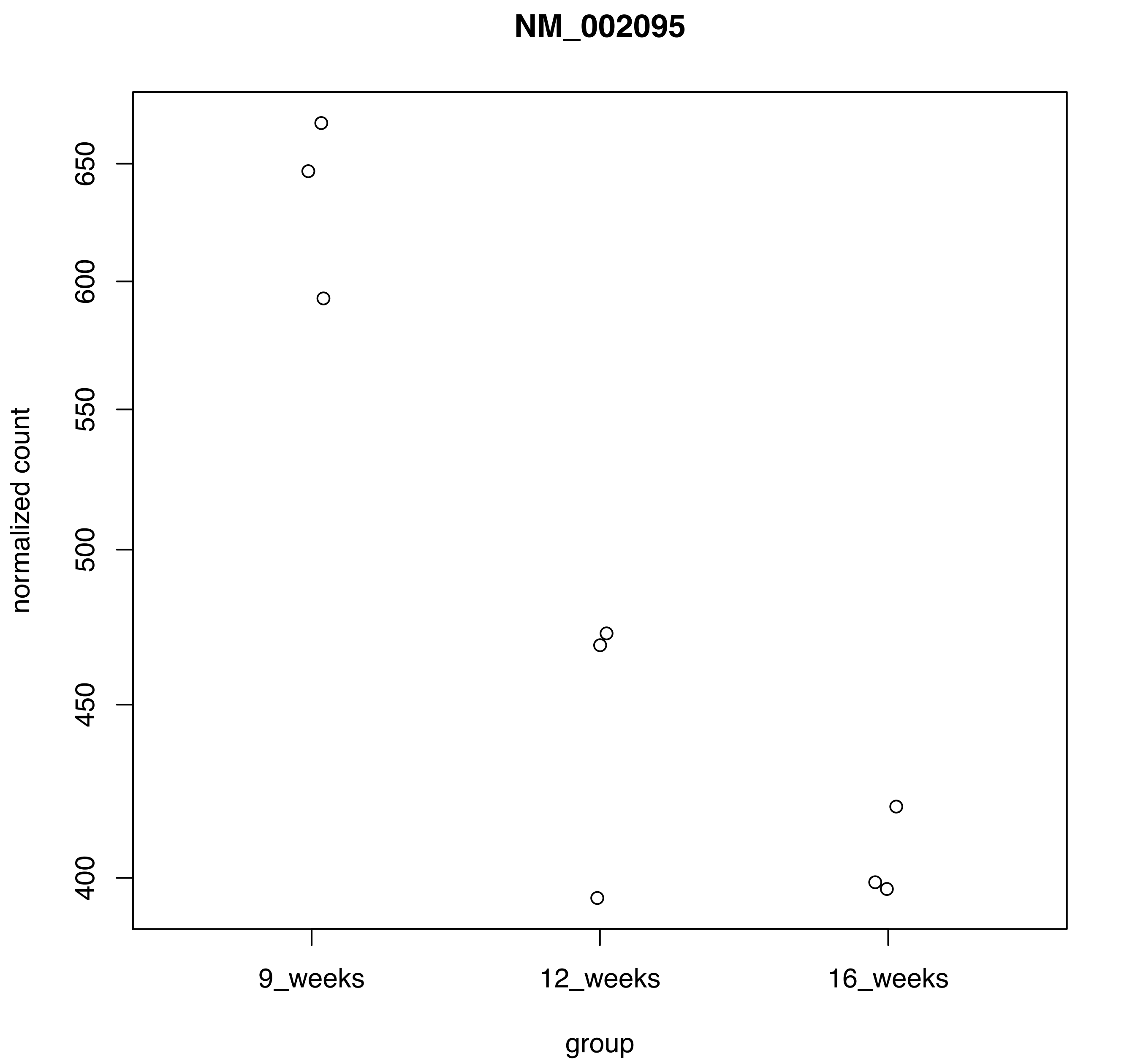
NM_002095
normalized count
650
600
550
500
450
400
9_weeks
12_weeks
16_weeks
group

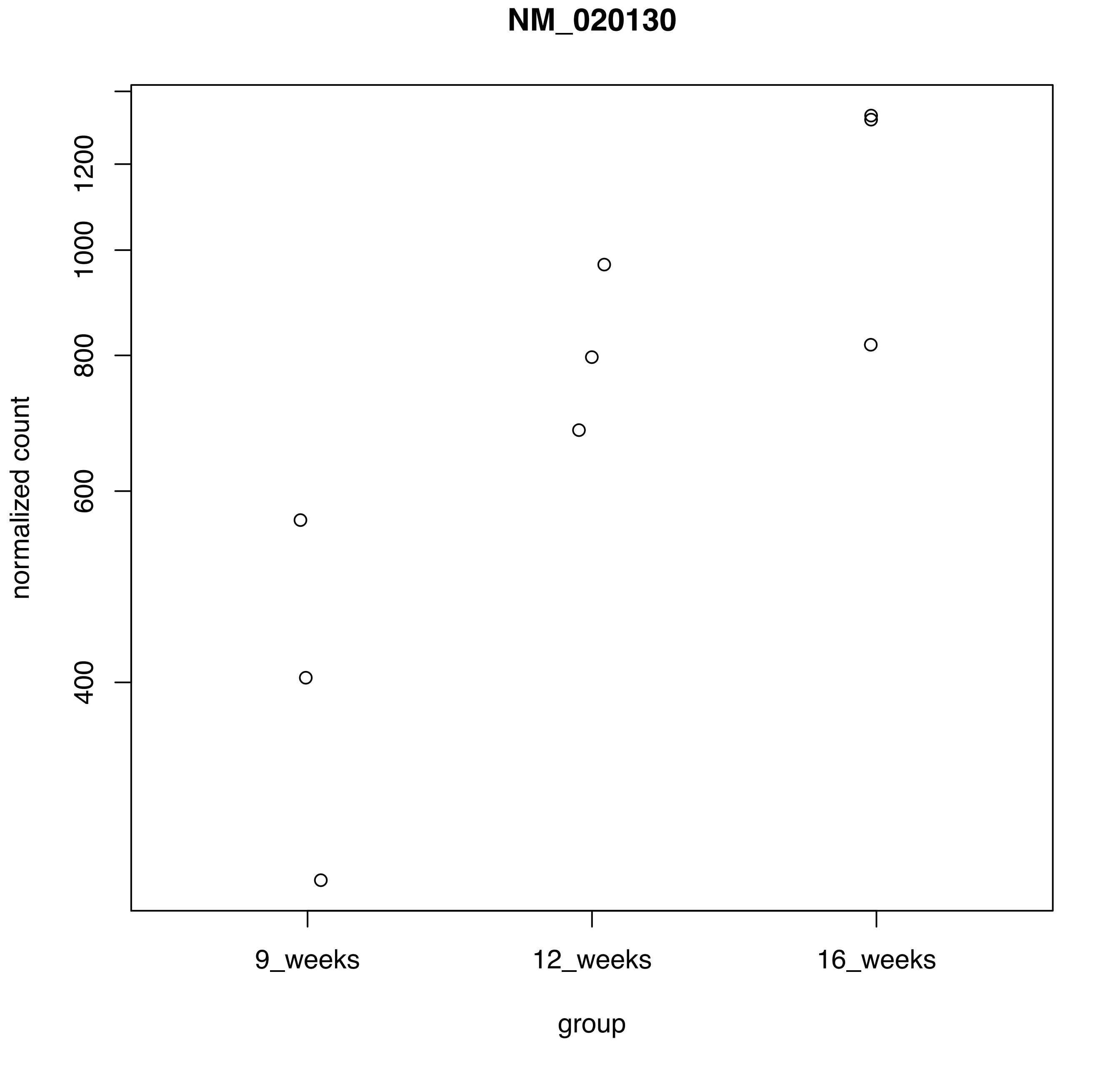
NM_020130
normalized count
1200
1000
800
600
400
9_weeks
12_weeks
16_weeks
group

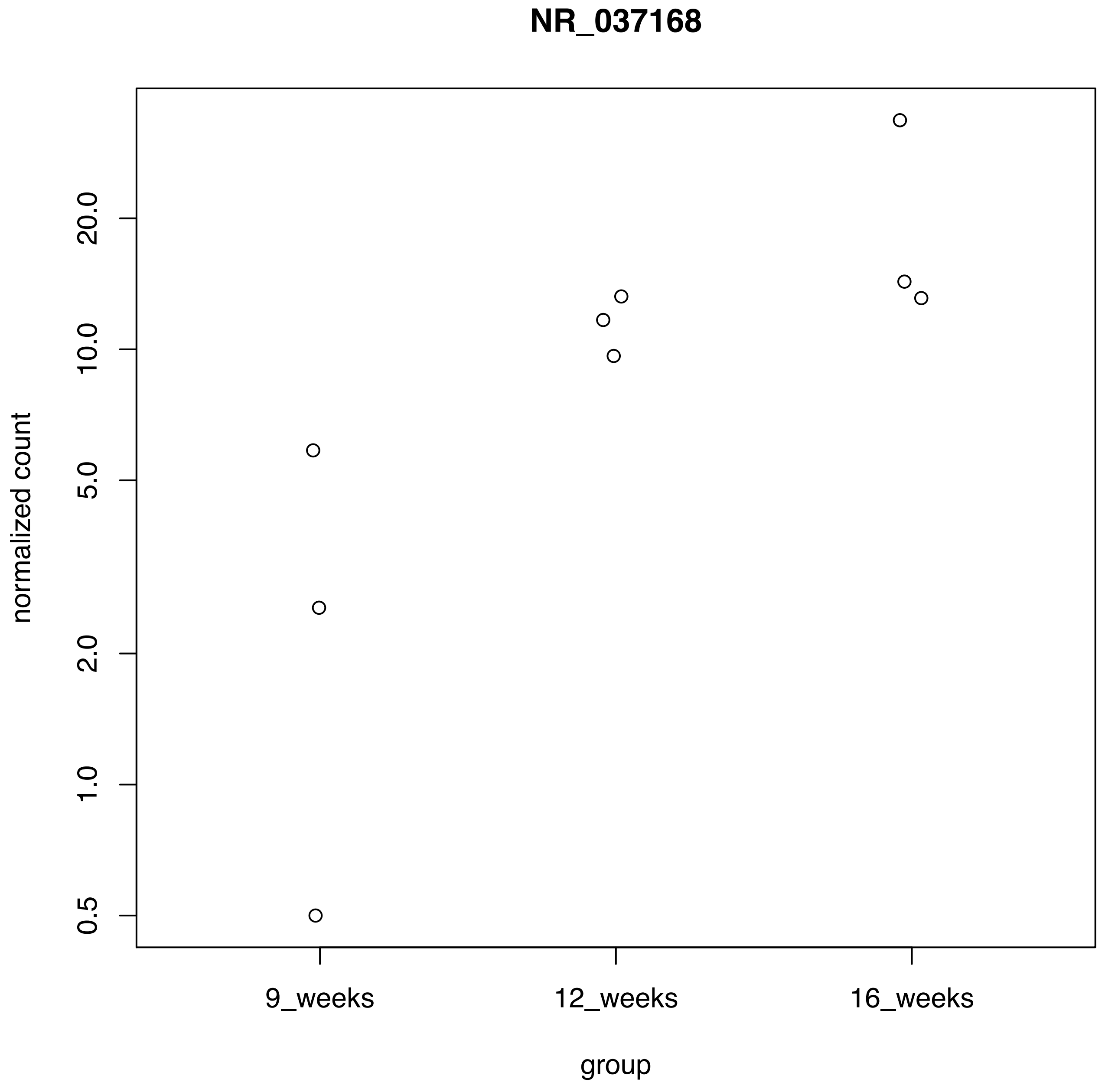
NR_037168
normalized count
0.5
1.0
2.0
5.0
10.0
20.0
9_weeks
12_weeks
16_weeks
group

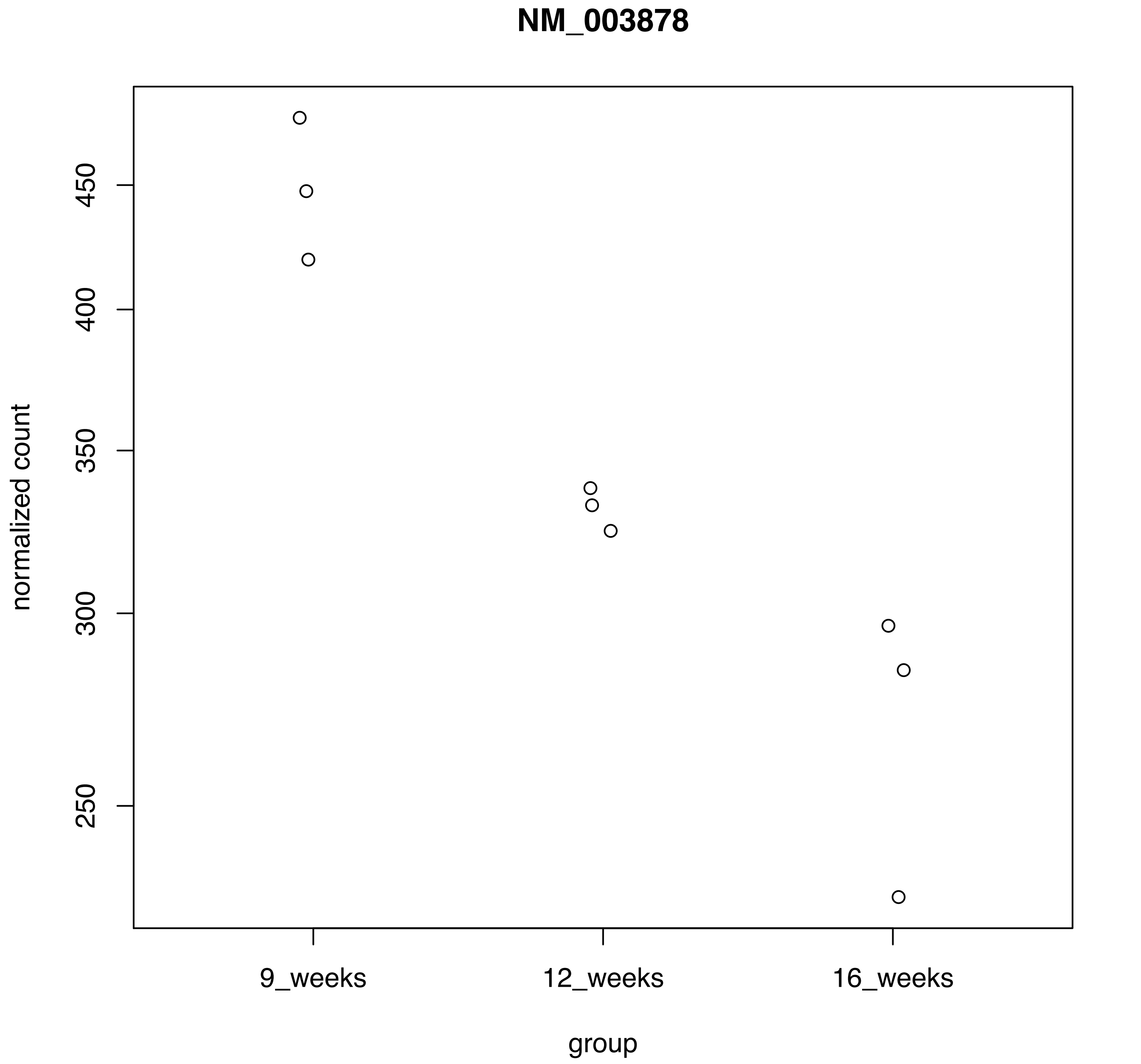
NM_003878
normalized count
250
300
350
400
450
9_weeks
12_weeks
16_weeks
group

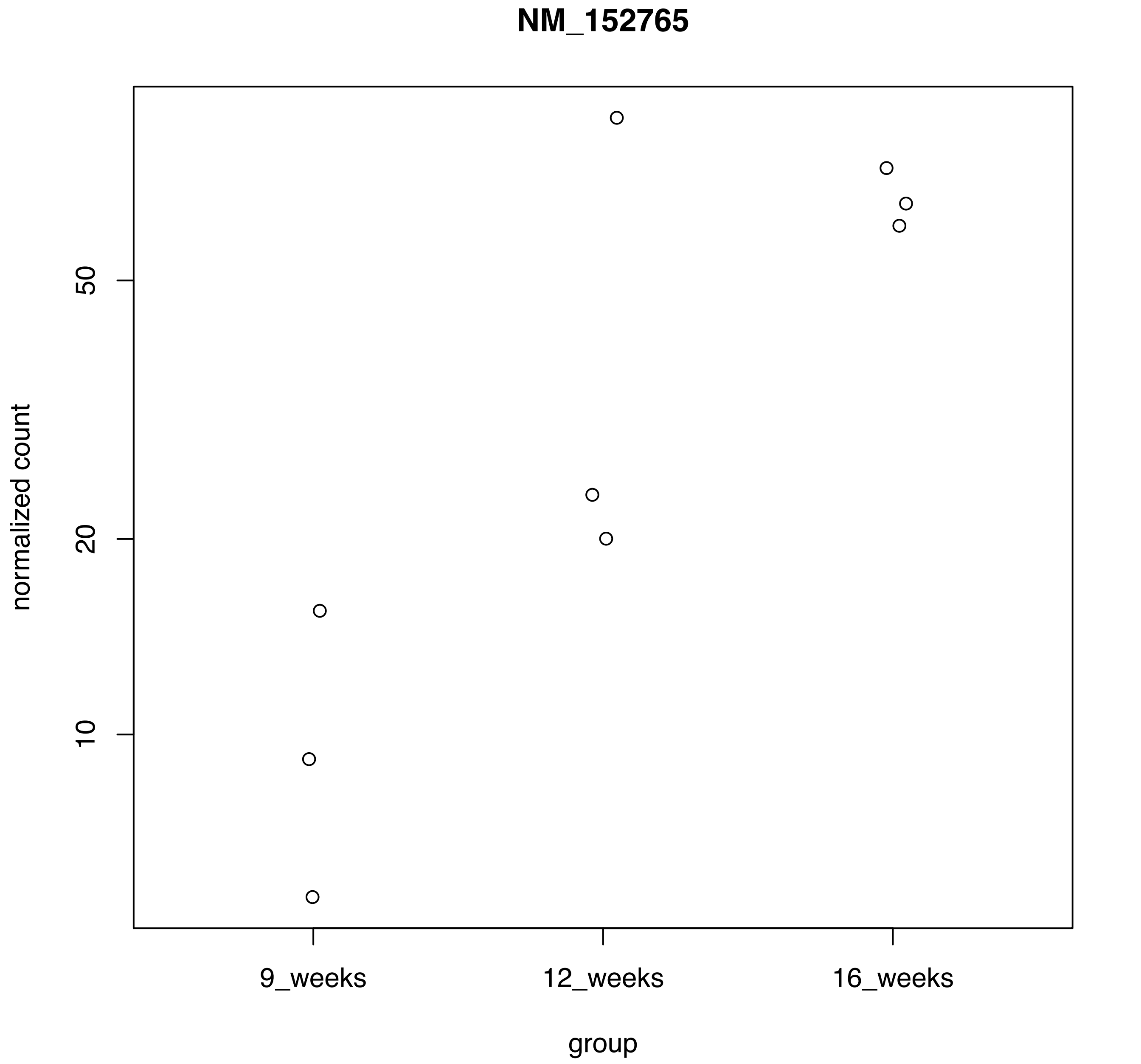
NM_152765
normalized count
50
20
10
9_weeks
12_weeks
16_weeks
group

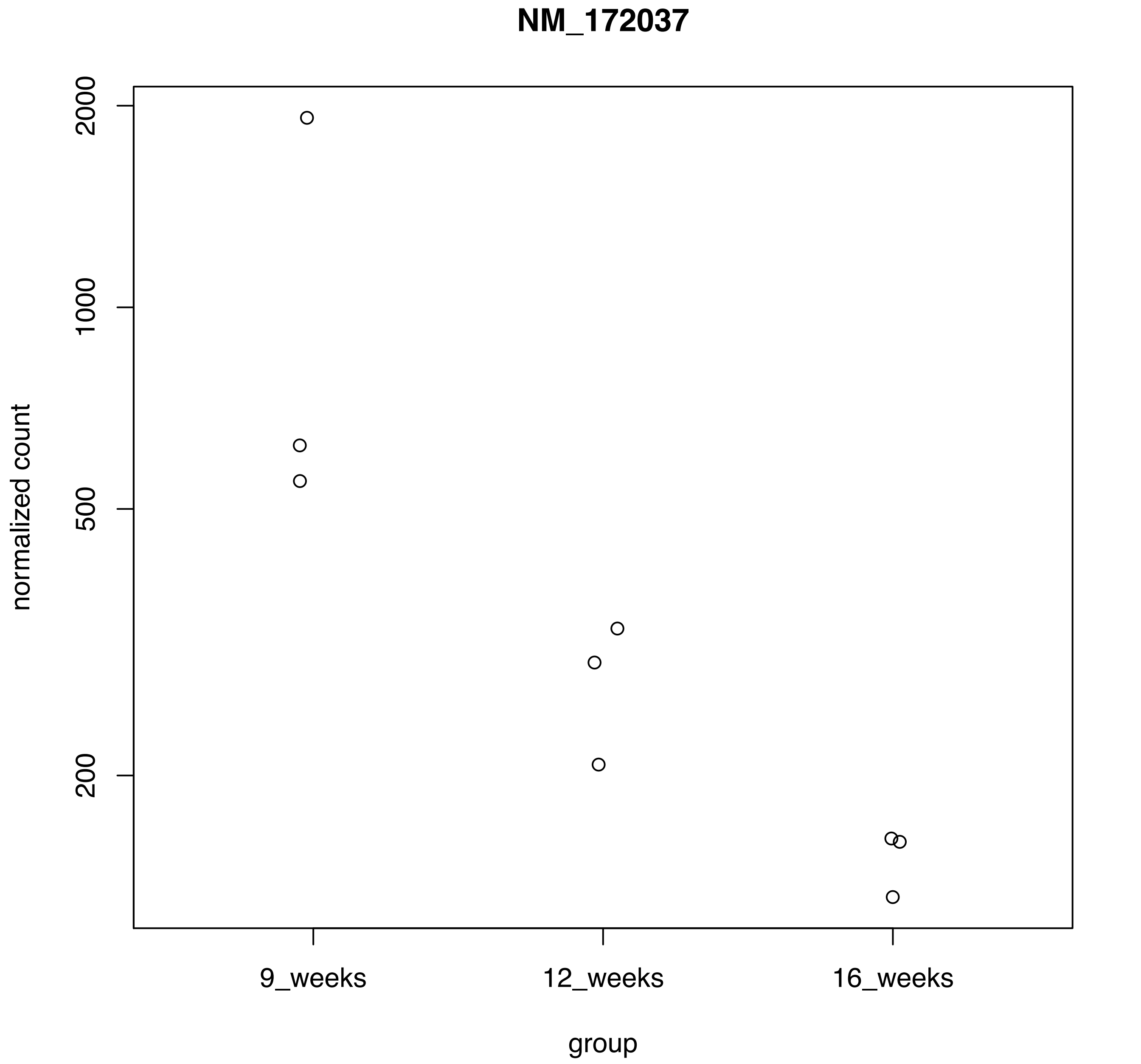
NM_172037
2000
1000
500
200
normalized count
9_weeks
12_weeks
16_weeks
group

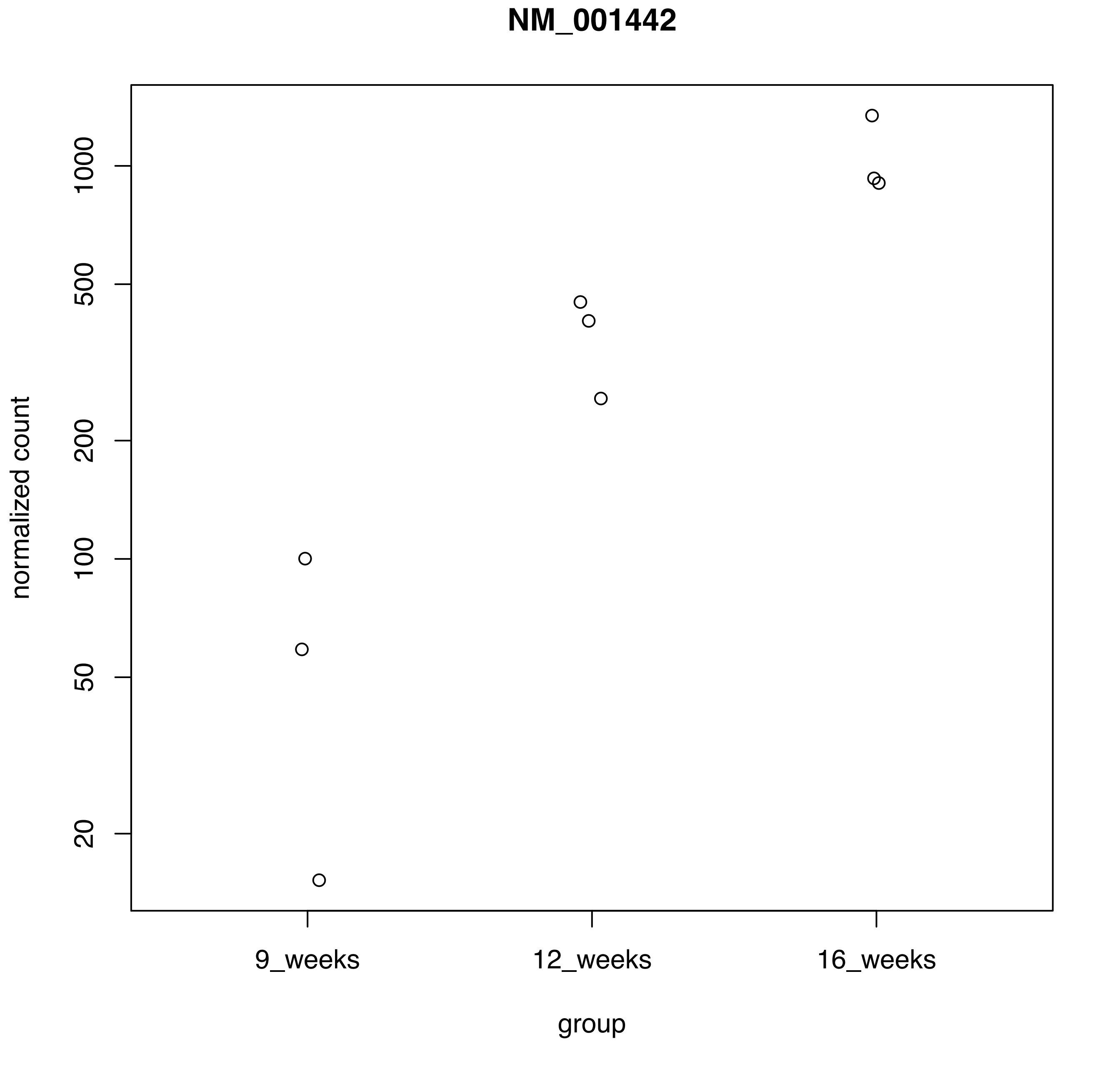
NM_001442
normalized count
1000
500
200
100
50
20
9_weeks
12_weeks
16_weeks
group

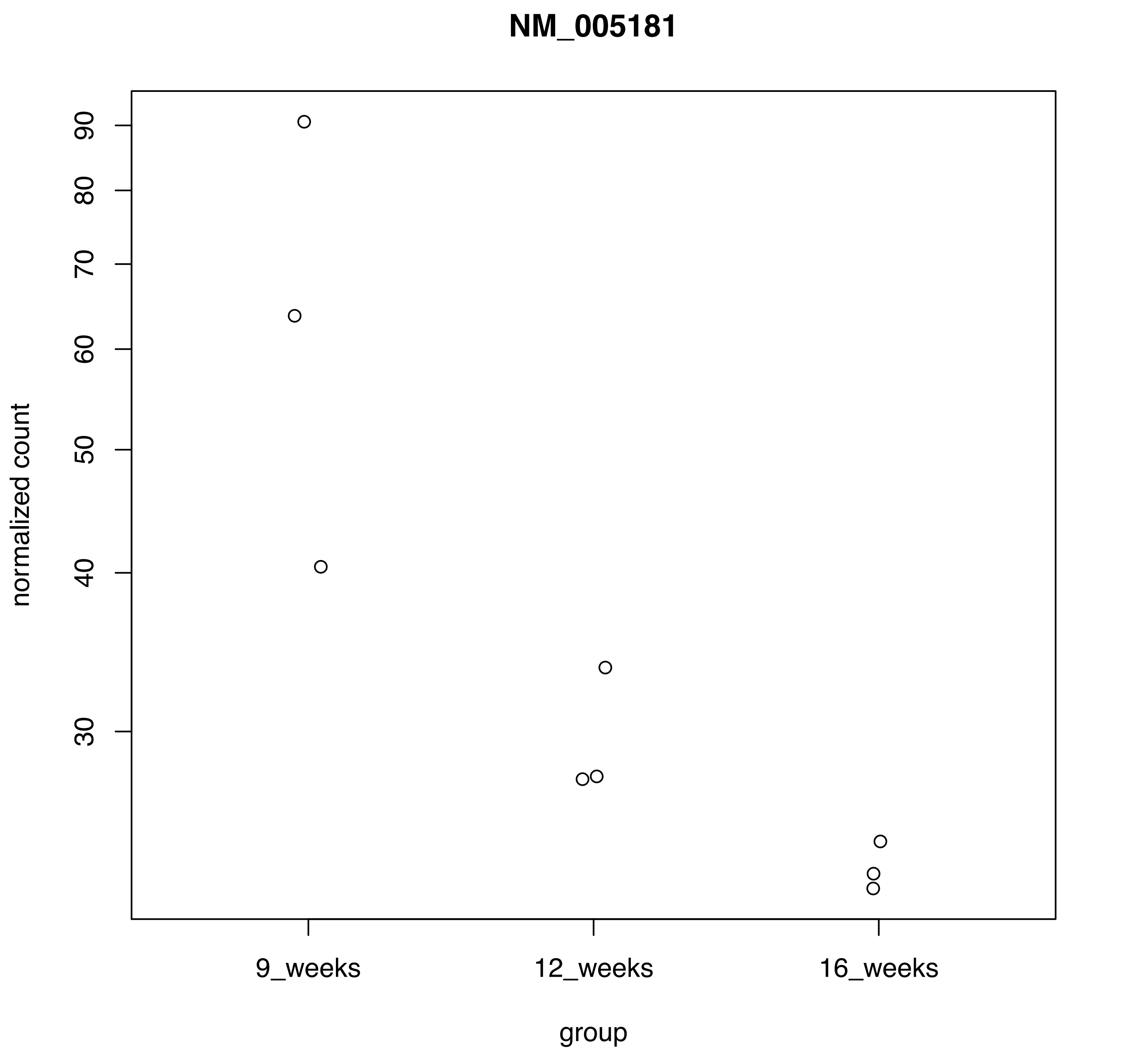
NM_005181
normalized count
90
80
70
60
50
40
30
9_weeks
12_weeks
16_weeks
group

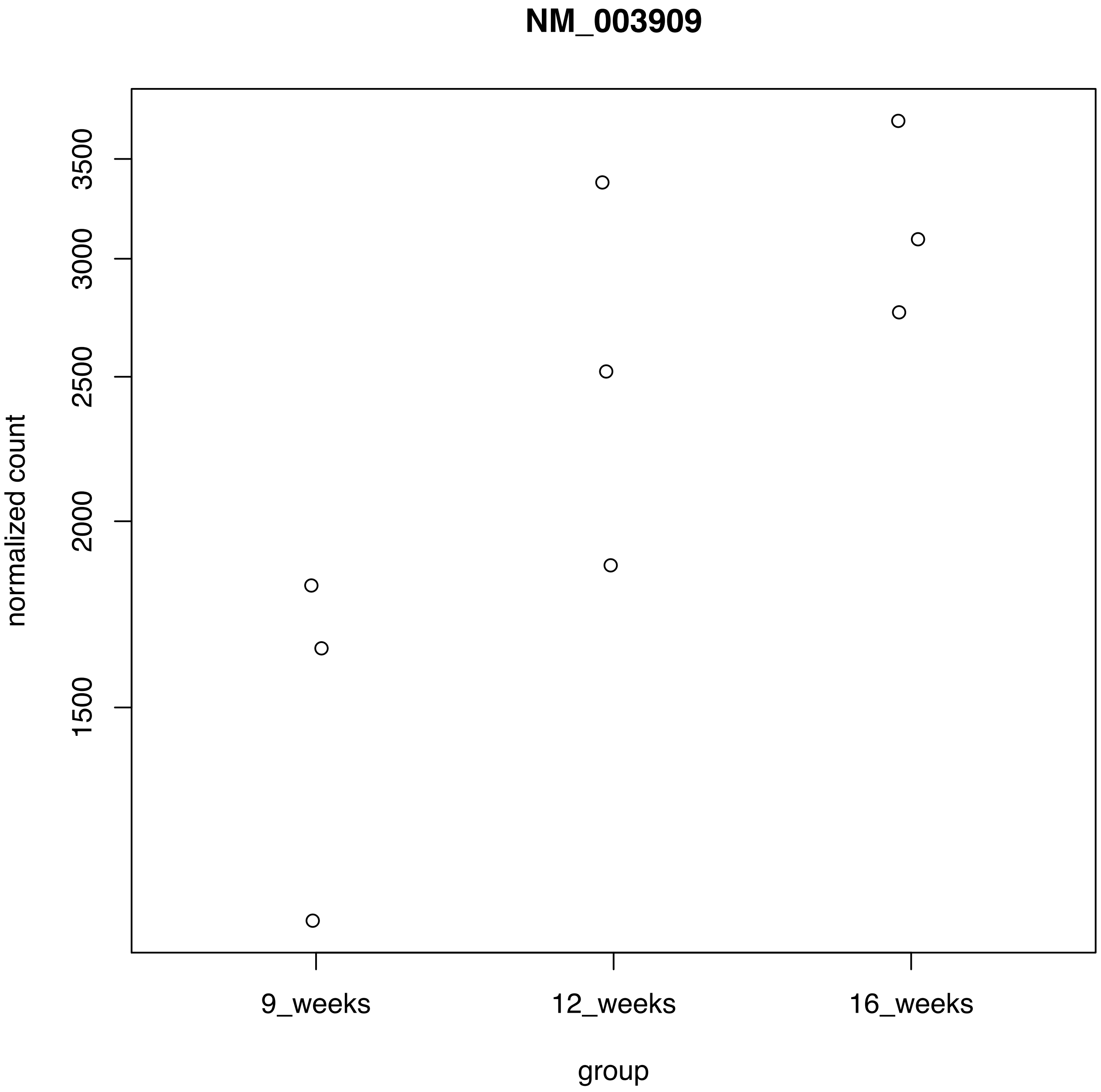
NM_003909
normalized count
1500
2000
2500
3000
3500
9_weeks
12_weeks
16_weeks
group

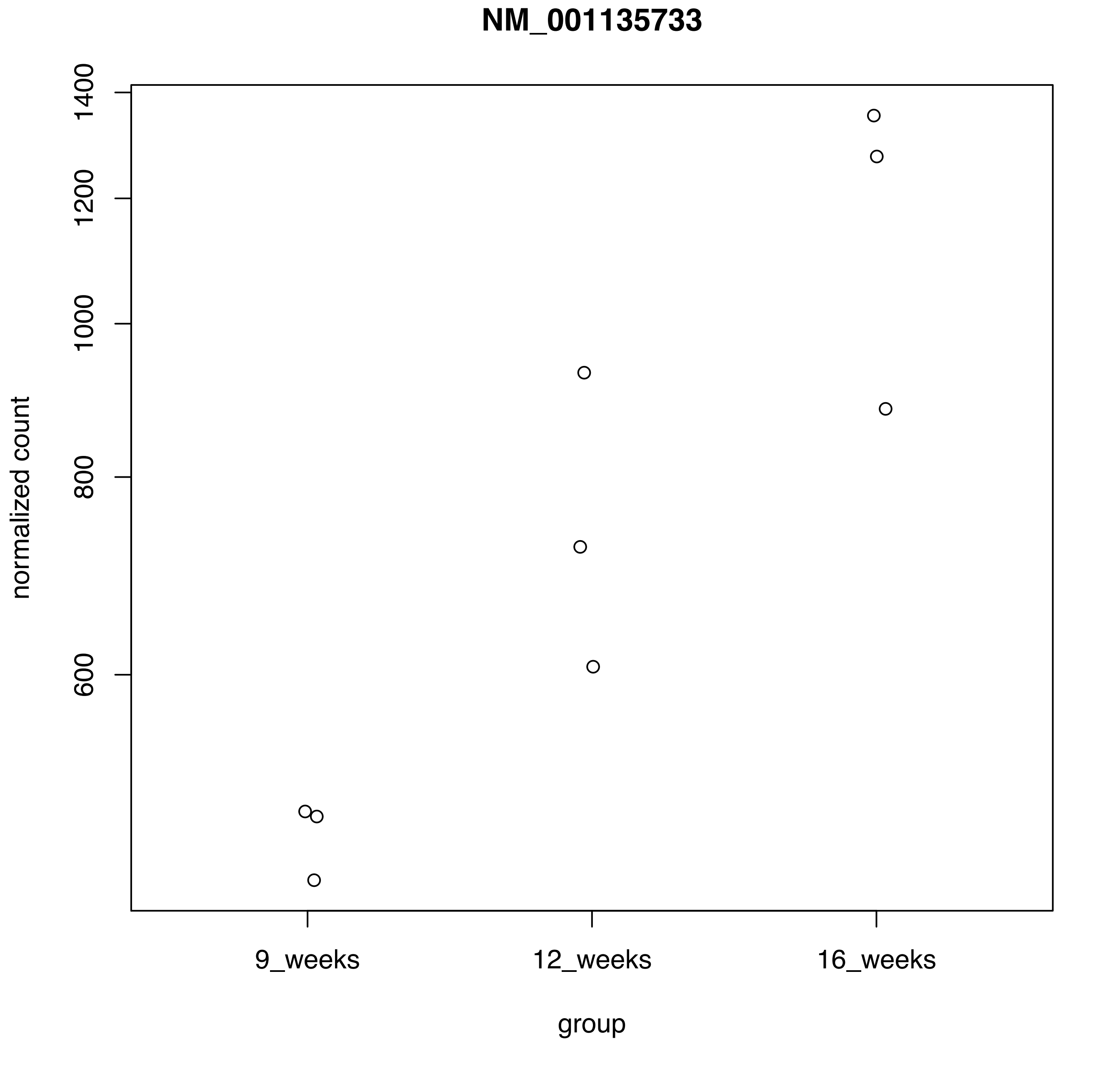
NM_001135733
normalized count
1400
1200
1000
800
600
9_weeks
12_weeks
16_weeks
group

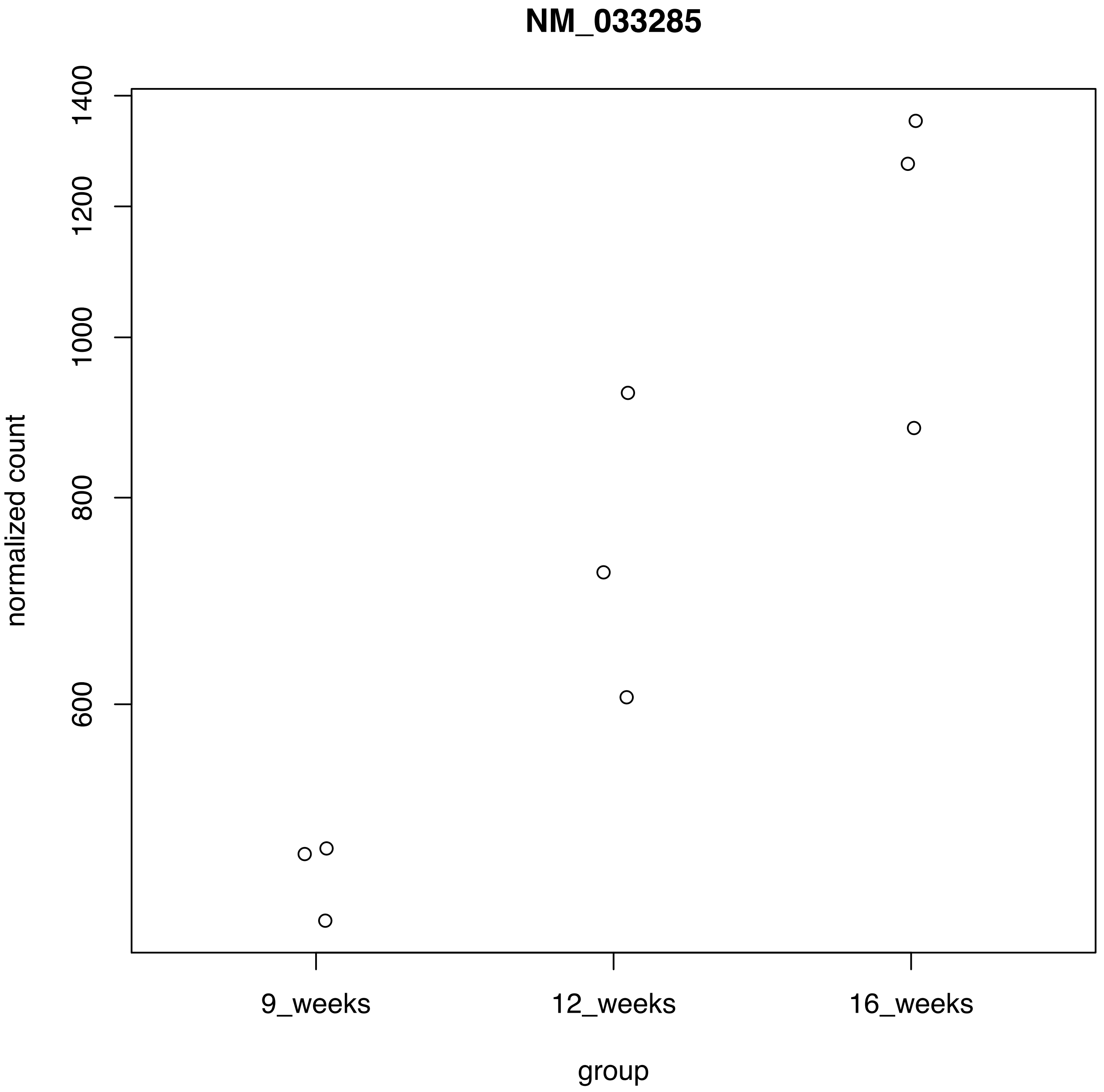
NM_033285
1400
1200
1000
800
600
normalized count
9_weeks
12_weeks
16_weeks
group

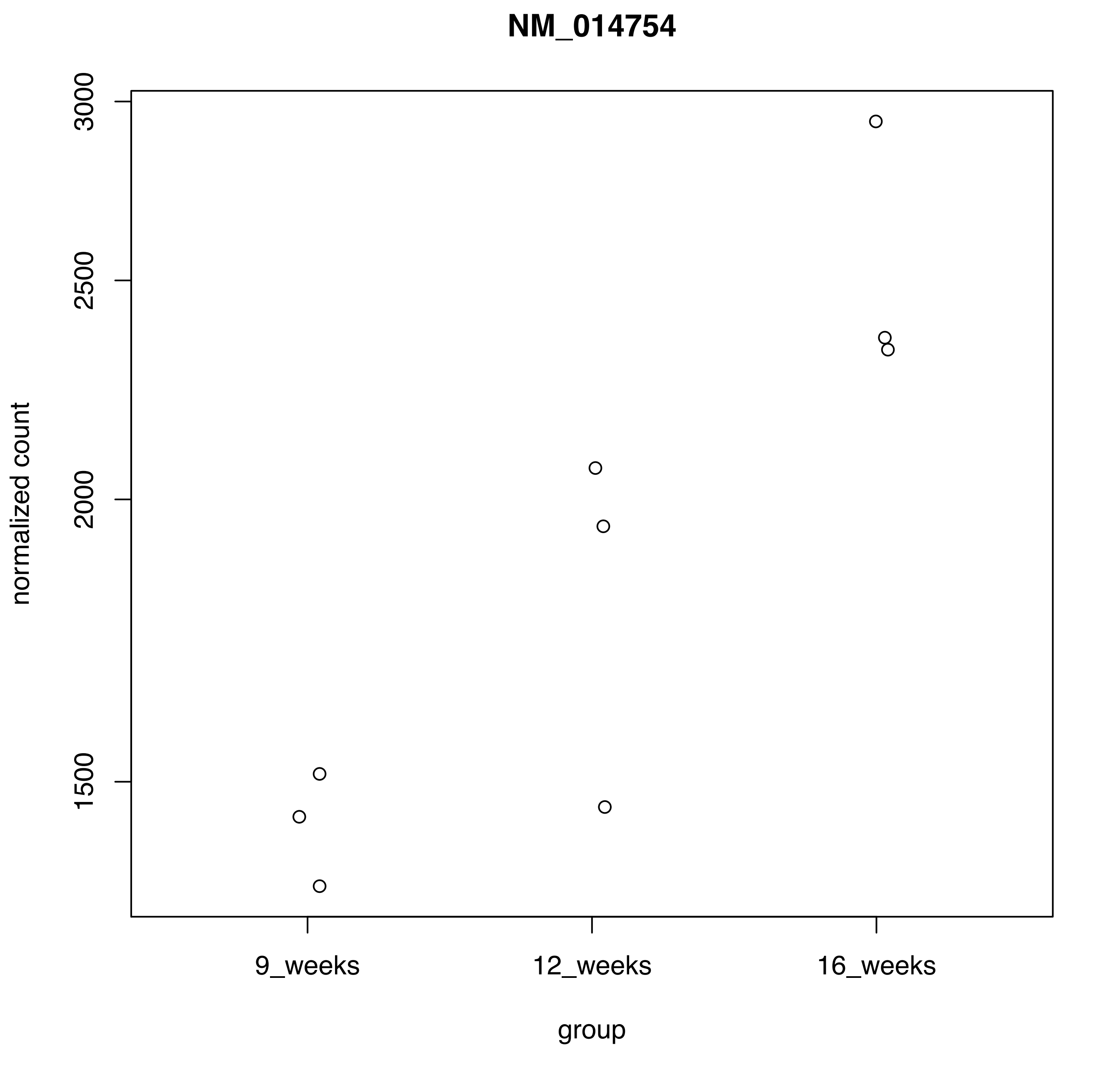
NM_014754
normalized count
3000
2500
2000
1500
9_weeks
12_weeks
16_weeks
group

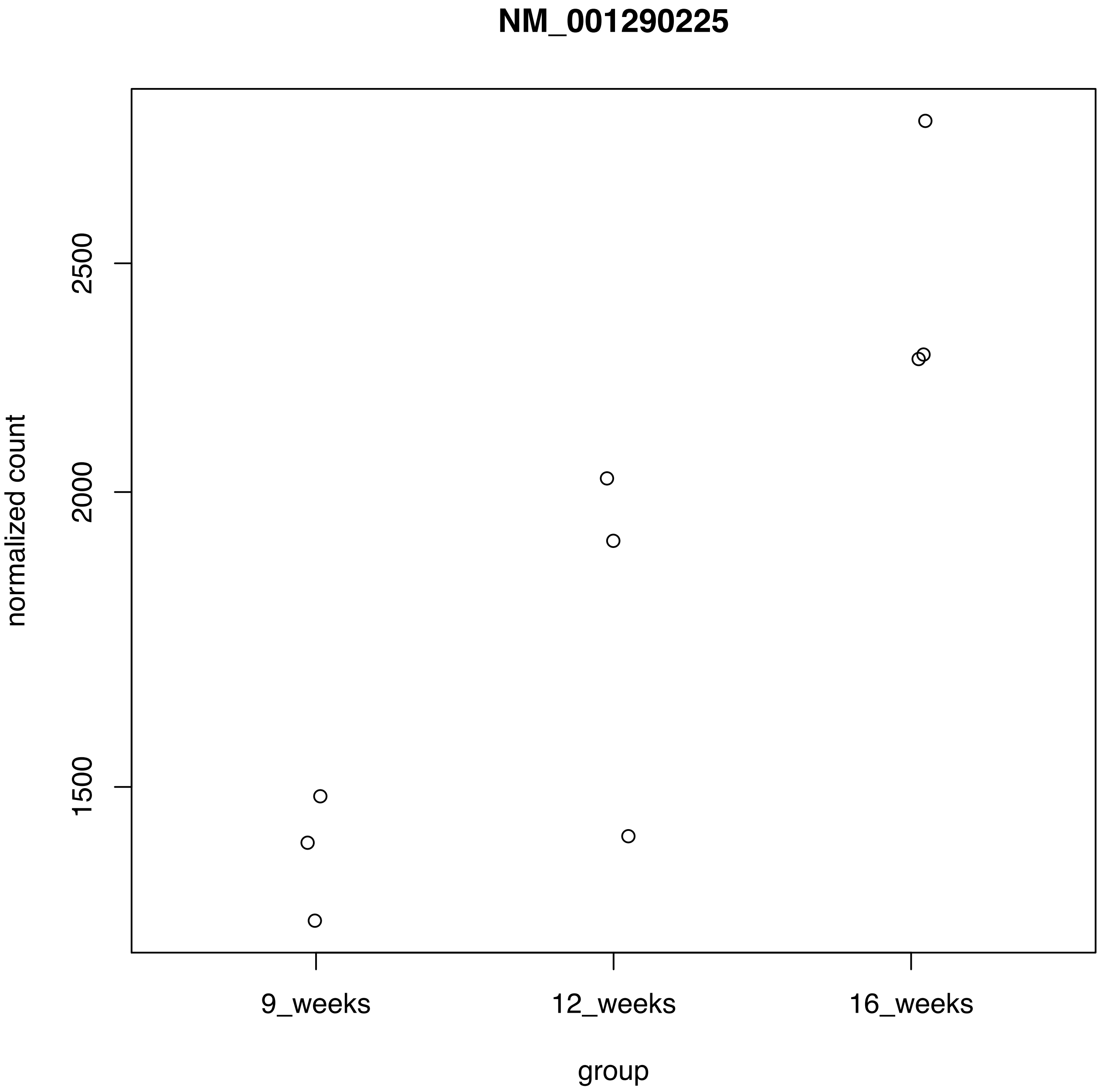
NM_001290225
normalized count
2500
2000
1500
9_weeks
12_weeks
16_weeks
group

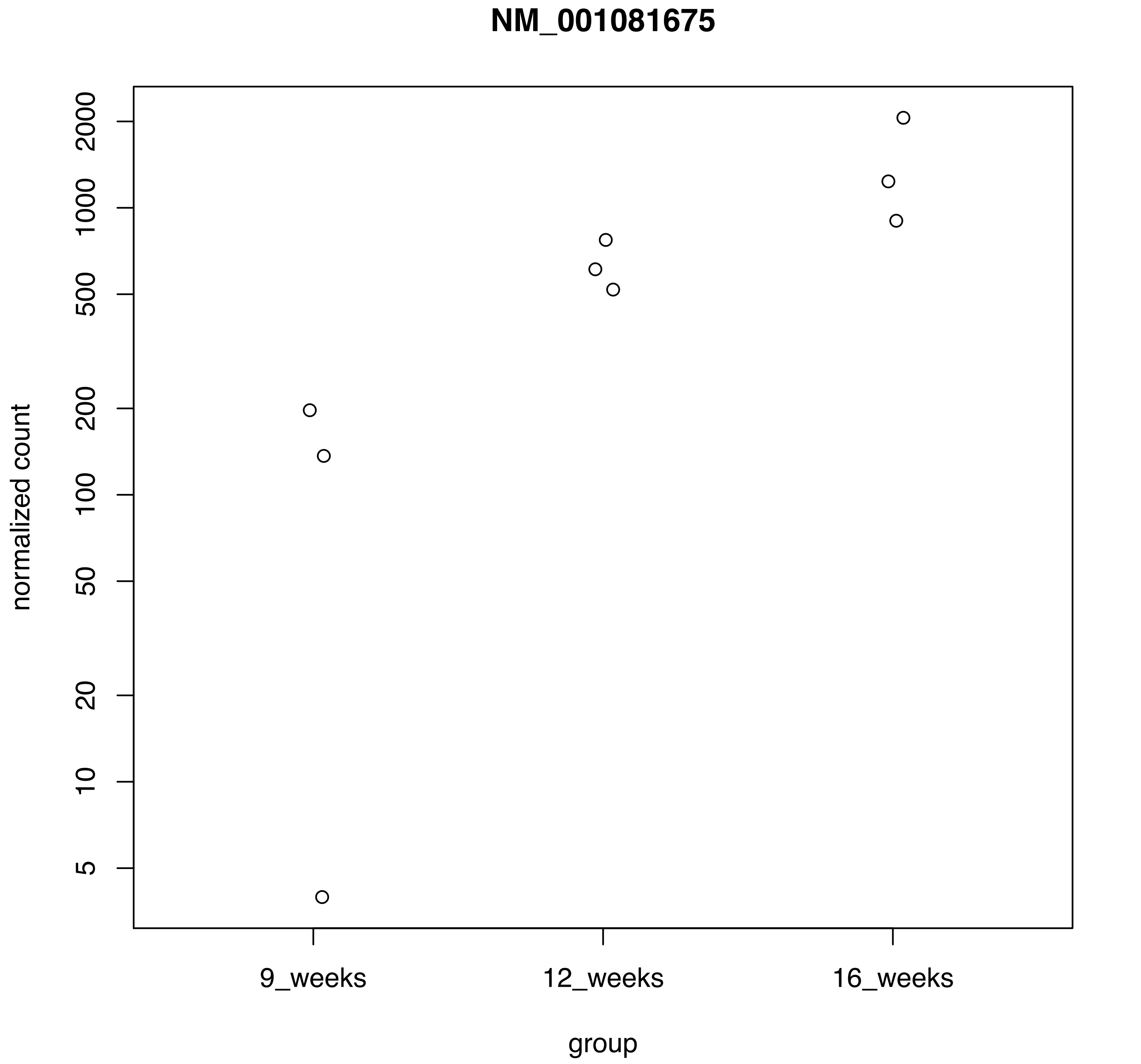
NM_001081675
normalized count
2000
1000
500
200
100
50
20
10
5
9_weeks
12_weeks
16_weeks
group

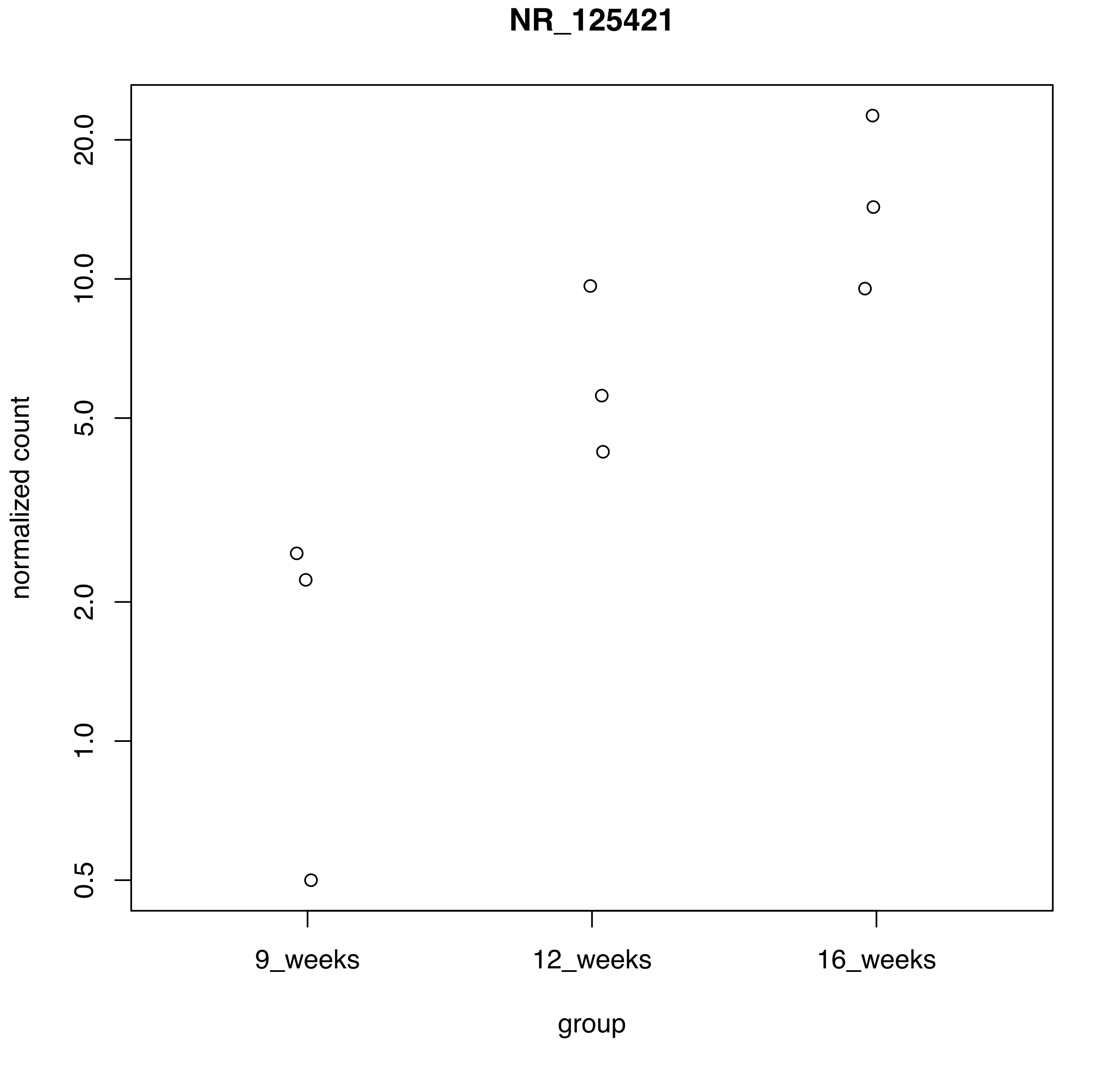
NR_125421
normalized count
20.0
10.0
5.0
2.0
1.0
0.5
9_weeks
12_weeks
16_weeks
group

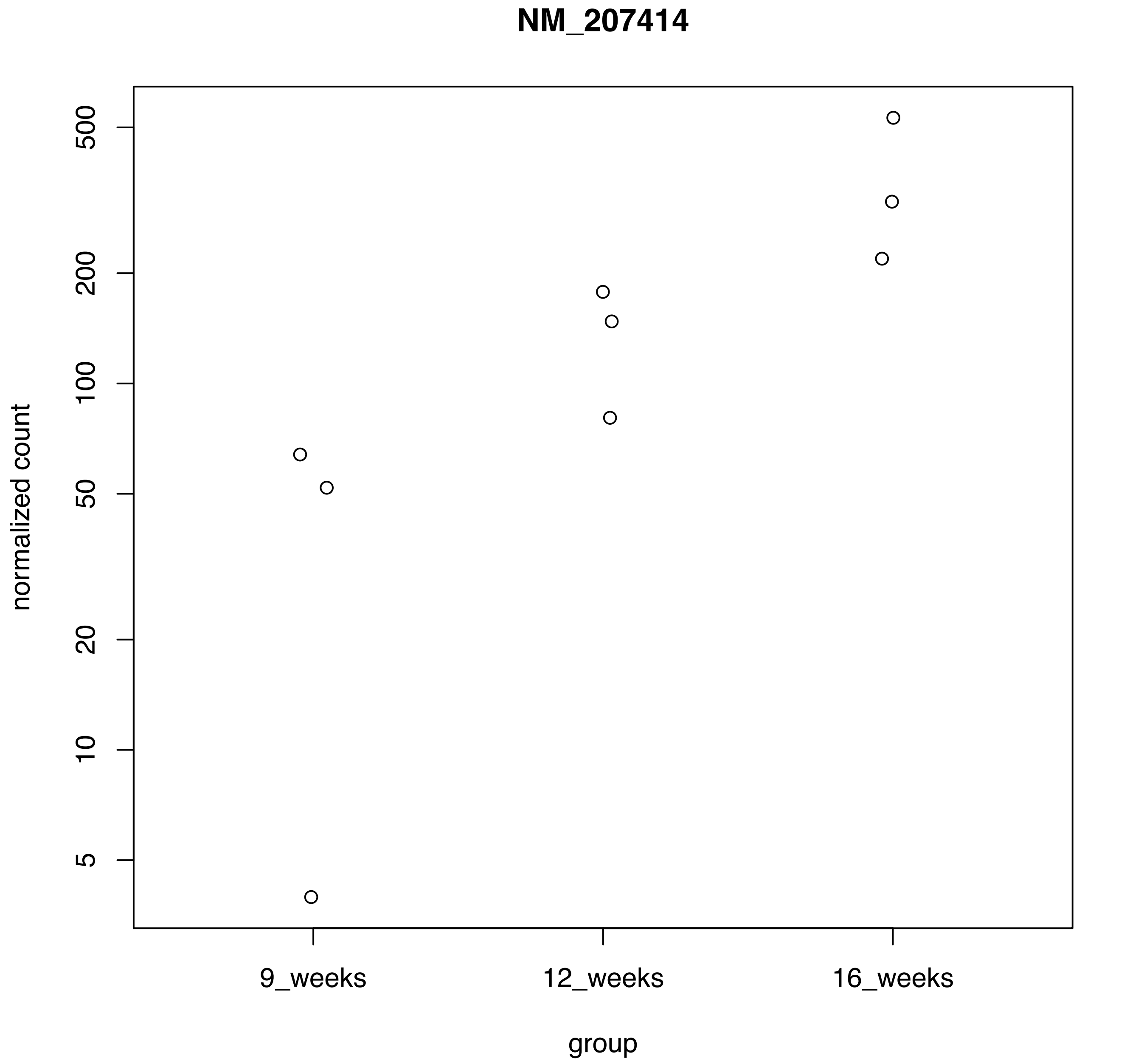
NM_207414
normalized count
500
200
100
50
20
10
5
9_weeks
12_weeks
16_weeks
group

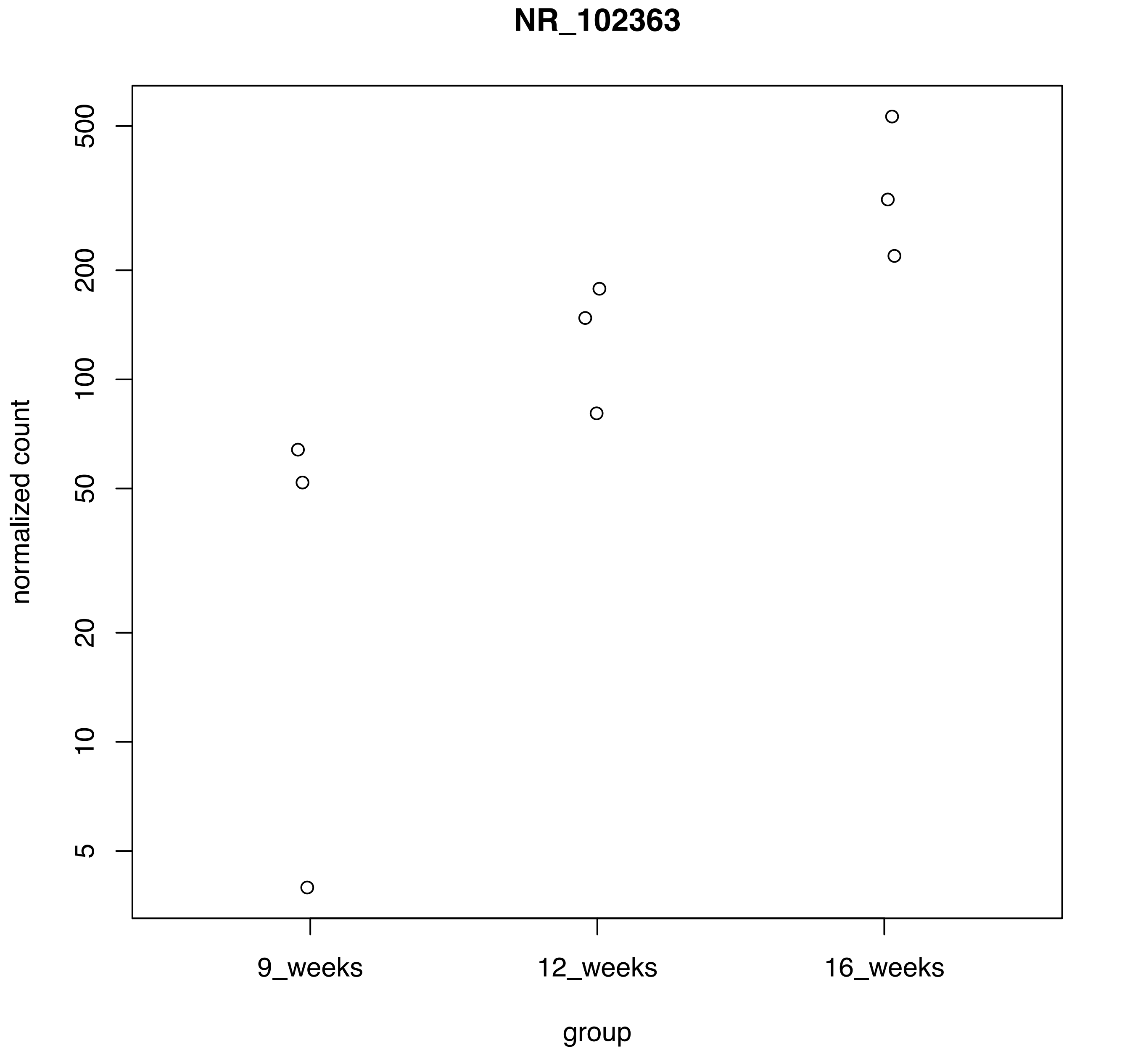
NR_102363
normalized count
500
200
100
50
20
10
5
9_weeks
12_weeks
16_weeks
group

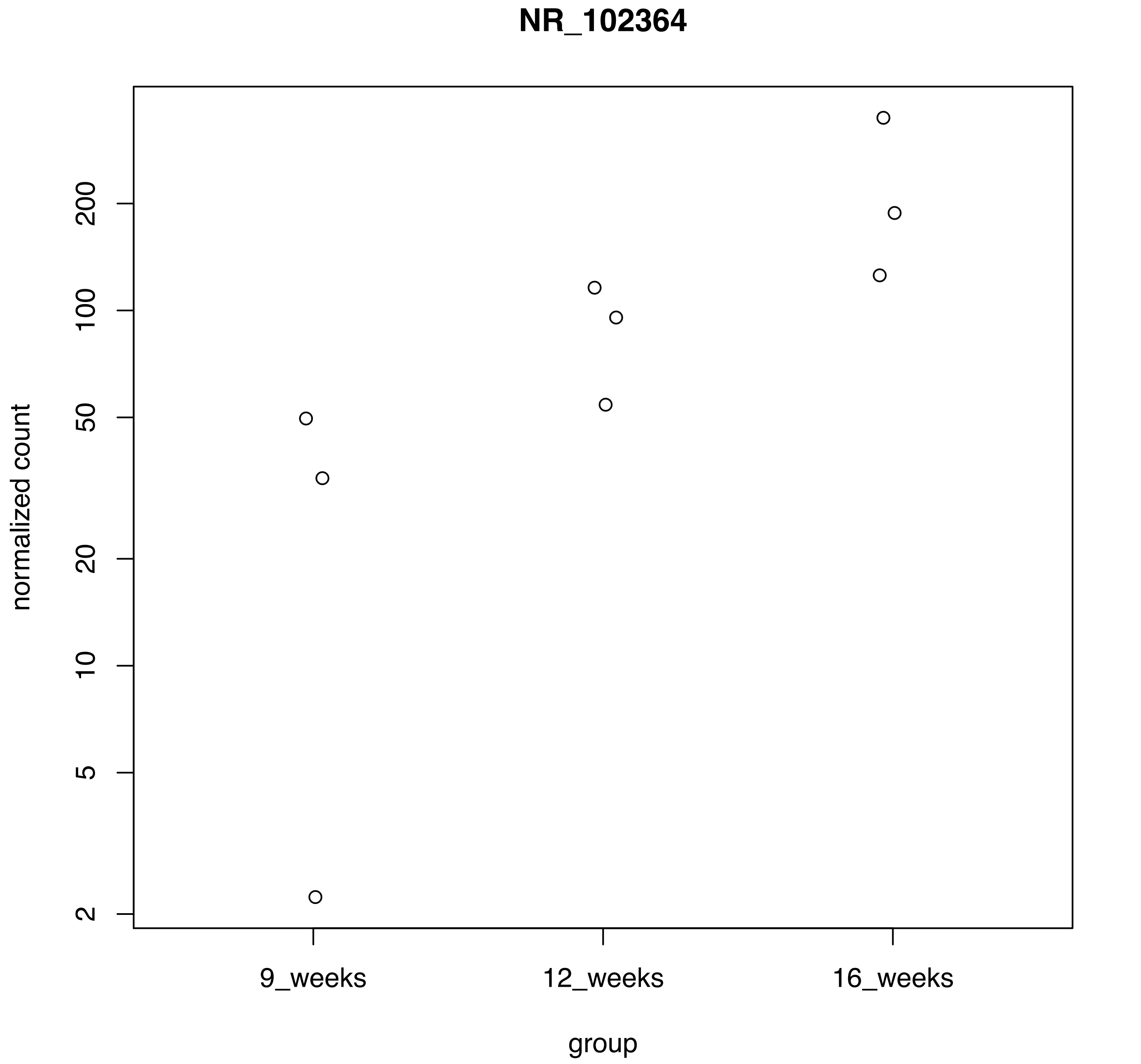
NR_102364
normalized count
200
100
50
20
10
5
2
9_weeks
12_weeks
16_weeks
group

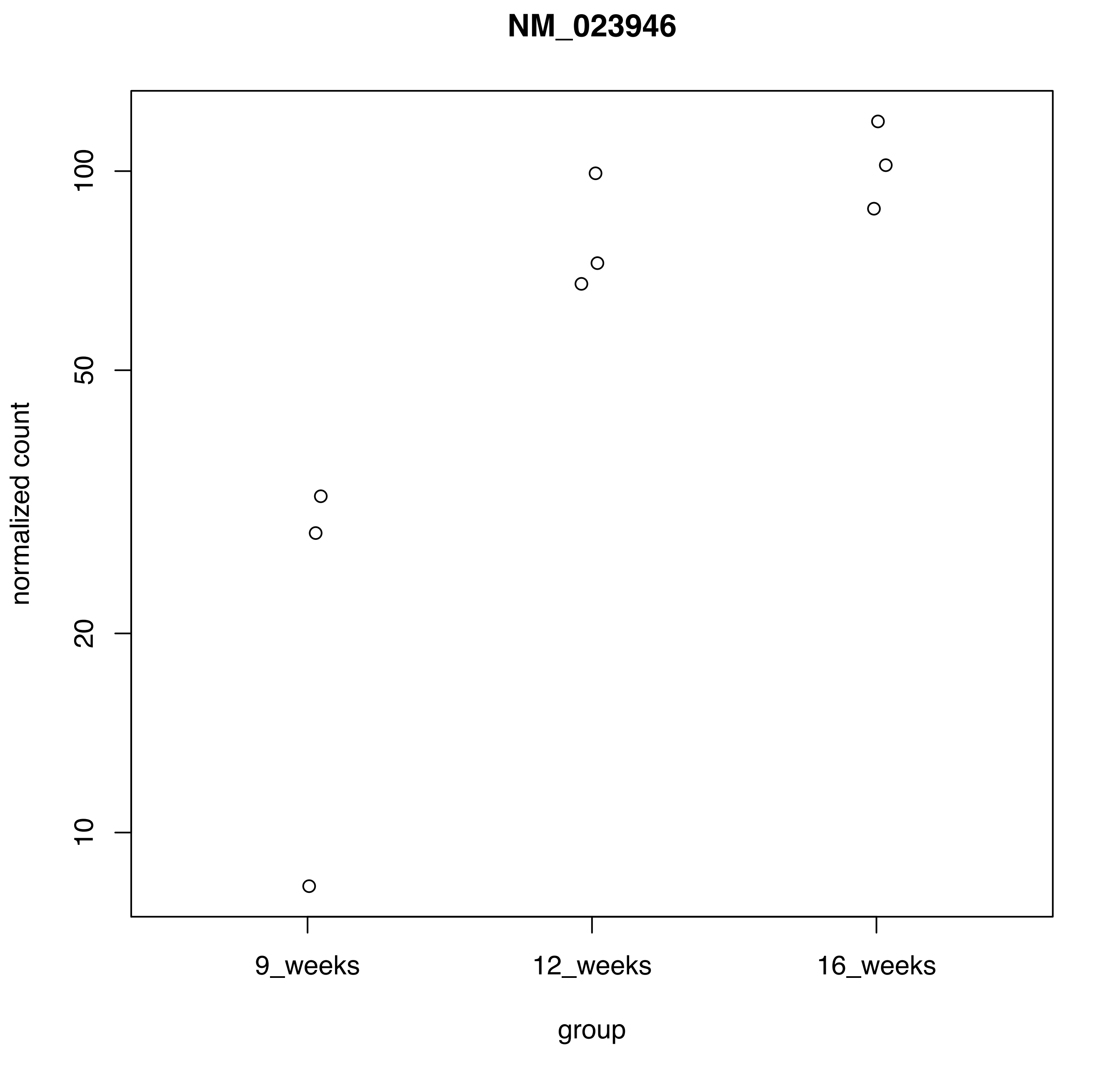
NM_023946
normalized count
100
50
20
10
9_weeks
12_weeks
16_weeks
group

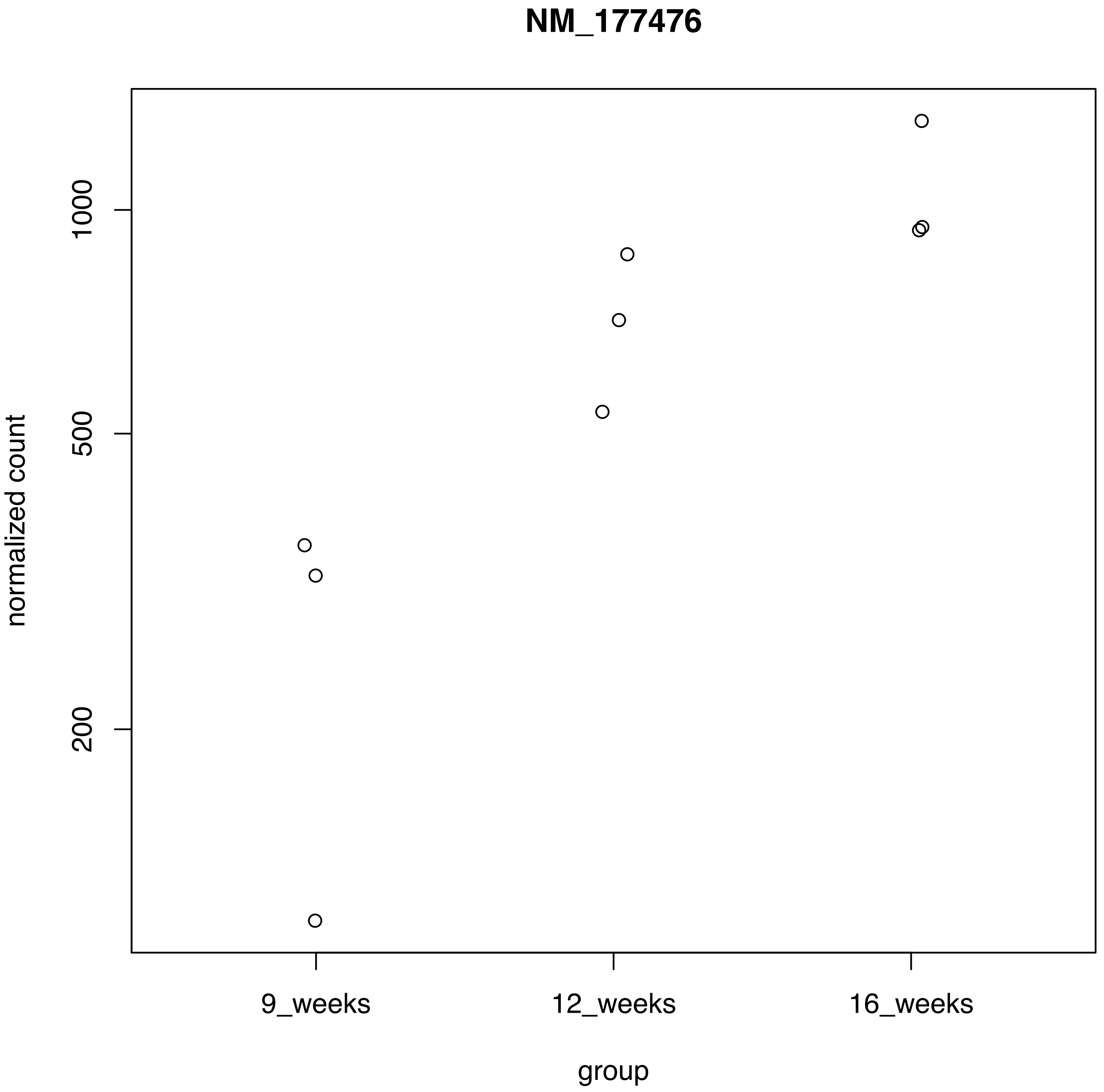
NM_177476
normalized count
1000
500
200
9_weeks
12_weeks
16_weeks
group

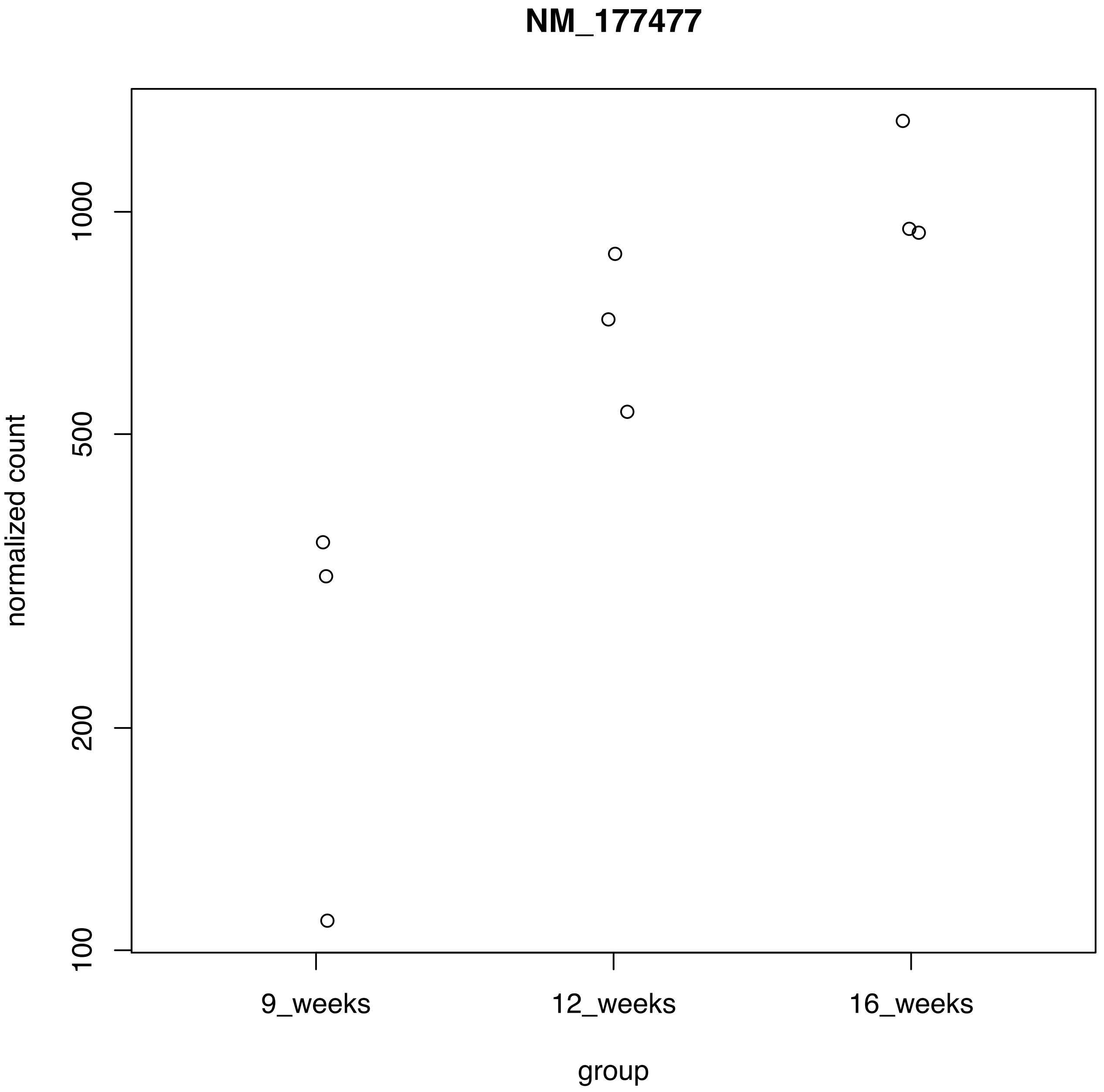

NM_177477
normalized count
1000
500
200
100
9_weeks
12_weeks
16_weeks
group

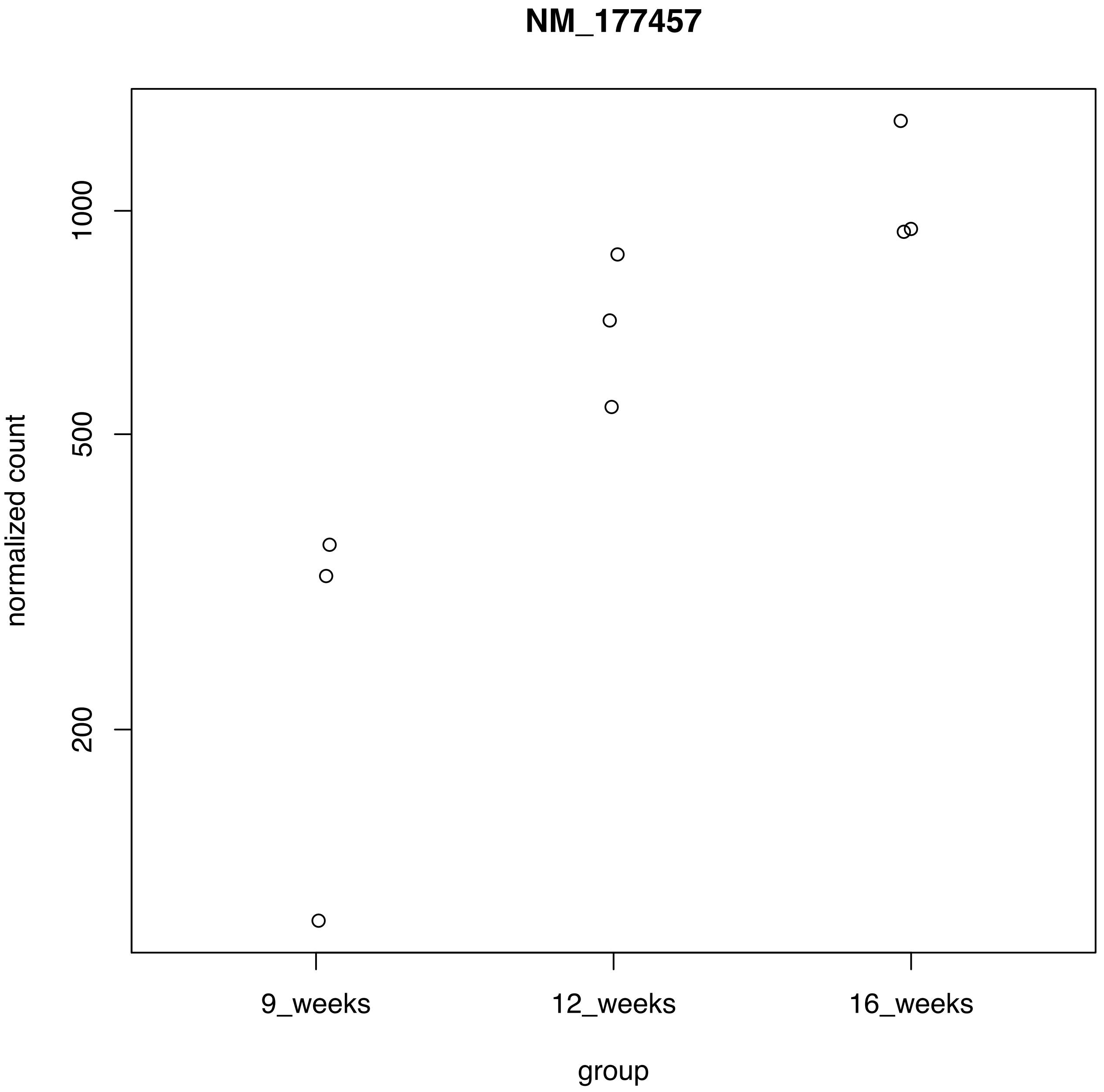

NM_177457
normalized count
1000
500
200
9_weeks
12_weeks
16_weeks
group

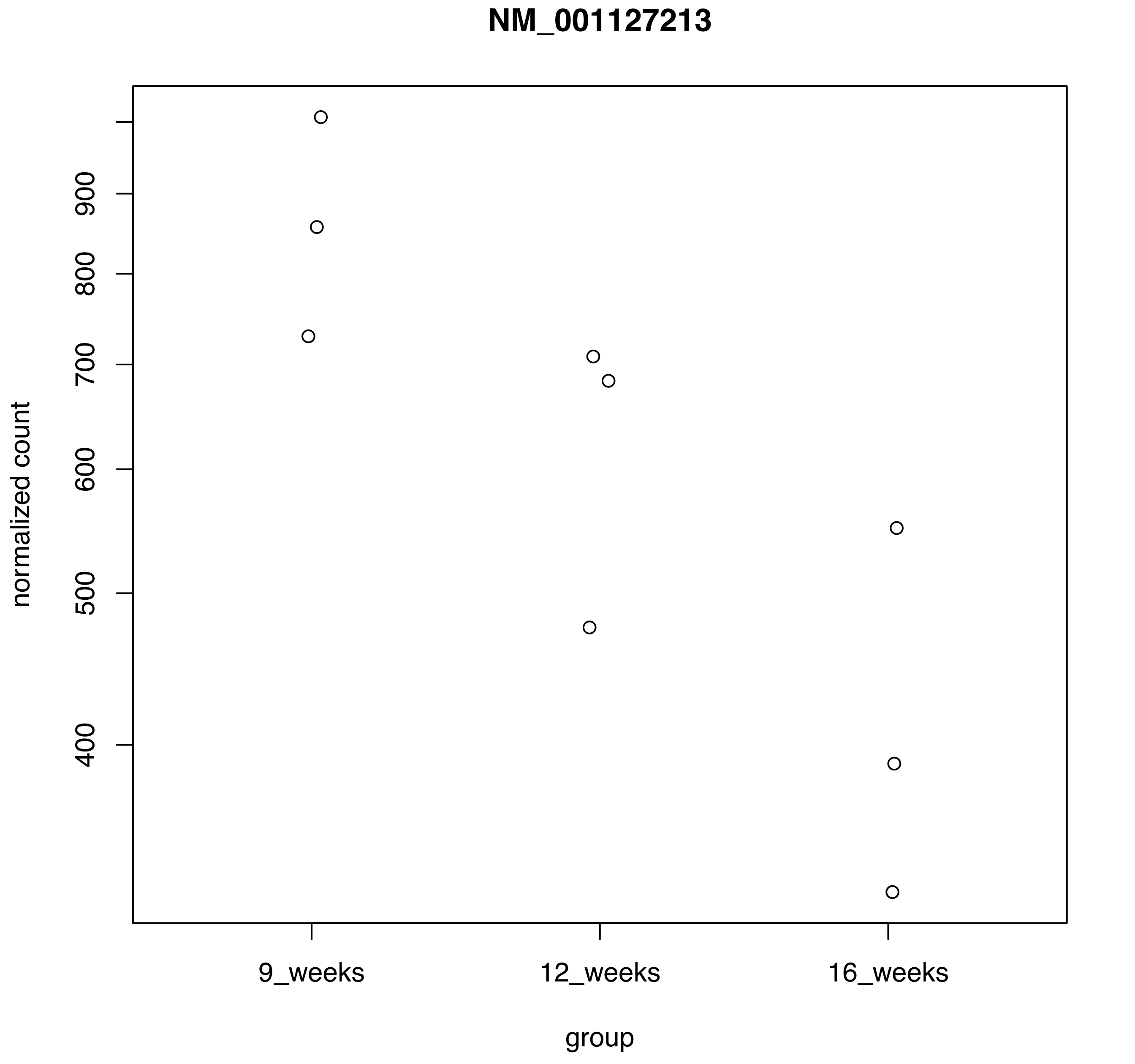
NM_001127213
normalized count
900
800
700
600
500
400
9_weeks
12_weeks
16_weeks
group

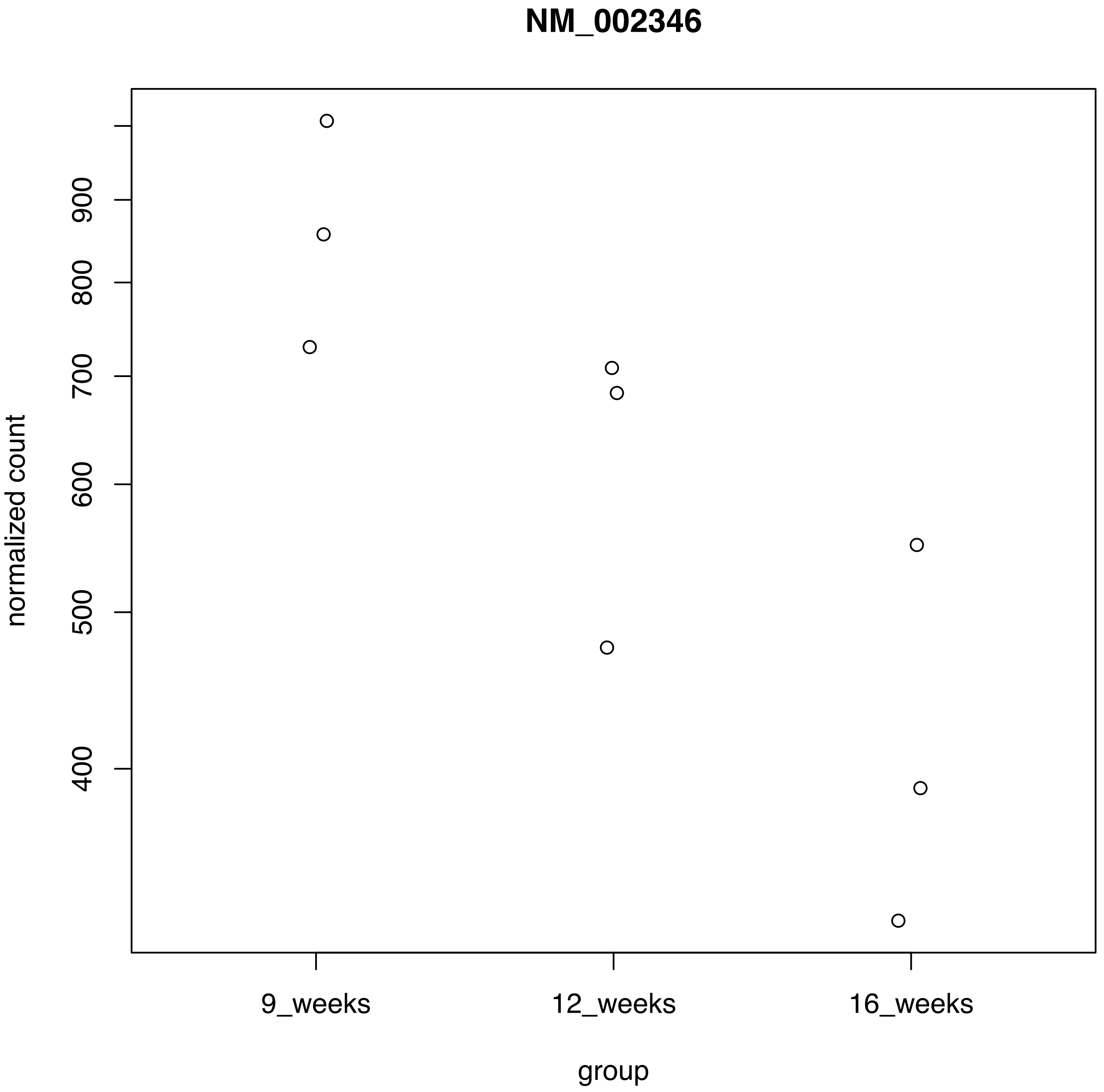
NM_002346
normalized count
900
800
700
600
500
400
9_weeks
12_weeks
16_weeks
group

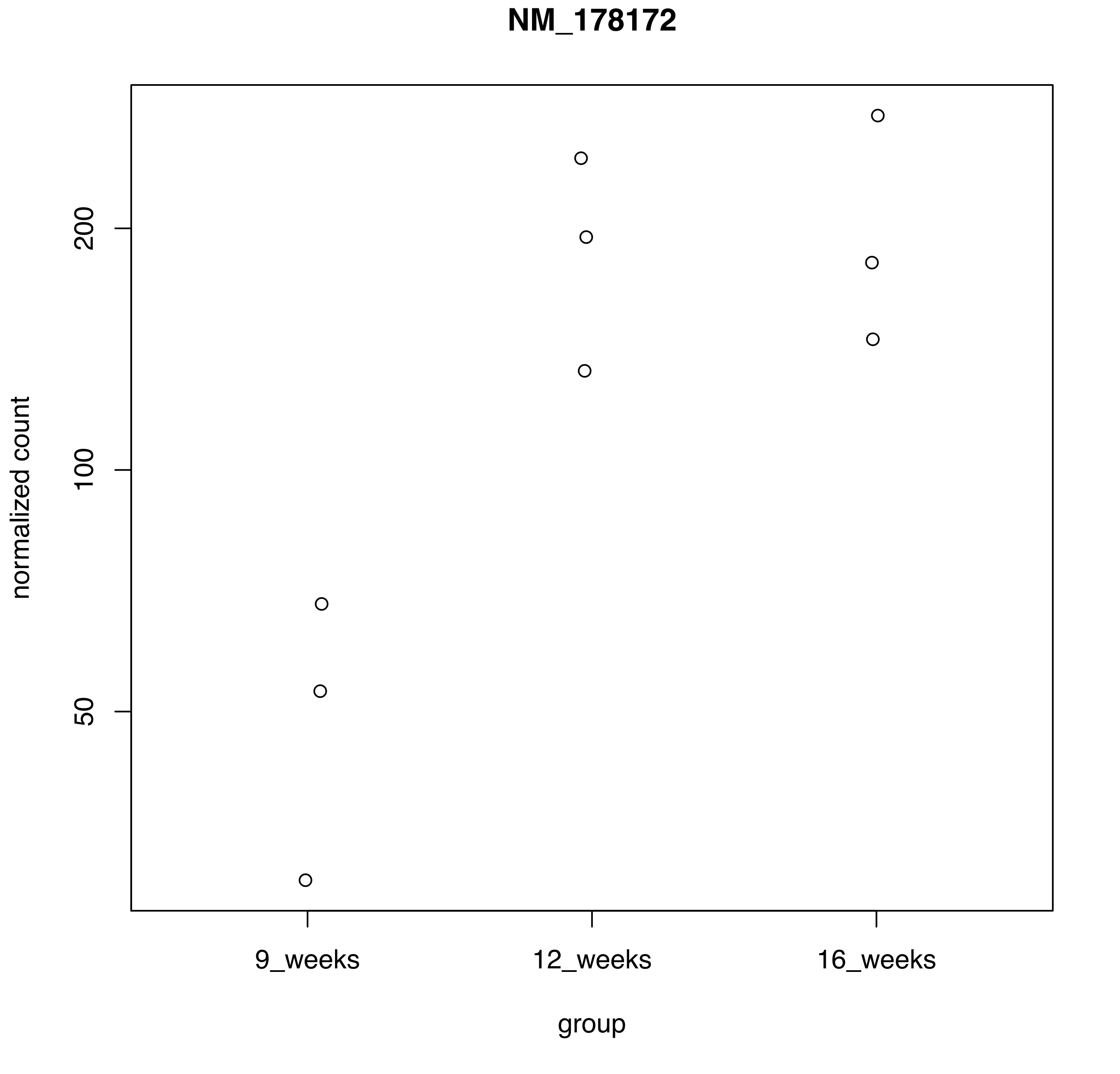
NM_178172
normalized count
200
100
50
9_weeks
12_weeks
16_weeks
group

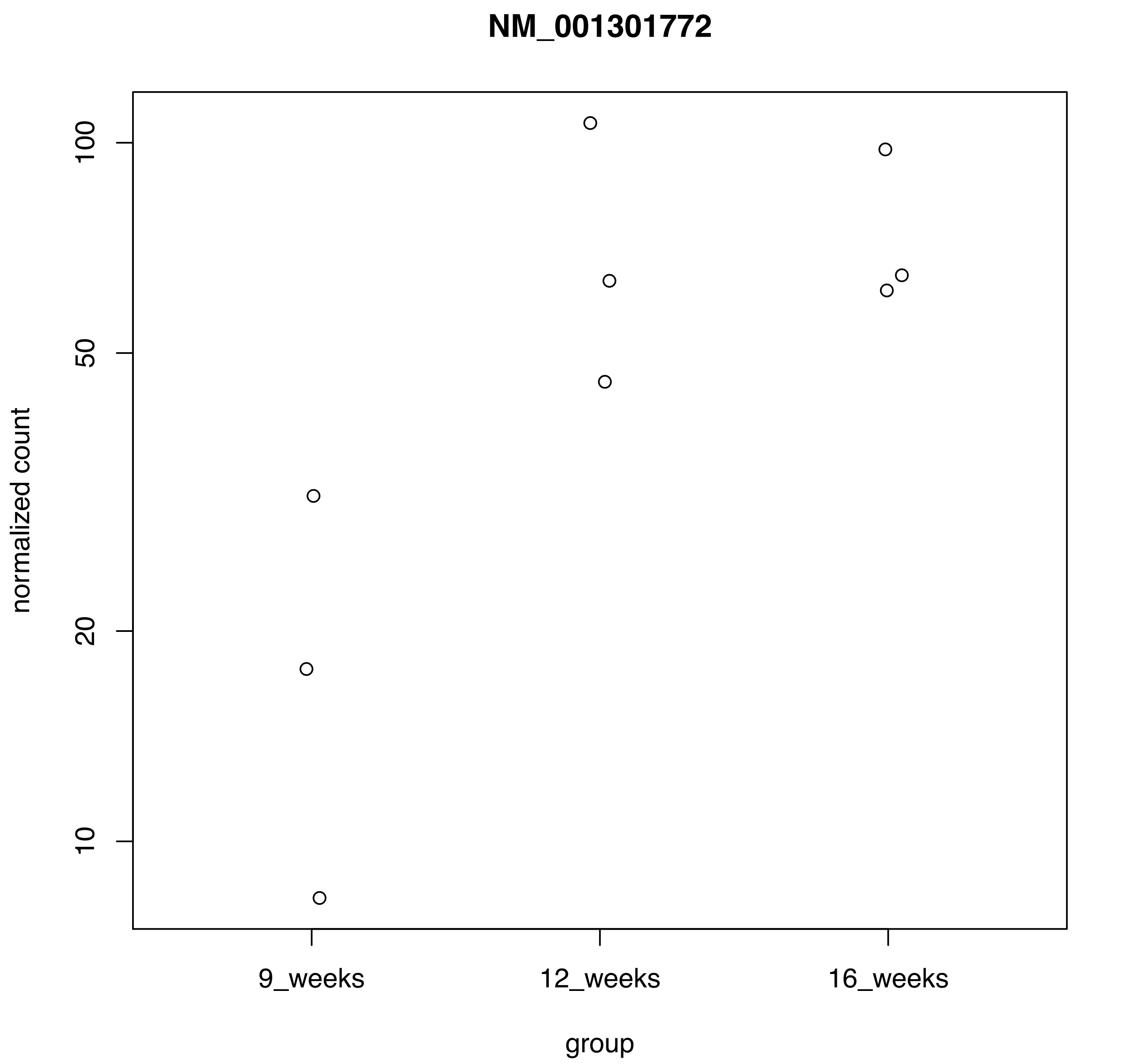
NM_001301772
normalized count
100
50
20
10
9_weeks
12_weeks
16_weeks
group

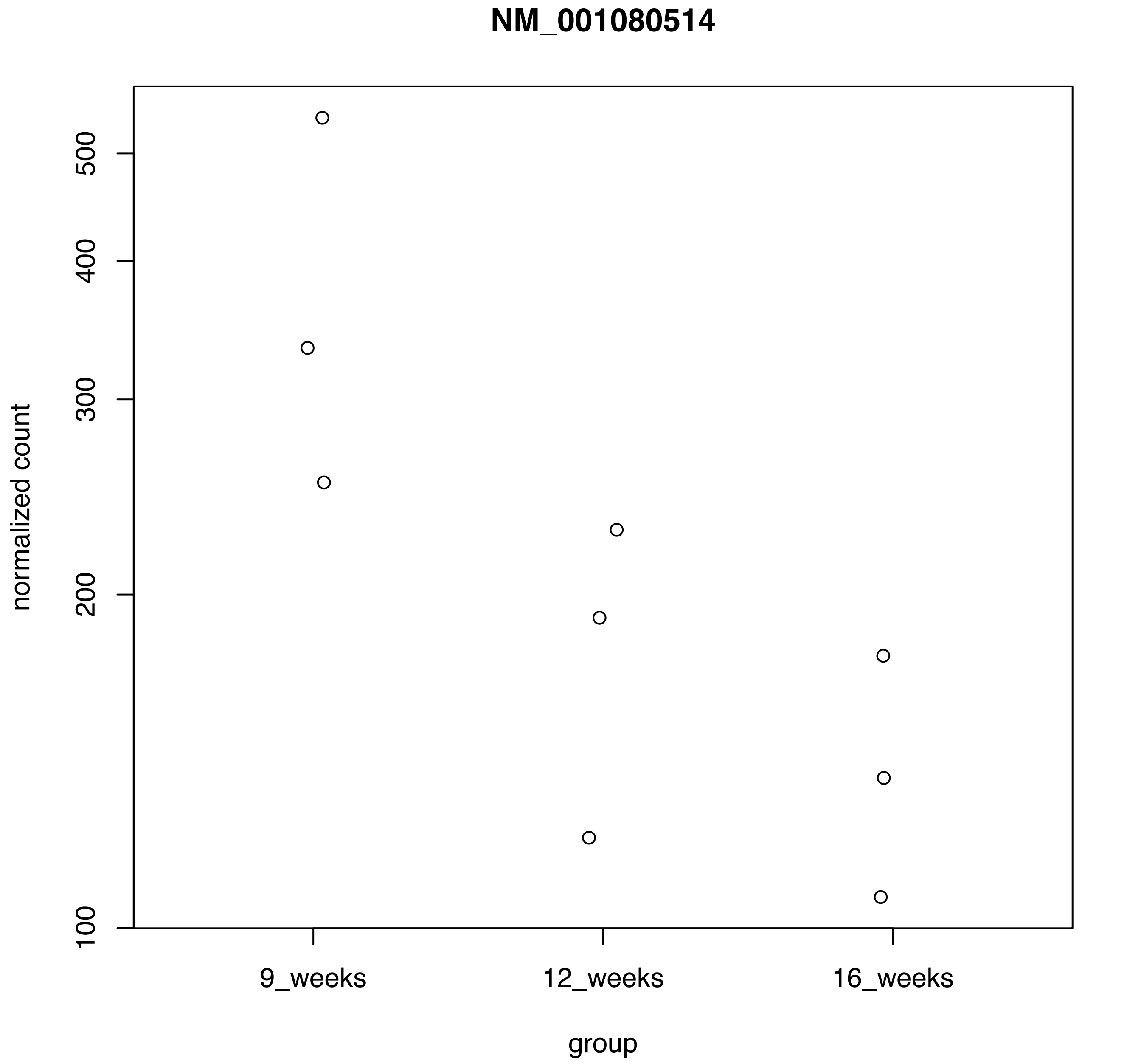
NM_001080514
normalized count
500
400
300
200
100
9_weeks
12_weeks
16_weeks
group

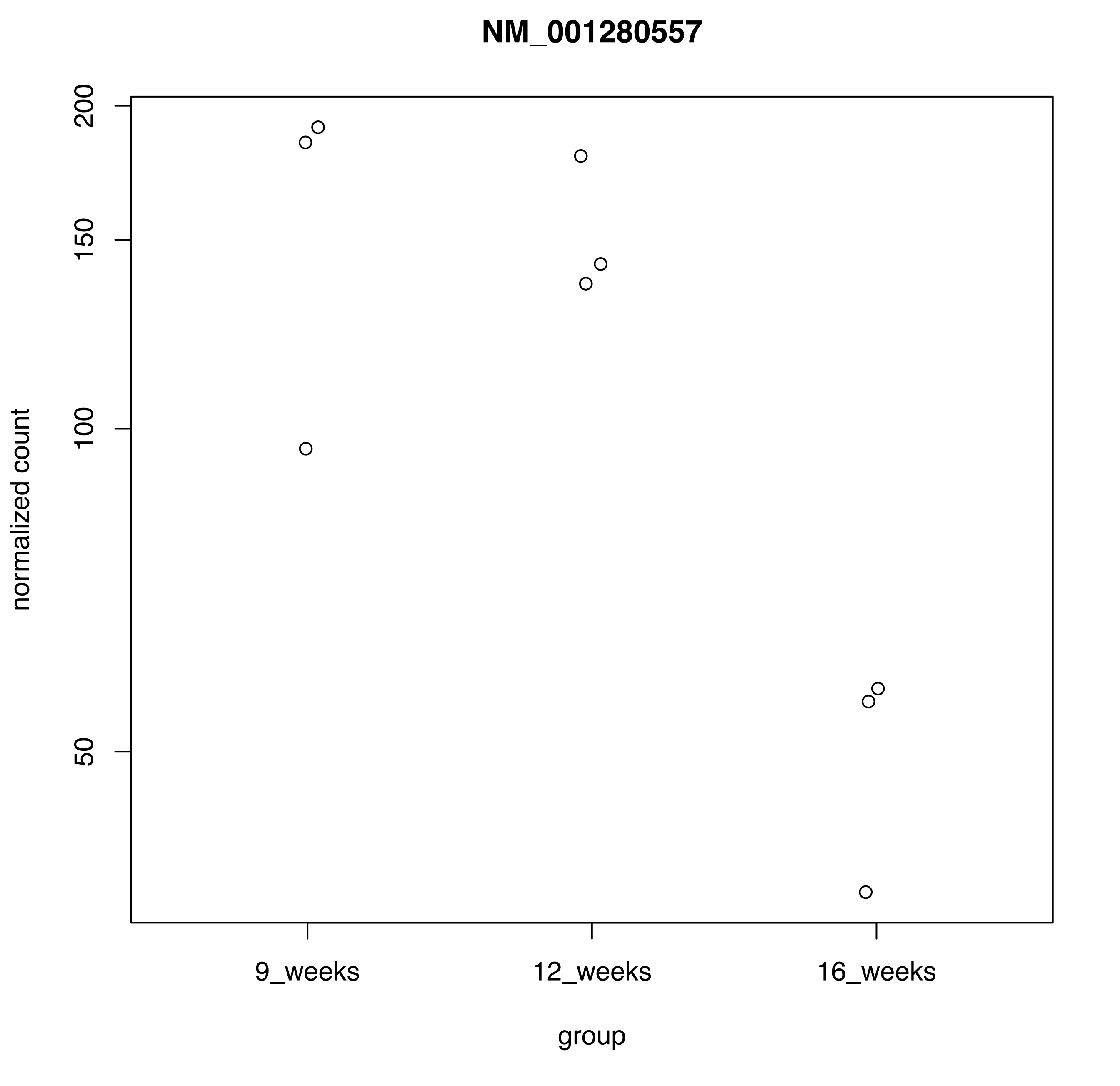
NM_001280557
normalized count
200
150
100
50
9_weeks
12_weeks
16_weeks
group

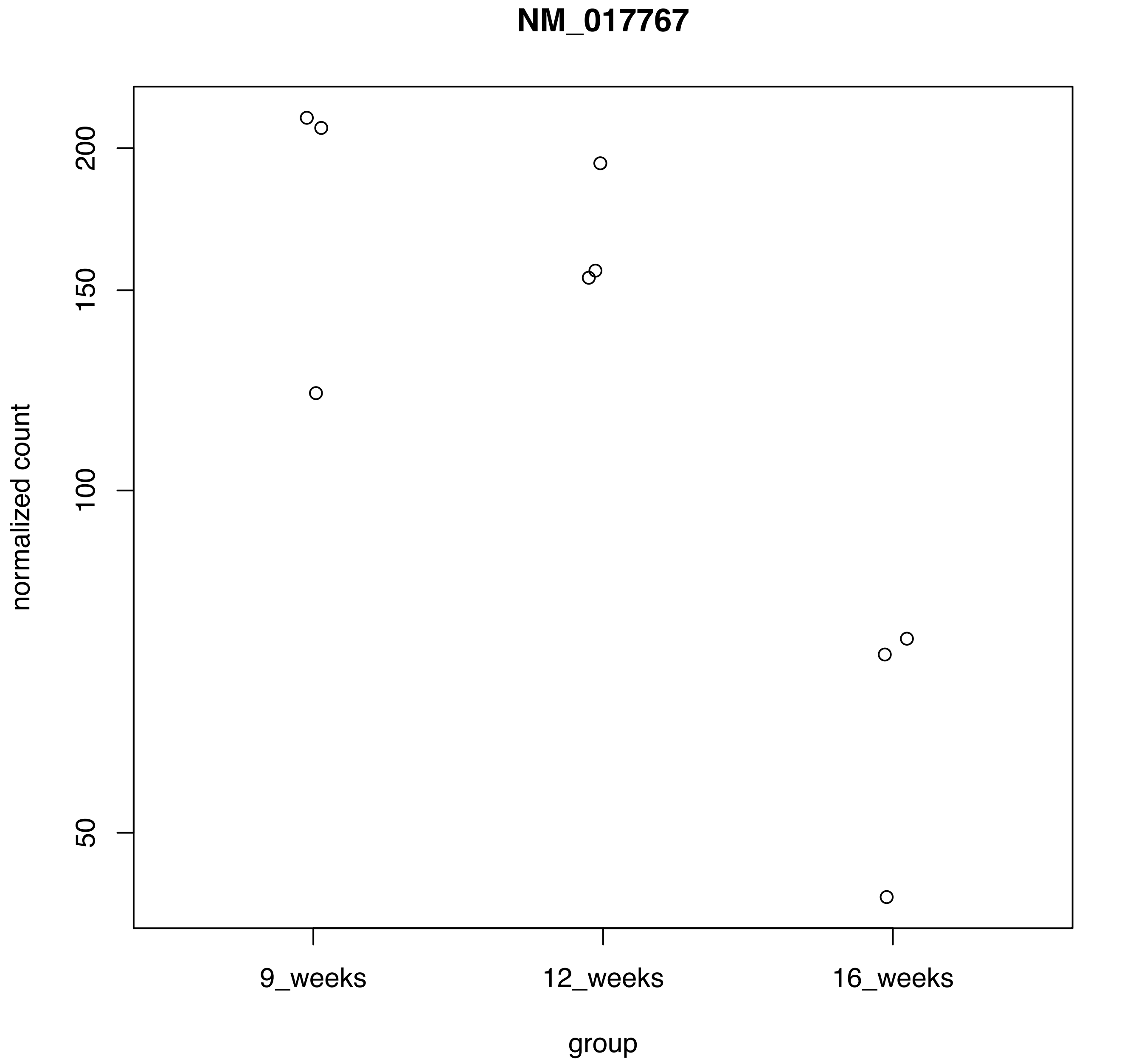

NM_017767
normalized count
200
150
100
50
9_weeks
12_weeks
16_weeks
group

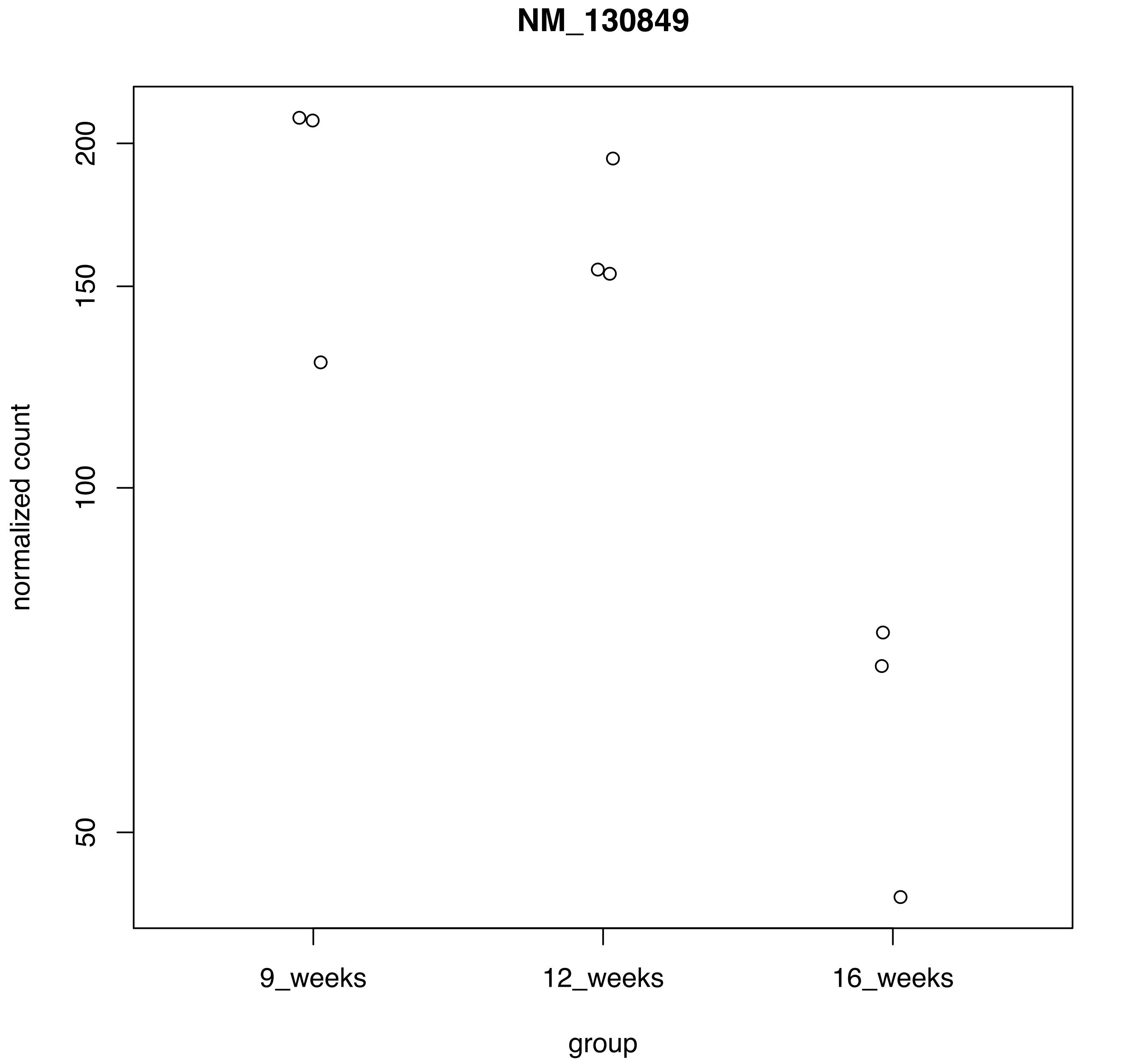
NM_130849
normalized count
200
150
100
50
9_weeks
12_weeks
16_weeks
group

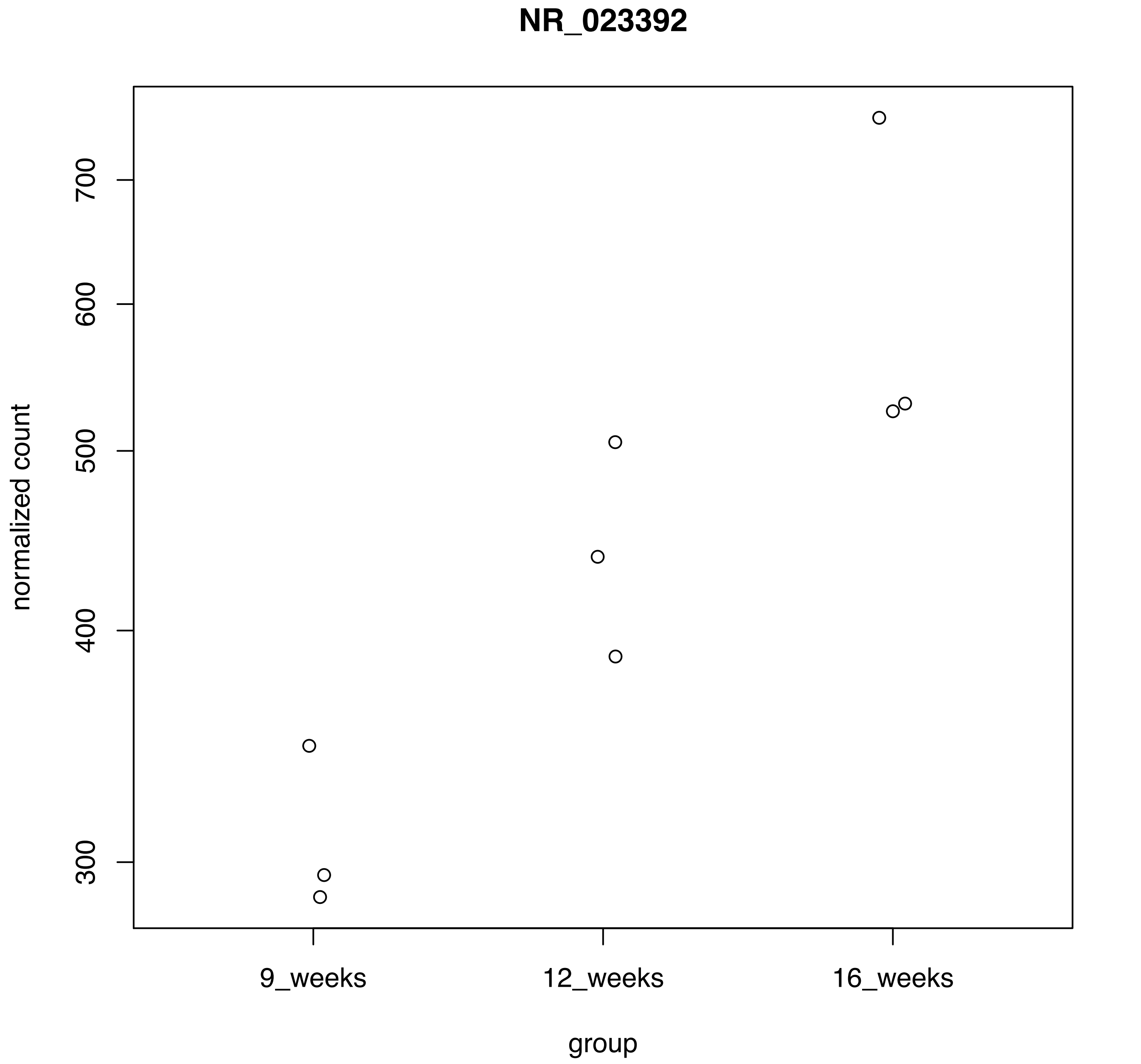
NR_023392
normalized count
300
400
500
600
700
9_weeks
12_weeks
16_weeks
group

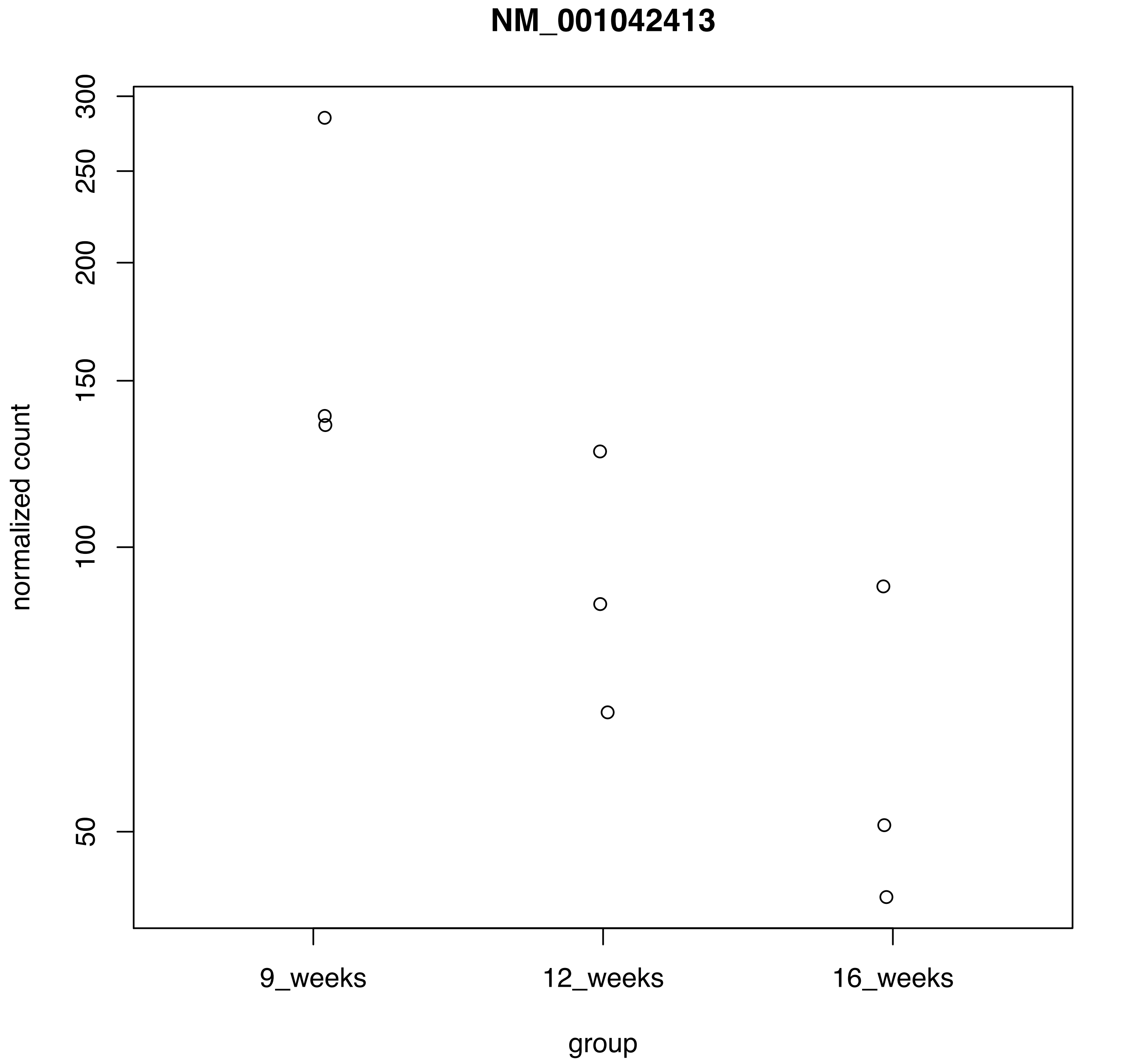
NM_001042413
normalized count
300
250
200
150
100
50
9_weeks
12_weeks
16_weeks
group

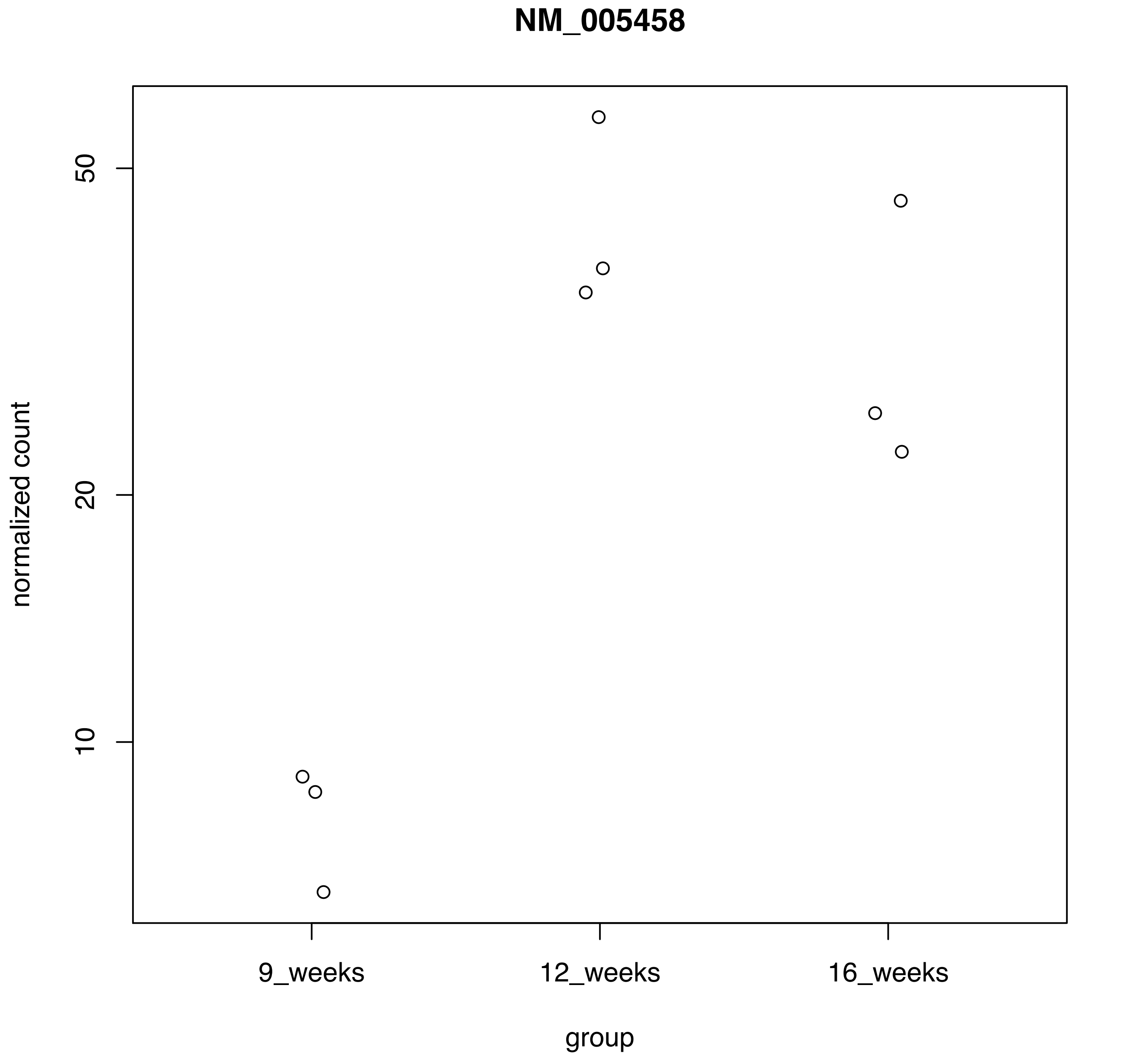
NM_005458
normalized count
50
20
10
9_weeks
12_weeks
16_weeks
group

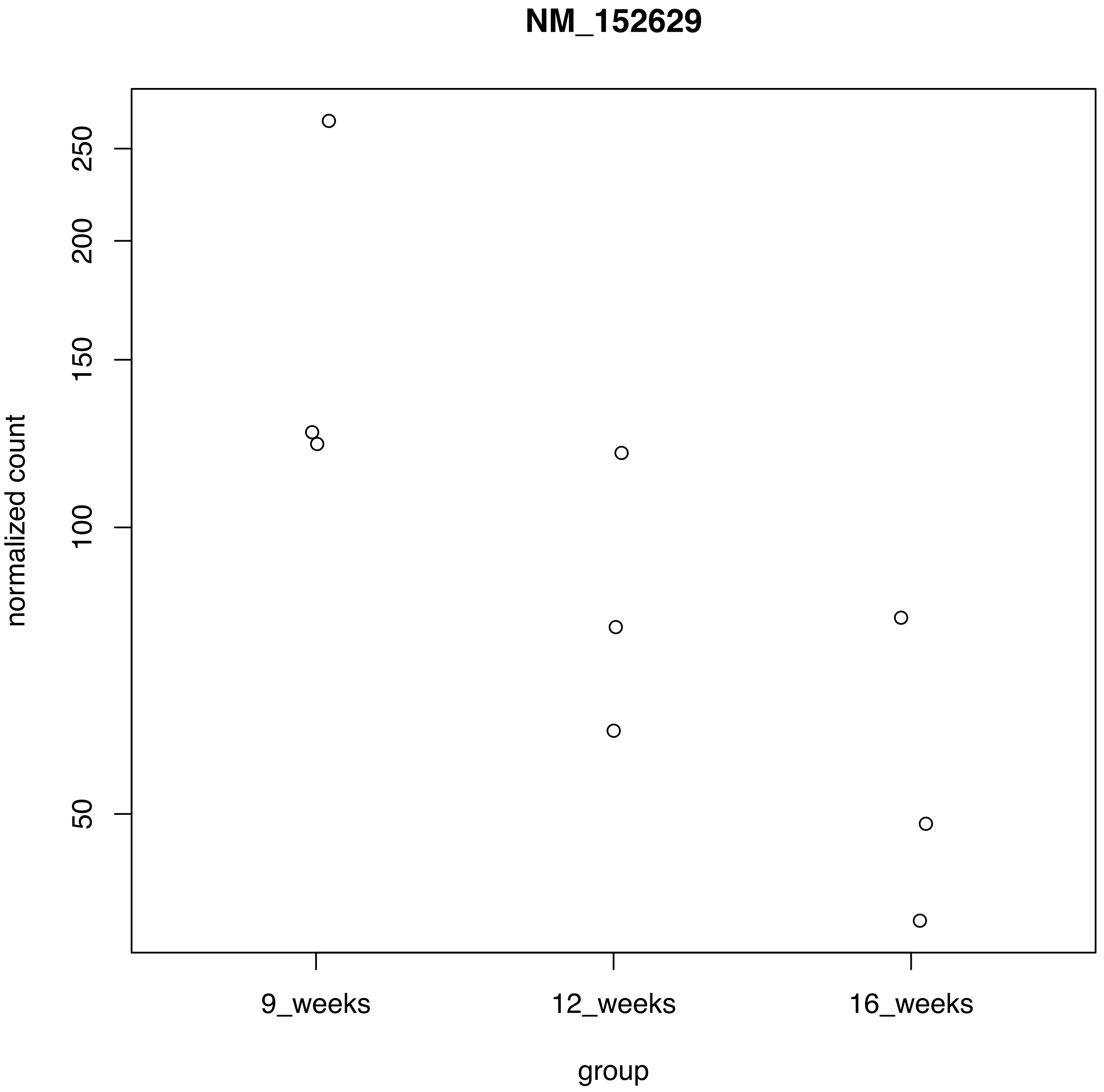
NM_152629
normalized count
250
200
150
100
50
9_weeks
12_weeks
16_weeks
group

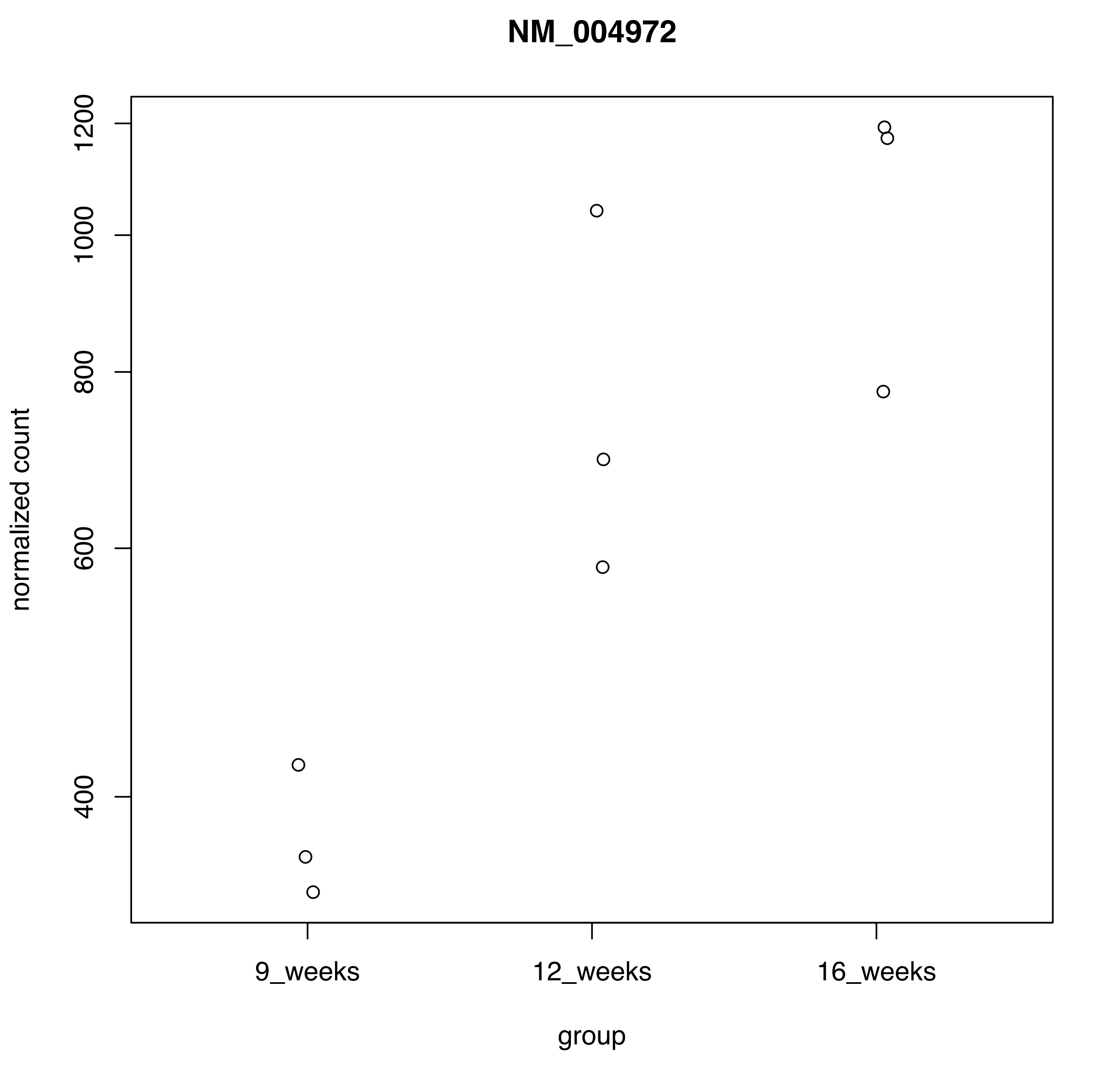
NM_004972
normalized count
1200
1000
800
600
400
9_weeks
12_weeks
16_weeks
group

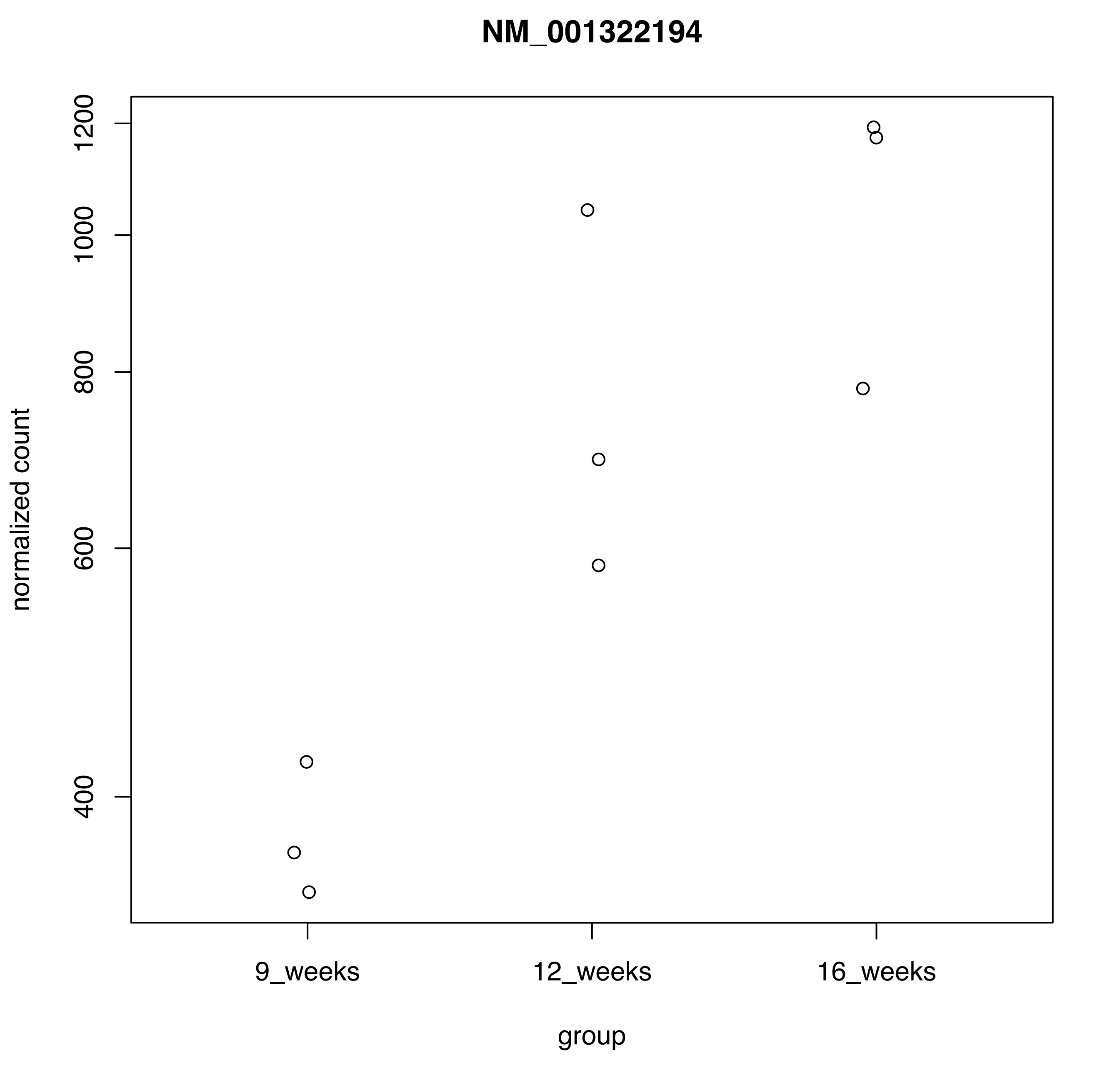
NM_001322194
normalized count
1200
1000
800
600
400
9_weeks
12_weeks
16_weeks
group

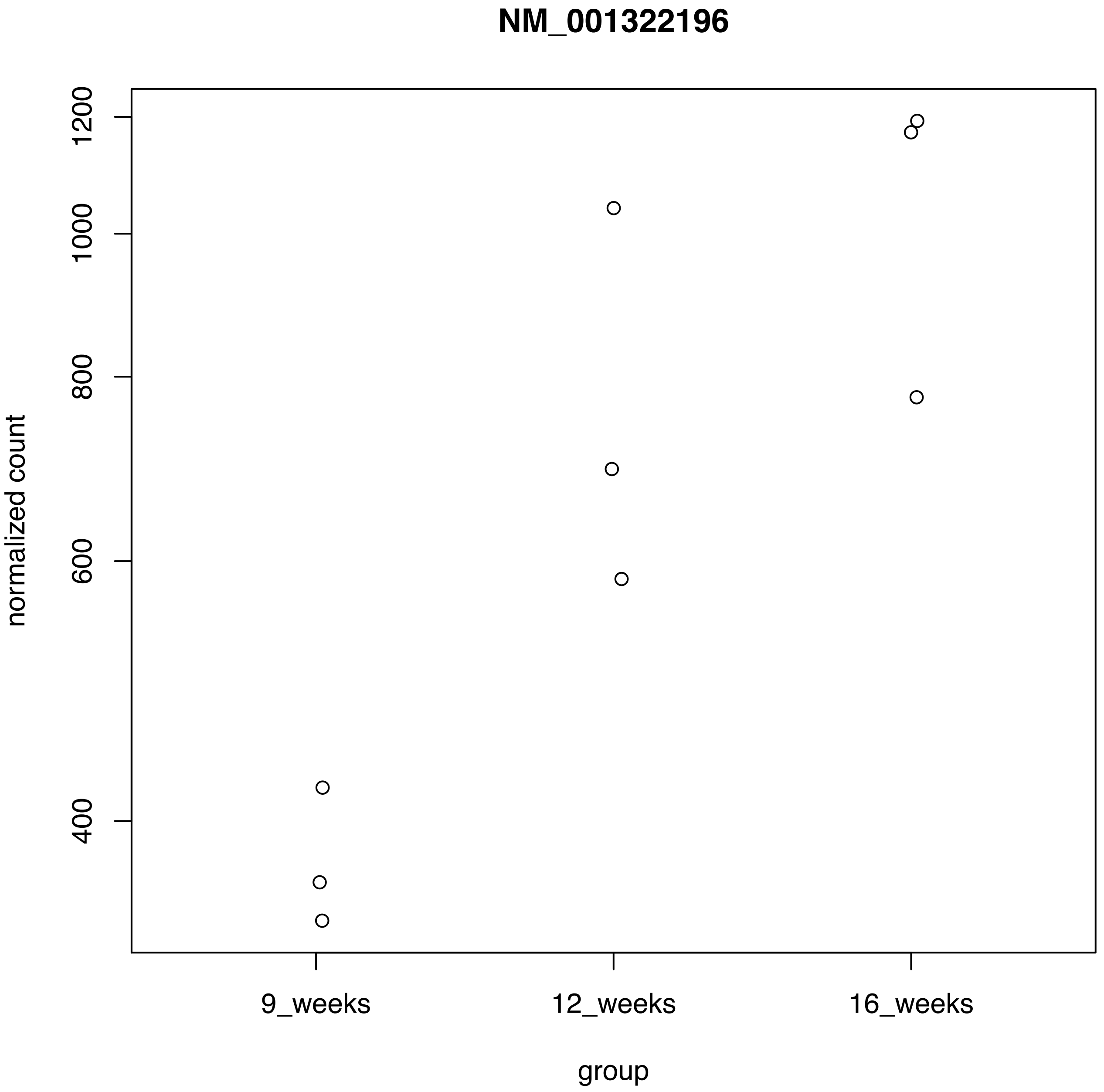

NM_001322196
normalized count
400
600
800
1000
1200
9_weeks
12_weeks
16_weeks
group

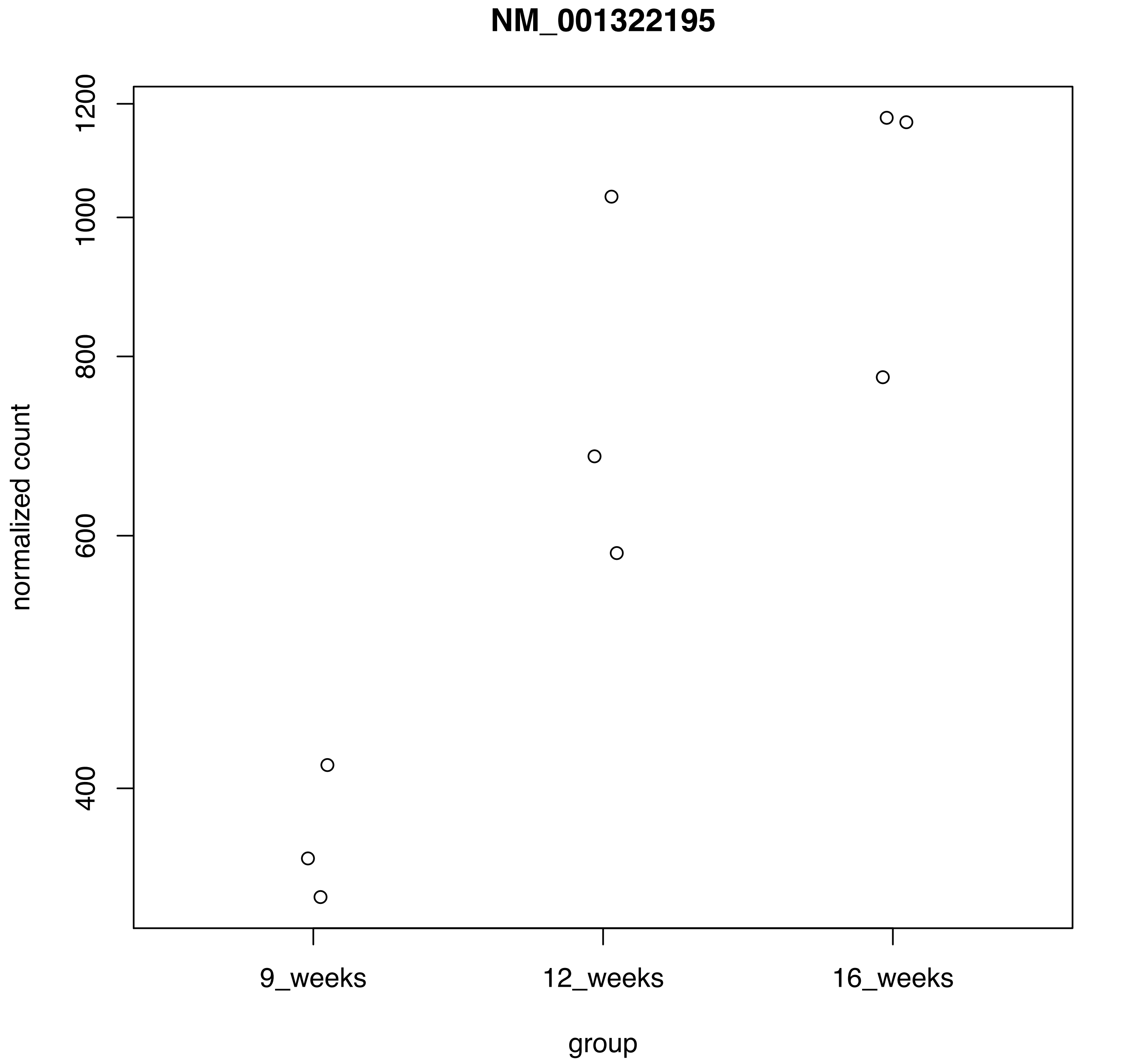
NM_001322195
normalized count
1200
1000
800
600
400
9_weeks
12_weeks
16_weeks
group

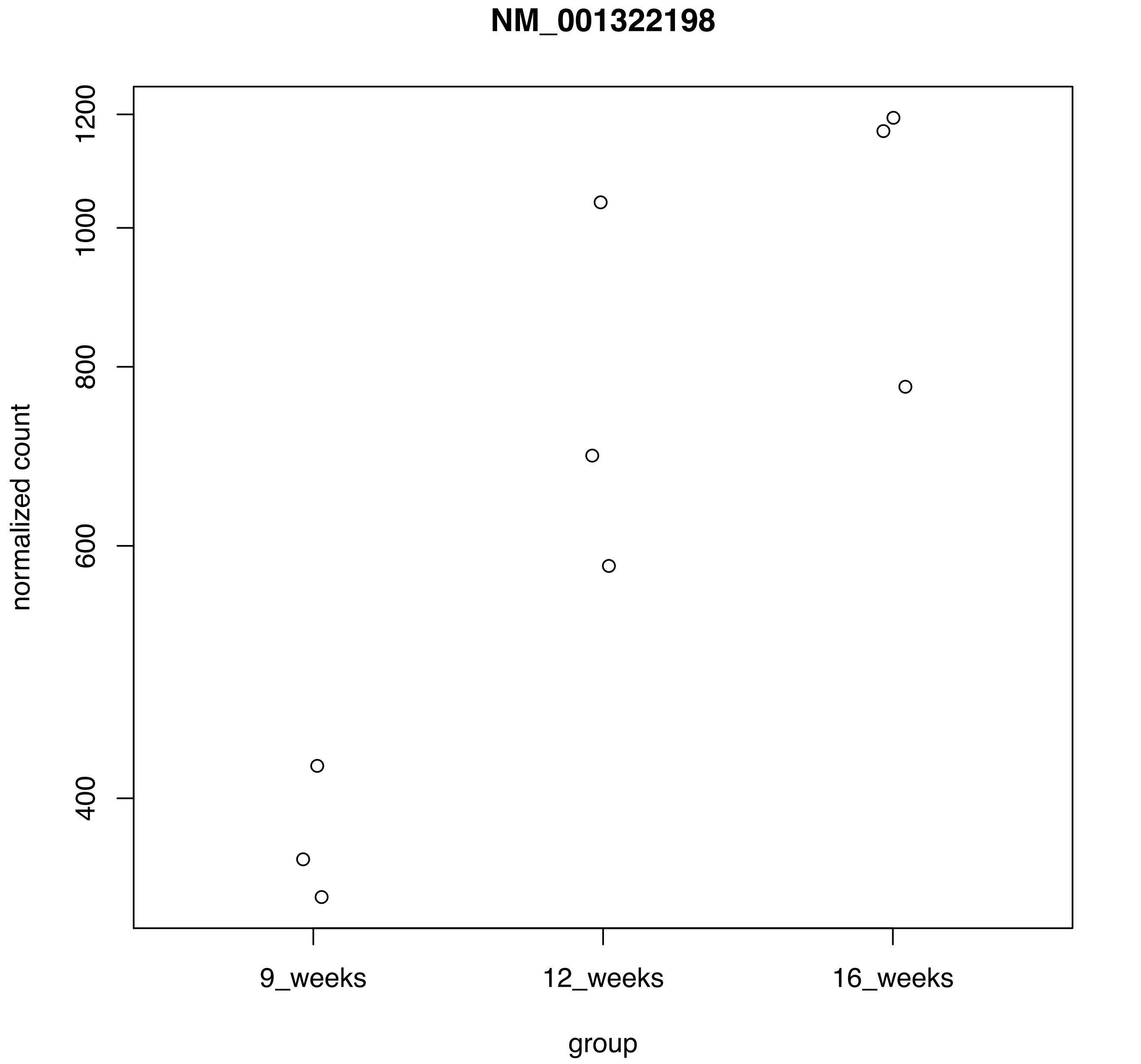
NM_001322198
normalized count
1200
1000
800
600
400
9_weeks
12_weeks
16_weeks
group

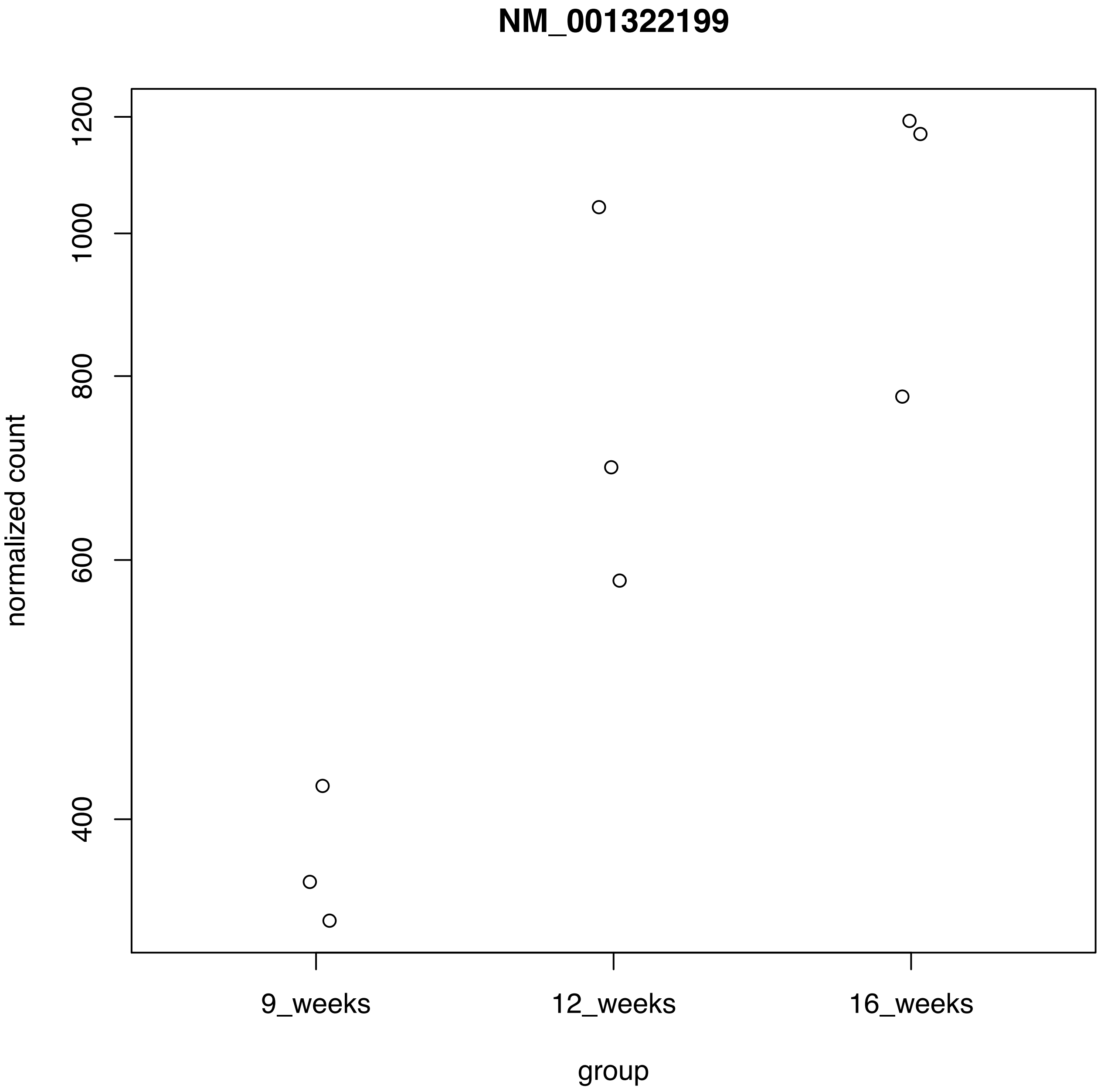

NM_001322199
normalized count
1200
1000
800
600
400
9_weeks
12_weeks
16_weeks
group

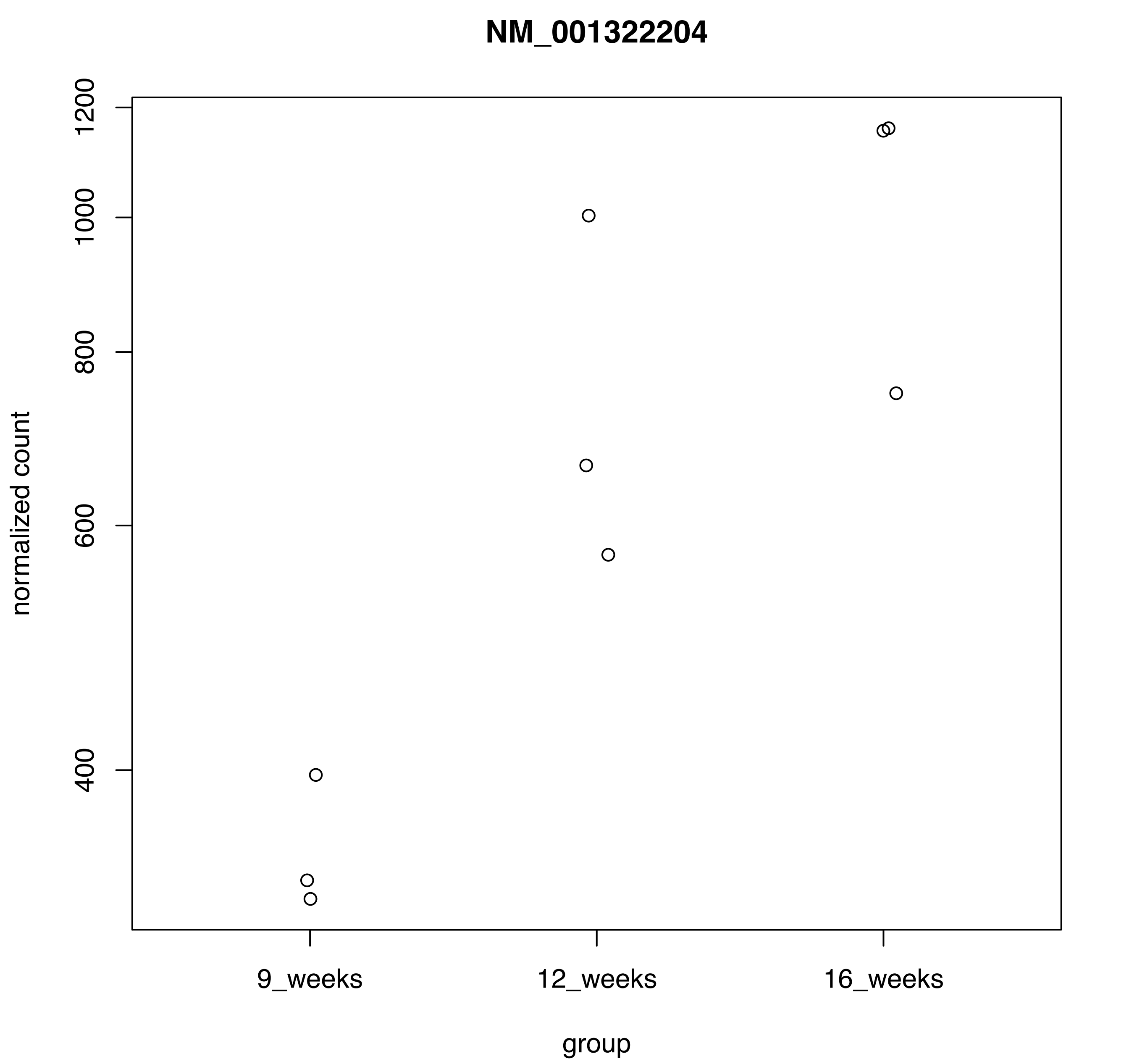
NM_001322204
normalized count
1200
1000
800
600
400
9_weeks
12_weeks
16_weeks
group

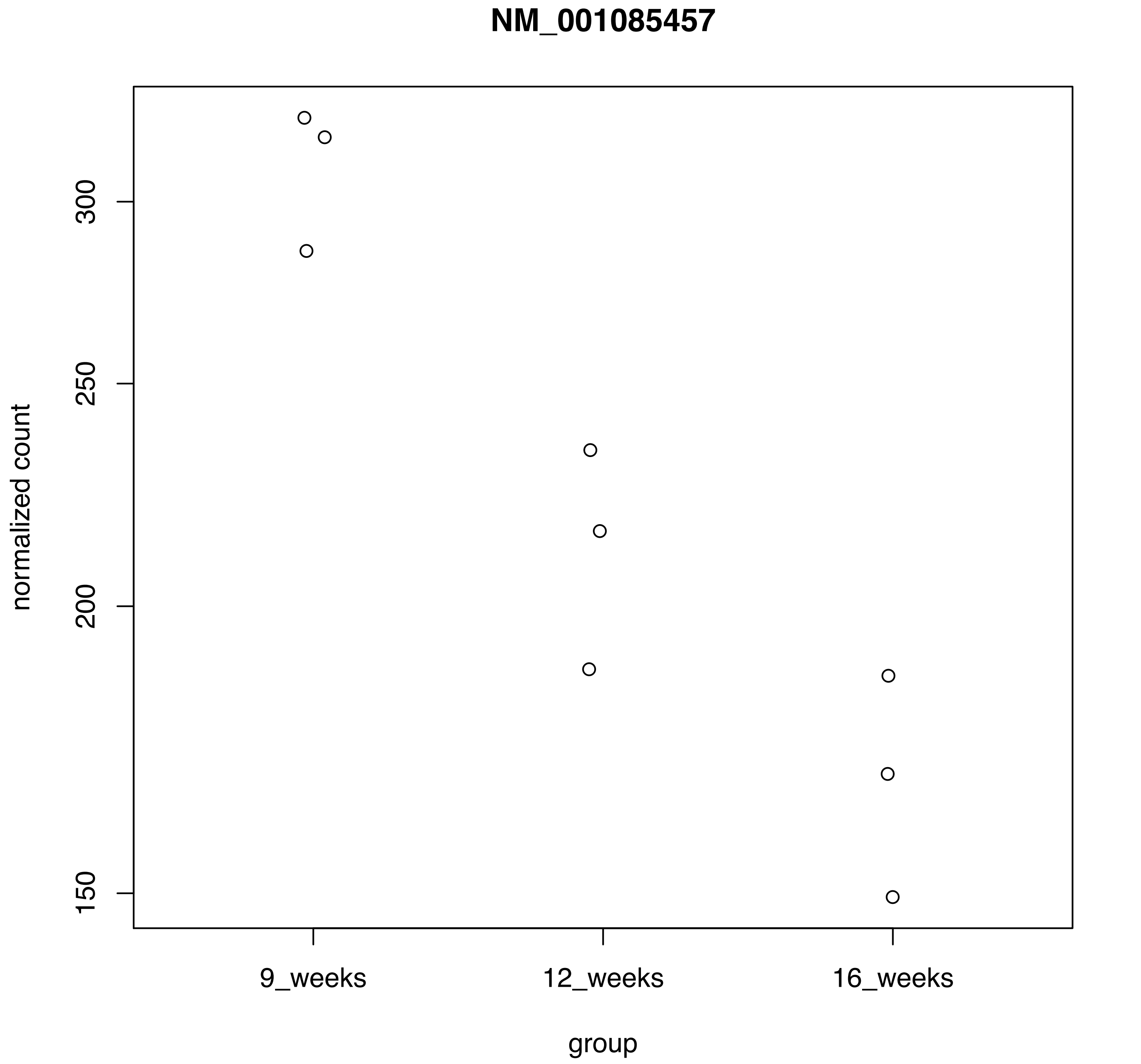
NM_001085457
normalized count
300
250
200
150
9_weeks
12_weeks
16_weeks
group

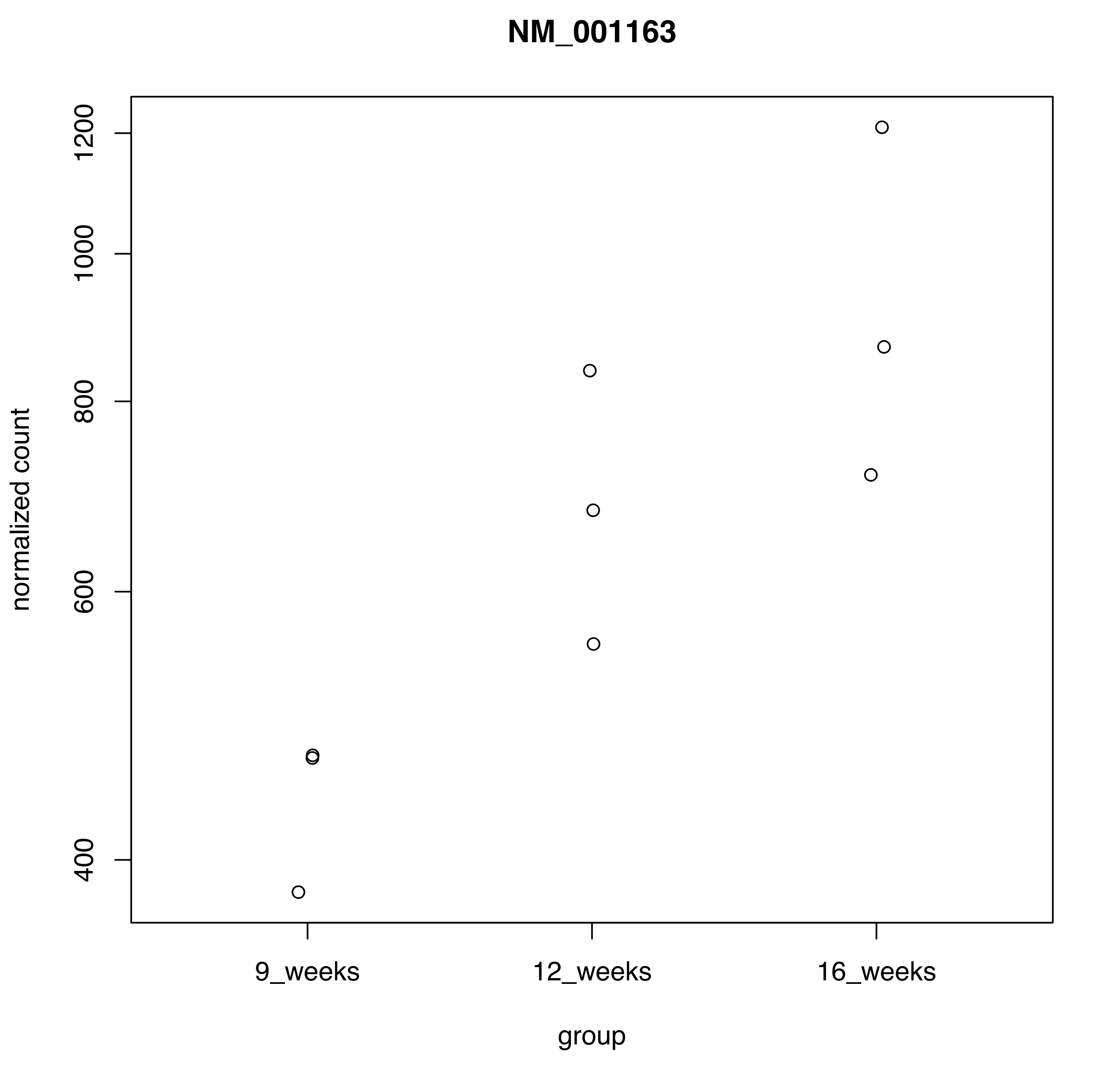
NM_001163
normalized count
400
600
800
1000
1200
9_weeks
12_weeks
16_weeks
group

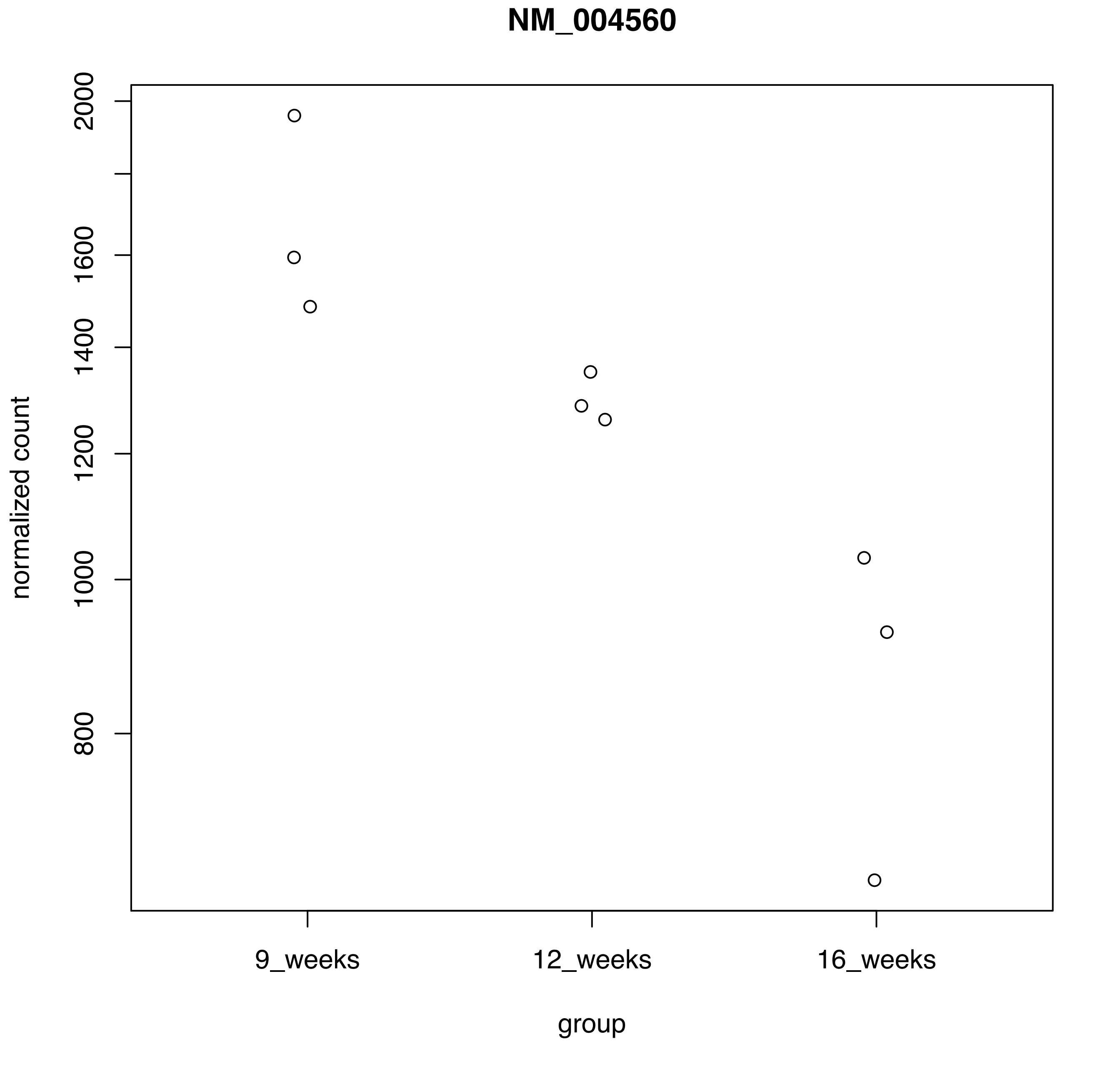
NM_004560
normalized count
2000
1600
1400
1200
1000
800
9_weeks
12_weeks
16_weeks
group

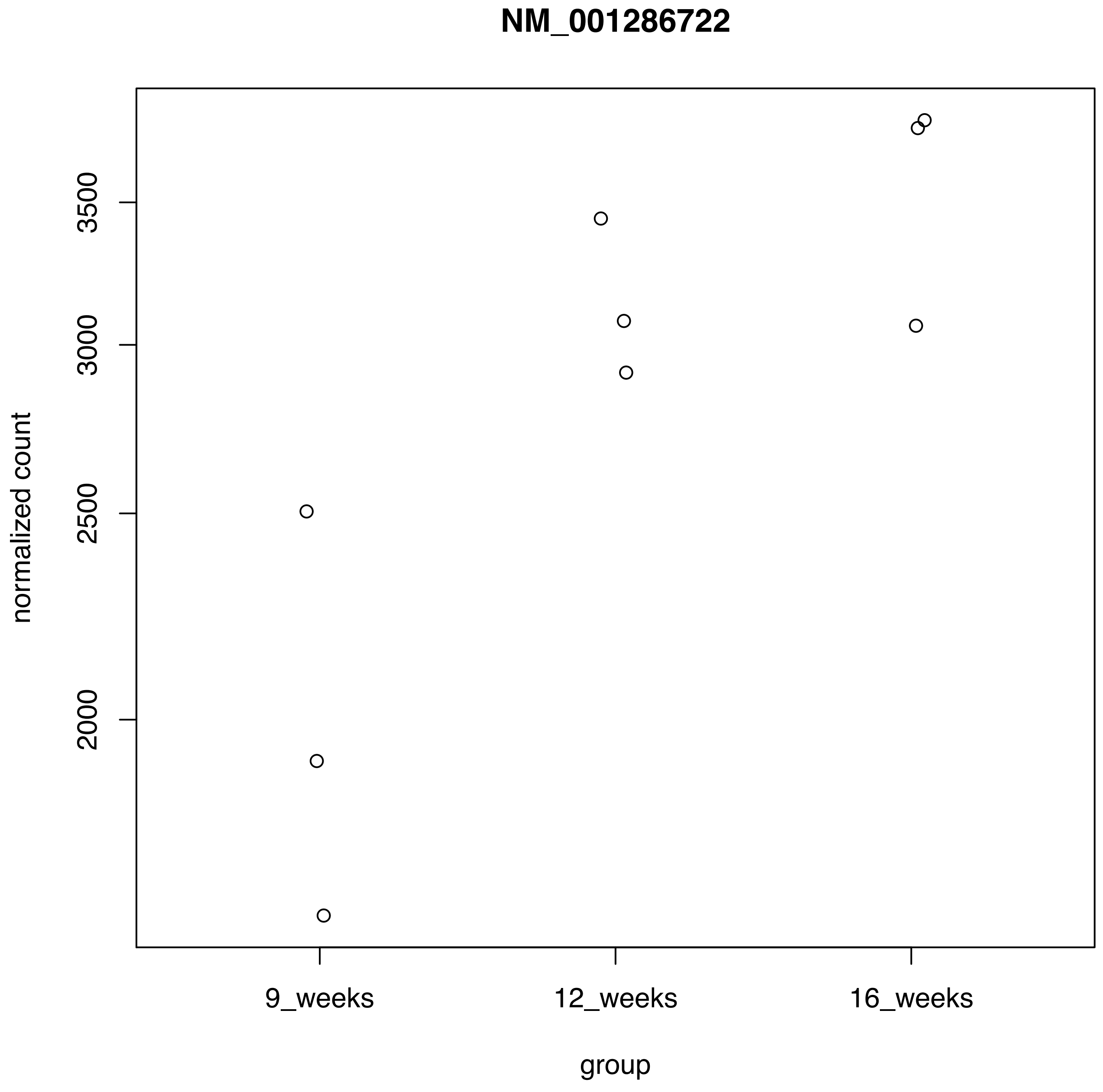
NM_001286722
normalized count
3500
3000
2500
2000
9_weeks
12_weeks
16_weeks
group

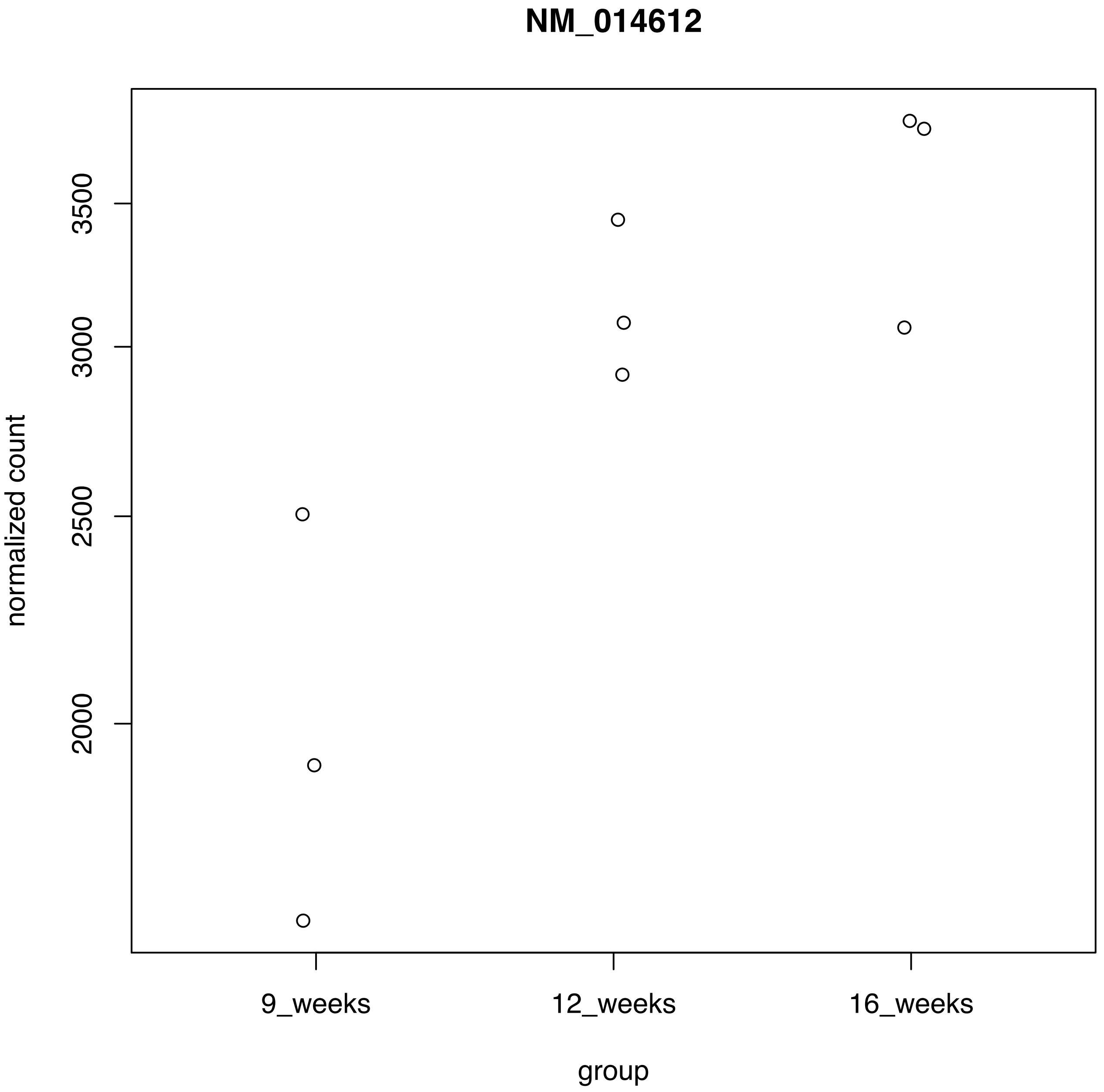
NM_014612
normalized count
3500
3000
2500
2000
9_weeks
12_weeks
16_weeks
group

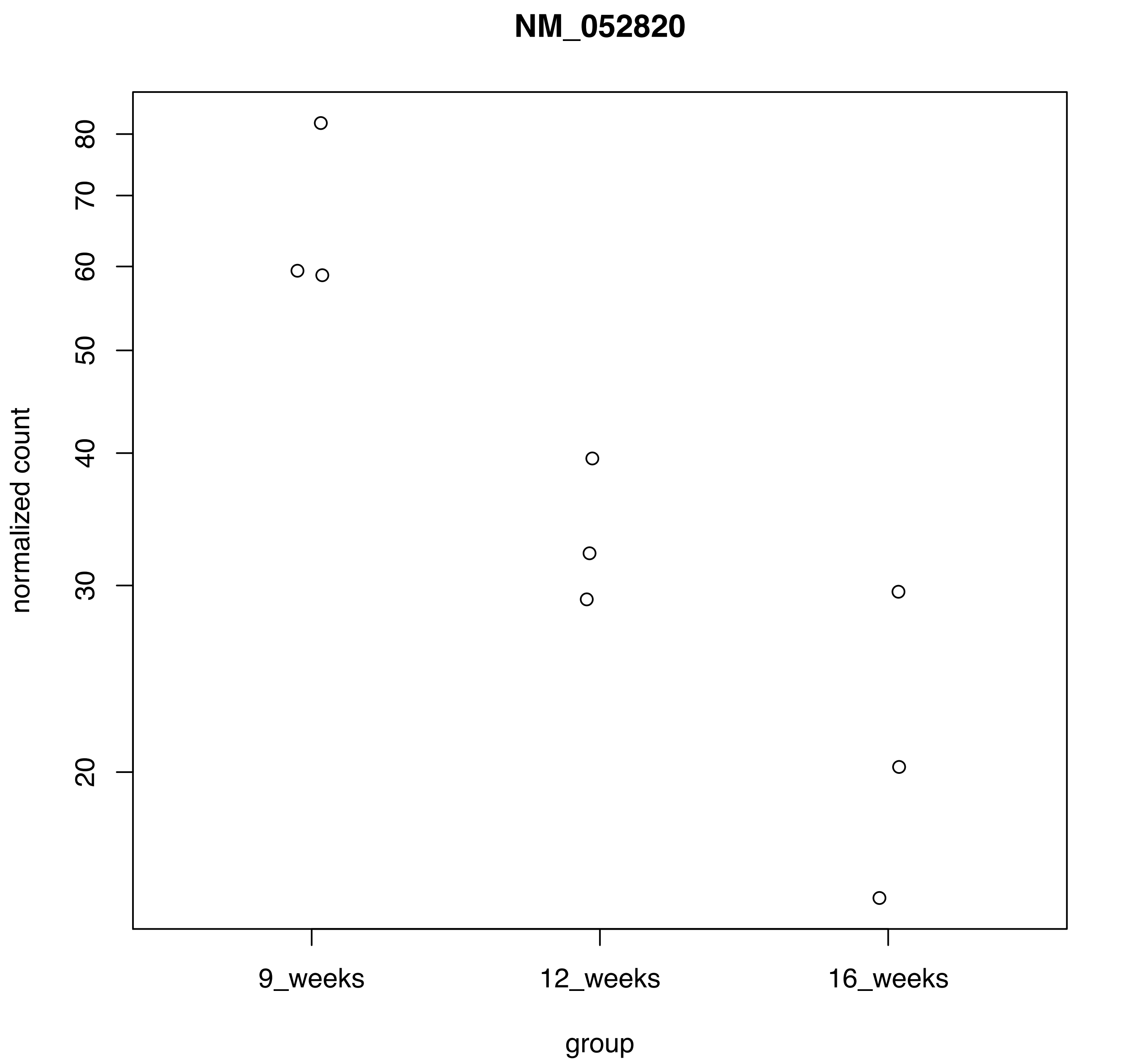
NM_052820
normalized count
80
70
60
50
40
30
20
9_weeks
12_weeks
16_weeks
group

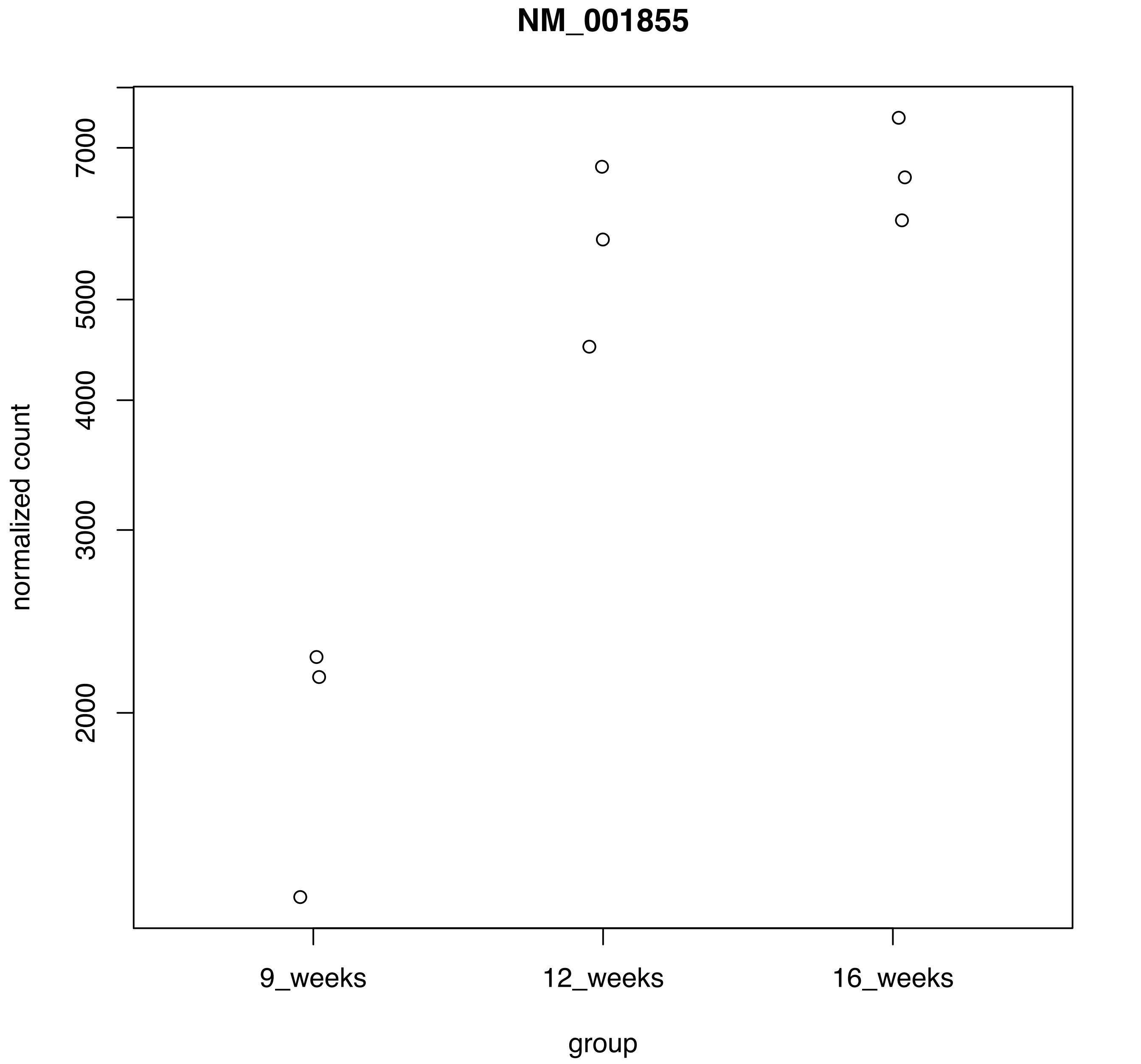

NM_001855
normalized count
7000
5000
4000
3000
2000
9_weeks
12_weeks
16_weeks
group

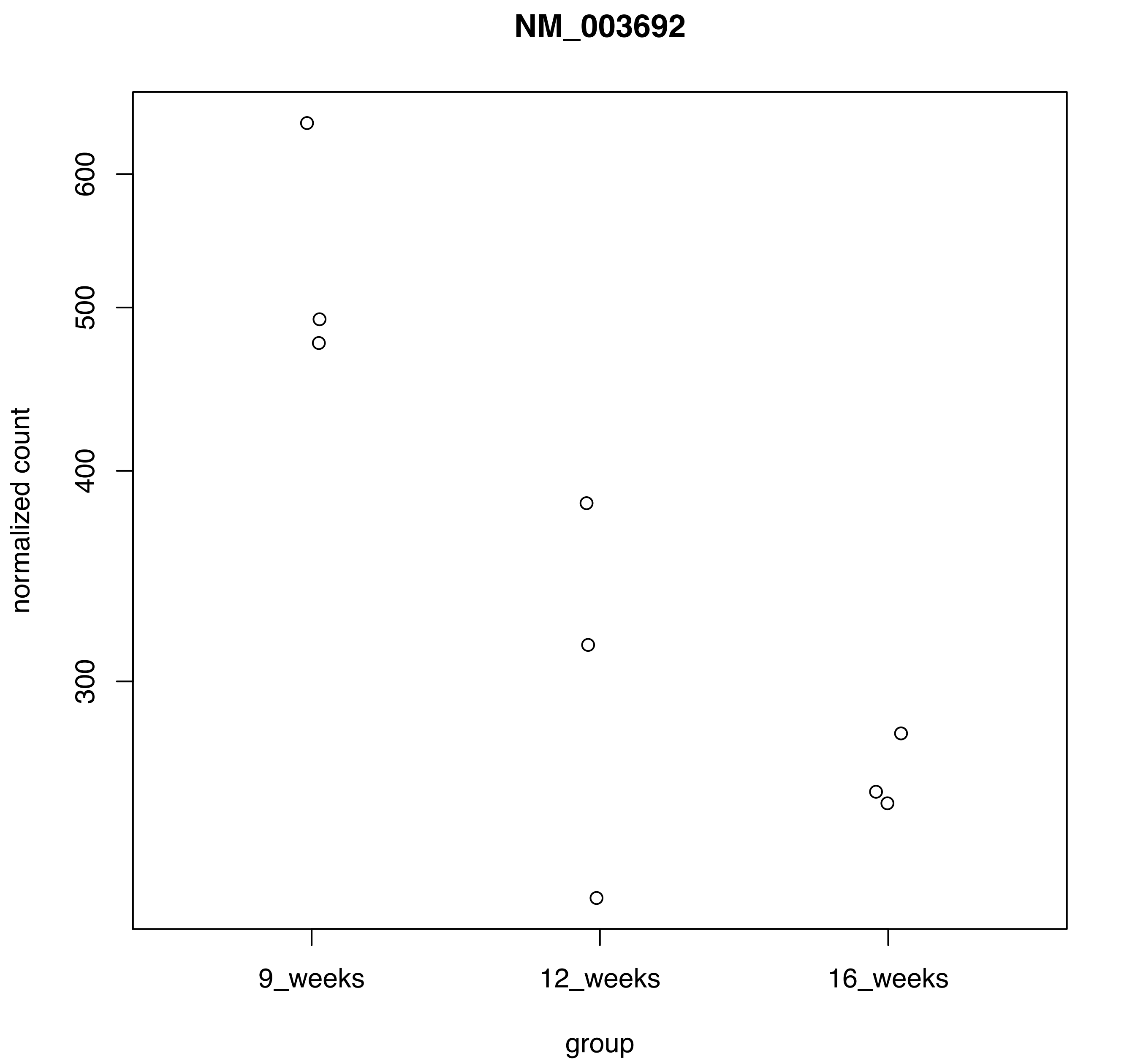
NM_003692
normalized count
600
500
400
300
9_weeks
12_weeks
16_weeks
group

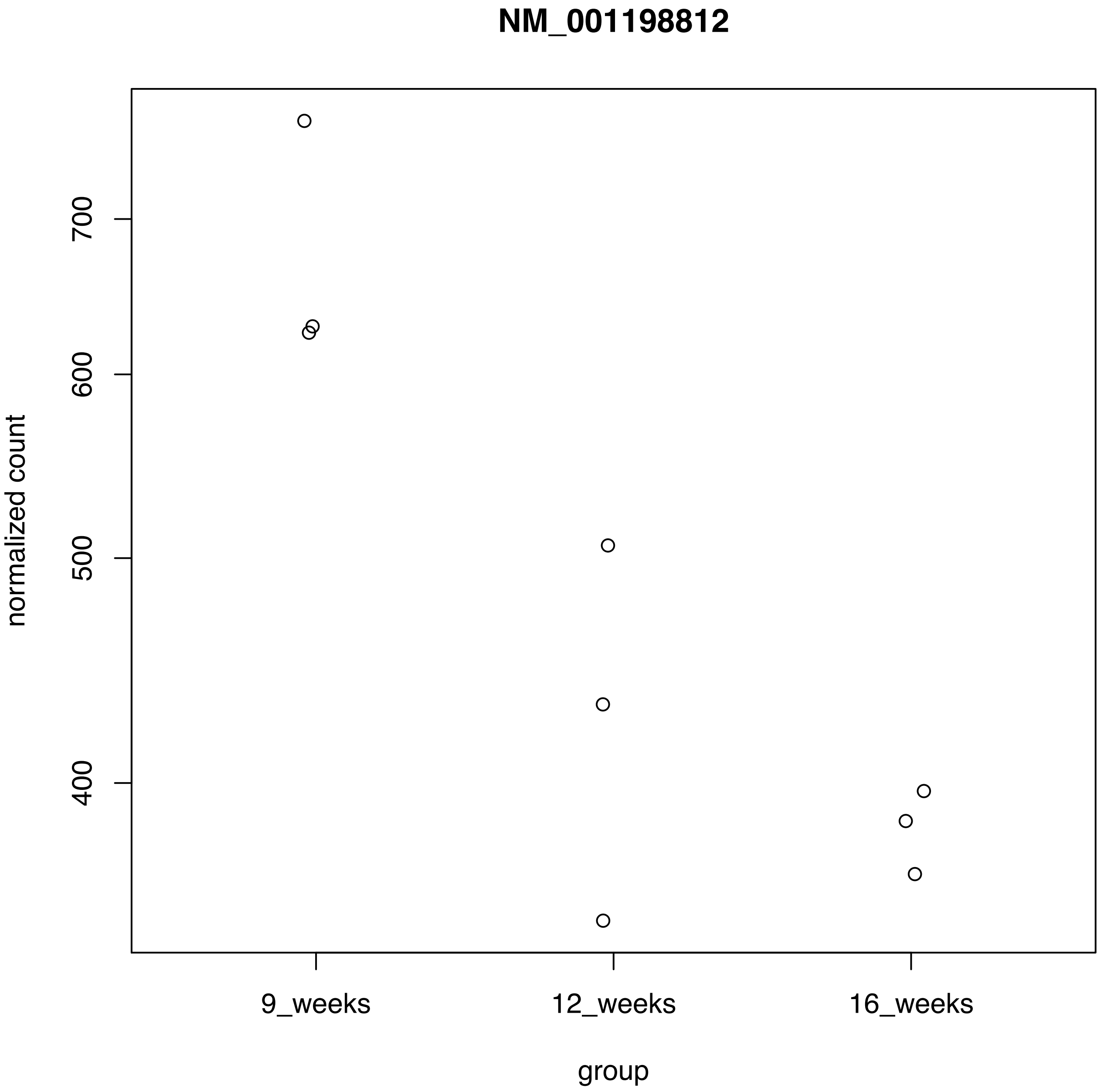

NM_001198812
normalized count
700
600
500
400
9_weeks
12_weeks
16_weeks
group

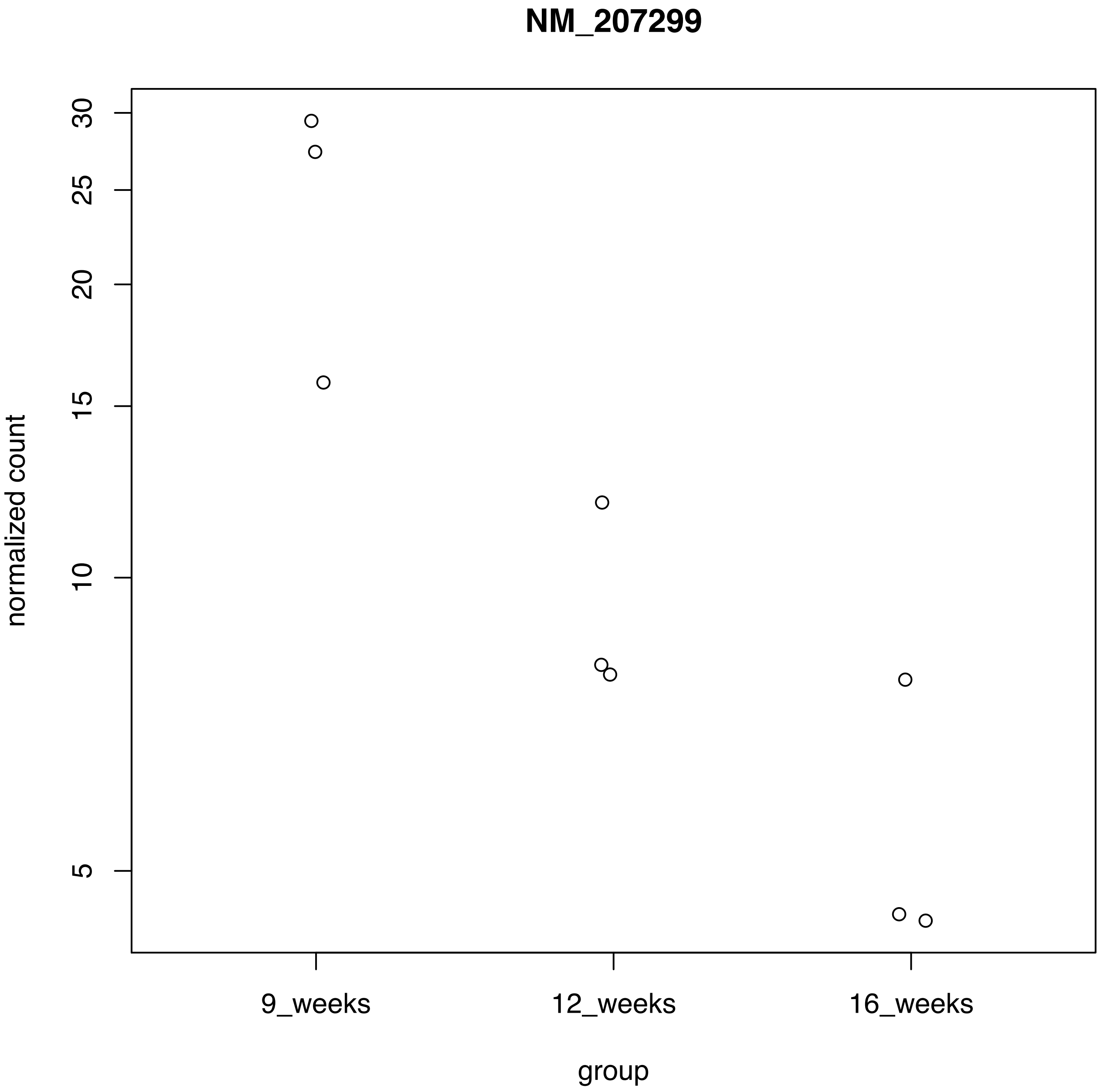
NM_207299
normalized count
30
25
20
15
10
5
9_weeks
12_weeks
16_weeks
group

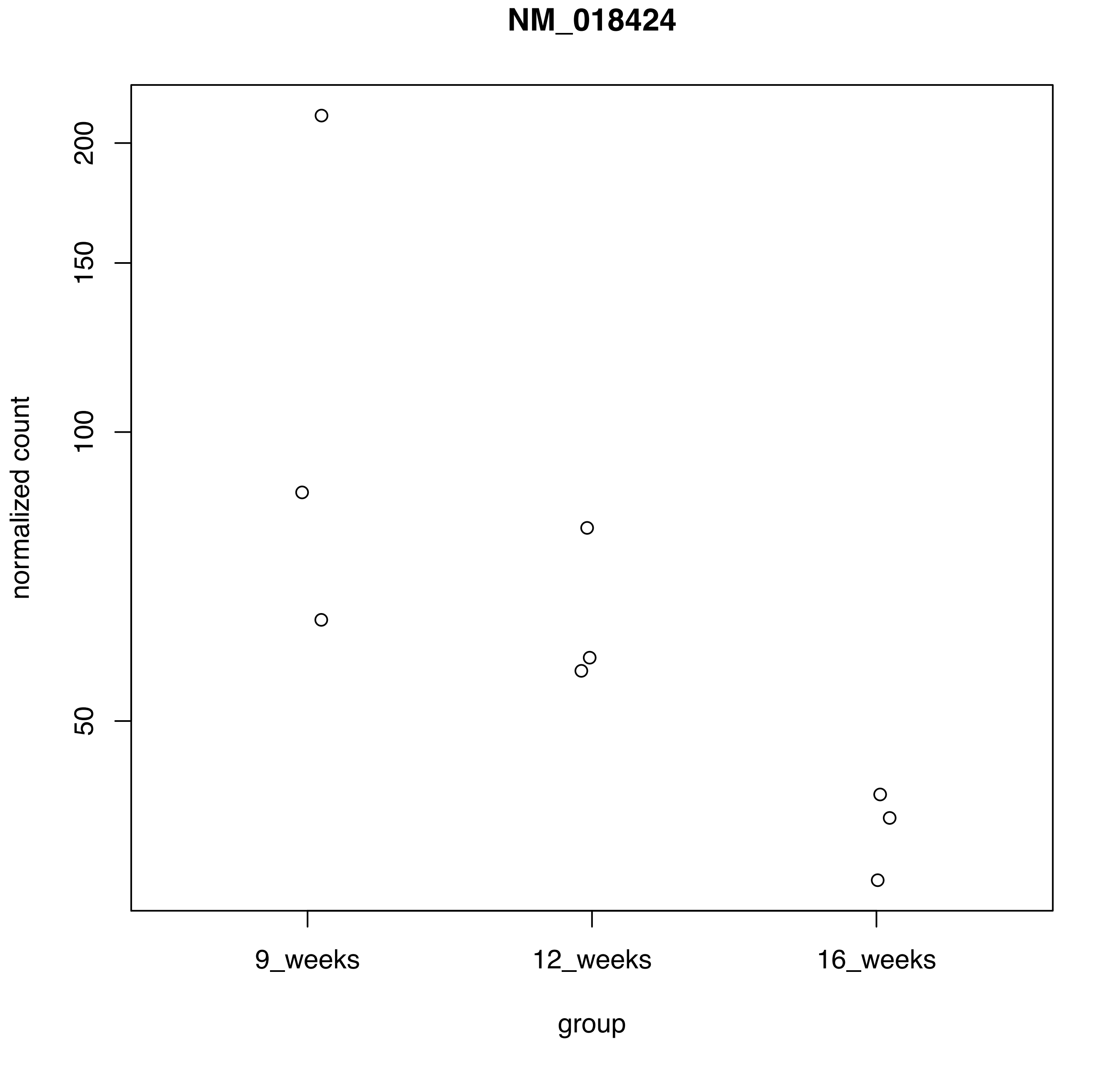
NM_018424
normalized count
200
150
100
50
9_weeks
12_weeks
16_weeks
group

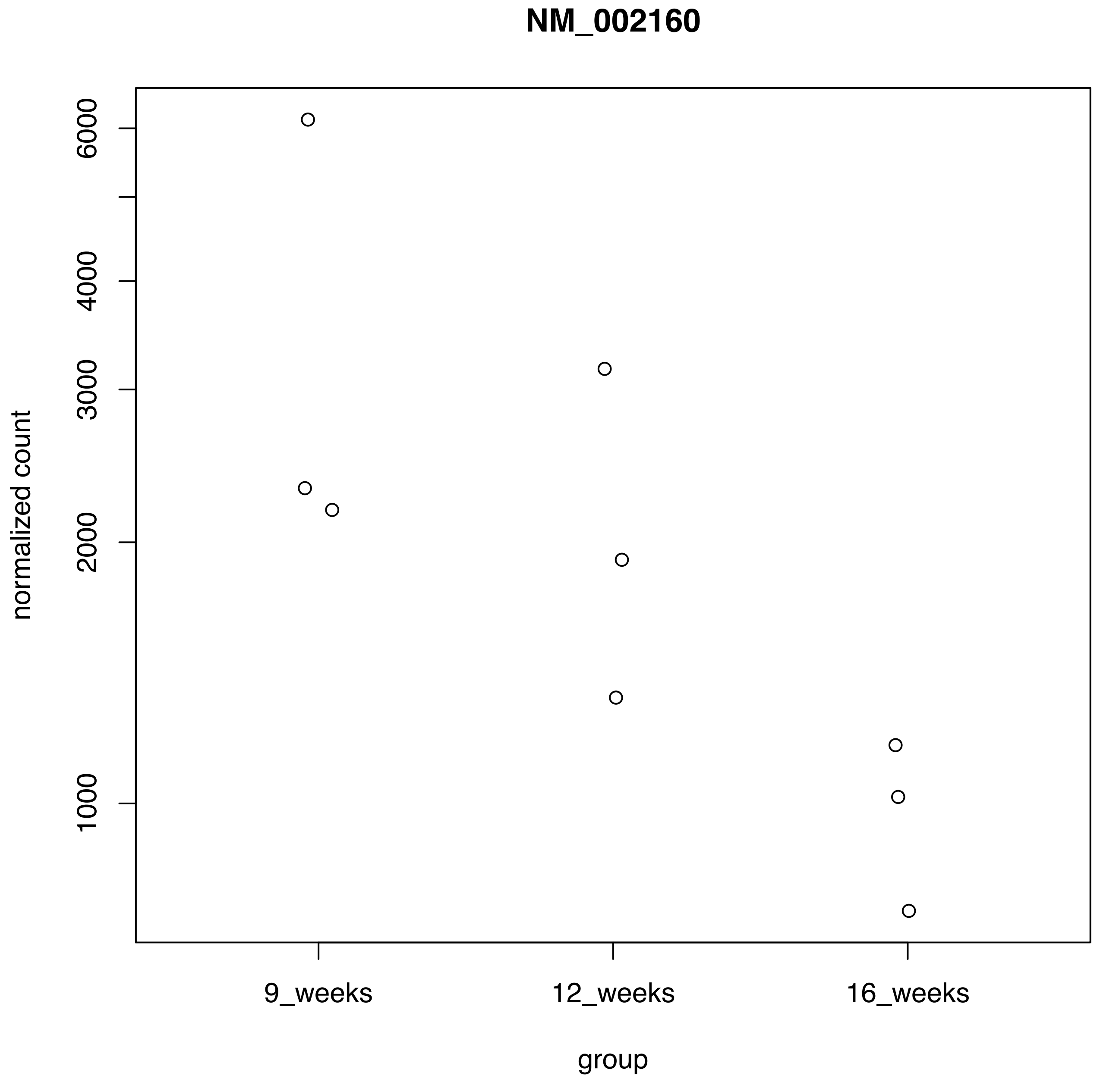
NM_002160
normalized count
6000
4000
3000
2000
1000
9_weeks
12_weeks
16_weeks
group

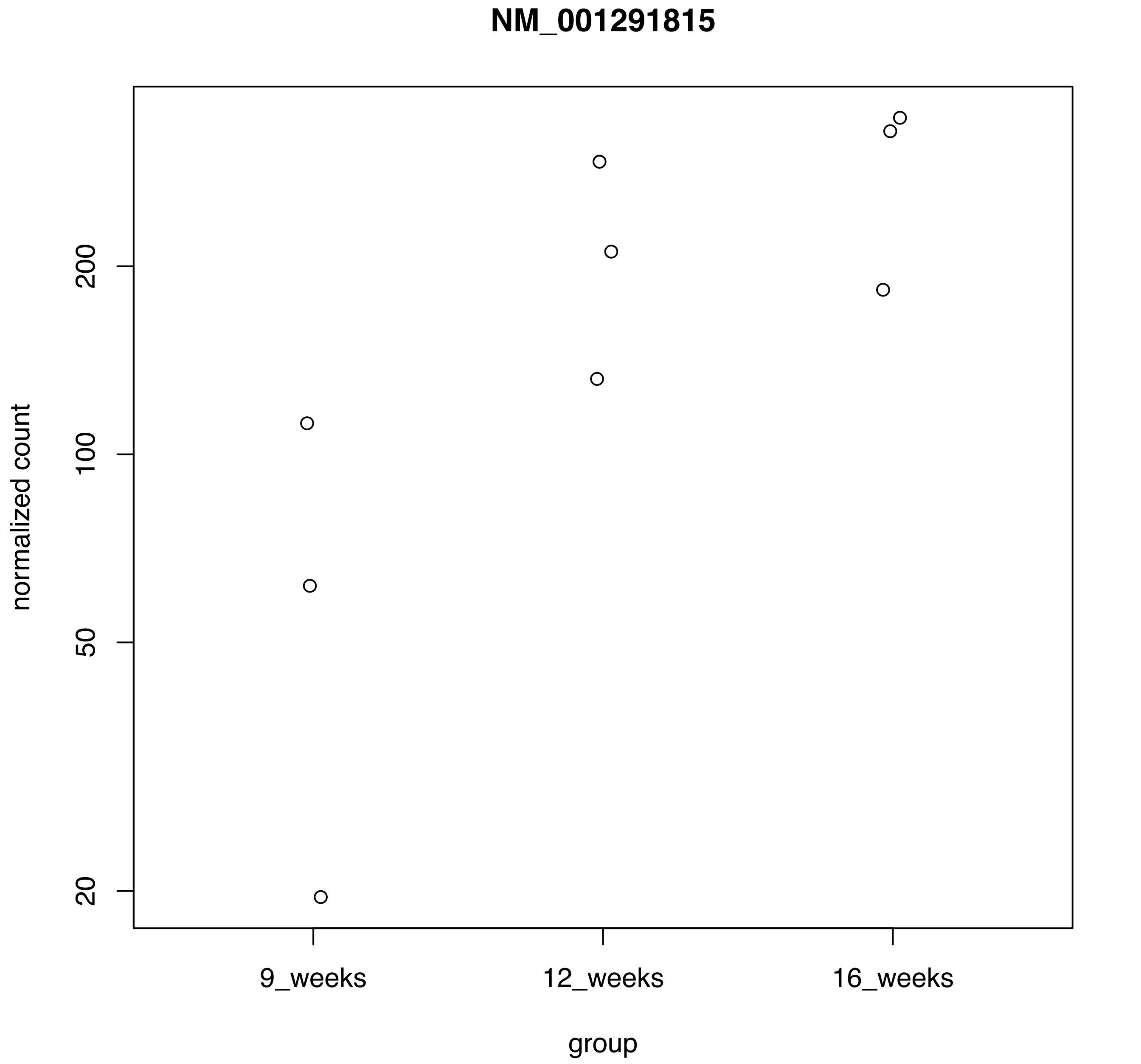

NM_001291815
normalized count
20
50
100
200
9_weeks
12_weeks
16_weeks
group

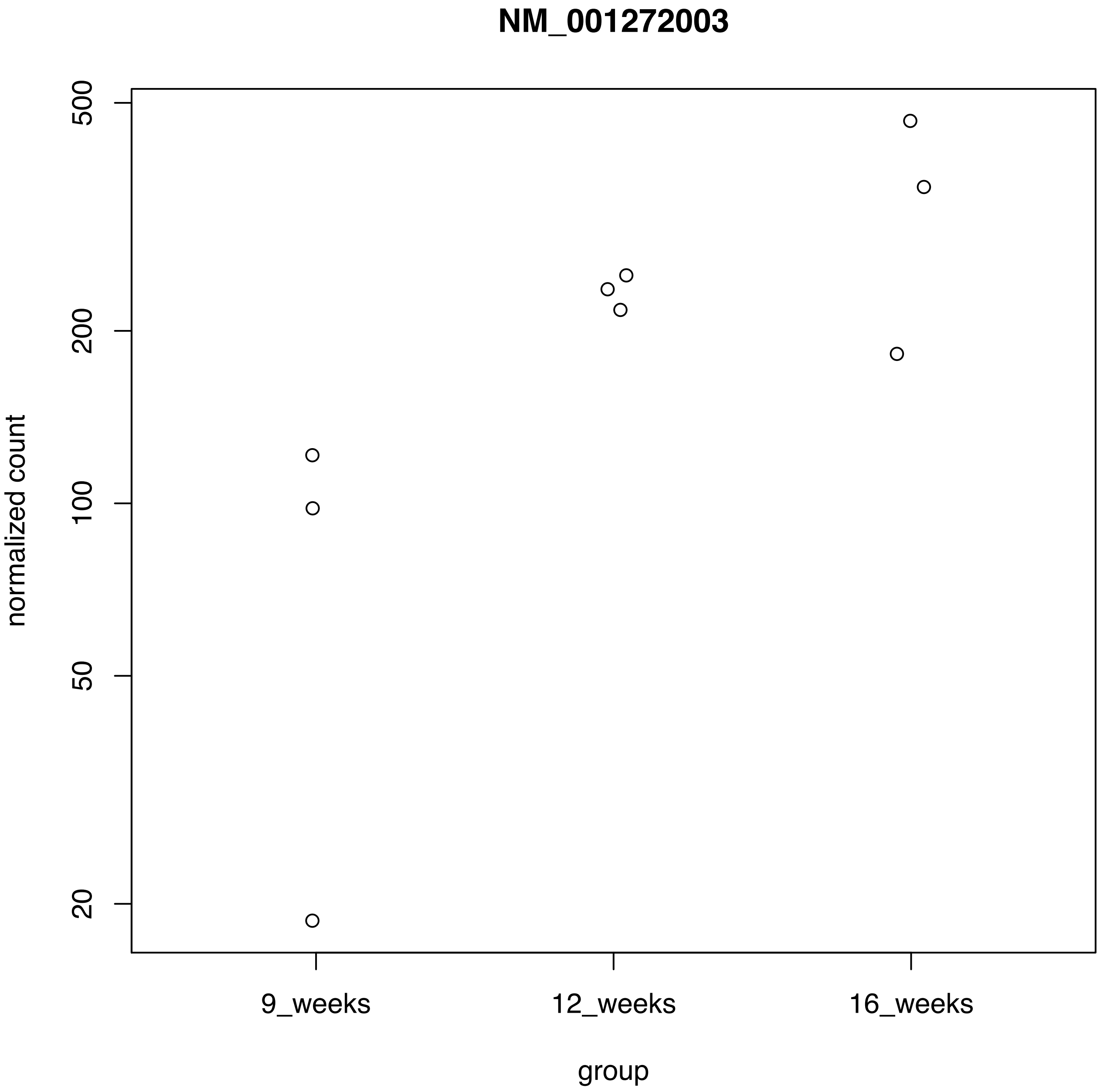
NM_001272003
normalized count
500
200
100
50
20
9_weeks
12_weeks
16_weeks
group

NM_020822

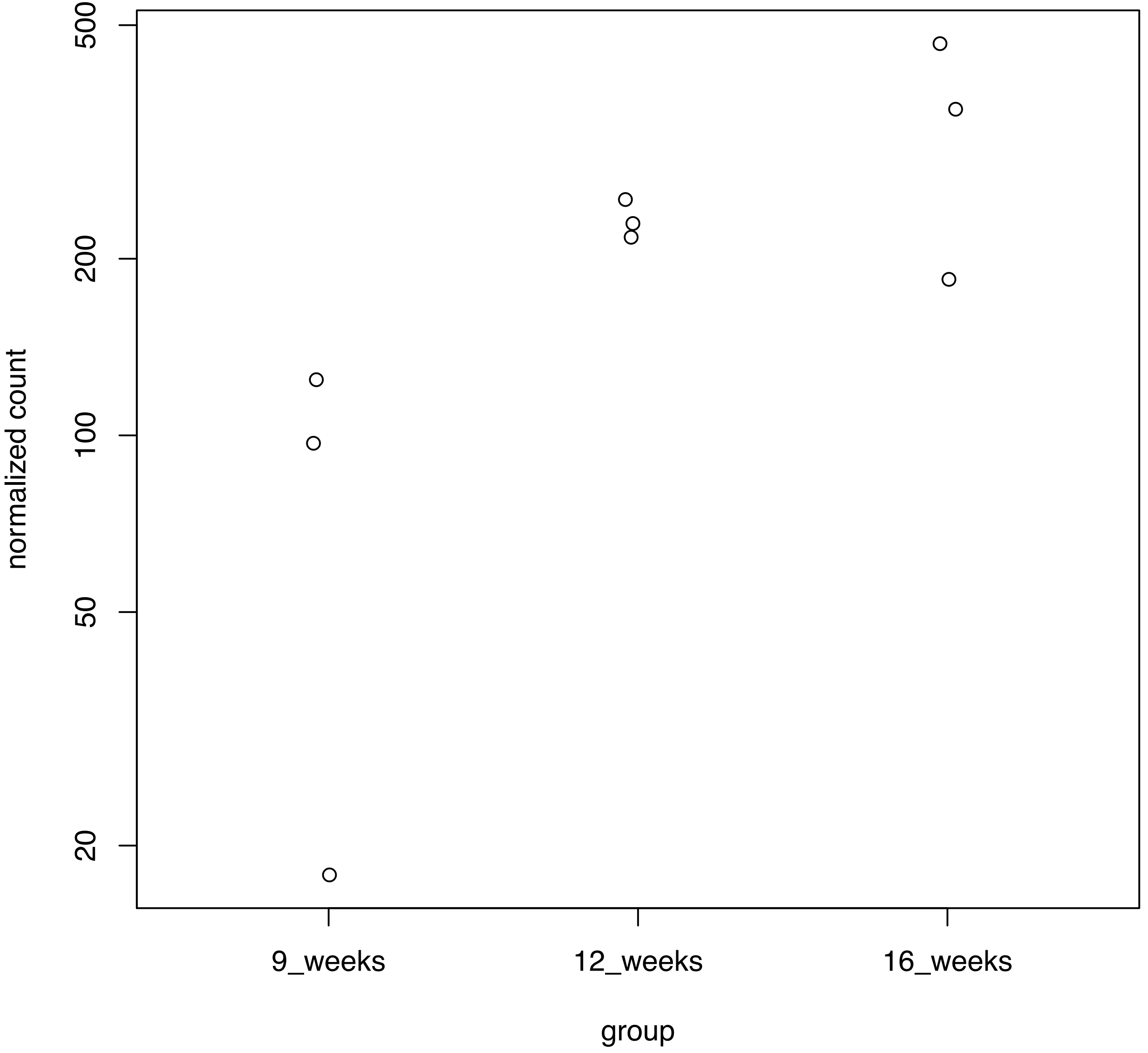

normalized count
500
200
100
50
20
9_weeks
12_weeks
16_weeks
group

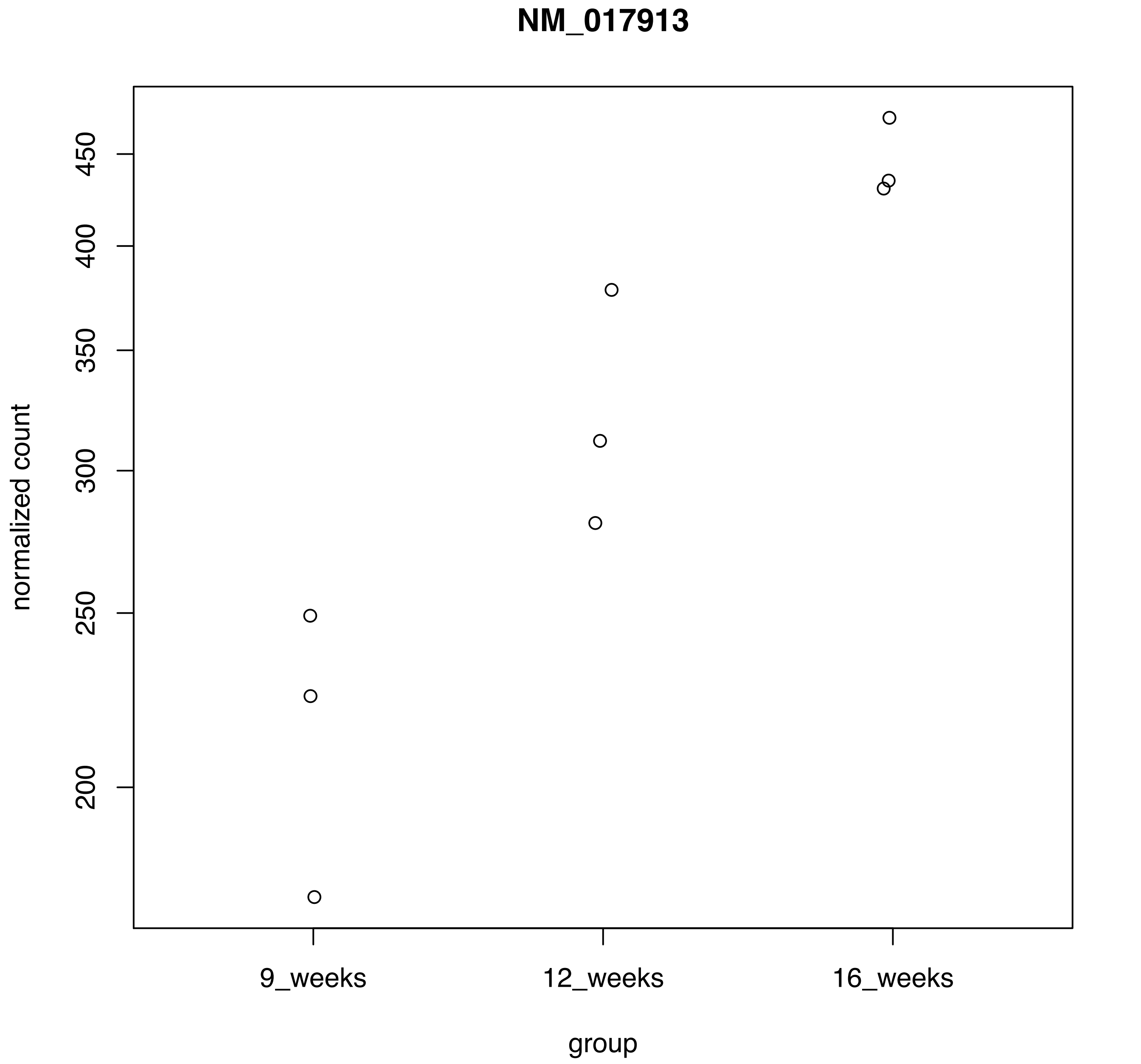

NM_017913
normalized count
450
400
350
300
250
200
9_weeks
12_weeks
16_weeks
group

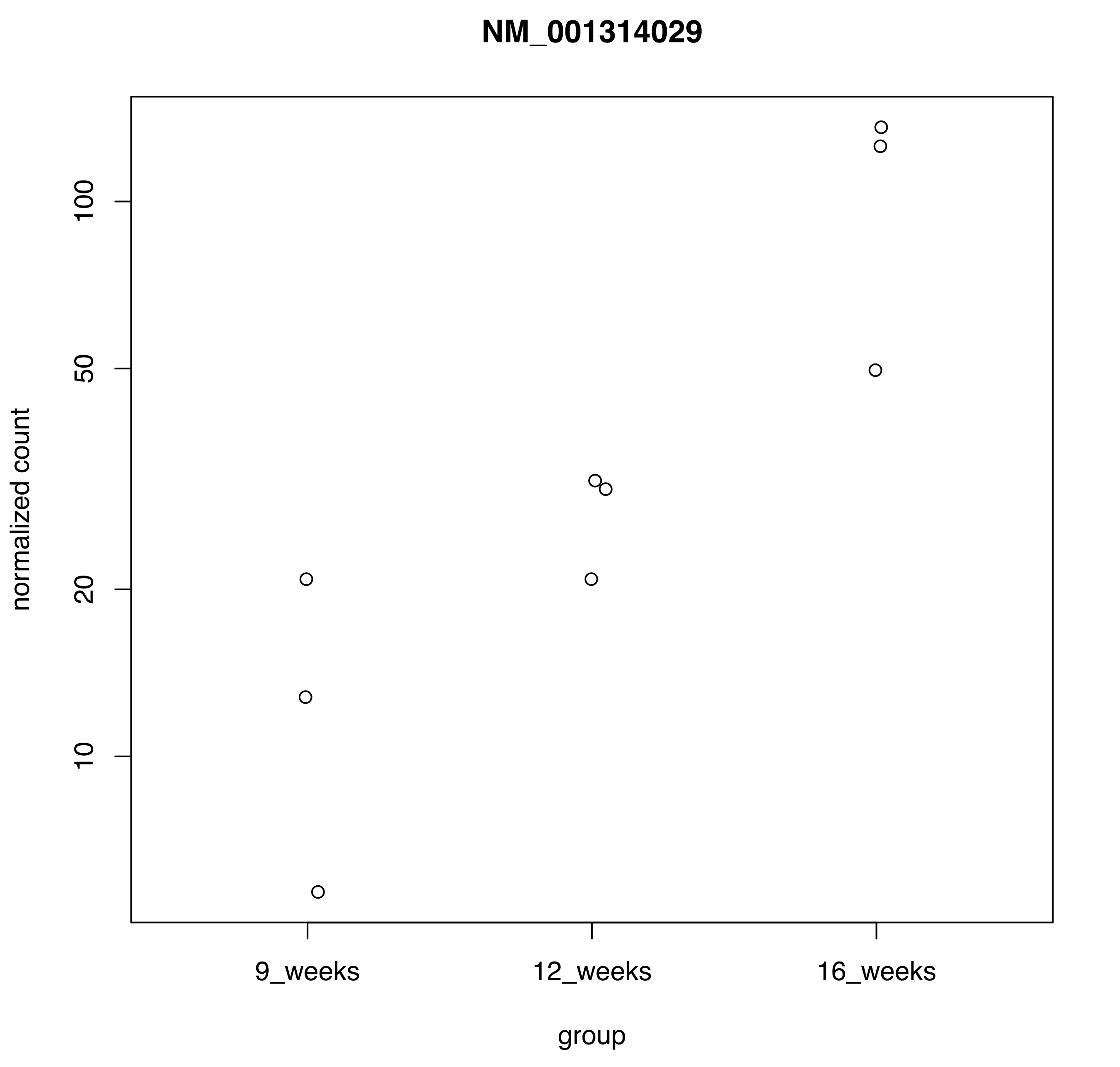
NM_001314029
normalized count
100
50
20
10
9_weeks
12_weeks
16_weeks
group

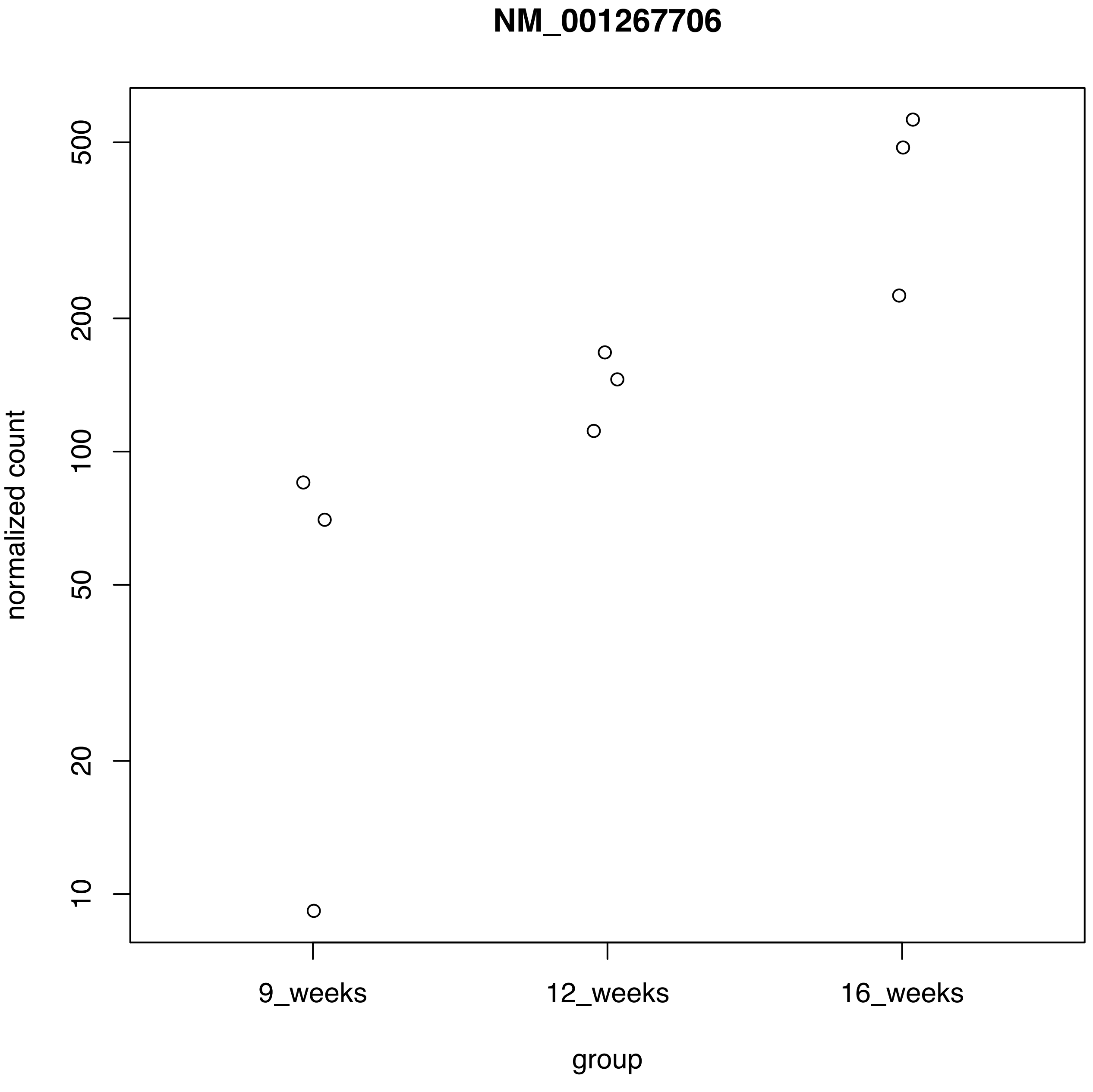

NM_001267706
normalized count
500
200
100
50
20
10
9_weeks
12_weeks
16_weeks
group

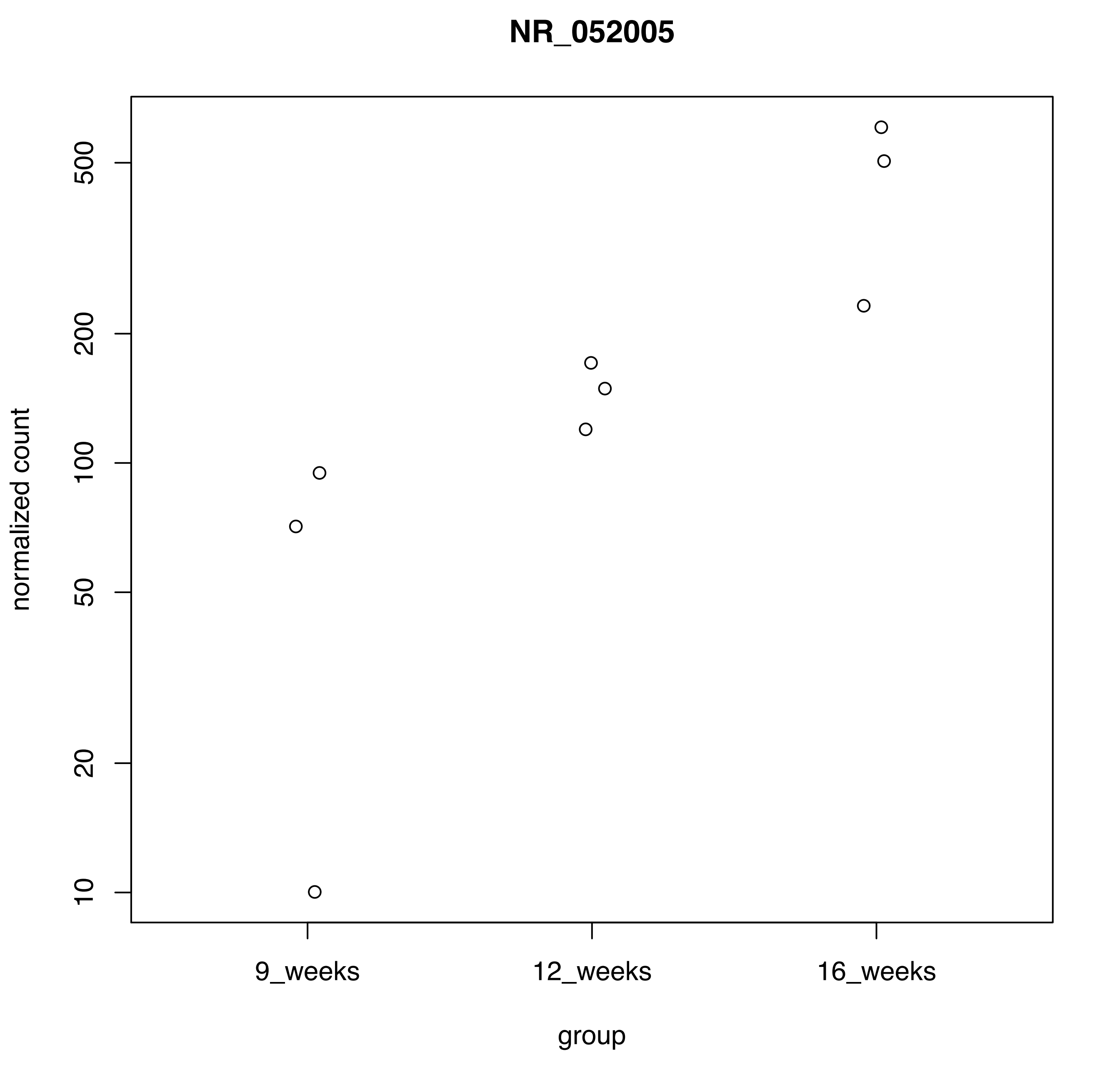
NR_052005
normalized count
500
200
100
50
20
10
9_weeks
12_weeks
16_weeks
group

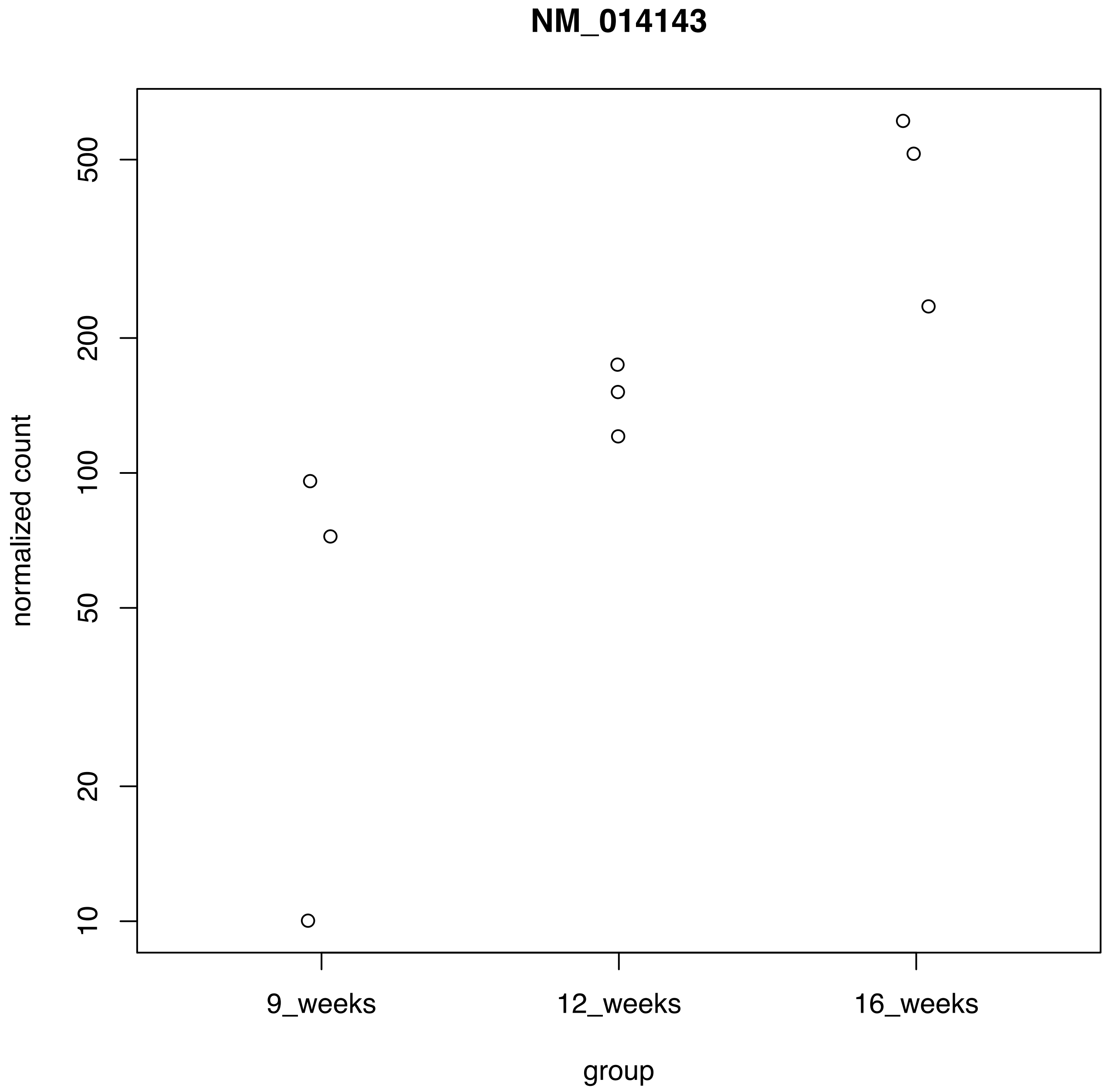
NM_014143
normalized count
500
200
100
50
20
10
9_weeks
12_weeks
16_weeks
group

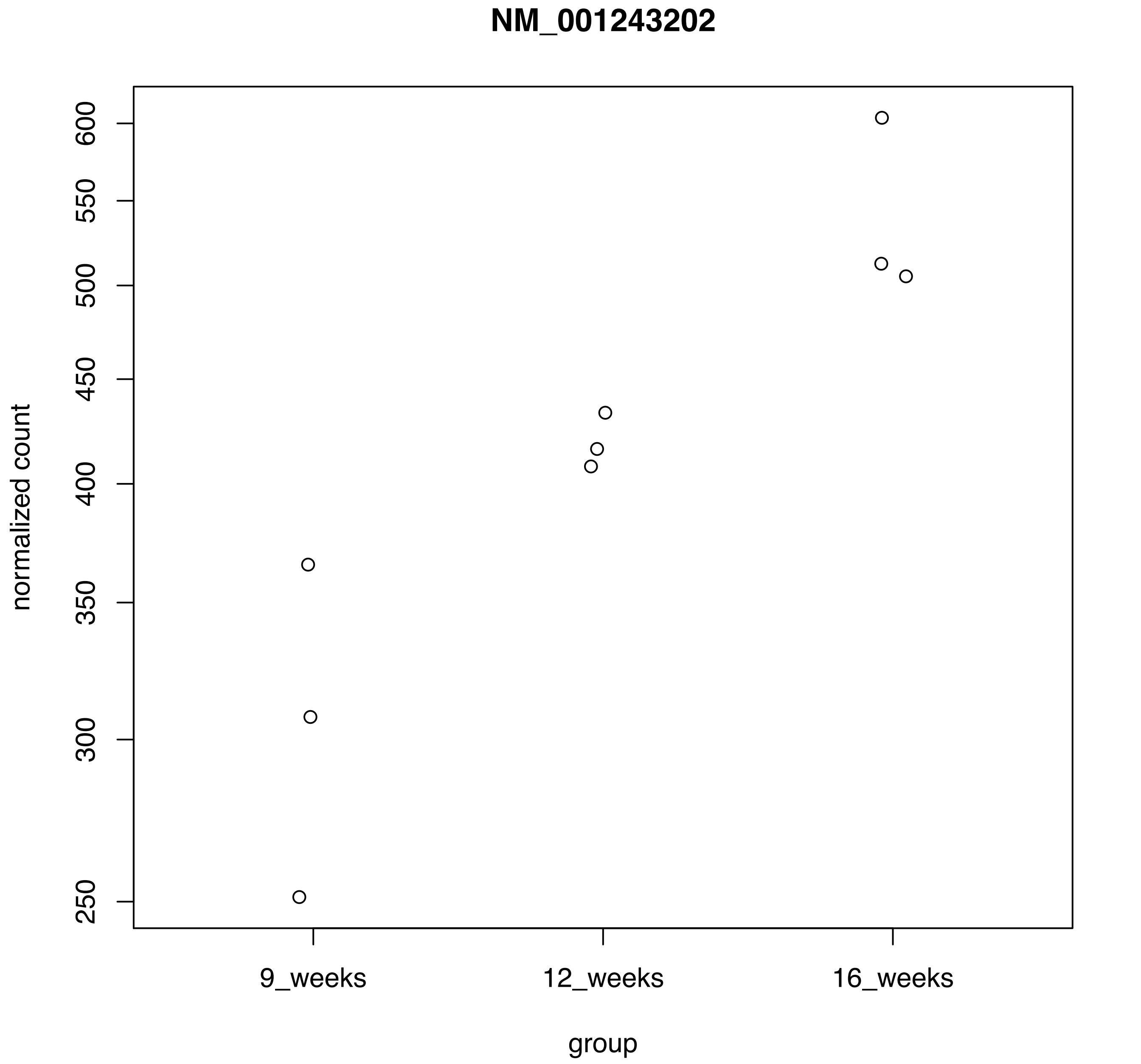

NM_001243202
normalized count
250
300
350
400
450
500
550
600
9_weeks
12_weeks
16_weeks
group

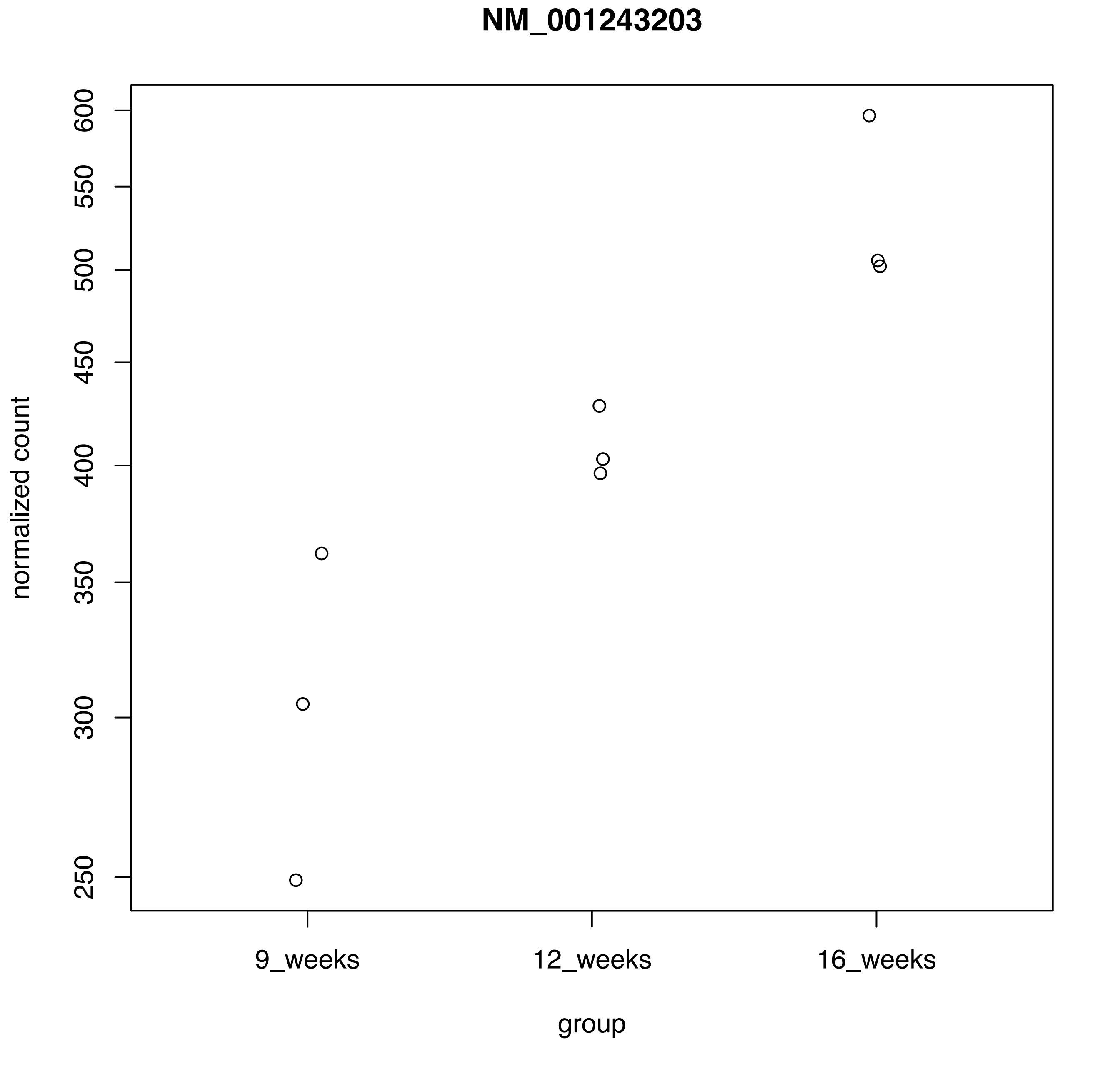
NM_001243203
normalized count
600
550
500
450
400
350
300
250
9_weeks
12_weeks
16_weeks
group

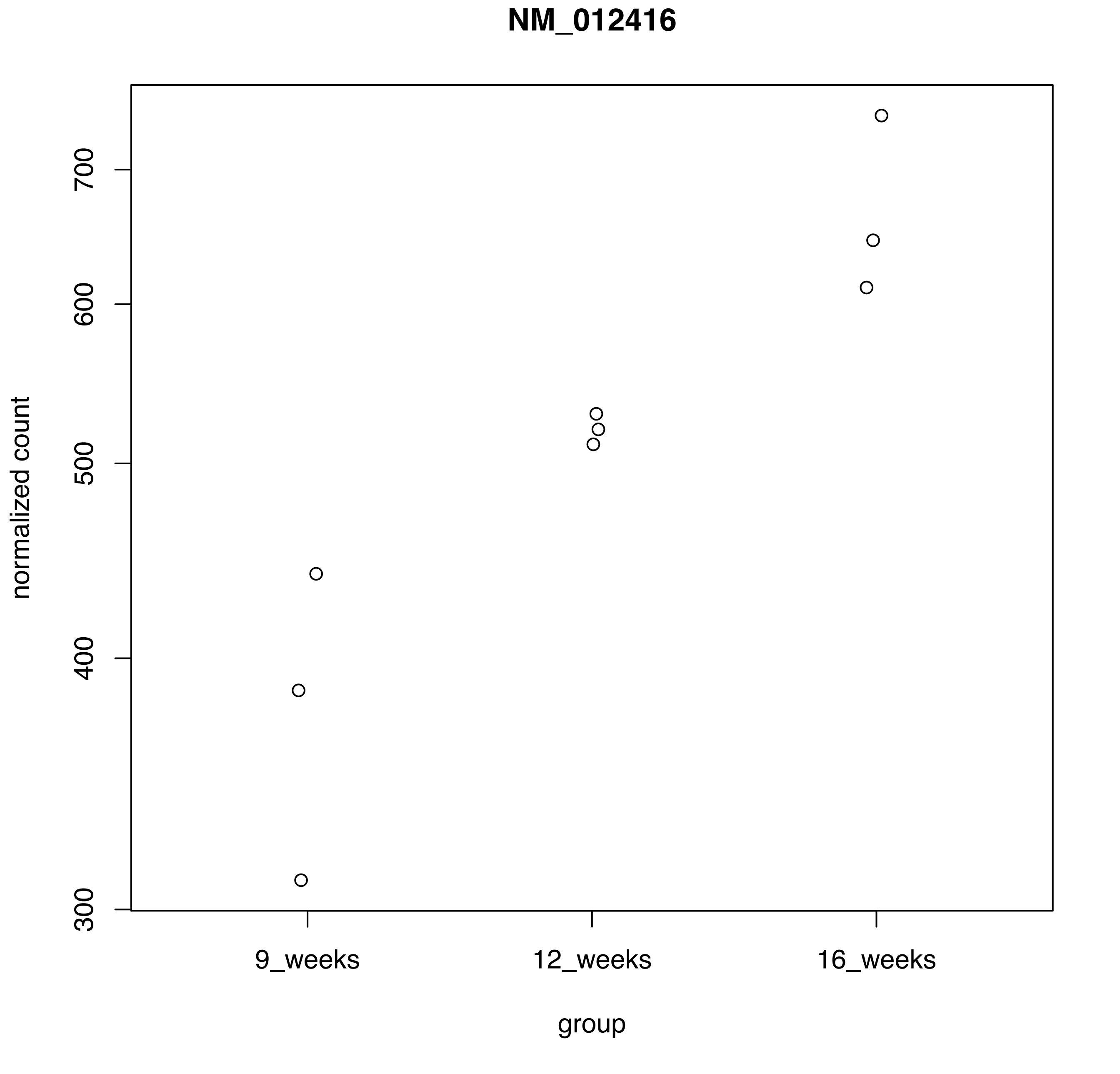
NM_012416
normalized count
700
600
500
400
300
9_weeks
12_weeks
16_weeks
group

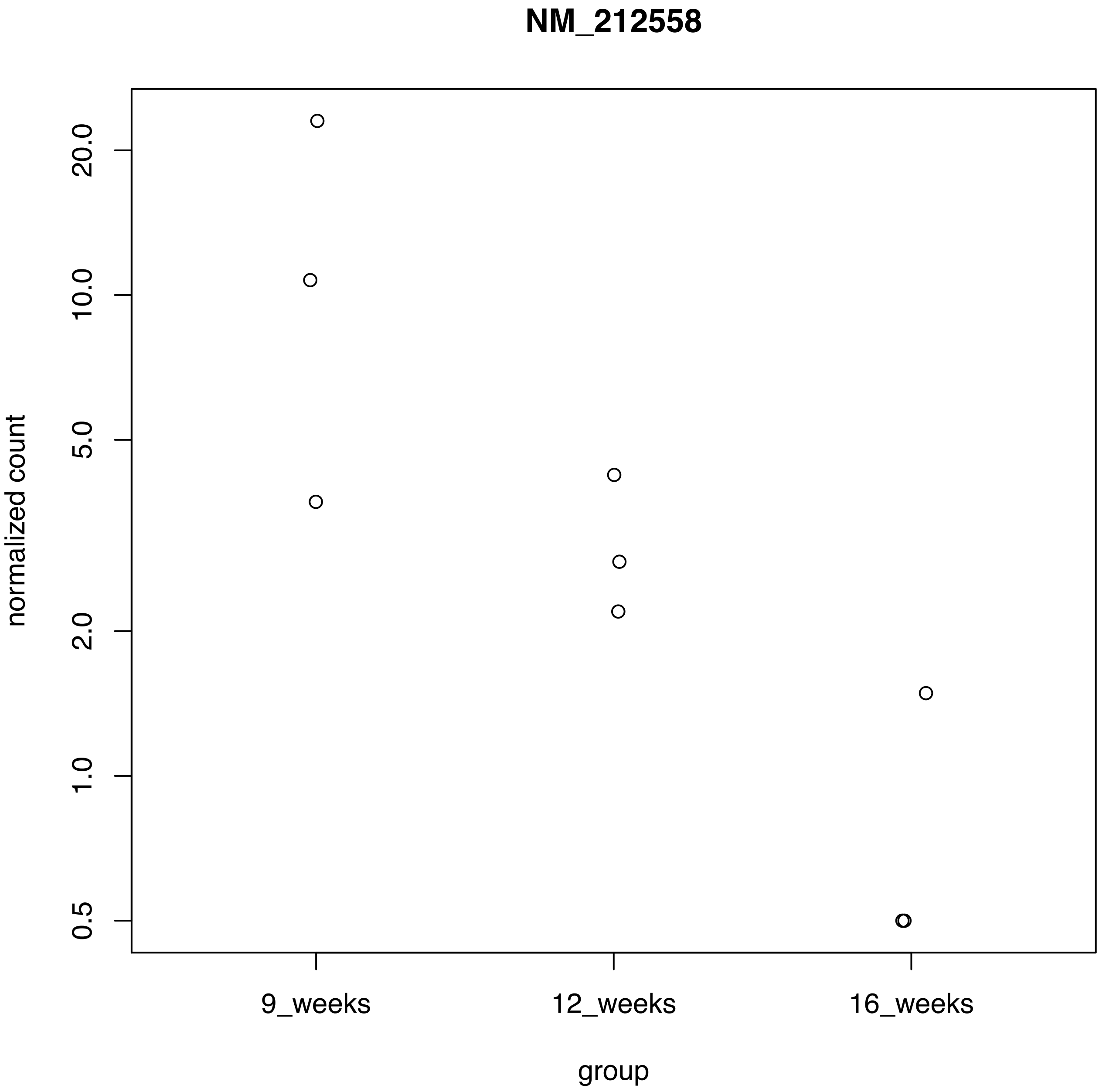

NM_212558
normalized count
20.0
10.0
5.0
2.0
1.0
0.5
9_weeks
12_weeks
16_weeks
group

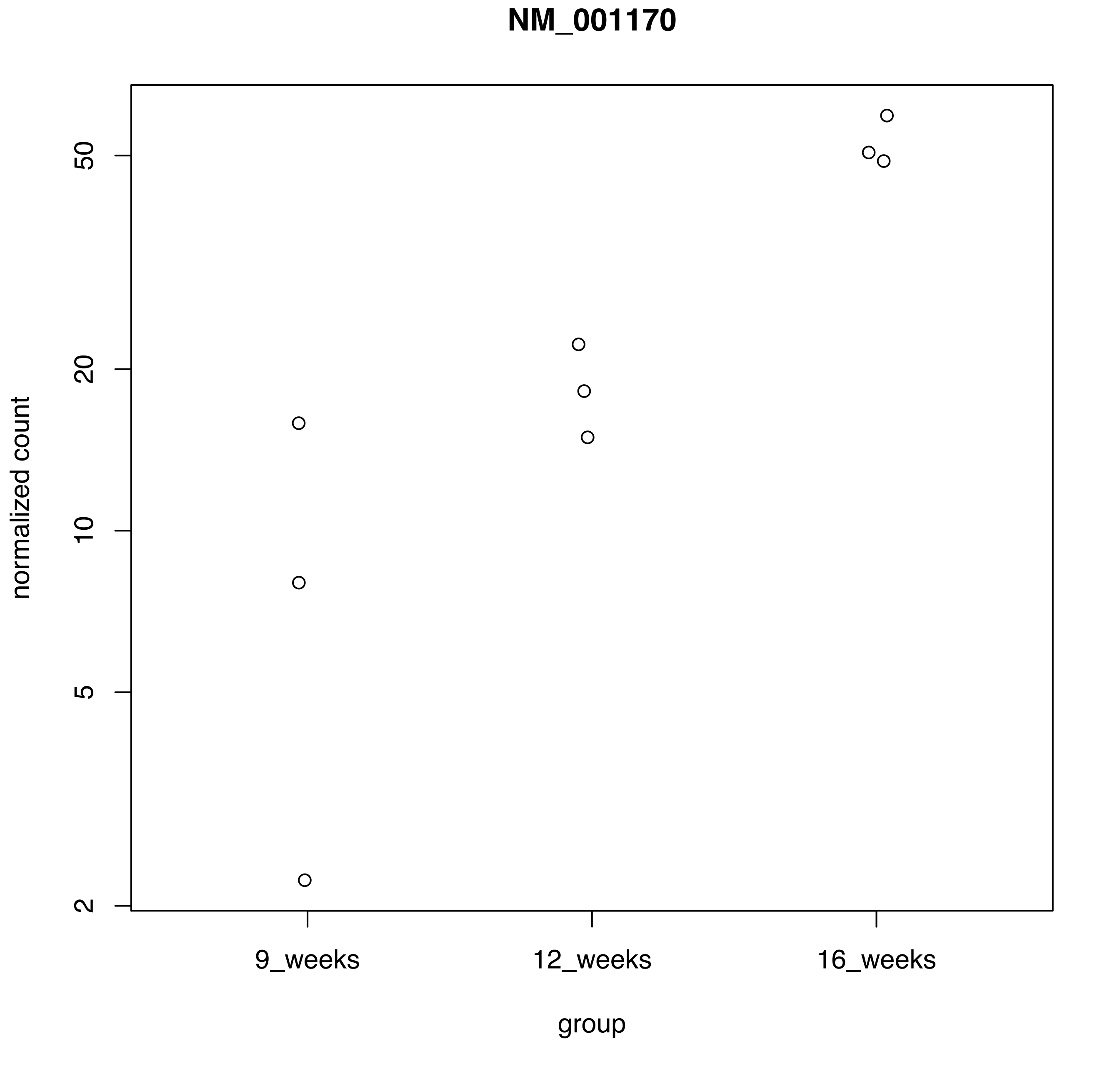
NM_001170
normalized count
50
20
10
5
2
9_weeks
12_weeks
16_weeks
group

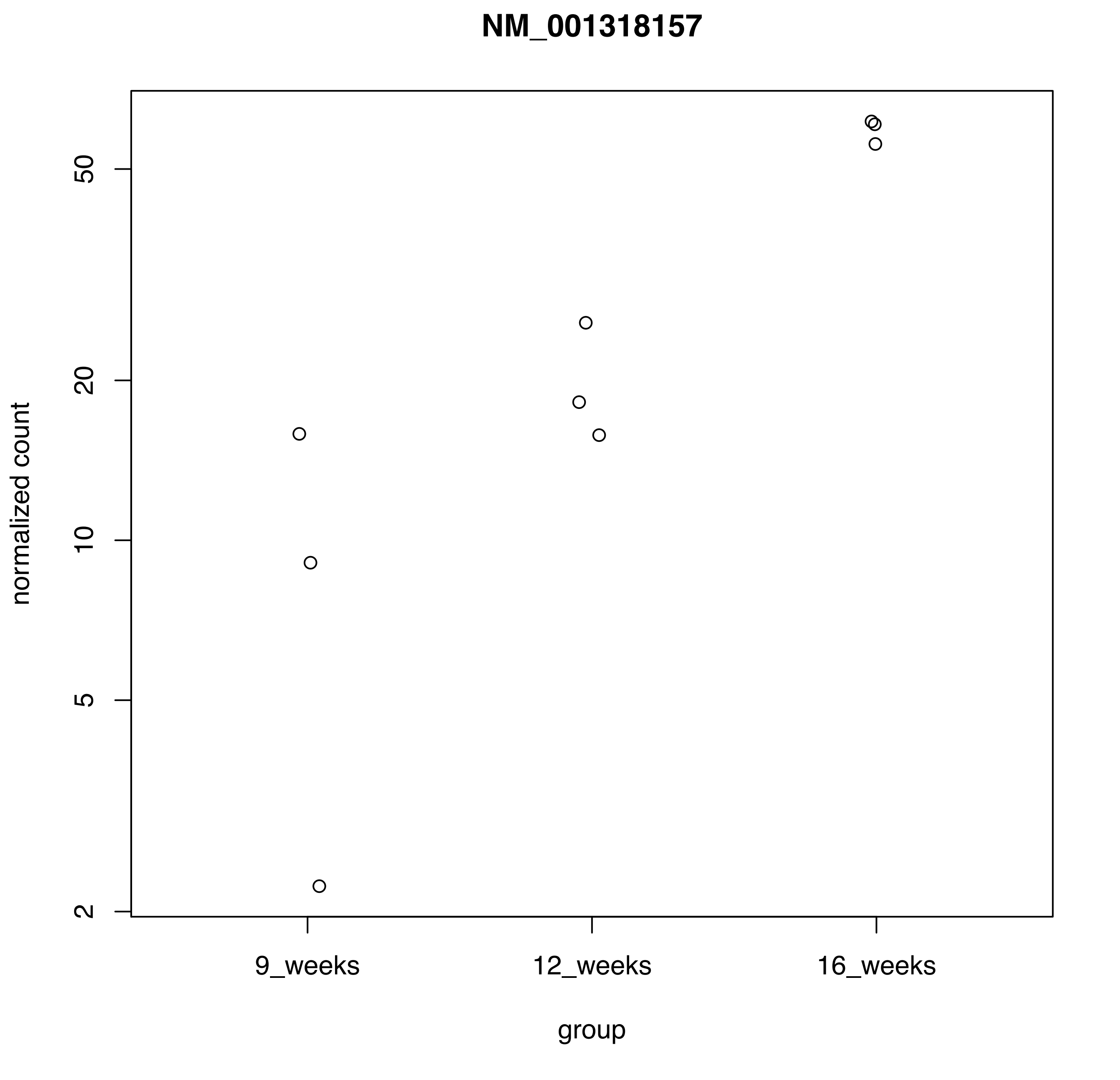
NM_001318157
normalized count
50
20
10
5
2
9_weeks
12_weeks
16_weeks
group

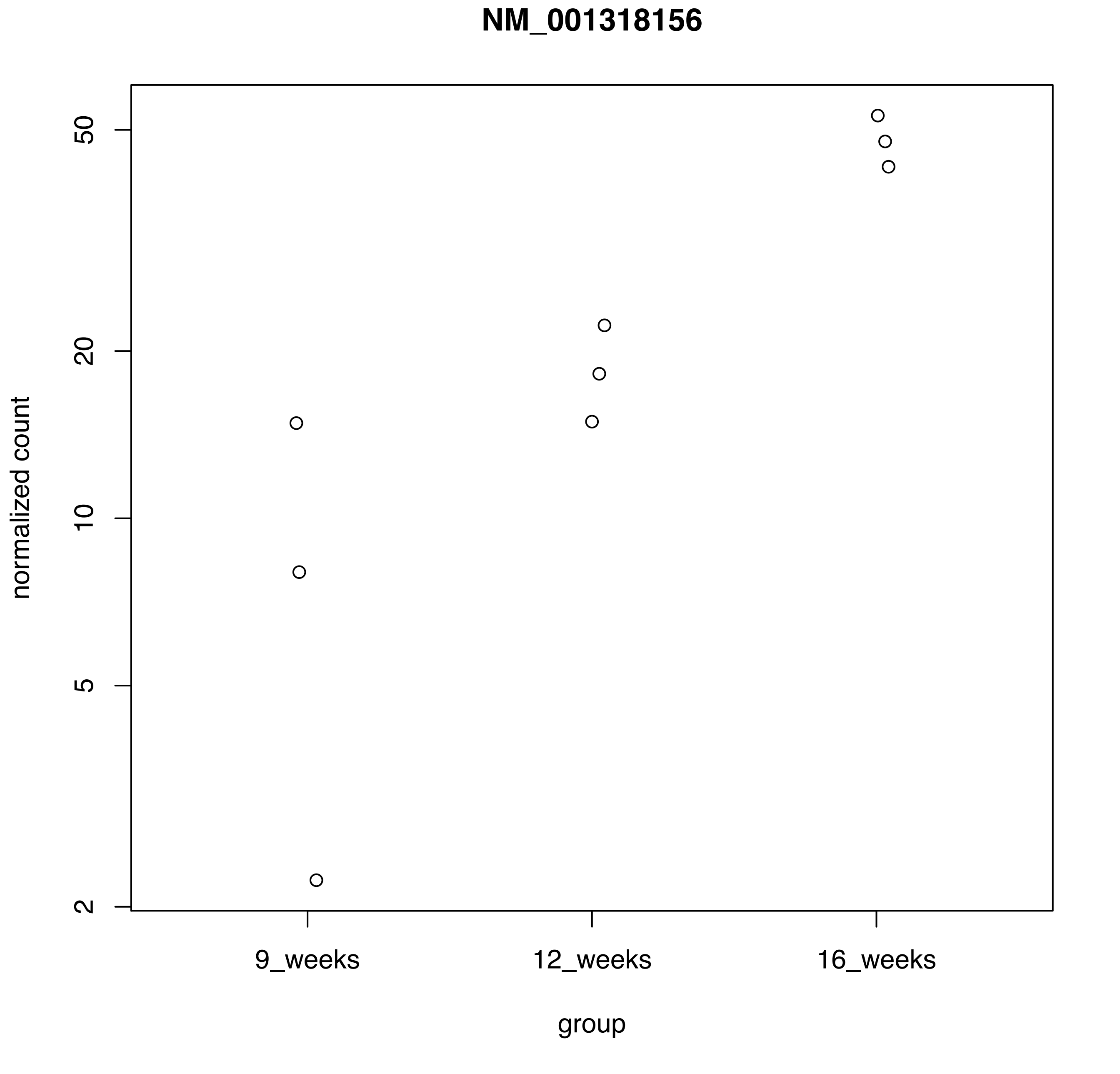

NM_001318156
normalized count
50
20
10
5
2
9_weeks
12_weeks
16_weeks
group

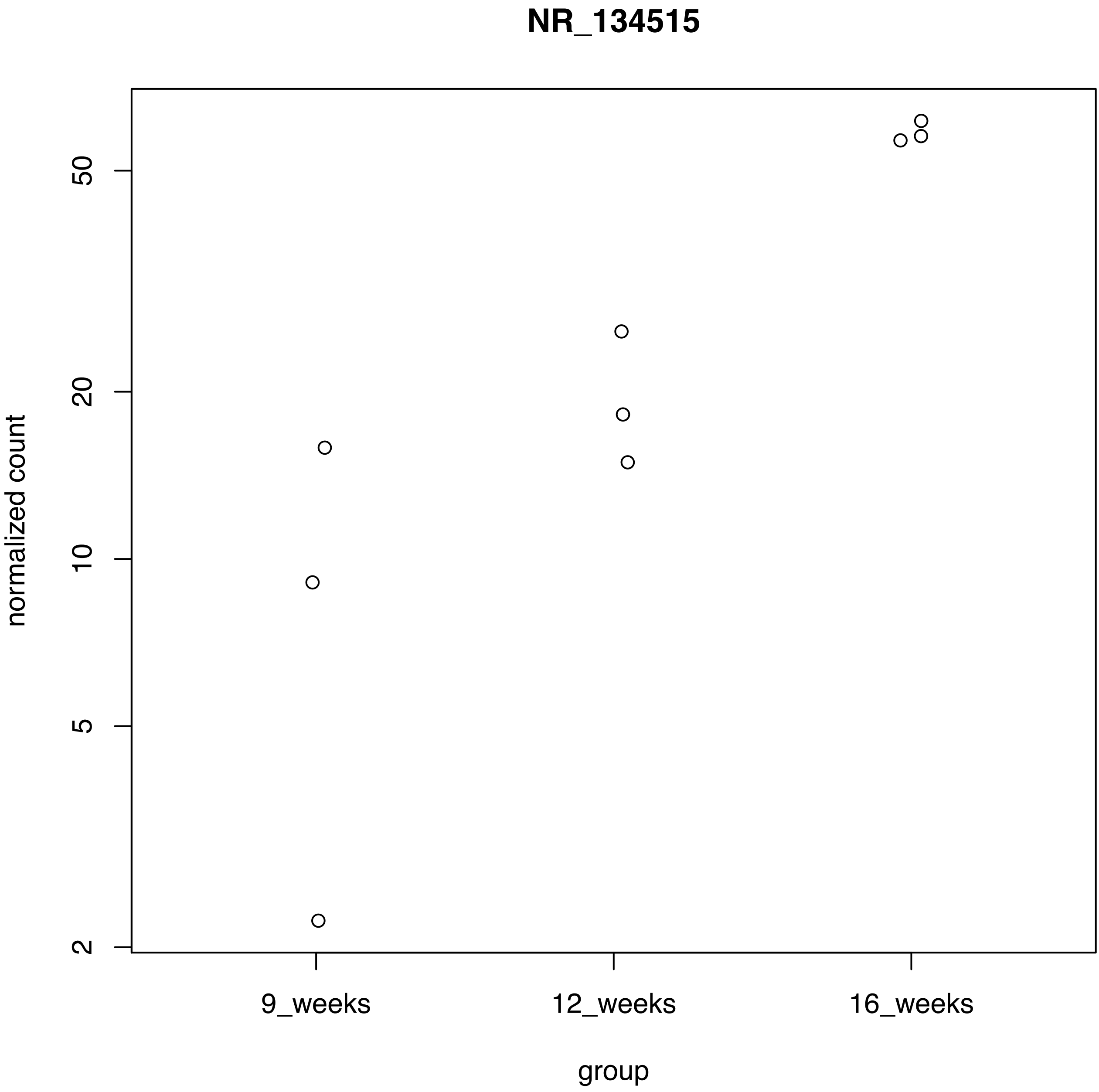
NR_134515
normalized count
50
20
10
5
2
9_weeks
12_weeks
16_weeks
group

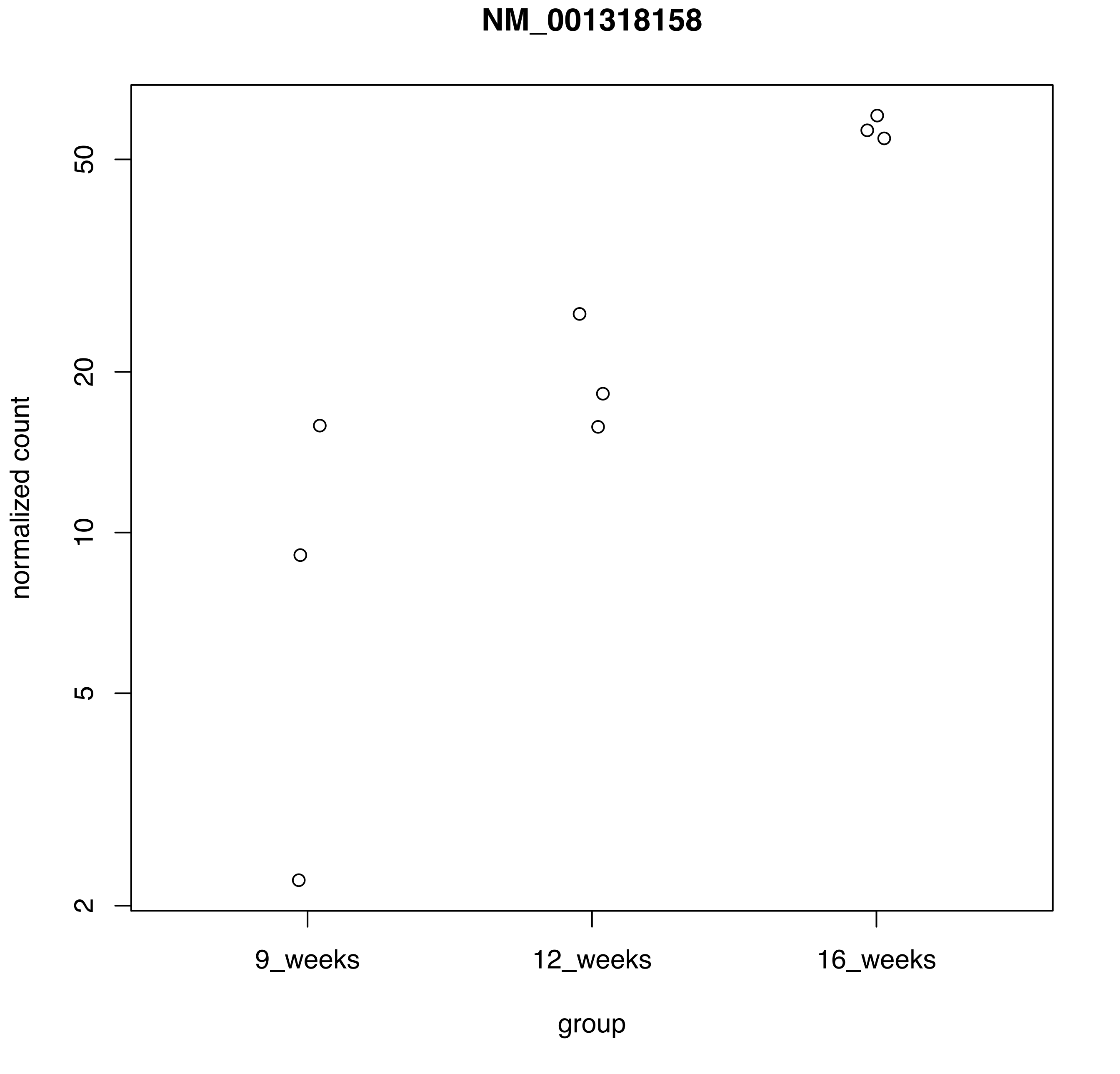
NM_001318158
normalized count
50
20
10
5
2
9_weeks
12_weeks
16_weeks
group

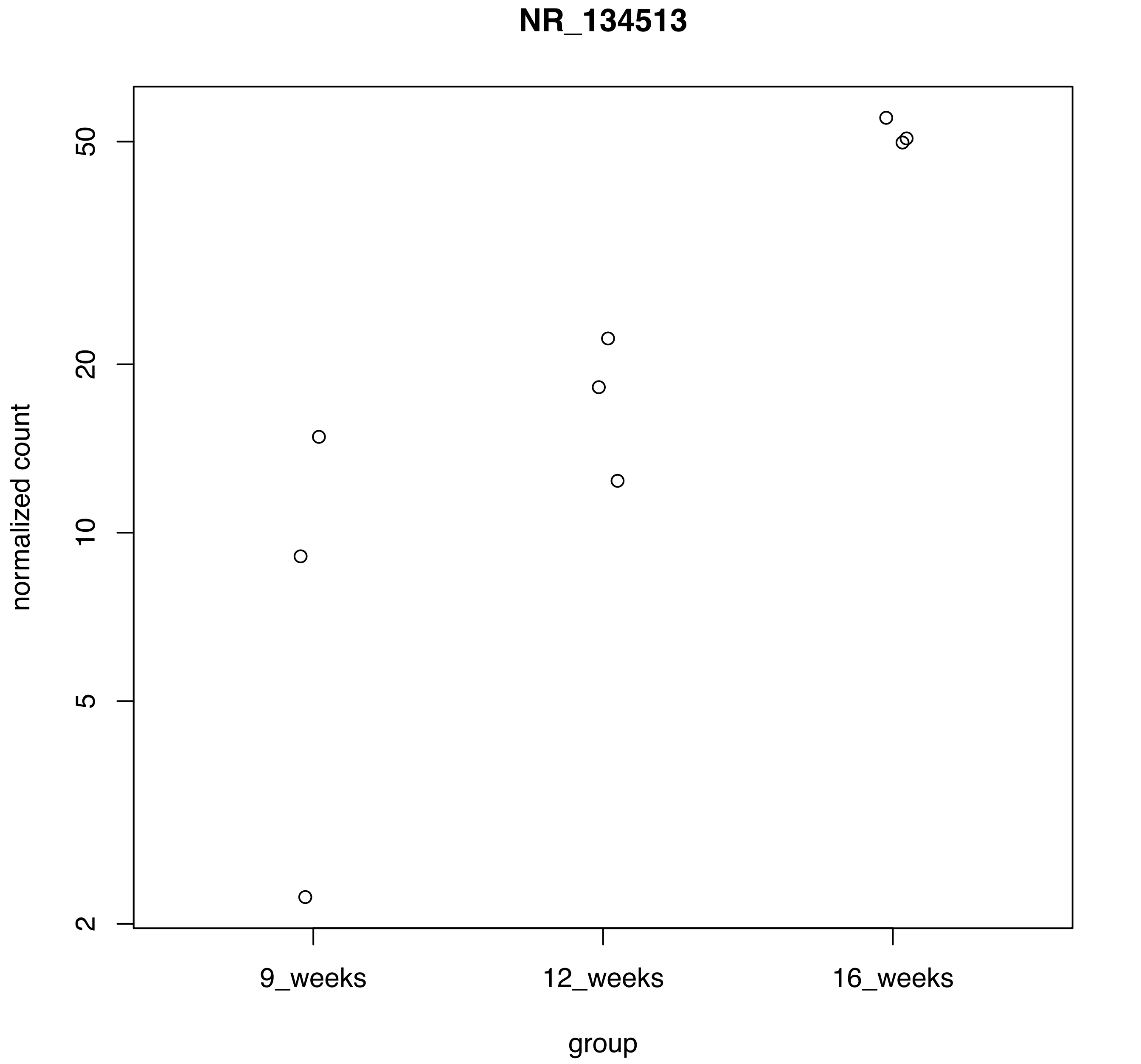
NR_134513
normalized count
50
20
10
5
2
9_weeks
12_weeks
16_weeks
group

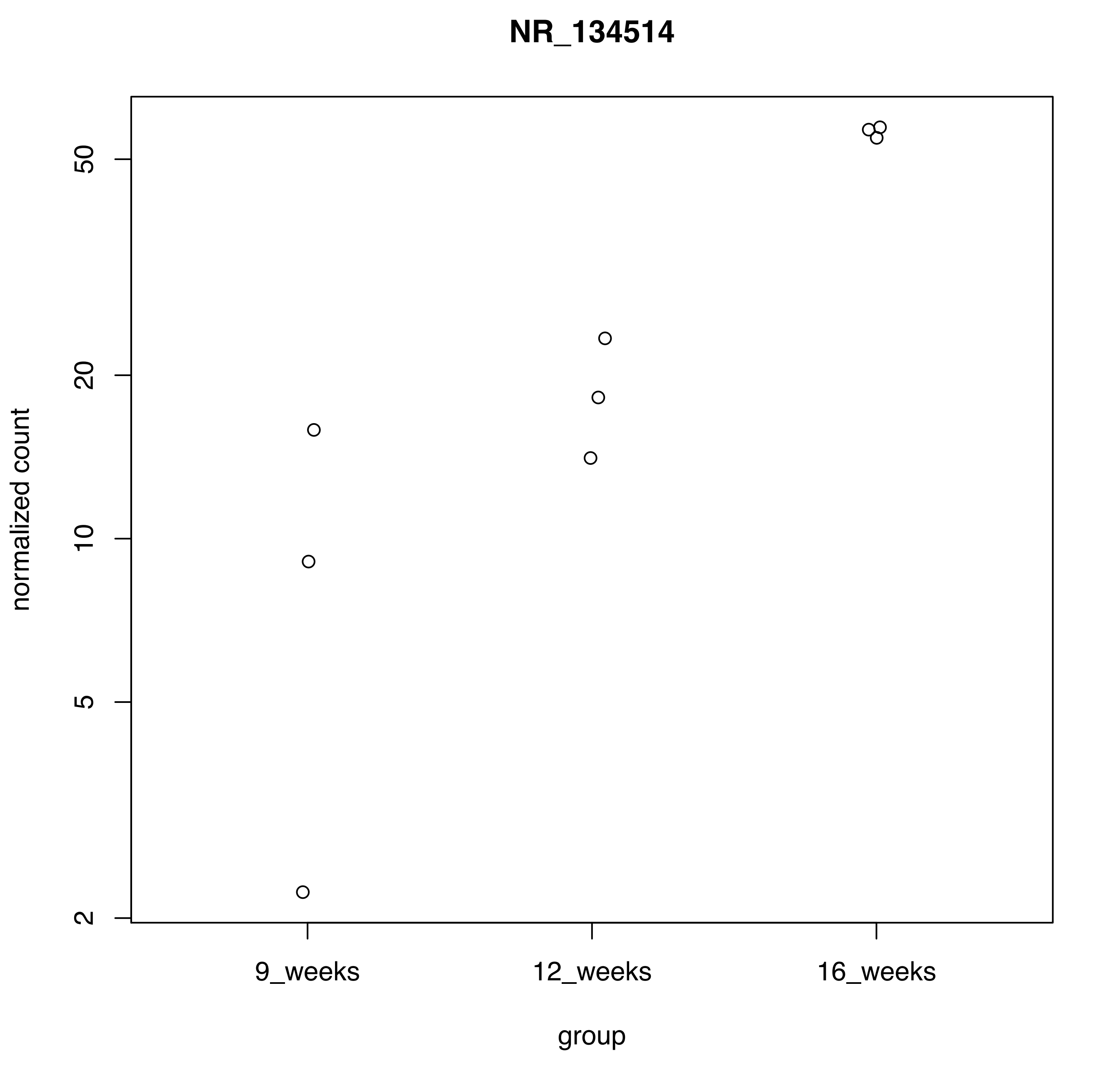

NR_134514
normalized count
2
5
10
20
50
9_weeks
12_weeks
16_weeks
group

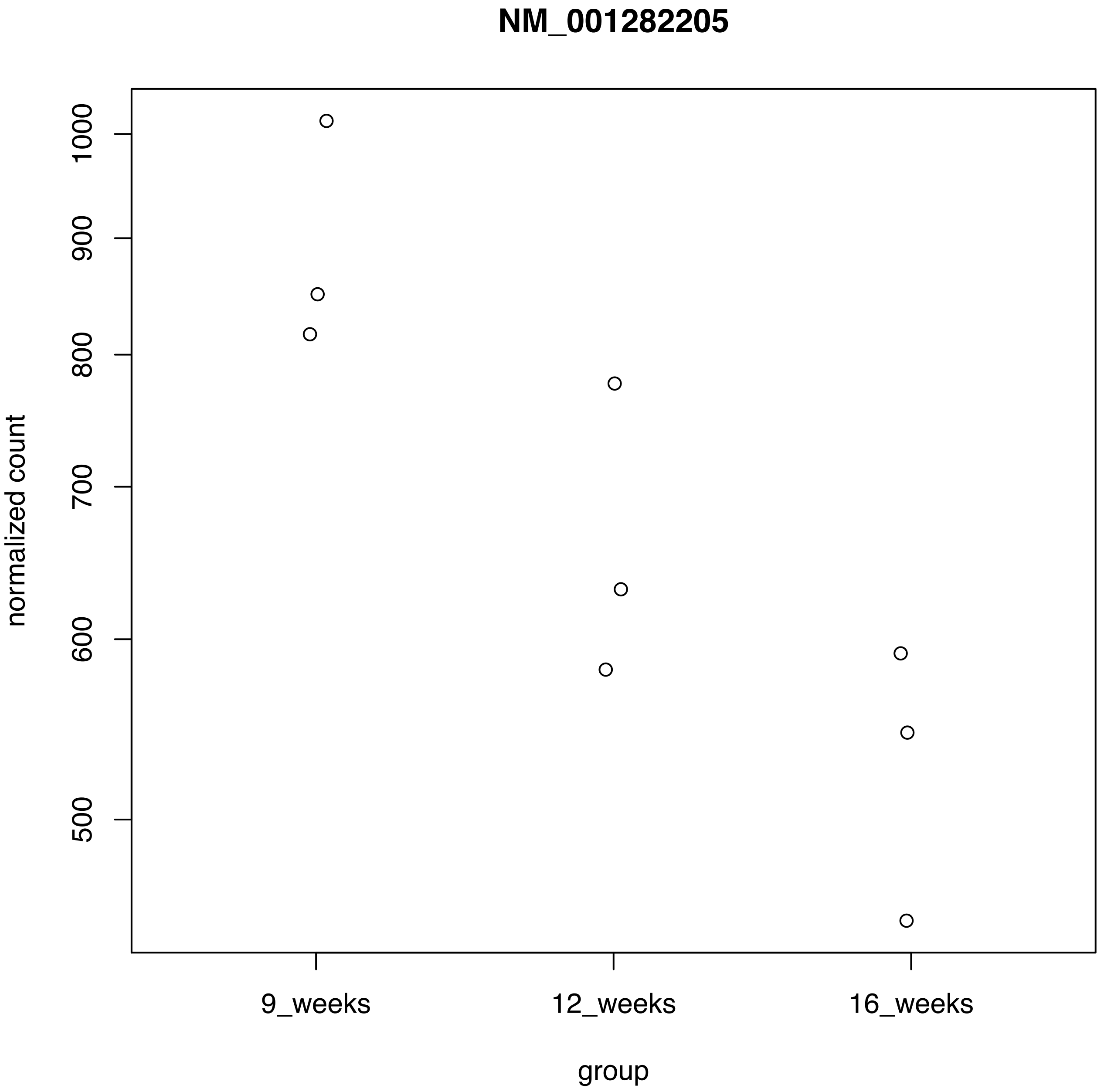
NM_001282205
normalized count
1000
900
800
700
600
500
9_weeks
12_weeks
16_weeks
group

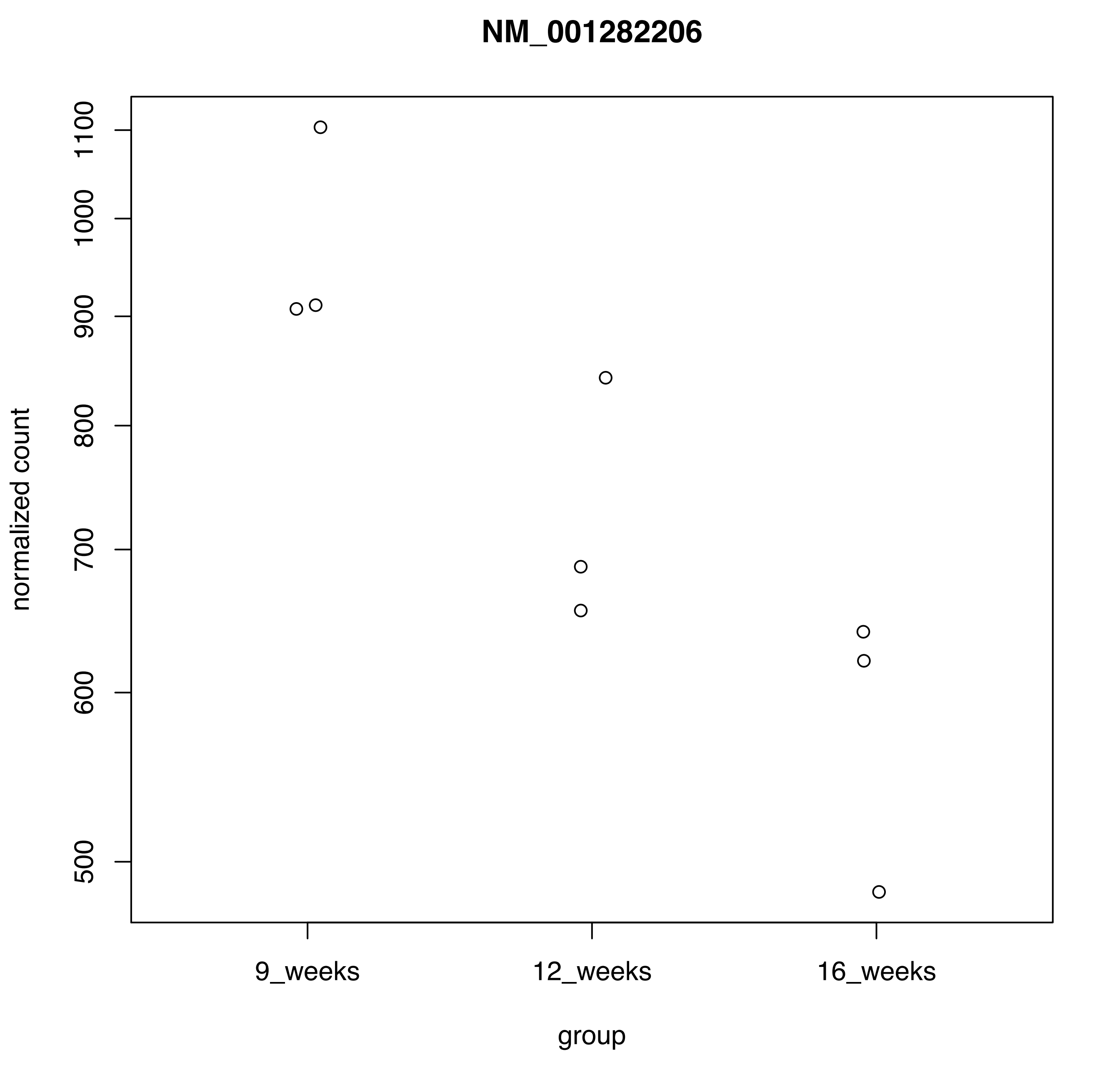
NM_001282206
normalized count
1100
1000
900
800
700
600
500
9_weeks
12_weeks
16_weeks
group

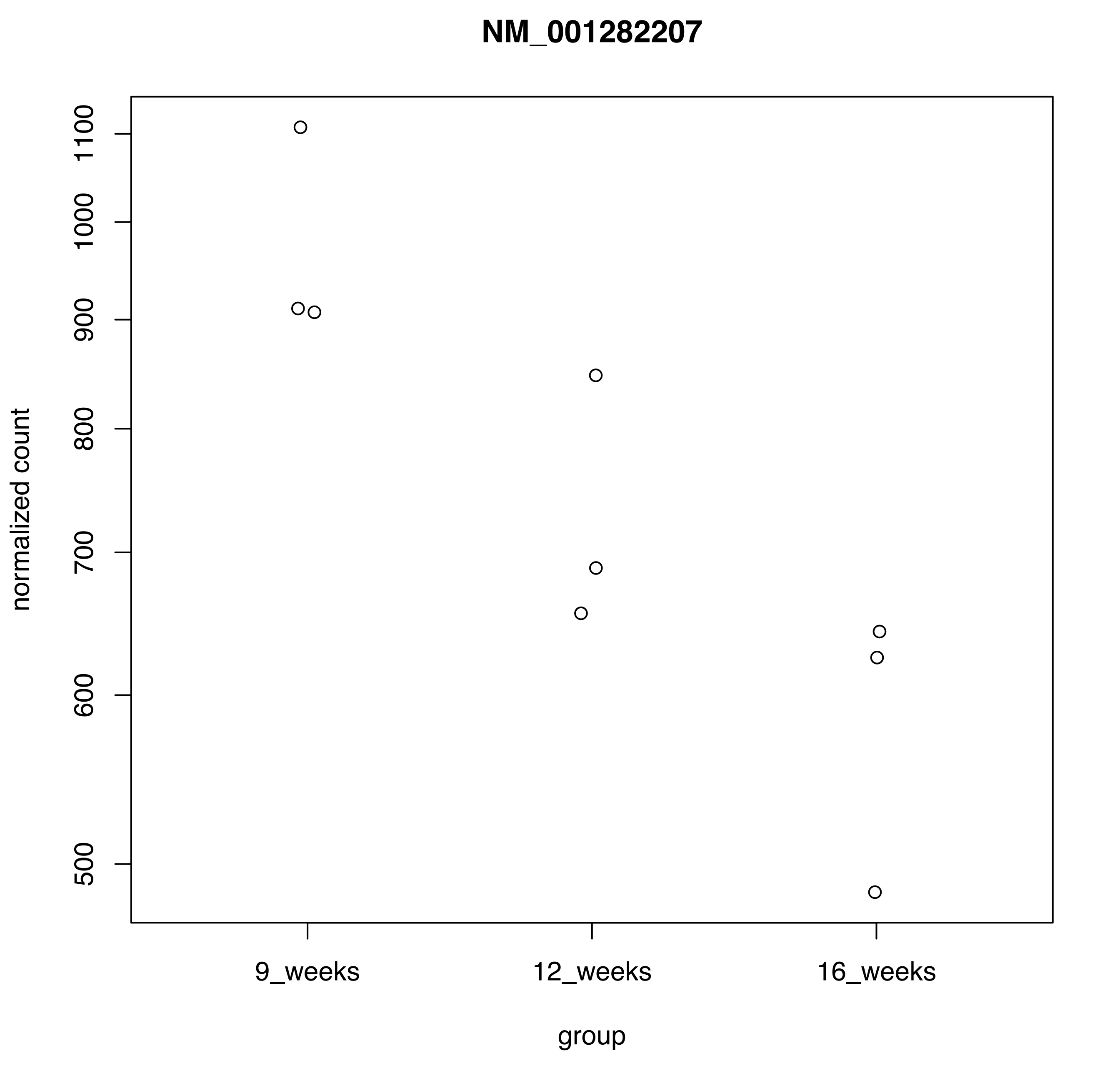

NM_001282207
normalized count
1100
1000
900
800
700
600
500
9_weeks
12_weeks
16_weeks
group

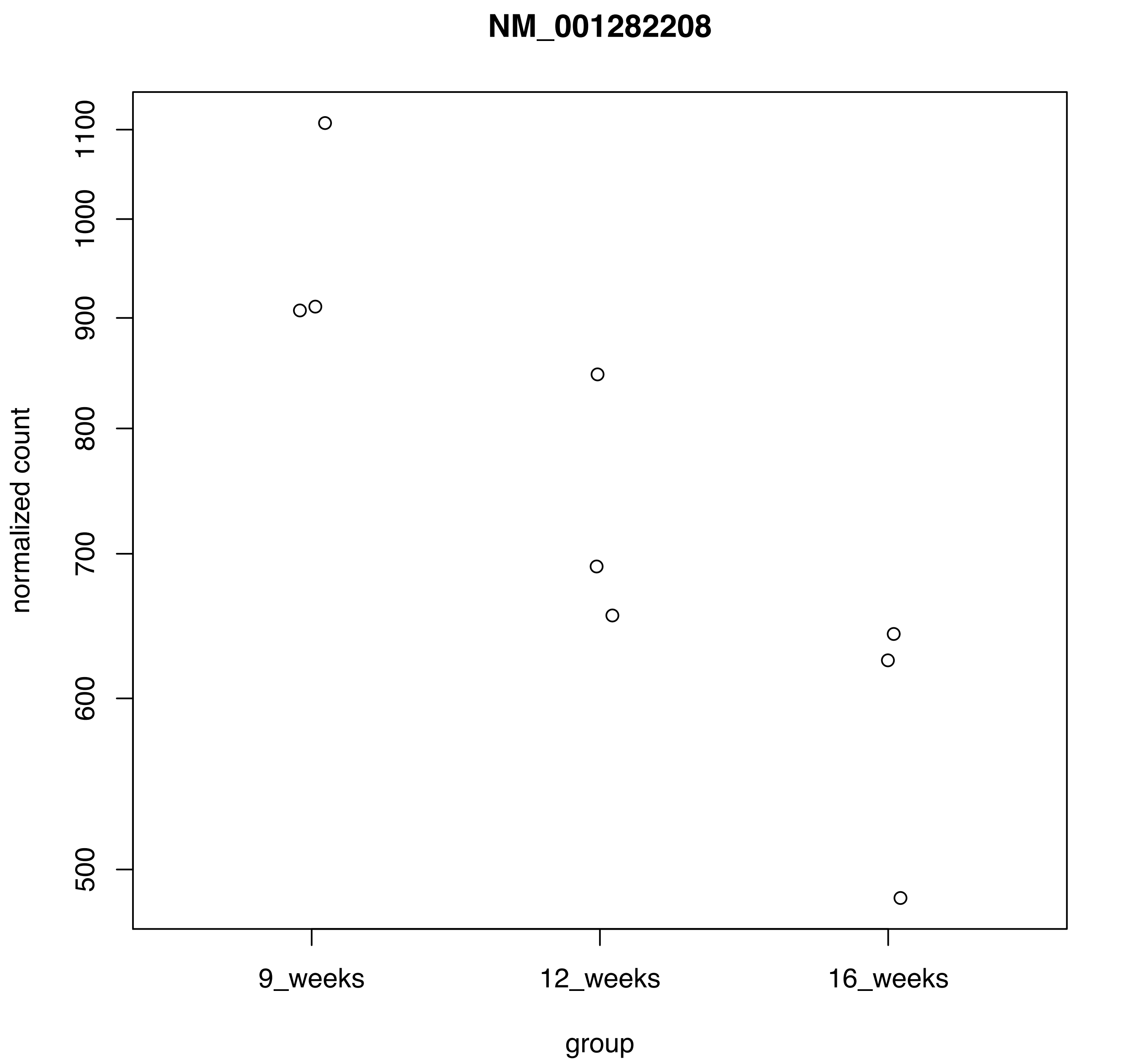

NM_001282208
normalized count
1100
1000
900
800
700
600
500
9_weeks
12_weeks
16_weeks
group

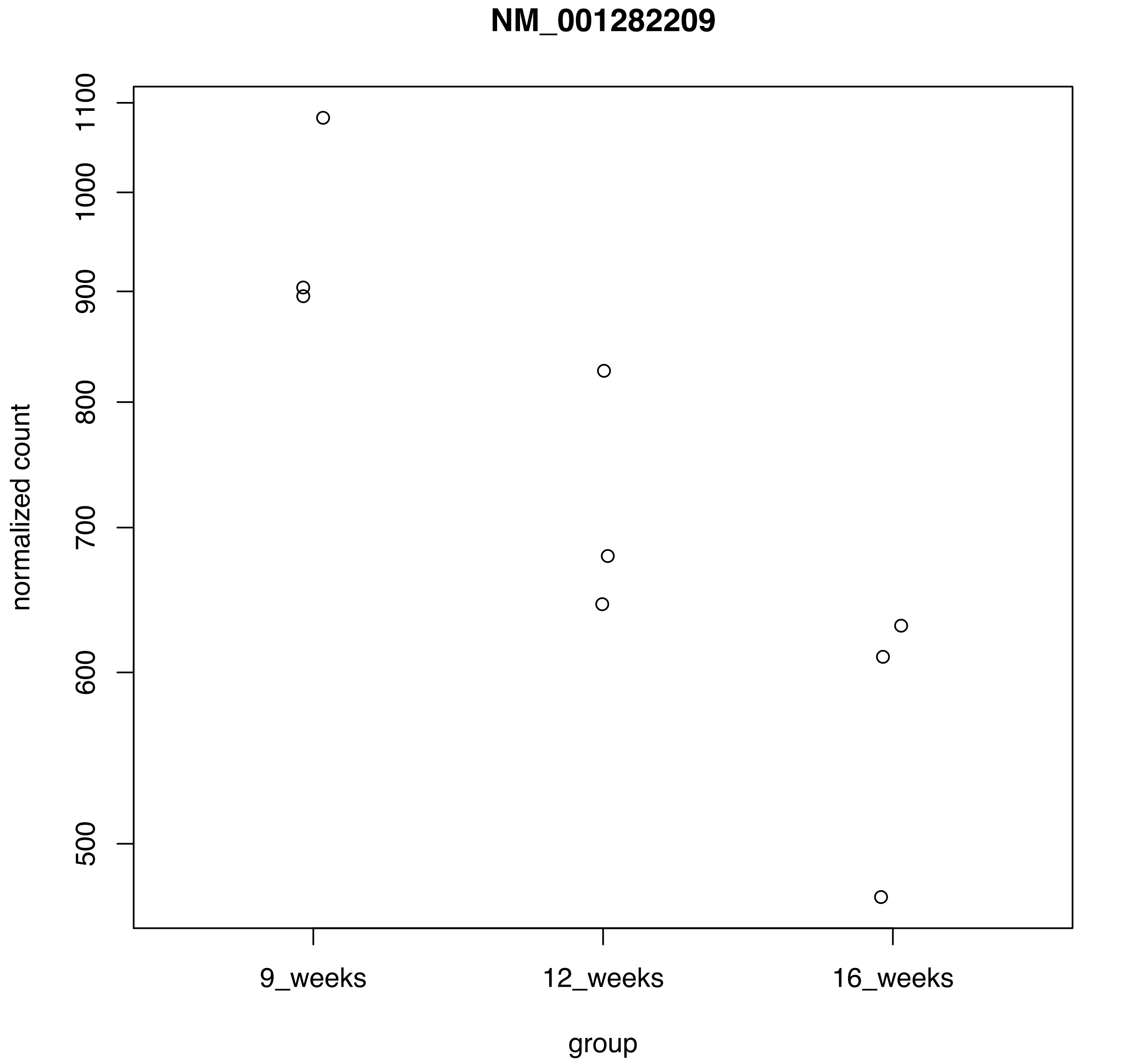
NM_001282209
normalized count
1100
1000
900
800
700
600
500
9_weeks
12_weeks
16_weeks
group

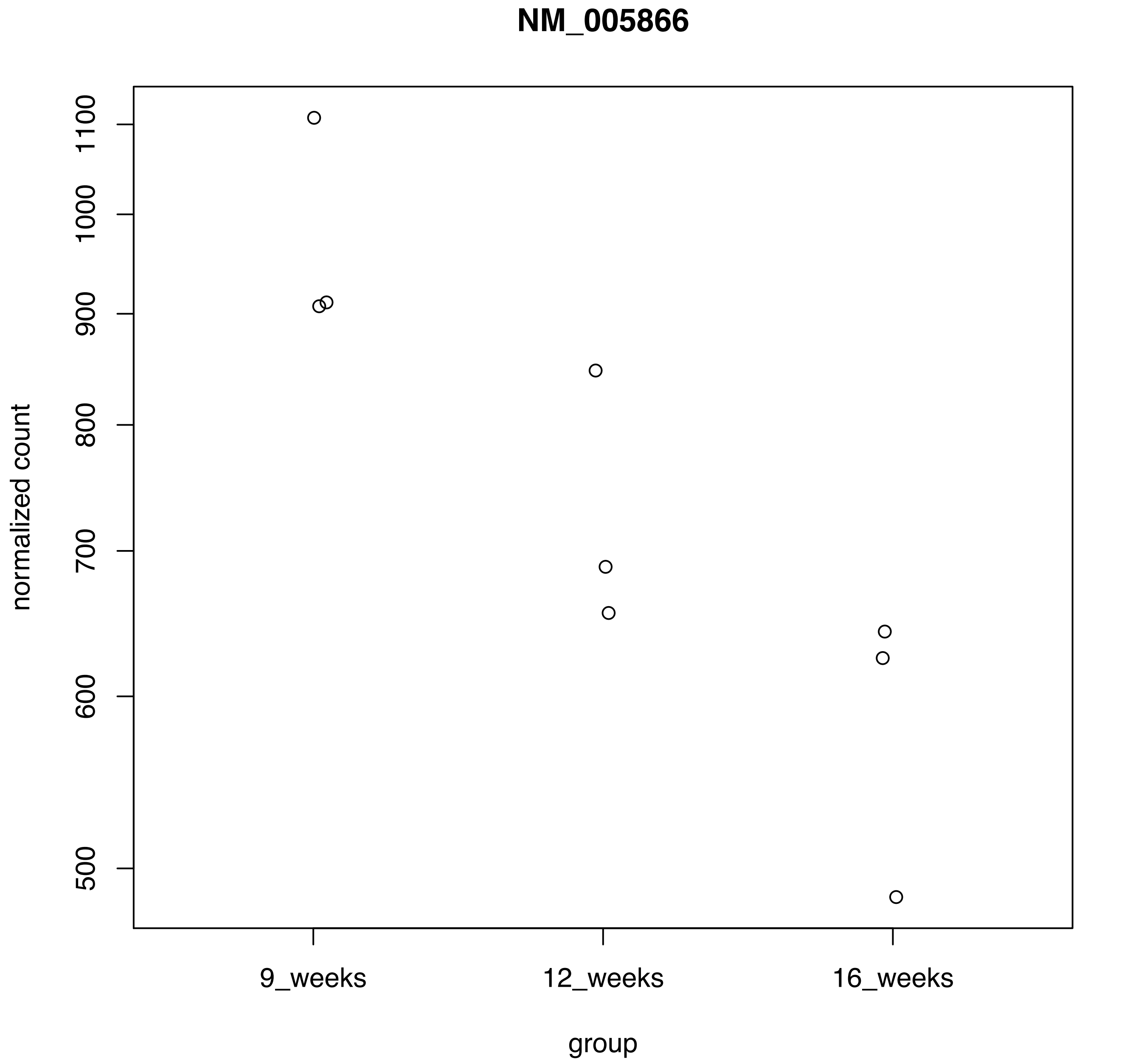

NM_005866
1100
1000
900
800
700
600
500
normalized count
9_weeks
12_weeks
16_weeks
group

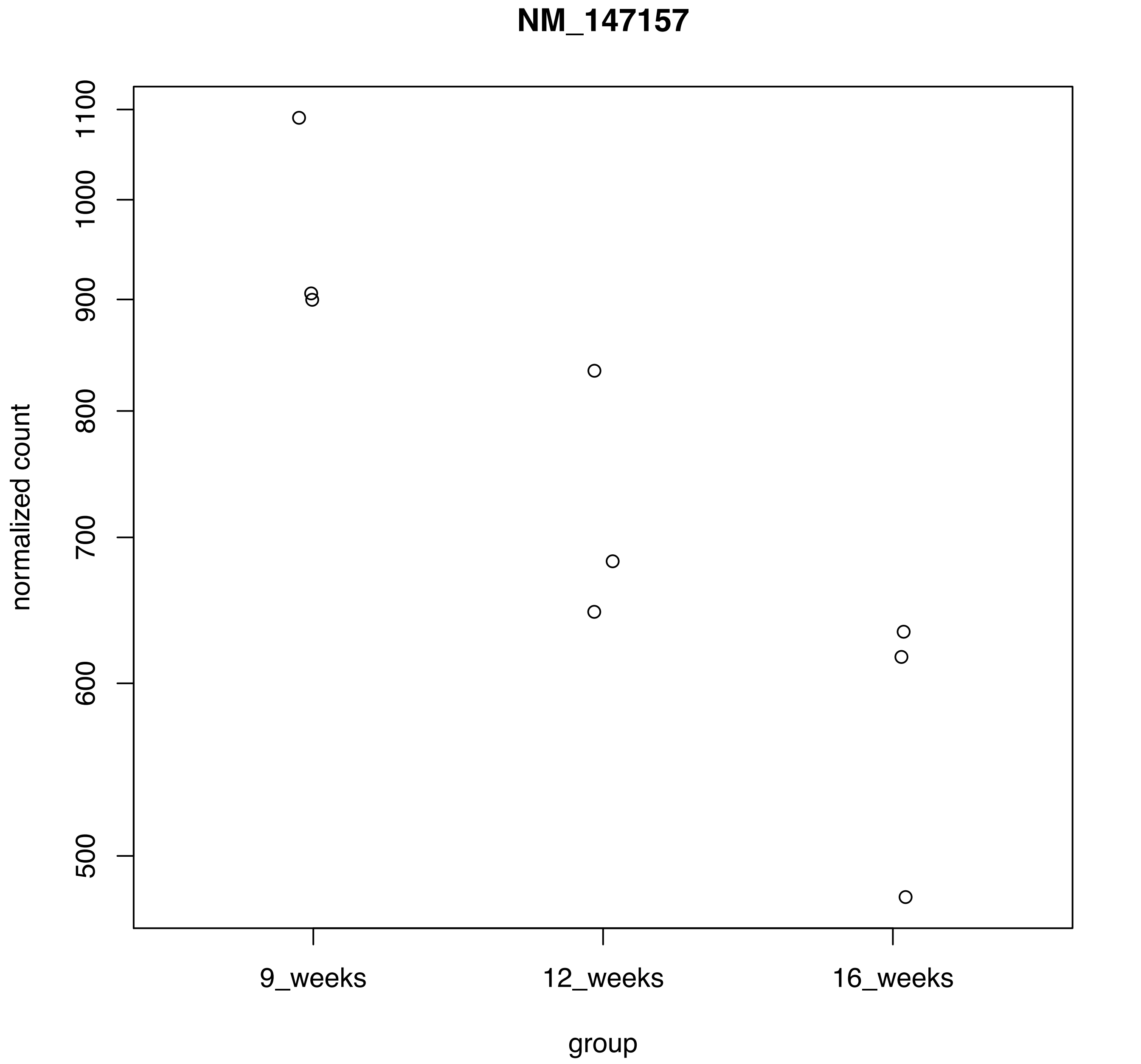
NM_147157
normalized count
1100
1000
900
800
700
600
500
9_weeks
12_weeks
16_weeks
group

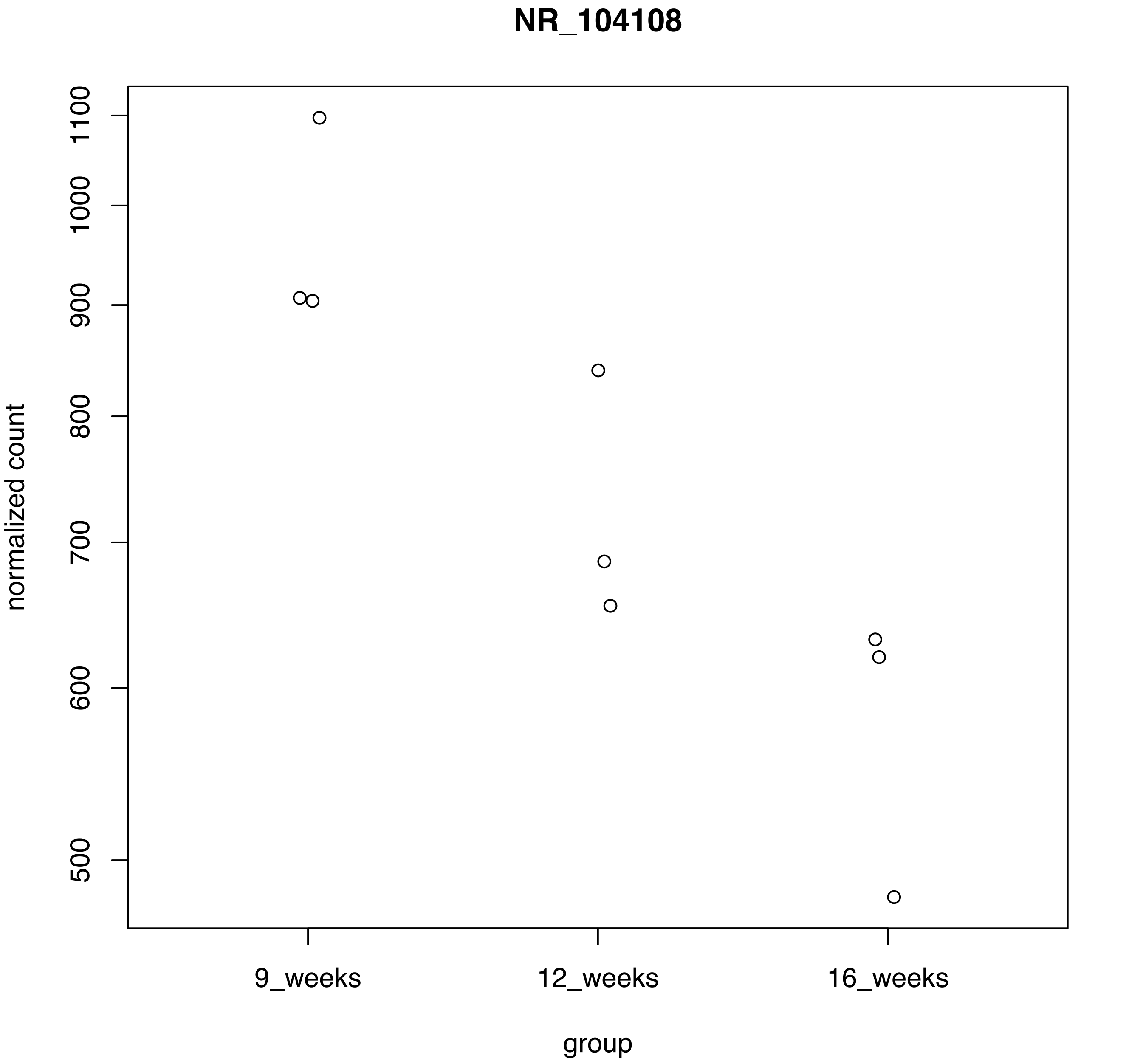
NR_104108
normalized count
1100
1000
900
800
700
600
500
9_weeks
12_weeks
16_weeks
group

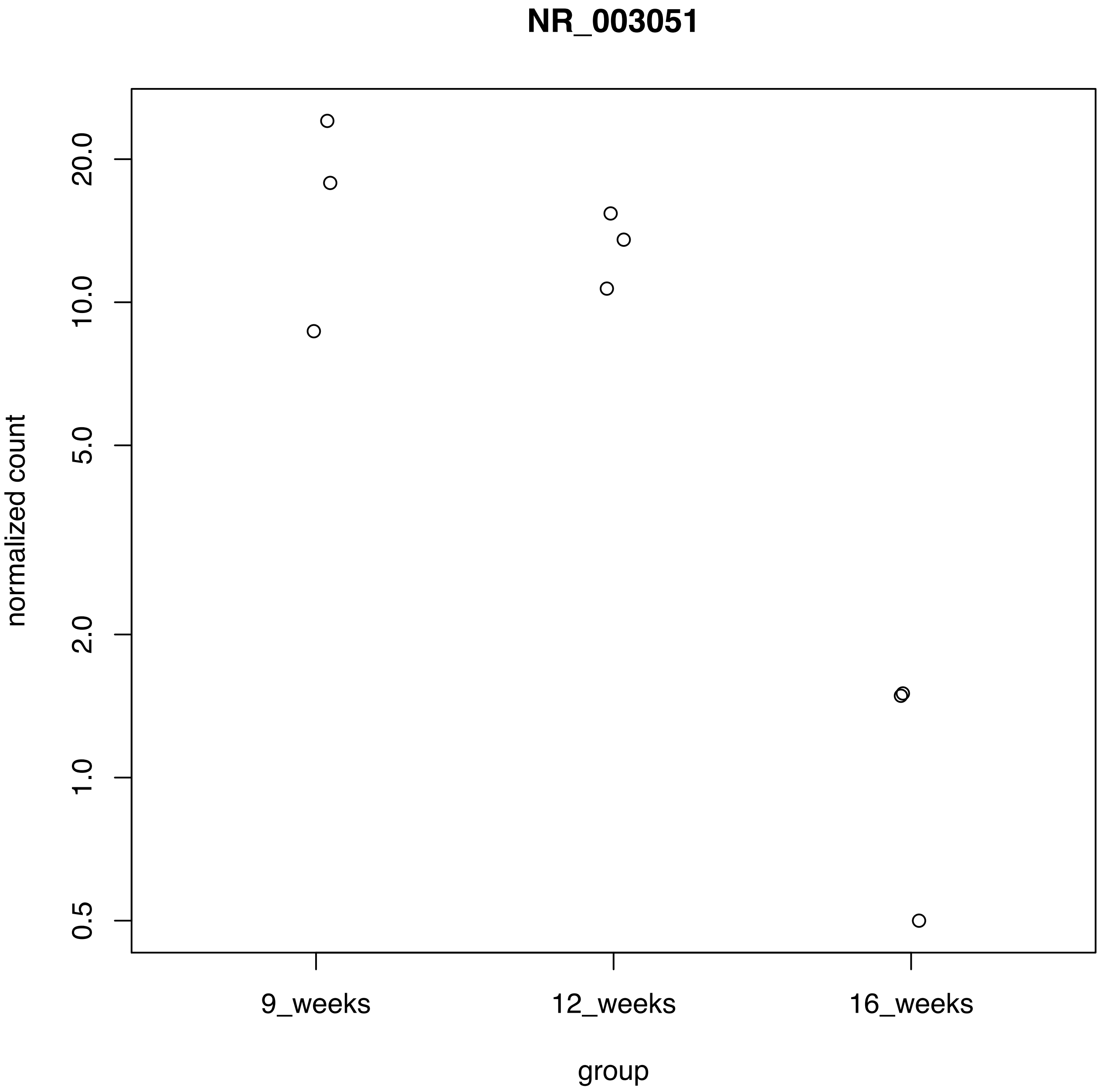
NR_003051
normalized count
20.0
10.0
5.0
2.0
1.0
0.5
9_weeks
12_weeks
16_weeks
group

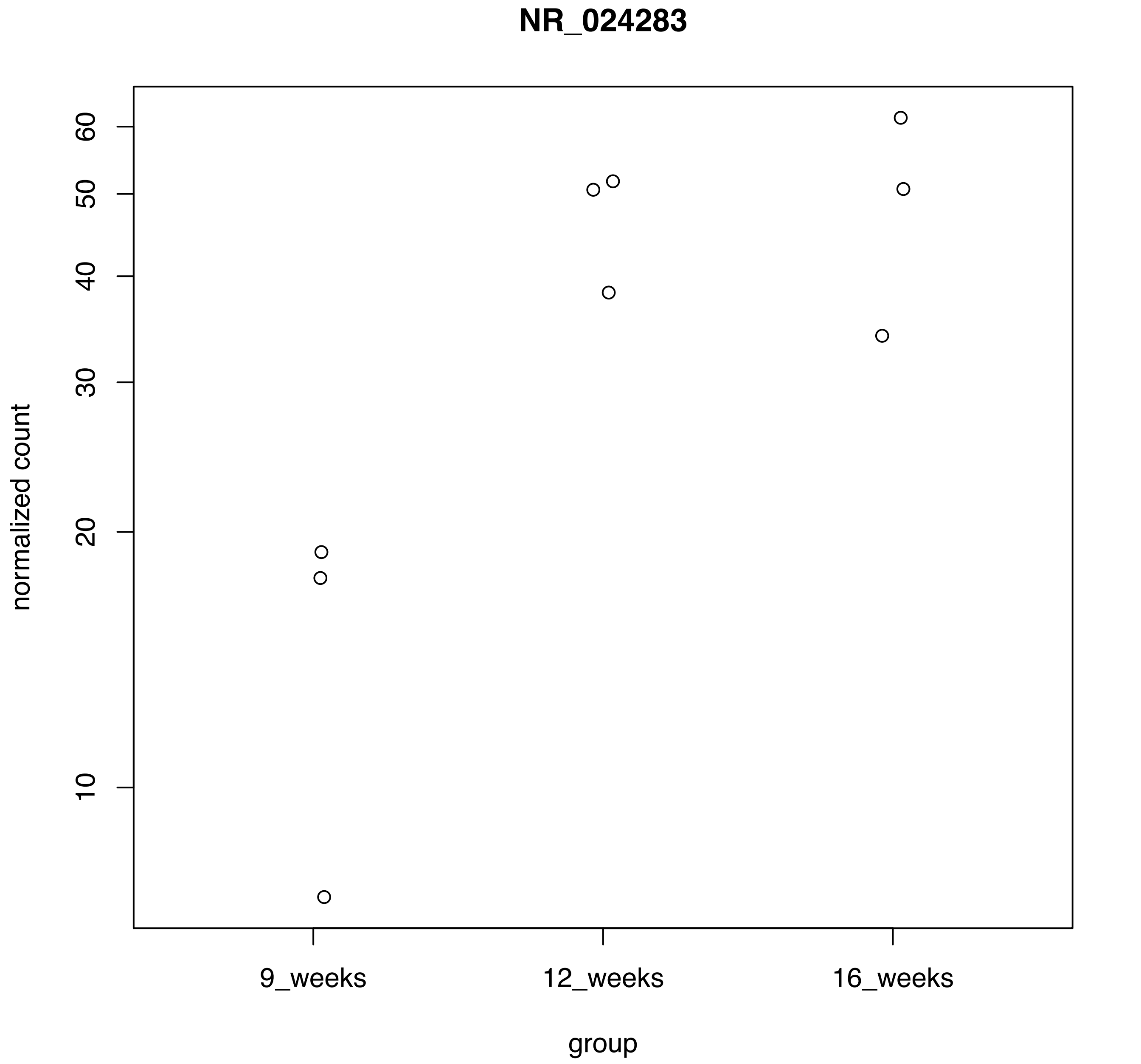

NR_024283
normalized count
10
20
30
40
50
60
9_weeks
12_weeks
16_weeks
group

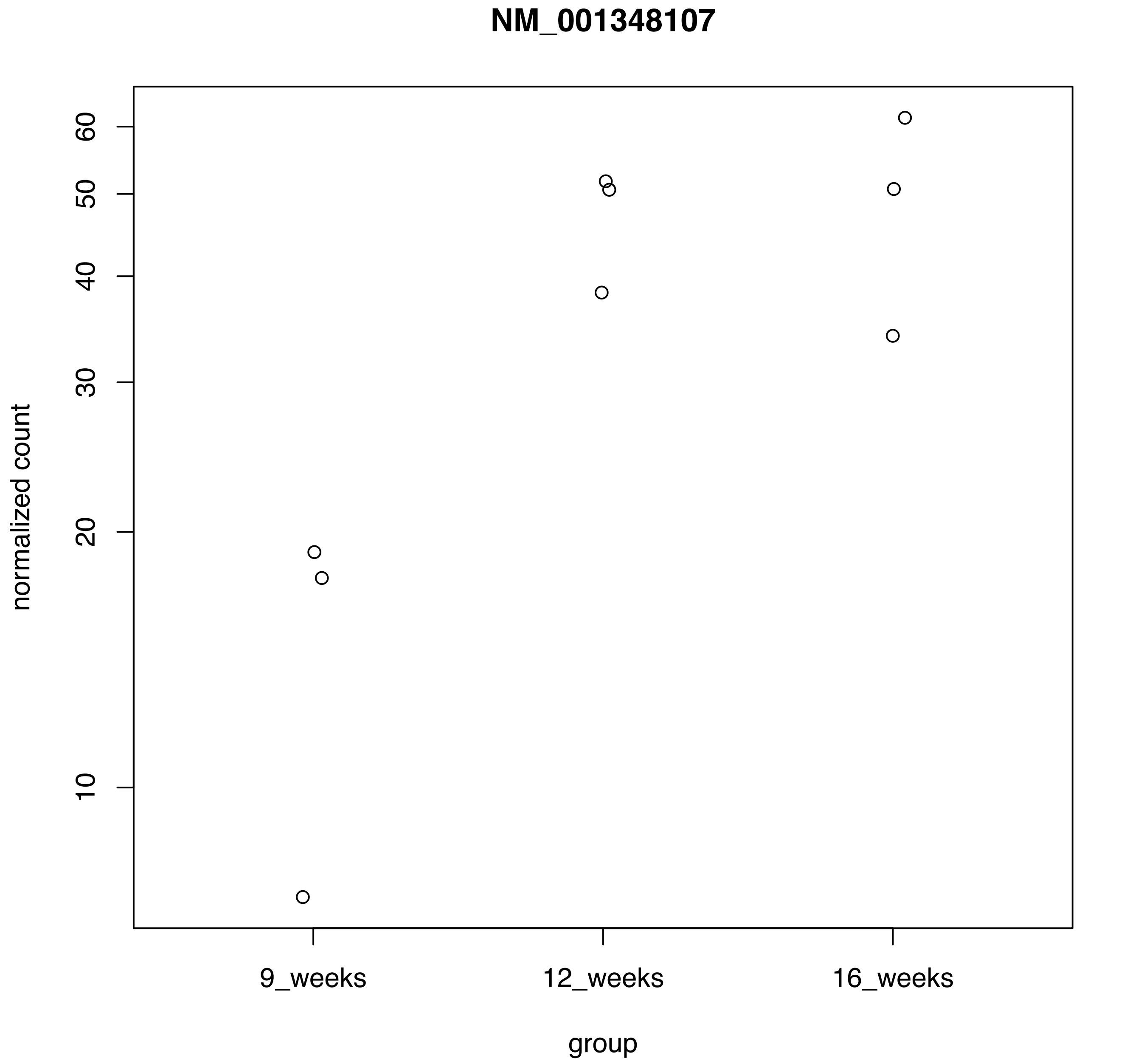
NM_001348107
normalized count
10
20
30
40
50
60
9_weeks
12_weeks
16_weeks
group

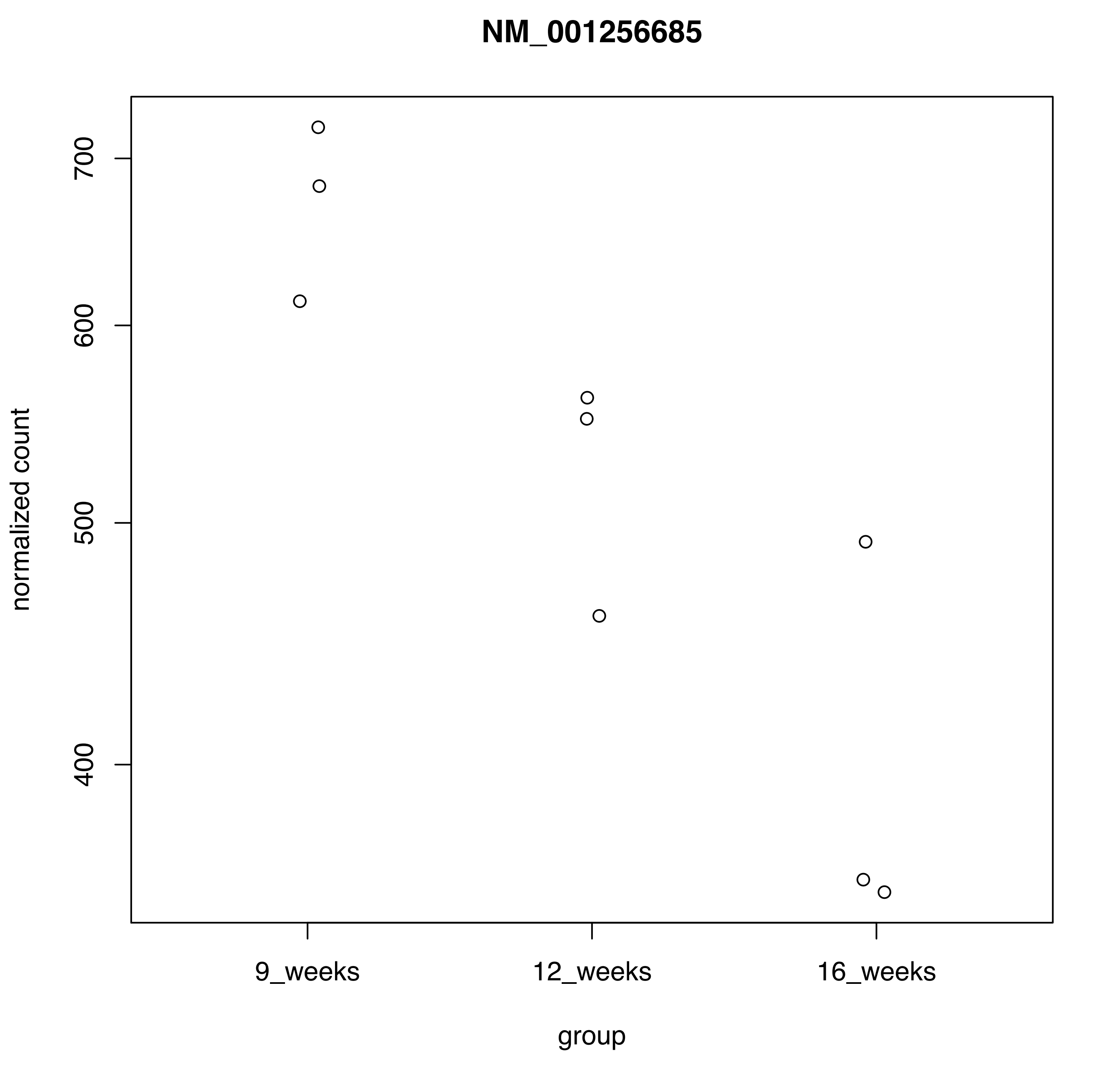
NM_001256685
normalized count
400
500
600
700
9_weeks
12_weeks
16_weeks
group

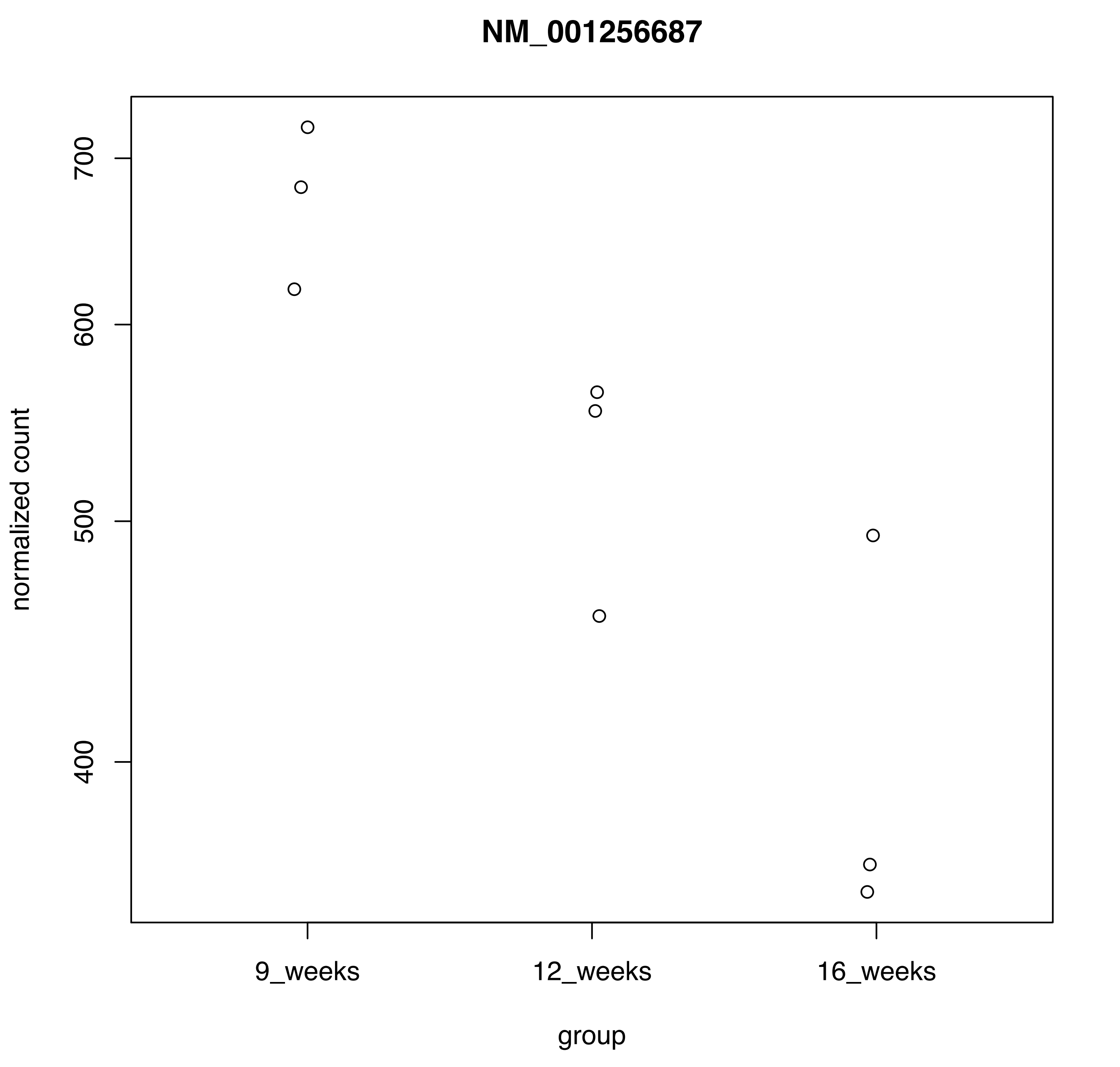
NM_001256687
normalized count
700
600
500
400
9_weeks
12_weeks
16_weeks
group

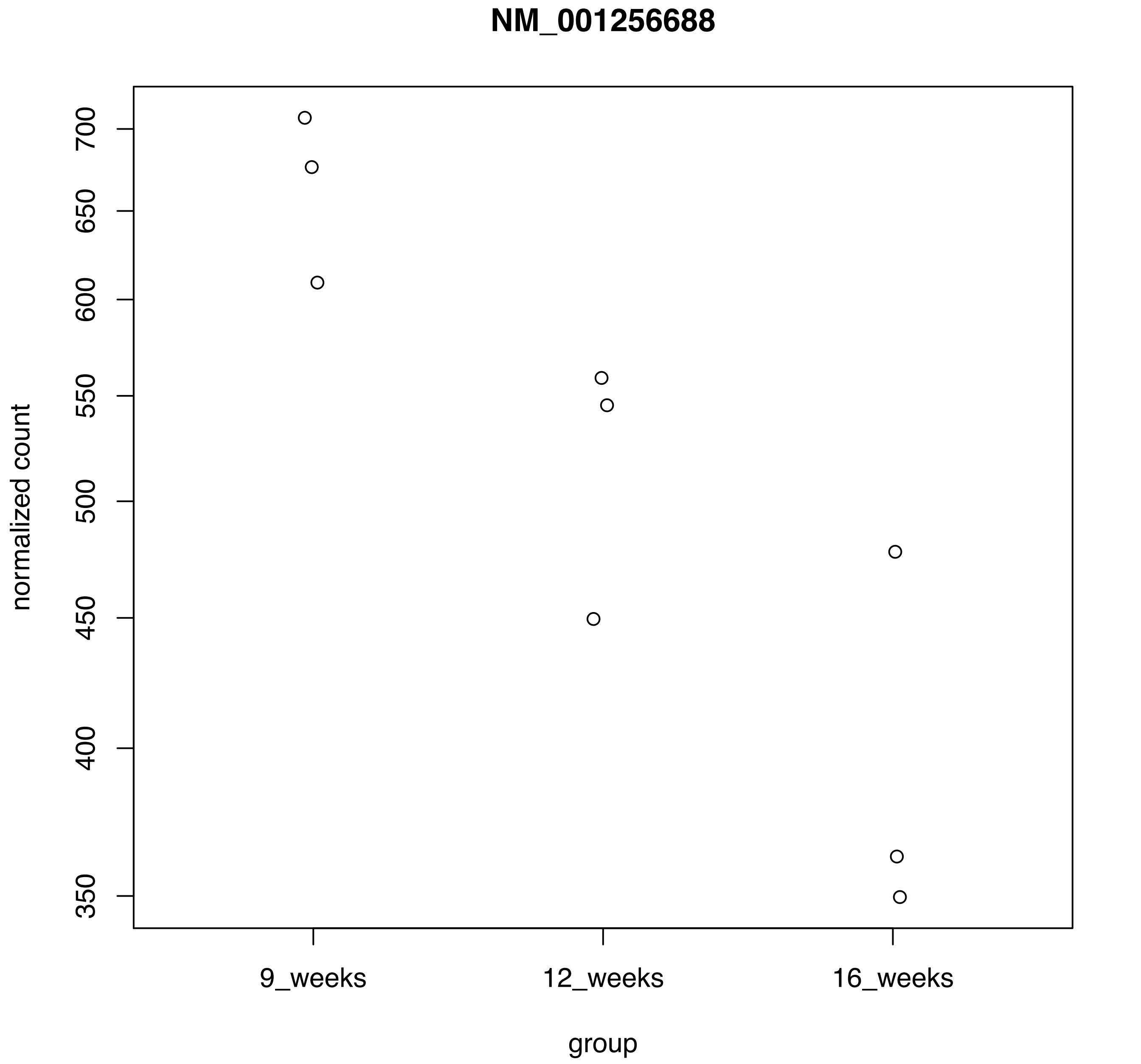
NM_001256688
normalized count
700
650
600
550
500
450
400
350
9_weeks
12_weeks
16_weeks
group

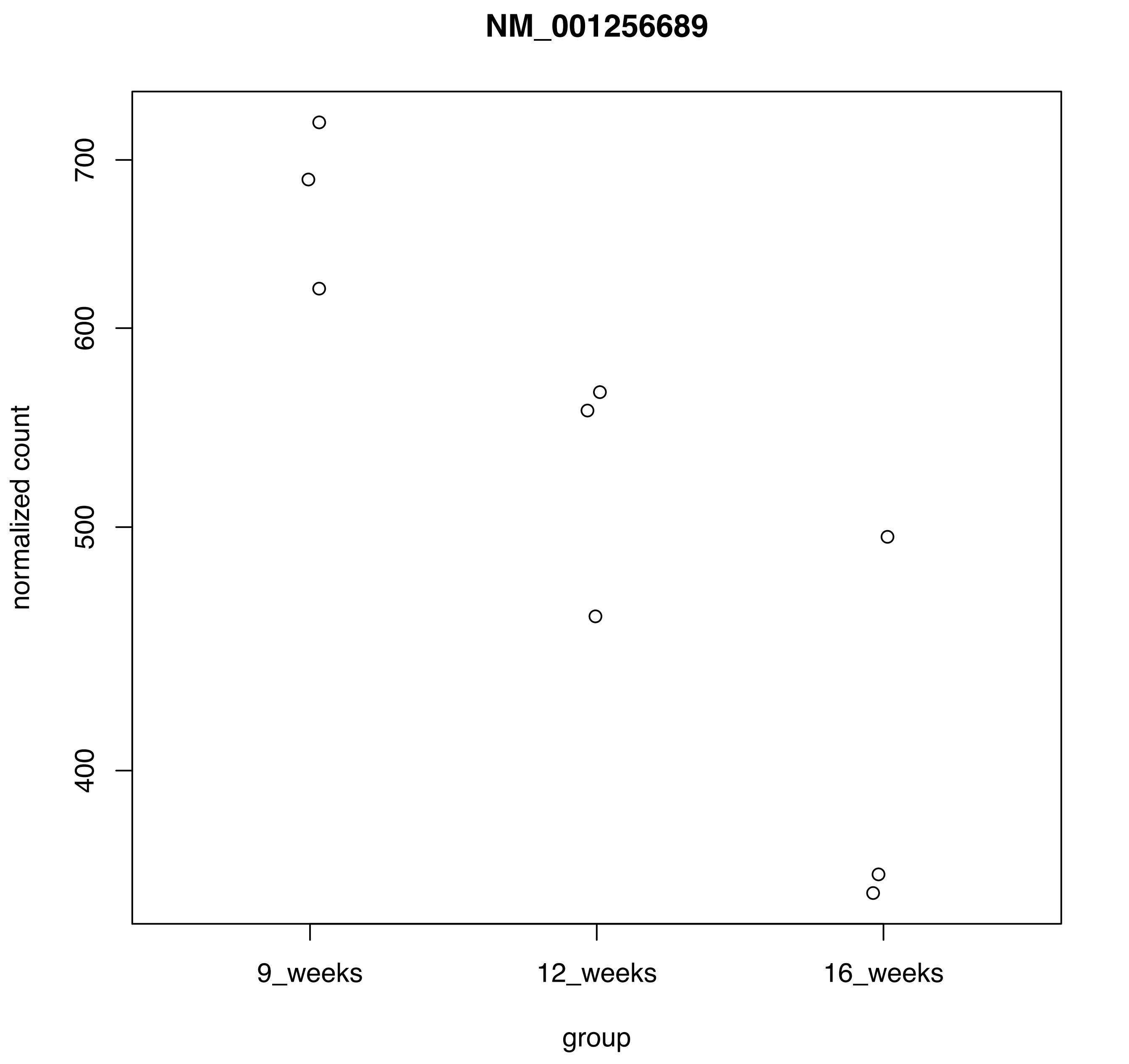
NM_001256689
700
600
500
400
normalized count
9_weeks
12_weeks
16_weeks
group

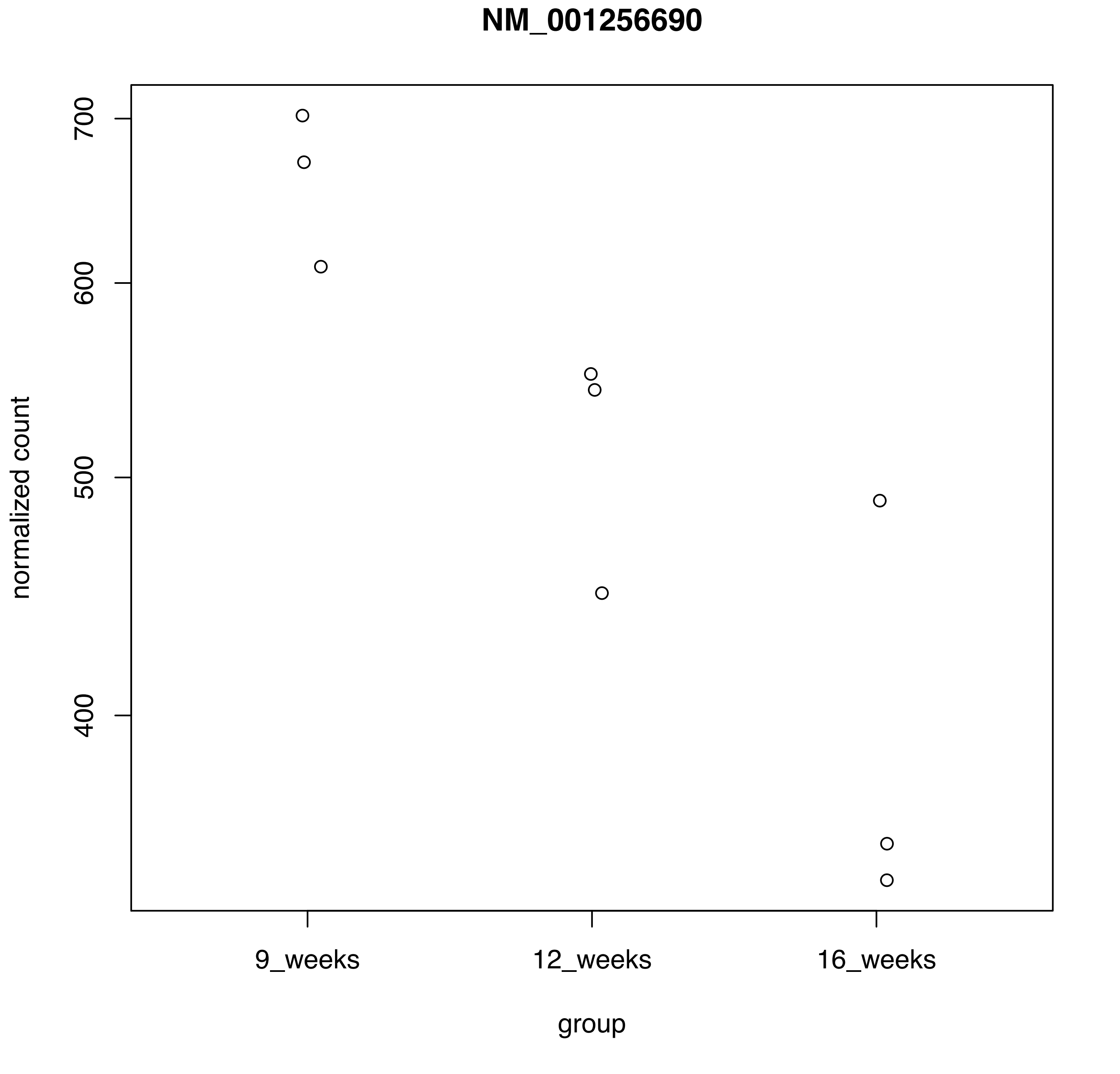
NM_001256690
normalized count
700
600
500
400
9_weeks
12_weeks
16_weeks
group

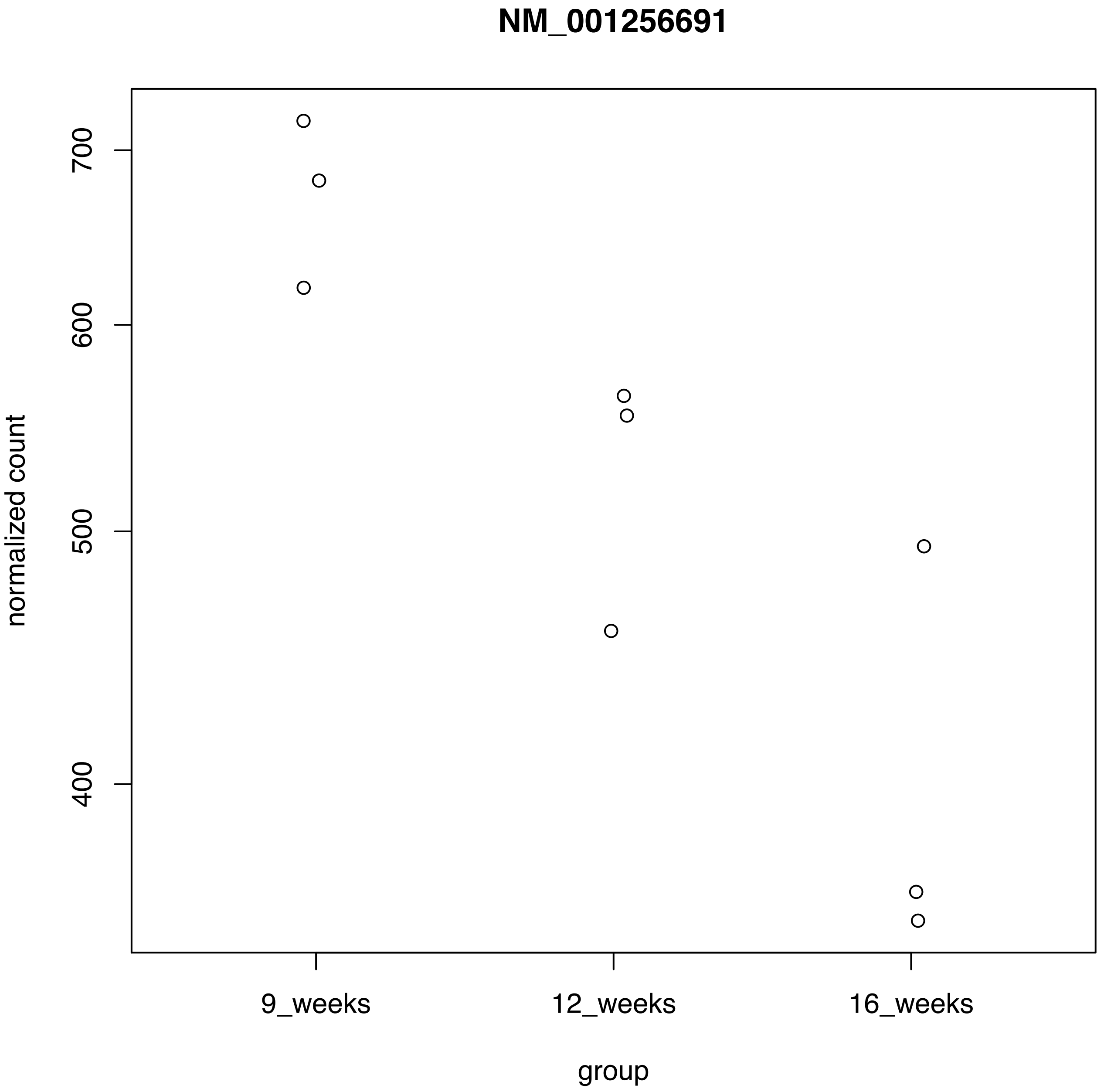
NM_001256691
normalized count
700
600
500
400
9_weeks
12_weeks
16_weeks
group

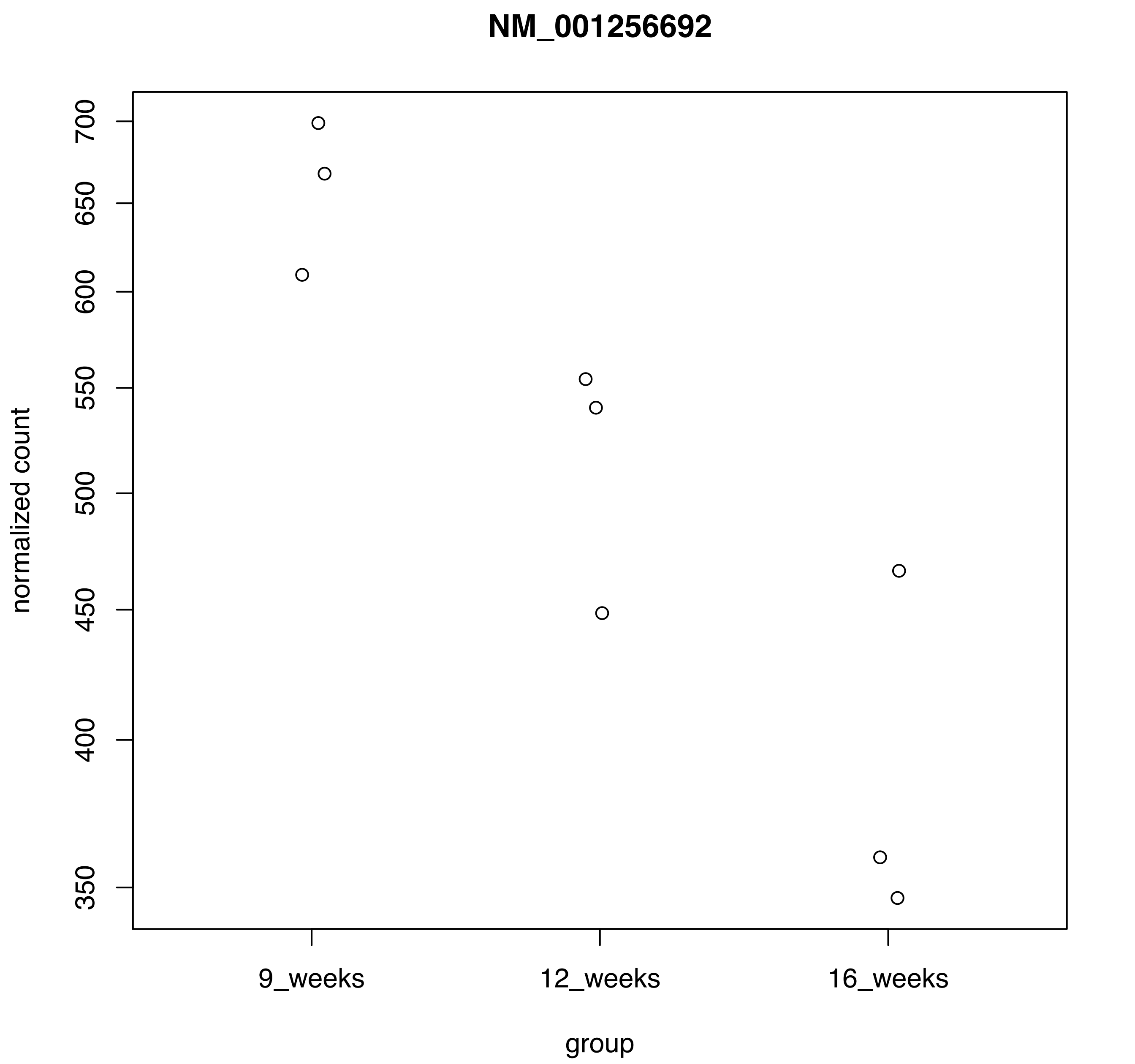

NM_001256692
normalized count
700
650
600
550
500
450
400
350
9_weeks
12_weeks
16_weeks
group

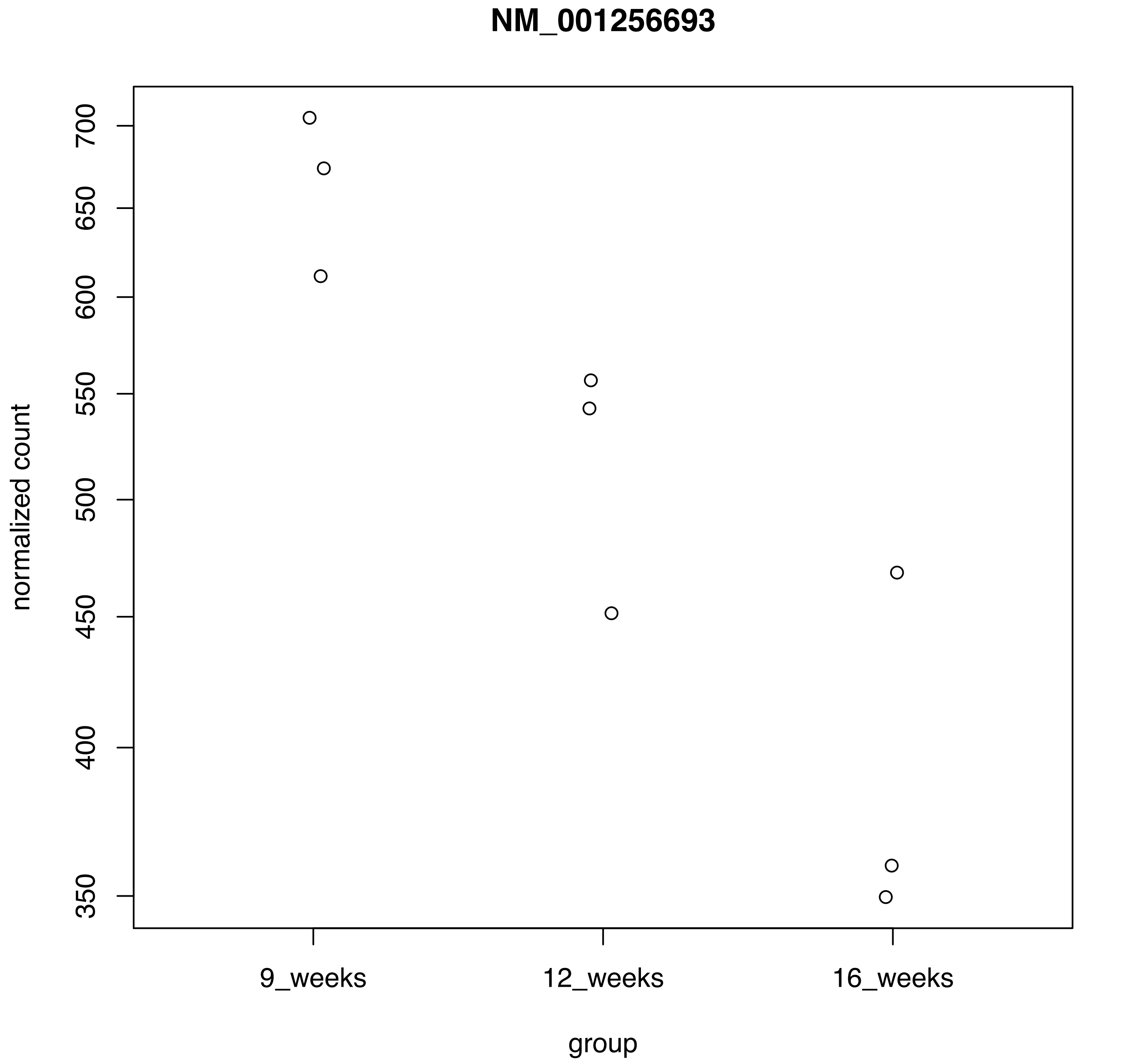
NM_001256693
normalized count
700
650
600
550
500
450
400
350
9_weeks
12_weeks
16_weeks
group

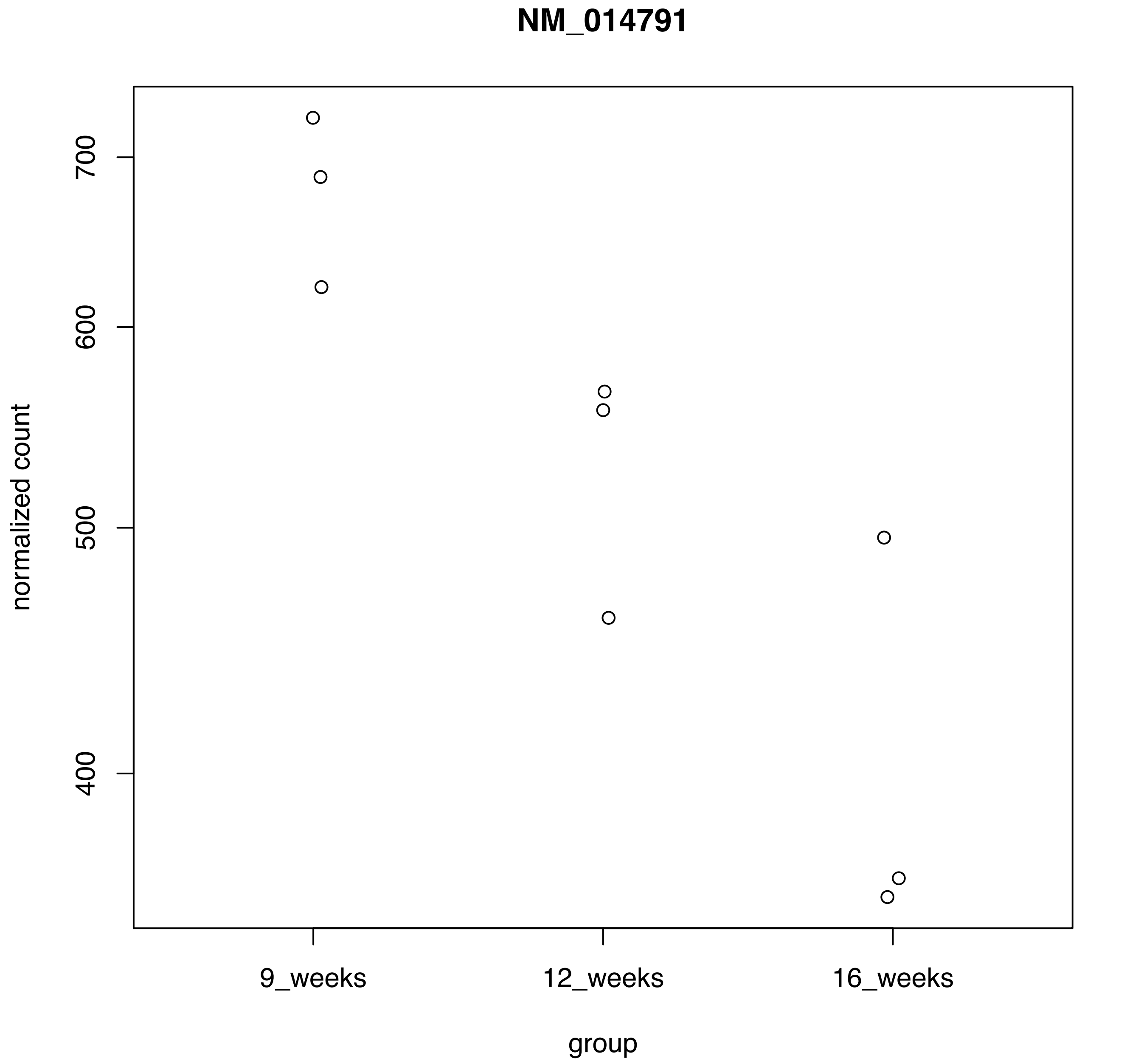
NM_014791
normalized count
700
600
500
400
9_weeks
12_weeks
16_weeks
group

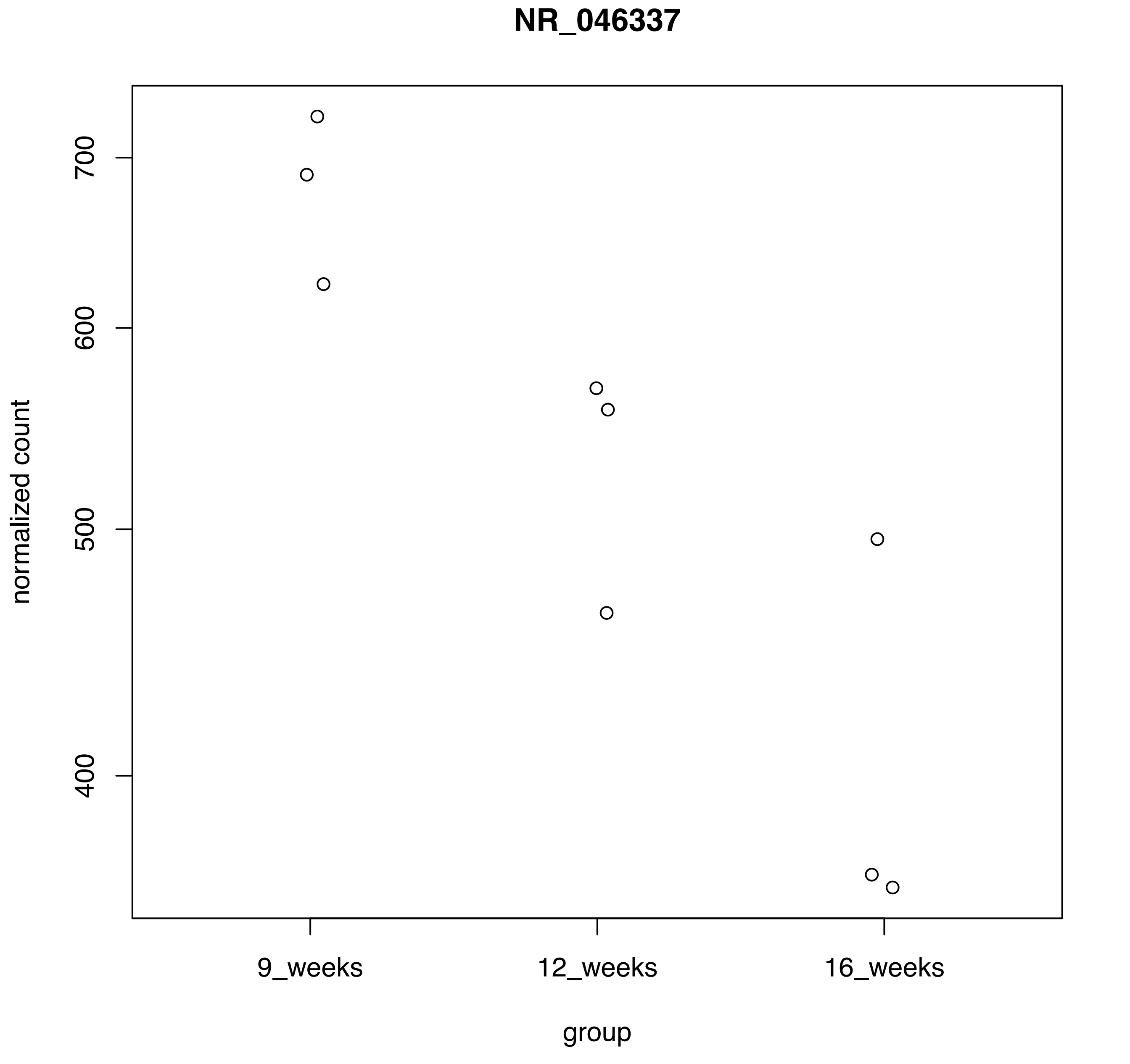
NR_046337
normalized count
700
600
500
400
9_weeks
12_weeks
16_weeks
group

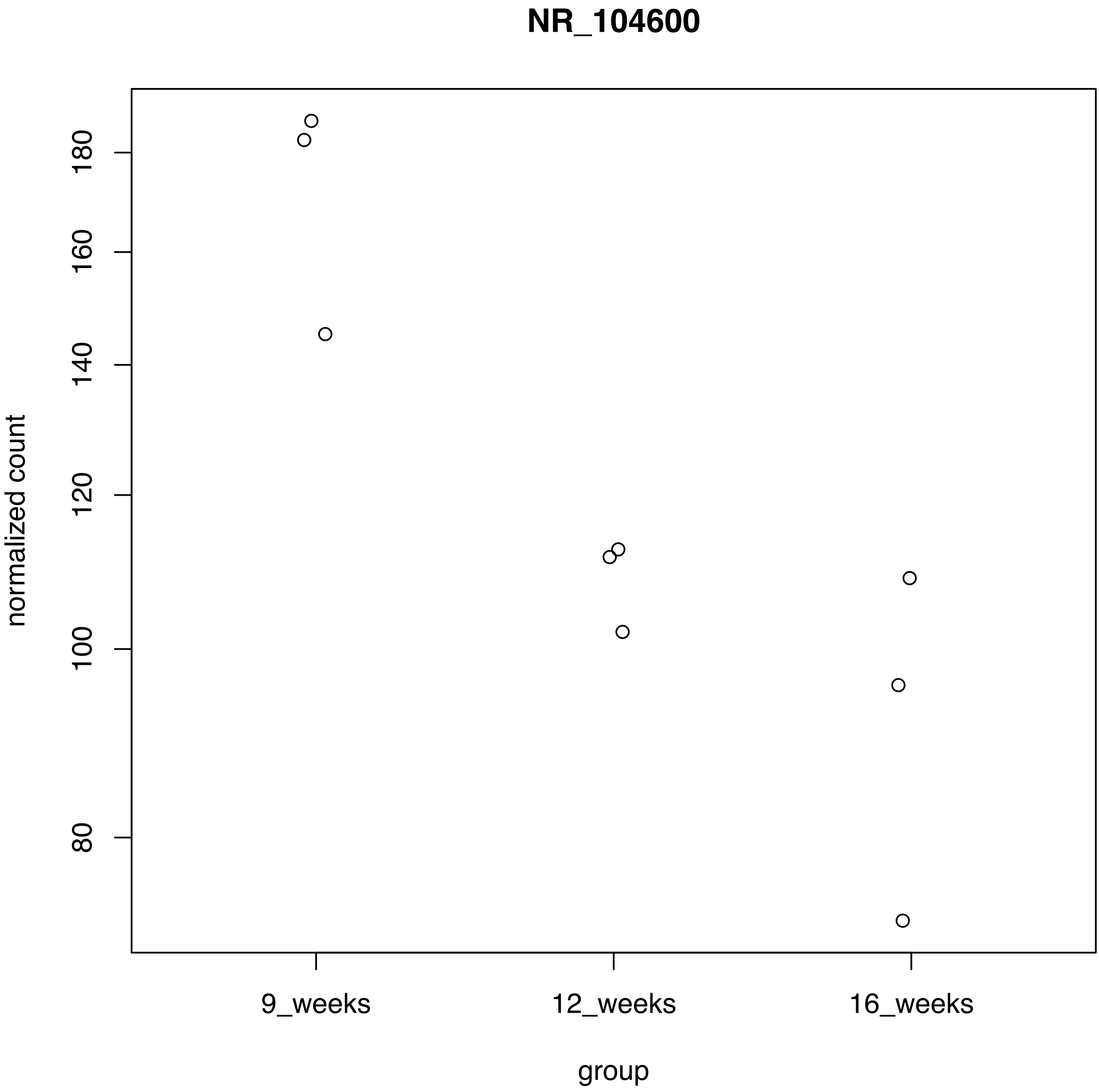

NR_104600
normalized count
180
160
140
120
100
80
9_weeks
12_weeks
16_weeks
group

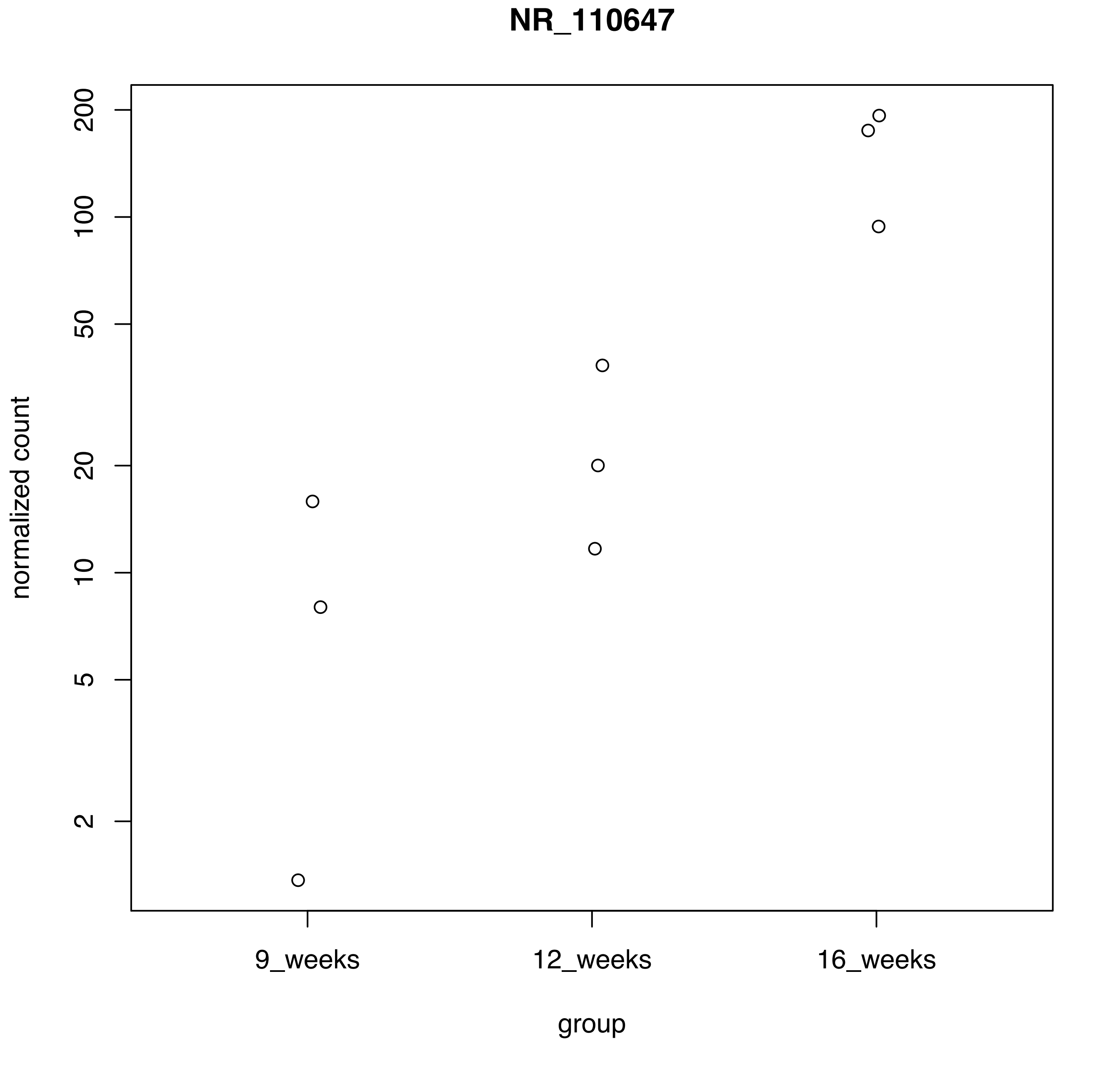
NR_110647
normalized count
200
100
50
20
10
5
2
9_weeks
12_weeks
16_weeks
group

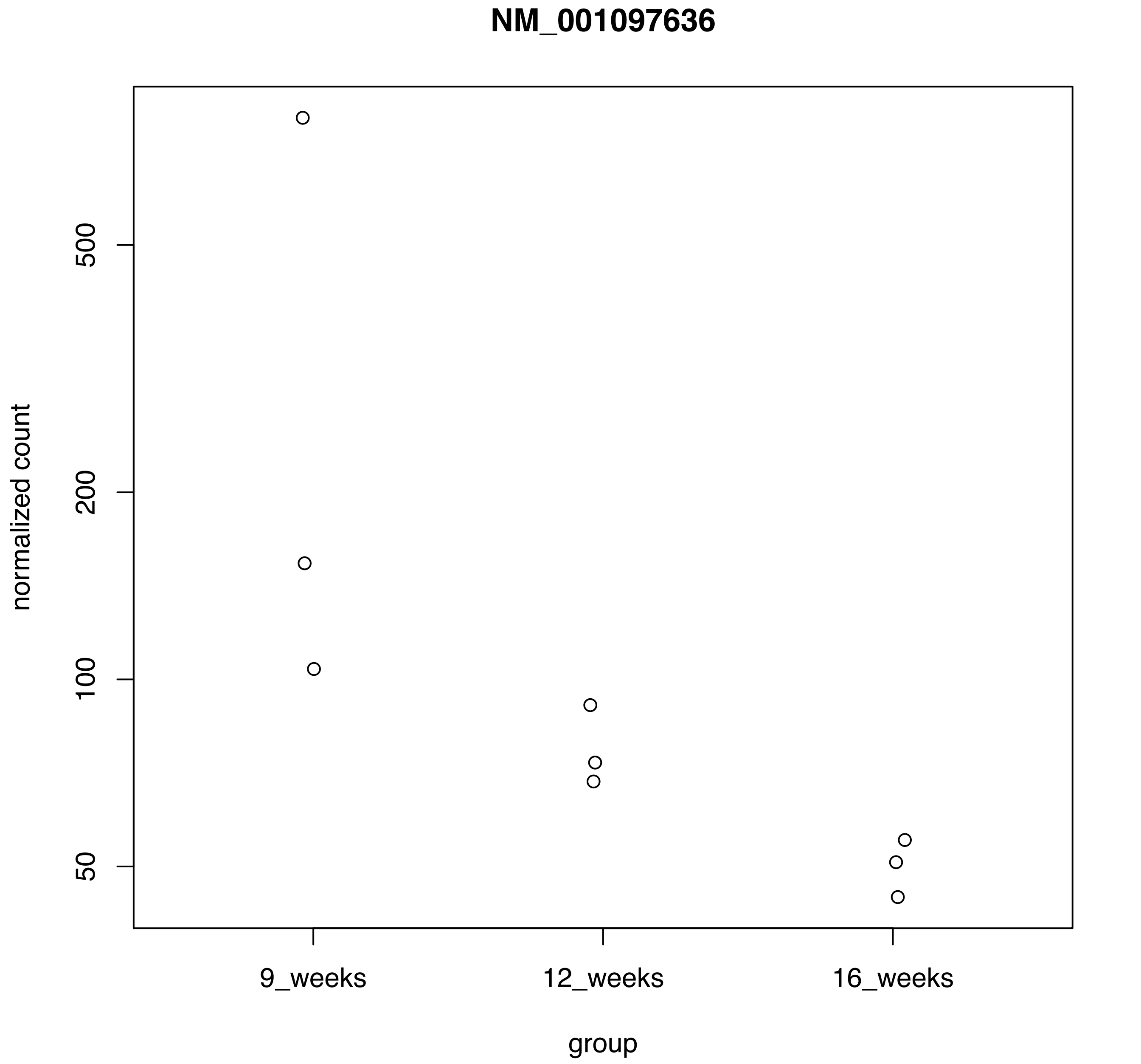

NM_001097636
normalized count
500
200
100
50
9_weeks
12_weeks
16_weeks
group

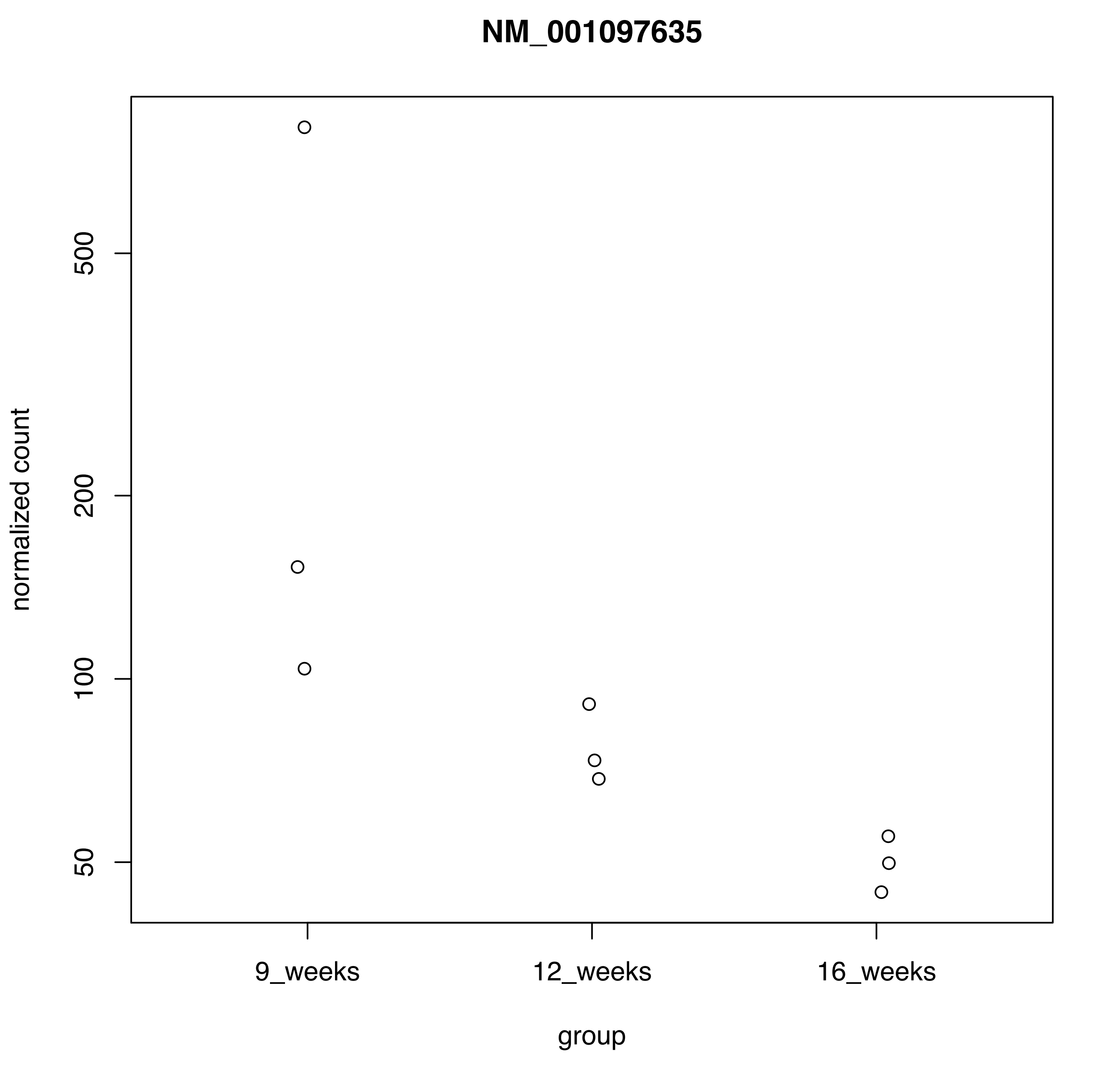
NM_001097635
normalized count
500
200
100
50
9_weeks
12_weeks
16_weeks
group

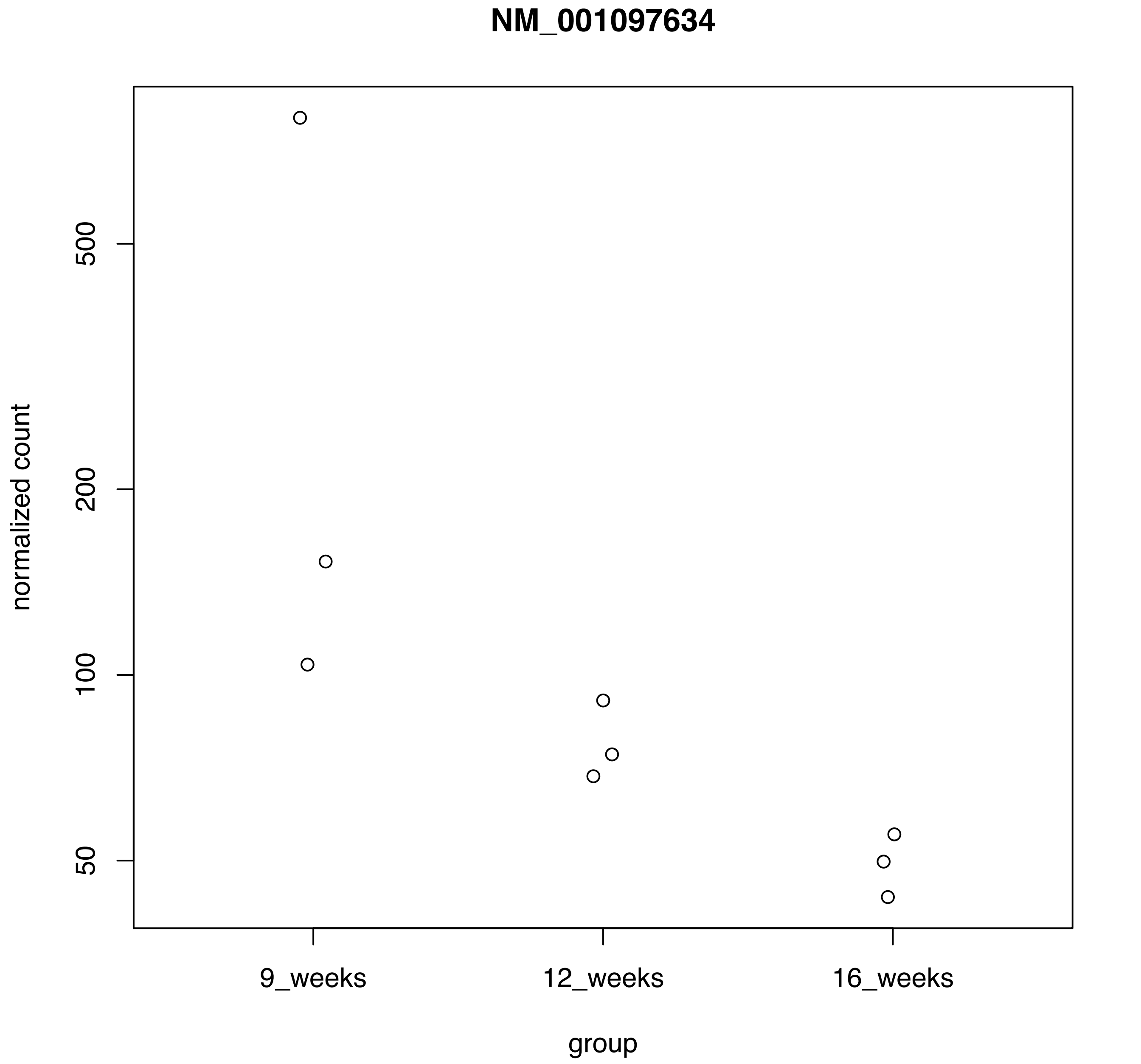
NM_001097634
normalized count
500
200
100
50
9_weeks
12_weeks
16_weeks
group

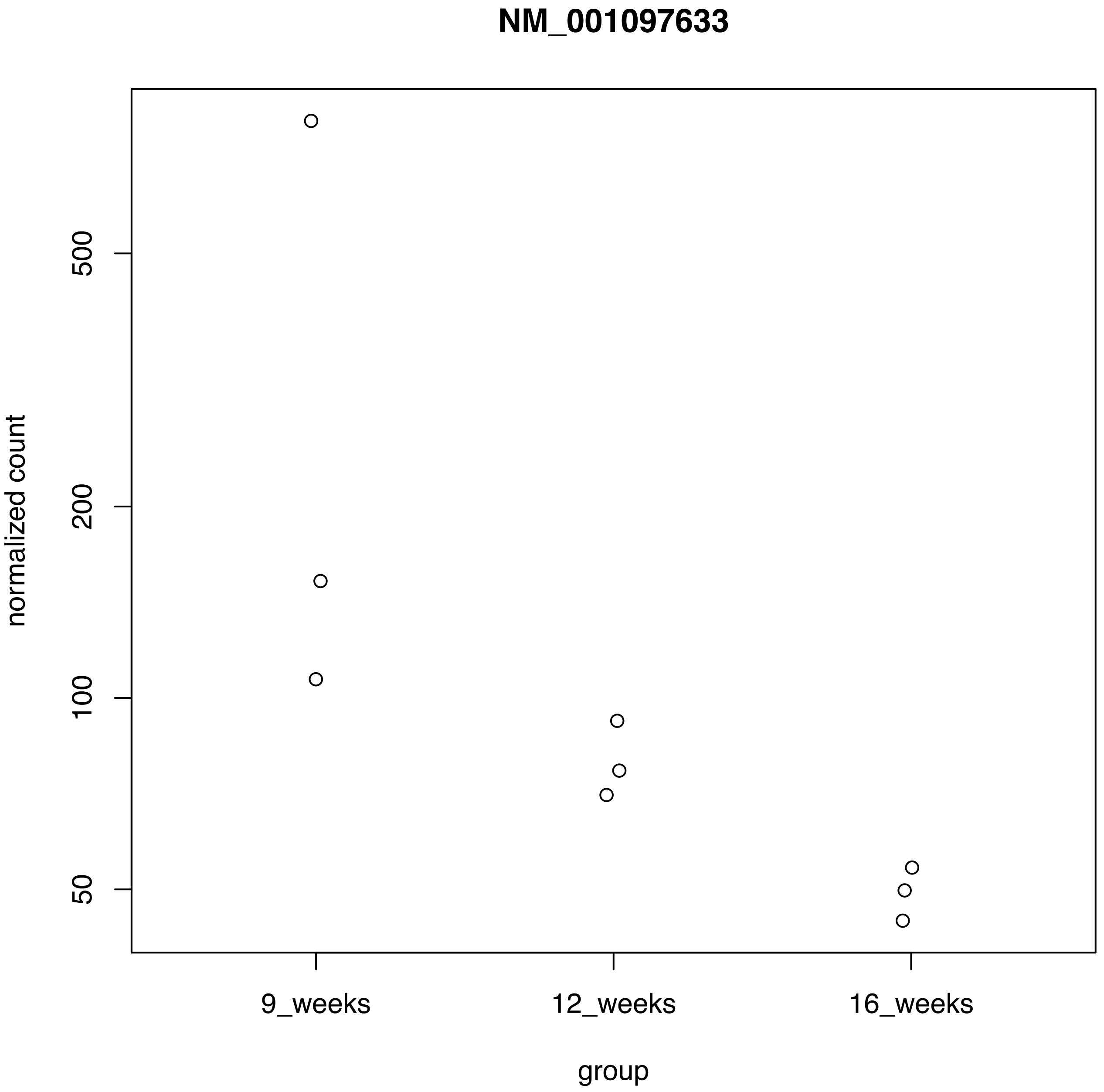
NM_001097633
normalized count
500
200
100
50
9_weeks
12_weeks
16_weeks
group

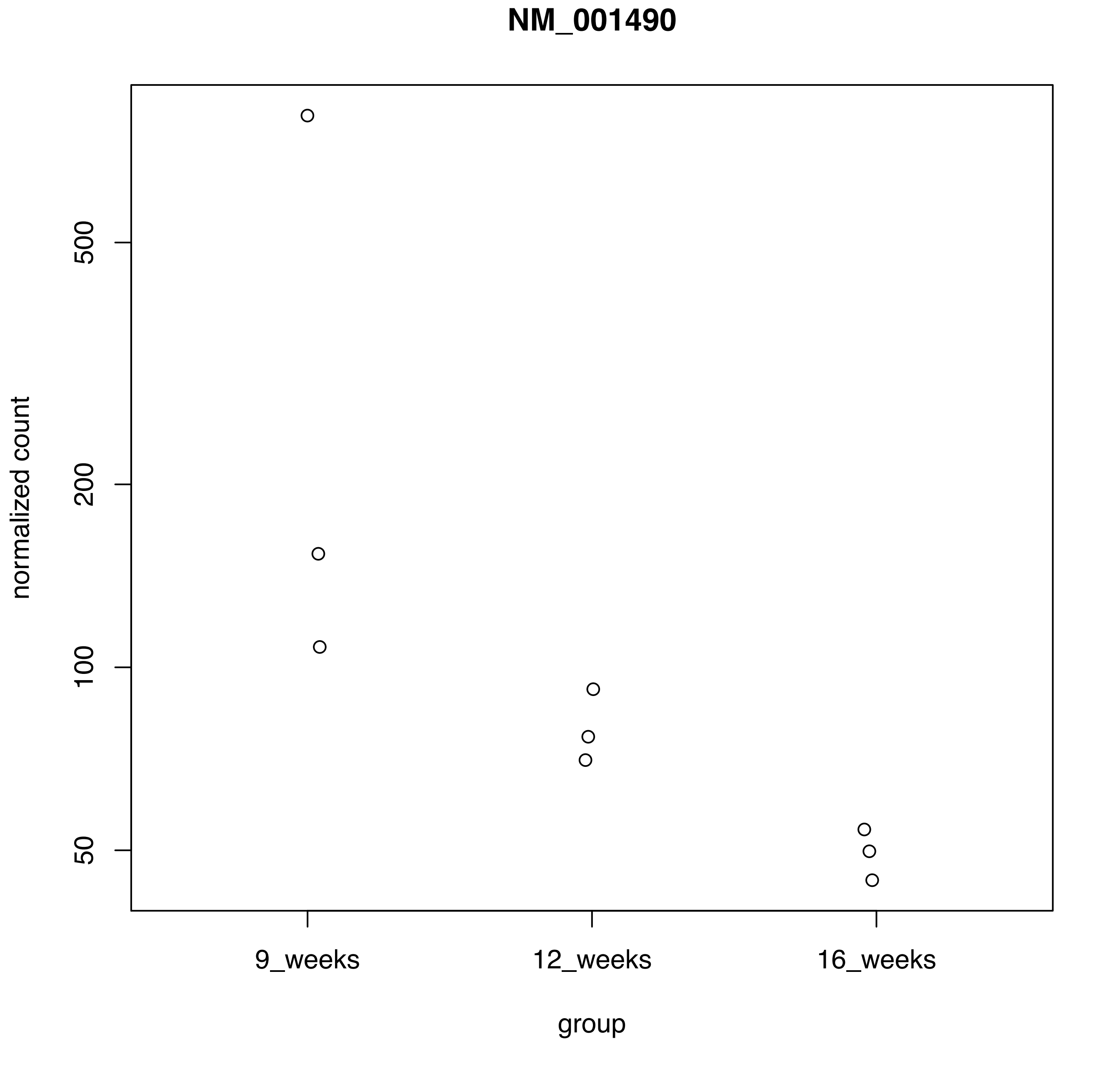
NM_001490
normalized count
500
200
100
50
9_weeks
12_weeks
16_weeks
group

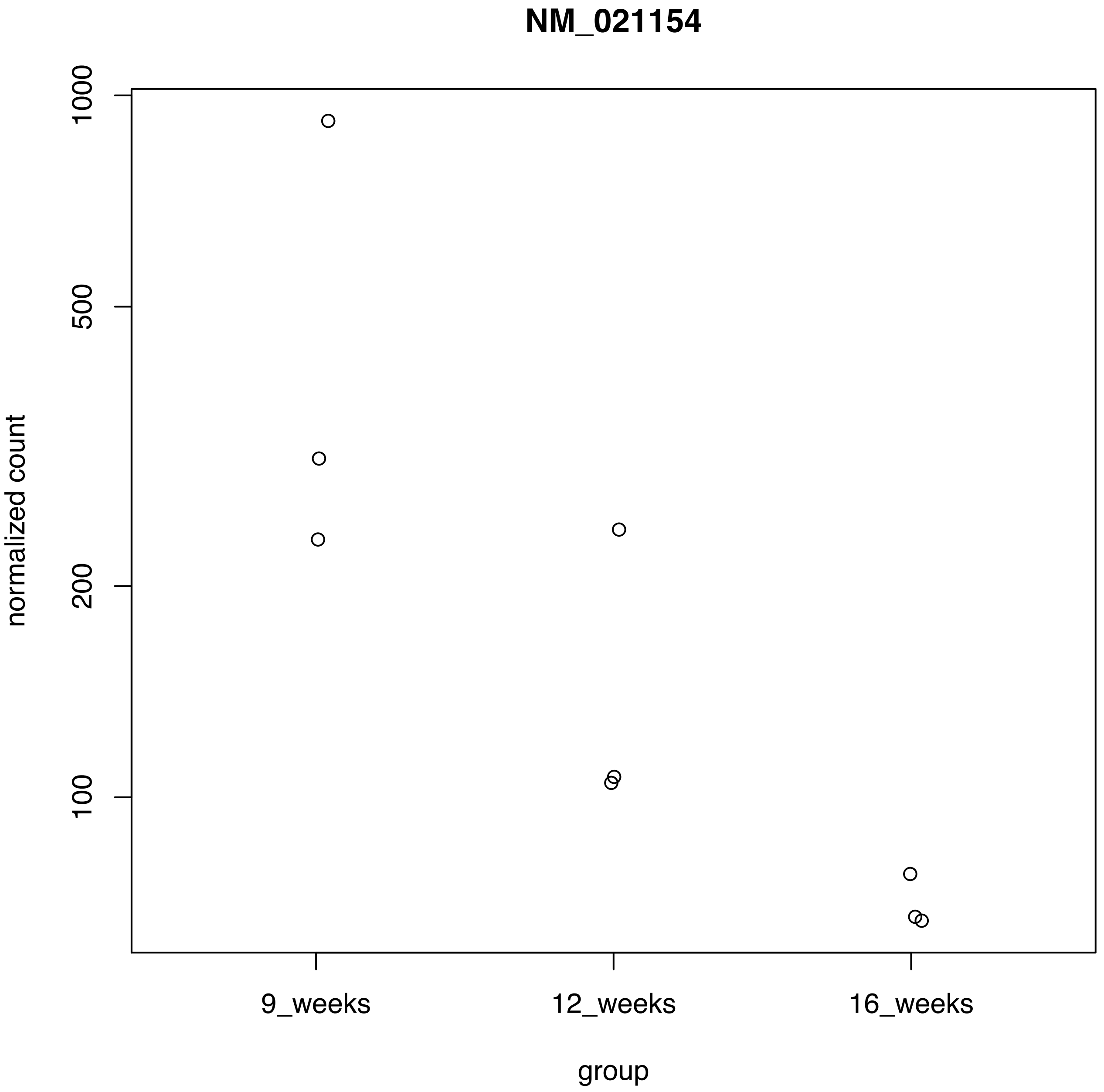
NM_021154
normalized count
1000
500
200
100
9_weeks
12_weeks
16_weeks
group

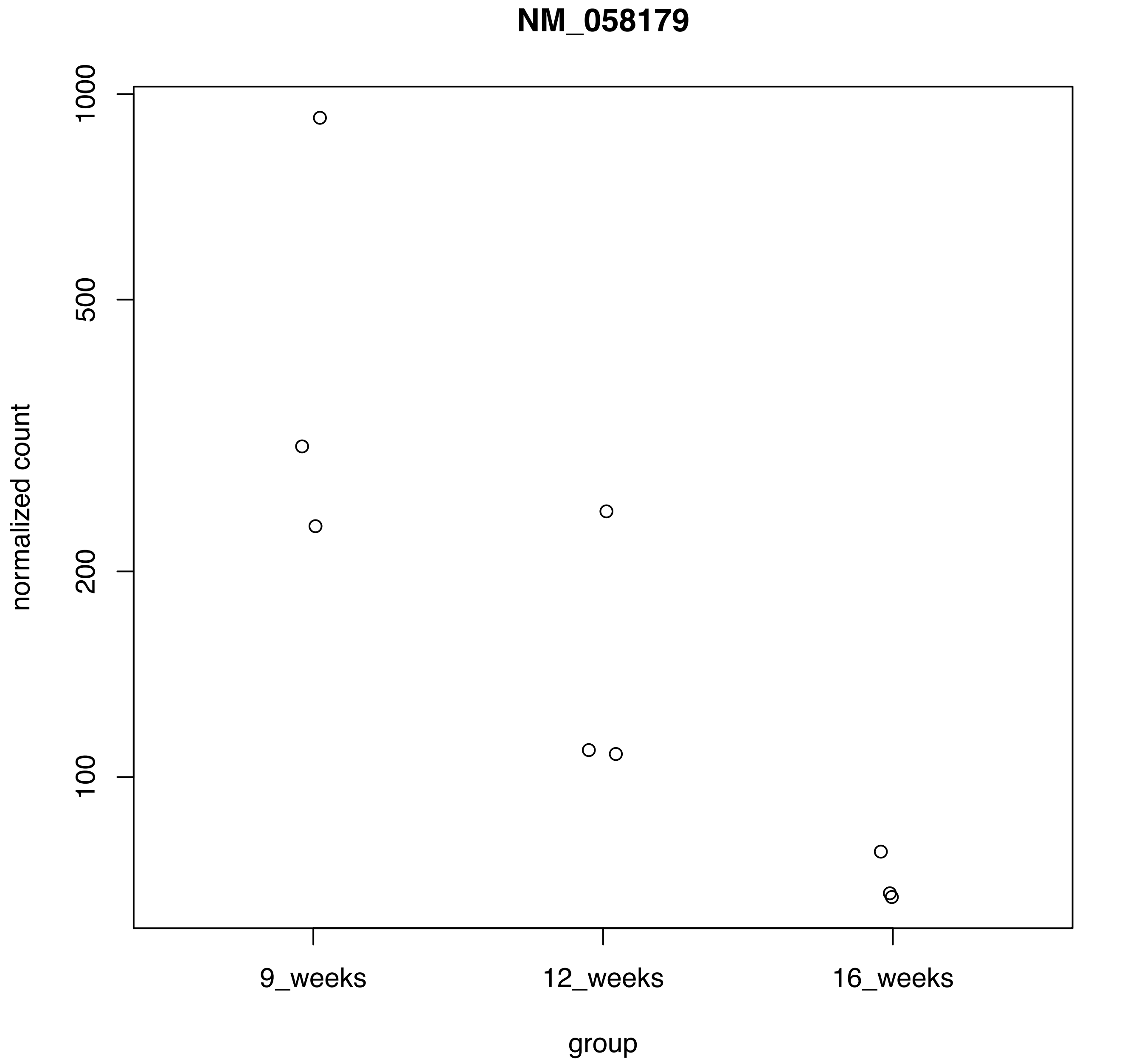
NM_058179
1000
500
200
100
normalized count
9_weeks
12_weeks
16_weeks
group

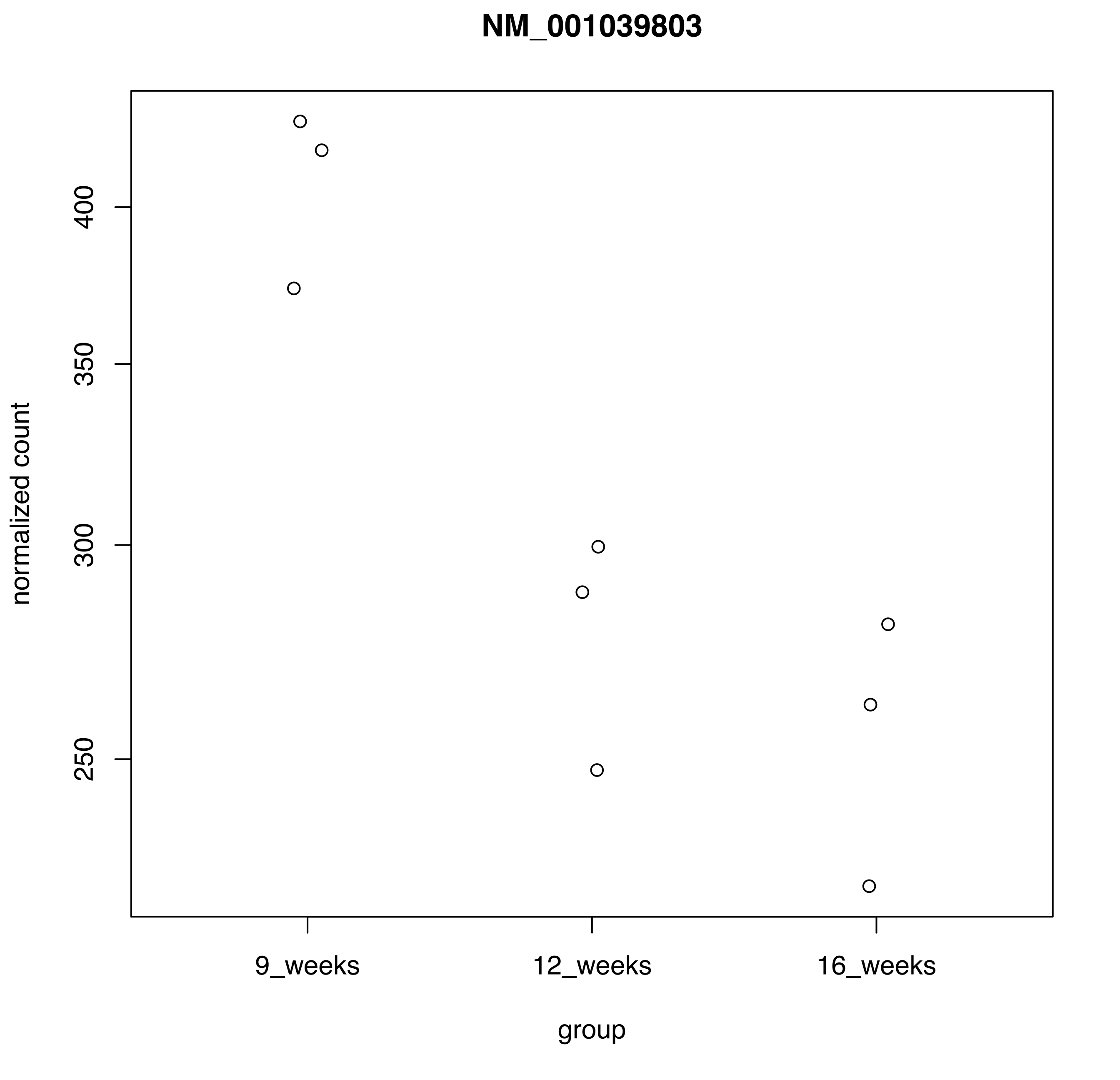
NM_001039803
normalized count
400
350
300
250
9_weeks
12_weeks
16_weeks
group

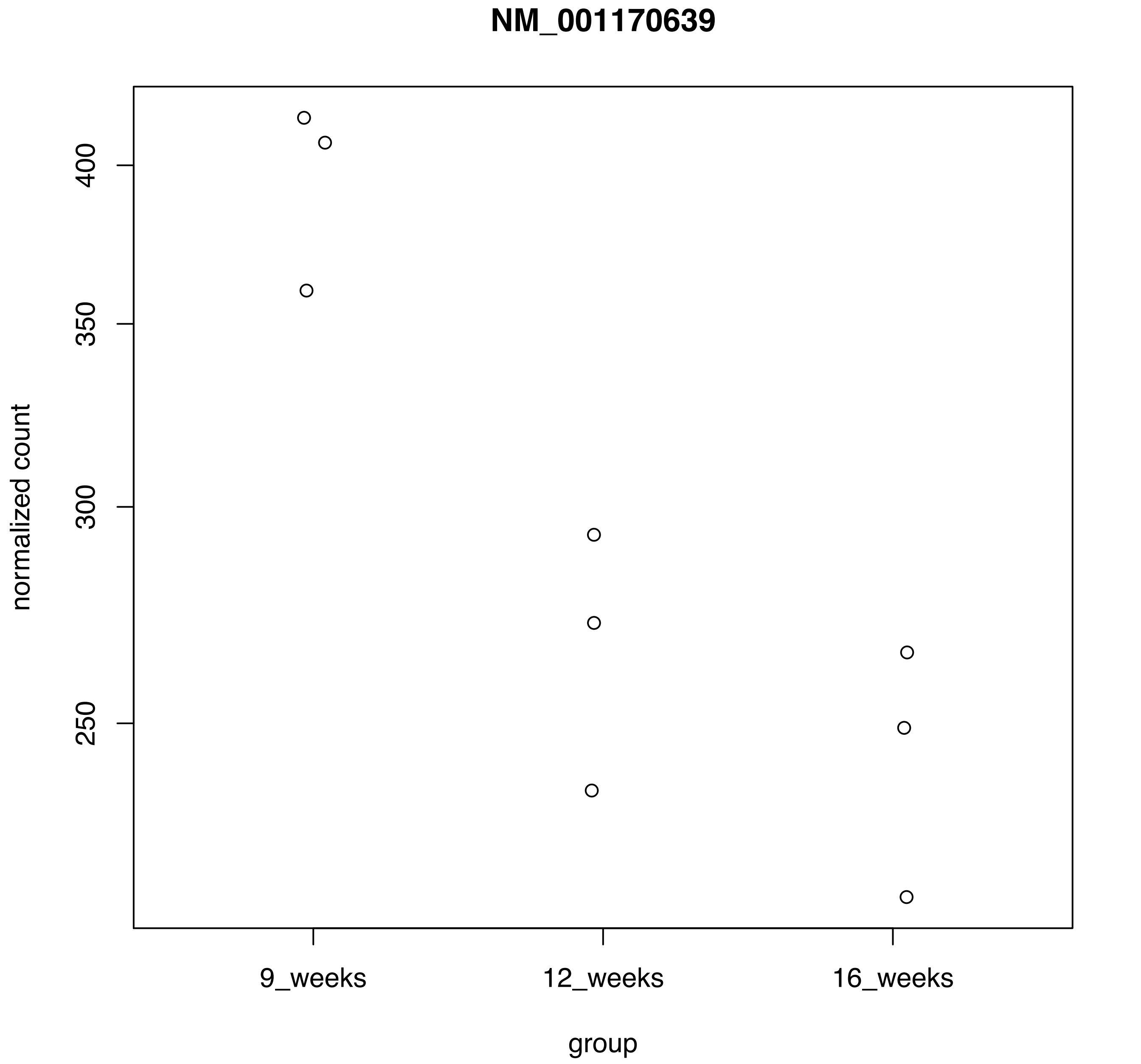

NM_001170639
normalized count
400
350
300
250
9_weeks
12_weeks
16_weeks
group

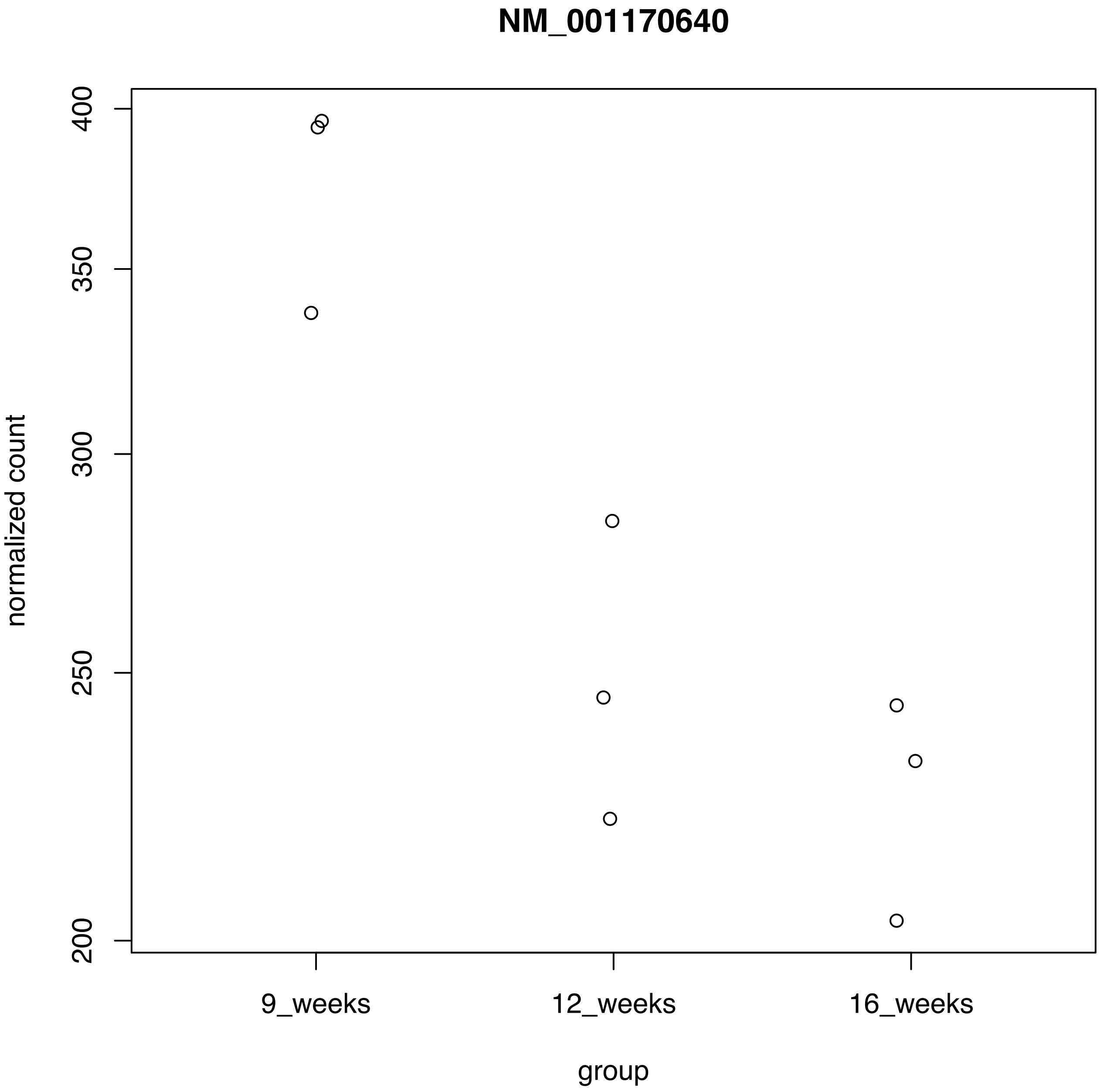
NM_001170640
normalized count
400
350
300
250
200
9_weeks
12_weeks
16_weeks
group

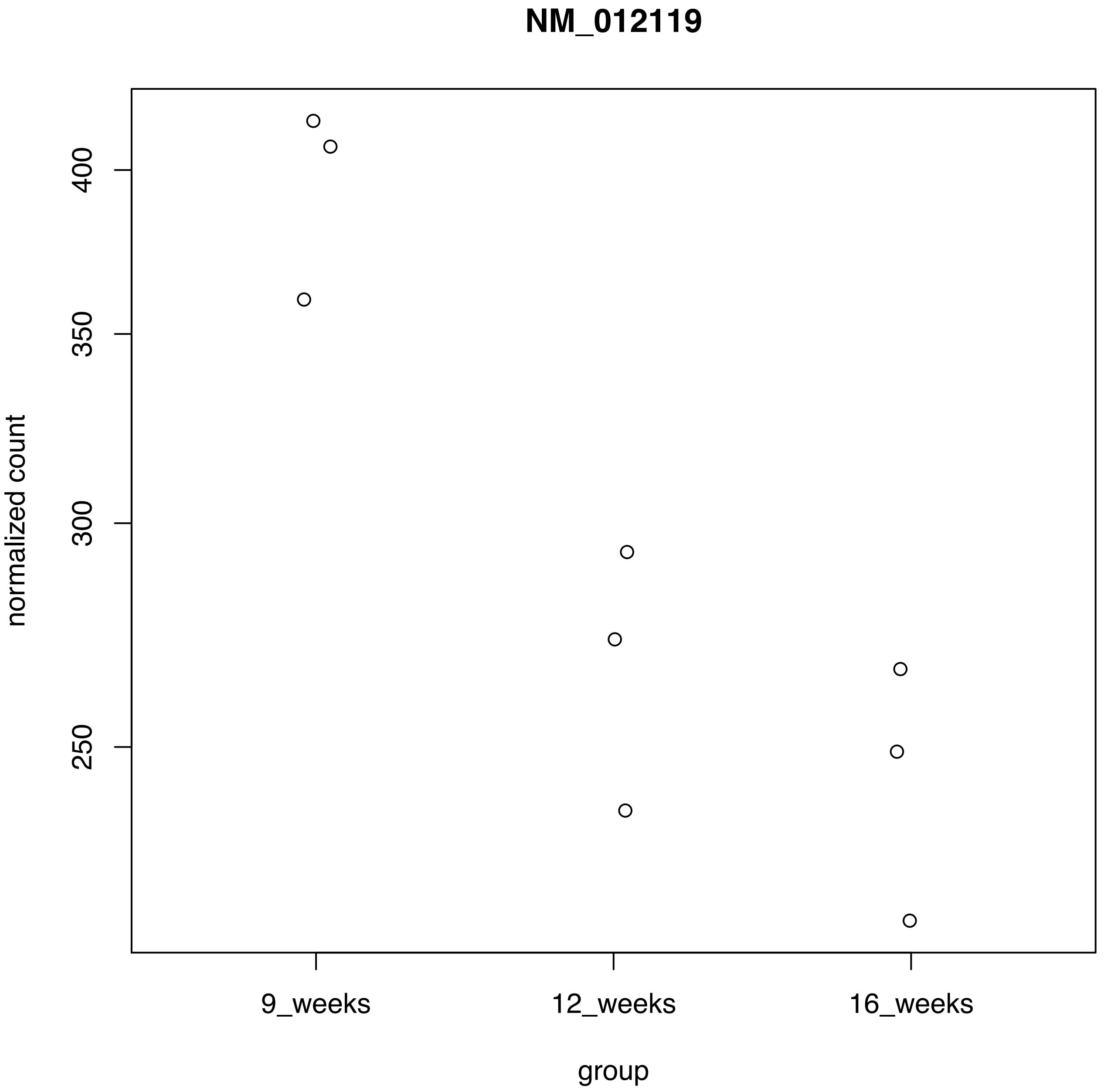

NM_012119
normalized count
400
350
300
250
9_weeks
12_weeks
16_weeks
group

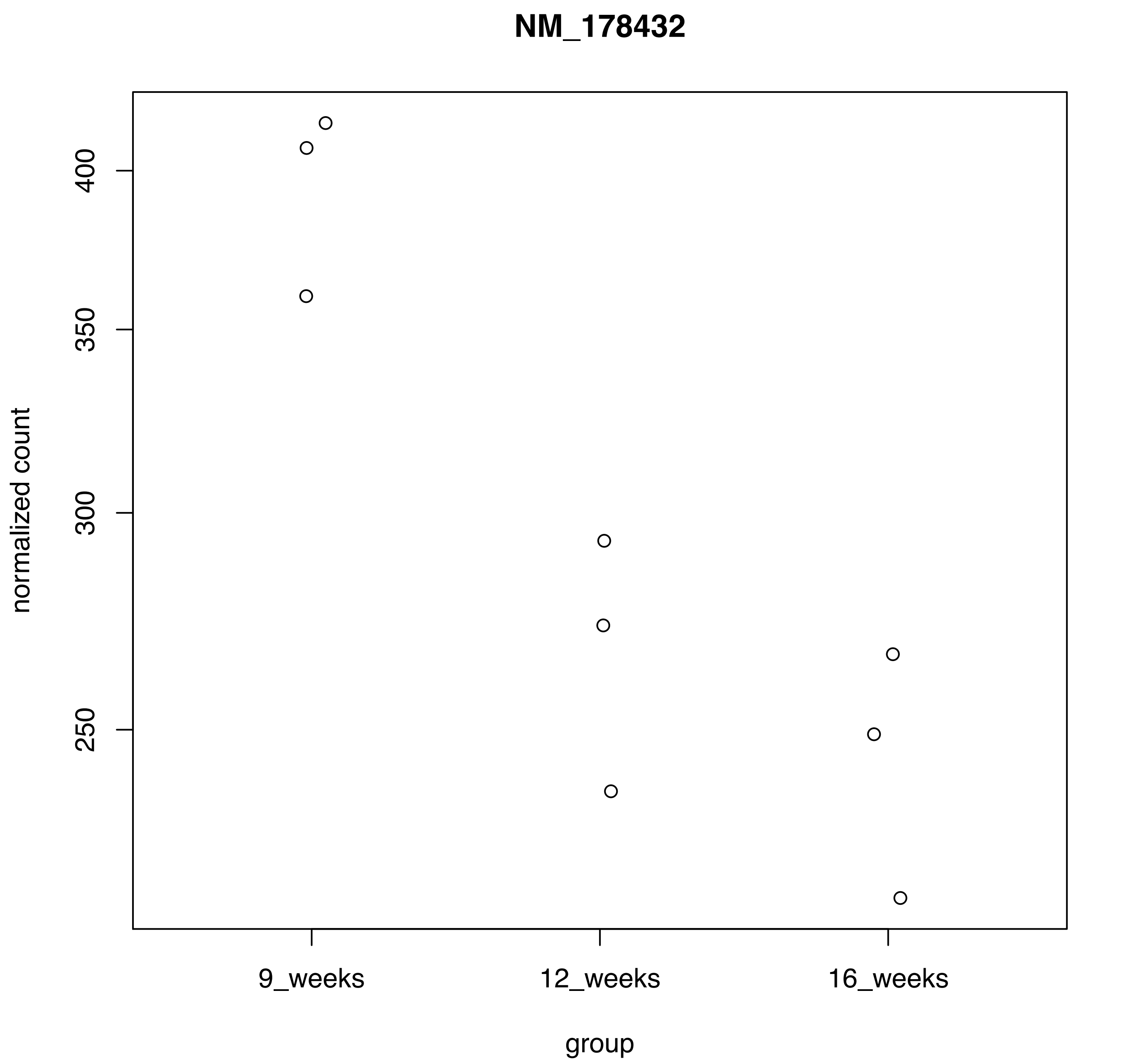
NM_178432
normalized count
400
350
300
250
9_weeks
12_weeks
16_weeks
group

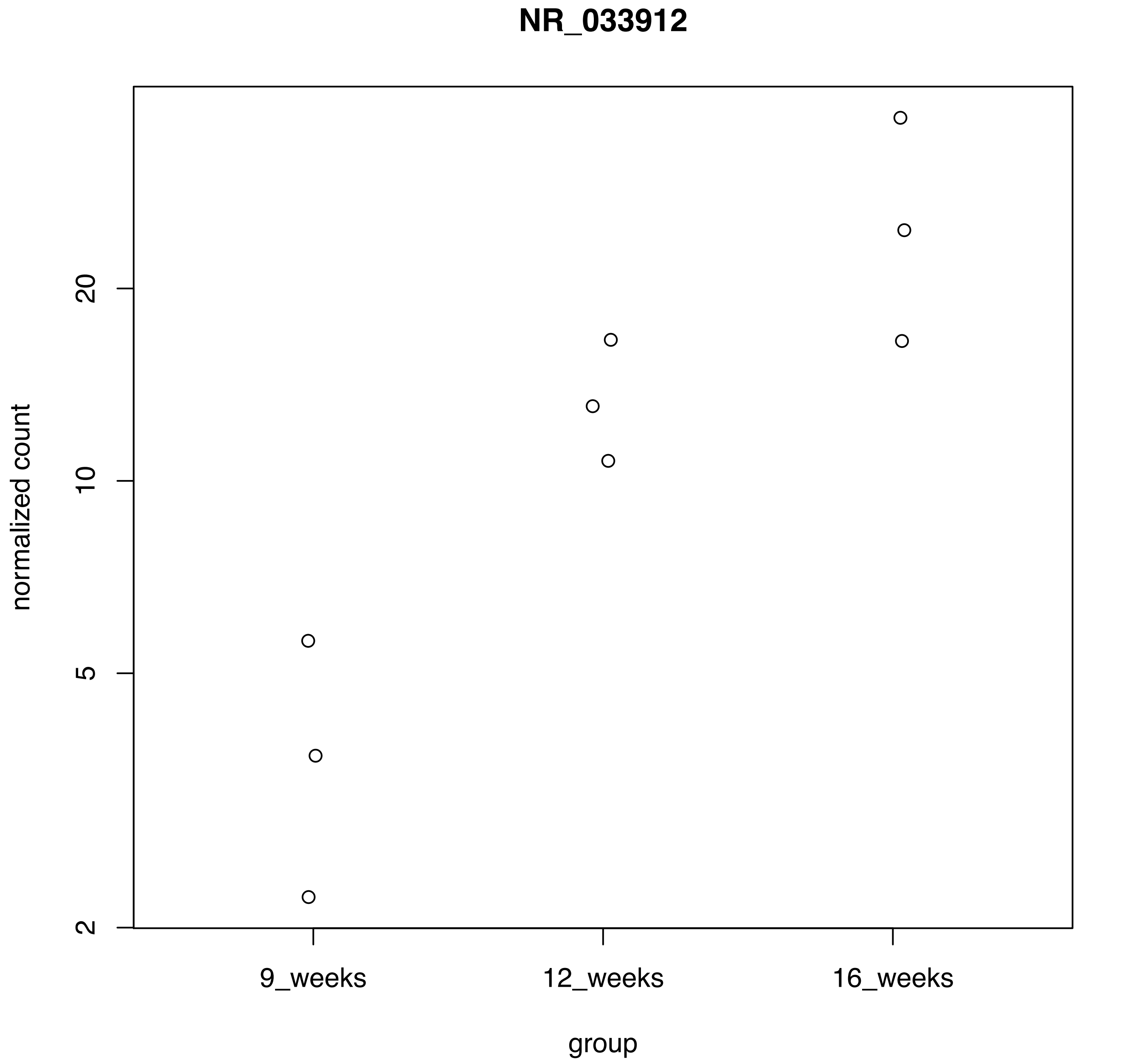

NR_033912
normalized count
20
10
5
2
9_weeks
12_weeks
16_weeks
group

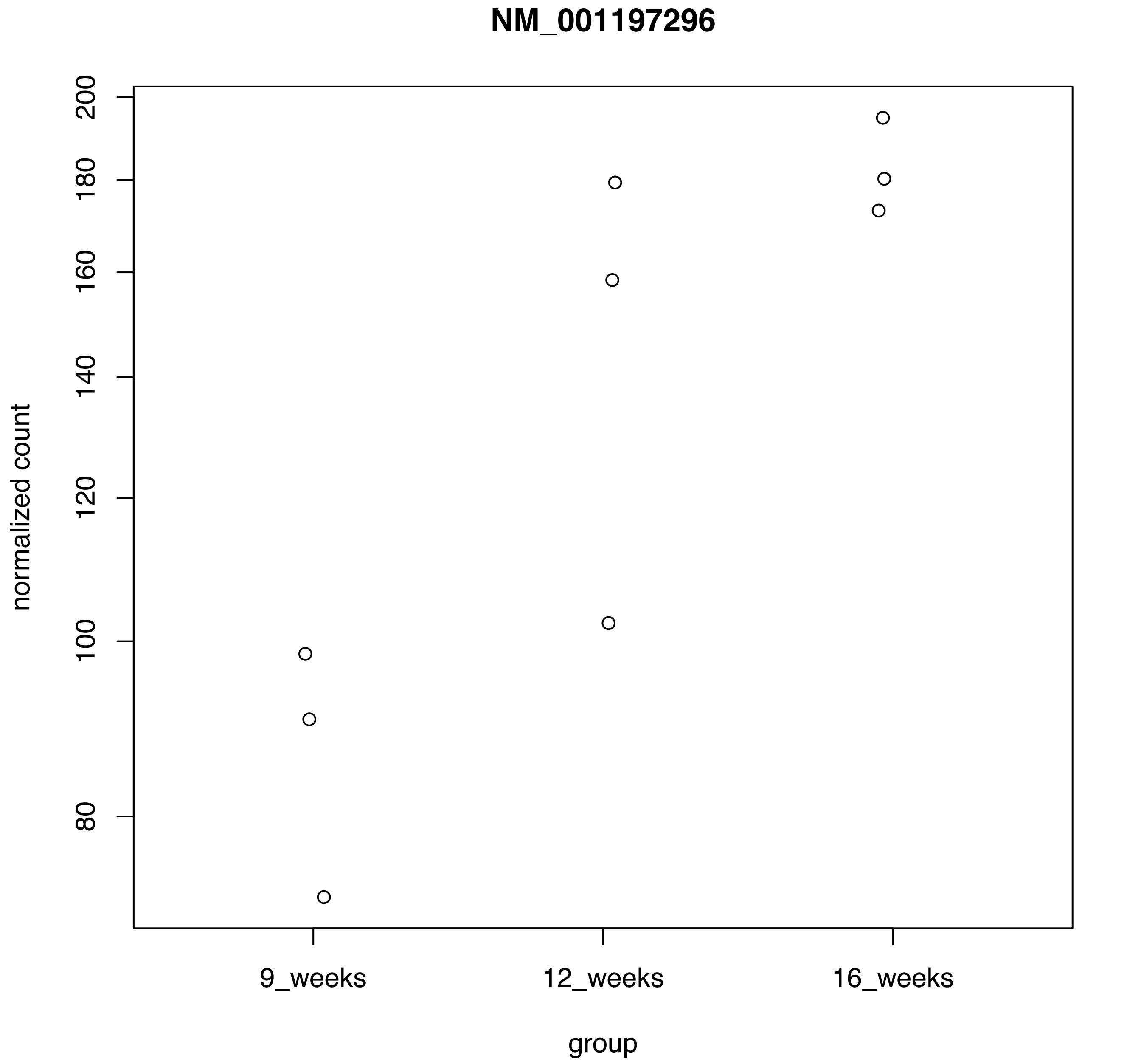

NM_001197296
normalized count
200
180
160
140
120
100
80
9_weeks
12_weeks
16_weeks
group

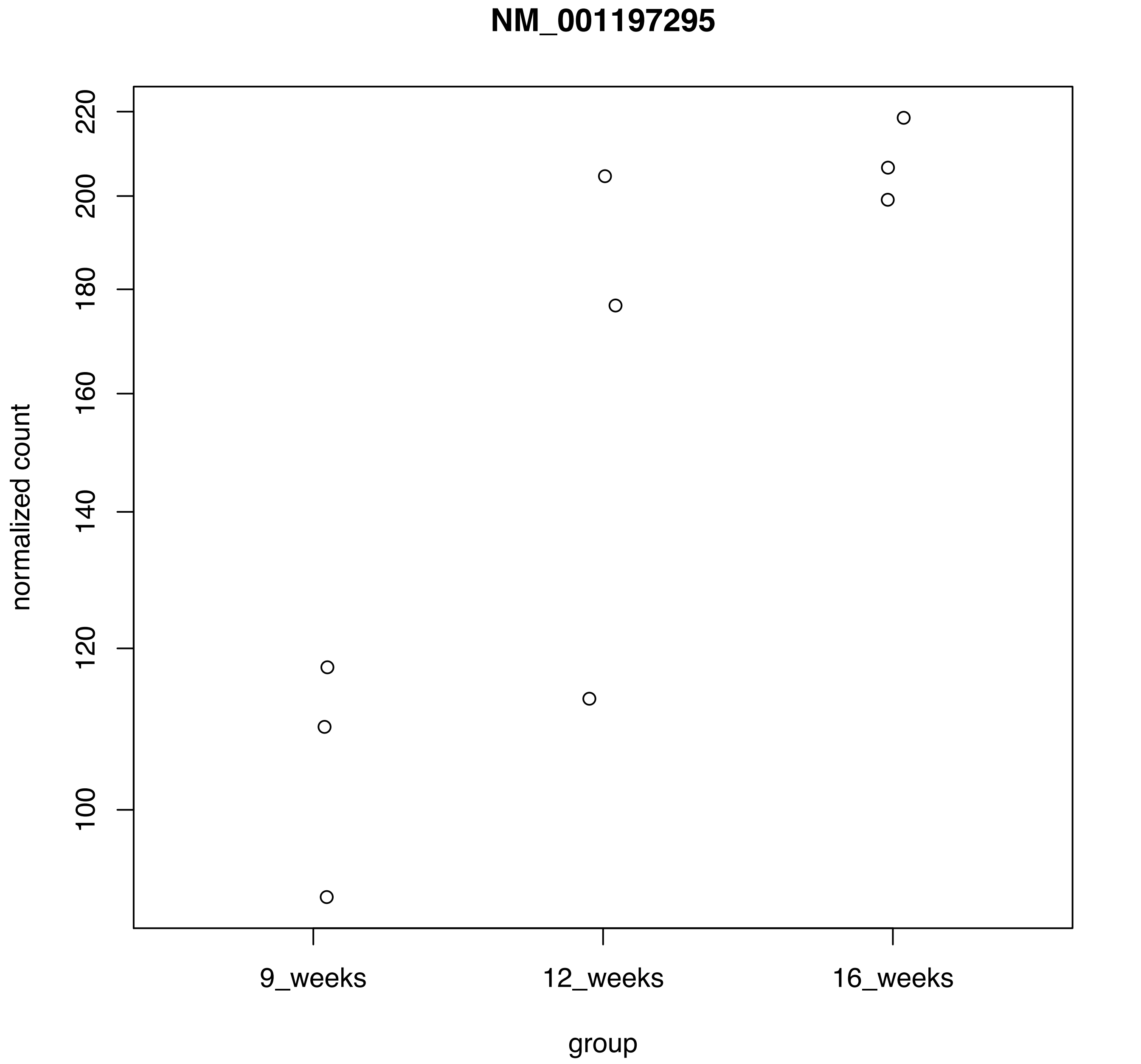
NM_001197295
normalized count
220
200
180
160
140
120
100
9_weeks
12_weeks
16_weeks
group

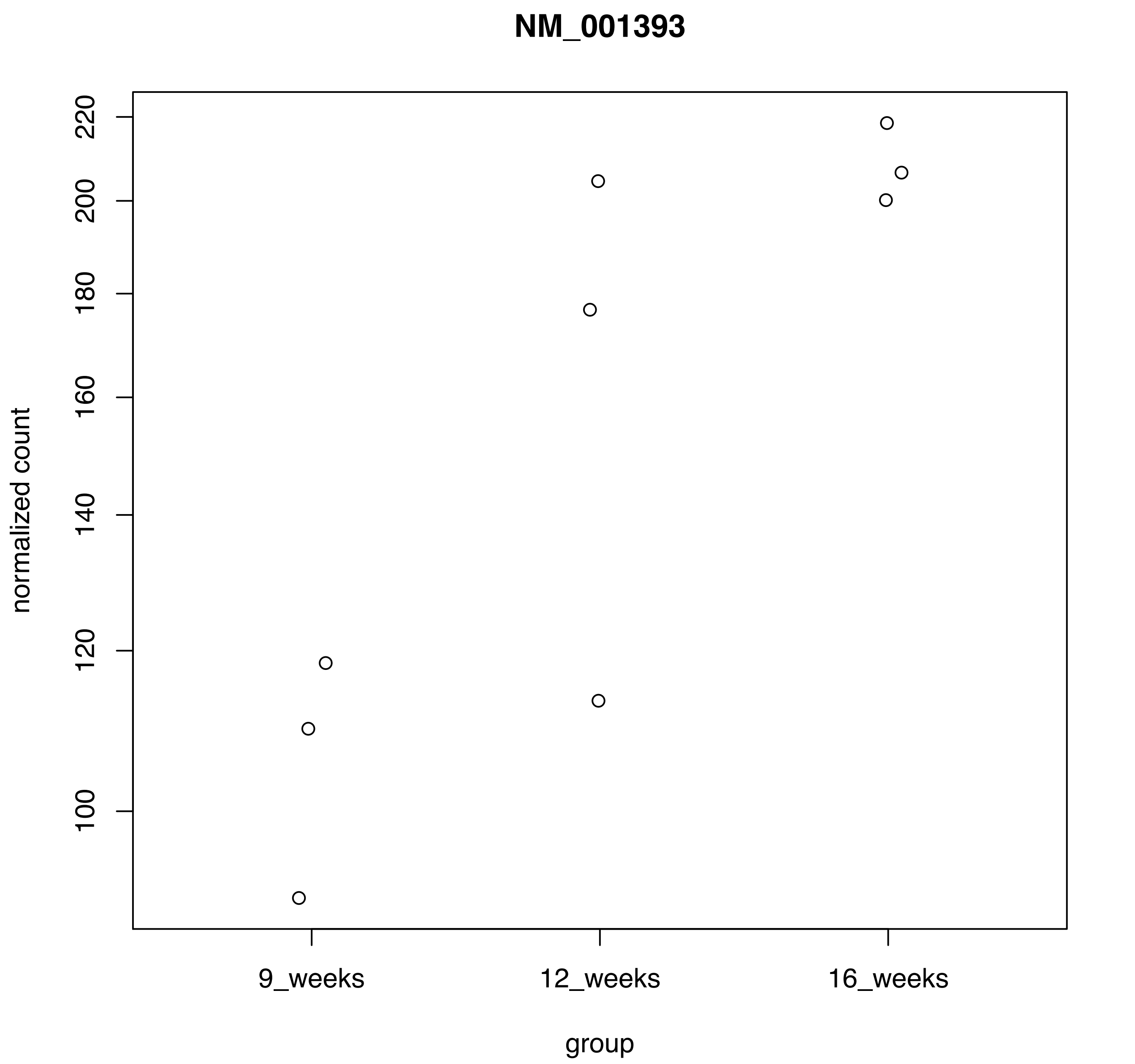
NM_001393
normalized count
100
120
140
160
180
200
220
9_weeks
12_weeks
16_weeks
group

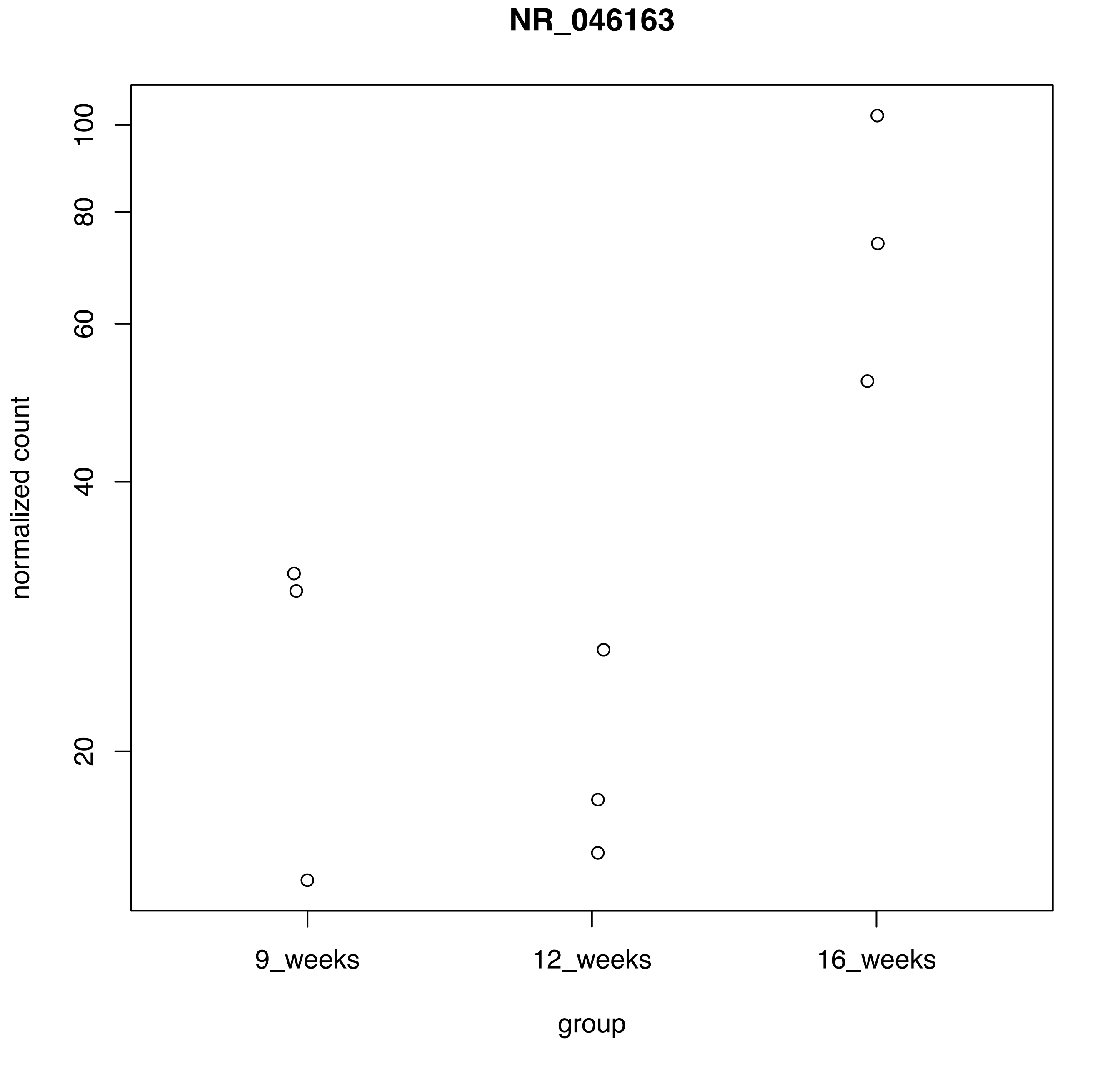
NR_046163
normalized count
100
80
60
40
20
9_weeks
12_weeks
16_weeks
group

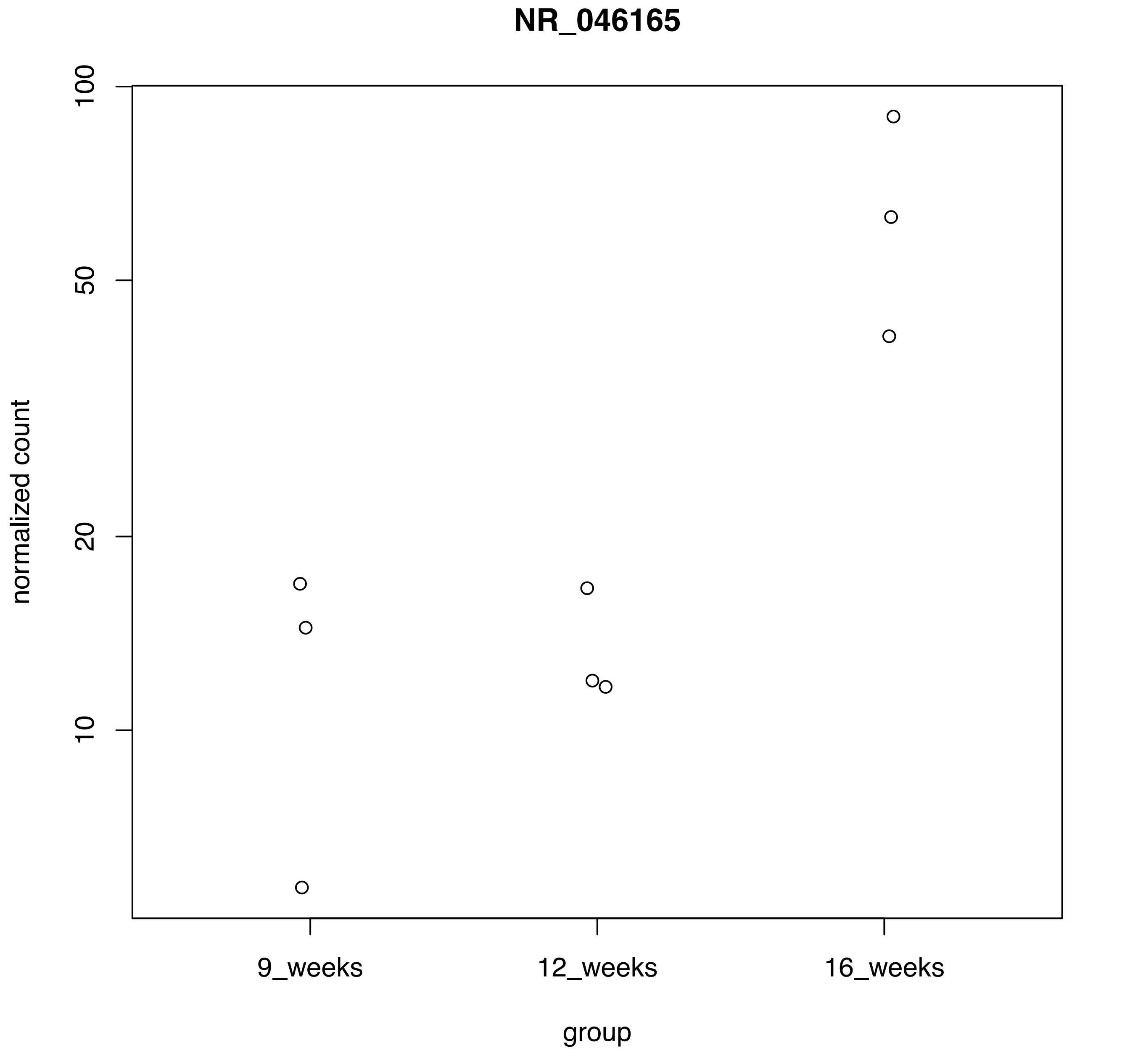
NR_046165
normalized count
100
50
20
10
9_weeks
12_weeks
16_weeks
group

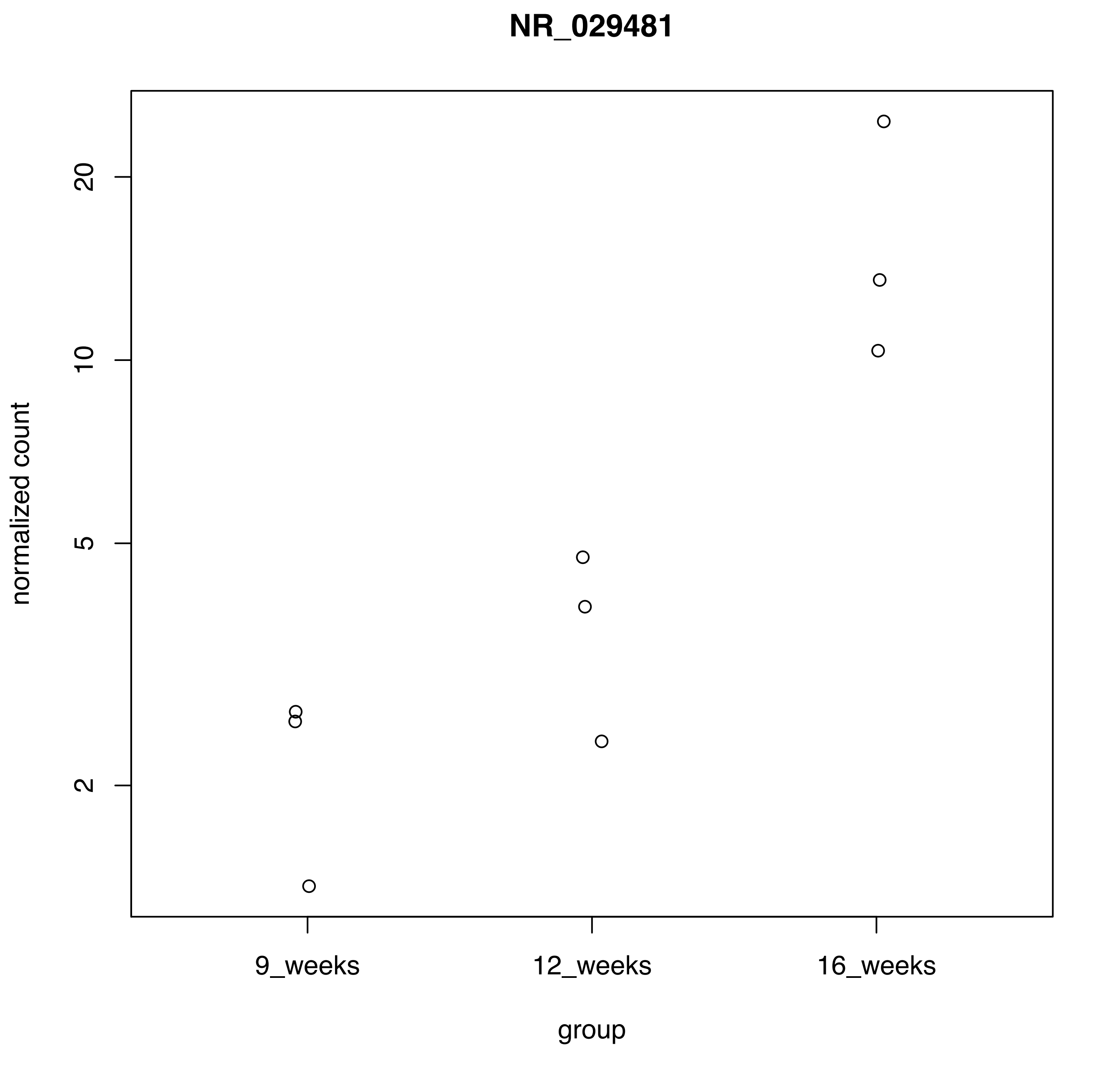
NR_029481
normalized count
20
10
5
2
9_weeks
12_weeks
16_weeks
group

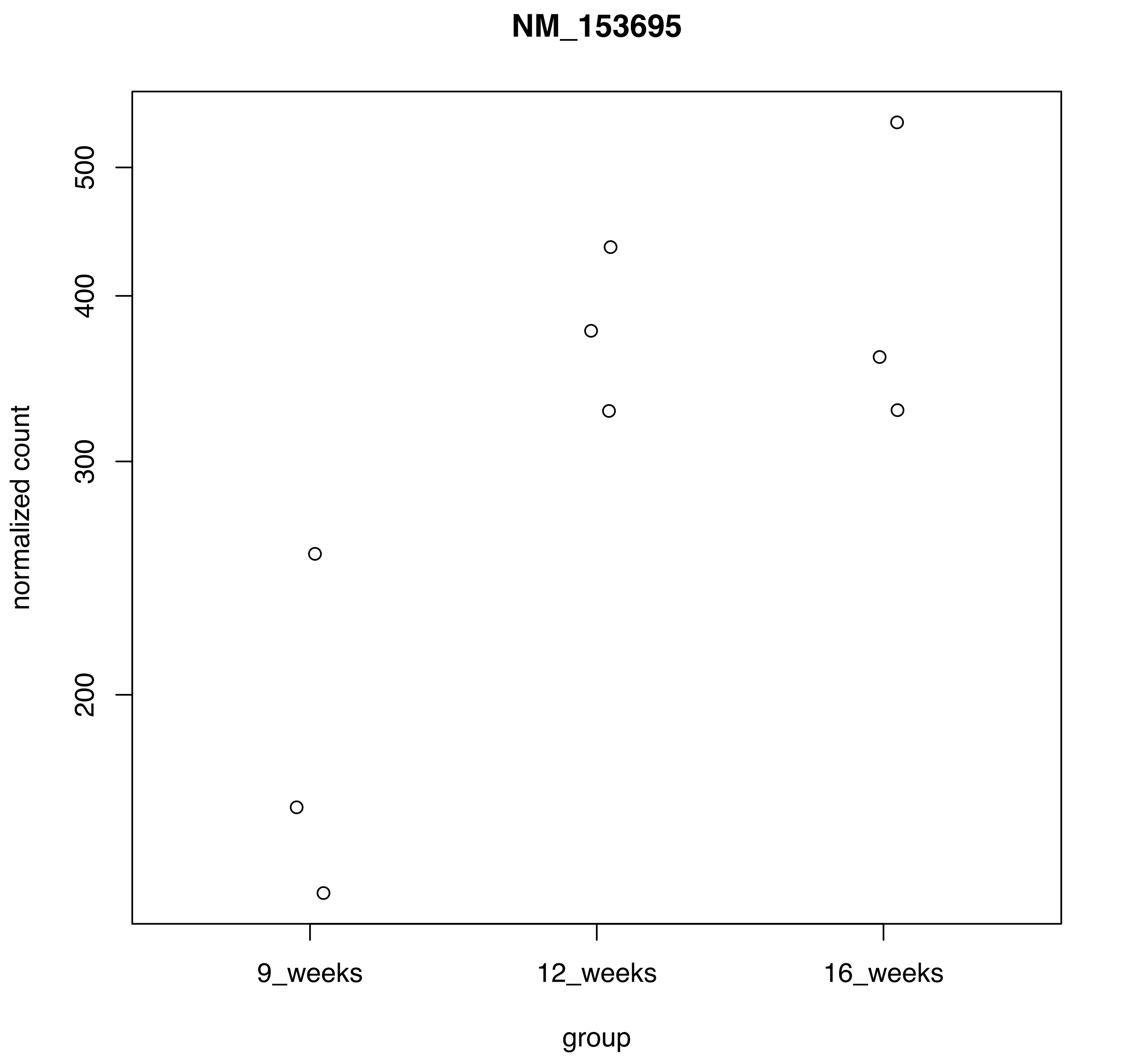
NM_153695
normalized count
500
400
300
200
9_weeks
12_weeks
16_weeks
group

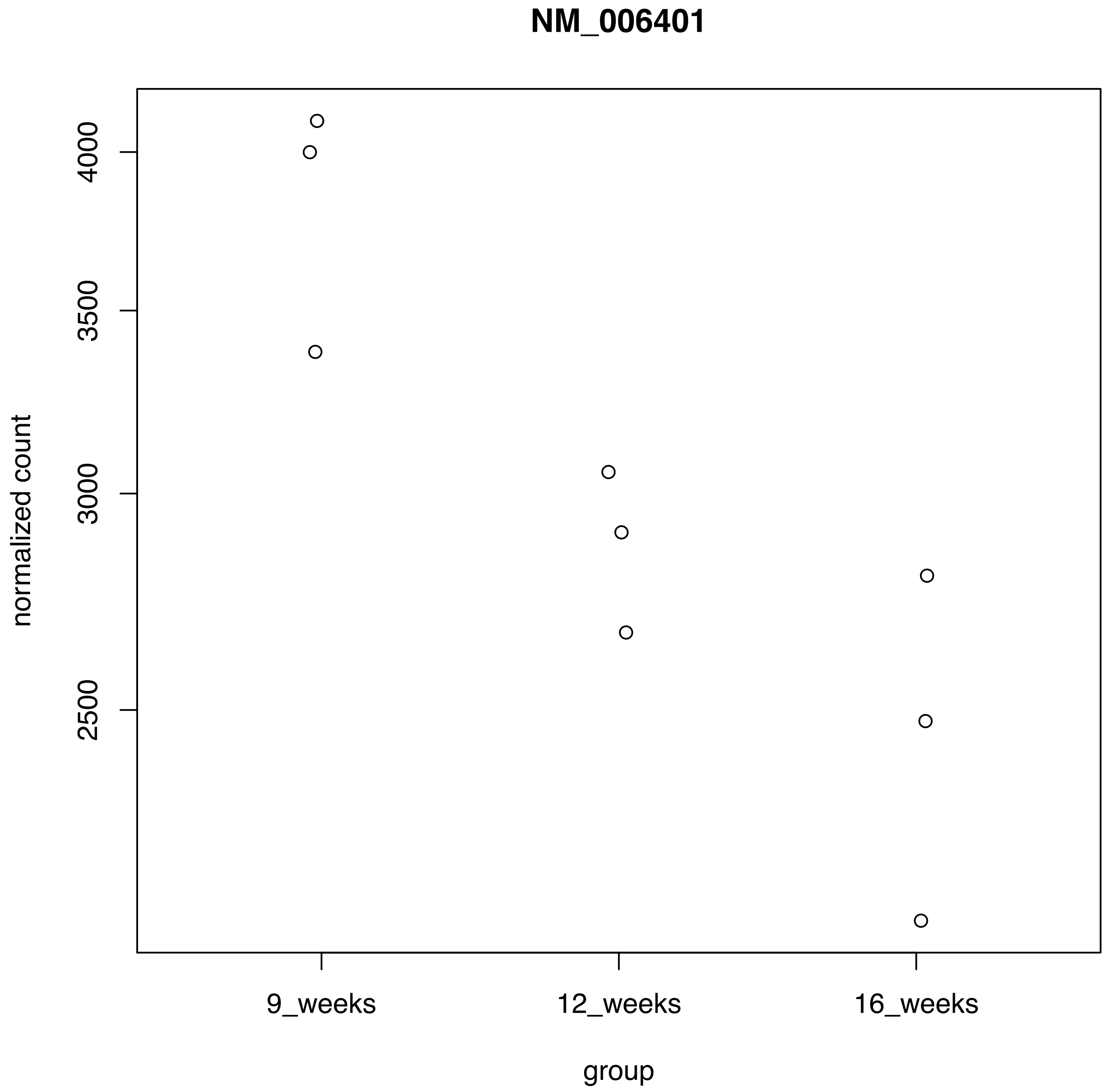
NM_006401
normalized count
4000
3500
3000
2500
9_weeks
12_weeks
16_weeks
group

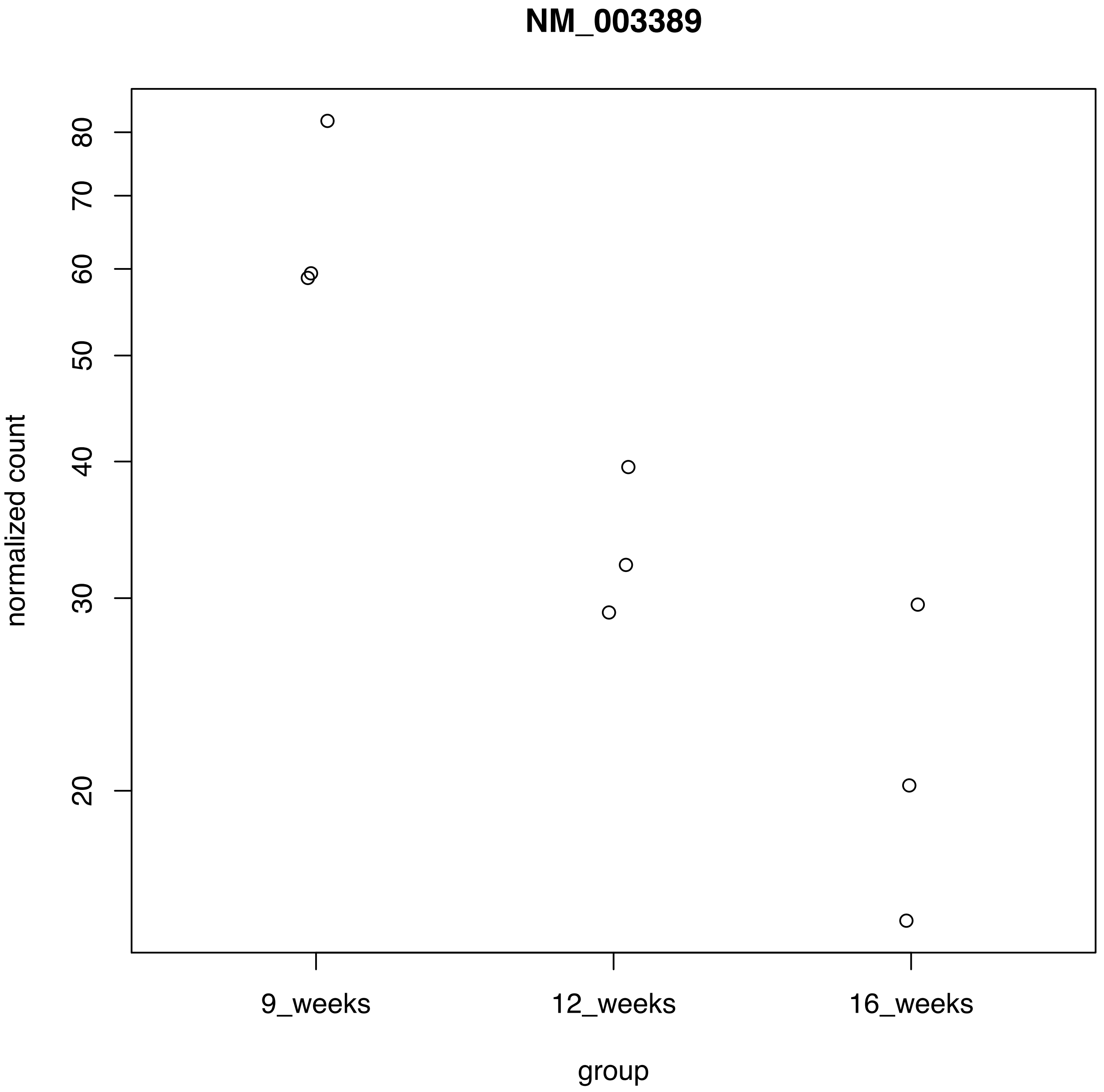
NM_003389
normalized count
80
70
60
50
40
30
20
9_weeks
12_weeks
16_weeks
group

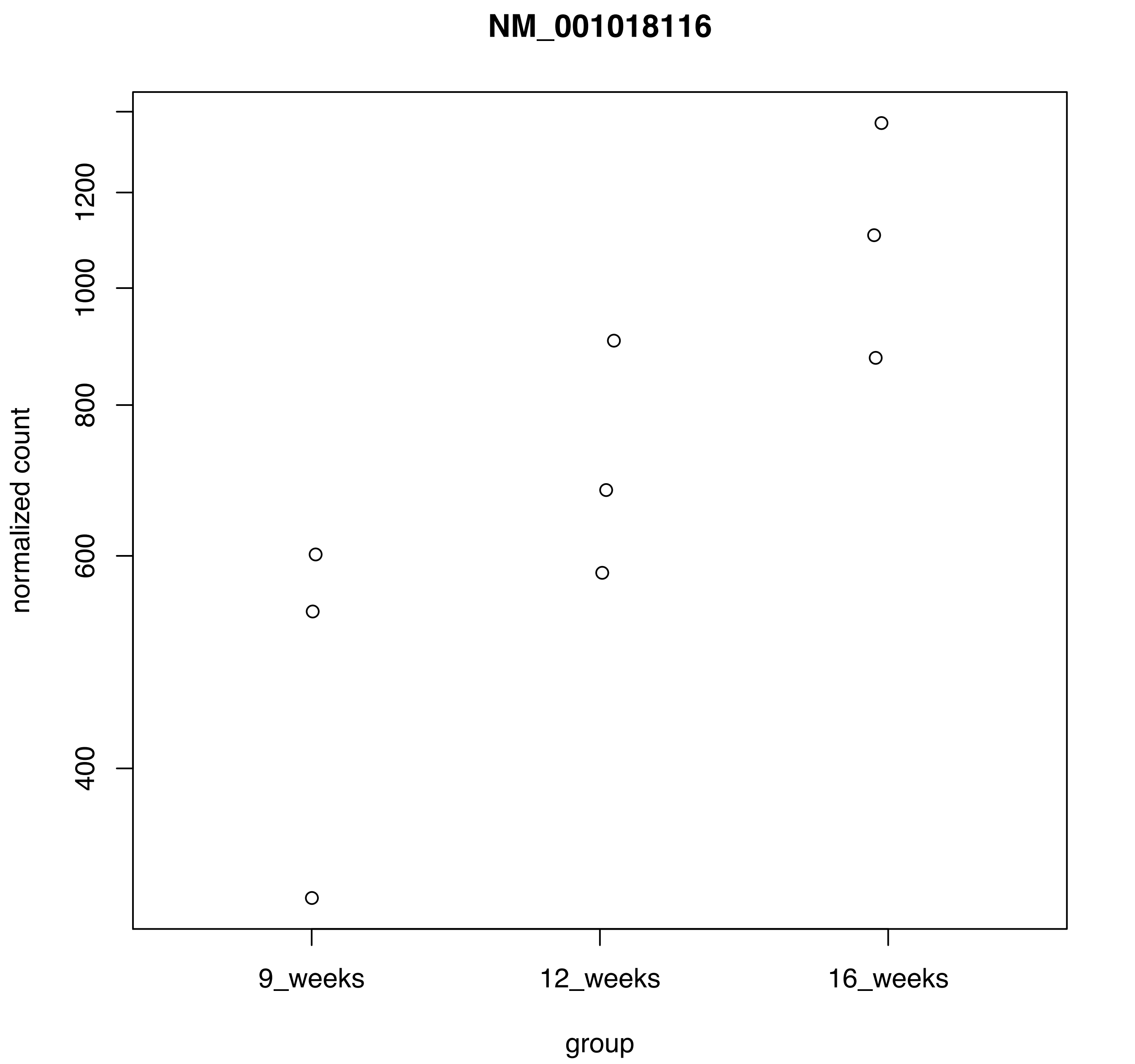
NM_001018116
normalized count
400
600
800
1000
1200
9_weeks
12_weeks
16_weeks
group

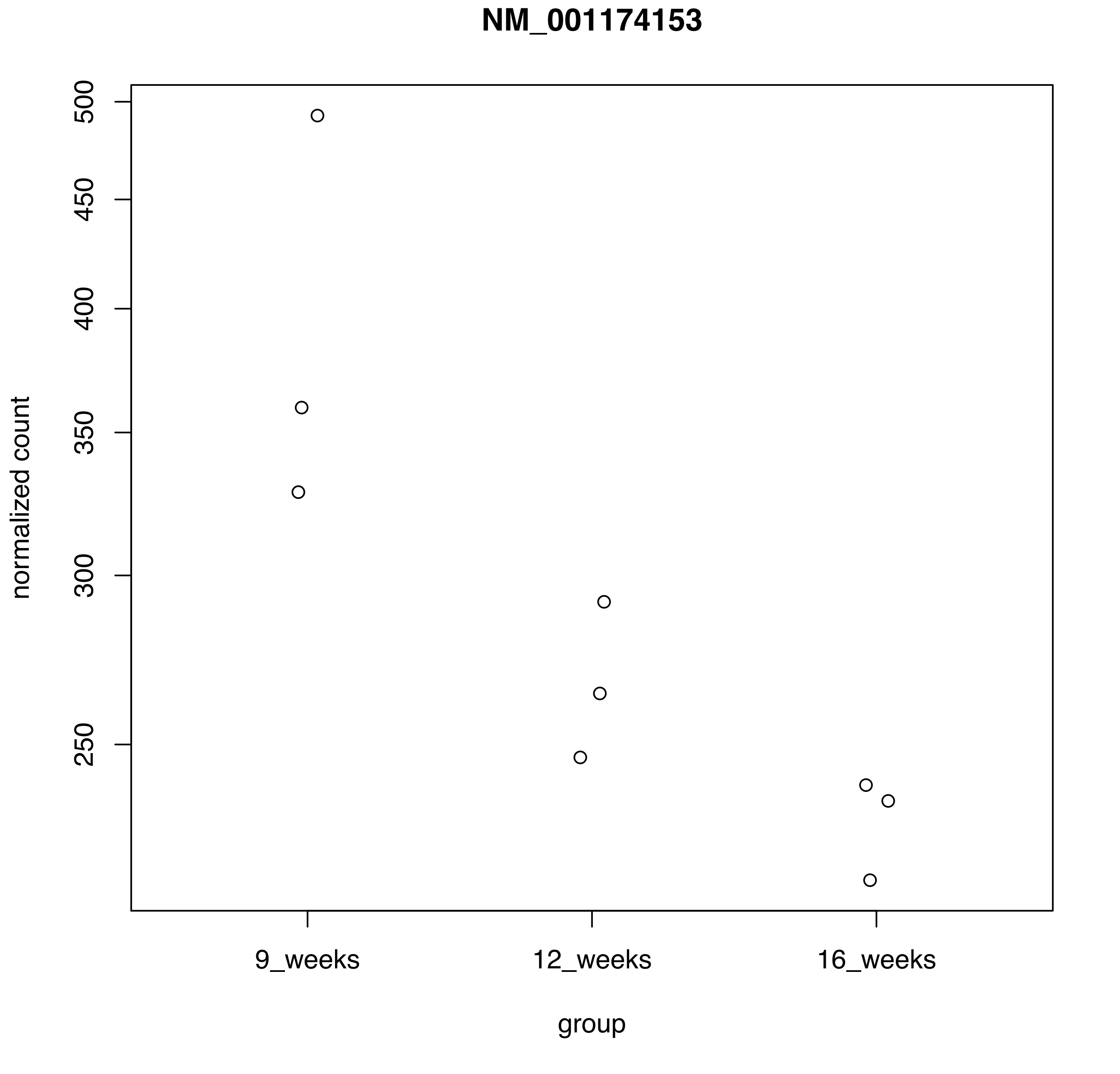
NM_001174153
normalized count
500
450
400
350
300
250
9_weeks
12_weeks
16_weeks
group

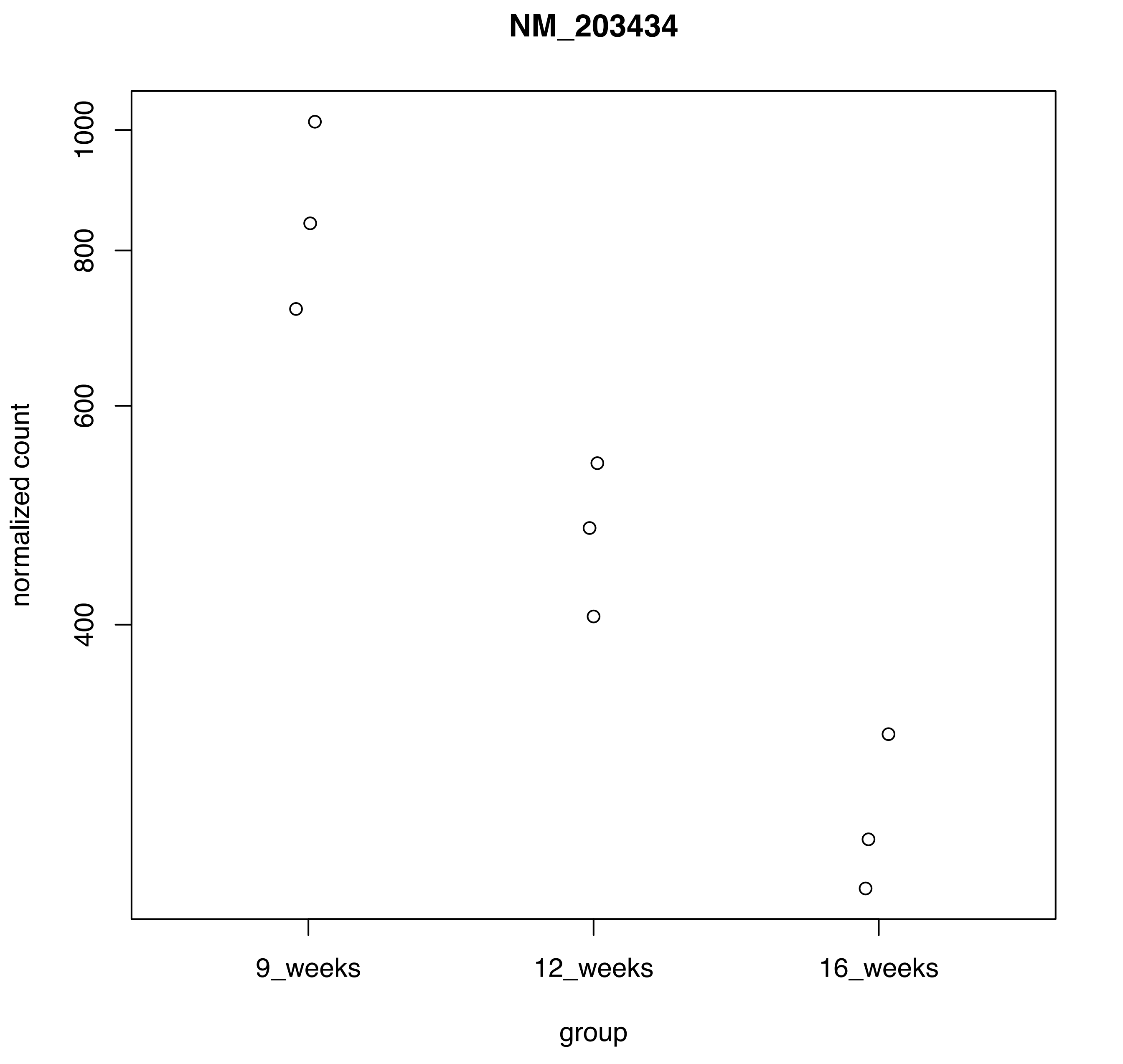

NM_203434
normalized count
1000
800
600
400
9_weeks
12_weeks
16_weeks
group

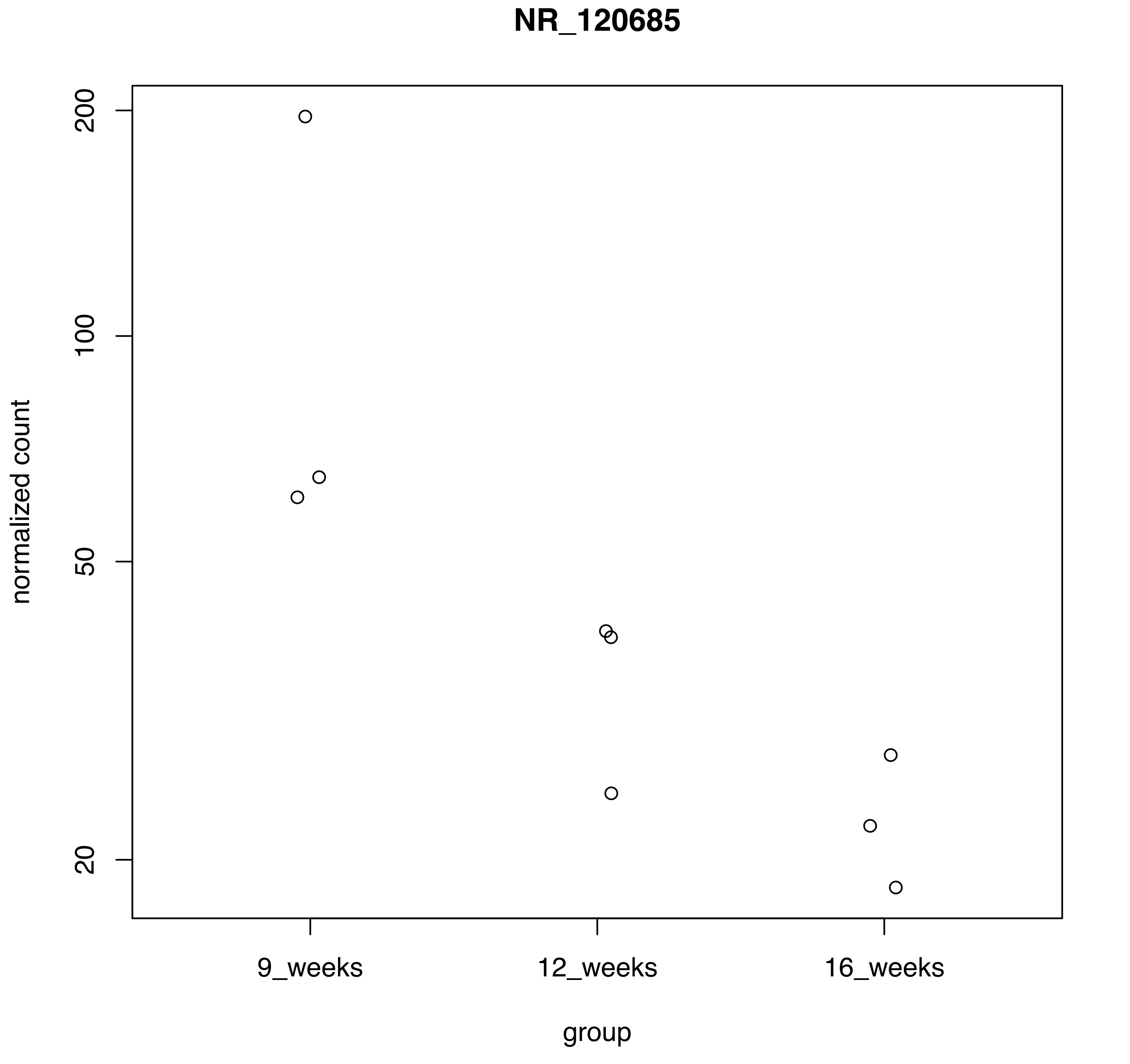
NR_120685
normalized count
200
100
50
20
9_weeks
12_weeks
16_weeks
group

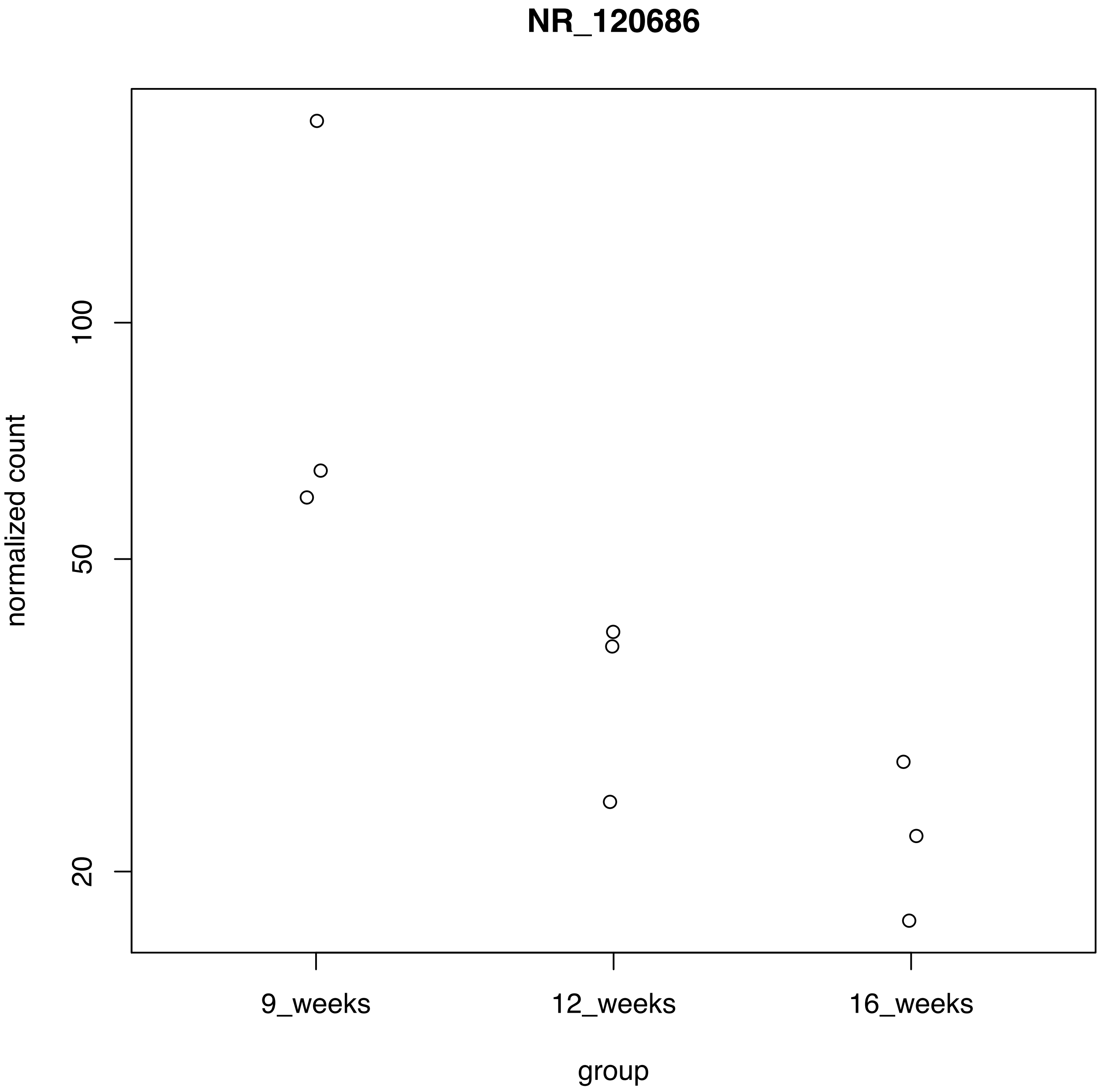
NR_120686
normalized count
100
50
20
9_weeks
12_weeks
16_weeks
group

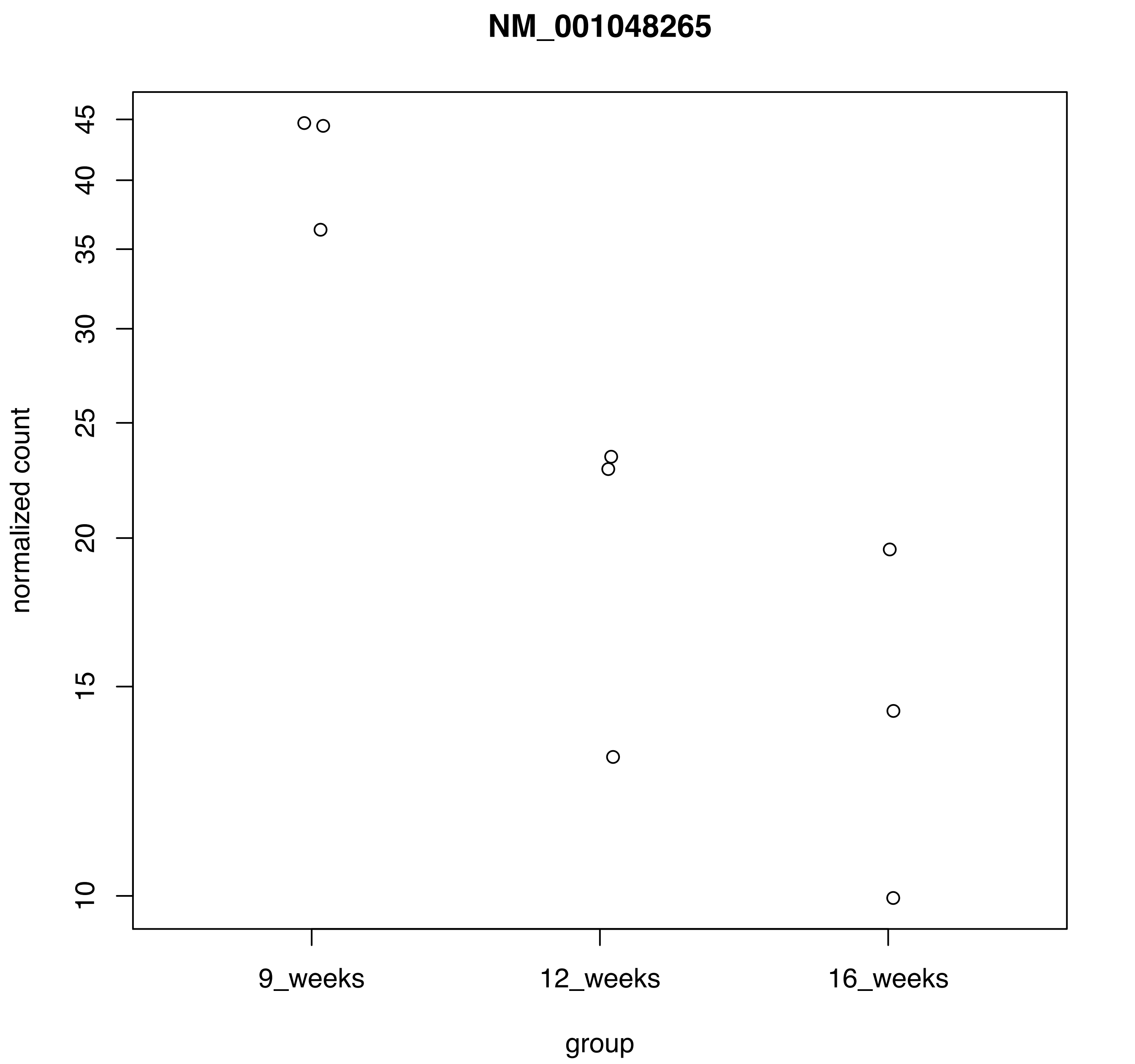
NM_001048265
normalized count
45
40
35
30
25
20
15
10
9_weeks
12_weeks
16_weeks
group

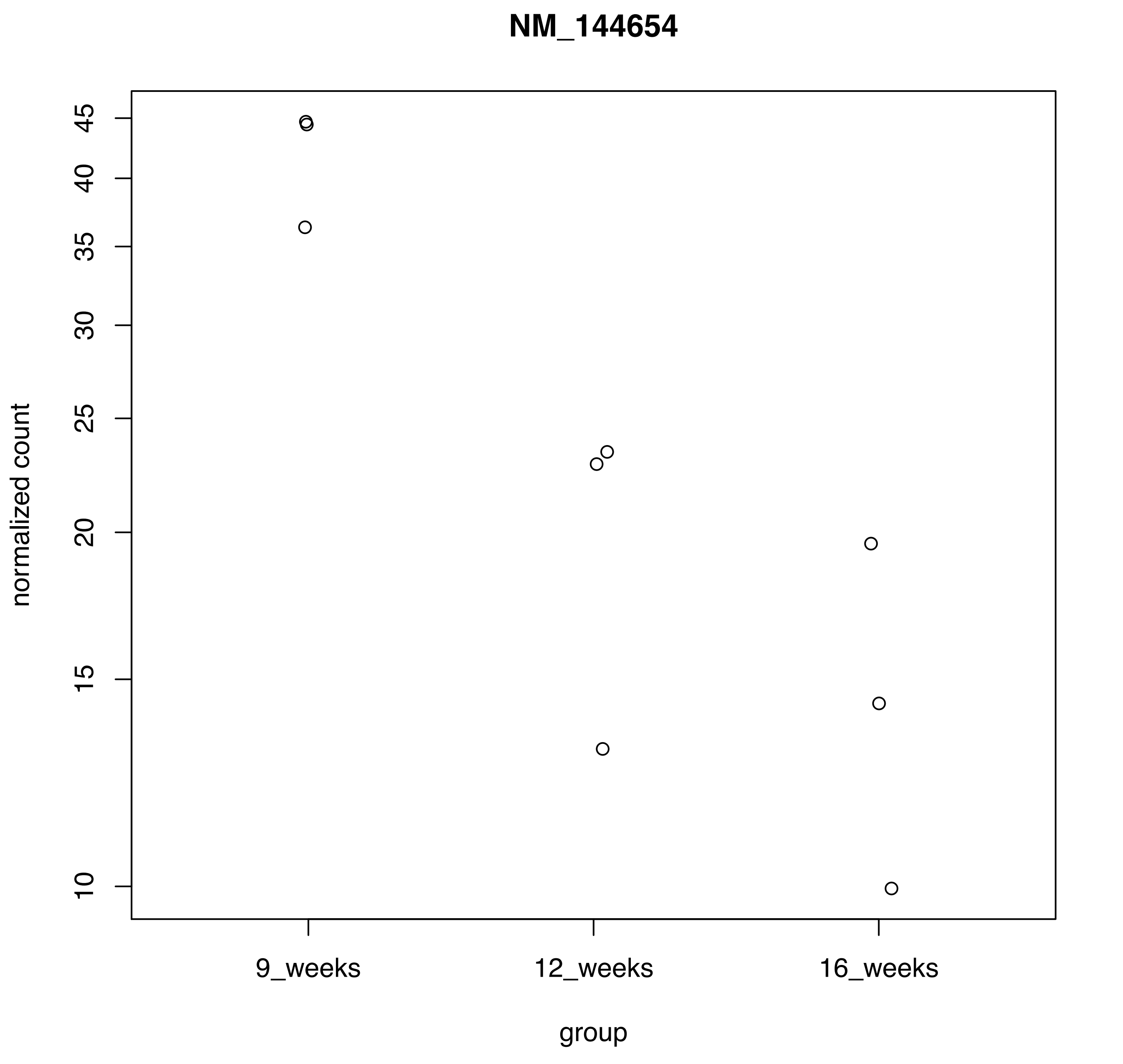
NM_144654
normalized count
45
40
35
30
25
20
15
10
9_weeks
12_weeks
16_weeks
group

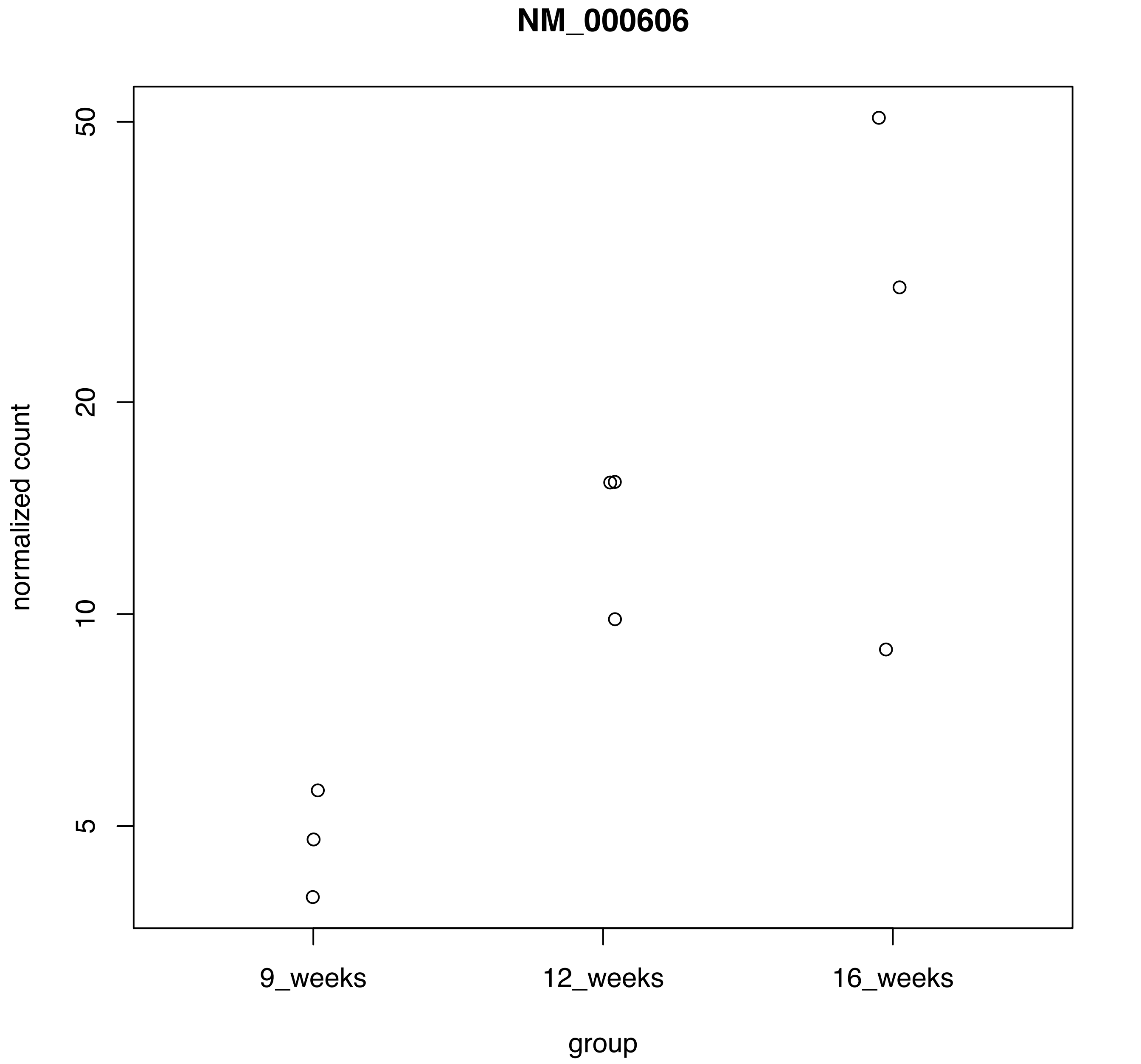
NM_000606
normalized count
50
20
10
5
9_weeks
12_weeks
16_weeks
group

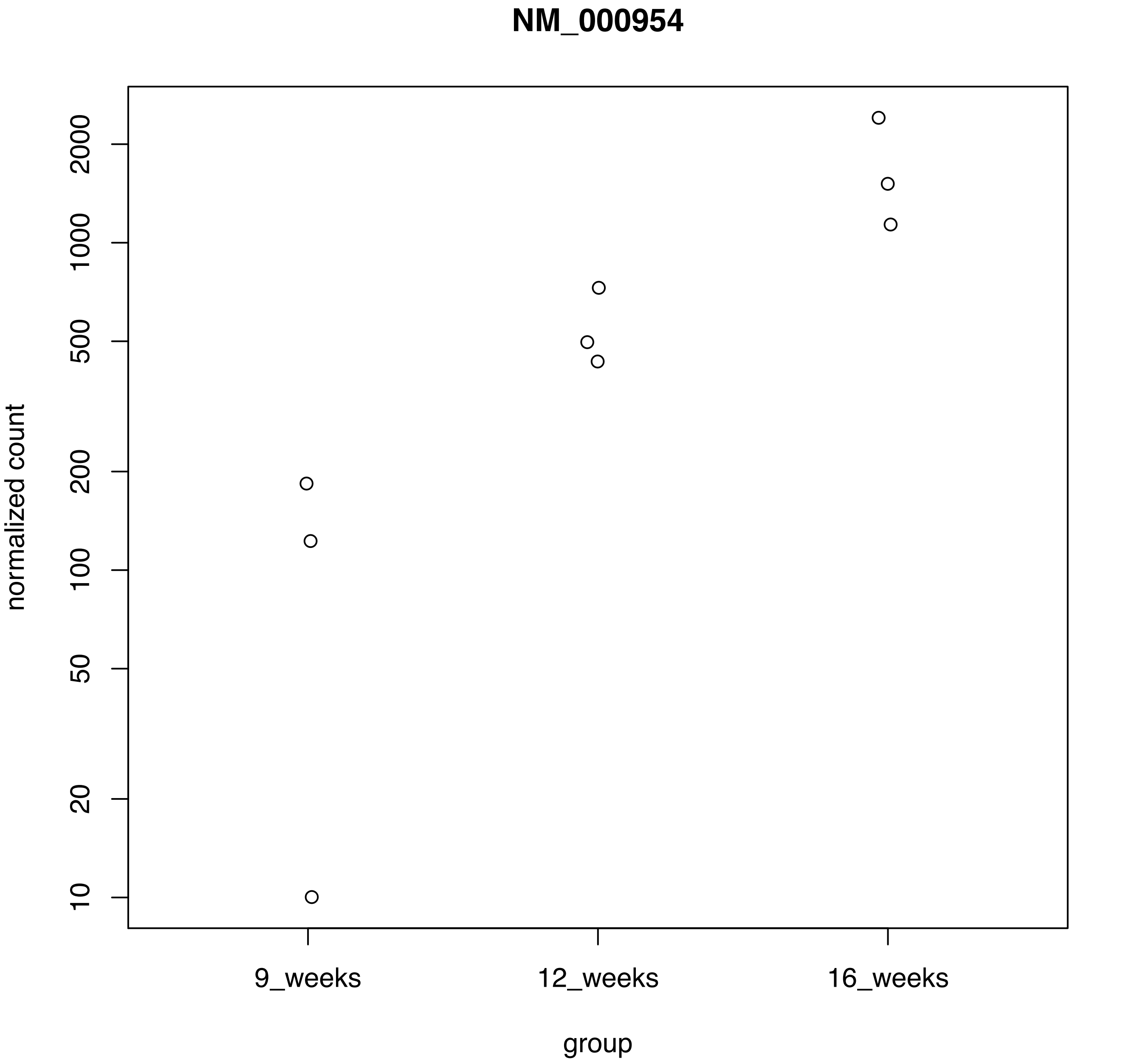
NM_000954
normalized count
2000
1000
500
200
100
50
20
10
9_weeks
12_weeks
16_weeks
group

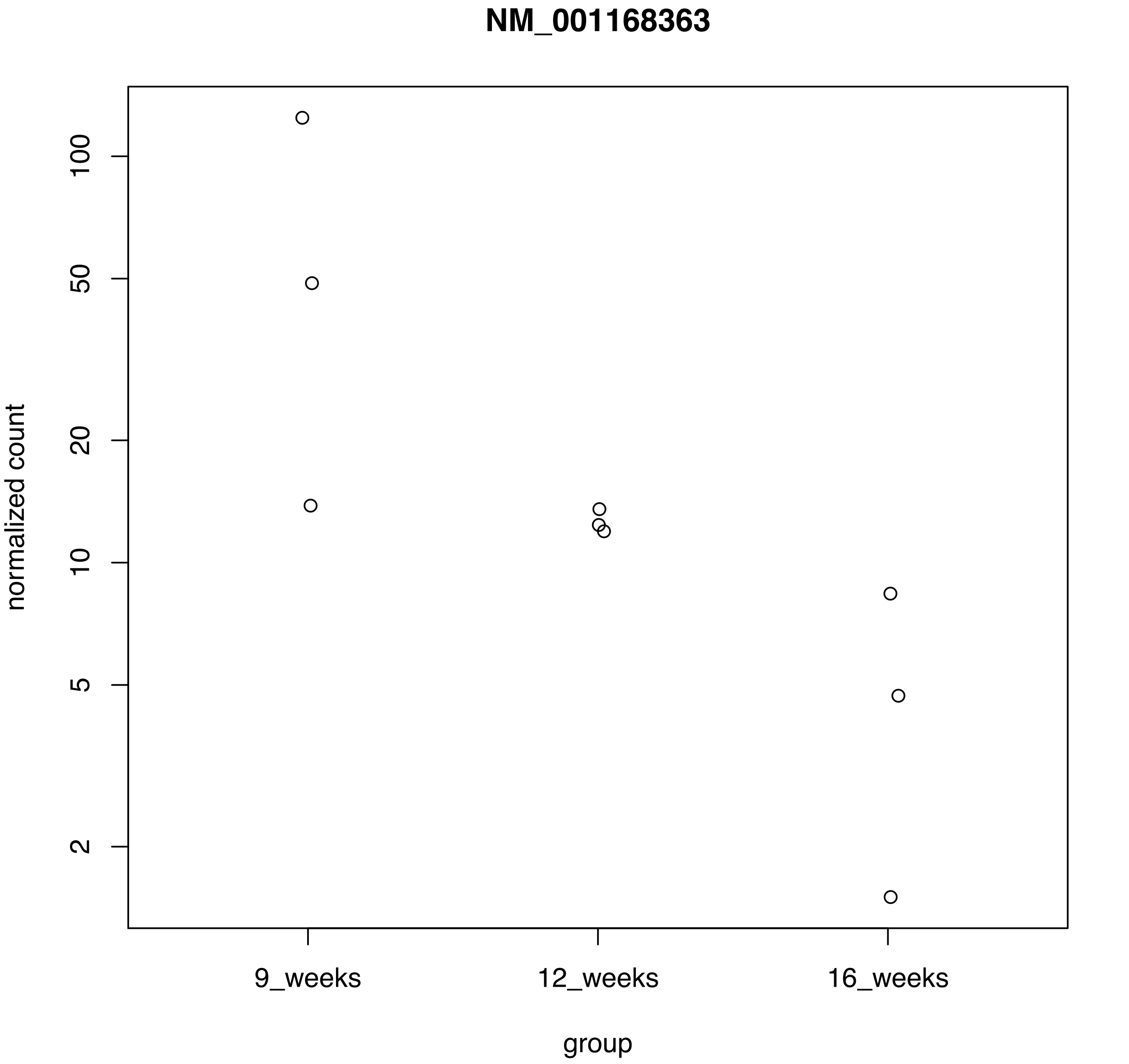
NM_001168363
100
50
20
10
5
2
normalized count
9_weeks
12_weeks
16_weeks
group

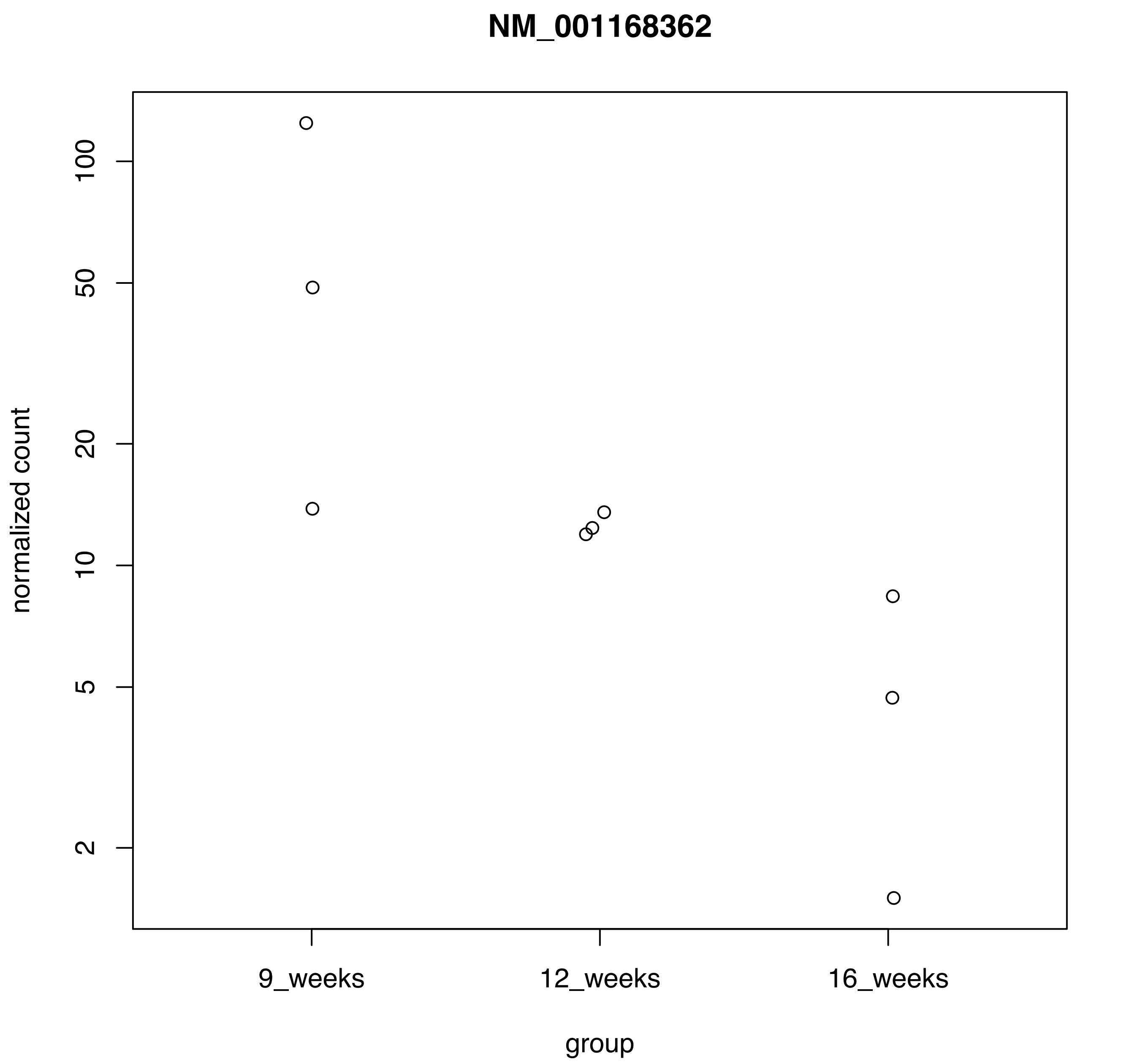
NM_001168362
normalized count
100
50
20
10
5
2
9_weeks
12_weeks
16_weeks
group

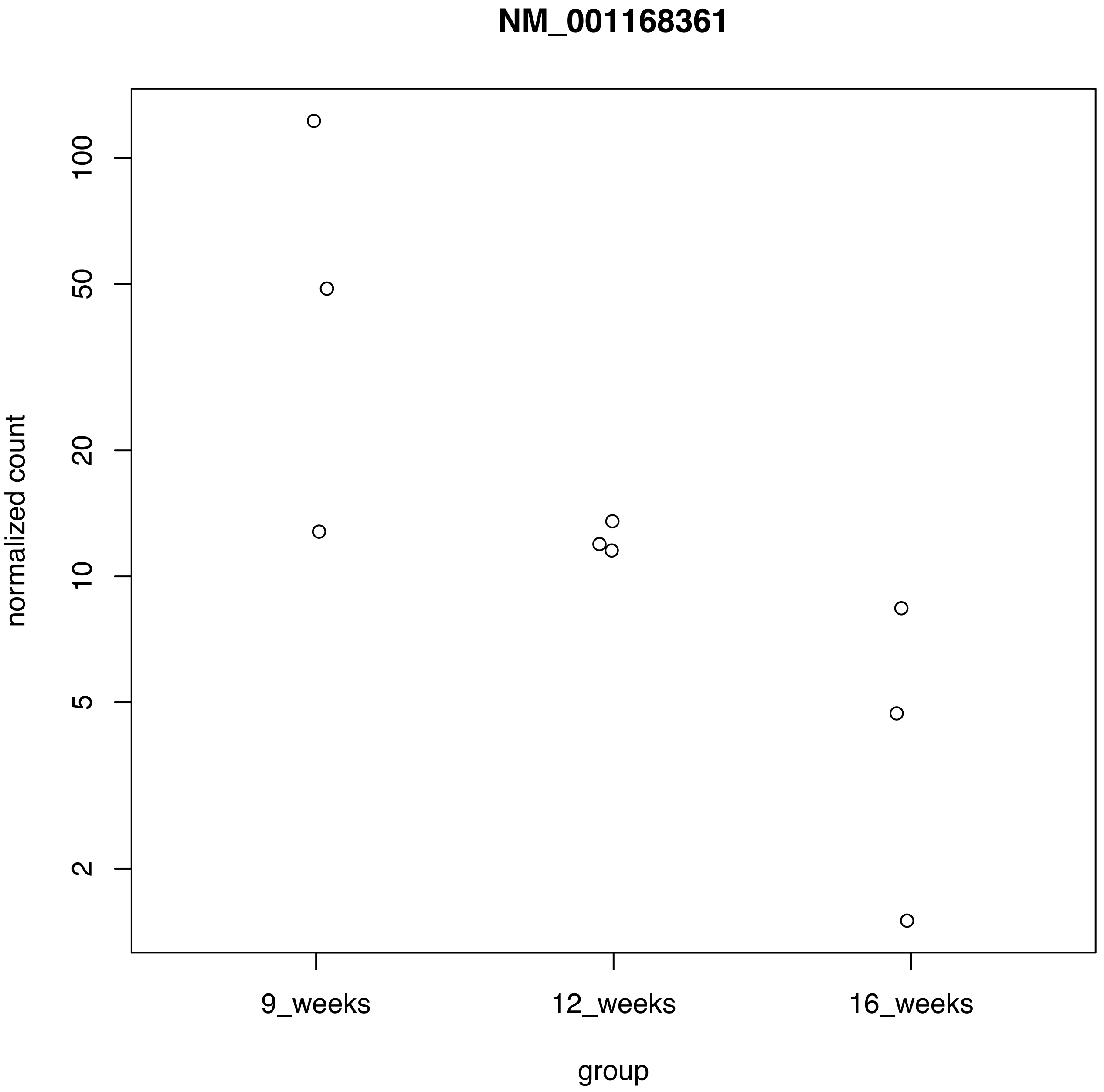

NM_001168361
normalized count
100
50
20
10
5
2
9_weeks
12_weeks
16_weeks
group

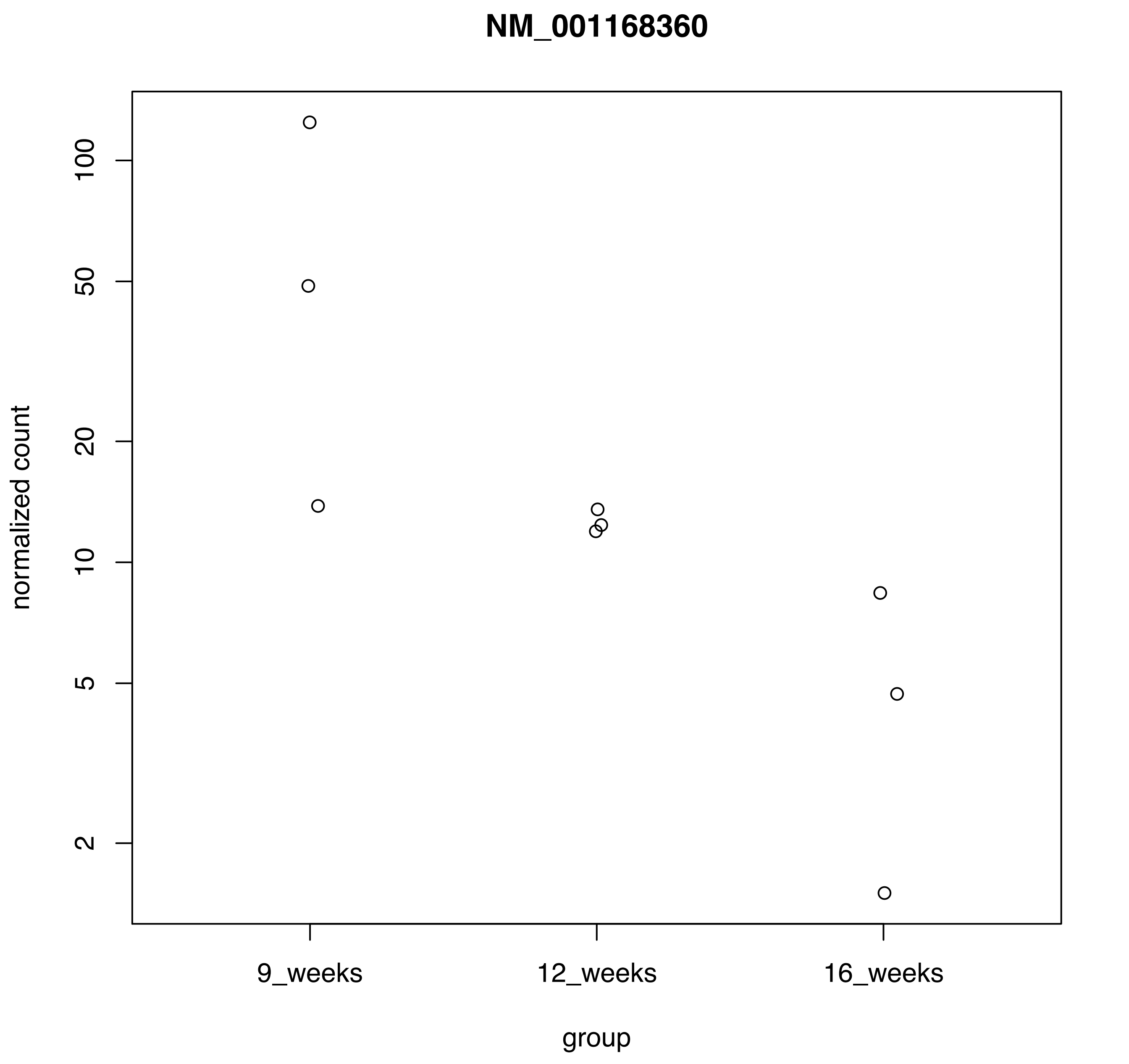
NM_001168360
normalized count
100
50
20
10
5
2
9_weeks
12_weeks
16_weeks
group

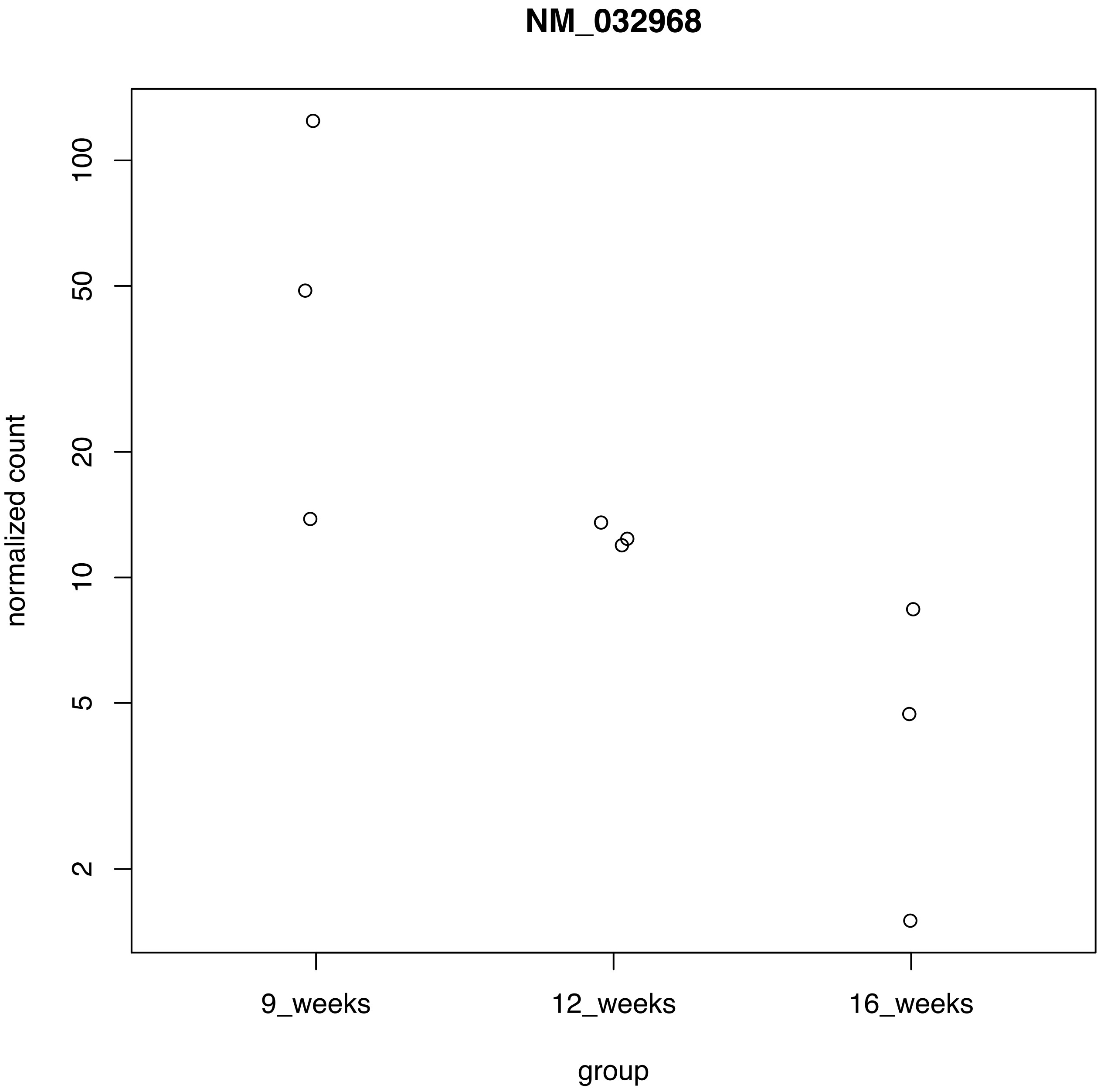
NM_032968
normalized count
100
50
20
10
5
2
9_weeks
12_weeks
16_weeks
group

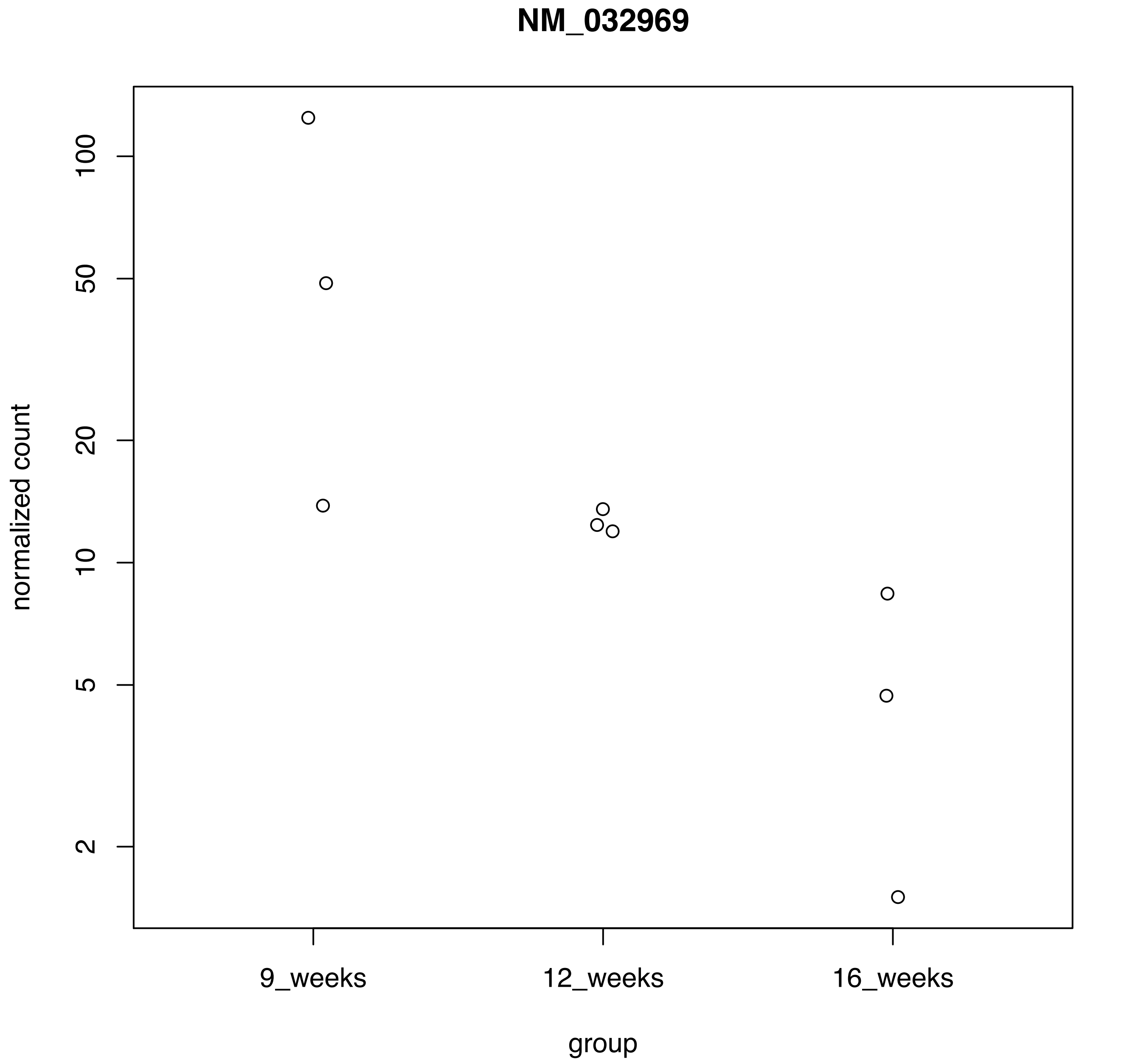

NM_032969
normalized count
100
50
20
10
5
2
9_weeks
12_weeks
16_weeks
group

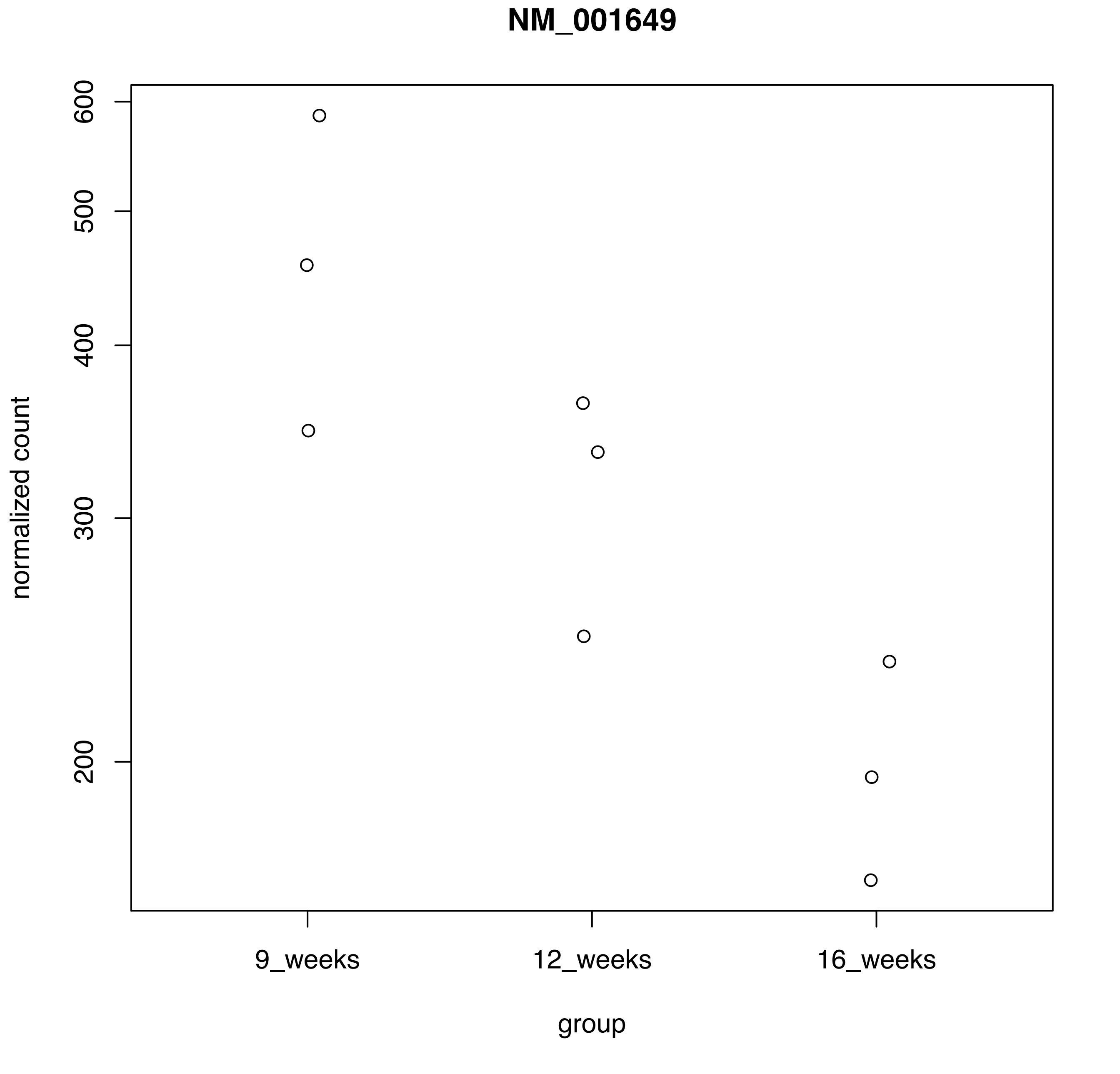

NM_001649
normalized count
600
500
400
300
200
9_weeks
12_weeks
16_weeks
group

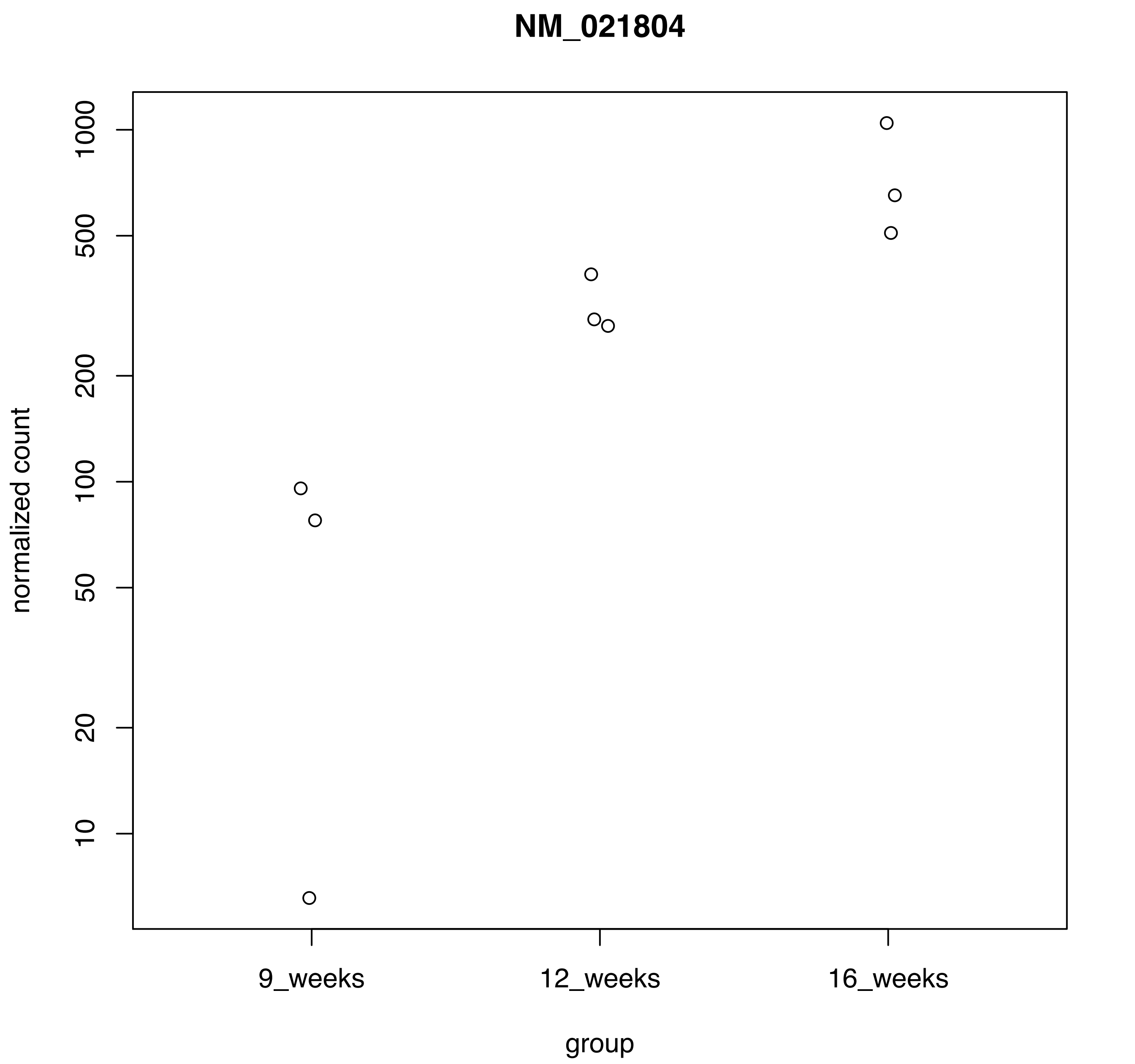
NM_021804
normalized count
1000
500
200
100
50
20
10
9_weeks
12_weeks
16_weeks
group

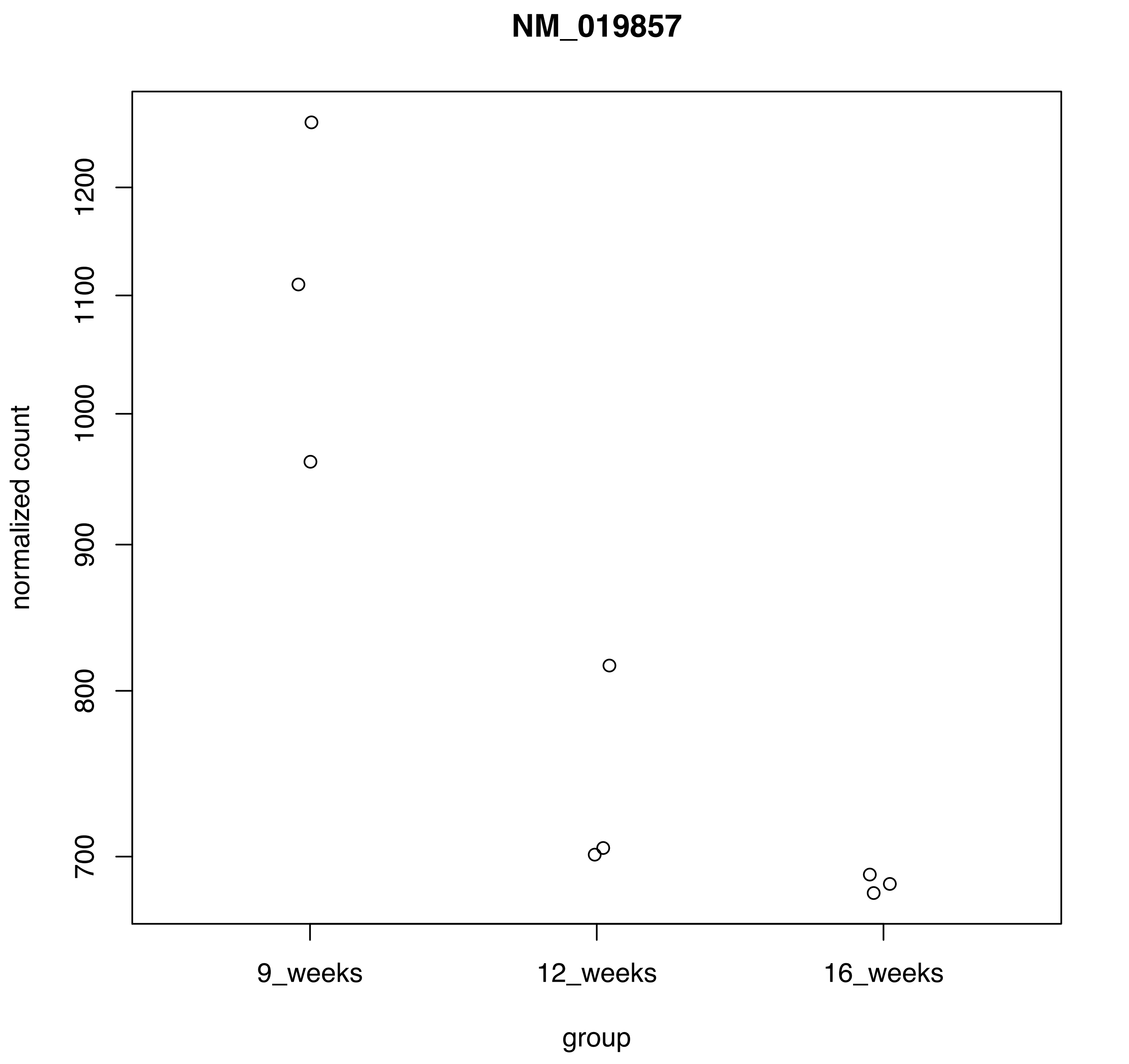
NM_019857
normalized count
1200
1100
1000
900
800
700
9_weeks
12_weeks
16_weeks
group

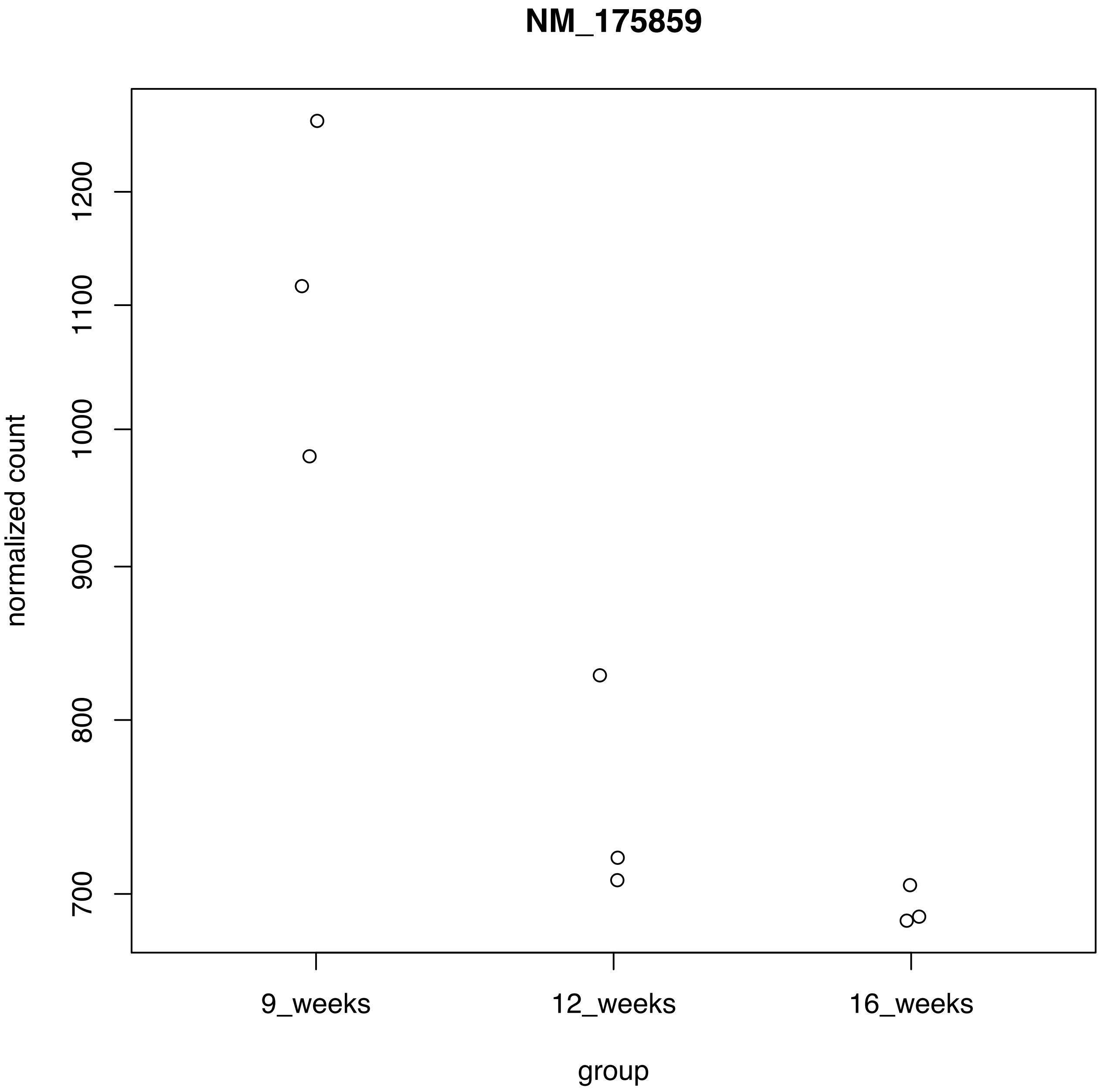
NM_175859
normalized count
1200
1100
1000
900
800
700
9_weeks
12_weeks
16_weeks
group

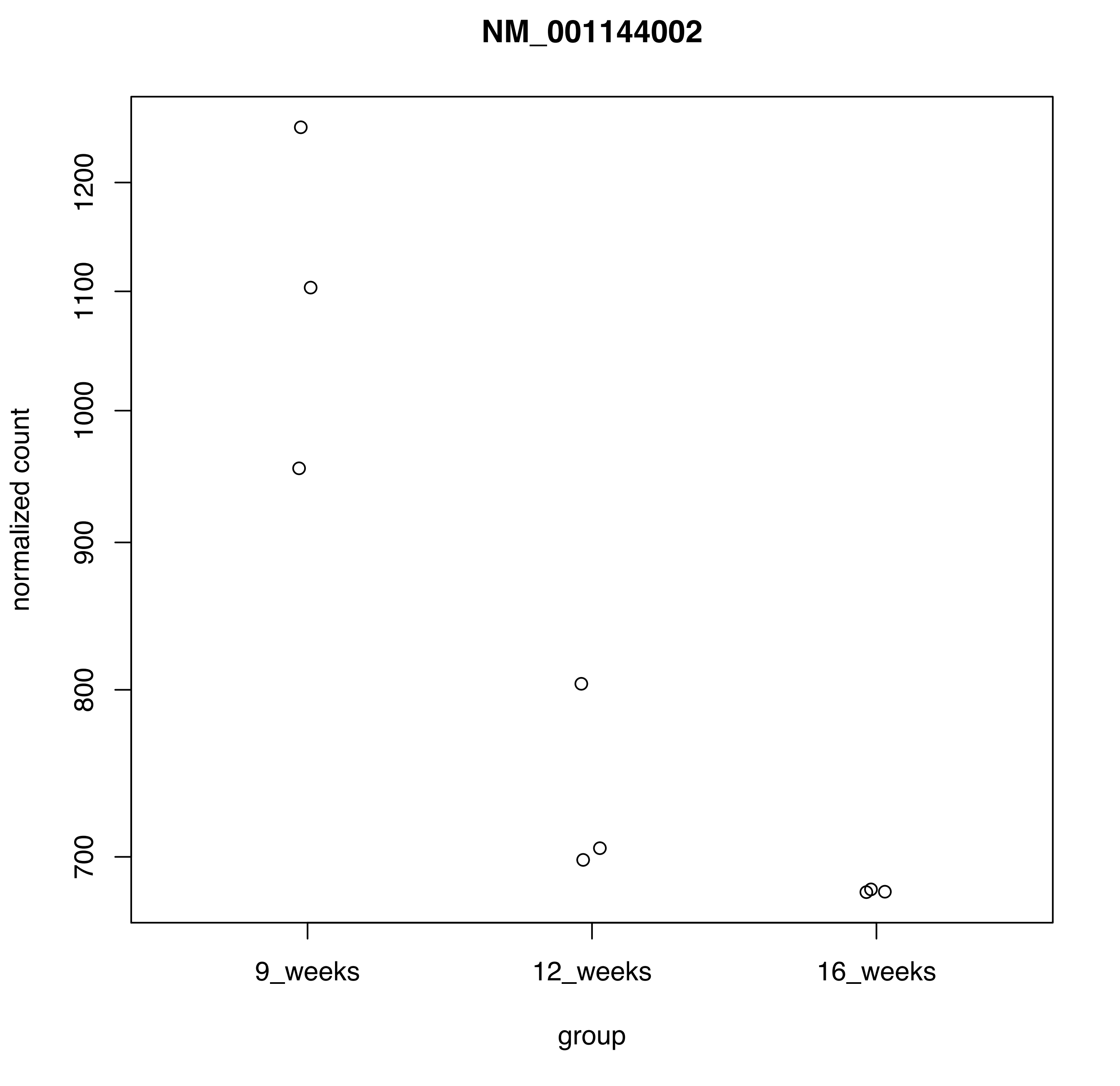

NM_001144002
normalized count
1200
1100
1000
900
800
700
9_weeks
12_weeks
16_weeks
group

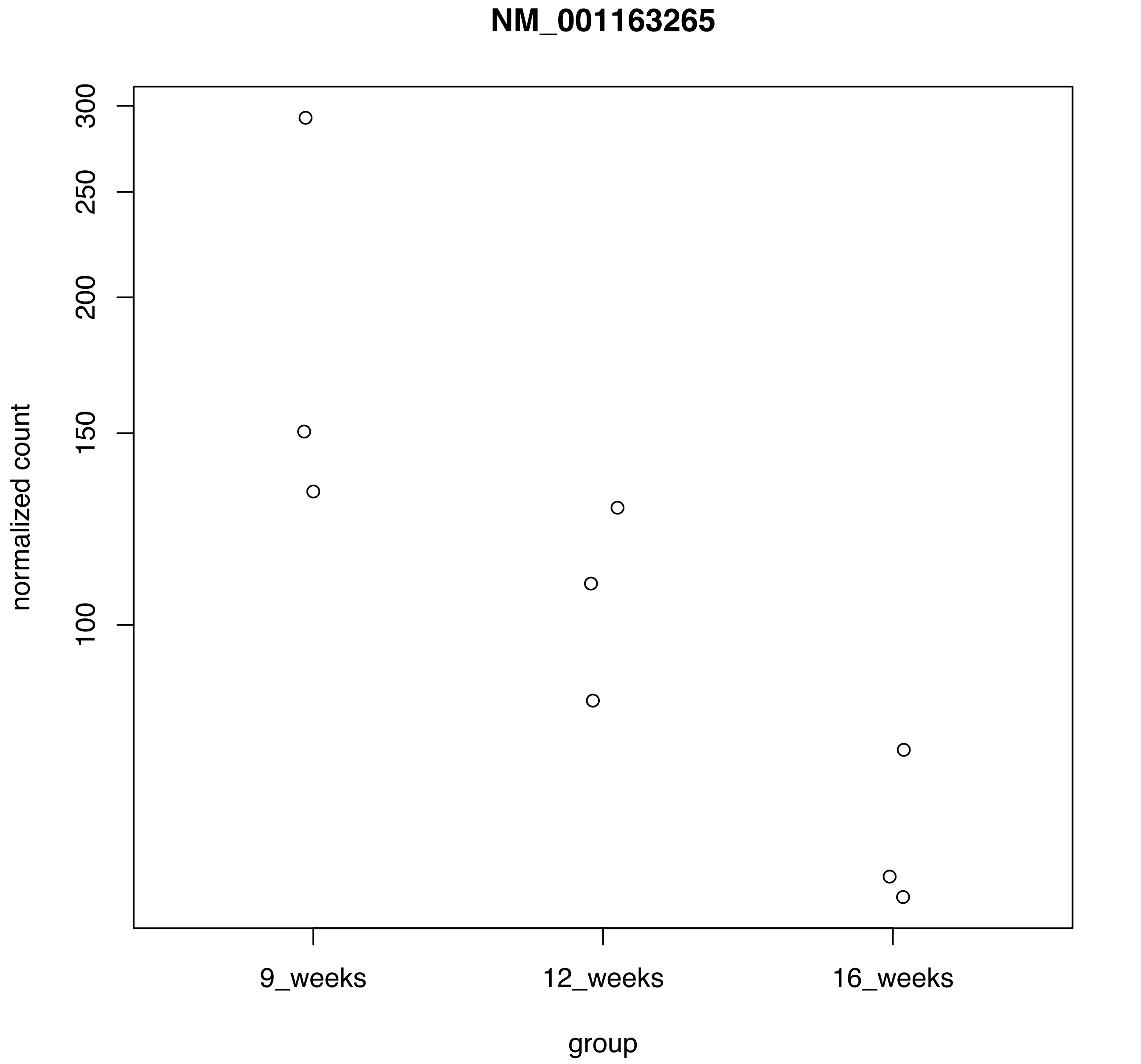

NM_001163265
normalized count
300
250
200
150
100
9_weeks
12_weeks
16_weeks
group

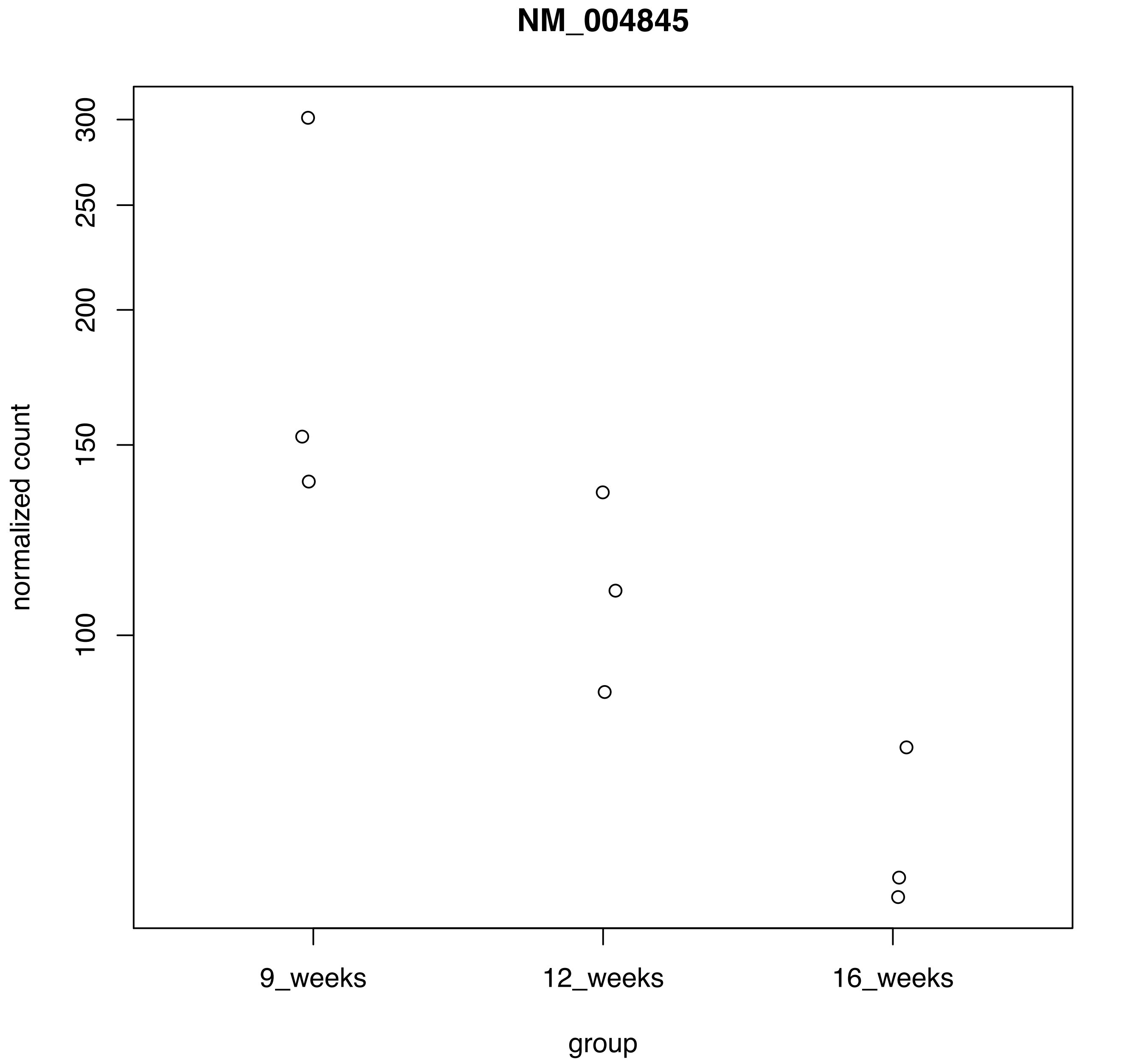
NM_004845
normalized count
300
250
200
150
100
9_weeks
12_weeks
16_weeks
group

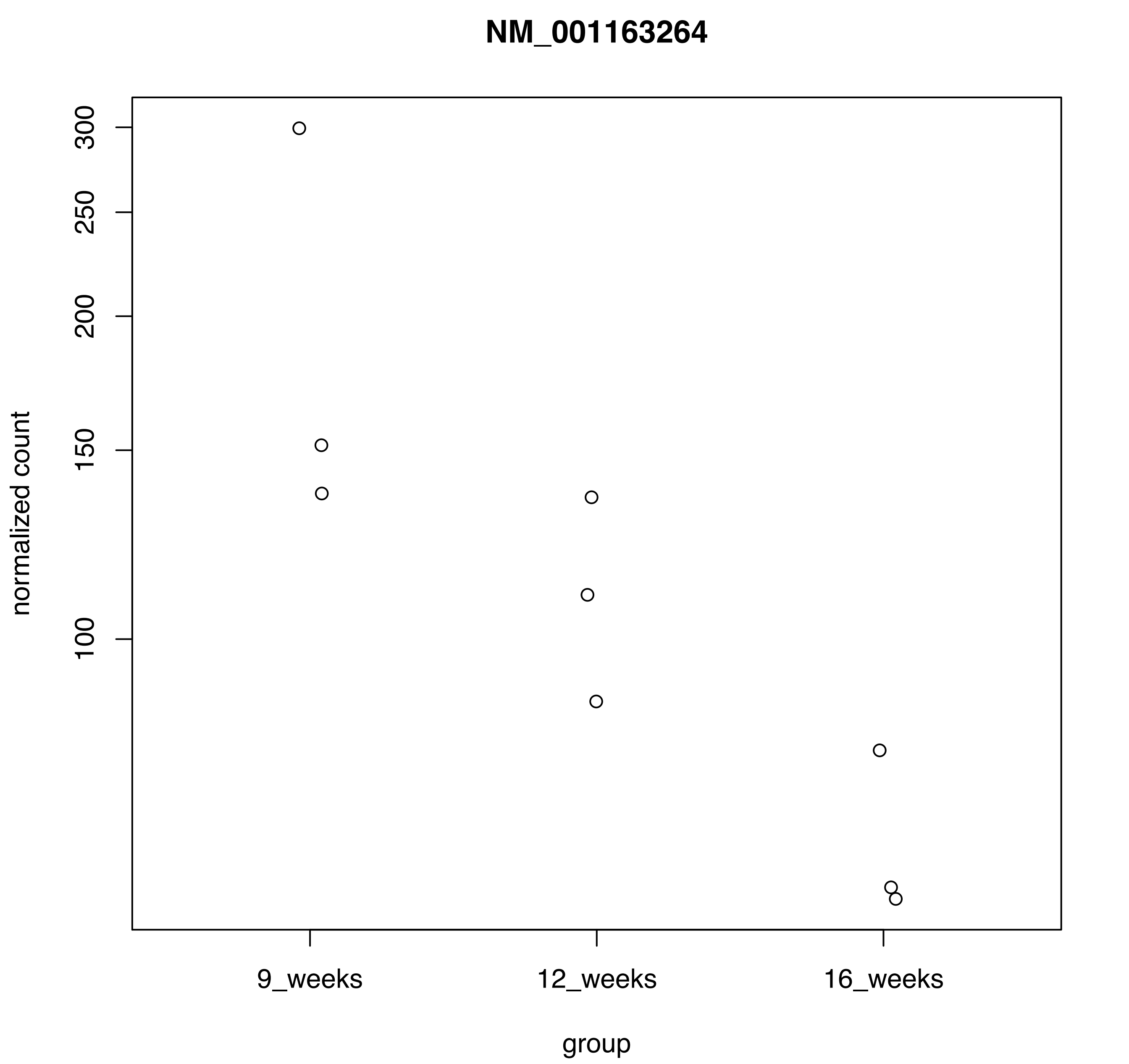
NM_001163264
normalized count
100
150
200
250
300
group
9_weeks
12_weeks
16_weeks

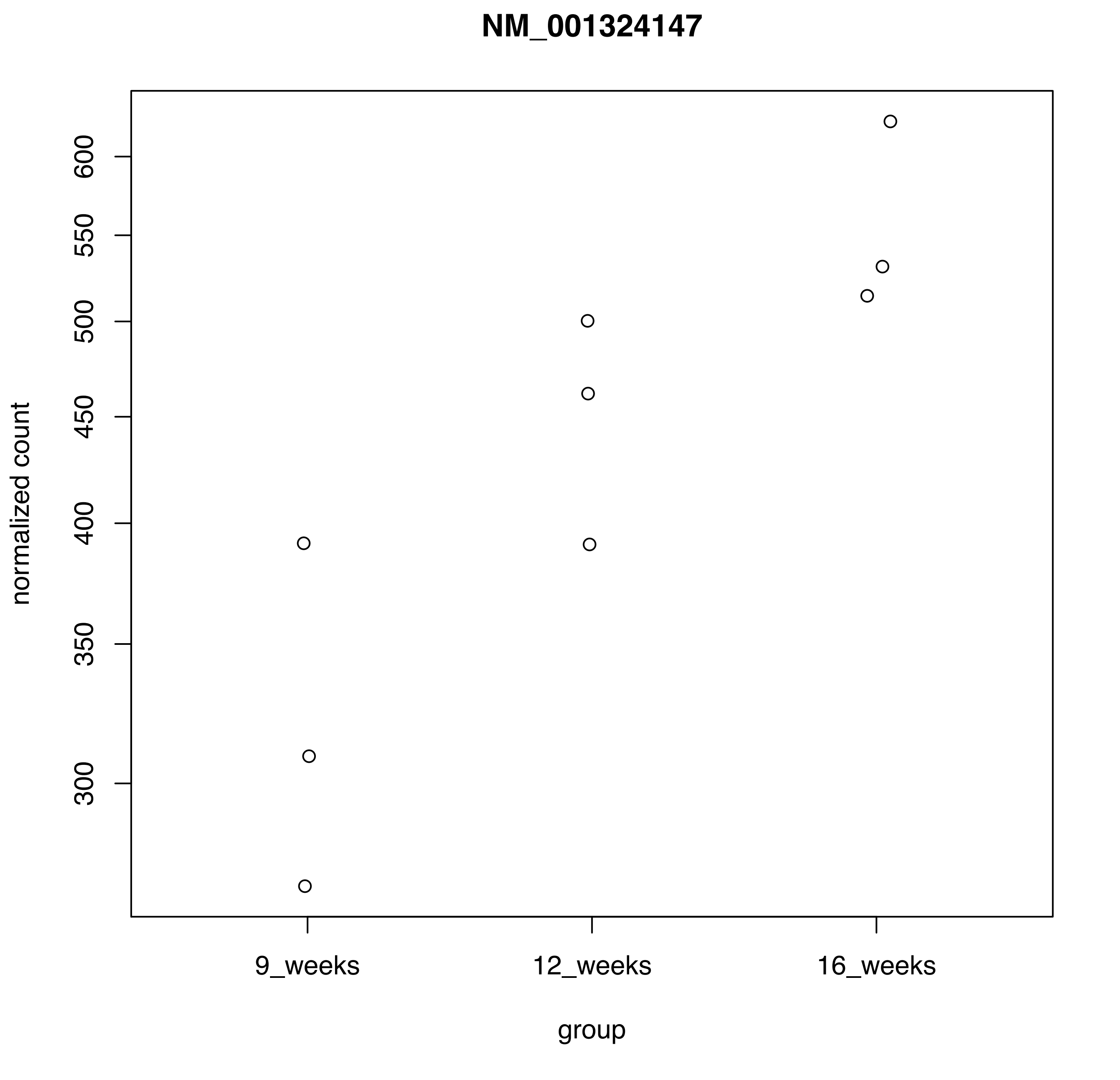
NM_001324147
normalized count
600
550
500
450
400
350
300
9_weeks
12_weeks
16_weeks
group

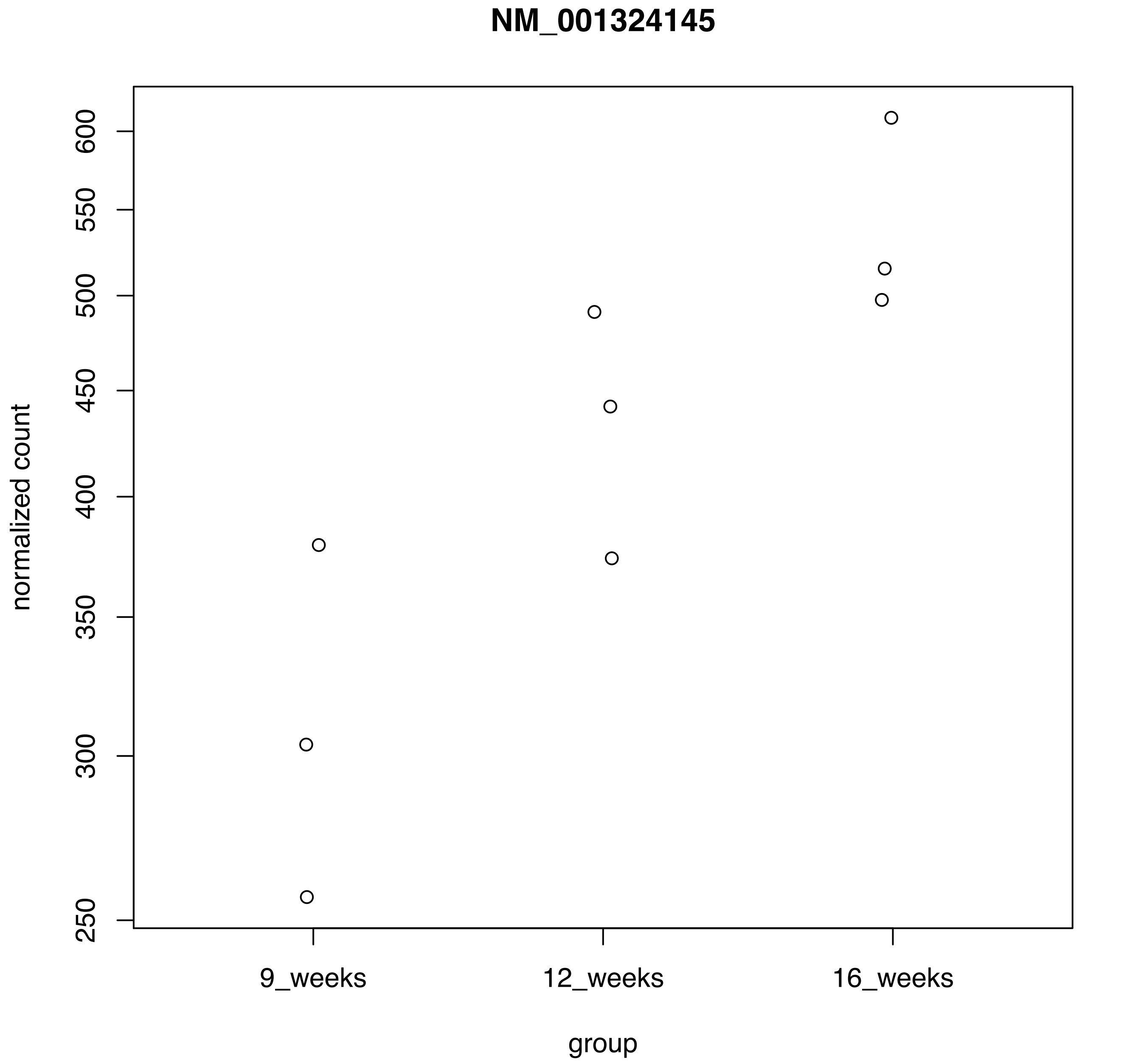
NM_001324145
normalized count
600
550
500
450
400
350
300
250
9_weeks
12_weeks
16_weeks
group

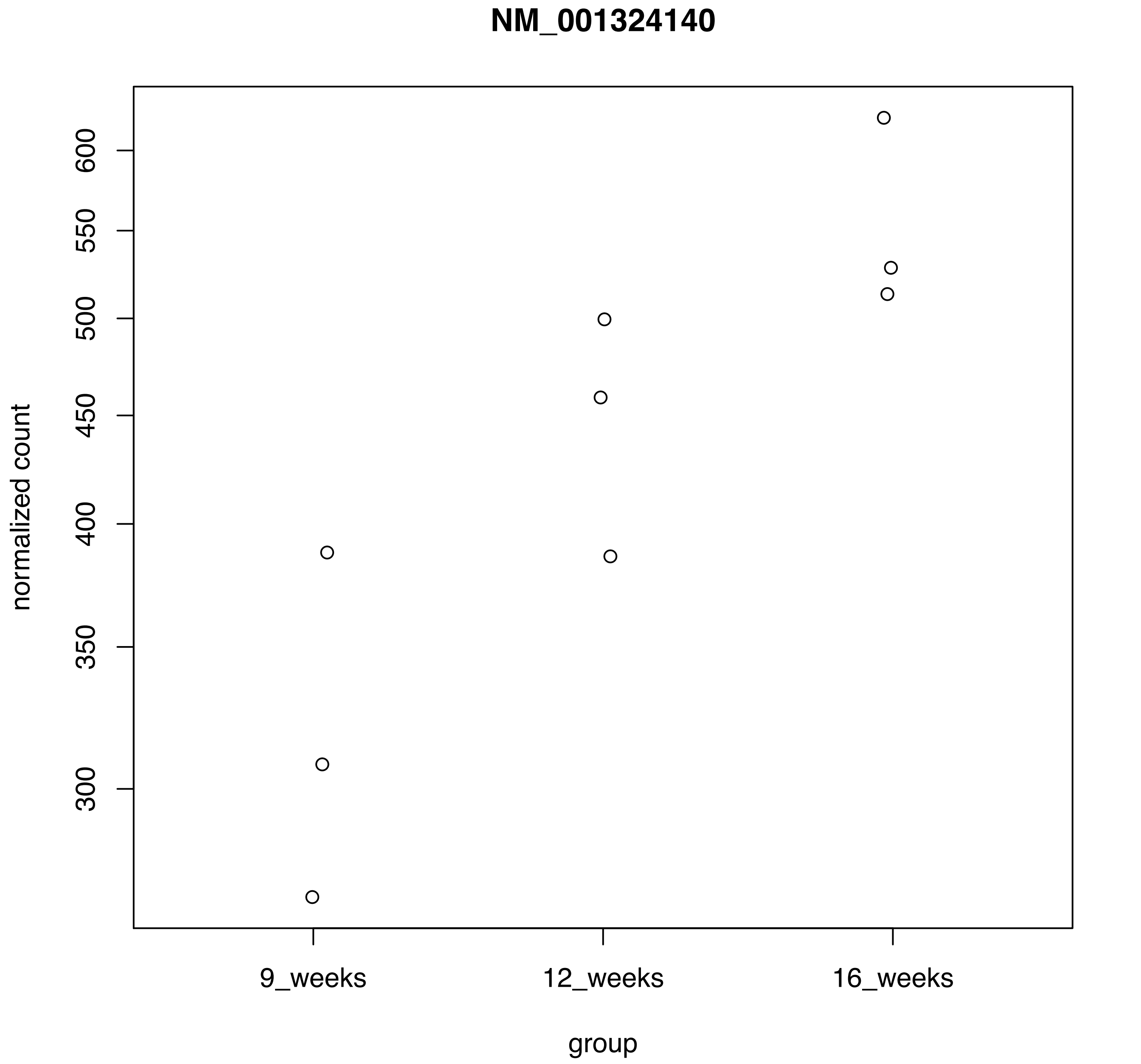
NM_001324140
normalized count
600
550
500
450
400
350
300
9_weeks
12_weeks
16_weeks
group

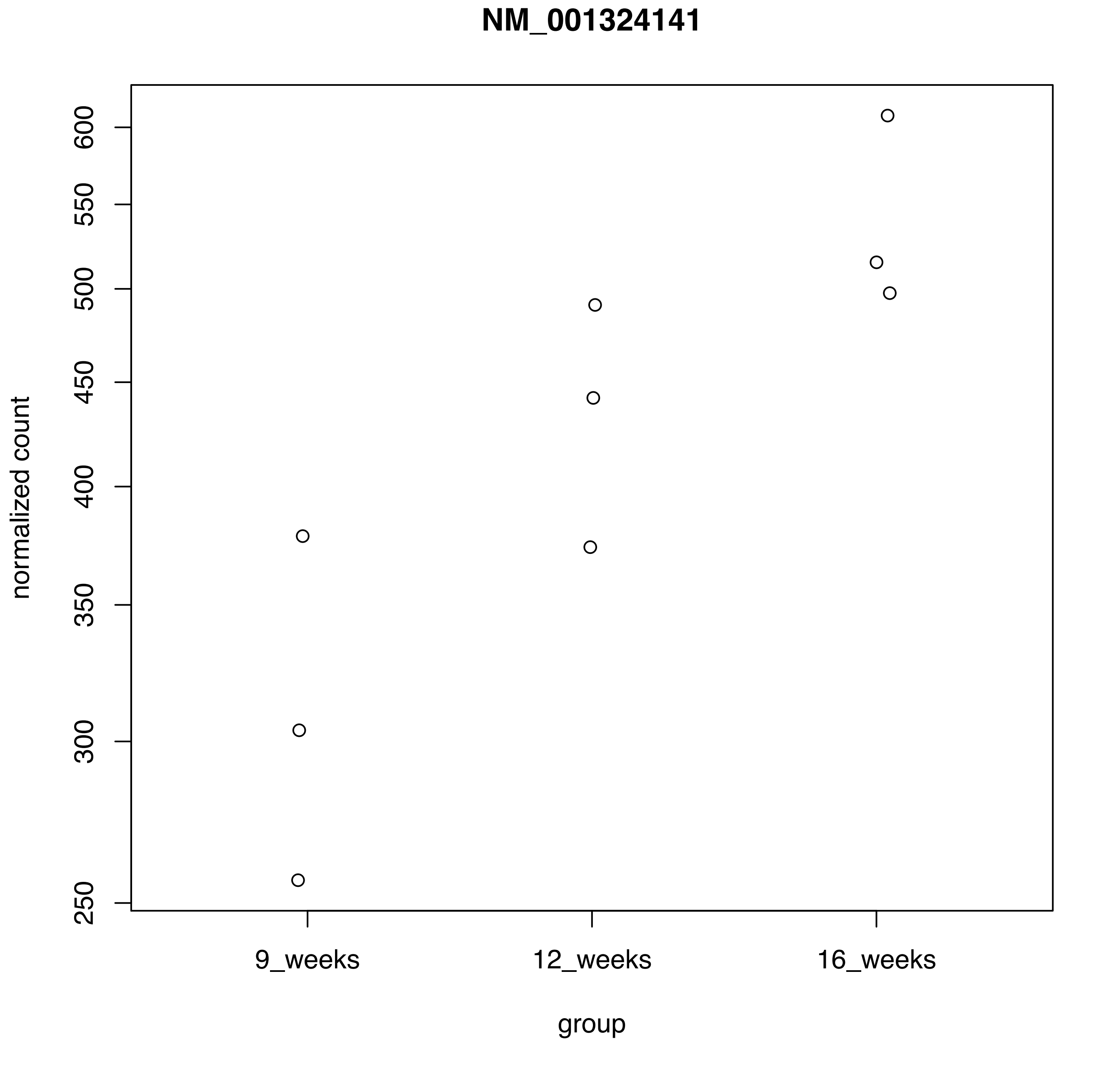
NM_001324141
normalized count
250
300
350
400
450
500
550
600
9_weeks
12_weeks
16_weeks
group

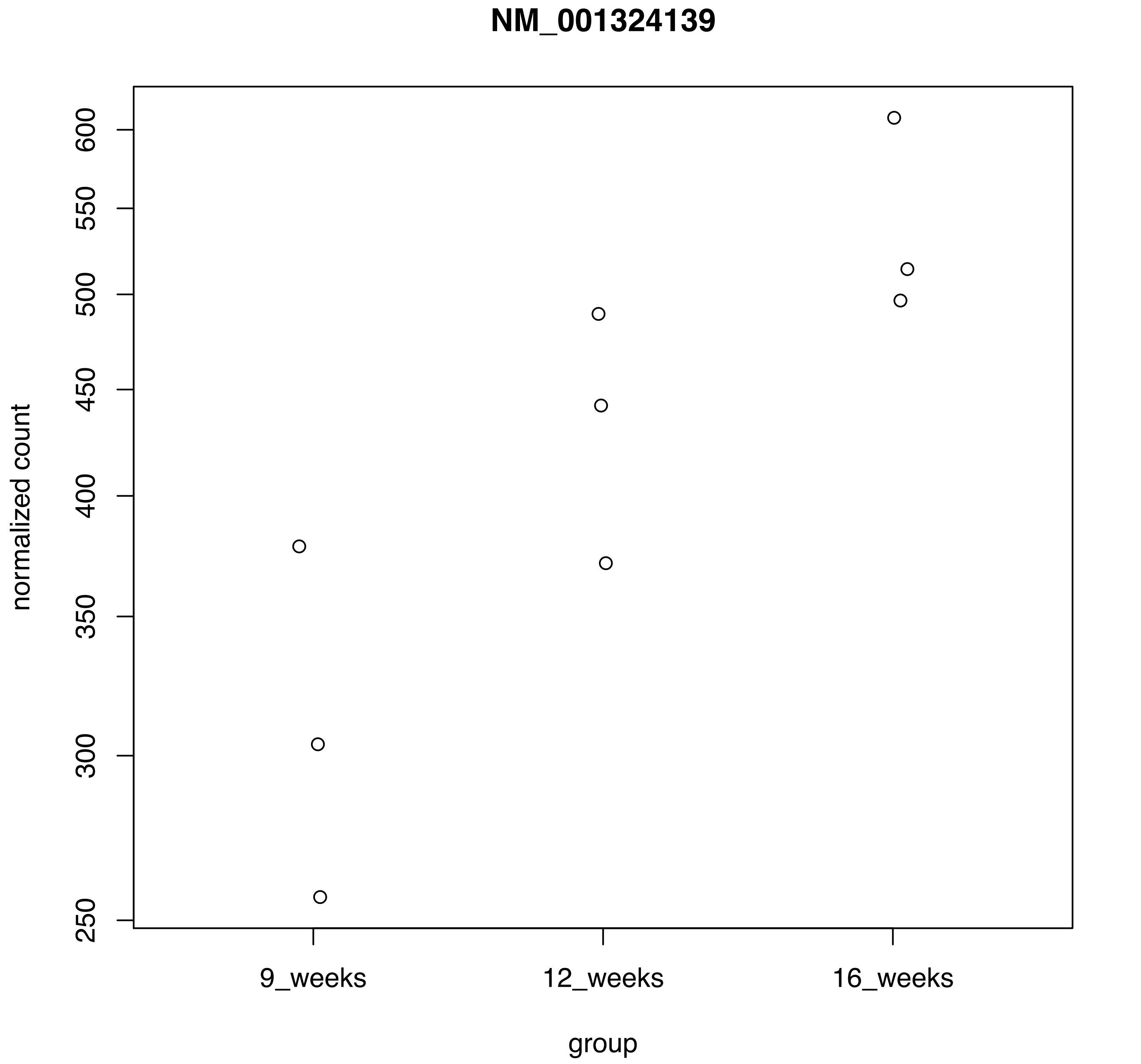
NM_001324139
normalized count
250
300
350
400
450
500
550
600
9_weeks
12_weeks
16_weeks
group

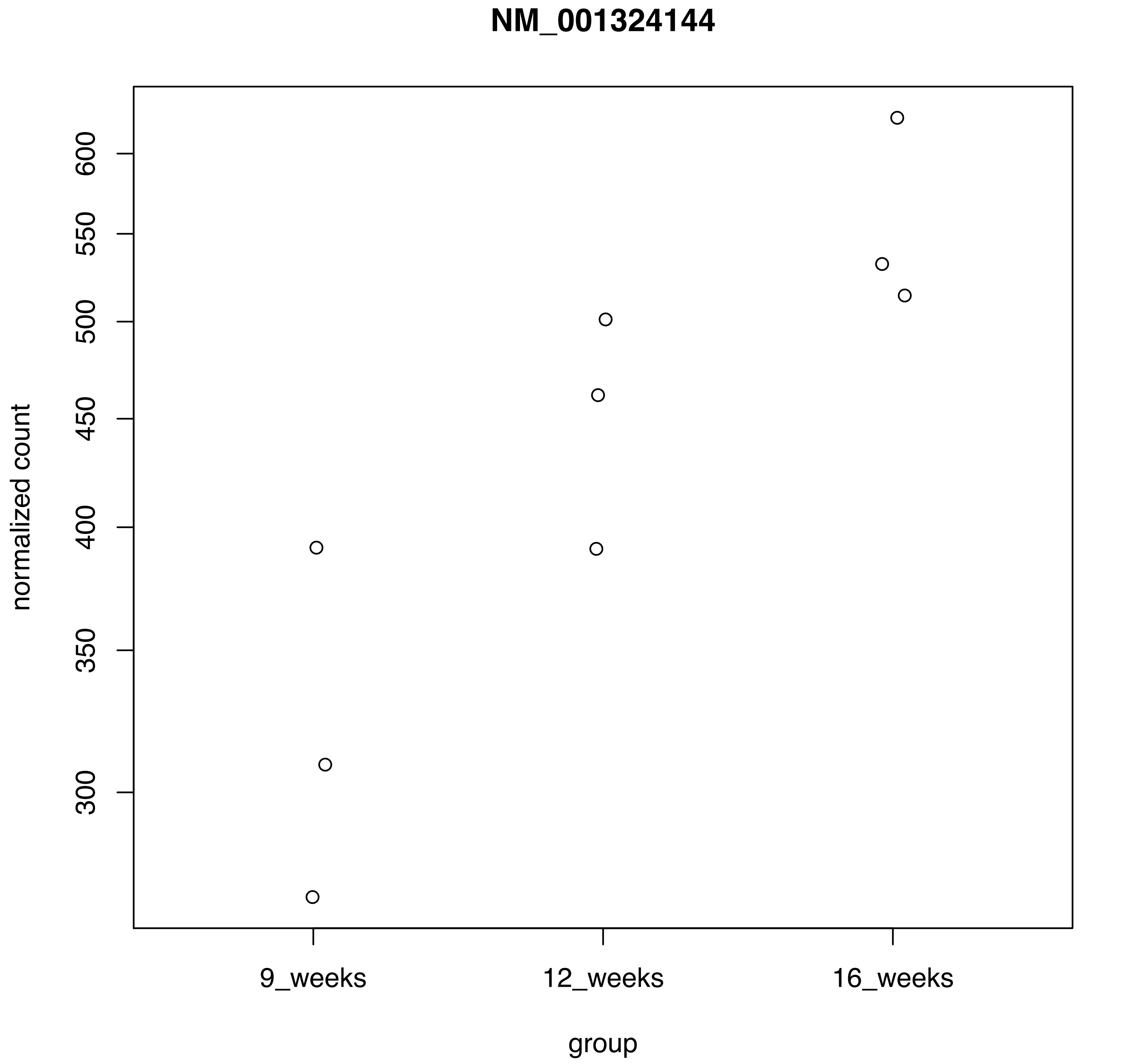
NM_001324144
normalized count
600
550
500
450
400
350
300
9_weeks
12_weeks
16_weeks
group

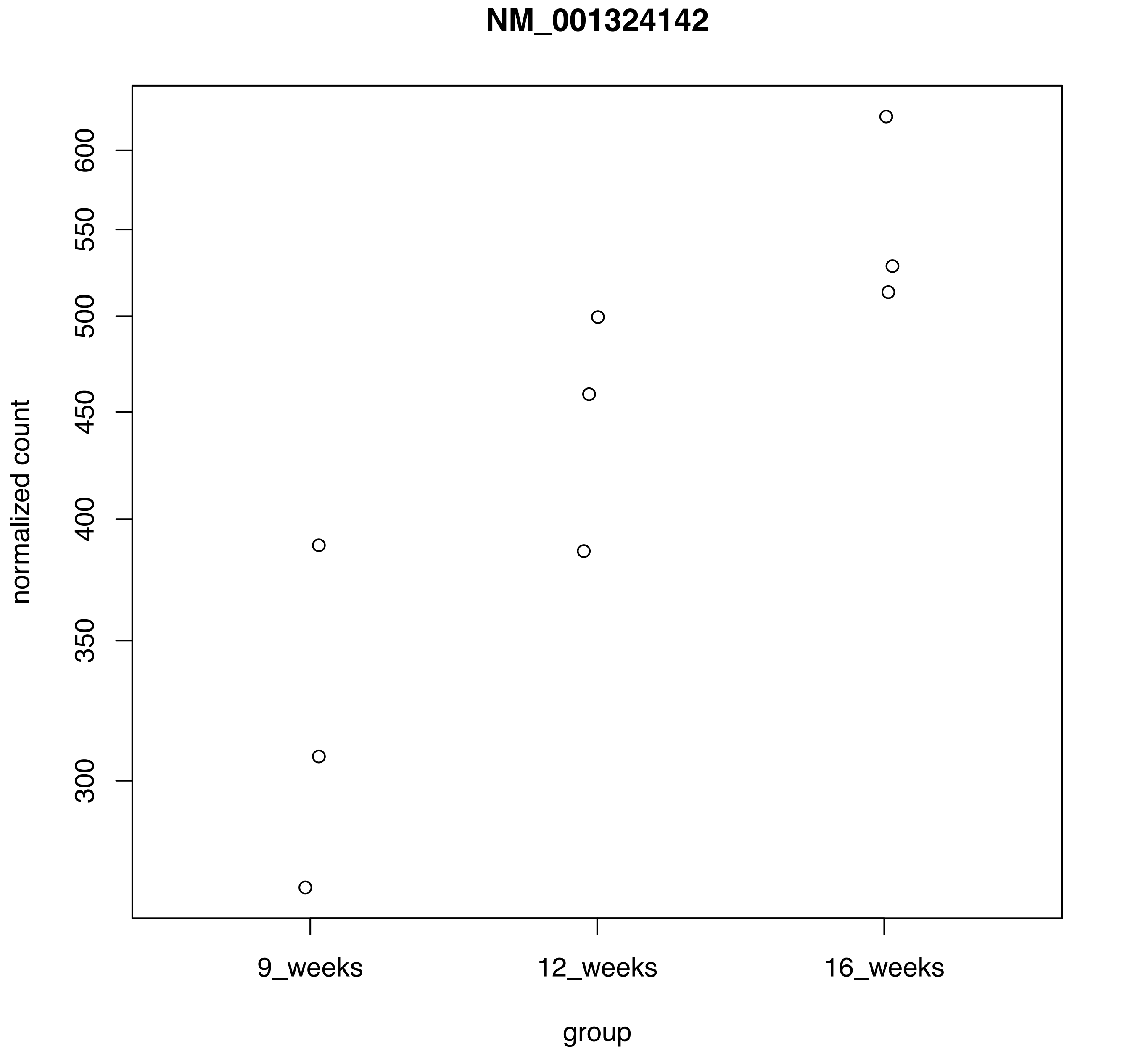

NM_001324142
normalized count
300
350
400
450
500
550
600
9_weeks
12_weeks
16_weeks
group

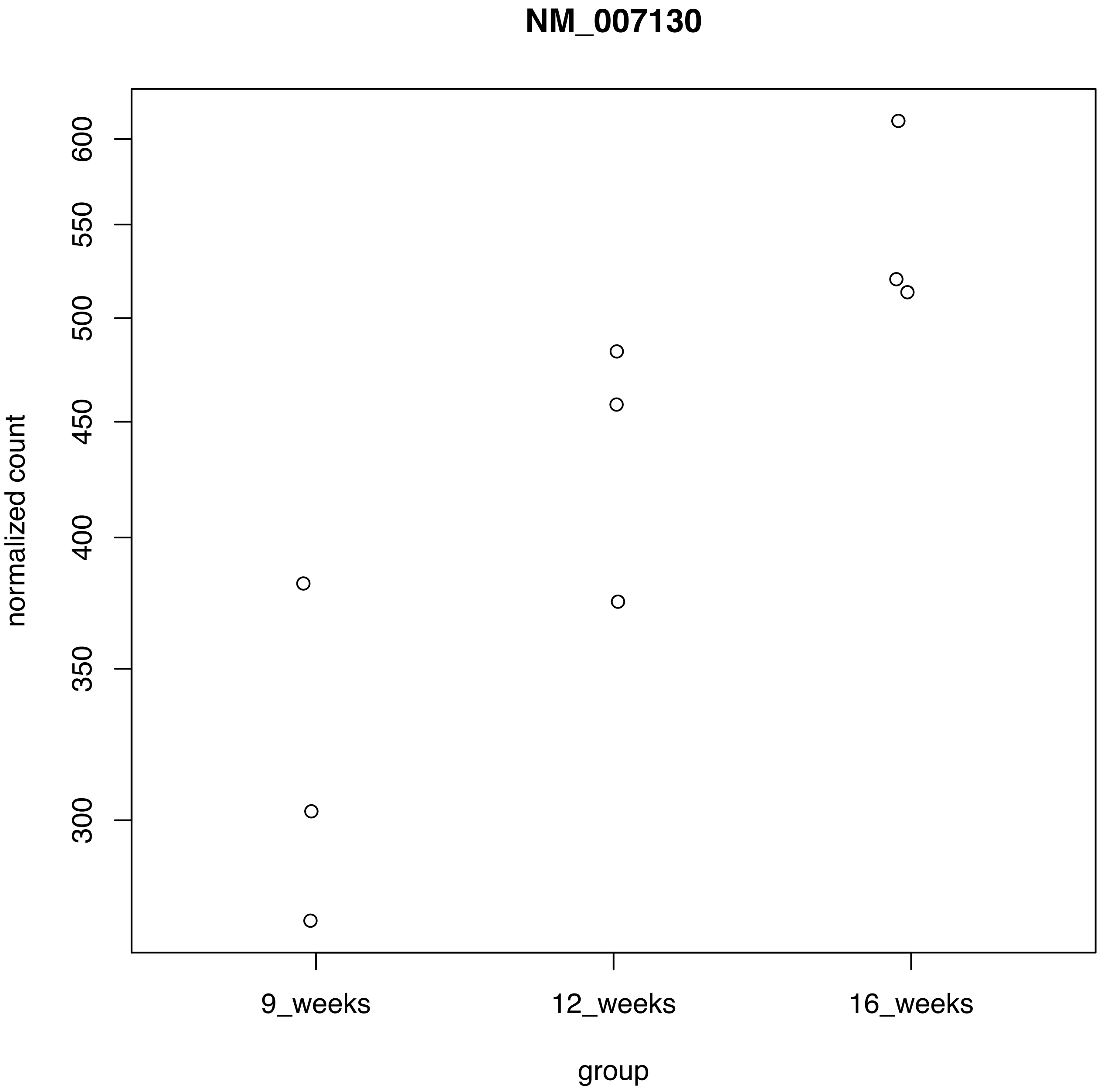
NM_007130
normalized count
600
550
500
450
400
350
300
9_weeks
12_weeks
16_weeks
group

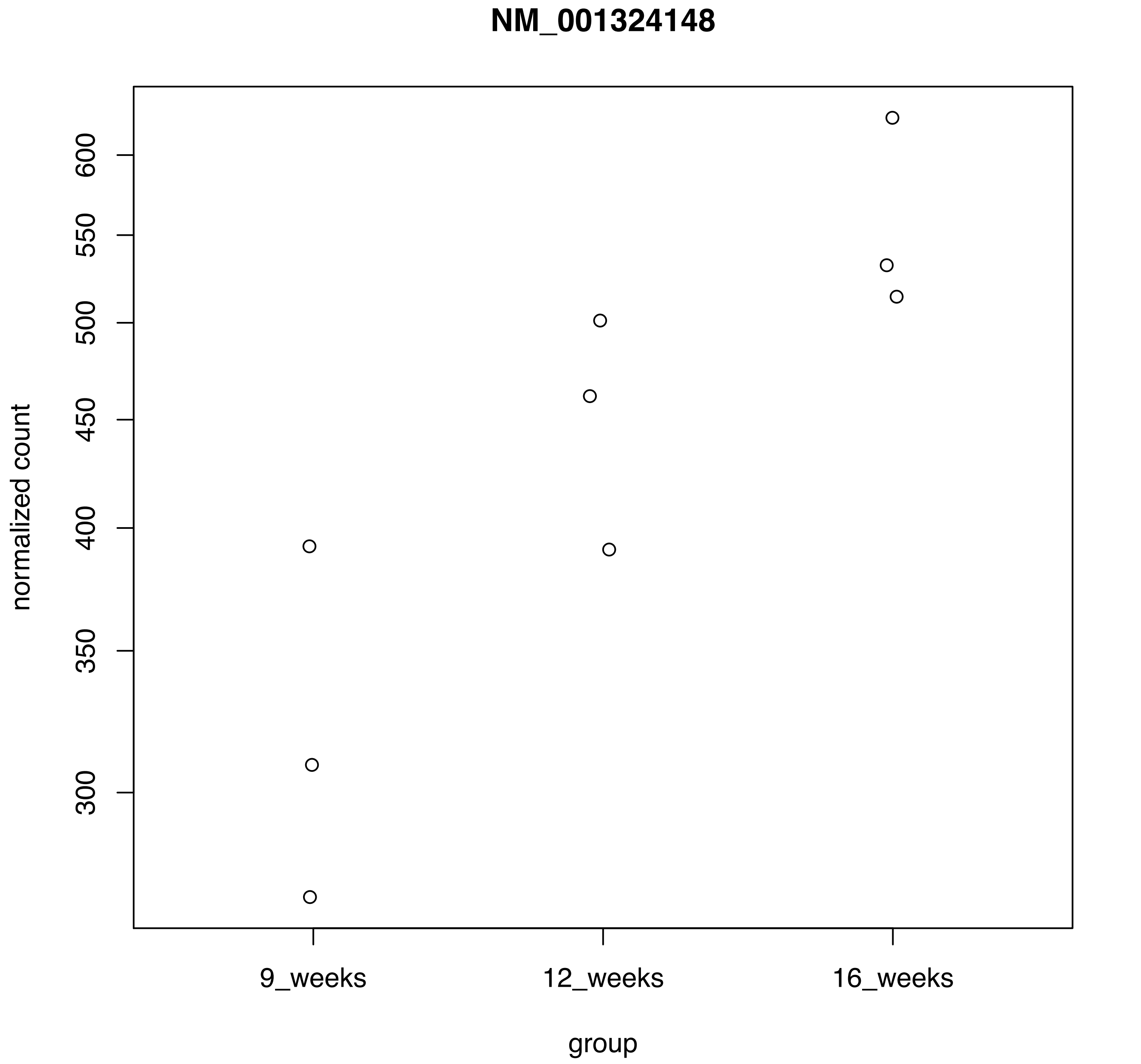
NM_001324148
normalized count
600
550
500
450
400
350
300
9_weeks
12_weeks
16_weeks
group

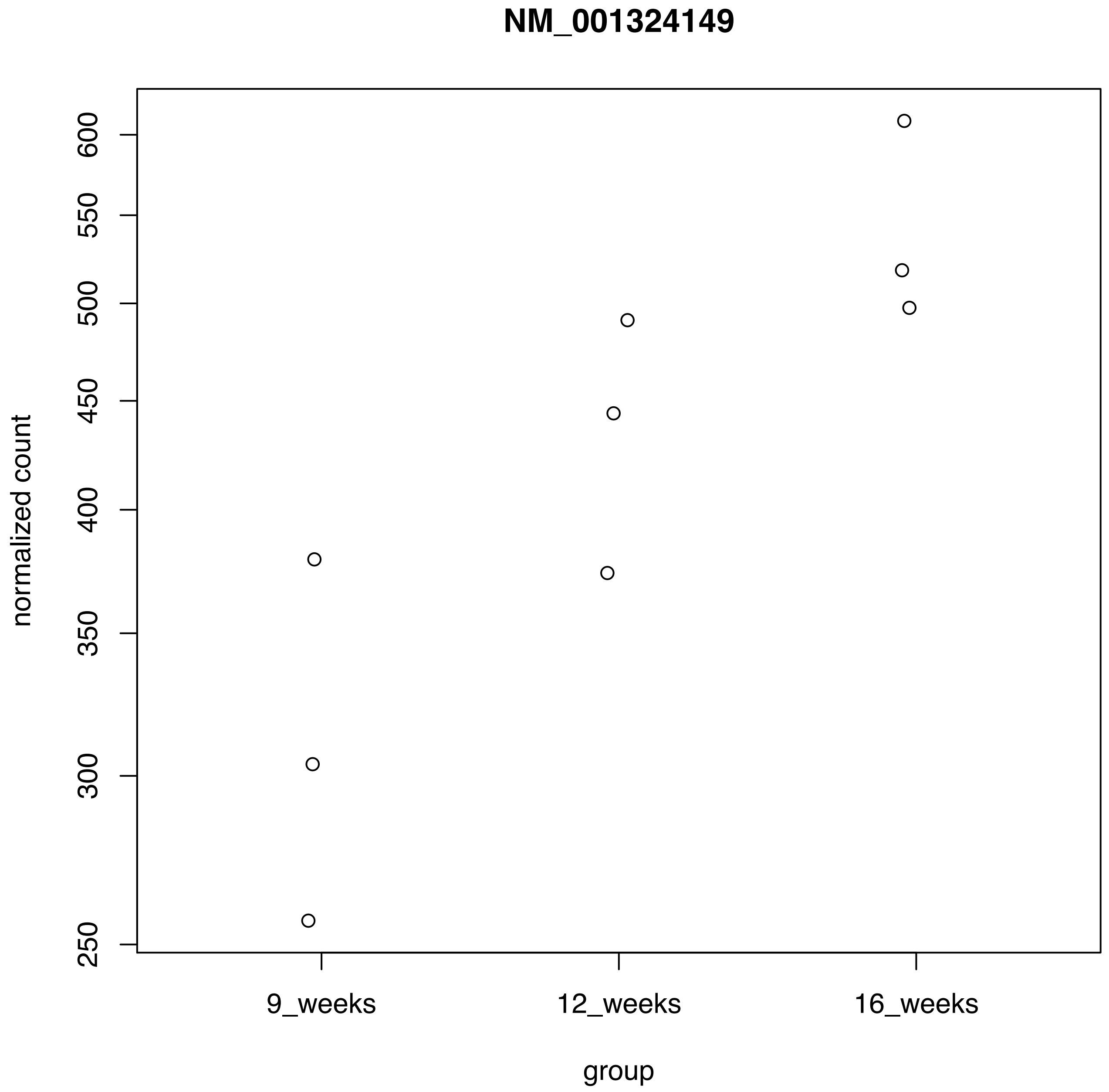

NM_001324149
normalized count
600
550
500
450
400
350
300
250
9_weeks
12_weeks
16_weeks
group

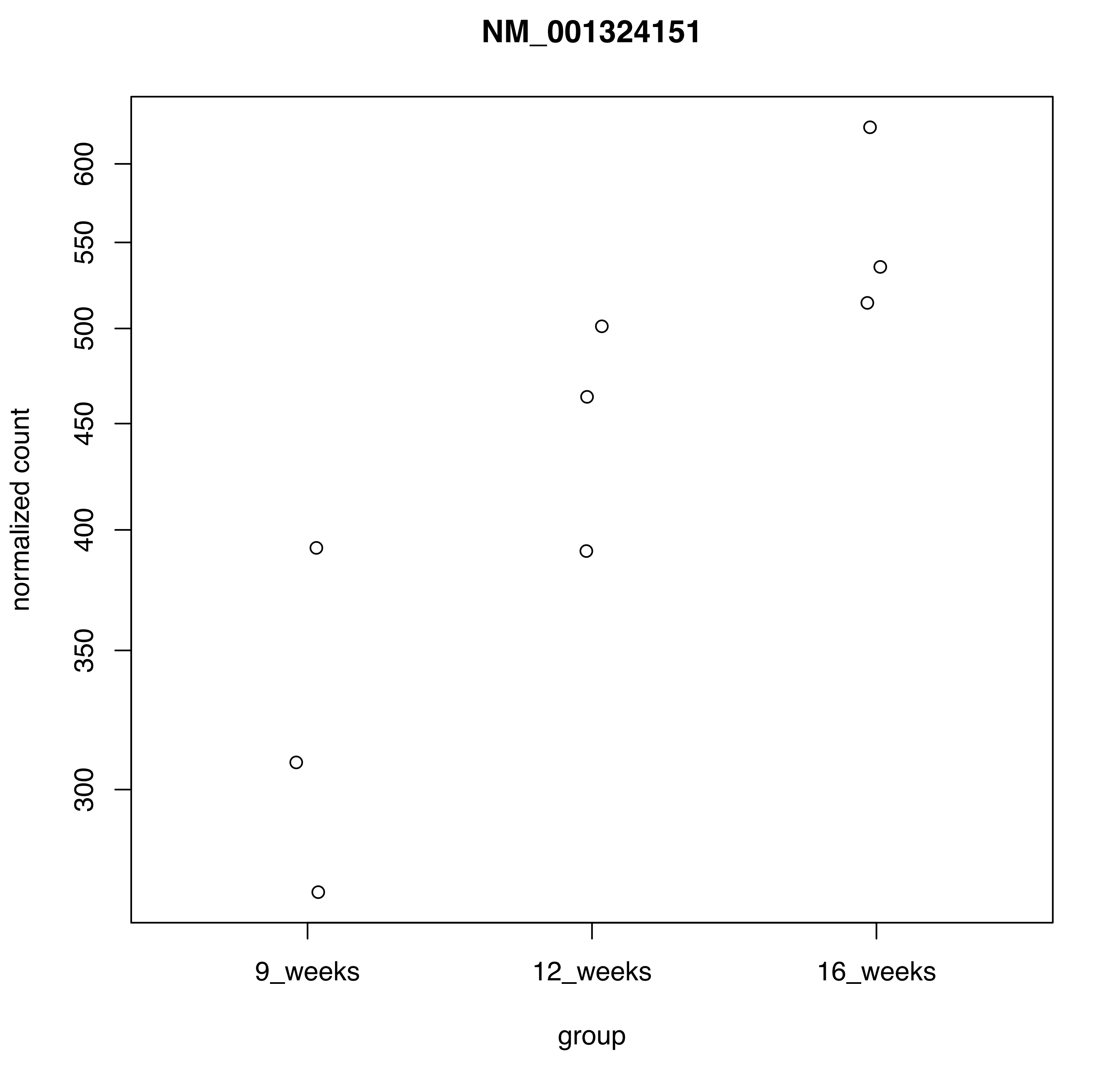

NM_001324151
normalized count
600
550
500
450
400
350
300
9_weeks
12_weeks
16_weeks
group

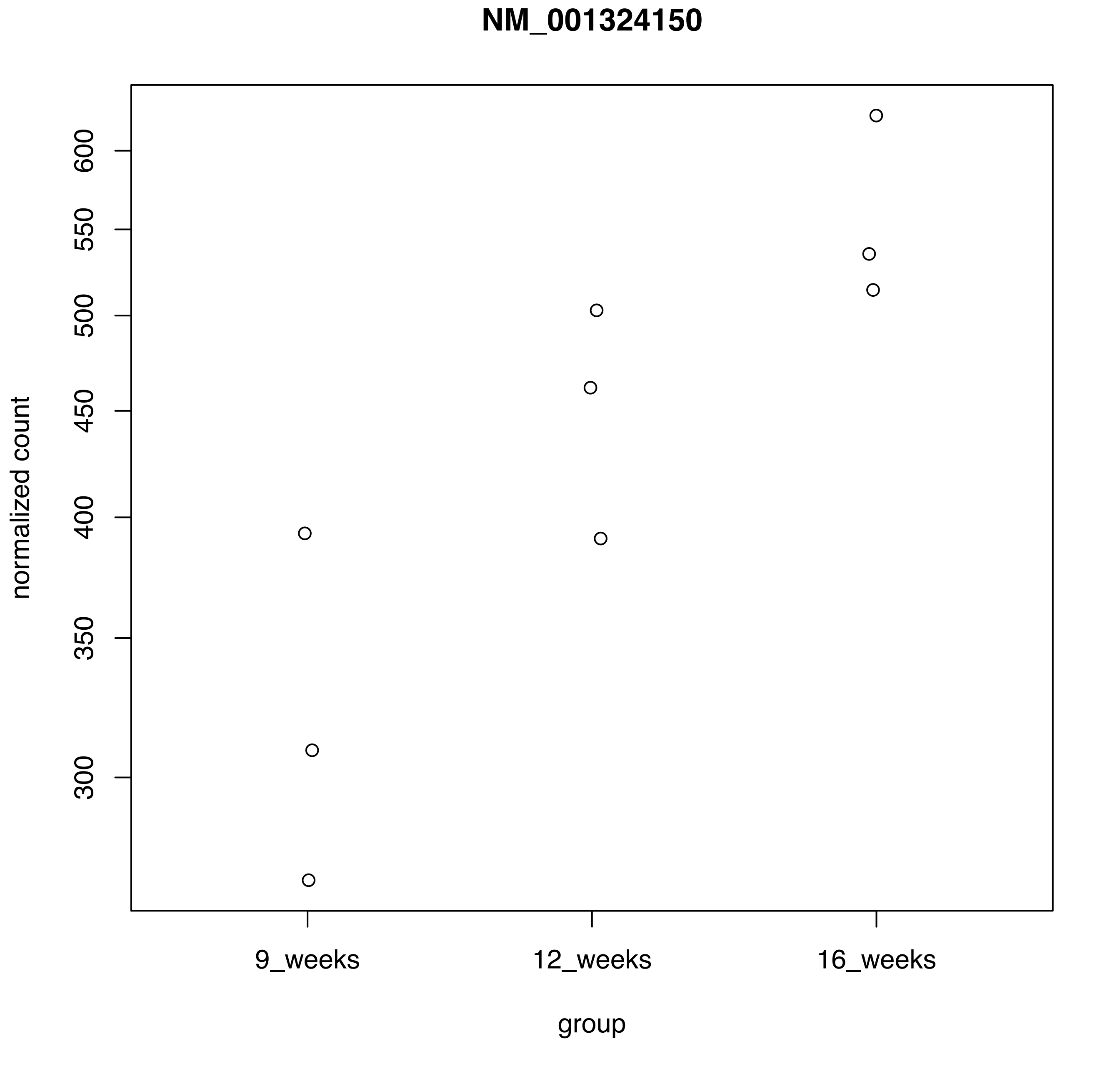
NM_001324150
normalized count
600
550
500
450
400
350
300
9_weeks
12_weeks
16_weeks
group

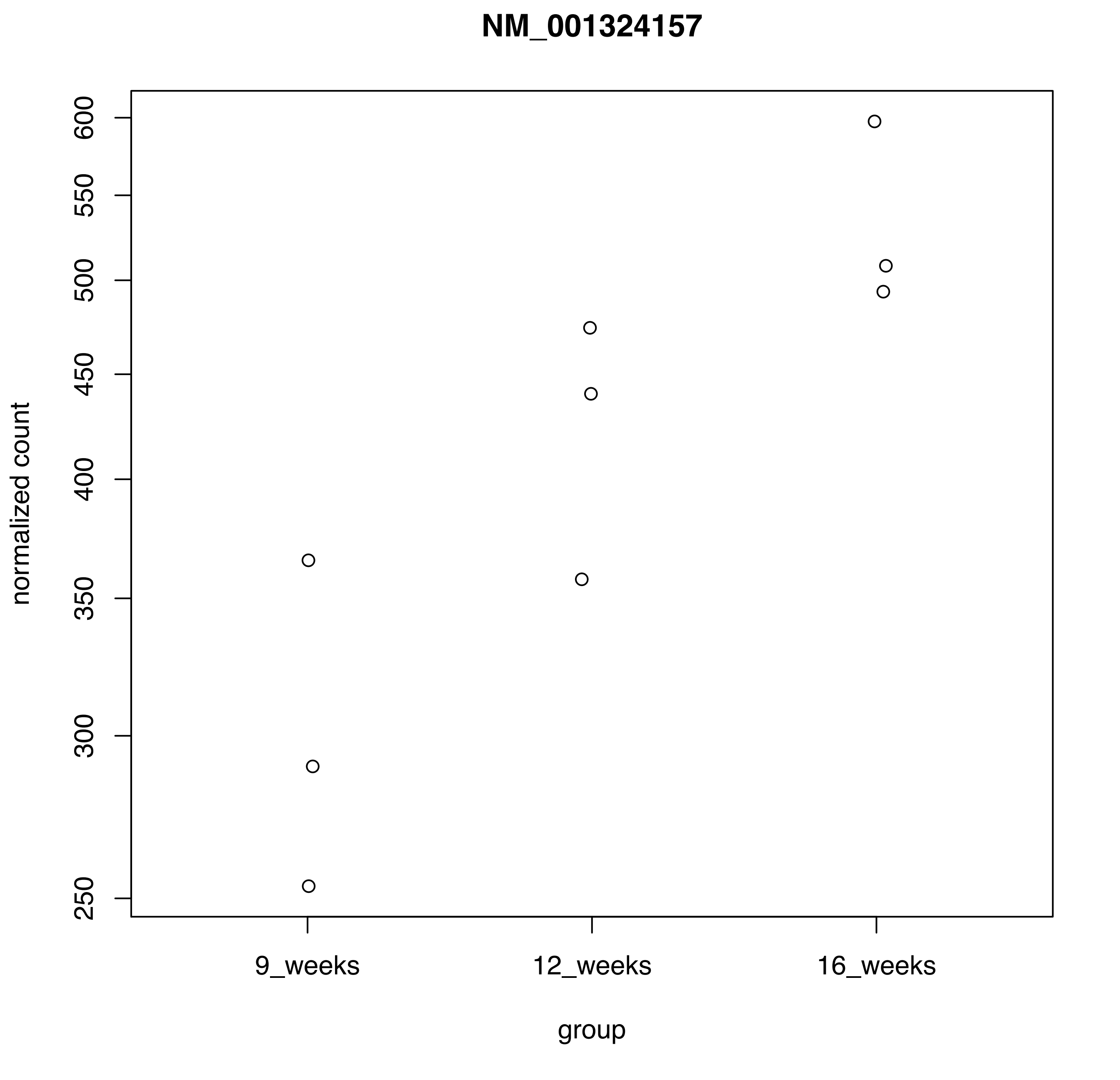
NM_001324157
normalized count
250
300
350
400
450
500
550
600
9_weeks
12_weeks
16_weeks
group

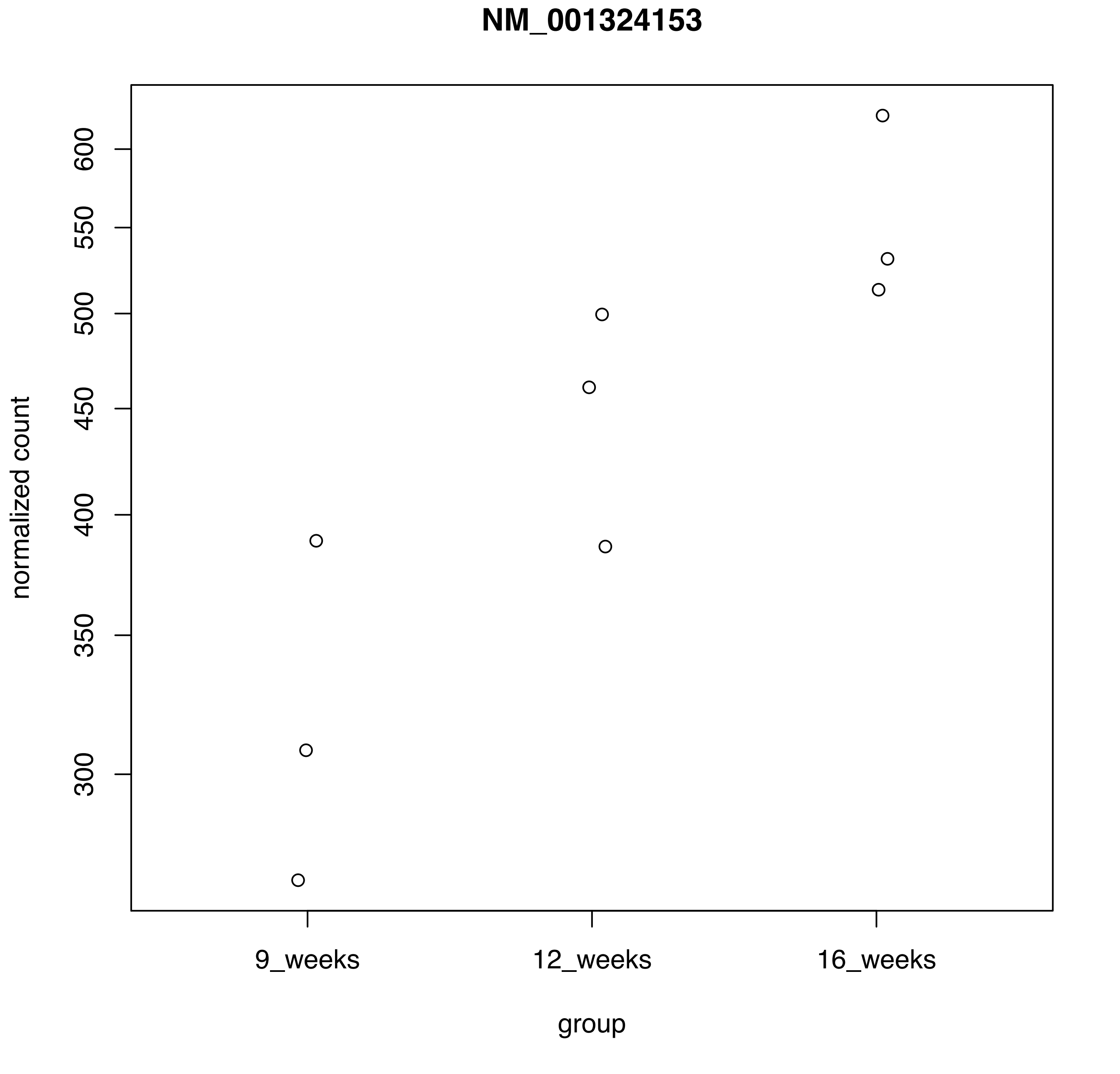
NM_001324153
normalized count
600
550
500
450
400
350
300
9_weeks
12_weeks
16_weeks
group

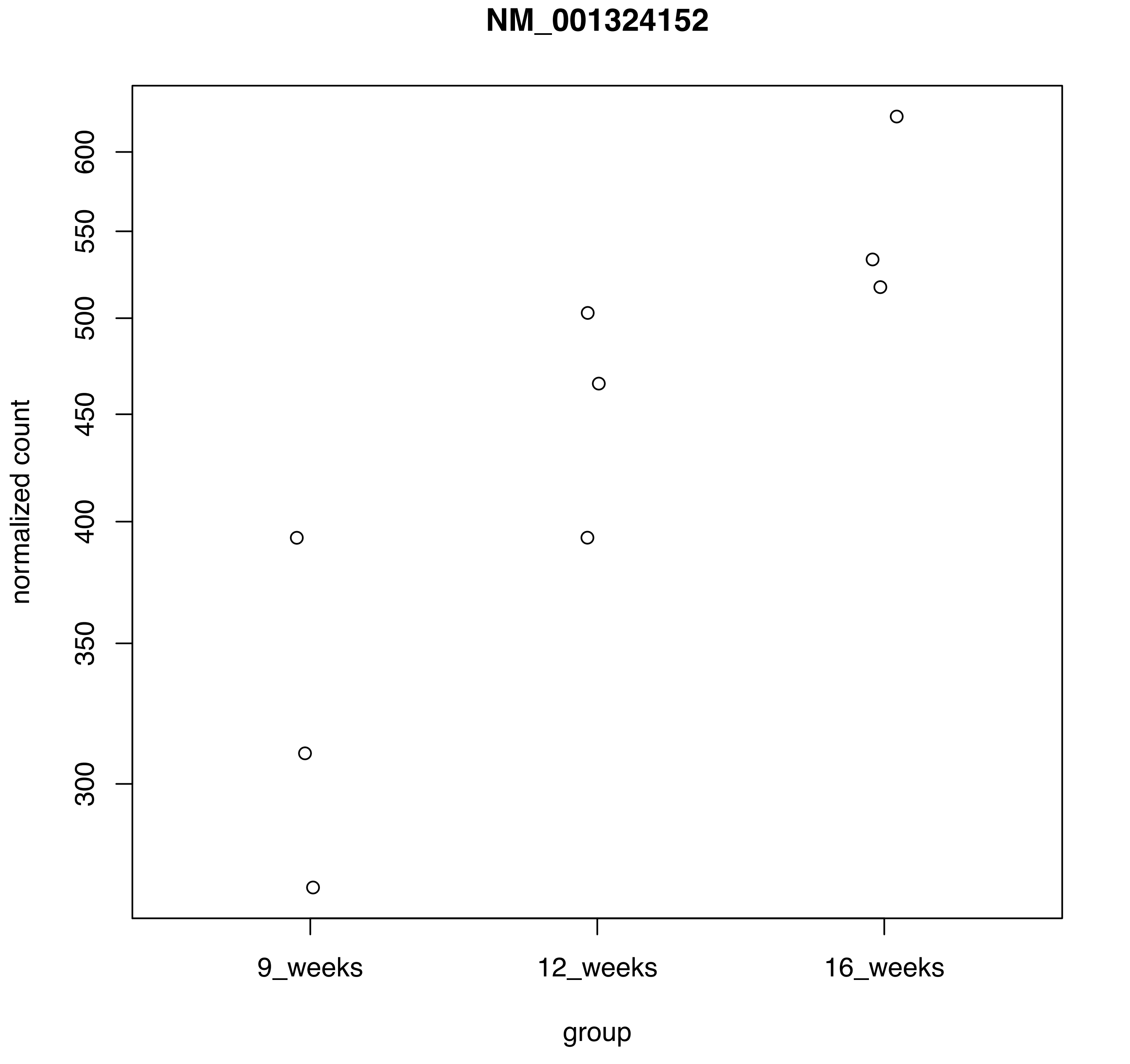
NM_001324152
normalized count
600
550
500
450
400
350
300
9_weeks
12_weeks
16_weeks
group

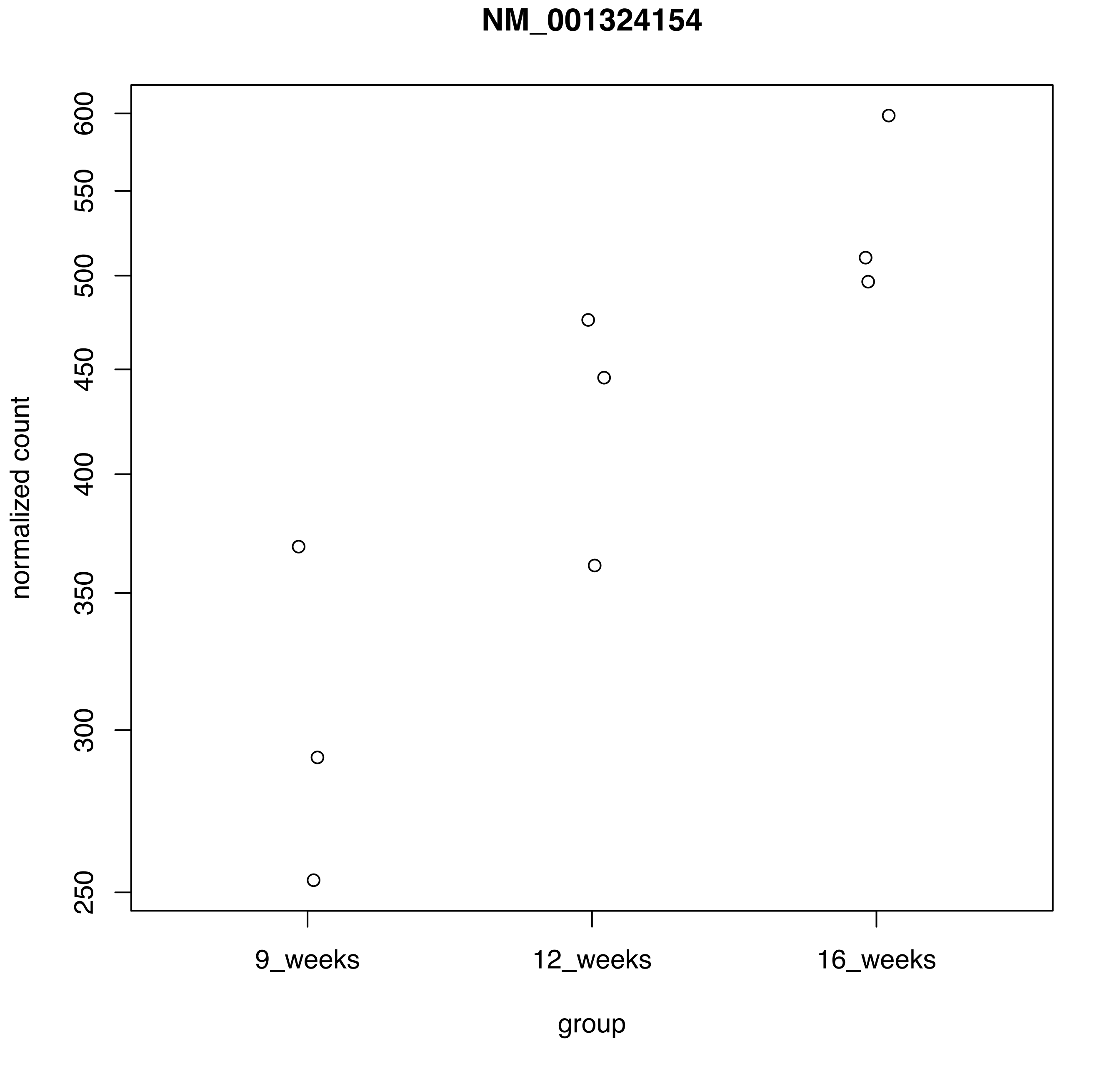

NM_001324154
normalized count
600
550
500
450
400
350
300
250
9_weeks
12_weeks
16_weeks
group

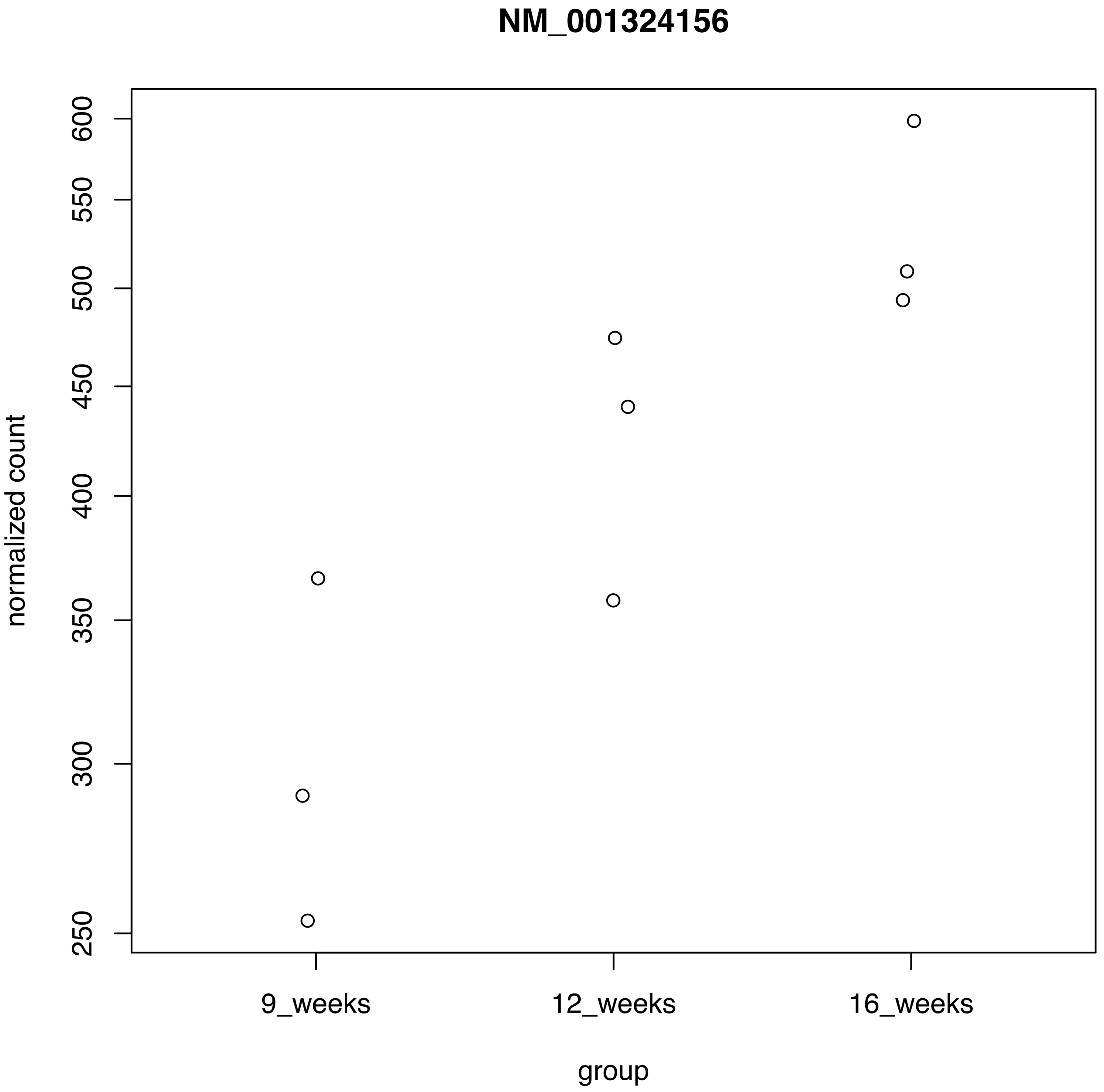

NM_001324156
normalized count
600
550
500
450
400
350
300
250
9_weeks
12_weeks
16_weeks
group

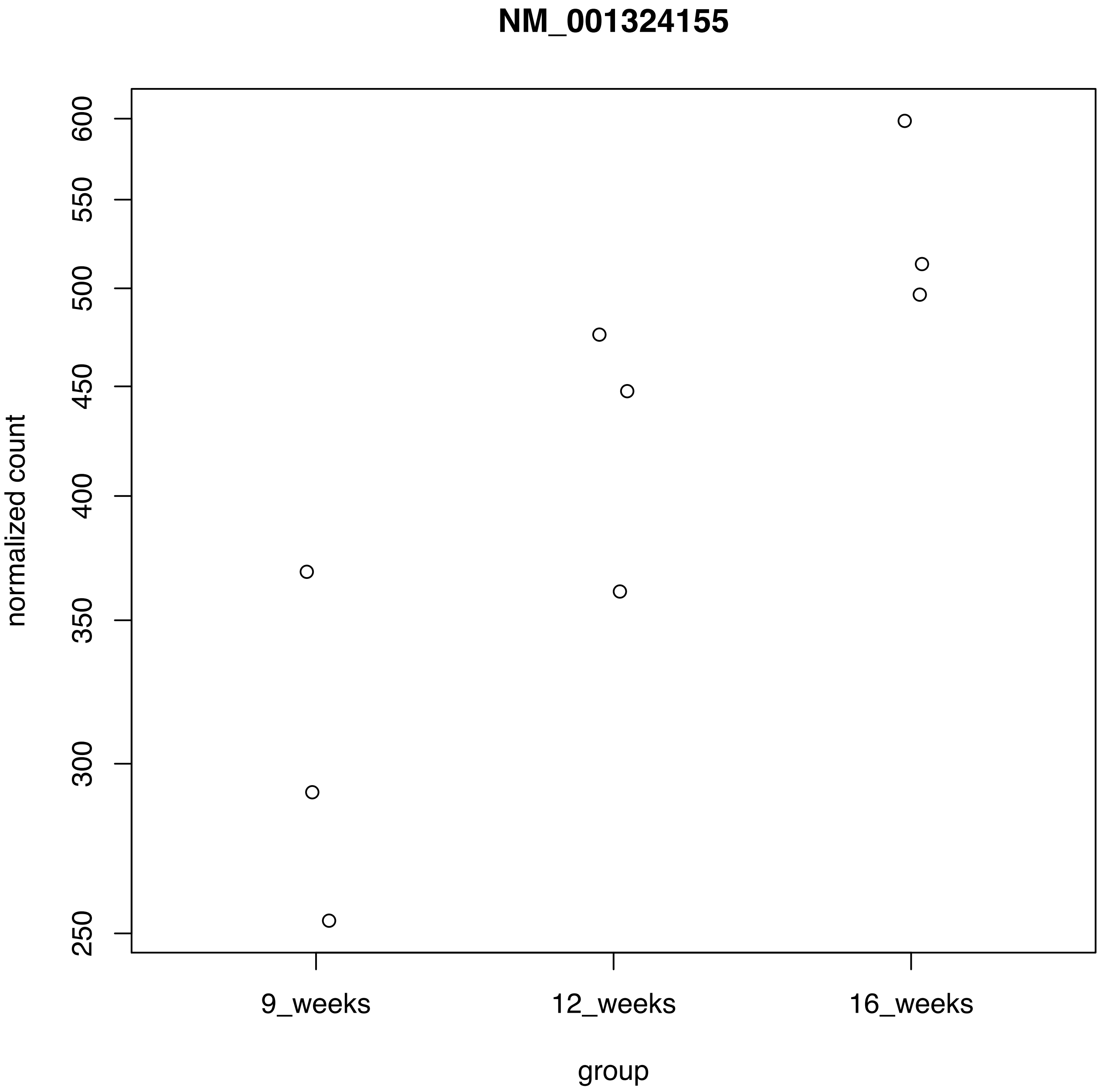
NM_001324155
normalized count
600
550
500
450
400
350
300
250
9_weeks
12_weeks
16_weeks
group

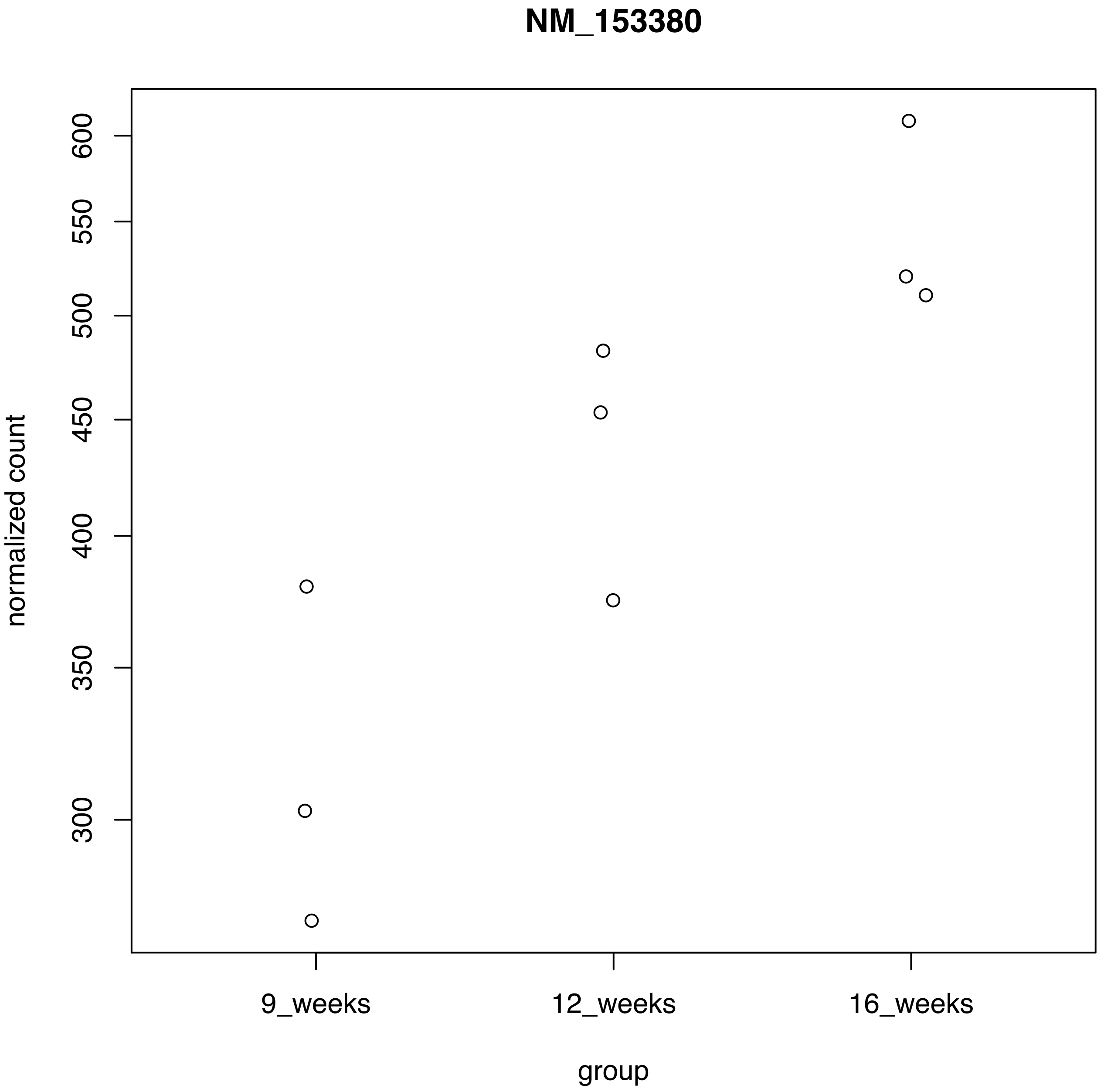
NM_153380
normalized count
300
350
400
450
500
550
600
9_weeks
12_weeks
16_weeks
group

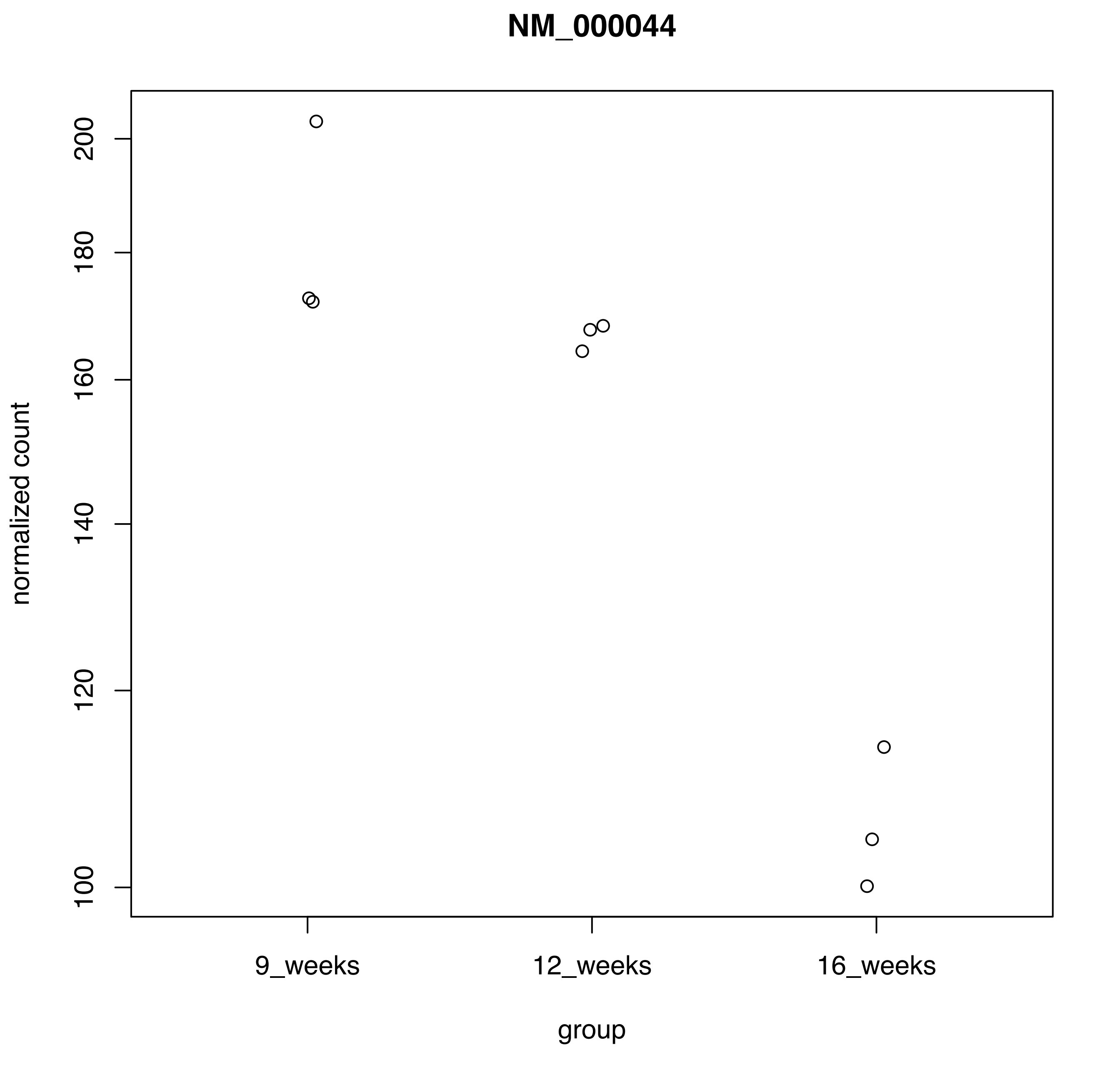
NM_000044
normalized count
200
180
160
140
120
100
9_weeks
12_weeks
16_weeks
group

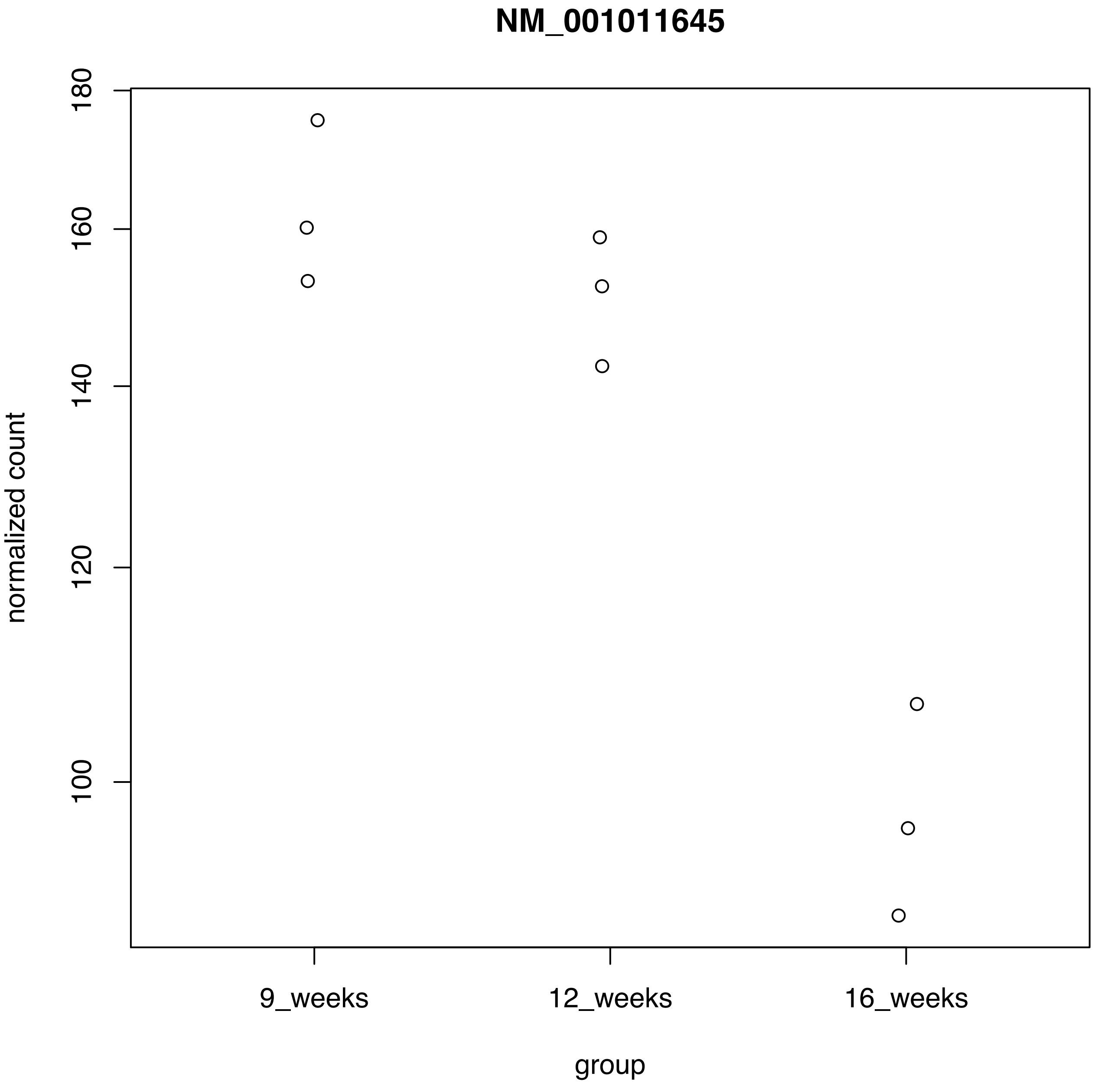

NM_001011645
180
160
140
120
100
normalized count
9_weeks
12_weeks
16_weeks
group

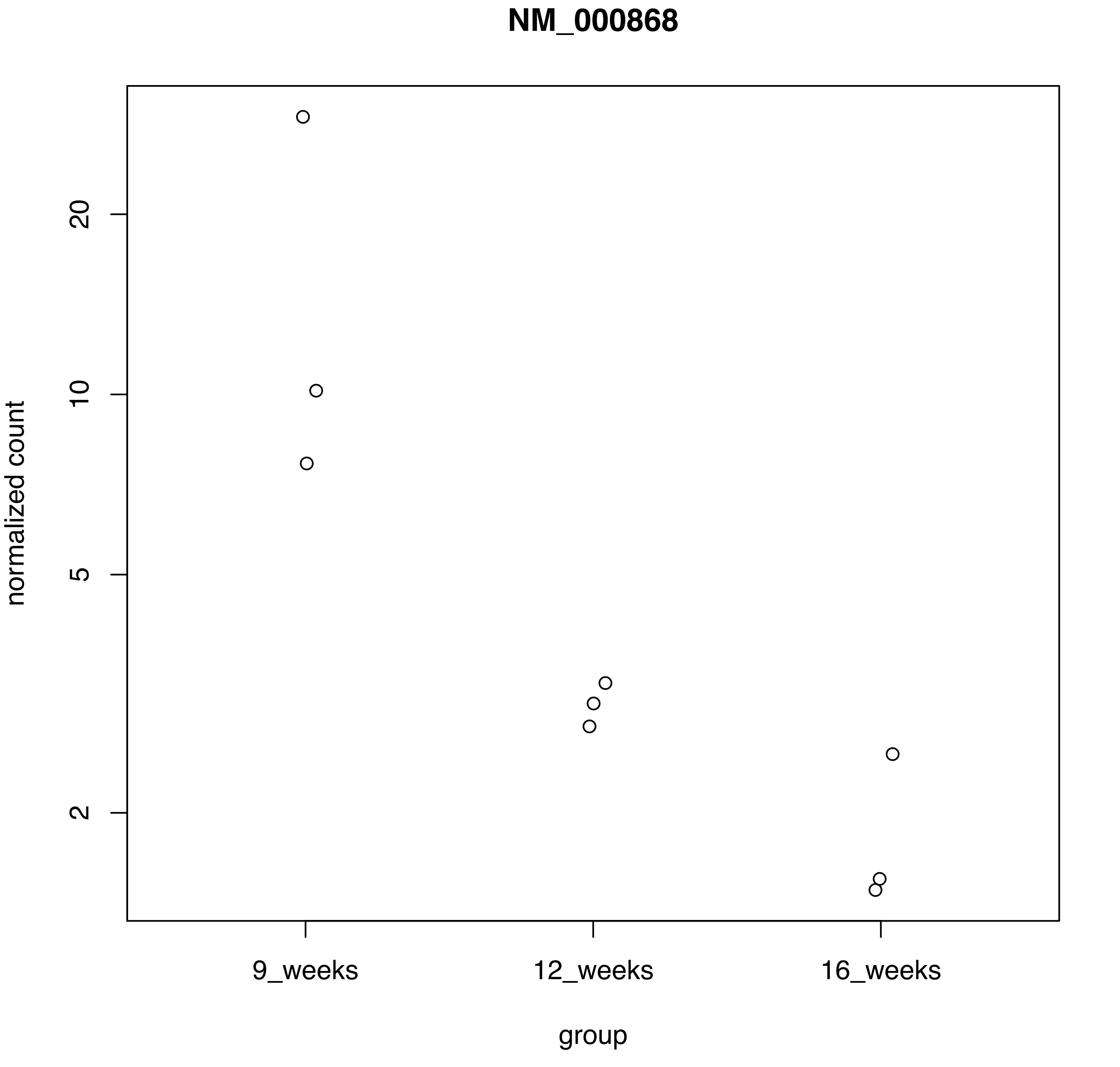
NM_000868
normalized count
20
10
5
2
9_weeks
12_weeks
16_weeks
group

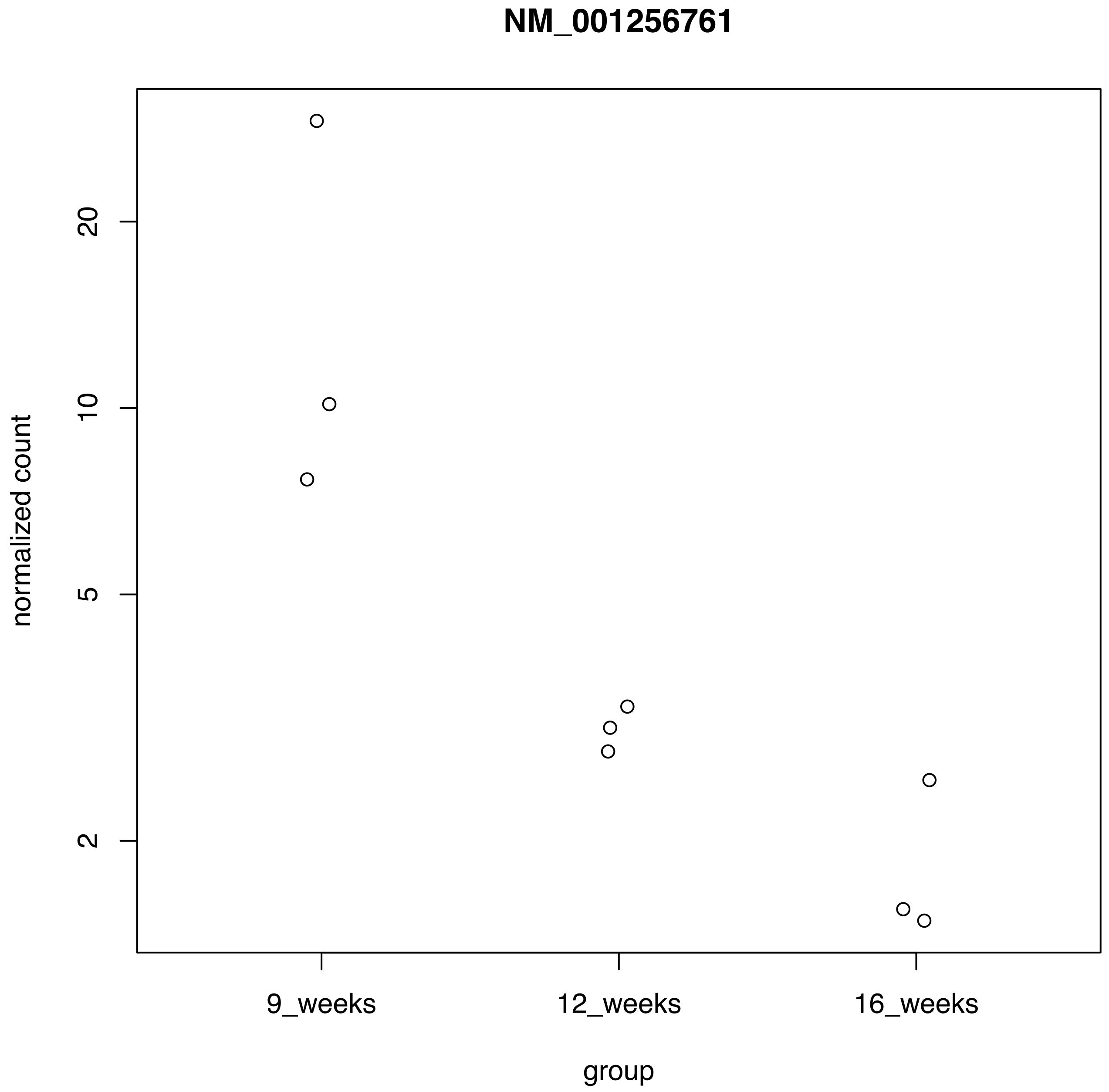
NM_001256761
normalized count
20
10
5
2
9_weeks
12_weeks
16_weeks
group

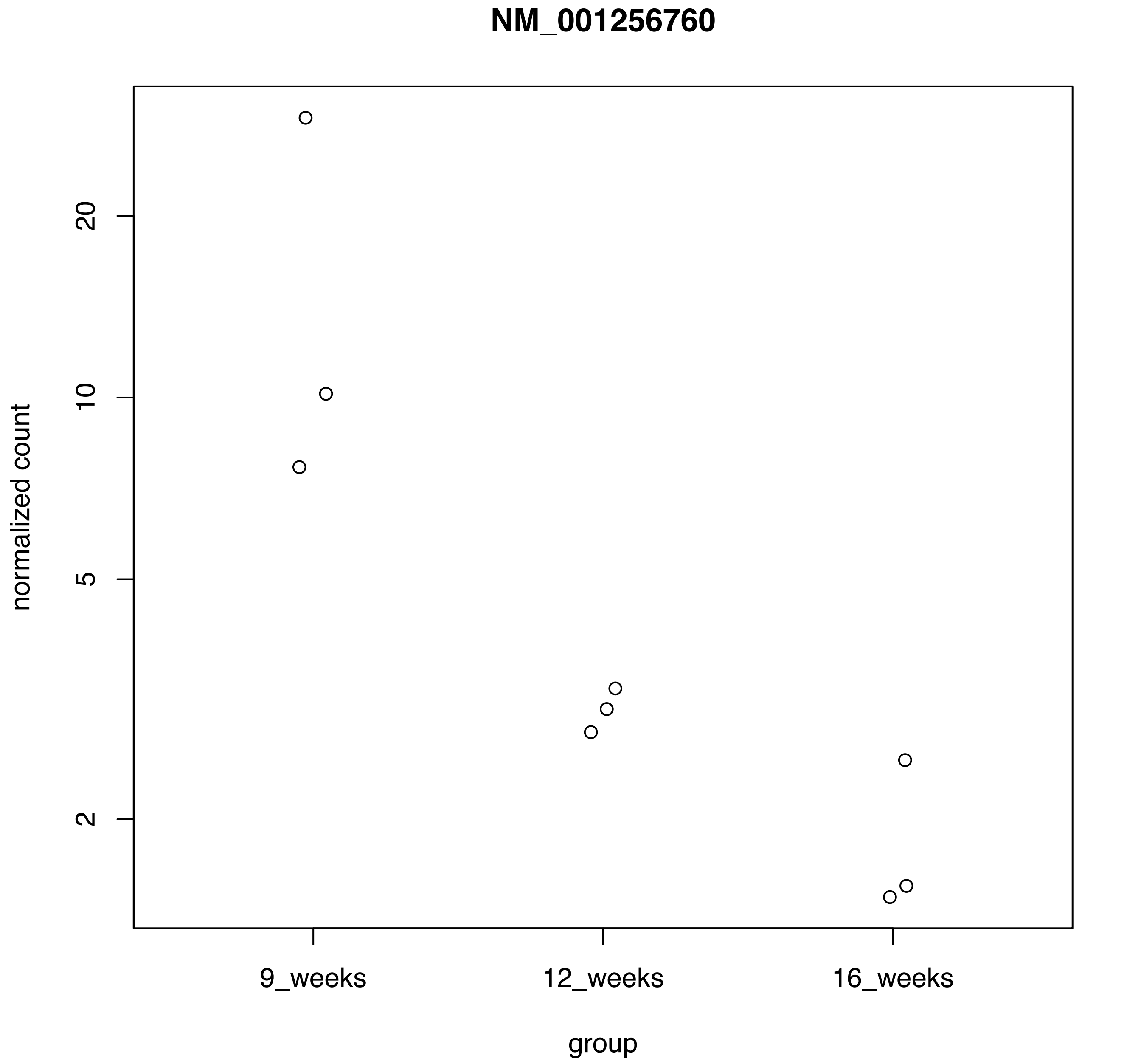

NM_001256760
normalized count
20
10
5
2
9_weeks
12_weeks
16_weeks
group

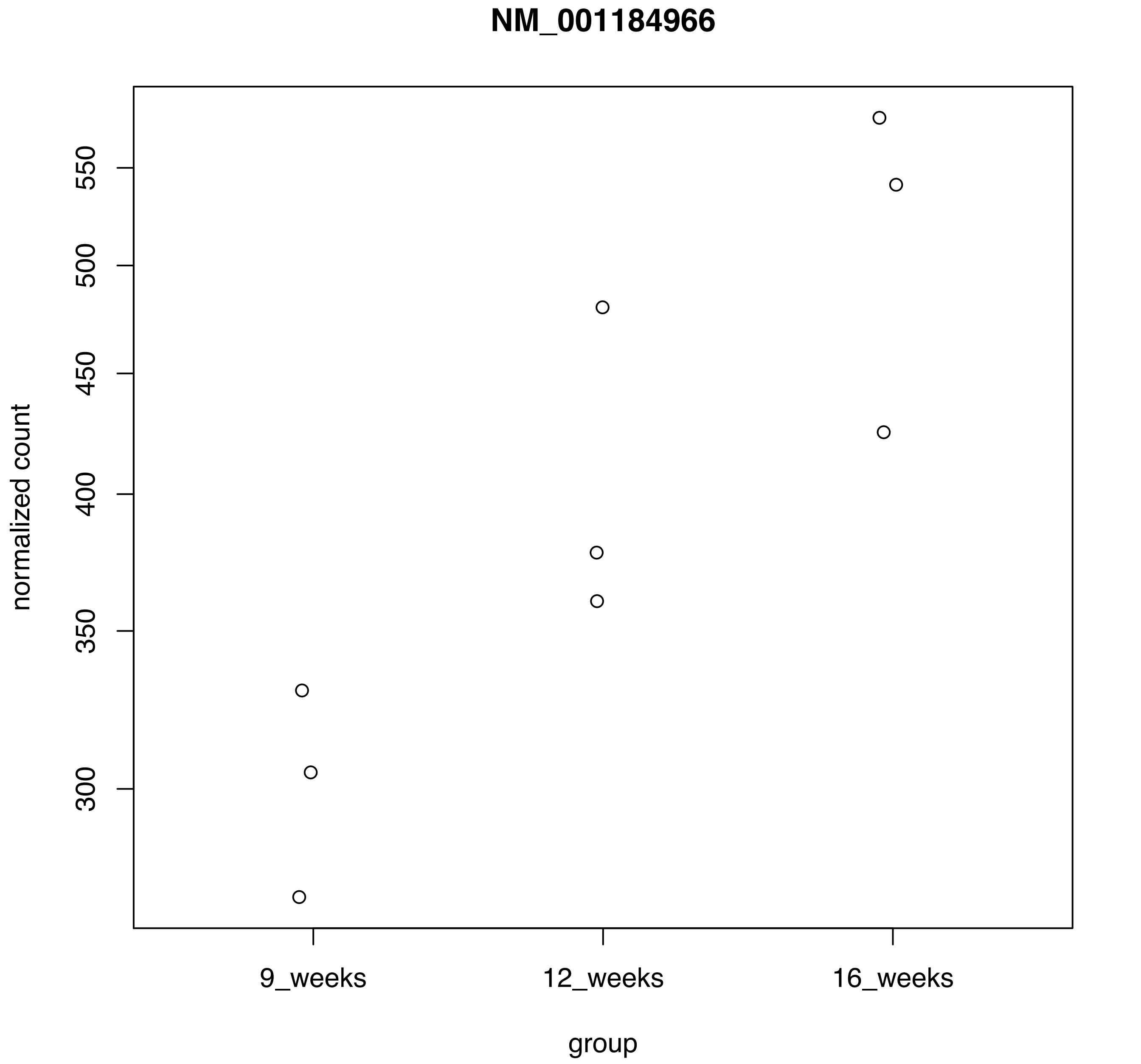
NM_001184966
normalized count
300
350
400
450
500
550
9_weeks
12_weeks
16_weeks
group

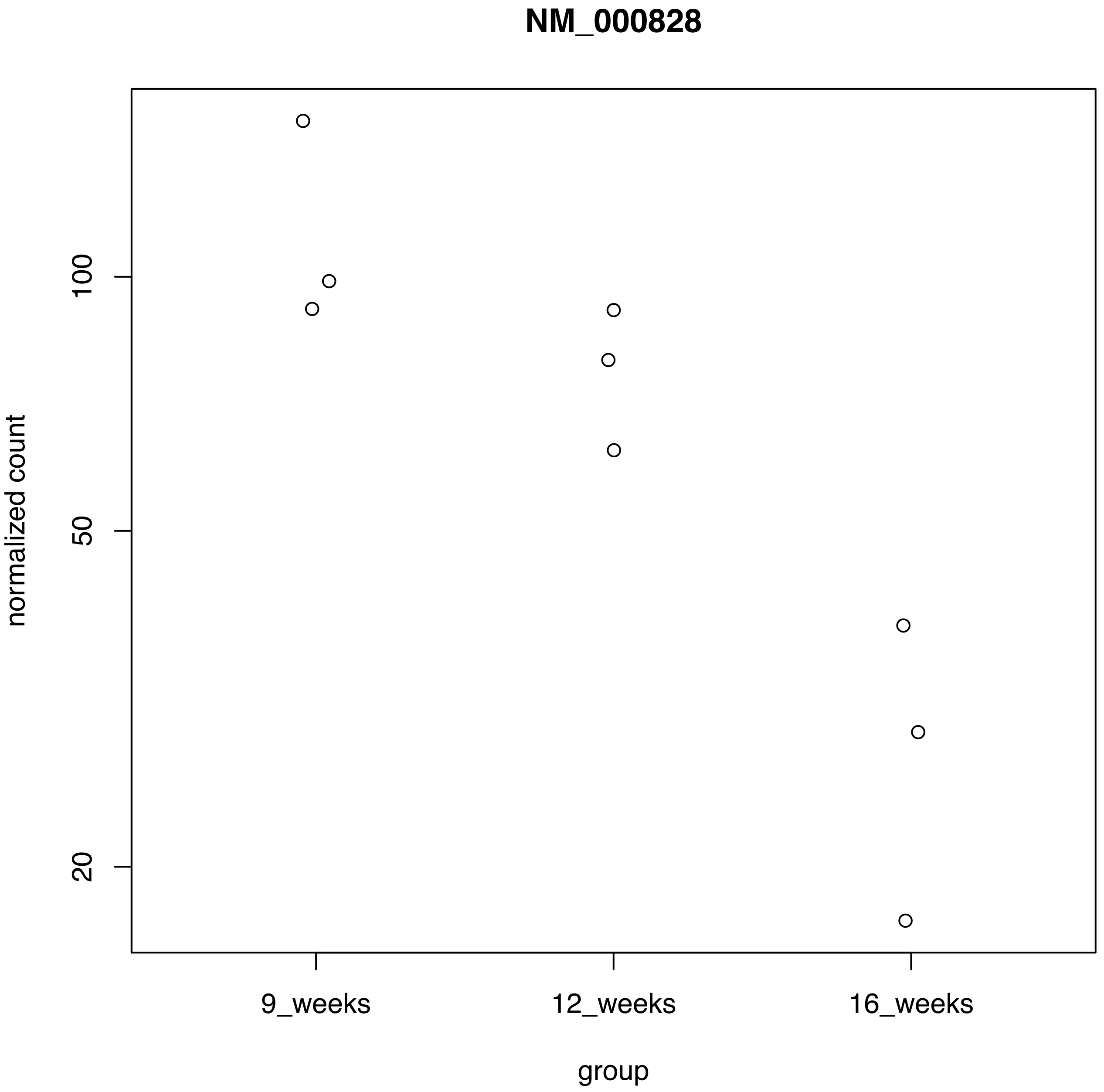
NM_000828
normalized count
100
50
20
9_weeks
12_weeks
16_weeks
group

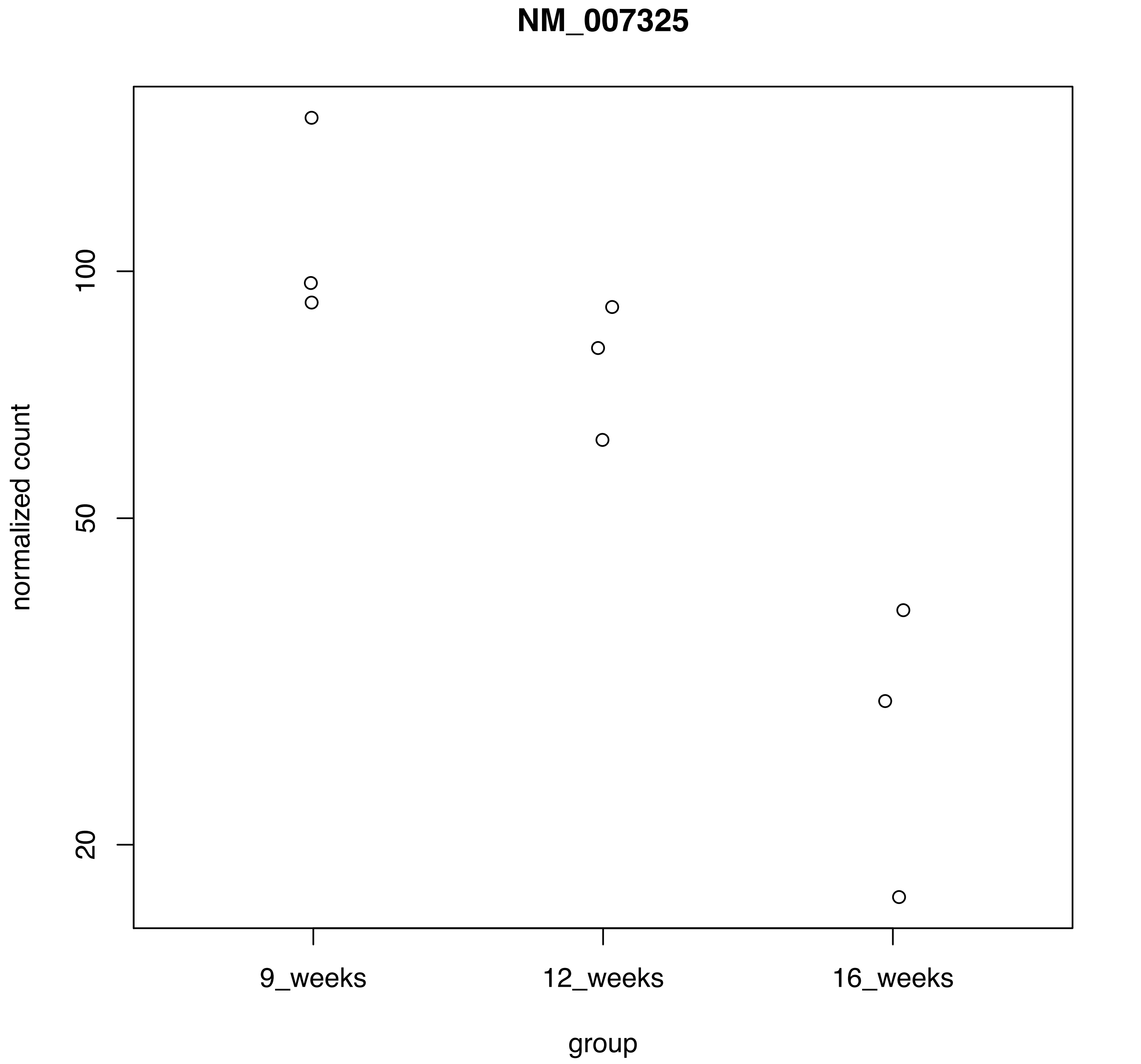
NM_007325
normalized count
100
50
20
9_weeks
12_weeks
16_weeks
group

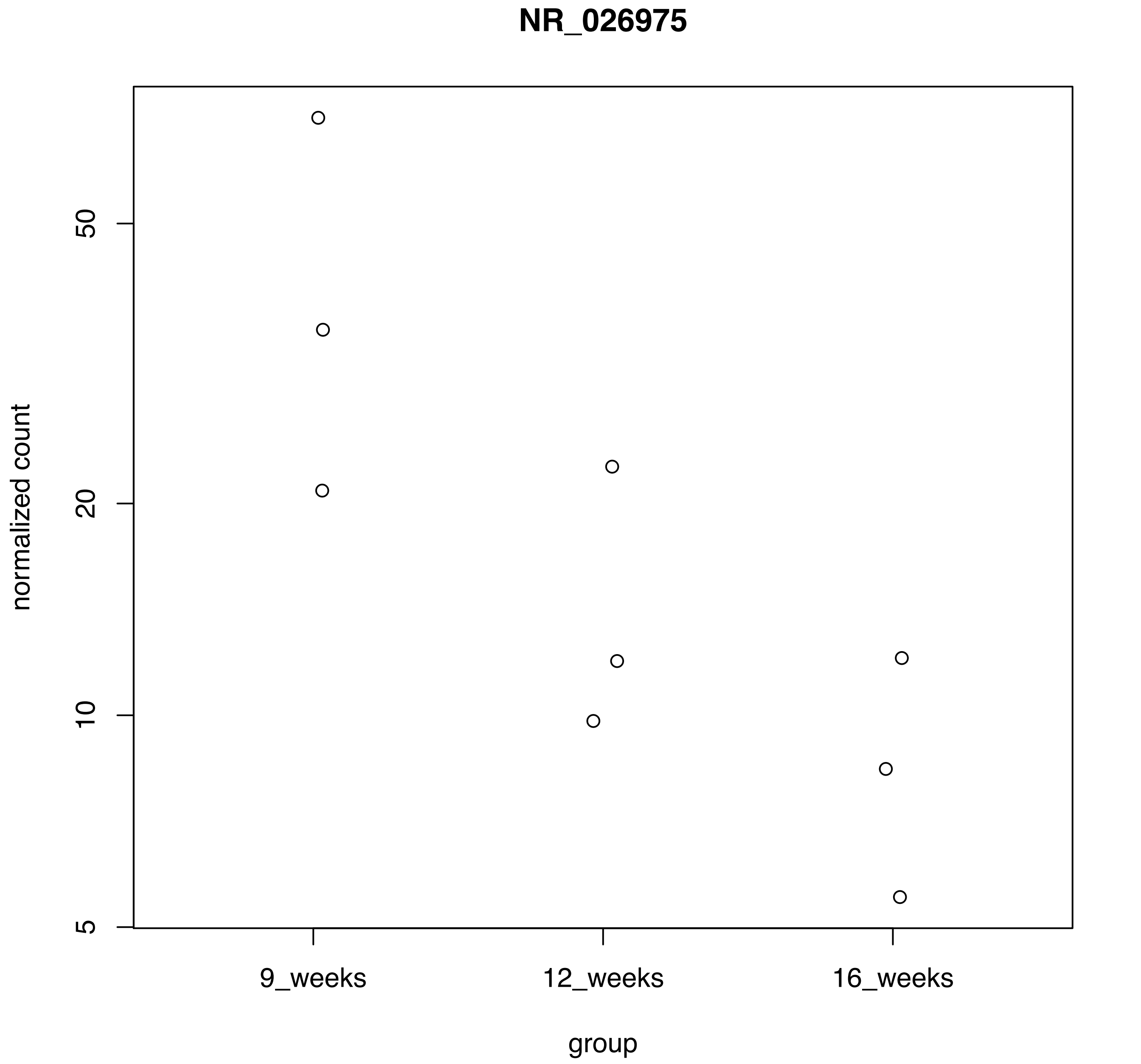
NR_026975
normalized count
50
20
10
5
9_weeks
12_weeks
16_weeks
group

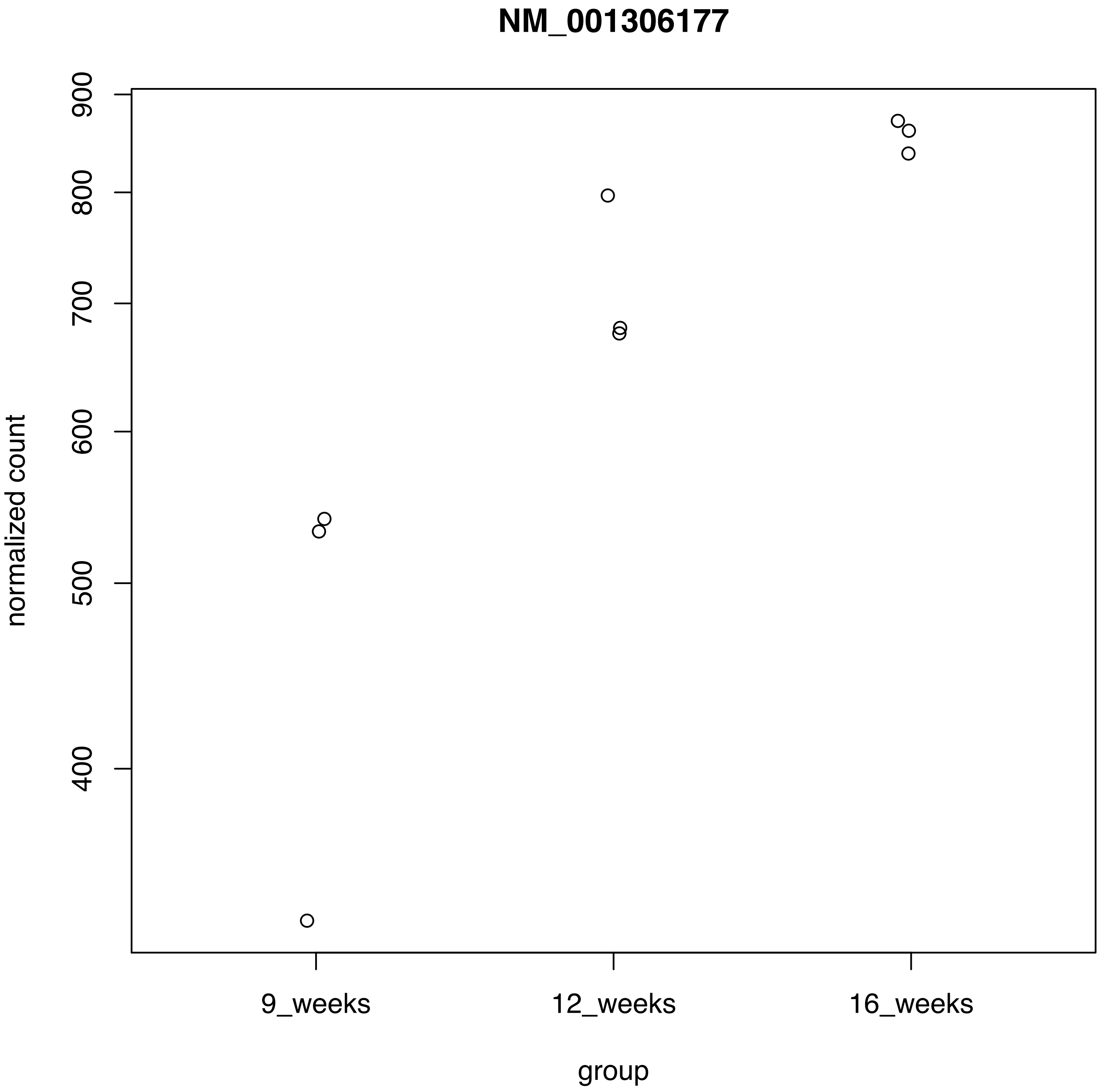
NM_001306177
normalized count
900
800
700
600
500
400
9_weeks
12_weeks
16_weeks
group

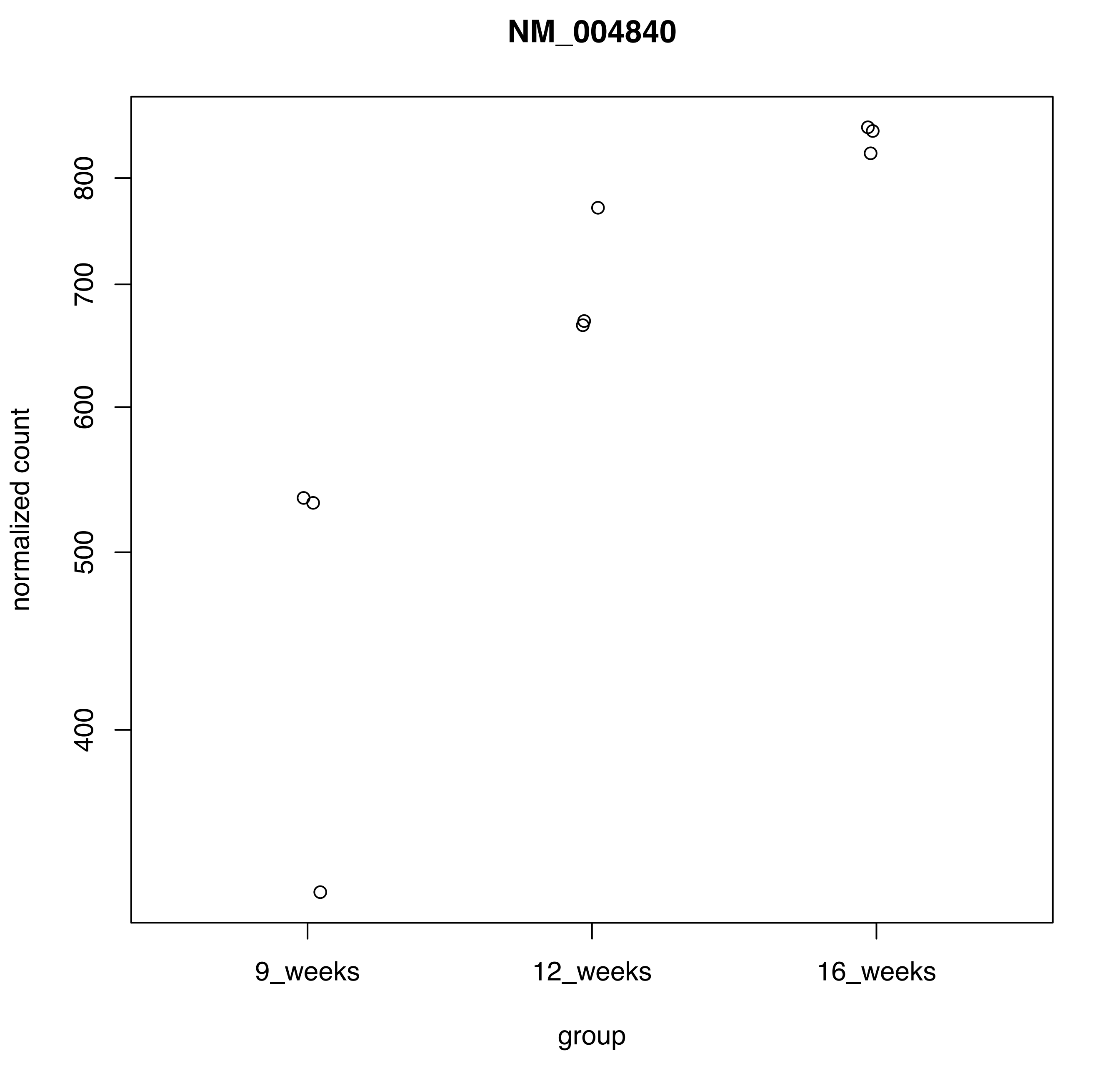
NM_004840
normalized count
800
700
600
500
400
9_weeks
12_weeks
16_weeks
group

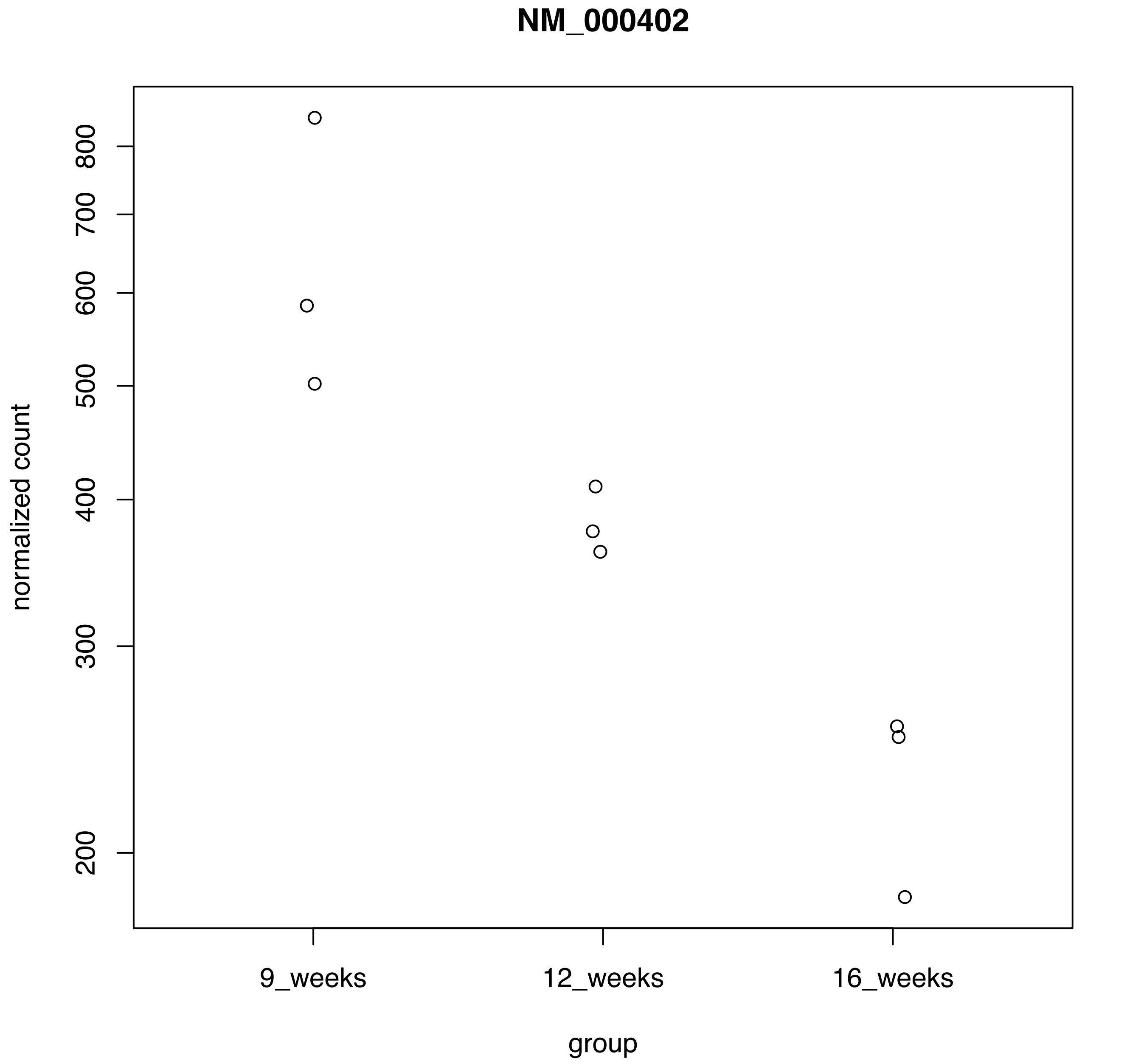

NM_000402
normalized count
800
700
600
500
400
300
200
9_weeks
12_weeks
16_weeks
group

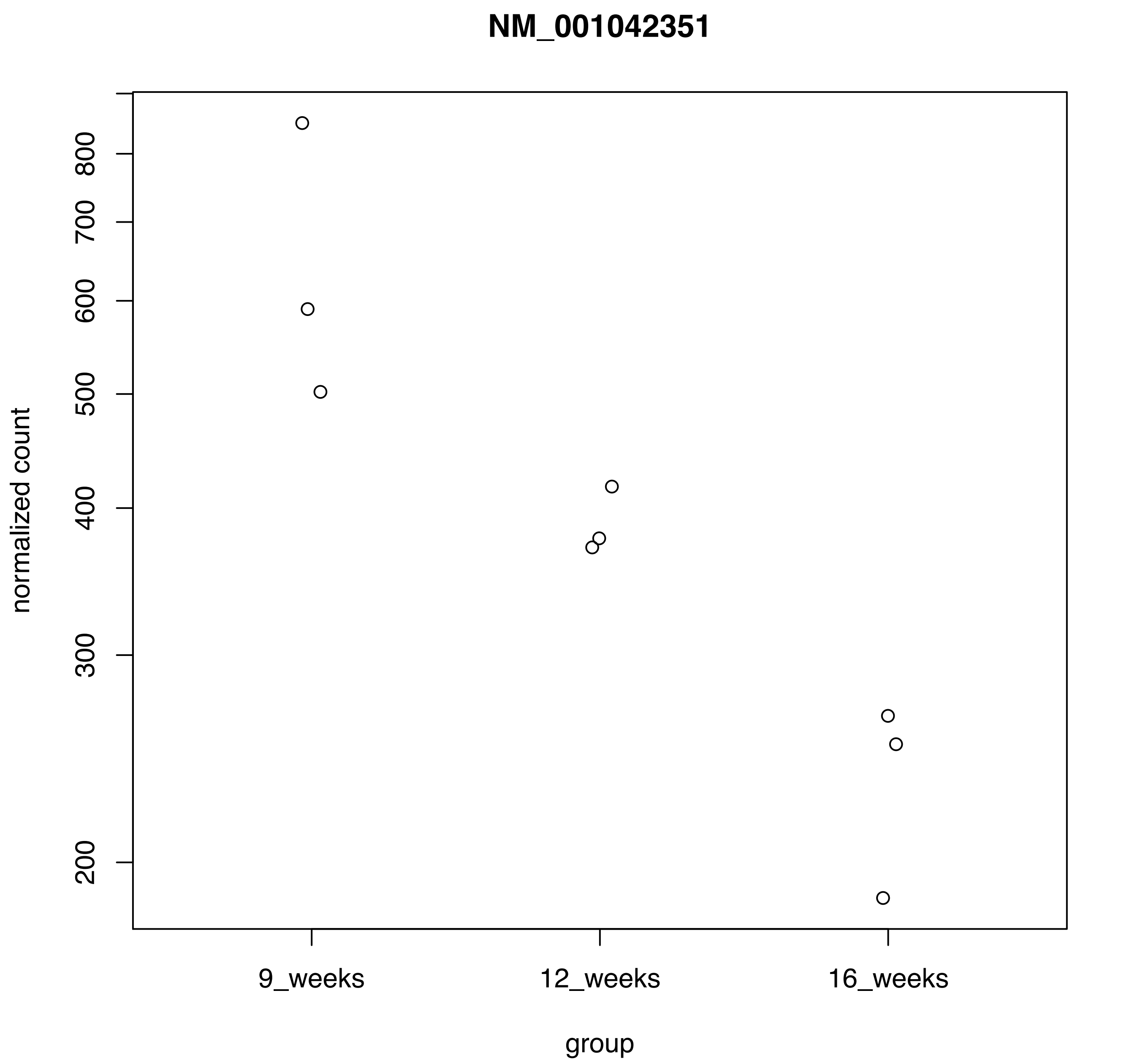

NM_001042351
normalized count
800
700
600
500
400
300
200
9_weeks
12_weeks
16_weeks
group

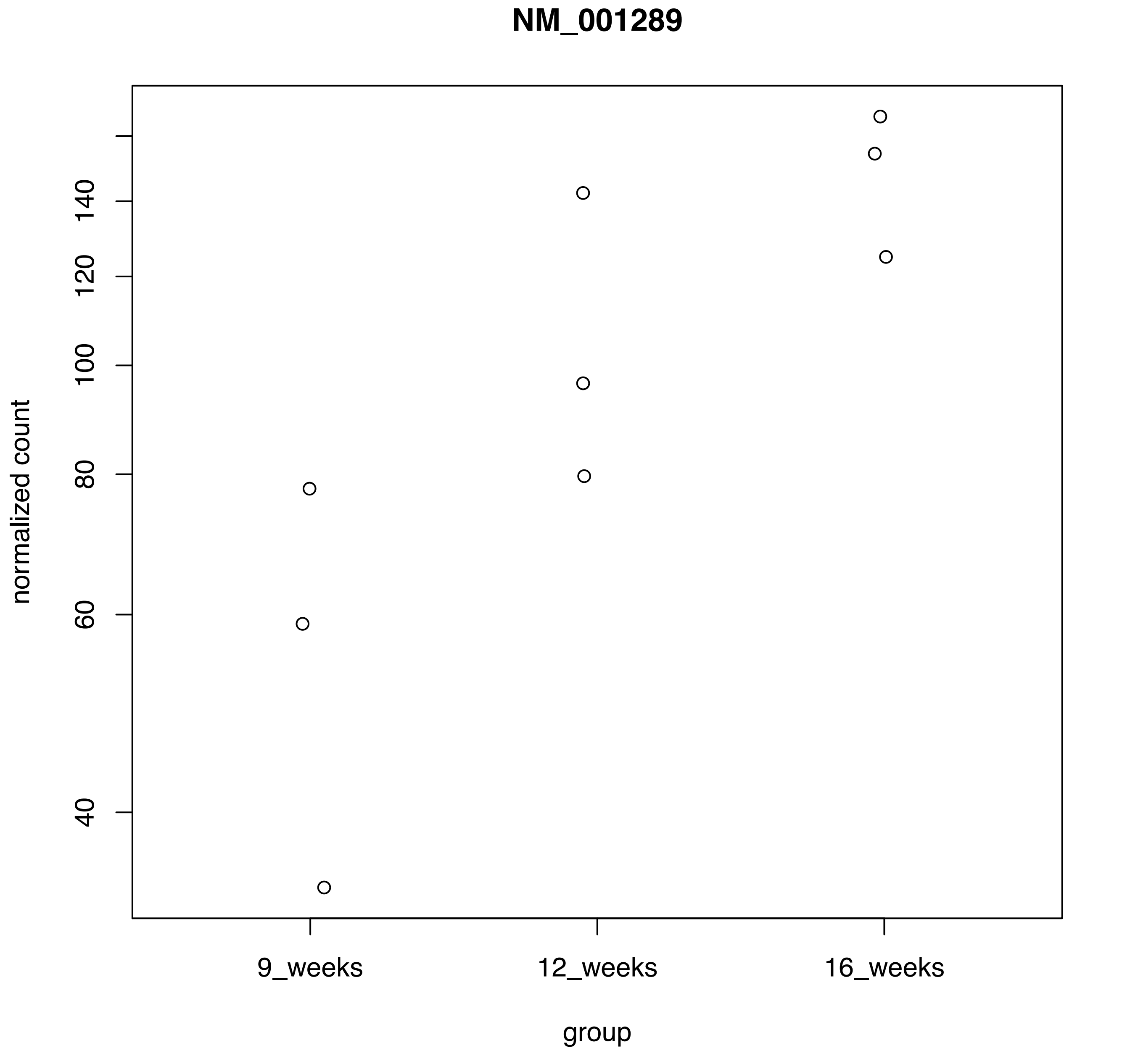
NM_001289
normalized count
140
120
100
80
60
40
9_weeks
12_weeks
16_weeks
group

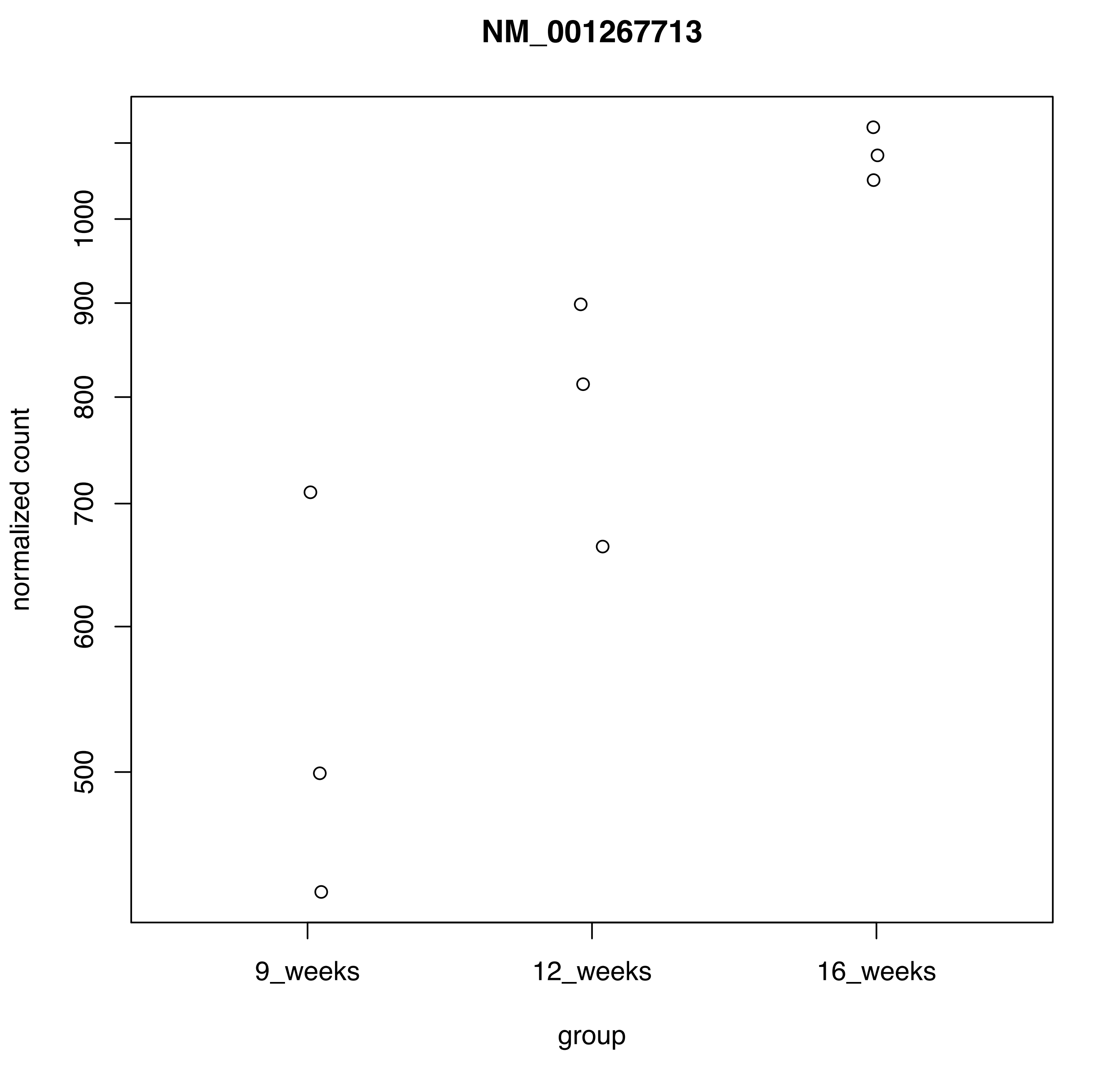
NM_001267713
normalized count
500
600
700
800
900
1000
9_weeks
12_weeks
16_weeks
group

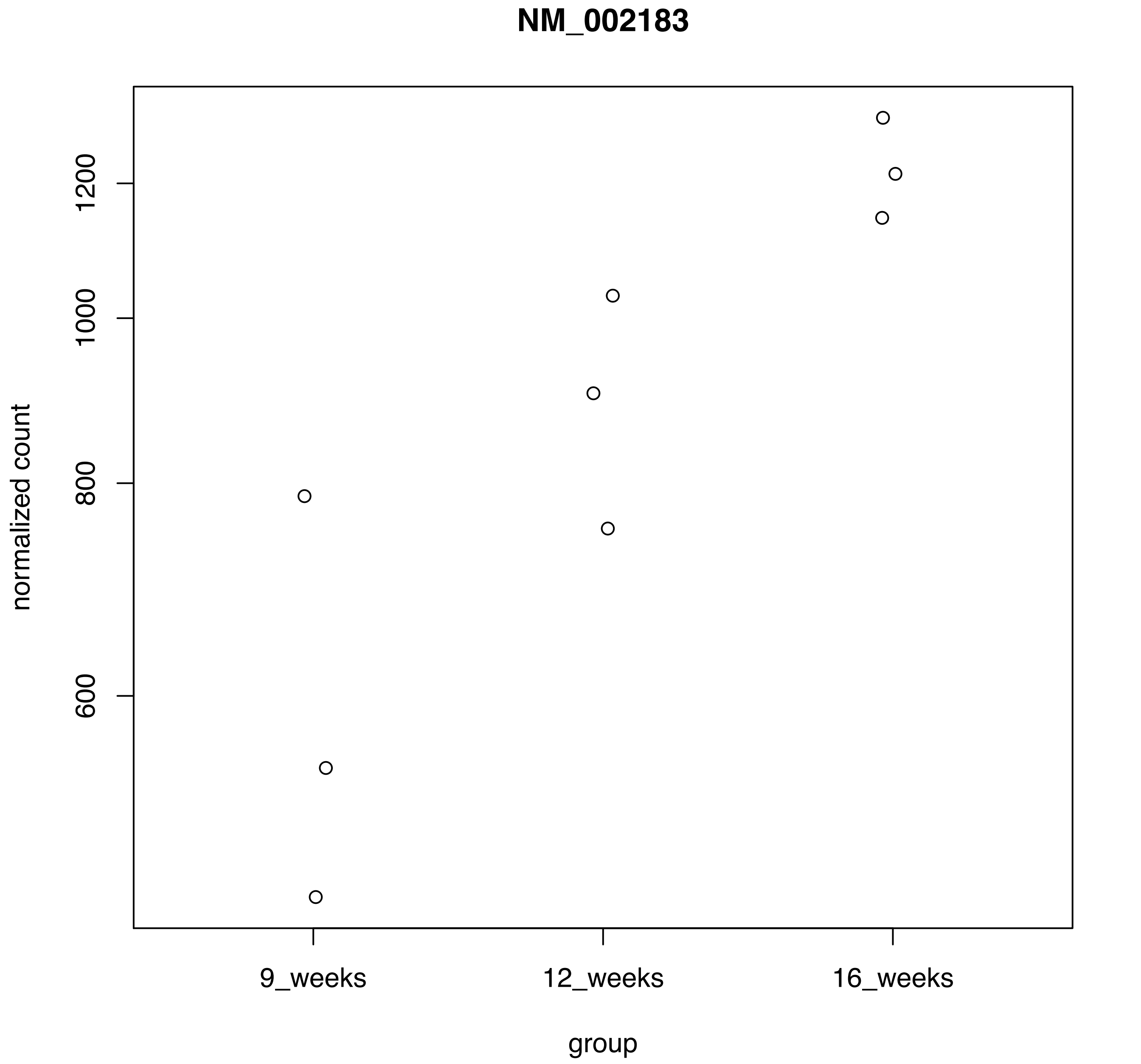
NM_002183
normalized count
600
800
1000
1200
9_weeks
12_weeks
16_weeks
group

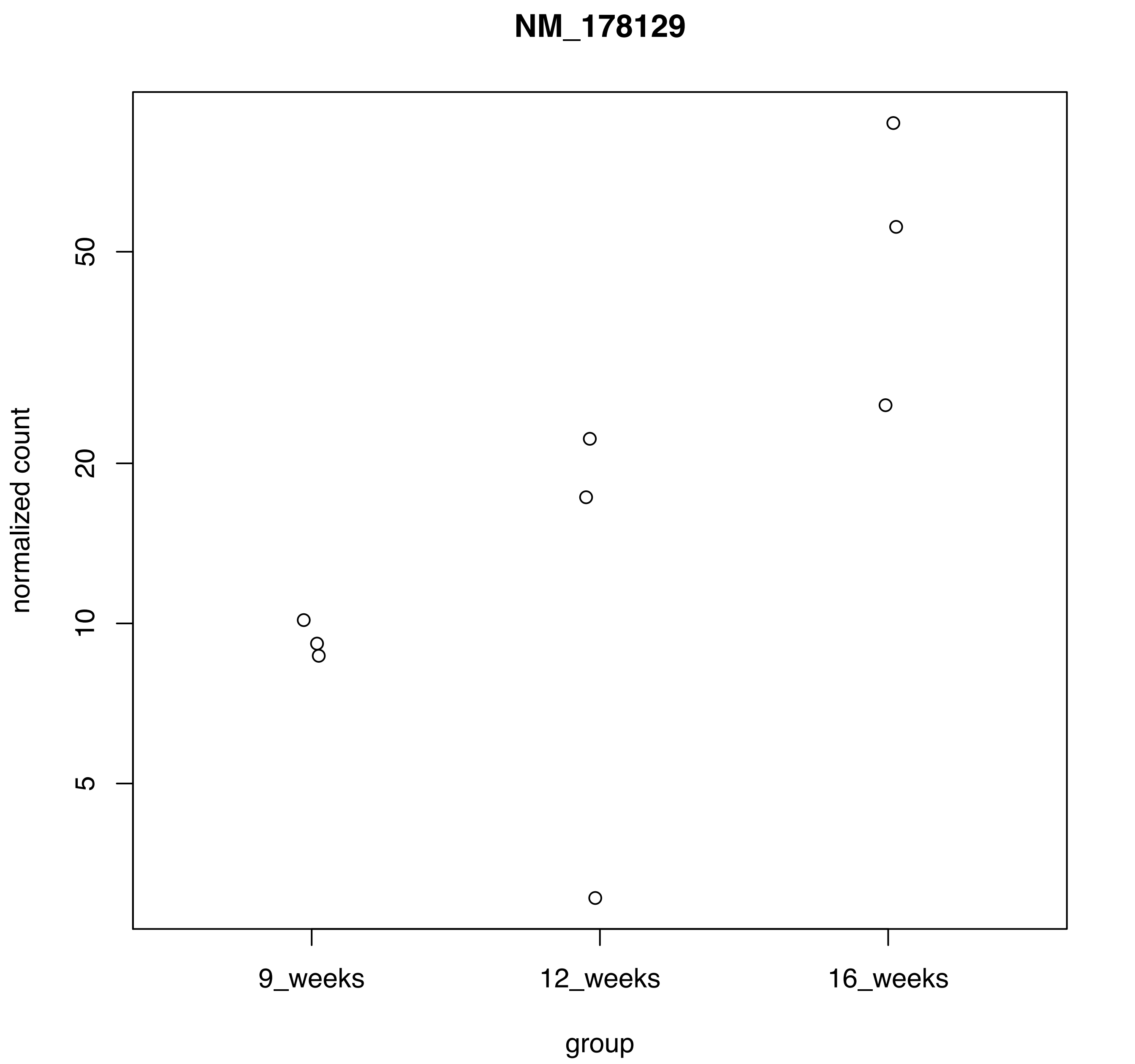
NM_178129
normalized count
50
20
10
5
9_weeks
12_weeks
16_weeks
group

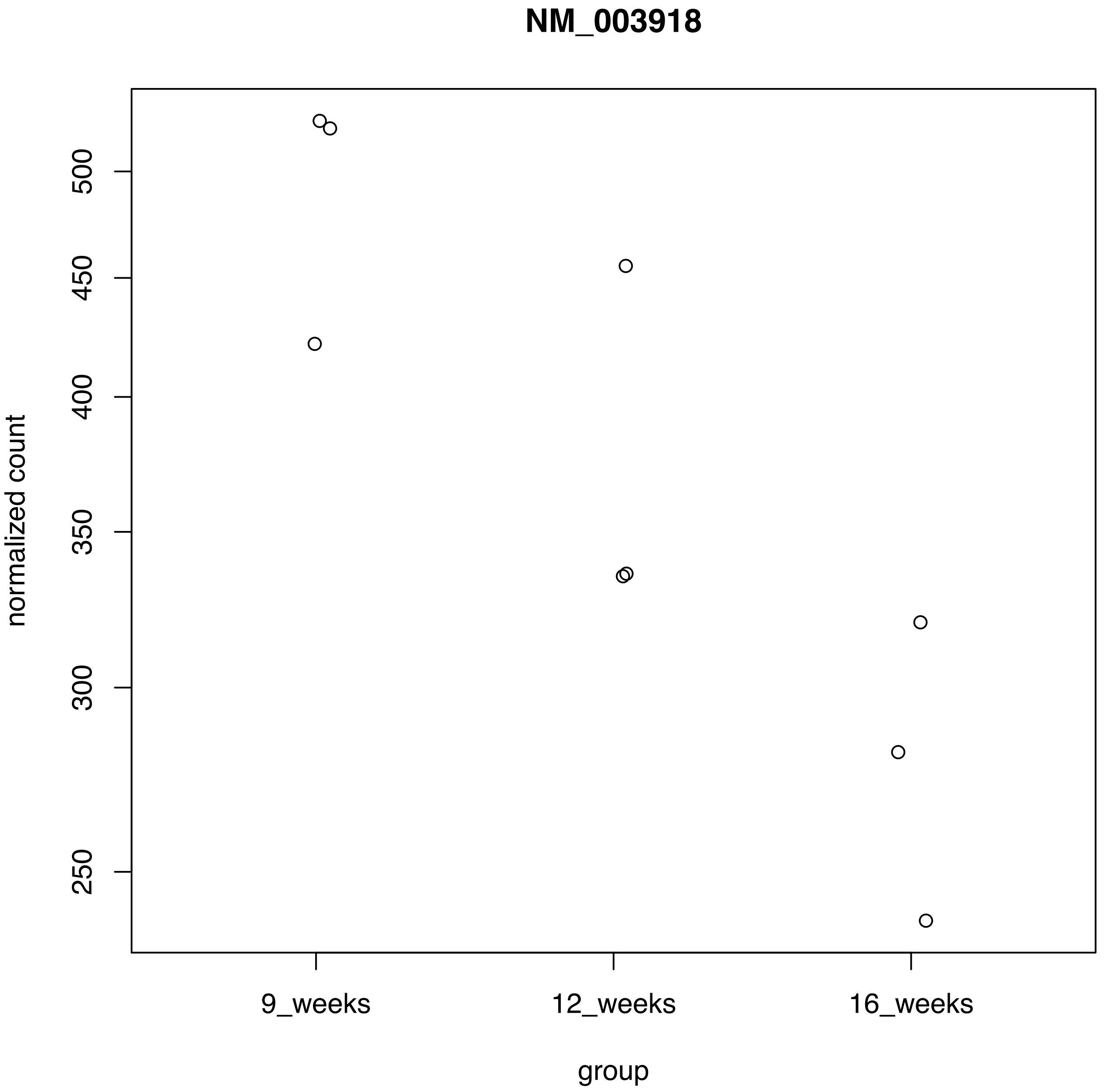
NM_003918
normalized count
250
300
350
400
450
500
9_weeks
12_weeks
16_weeks
group

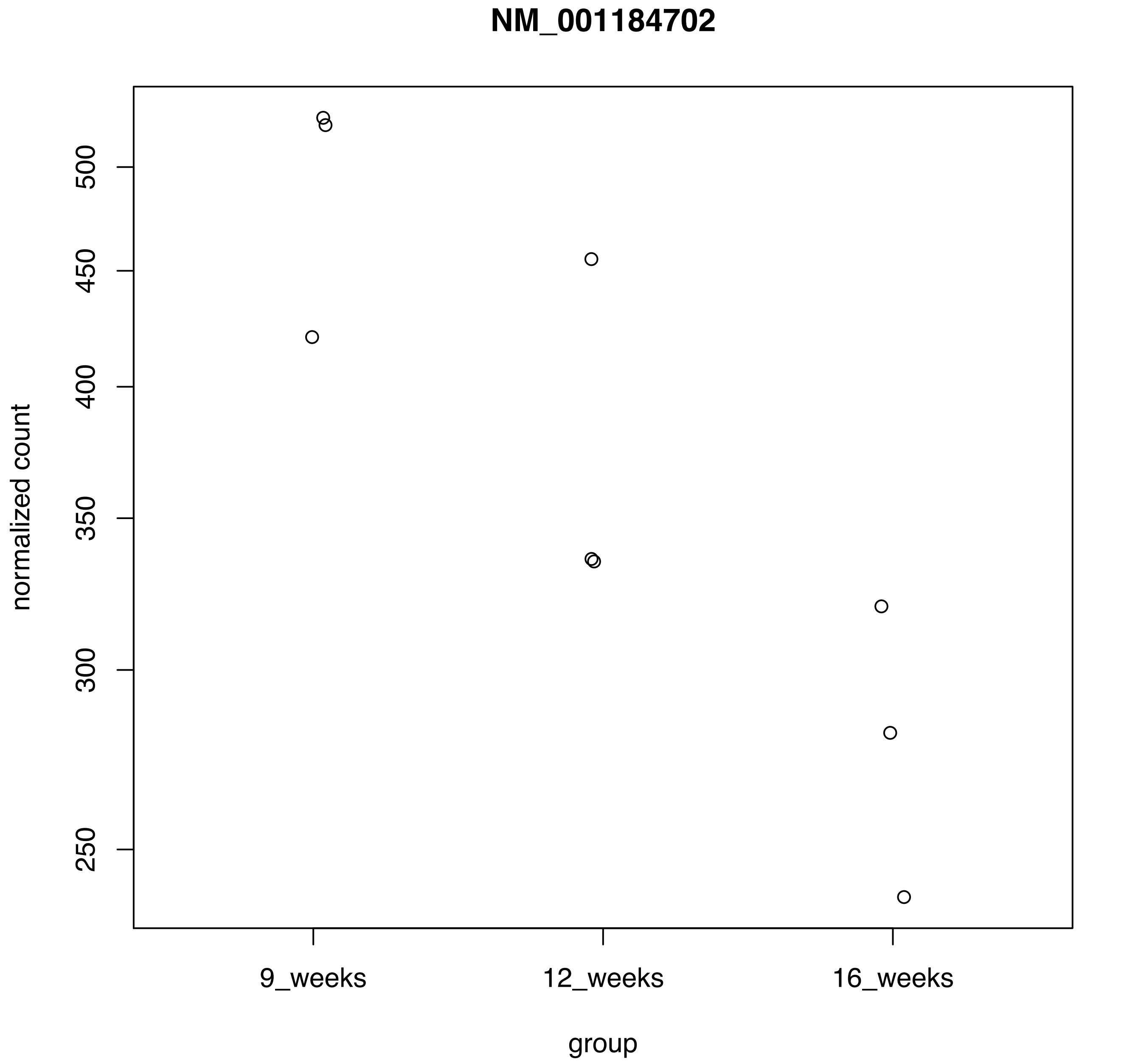
NM_001184702
normalized count
500
450
400
350
300
250
9_weeks
12_weeks
16_weeks
group

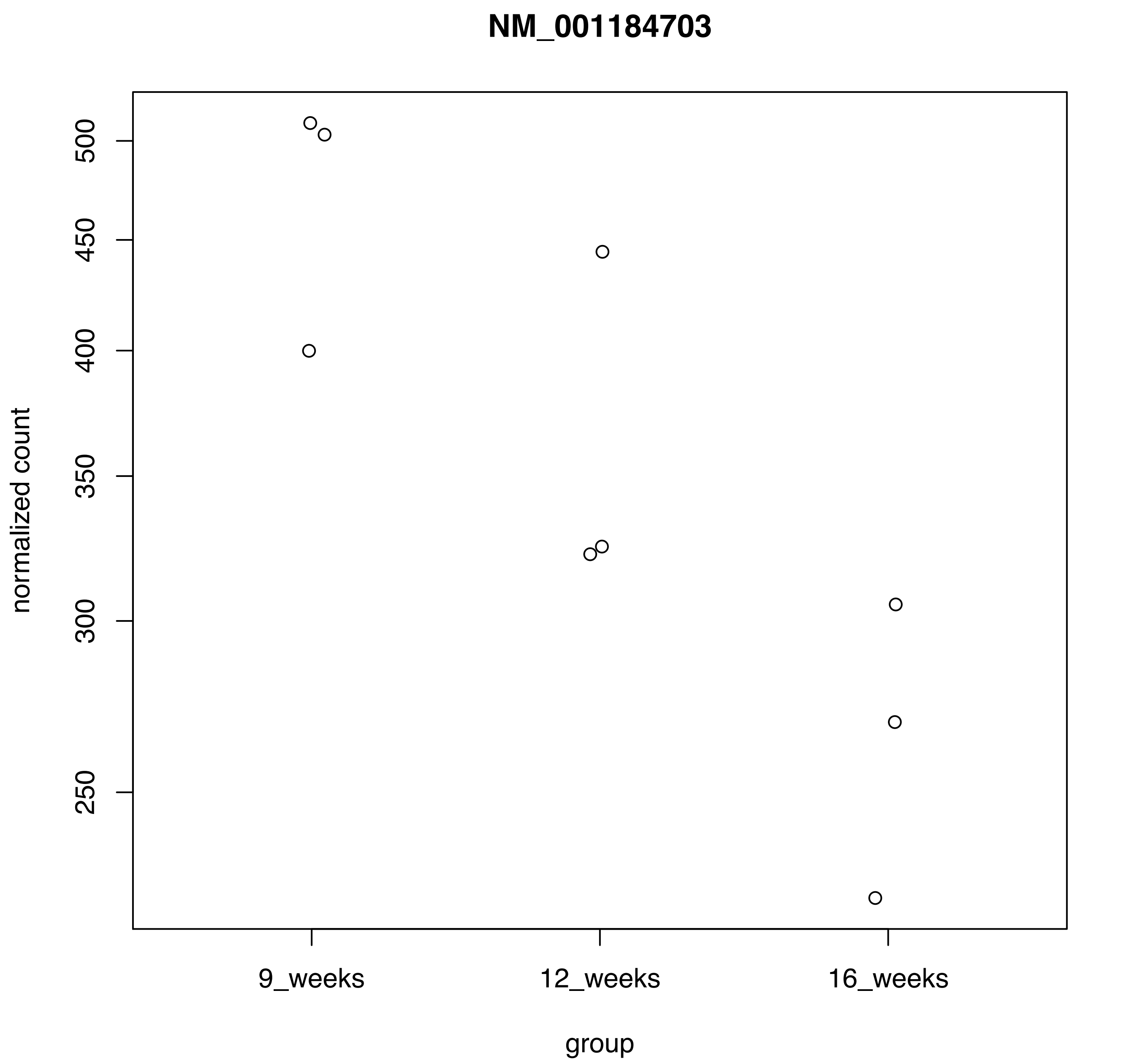
NM_001184703
normalized count
500
450
400
350
300
250
9_weeks
12_weeks
16_weeks
group

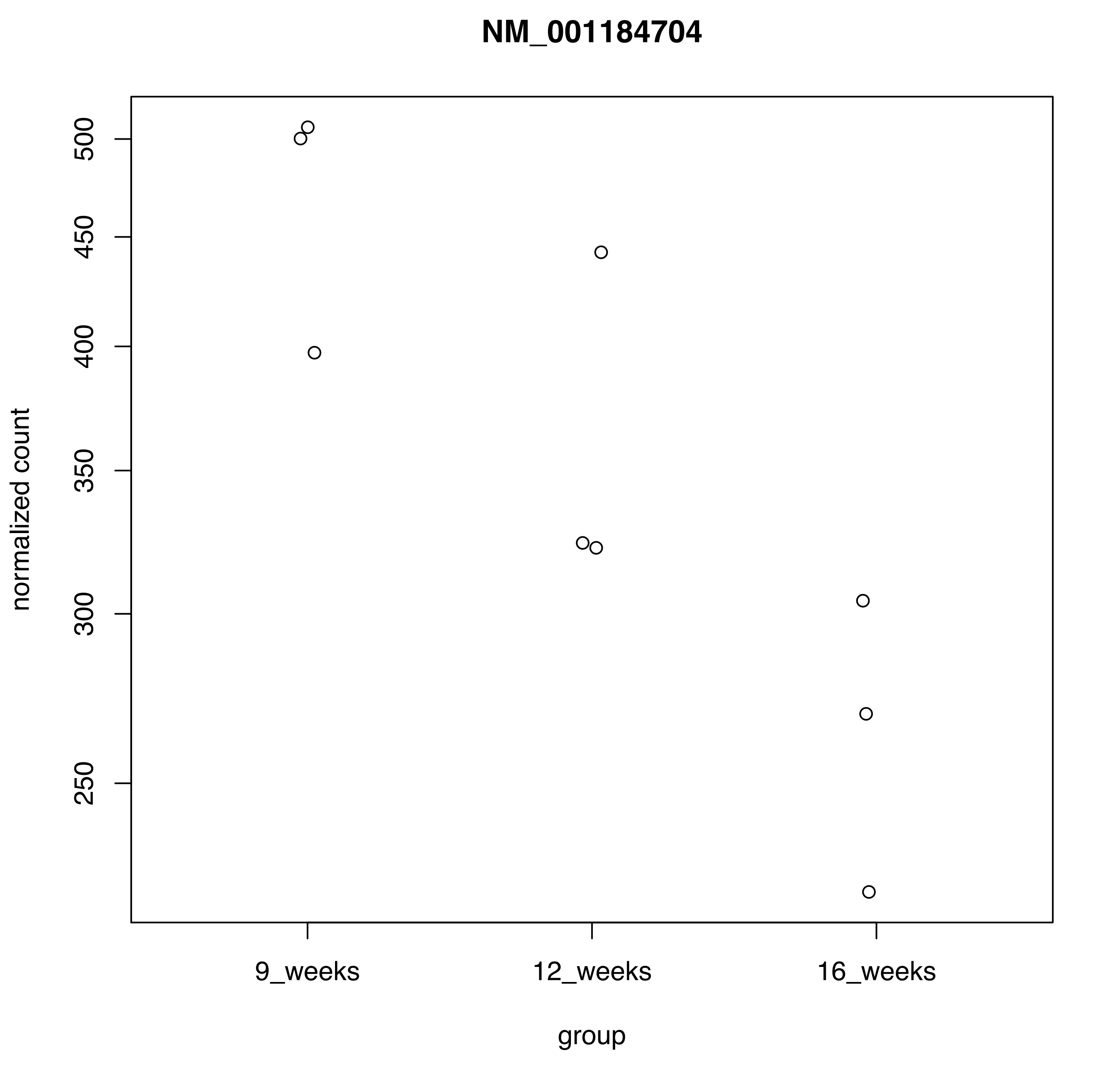

NM_001184704
normalized count
250
300
350
400
450
500
9_weeks
12_weeks
16_weeks
group

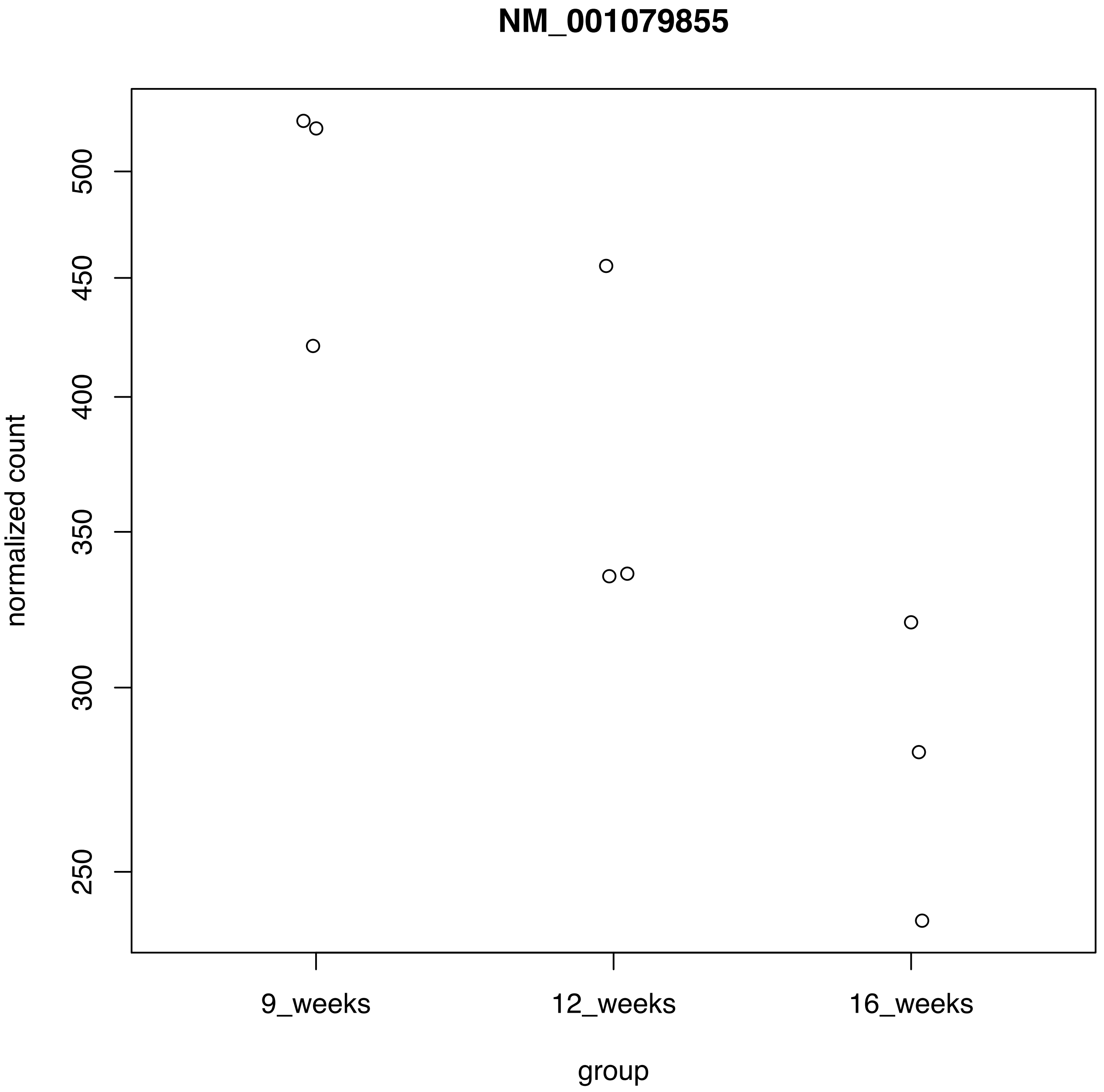
NM_001079855
normalized count
500
450
400
350
300
250
9_weeks
12_weeks
16_weeks
group

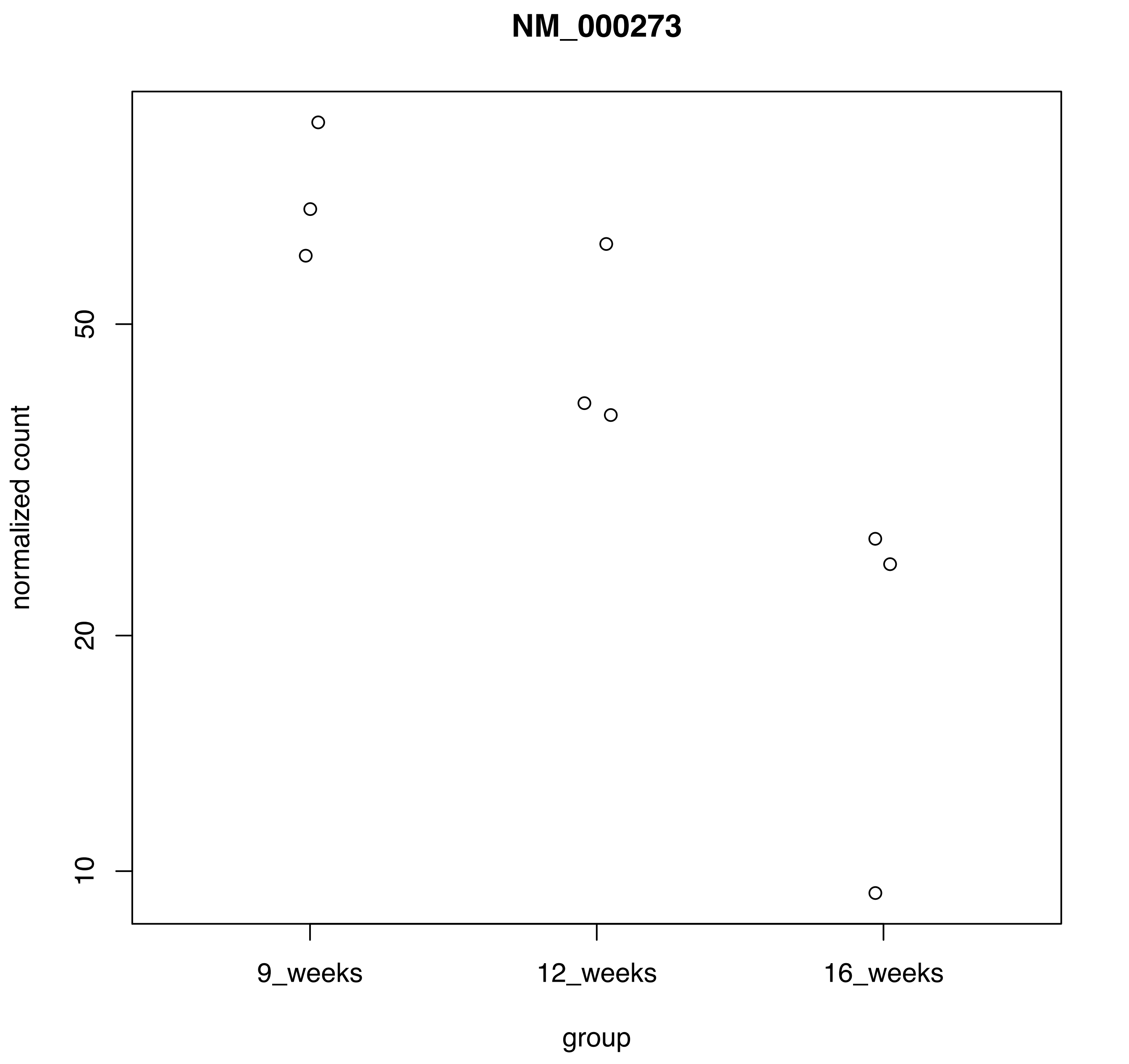
NM_000273
normalized count
50
20
10
9_weeks
12_weeks
16_weeks
group

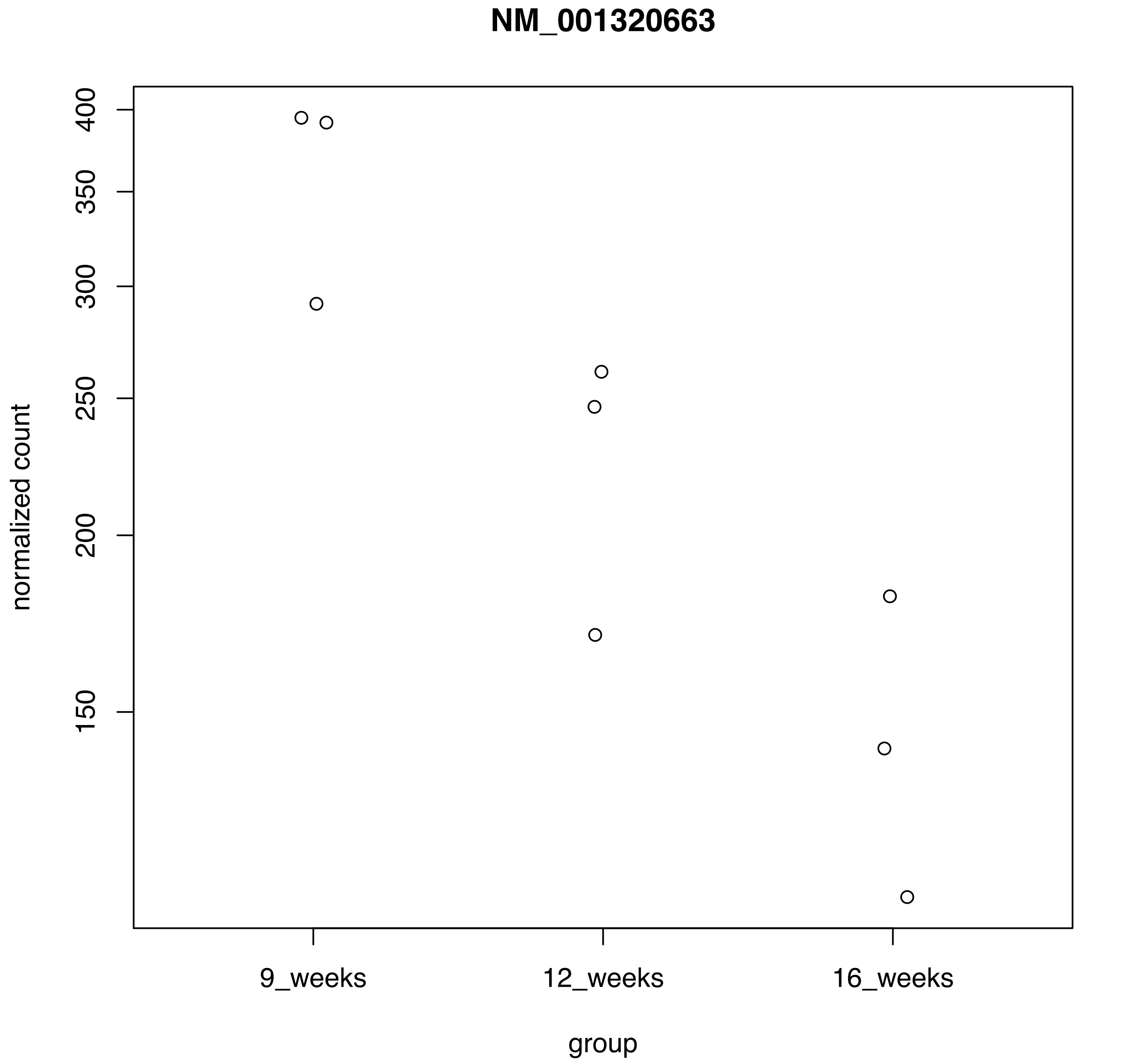

NM_001320663
normalized count
400
350
300
250
200
150
9_weeks
12_weeks
16_weeks
group

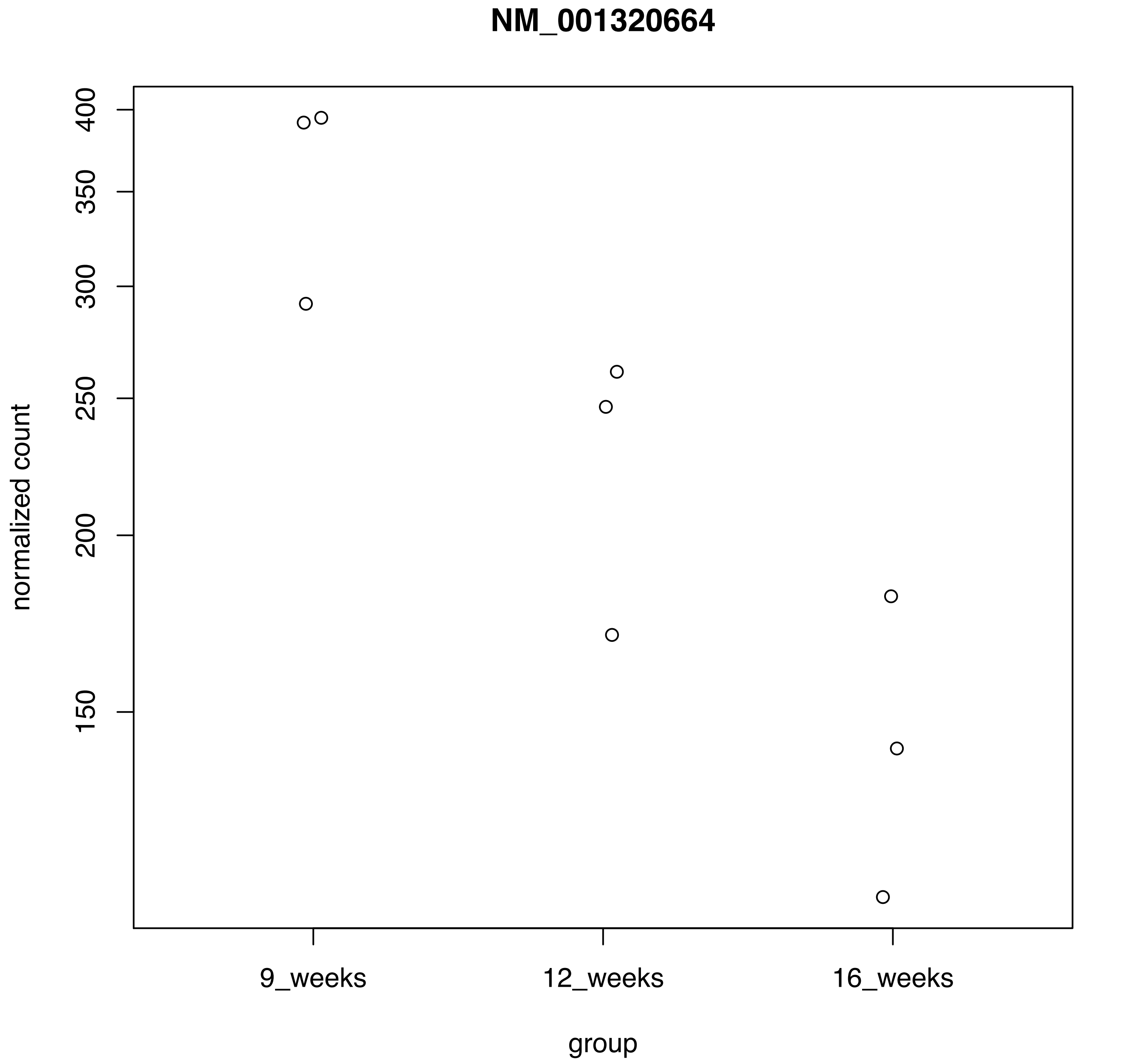

NM_001320664
normalized count
400
350
300
250
200
150
9_weeks
12_weeks
16_weeks
group

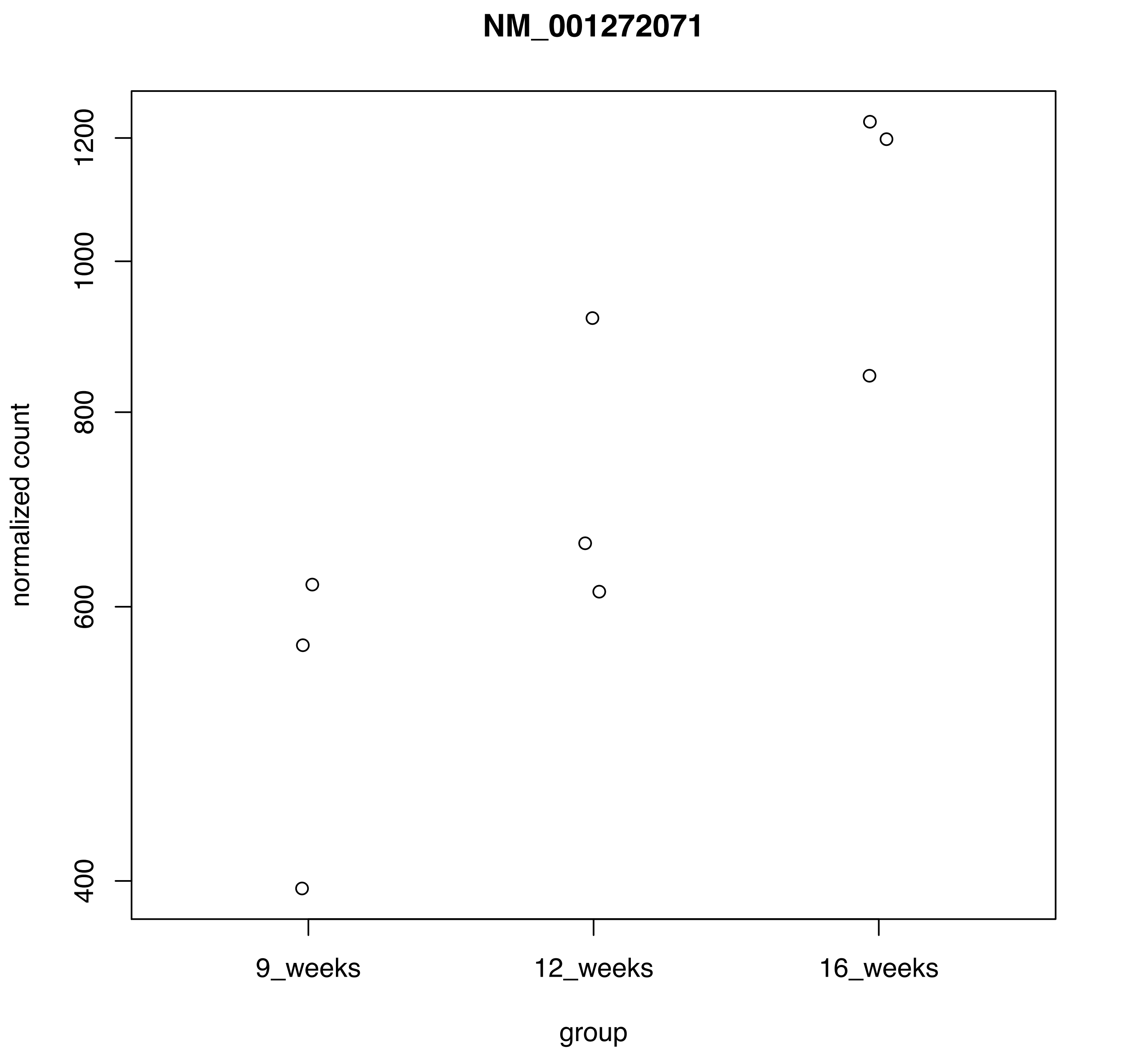
NM_001272071
normalized count
400
600
800
1000
1200
9_weeks
12_weeks
16_weeks
group

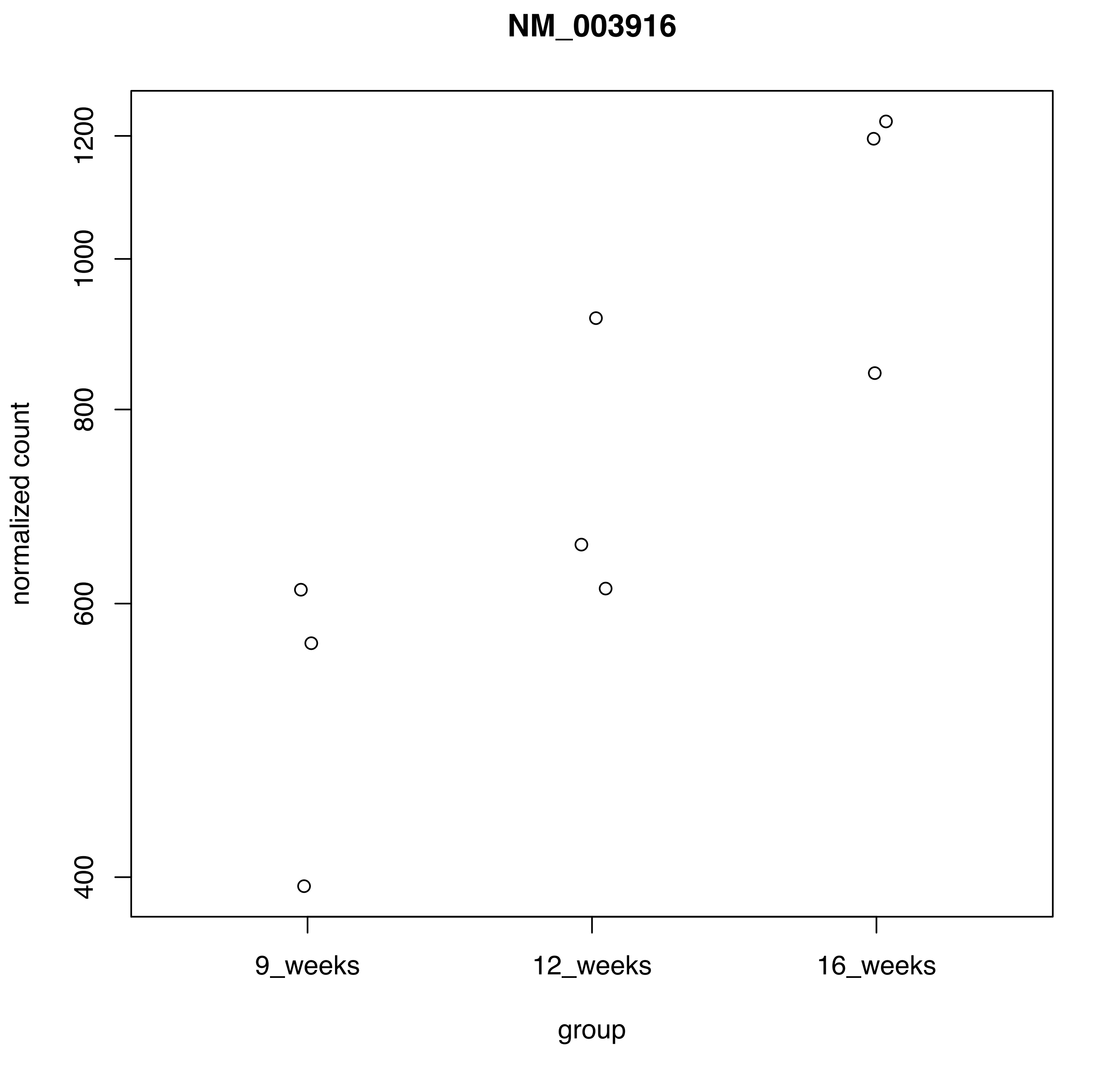

NM_003916
normalized count
400
600
800
1000
1200
9_weeks
12_weeks
16_weeks
group

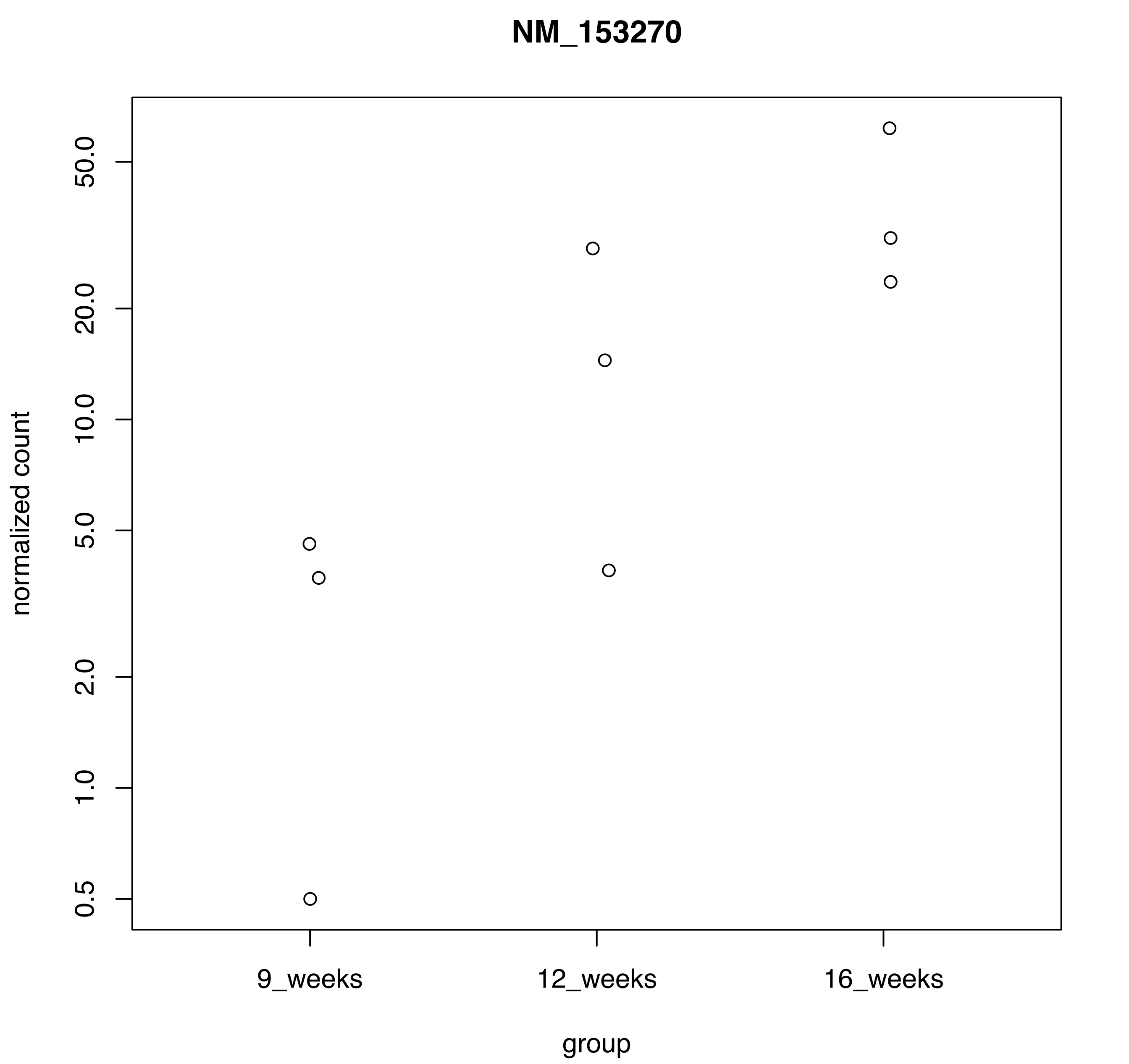

NM_153270
normalized count
50.0
20.0
10.0
5.0
2.0
1.0
0.5
9_weeks
12_weeks
16_weeks
group

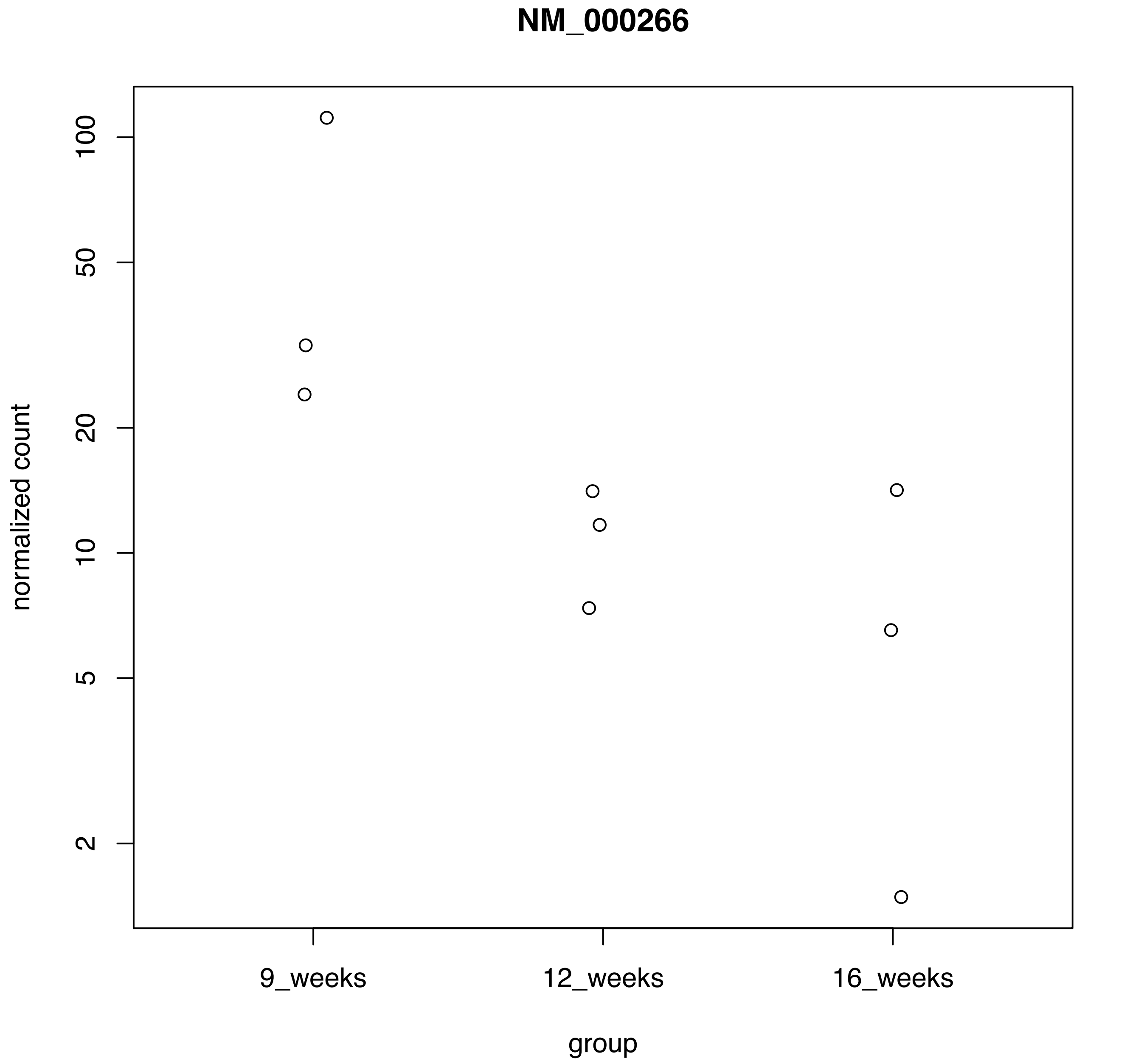

NM_000266
normalized count
100
50
20
10
5
2
9_weeks
12_weeks
16_weeks
group

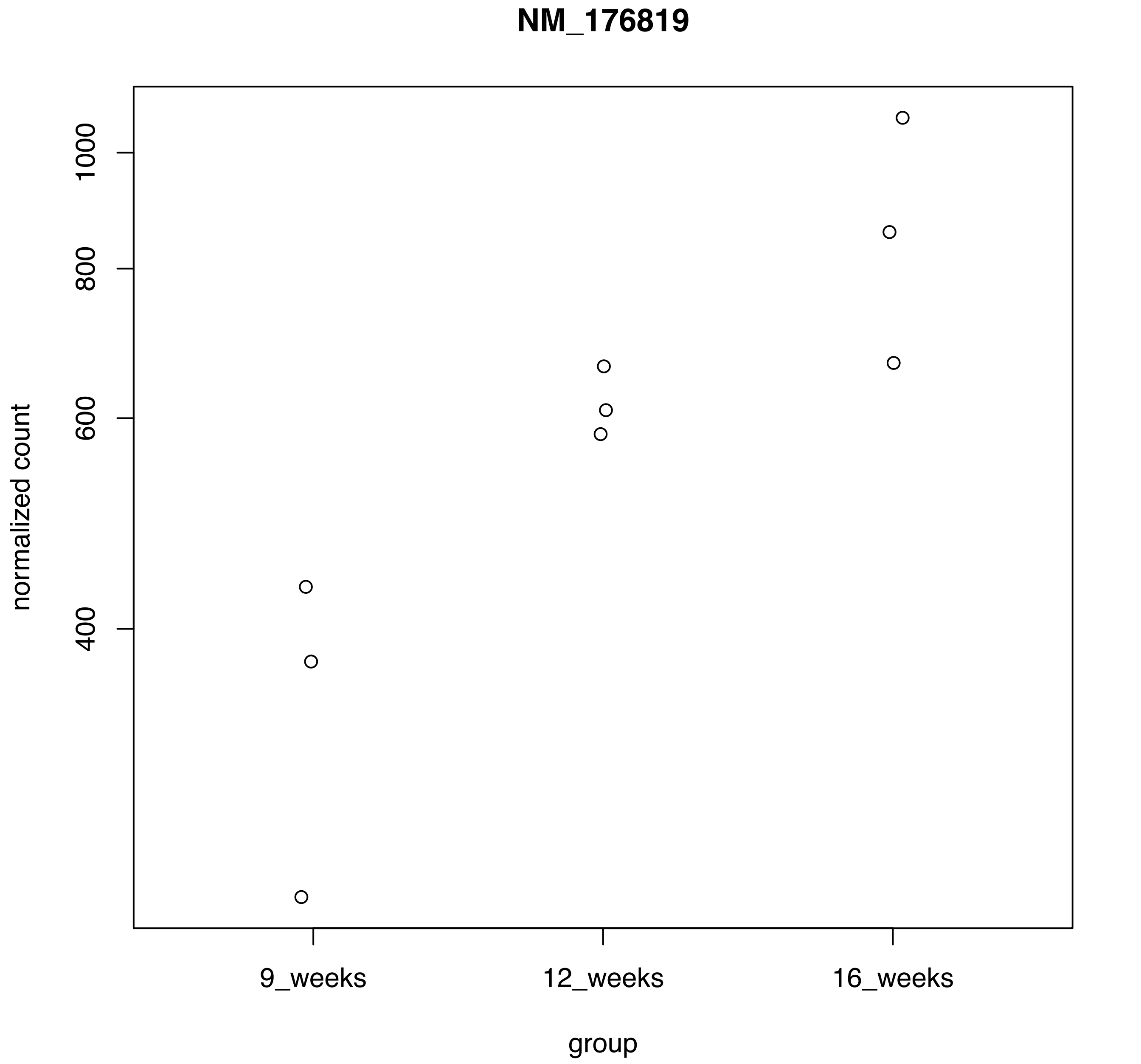

NM_176819
normalized count
1000
800
600
400
9_weeks
12_weeks
16_weeks
group

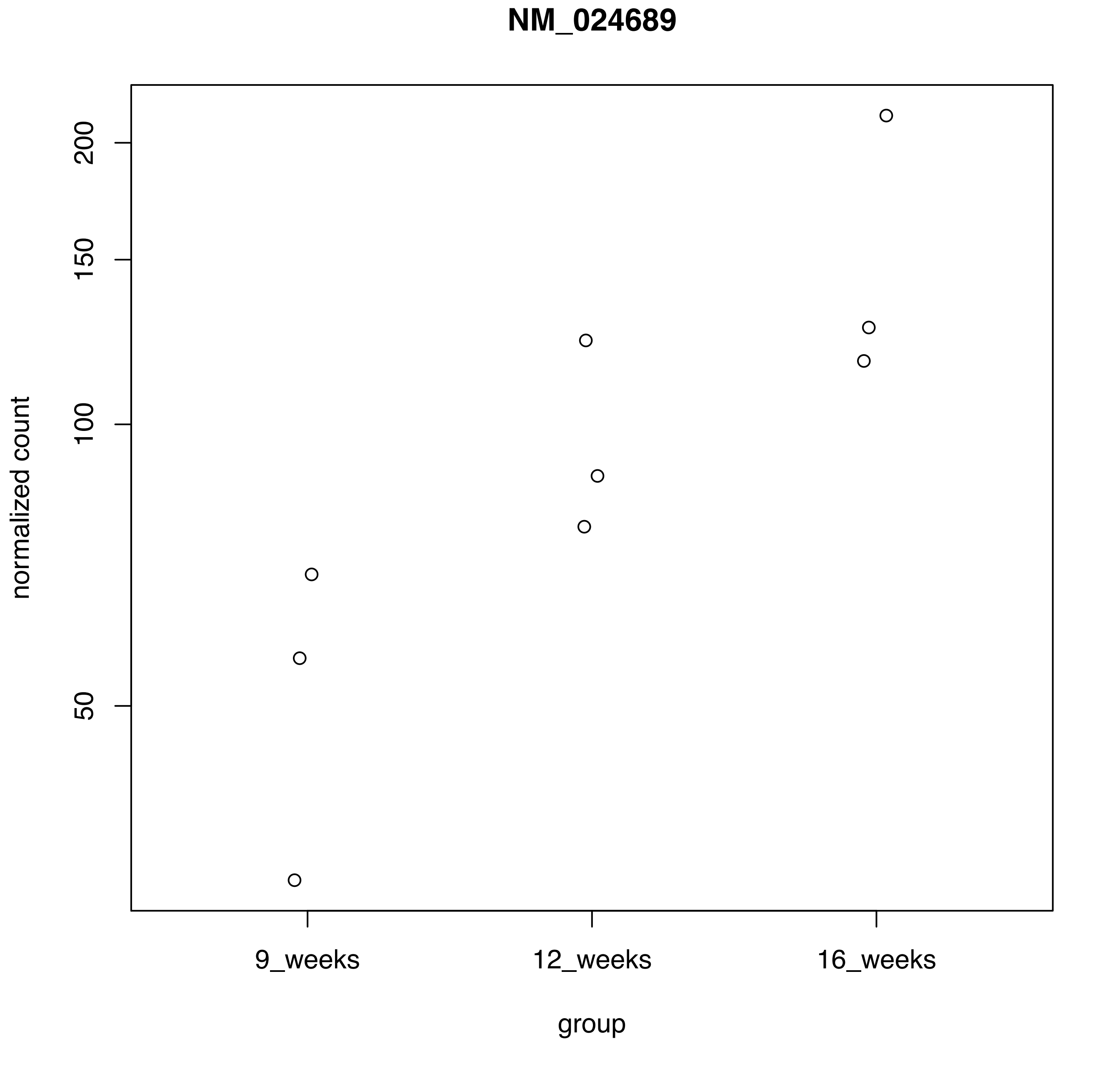

NM_024689
normalized count
200
150
100
50
9_weeks
12_weeks
16_weeks
group

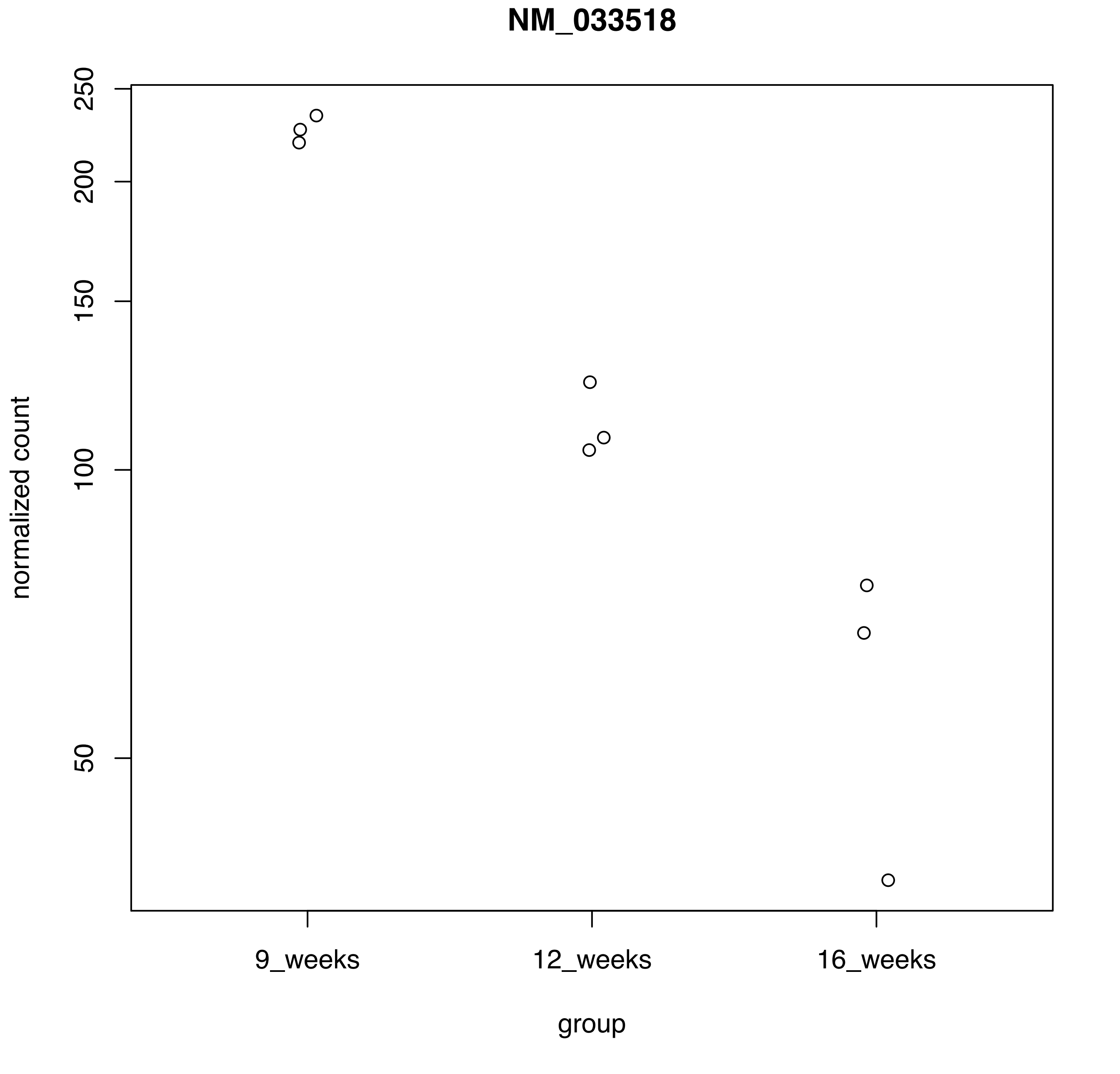
NM_033518
normalized count
250
200
150
100
50
9_weeks
12_weeks
16_weeks
group

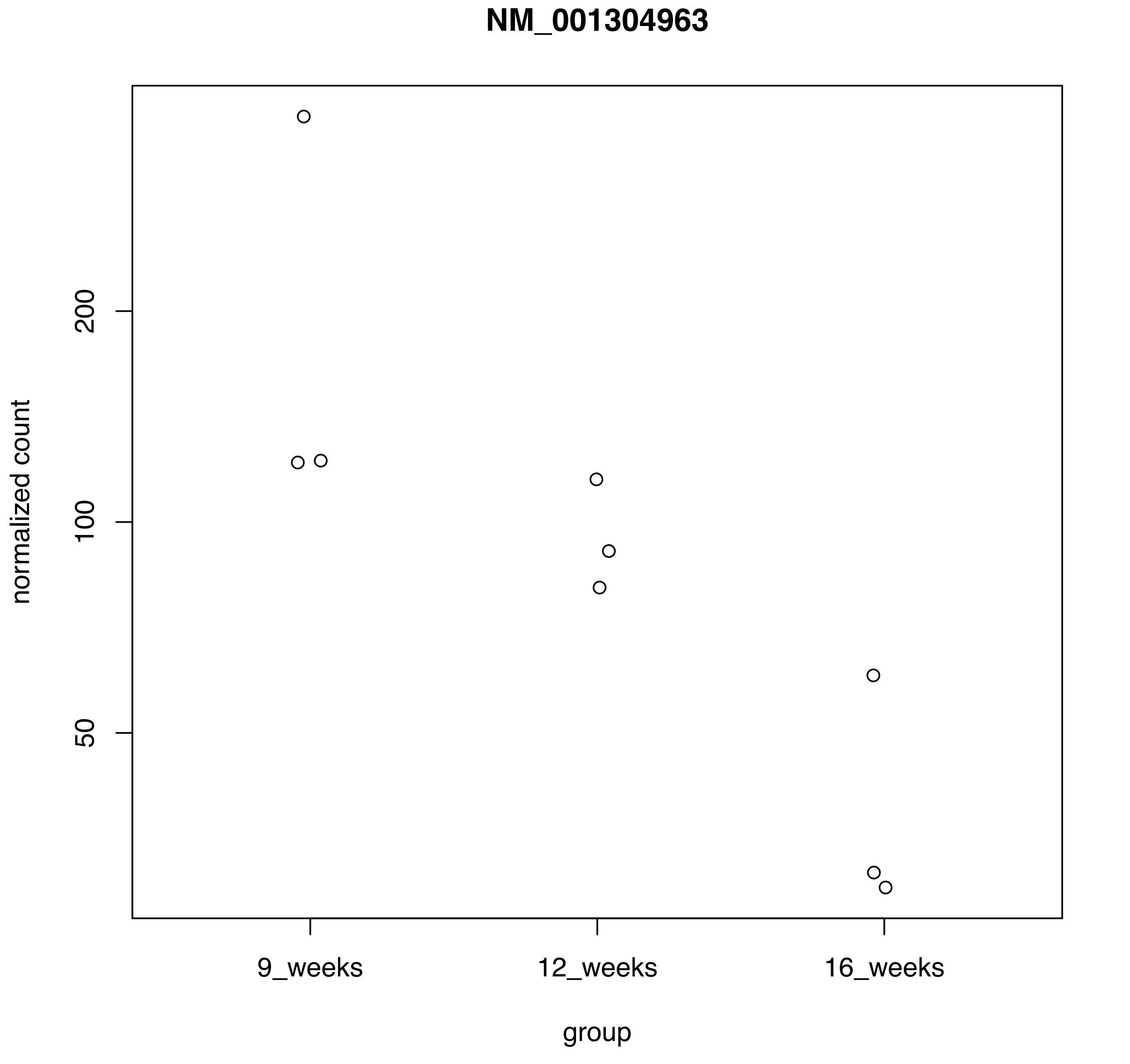
NM_001304963
normalized count
200
100
50
9_weeks
12_weeks
16_weeks
group

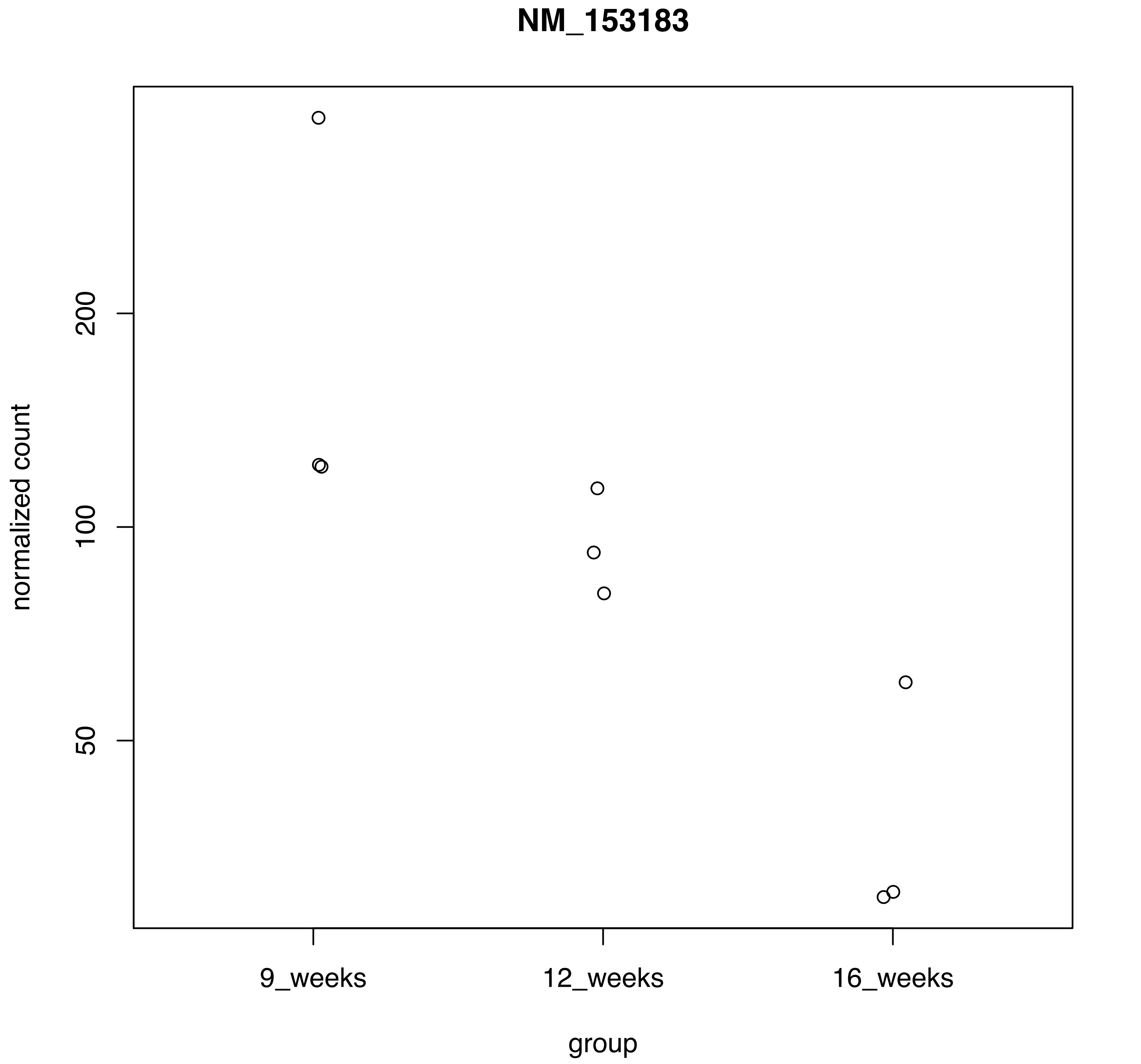
NM_153183
normalized count
200
100
50
9_weeks
12_weeks
16_weeks
group

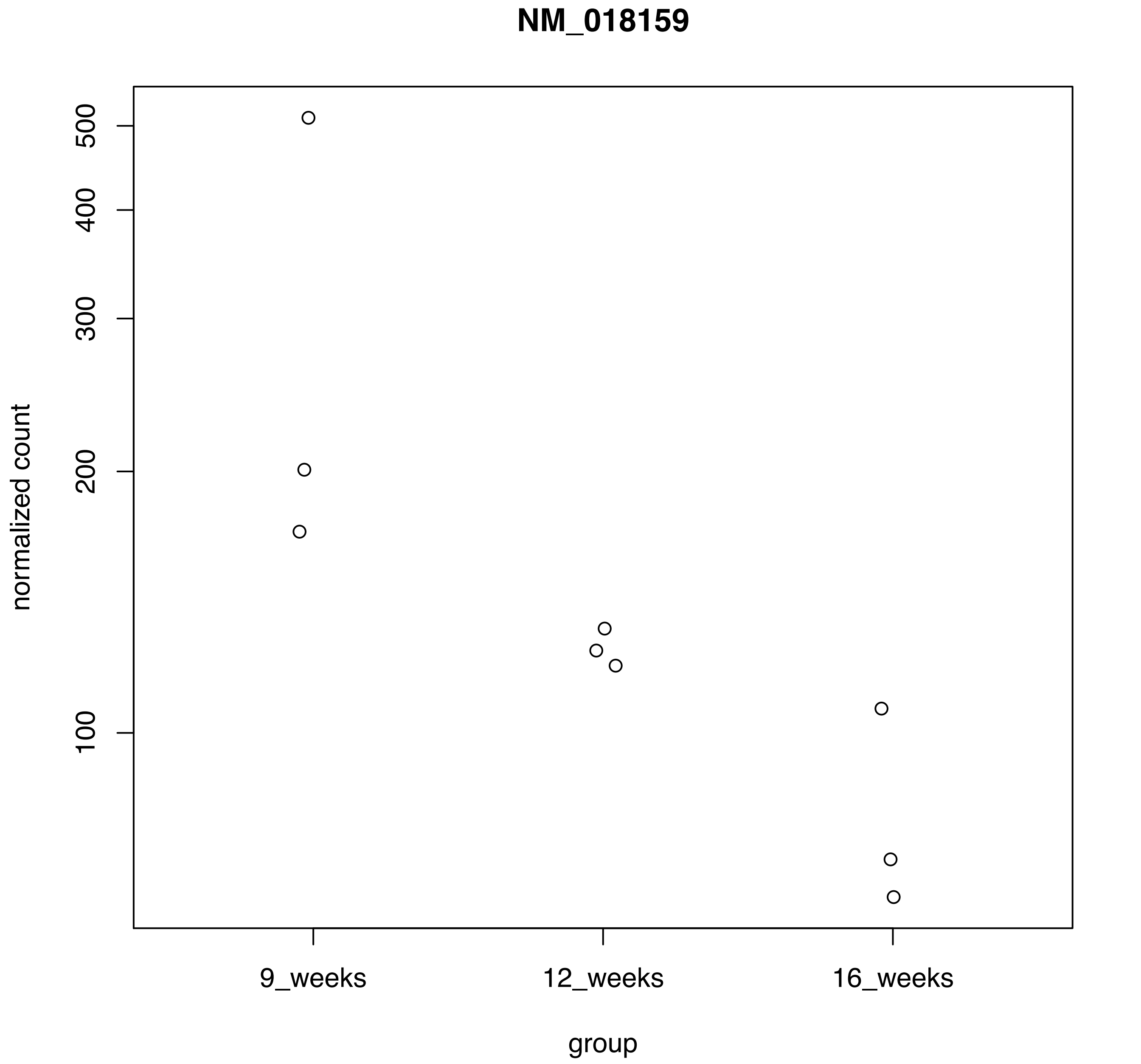
NM_018159
normalized count
100
200
300
400
500
9_weeks
12_weeks
16_weeks
group

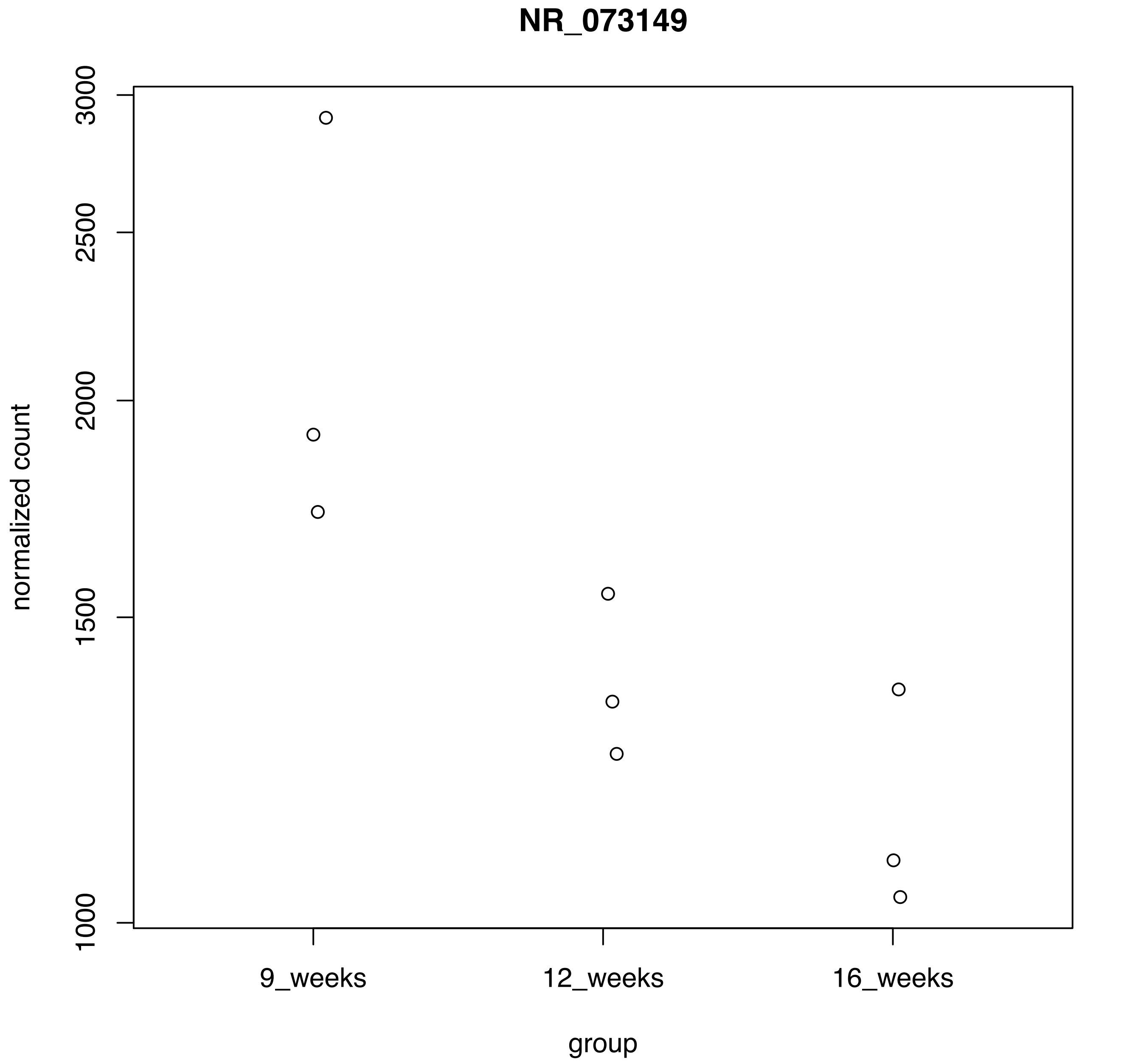
NR_073149
normalized count
3000
2500
2000
1500
1000
9_weeks
12_weeks
16_weeks
group

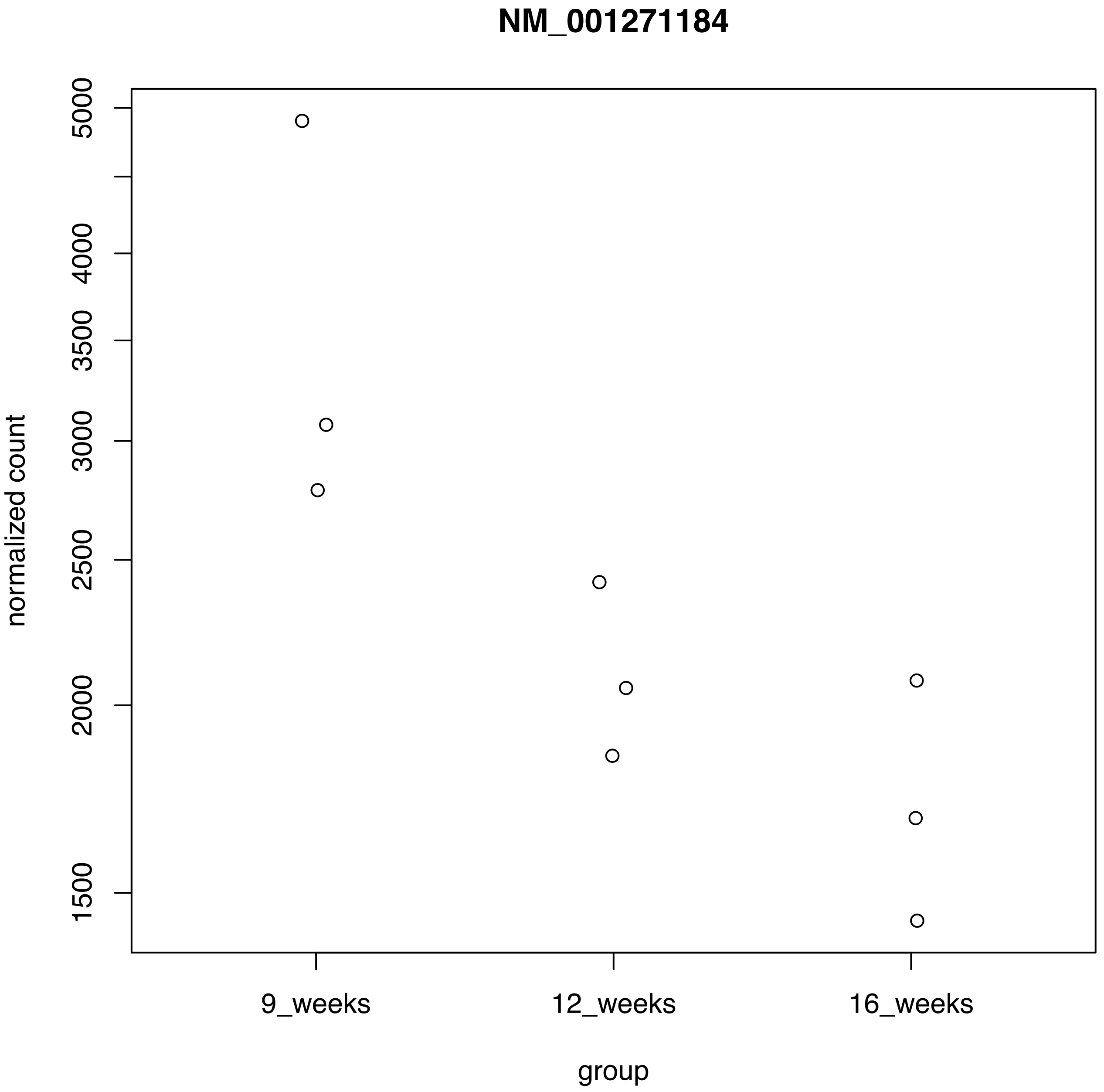
NM_001271184
normalized count
5000
4000
3500
3000
2500
2000
1500
9_weeks
12_weeks
16_weeks
group

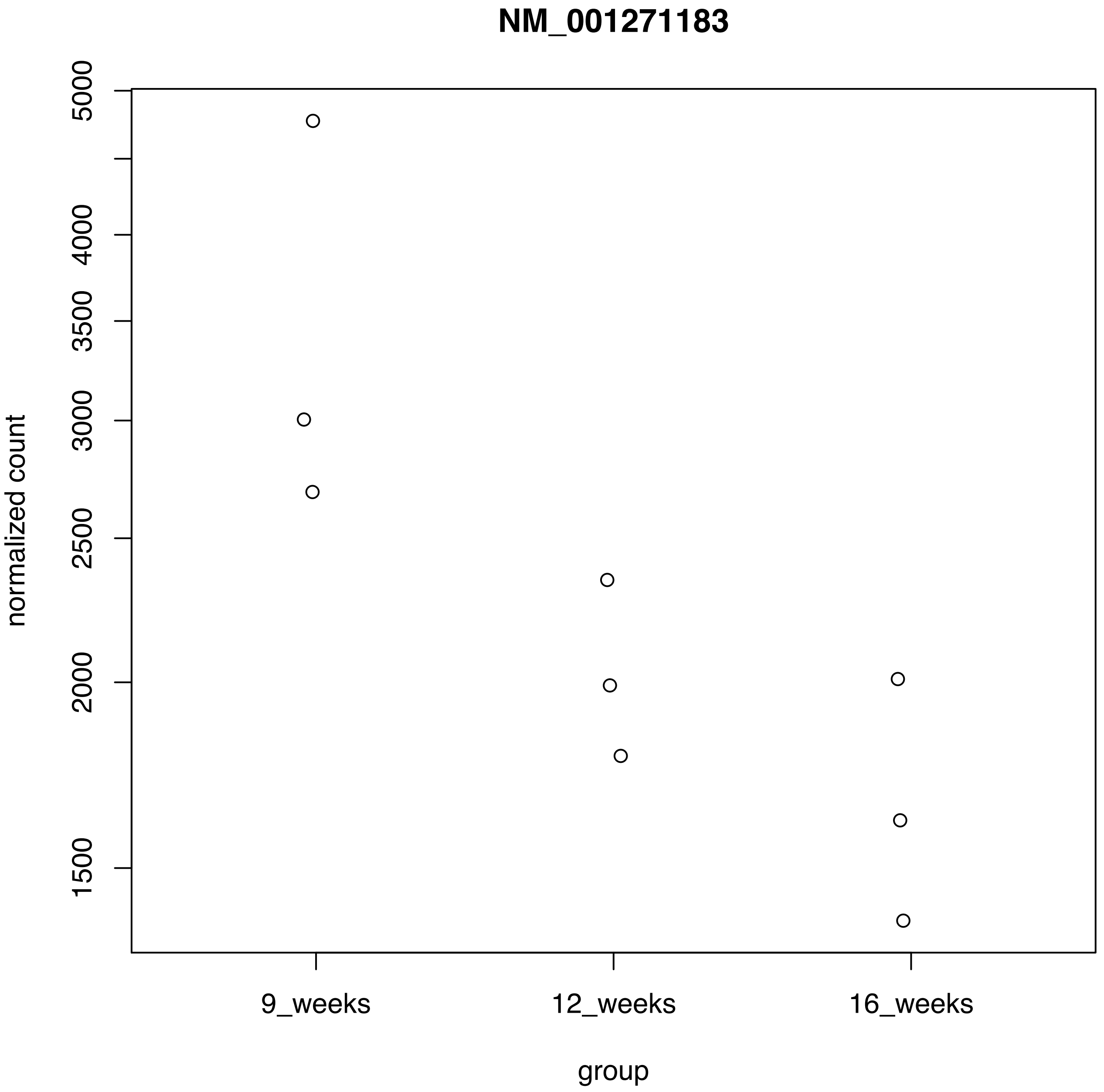
NM_001271183
normalized count
5000
4000
3500
3000
2500
2000
1500
9_weeks
12_weeks
16_weeks
group

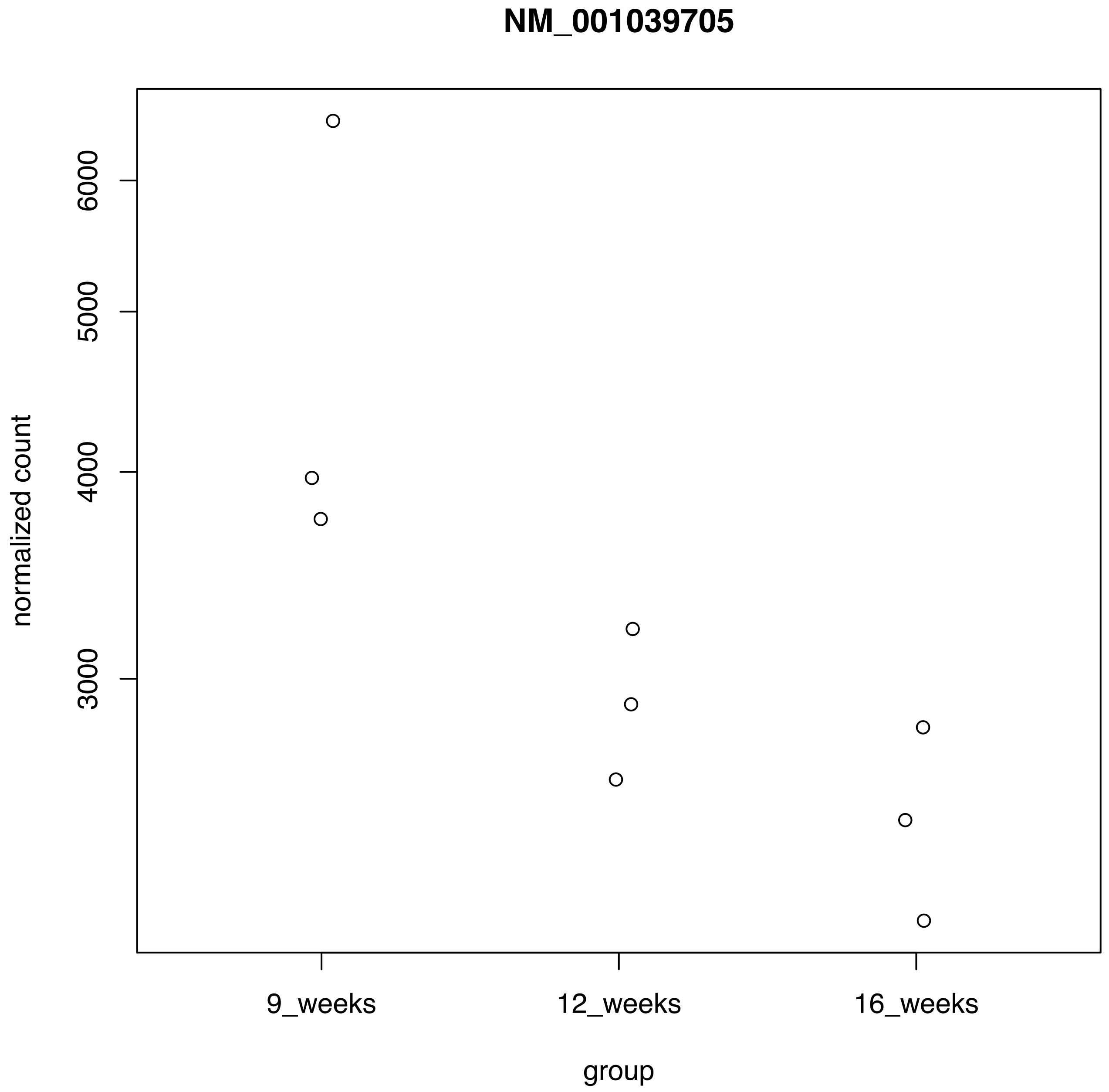
NM_001039705
normalized count
6000
5000
4000
3000
9_weeks
12_weeks
16_weeks
group

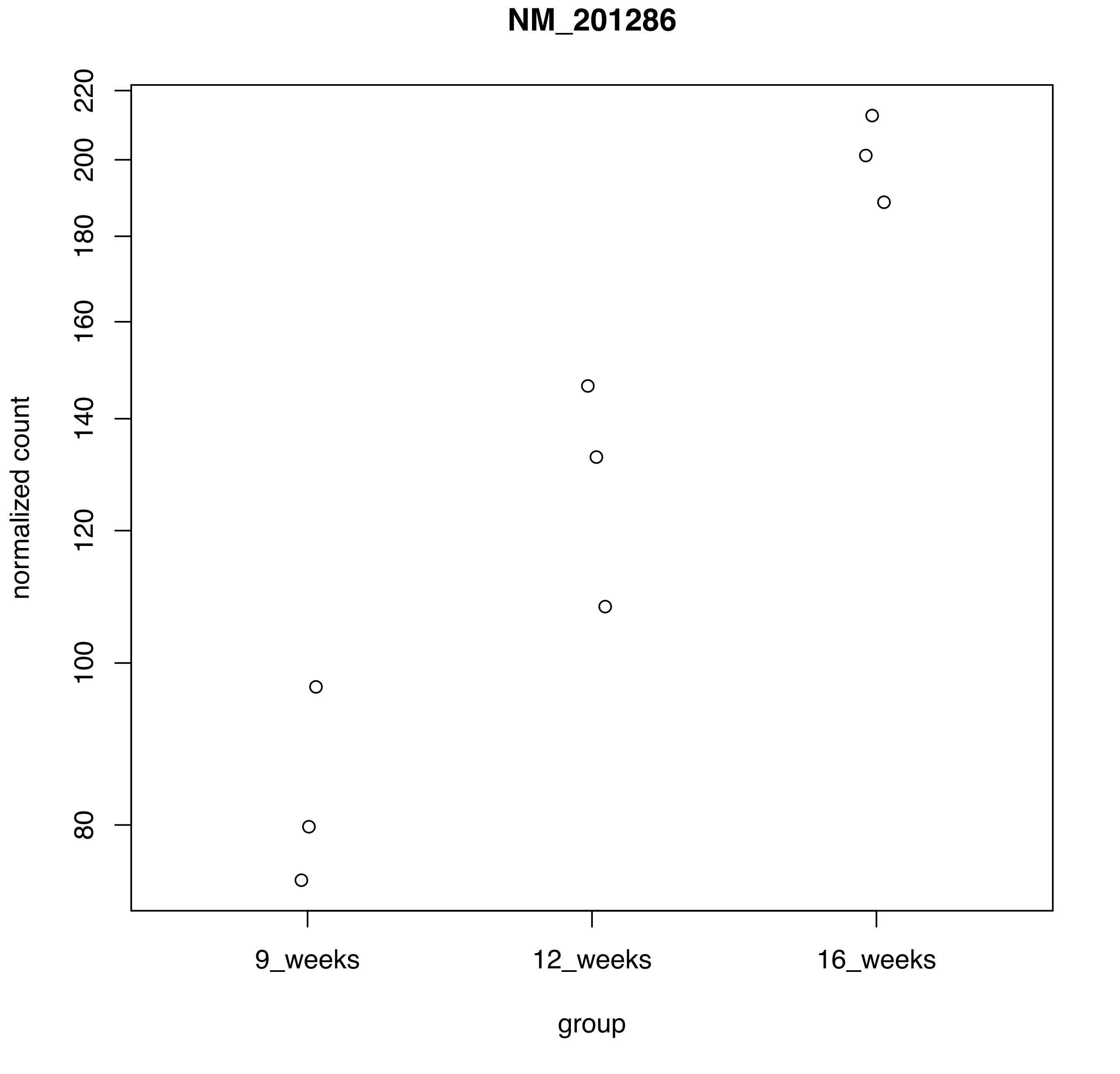
NM_201286
normalized count
220
200
180
160
140
120
100
80
9_weeks
12_weeks
16_weeks
group

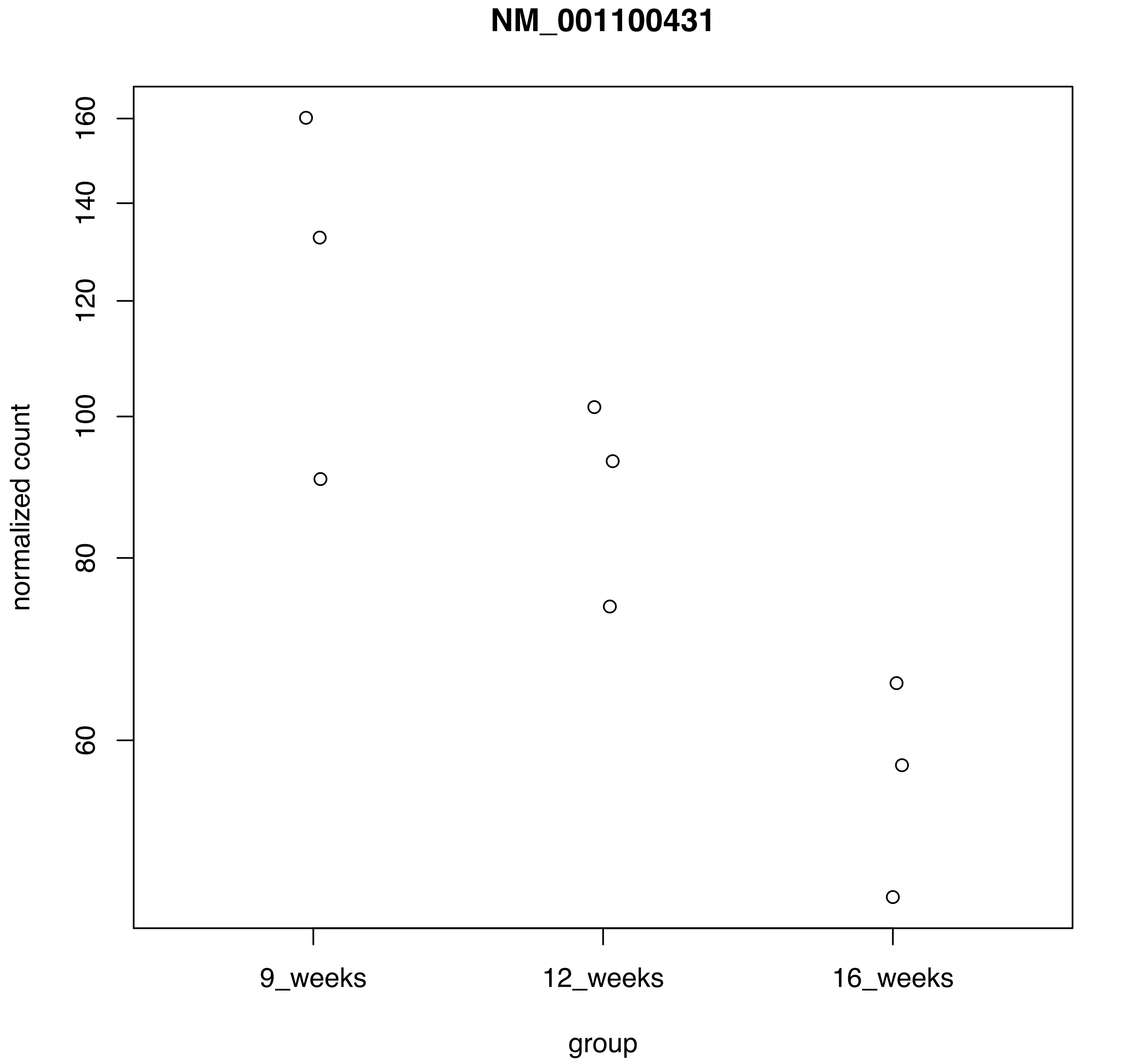
NM_001100431
normalized count
160
140
120
100
80
60
9_weeks
12_weeks
16_weeks
group

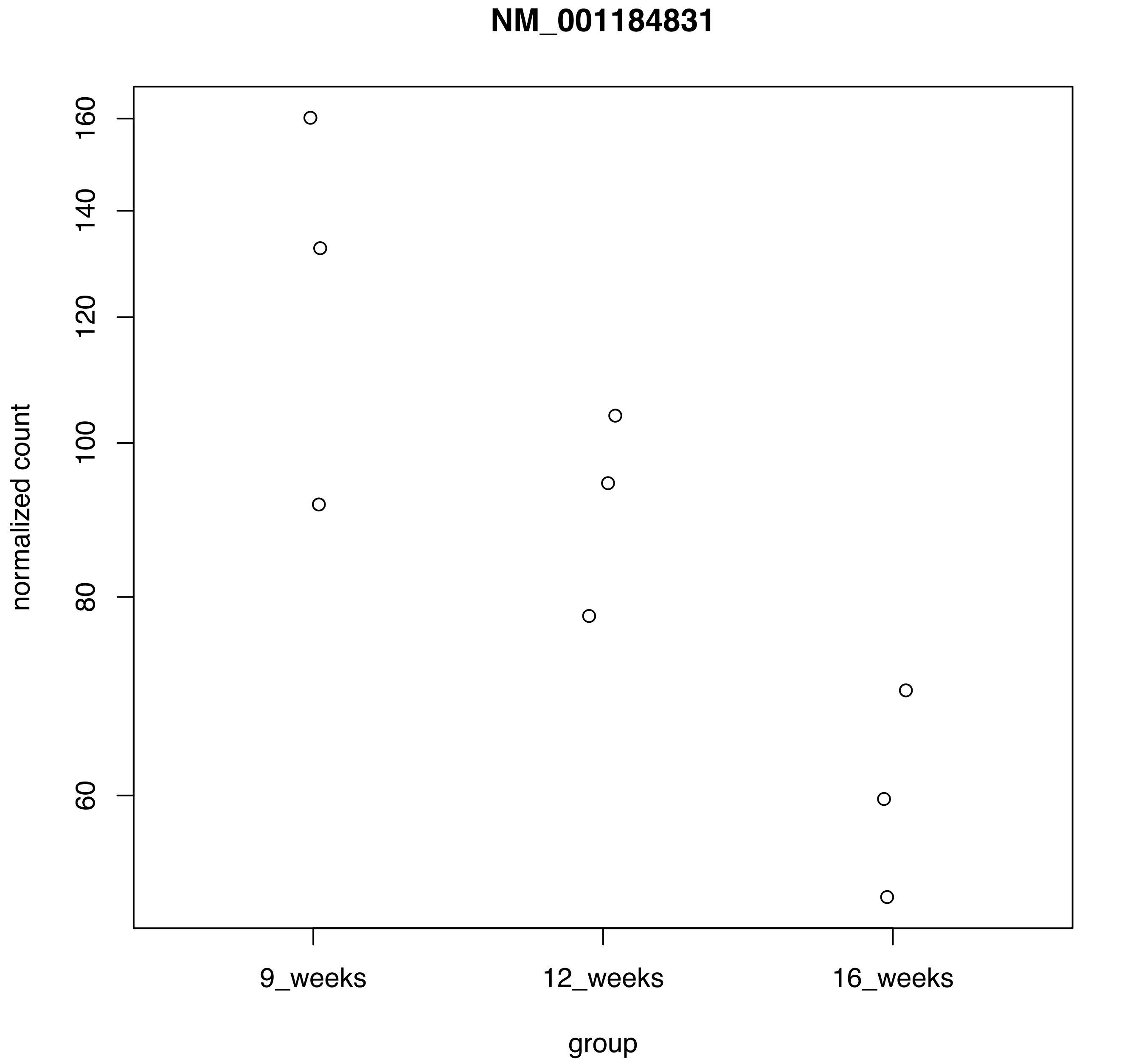
NM_001184831
normalized count
160
140
120
100
80
60
9_weeks
12_weeks
16_weeks
group

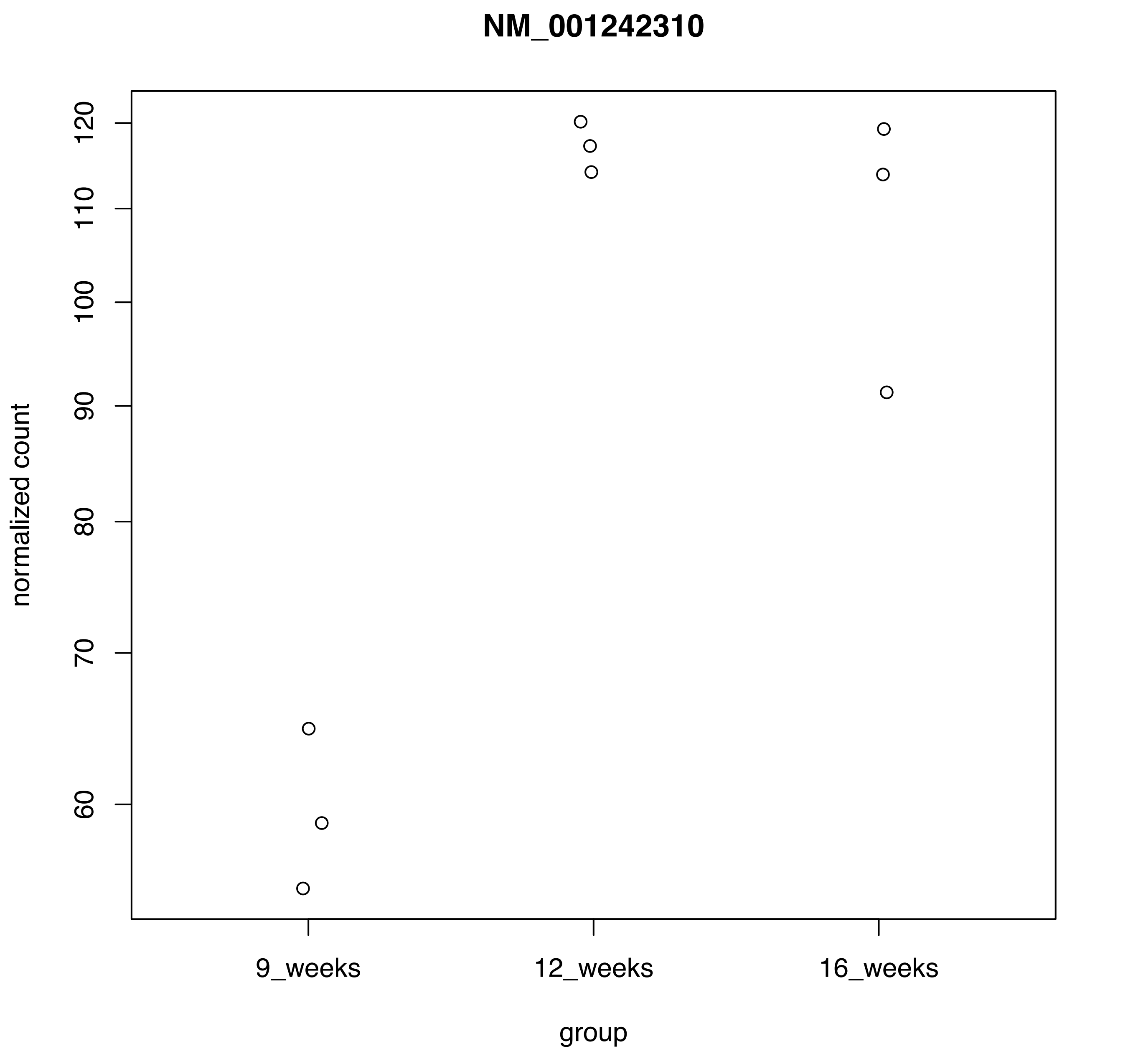
NM_001242310
normalized count
group
9_weeks
12_weeks
16_weeks
60
70
80
90
100
110
120

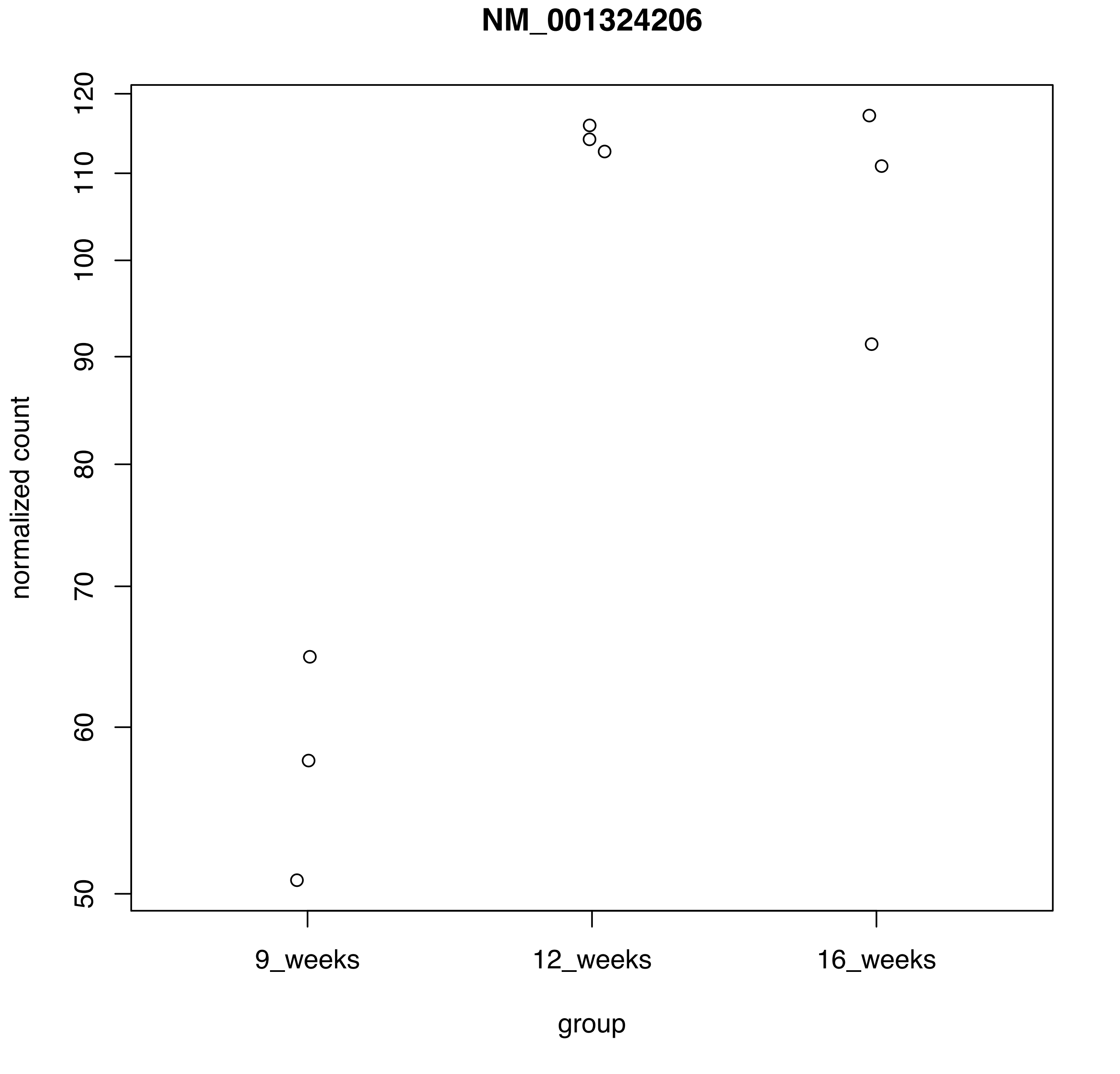
NM_001324206
normalized count
120
110
100
90
80
70
60
50
9_weeks
12_weeks
16_weeks
group

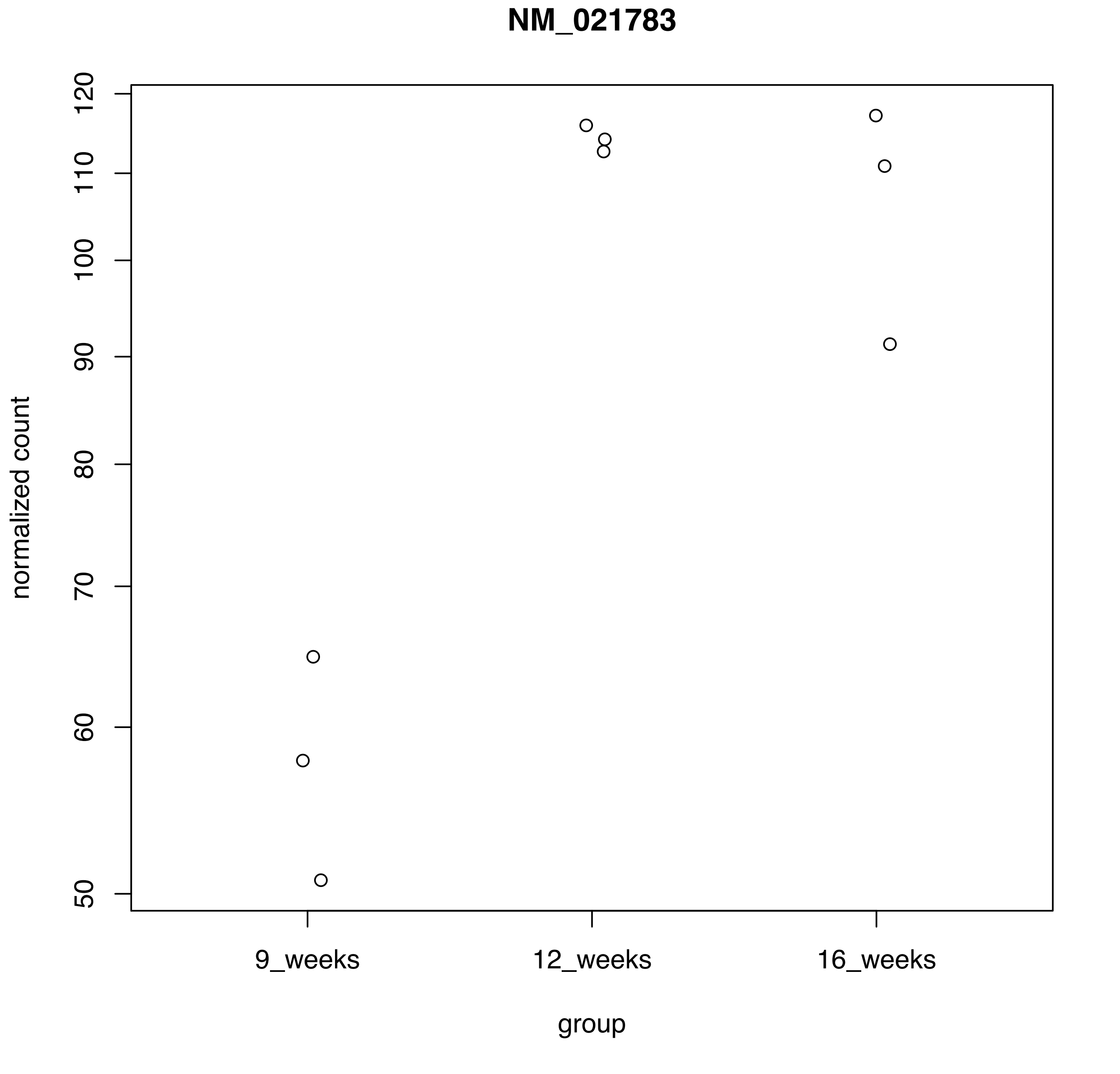
NM_021783
normalized count
120
110
100
90
80
70
60
50
9_weeks
12_weeks
16_weeks
group

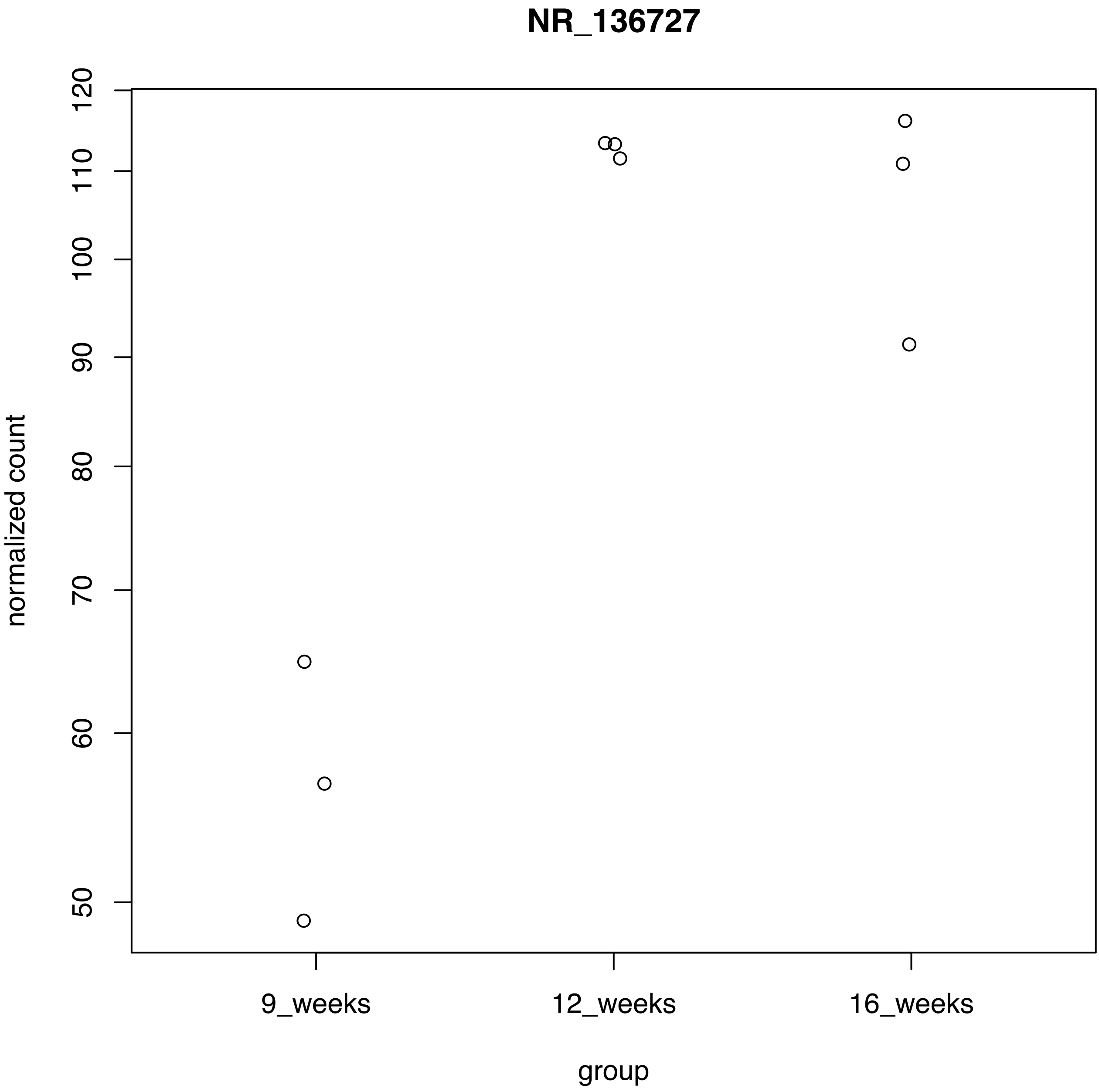
NR_136727
normalized count
120
110
100
90
80
70
60
50
9_weeks
12_weeks
16_weeks
group

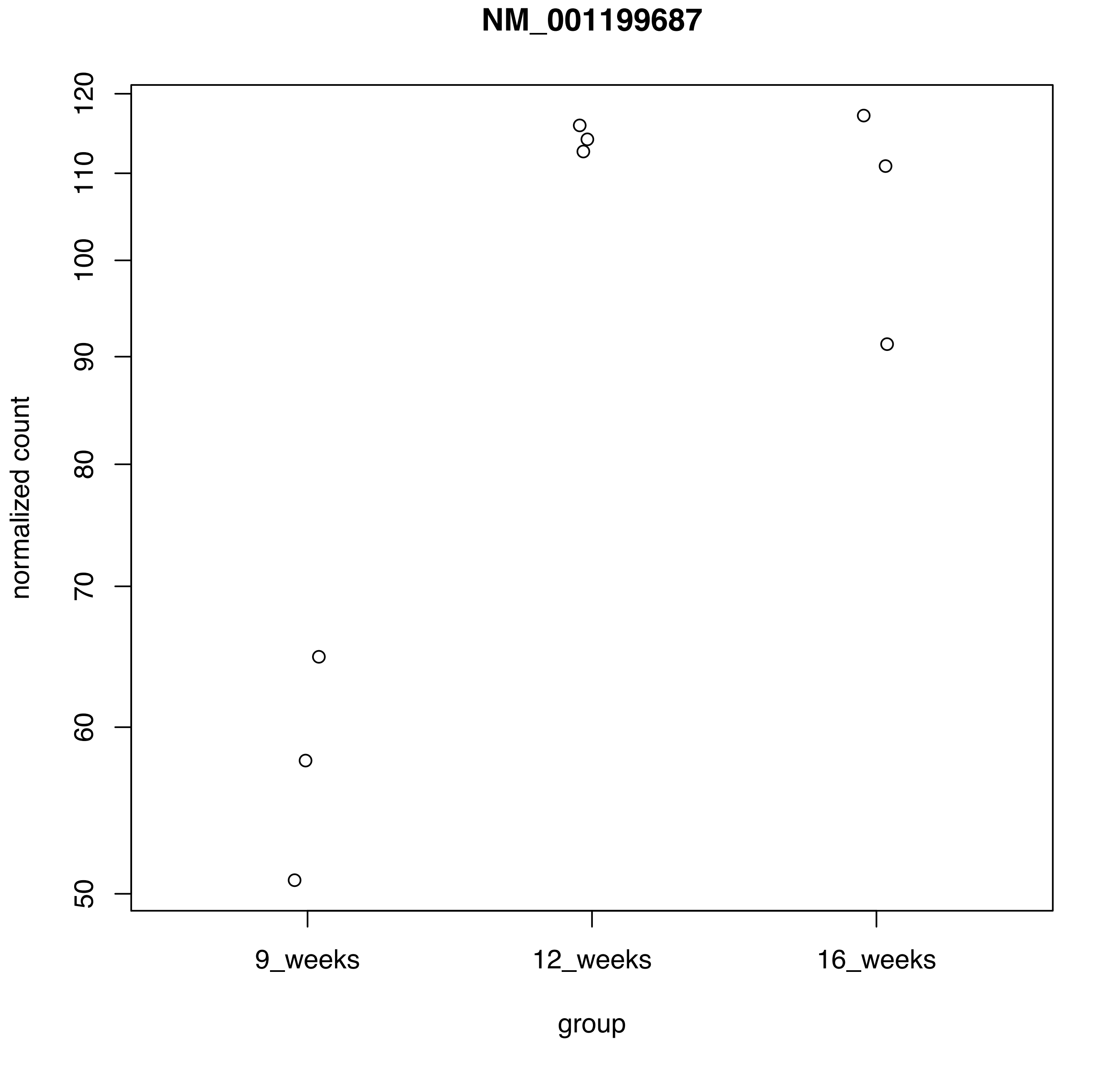
NM_001199687
normalized count
120
110
100
90
80
70
60
50
9_weeks
12_weeks
16_weeks
group

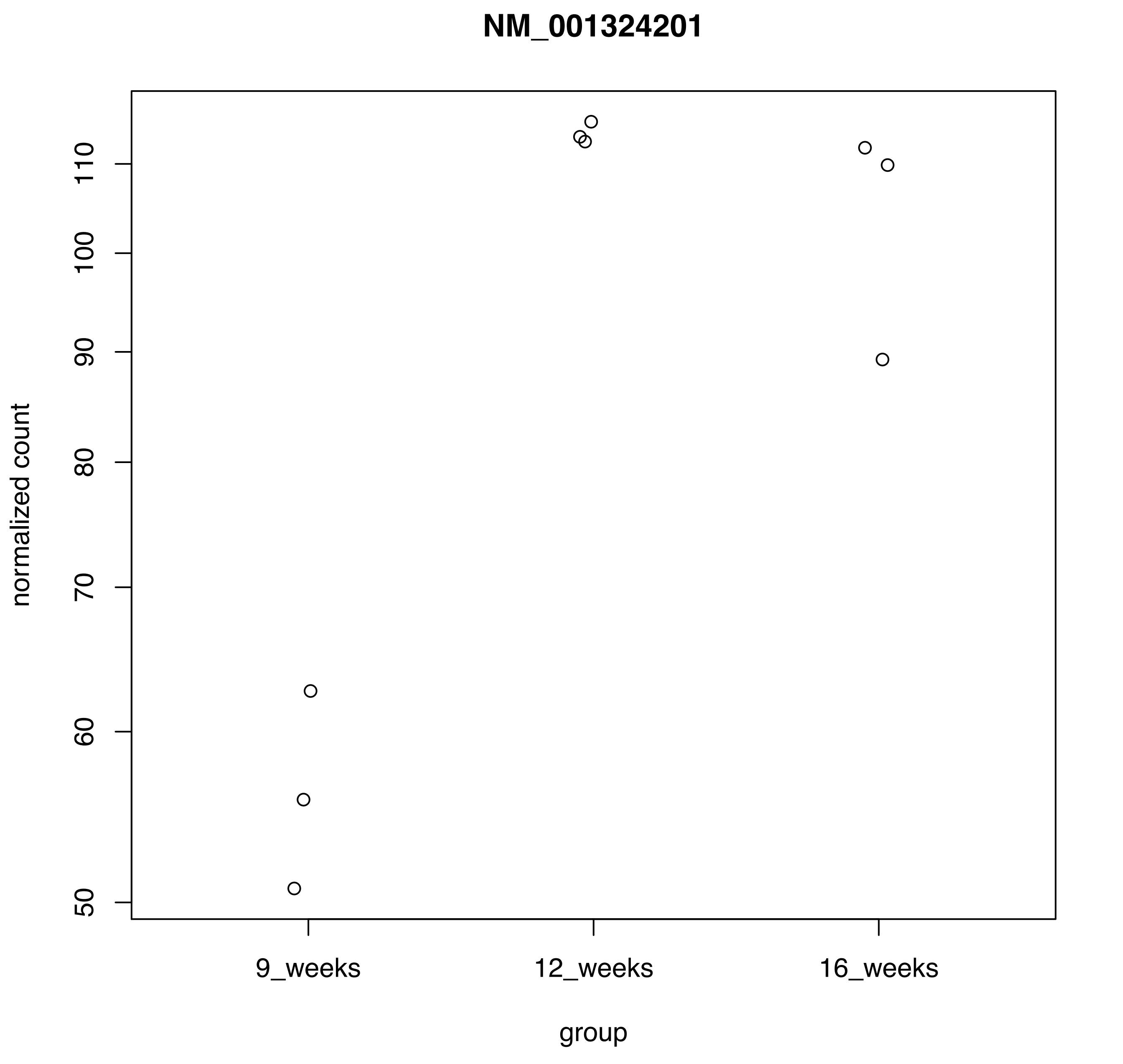
NM_001324201
normalized count
50
60
70
80
90
100
110
9_weeks
12_weeks
16_weeks
group

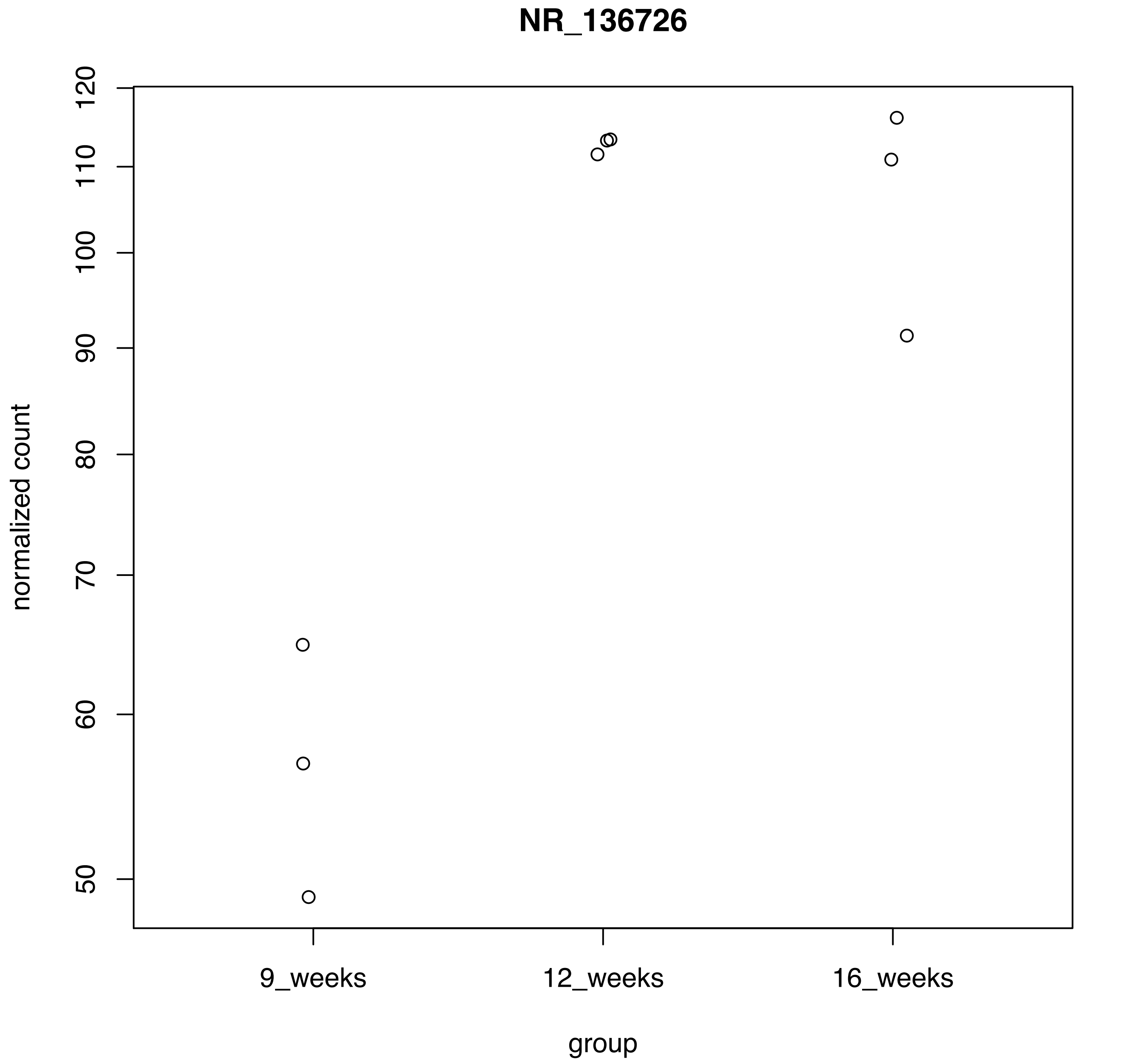

NR_136726
normalized count
120
110
100
90
80
70
60
50
9_weeks
12_weeks
16_weeks
group

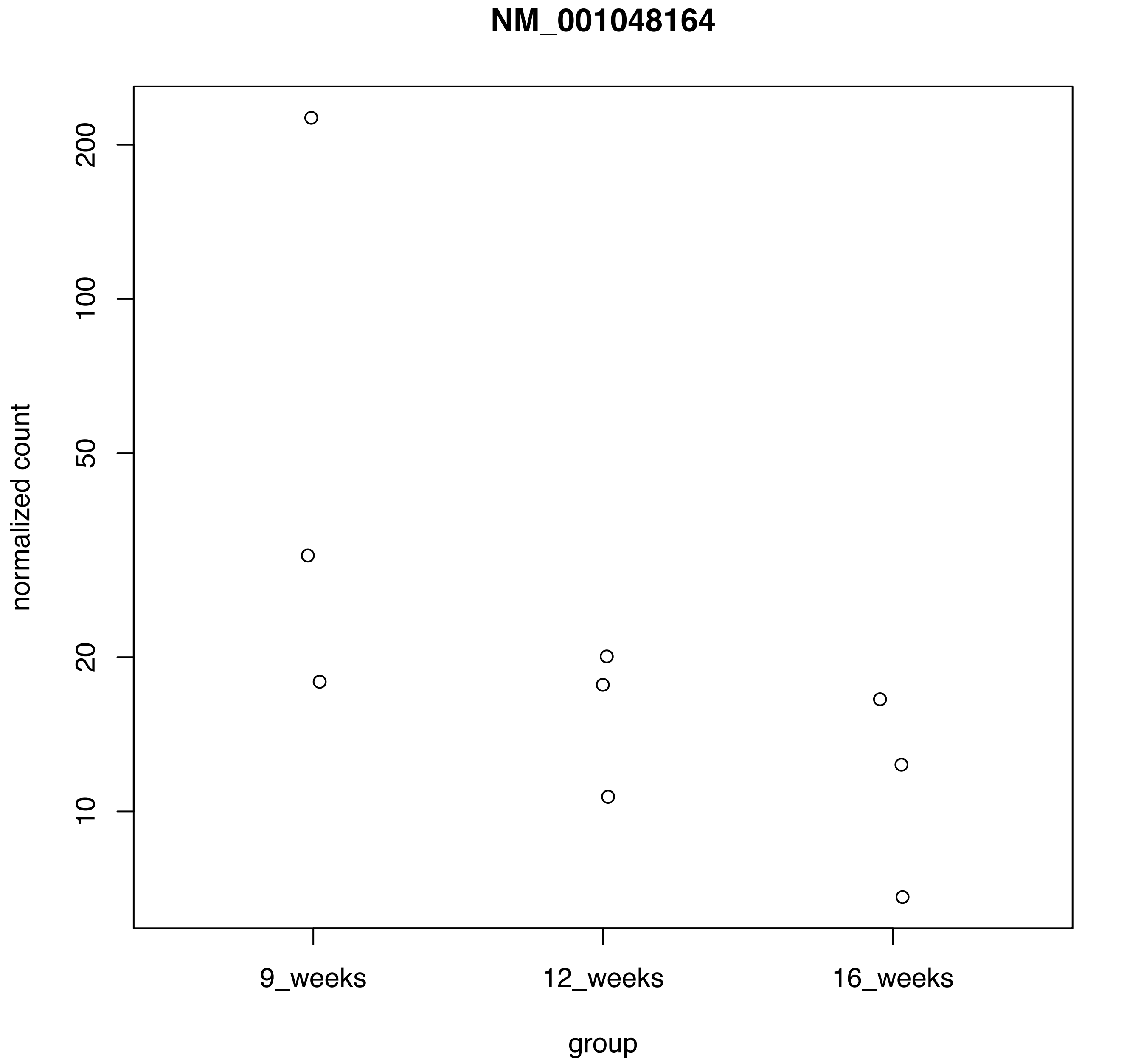
NM_001048164
normalized count
200
100
50
20
10
9_weeks
12_weeks
16_weeks
group

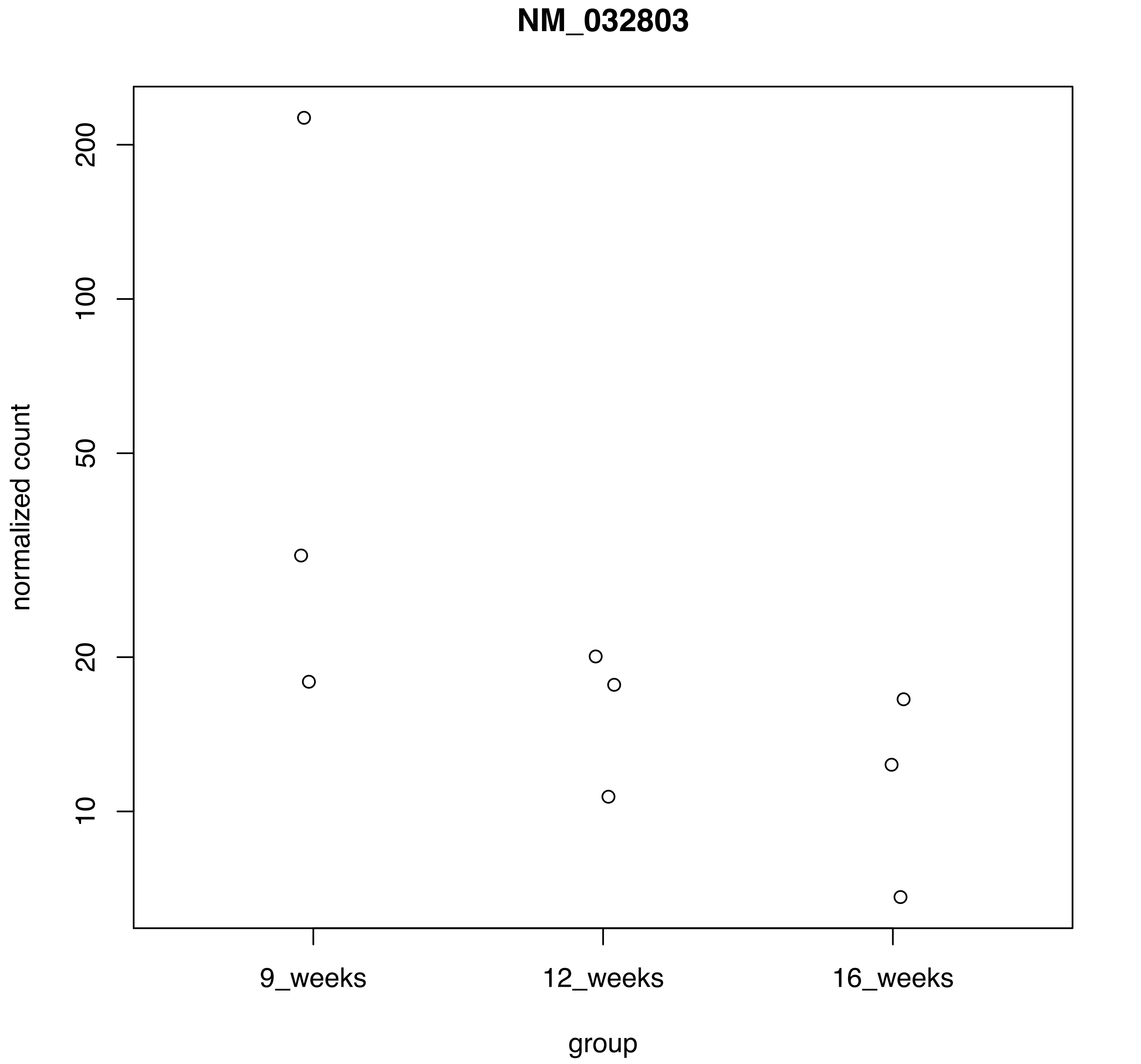
NM_032803
normalized count
200
100
50
20
10
9_weeks
12_weeks
16_weeks
group

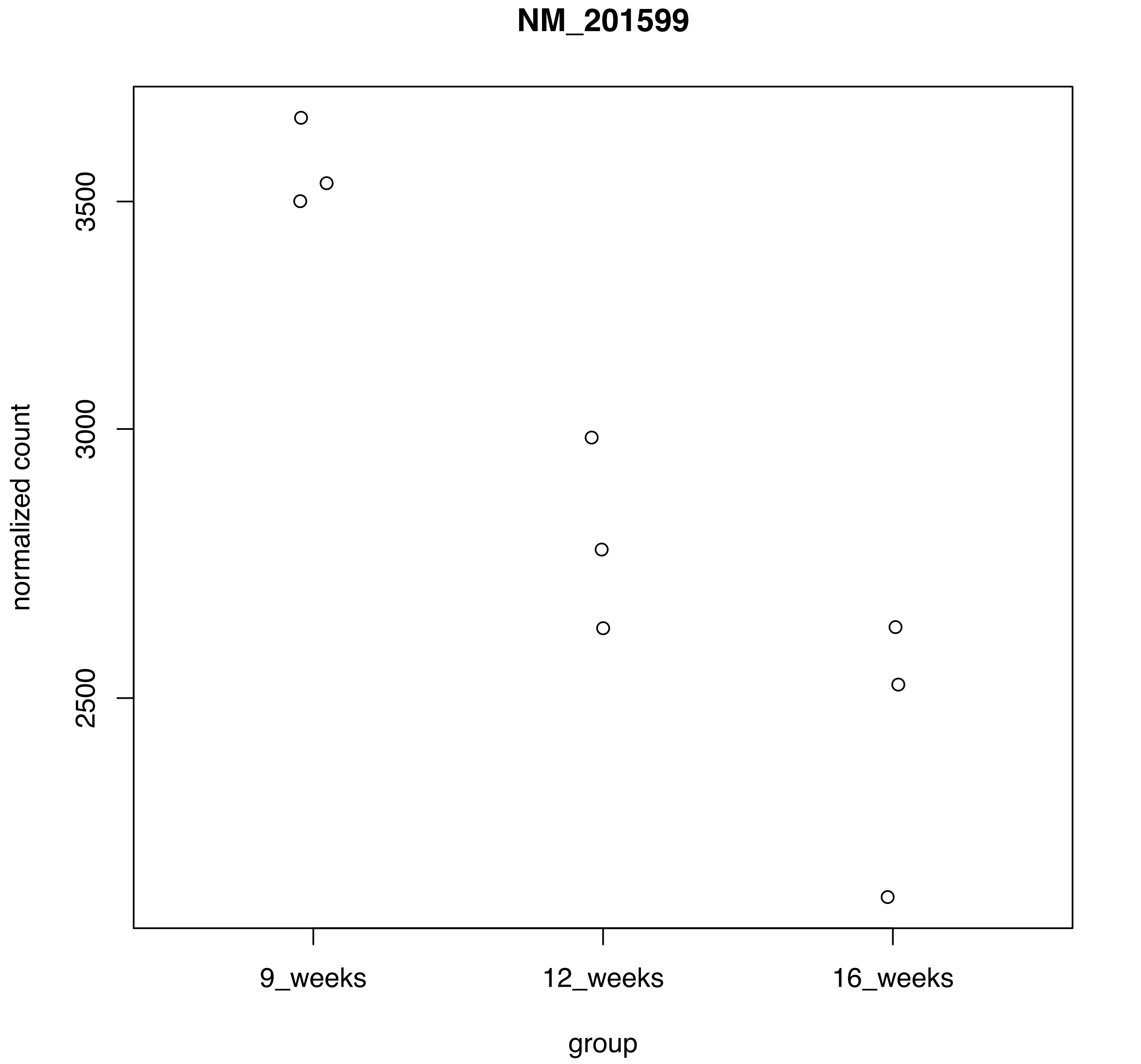

NM_201599
normalized count
3500
3000
2500
9_weeks
12_weeks
16_weeks
group

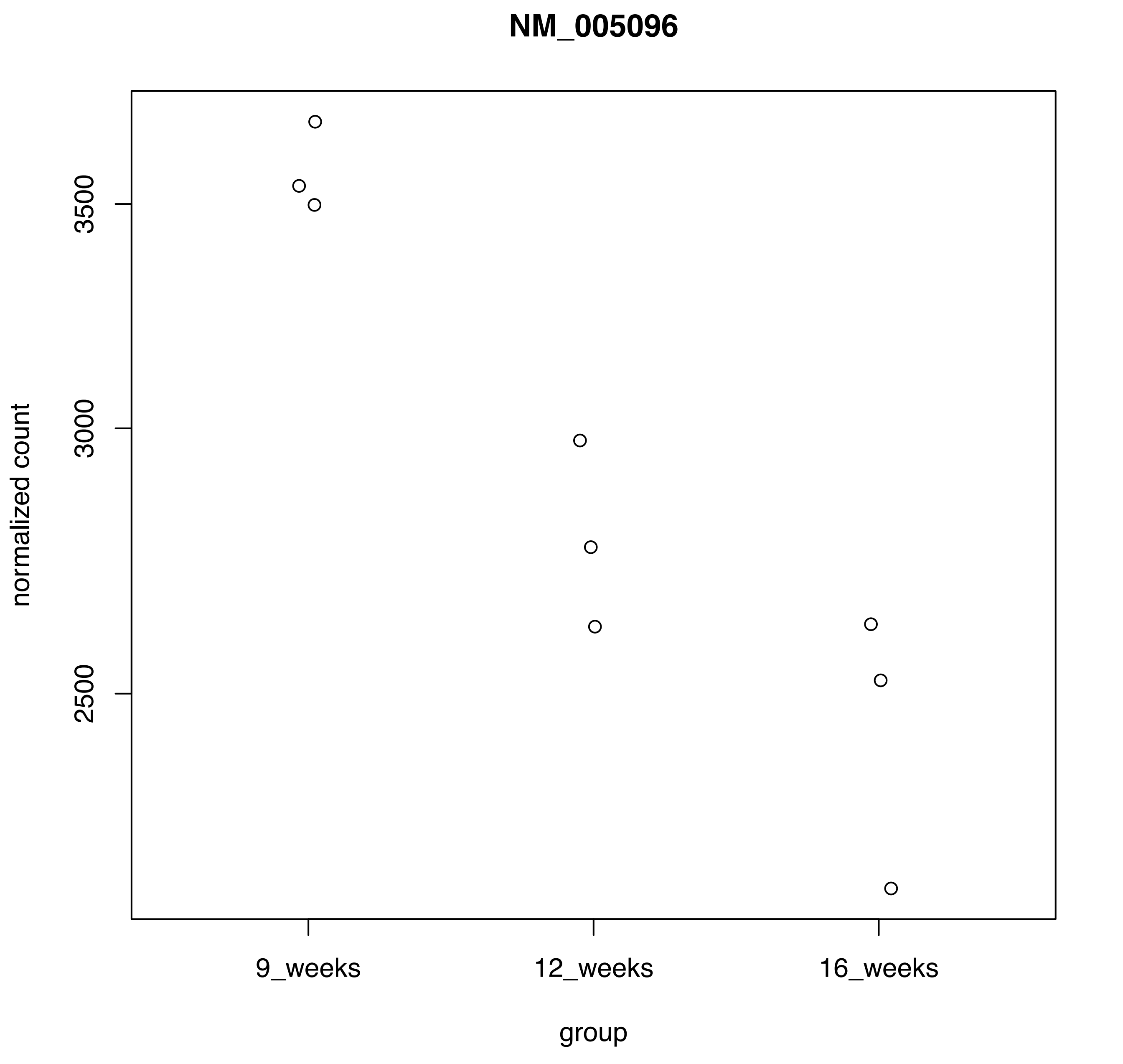
NM_005096
normalized count
3500
3000
2500
9_weeks
12_weeks
16_weeks
group

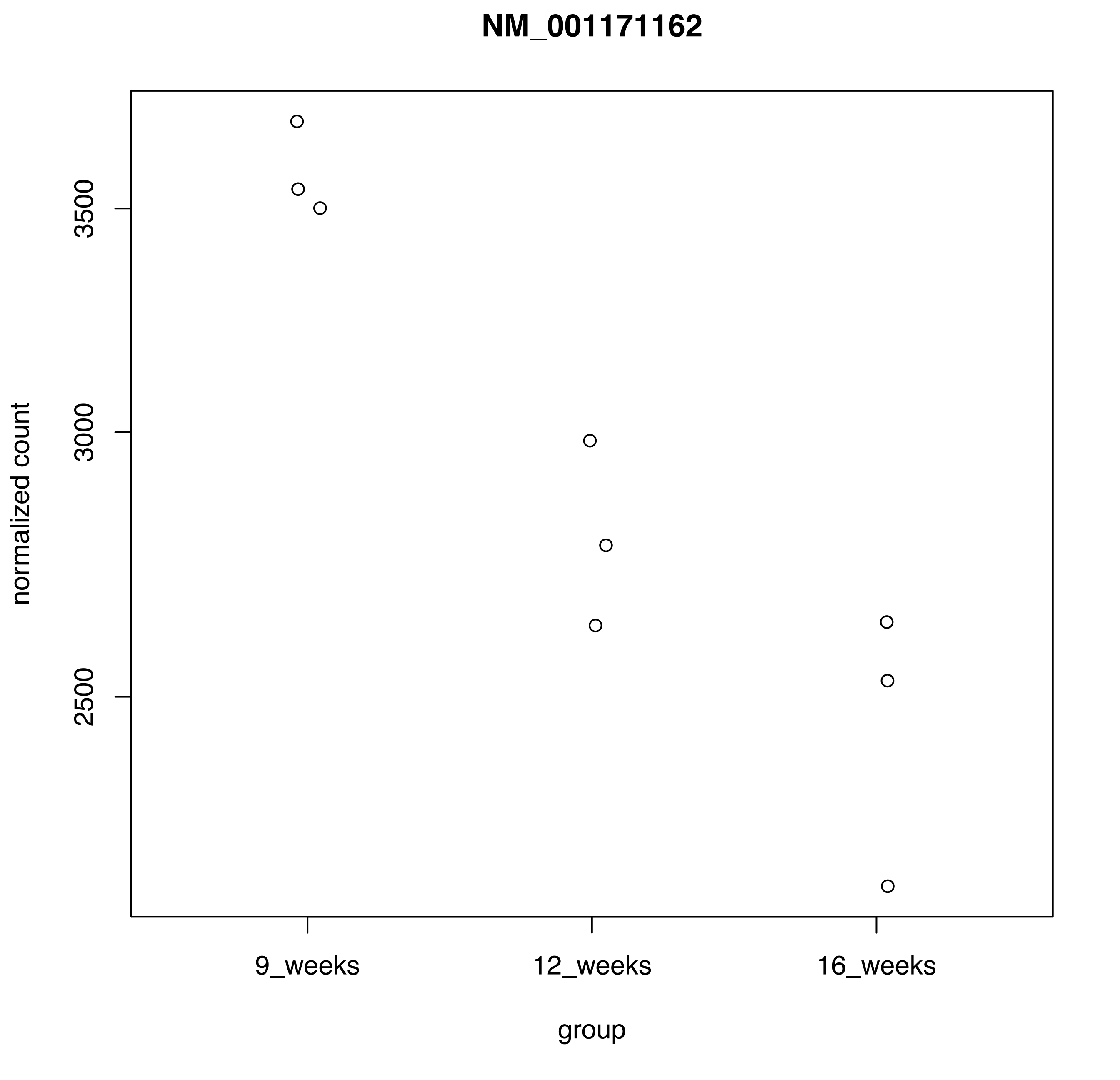

NM_001171162
normalized count
2500
3000
3500
9_weeks
12_weeks
16_weeks
group

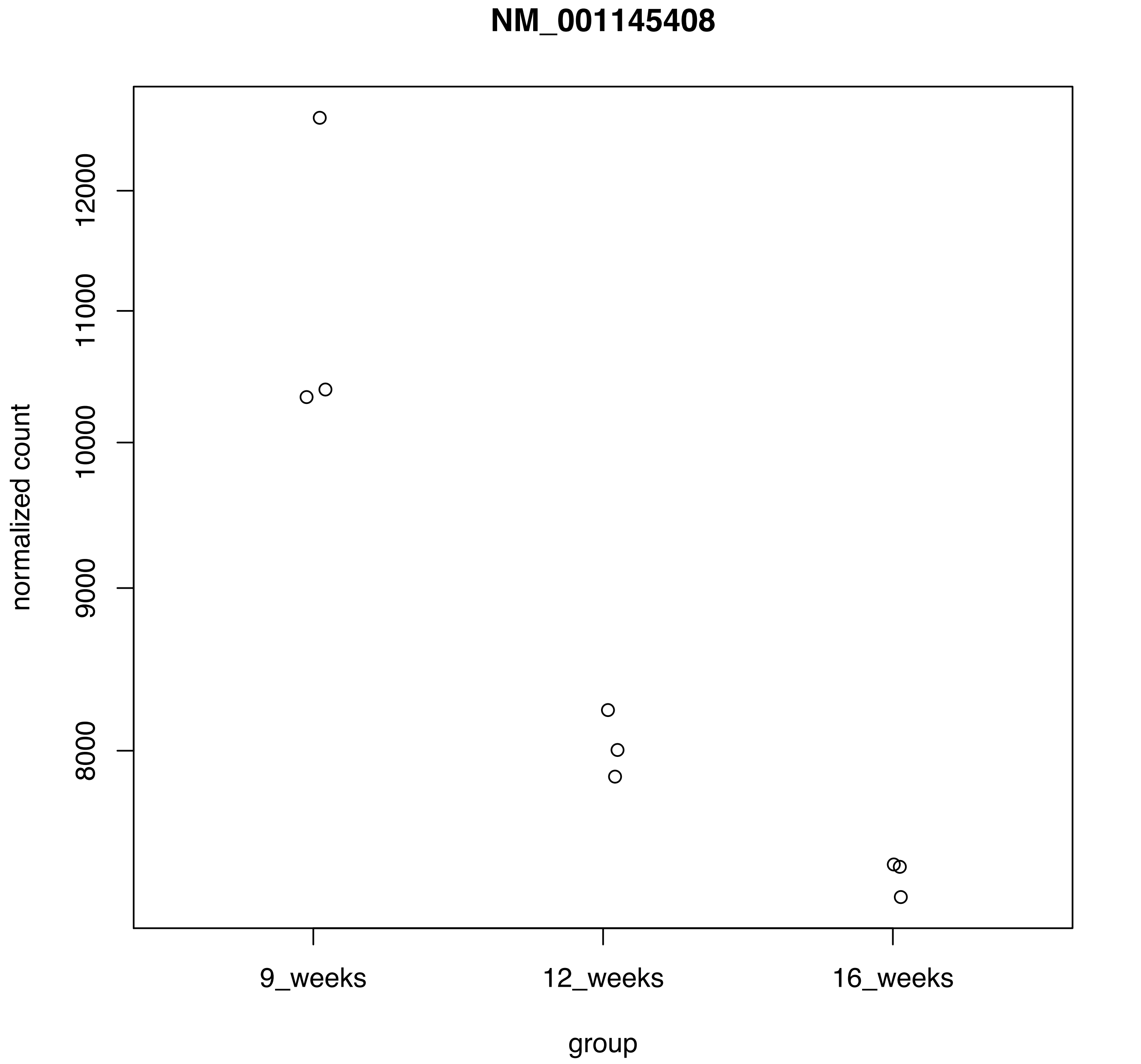

NM_001145408
normalized count
12000
11000
10000
9000
8000
9_weeks
12_weeks
16_weeks
group

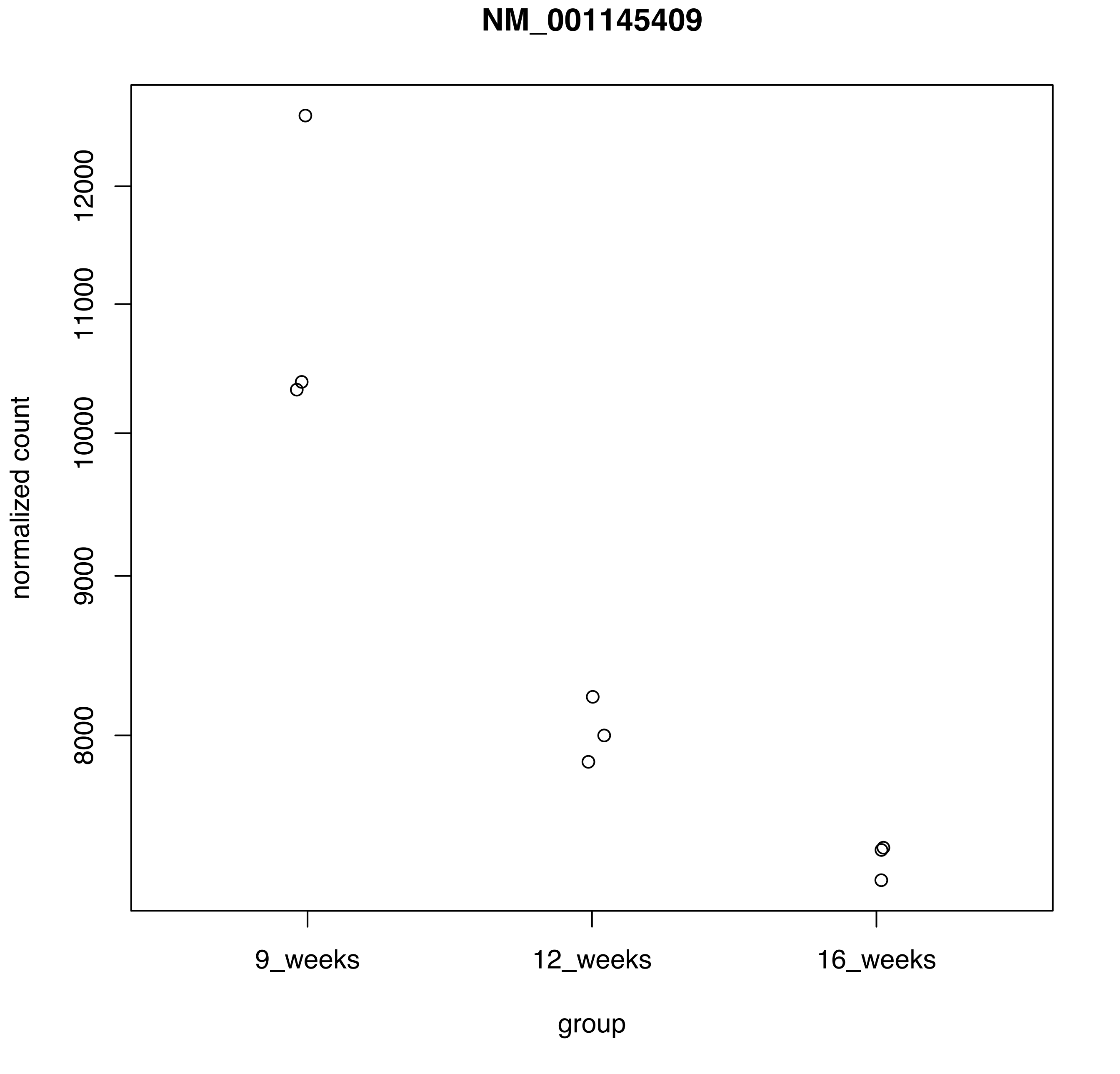
NM_001145409
normalized count
8000
9000
10000
11000
12000
9_weeks
12_weeks
16_weeks
group

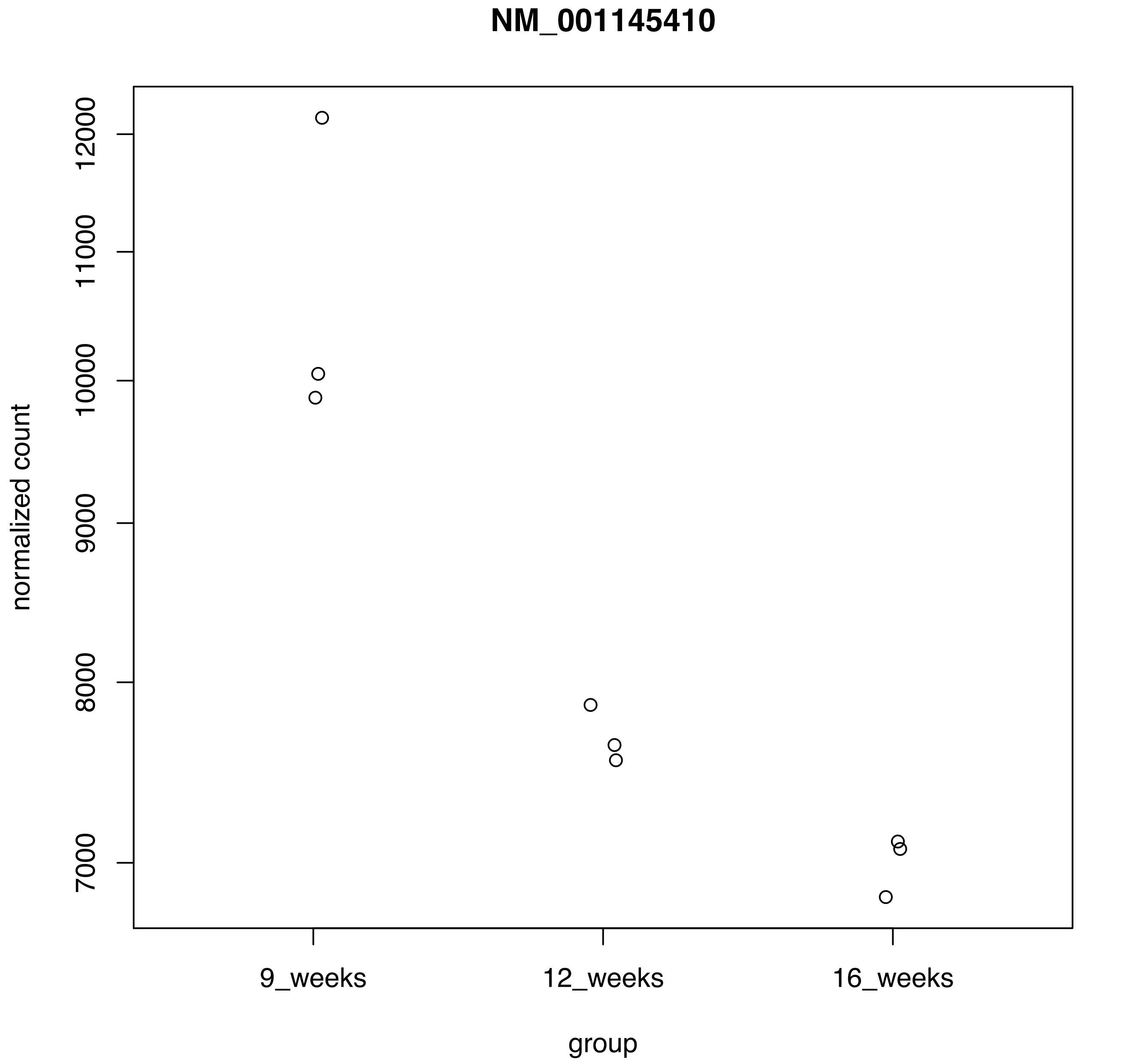
NM_001145410
normalized count
12000
11000
10000
9000
8000
7000
9_weeks
12_weeks
16_weeks
group

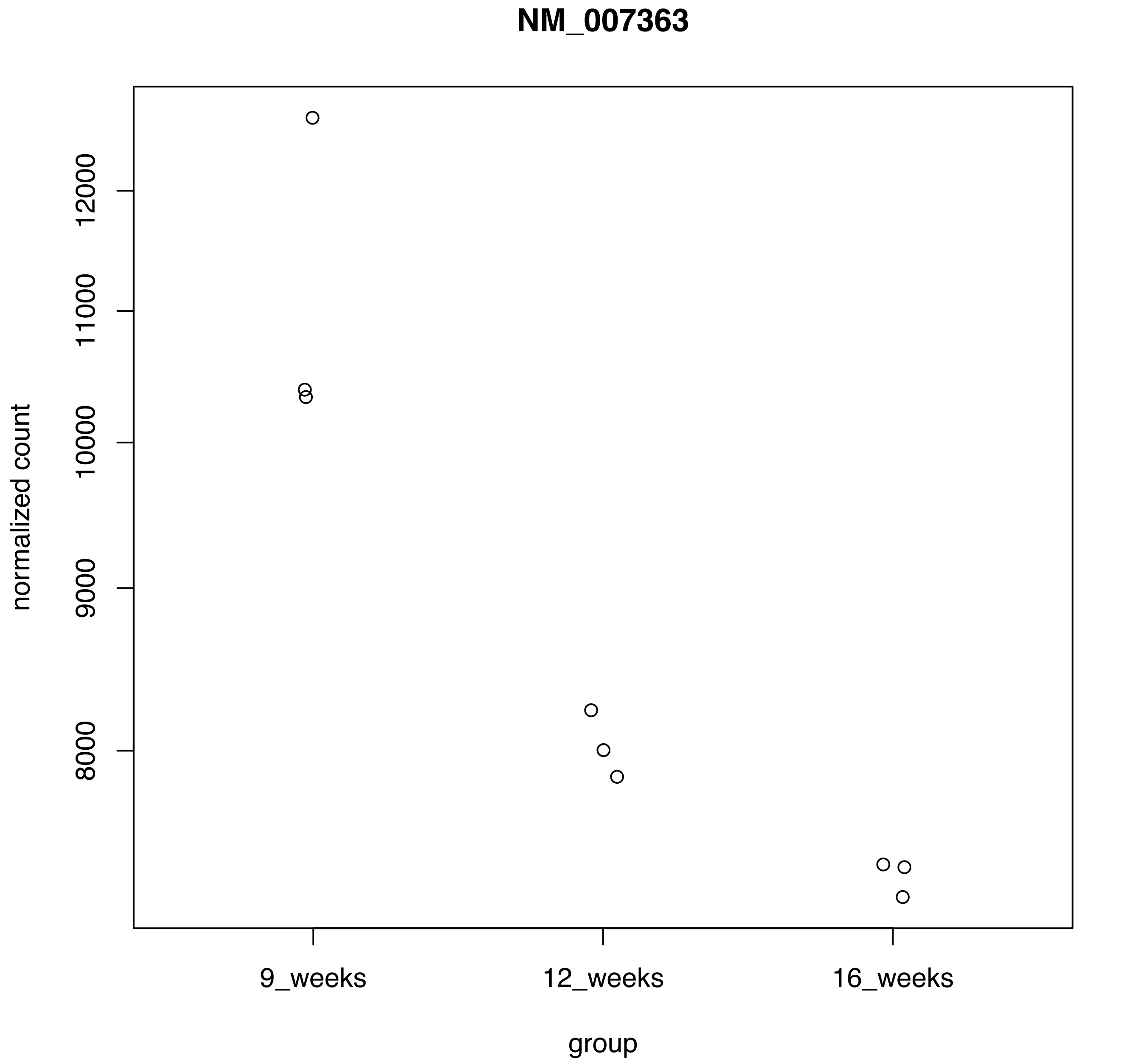
NM_007363
normalized count
12000
11000
10000
9000
8000
9_weeks
12_weeks
16_weeks
group

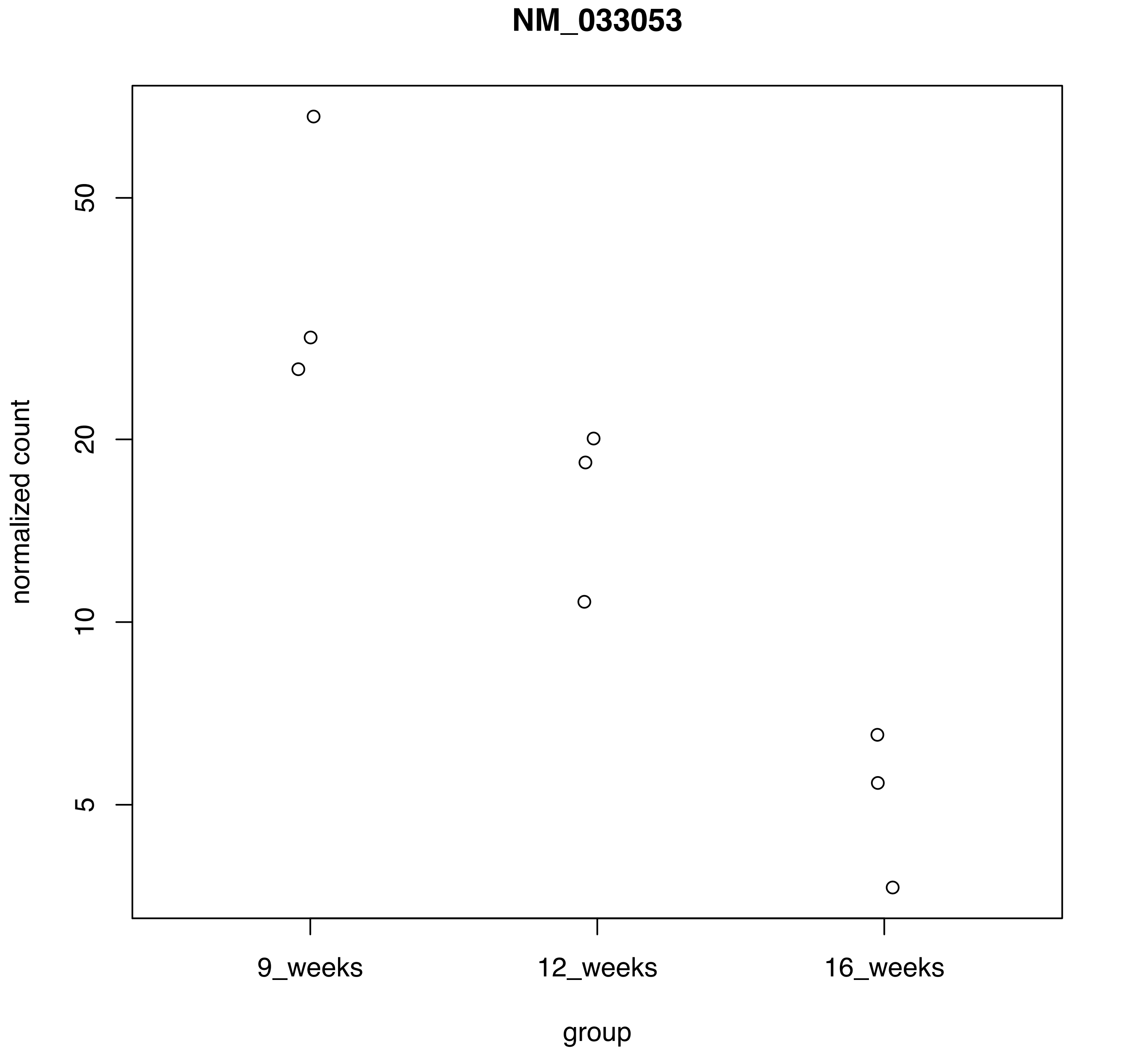
NM_033053
normalized count
50
20
10
5
9_weeks
12_weeks
16_weeks
group

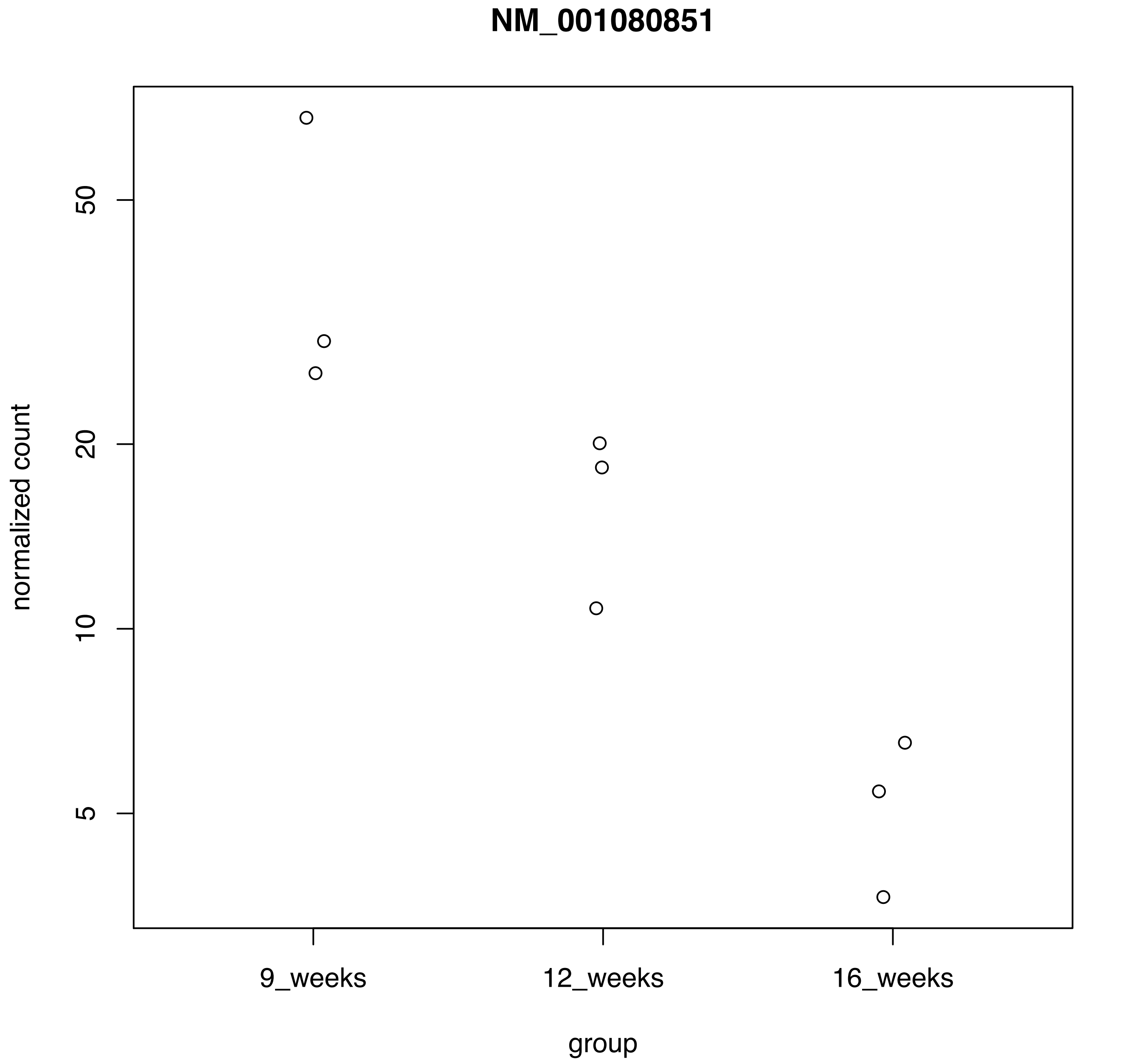
NM_001080851
normalized count
50
20
10
5
9_weeks
12_weeks
16_weeks
group

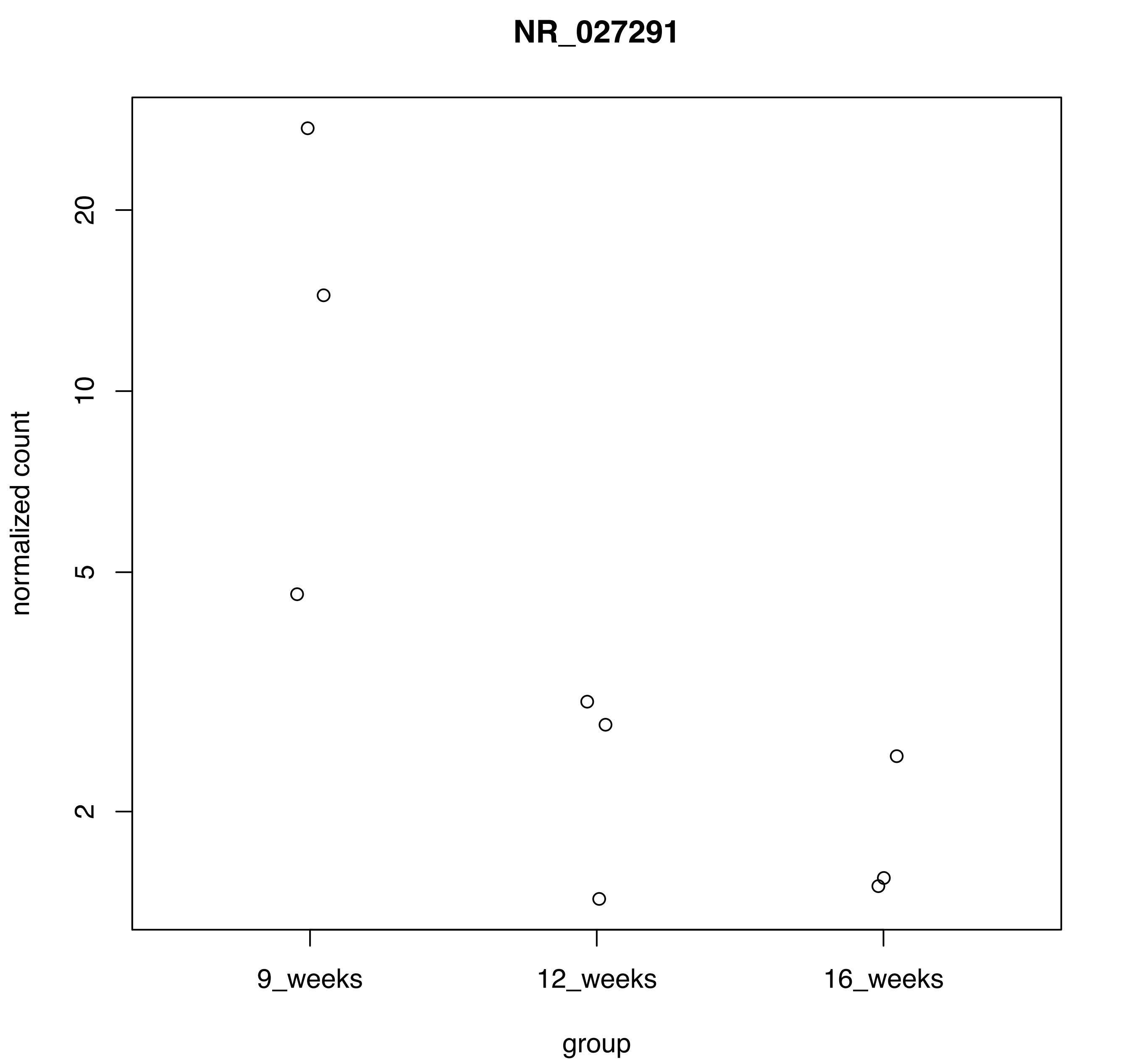
NR_027291
normalized count
20
10
5
2
9_weeks
12_weeks
16_weeks
group

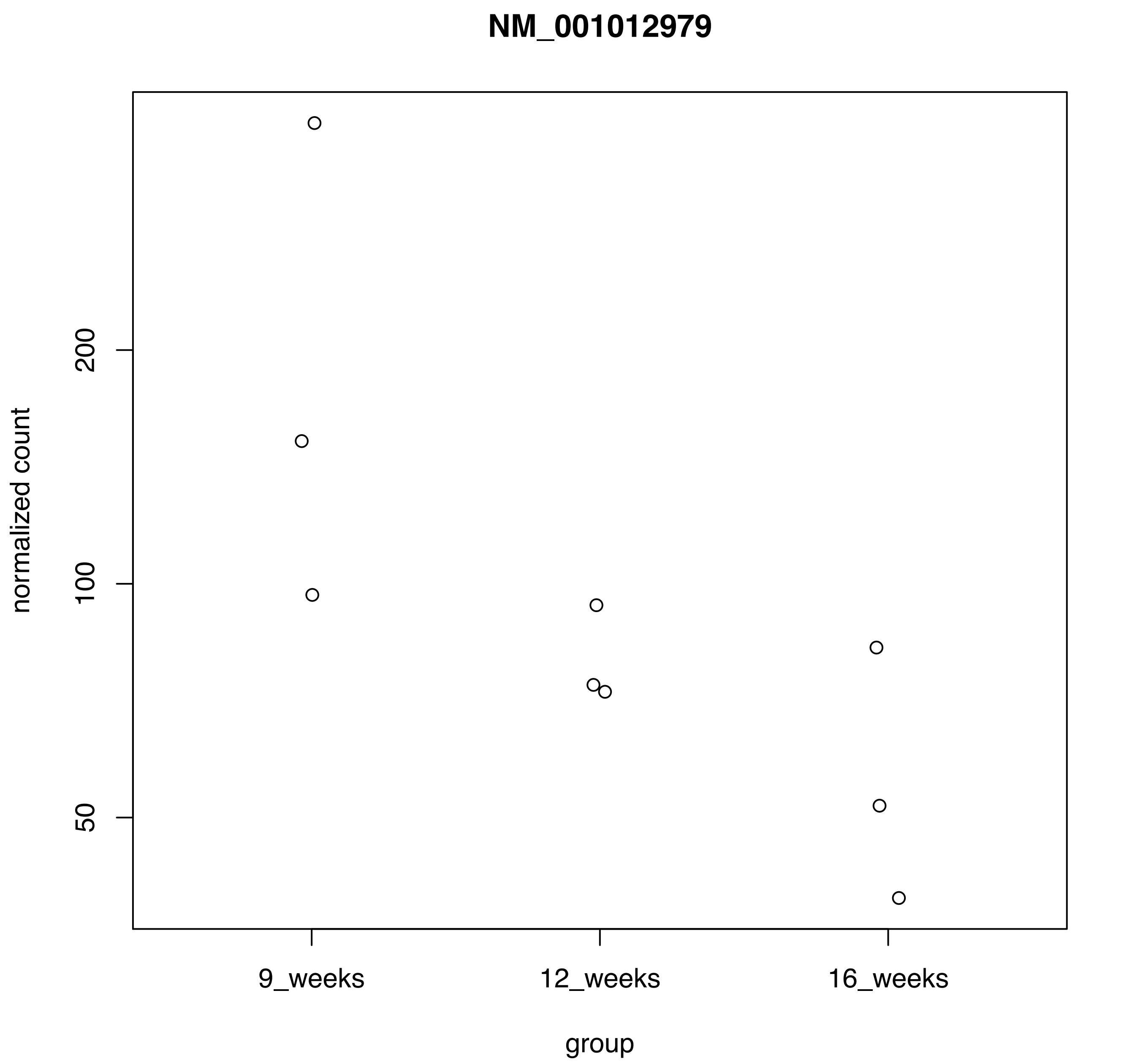
NM_001012979
normalized count
200
100
50
9_weeks
12_weeks
16_weeks
group

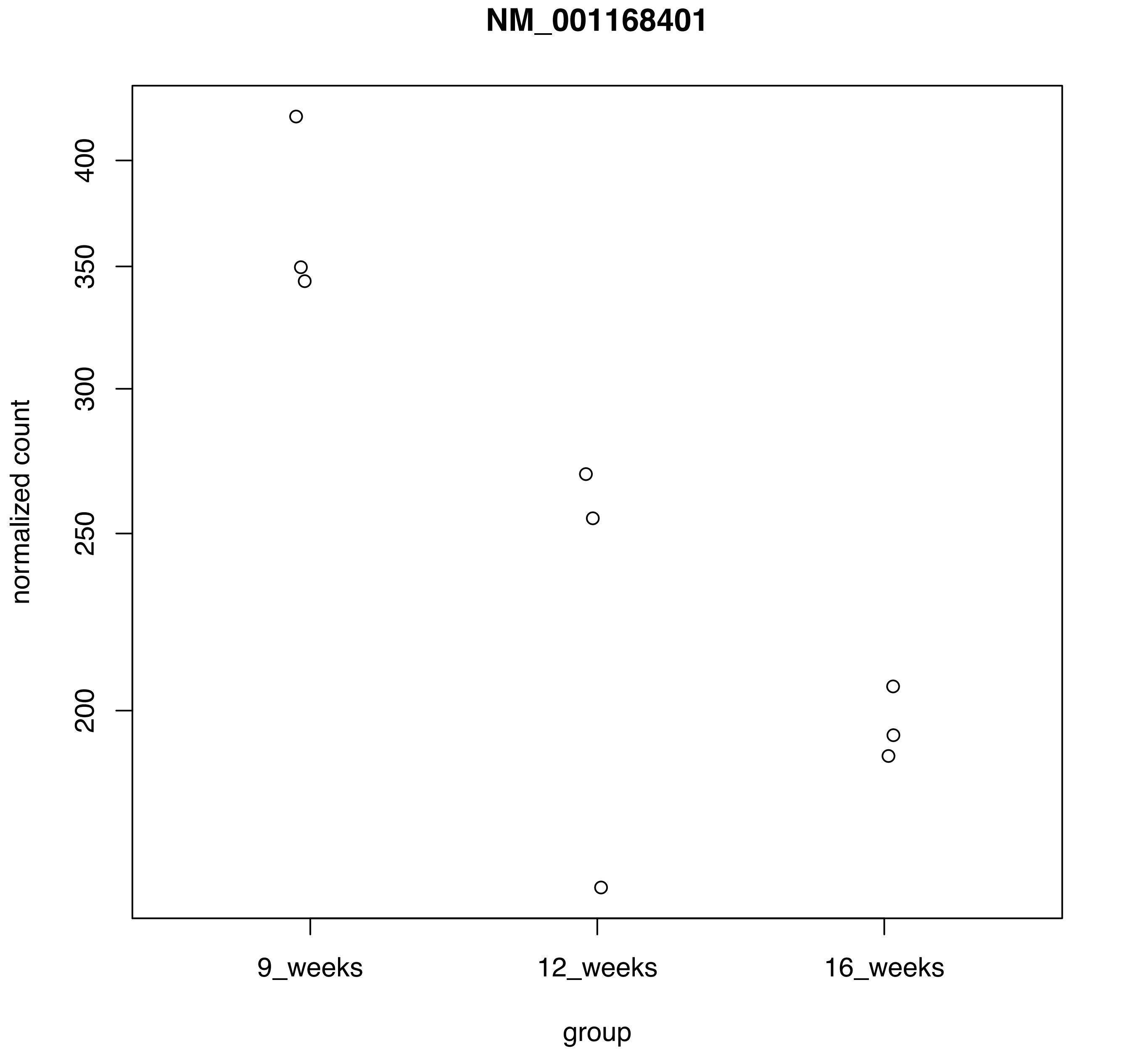
NM_001168401
normalized count
400
350
300
250
200
9_weeks
12_weeks
16_weeks
group

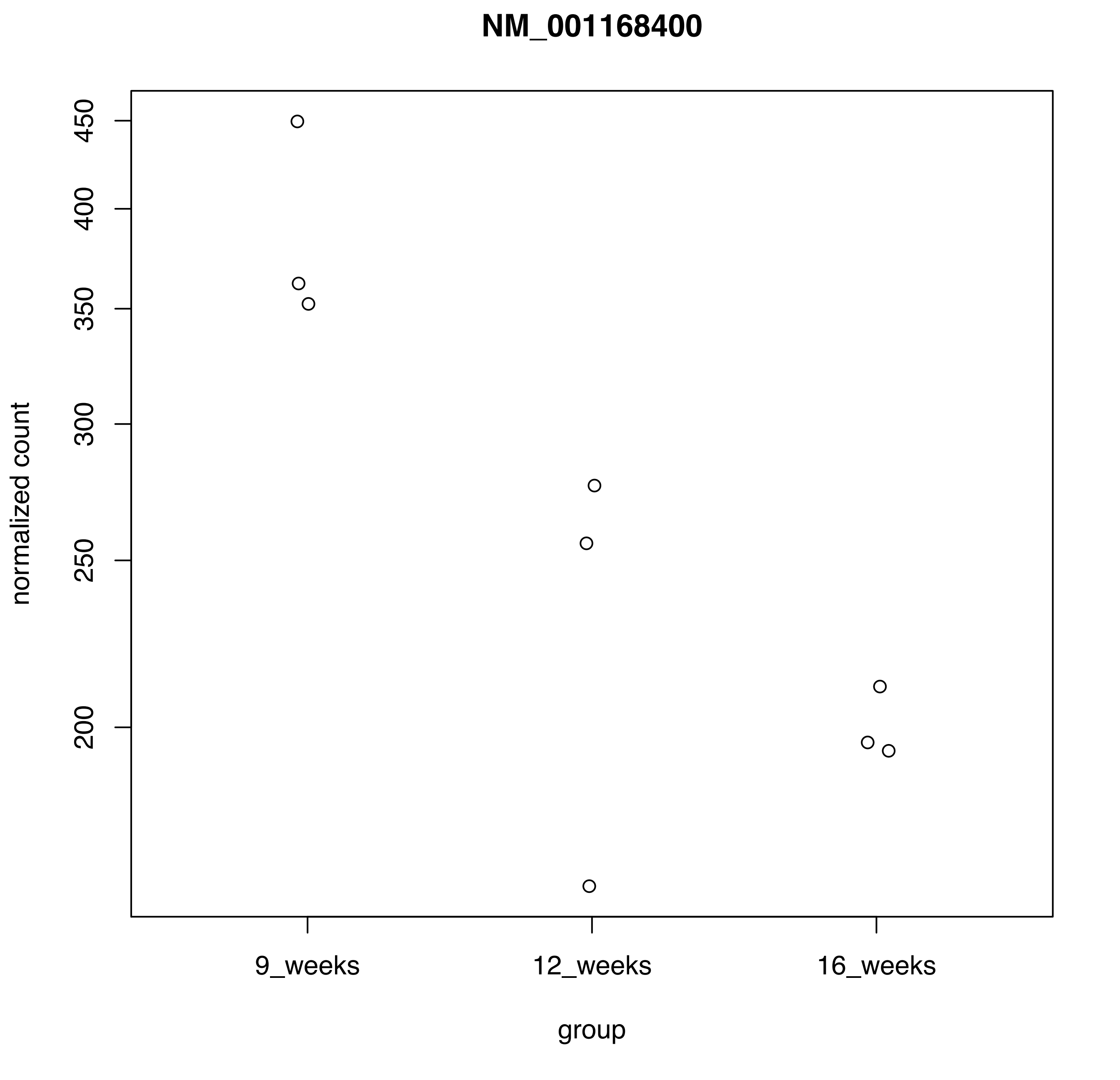
NM_001168400
normalized count
450
400
350
300
250
200
9_weeks
12_weeks
16_weeks
group

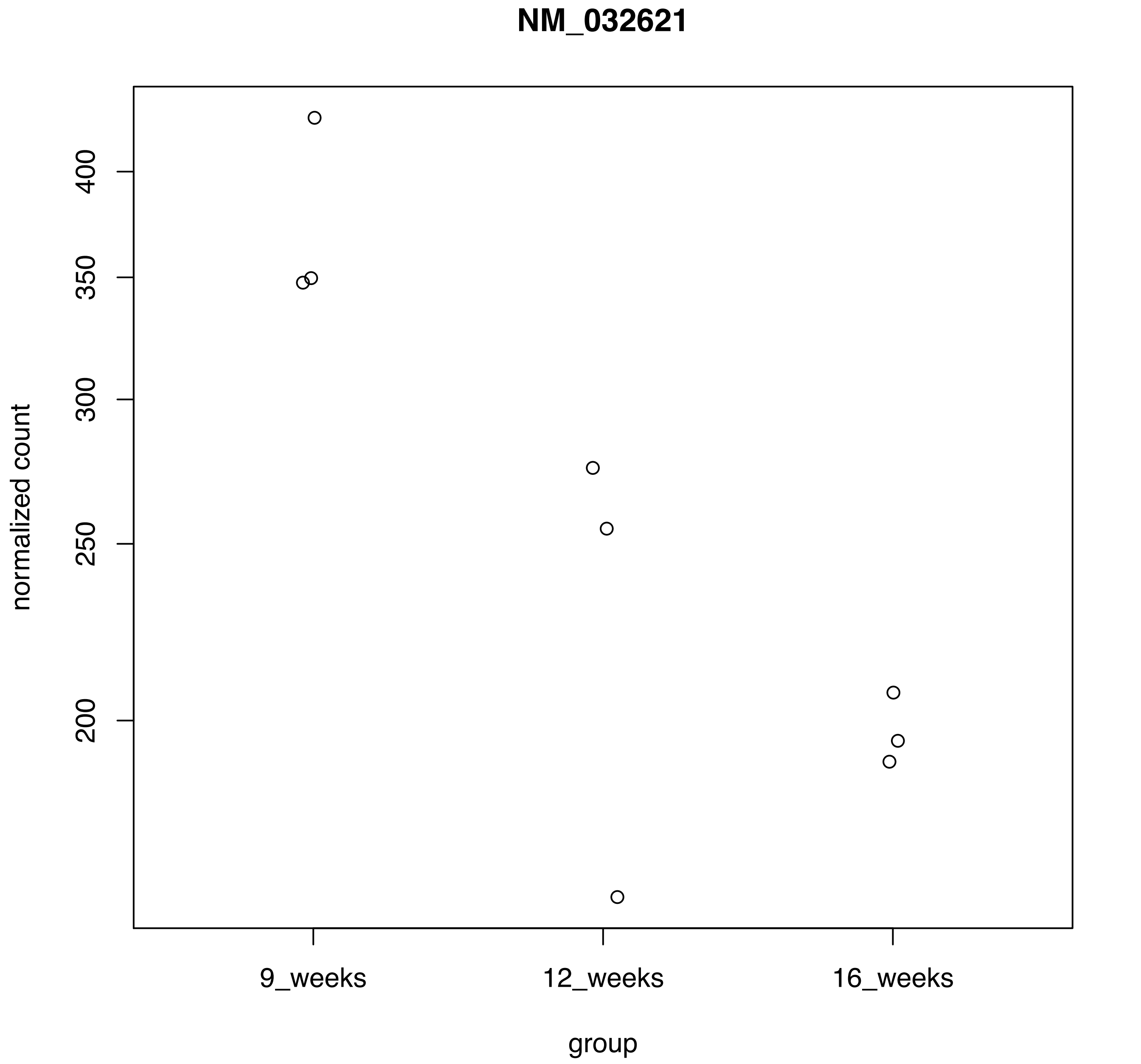

NM_032621
400
350
300
250
200
normalized count
9_weeks
12_weeks
16_weeks
group

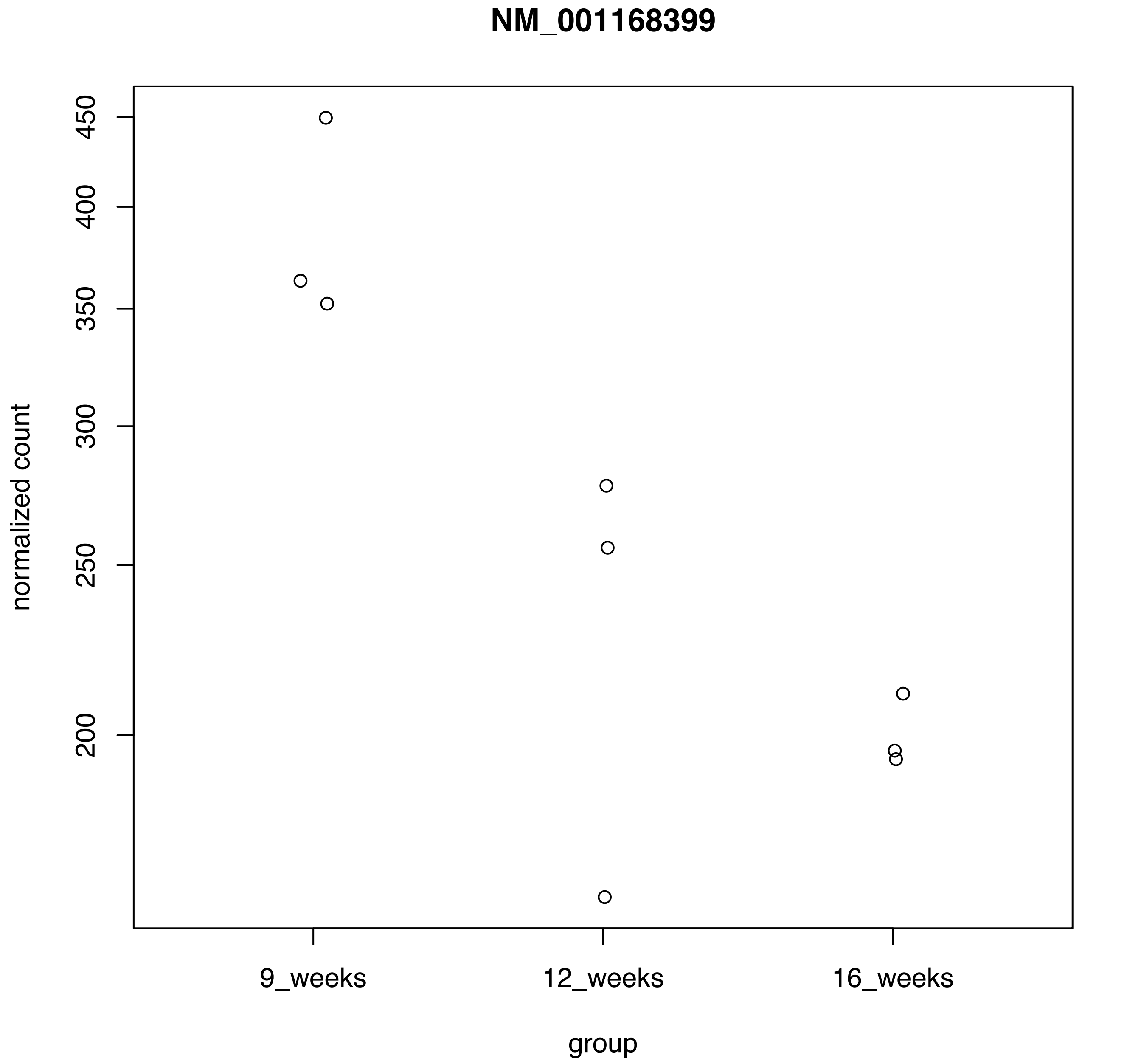
NM_001168399
normalized count
450
400
350
300
250
200
9_weeks
12_weeks
16_weeks
group

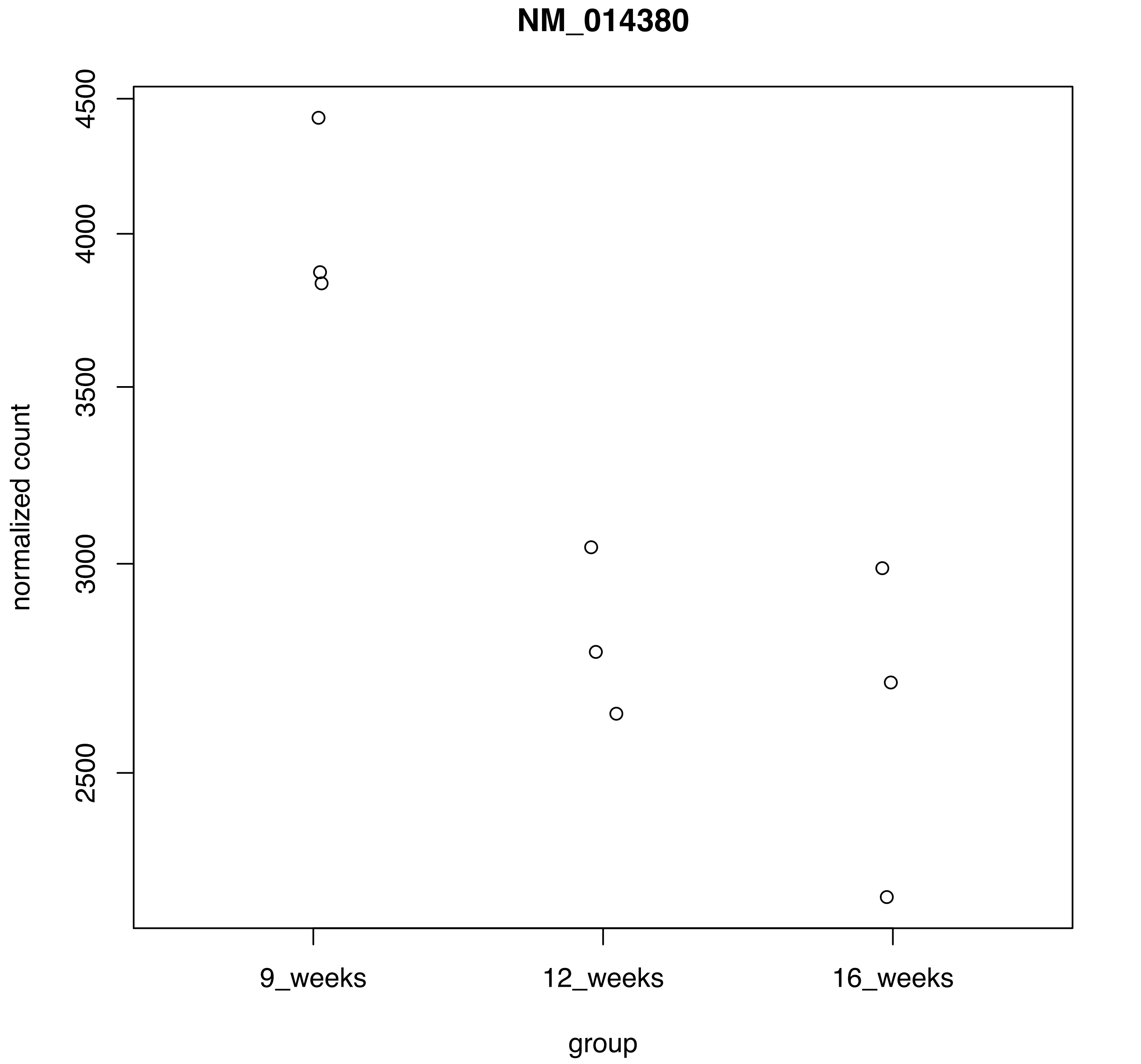
NM_014380
normalized count
4500
4000
3500
3000
2500
9_weeks
12_weeks
16_weeks
group

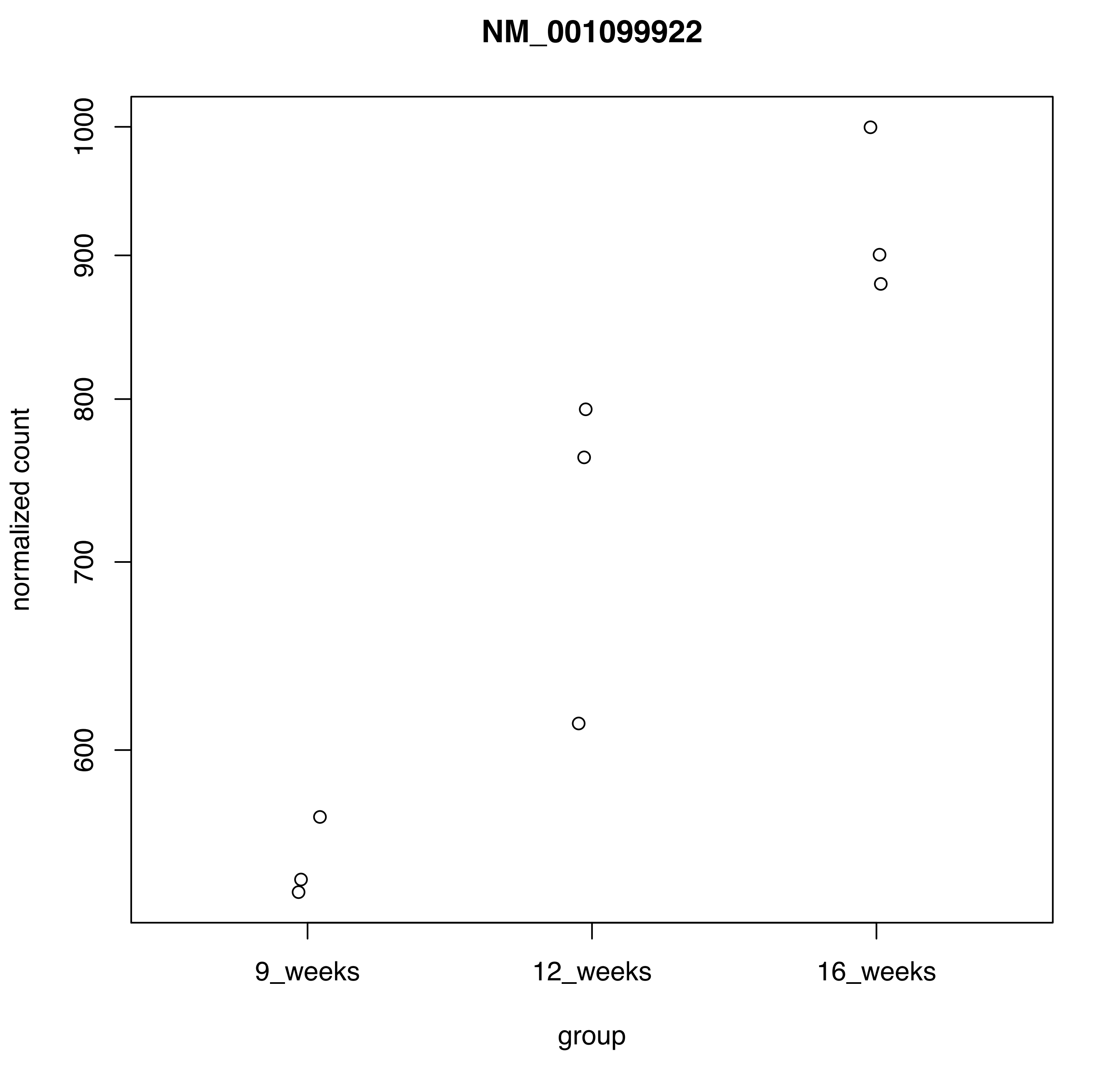

NM_001099922
normalized count
1000
900
800
700
600
9_weeks
12_weeks
16_weeks
group

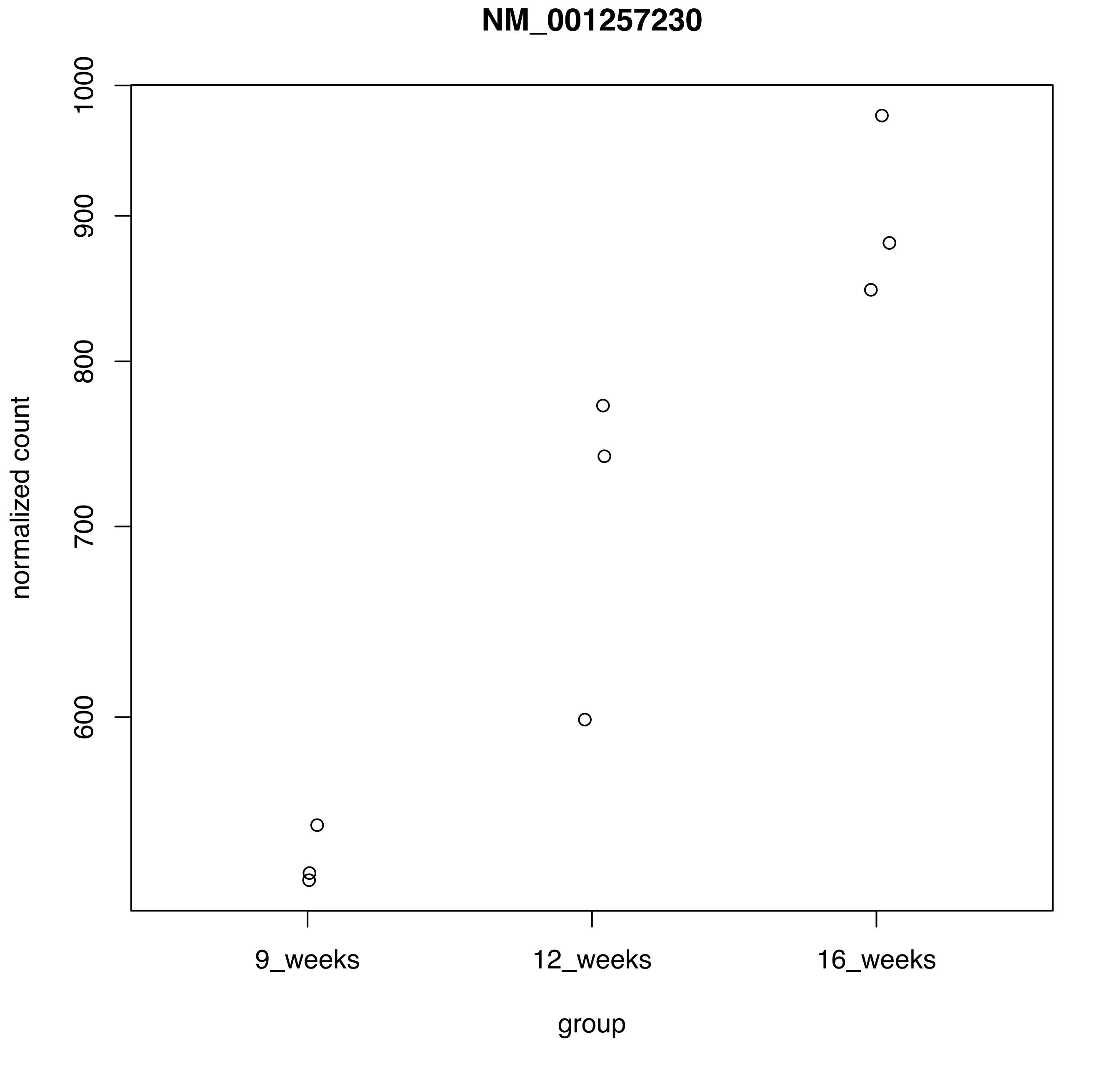
NM_001257230
normalized count
1000
900
800
700
600
9_weeks
12_weeks
16_weeks
group

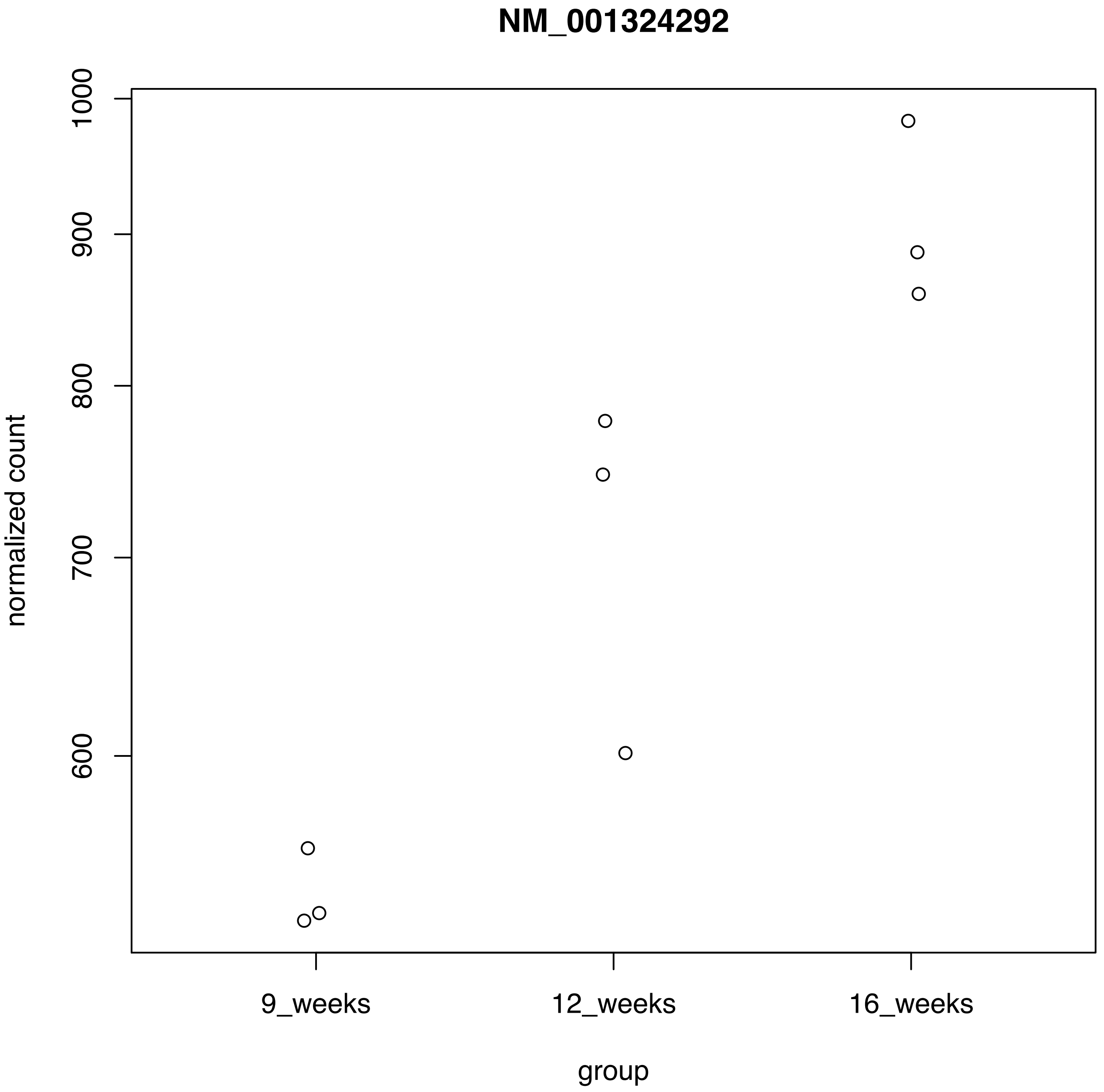

NM_001324292
normalized count
1000
900
800
700
600
9_weeks
12_weeks
16_weeks
group

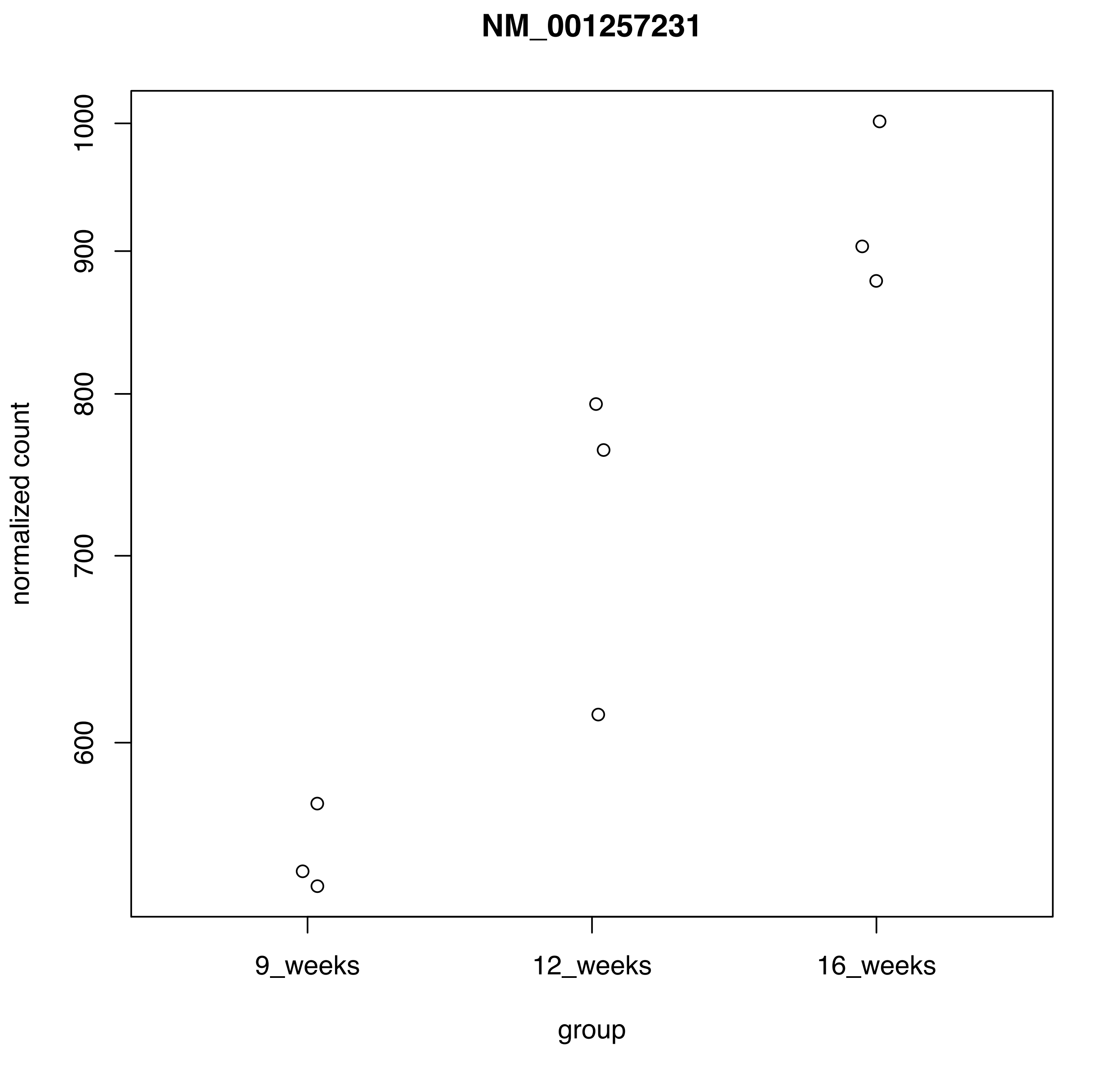
NM_001257231
normalized count
1000
900
800
700
600
9_weeks
12_weeks
16_weeks
group

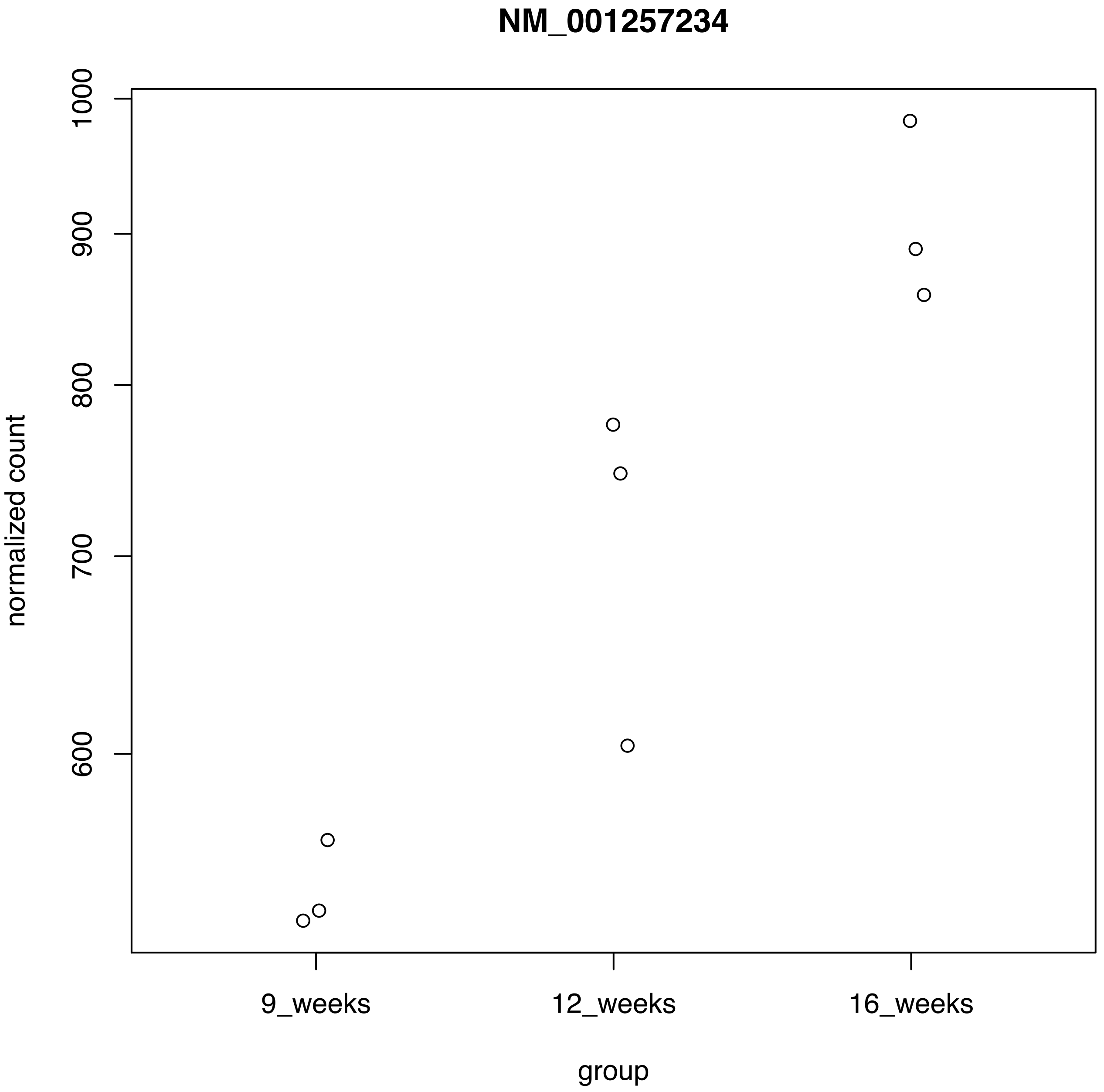

NM_001257234
normalized count
1000
900
800
700
600
9_weeks
12_weeks
16_weeks
group

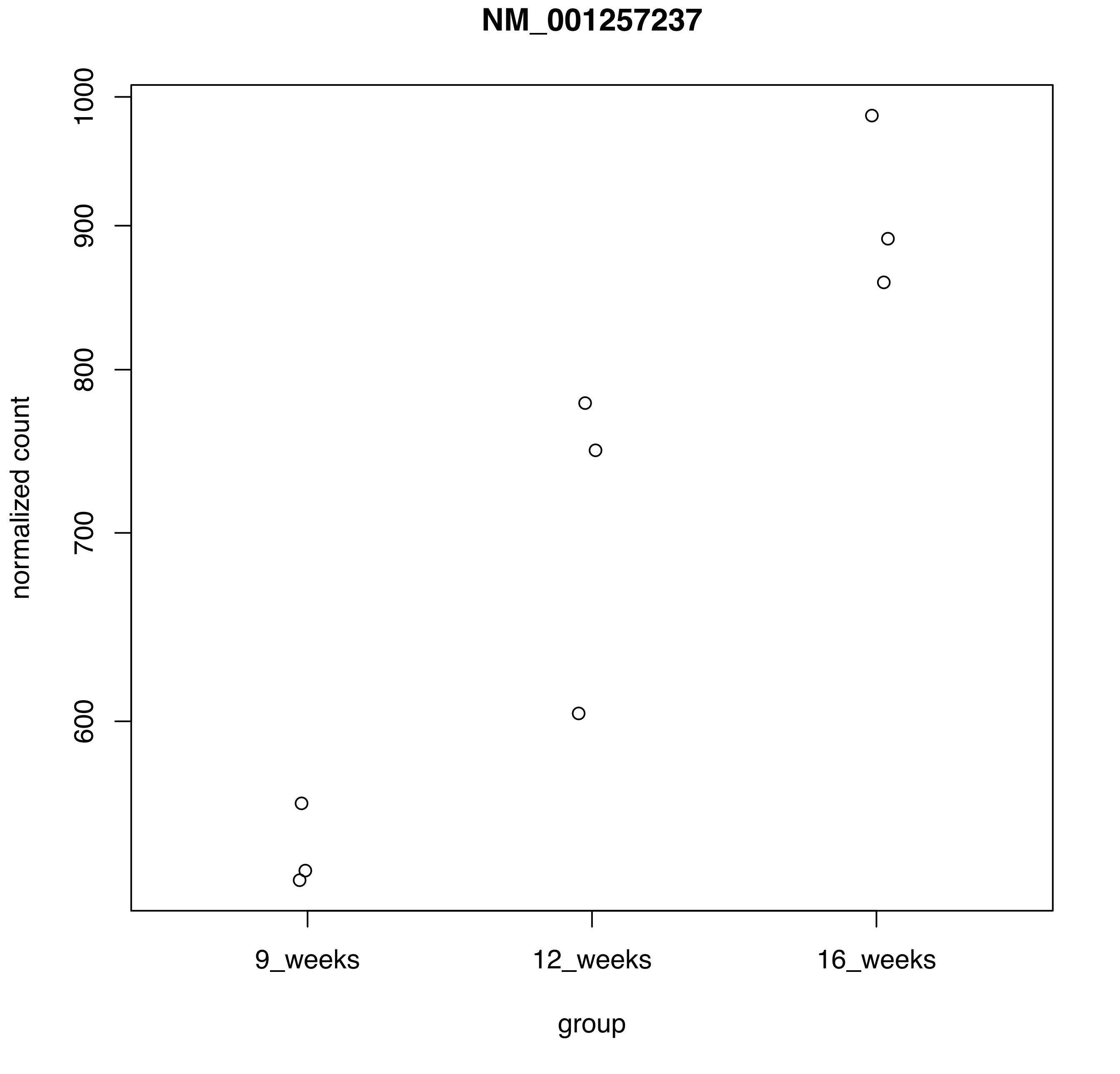
NM_001257237
normalized count
1000
900
800
700
600
9_weeks
12_weeks
16_weeks
group

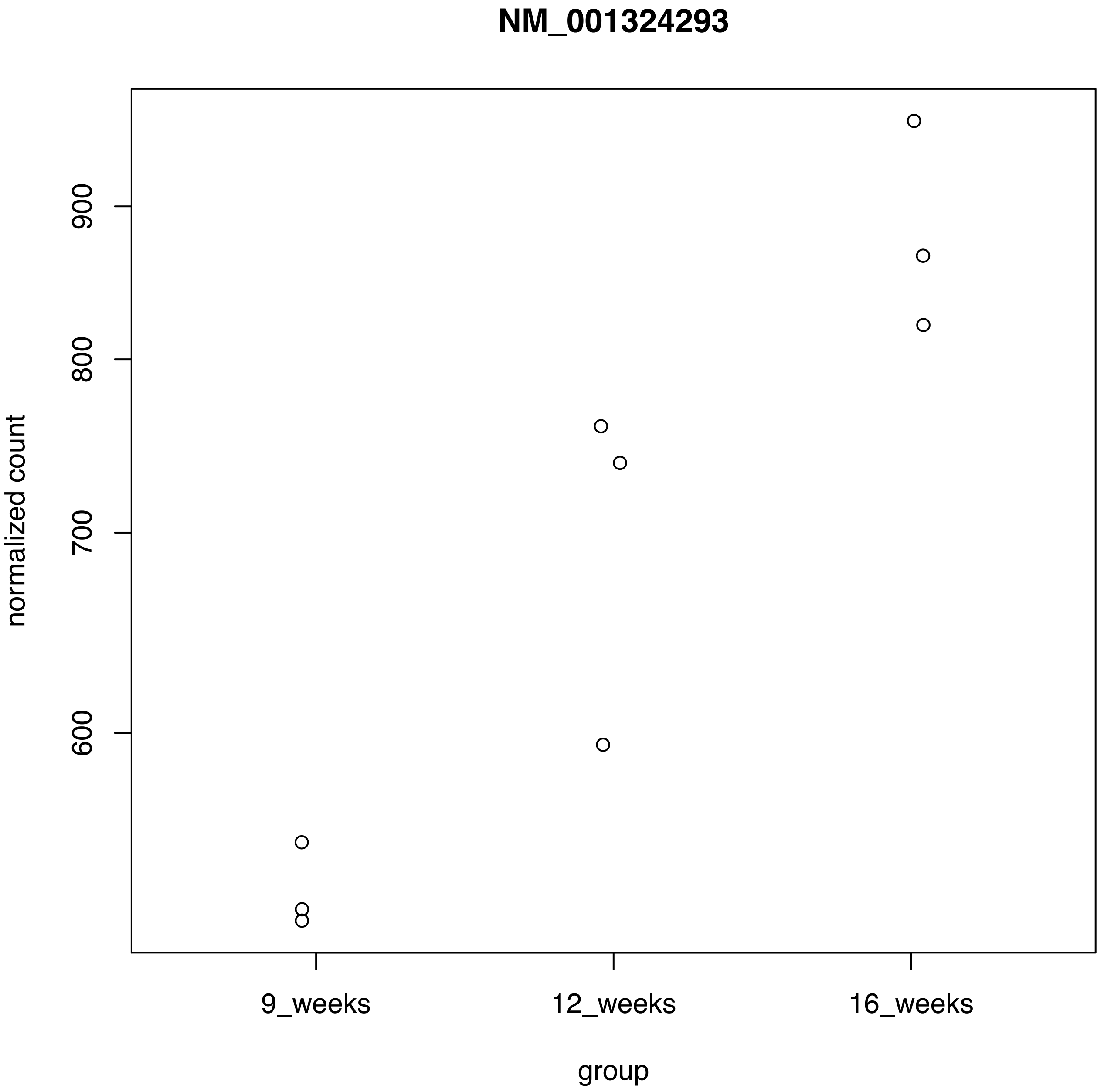
NM_001324293
normalized count
600
700
800
900
9_weeks
12_weeks
16_weeks
group

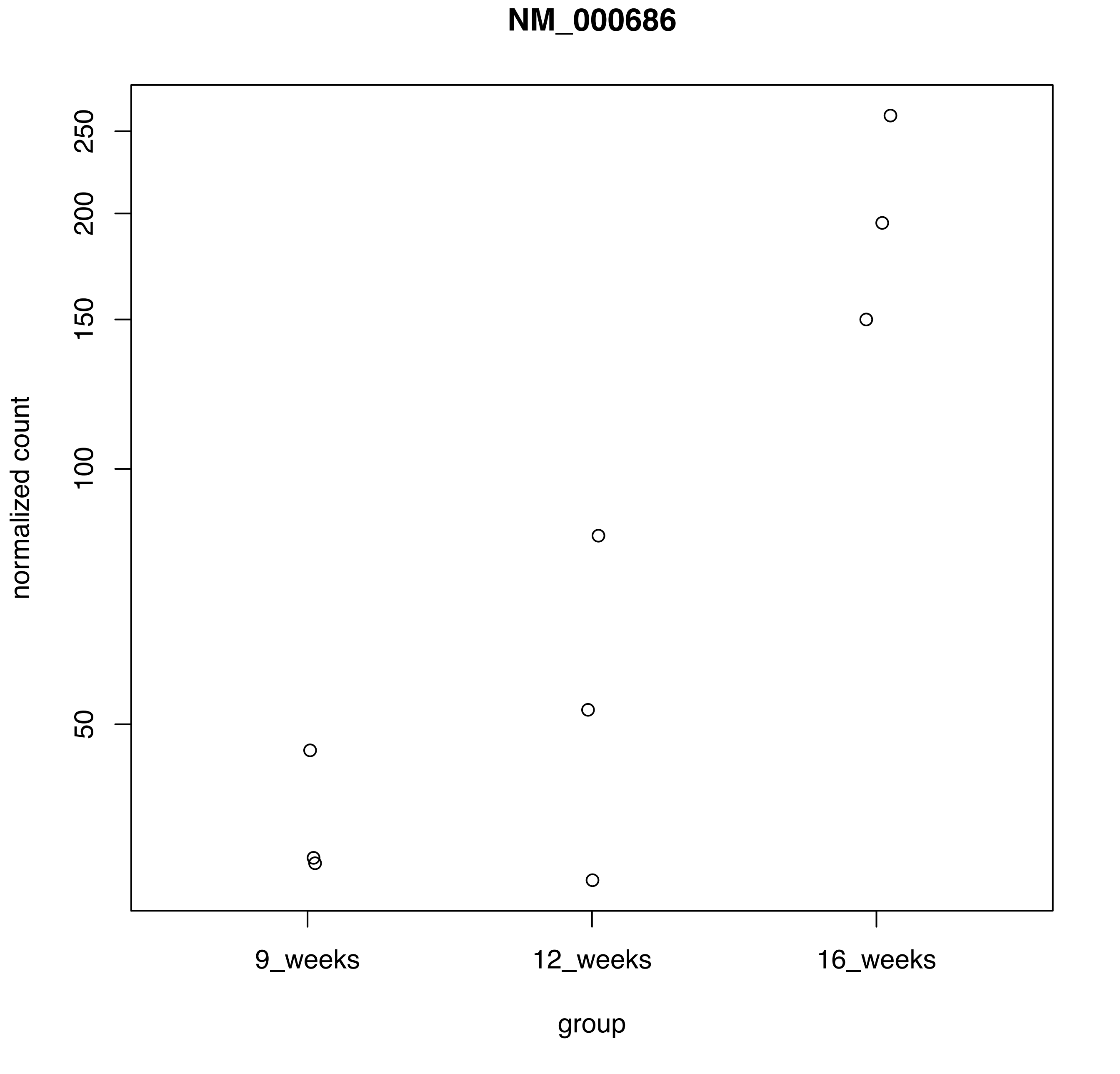
NM_000686
normalized count
250
200
150
100
50
9_weeks
12_weeks
16_weeks
group

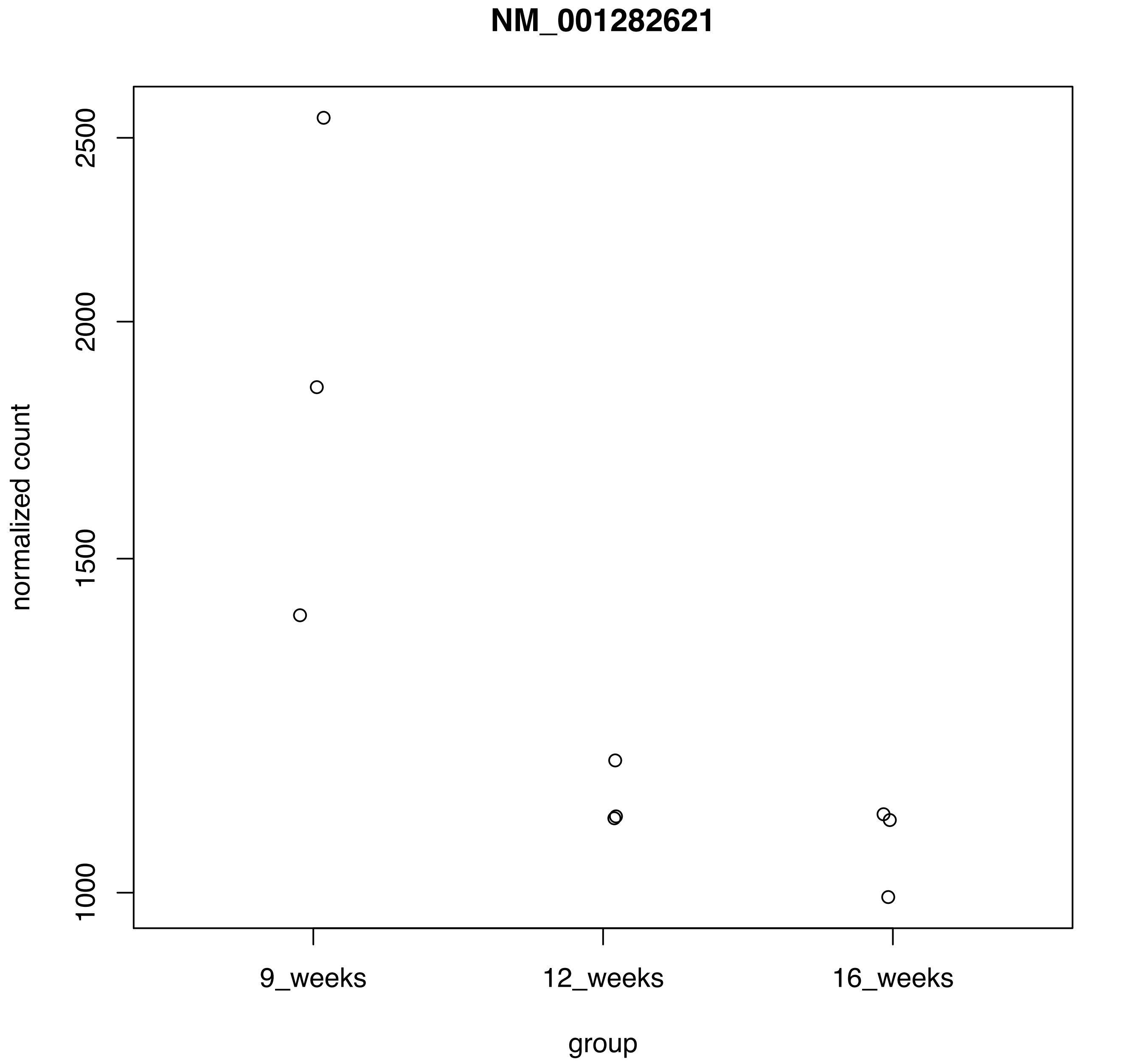
NM_001282621
normalized count
2500
2000
1500
1000
9_weeks
12_weeks
16_weeks
group

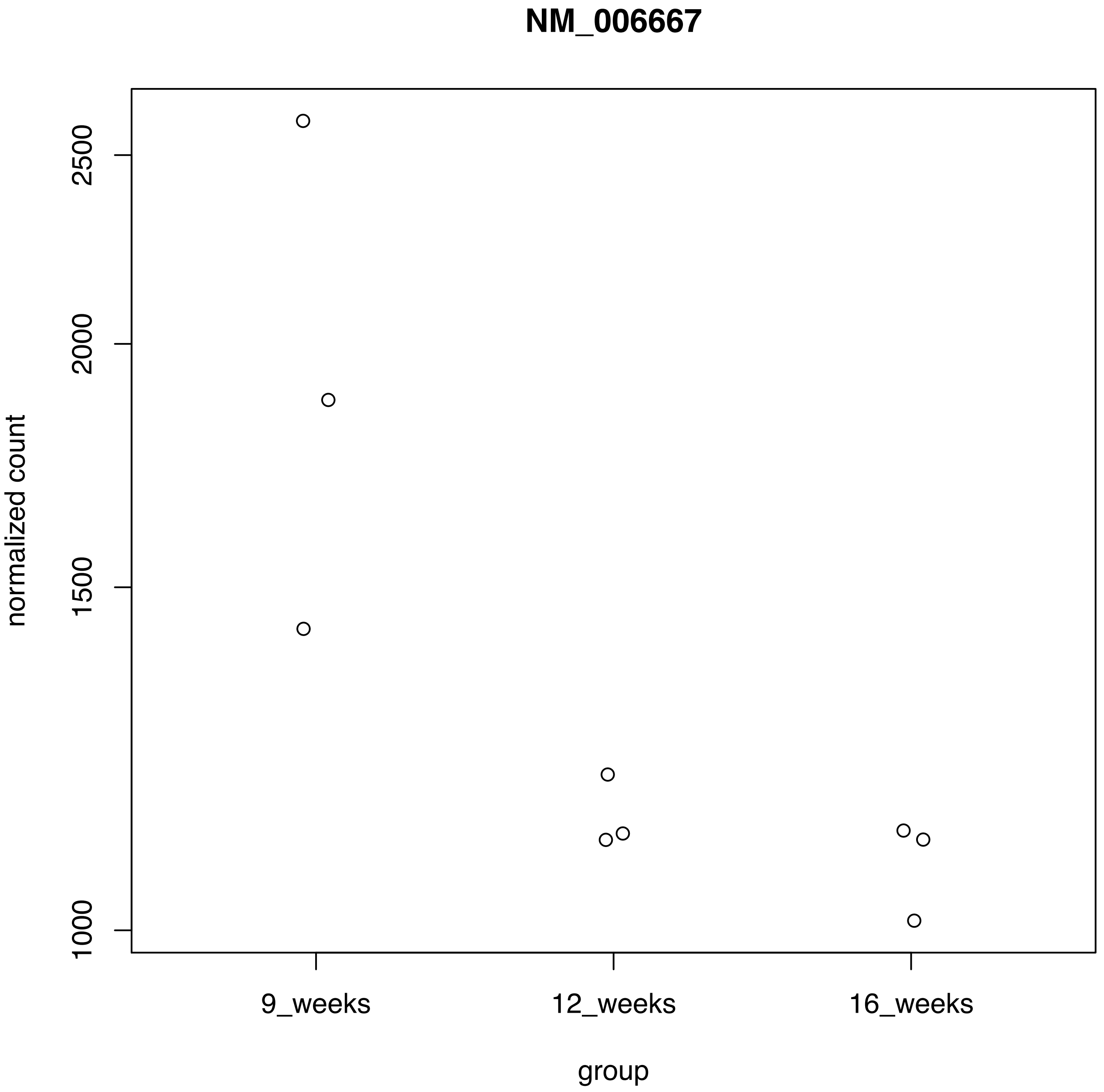
NM_006667
normalized count
2500
2000
1500
1000
9_weeks
12_weeks
16_weeks
group

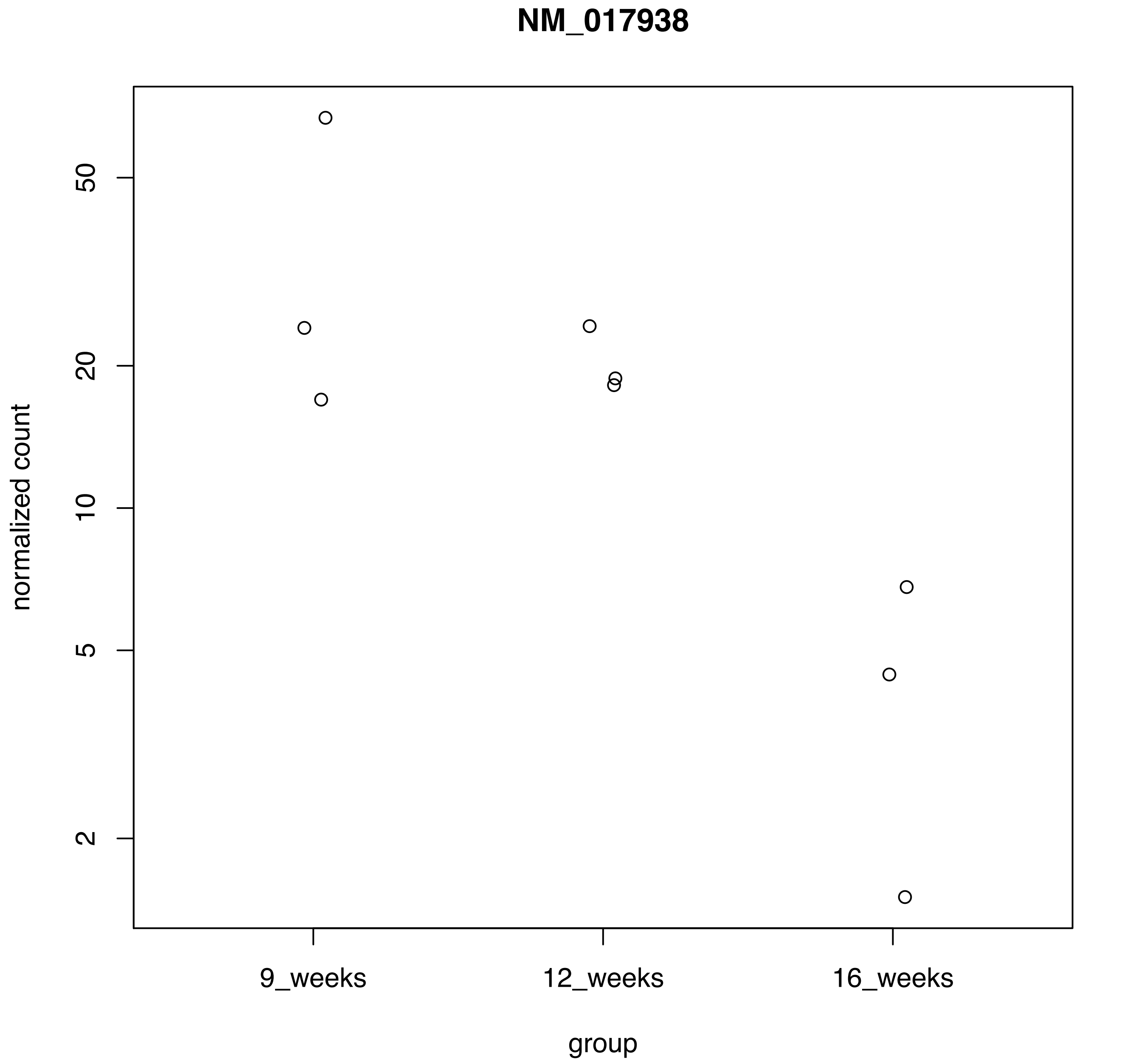
NM_017938
normalized count
50
20
10
5
2
9_weeks
12_weeks
16_weeks
group

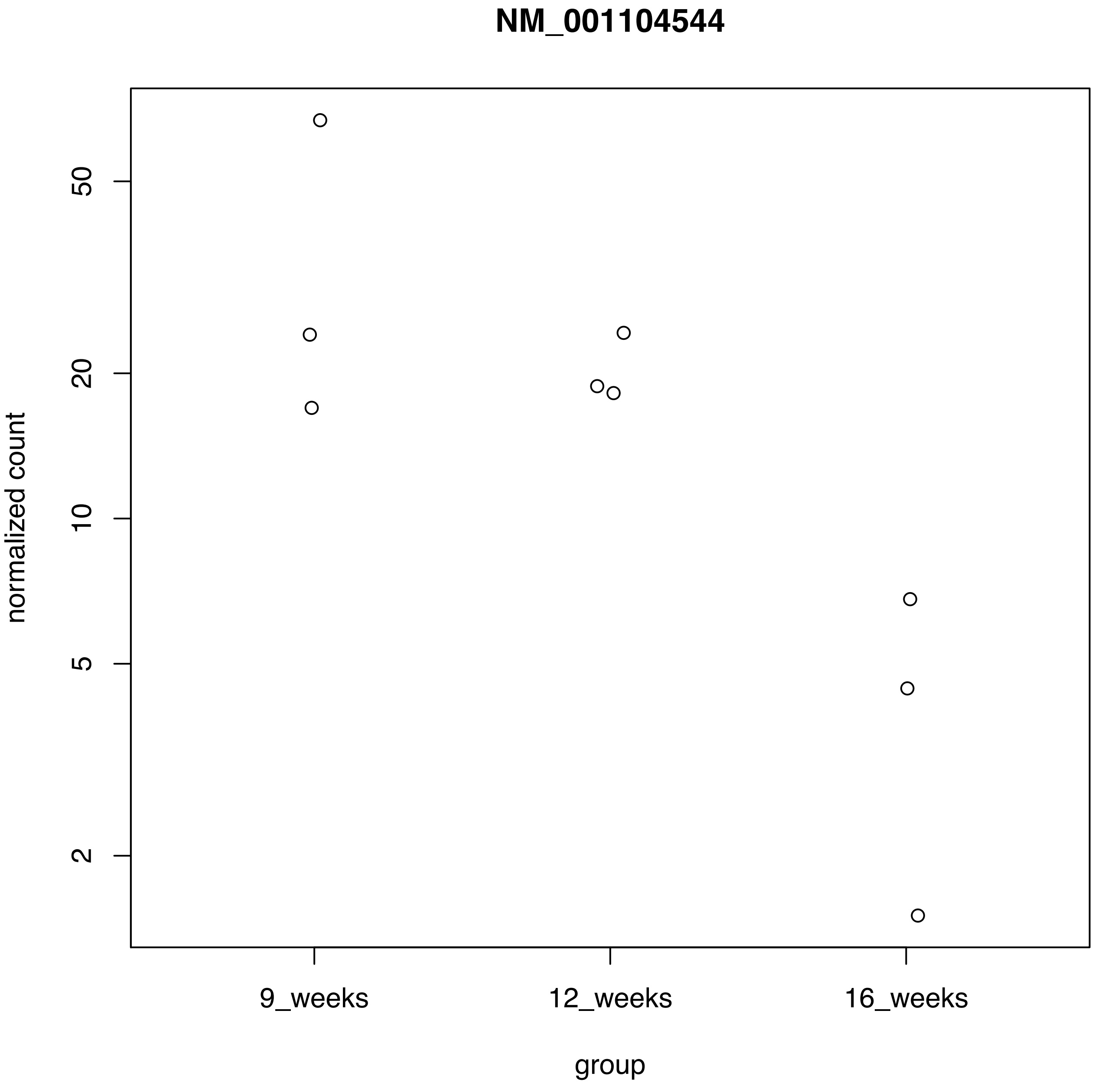
NM_001104544
normalized count
50
20
10
5
2
9_weeks
12_weeks
16_weeks
group

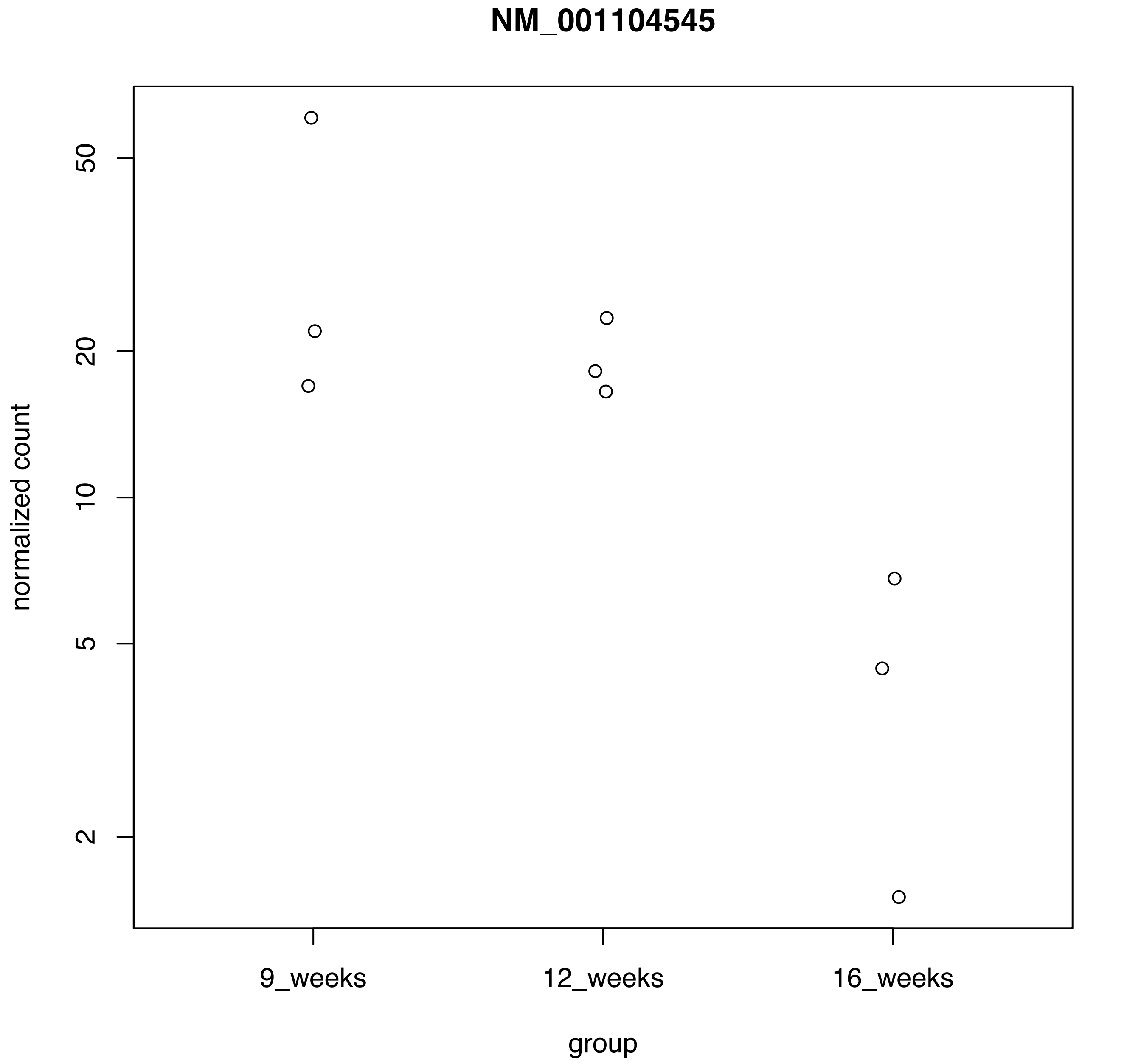

NM_001104545
normalized count
50
20
10
5
2
9_weeks
12_weeks
16_weeks
group

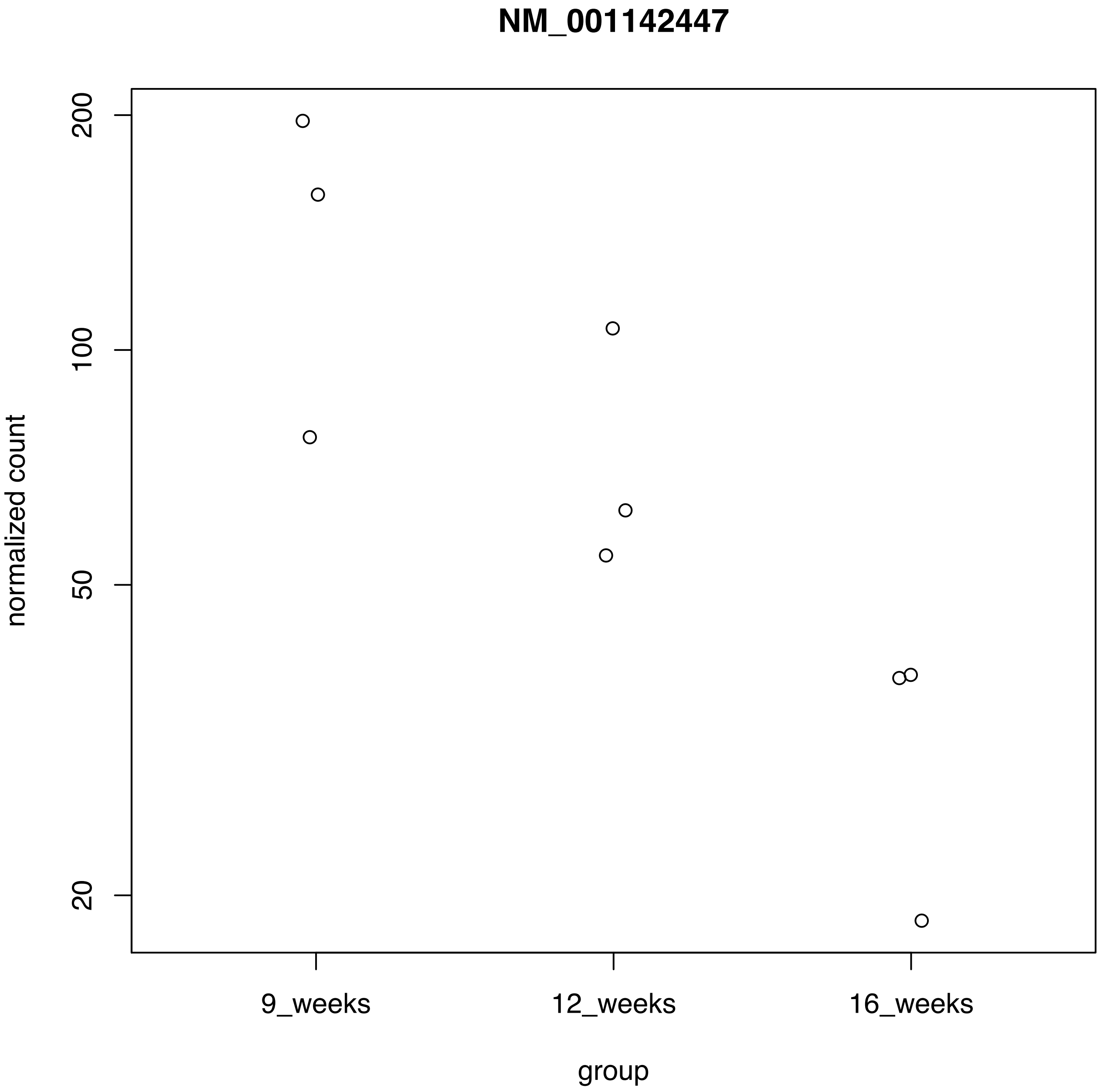
NM_001142447
normalized count
200
100
50
20
9_weeks
12_weeks
16_weeks
group

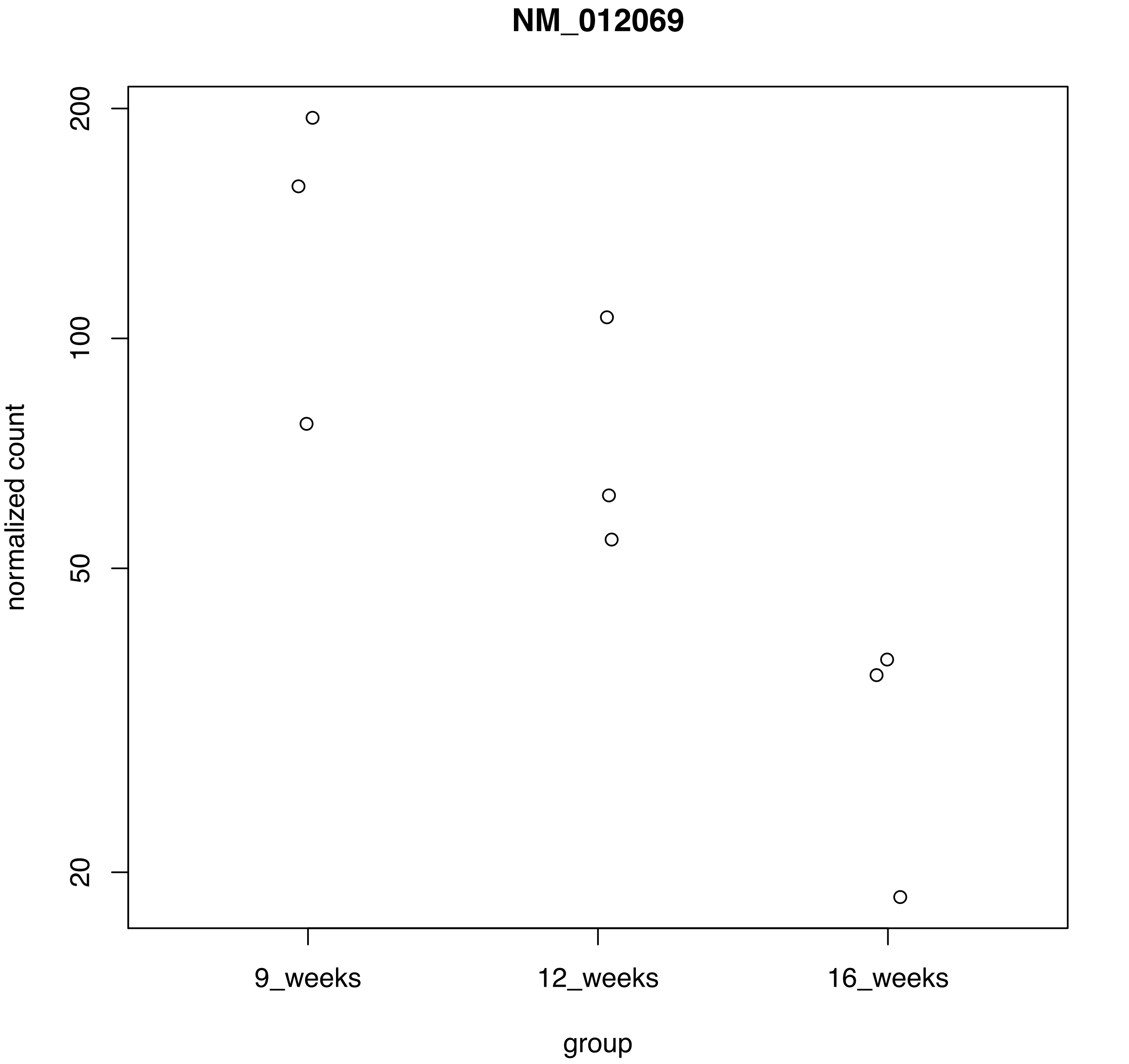

NM_012069
normalized count
200
100
50
20
9_weeks
12_weeks
16_weeks
group

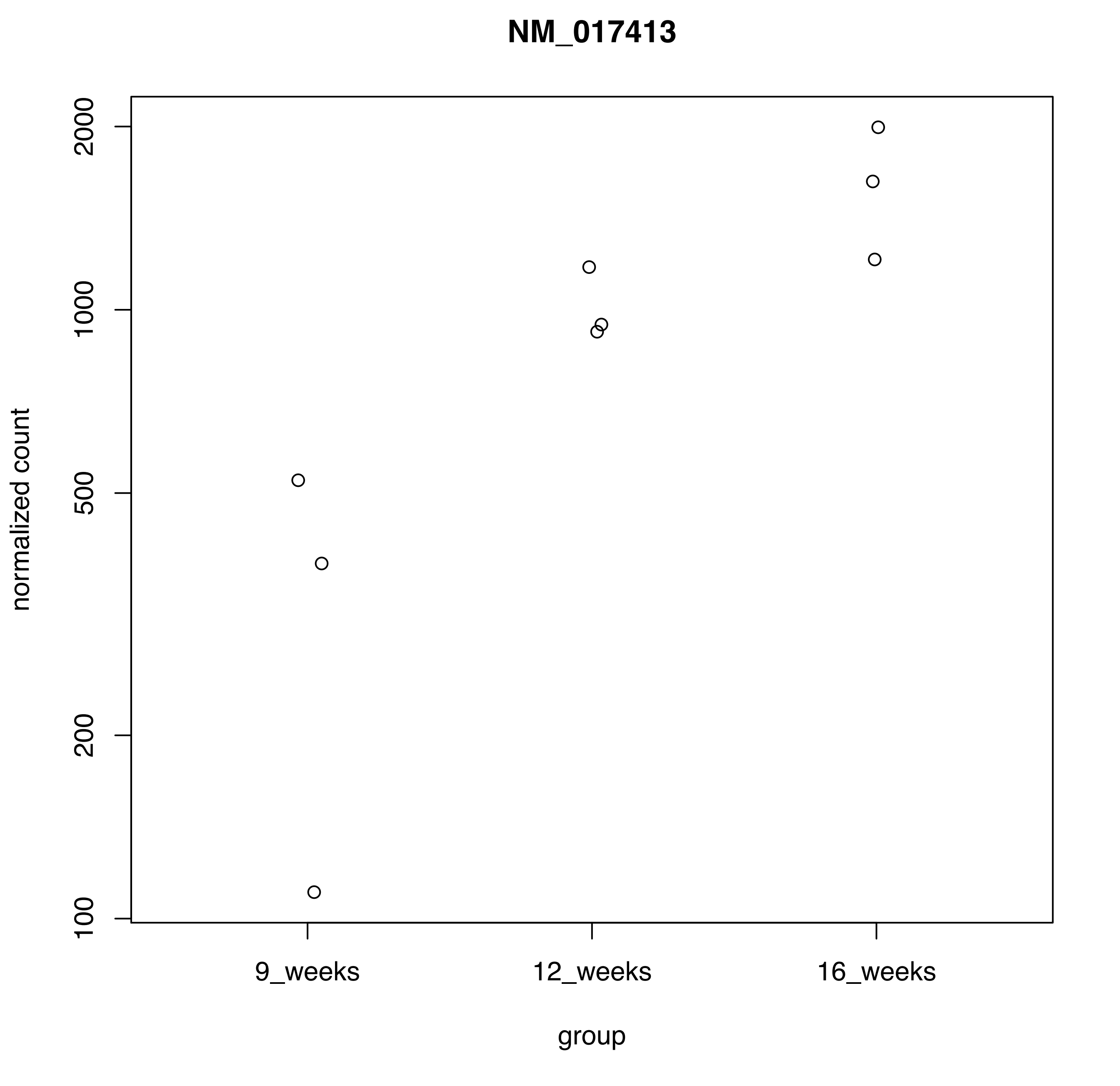

NM_017413
normalized count
2000
1000
500
200
100
9_weeks
12_weeks
16_weeks
group

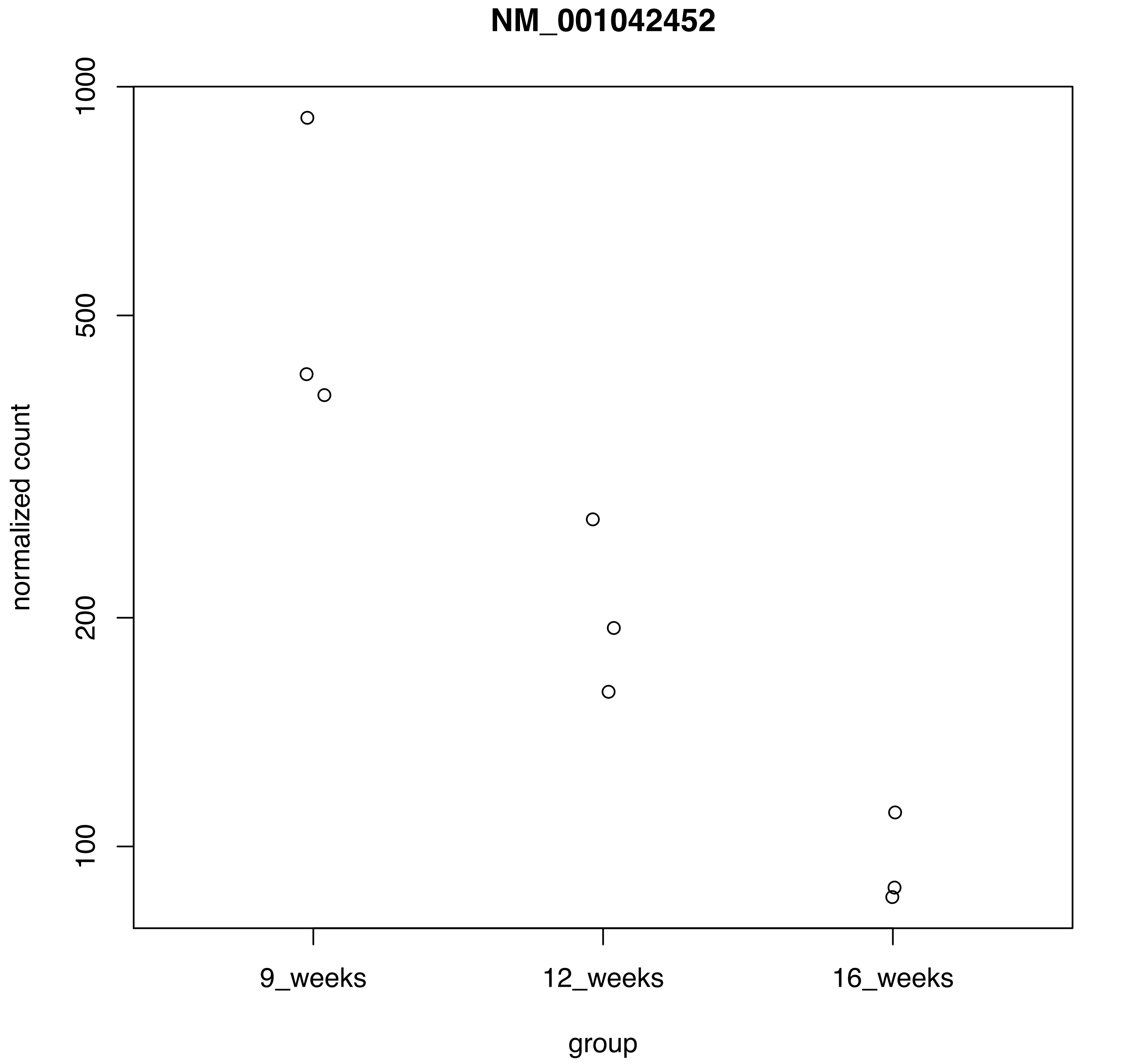
NM_001042452
1000
500
200
100
normalized count
9_weeks
12_weeks
16_weeks
group

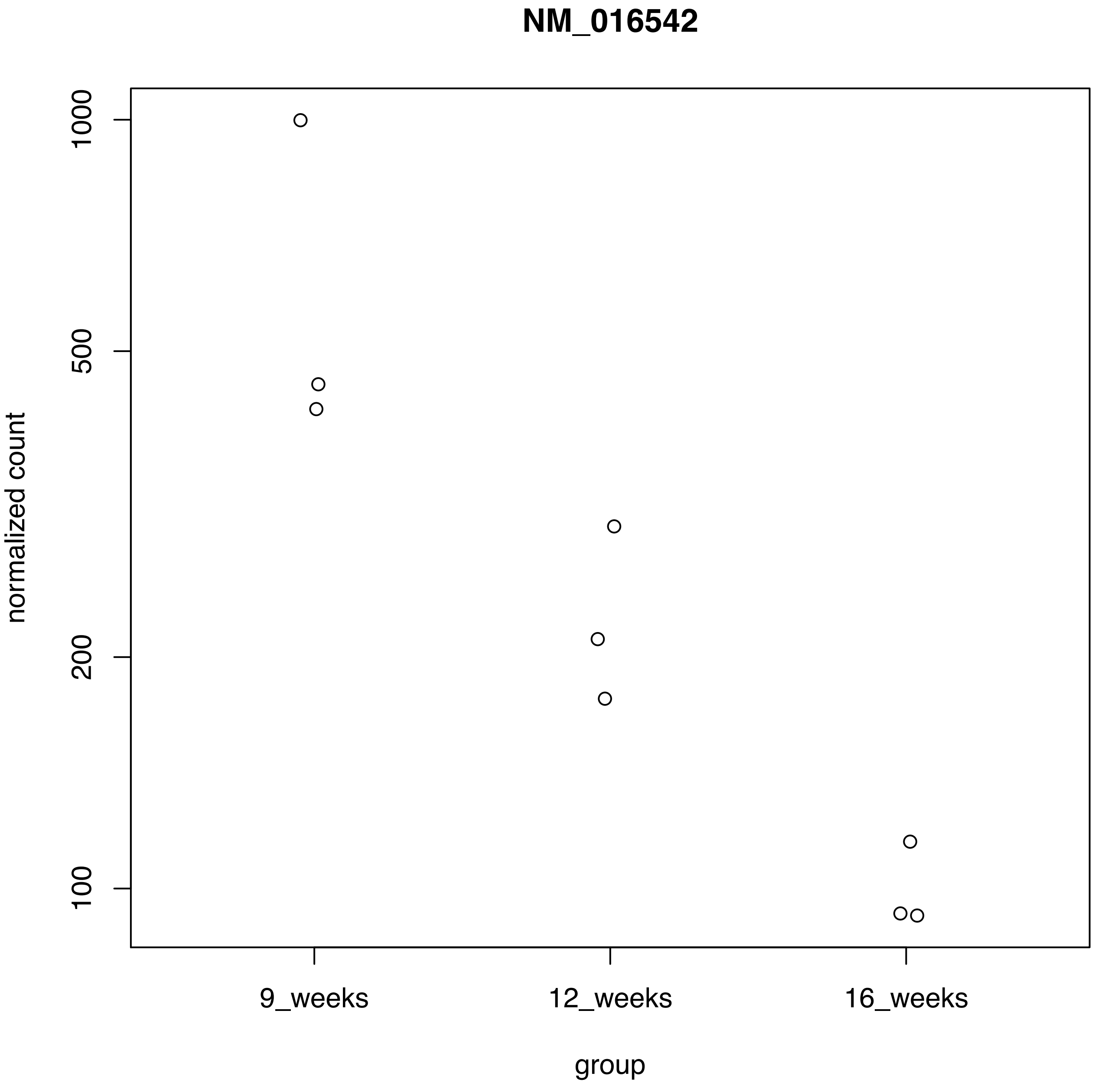
NM_016542
1000
500
200
100
normalized count
9_weeks
12_weeks
16_weeks
group

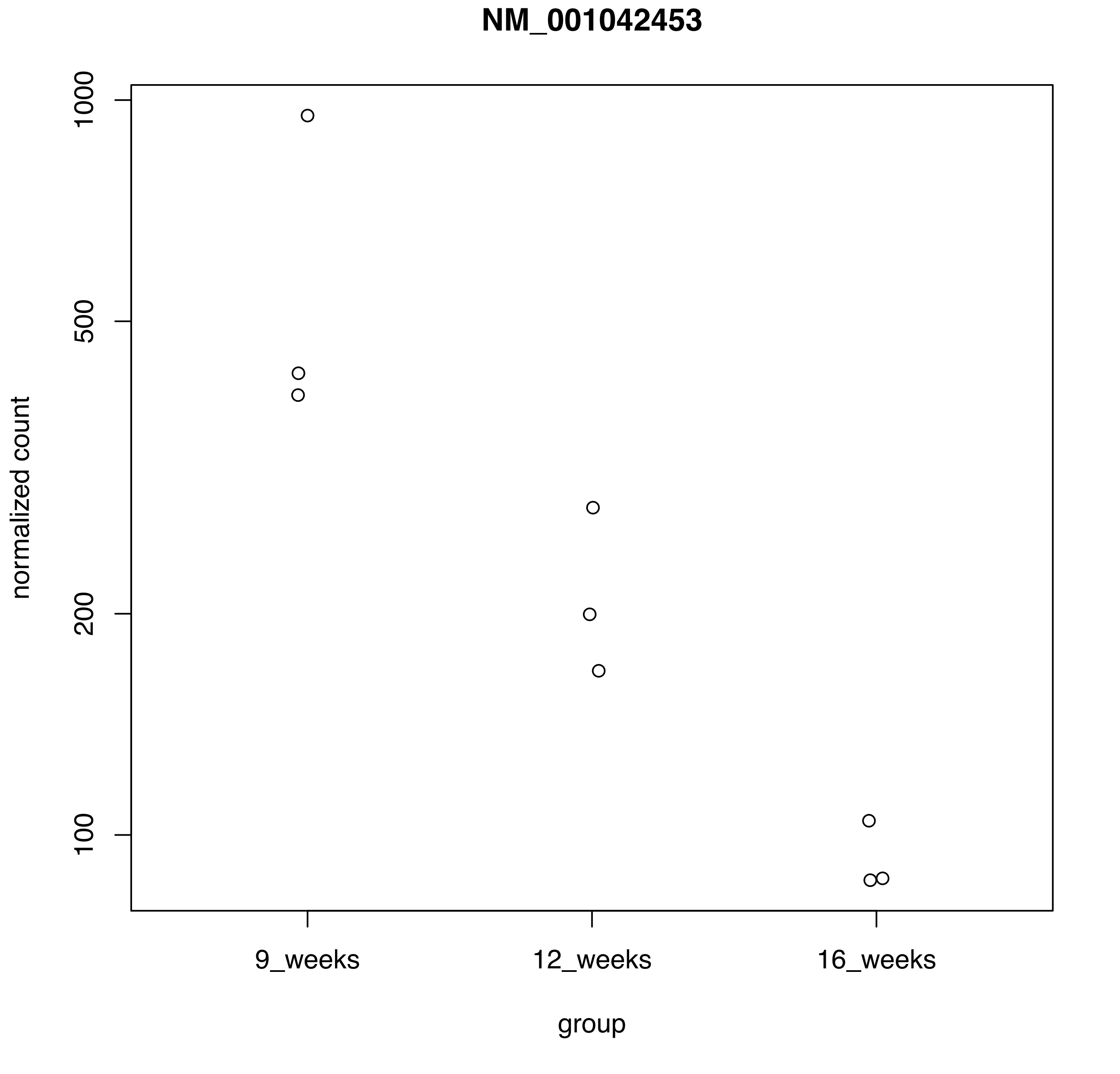
NM_001042453
1000
500
200
100
normalized count
9_weeks
12_weeks
16_weeks
group

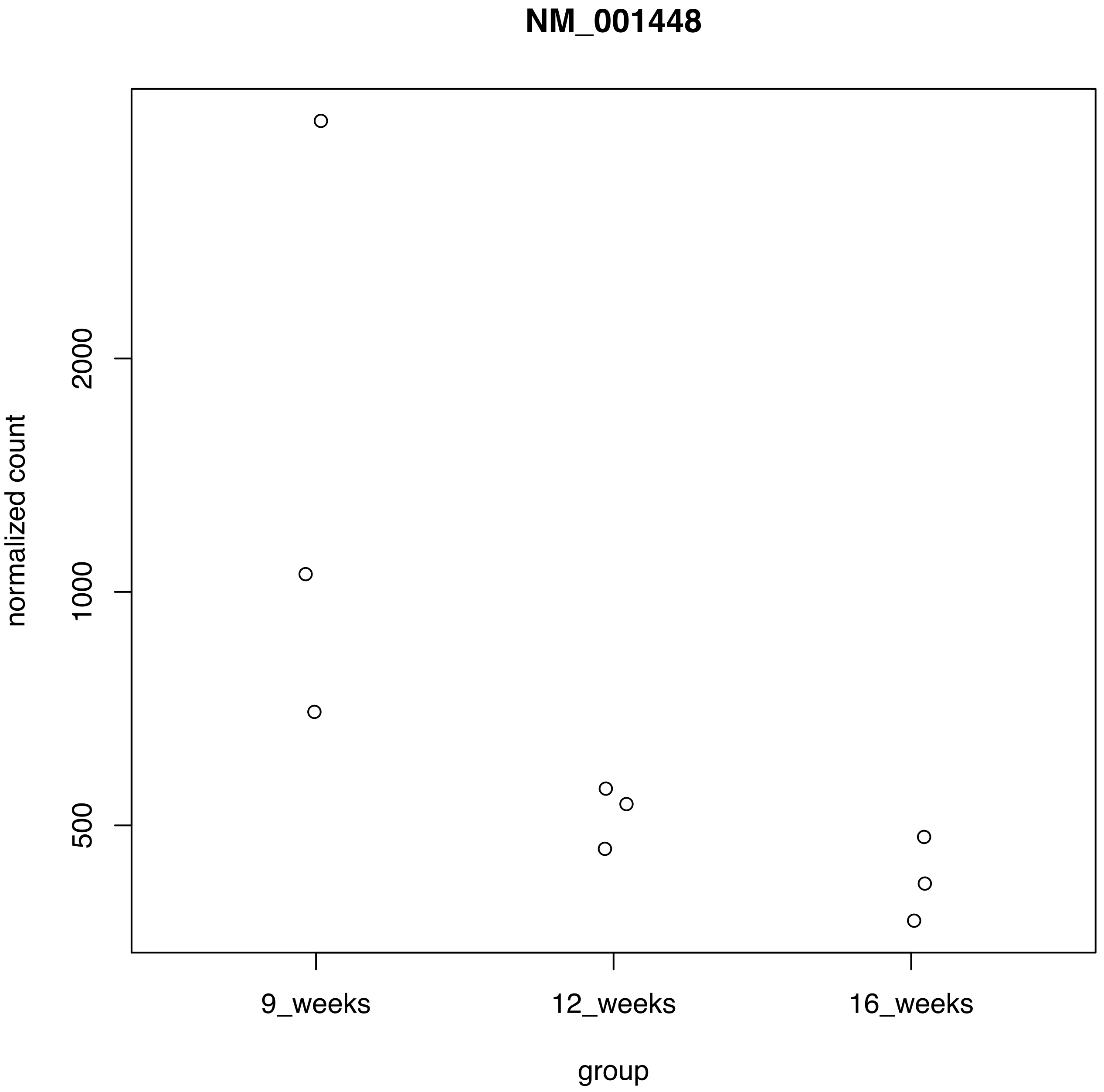

NM_001448
normalized count
2000
1000
500
9_weeks
12_weeks
16_weeks
group

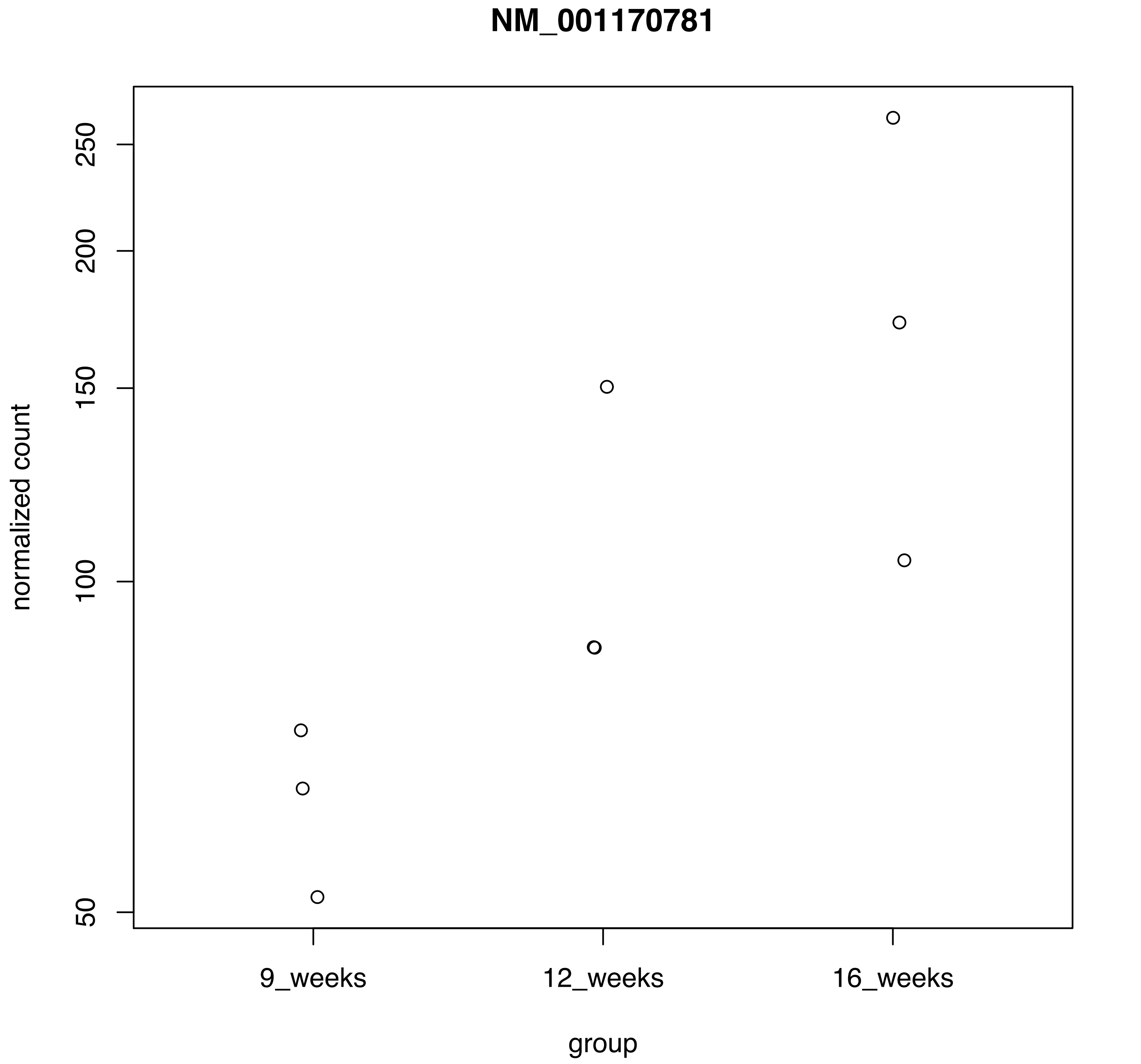

NM_001170781
normalized count
50
100
150
200
250
9_weeks
12_weeks
16_weeks
group

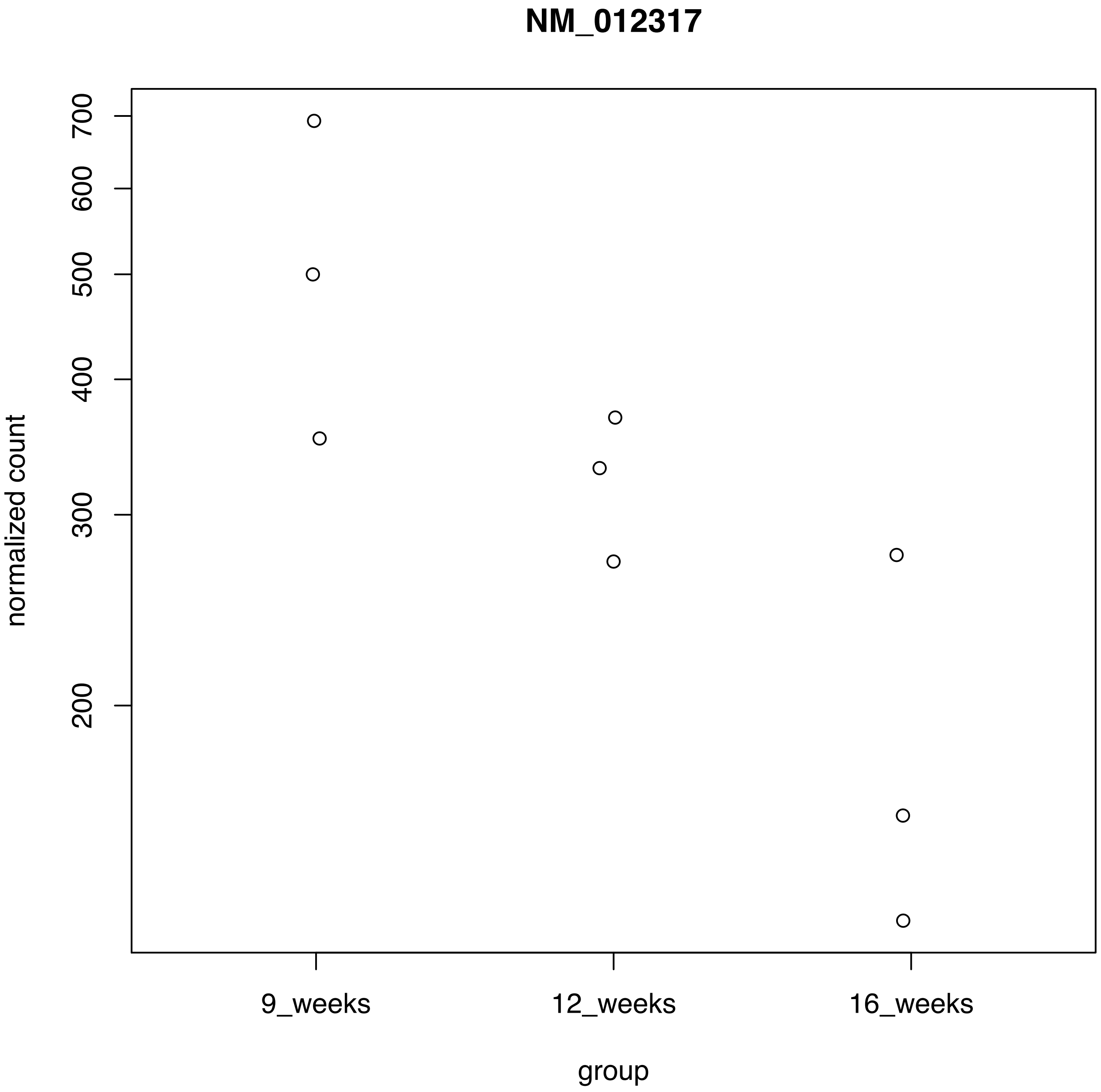
NM_012317
normalized count
700
600
500
400
300
200
9_weeks
12_weeks
16_weeks
group

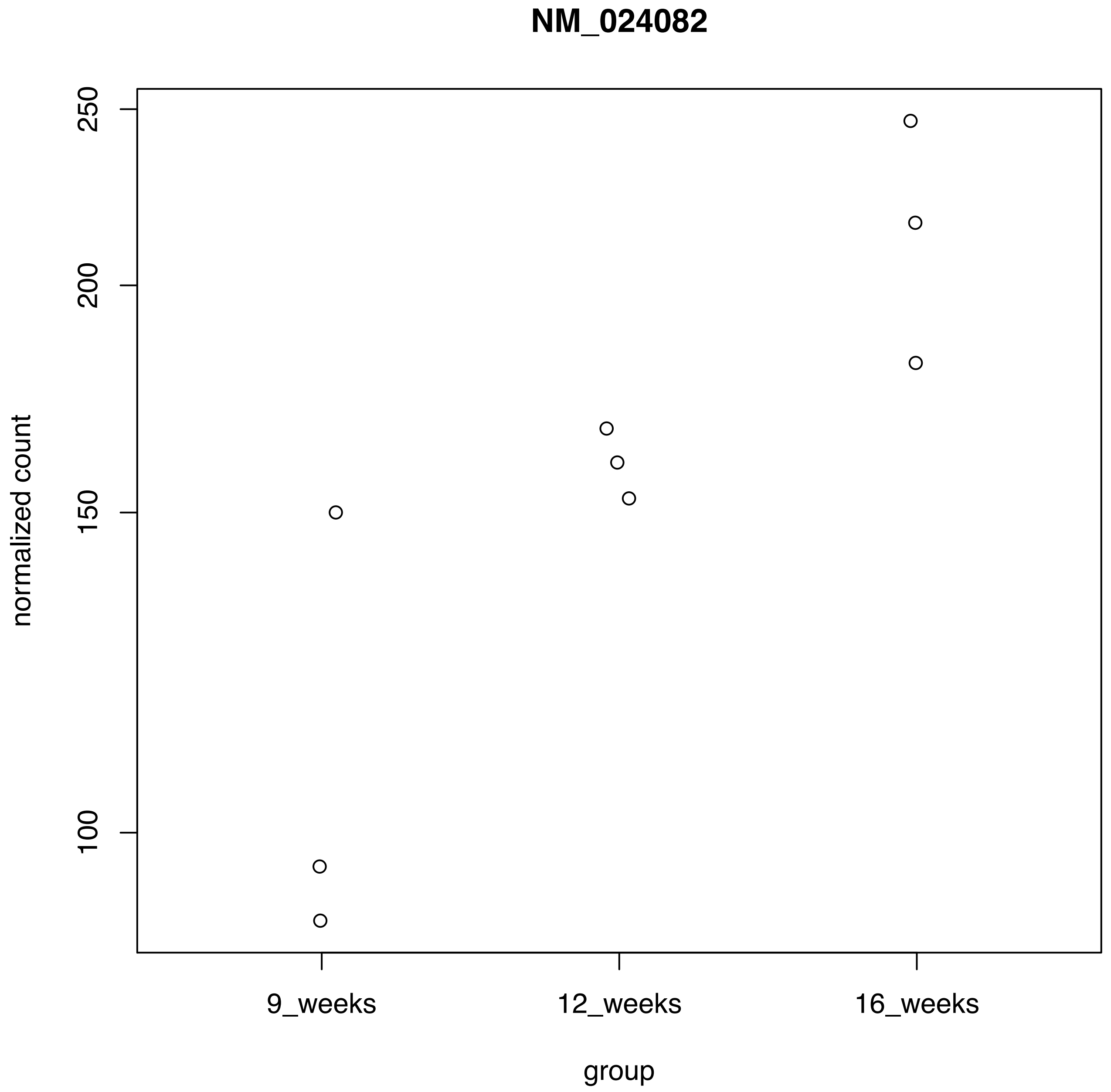
NM_024082
normalized count
250
200
150
100
9_weeks
12_weeks
16_weeks
group

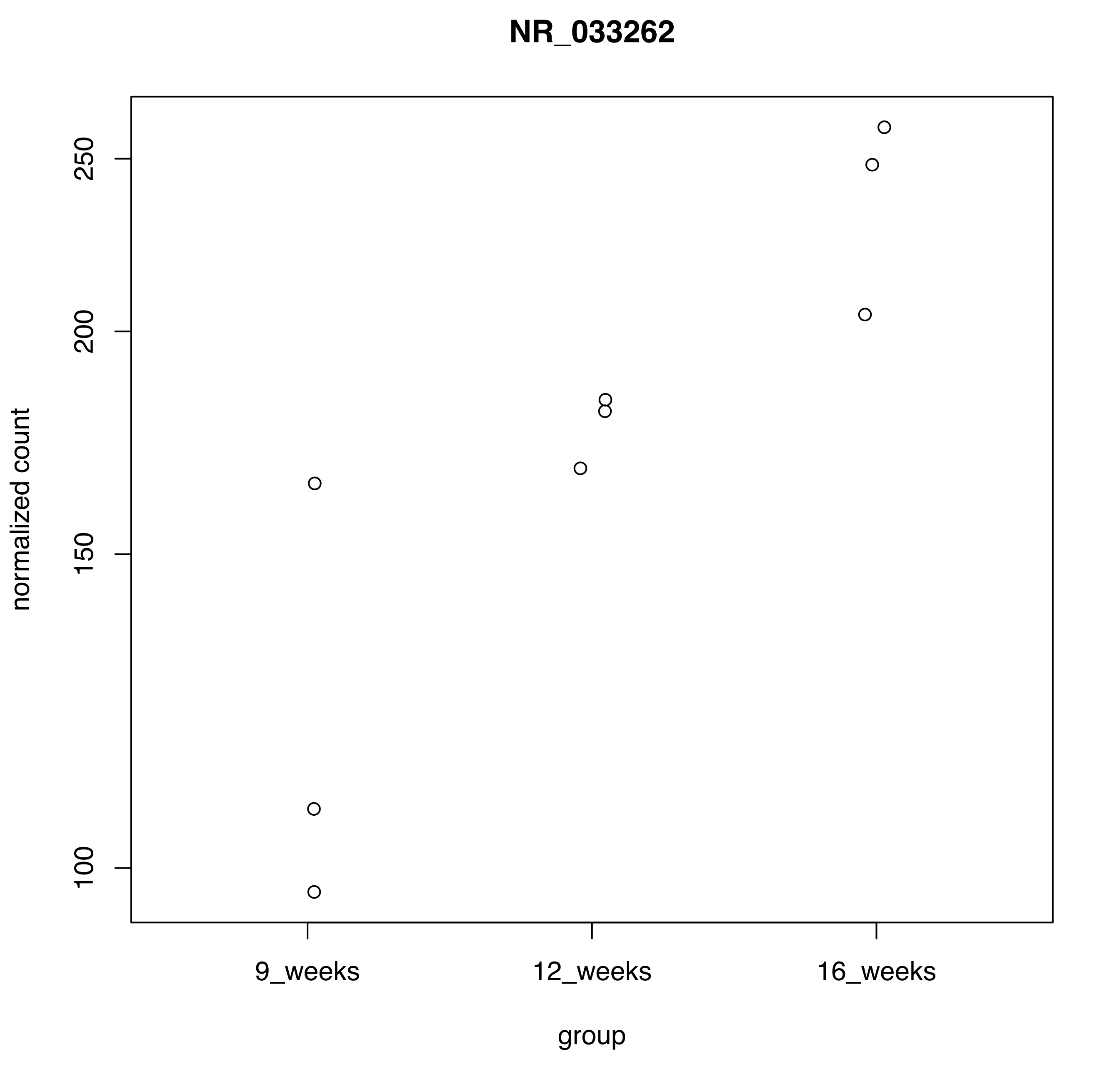
NR_033262
normalized count
250
200
150
100
9_weeks
12_weeks
16_weeks
group

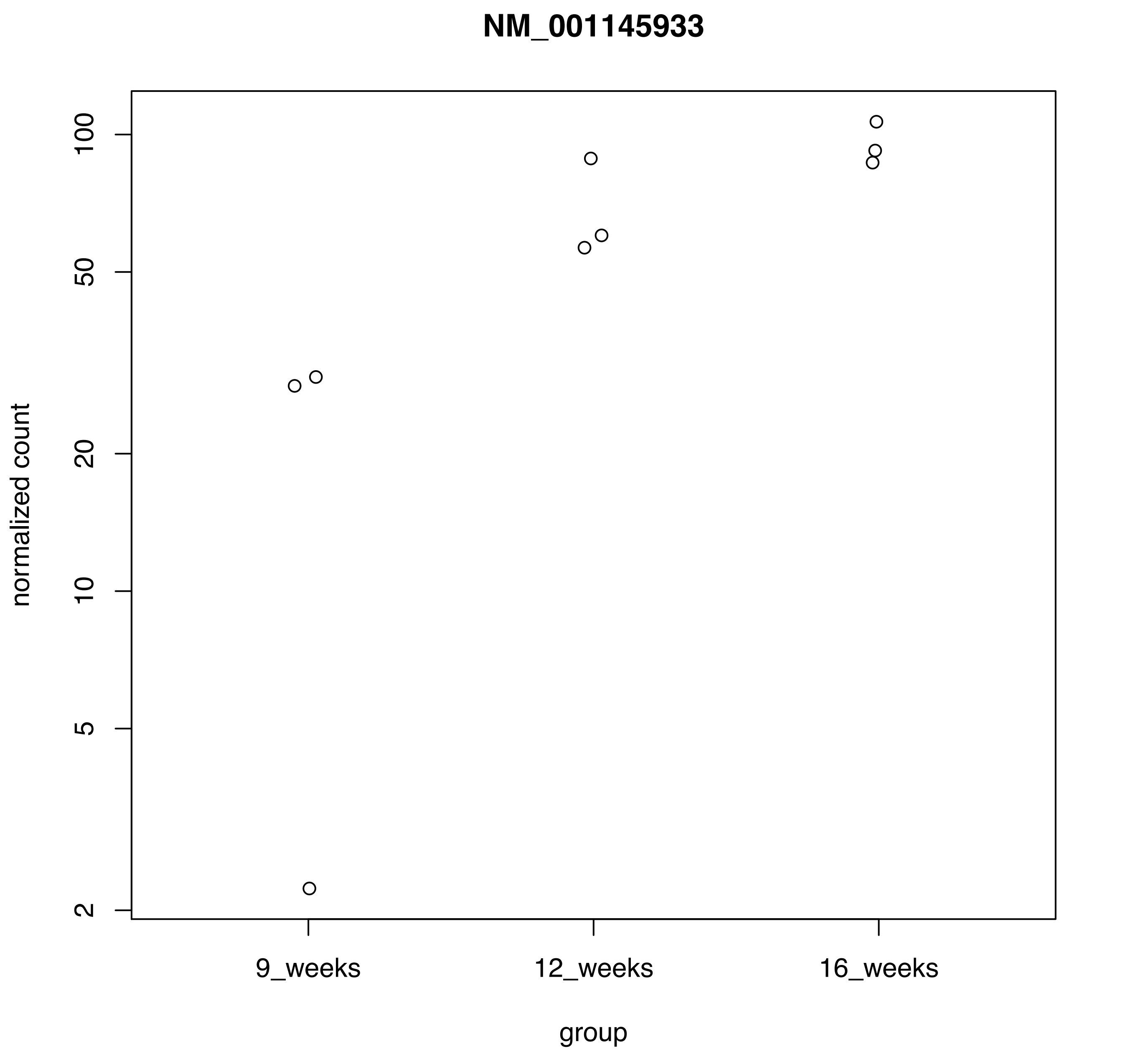

NM_001145933
normalized count
100
50
20
10
5
2
9_weeks
12_weeks
16_weeks
group

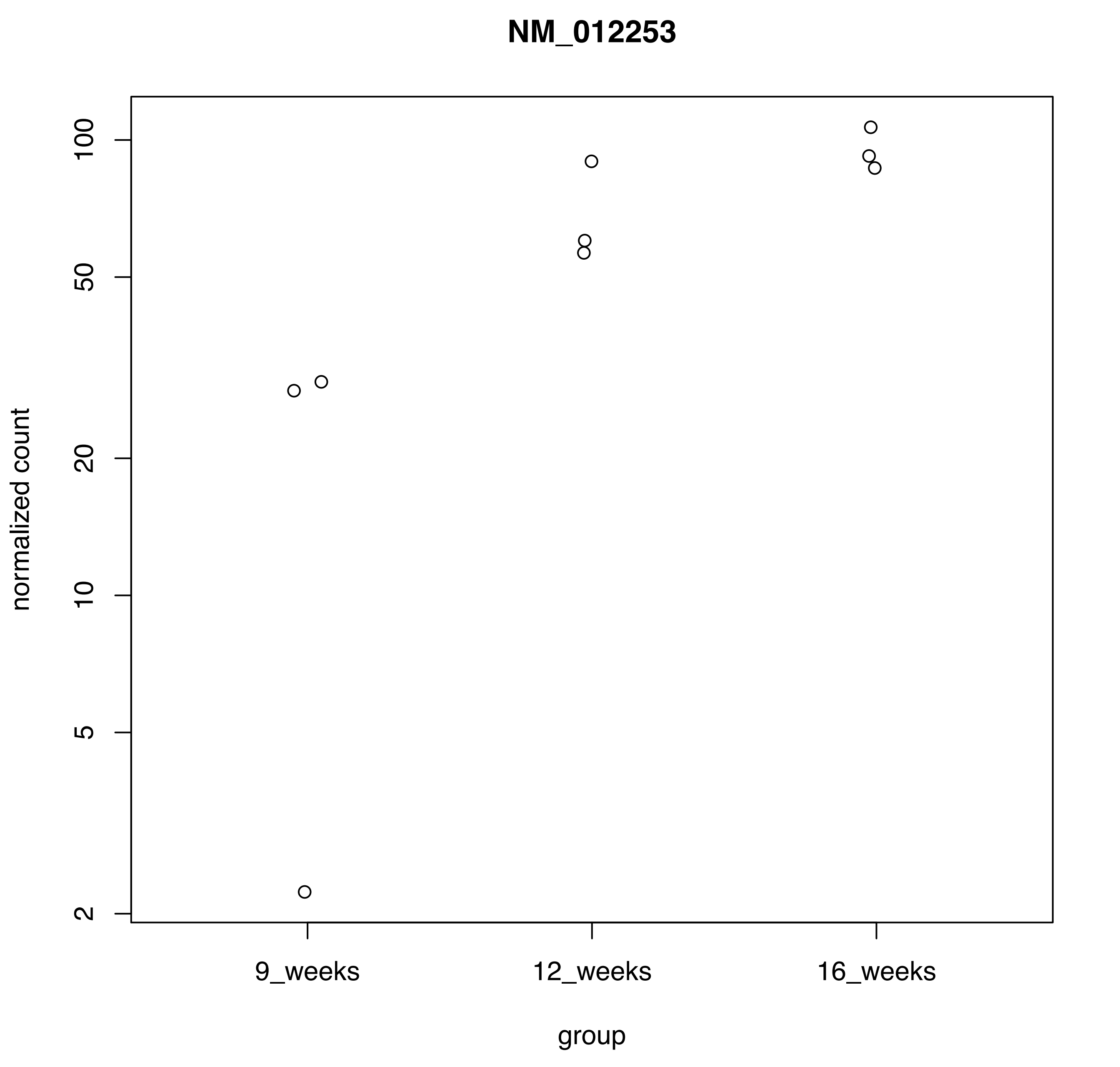
NM_012253
normalized count
100
50
20
10
5
2
9_weeks
12_weeks
16_weeks
group

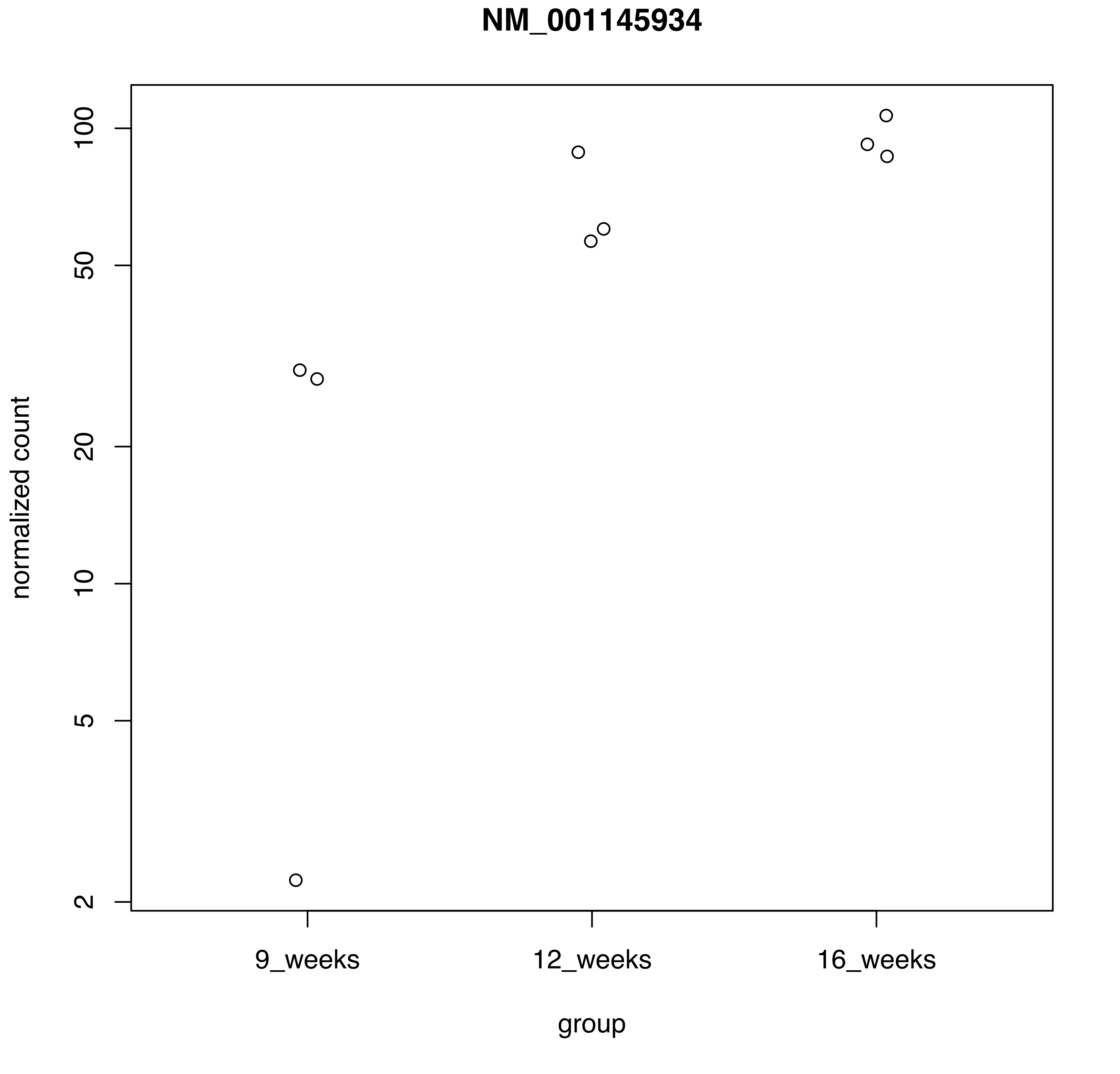
NM_001145934
normalized count
100
50
20
10
5
2
9_weeks
12_weeks
16_weeks
group

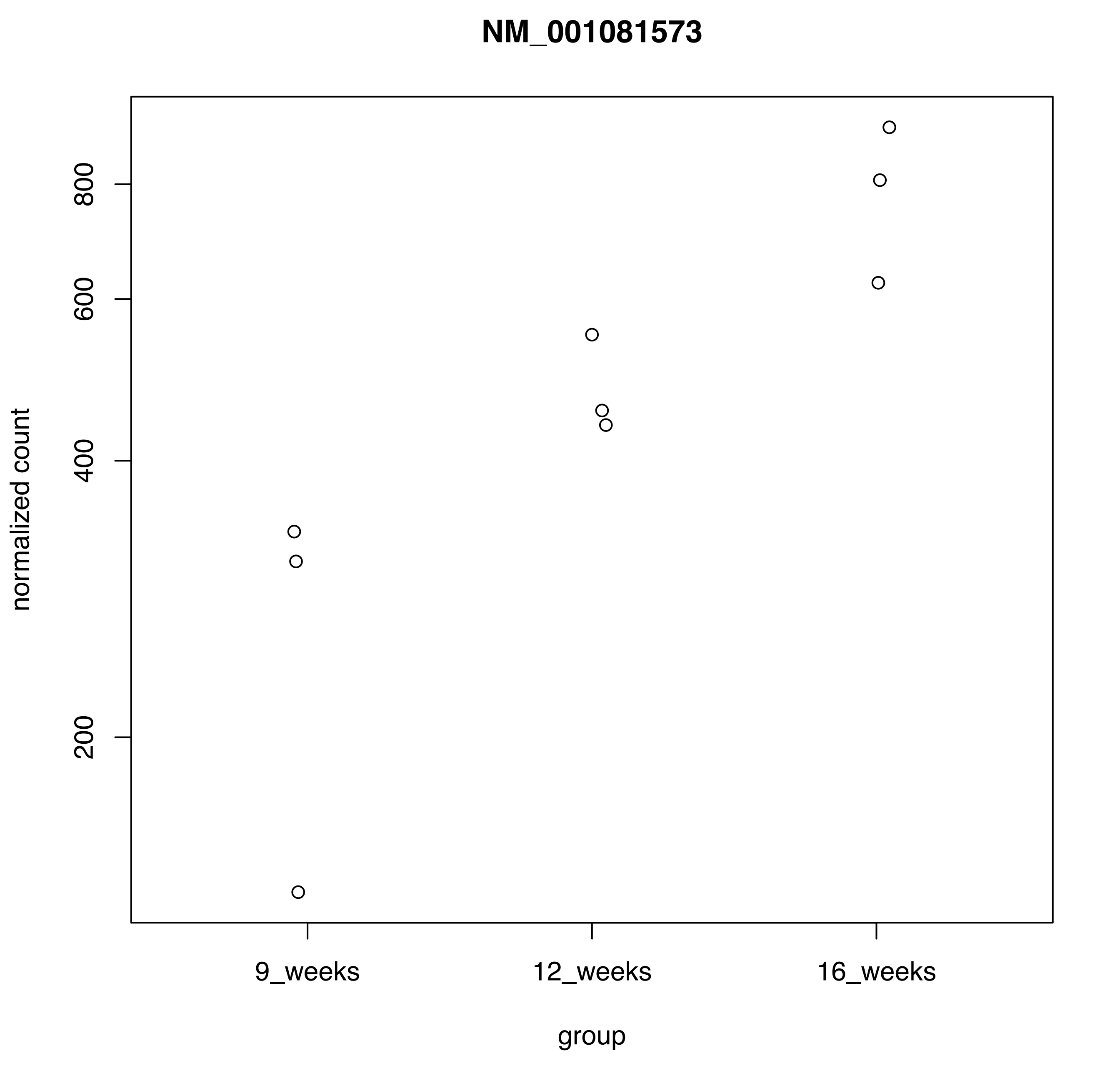
NM_001081573
normalized count
200
400
600
800
9_weeks
12_weeks
16_weeks
group

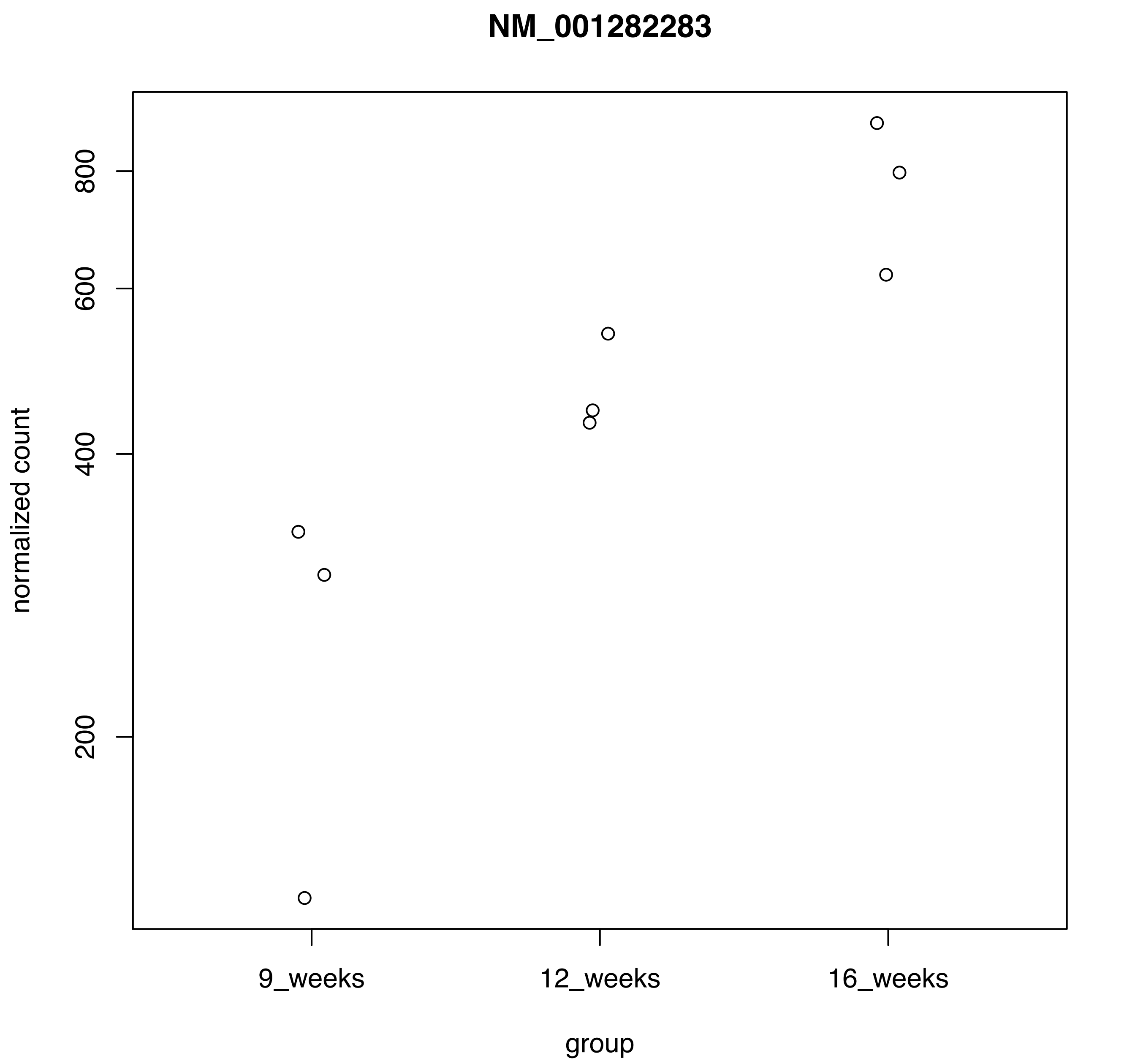
NM_001282283
normalized count
800
600
400
200
9_weeks
12_weeks
16_weeks
group

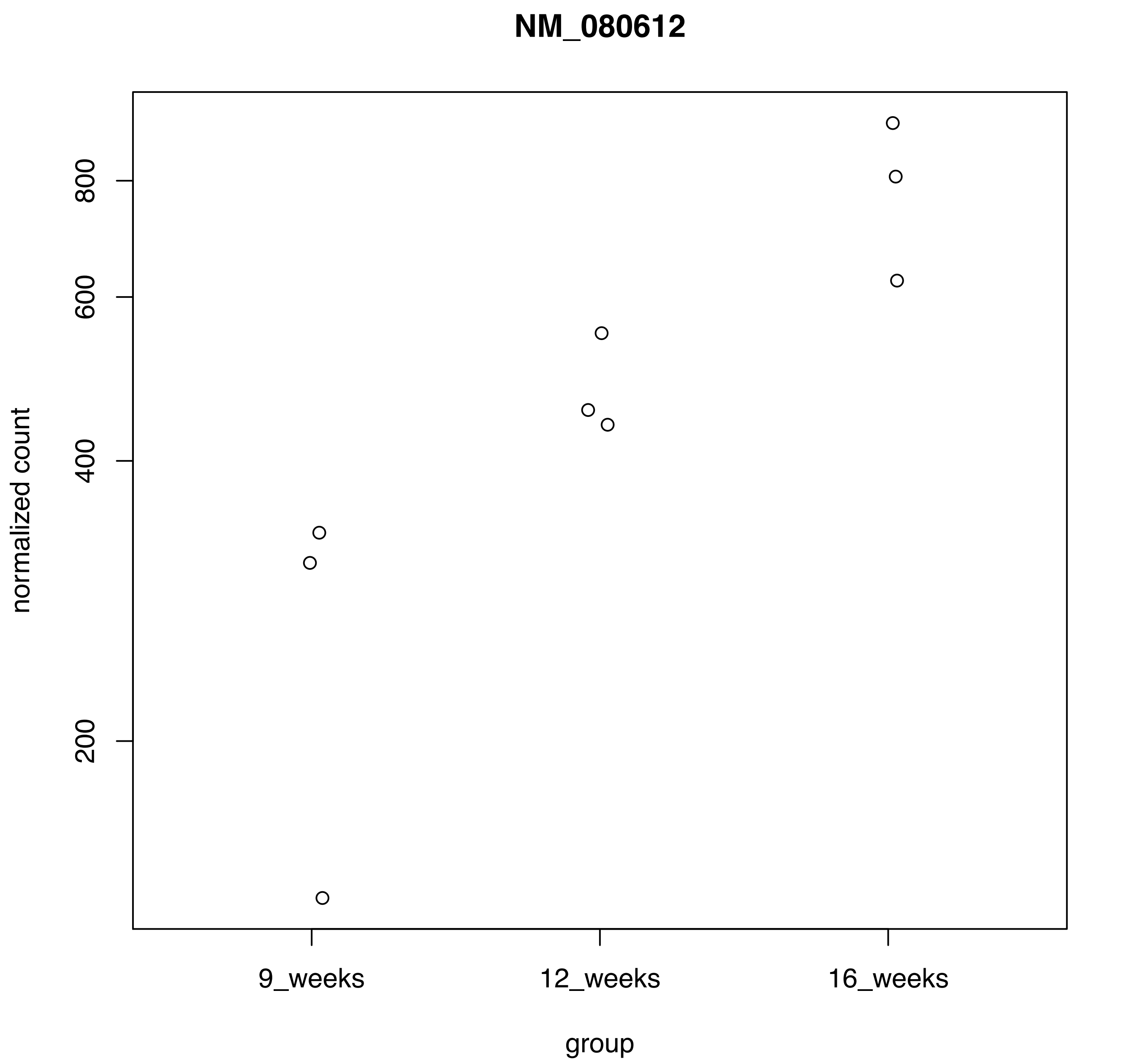
NM_080612
normalized count
200
400
600
800
9_weeks
12_weeks
16_weeks
group

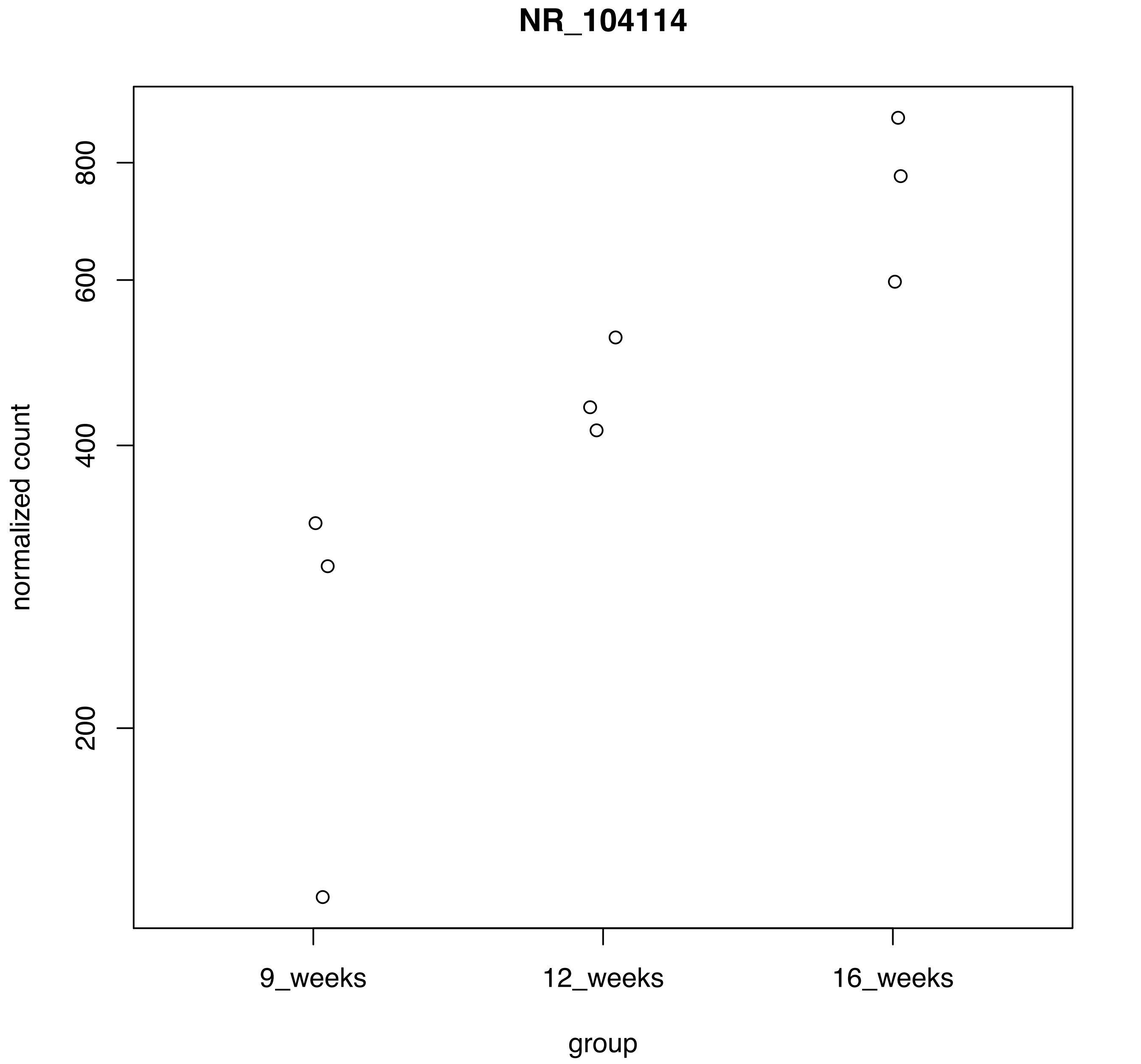

NR_104114
normalized count
800
600
400
200
9_weeks
12_weeks
16_weeks
group

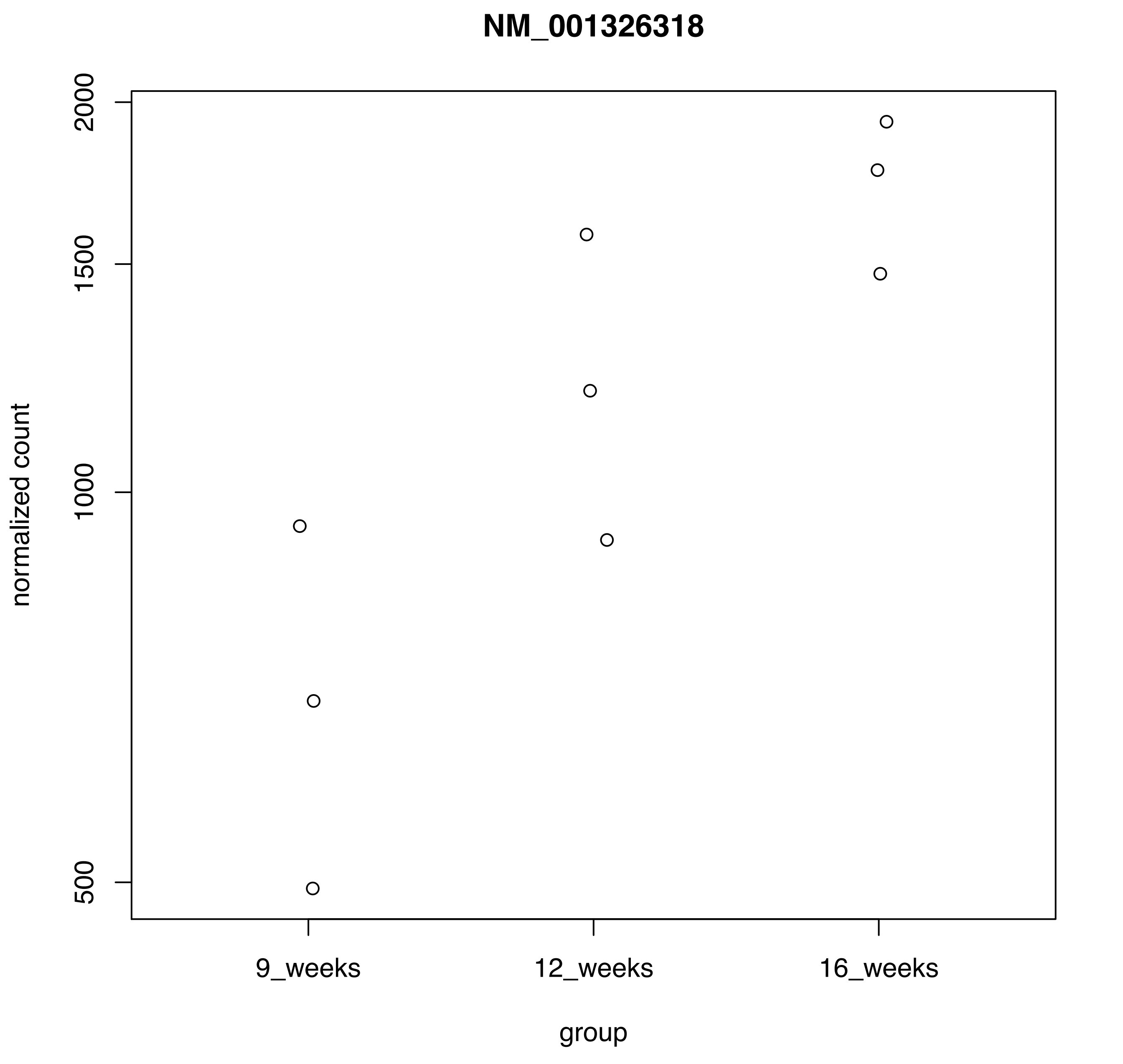

NM_001326318
normalized count
2000
1500
1000
500
9_weeks
12_weeks
16_weeks
group

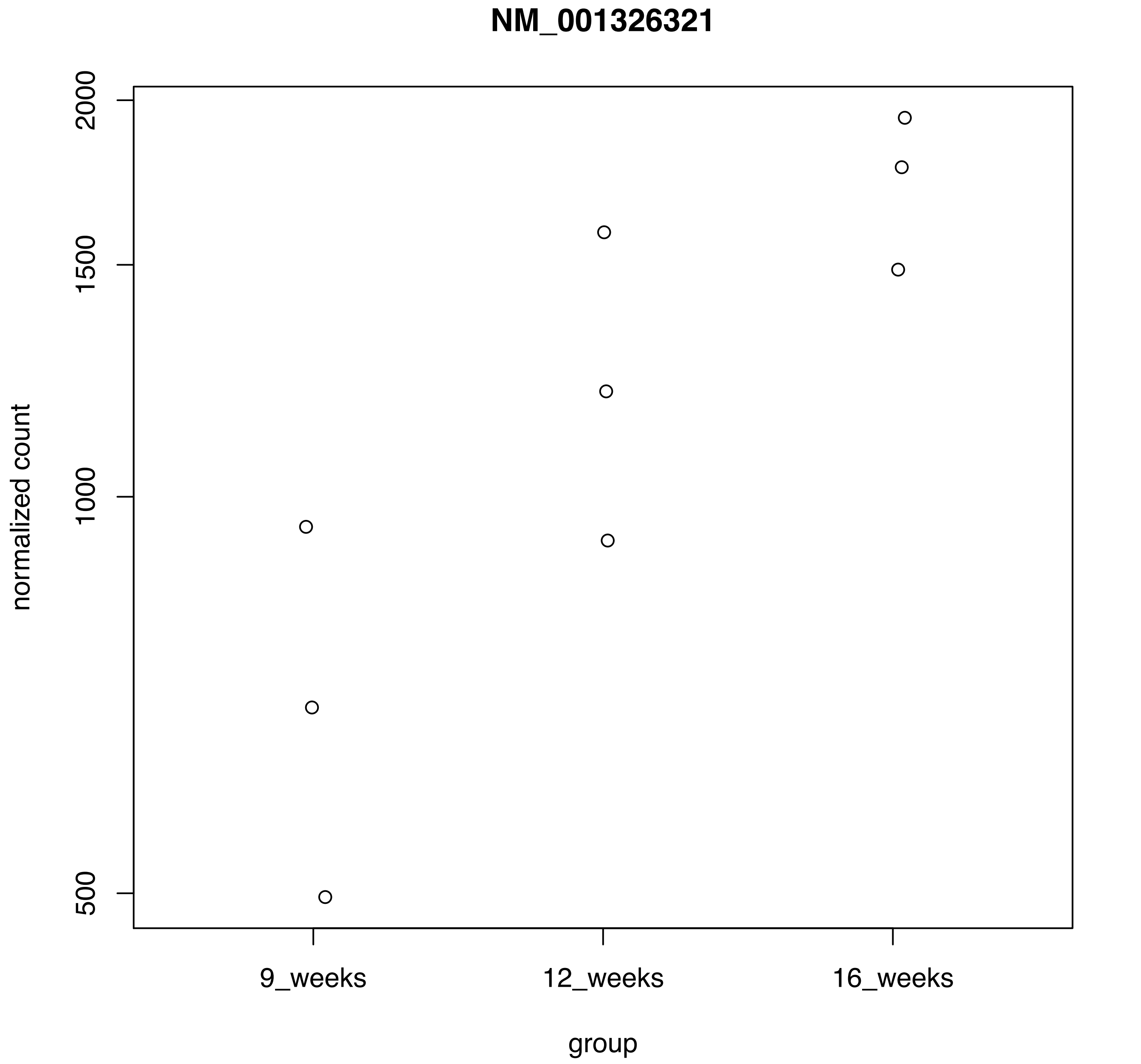
NM_001326321
normalized count
2000
1500
1000
500
9_weeks
12_weeks
16_weeks
group

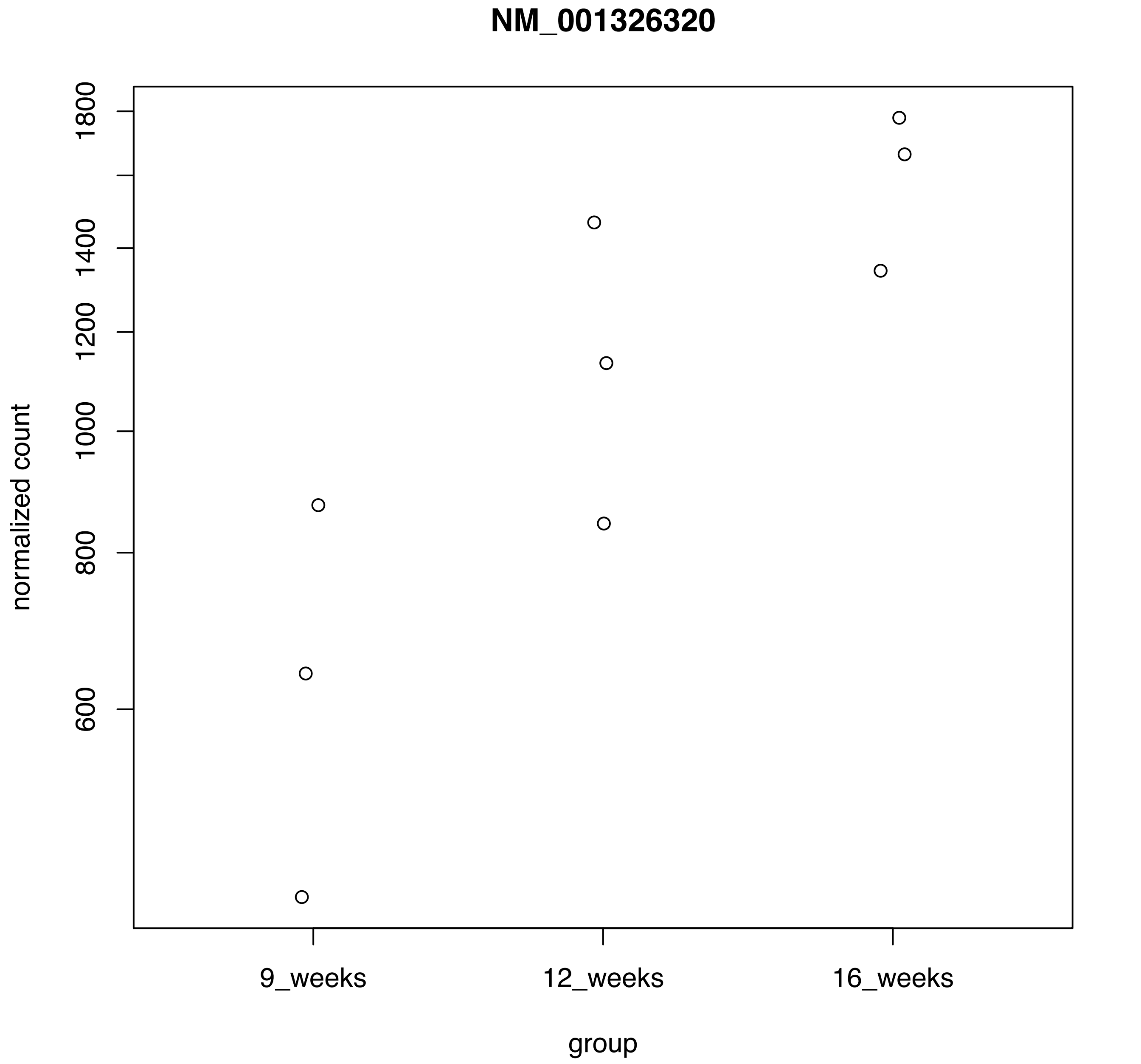
NM_001326320
normalized count
600
800
1000
1200
1400
1800
9_weeks
12_weeks
16_weeks
group

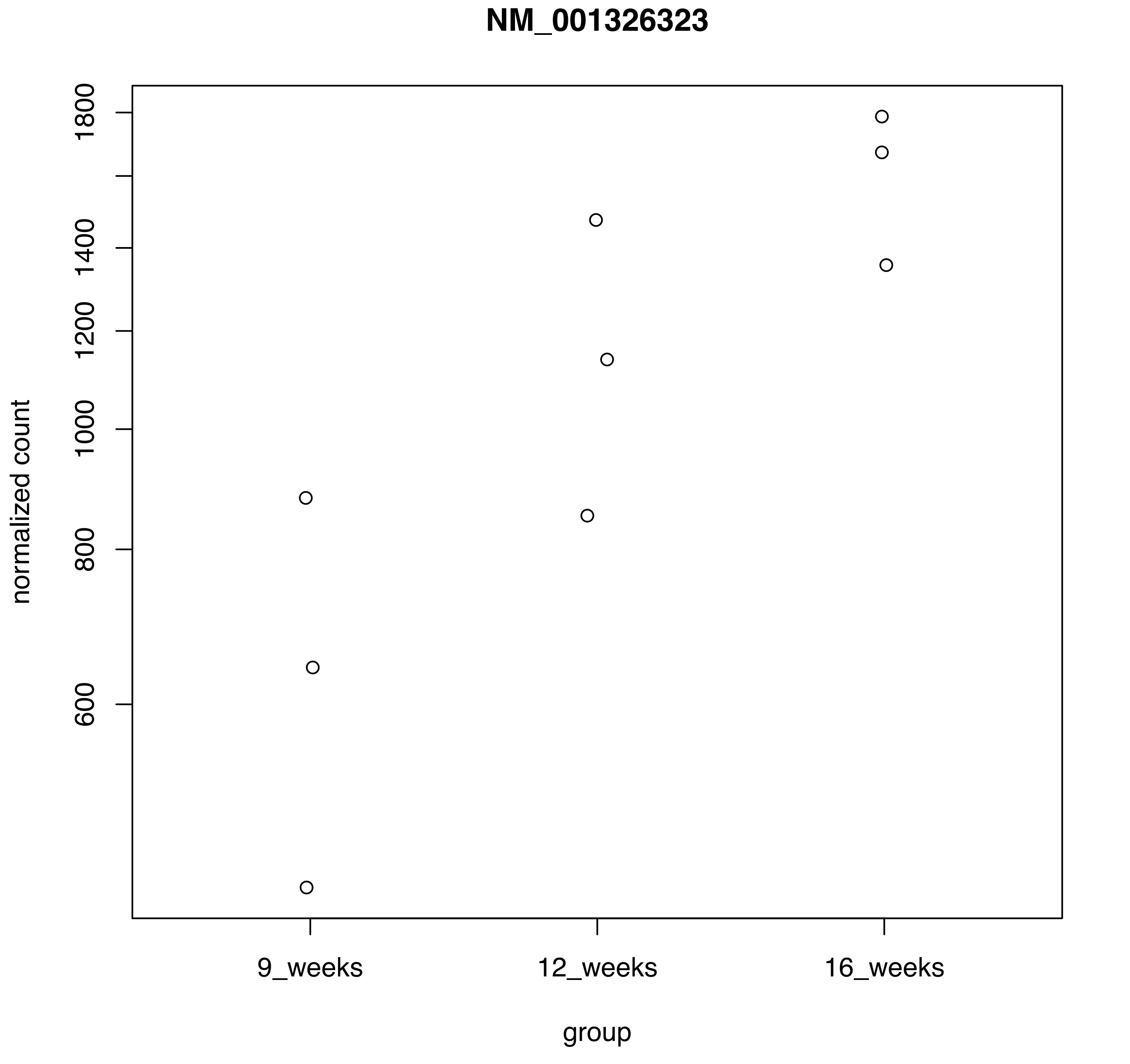

NM_001326323
normalized count
1800
1400
1200
1000
800
600
9_weeks
12_weeks
16_weeks
group

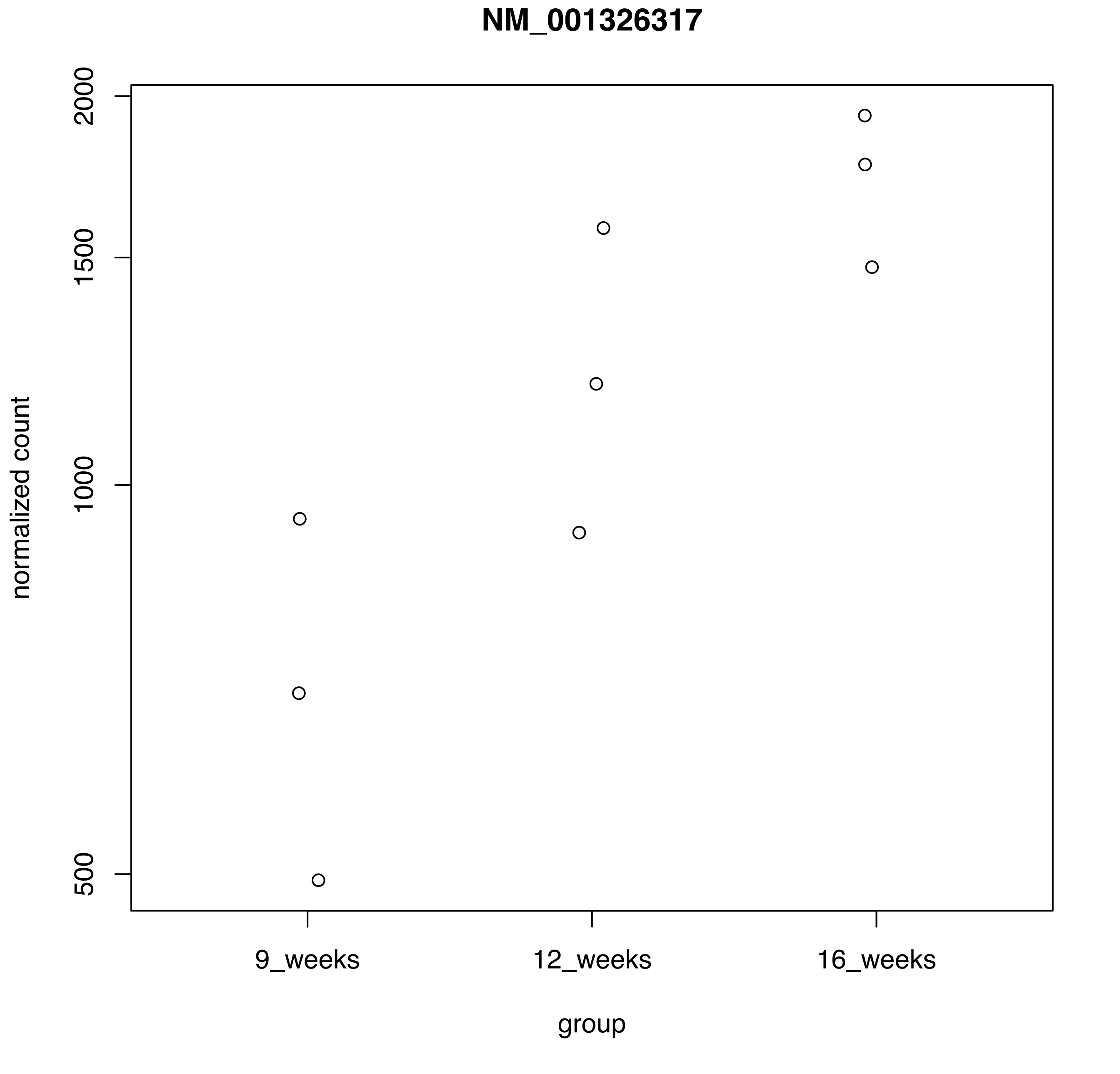

NM_001326317
normalized count
2000
1500
1000
500
9_weeks
12_weeks
16_weeks
group

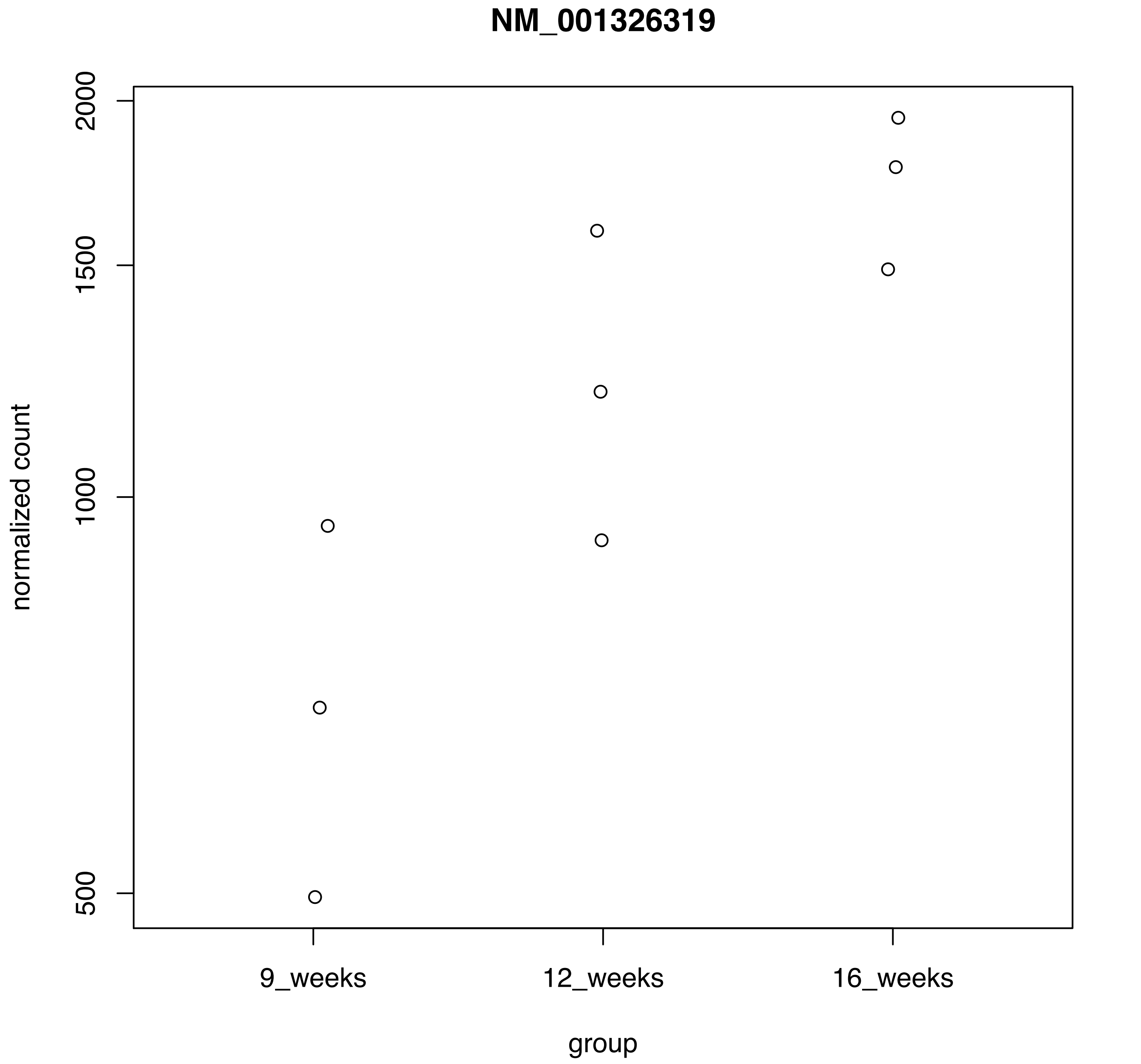
NM_001326319
normalized count
500
1000
1500
2000
9_weeks
12_weeks
16_weeks
group

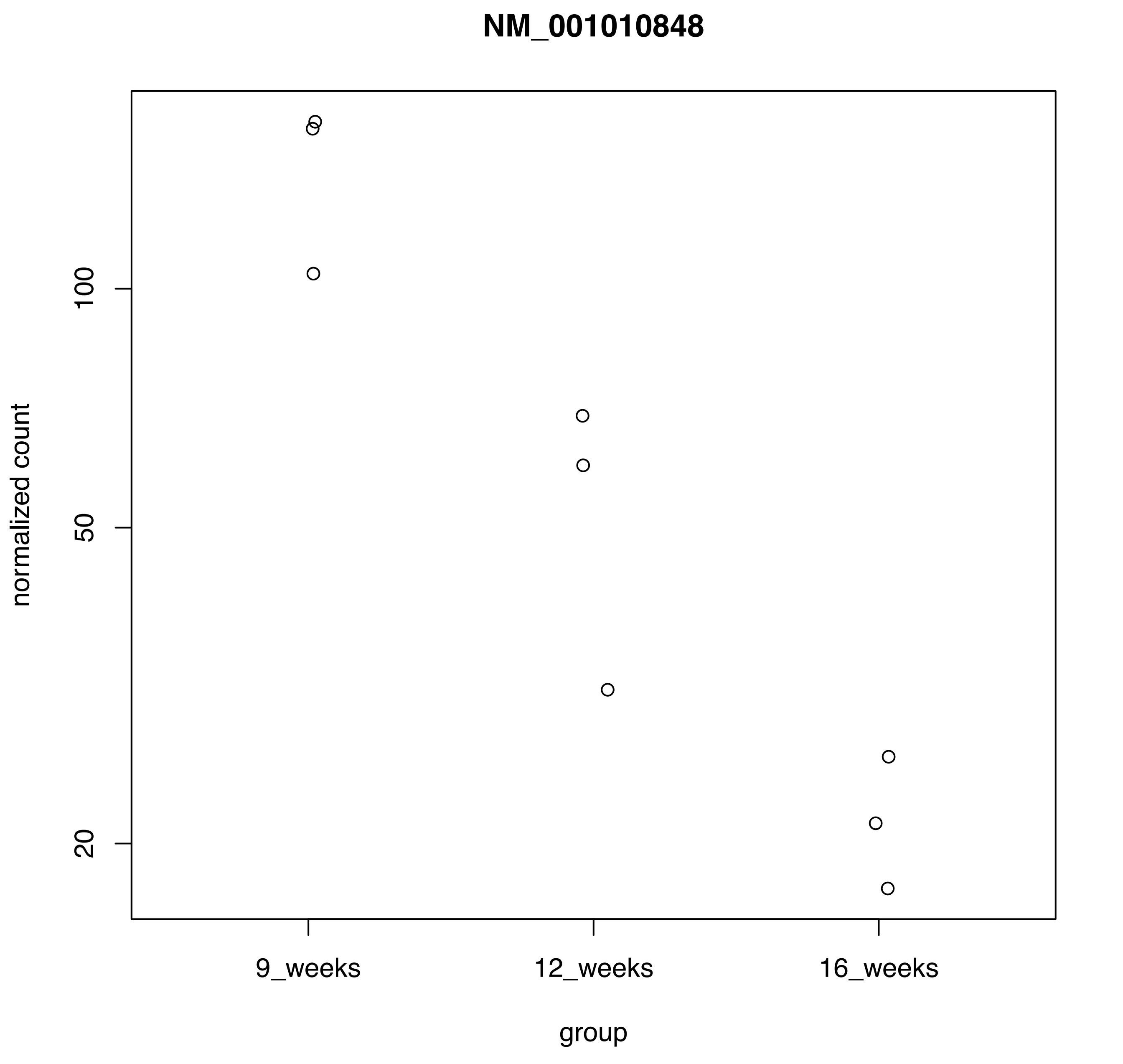

NM_001010848
normalized count
100
50
20
9_weeks
12_weeks
16_weeks
group

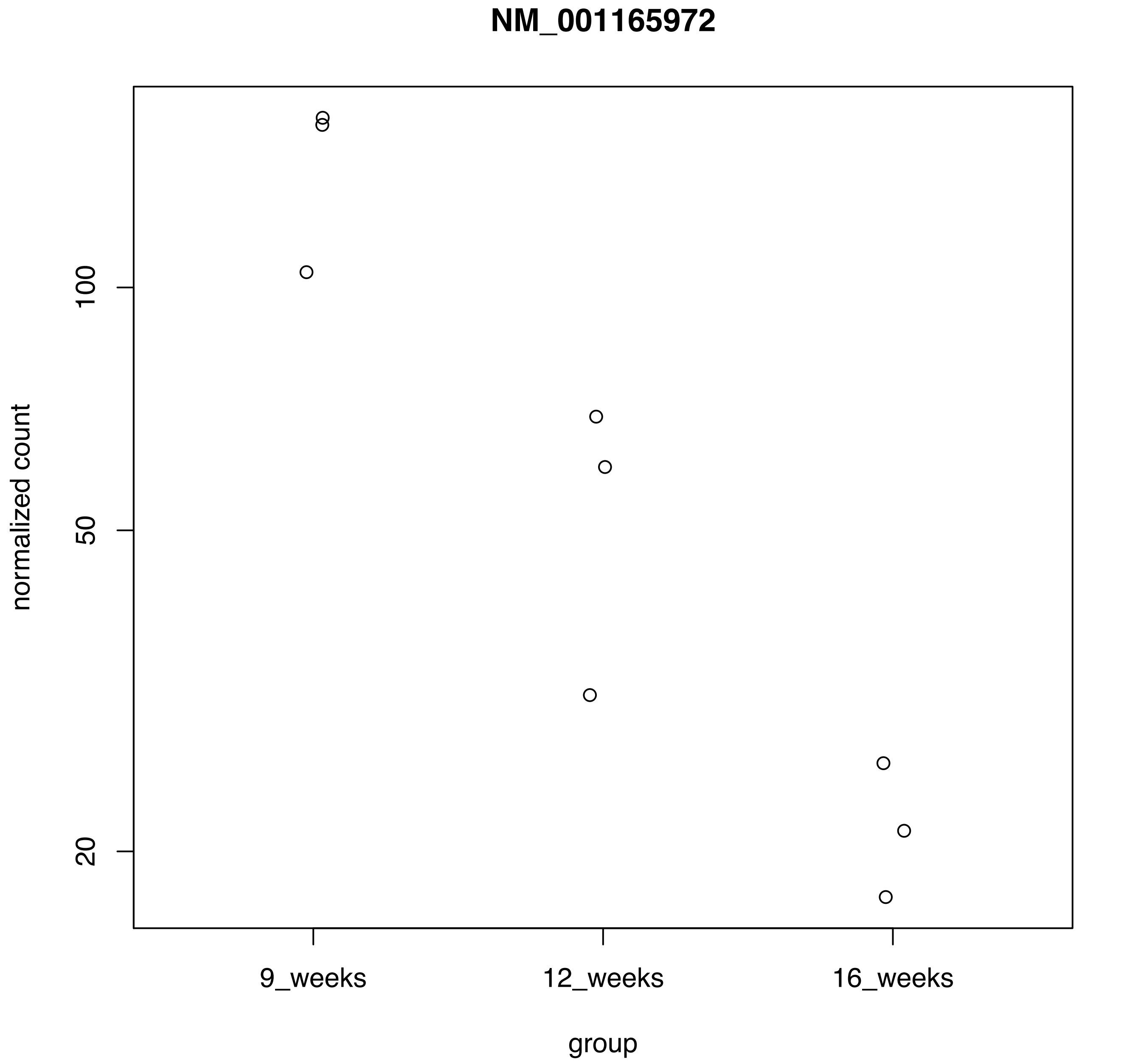
NM_001165972
normalized count
100
50
20
9_weeks
12_weeks
16_weeks
group

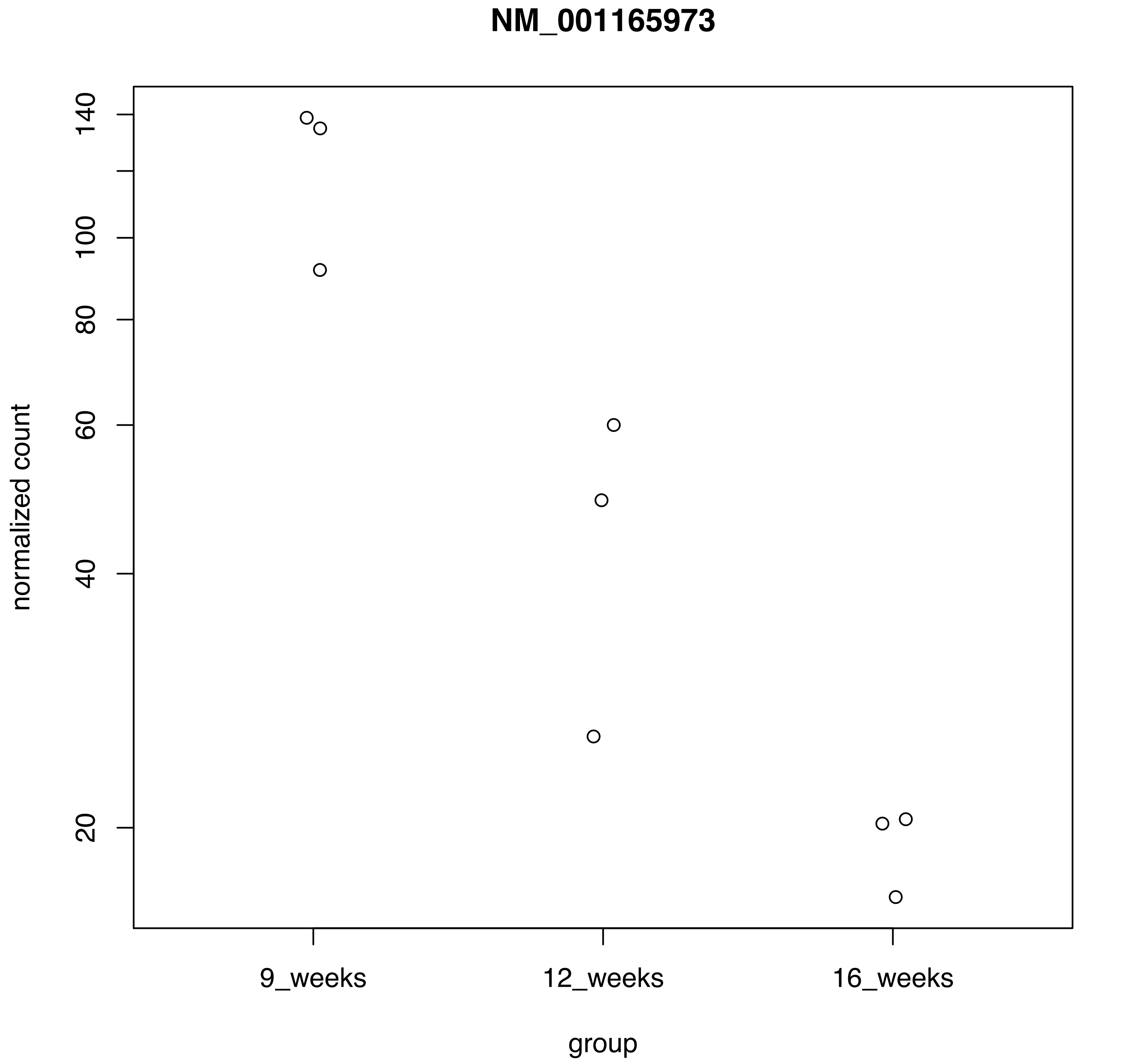
NM_001165973
normalized count
140
100
80
60
40
20
9_weeks
12_weeks
16_weeks
group

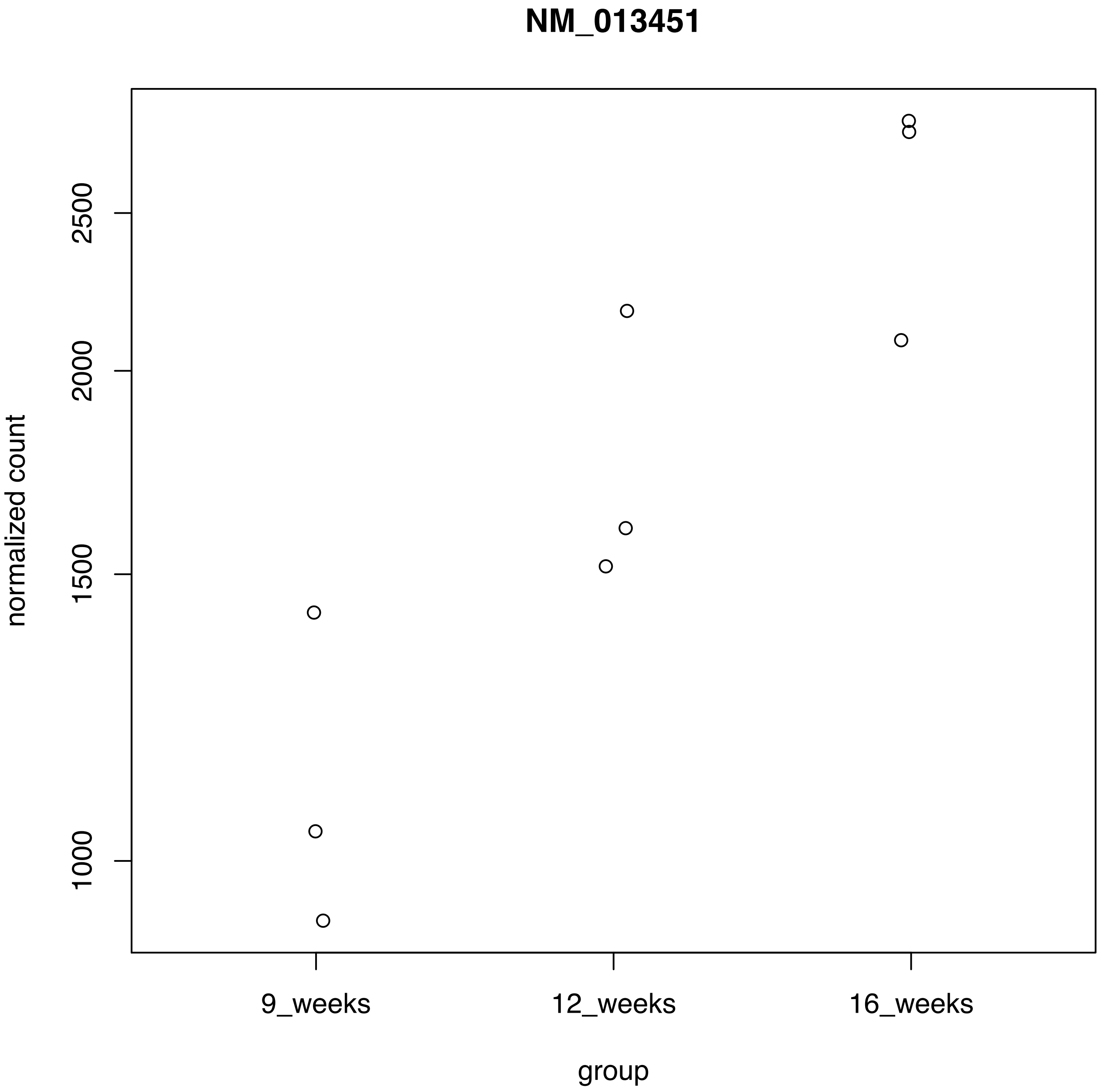
NM_013451
normalized count
1000
1500
2000
2500
9_weeks
12_weeks
16_weeks
group

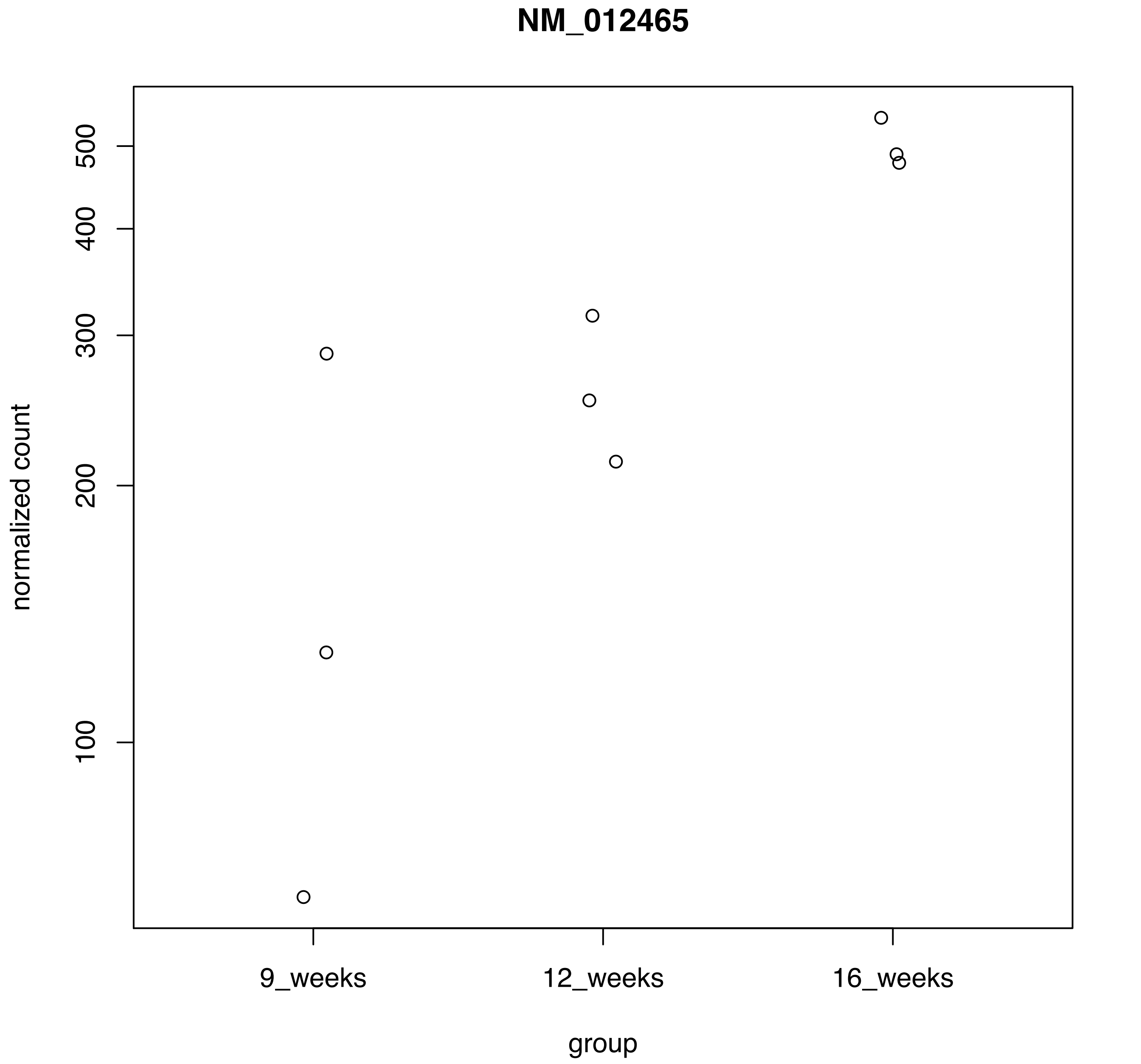
NM_012465
normalized count
100
200
300
400
500
group
9_weeks
12_weeks
16_weeks

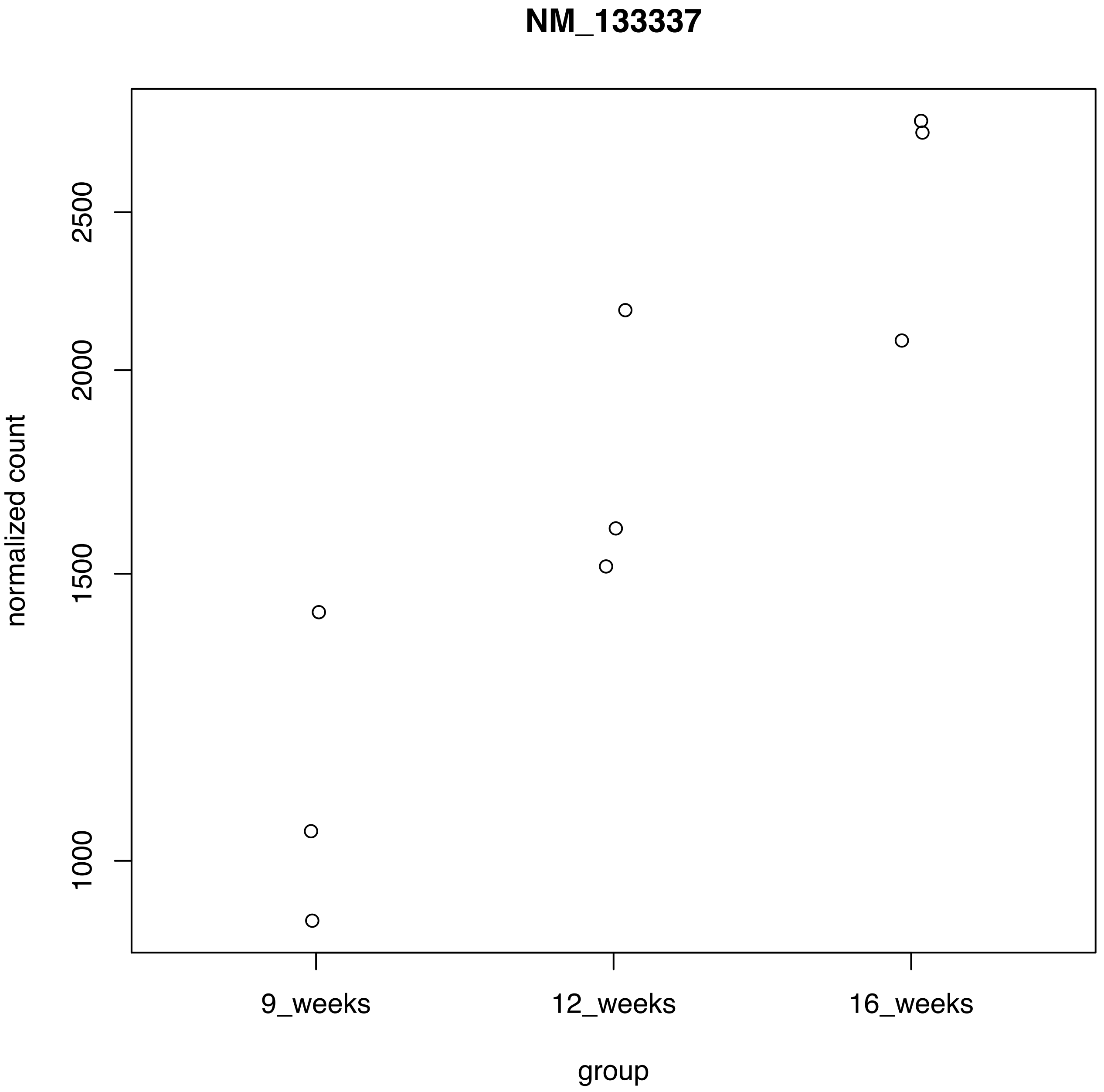

NM_133337
normalized count
1000
1500
2000
2500
9_weeks
12_weeks
16_weeks
group

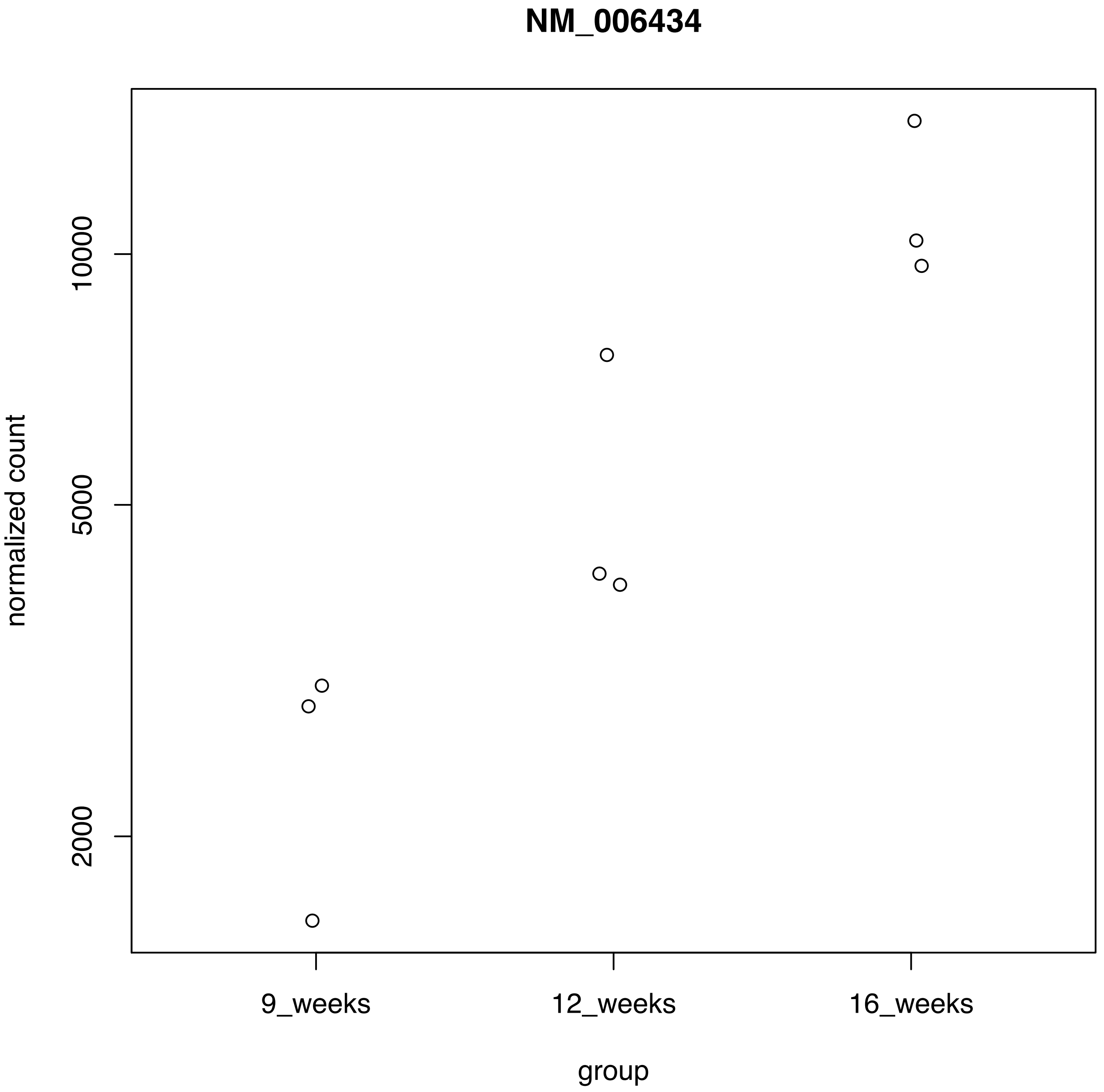

NM_006434
normalized count
10000
5000
2000
9_weeks
12_weeks
16_weeks
group

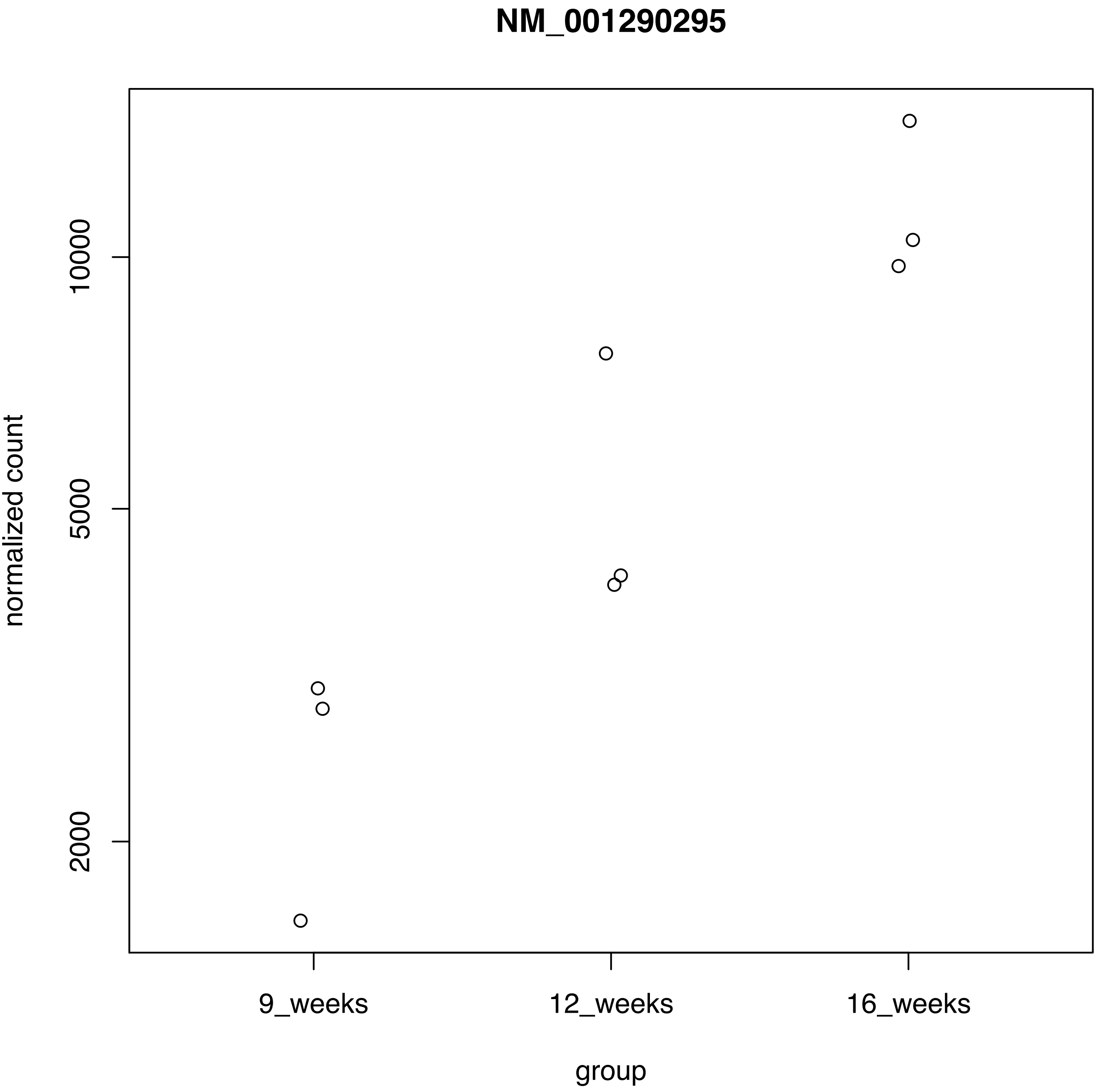
NM_001290295
normalized count
10000
5000
2000
9_weeks
12_weeks
16_weeks
group

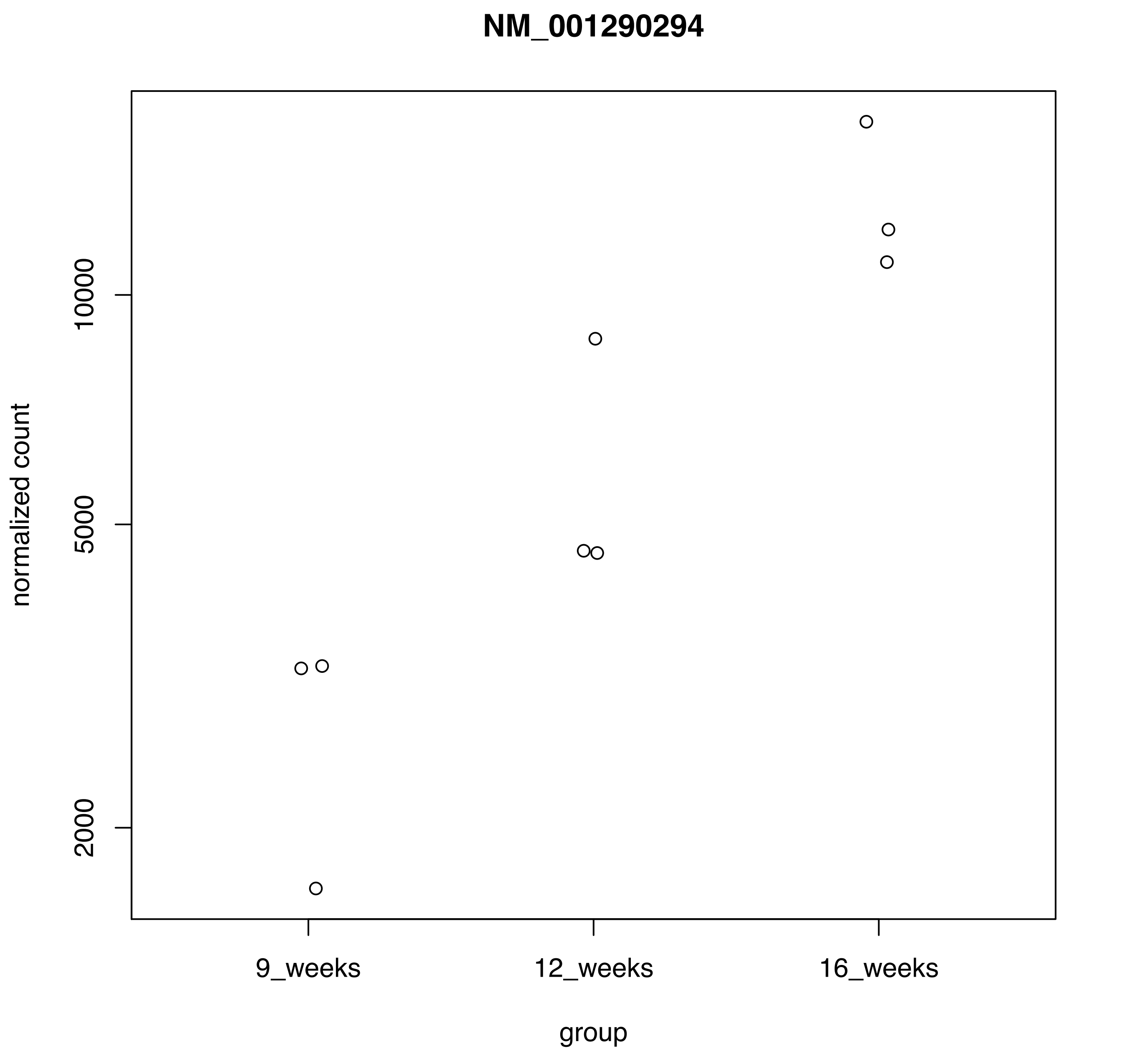
NM_001290294
normalized count
10000
5000
2000
9_weeks
12_weeks
16_weeks
group

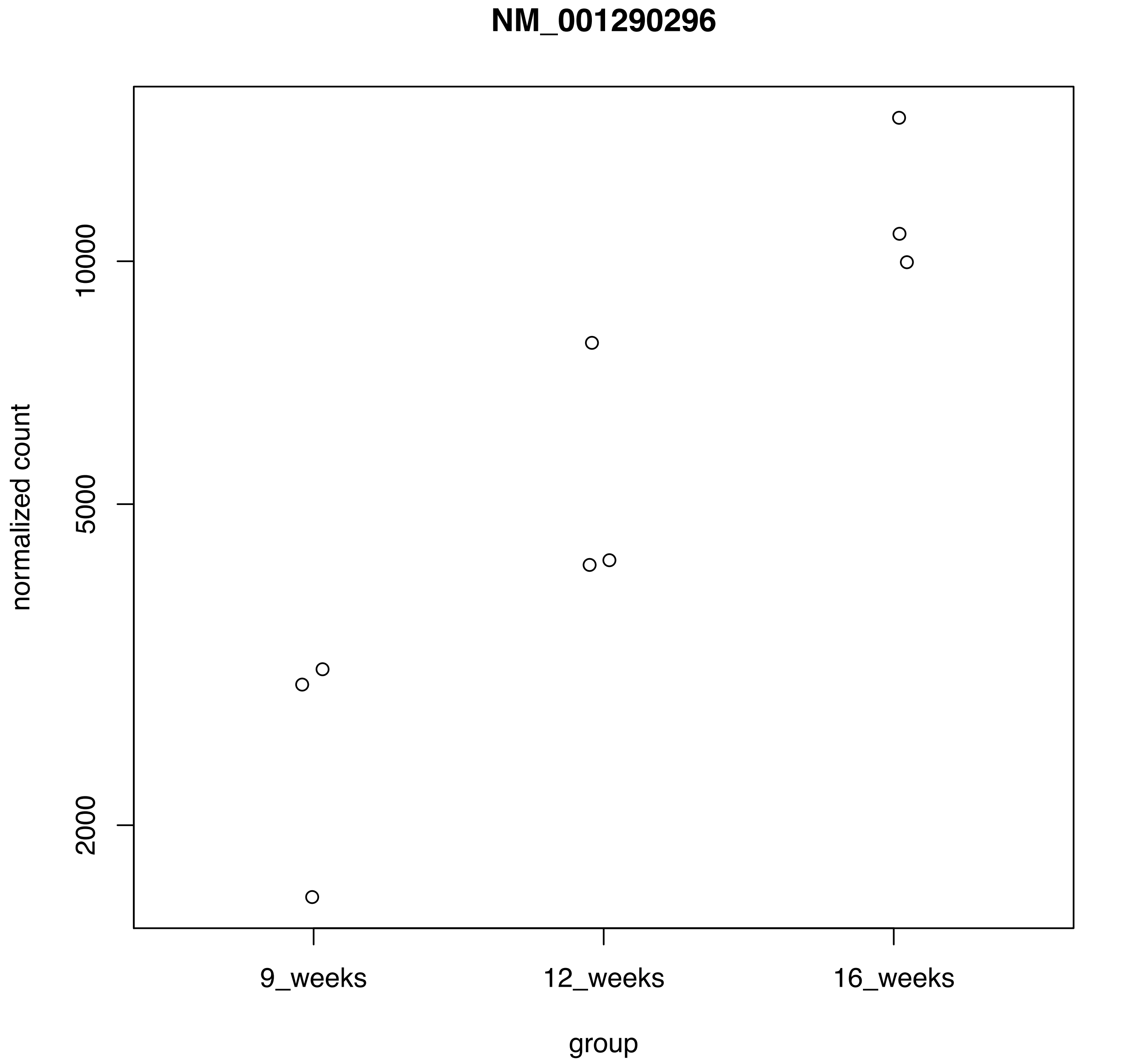

NM_001290296
normalized count
10000
5000
2000
9_weeks
12_weeks
16_weeks
group

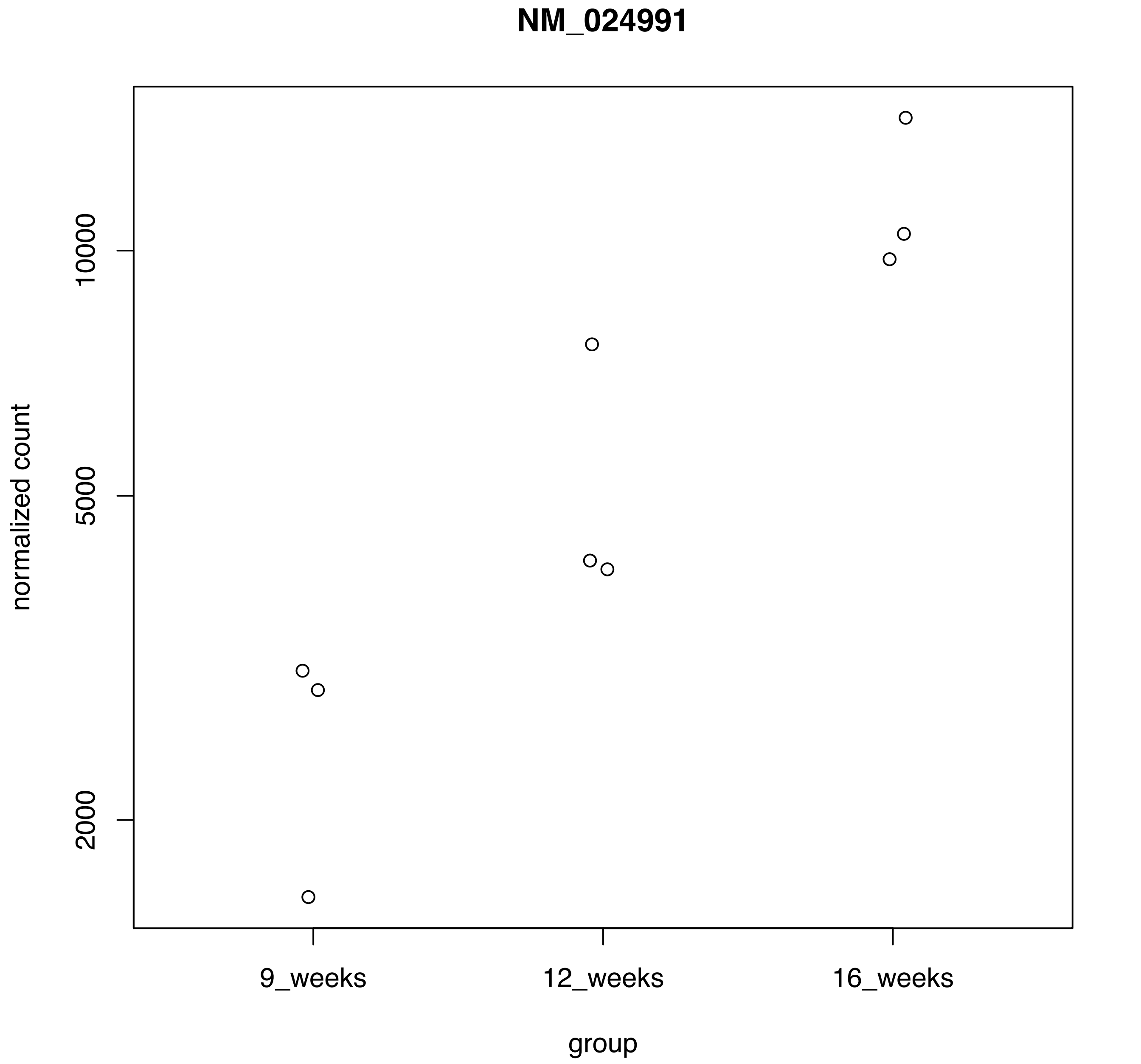
NM_024991
normalized count
10000
5000
2000
9_weeks
12_weeks
16_weeks
group

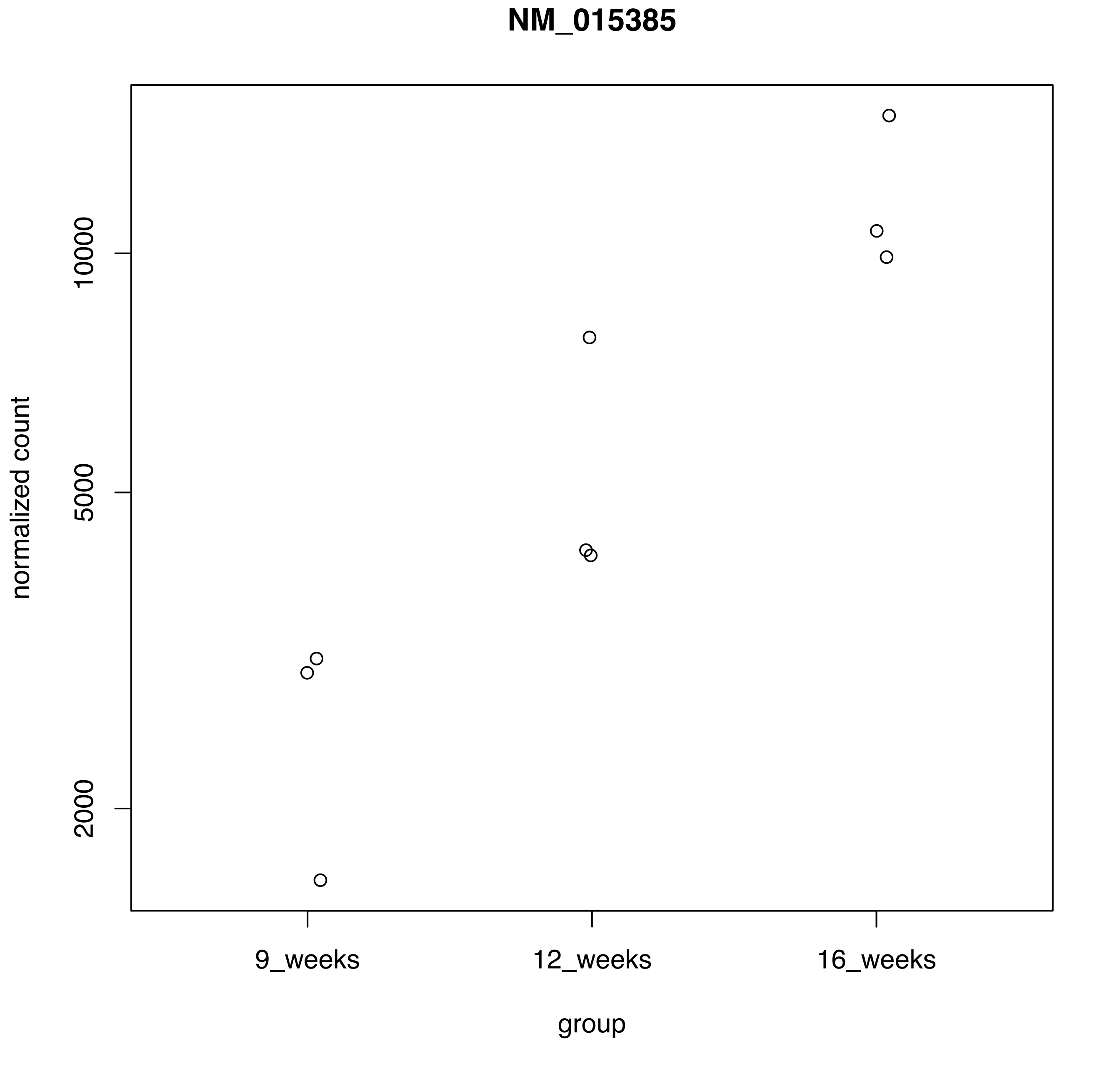

NM_015385
normalized count
10000
5000
2000
9_weeks
12_weeks
16_weeks
group

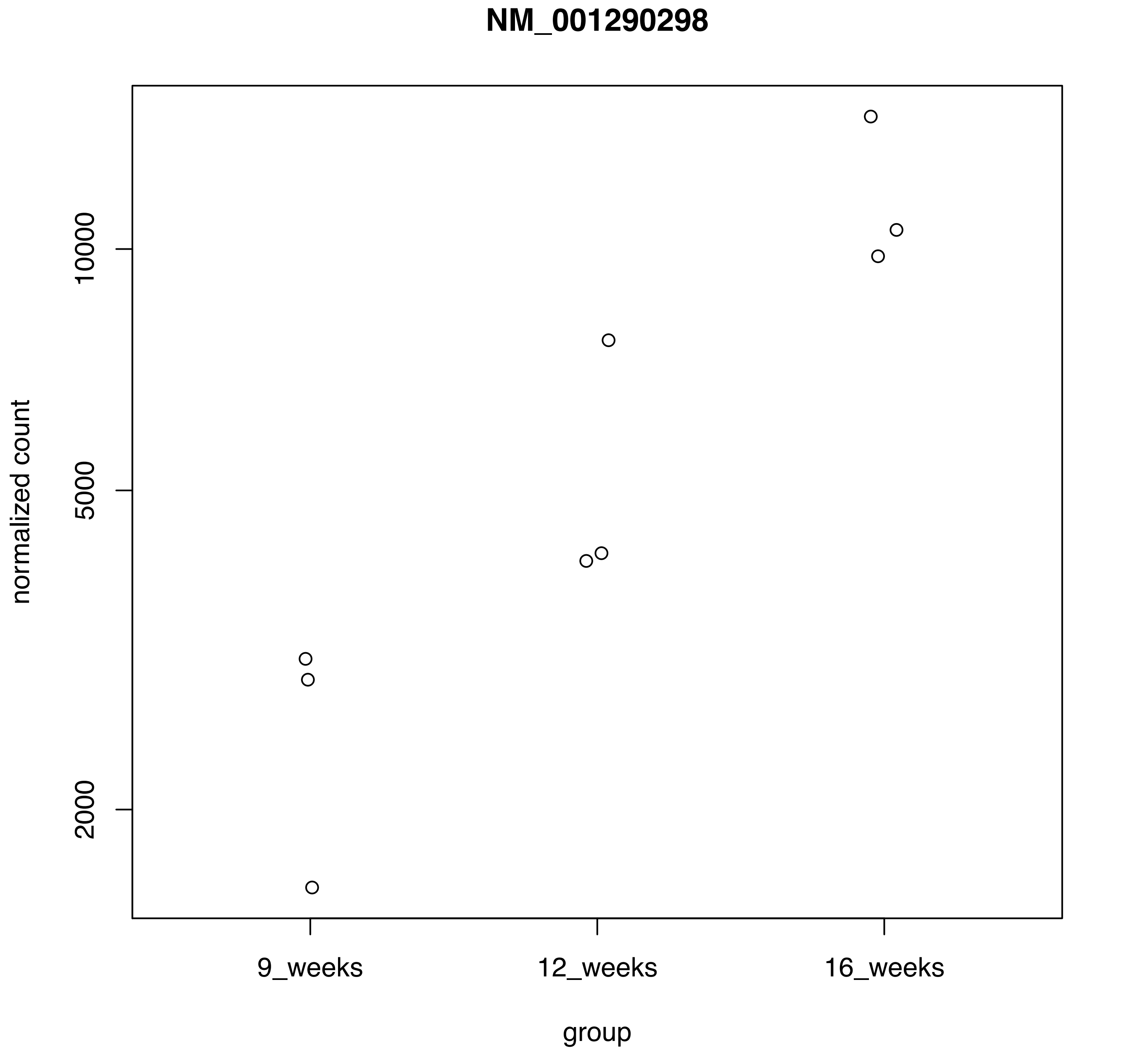

NM_001290298
normalized count
10000
5000
2000
9_weeks
12_weeks
16_weeks
group

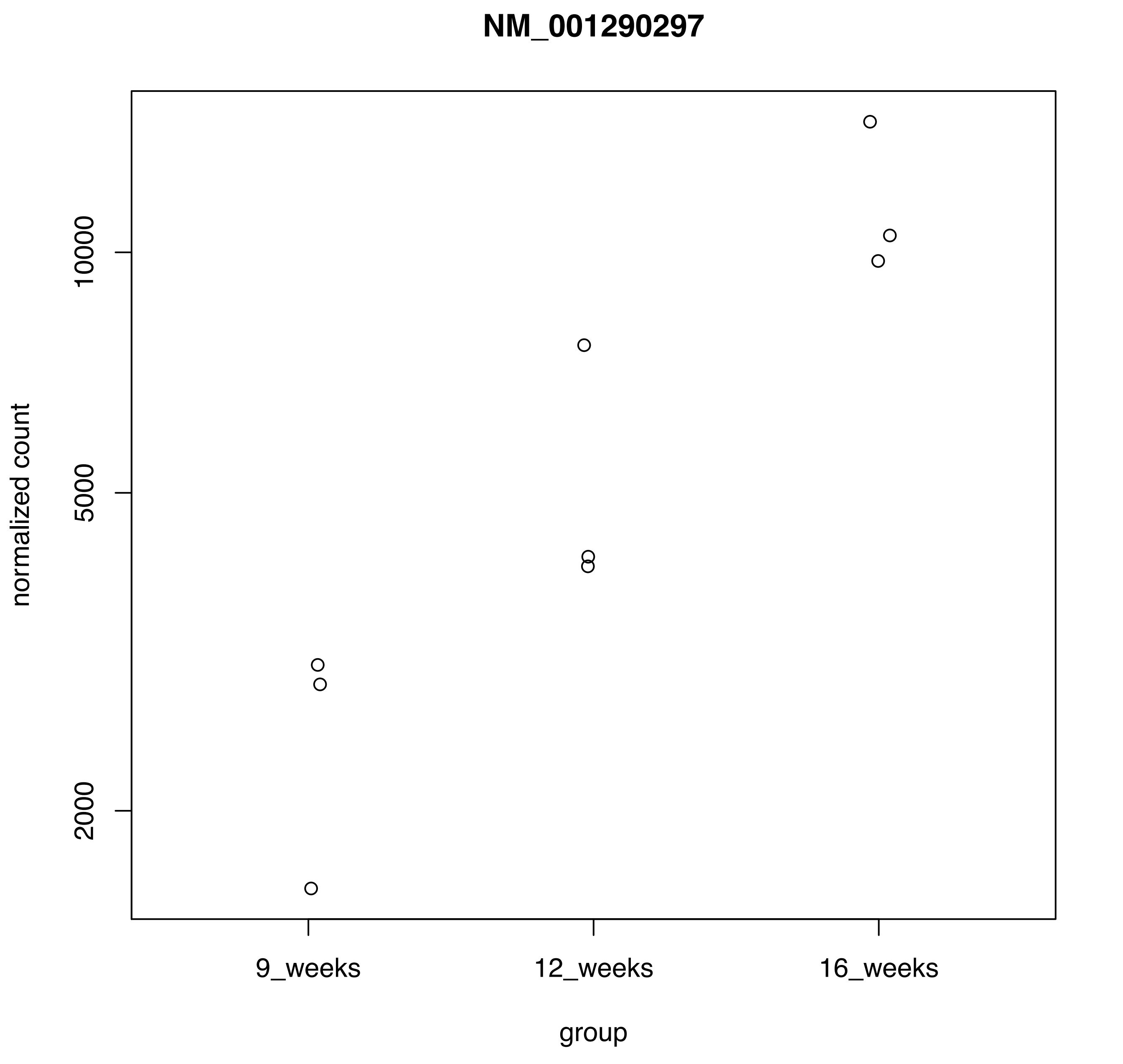
NM_001290297
normalized count
10000
5000
2000
9_weeks
12_weeks
16_weeks
group

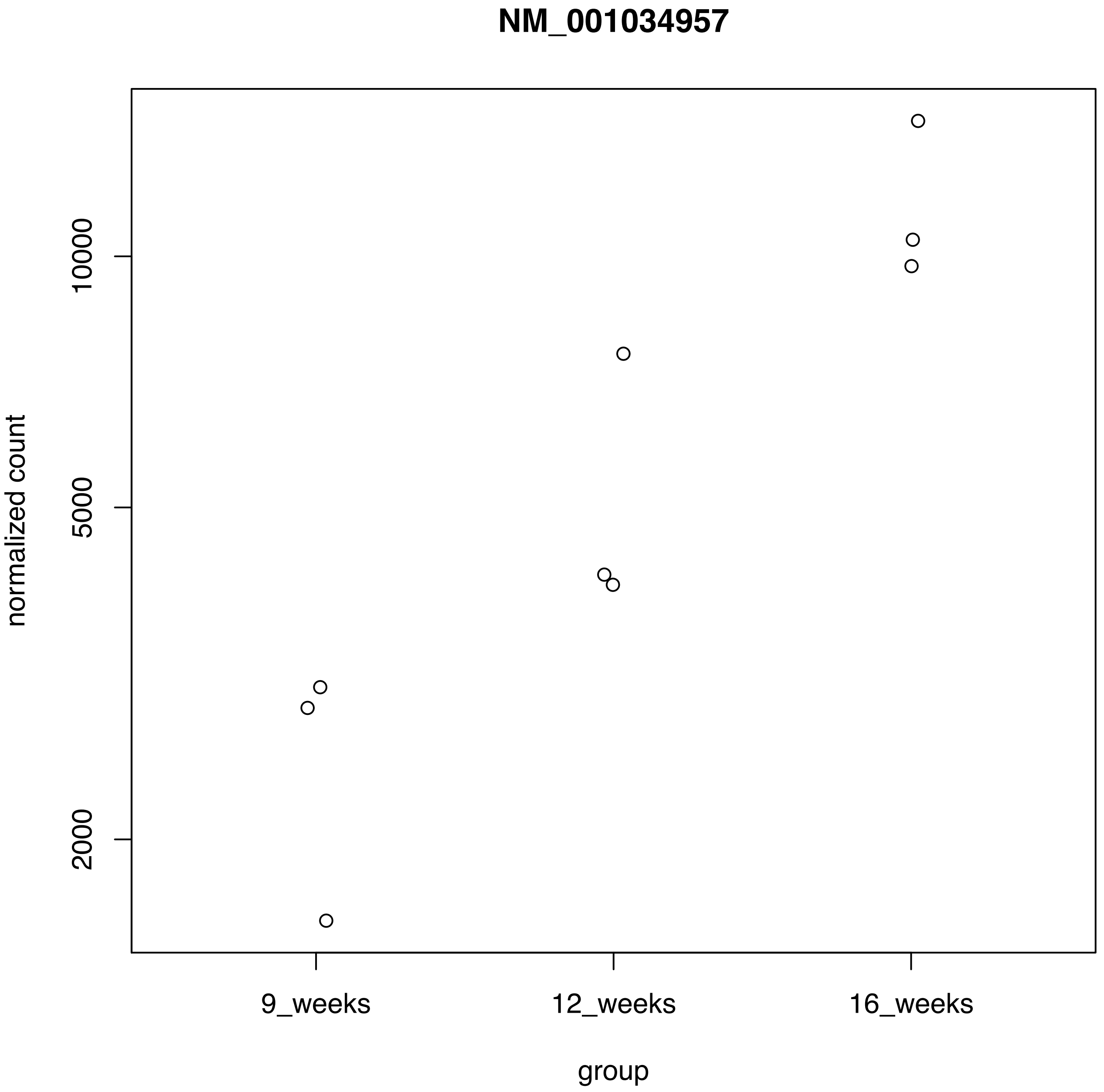
NM_001034957
normalized count
10000
5000
2000
9_weeks
12_weeks
16_weeks
group

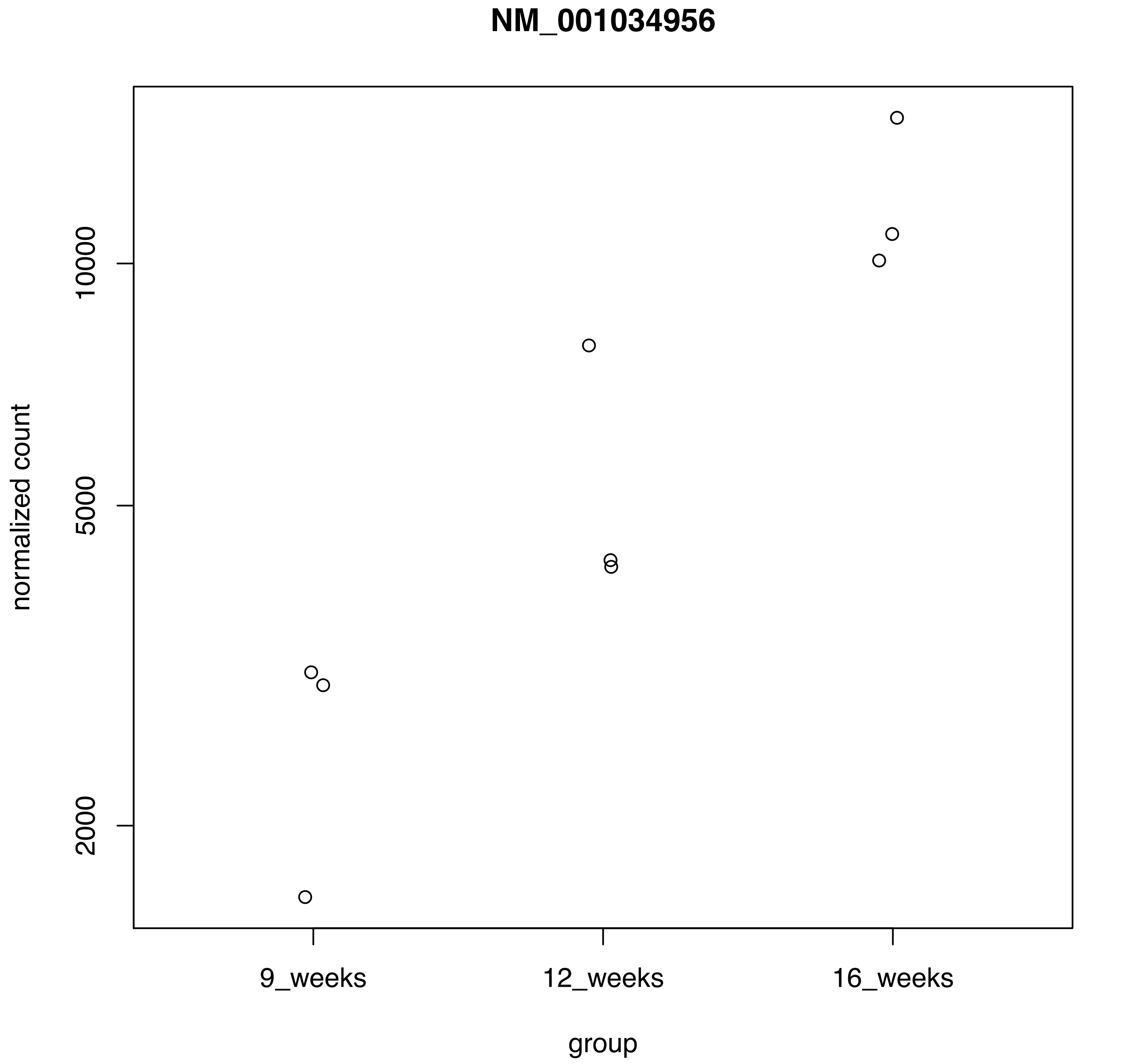

NM_001034956
normalized count
10000
5000
2000
9_weeks
12_weeks
16_weeks
group

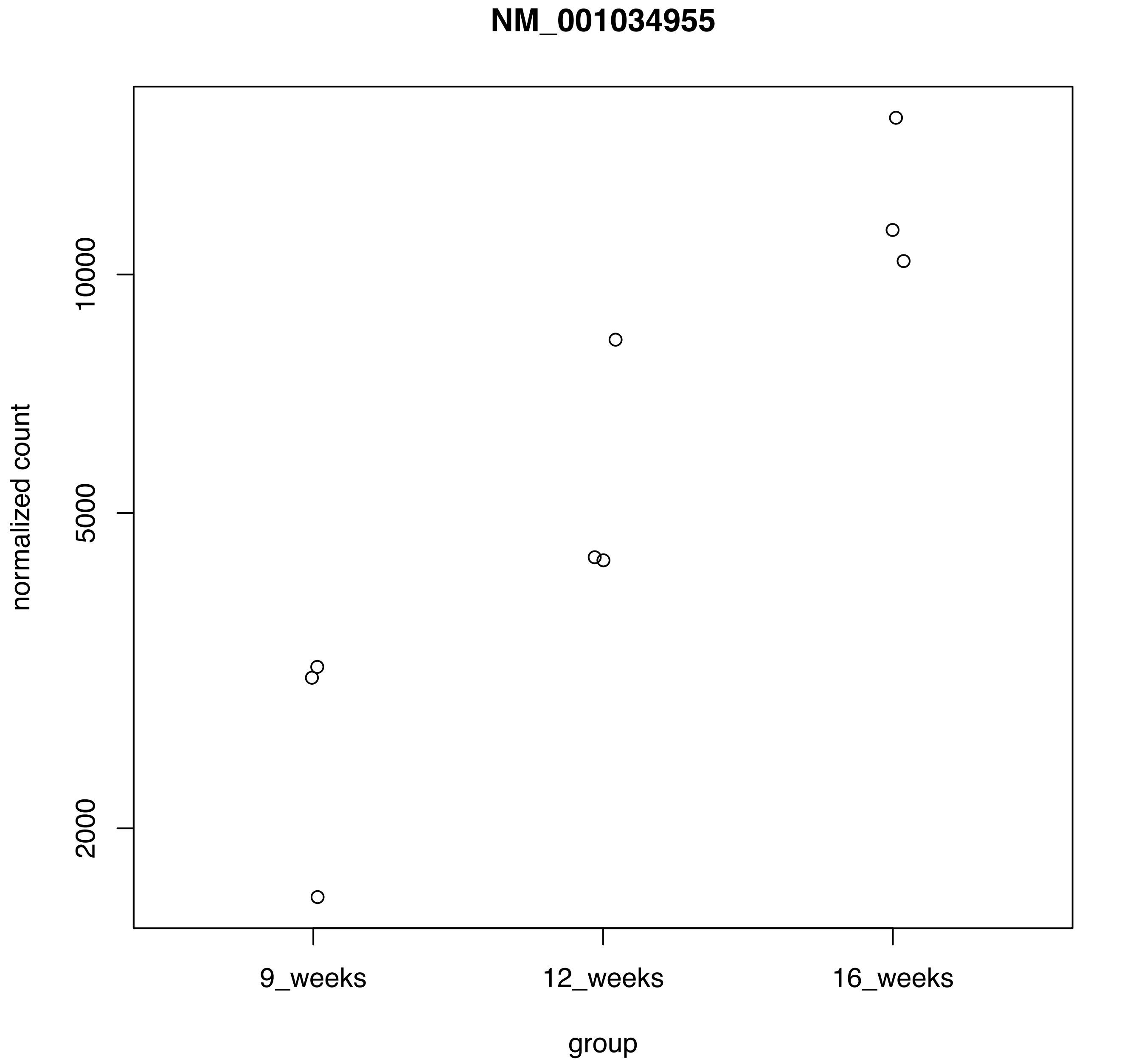
NM_001034955
normalized count
10000
5000
2000
9_weeks
12_weeks
16_weeks
group

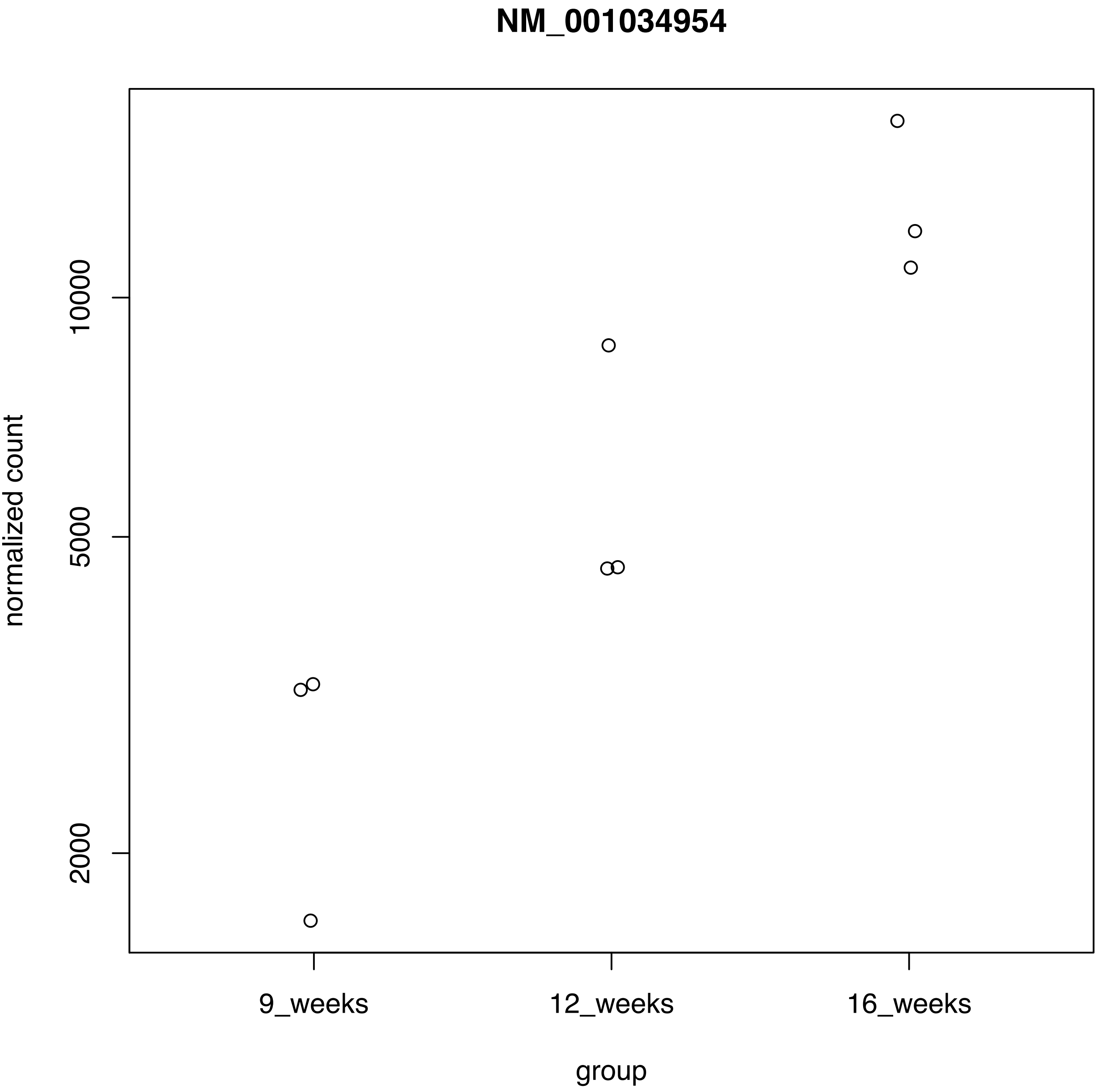
NM_001034954
normalized count
10000
5000
2000
9_weeks
12_weeks
16_weeks
group

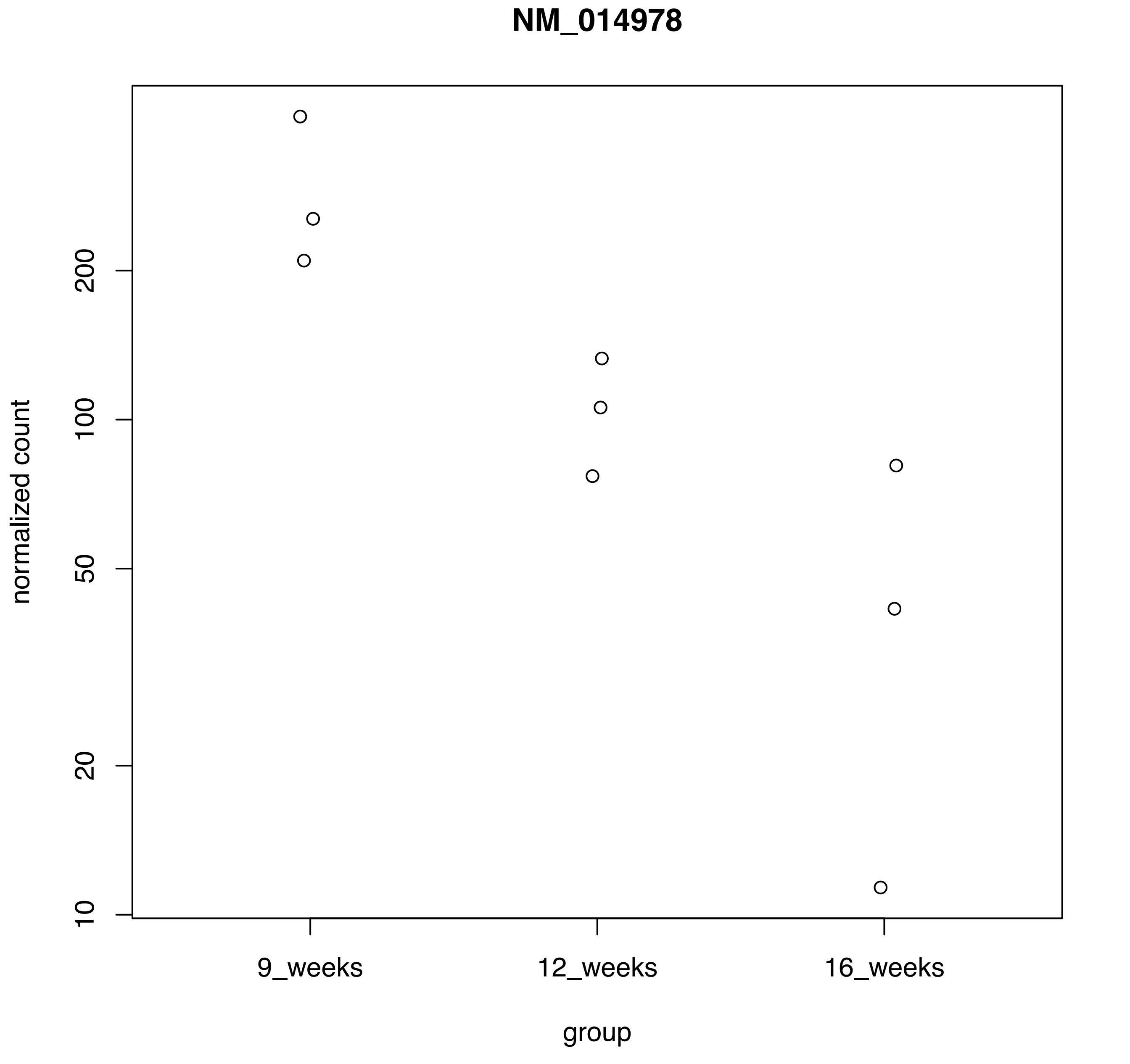

NM_014978
normalized count
10
20
50
100
200
9_weeks
12_weeks
16_weeks
group

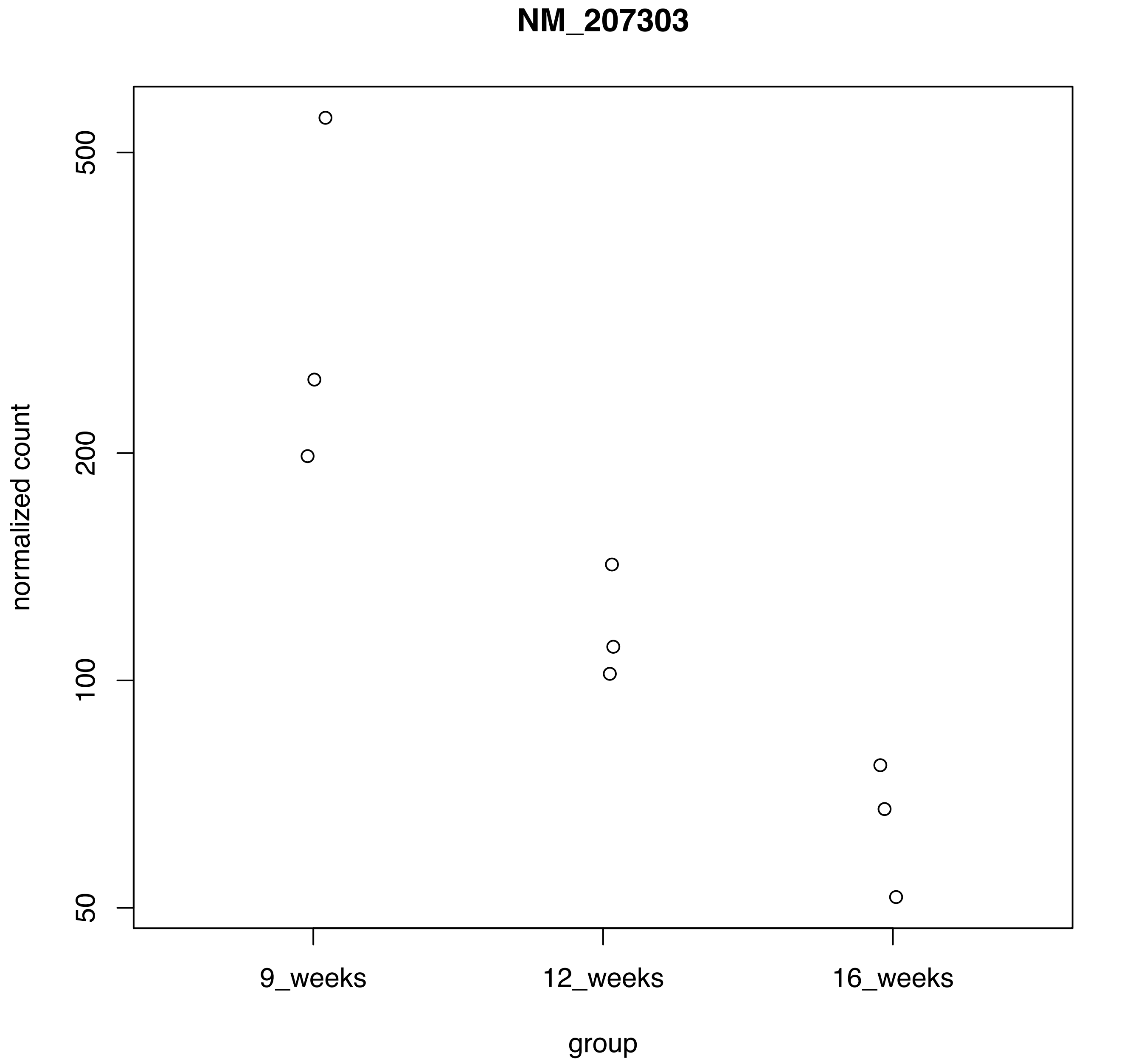
NM_207303
normalized count
500
200
100
50
9_weeks
12_weeks
16_weeks
group

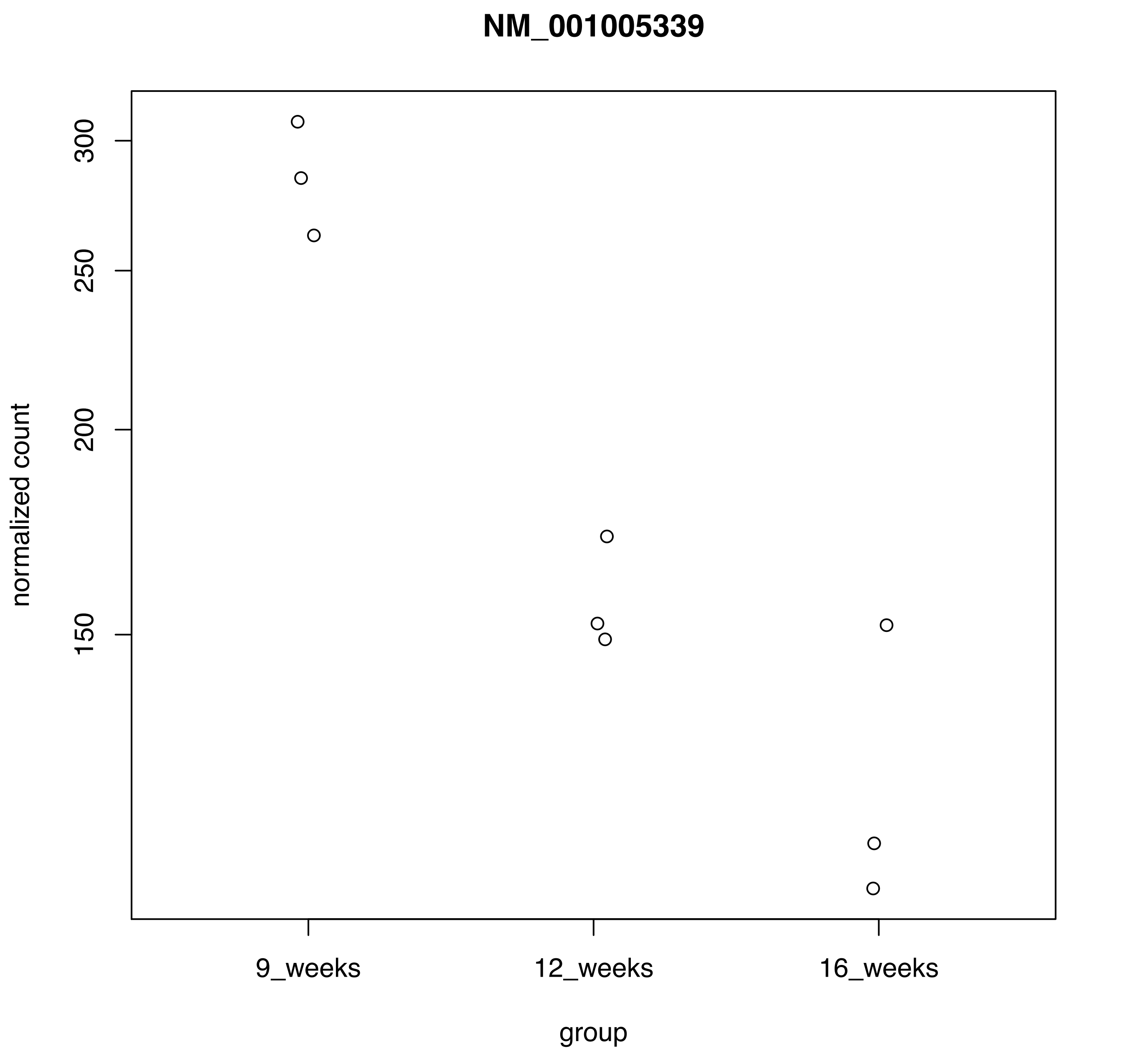

NM_001005339
normalized count
300
250
200
150
9_weeks
12_weeks
16_weeks
group

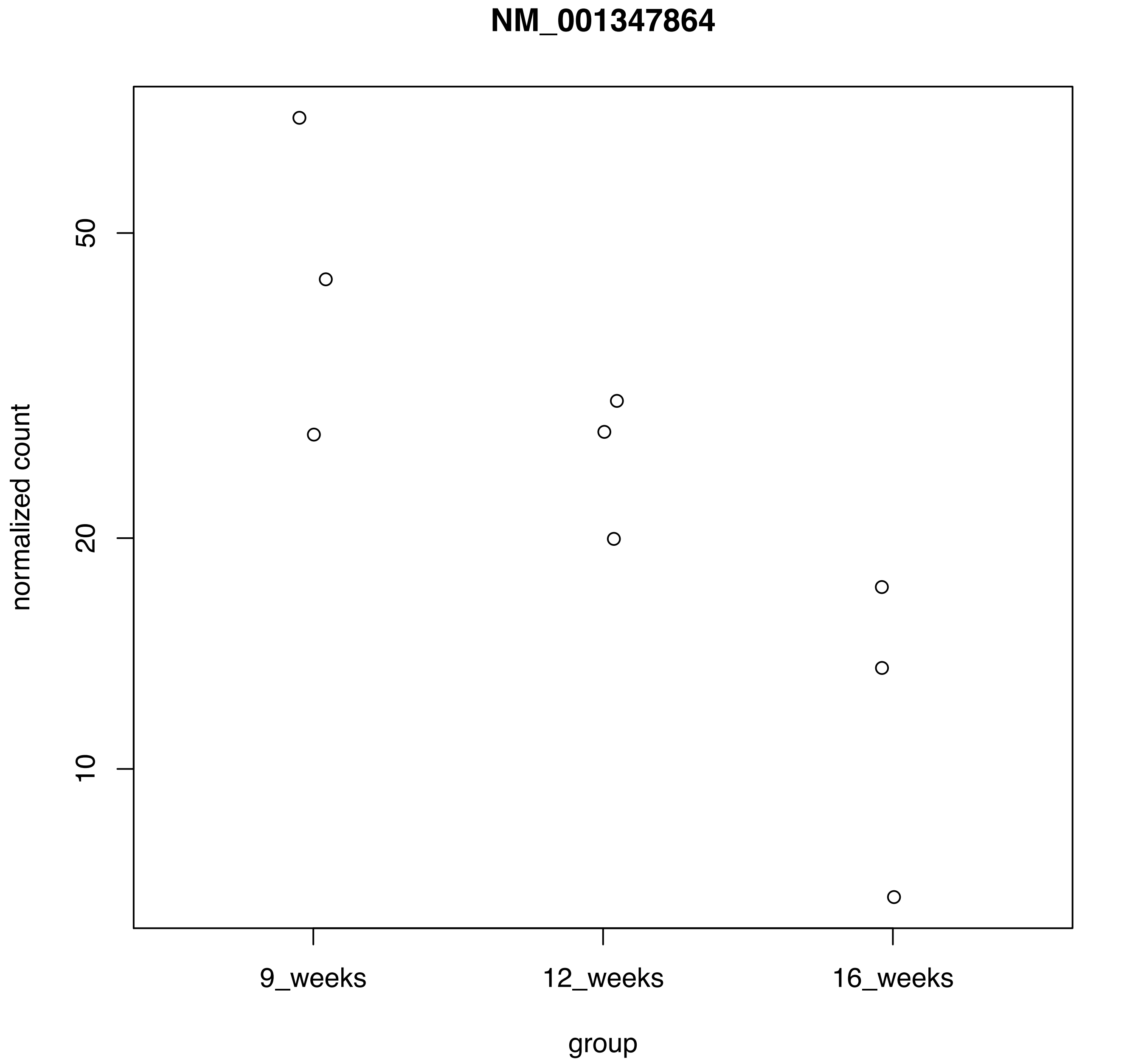
NM_001347864
normalized count
50
20
10
9_weeks
12_weeks
16_weeks
group

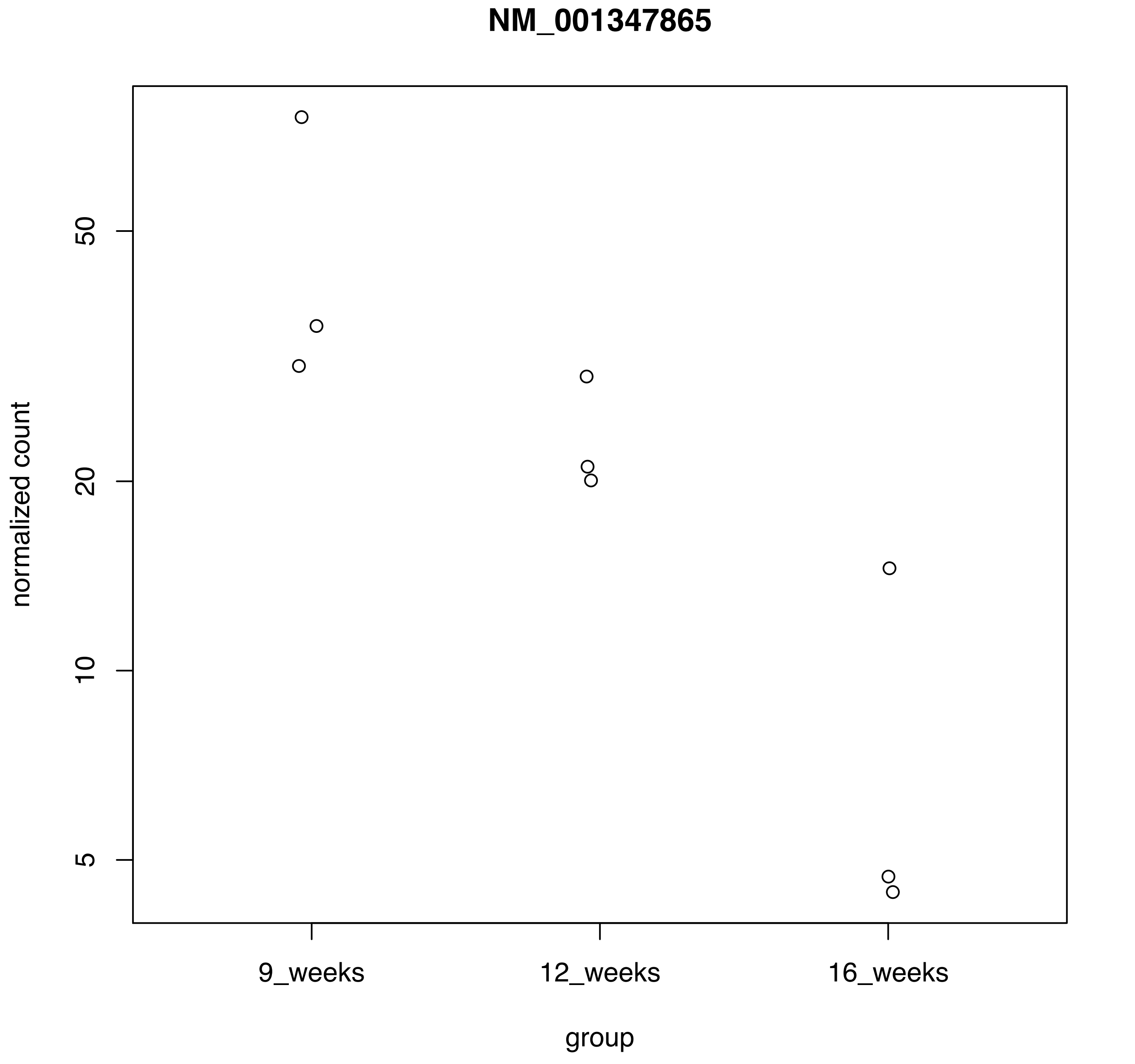
NM_001347865
normalized count
50
20
10
5
9_weeks
12_weeks
16_weeks
group

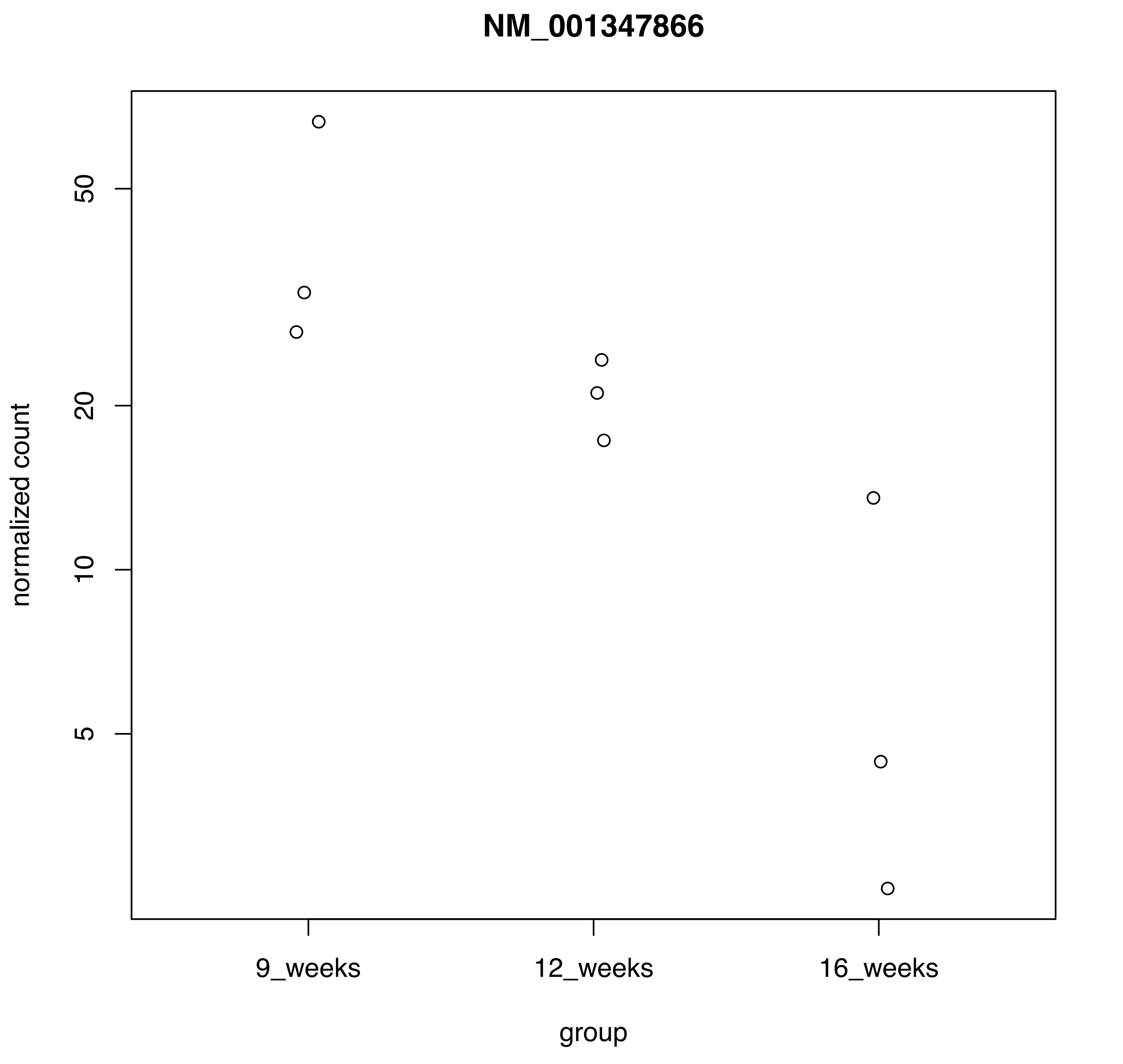
NM_001347866
normalized count
50
20
10
5
9_weeks
12_weeks
16_weeks
group

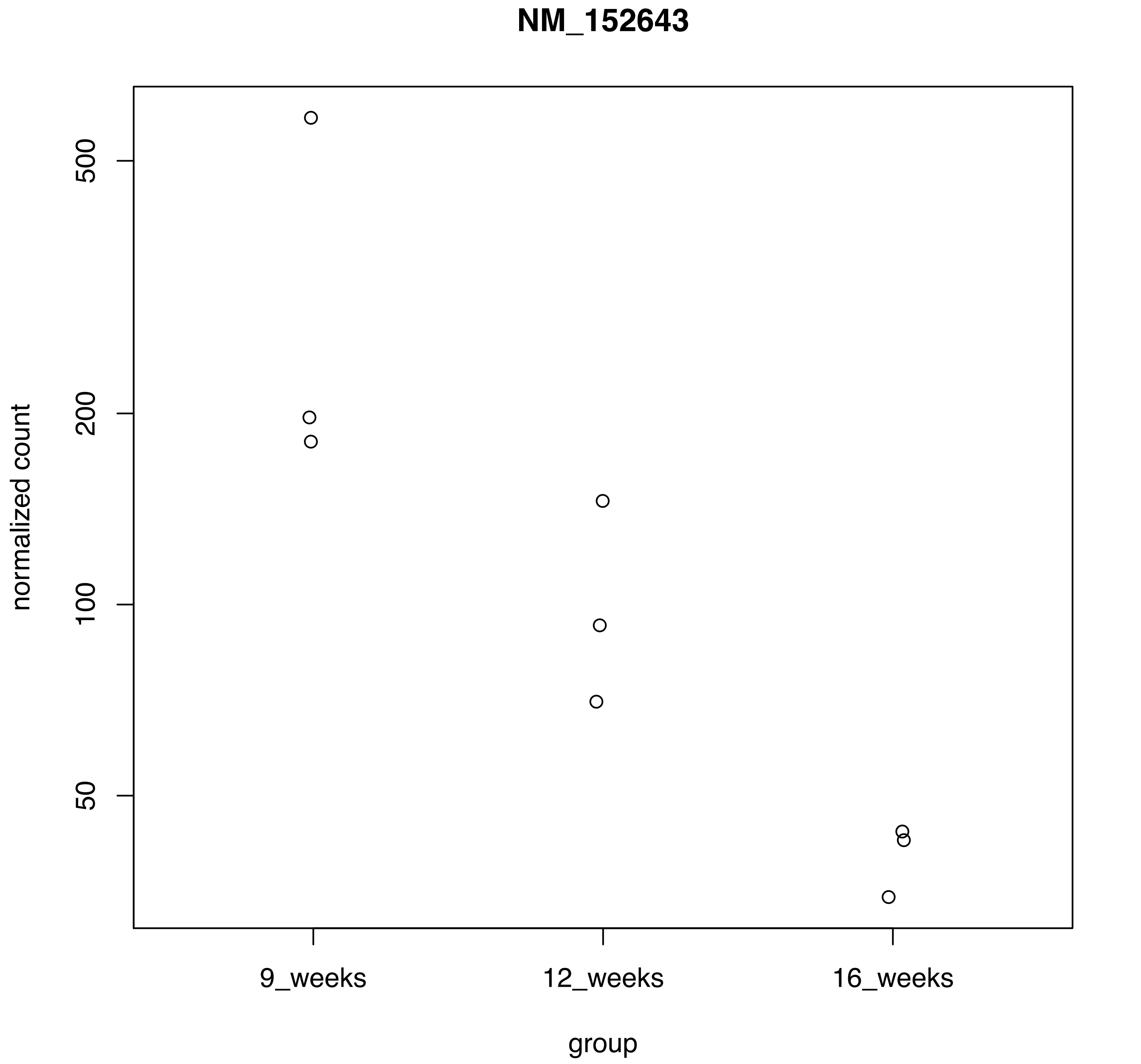

NM_152643
500
200
100
50
normalized count
9_weeks
12_weeks
16_weeks
group

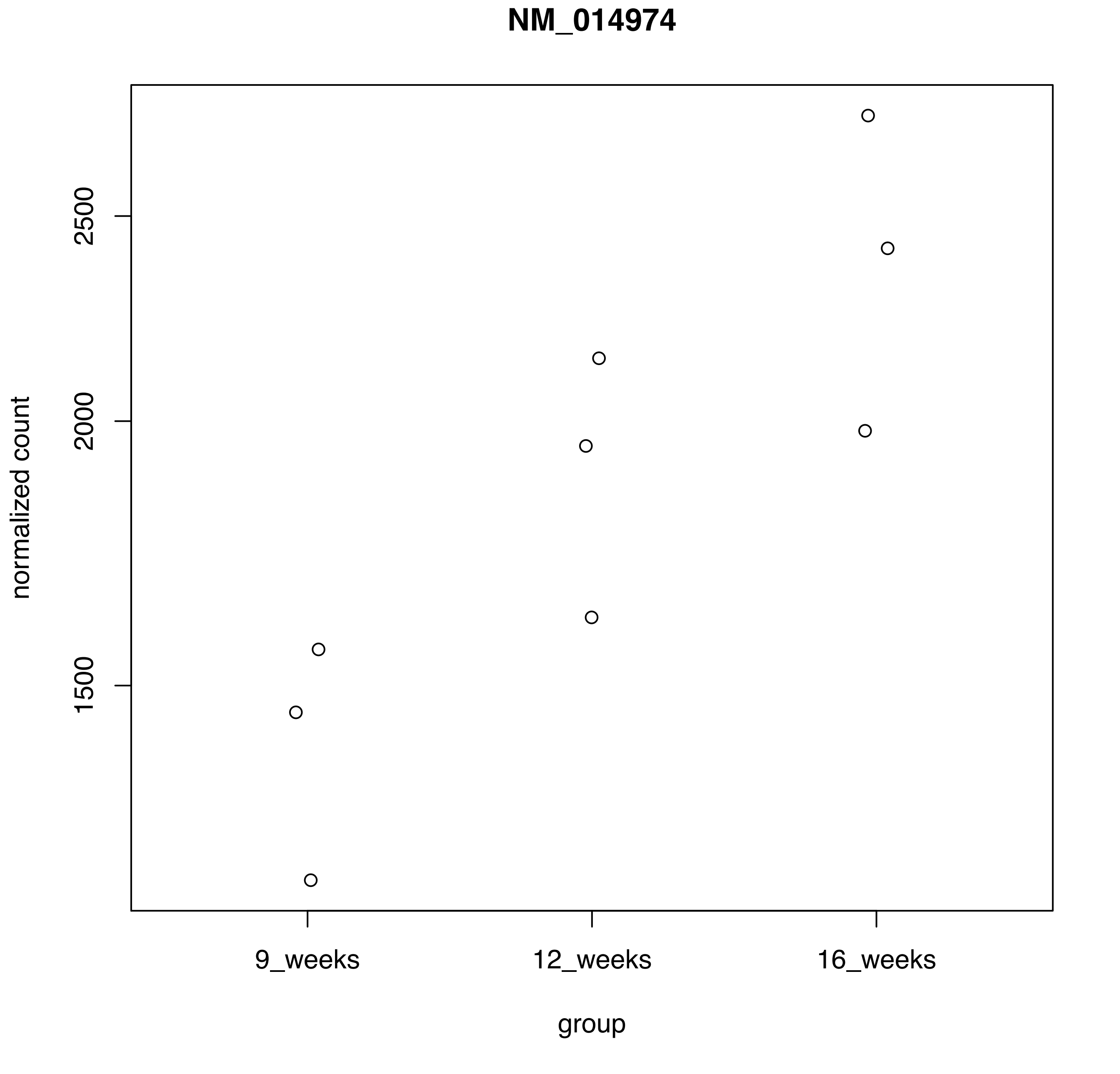
NM_014974
normalized count
2500
2000
1500
9_weeks
12_weeks
16_weeks
group

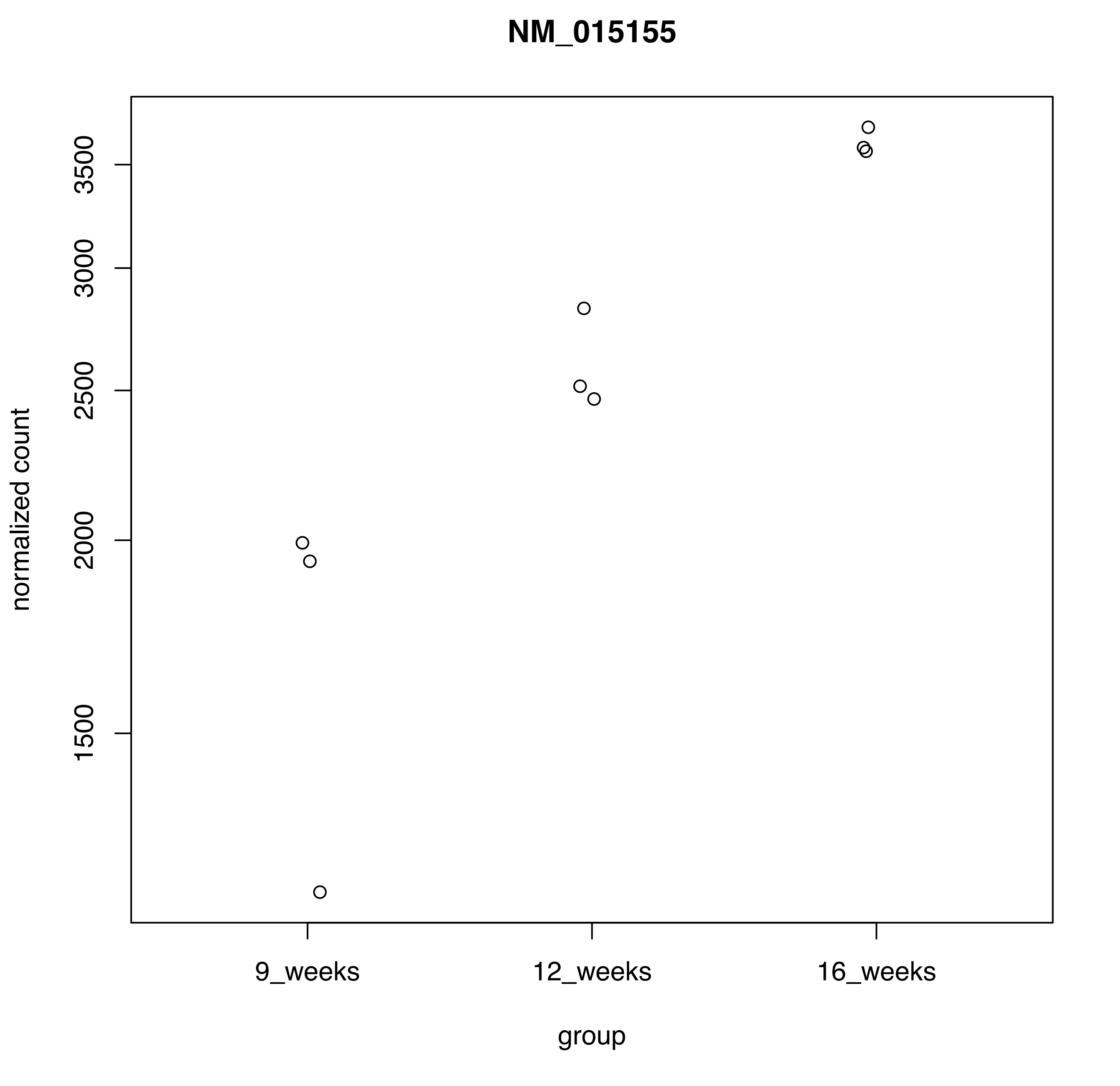
NM_015155
normalized count
3500
3000
2500
2000
1500
9_weeks
12_weeks
16_weeks
group

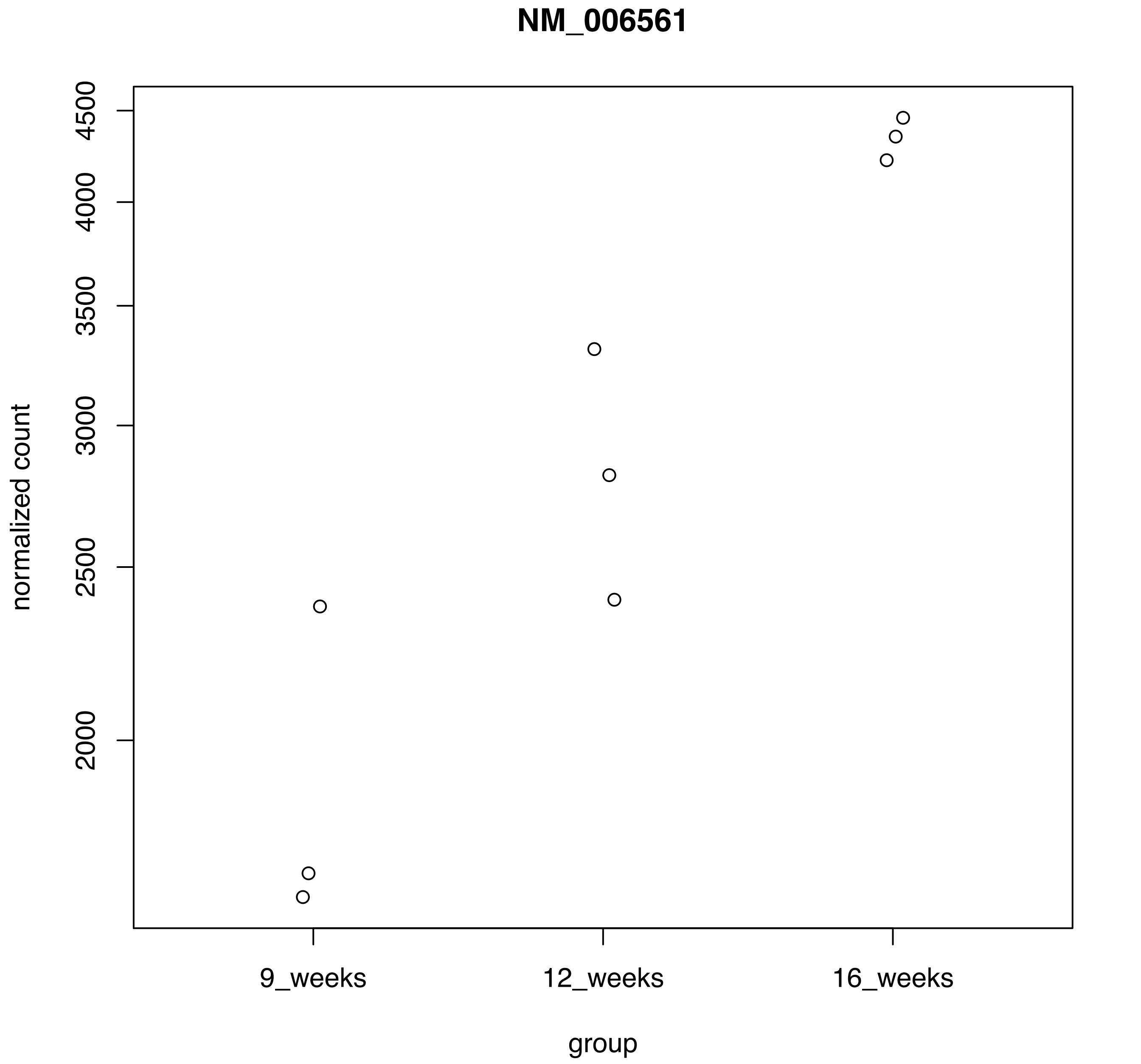
NM_006561
normalized count
4500
4000
3500
3000
2500
2000
9_weeks
12_weeks
16_weeks
group

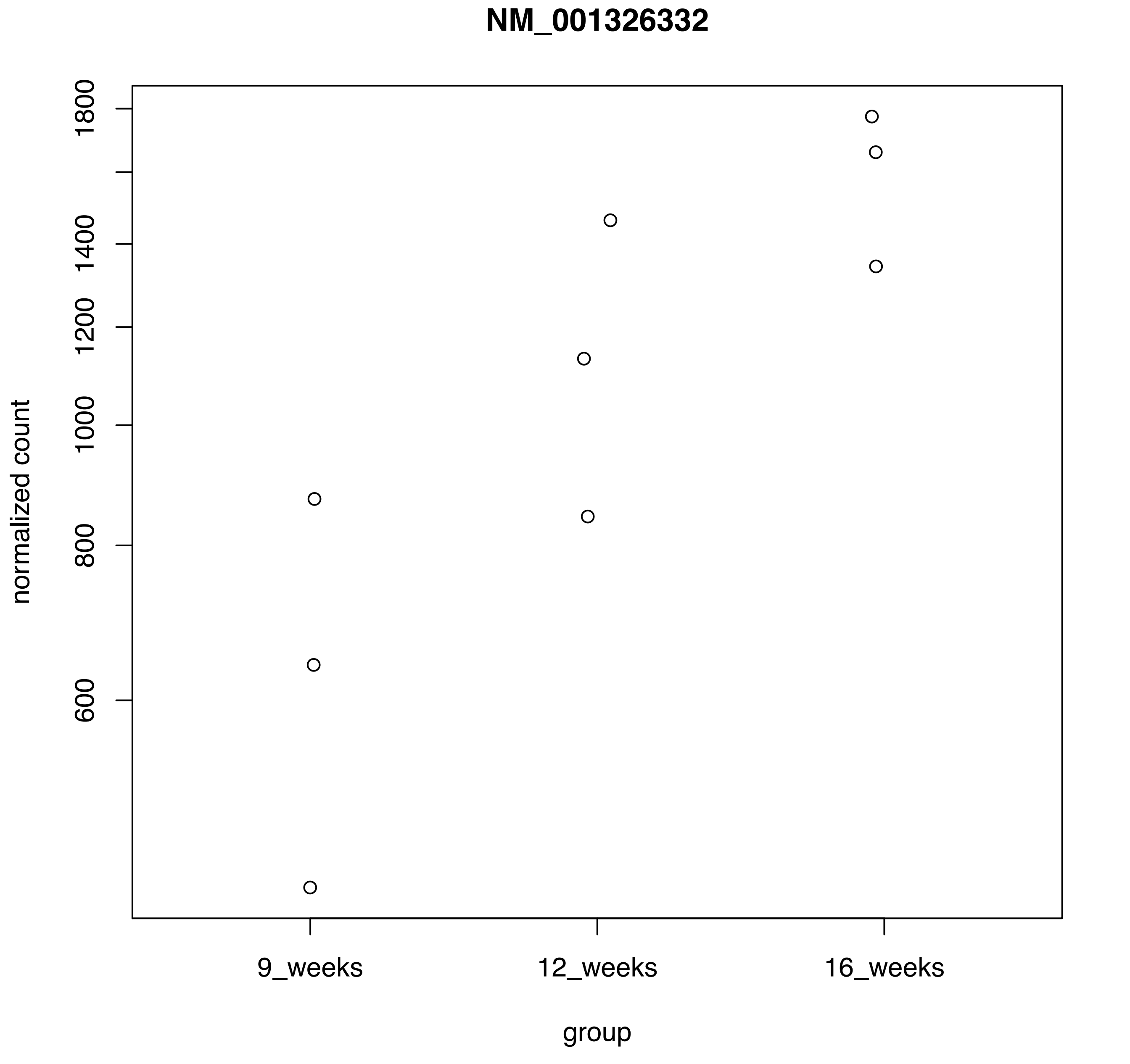
NM_001326332
normalized count
600
800
1000
1200
1400
1800
9_weeks
12_weeks
16_weeks
group

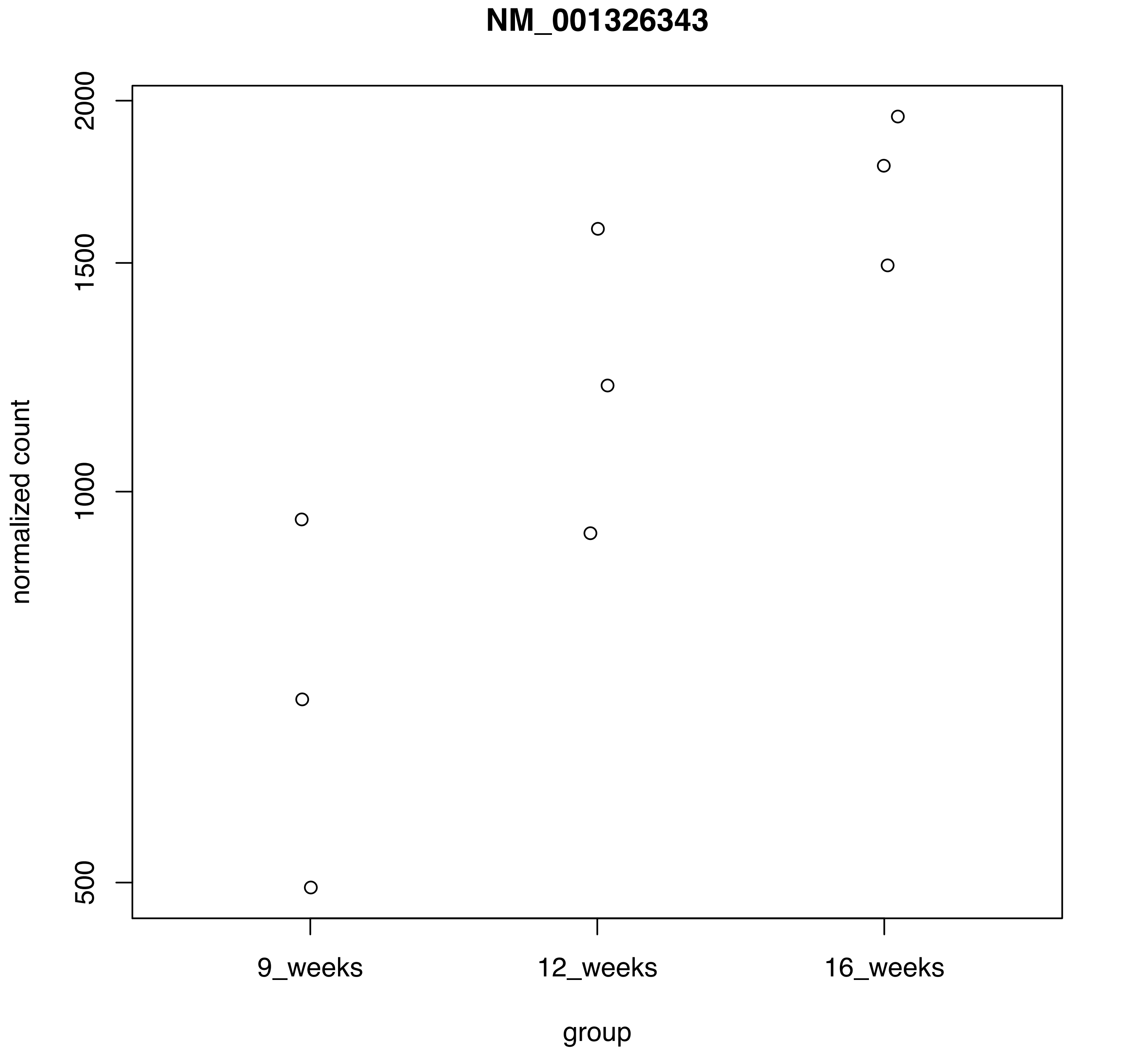
NM_001326343
normalized count
2000
1500
1000
500
9_weeks
12_weeks
16_weeks
group

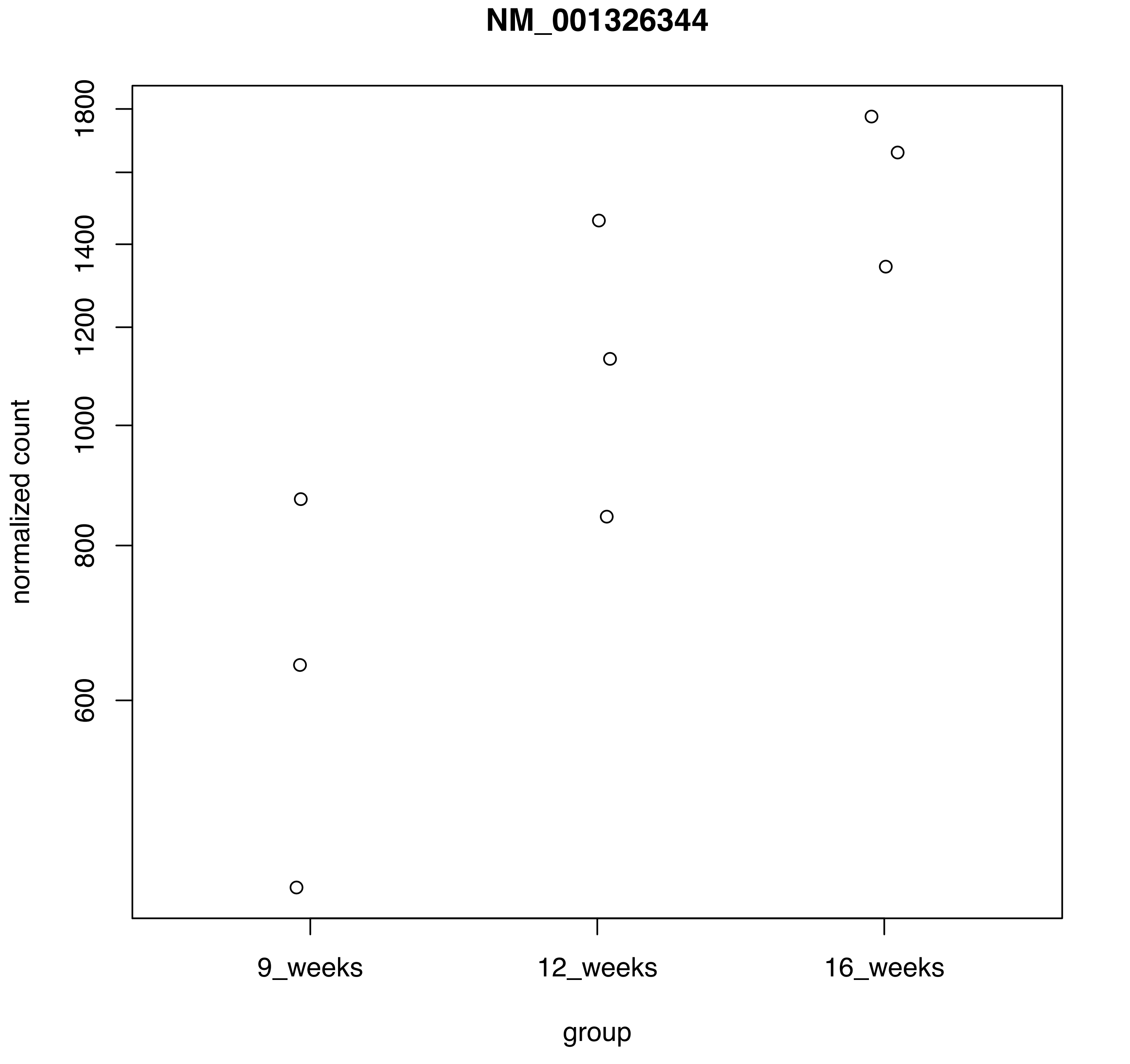
NM_001326344
normalized count
1800
1400
1200
1000
800
600
9_weeks
12_weeks
16_weeks
group

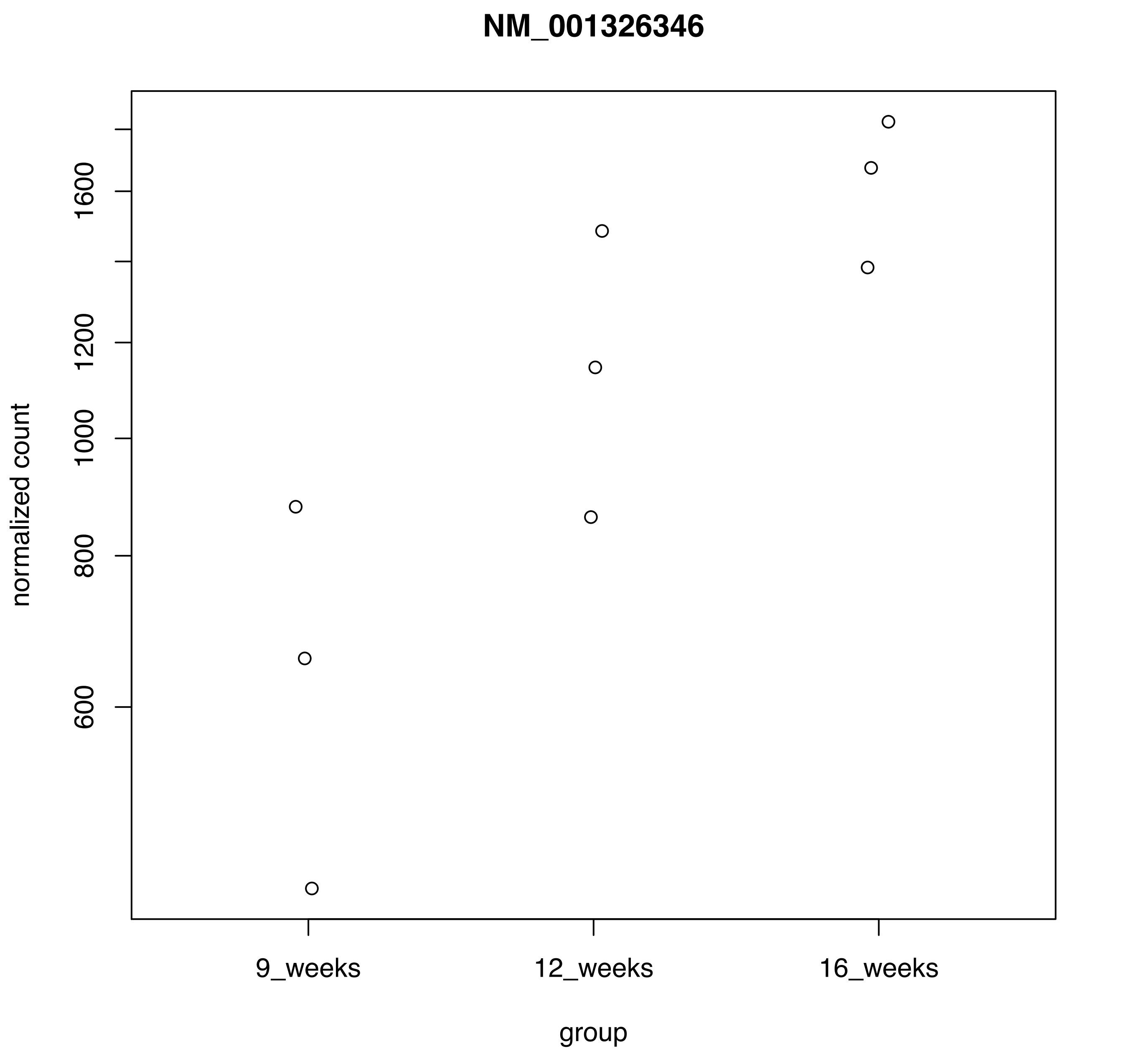

NM_001326346
normalized count
1600
1200
1000
800
600
9_weeks
12_weeks
16_weeks
group

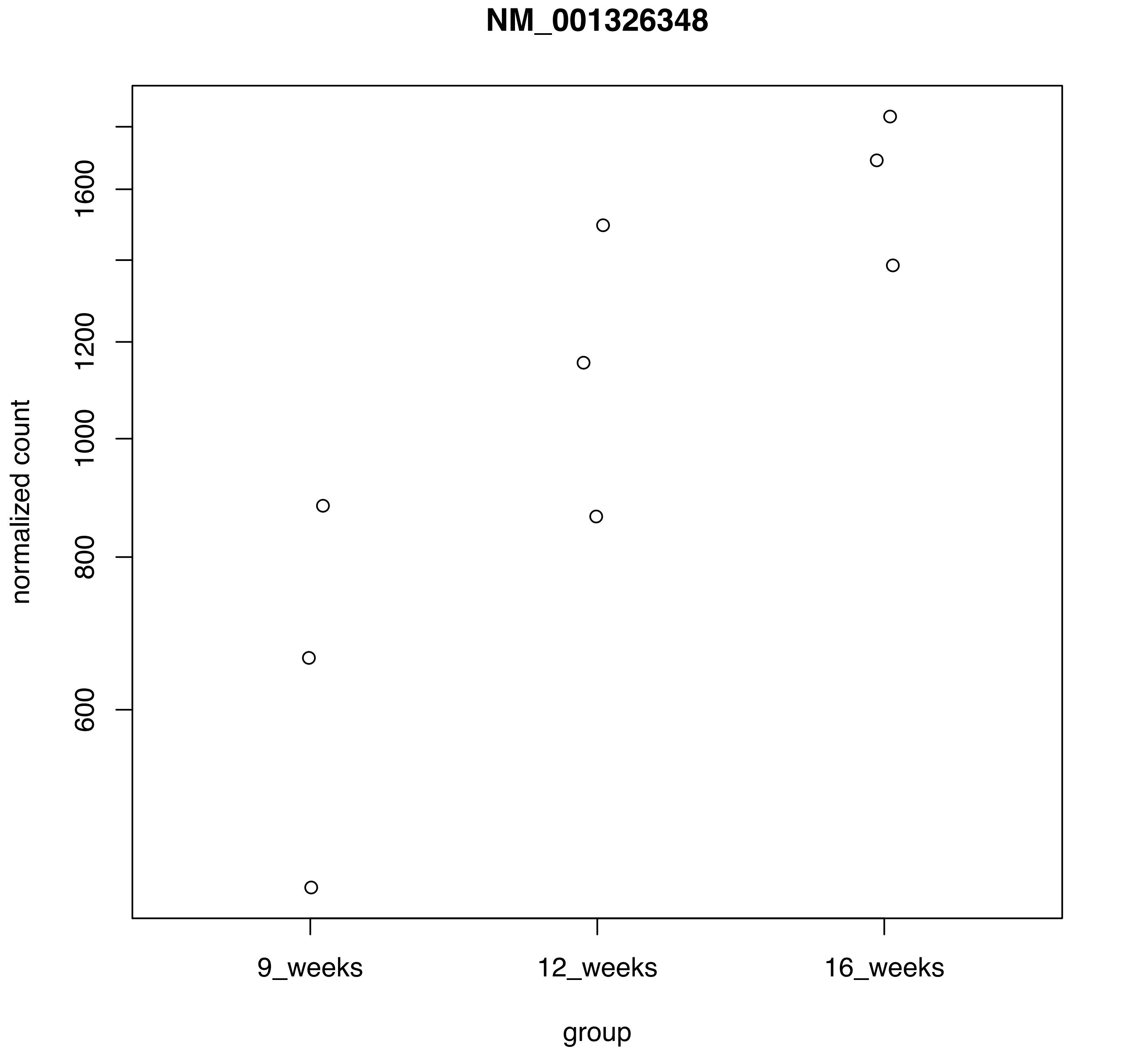
NM_001326348
normalized count
600
800
1000
1200
1600
9_weeks
12_weeks
16_weeks
group

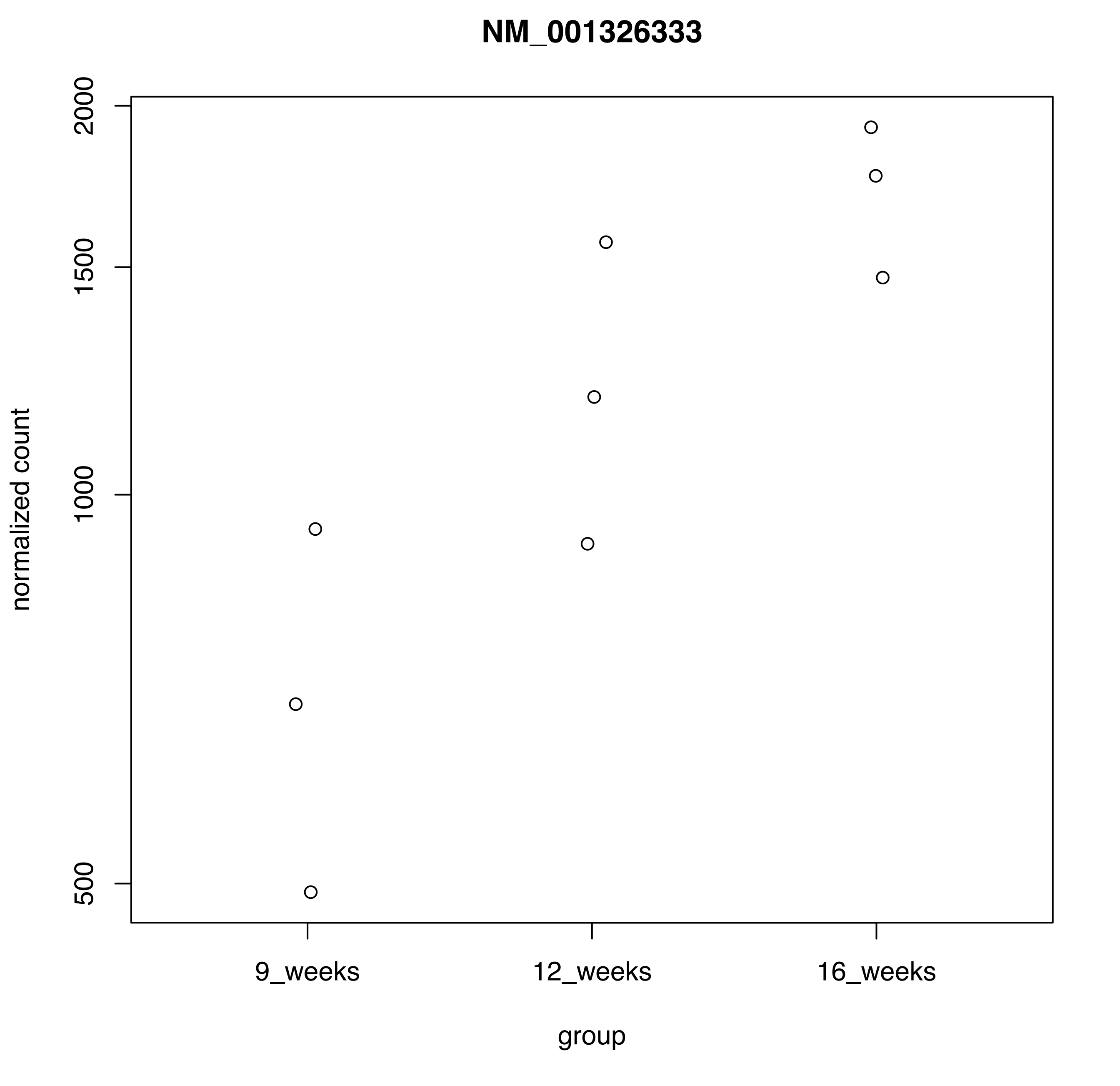

NM_001326333
normalized count
2000
1500
1000
500
9_weeks
12_weeks
16_weeks
group

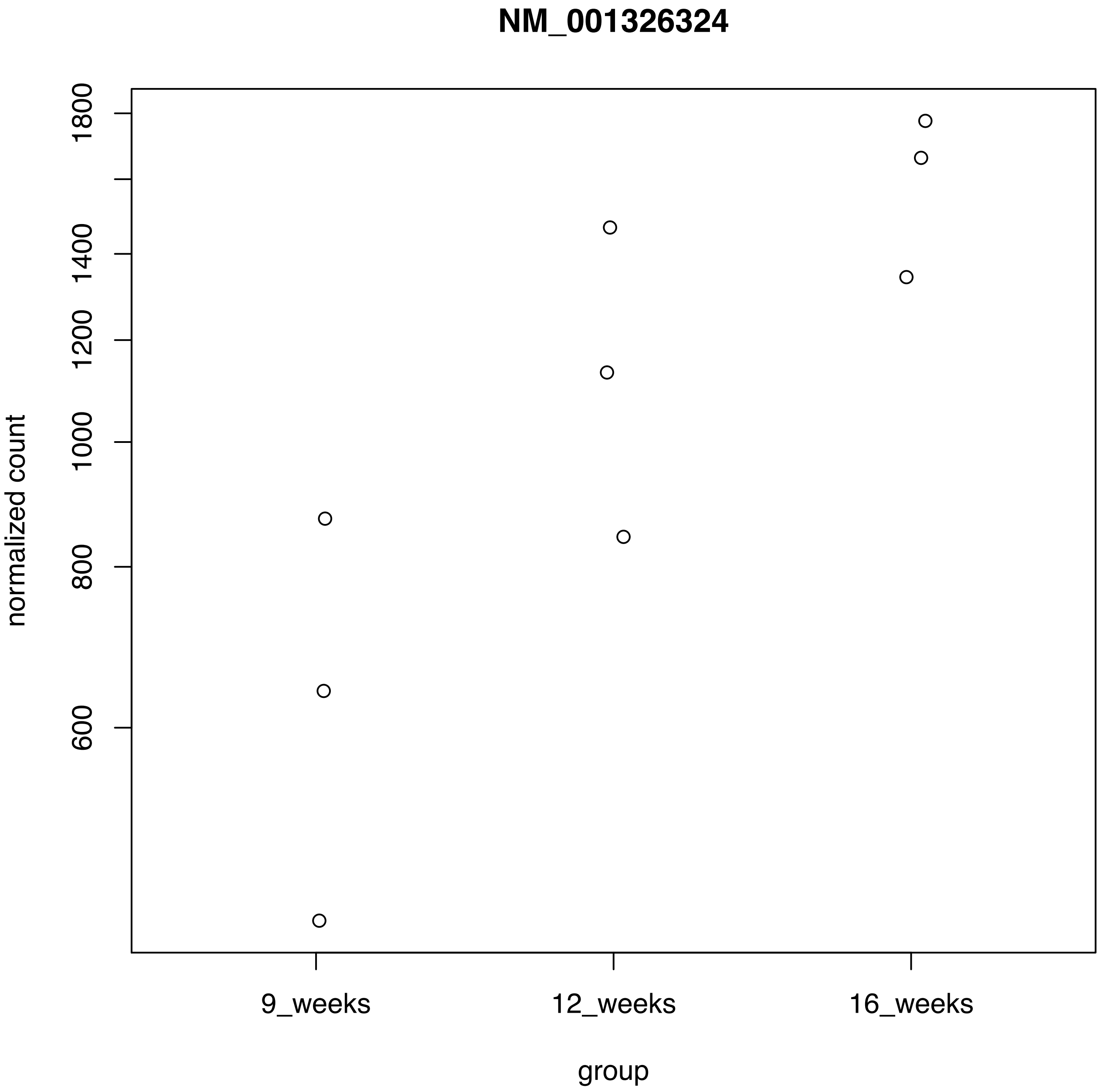
NM_001326324
normalized count
600
800
1000
1200
1400
1800
9_weeks
12_weeks
16_weeks
group

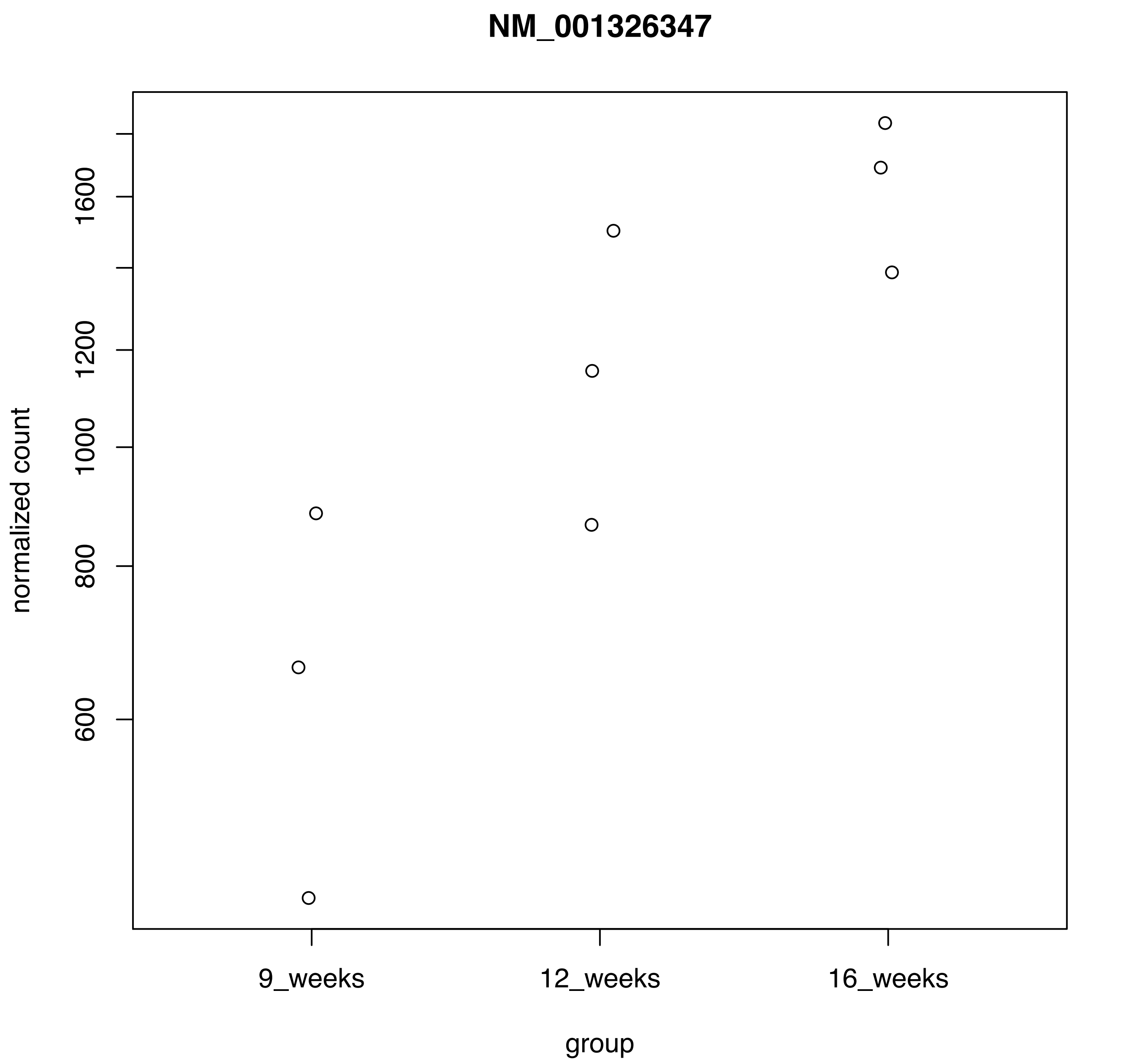
NM_001326347
normalized count
600
800
1000
1200
1600
9_weeks
12_weeks
16_weeks
group

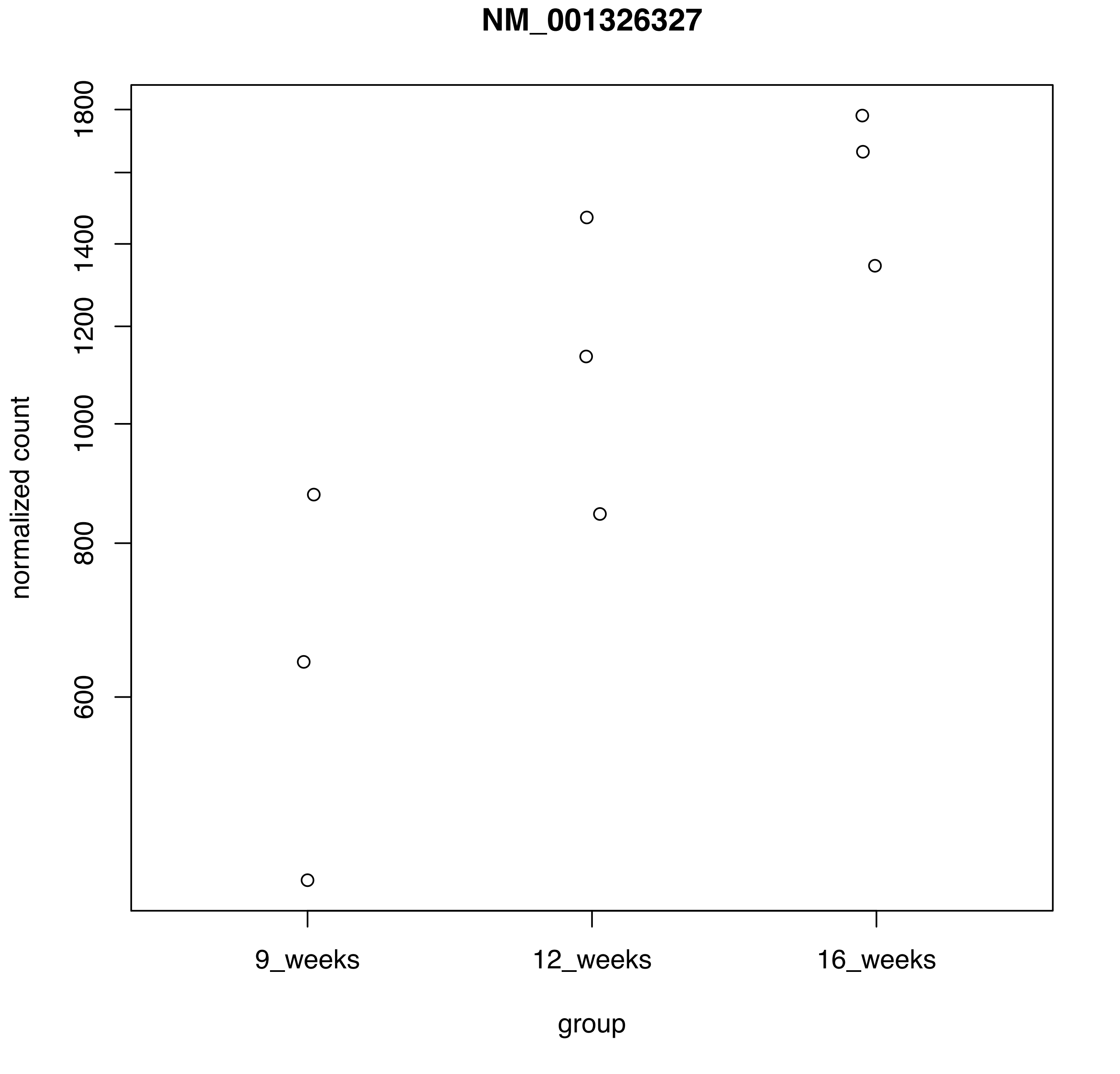

NM_001326327
normalized count
600
800
1000
1200
1400
1800
9_weeks
12_weeks
16_weeks
group

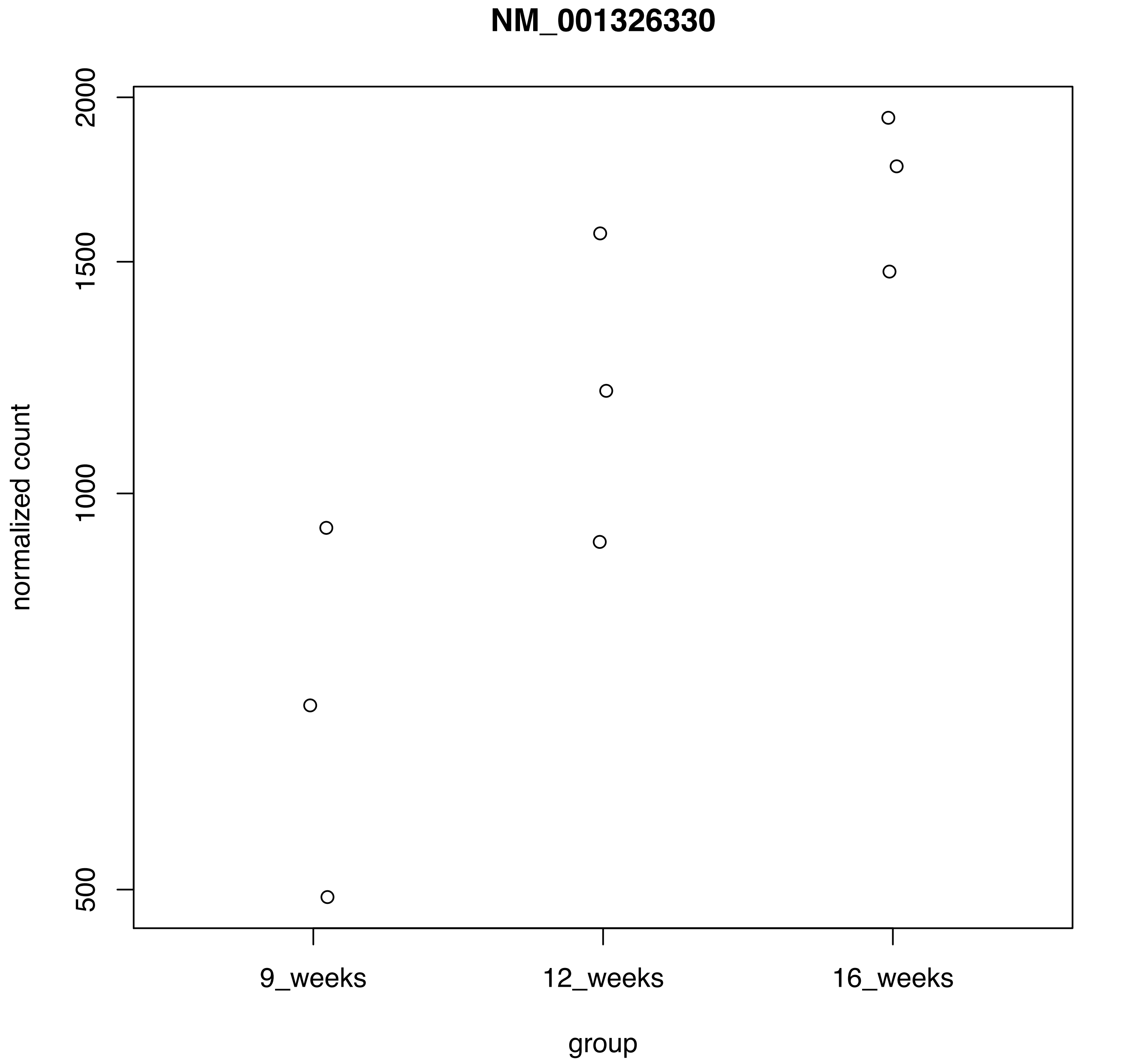
NM_001326330
normalized count
2000
1500
1000
500
9_weeks
12_weeks
16_weeks
group

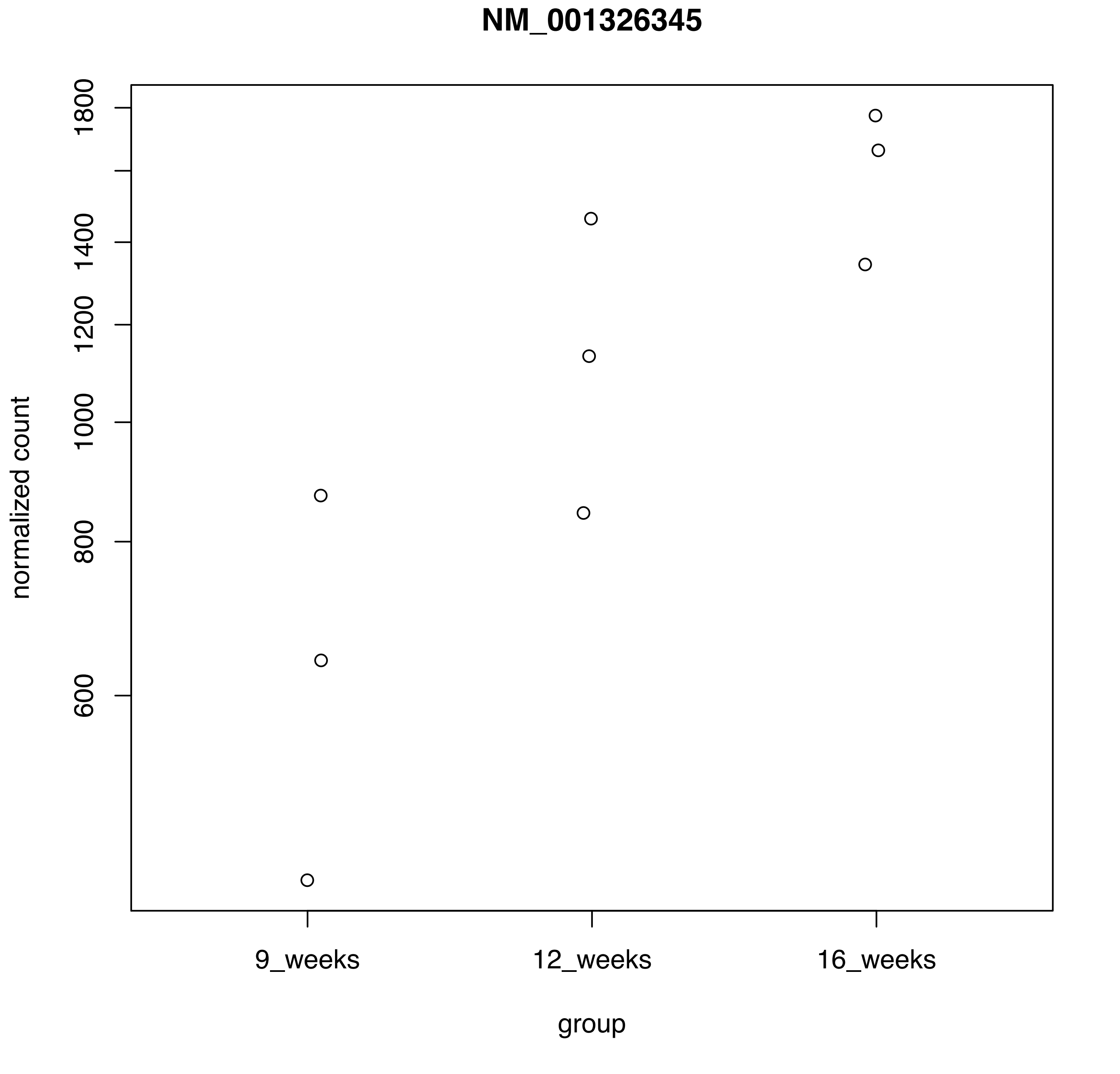
NM_001326345
normalized count
600
800
1000
1200
1400
1800
group
9_weeks
12_weeks
16_weeks

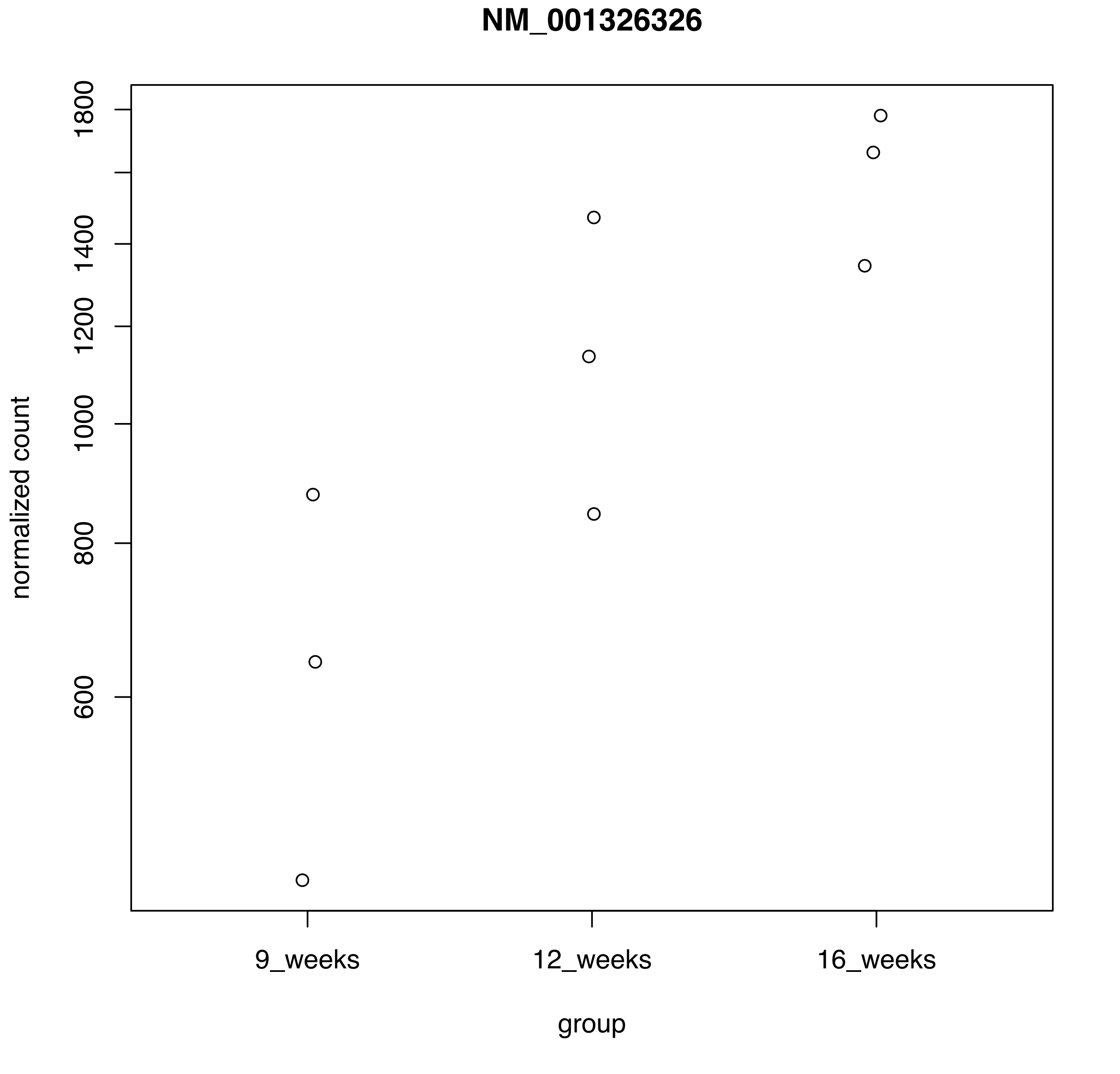
NM_001326326
normalized count
1800
1400
1200
1000
800
600
9_weeks
12_weeks
16_weeks
group

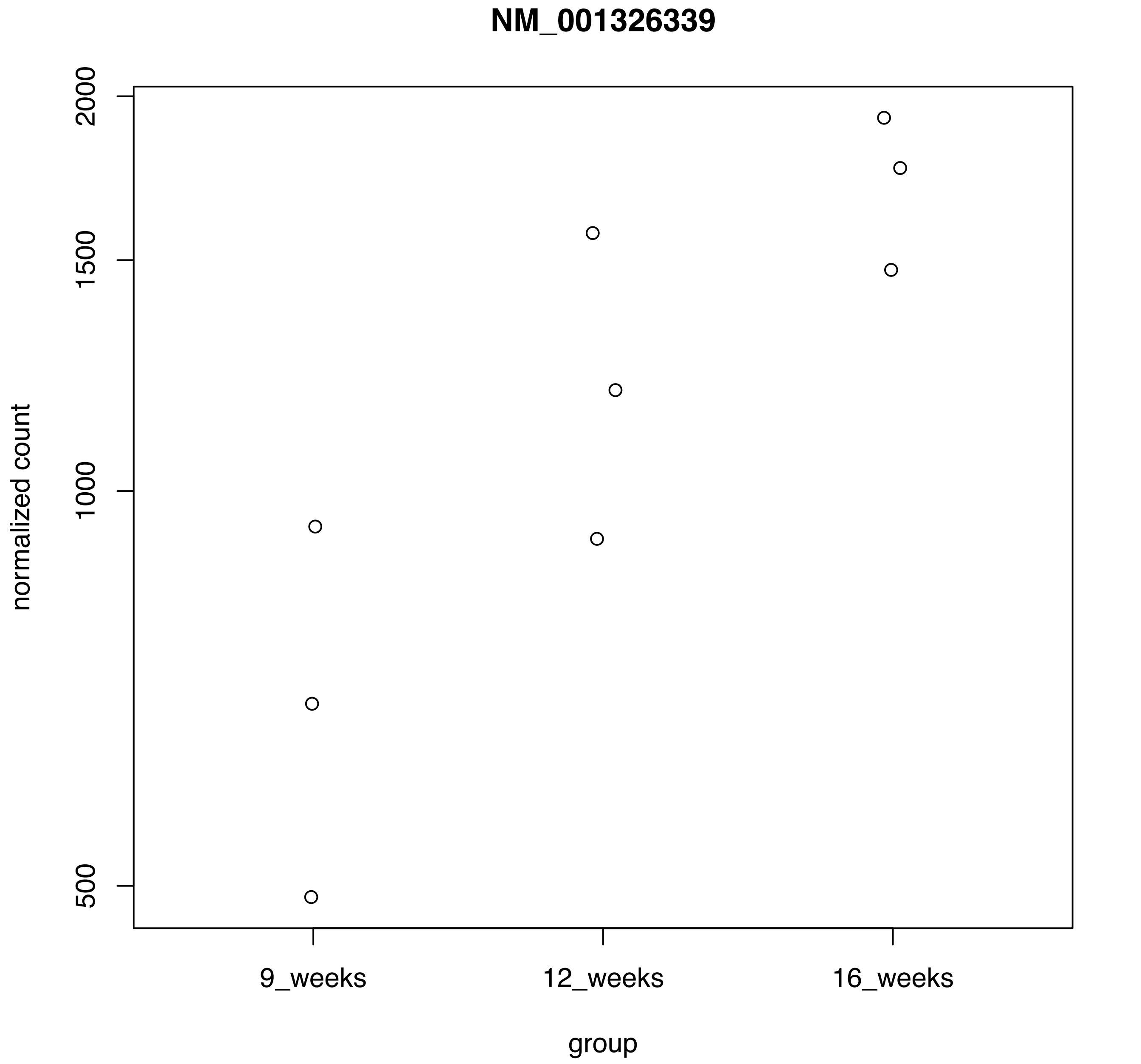
NM_001326339
normalized count
2000
1500
1000
500
9_weeks
12_weeks
16_weeks
group

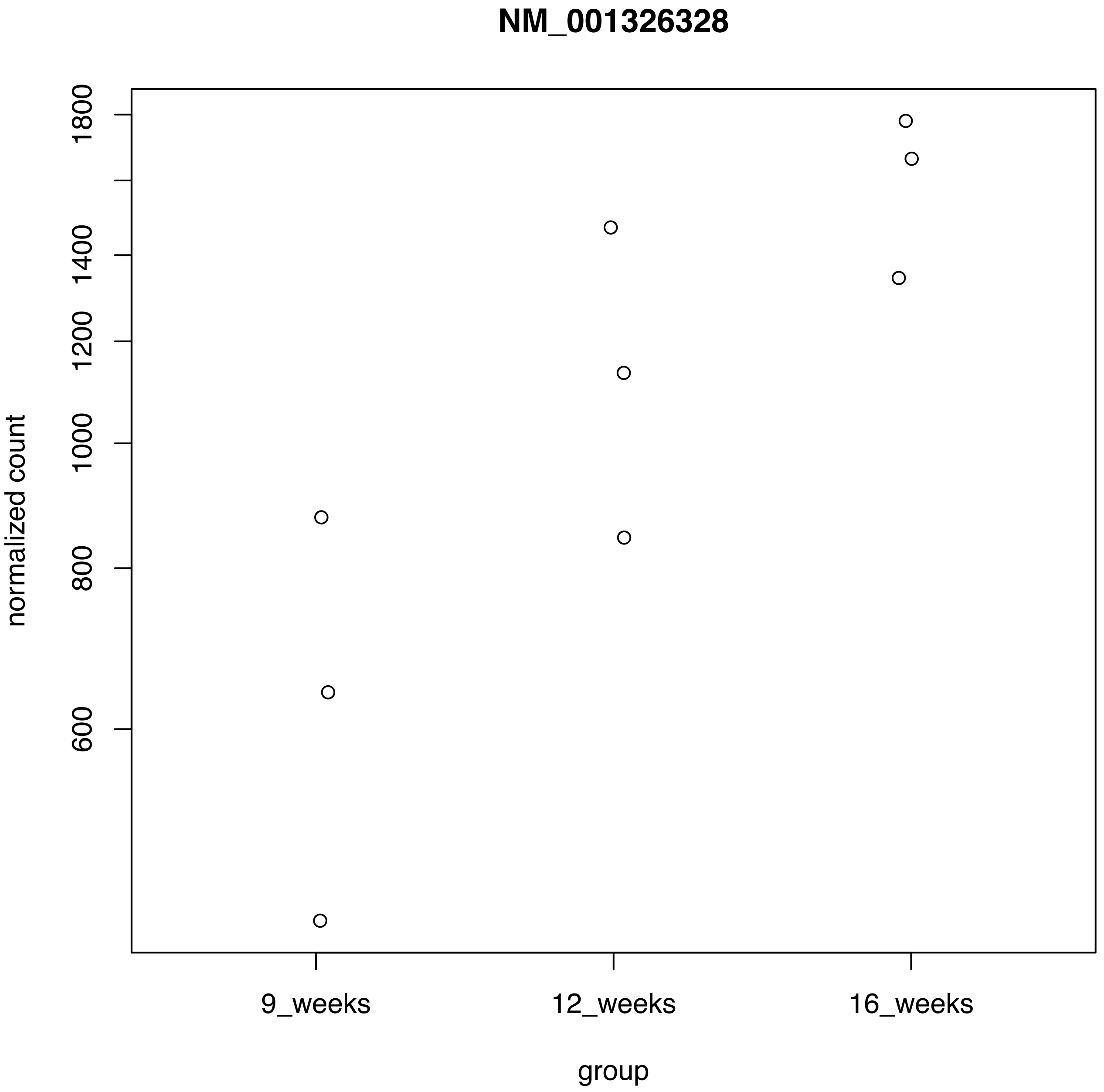
NM_001326328
normalized count
1800
1400
1200
1000
800
600
9_weeks
12_weeks
16_weeks
group

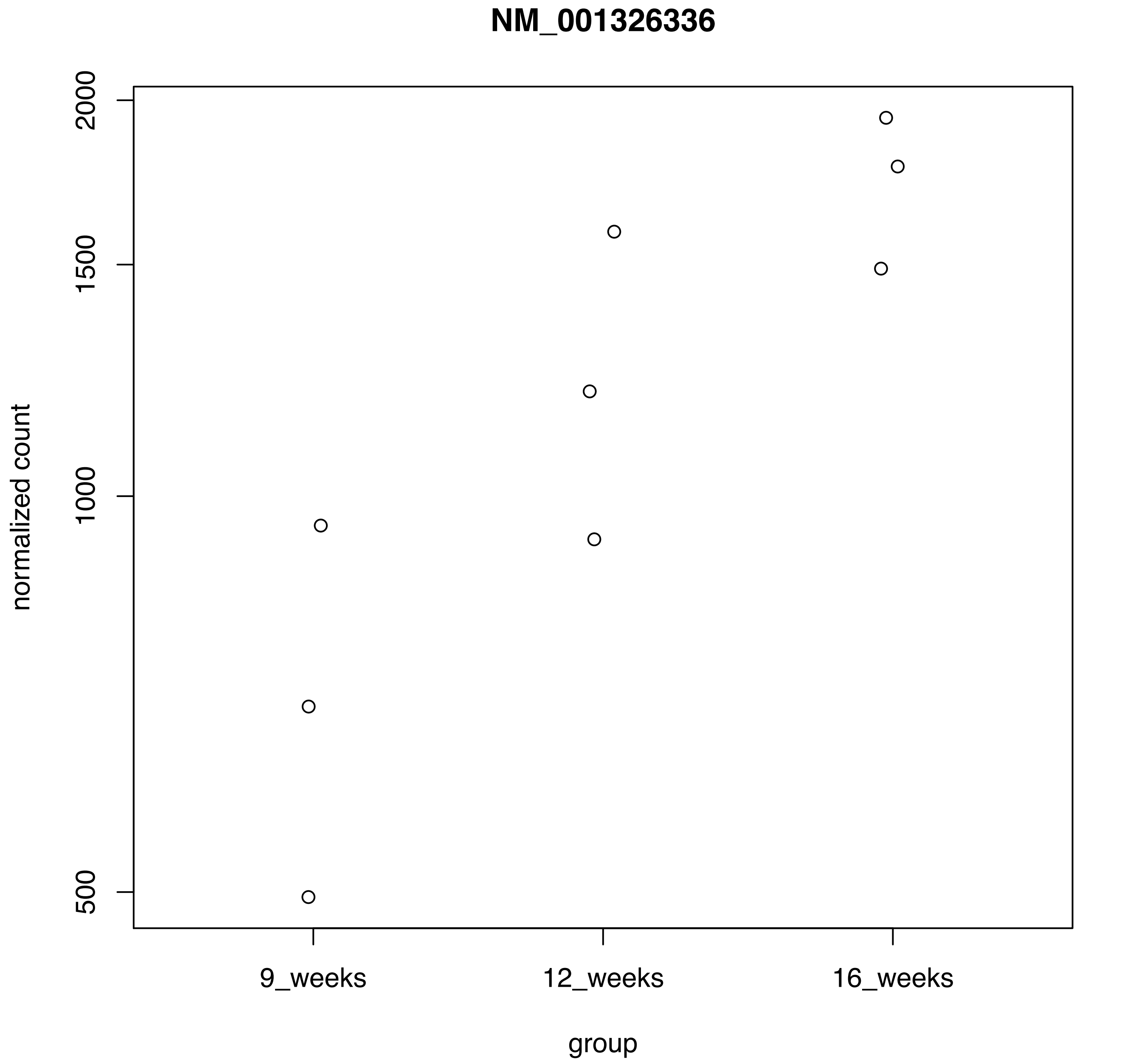
NM_001326336
normalized count
2000
1500
1000
500
9_weeks
12_weeks
16_weeks
group

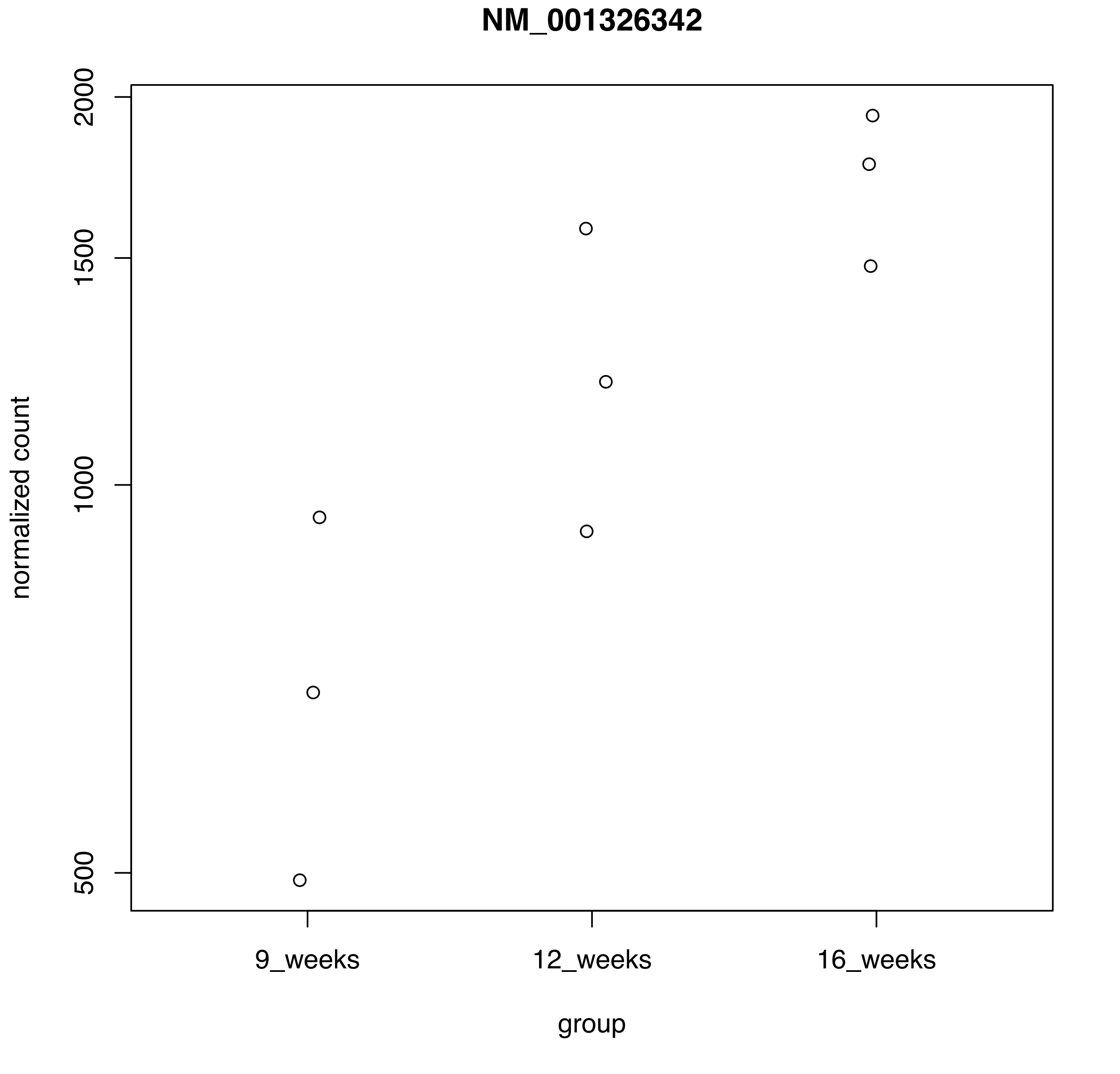
NM_001326342
normalized count
2000
1500
1000
500
9_weeks
12_weeks
16_weeks
group

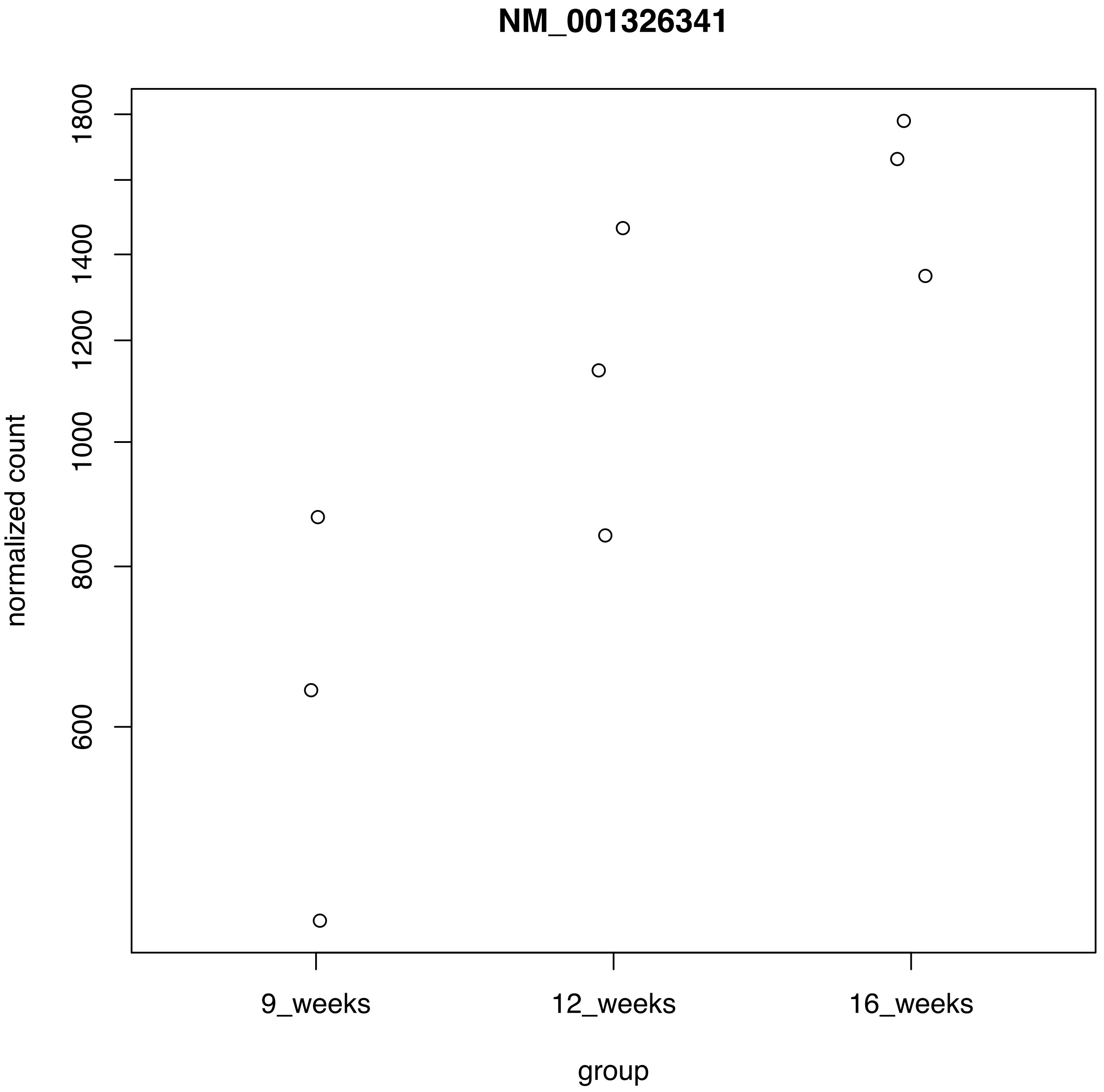
NM_001326341
normalized count
1800
1400
1200
1000
800
600
9_weeks
12_weeks
16_weeks
group

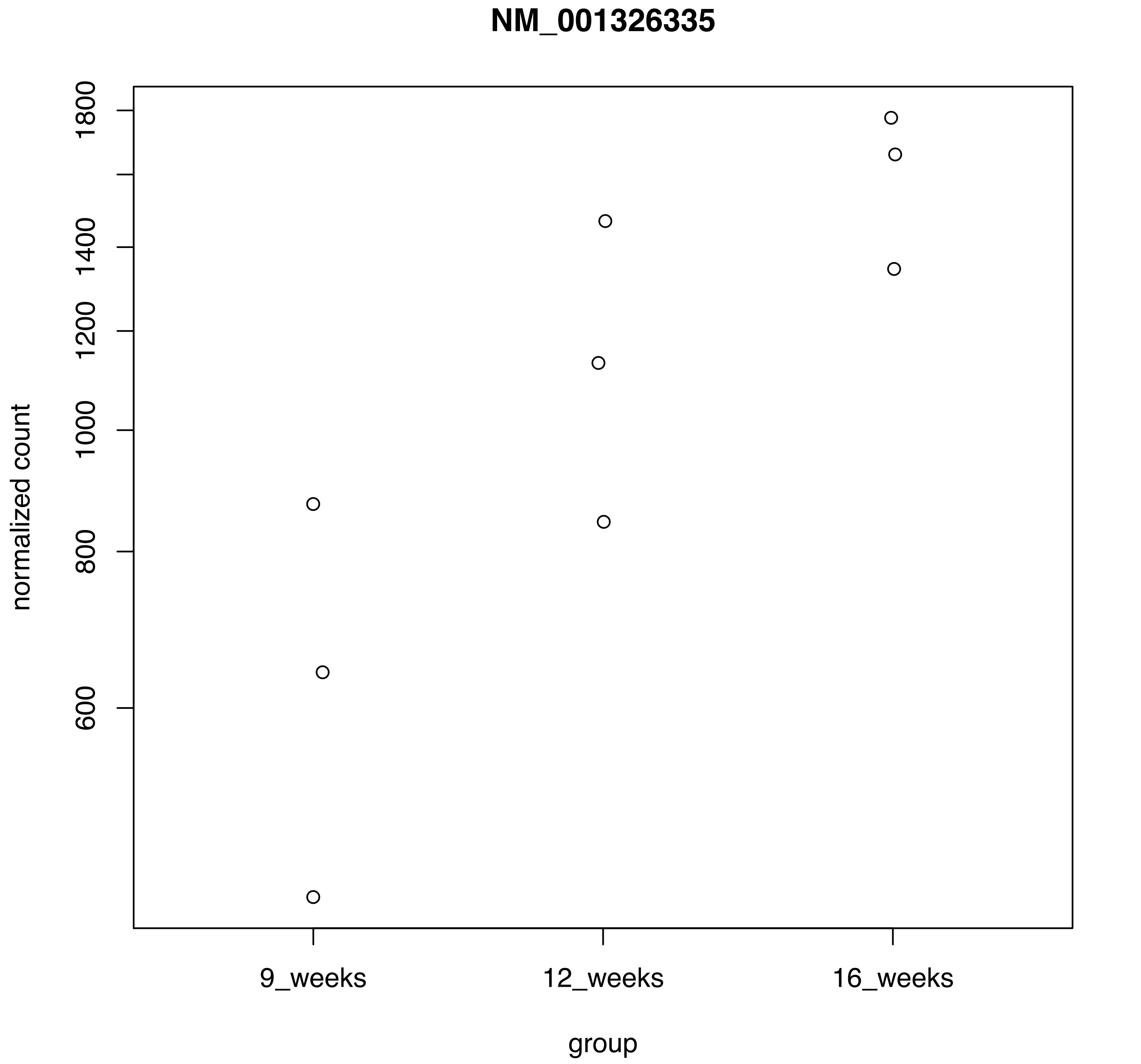
NM_001326335
normalized count
1800
1400
1200
1000
800
600
9_weeks
12_weeks
16_weeks
group

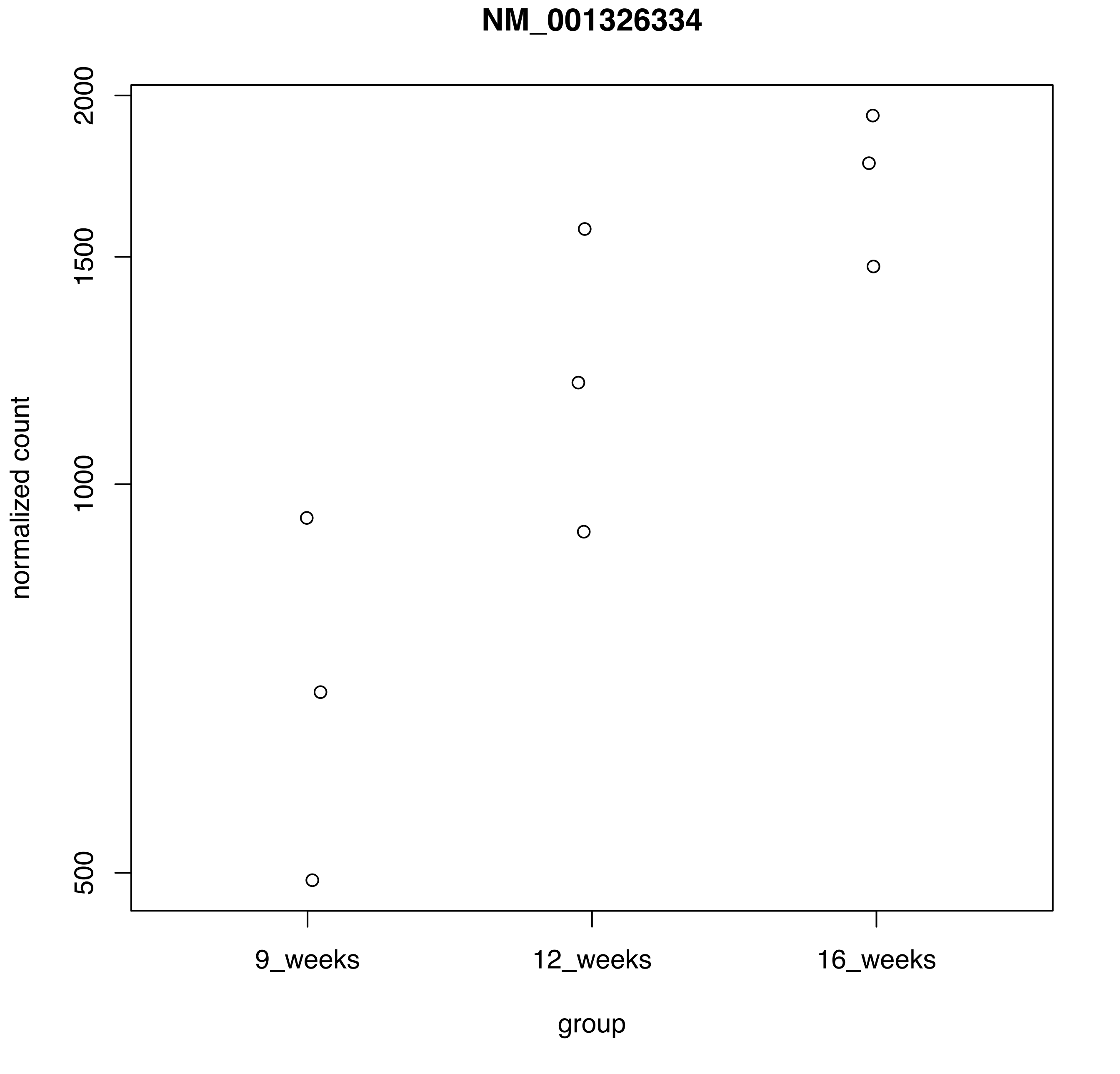
NM_001326334
normalized count
2000
1500
1000
500
9_weeks
12_weeks
16_weeks
group

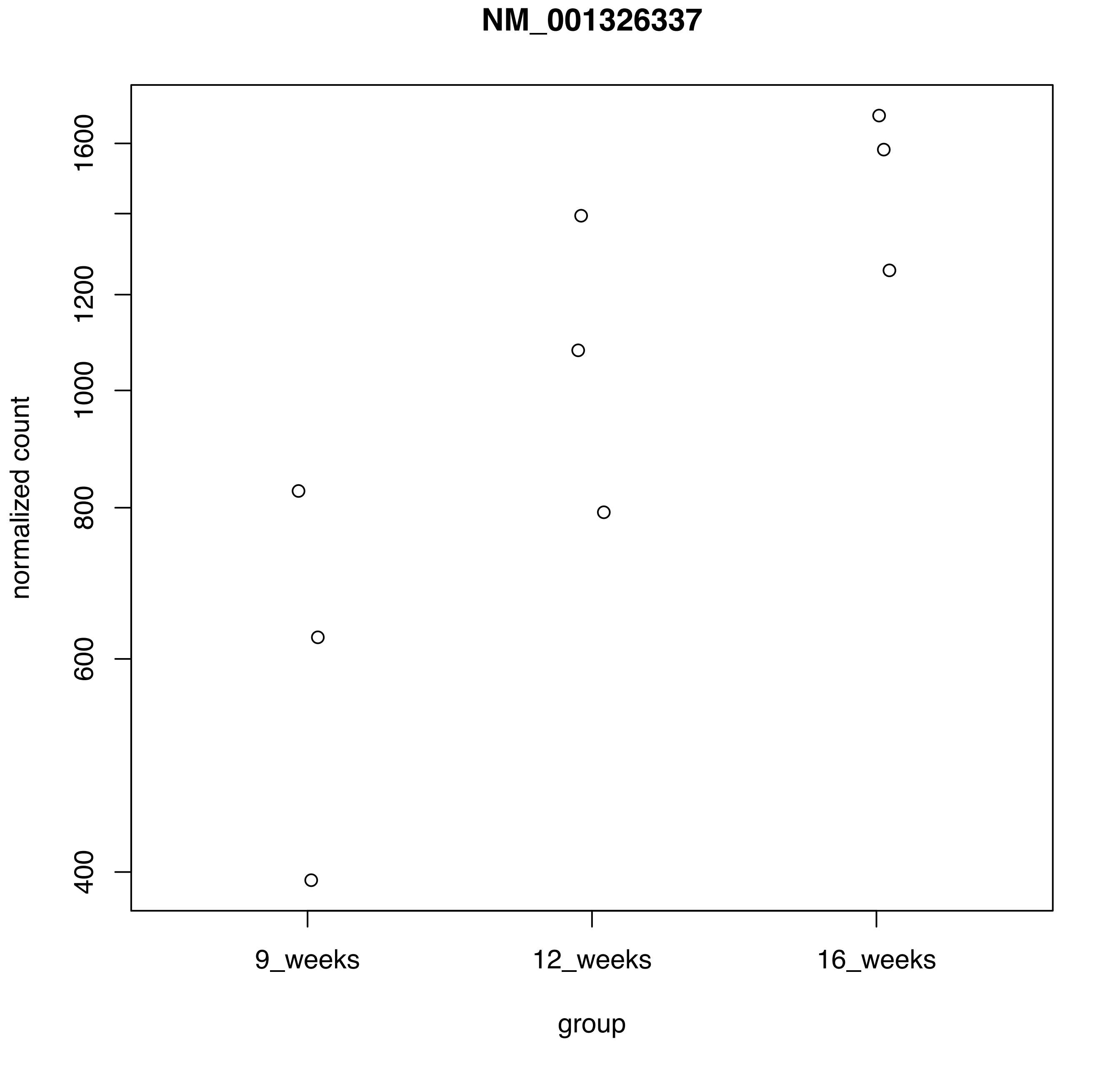
NM_001326337
normalized count
1600
1200
1000
800
600
400
9_weeks
12_weeks
16_weeks
group

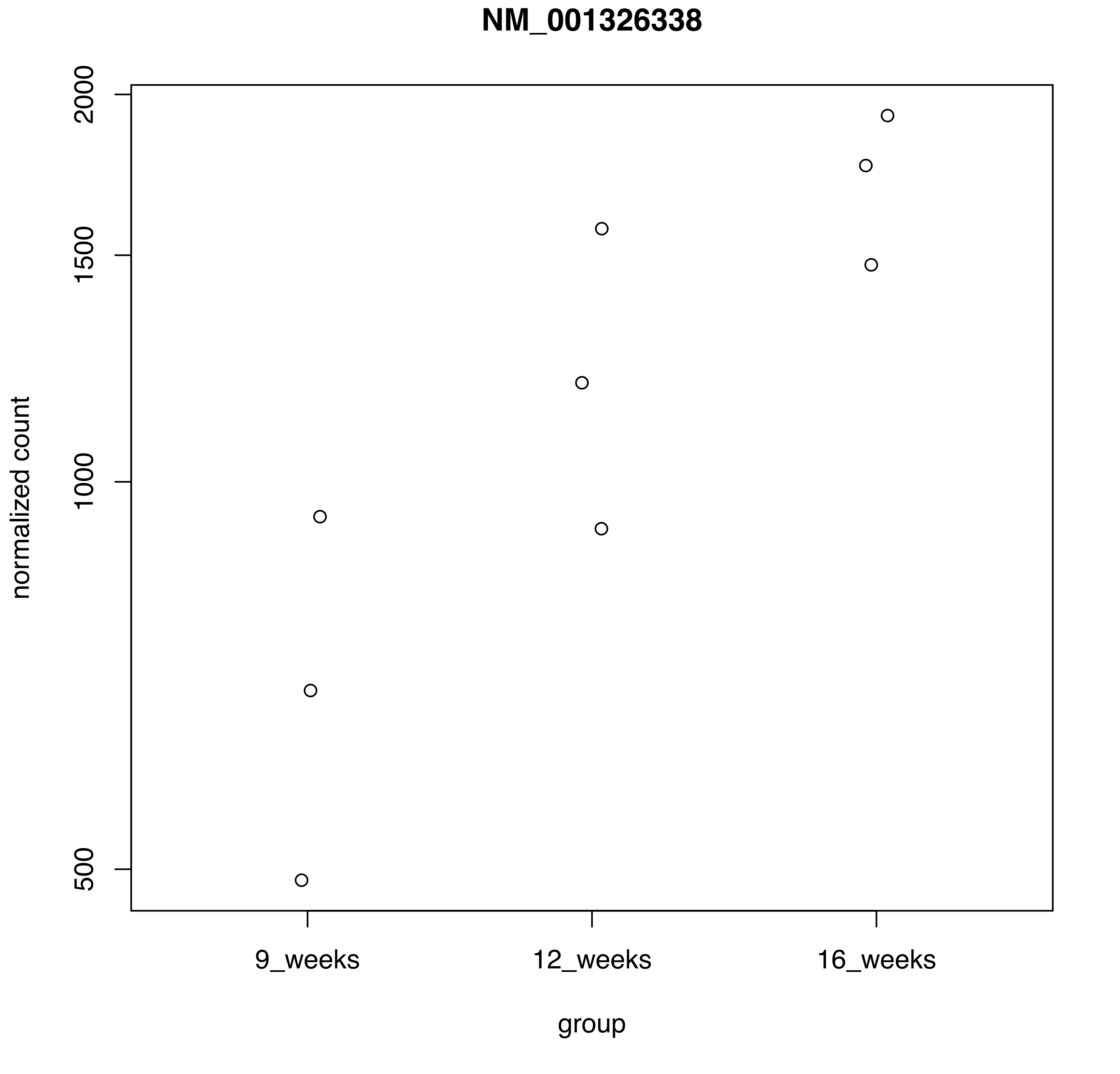
NM_001326338
normalized count
2000
1500
1000
500
9_weeks
12_weeks
16_weeks
group

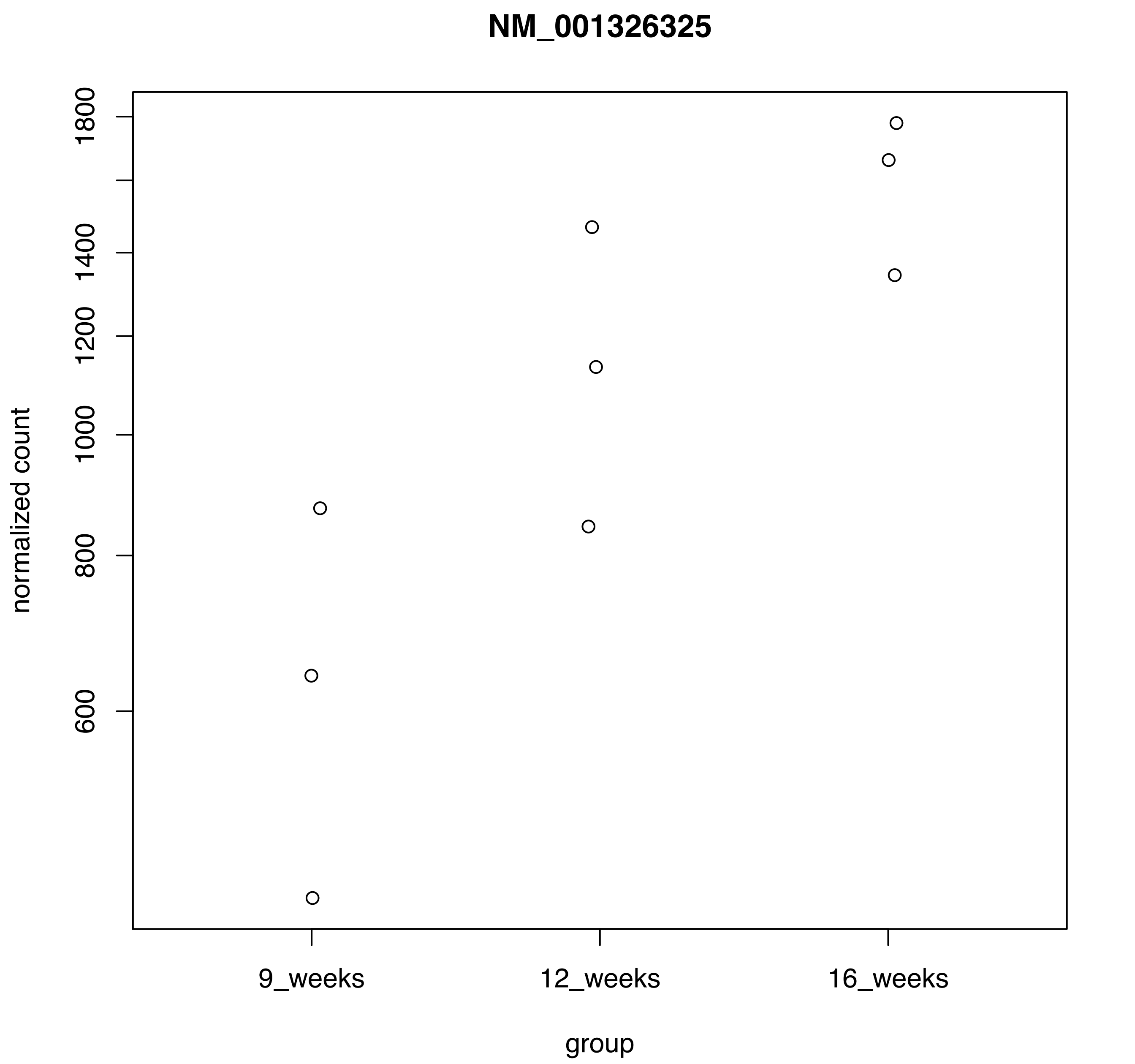

NM_001326325
normalized count
1800
1400
1200
1000
800
600
9_weeks
12_weeks
16_weeks
group

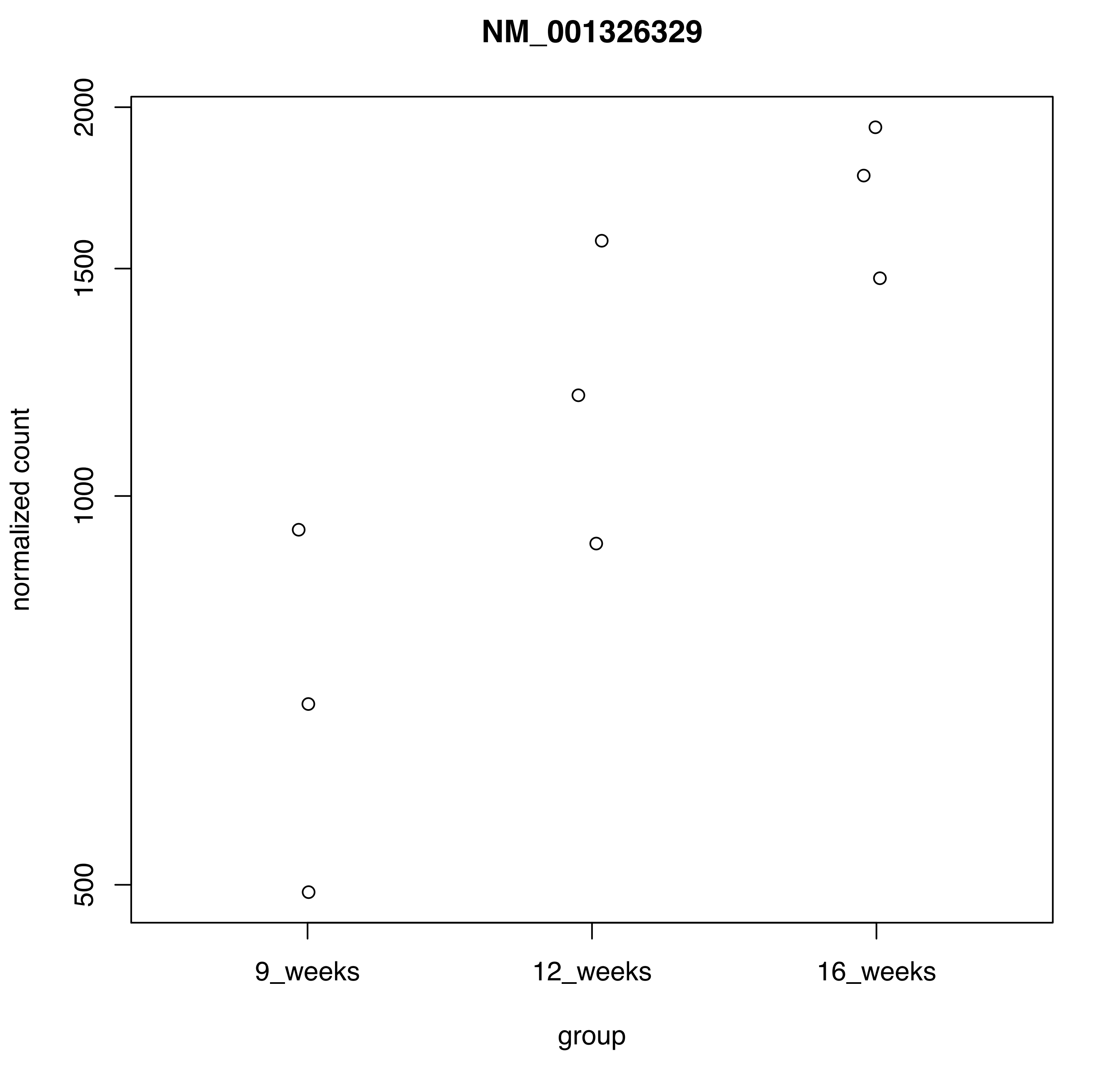
NM_001326329
normalized count
2000
1500
1000
500
9_weeks
12_weeks
16_weeks
group

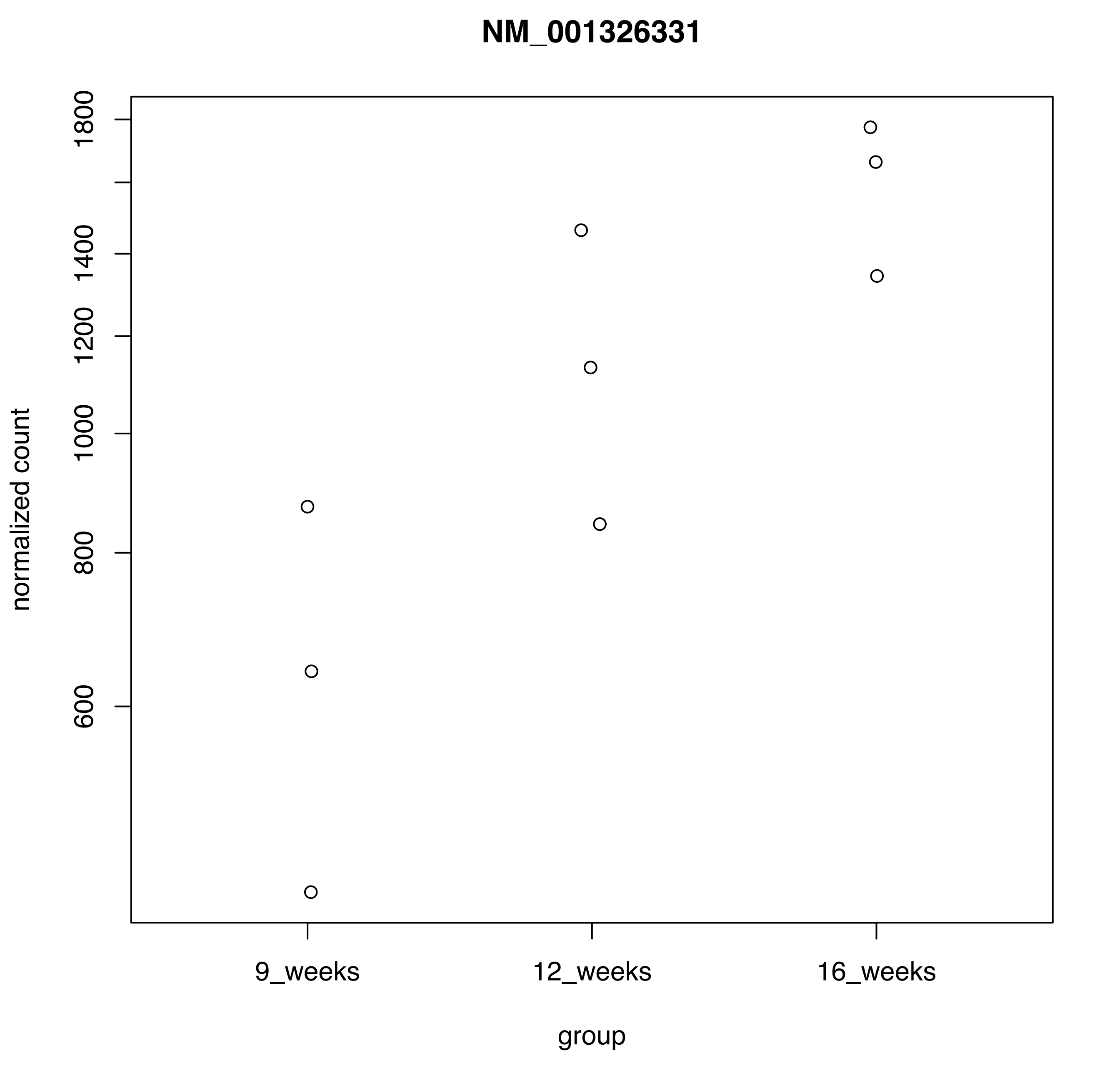
NM_001326331
normalized count
600
800
1000
1200
1400
1800
9_weeks
12_weeks
16_weeks
group

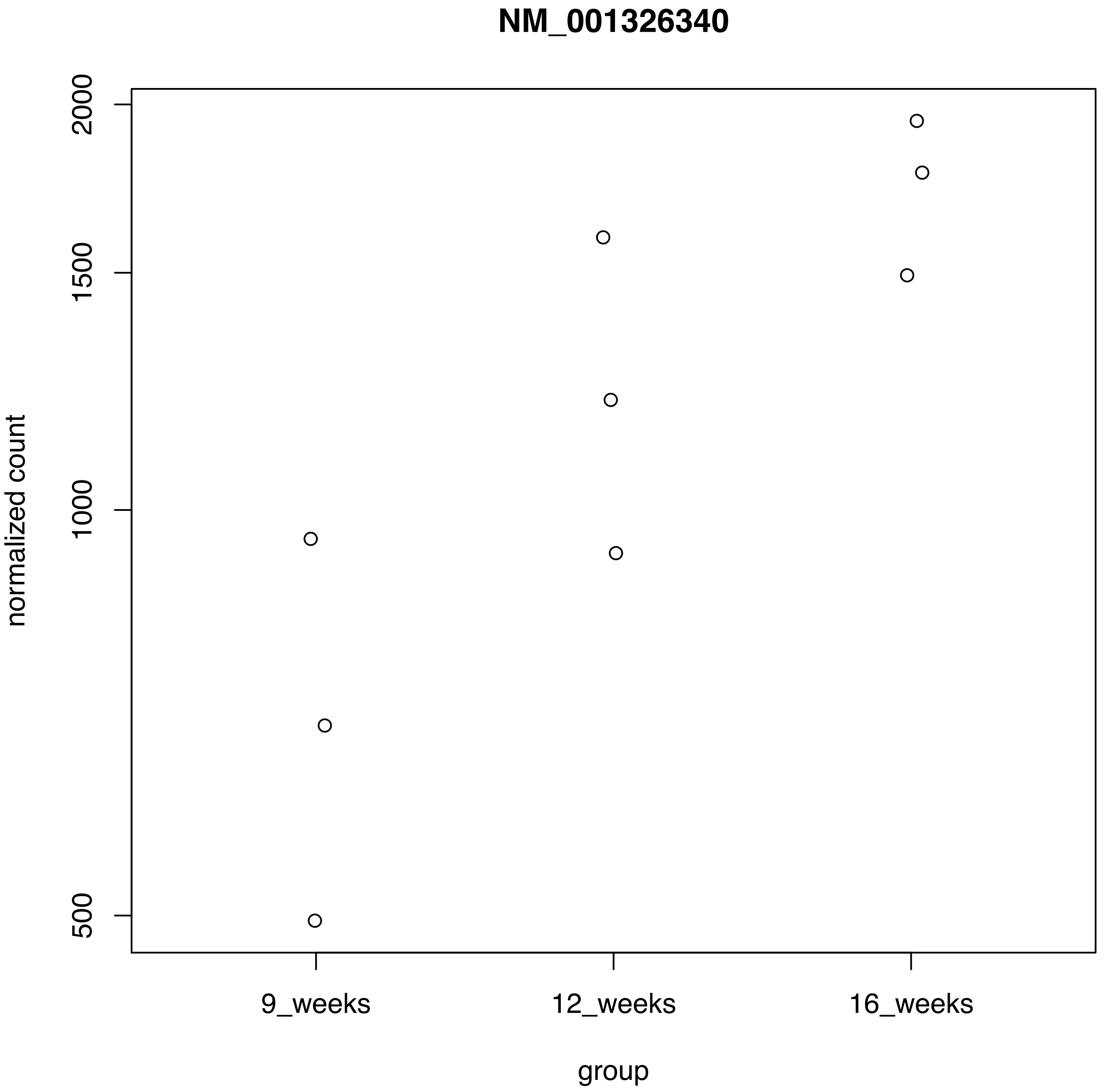

NM_001326340
normalized count
2000
1500
1000
500
9_weeks
12_weeks
16_weeks
group

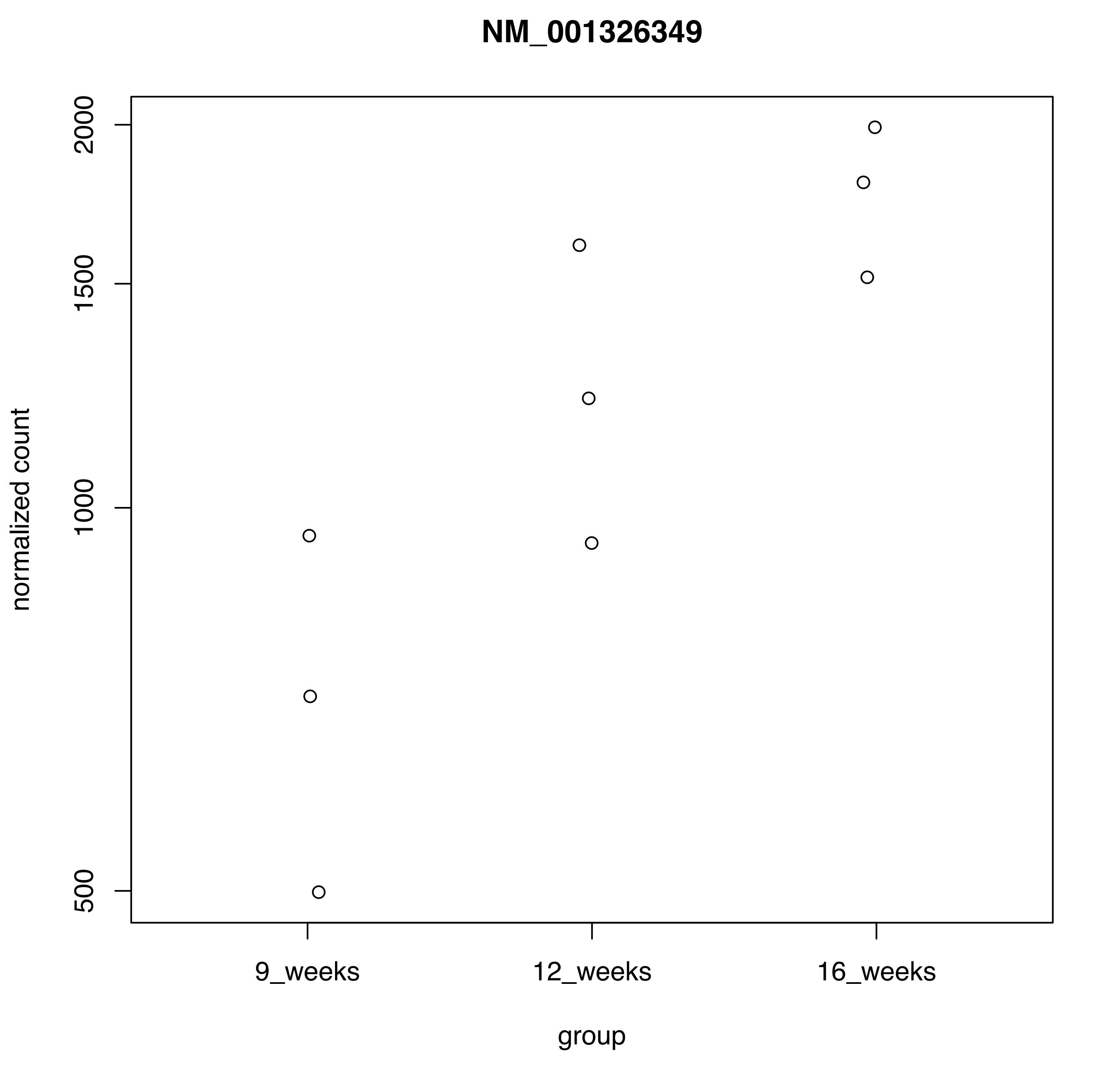
NM_001326349
normalized count
2000
1500
1000
500
9_weeks
12_weeks
16_weeks
group

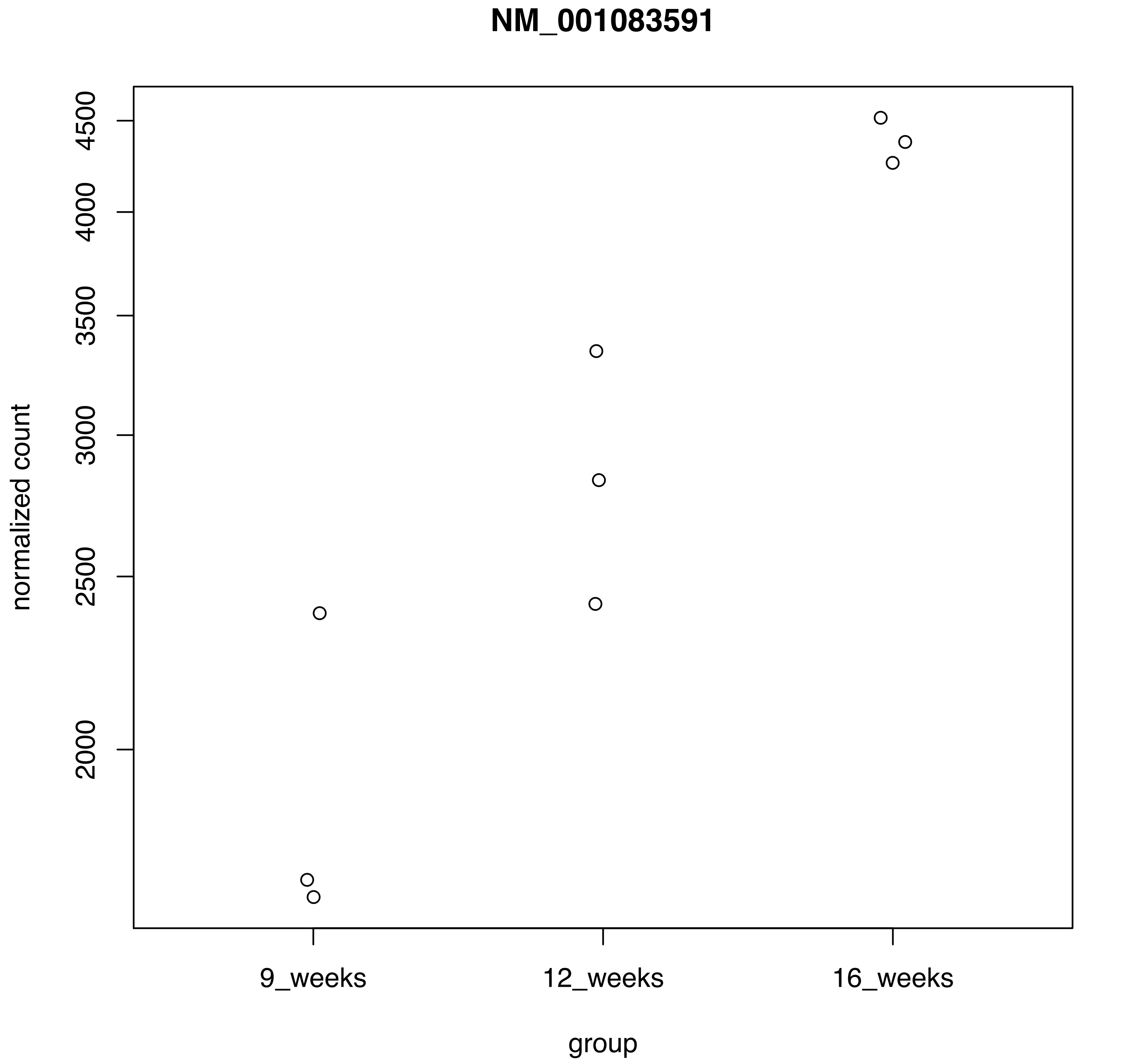

NM_001083591
normalized count
4500
4000
3500
3000
2500
2000
9_weeks
12_weeks
16_weeks
group

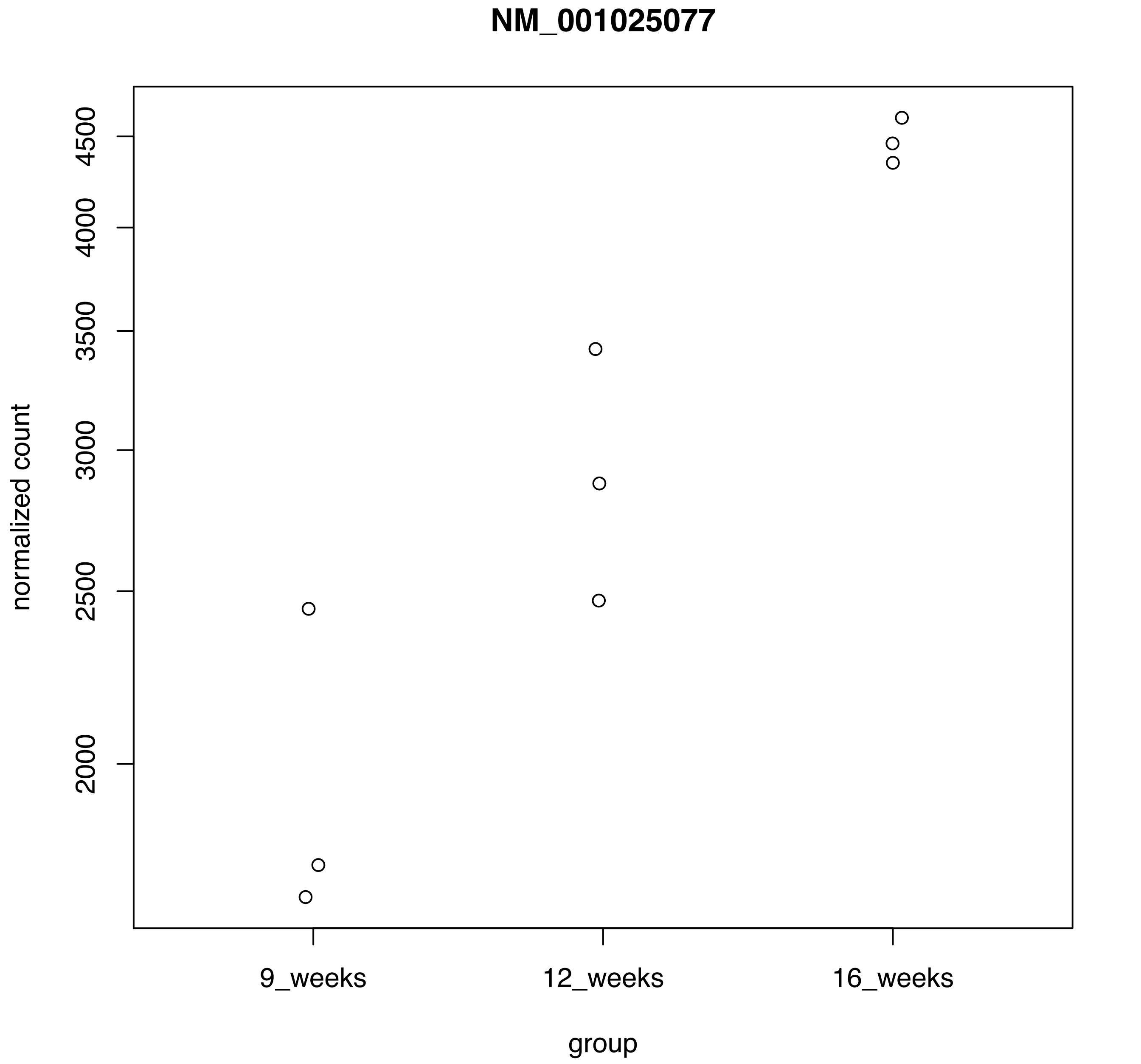
NM_001025077
normalized count
4500
4000
3500
3000
2500
2000
9_weeks
12_weeks
16_weeks
group

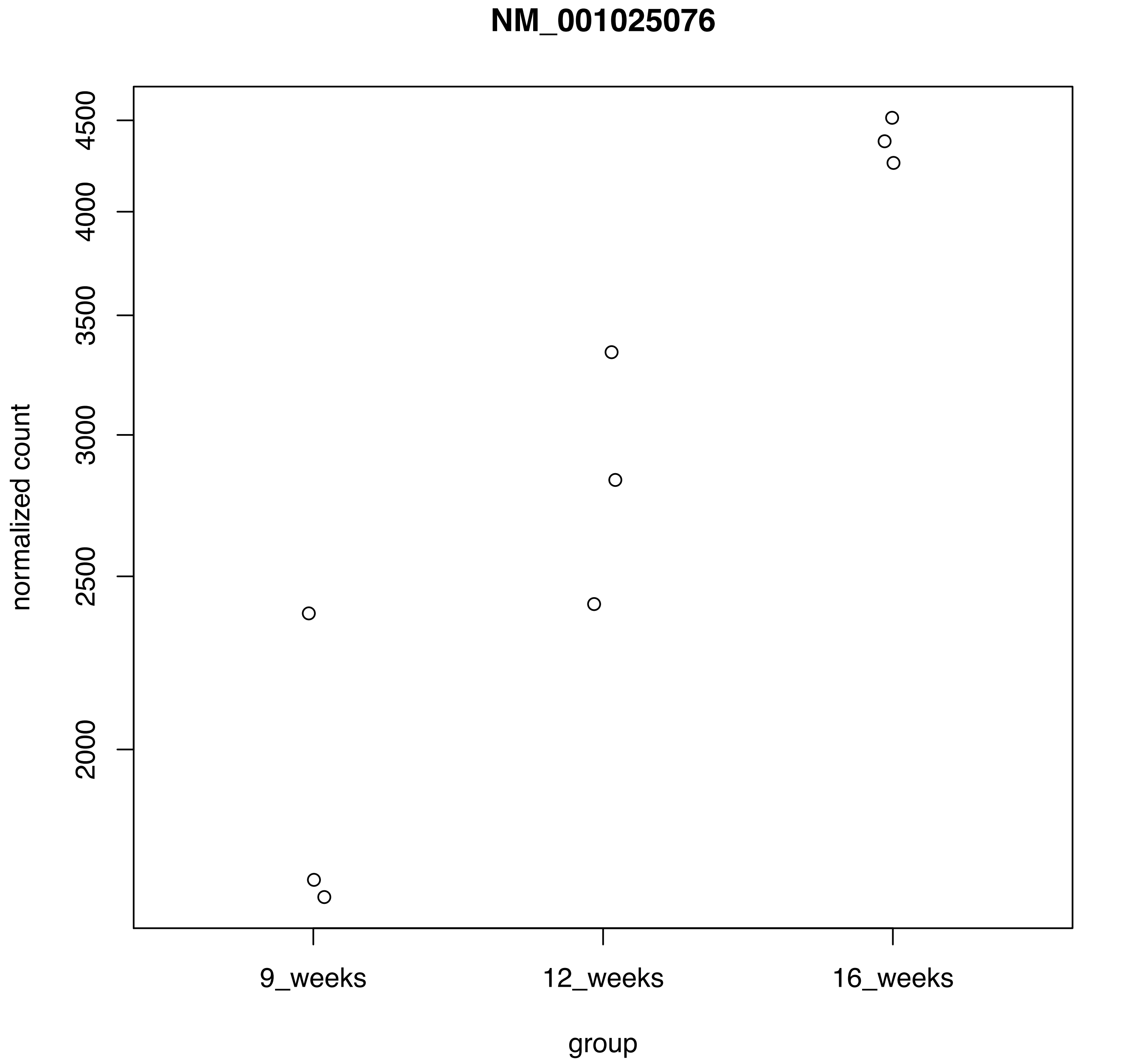
NM_001025076
normalized count
4500
4000
3500
3000
2500
2000
9_weeks
12_weeks
16_weeks
group

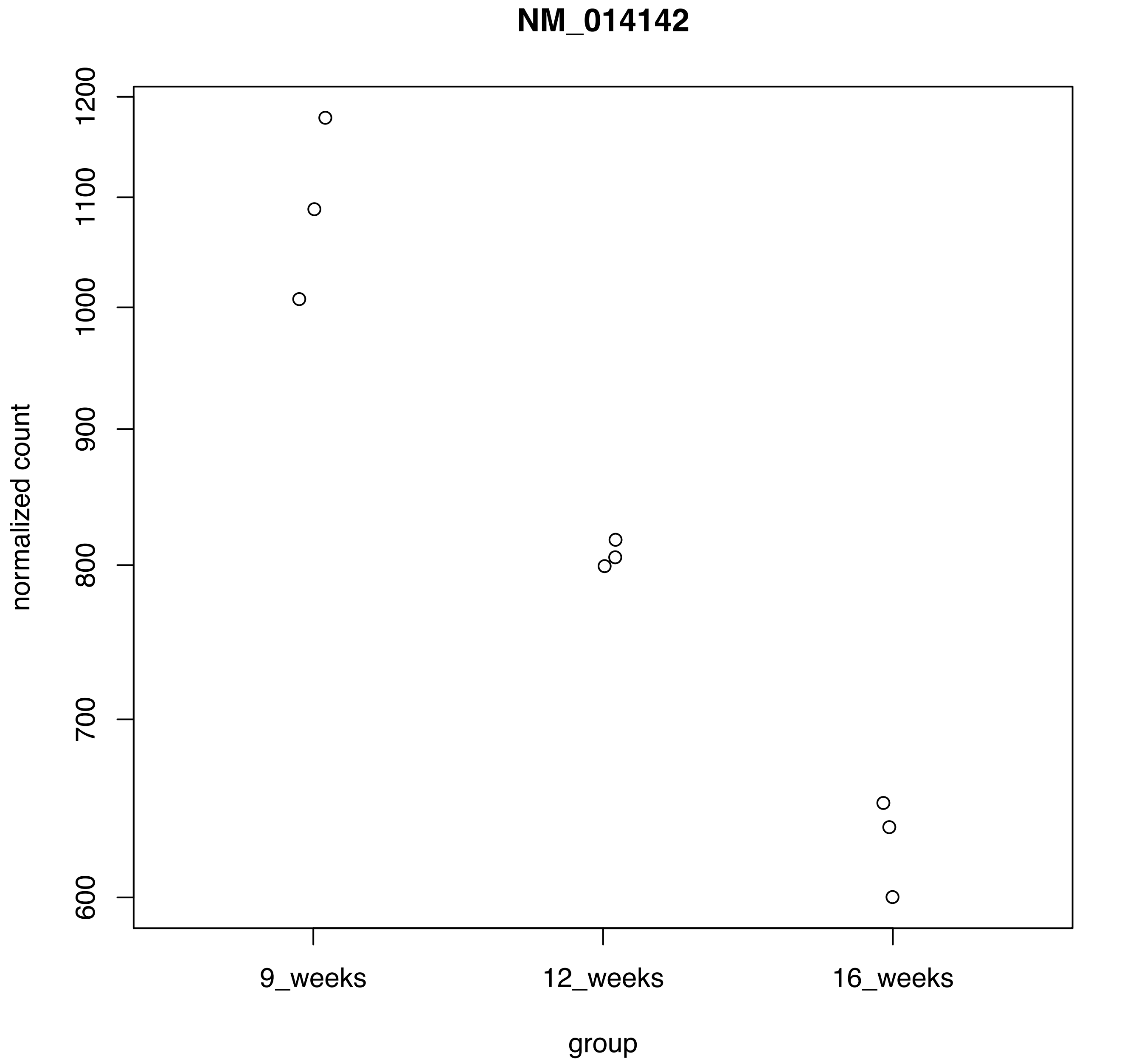

NM_014142
normalized count
1200
1100
1000
900
800
700
600
9_weeks
12_weeks
16_weeks
group

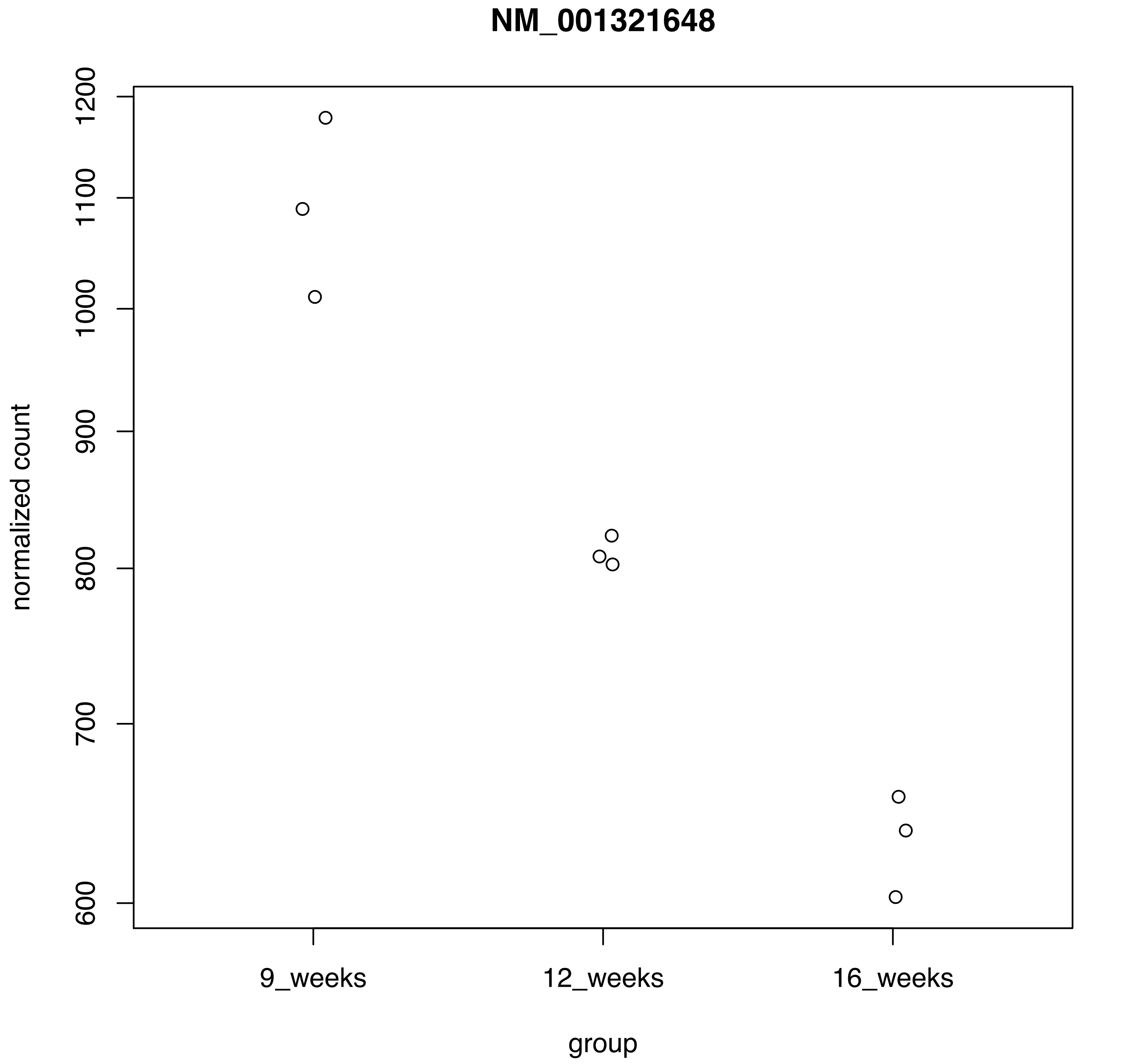

NM_001321648
normalized count
1200
1100
1000
900
800
700
600
9_weeks
12_weeks
16_weeks
group

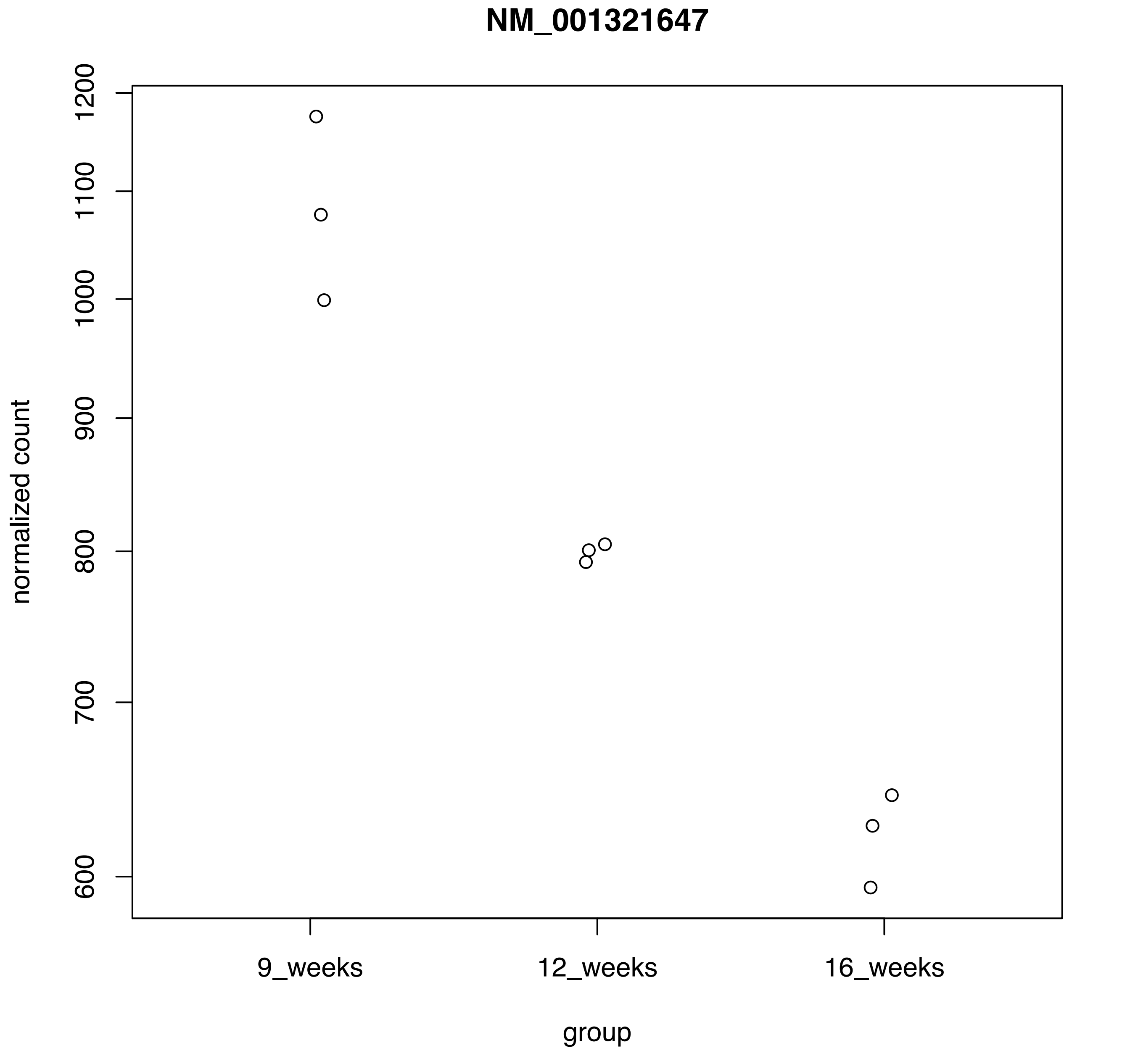
NM_001321647
normalized count
1200
1100
1000
900
800
700
600
9_weeks
12_weeks
16_weeks
group

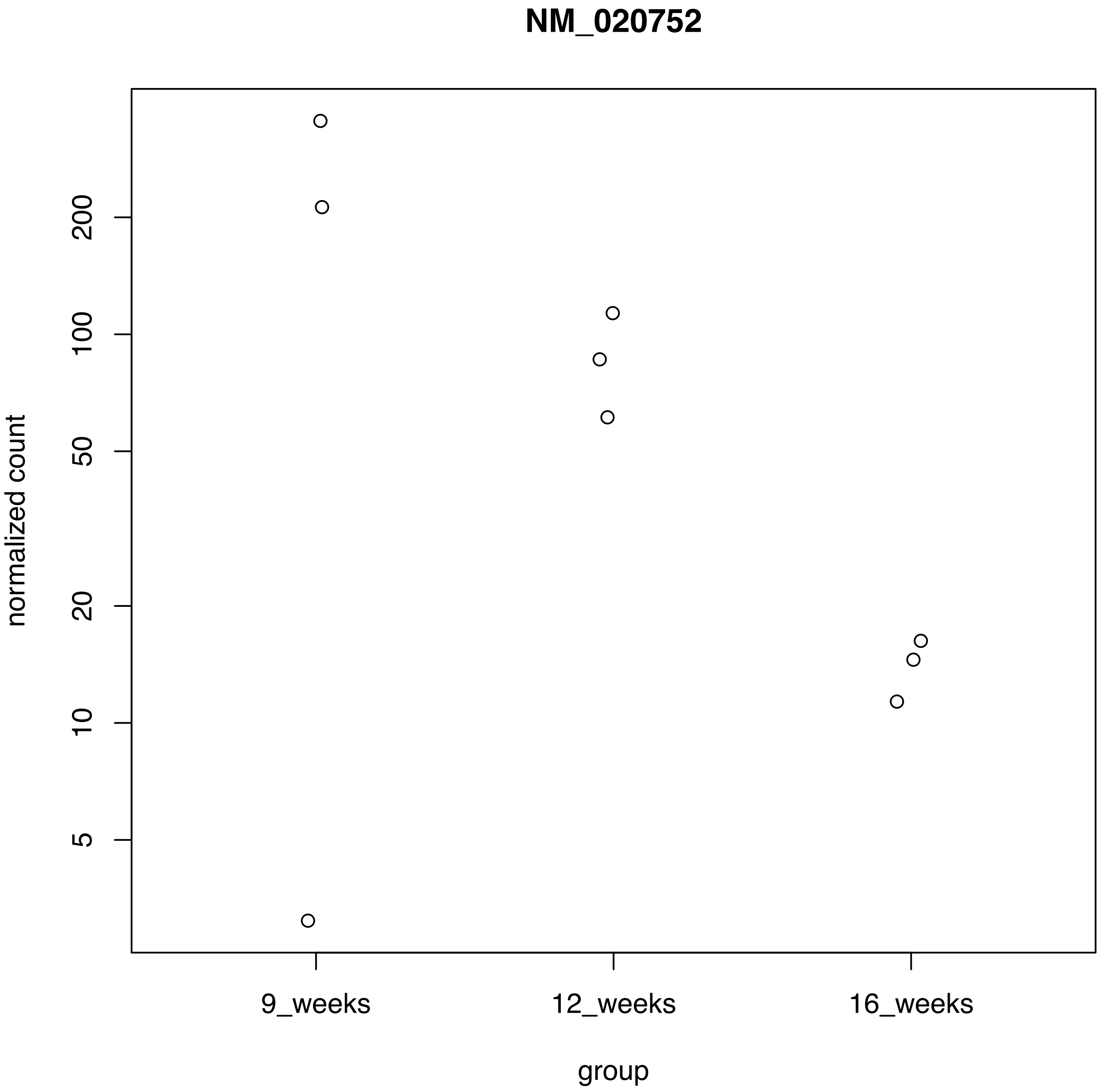
NM_020752
normalized count
200
100
50
20
10
5
9_weeks
12_weeks
16_weeks
group

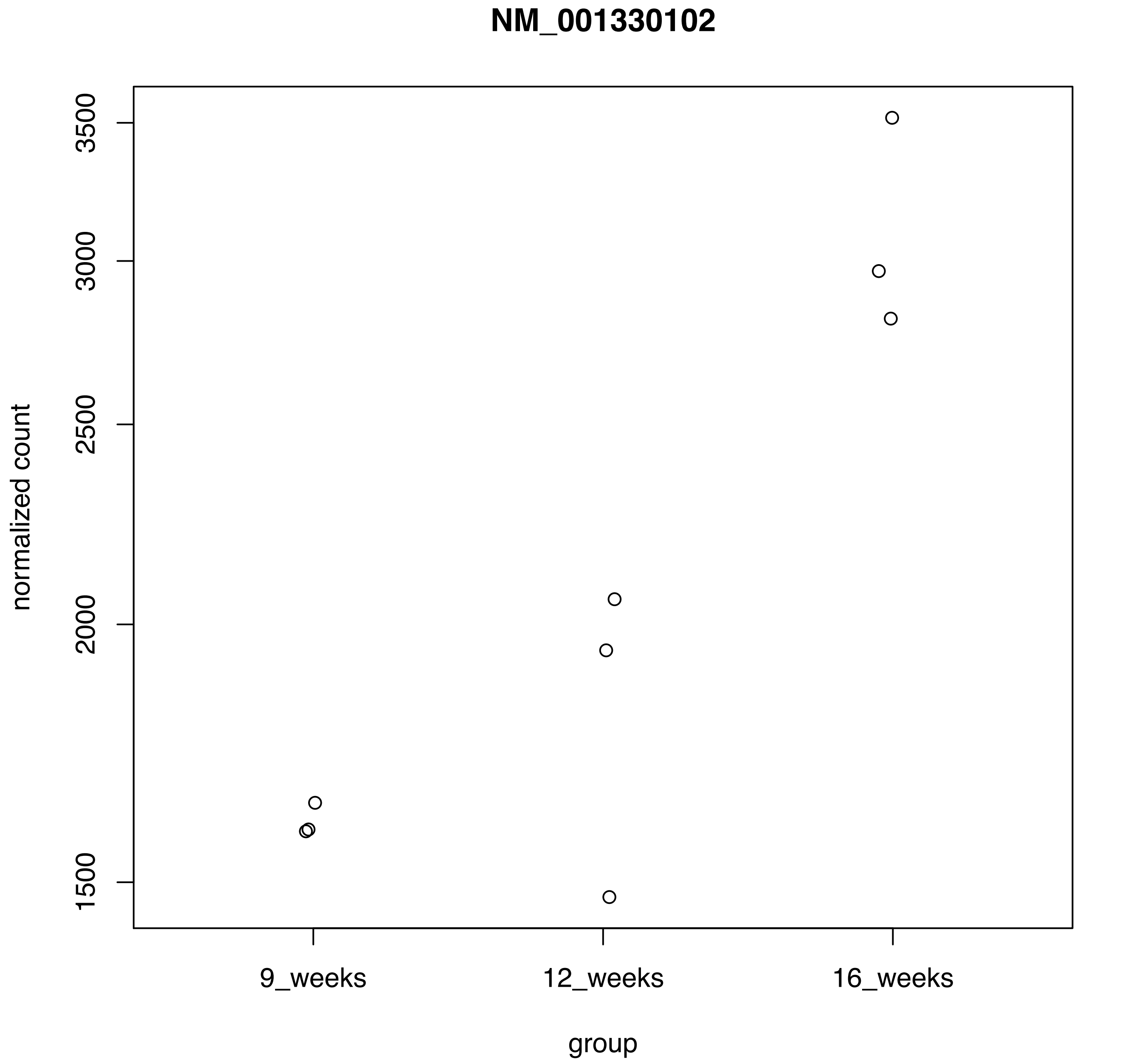

NM_001330102
normalized count
3500
3000
2500
2000
1500
9_weeks
12_weeks
16_weeks
group

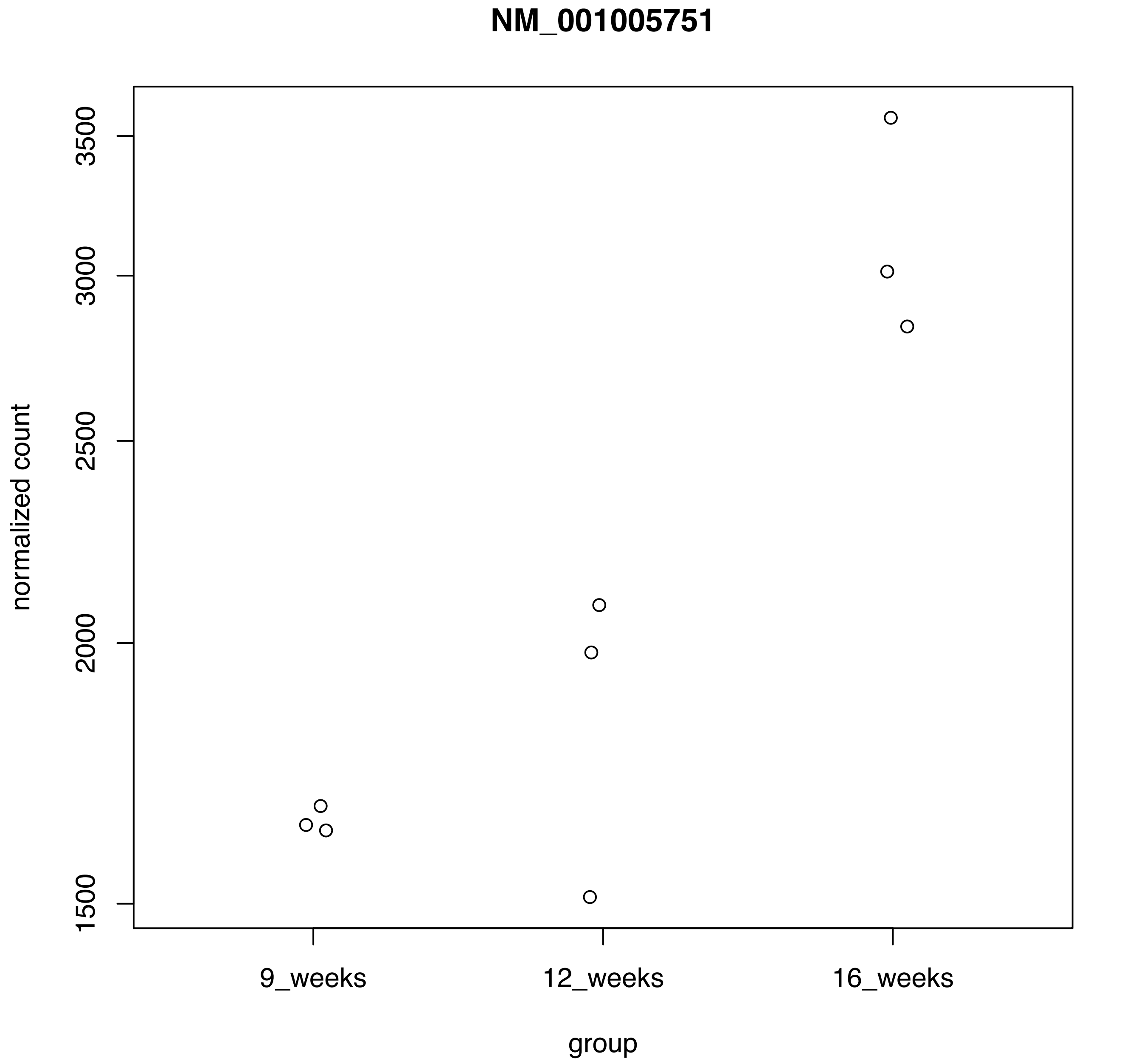
NM_001005751
normalized count
3500
3000
2500
2000
1500
9_weeks
12_weeks
16_weeks
group

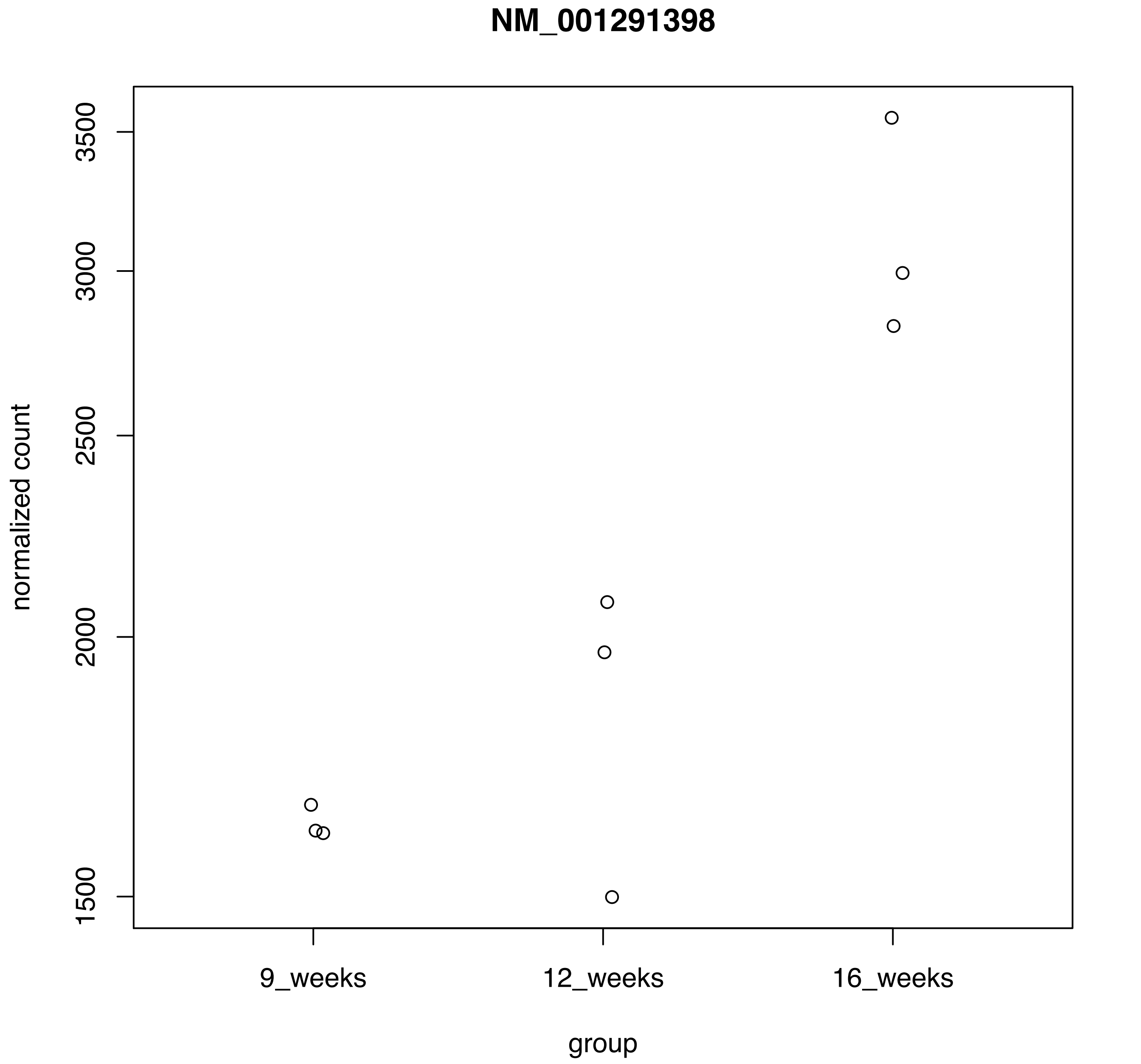
NM_001291398
normalized count
1500
2000
2500
3000
3500
9_weeks
12_weeks
16_weeks
group

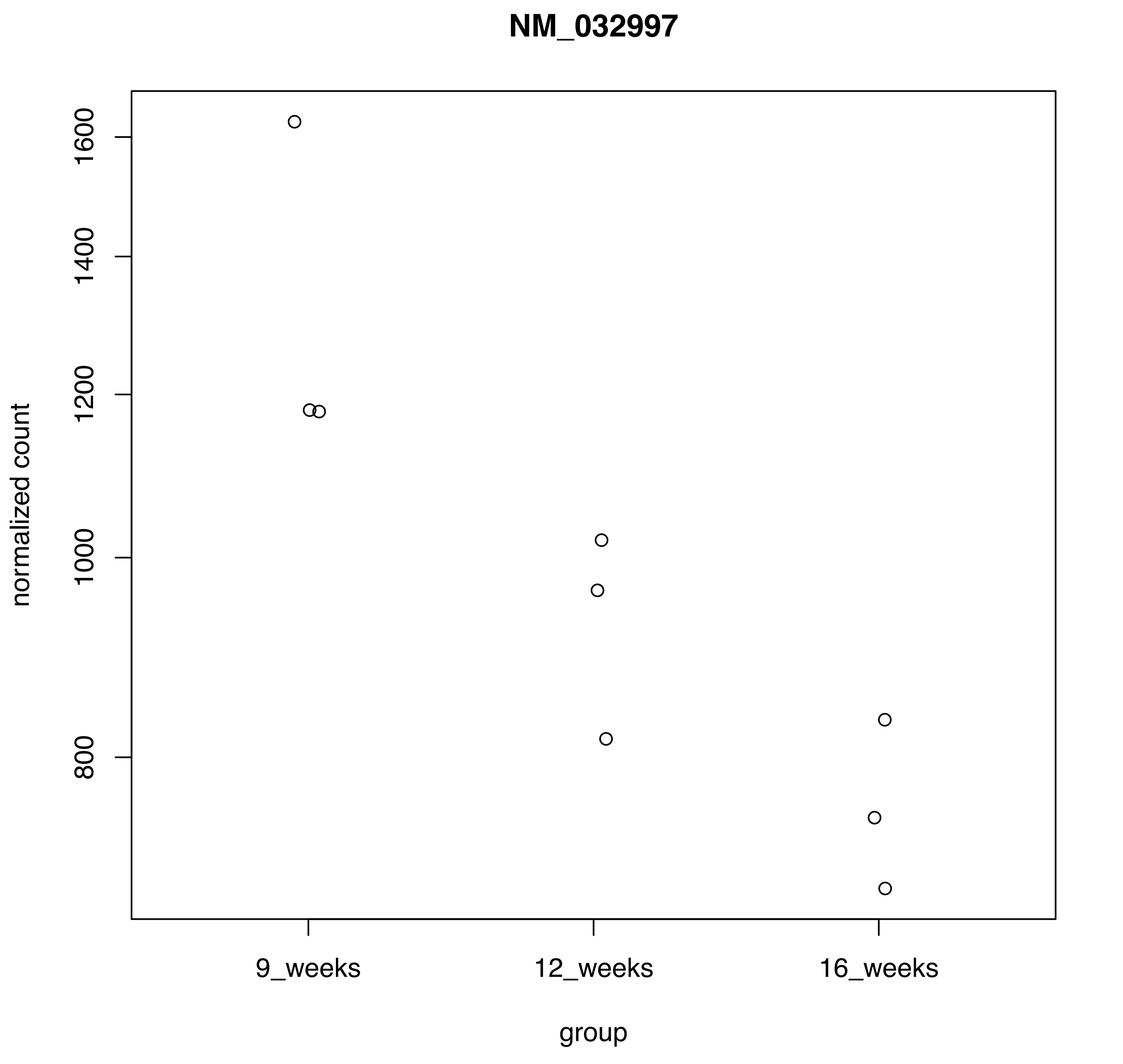
NM_032997
normalized count
1600
1400
1200
1000
800
9_weeks
12_weeks
16_weeks
group

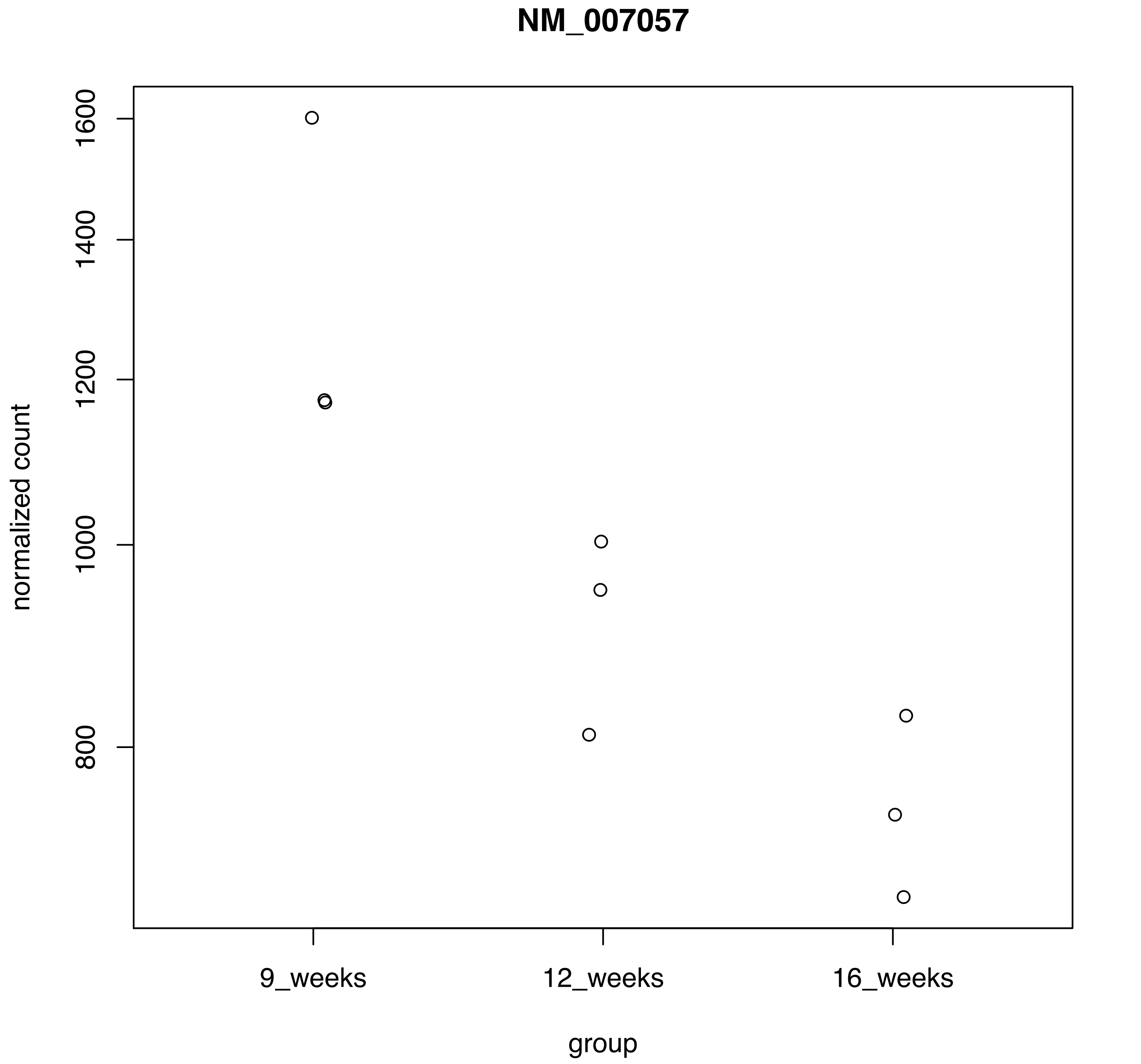
NM_007057
normalized count
1600
1400
1200
1000
800
9_weeks
12_weeks
16_weeks
group

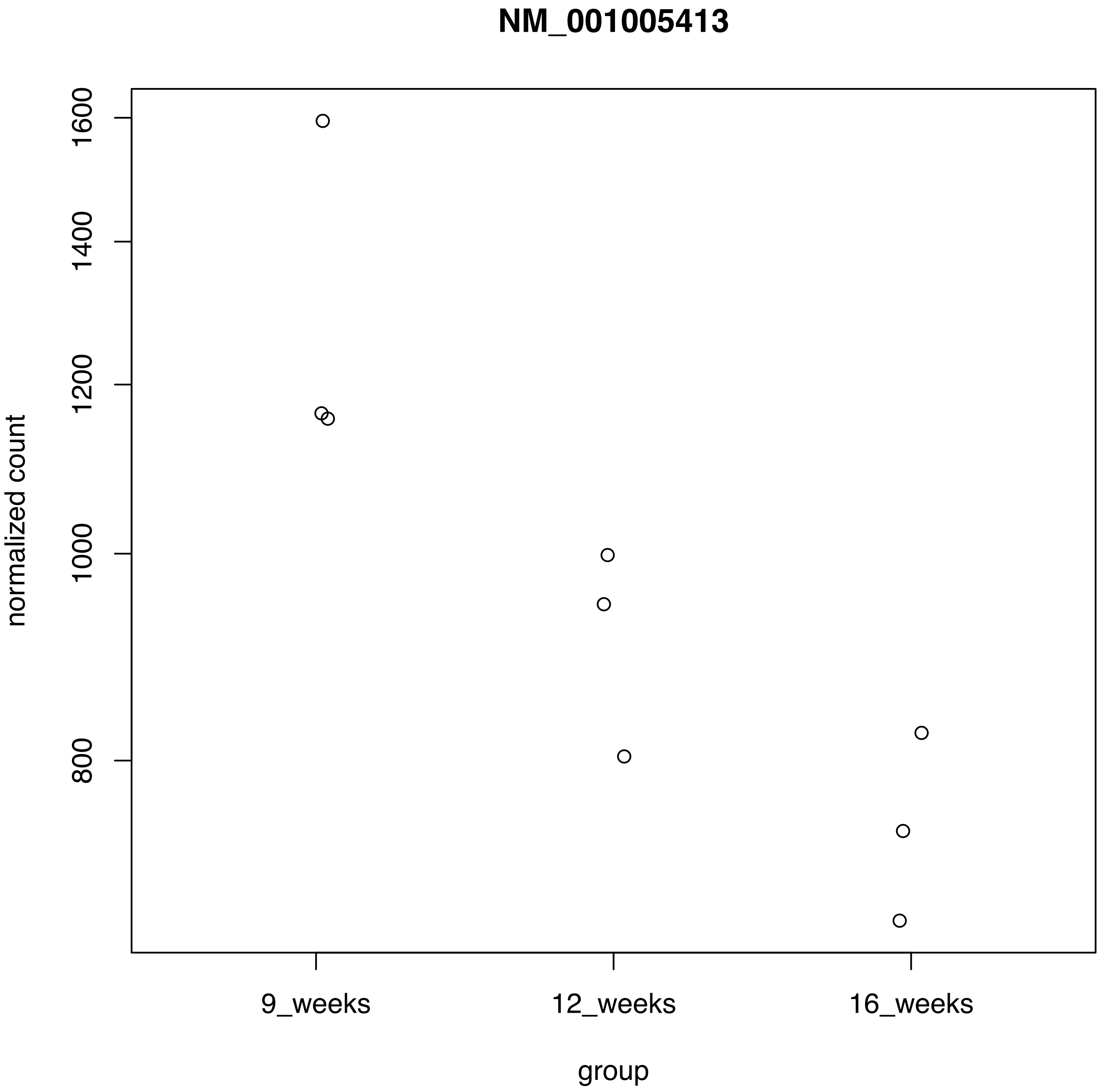
NM_001005413
normalized count
1600
1400
1200
1000
800
9_weeks
12_weeks
16_weeks
group

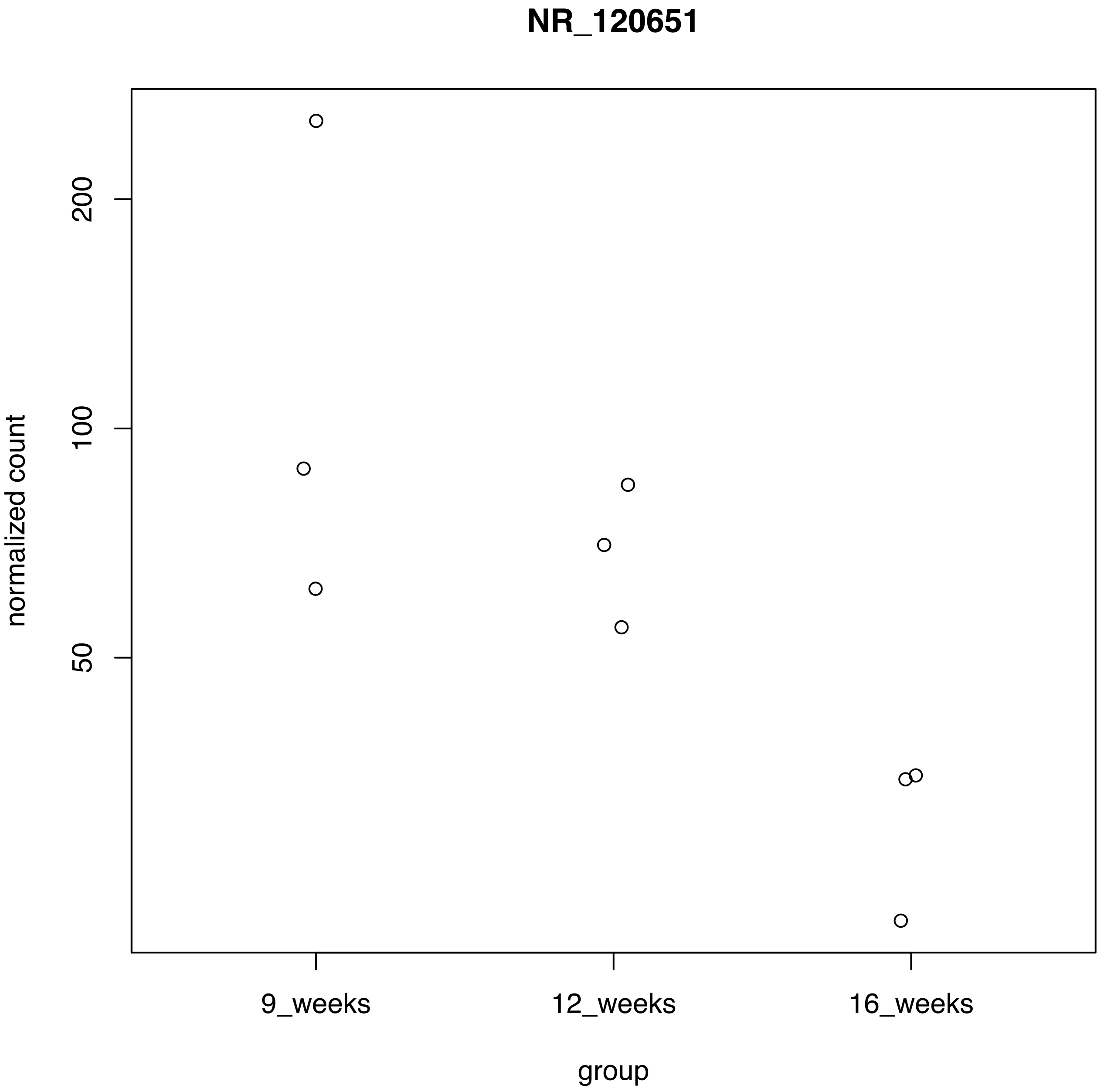

NR_120651
normalized count
200
100
50
9_weeks
12_weeks
16_weeks
group

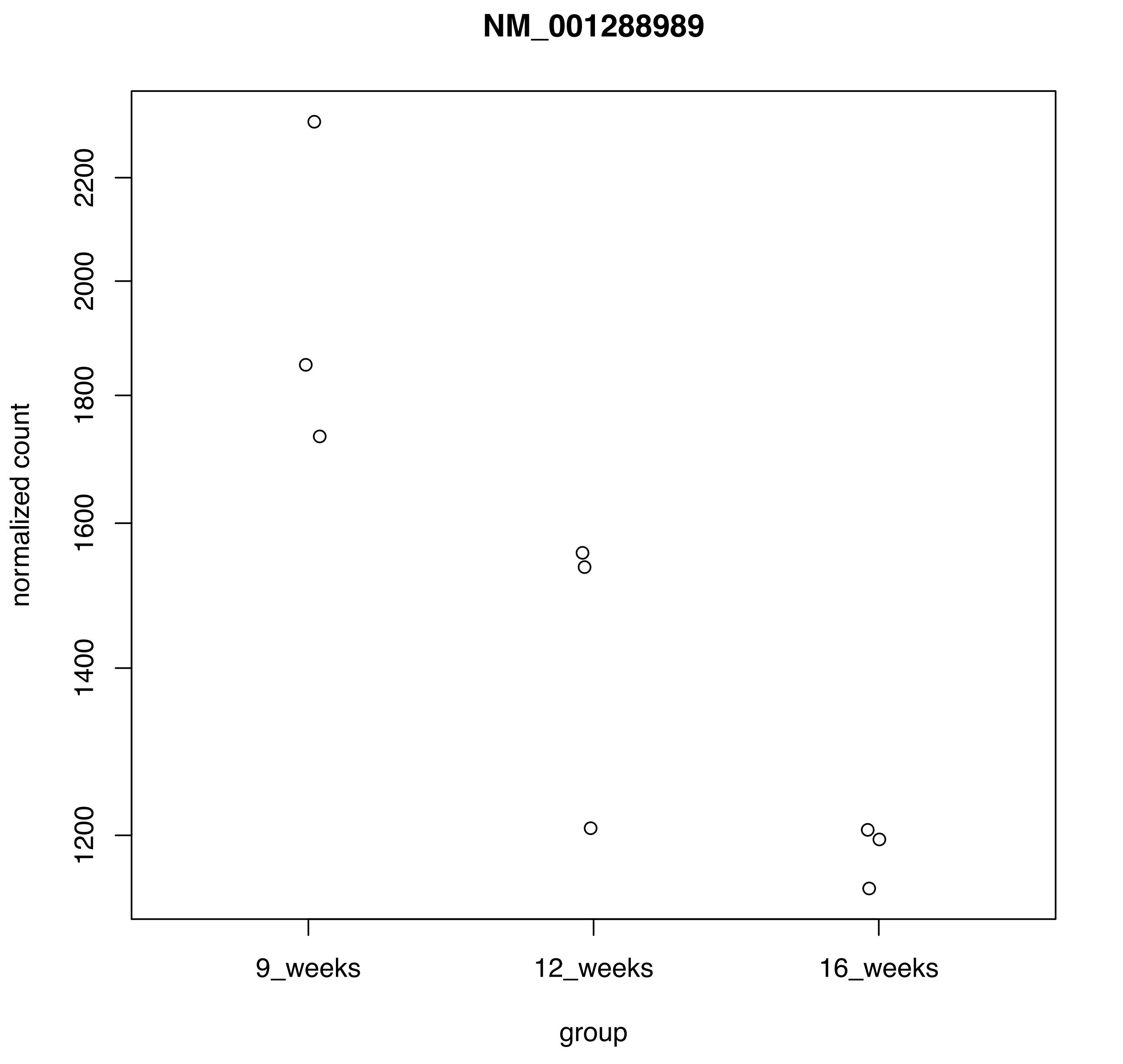
NM_001288989
normalized count
2200
2000
1800
1600
1400
1200
9_weeks
12_weeks
16_weeks
group

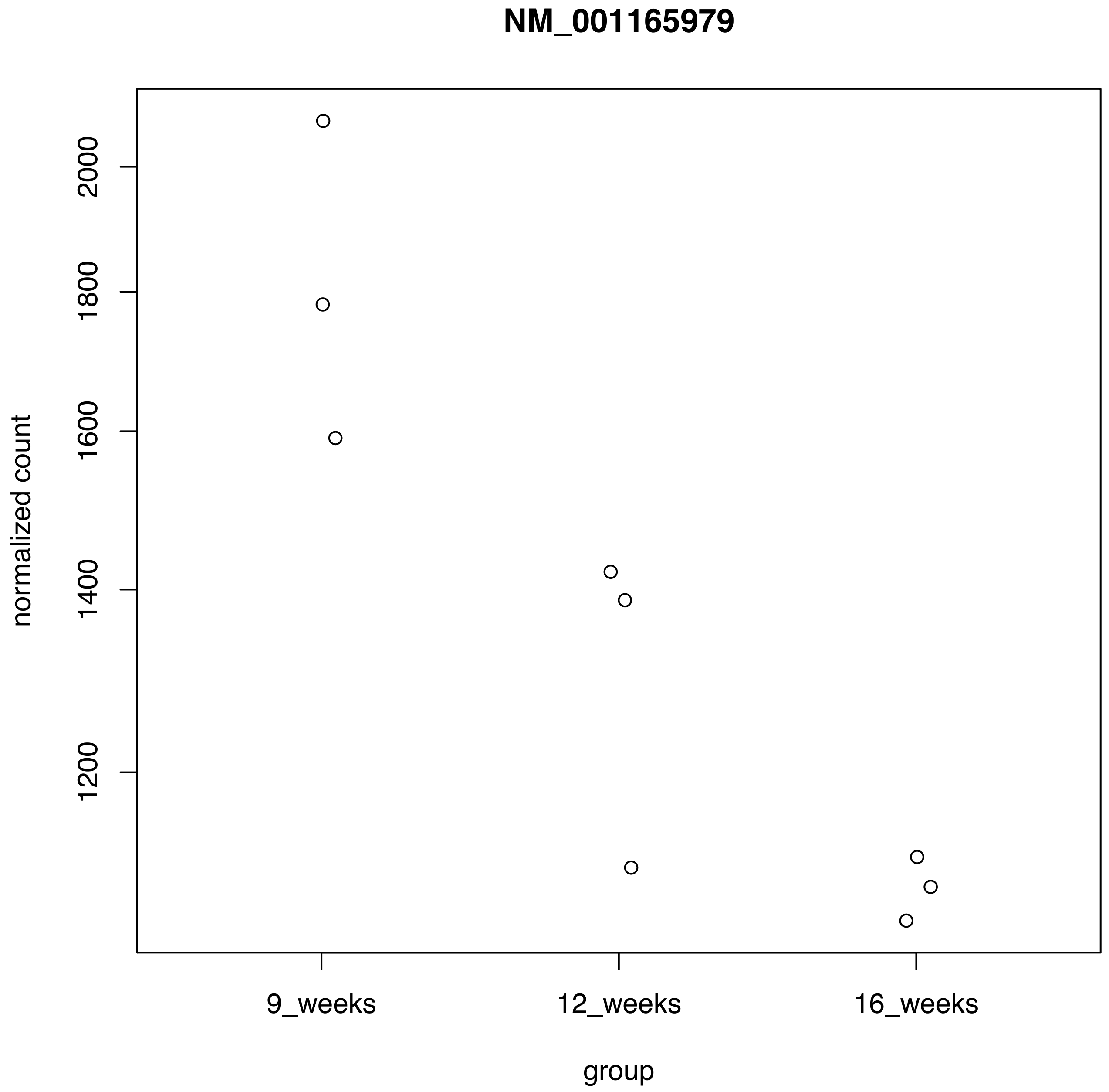
NM_001165979
2000
1800
1600
1400
1200
normalized count
9_weeks
12_weeks
16_weeks
group

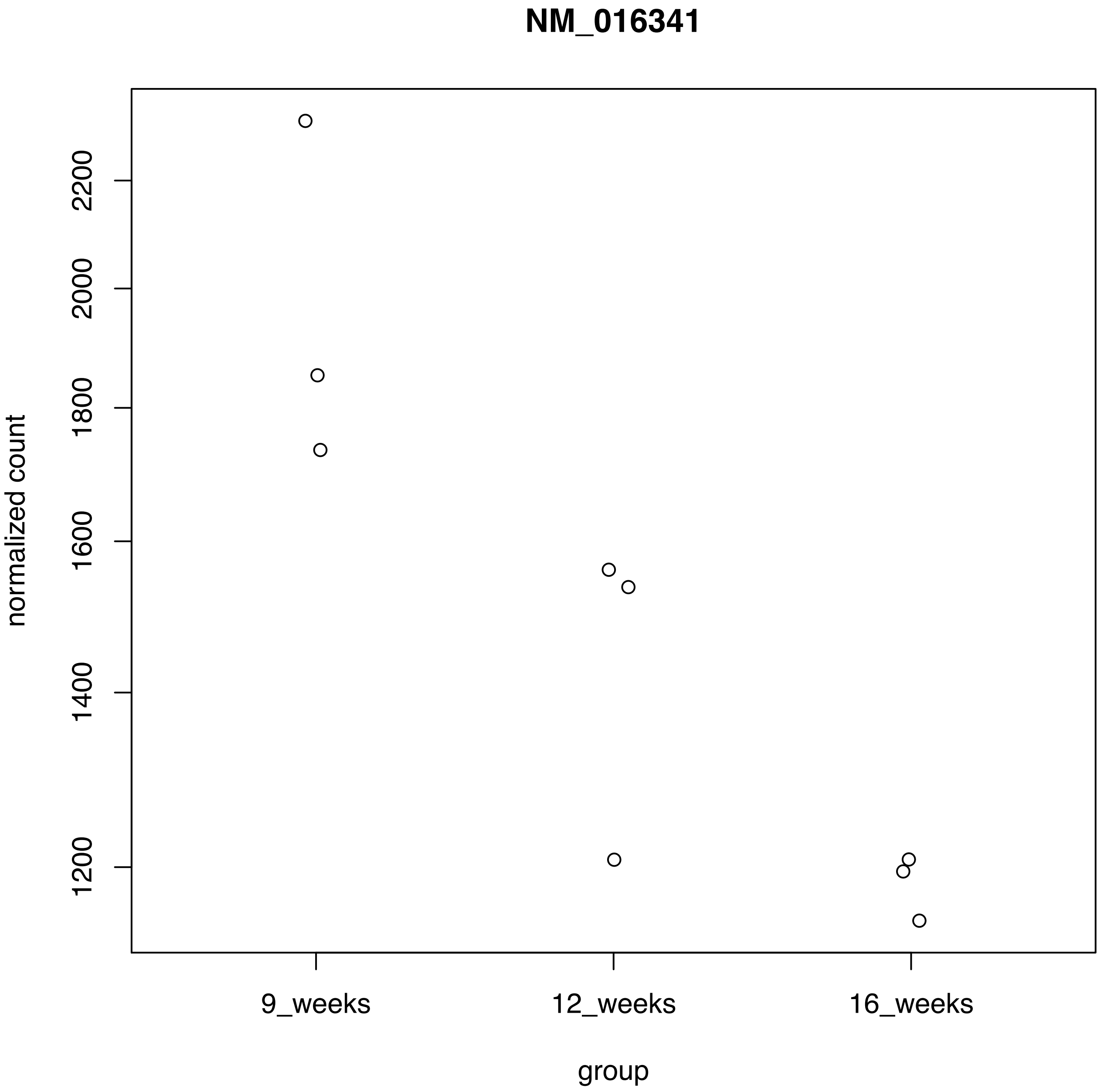
NM_016341
normalized count
2200
2000
1800
1600
1400
1200
9_weeks
12_weeks
16_weeks
group

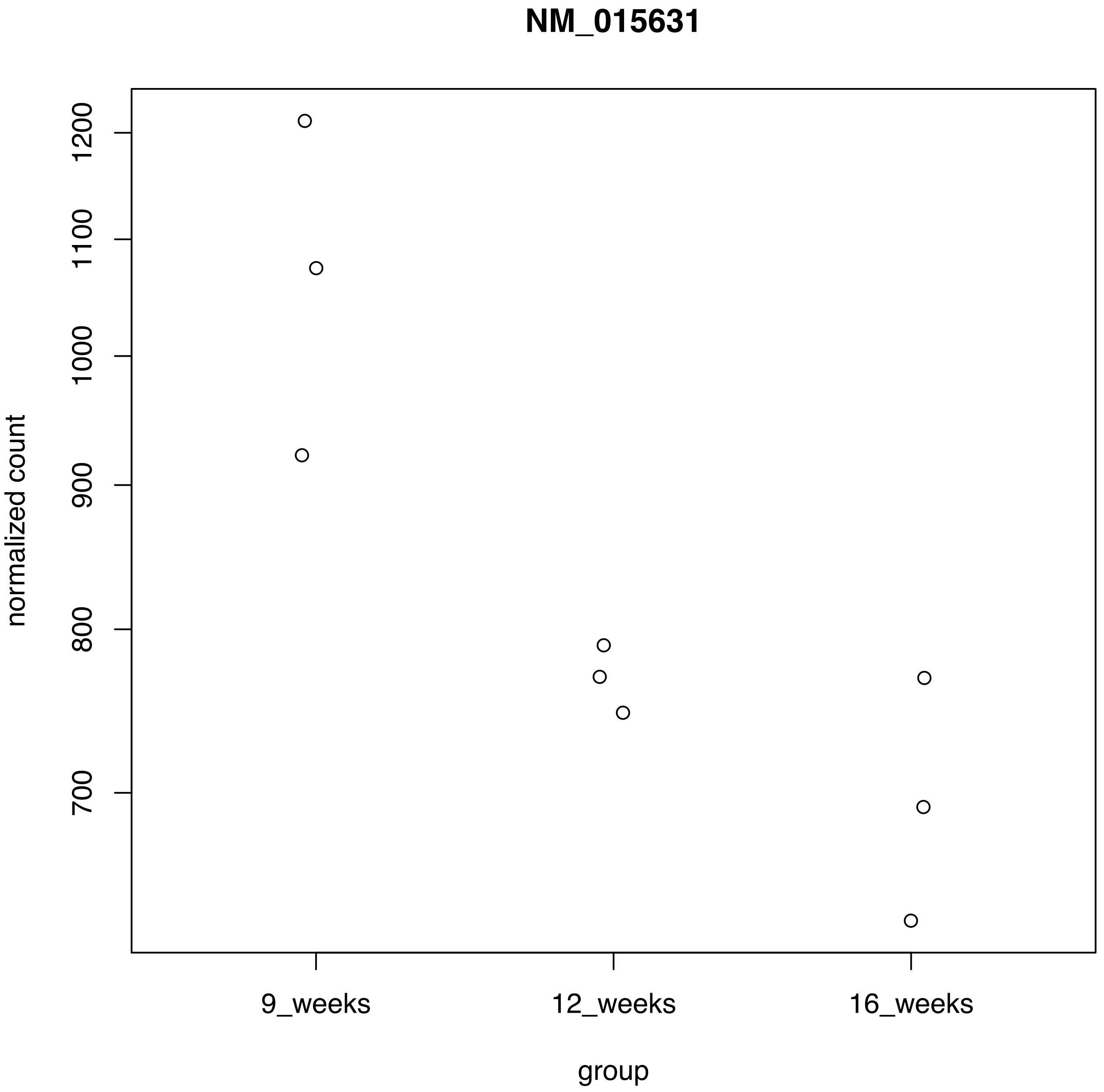
NM_015631
normalized count
1200
1100
1000
900
800
700
9_weeks
12_weeks
16_weeks
group

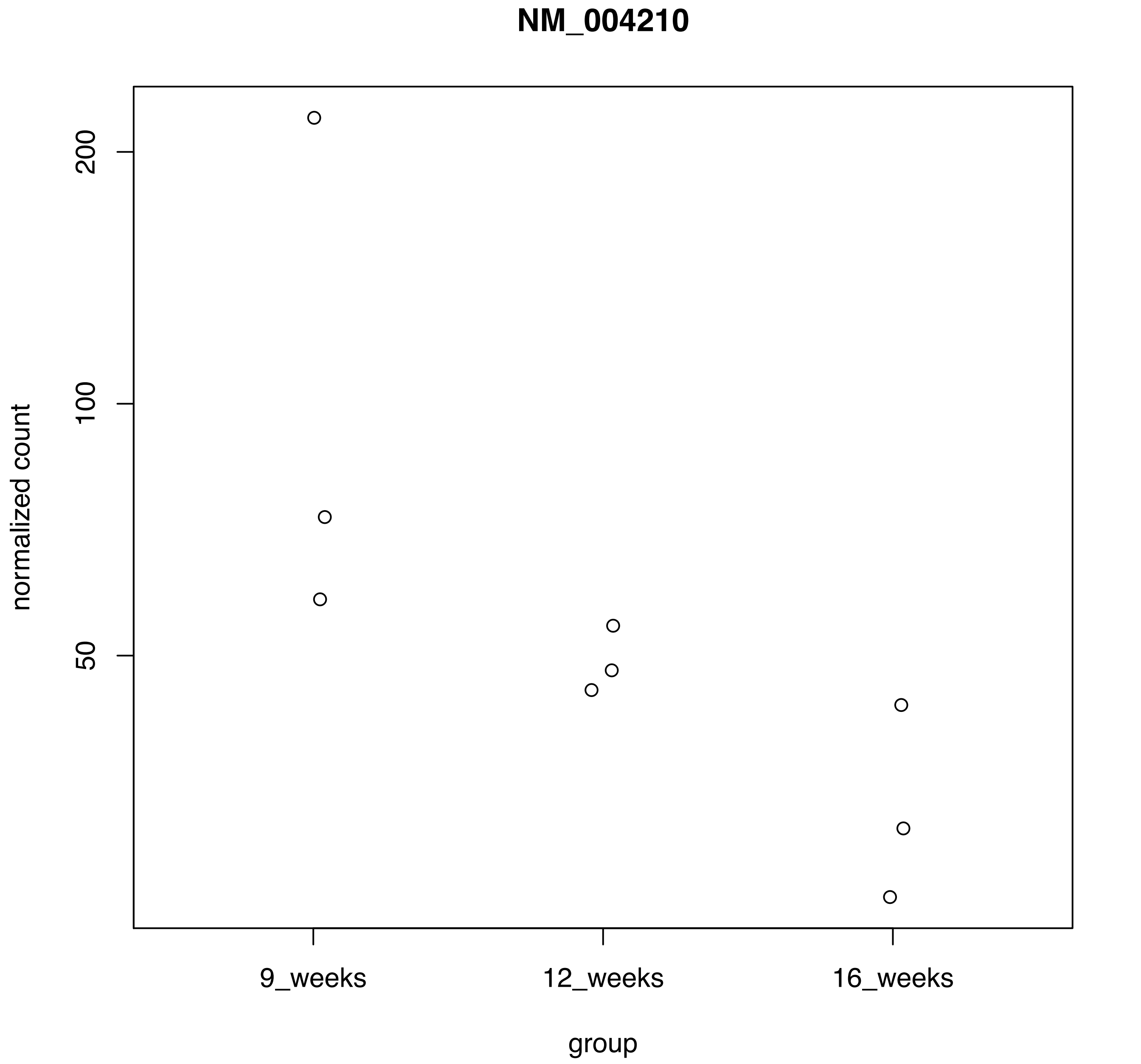
NM_004210
normalized count
200
100
50
9_weeks
12_weeks
16_weeks
group

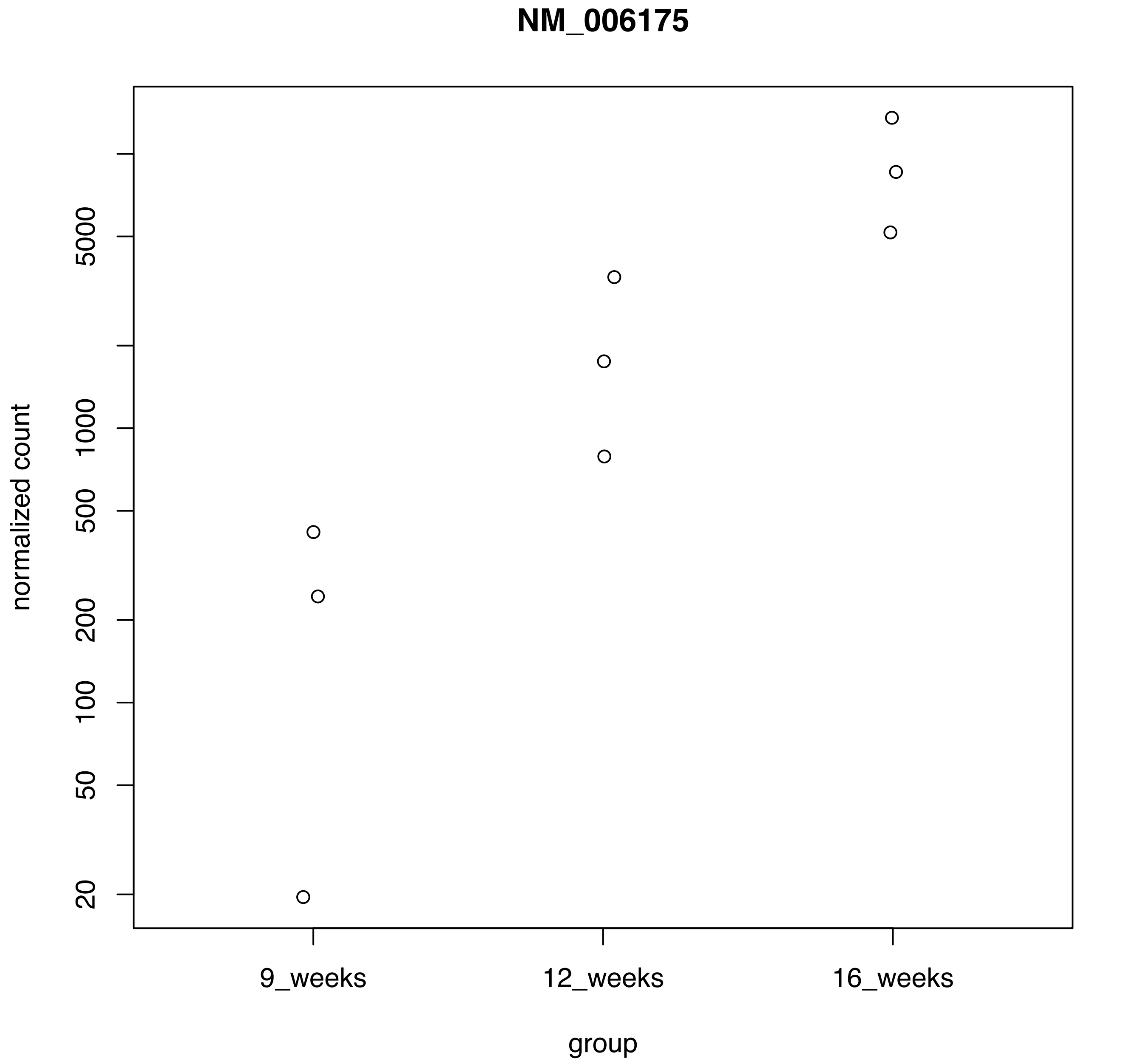
NM_006175
normalized count
5000
1000
500
200
100
50
20
9_weeks
12_weeks
16_weeks
group

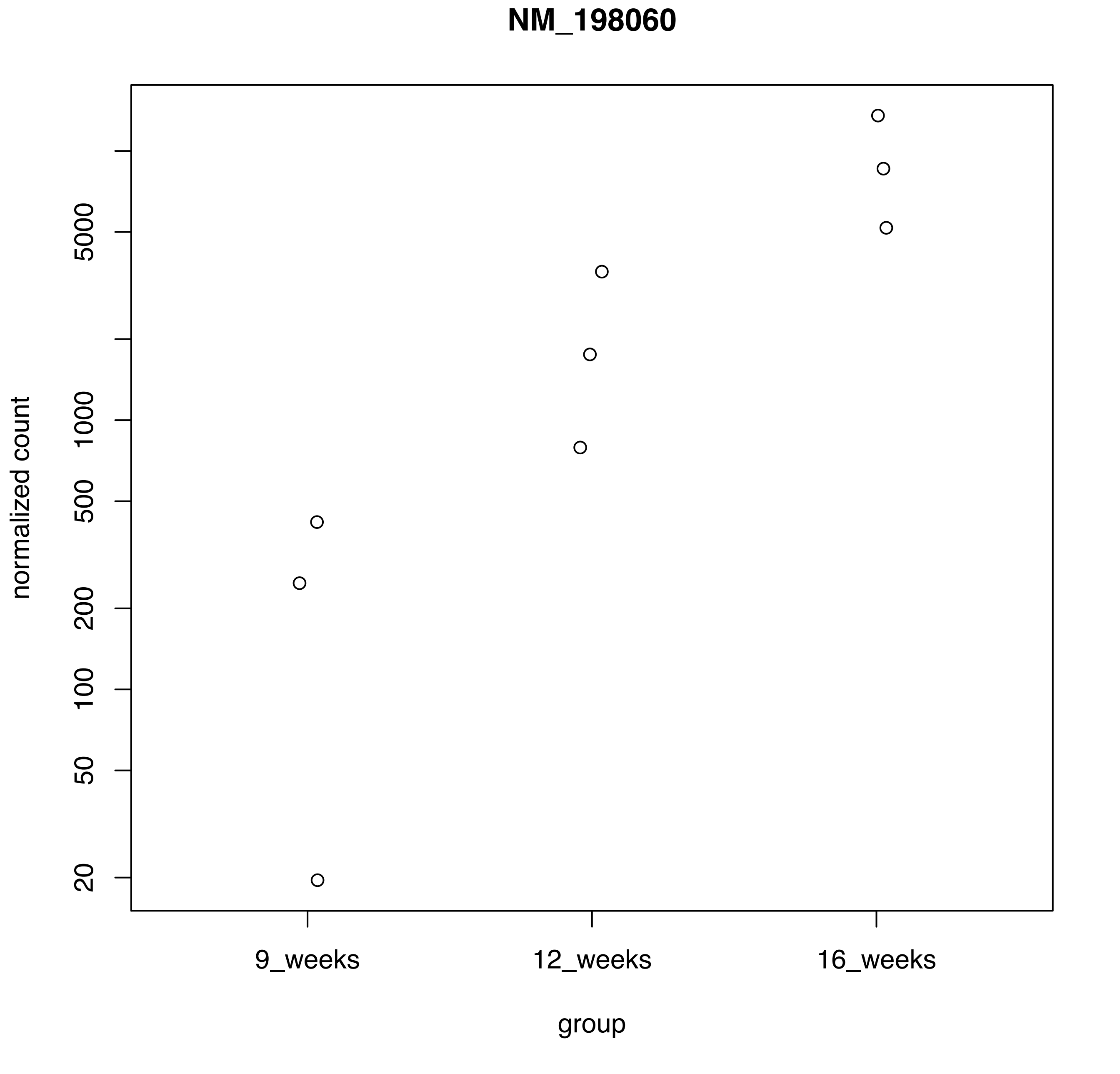

NM_198060
normalized count
5000
1000
500
200
100
50
20
9_weeks
12_weeks
16_weeks
group

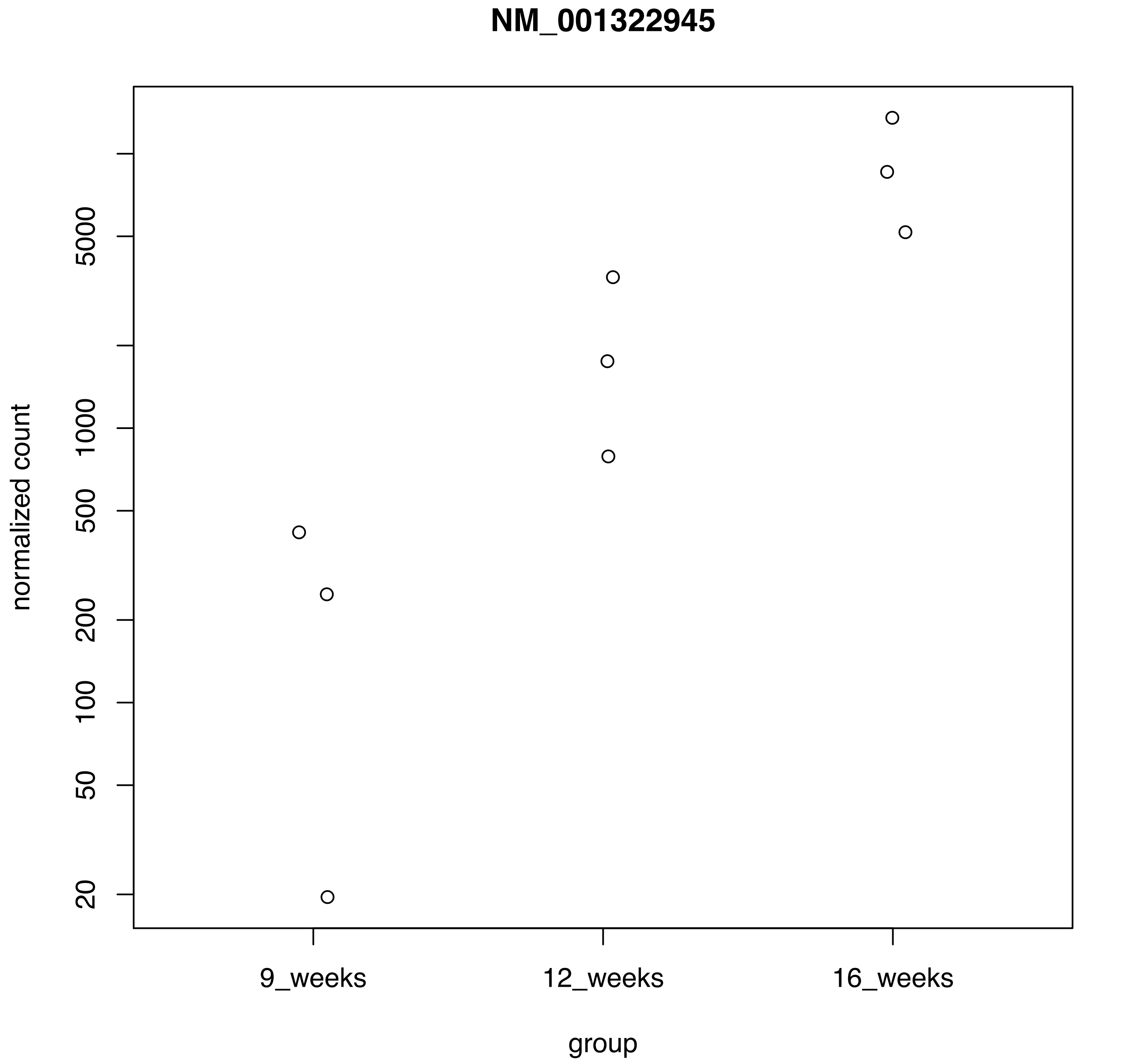

NM_001322945
normalized count
5000
1000
500
200
100
50
20
9_weeks
12_weeks
16_weeks
group

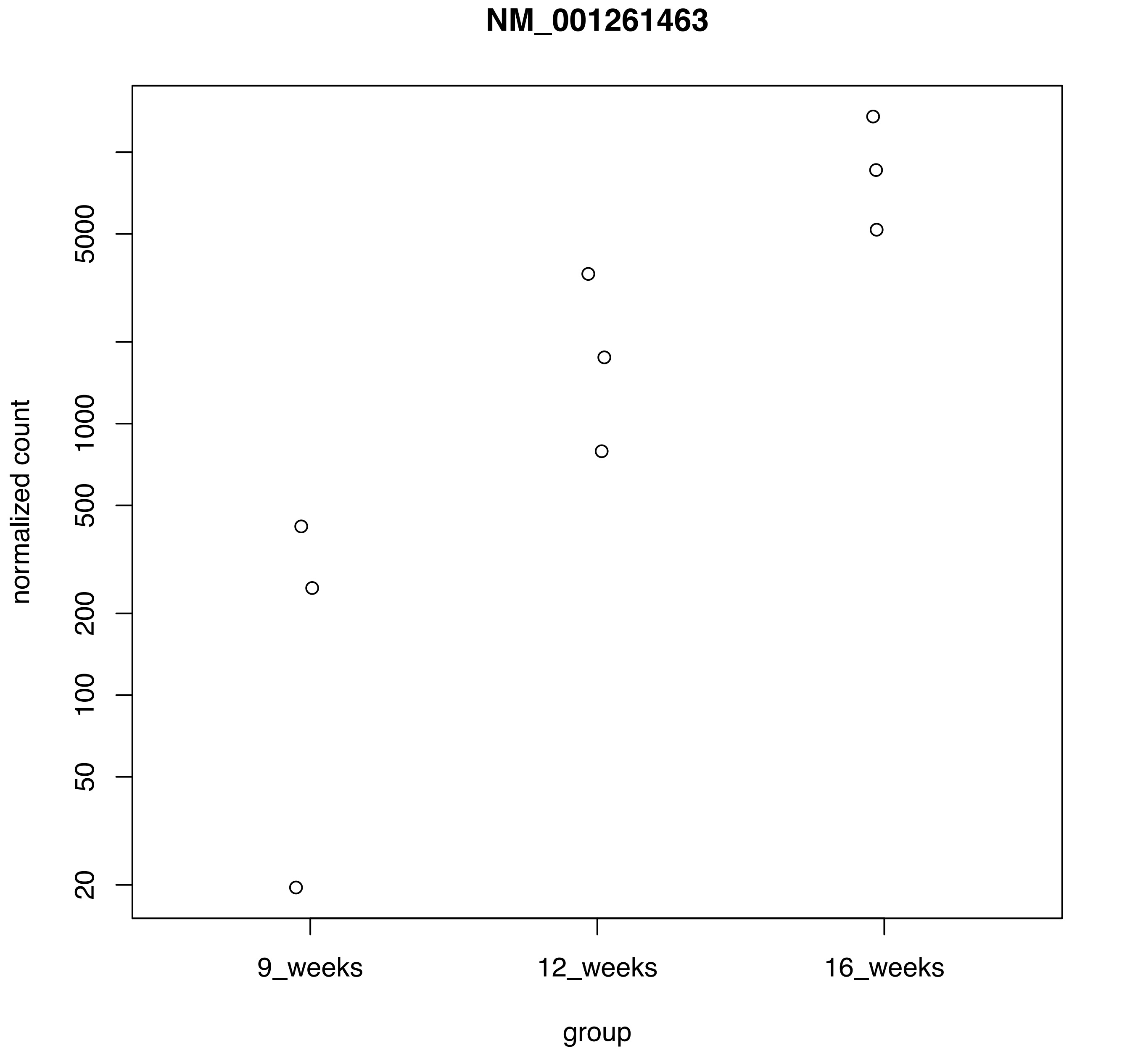
NM_001261463
normalized count
5000
1000
500
200
100
50
20
9_weeks
12_weeks
16_weeks
group

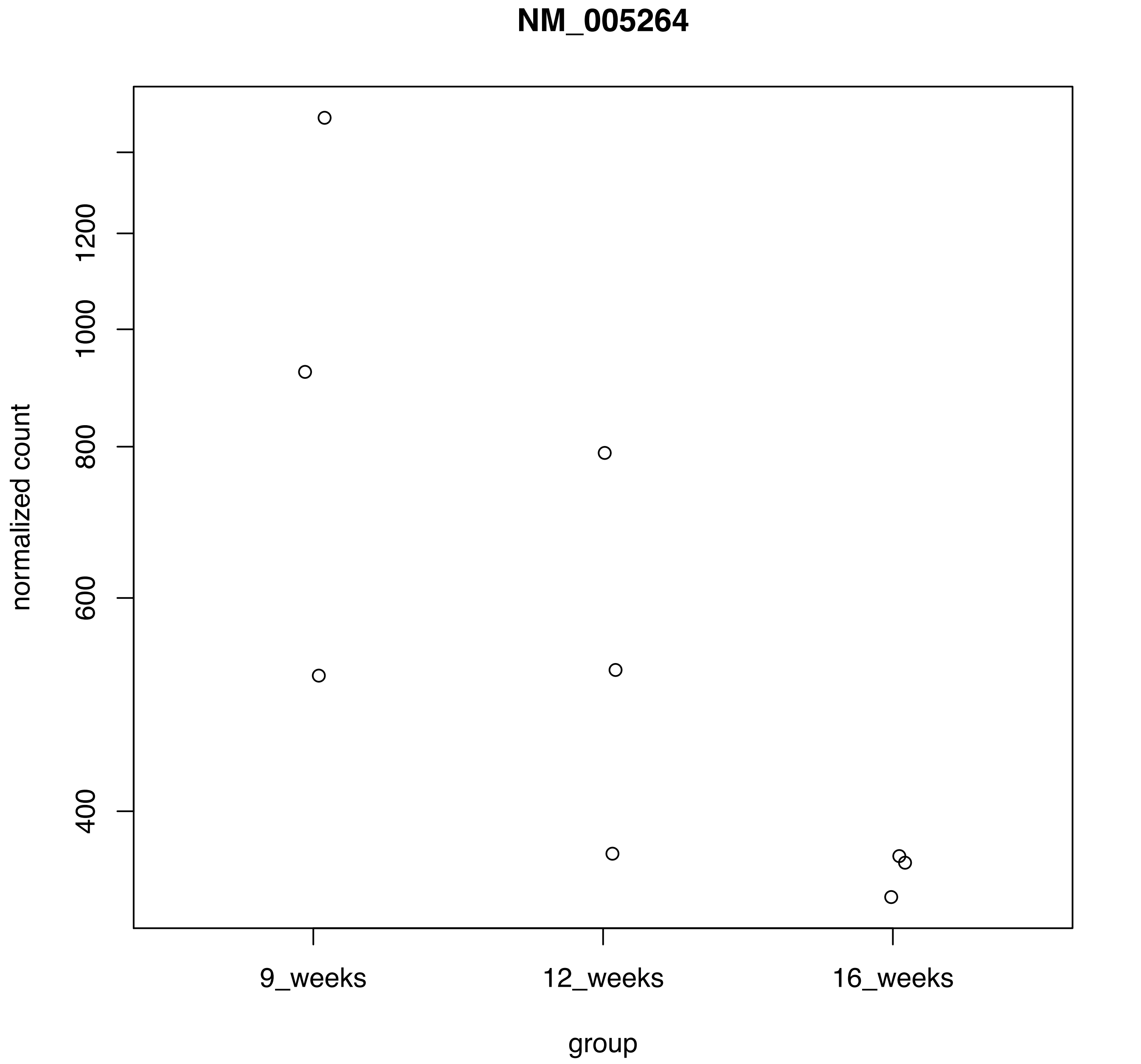
NM_005264
normalized count
400
600
800
1000
1200
9_weeks
12_weeks
16_weeks
group

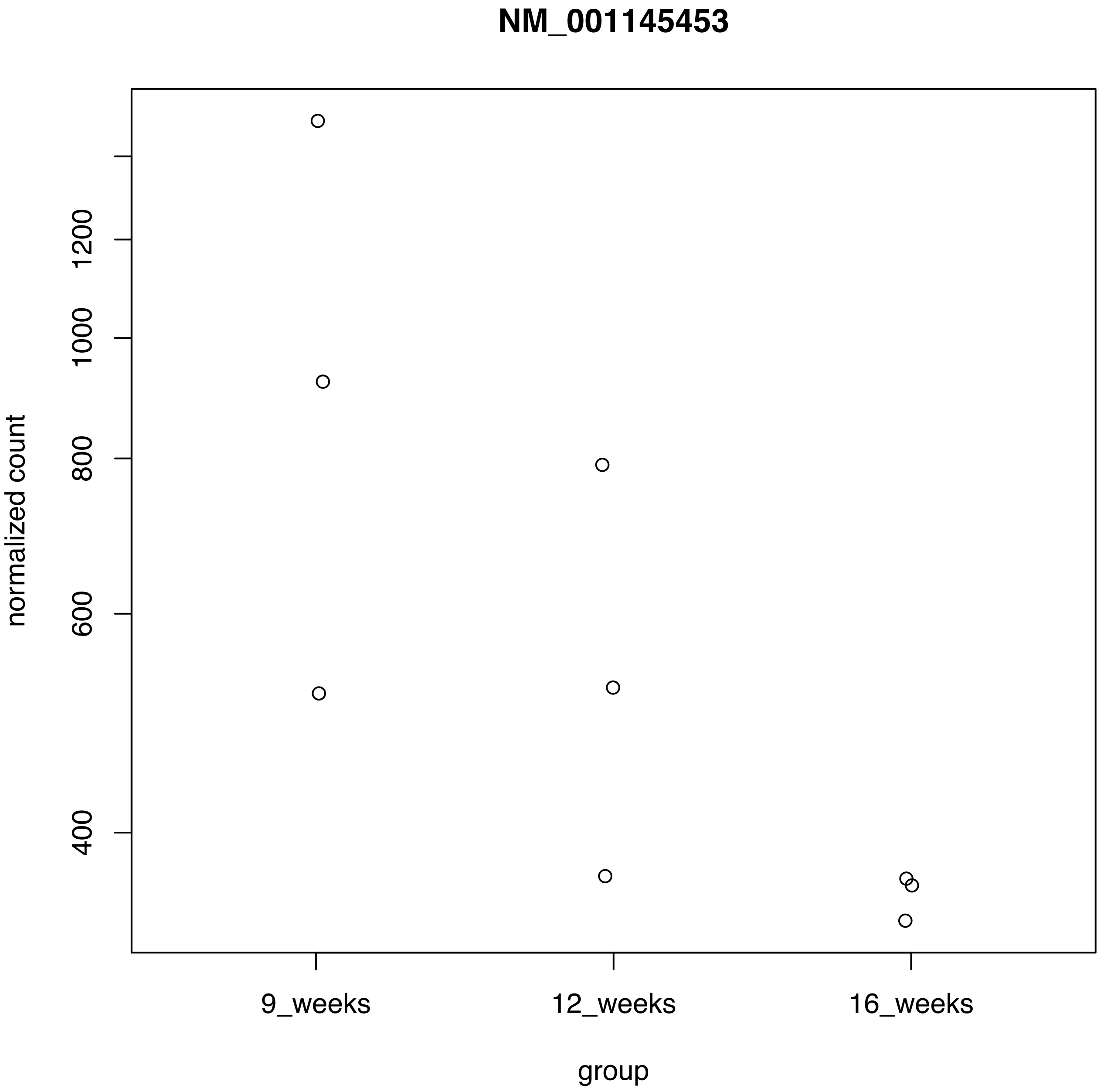
NM_001145453
normalized count
400
600
800
1000
1200
9_weeks
12_weeks
16_weeks
group

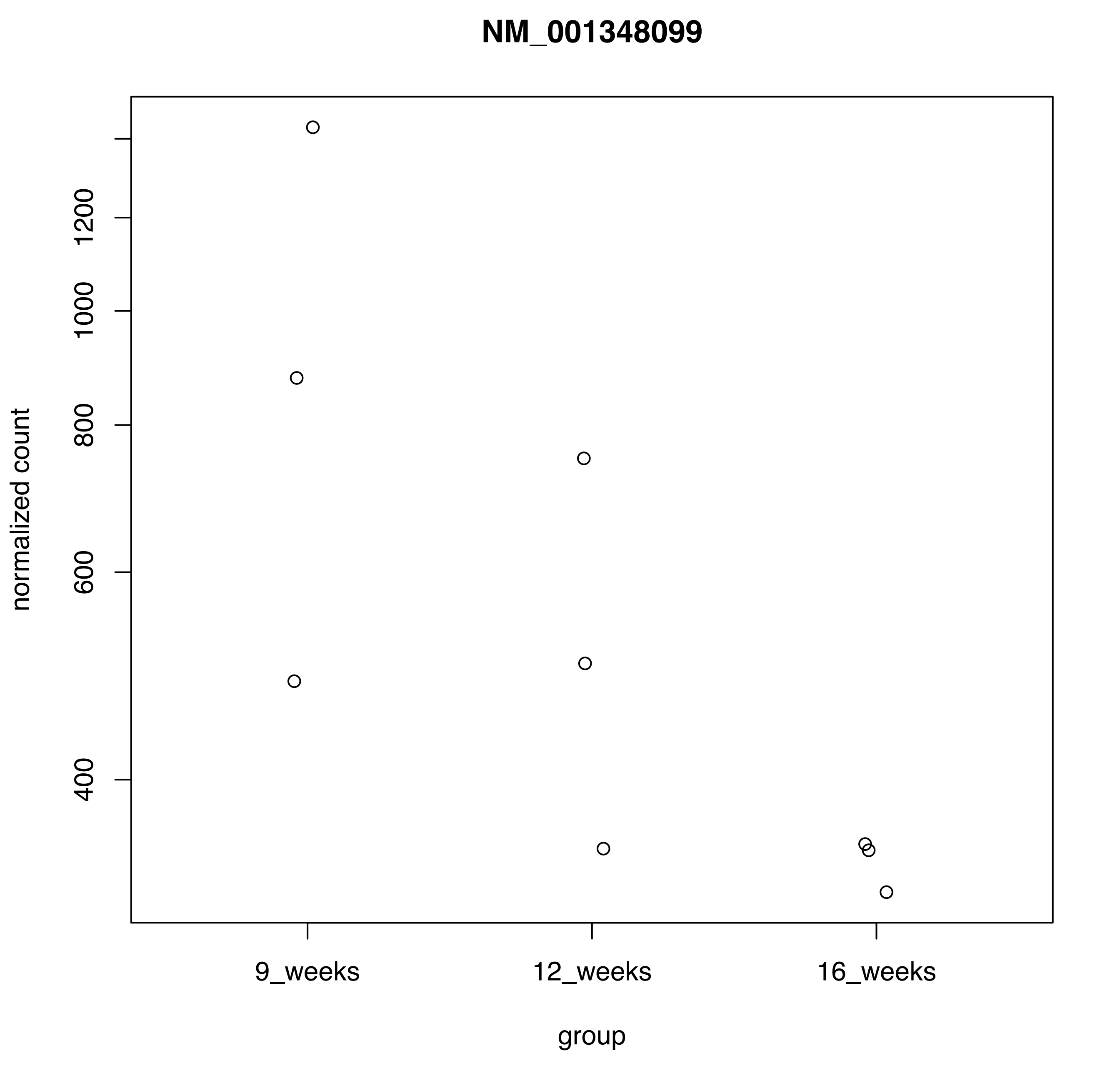

NM_001348099
normalized count
400
600
800
1000
1200
9_weeks
12_weeks
16_weeks
group

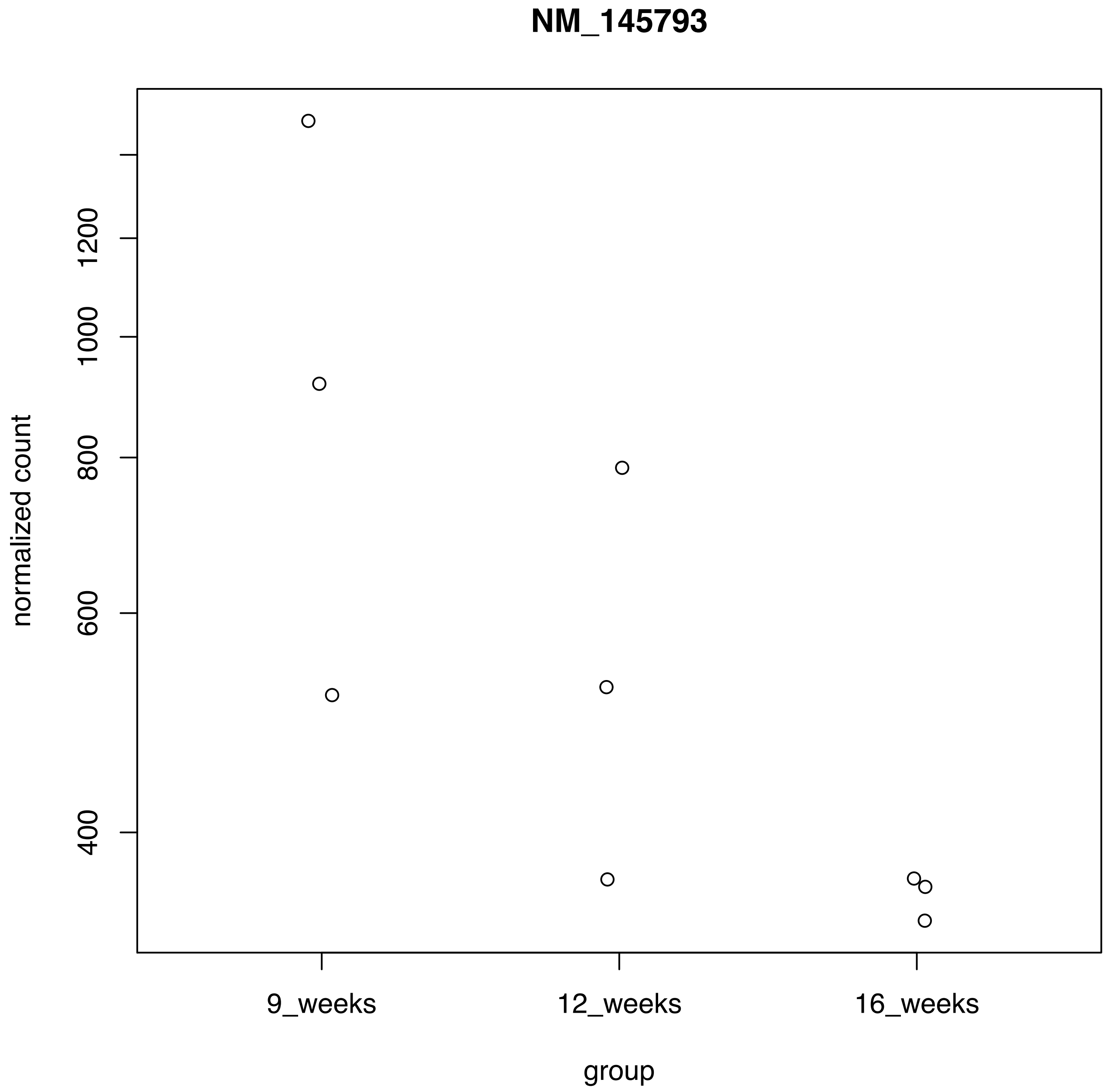

NM_145793
normalized count
400
600
800
1000
1200
9_weeks
12_weeks
16_weeks
group

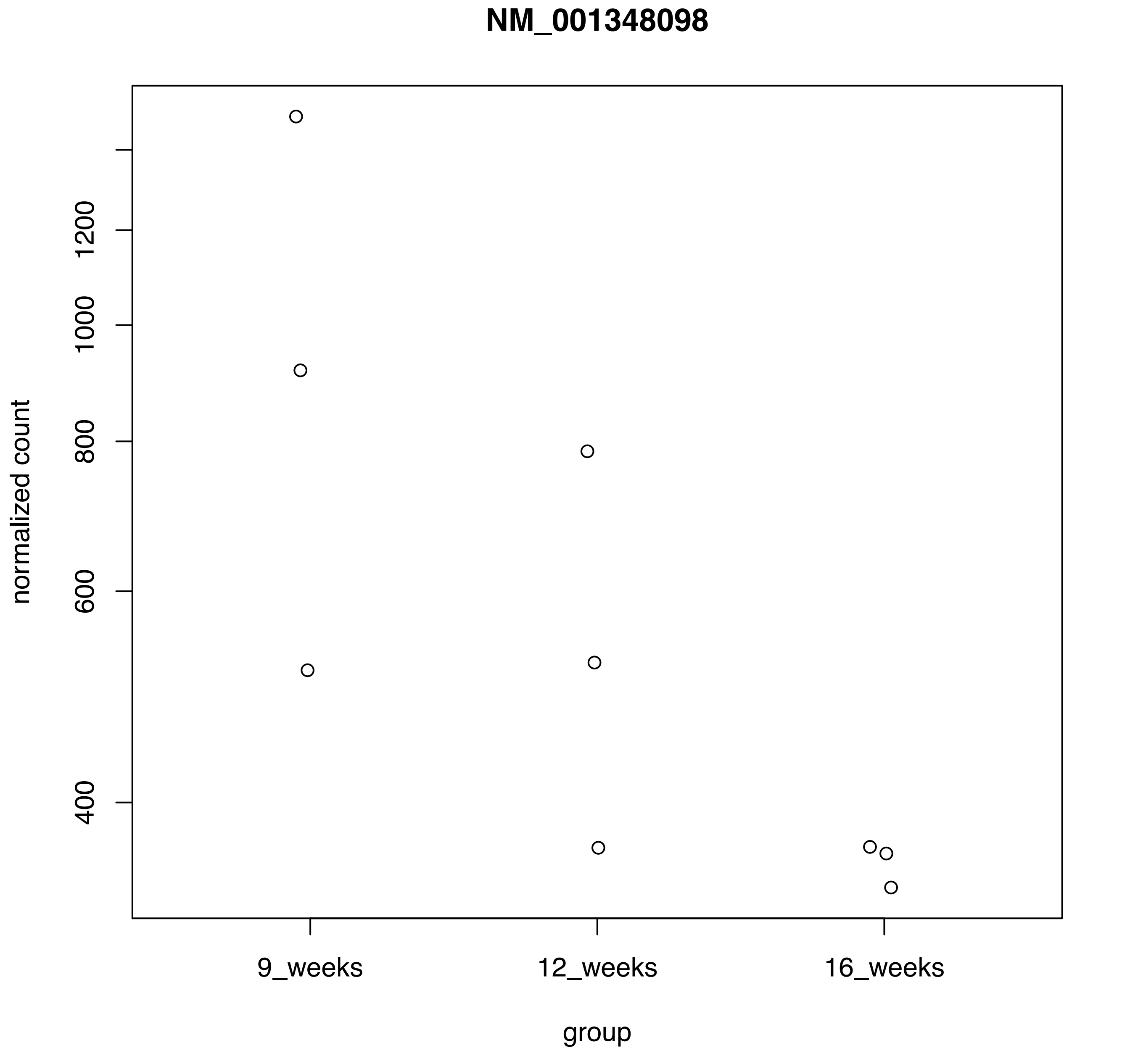

NM_001348098
normalized count
1200
1000
800
600
400
9_weeks
12_weeks
16_weeks
group

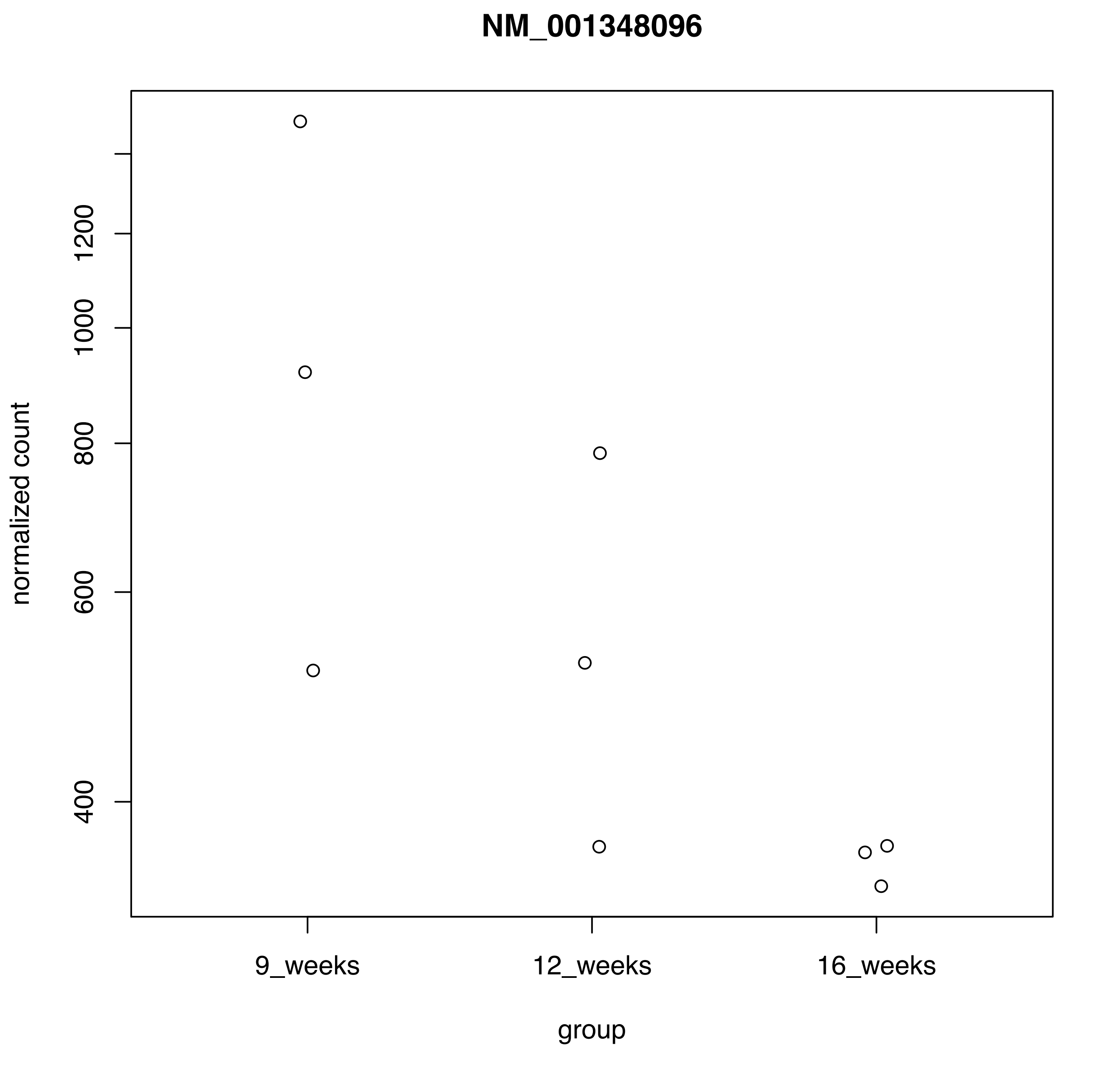
NM_001348096
normalized count
400
600
800
1000
1200
9_weeks
12_weeks
16_weeks
group

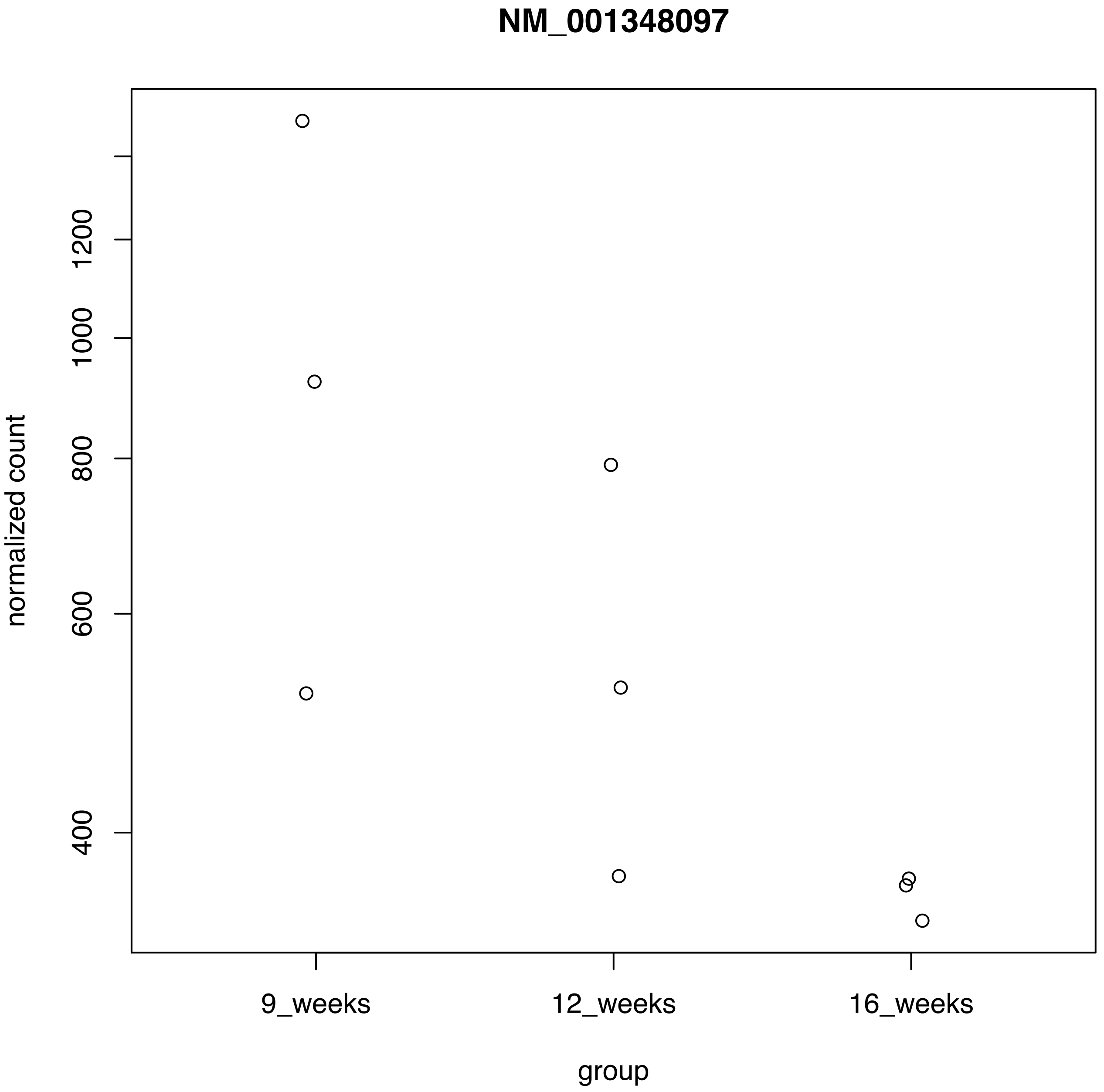

NM_001348097
normalized count
400
600
800
1000
1200
9_weeks
12_weeks
16_weeks
group

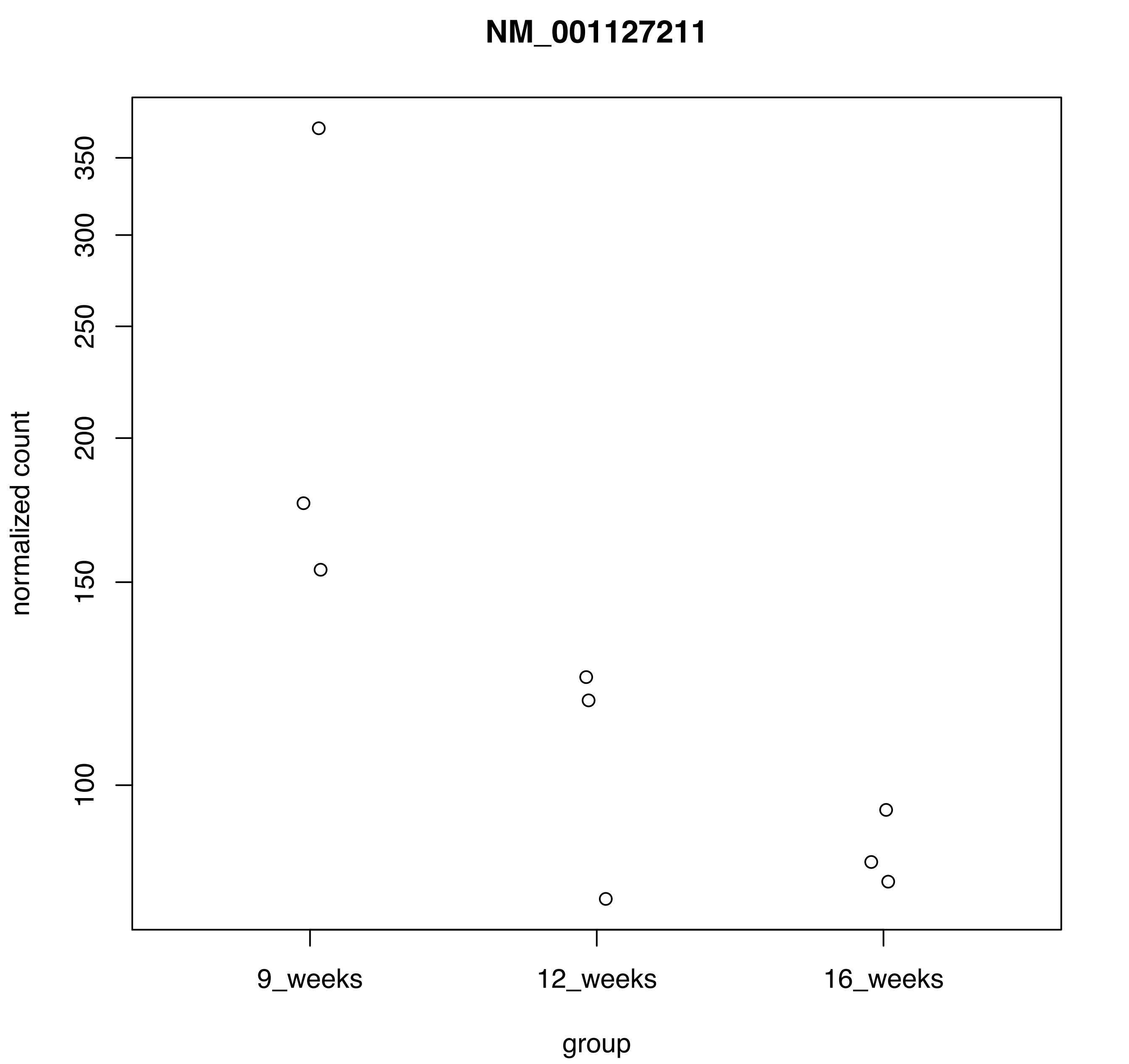
NM_001127211
normalized count
100
150
200
250
300
350
9_weeks
12_weeks
16_weeks
group

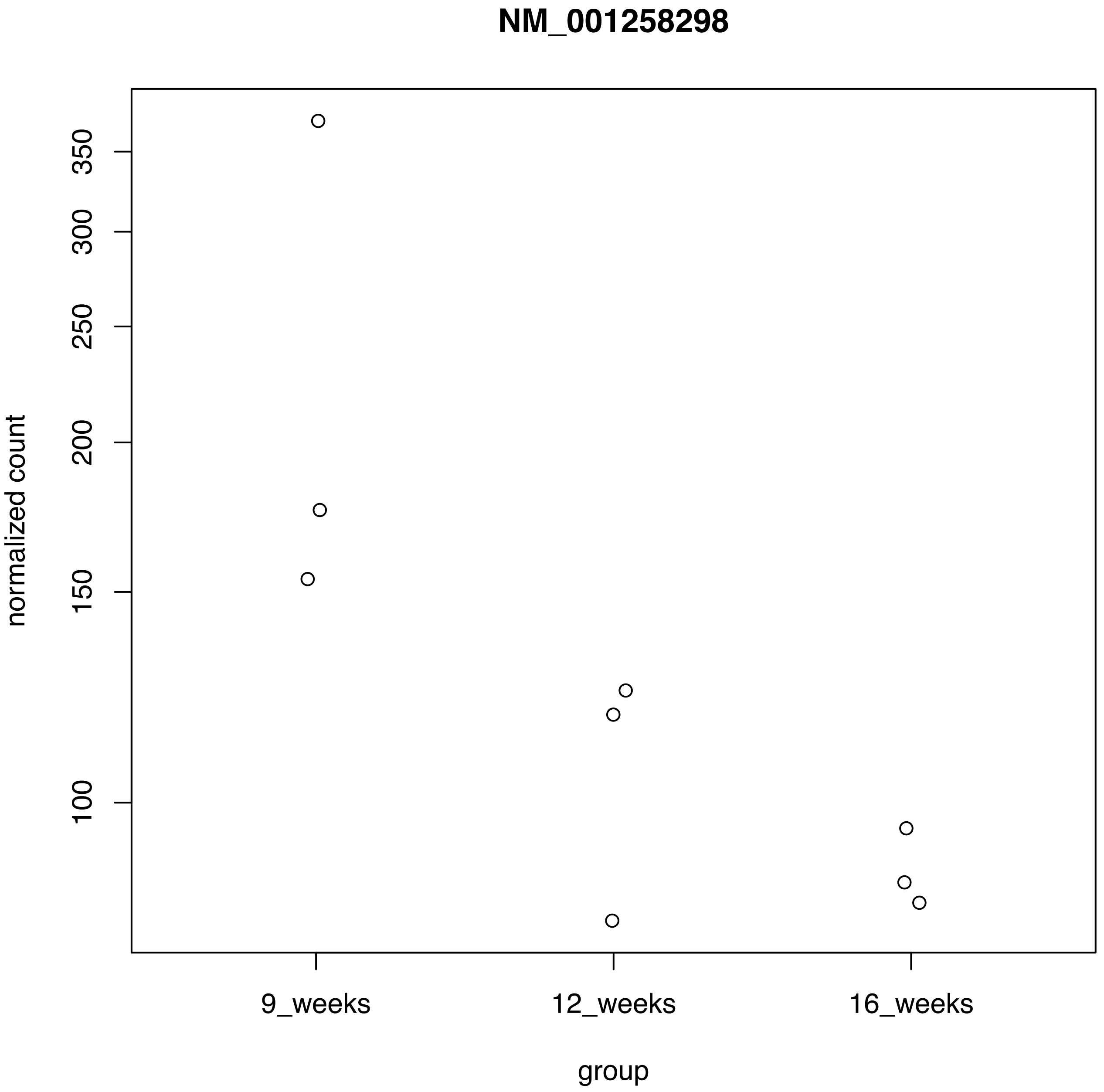
NM_001258298
normalized count
100
150
200
250
300
350
9_weeks
12_weeks
16_weeks
group

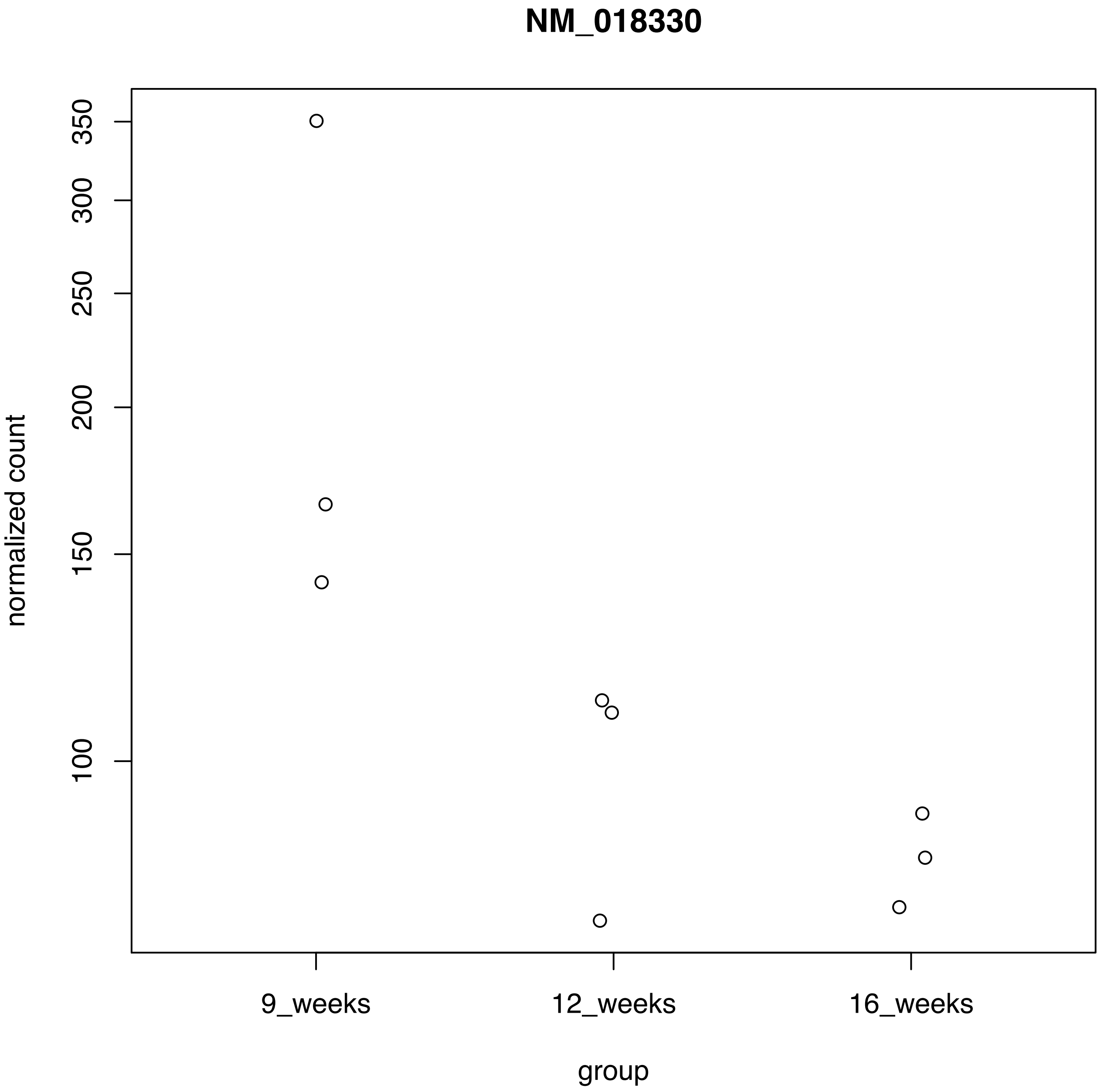
NM_018330
normalized count
350
300
250
200
150
100
9_weeks
12_weeks
16_weeks
group

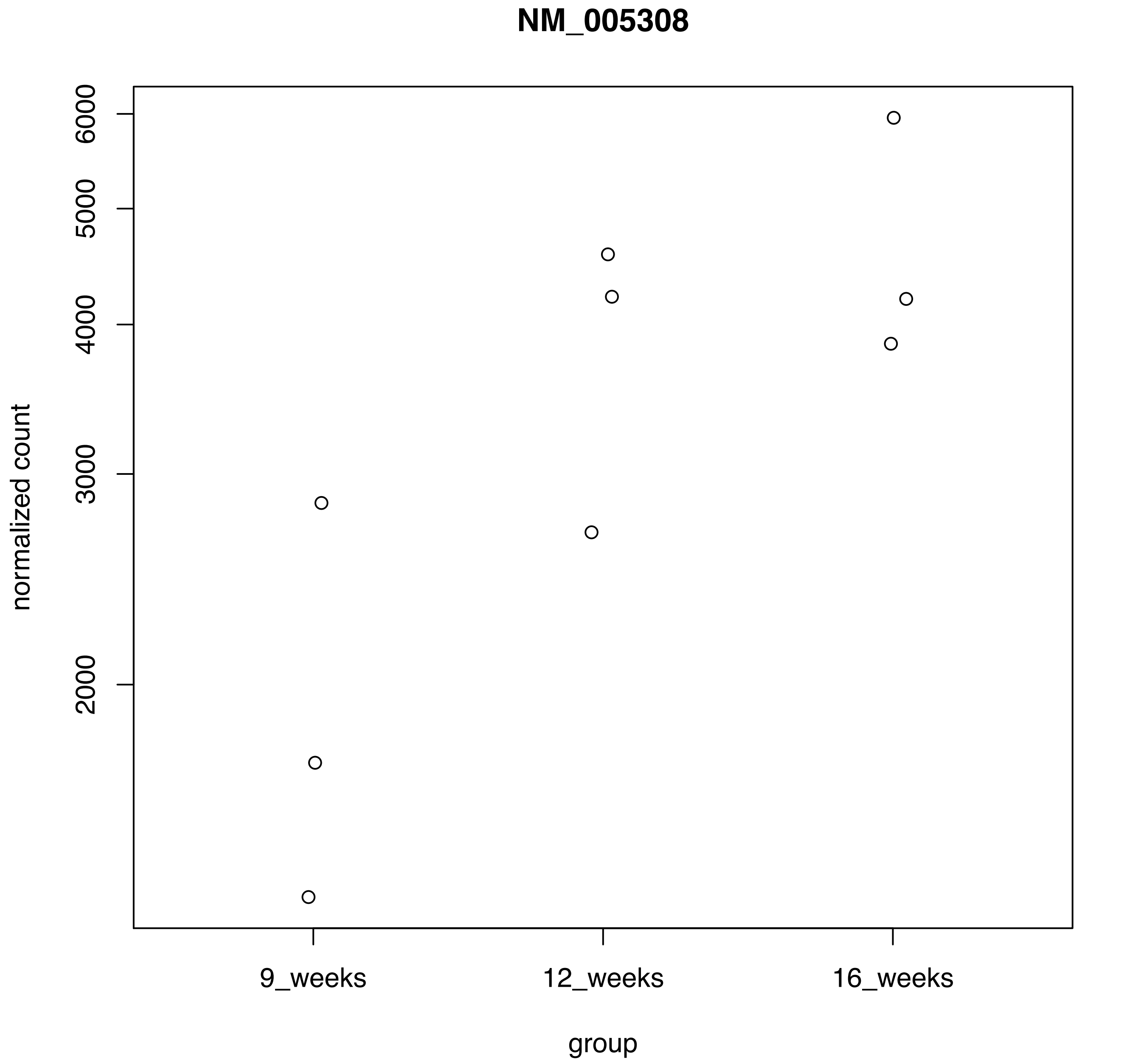
NM_005308
normalized count
6000
5000
4000
3000
2000
9_weeks
12_weeks
16_weeks
group

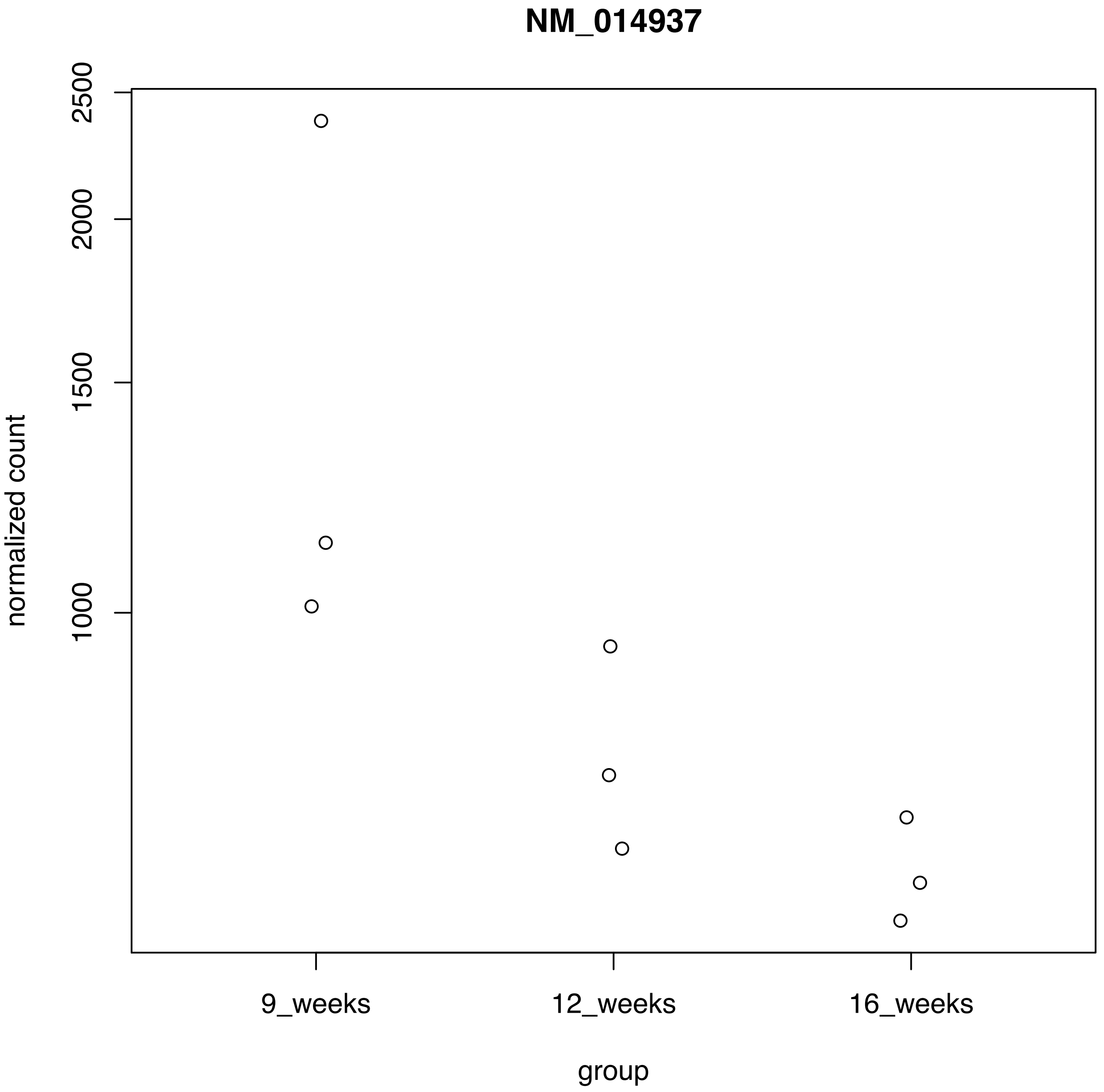
NM_014937
normalized count
2500
2000
1500
1000
9_weeks
12_weeks
16_weeks
group

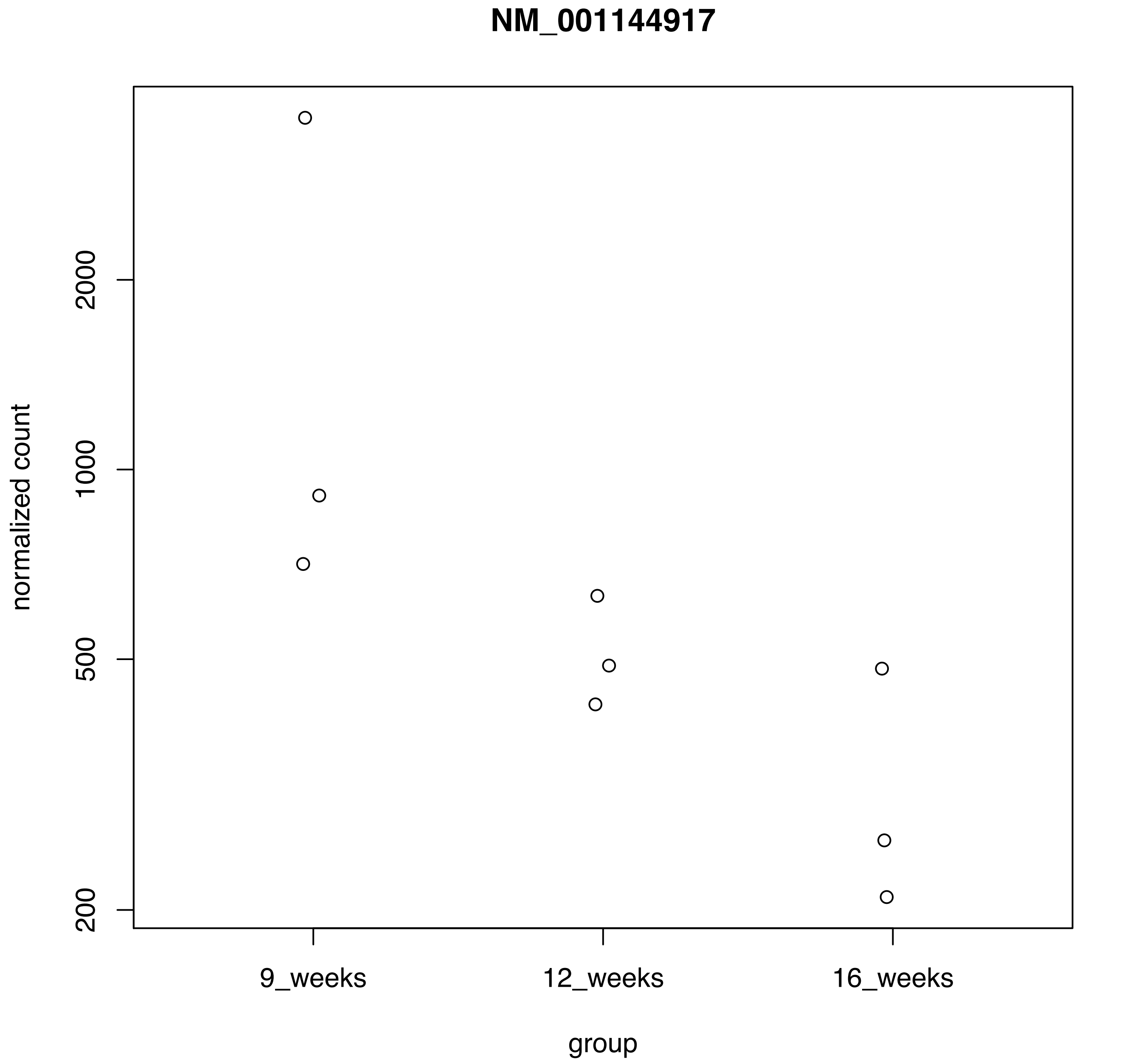
NM_001144917
normalized count
2000
1000
500
200
9_weeks
12_weeks
16_weeks
group

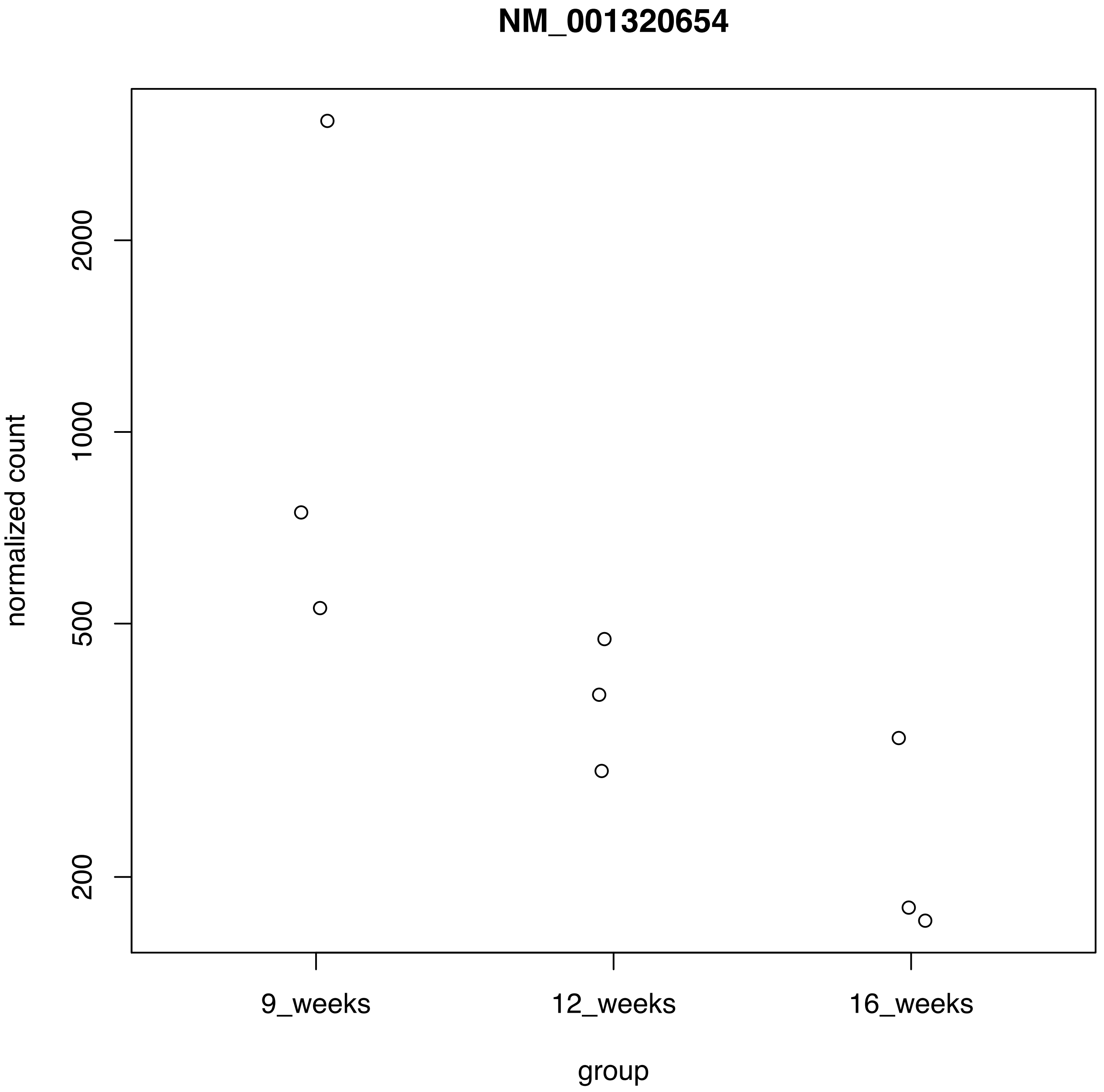
NM_001320654
normalized count
2000
1000
500
200
9_weeks
12_weeks
16_weeks
group

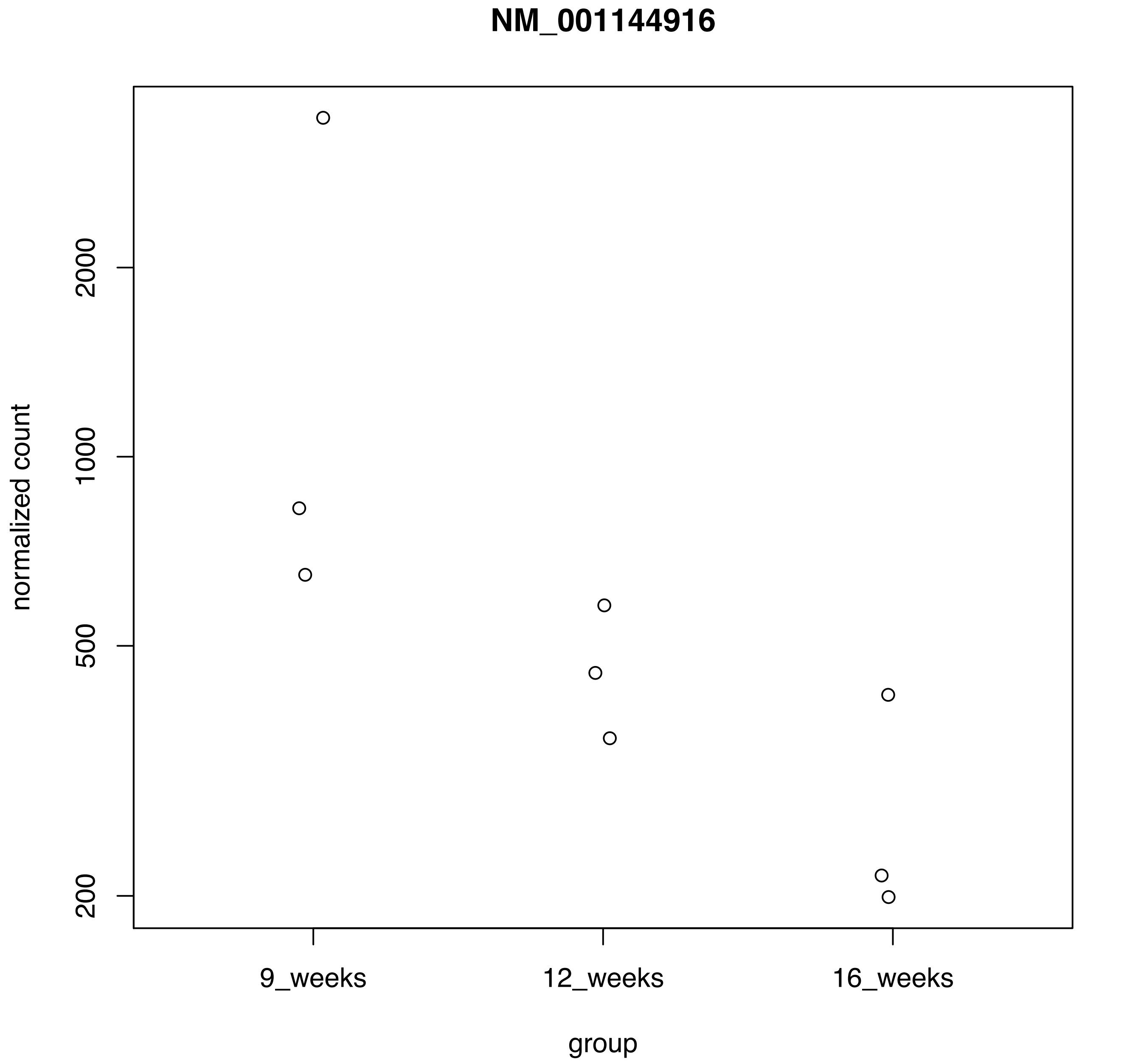

NM_001144916
normalized count
2000
1000
500
200
9_weeks
12_weeks
16_weeks
group

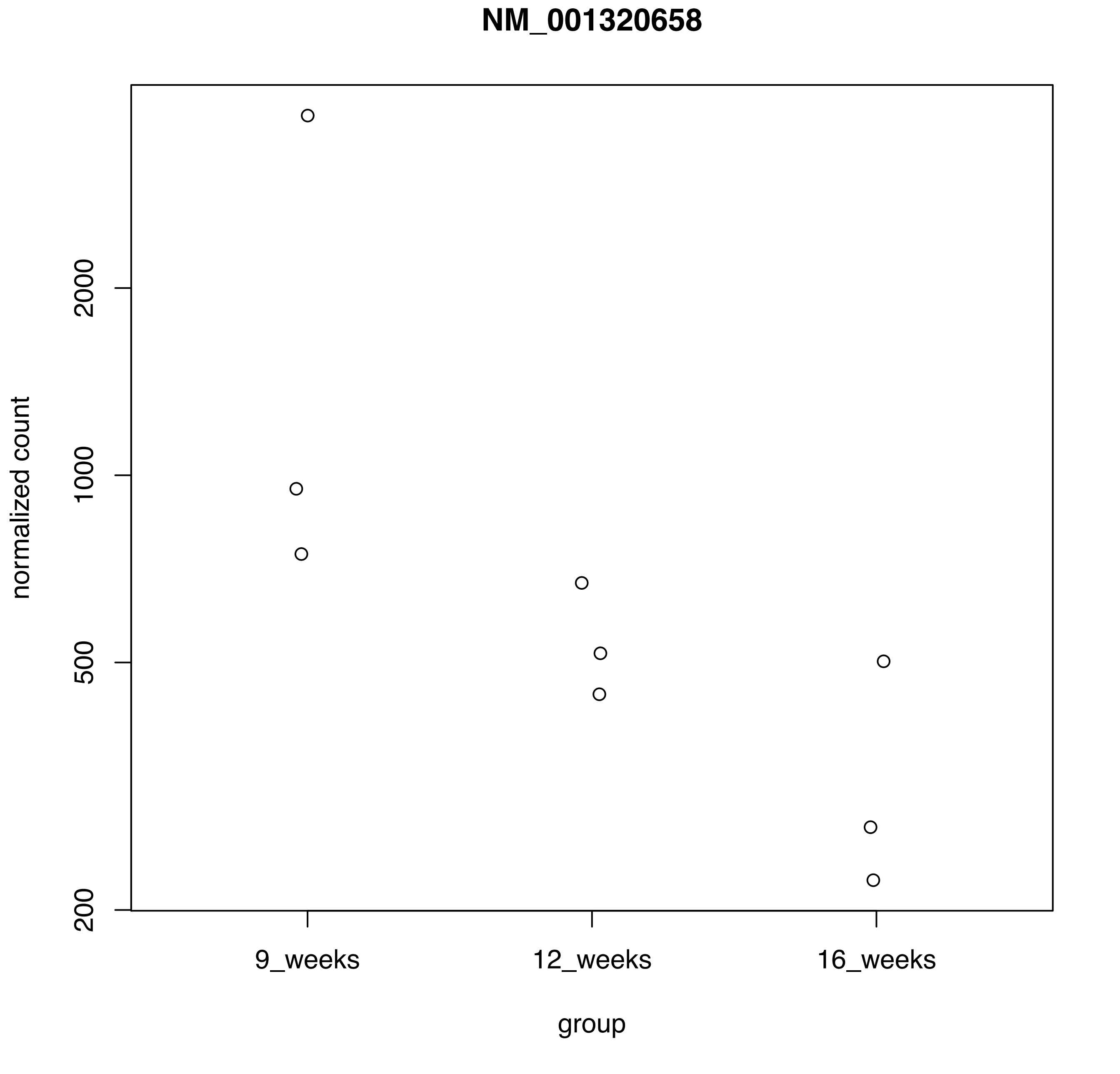
NM_001320658
normalized count
2000
1000
500
200
9_weeks
12_weeks
16_weeks
group

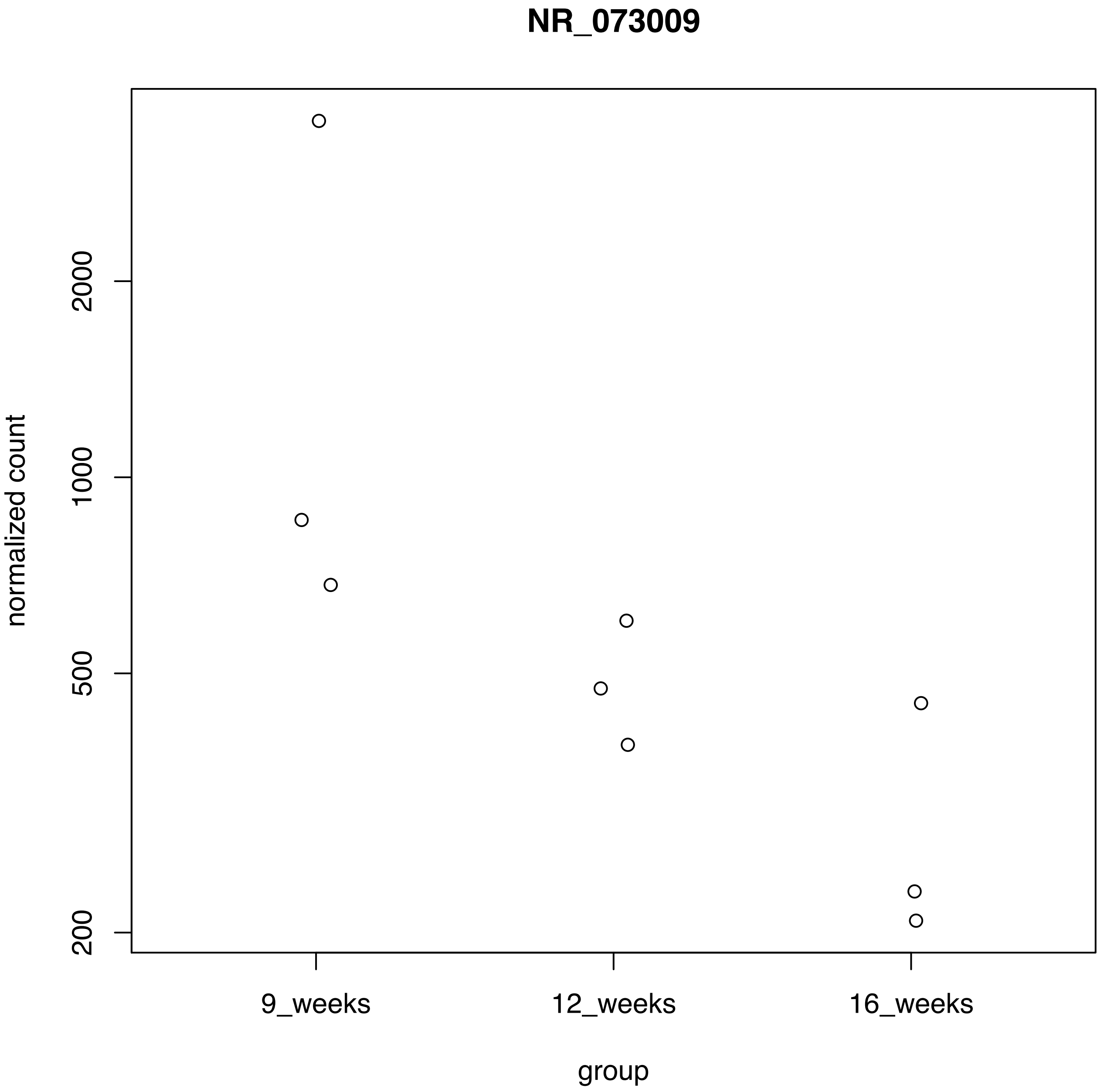
NR_073009
normalized count
2000
1000
500
200
9_weeks
12_weeks
16_weeks
group

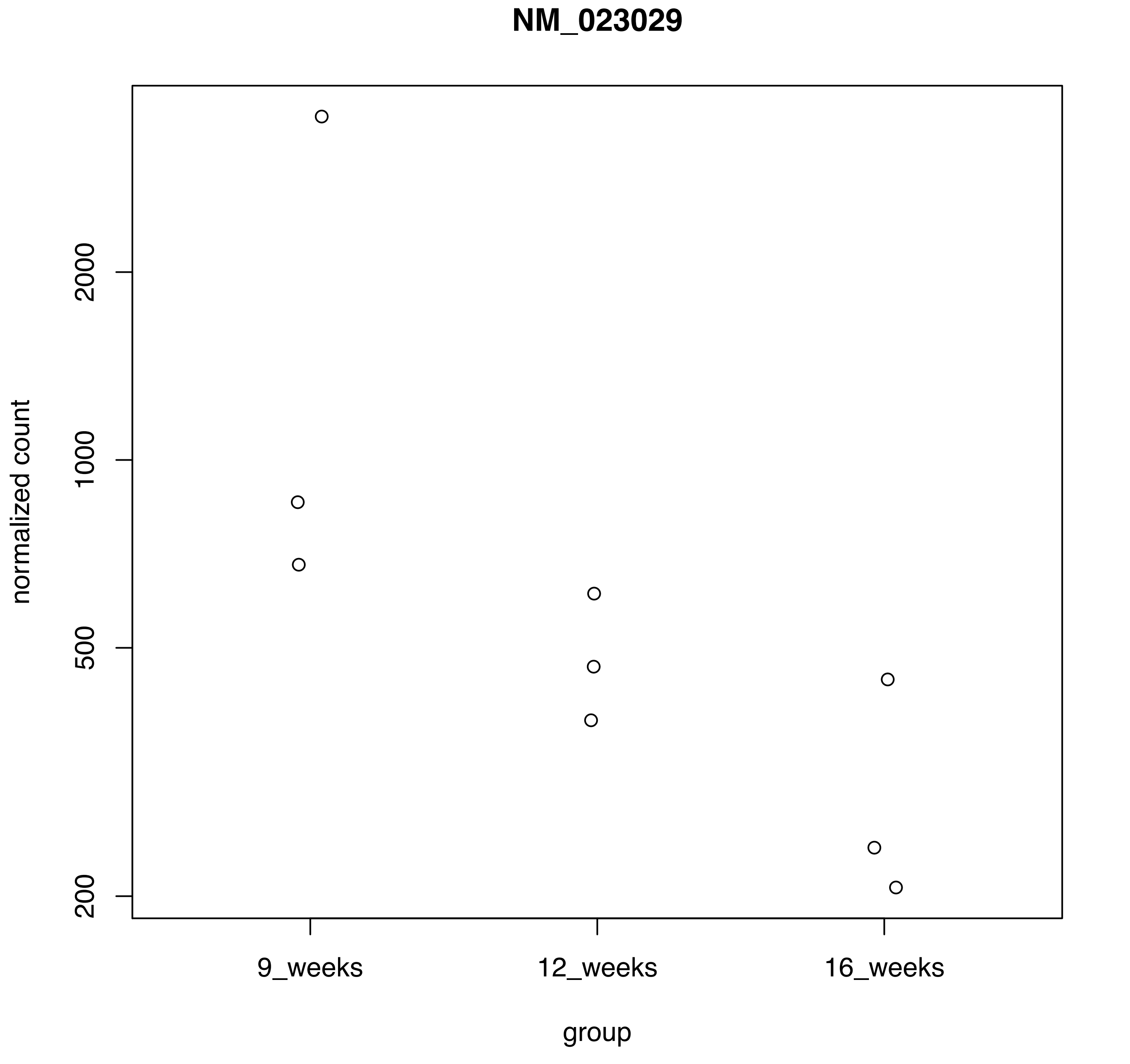
NM_023029
normalized count
2000
1000
500
200
9_weeks
12_weeks
16_weeks
group

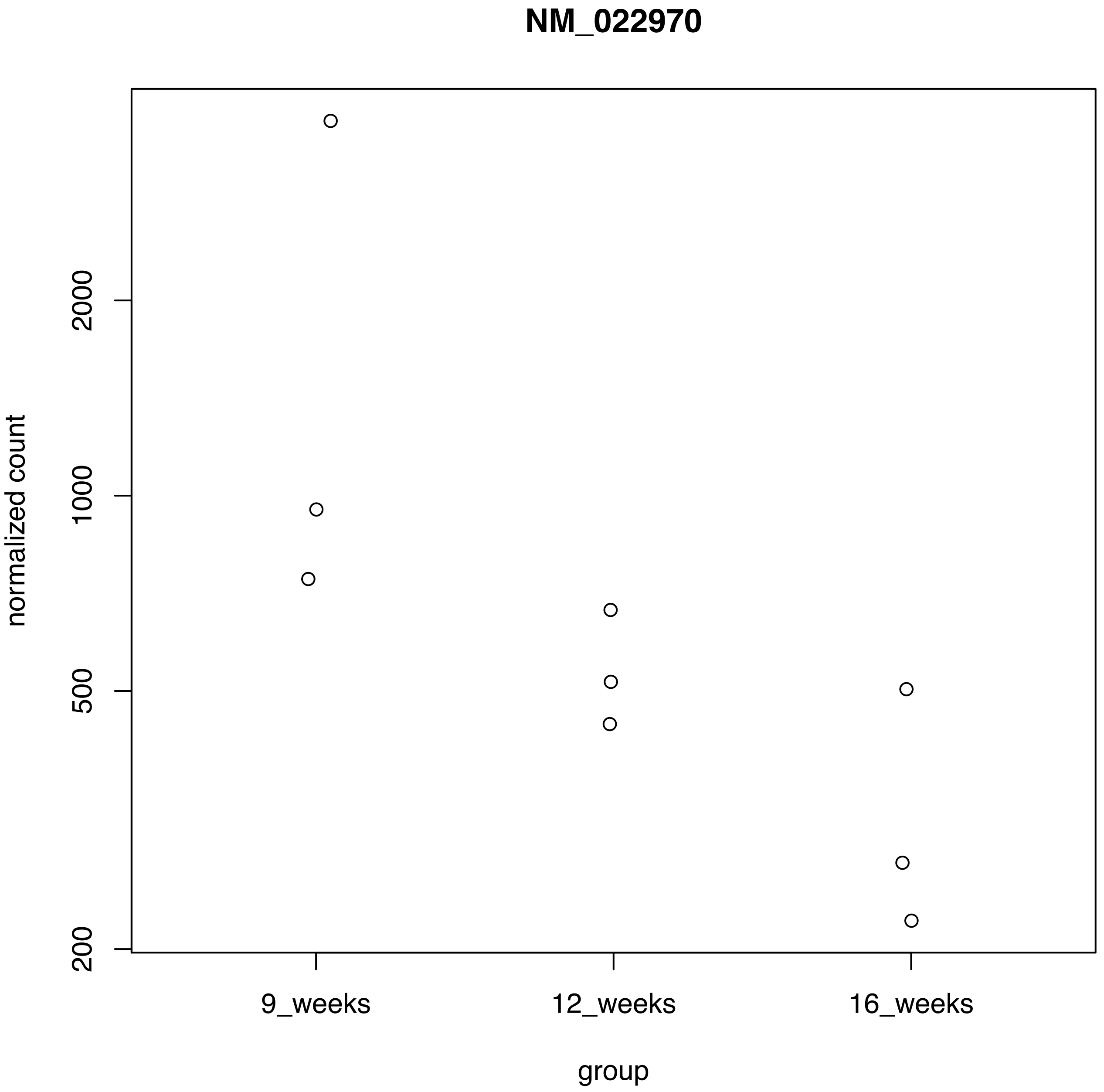
NM_022970
normalized count
2000
1000
500
200
9_weeks
12_weeks
16_weeks
group

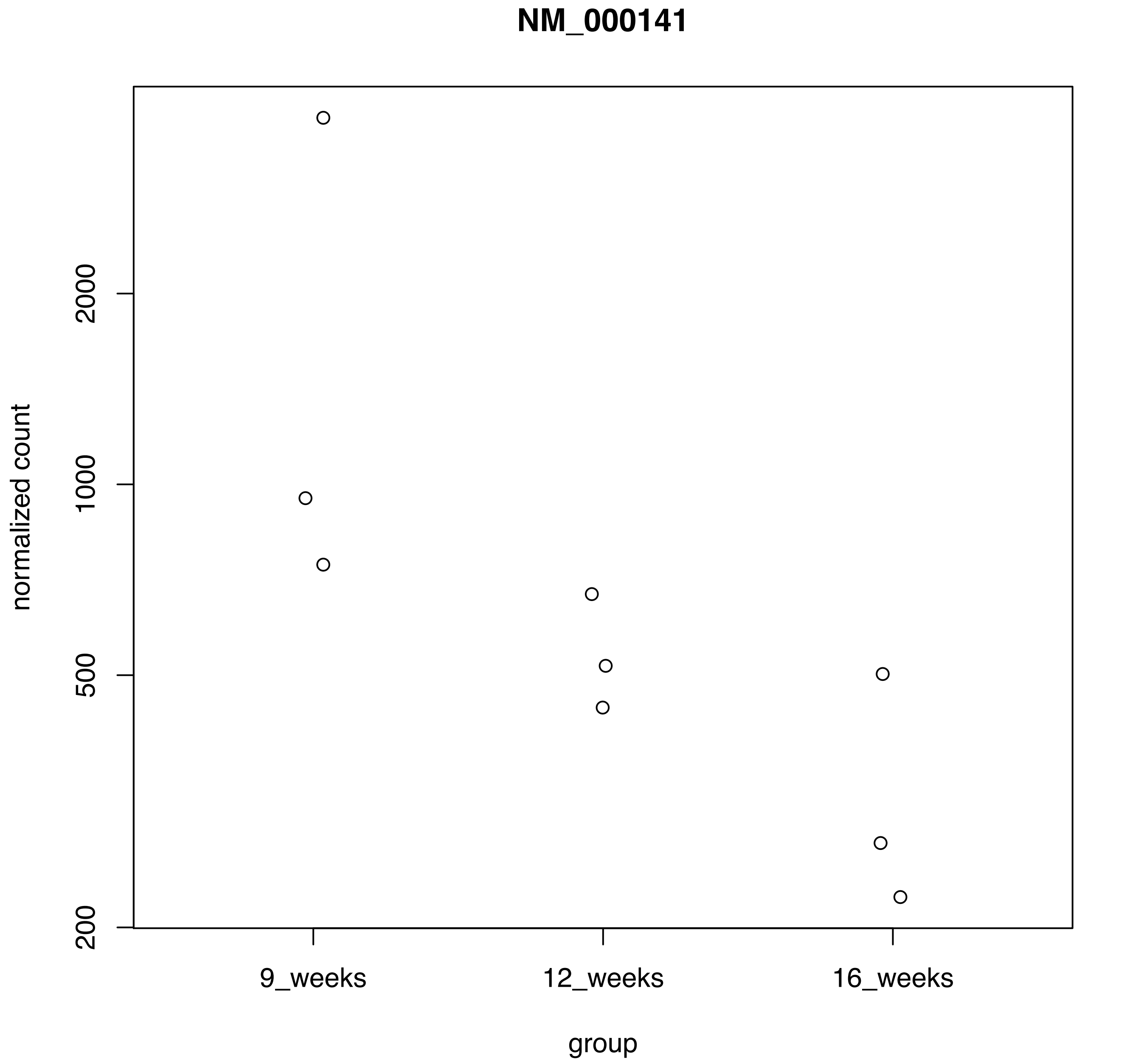
NM_000141
normalized count
2000
1000
500
200
9_weeks
12_weeks
16_weeks
group

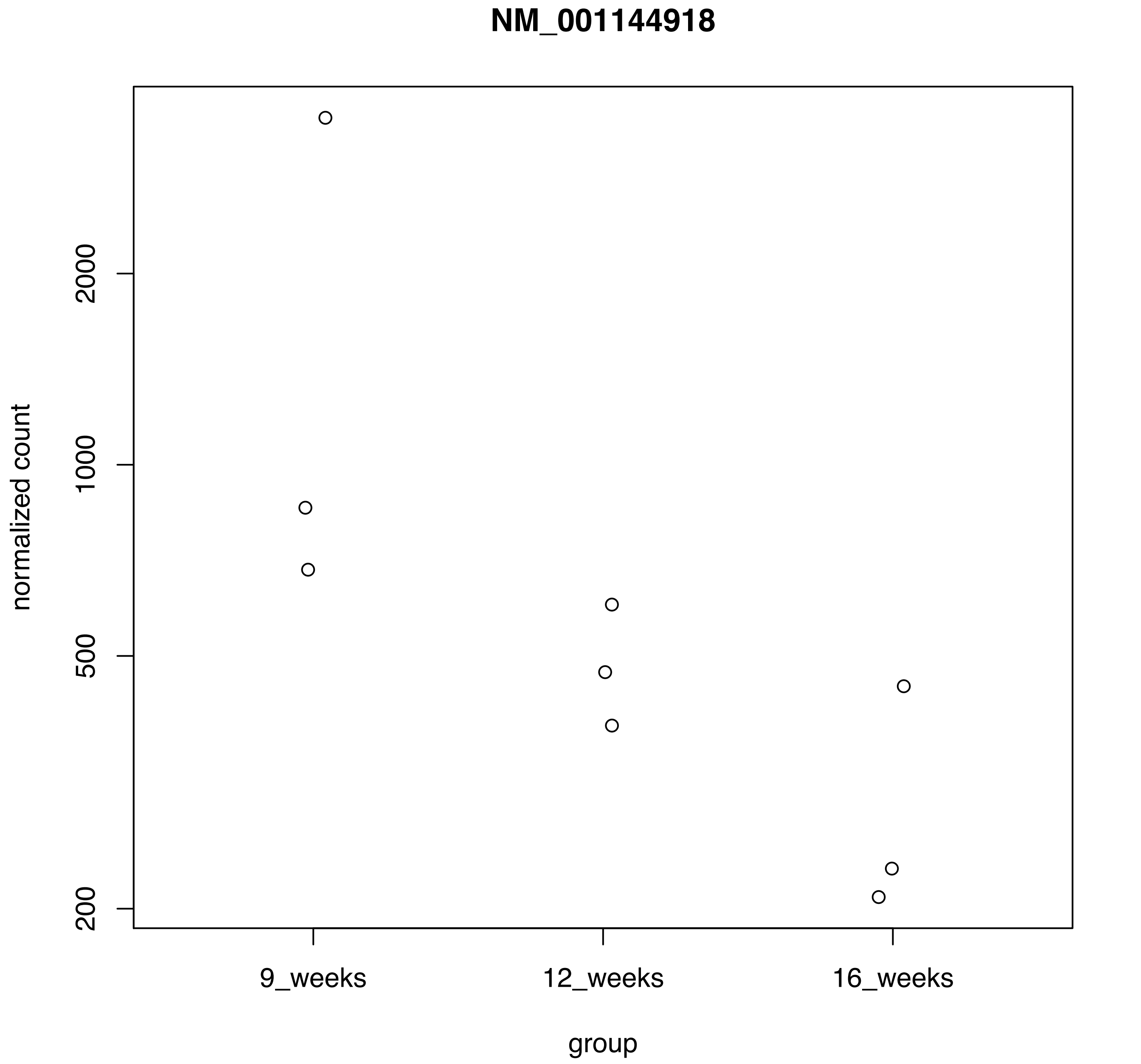
NM_001144918
normalized count
2000
1000
500
200
9_weeks
12_weeks
16_weeks
group

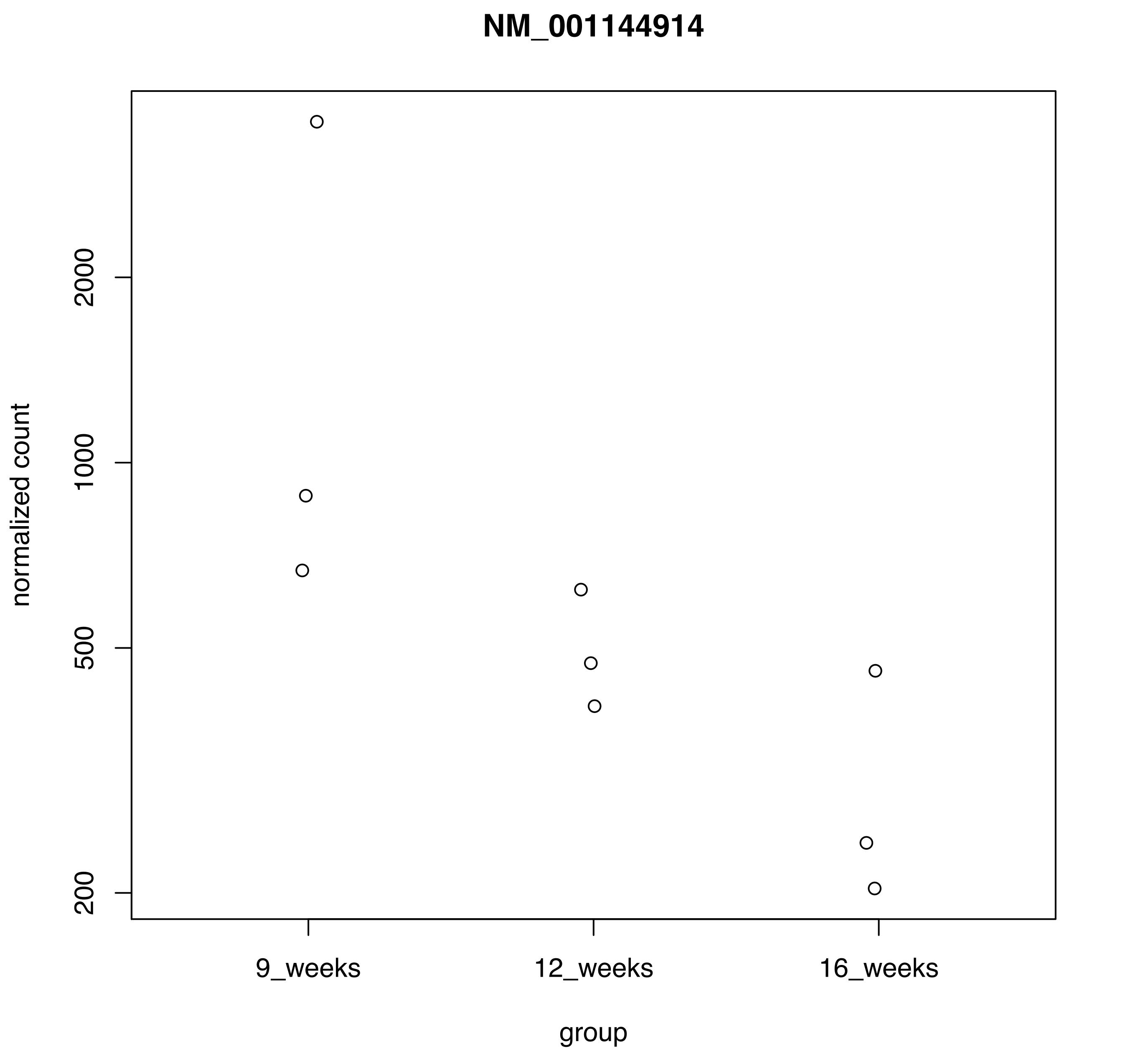
NM_001144914
normalized count
2000
1000
500
200
9_weeks
12_weeks
16_weeks
group

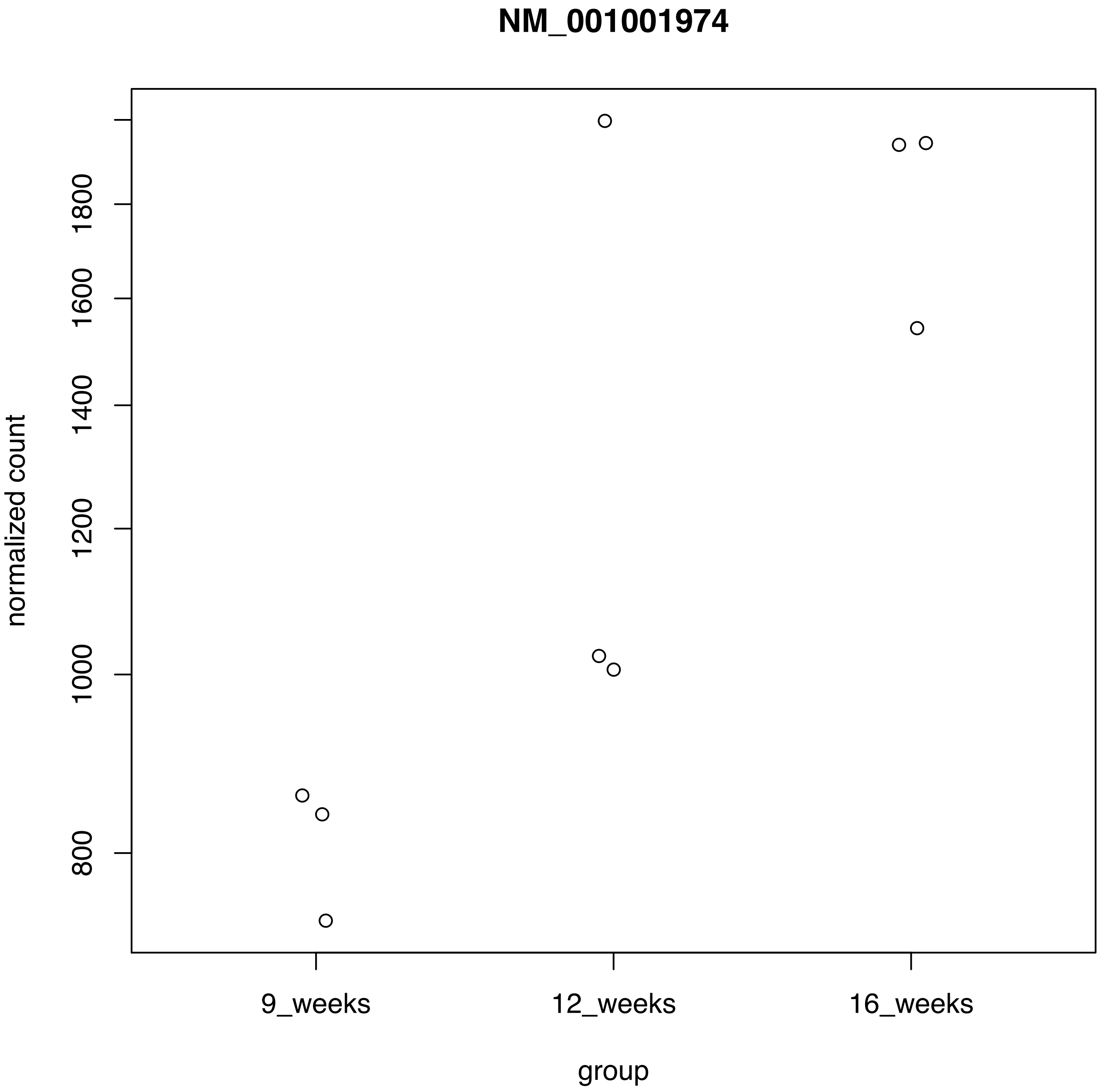
NM_001001974
normalized count
800
1000
1200
1400
1600
1800
9_weeks
12_weeks
16_weeks
group

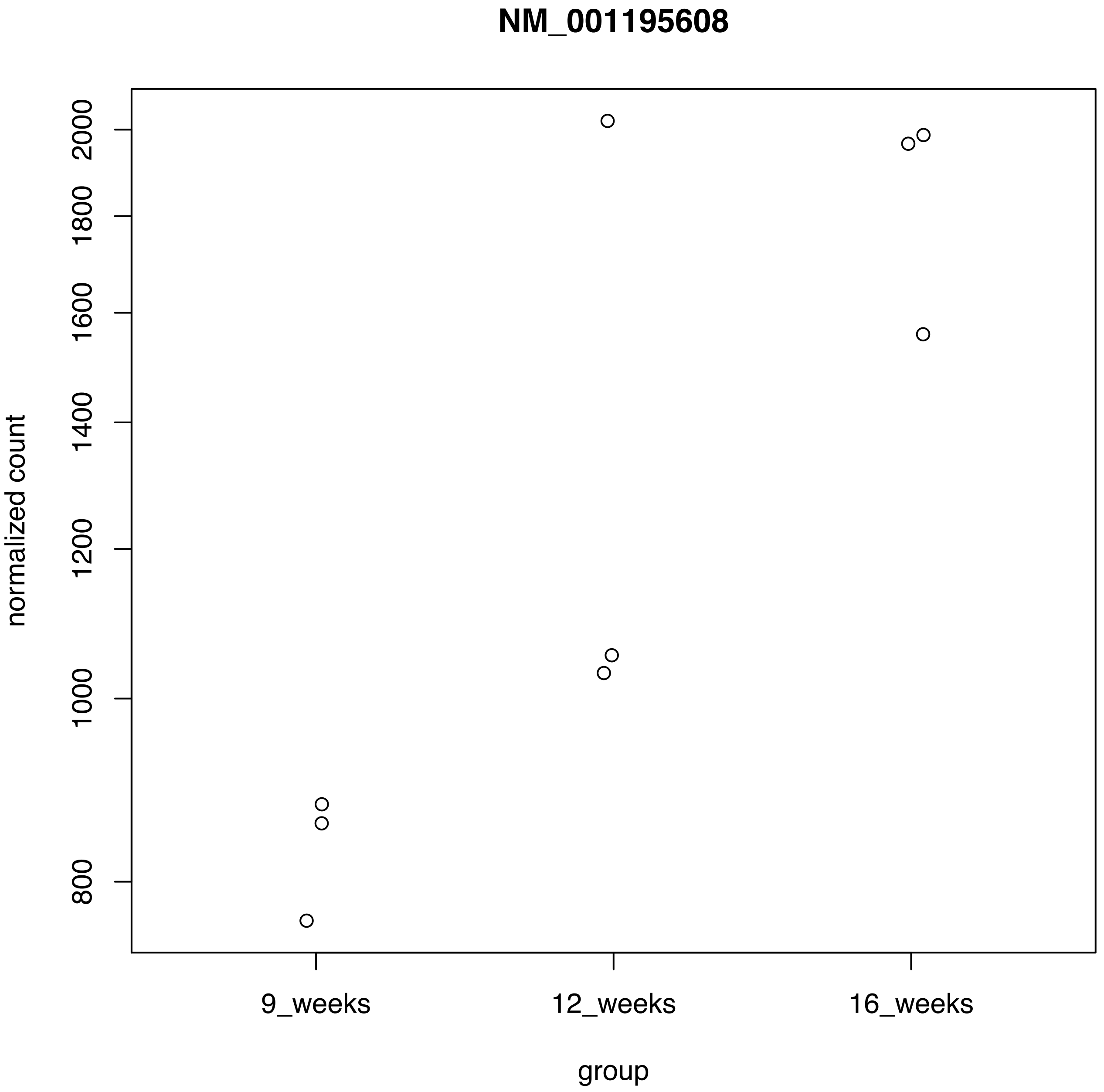
NM_001195608
normalized count
2000
1800
1600
1400
1200
1000
800
9_weeks
12_weeks
16_weeks
group

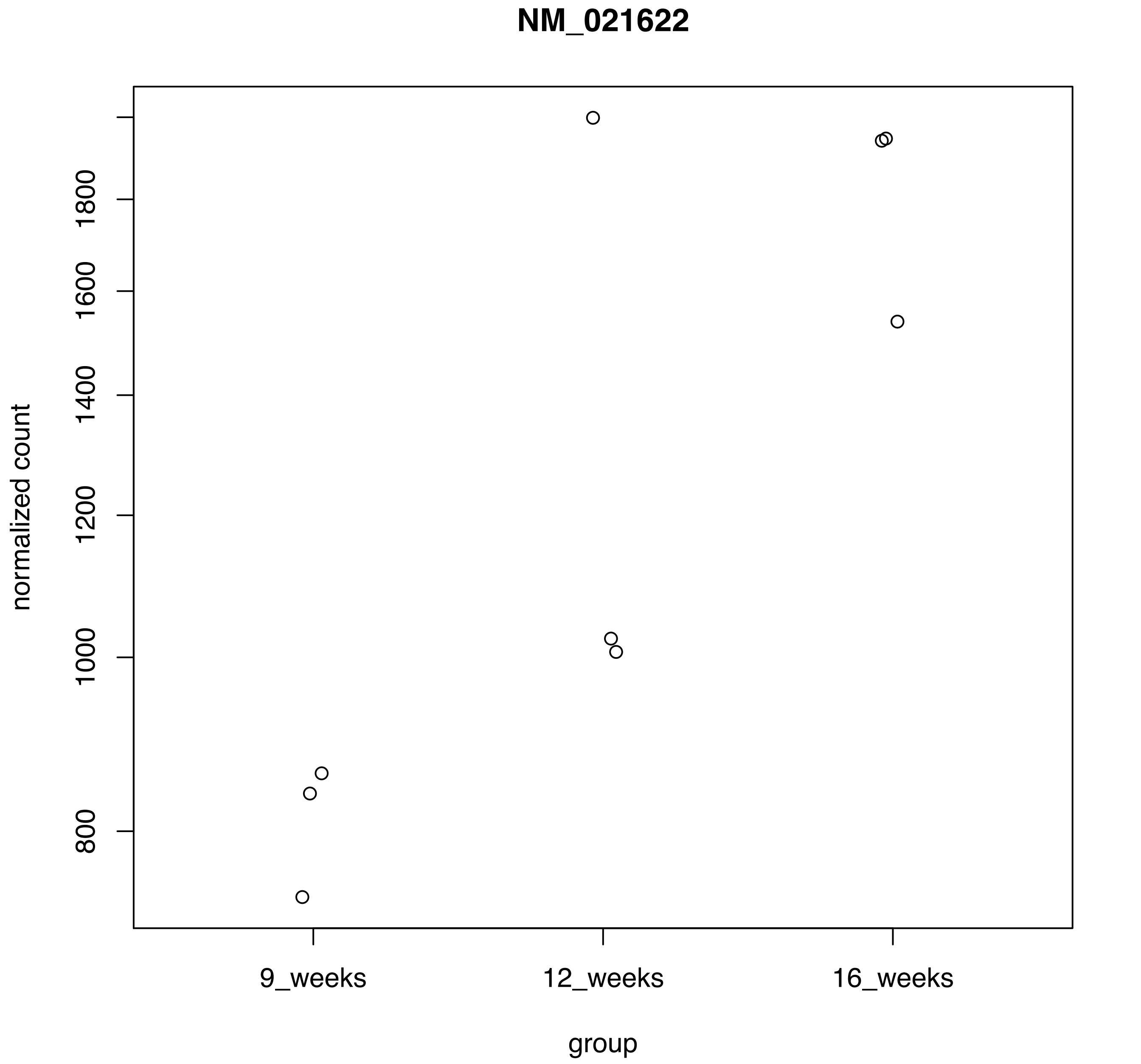
NM_021622
normalized count
800
1000
1200
1400
1600
1800
9_weeks
12_weeks
16_weeks
group

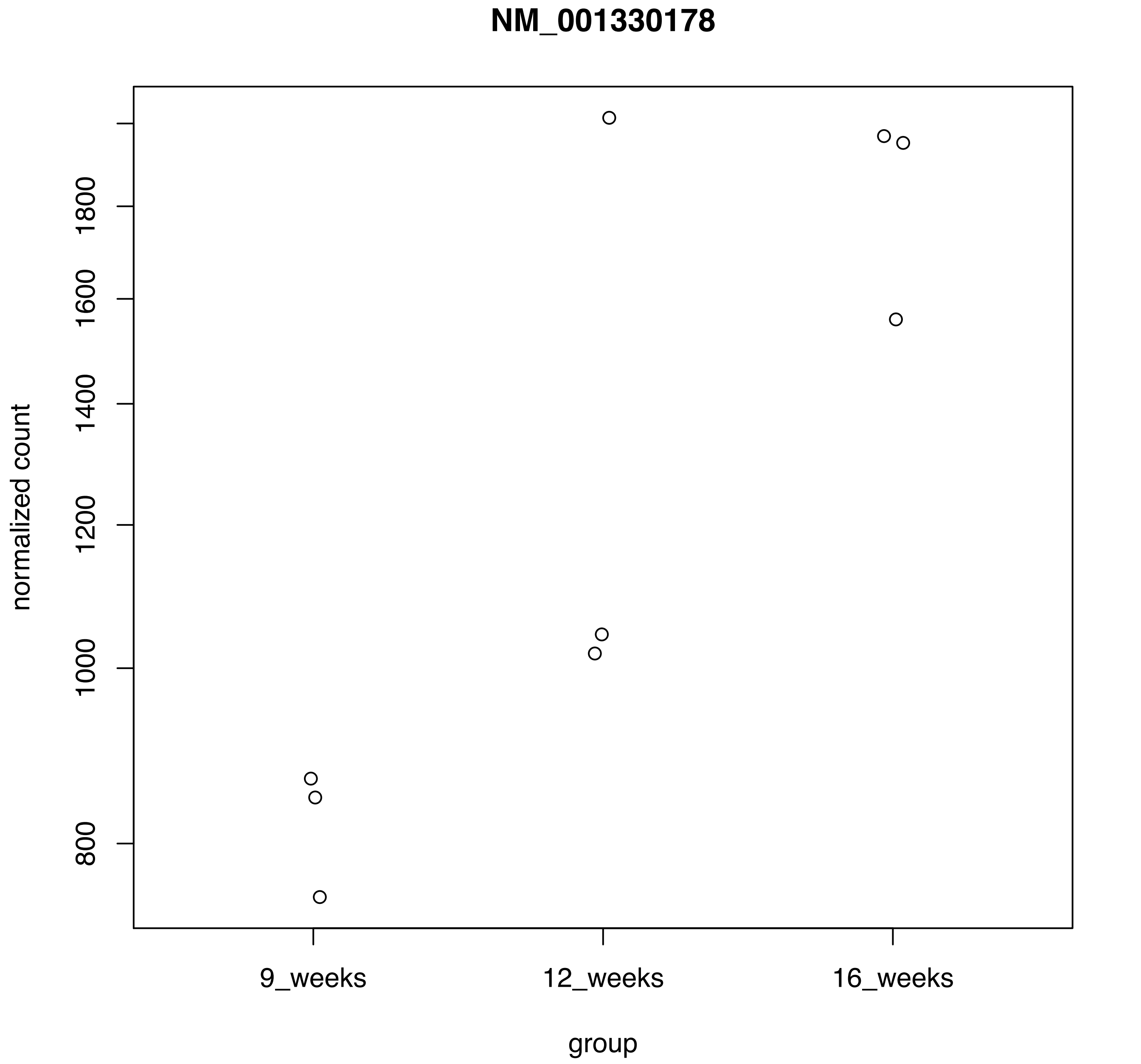
NM_001330178
normalized count
1800
1600
1400
1200
1000
800
9_weeks
12_weeks
16_weeks
group

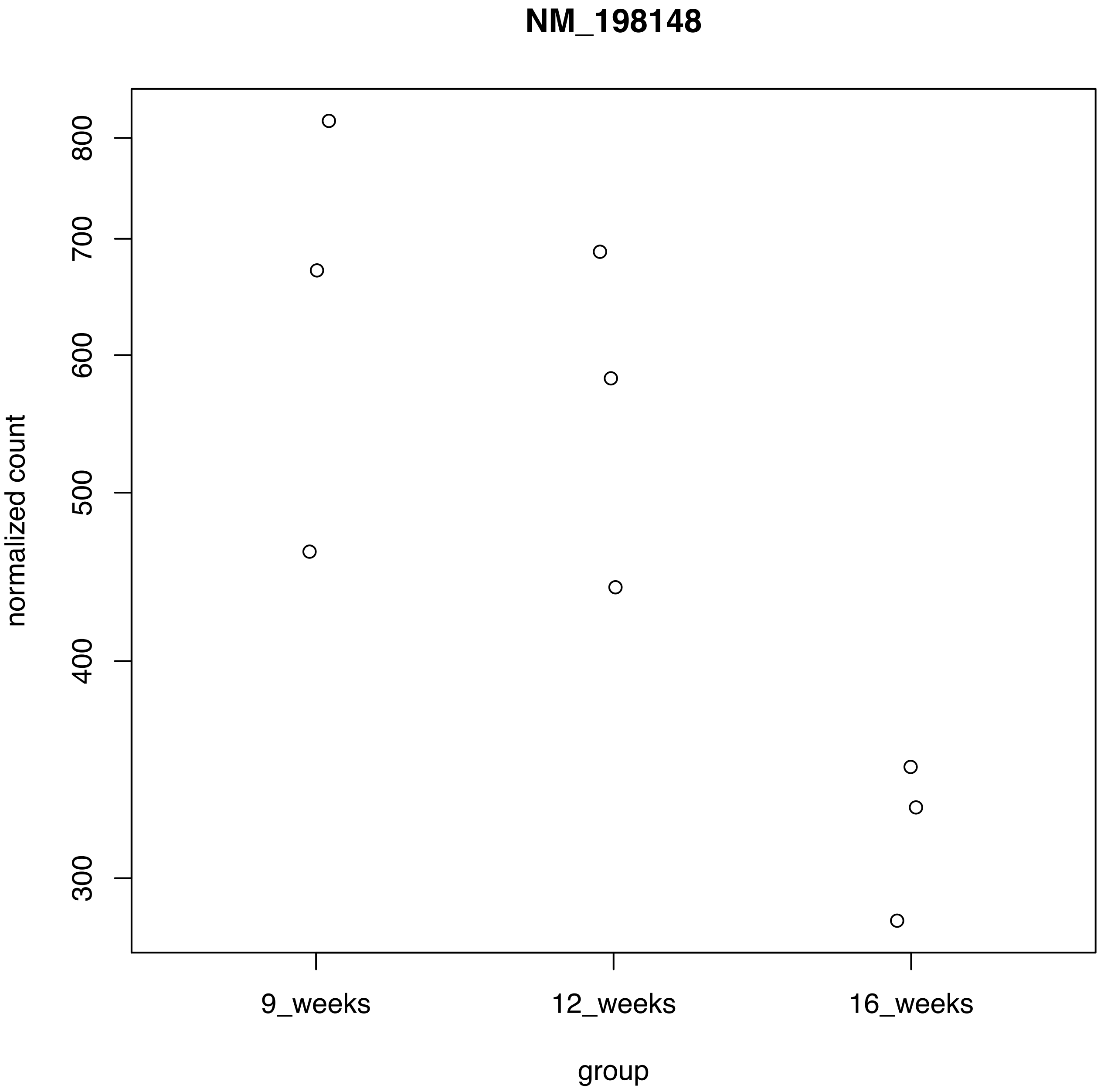
NM_198148
normalized count
800
700
600
500
400
300
9_weeks
12_weeks
16_weeks
group

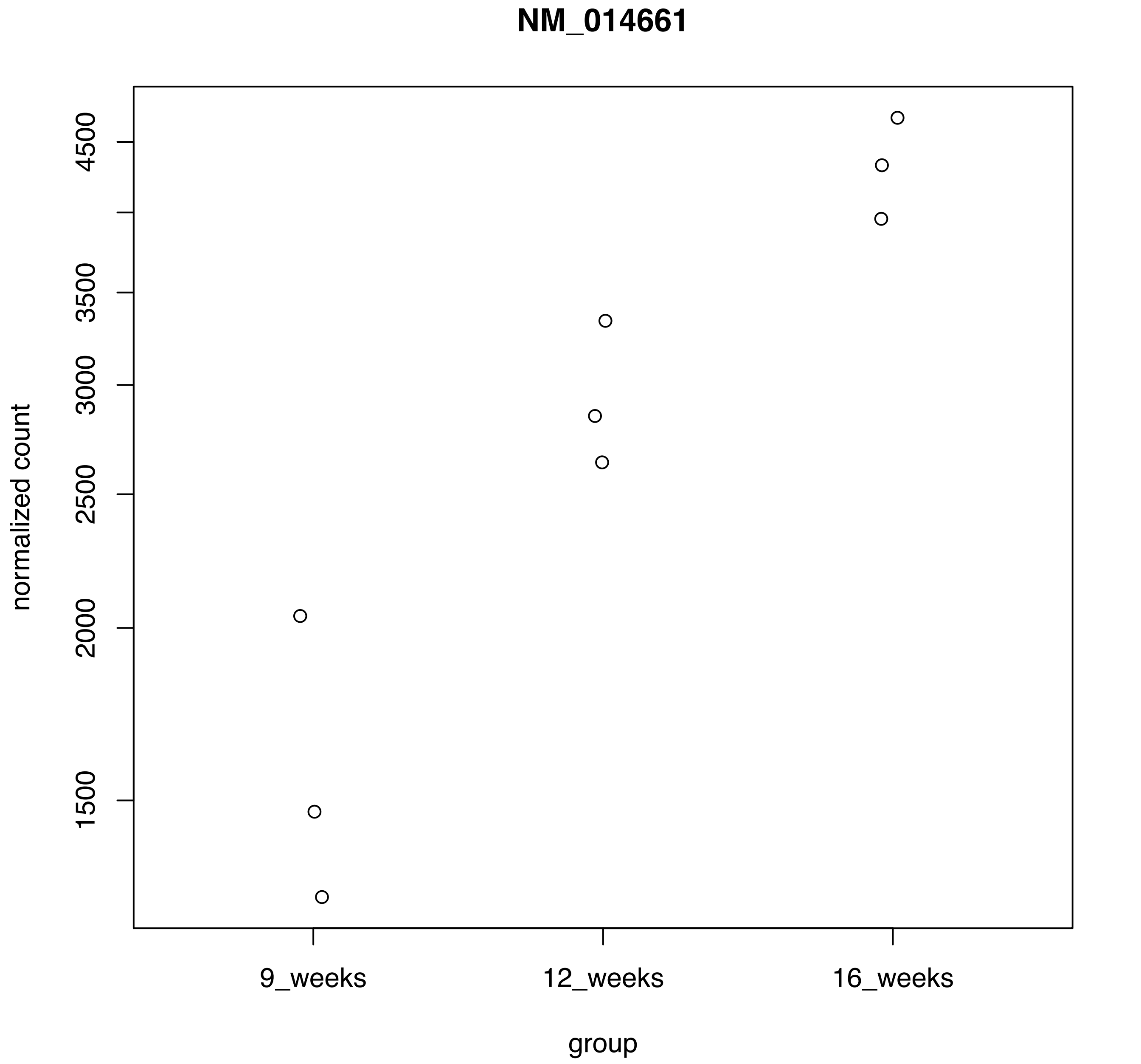
NM_014661
normalized count
1500
2000
2500
3000
3500
4500
9_weeks
12_weeks
16_weeks
group

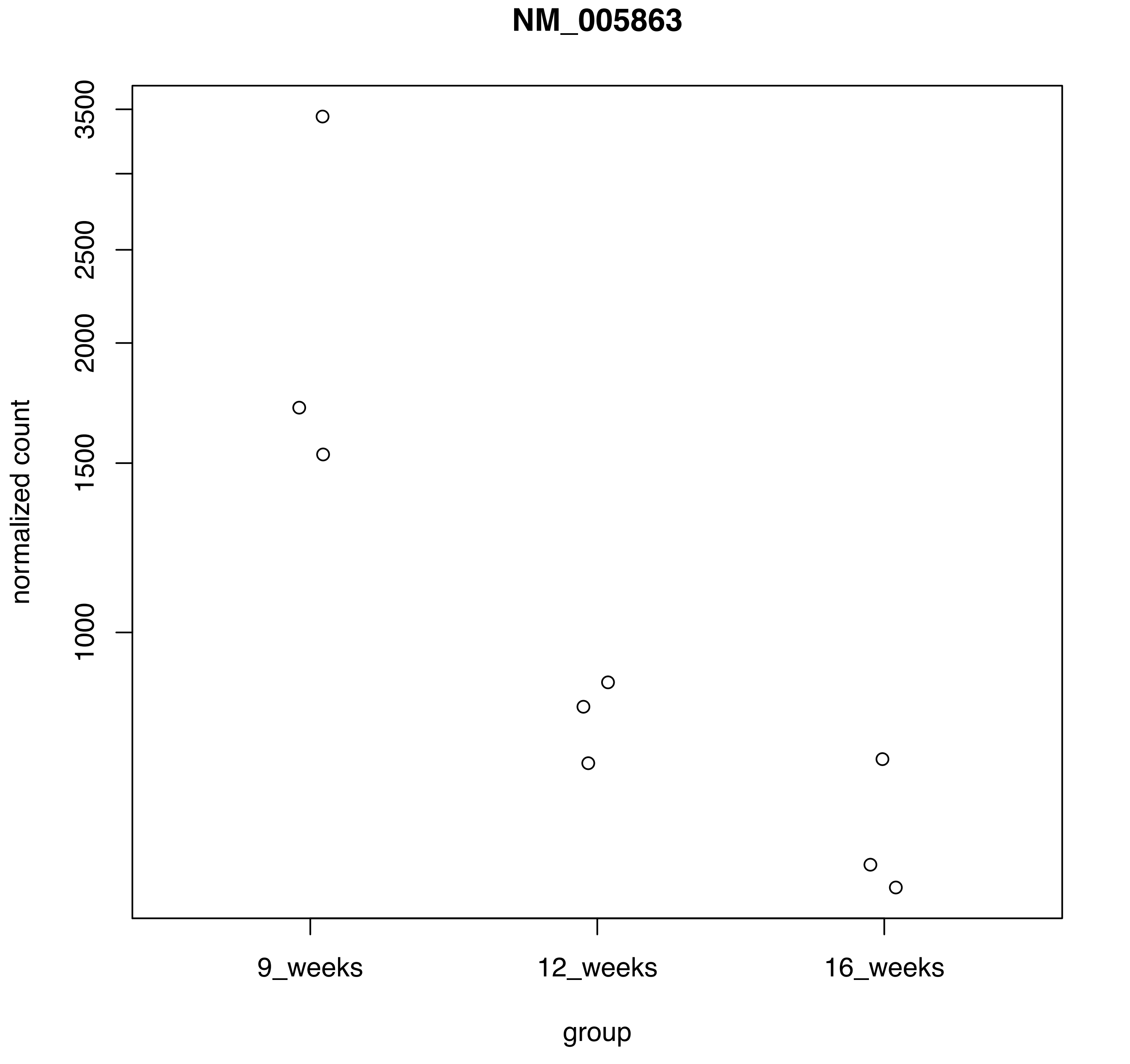

NM_005863
normalized count
3500
2500
2000
1500
1000
9_weeks
12_weeks
16_weeks
group

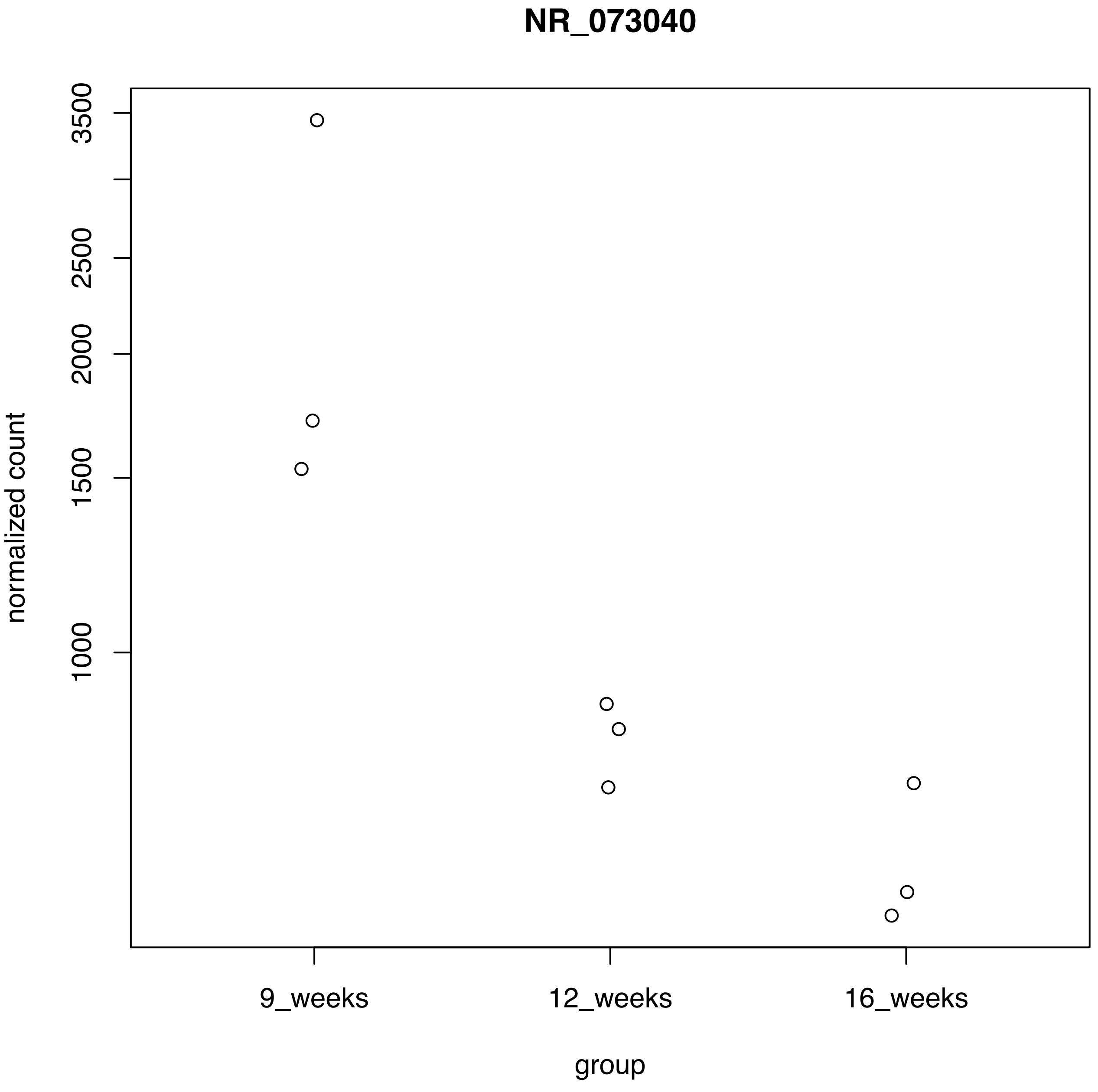
NR_073040
normalized count
3500
2500
2000
1500
1000
9_weeks
12_weeks
16_weeks
group

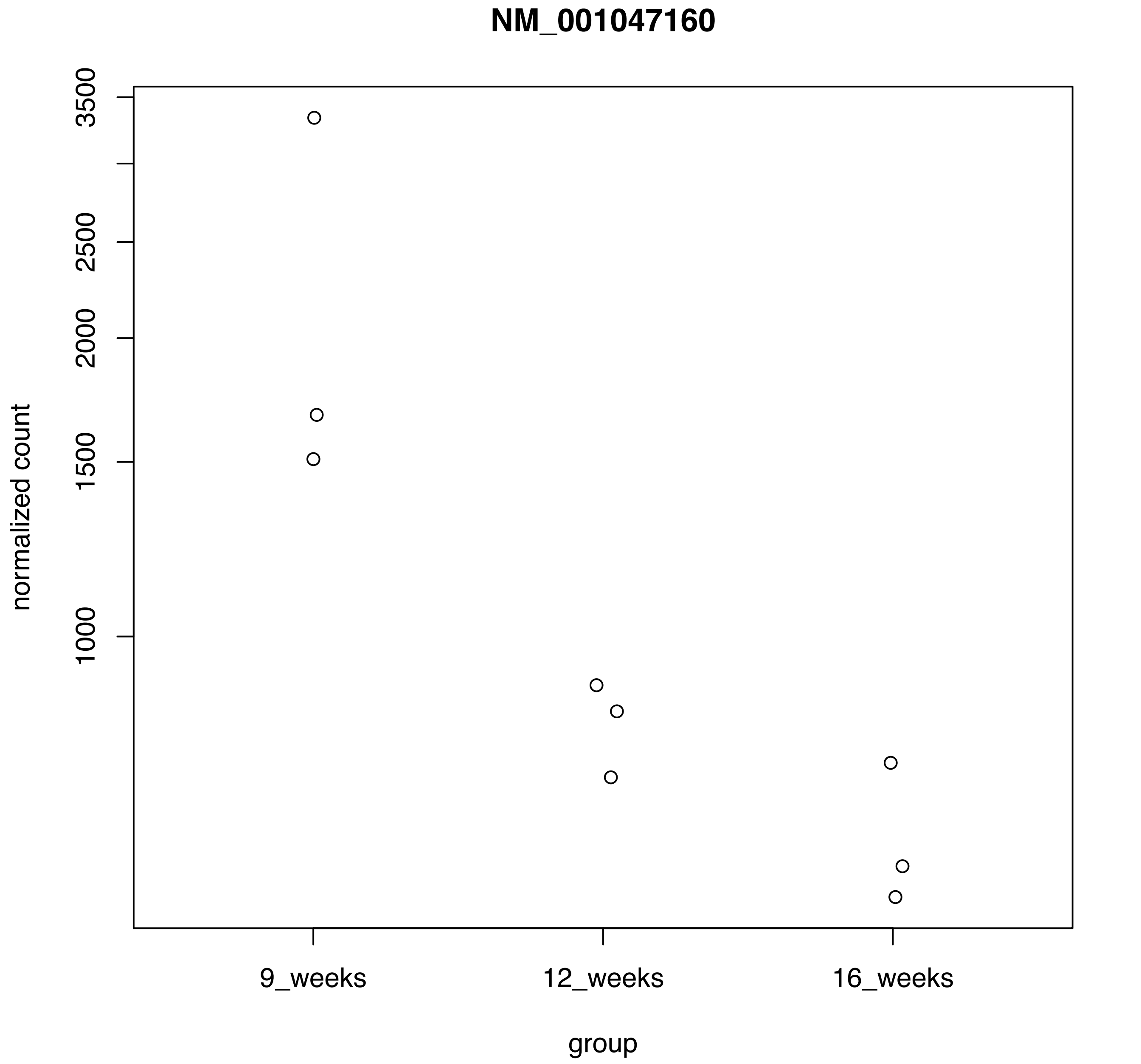
NM_001047160
normalized count
3500
2500
2000
1500
1000
9_weeks
12_weeks
16_weeks
group

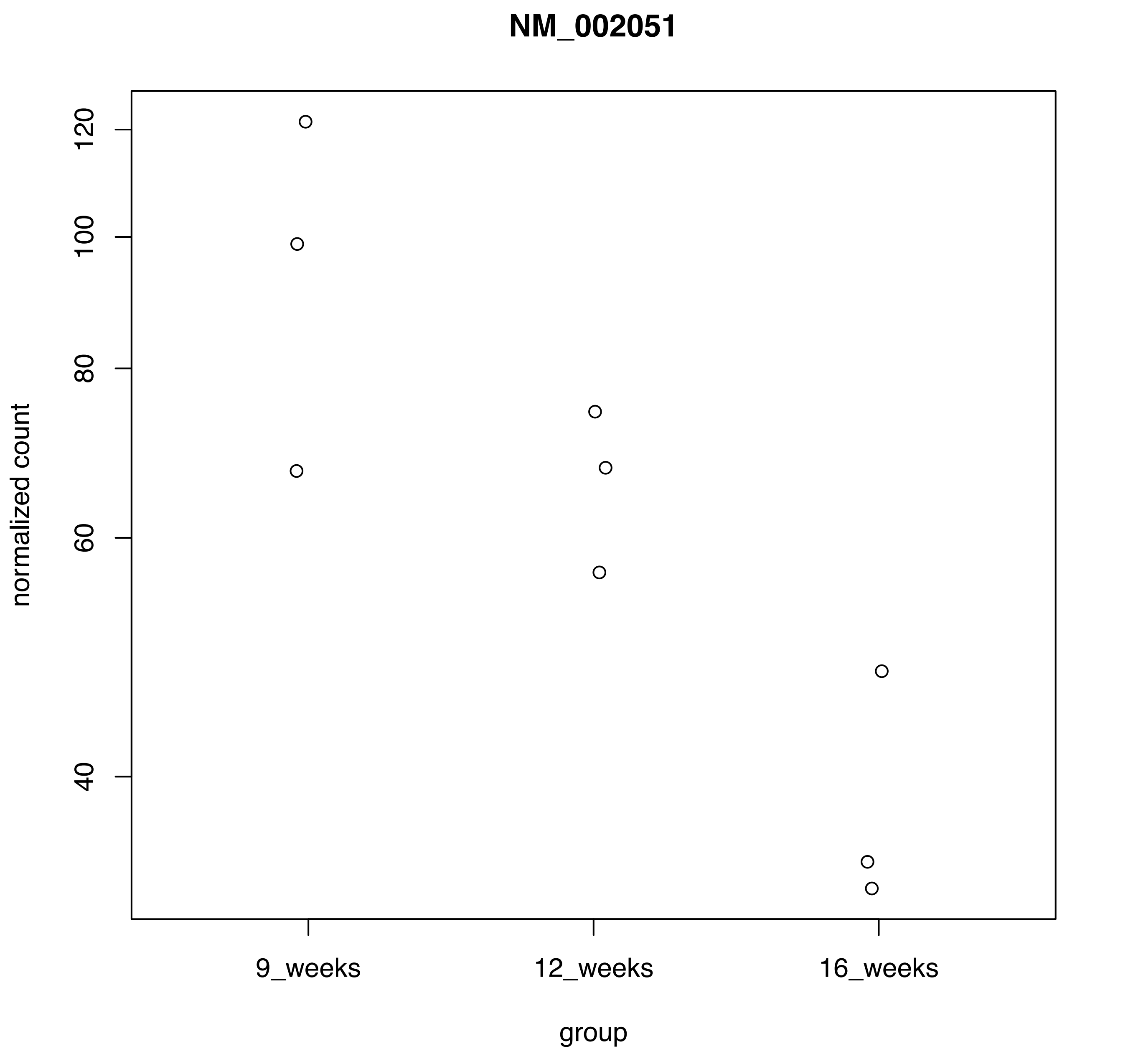
NM_002051
normalized count
120
100
80
60
40
9_weeks
12_weeks
16_weeks
group

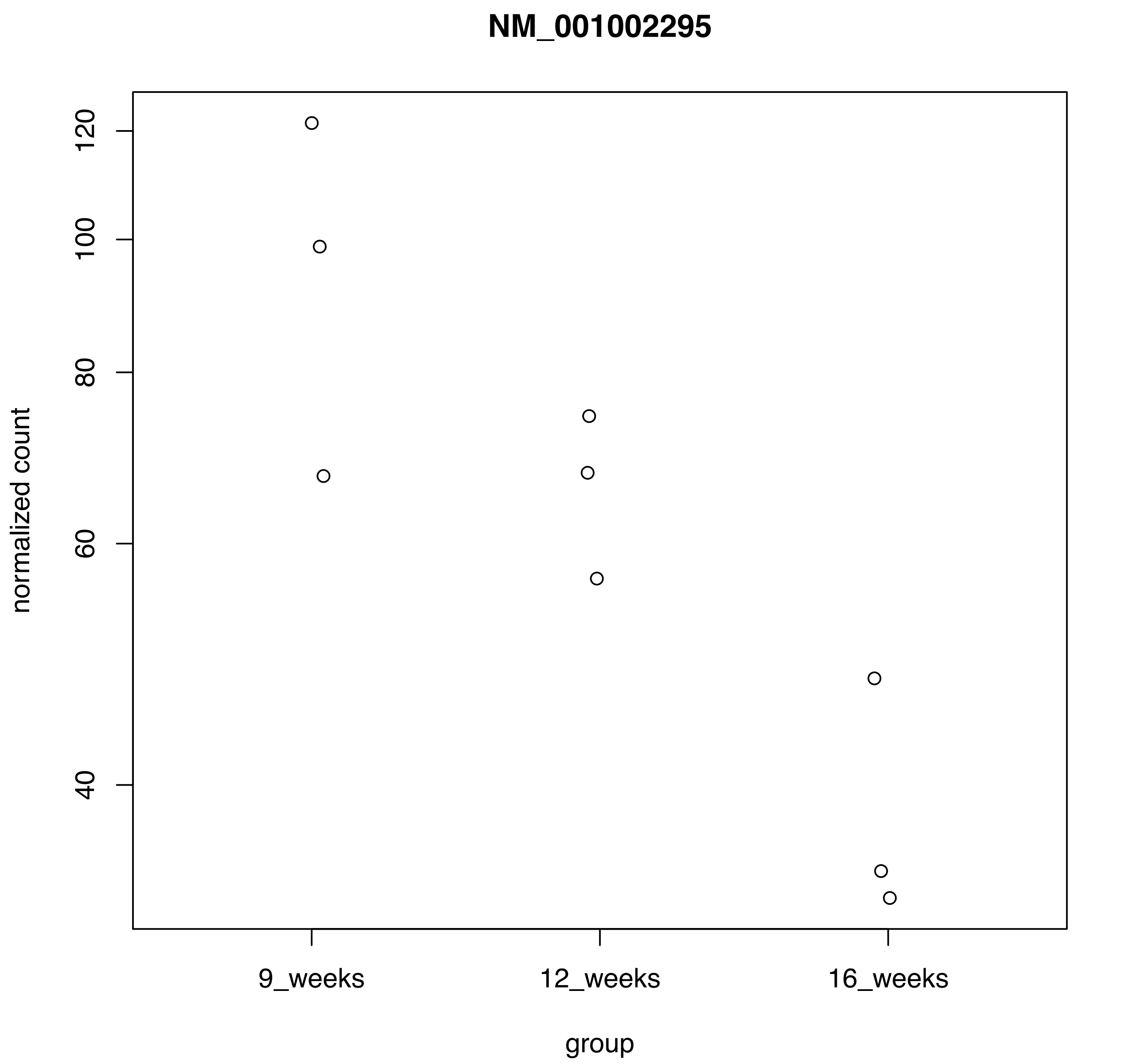
NM_001002295
normalized count
120
100
80
60
40
9_weeks
12_weeks
16_weeks
group

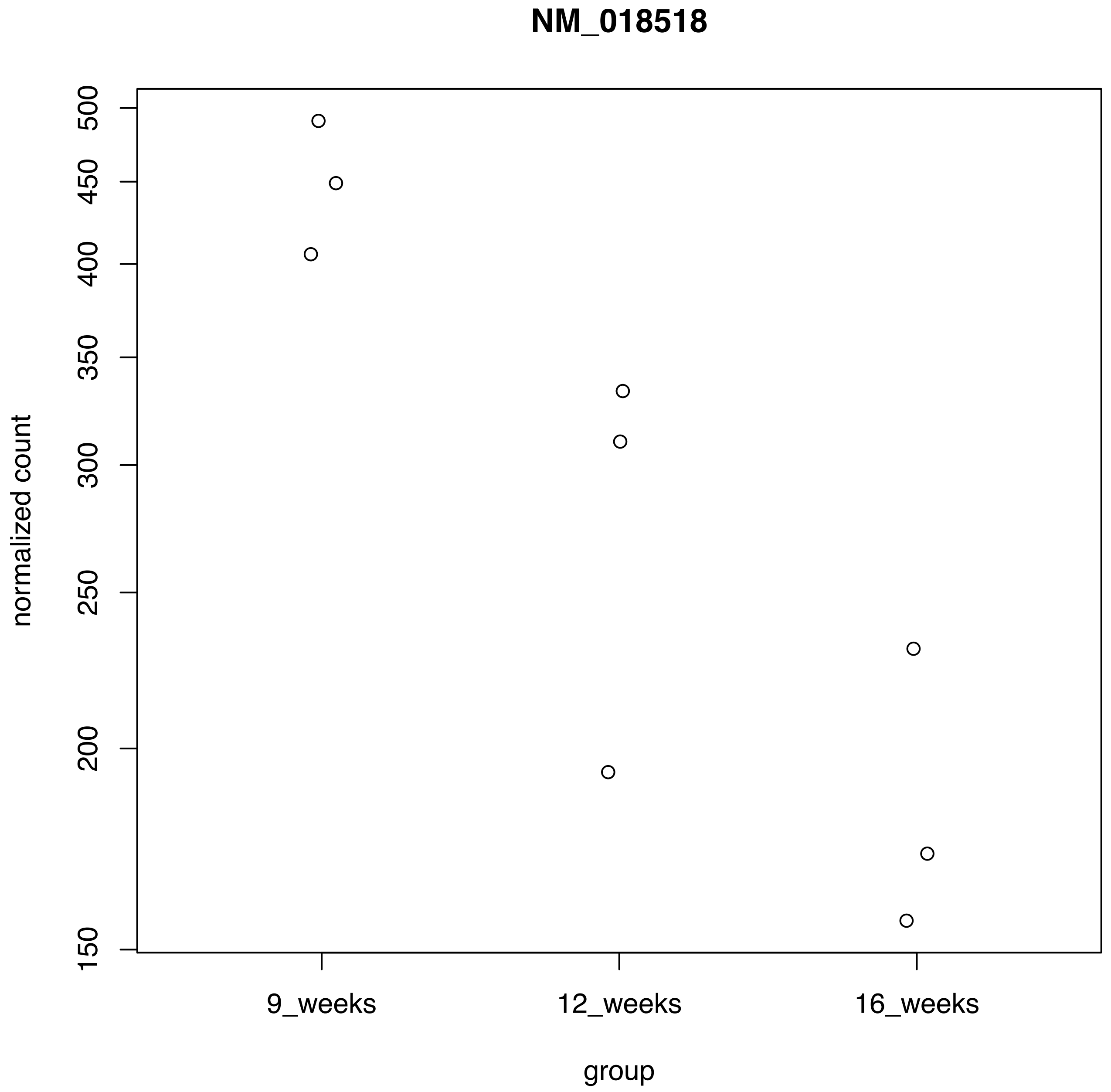
NM_018518
normalized count
500
450
400
350
300
250
200
150
9_weeks
12_weeks
16_weeks
group

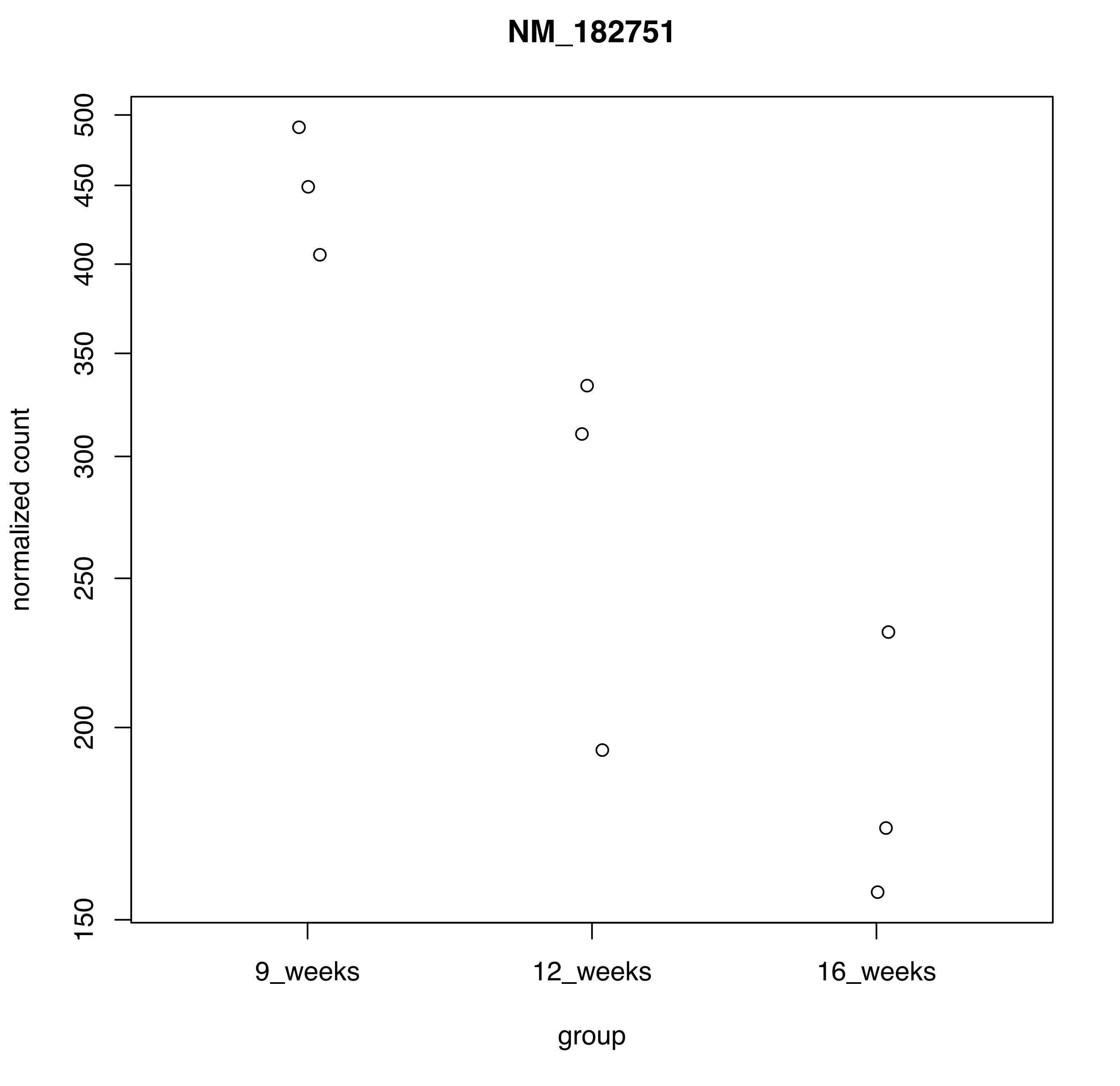

NM_182751
normalized count
500
450
400
350
300
250
200
150
9_weeks
12_weeks
16_weeks
group

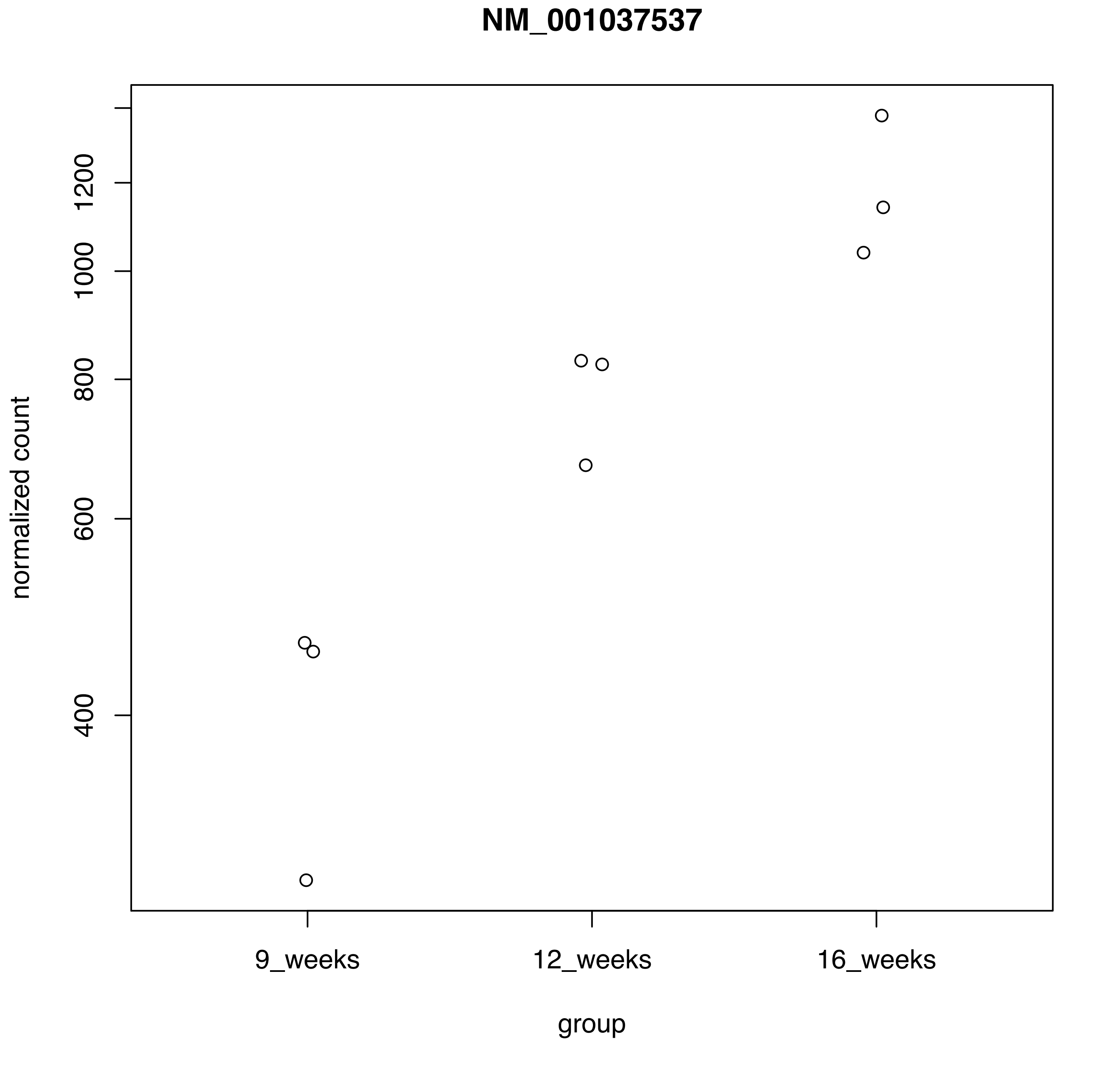

NM_001037537
normalized count
400
600
800
1000
1200
9_weeks
12_weeks
16_weeks
group

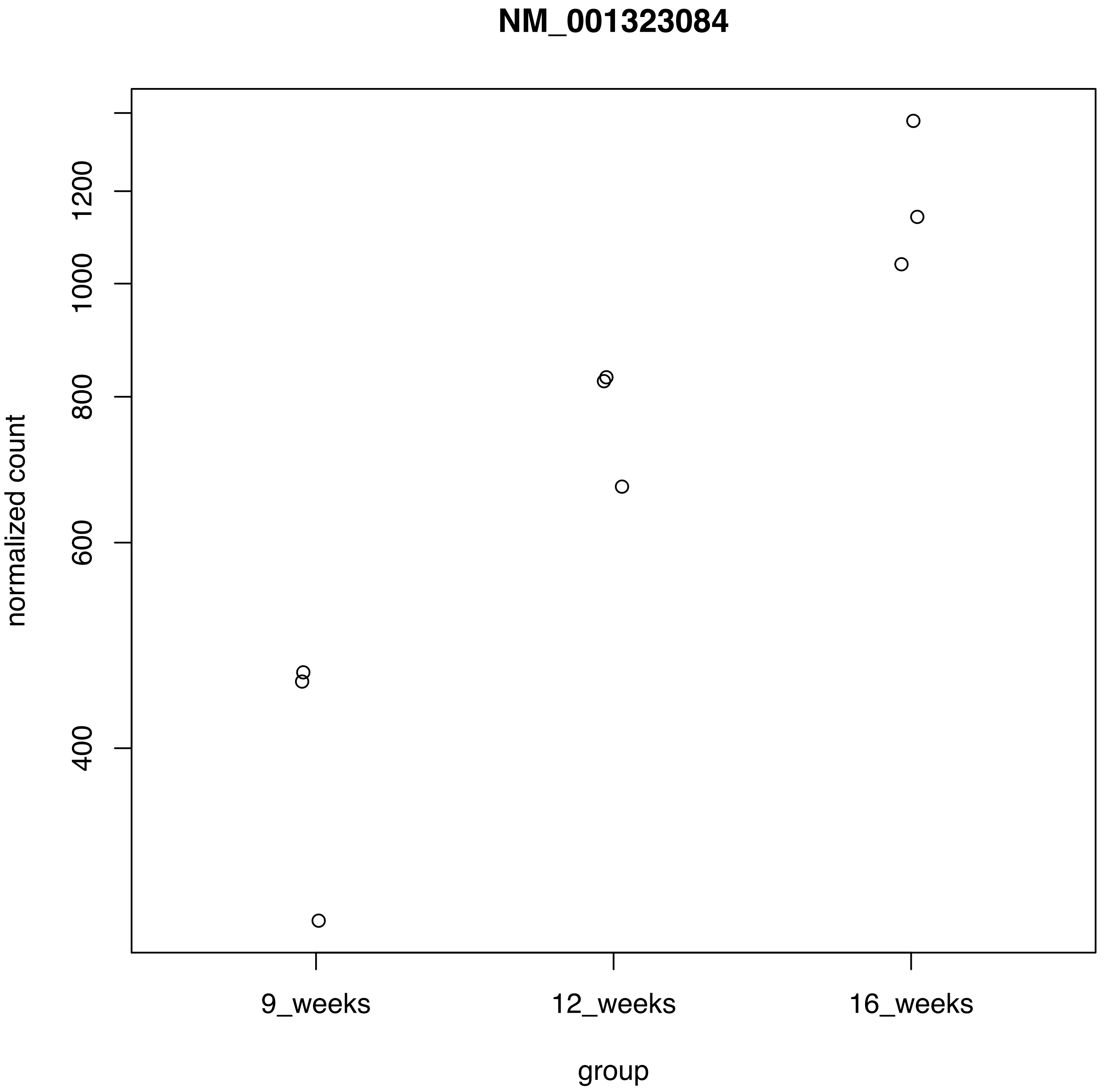
NM_001323084
normalized count
400
600
800
1000
1200
9_weeks
12_weeks
16_weeks
group

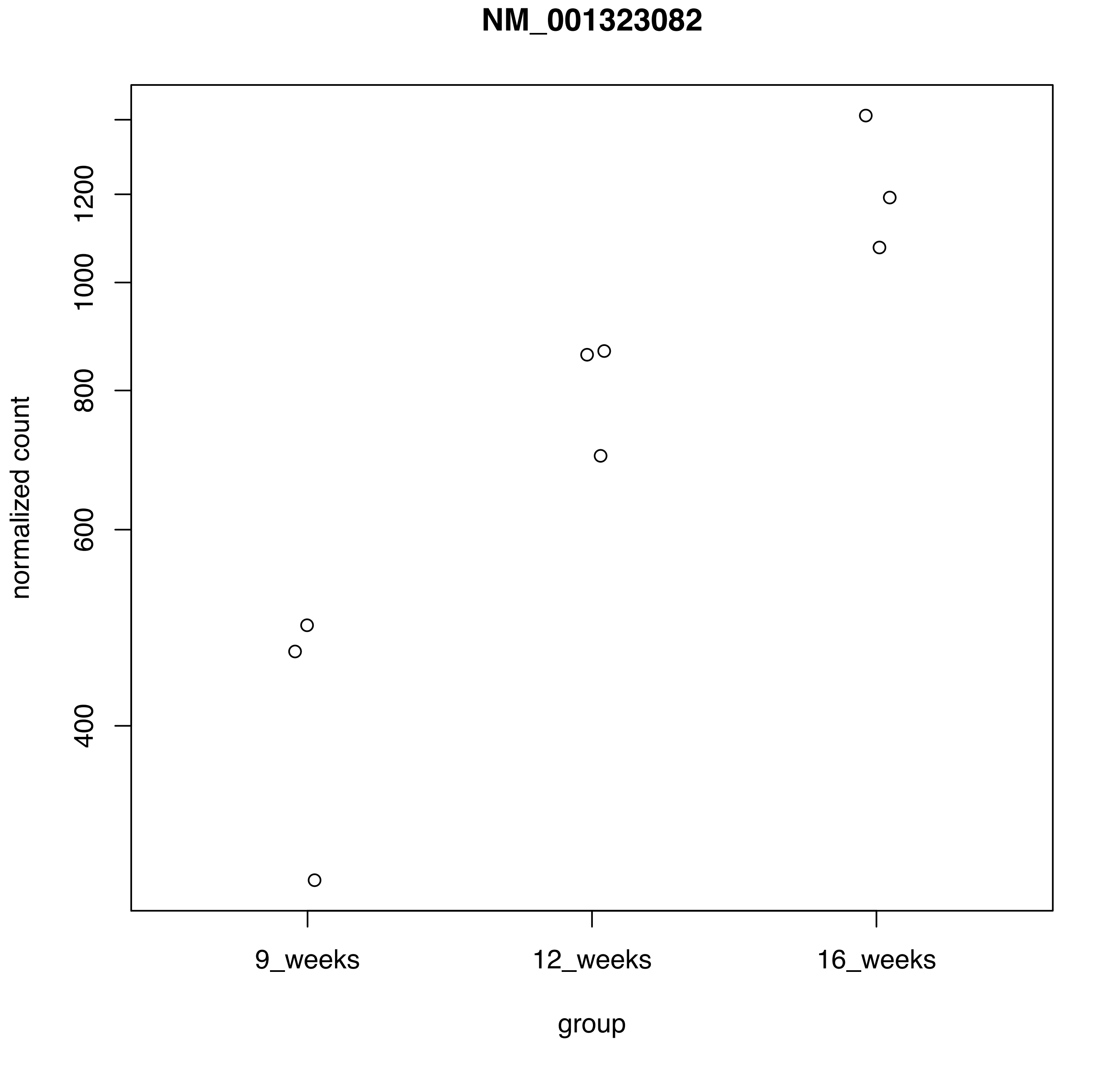
NM_001323082
normalized count
400
600
800
1000
1200
9_weeks
12_weeks
16_weeks
group

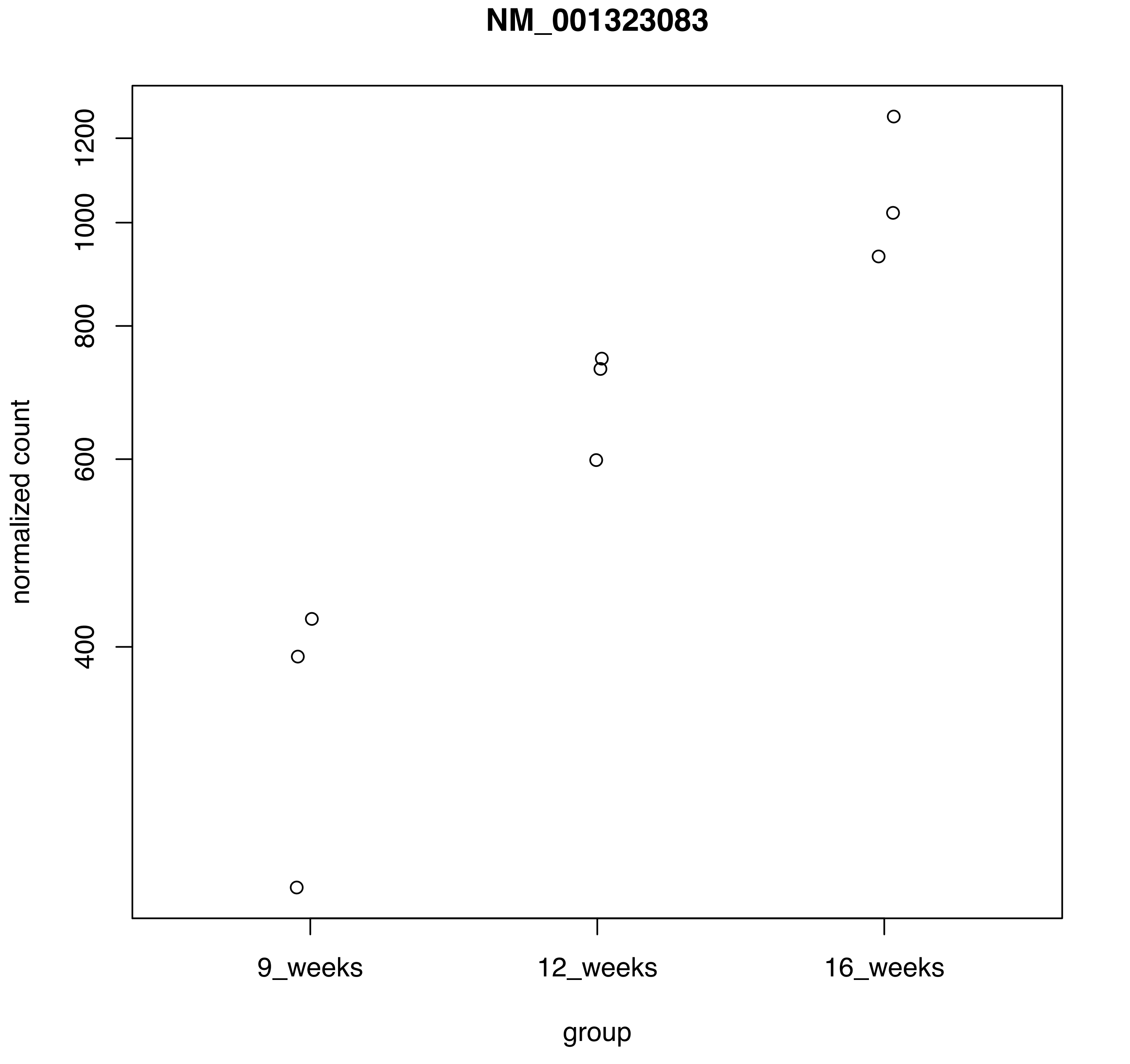

NM_001323083
normalized count
1200
1000
800
600
400
9_weeks
12_weeks
16_weeks
group

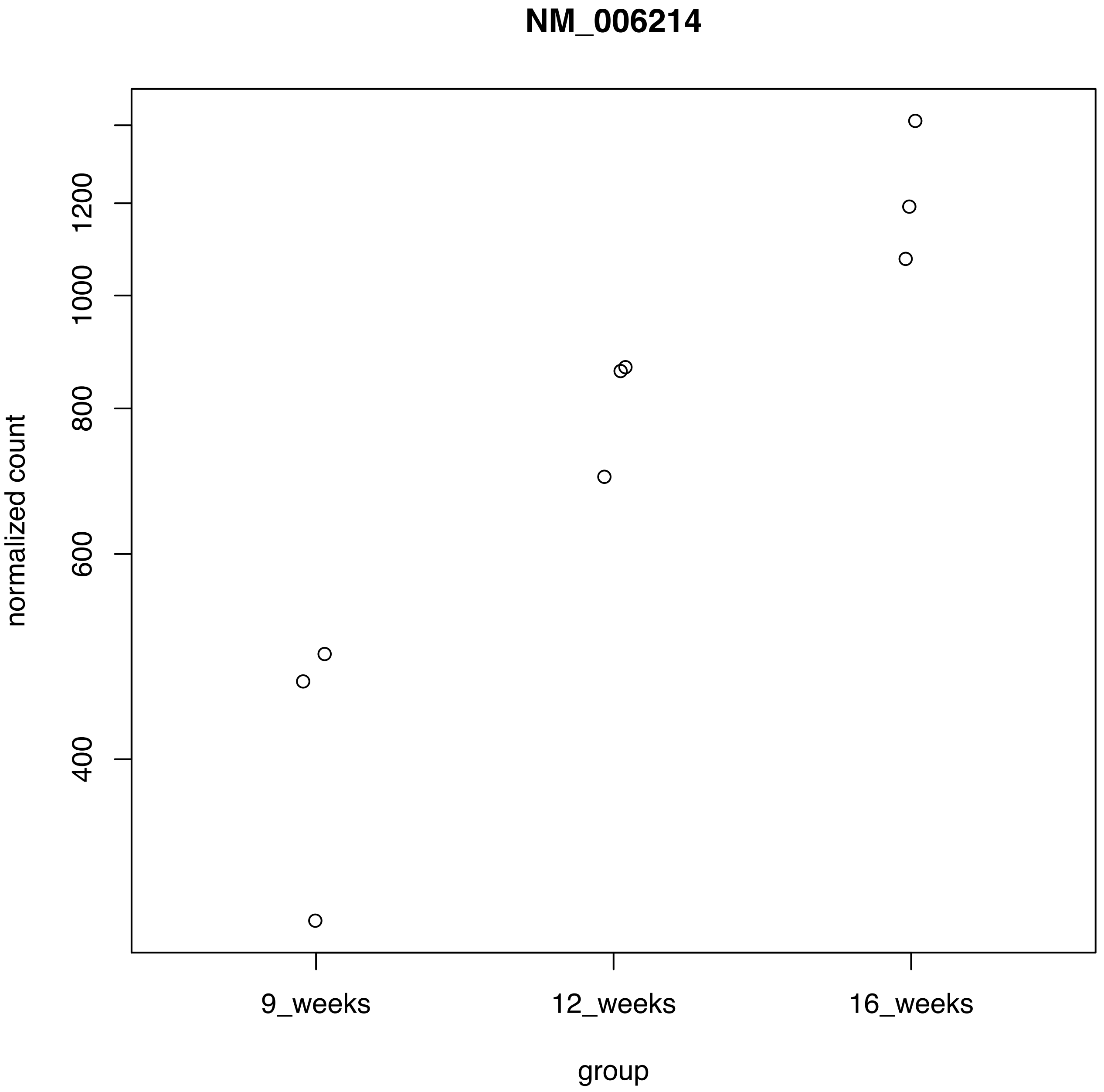

NM_006214
normalized count
400
600
800
1000
1200
9_weeks
12_weeks
16_weeks
group

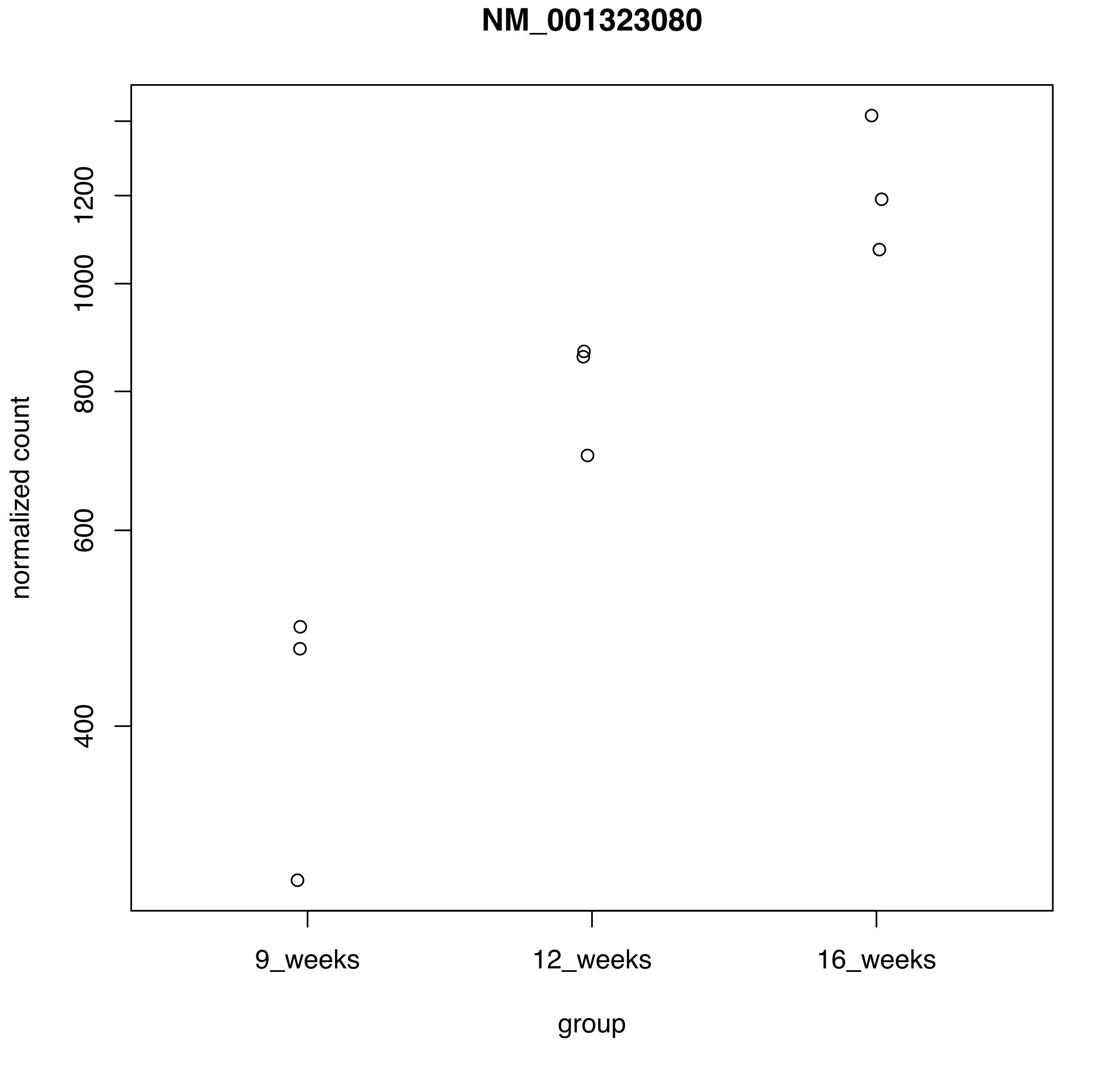

NM_001323080
normalized count
400
600
800
1000
1200
9_weeks
12_weeks
16_weeks
group

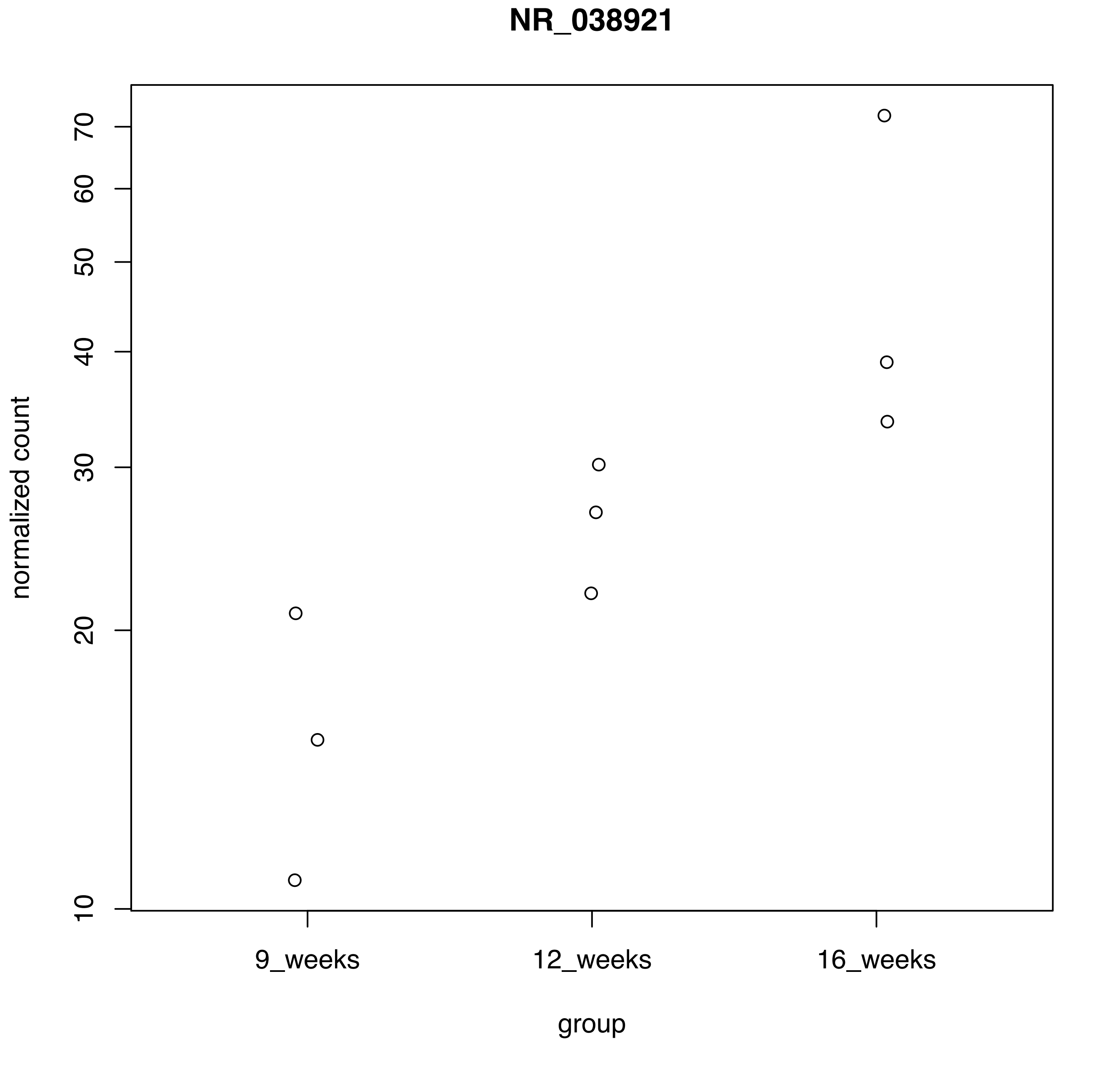

NR_038921
normalized count
10
20
30
40
50
60
70
9_weeks
12_weeks
16_weeks
group

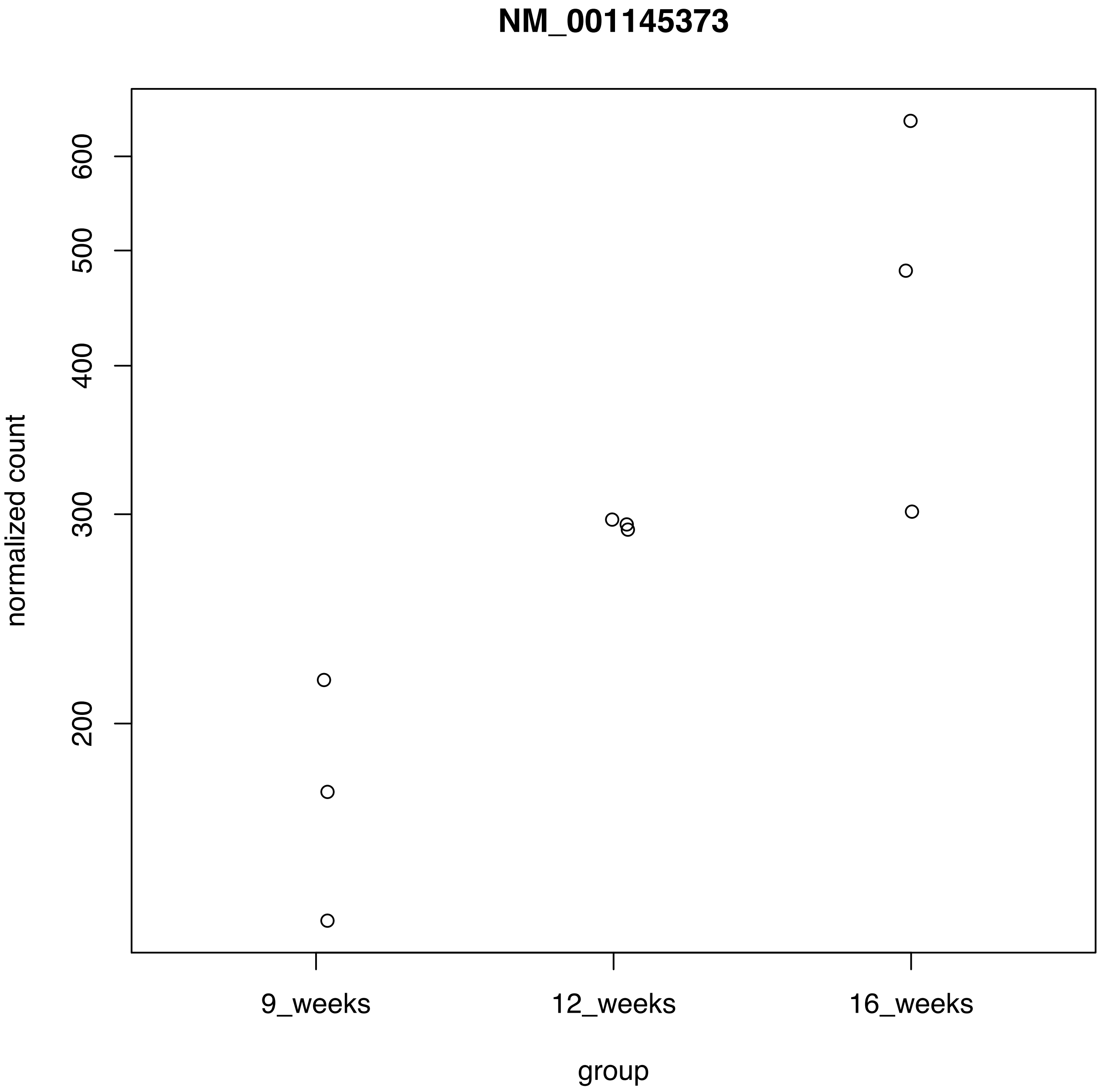
NM_001145373
normalized count
600
500
400
300
200
9_weeks
12_weeks
16_weeks
group

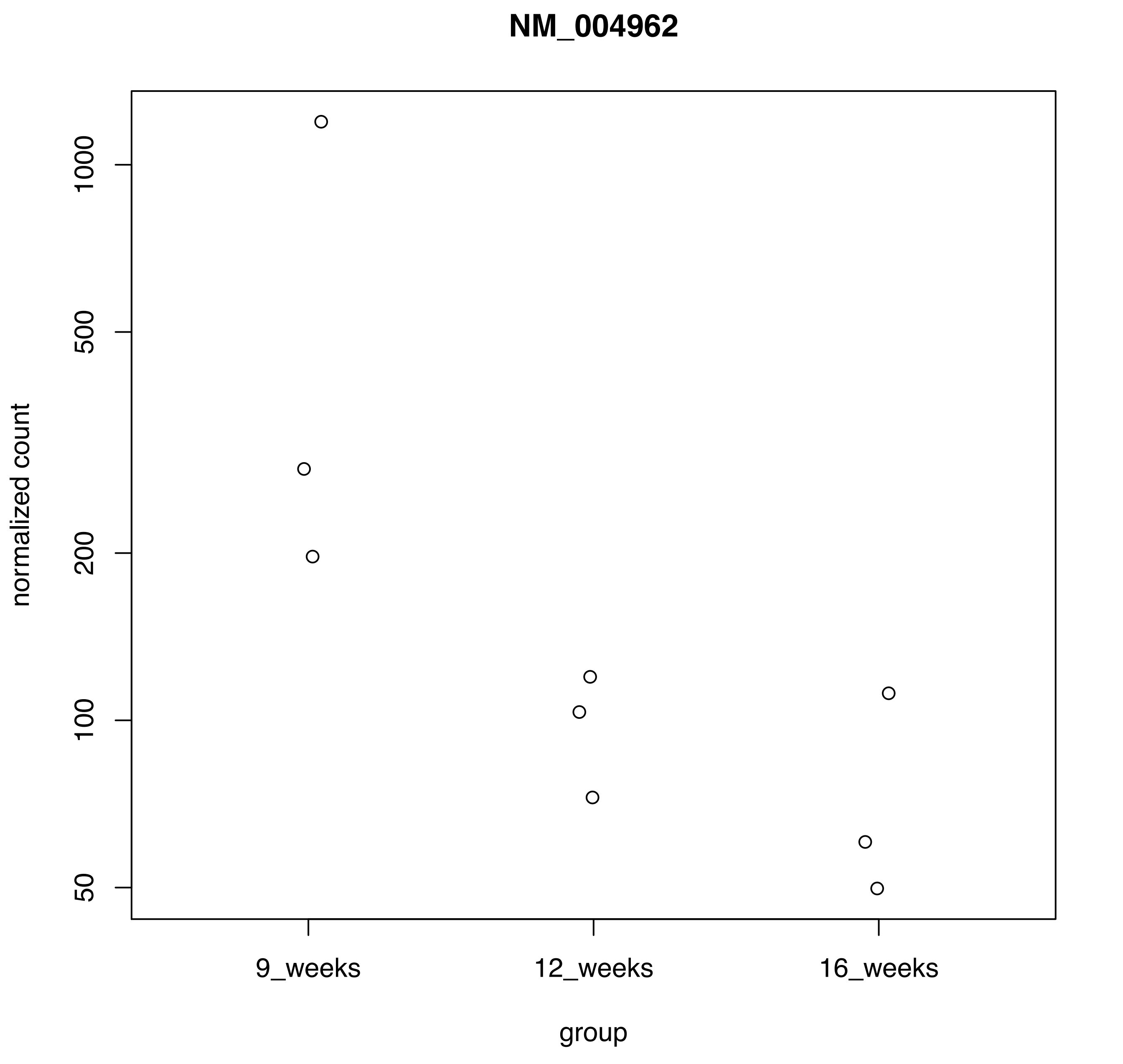

NM_004962
normalized count
1000
500
200
100
50
9_weeks
12_weeks
16_weeks
group

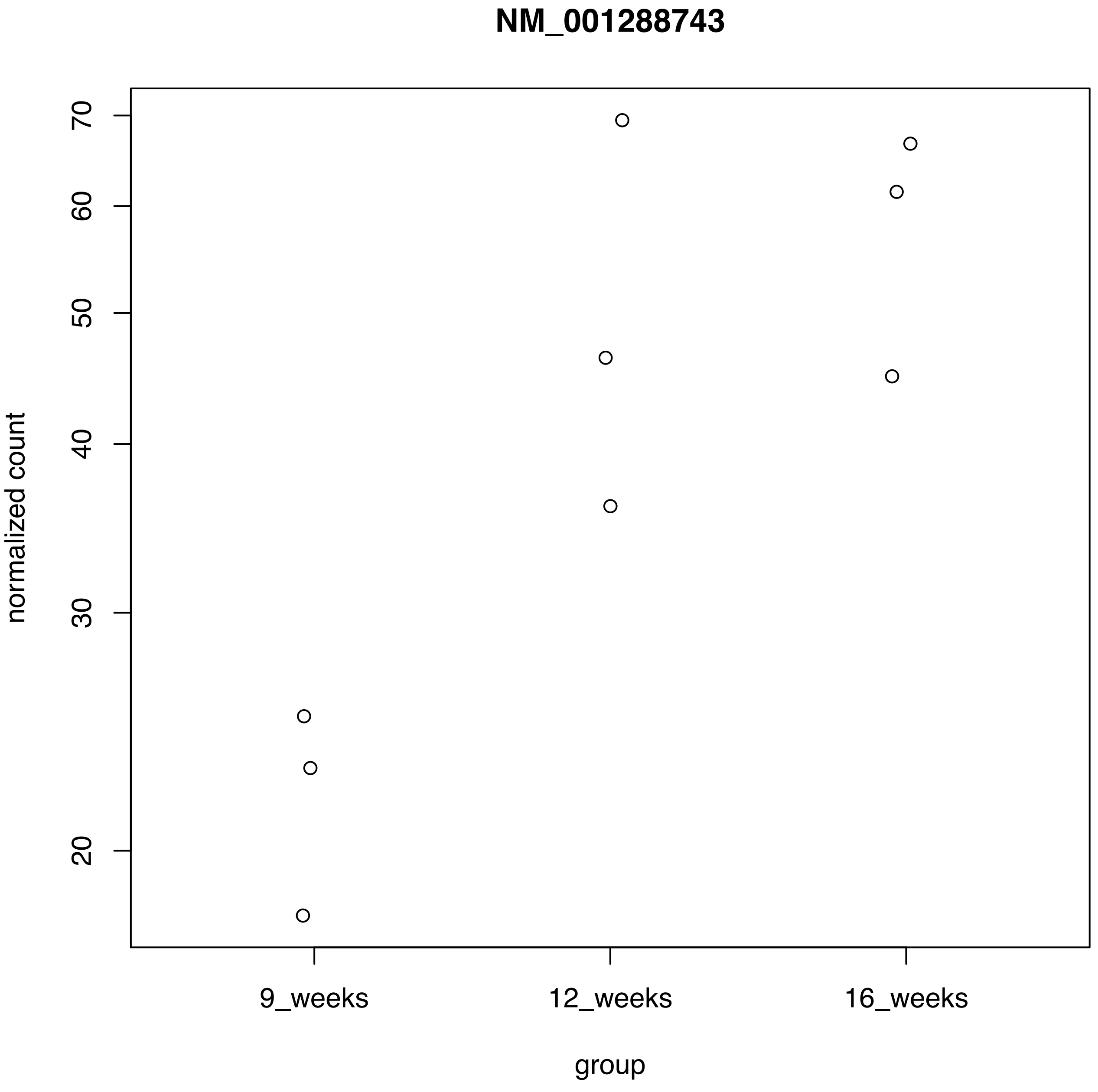
NM_001288743
normalized count
20
30
40
50
60
70
9_weeks
12_weeks
16_weeks
group

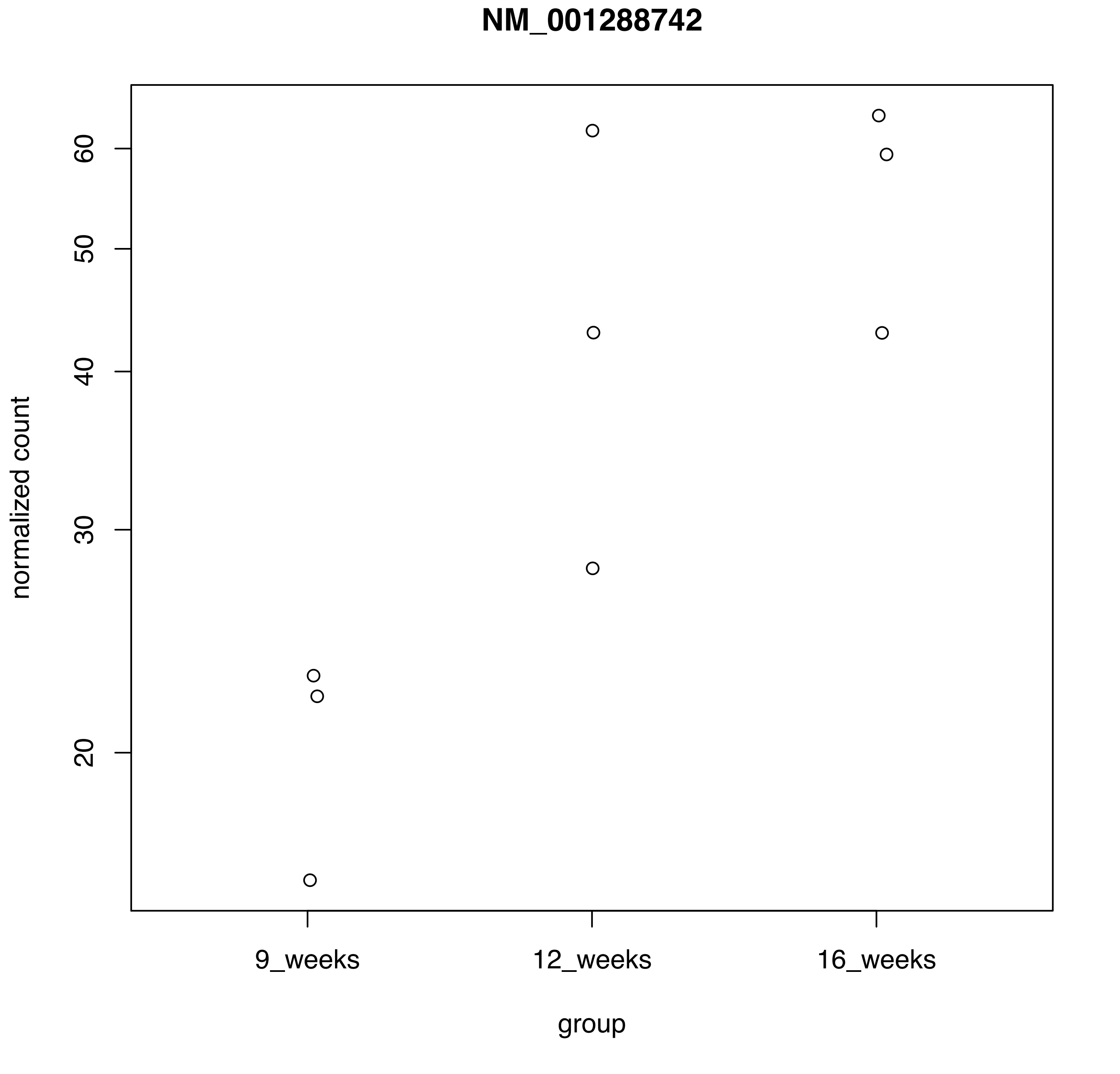
NM_001288742
normalized count
20
30
40
50
60
9_weeks
12_weeks
16_weeks
group

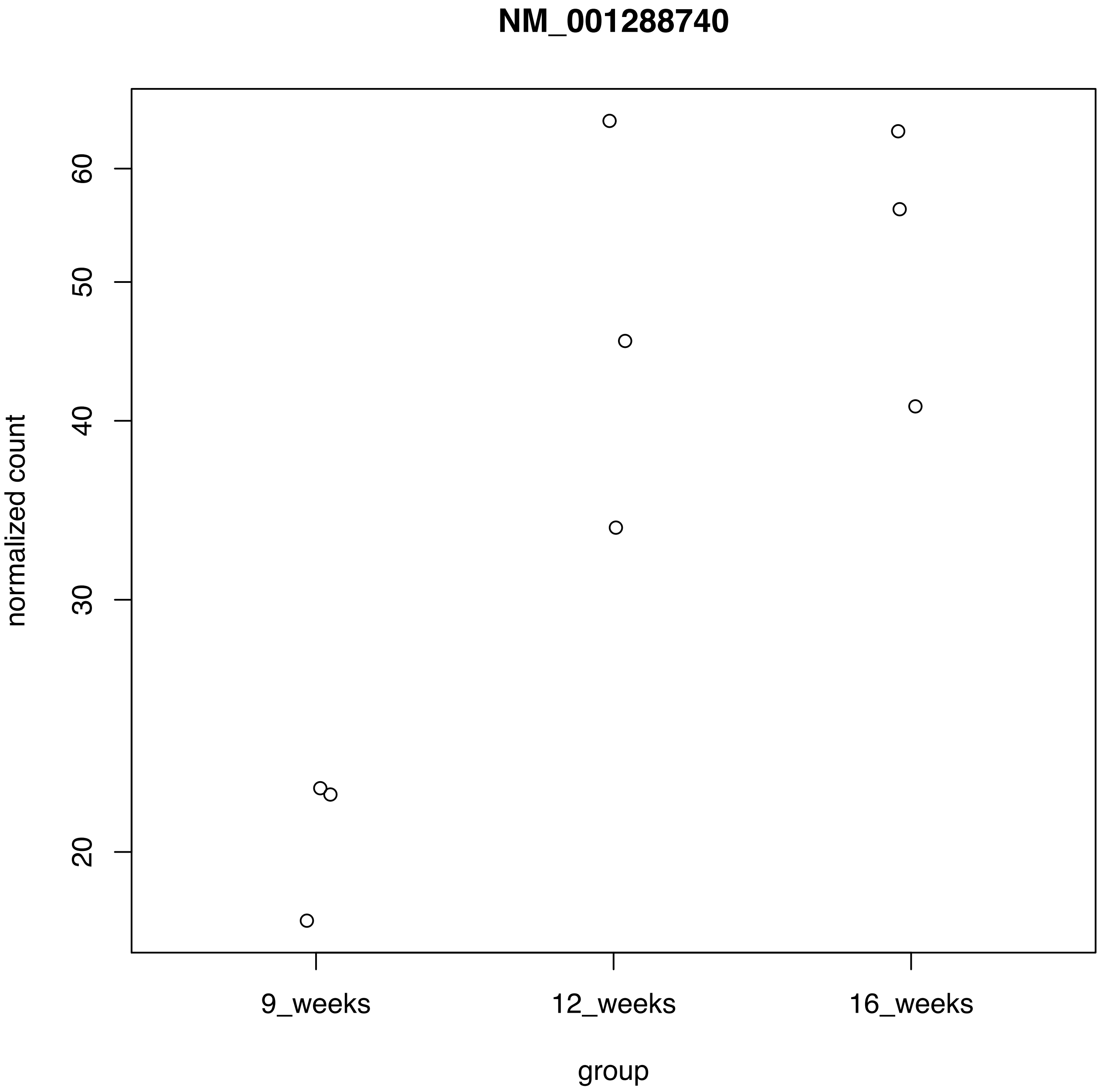
NM_001288740
normalized count
20
30
40
50
60
9_weeks
12_weeks
16_weeks
group

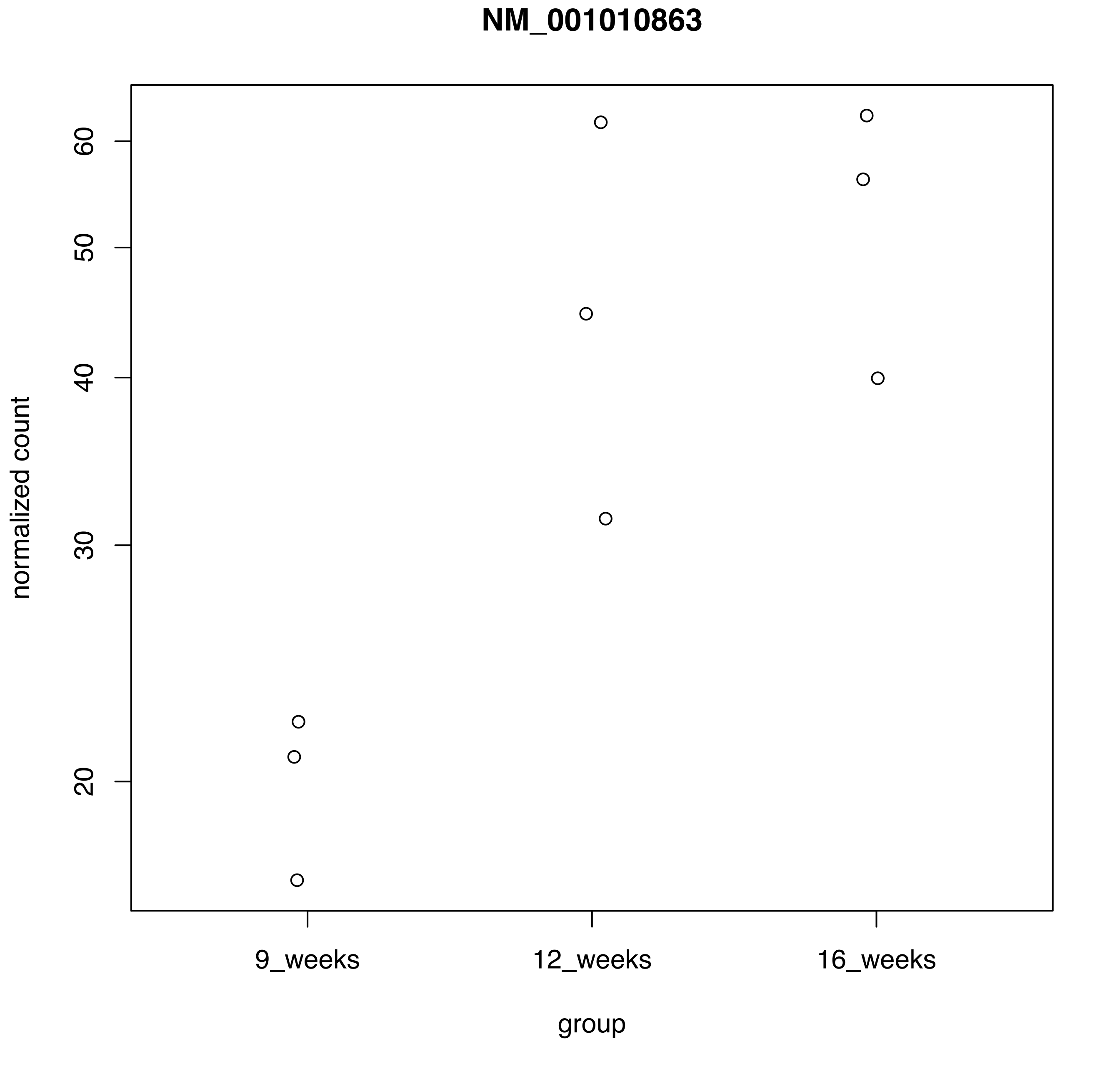
NM_001010863
normalized count
60
50
40
30
20
9_weeks
12_weeks
16_weeks
group

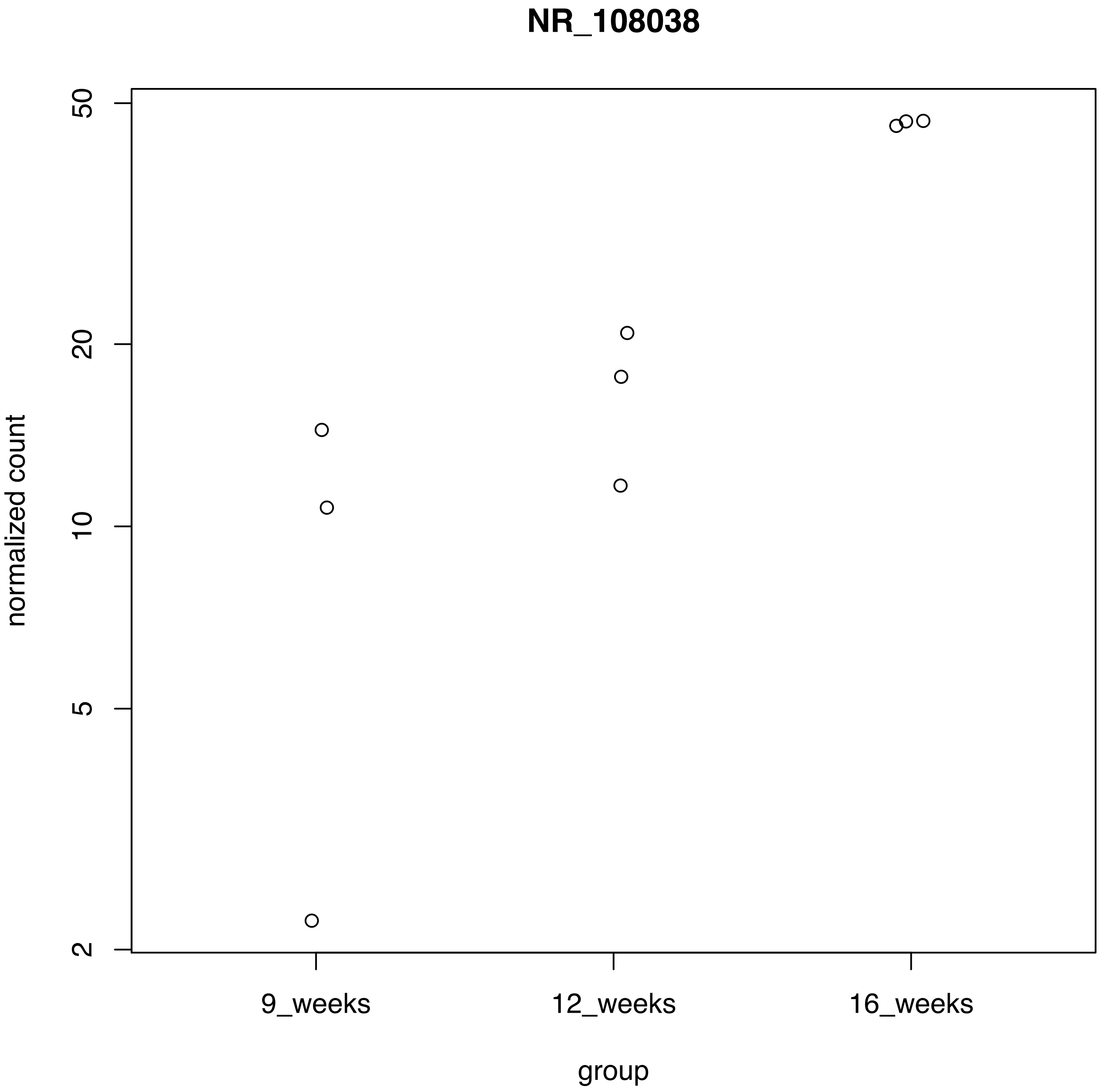
NR_108038
normalized count
50
20
10
5
2
9_weeks
12_weeks
16_weeks
group

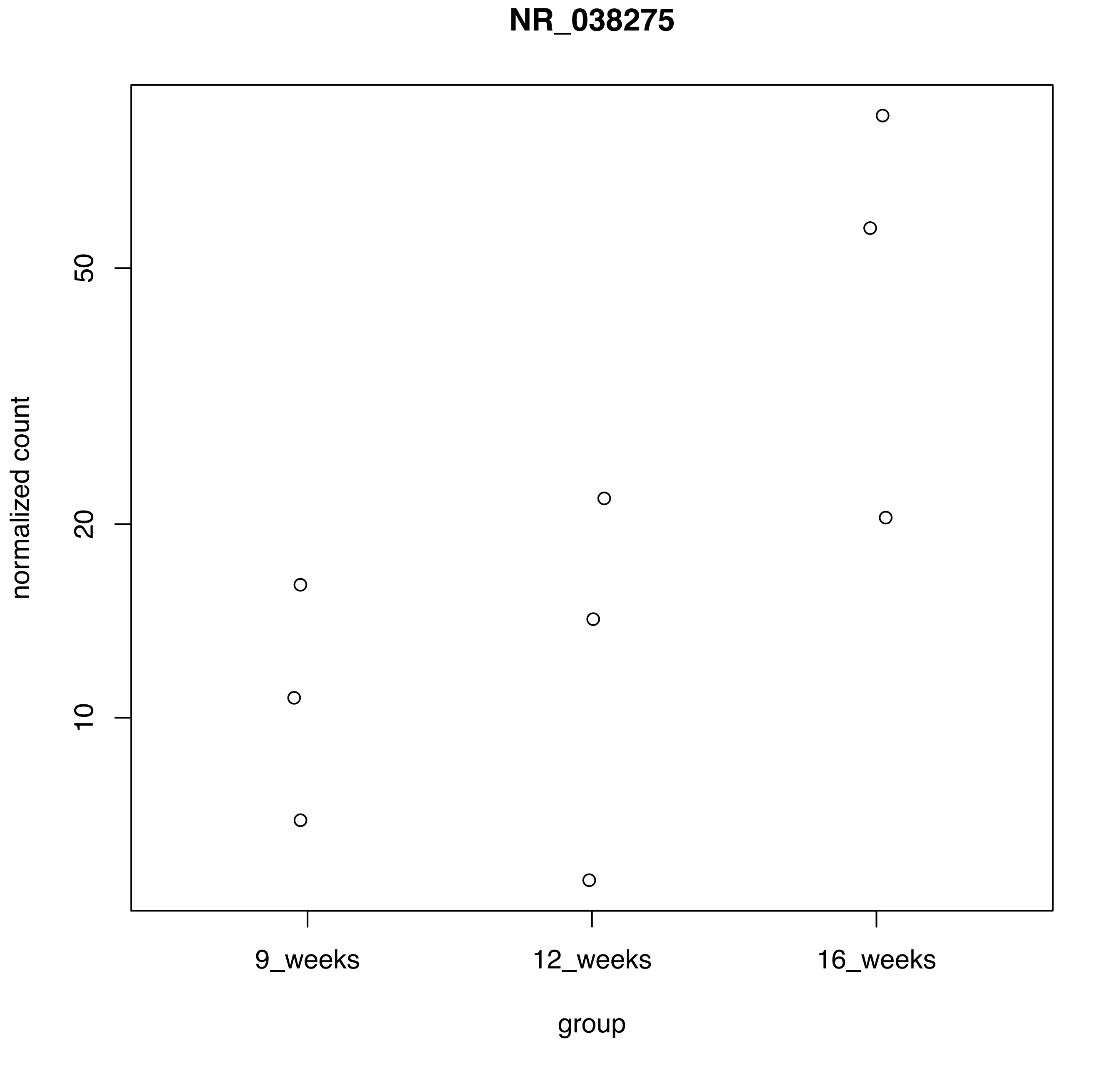
NR_038275
normalized count
50
20
10
9_weeks
12_weeks
16_weeks
group

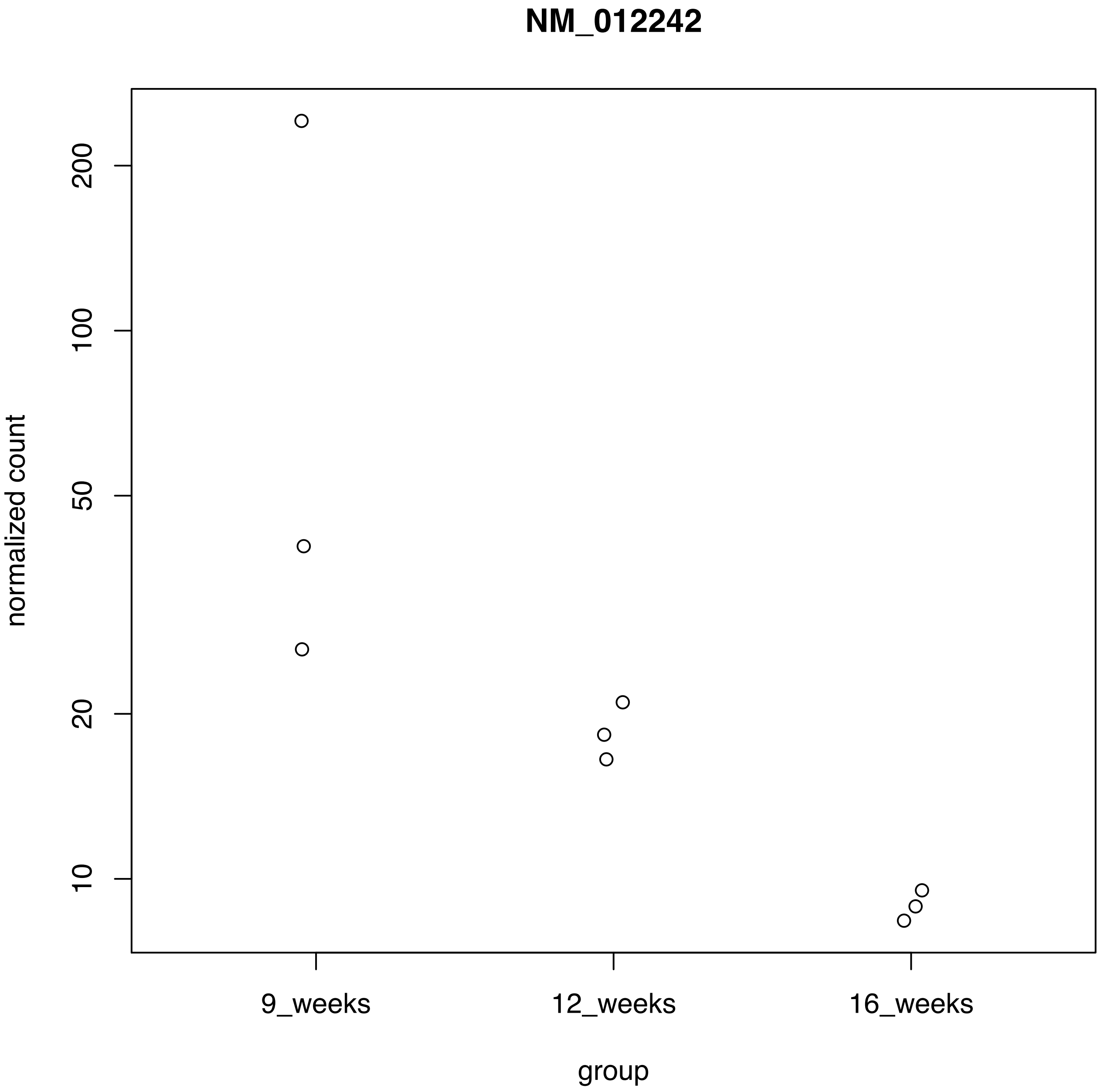

NM_012242
normalized count
200
100
50
20
10
9_weeks
12_weeks
16_weeks
group

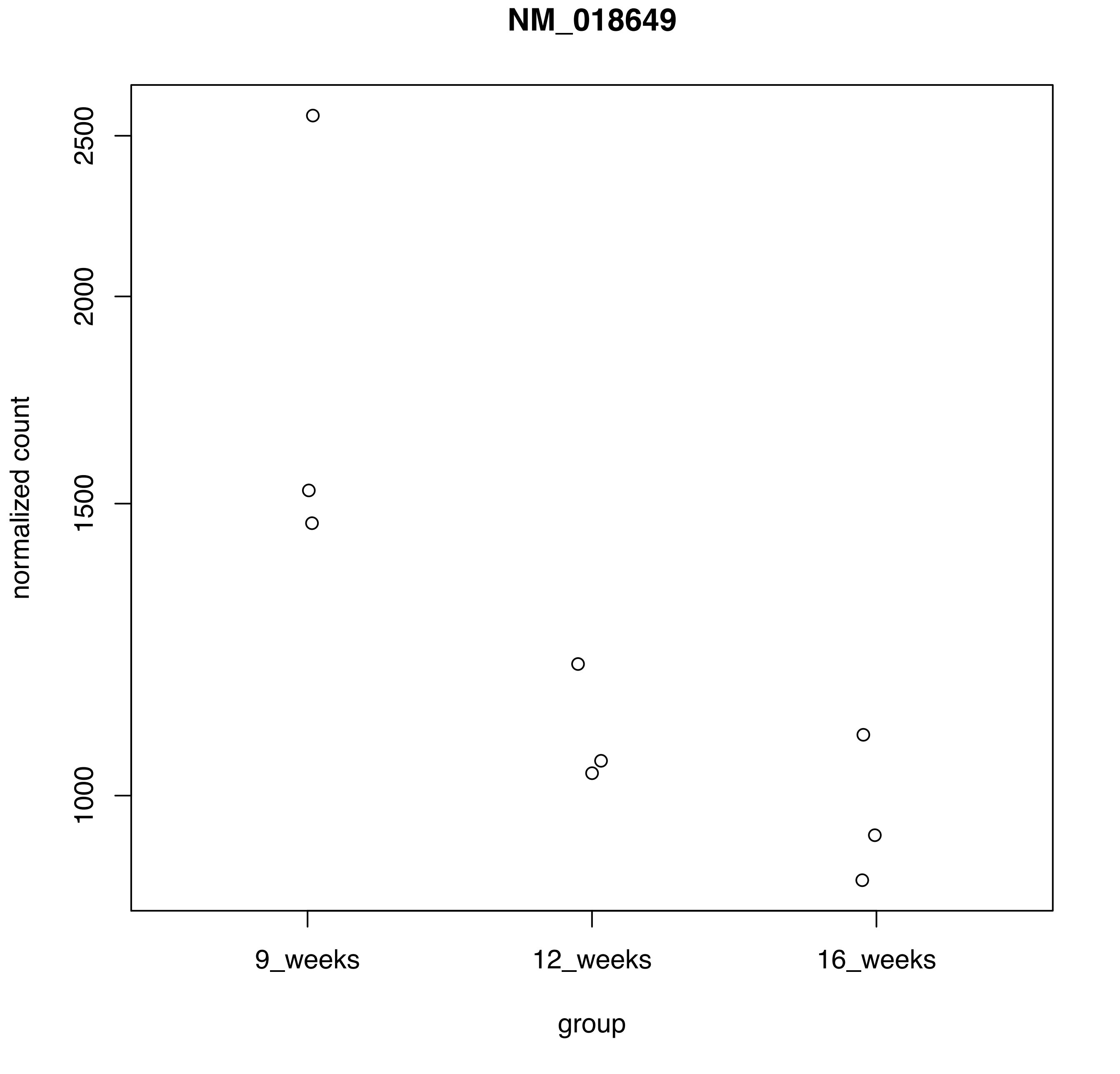
NM_018649
normalized count
2500
2000
1500
1000
9_weeks
12_weeks
16_weeks
group

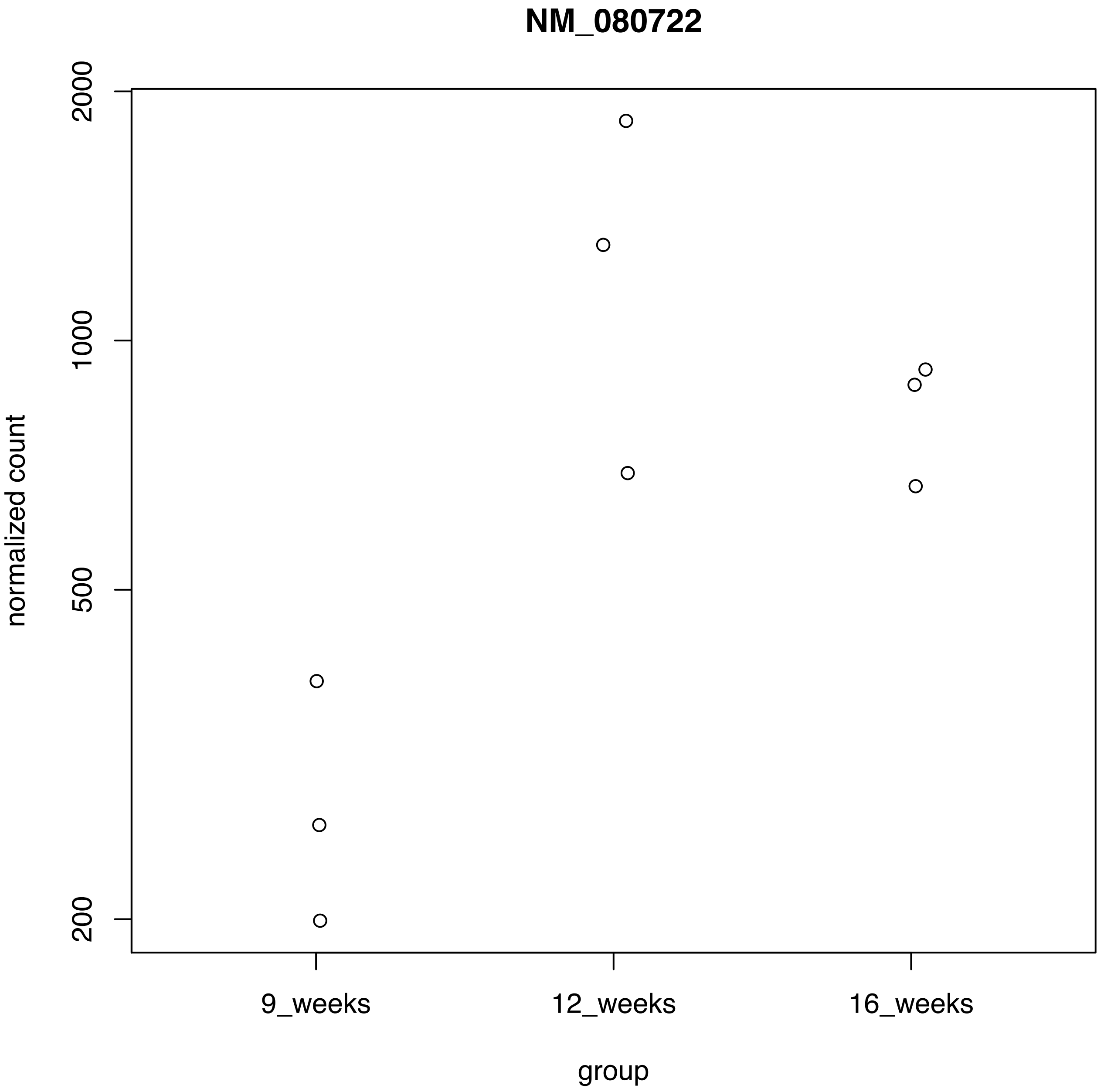

NM_080722
normalized count
2000
1000
500
200
9_weeks
12_weeks
16_weeks
group

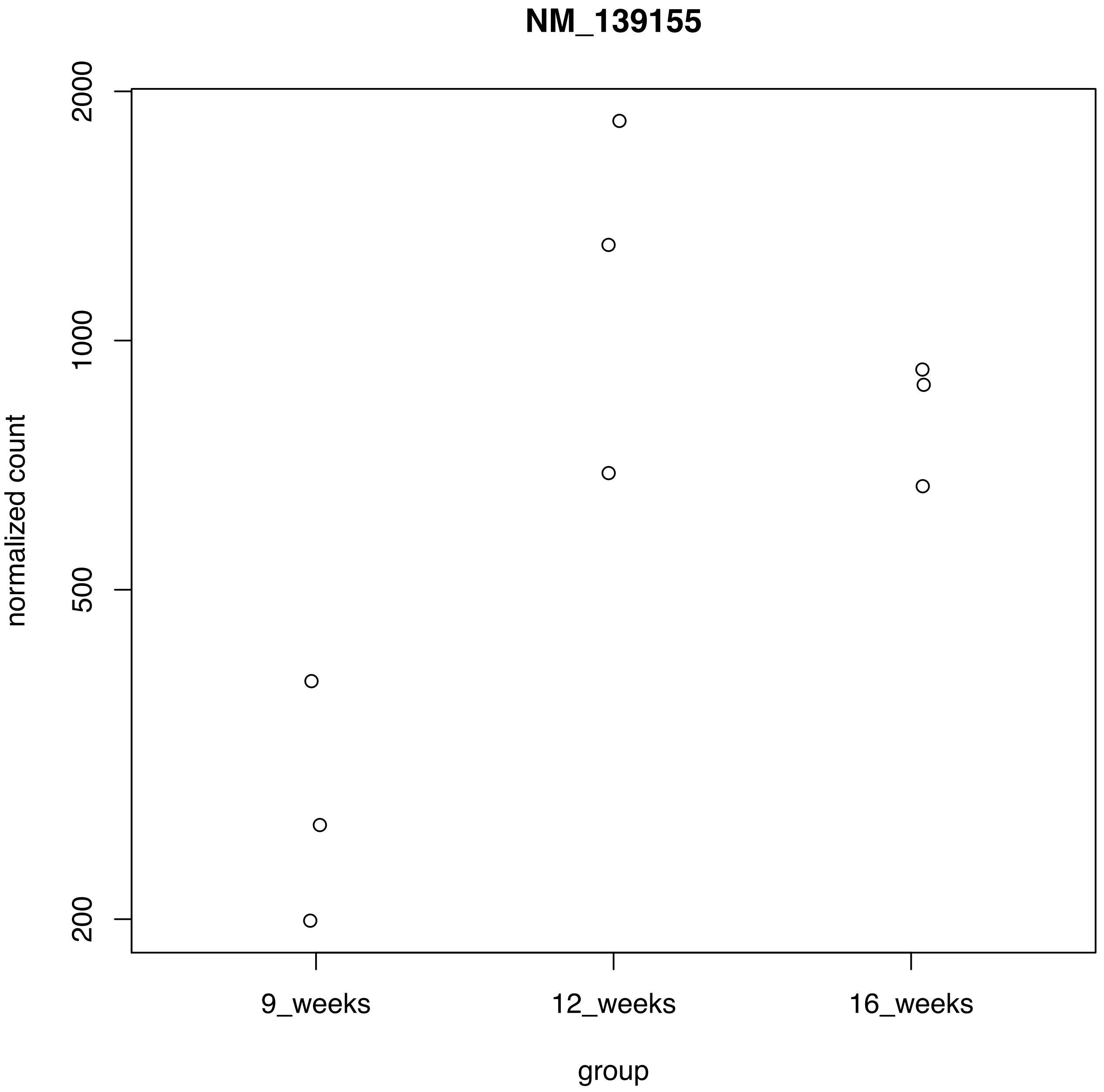
NM_139155
normalized count
2000
1000
500
200
9_weeks
12_weeks
16_weeks
group

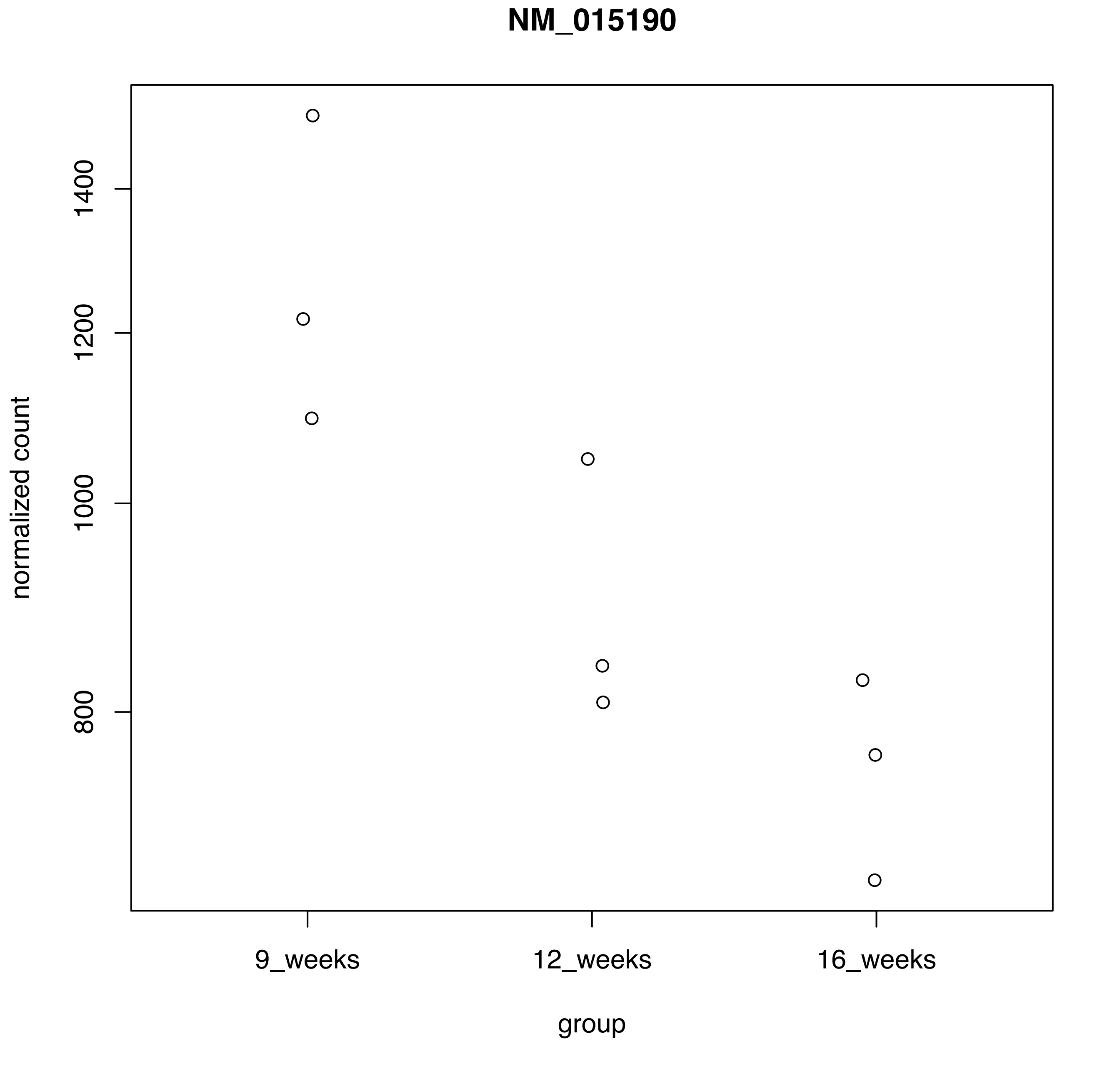

NM_015190
normalized count
1400
1200
1000
800
9_weeks
12_weeks
16_weeks
group

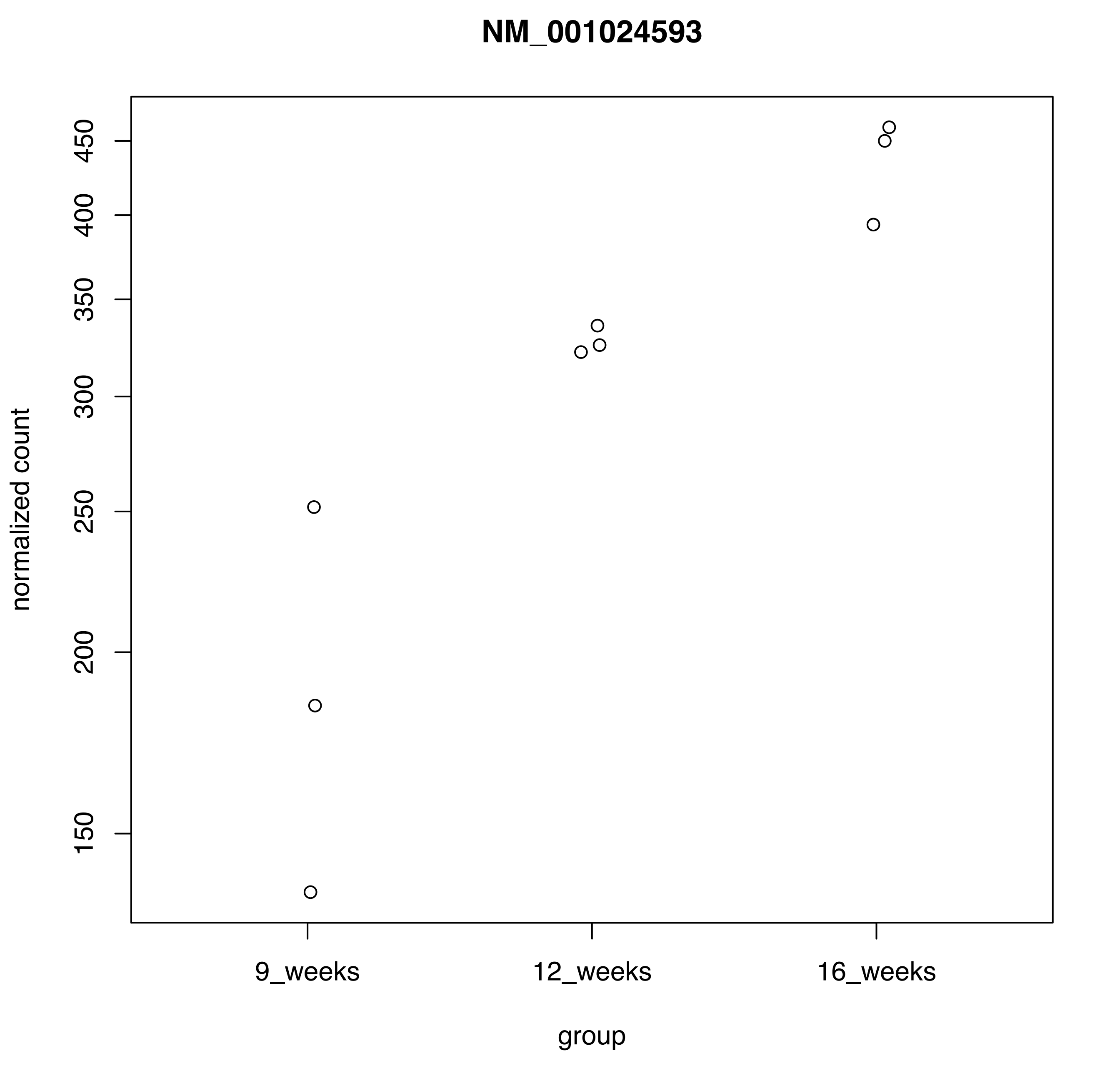
NM_001024593
normalized count
450
400
350
300
250
200
150
9_weeks
12_weeks
16_weeks
group

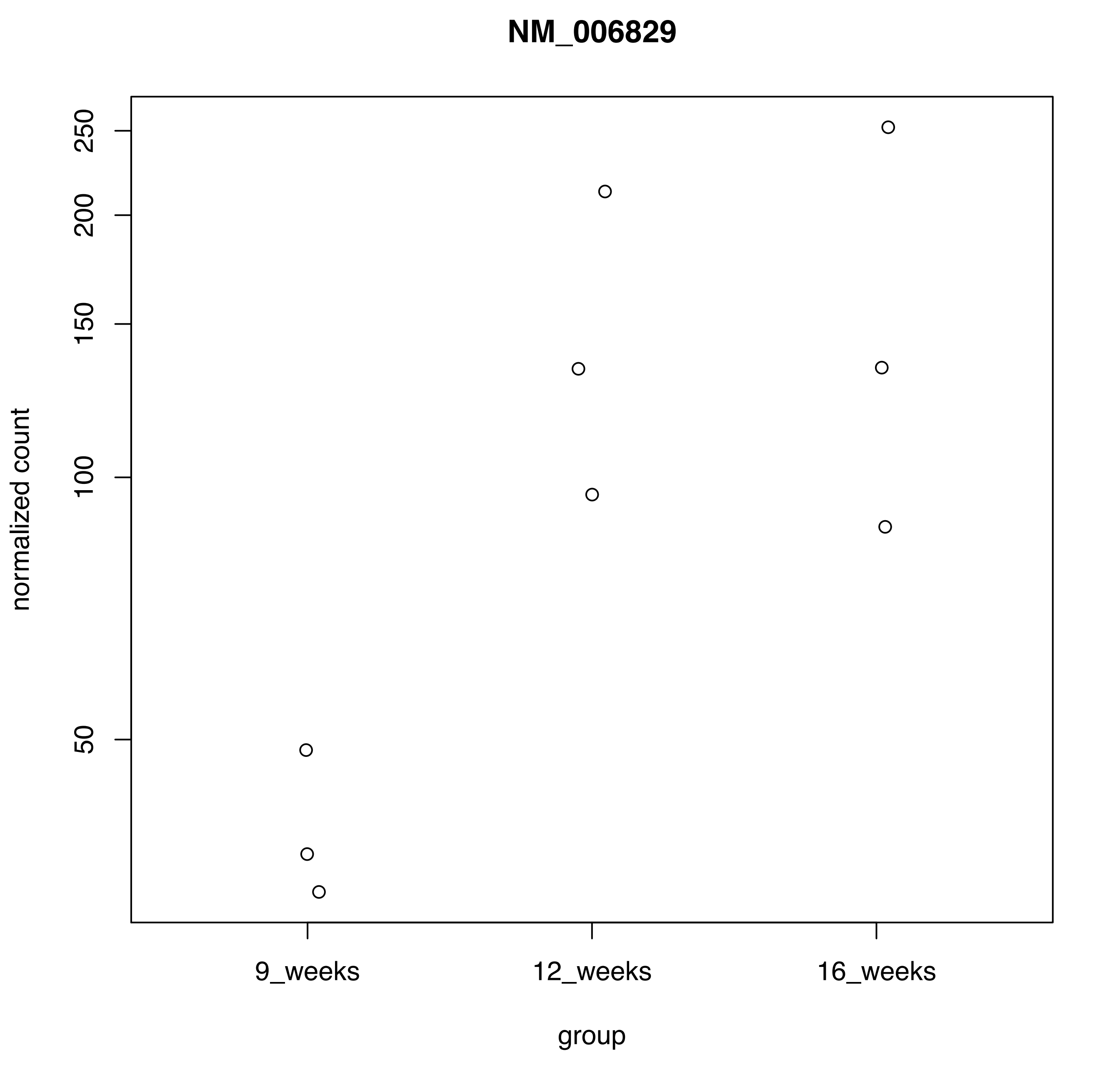
NM_006829
normalized count
250
200
150
100
50
9_weeks
12_weeks
16_weeks
group

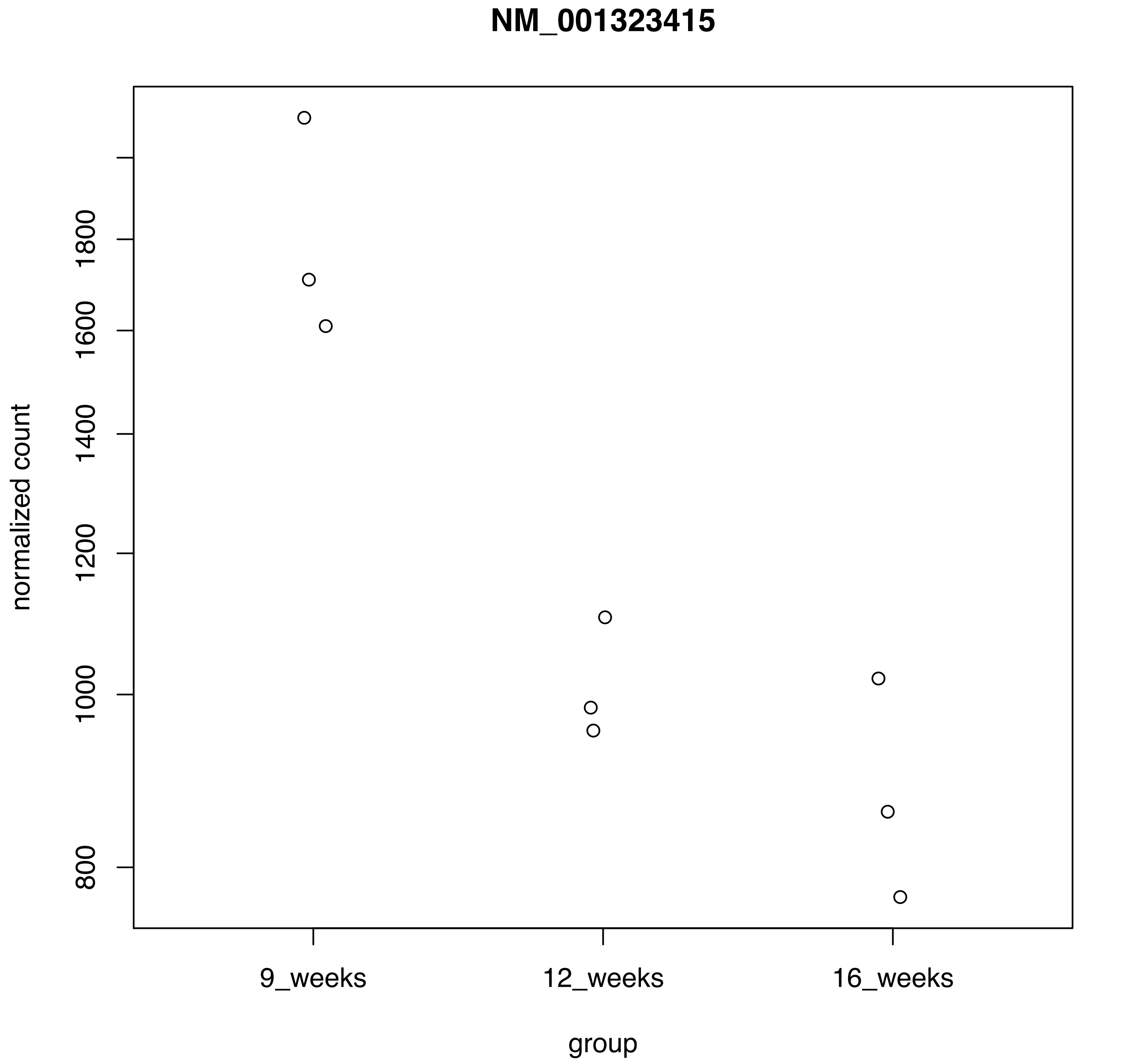
NM_001323415
normalized count
800
1000
1200
1400
1600
1800
9_weeks
12_weeks
16_weeks
group

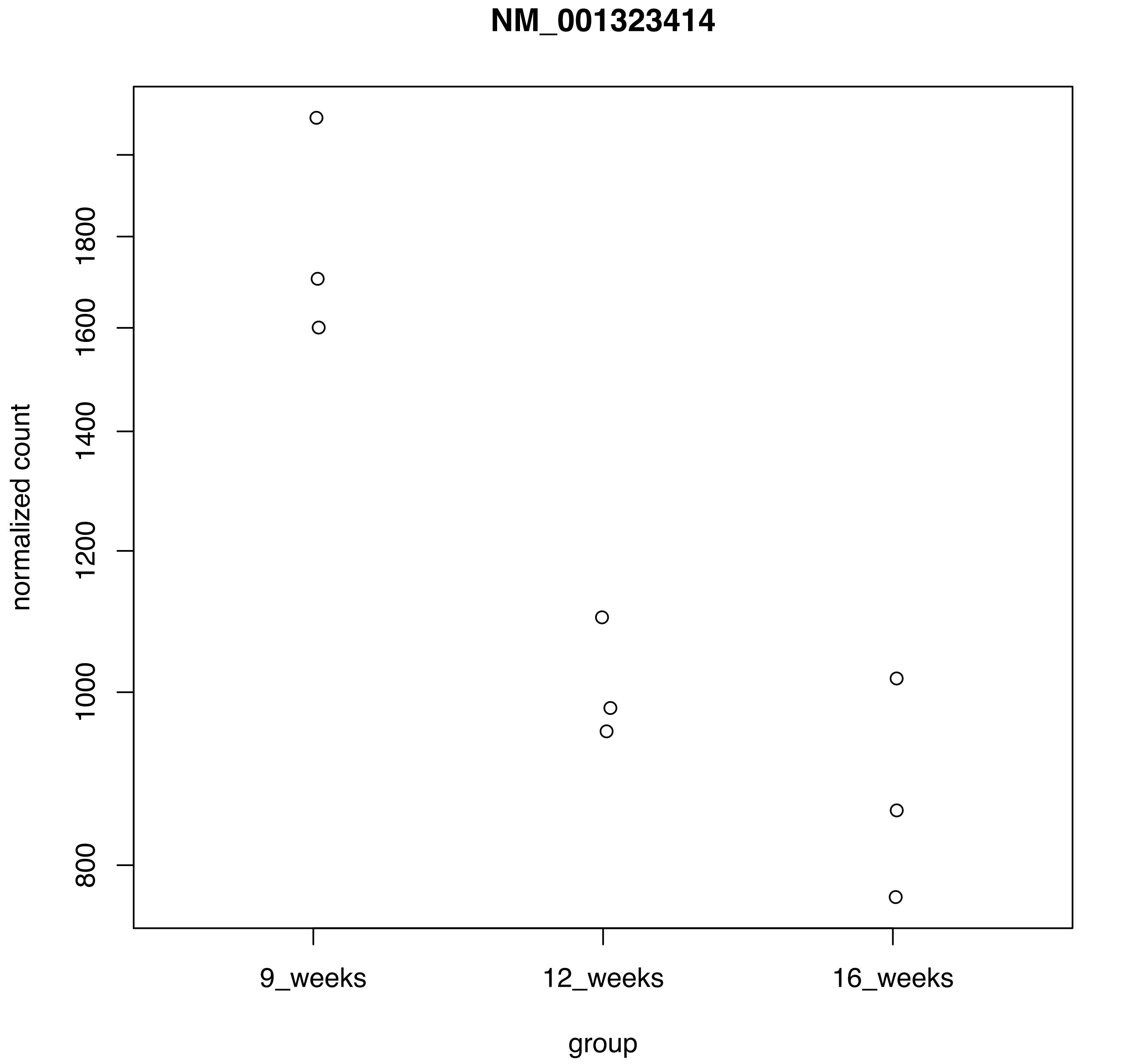
NM_001323414
normalized count
800
1000
1200
1400
1600
1800
9_weeks
12_weeks
16_weeks
group

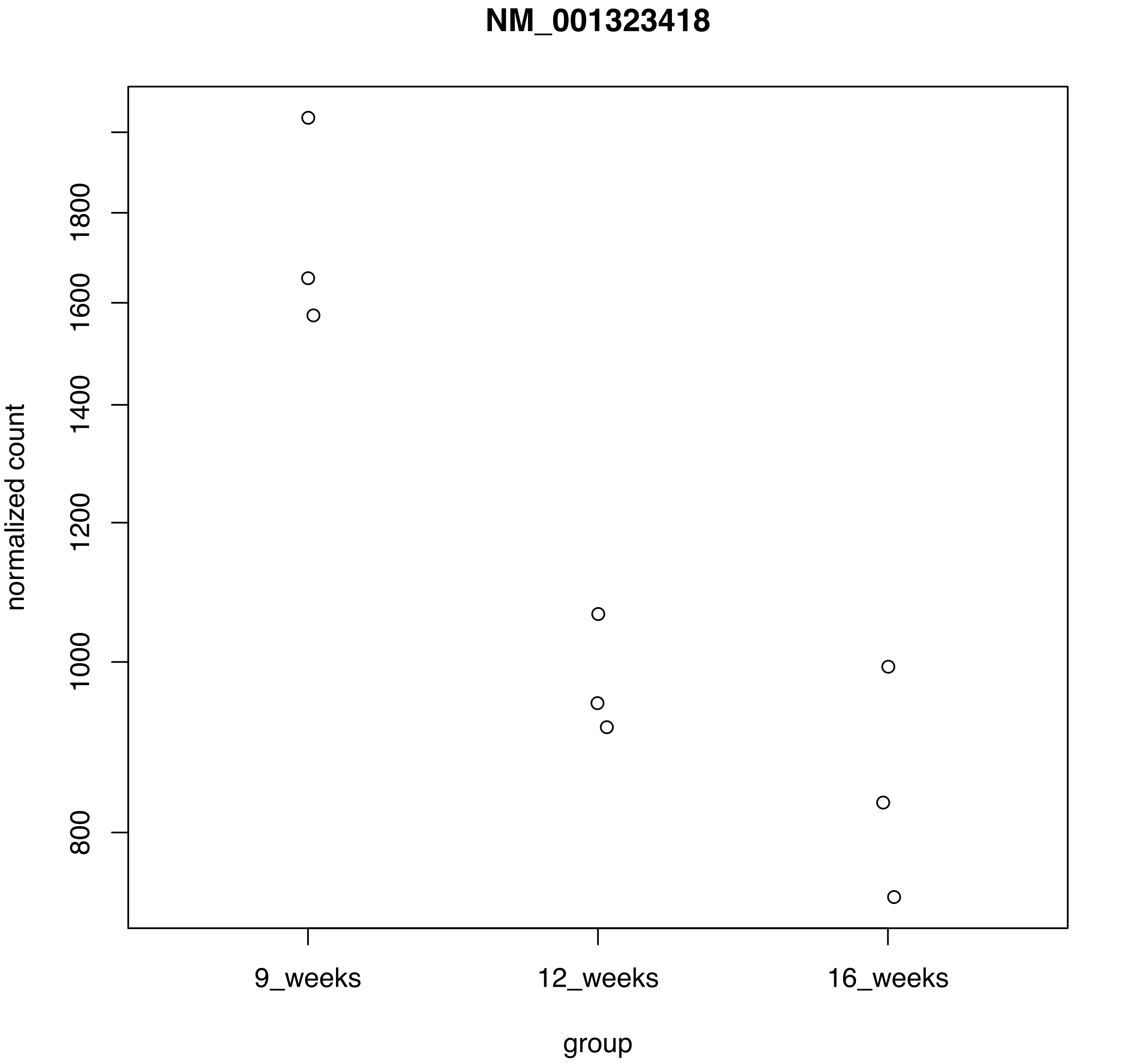
NM_001323418
normalized count
800
1000
1200
1400
1600
1800
9_weeks
12_weeks
16_weeks
group

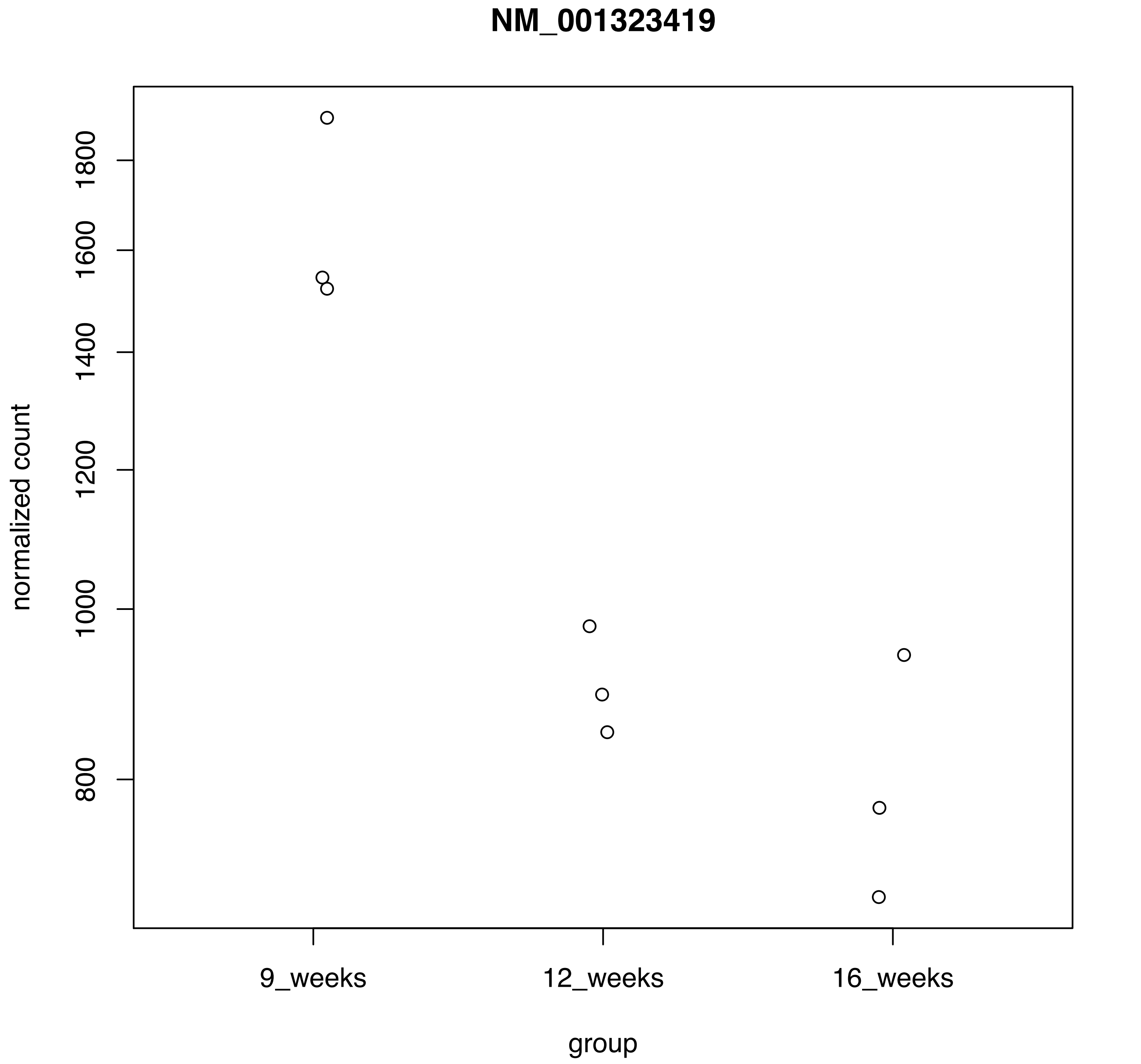
NM_001323419
normalized count
800
1000
1200
1400
1600
1800
9_weeks
12_weeks
16_weeks
group

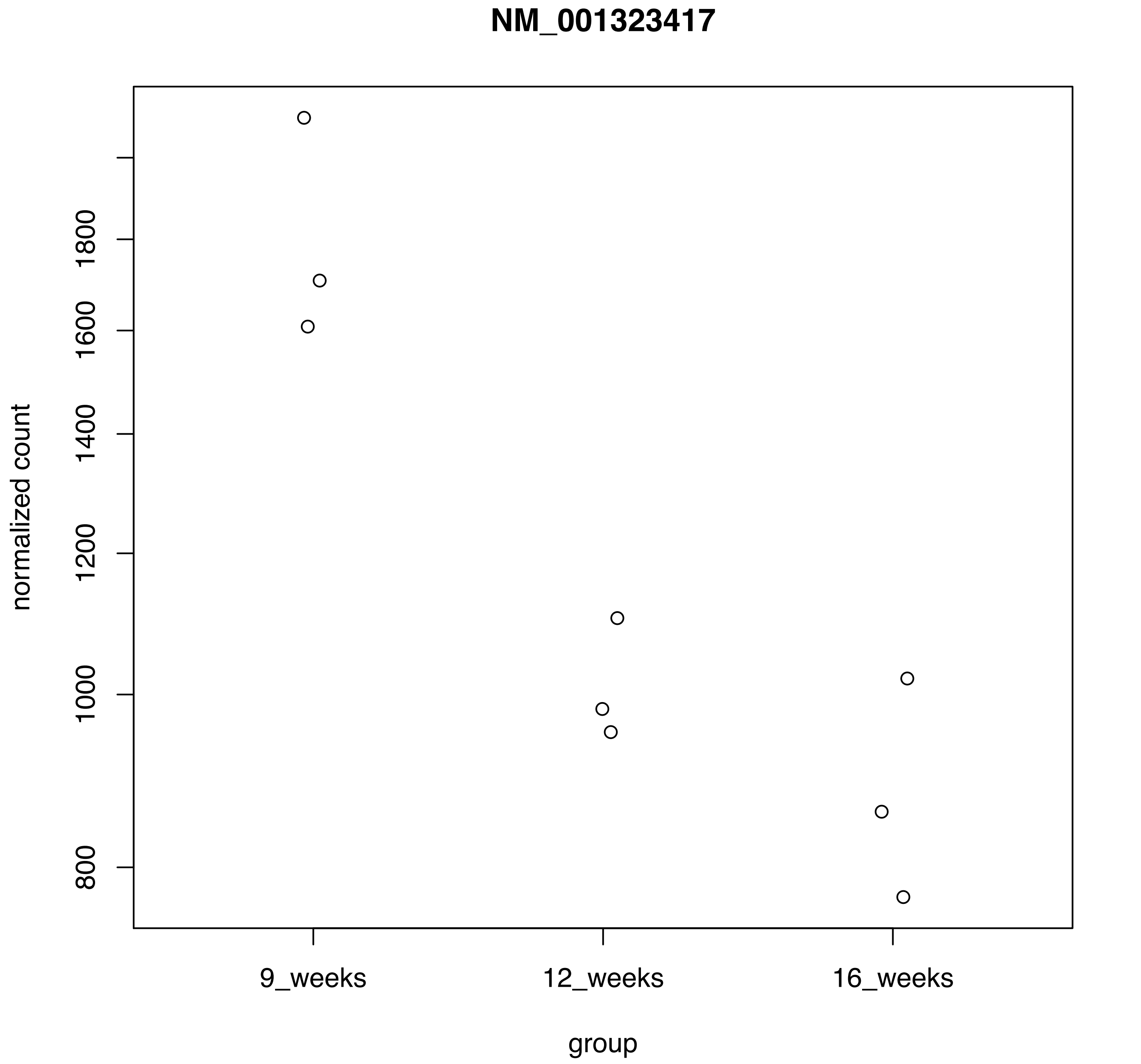
NM_001323417
normalized count
800
1000
1200
1400
1600
1800
9_weeks
12_weeks
16_weeks
group

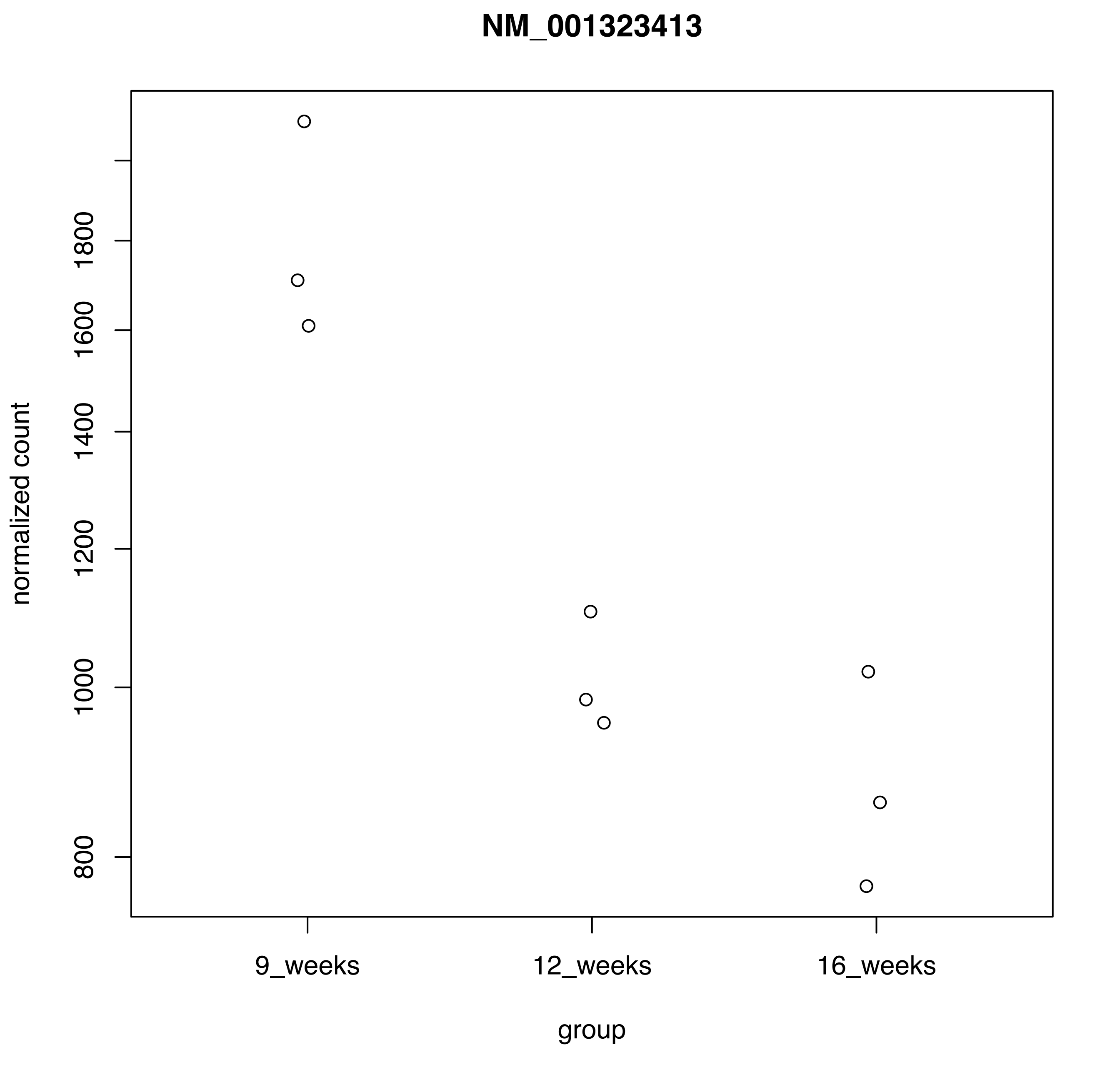

NM_001323413
normalized count
800
1000
1200
1400
1600
1800
9_weeks
12_weeks
16_weeks
group

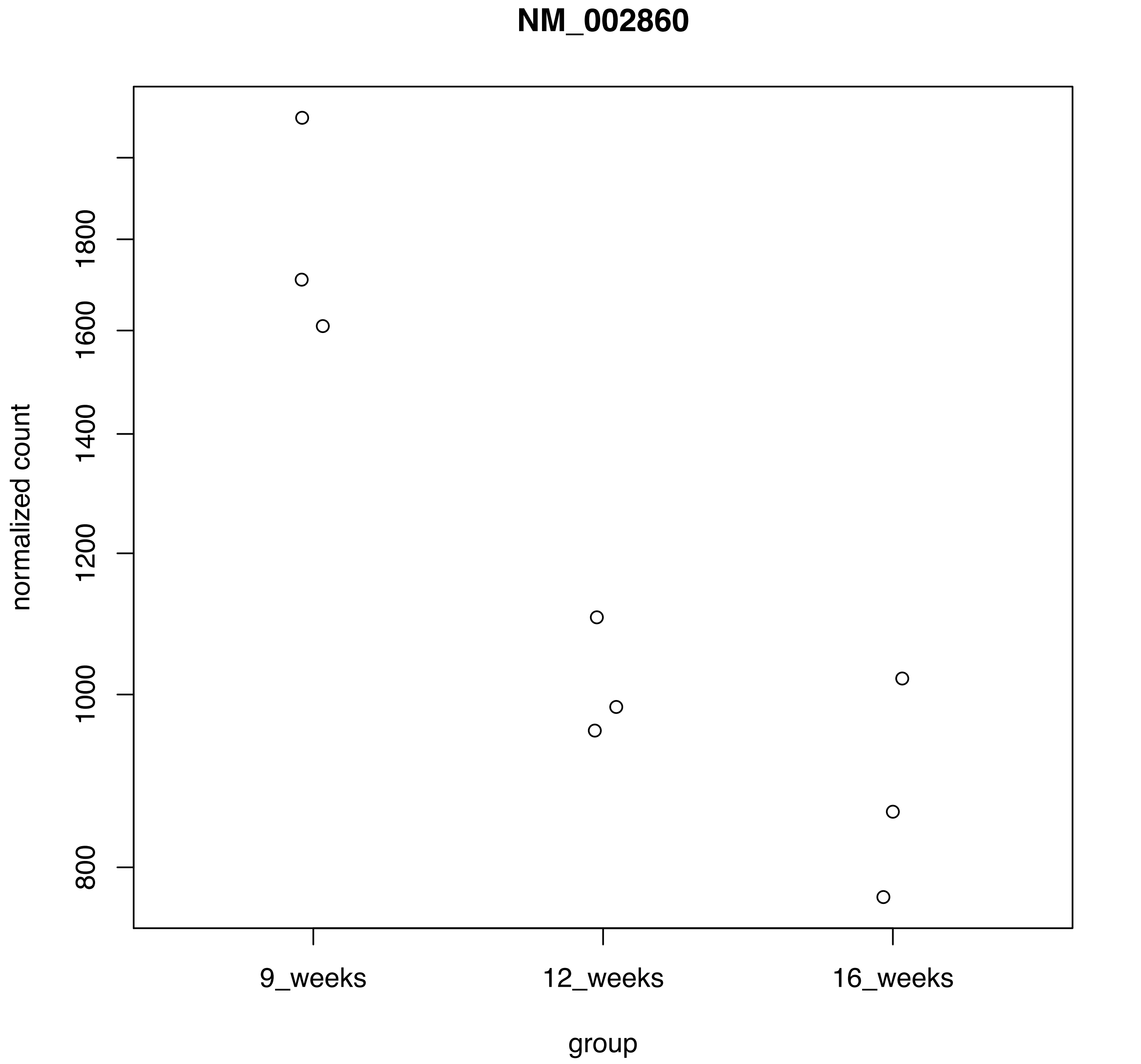
NM_002860
normalized count
800
1000
1200
1400
1600
1800
9_weeks
12_weeks
16_weeks
group

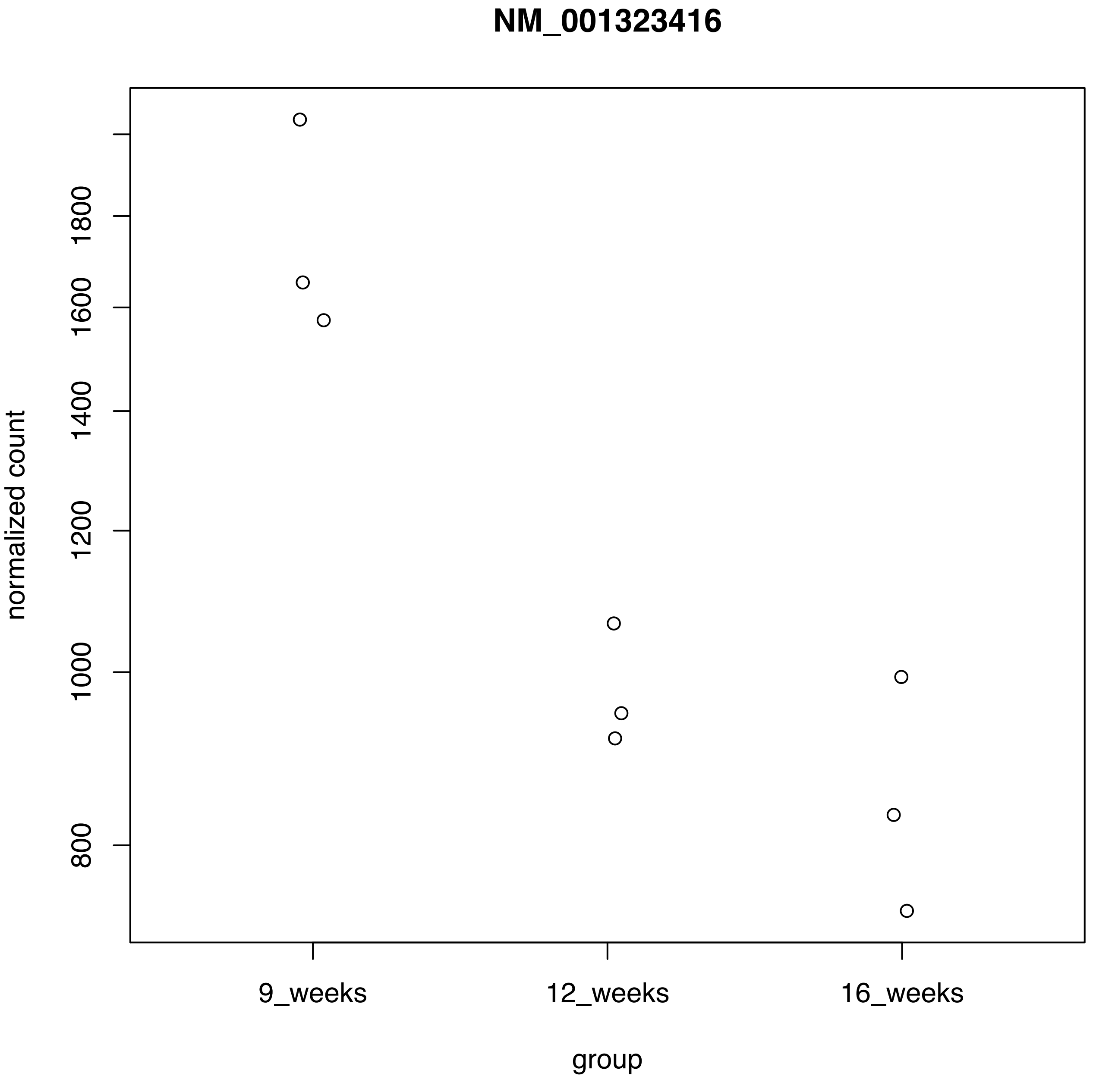
NM_001323416
normalized count
800
1000
1200
1400
1600
1800
9_weeks
12_weeks
16_weeks
group

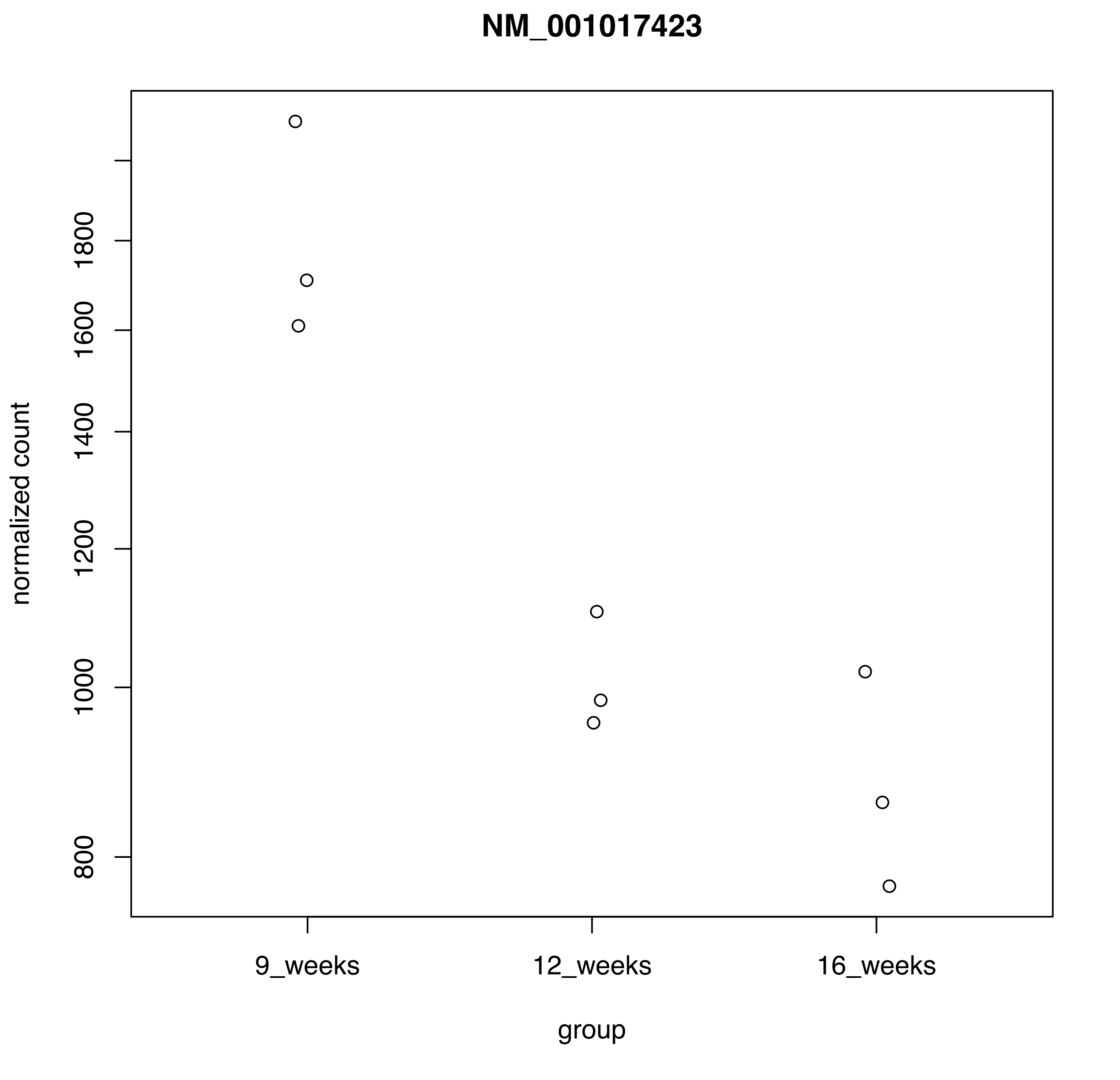
NM_001017423
normalized count
800
1000
1200
1400
1600
1800
9_weeks
12_weeks
16_weeks
group

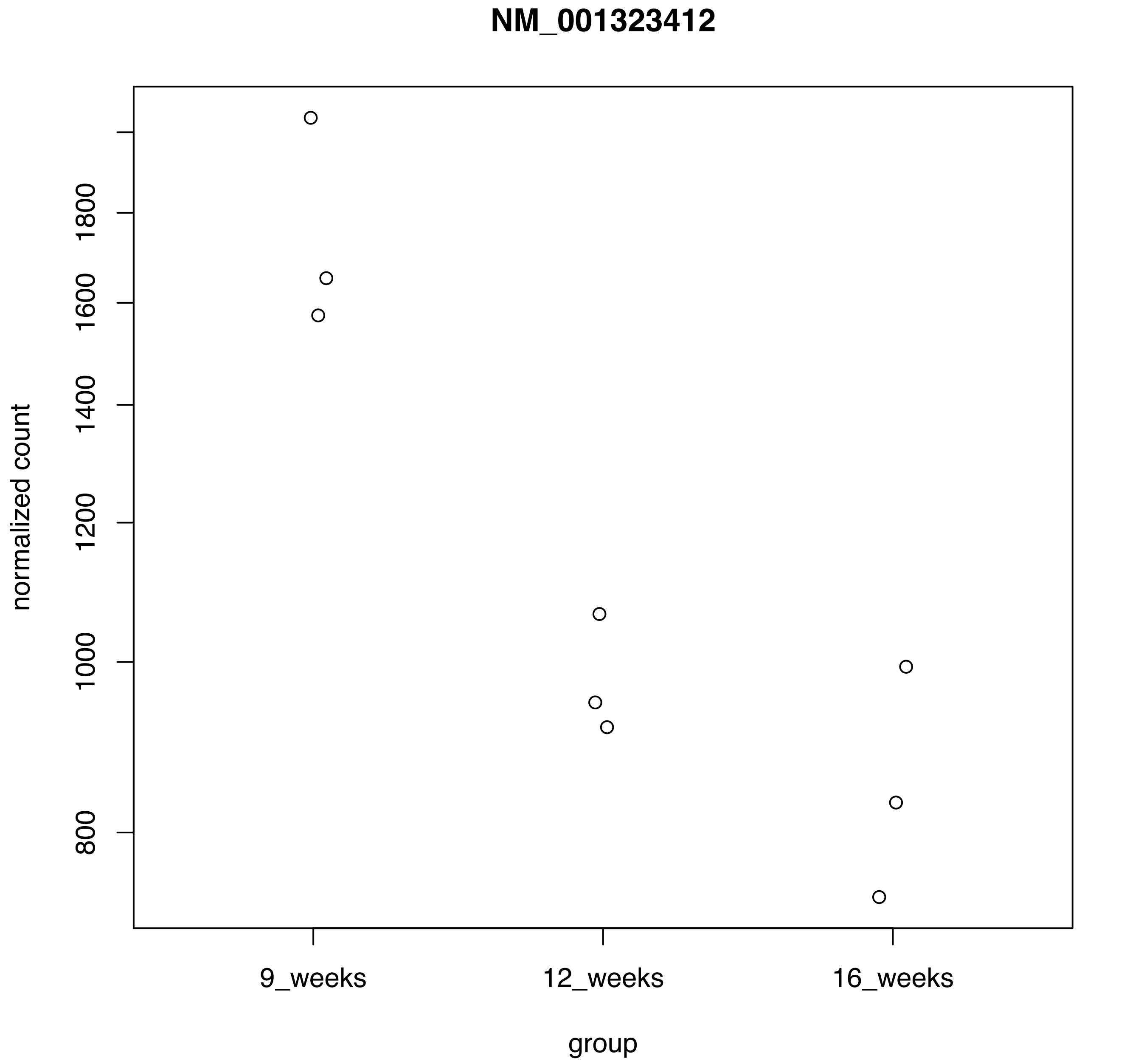
NM_001323412
normalized count
800
1000
1200
1400
1600
1800
9_weeks
12_weeks
16_weeks
group

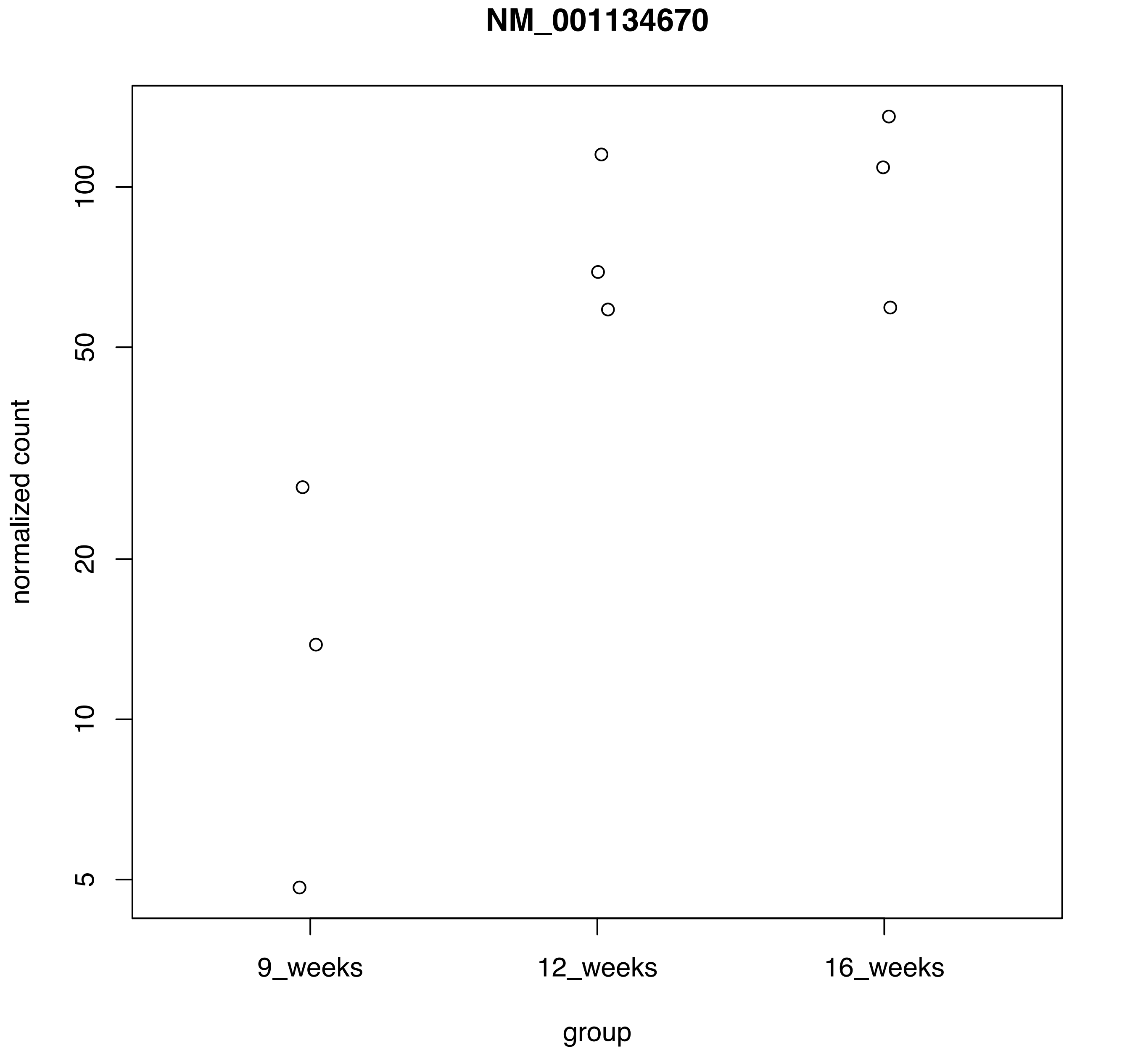

NM_001134670
normalized count
100
50
20
10
5
9_weeks
12_weeks
16_weeks
group

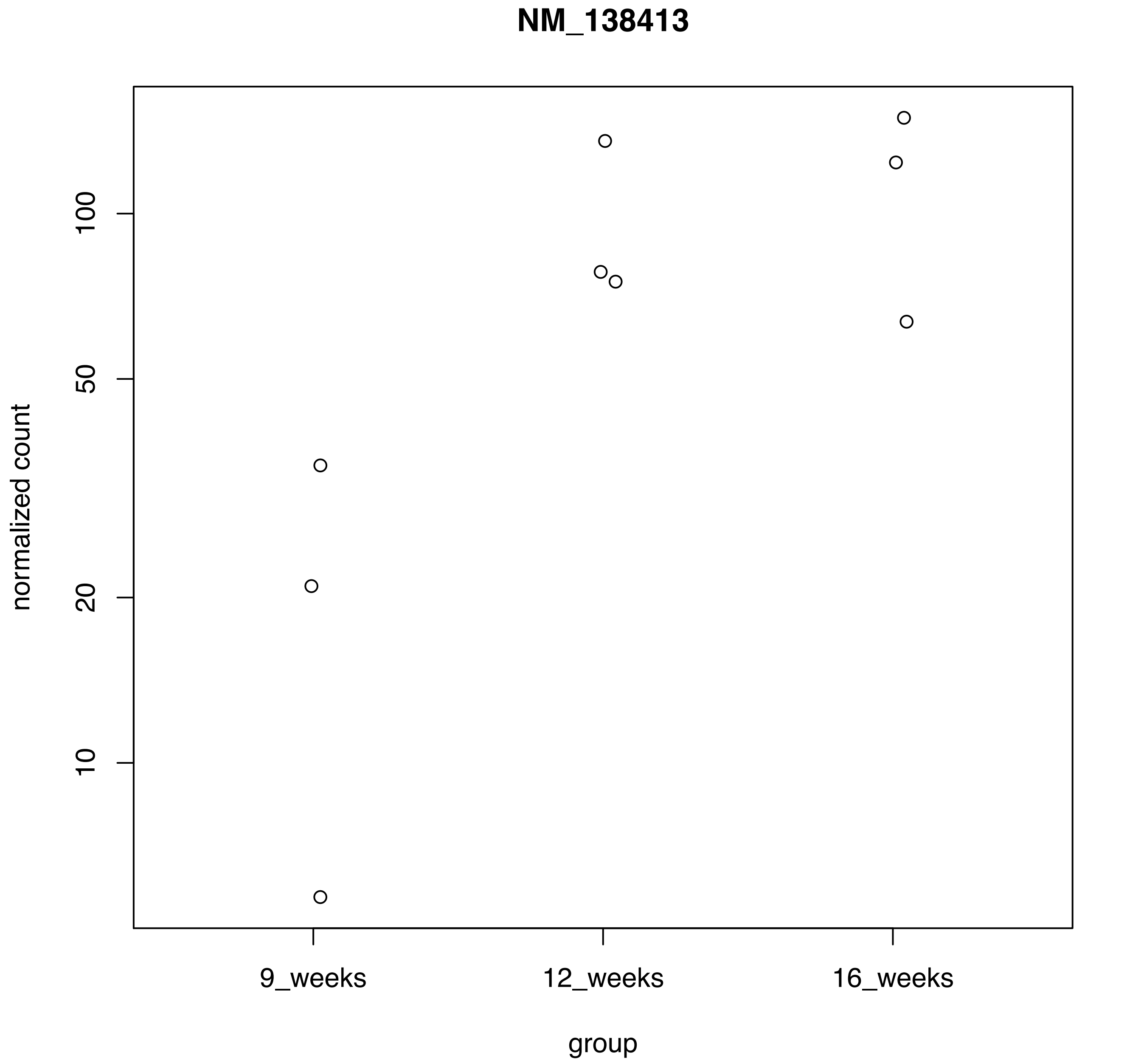

NM_138413
normalized count
100
50
20
10
9_weeks
12_weeks
16_weeks
group

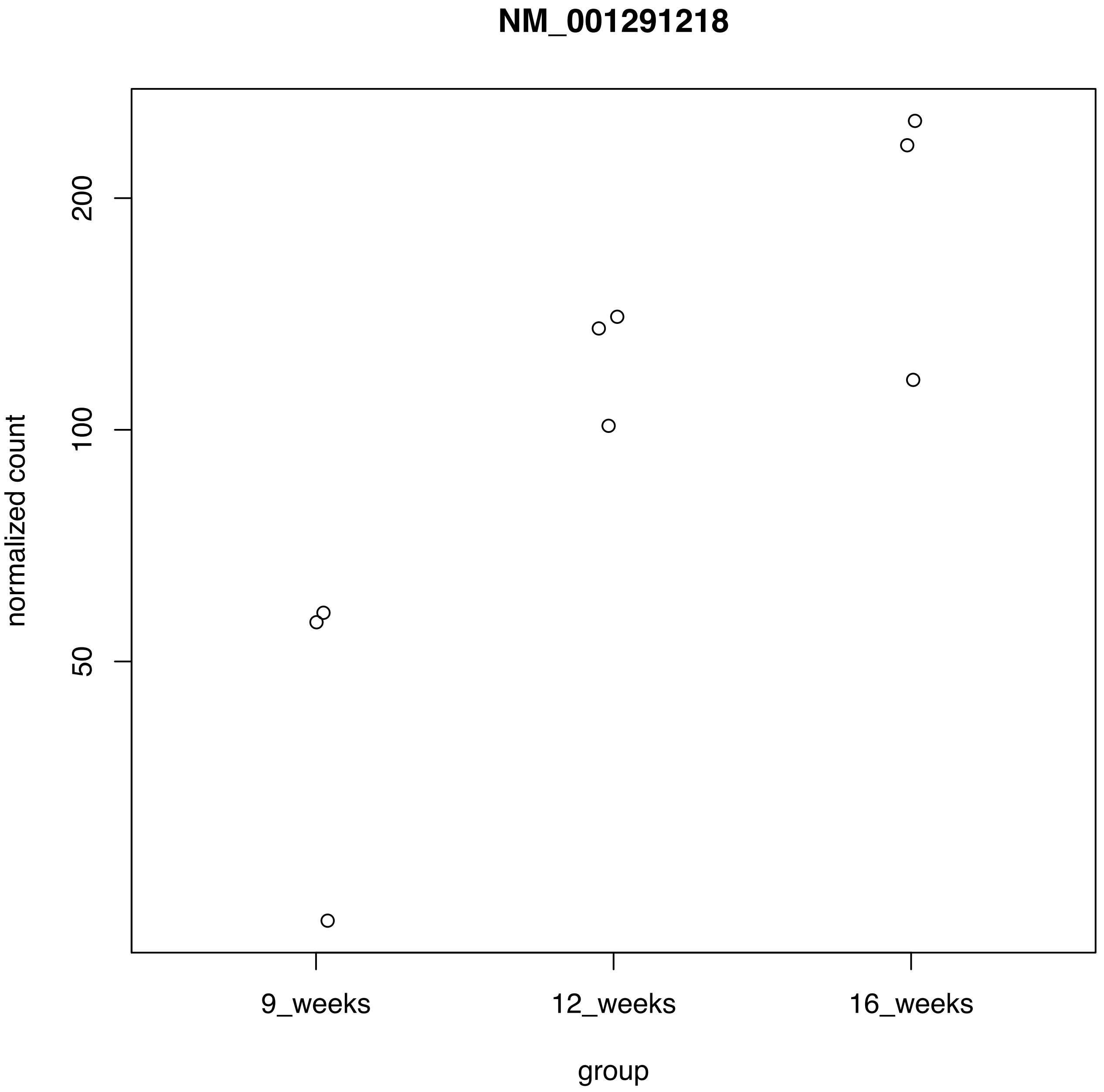

NM_001291218
normalized count
200
100
50
9_weeks
12_weeks
16_weeks
group

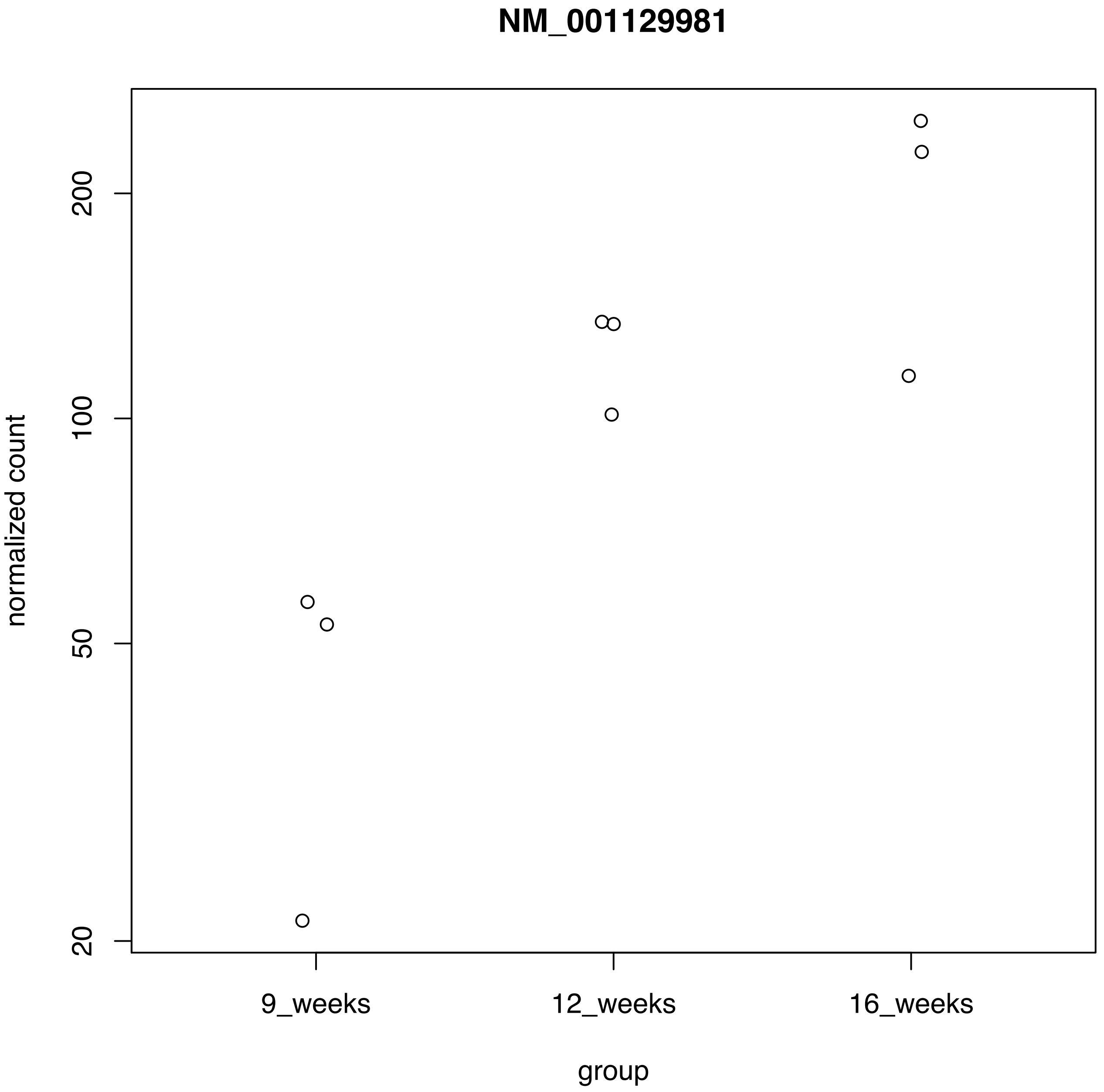
NM_001129981
normalized count
20
50
100
200
9_weeks
12_weeks
16_weeks
group

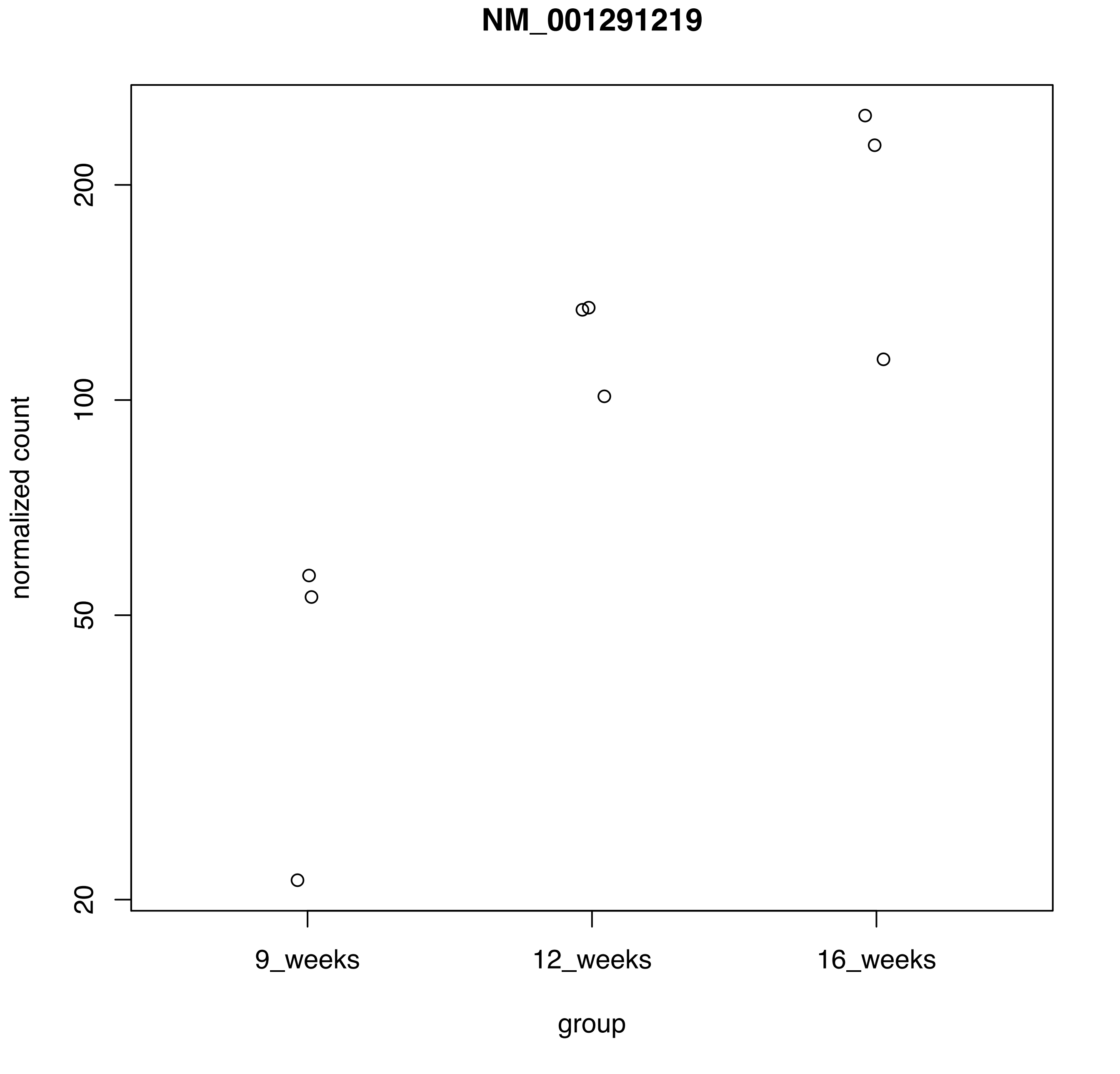
NM_001291219
normalized count
200
100
50
20
9_weeks
12_weeks
16_weeks
group

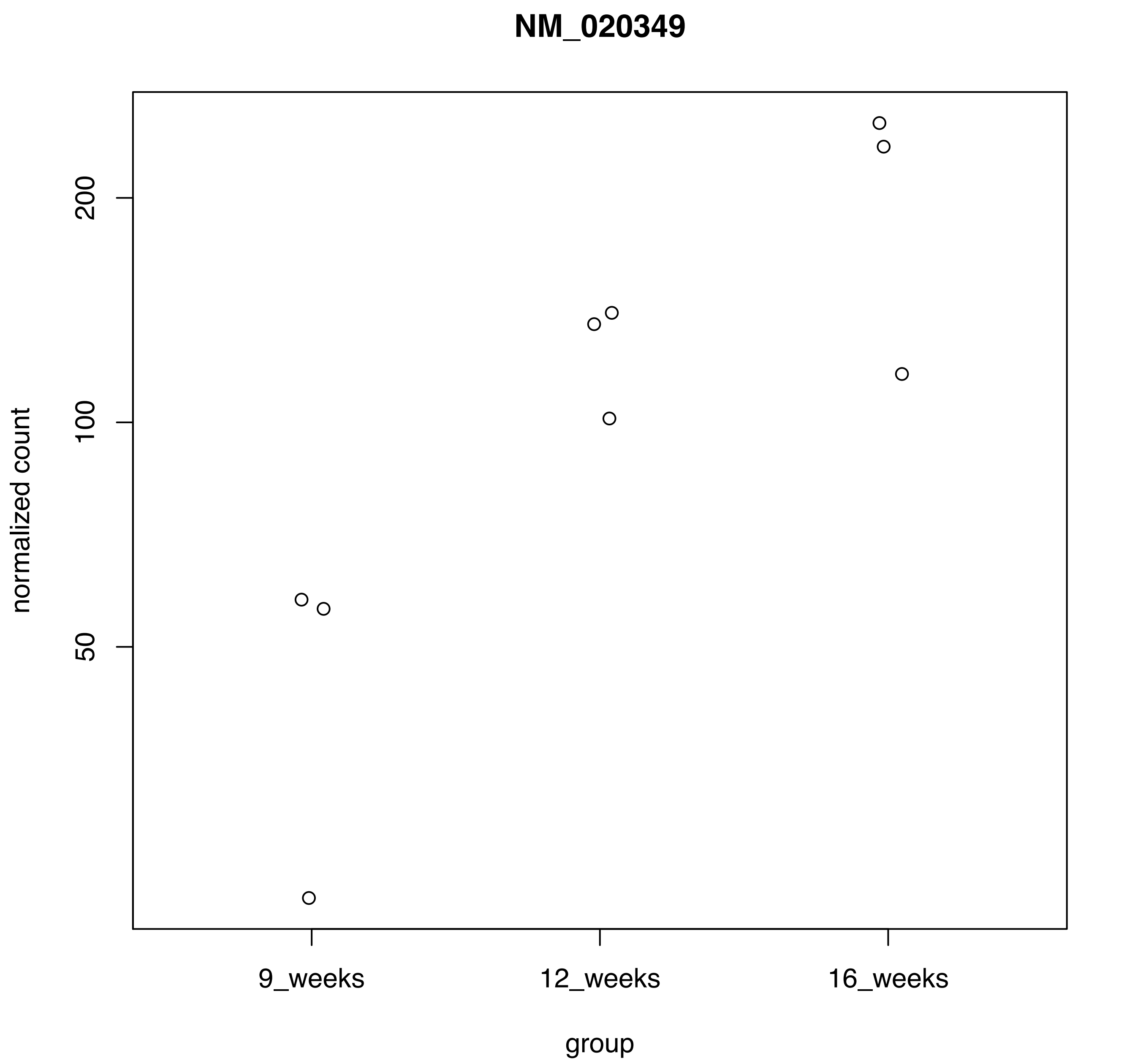

NM_020349
normalized count
200
100
50
9_weeks
12_weeks
16_weeks
group

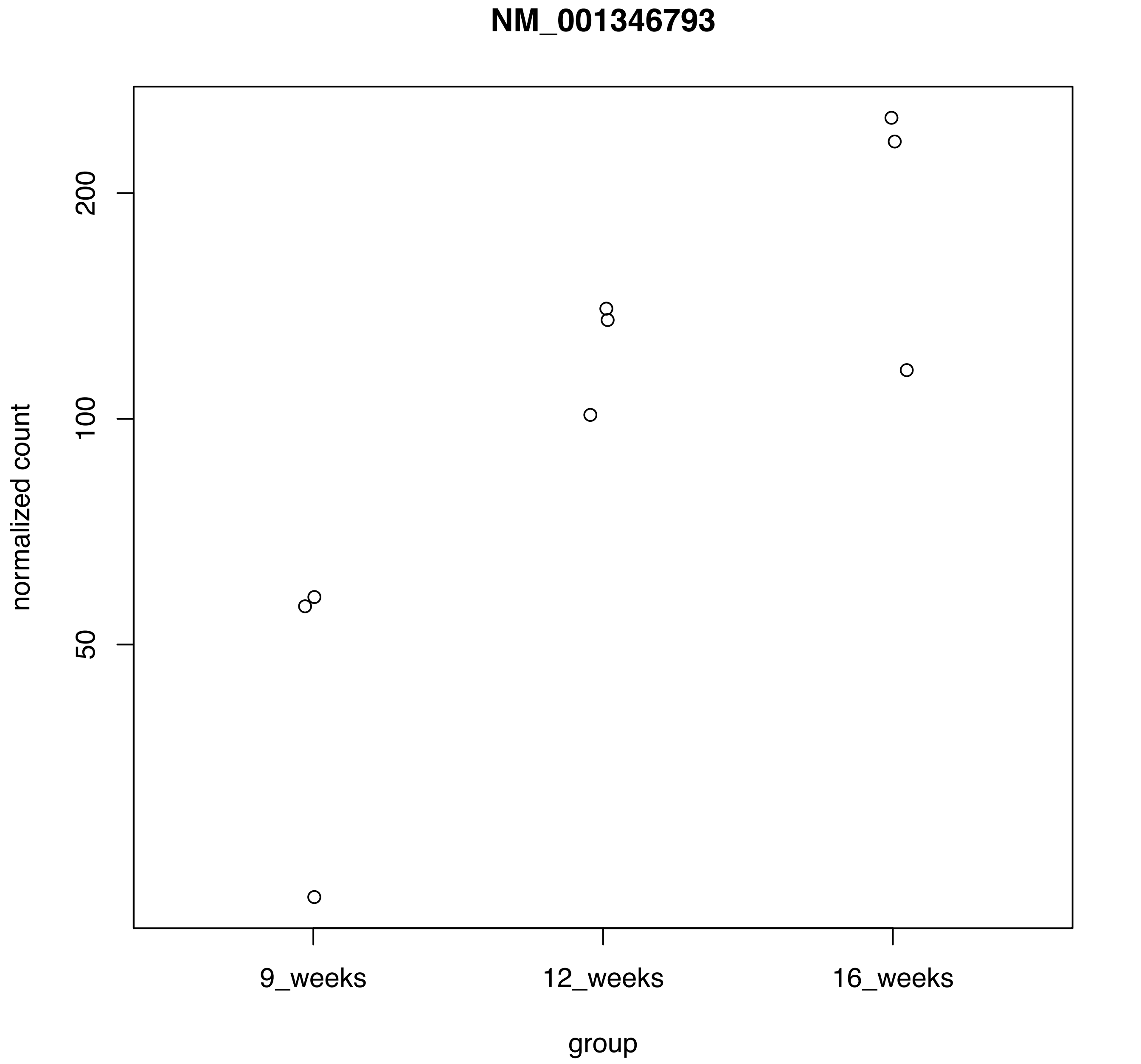
NM_001346793
normalized count
200
100
50
9_weeks
12_weeks
16_weeks
group

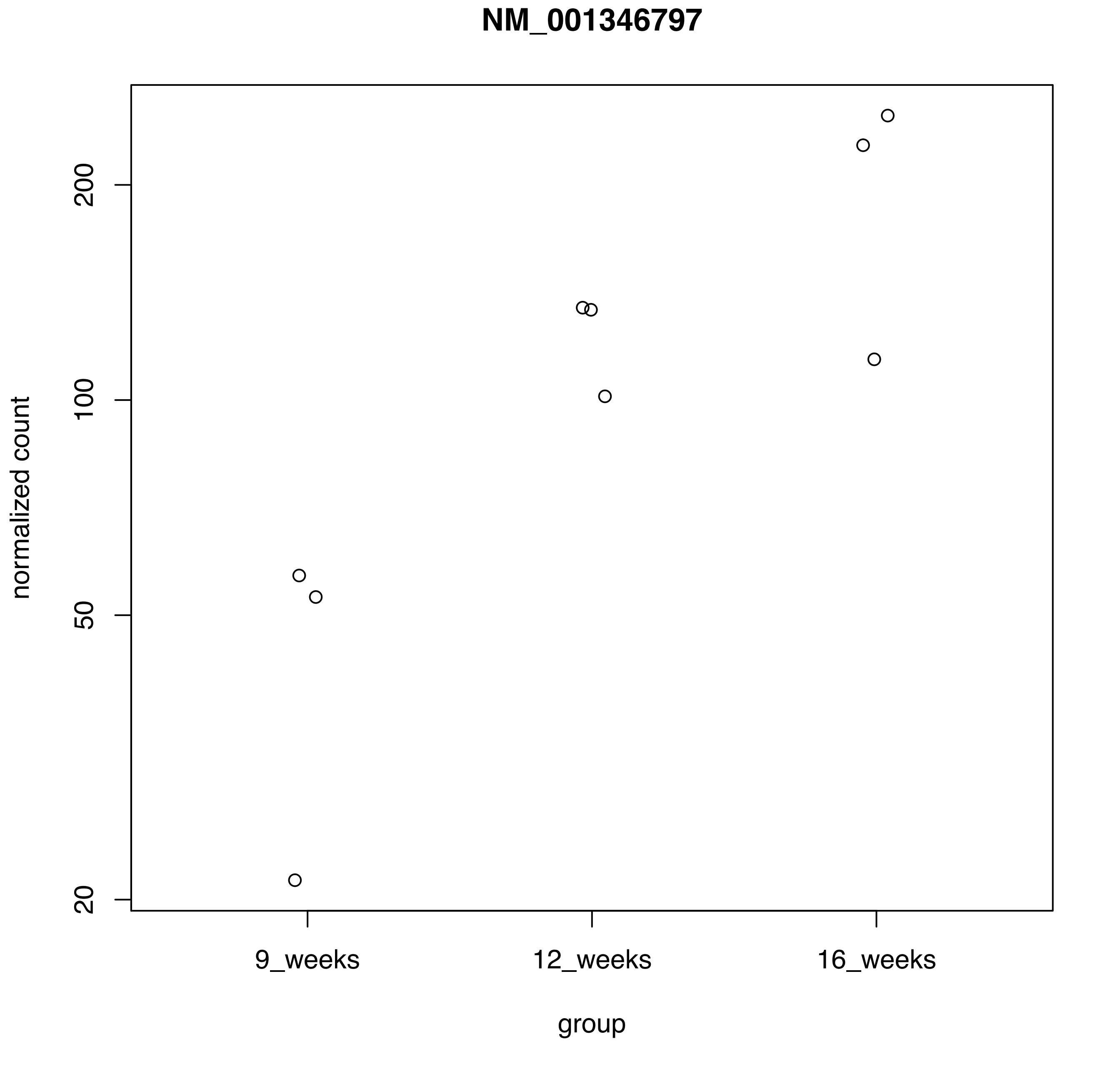

NM_001346797
normalized count
200
100
50
20
9_weeks
12_weeks
16_weeks
group

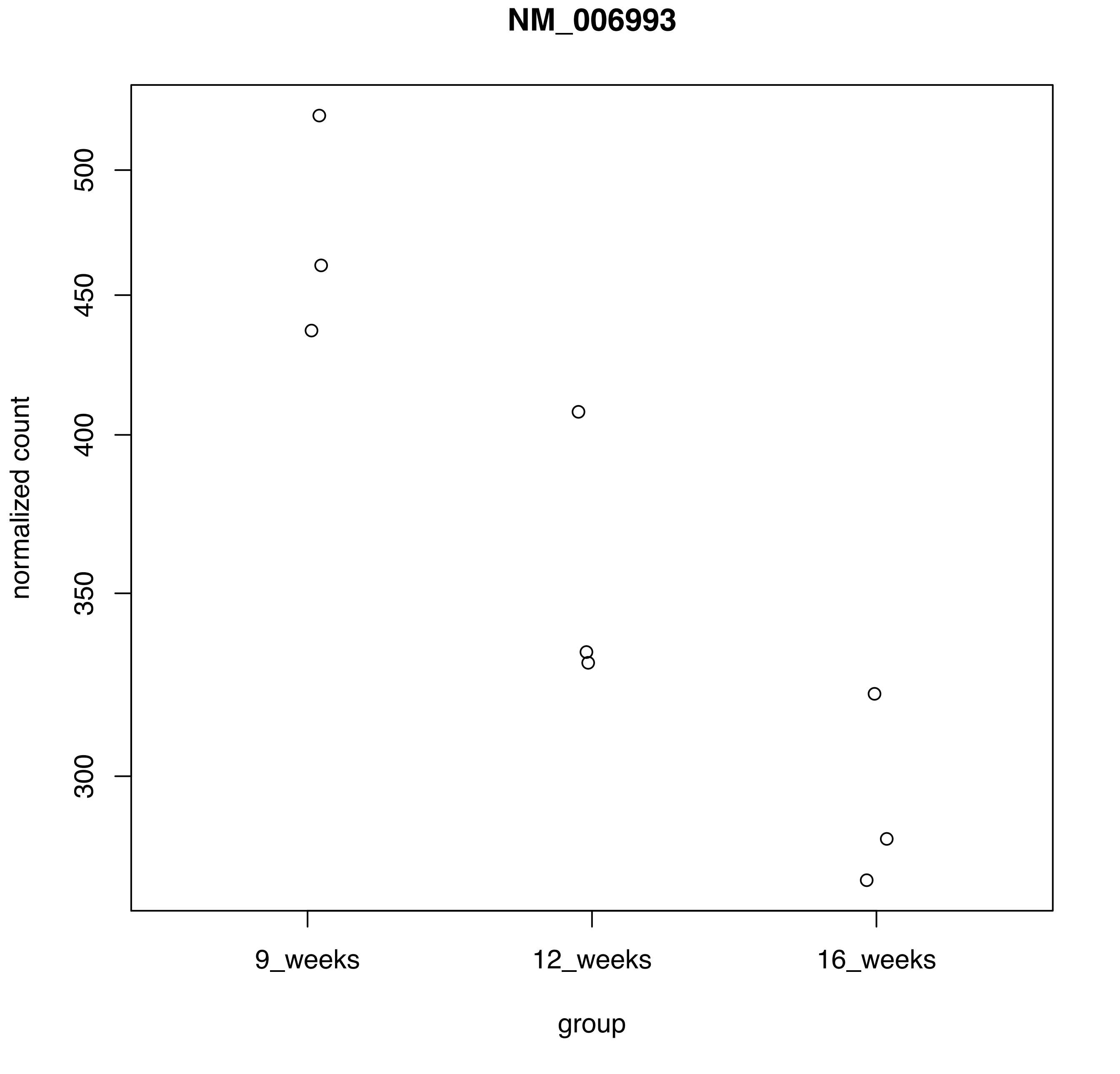
NM_006993
normalized count
500
450
400
350
300
9_weeks
12_weeks
16_weeks
group

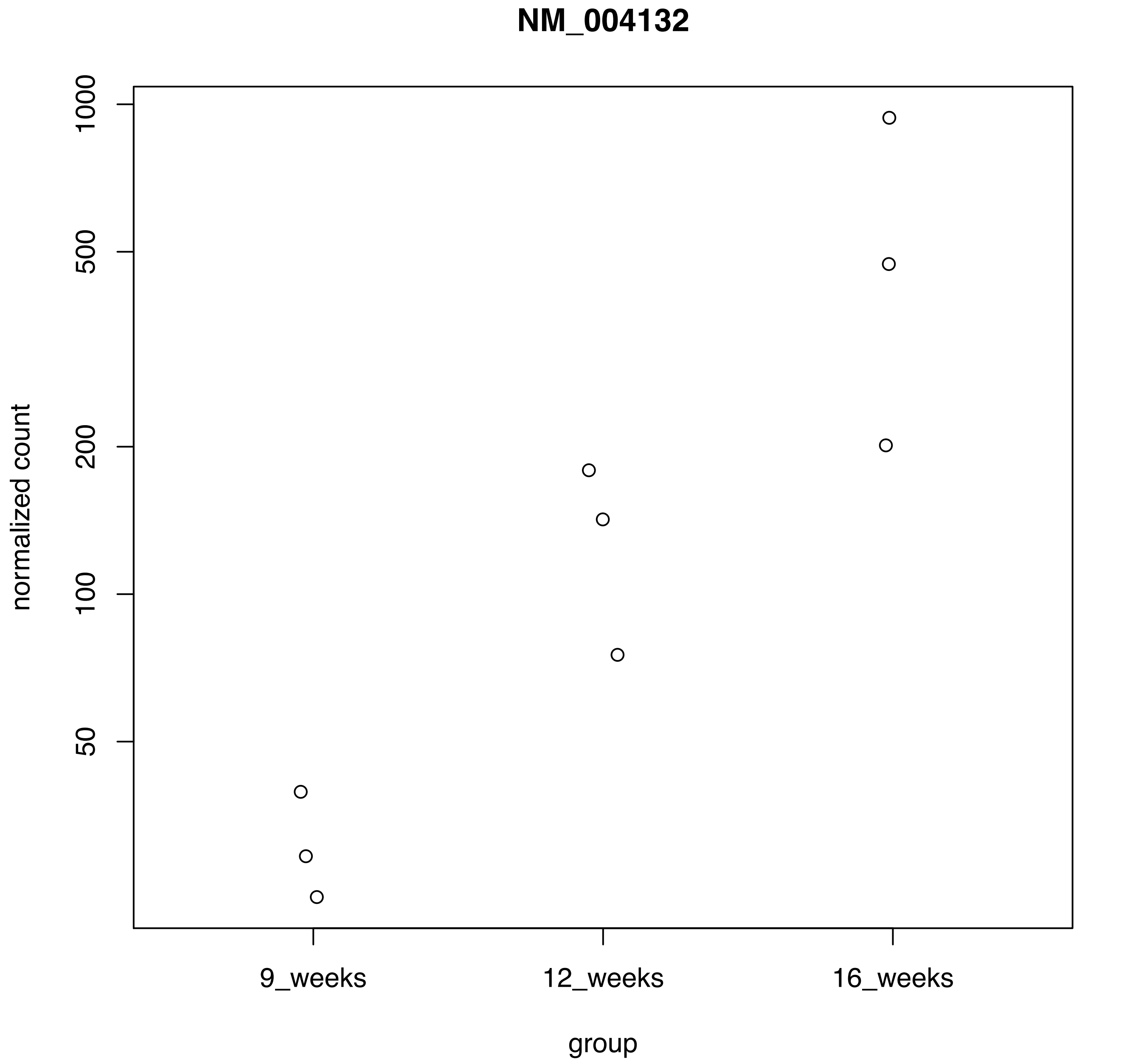
NM_004132
normalized count
1000
500
200
100
50
9_weeks
12_weeks
16_weeks
group

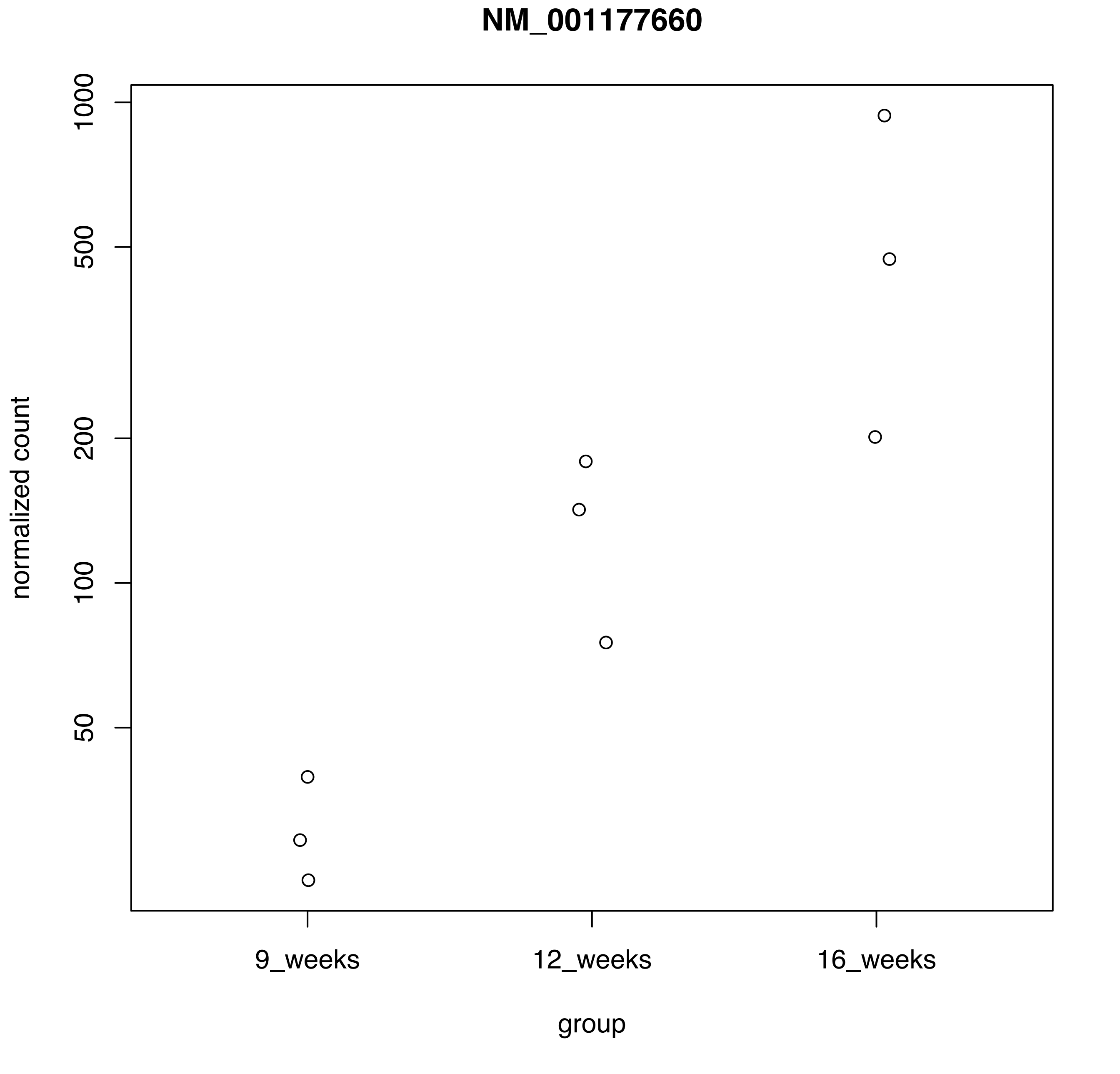

NM_001177660
normalized count
1000
500
200
100
50
9_weeks
12_weeks
16_weeks
group

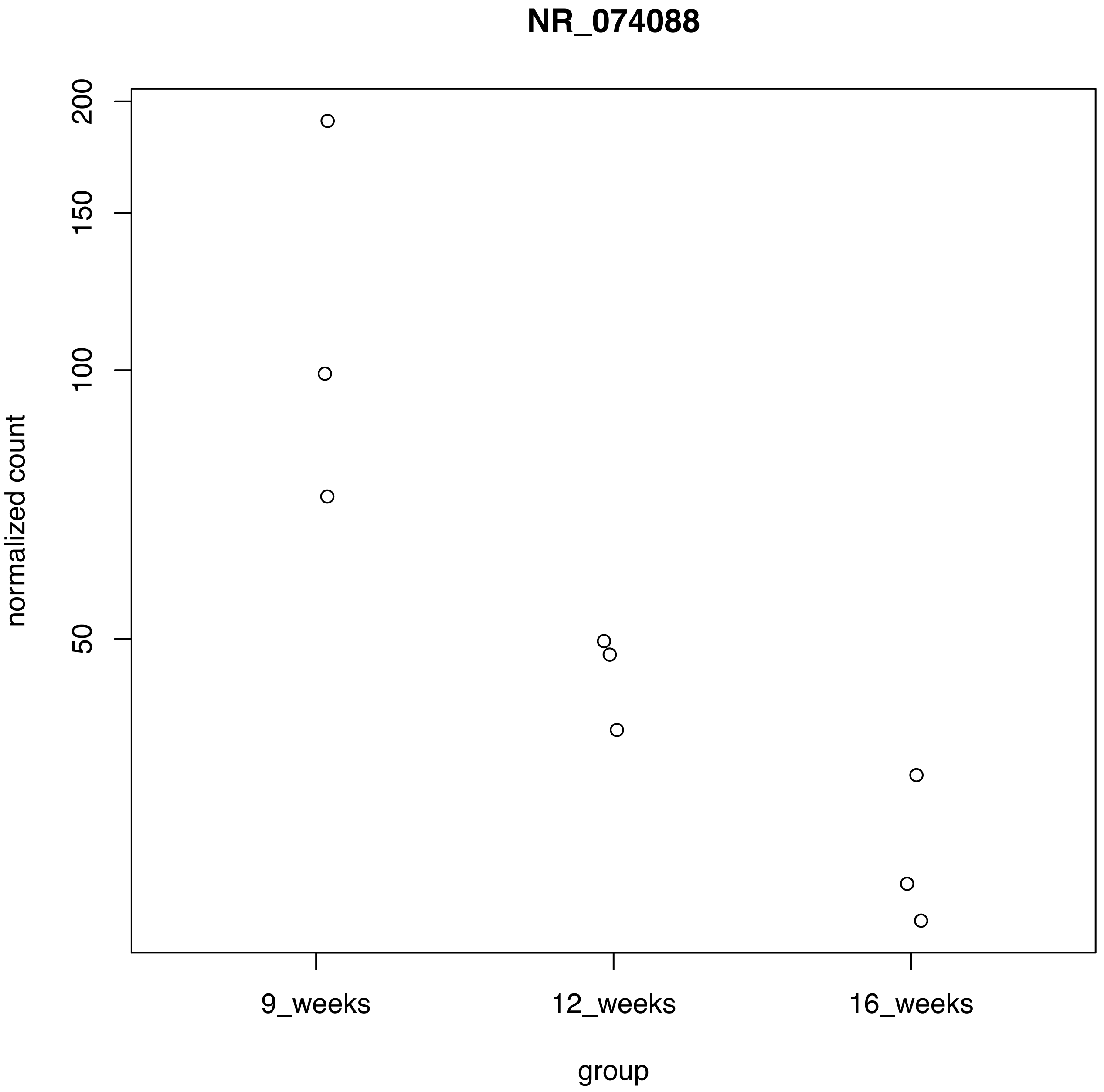
NR_074088
normalized count
200
150
100
50
9_weeks
12_weeks
16_weeks
group

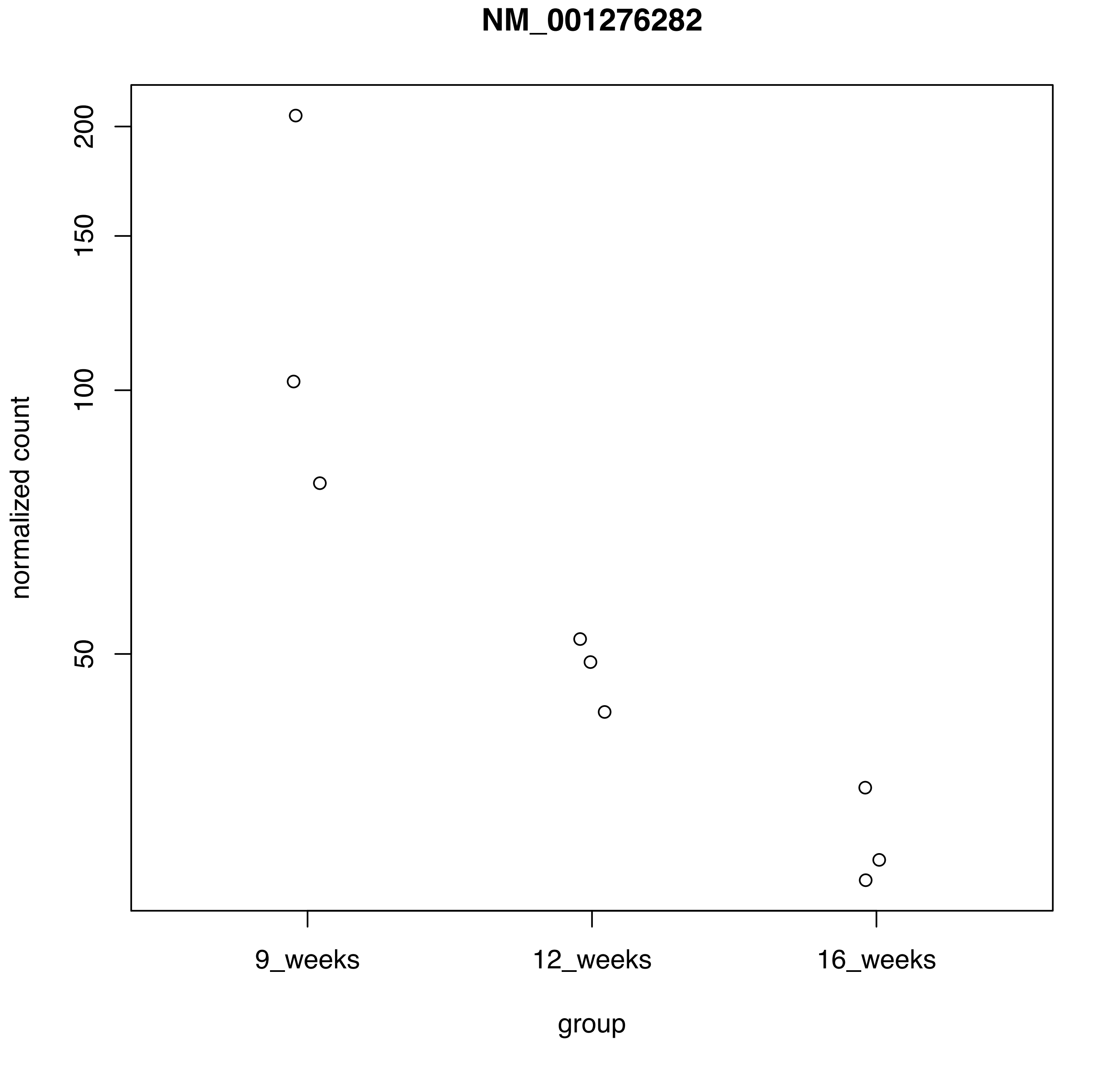

NM_001276282
normalized count
200
150
100
50
9_weeks
12_weeks
16_weeks
group

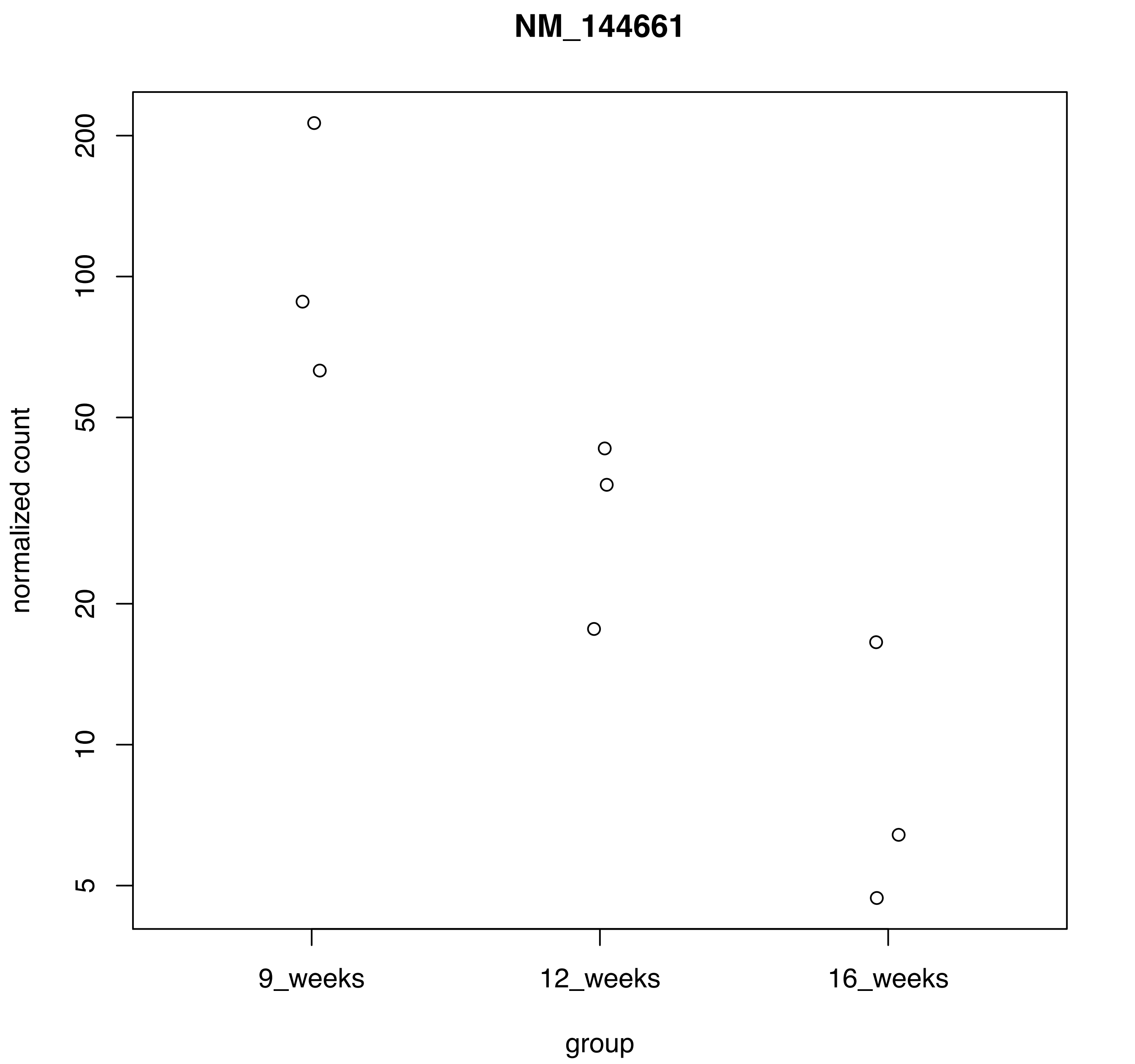
NM_144661
normalized count
200
100
50
20
10
5
9_weeks
12_weeks
16_weeks
group

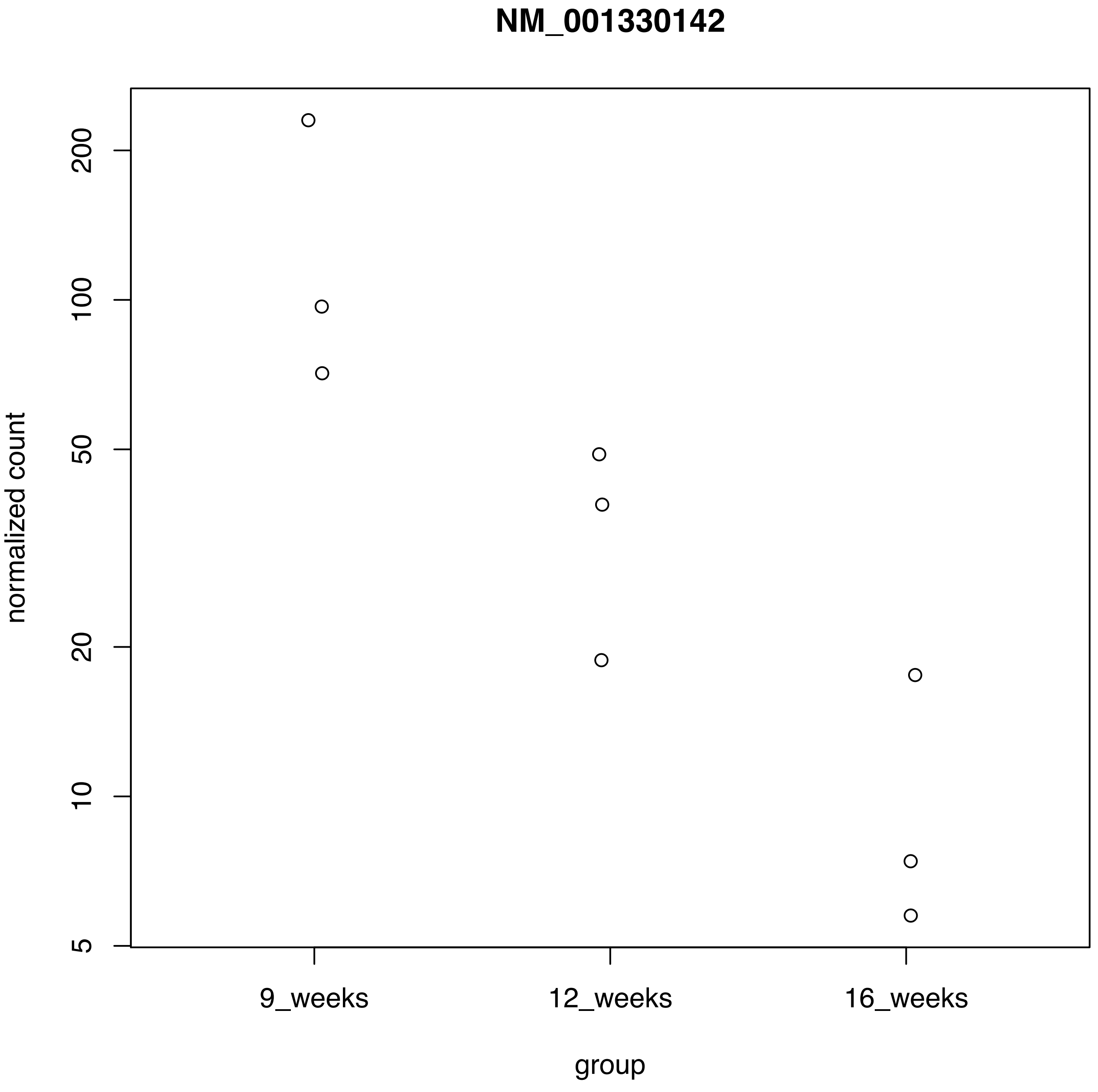
NM_001330142
normalized count
200
100
50
20
10
5
9_weeks
12_weeks
16_weeks
group

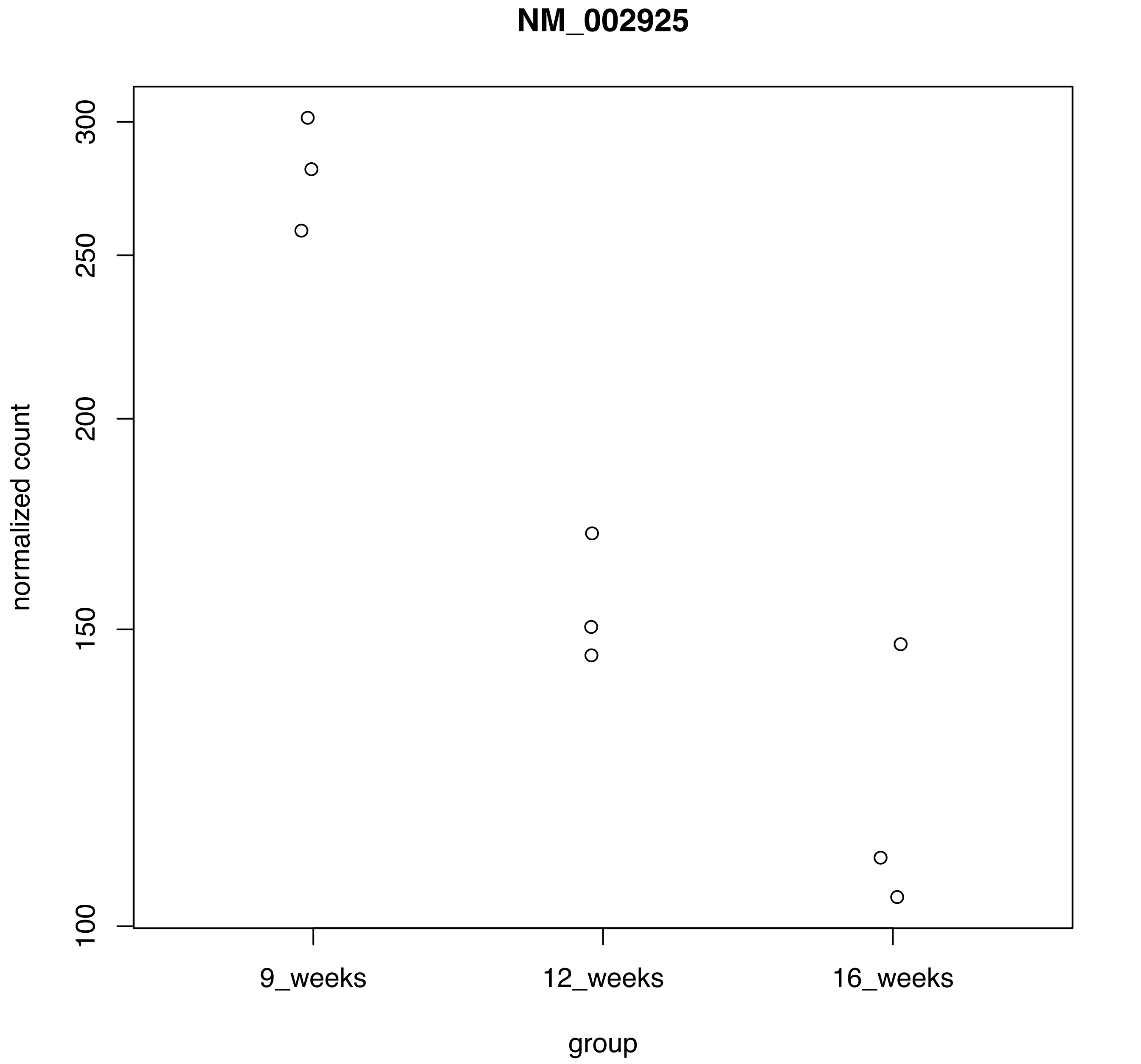
NM_002925
normalized count
300
250
200
150
100
9_weeks
12_weeks
16_weeks
group

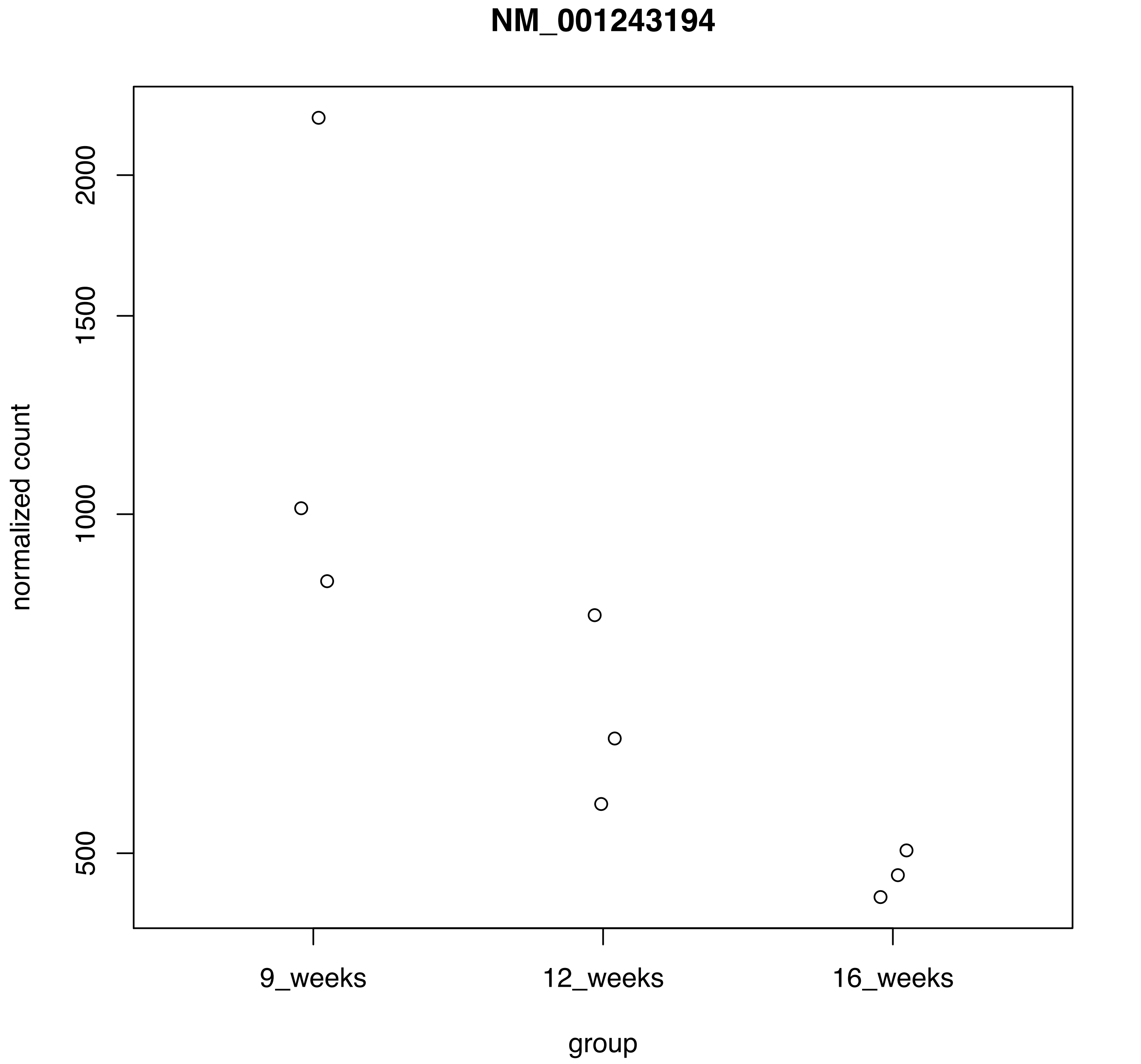
NM_001243194
normalized count
2000
1500
1000
500
9_weeks
12_weeks
16_weeks
group

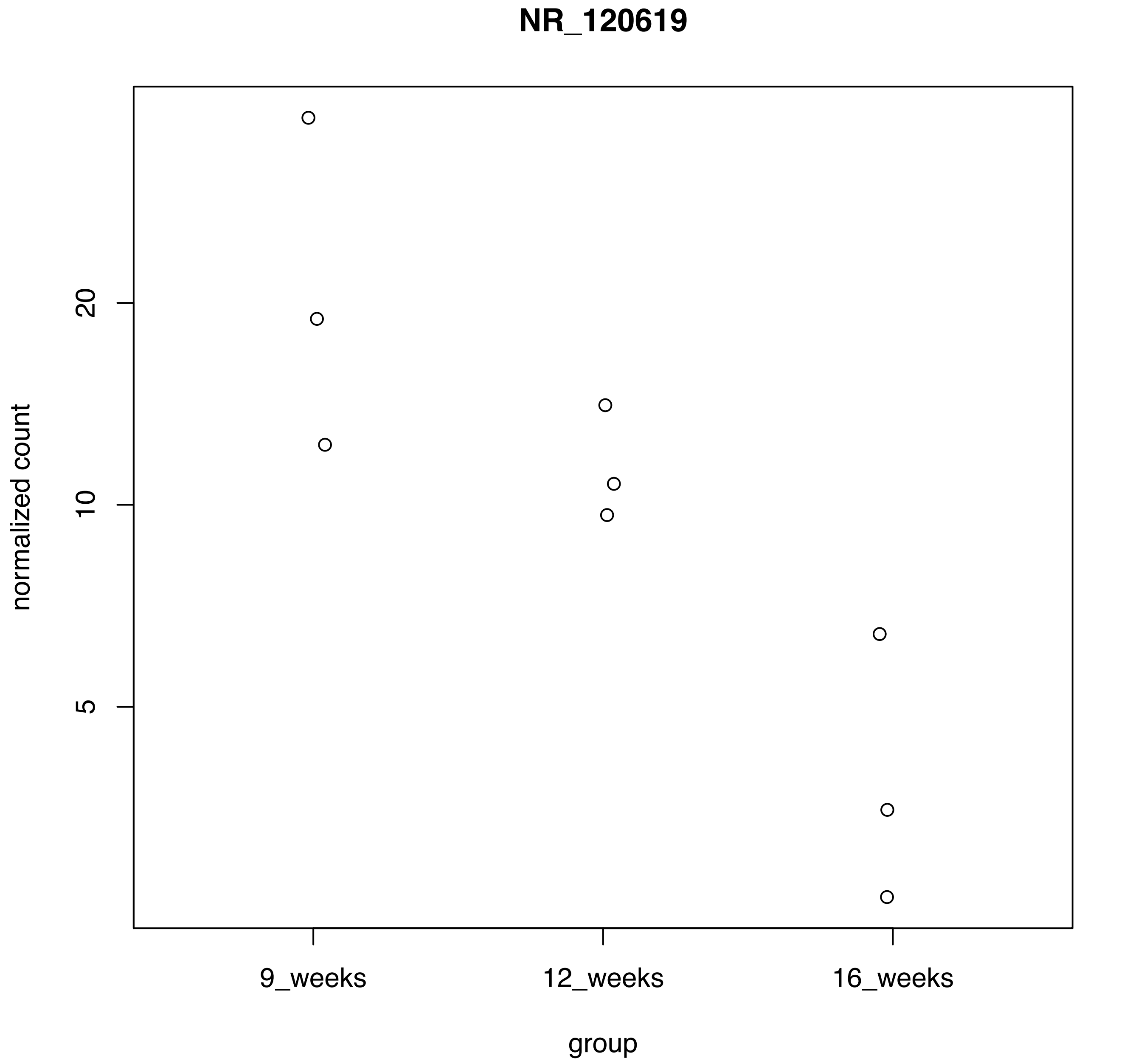
NR_120619
normalized count
20
10
5
9_weeks
12_weeks
16_weeks
group

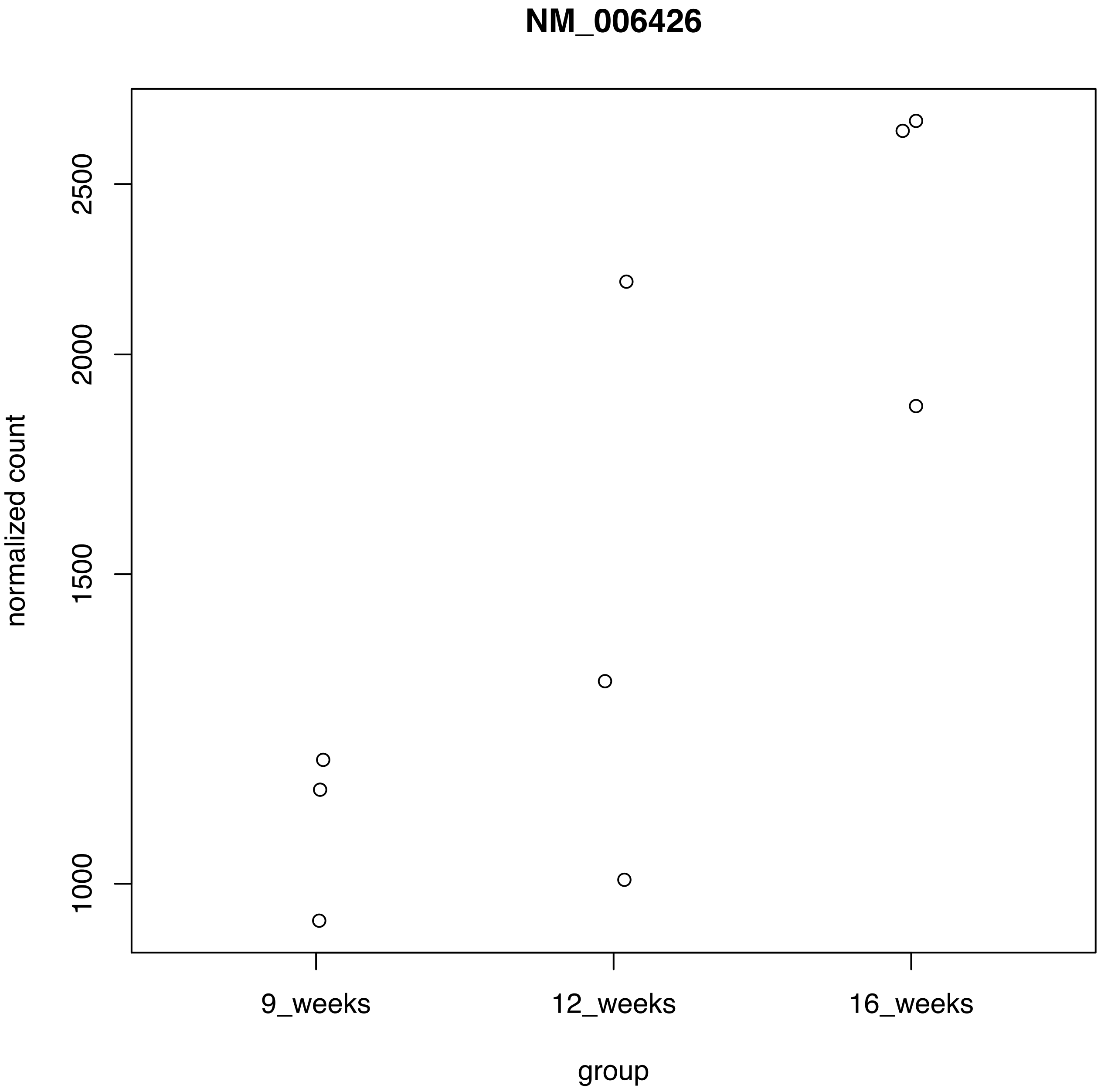
NM_006426
normalized count
1000
1500
2000
2500
9_weeks
12_weeks
16_weeks
group

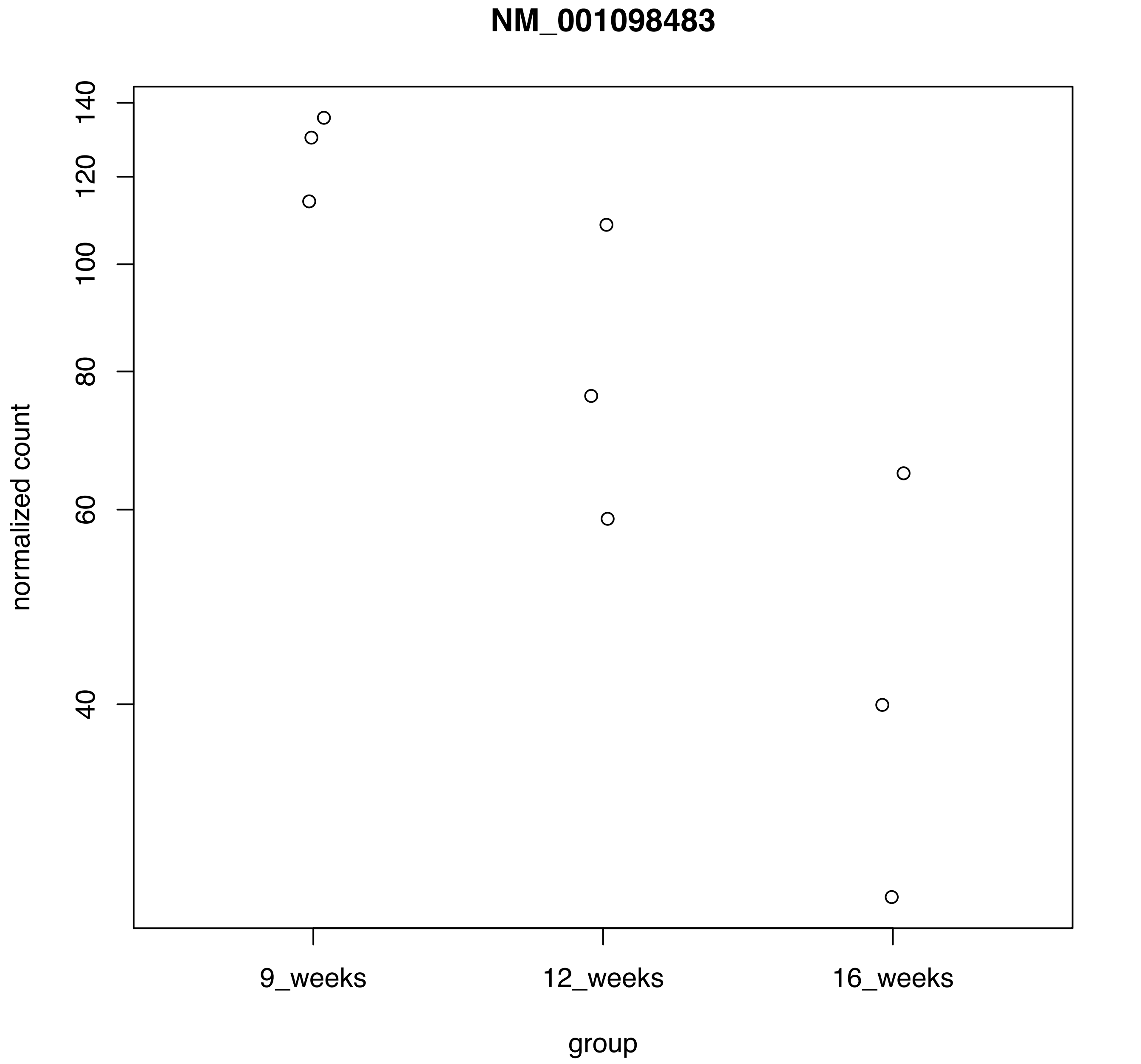

NM_001098483
normalized count
140
120
100
80
60
40
9_weeks
12_weeks
16_weeks
group

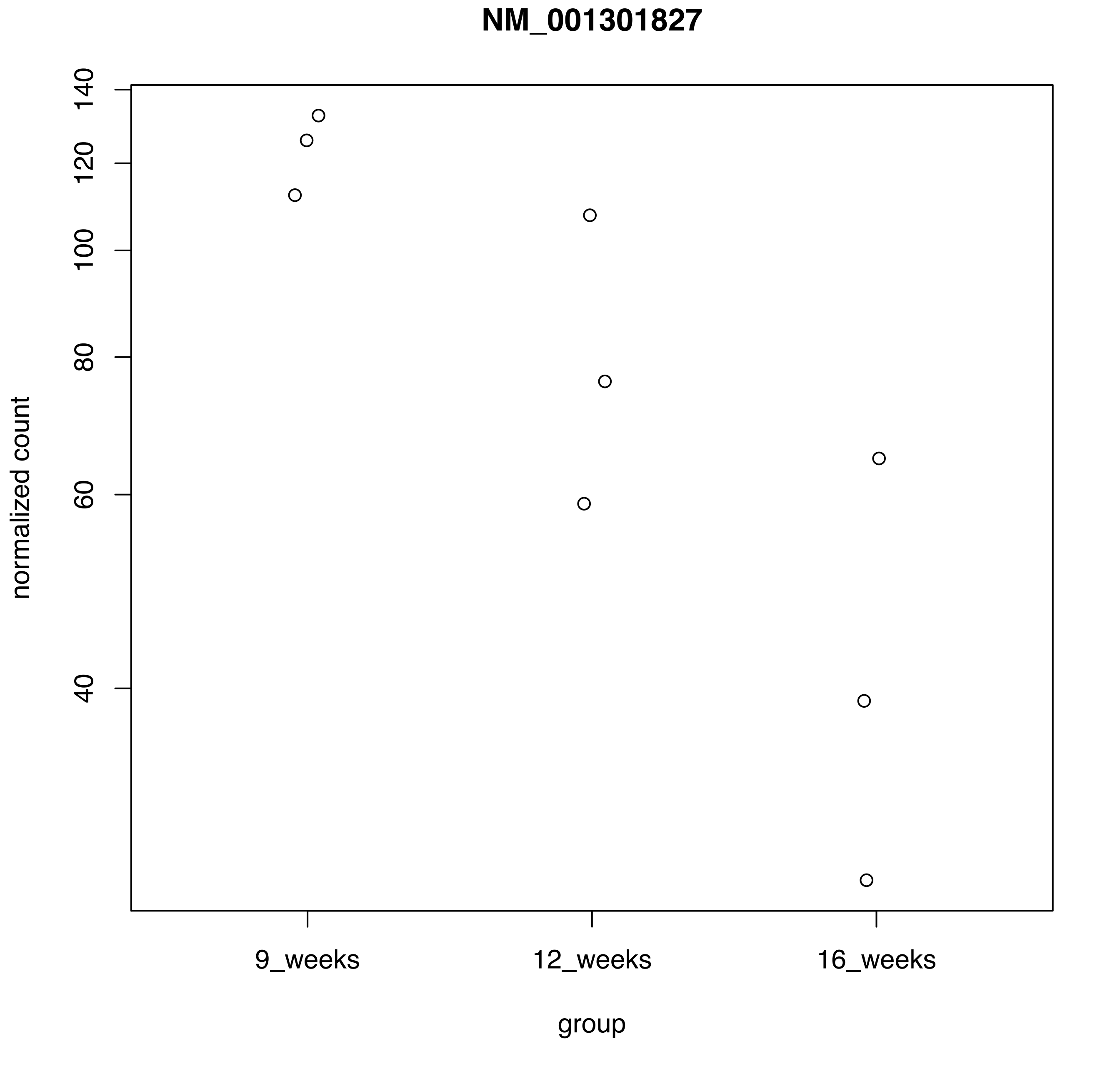

NM_001301827
normalized count
140
120
100
80
60
40
9_weeks
12_weeks
16_weeks
group

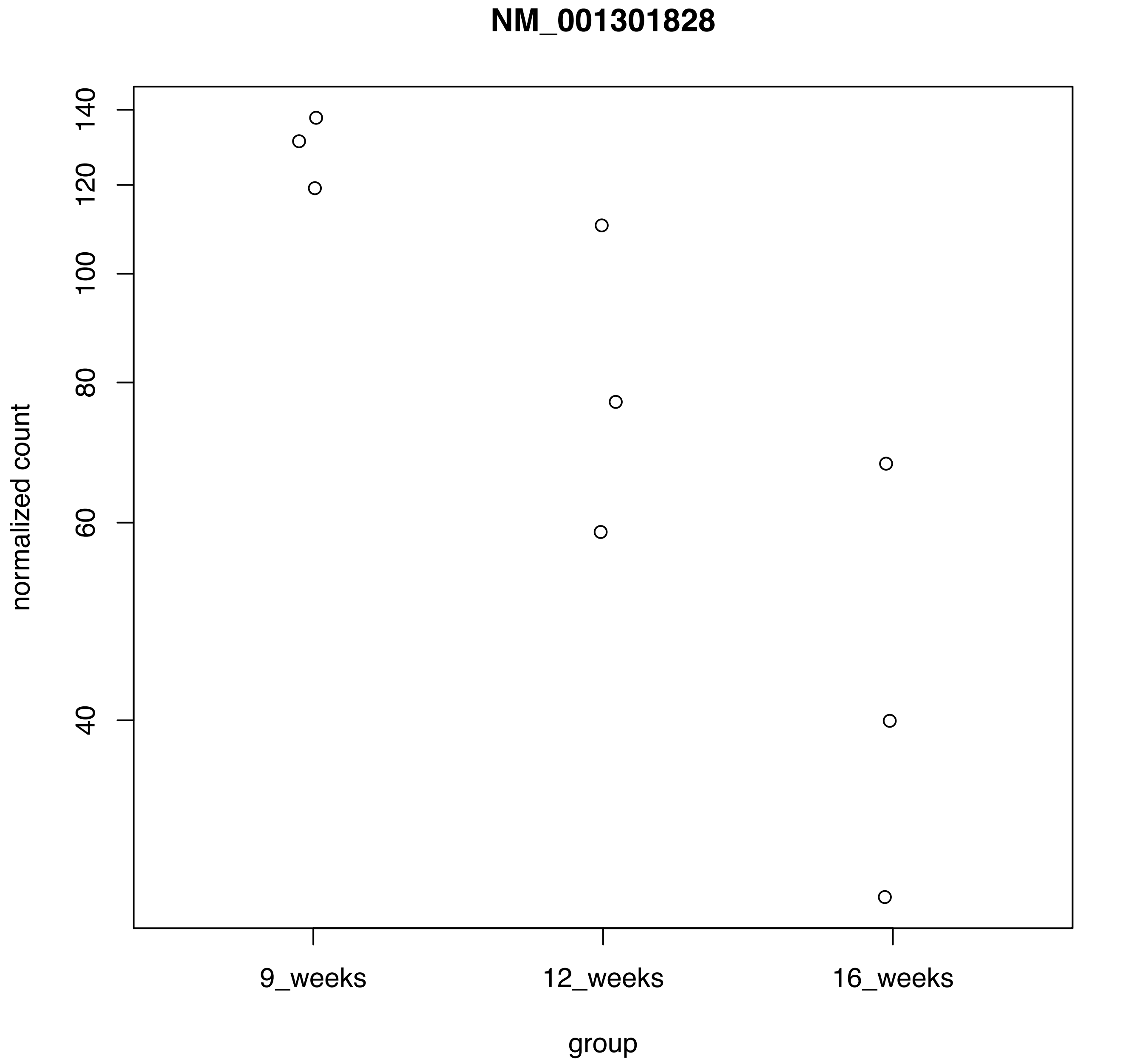

NM_001301828
normalized count
140
120
100
80
60
40
9_weeks
12_weeks
16_weeks
group

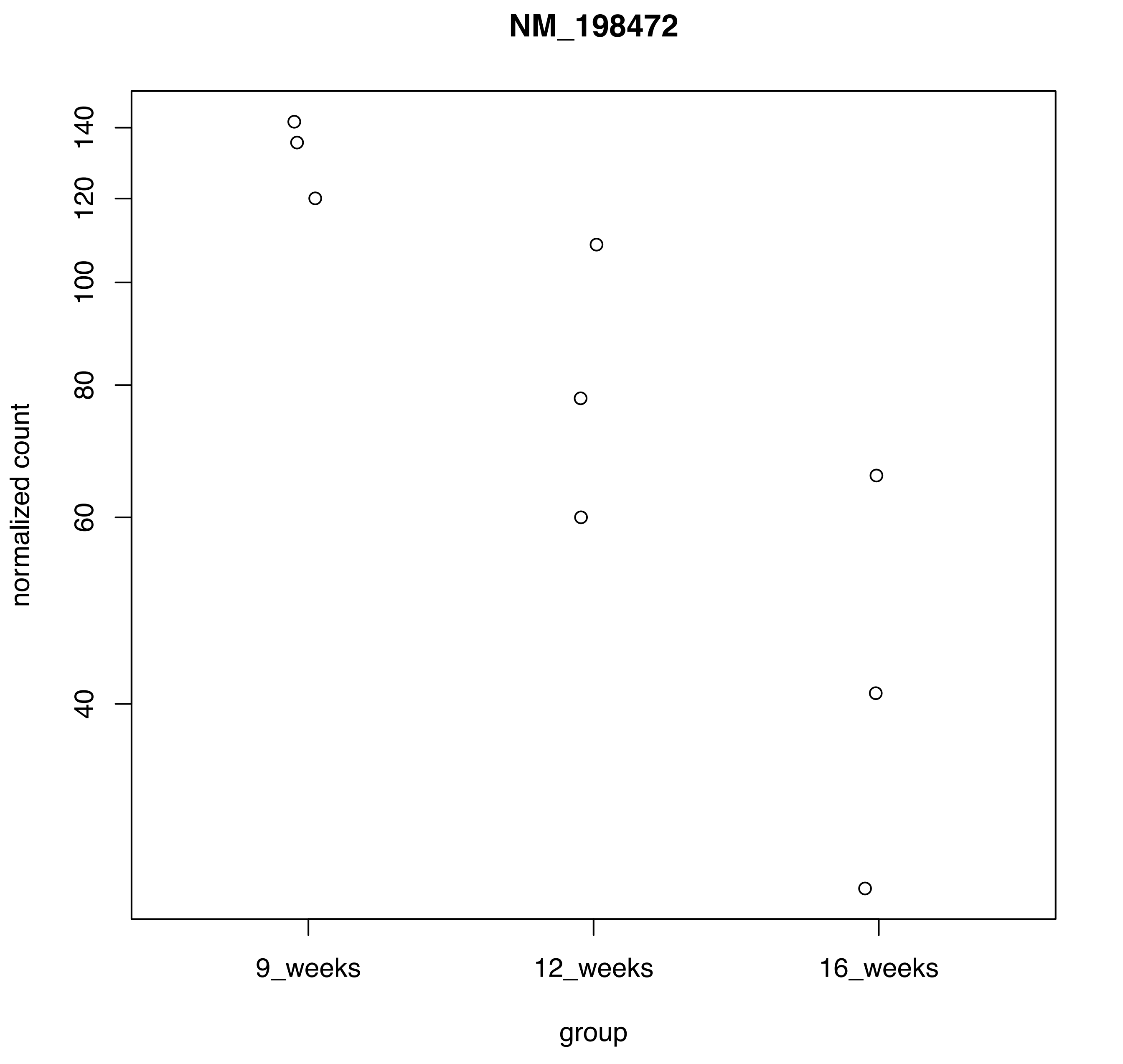
NM_198472
normalized count
140
120
100
80
60
40
9_weeks
12_weeks
16_weeks
group

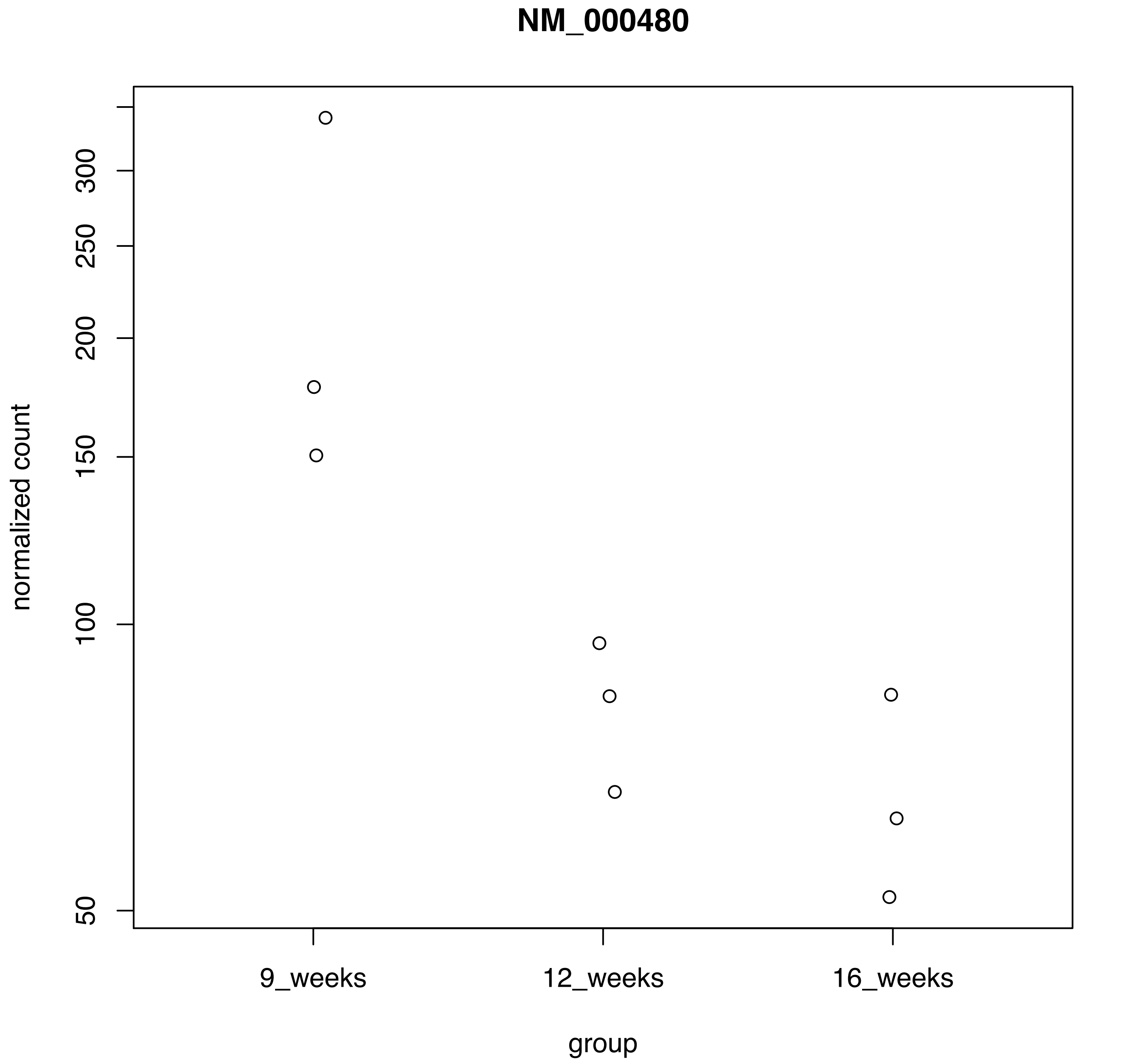

NM_000480
normalized count
300
250
200
150
100
50
9_weeks
12_weeks
16_weeks
group

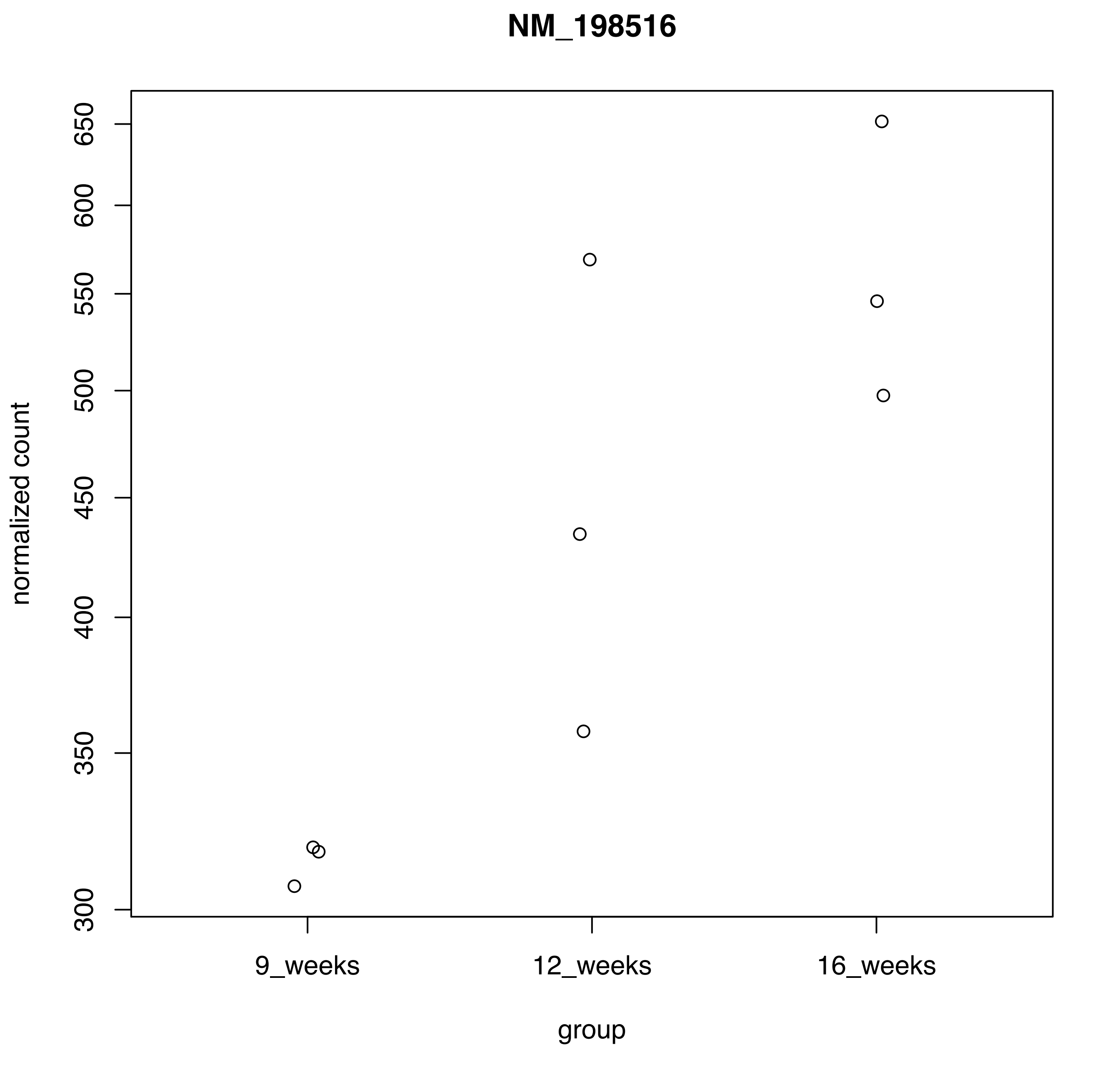
NM_198516
normalized count
300
350
400
450
500
550
600
650
9_weeks
12_weeks
16_weeks
group

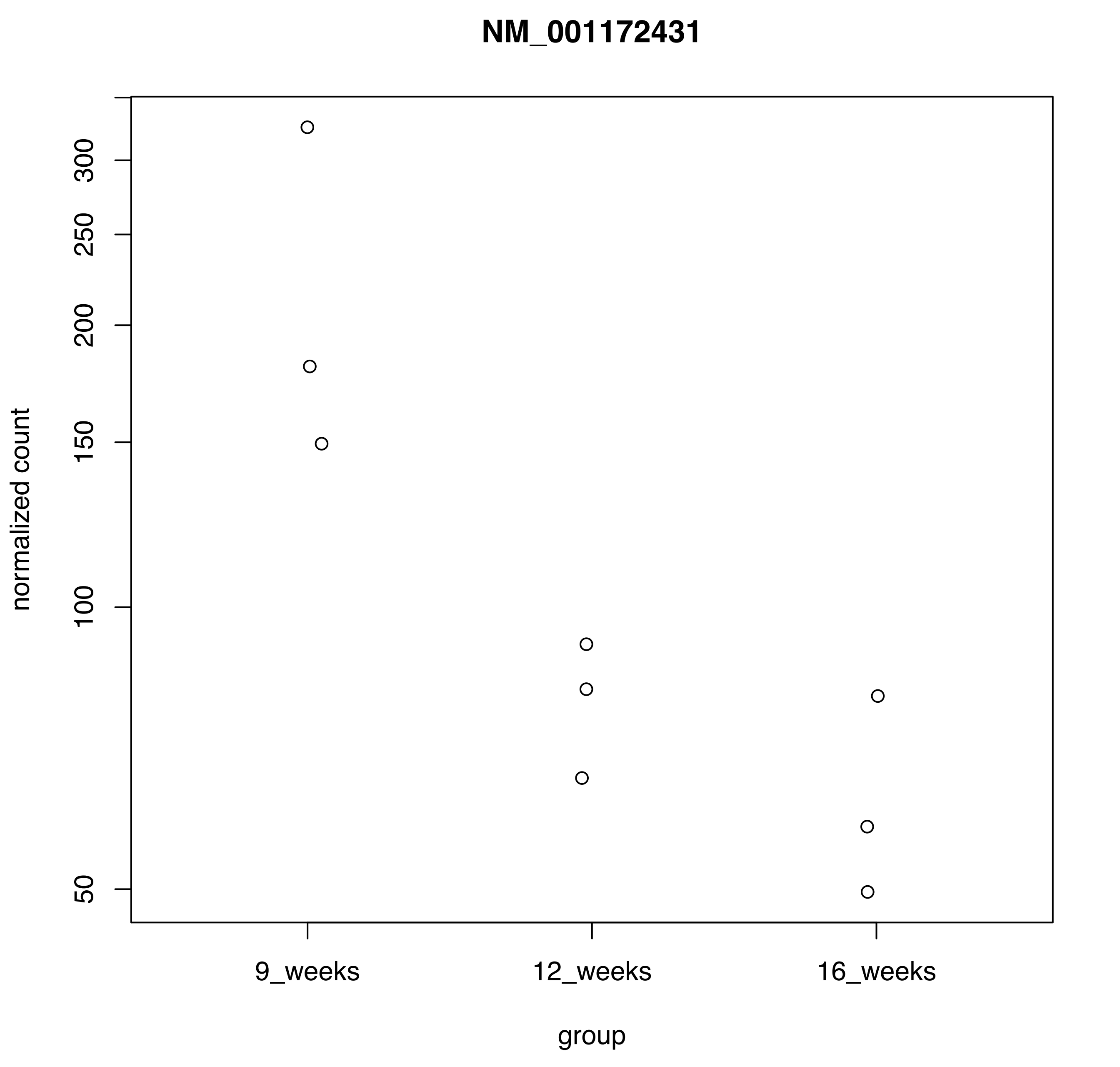

NM_001172431
normalized count
300
250
200
150
100
50
9_weeks
12_weeks
16_weeks
group

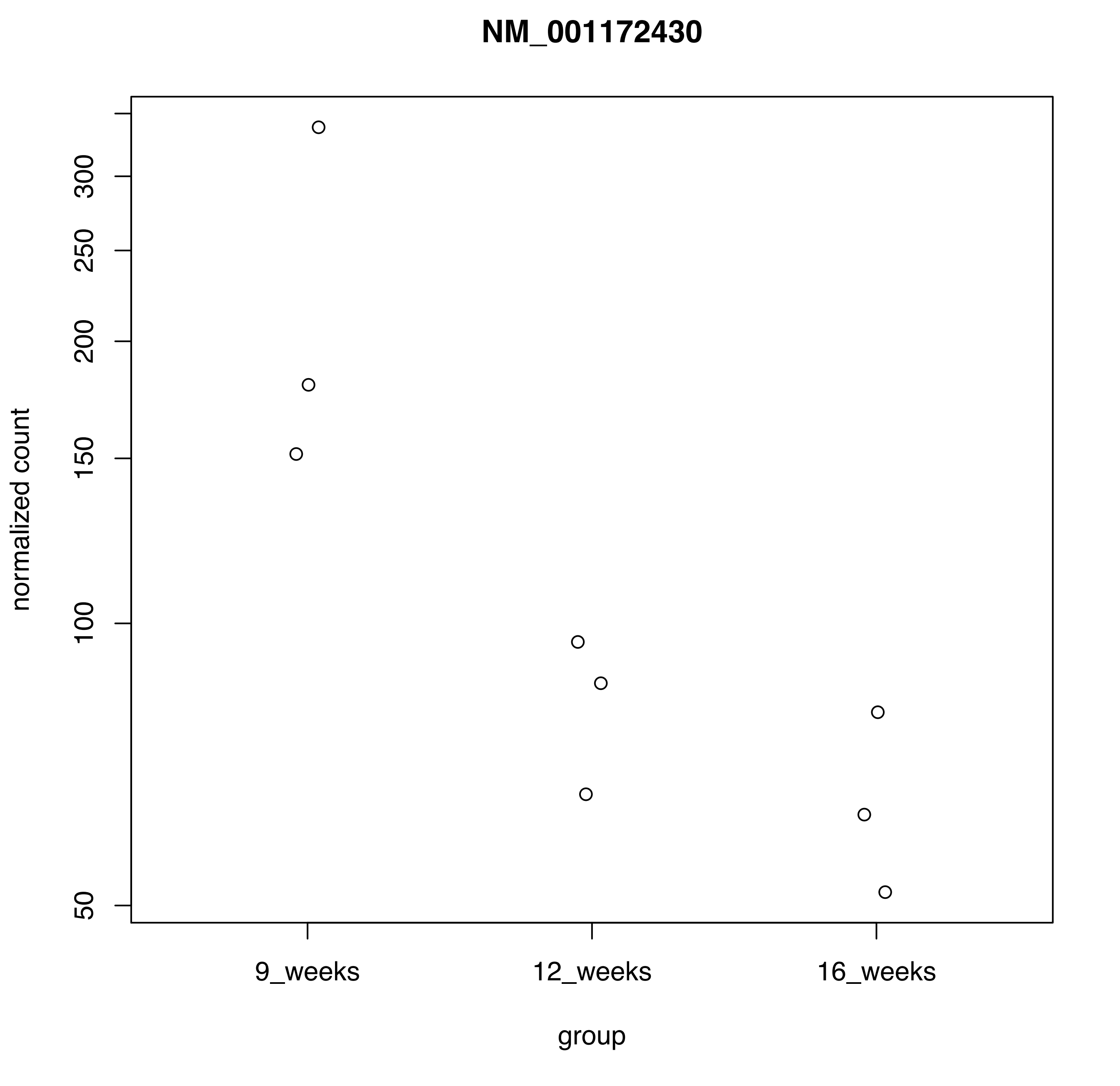
NM_001172430
normalized count
300
250
200
150
100
50
9_weeks
12_weeks
16_weeks
group

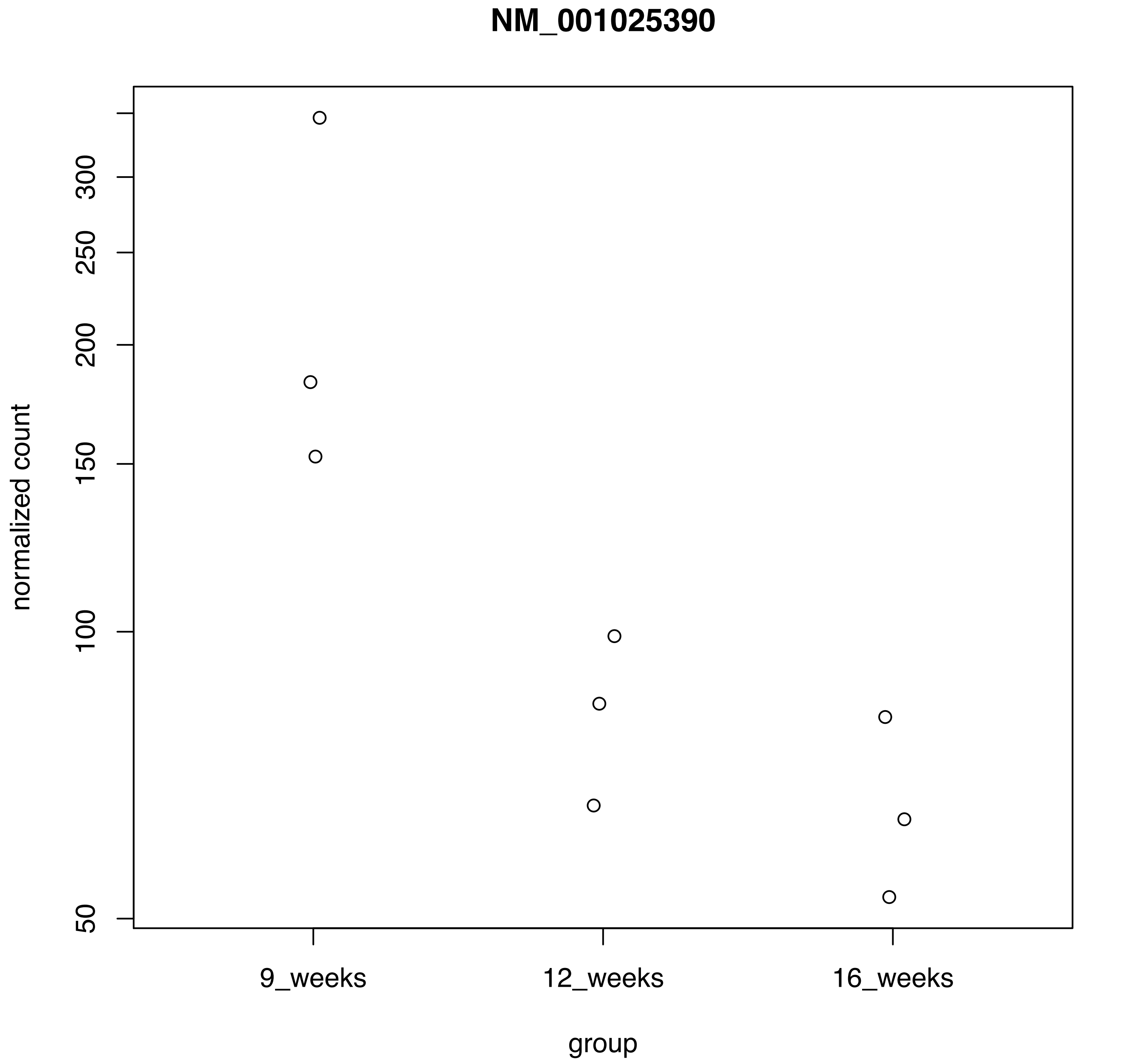
NM_001025390
normalized count
50
100
150
200
250
300
9_weeks
12_weeks
16_weeks
group

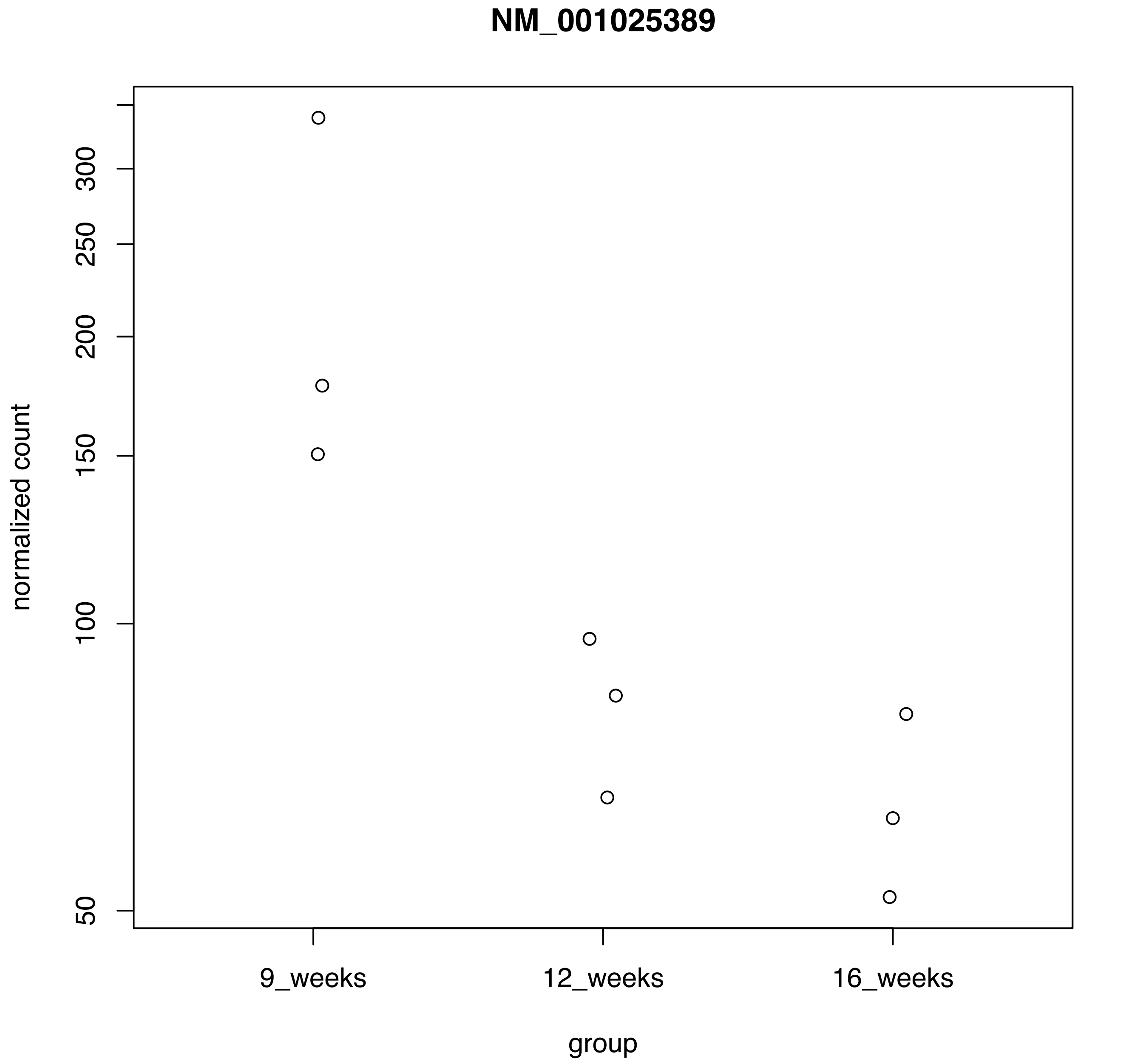

NM_001025389
normalized count
300
250
200
150
100
50
9_weeks
12_weeks
16_weeks
group

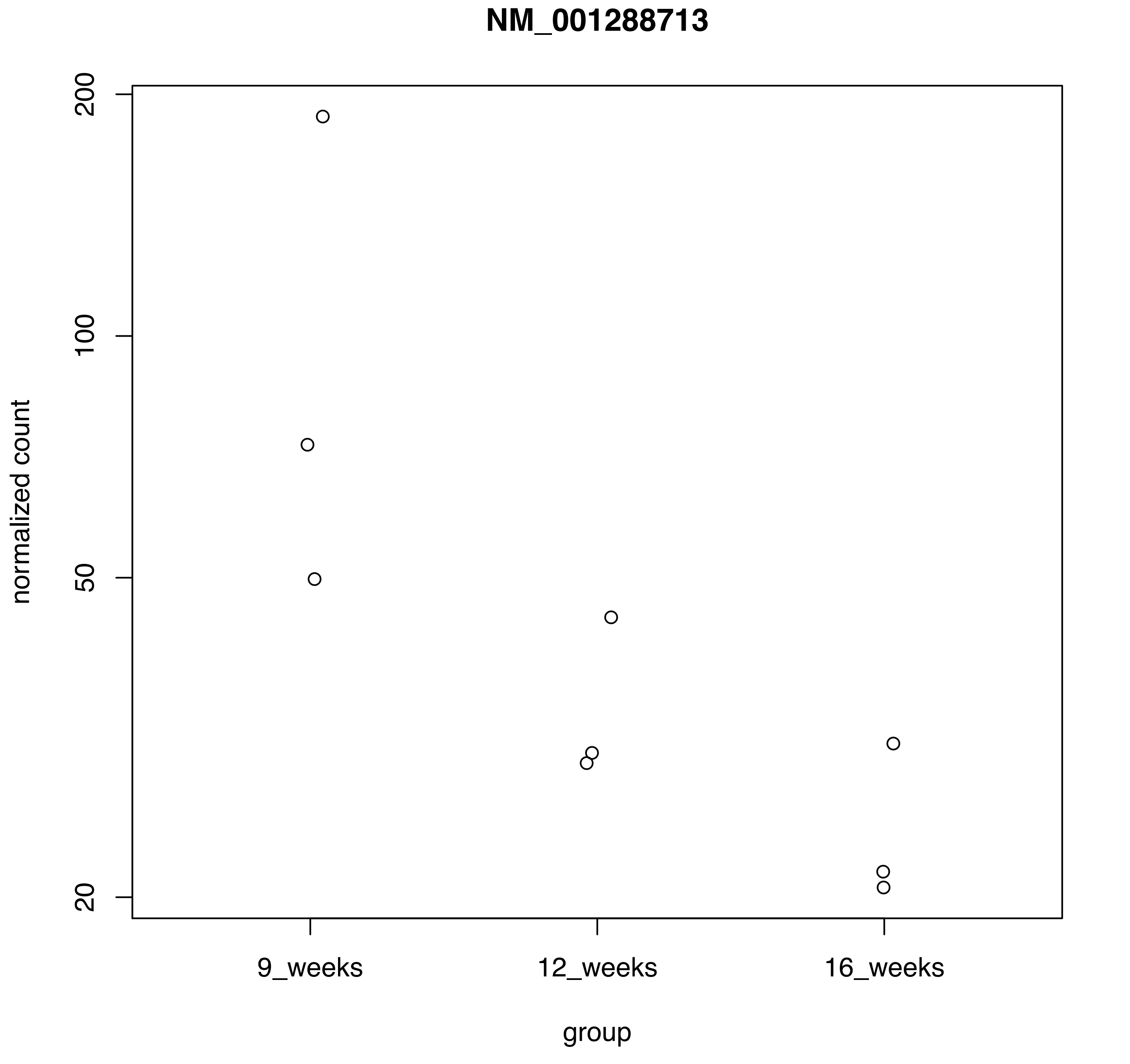

NM_001288713
normalized count
200
100
50
20
9_weeks
12_weeks
16_weeks
group

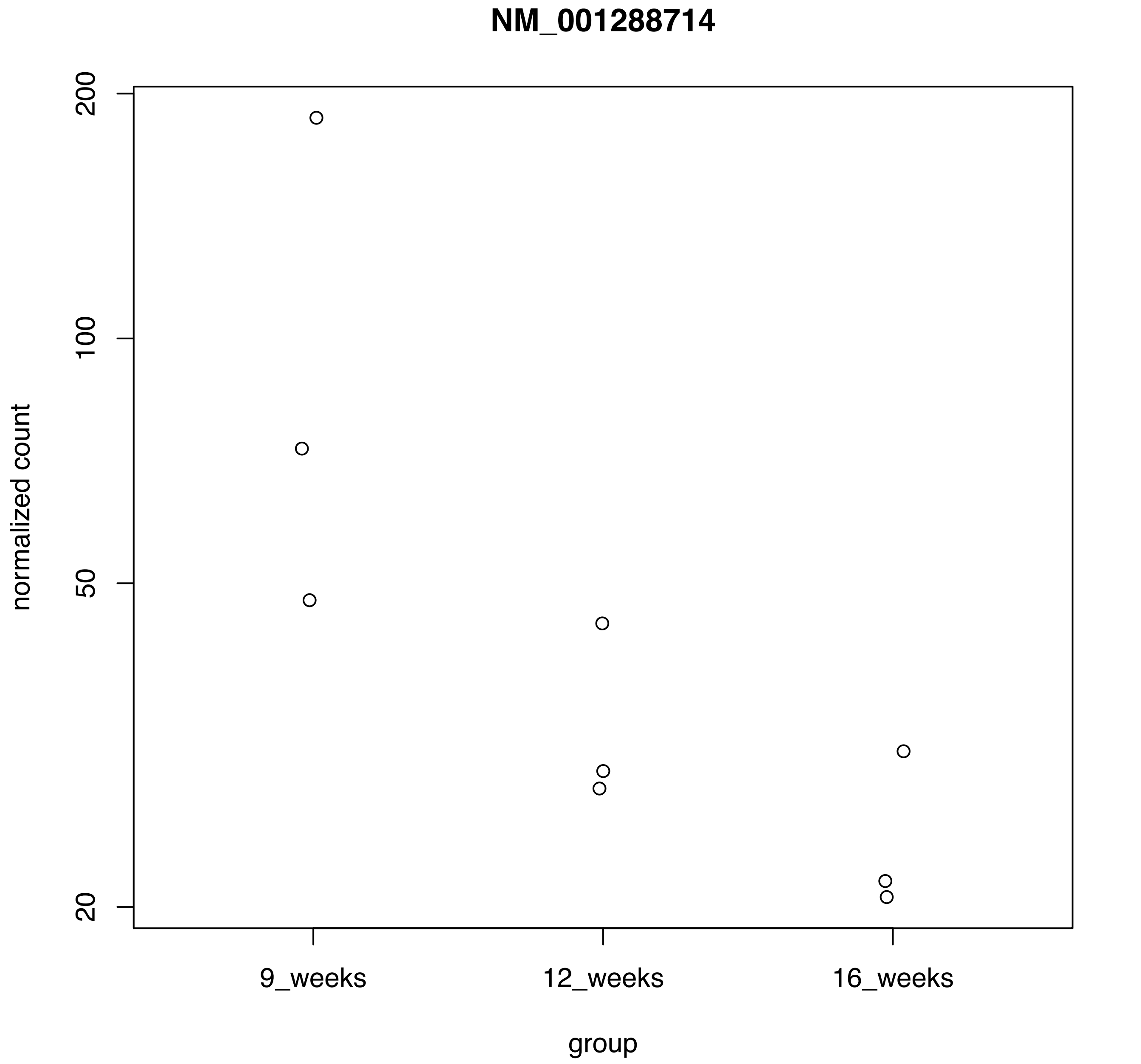
NM_001288714
normalized count
200
100
50
20
9_weeks
12_weeks
16_weeks
group

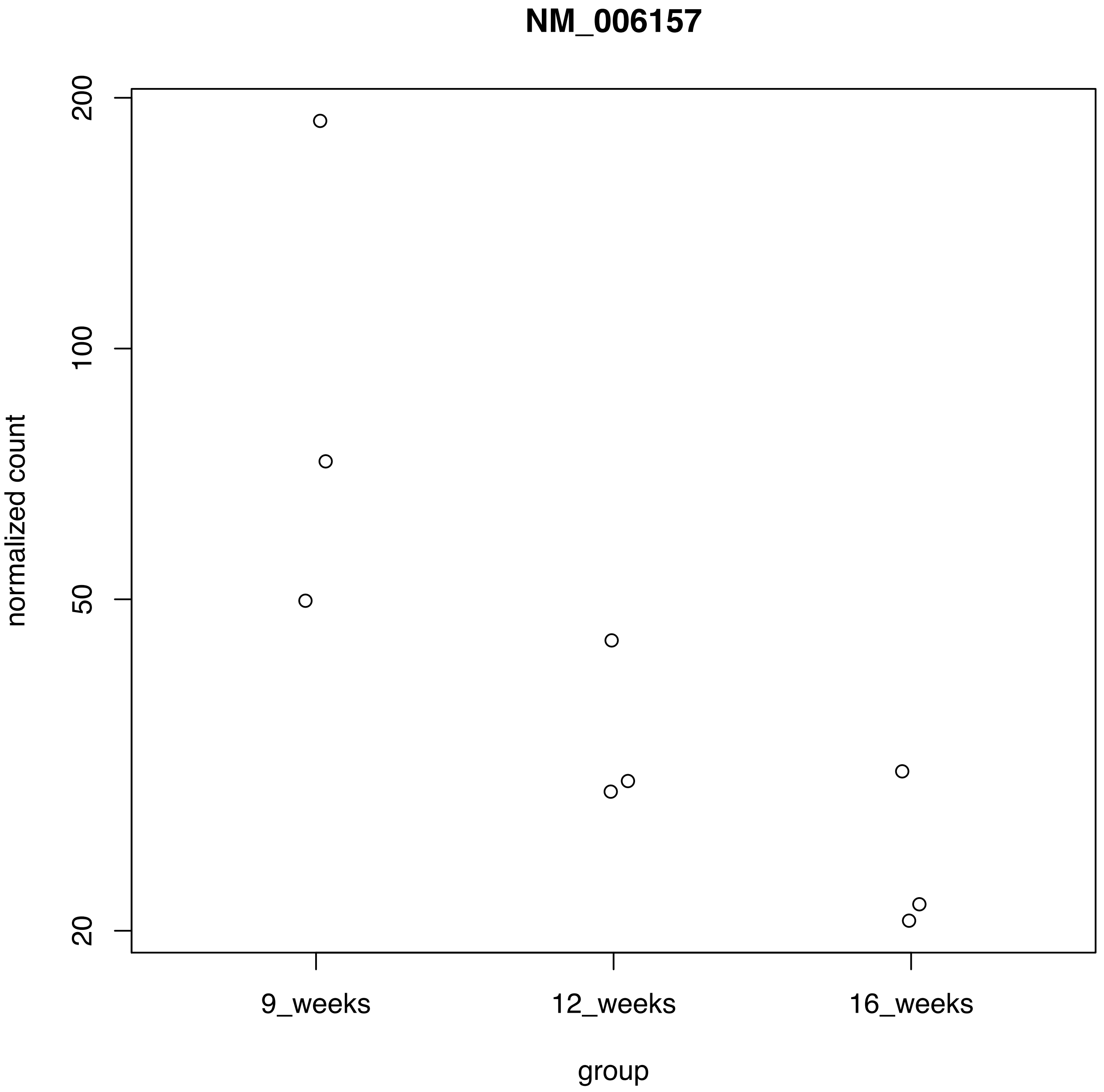

NM_006157
200
100
50
20
normalized count
9_weeks
12_weeks
16_weeks
group

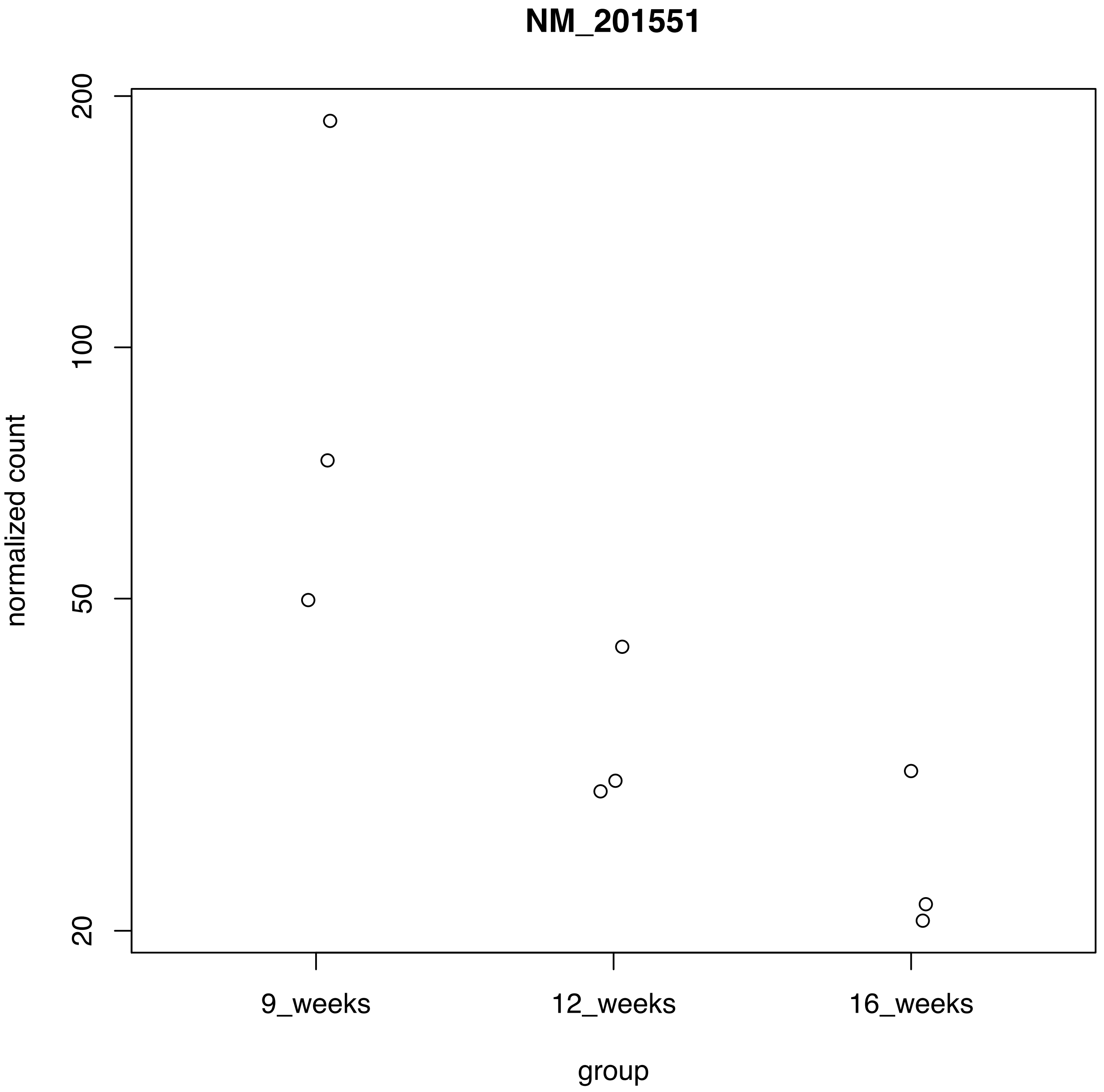
NM_201551
normalized count
200
100
50
20
9_weeks
12_weeks
16_weeks
group

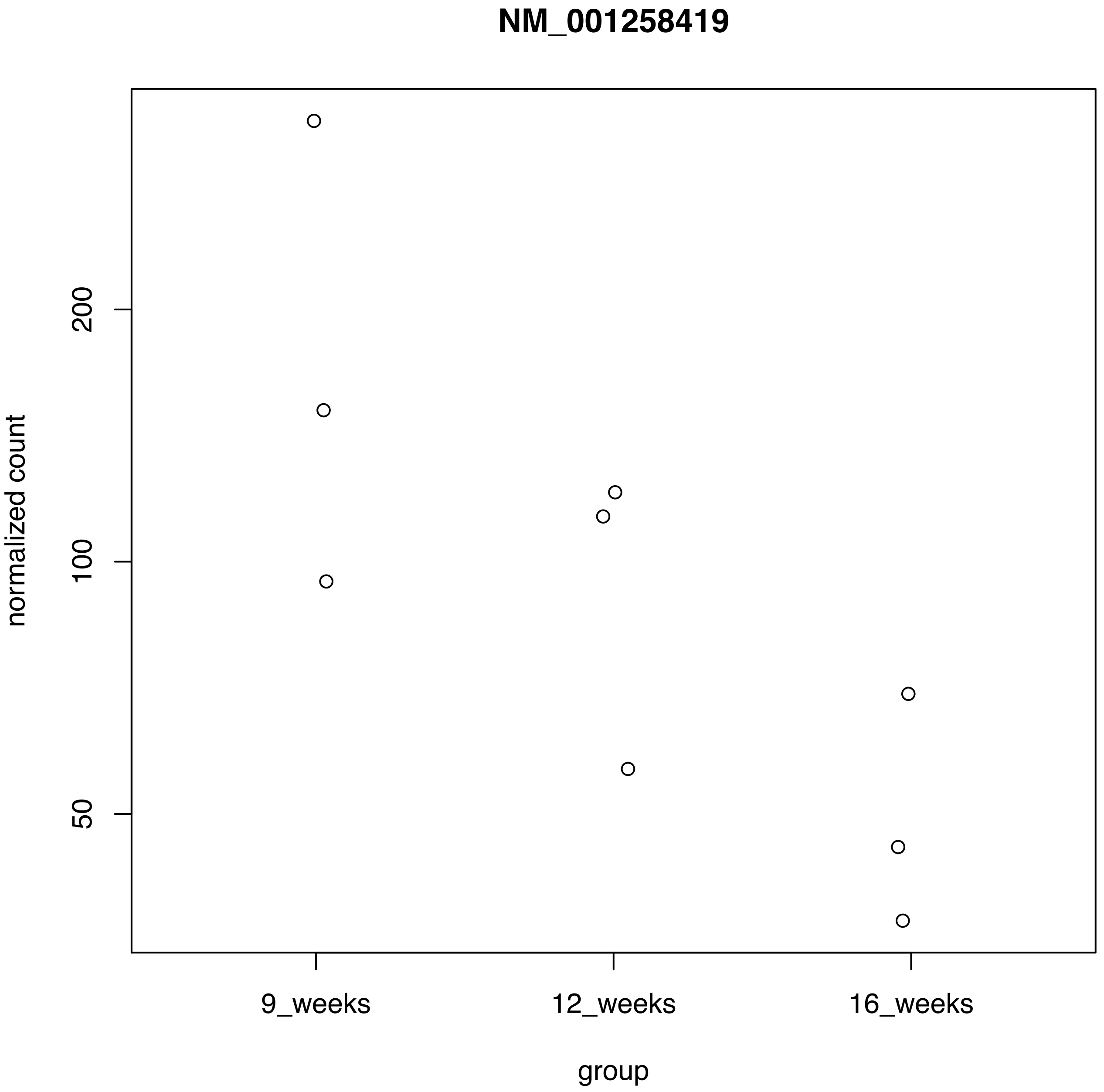
NM_001258419
normalized count
200
100
50
9_weeks
12_weeks
16_weeks
group

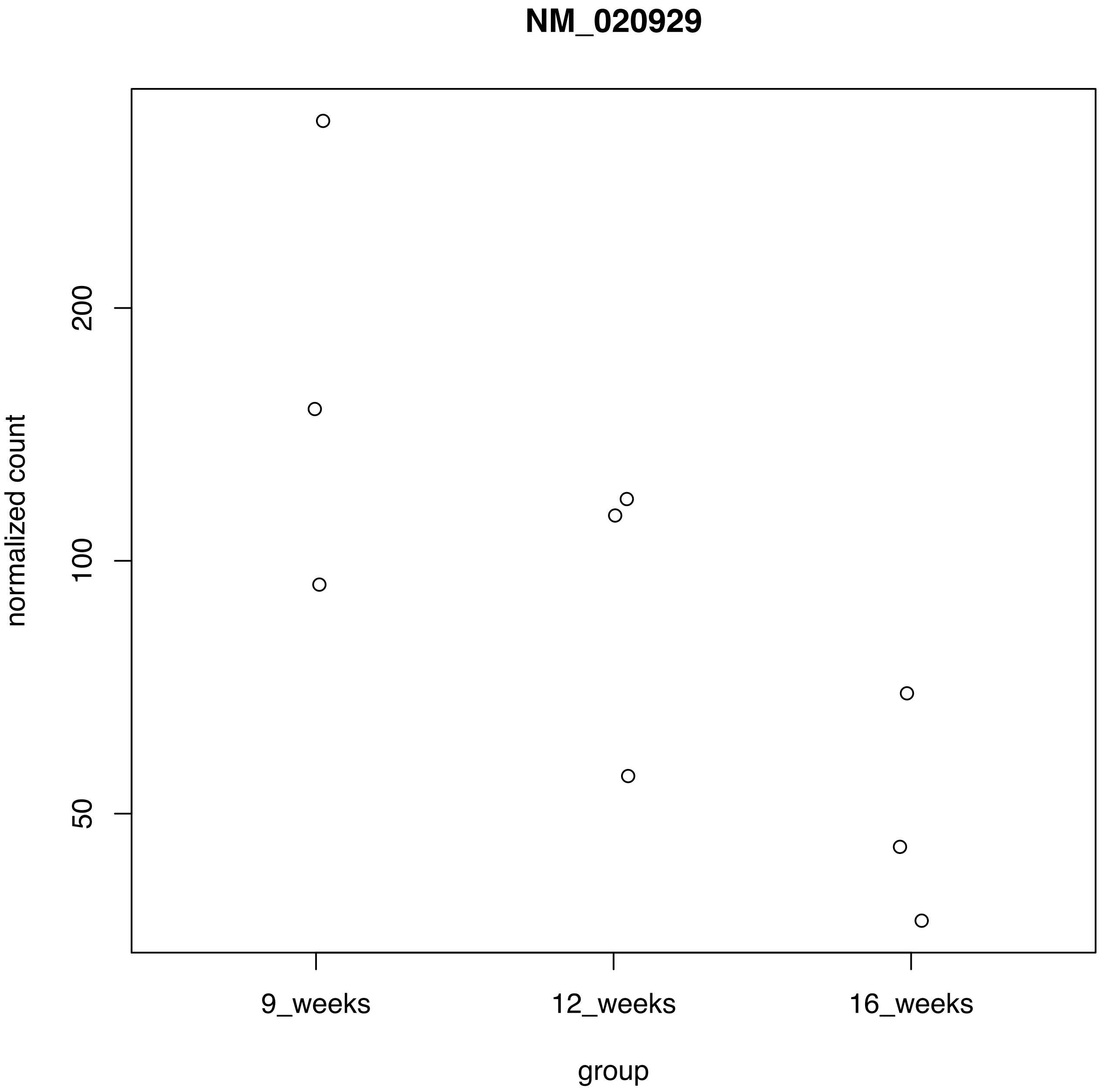
NM_020929
normalized count
200
100
50
9_weeks
12_weeks
16_weeks
group

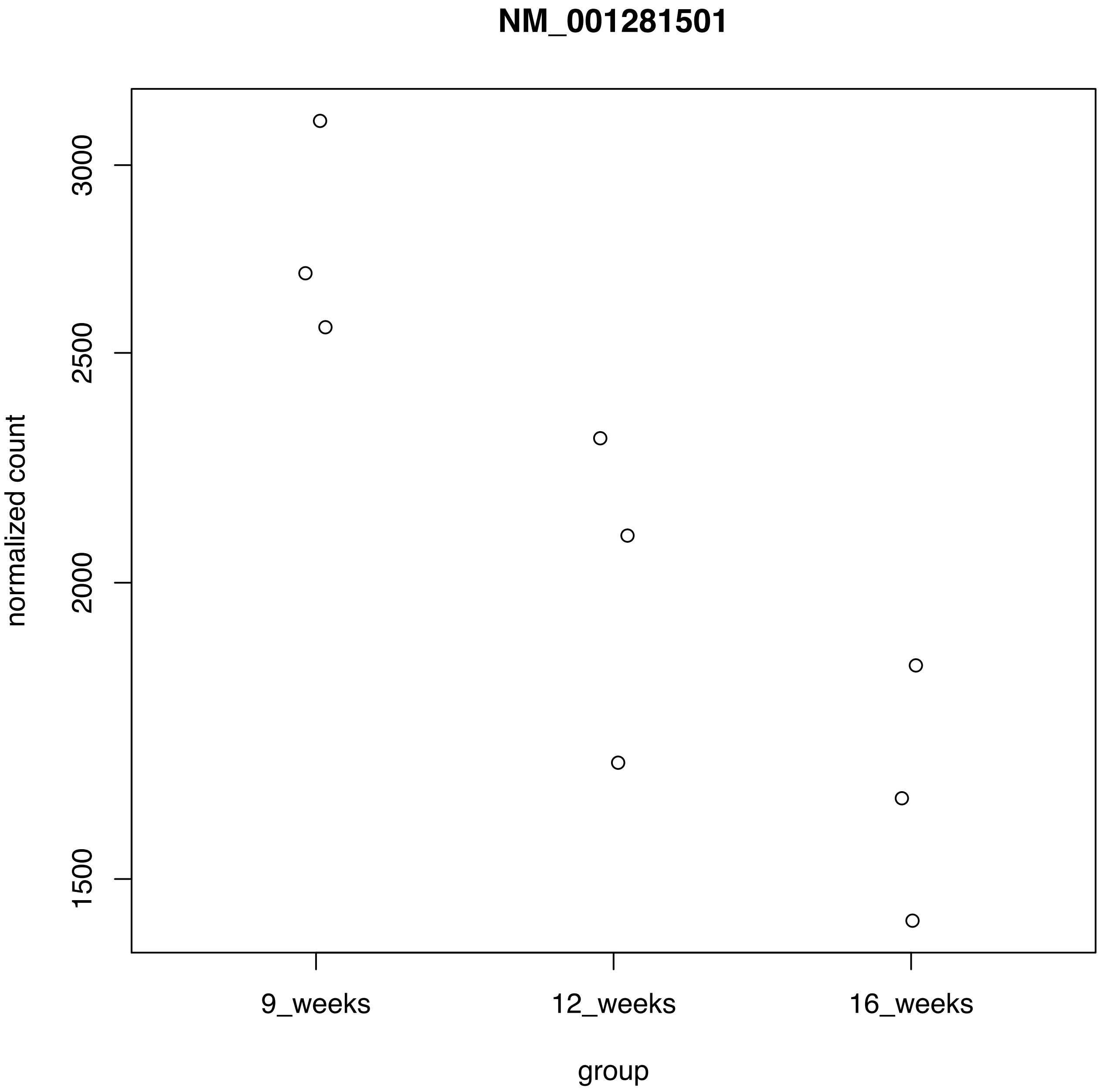
NM_001281501
normalized count
3000
2500
2000
1500
9_weeks
12_weeks
16_weeks
group

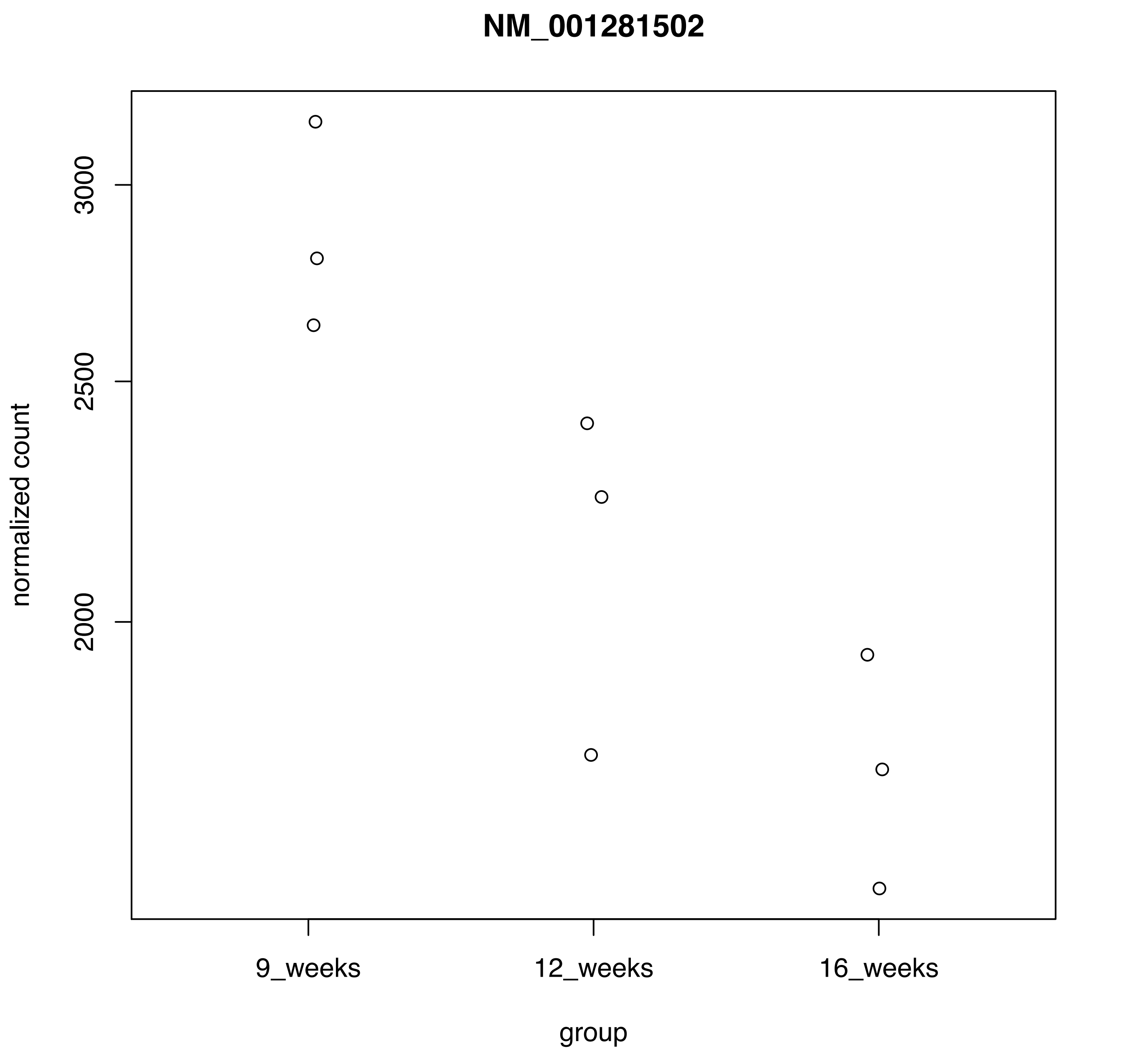
NM_001281502
normalized count
3000
2500
2000
9_weeks
12_weeks
16_weeks
group

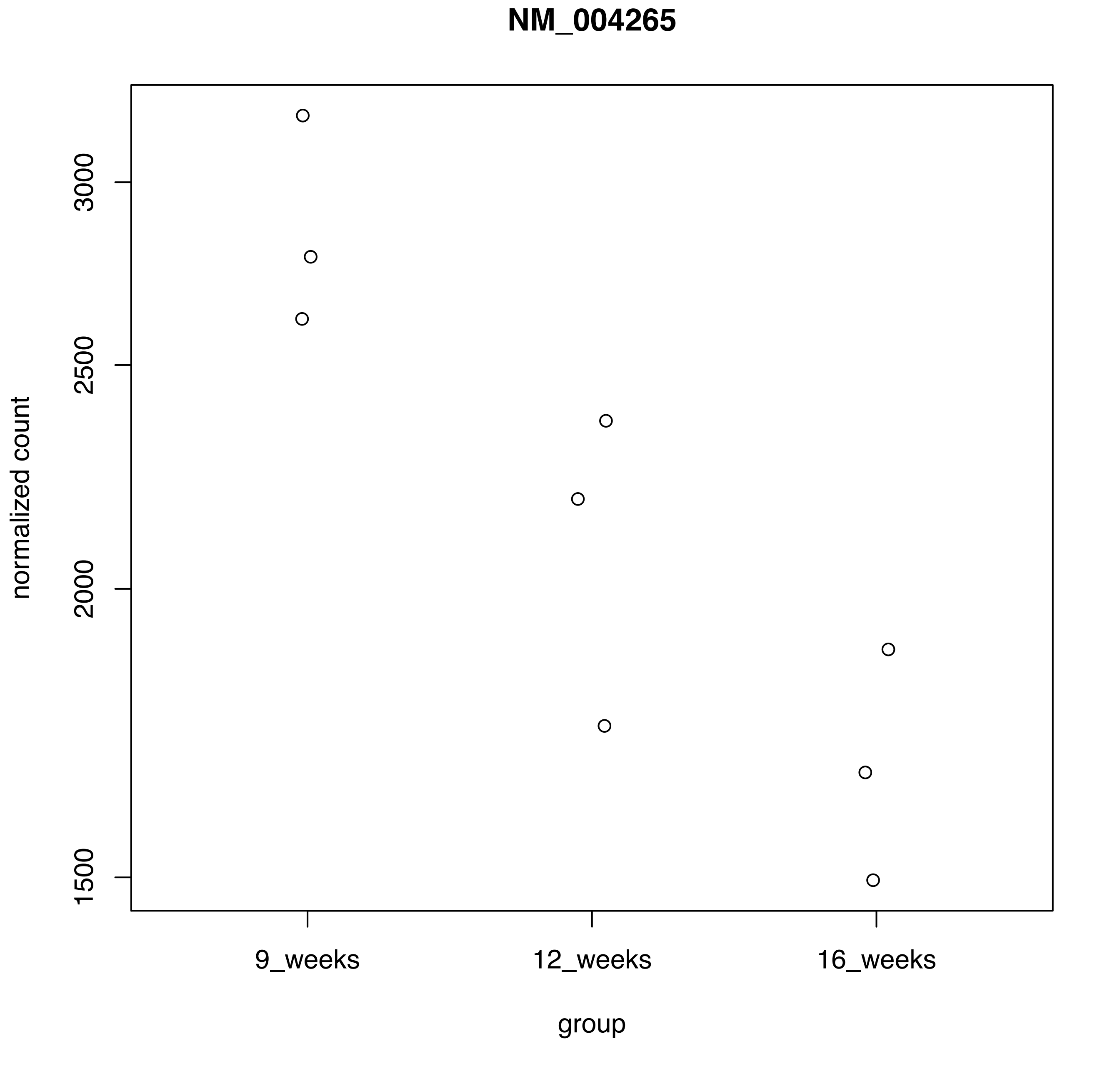
NM_004265
normalized count
3000
2500
2000
1500
9_weeks
12_weeks
16_weeks
group

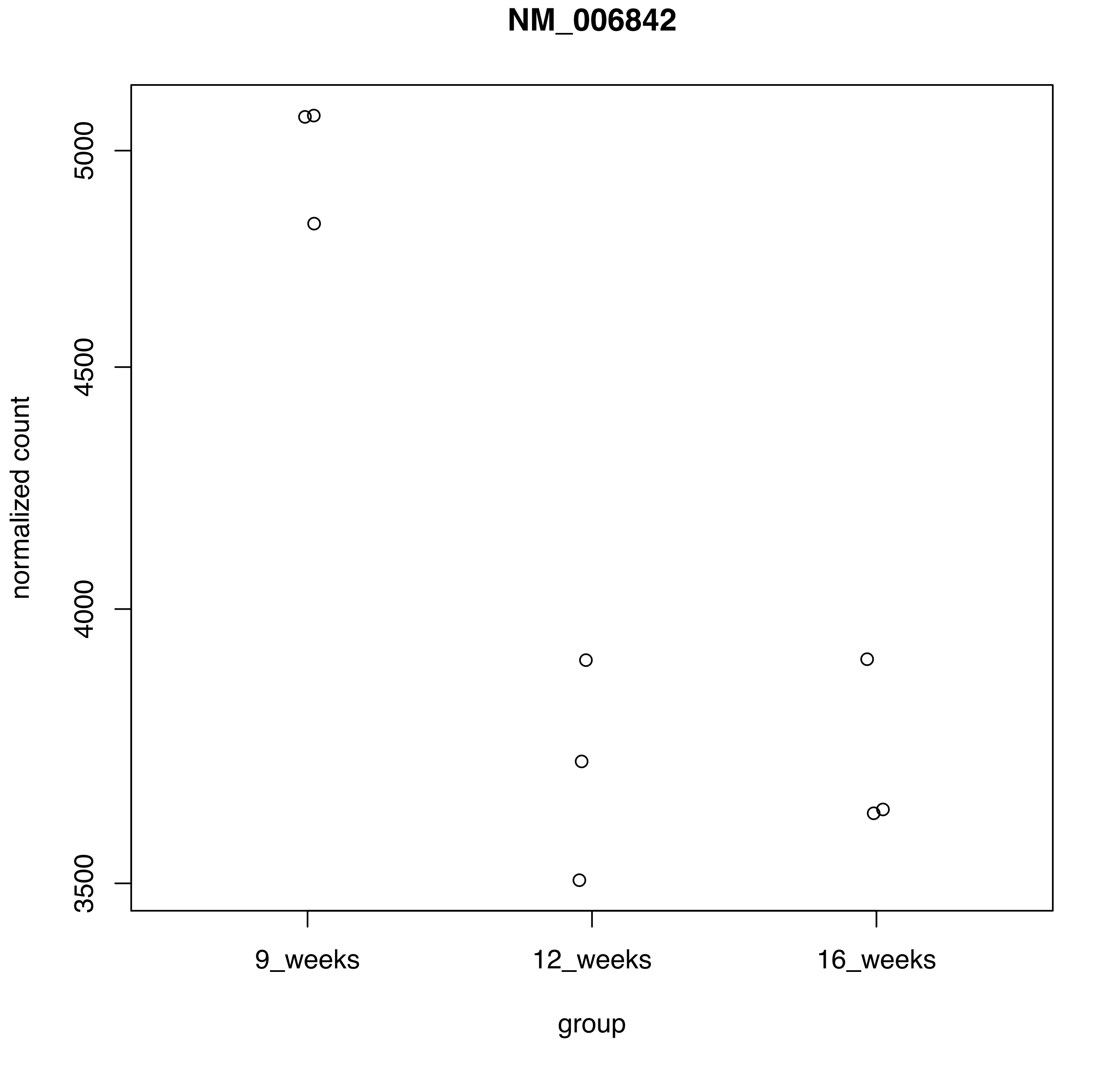
NM_006842
normalized count
5000
4500
4000
3500
9_weeks
12_weeks
16_weeks
group

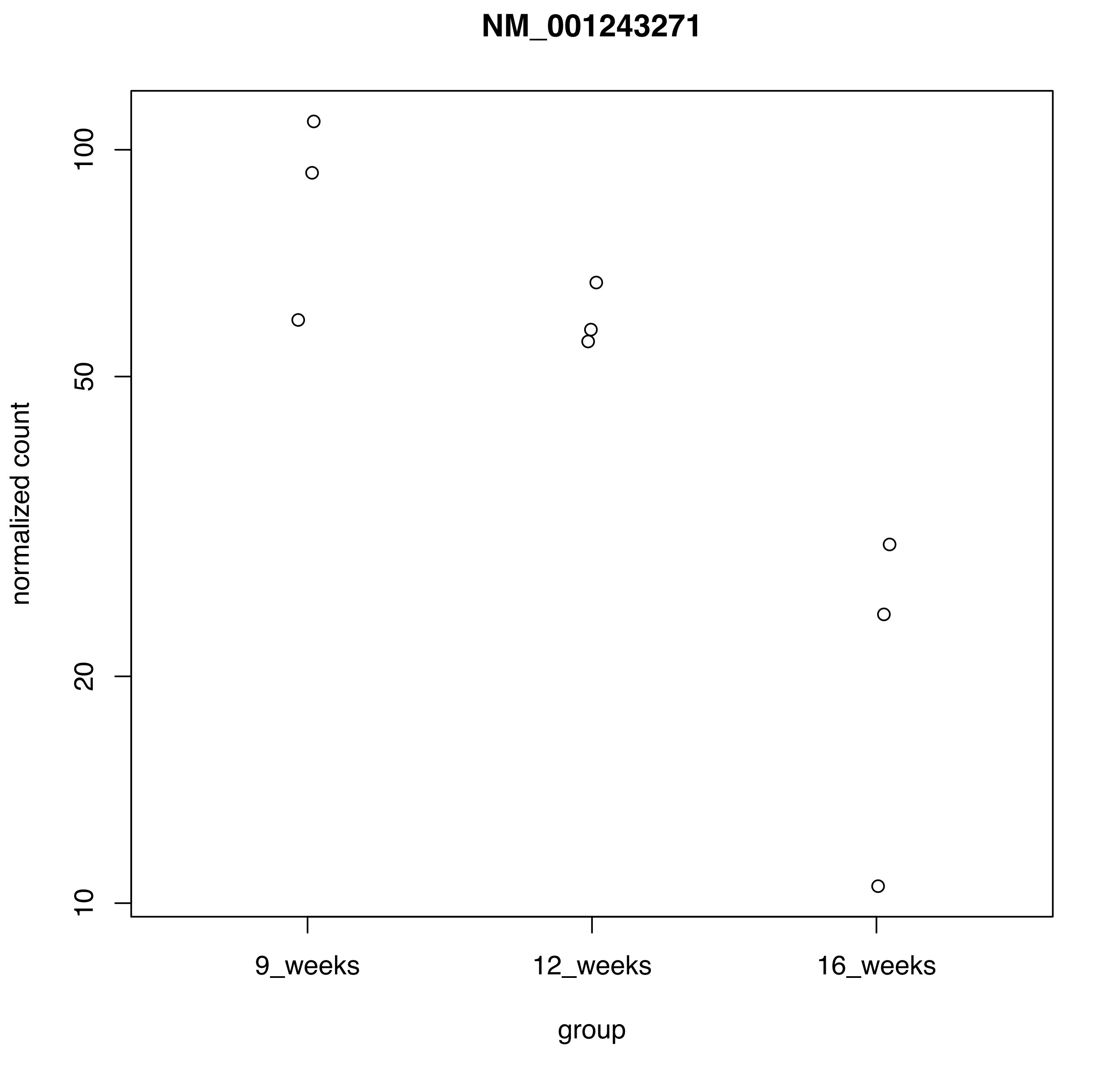

NM_001243271
normalized count
100
50
20
10
9_weeks
12_weeks
16_weeks
group

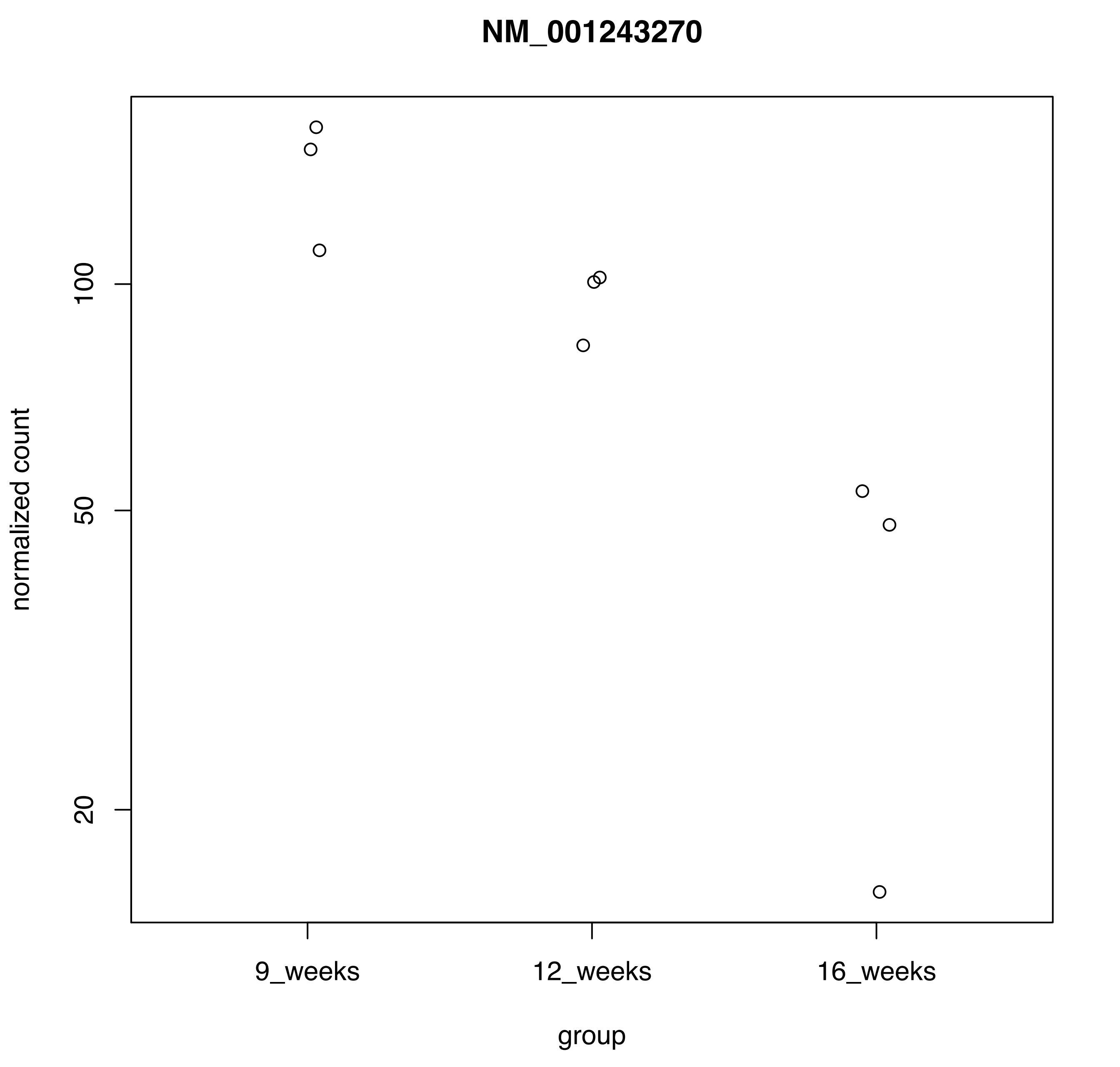

NM_001243270
normalized count
100
50
20
9_weeks
12_weeks
16_weeks
group

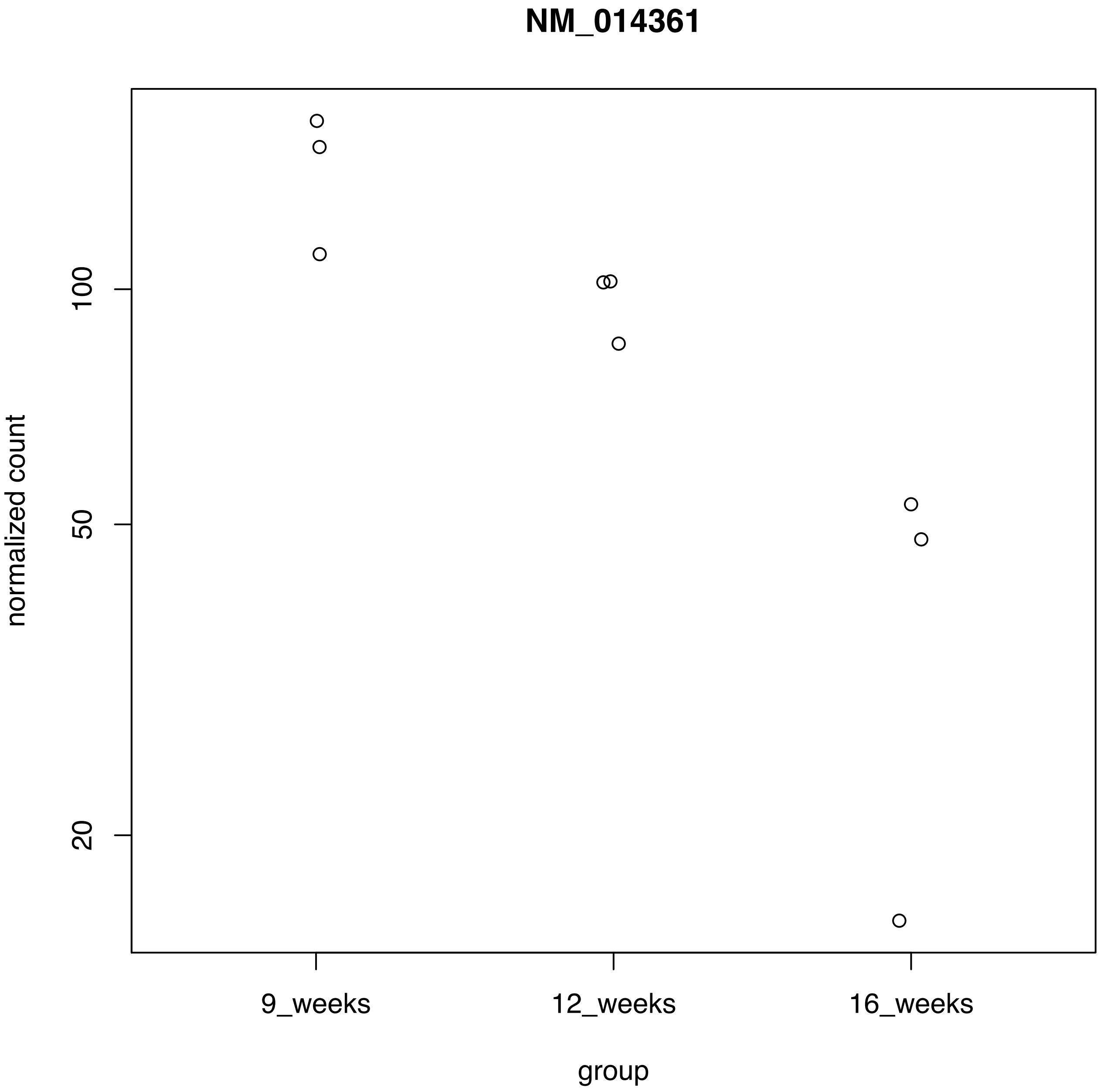

NM_014361
normalized count
100
50
20
9_weeks
12_weeks
16_weeks
group

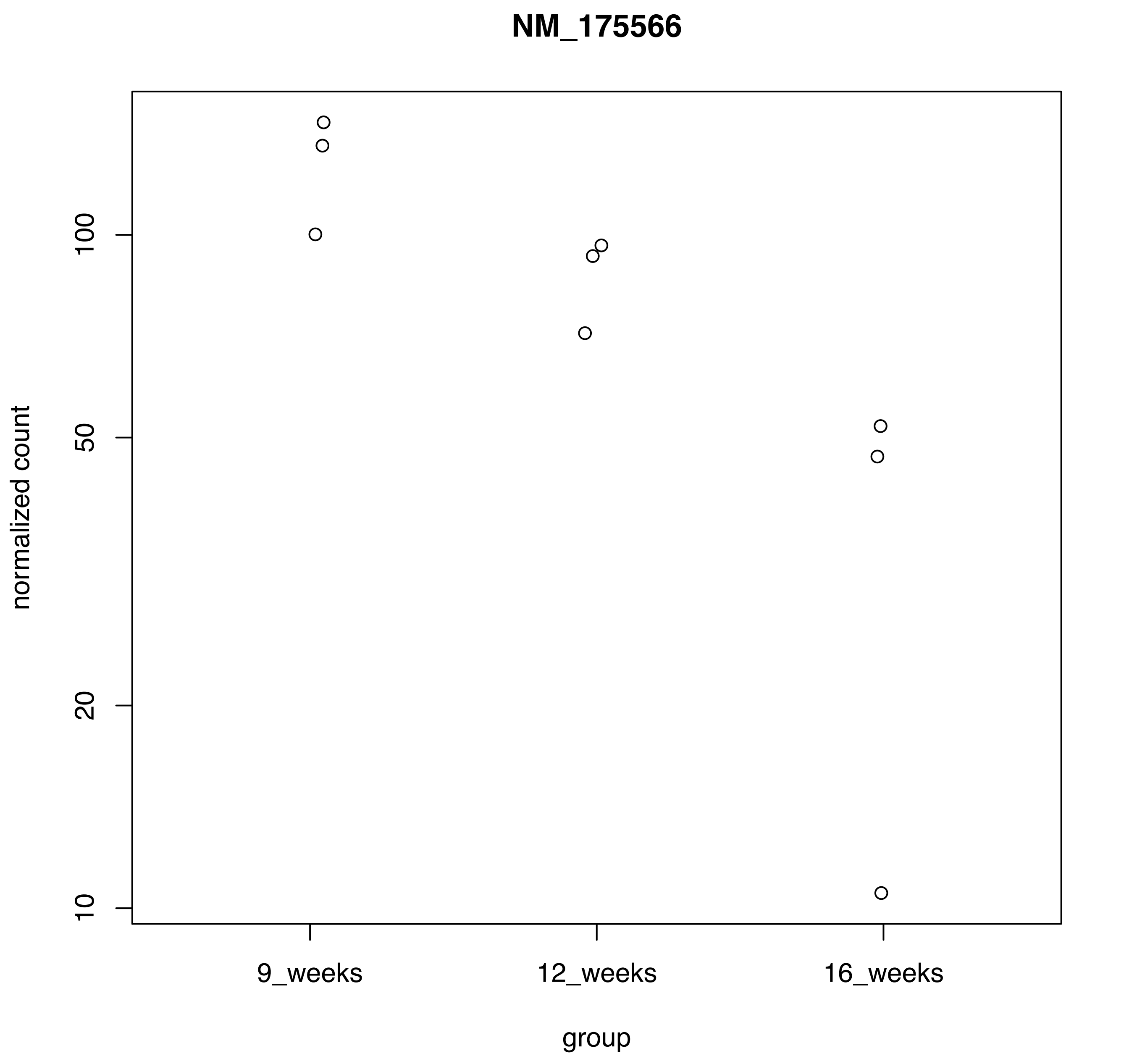

NM_175566
normalized count
100
50
20
10
9_weeks
12_weeks
16_weeks
group

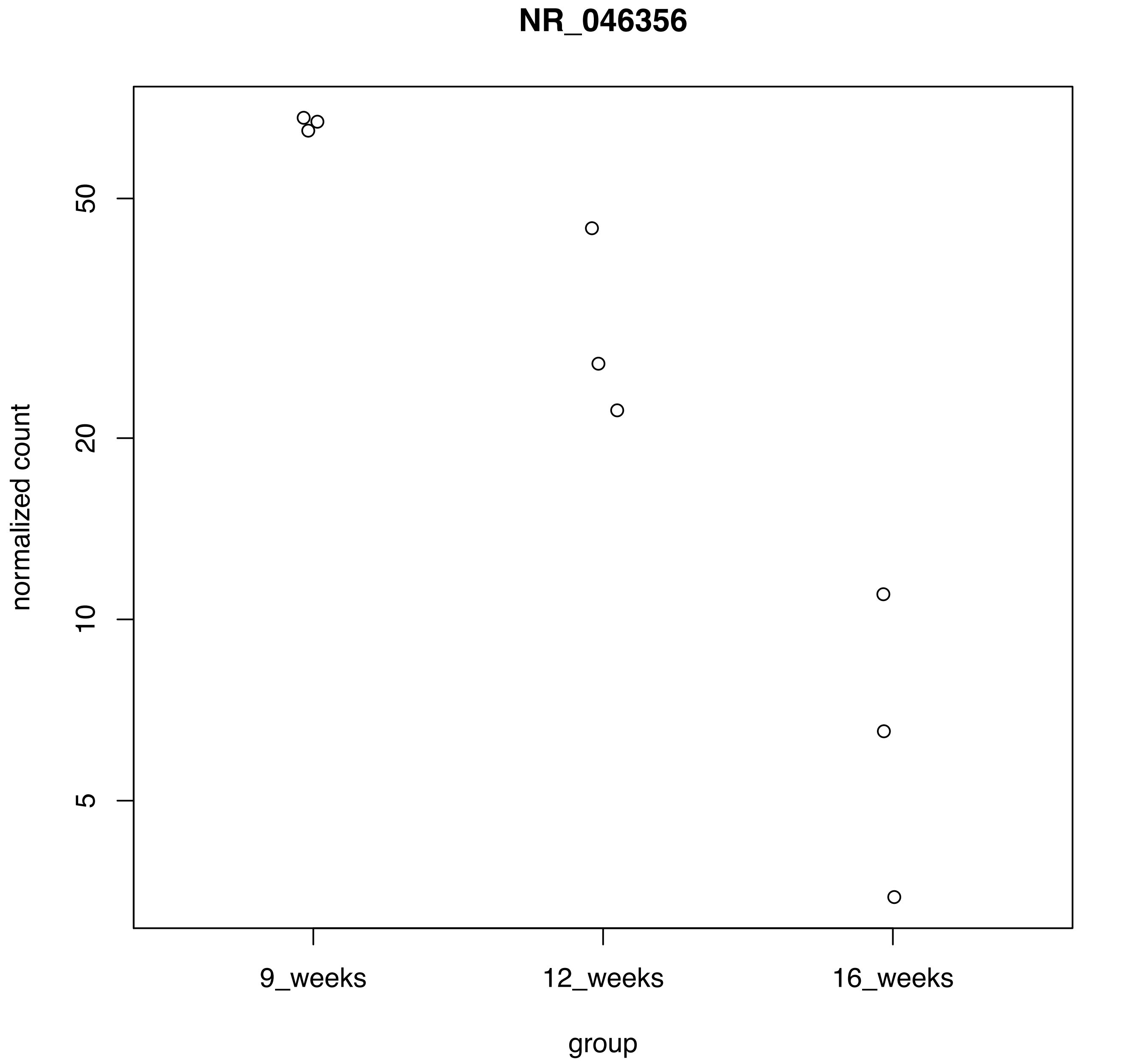

NR_046356
normalized count
50
20
10
5
9_weeks
12_weeks
16_weeks
group

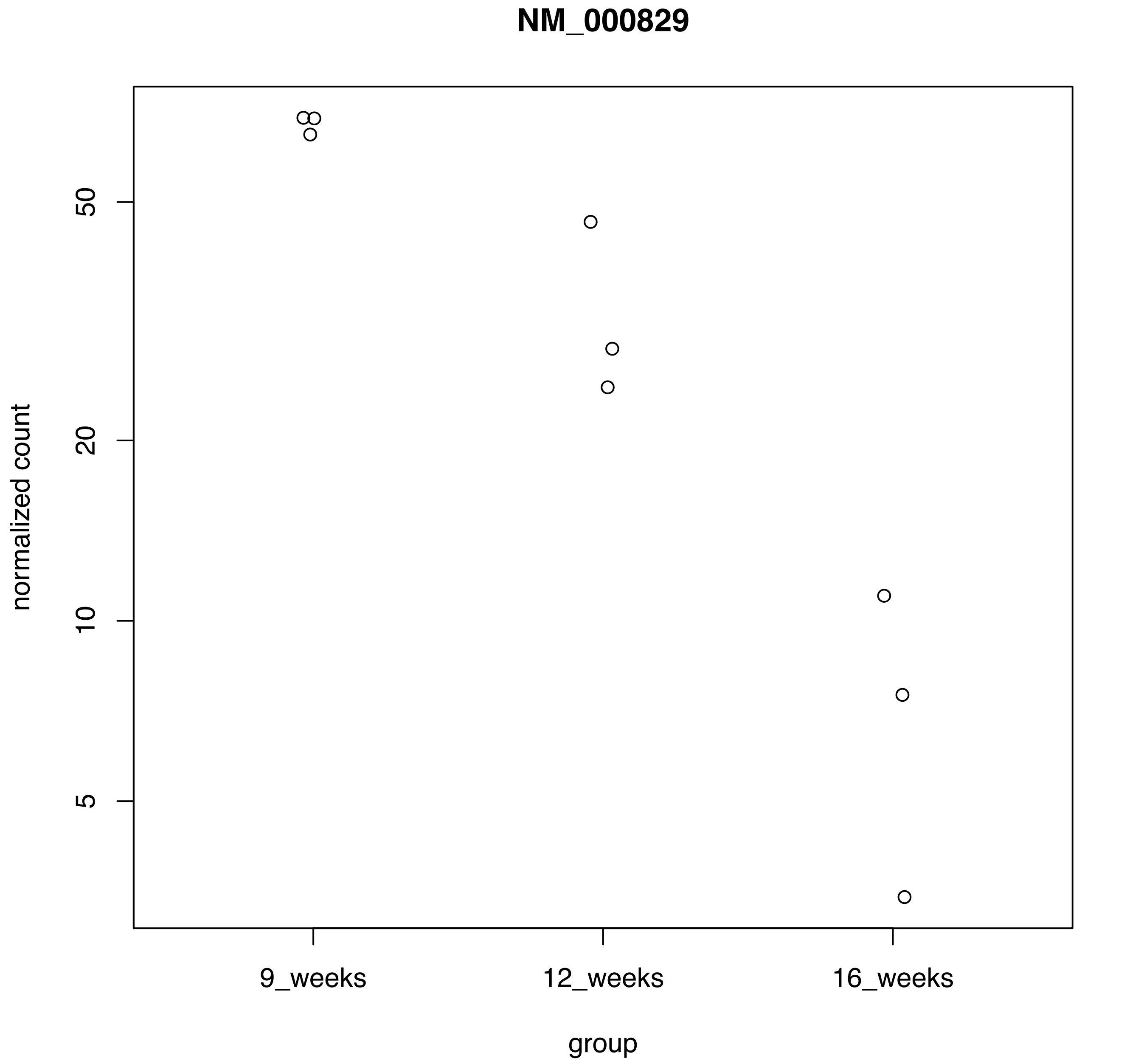

NM_000829
normalized count
50
20
10
5
9_weeks
12_weeks
16_weeks
group

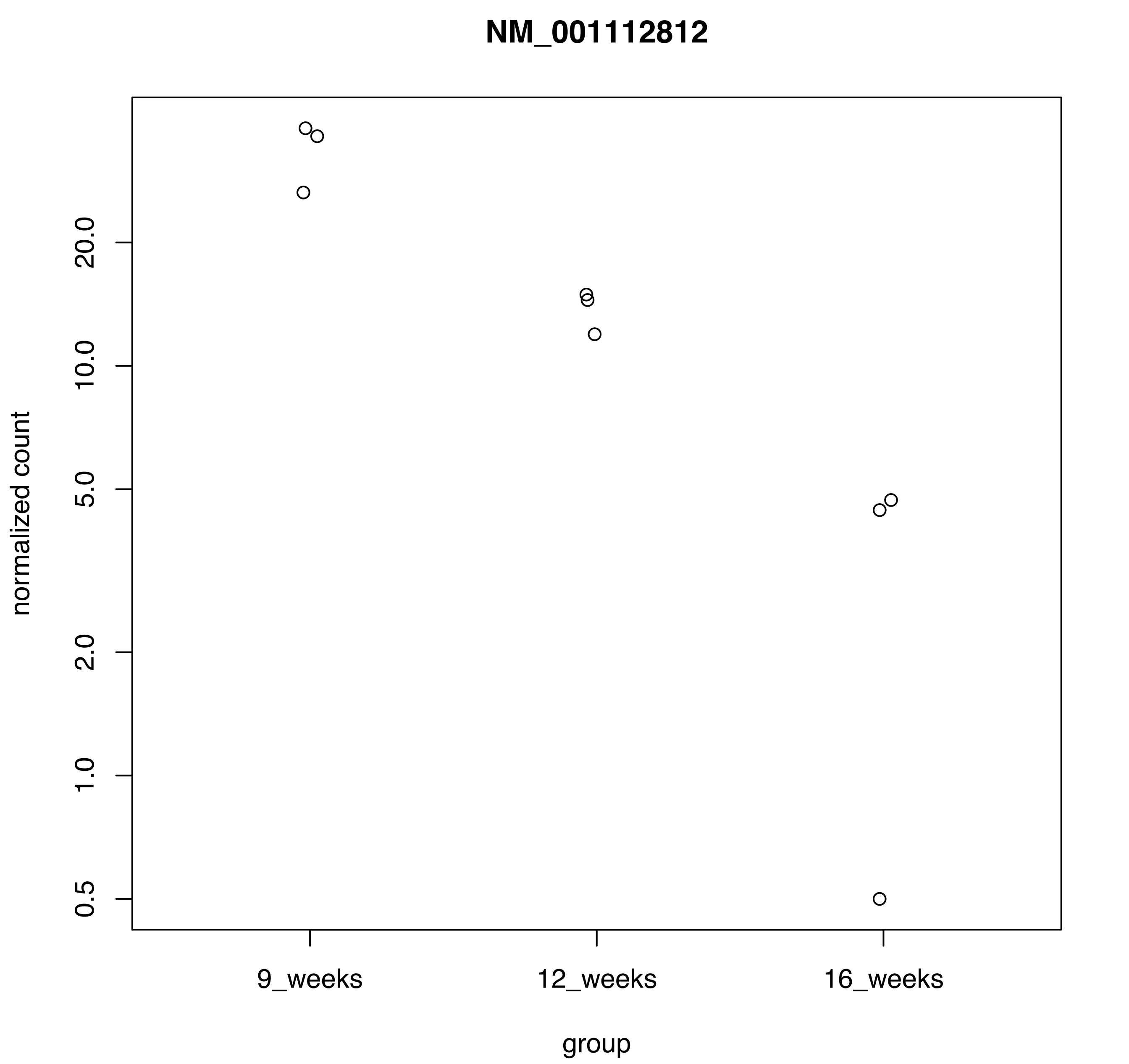
NM_001112812
normalized count
20.0
10.0
5.0
2.0
1.0
0.5
9_weeks
12_weeks
16_weeks
group

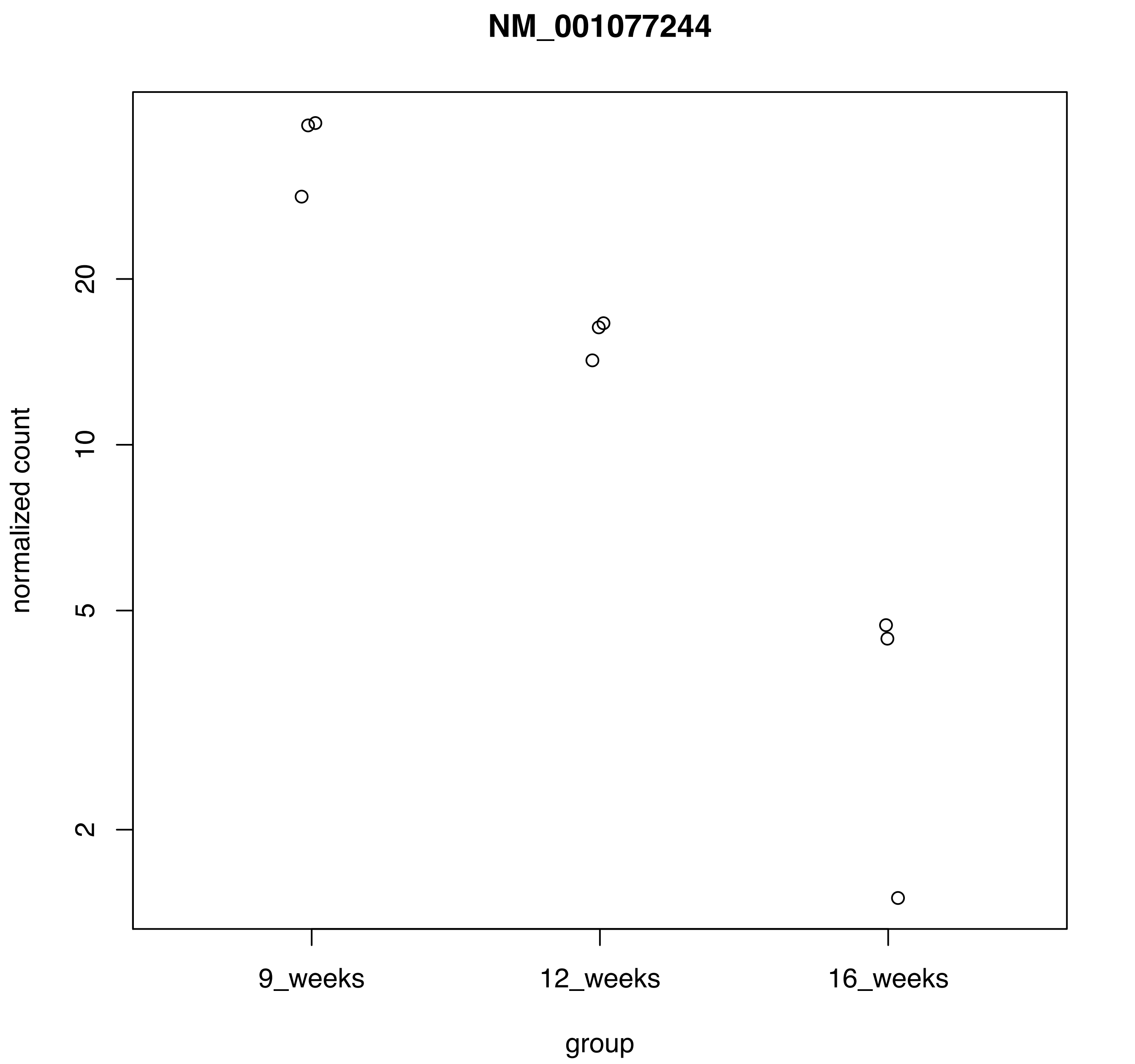
NM_001077244
normalized count
20
10
5
2
9_weeks
12_weeks
16_weeks
group

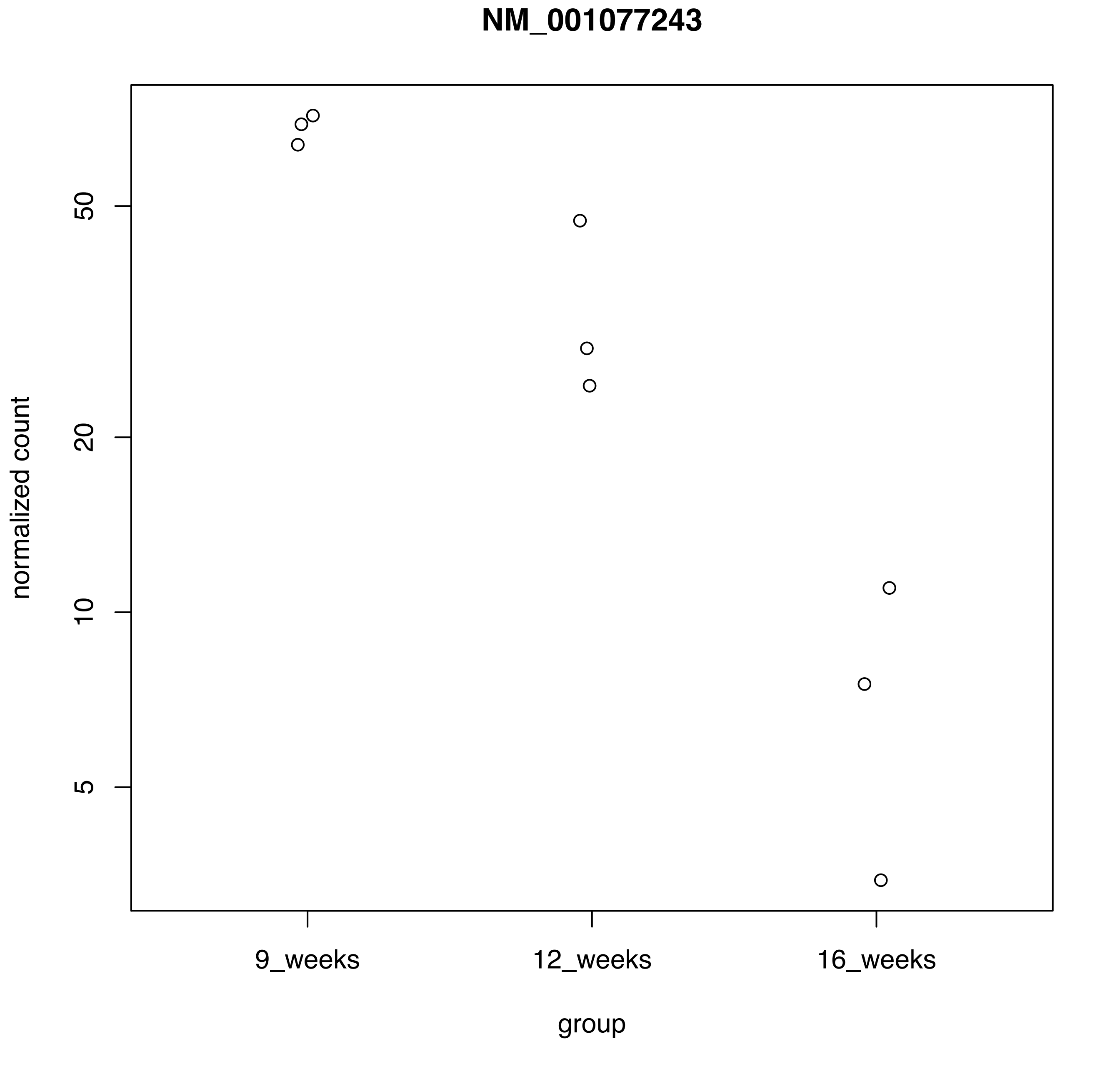
NM_001077243
normalized count
50
20
10
5
9_weeks
12_weeks
16_weeks
group

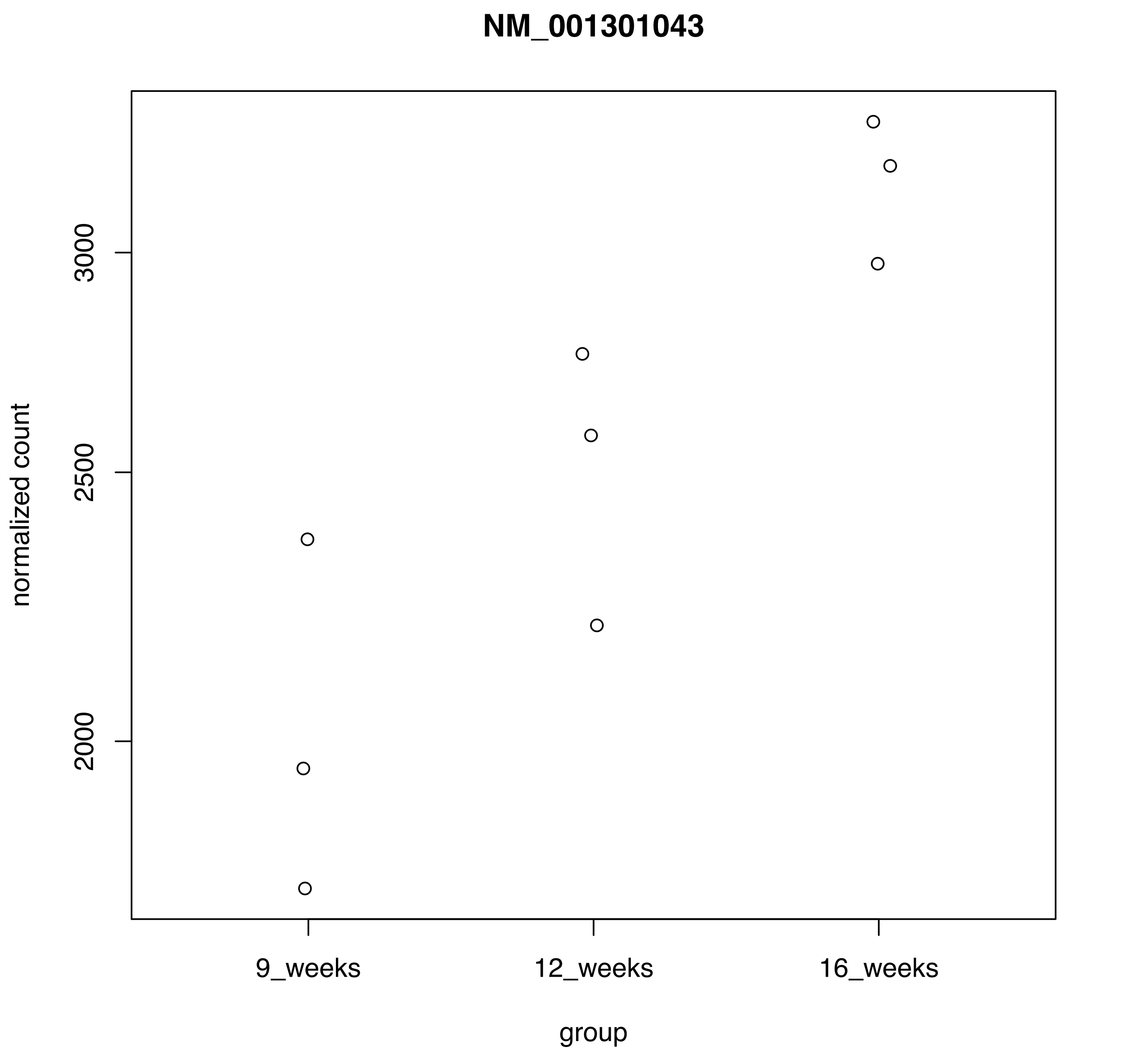
NM_001301043
normalized count
3000
2500
2000
9_weeks
12_weeks
16_weeks
group

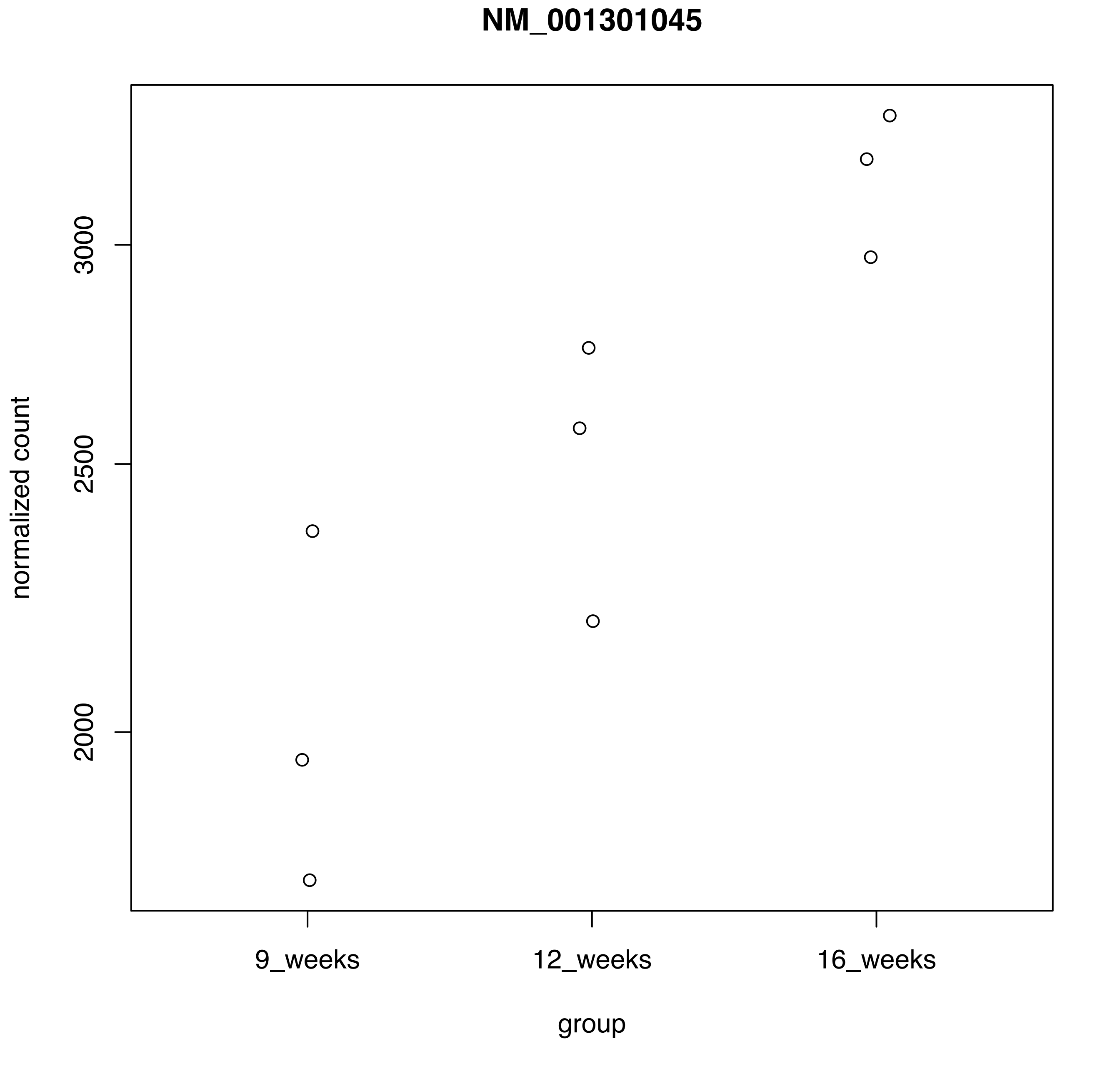

NM_001301045
normalized count
3000
2500
2000
9_weeks
12_weeks
16_weeks
group

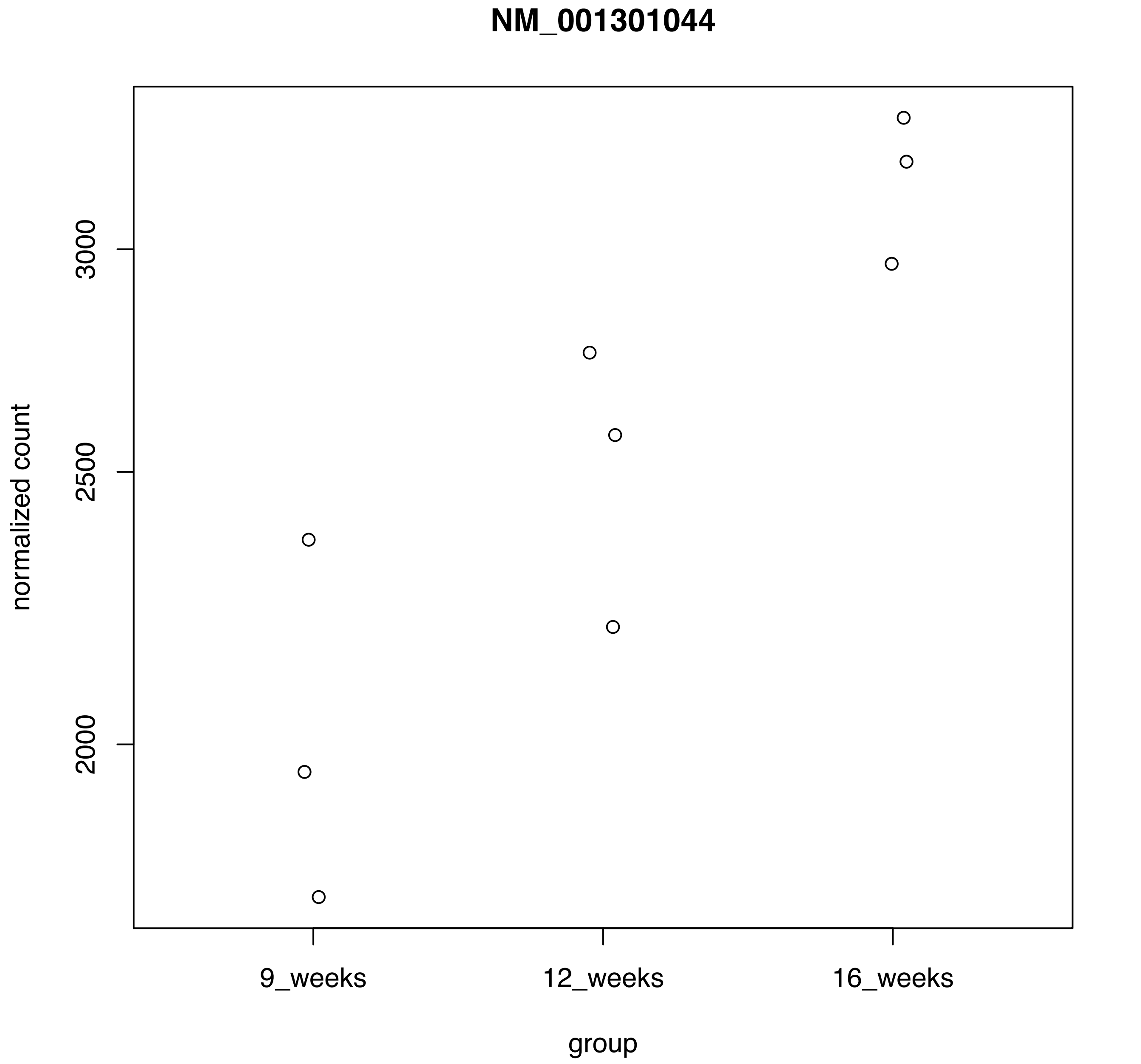
NM_001301044
normalized count
2000
2500
3000
9_weeks
12_weeks
16_weeks
group

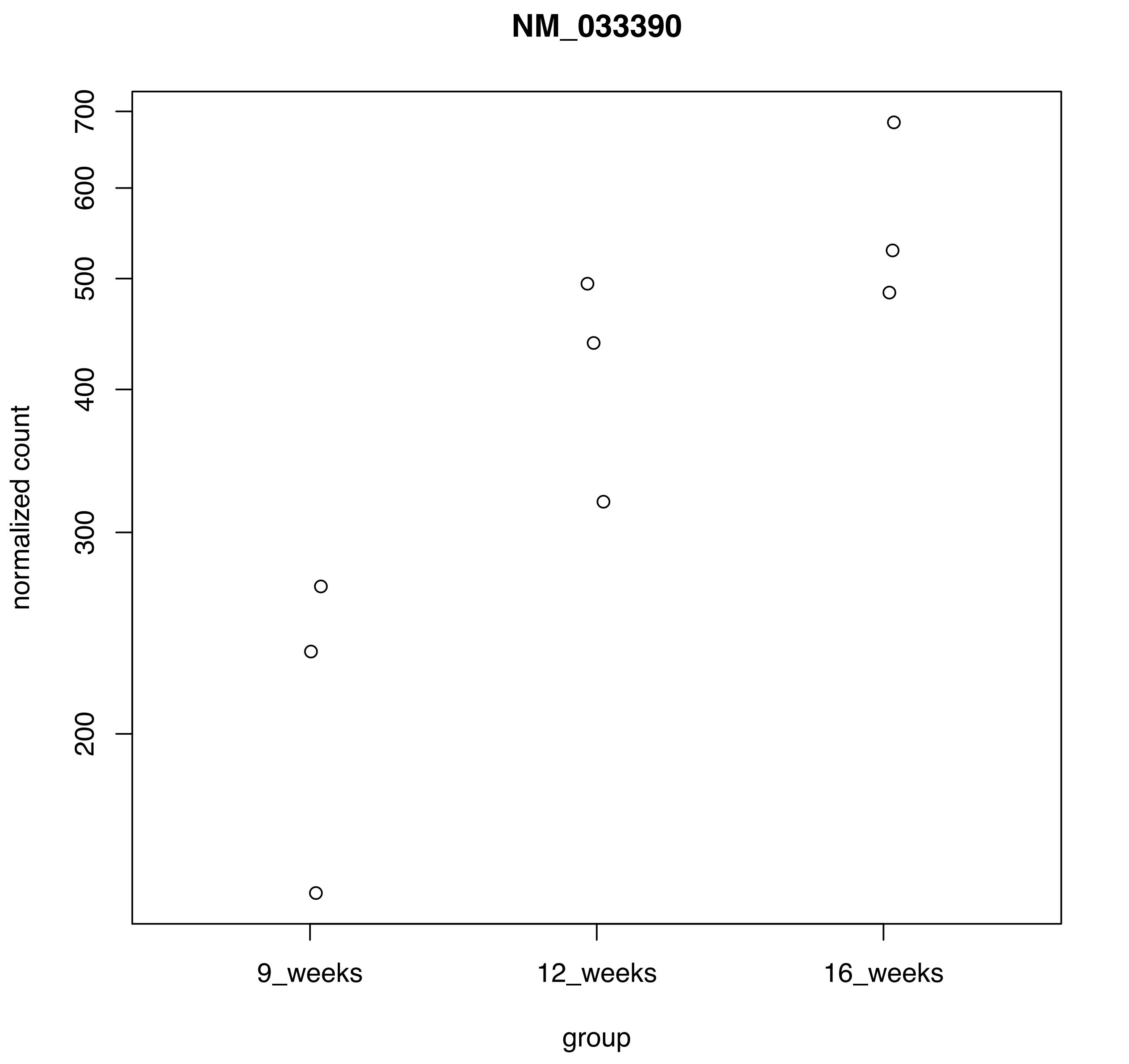
NM_033390
normalized count
700
600
500
400
300
200
9_weeks
12_weeks
16_weeks
group

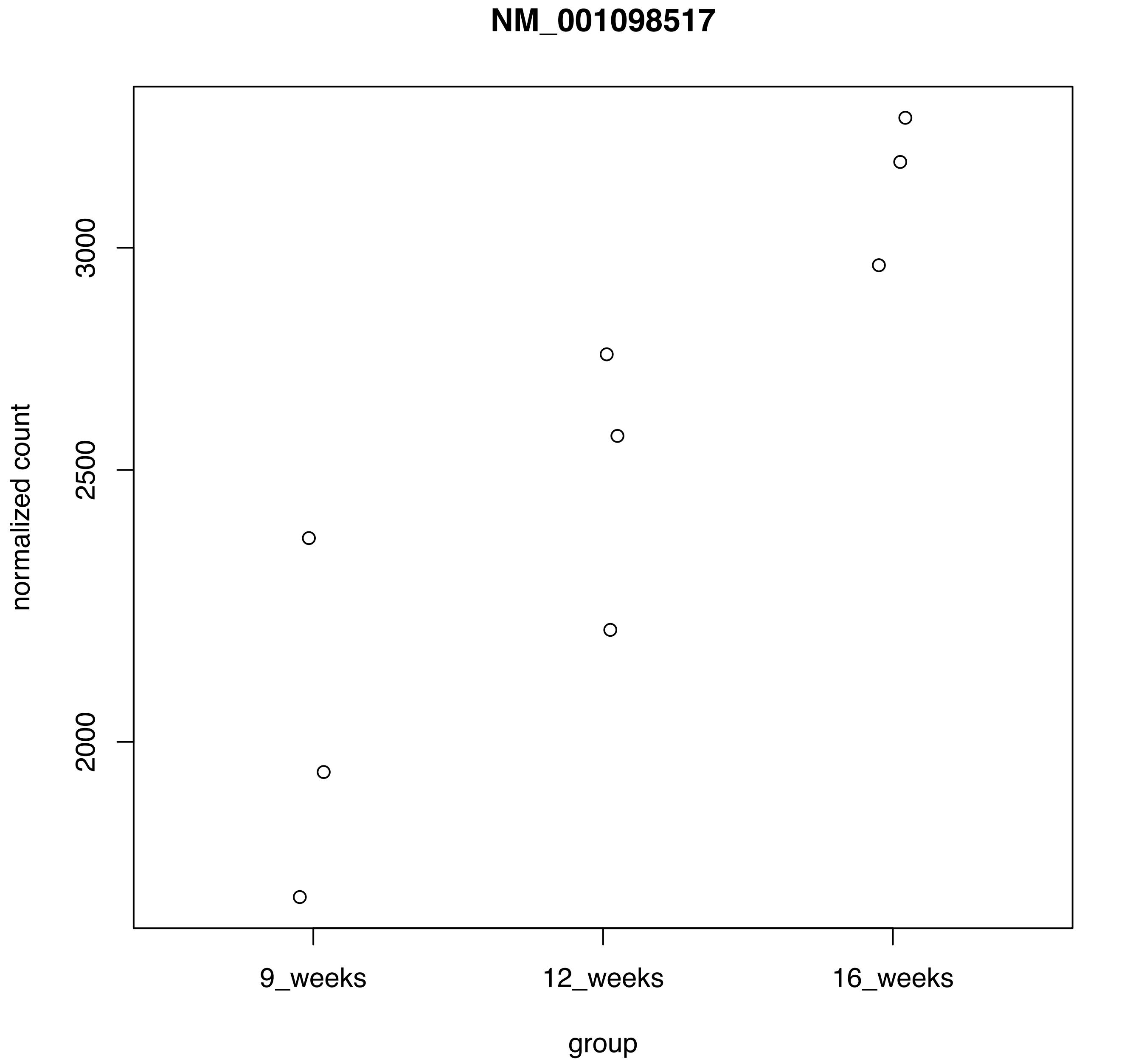

NM_001098517
normalized count
2000
2500
3000
9_weeks
12_weeks
16_weeks
group

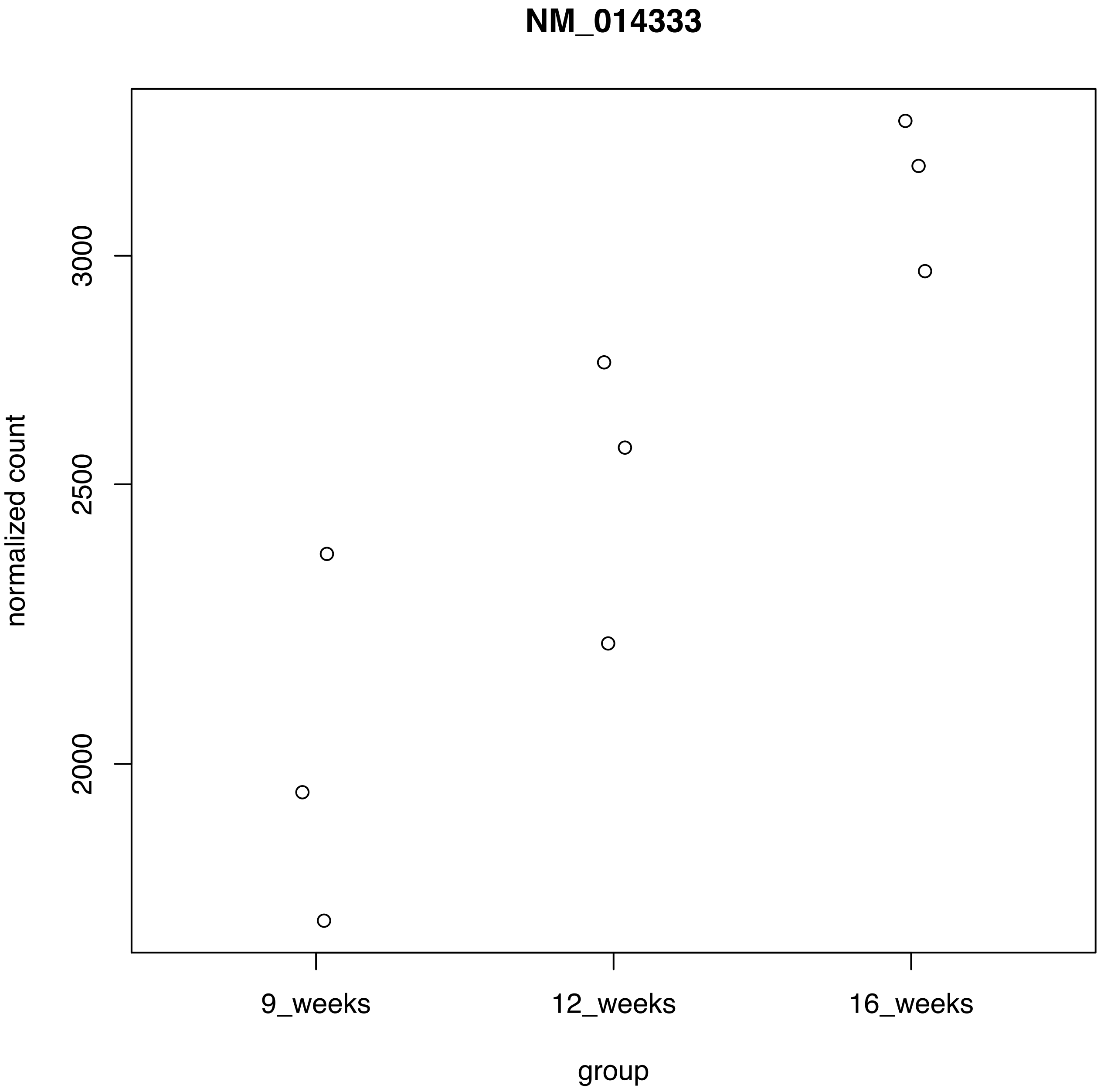
NM_014333
normalized count
2000
2500
3000
9_weeks
12_weeks
16_weeks
group

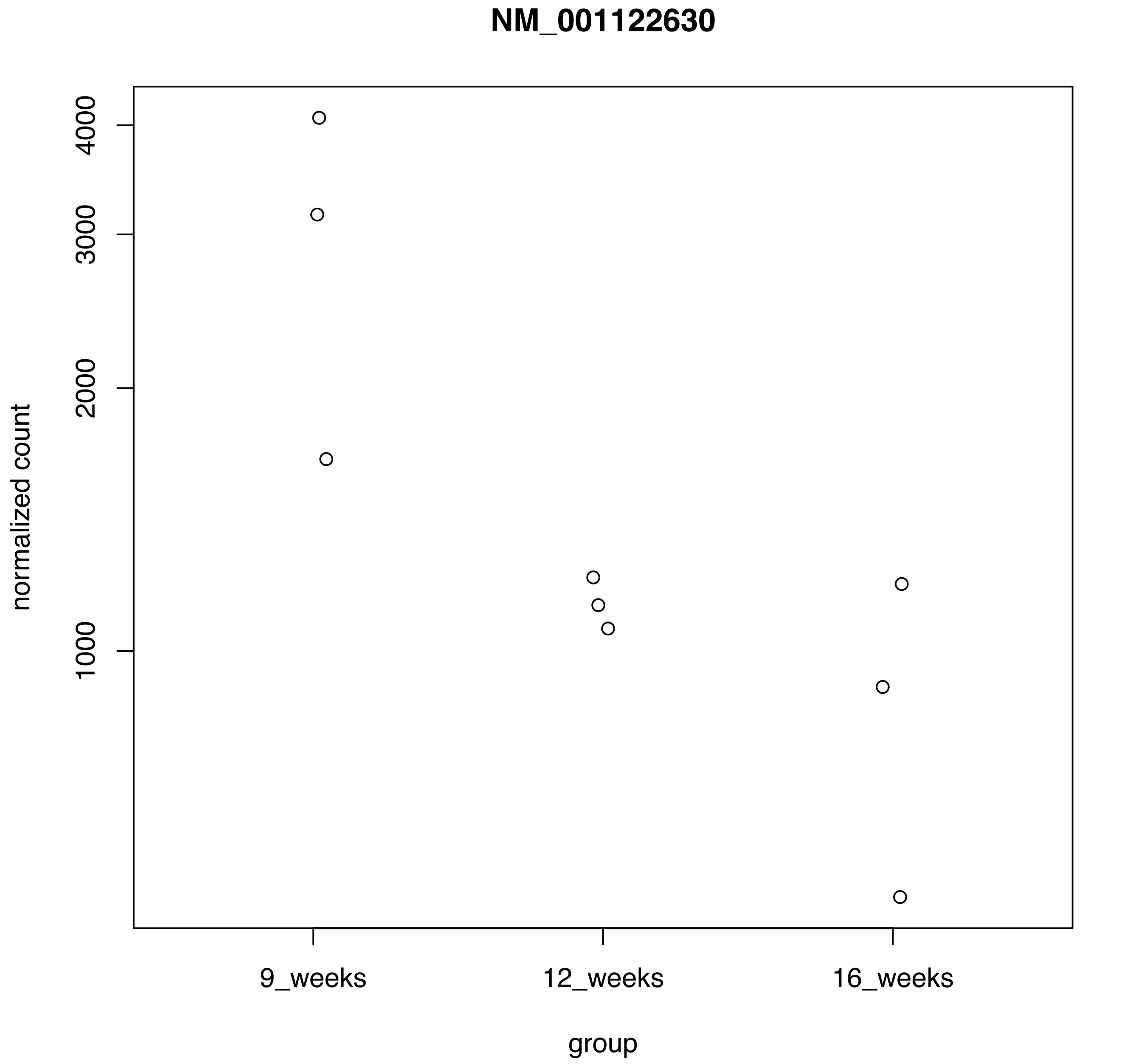
NM_001122630
normalized count
1000
2000
3000
4000
9_weeks
12_weeks
16_weeks
group

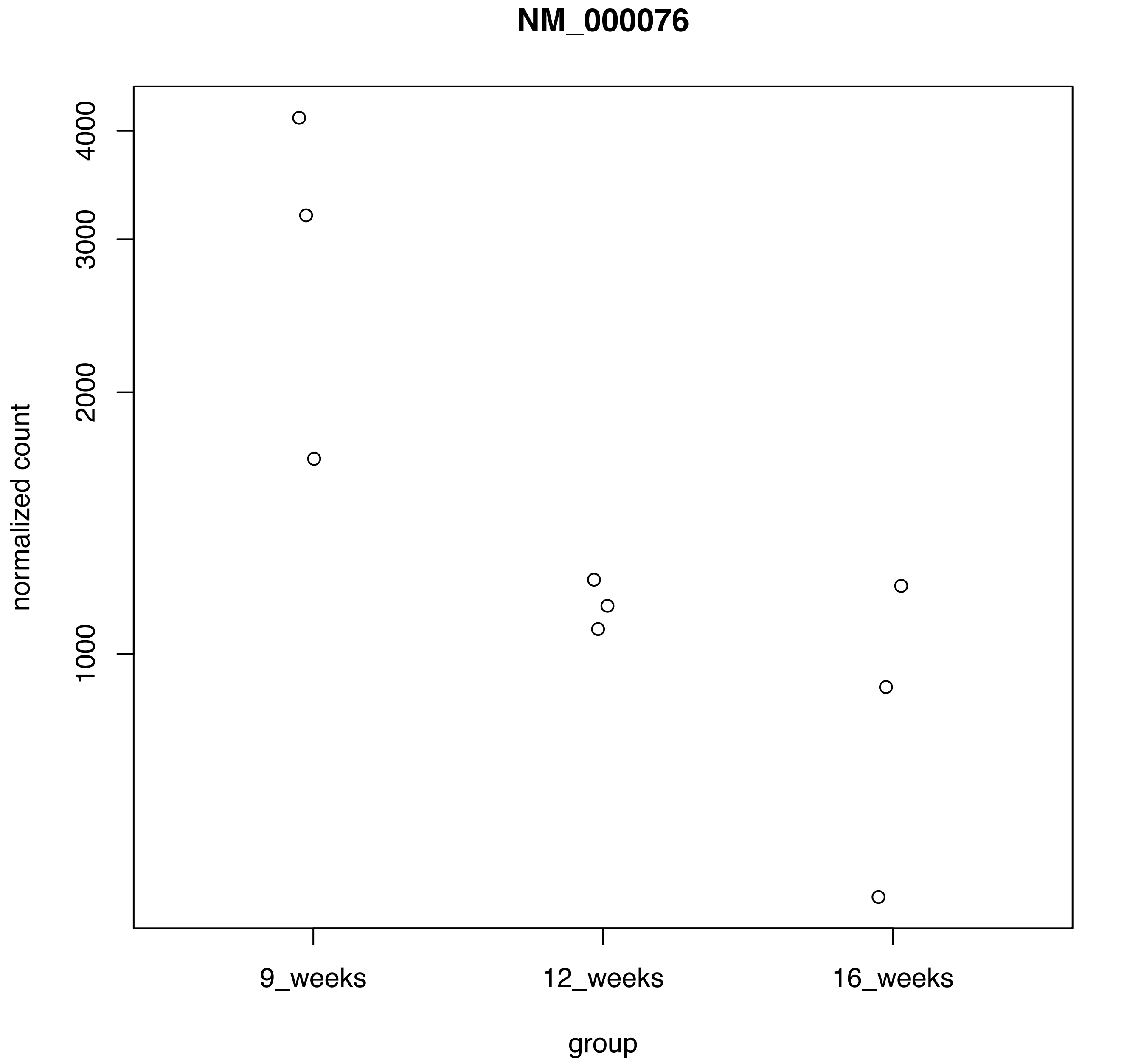

NM_000076
normalized count
4000
3000
2000
1000
9_weeks
12_weeks
16_weeks
group

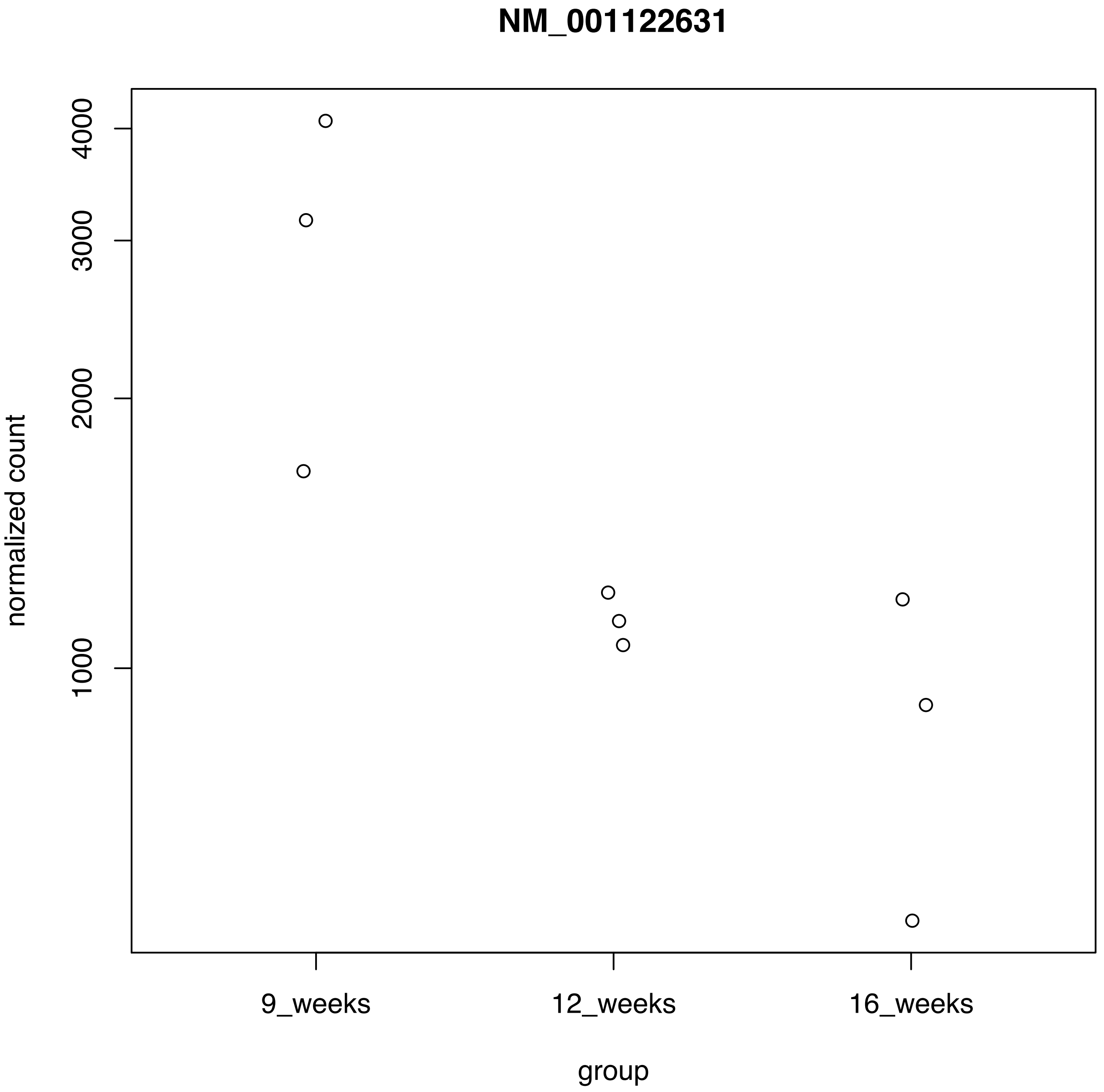
NM_001122631
normalized count
4000
3000
2000
1000
9_weeks
12_weeks
16_weeks
group

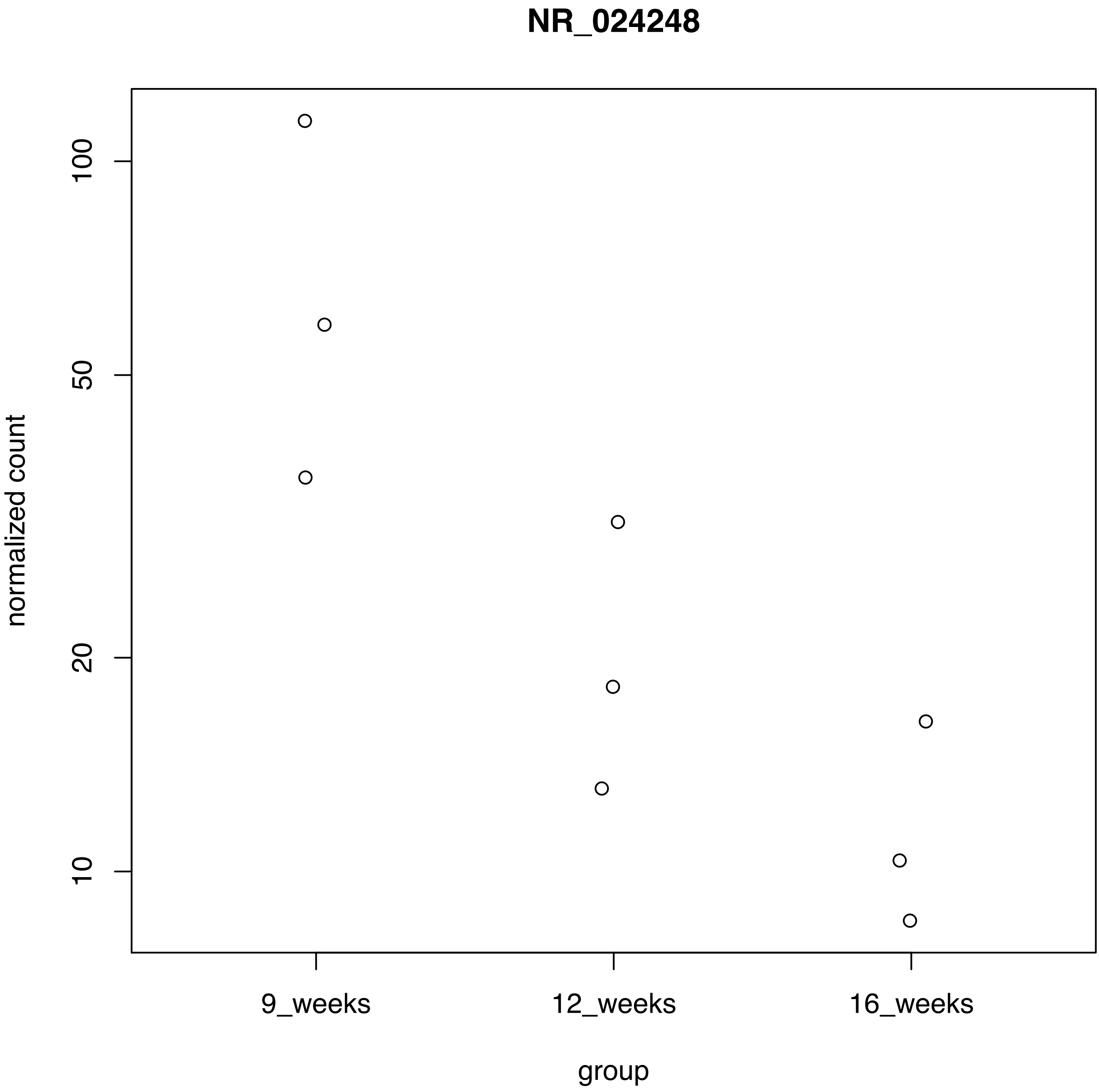
NR_024248
normalized count
100
50
20
10
9_weeks
12_weeks
16_weeks
group

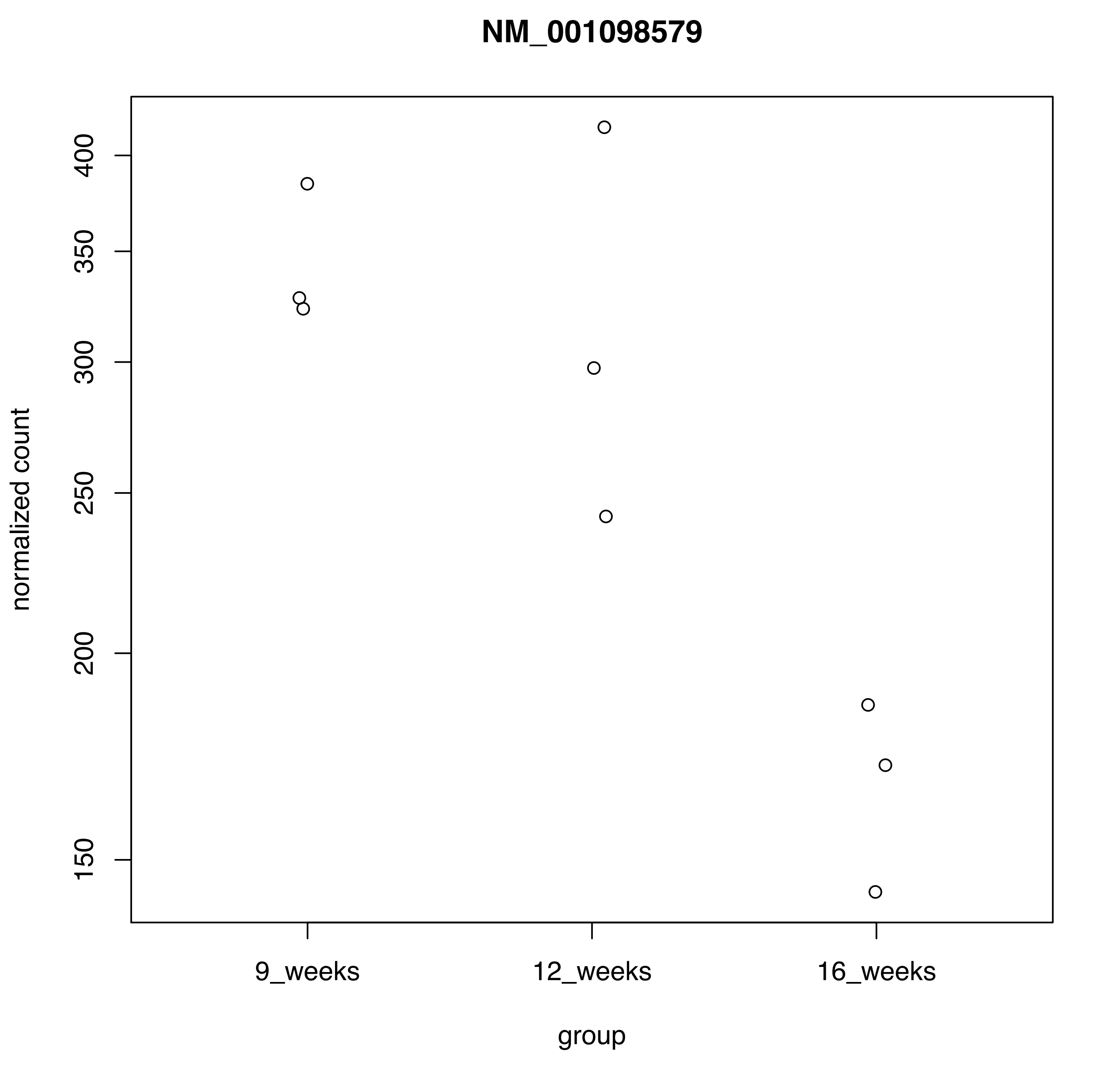

NM_001098579
normalized count
400
350
300
250
200
150
9_weeks
12_weeks
16_weeks
group

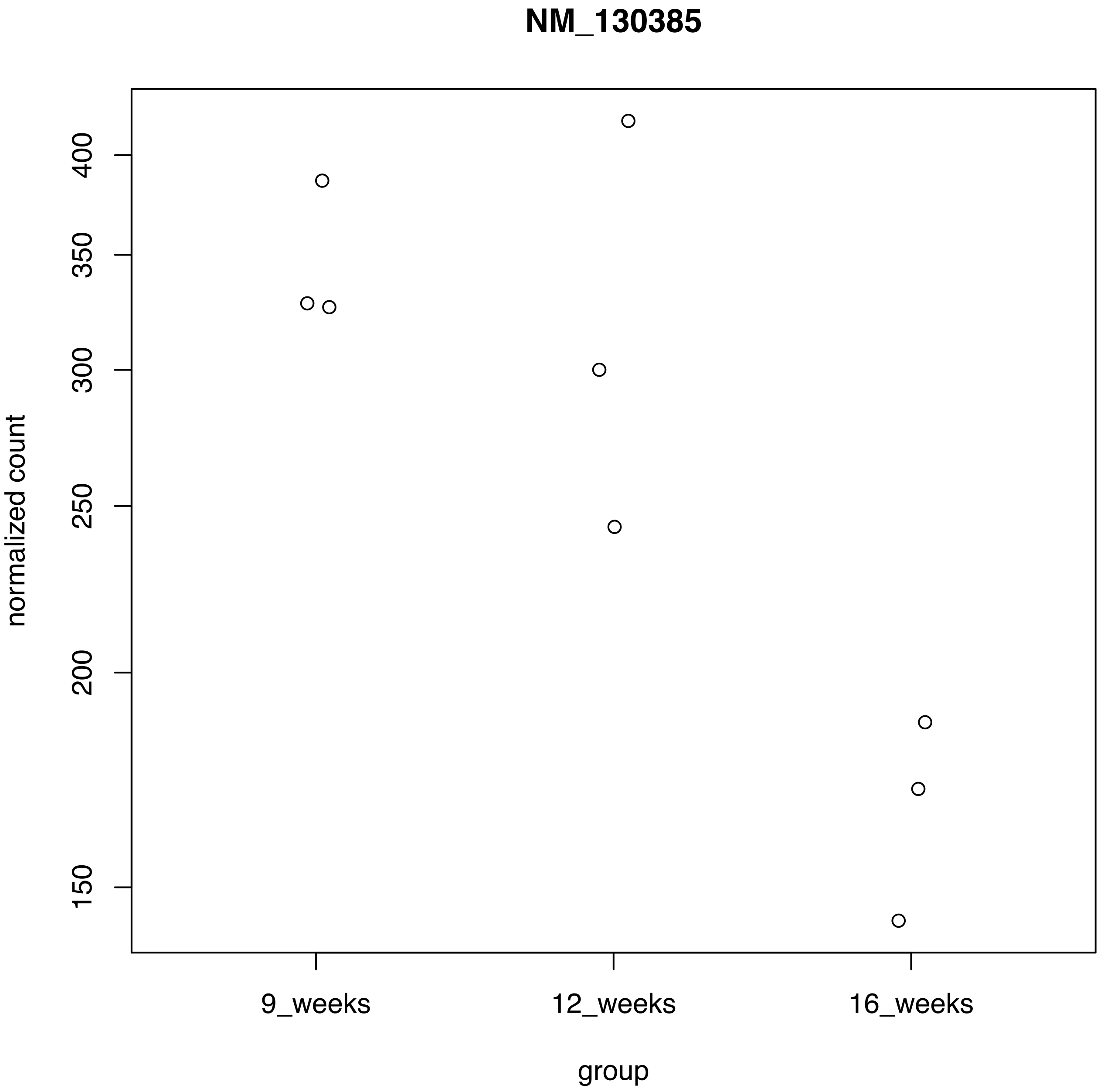

NM_130385
normalized count
400
350
300
250
200
150
9_weeks
12_weeks
16_weeks
group

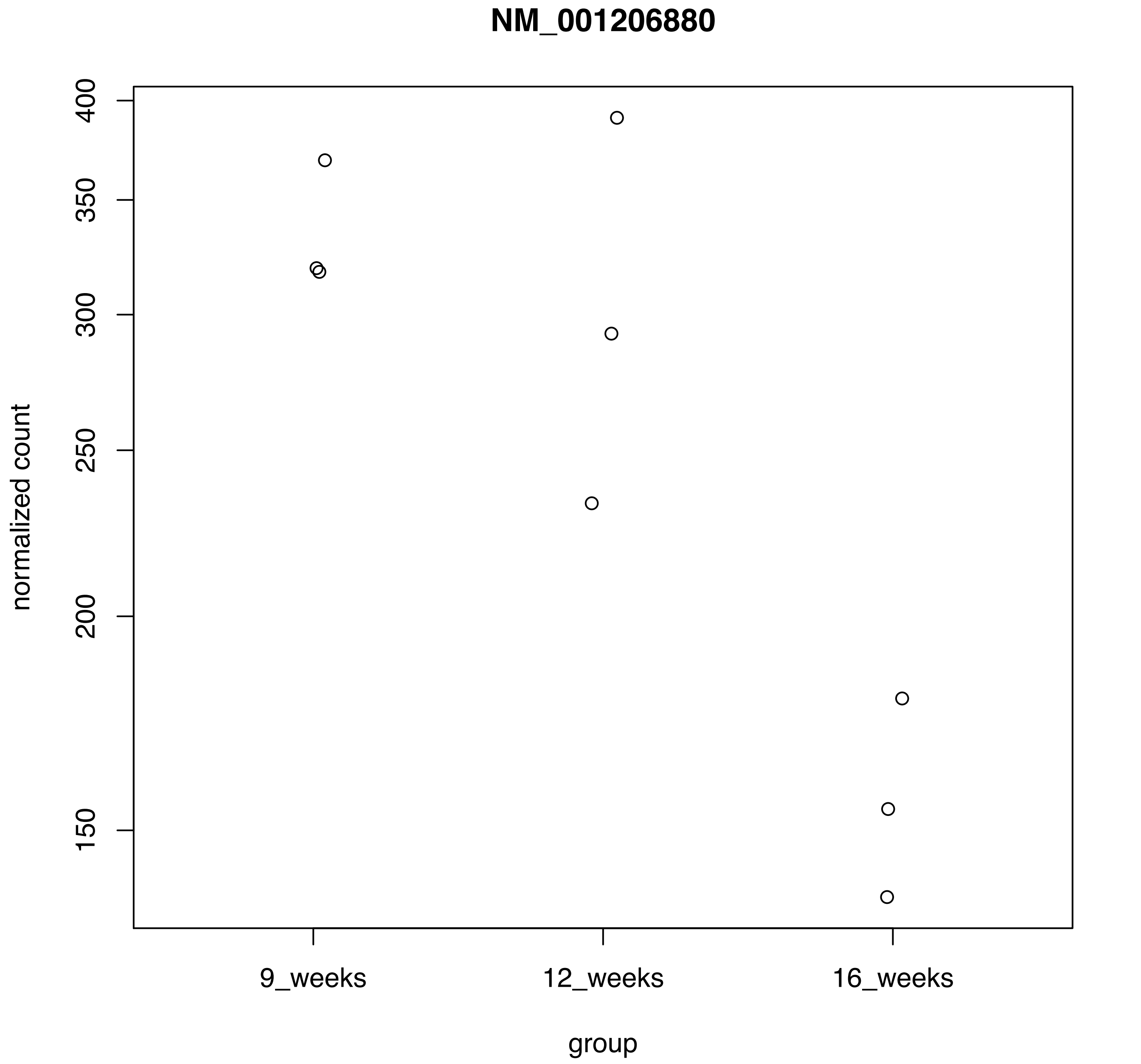

NM_001206880
400
350
300
250
200
150
normalized count
9_weeks
12_weeks
16_weeks
group

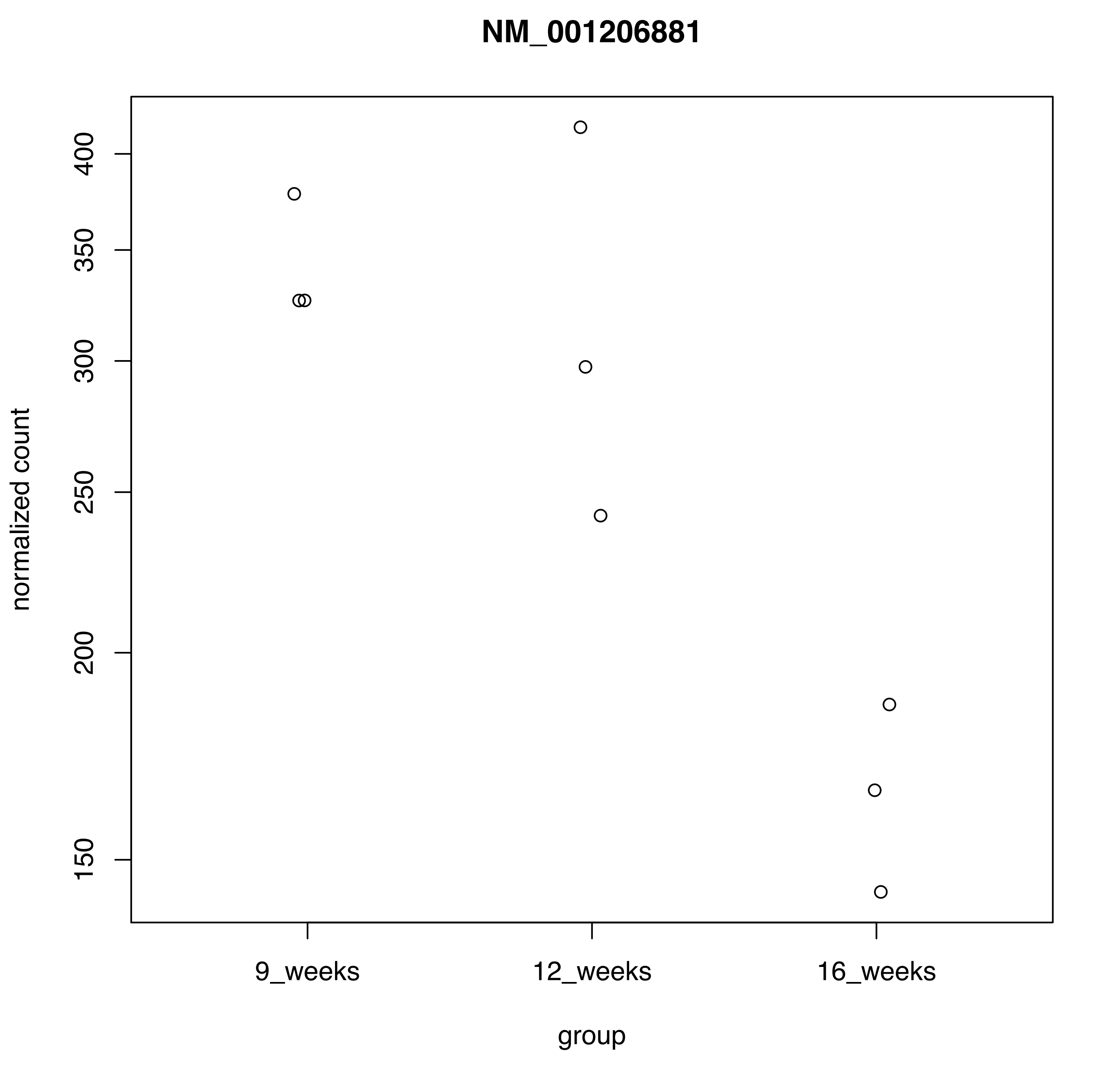
NM_001206881
normalized count
400
350
300
250
200
150
9_weeks
12_weeks
16_weeks
group

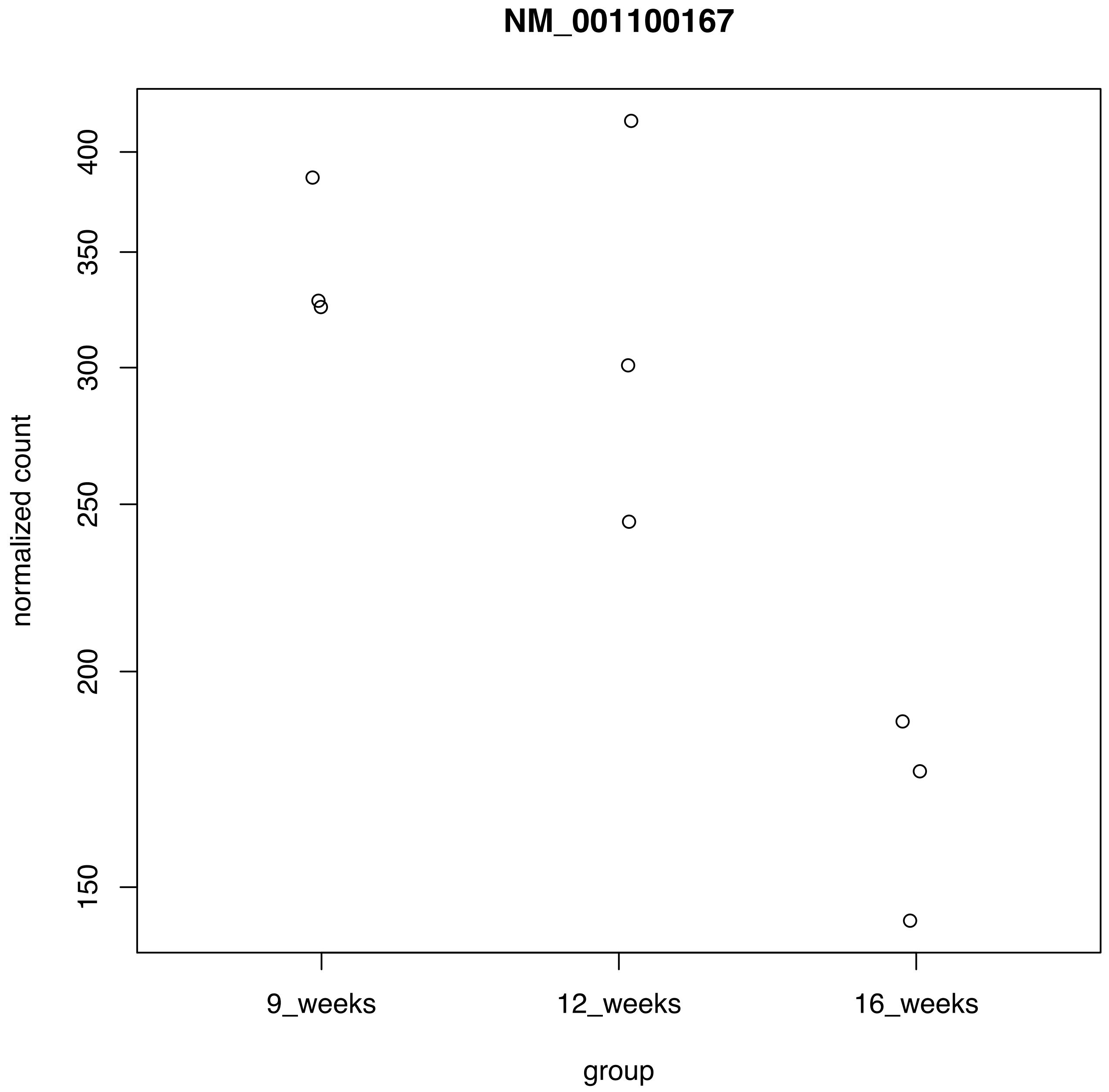
NM_001100167
normalized count
400
350
300
250
200
150
9_weeks
12_weeks
16_weeks
group

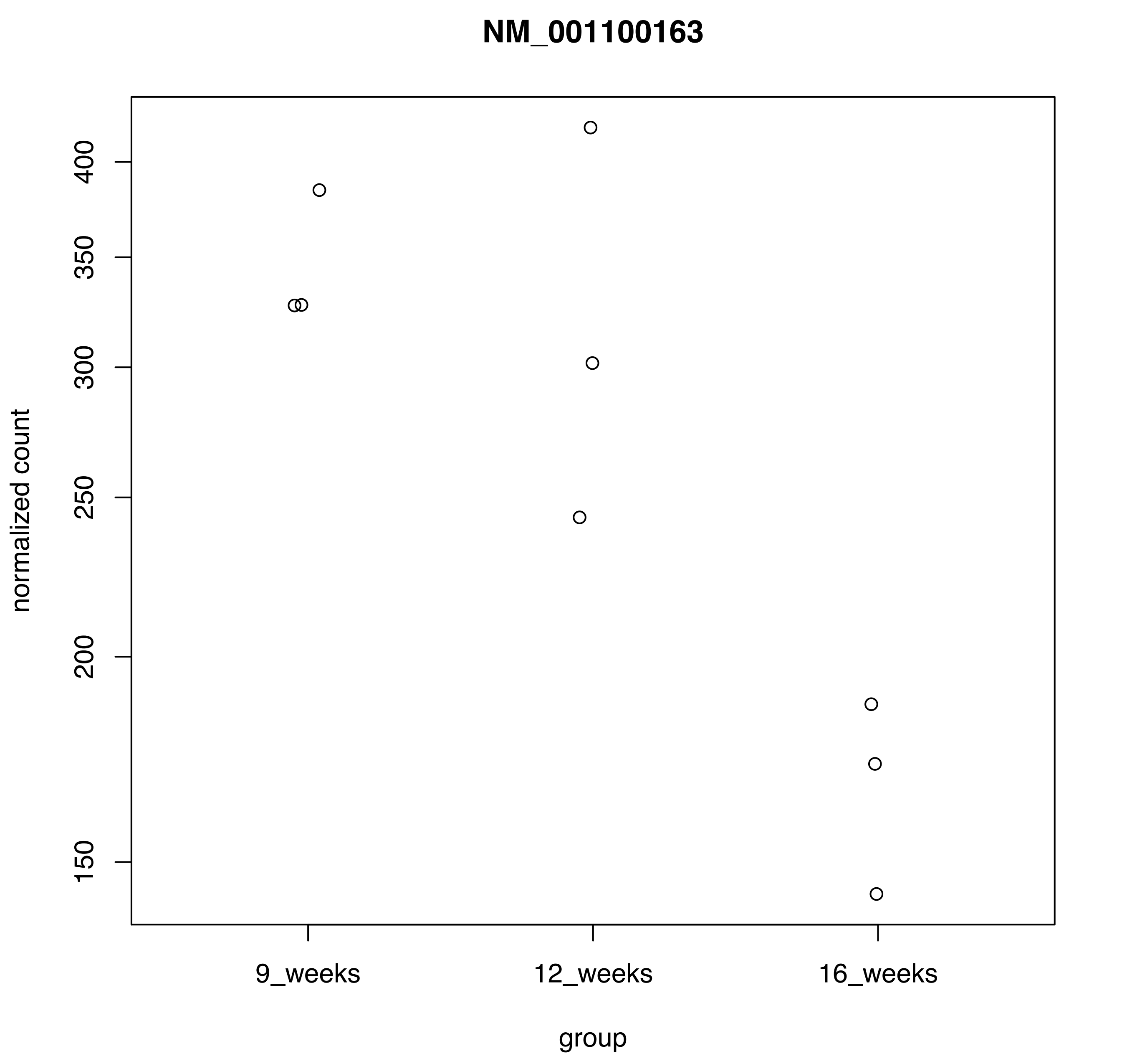
NM_001100163
normalized count
400
350
300
250
200
150
9_weeks
12_weeks
16_weeks
group

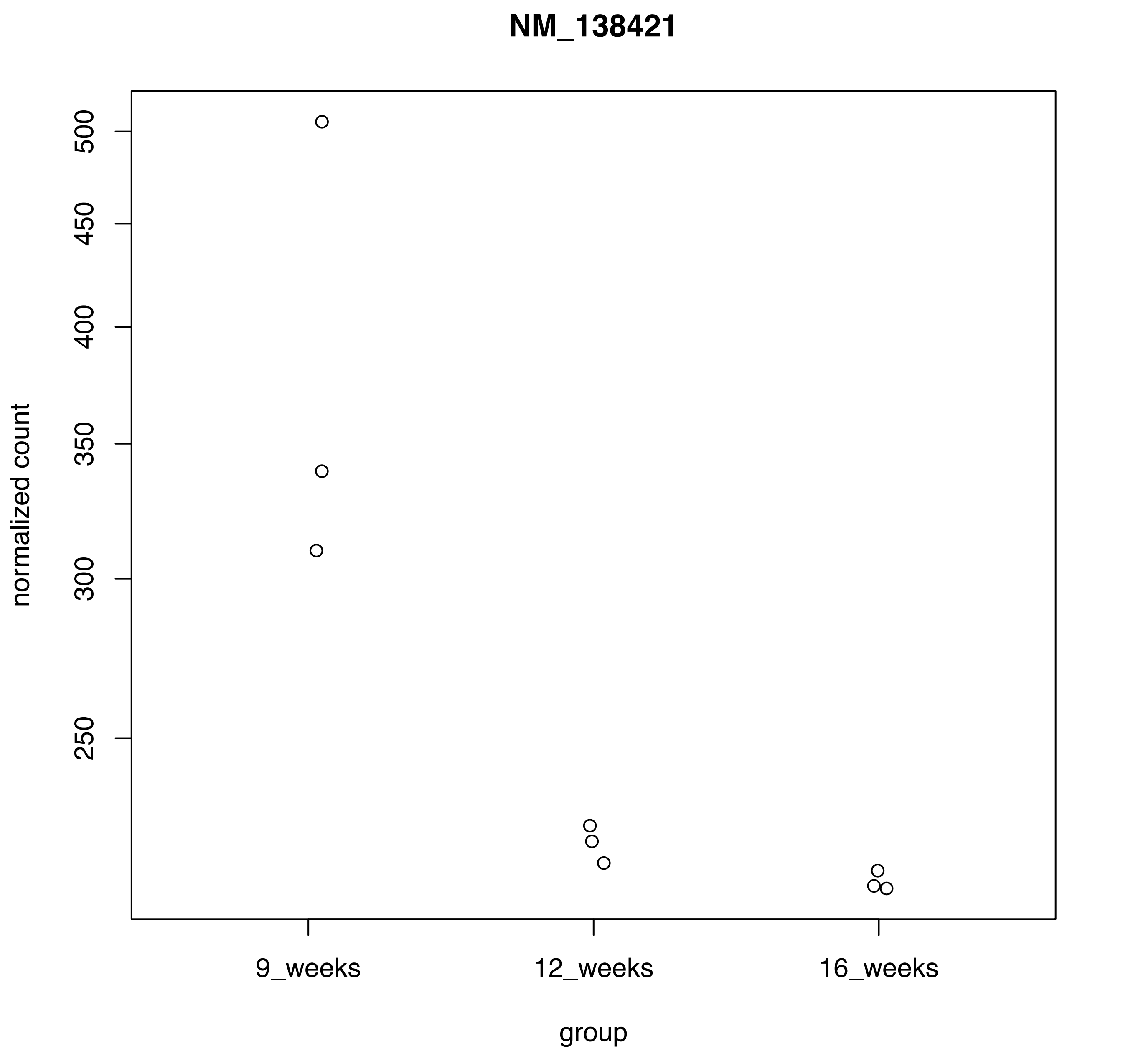
NM_138421
normalized count
500
450
400
350
300
250
9_weeks
12_weeks
16_weeks
group

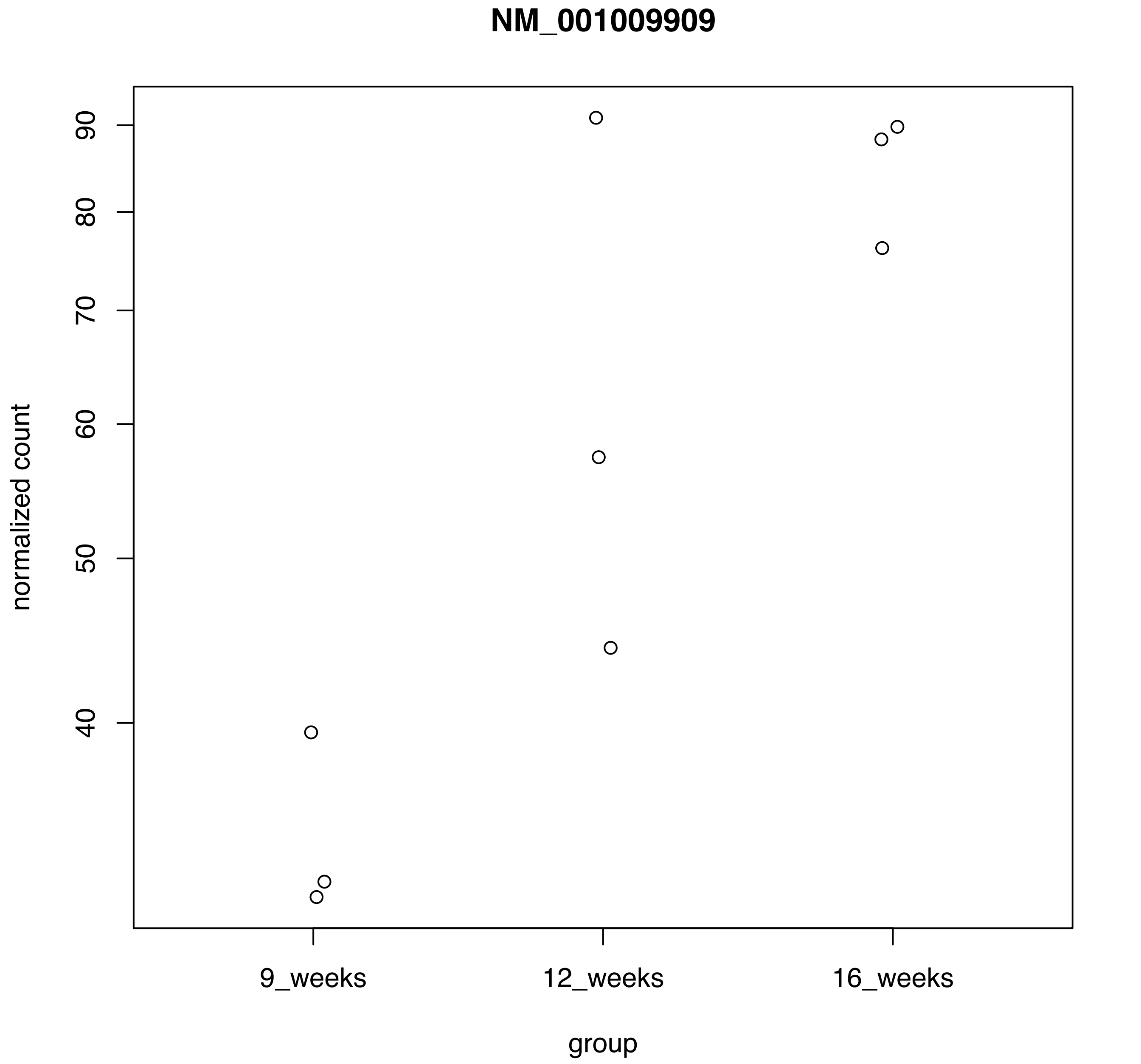
NM_001009909
normalized count
90
80
70
60
50
40
9_weeks
12_weeks
16_weeks
group

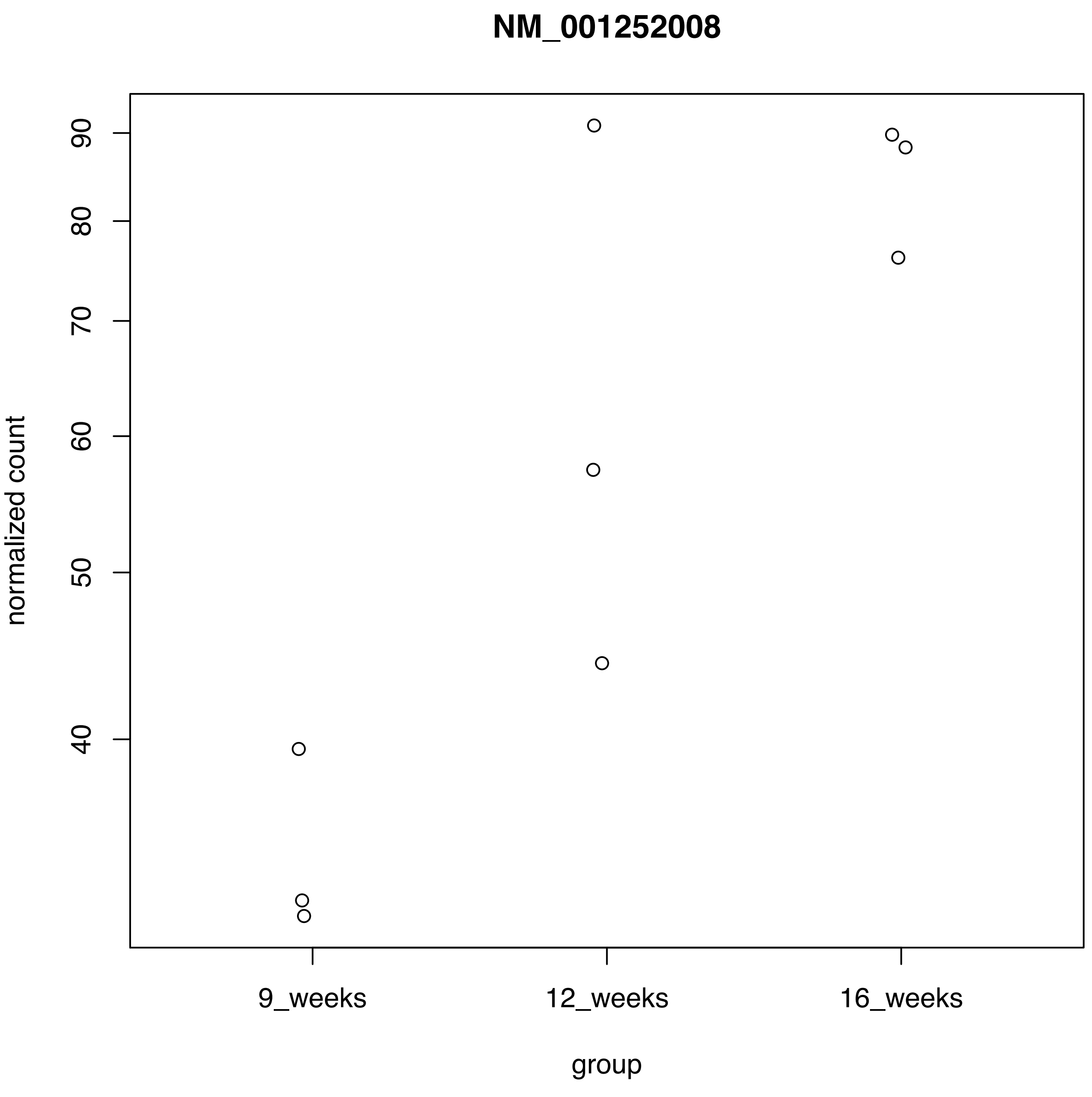
NM_001252008
normalized count
90
80
70
60
50
40
9_weeks
12_weeks
16_weeks
group

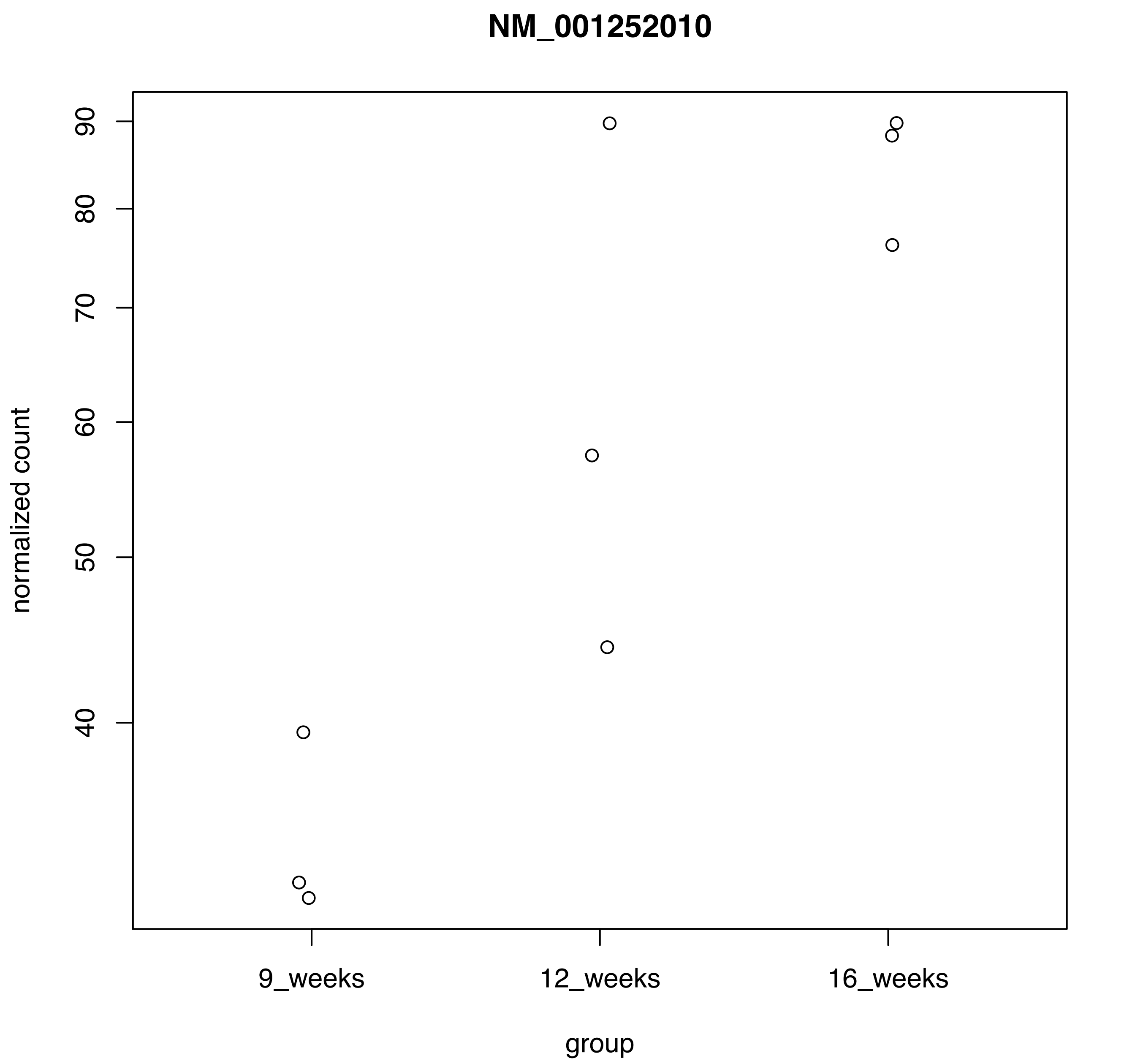

NM_001252010
normalized count
90
80
70
60
50
40
9_weeks
12_weeks
16_weeks
group

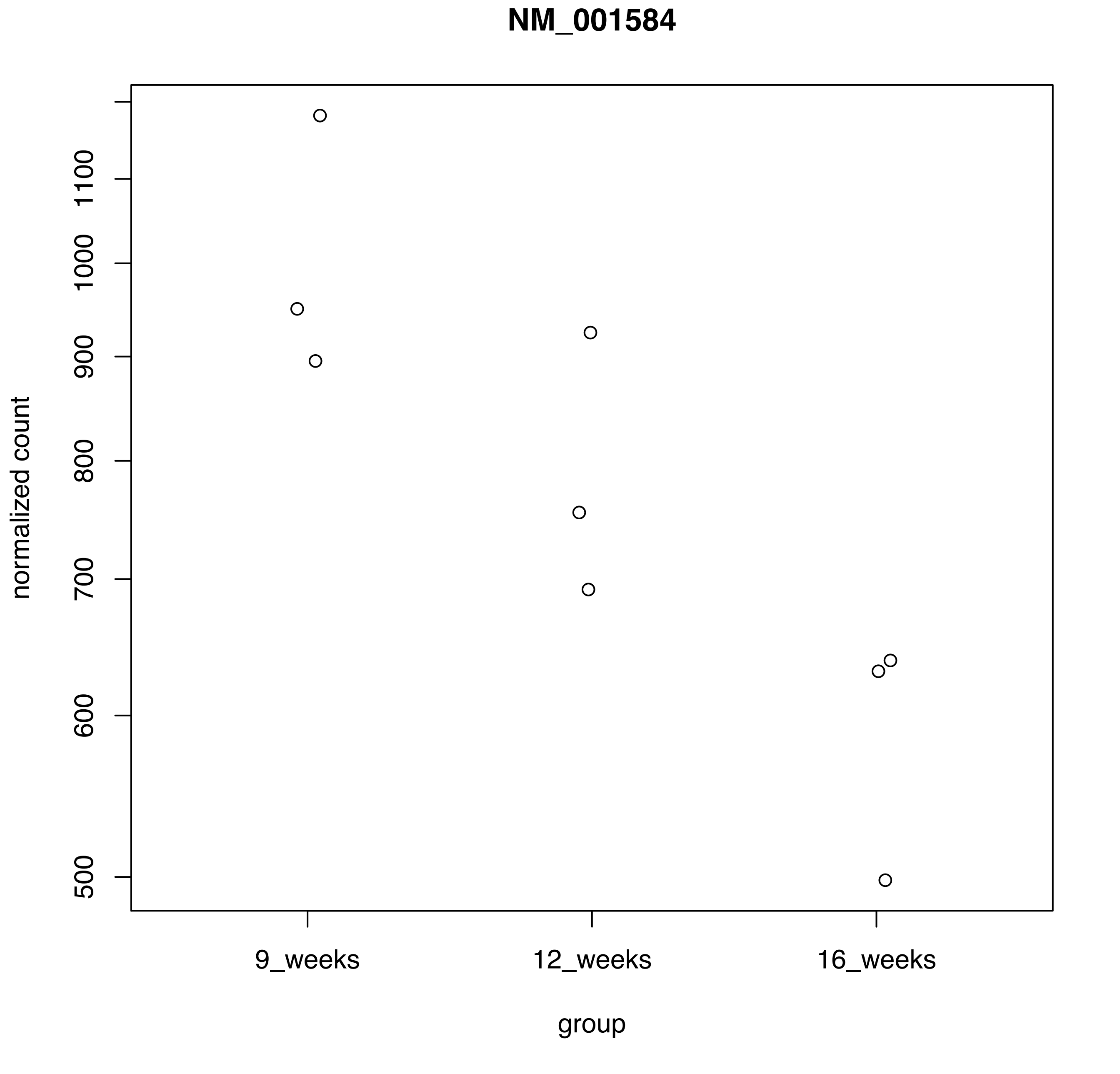
NM_001584
normalized count
1100
1000
900
800
700
600
500
9_weeks
12_weeks
16_weeks
group

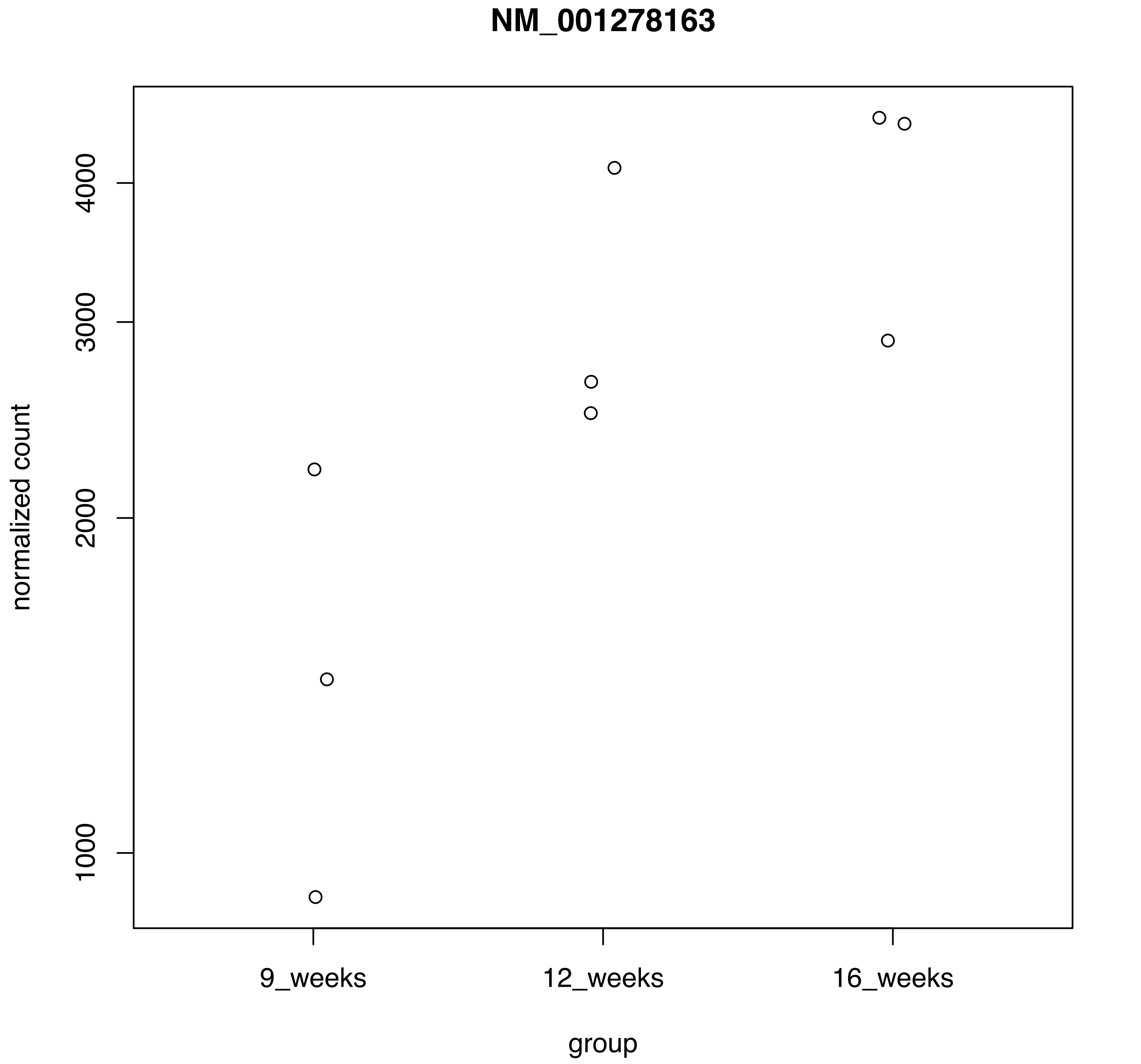
NM_001278163
normalized count
1000
2000
3000
4000
9_weeks
12_weeks
16_weeks
group

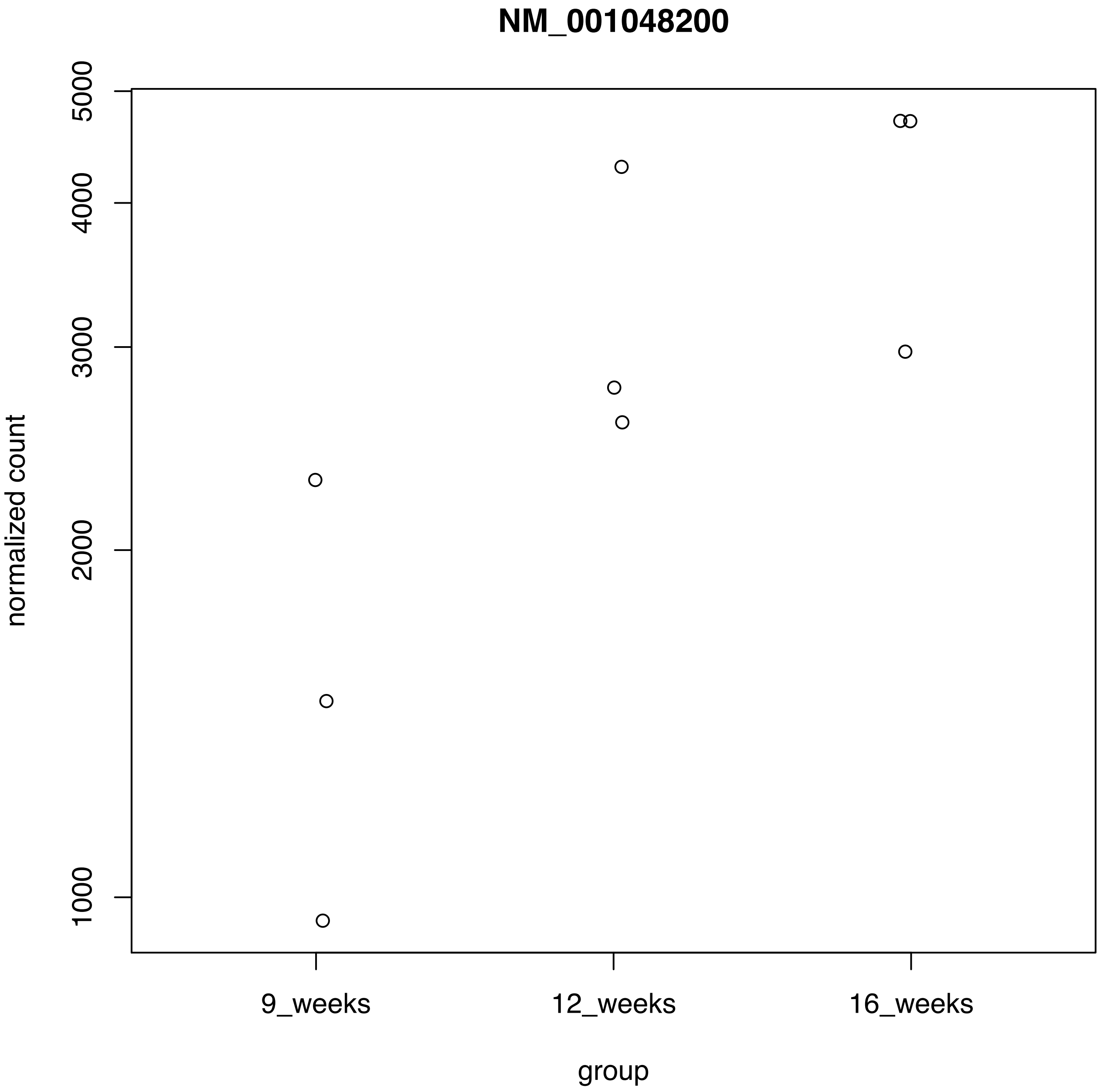
NM_001048200
normalized count
5000
4000
3000
2000
1000
9_weeks
12_weeks
16_weeks
group

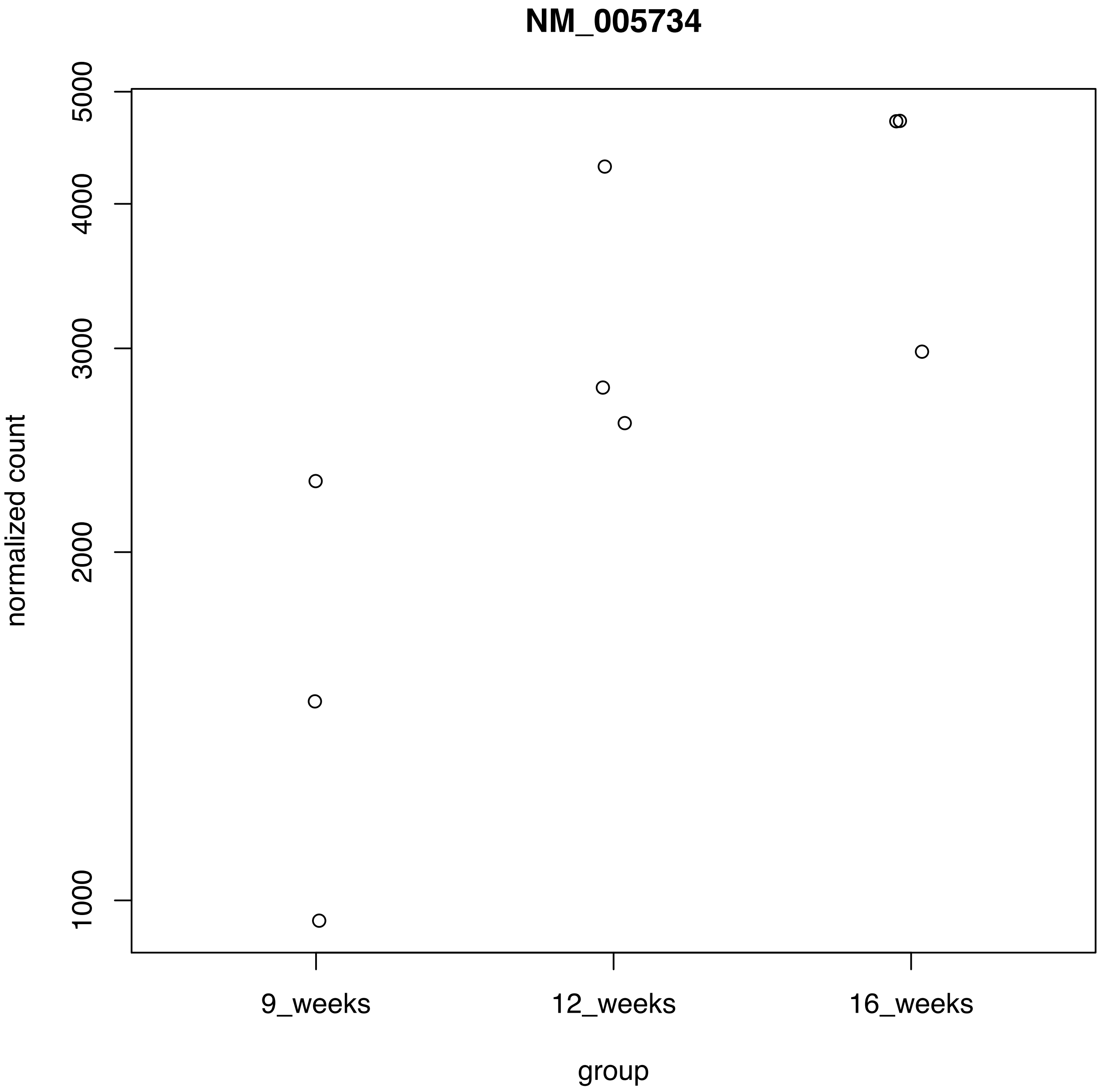
NM_005734
normalized count
5000
4000
3000
2000
1000
9_weeks
12_weeks
16_weeks
group

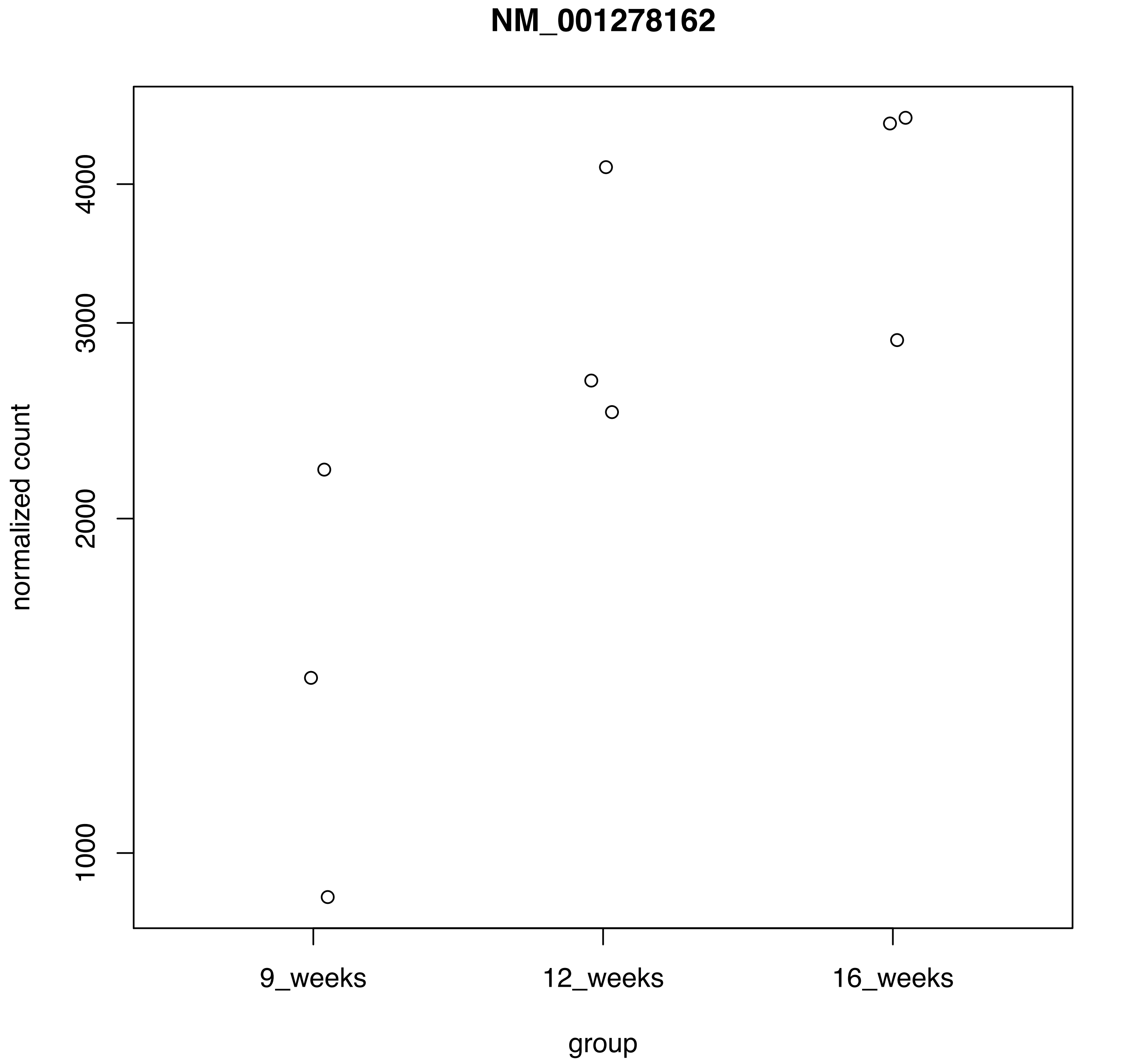
NM_001278162
normalized count
4000
3000
2000
1000
9_weeks
12_weeks
16_weeks
group

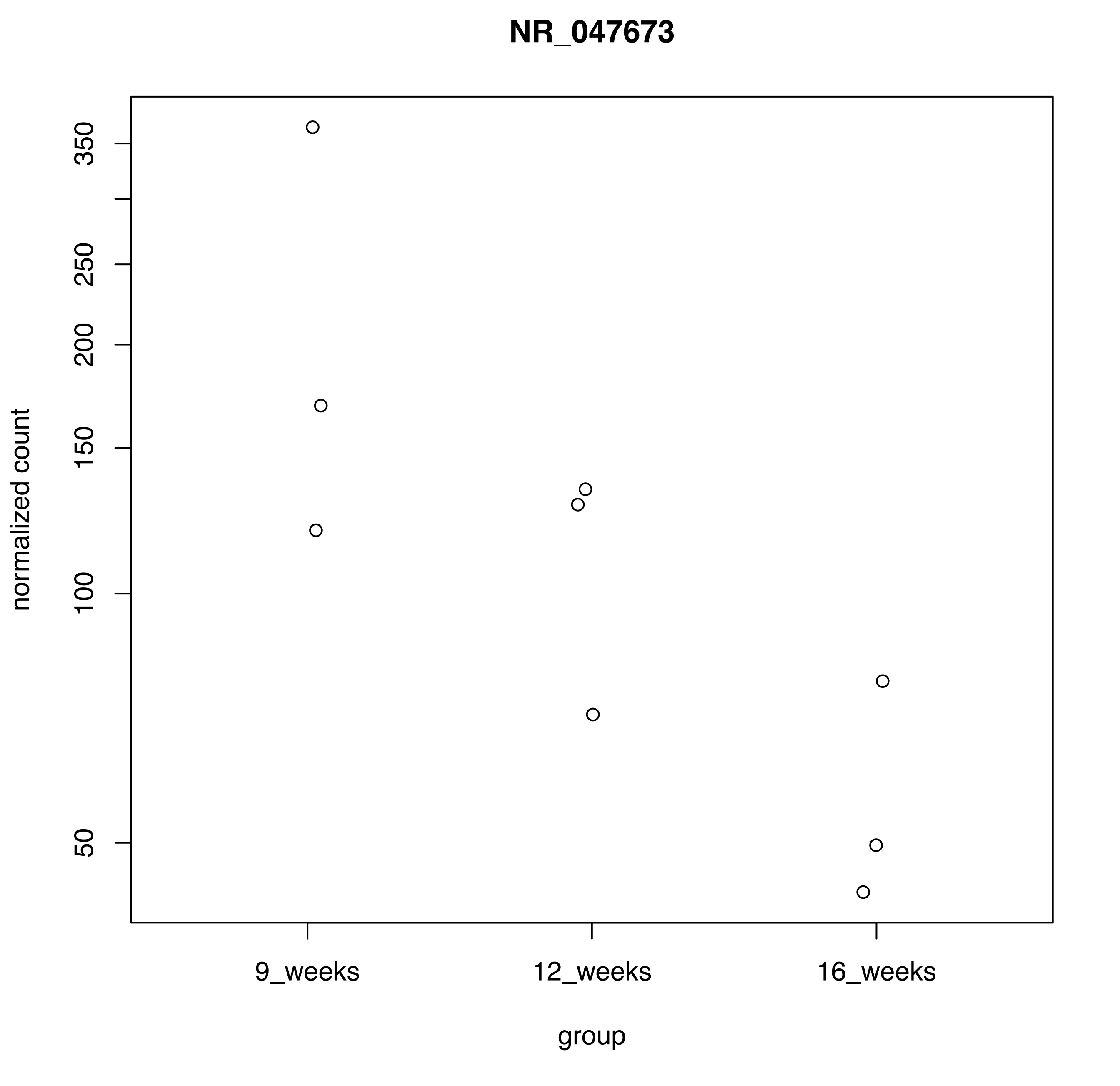
NR_047673
normalized count
50
100
150
200
250
350
9_weeks
12_weeks
16_weeks
group

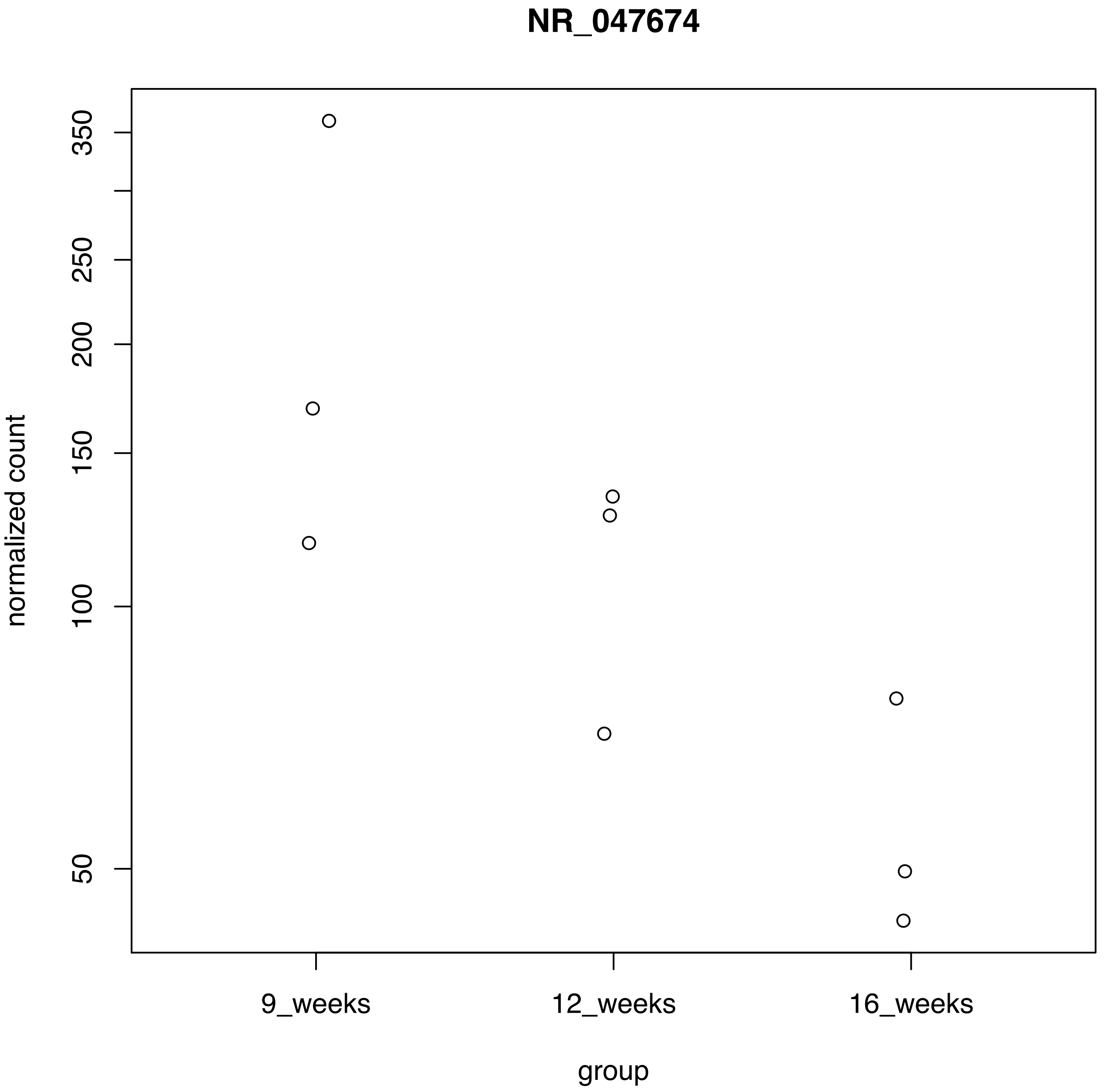
NR_047674
normalized count
350
250
200
150
100
50
9_weeks
12_weeks
16_weeks
group

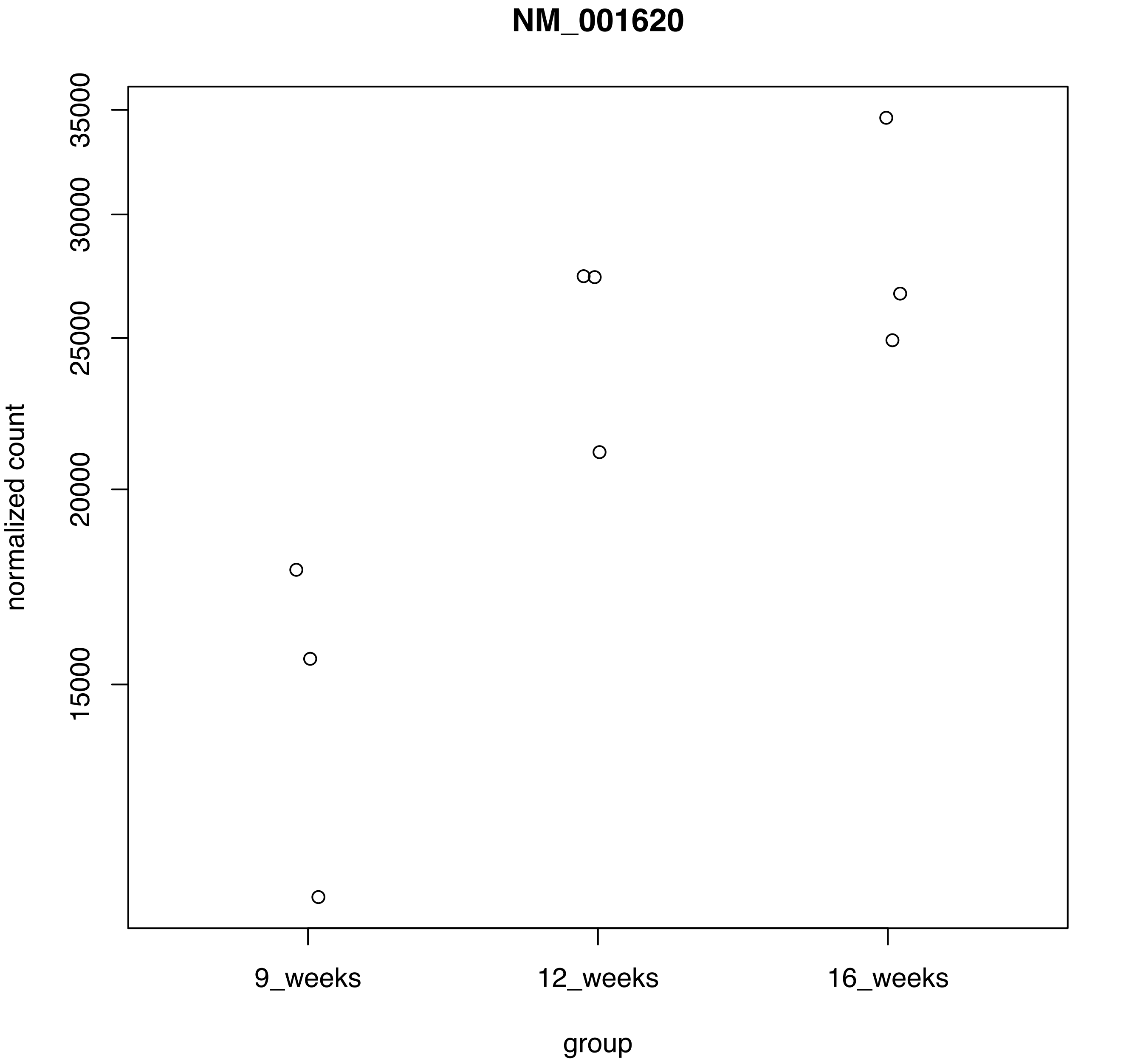
NM_001620
normalized count
15000
20000
25000
30000
35000
9_weeks
12_weeks
16_weeks
group

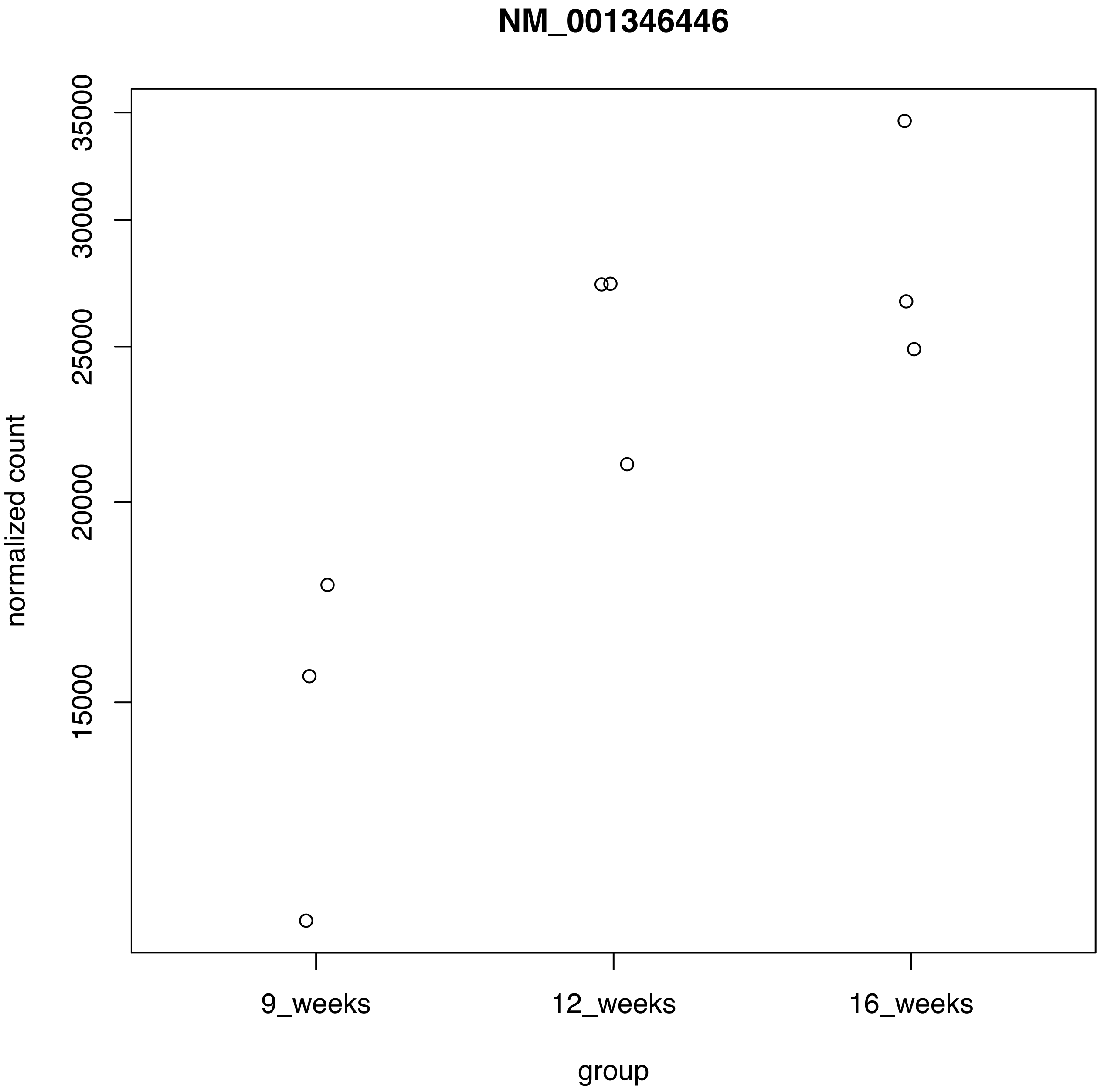
NM_001346446
normalized count
35000
30000
25000
20000
15000
9_weeks
12_weeks
16_weeks
group

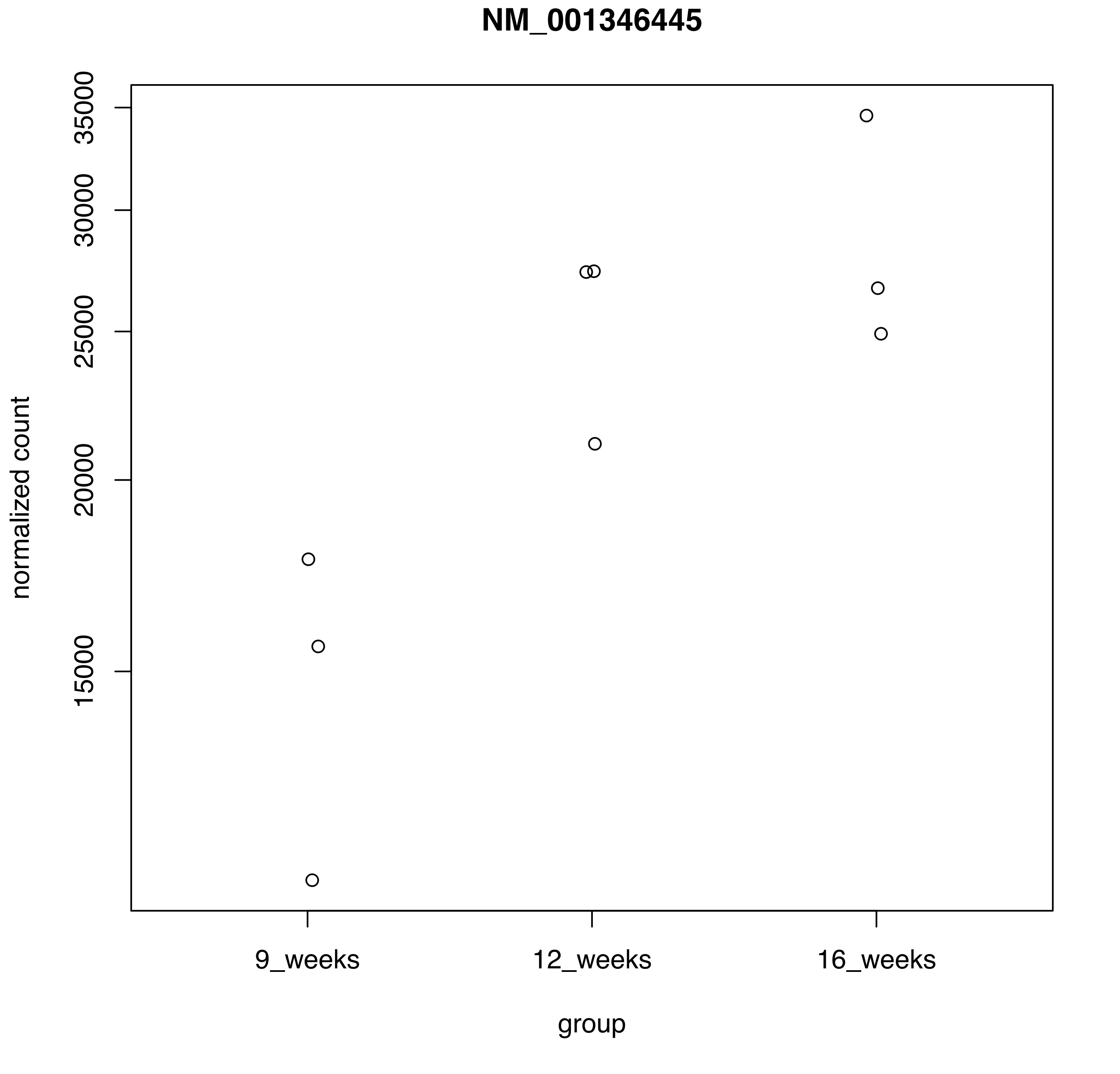

NM_001346445
normalized count
35000
30000
25000
20000
15000
9_weeks
12_weeks
16_weeks
group

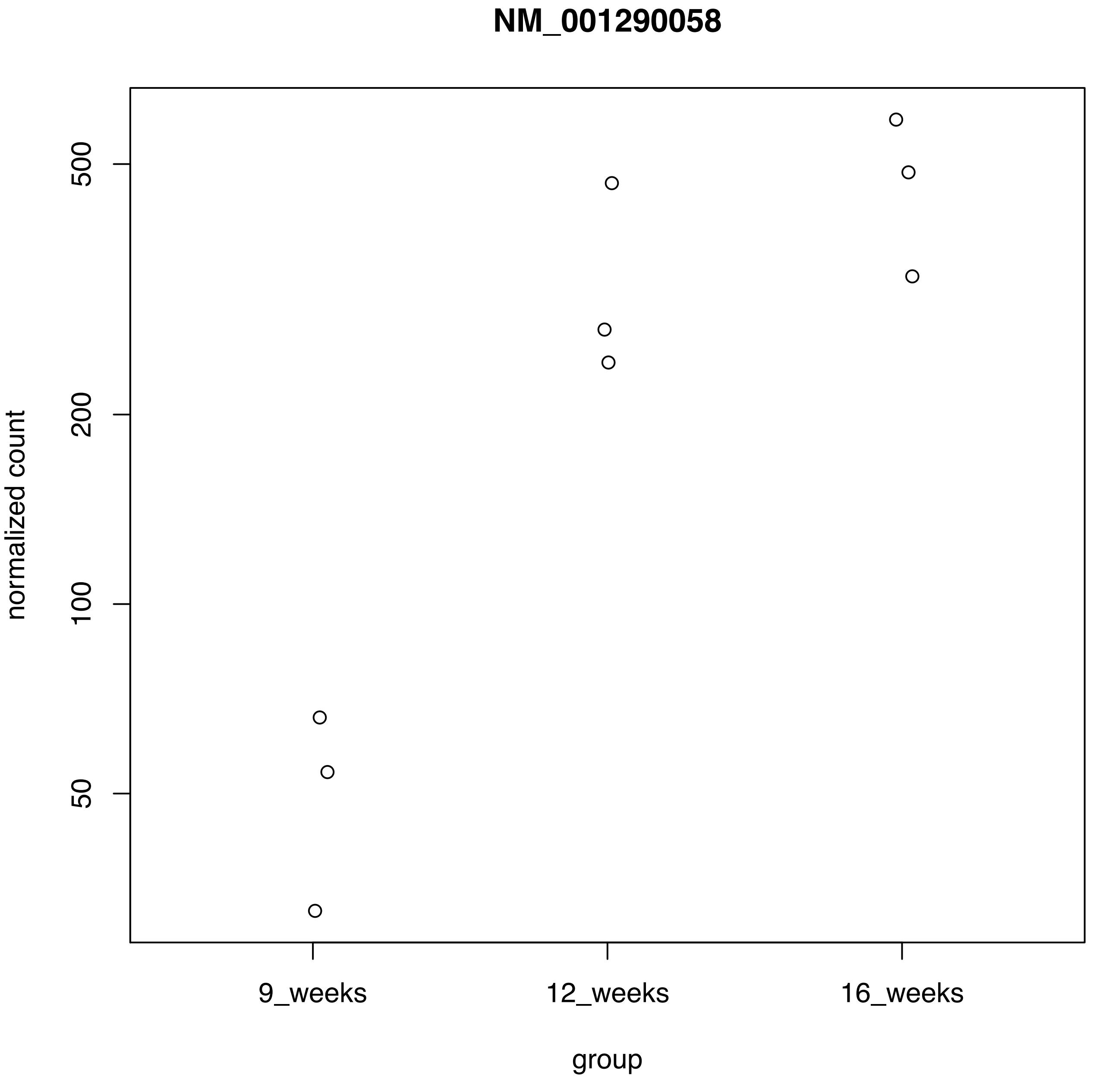
NM_001290058
normalized count
500
200
100
50
9_weeks
12_weeks
16_weeks
group

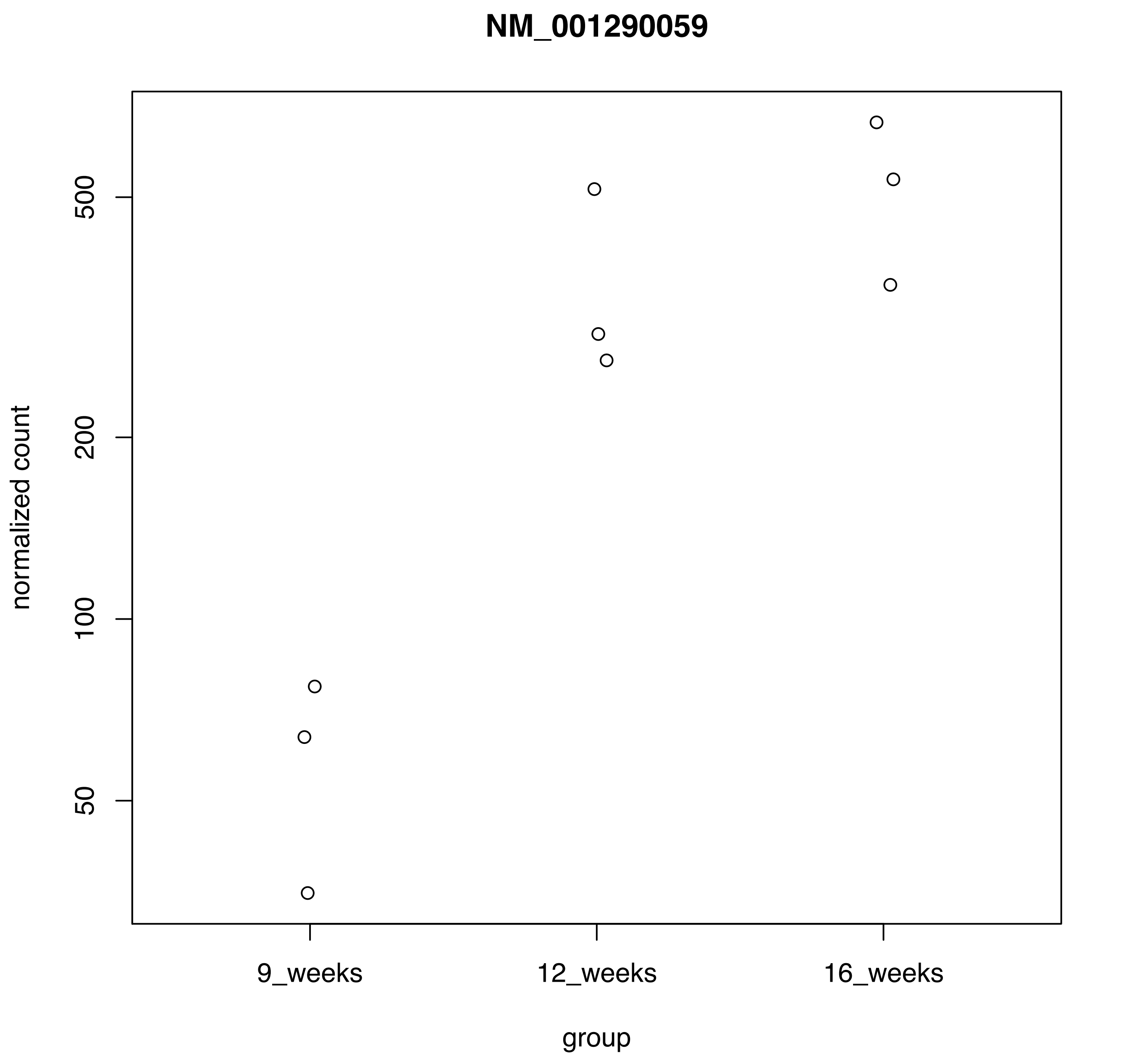
NM_001290059
normalized count
500
200
100
50
9_weeks
12_weeks
16_weeks
group

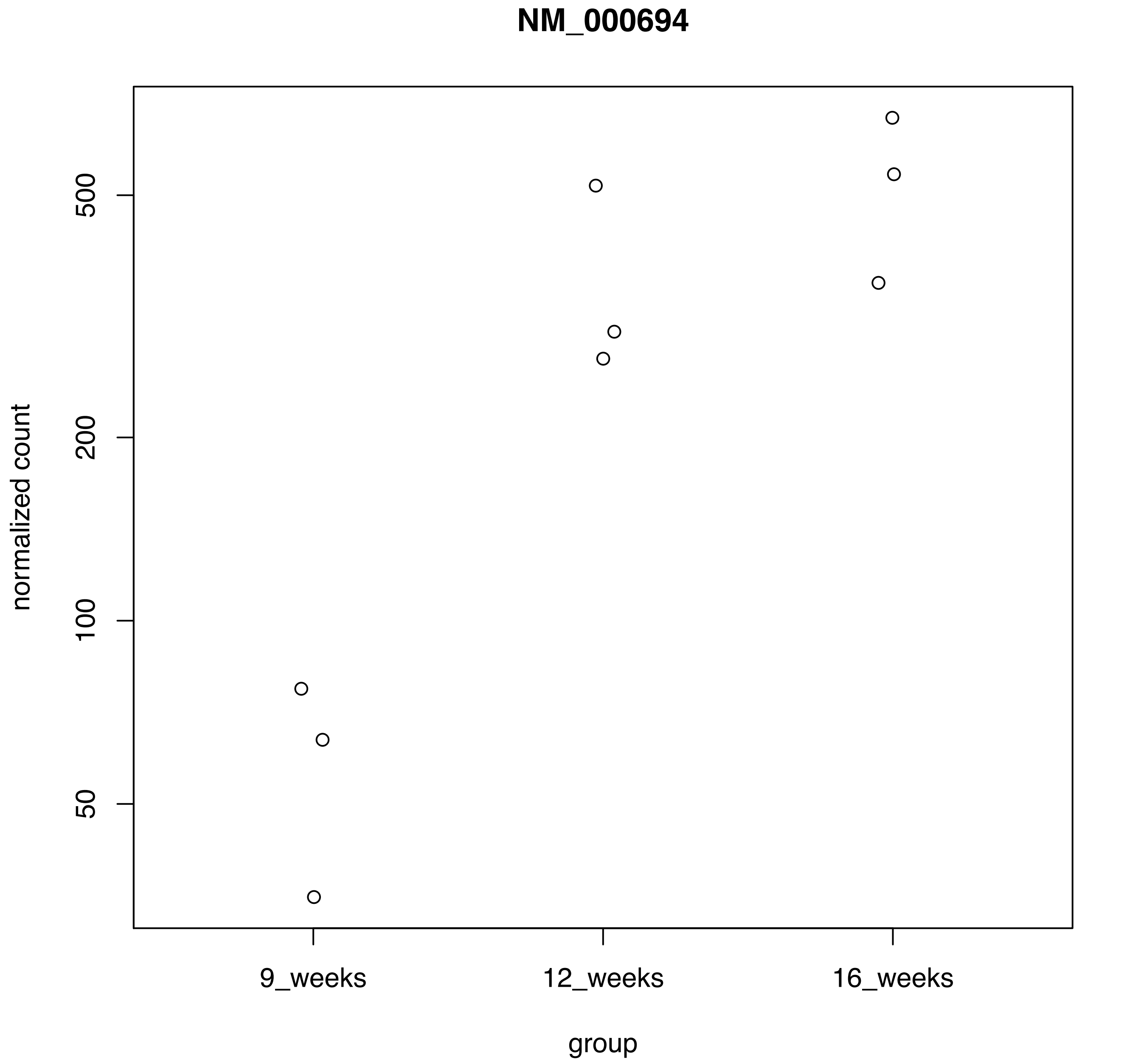
NM_000694
normalized count
500
200
100
50
9_weeks
12_weeks
16_weeks
group

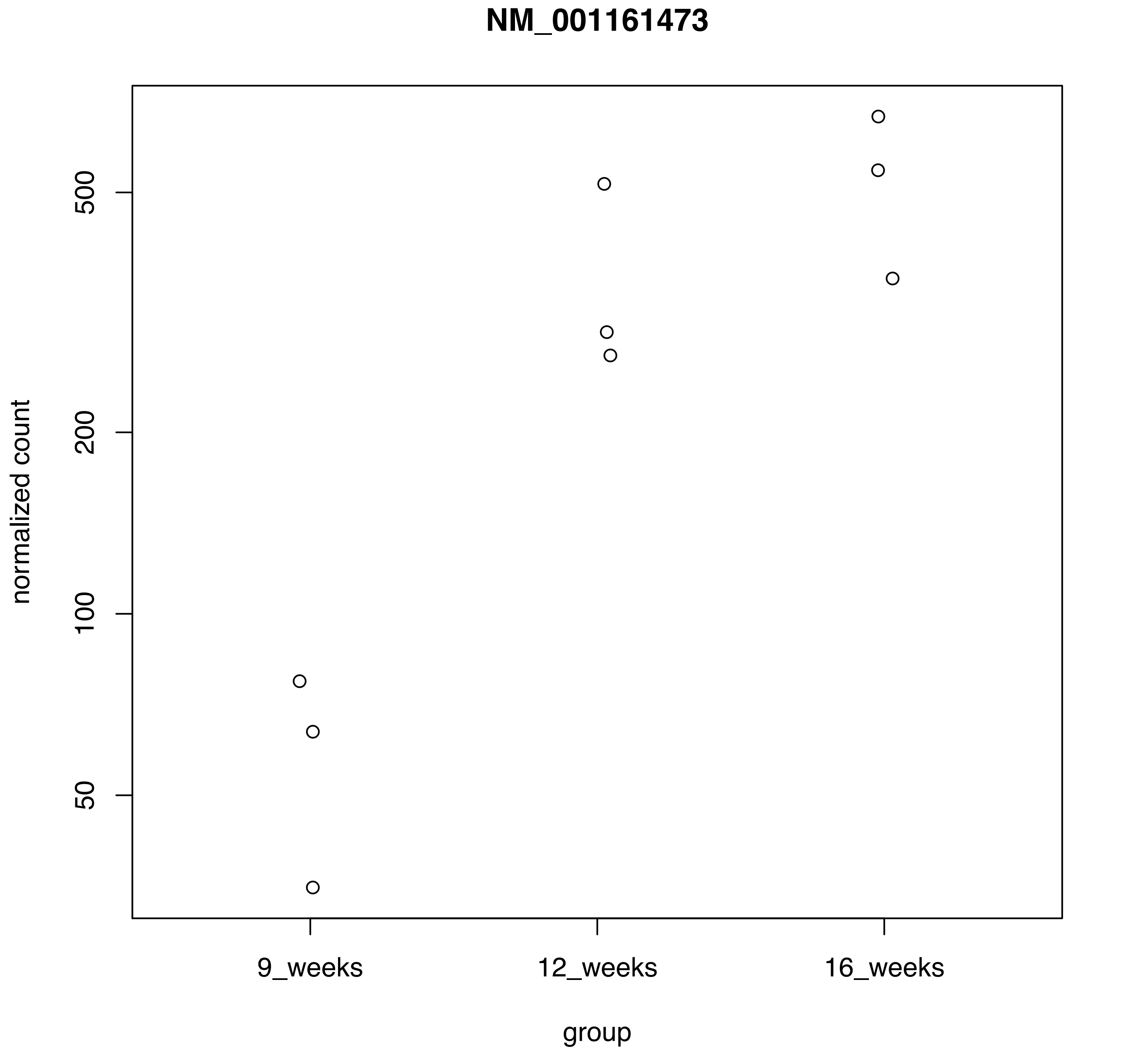
NM_001161473
normalized count
500
200
100
50
9_weeks
12_weeks
16_weeks
group

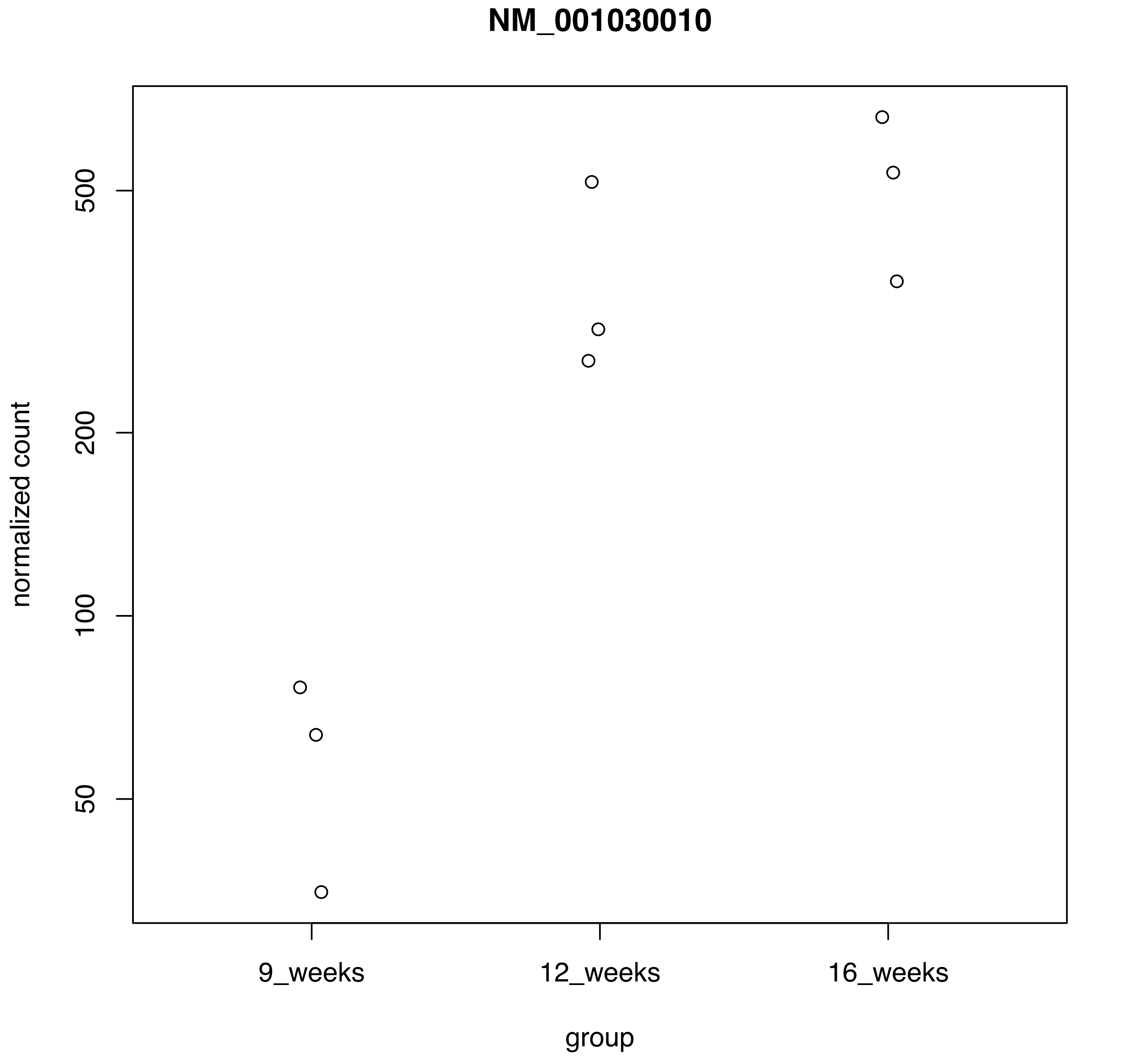

NM_001030010
normalized count
500
200
100
50
9_weeks
12_weeks
16_weeks
group

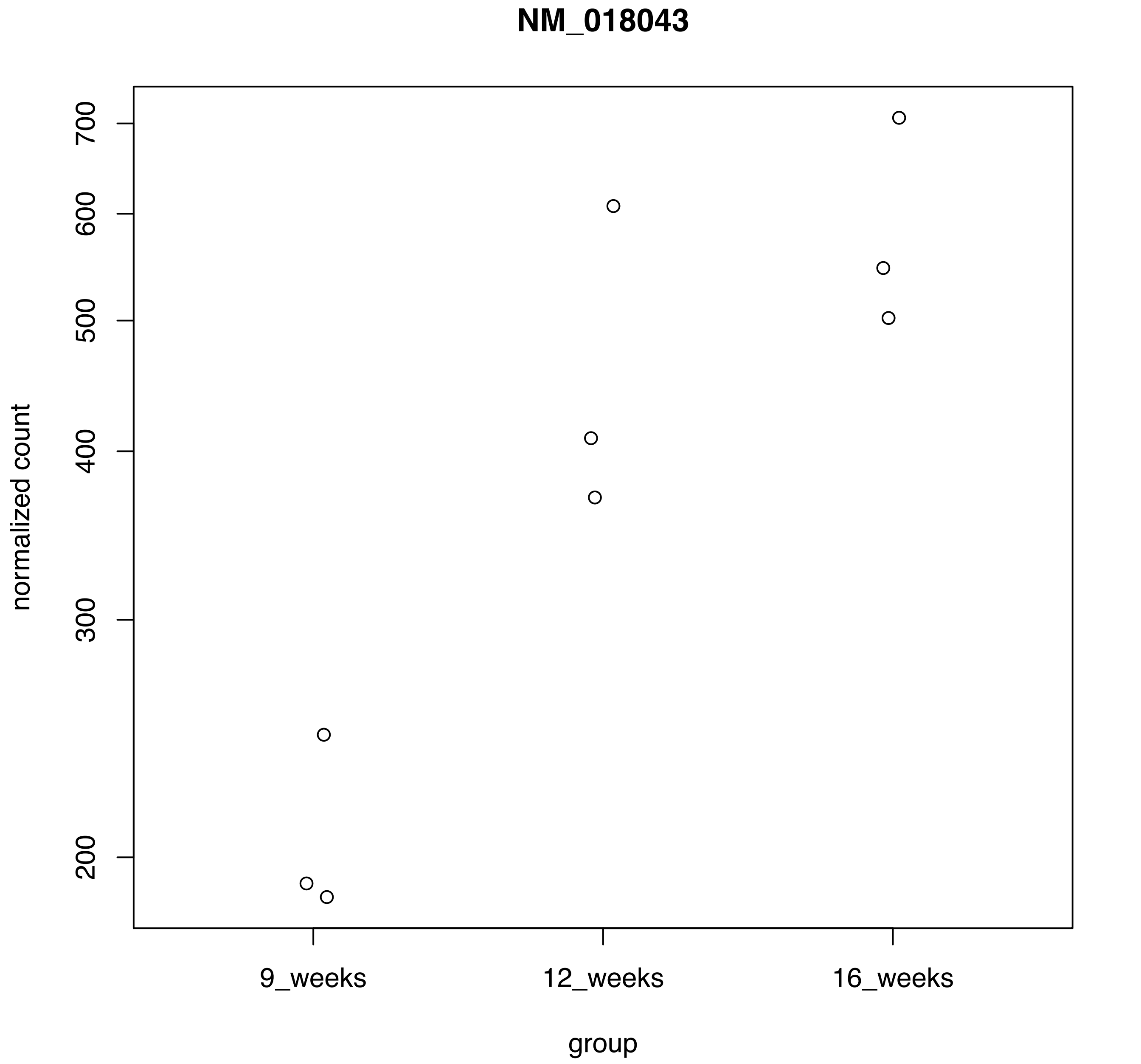

NM_018043
normalized count
700
600
500
400
300
200
9_weeks
12_weeks
16_weeks
group

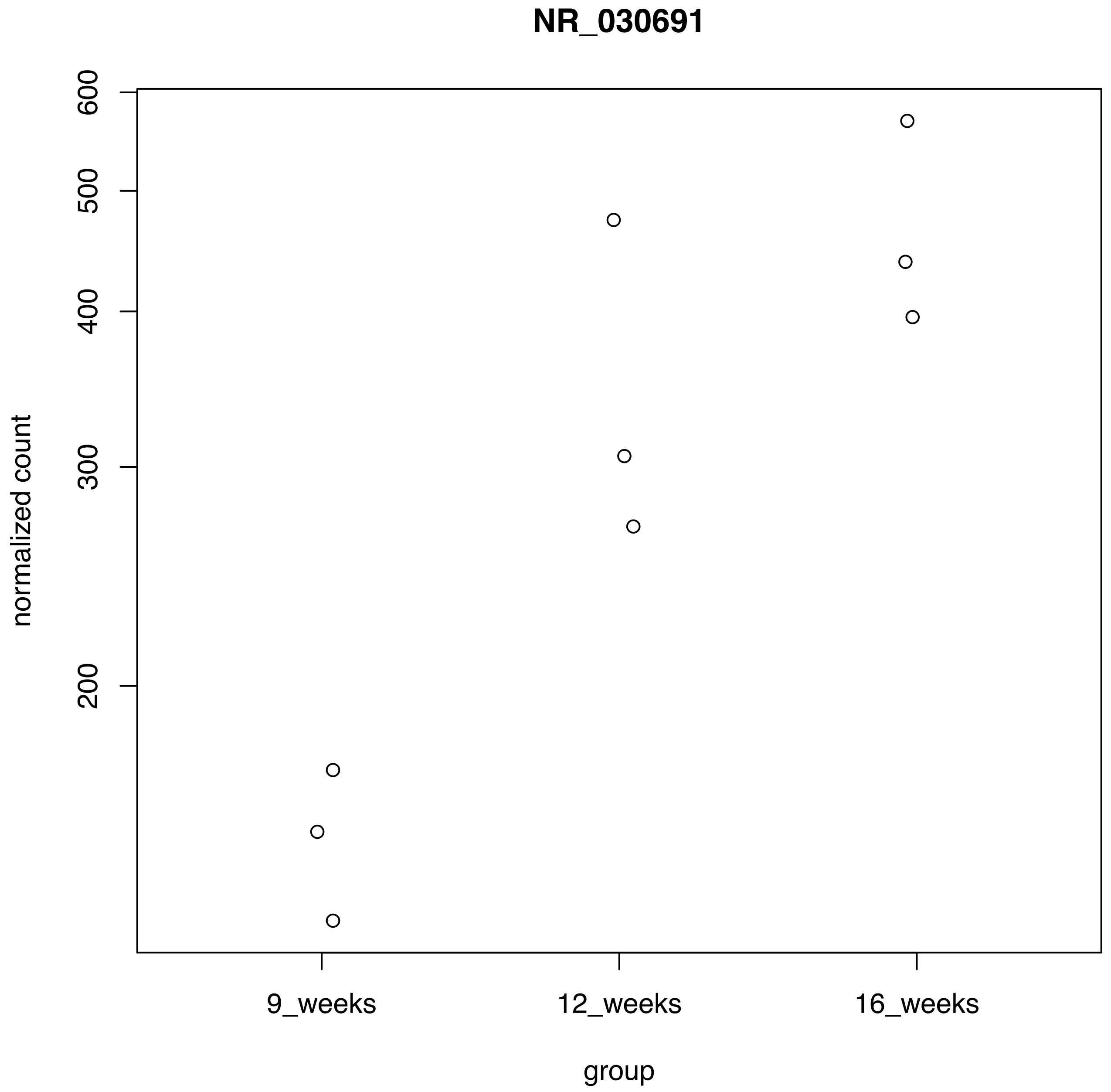
NR_030691
normalized count
600
500
400
300
200
9_weeks
12_weeks
16_weeks
group

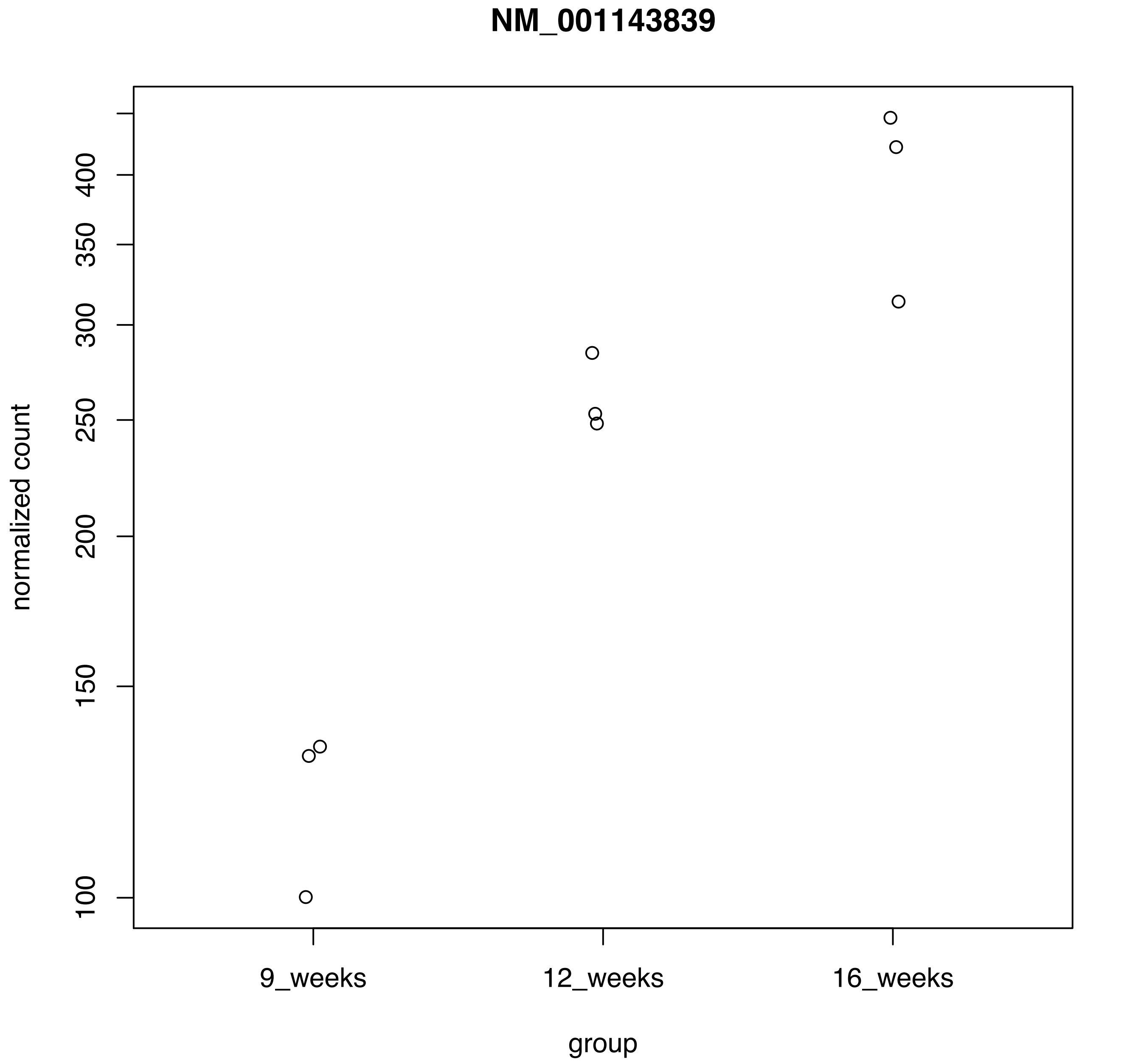
NM_001143839
normalized count
100
150
200
250
300
350
400
9_weeks
12_weeks
16_weeks
group

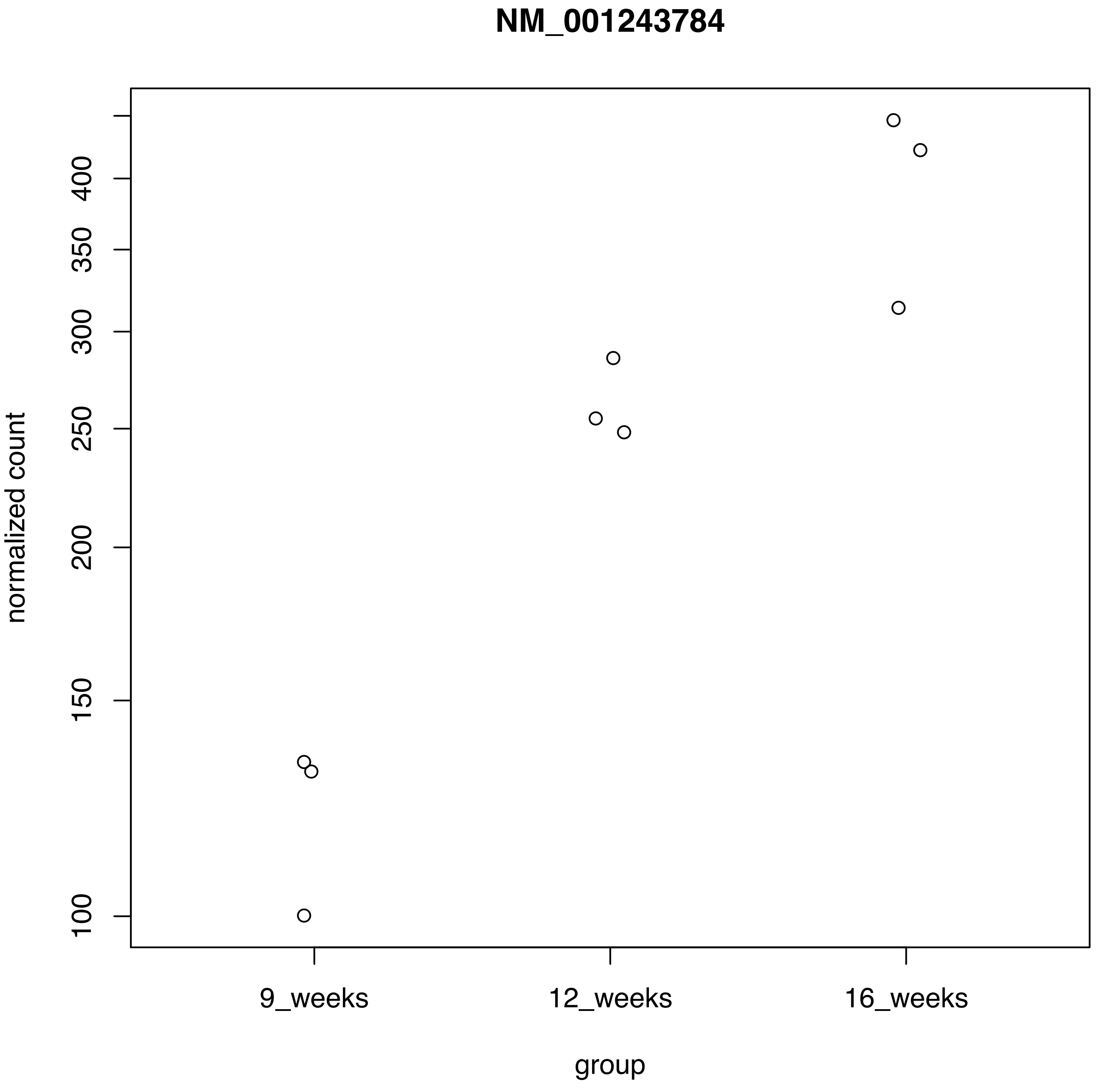
NM_001243784
normalized count
100
150
200
250
300
350
400
9_weeks
12_weeks
16_weeks
group

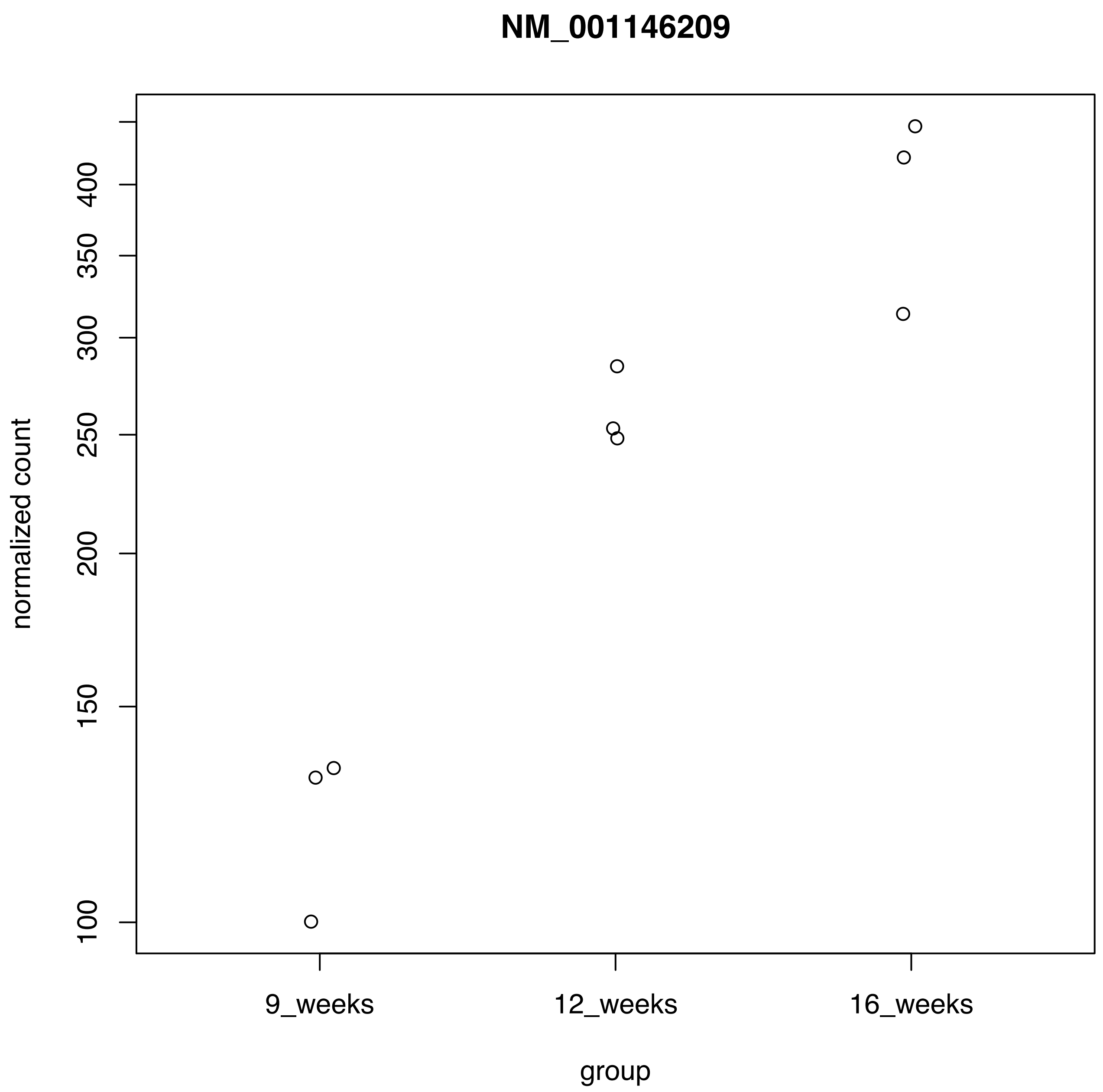

NM_001146209
normalized count
100
150
200
250
300
350
400
9_weeks
12_weeks
16_weeks
group

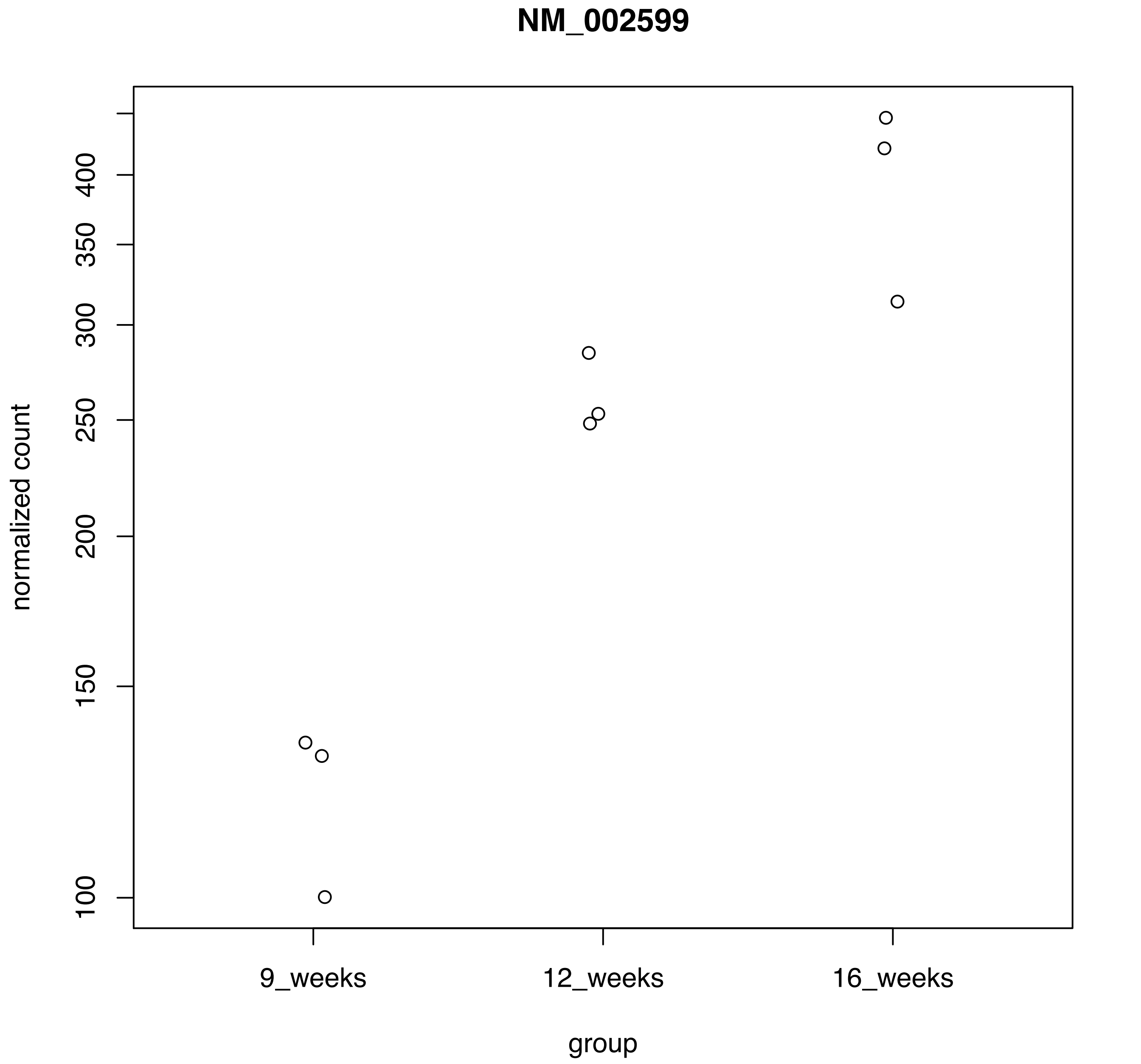
NM_002599
normalized count
100
150
200
250
300
350
400
9_weeks
12_weeks
16_weeks
group

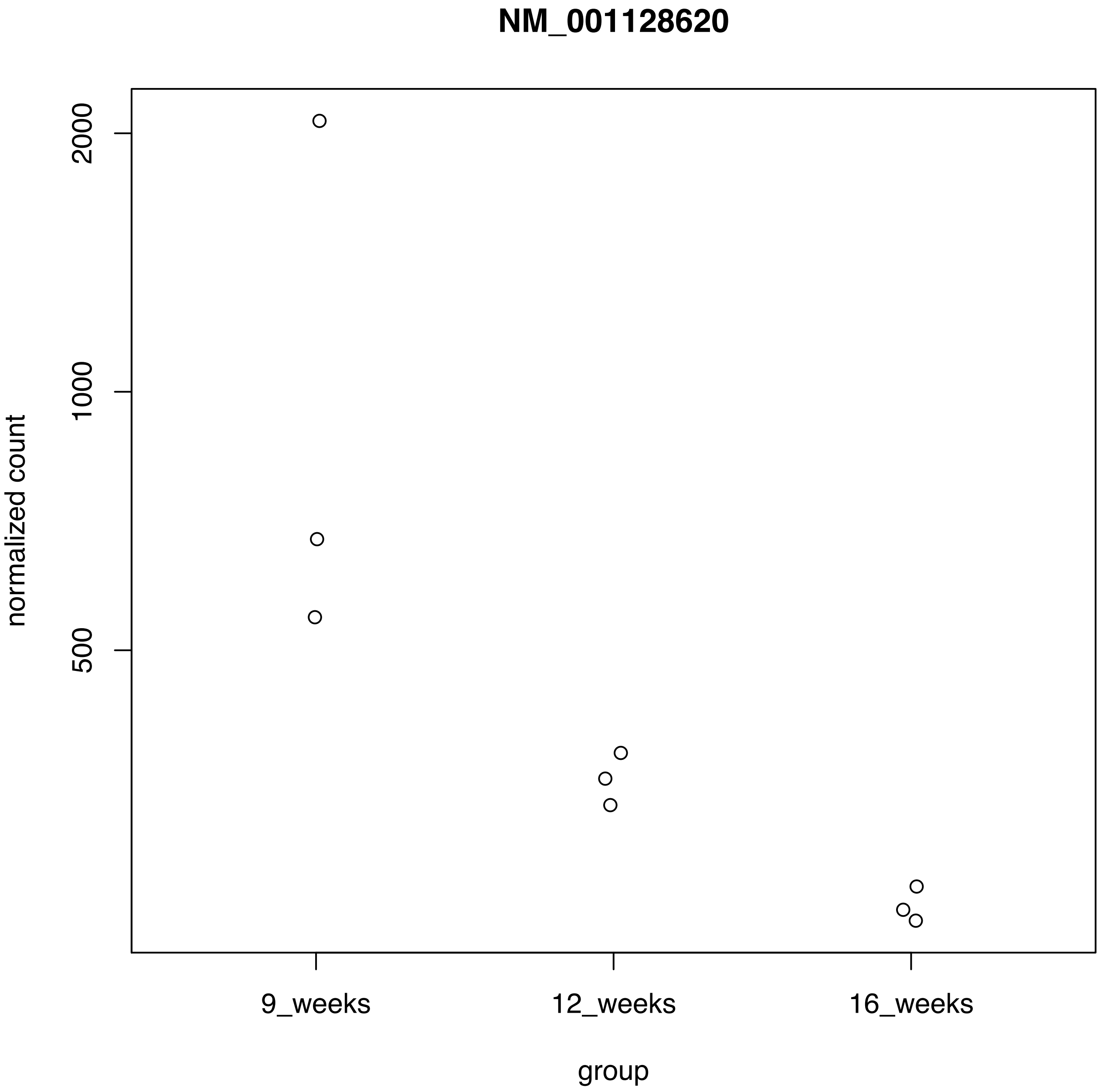
NM_001128620
normalized count
2000
1000
500
9_weeks
12_weeks
16_weeks
group

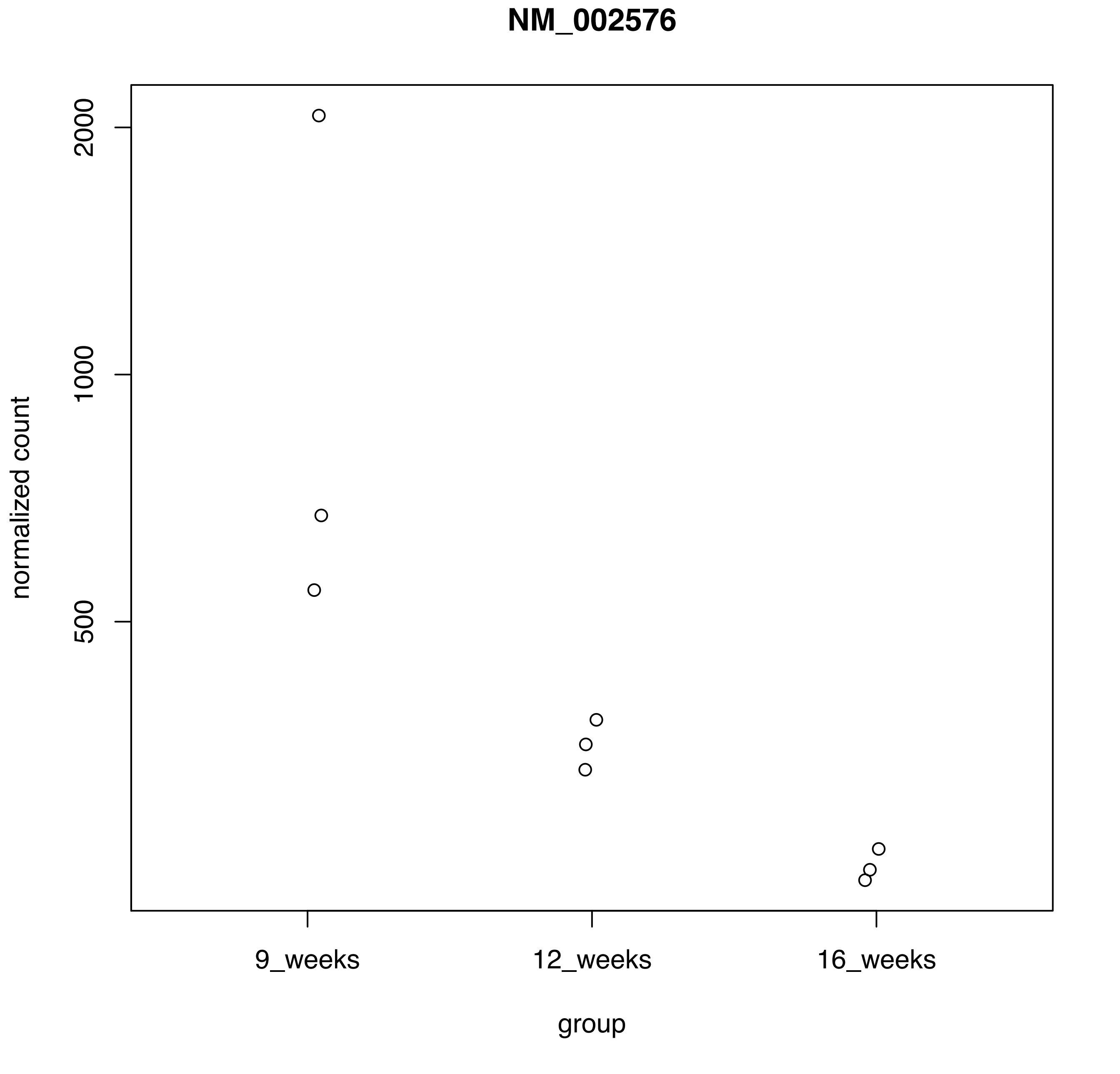

NM_002576
normalized count
2000
1000
500
9_weeks
12_weeks
16_weeks
group

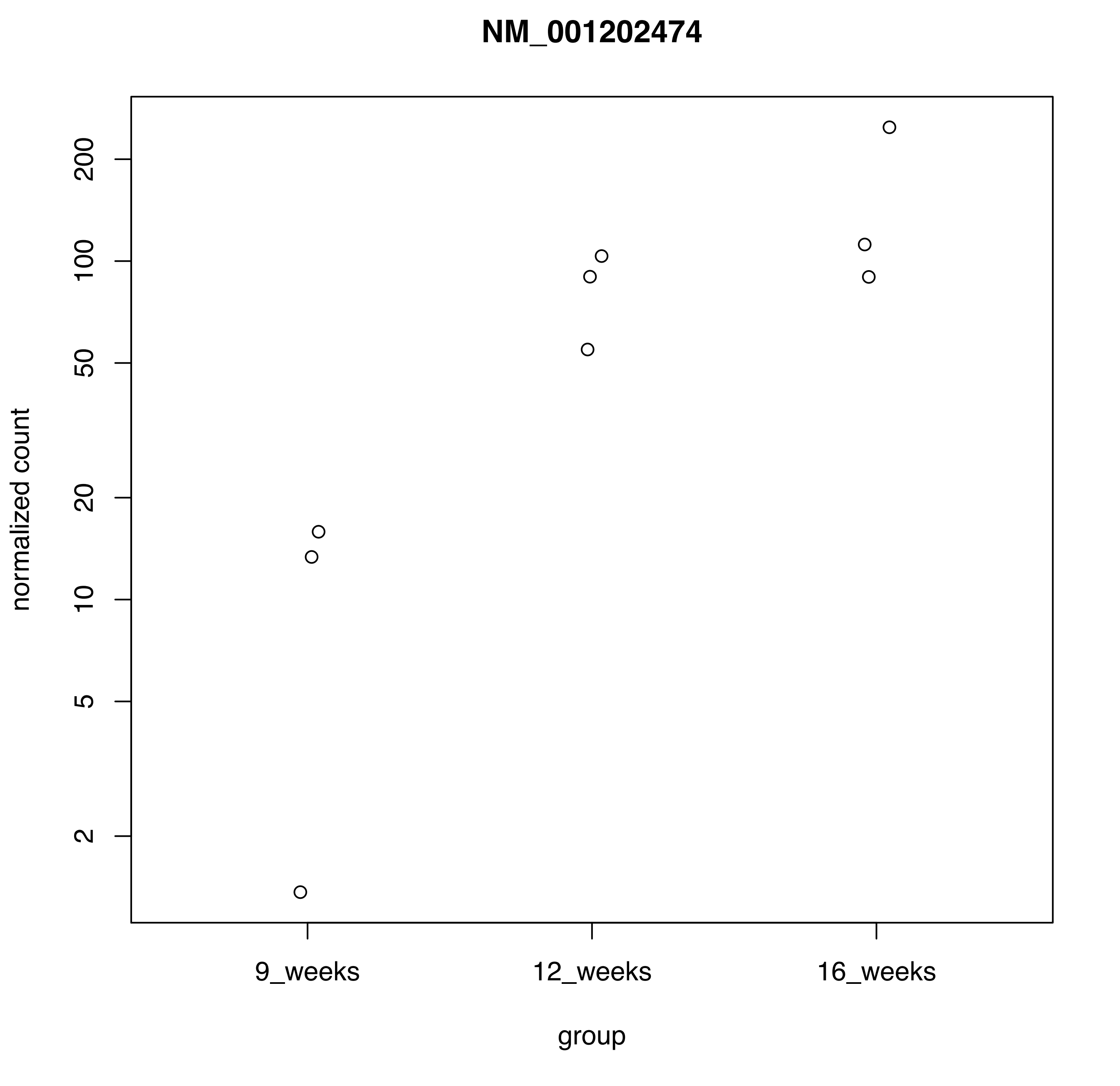
NM_001202474
normalized count
200
100
50
20
10
5
2
9_weeks
12_weeks
16_weeks
group

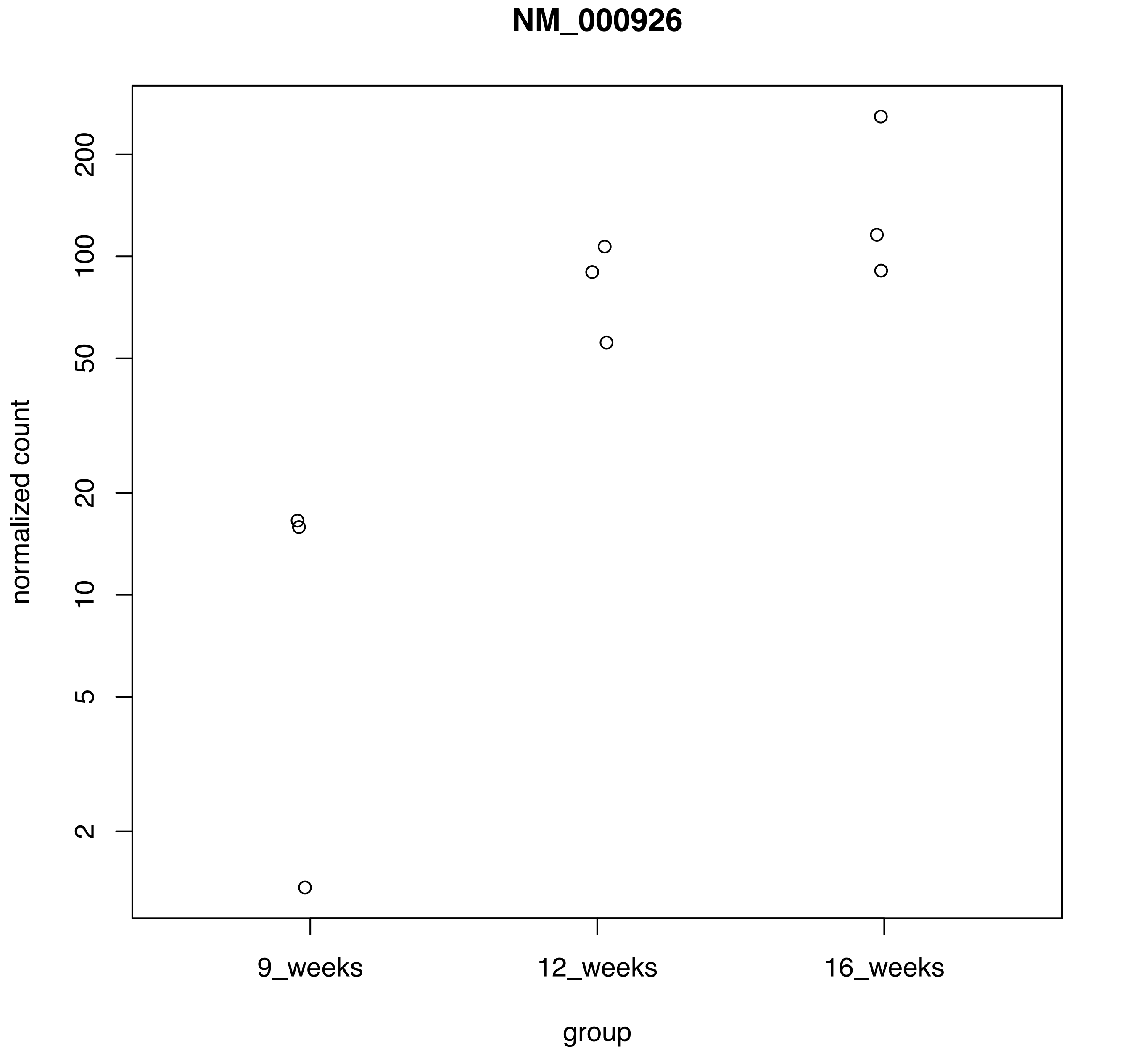
NM_000926
normalized count
200
100
50
20
10
5
2
9_weeks
12_weeks
16_weeks
group

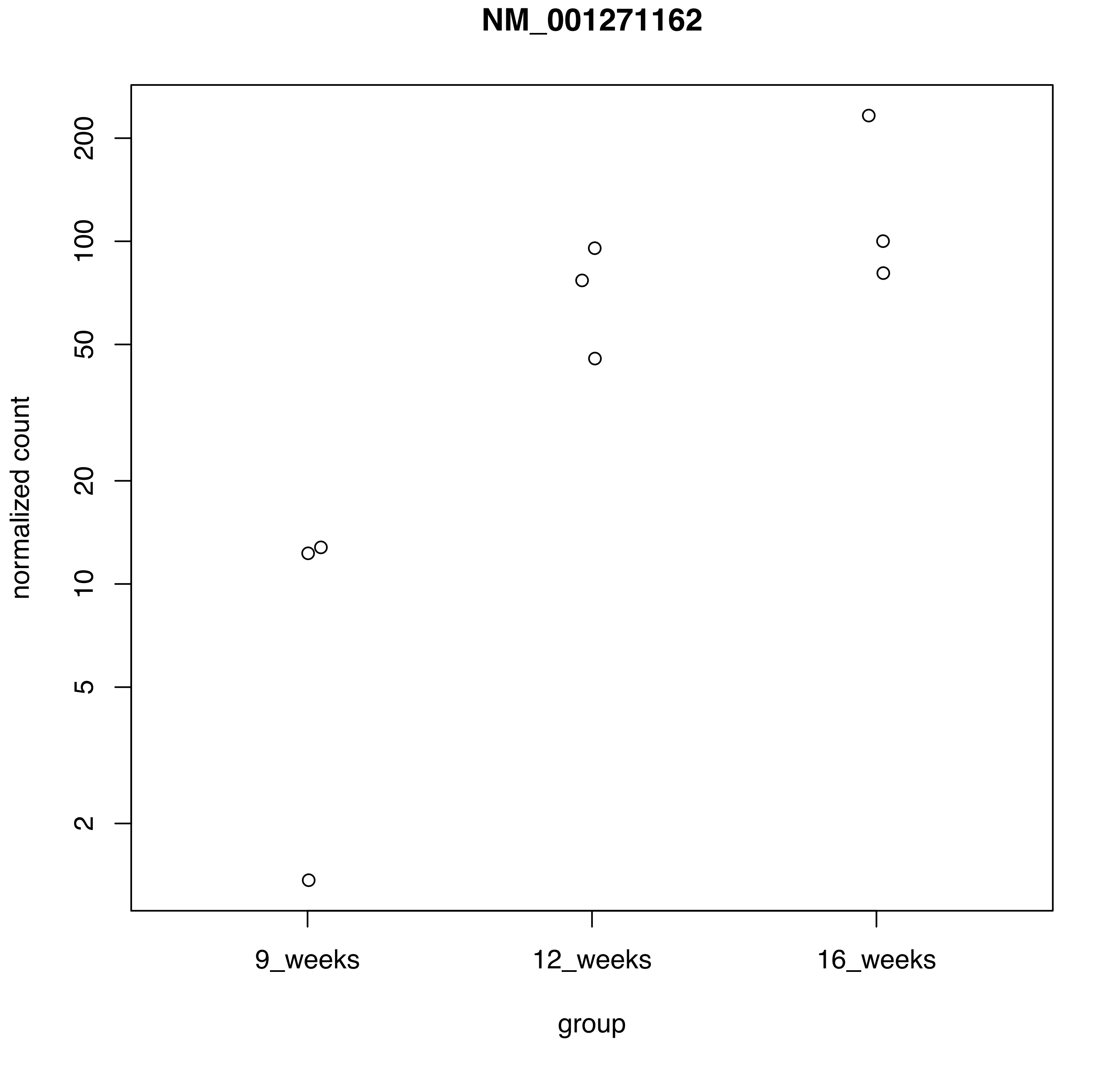
NM_001271162
normalized count
200
100
50
20
10
5
2
9_weeks
12_weeks
16_weeks
group

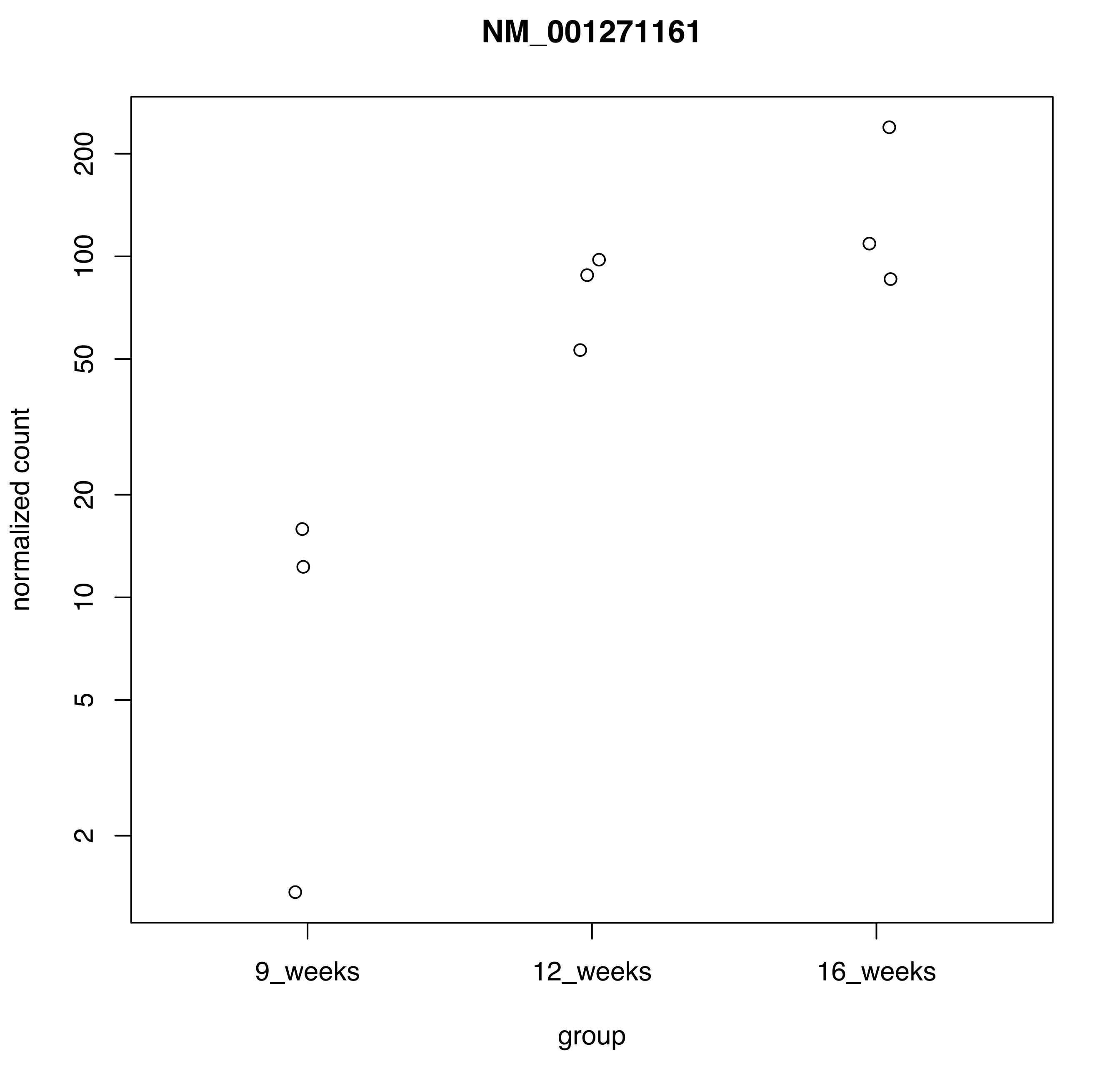
NM_001271161
normalized count
200
100
50
20
10
5
2
9_weeks
12_weeks
16_weeks
group

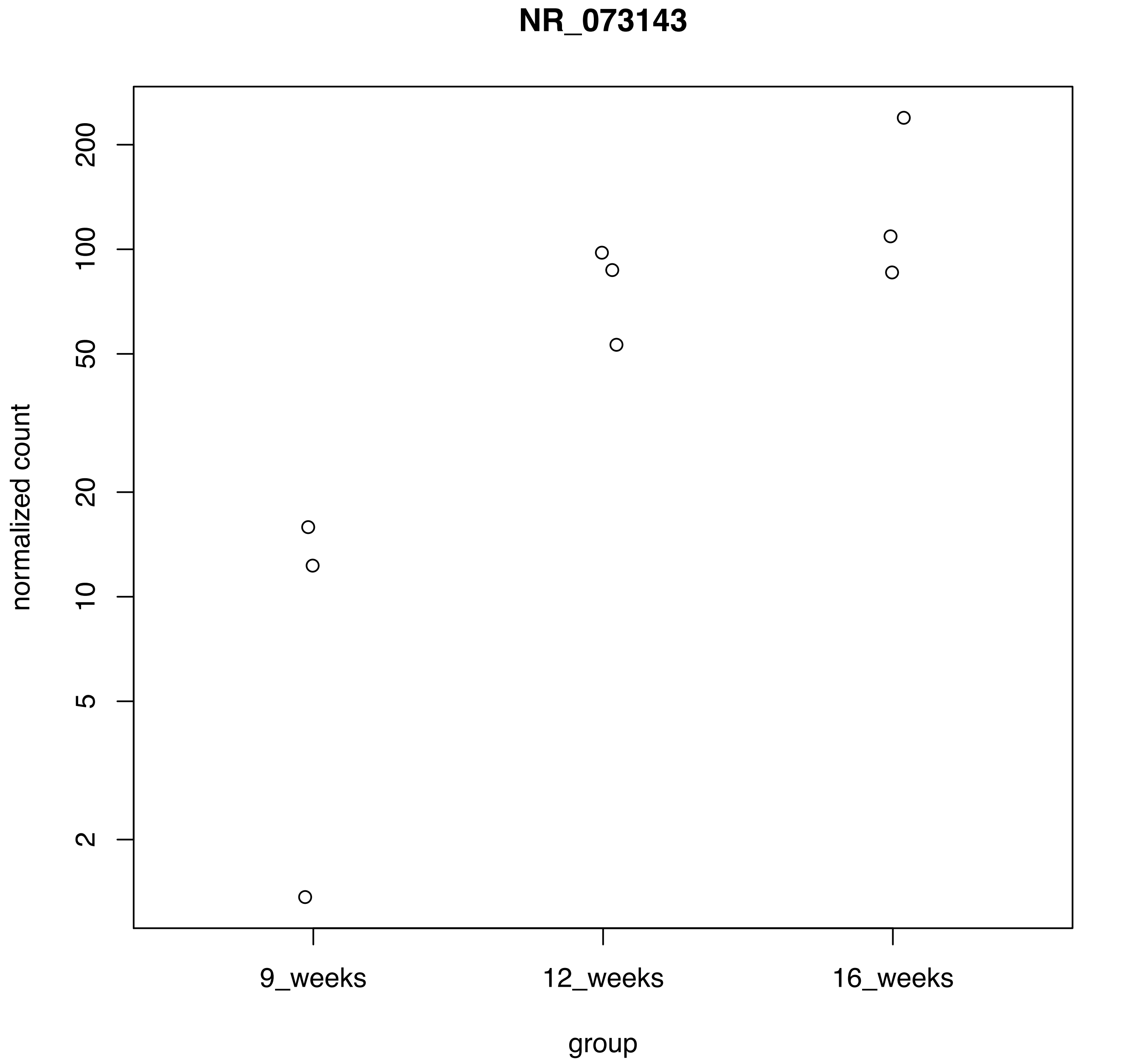
NR_073143
normalized count
200
100
50
20
10
5
2
9_weeks
12_weeks
16_weeks
group

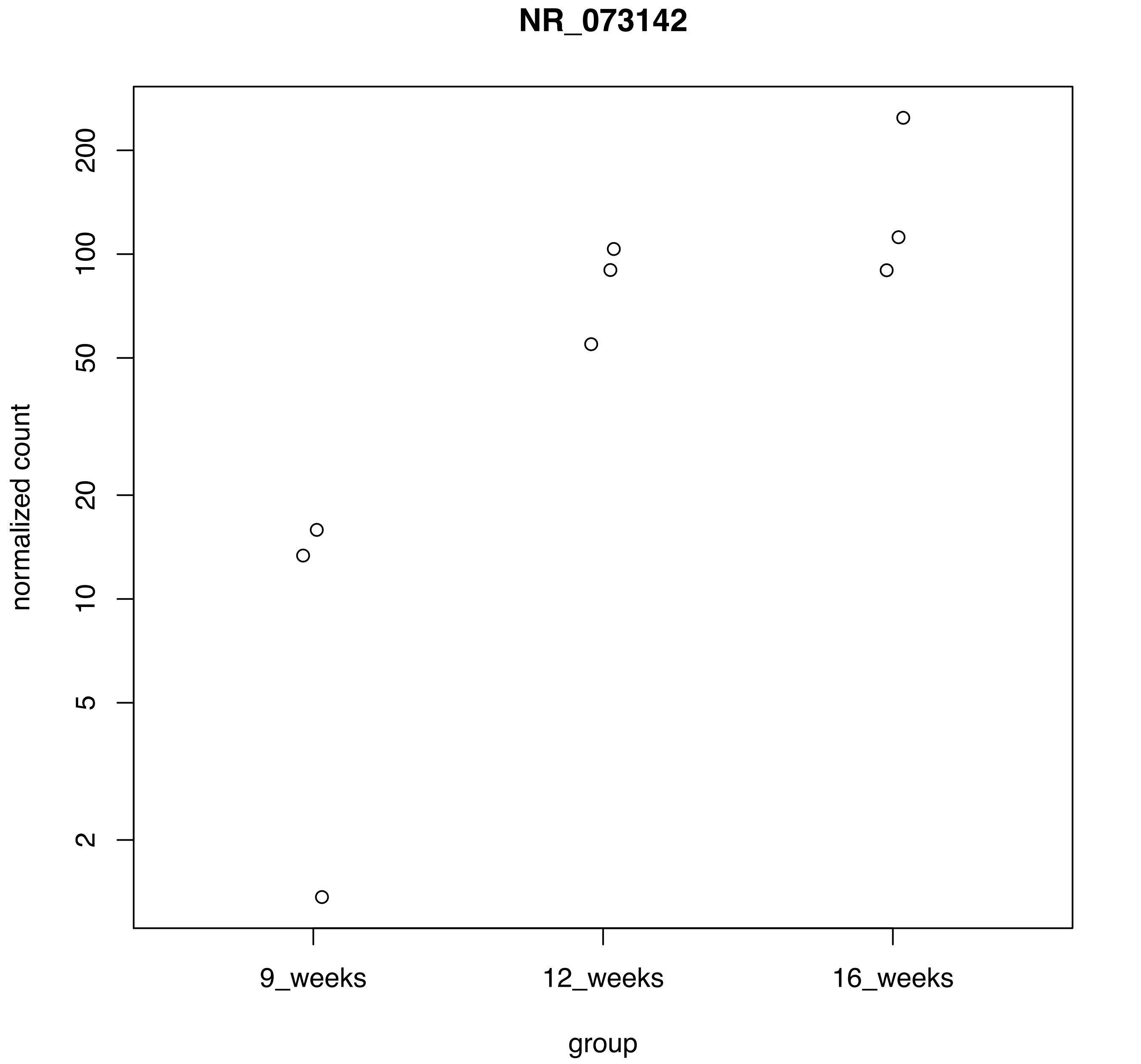
NR_073142
normalized count
200
100
50
20
10
5
2
9_weeks
12_weeks
16_weeks
group

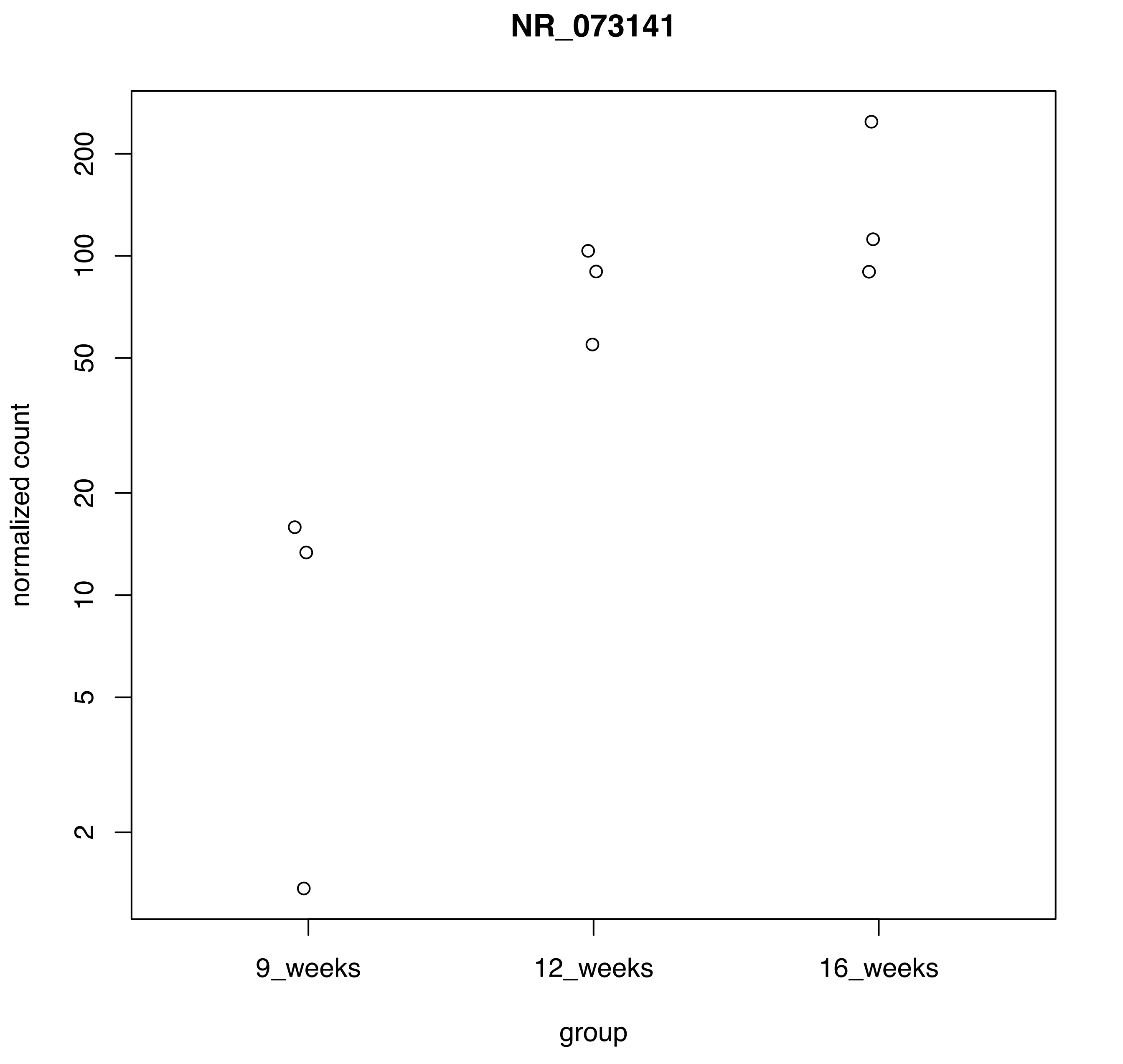
NR_073141
normalized count
200
100
50
20
10
5
2
9_weeks
12_weeks
16_weeks
group

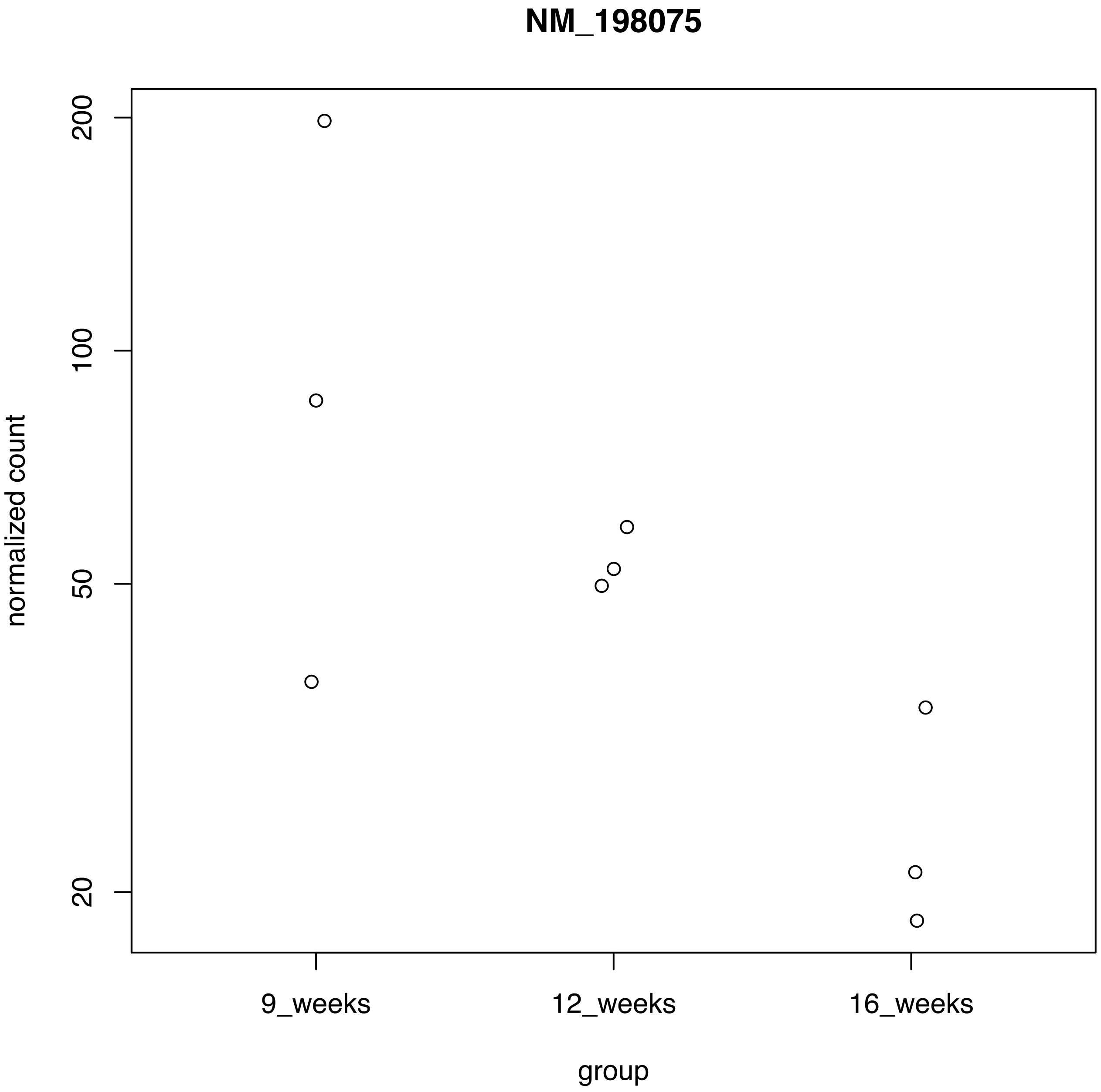
NM_198075
normalized count
200
100
50
20
9_weeks
12_weeks
16_weeks
group

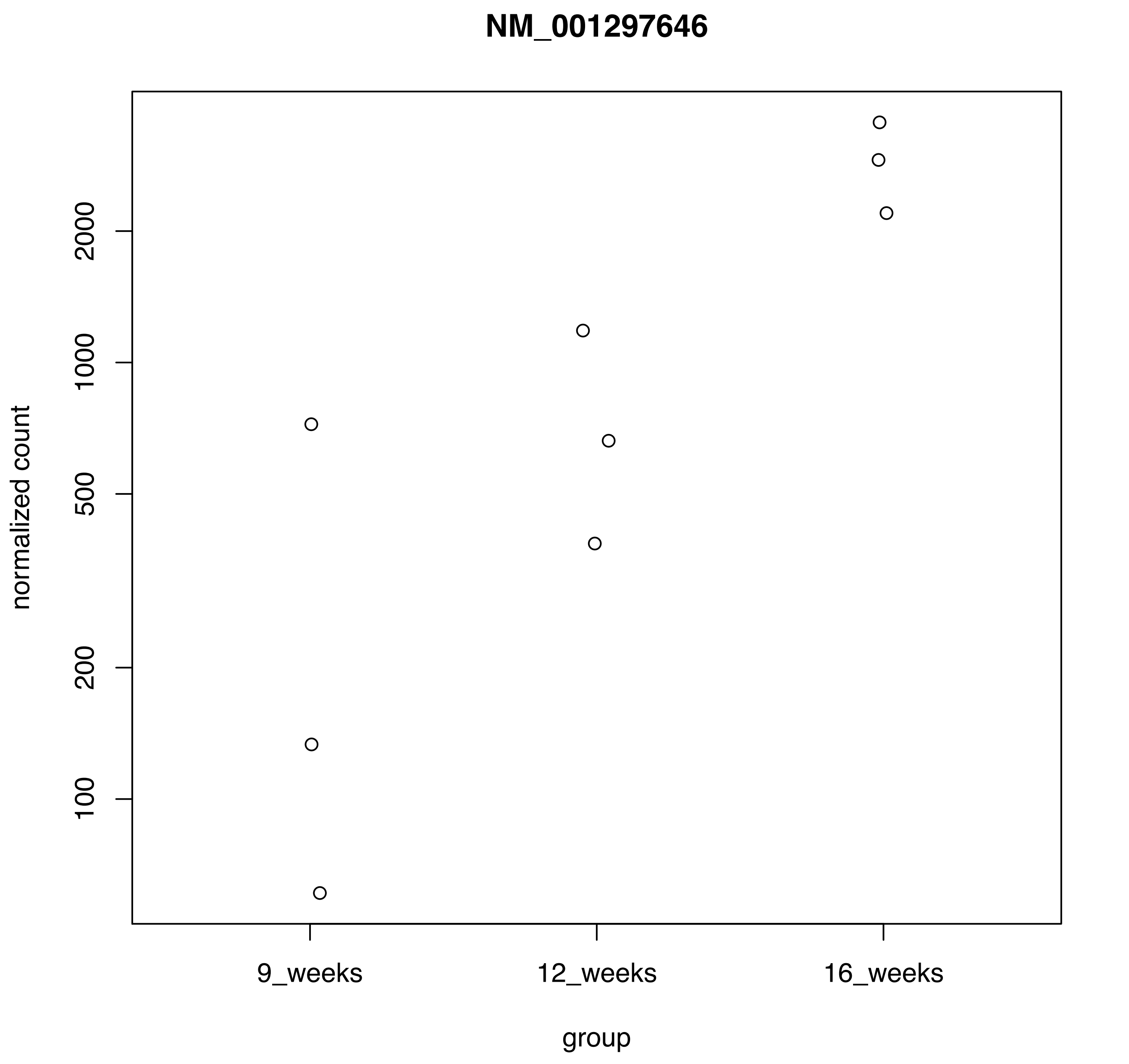
NM_001297646
normalized count
2000
1000
500
200
100
9_weeks
12_weeks
16_weeks
group

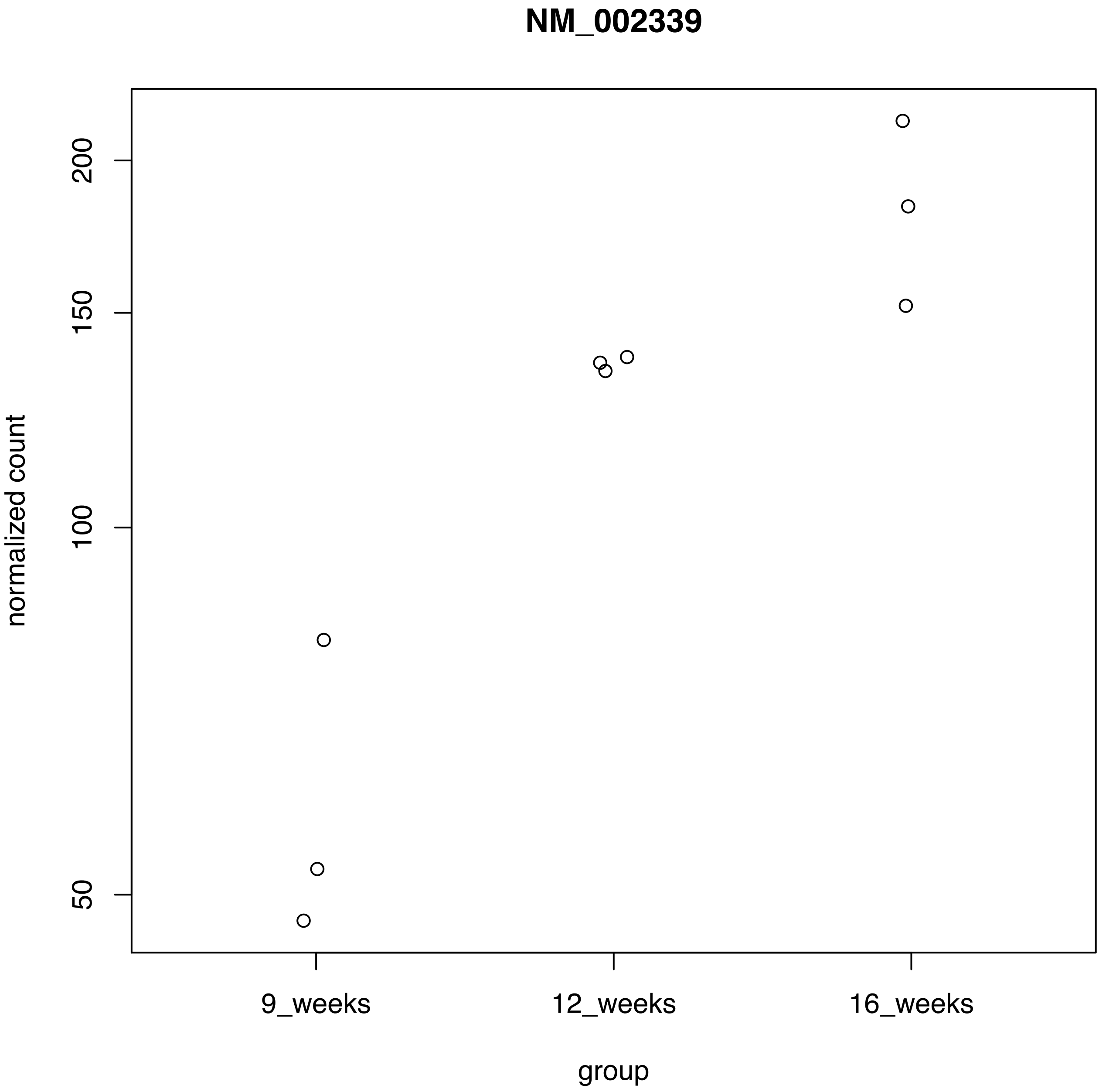
NM_002339
normalized count
200
150
100
50
9_weeks
12_weeks
16_weeks
group

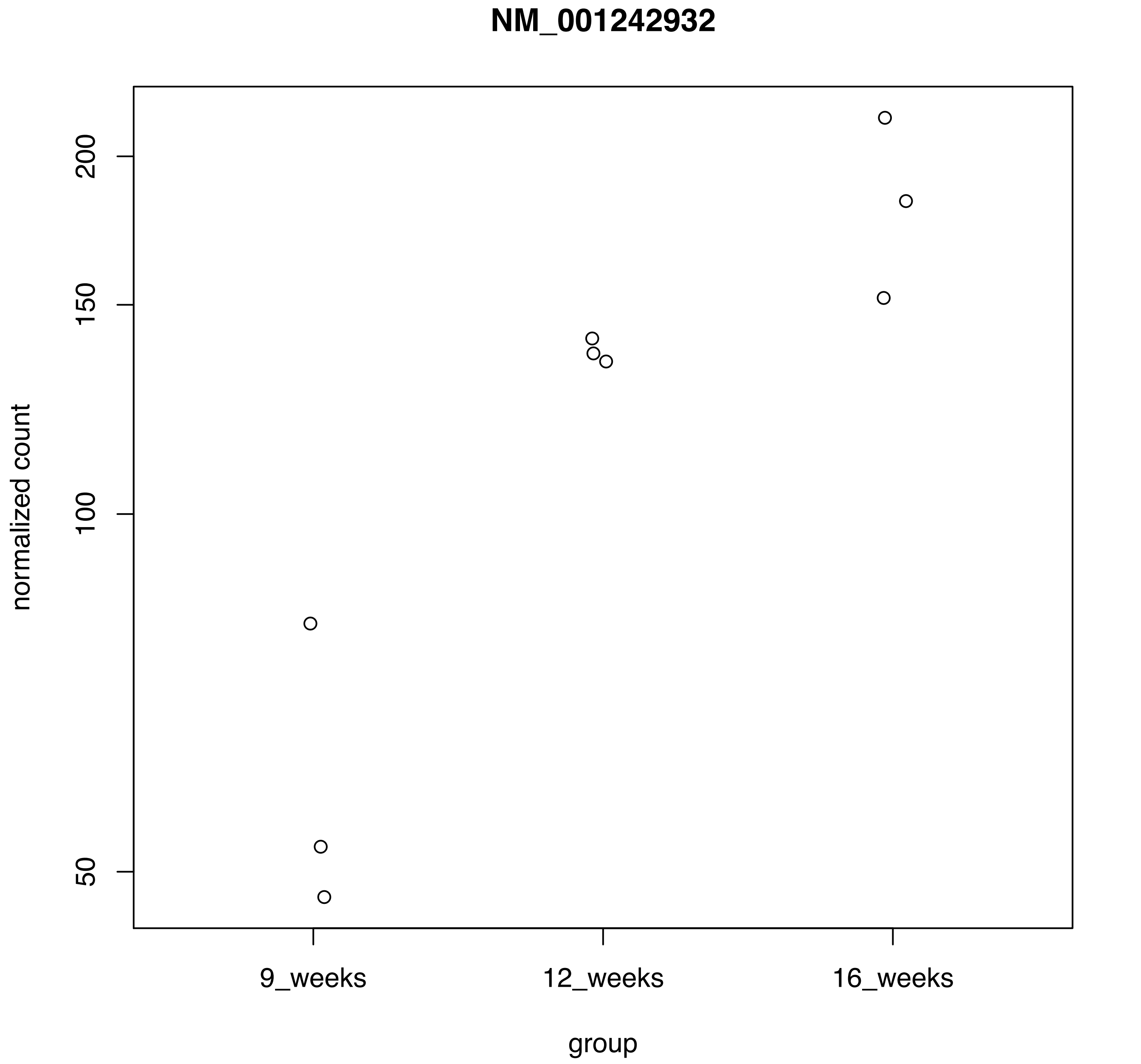
NM_001242932
normalized count
200
150
100
50
9_weeks
12_weeks
16_weeks
group

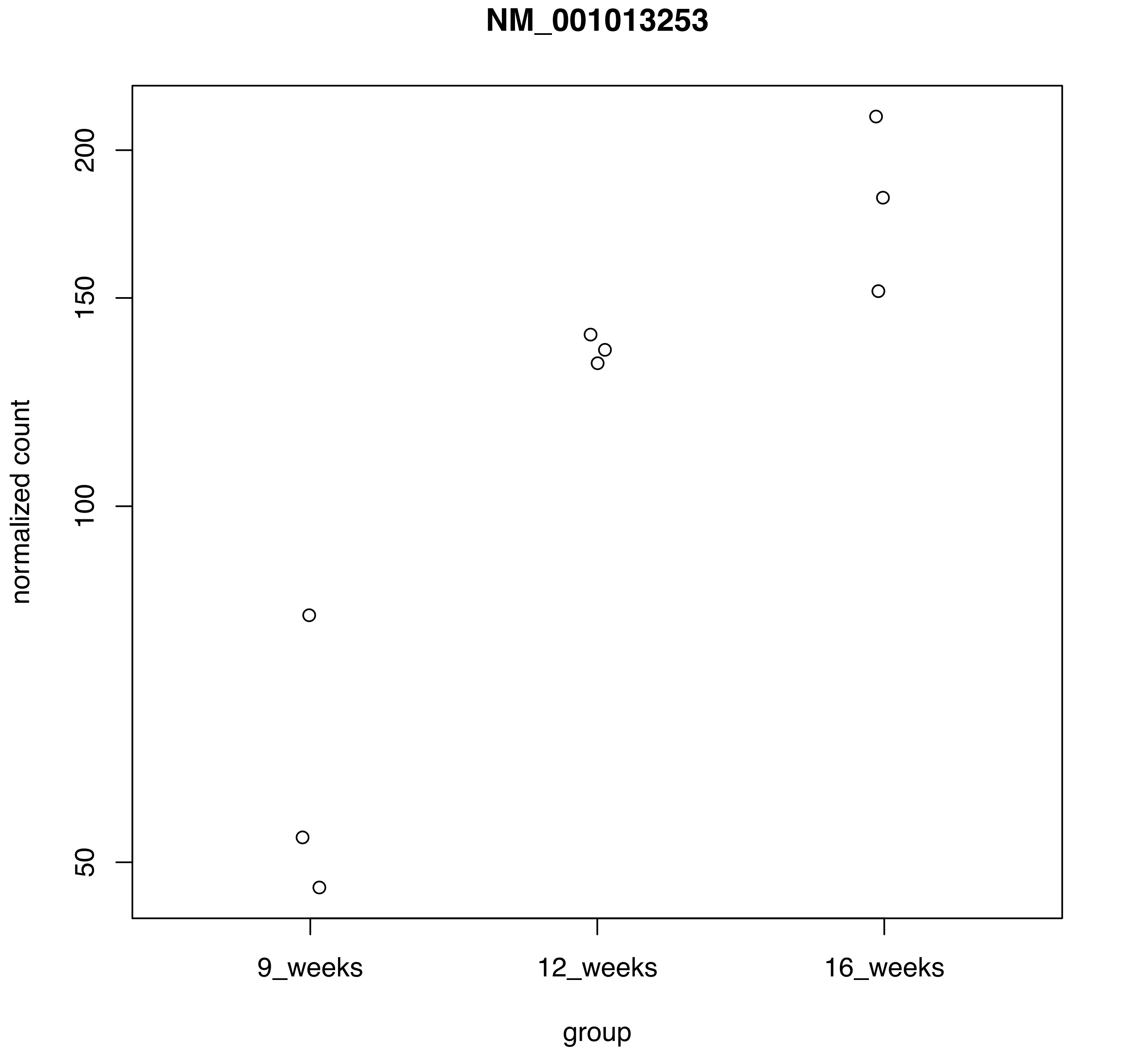
NM_001013253
normalized count
200
150
100
50
9_weeks
12_weeks
16_weeks
group

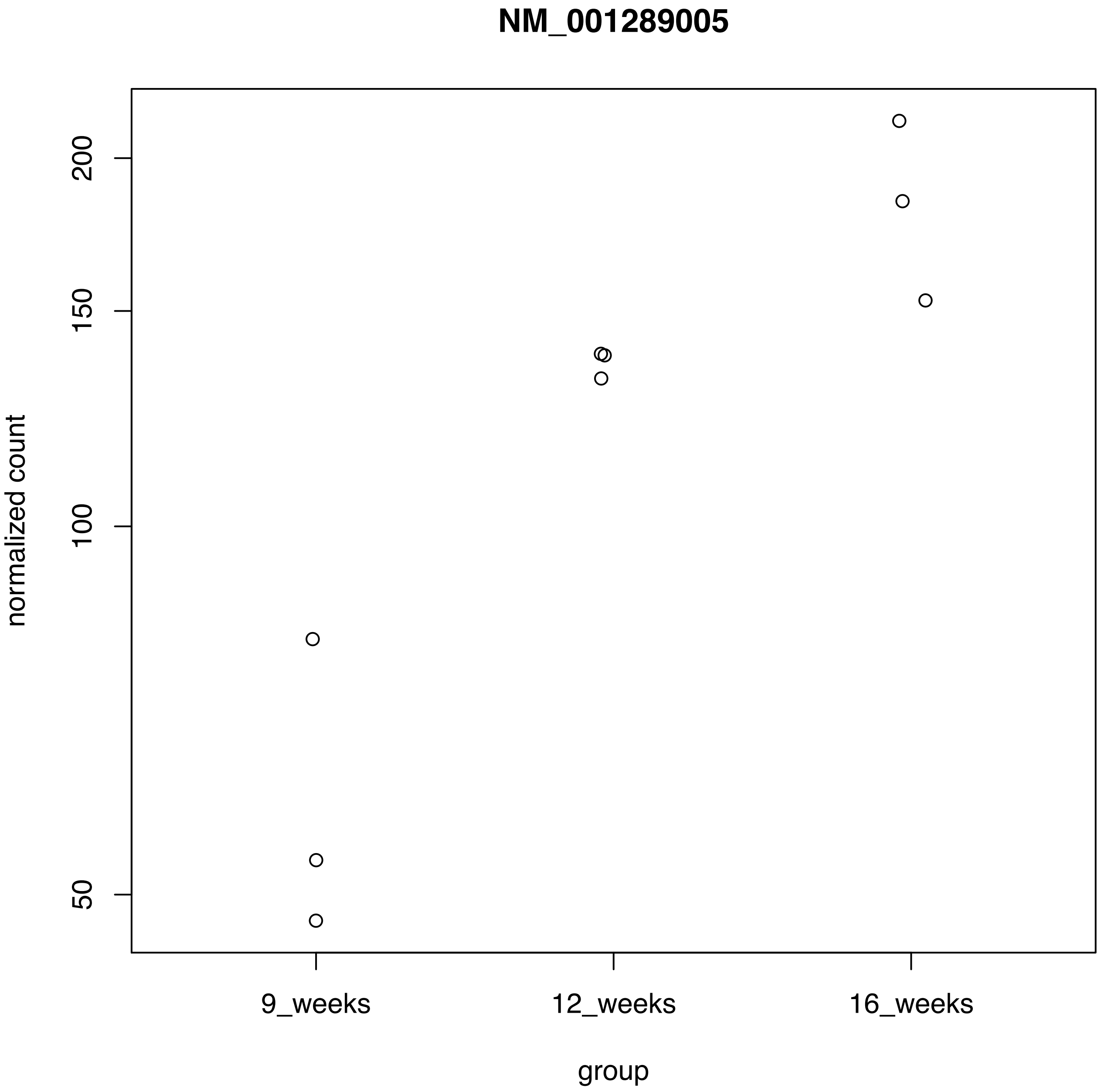
NM_001289005
normalized count
50
100
150
200
group
9_weeks
12_weeks
16_weeks

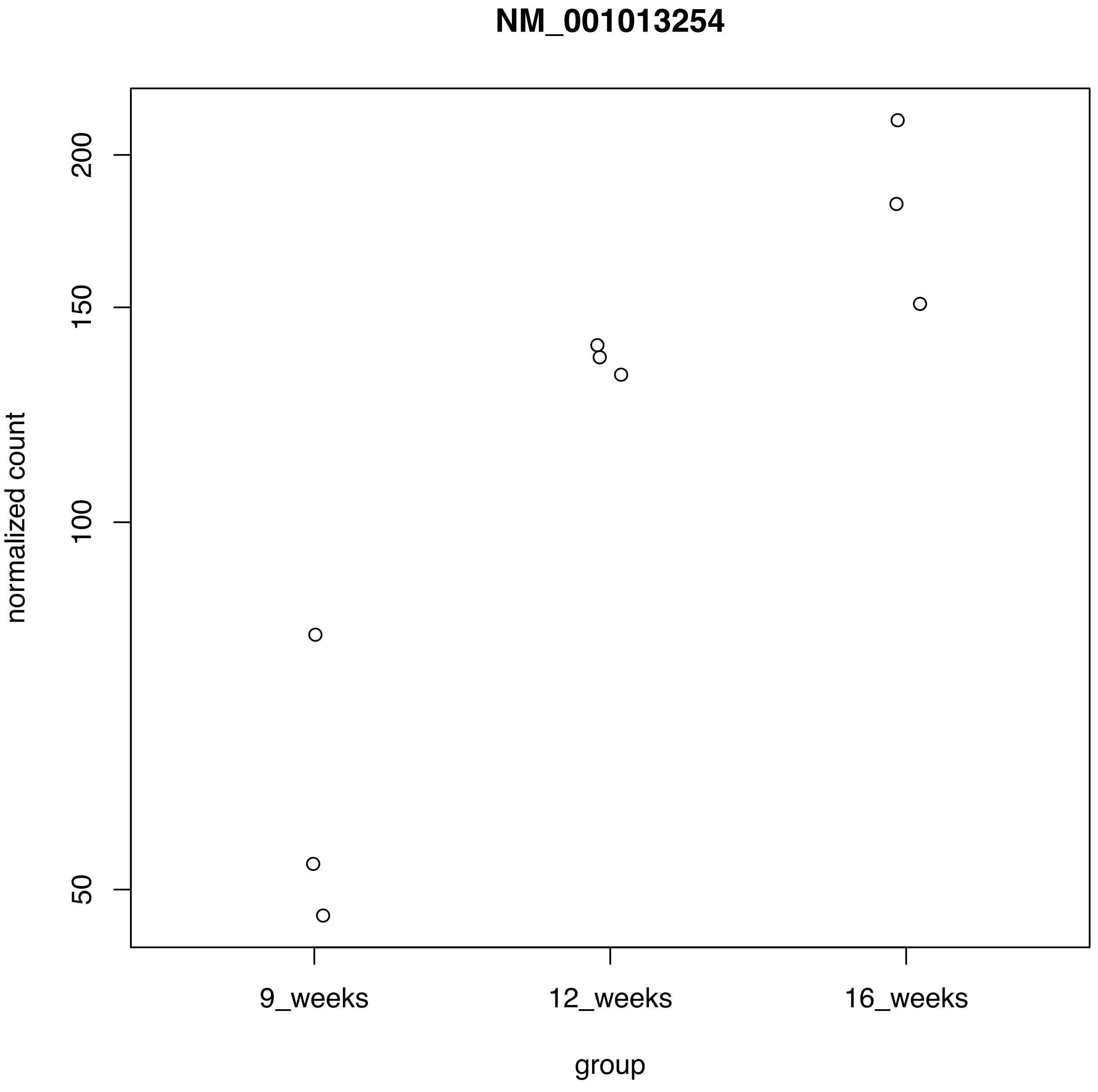
NM_001013254
normalized count
200
150
100
50
9_weeks
12_weeks
16_weeks
group

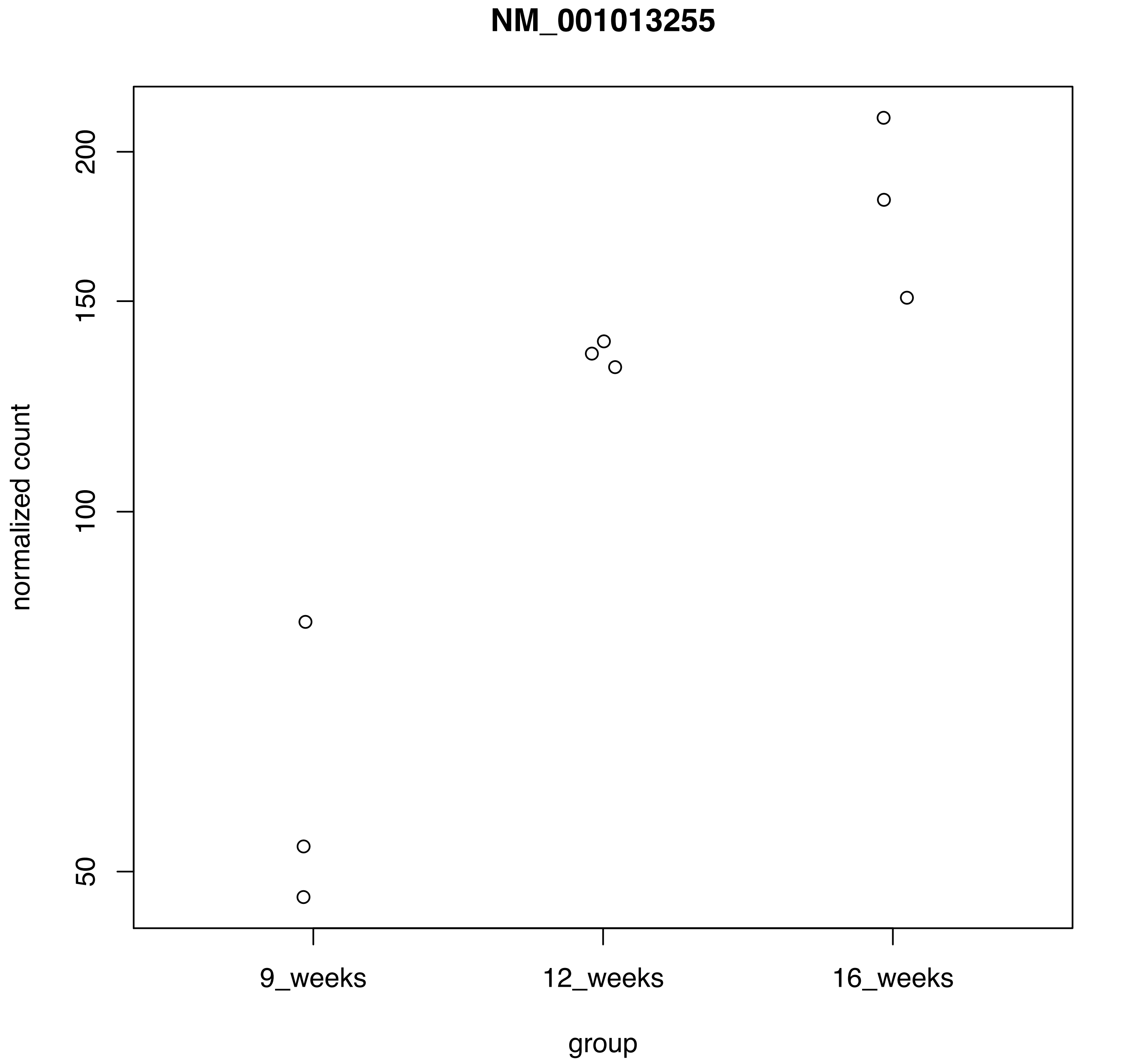
NM_001013255
normalized count
200
150
100
50
9_weeks
12_weeks
16_weeks
group

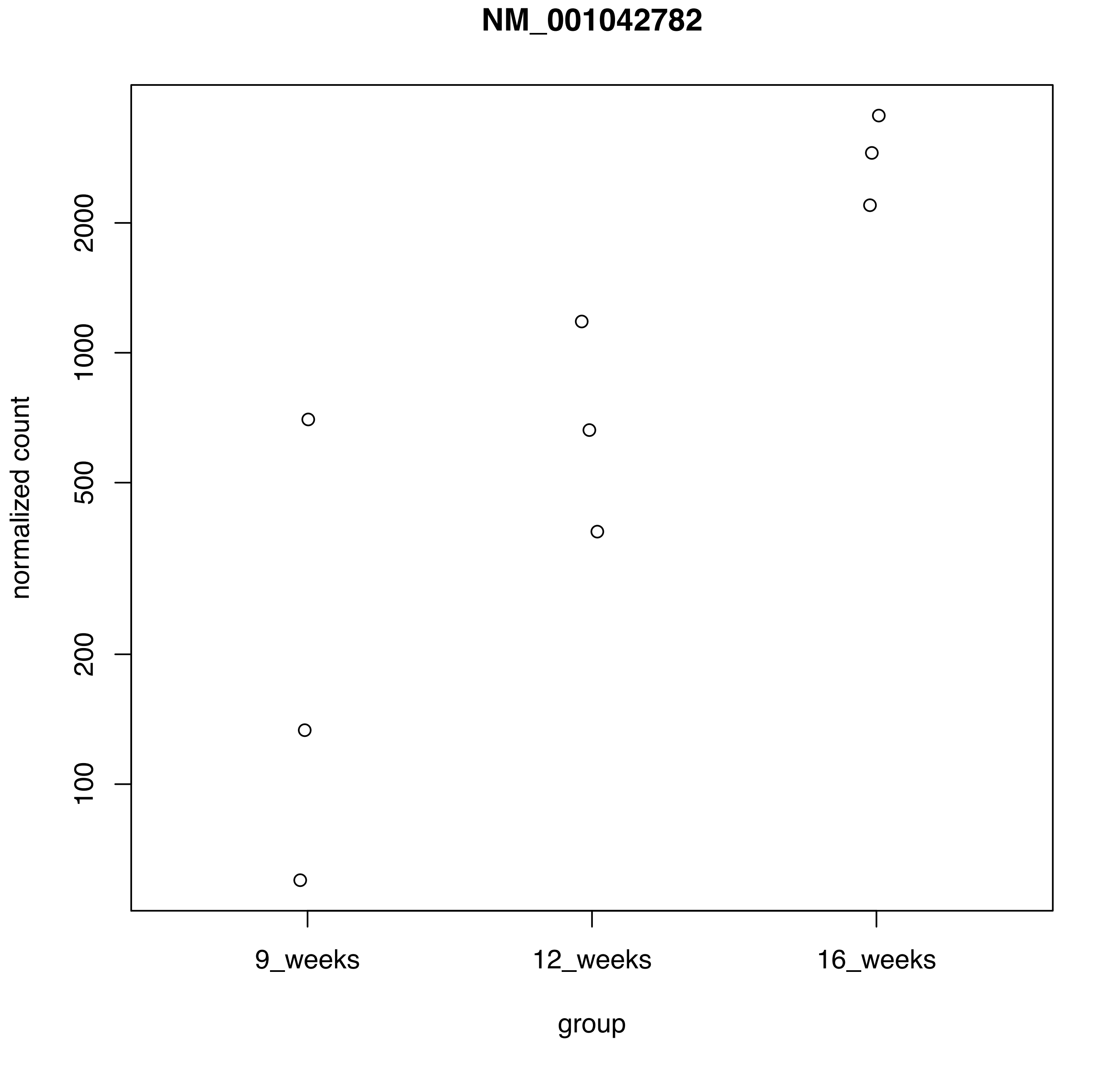
NM_001042782
normalized count
2000
1000
500
200
100
9_weeks
12_weeks
16_weeks
group

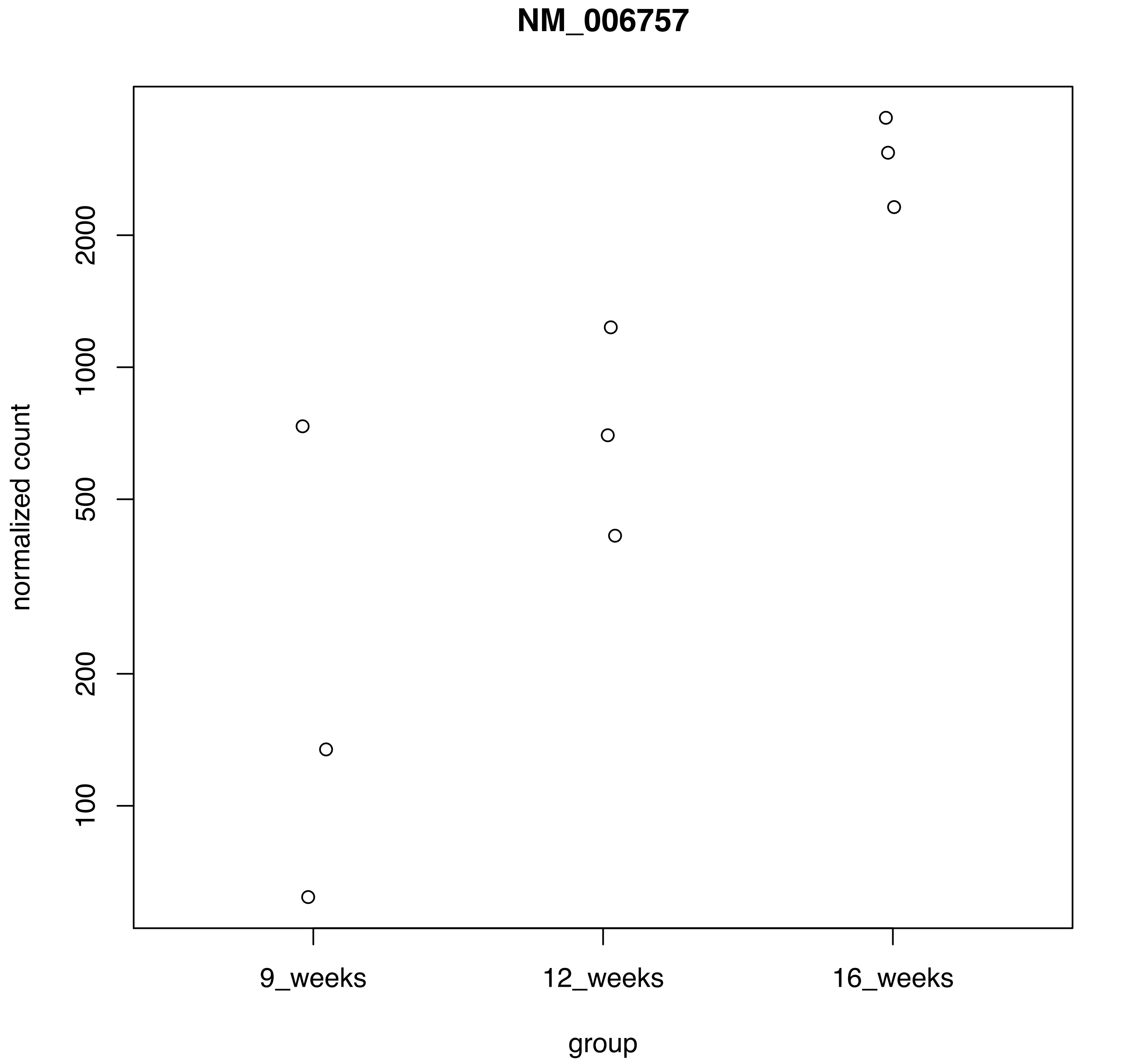

NM_006757
normalized count
2000
1000
500
200
100
9_weeks
12_weeks
16_weeks
group

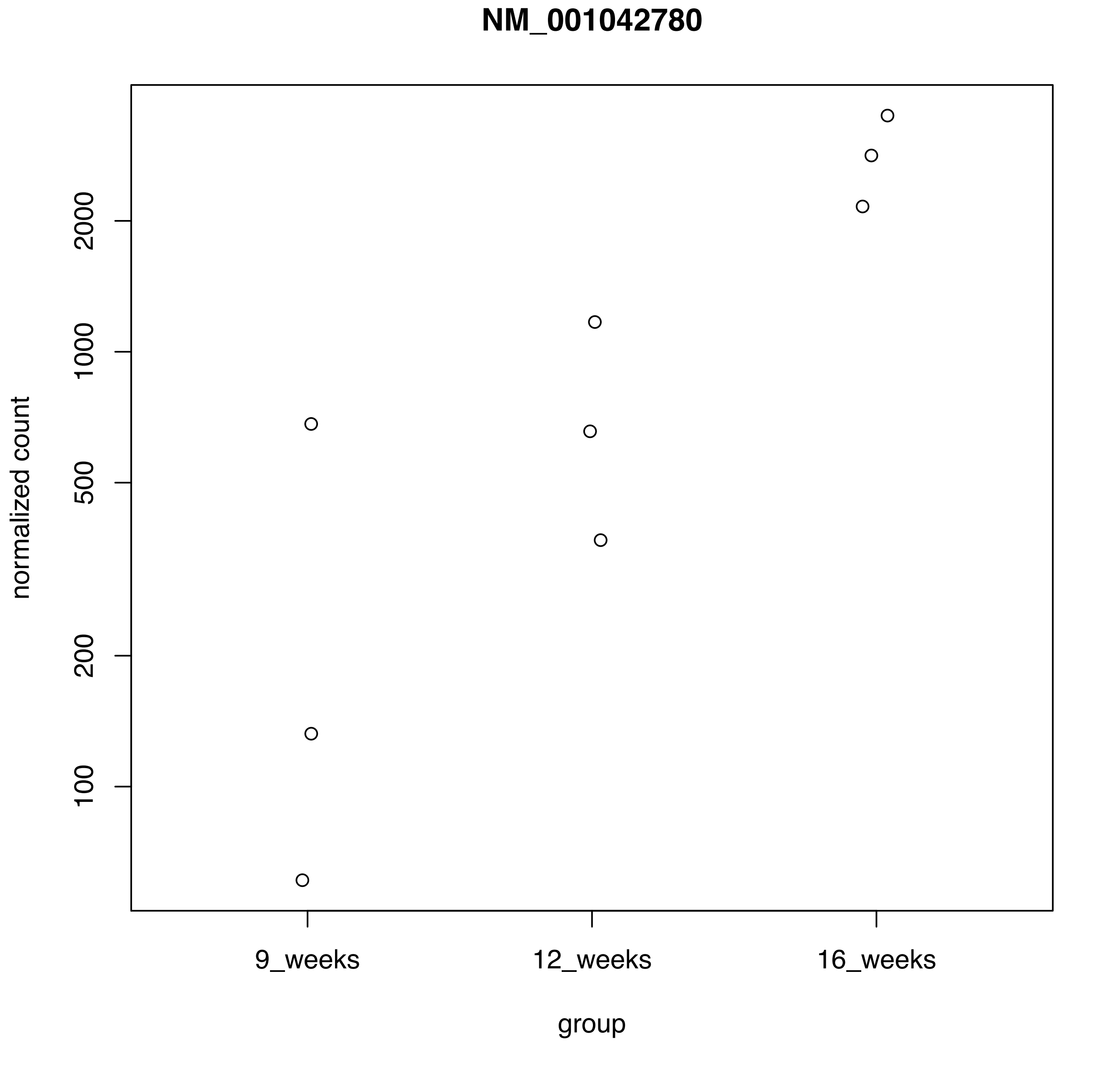

NM_001042780
normalized count
2000
1000
500
200
100
9_weeks
12_weeks
16_weeks
group

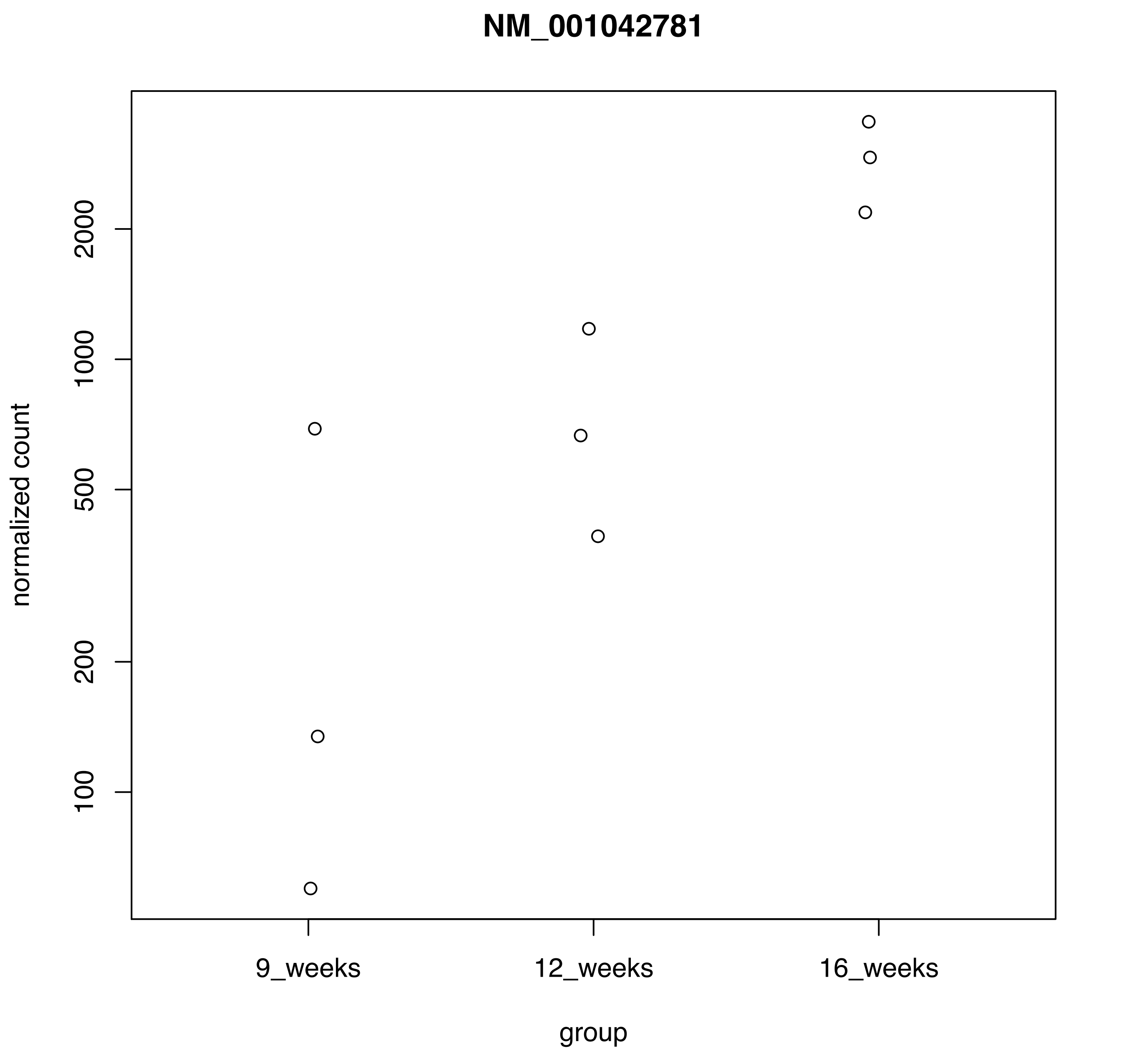
NM_001042781
normalized count
100
200
500
1000
2000
group
9_weeks
12_weeks
16_weeks

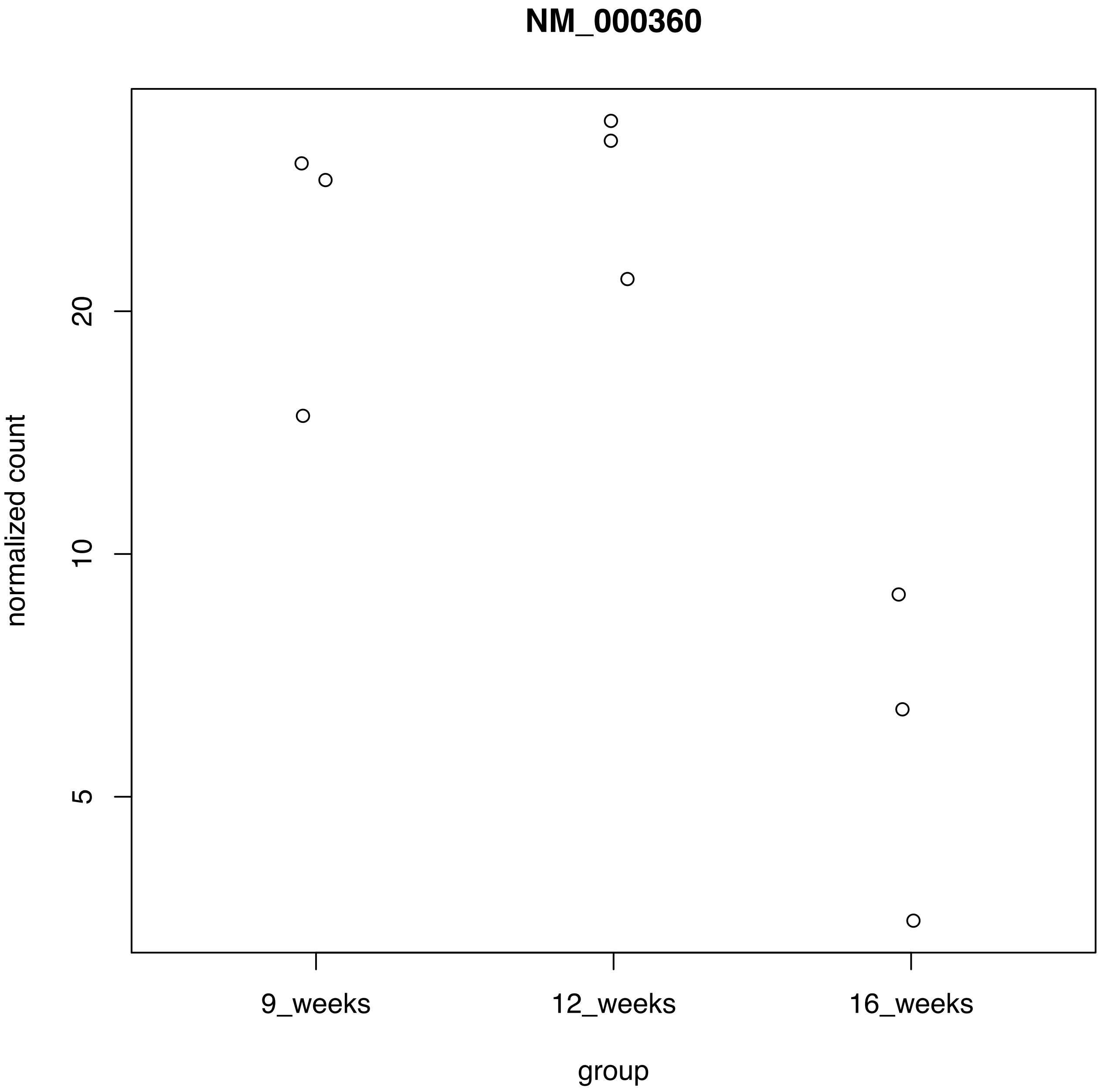
NM_000360
normalized count
20
10
5
9_weeks
12_weeks
16_weeks
group

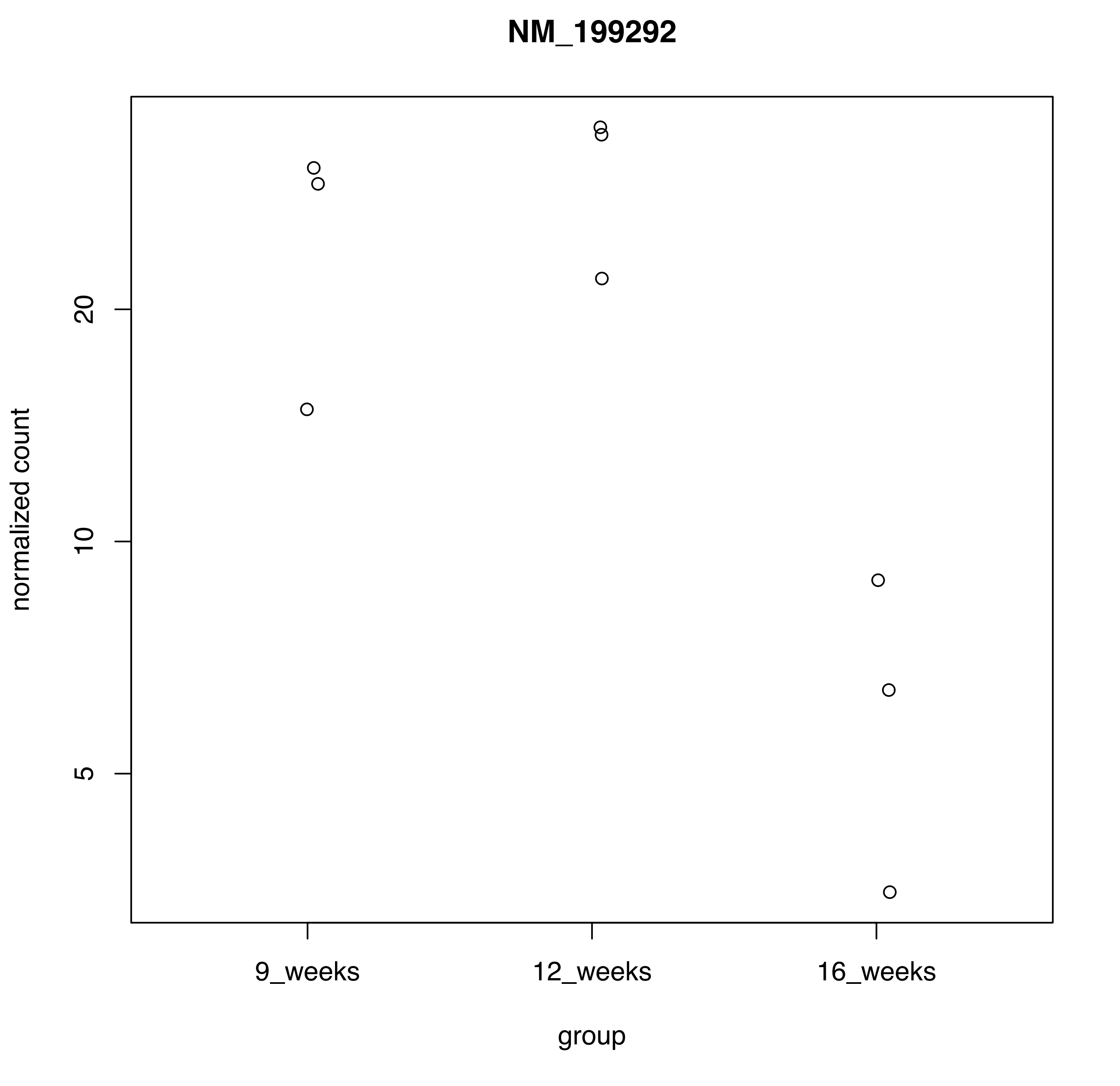

NM_199292
normalized count
20
10
5
9_weeks
12_weeks
16_weeks
group

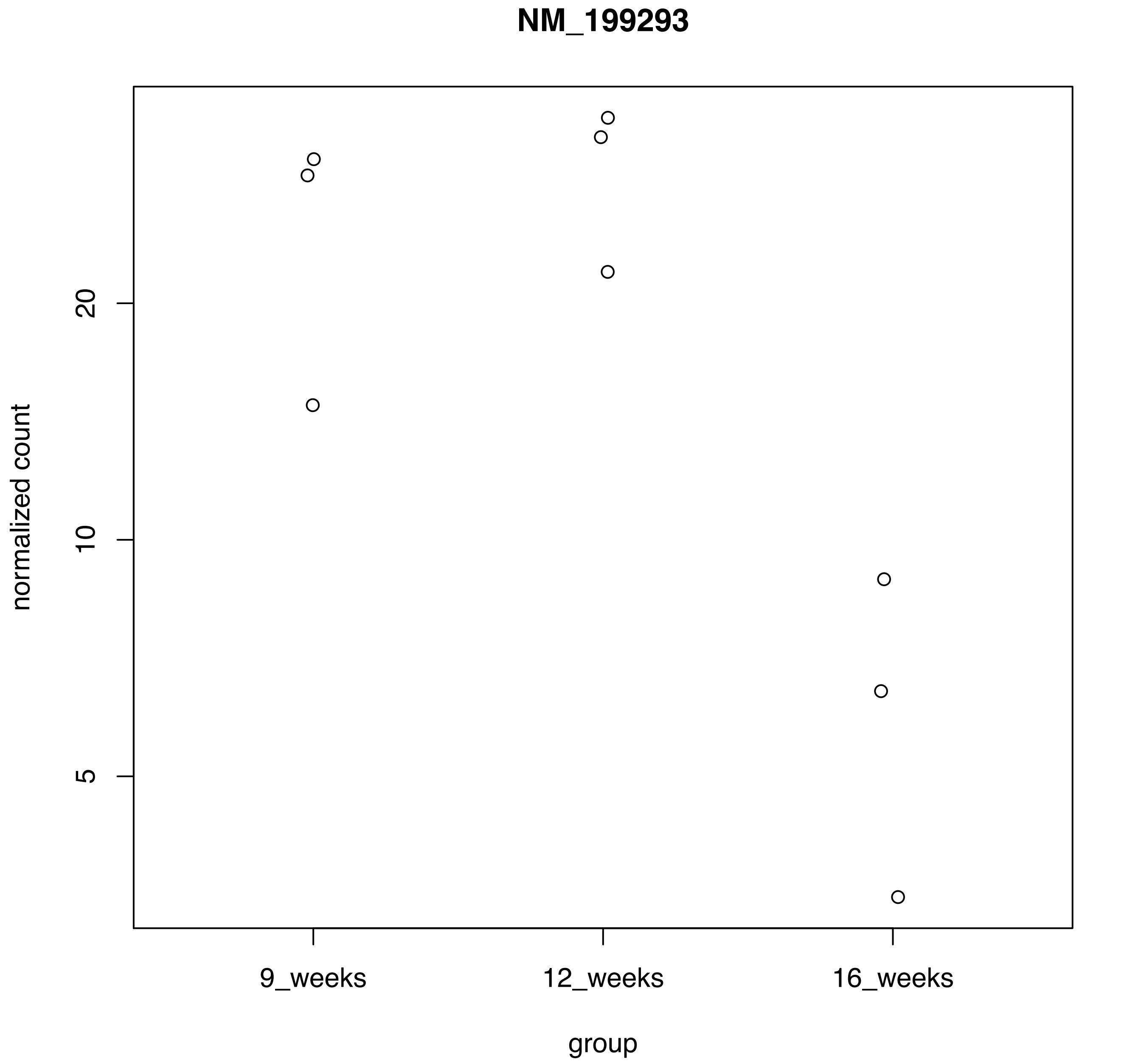
NM_199293
normalized count
5
10
20
9_weeks
12_weeks
16_weeks
group

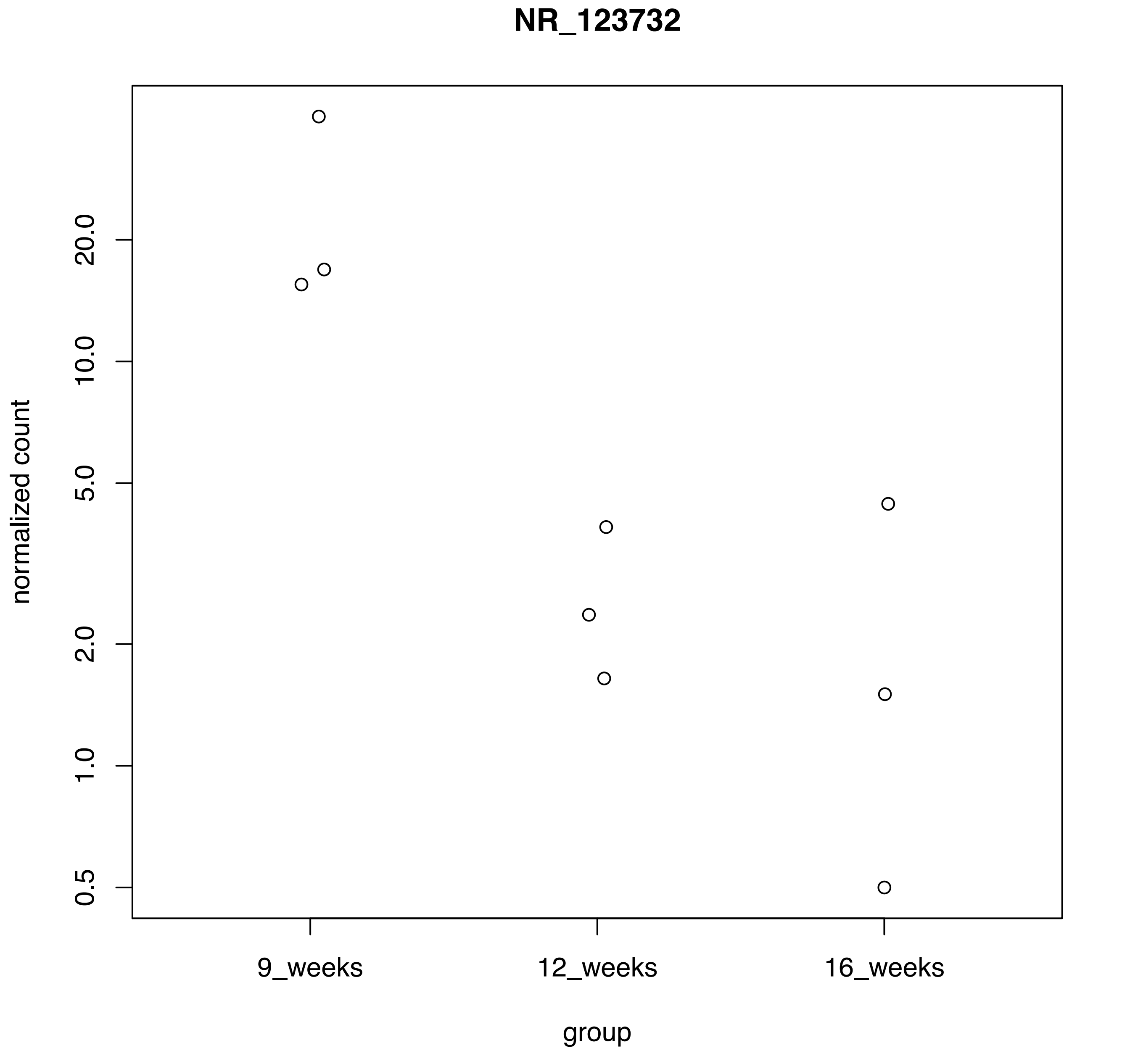

NR_123732
normalized count
20.0
10.0
5.0
2.0
1.0
0.5
9_weeks
12_weeks
16_weeks
group

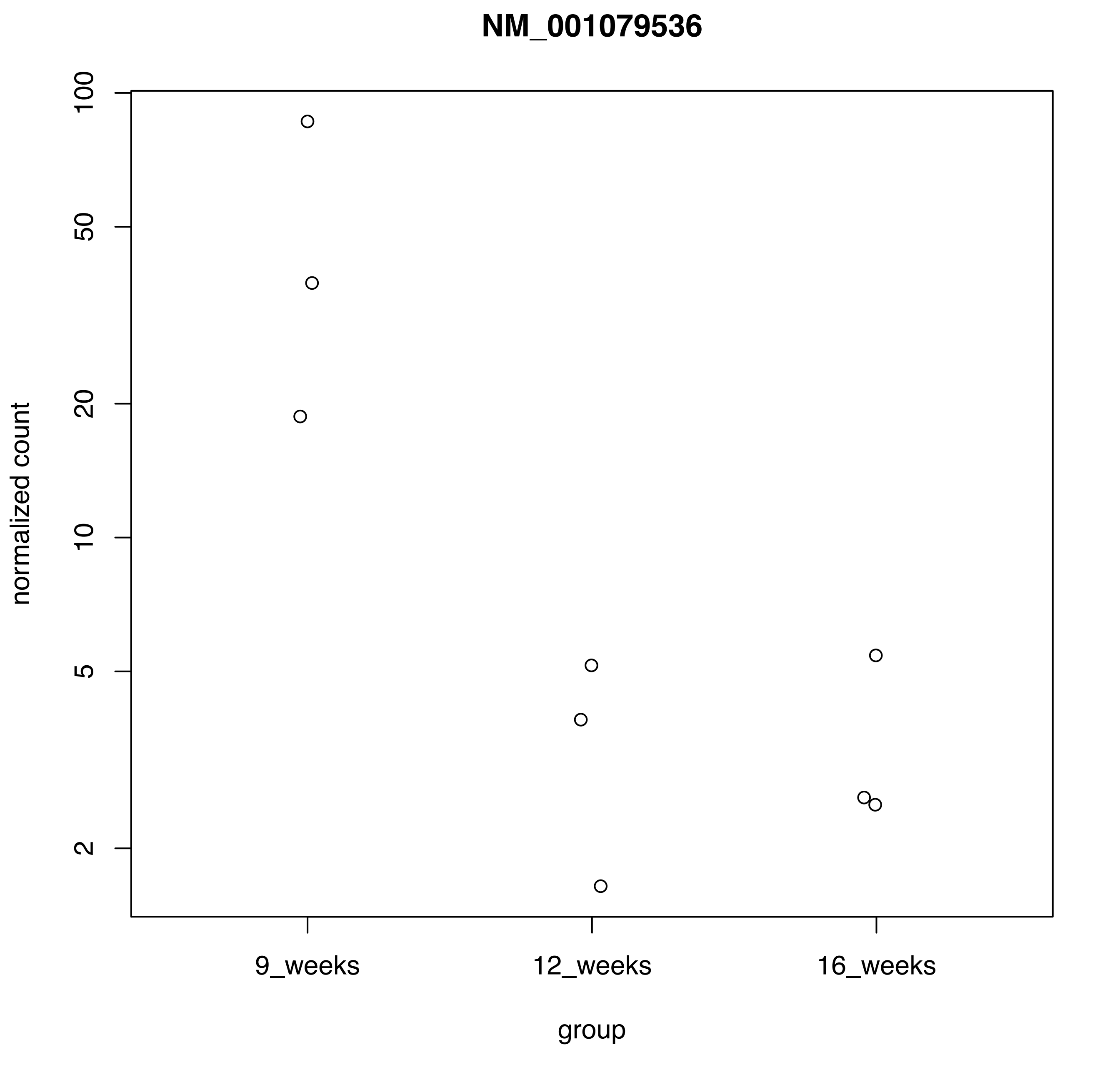
NM_001079536
normalized count
100
50
20
10
5
2
9_weeks
12_weeks
16_weeks
group

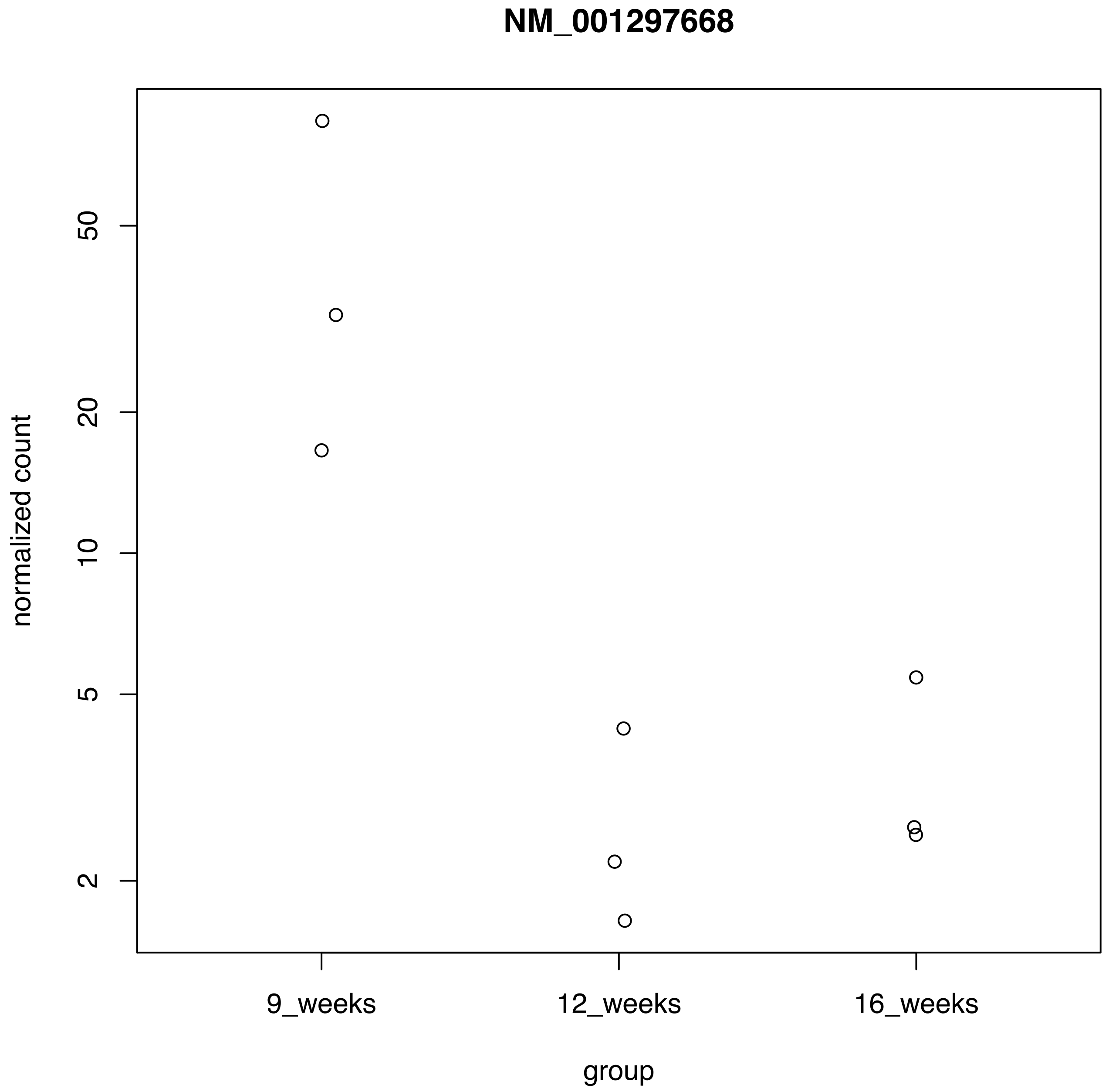
NM_001297668
normalized count
50
20
10
5
2
9_weeks
12_weeks
16_weeks
group

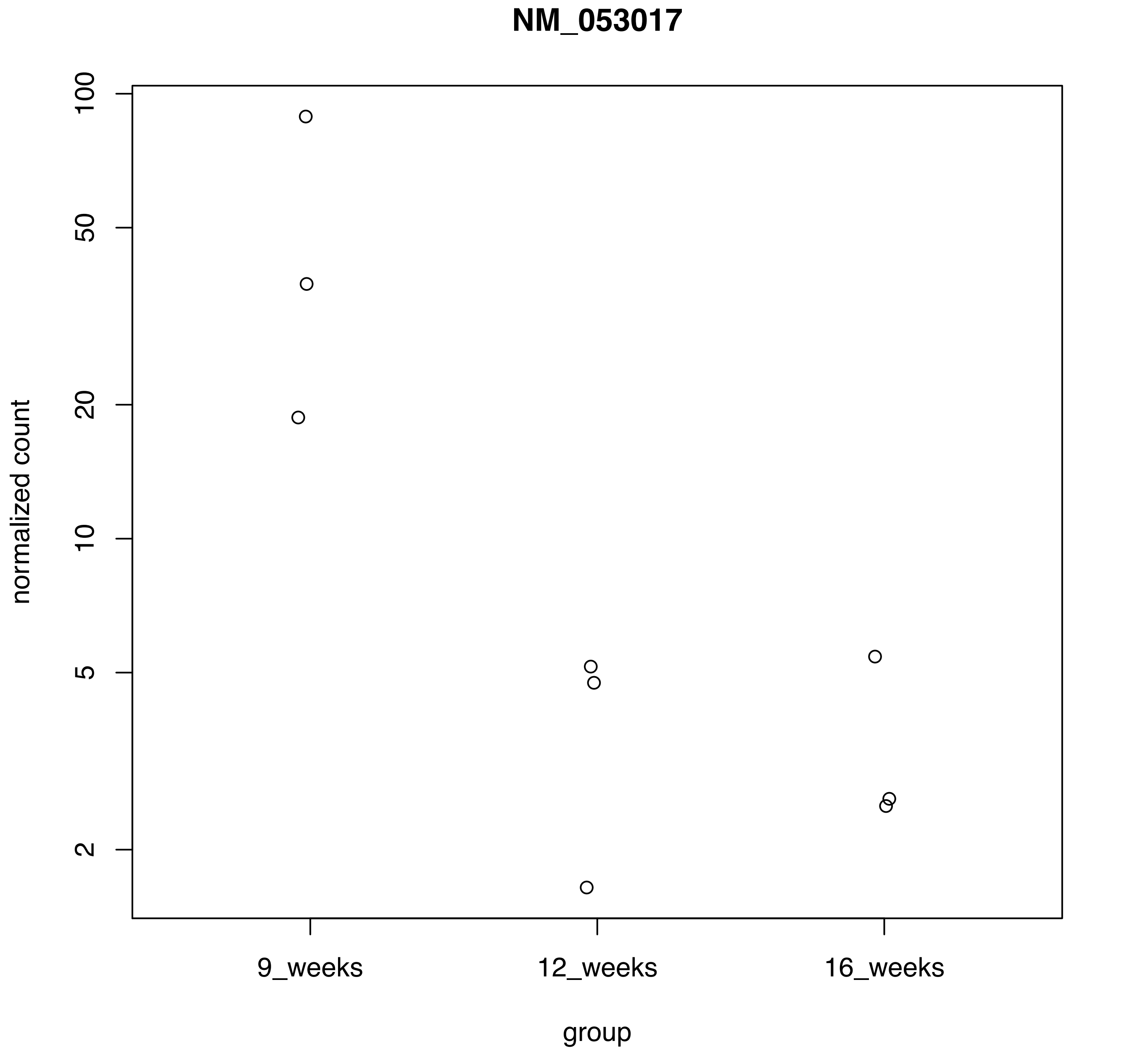
NM_053017
normalized count
100
50
20
10
5
2
9_weeks
12_weeks
16_weeks
group

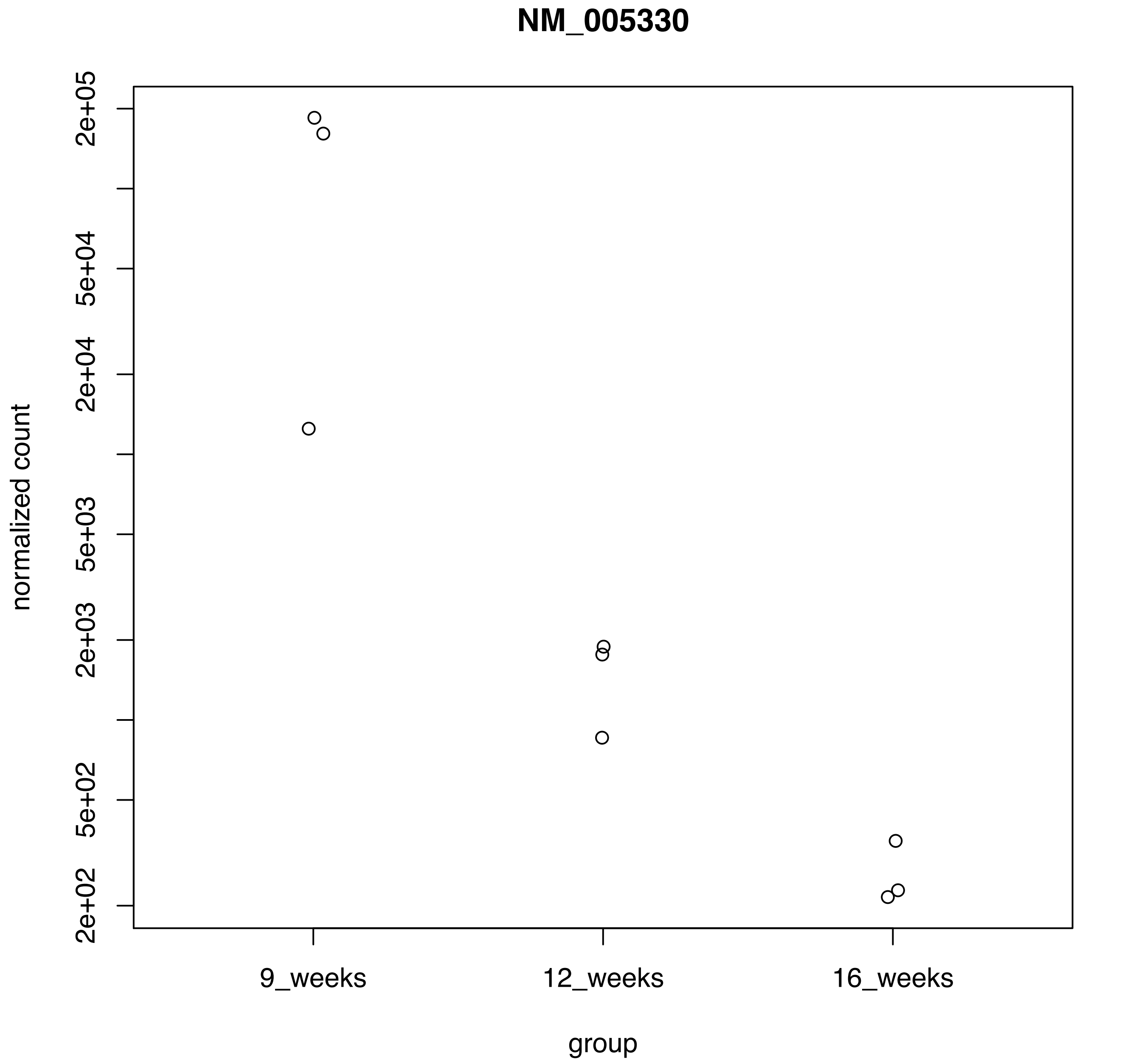
NM_005330
normalized count
2e+05
5e+04
2e+04
5e+03
2e+03
5e+02
2e+02
9_weeks
12_weeks
16_weeks
group

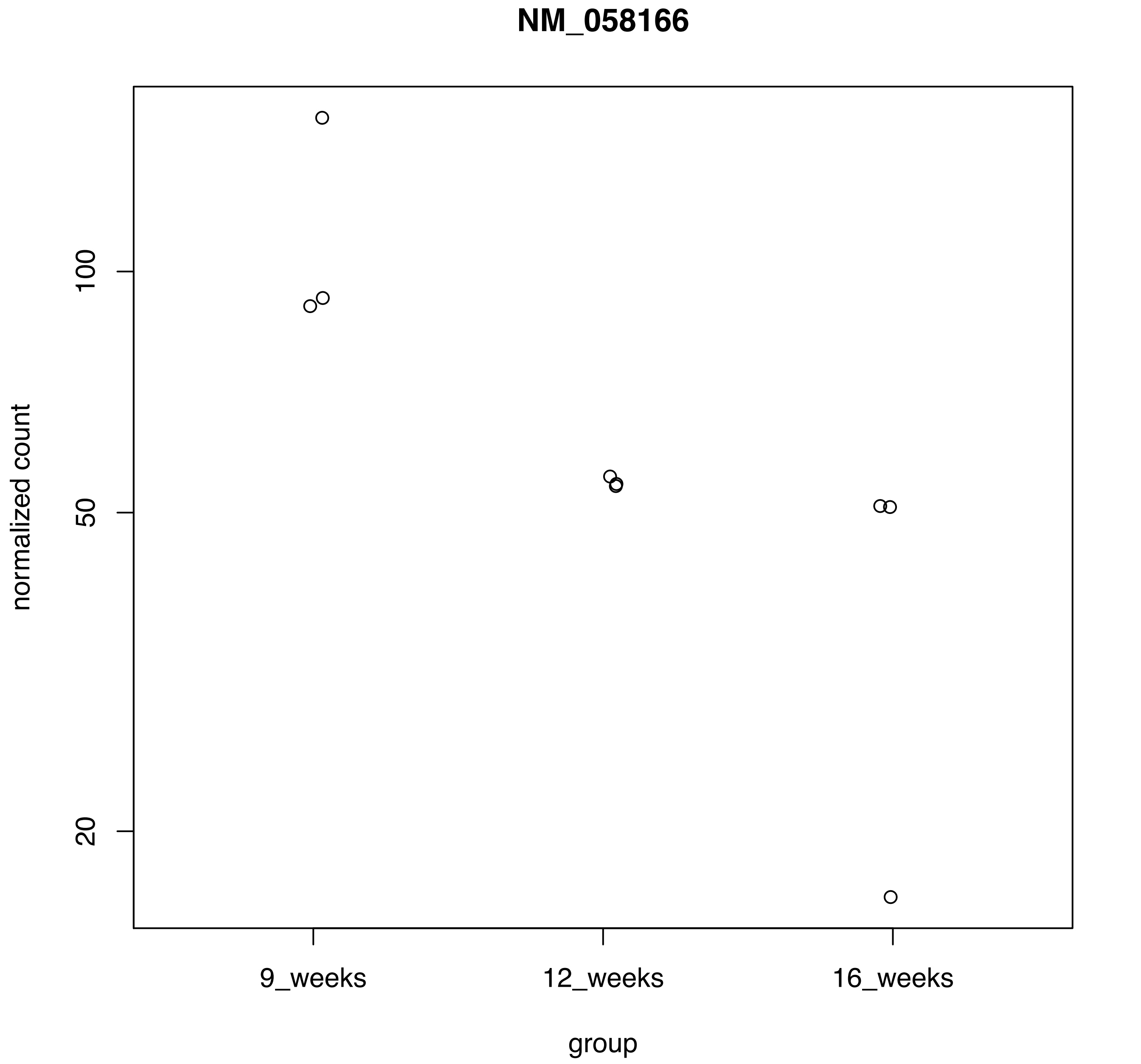

NM_058166
100
50
20
normalized count
9_weeks
12_weeks
16_weeks
group

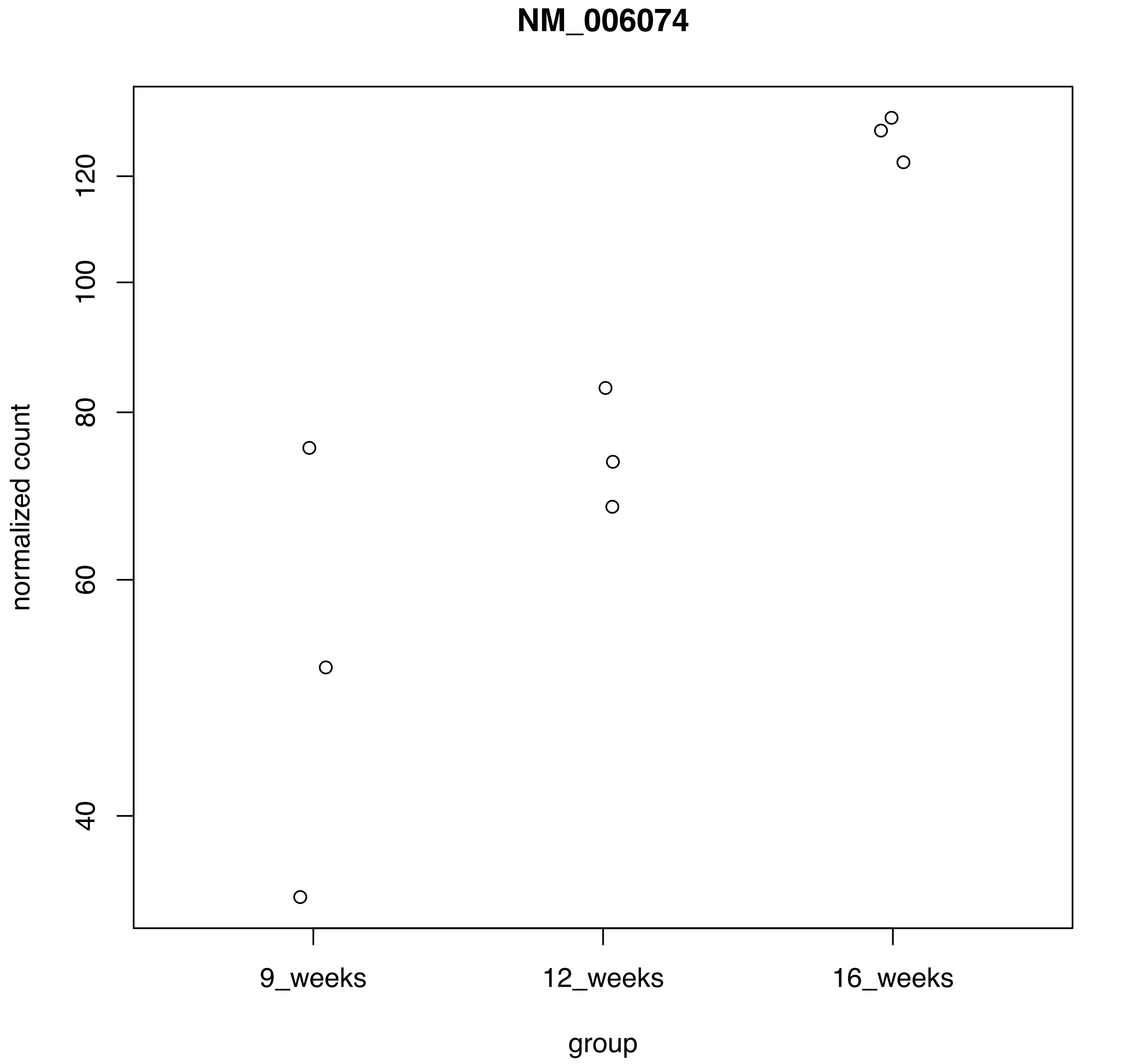
NM_006074
normalized count
40
60
80
100
120
9_weeks
12_weeks
16_weeks
group

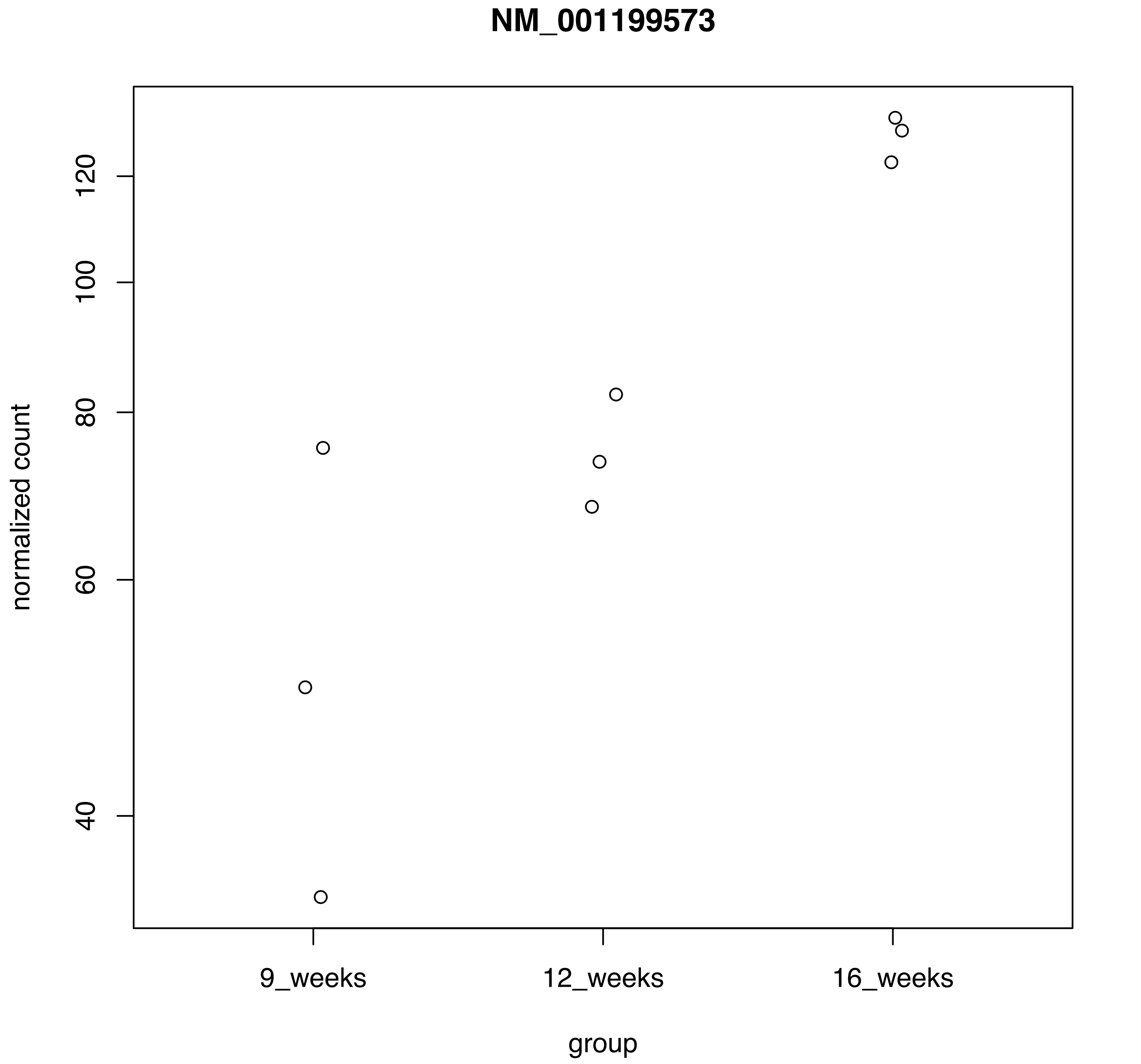
NM_001199573
normalized count
120
100
80
60
40
9_weeks
12_weeks
16_weeks
group

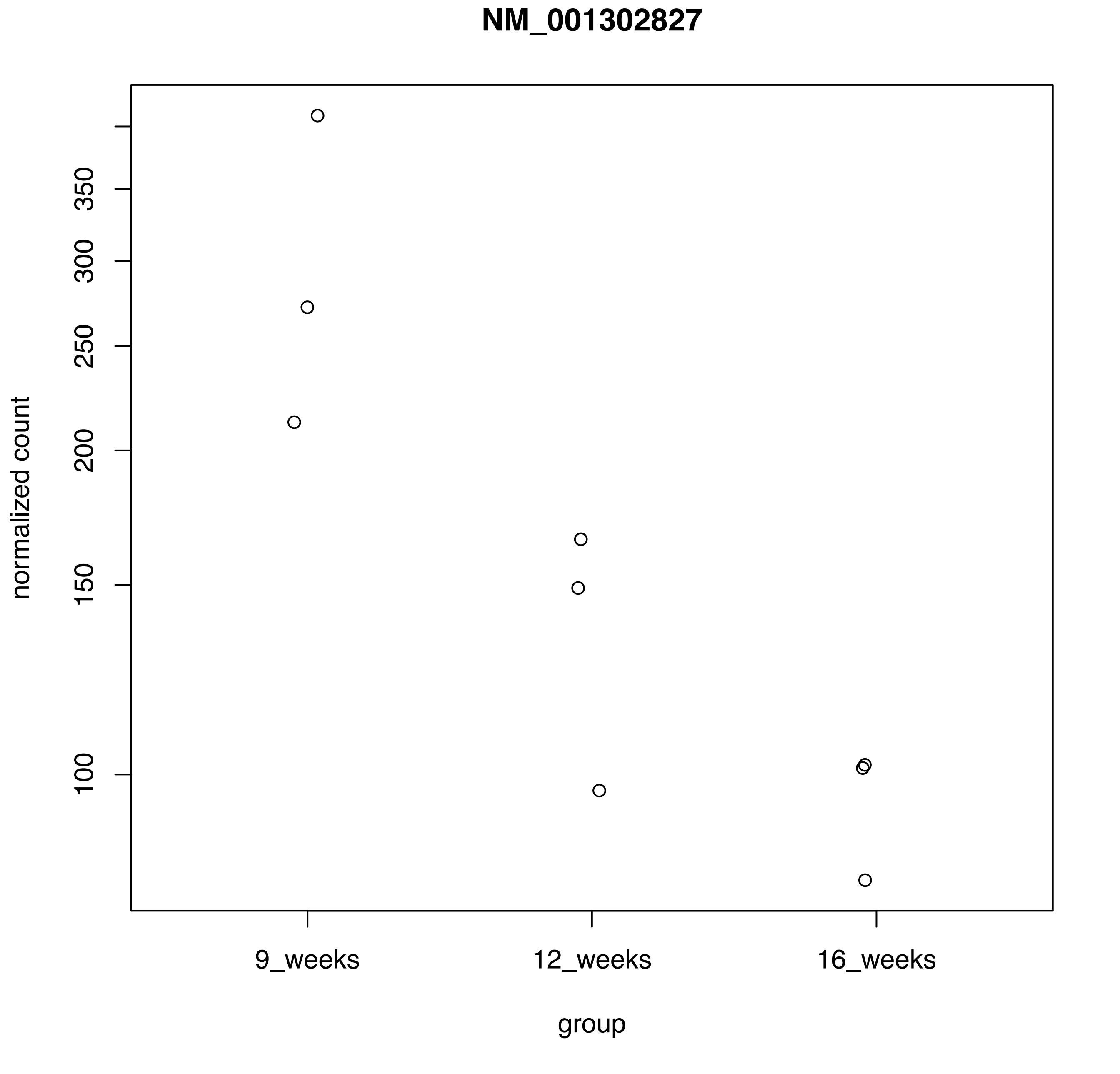
NM_001302827
normalized count
350
300
250
200
150
100
9_weeks
12_weeks
16_weeks
group

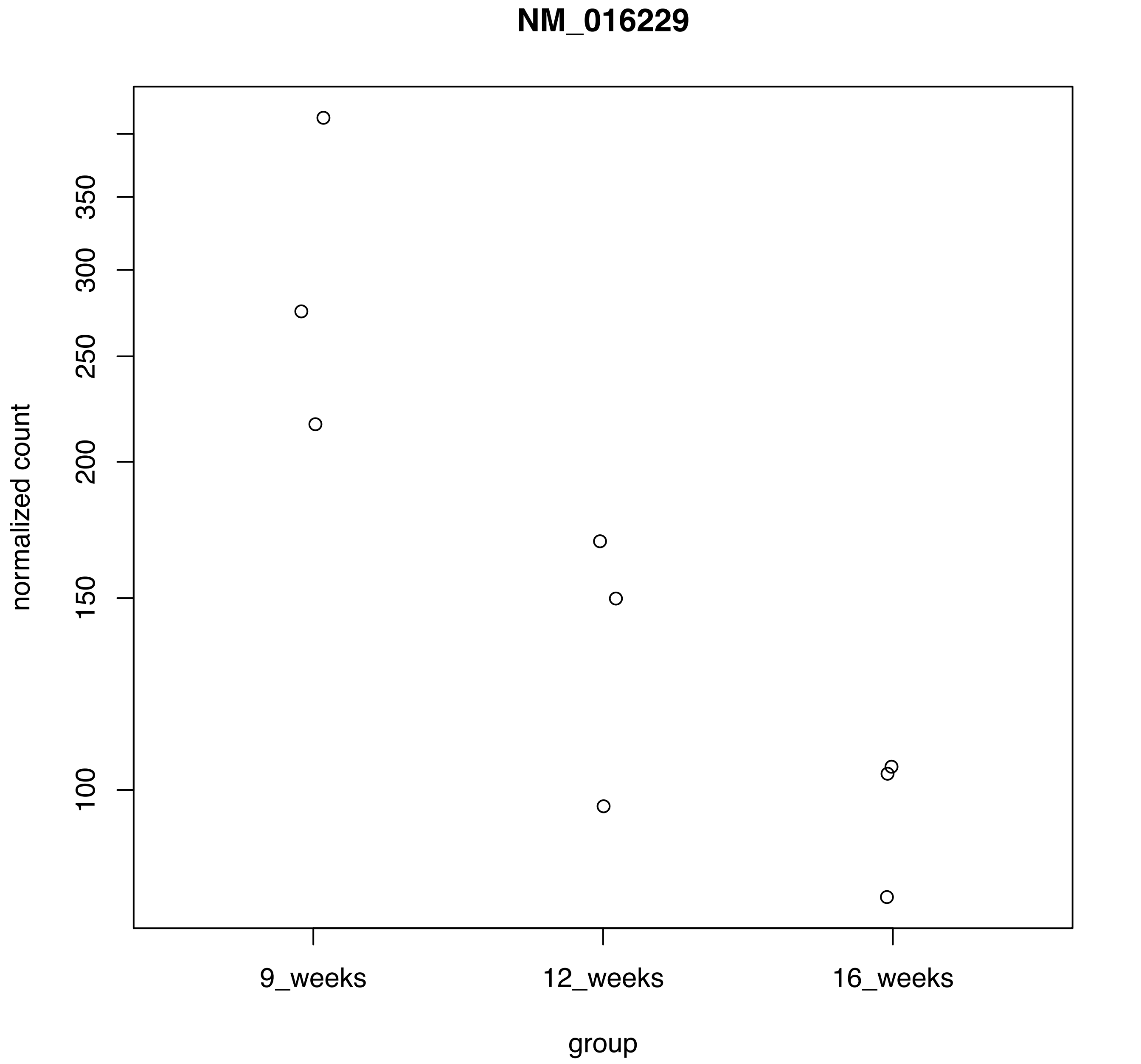
NM_016229
normalized count
100
150
200
250
300
350
group
9_weeks
12_weeks
16_weeks

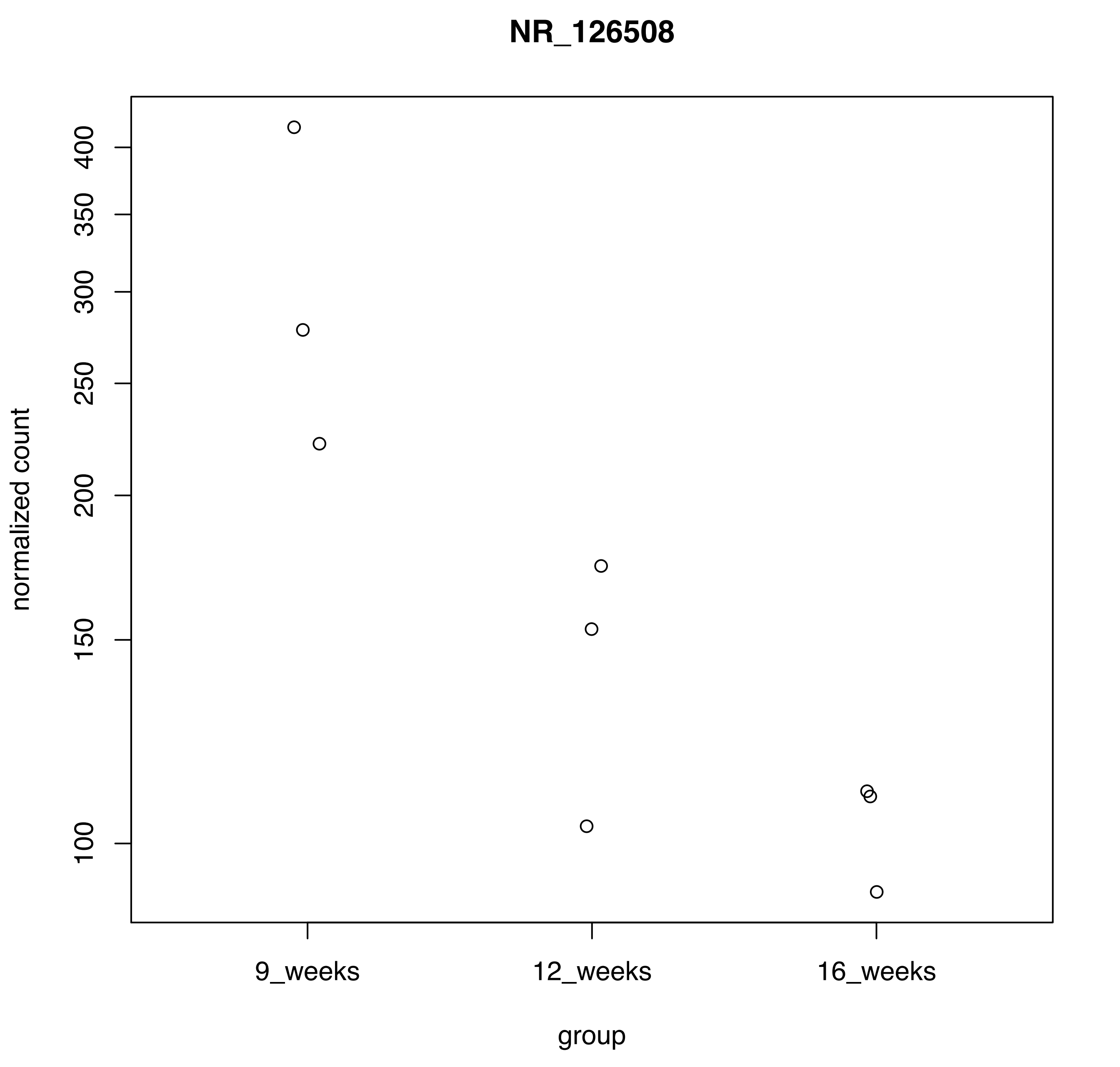
NR_126508
normalized count
400
350
300
250
200
150
100
9_weeks
12_weeks
16_weeks
group

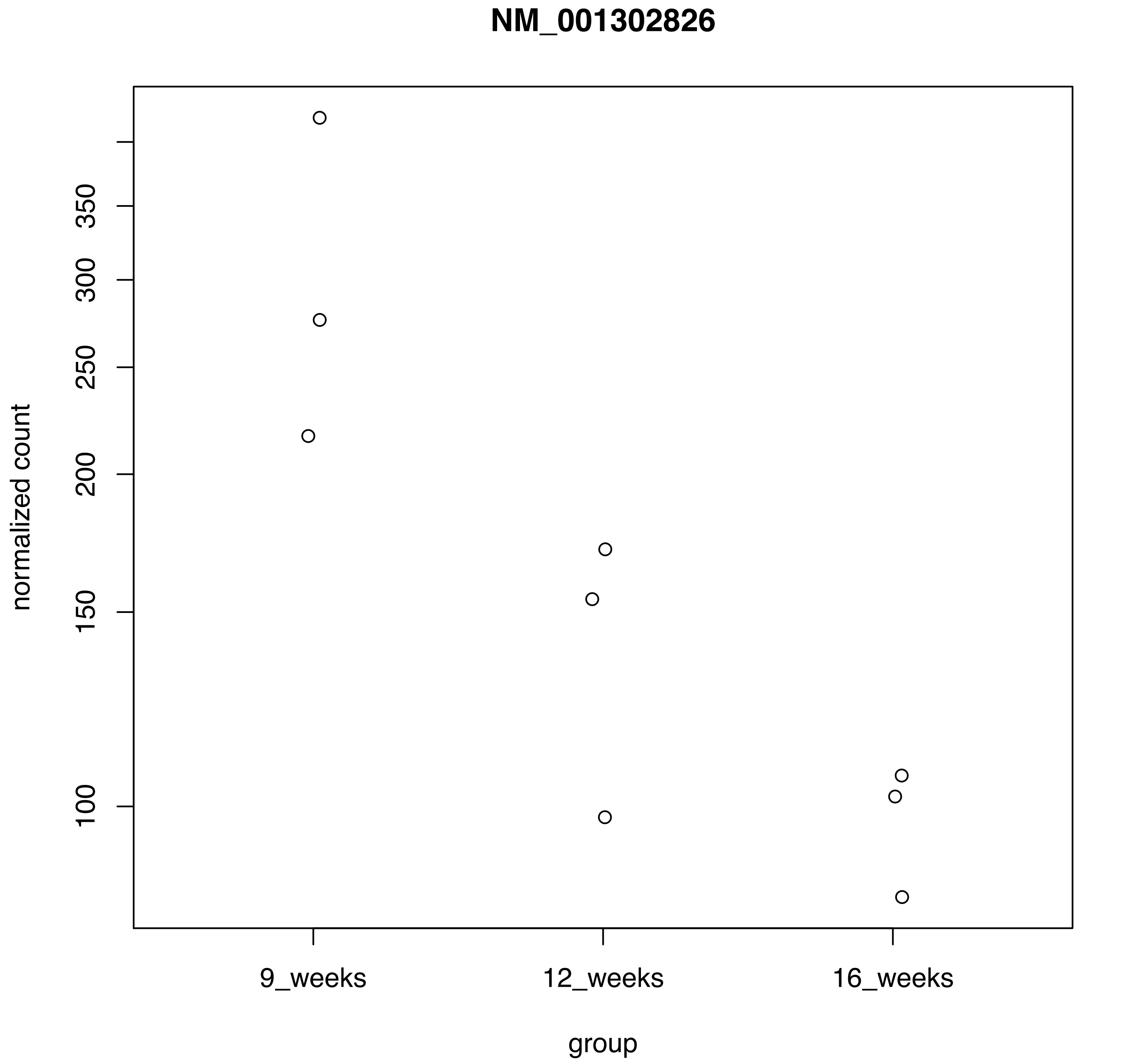
NM_001302826
normalized count
100
150
200
250
300
350
9_weeks
12_weeks
16_weeks
group

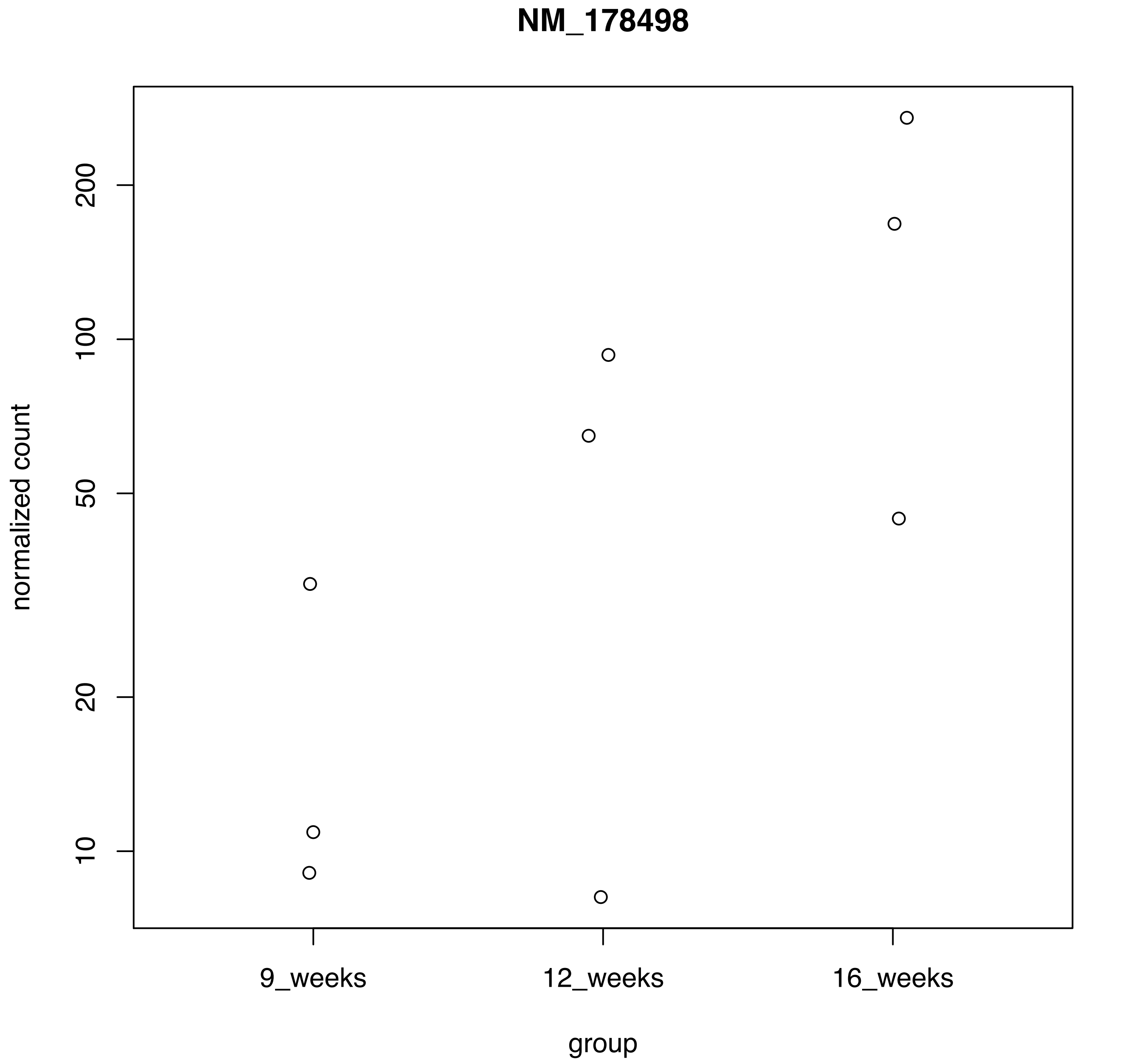
NM_178498
normalized count
10
20
50
100
200
9_weeks
12_weeks
16_weeks
group

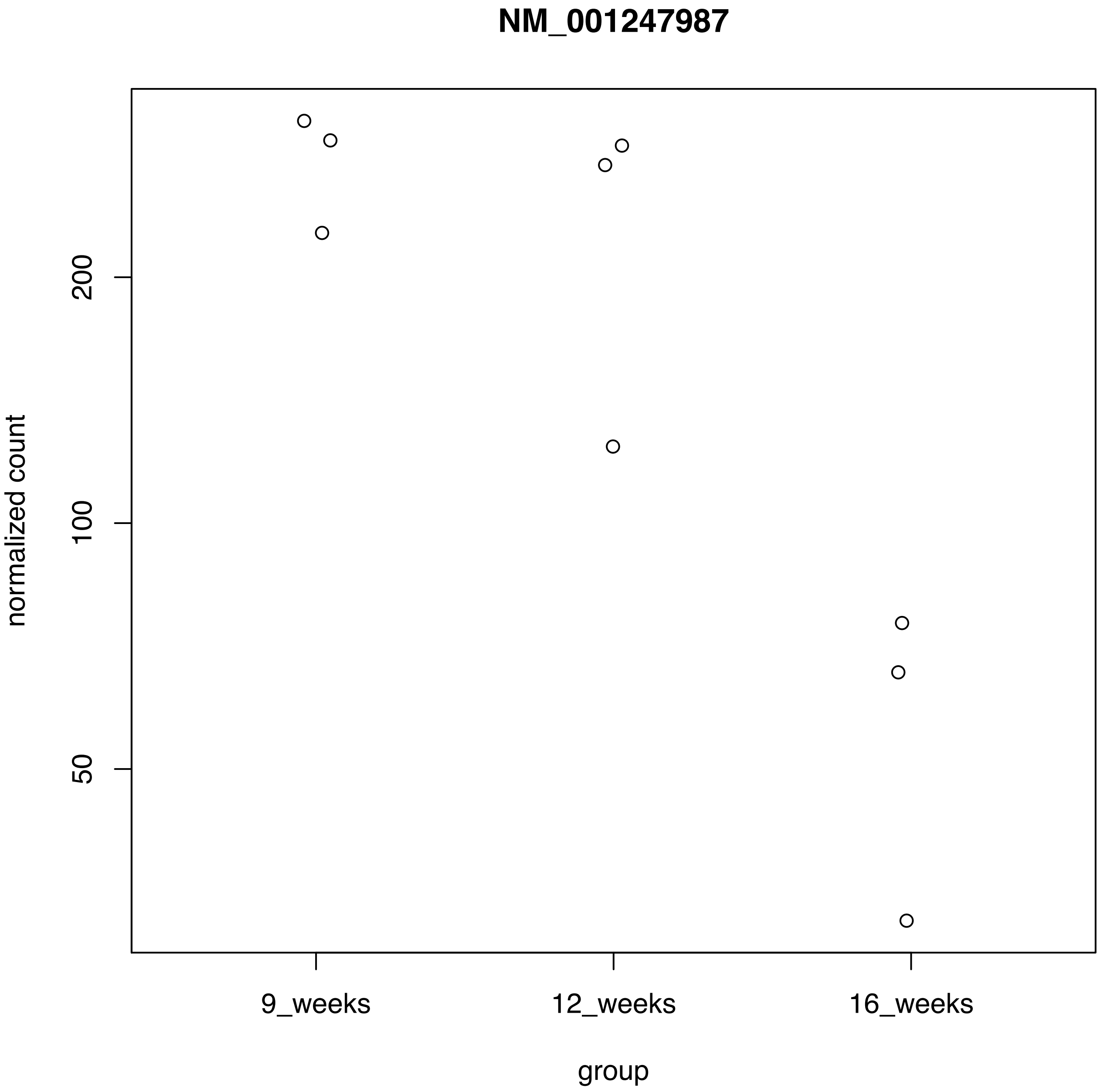
NM_001247987
normalized count
200
100
50
9_weeks
12_weeks
16_weeks
group

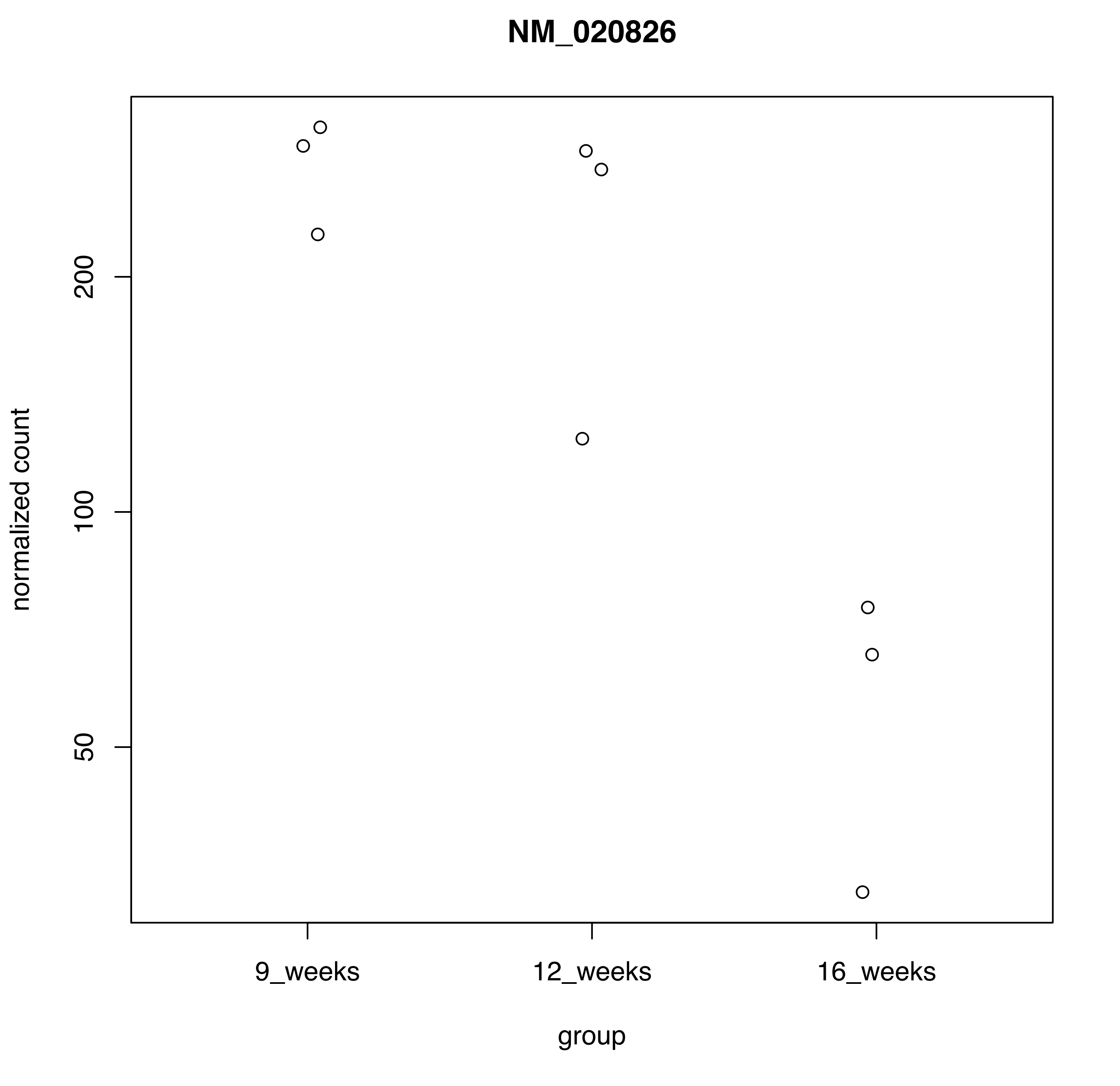
NM_020826
normalized count
200
100
50
9_weeks
12_weeks
16_weeks
group

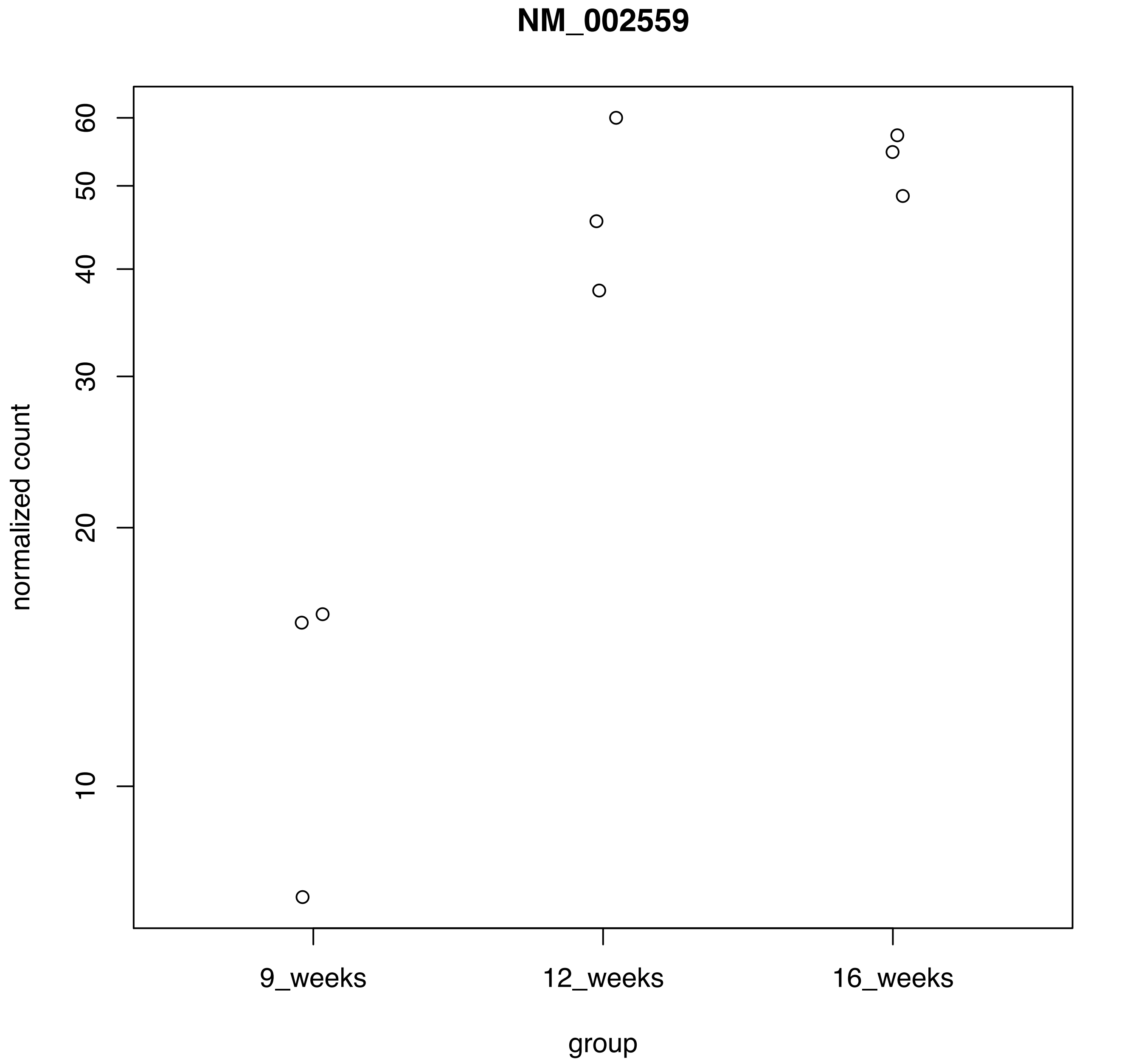

NM_002559
normalized count
60
50
40
30
20
10
9_weeks
12_weeks
16_weeks
group

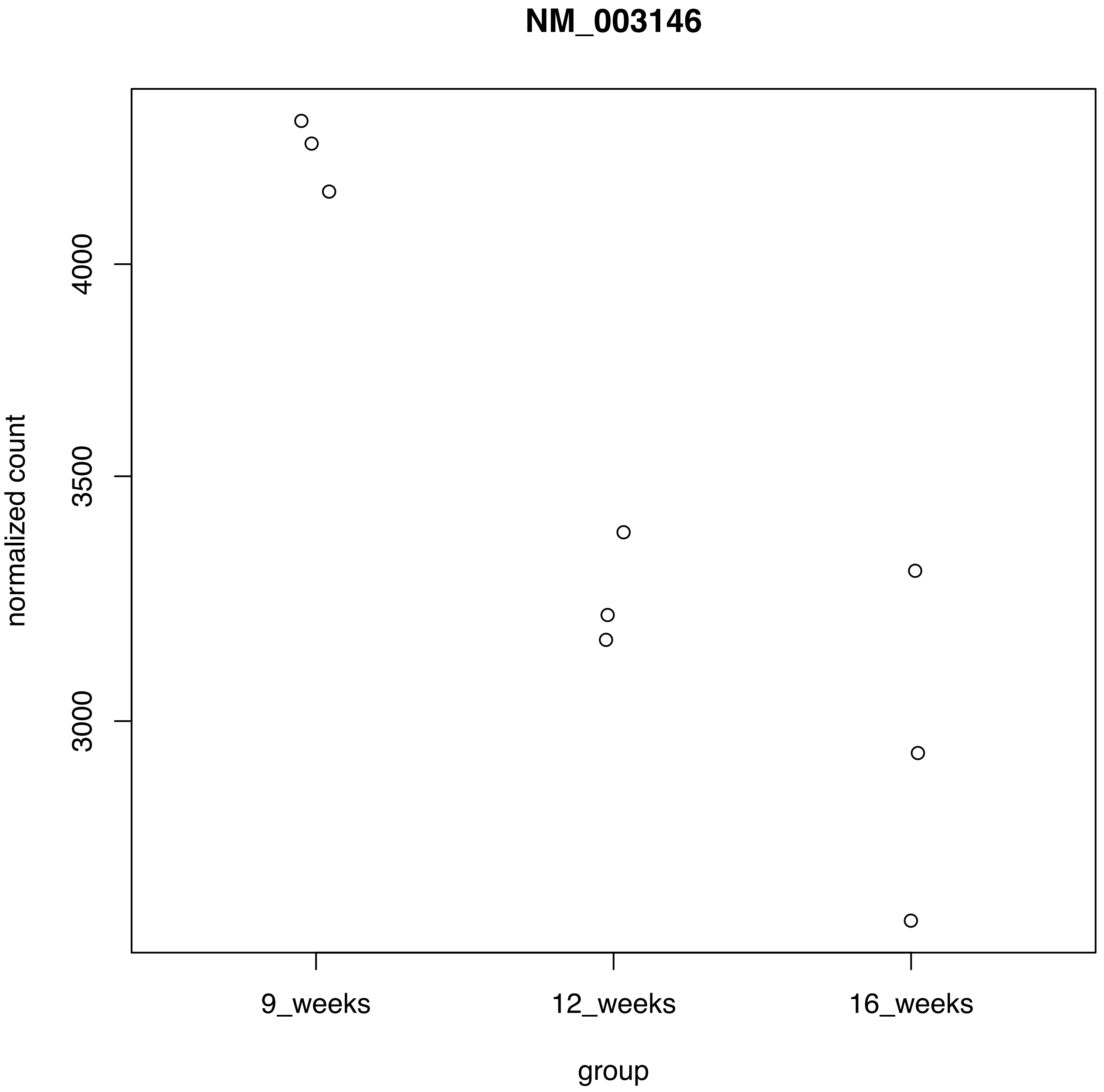
NM_003146
normalized count
4000
3500
3000
9_weeks
12_weeks
16_weeks
group

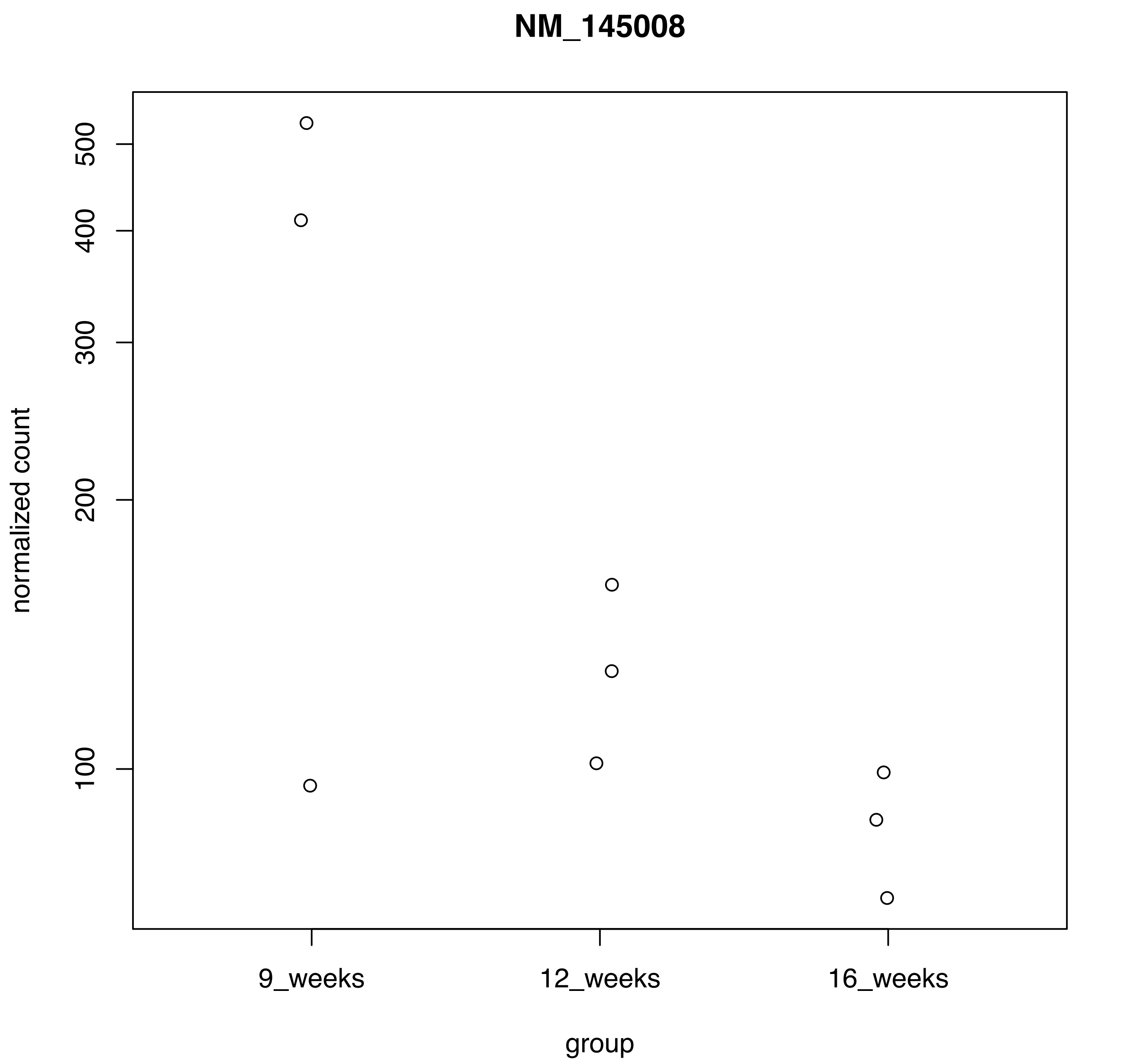
NM_145008
normalized count
500
400
300
200
100
9_weeks
12_weeks
16_weeks
group

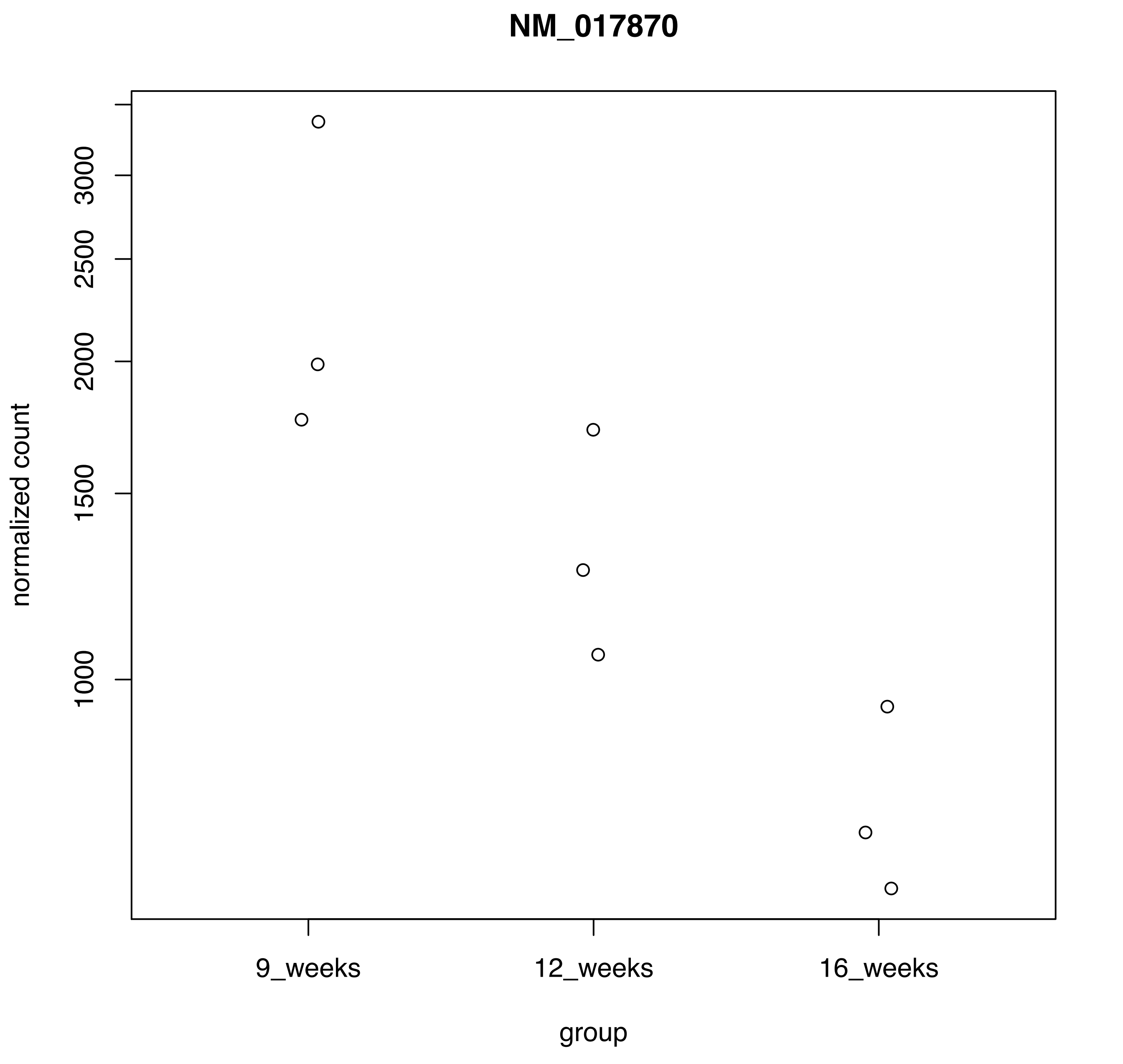
NM_017870
normalized count
1000
1500
2000
2500
3000
9_weeks
12_weeks
16_weeks
group

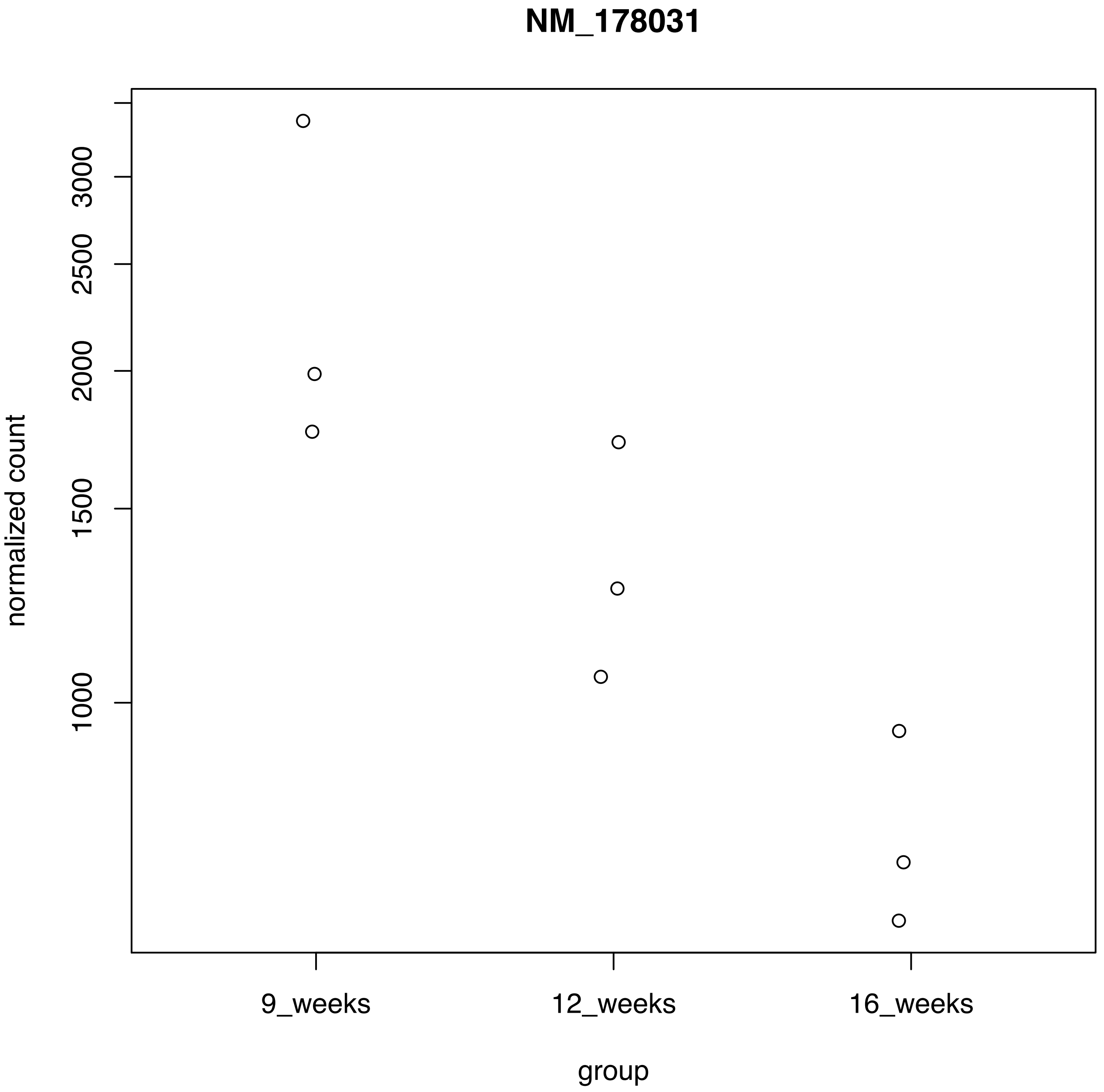
NM_178031
normalized count
1000
1500
2000
2500
3000
9_weeks
12_weeks
16_weeks
group

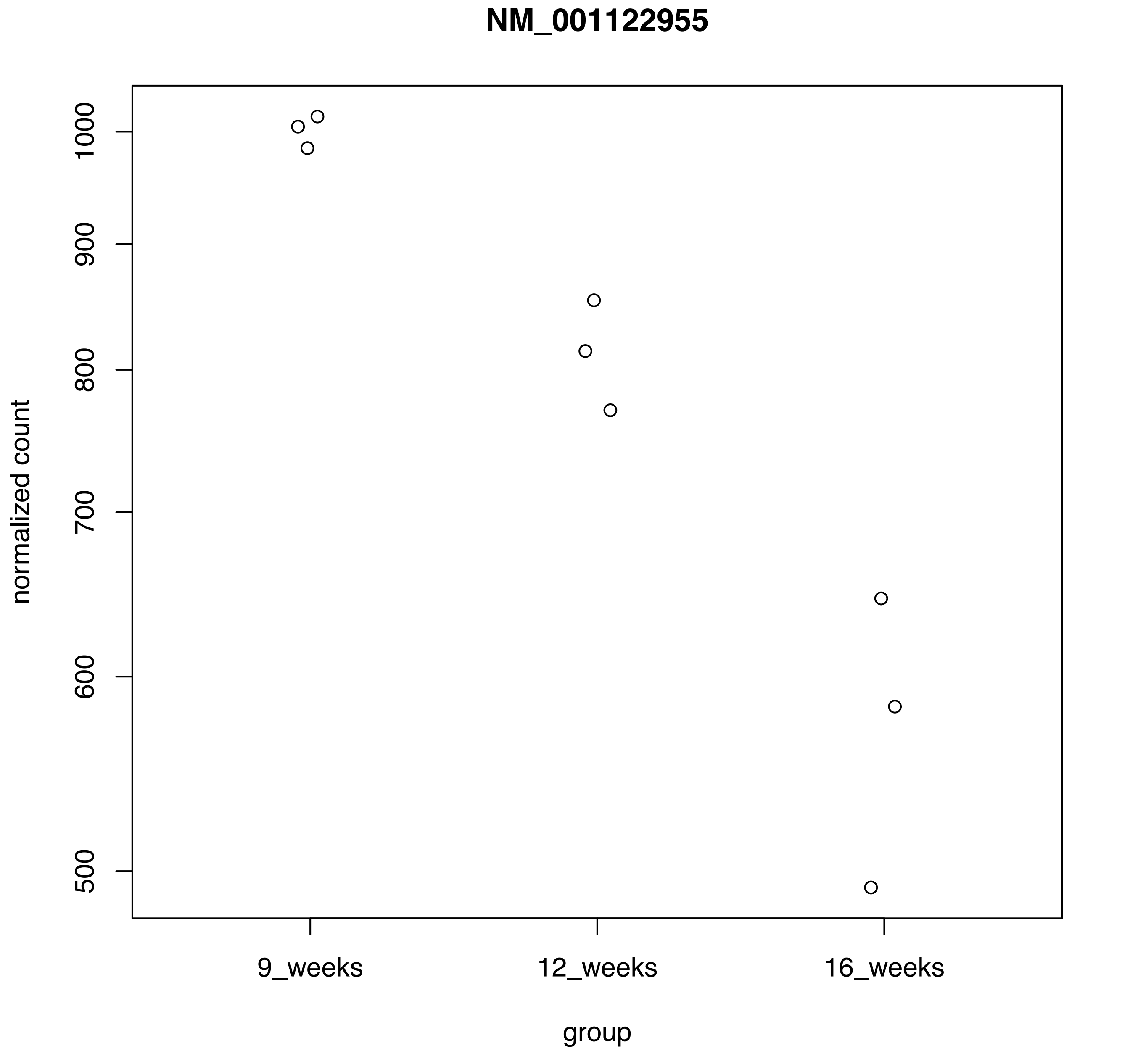
NM_001122955
normalized count
1000
900
800
700
600
500
9_weeks
12_weeks
16_weeks
group

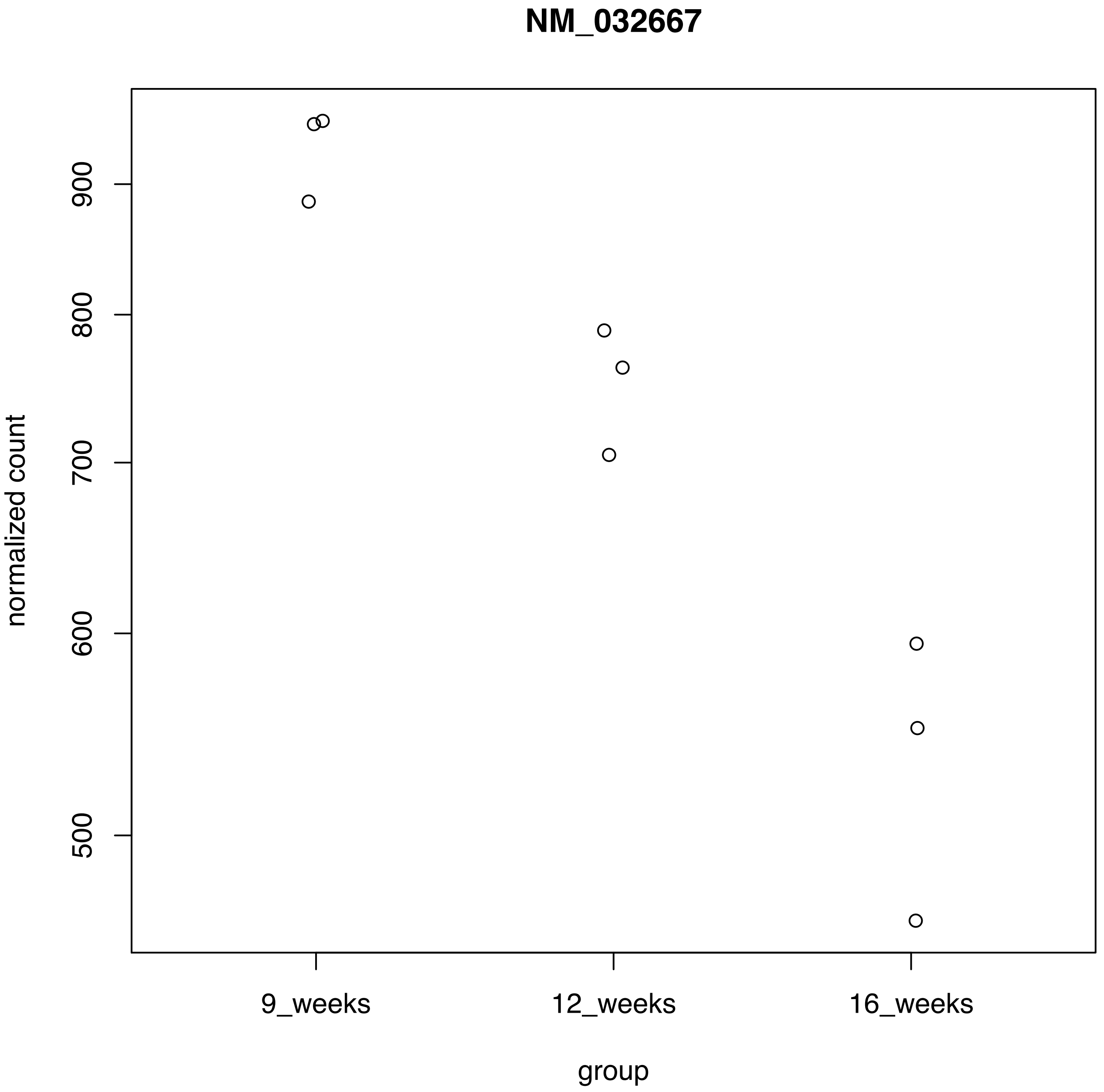
NM_032667
normalized count
900
800
700
600
500
9_weeks
12_weeks
16_weeks
group

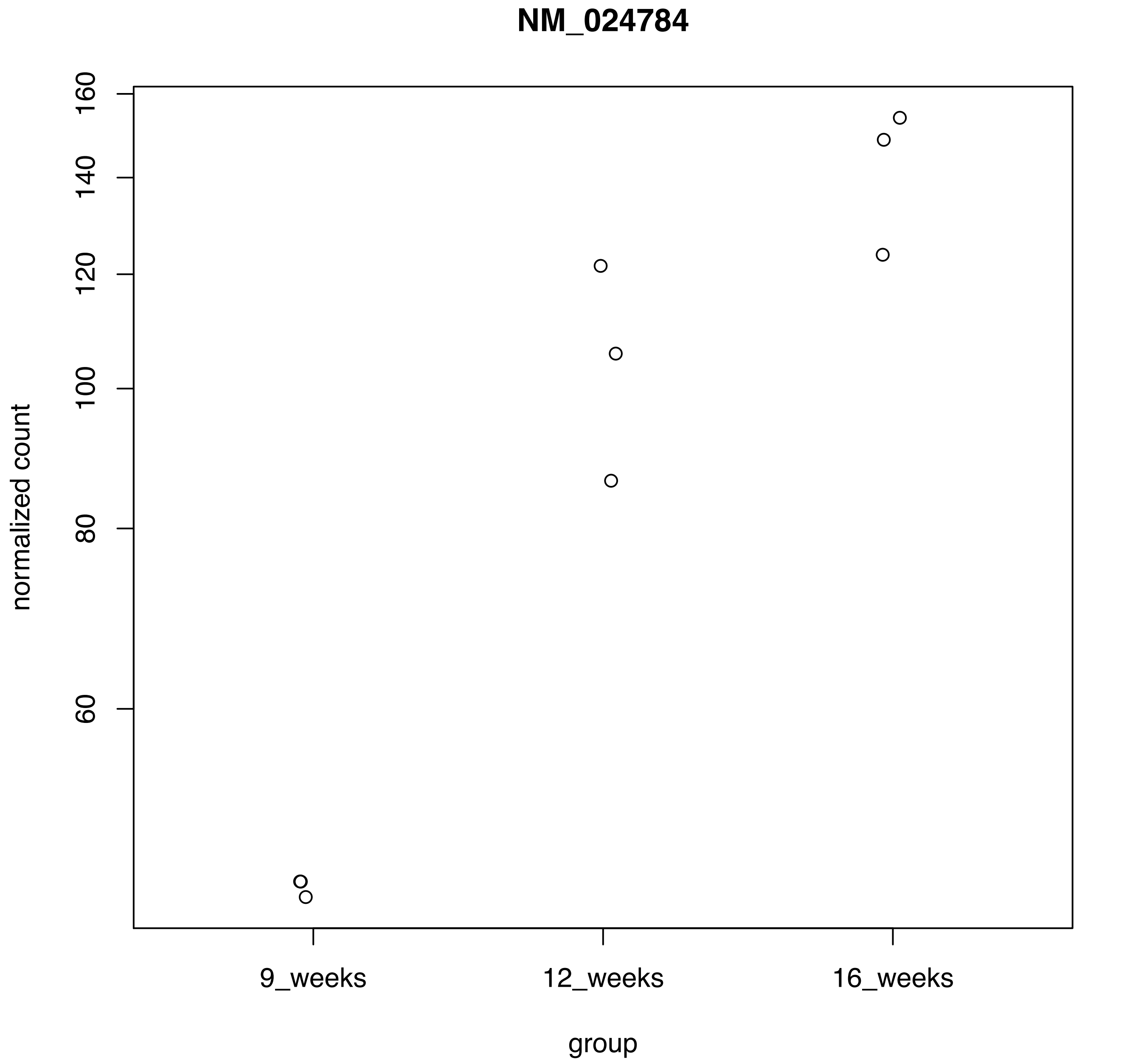

NM_024784
normalized count
160
140
120
100
80
60
9_weeks
12_weeks
16_weeks
group

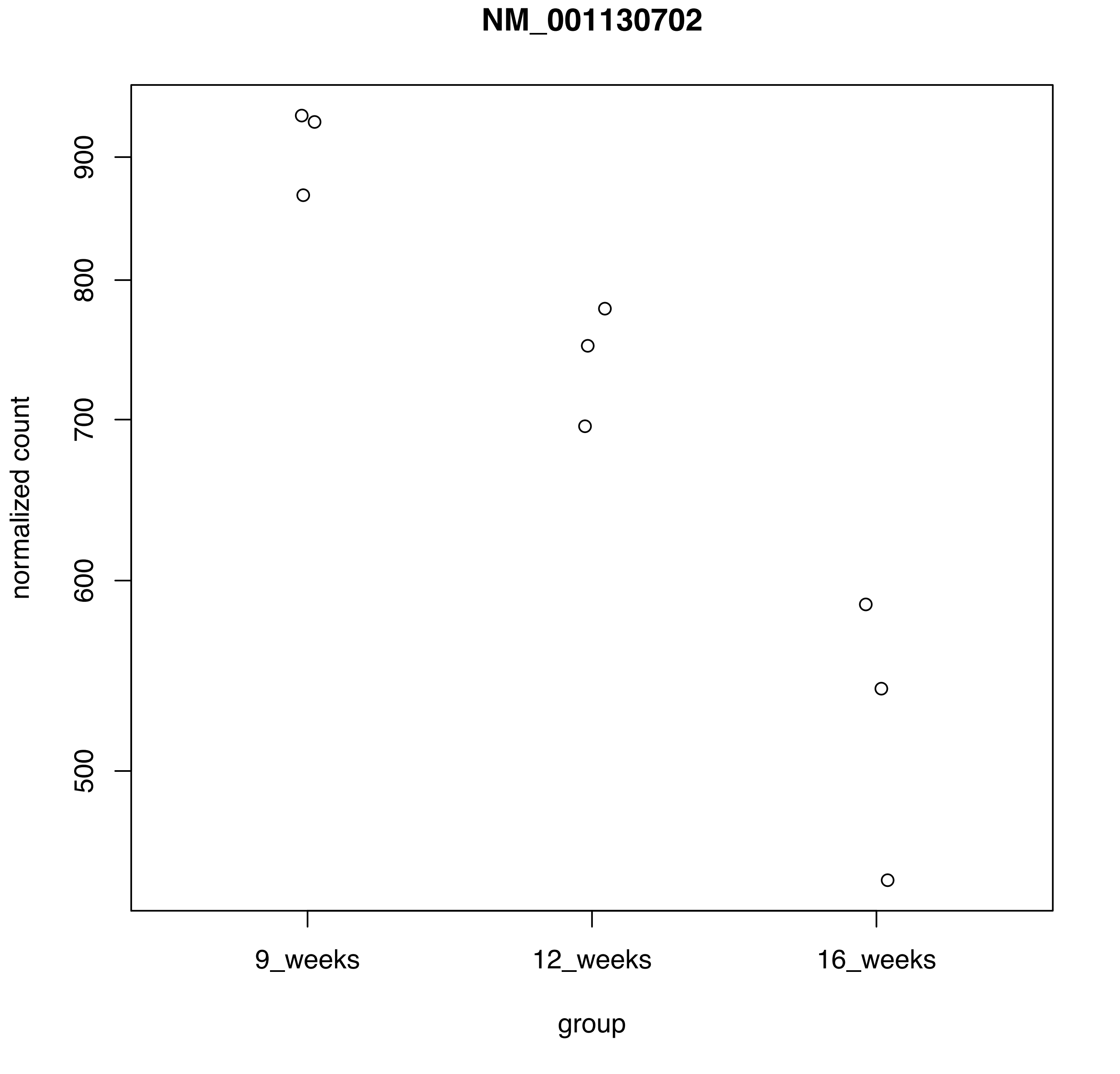
NM_001130702
normalized count
900
800
700
600
500
9_weeks
12_weeks
16_weeks
group

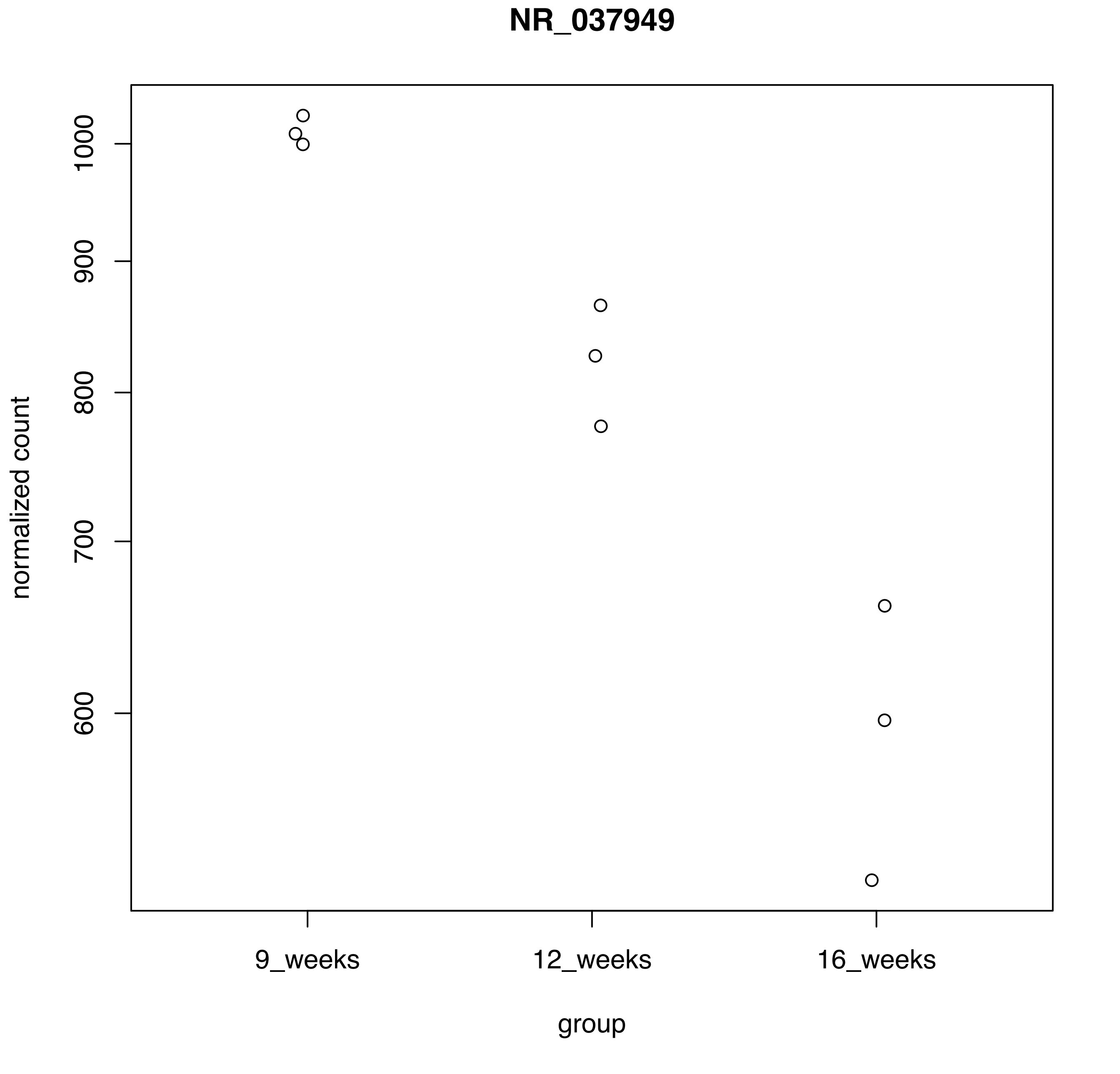

NR_037949
normalized count
1000
900
800
700
600
9_weeks
12_weeks
16_weeks
group

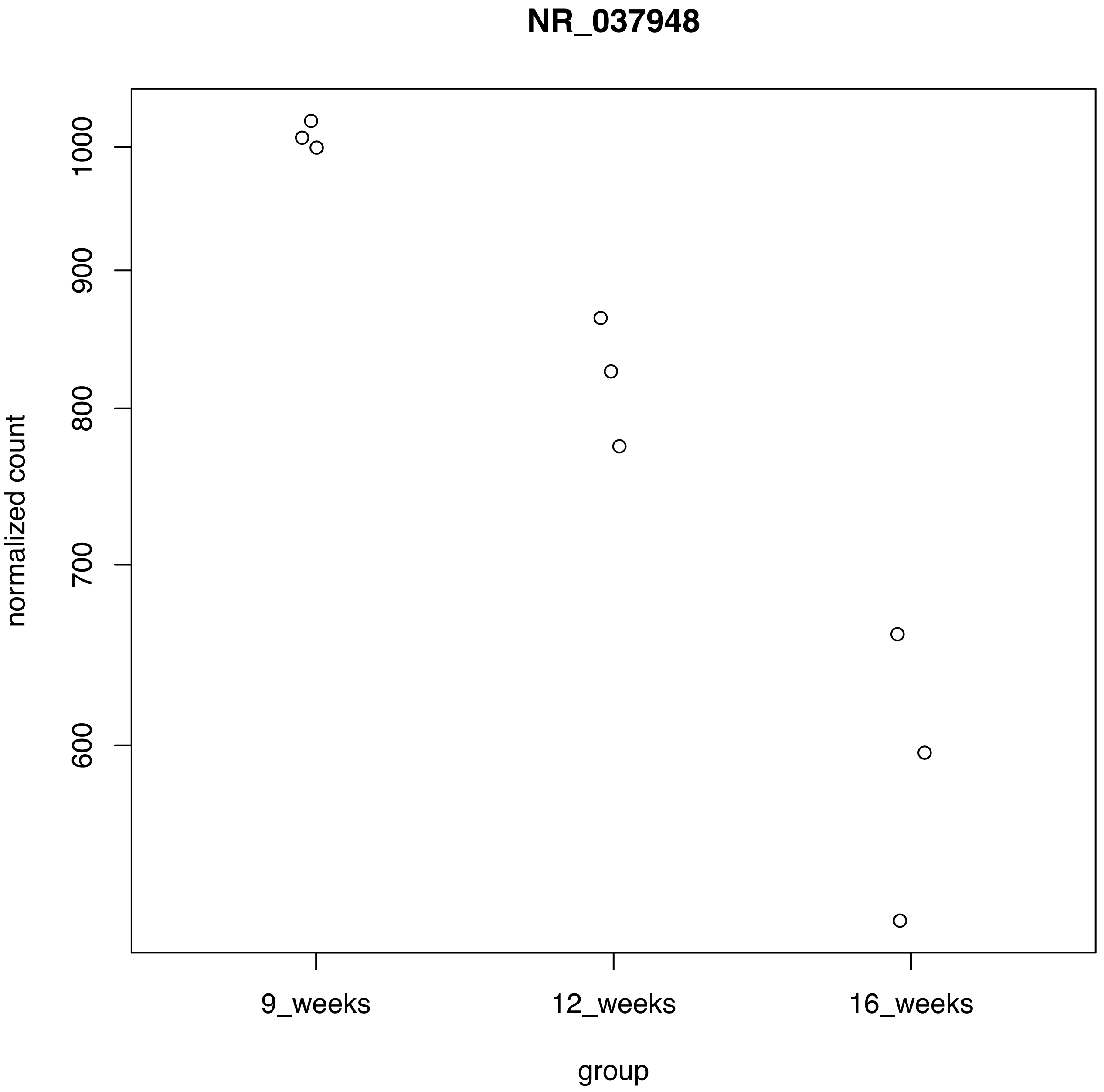
NR_037948
normalized count
1000
900
800
700
600
9_weeks
12_weeks
16_weeks
group

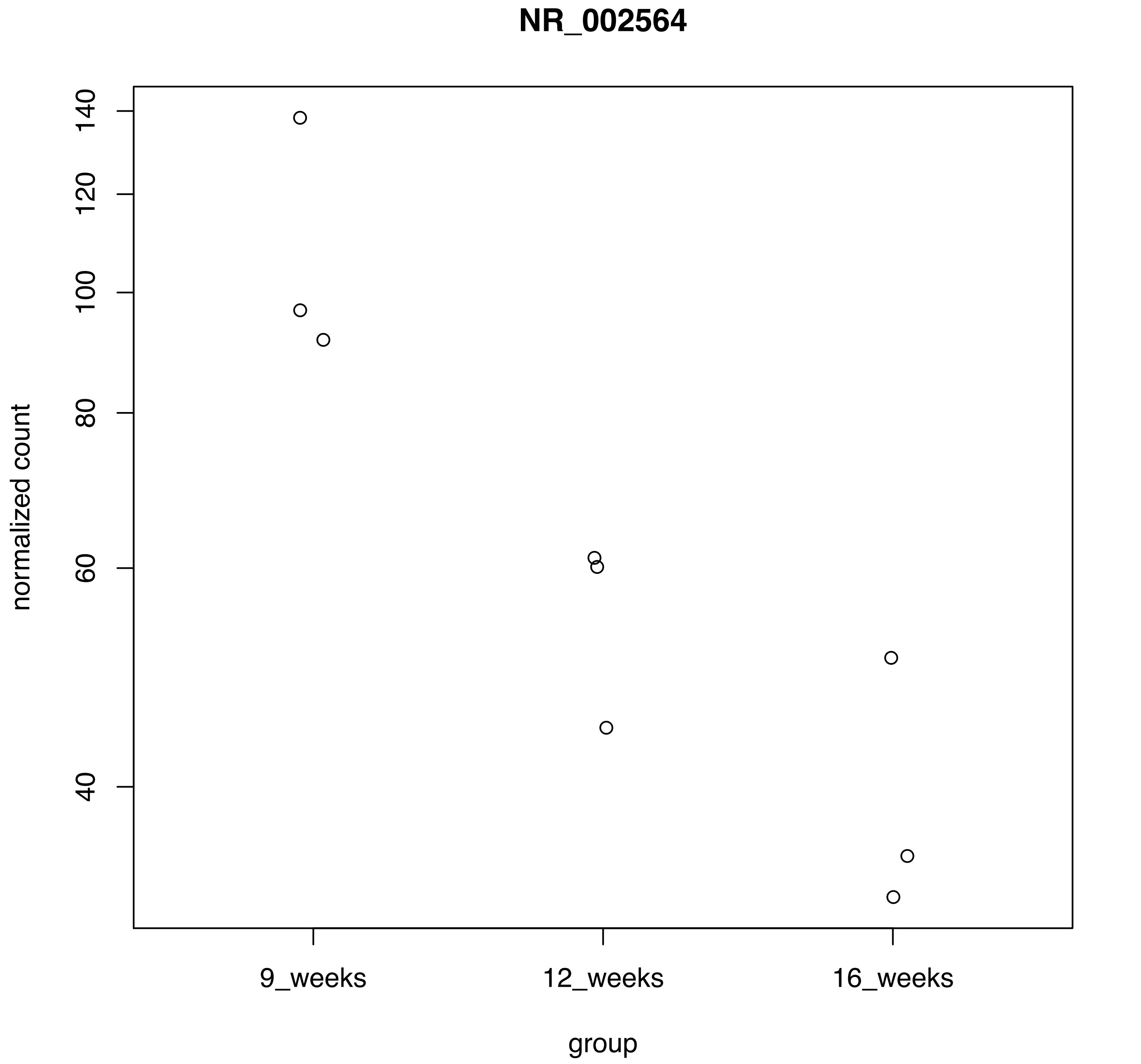

NR_002564
normalized count
140
120
100
80
60
40
9_weeks
12_weeks
16_weeks
group

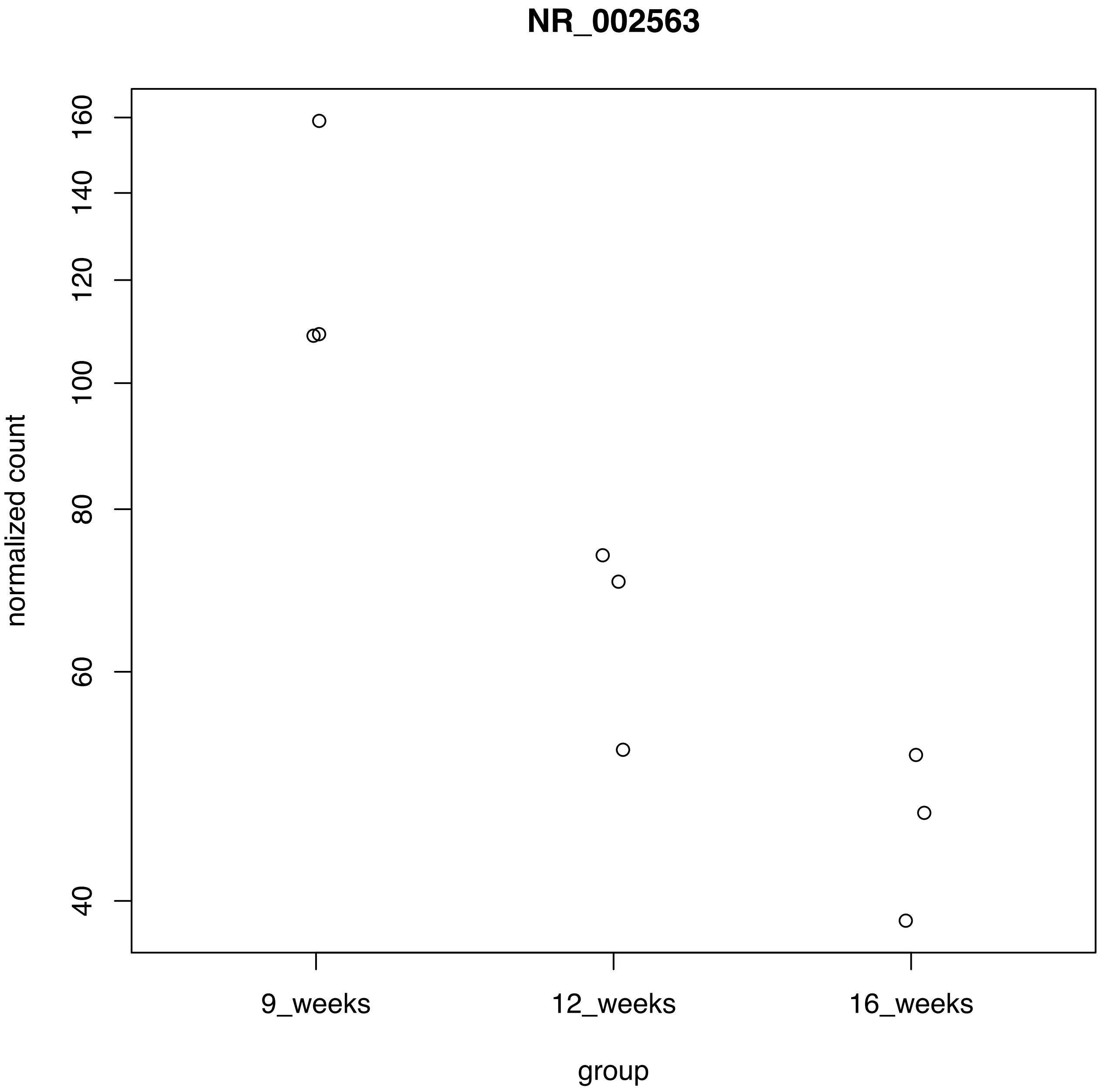
NR_002563
normalized count
160
140
120
100
80
60
40
9_weeks
12_weeks
16_weeks
group

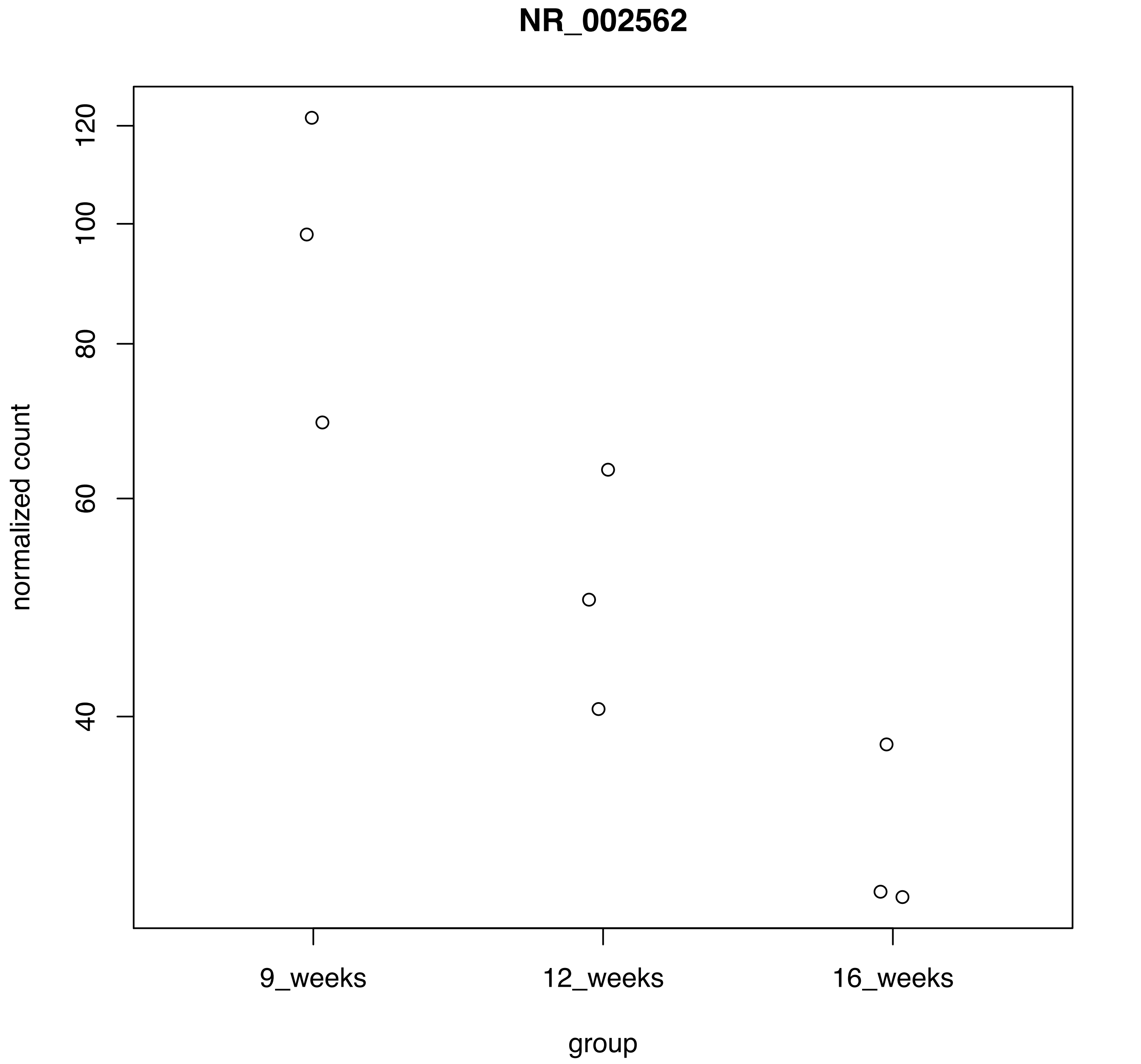

NR_002562
120
100
80
60
40
normalized count
9_weeks
12_weeks
16_weeks
group

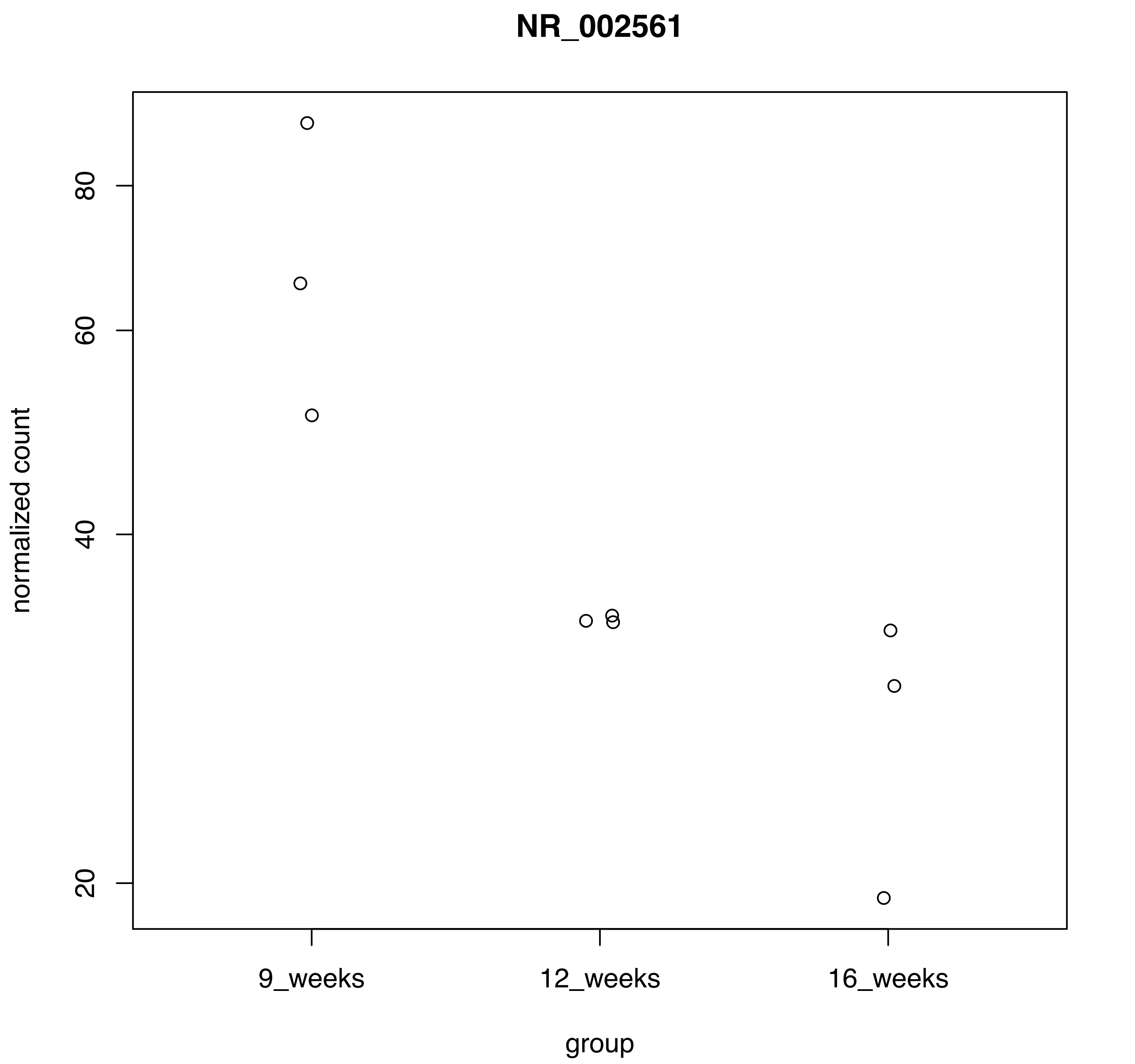
NR_002561
normalized count
20
40
60
80
9_weeks
12_weeks
16_weeks
group

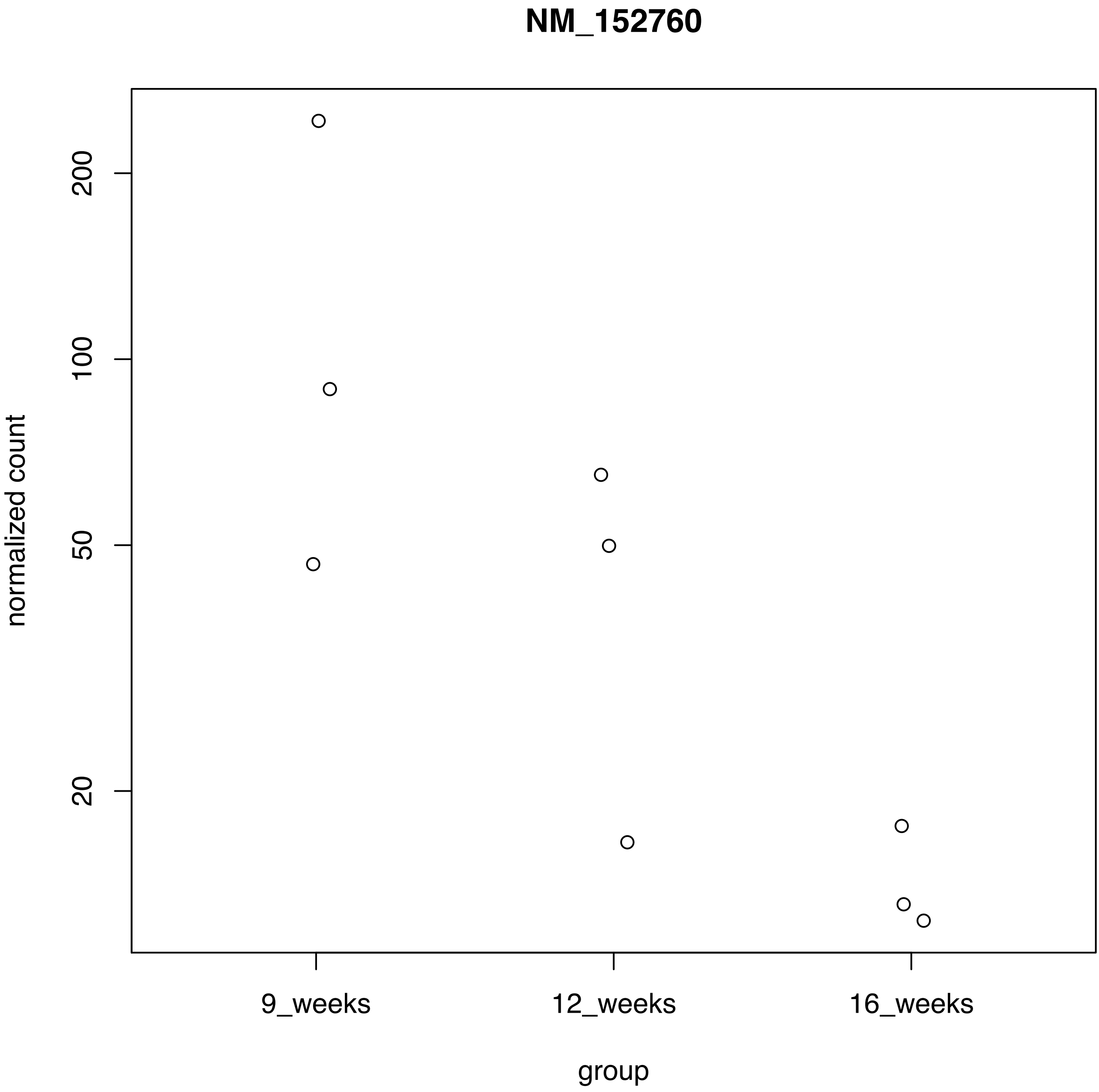
NM_152760
normalized count
200
100
50
20
9_weeks
12_weeks
16_weeks
group

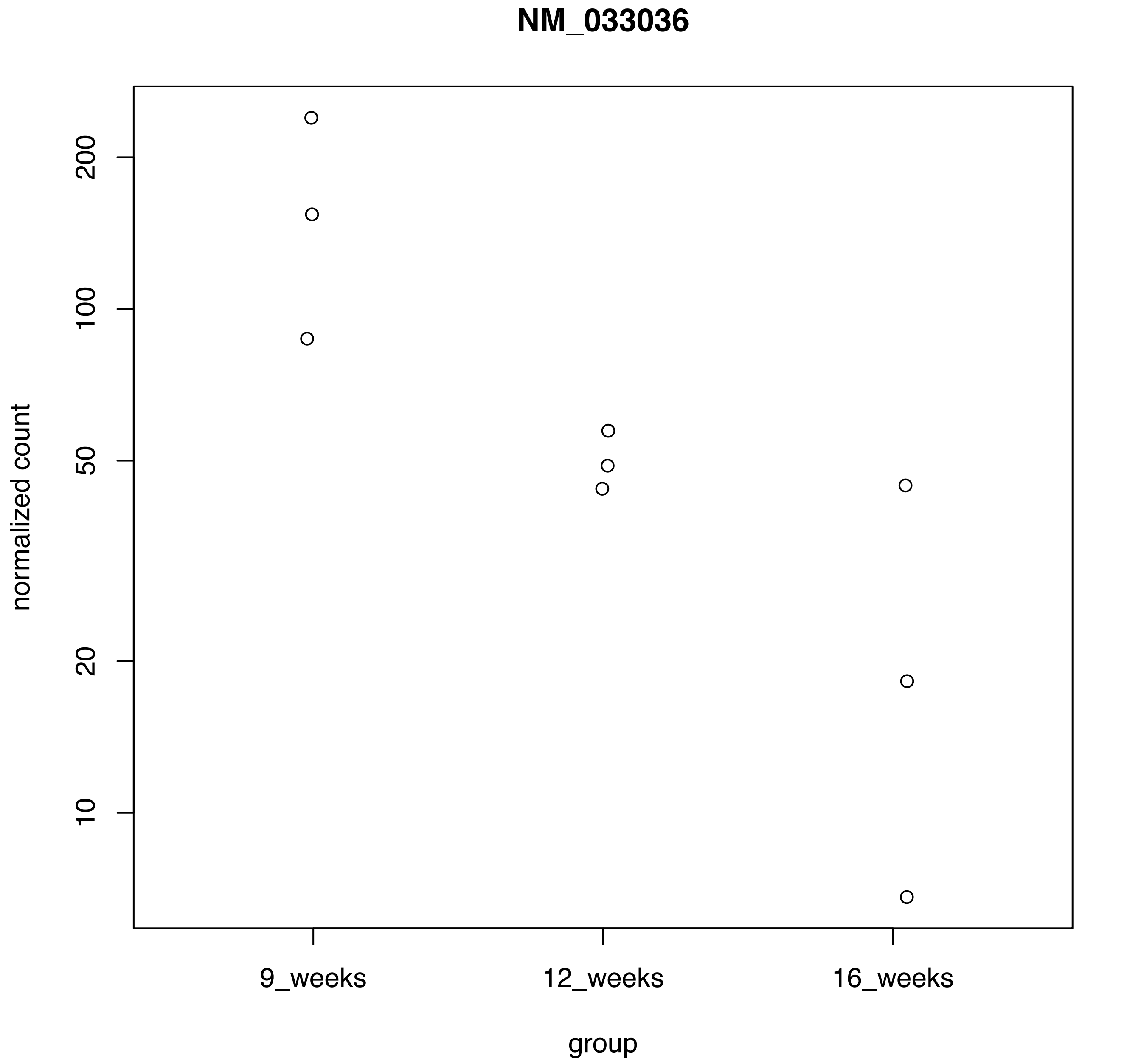
NM_033036
normalized count
200
100
50
20
10
9_weeks
12_weeks
16_weeks
group

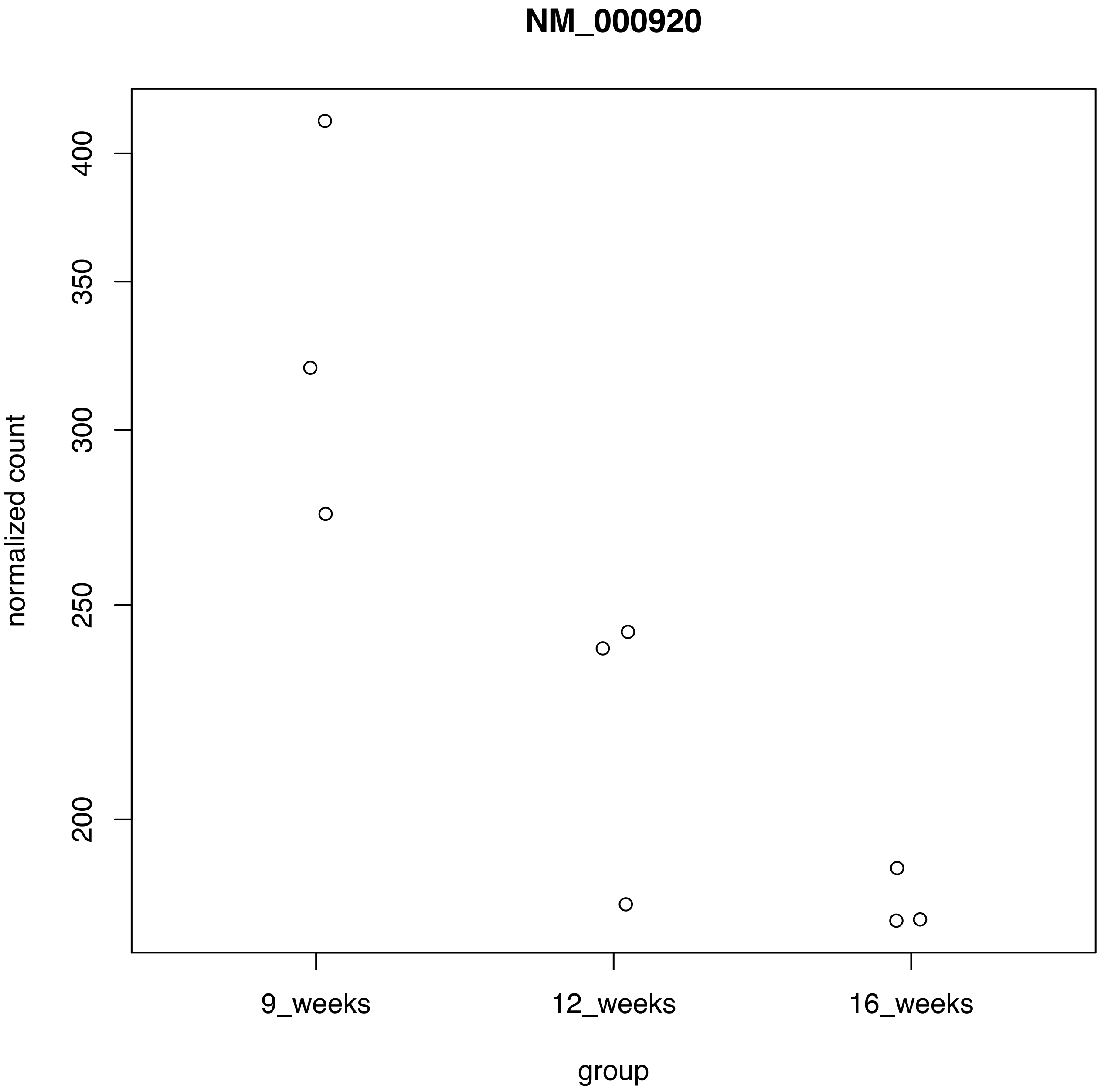
NM_000920
normalized count
400
350
300
250
200
9_weeks
12_weeks
16_weeks
group

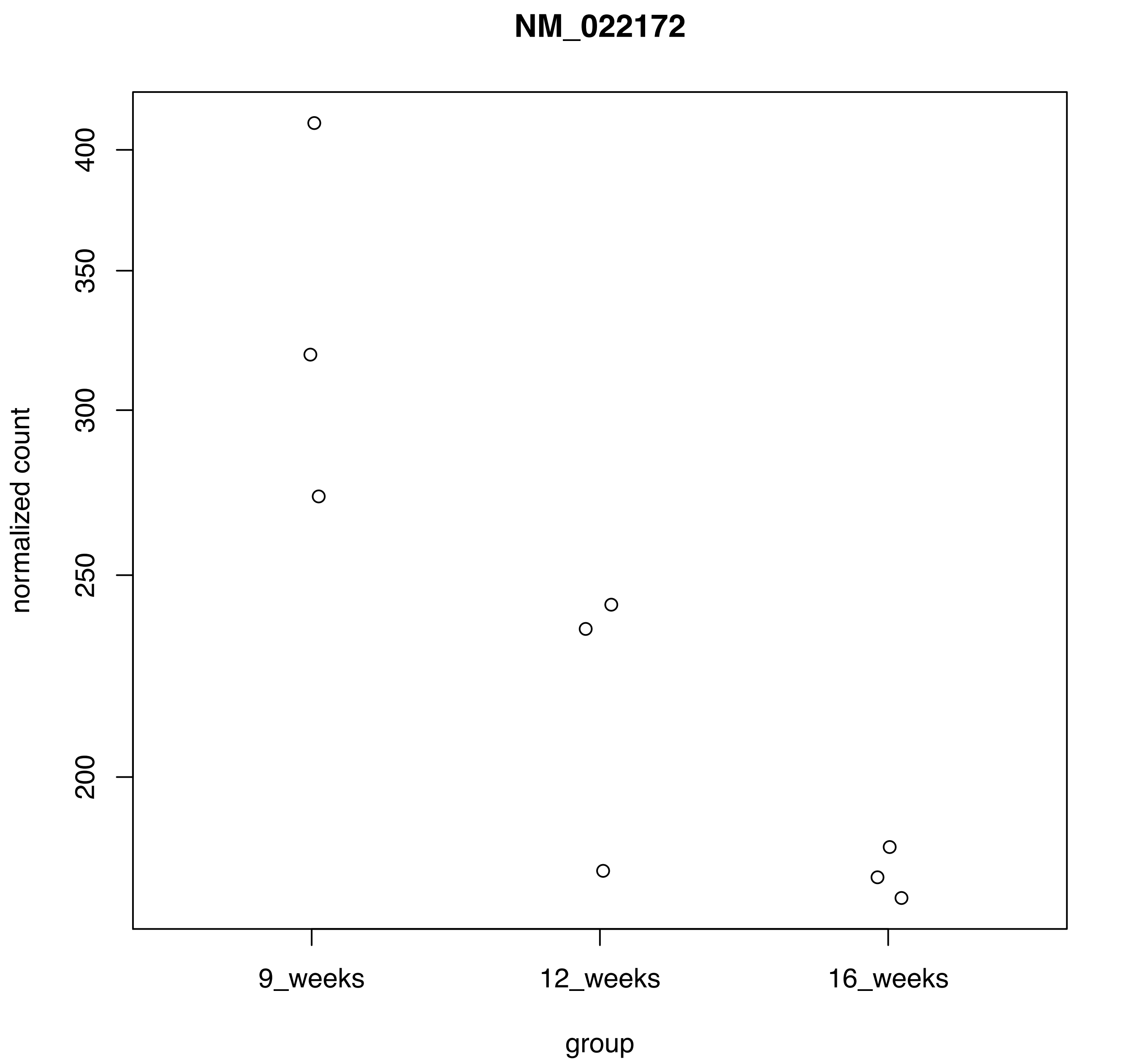
NM_022172
normalized count
400
350
300
250
200
9_weeks
12_weeks
16_weeks
group

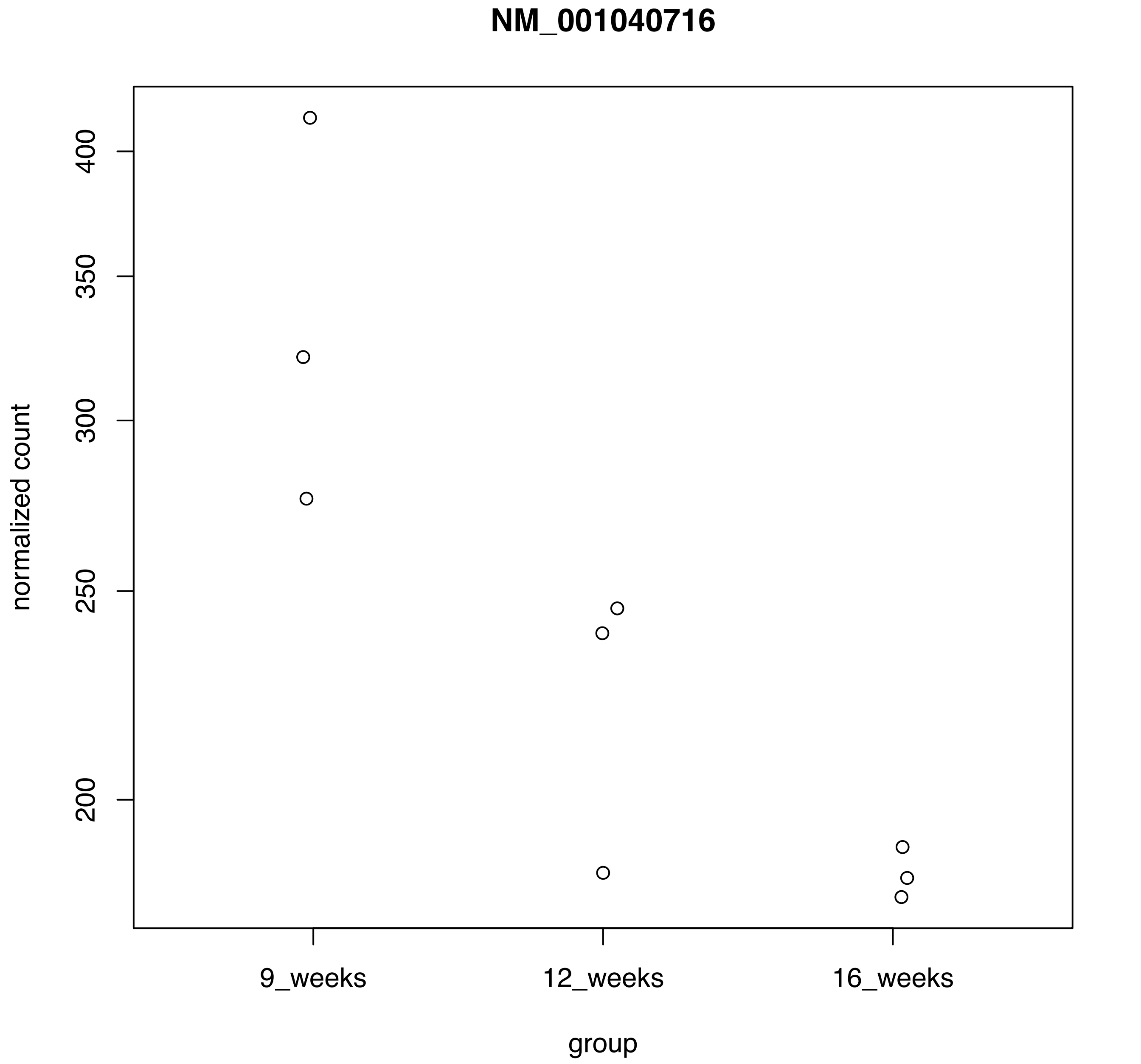

NM_001040716
normalized count
400
350
300
250
200
9_weeks
12_weeks
16_weeks
group

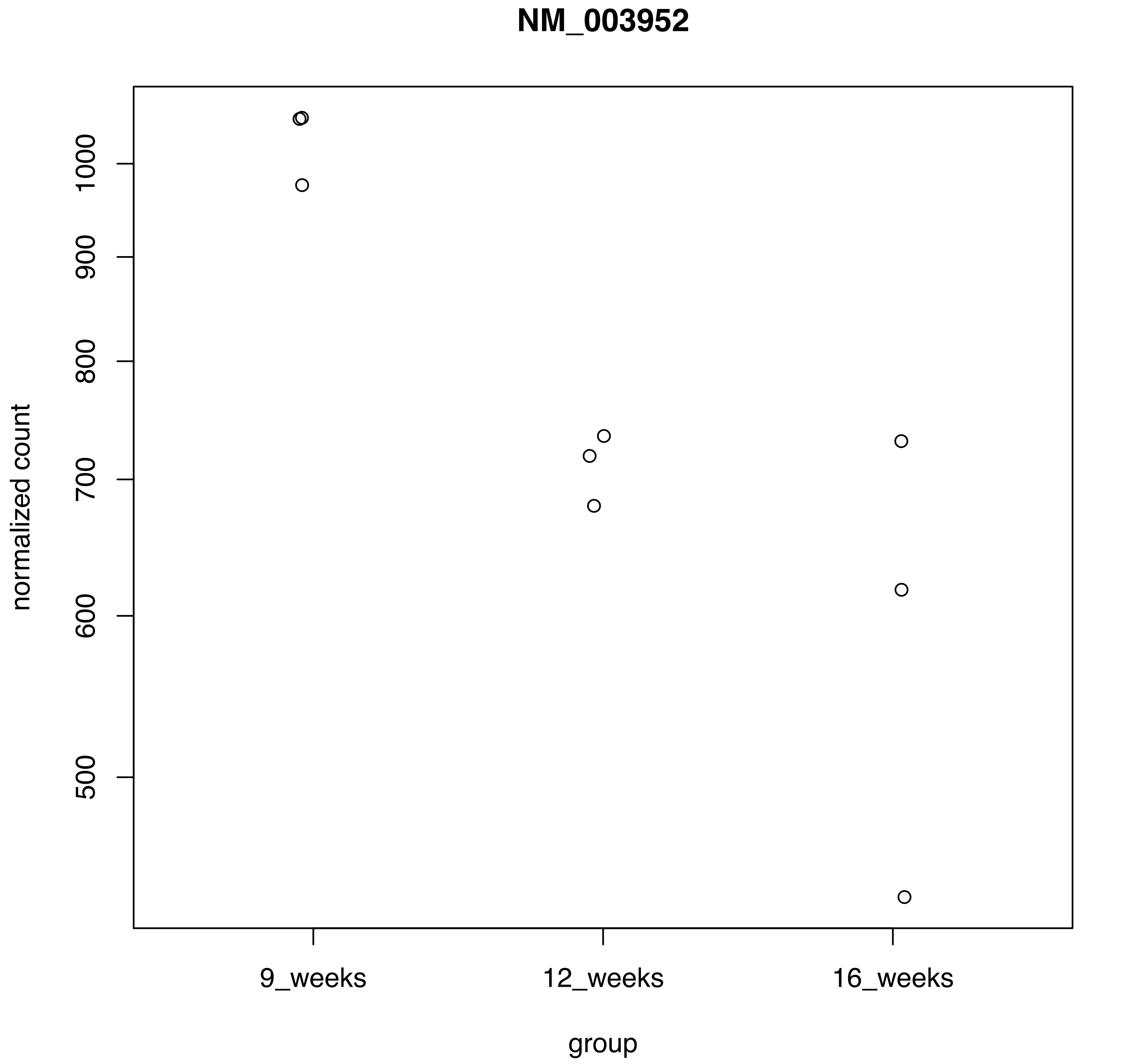

NM_003952
normalized count
1000
900
800
700
600
500
9_weeks
12_weeks
16_weeks
group

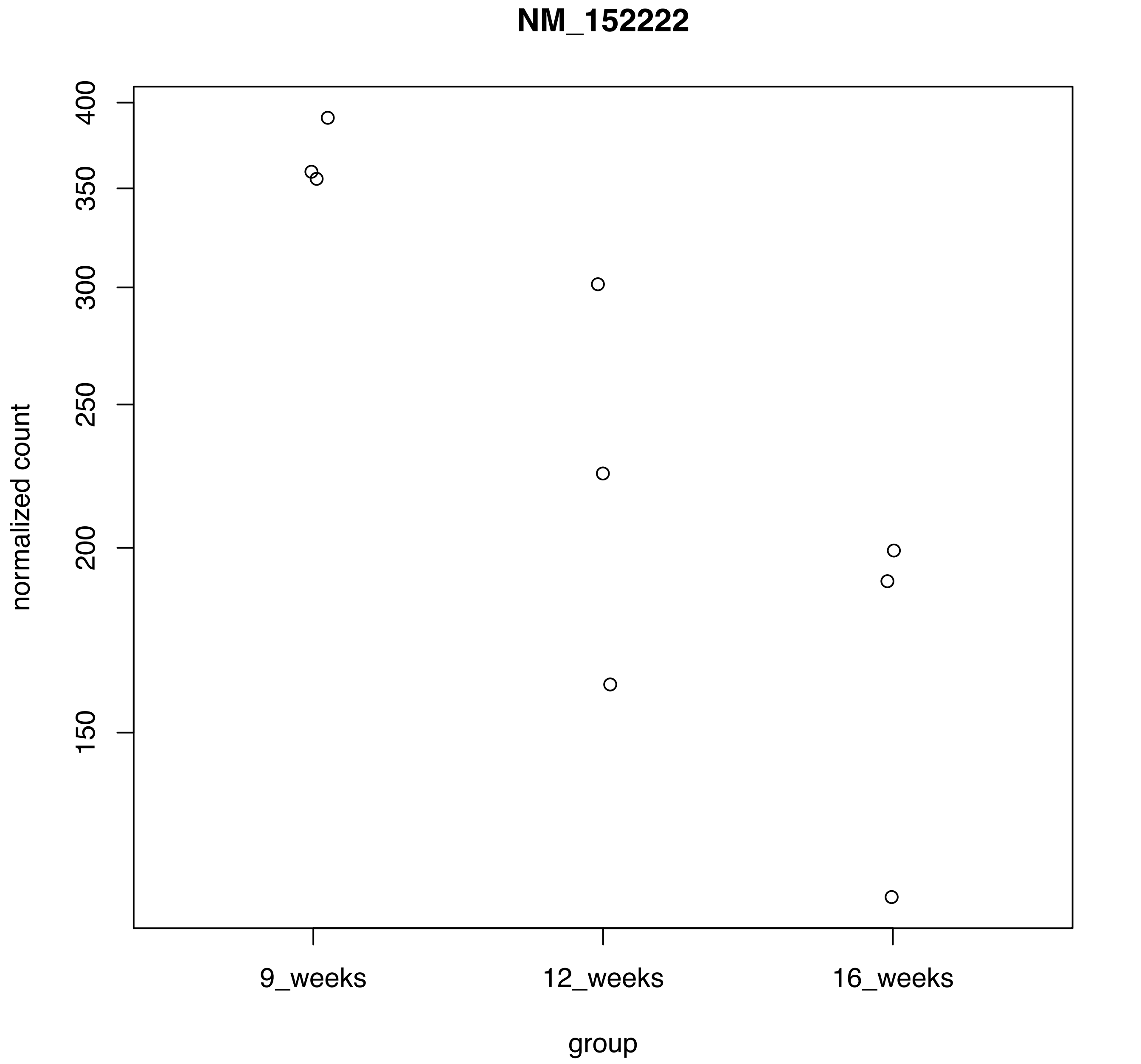
NM_152222
normalized count
400
350
300
250
200
150
9_weeks
12_weeks
16_weeks
group

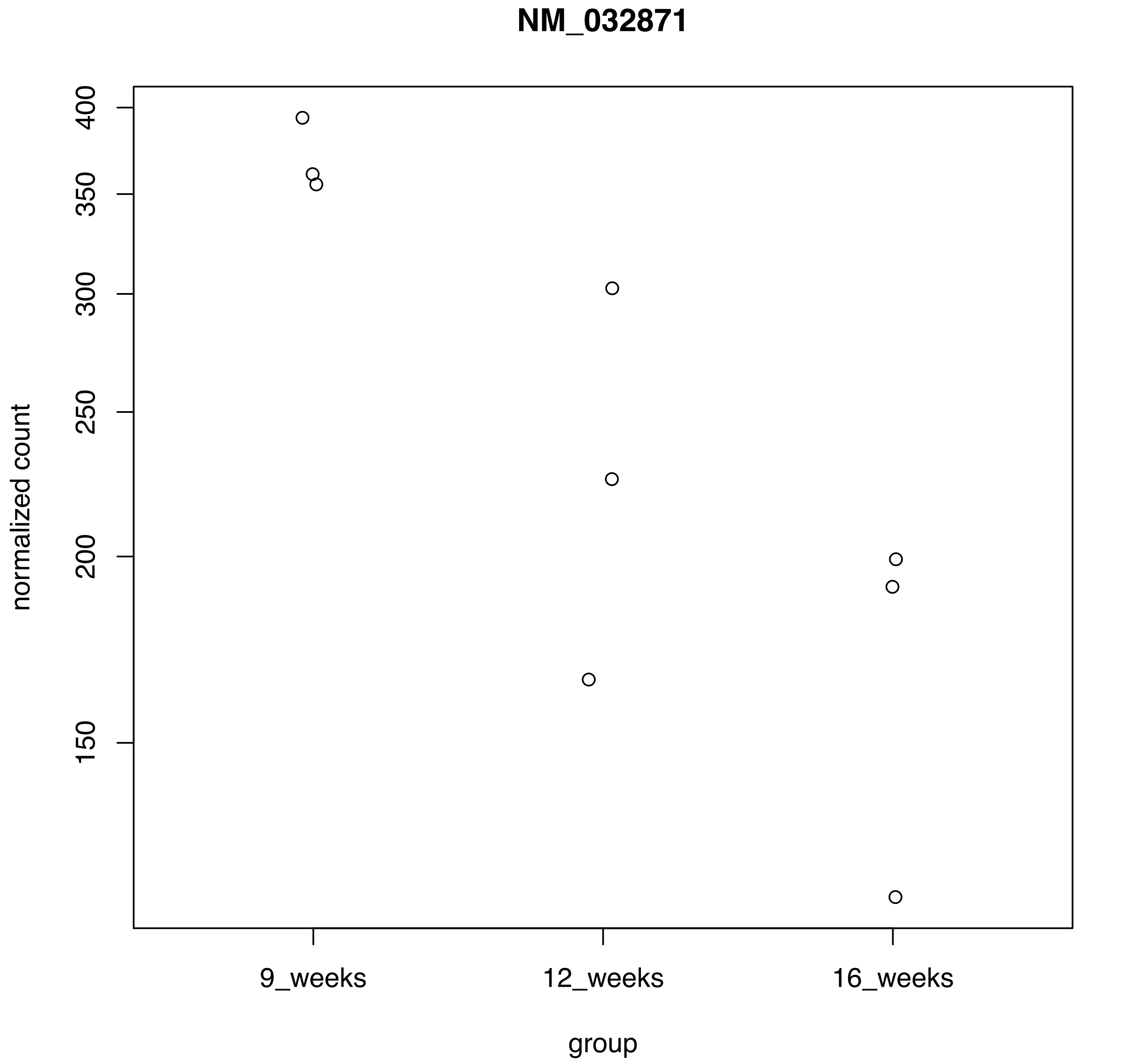
NM_032871
normalized count
400
350
300
250
200
150
9_weeks
12_weeks
16_weeks
group

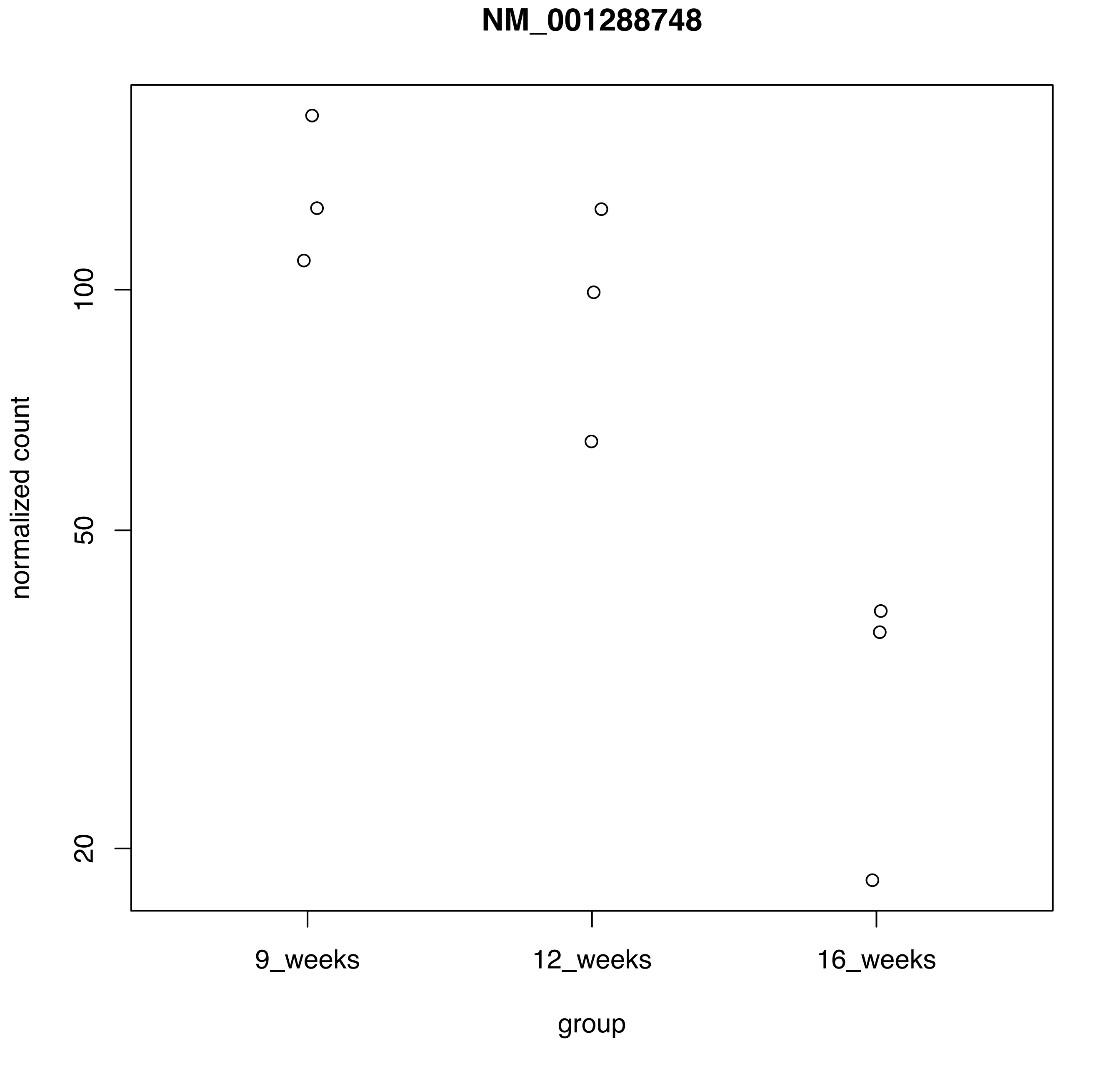
NM_001288748
normalized count
100
50
20
9_weeks
12_weeks
16_weeks
group

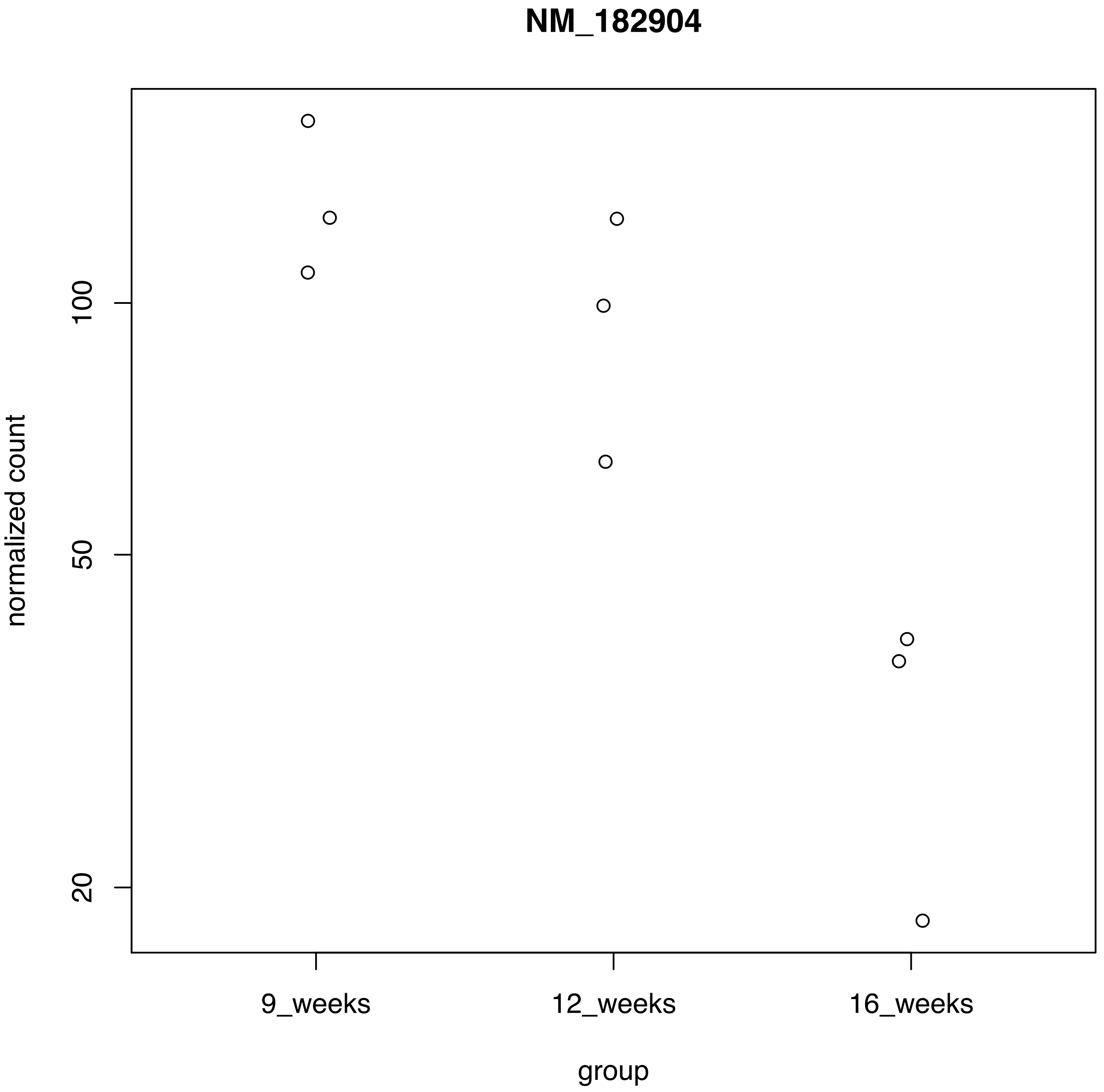
NM_182904
normalized count
100
50
20
9_weeks
12_weeks
16_weeks
group

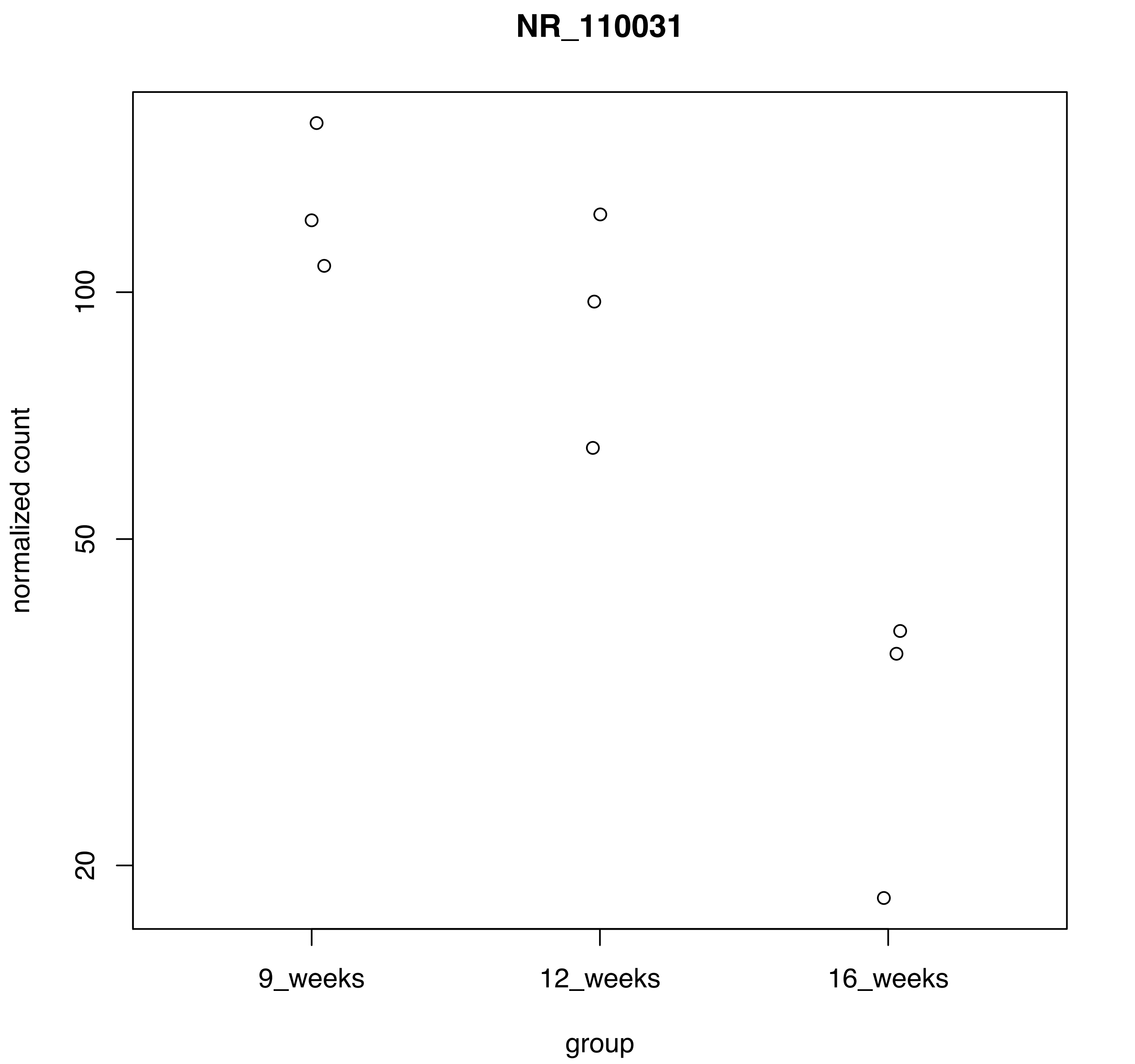
NR_110031
normalized count
100
50
20
9_weeks
12_weeks
16_weeks
group

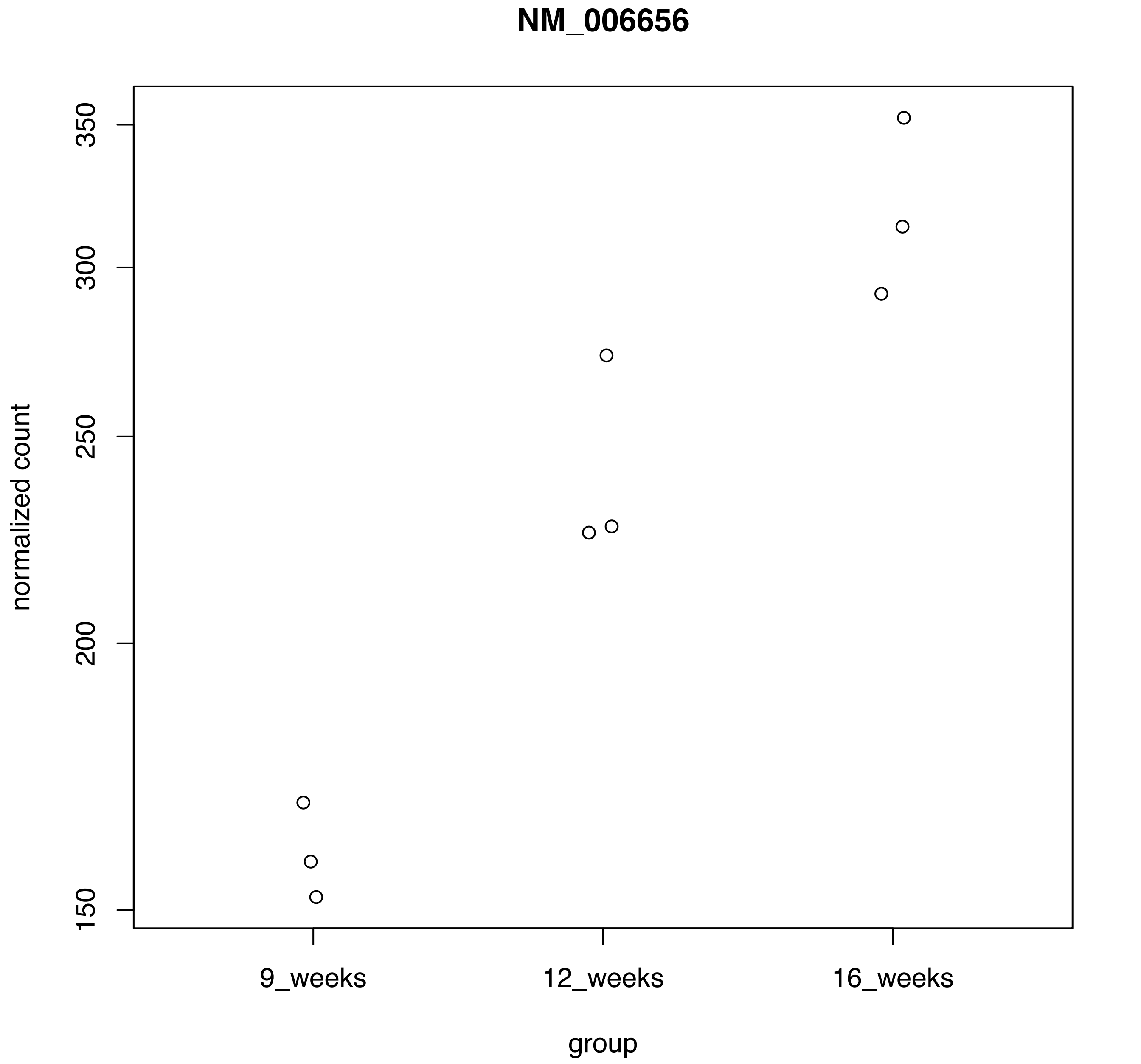
NM_006656
normalized count
350
300
250
200
150
9_weeks
12_weeks
16_weeks
group

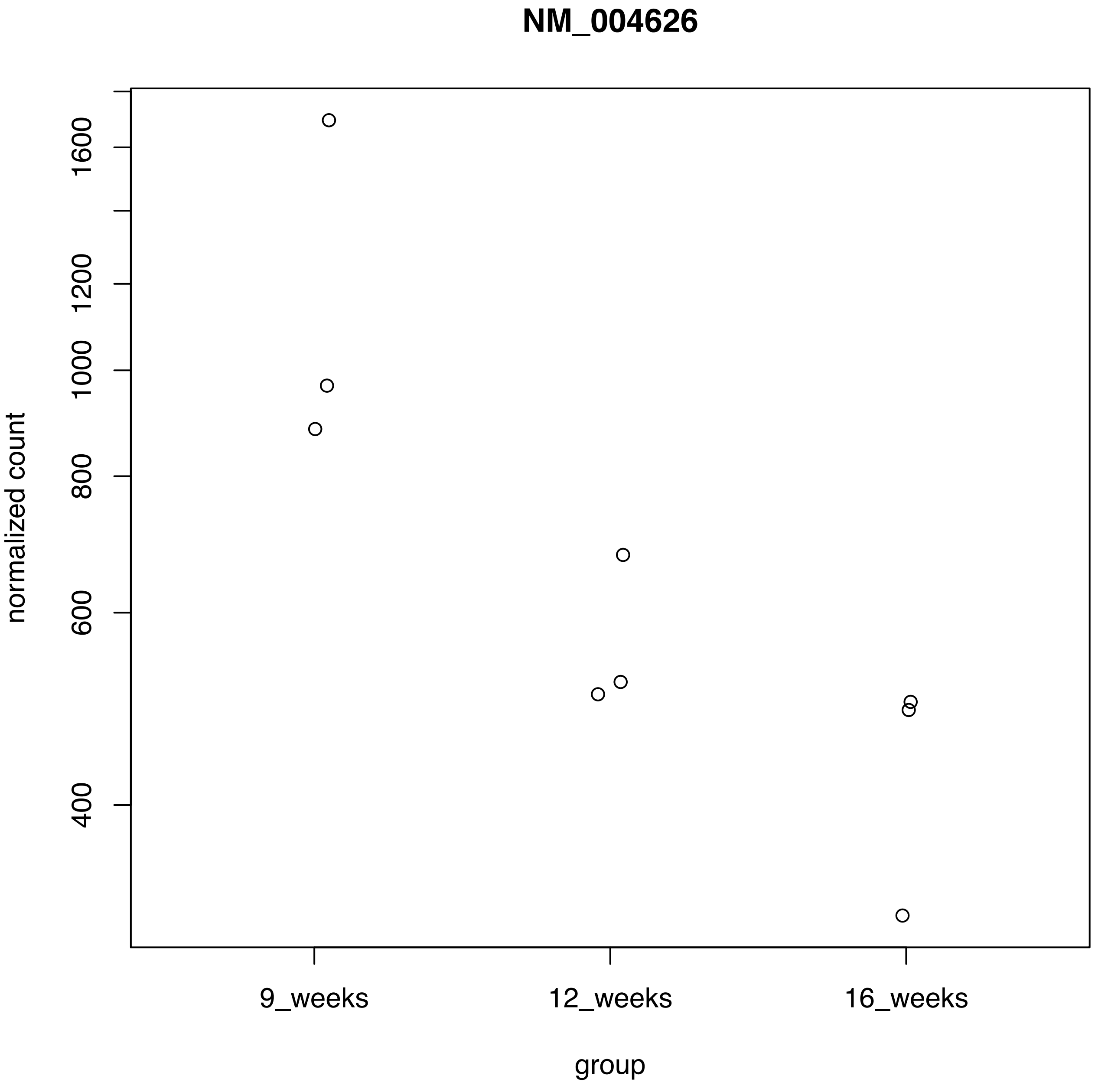
NM_004626
normalized count
1600
1200
1000
800
600
400
9_weeks
12_weeks
16_weeks
group

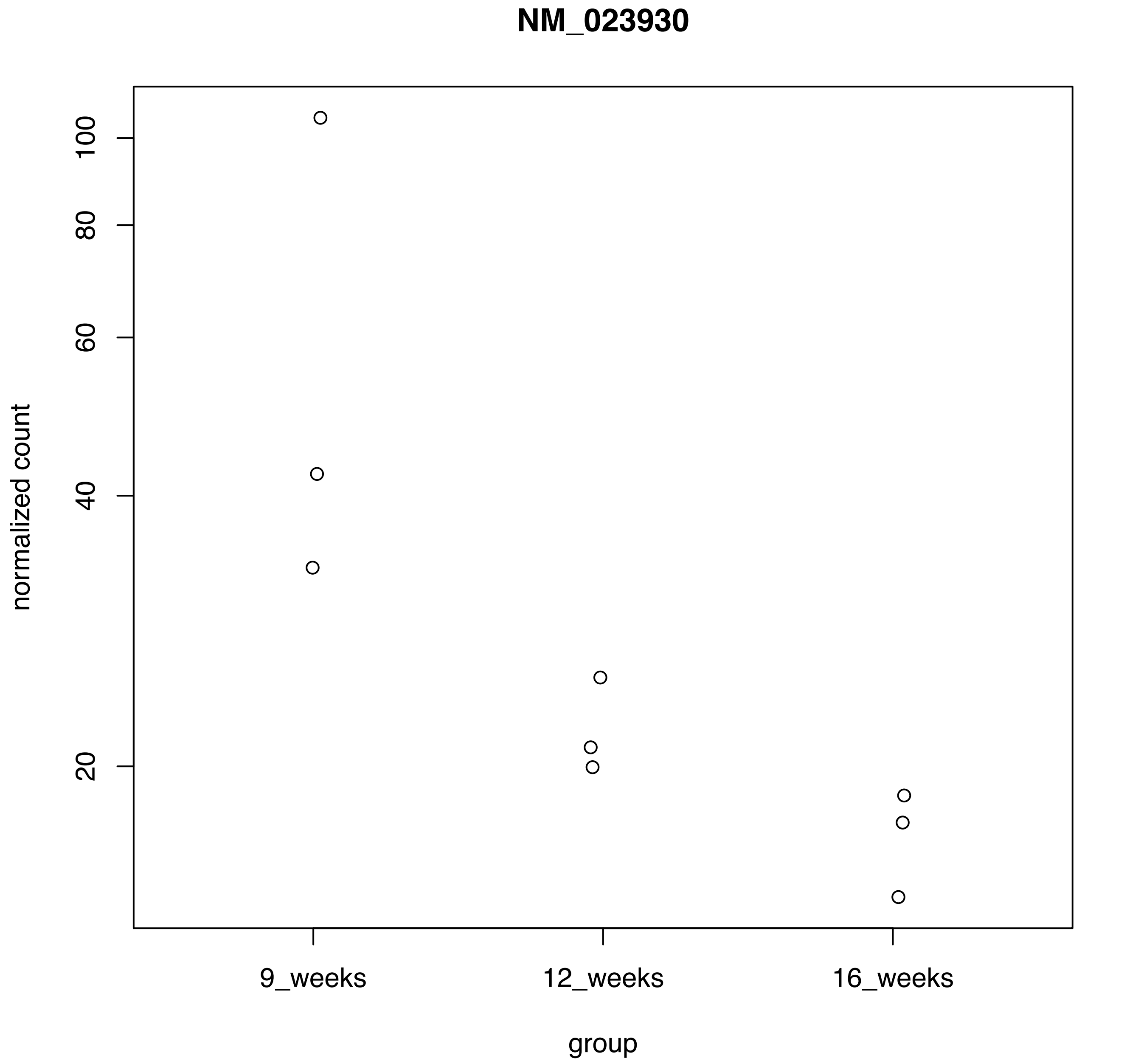
NM_023930
normalized count
100
80
60
40
20
9_weeks
12_weeks
16_weeks
group

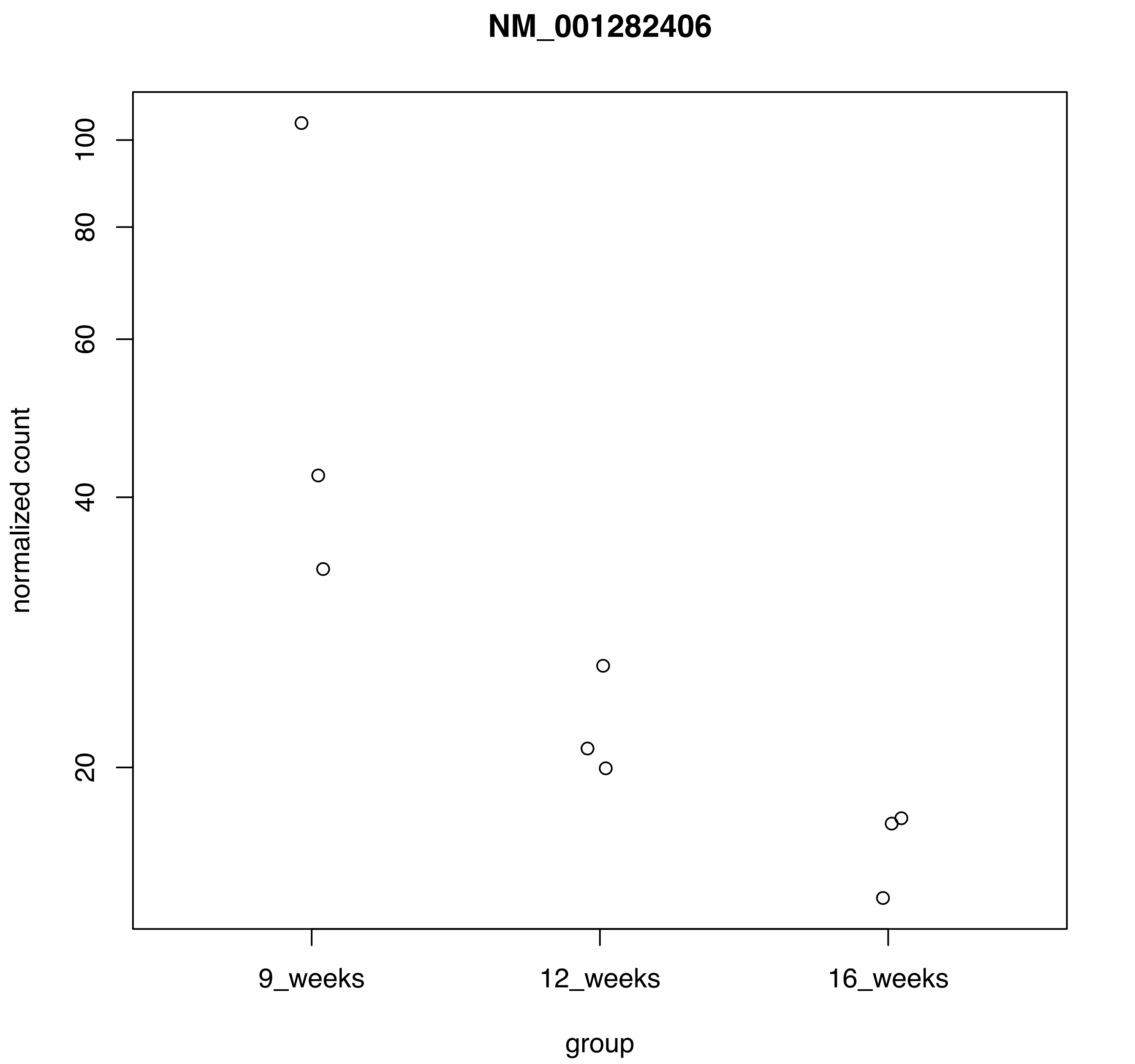

NM_001282406
normalized count
100
80
60
40
20
9_weeks
12_weeks
16_weeks
group

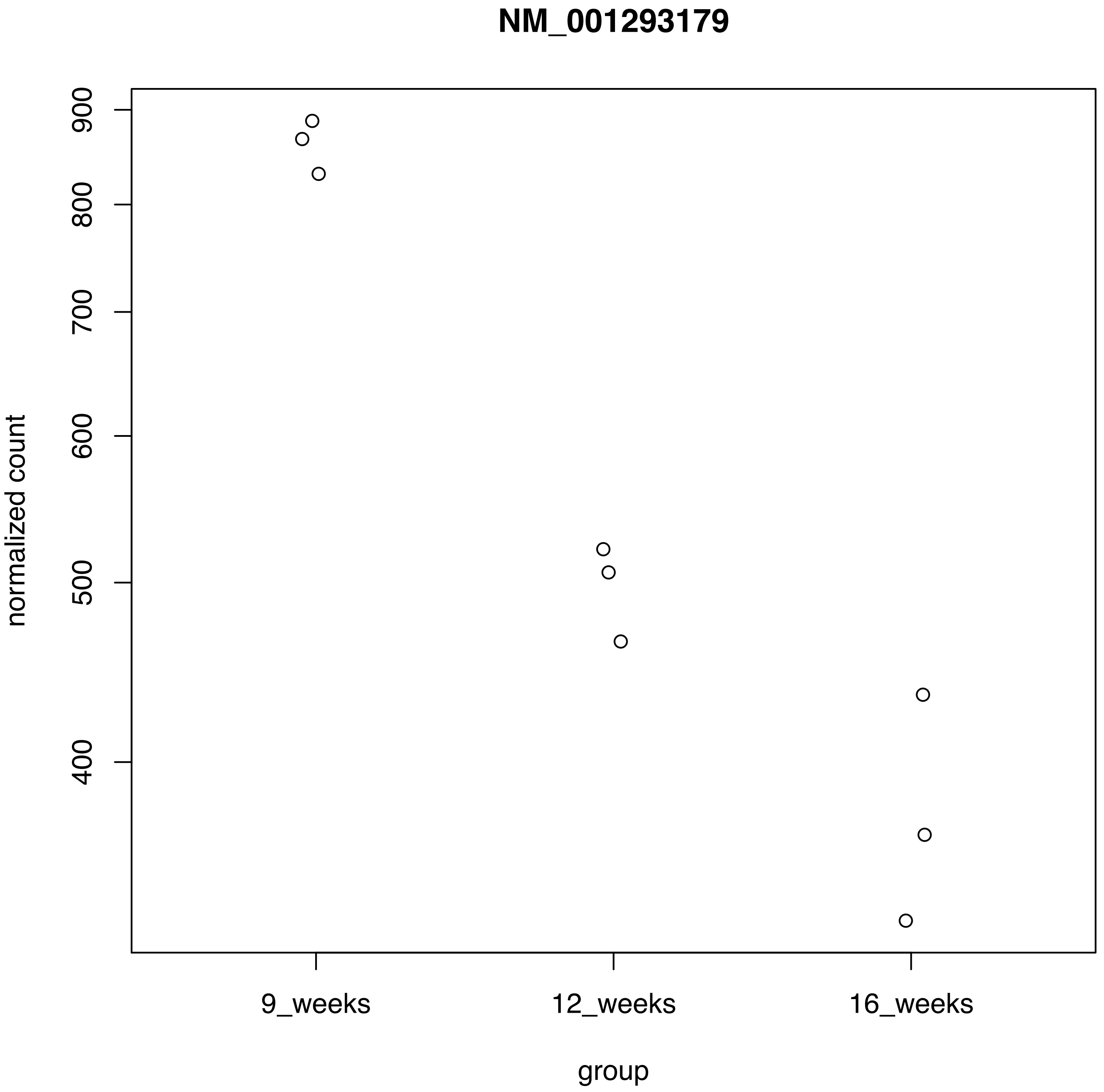

NM_001293179
normalized count
900
800
700
600
500
400
9_weeks
12_weeks
16_weeks
group

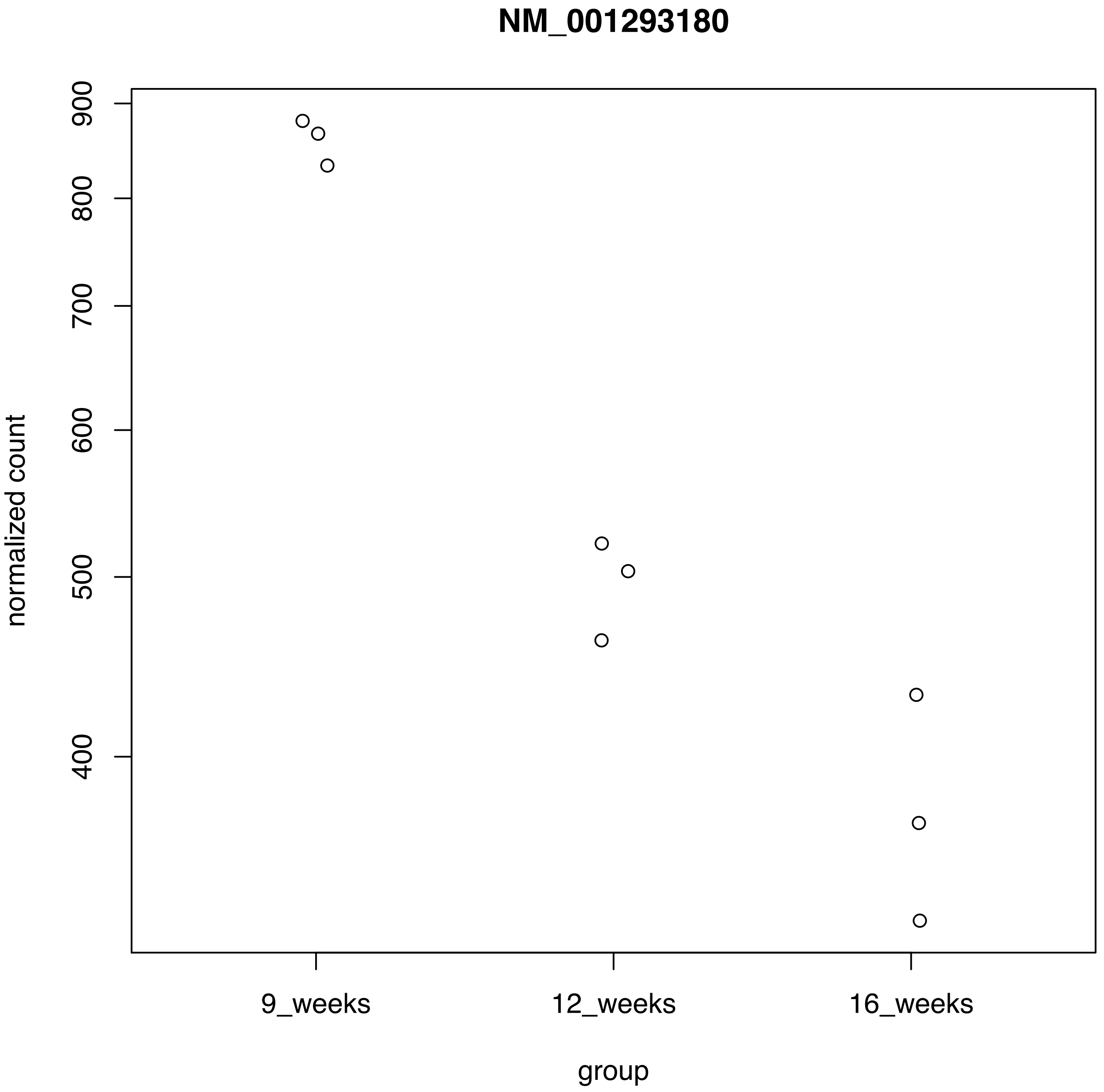
NM_001293180
normalized count
900
800
700
600
500
400
group
9_weeks
12_weeks
16_weeks

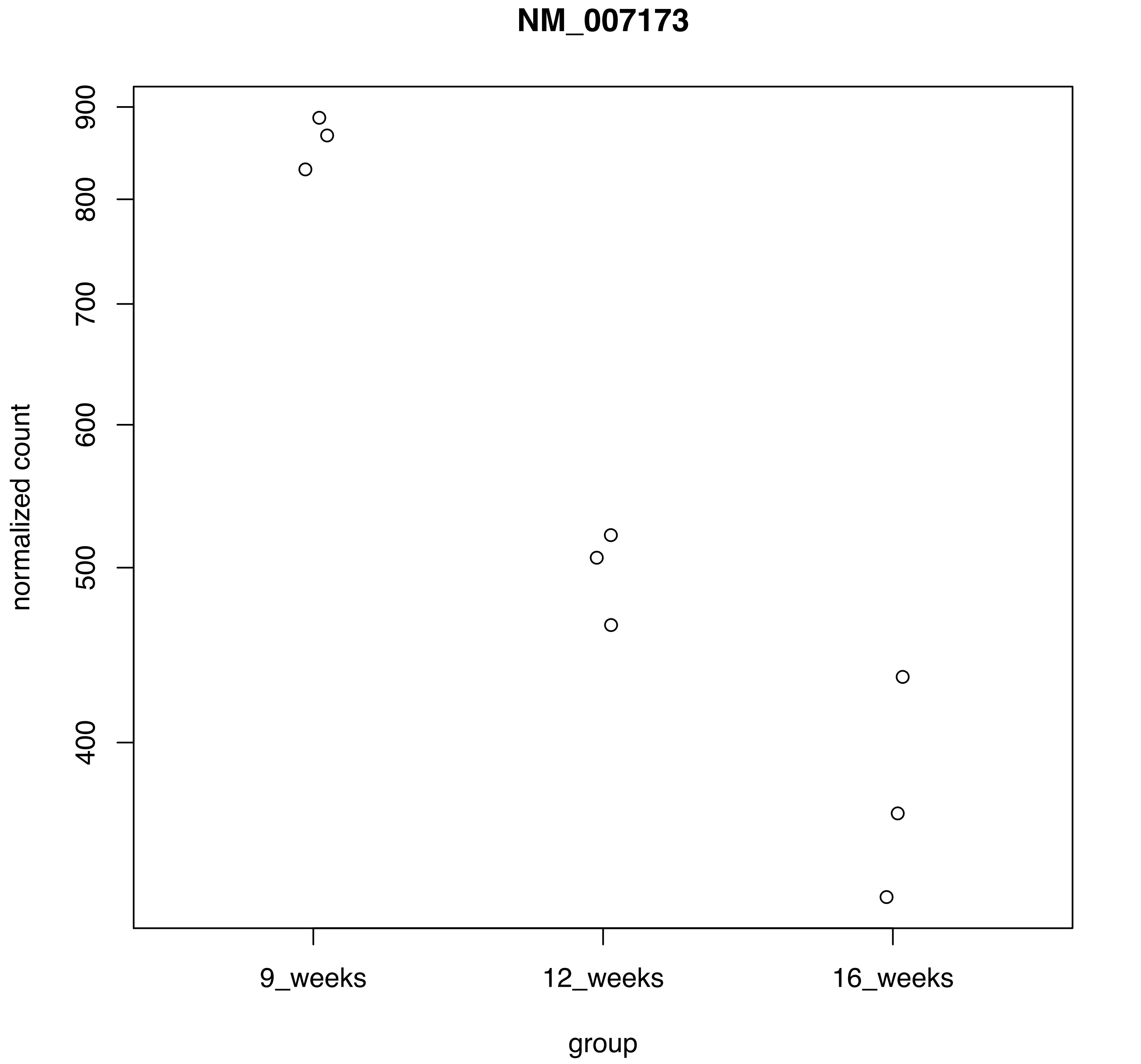
NM_007173
normalized count
900
800
700
600
500
400
9_weeks
12_weeks
16_weeks
group

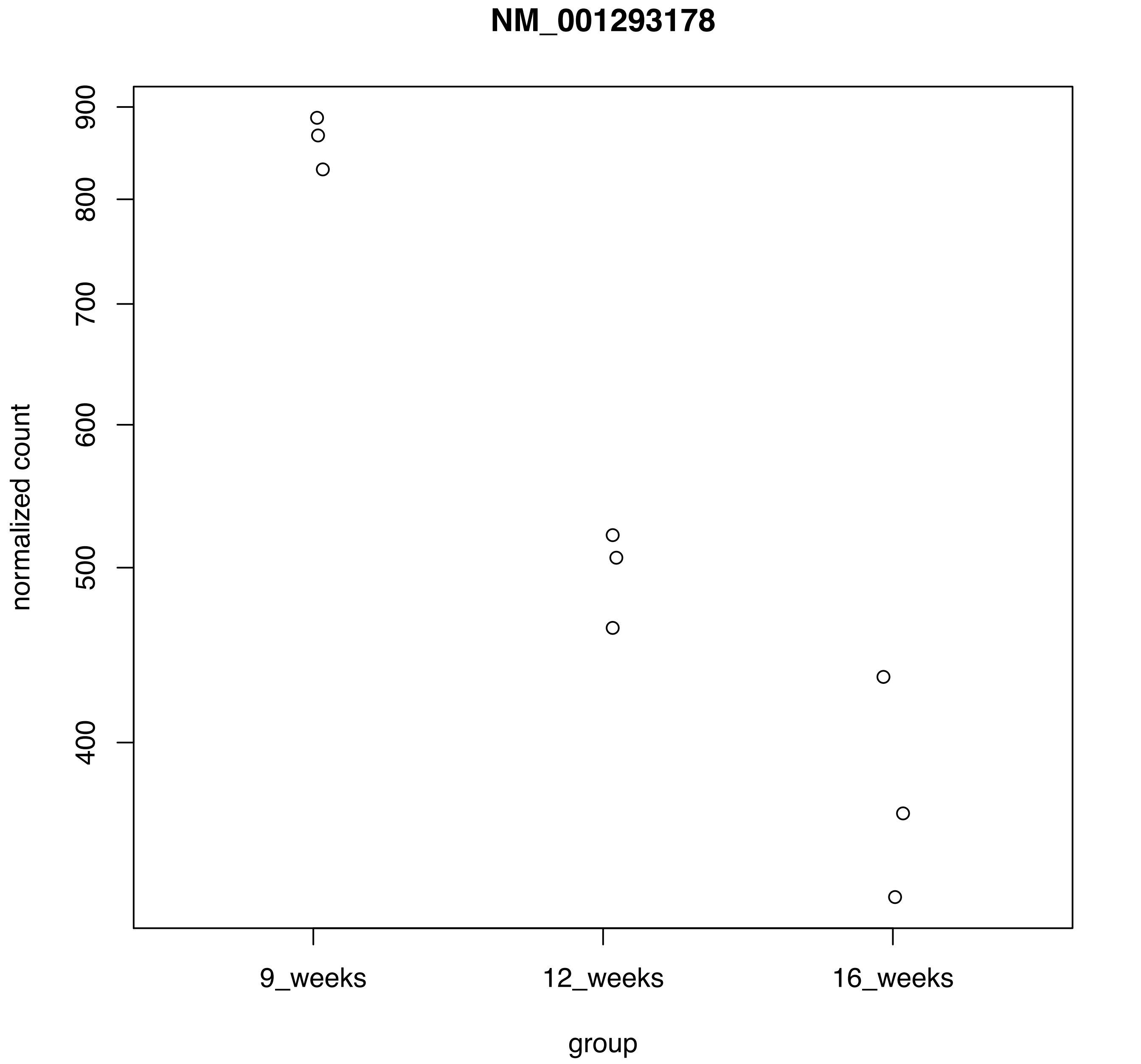
NM_001293178
normalized count
900
800
700
600
500
400
9_weeks
12_weeks
16_weeks
group

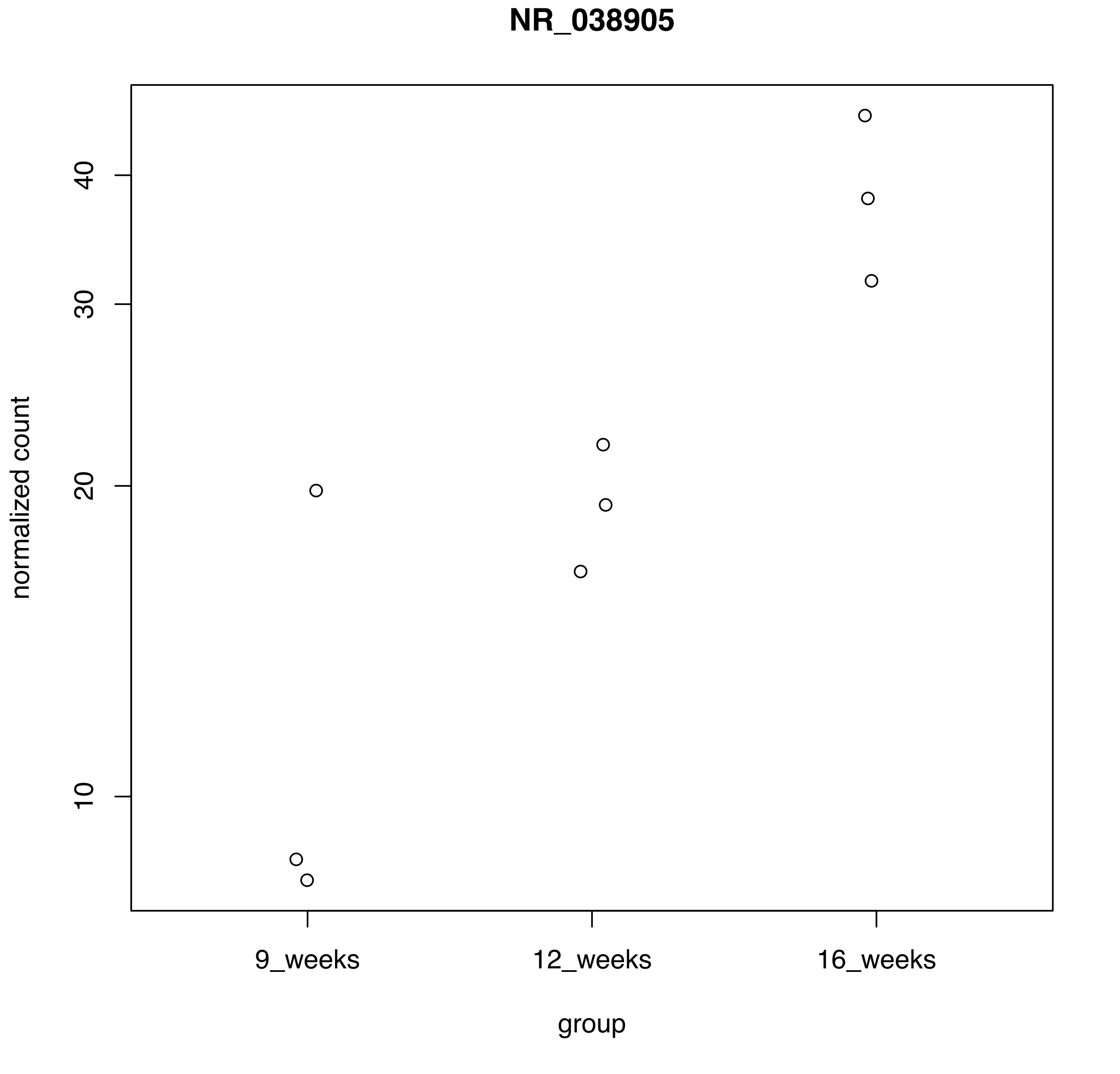

NR_038905
normalized count
10
20
30
40
9_weeks
12_weeks
16_weeks
group

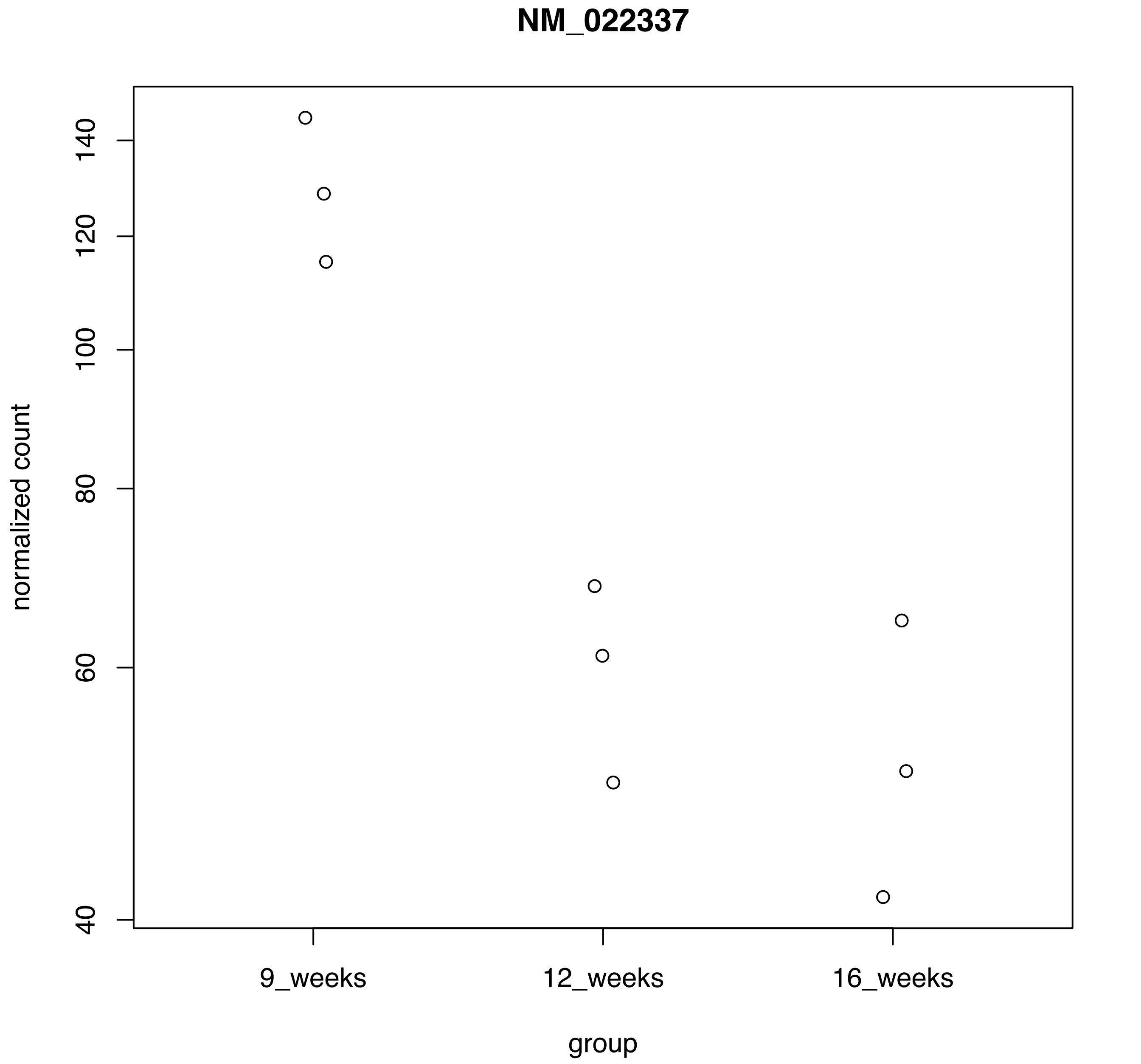

NM_022337
normalized count
140
120
100
80
60
40
9_weeks
12_weeks
16_weeks
group

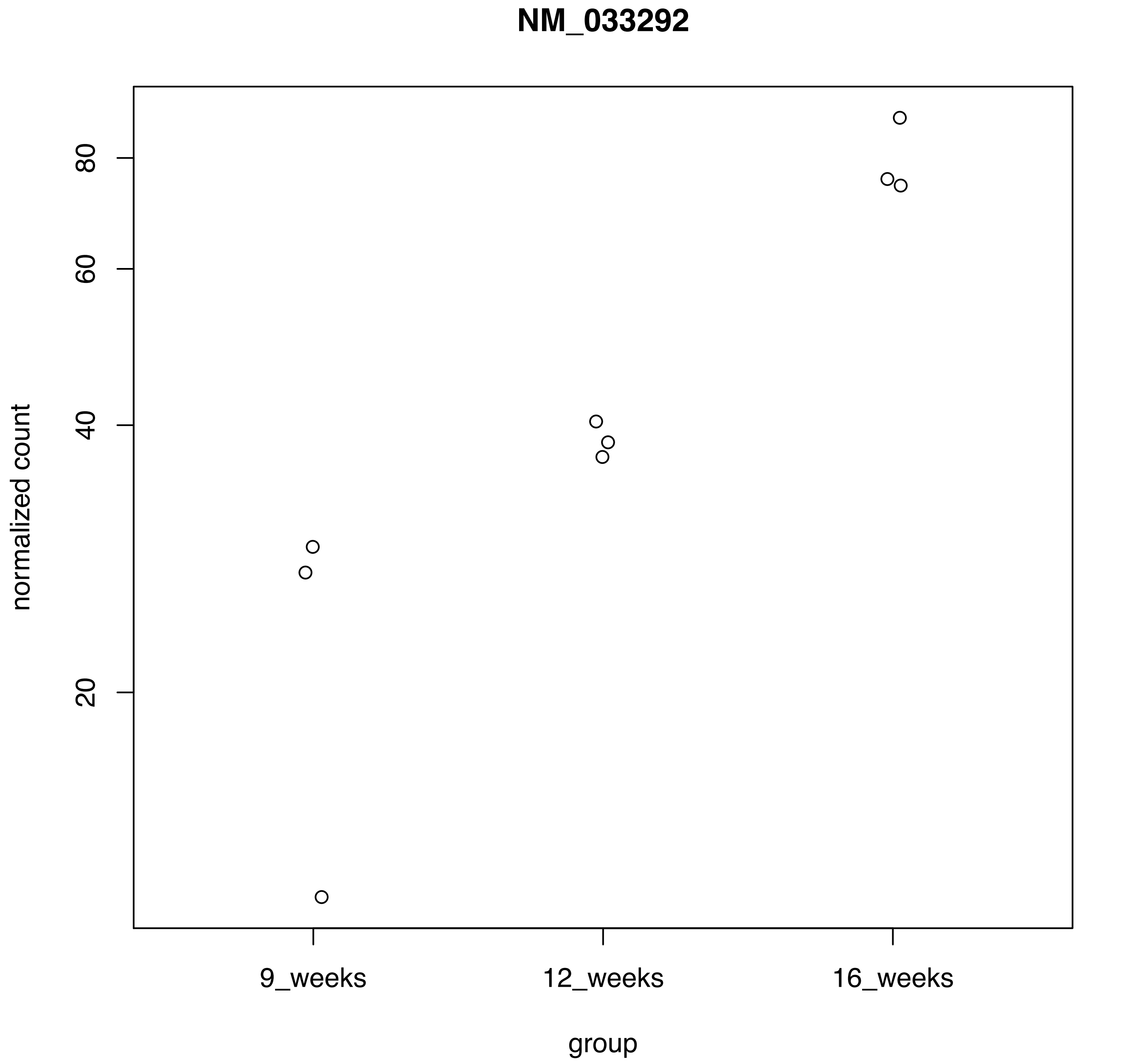
NM_033292
80
60
40
20
normalized count
9_weeks
12_weeks
16_weeks
group

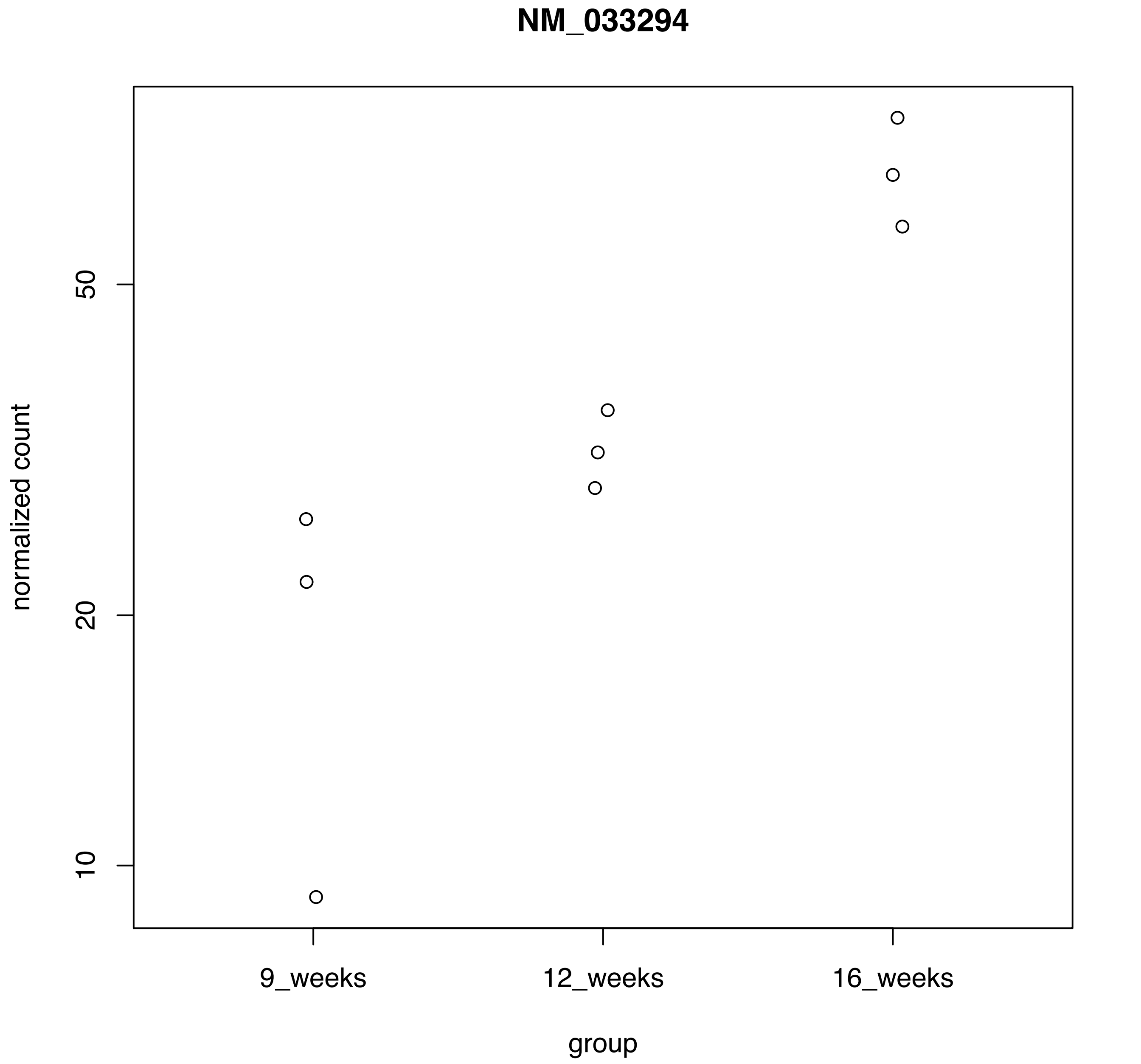
NM_033294
normalized count
50
20
10
9_weeks
12_weeks
16_weeks
group

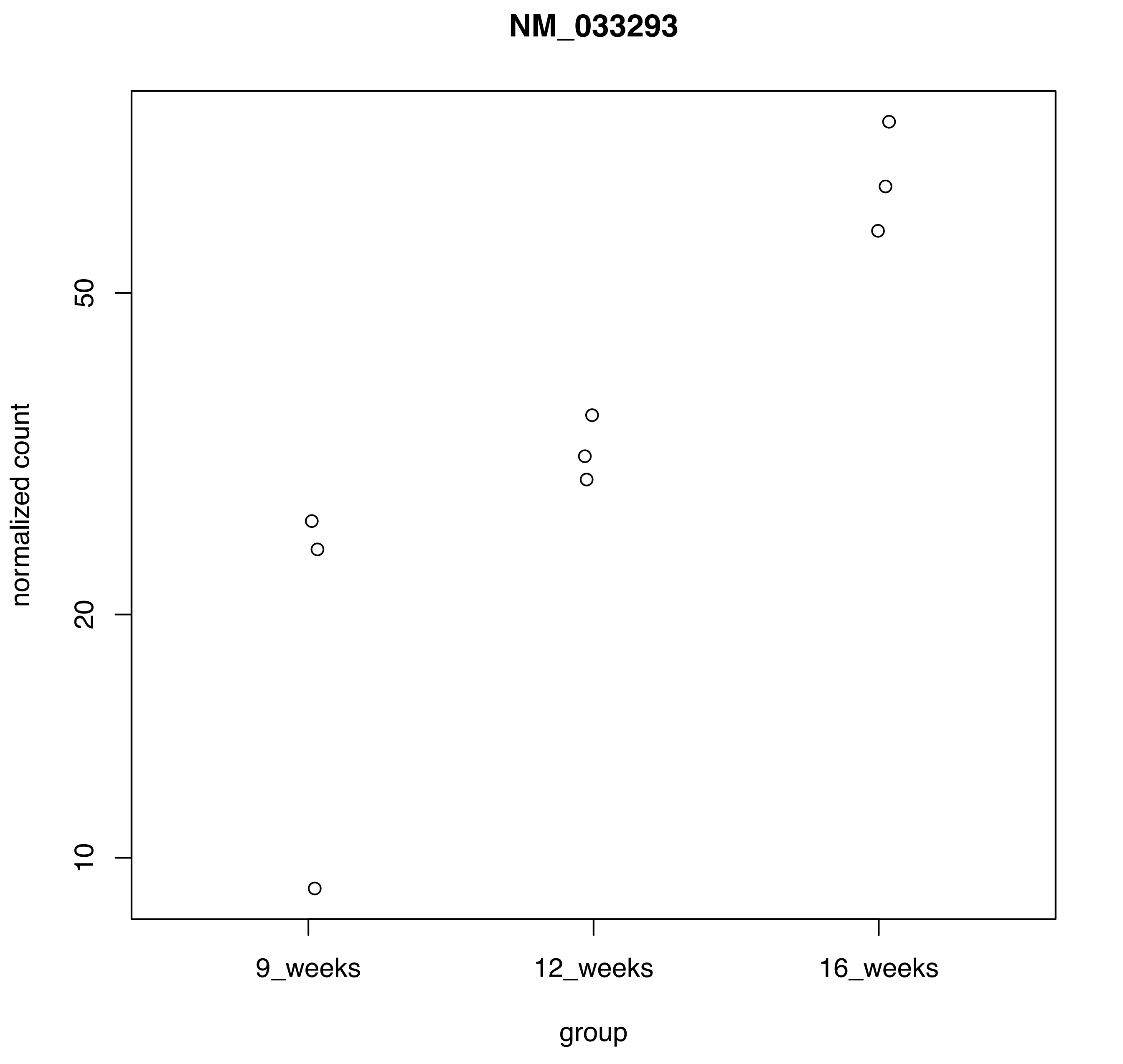
NM_033293
normalized count
50
20
10
9_weeks
12_weeks
16_weeks
group

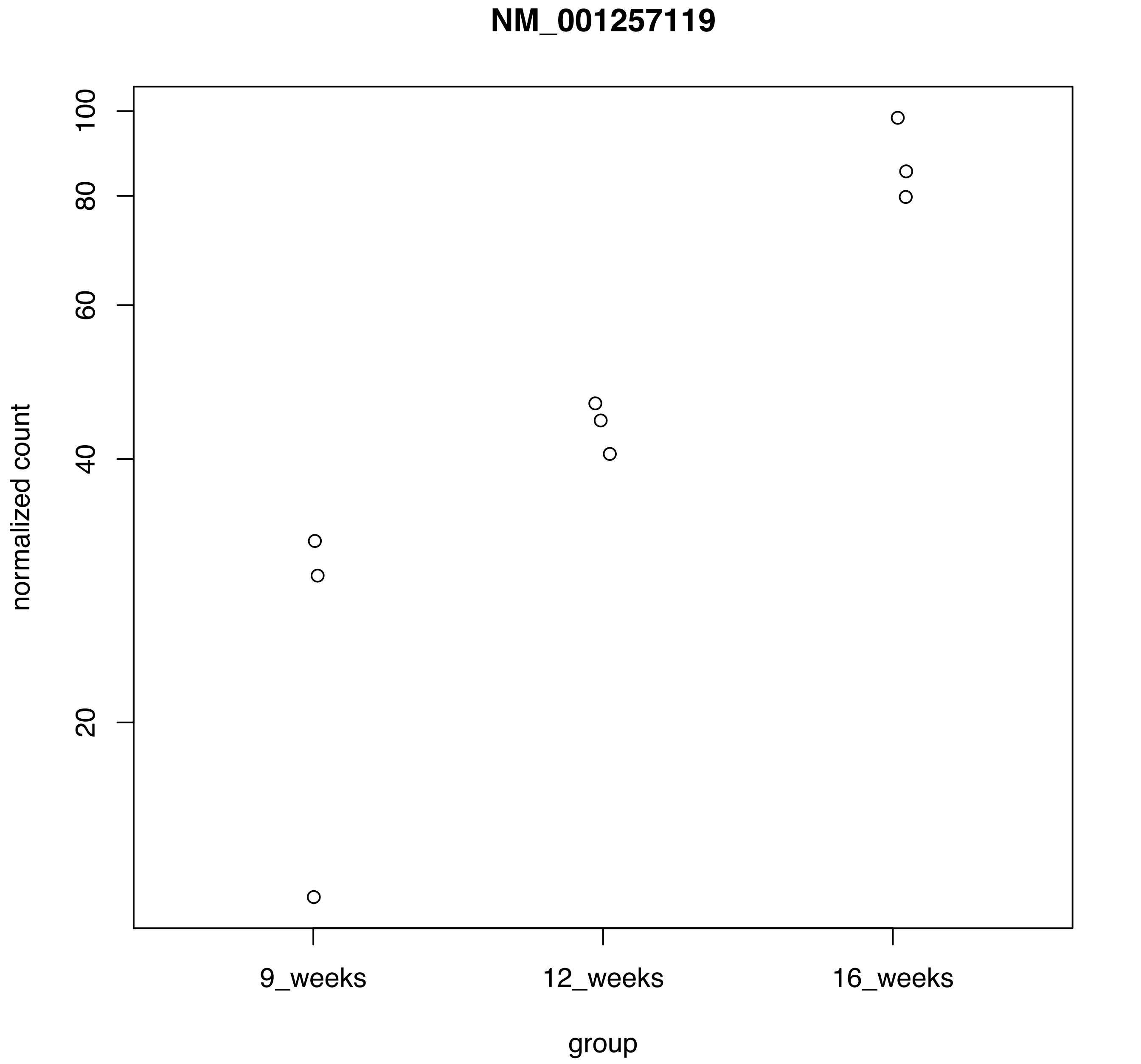
NM_001257119
normalized count
100
80
60
40
20
9_weeks
12_weeks
16_weeks
group

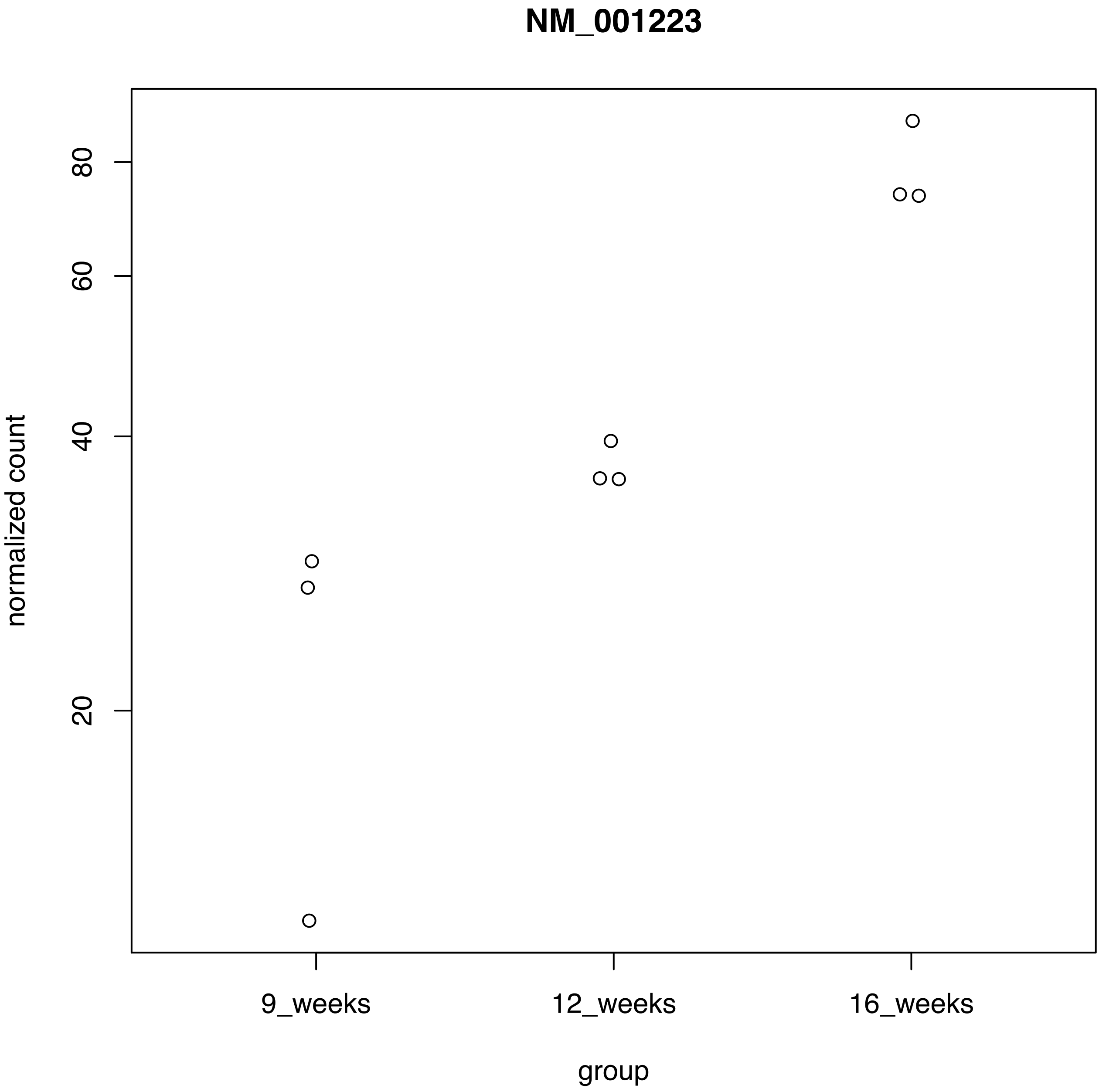
NM_001223
normalized count
20
40
60
80
9_weeks
12_weeks
16_weeks
group

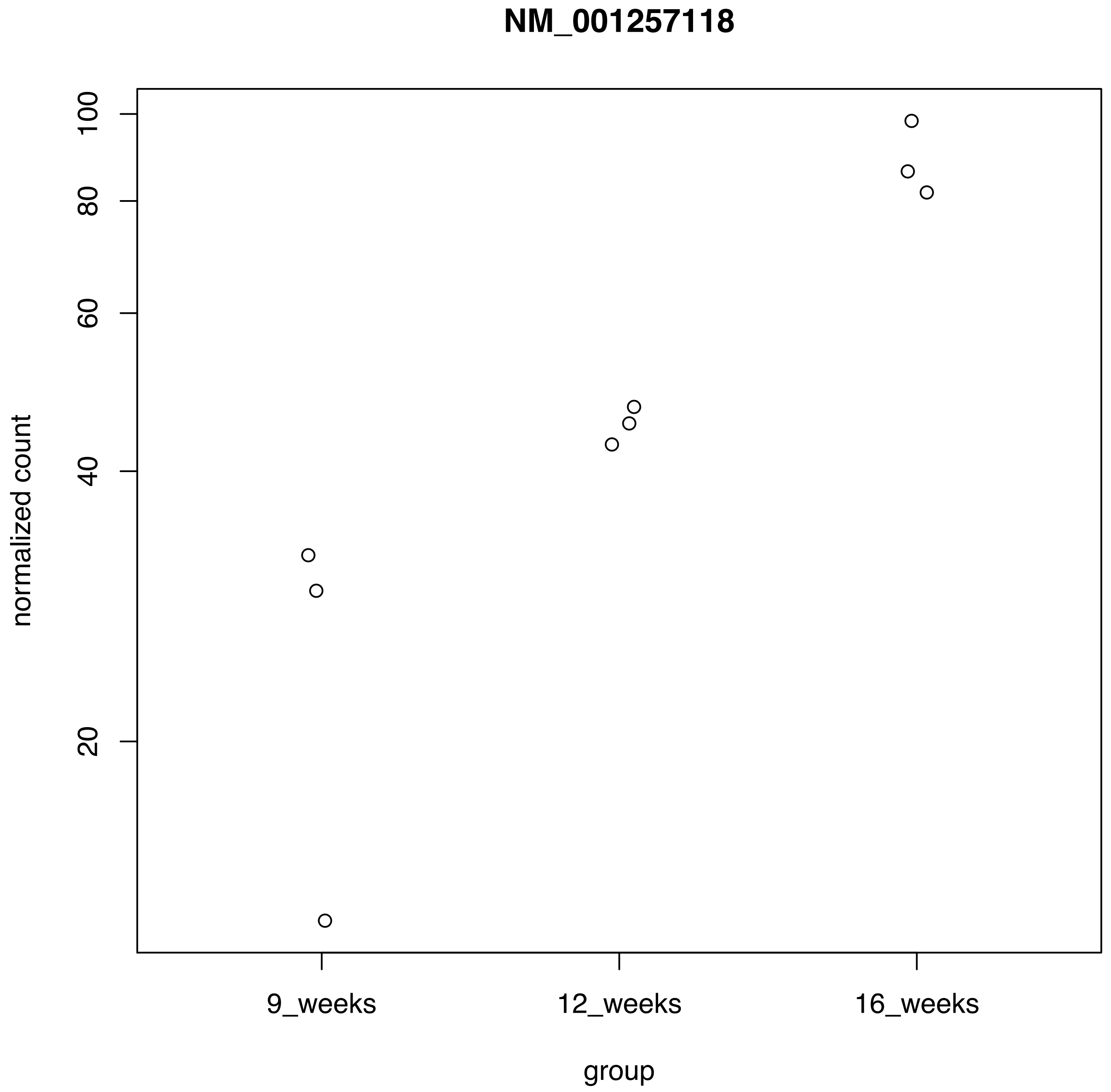
NM_001257118
normalized count
100
80
60
40
20
9_weeks
12_weeks
16_weeks
group

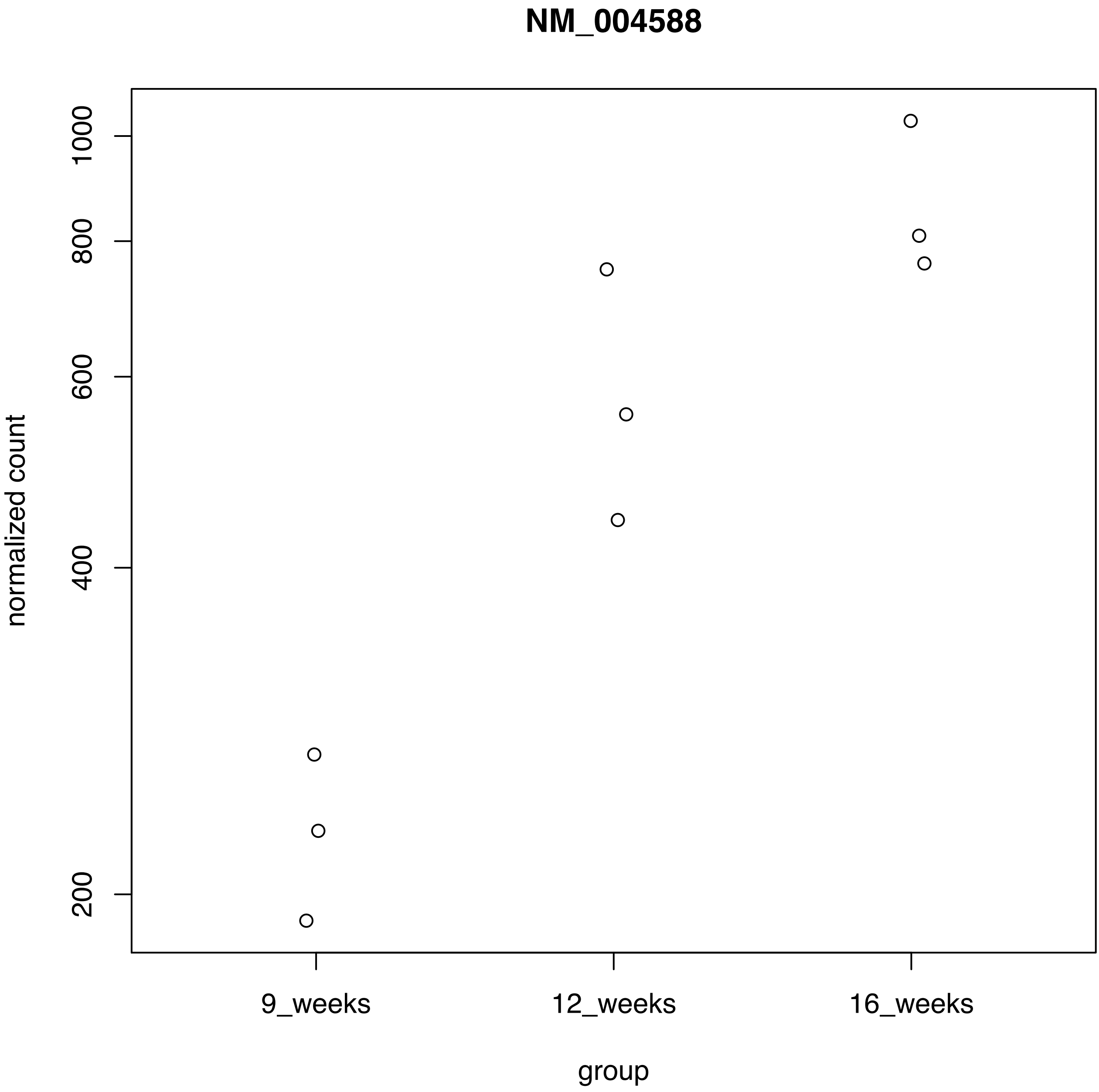
NM_004588
normalized count
1000
800
600
400
200
9_weeks
12_weeks
16_weeks
group

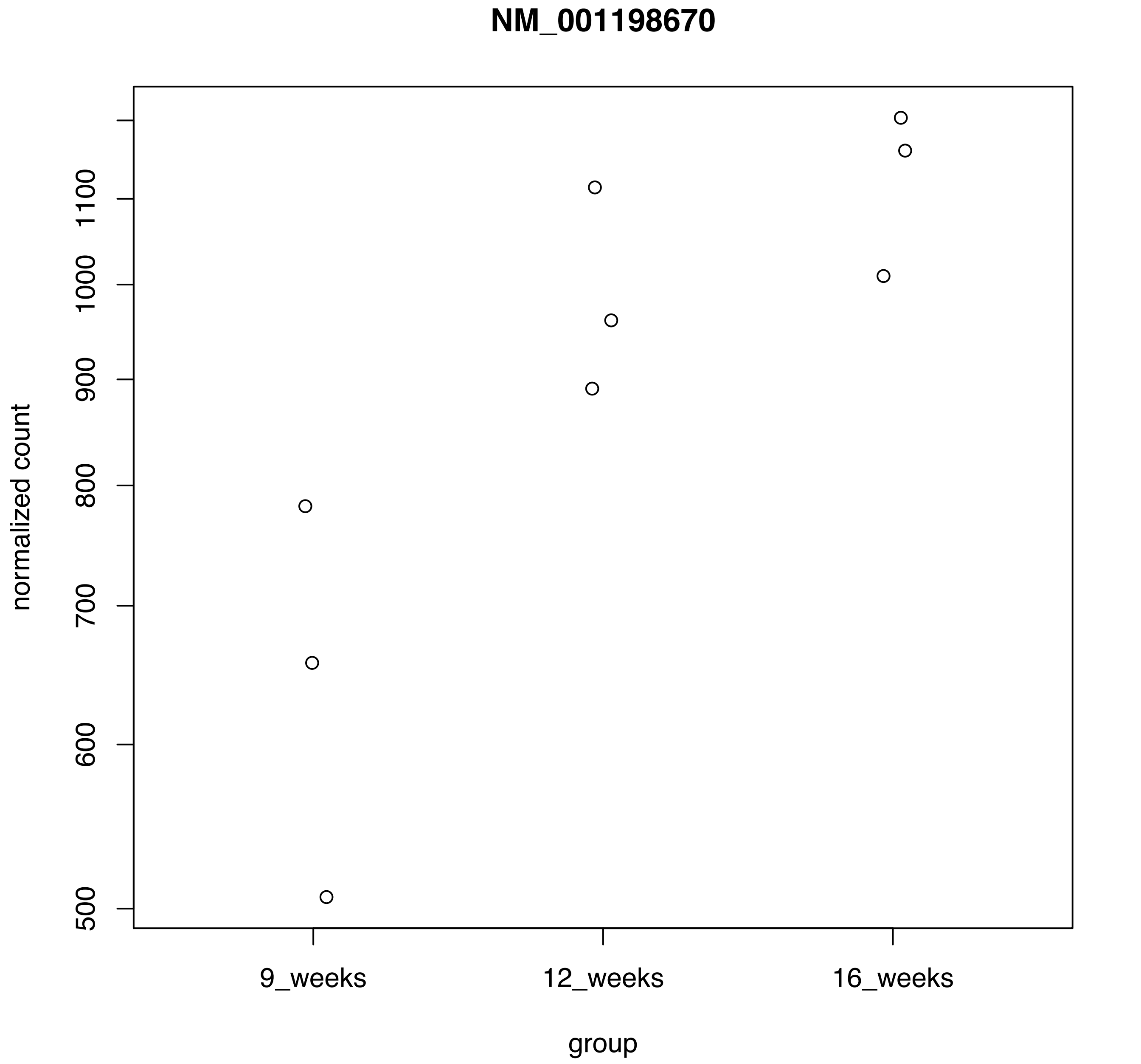

NM_001198670
normalized count
500
600
700
800
900
1000
1100
9_weeks
12_weeks
16_weeks
group

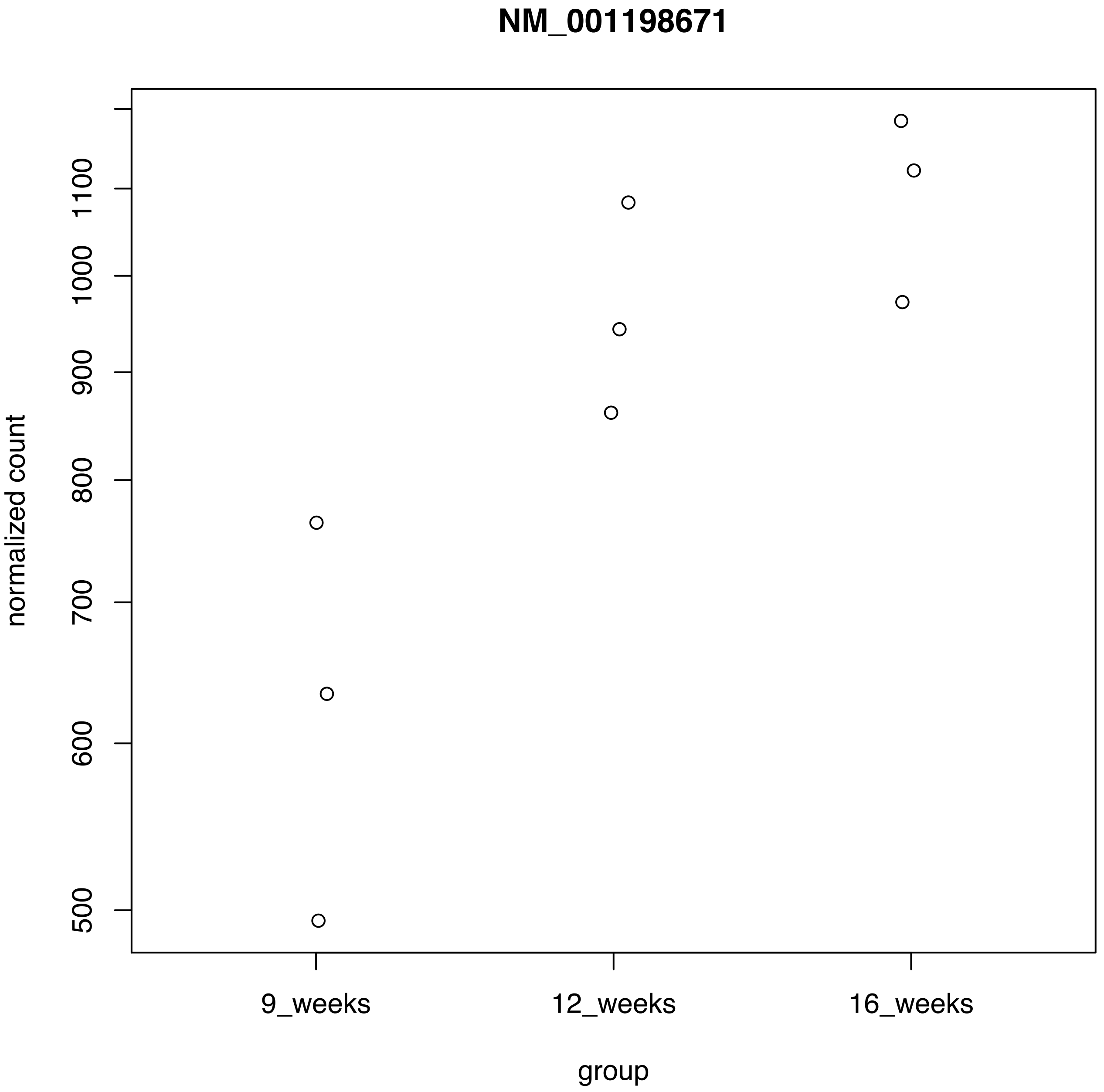
NM_001198671
normalized count
500
600
700
800
900
1000
1100
9_weeks
12_weeks
16_weeks
group

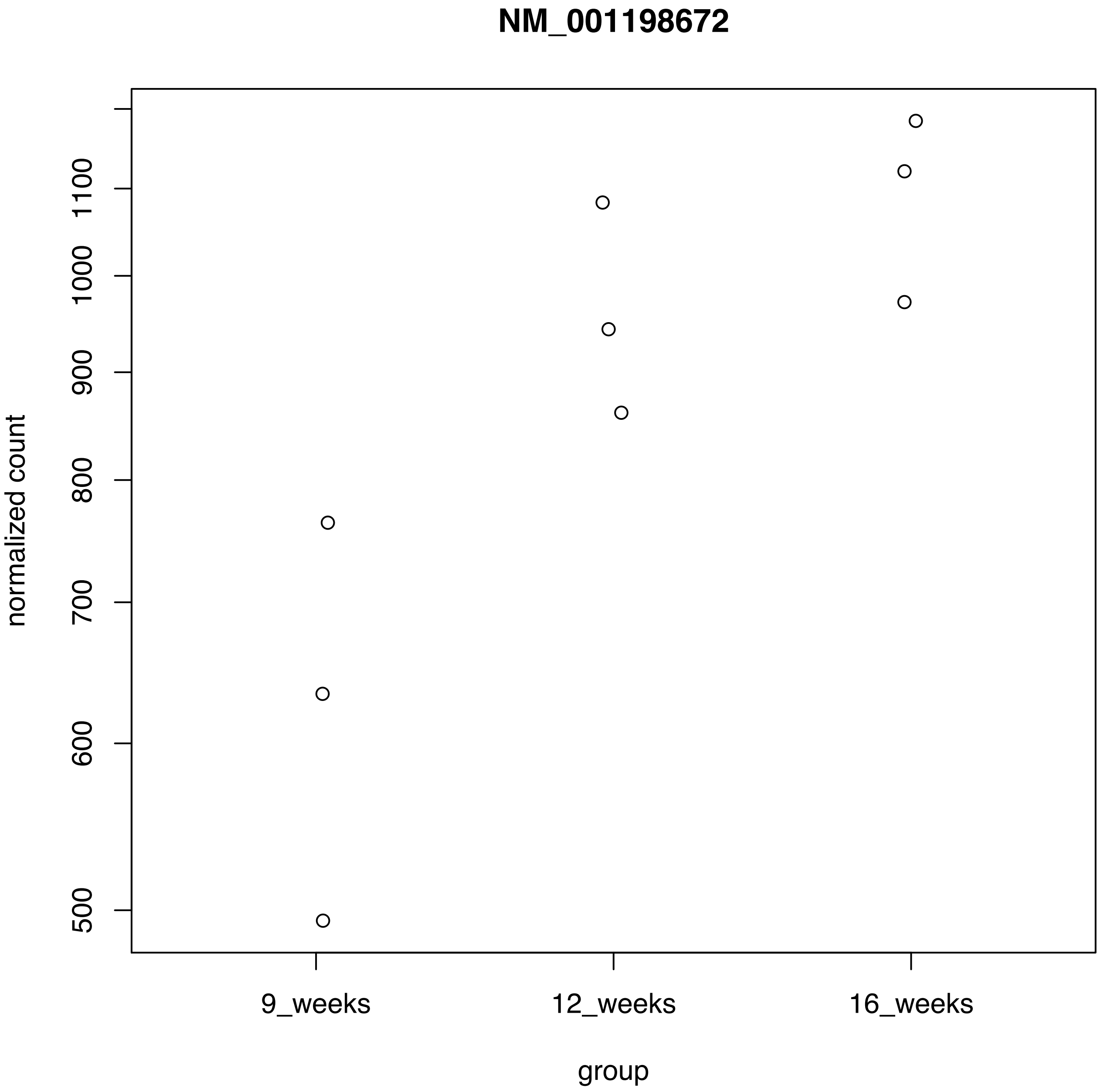

NM_001198672
normalized count
500
600
700
800
900
1000
1100
9_weeks
12_weeks
16_weeks
group

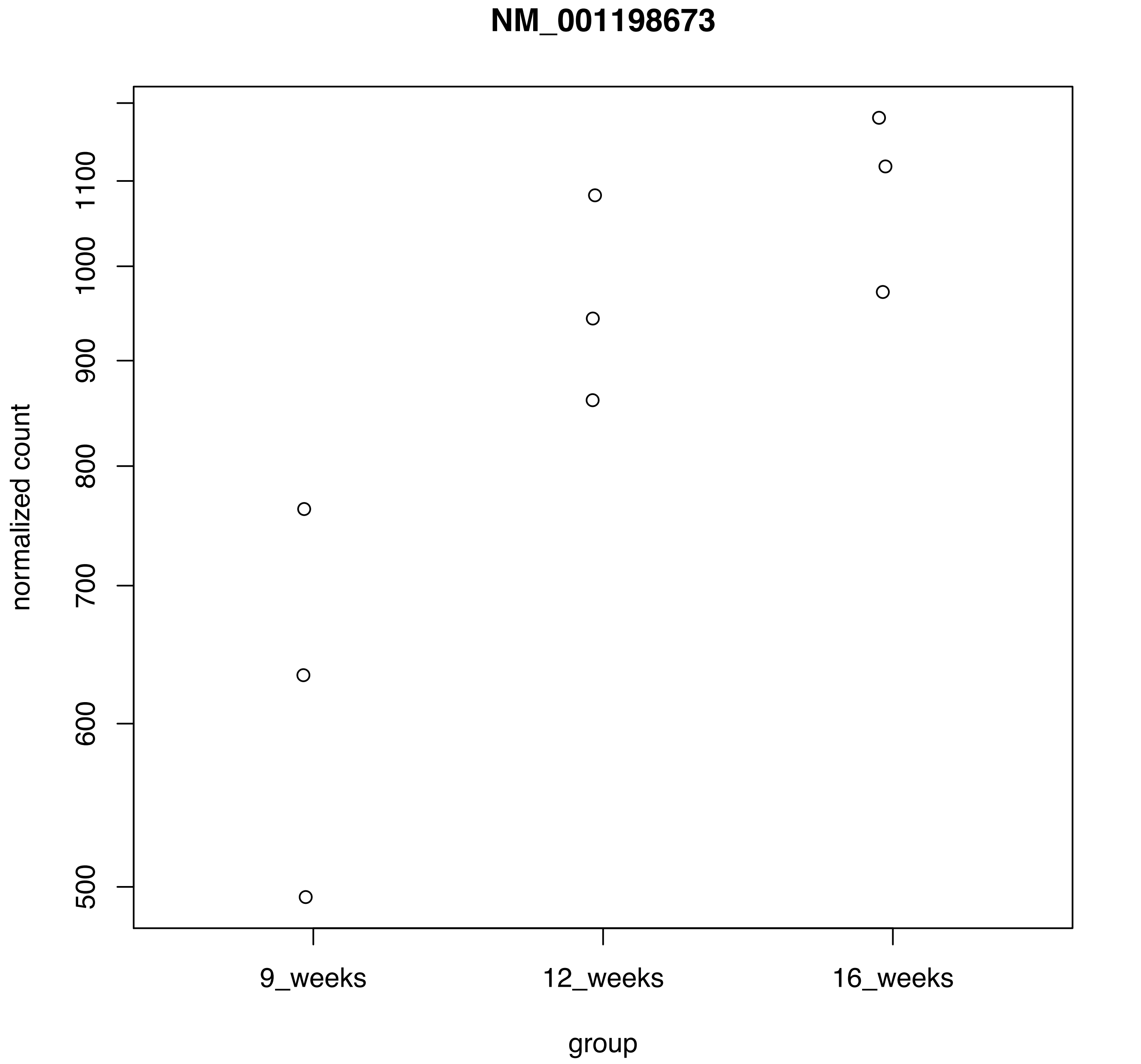
NM_001198673
normalized count
1100
1000
900
800
700
600
500
9_weeks
12_weeks
16_weeks
group

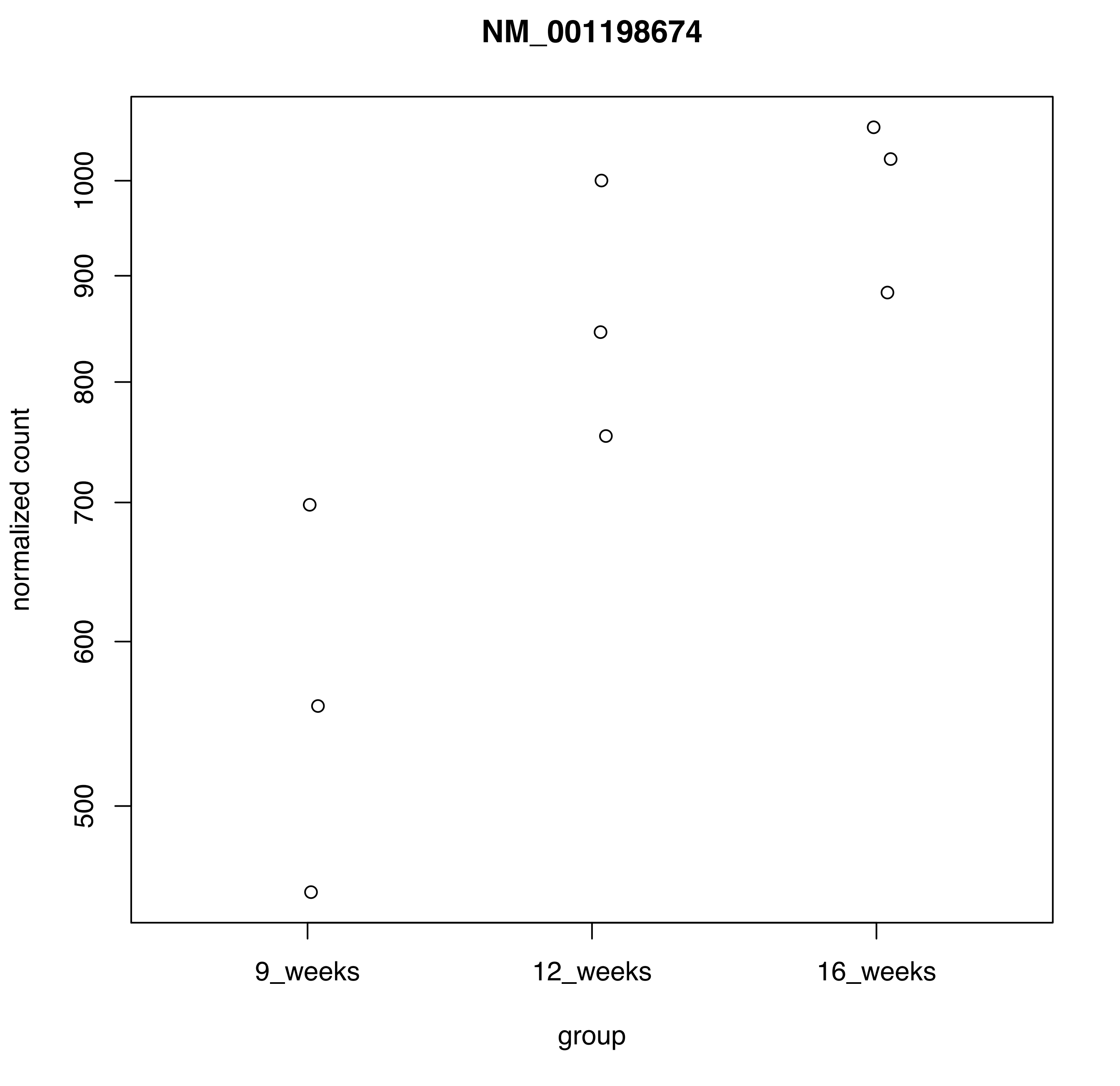
NM_001198674
normalized count
500
600
700
800
900
1000
9_weeks
12_weeks
16_weeks
group

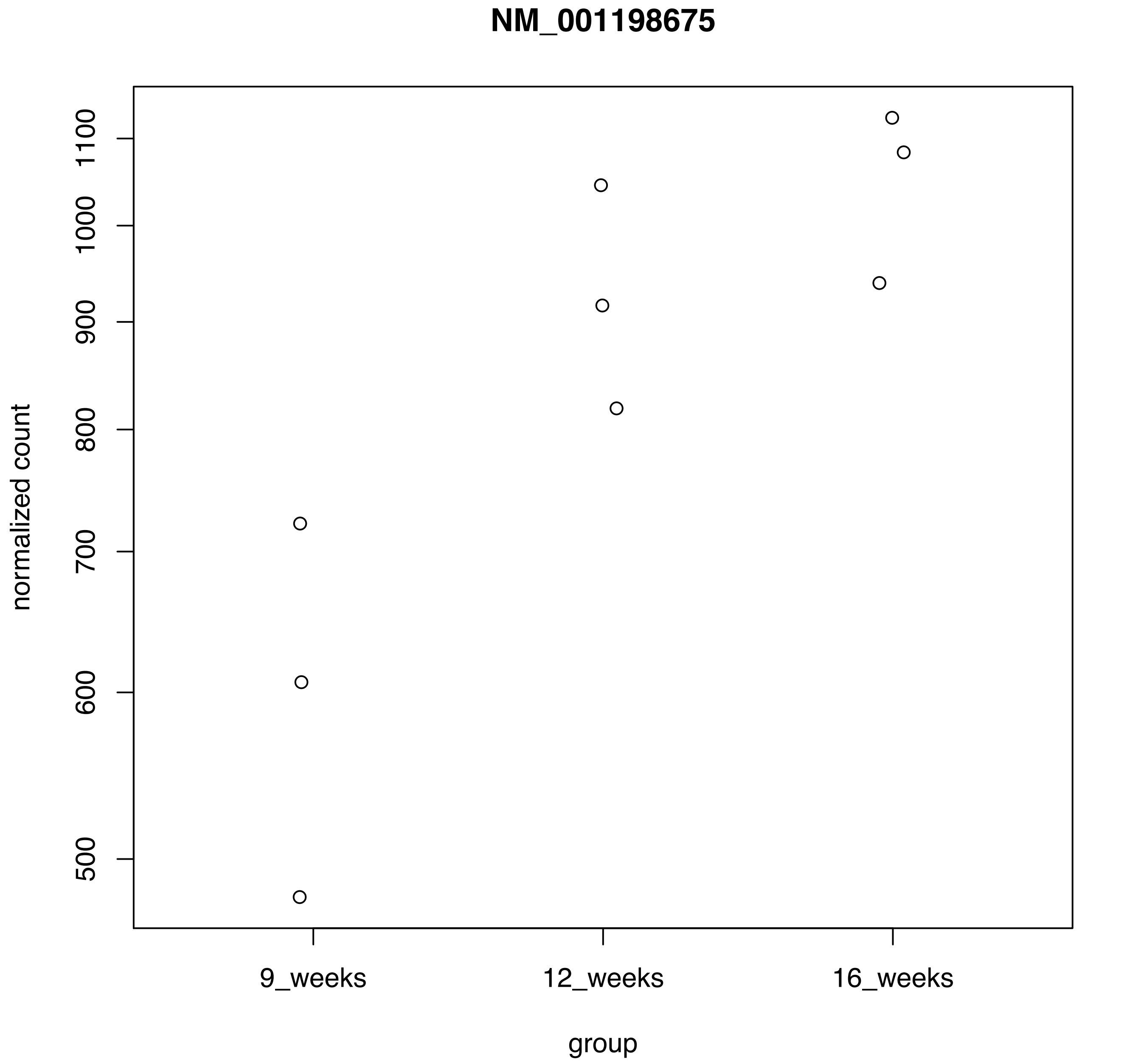

NM_001198675
normalized count
500
600
700
800
900
1000
1100
9_weeks
12_weeks
16_weeks
group

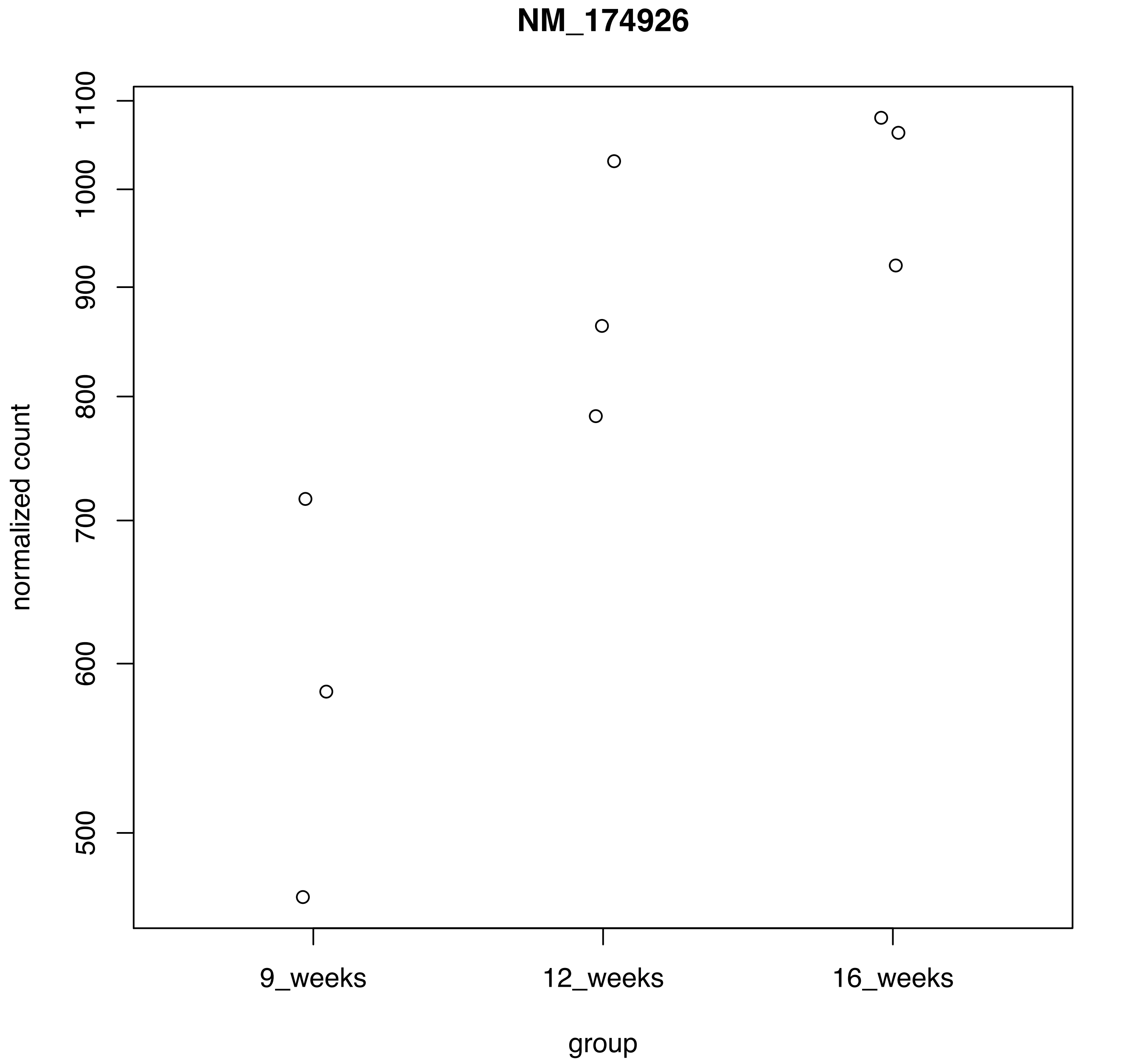
NM_174926
normalized count
1100
1000
900
800
700
600
500
9_weeks
12_weeks
16_weeks
group

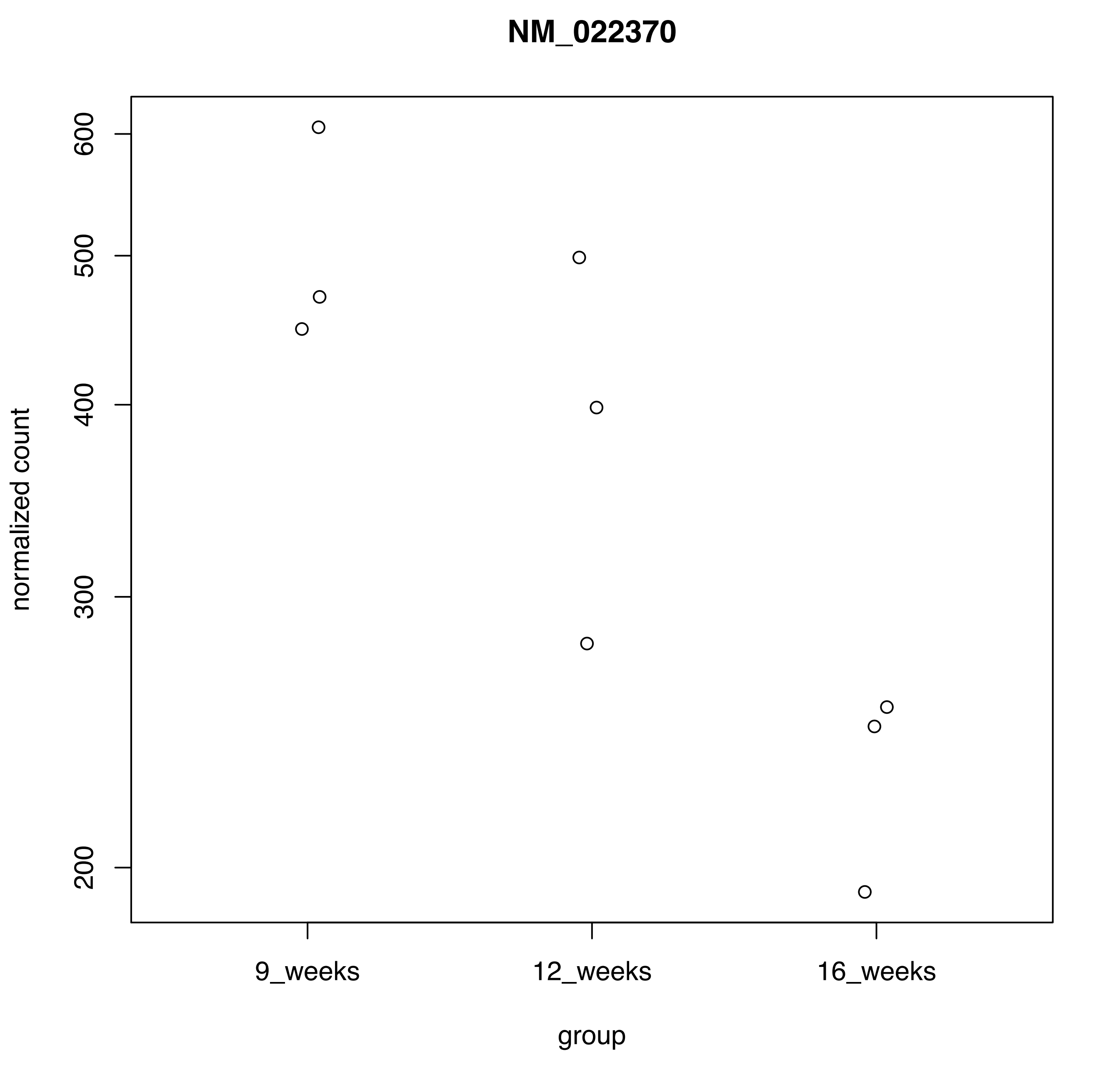
NM_022370
normalized count
600
500
400
300
200
9_weeks
12_weeks
16_weeks
group

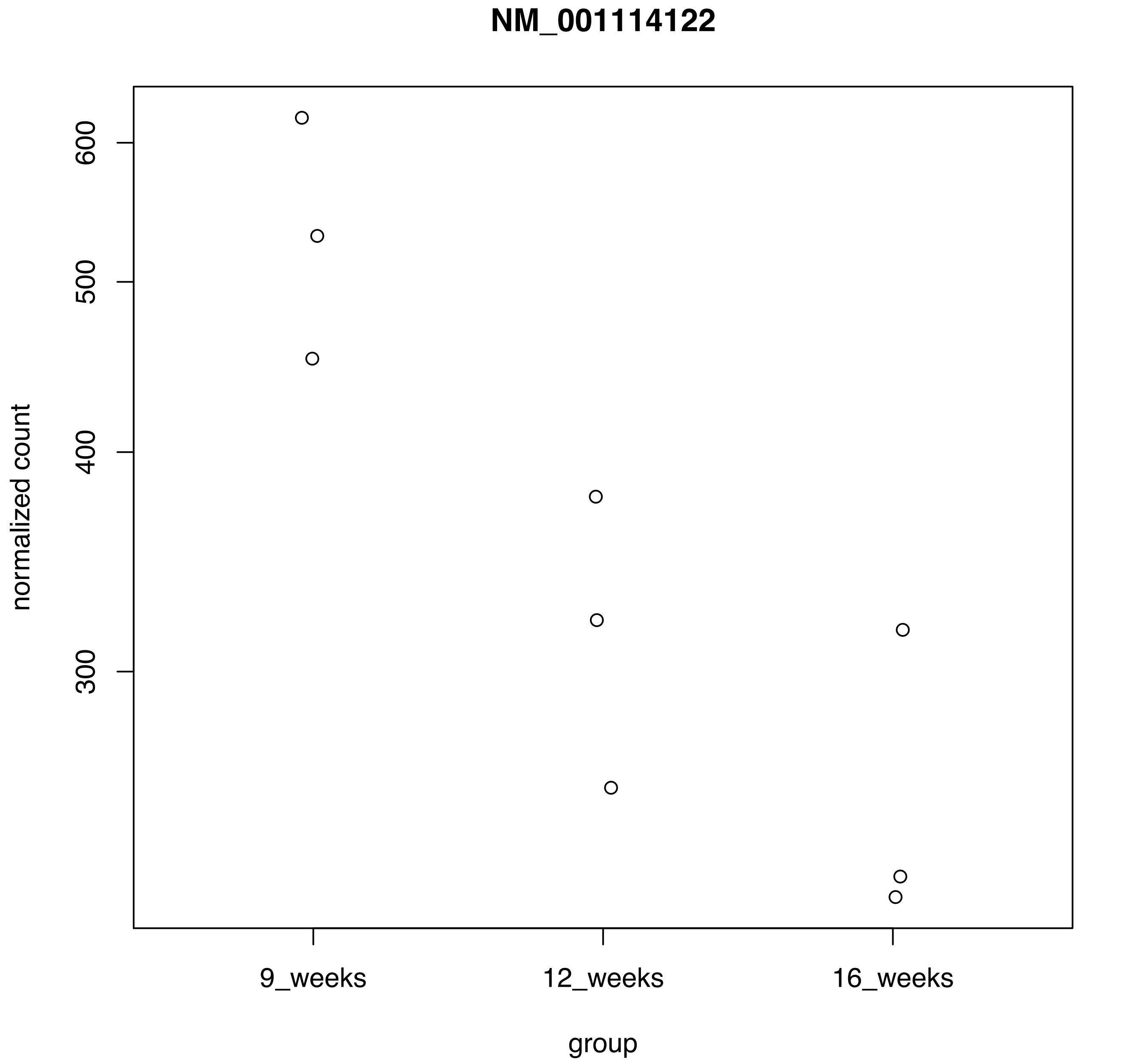

NM_001114122
normalized count
600
500
400
300
9_weeks
12_weeks
16_weeks
group

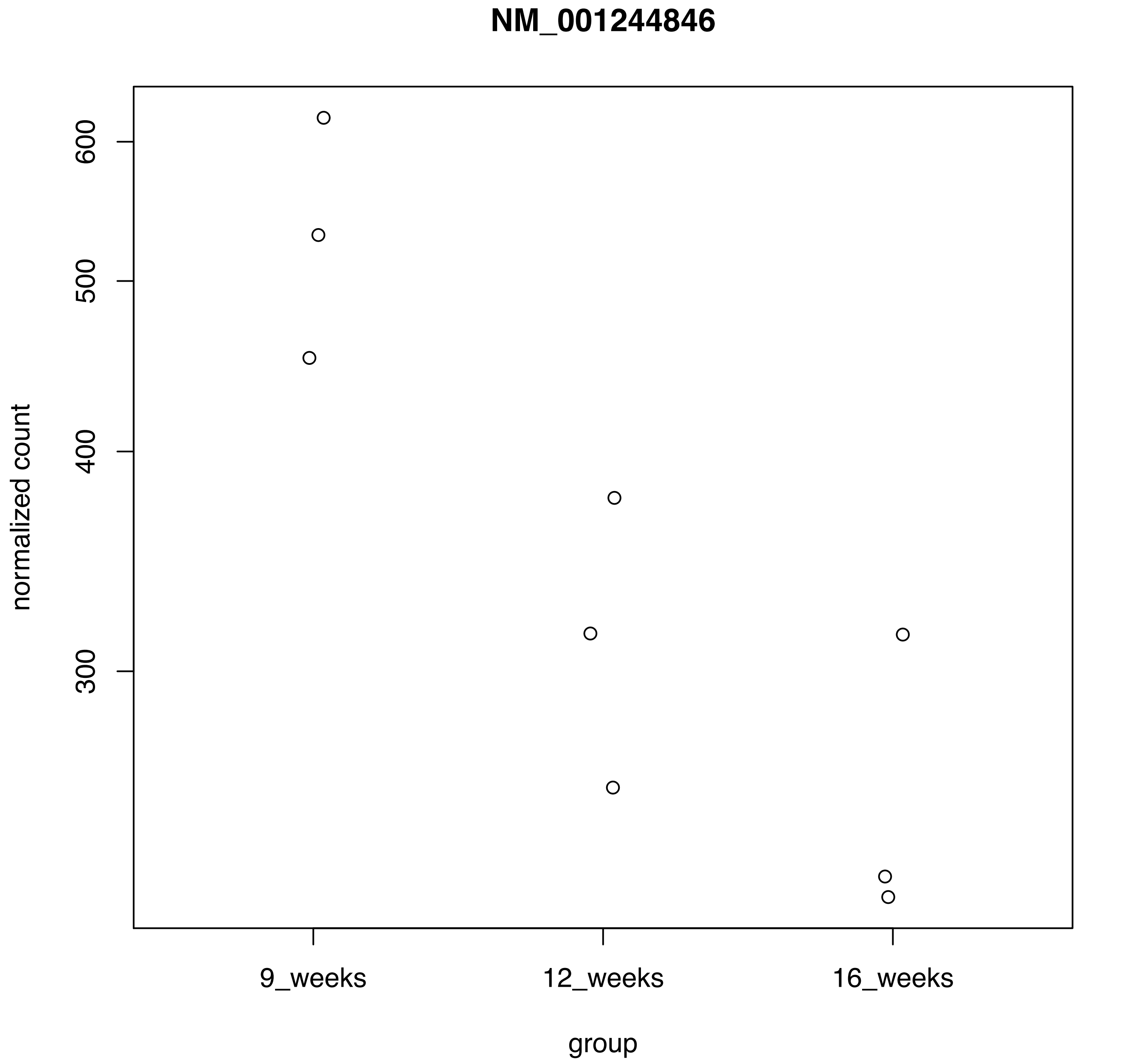
NM_001244846
normalized count
600
500
400
300
9_weeks
12_weeks
16_weeks
group

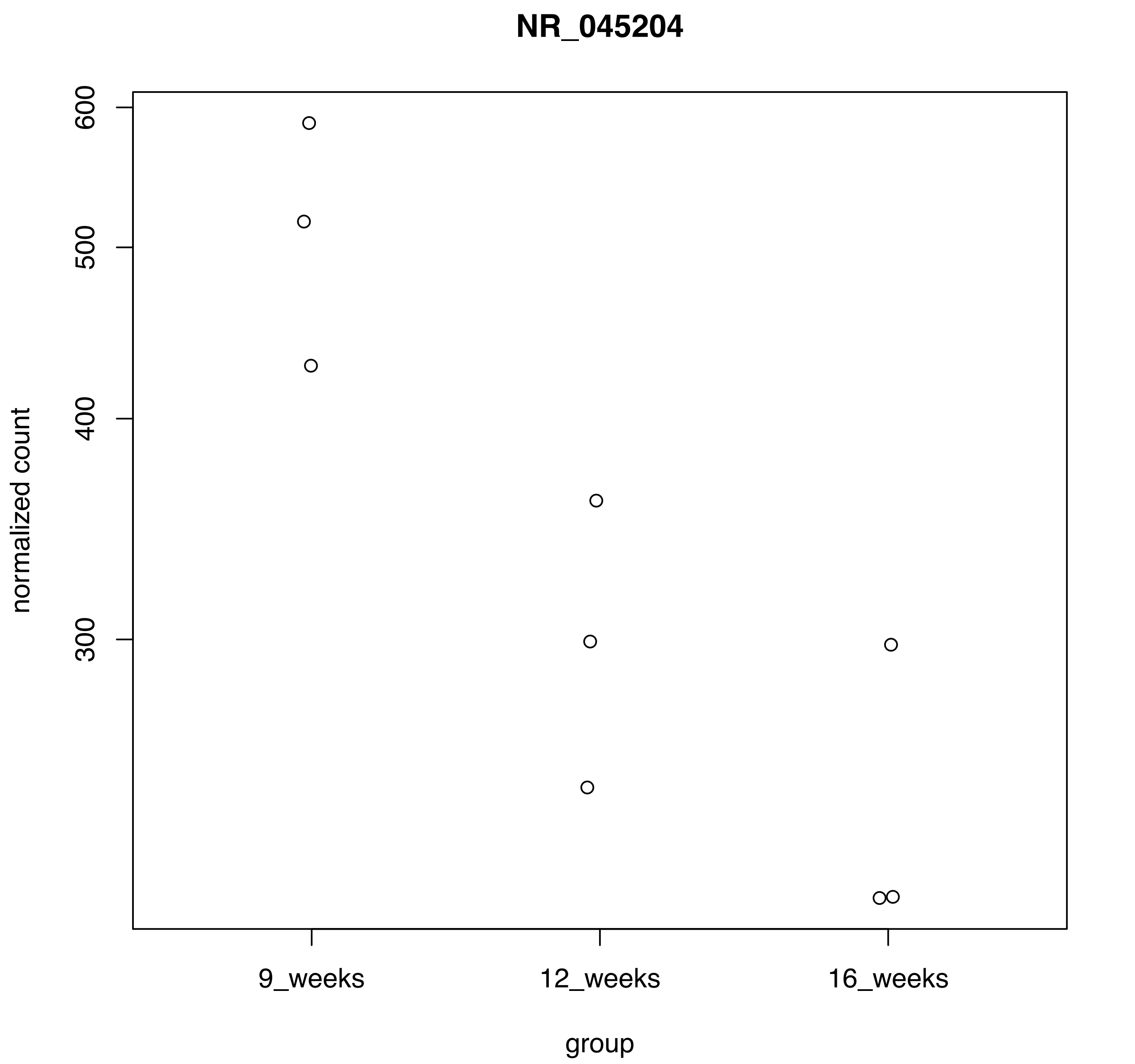
NR_045204
normalized count
600
500
400
300
9_weeks
12_weeks
16_weeks
group

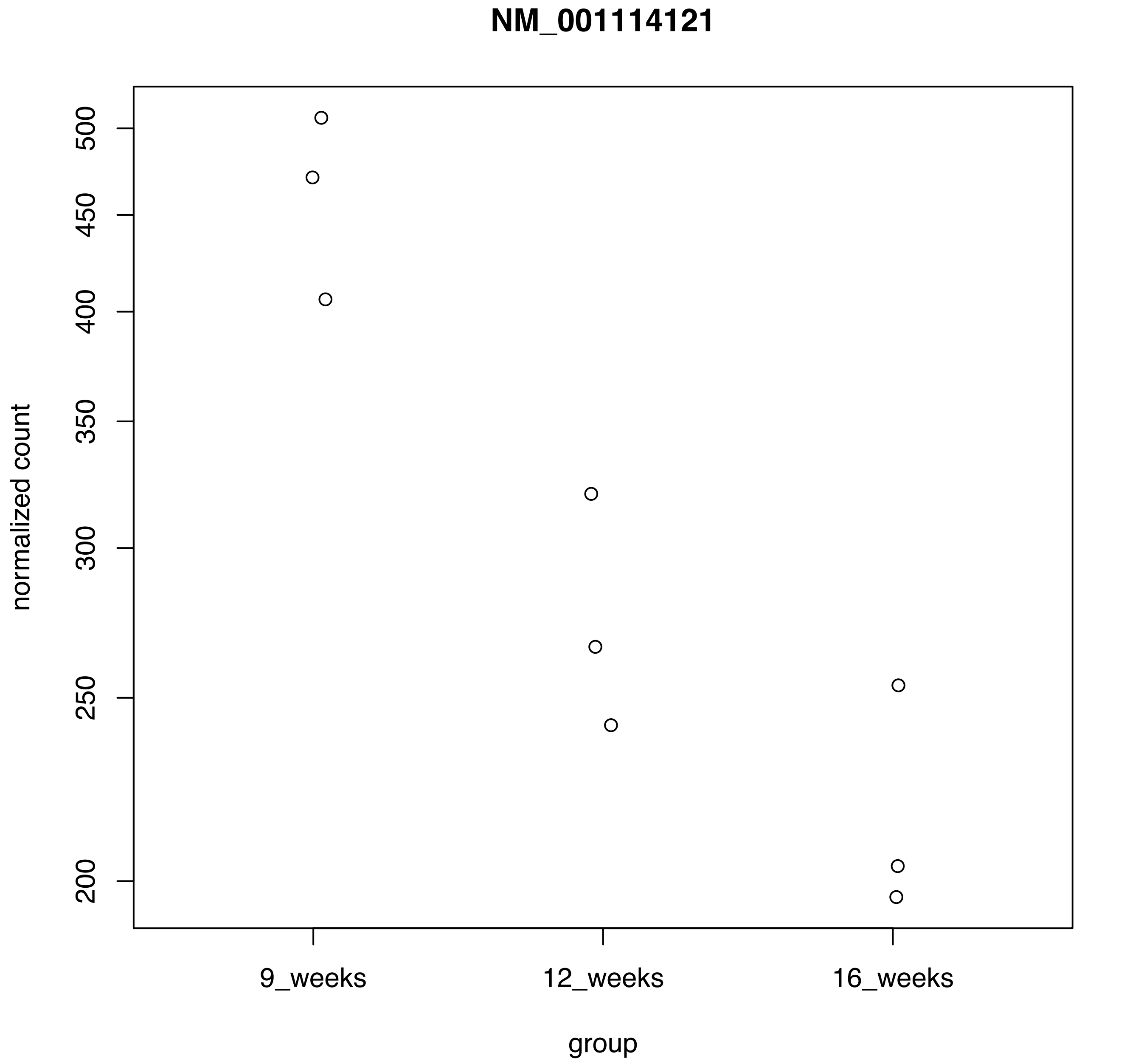
NM_001114121
normalized count
500
450
400
350
300
250
200
9_weeks
12_weeks
16_weeks
group

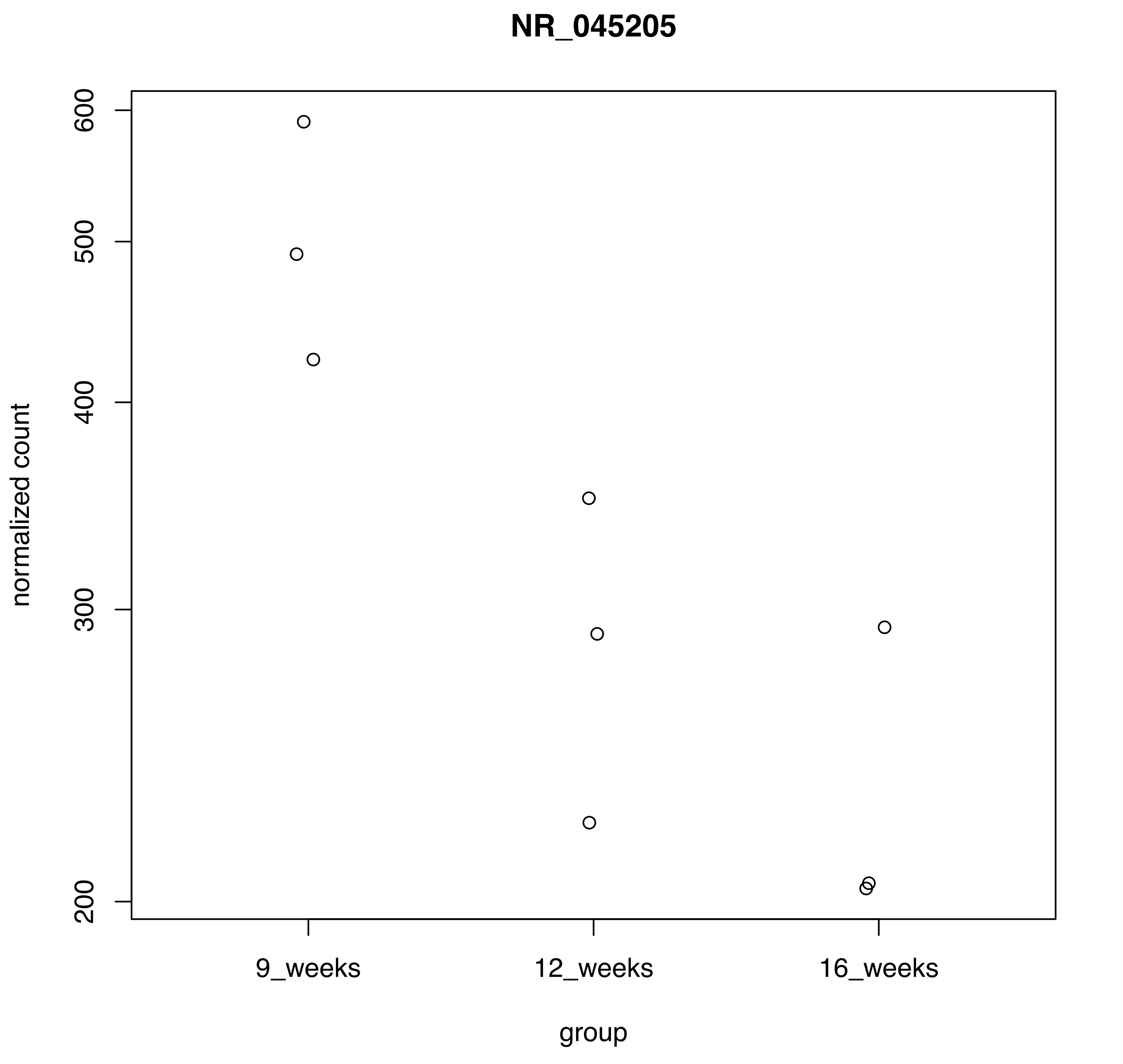
NR_045205
normalized count
600
500
400
300
200
9_weeks
12_weeks
16_weeks
group

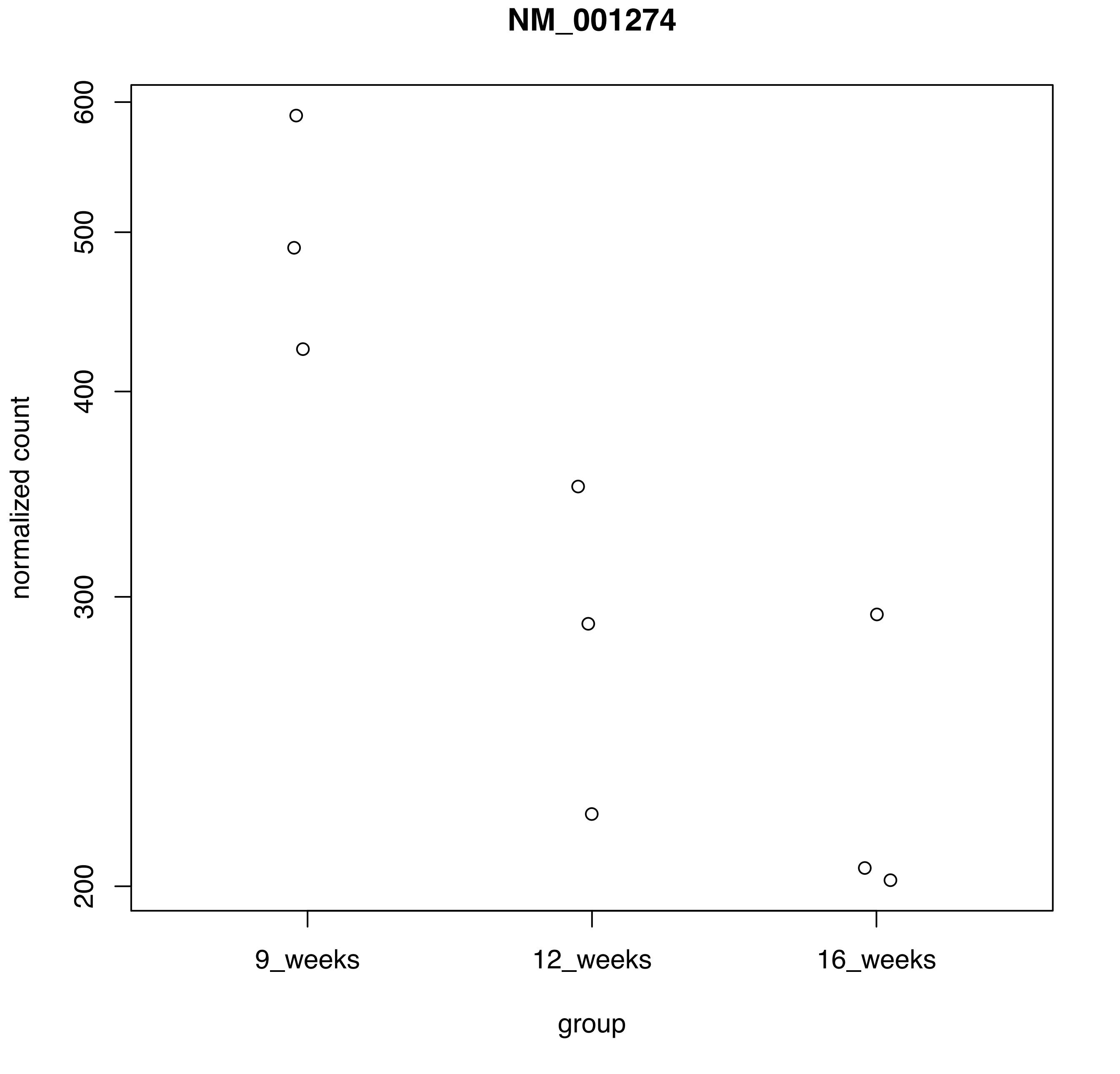
NM_001274
normalized count
600
500
400
300
200
9_weeks
12_weeks
16_weeks
group

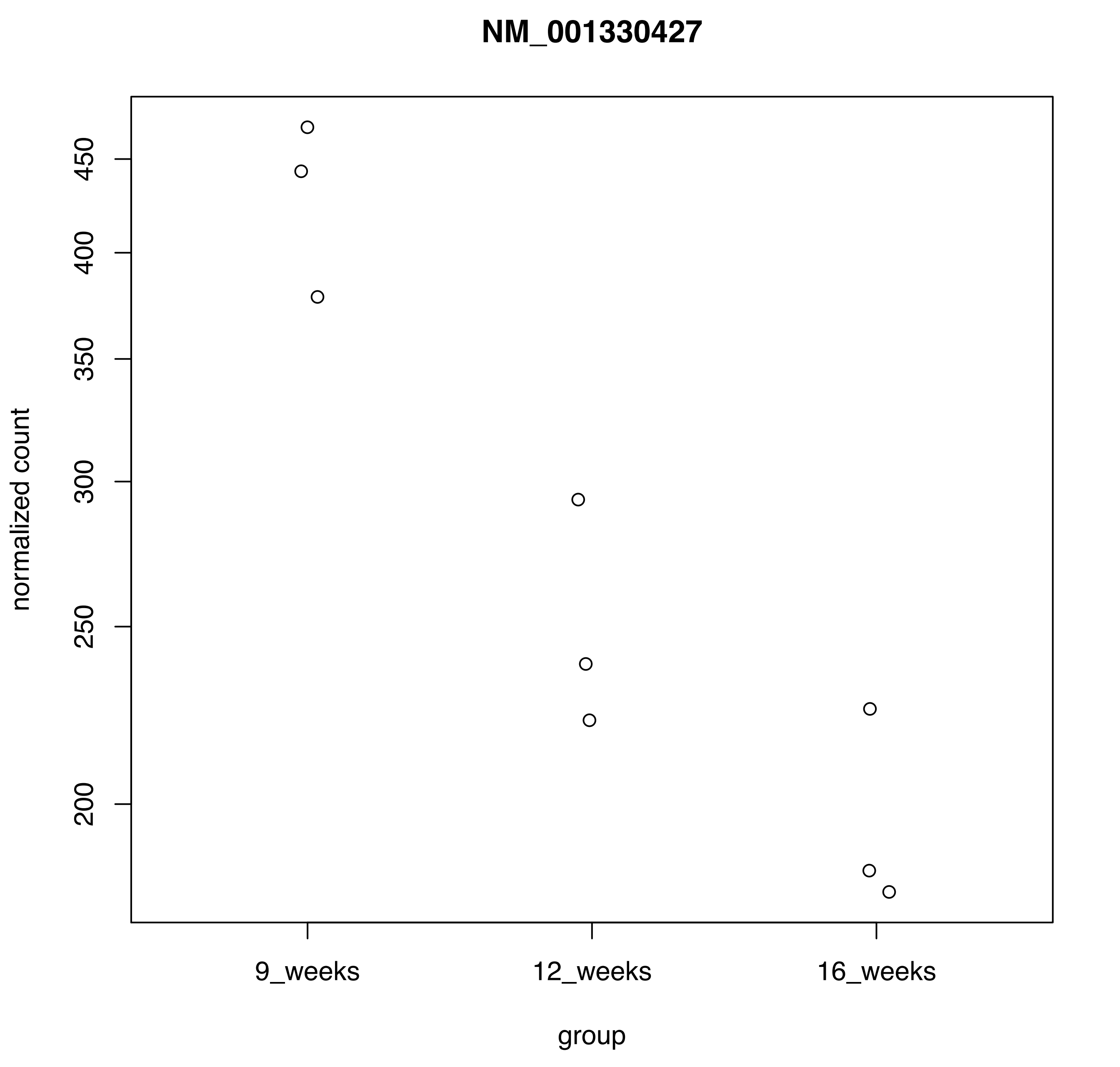
NM_001330427
normalized count
450
400
350
300
250
200
9_weeks
12_weeks
16_weeks
group

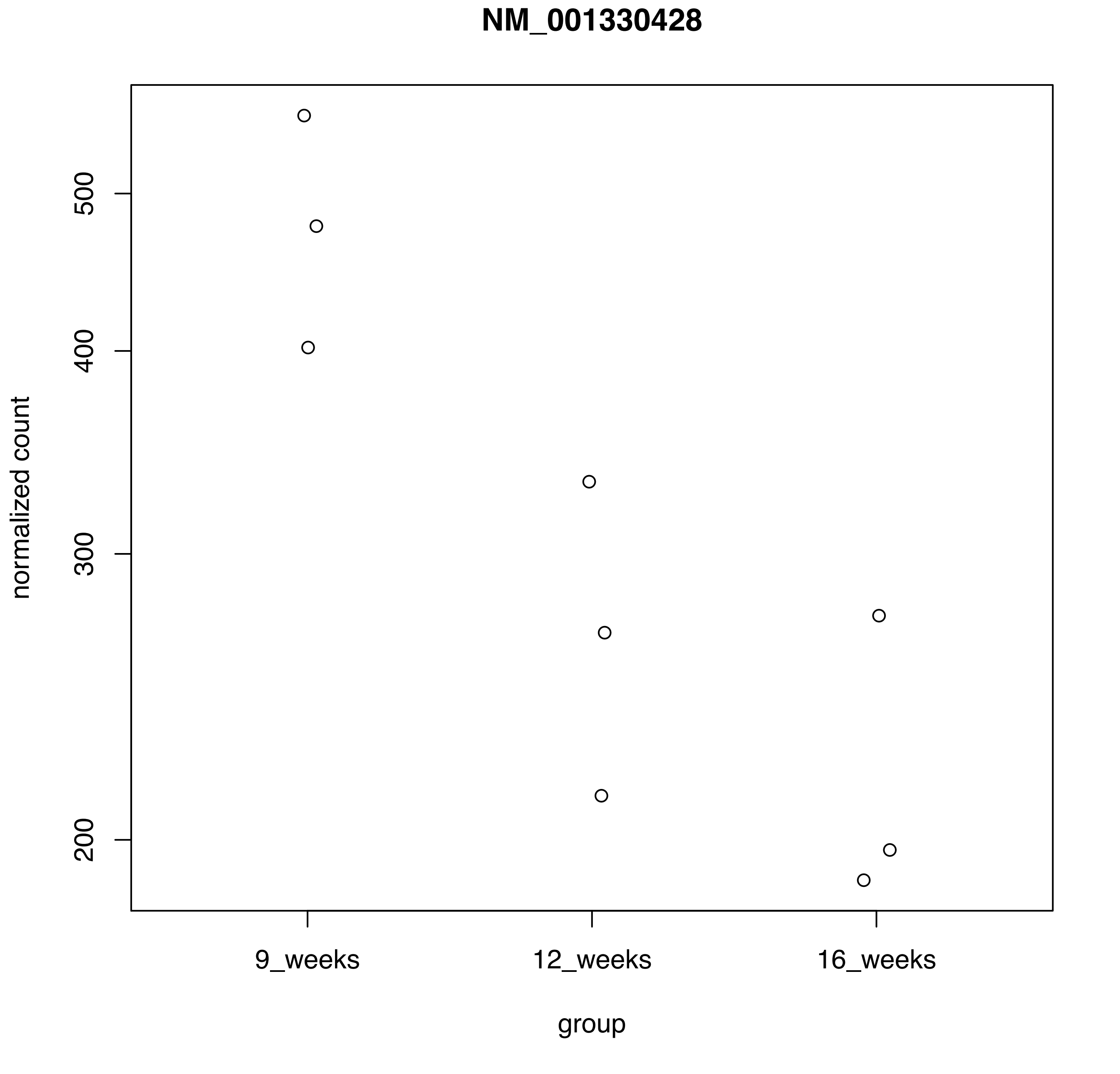

NM_001330428
normalized count
200
300
400
500
9_weeks
12_weeks
16_weeks
group

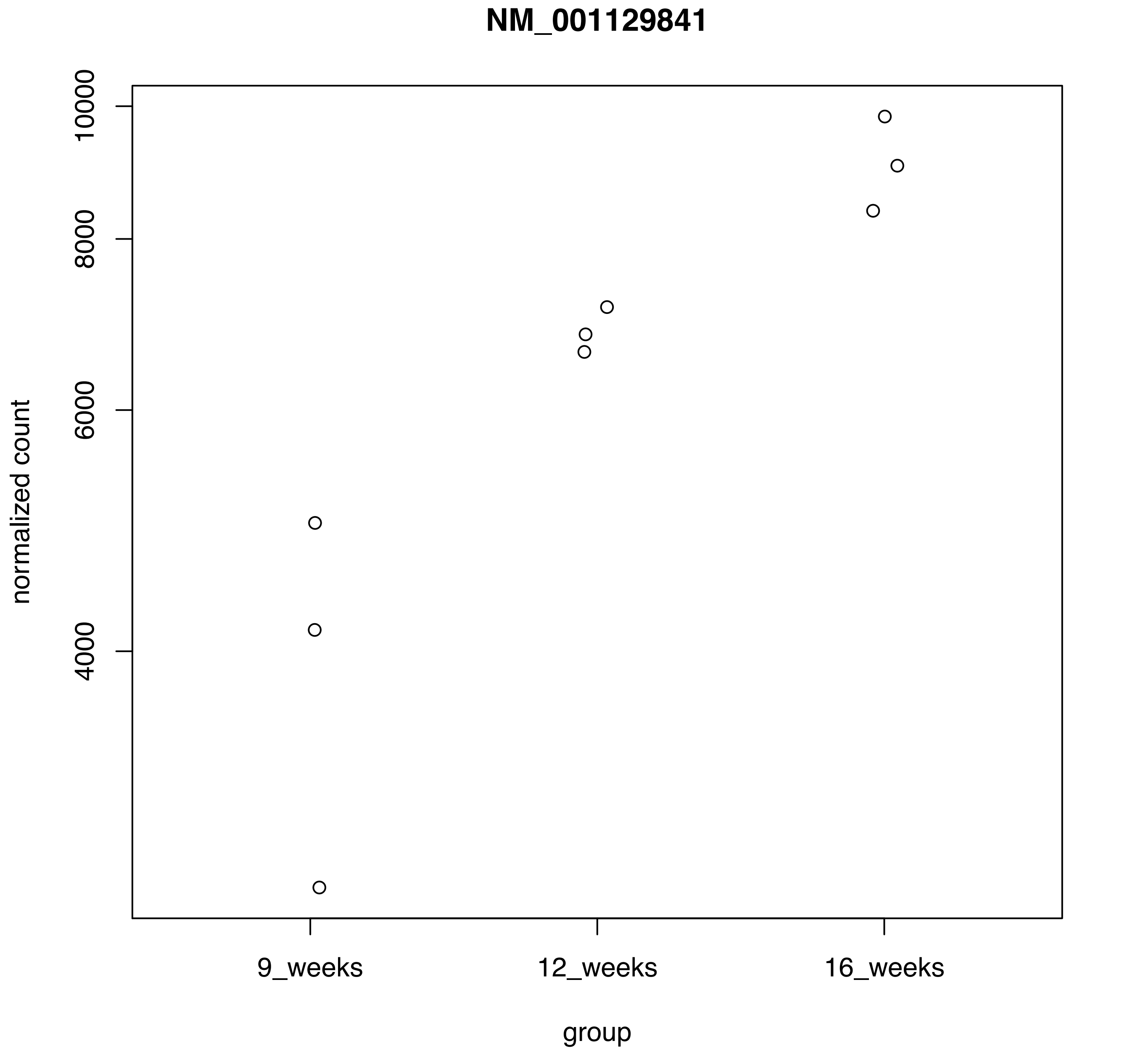
NM_001129841
normalized count
10000
8000
6000
4000
9_weeks
12_weeks
16_weeks
group

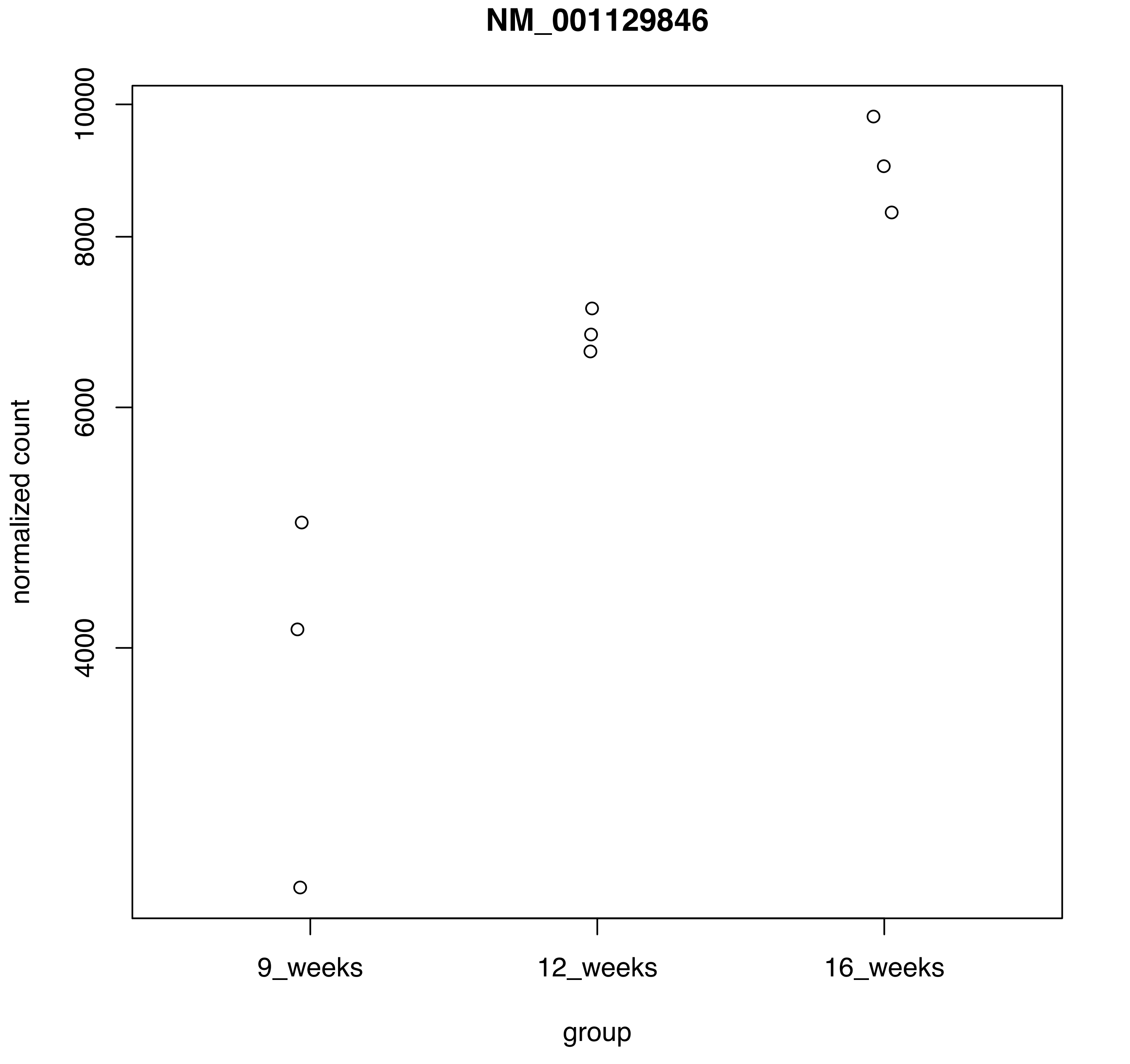
NM_001129846
normalized count
10000
8000
6000
4000
9_weeks
12_weeks
16_weeks
group

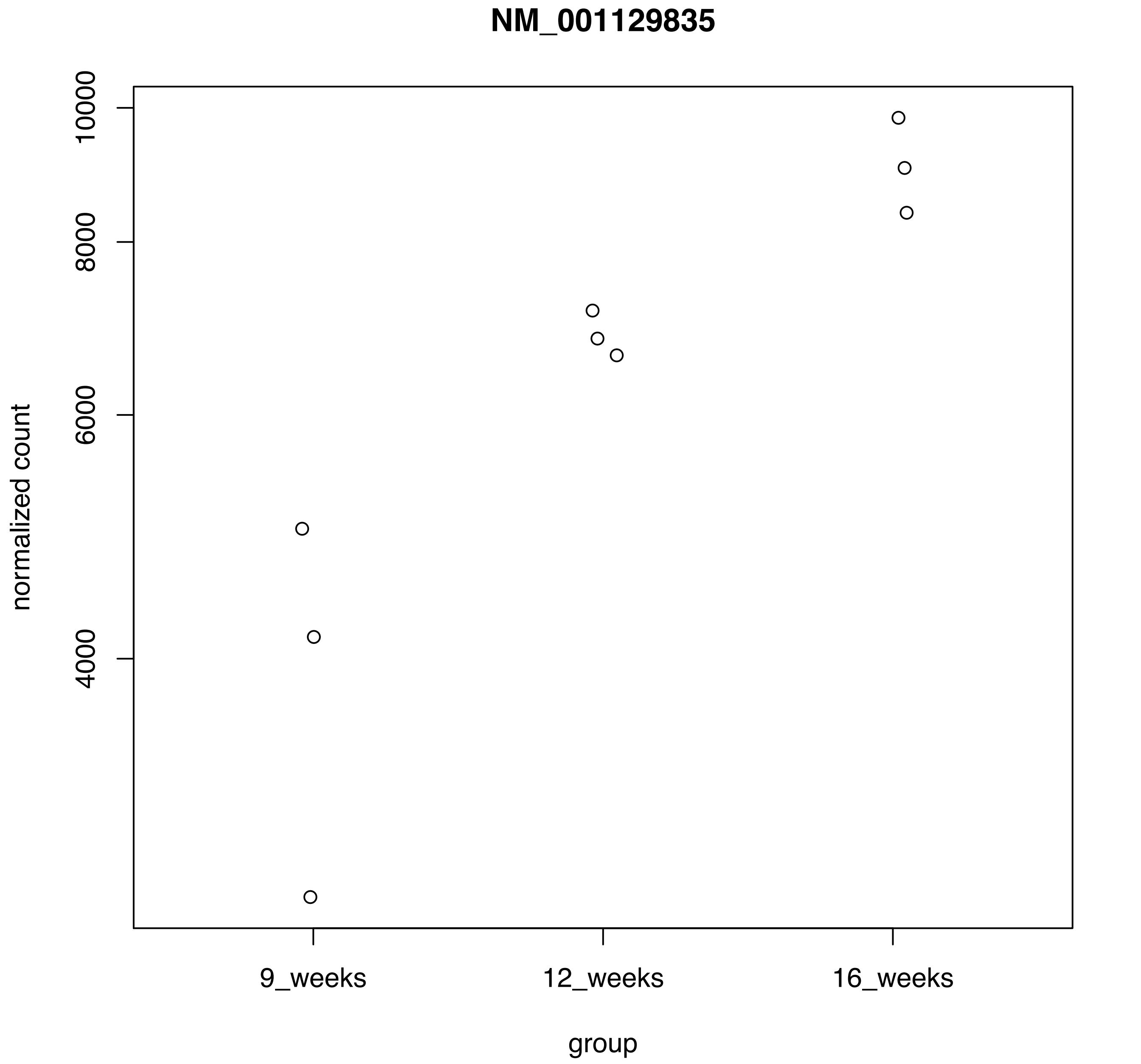
NM_001129835
normalized count
10000
8000
6000
4000
9_weeks
12_weeks
16_weeks
group

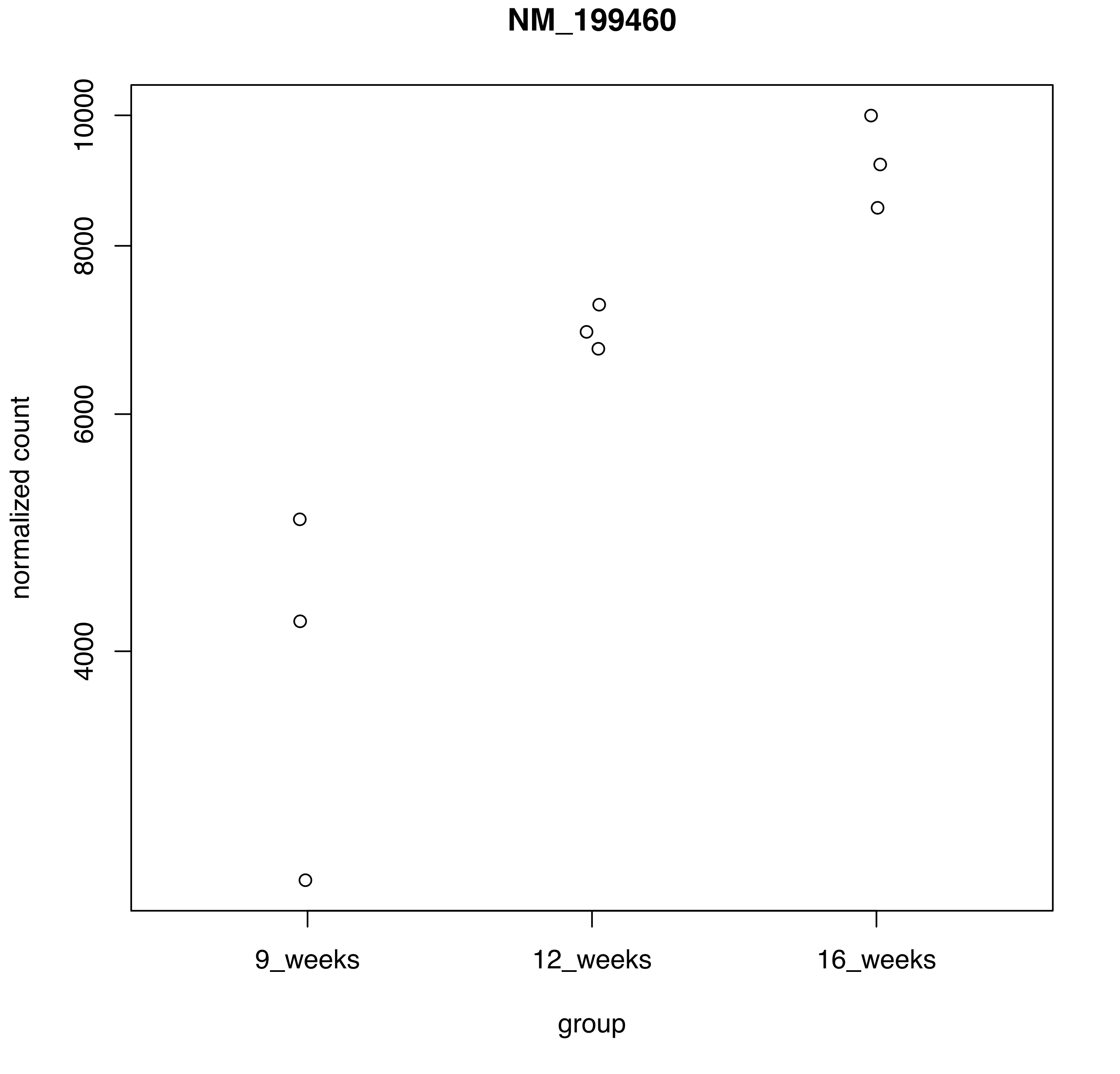
NM_199460
normalized count
10000
8000
6000
4000
9_weeks
12_weeks
16_weeks
group

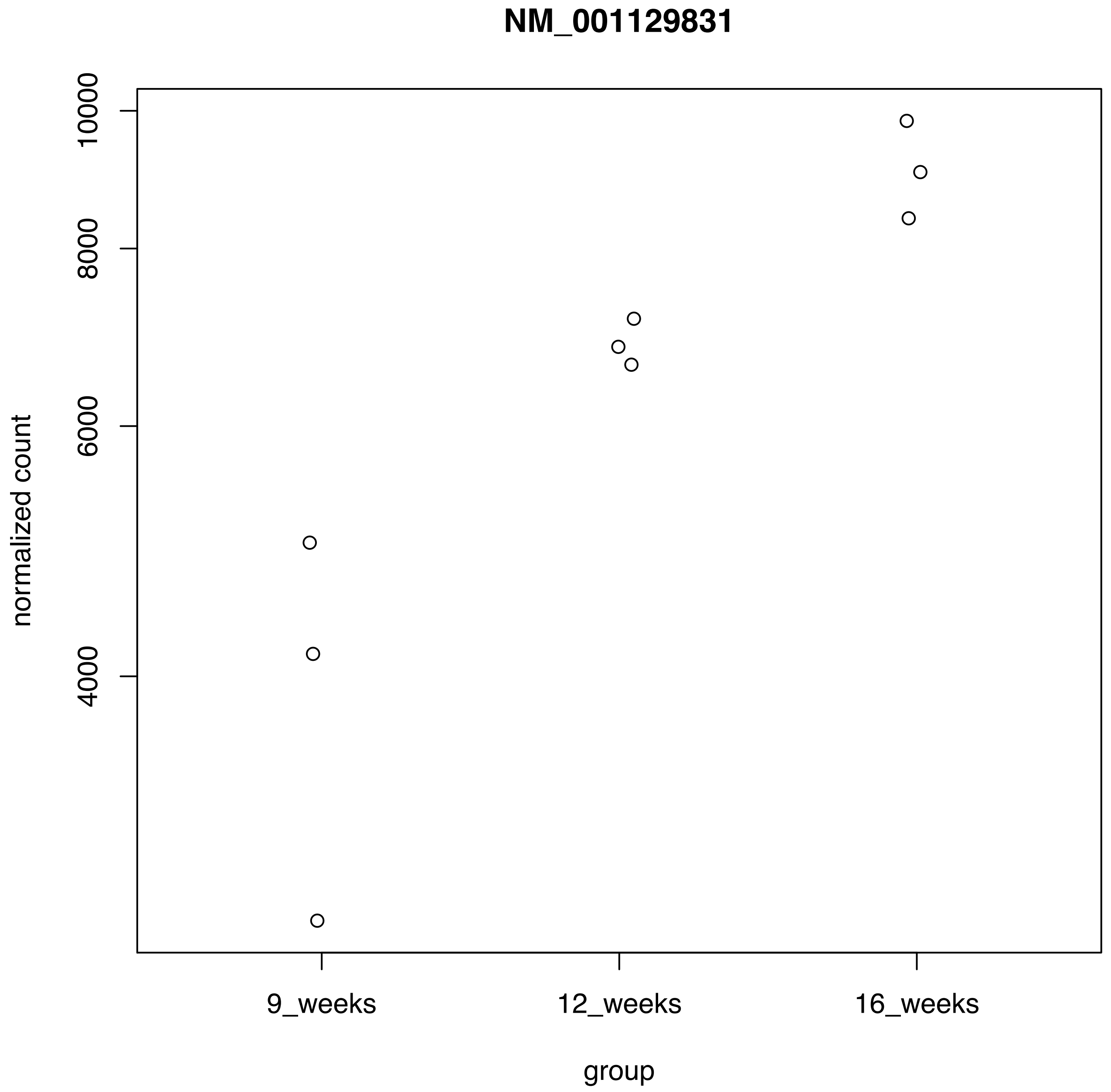
NM_001129831
normalized count
10000
8000
6000
4000
9_weeks
12_weeks
16_weeks
group

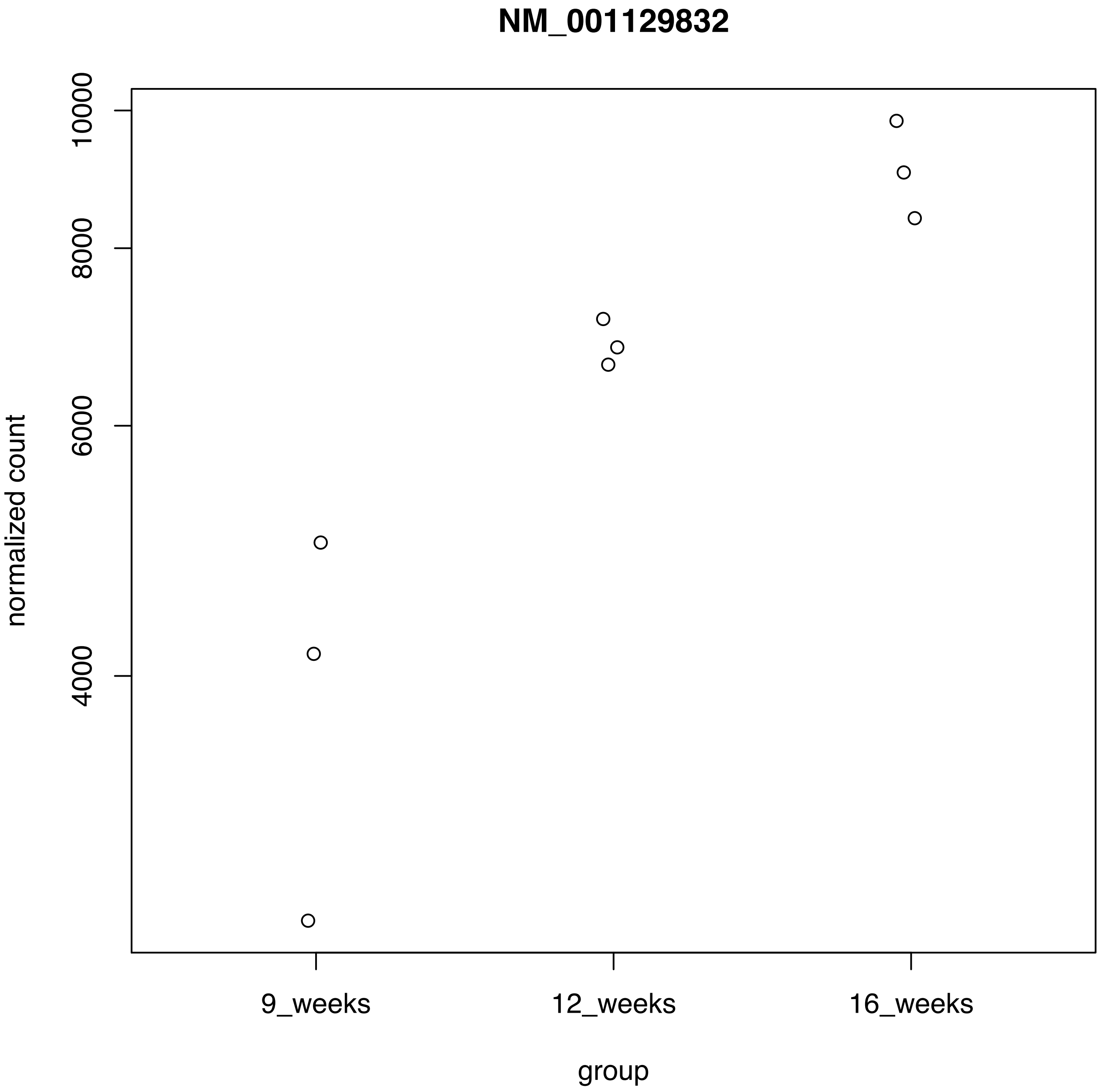
NM_001129832
normalized count
10000
8000
6000
4000
9_weeks
12_weeks
16_weeks
group

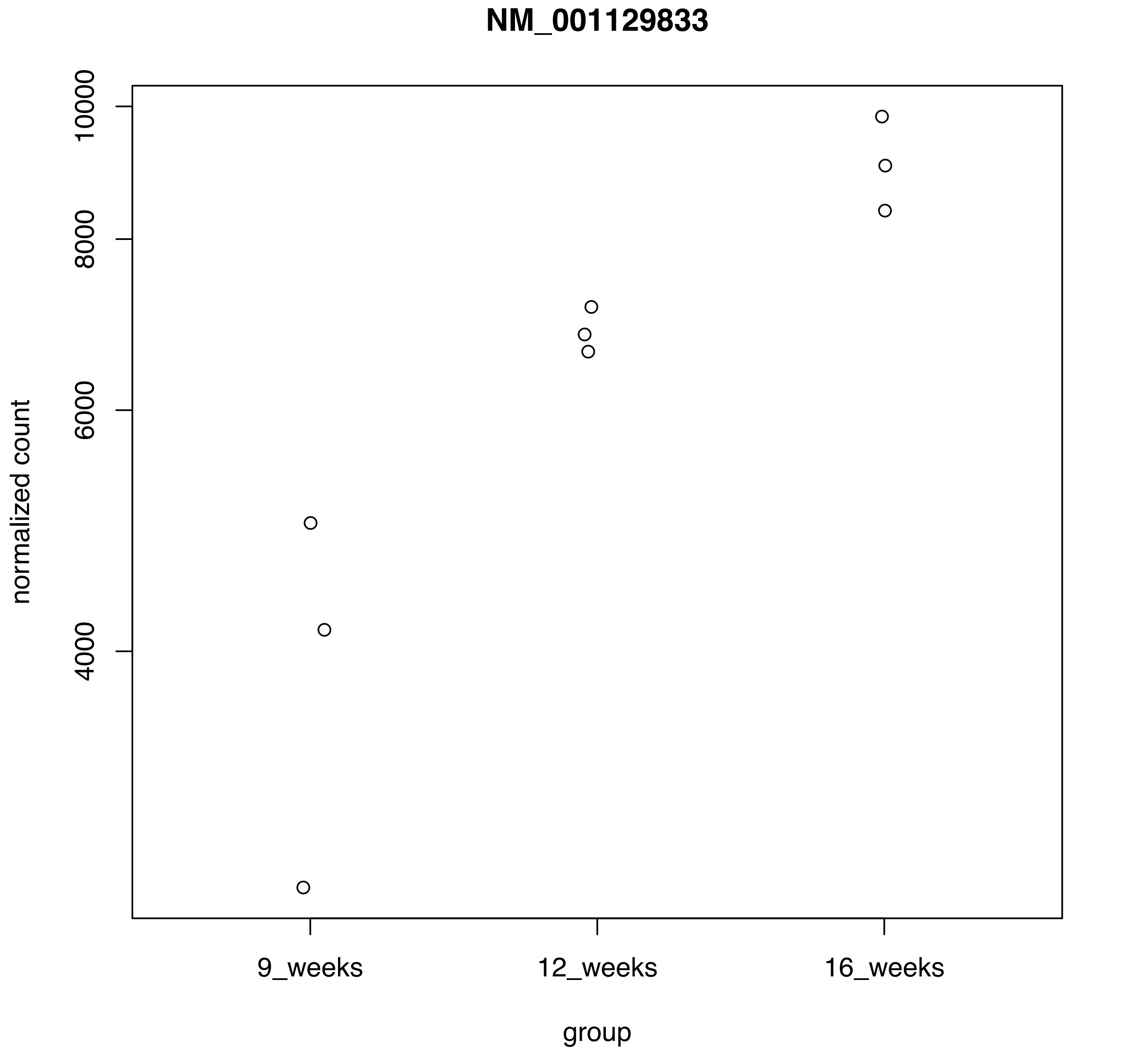
NM_001129833
normalized count
10000
8000
6000
4000
9_weeks
12_weeks
16_weeks
group

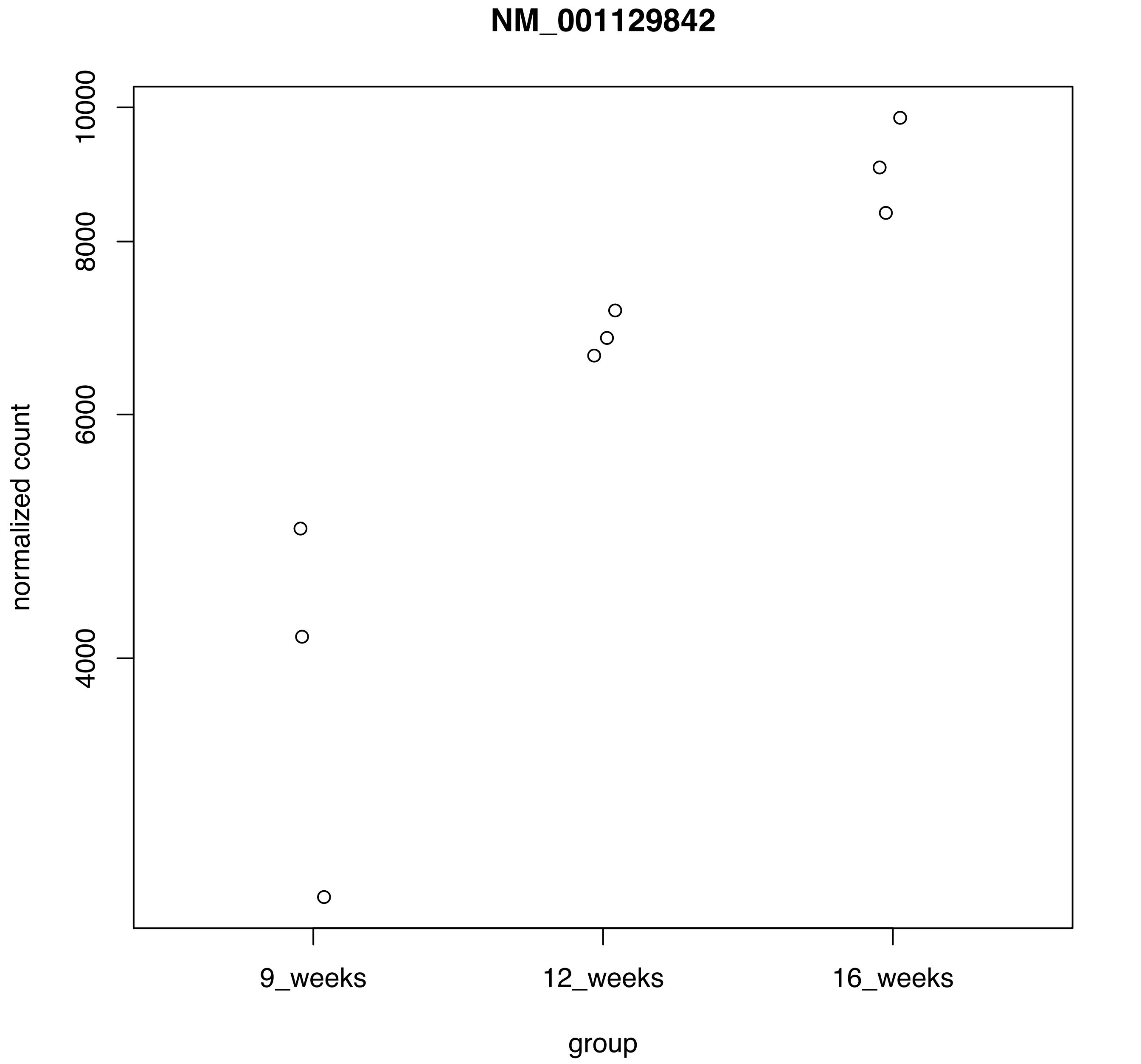
NM_001129842
normalized count
10000
8000
6000
4000
9_weeks
12_weeks
16_weeks
group

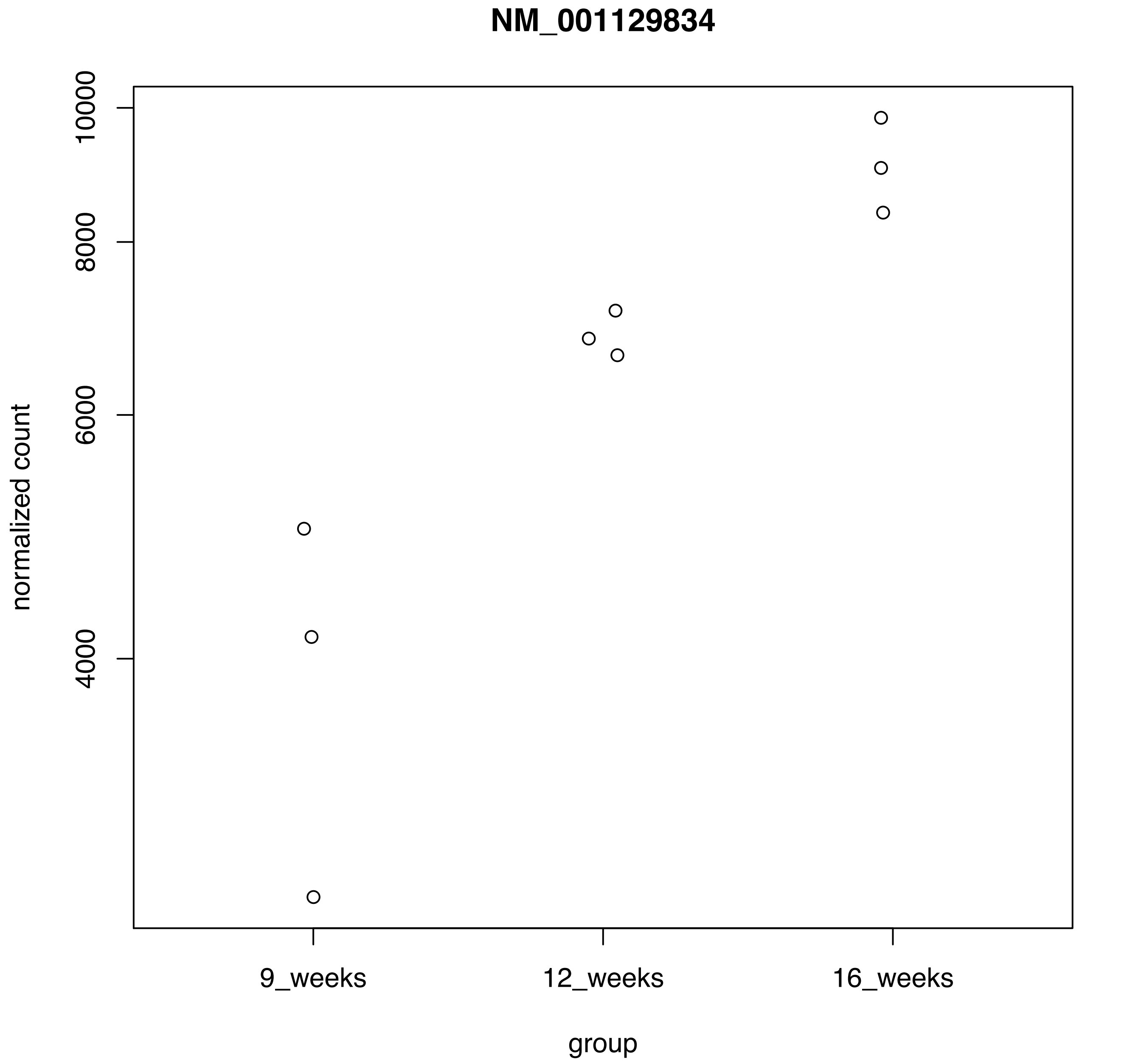
NM_001129834
normalized count
10000
8000
6000
4000
9_weeks
12_weeks
16_weeks
group

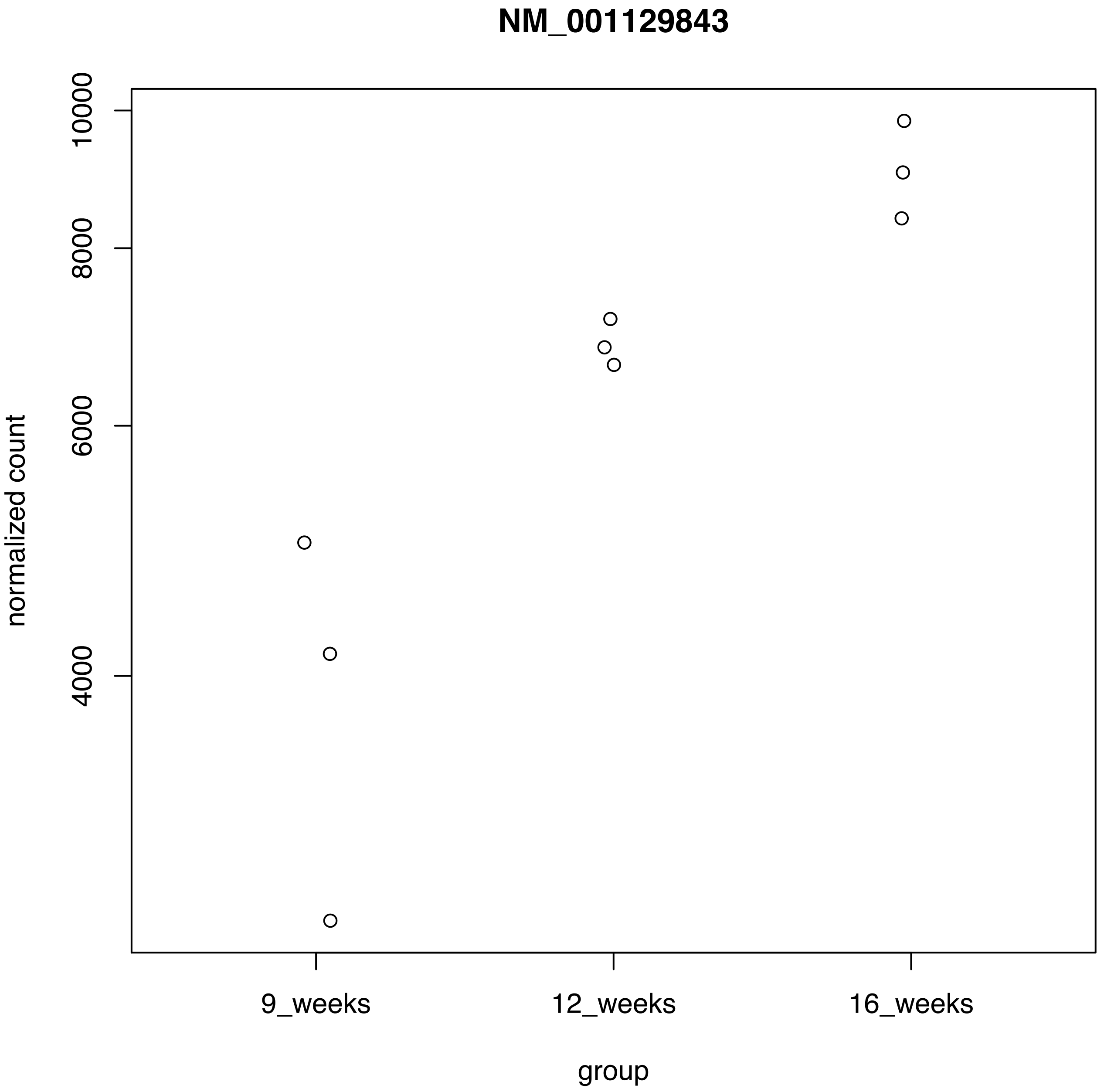
NM_001129843
normalized count
10000
8000
6000
4000
9_weeks
12_weeks
16_weeks
group

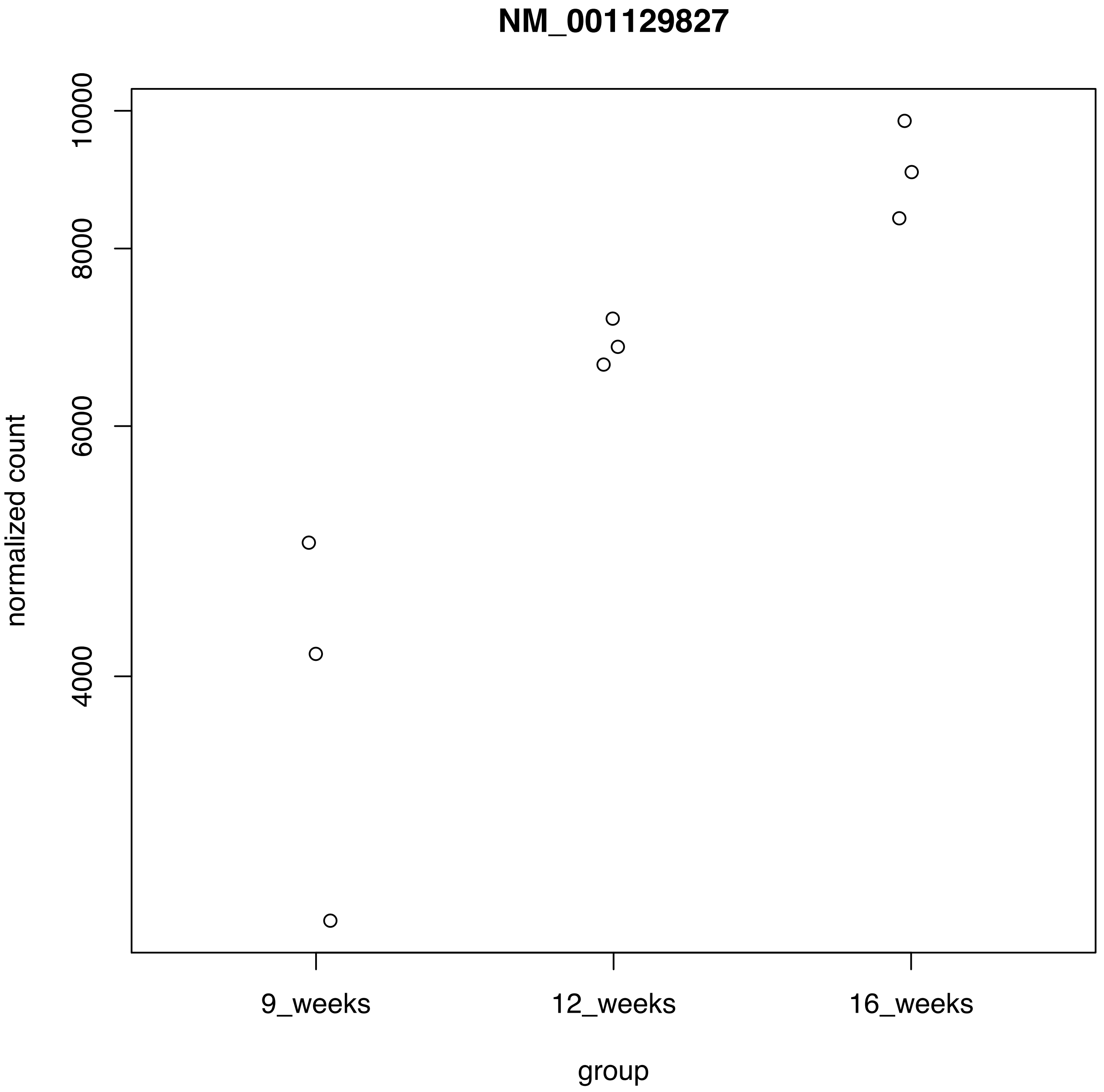
NM_001129827
normalized count
10000
8000
6000
4000
9_weeks
12_weeks
16_weeks
group

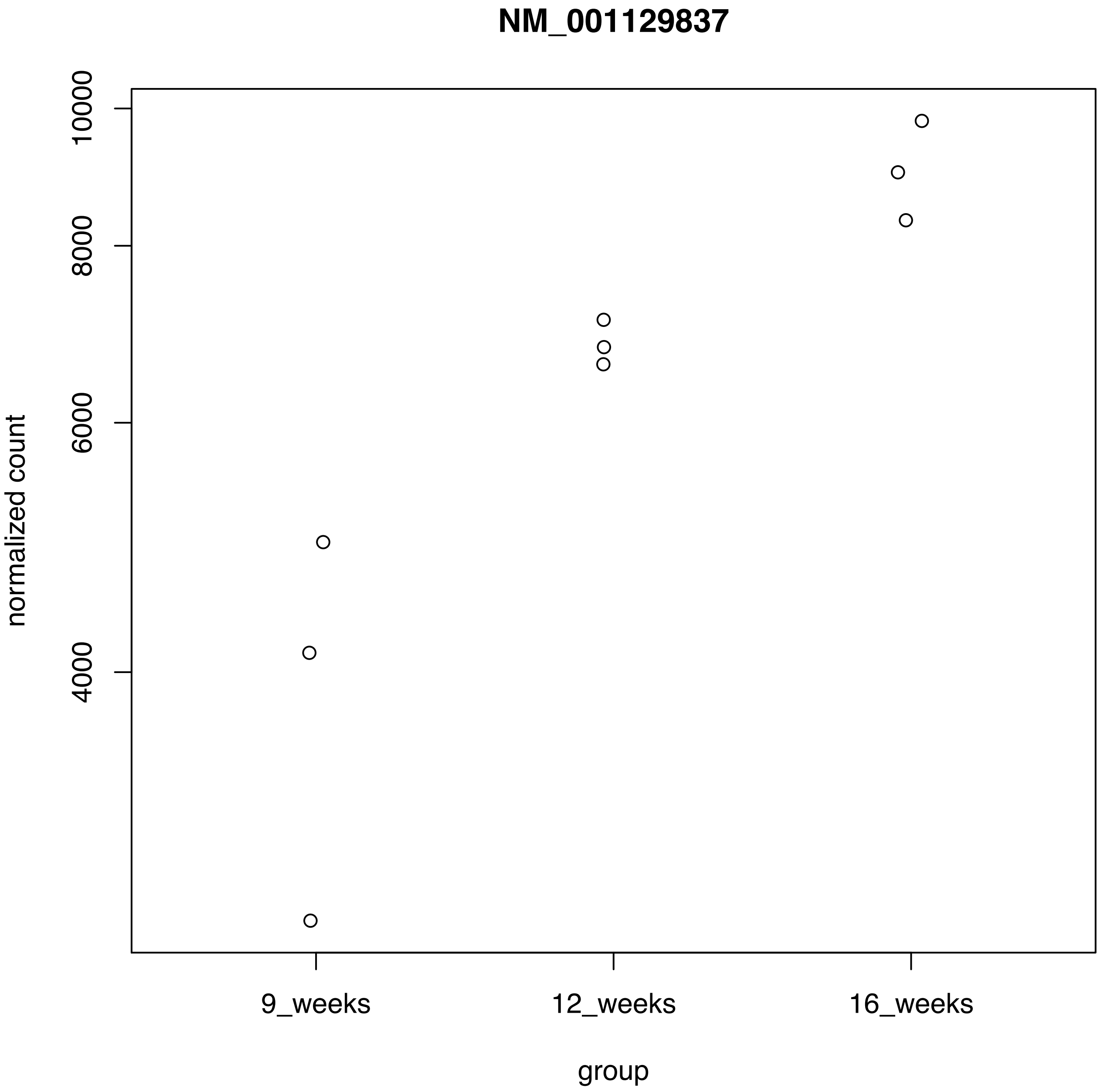
NM_001129837
normalized count
10000
8000
6000
4000
9_weeks
12_weeks
16_weeks
group

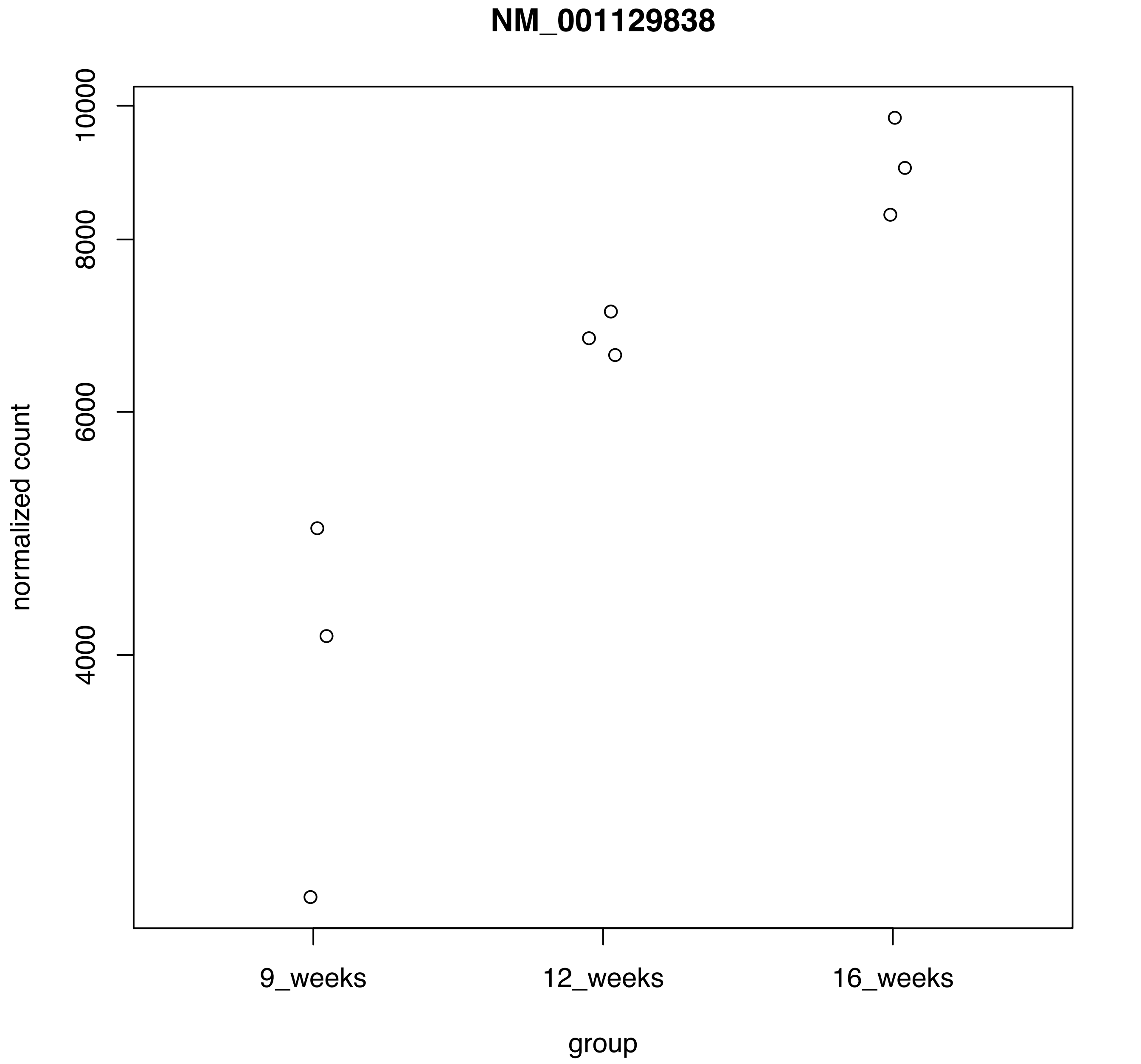
NM_001129838
normalized count
10000
8000
6000
4000
9_weeks
12_weeks
16_weeks
group

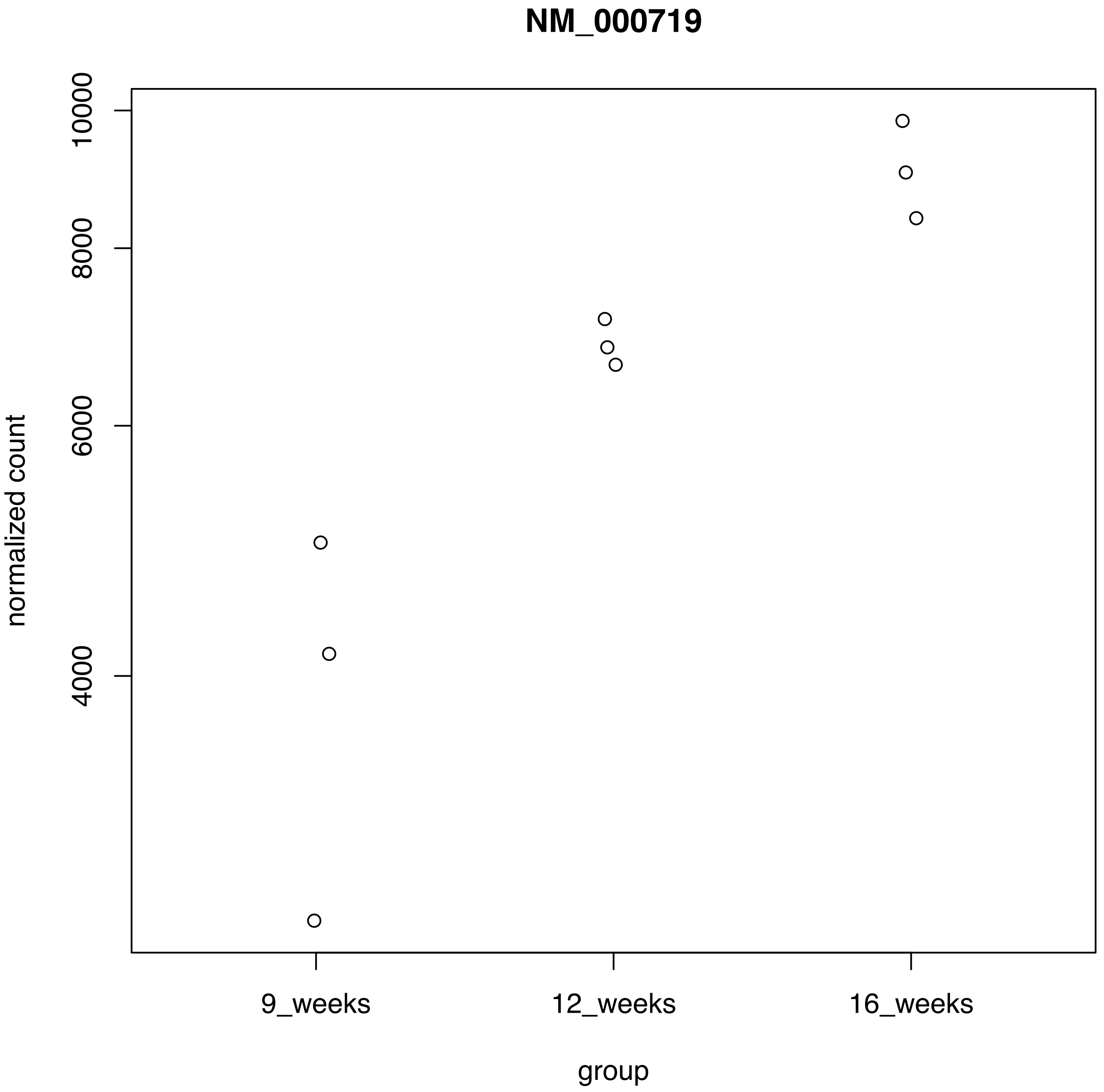
NM_000719
normalized count
10000
8000
6000
4000
9_weeks
12_weeks
16_weeks
group

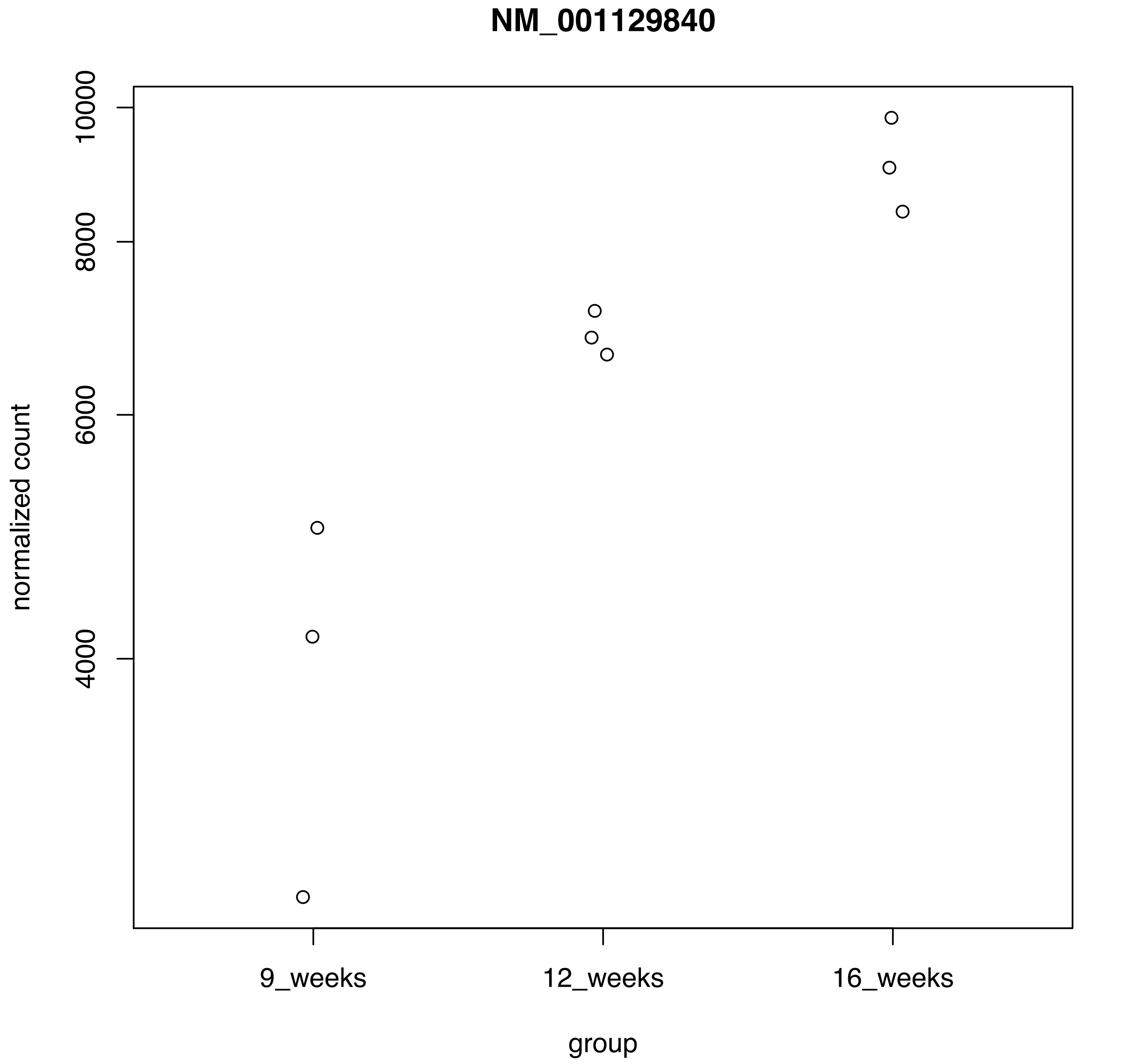
NM_001129840
normalized count
10000
8000
6000
4000
9_weeks
12_weeks
16_weeks
group

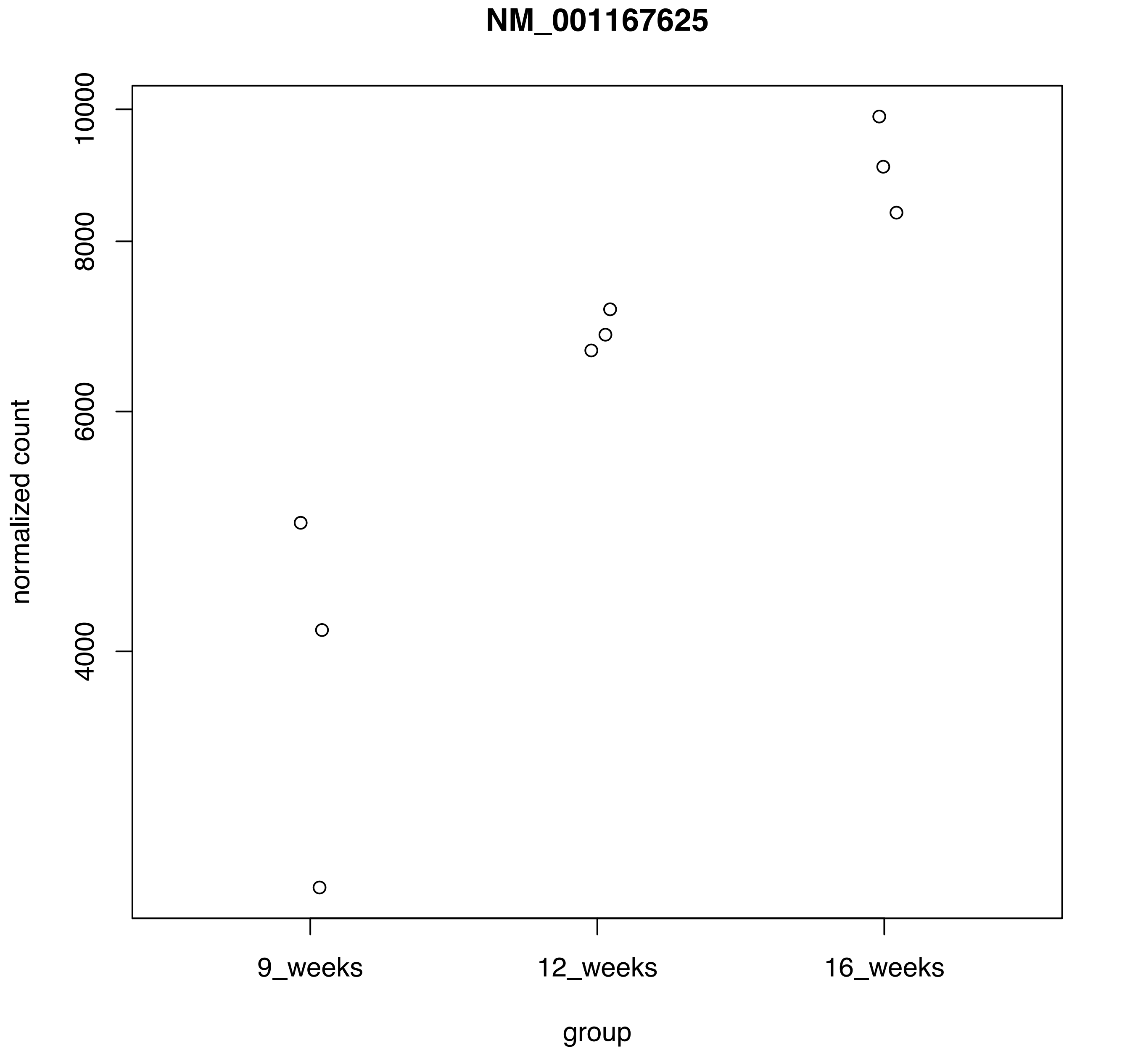
NM_001167625
normalized count
10000
8000
6000
4000
9_weeks
12_weeks
16_weeks
group

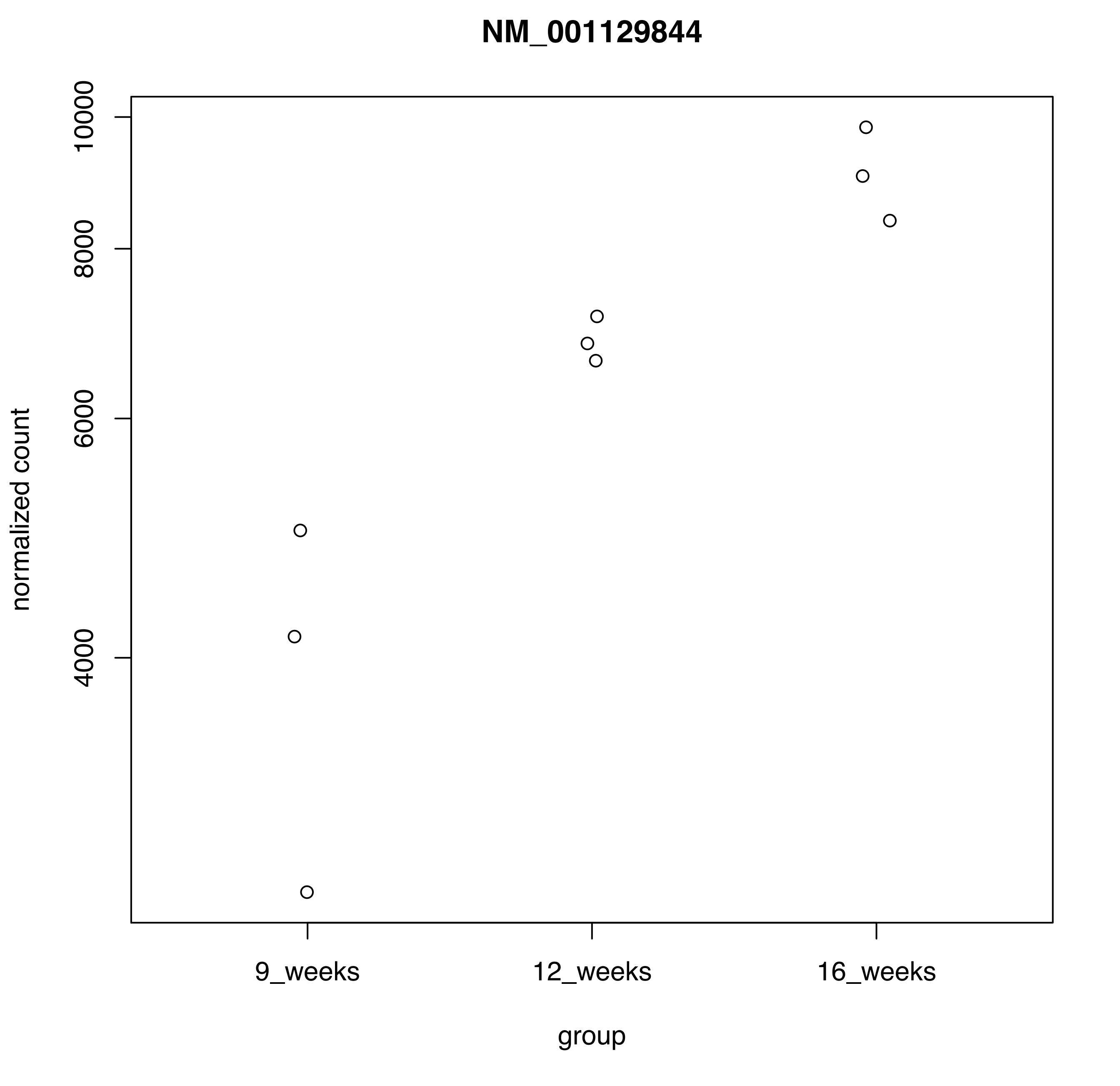
NM_001129844
normalized count
10000
8000
6000
4000
9_weeks
12_weeks
16_weeks
group

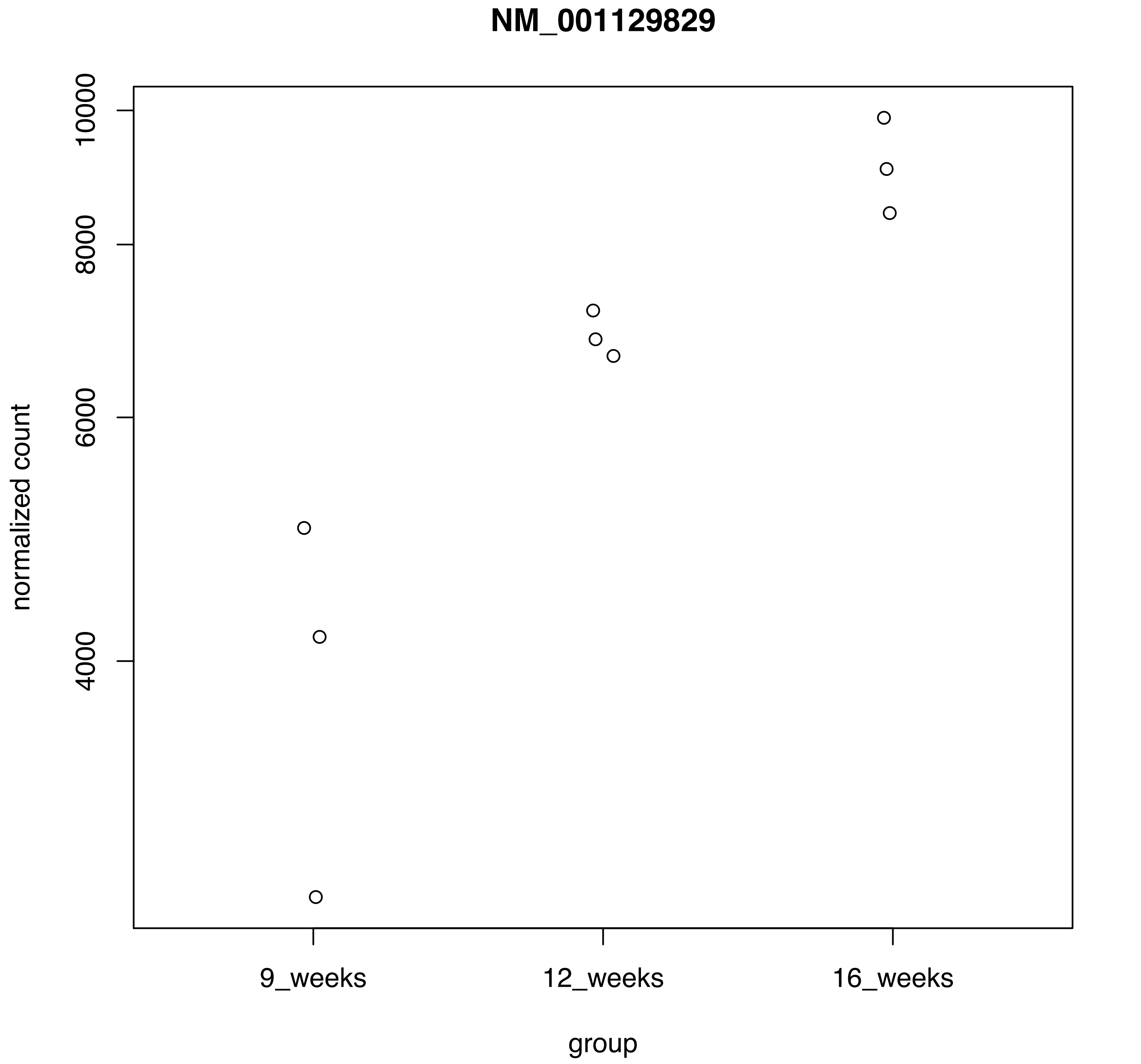

NM_001129829
normalized count
10000
8000
6000
4000
9_weeks
12_weeks
16_weeks
group

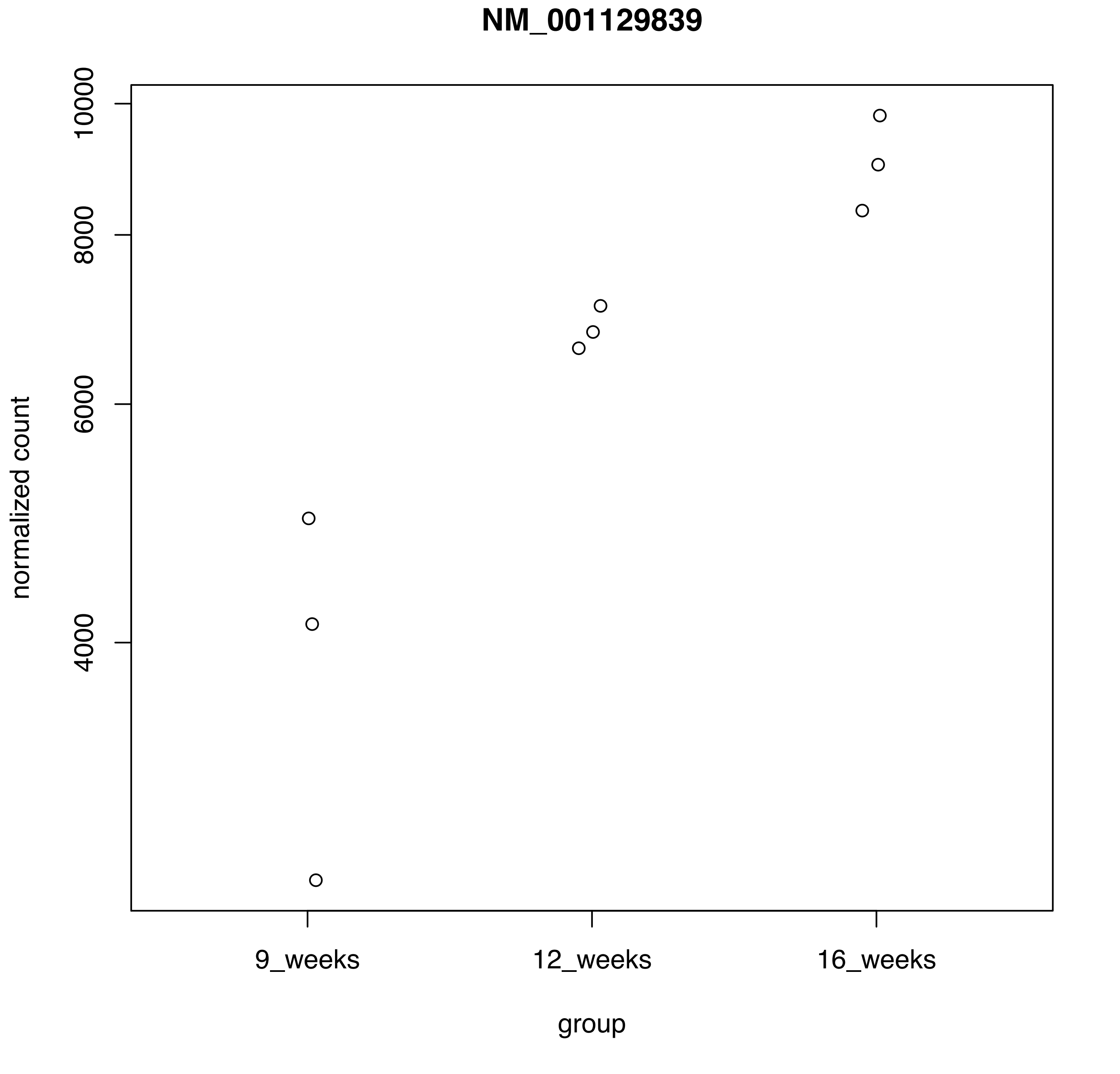
NM_001129839
normalized count
10000
8000
6000
4000
9_weeks
12_weeks
16_weeks
group

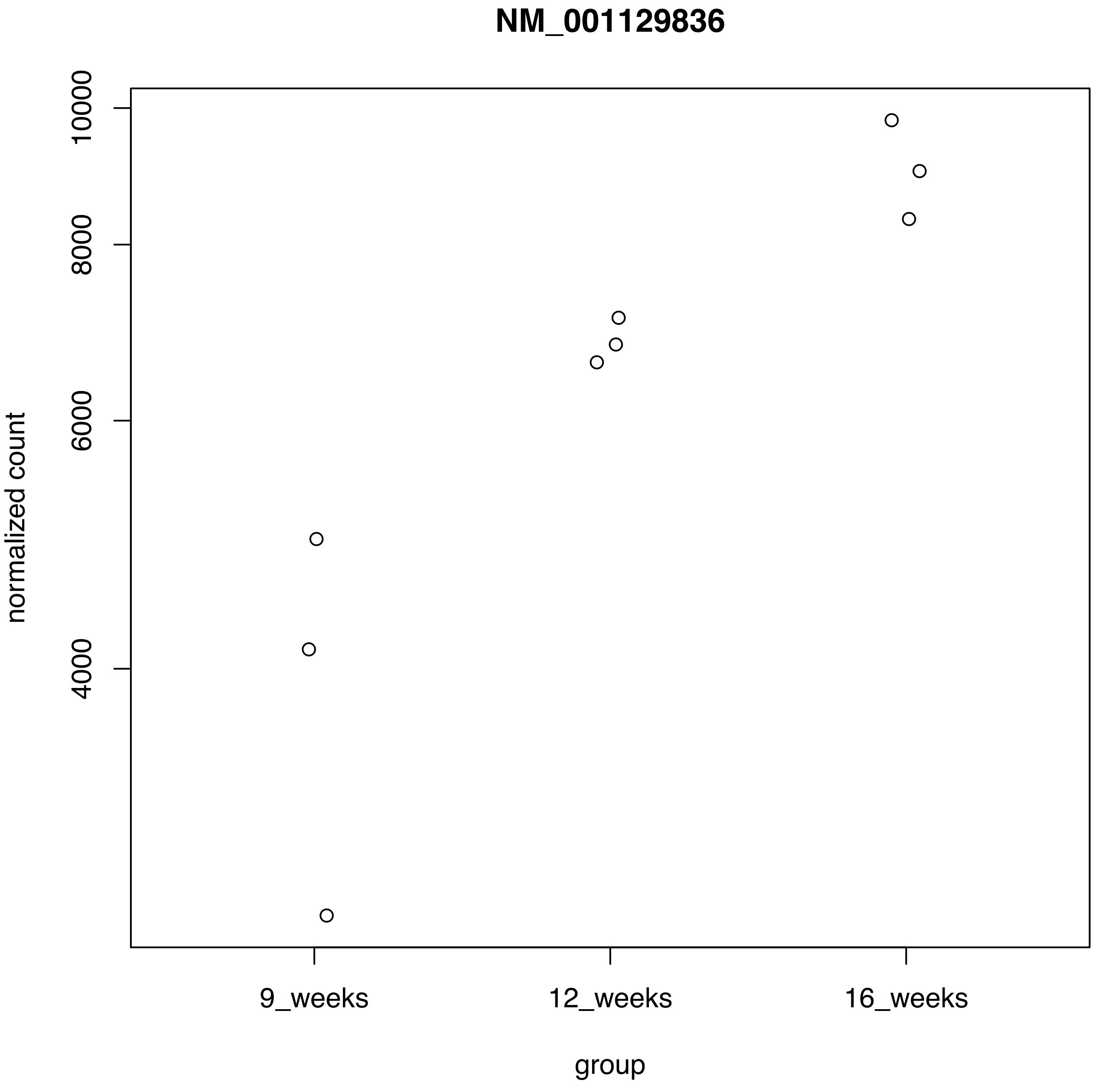
NM_001129836
normalized count
10000
8000
6000
4000
9_weeks
12_weeks
16_weeks
group

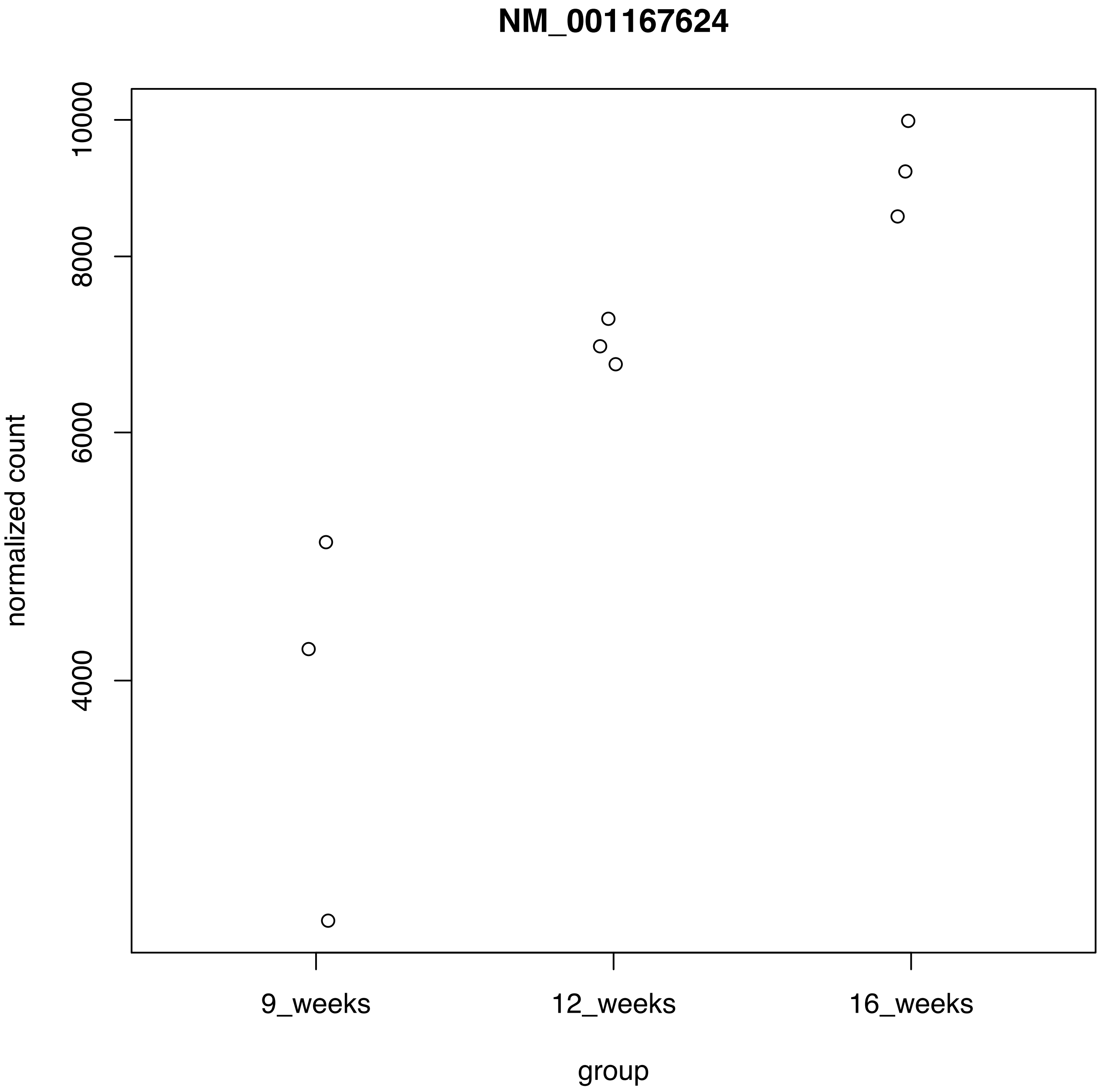

NM_001167624
normalized count
10000
8000
6000
4000
9_weeks
12_weeks
16_weeks
group

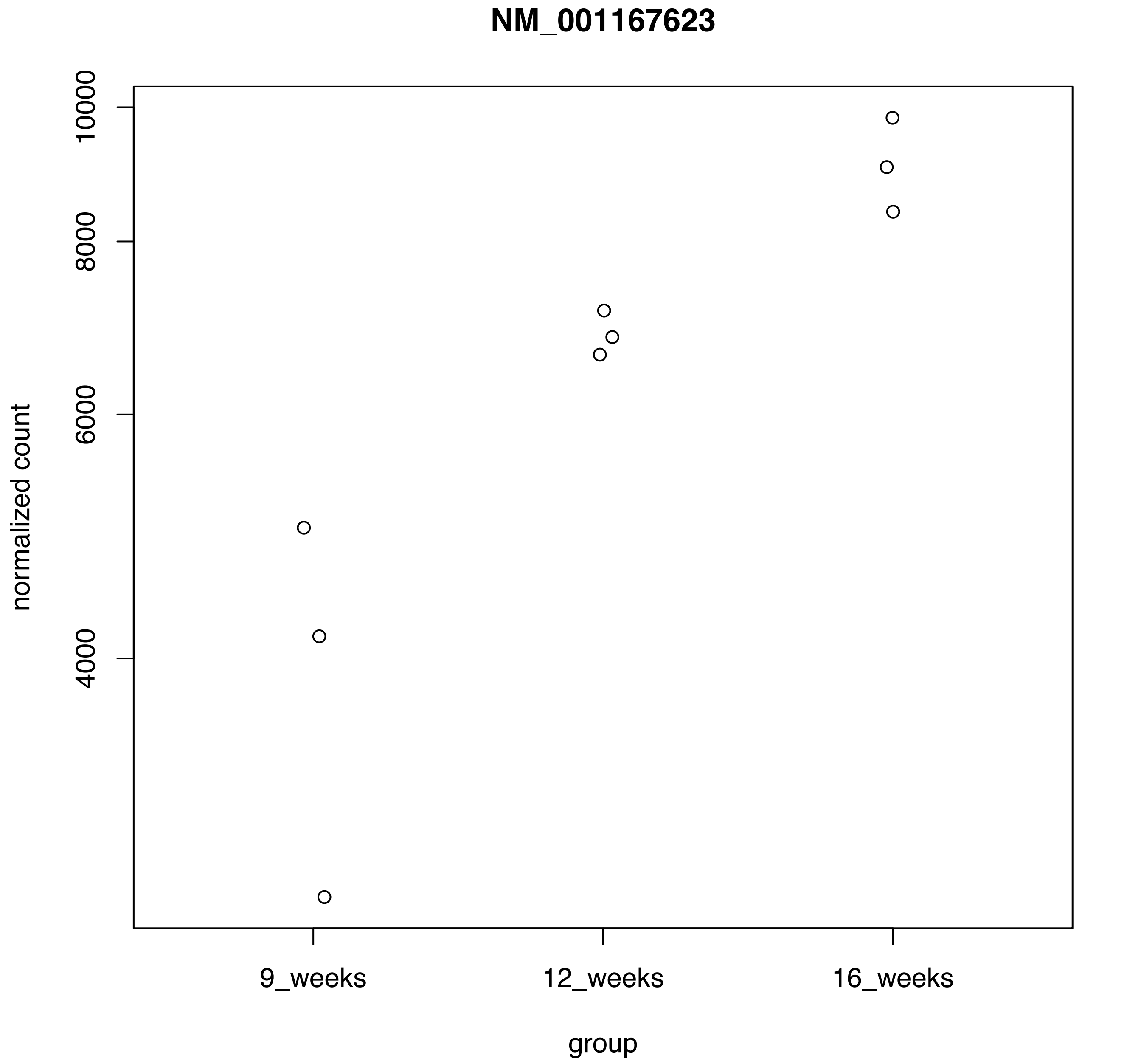
NM_001167623
normalized count
10000
8000
6000
4000
9_weeks
12_weeks
16_weeks
group

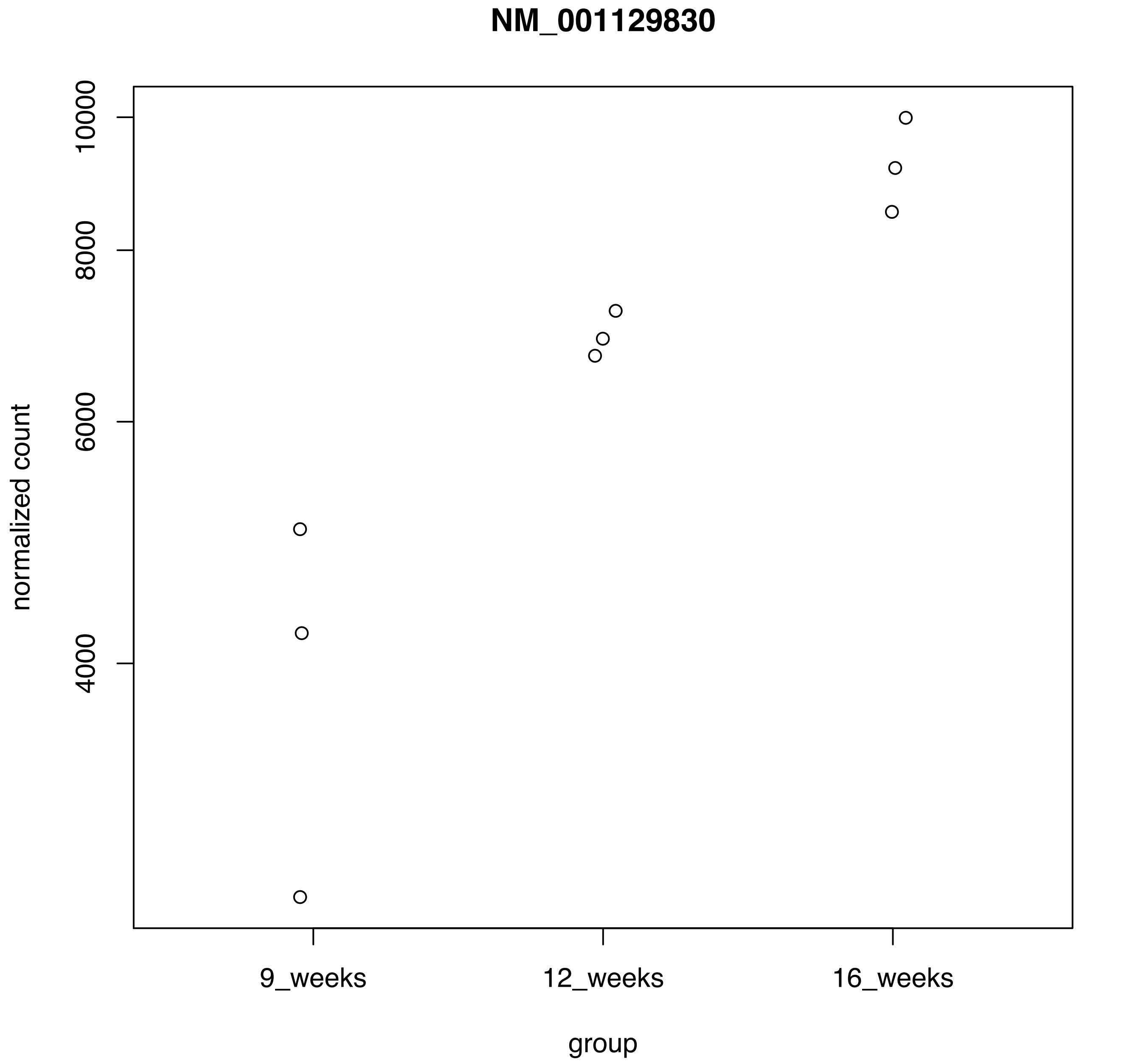

NM_001129830
normalized count
10000
8000
6000
4000
9_weeks
12_weeks
16_weeks
group

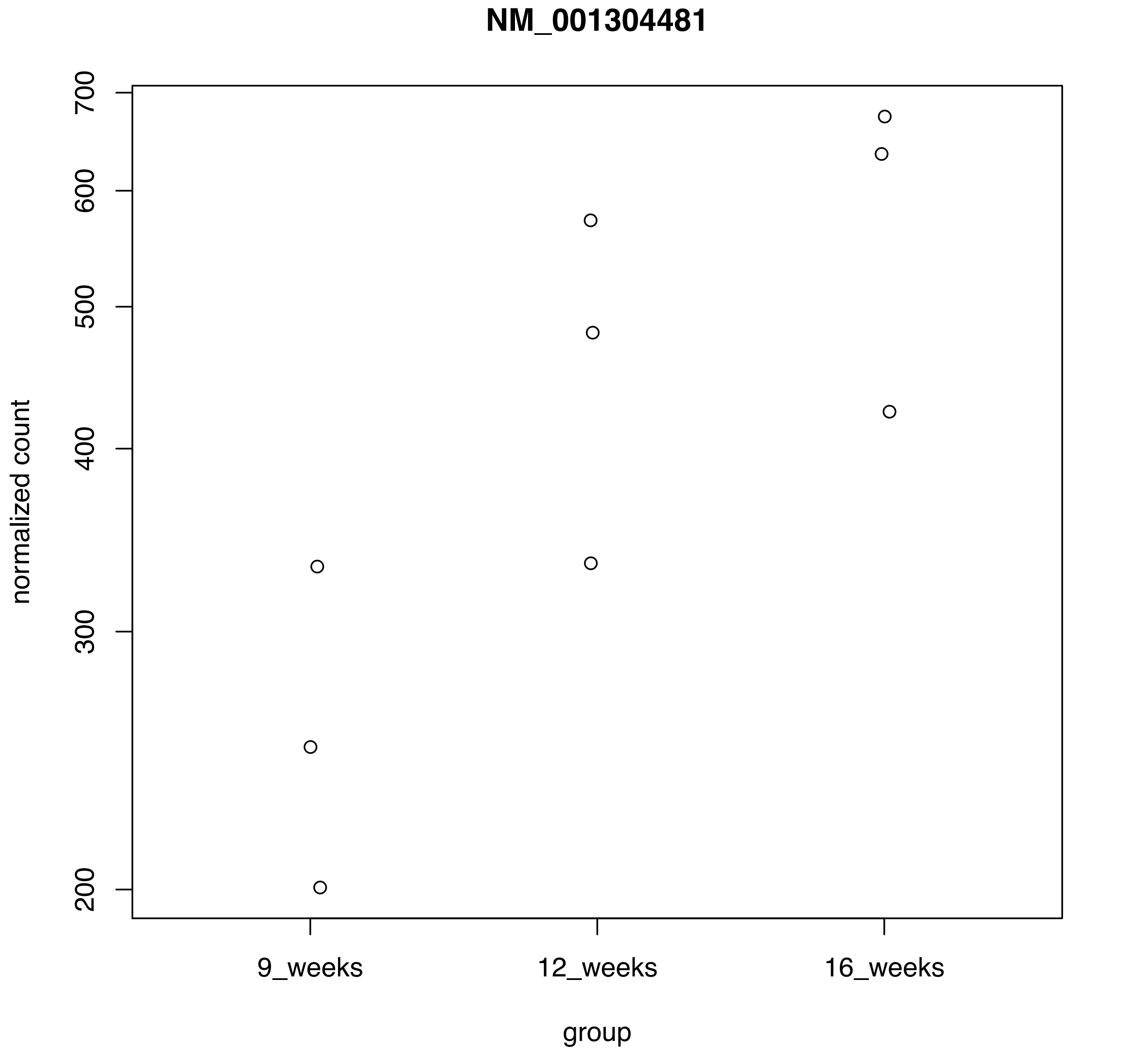
NM_001304481
normalized count
200
300
400
500
600
700
9_weeks
12_weeks
16_weeks
group

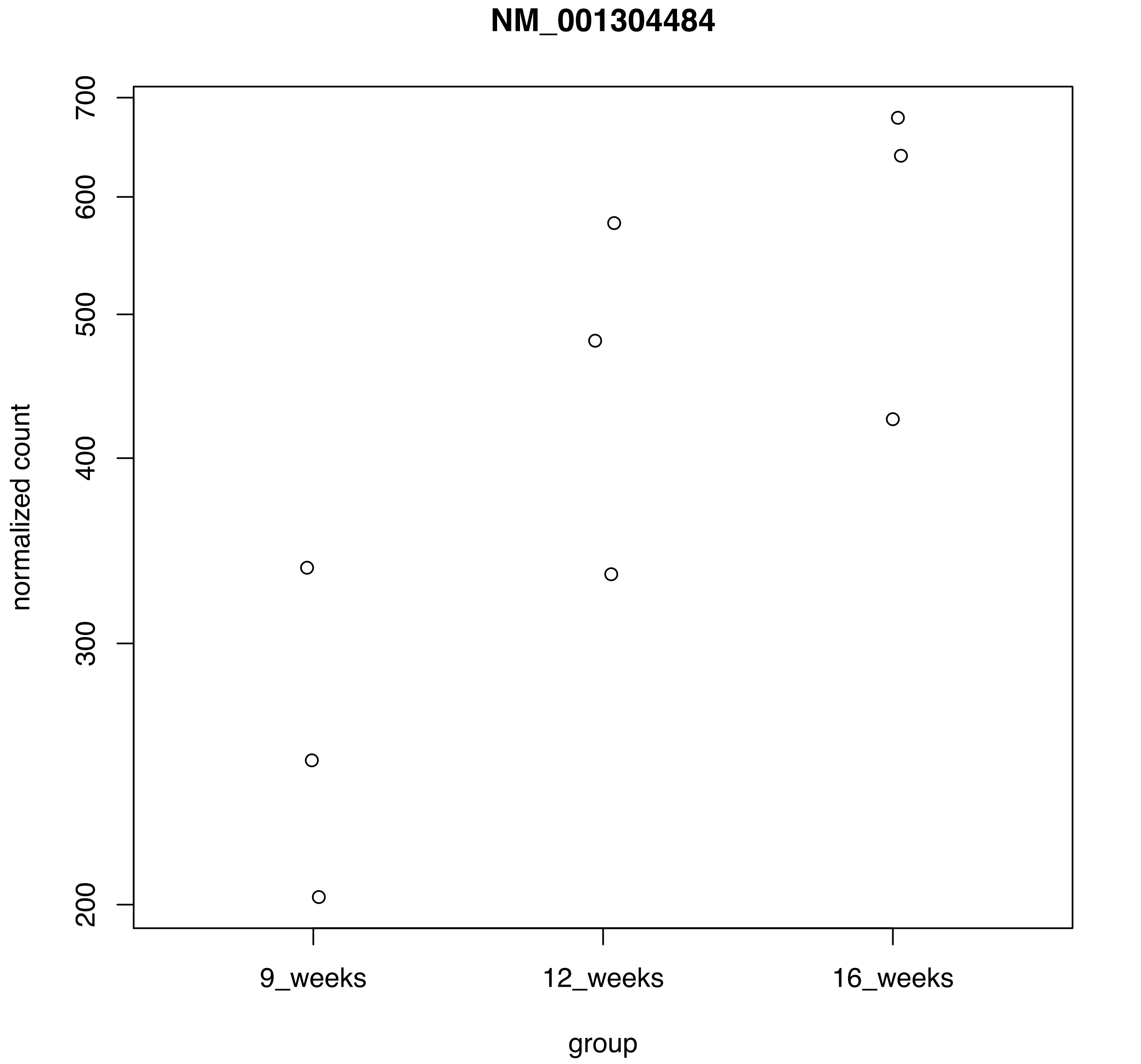
NM_001304484
normalized count
700
600
500
400
300
200
9_weeks
12_weeks
16_weeks
group

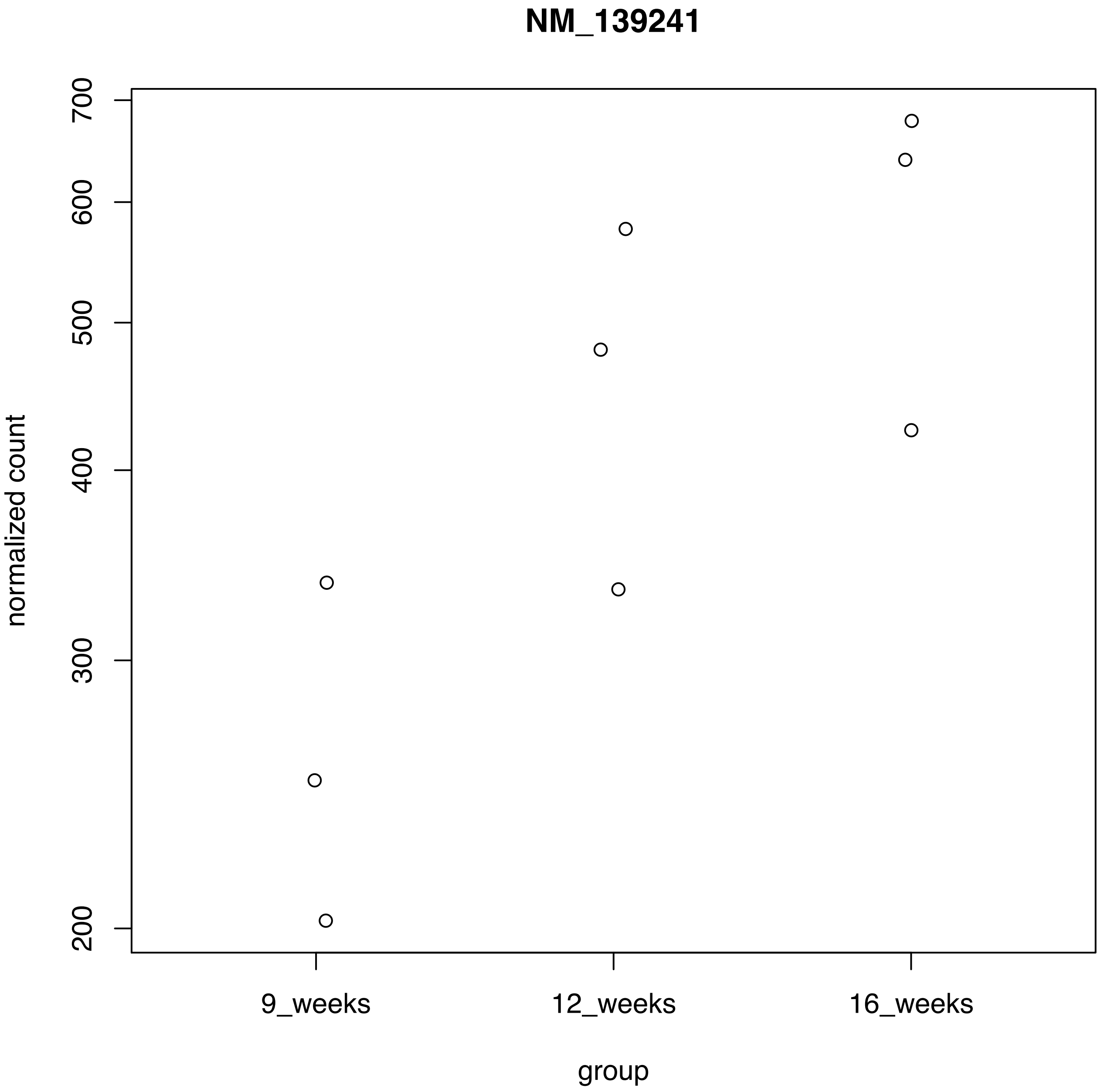
NM_139241
normalized count
700
600
500
400
300
200
9_weeks
12_weeks
16_weeks
group

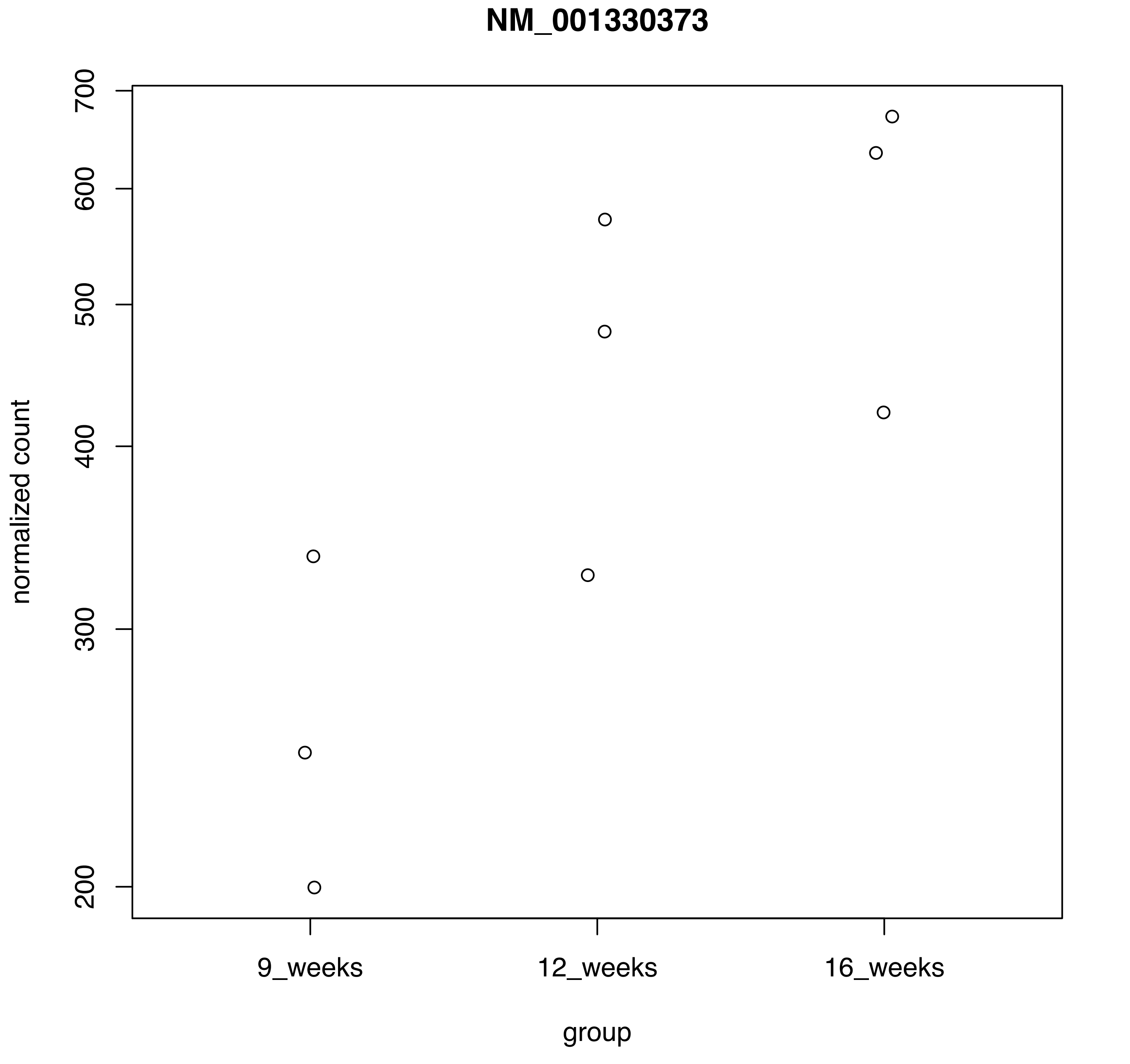
NM_001330373
normalized count
700
600
500
400
300
200
9_weeks
12_weeks
16_weeks
group

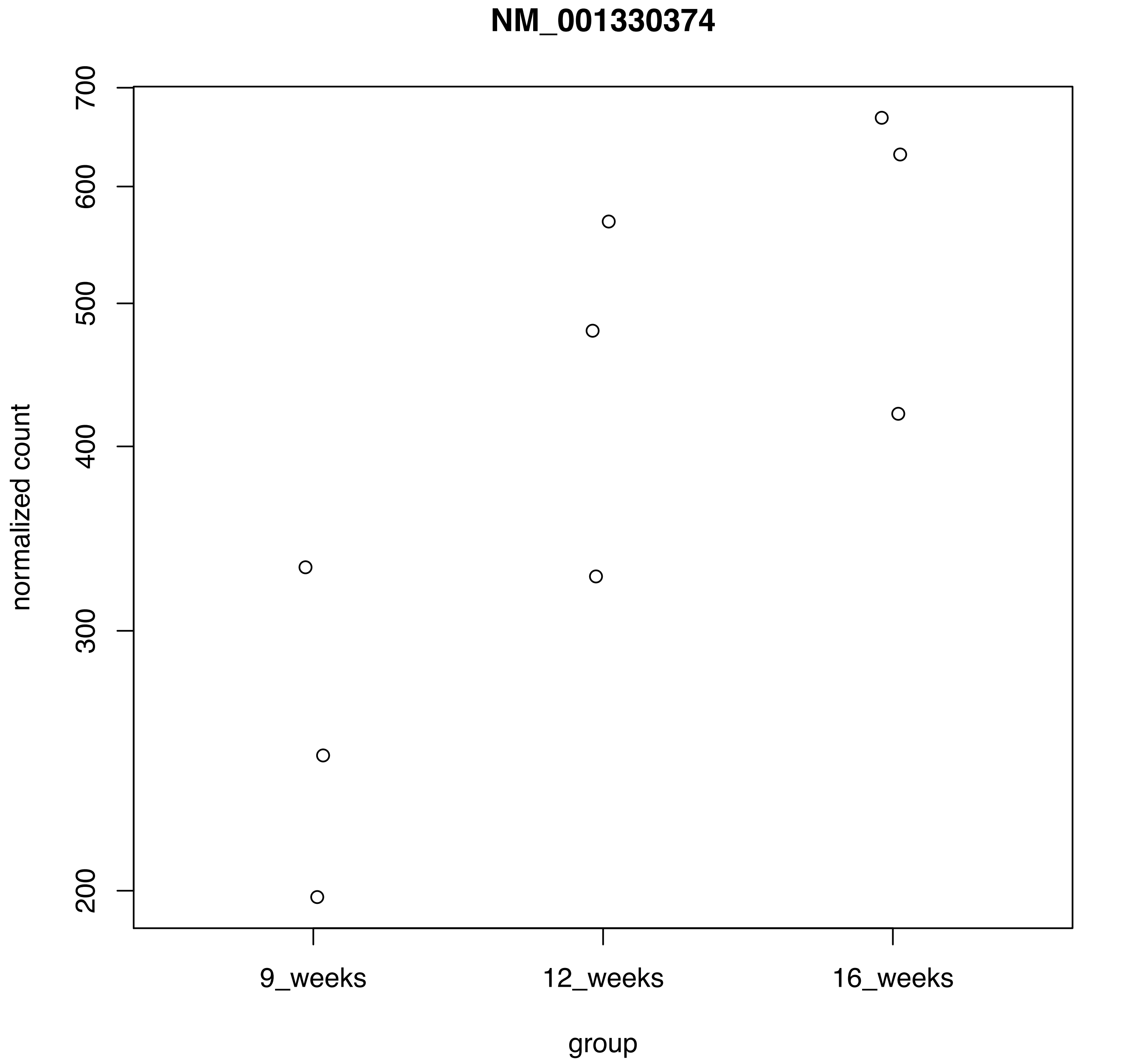

NM_001330374
normalized count
700
600
500
400
300
200
9_weeks
12_weeks
16_weeks
group

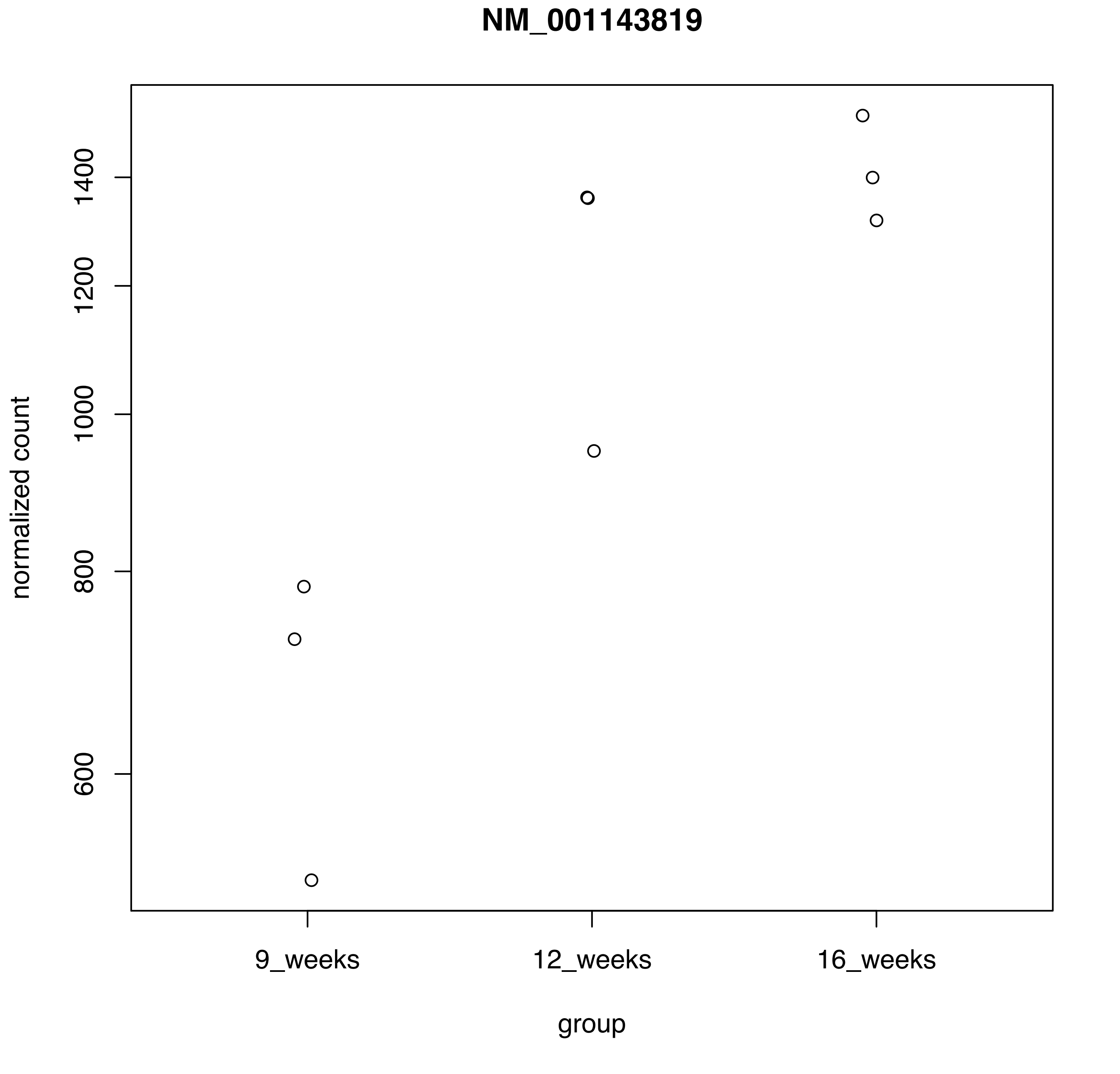
NM_001143819
normalized count
600
800
1000
1200
1400
9_weeks
12_weeks
16_weeks
group

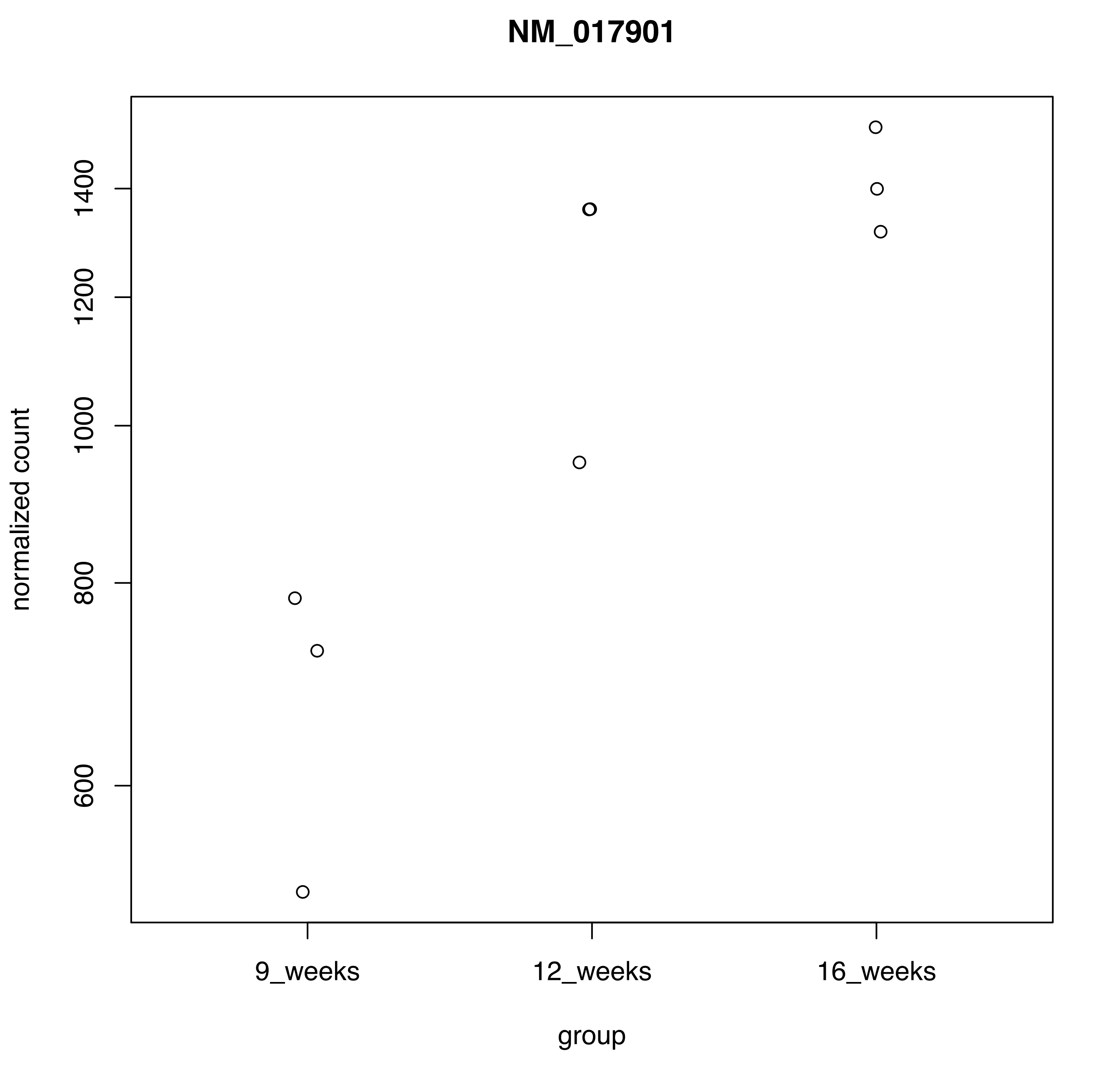
NM_017901
normalized count
600
800
1000
1200
1400
9_weeks
12_weeks
16_weeks
group

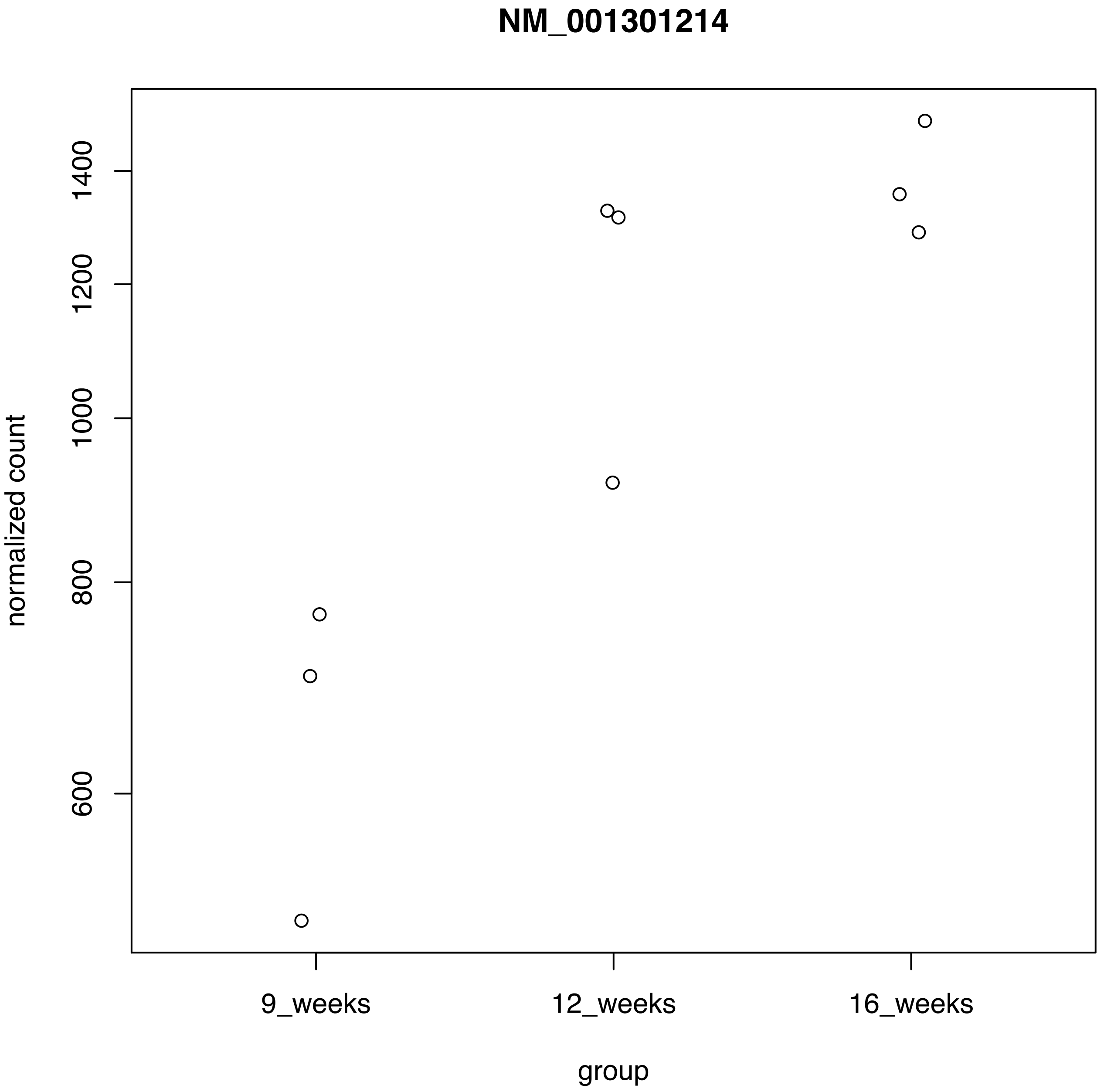
NM_001301214
normalized count
600
800
1000
1200
1400
9_weeks
12_weeks
16_weeks
group

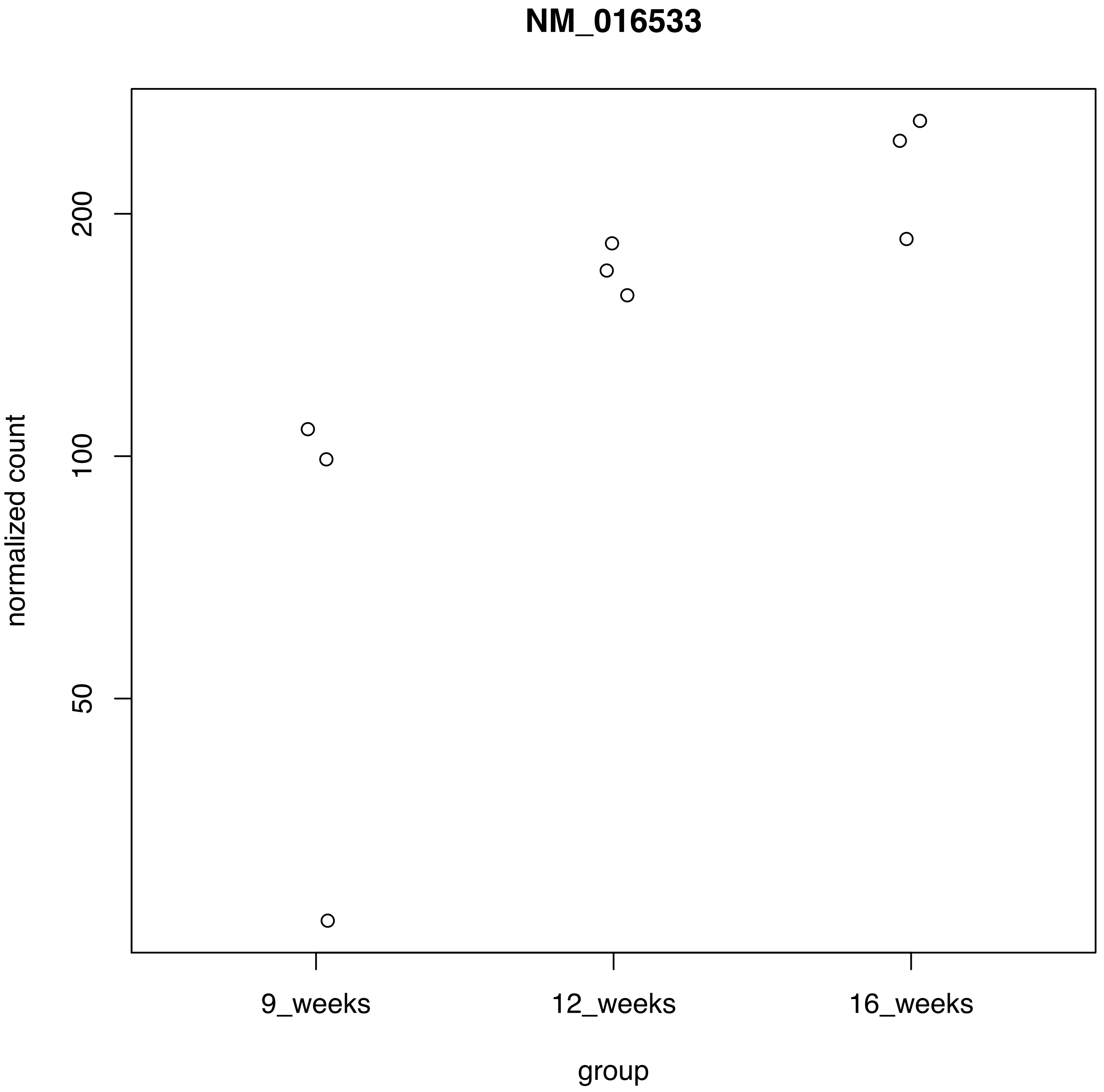
NM_016533
normalized count
200
100
50
9_weeks
12_weeks
16_weeks
group

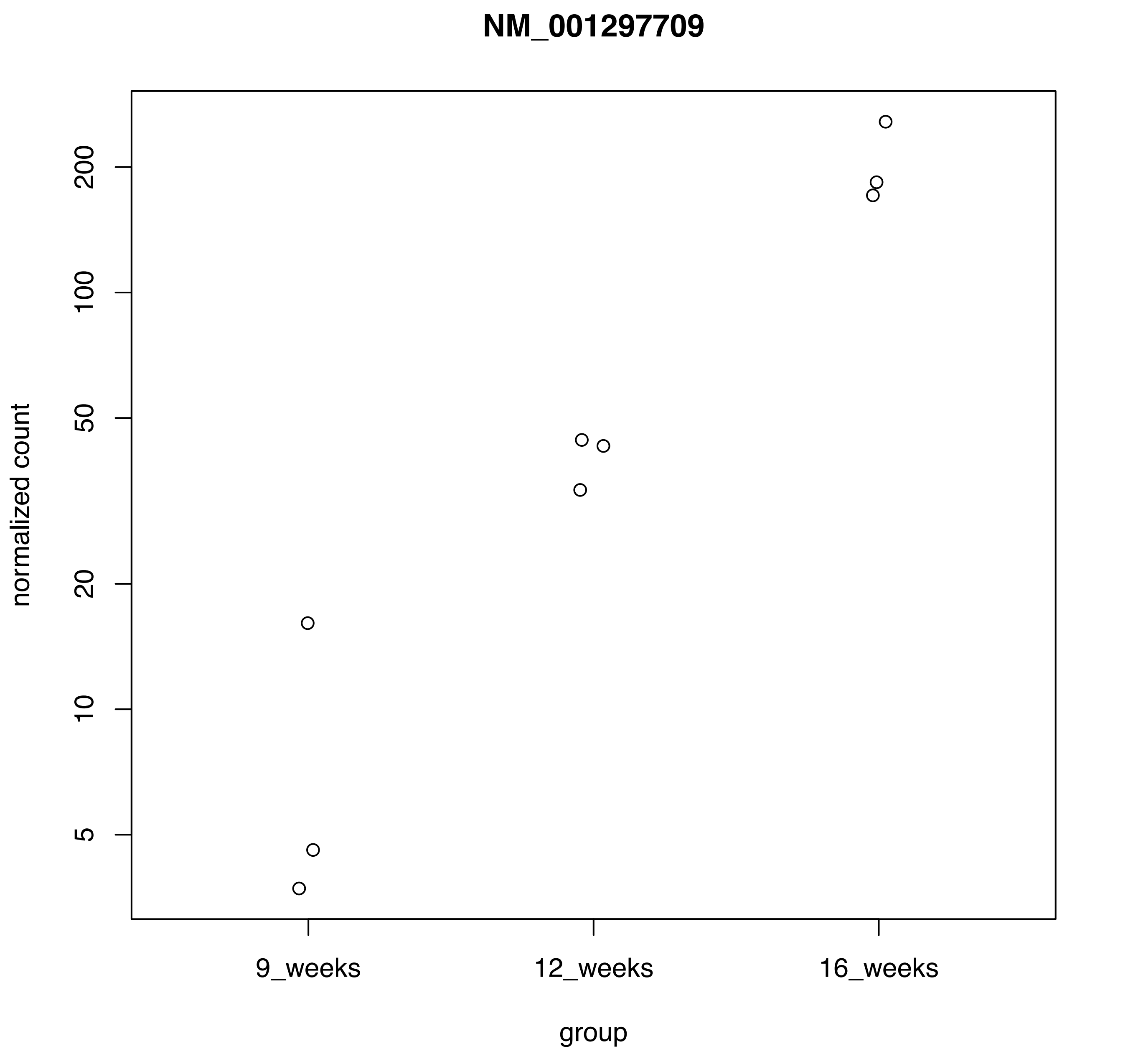

NM_001297709
normalized count
200
100
50
20
10
5
9_weeks
12_weeks
16_weeks
group

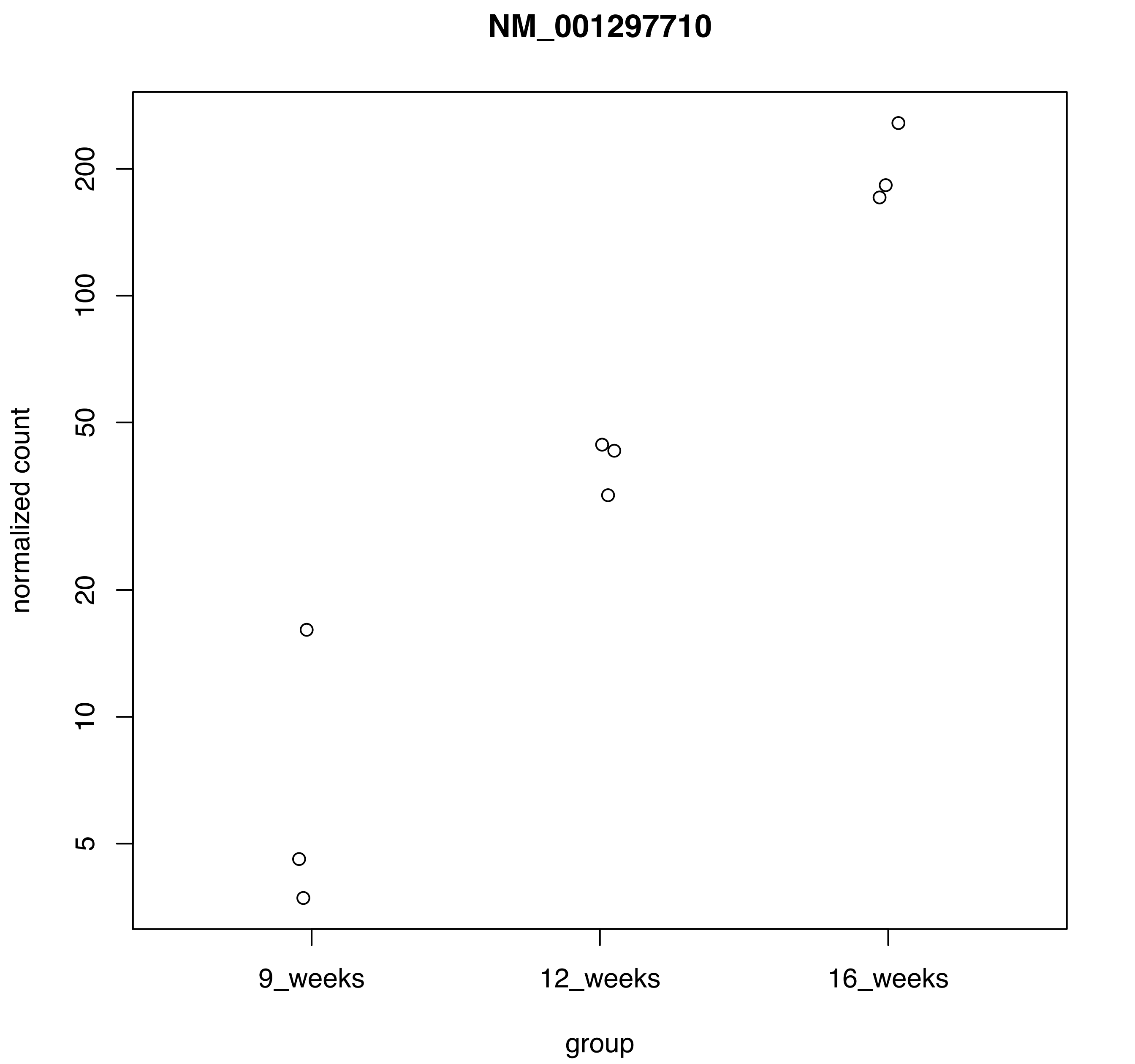

NM_001297710
normalized count
200
100
50
20
10
5
9_weeks
12_weeks
16_weeks
group

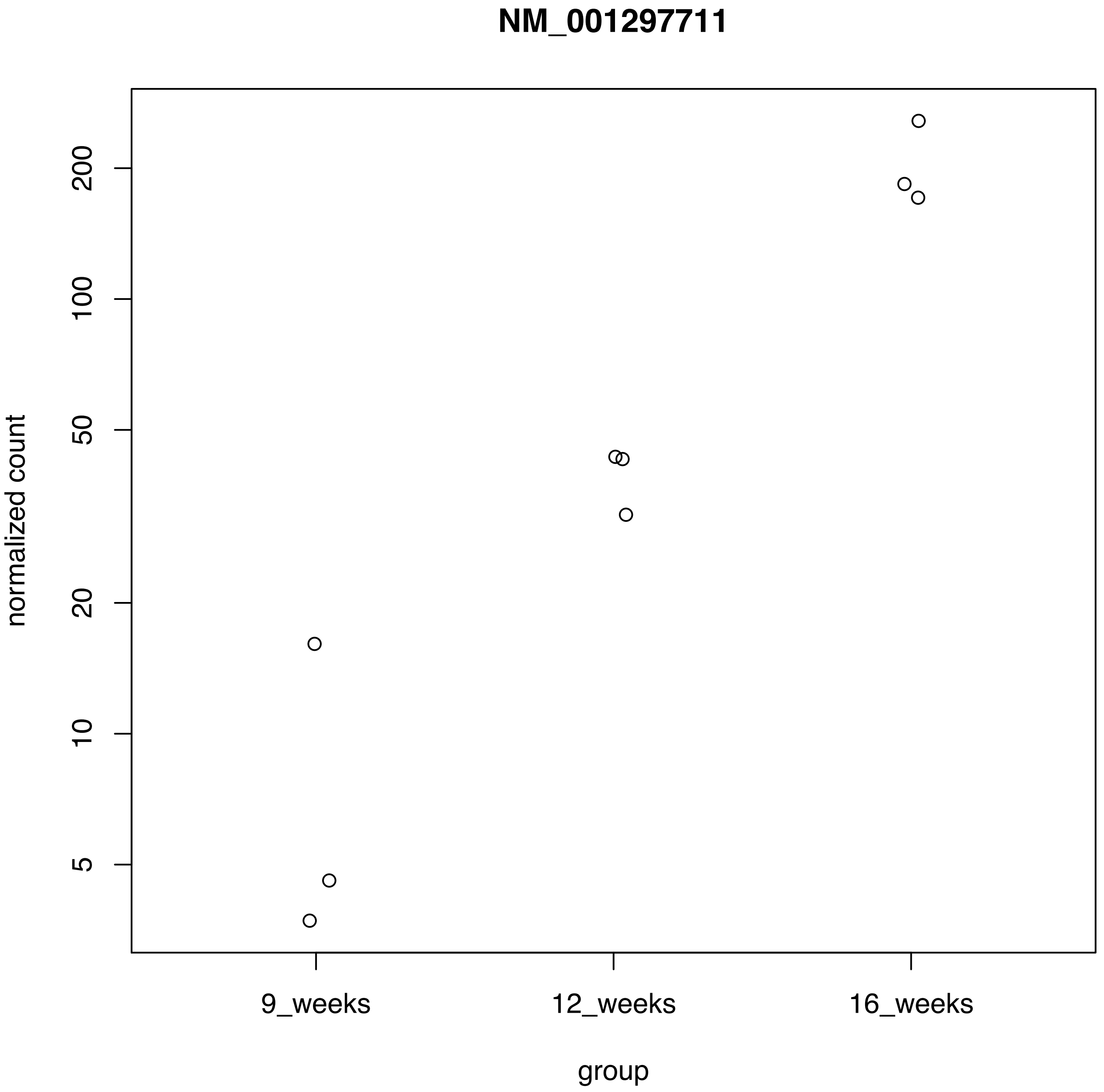
NM_001297711
normalized count
200
100
50
20
10
5
9_weeks
12_weeks
16_weeks
group

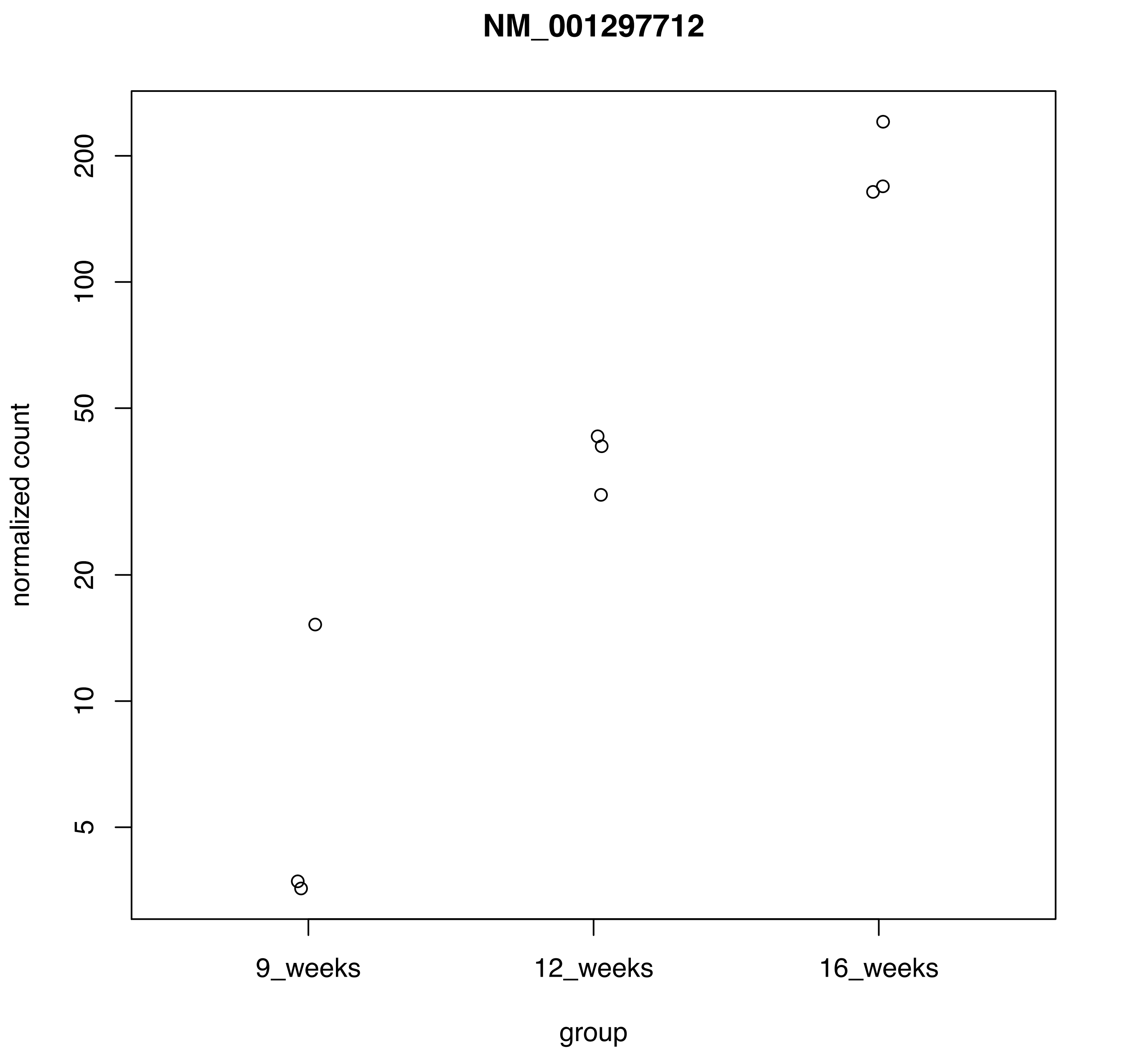
NM_001297712
normalized count
200
100
50
20
10
5
9_weeks
12_weeks
16_weeks
group

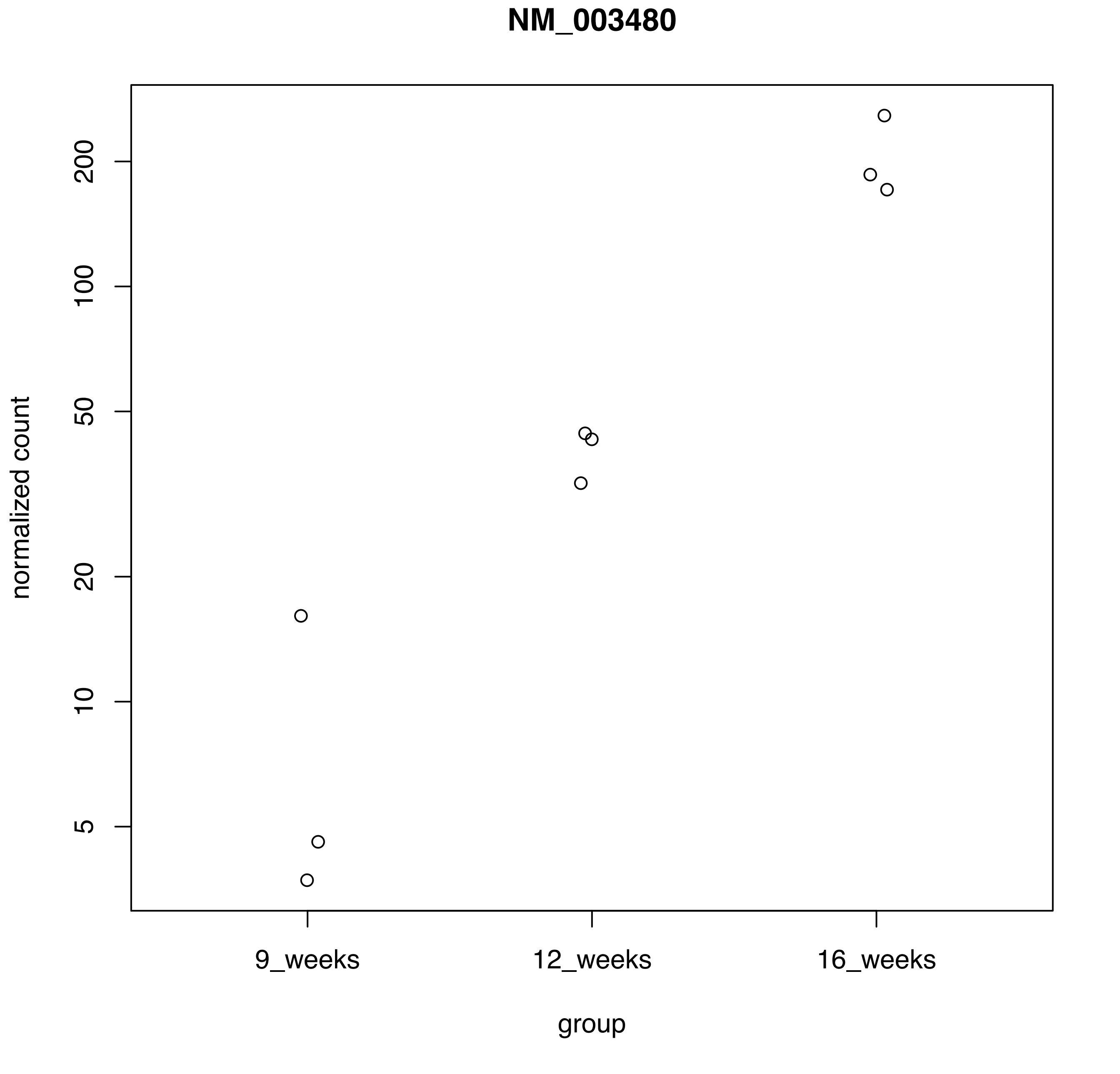

NM_003480
normalized count
200
100
50
20
10
5
9_weeks
12_weeks
16_weeks
group

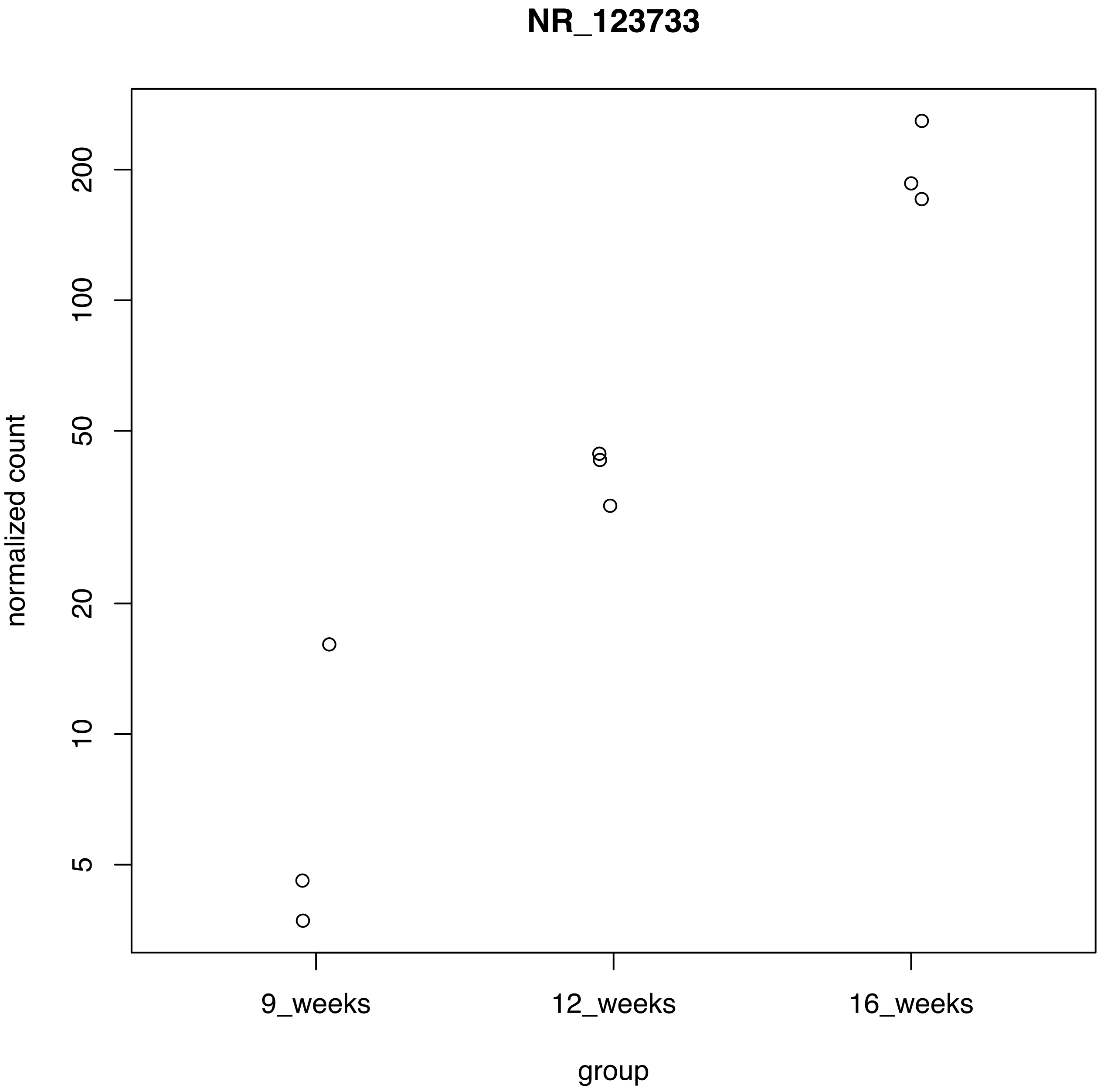
NR_123733
normalized count
200
100
50
20
10
5
9_weeks
12_weeks
16_weeks
group

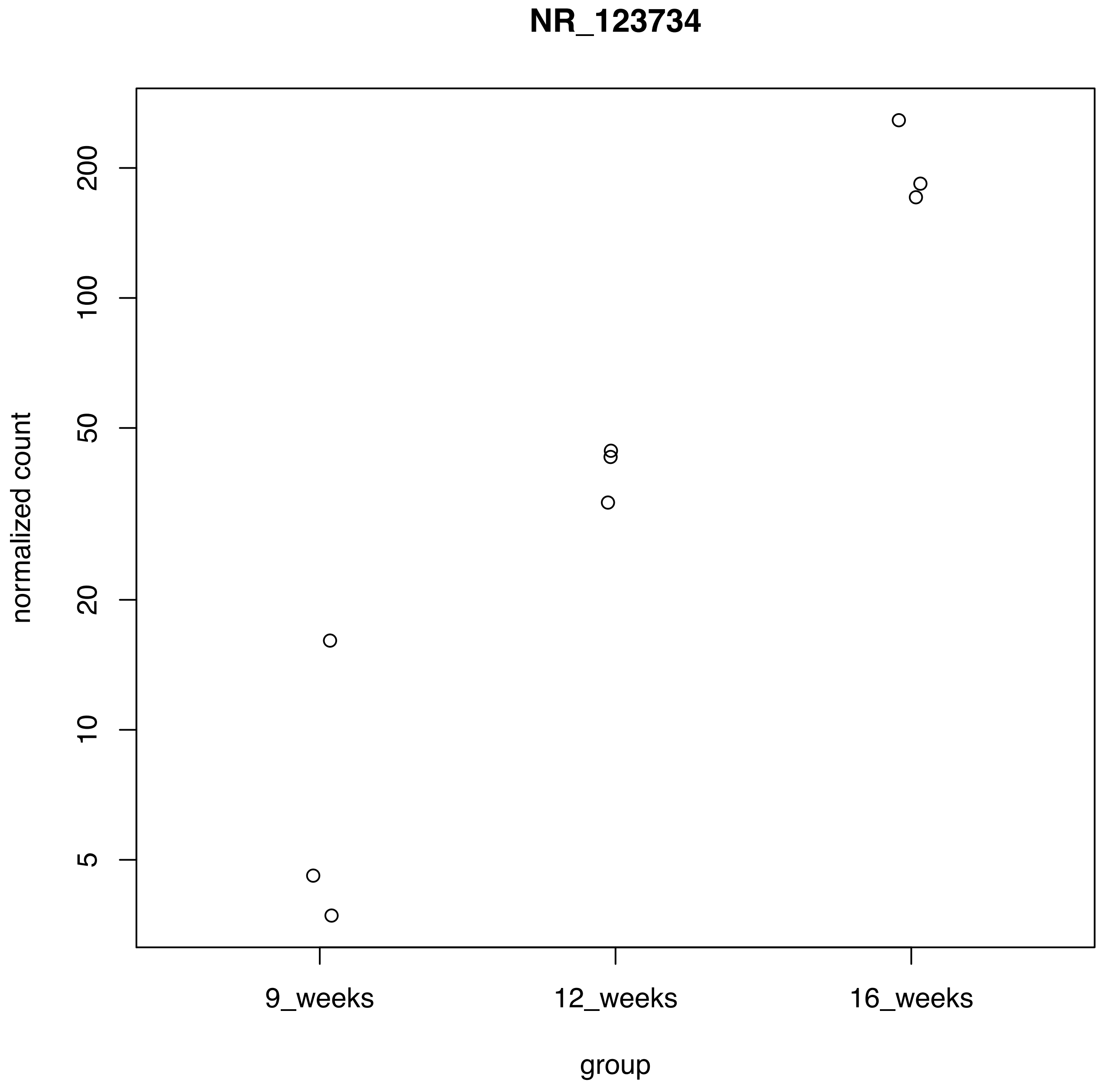
NR_123734
normalized count
200
100
50
20
10
5
9_weeks
12_weeks
16_weeks
group

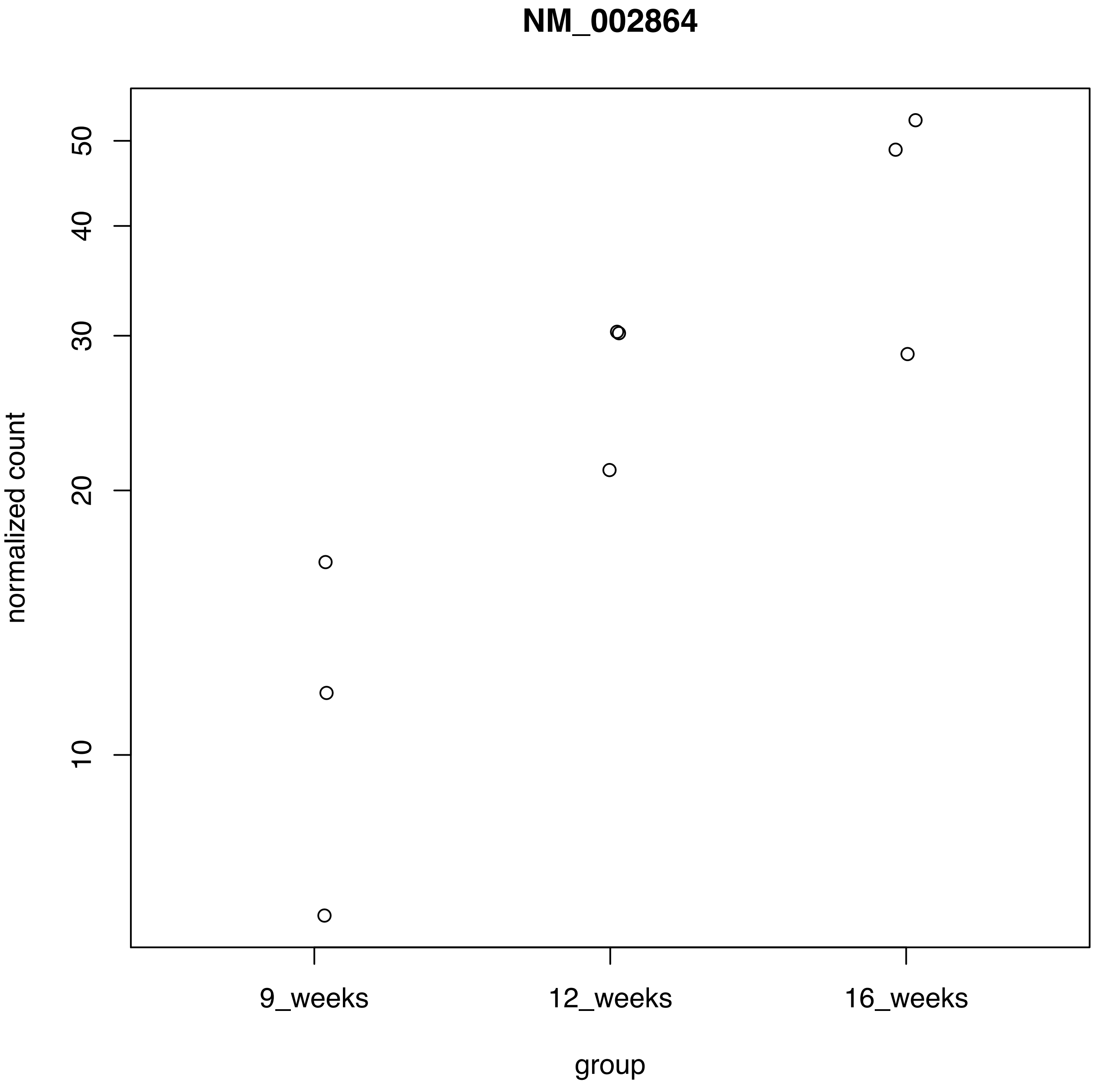
NM_002864
normalized count
10
20
30
40
50
9_weeks
12_weeks
16_weeks
group

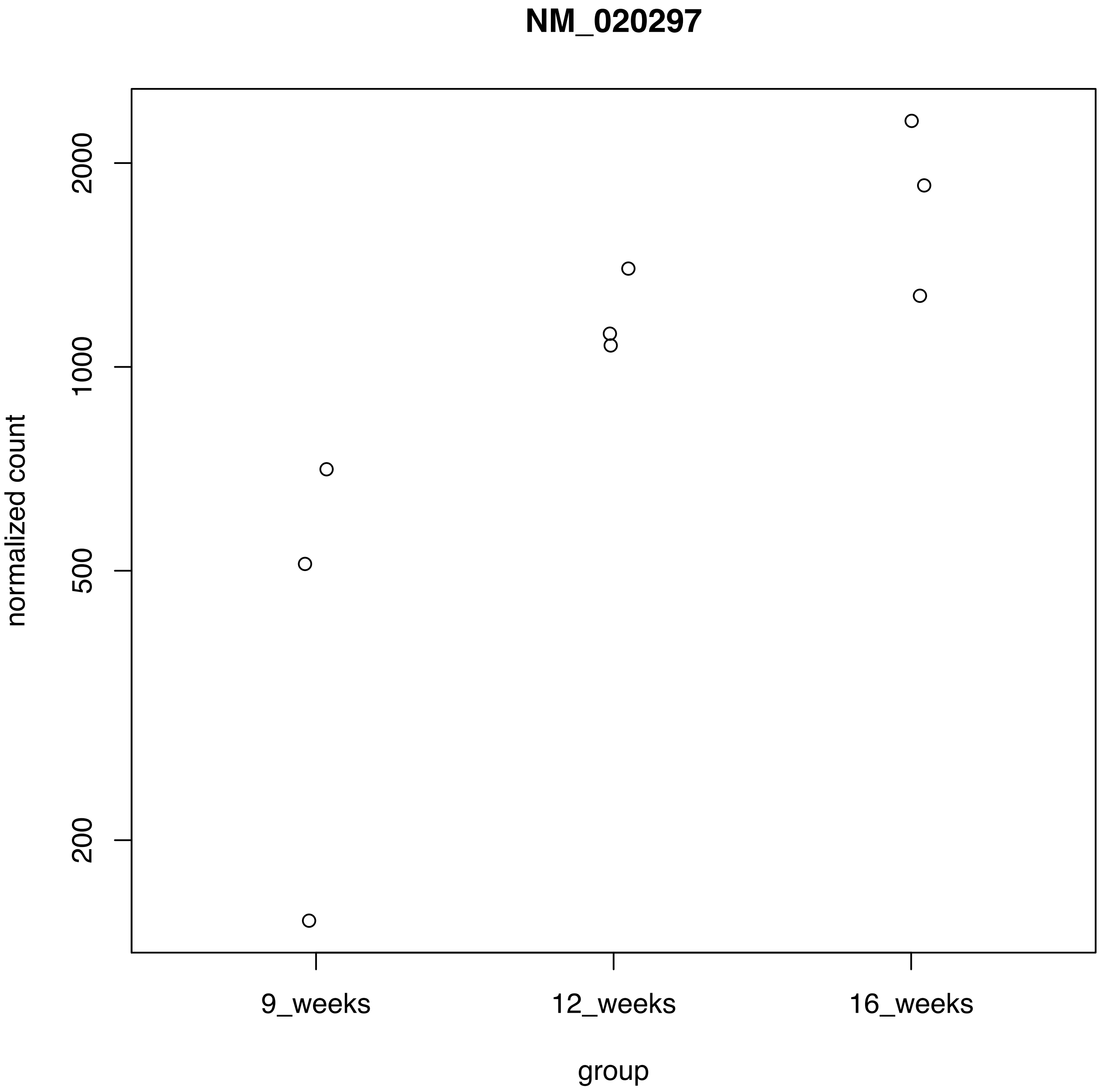
NM_020297
normalized count
2000
1000
500
200
9_weeks
12_weeks
16_weeks
group

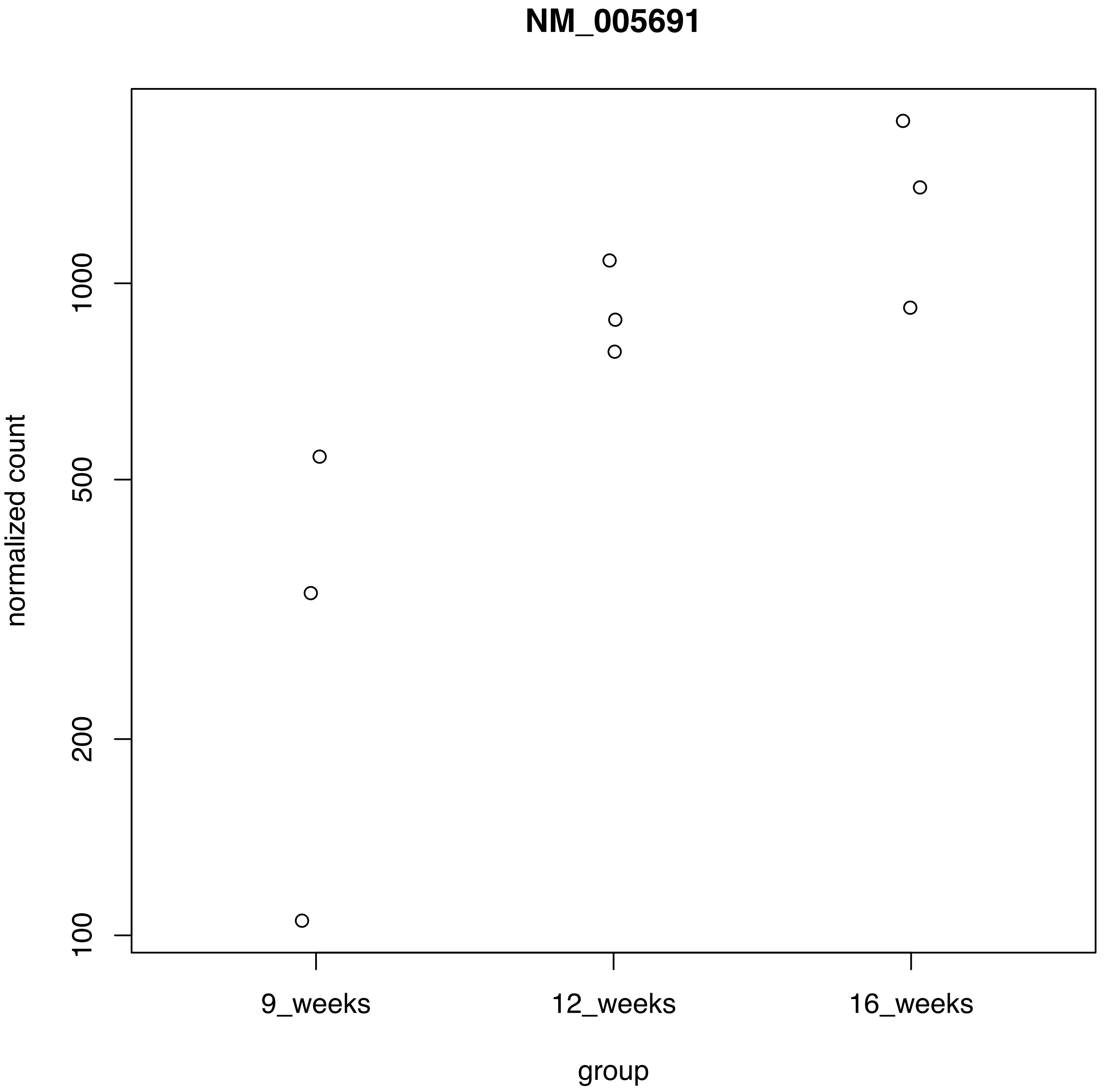
NM_005691
normalized count
1000
500
200
100
9_weeks
12_weeks
16_weeks
group

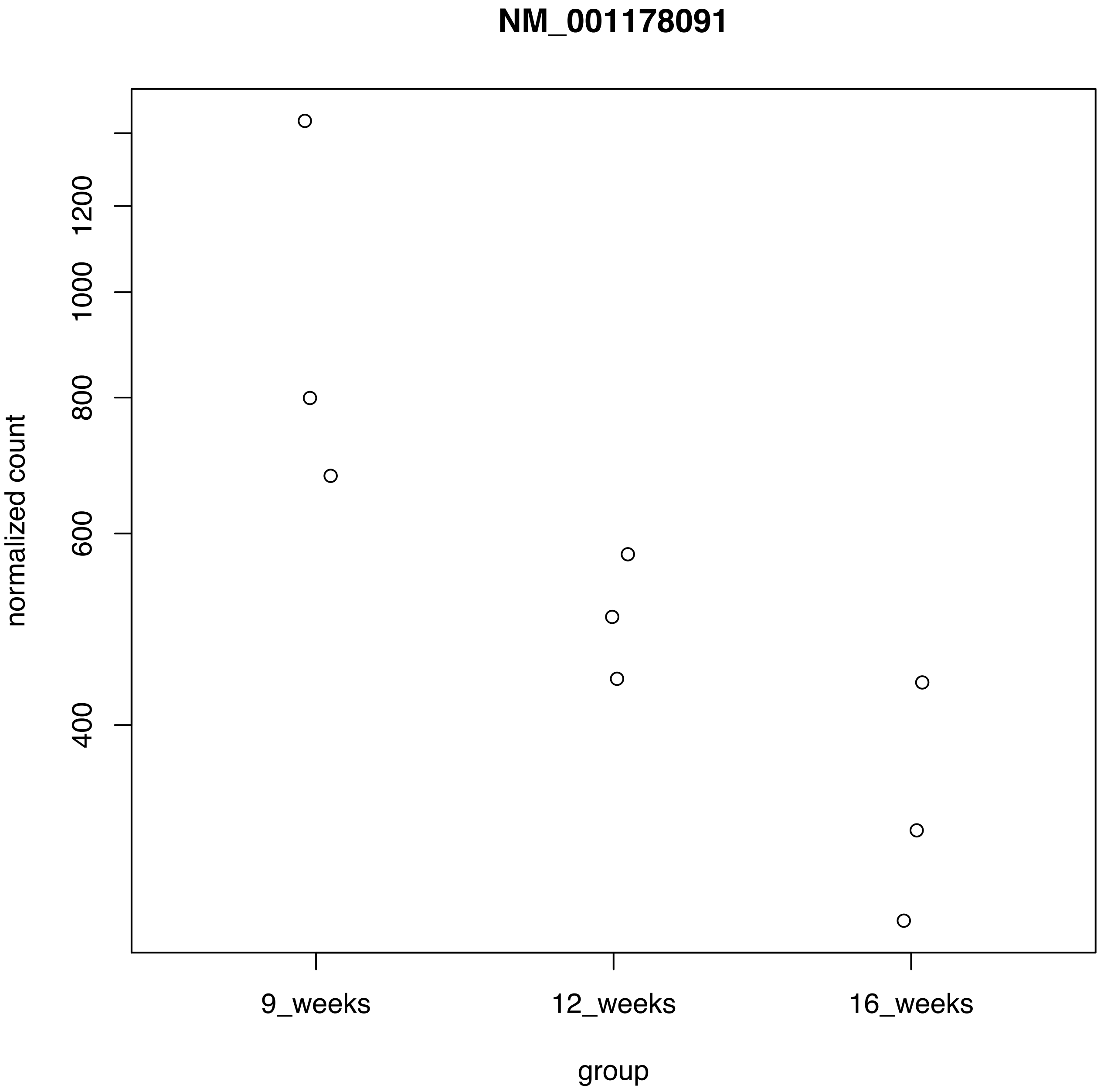
NM_001178091
normalized count
400
600
800
1000
1200
9_weeks
12_weeks
16_weeks
group

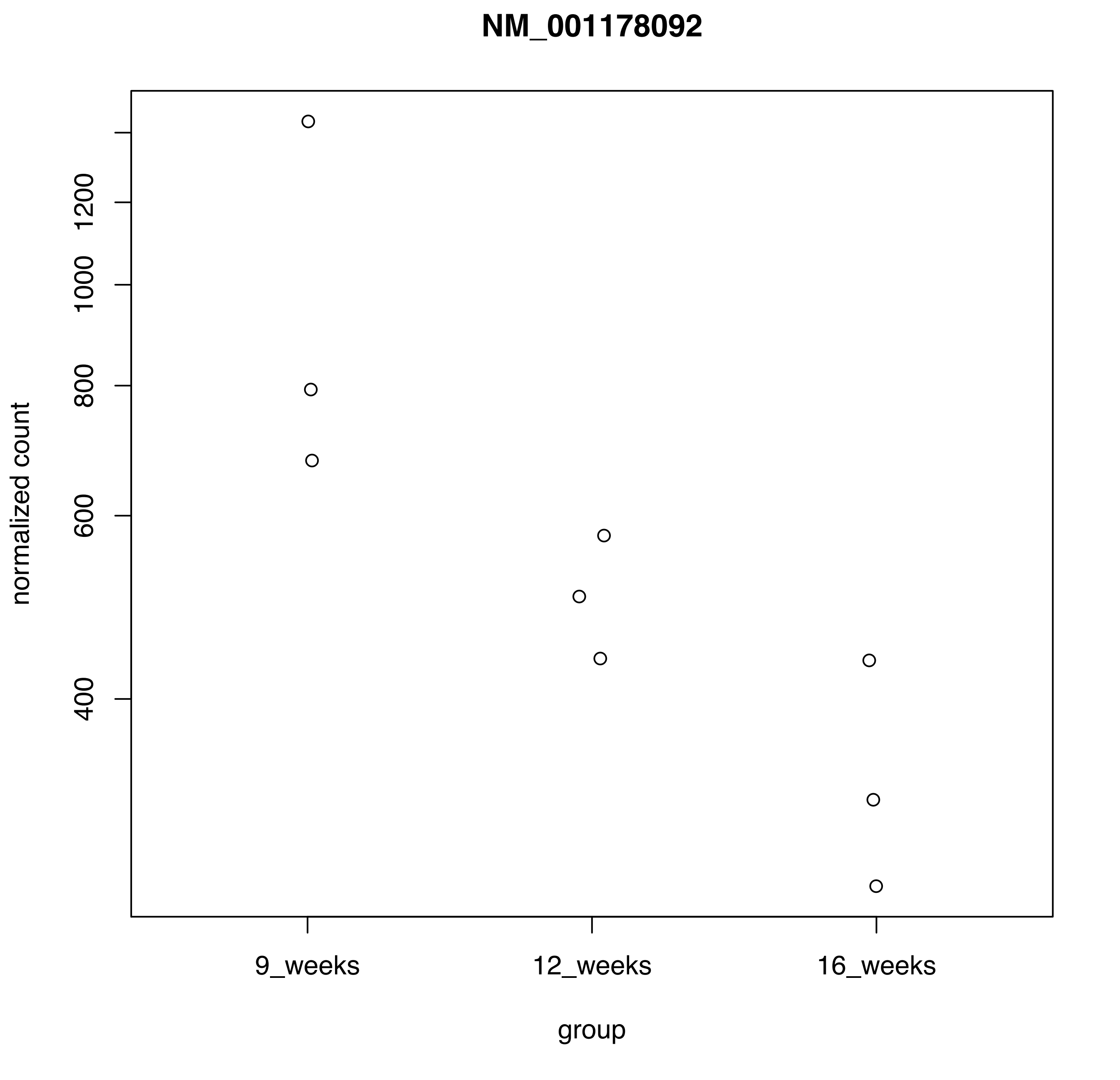
NM_001178092
normalized count
400
600
800
1000
1200
9_weeks
12_weeks
16_weeks
group

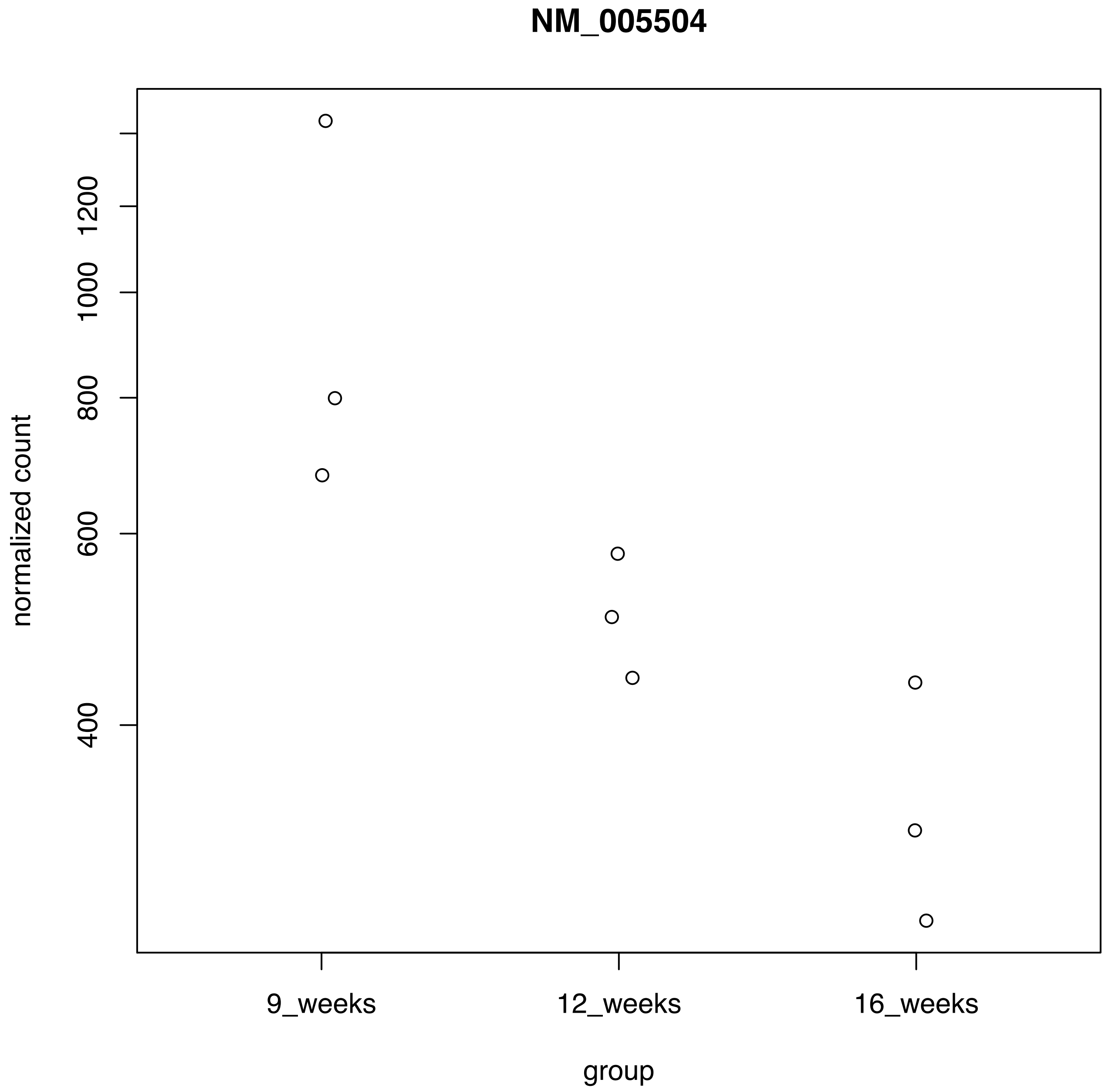
NM_005504
normalized count
400
600
800
1000
1200
9_weeks
12_weeks
16_weeks
group

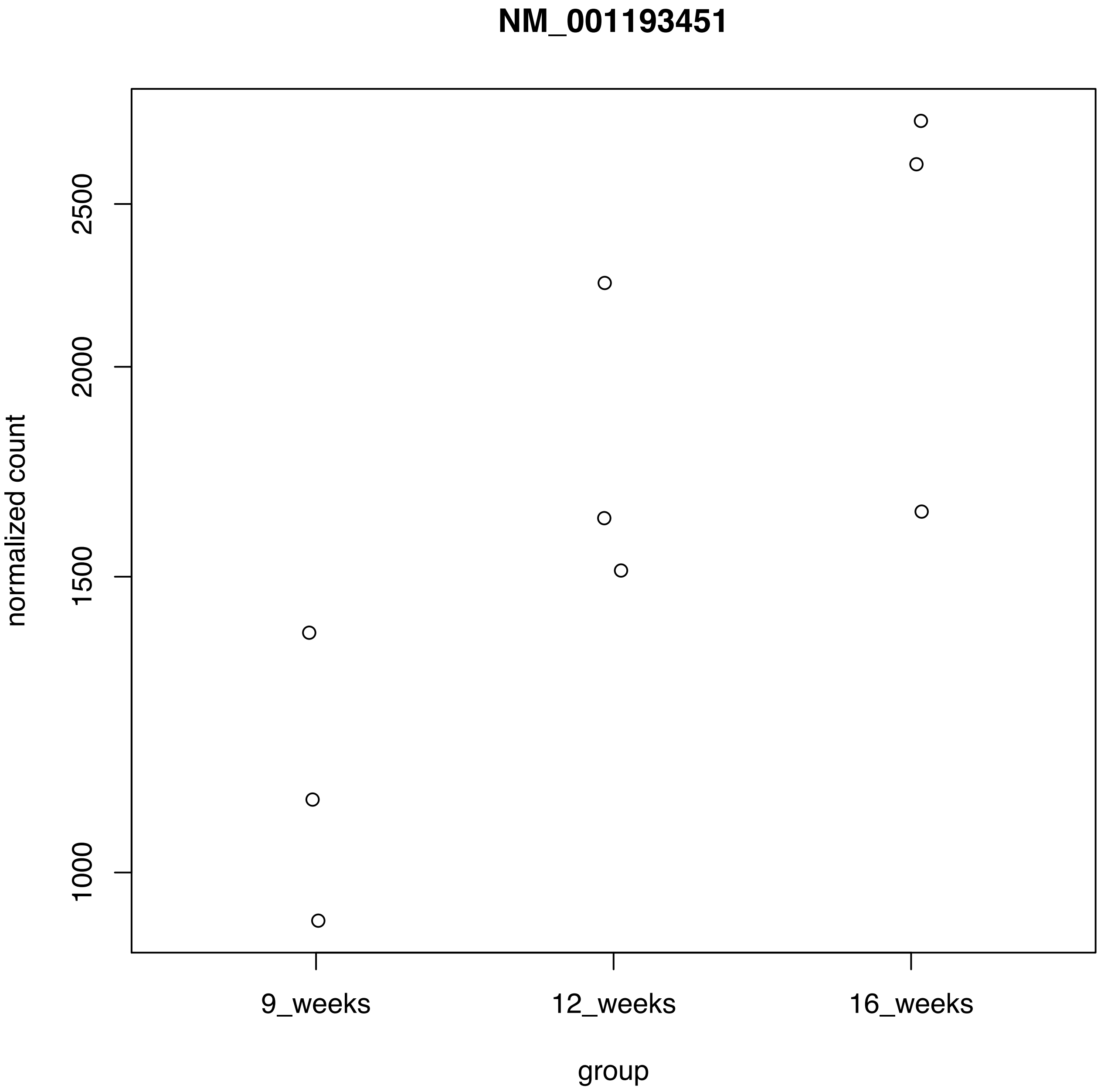

NM_001193451
normalized count
1000
1500
2000
2500
9_weeks
12_weeks
16_weeks
group

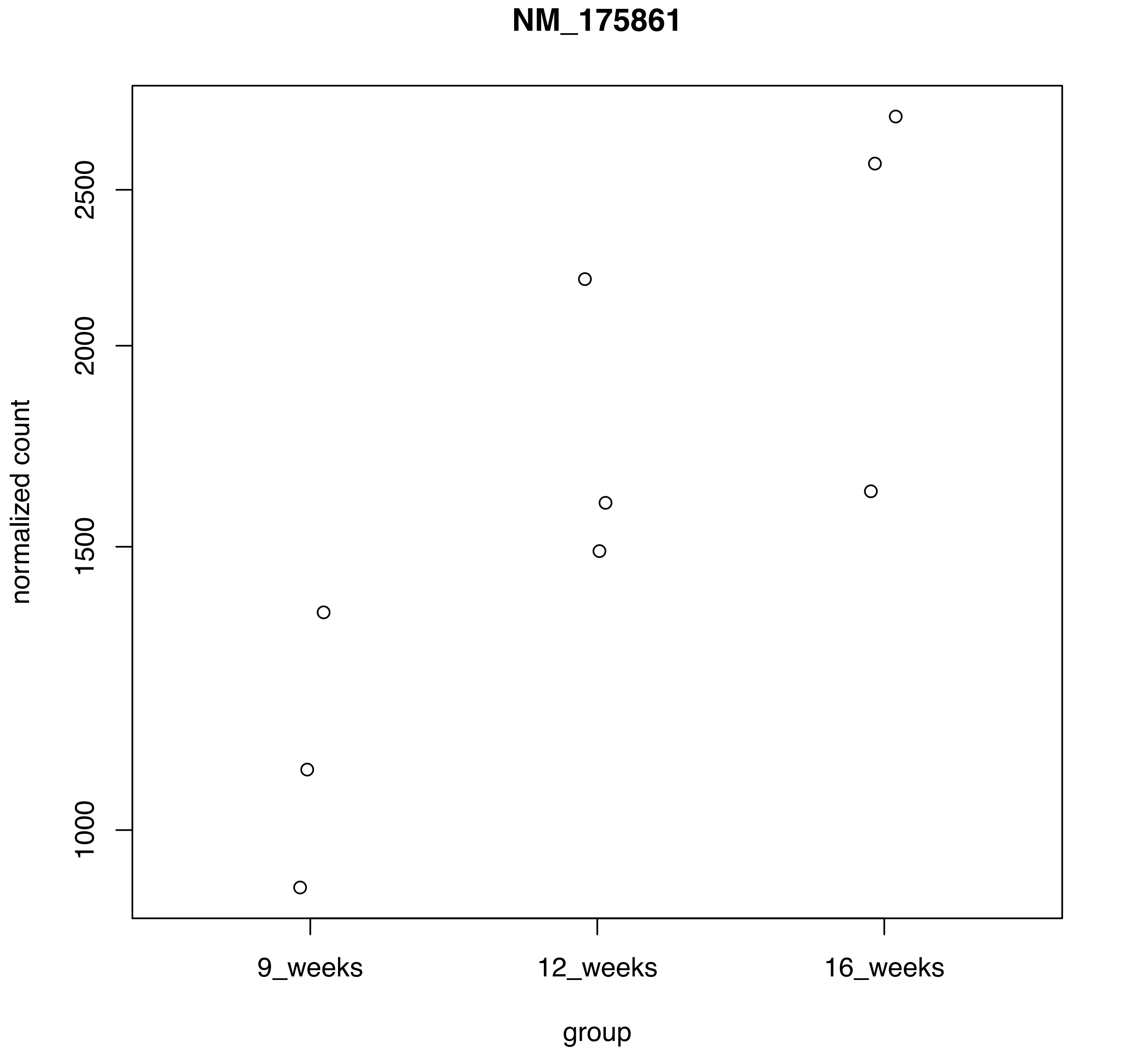

NM_175861
normalized count
2500
2000
1500
1000
9_weeks
12_weeks
16_weeks
group

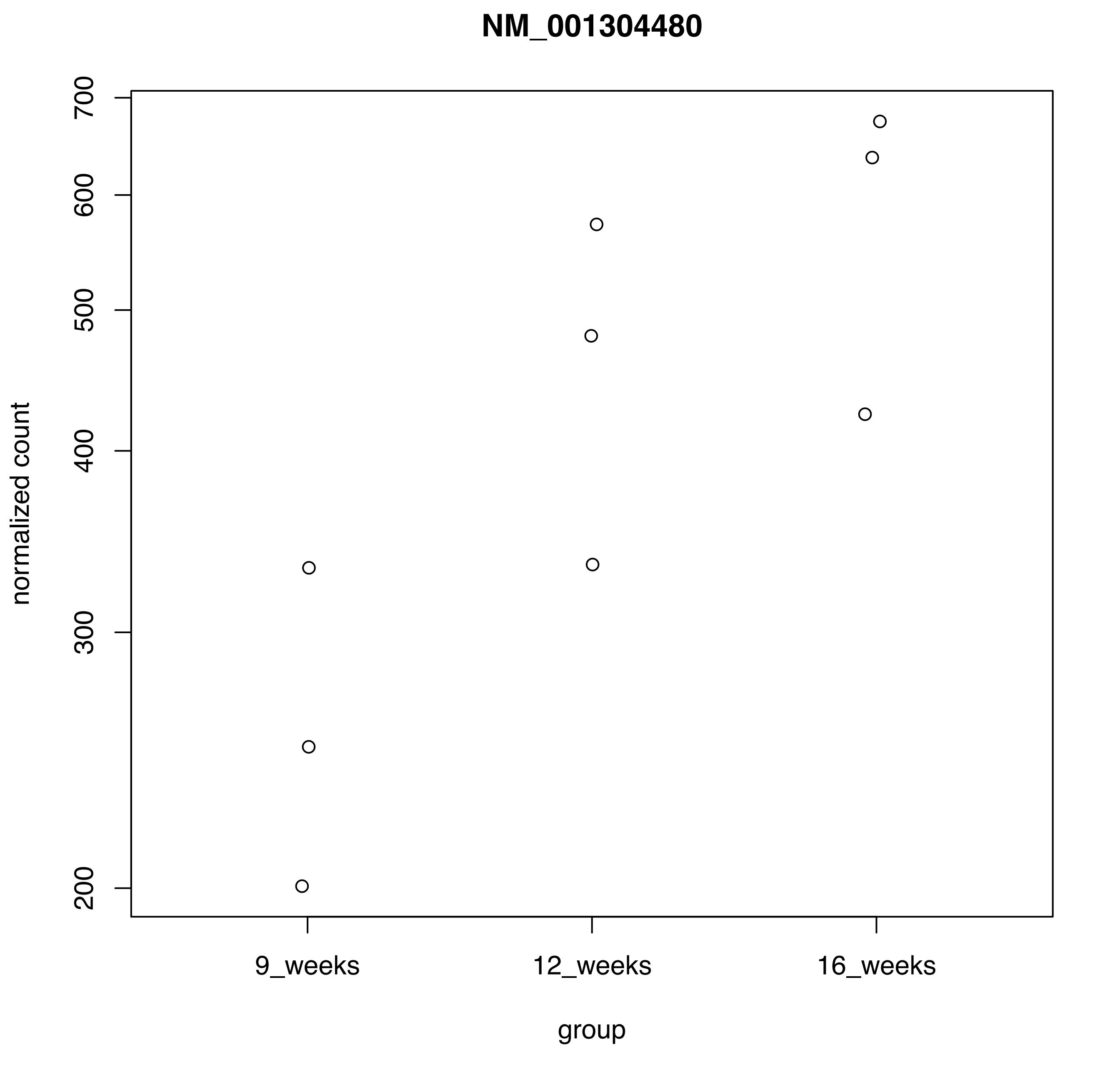
NM_001304480
normalized count
200
300
400
500
600
700
9_weeks
12_weeks
16_weeks
group

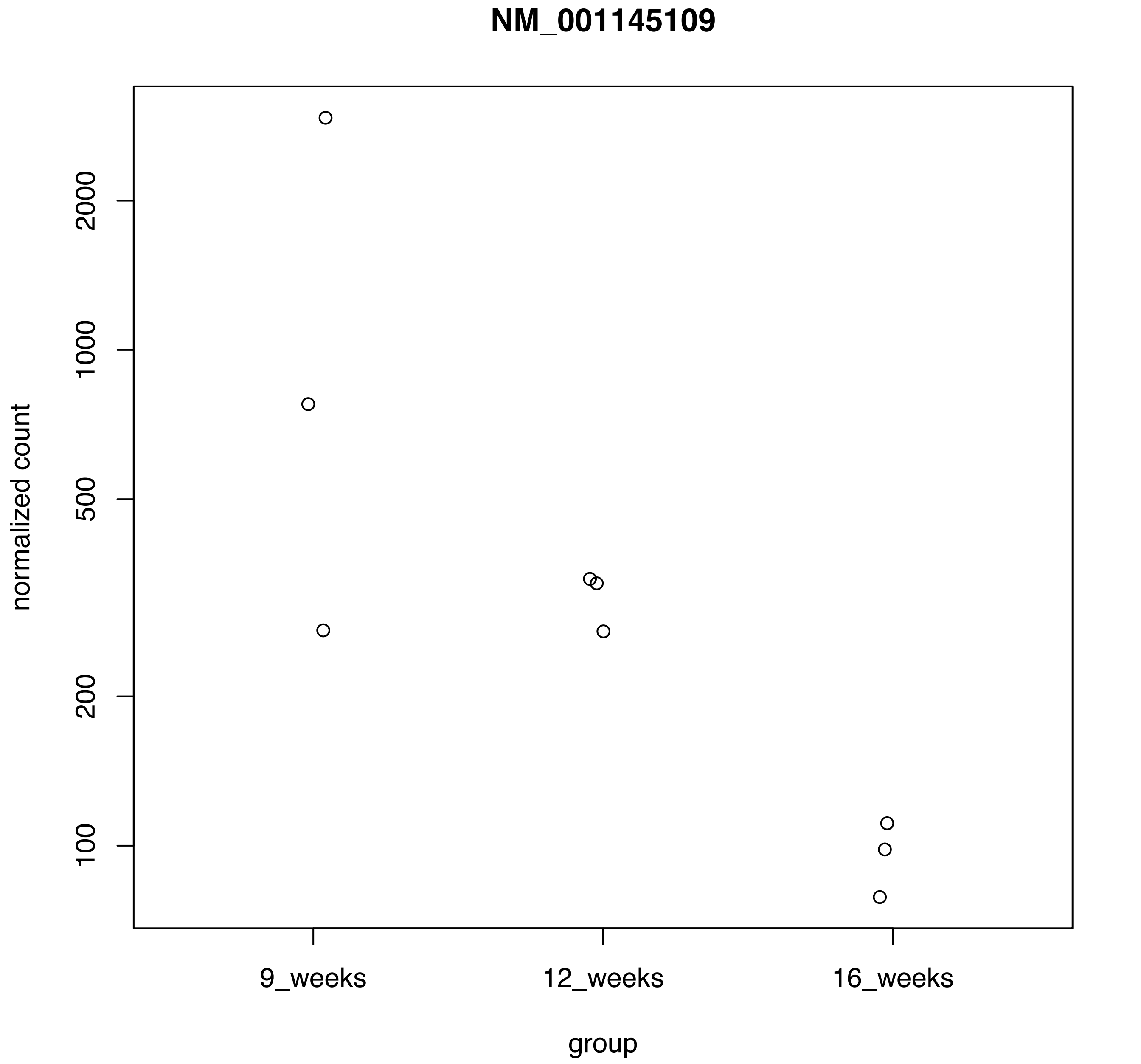
NM_001145109
normalized count
2000
1000
500
200
100
9_weeks
12_weeks
16_weeks
group

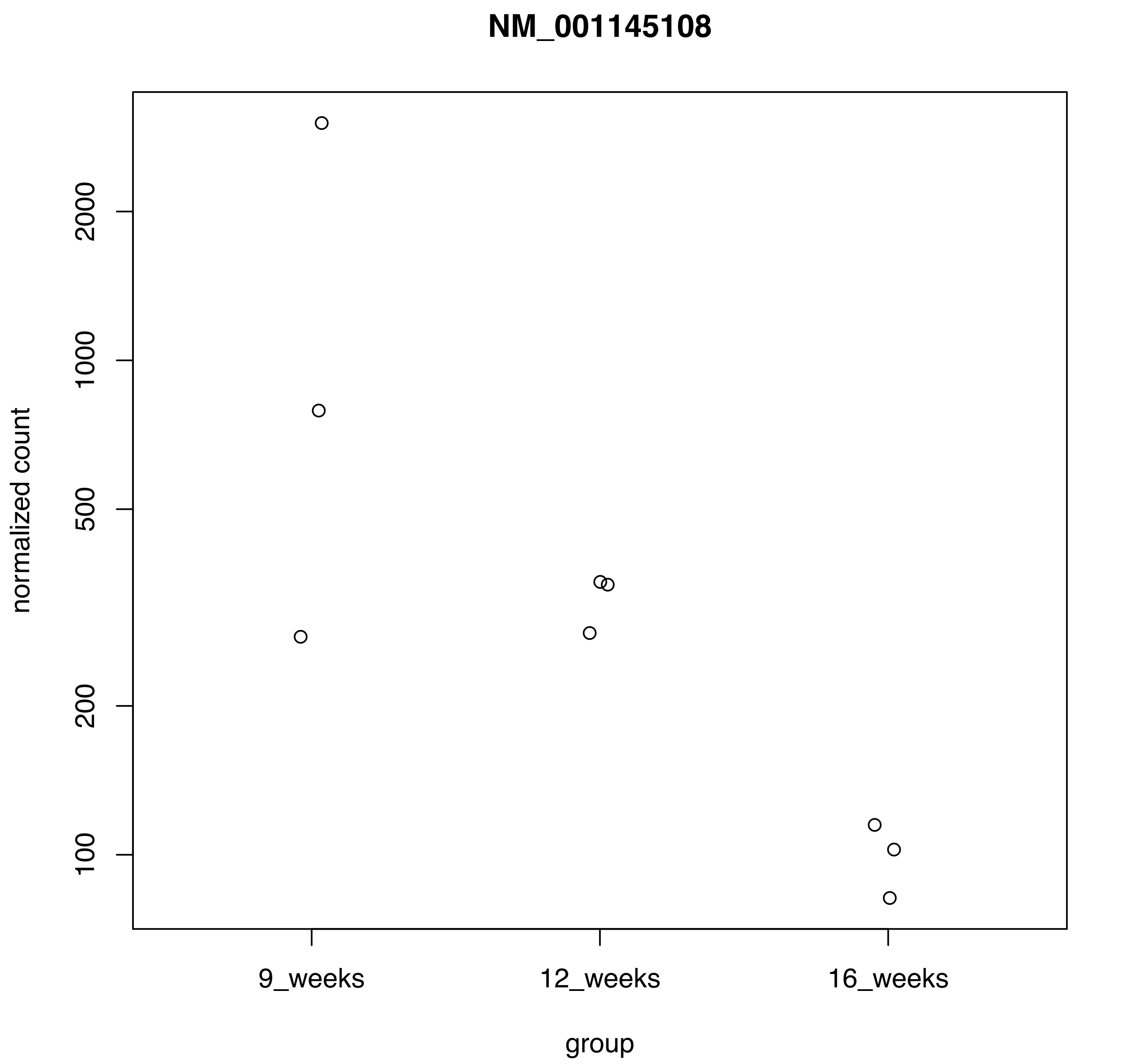
NM_001145108
normalized count
2000
1000
500
200
100
9_weeks
12_weeks
16_weeks
group

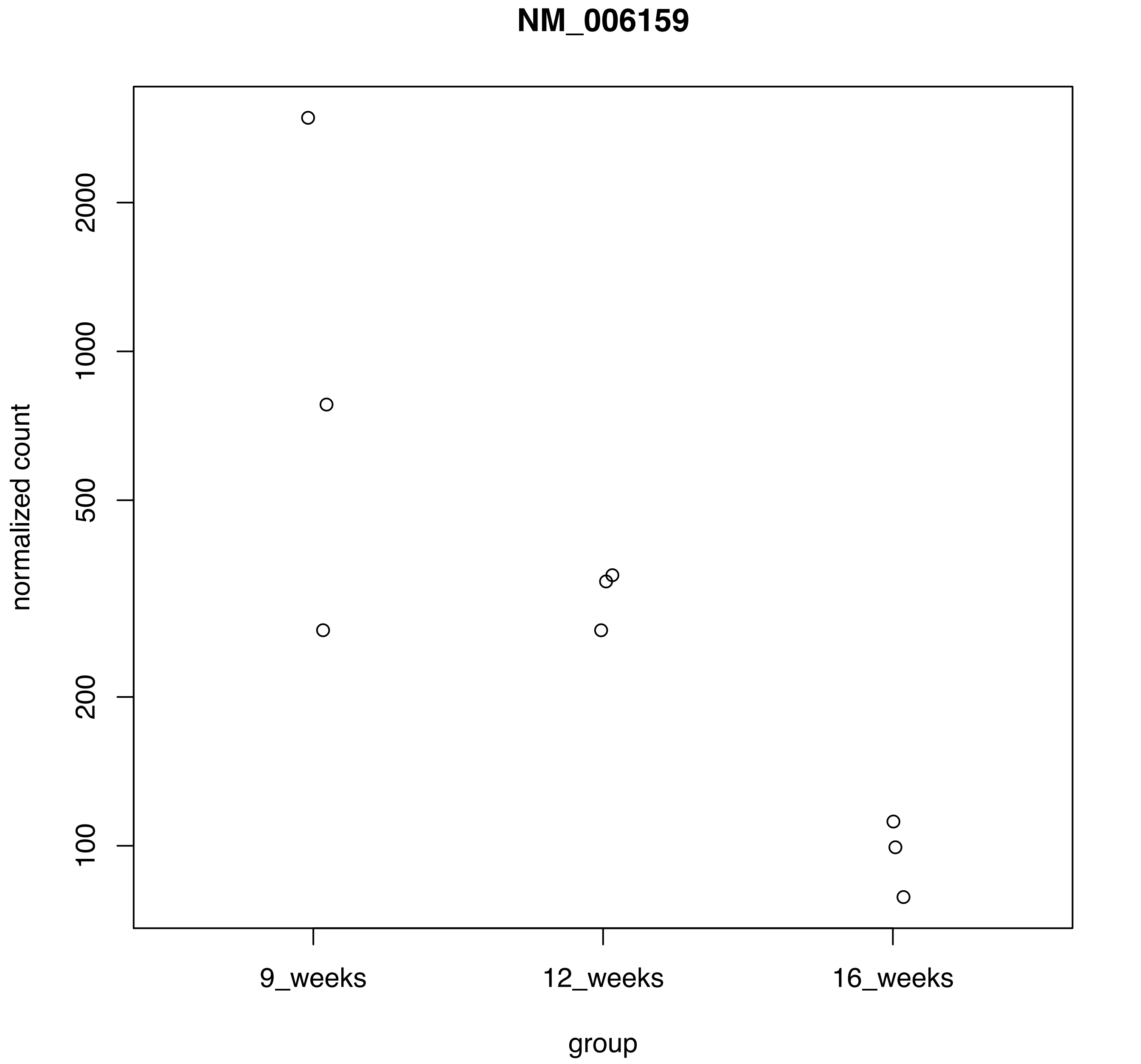
NM_006159
normalized count
2000
1000
500
200
100
9_weeks
12_weeks
16_weeks
group

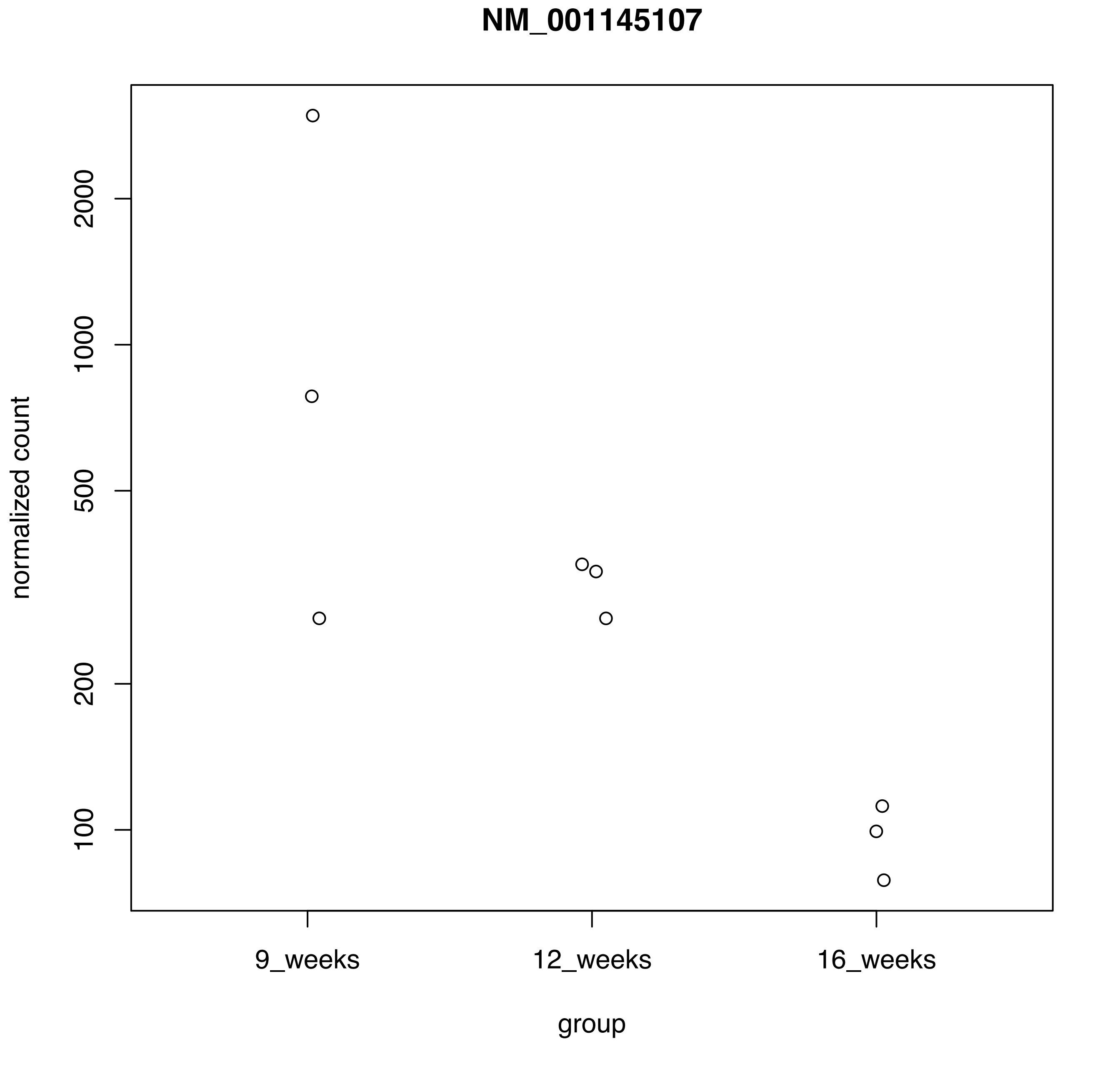

NM_001145107
2000
1000
500
200
100
normalized count
9_weeks
12_weeks
16_weeks
group

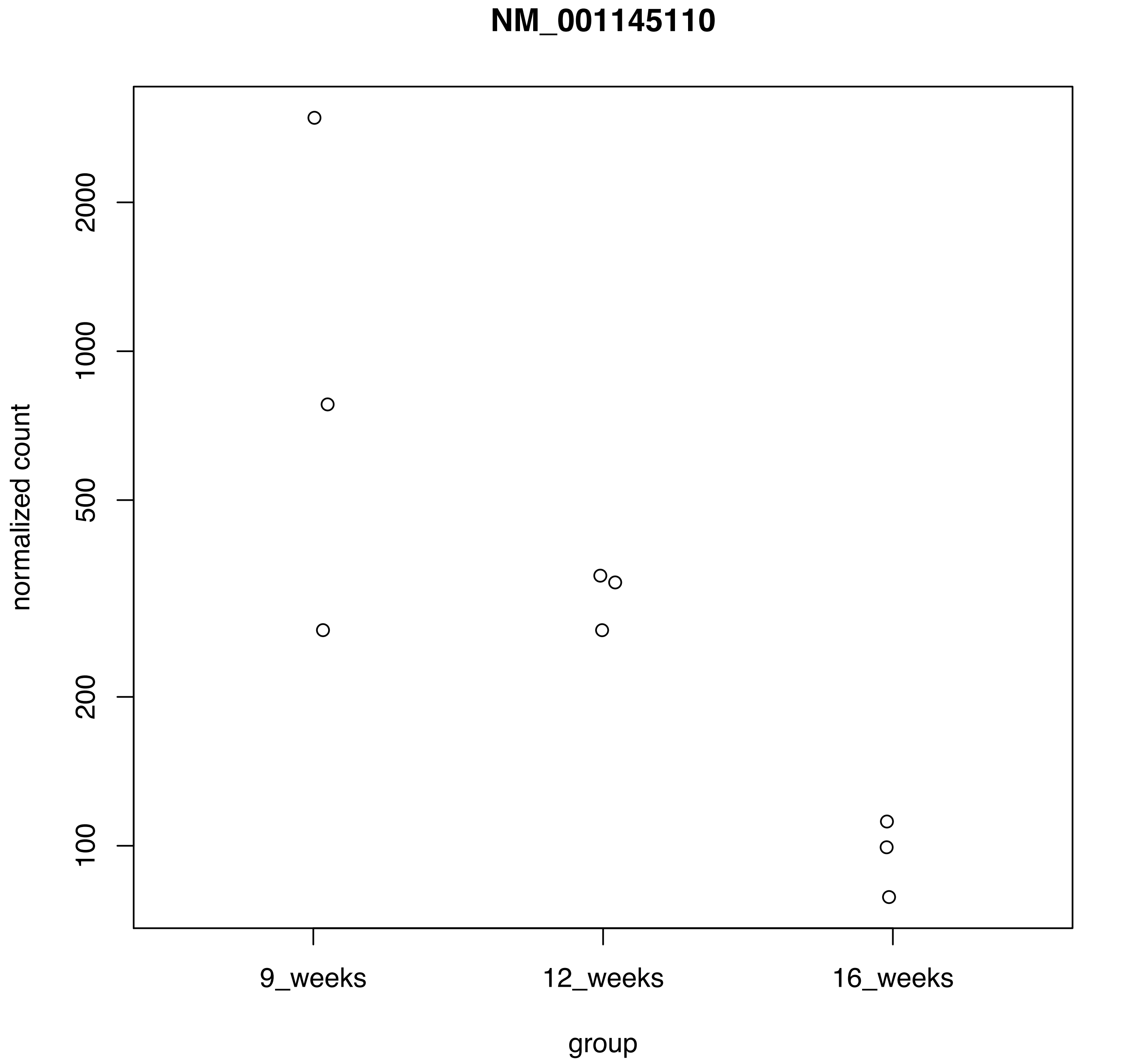

NM_001145110
normalized count
2000
1000
500
200
100
9_weeks
12_weeks
16_weeks
group

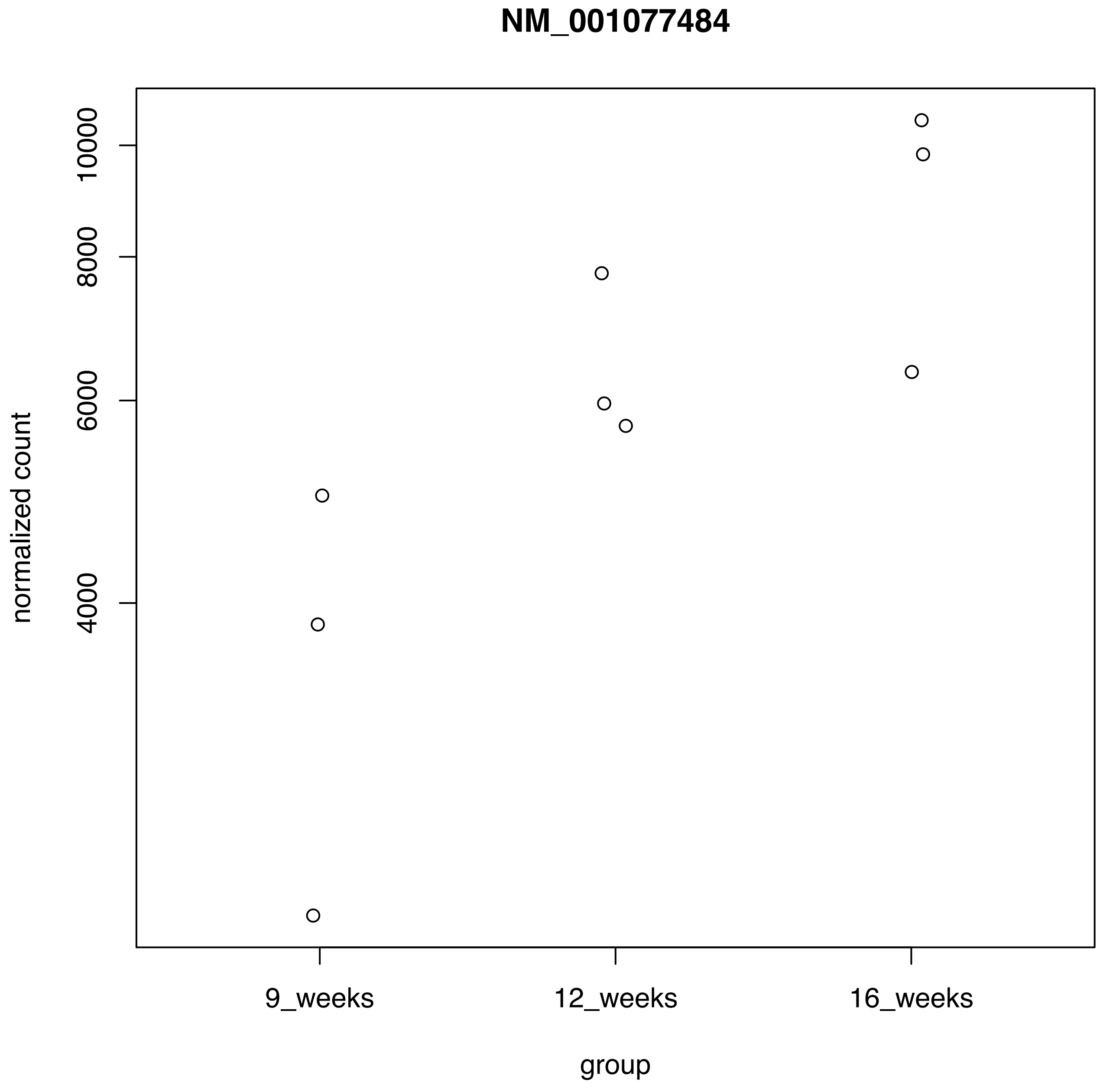
NM_001077484
normalized count
10000
8000
6000
4000
9_weeks
12_weeks
16_weeks
group

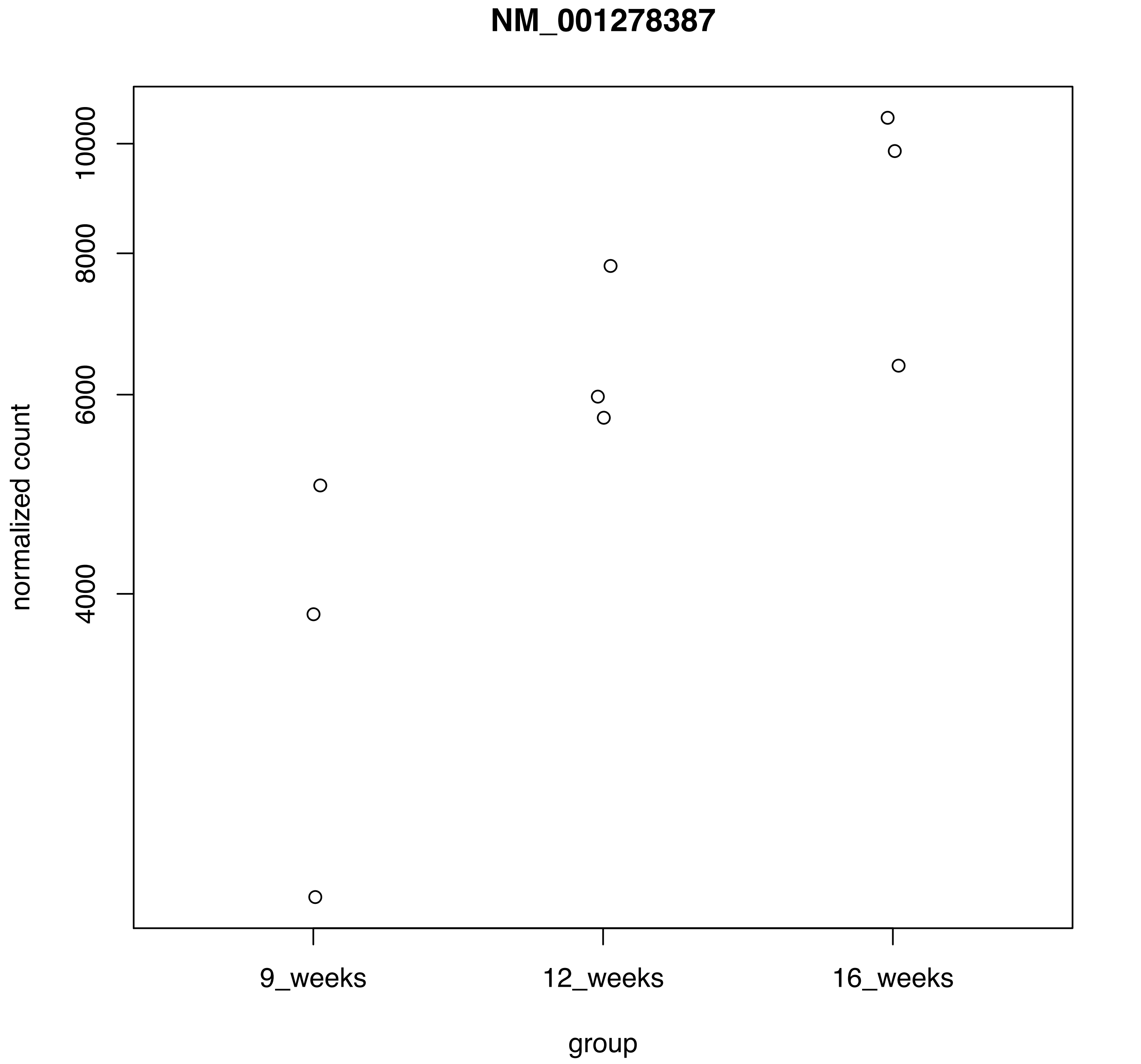
NM_001278387
normalized count
4000
6000
8000
10000
9_weeks
12_weeks
16_weeks
group

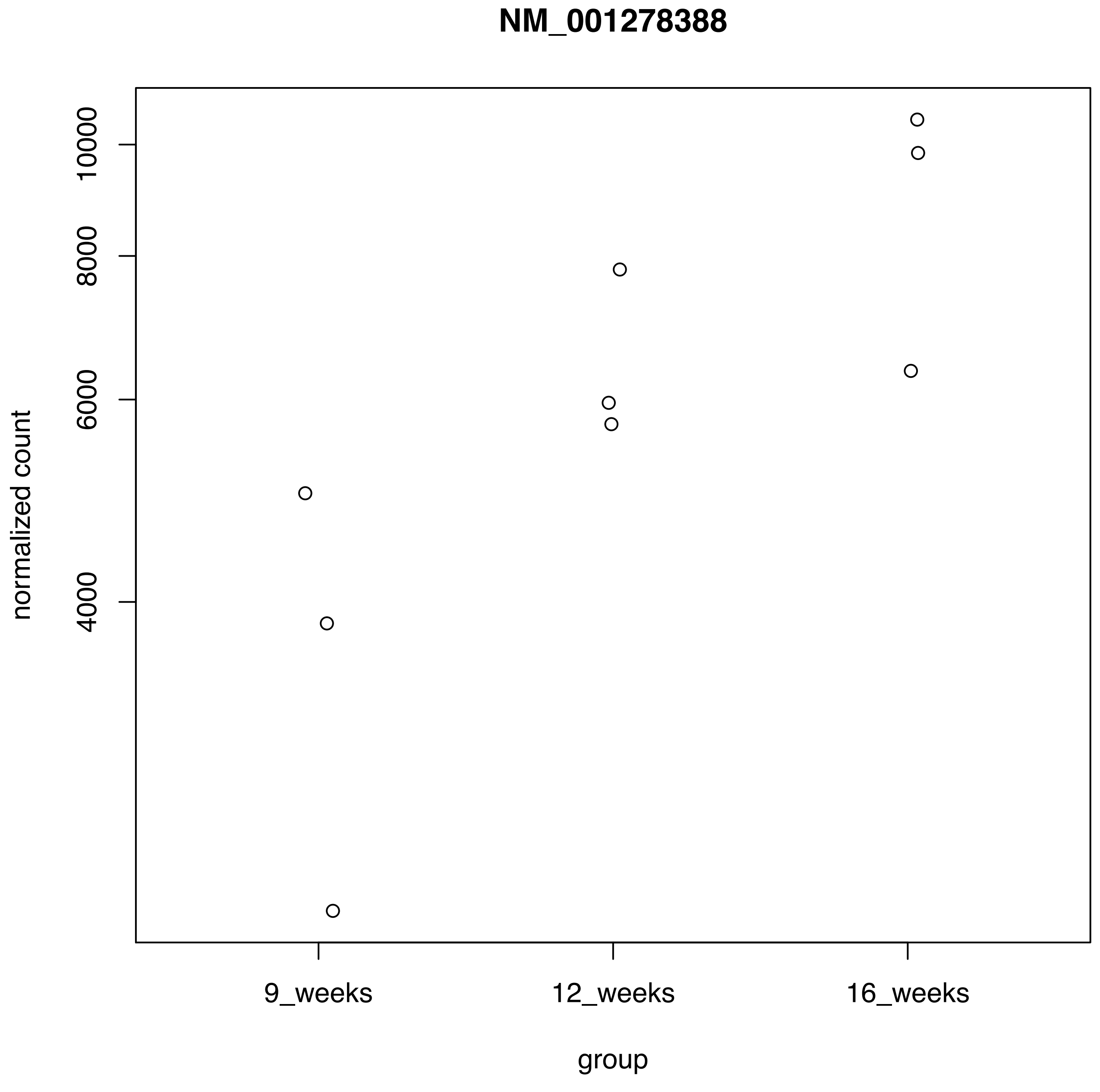
NM_001278388
normalized count
10000
8000
6000
4000
9_weeks
12_weeks
16_weeks
group

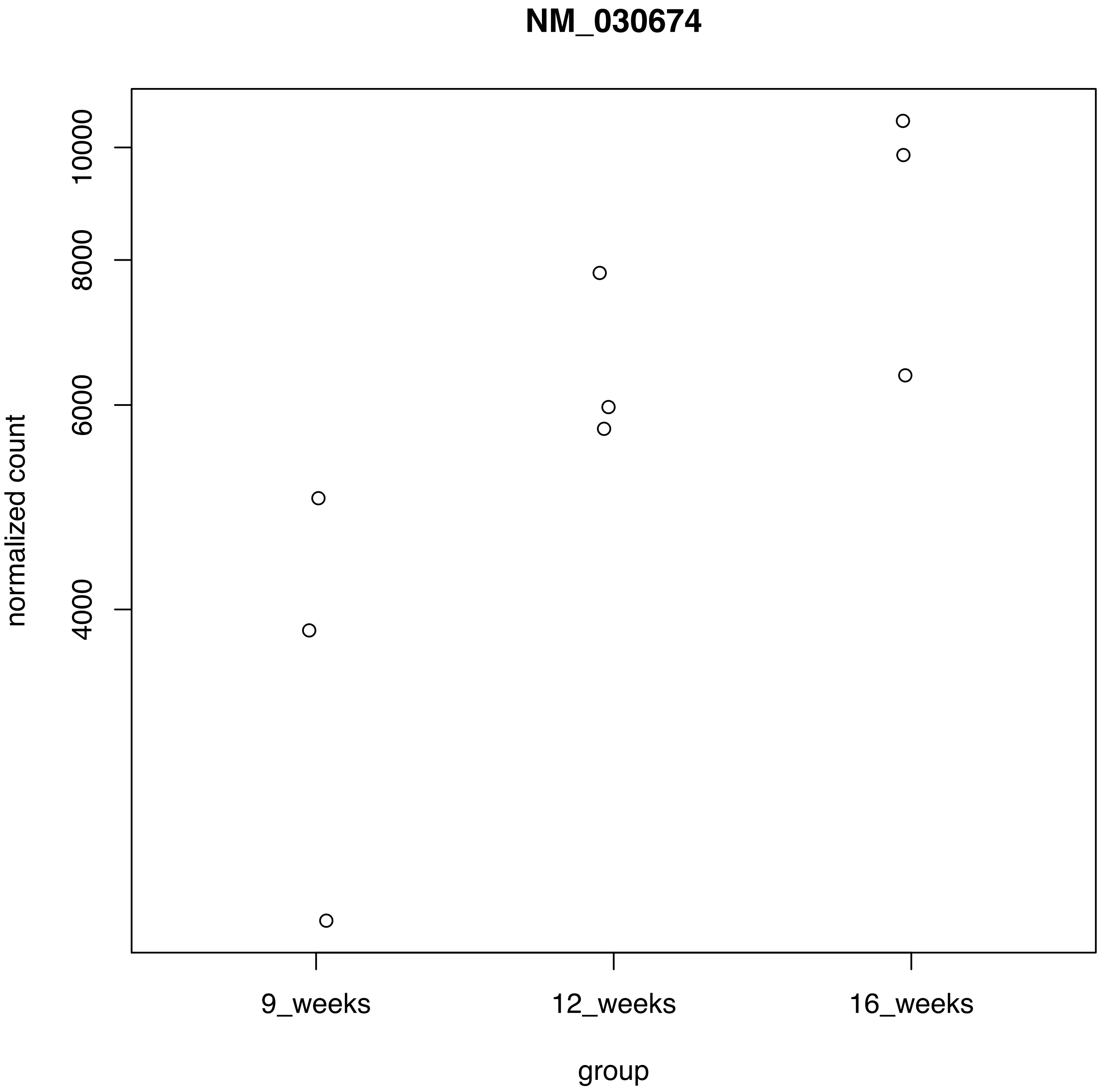
NM_030674
normalized count
10000
8000
6000
4000
9_weeks
12_weeks
16_weeks
group

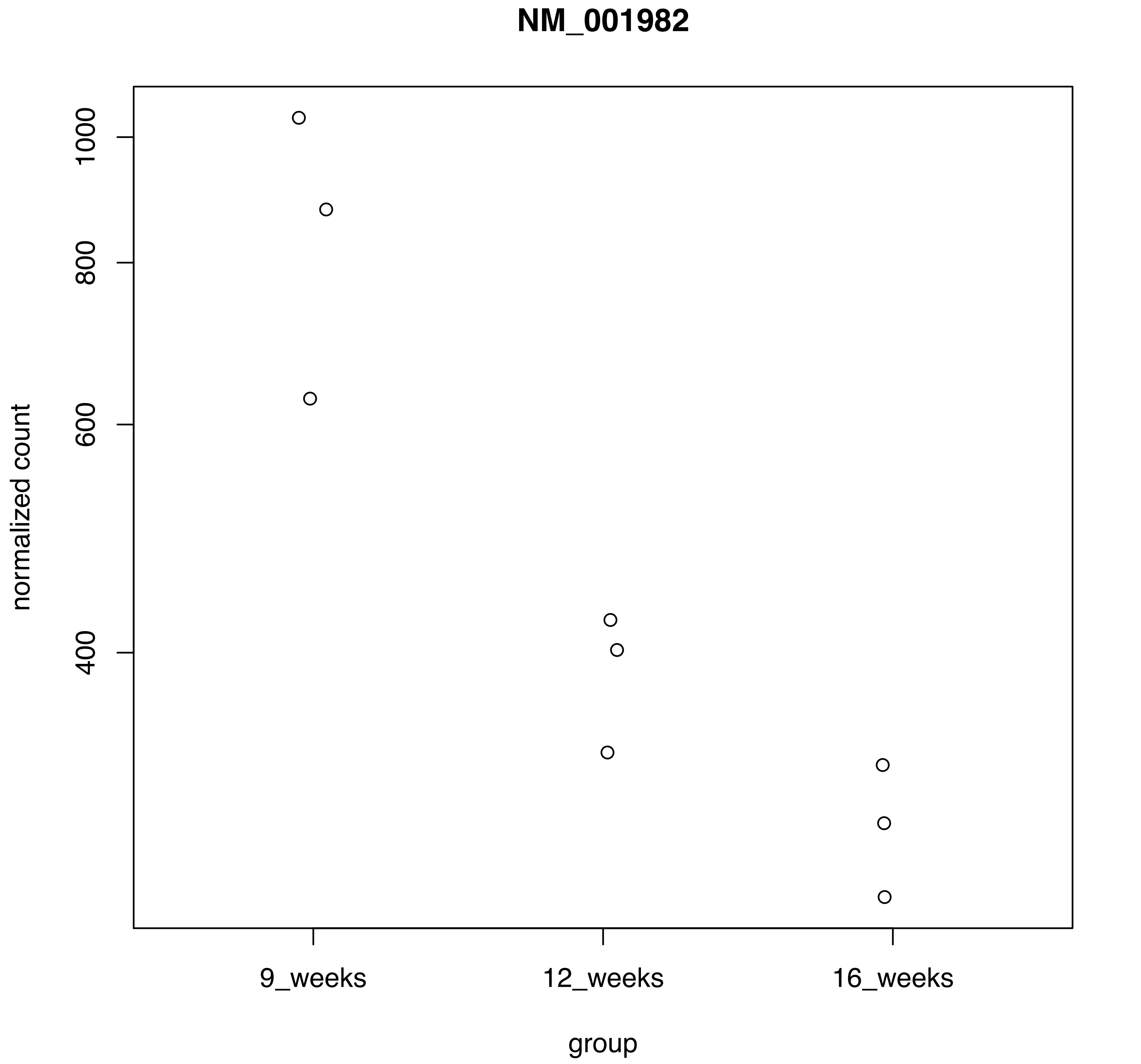
NM_001982
normalized count
1000
800
600
400
9_weeks
12_weeks
16_weeks
group

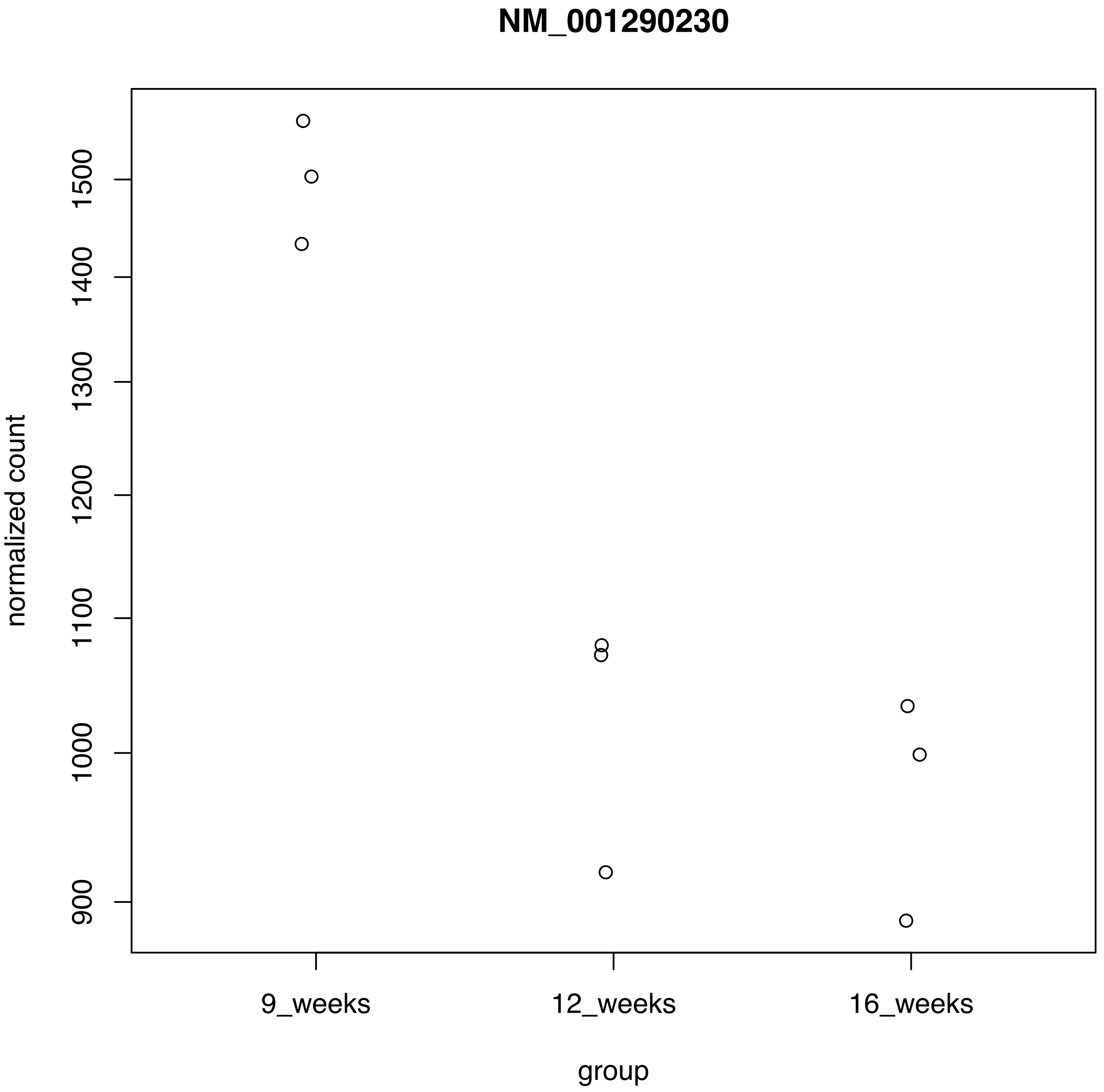
NM_001290230
normalized count
900
1000
1100
1200
1300
1400
1500
9_weeks
12_weeks
16_weeks
group

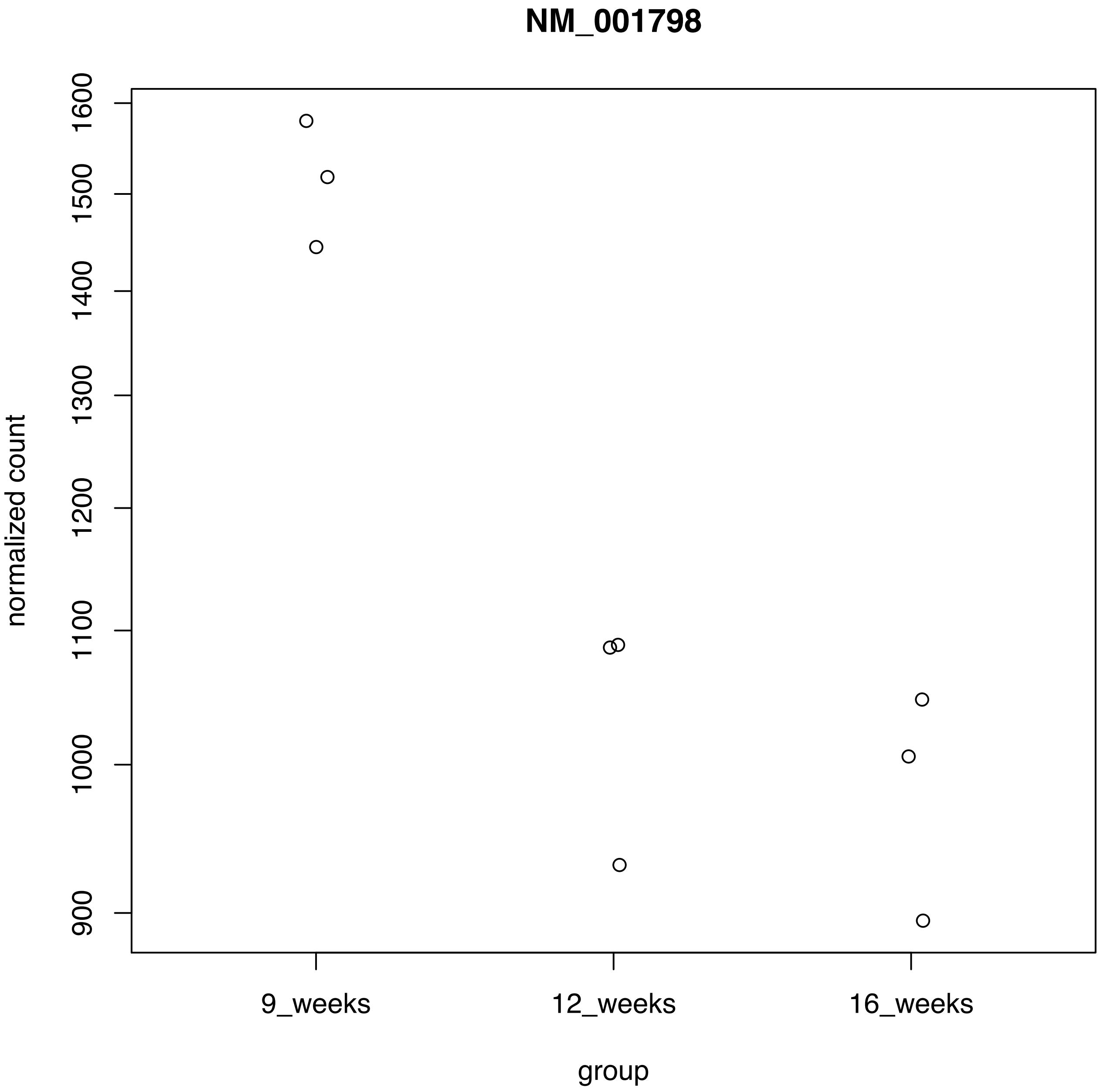

NM_001798
normalized count
1600
1500
1400
1300
1200
1100
1000
900
9_weeks
12_weeks
16_weeks
group

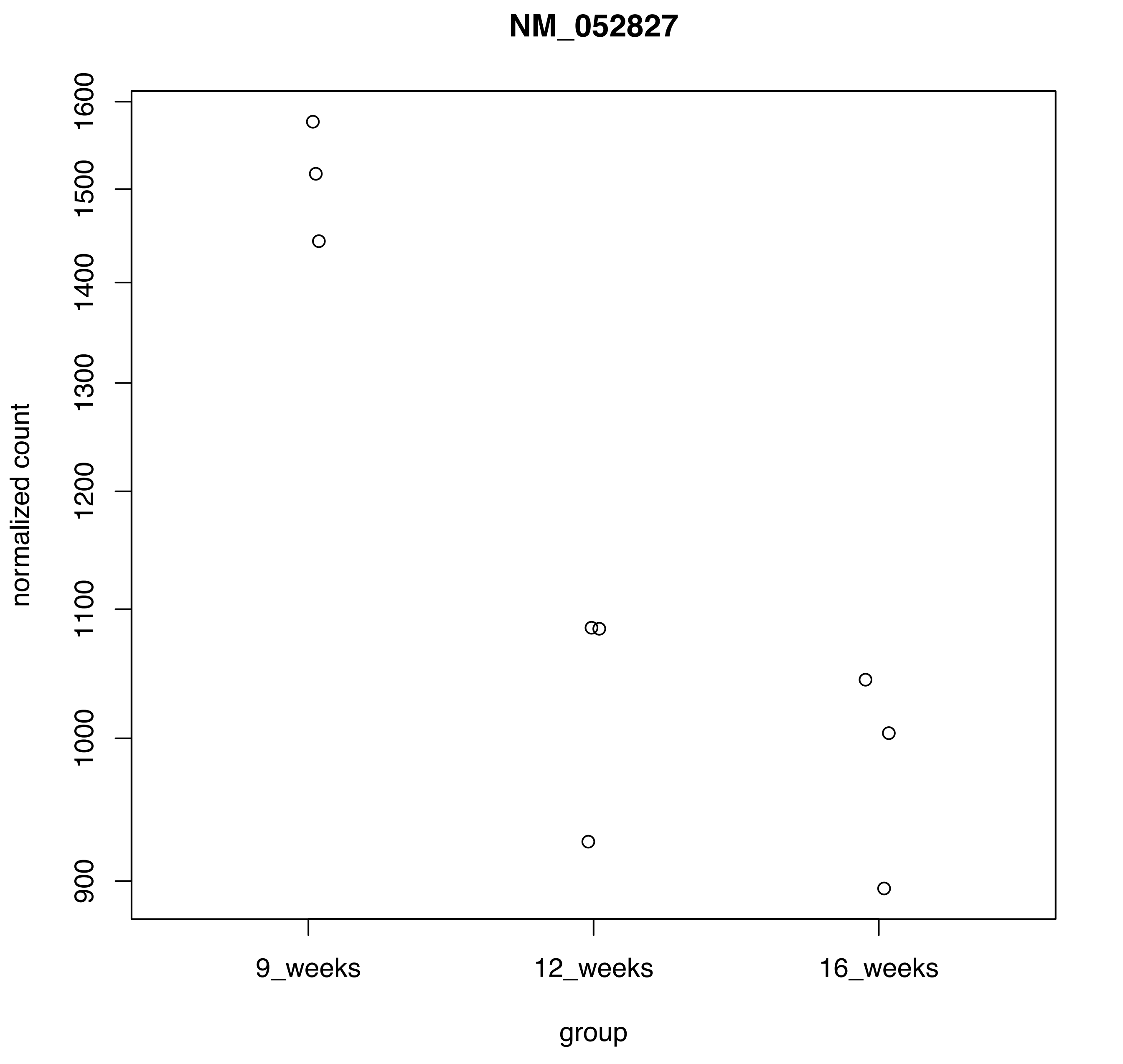
NM_052827
normalized count
1600
1500
1400
1300
1200
1100
1000
900
9_weeks
12_weeks
16_weeks
group

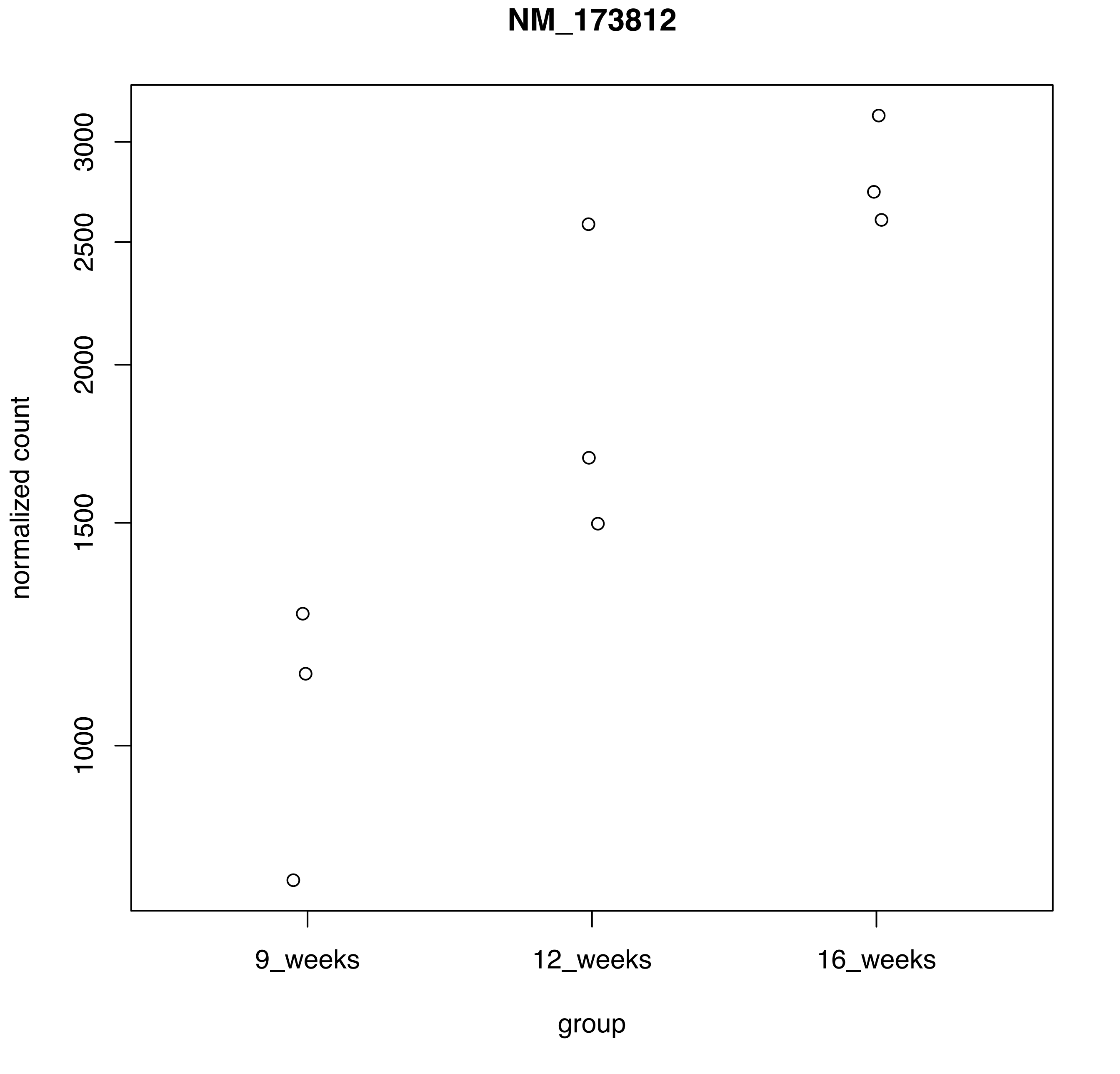
NM_173812
normalized count
3000
2500
2000
1500
1000
9_weeks
12_weeks
16_weeks
group

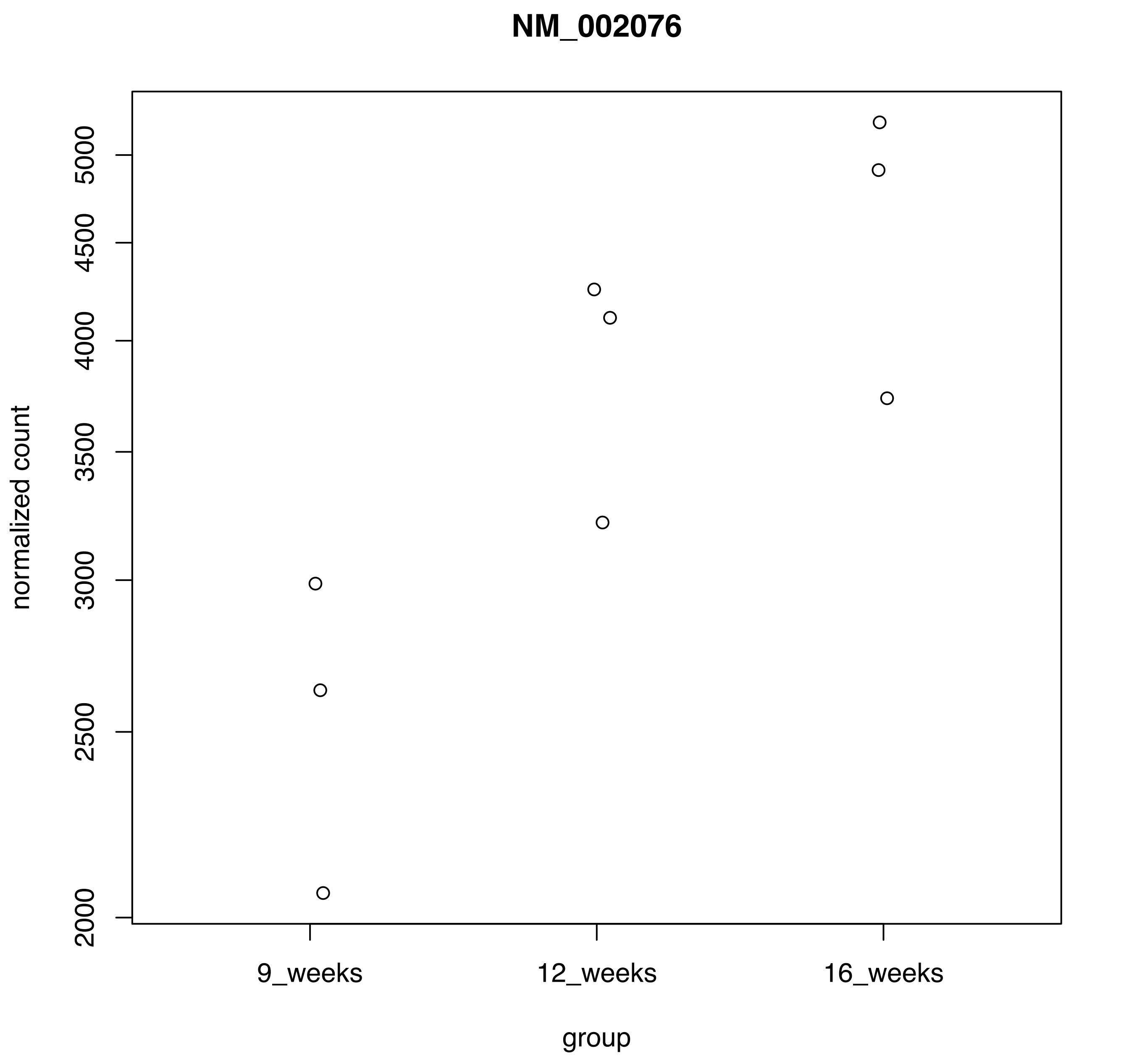
NM_002076
normalized count
5000
4500
4000
3500
3000
2500
2000
9_weeks
12_weeks
16_weeks
group

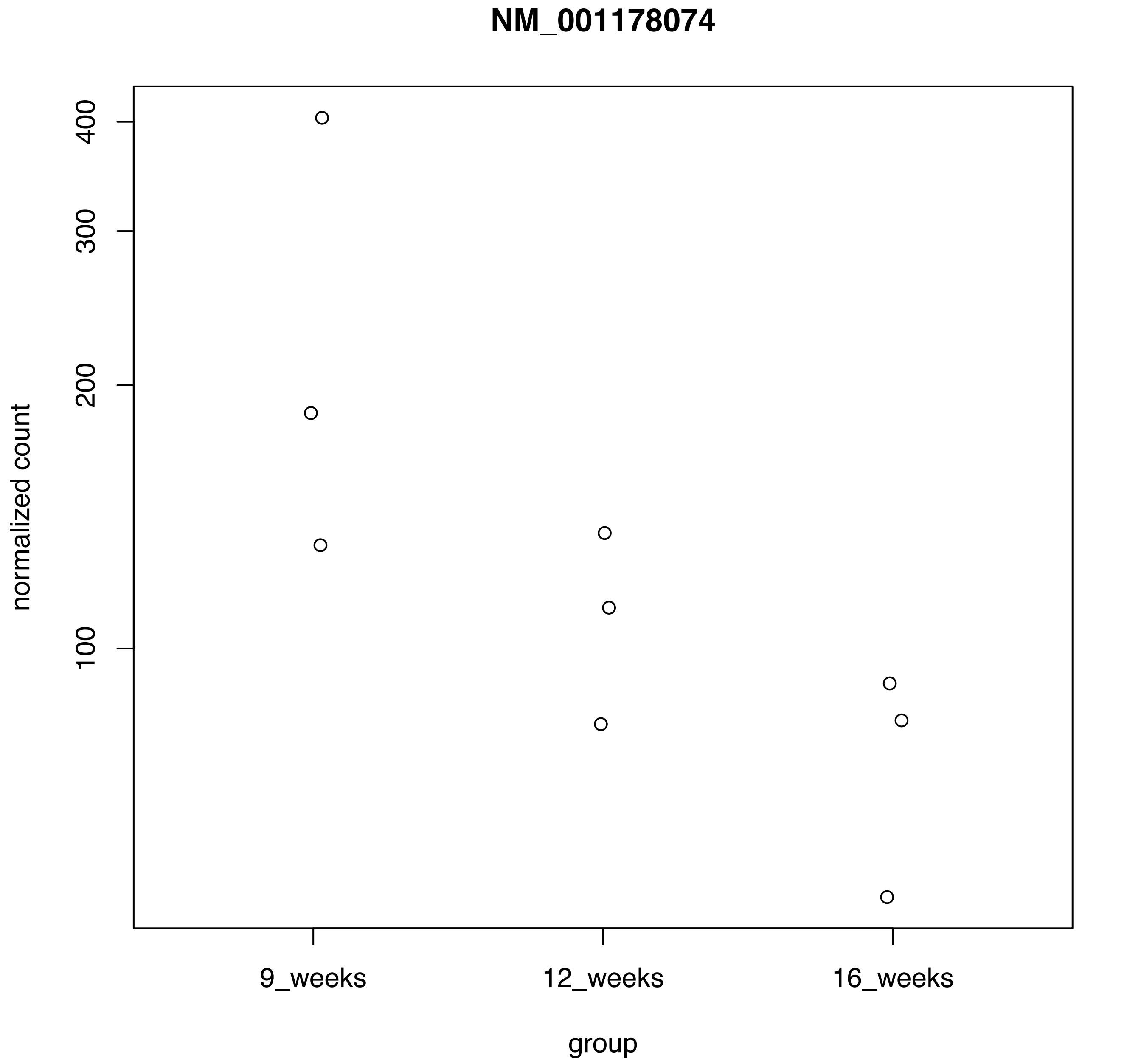

NM_001178074
normalized count
400
300
200
100
9_weeks
12_weeks
16_weeks
group

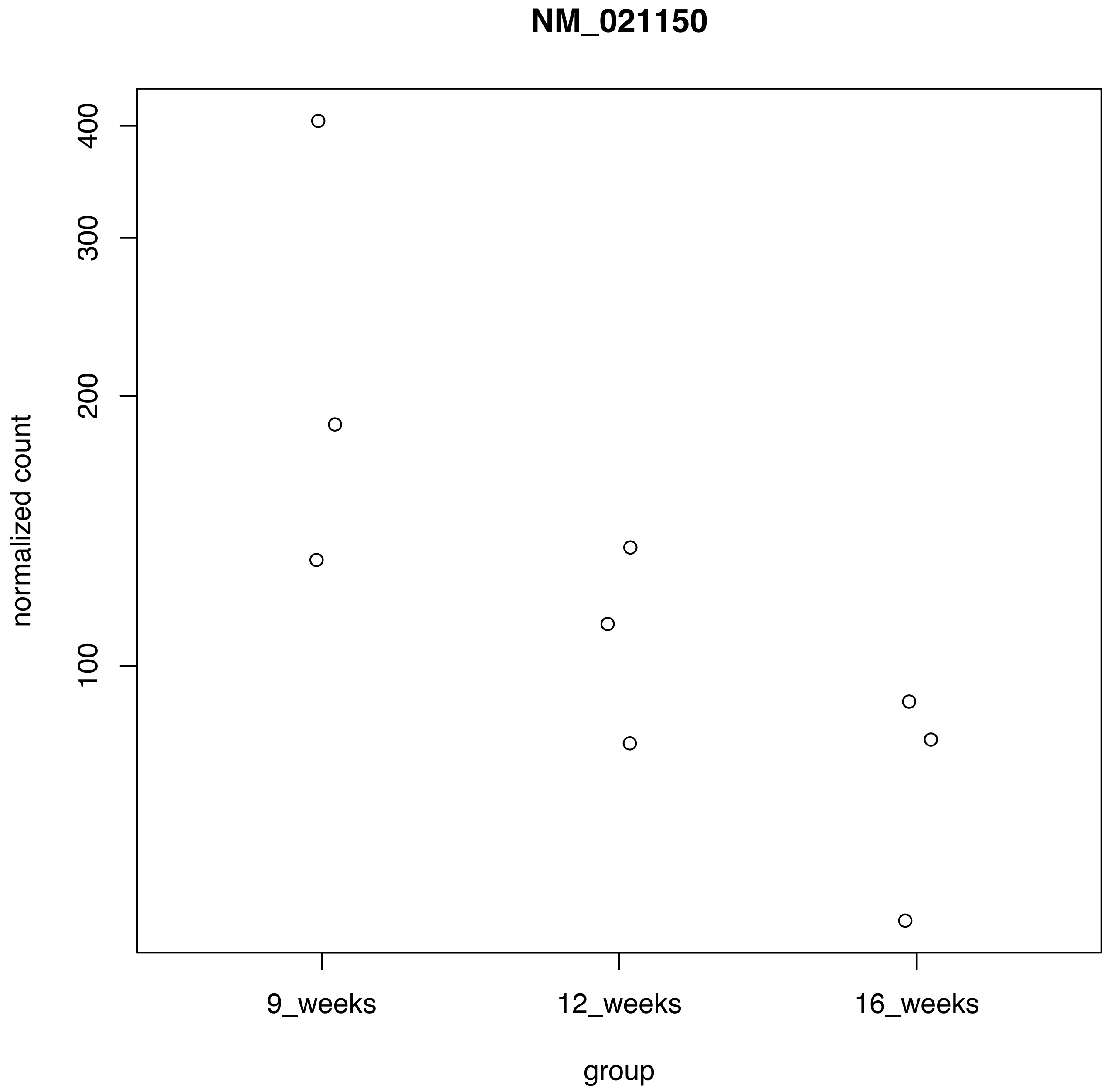
NM_021150
normalized count
400
300
200
100
9_weeks
12_weeks
16_weeks
group

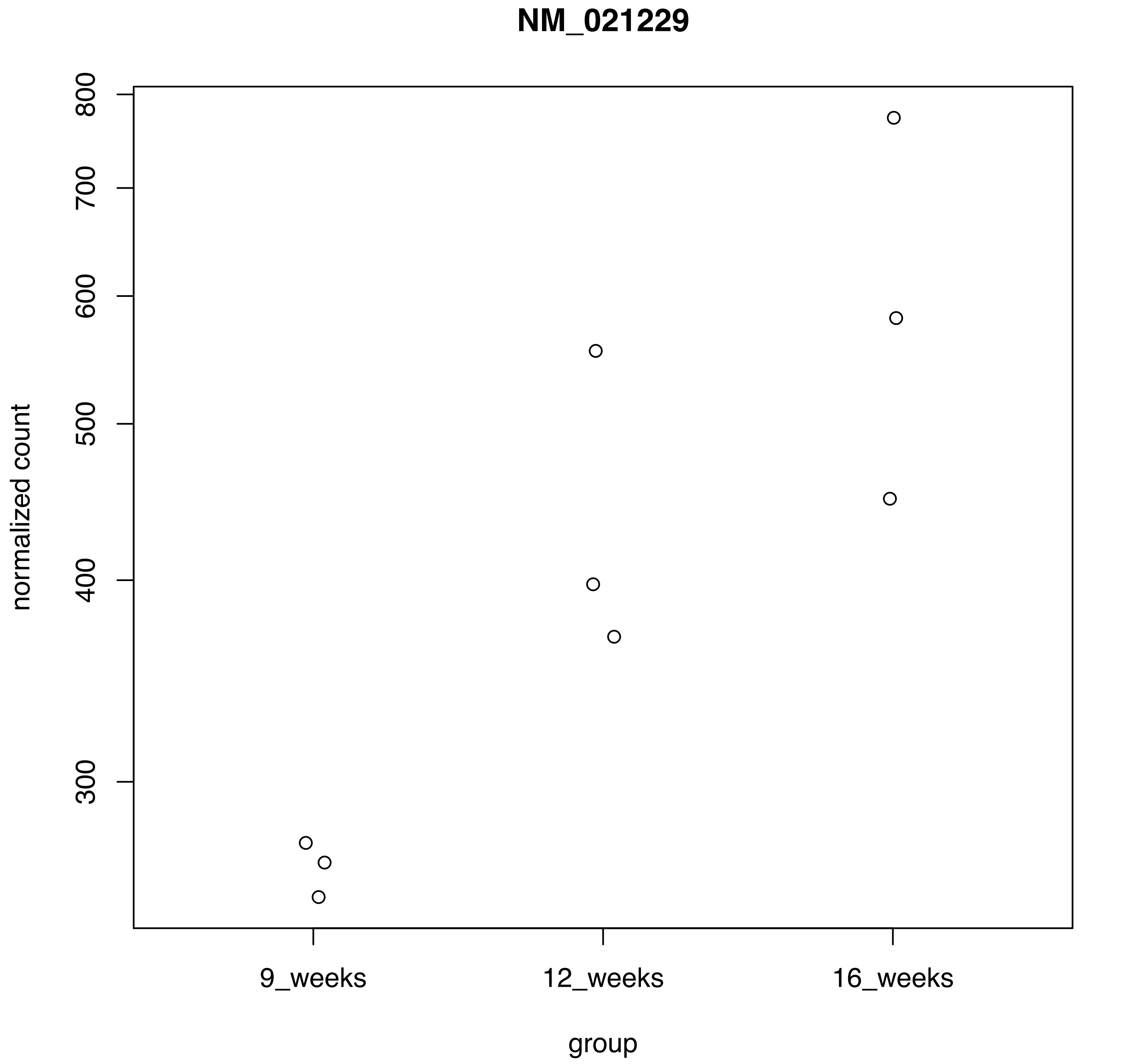

NM_021229
normalized count
800
700
600
500
400
300
9_weeks
12_weeks
16_weeks
group

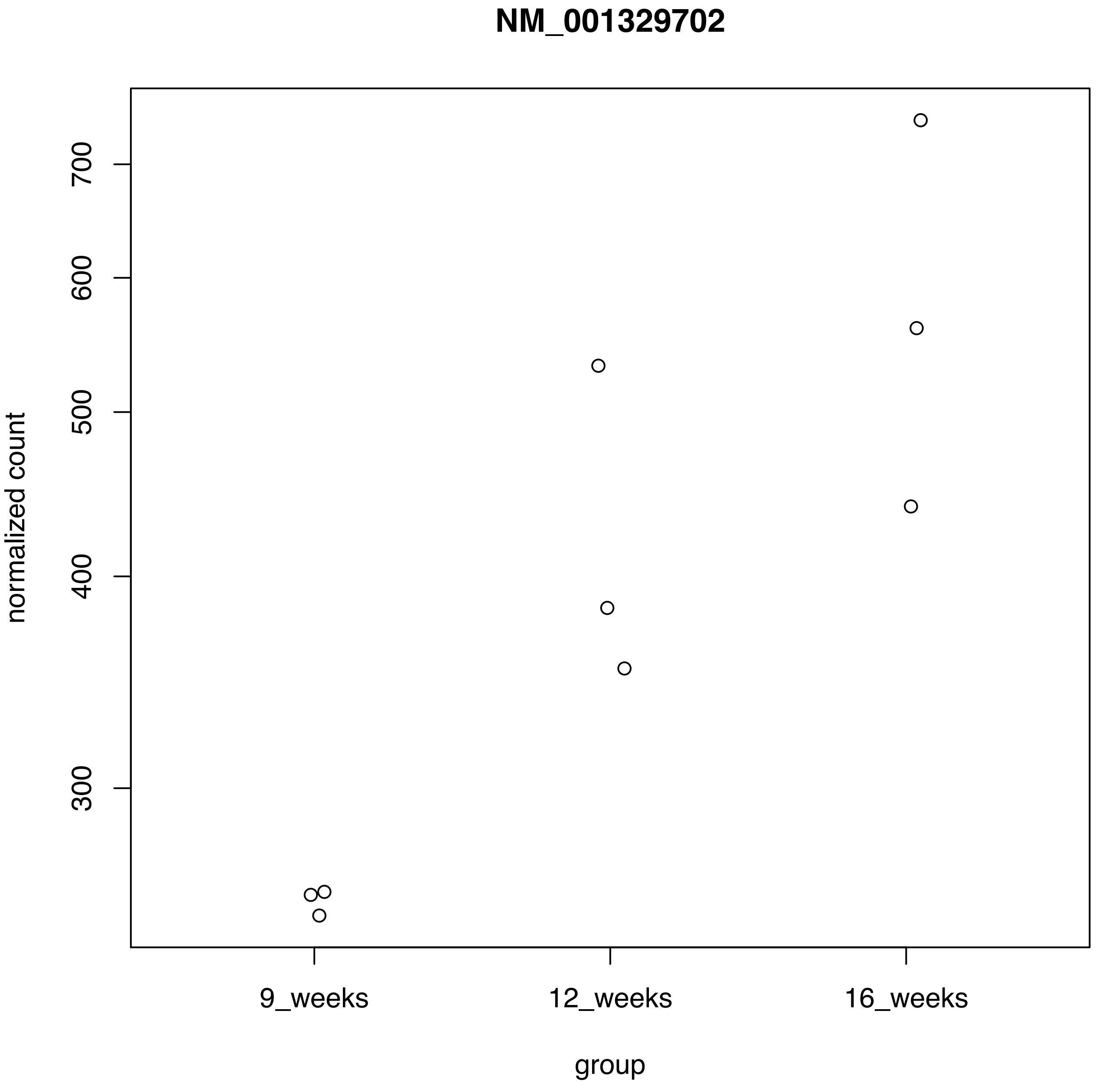
NM_001329702
normalized count
300
400
500
600
700
group
9_weeks
12_weeks
16_weeks

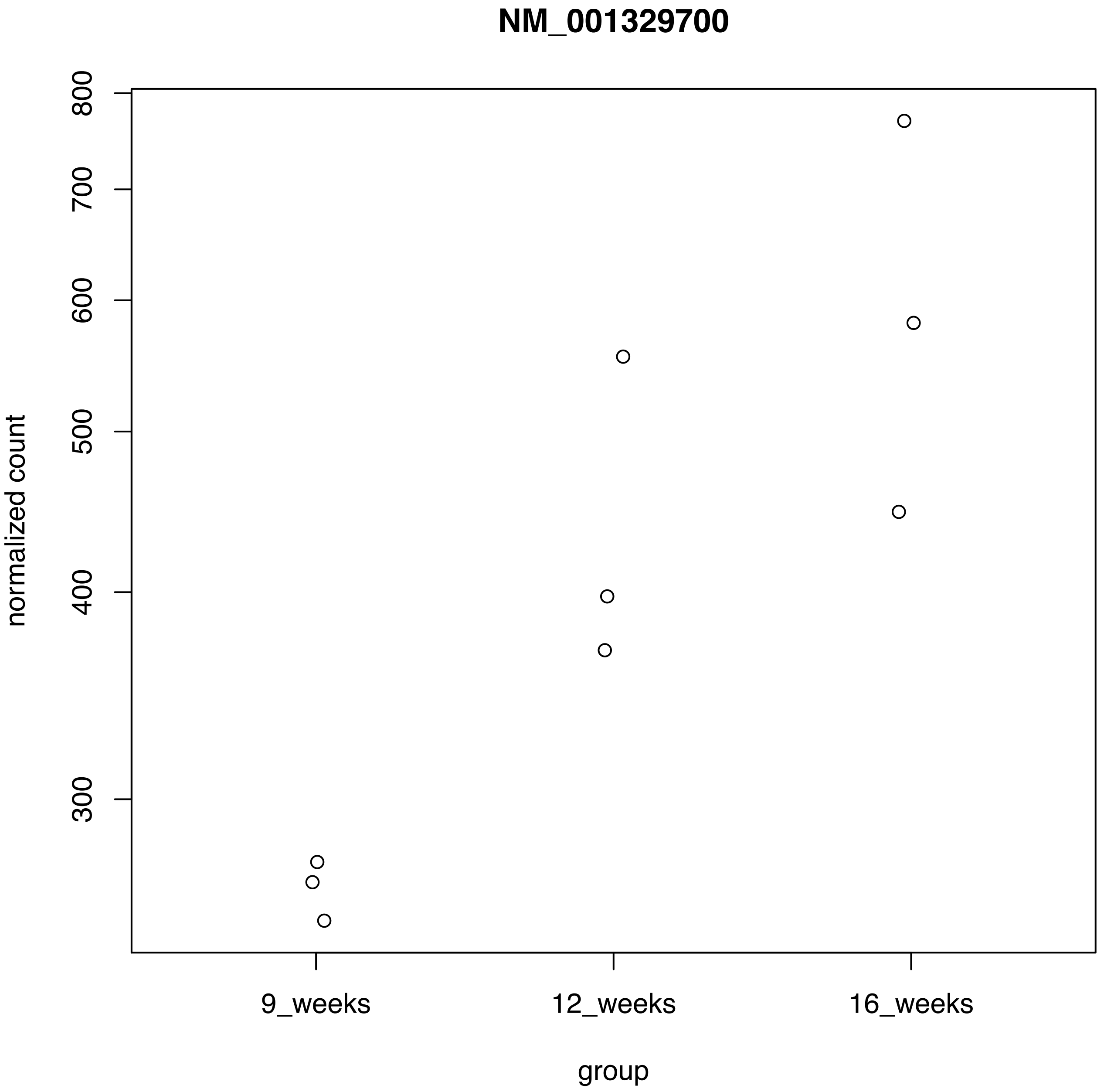

NM_001329700
normalized count
800
700
600
500
400
300
9_weeks
12_weeks
16_weeks
group

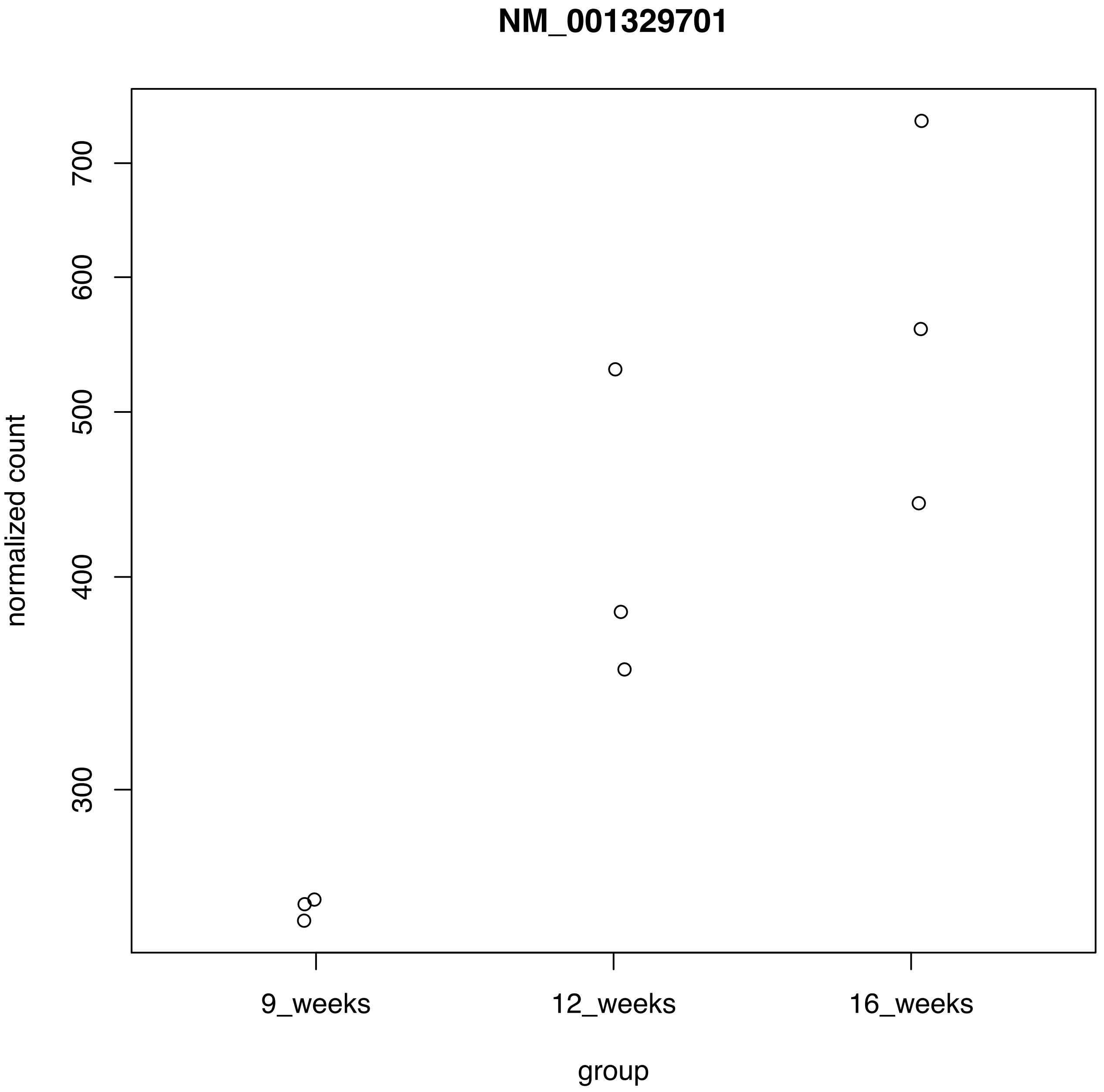

NM_001329701
normalized count
300
400
500
600
700
9_weeks
12_weeks
16_weeks
group

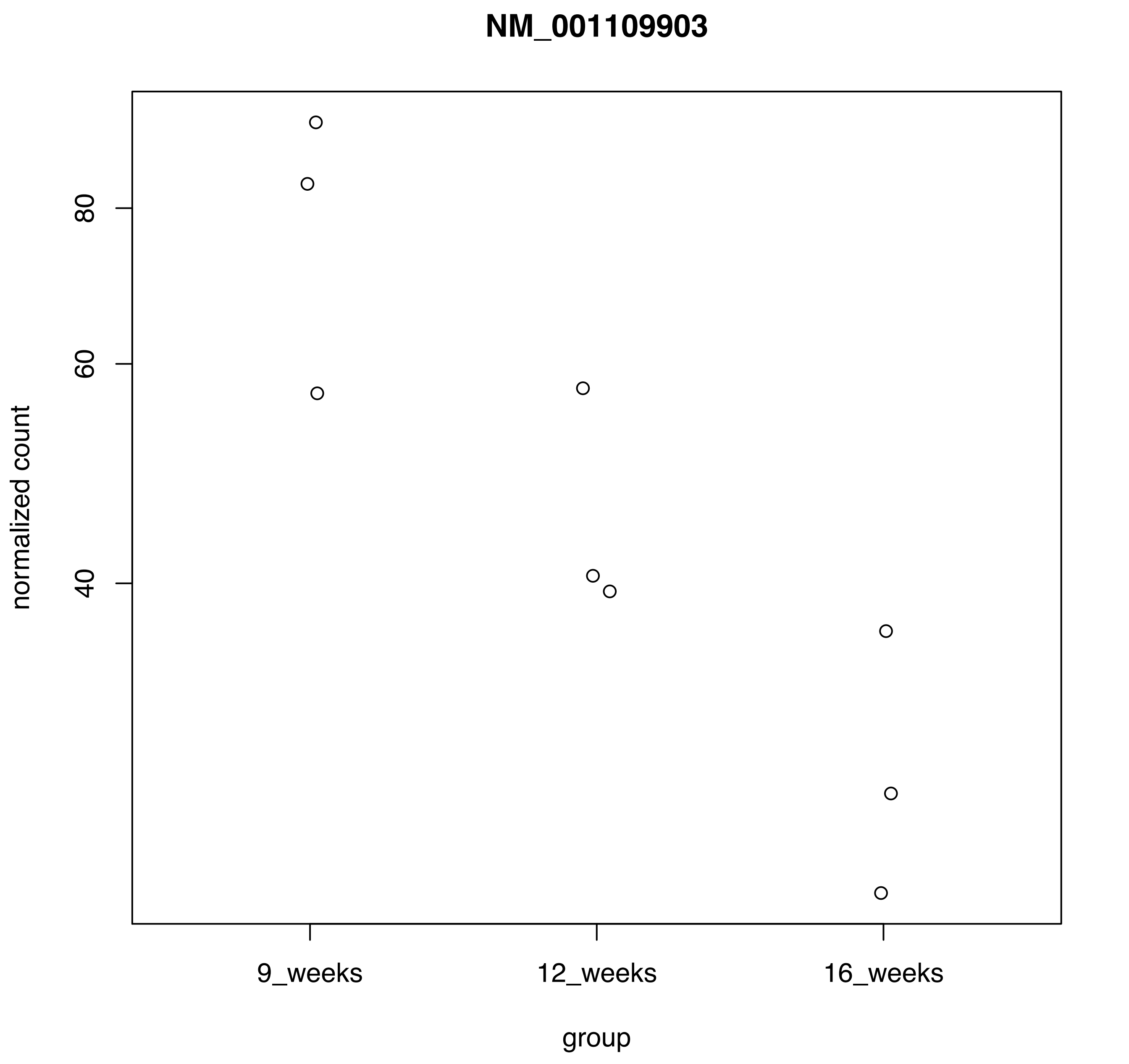
NM_001109903
80
60
40
normalized count
9_weeks
12_weeks
16_weeks
group

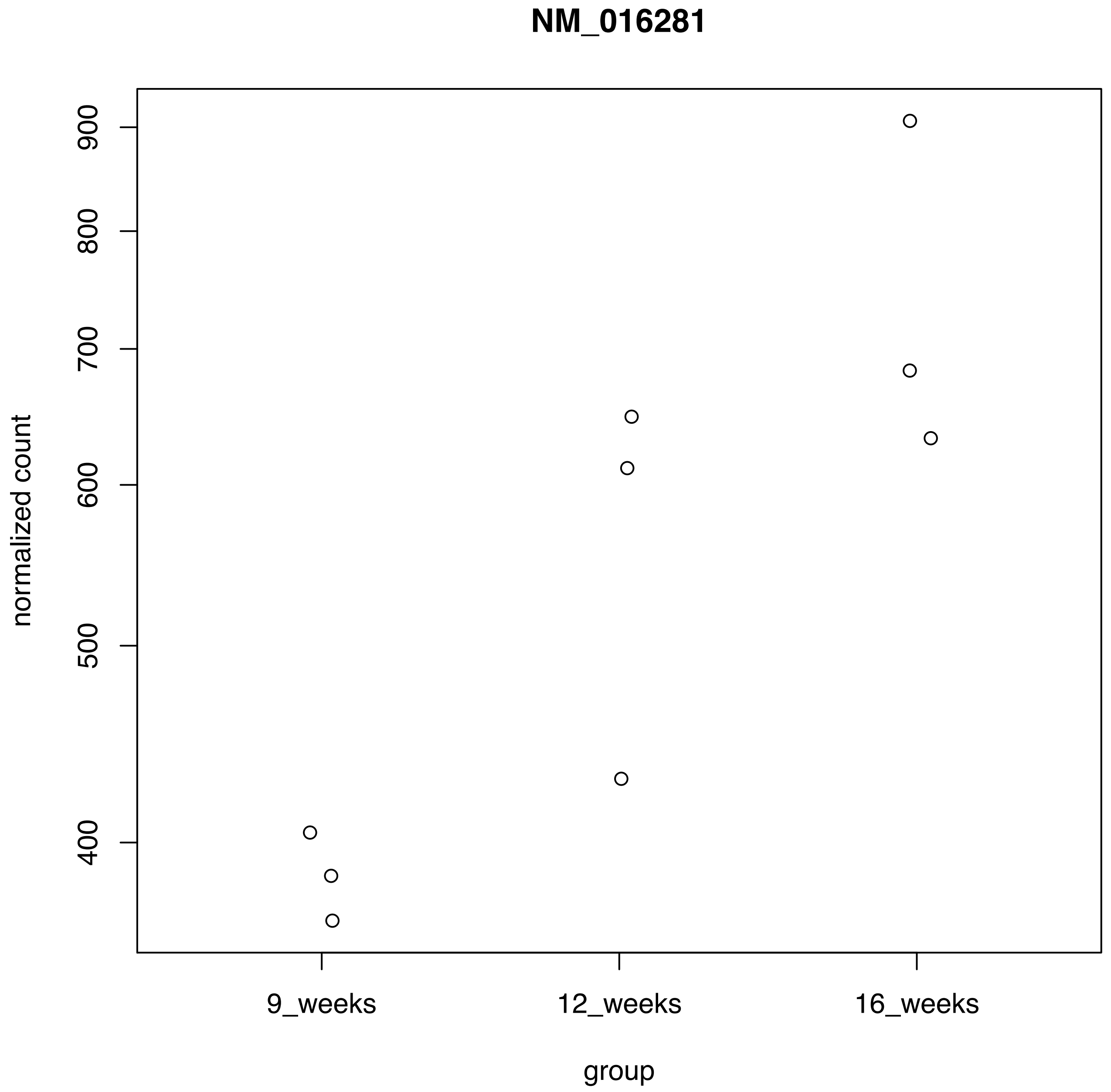
NM_016281
normalized count
400
500
600
700
800
900
group
9_weeks
12_weeks
16_weeks

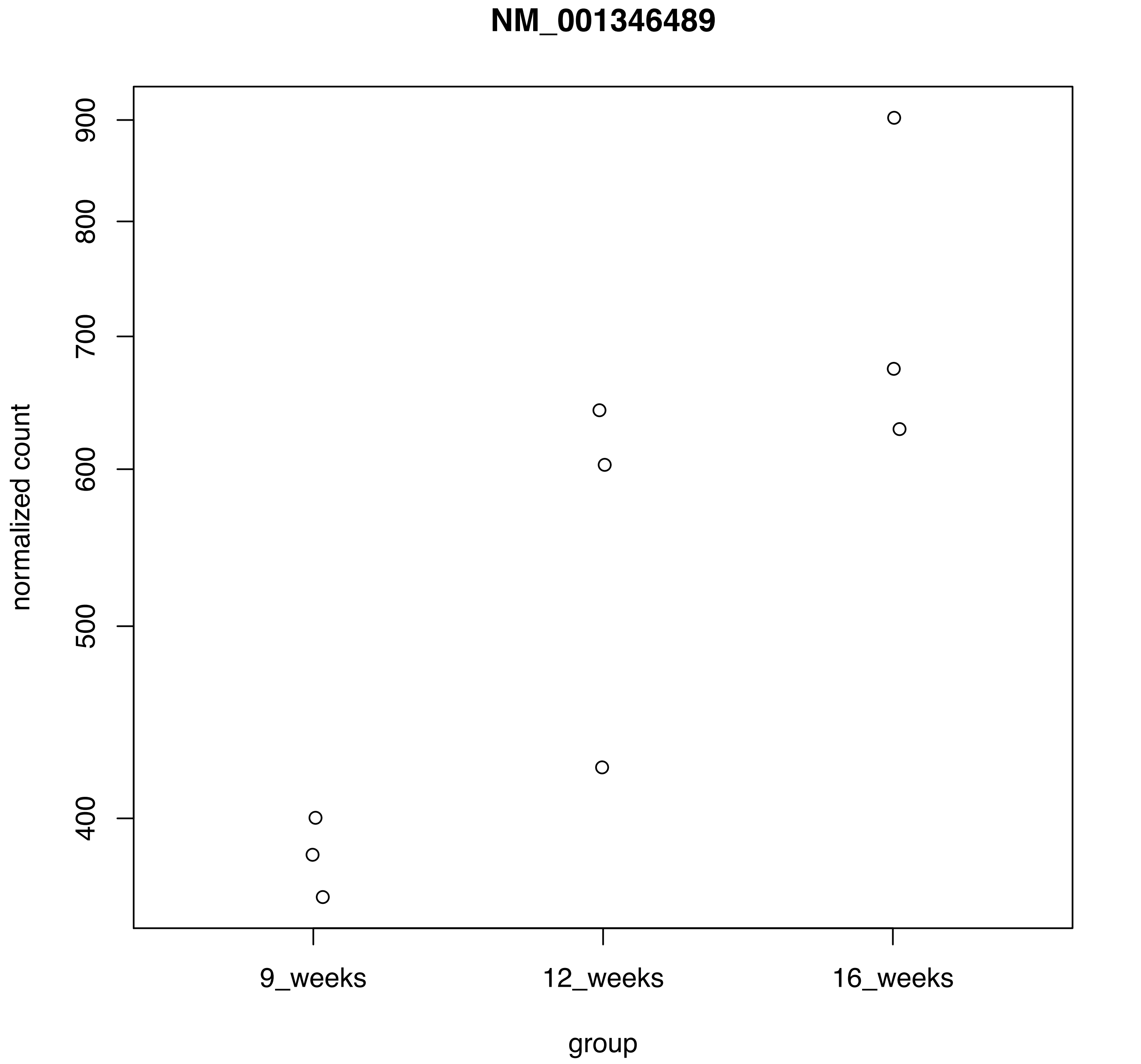

NM_001346489
normalized count
900
800
700
600
500
400
9_weeks
12_weeks
16_weeks
group

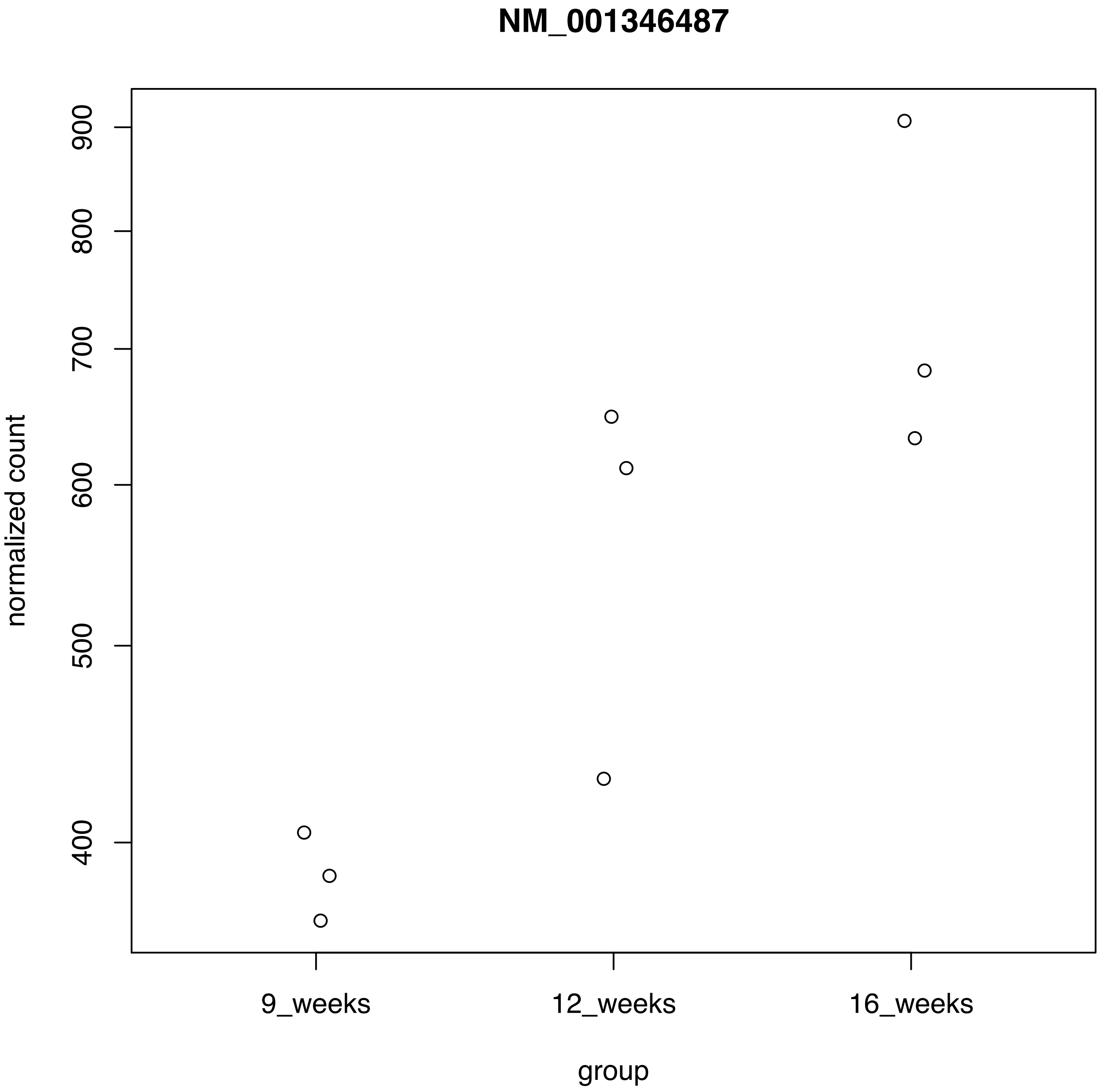

NM_001346487
normalized count
900
800
700
600
500
400
9_weeks
12_weeks
16_weeks
group

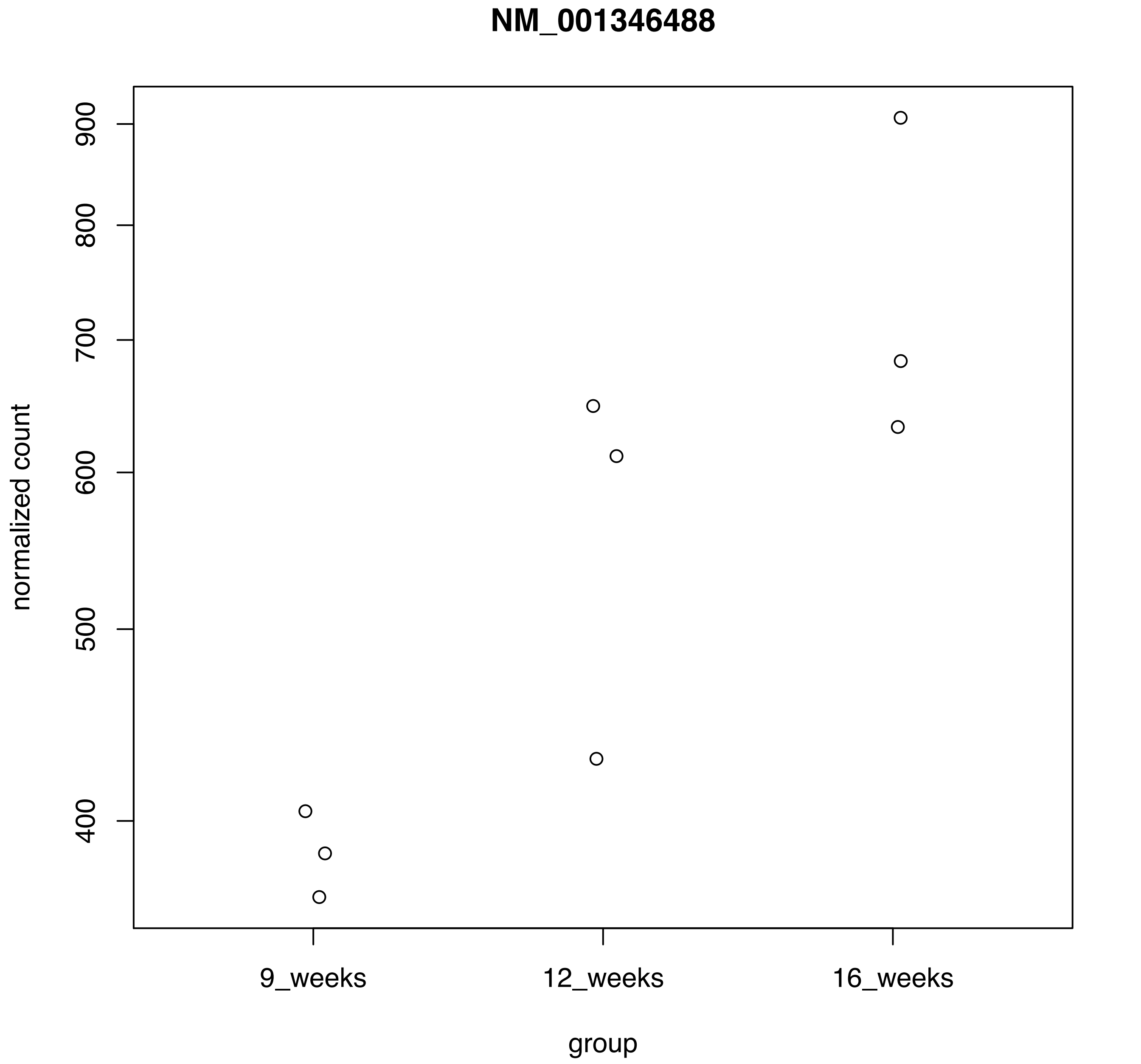
NM_001346488
normalized count
900
800
700
600
500
400
9_weeks
12_weeks
16_weeks
group

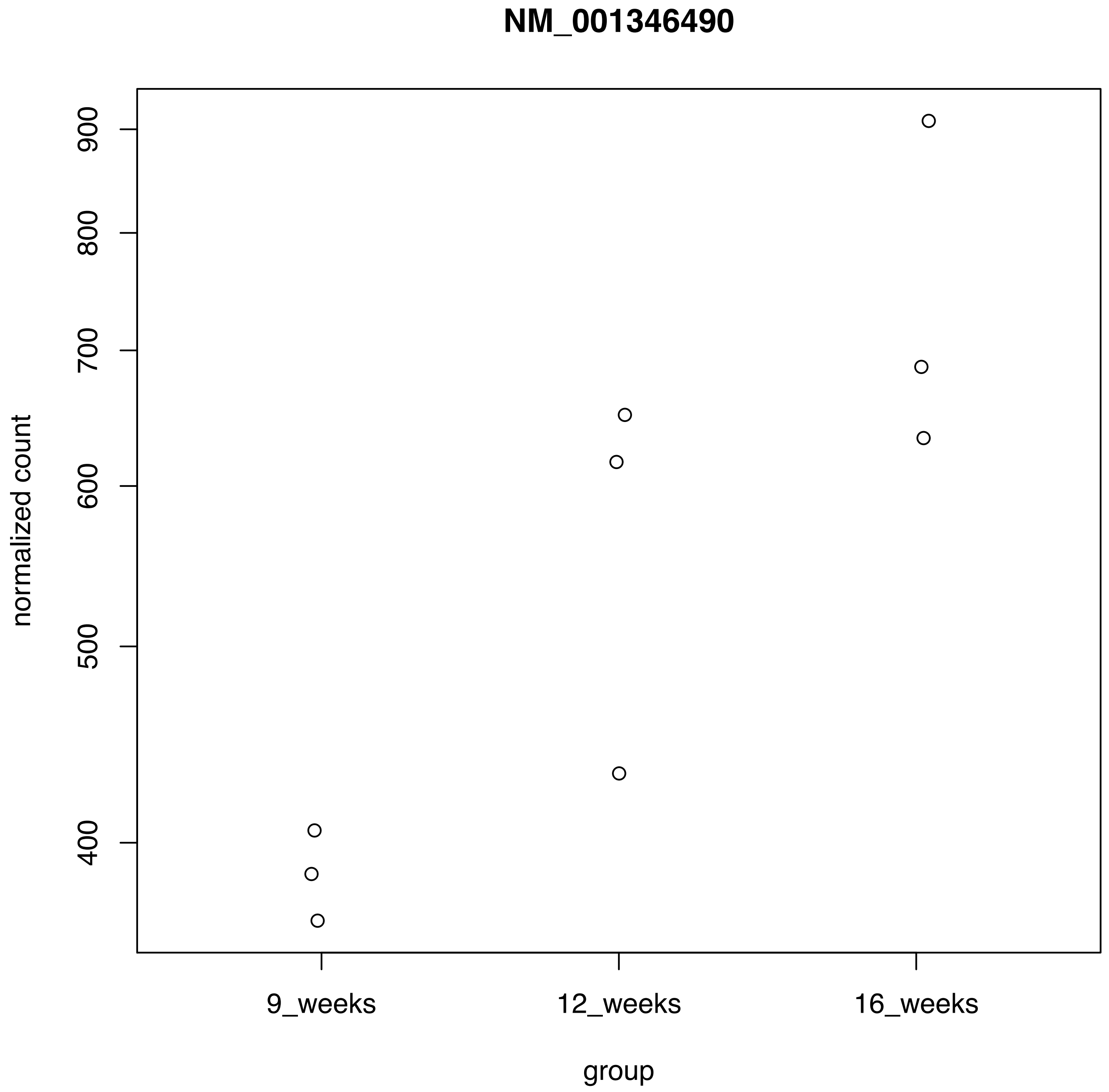
NM_001346490
normalized count
900
800
700
600
500
400
9_weeks
12_weeks
16_weeks
group

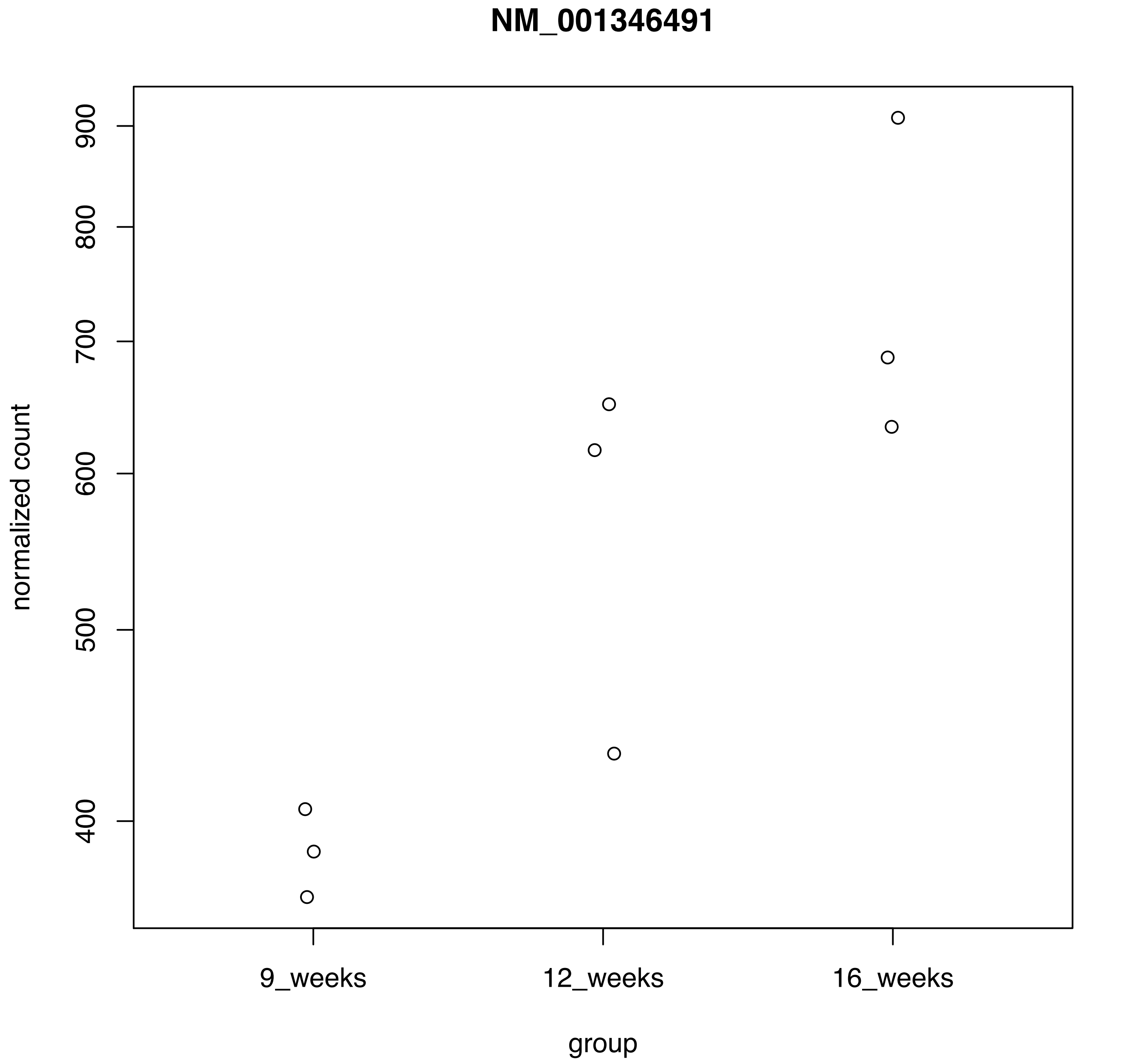
NM_001346491
normalized count
900
800
700
600
500
400
9_weeks
12_weeks
16_weeks
group

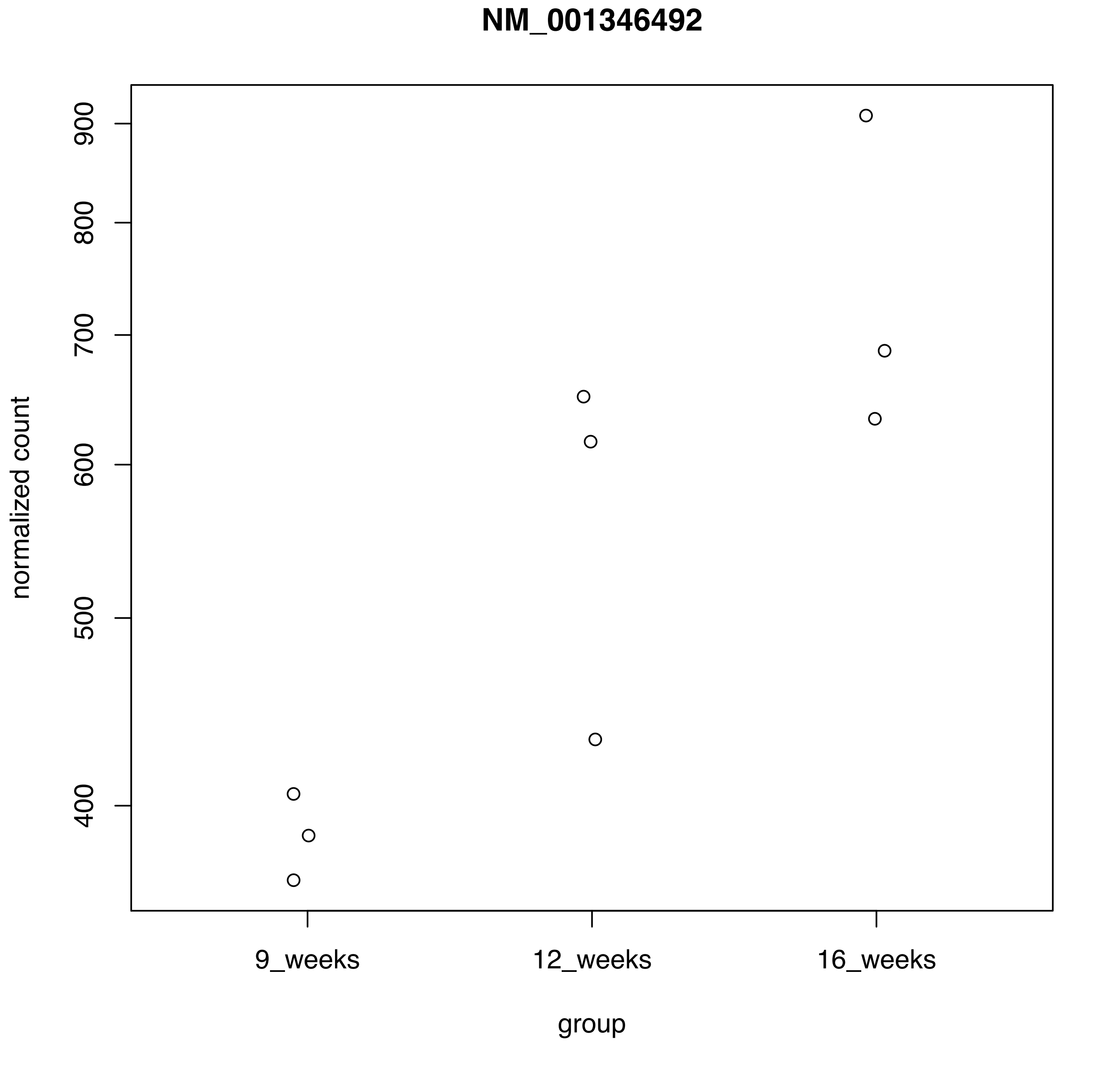

NM_001346492
normalized count
900
800
700
600
500
400
9_weeks
12_weeks
16_weeks
group

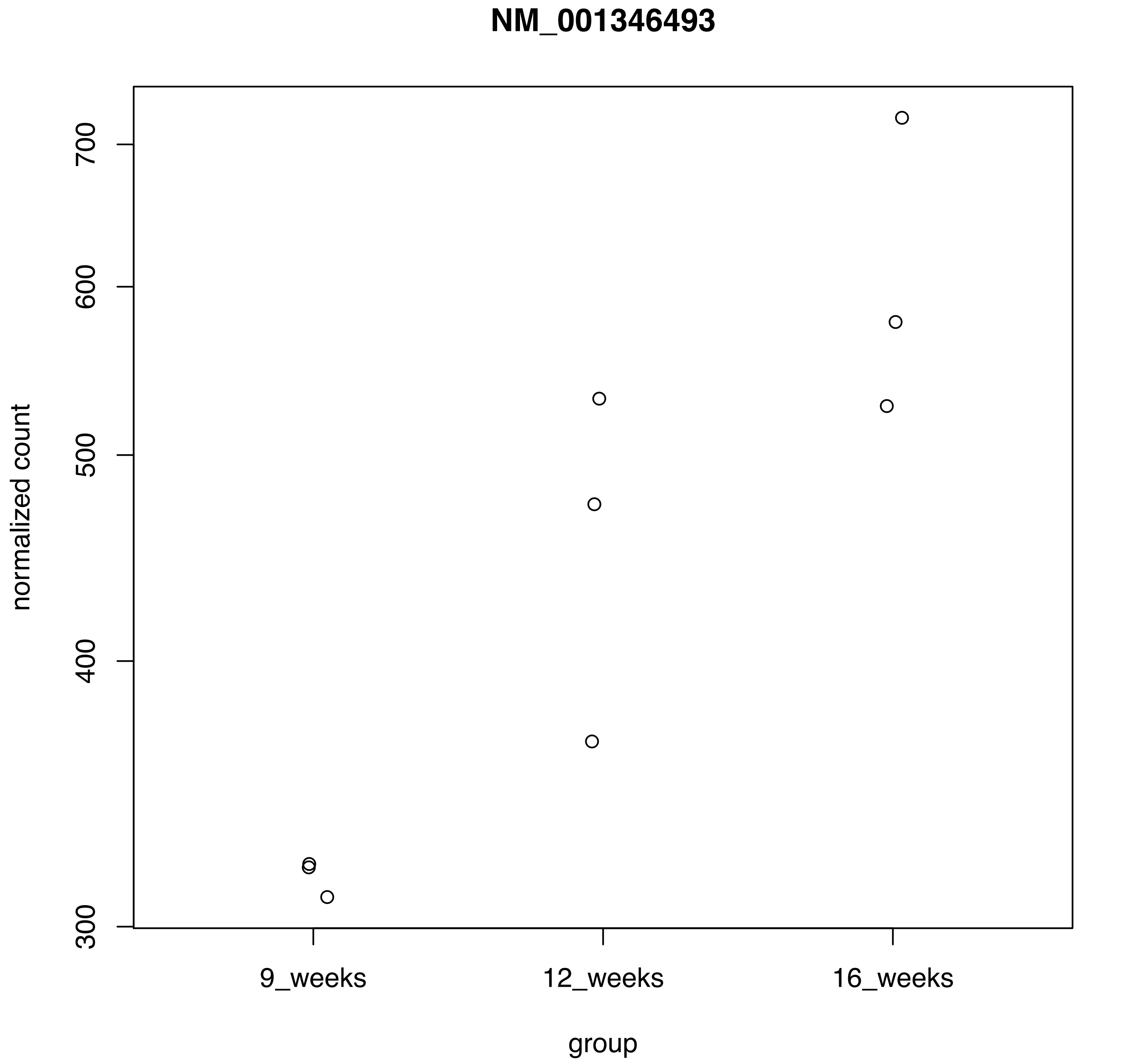
NM_001346493
normalized count
300
400
500
600
700
9_weeks
12_weeks
16_weeks
group

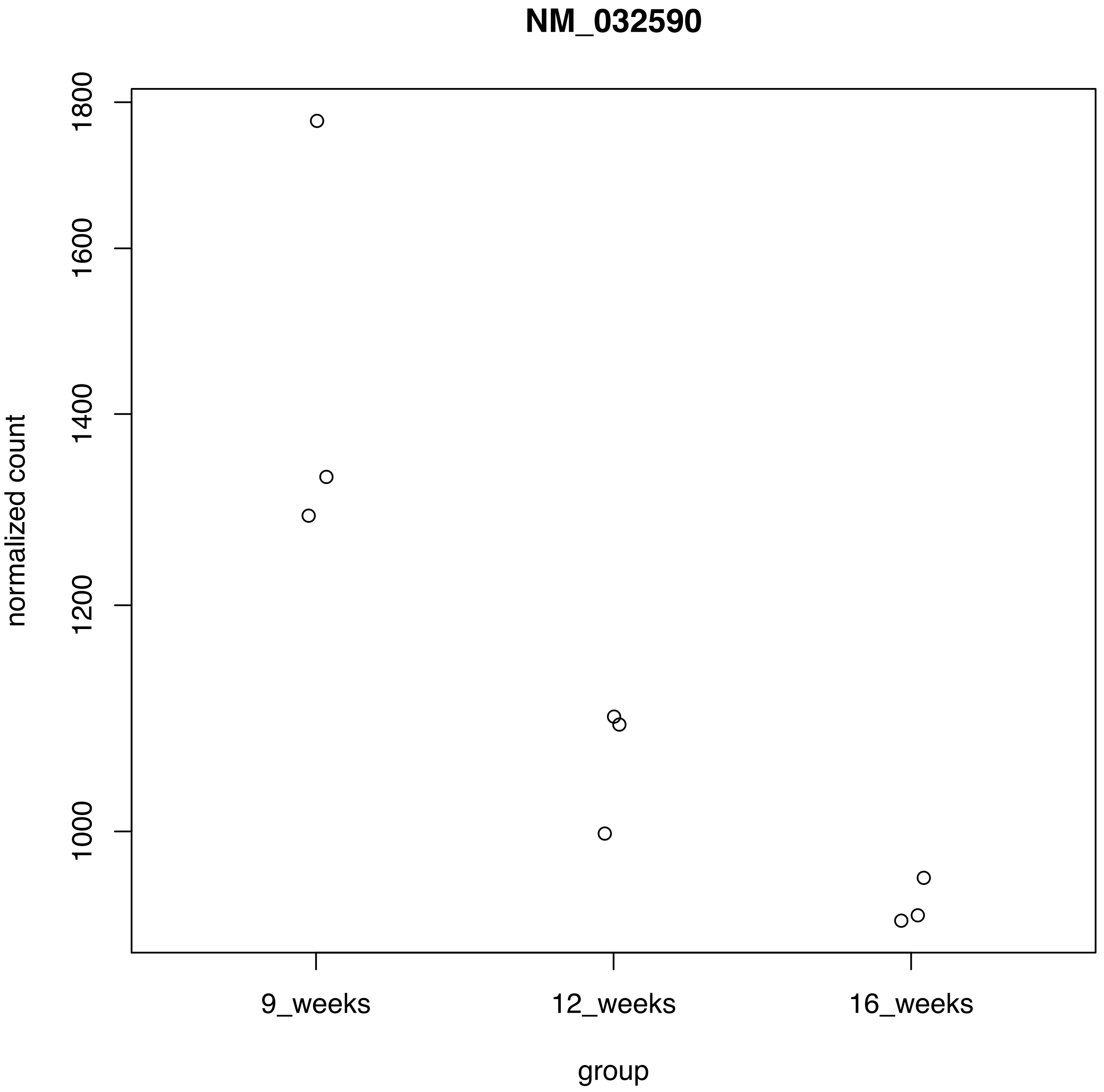
NM_032590
normalized count
1800
1600
1400
1200
1000
9_weeks
12_weeks
16_weeks
group

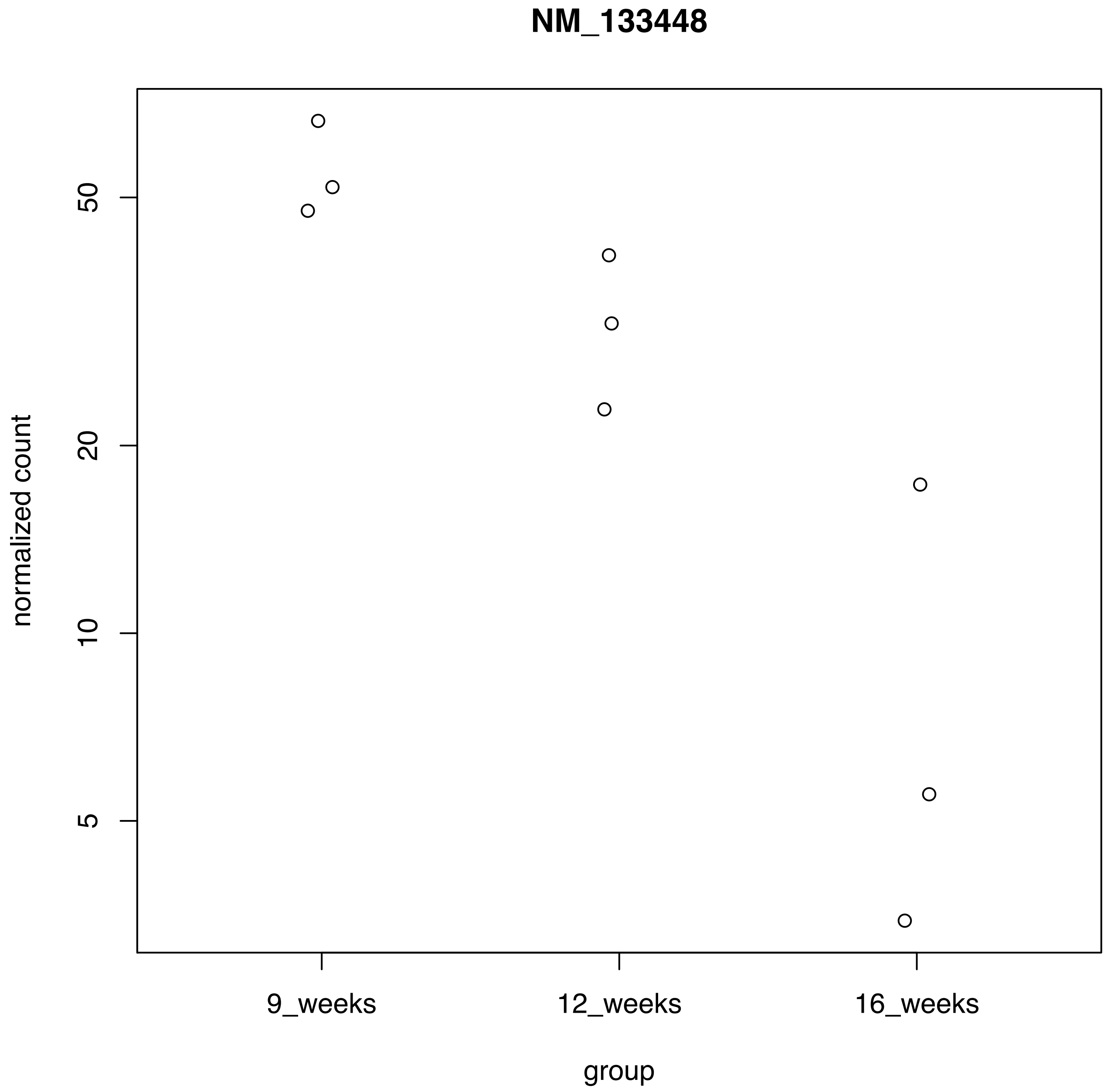
NM_133448
normalized count
50
20
10
5
9_weeks
12_weeks
16_weeks
group

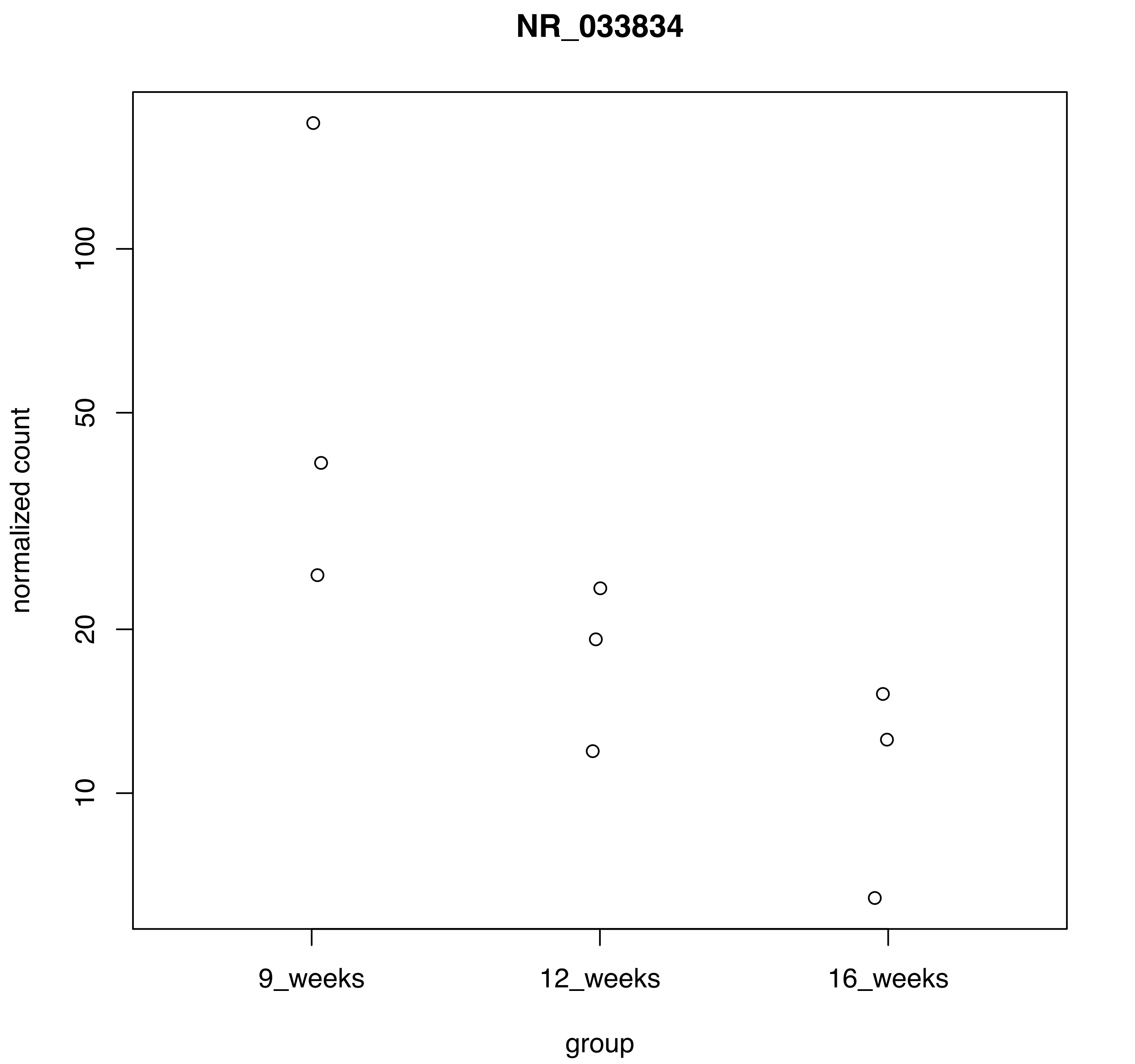
NR_033834
normalized count
100
50
20
10
9_weeks
12_weeks
16_weeks
group

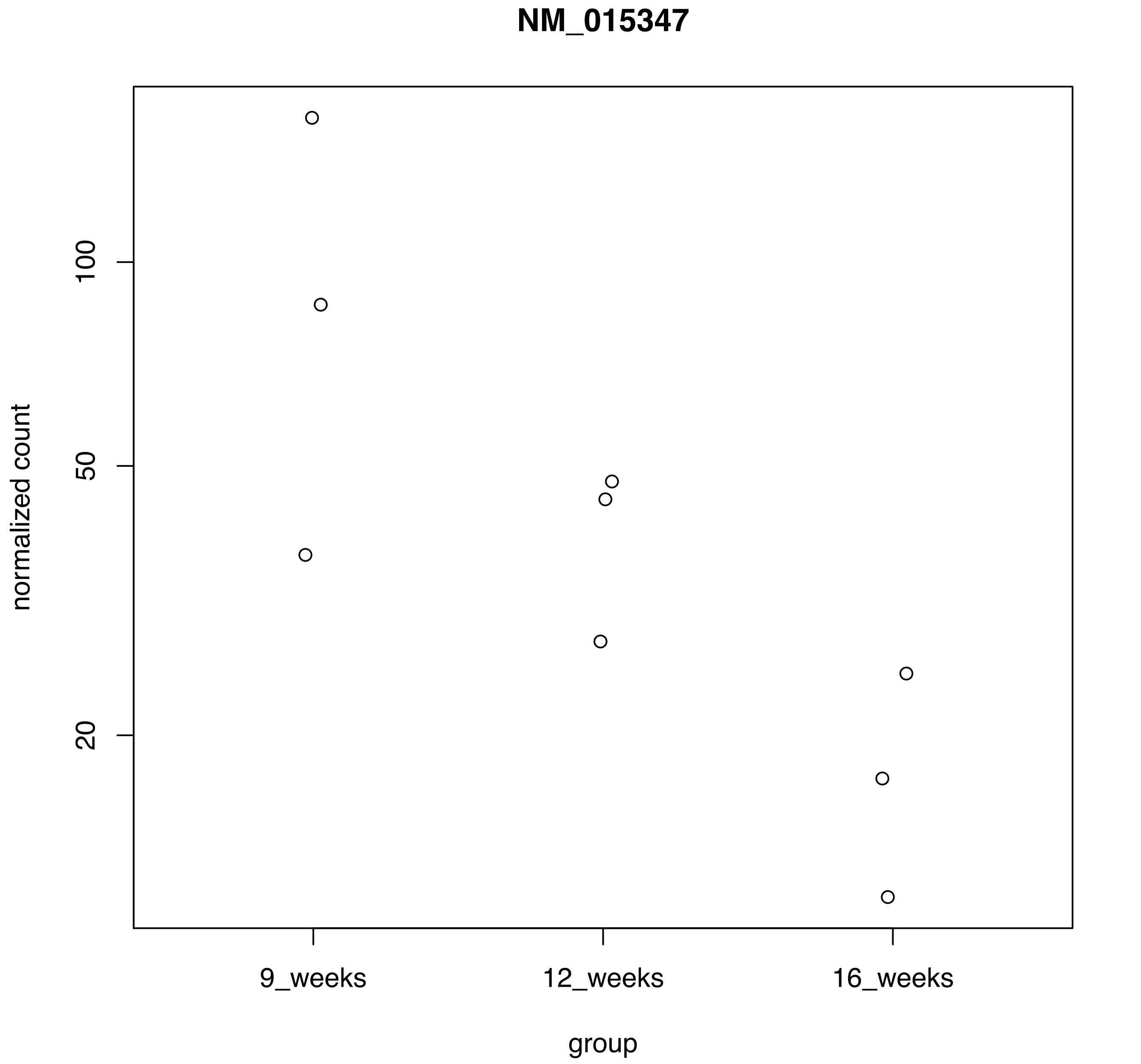
NM_015347
normalized count
100
50
20
9_weeks
12_weeks
16_weeks
group

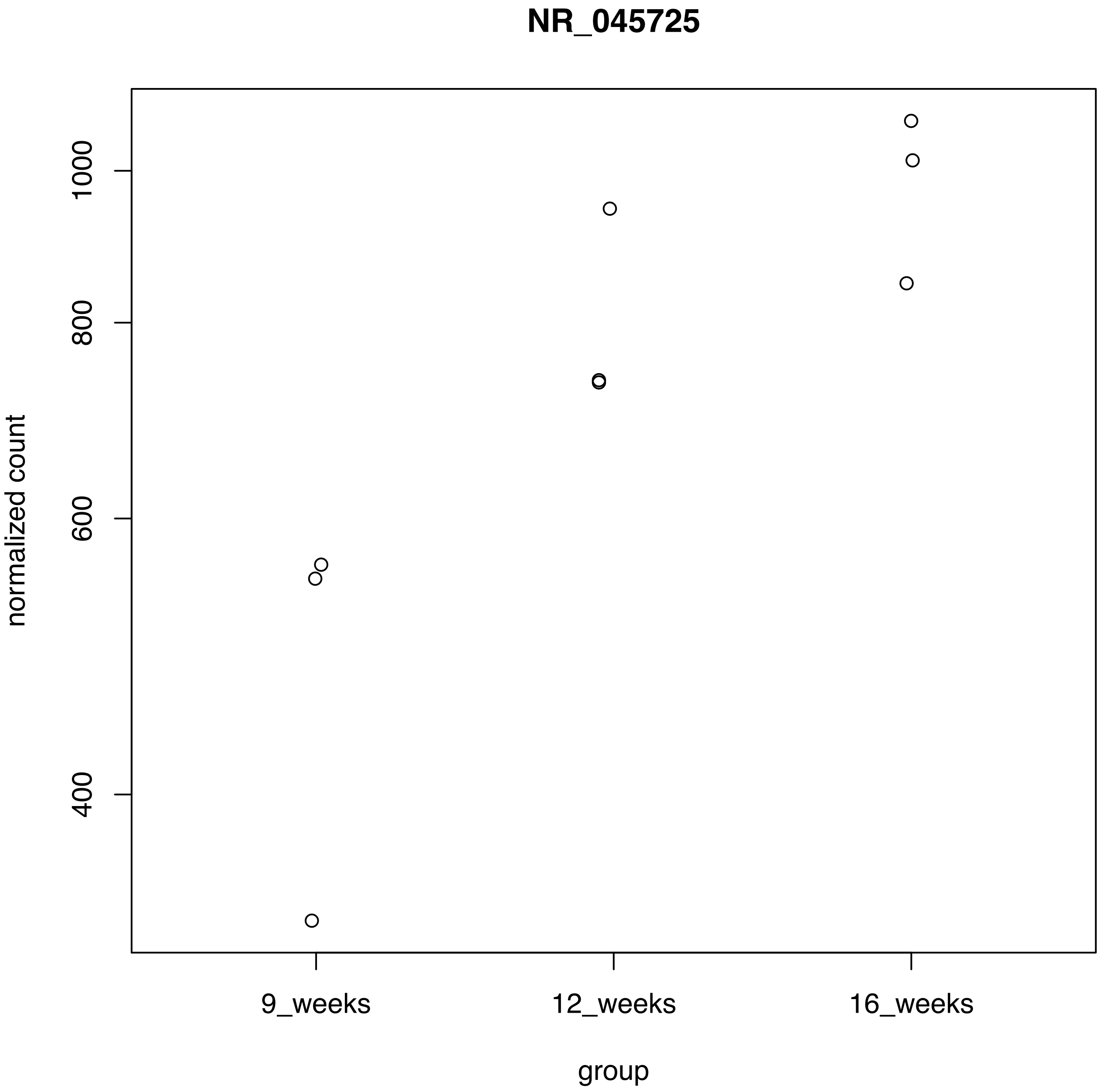

NR_045725
normalized count
400
600
800
1000
9_weeks
12_weeks
16_weeks
group

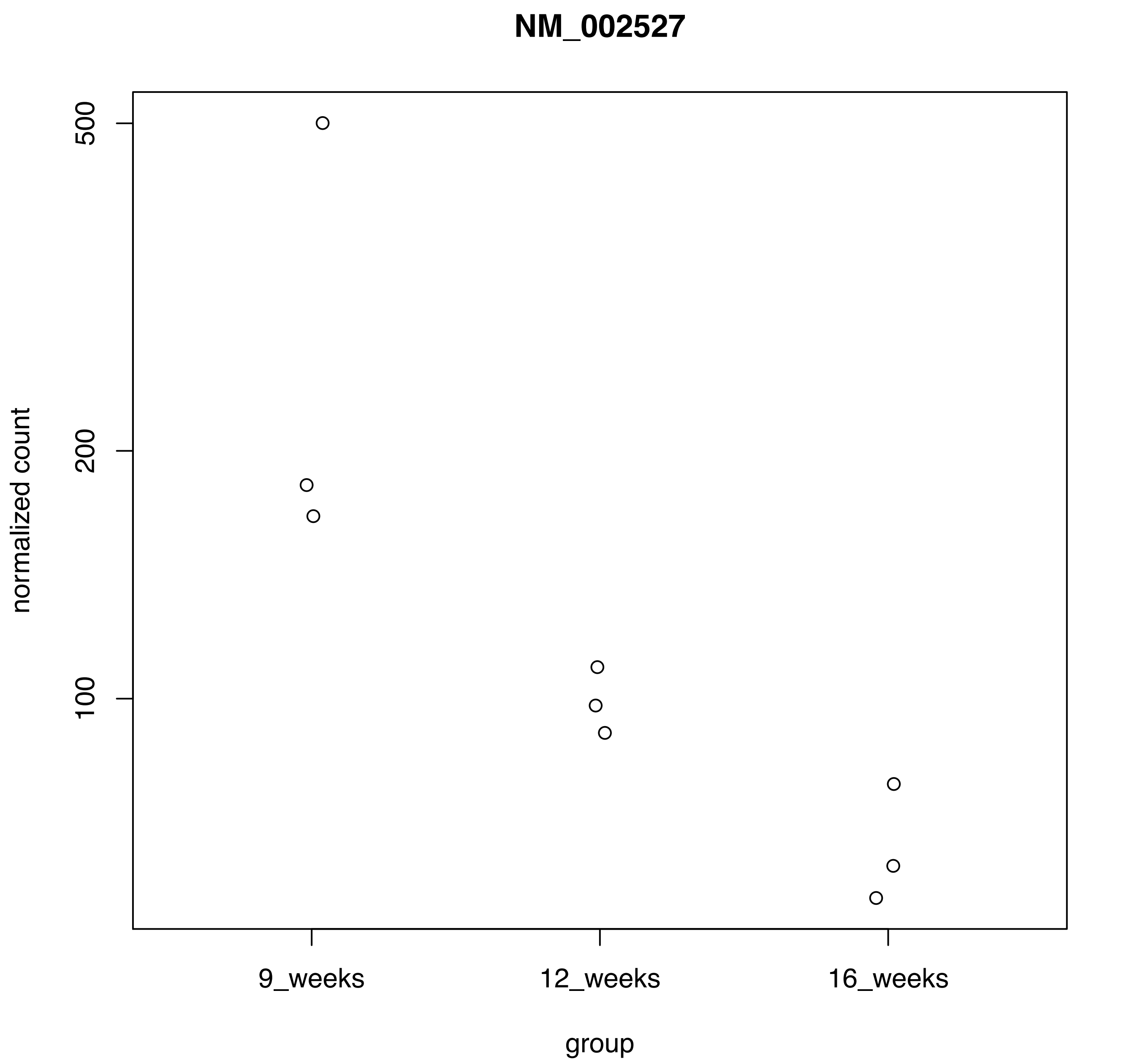
NM_002527
normalized count
500
200
100
9_weeks
12_weeks
16_weeks
group

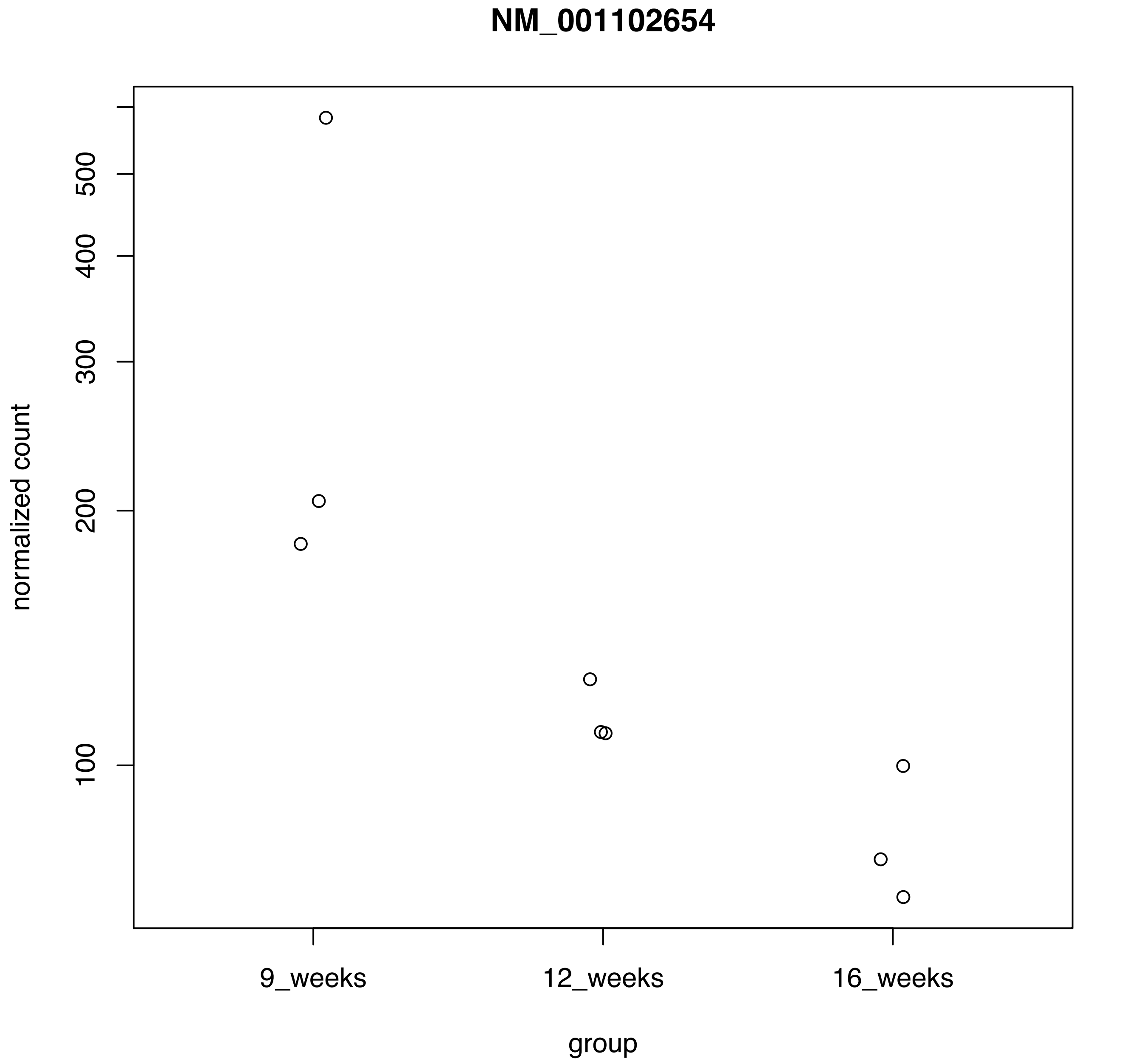
NM_001102654
normalized count
500
400
300
200
100
9_weeks
12_weeks
16_weeks
group

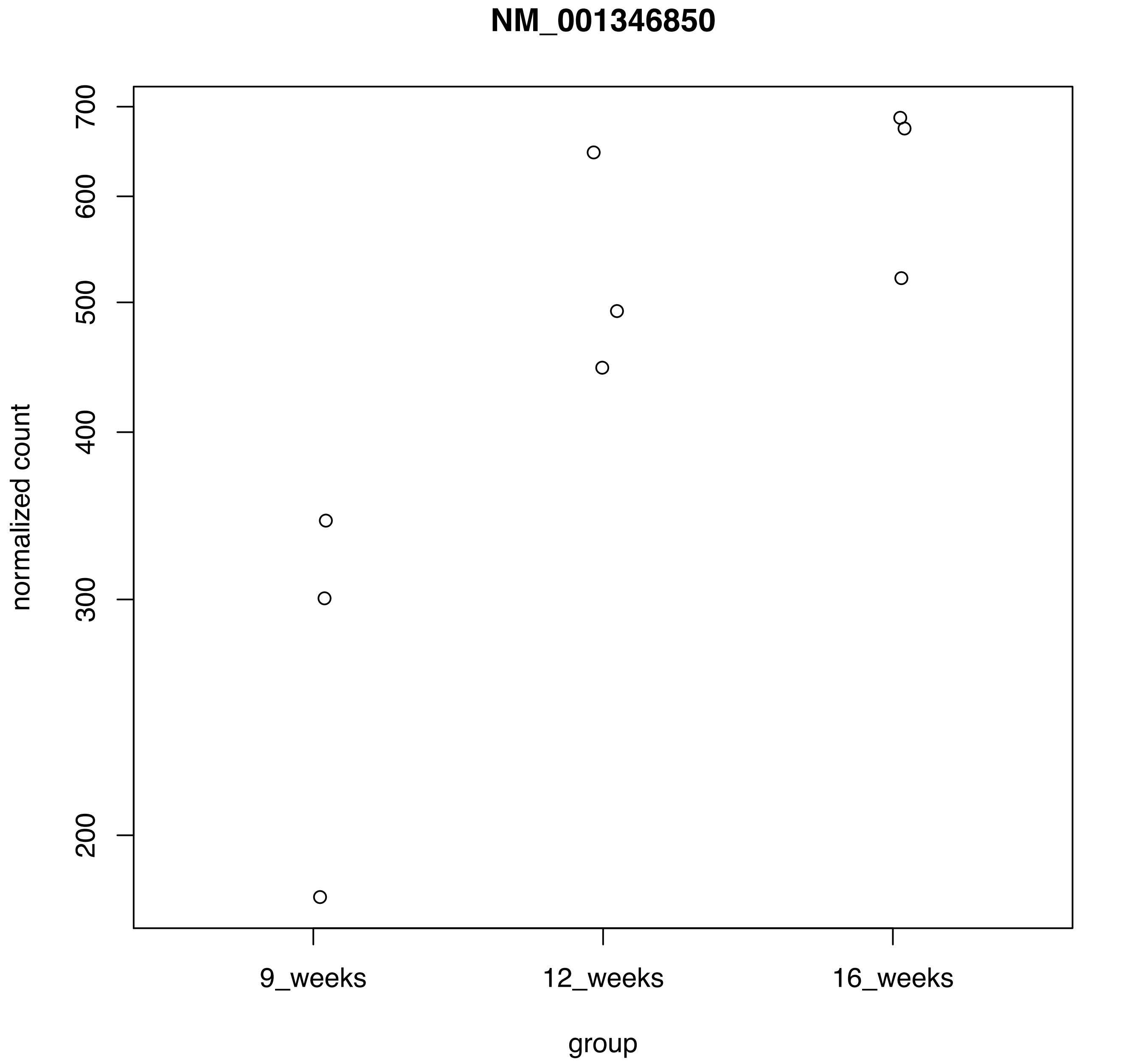
NM_001346850
normalized count
700
600
500
400
300
200
9_weeks
12_weeks
16_weeks
group

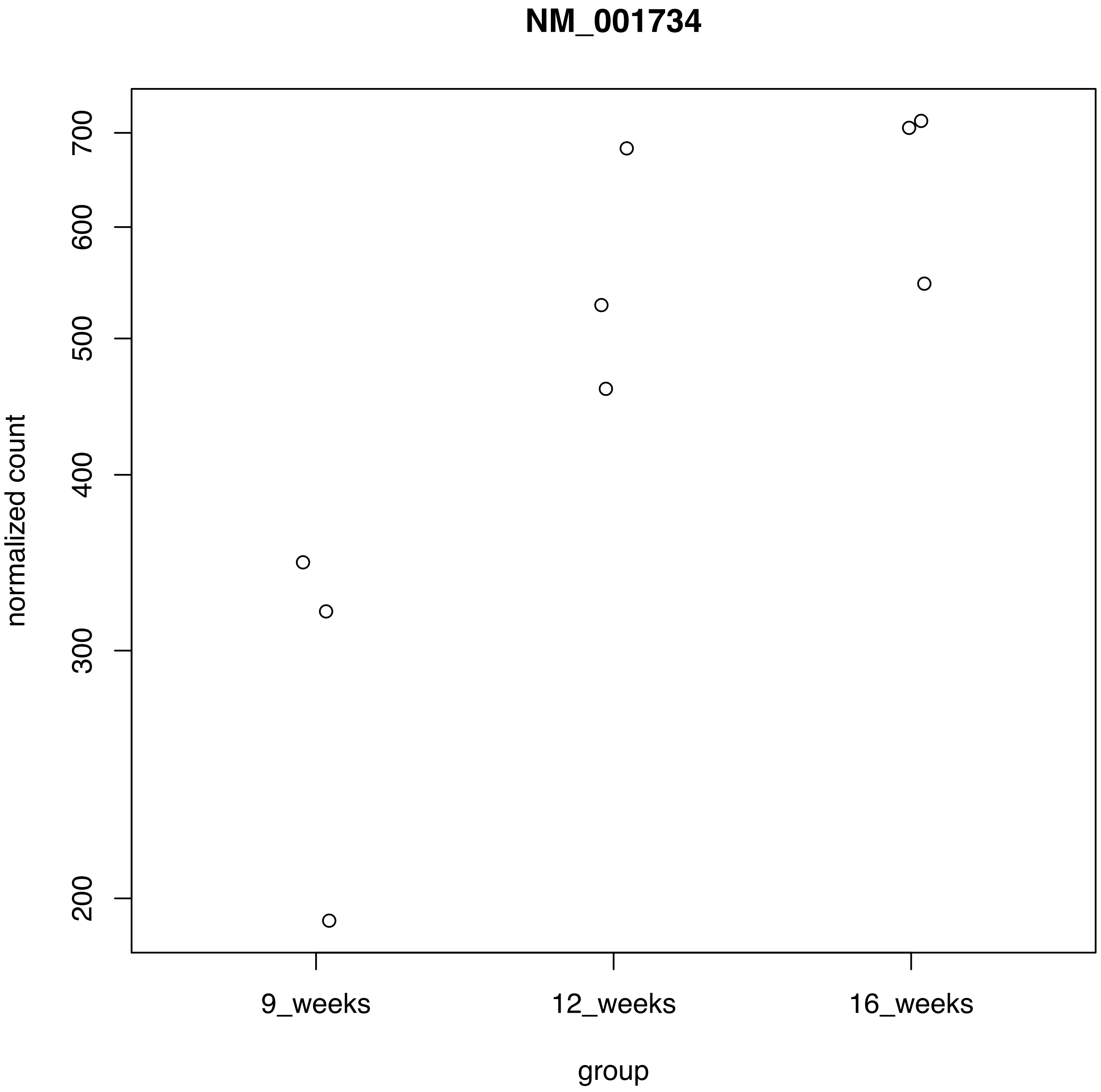
NM_001734
normalized count
700
600
500
400
300
200
9_weeks
12_weeks
16_weeks
group

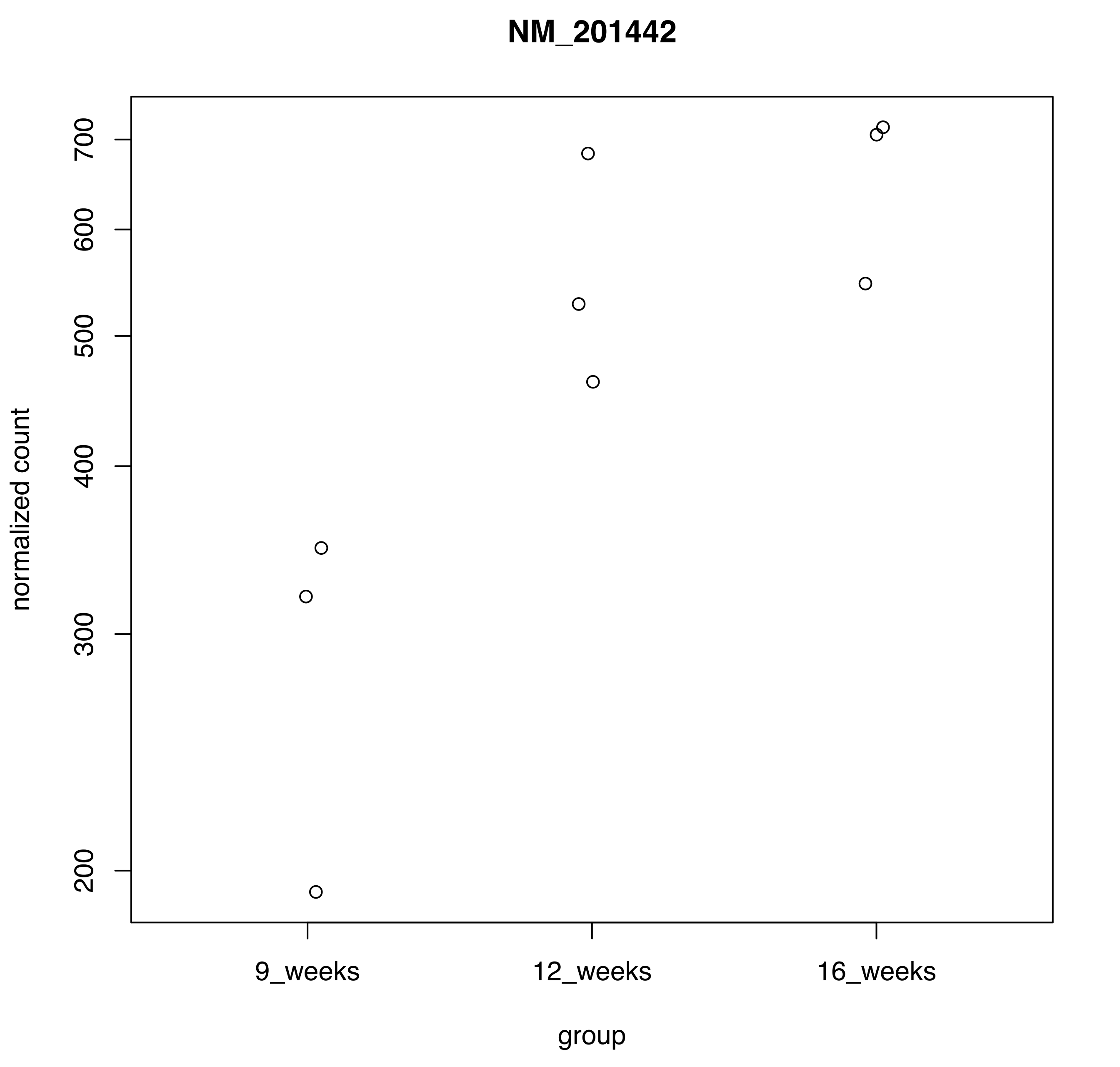
NM_201442
normalized count
200
300
400
500
600
700
9_weeks
12_weeks
16_weeks
group

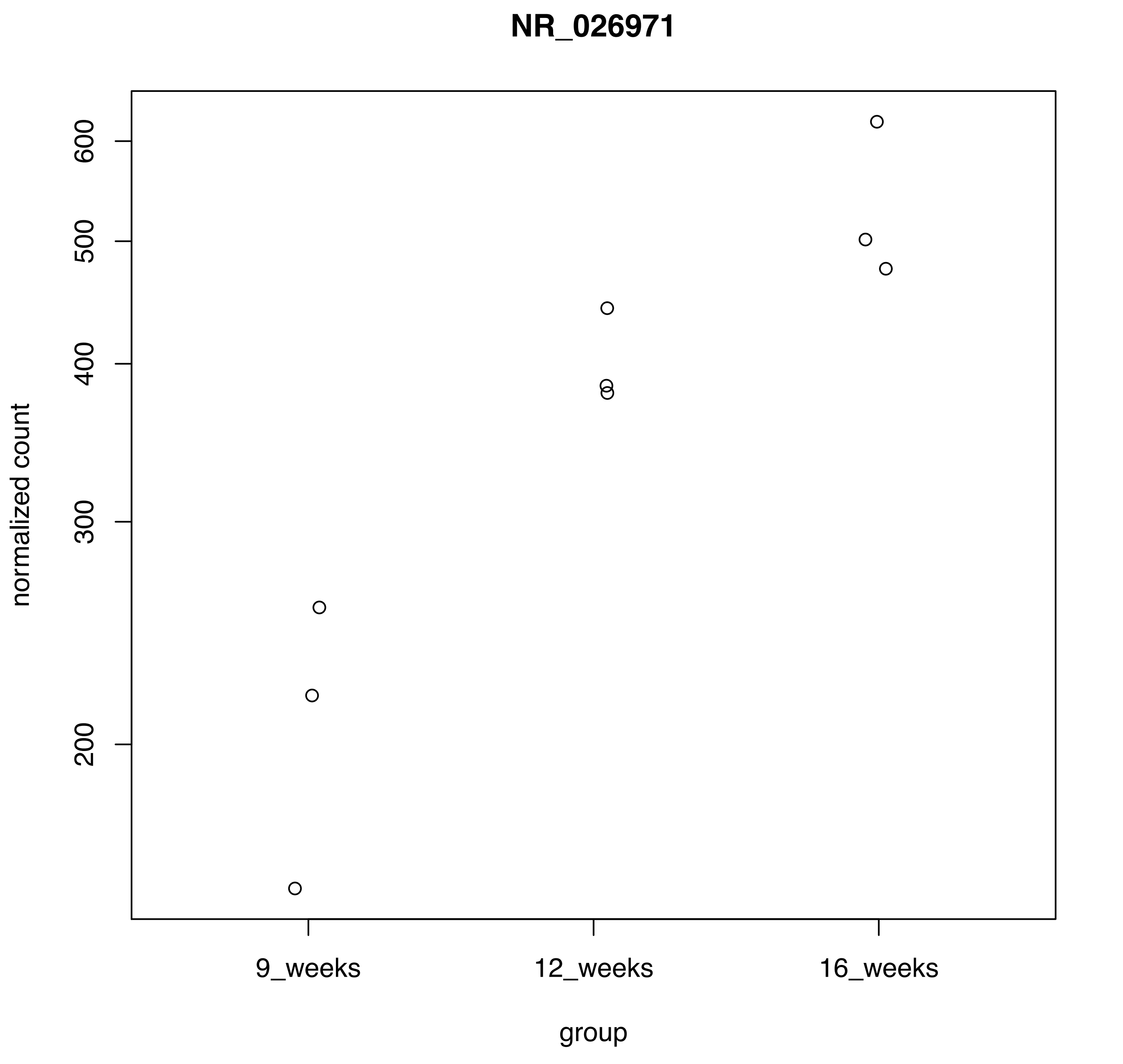
NR_026971
normalized count
200
300
400
500
600
9_weeks
12_weeks
16_weeks
group

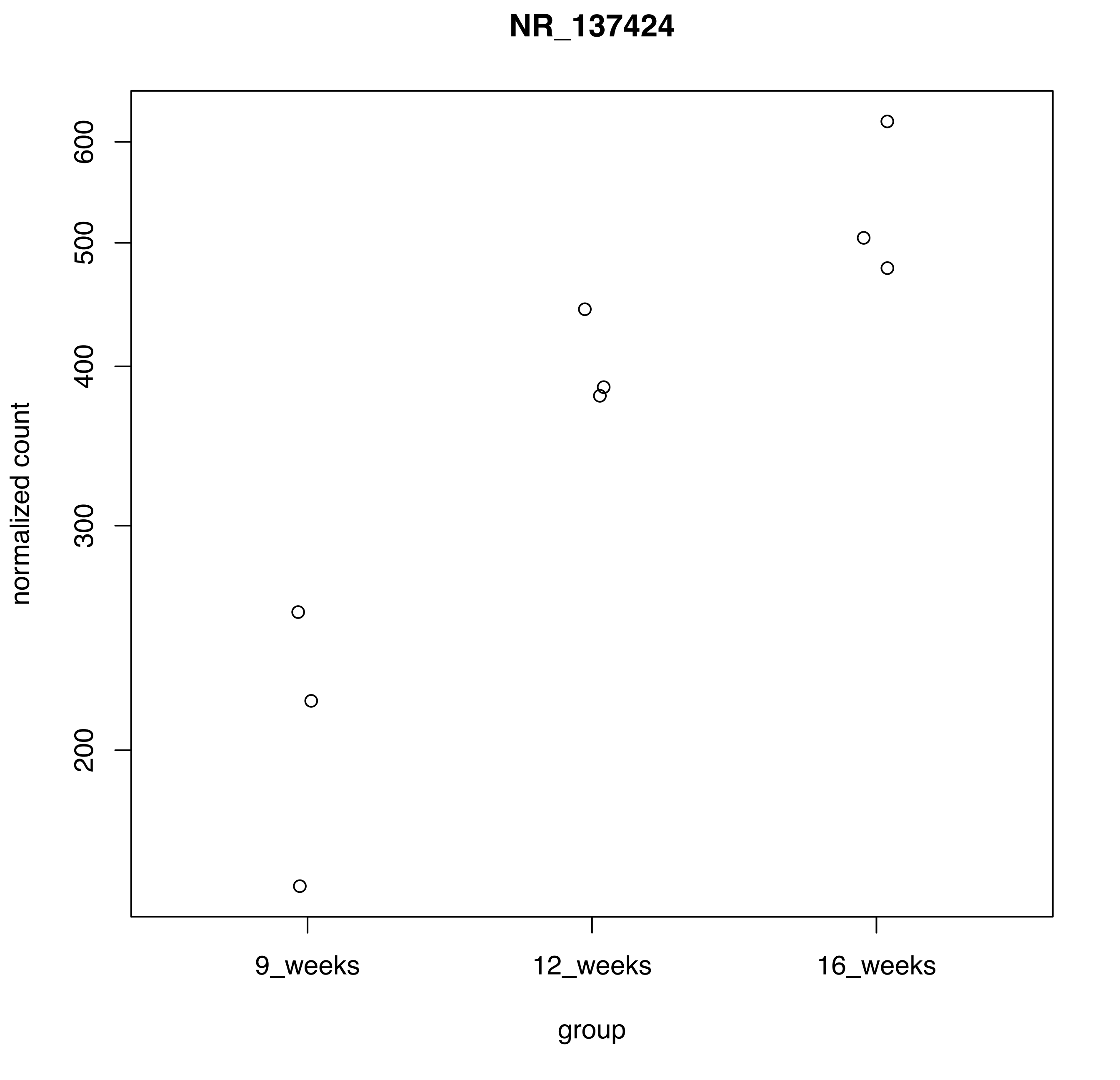
NR_137424
normalized count
600
500
400
300
200
9_weeks
12_weeks
16_weeks
group

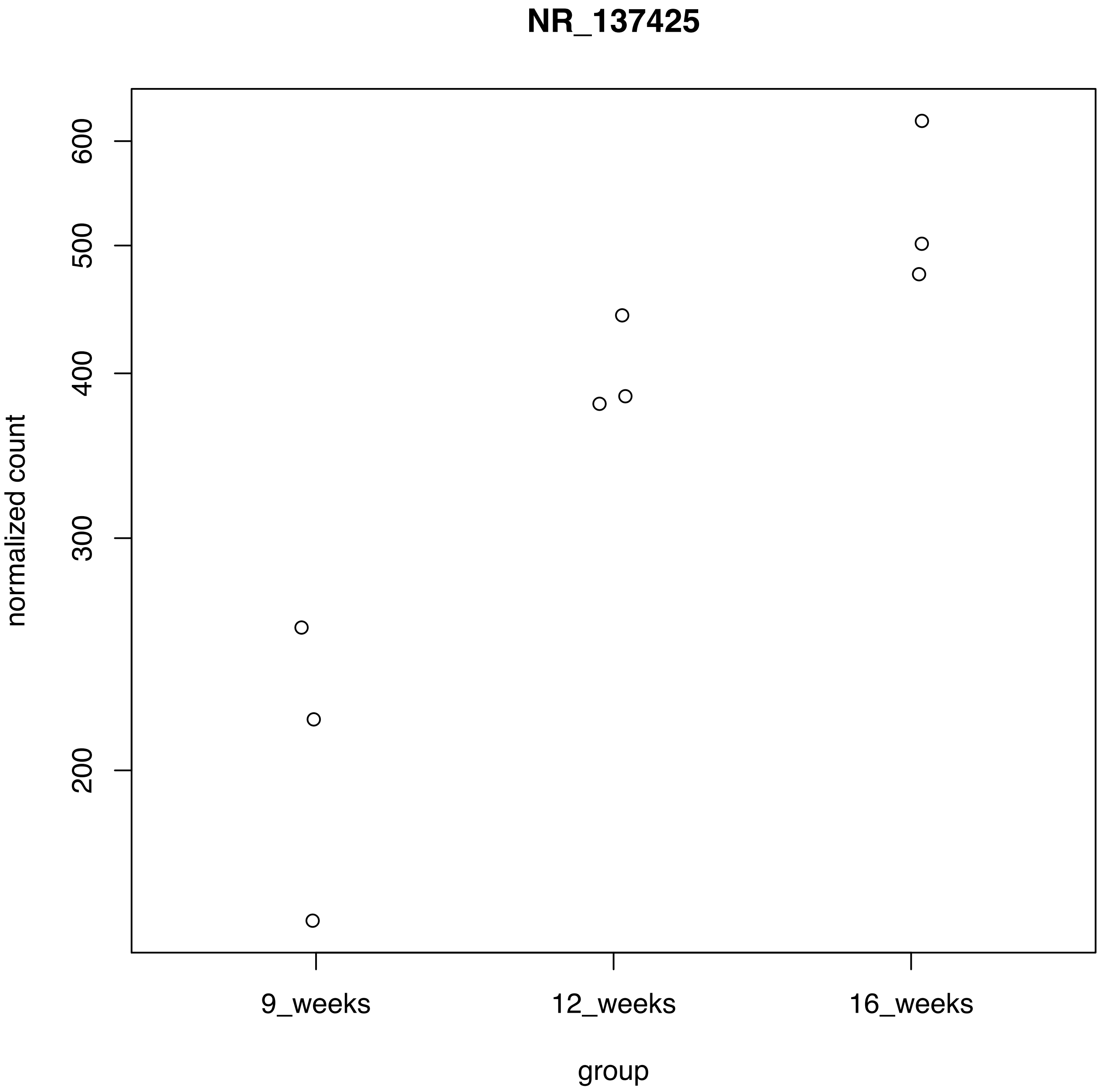
NR_137425
600
500
400
300
200
normalized count
9_weeks
12_weeks
16_weeks
group

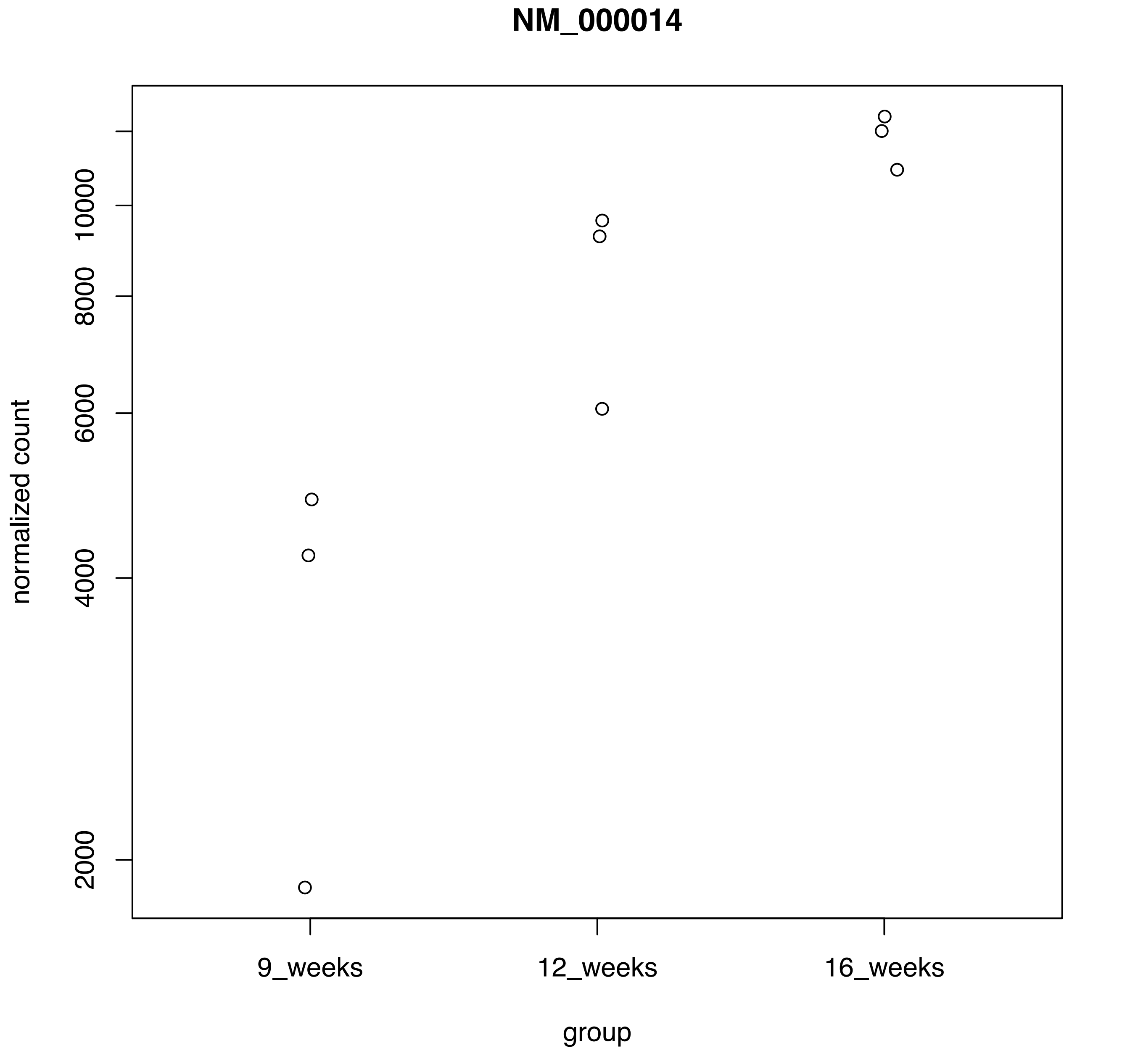

NM_000014
normalized count
2000
4000
6000
8000
10000
9_weeks
12_weeks
16_weeks
group

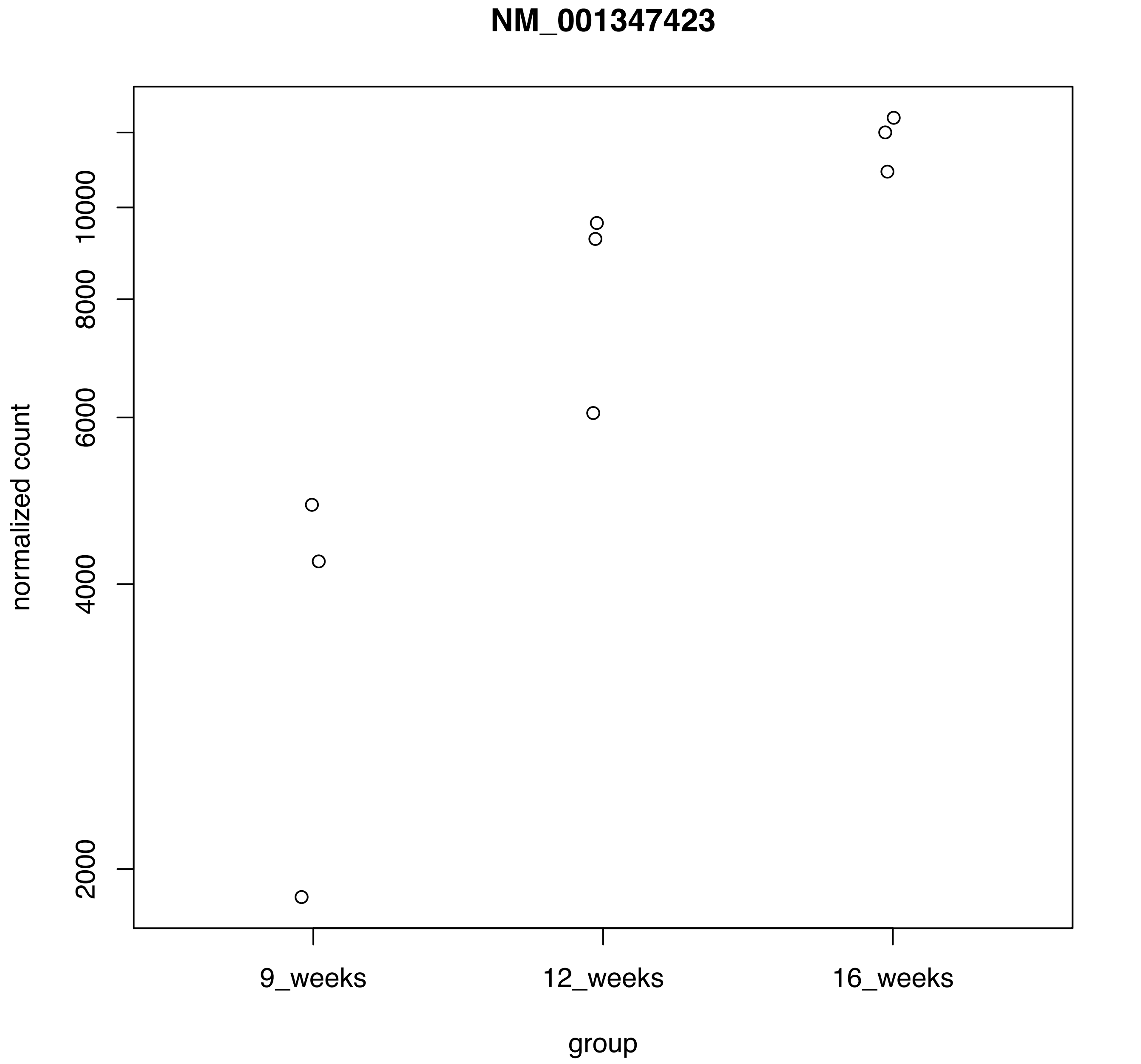
NM_001347423
normalized count
2000
4000
6000
8000
10000
9_weeks
12_weeks
16_weeks
group

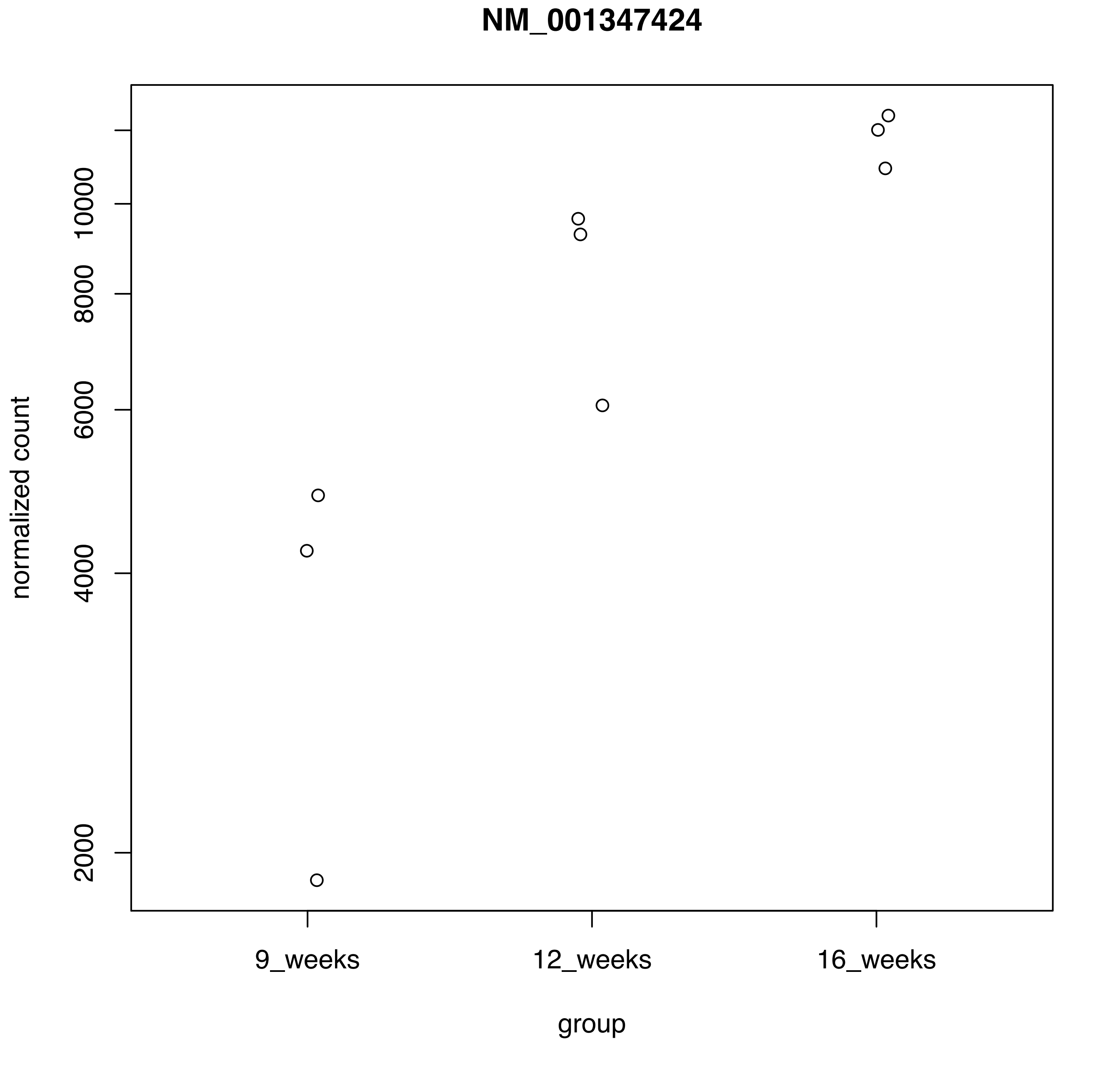
NM_001347424
normalized count
2000
4000
6000
8000
10000
9_weeks
12_weeks
16_weeks
group

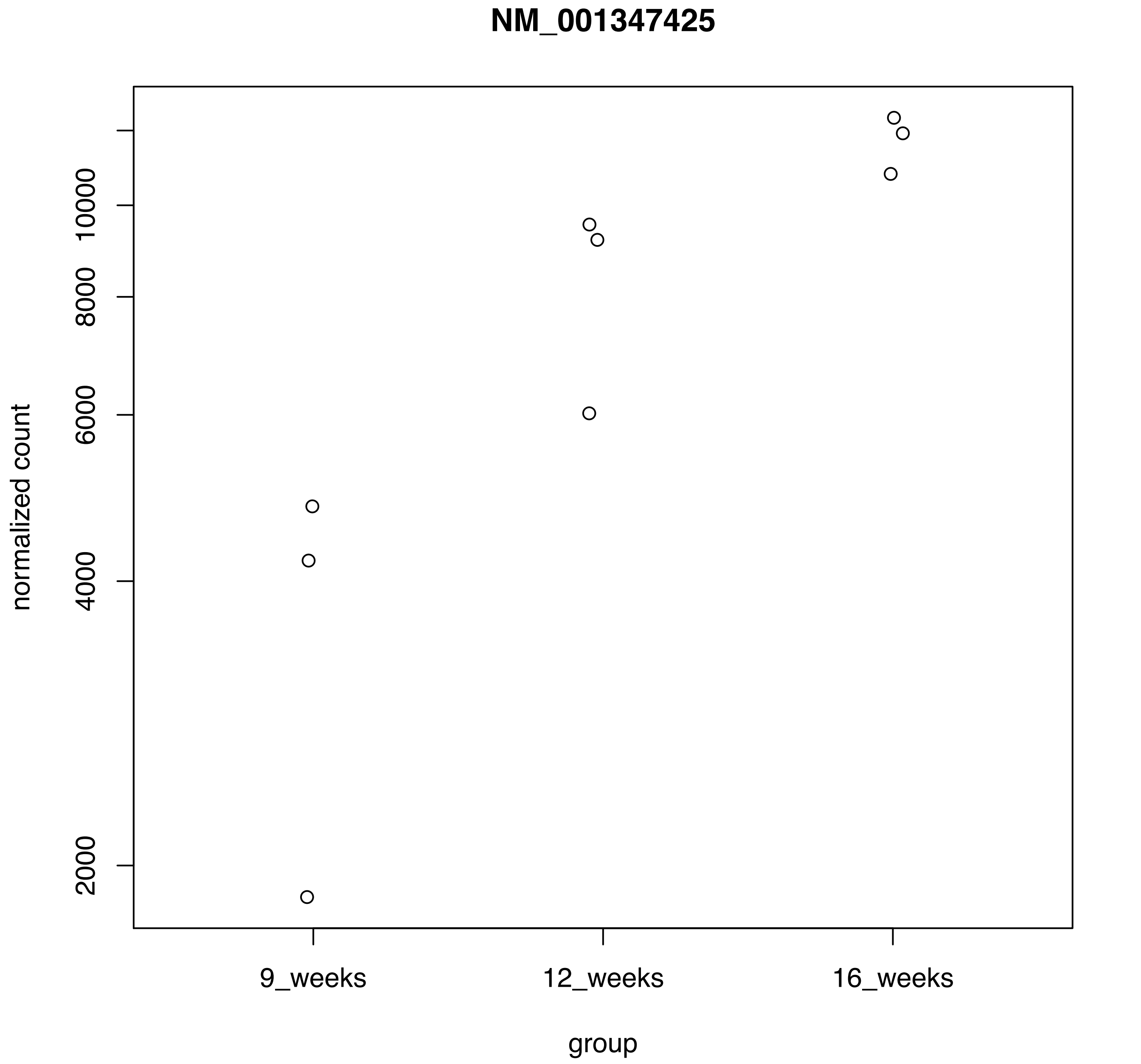
NM_001347425
normalized count
2000
4000
6000
8000
10000
9_weeks
12_weeks
16_weeks
group

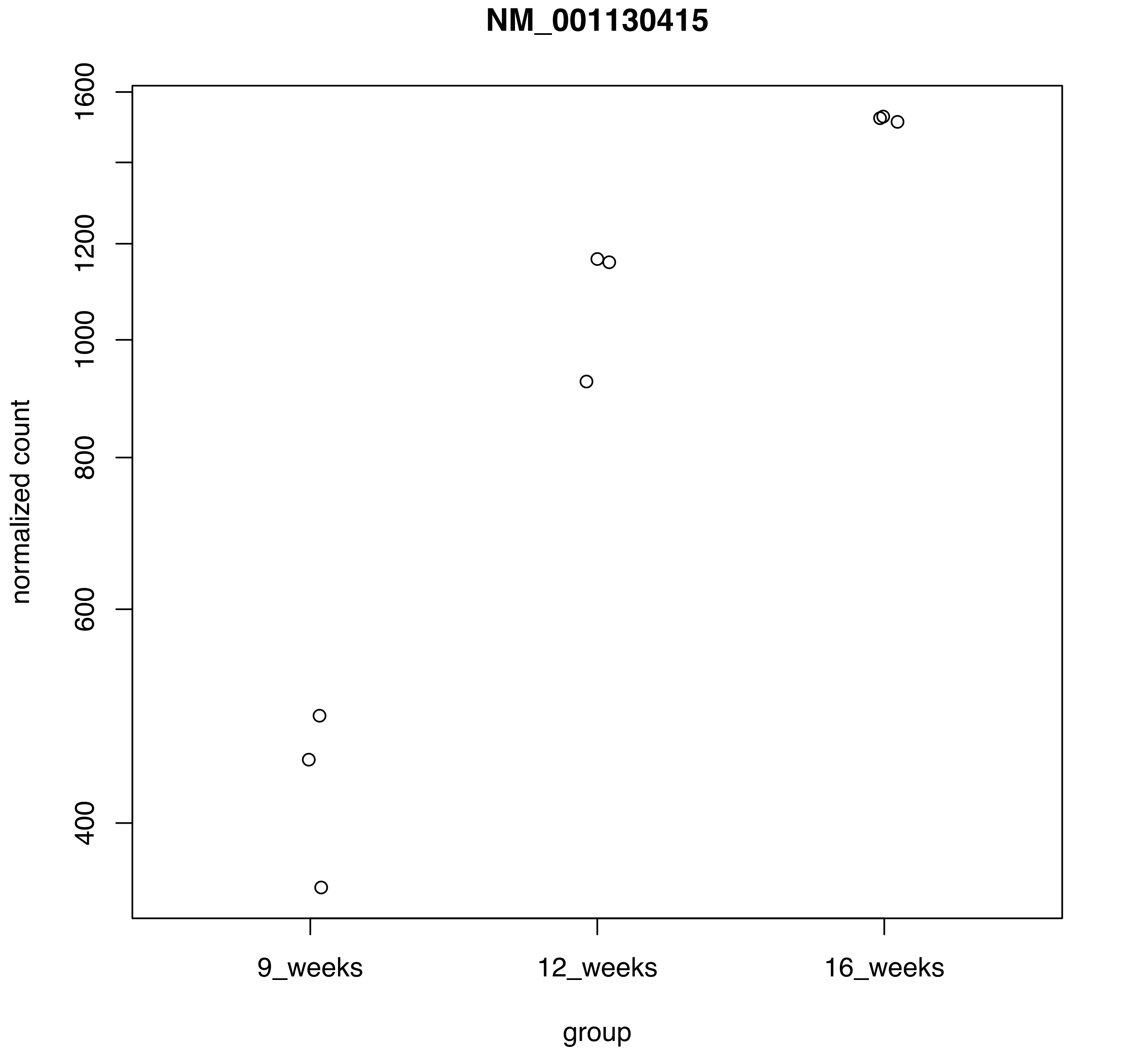

NM_001130415
normalized count
400
600
800
1000
1200
1600
9_weeks
12_weeks
16_weeks
group

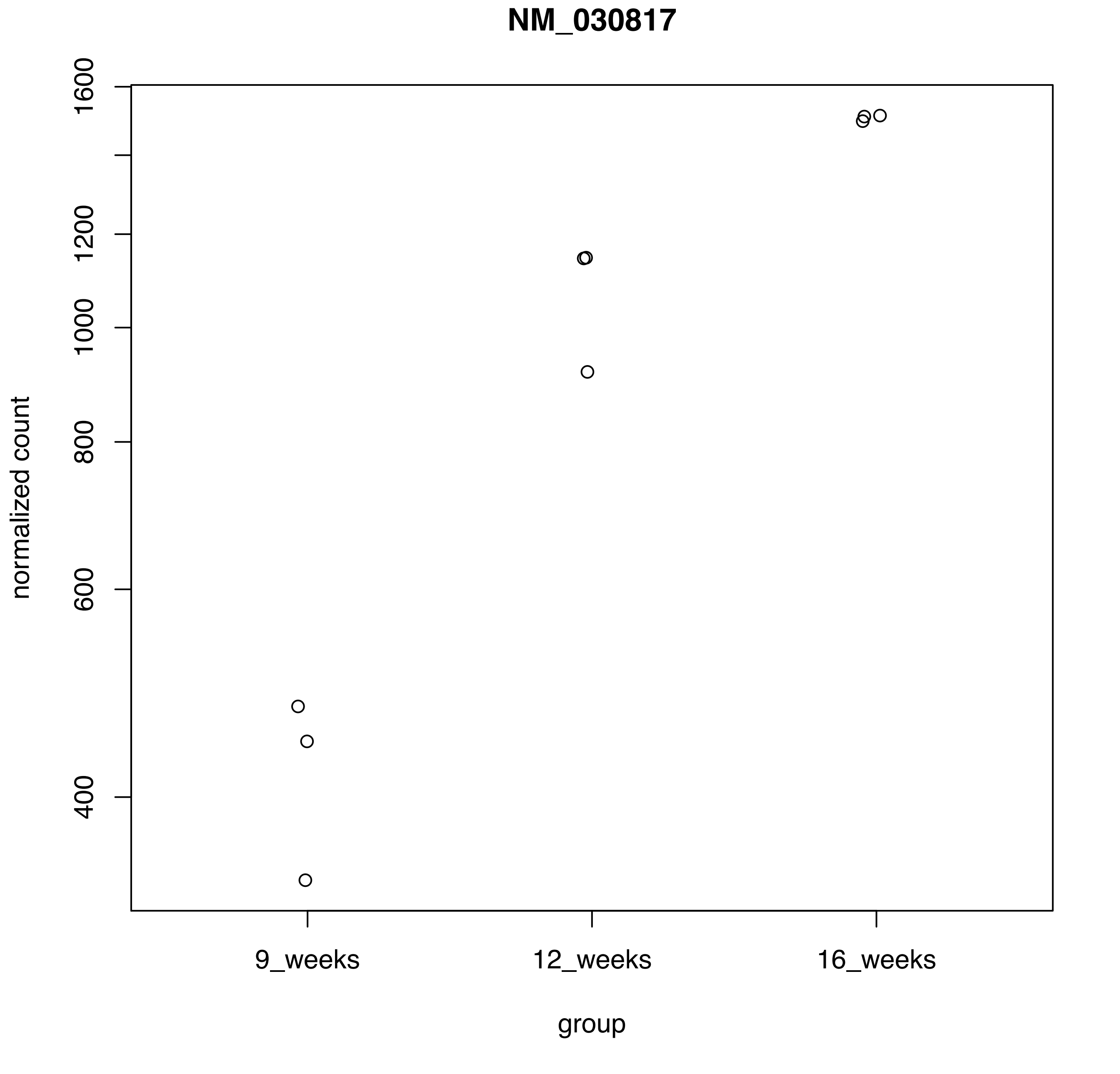
NM_030817
normalized count
400
600
800
1000
1200
1600
9_weeks
12_weeks
16_weeks
group

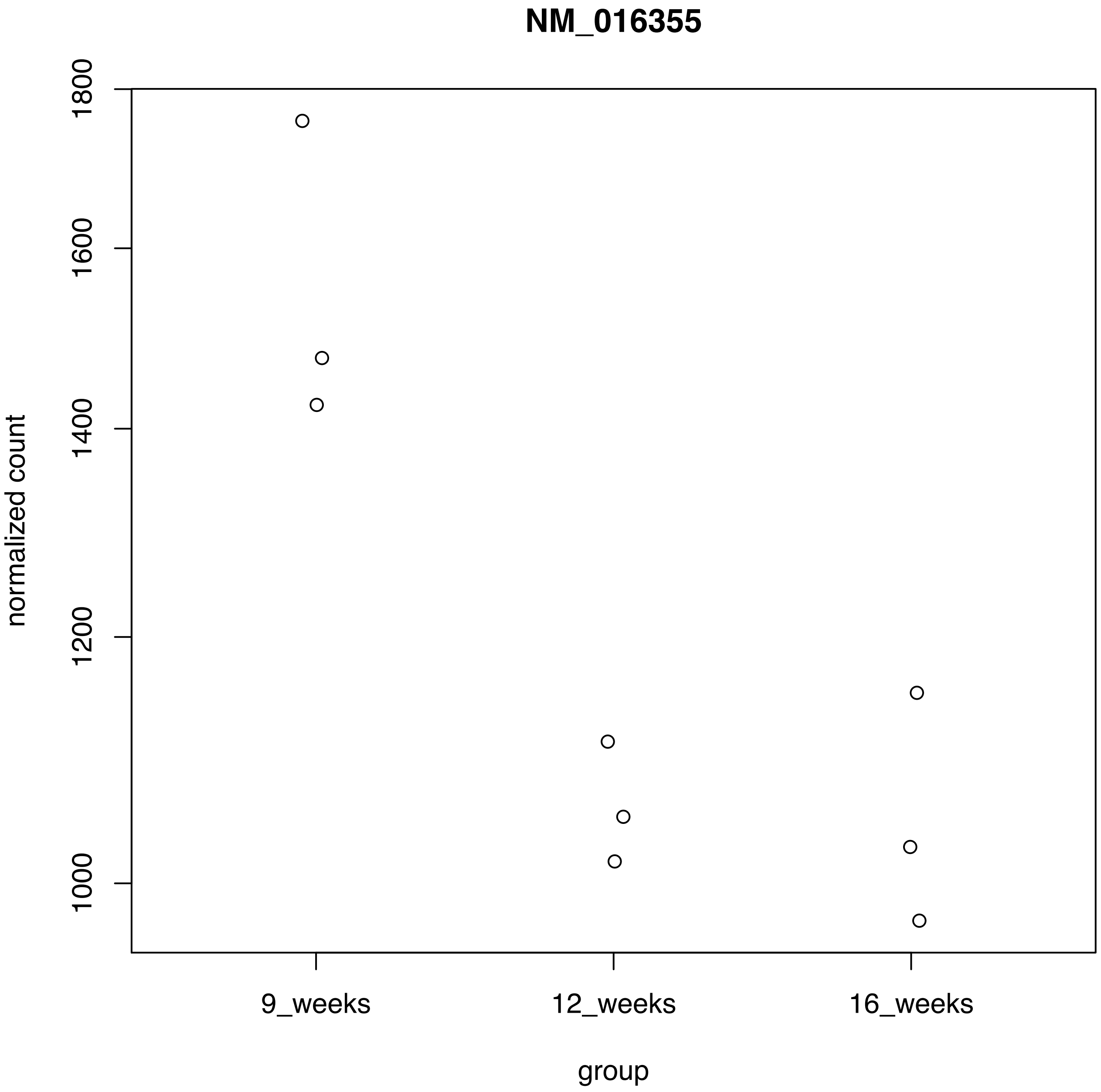
NM_016355
normalized count
1800
1600
1400
1200
1000
9_weeks
12_weeks
16_weeks
group

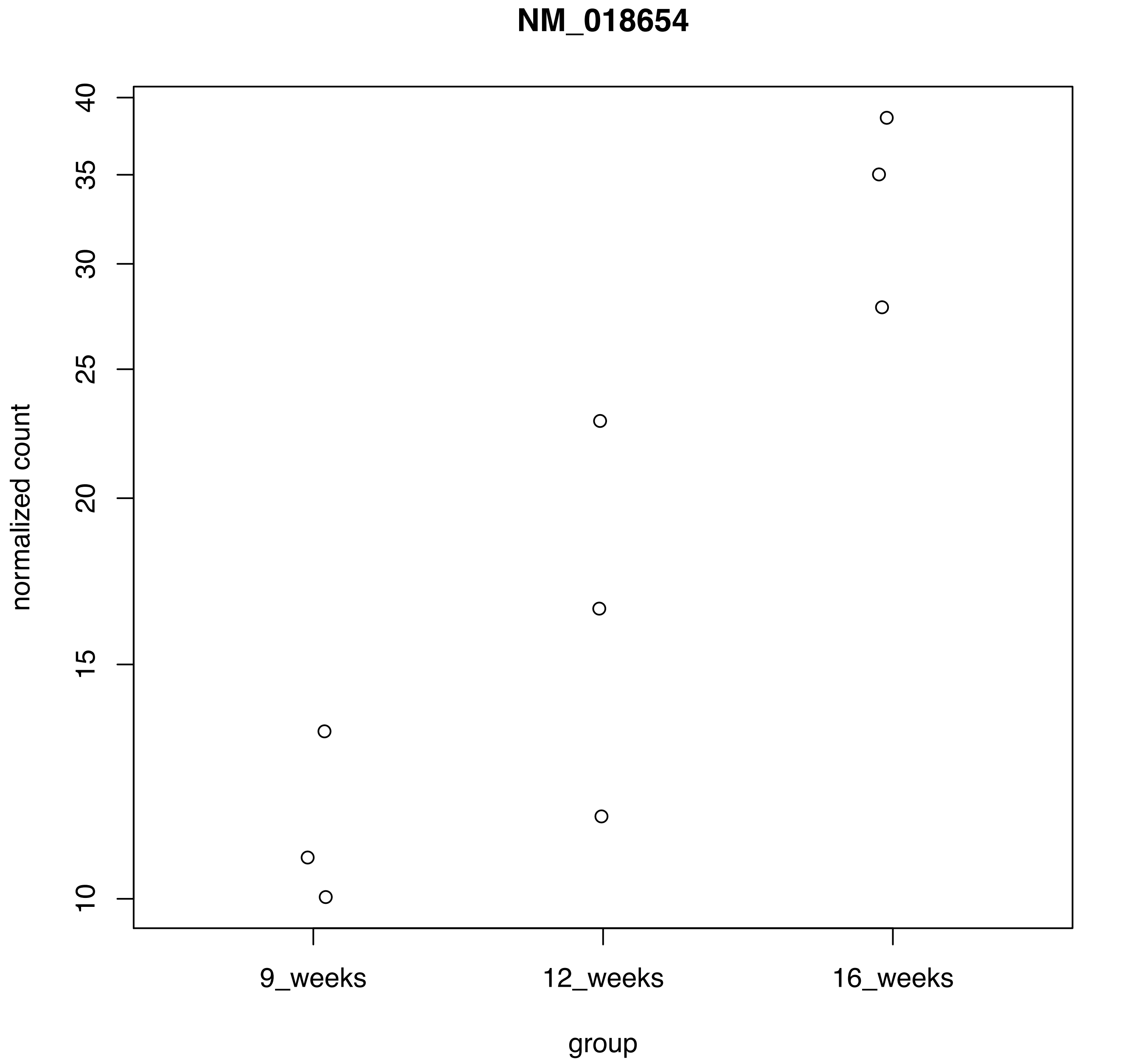
NM_018654
normalized count
40
35
30
25
20
15
10
9_weeks
12_weeks
16_weeks
group

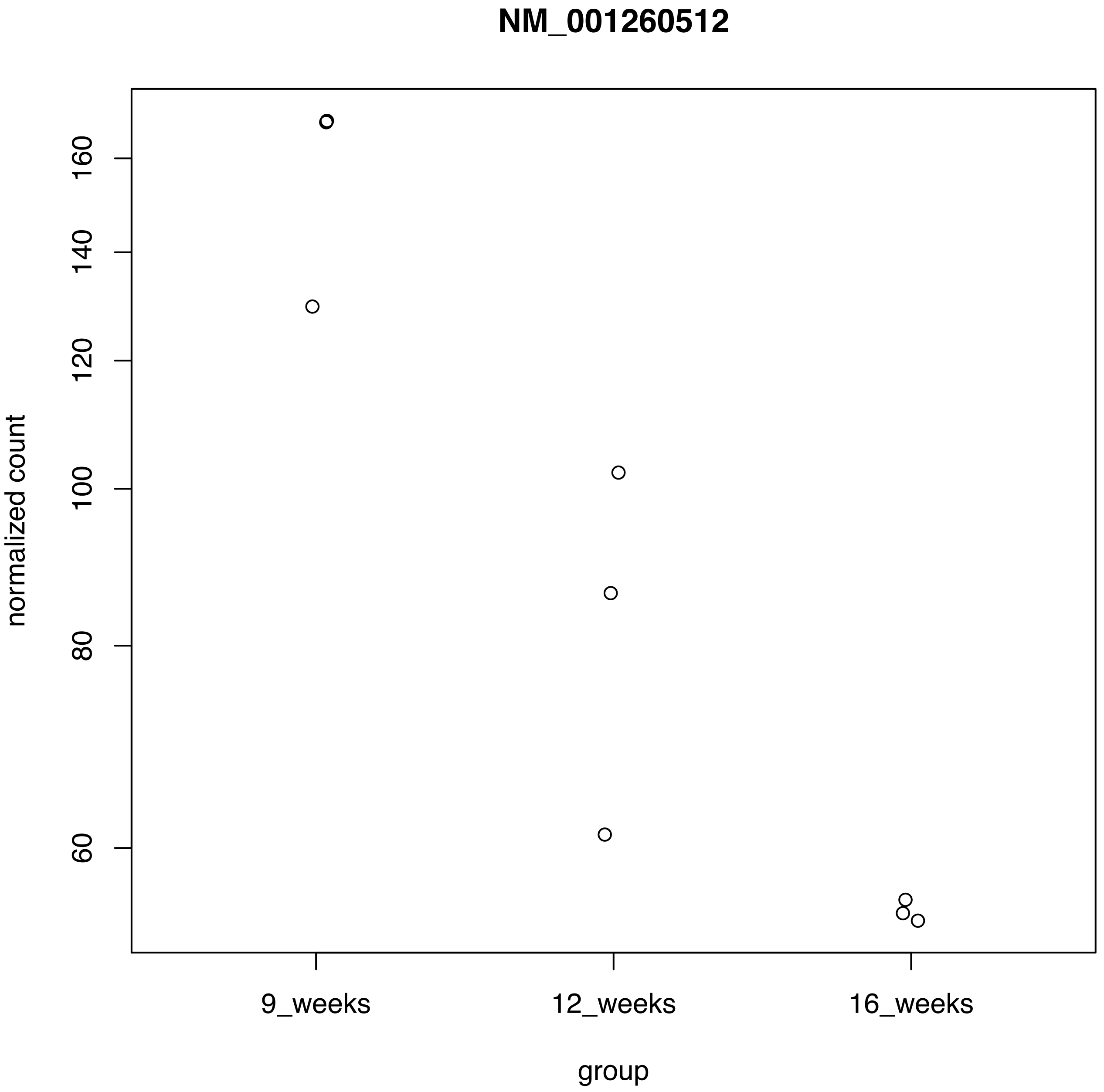
NM_001260512
normalized count
160
140
120
100
80
60
9_weeks
12_weeks
16_weeks
group

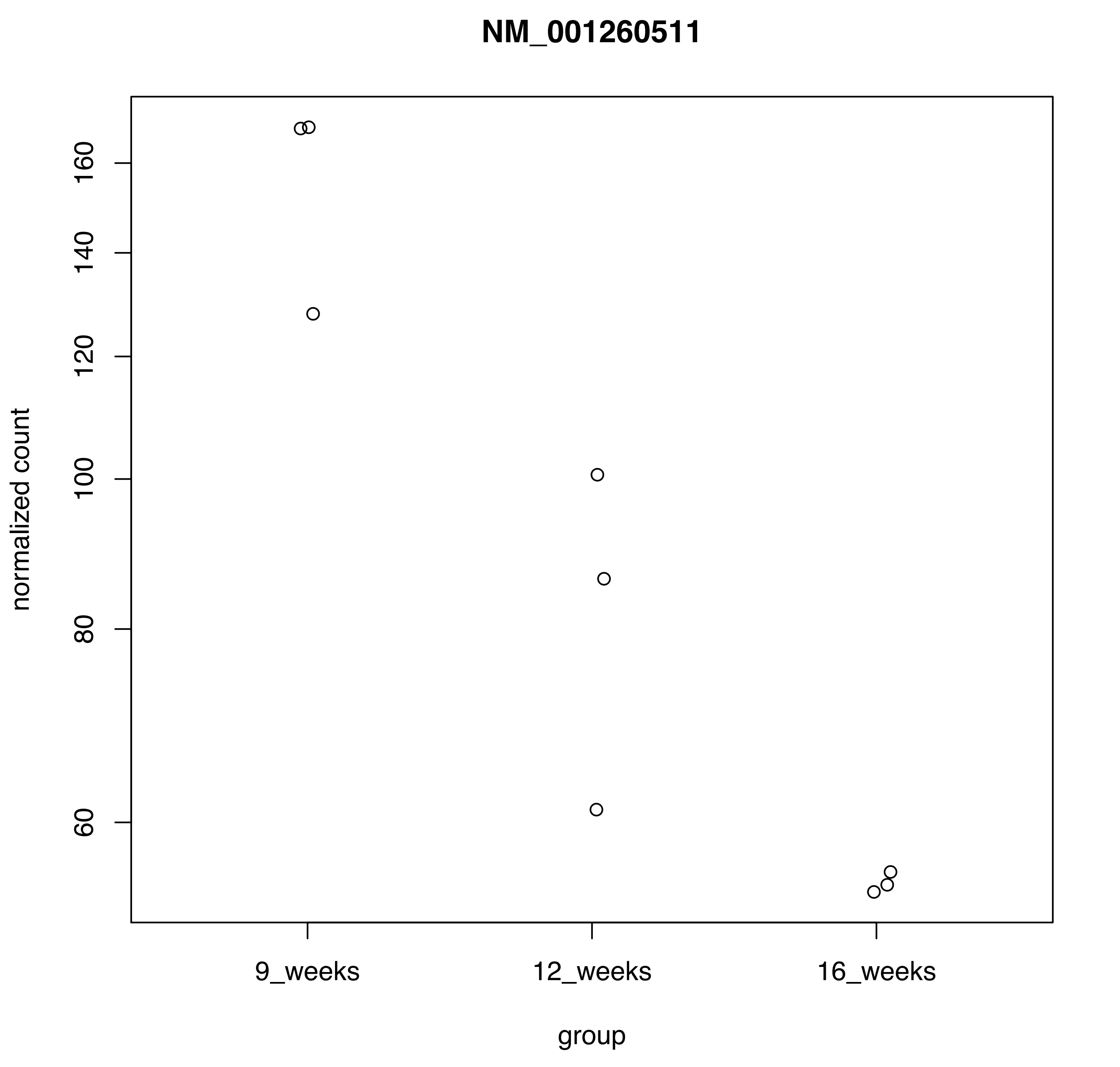

NM_001260511
normalized count
60
80
100
120
140
160
9_weeks
12_weeks
16_weeks
group

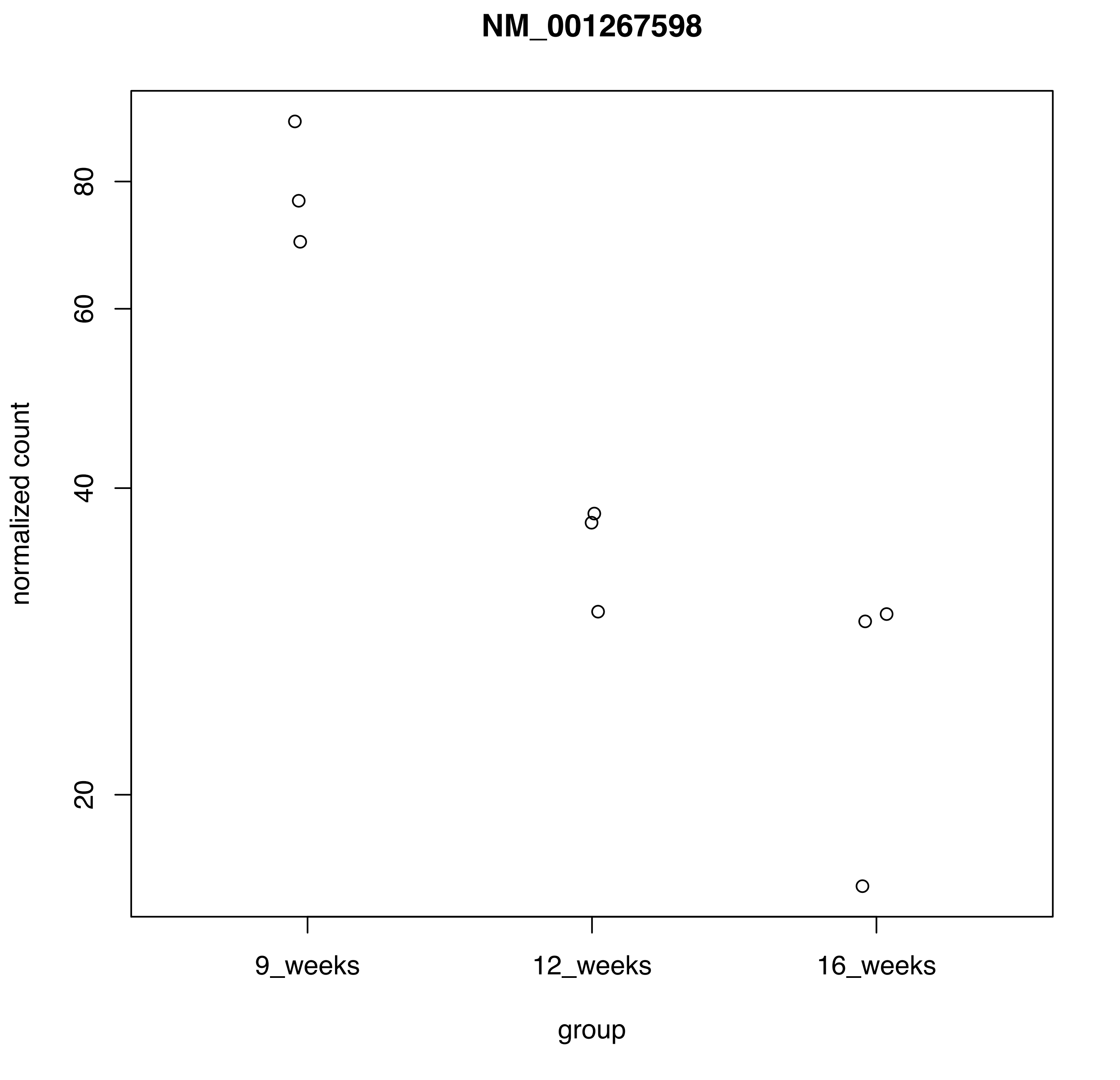
NM_001267598
normalized count
20
40
60
80
9_weeks
12_weeks
16_weeks
group

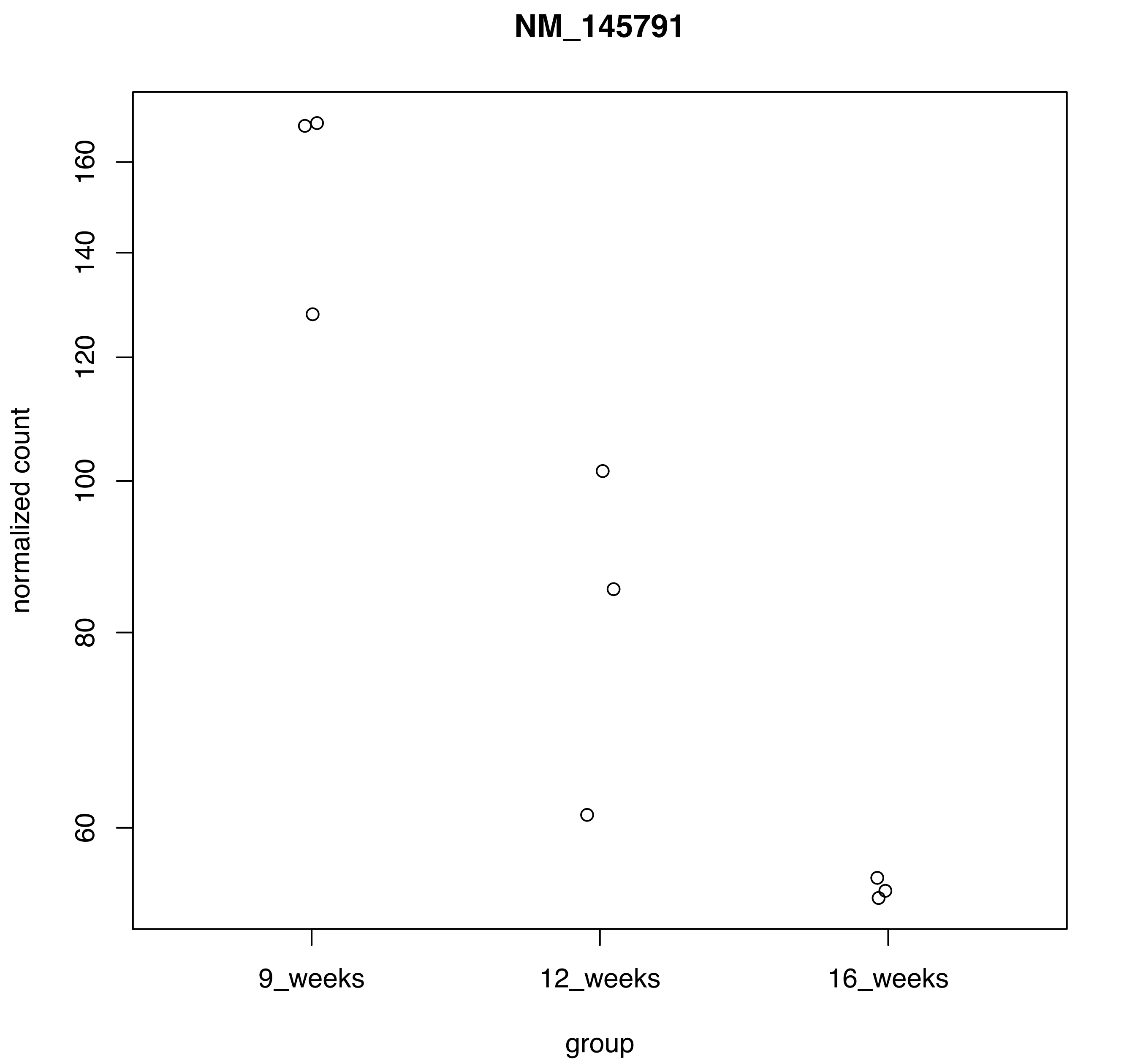

NM_145791
normalized count
160
140
120
100
80
60
9_weeks
12_weeks
16_weeks
group

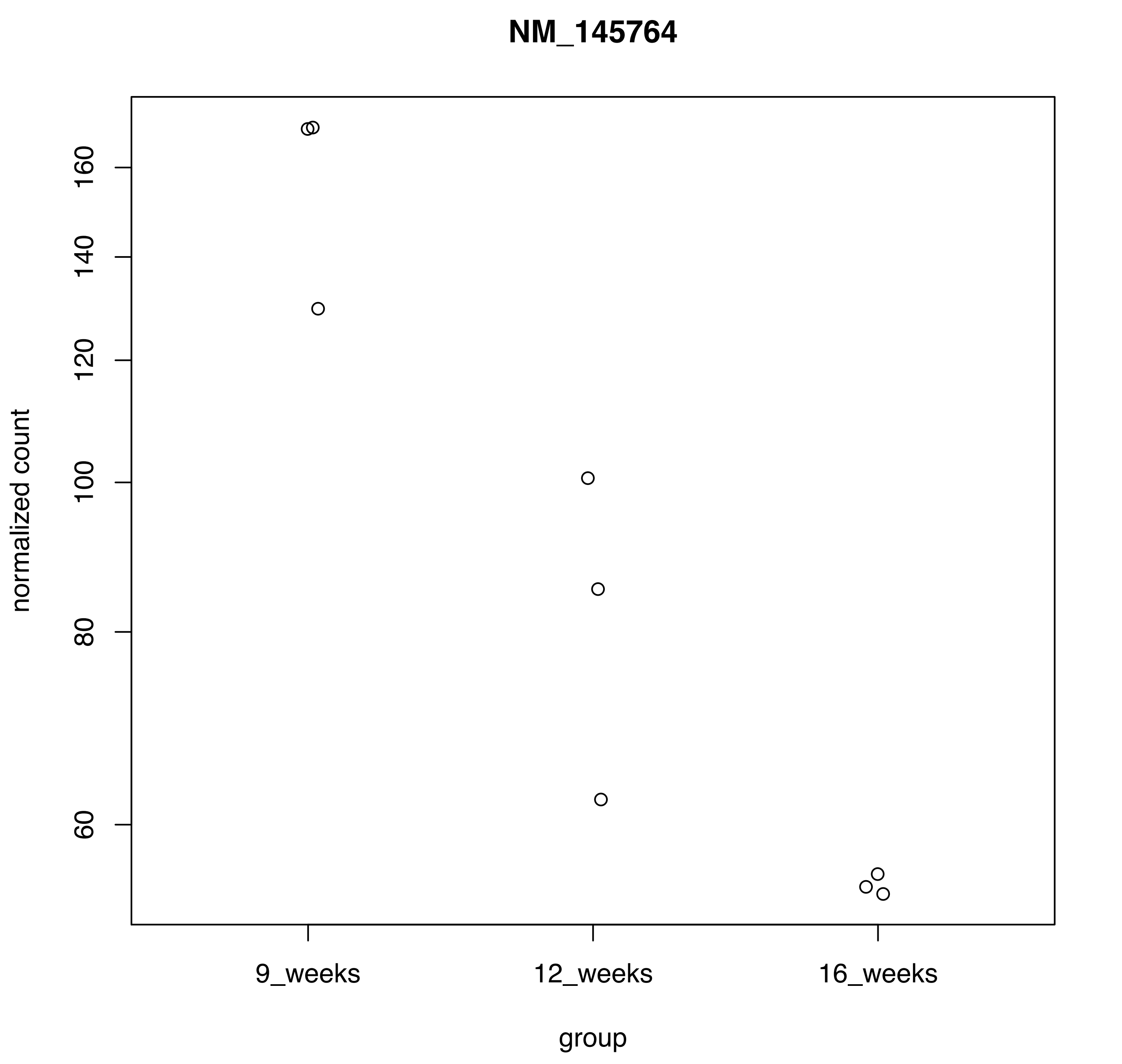

NM_145764
normalized count
160
140
120
100
80
60
9_weeks
12_weeks
16_weeks
group

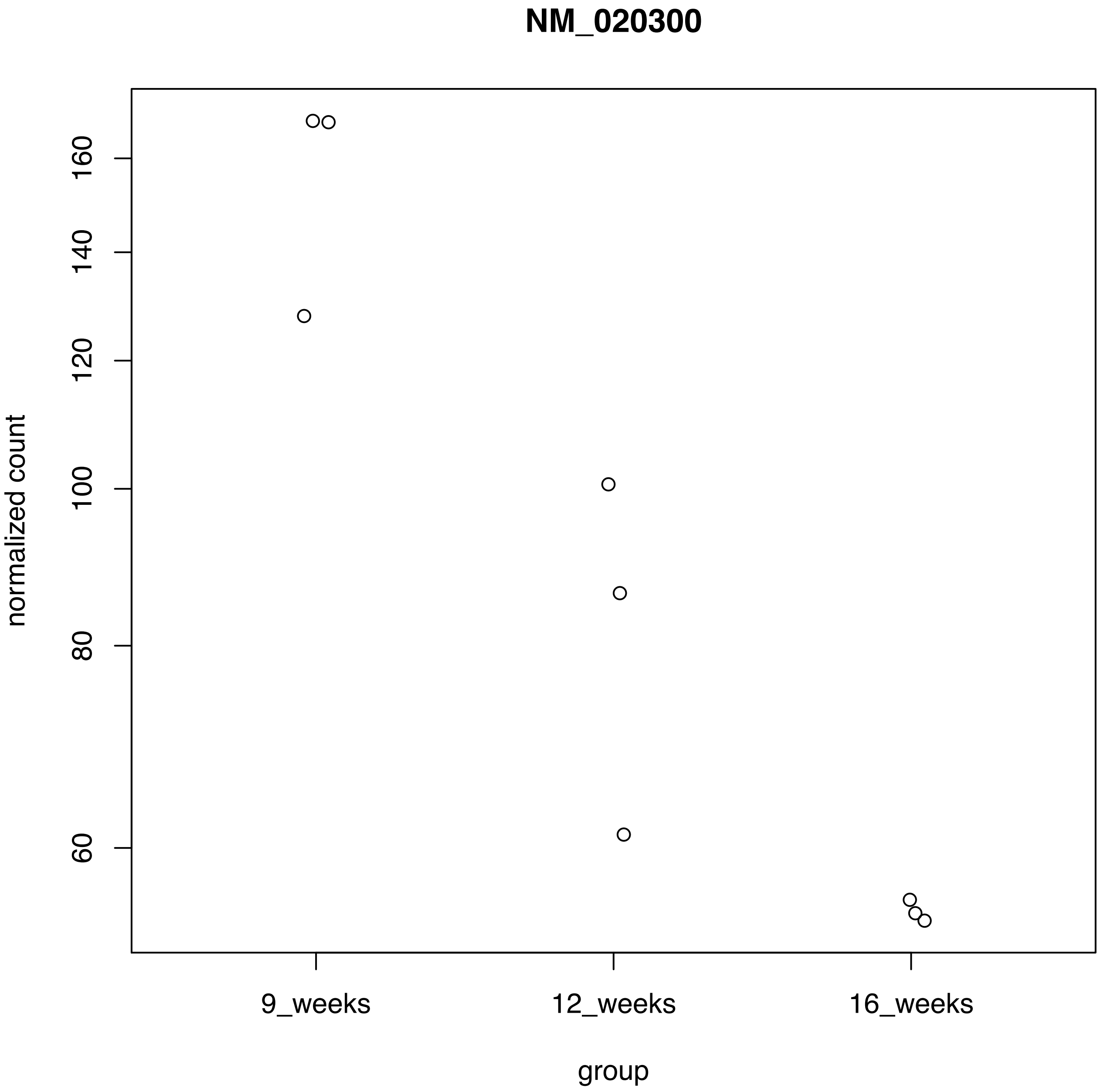
NM_020300
normalized count
160
140
120
100
80
60
9_weeks
12_weeks
16_weeks
group

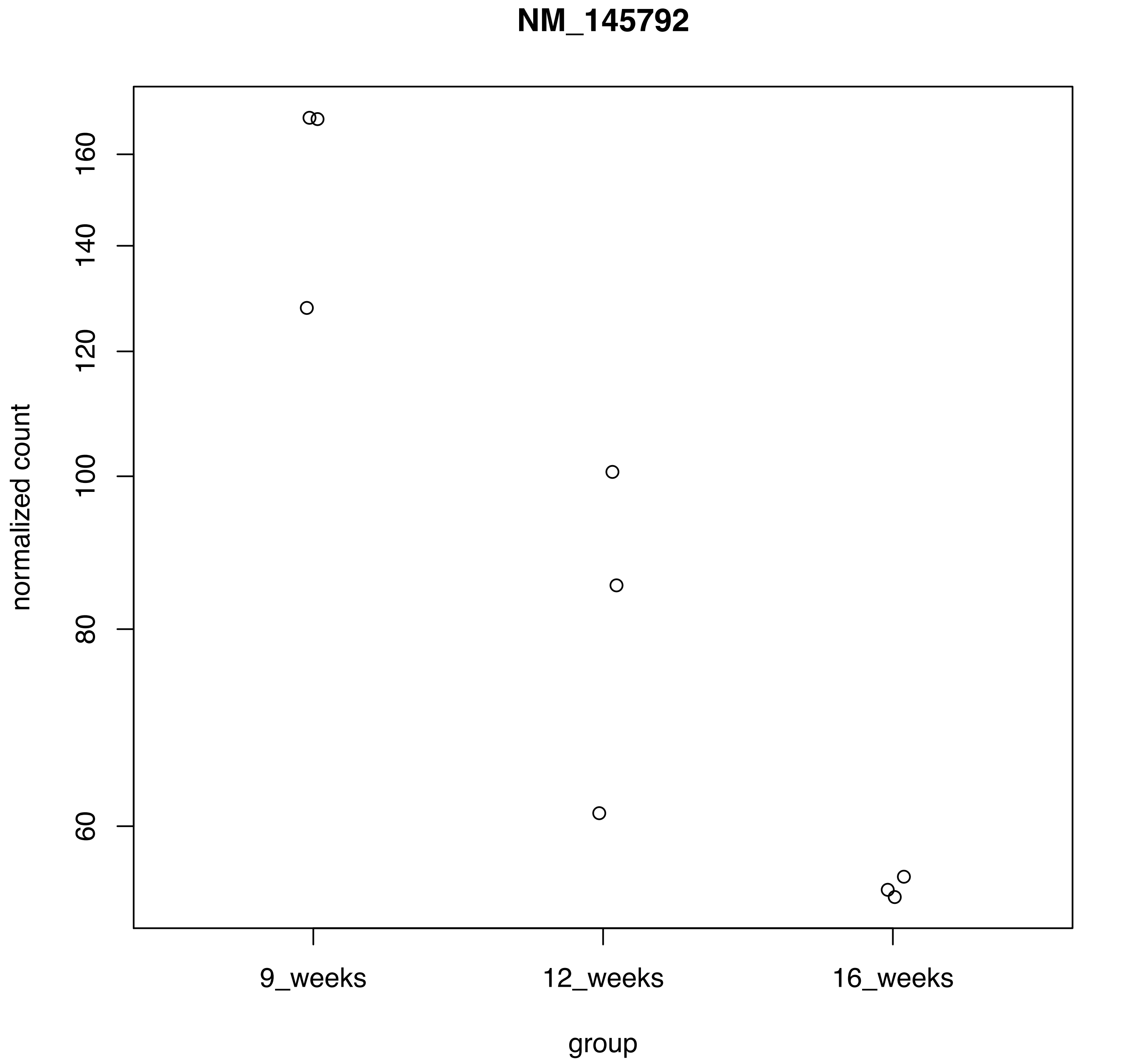
NM_145792
normalized count
160
140
120
100
80
60
9_weeks
12_weeks
16_weeks
group

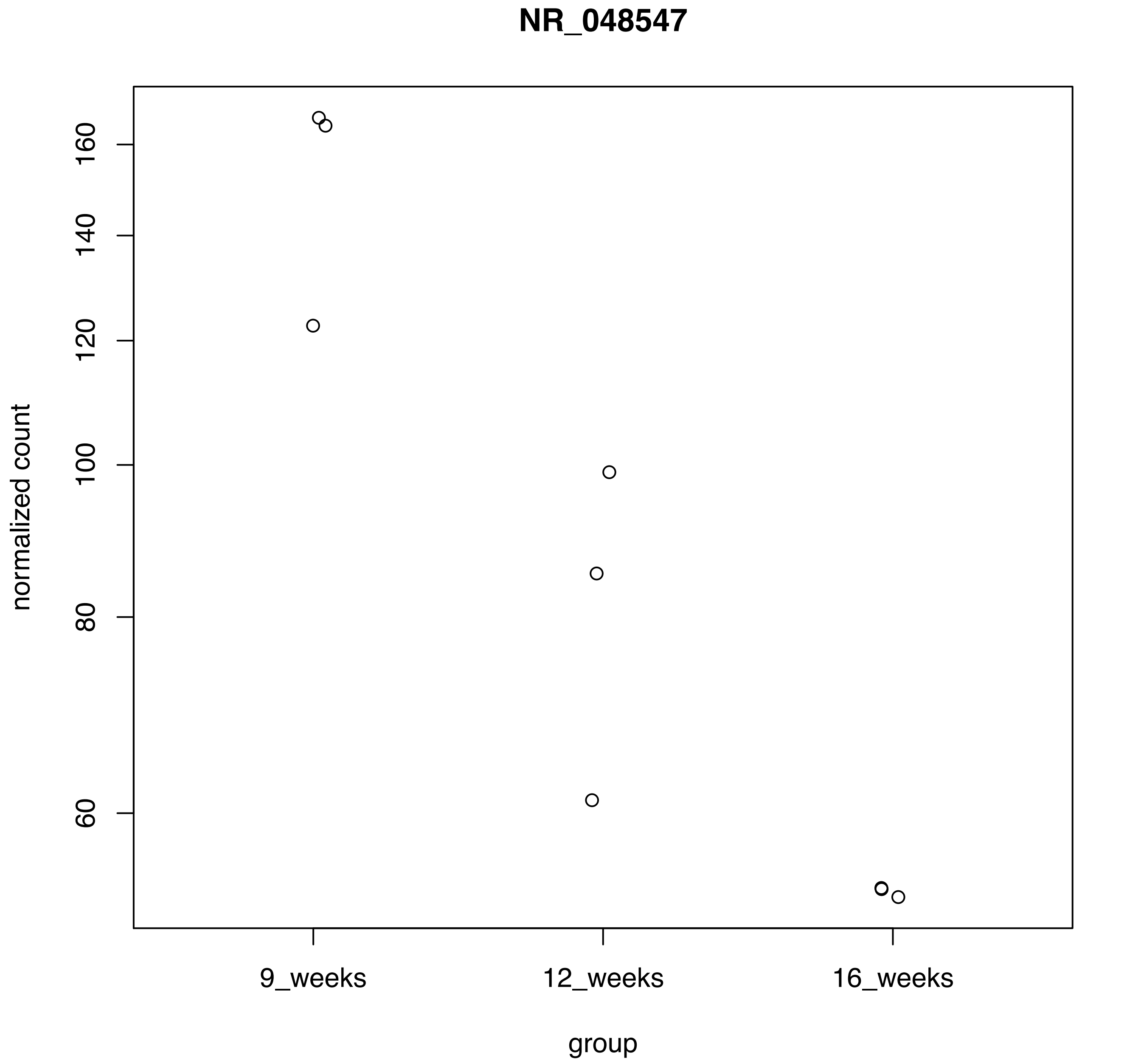
NR_048547
normalized count
160
140
120
100
80
60
9_weeks
12_weeks
16_weeks
group

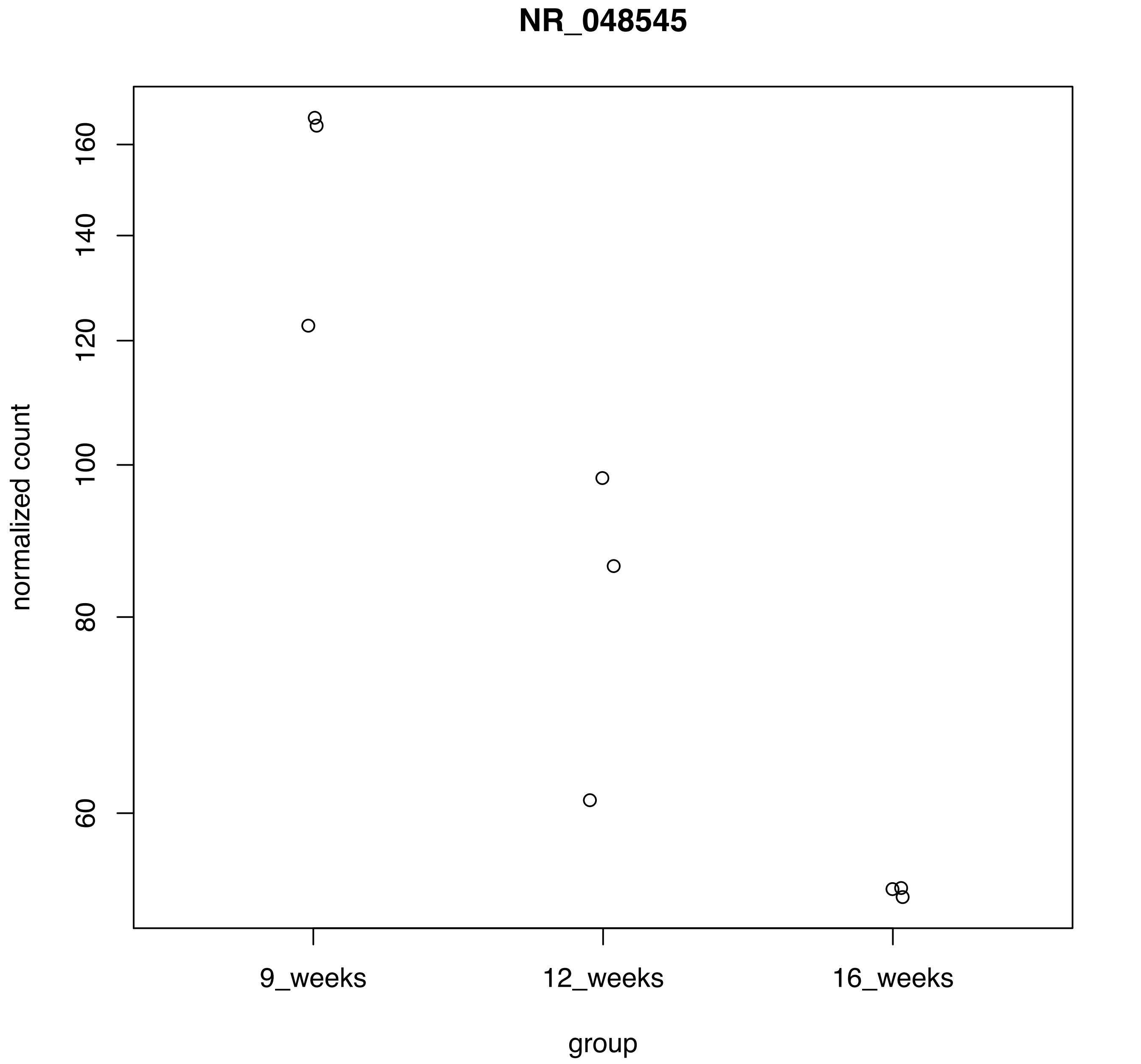

NR_048545
normalized count
60
80
100
120
140
160
9_weeks
12_weeks
16_weeks
group

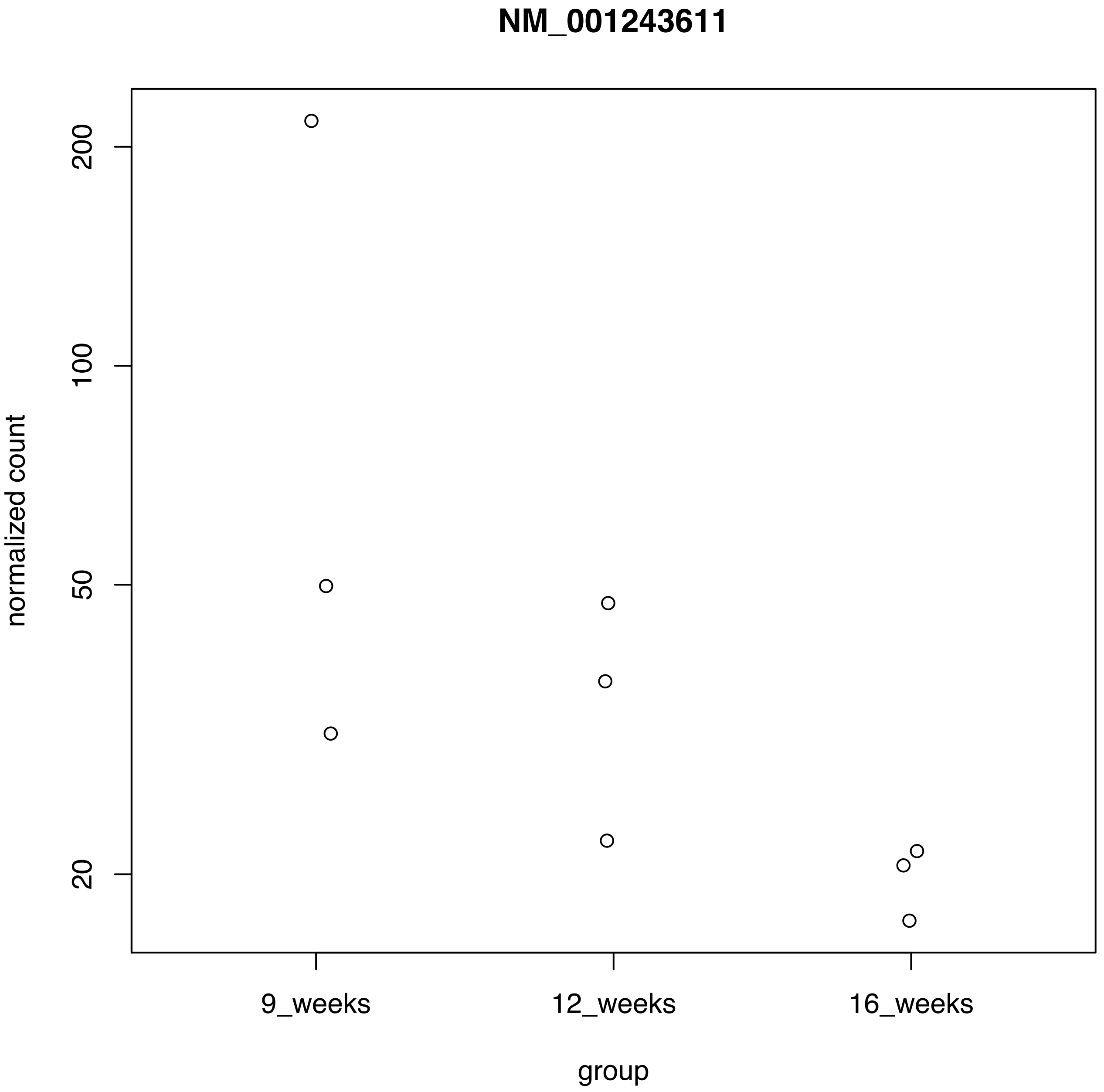
NM_001243611
normalized count
200
100
50
20
9_weeks
12_weeks
16_weeks
group

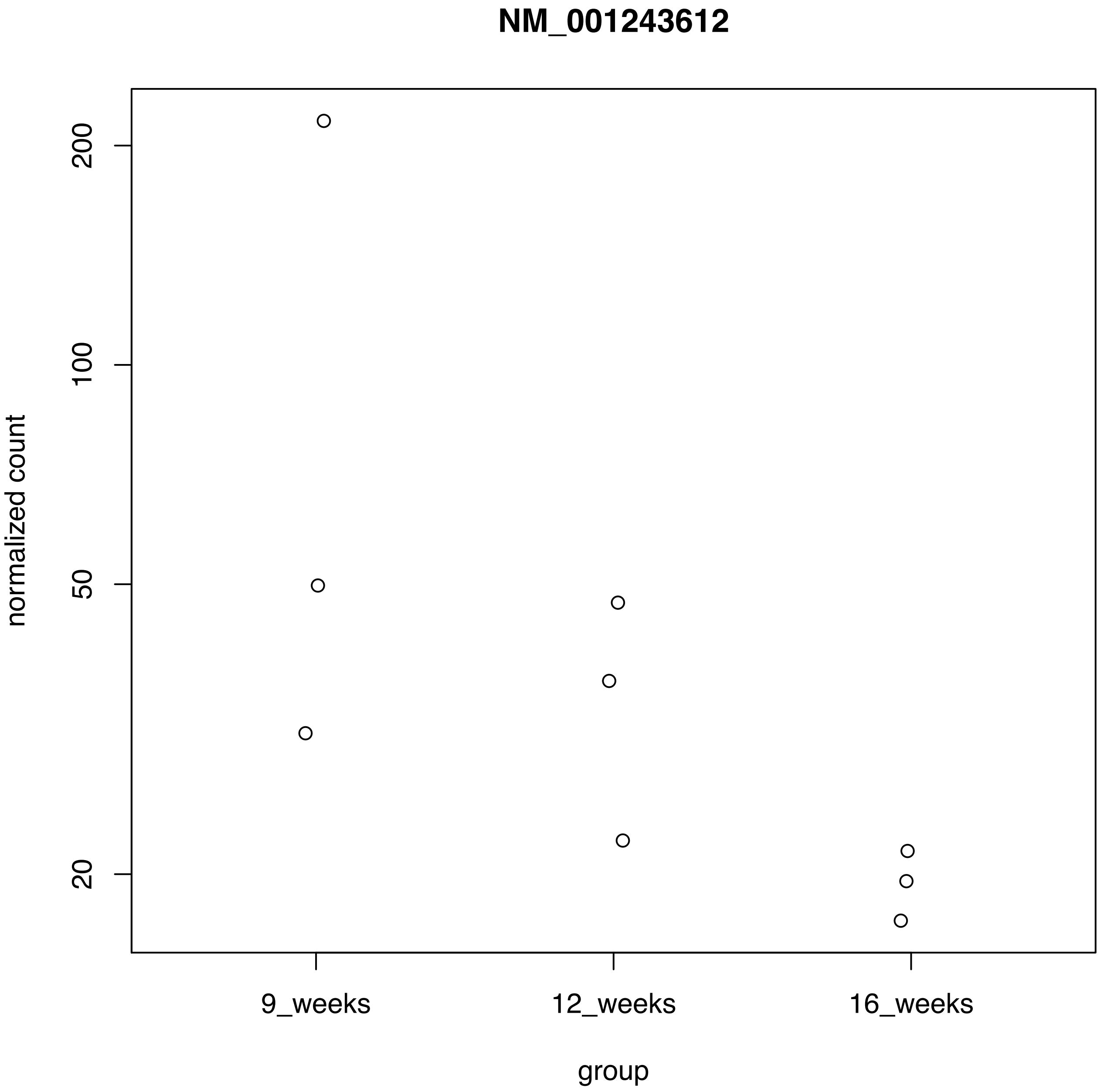
NM_001243612
normalized count
200
100
50
20
9_weeks
12_weeks
16_weeks
group

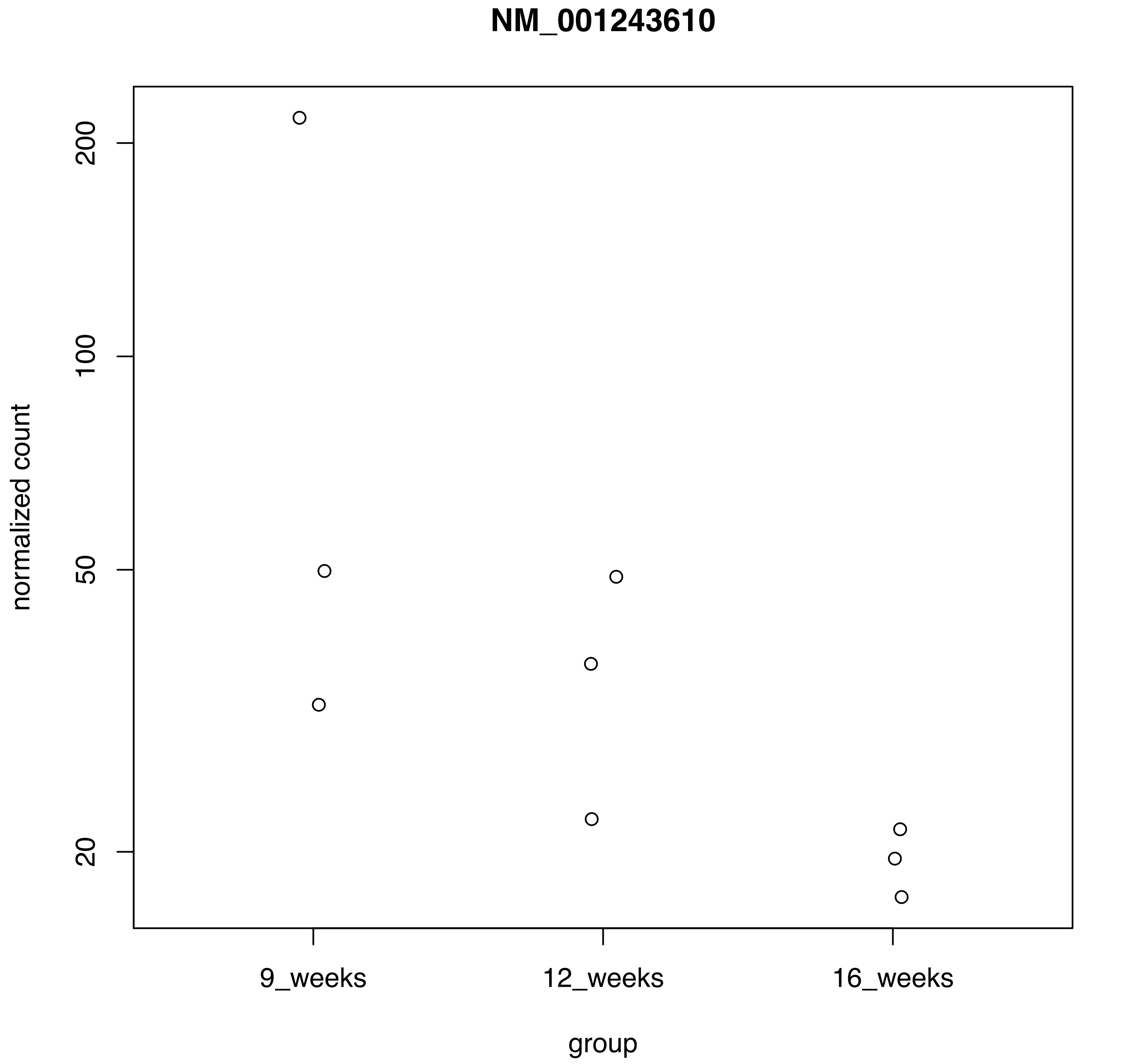

NM_001243610
normalized count
200
100
50
20
9_weeks
12_weeks
16_weeks
group

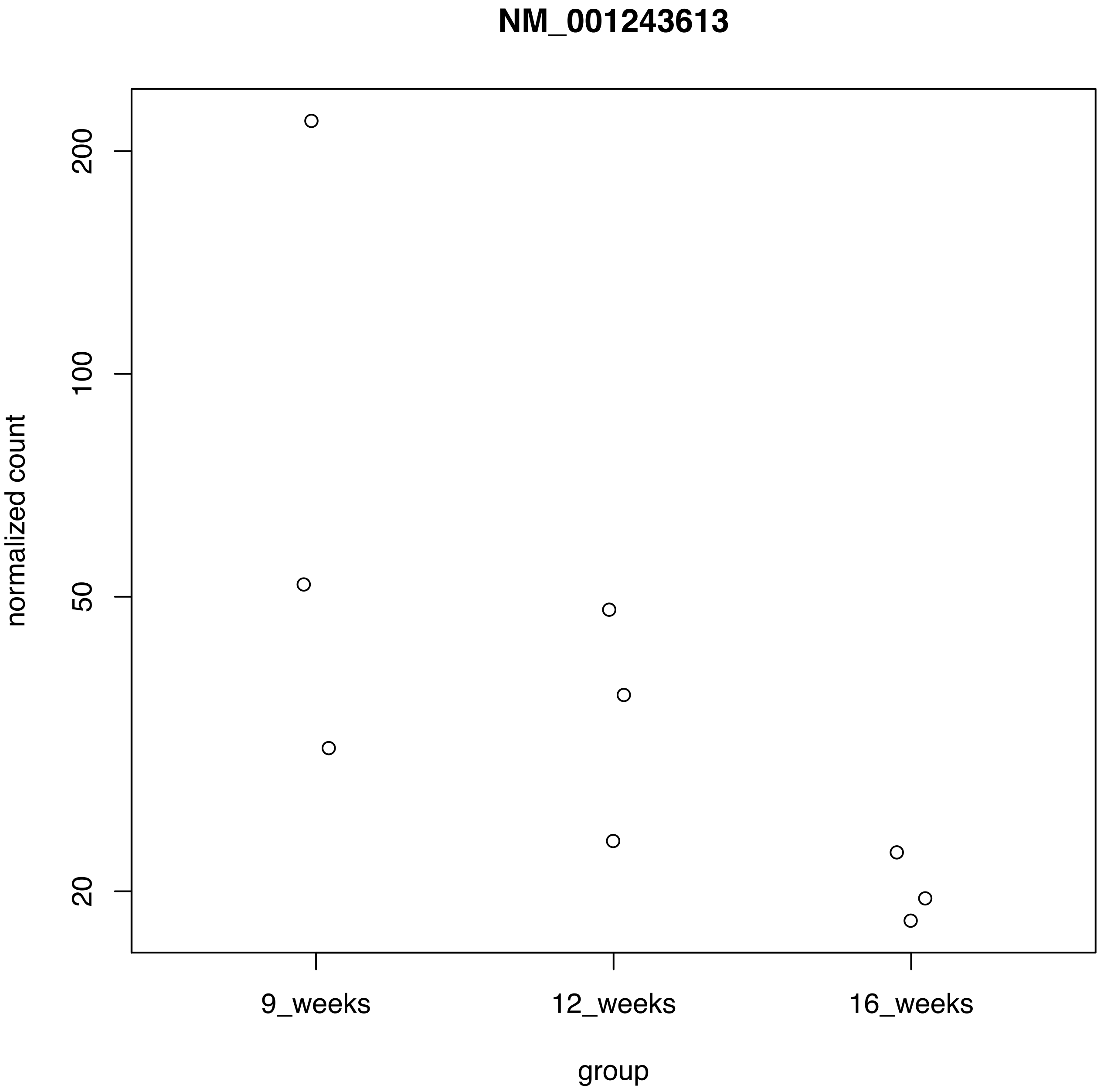

NM_001243613
normalized count
200
100
50
20
9_weeks
12_weeks
16_weeks
group

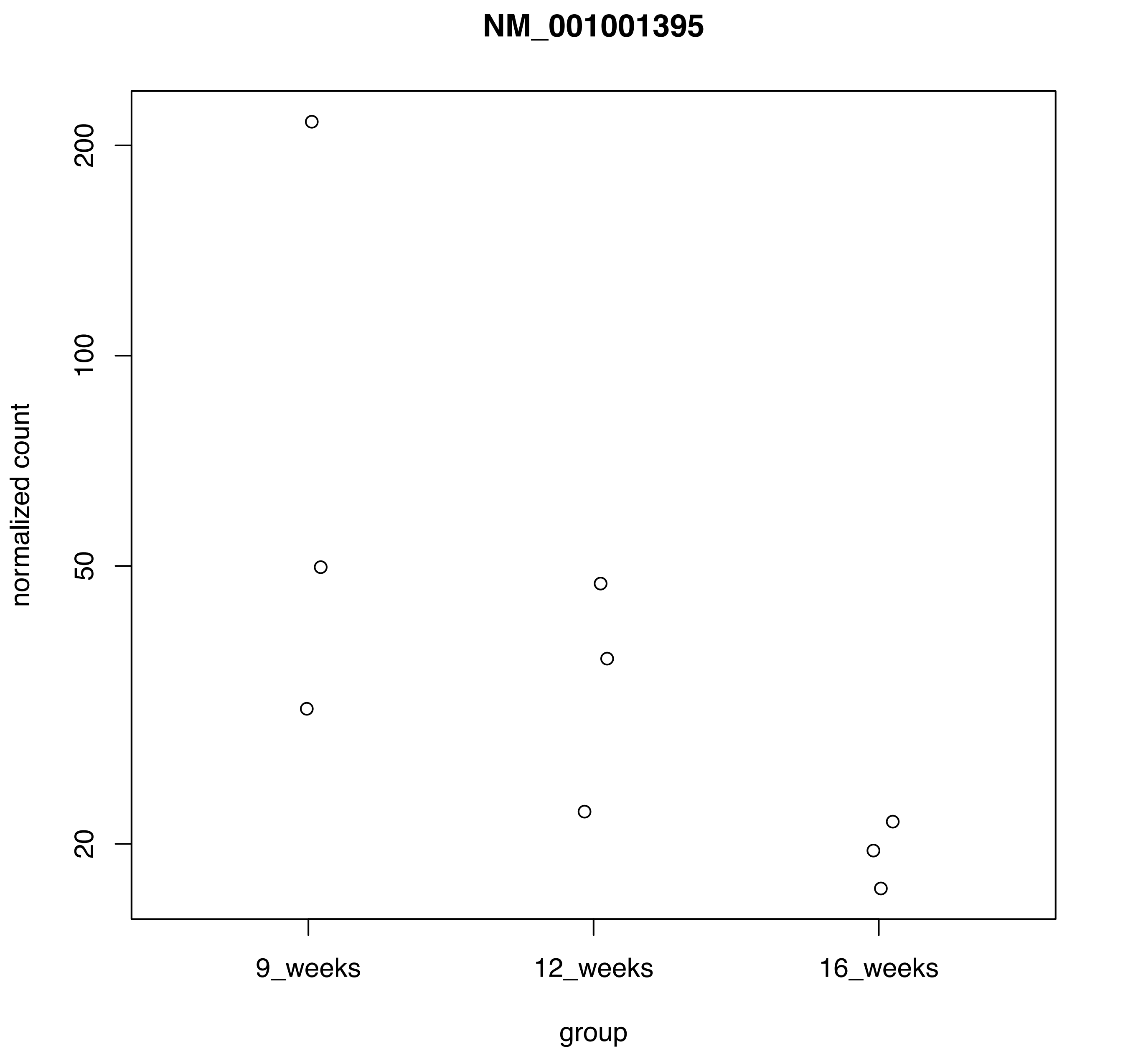
NM_001001395
normalized count
200
100
50
20
9_weeks
12_weeks
16_weeks
group

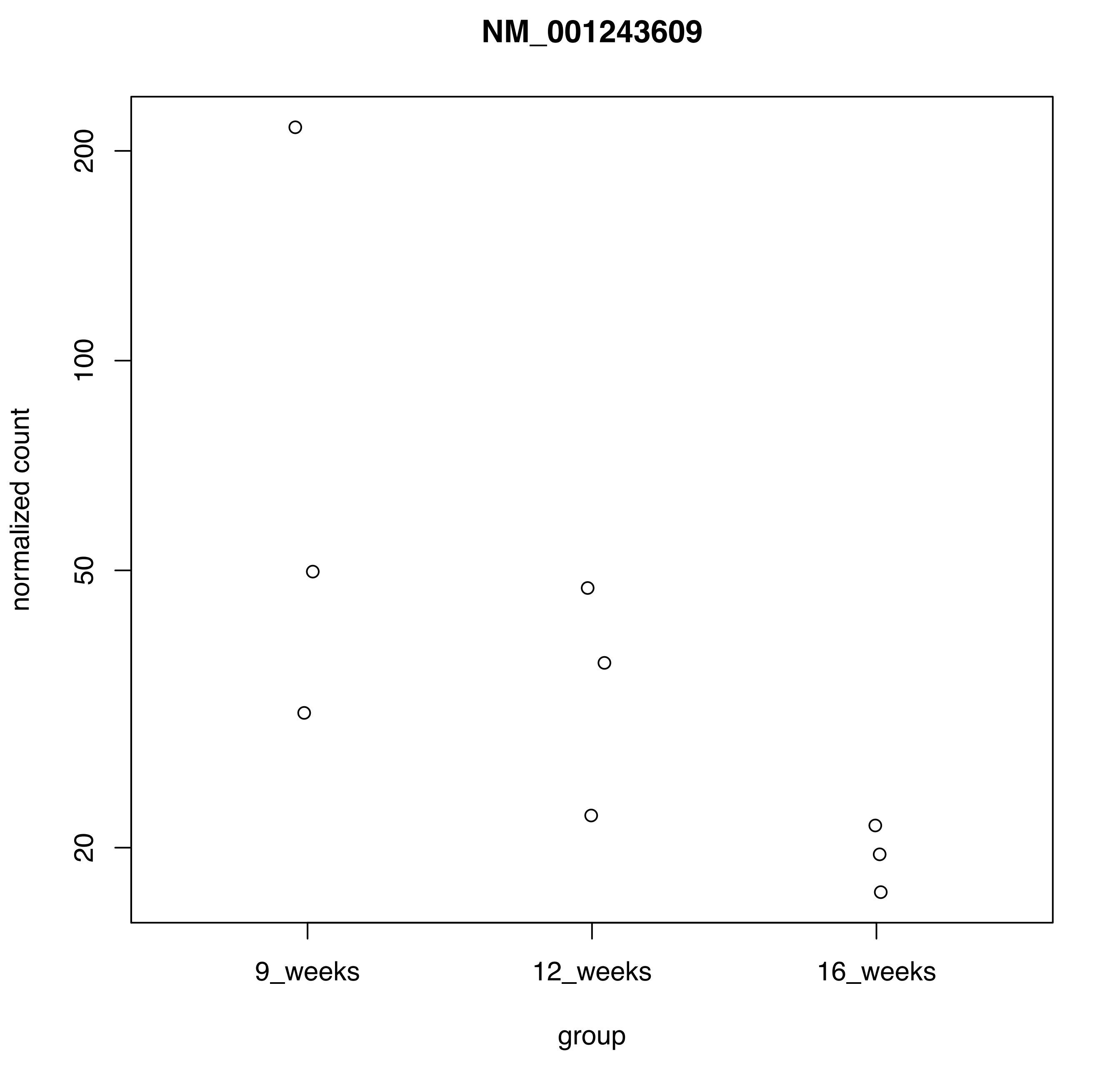
NM_001243609
normalized count
200
100
50
20
9_weeks
12_weeks
16_weeks
group

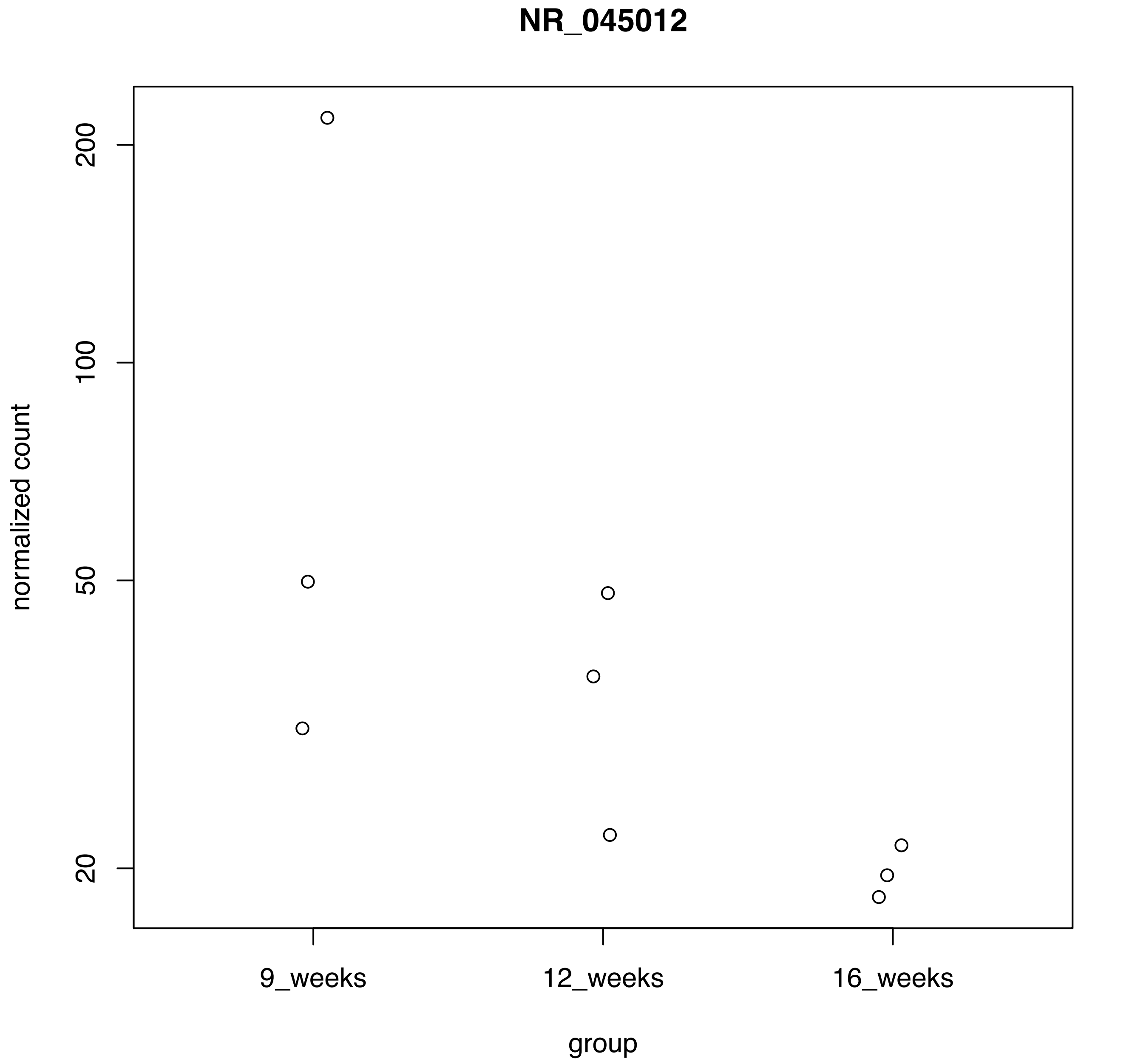
NR_045012
normalized count
200
100
50
20
9_weeks
12_weeks
16_weeks
group

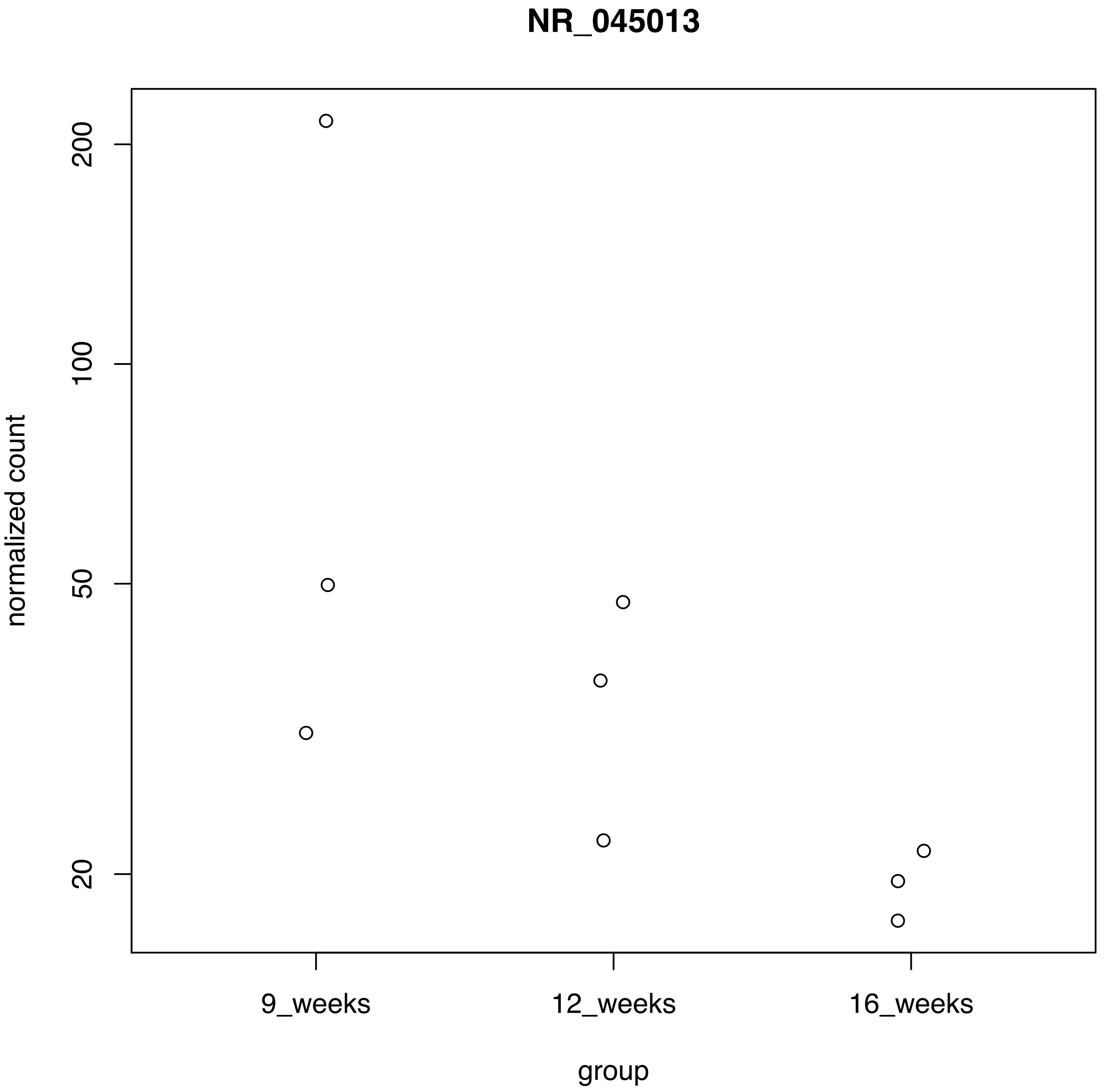
NR_045013
normalized count
200
100
50
20
9_weeks
12_weeks
16_weeks
group

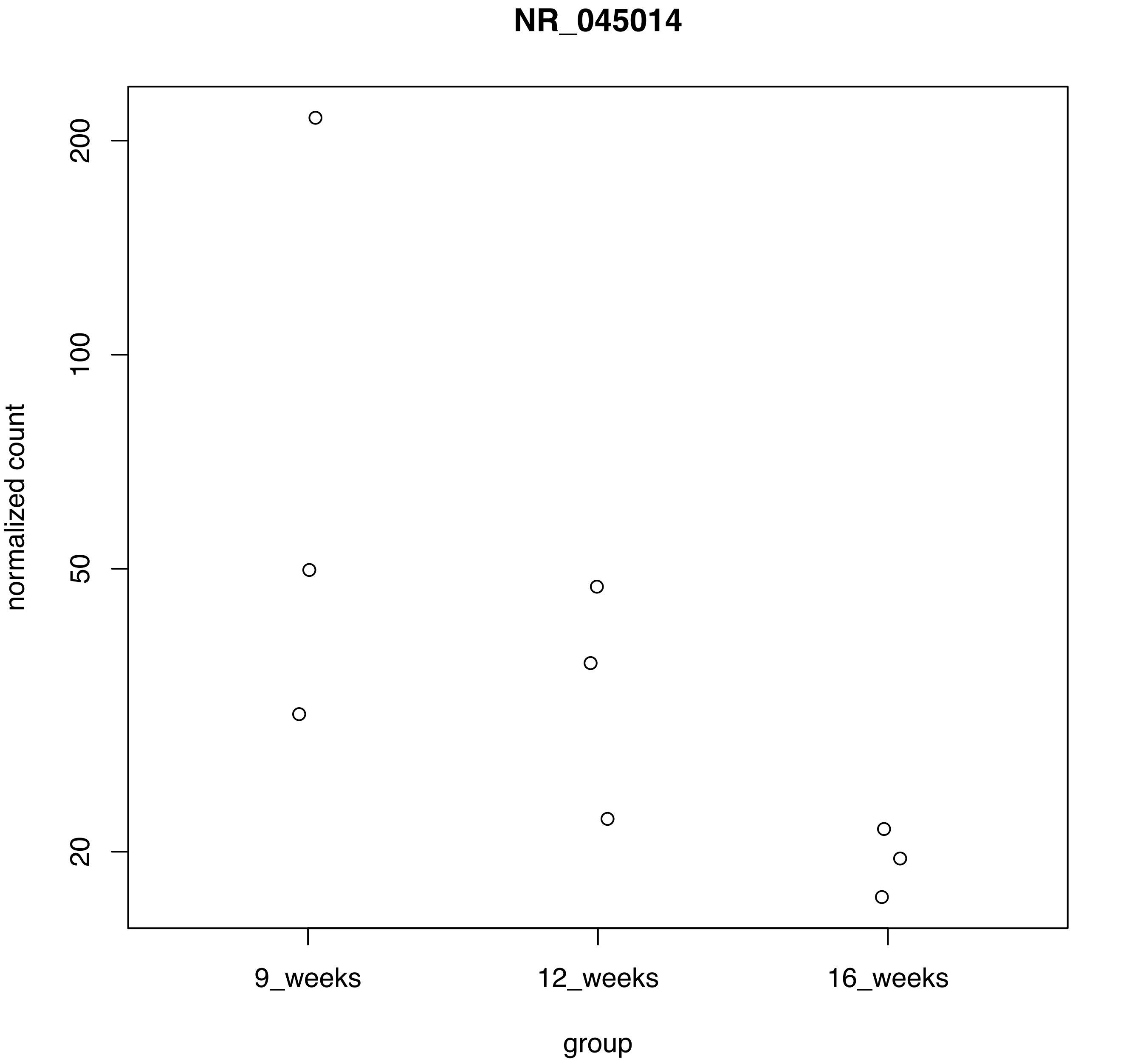
NR_045014
normalized count
200
100
50
20
9_weeks
12_weeks
16_weeks
group

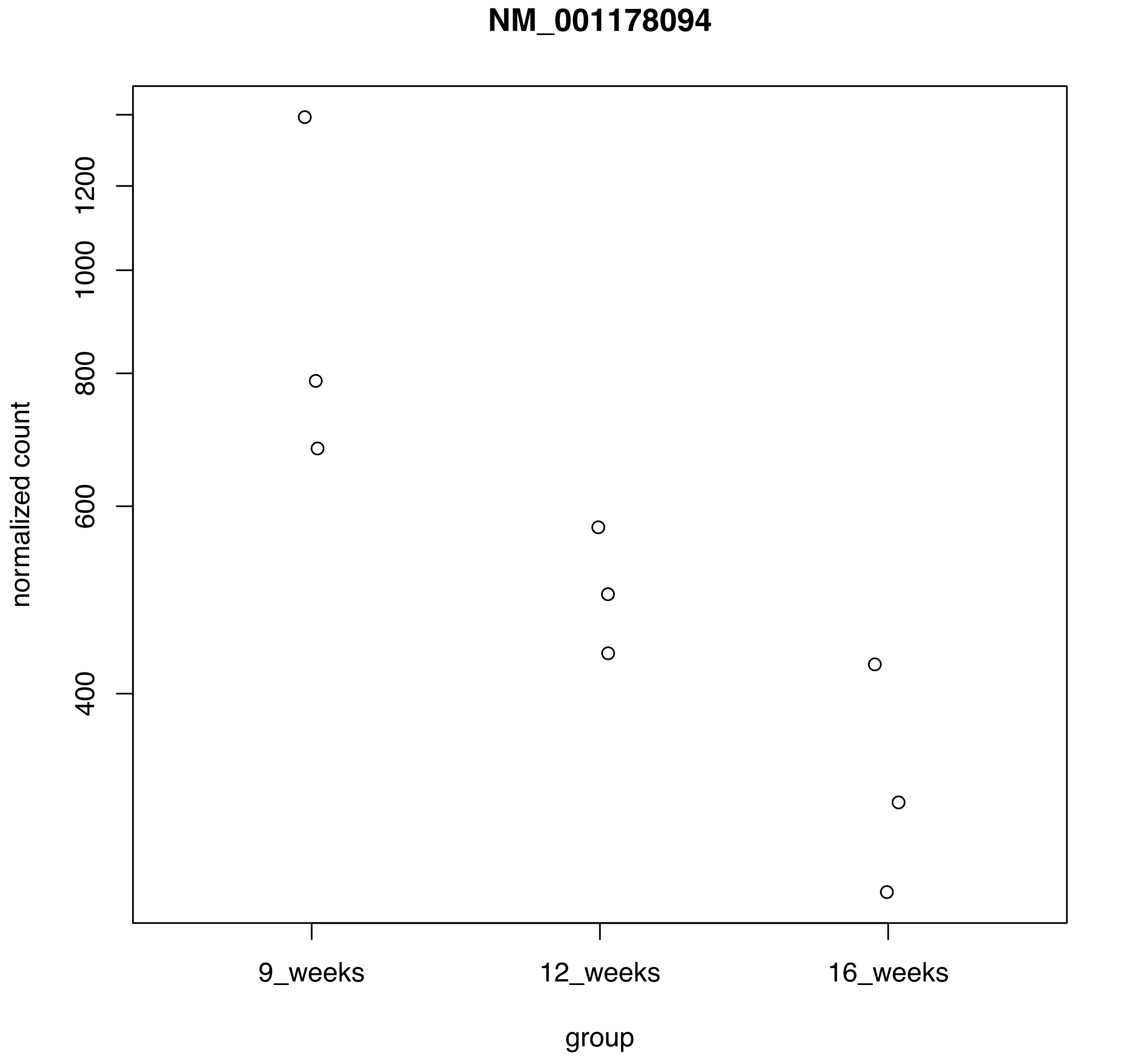

NM_001178094
normalized count
400
600
800
1000
1200
9_weeks
12_weeks
16_weeks
group

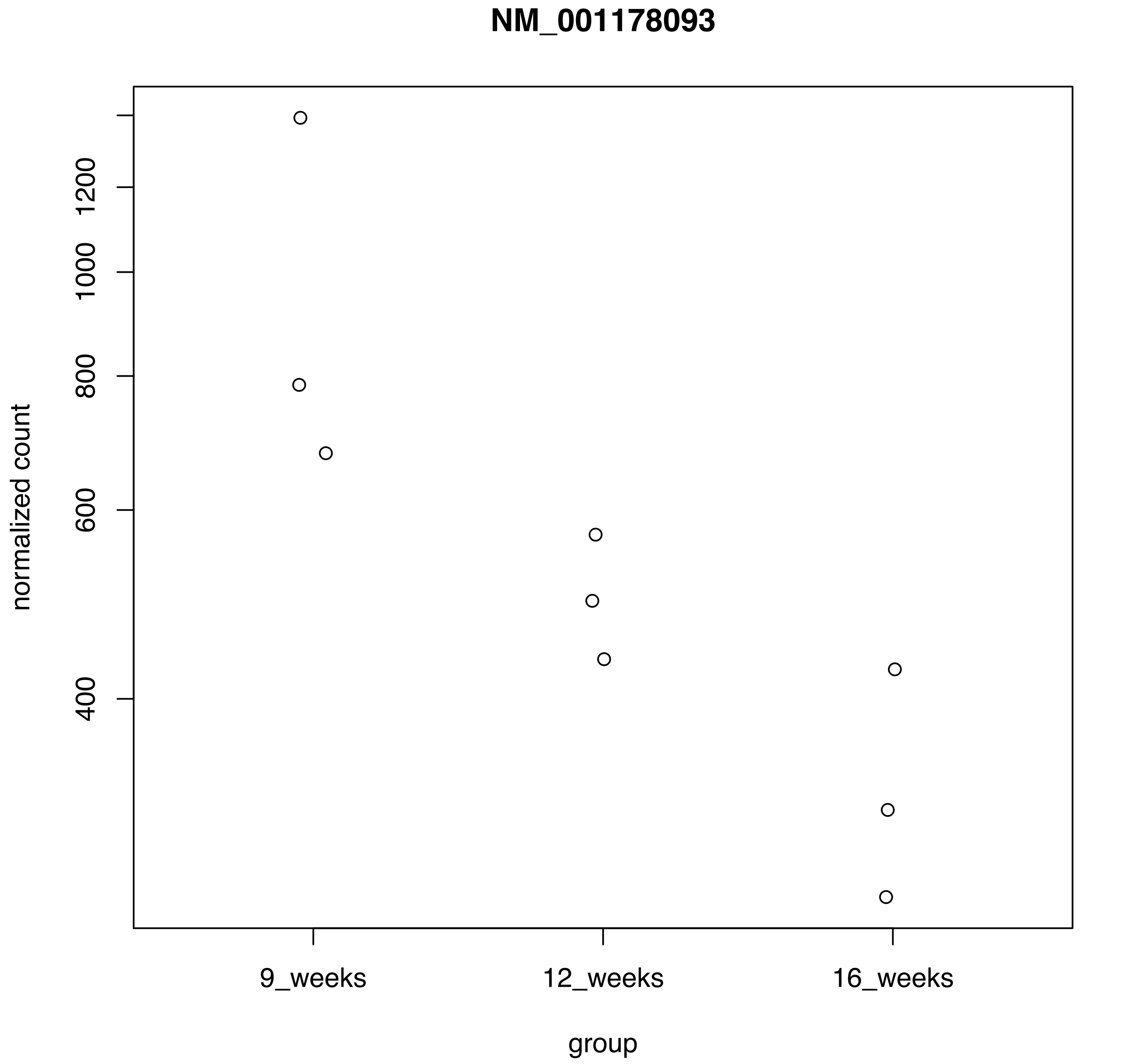

NM_001178093
normalized count
400
600
800
1000
1200
9_weeks
12_weeks
16_weeks
group

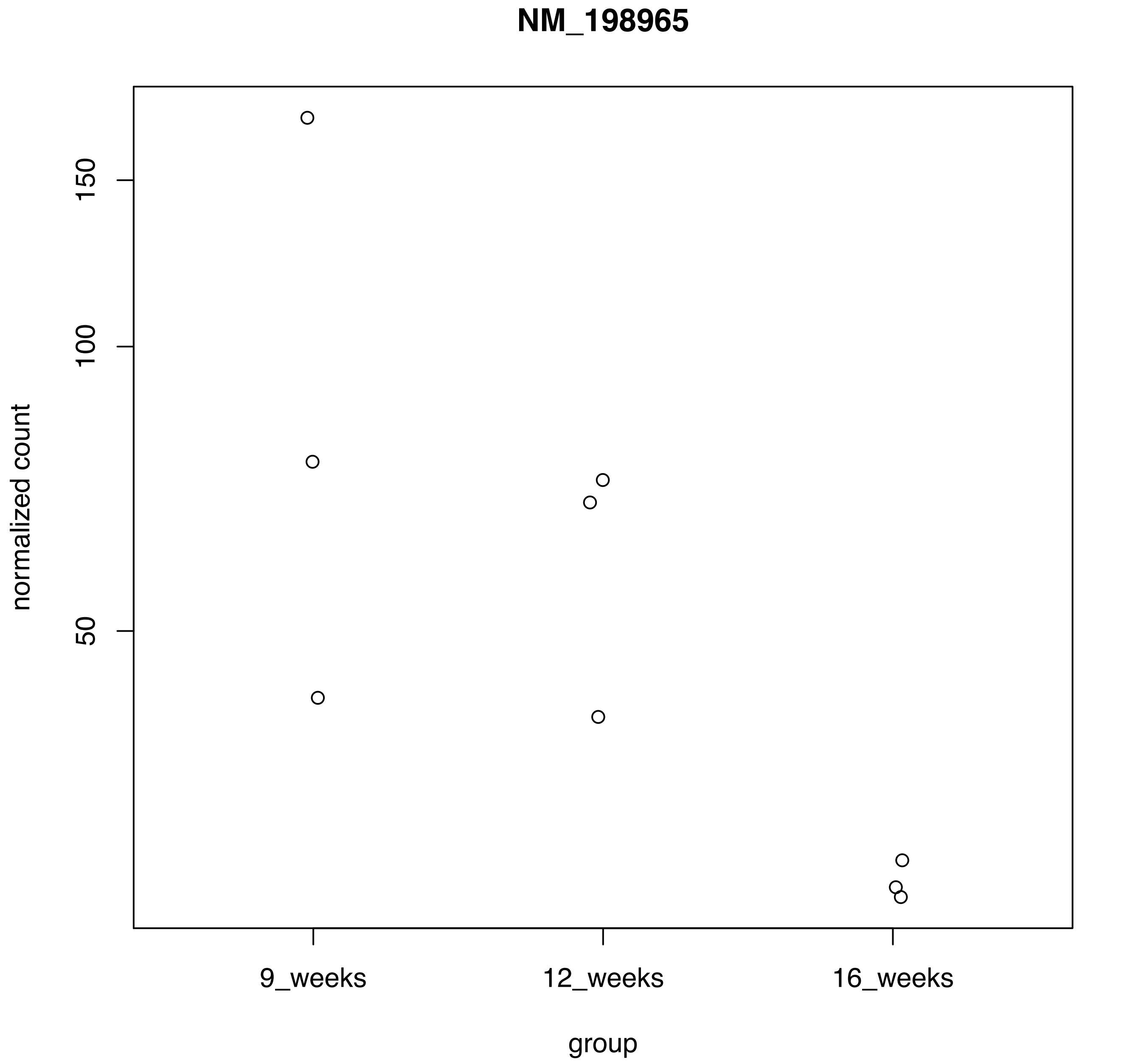
NM_198965
normalized count
150
100
50
9_weeks
12_weeks
16_weeks
group

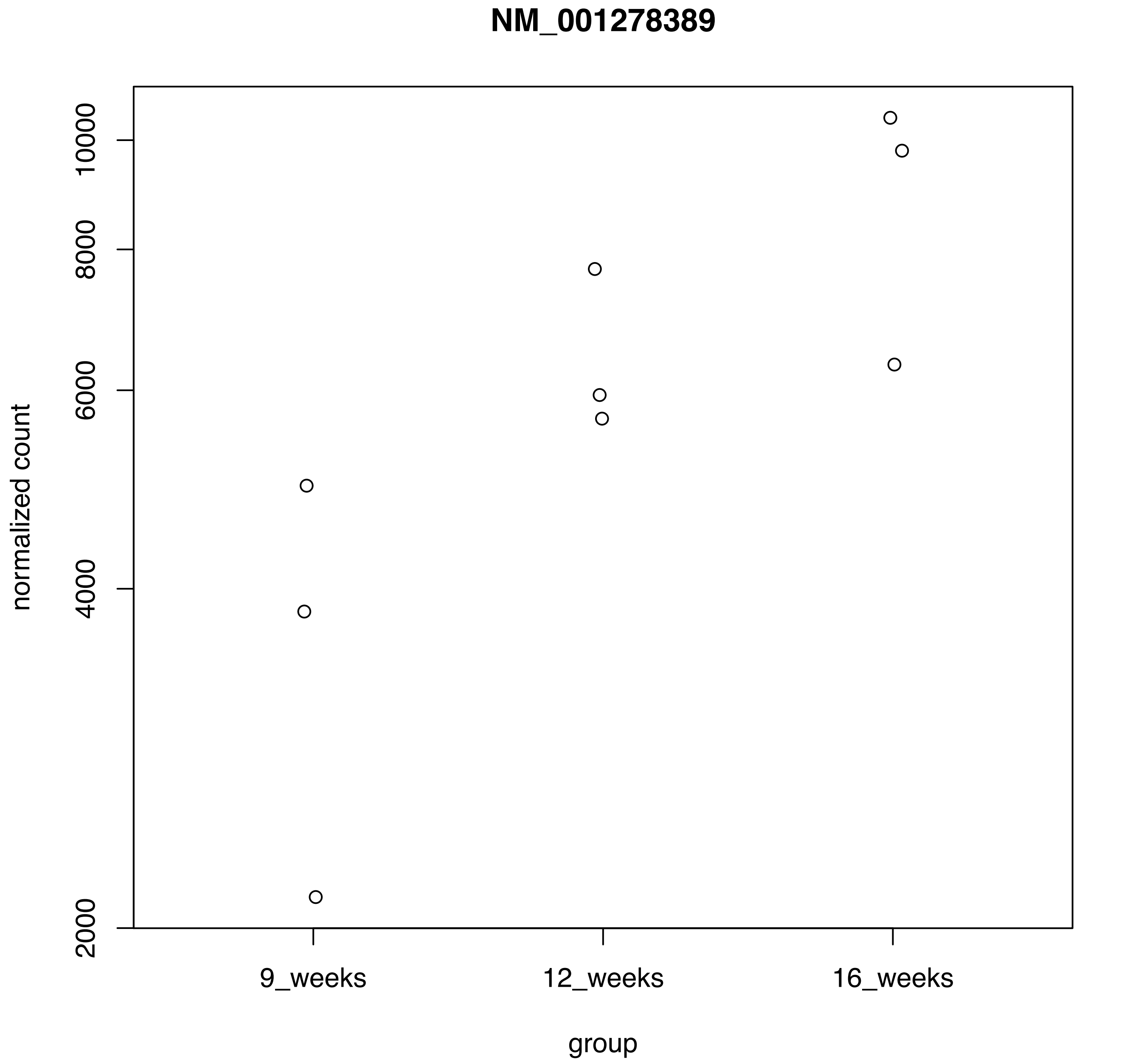
NM_001278389
normalized count
10000
8000
6000
4000
2000
9_weeks
12_weeks
16_weeks
group

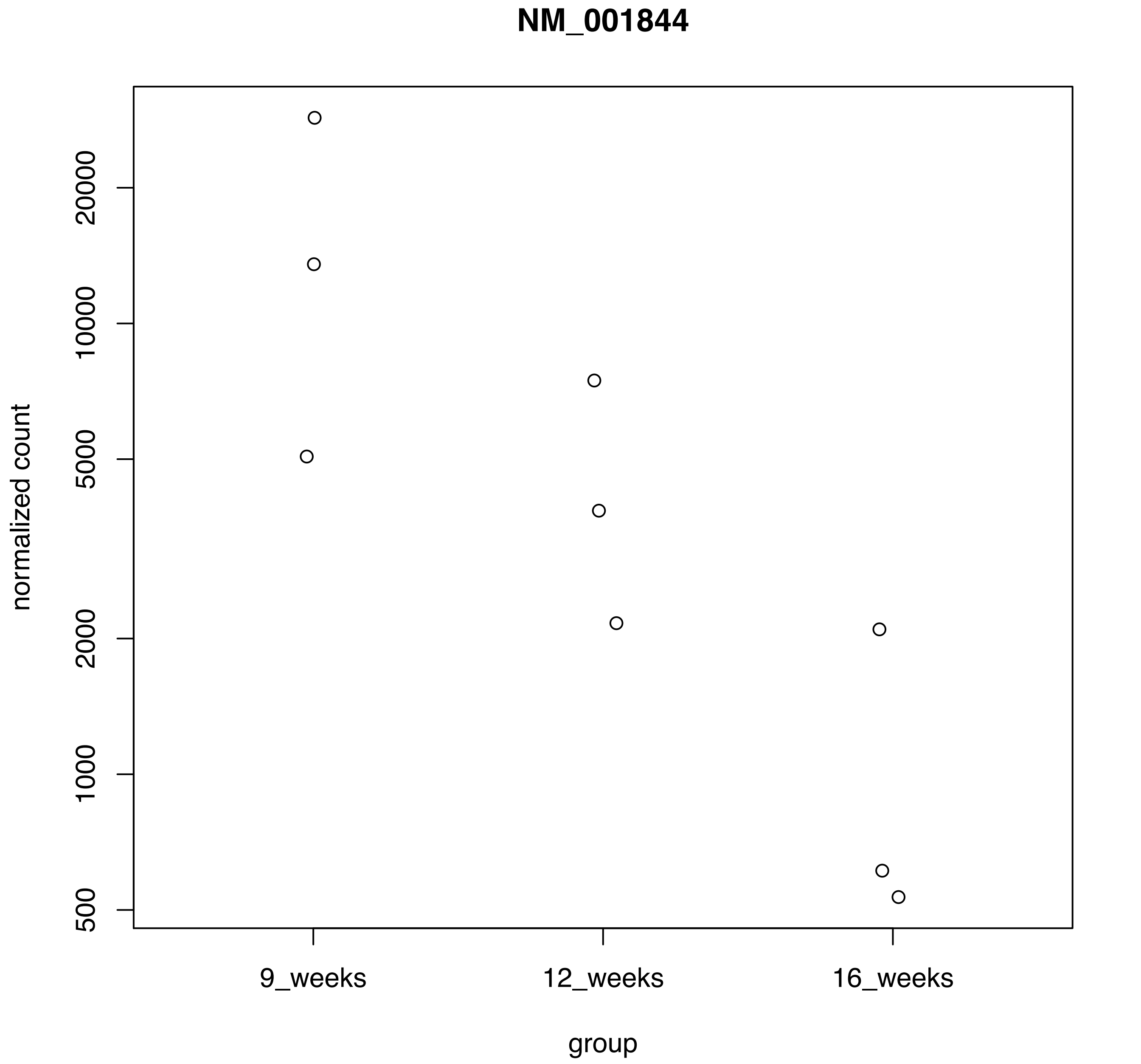
NM_001844
normalized count
20000
10000
5000
2000
1000
500
9_weeks
12_weeks
16_weeks
group

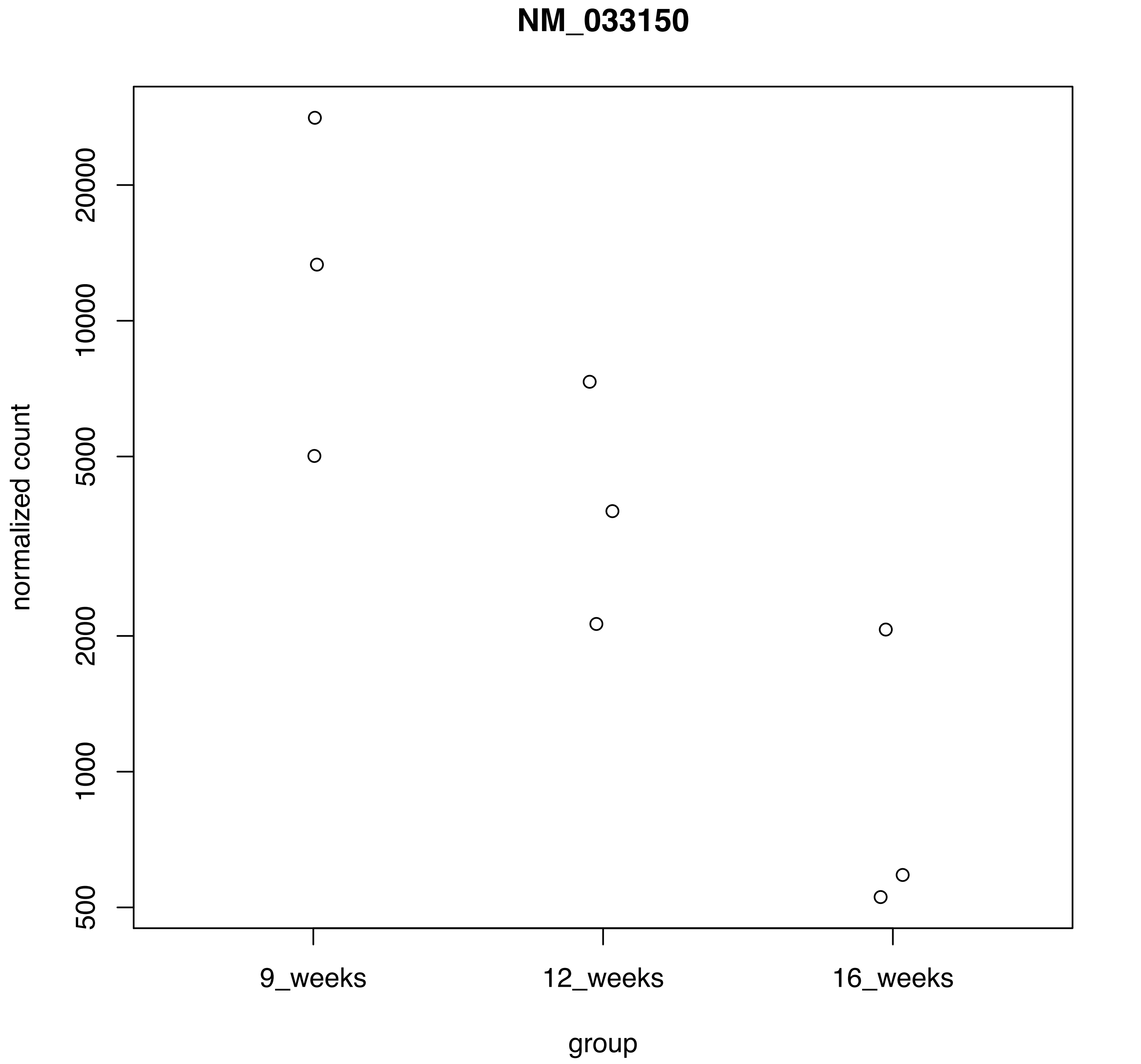
NM_033150
normalized count
20000
10000
5000
2000
1000
500
9_weeks
12_weeks
16_weeks
group

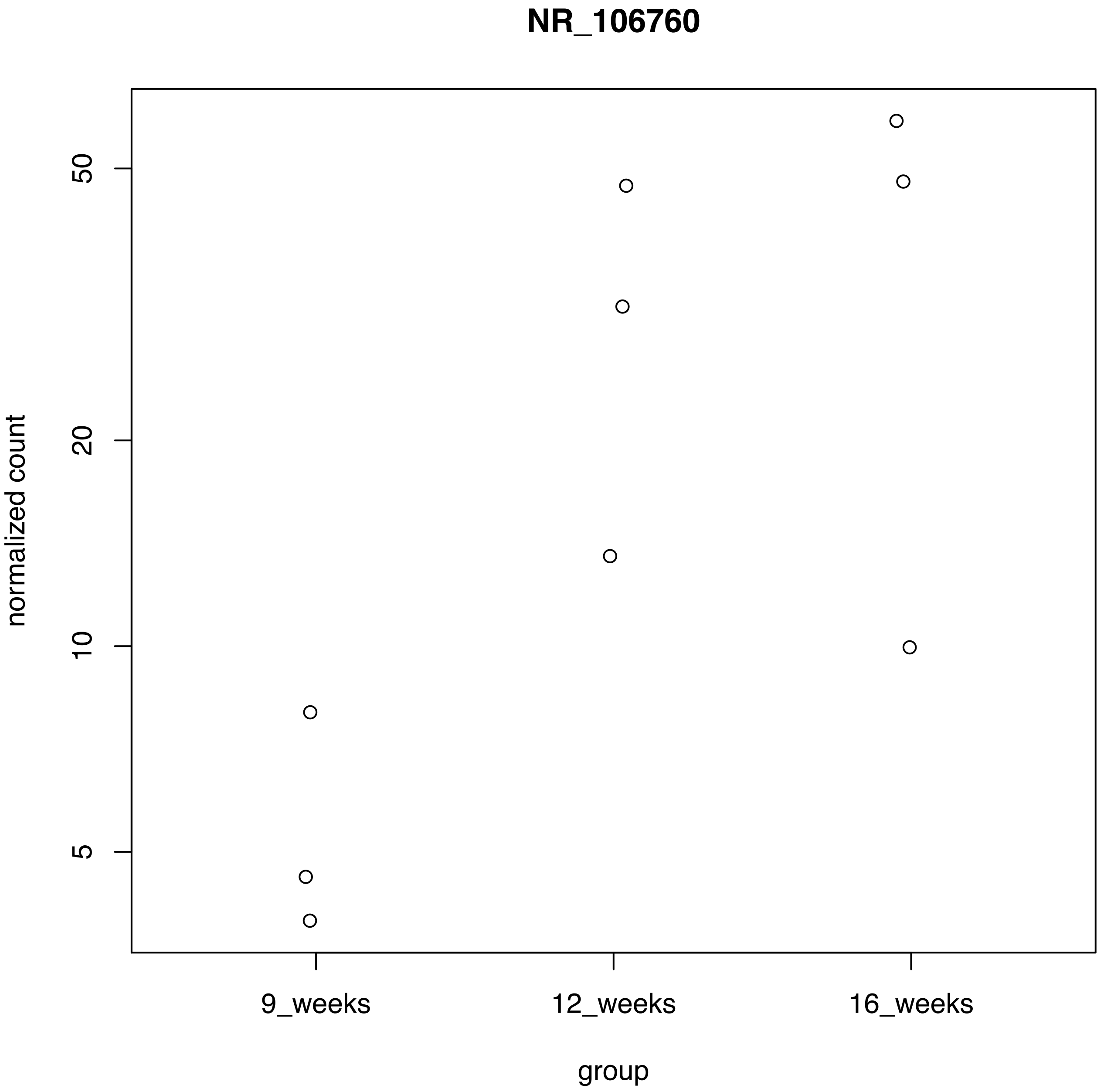
NR_106760
normalized count
50
20
10
5
9_weeks
12_weeks
16_weeks
group

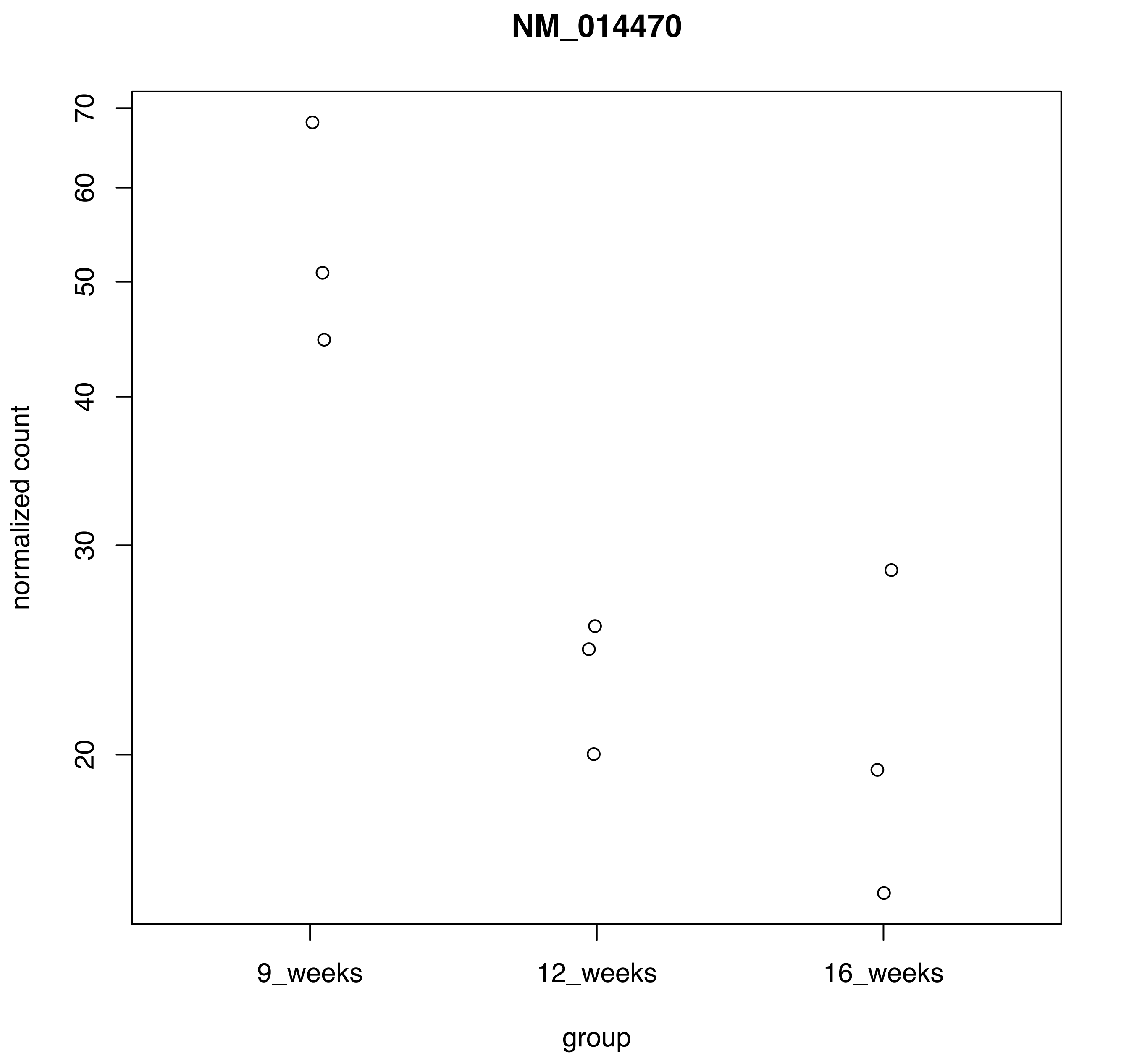
NM_014470
normalized count
70
60
50
40
30
20
9_weeks
12_weeks
16_weeks
group

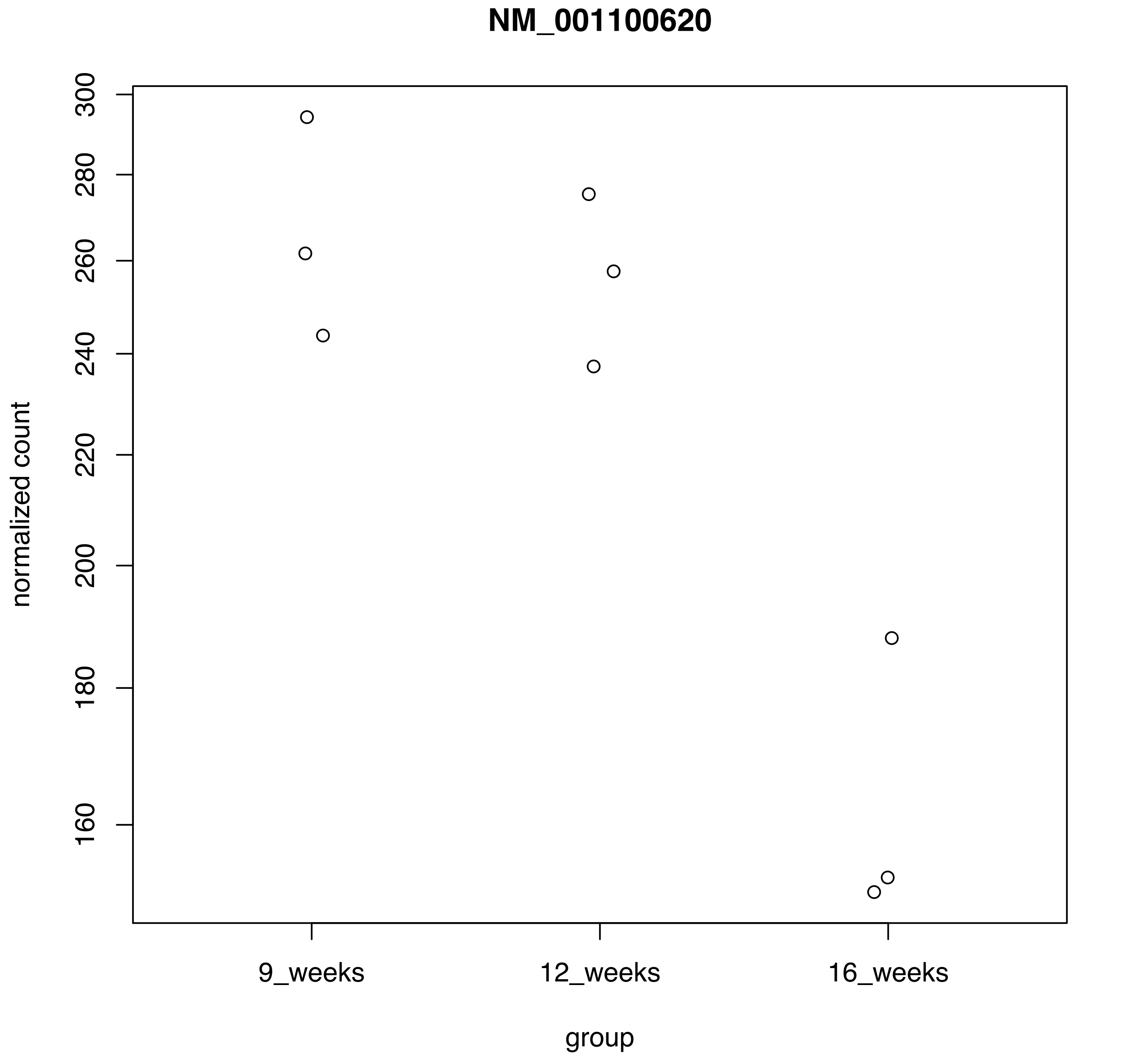
NM_001100620
normalized count
300
280
260
240
220
200
180
160
9_weeks
12_weeks
16_weeks
group

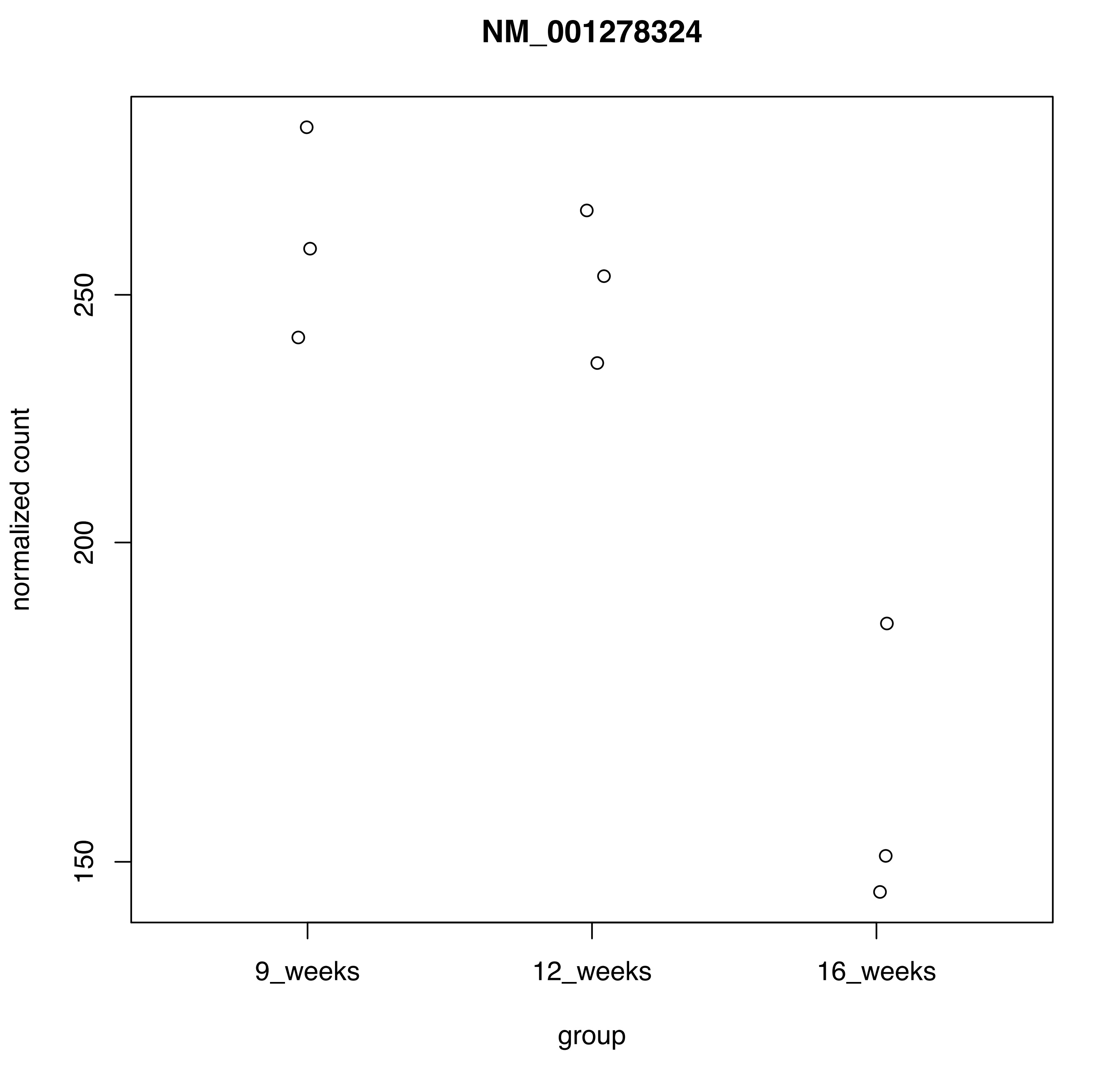
NM_001278324
normalized count
250
200
150
9_weeks
12_weeks
16_weeks
group

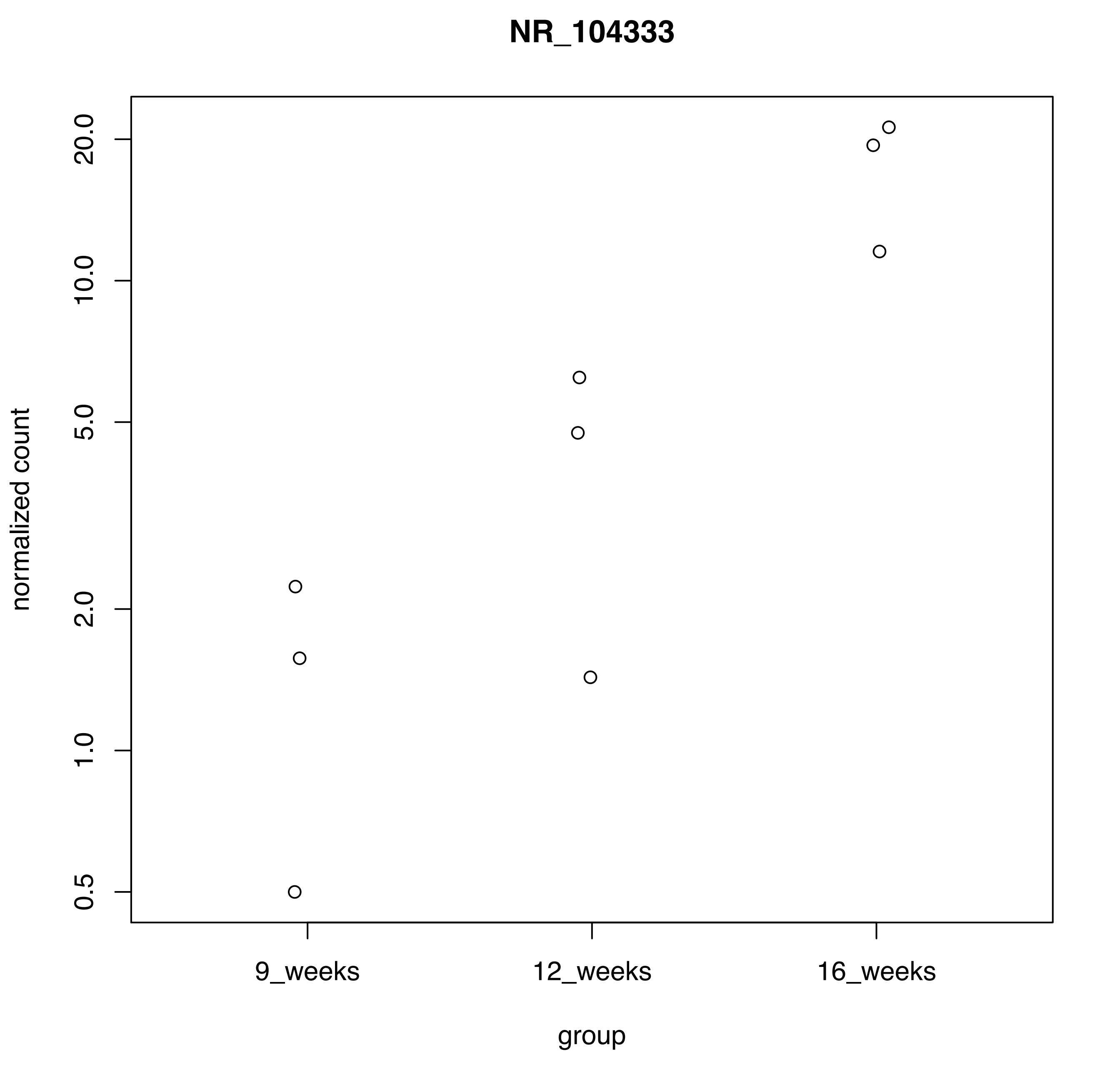

NR_104333
normalized count
20.0
10.0
5.0
2.0
1.0
0.5
9_weeks
12_weeks
16_weeks
group

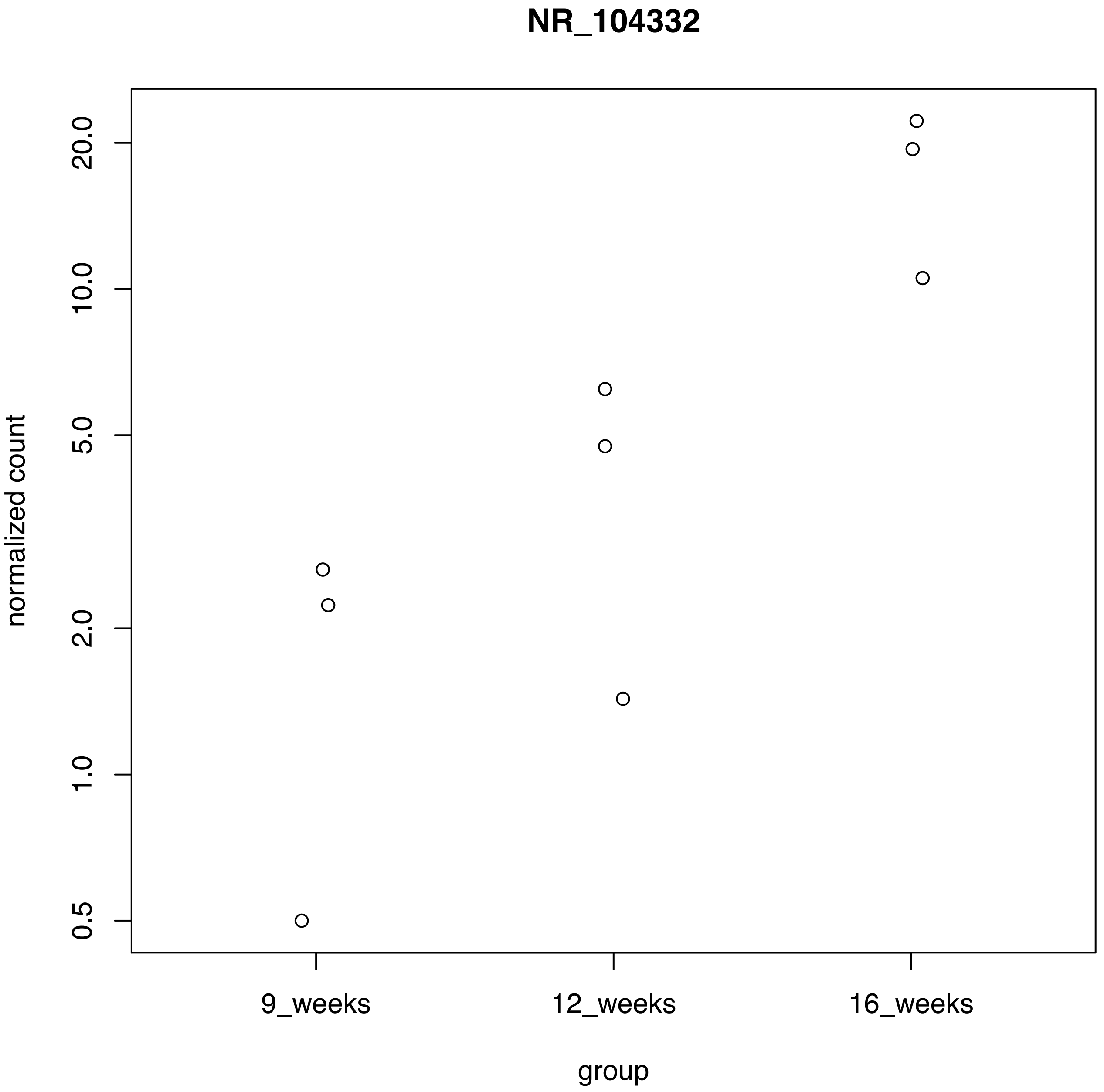
NR_104332
normalized count
0.5
1.0
2.0
5.0
10.0
20.0
9_weeks
12_weeks
16_weeks
group

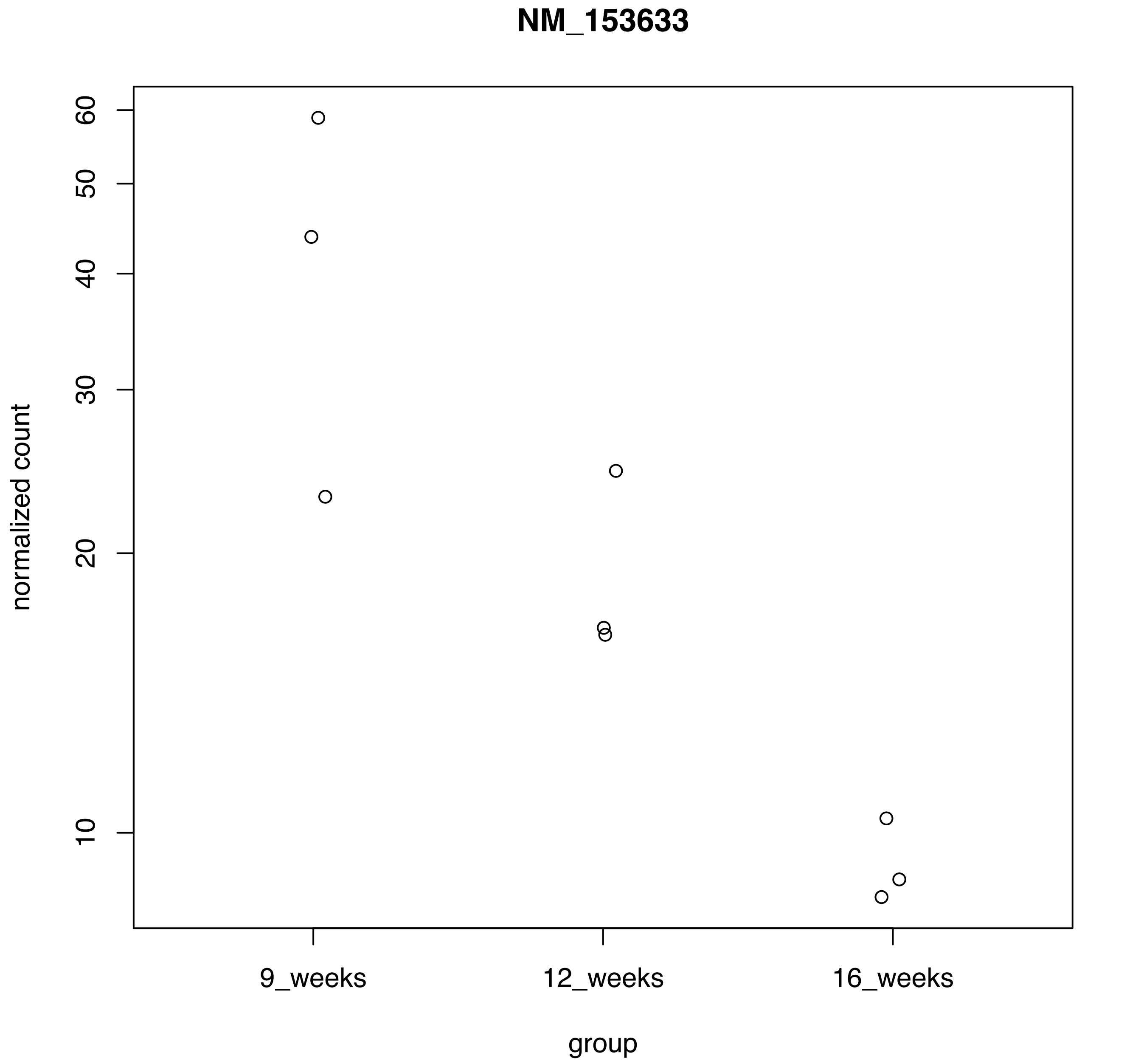
NM_153633
normalized count
60
50
40
30
20
10
9_weeks
12_weeks
16_weeks
group

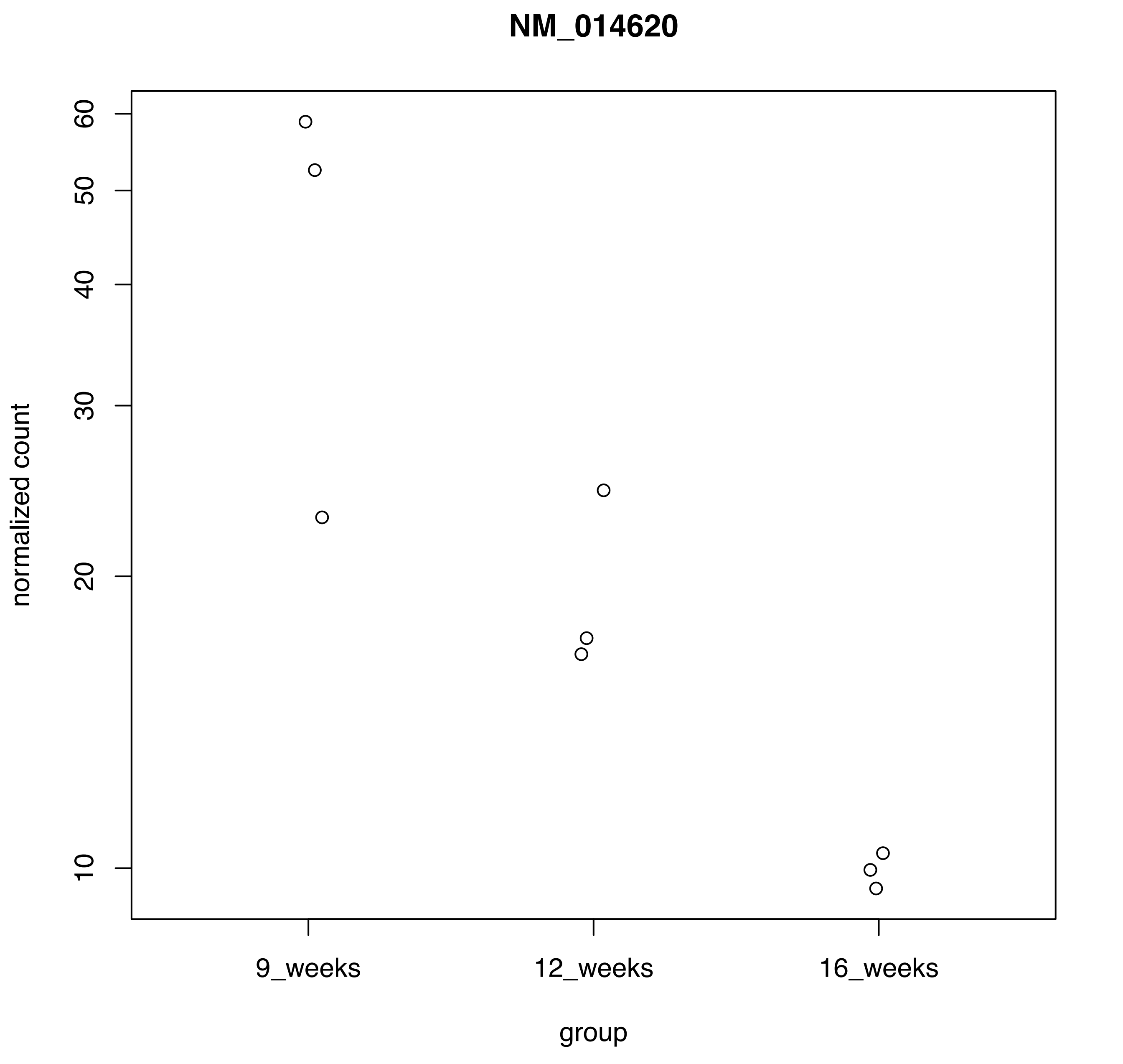
NM_014620
normalized count
60
50
40
30
20
10
9_weeks
12_weeks
16_weeks
group

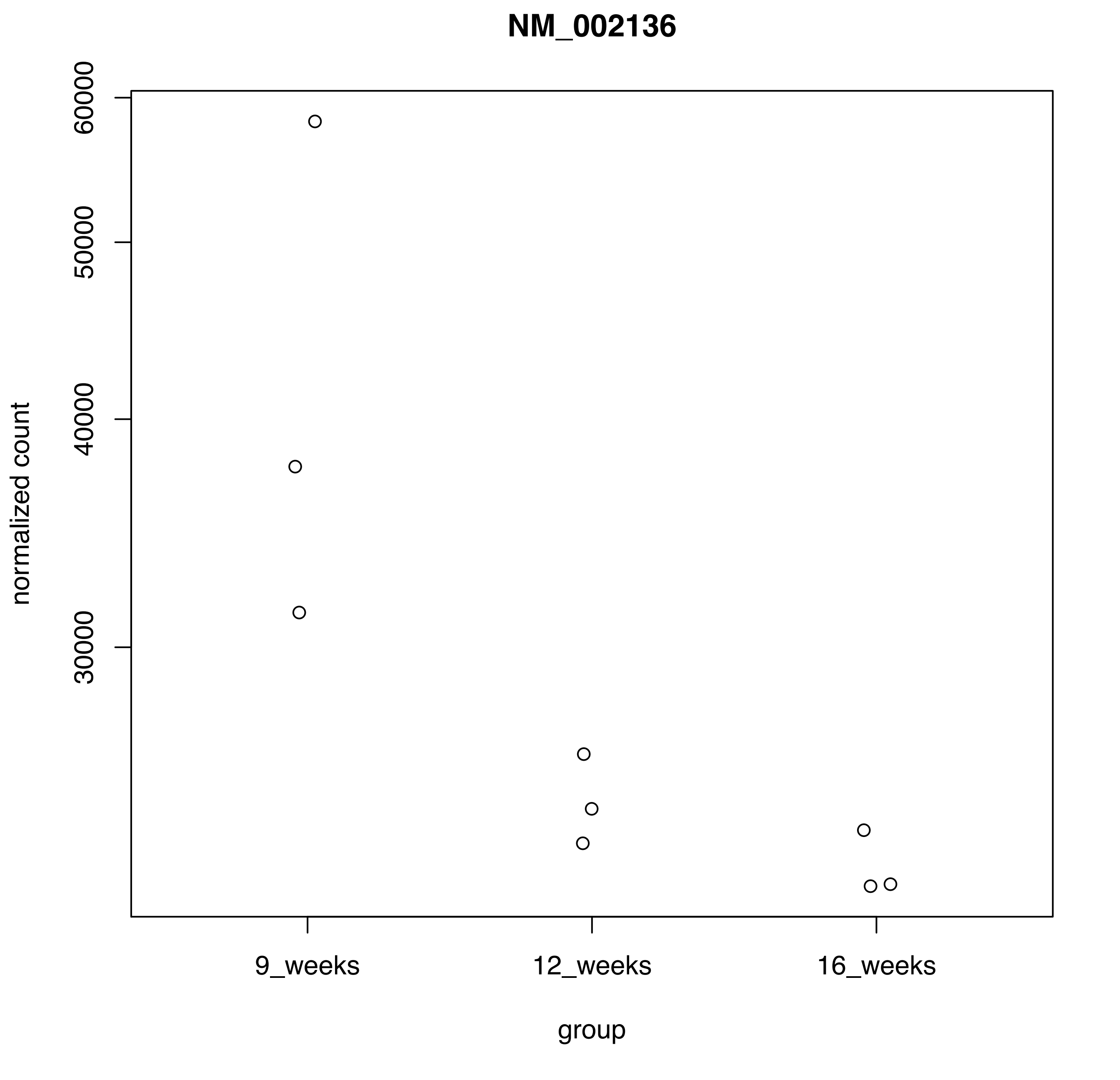

NM_002136
normalized count
60000
50000
40000
30000
9_weeks
12_weeks
16_weeks
group

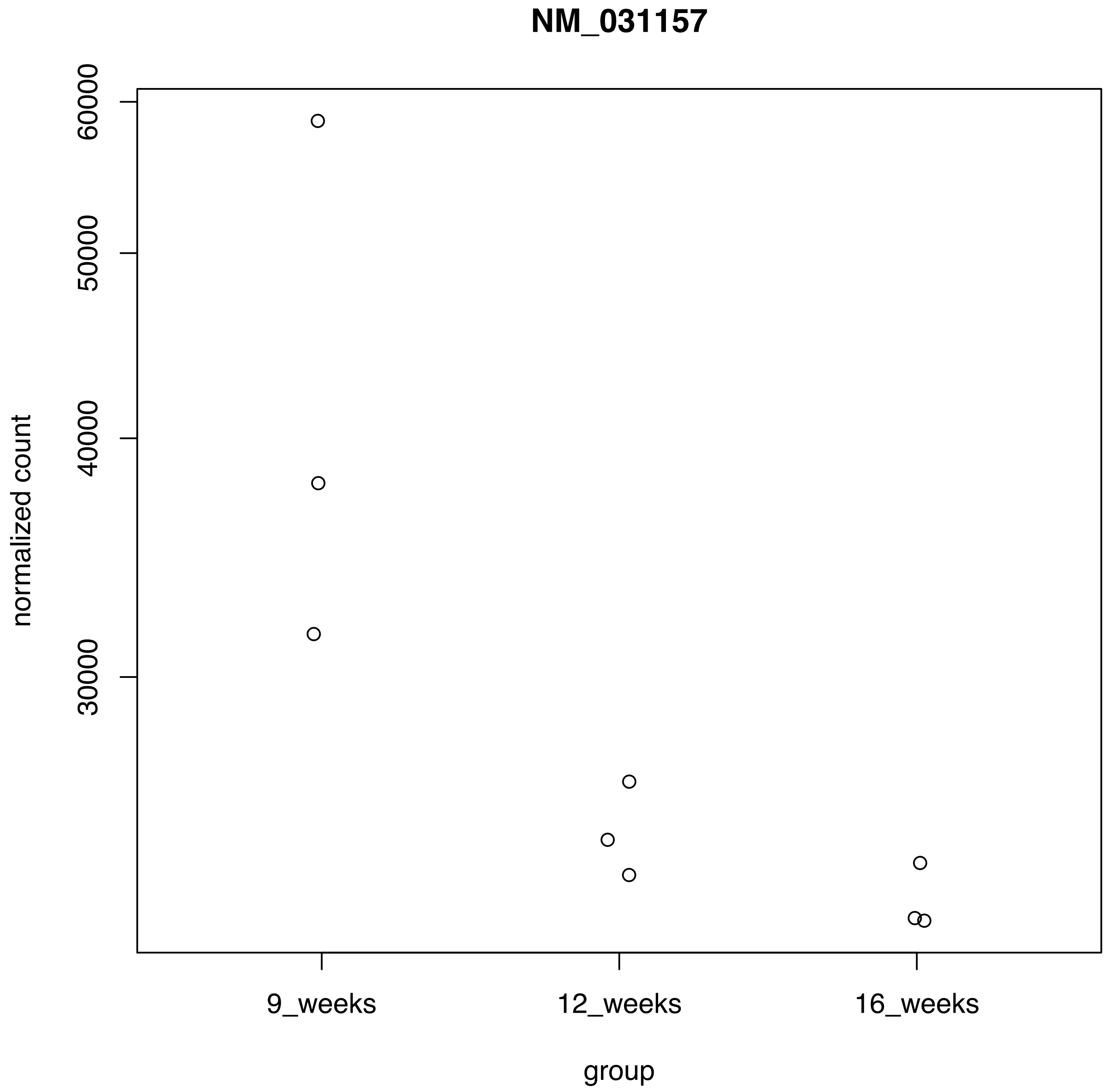

NM_031157
normalized count
60000
50000
40000
30000
9_weeks
12_weeks
16_weeks
group

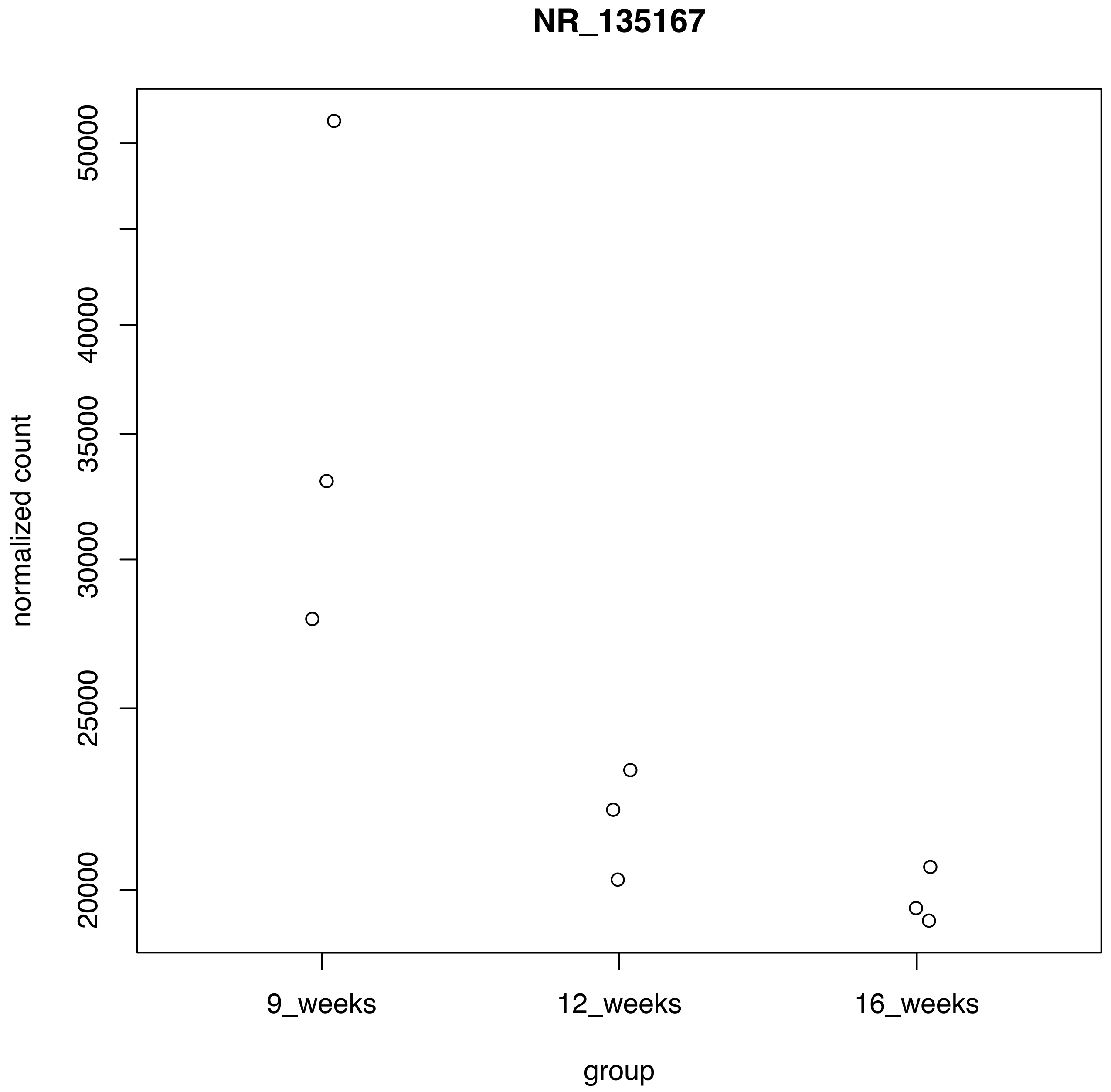
NR_135167
normalized count
50000
40000
35000
30000
25000
20000
9_weeks
12_weeks
16_weeks
group

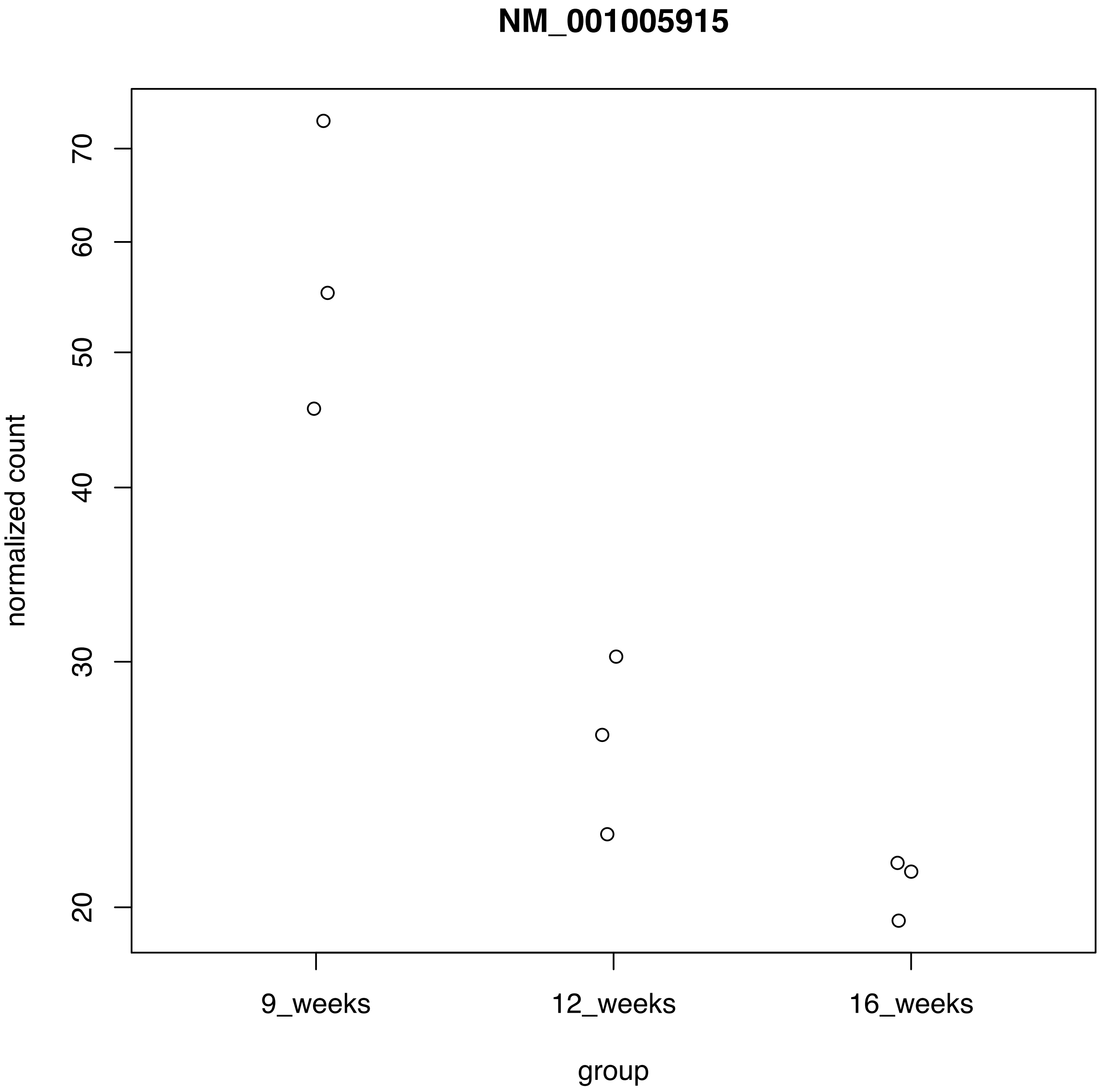

NM_001005915
normalized count
20
30
40
50
60
70
9_weeks
12_weeks
16_weeks
group

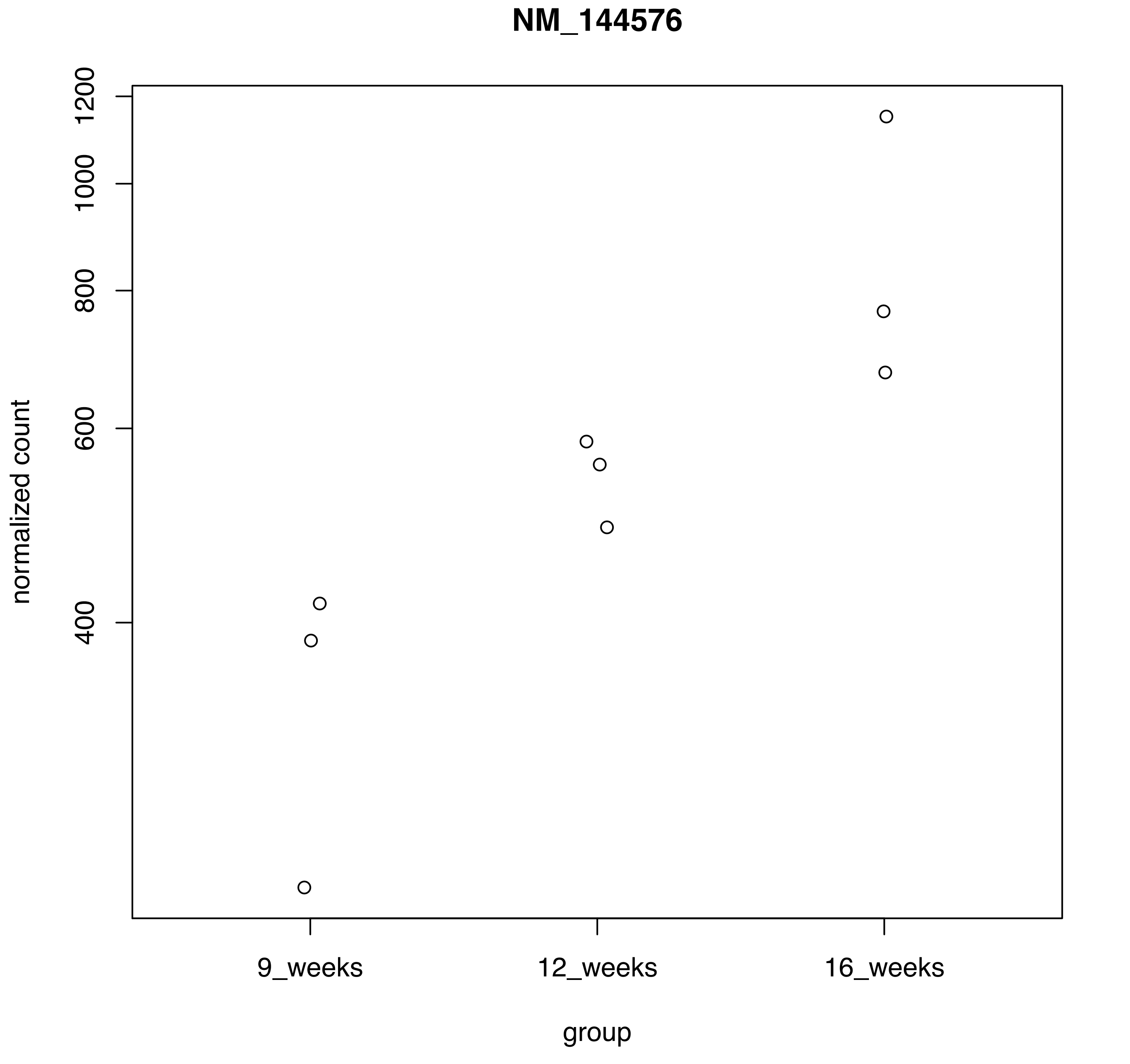
NM_144576
normalized count
1200
1000
800
600
400
group
9_weeks
12_weeks
16_weeks

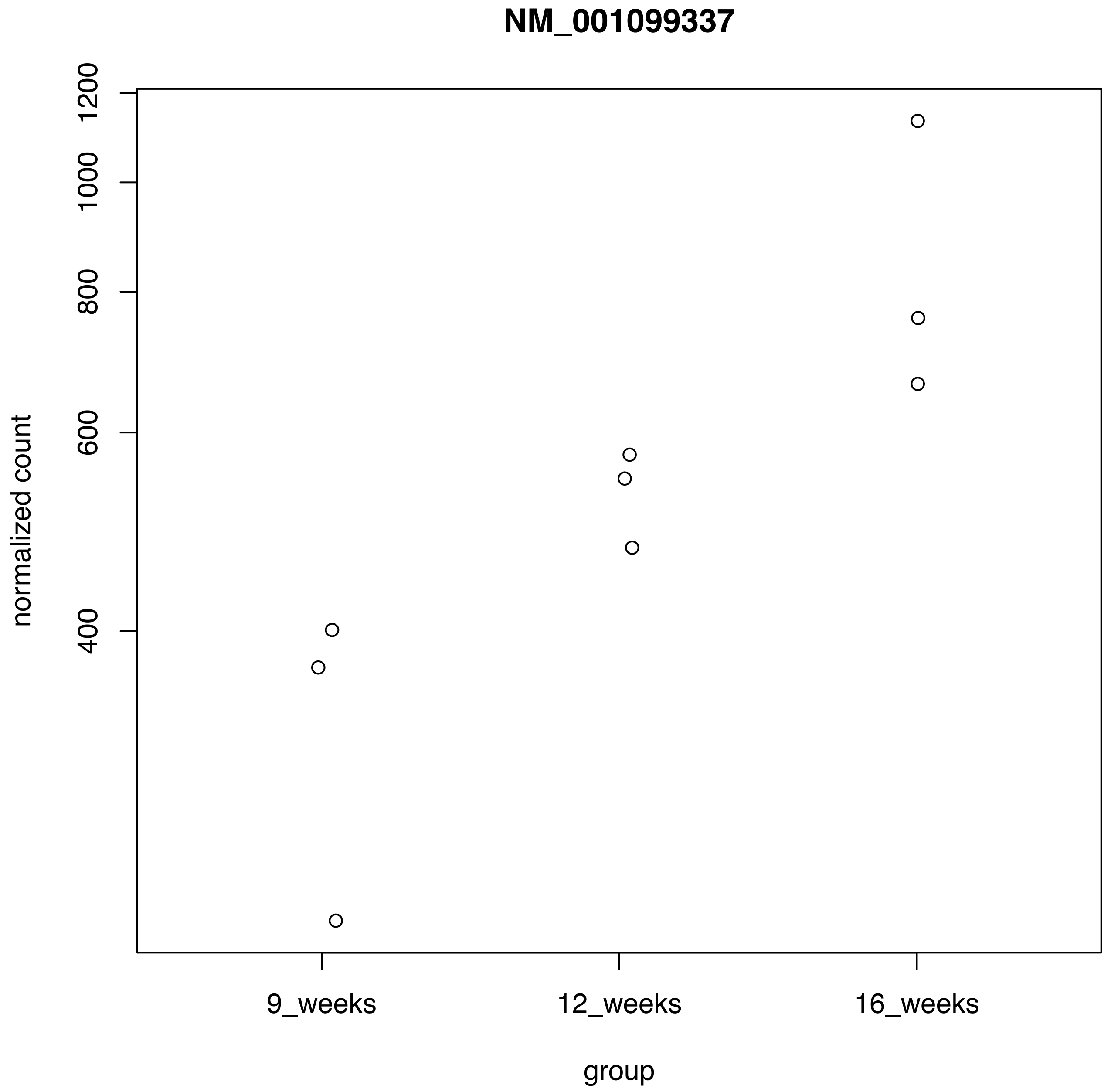
NM_001099337
normalized count
1200
1000
800
600
400
9_weeks
12_weeks
16_weeks
group

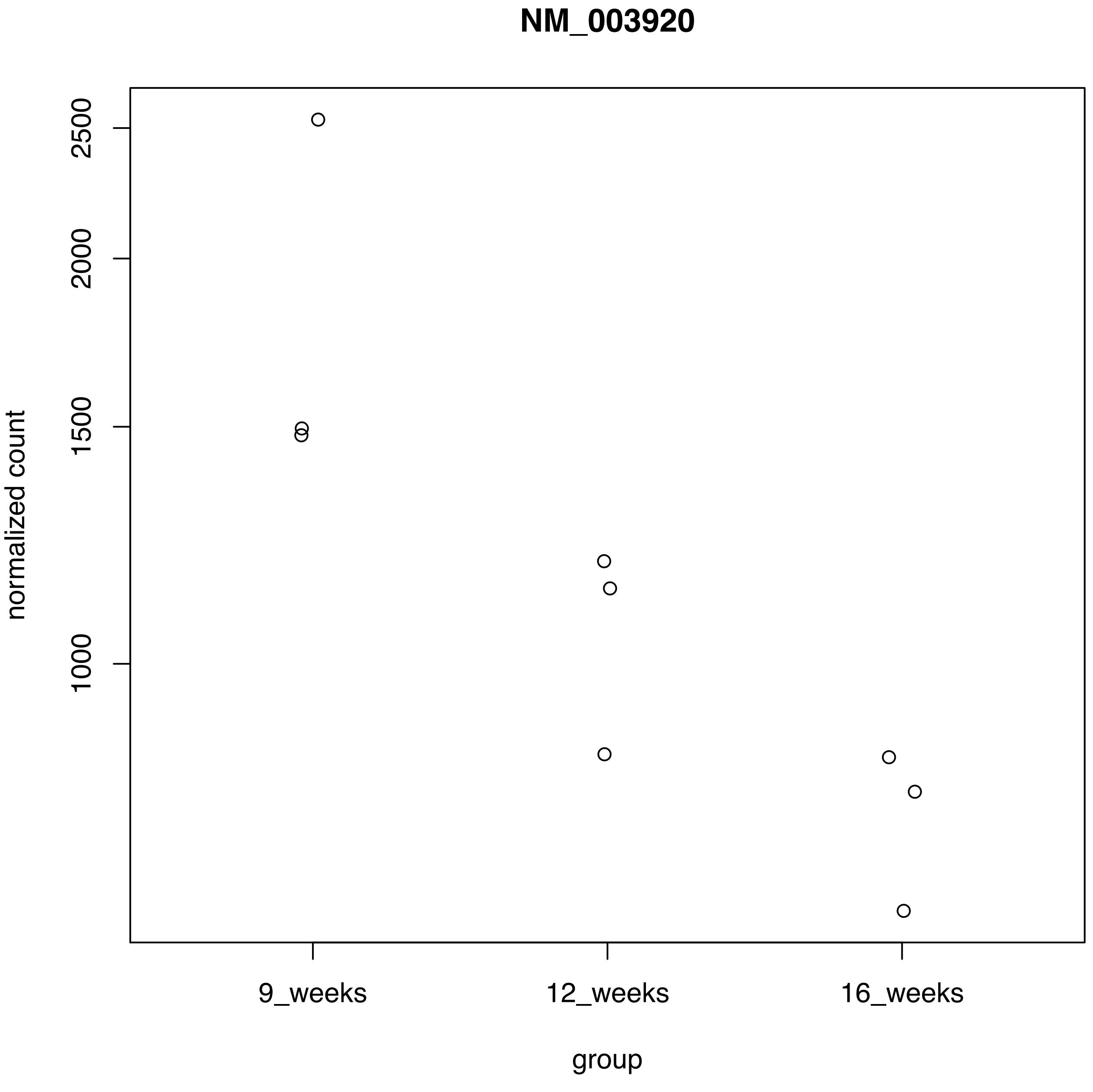
NM_003920
normalized count
2500
2000
1500
1000
9_weeks
12_weeks
16_weeks
group

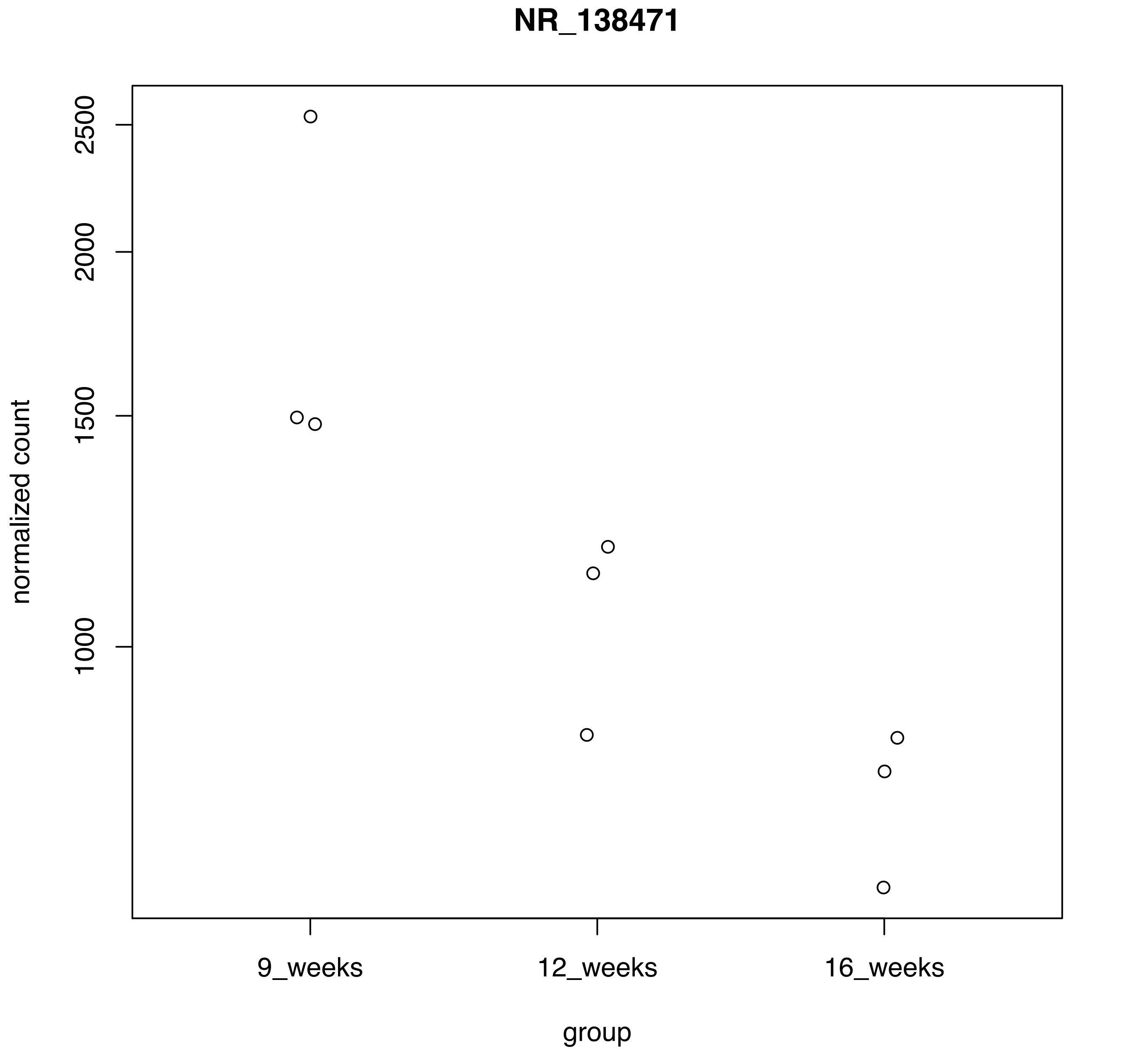
NR_138471
normalized count
2500
2000
1500
1000
9_weeks
12_weeks
16_weeks
group

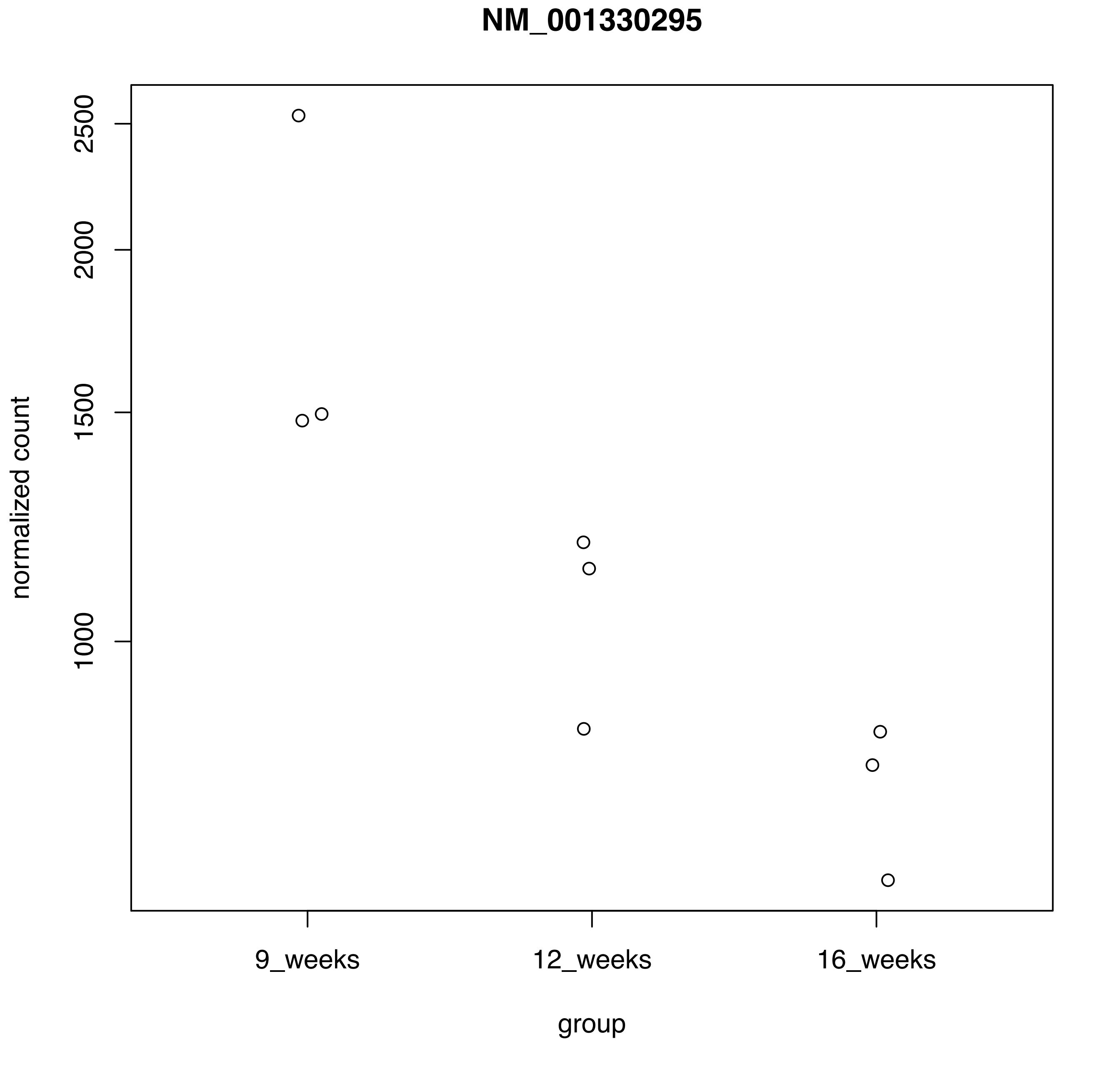
NM_001330295
normalized count
2500
2000
1500
1000
9_weeks
12_weeks
16_weeks
group

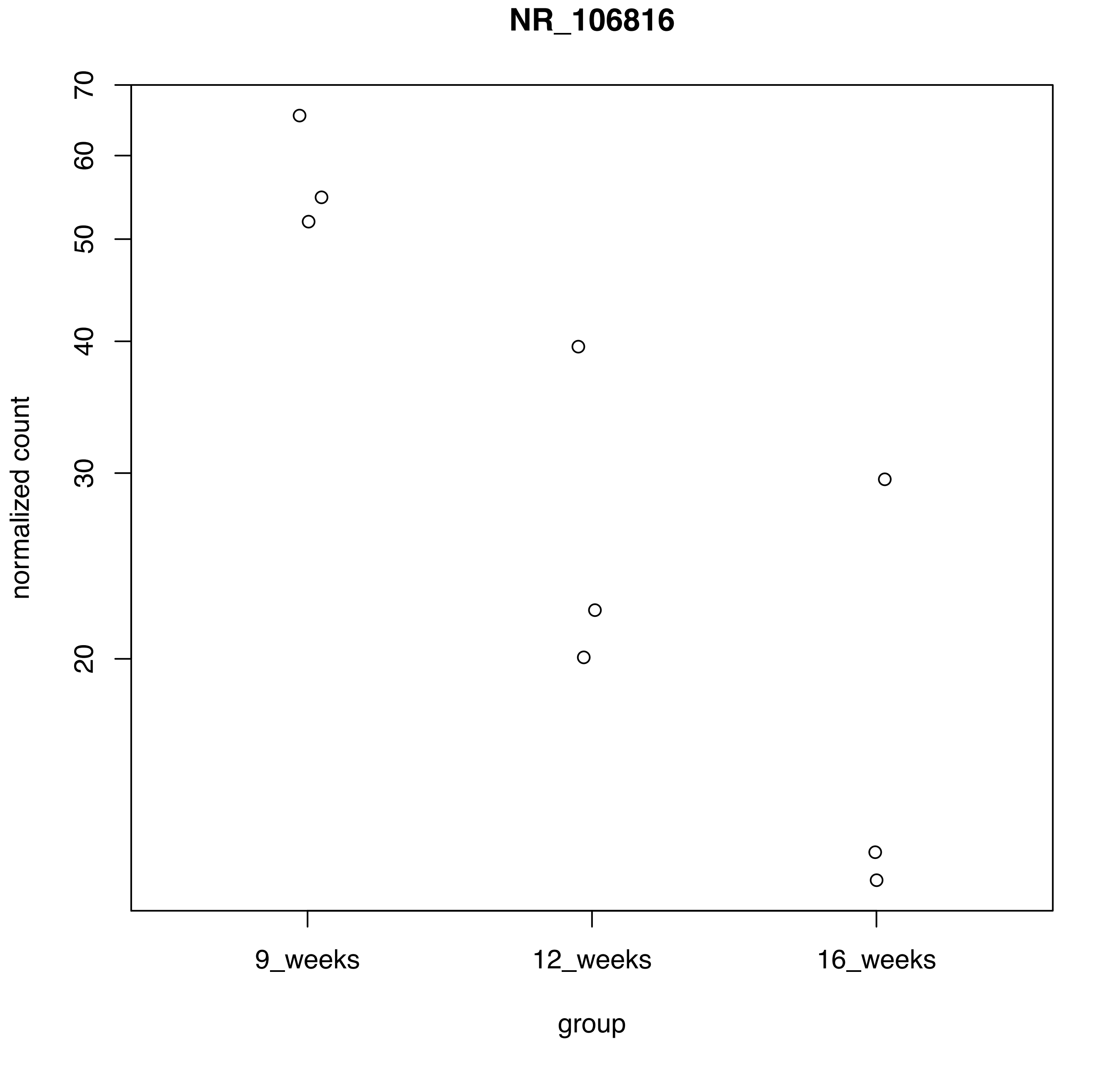
NR_106816
normalized count
70
60
50
40
30
20
9_weeks
12_weeks
16_weeks
group

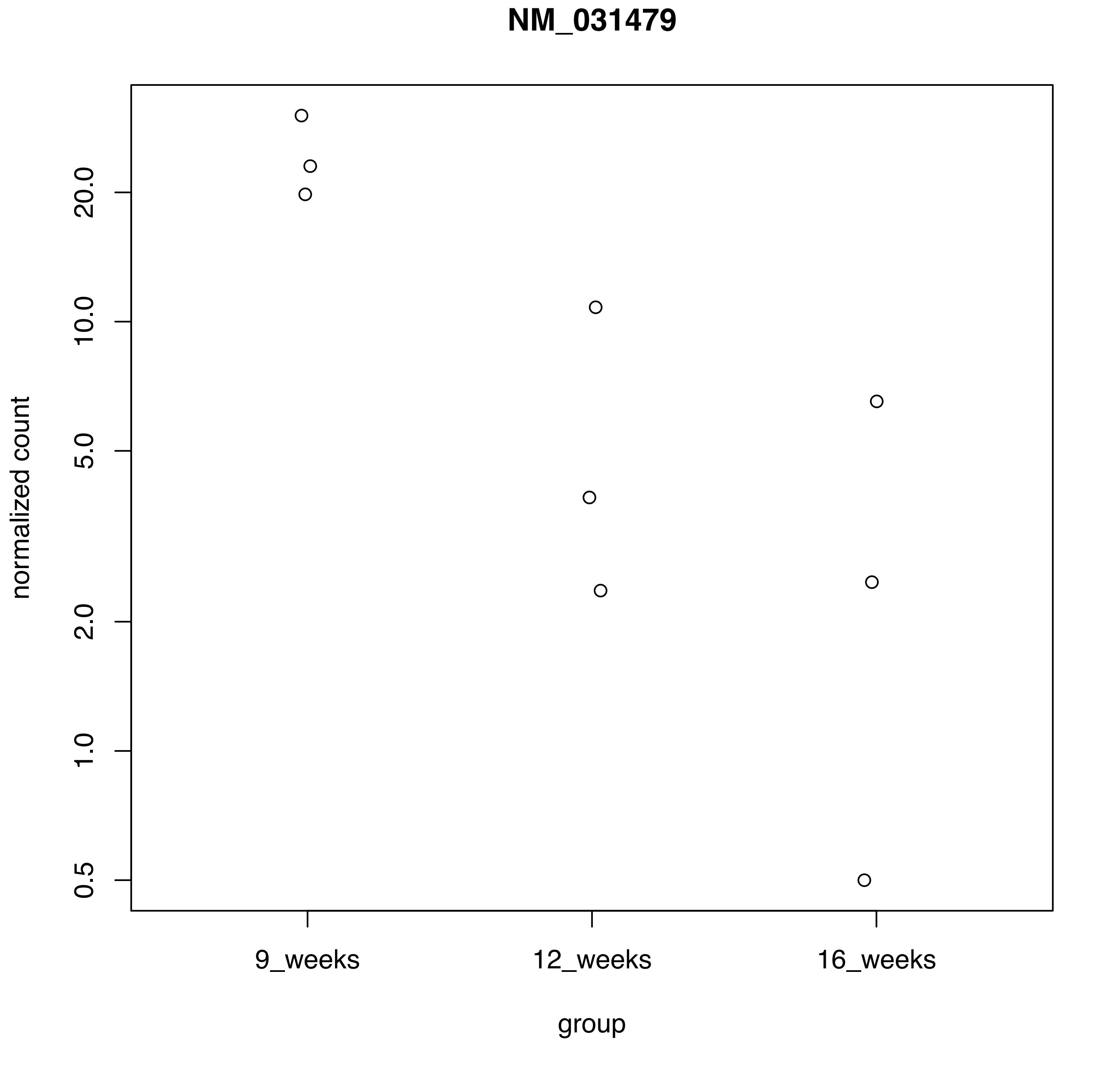
NM_031479
normalized count
20.0
10.0
5.0
2.0
1.0
0.5
9_weeks
12_weeks
16_weeks
group

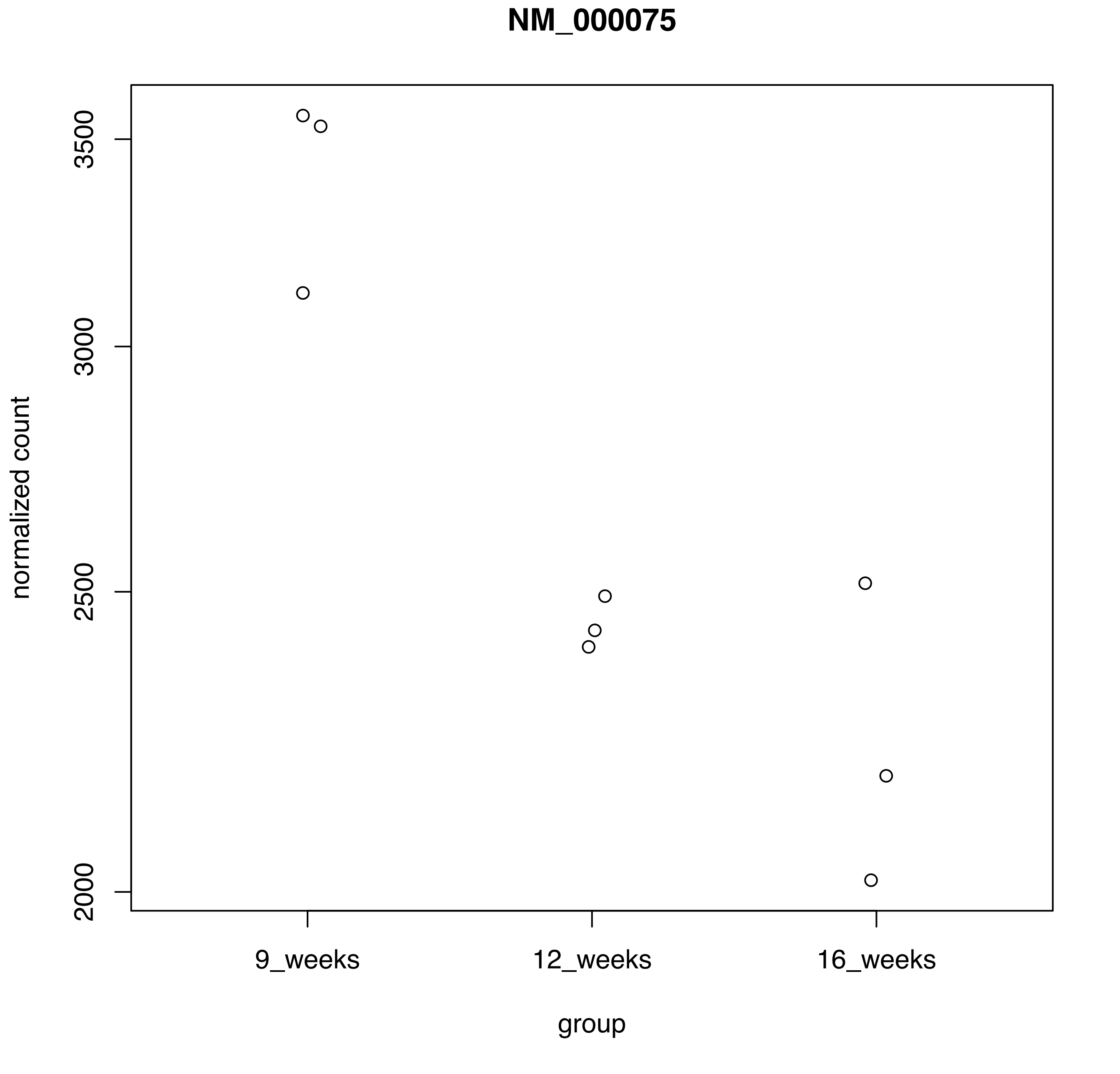
NM_000075
normalized count
3500
3000
2500
2000
9_weeks
12_weeks
16_weeks
group

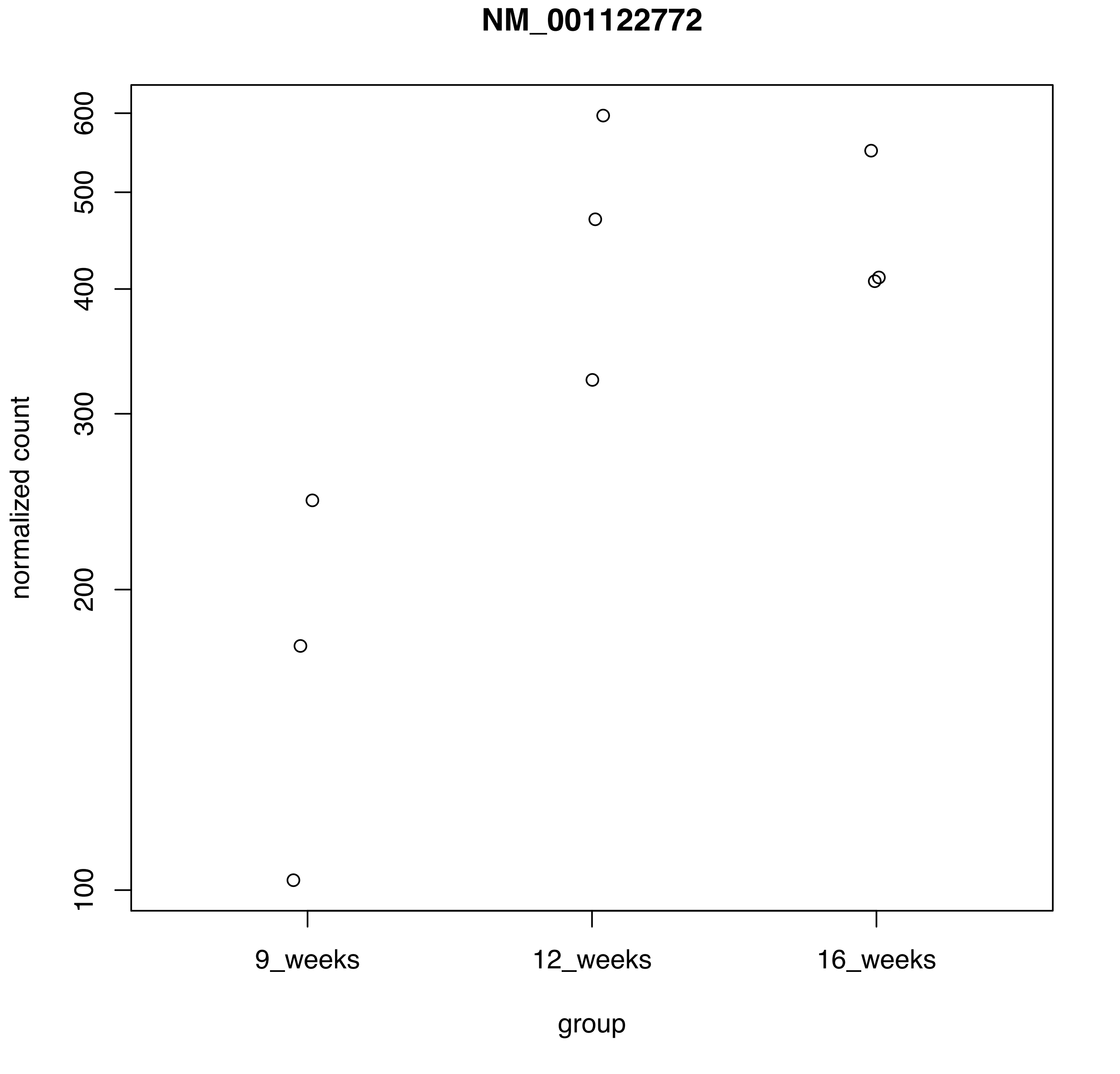
NM_001122772
normalized count
100
200
300
400
500
600
9_weeks
12_weeks
16_weeks
group

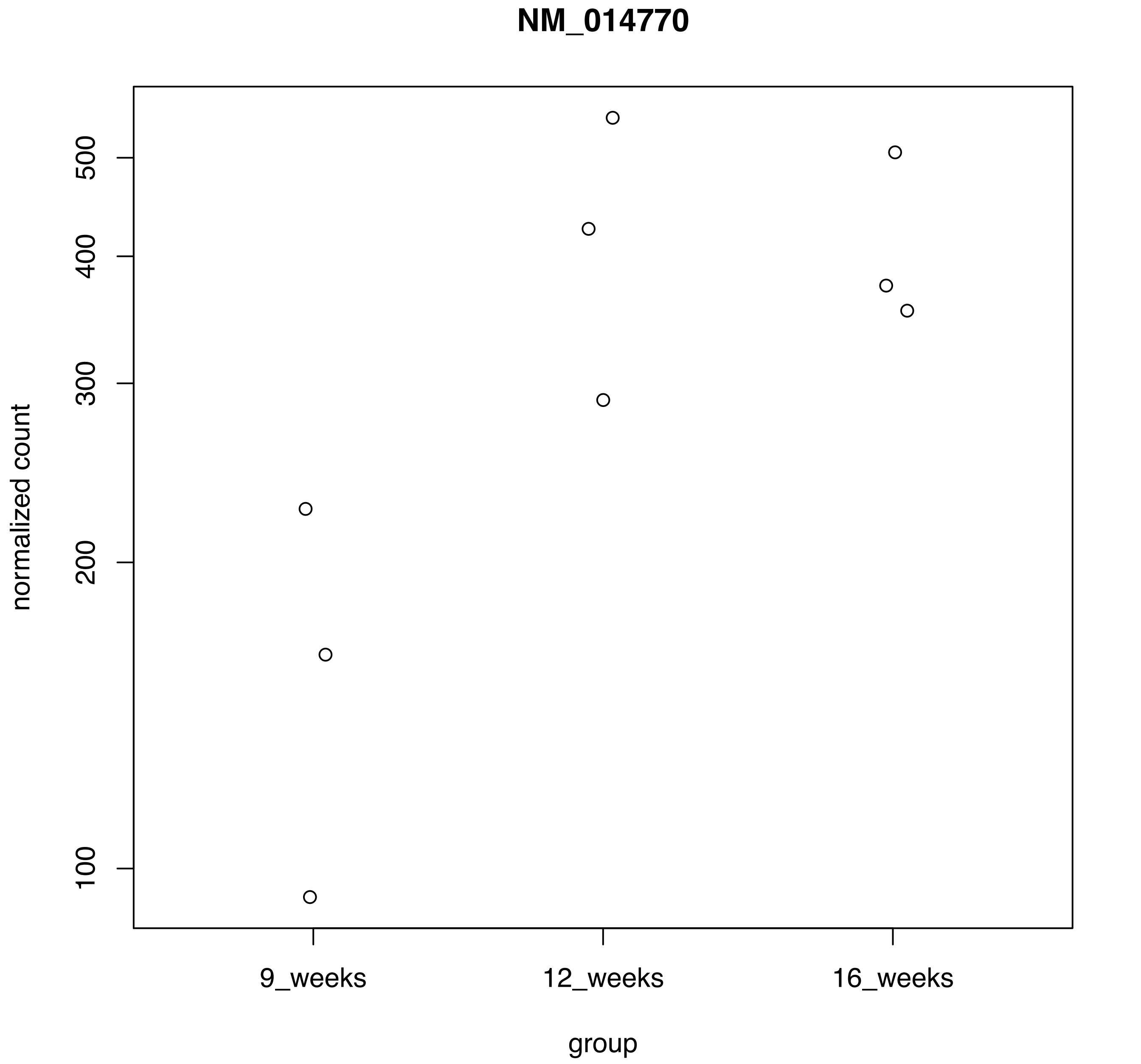
NM_014770
normalized count
500
400
300
200
100
9_weeks
12_weeks
16_weeks
group

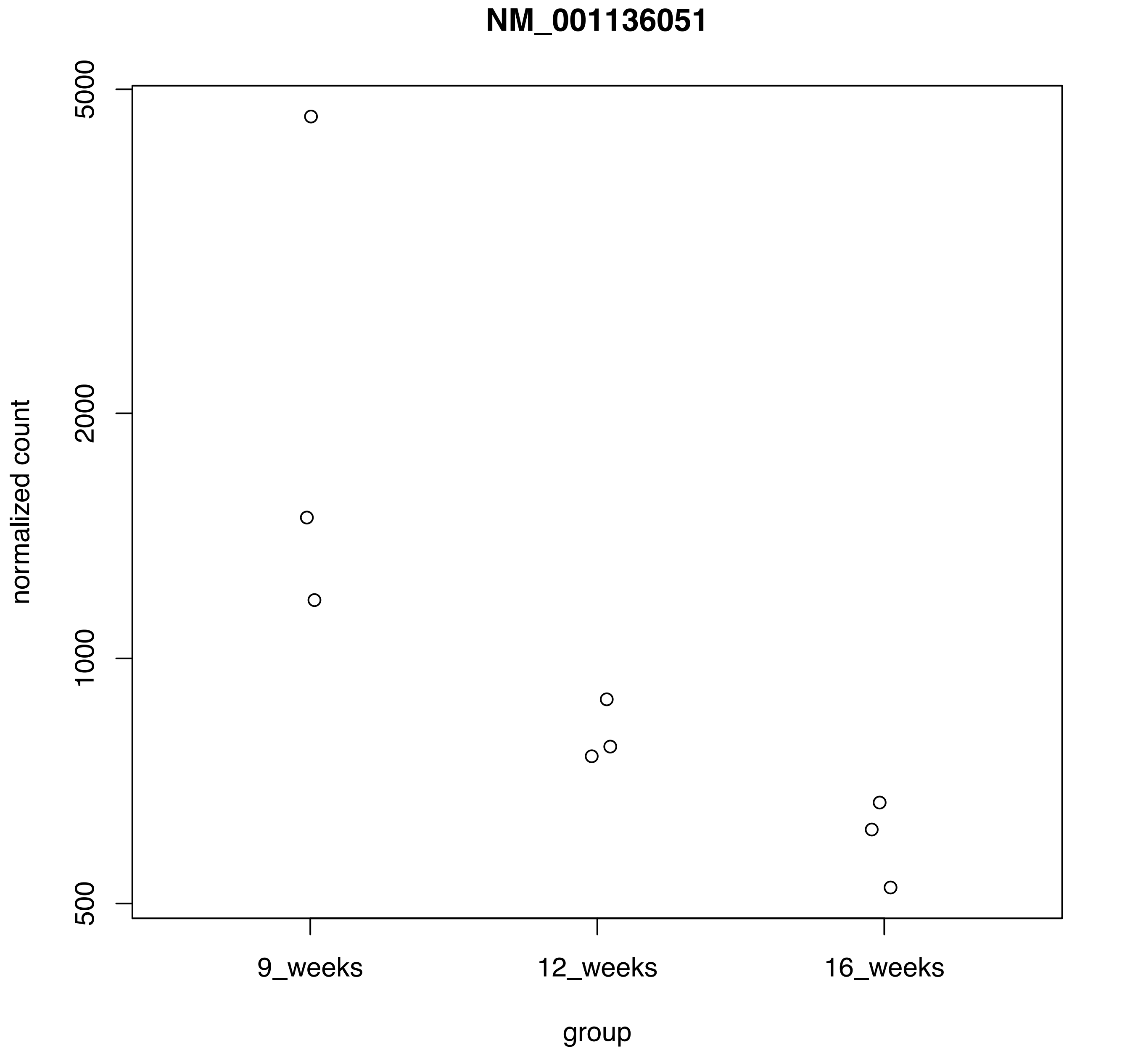

NM_001136051
5000
2000
1000
500
normalized count
9_weeks
12_weeks
16_weeks
group

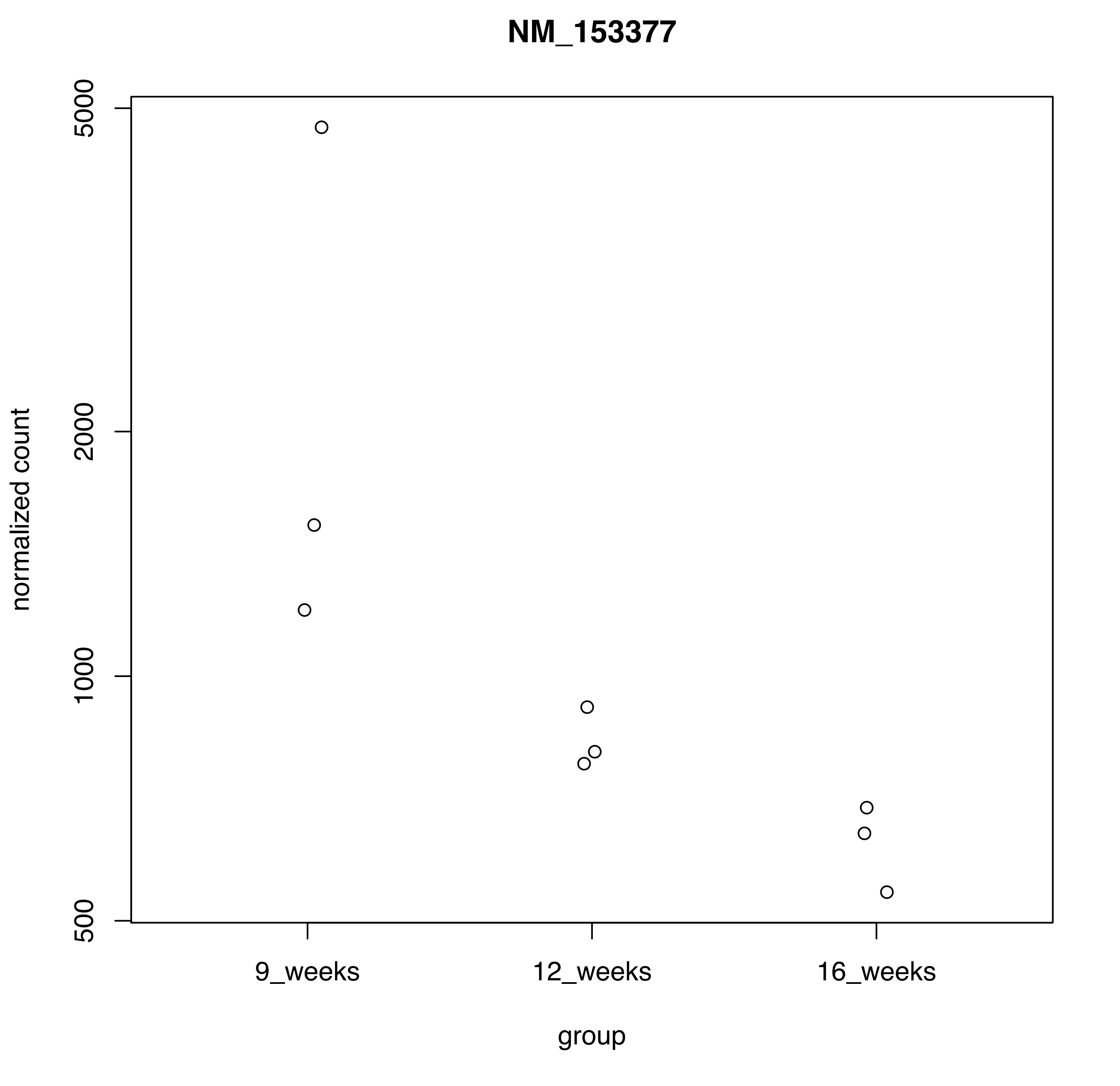
NM_153377
normalized count
5000
2000
1000
500
9_weeks
12_weeks
16_weeks
group

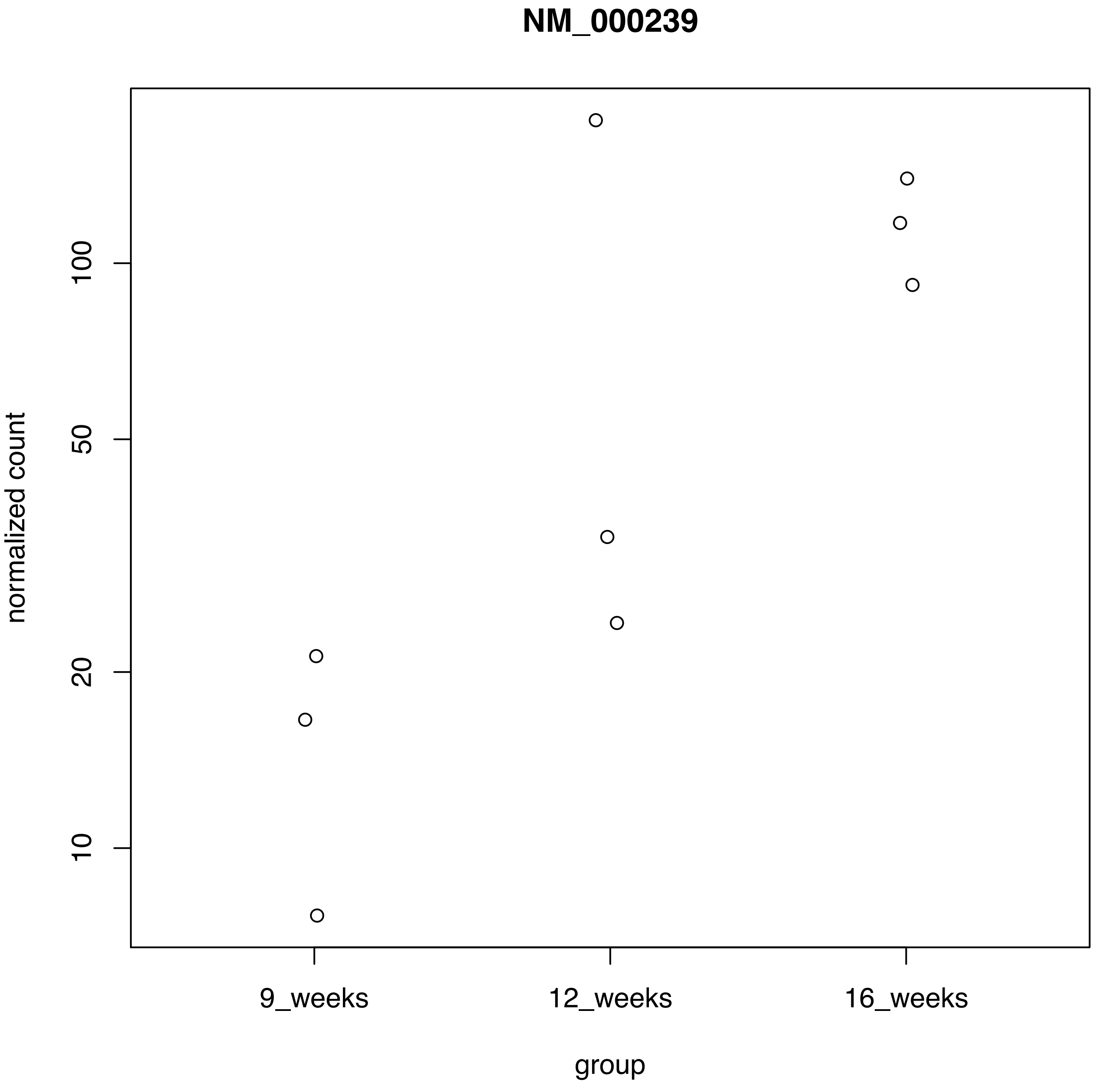
NM_000239
normalized count
100
50
20
10
9_weeks
12_weeks
16_weeks
group

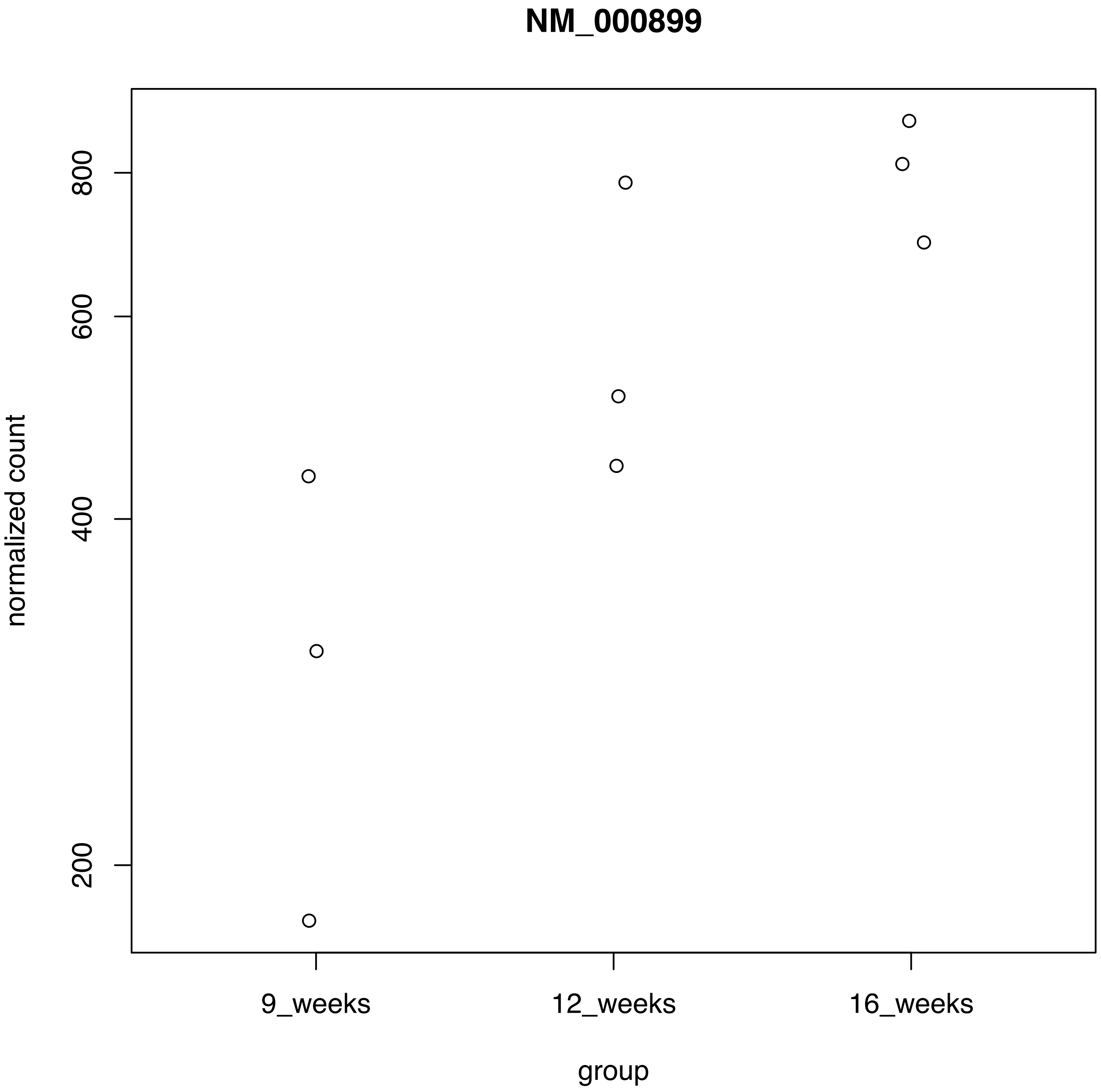

NM_000899
normalized count
200
400
600
800
9_weeks
12_weeks
16_weeks
group

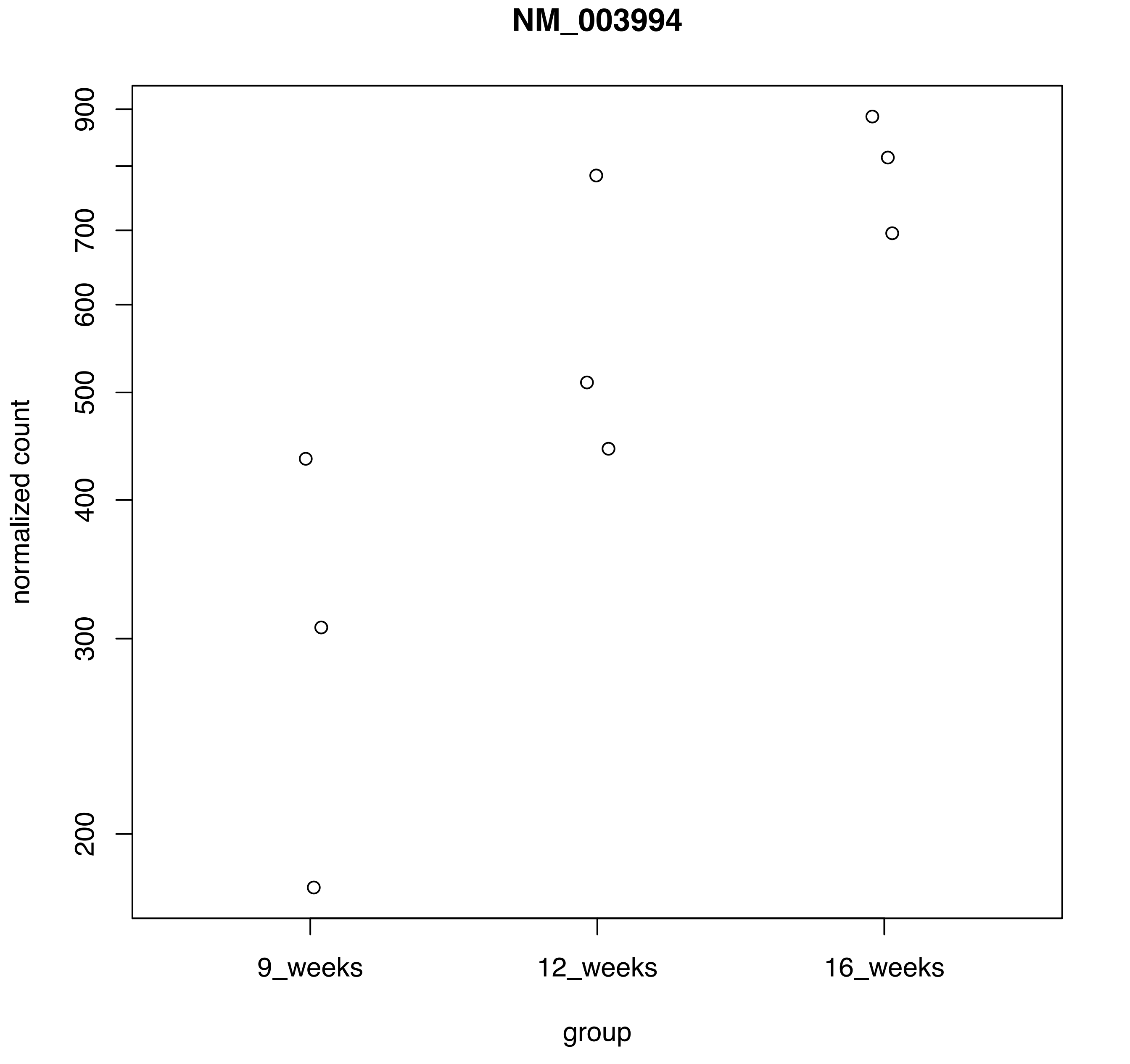
NM_003994
normalized count
900
700
600
500
400
300
200
9_weeks
12_weeks
16_weeks
group

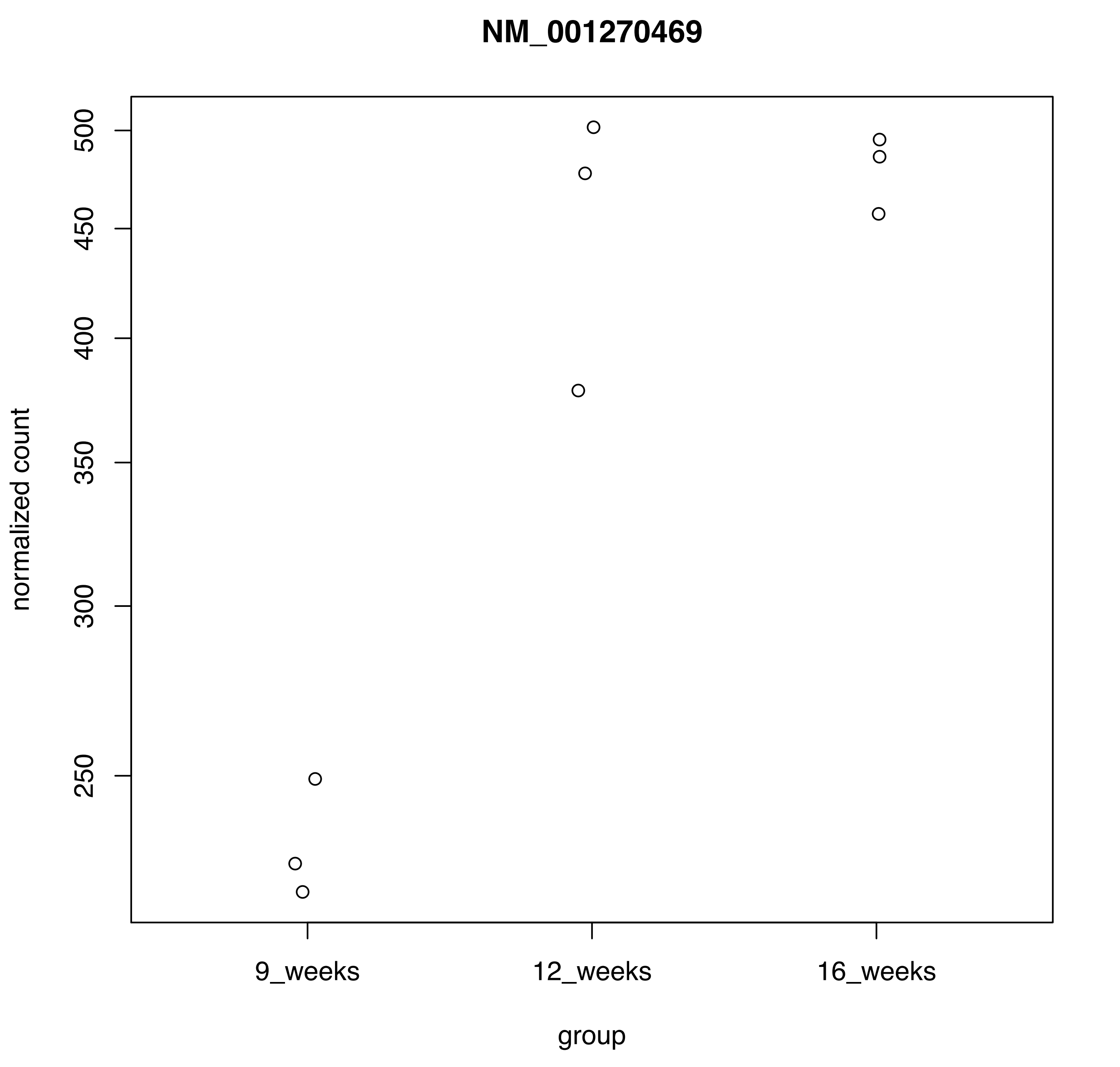

NM_001270469
normalized count
250
300
350
400
450
500
9_weeks
12_weeks
16_weeks
group

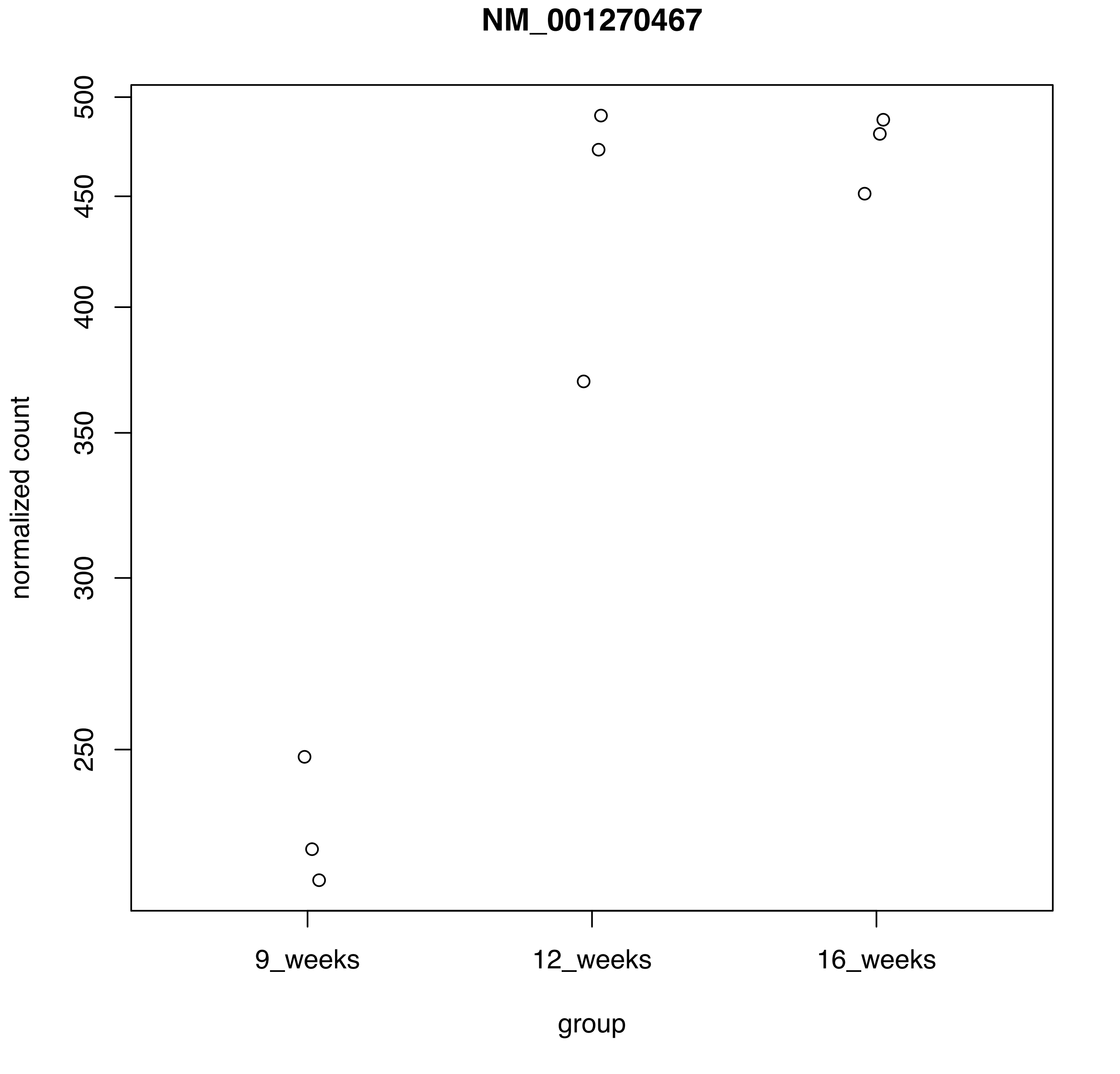
NM_001270467
normalized count
500
450
400
350
300
250
9_weeks
12_weeks
16_weeks
group

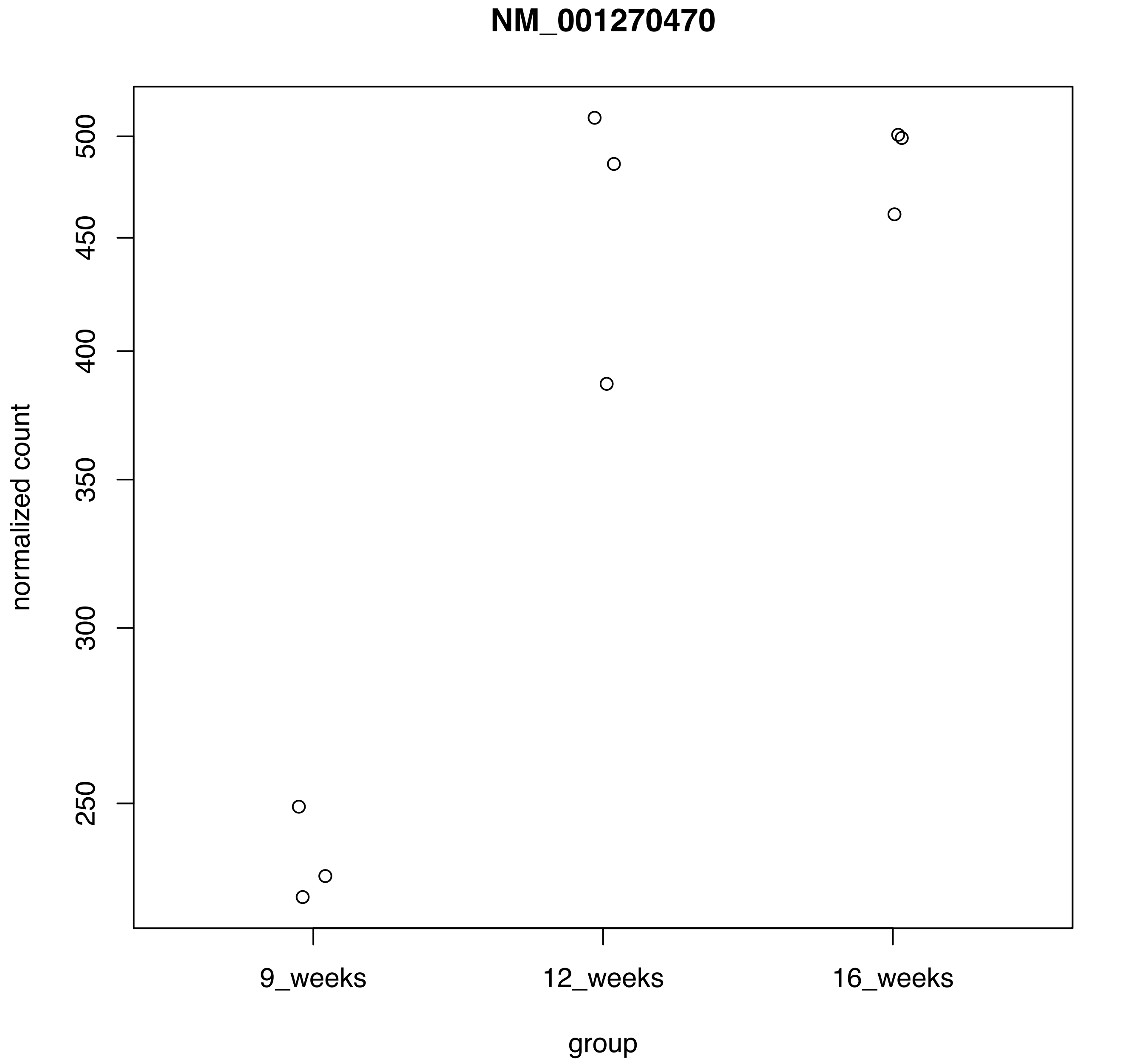
NM_001270470
normalized count
group
9_weeks
12_weeks
16_weeks
250
300
350
400
450
500

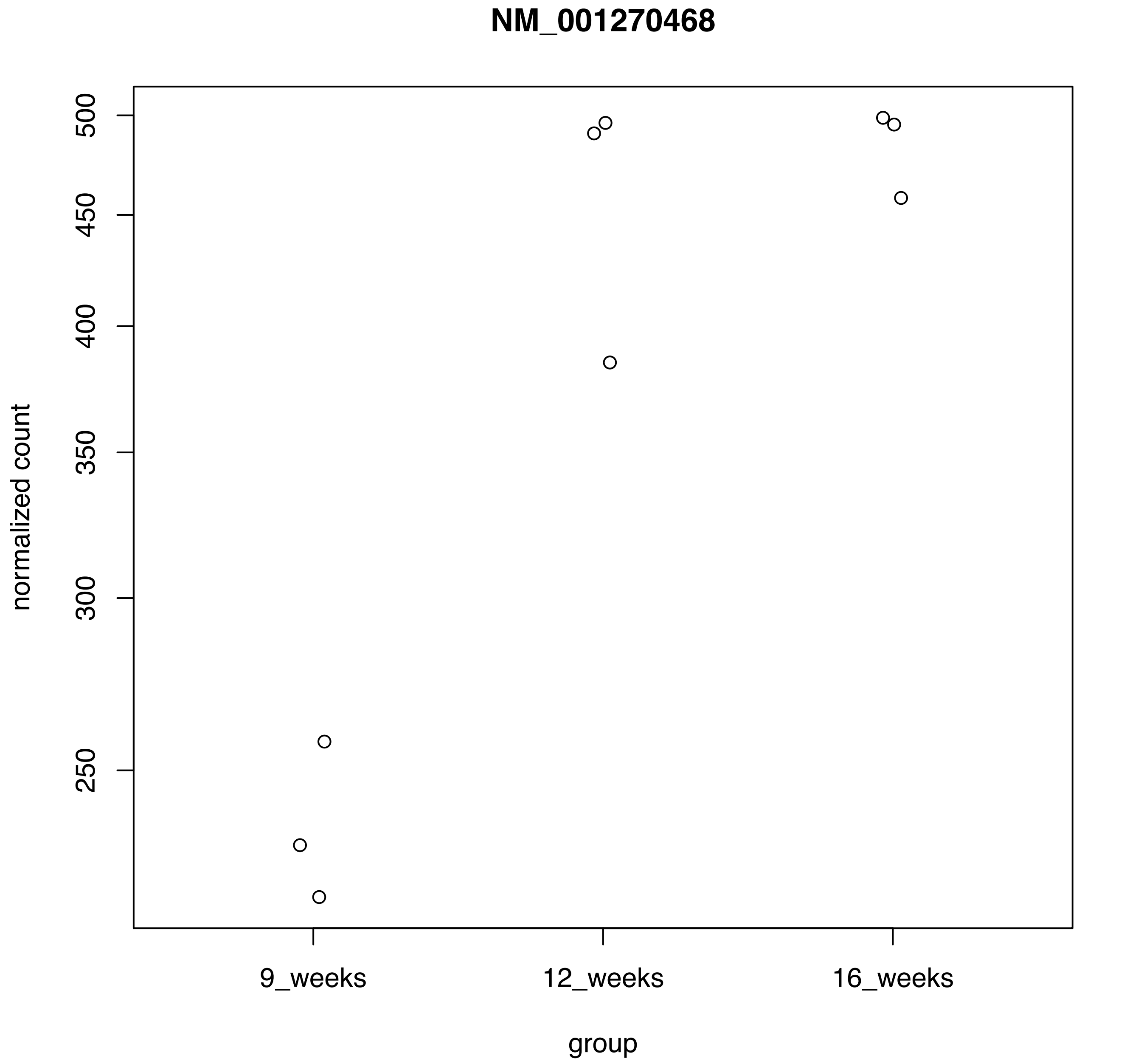
NM_001270468
normalized count
500
450
400
350
300
250
9_weeks
12_weeks
16_weeks
group

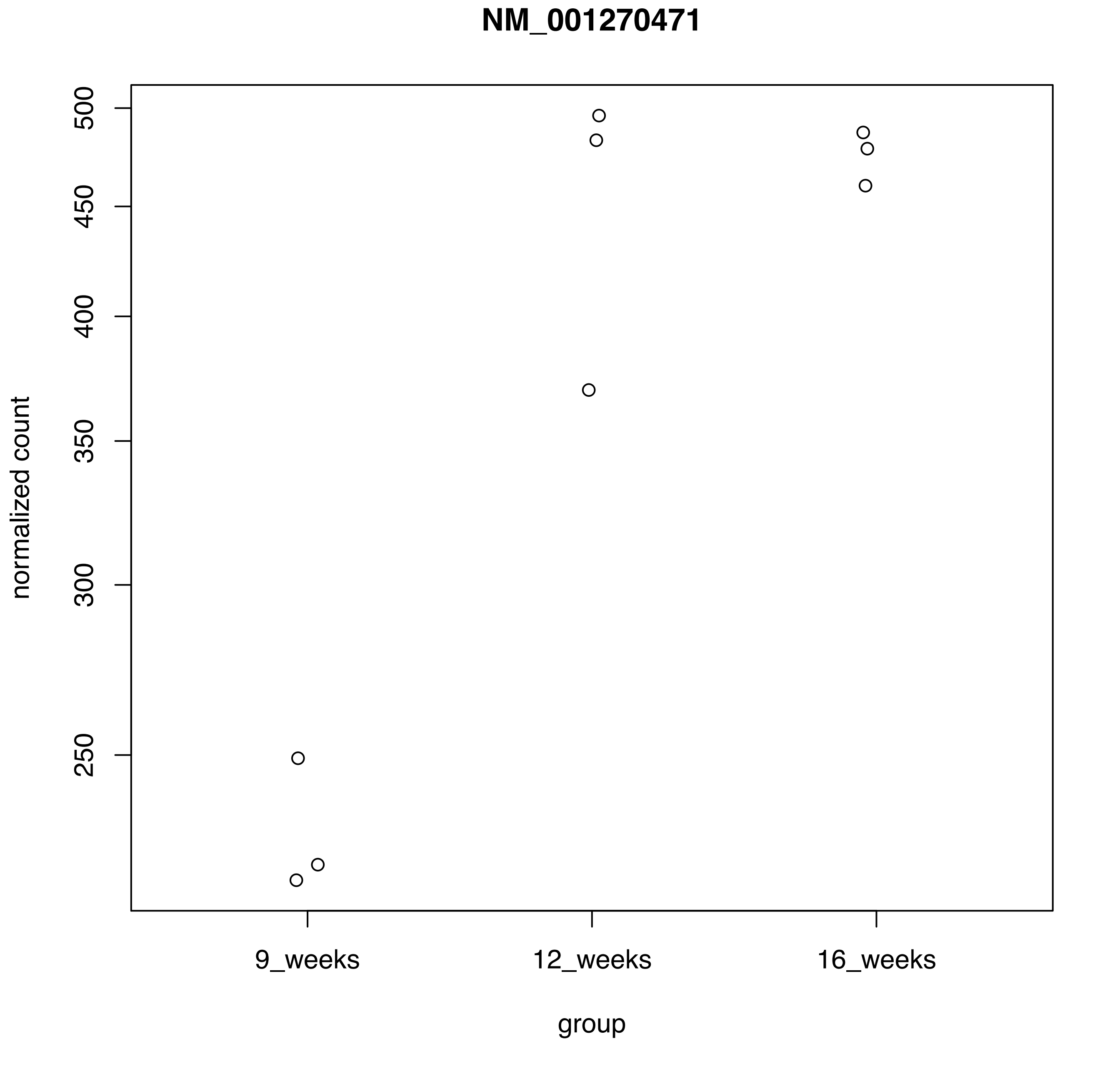
NM_001270471
normalized count
500
450
400
350
300
250
9_weeks
12_weeks
16_weeks
group

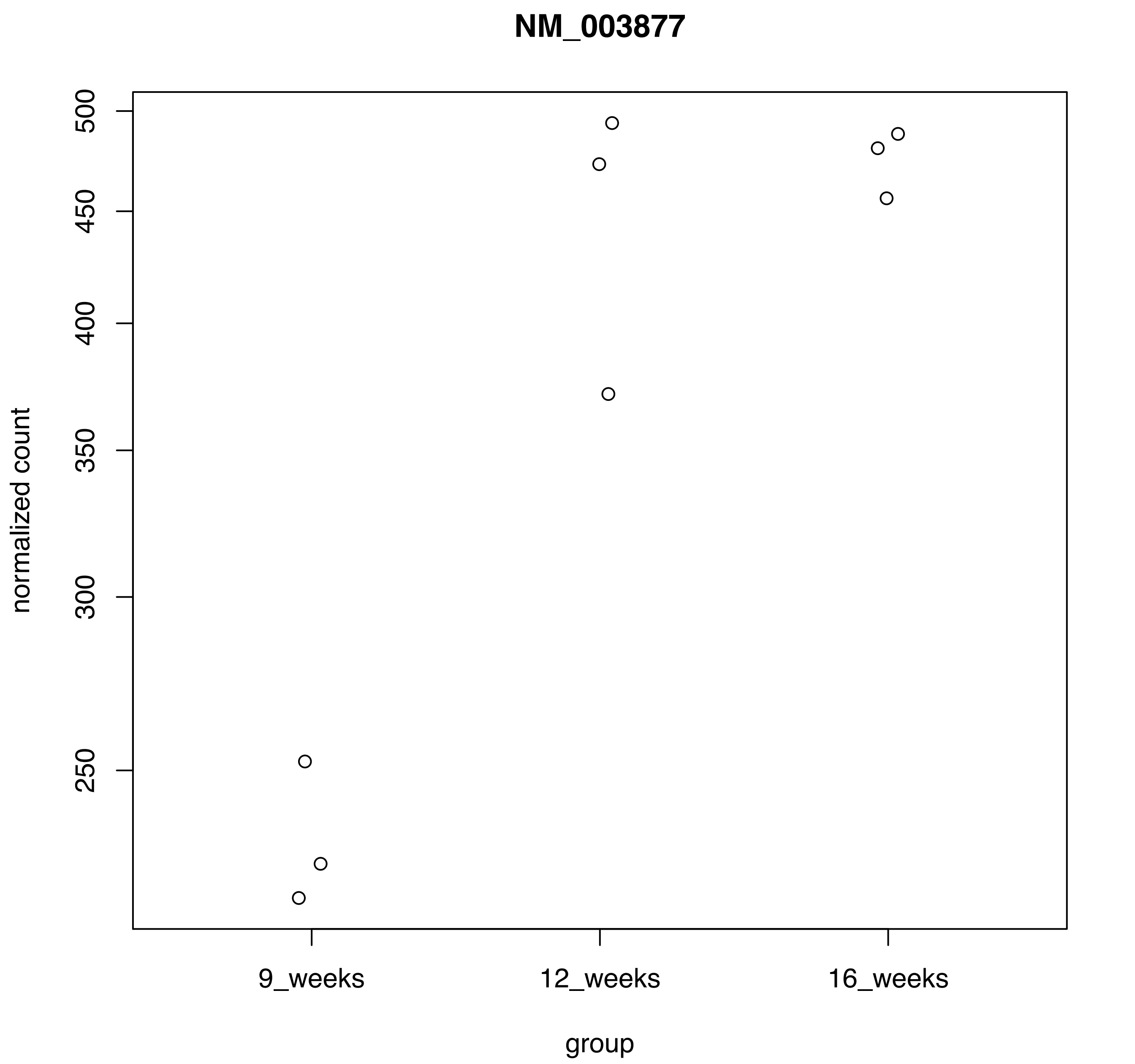
NM_003877
normalized count
500
450
400
350
300
250
9_weeks
12_weeks
16_weeks
group

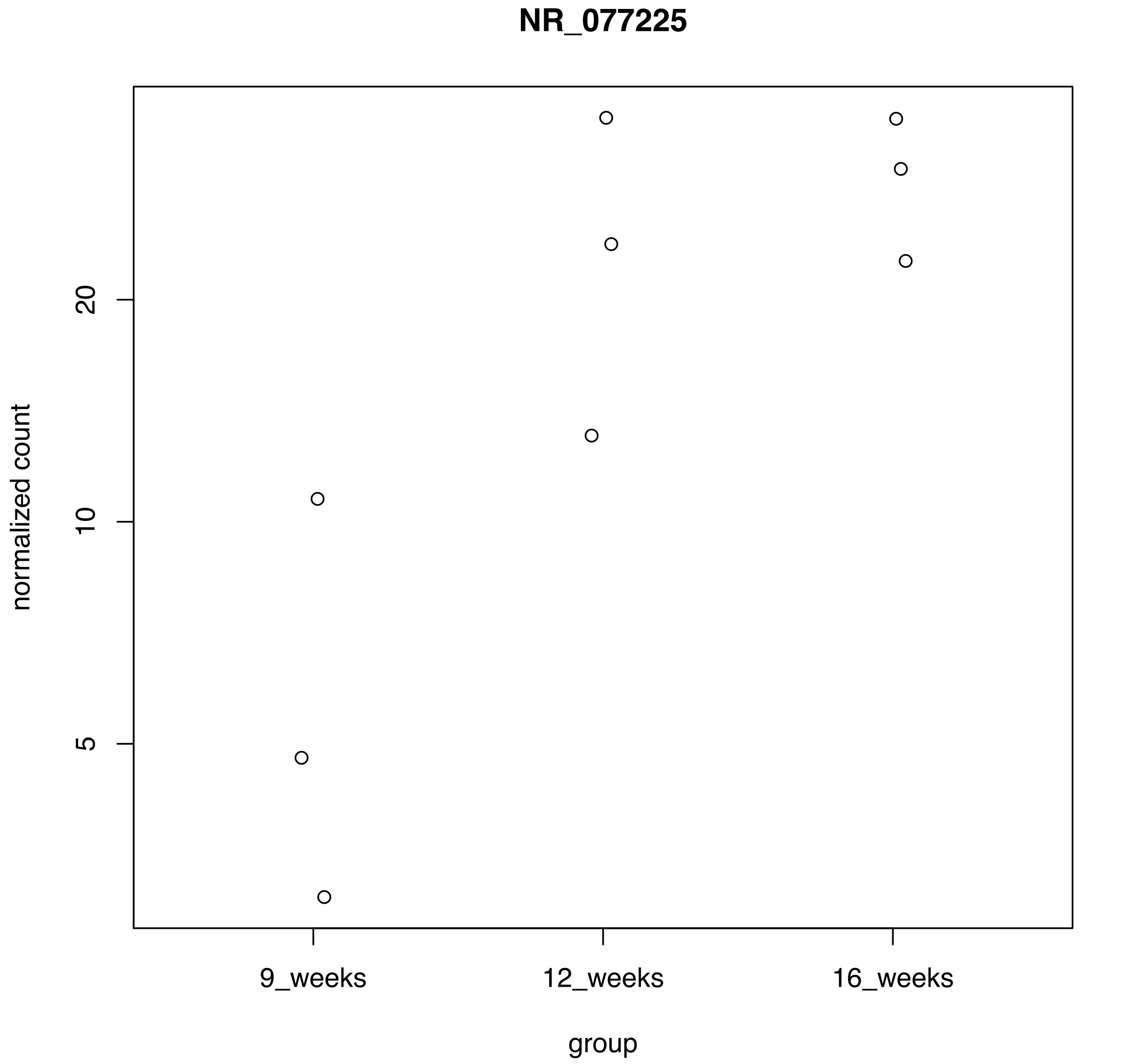
NR_077225
normalized count
5
10
20
9_weeks
12_weeks
16_weeks
group

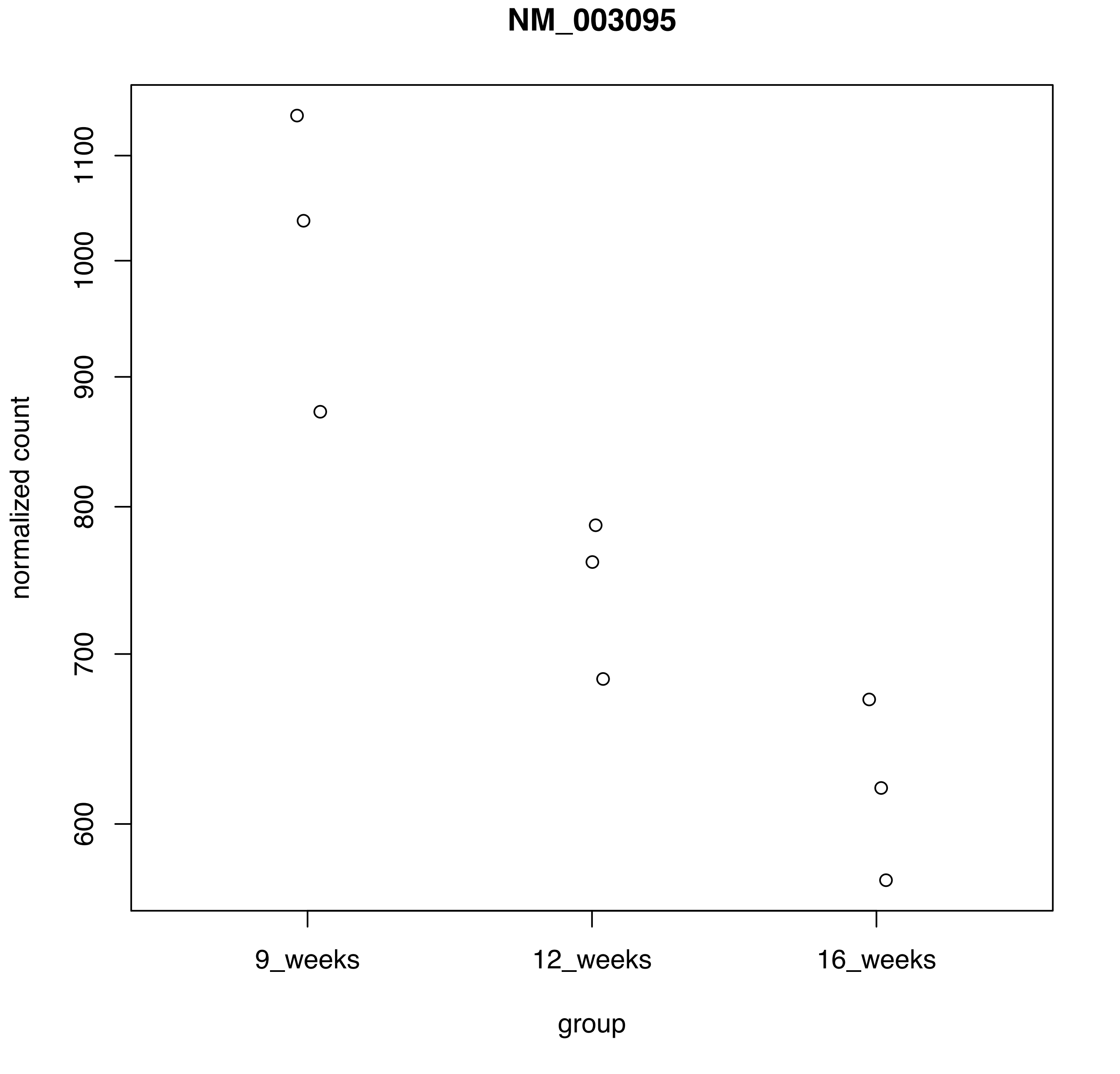

NM_003095
normalized count
1100
1000
900
800
700
600
9_weeks
12_weeks
16_weeks
group

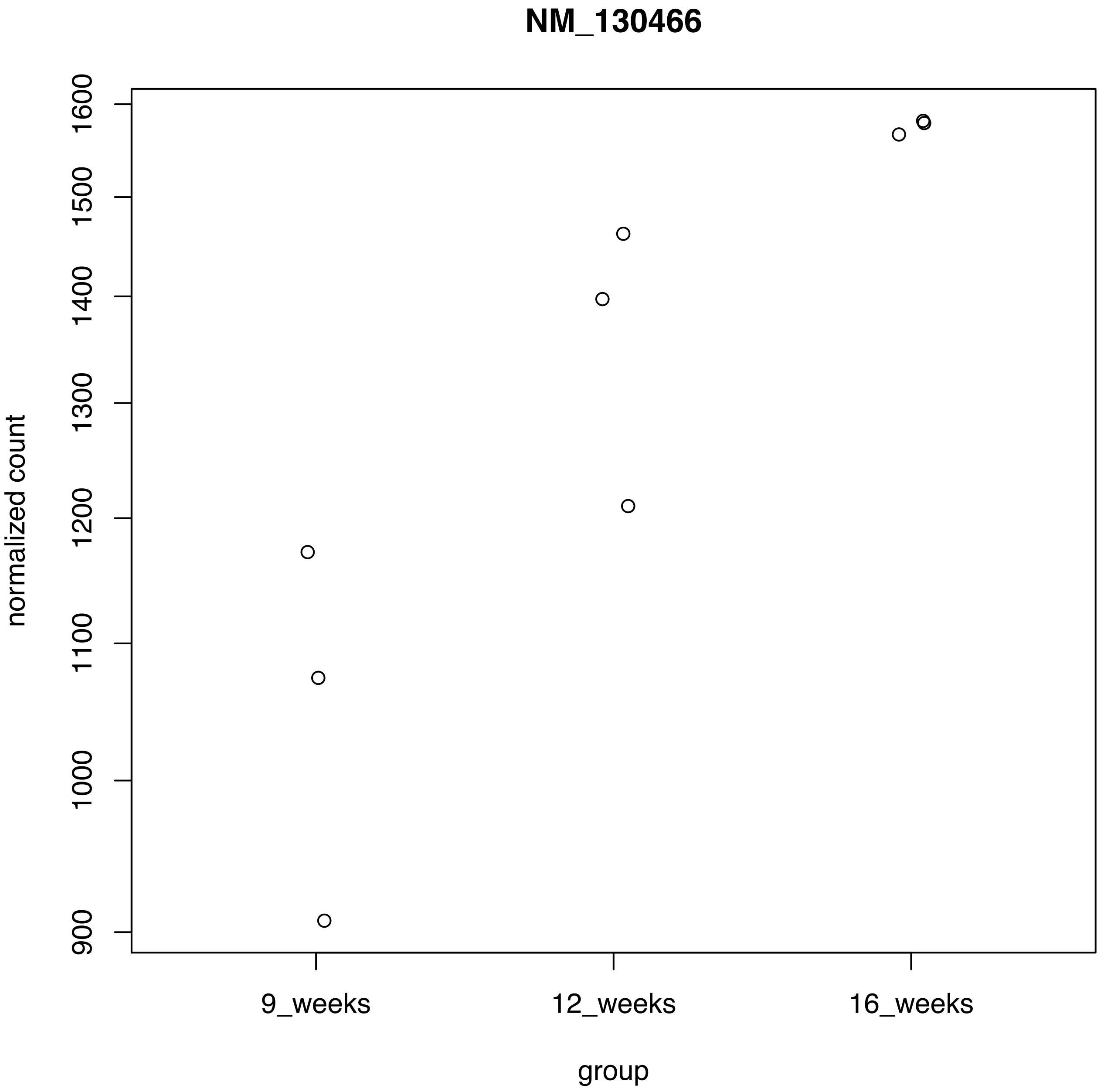
NM_130466
normalized count
900
1000
1100
1200
1300
1400
1500
1600
9_weeks
12_weeks
16_weeks
group

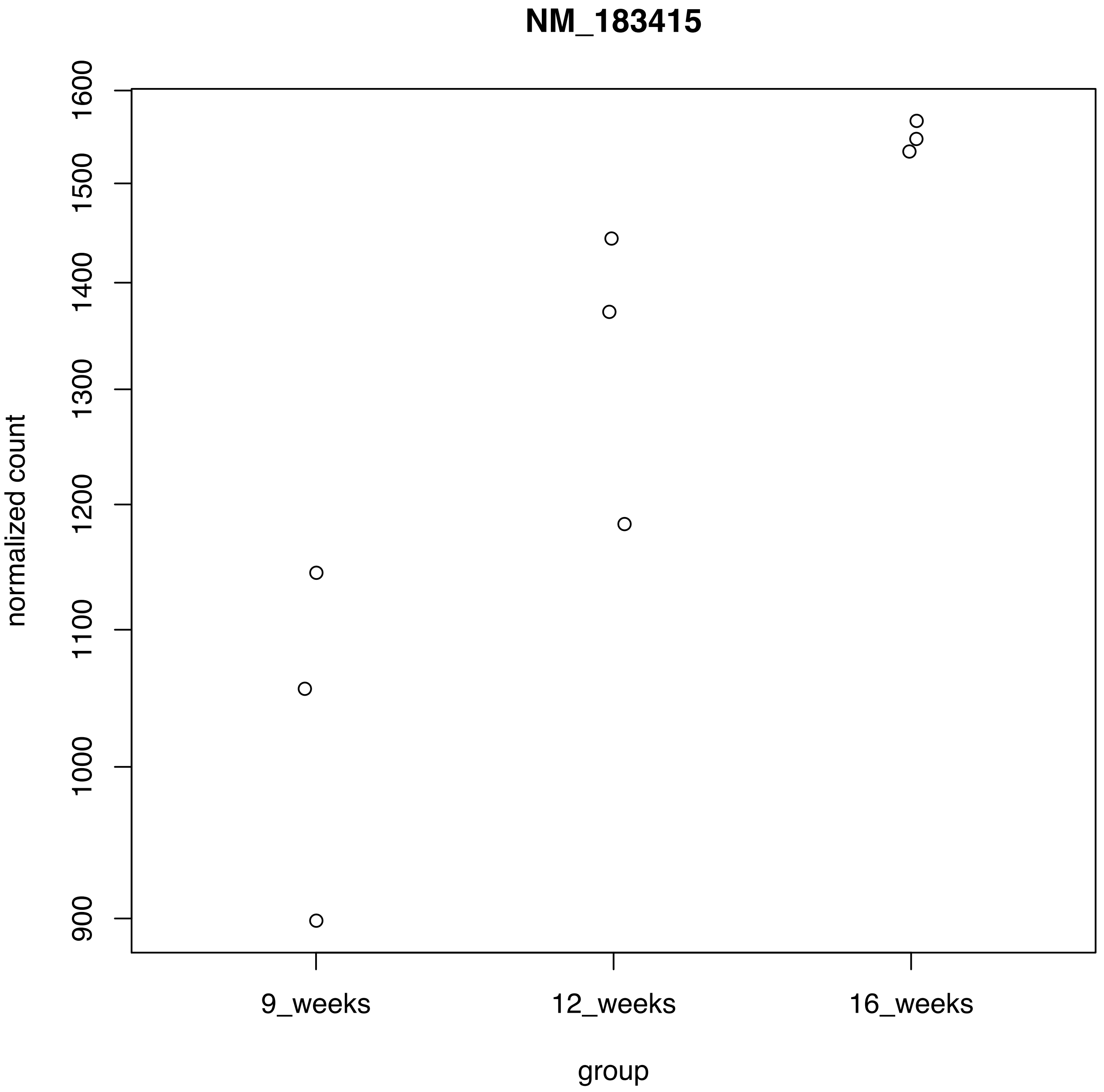

NM_183415
normalized count
900
1000
1100
1200
1300
1400
1500
1600
9_weeks
12_weeks
16_weeks
group

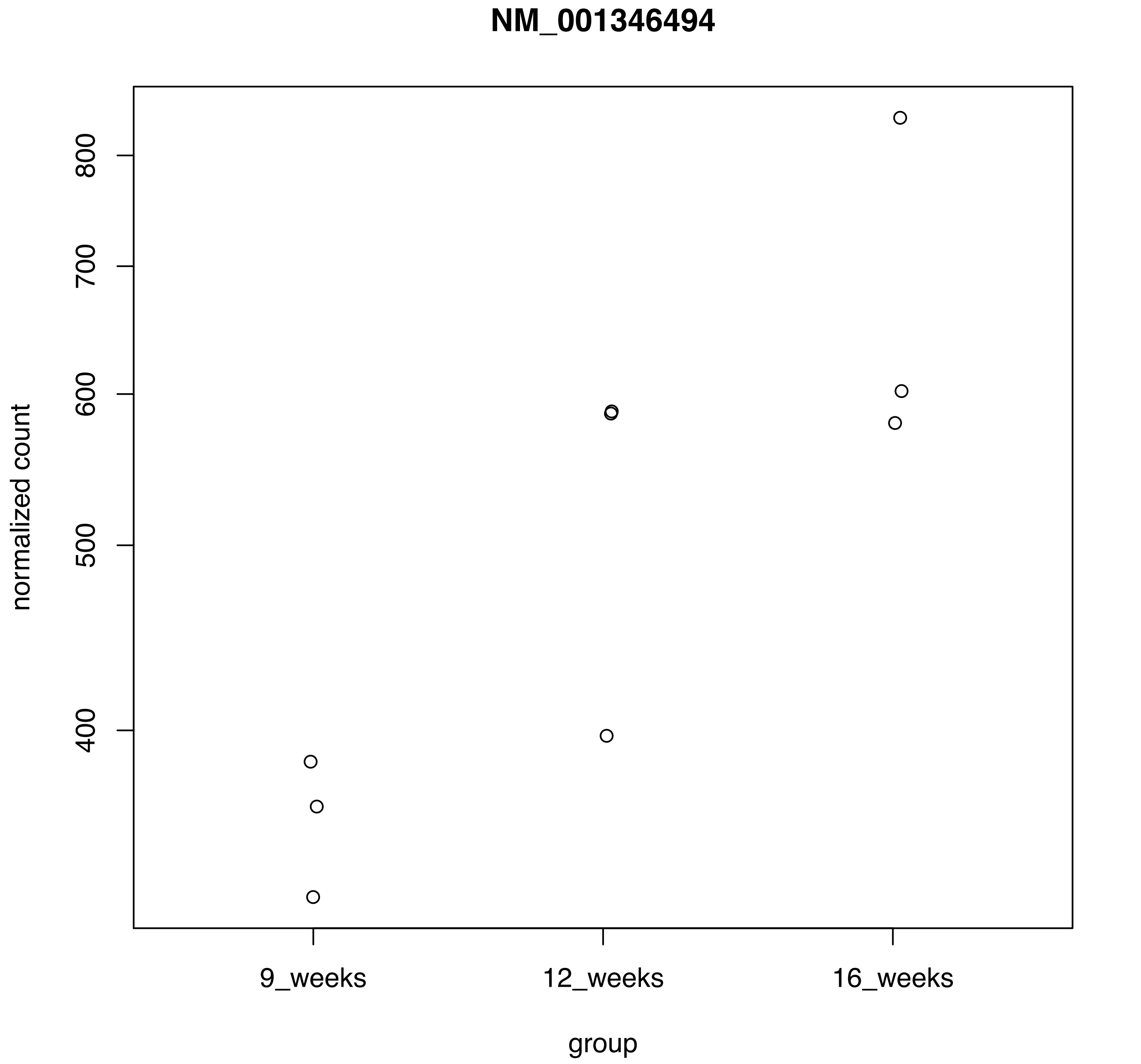

NM_001346494
normalized count
800
700
600
500
400
9_weeks
12_weeks
16_weeks
group

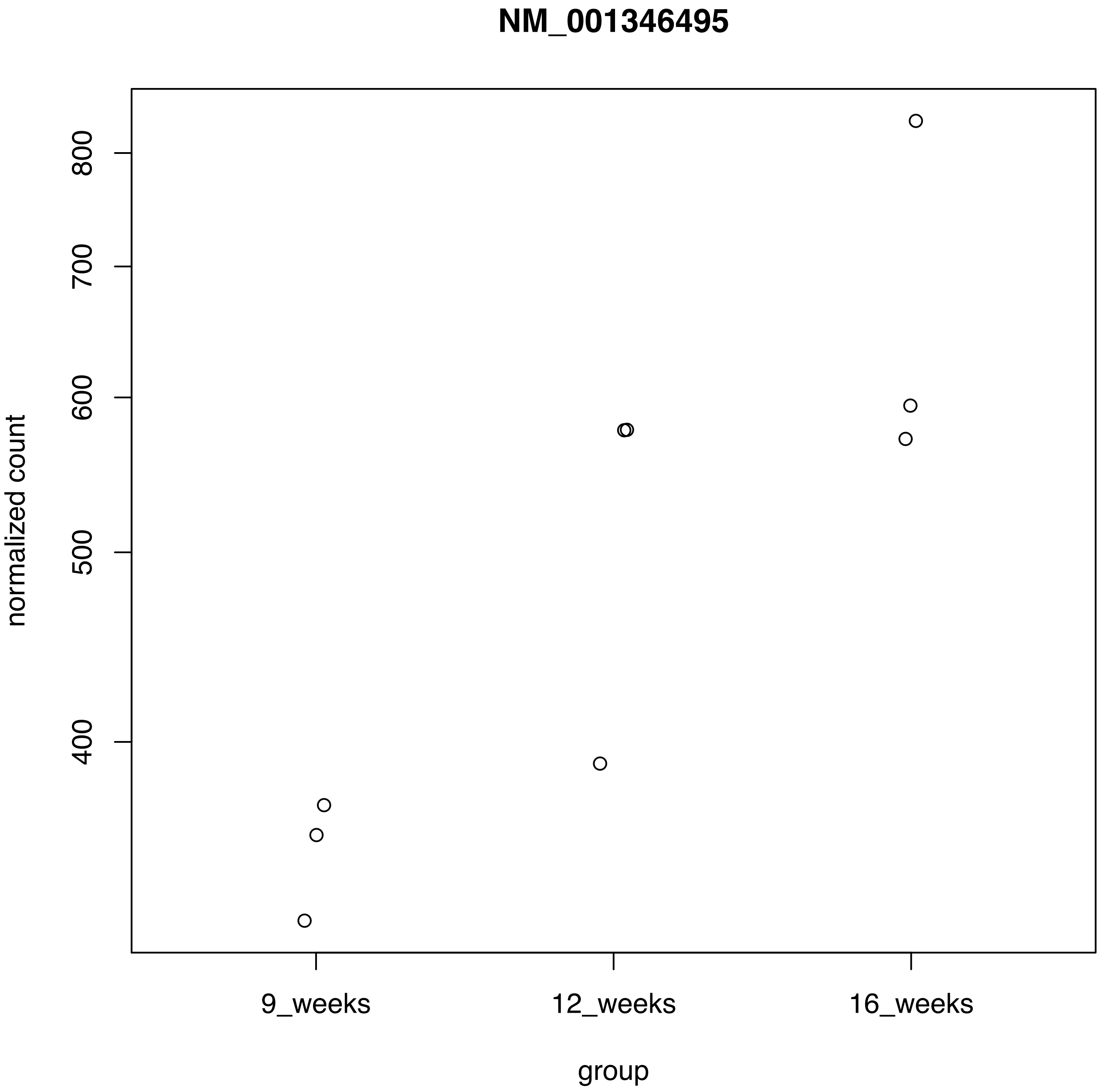
NM_001346495
normalized count
400
500
600
700
800
9_weeks
12_weeks
16_weeks
group

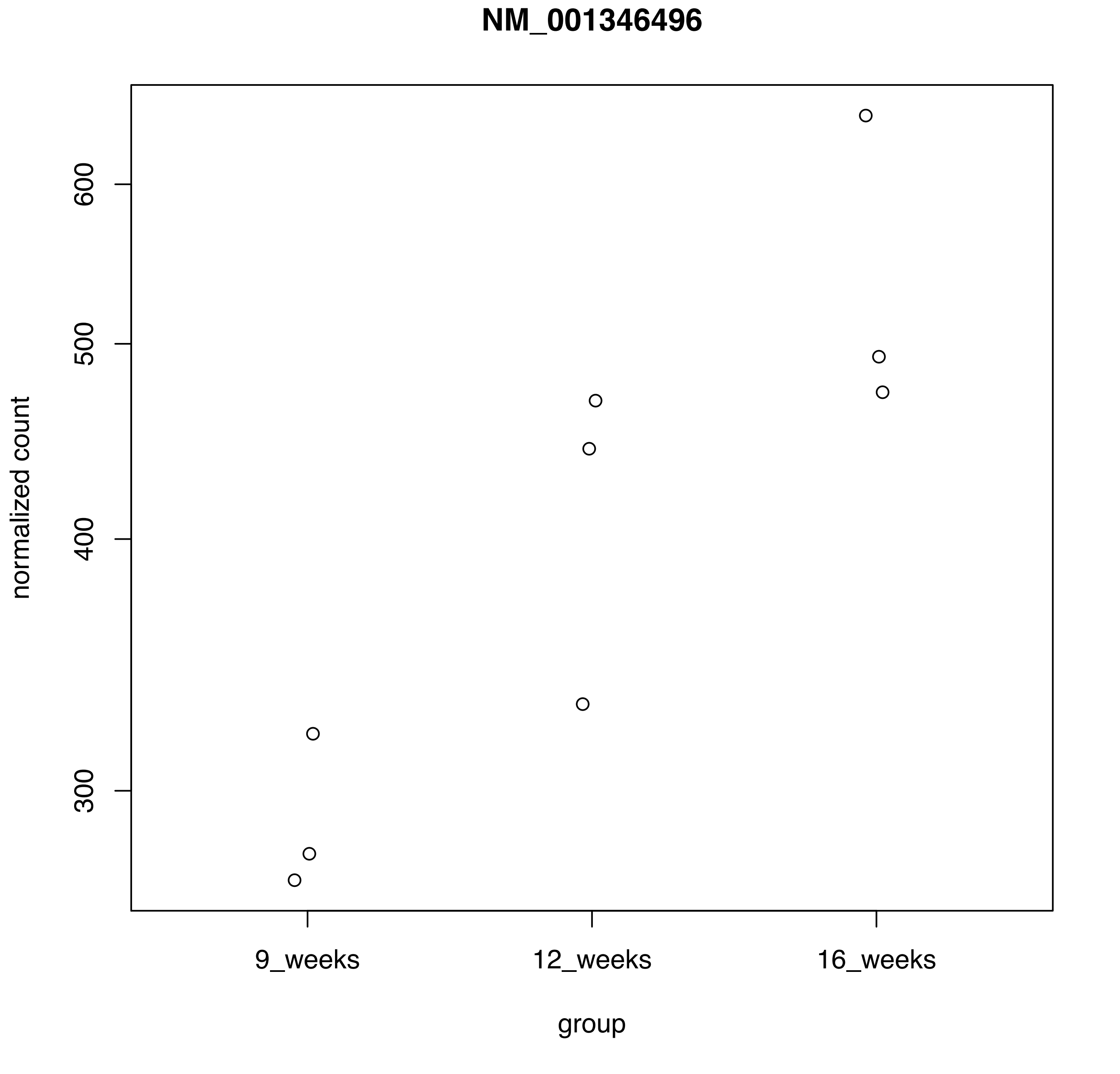

NM_001346496
normalized count
600
500
400
300
9_weeks
12_weeks
16_weeks
group

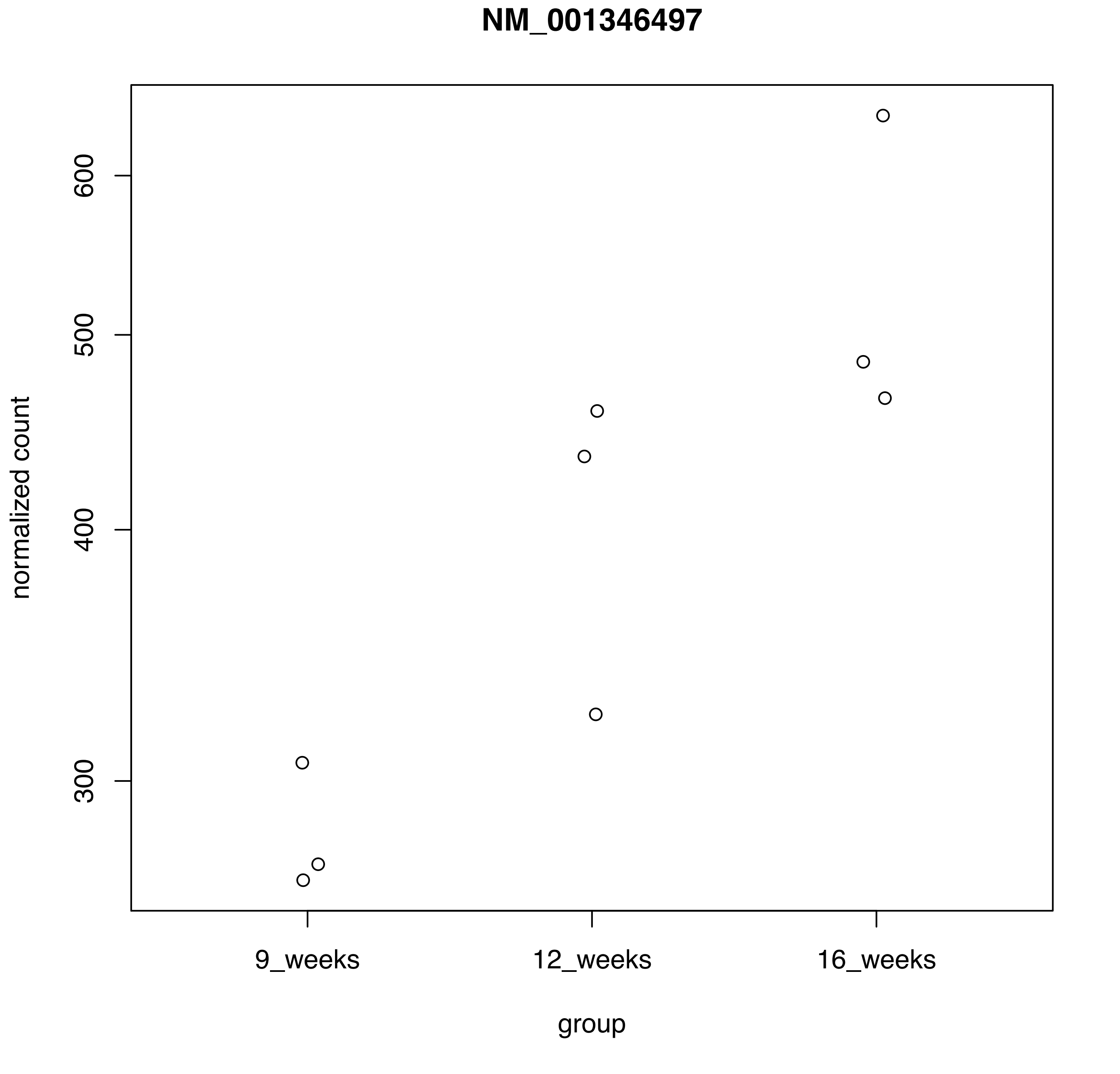

NM_001346497
normalized count
600
500
400
300
9_weeks
12_weeks
16_weeks
group

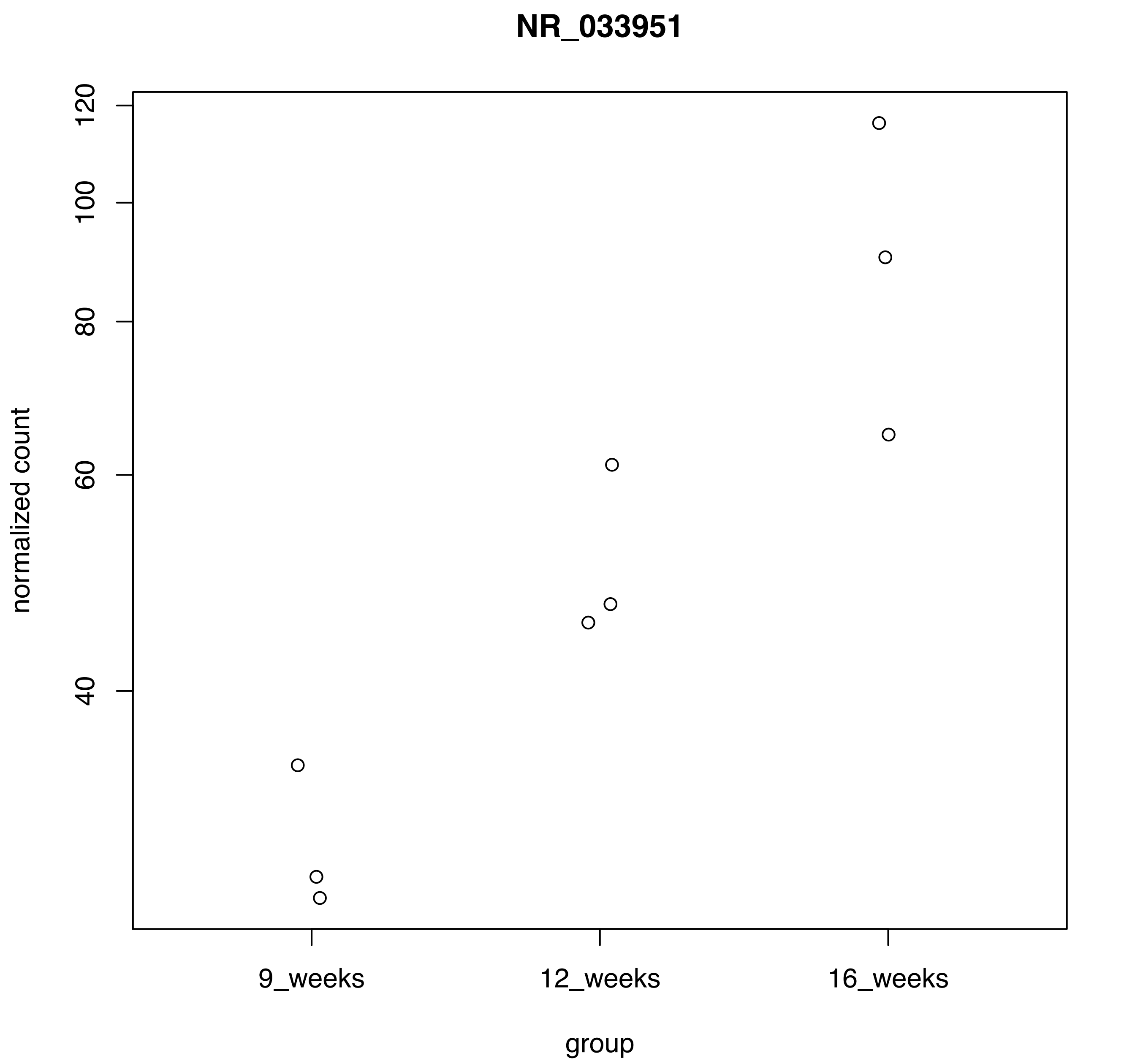
NR_033951
normalized count
120
100
80
60
40
9_weeks
12_weeks
16_weeks
group

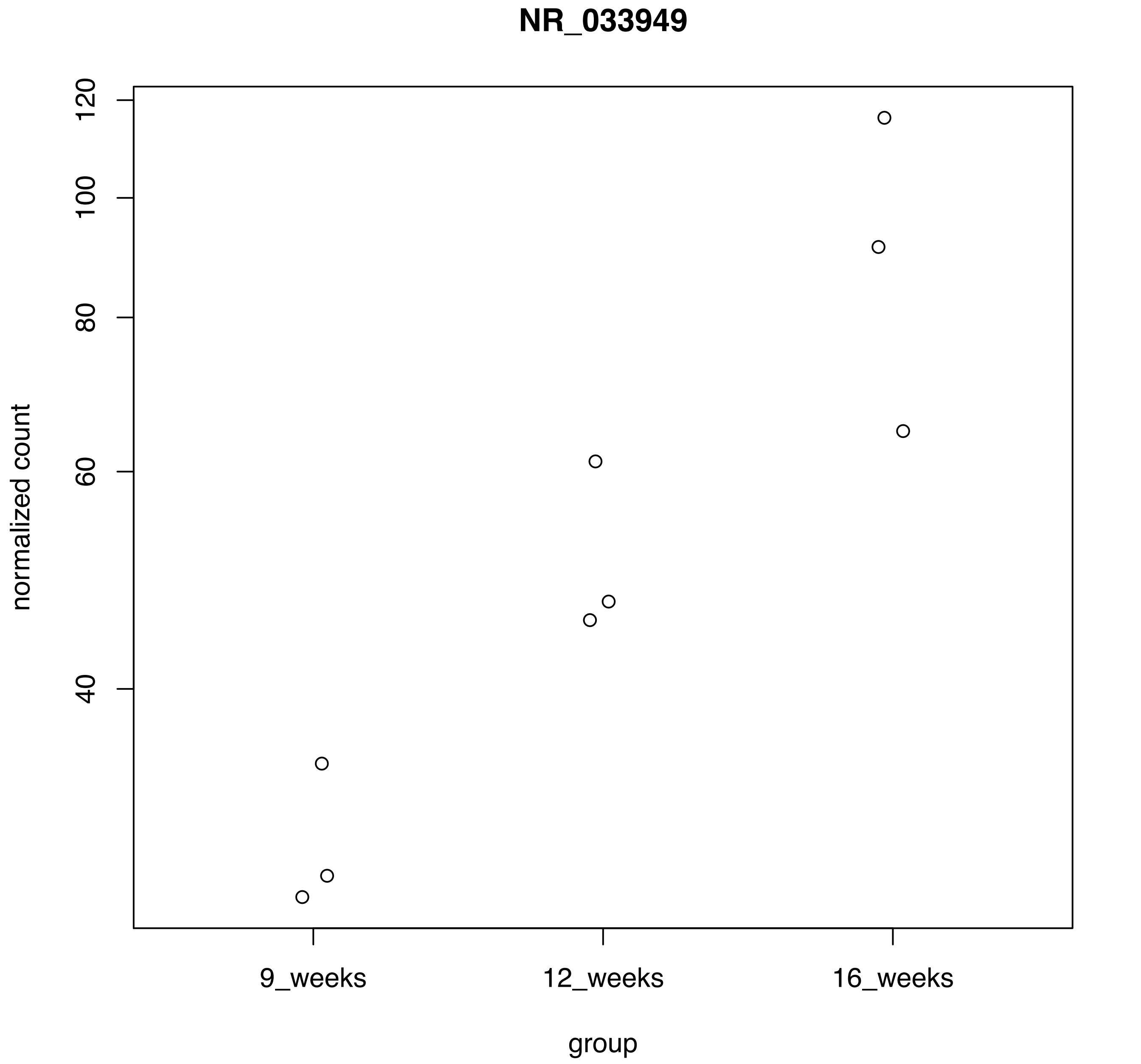
NR_033949
normalized count
120
100
80
60
40
9_weeks
12_weeks
16_weeks
group

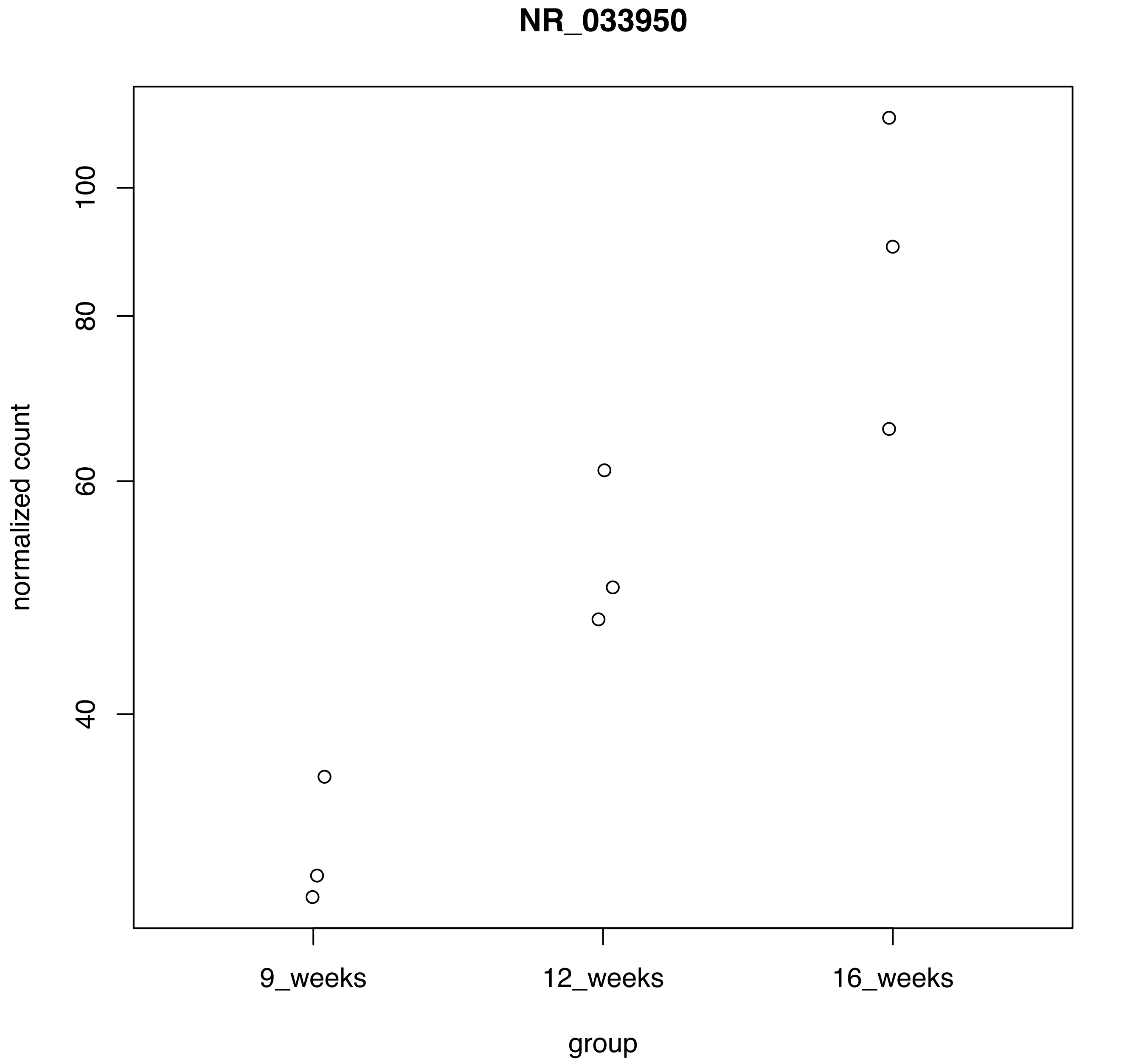
NR_033950
normalized count
100
80
60
40
9_weeks
12_weeks
16_weeks
group

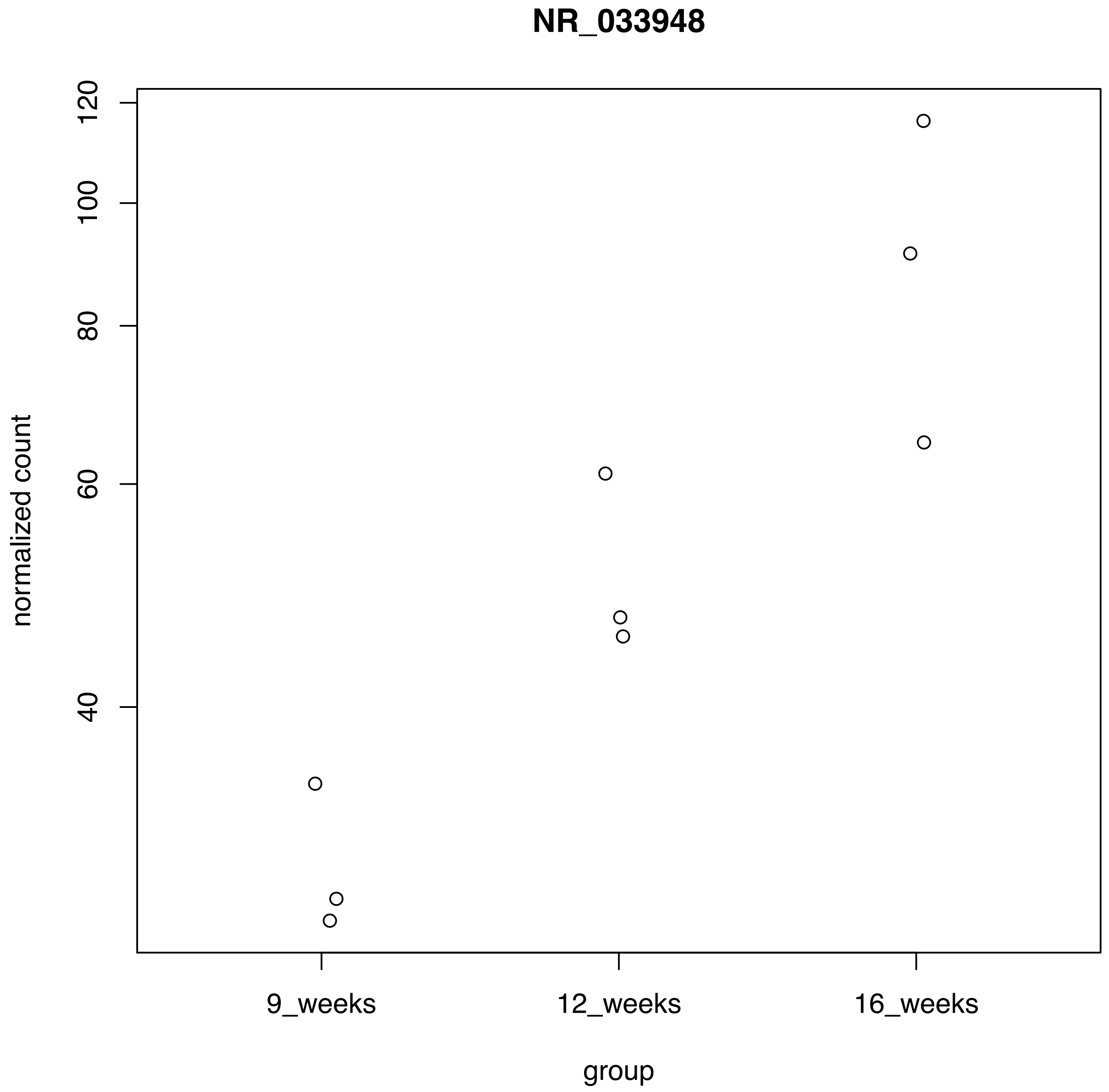
NR_033948
normalized count
120
100
80
60
40
9_weeks
12_weeks
16_weeks
group

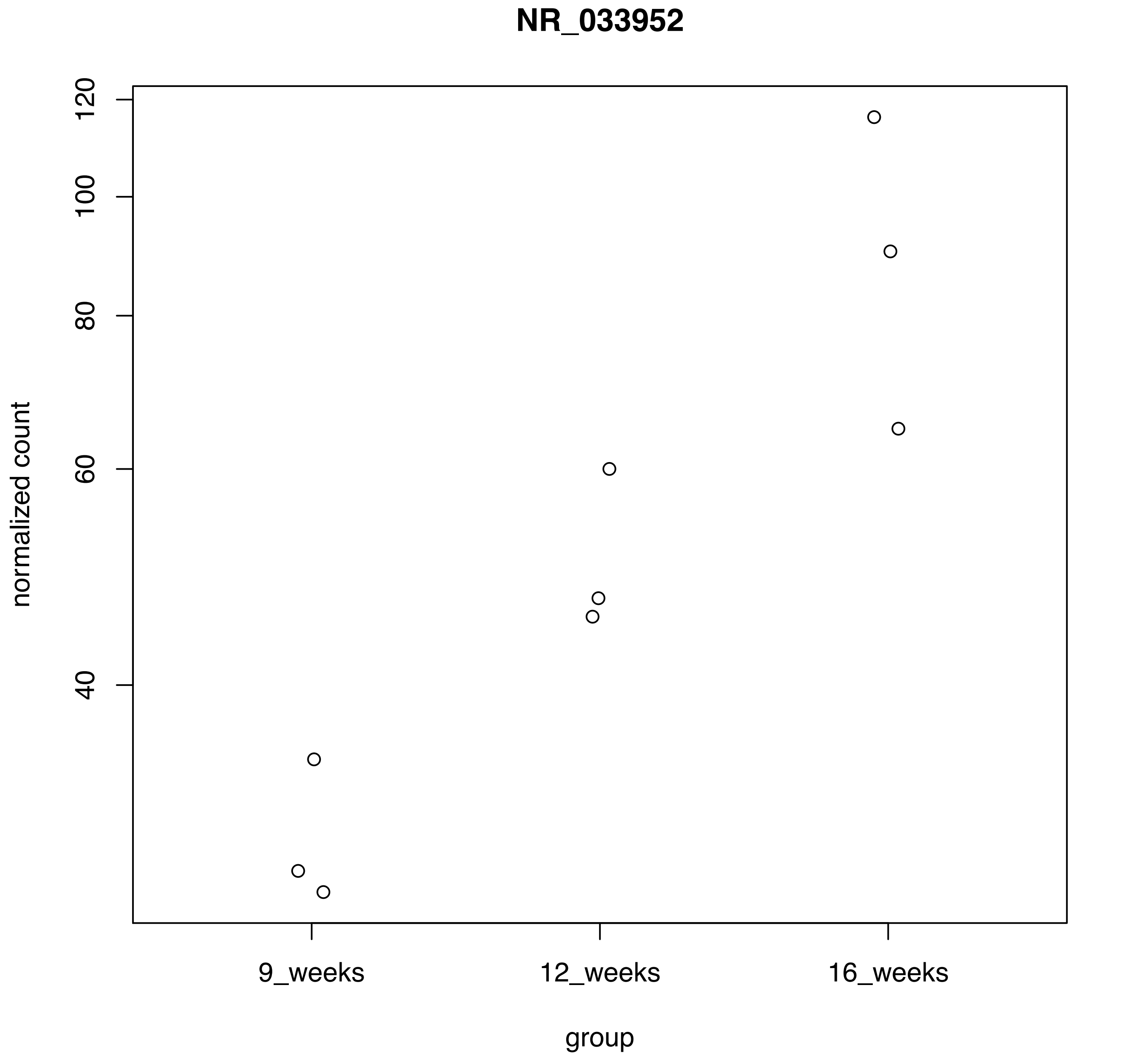
NR_033952
normalized count
120
100
80
60
40
9_weeks
12_weeks
16_weeks
group

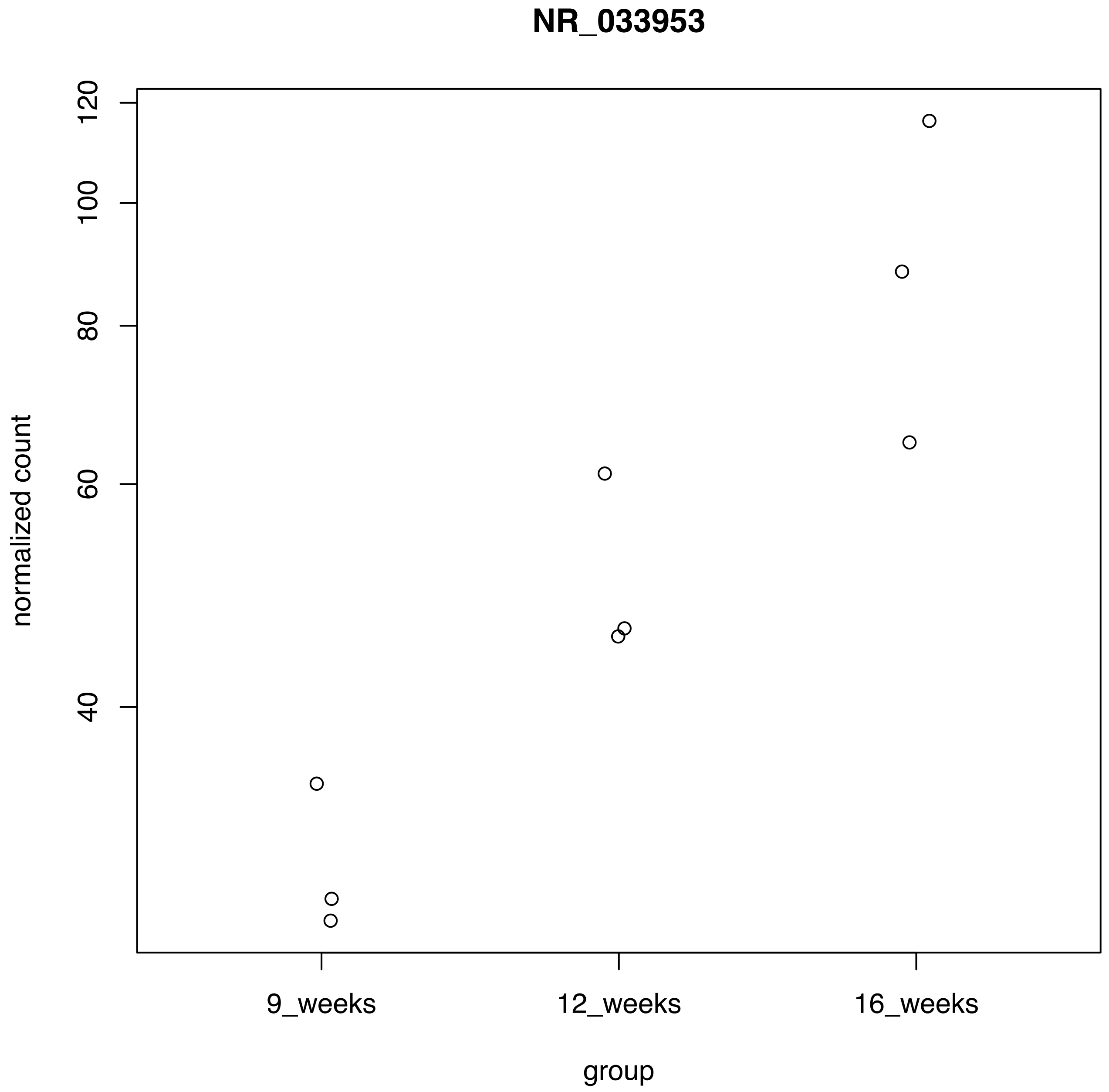
NR_033953
normalized count
120
100
80
60
40
9_weeks
12_weeks
16_weeks
group

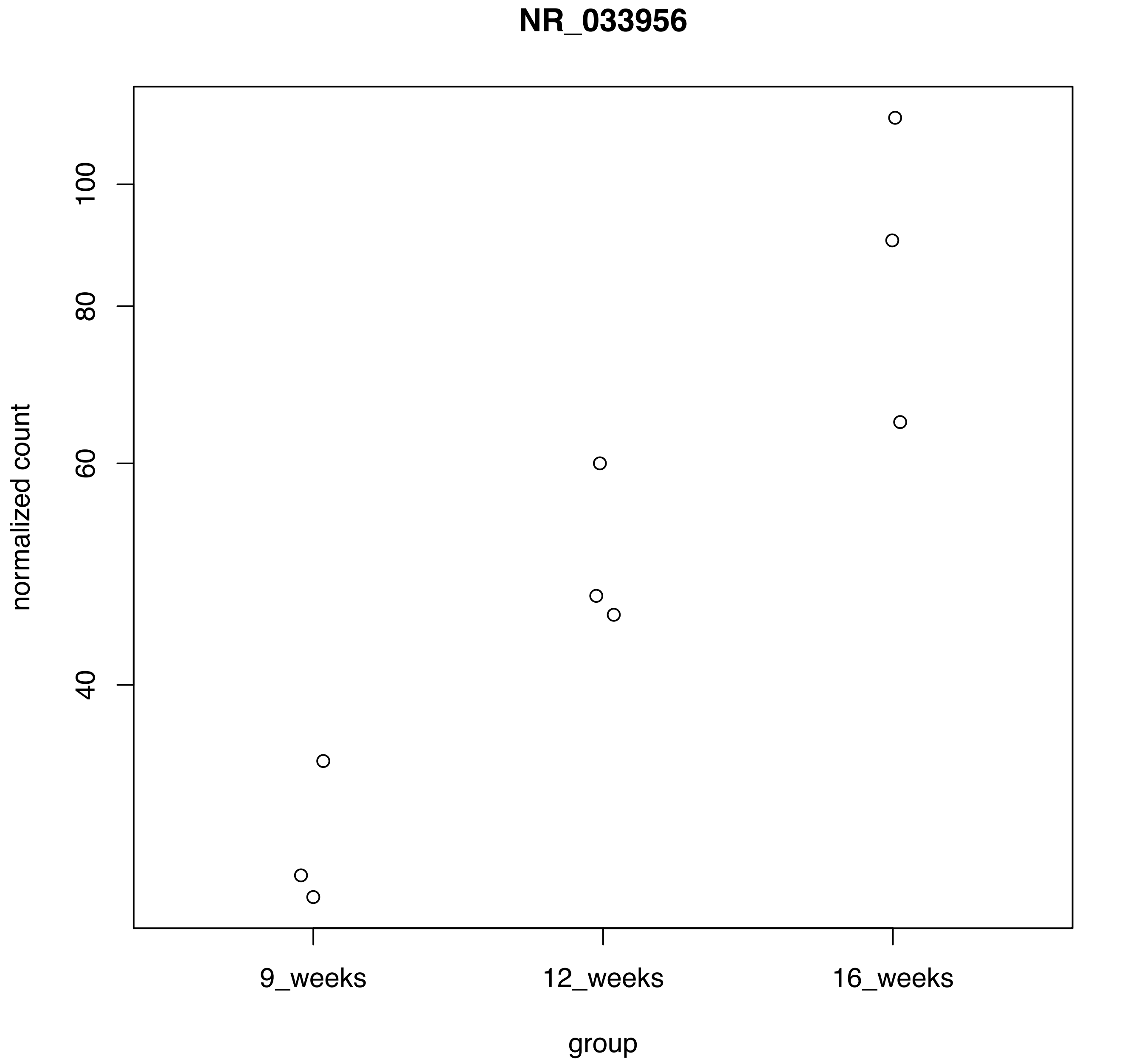
NR_033956
normalized count
100
80
60
40
9_weeks
12_weeks
16_weeks
group

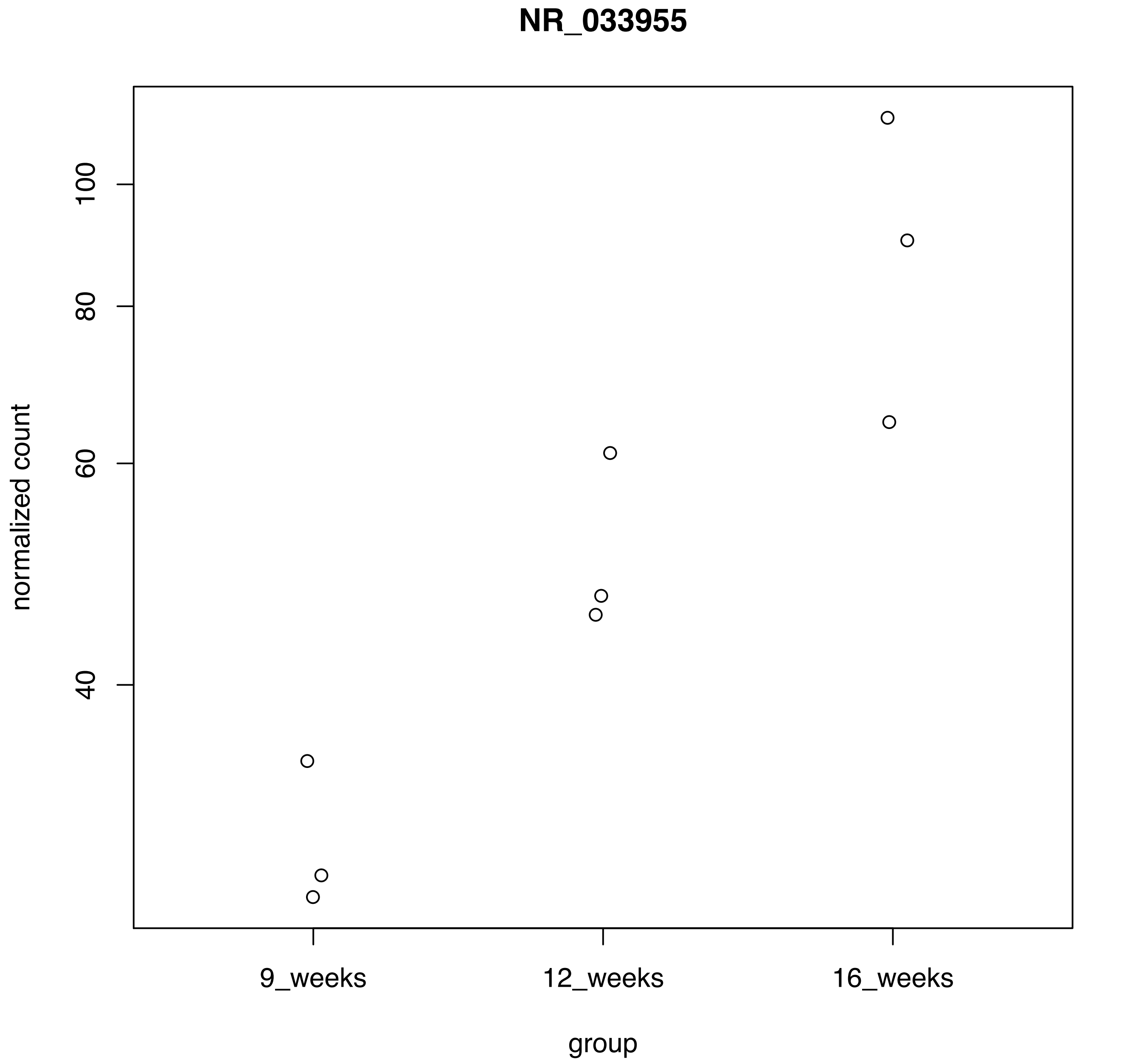

NR_033955
100
80
60
40
normalized count
9_weeks
12_weeks
16_weeks
group

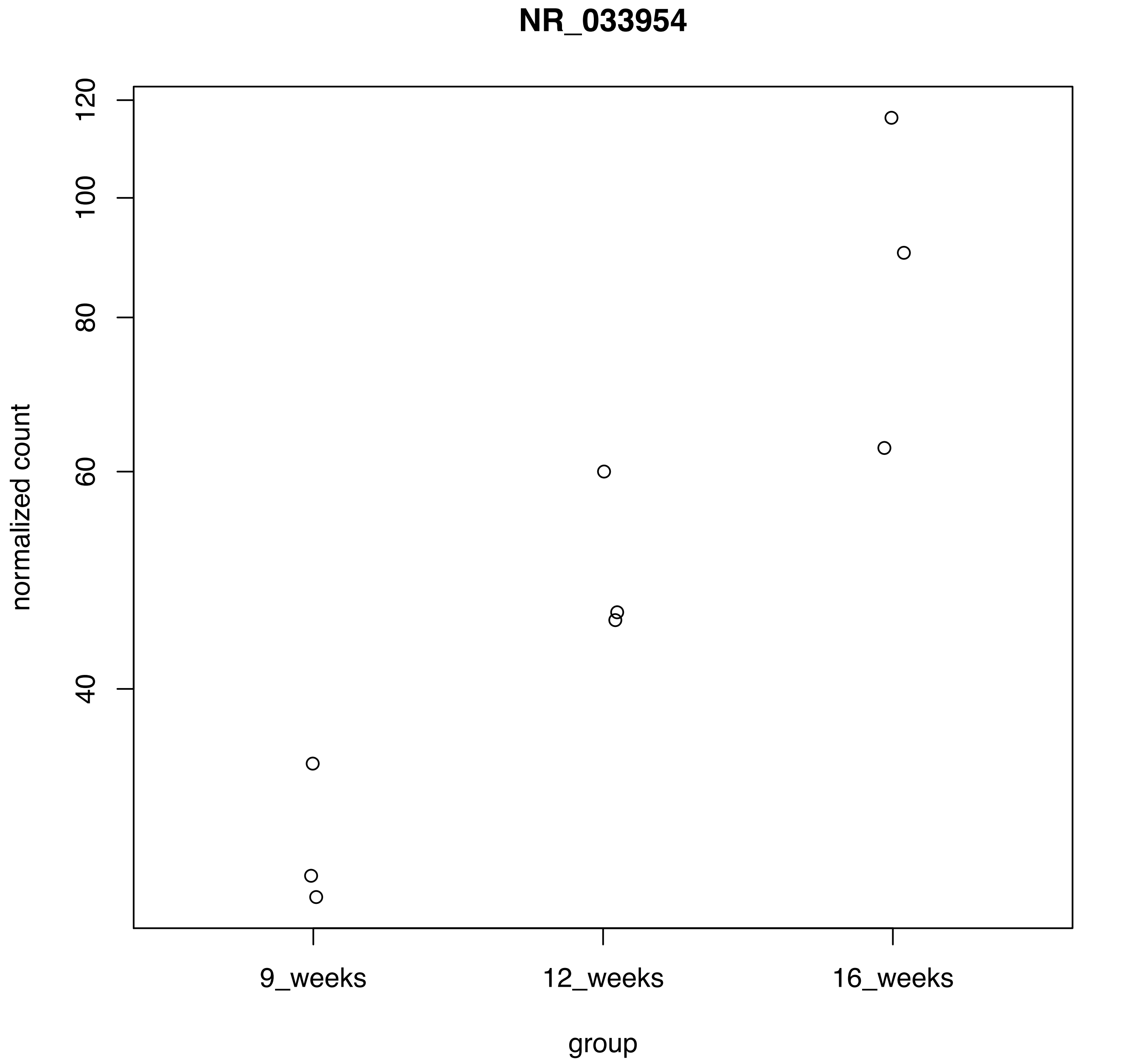
NR_033954
normalized count
120
100
80
60
40
9_weeks
12_weeks
16_weeks
group

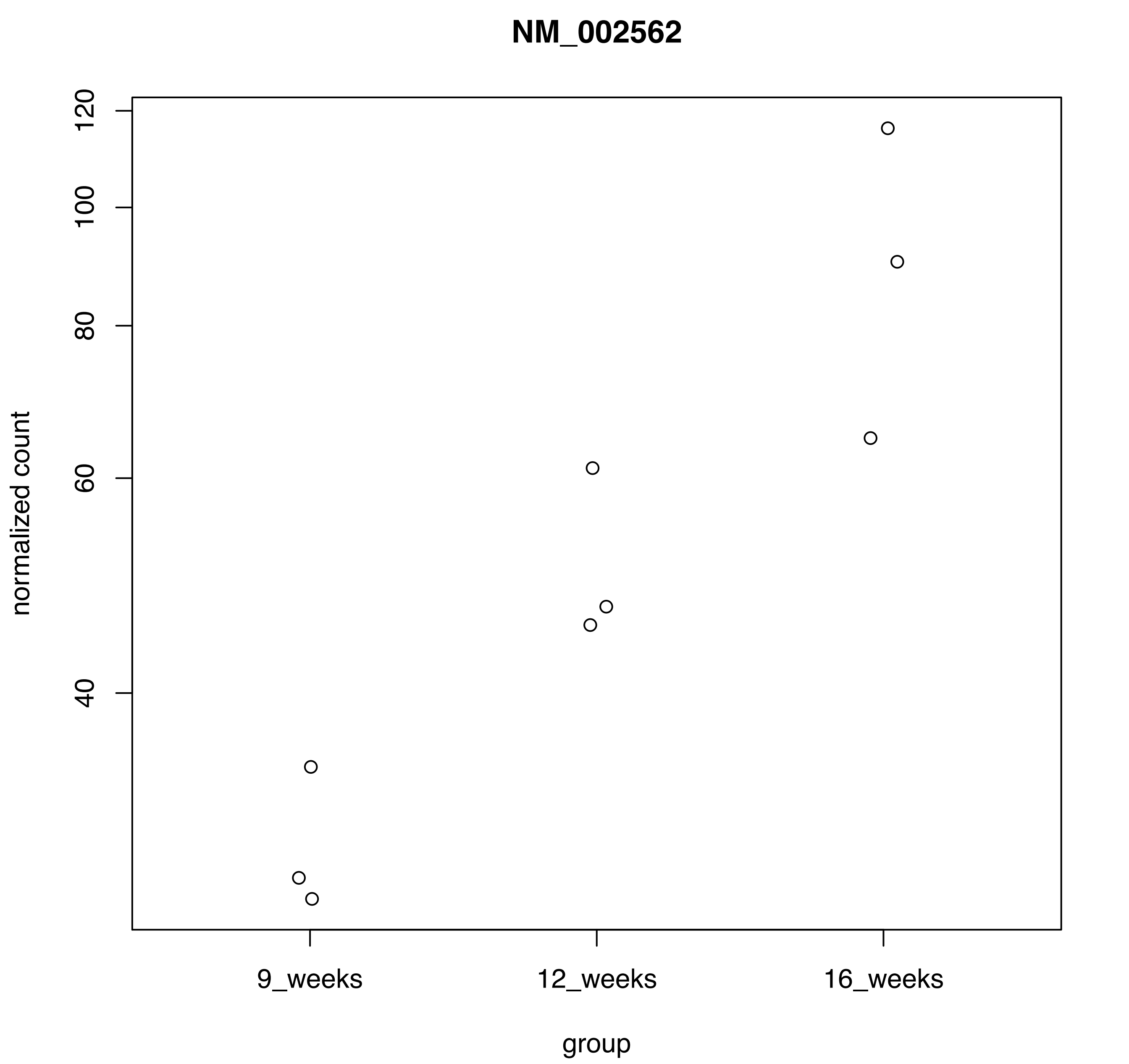
NM_002562
normalized count
120
100
80
60
40
9_weeks
12_weeks
16_weeks
group

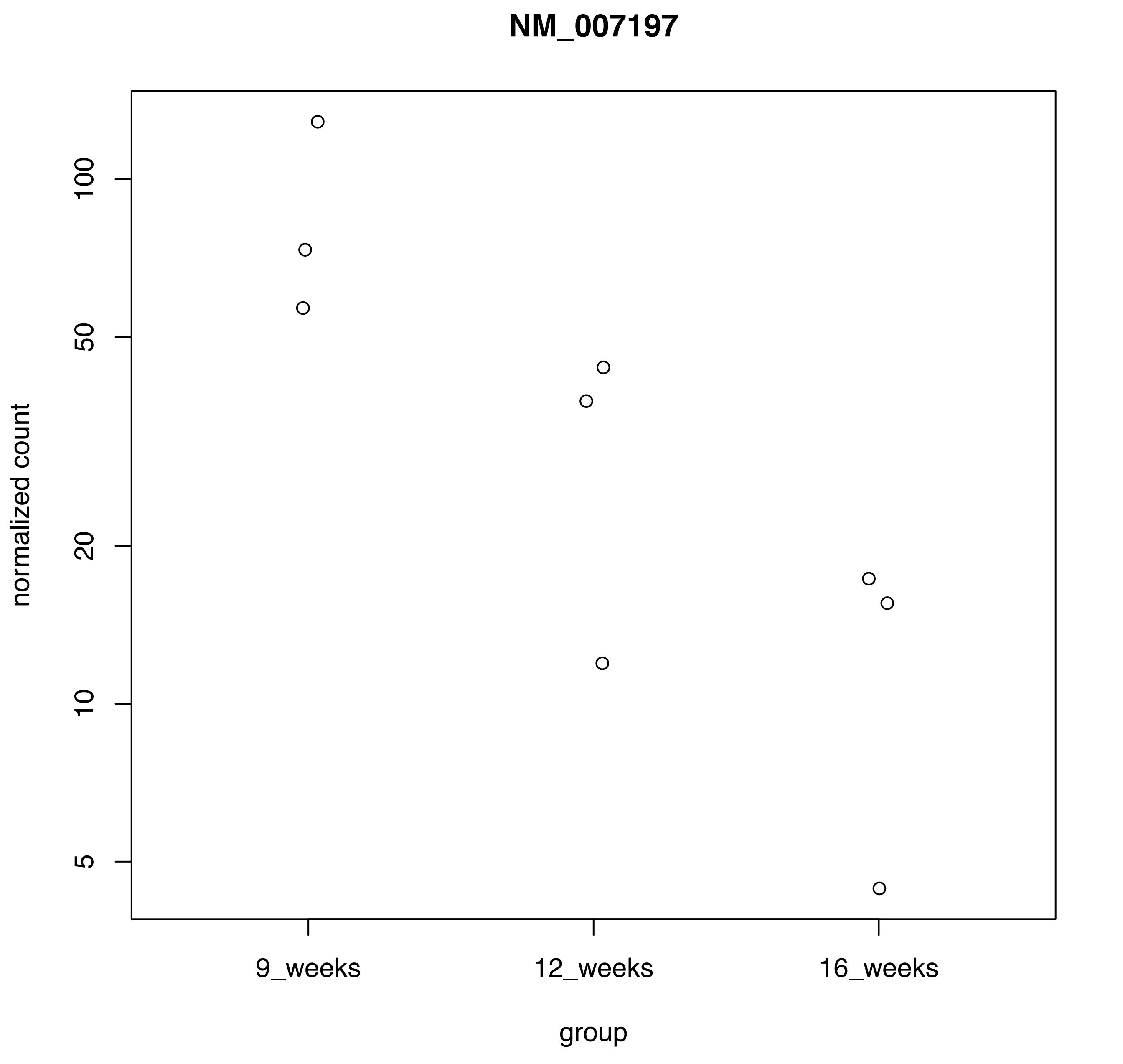
NM_007197
normalized count
100
50
20
10
5
9_weeks
12_weeks
16_weeks
group

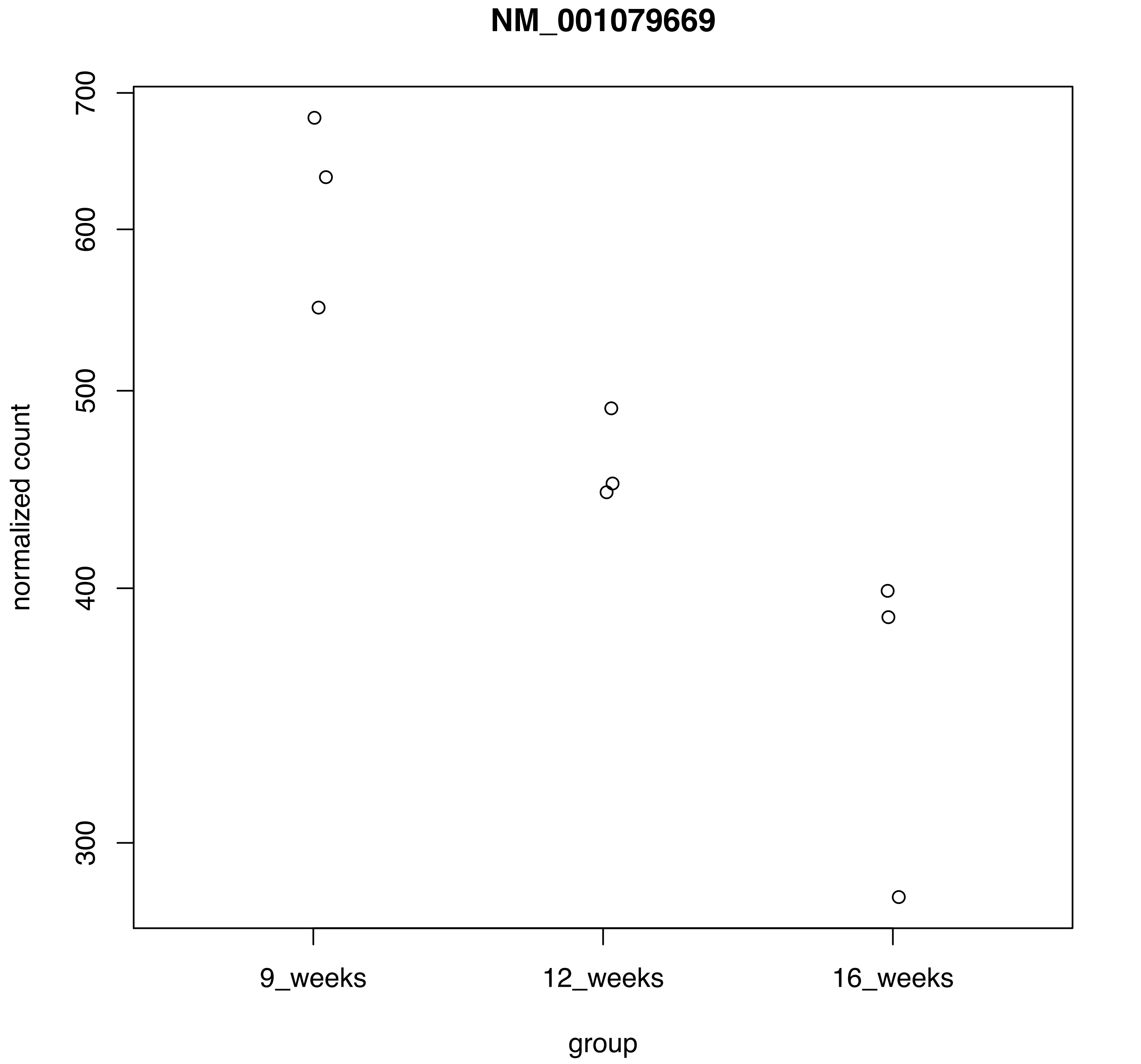
NM_001079669
normalized count
700
600
500
400
300
9_weeks
12_weeks
16_weeks
group

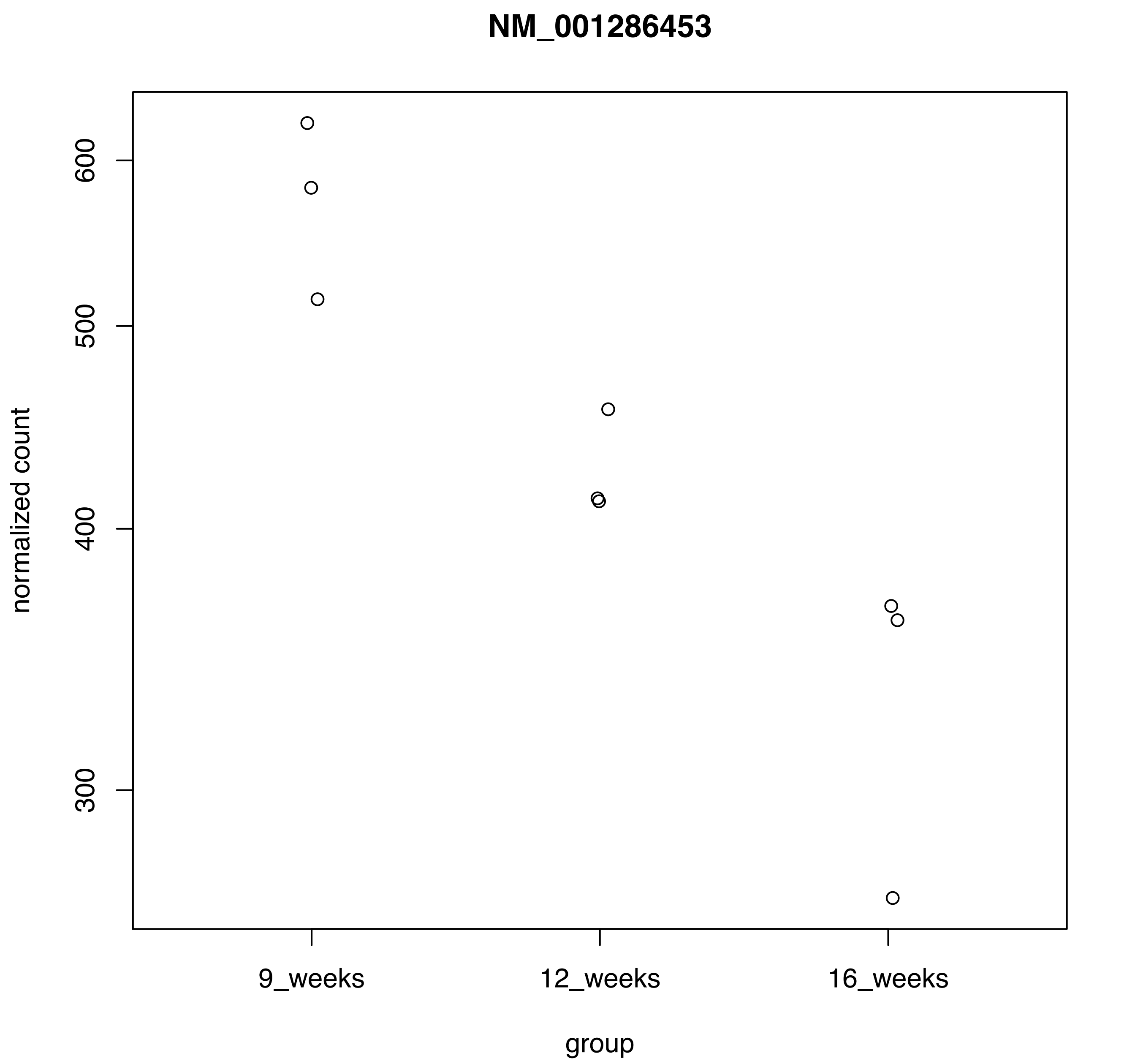
NM_001286453
normalized count
600
500
400
300
9_weeks
12_weeks
16_weeks
group

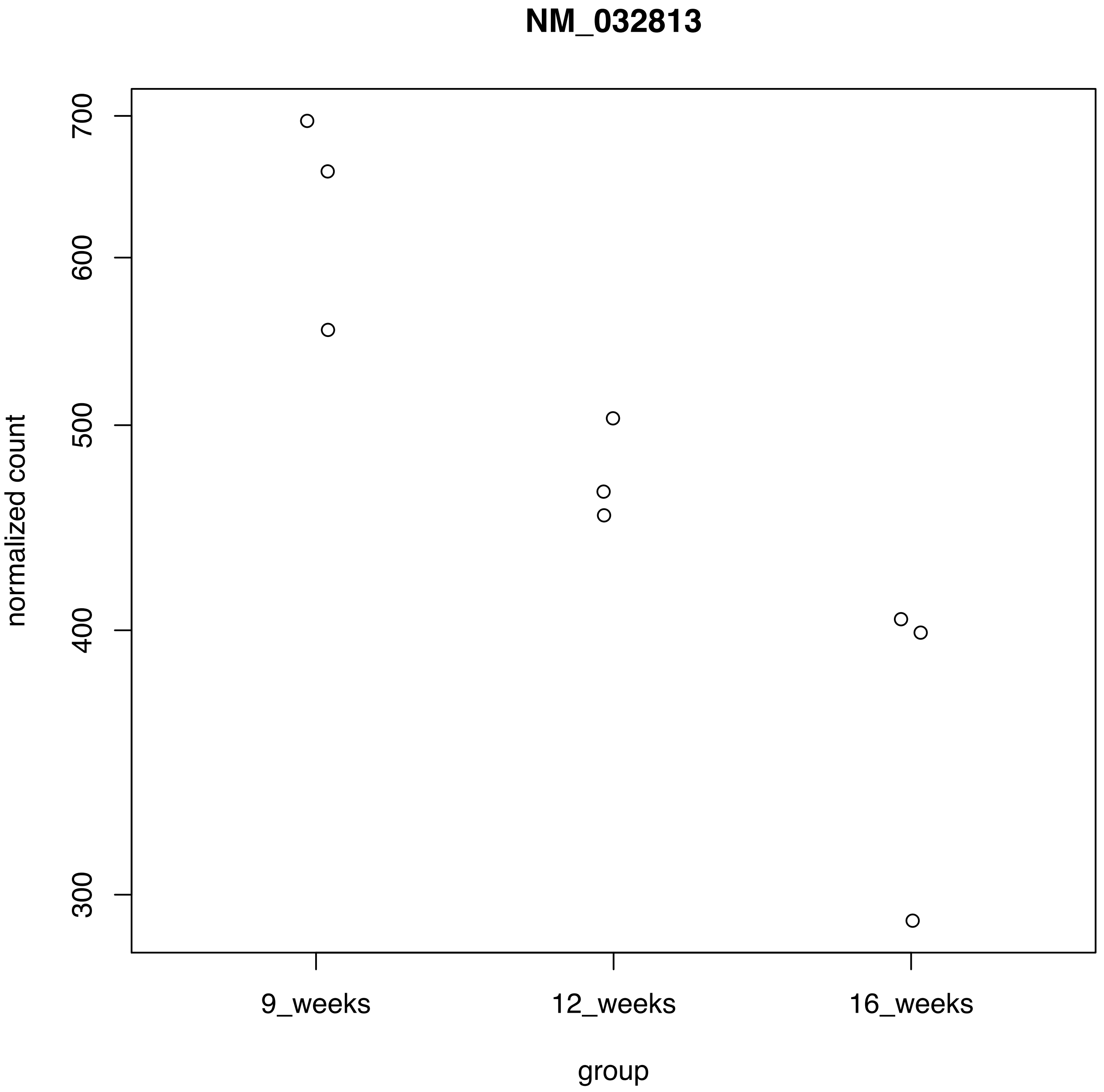
NM_032813
normalized count
700
600
500
400
300
9_weeks
12_weeks
16_weeks
group

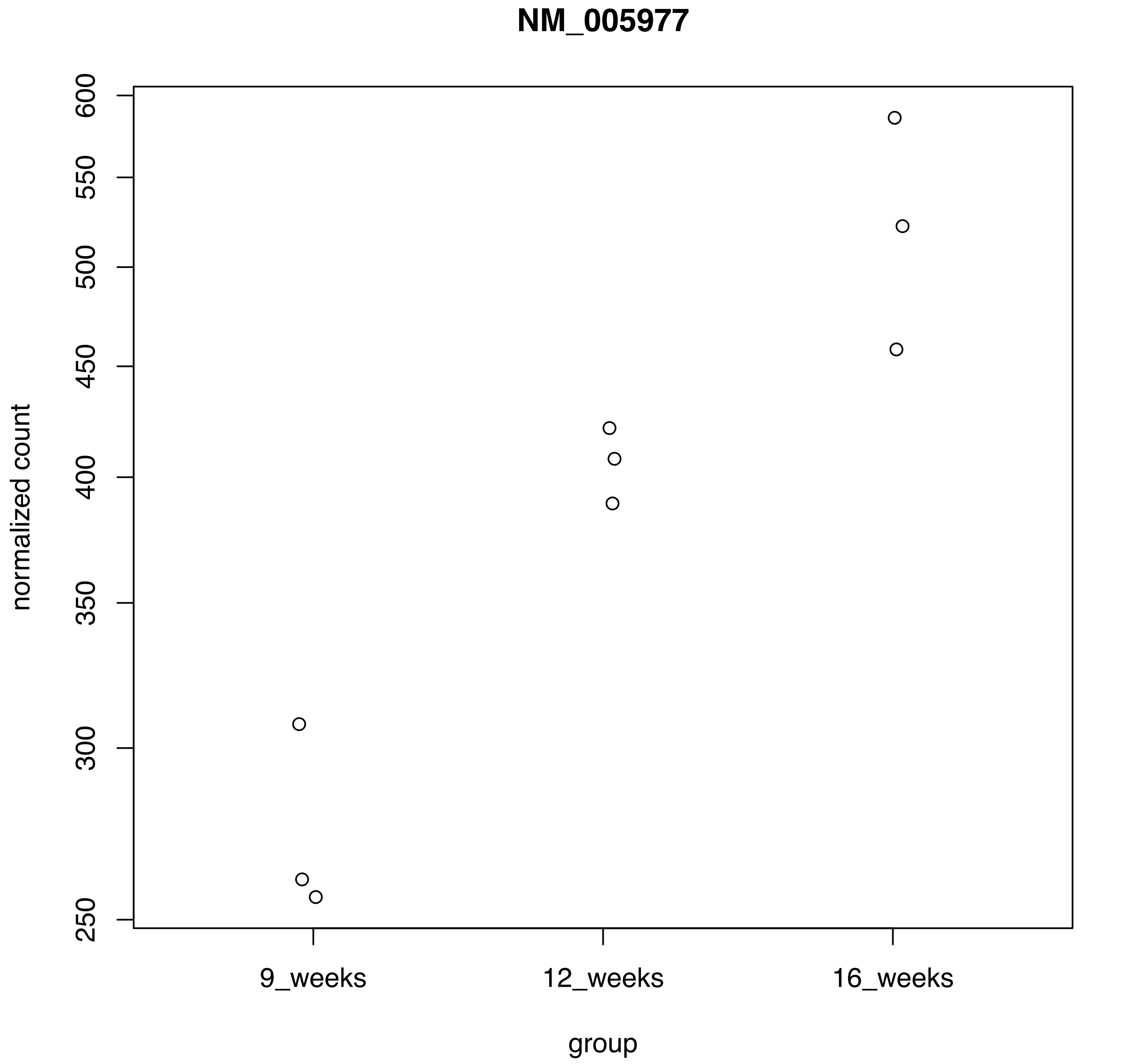

NM_005977
600
550
500
450
400
350
300
250
normalized count
9_weeks
12_weeks
16_weeks
group

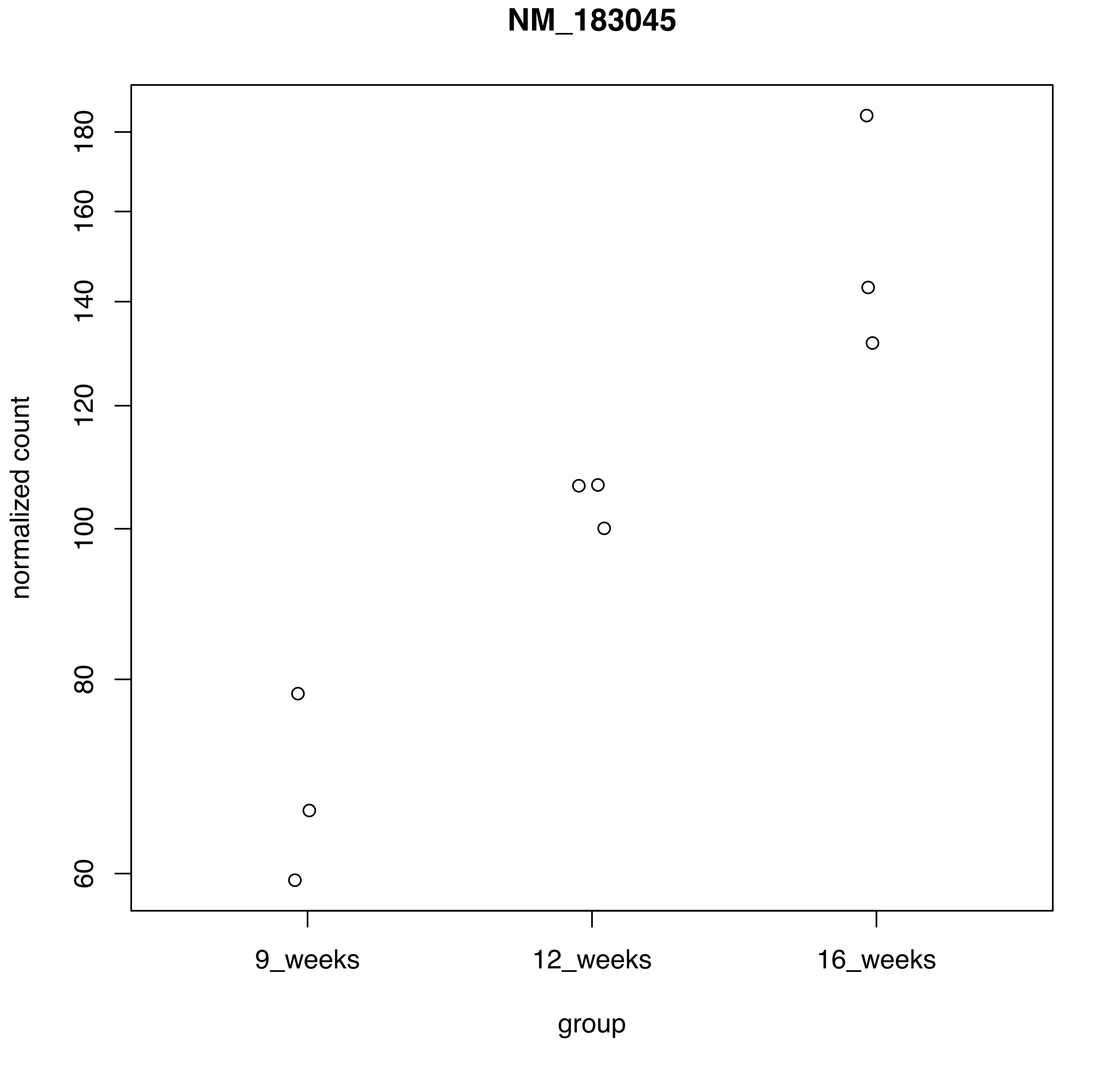

NM_183045
normalized count
60
80
100
120
140
160
180
9_weeks
12_weeks
16_weeks
group

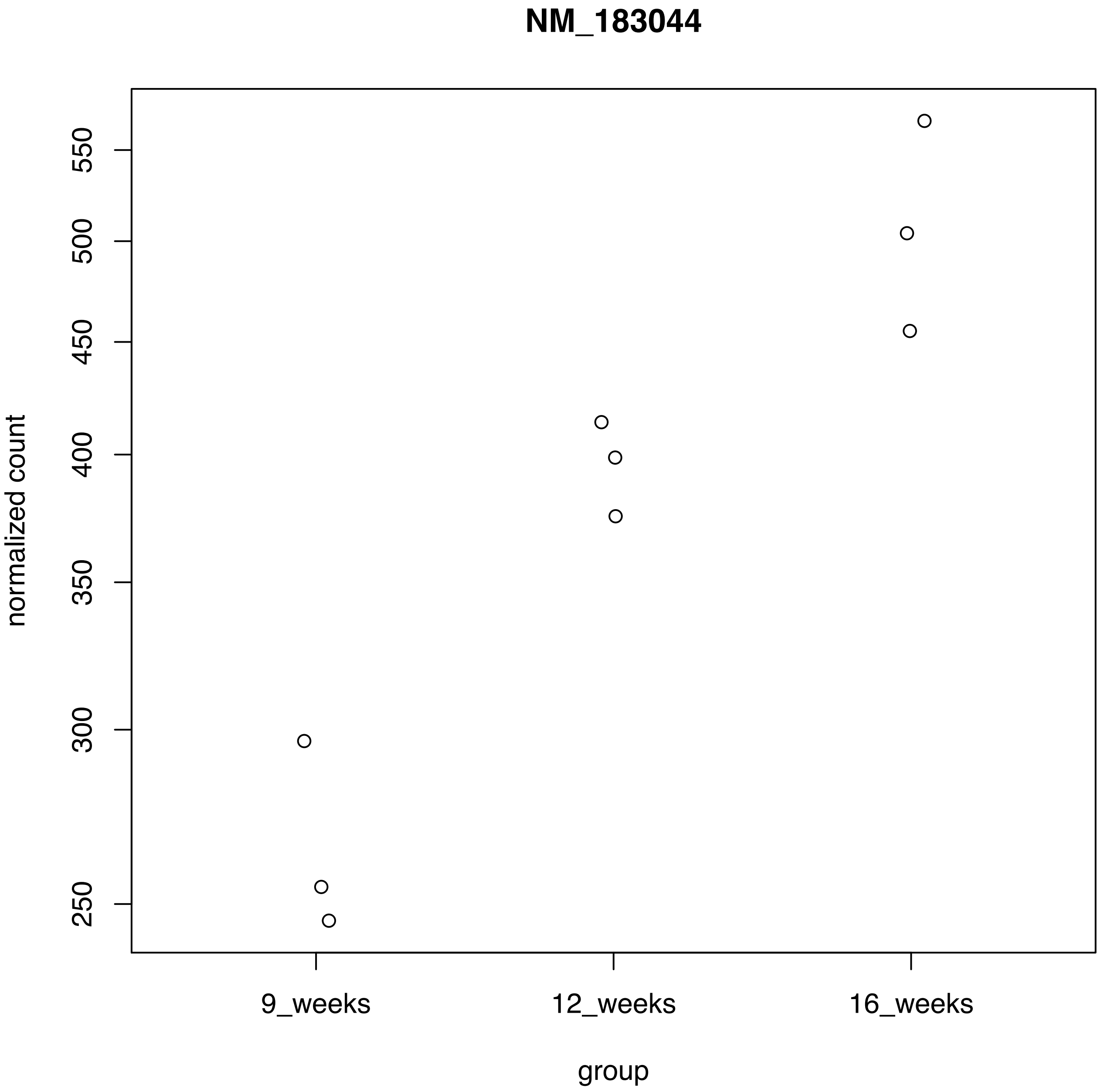

NM_183044
normalized count
550
500
450
400
350
300
250
9_weeks
12_weeks
16_weeks
group

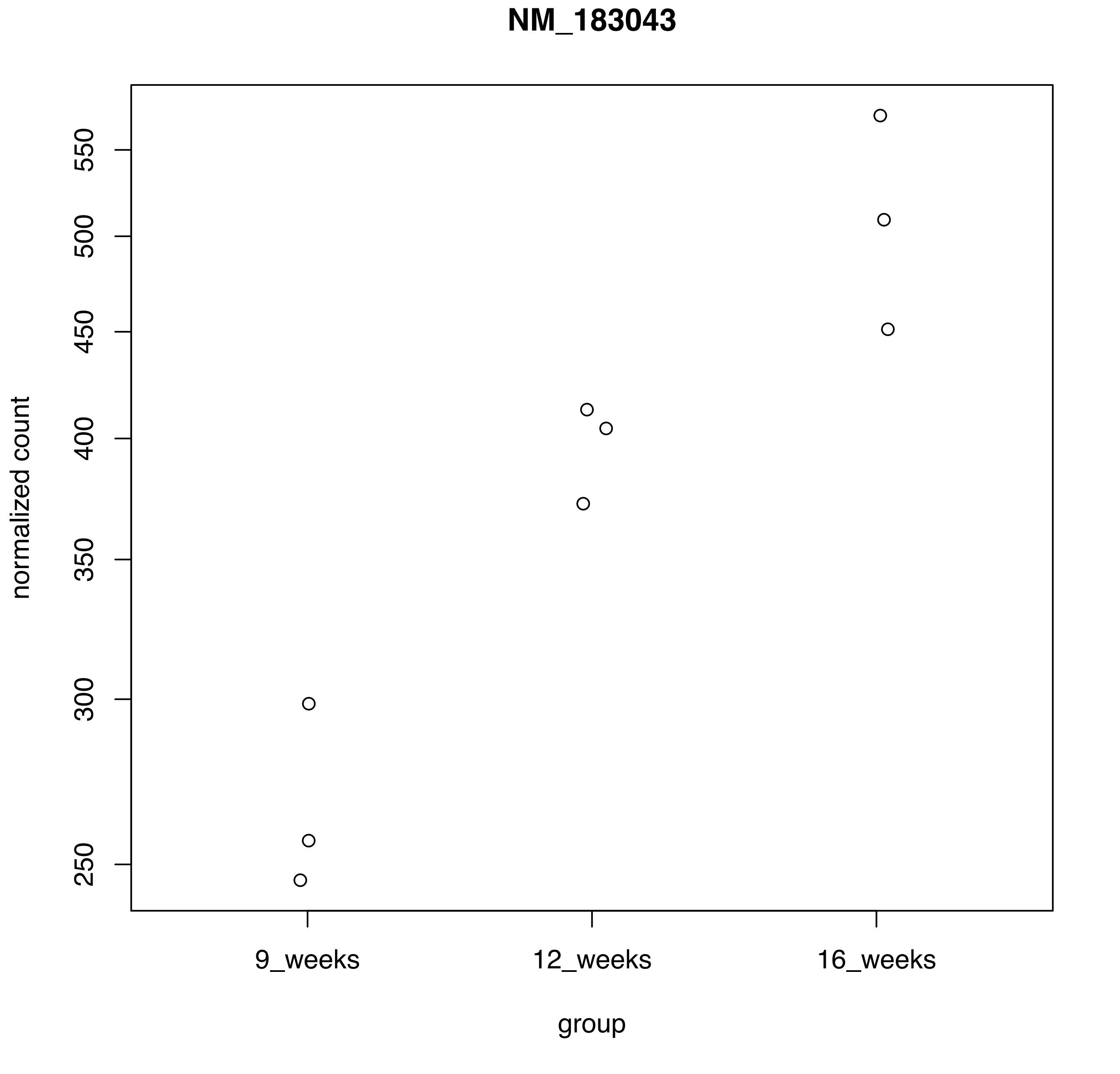
NM_183043
normalized count
550
500
450
400
350
300
250
9_weeks
12_weeks
16_weeks
group

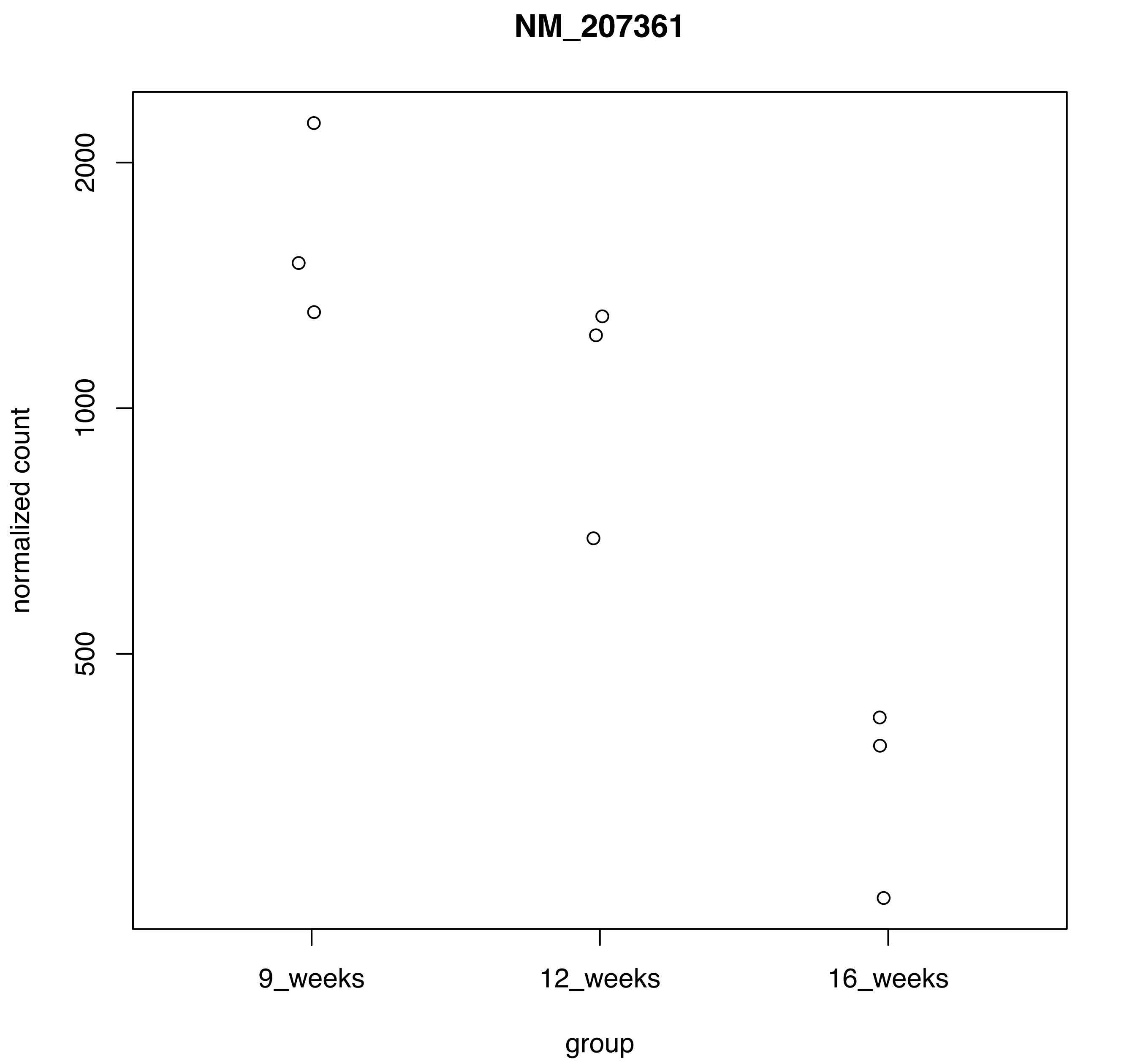
NM_207361
normalized count
2000
1000
500
9_weeks
12_weeks
16_weeks
group

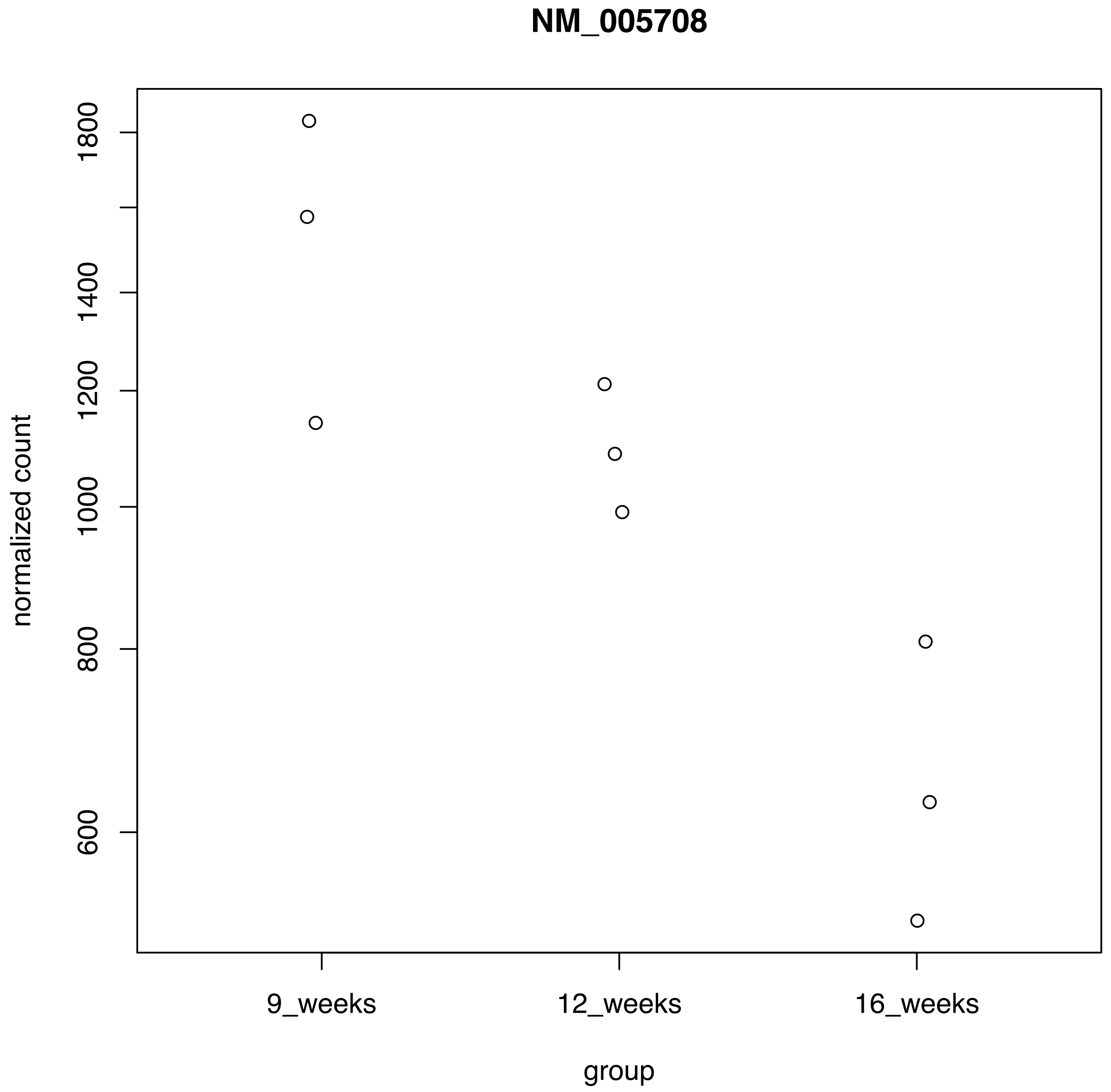
NM_005708
normalized count
600
800
1000
1200
1400
1800
9_weeks
12_weeks
16_weeks
group

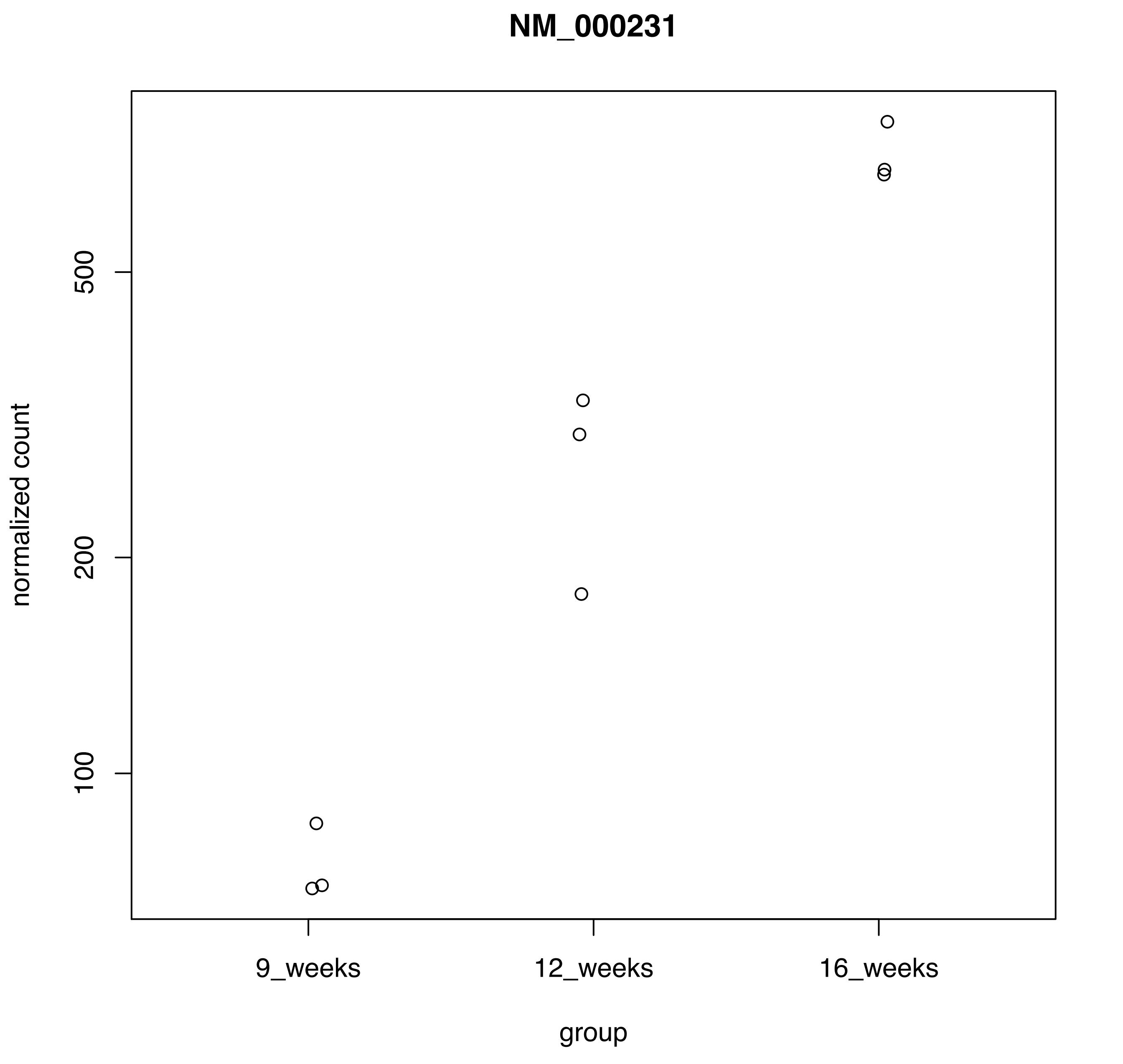
NM_000231
normalized count
500
200
100
9_weeks
12_weeks
16_weeks
group

NM_006646

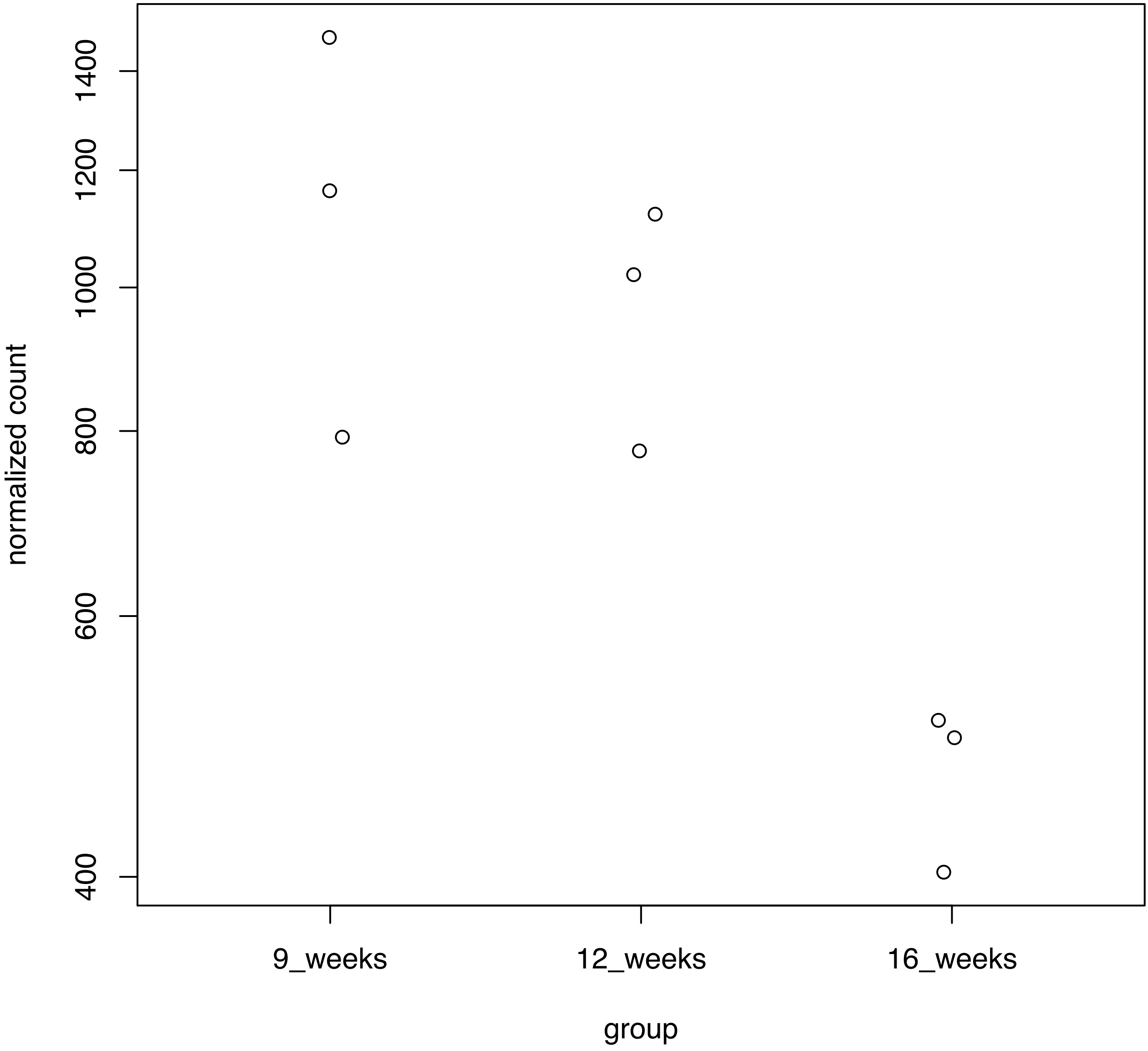

normalized count
1400
1200
1000
800
600
400
9_weeks
12_weeks
16_weeks
group

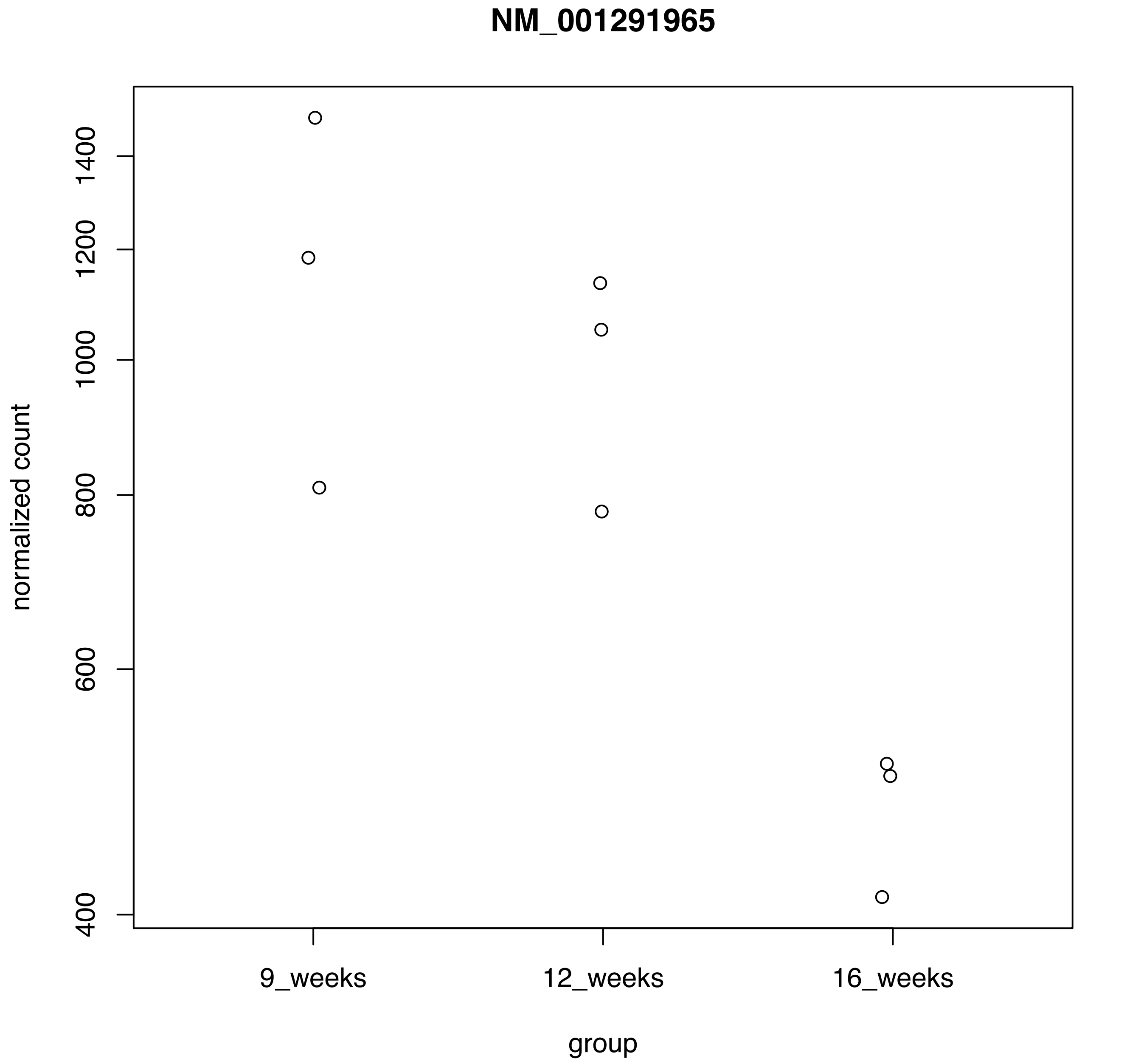
NM_001291965
normalized count
1400
1200
1000
800
600
400
9_weeks
12_weeks
16_weeks
group

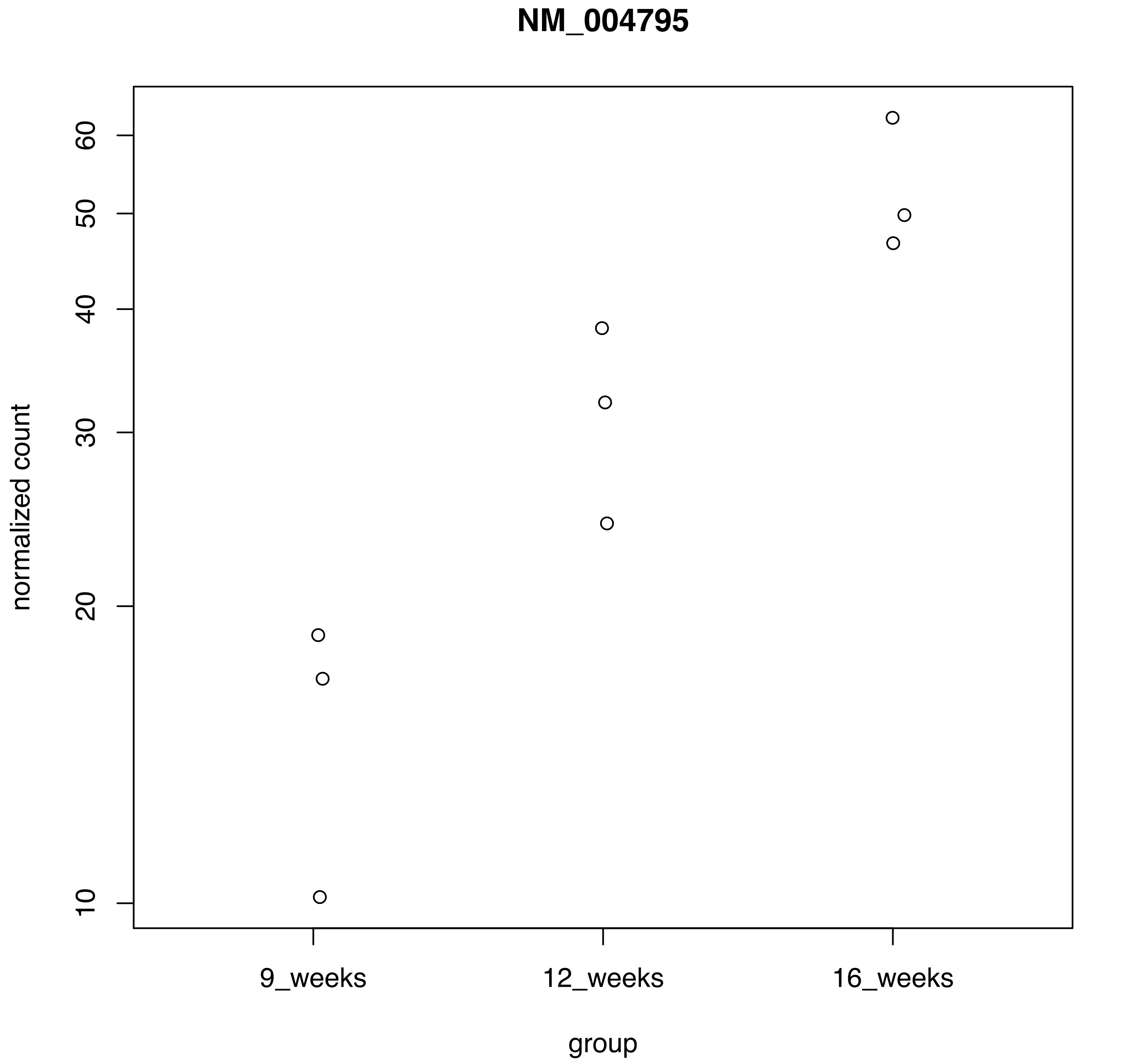
NM_004795
normalized count
60
50
40
30
20
10
9_weeks
12_weeks
16_weeks
group

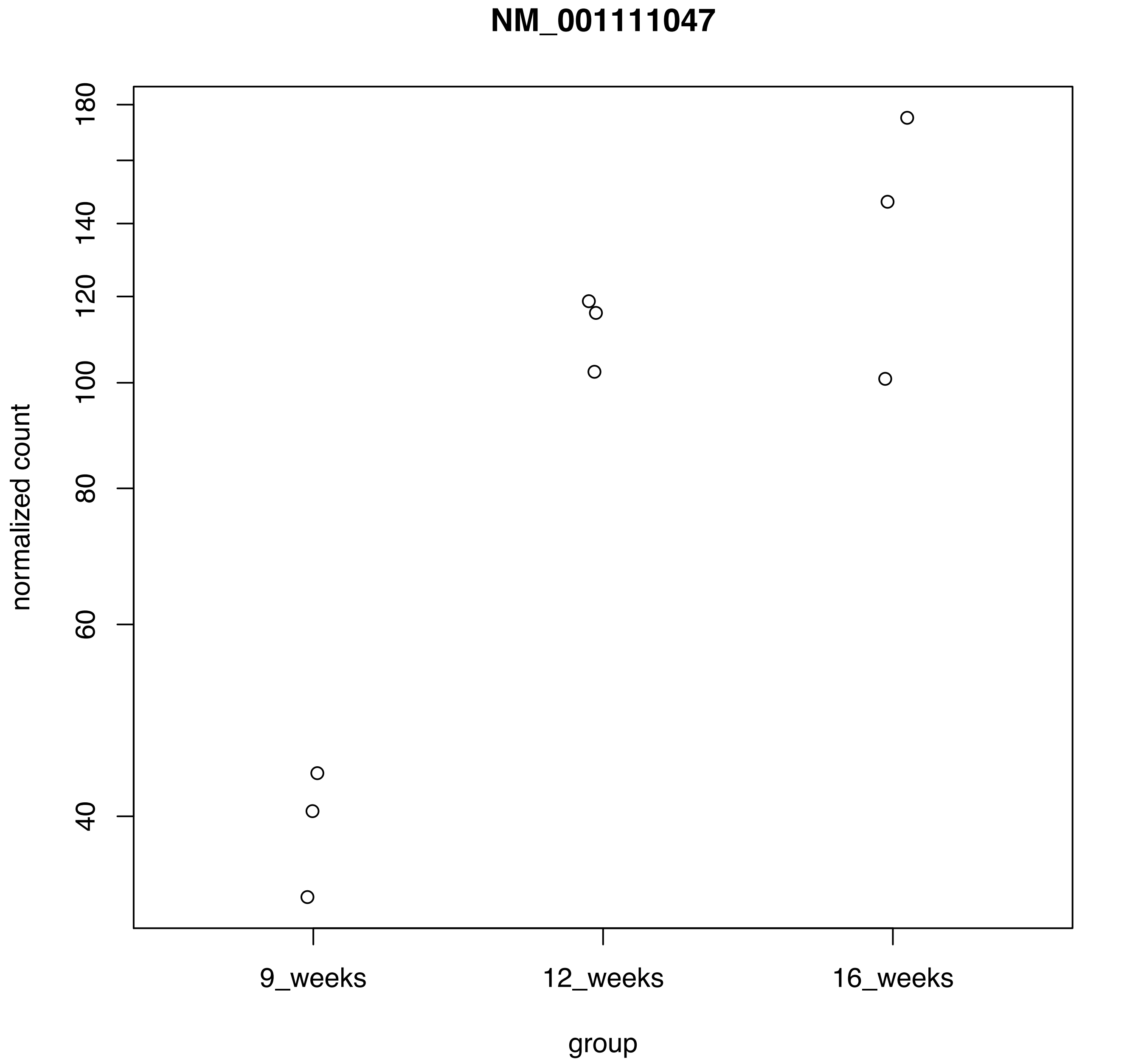
NM_001111047
normalized count
180
140
120
100
80
60
40
9_weeks
12_weeks
16_weeks
group

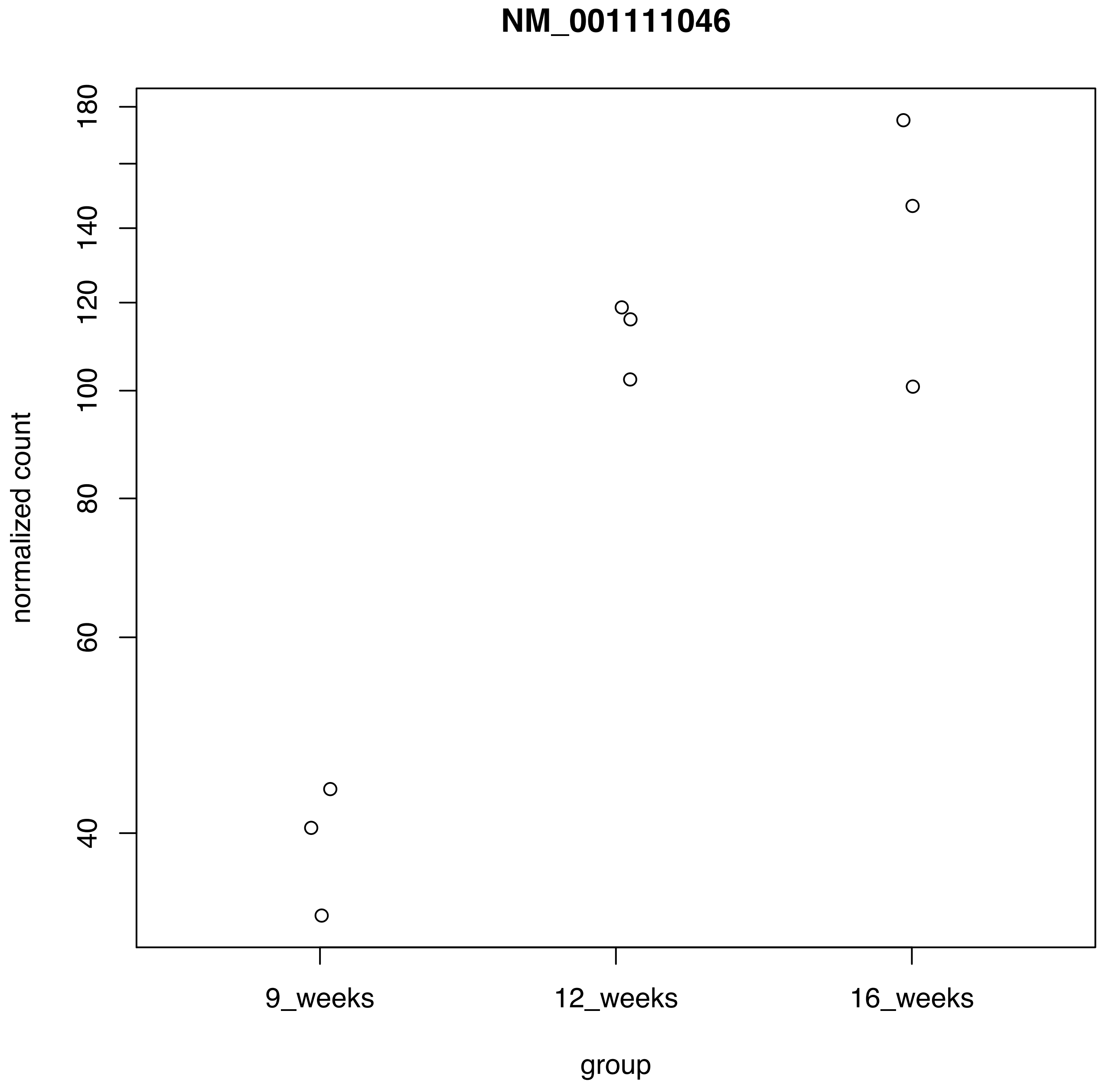
NM_001111046
180
140
120
100
80
60
40
normalized count
9_weeks
12_weeks
16_weeks
group

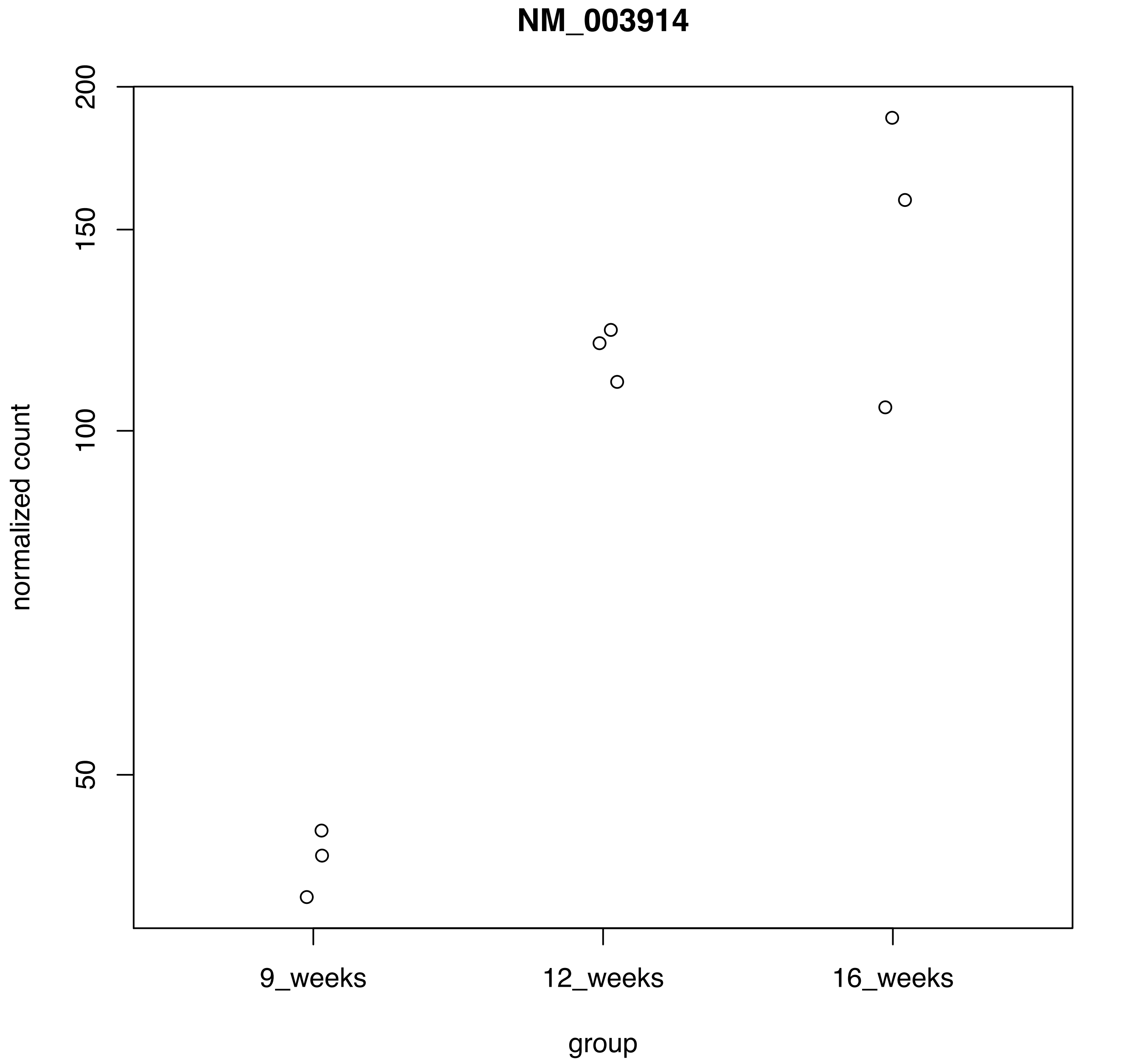
NM_003914
normalized count
200
150
100
50
9_weeks
12_weeks
16_weeks
group

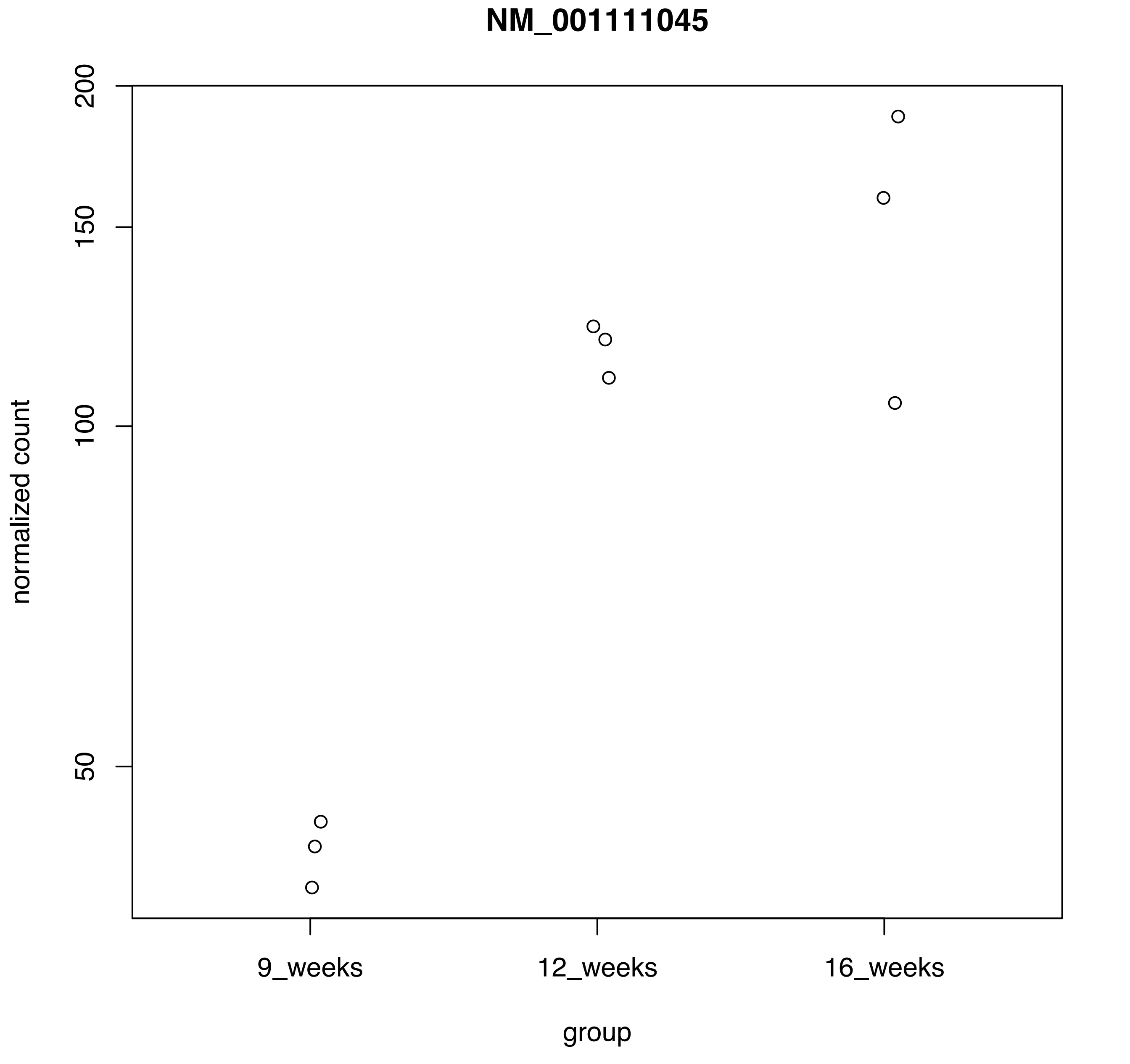
NM_001111045
normalized count
200
150
100
50
9_weeks
12_weeks
16_weeks
group

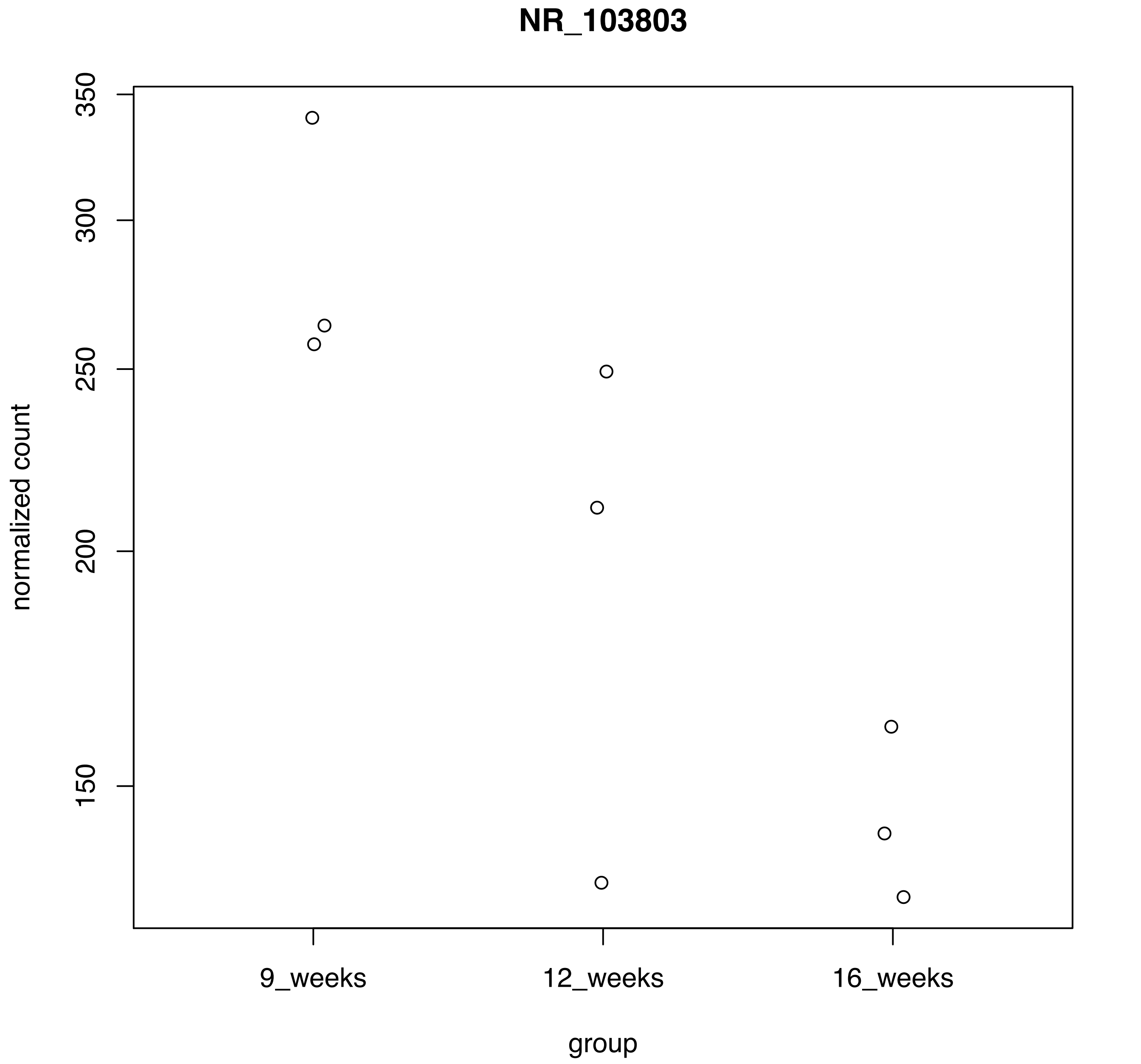

NR_103803
normalized count
350
300
250
200
150
9_weeks
12_weeks
16_weeks
group

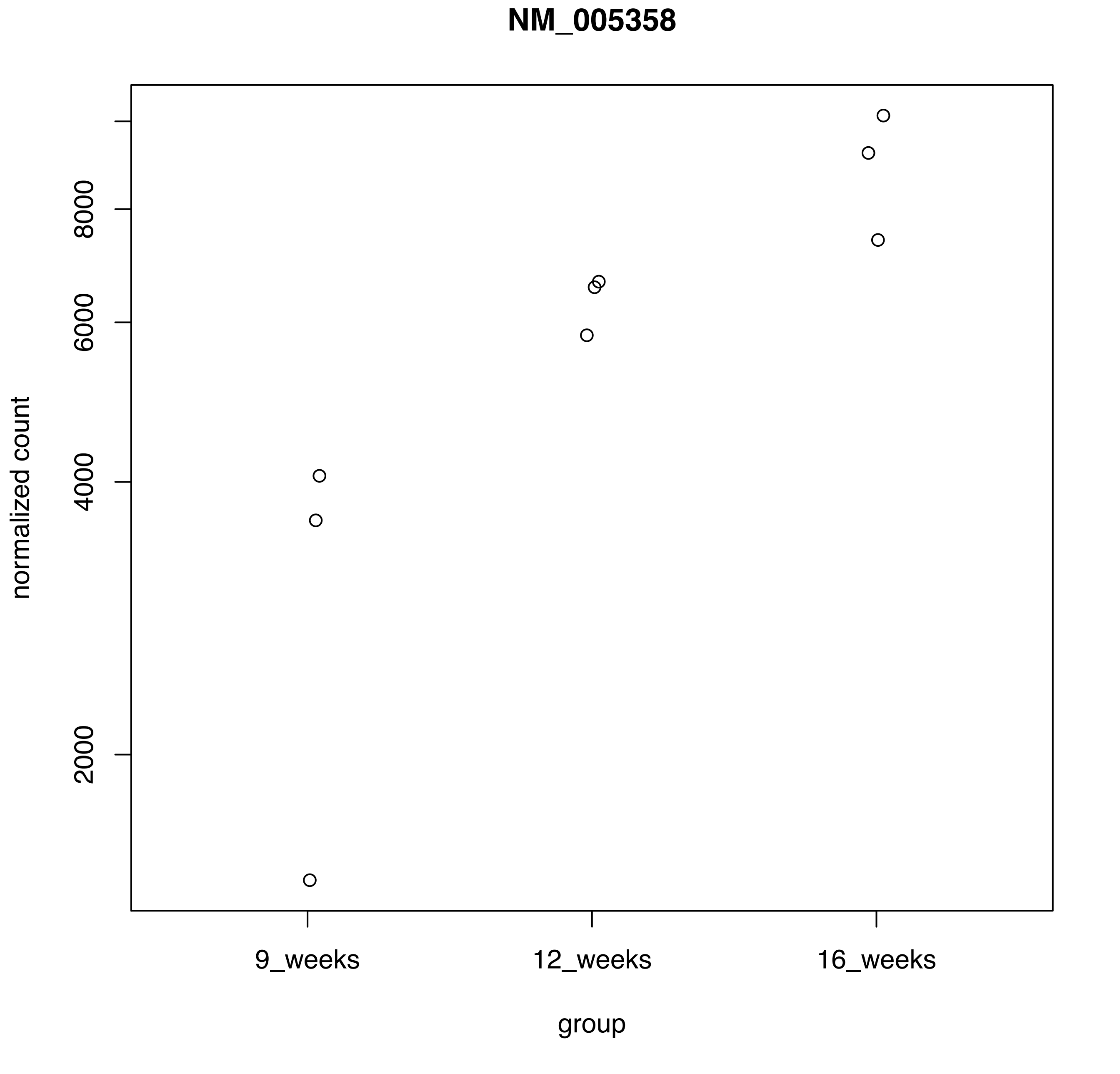
NM_005358
normalized count
2000
4000
6000
8000
9_weeks
12_weeks
16_weeks
group

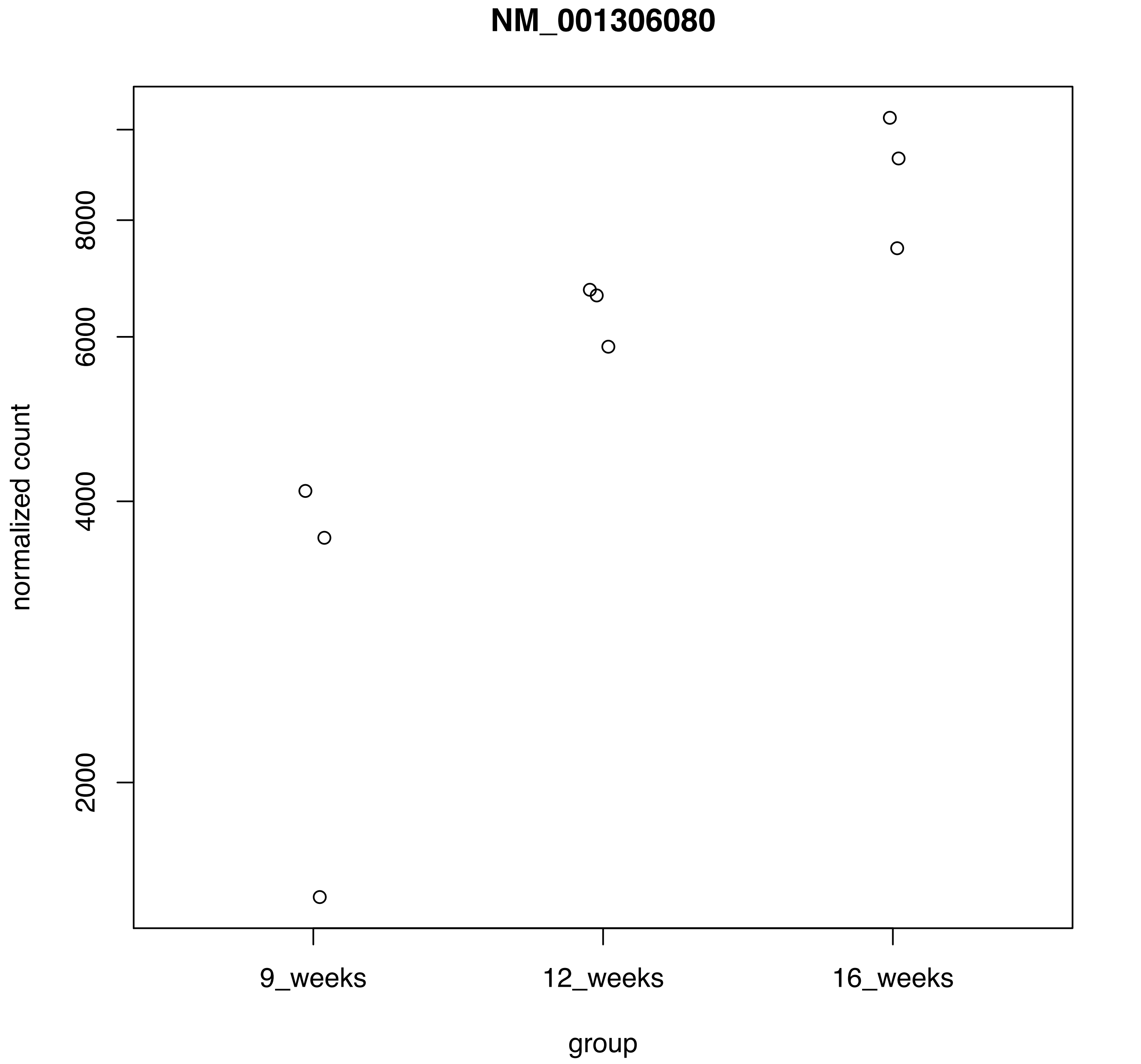
NM_001306080
normalized count
8000
6000
4000
2000
9_weeks
12_weeks
16_weeks
group

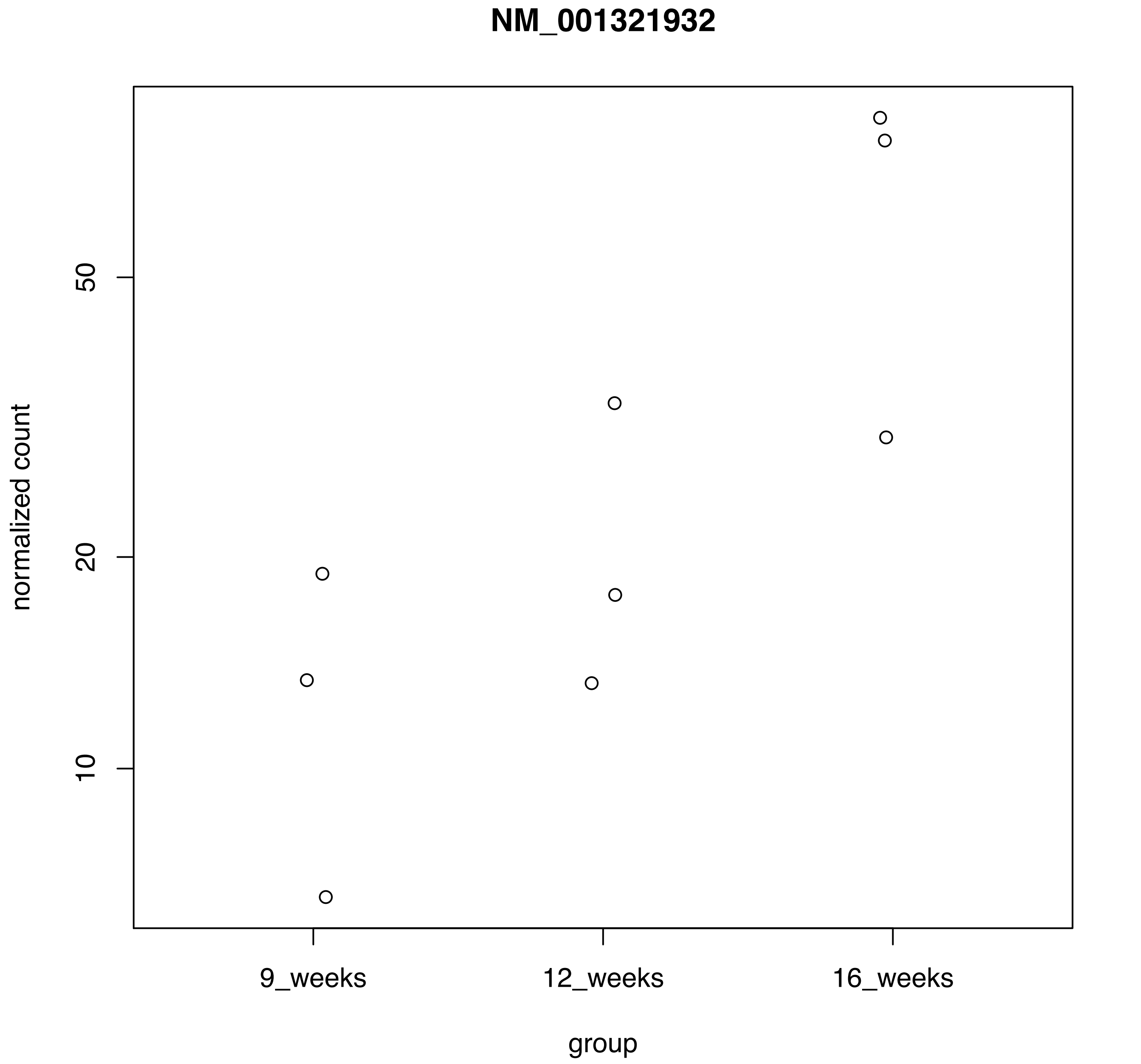
NM_001321932
normalized count
50
20
10
9_weeks
12_weeks
16_weeks
group

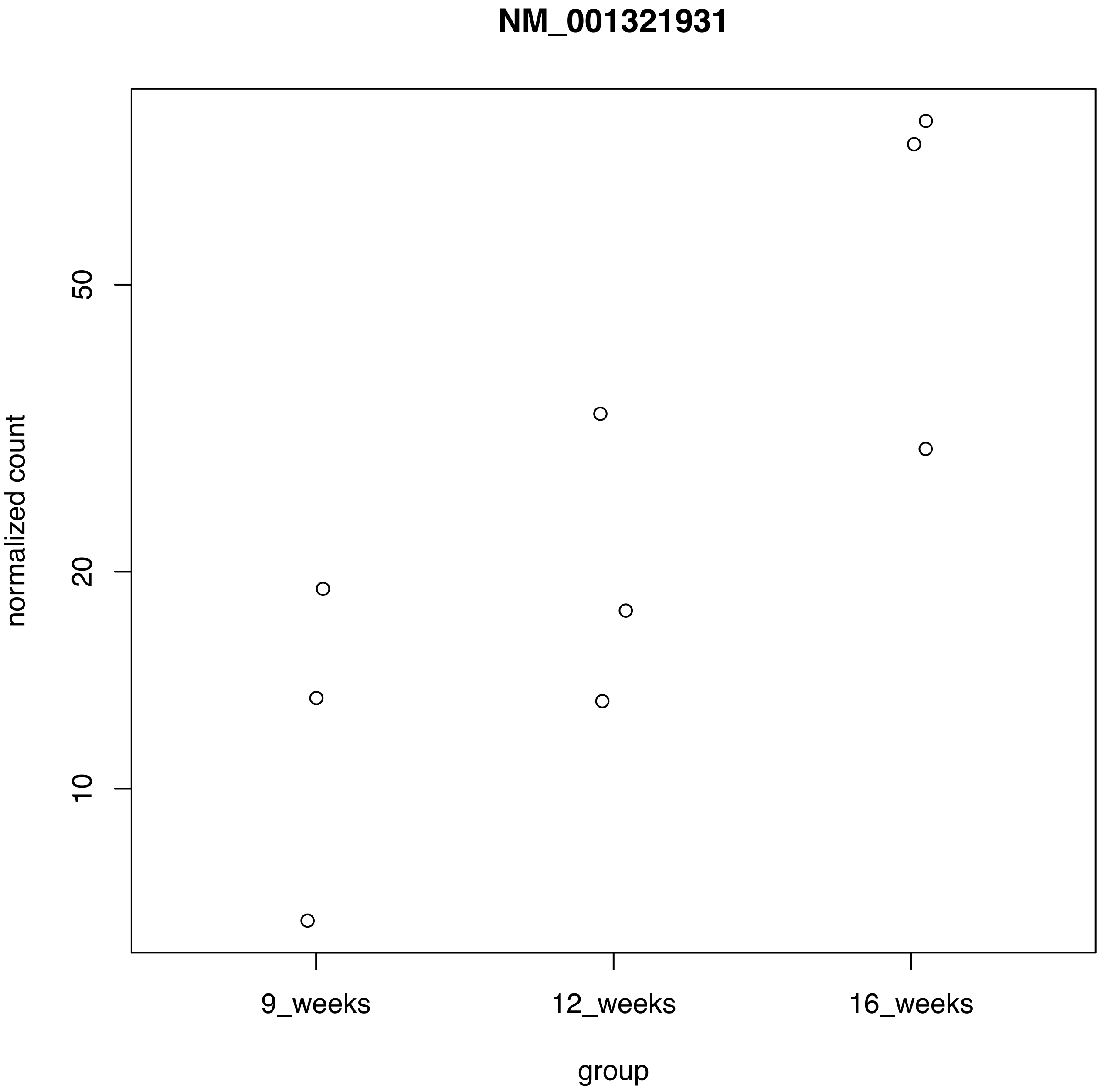
NM_001321931
normalized count
50
20
10
9_weeks
12_weeks
16_weeks
group

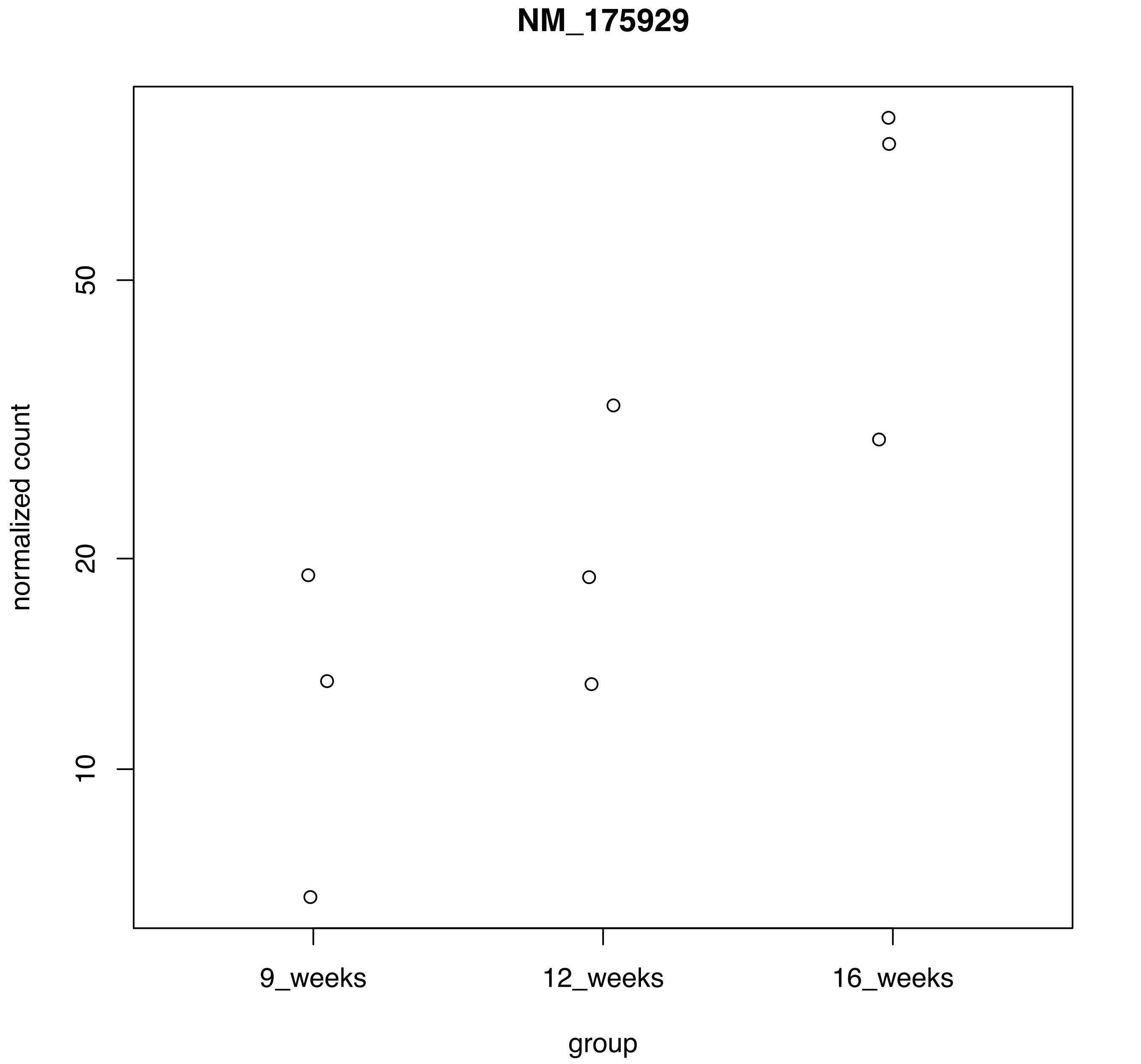
NM_175929
normalized count
50
20
10
9_weeks
12_weeks
16_weeks
group

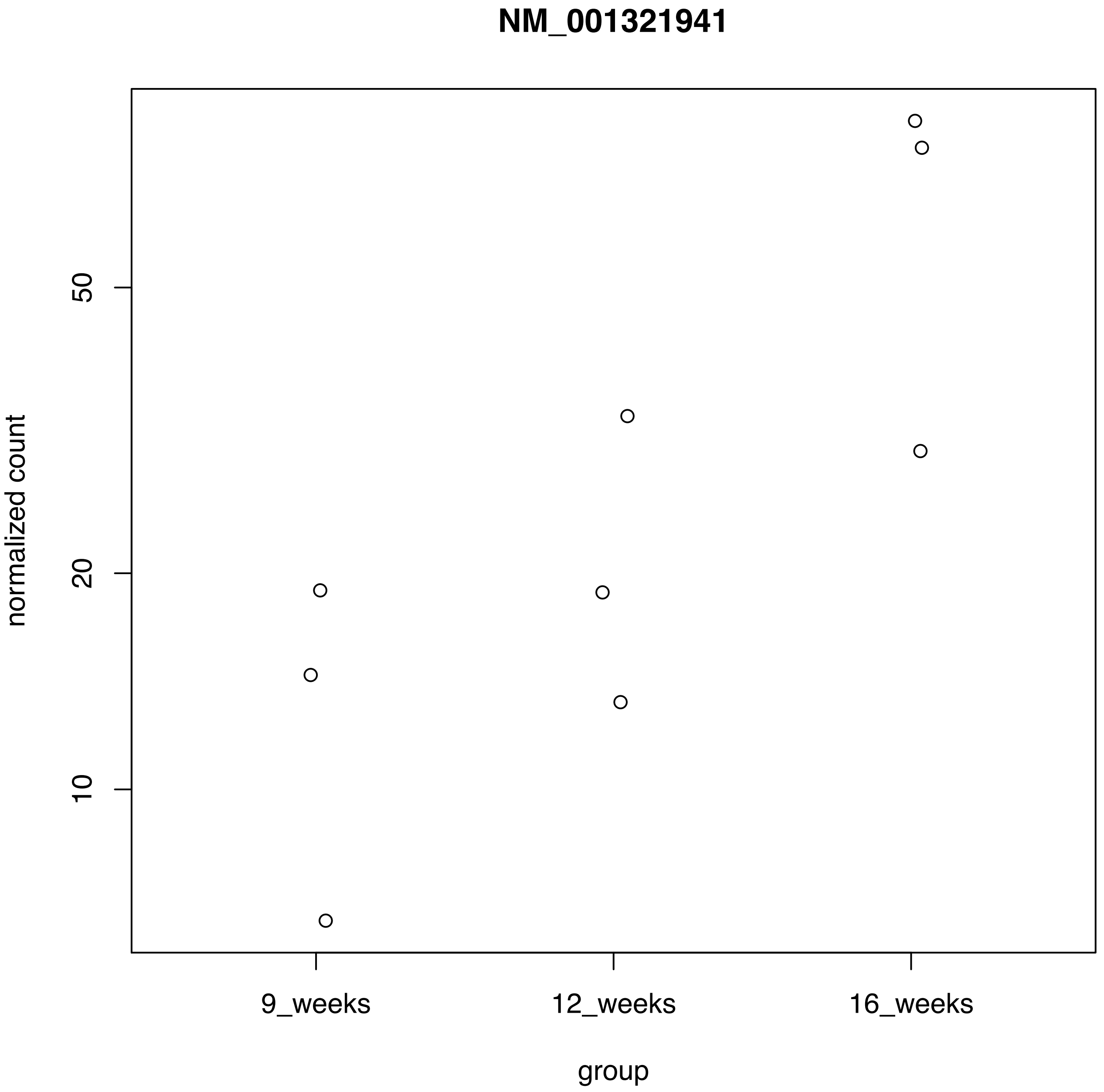
NM_001321941
normalized count
50
20
10
9_weeks
12_weeks
16_weeks
group

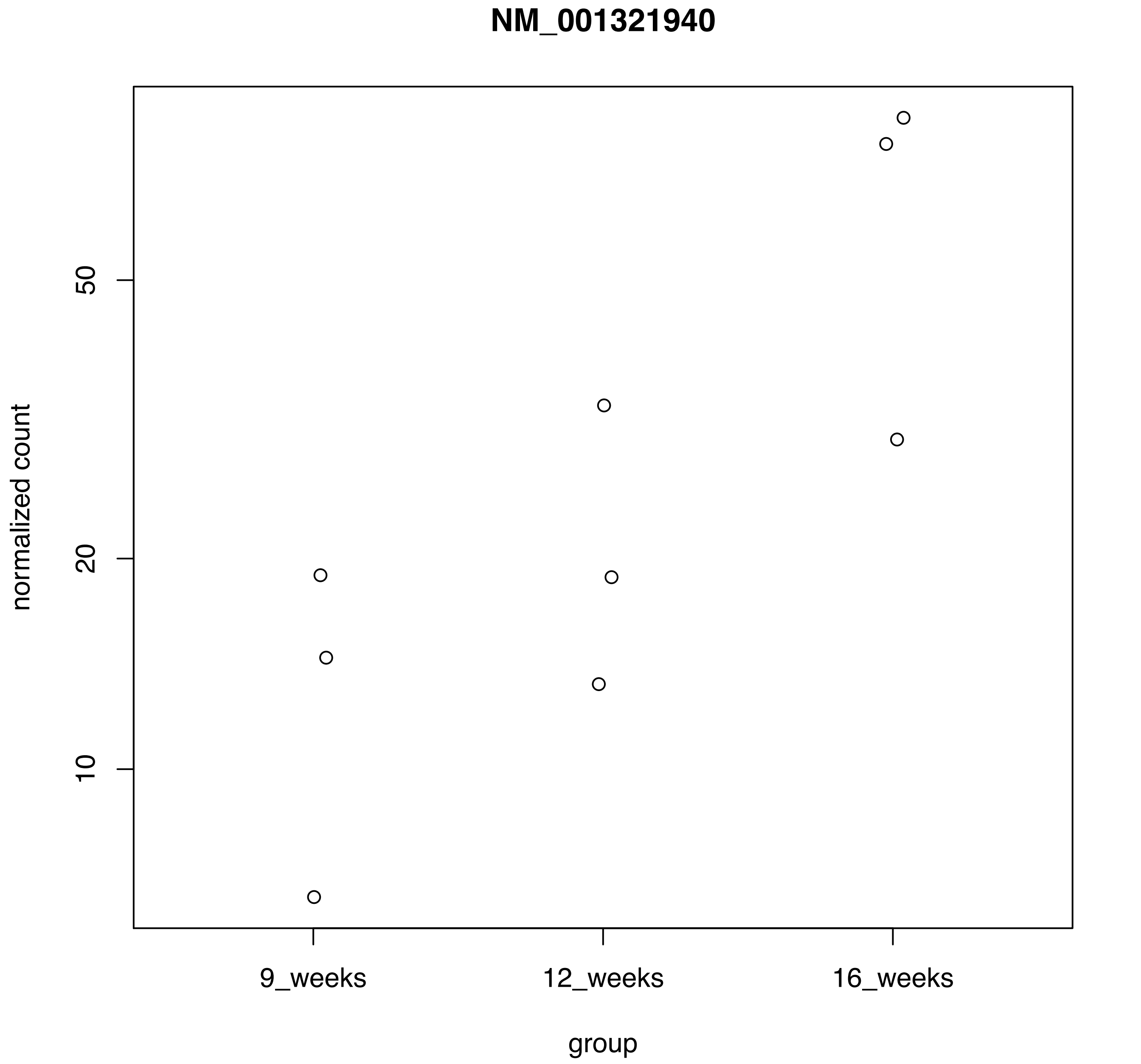

NM_001321940
normalized count
50
20
10
9_weeks
12_weeks
16_weeks
group

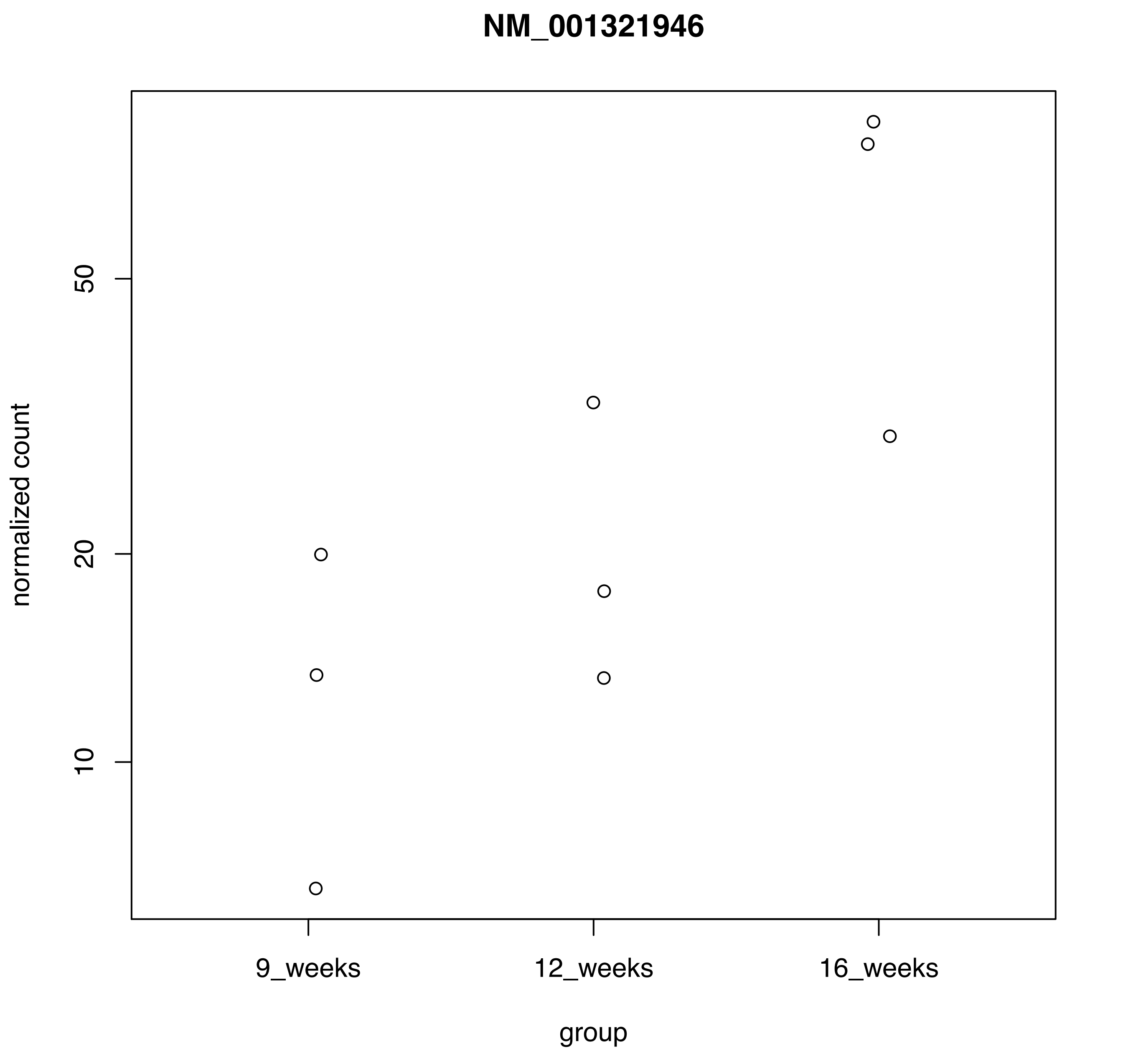
NM_001321946
normalized count
50
20
10
9_weeks
12_weeks
16_weeks
group

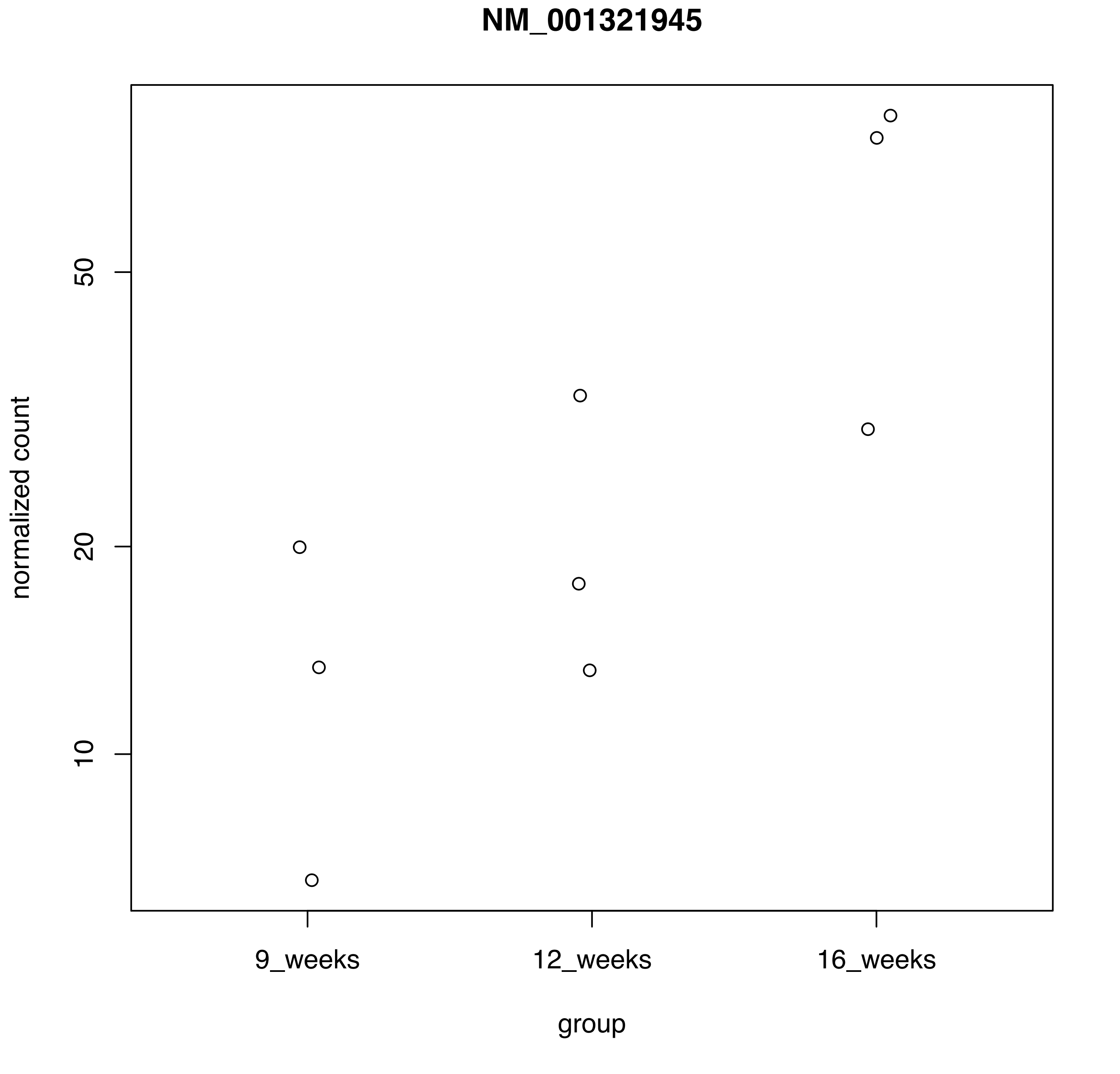

NM_001321945
normalized count
50
20
10
9_weeks
12_weeks
16_weeks
group

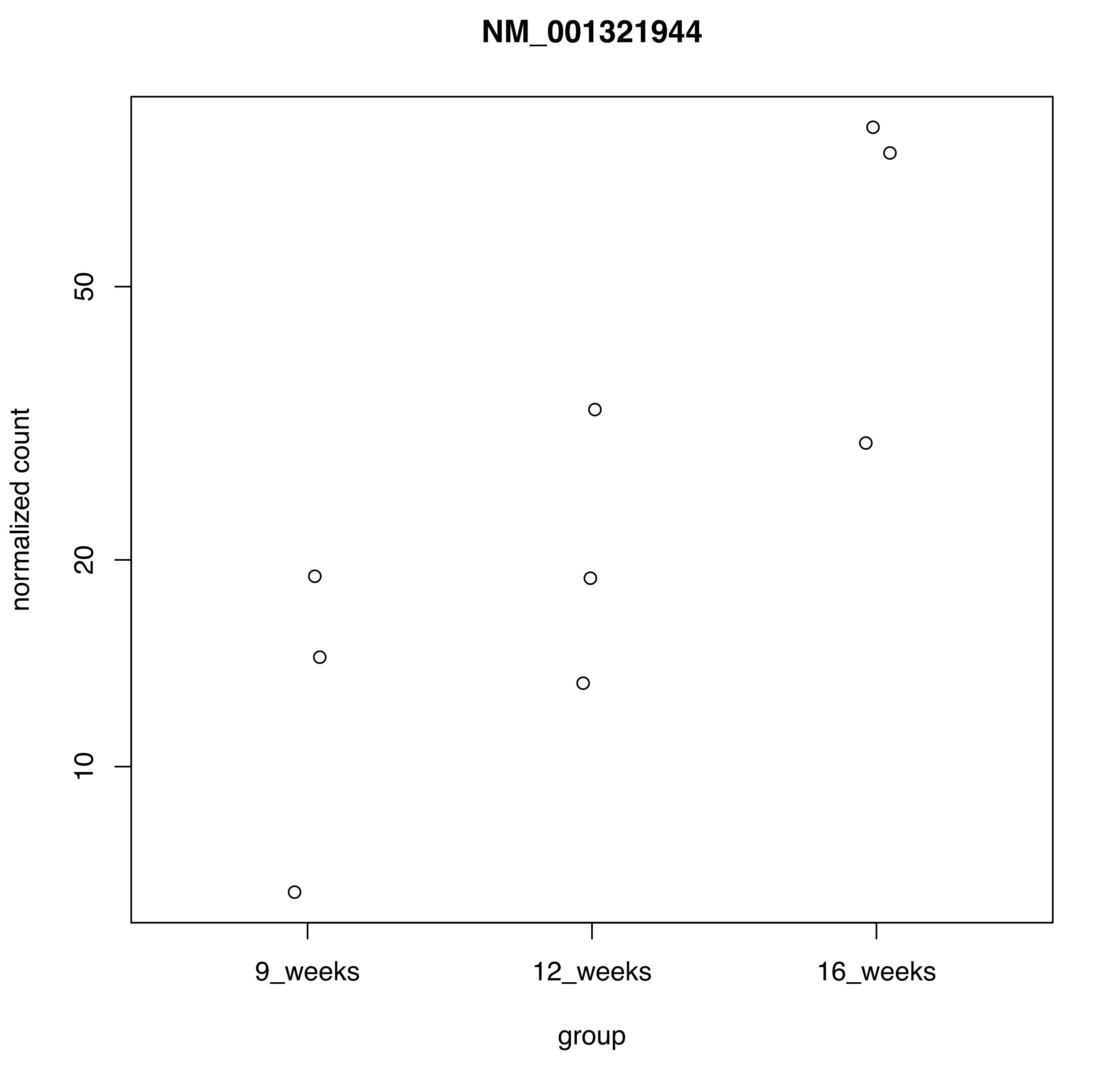
NM_001321944
normalized count
50
20
10
9_weeks
12_weeks
16_weeks
group

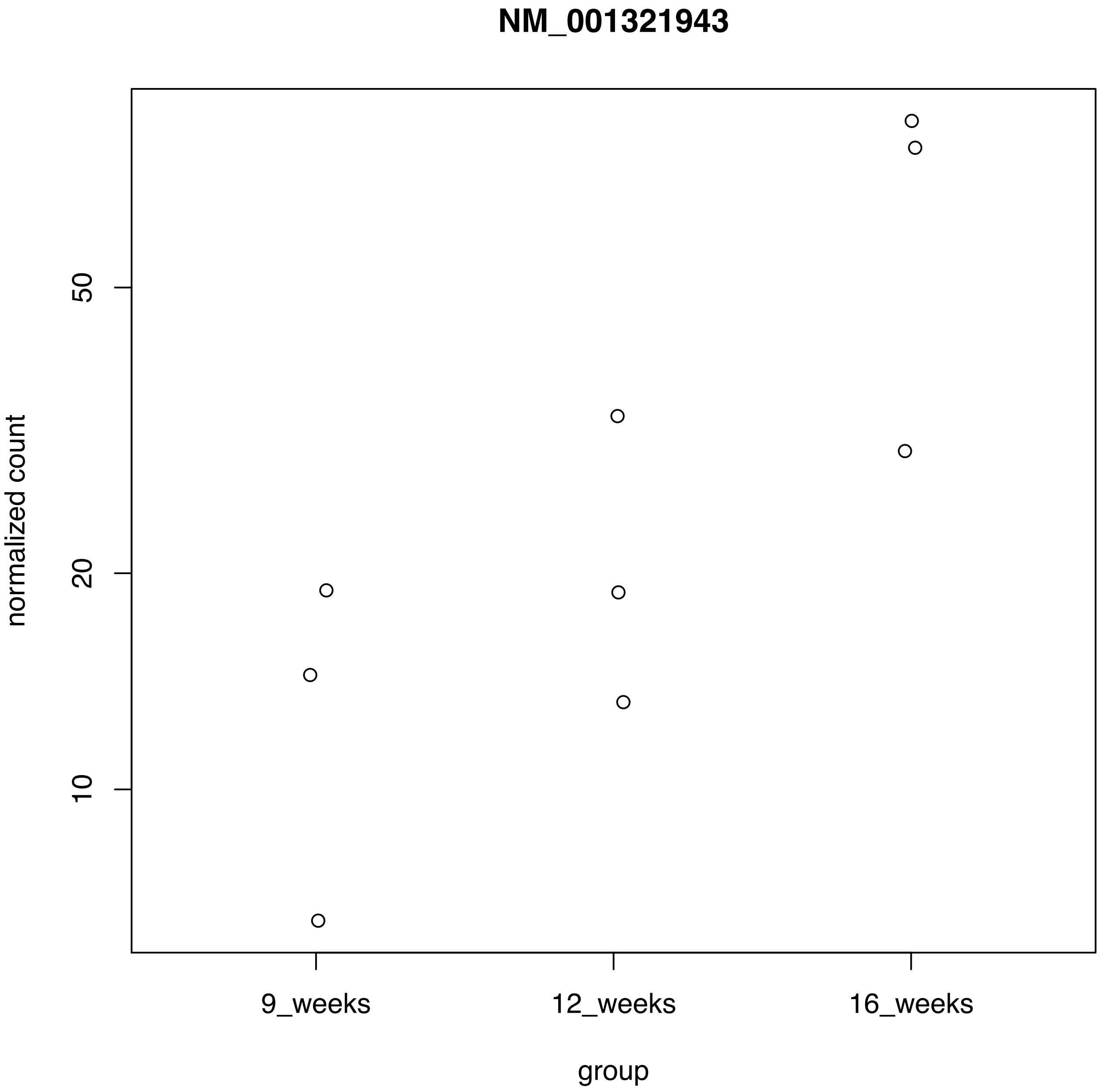
NM_001321943
normalized count
50
20
10
9_weeks
12_weeks
16_weeks
group

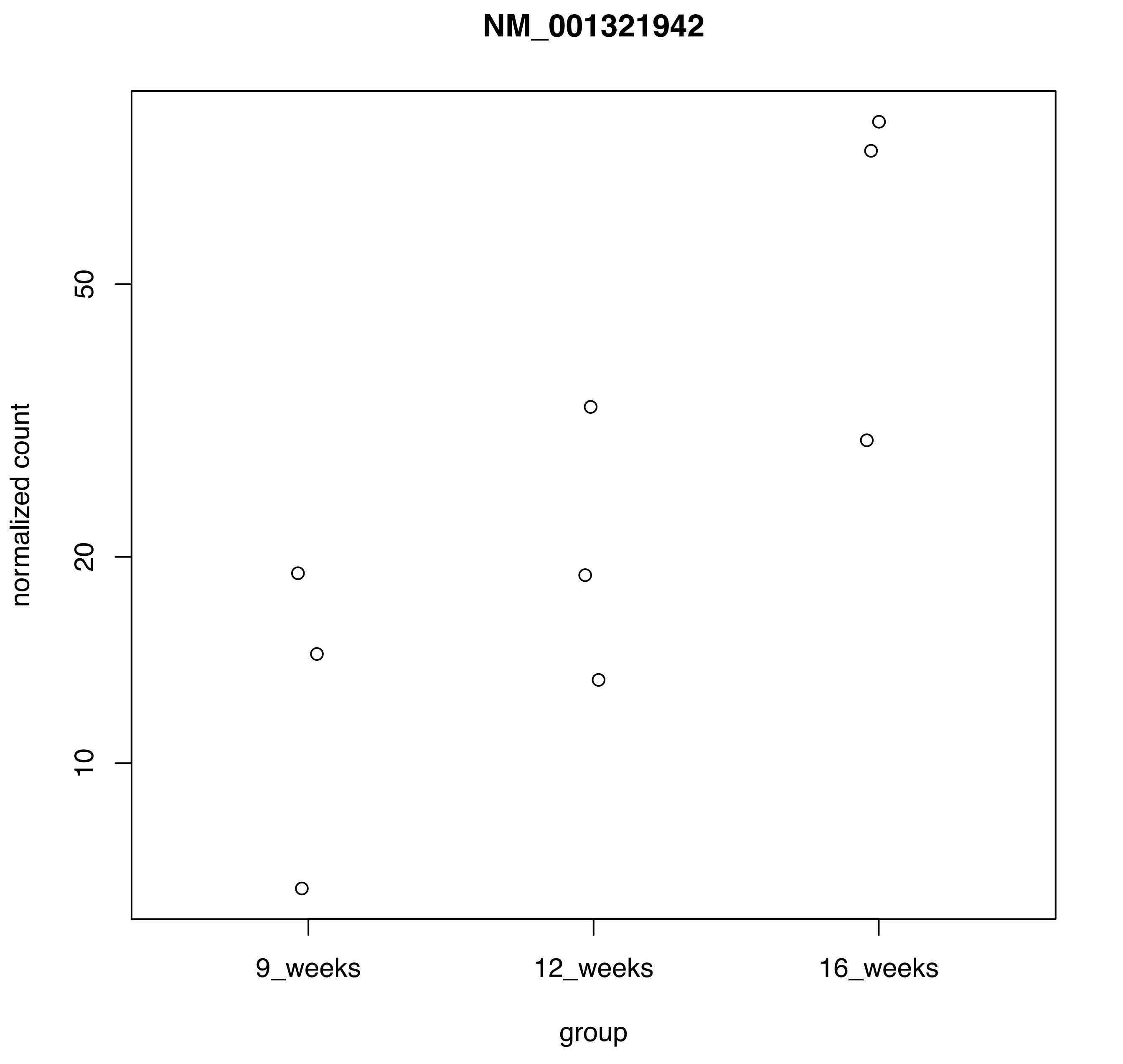

NM_001321942
normalized count
50
20
10
9_weeks
12_weeks
16_weeks
group

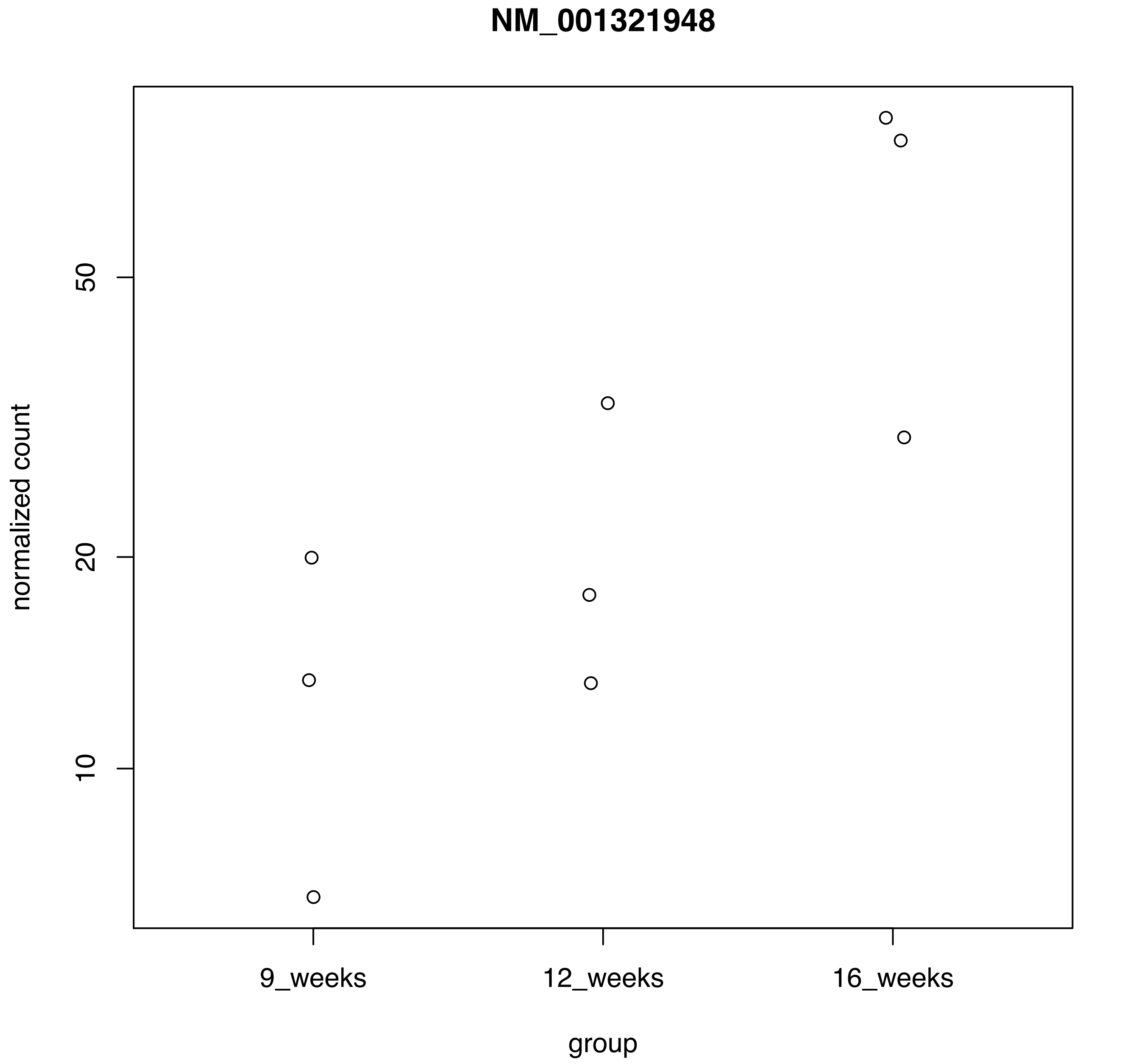
NM_001321948
normalized count
50
20
10
9_weeks
12_weeks
16_weeks
group

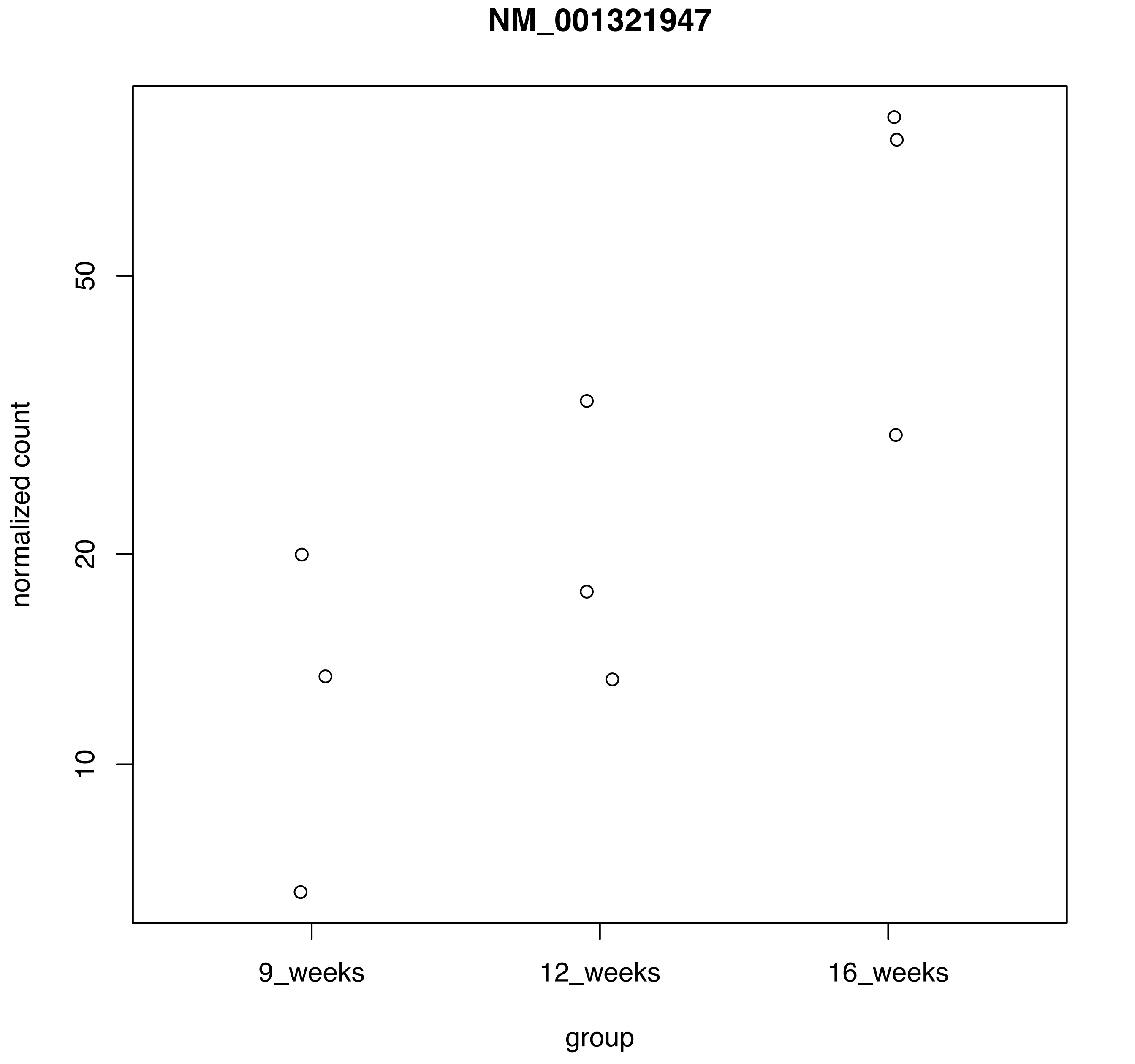
NM_001321947
normalized count
50
20
10
9_weeks
12_weeks
16_weeks
group

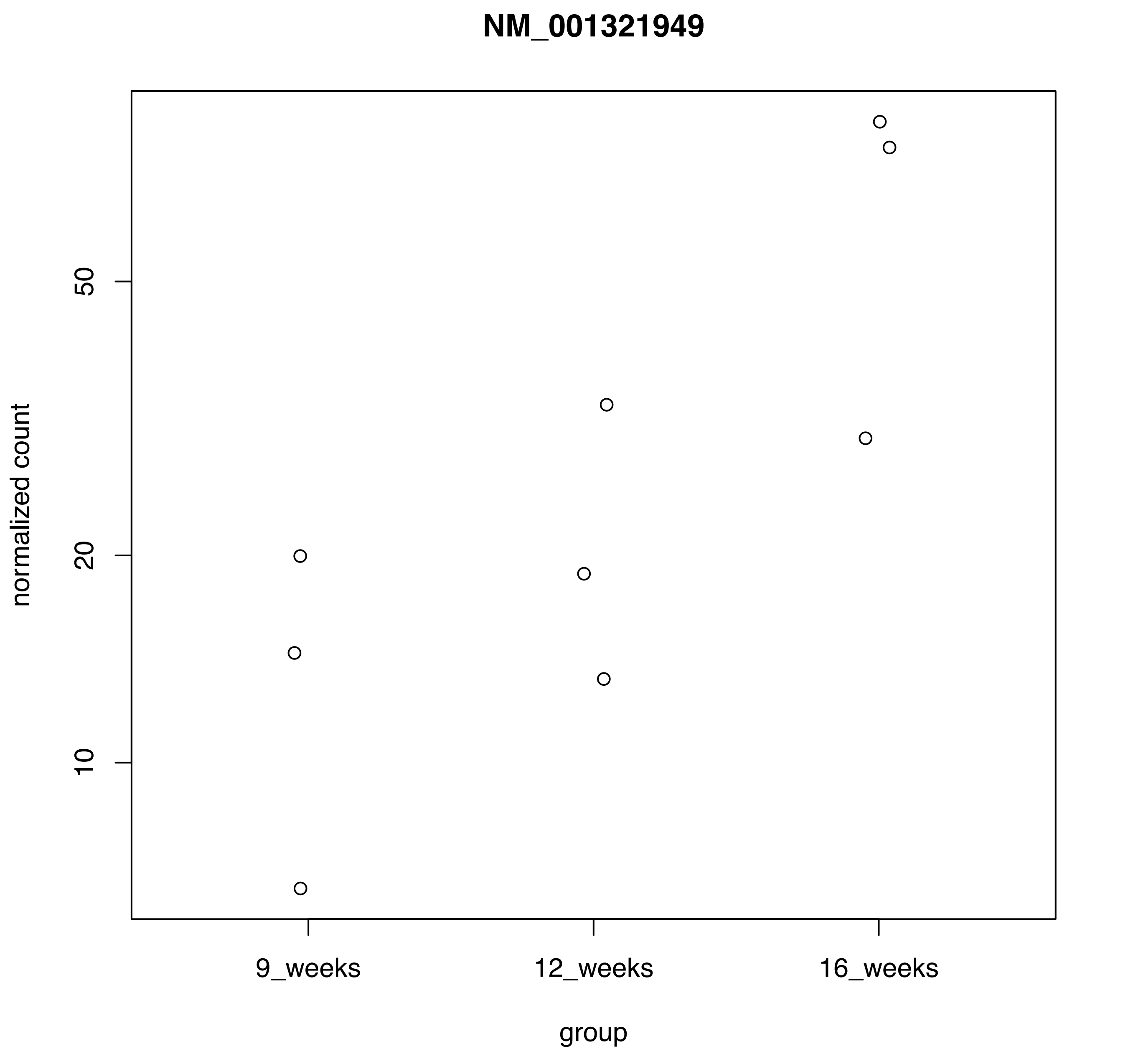
NM_001321949
normalized count
50
20
10
9_weeks
12_weeks
16_weeks
group

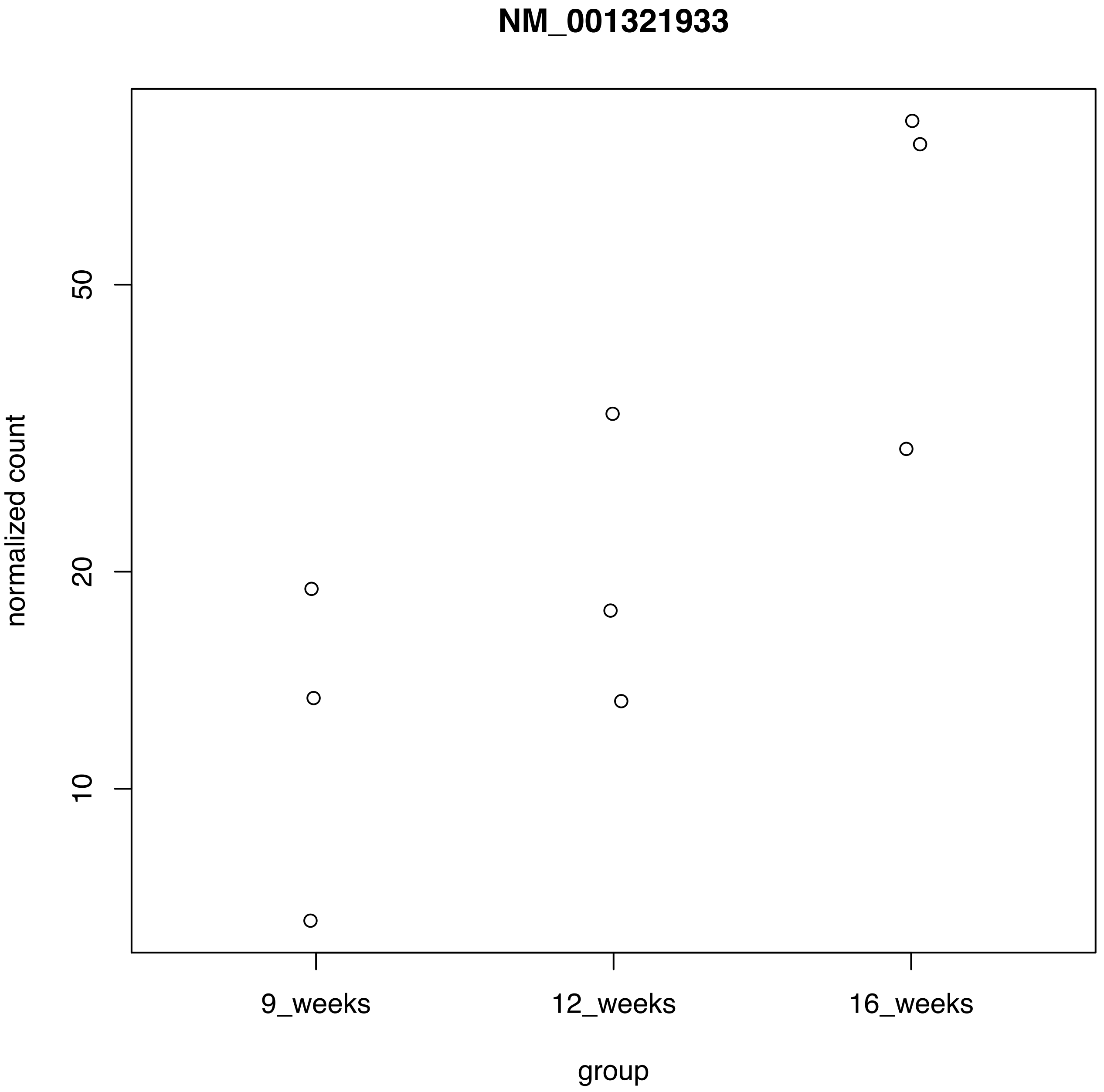
NM_001321933
normalized count
50
20
10
9_weeks
12_weeks
16_weeks
group

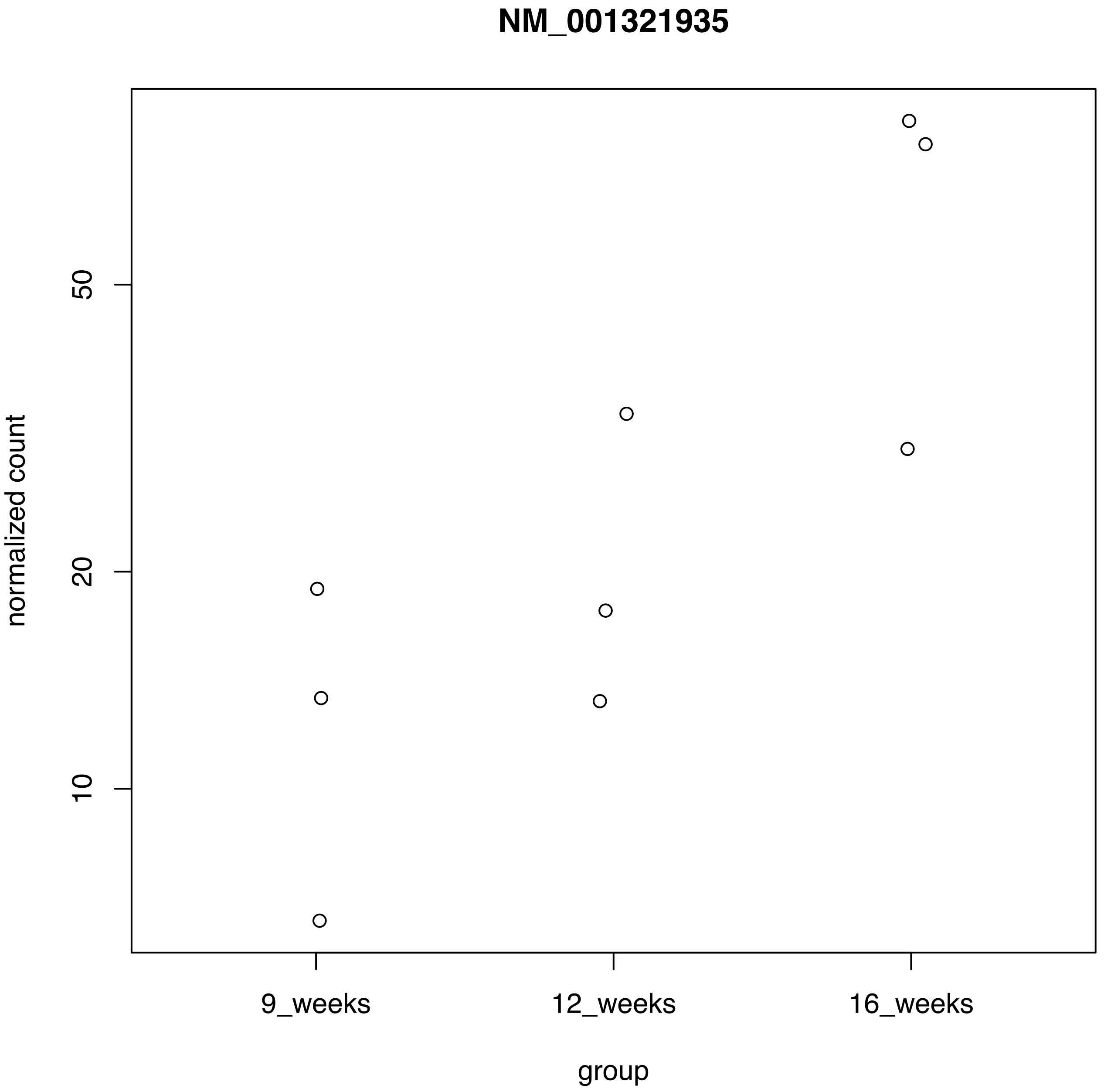
NM_001321935
normalized count
50
20
10
9_weeks
12_weeks
16_weeks
group

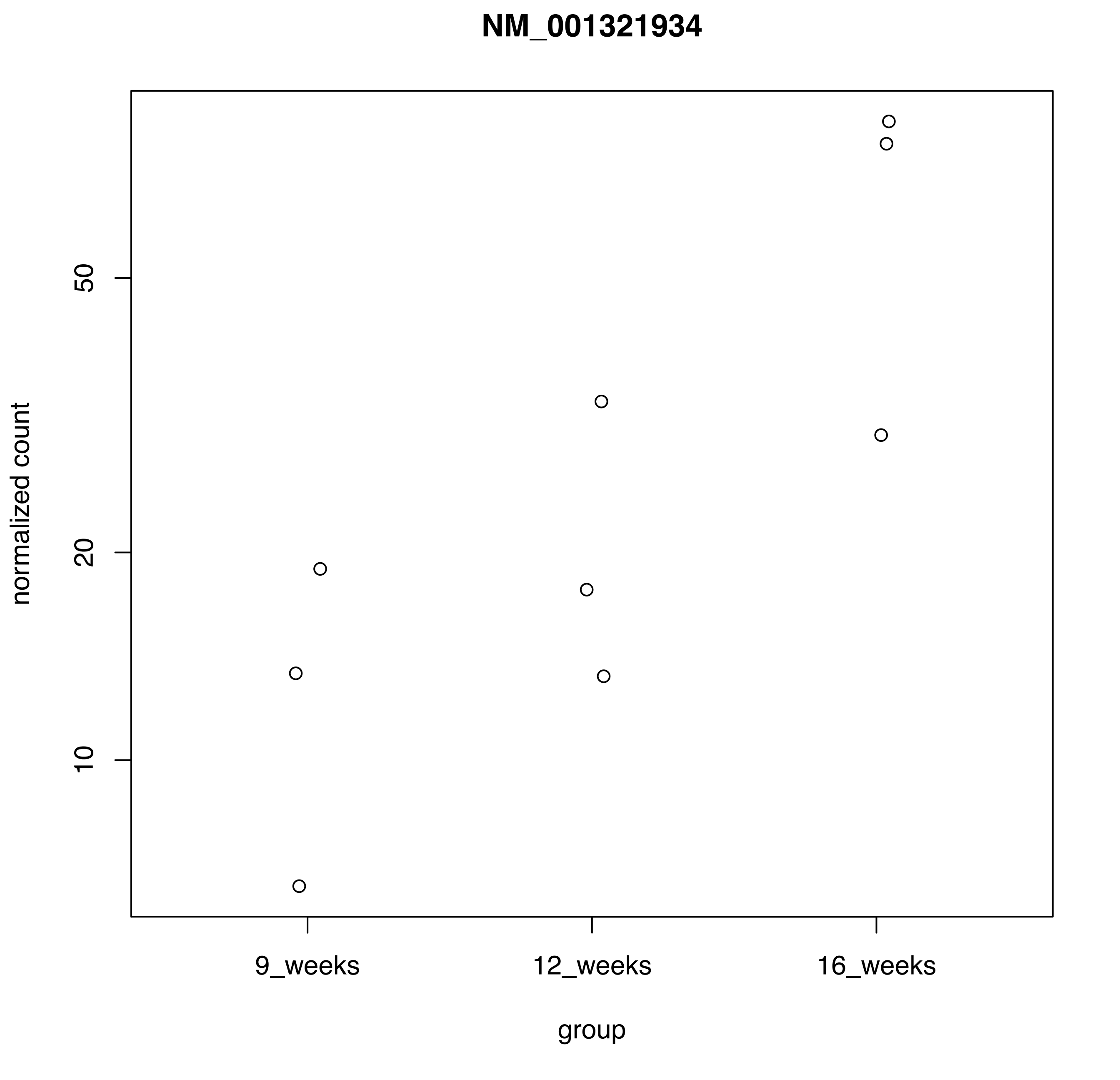
NM_001321934
normalized count
50
20
10
9_weeks
12_weeks
16_weeks
group

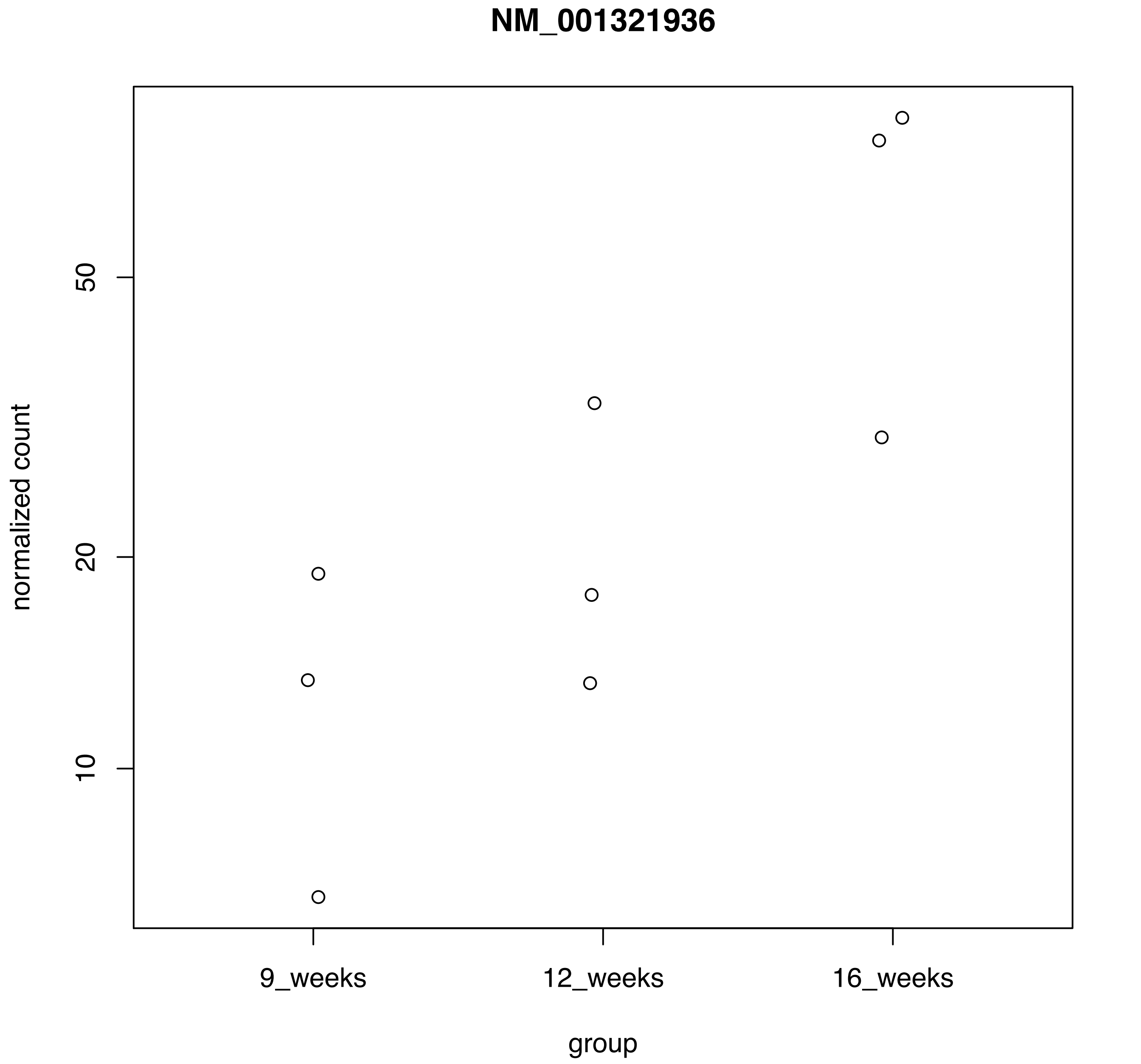
NM_001321936
normalized count
50
20
10
9_weeks
12_weeks
16_weeks
group

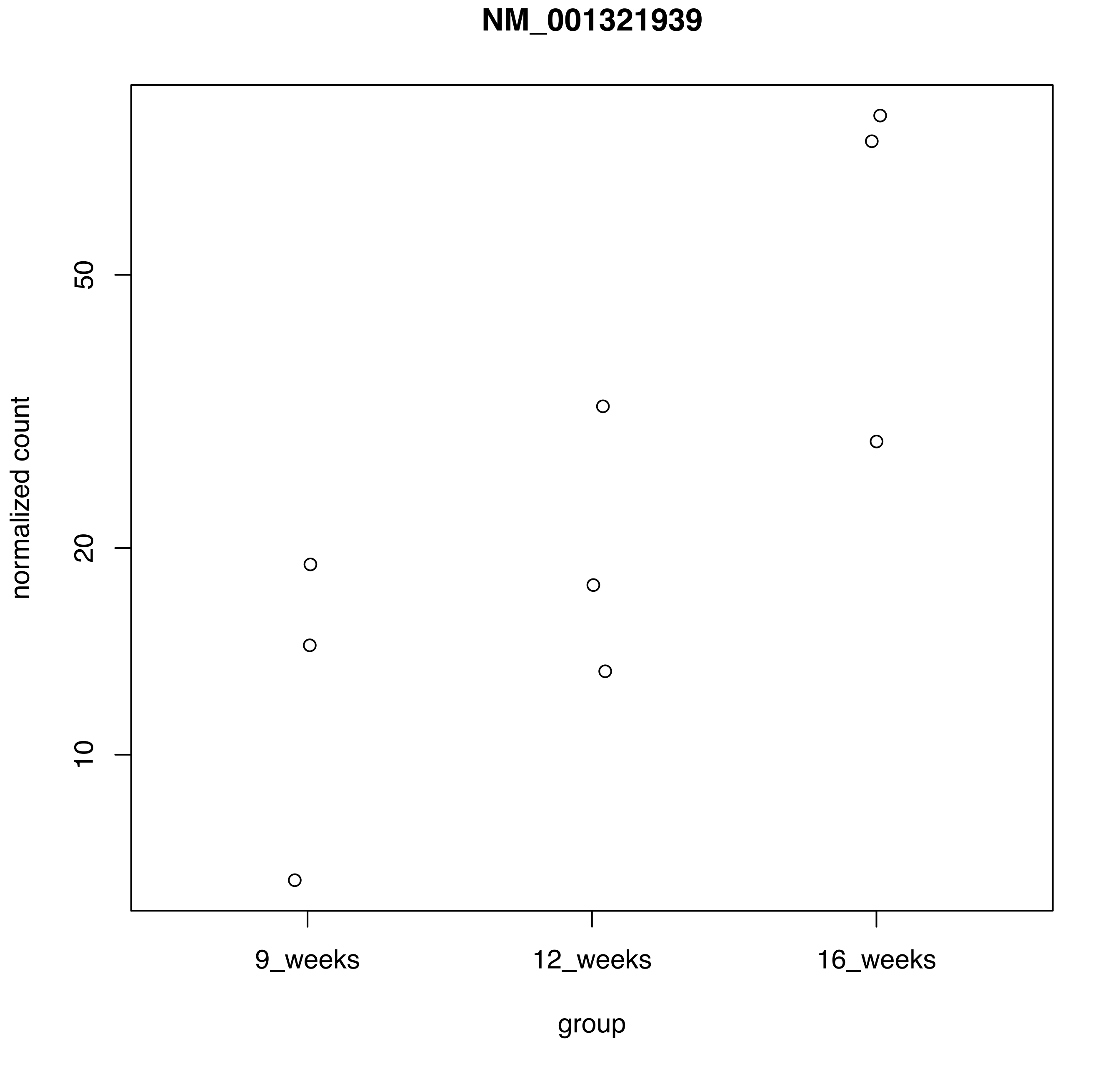

NM_001321939
normalized count
50
20
10
9_weeks
12_weeks
16_weeks
group

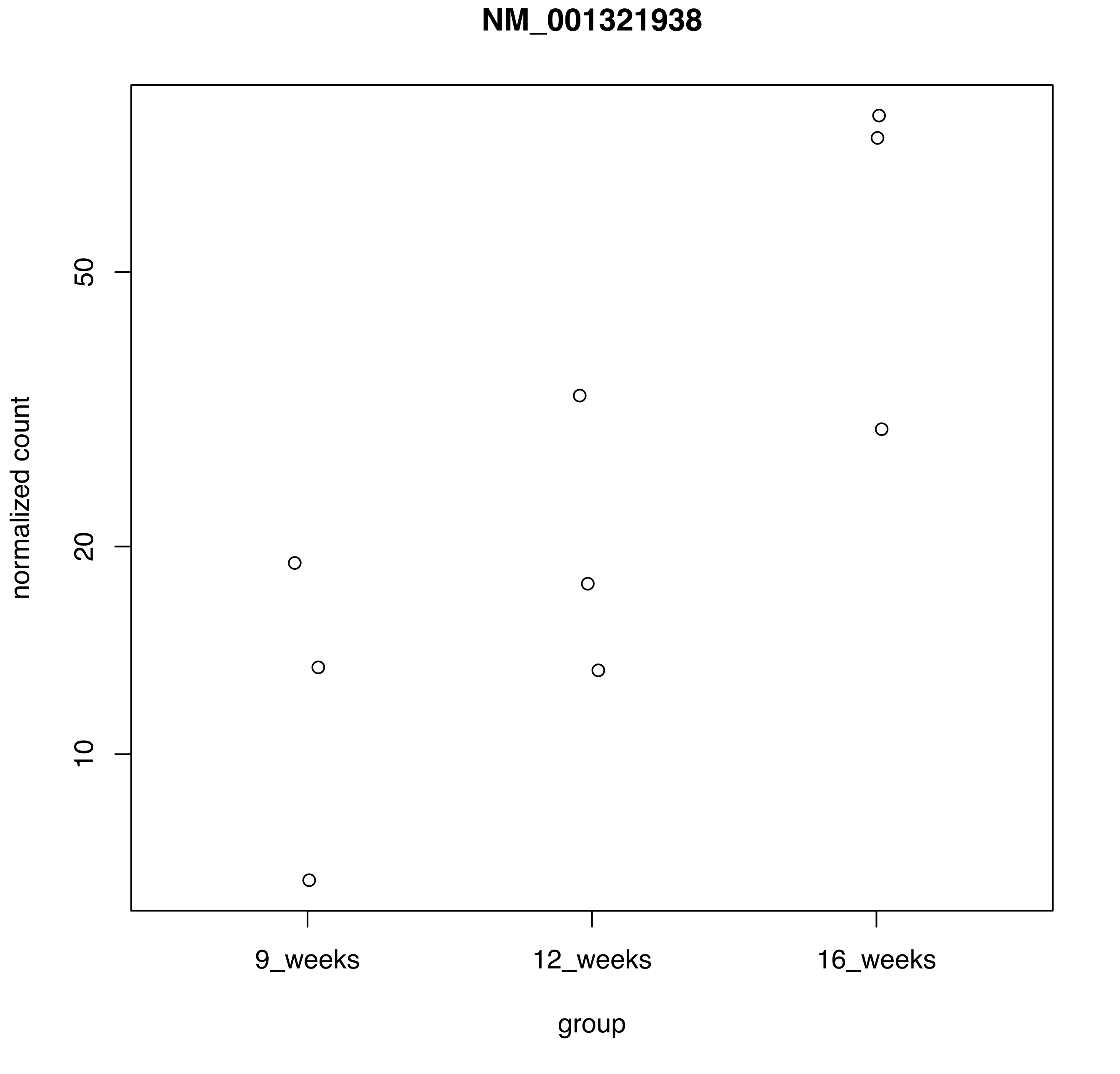
NM_001321938
normalized count
50
20
10
9_weeks
12_weeks
16_weeks
group

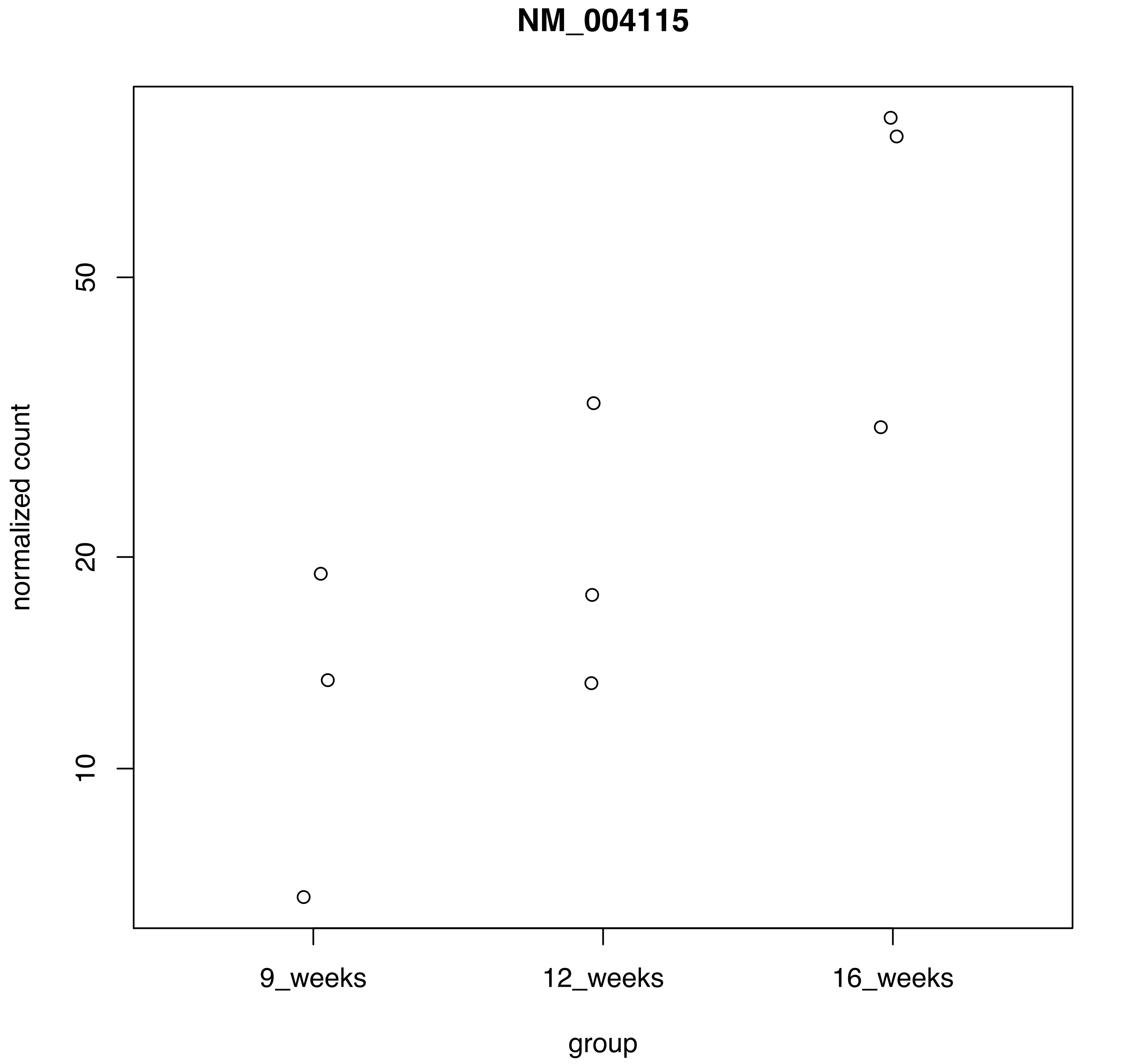
NM_004115
normalized count
50
20
10
9_weeks
12_weeks
16_weeks
group

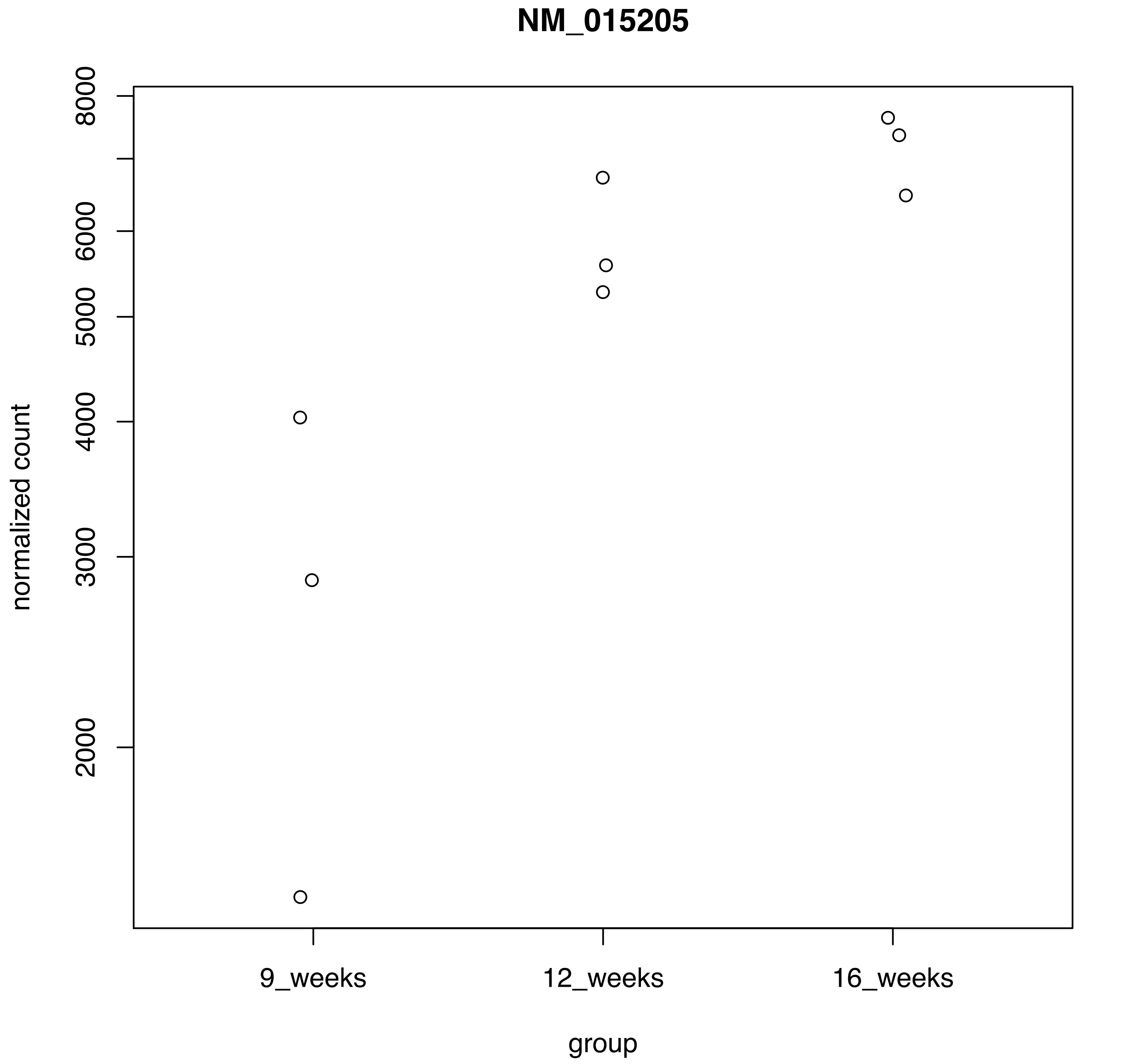

NM_015205
normalized count
8000
6000
5000
4000
3000
2000
9_weeks
12_weeks
16_weeks
group

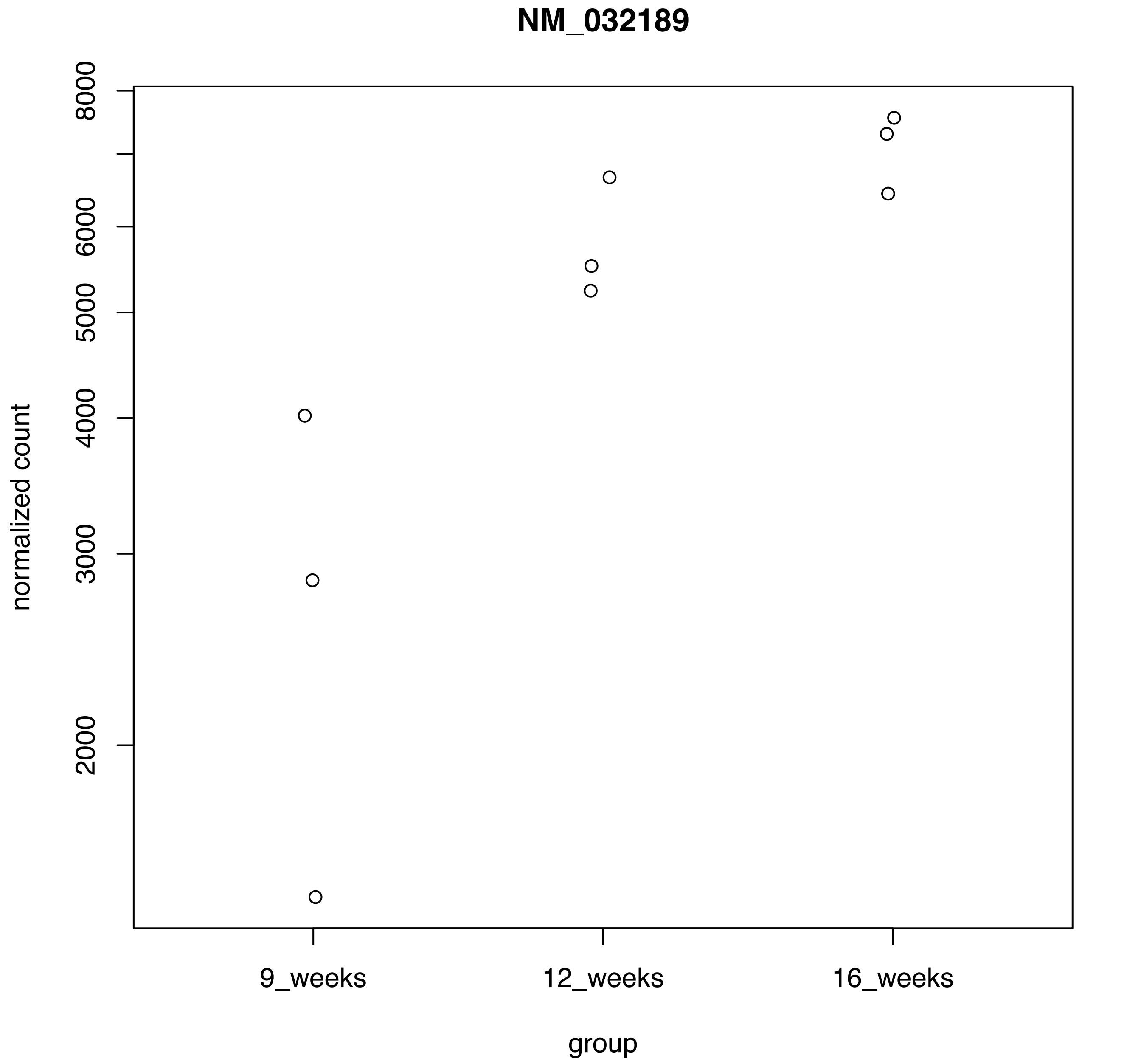
NM_032189
normalized count
8000
6000
5000
4000
3000
2000
9_weeks
12_weeks
16_weeks
group

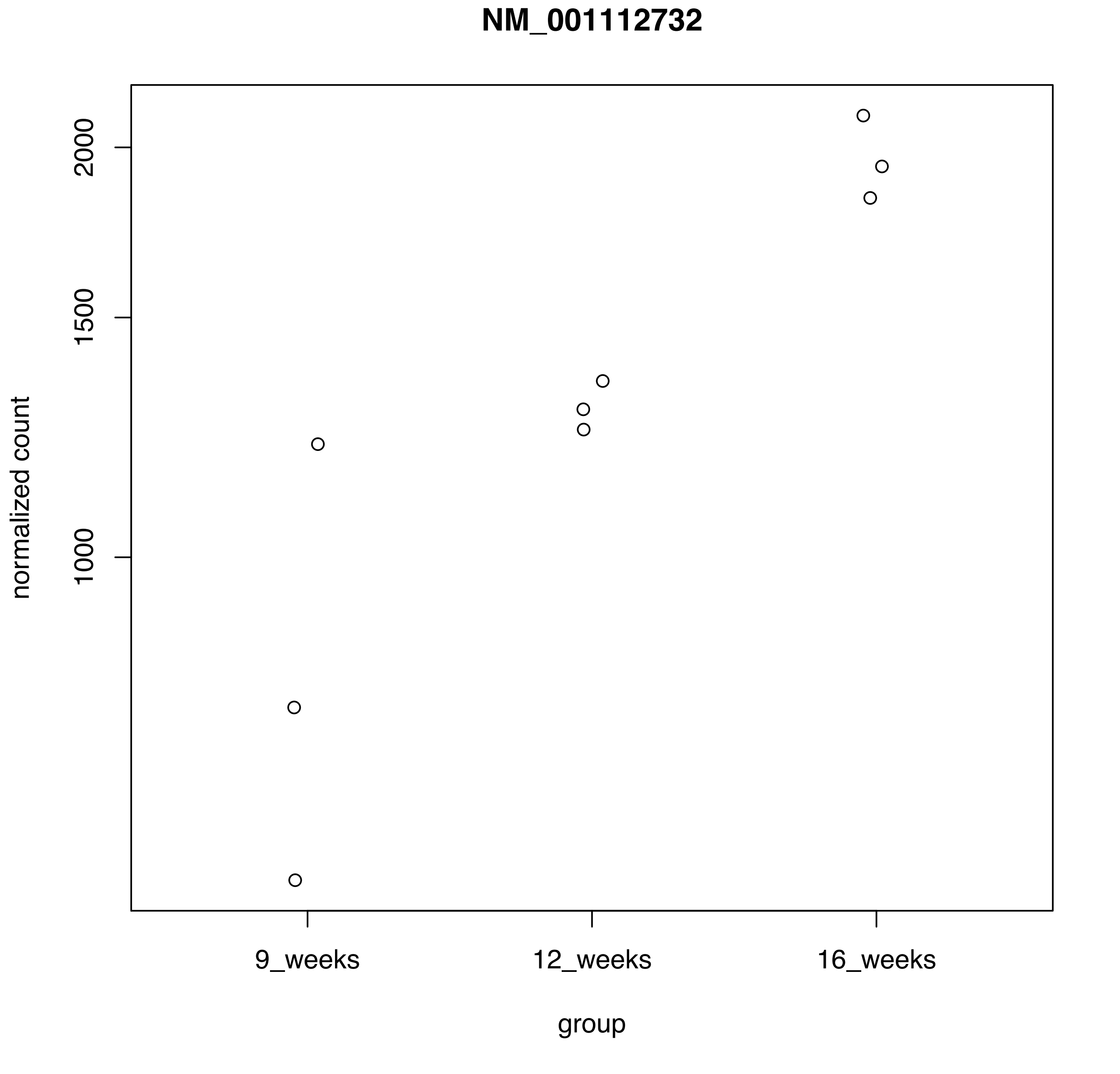
NM_001112732
normalized count
2000
1500
1000
9_weeks
12_weeks
16_weeks
group

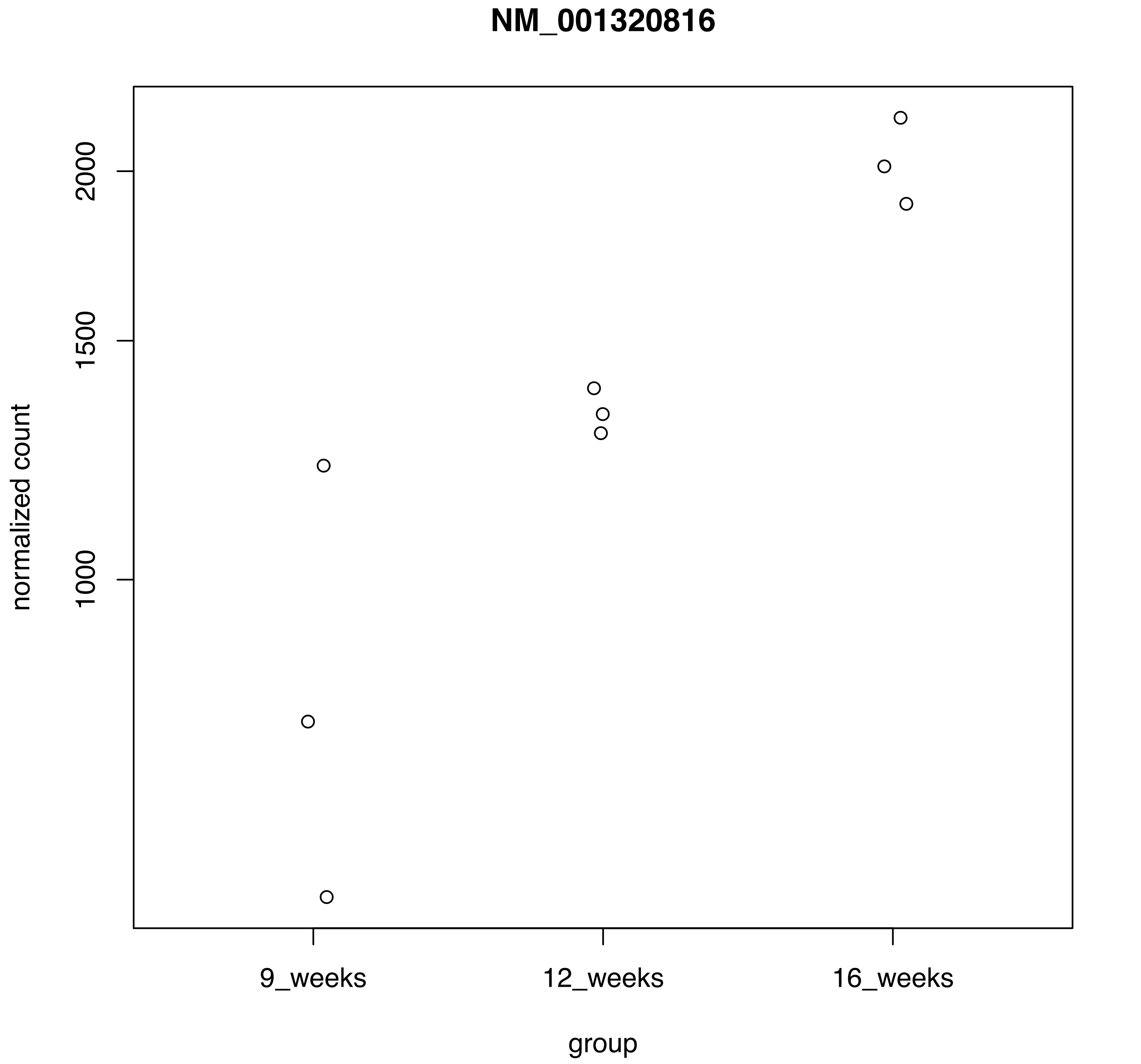
NM_001320816
normalized count
2000
1500
1000
9_weeks
12_weeks
16_weeks
group

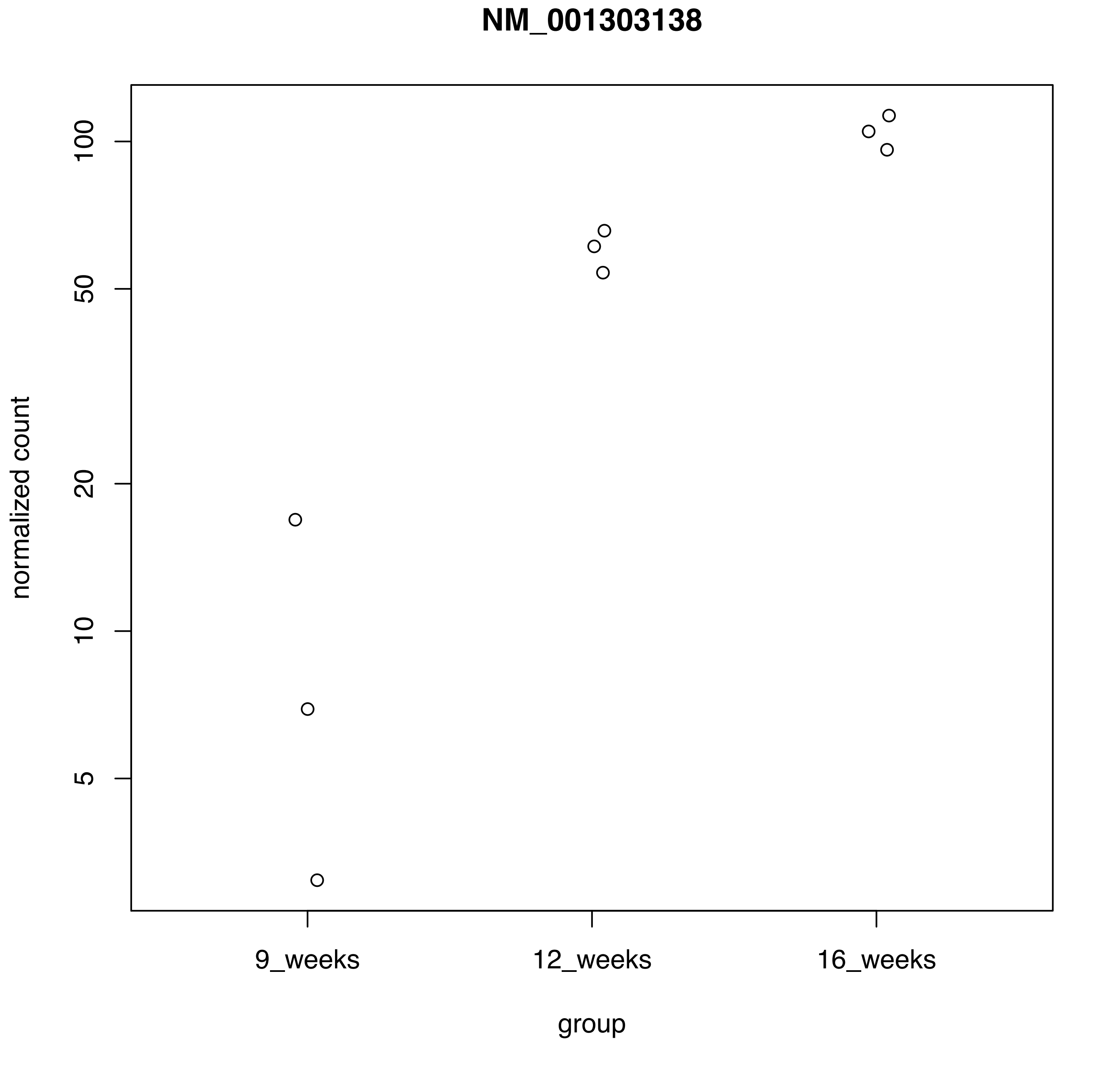
NM_001303138
normalized count
100
50
20
10
5
9_weeks
12_weeks
16_weeks
group

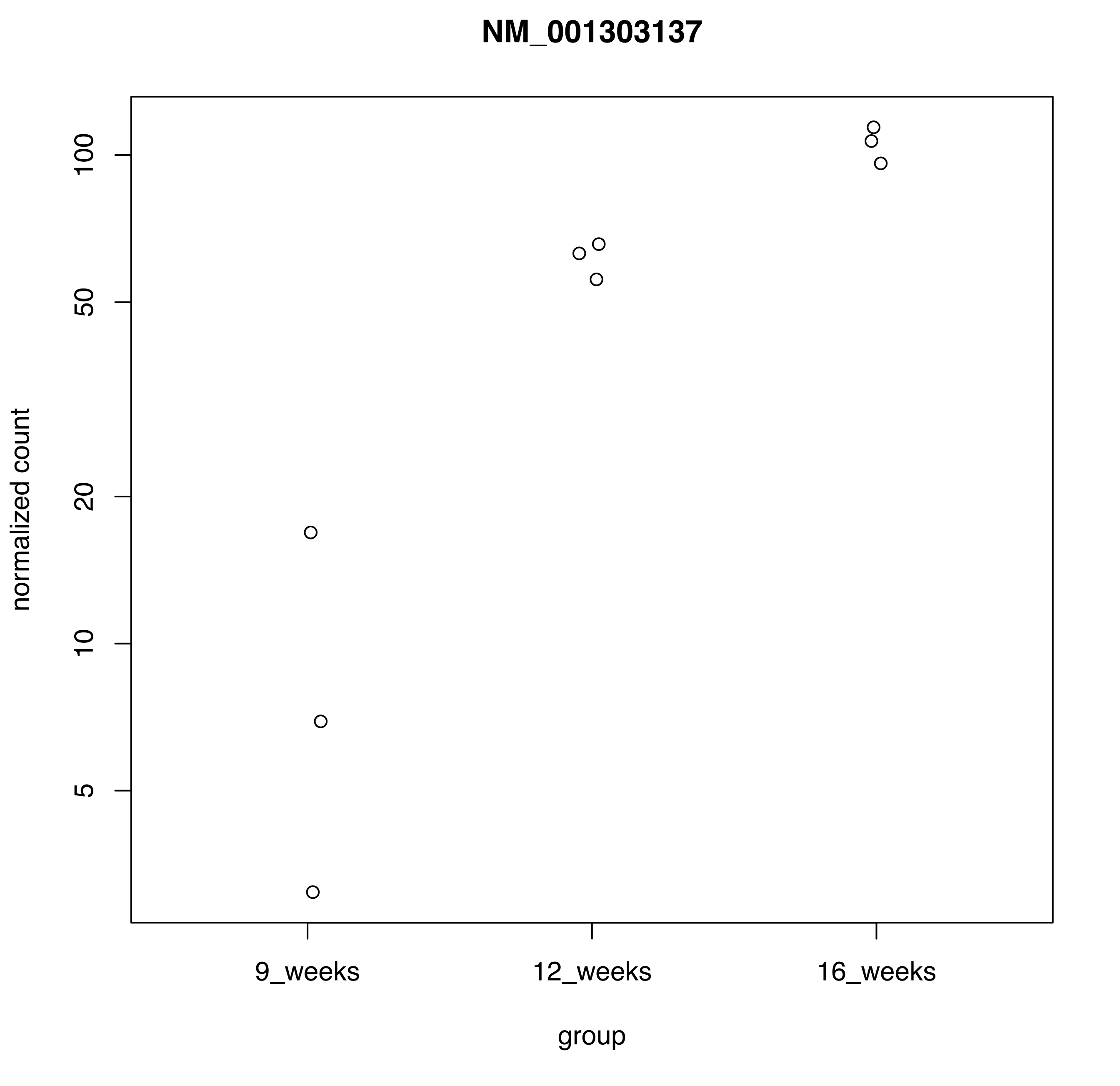
NM_001303137
normalized count
100
50
20
10
5
9_weeks
12_weeks
16_weeks
group

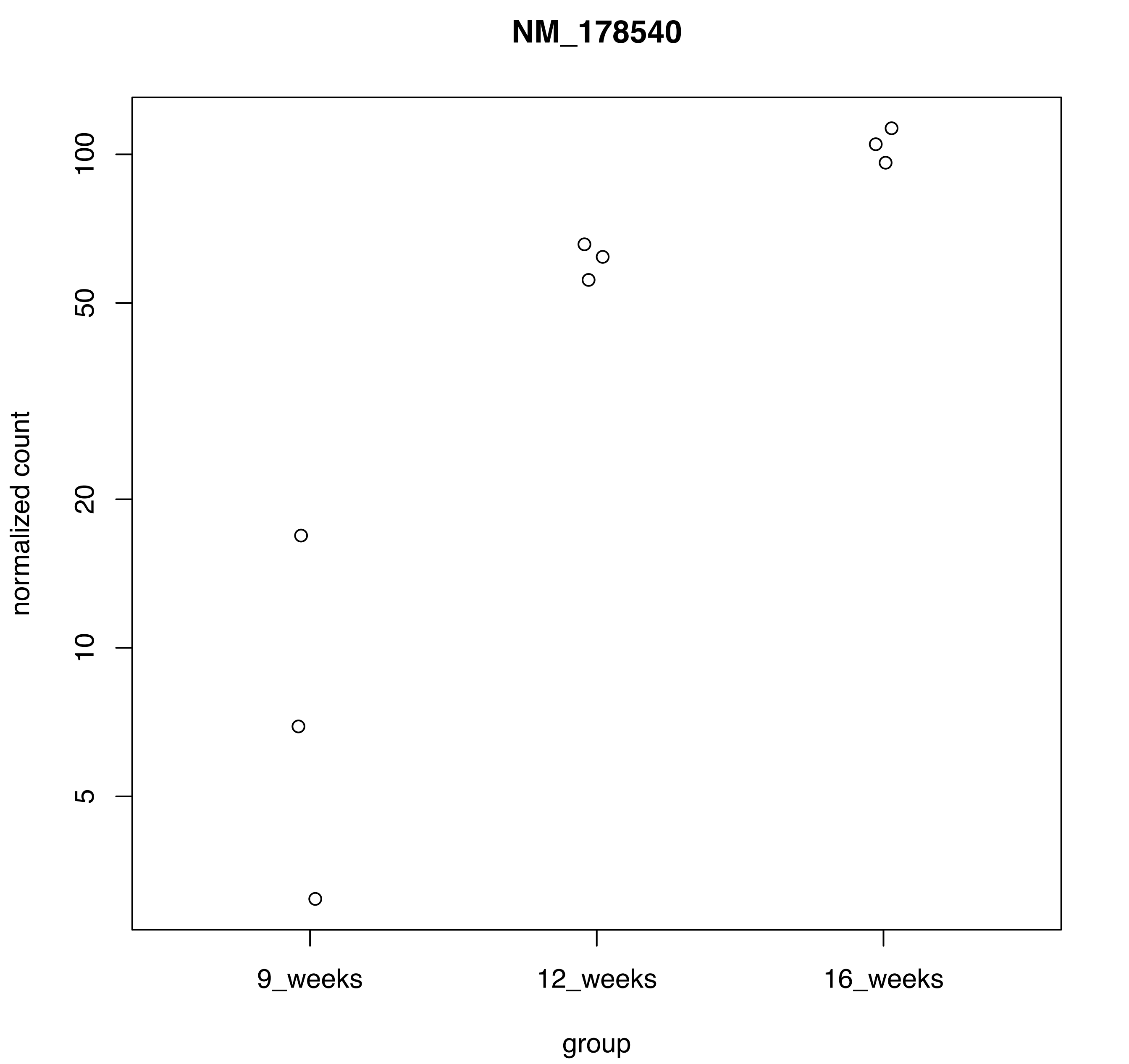
NM_178540
normalized count
100
50
20
10
5
9_weeks
12_weeks
16_weeks
group

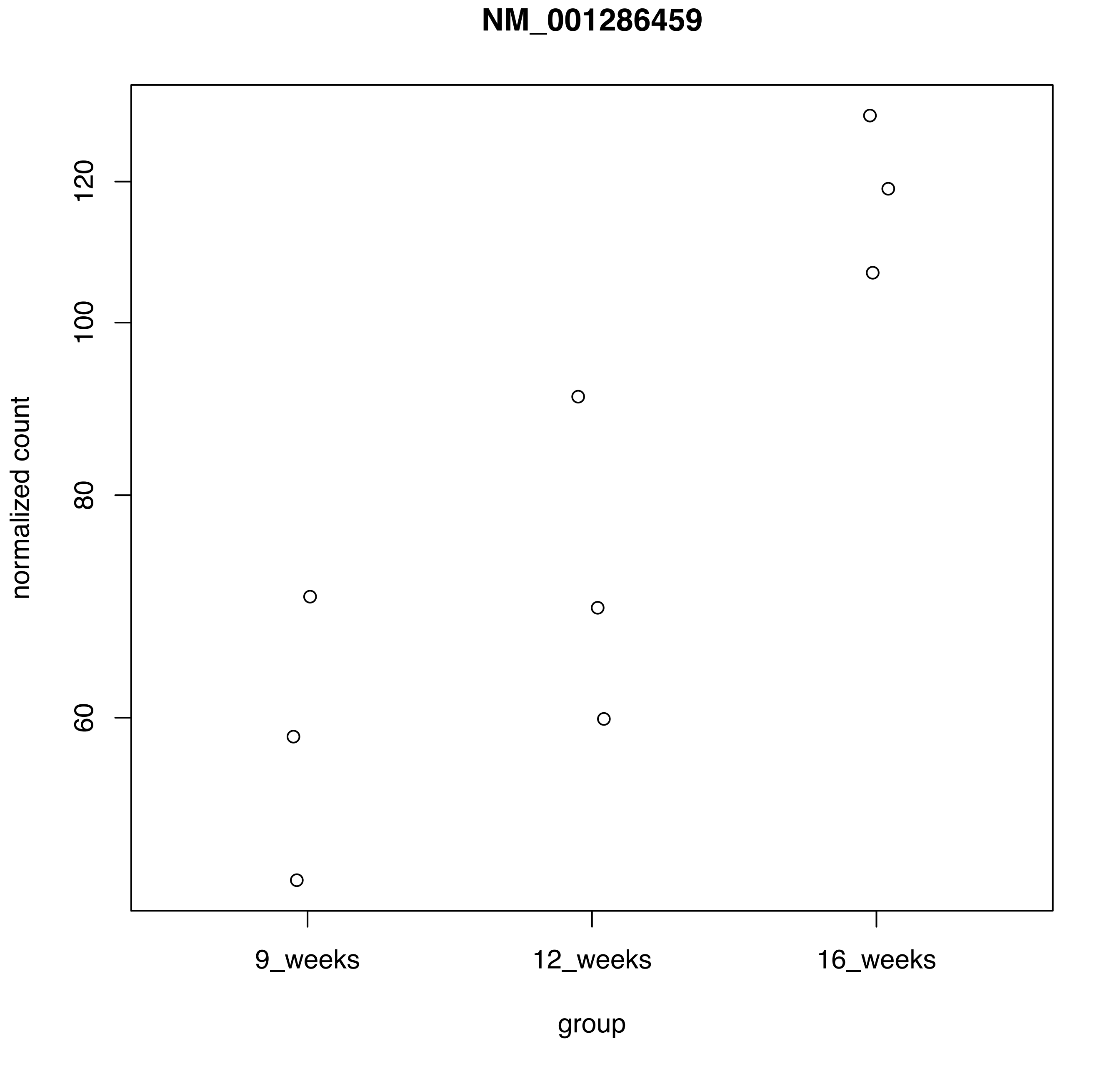

NM_001286459
normalized count
60
80
100
120
9_weeks
12_weeks
16_weeks
group

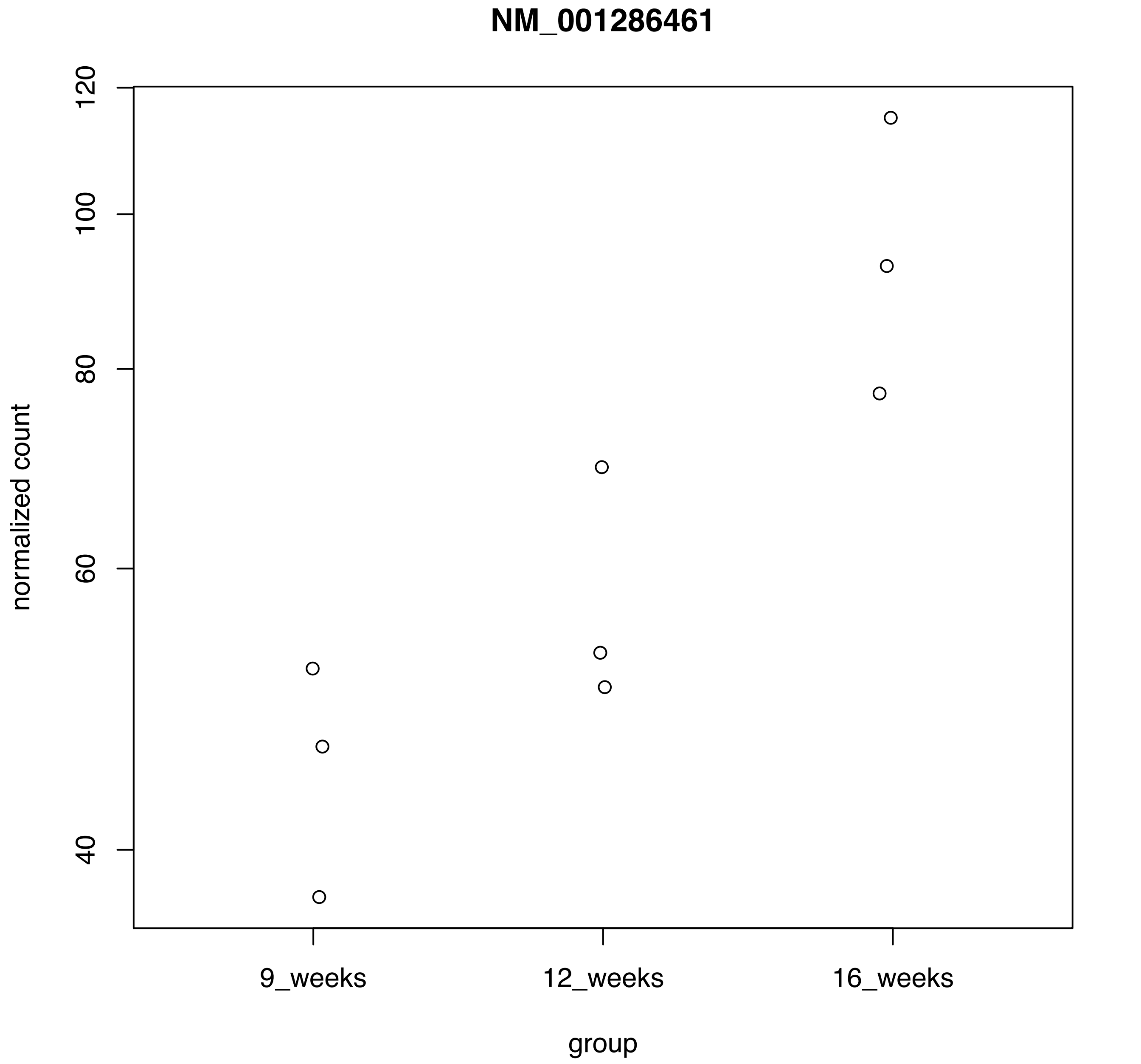
NM_001286461
normalized count
120
100
80
60
40
9_weeks
12_weeks
16_weeks
group

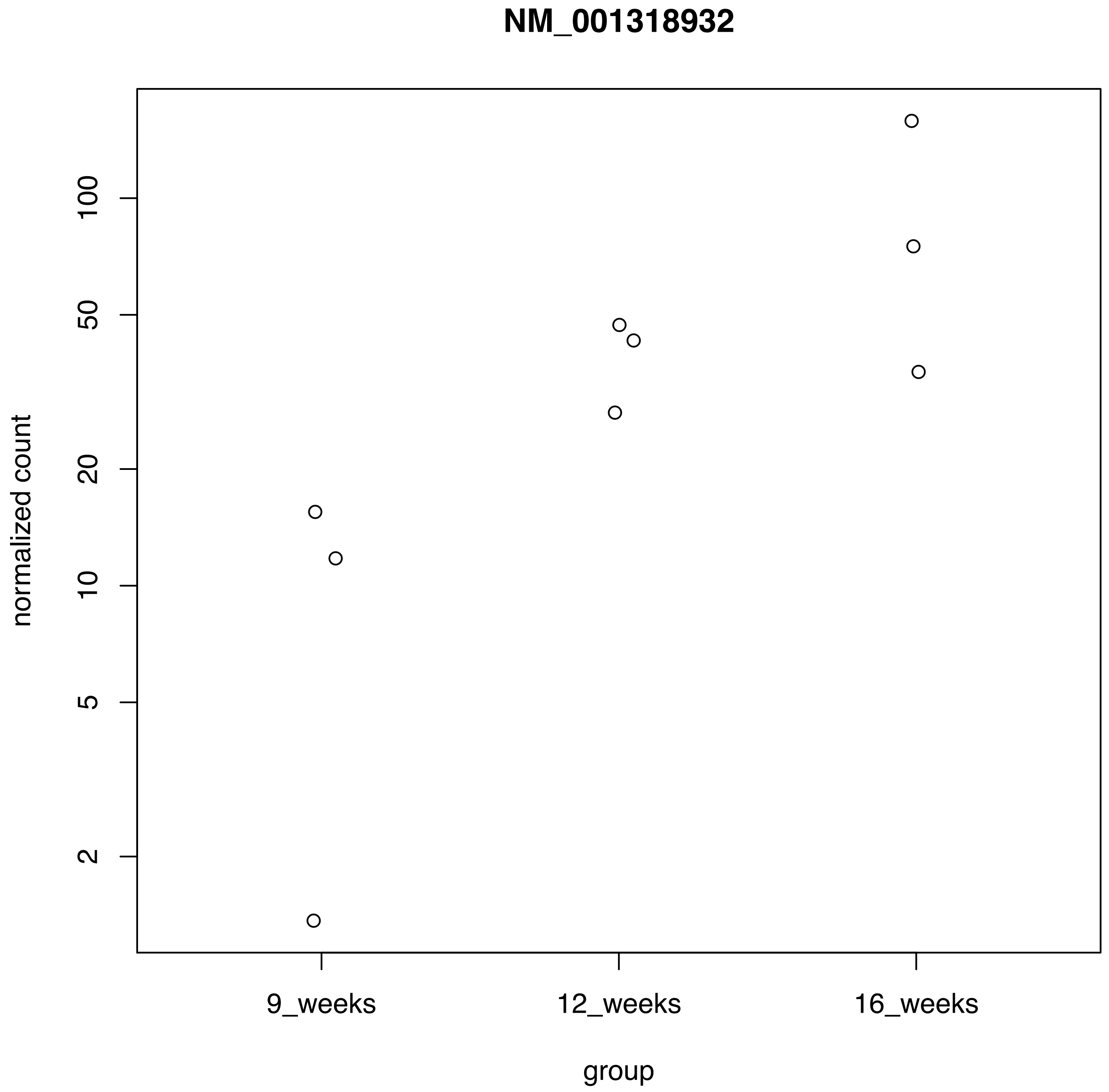

NM_001318932
normalized count
100
50
20
10
5
2
9_weeks
12_weeks
16_weeks
group

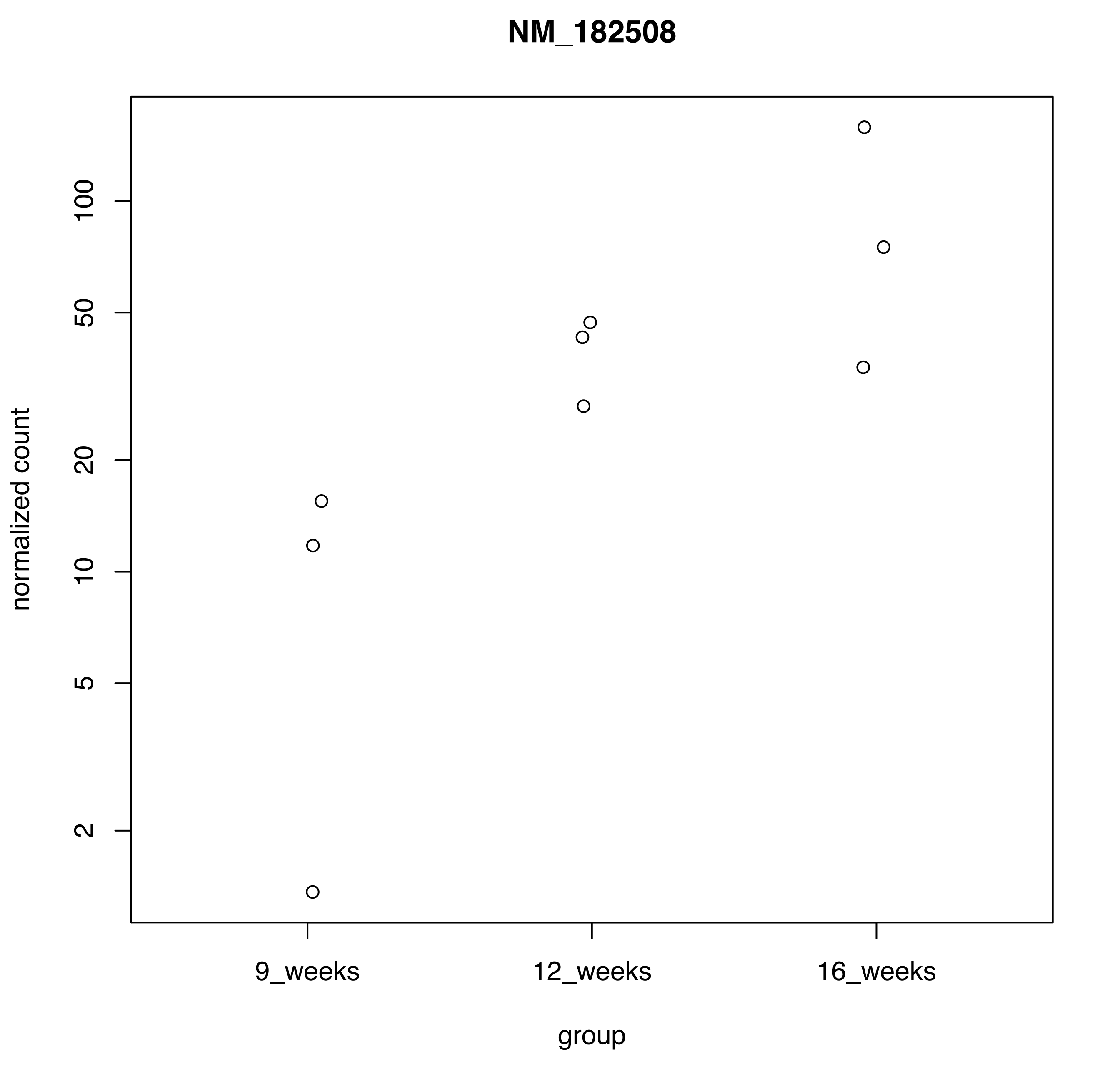
NM_182508
normalized count
100
50
20
10
5
2
9_weeks
12_weeks
16_weeks
group

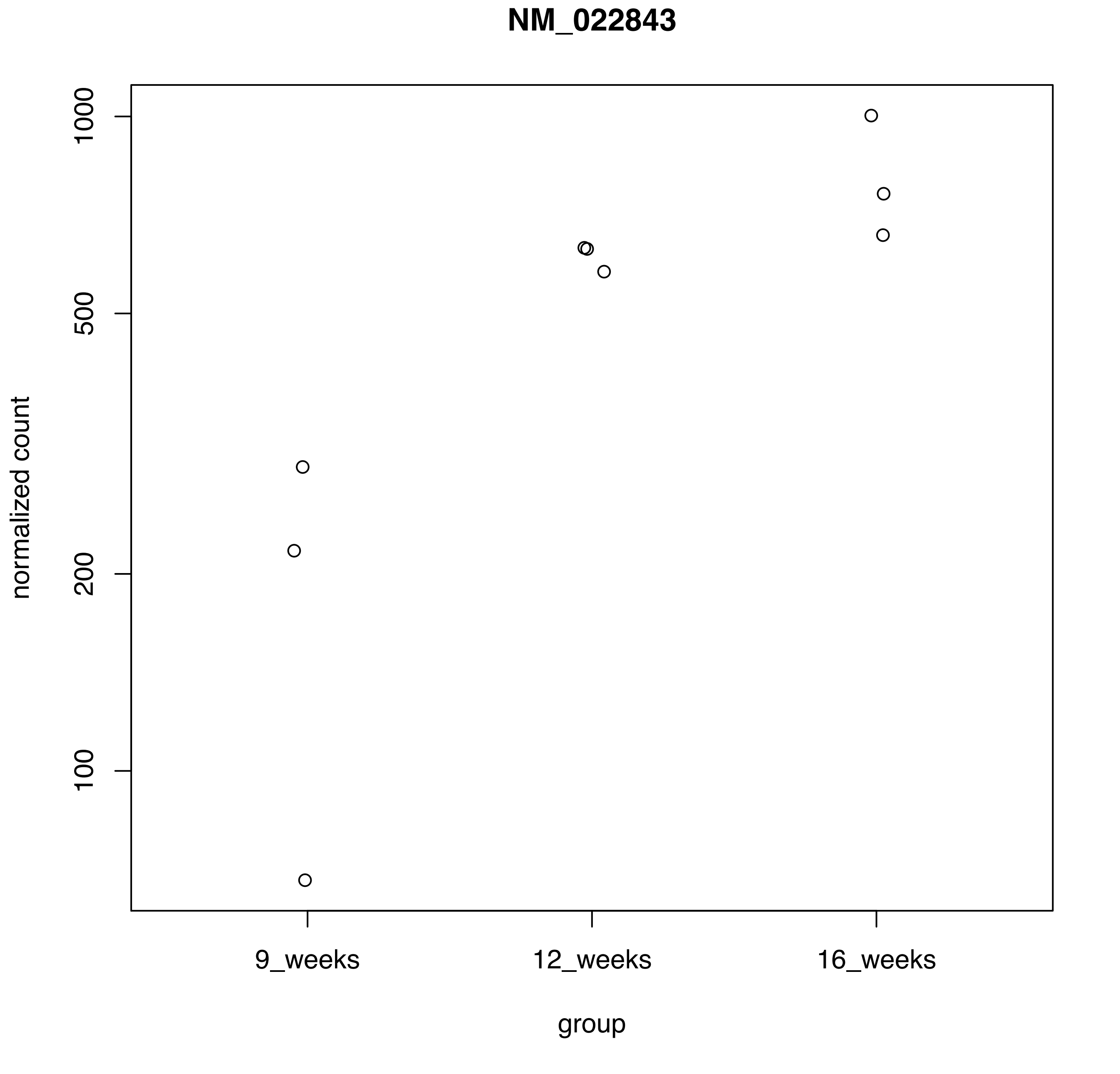
NM_022843
normalized count
1000
500
200
100
9_weeks
12_weeks
16_weeks
group

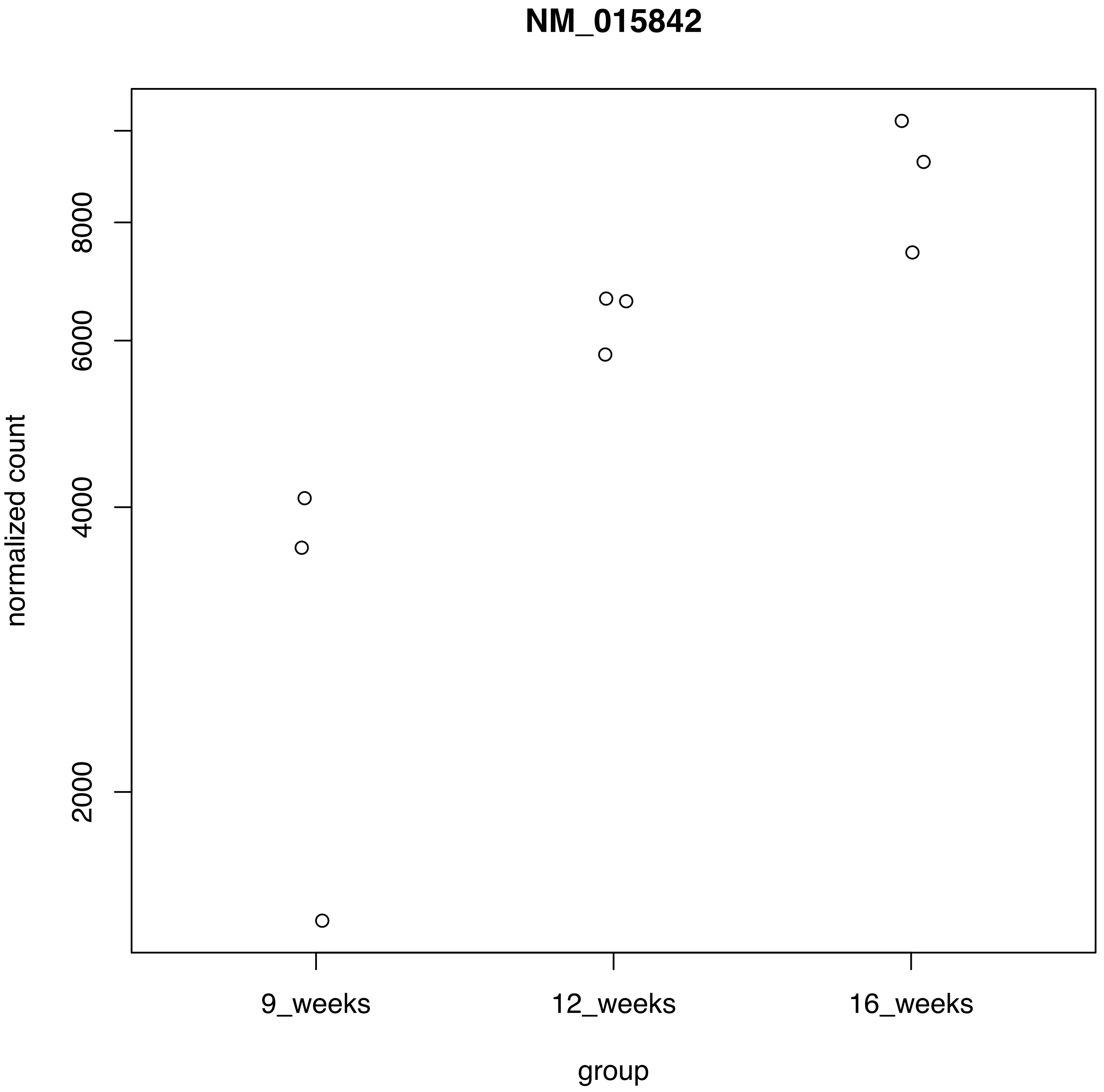
NM_015842
normalized count
2000
4000
6000
8000
9_weeks
12_weeks
16_weeks
group

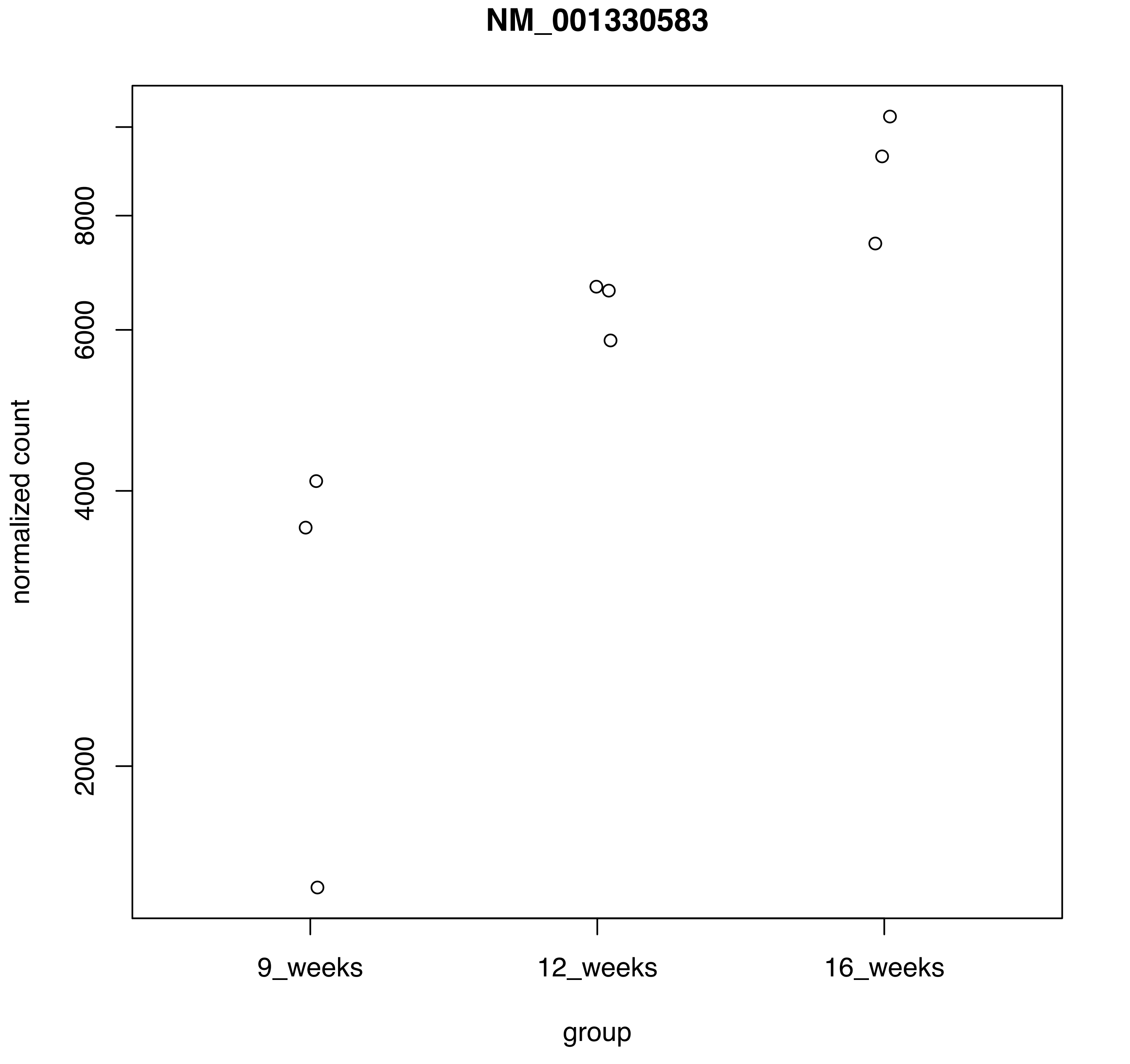
NM_001330583
normalized count
2000
4000
6000
8000
9_weeks
12_weeks
16_weeks
group

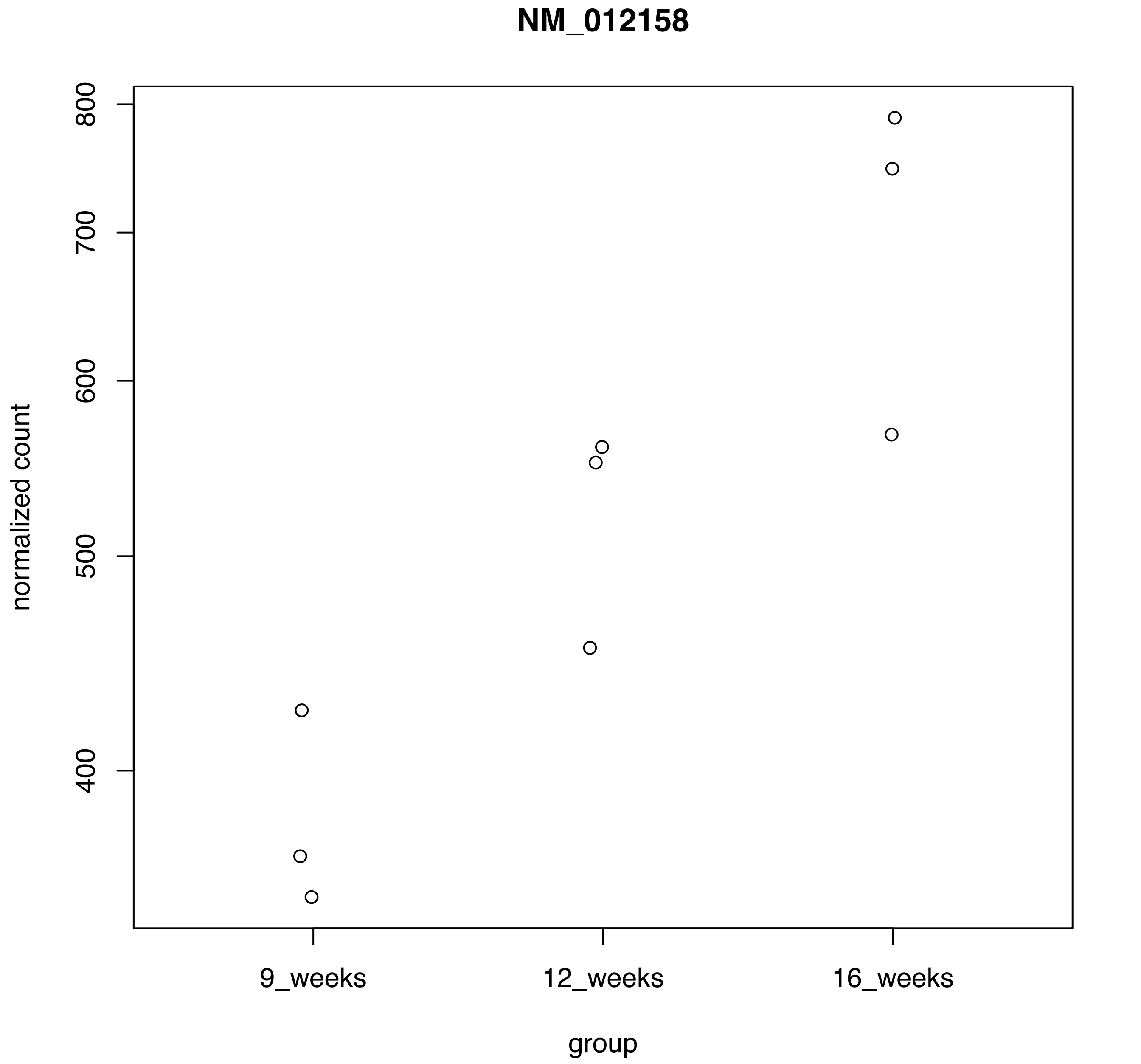

NM_012158
normalized count
800
700
600
500
400
9_weeks
12_weeks
16_weeks
group

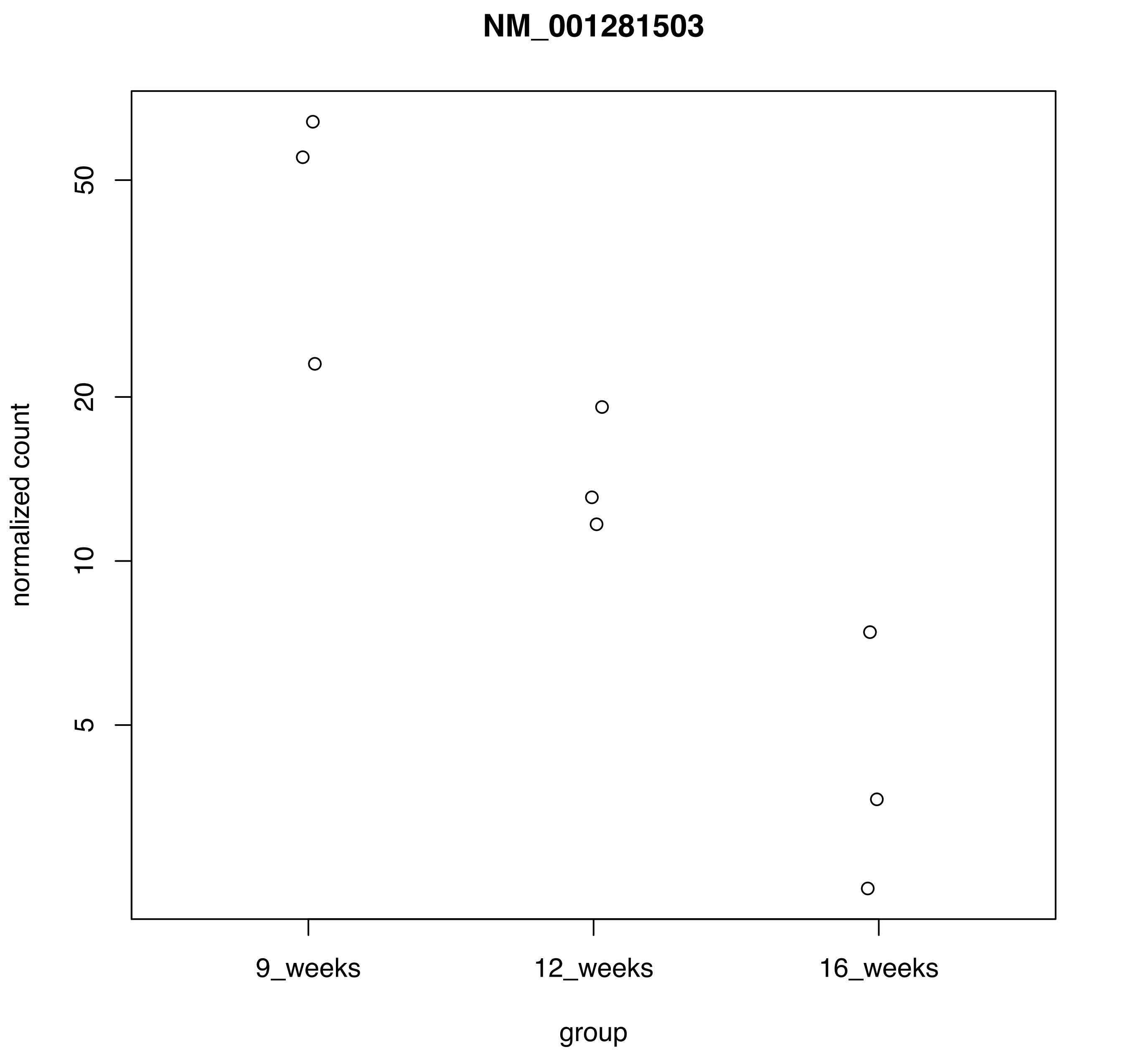
NM_001281503
normalized count
50
20
10
5
9_weeks
12_weeks
16_weeks
group

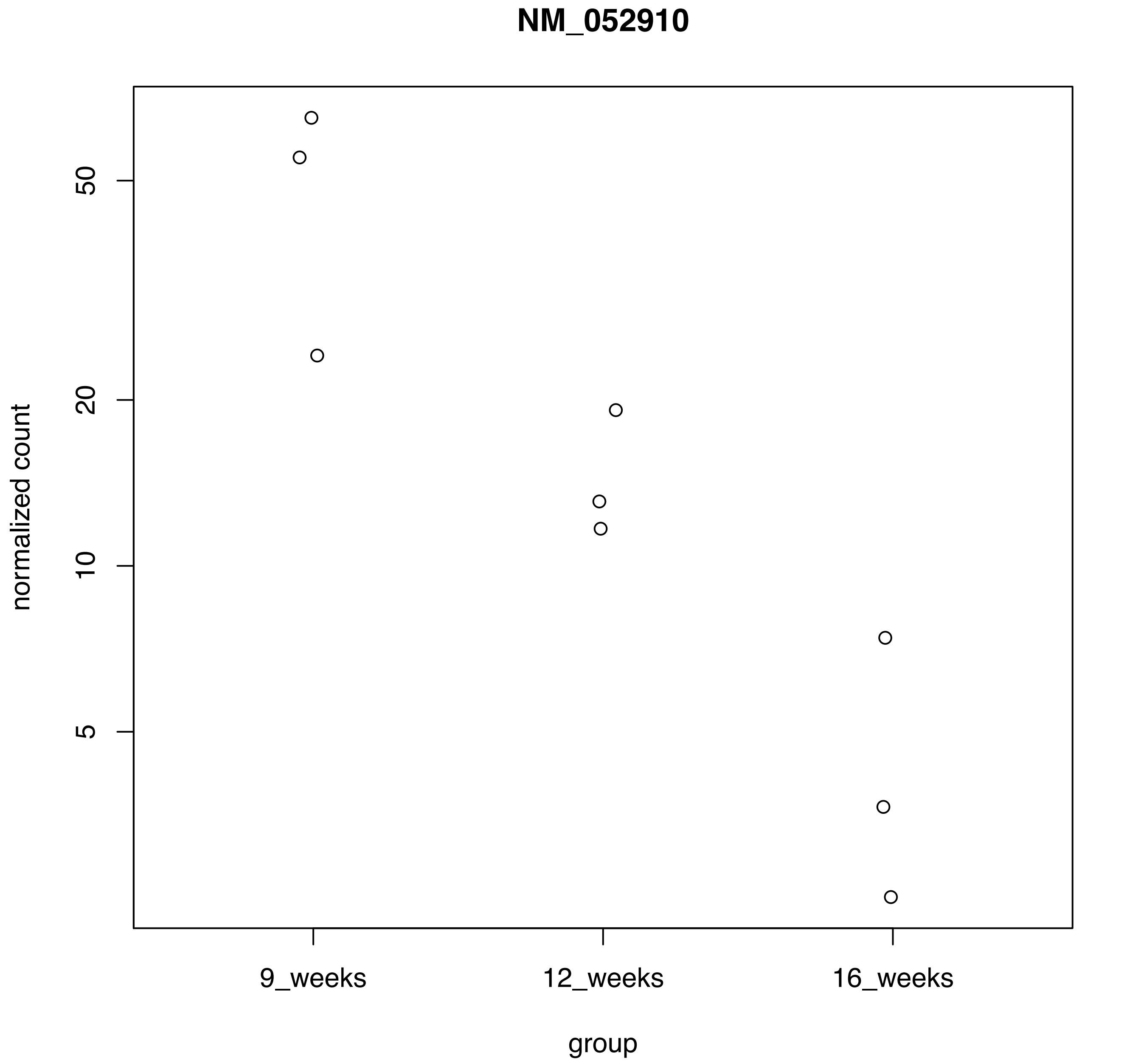
NM_052910
normalized count
50
20
10
5
9_weeks
12_weeks
16_weeks
group

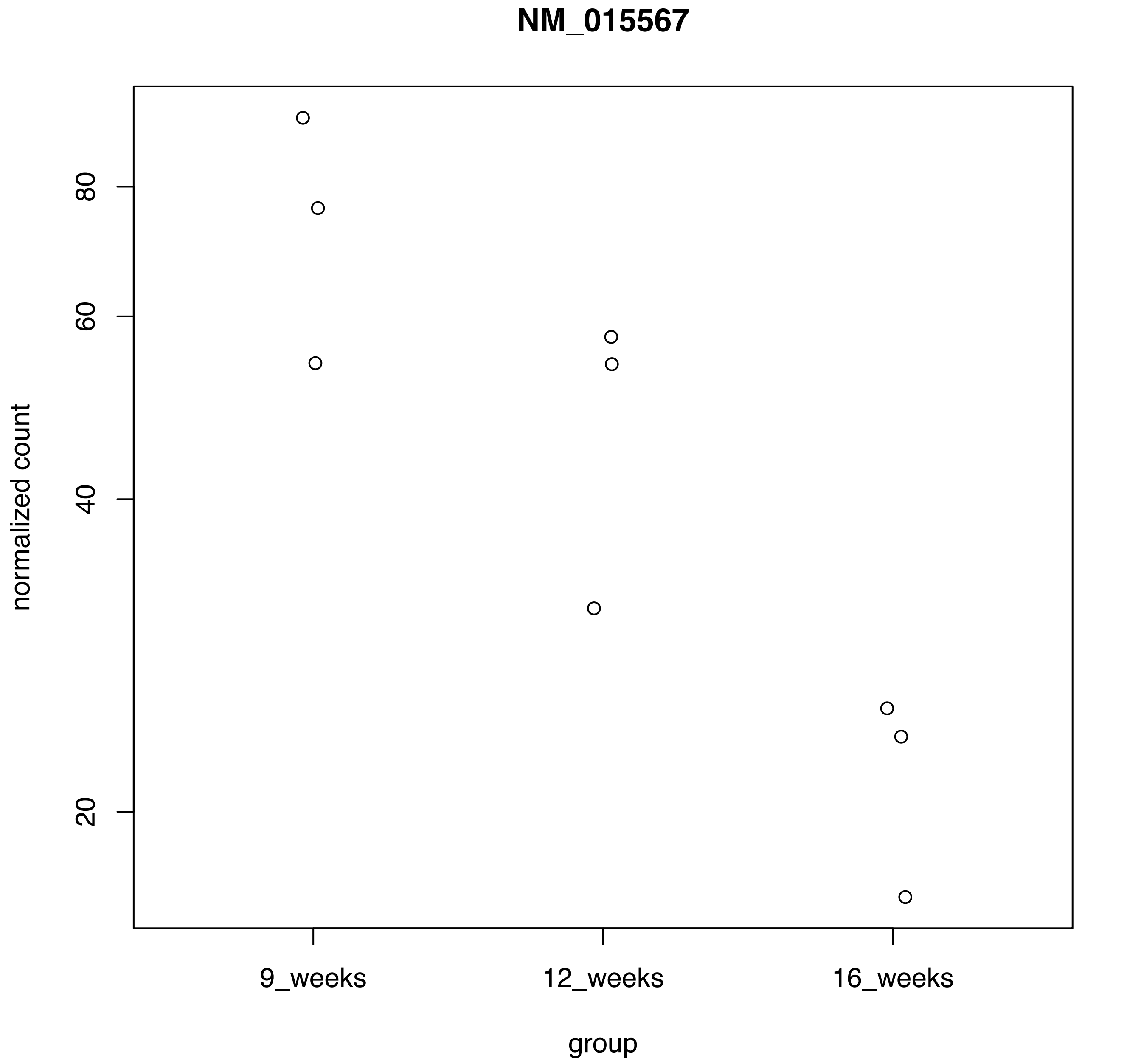
NM_015567
normalized count
20
40
60
80
9_weeks
12_weeks
16_weeks
group

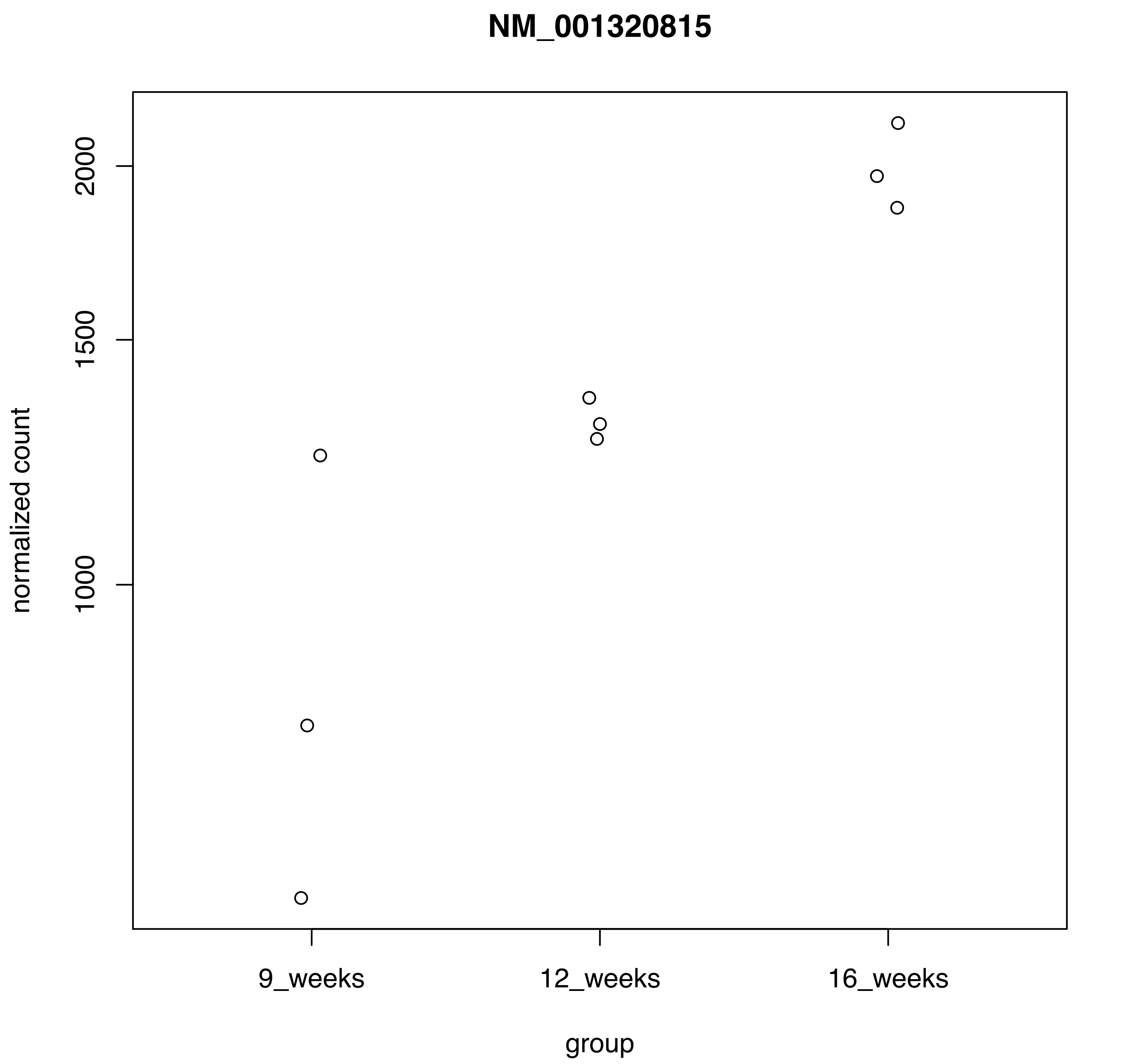

NM_001320815
normalized count
2000
1500
1000
9_weeks
12_weeks
16_weeks
group

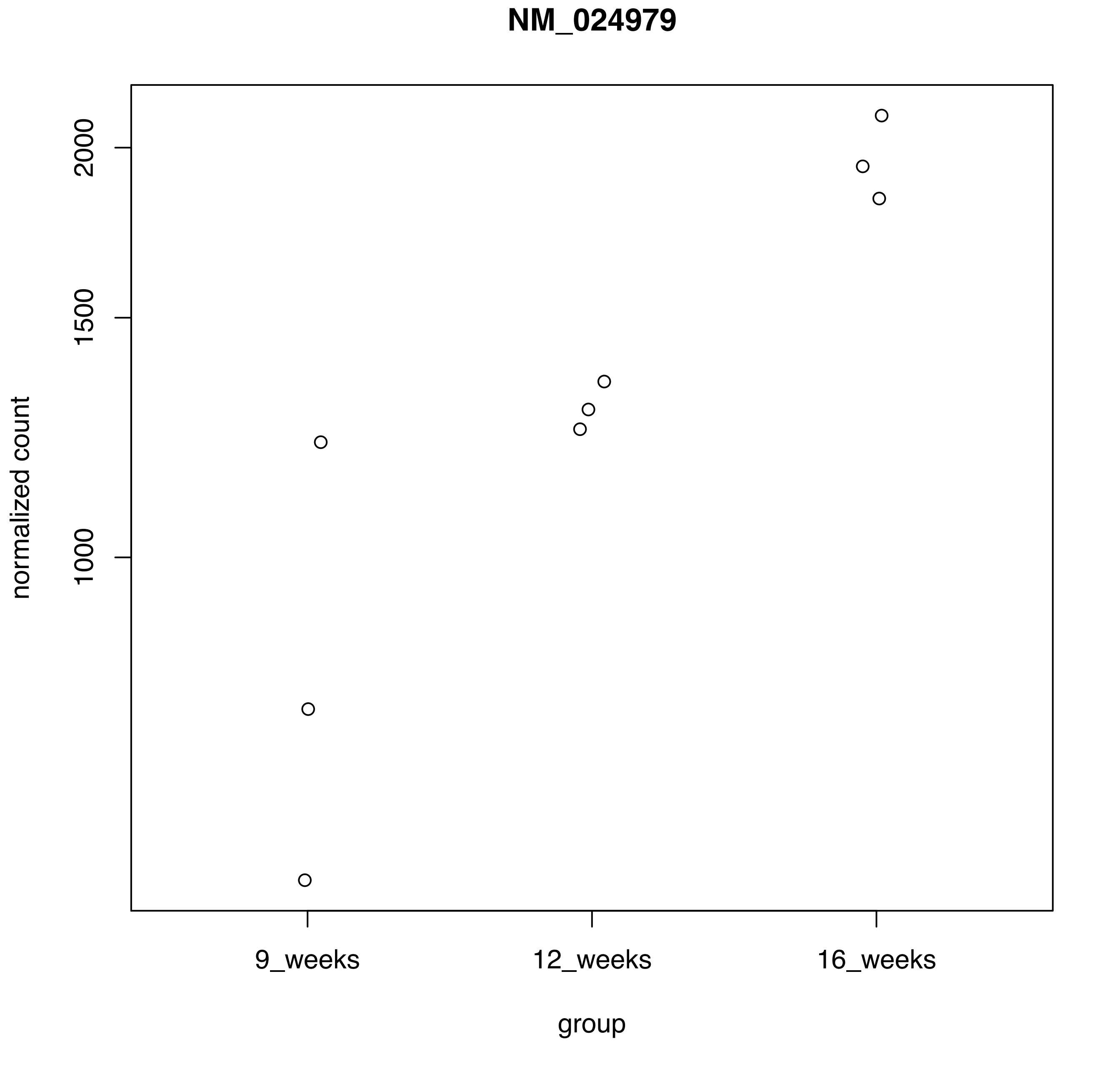
NM_024979
normalized count
2000
1500
1000
9_weeks
12_weeks
16_weeks
group

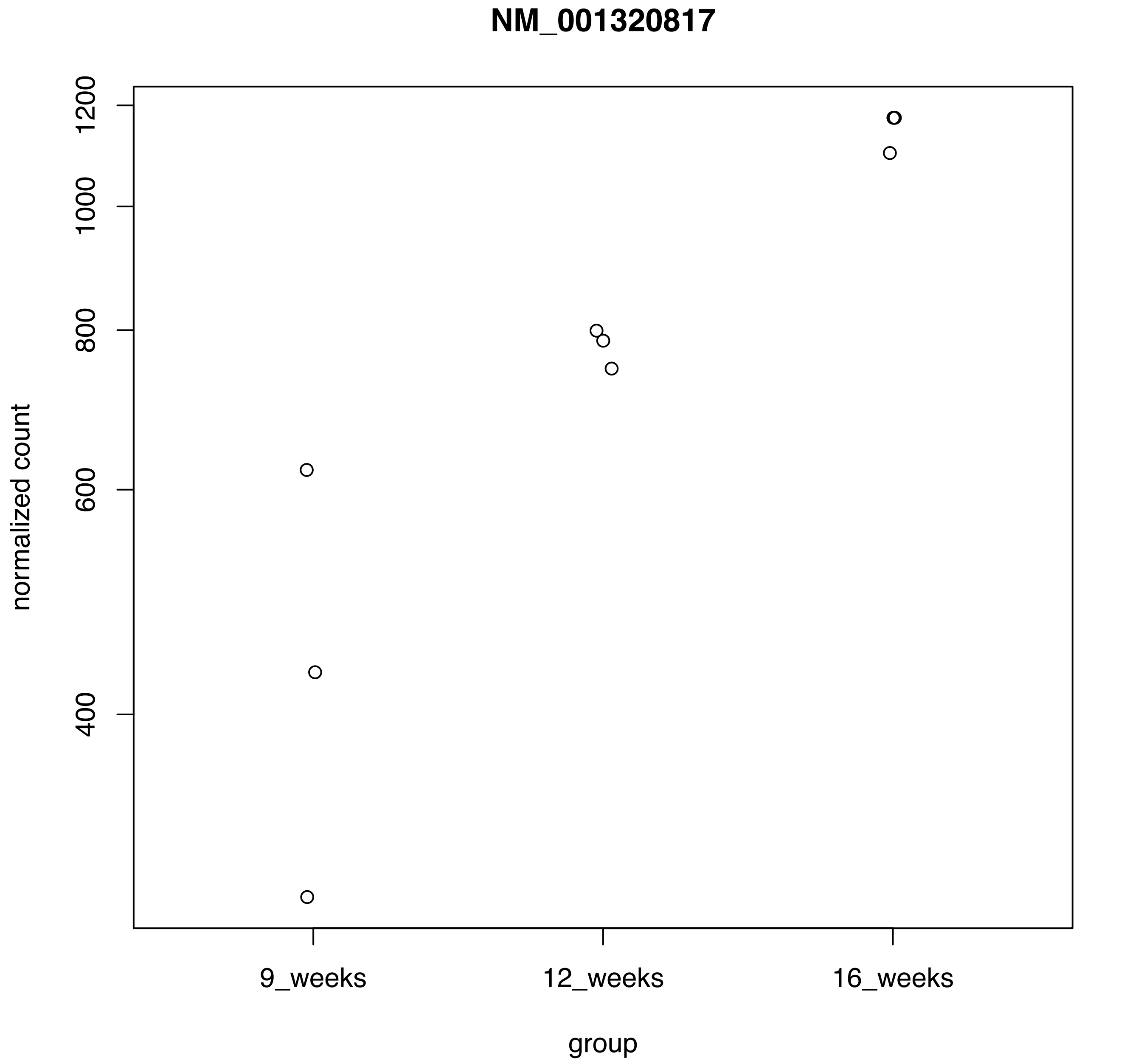
NM_001320817
normalized count
400
600
800
1000
1200
group
9_weeks
12_weeks
16_weeks

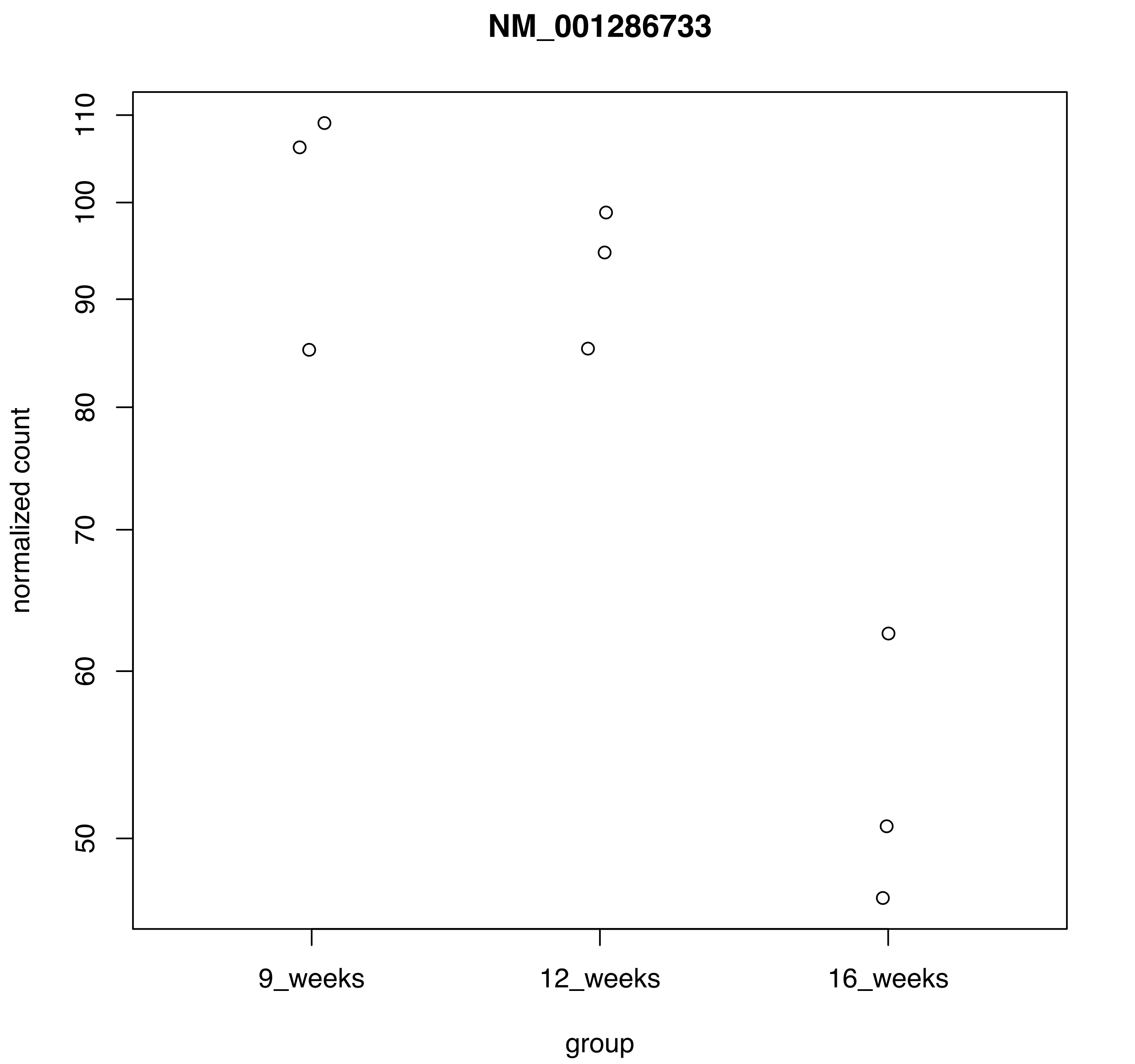
NM_001286733
normalized count
110
100
90
80
70
60
50
9_weeks
12_weeks
16_weeks
group

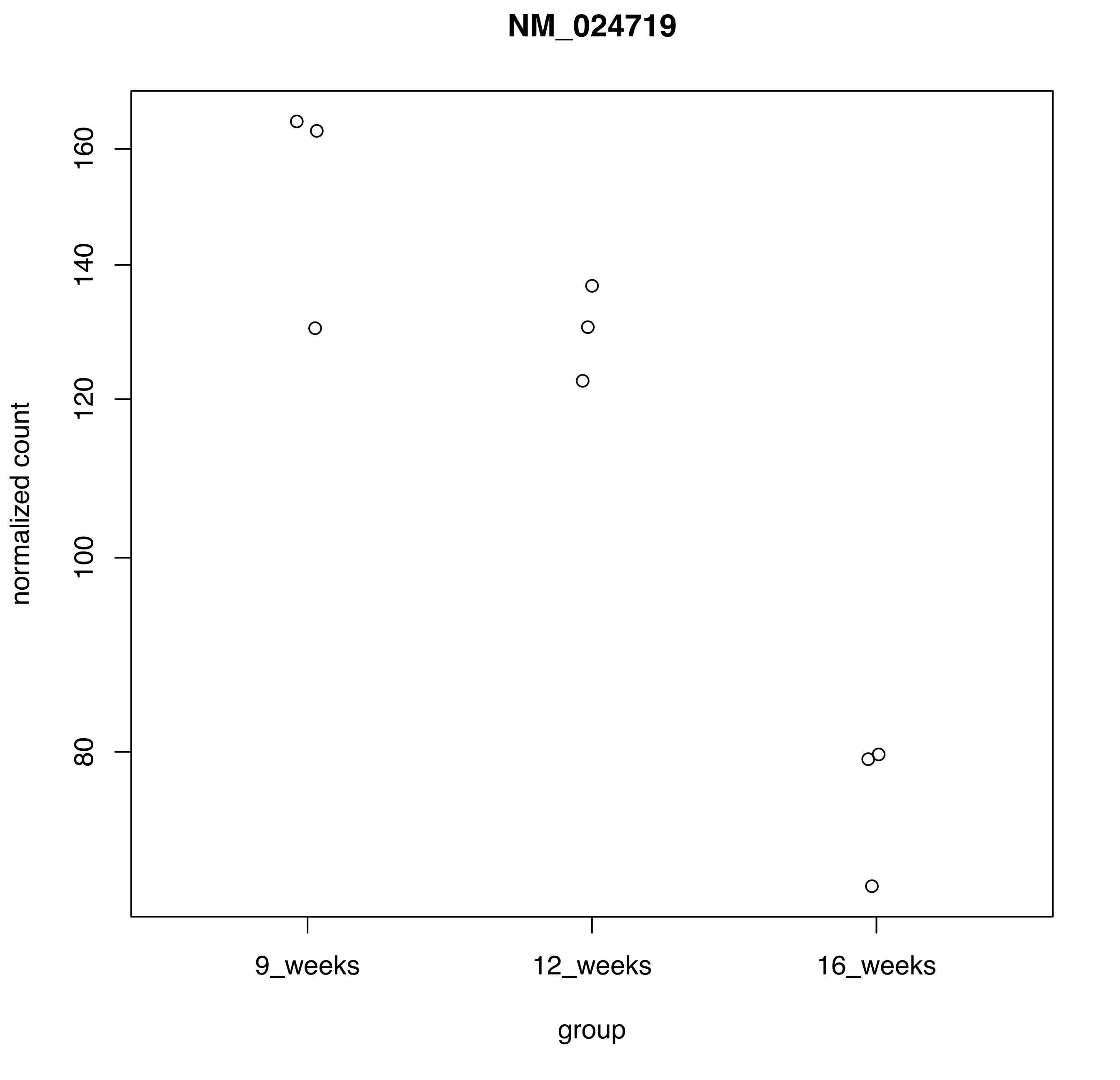
NM_024719
normalized count
160
140
120
100
80
9_weeks
12_weeks
16_weeks
group

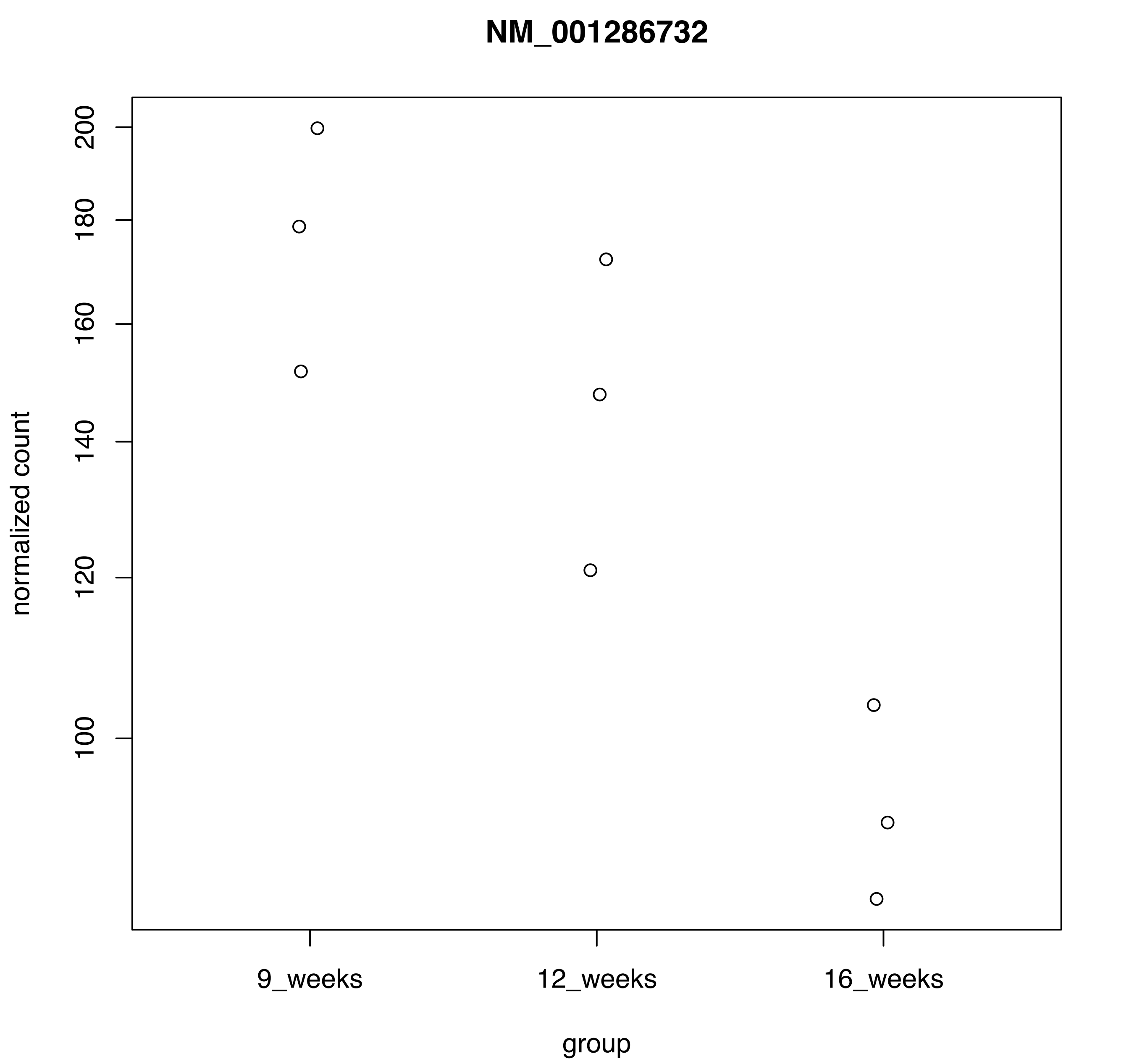
NM_001286732
normalized count
200
180
160
140
120
100
9_weeks
12_weeks
16_weeks
group

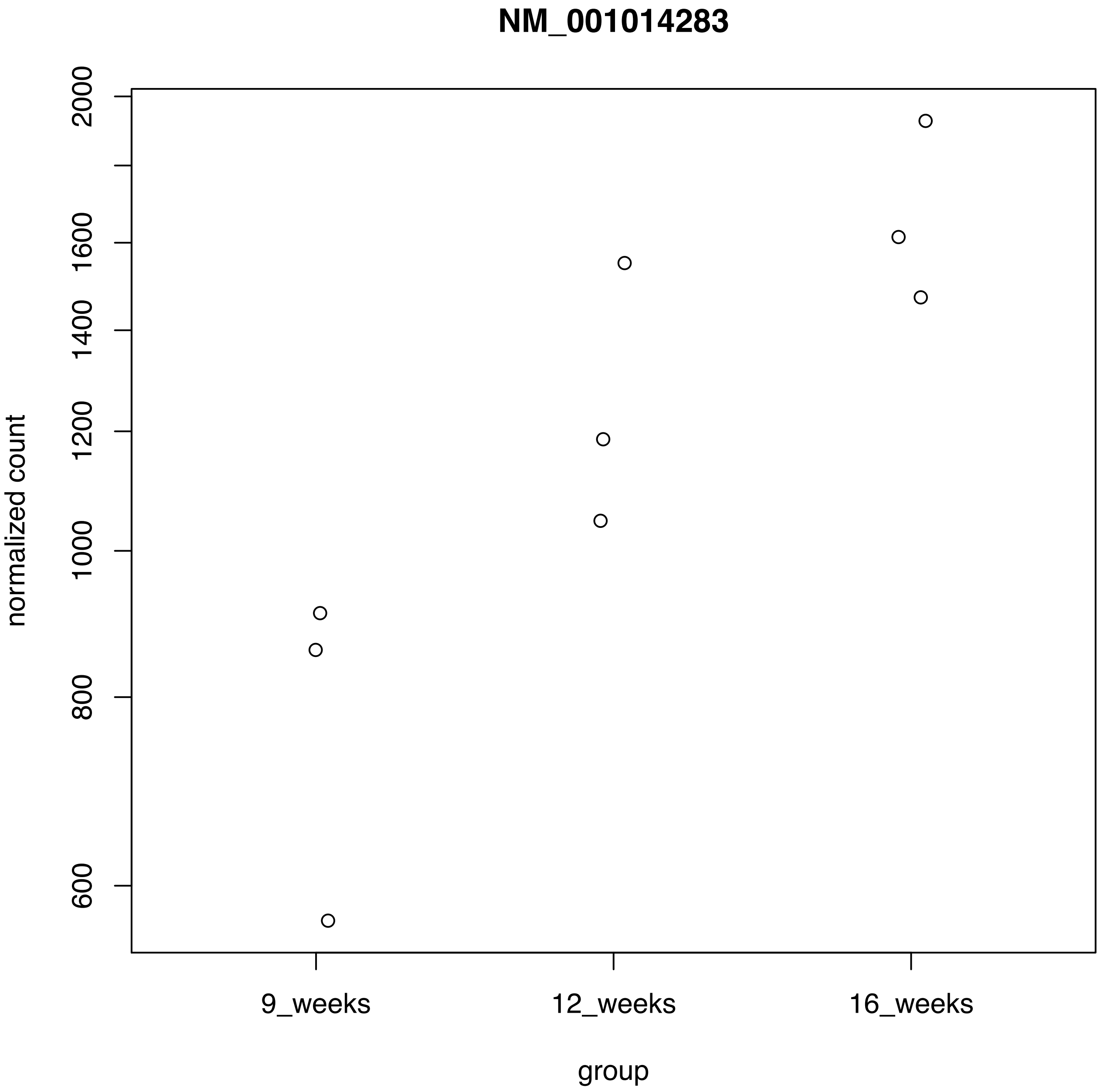
NM_001014283
normalized count
2000
1600
1400
1200
1000
800
600
9_weeks
12_weeks
16_weeks
group

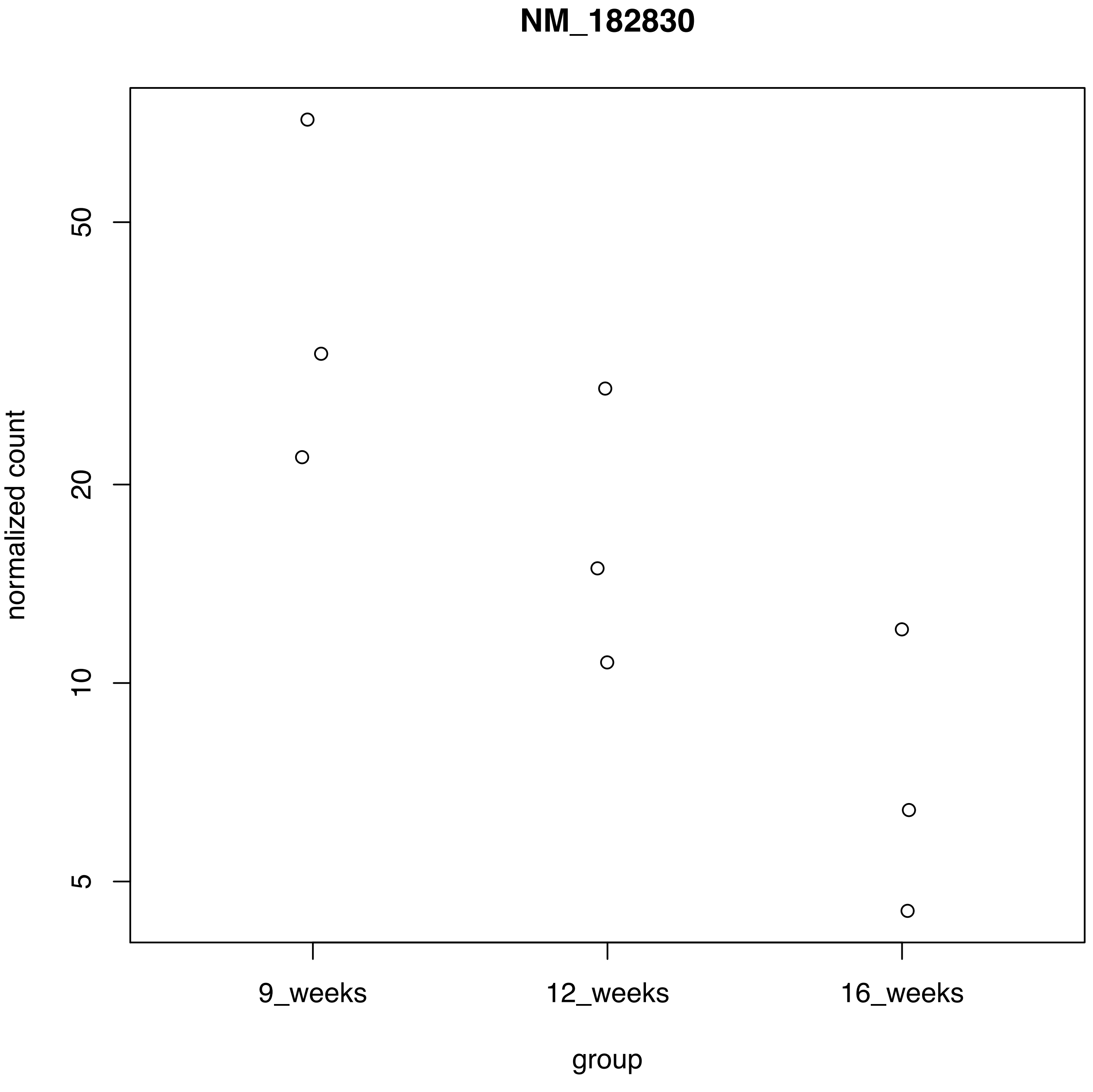
NM_182830
normalized count
50
20
10
5
9_weeks
12_weeks
16_weeks
group

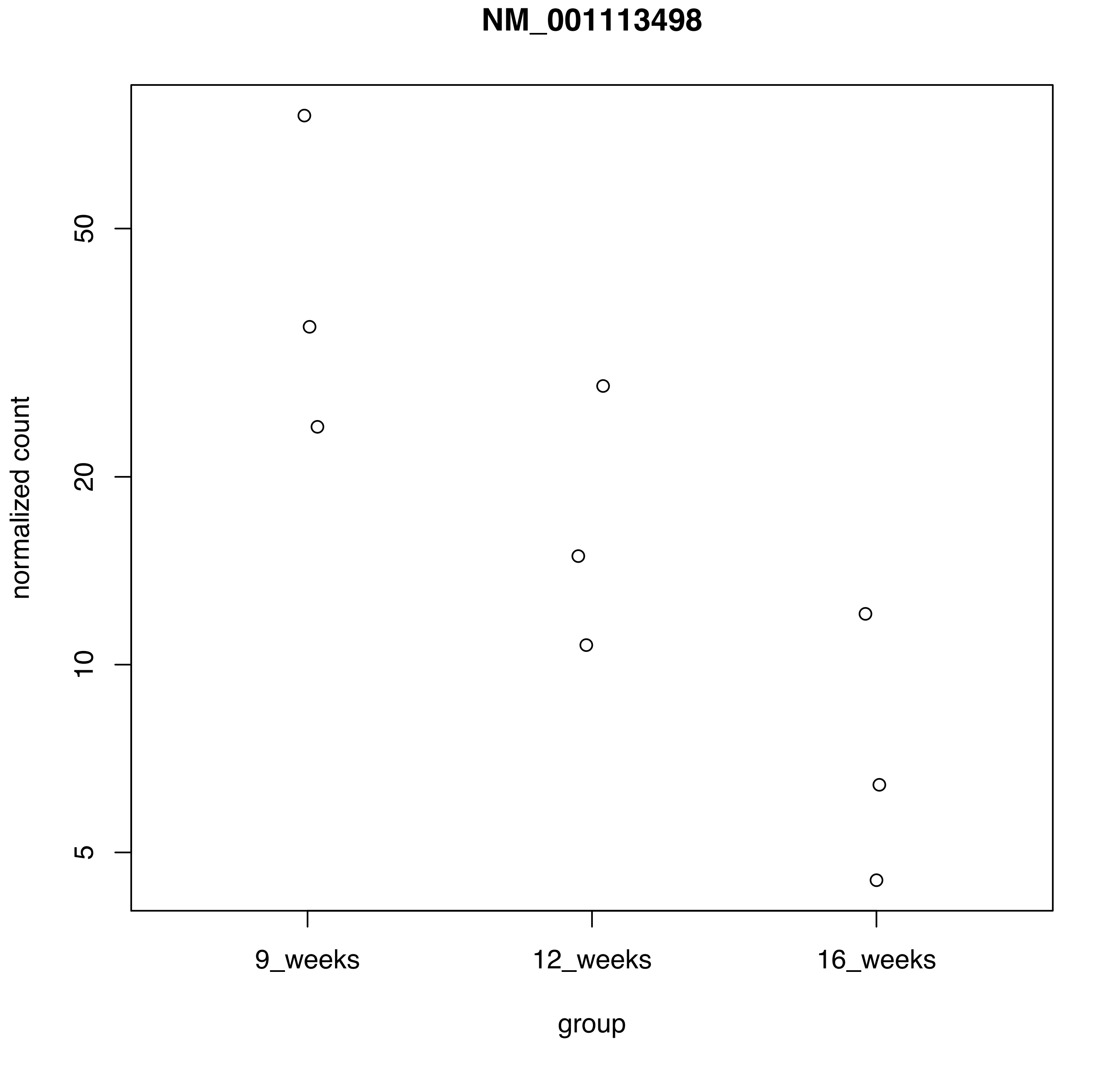
NM_001113498
normalized count
50
20
10
5
9_weeks
12_weeks
16_weeks
group

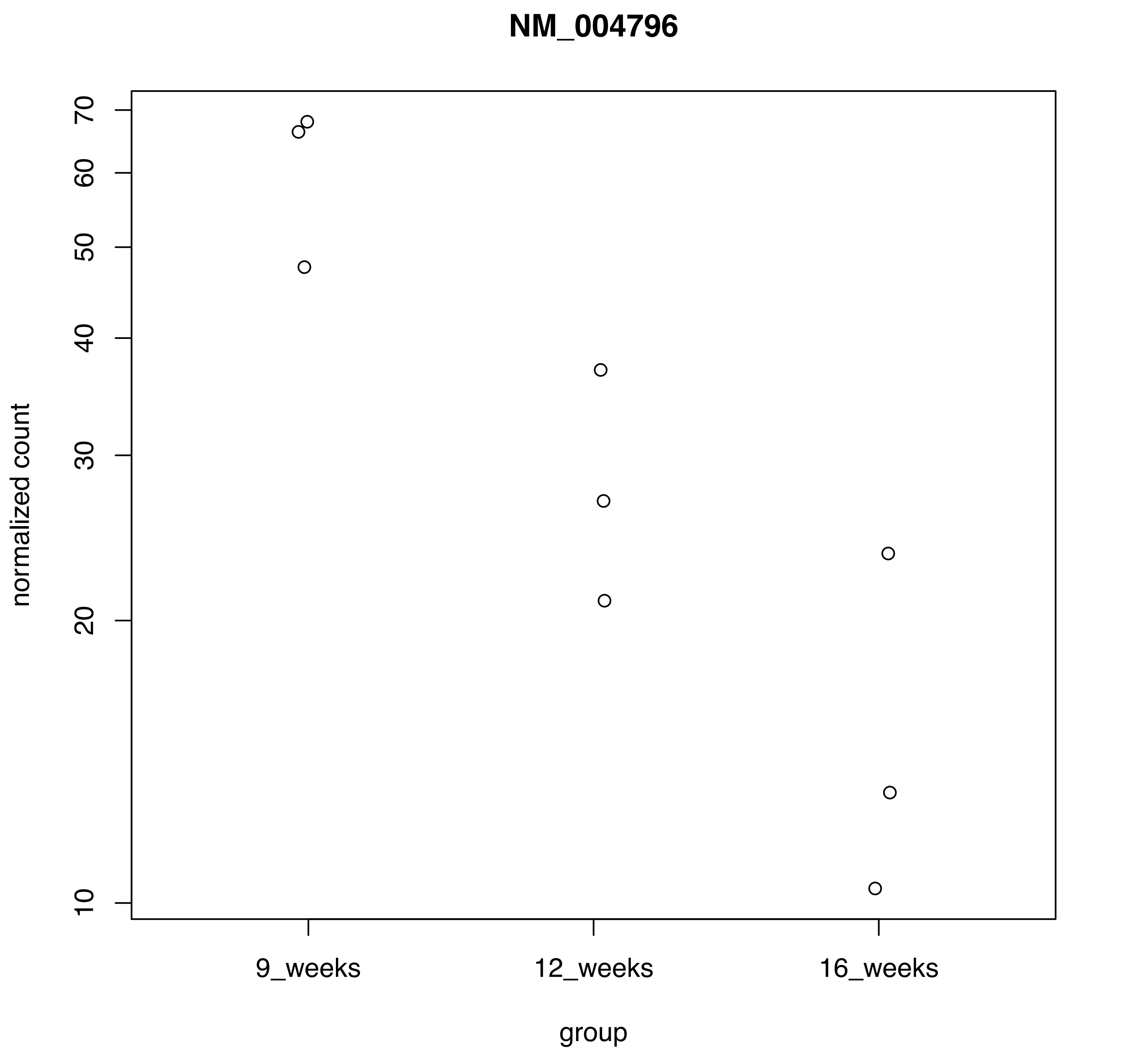
NM_004796
normalized count
70
60
50
40
30
20
10
9_weeks
12_weeks
16_weeks
group

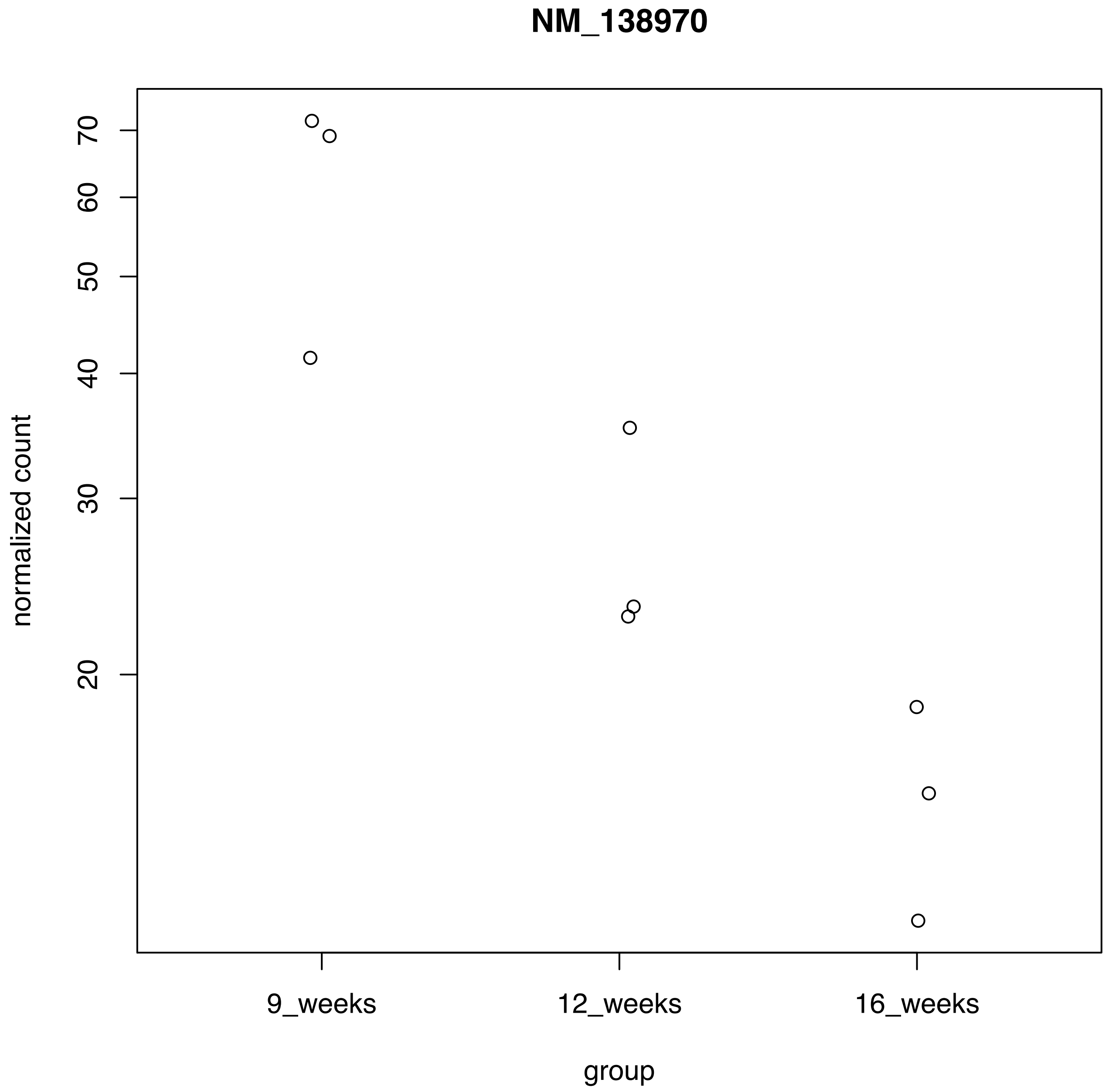

NM_138970
70
60
50
40
30
20
normalized count
9_weeks
12_weeks
16_weeks
group

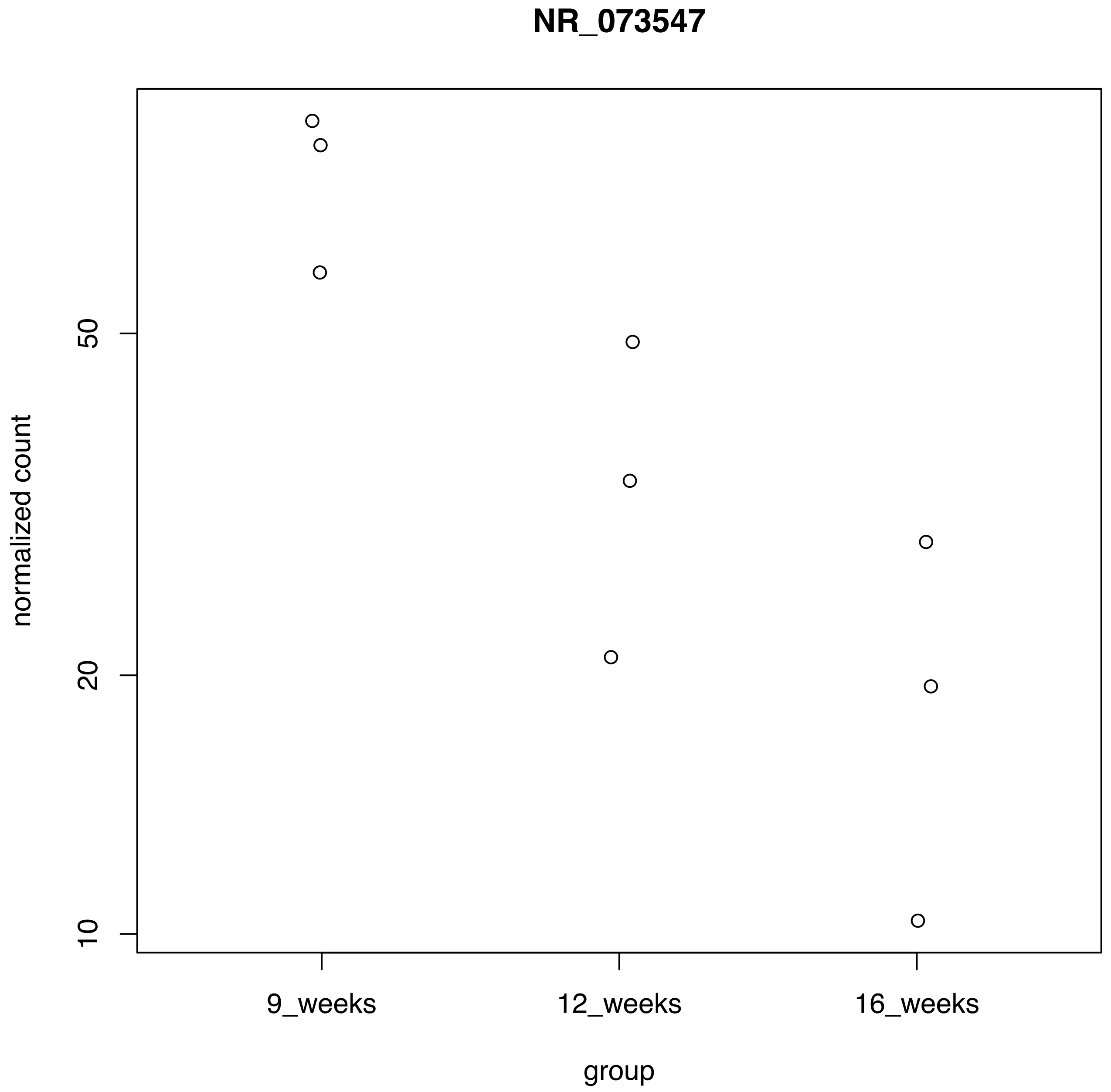

NR_073547
normalized count
50
20
10
9_weeks
12_weeks
16_weeks
group

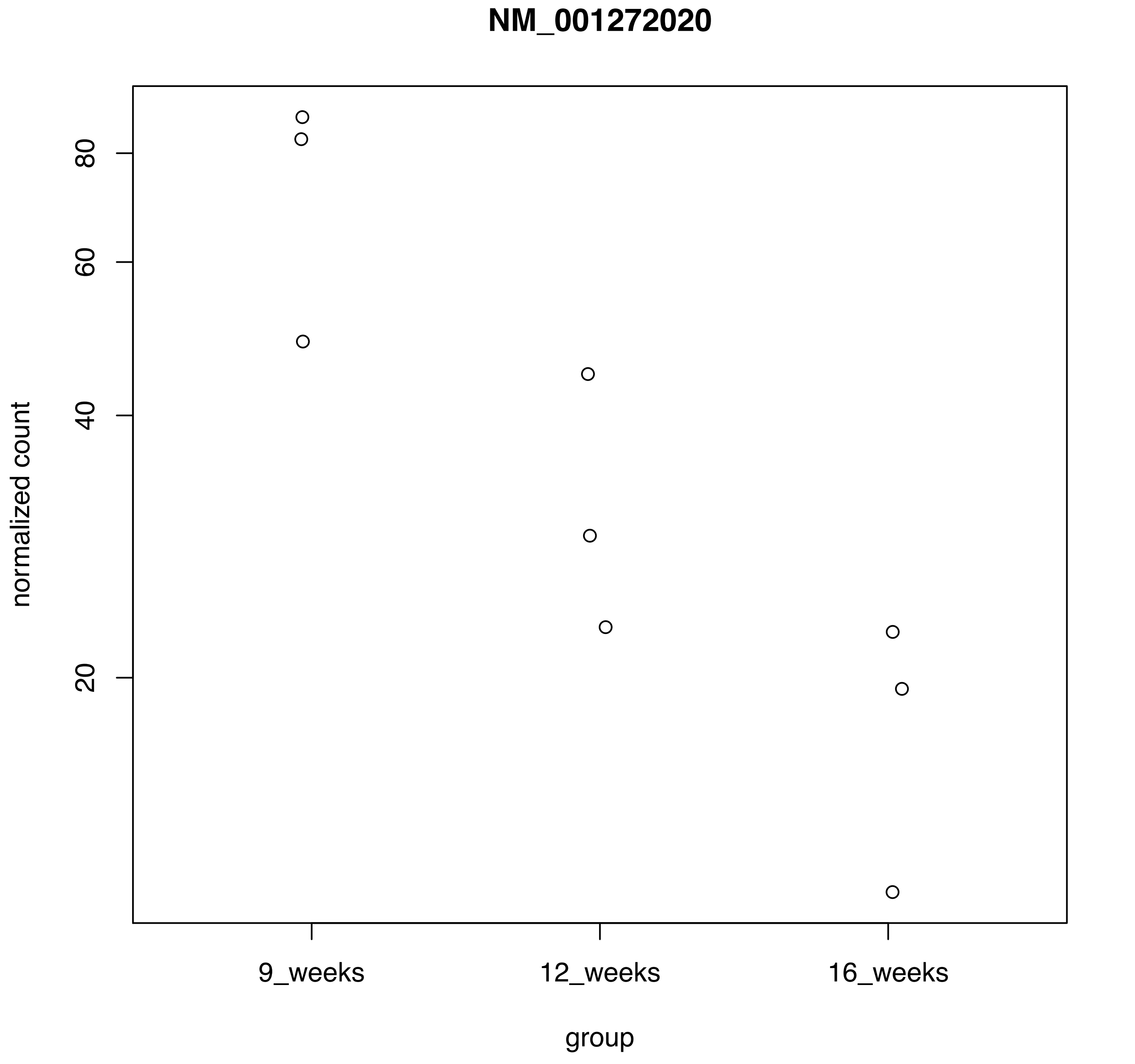

NM_001272020
normalized count
80
60
40
20
9_weeks
12_weeks
16_weeks
group

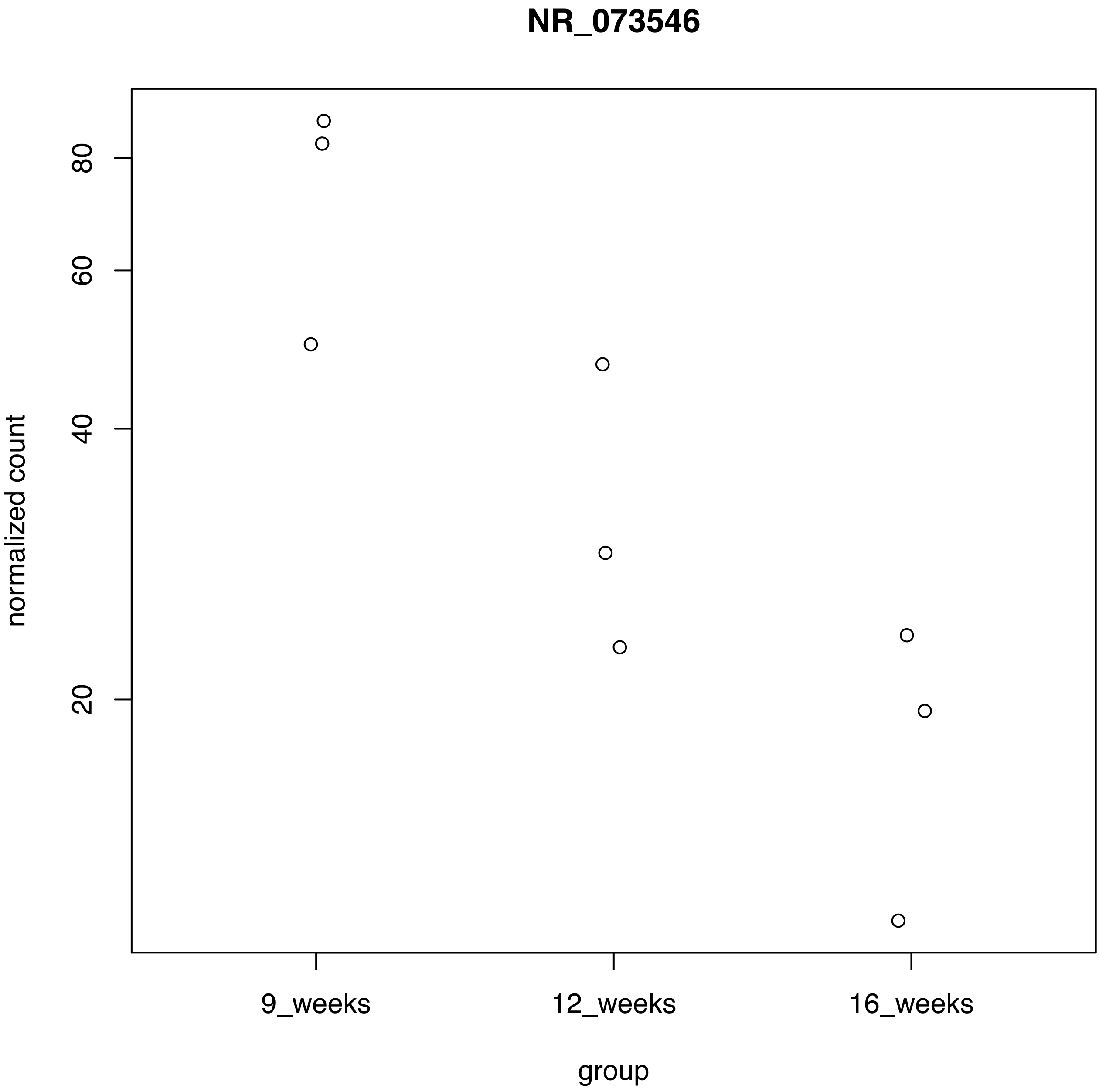
NR_073546
normalized count
80
60
40
20
9_weeks
12_weeks
16_weeks
group

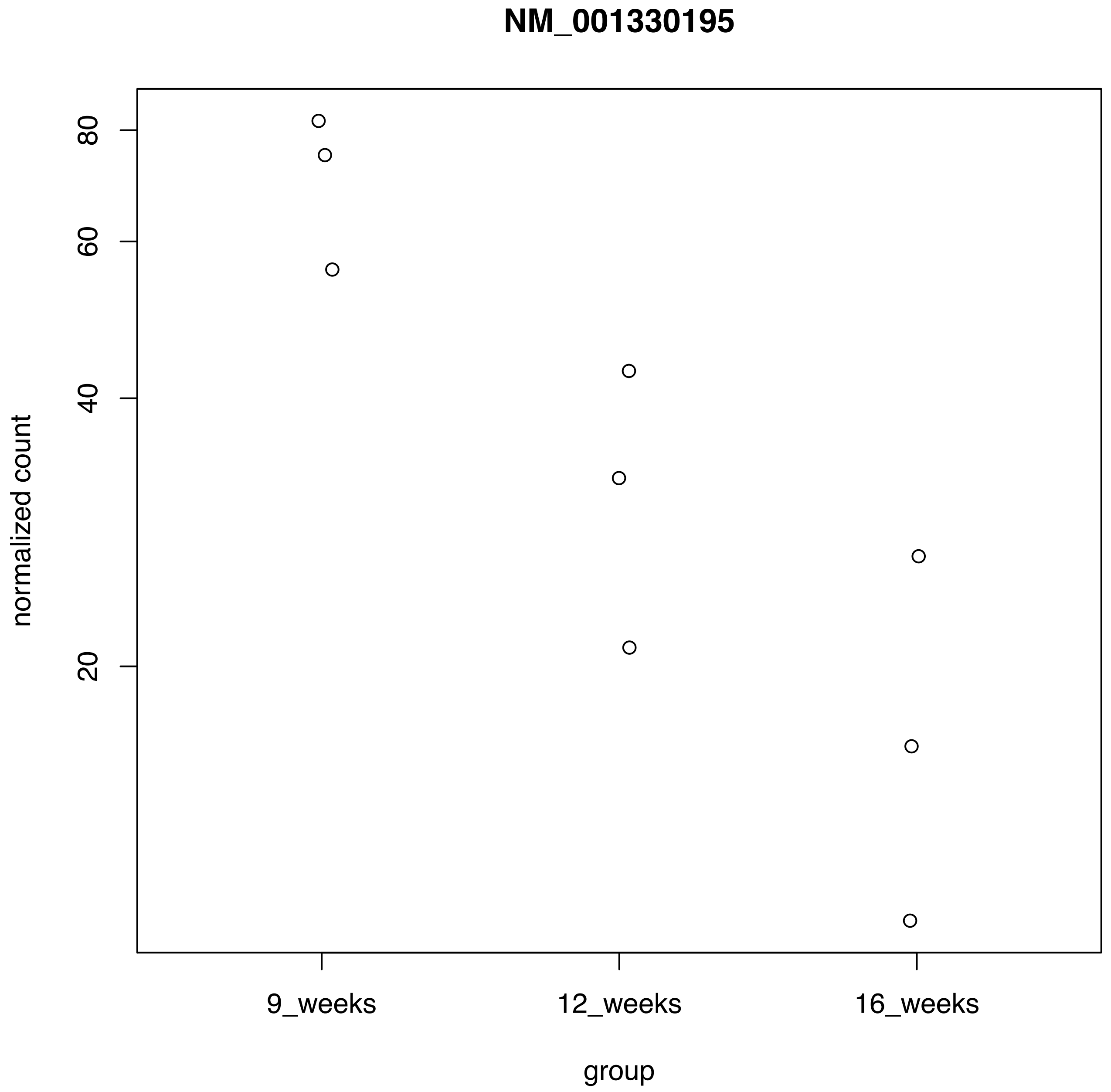
NM_001330195
normalized count
20
40
60
80
9_weeks
12_weeks
16_weeks
group

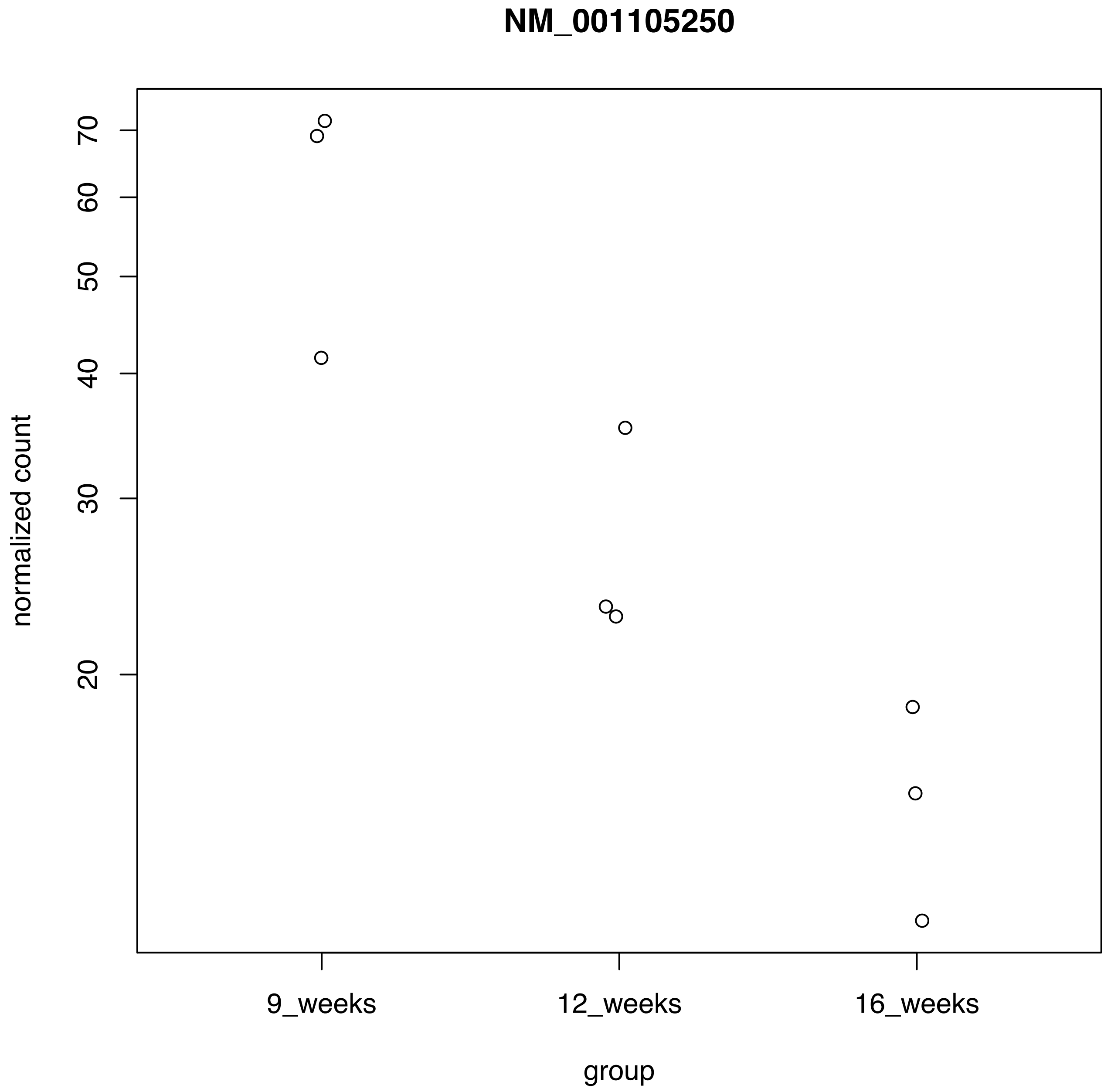

NM_001105250
normalized count
70
60
50
40
30
20
9_weeks
12_weeks
16_weeks
group

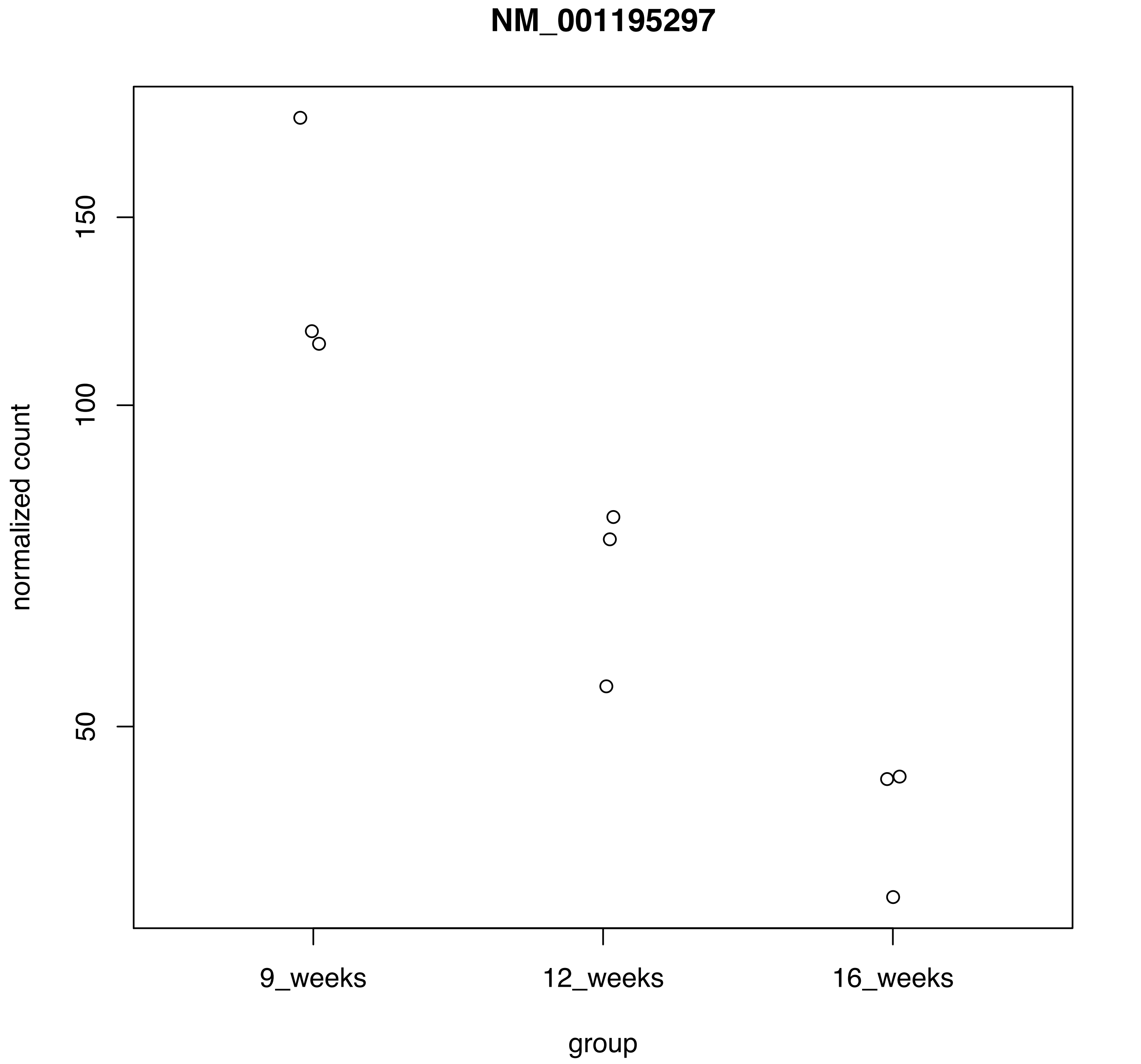
NM_001195297
normalized count
150
100
50
9_weeks
12_weeks
16_weeks
group

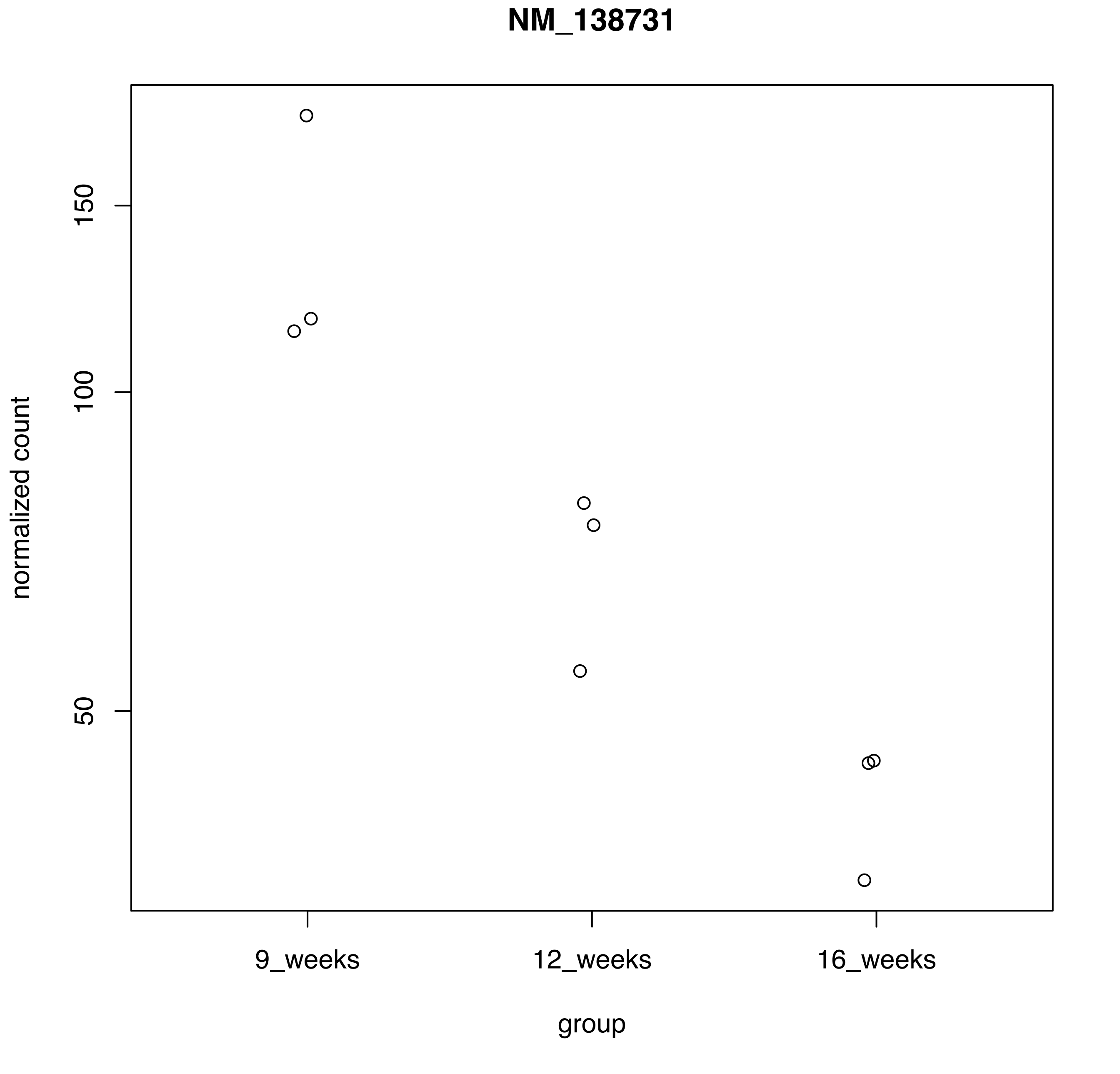

NM_138731
normalized count
150
100
50
9_weeks
12_weeks
16_weeks
group

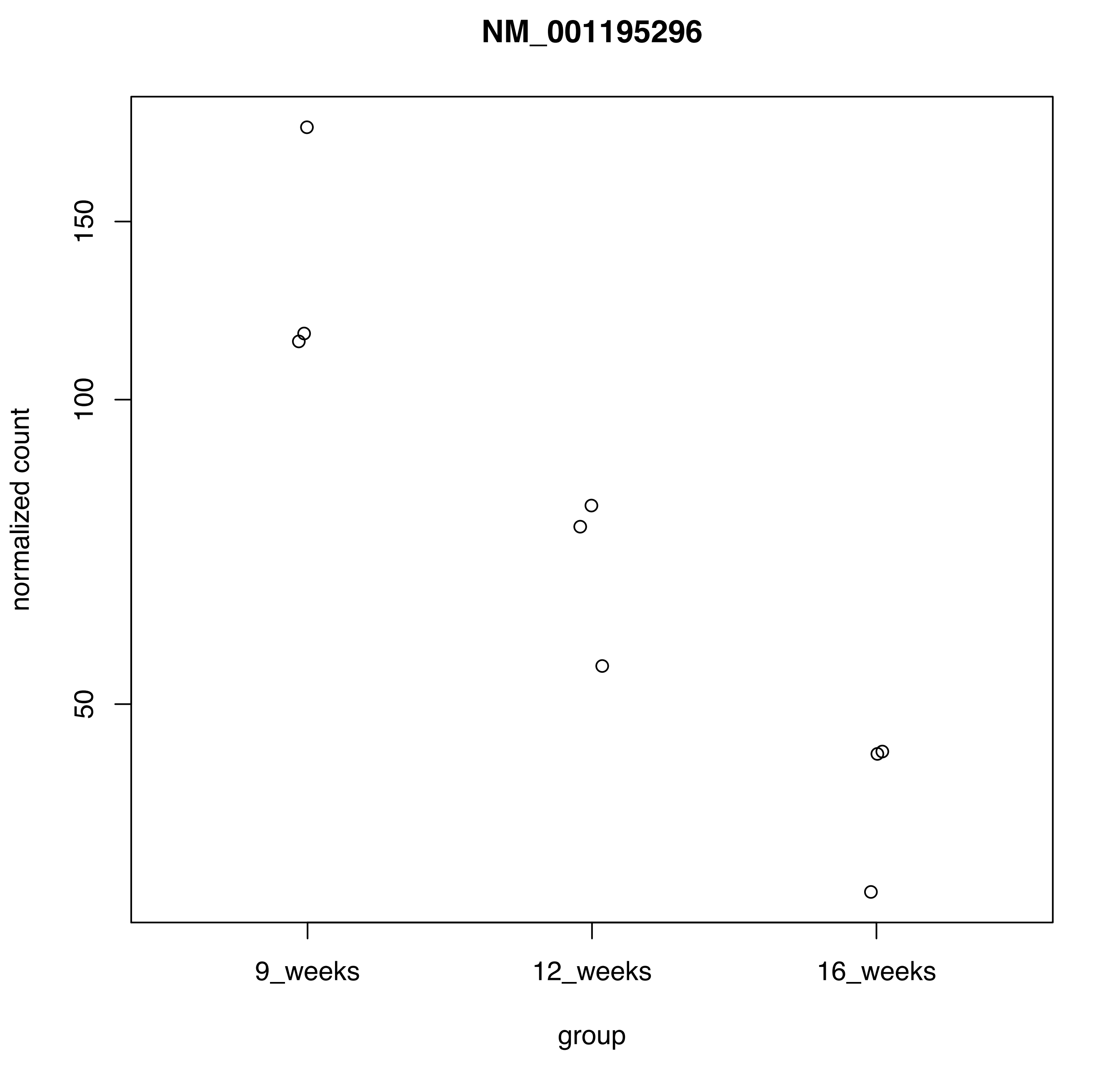
NM_001195296
normalized count
150
100
50
9_weeks
12_weeks
16_weeks
group

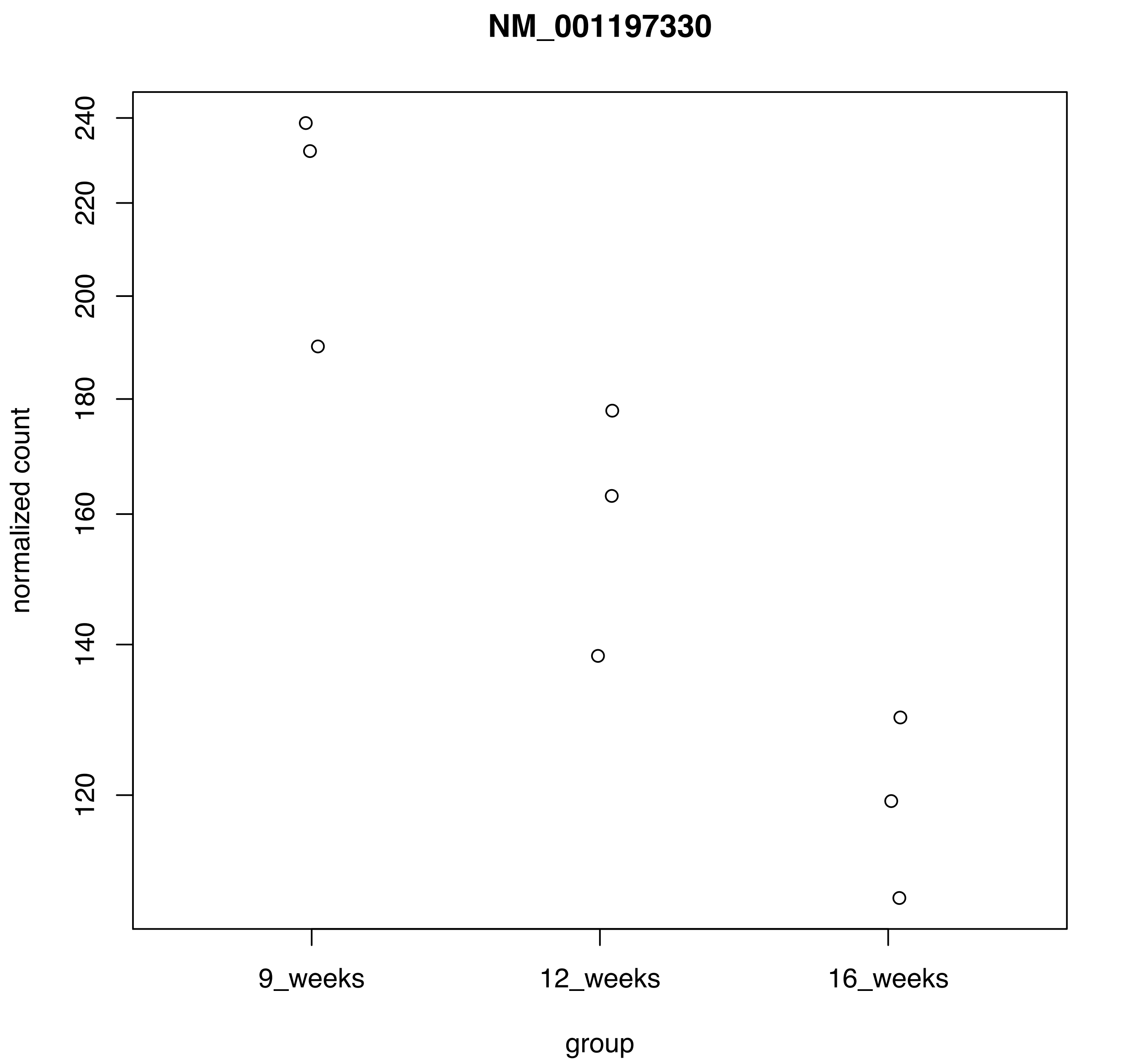
NM_001197330
normalized count
120
140
160
180
200
220
240
9_weeks
12_weeks
16_weeks
group

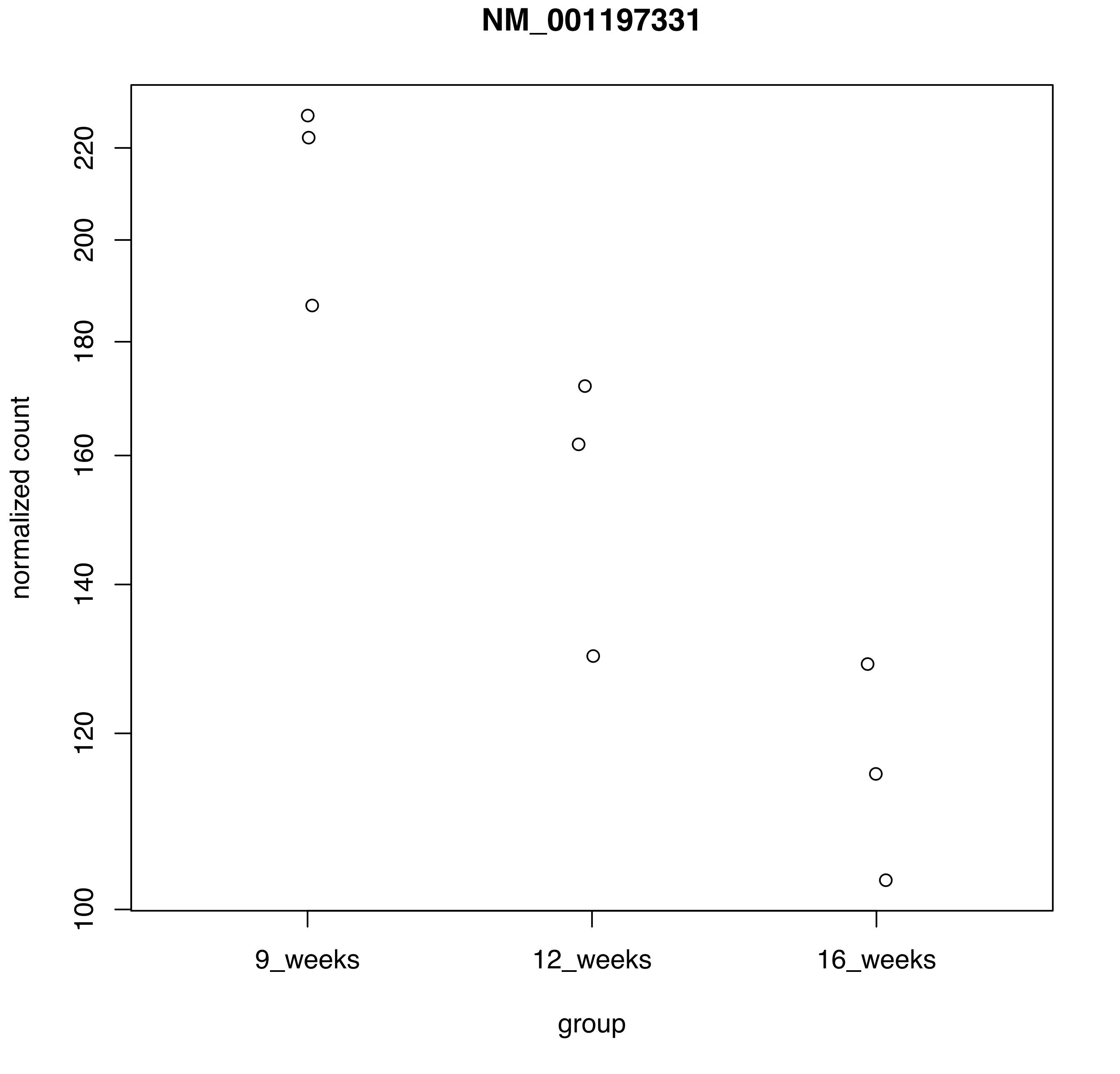
NM_001197331
normalized count
220
200
180
160
140
120
100
9_weeks
12_weeks
16_weeks
group

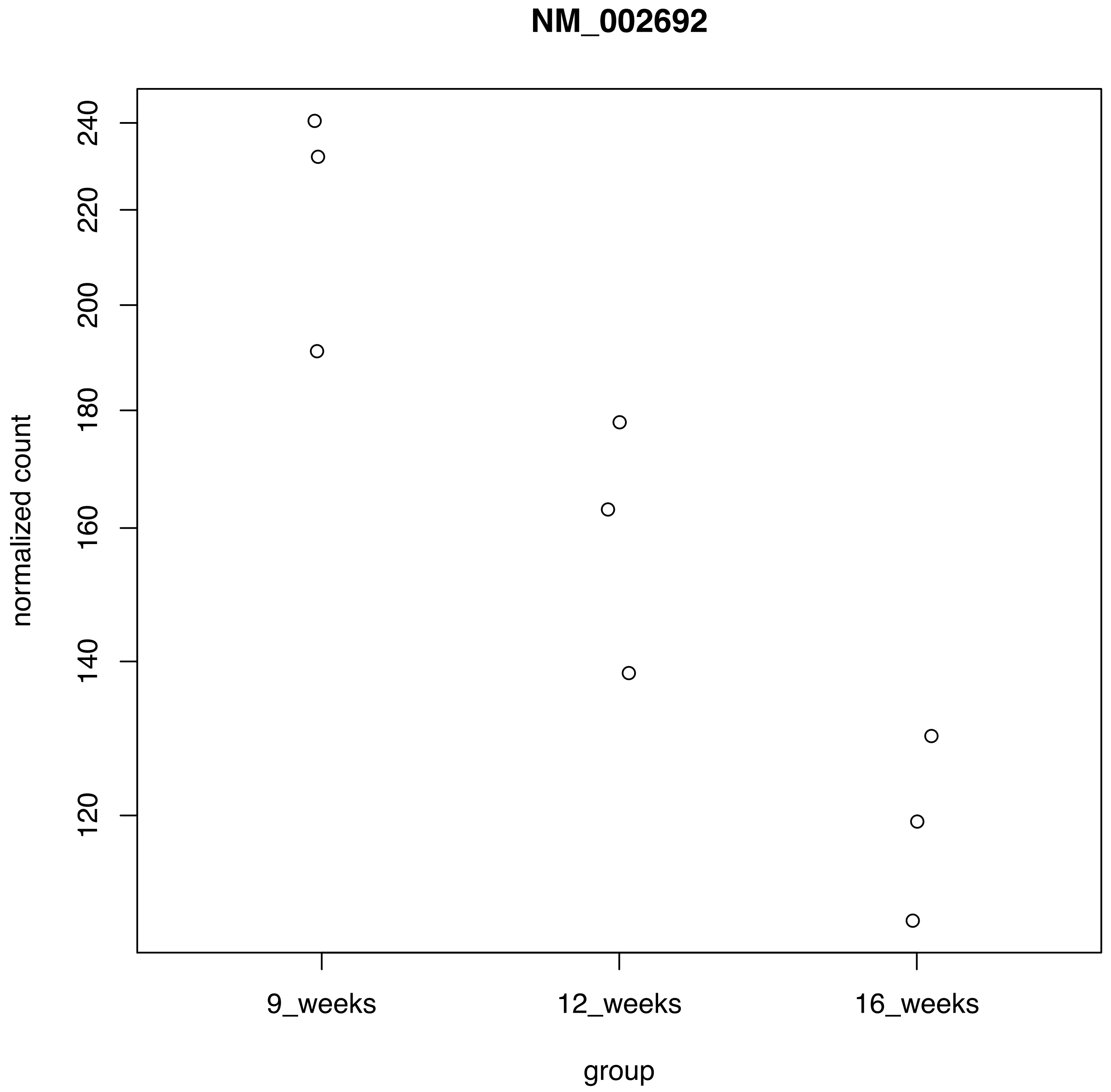
NM_002692
normalized count
240
220
200
180
160
140
120
9_weeks
12_weeks
16_weeks
group

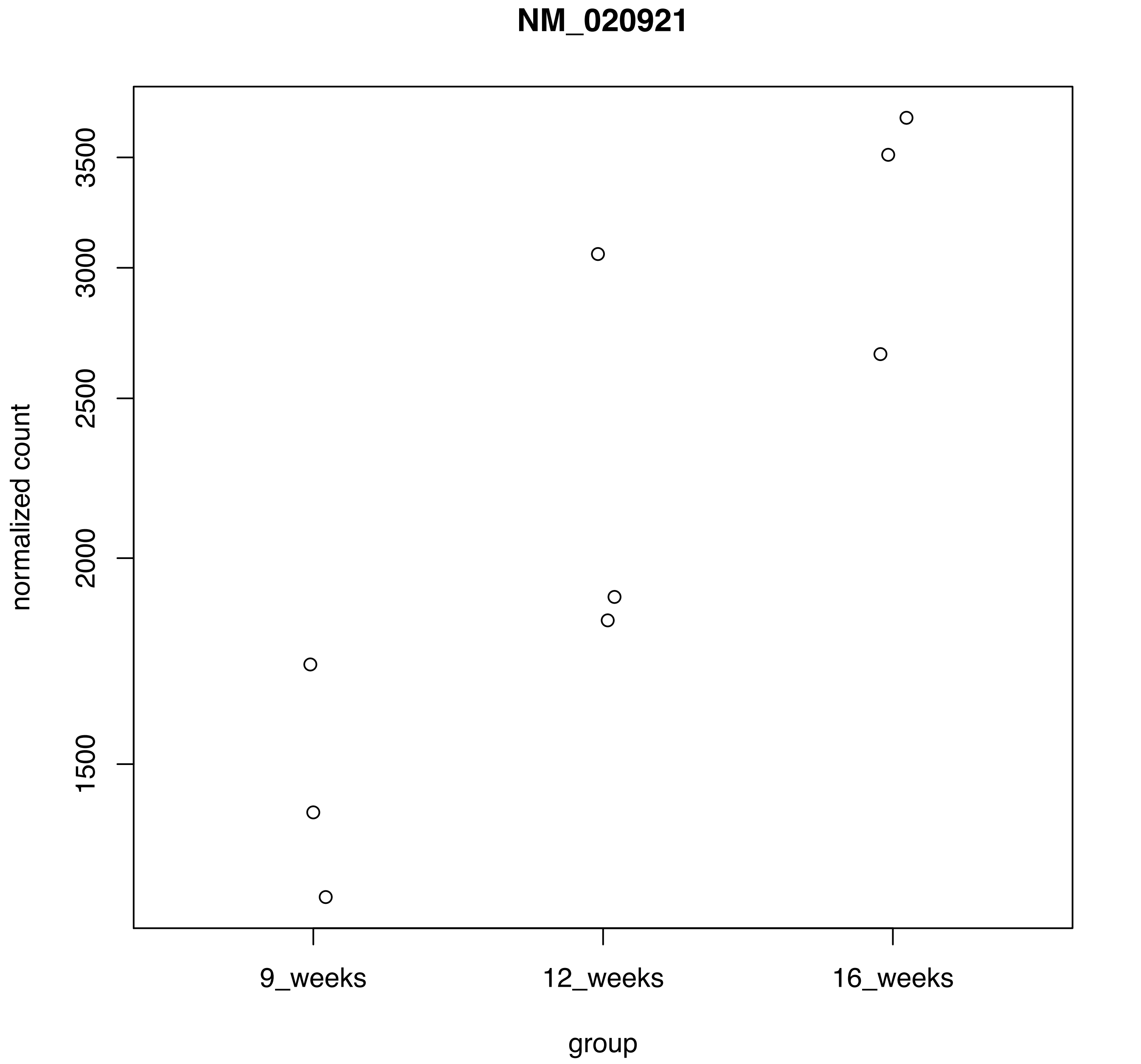
NM_020921
normalized count
1500
2000
2500
3000
3500
9_weeks
12_weeks
16_weeks
group

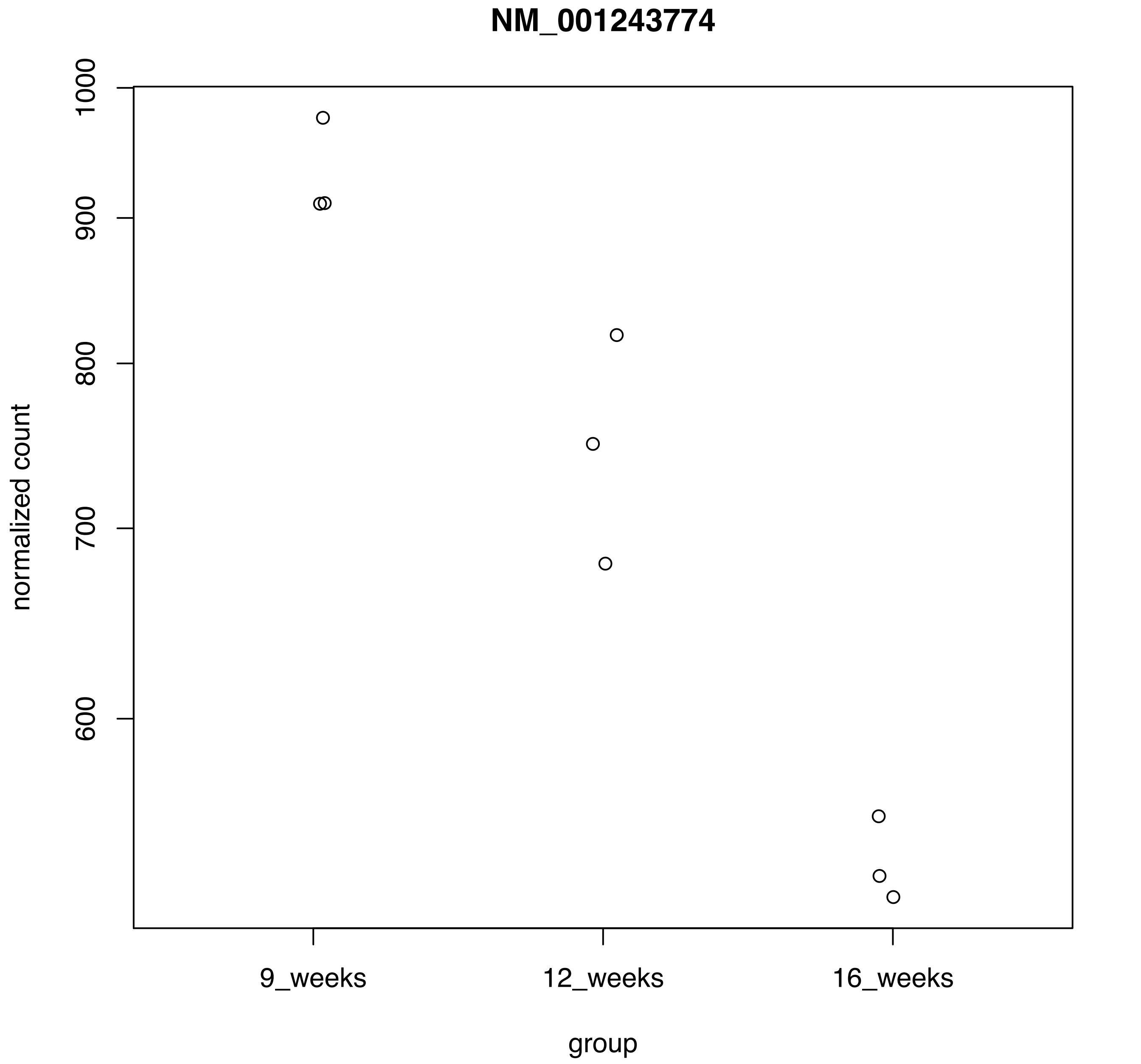
NM_001243774
normalized count
1000
900
800
700
600
9_weeks
12_weeks
16_weeks
group

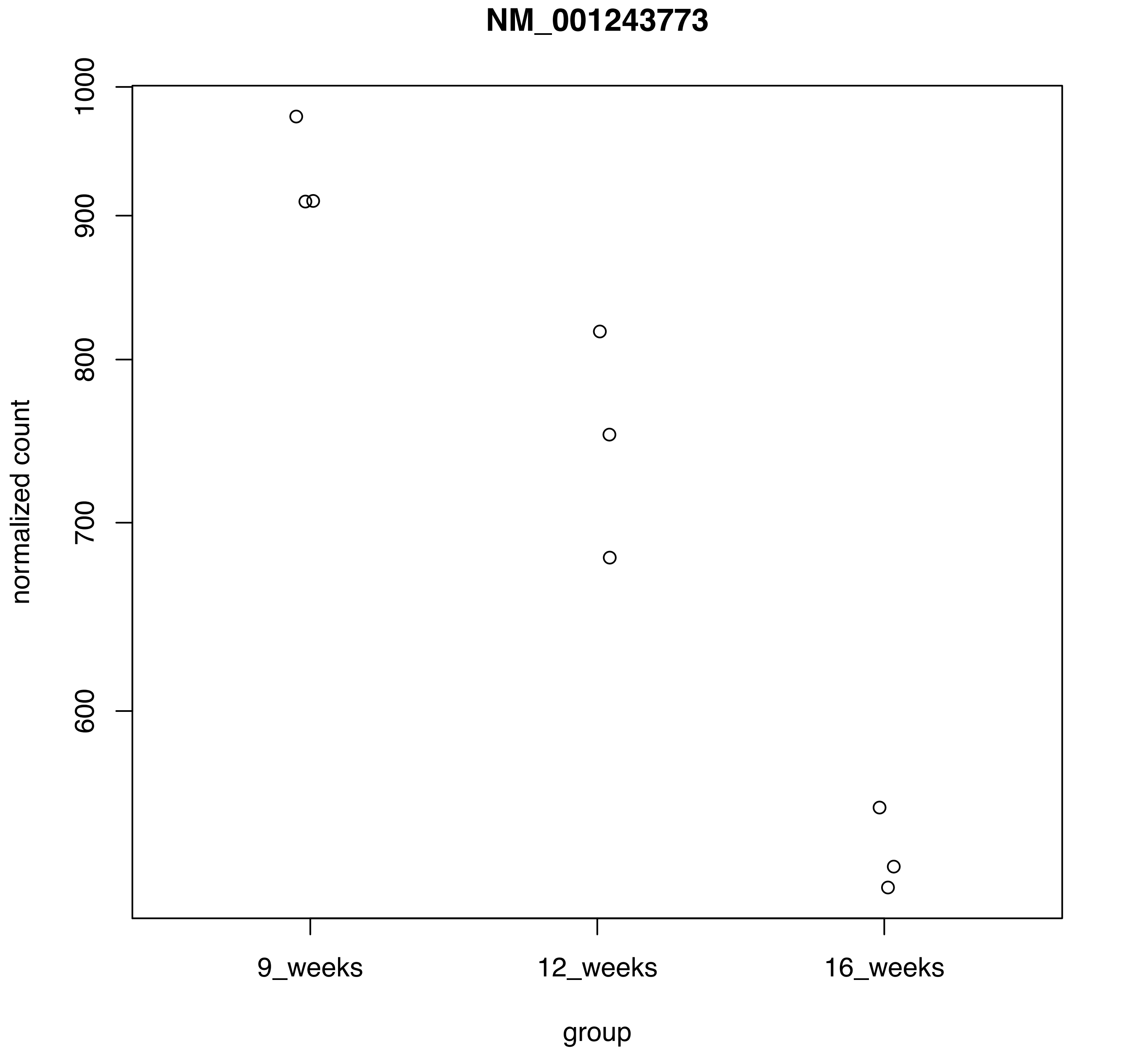
NM_001243773
normalized count
1000
900
800
700
600
9_weeks
12_weeks
16_weeks
group

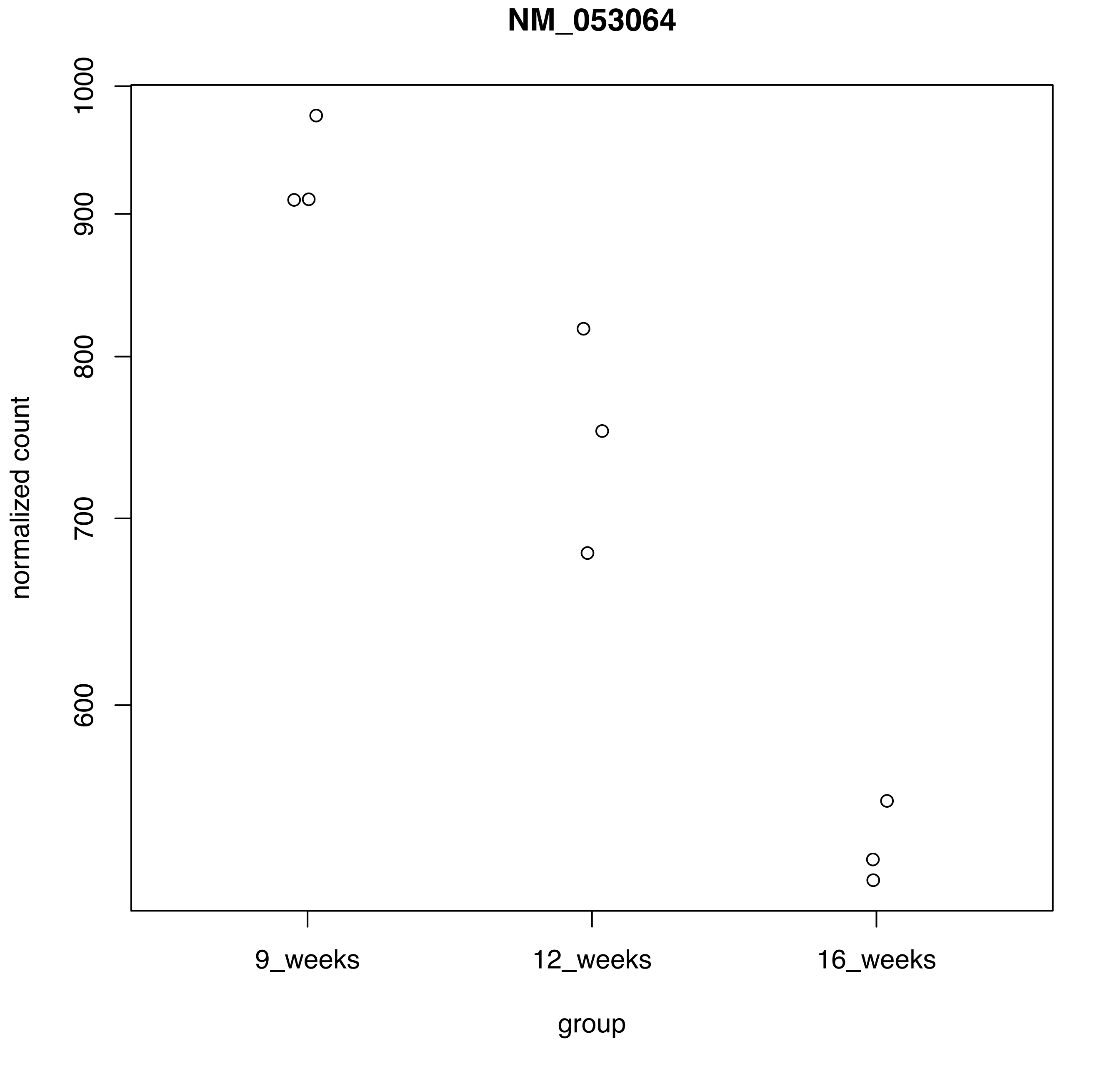

NM_053064
normalized count
1000
900
800
700
600
9_weeks
12_weeks
16_weeks
group

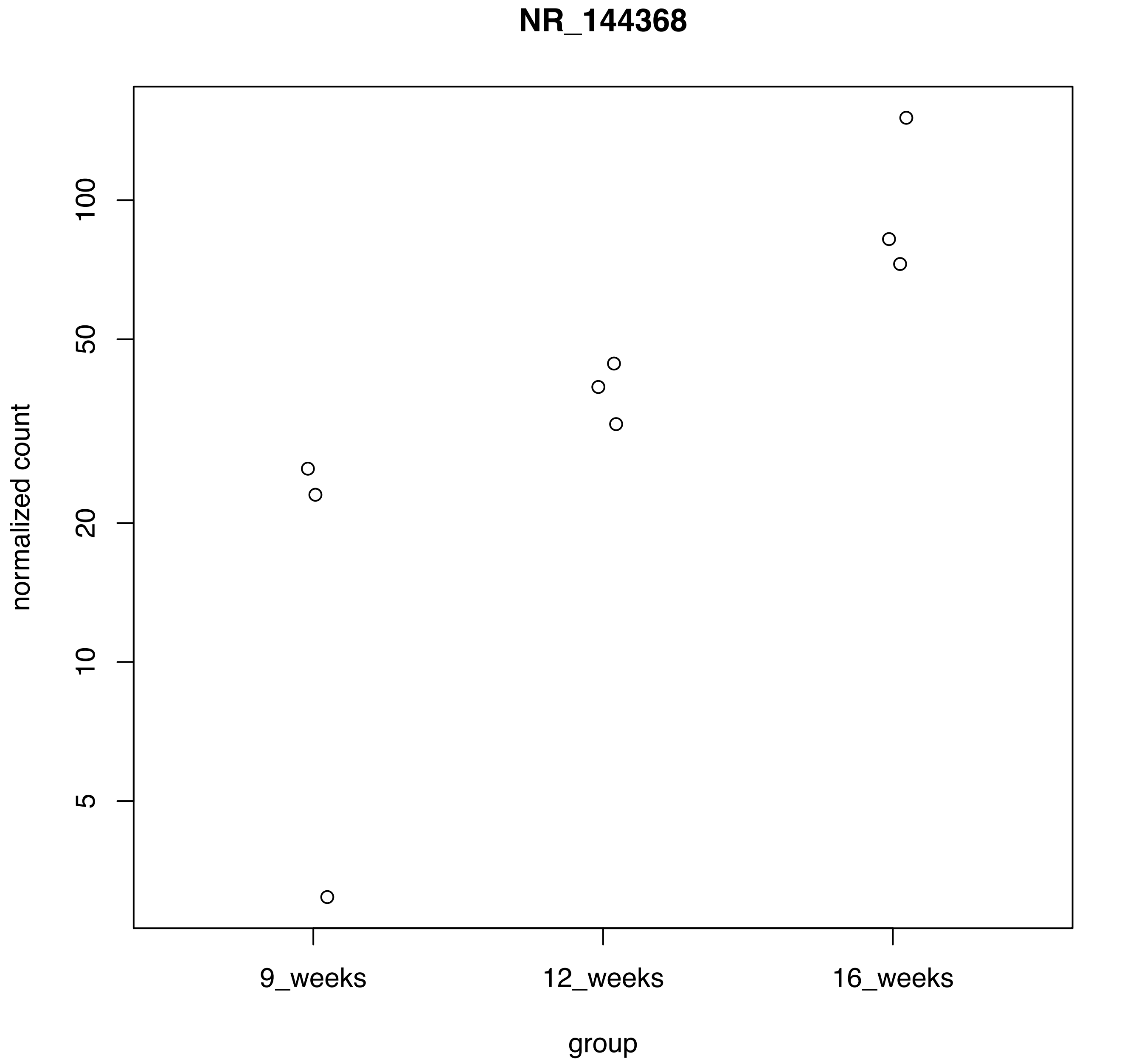

NR_144368
normalized count
100
50
20
10
5
9_weeks
12_weeks
16_weeks
group

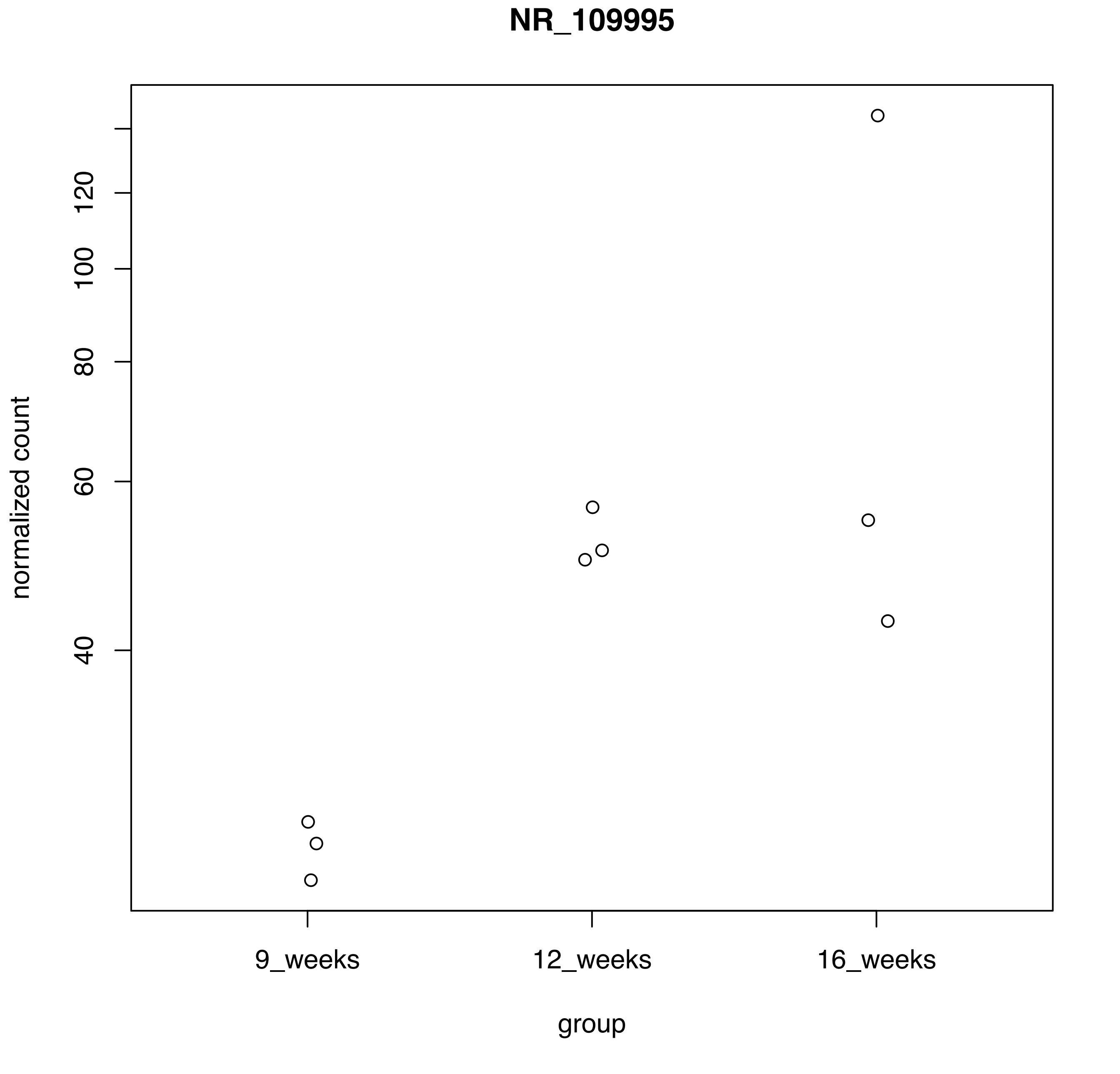

NR_109995
normalized count
120
100
80
60
40
9_weeks
12_weeks
16_weeks
group

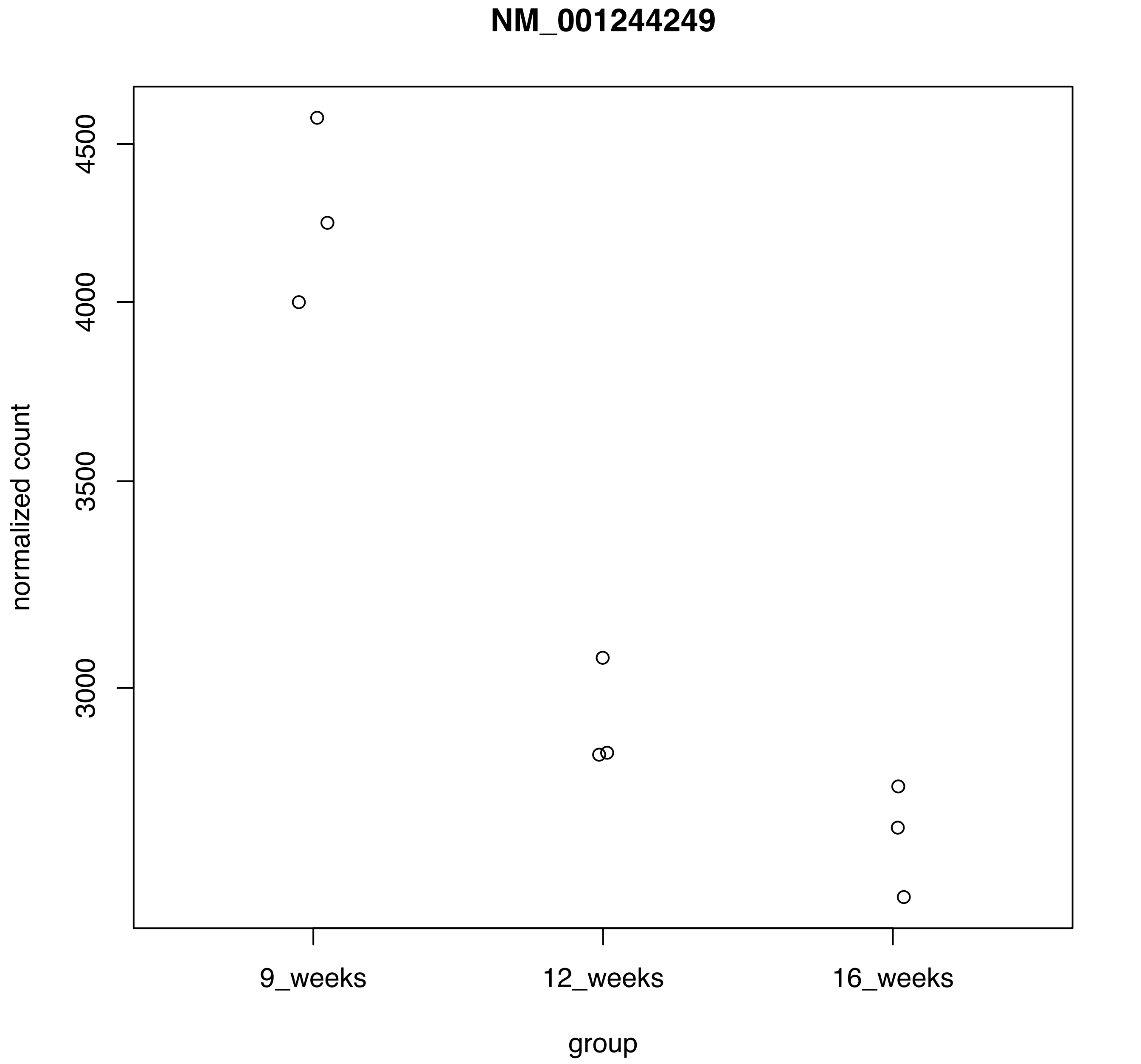

NM_001244249
normalized count
4500
4000
3500
3000
9_weeks
12_weeks
16_weeks
group

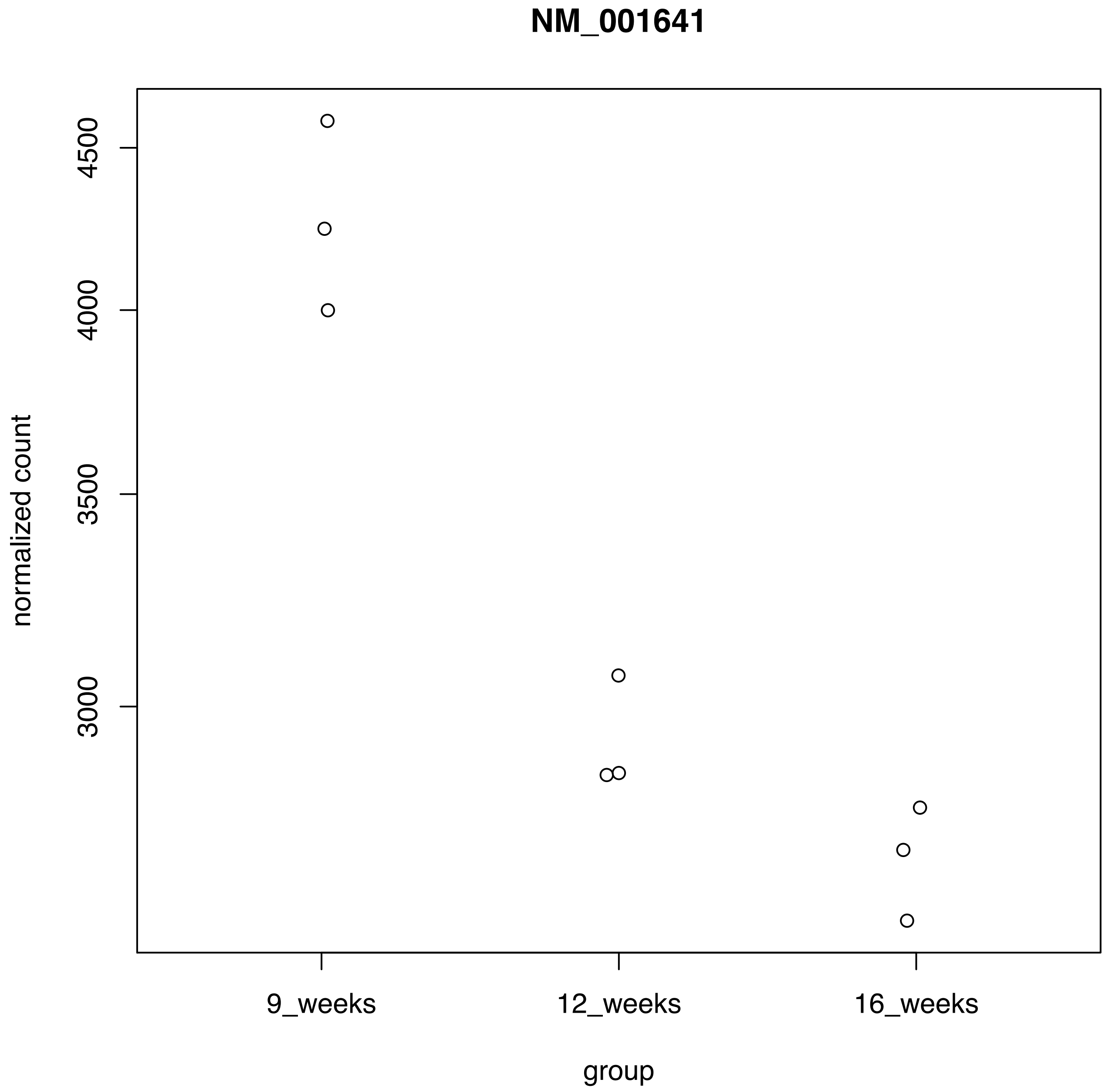
NM_001641
normalized count
4500
4000
3500
3000
9_weeks
12_weeks
16_weeks
group

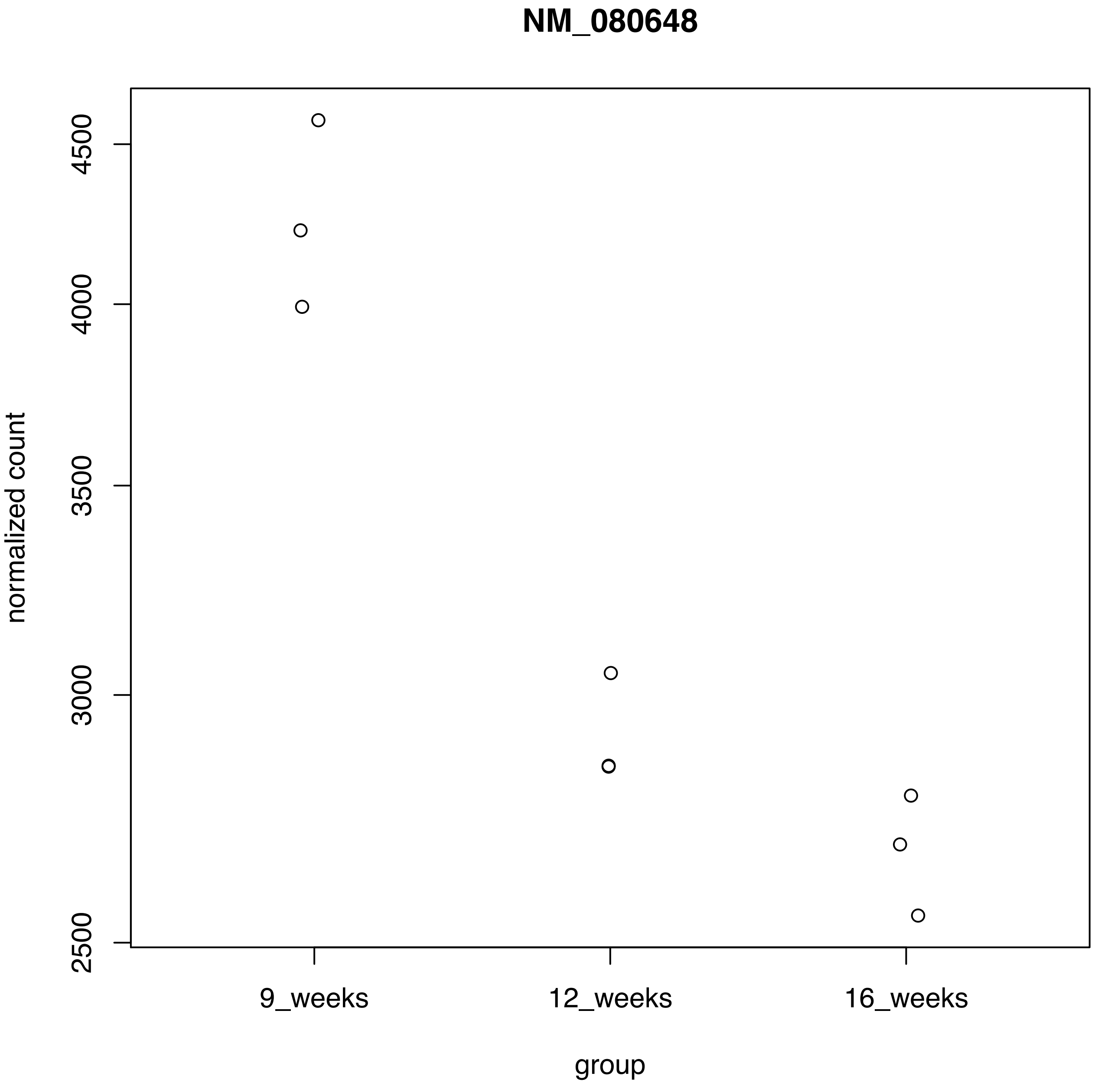

NM_080648
normalized count
4500
4000
3500
3000
2500
9_weeks
12_weeks
16_weeks
group

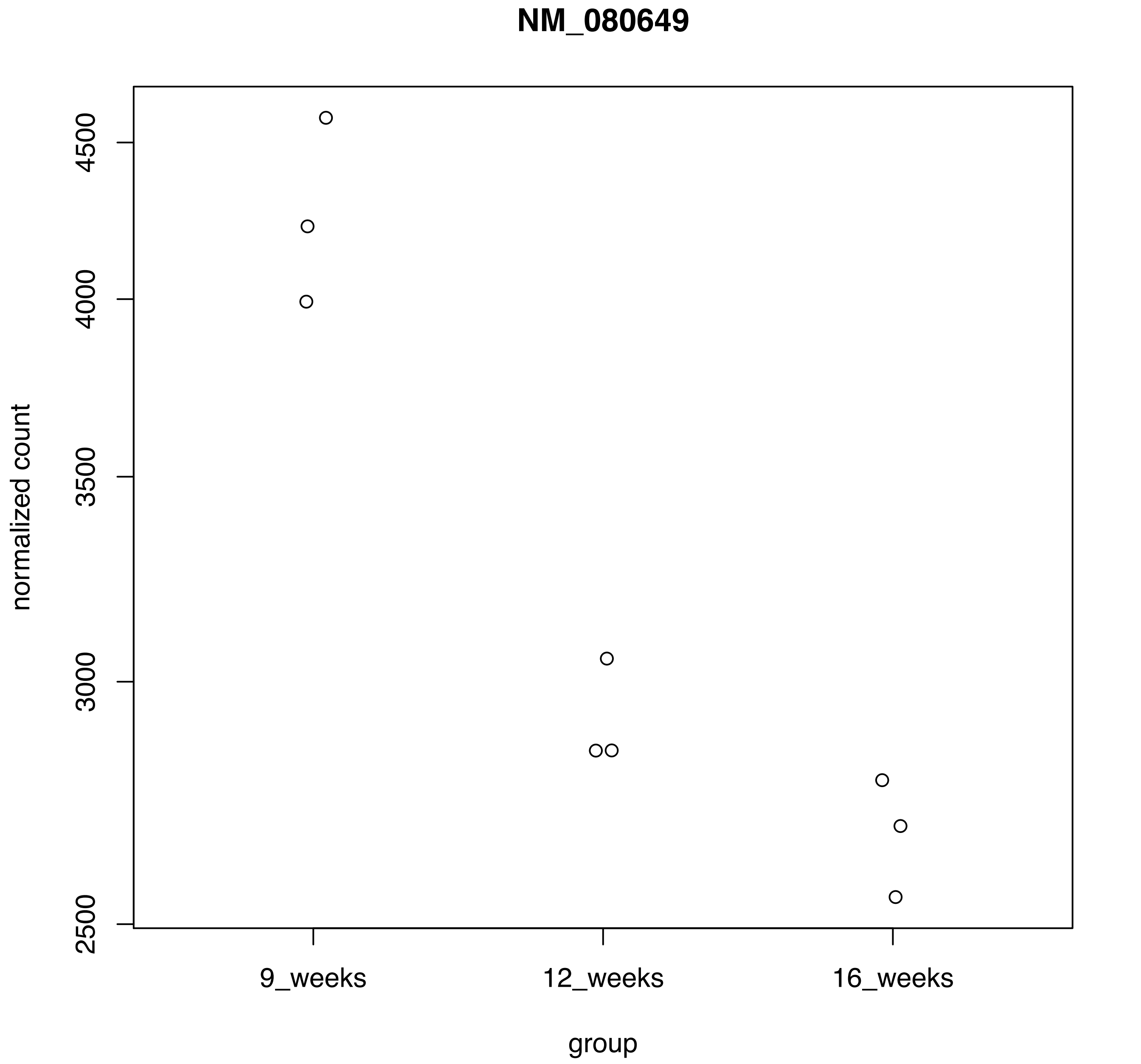
NM_080649
normalized count
4500
4000
3500
3000
2500
9_weeks
12_weeks
16_weeks
group

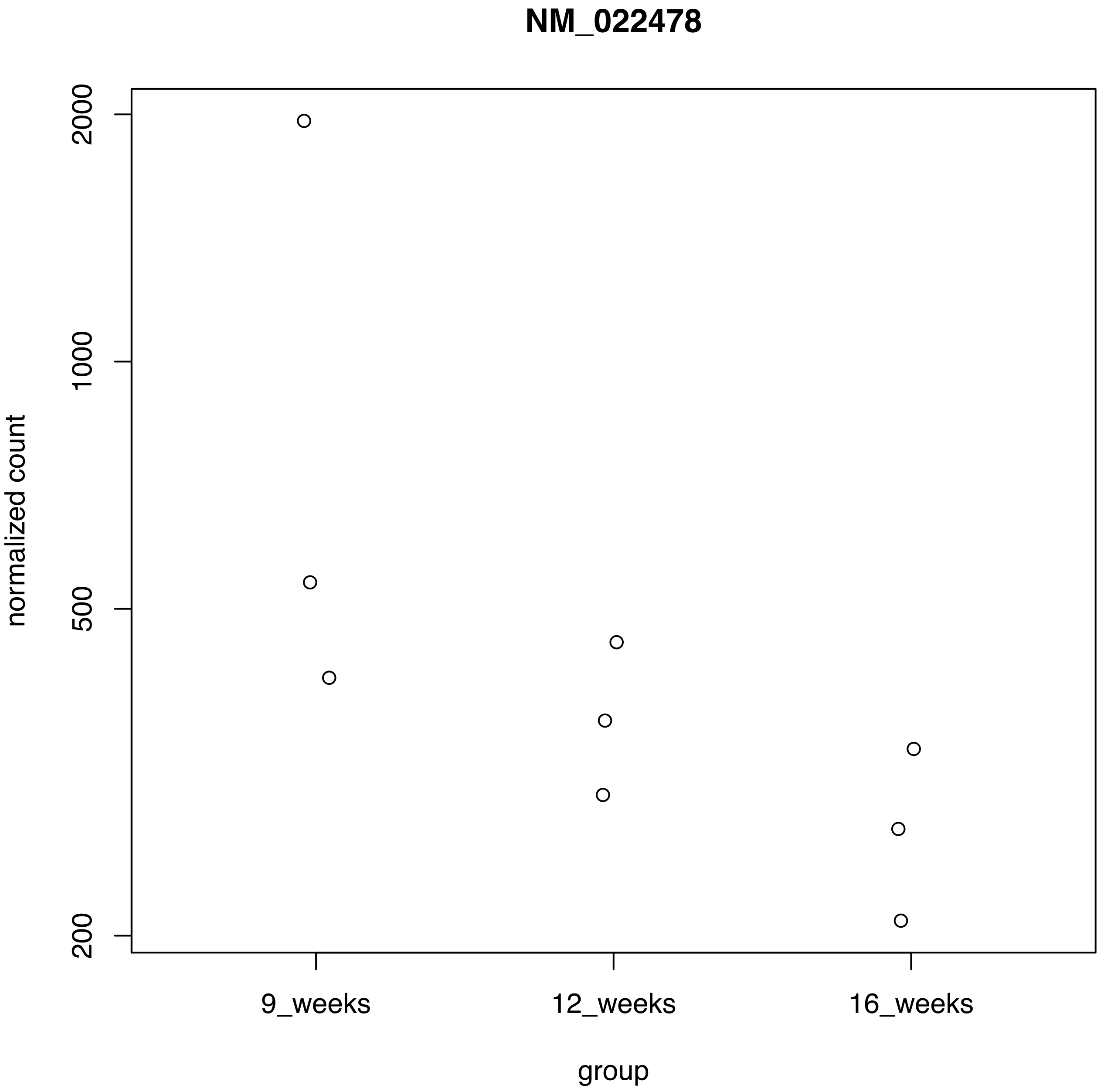
NM_022478
2000
1000
500
200
normalized count
9_weeks
12_weeks
16_weeks
group

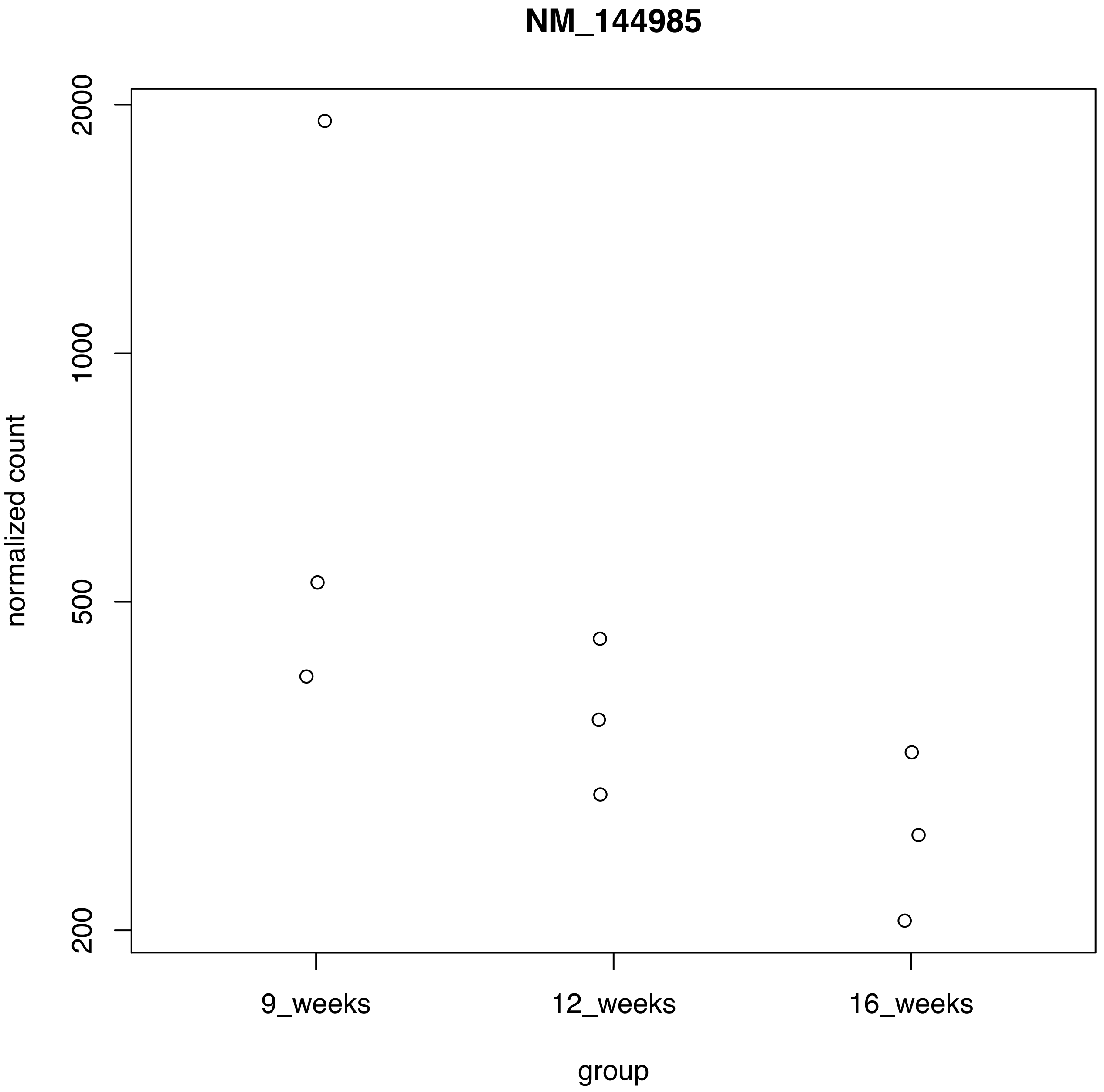
NM_144985
normalized count
2000
1000
500
200
9_weeks
12_weeks
16_weeks
group

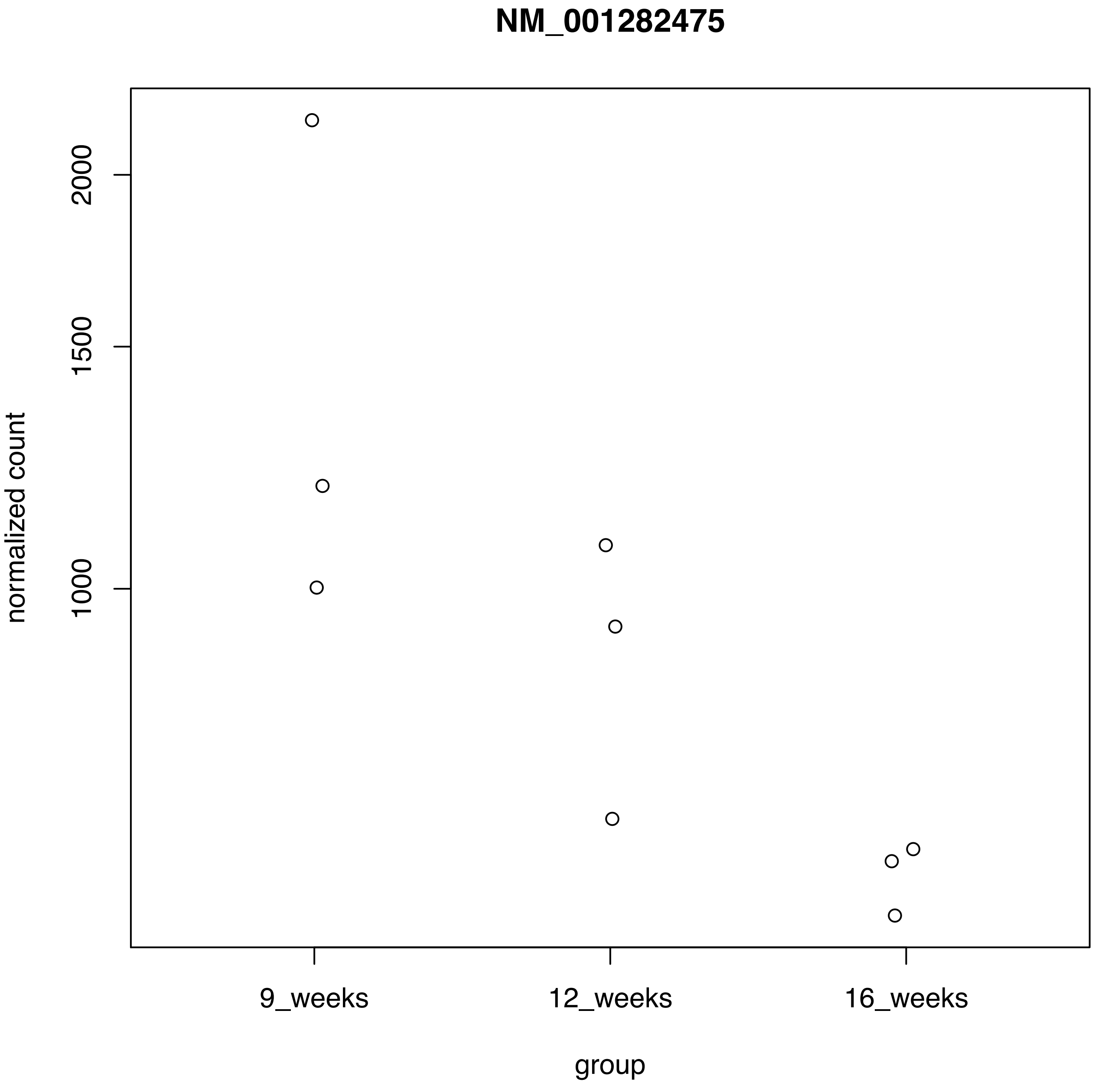
NM_001282475
normalized count
2000
1500
1000
9_weeks
12_weeks
16_weeks
group

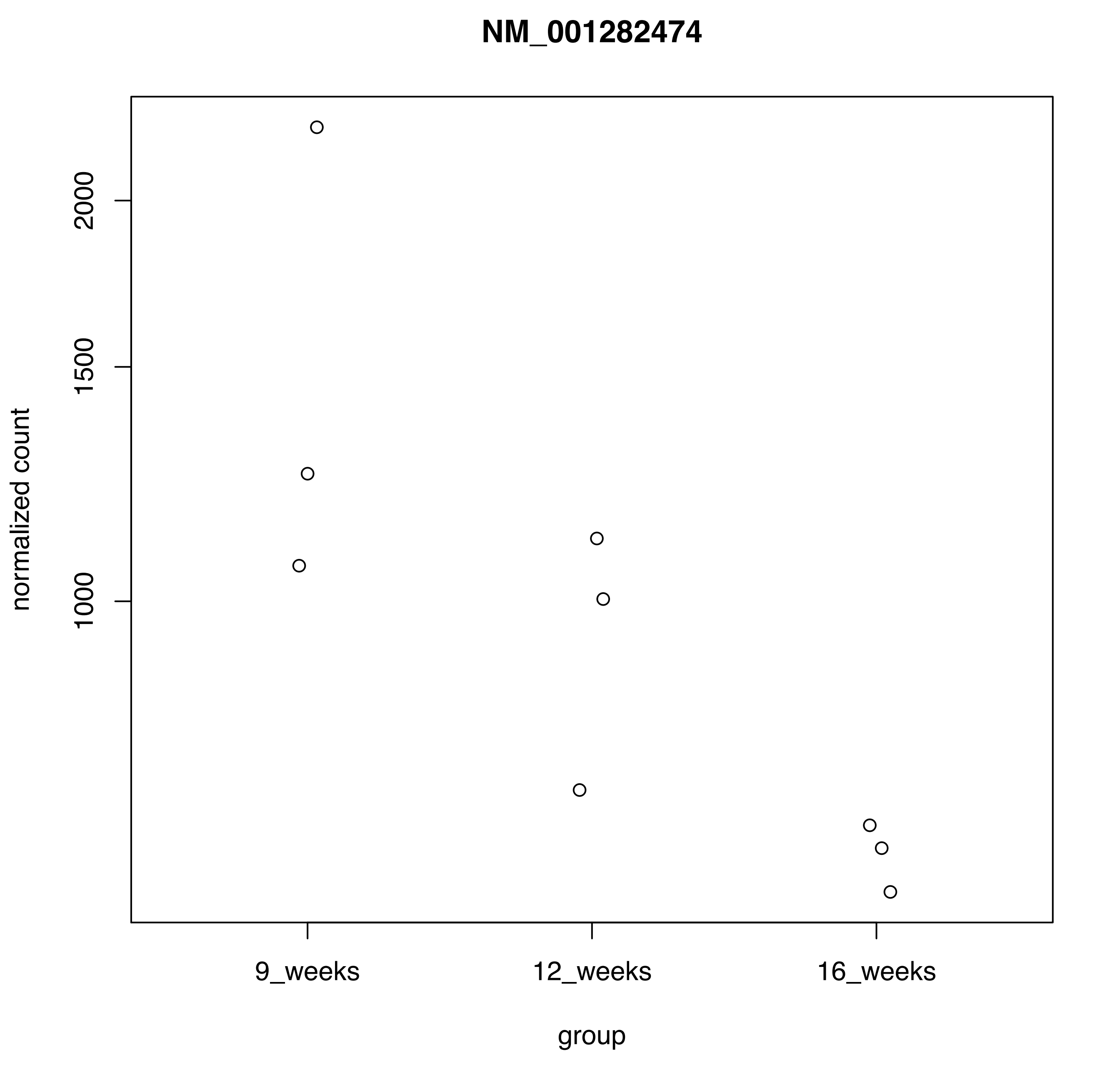

NM_001282474
normalized count
2000
1500
1000
9_weeks
12_weeks
16_weeks
group

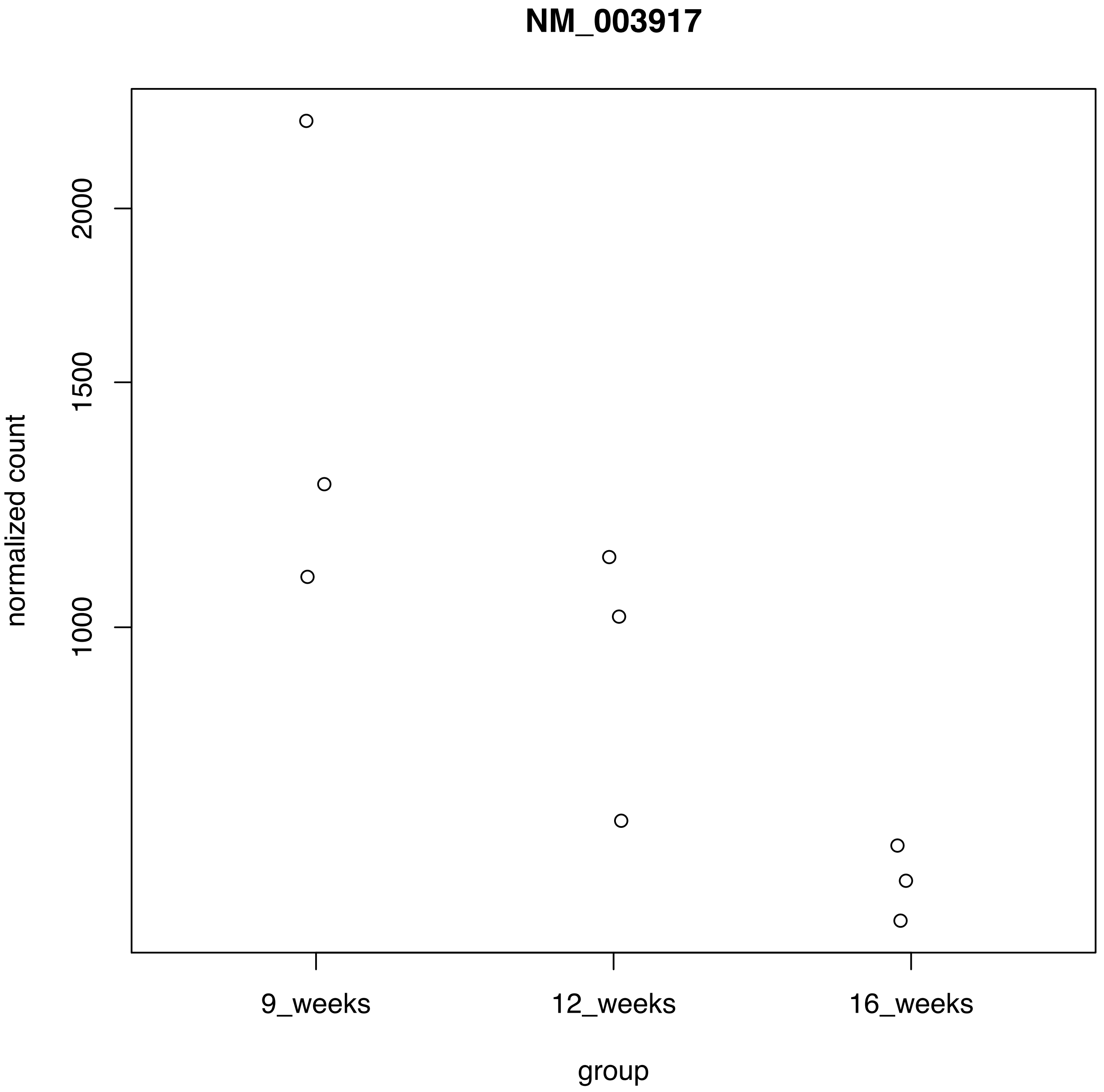

NM_003917
normalized count
2000
1500
1000
9_weeks
12_weeks
16_weeks
group

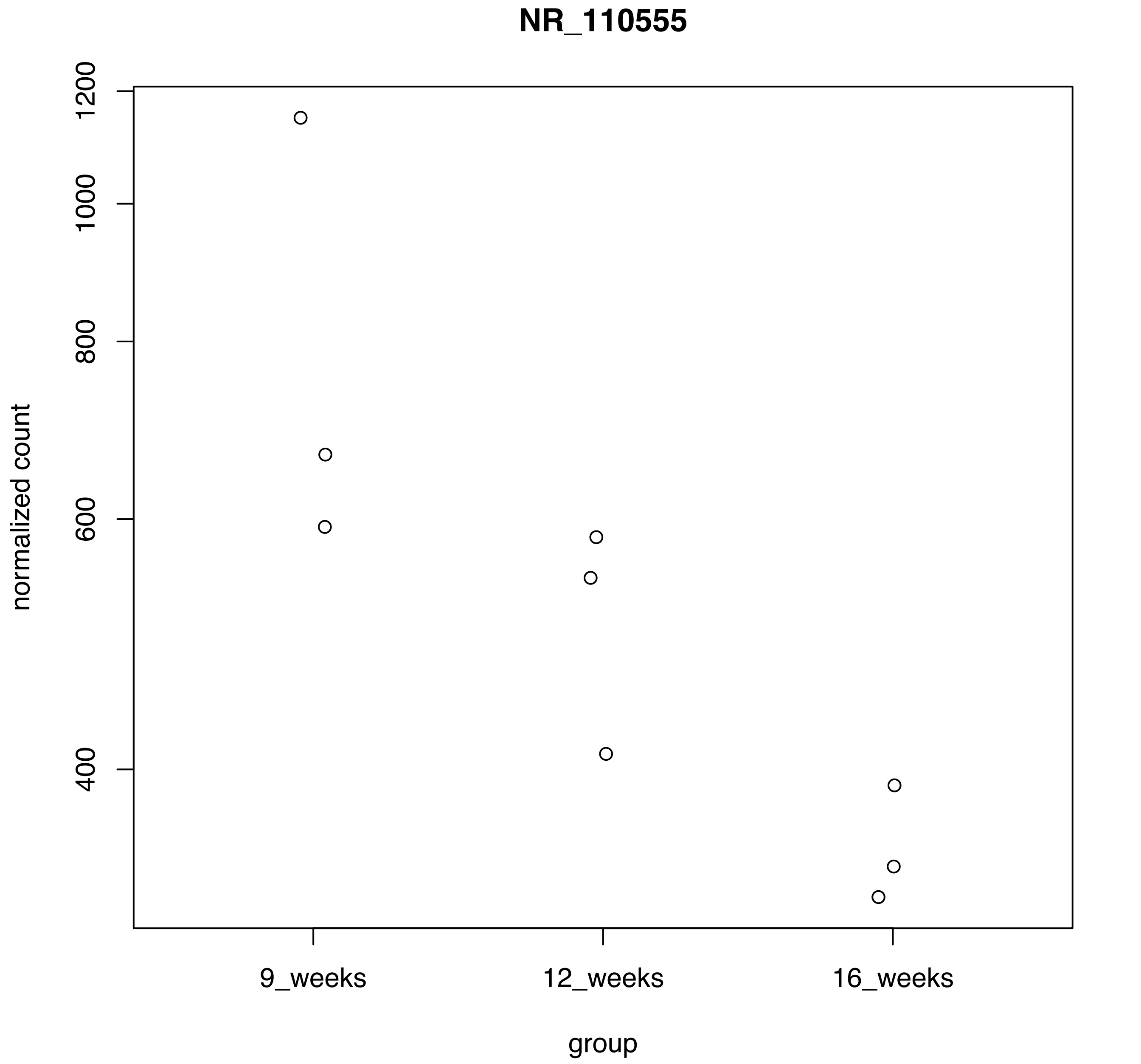
NR_110555
normalized count
1200
1000
800
600
400
9_weeks
12_weeks
16_weeks
group

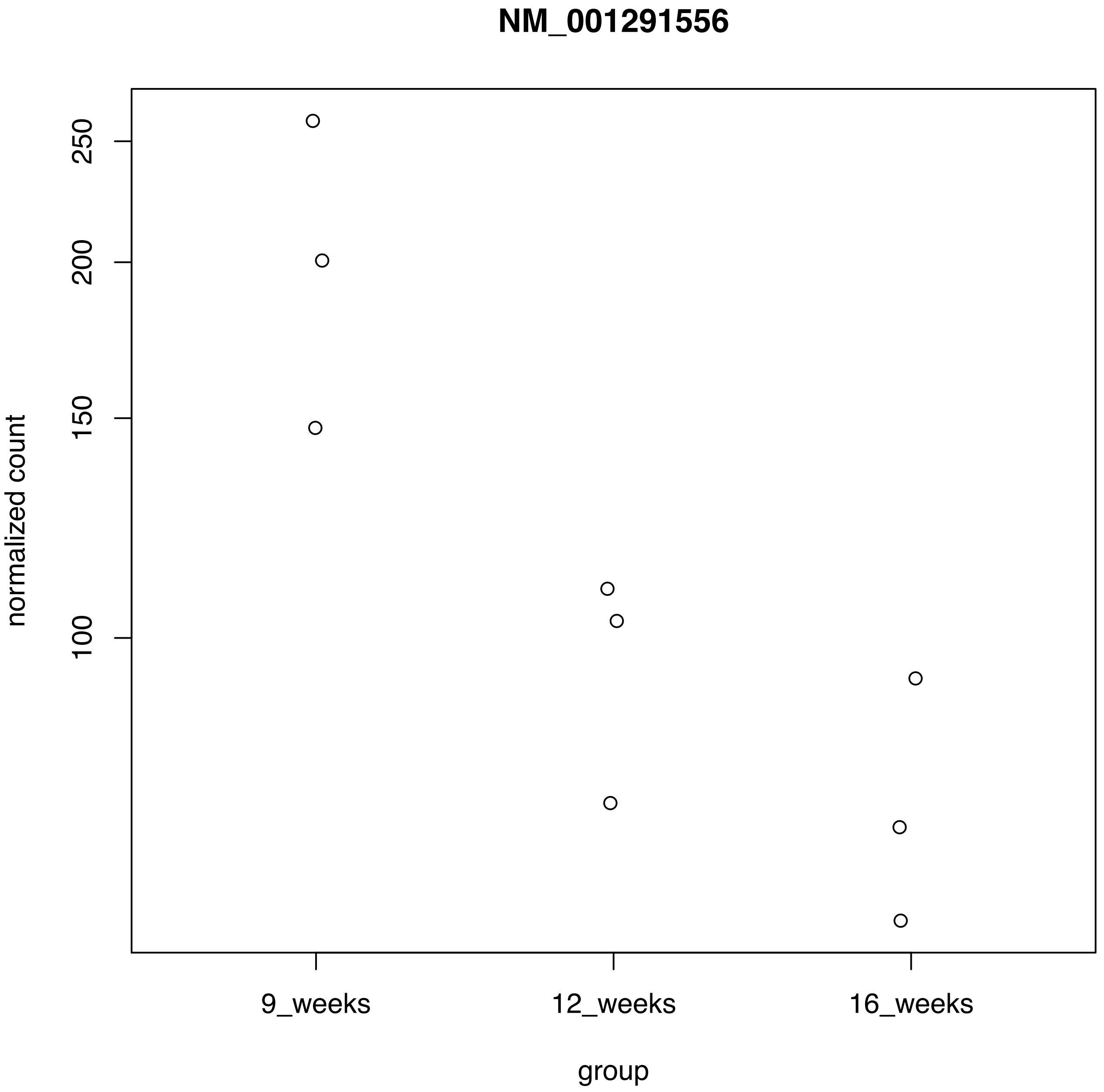
NM_001291556
normalized count
250
200
150
100
9_weeks
12_weeks
16_weeks
group

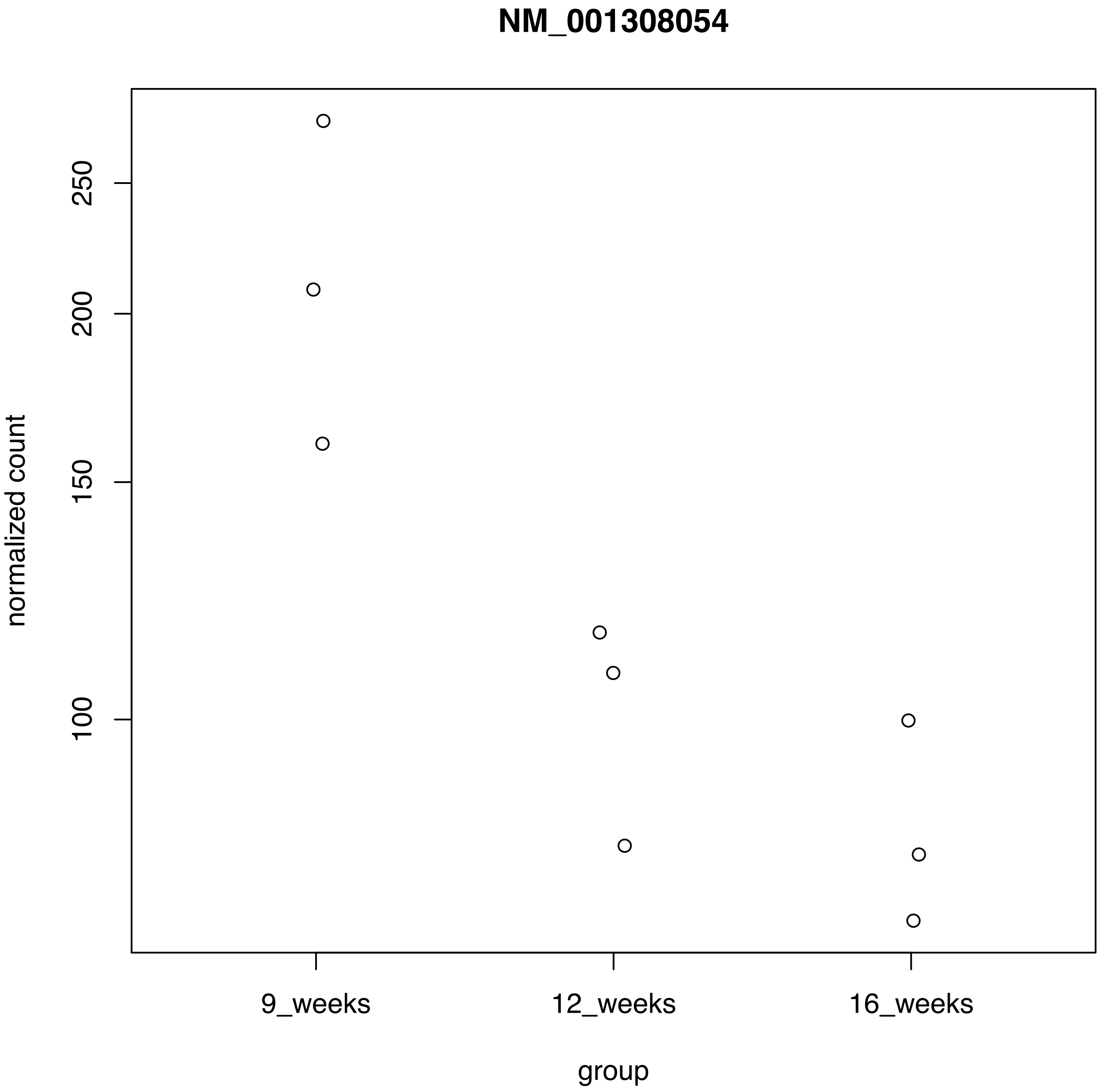
NM_001308054
normalized count
250
200
150
100
9_weeks
12_weeks
16_weeks
group

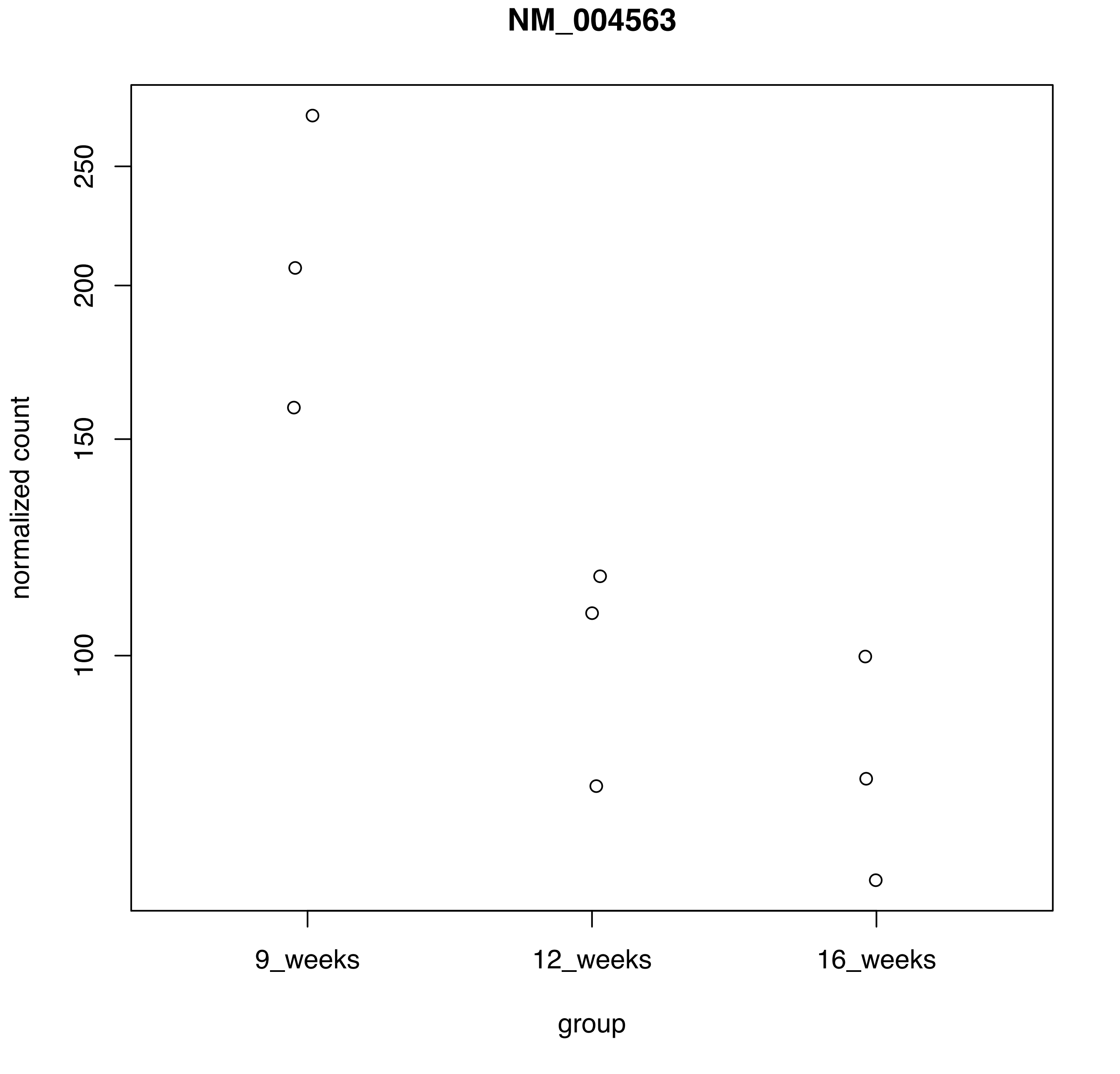
NM_004563
normalized count
100
150
200
250
9_weeks
12_weeks
16_weeks
group

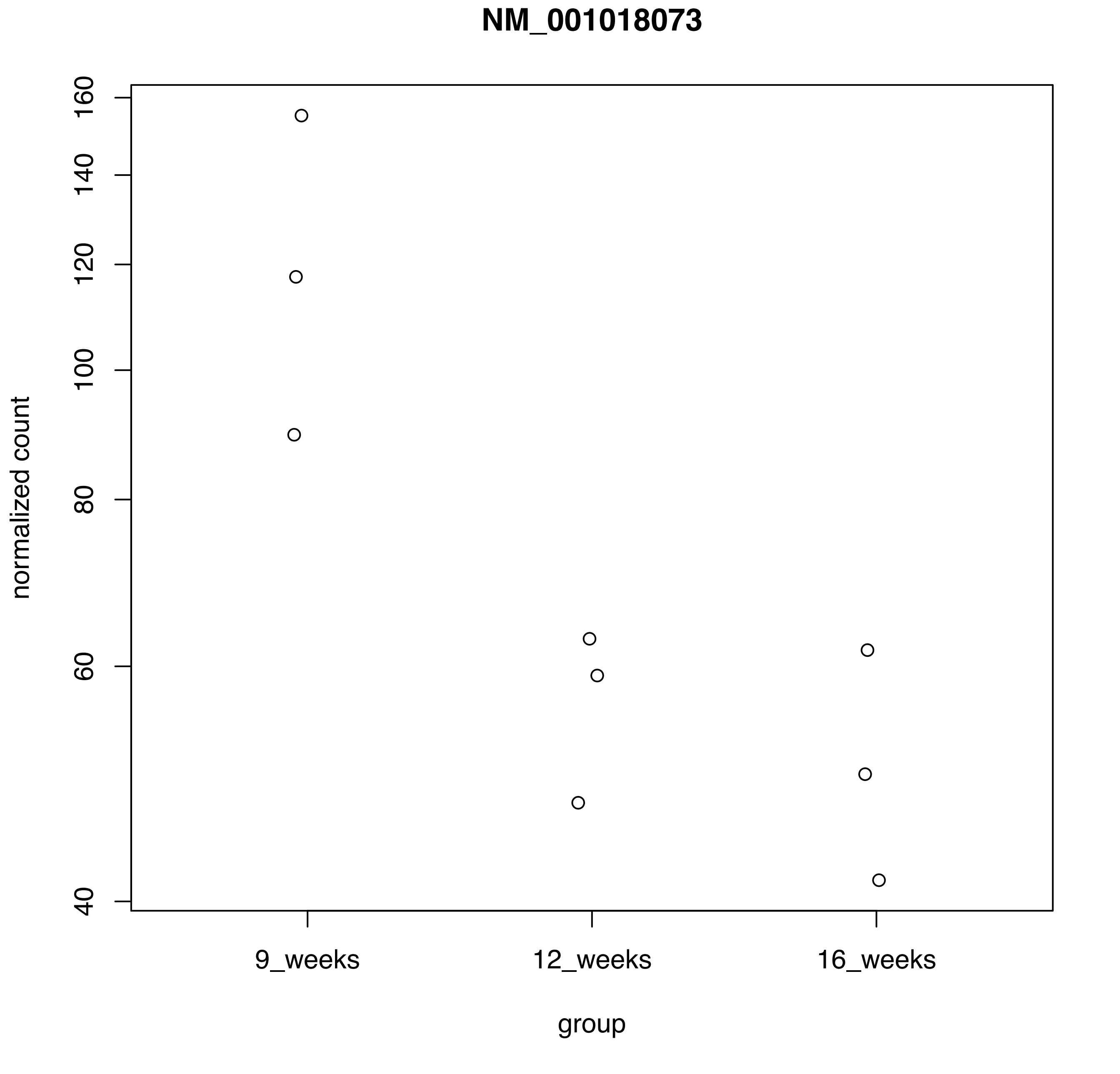
NM_001018073
normalized count
160
140
120
100
80
60
40
9_weeks
12_weeks
16_weeks
group

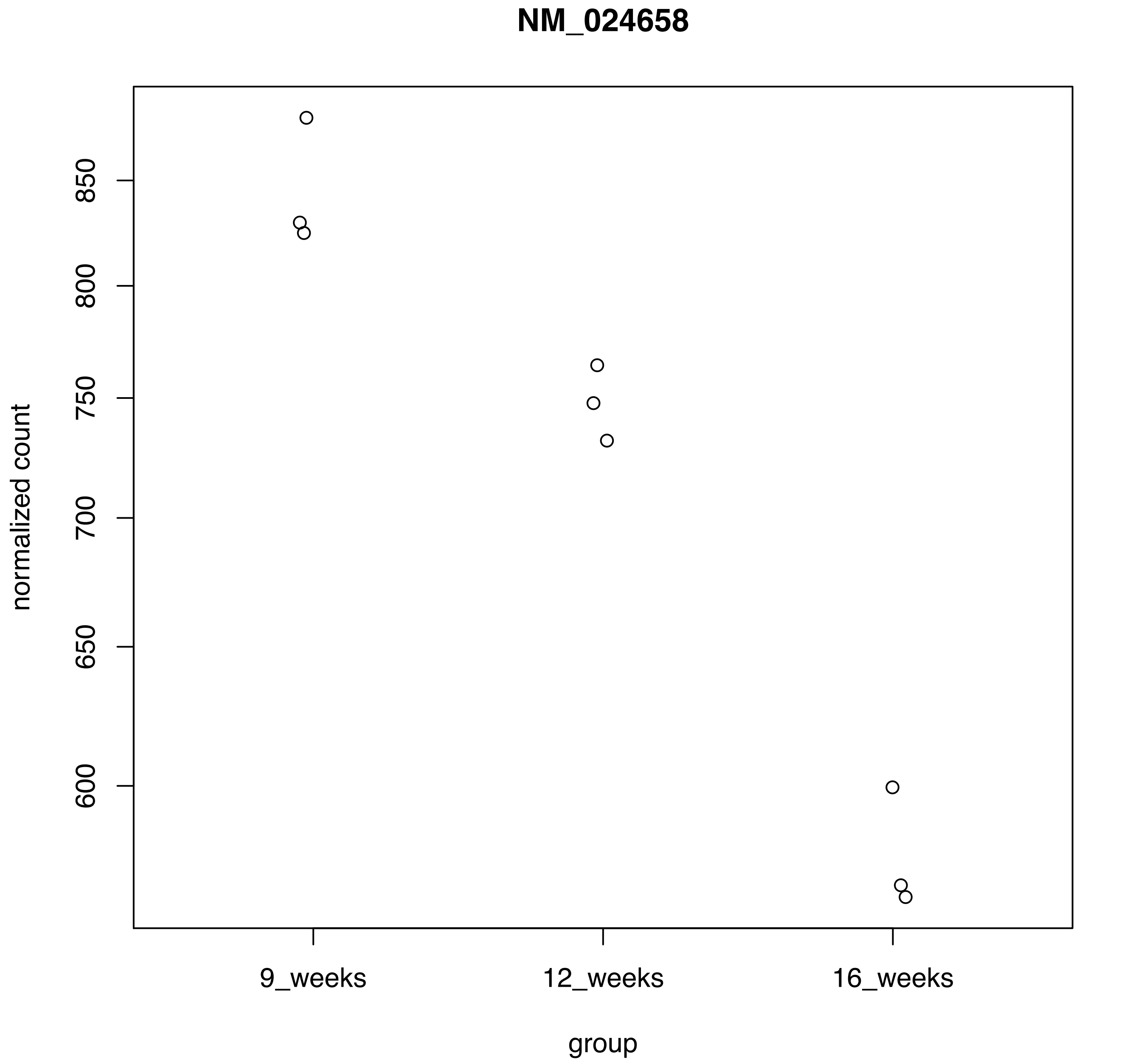
NM_024658
normalized count
850
800
750
700
650
600
9_weeks
12_weeks
16_weeks
group

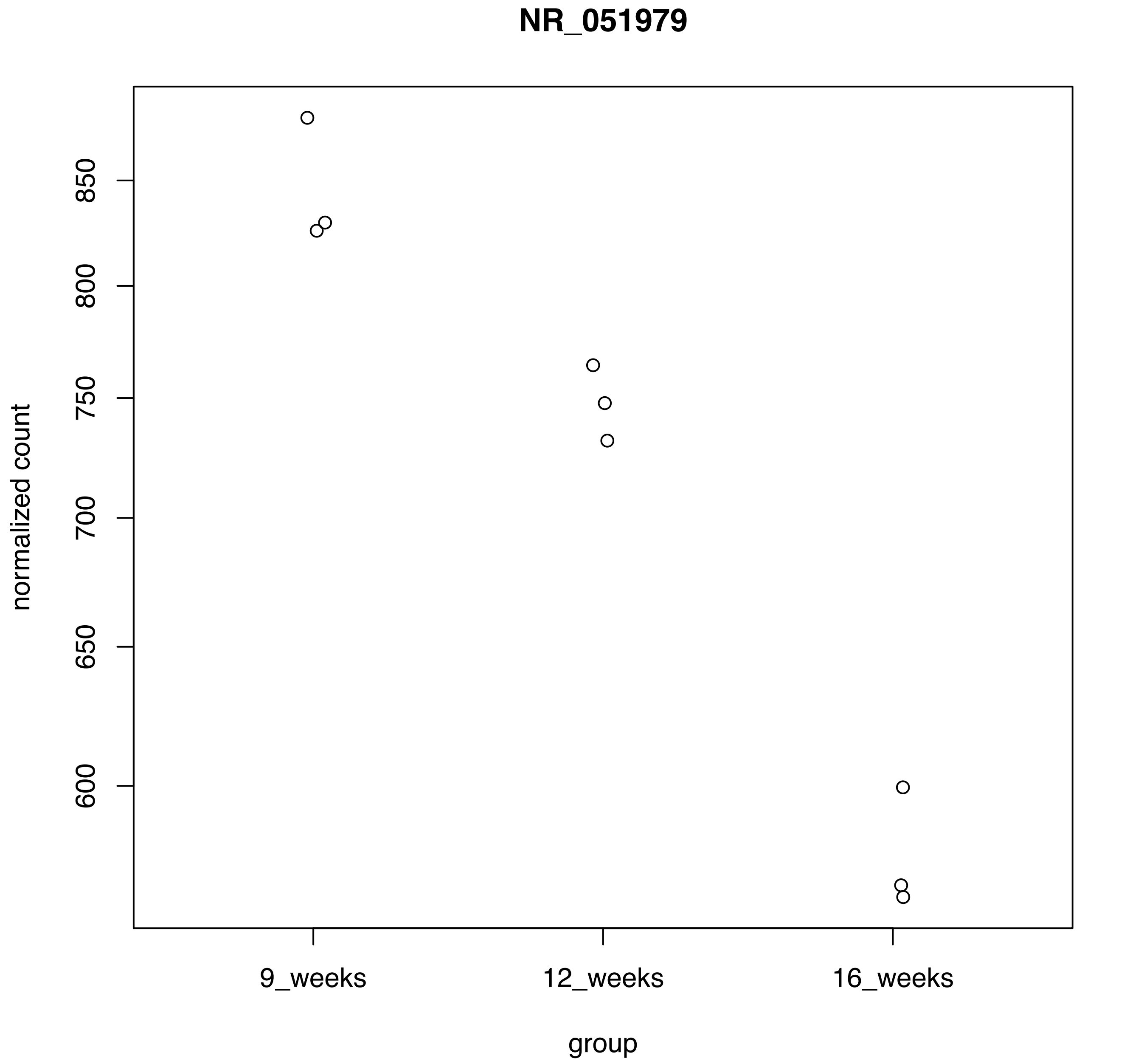

NR_051979
normalized count
600
650
700
750
800
850
9_weeks
12_weeks
16_weeks
group

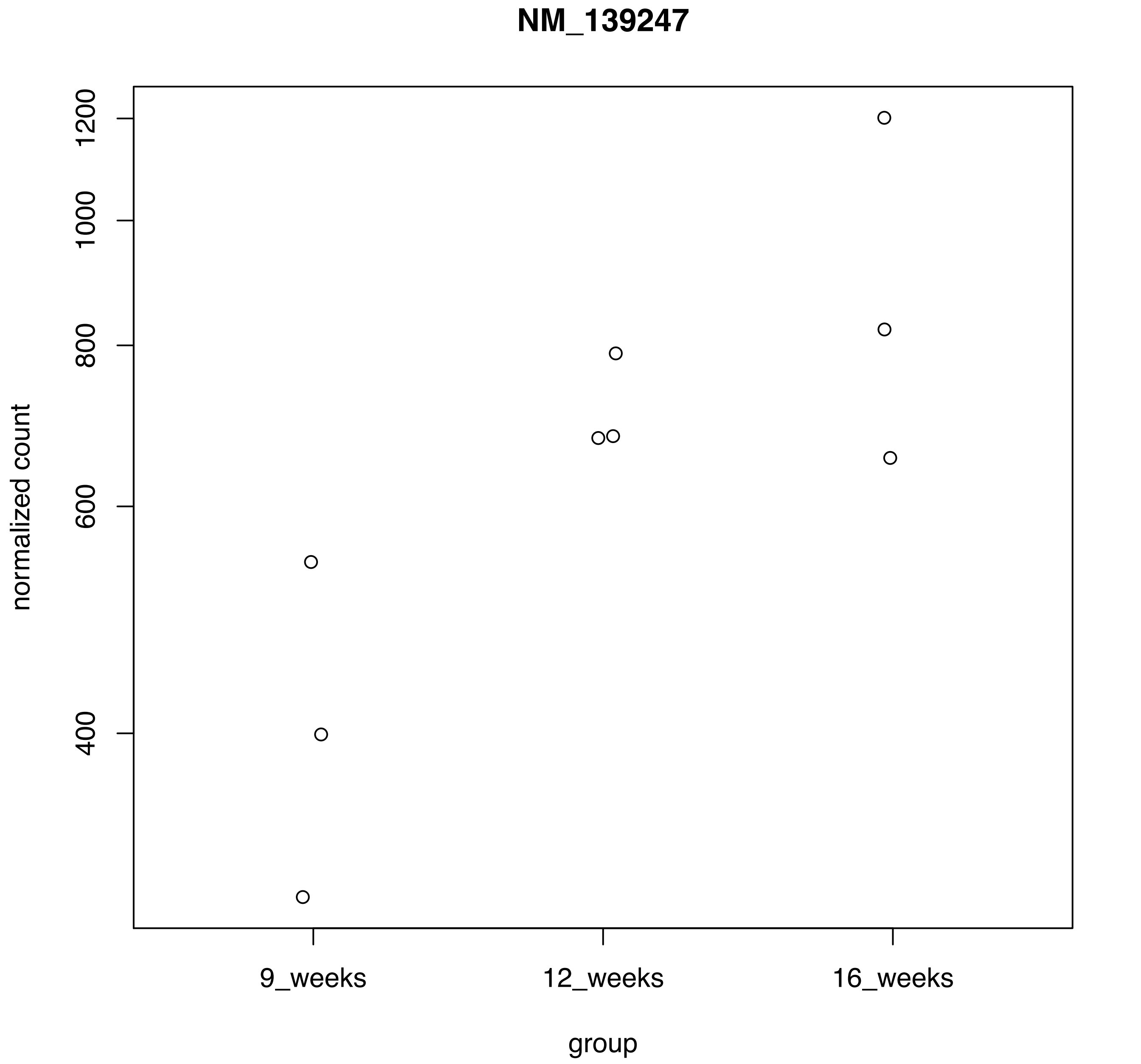
NM_139247
normalized count
400
600
800
1000
1200
9_weeks
12_weeks
16_weeks
group

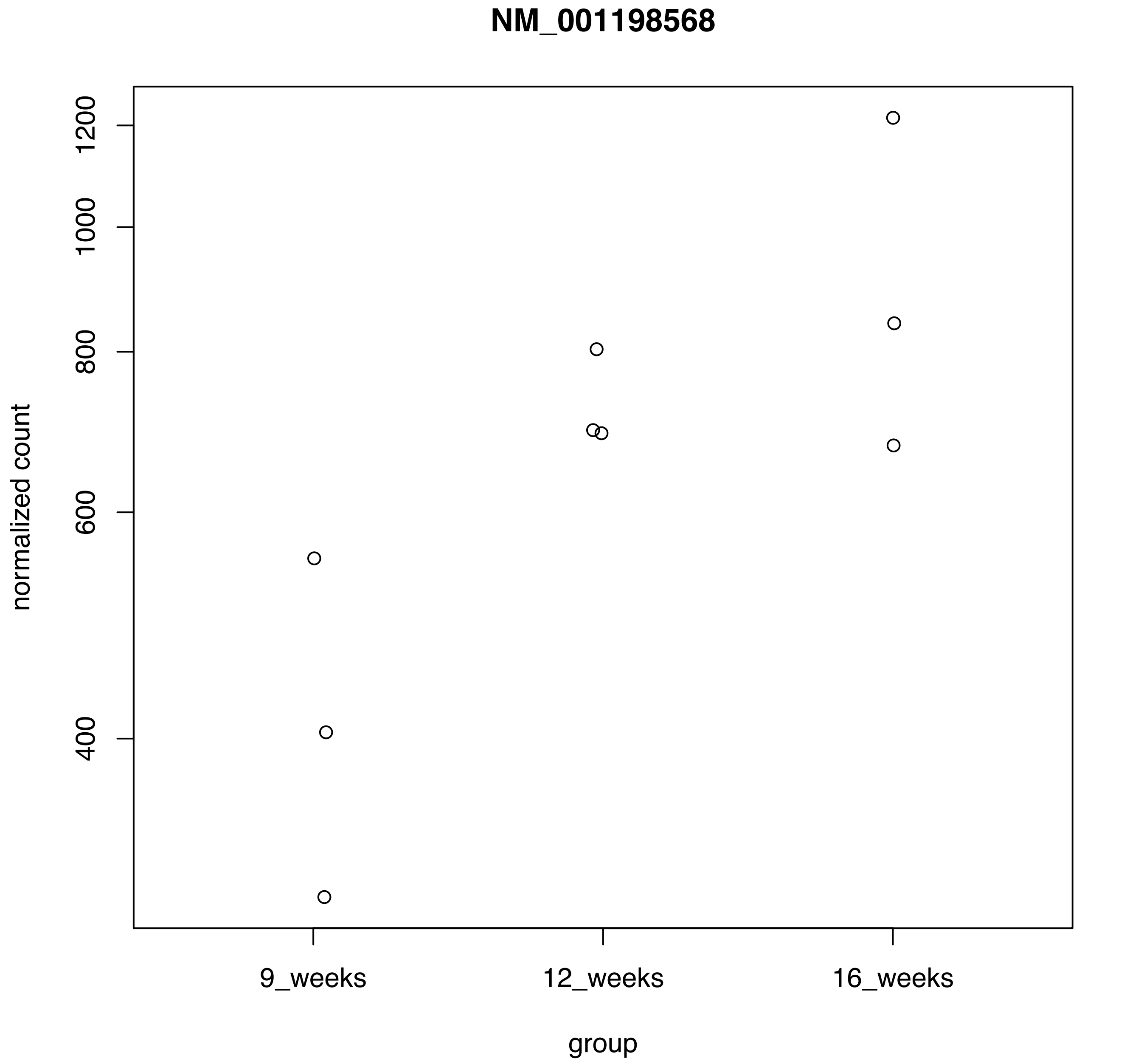
NM_001198568
normalized count
1200
1000
800
600
400
9_weeks
12_weeks
16_weeks
group

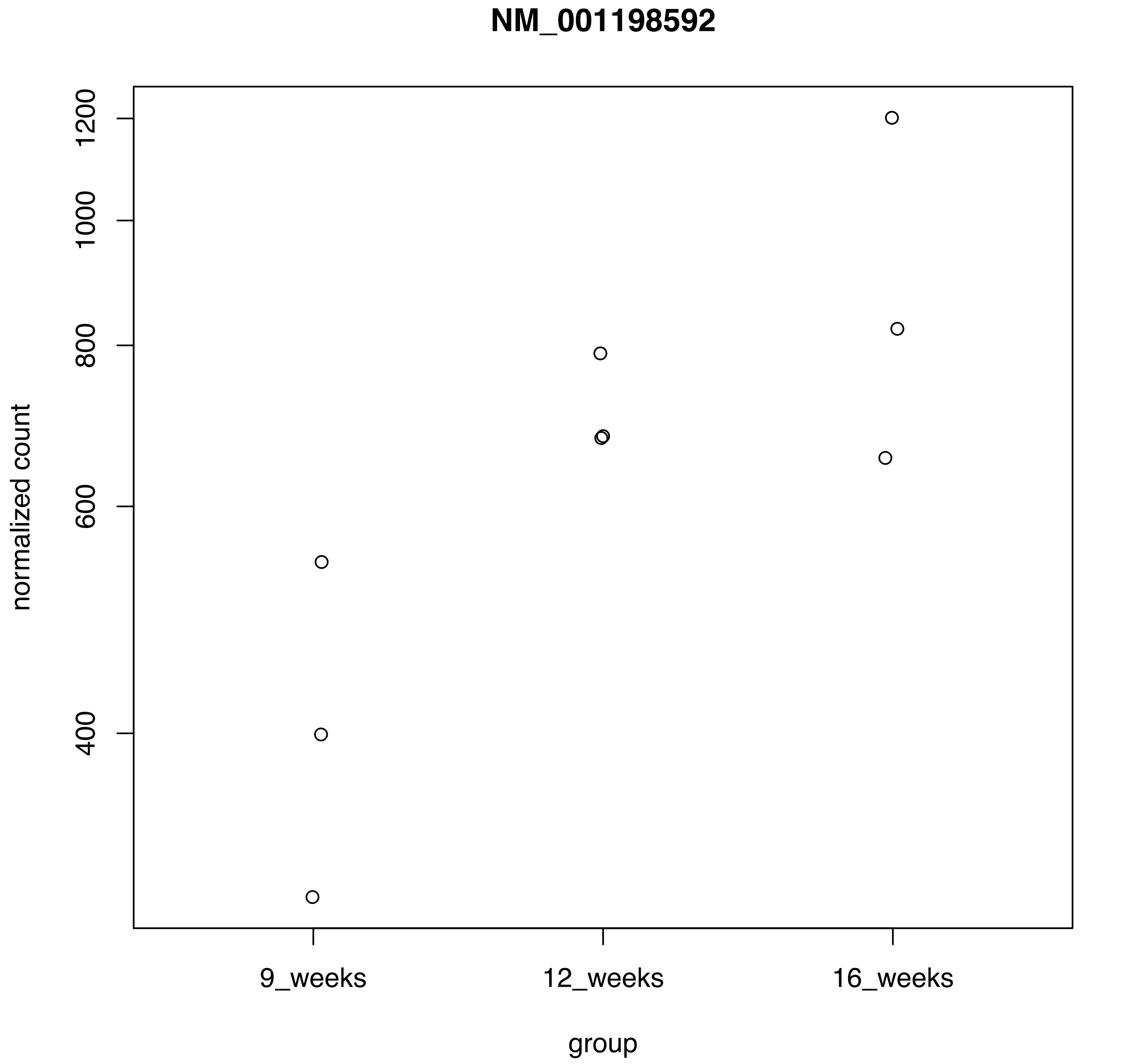
NM_001198592
normalized count
1200
1000
800
600
400
9_weeks
12_weeks
16_weeks
group

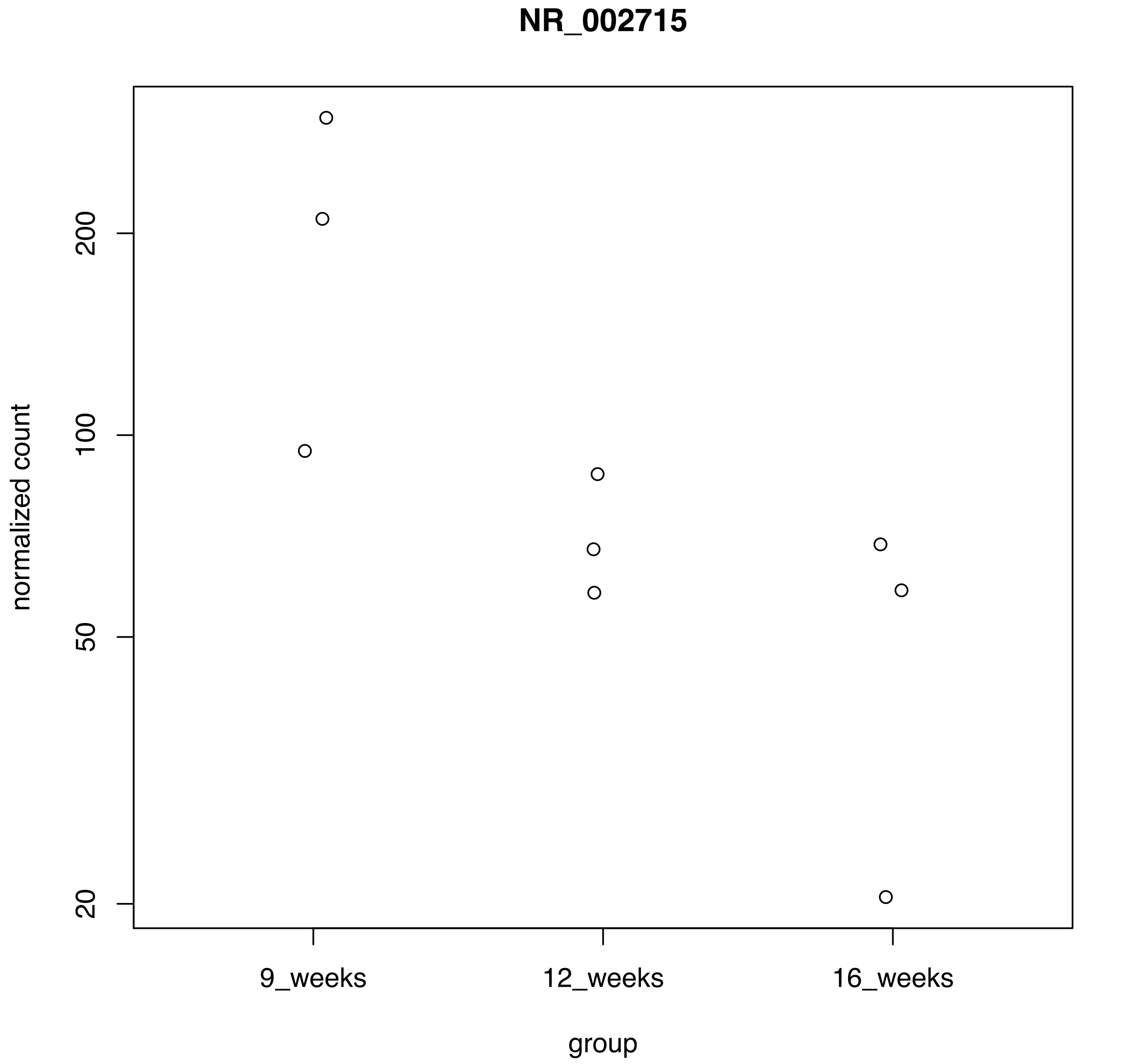

NR_002715
normalized count
200
100
50
20
9_weeks
12_weeks
16_weeks
group

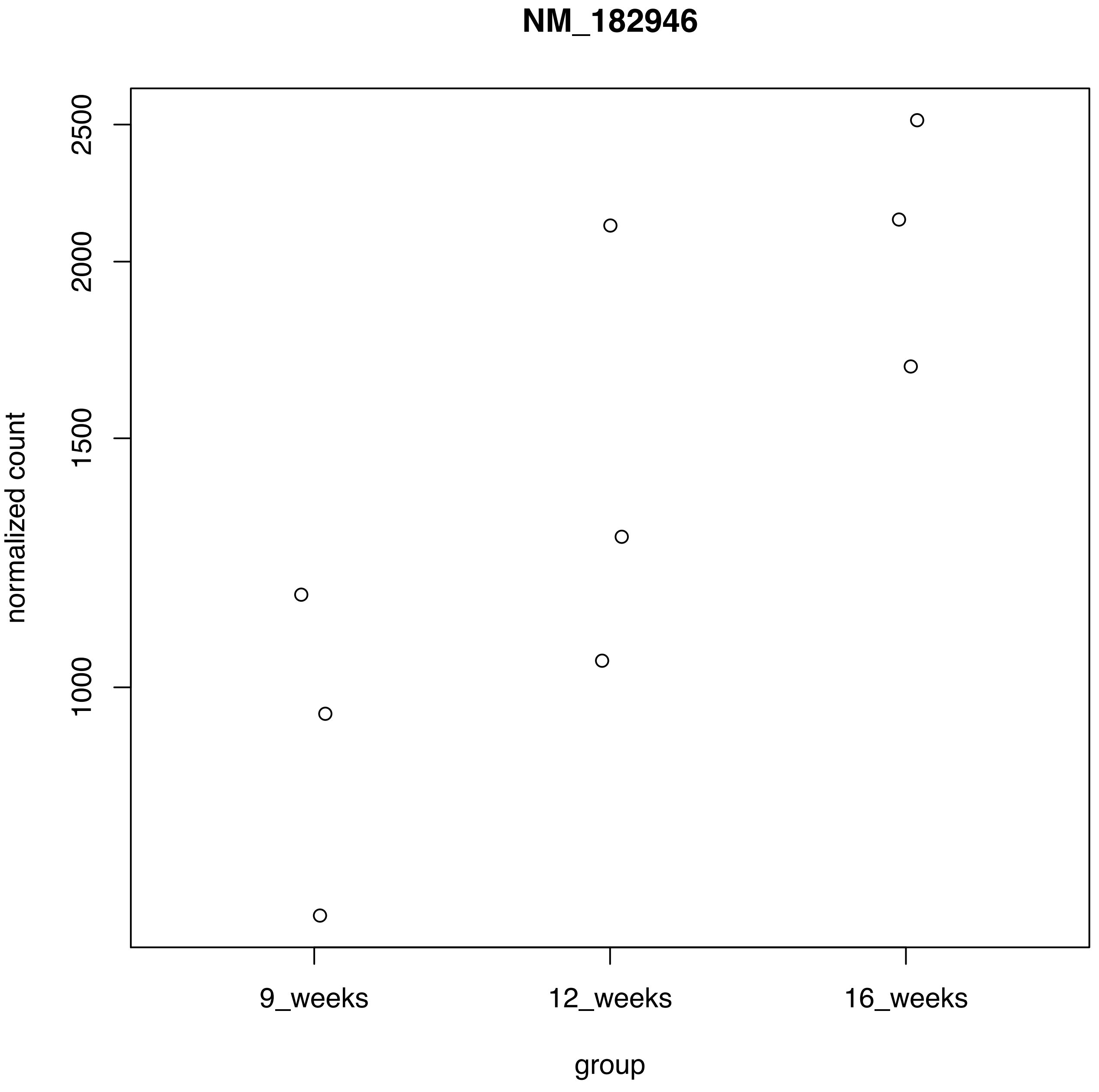
NM_182946
normalized count
2500
2000
1500
1000
9_weeks
12_weeks
16_weeks
group

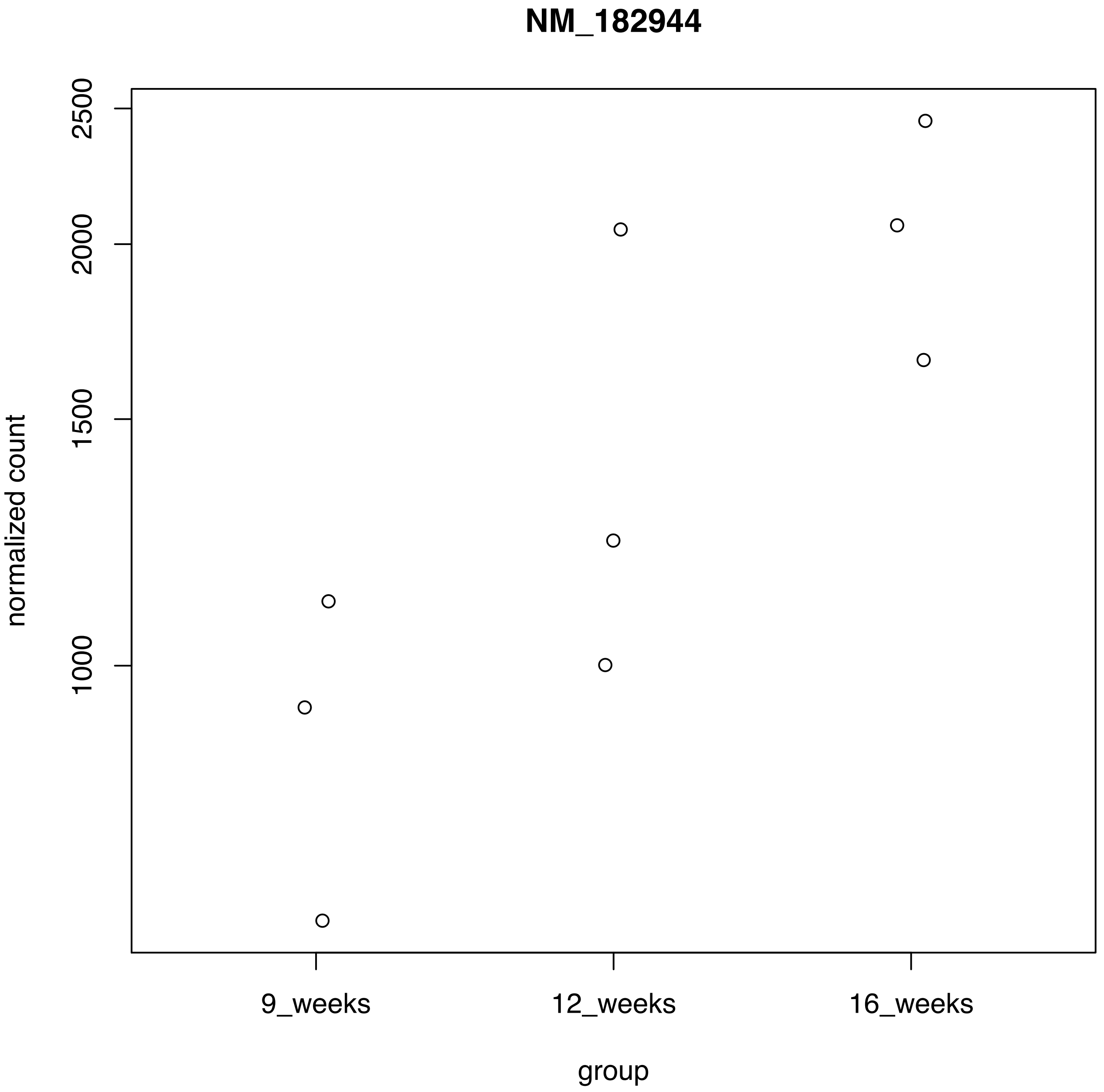
NM_182944
normalized count
2500
2000
1500
1000
9_weeks
12_weeks
16_weeks
group

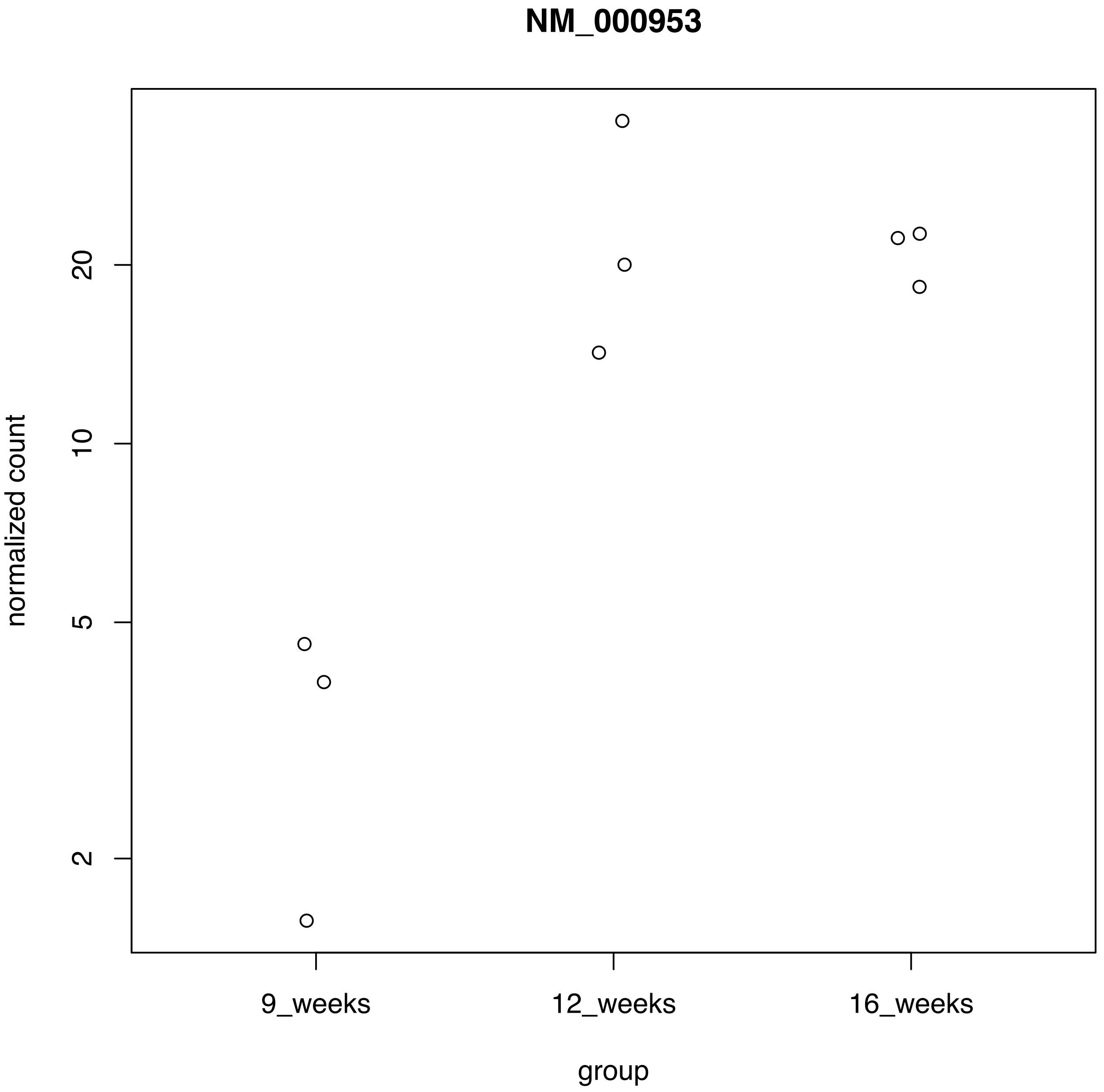
NM_000953
normalized count
2
5
10
20
9_weeks
12_weeks
16_weeks
group

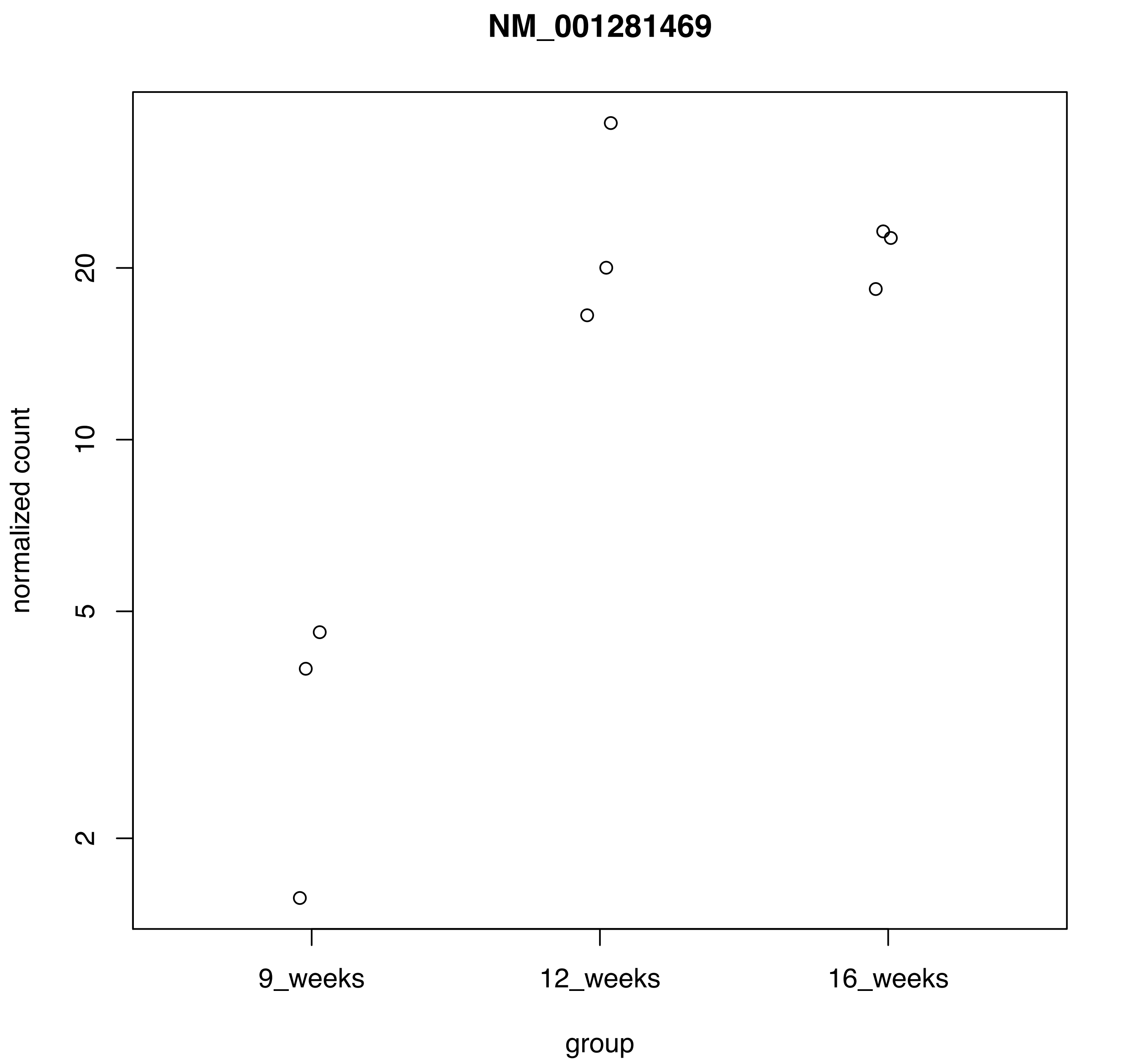
NM_001281469
normalized count
2
5
10
20
9_weeks
12_weeks
16_weeks
group

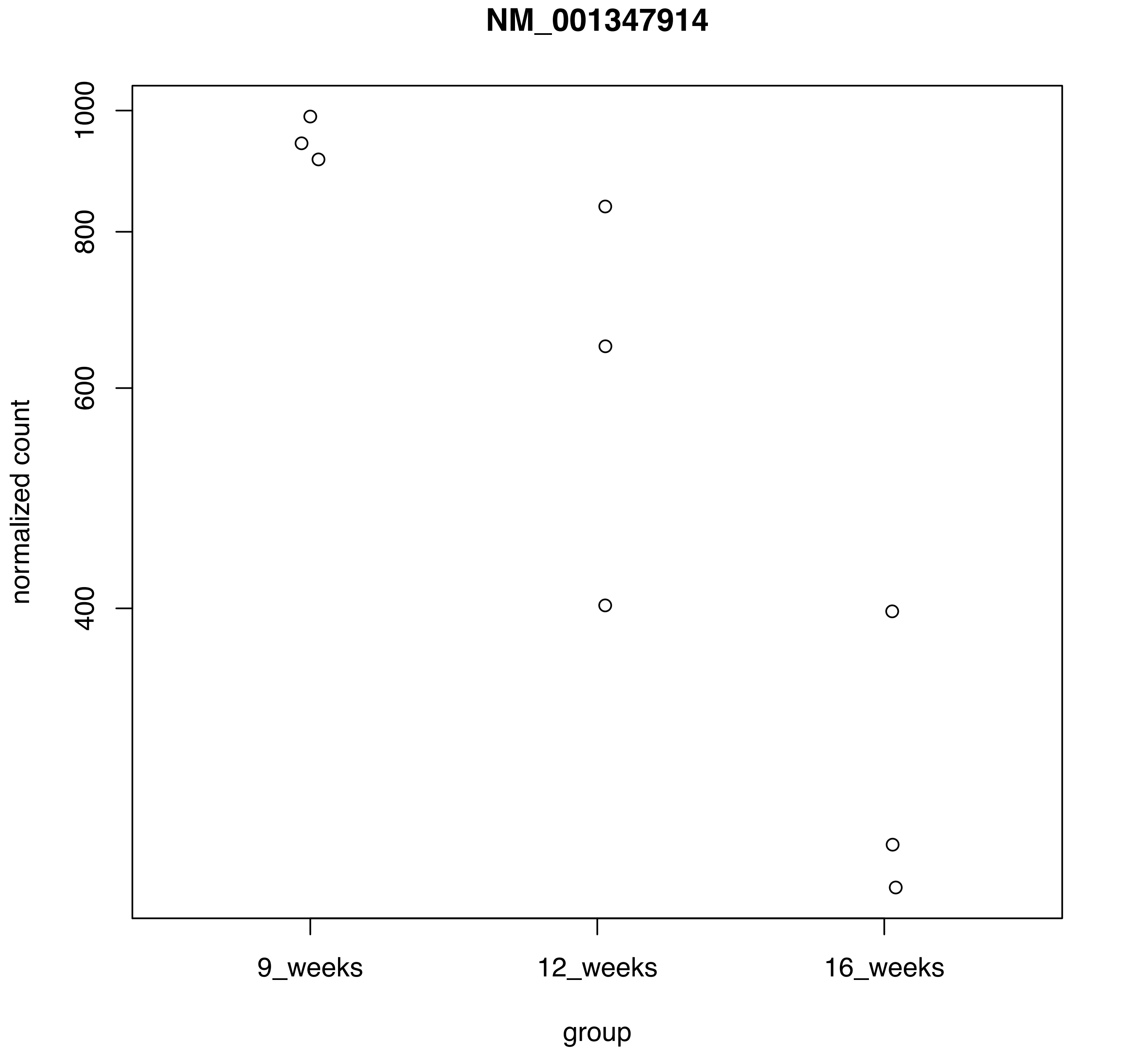
NM_001347914
normalized count
1000
800
600
400
9_weeks
12_weeks
16_weeks
group

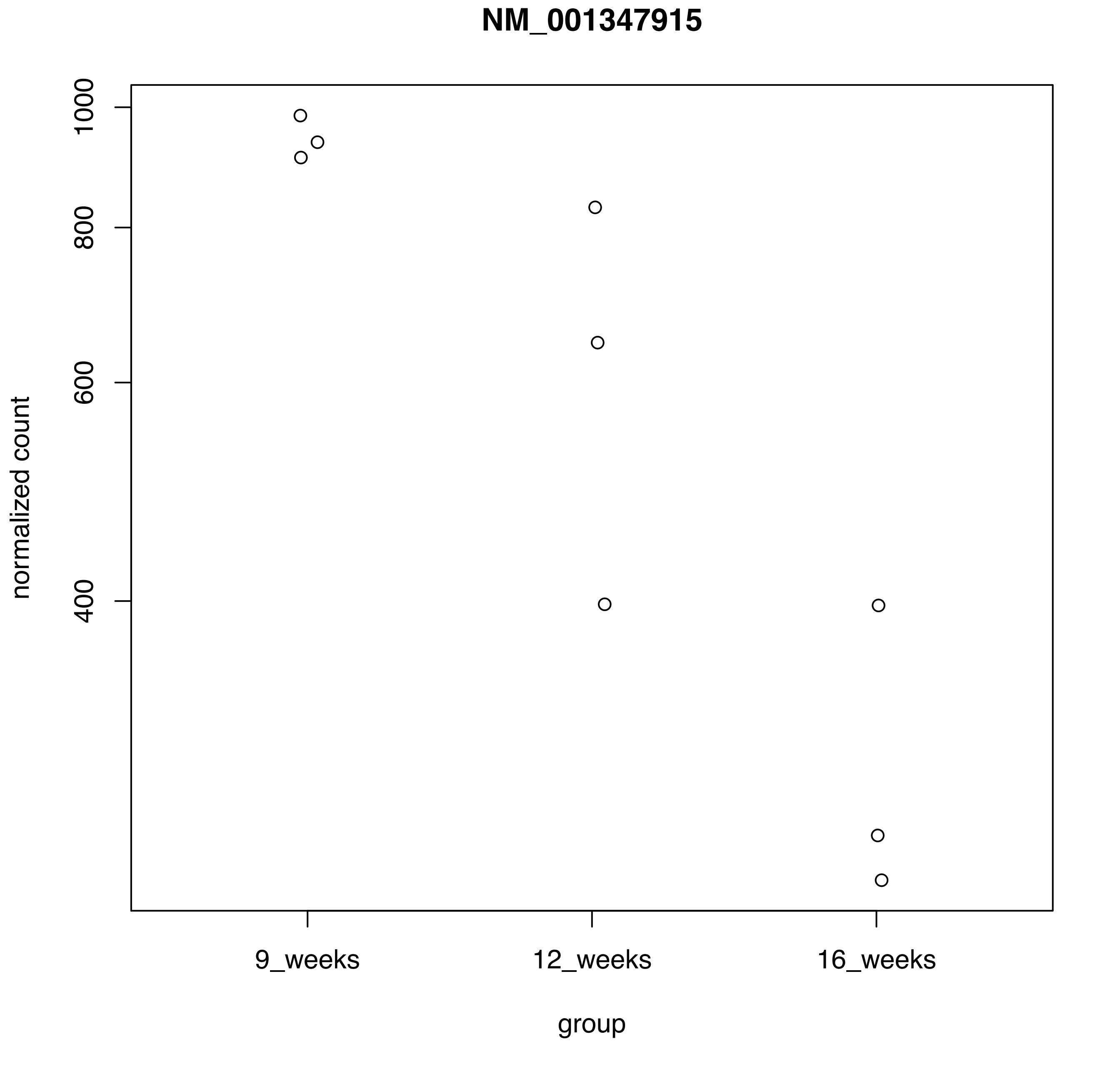

NM_001347915
normalized count
1000
800
600
400
9_weeks
12_weeks
16_weeks
group

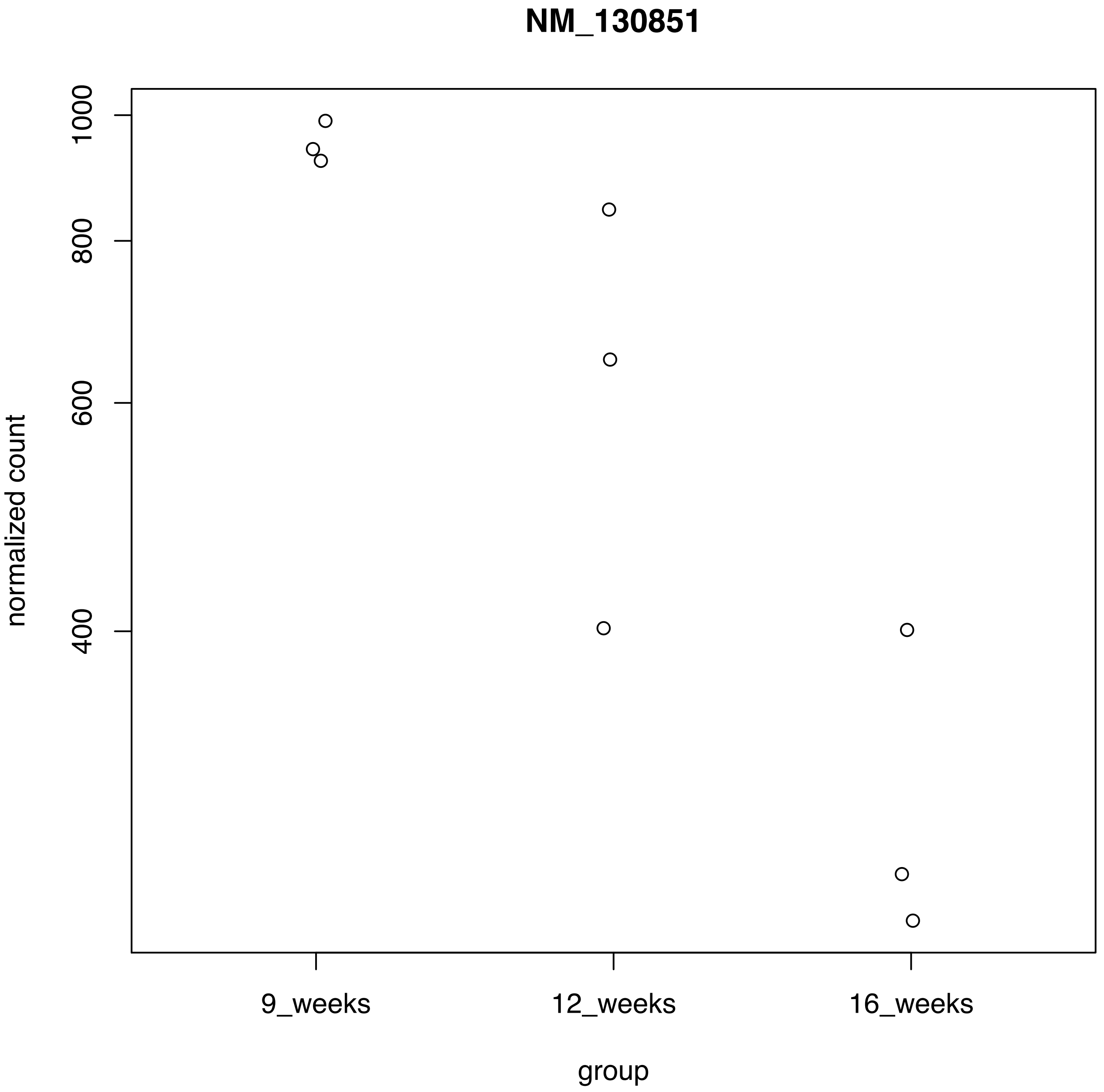
NM_130851
normalized count
1000
800
600
400
9_weeks
12_weeks
16_weeks
group

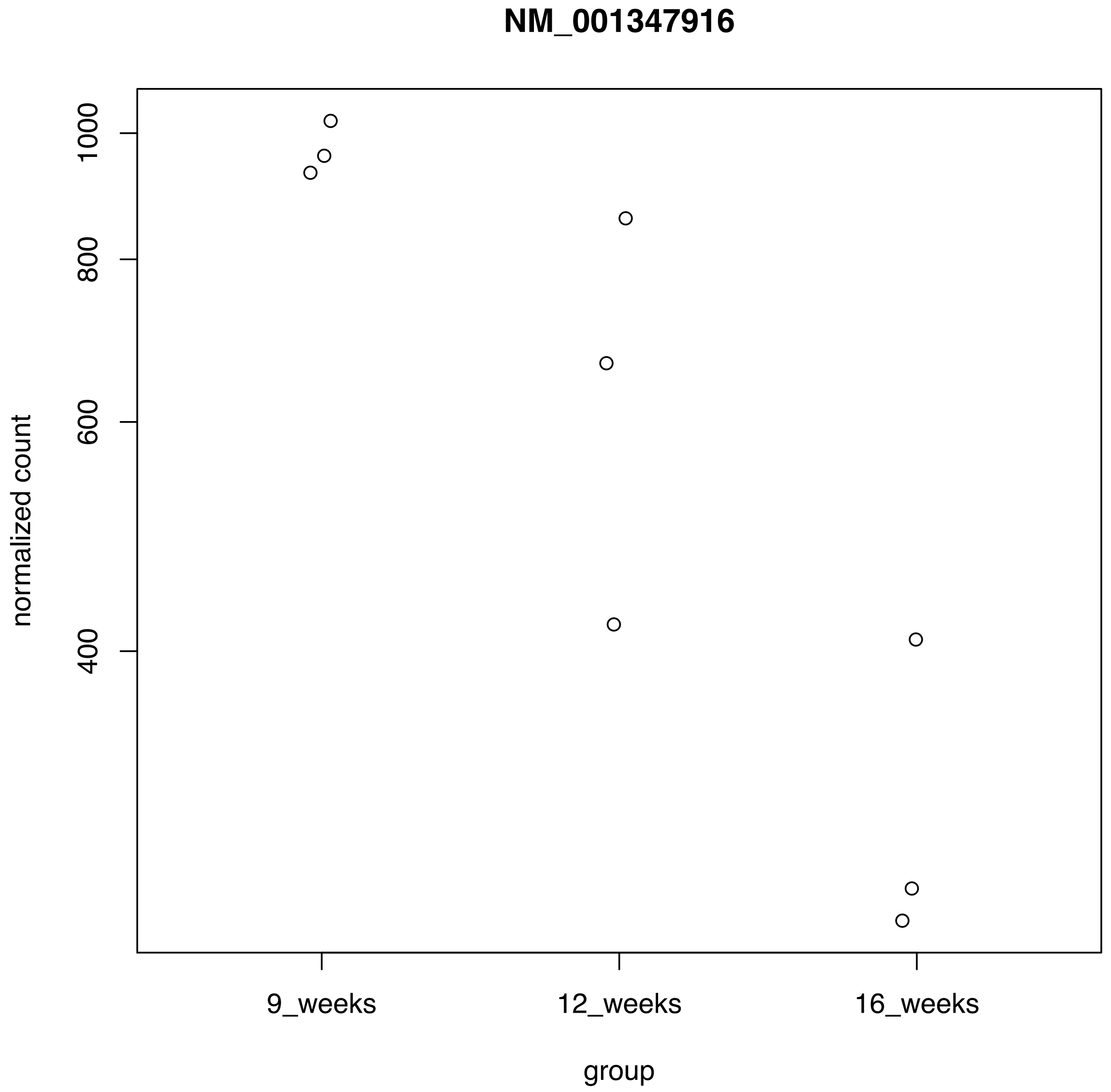

NM_001347916
normalized count
1000
800
600
400
9_weeks
12_weeks
16_weeks
group

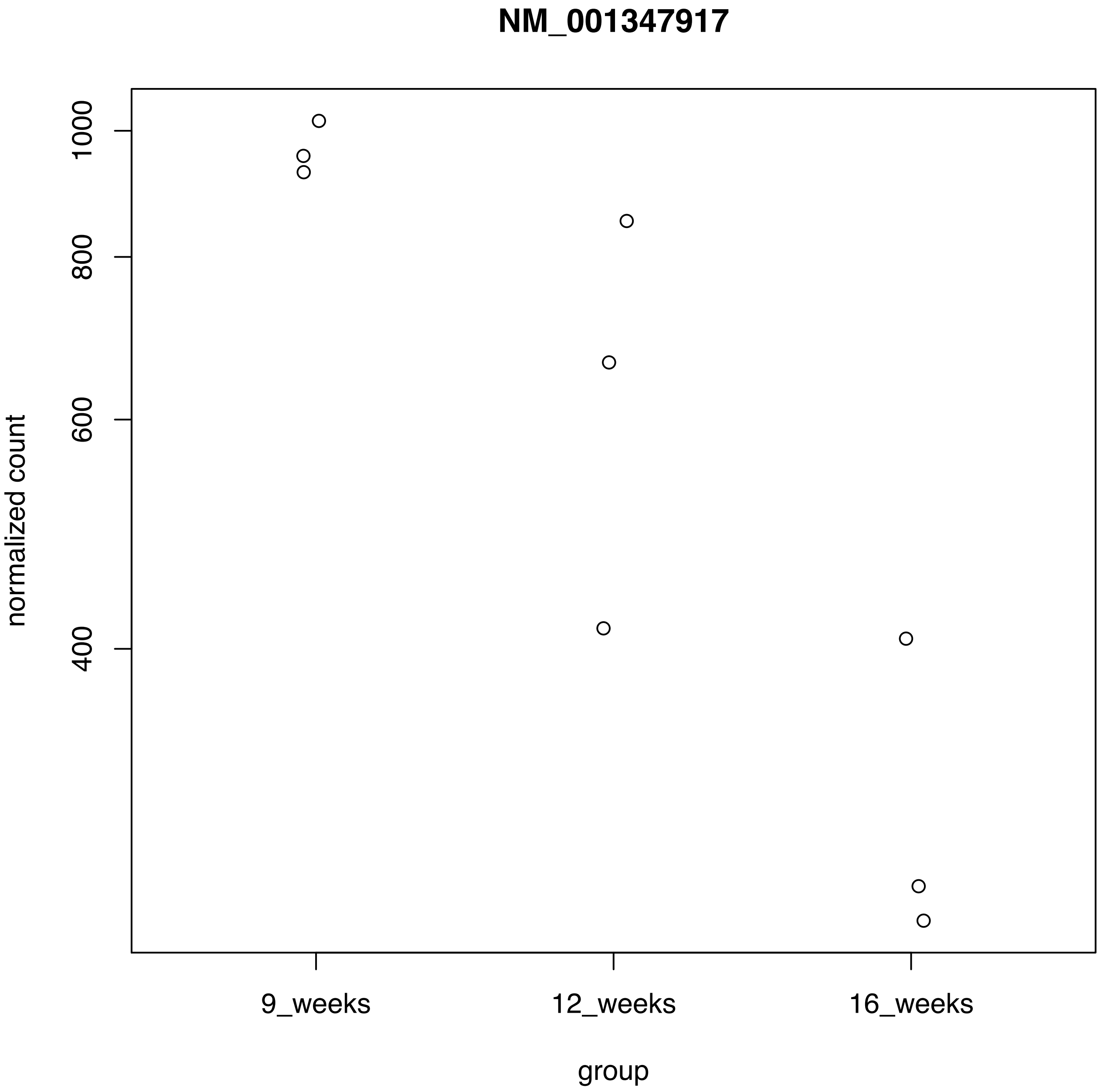
NM_001347917
normalized count
1000
800
600
400
9_weeks
12_weeks
16_weeks
group

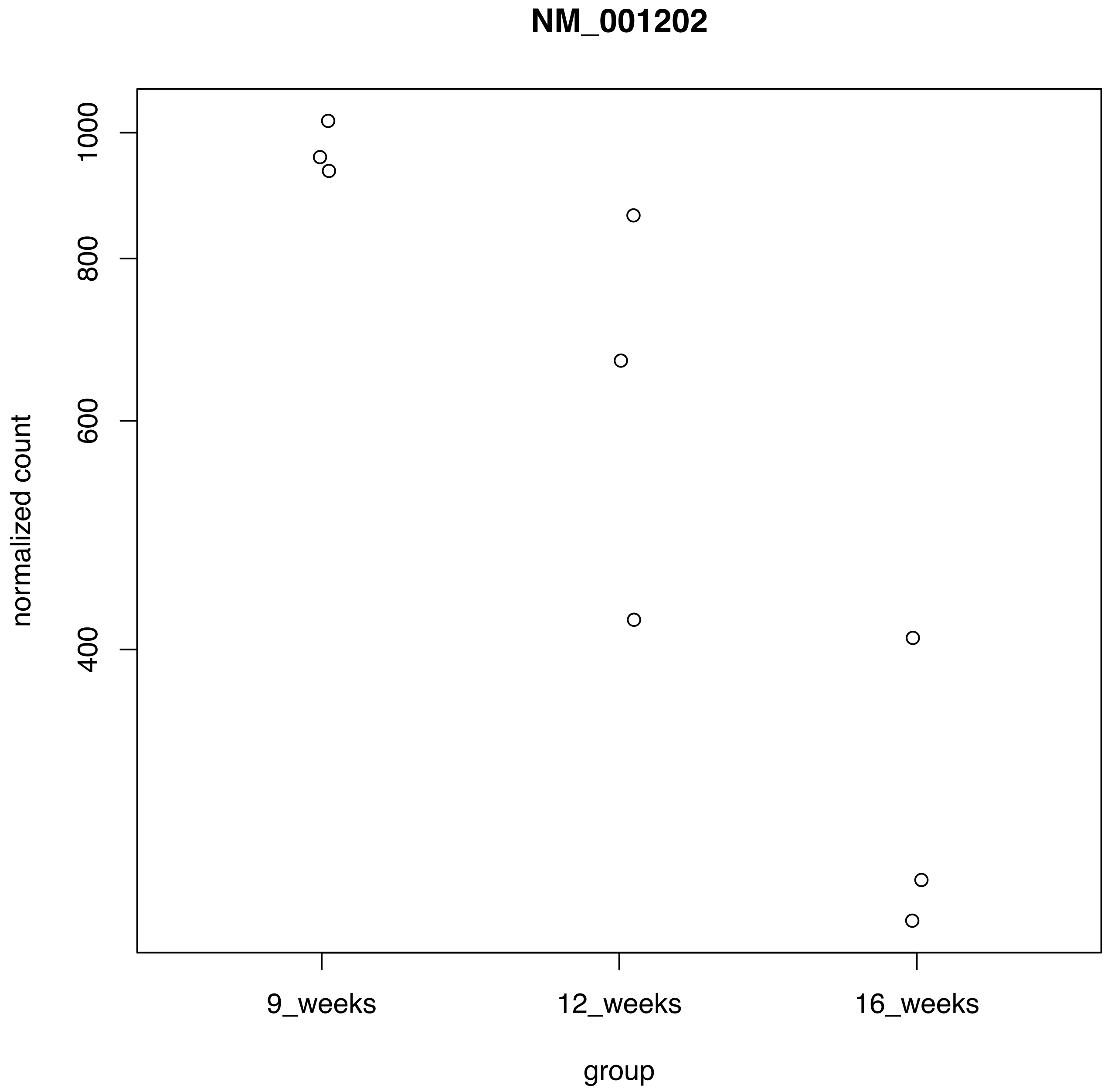

NM_001202
normalized count
1000
800
600
400
9_weeks
12_weeks
16_weeks
group

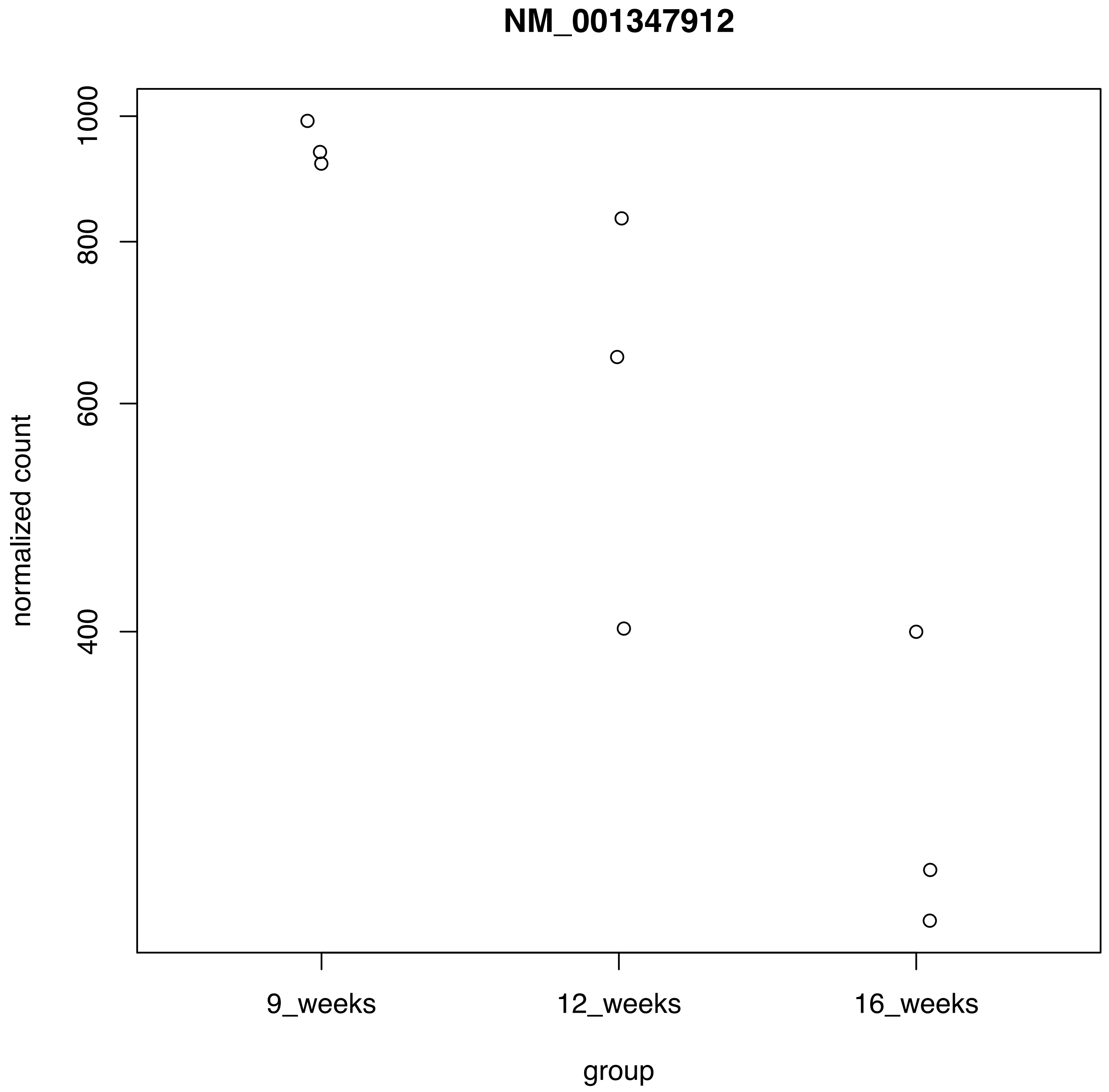
NM_001347912
normalized count
1000
800
600
400
9_weeks
12_weeks
16_weeks
group

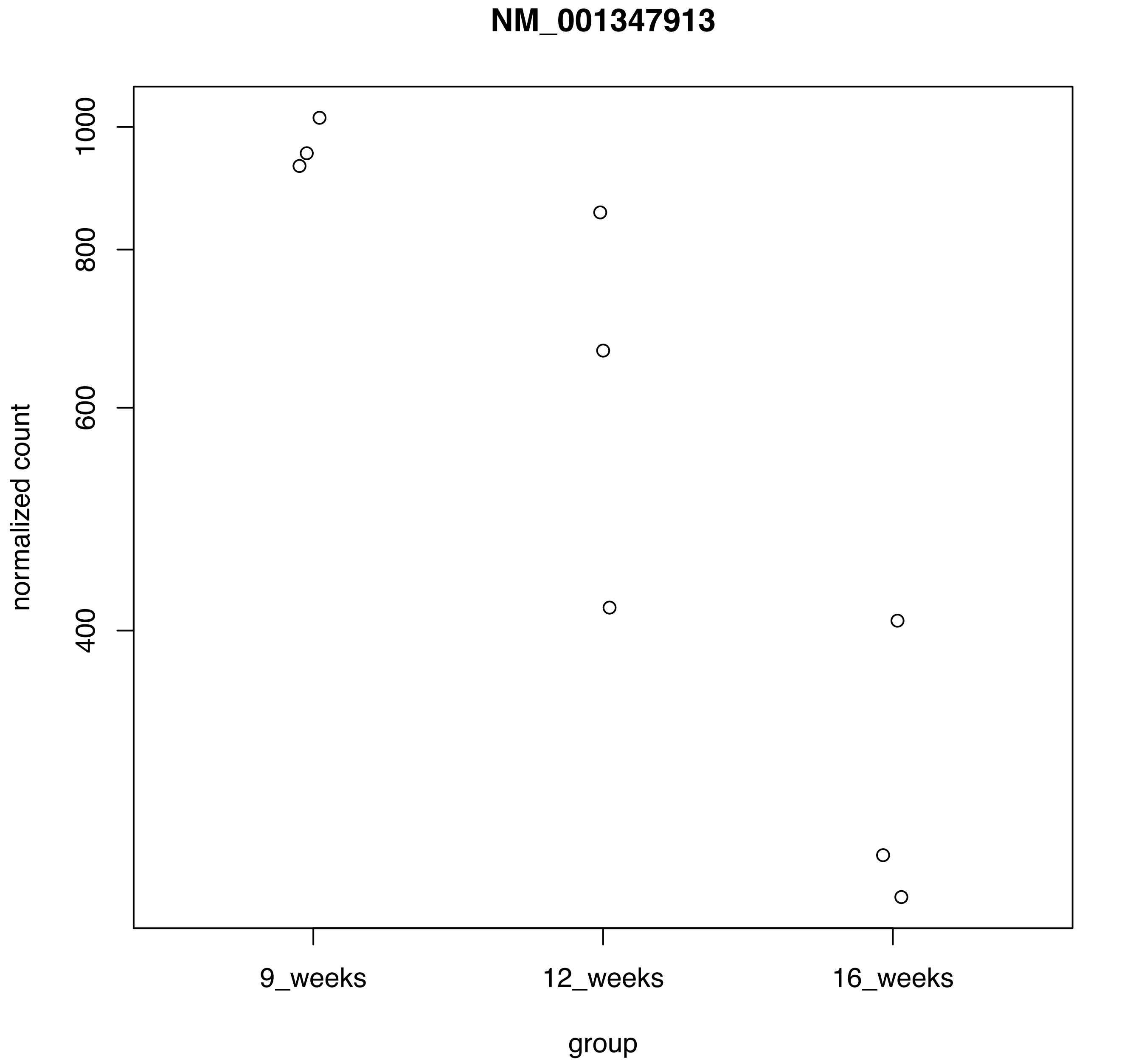
NM_001347913
normalized count
1000
800
600
400
9_weeks
12_weeks
16_weeks
group

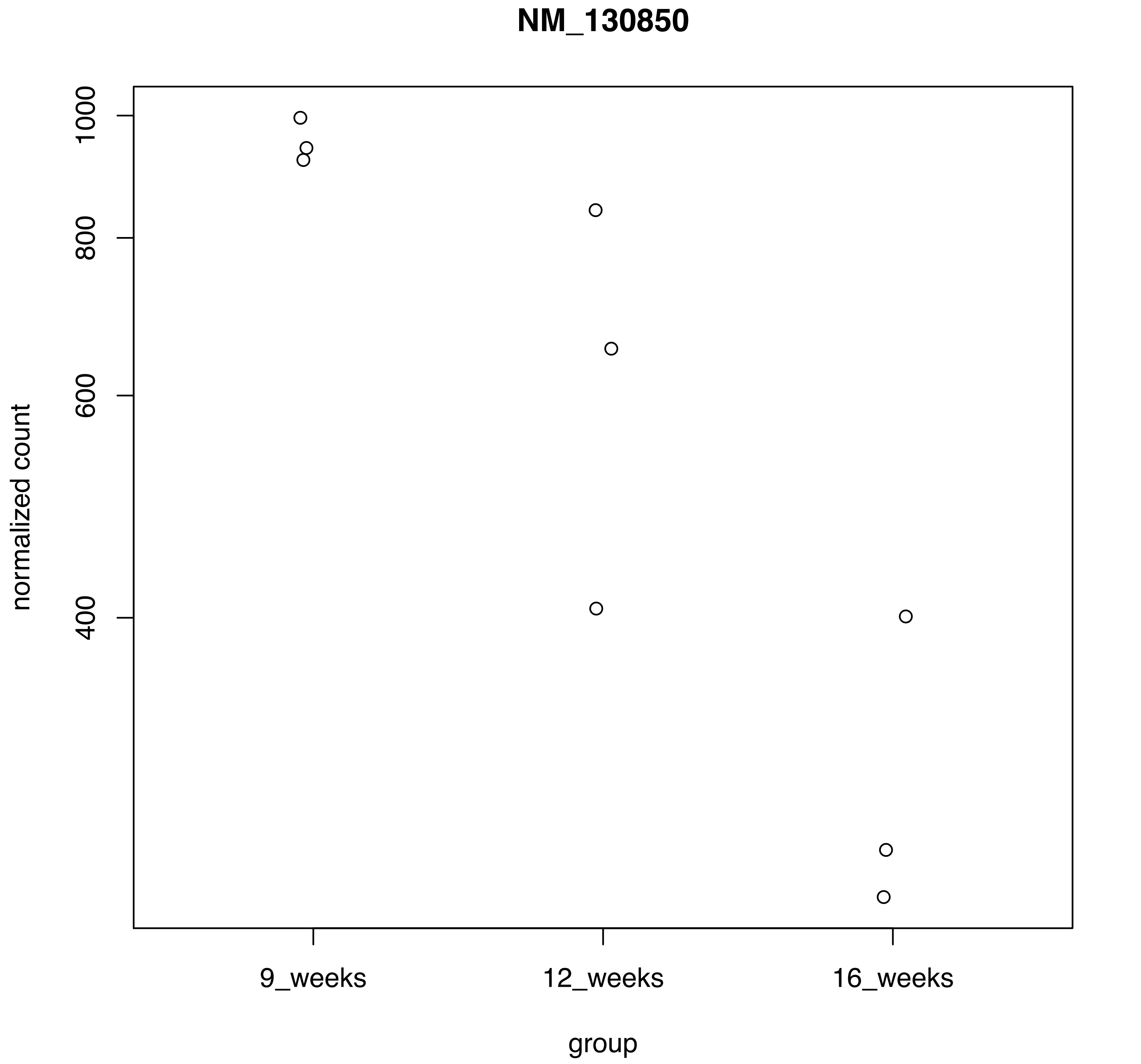
NM_130850
normalized count
1000
800
600
400
9_weeks
12_weeks
16_weeks
group

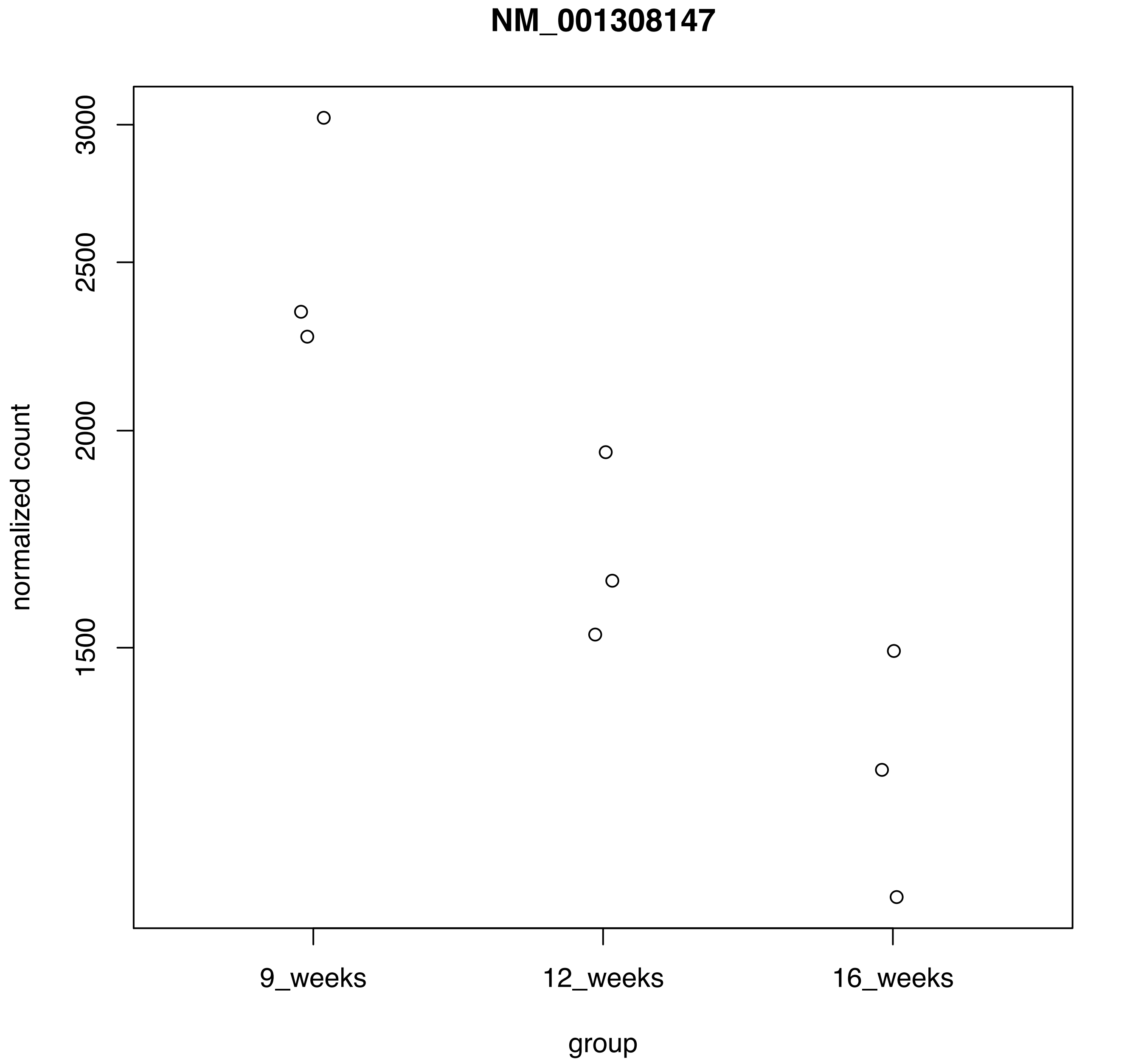
NM_001308147
normalized count
3000
2500
2000
1500
9_weeks
12_weeks
16_weeks
group

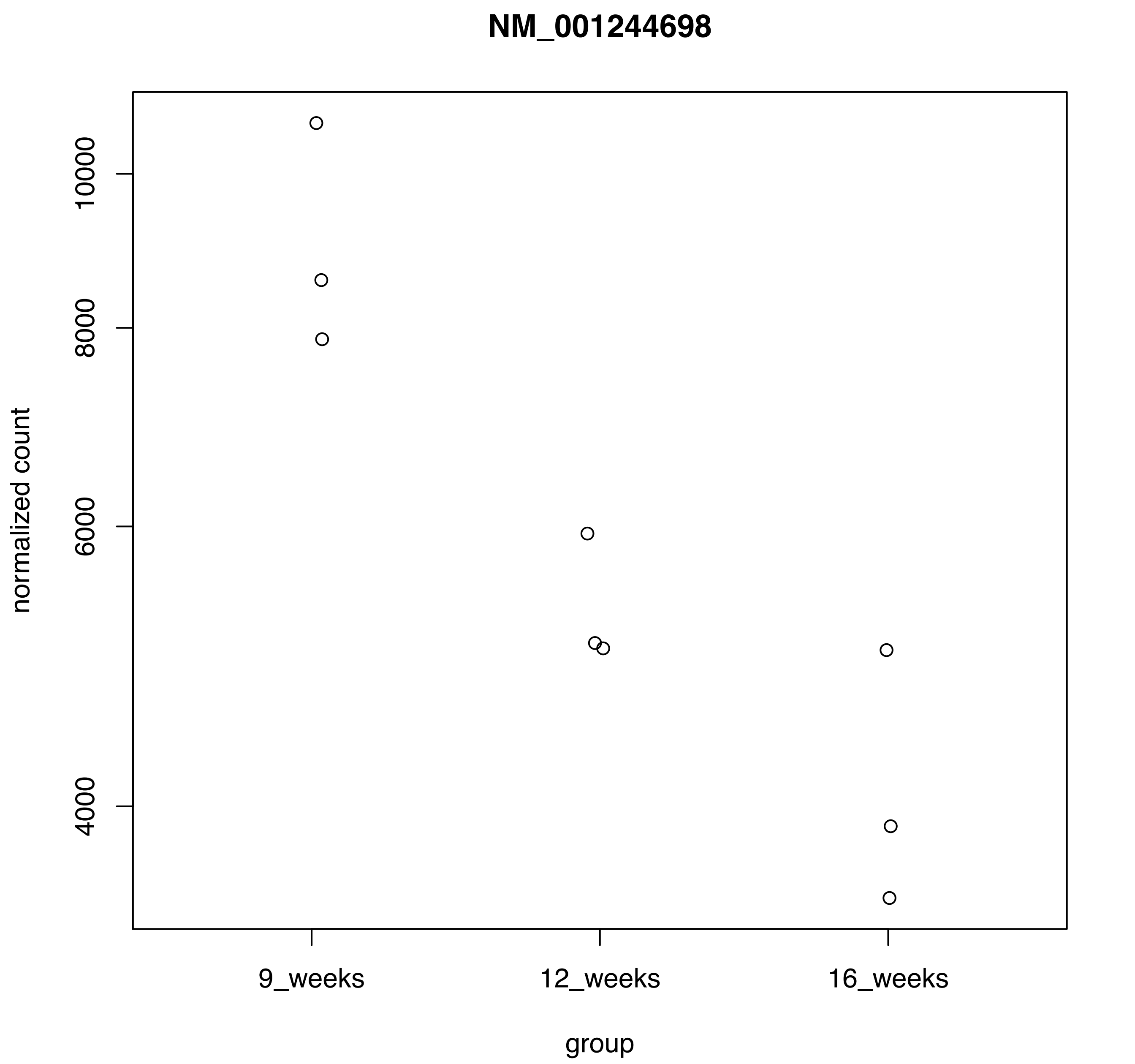
NM_001244698
normalized count
10000
8000
6000
4000
9_weeks
12_weeks
16_weeks
group

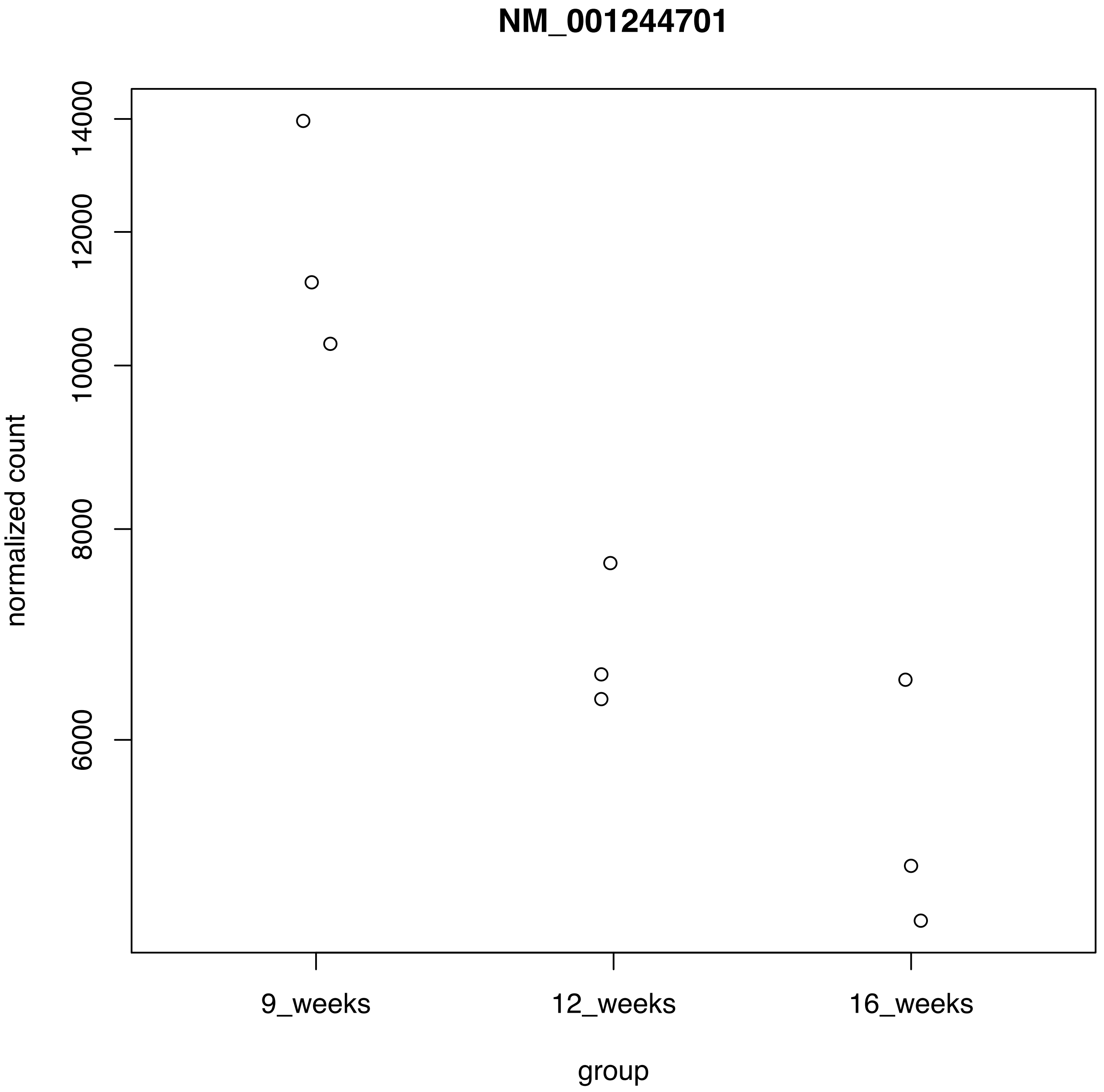
NM_001244701
normalized count
14000
12000
10000
8000
6000
9_weeks
12_weeks
16_weeks
group

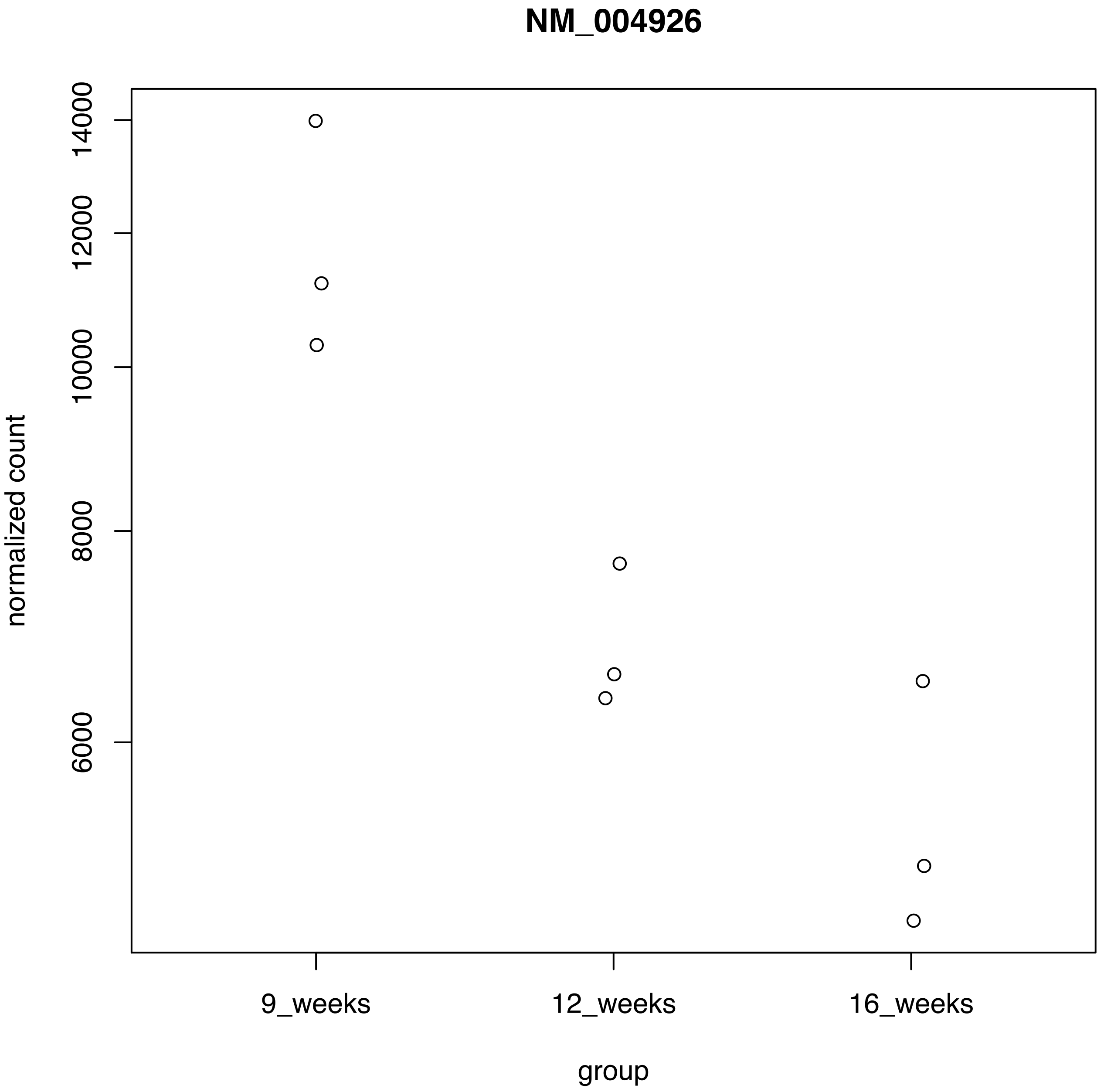
NM_004926
normalized count
6000
8000
10000
12000
14000
9_weeks
12_weeks
16_weeks
group

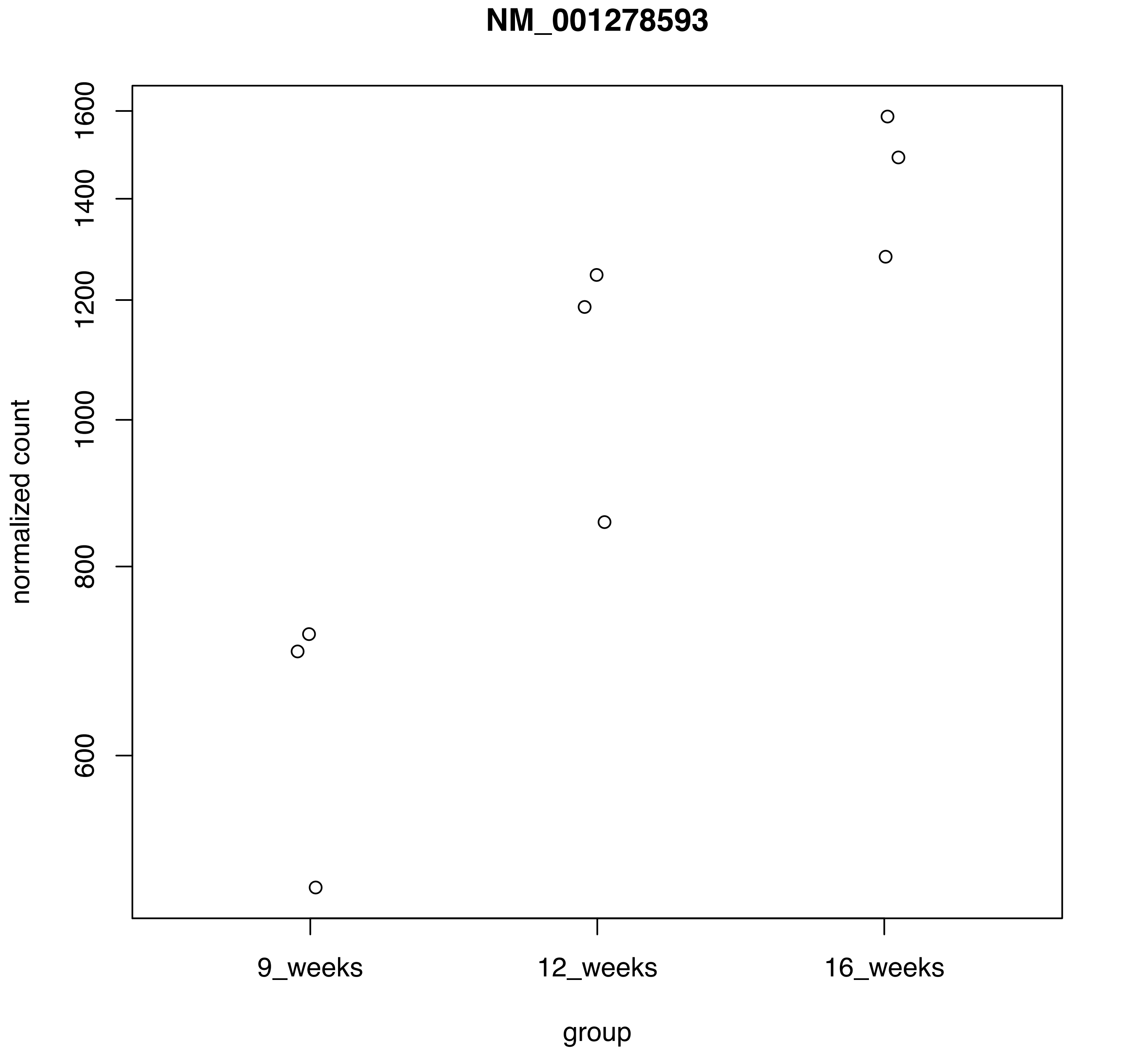

NM_001278593
normalized count
600
800
1000
1200
1400
1600
9_weeks
12_weeks
16_weeks
group

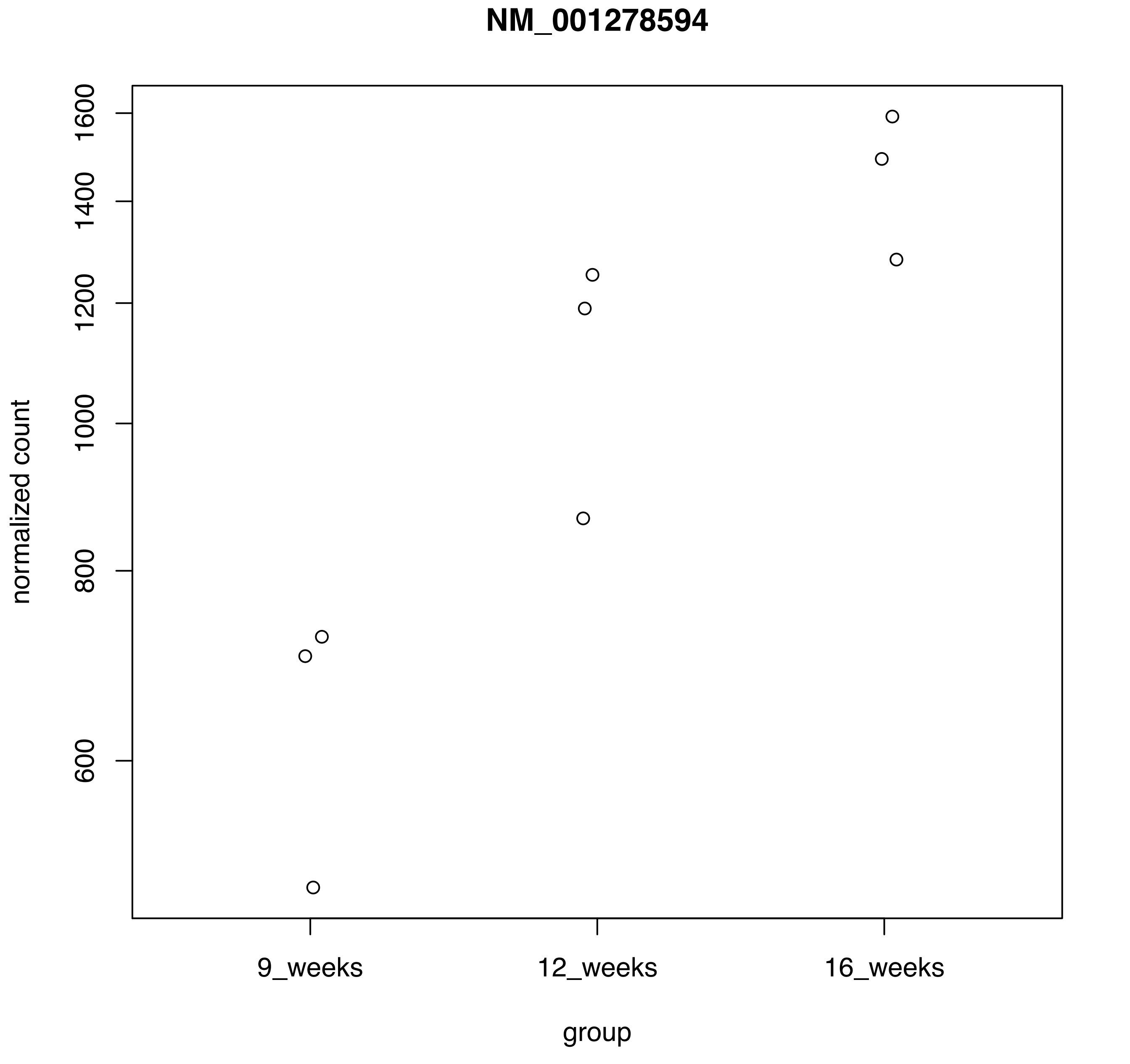
NM_001278594
normalized count
1600
1400
1200
1000
800
600
9_weeks
12_weeks
16_weeks
group

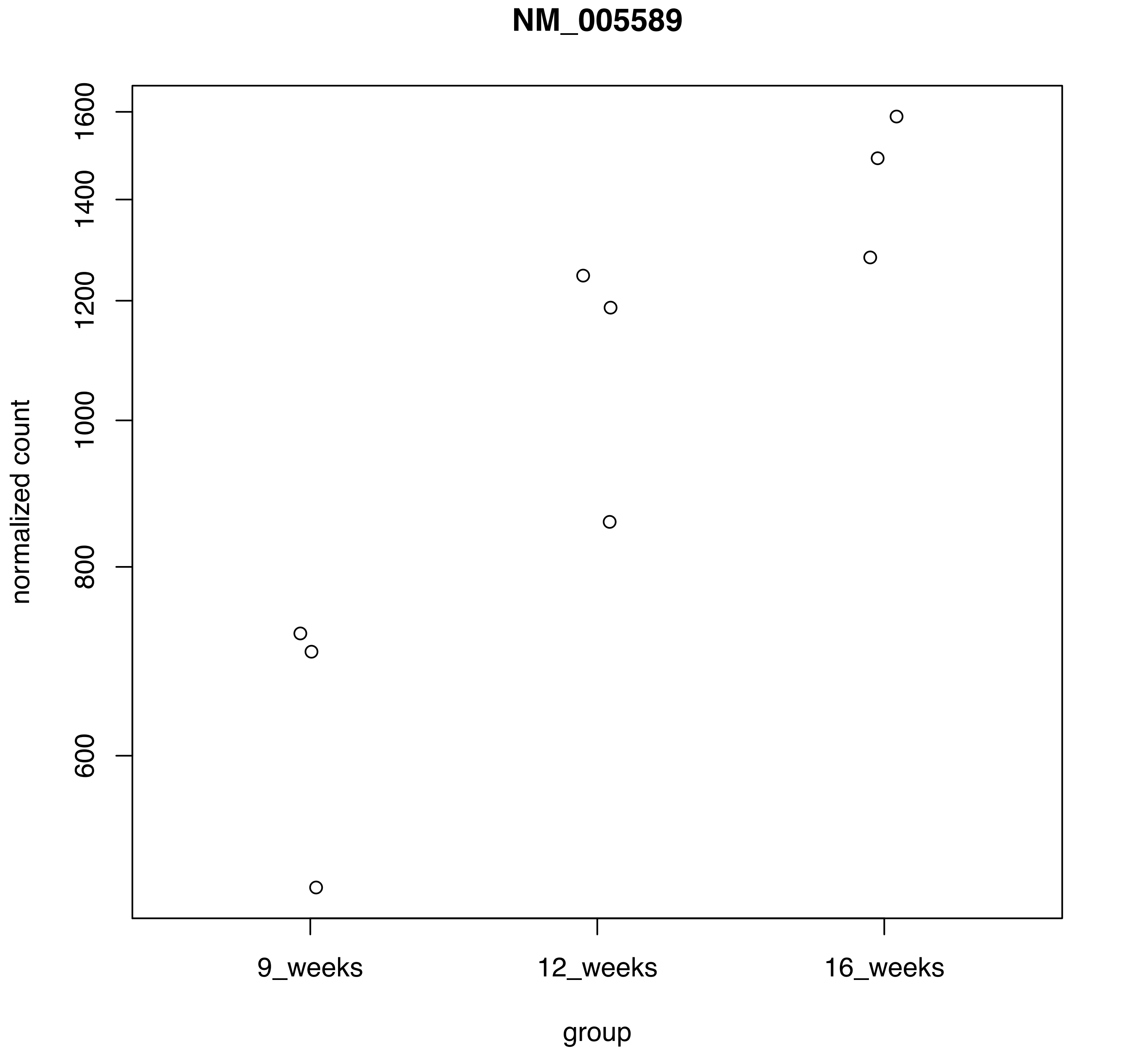
NM_005589
normalized count
1600
1400
1200
1000
800
600
9_weeks
12_weeks
16_weeks
group

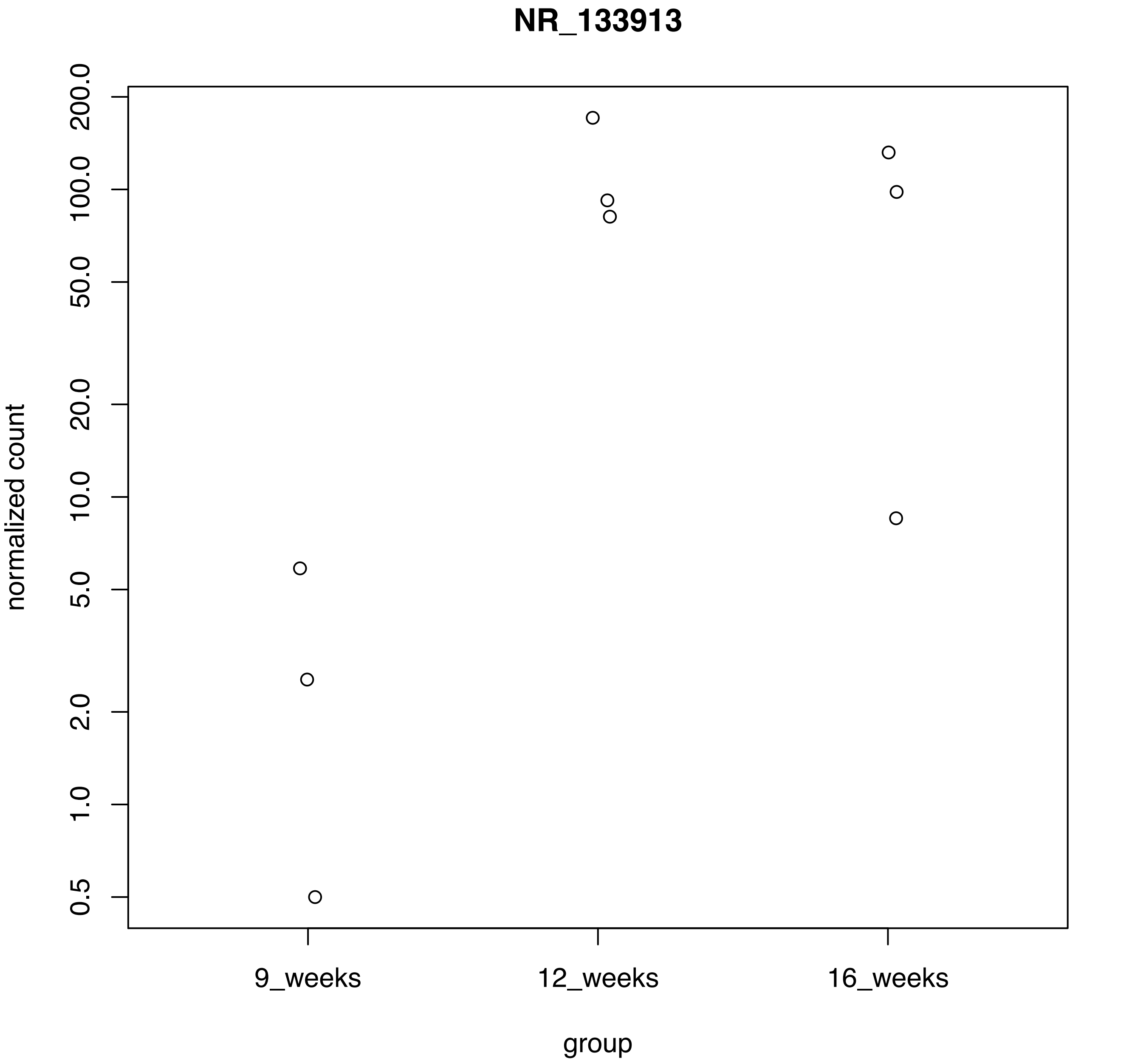
NR_133913
normalized count
200.0
100.0
50.0
20.0
10.0
5.0
2.0
1.0
0.5
9_weeks
12_weeks
16_weeks
group

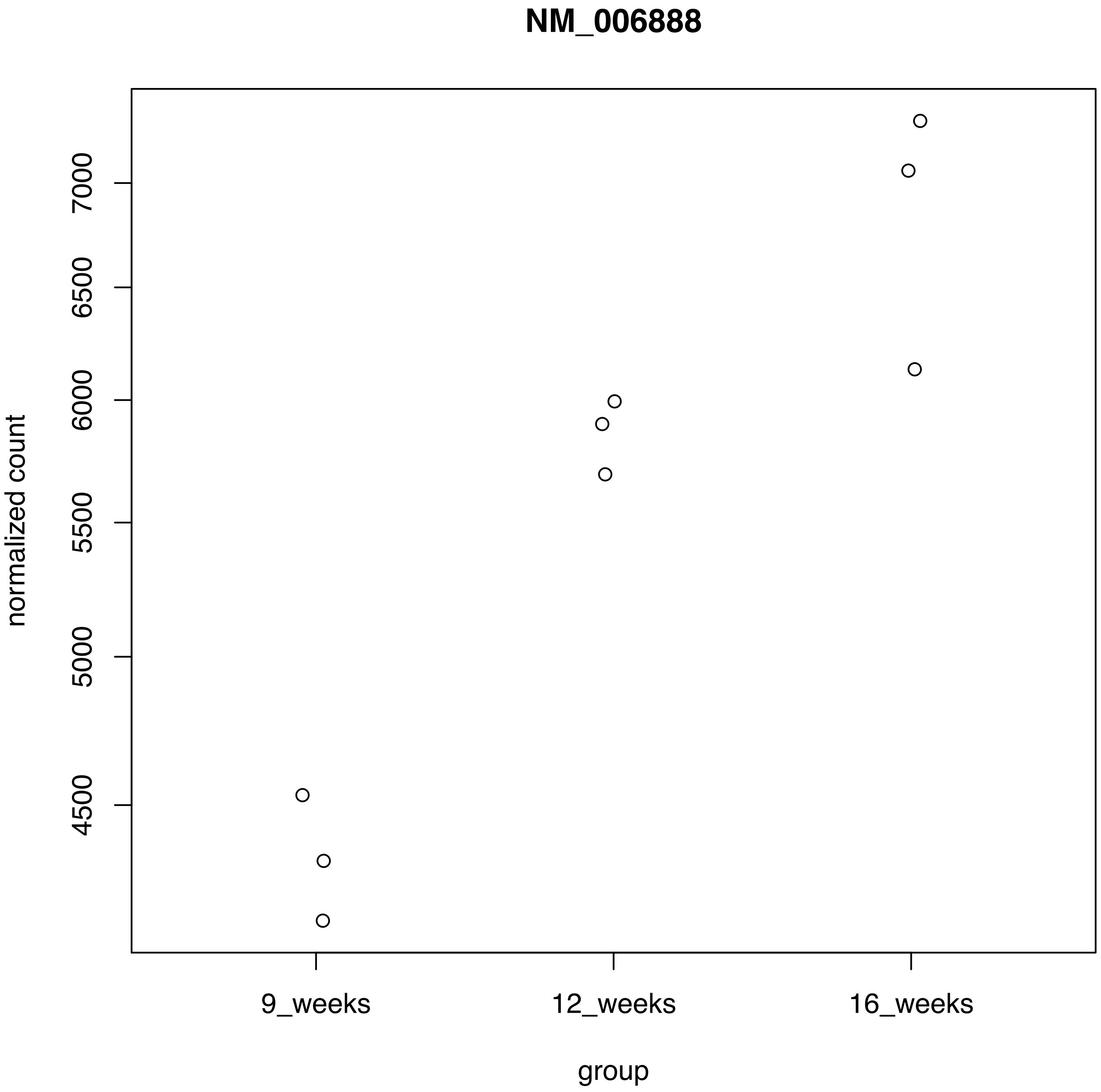
NM_006888
normalized count
4500
5000
5500
6000
6500
7000
group
9_weeks
12_weeks
16_weeks

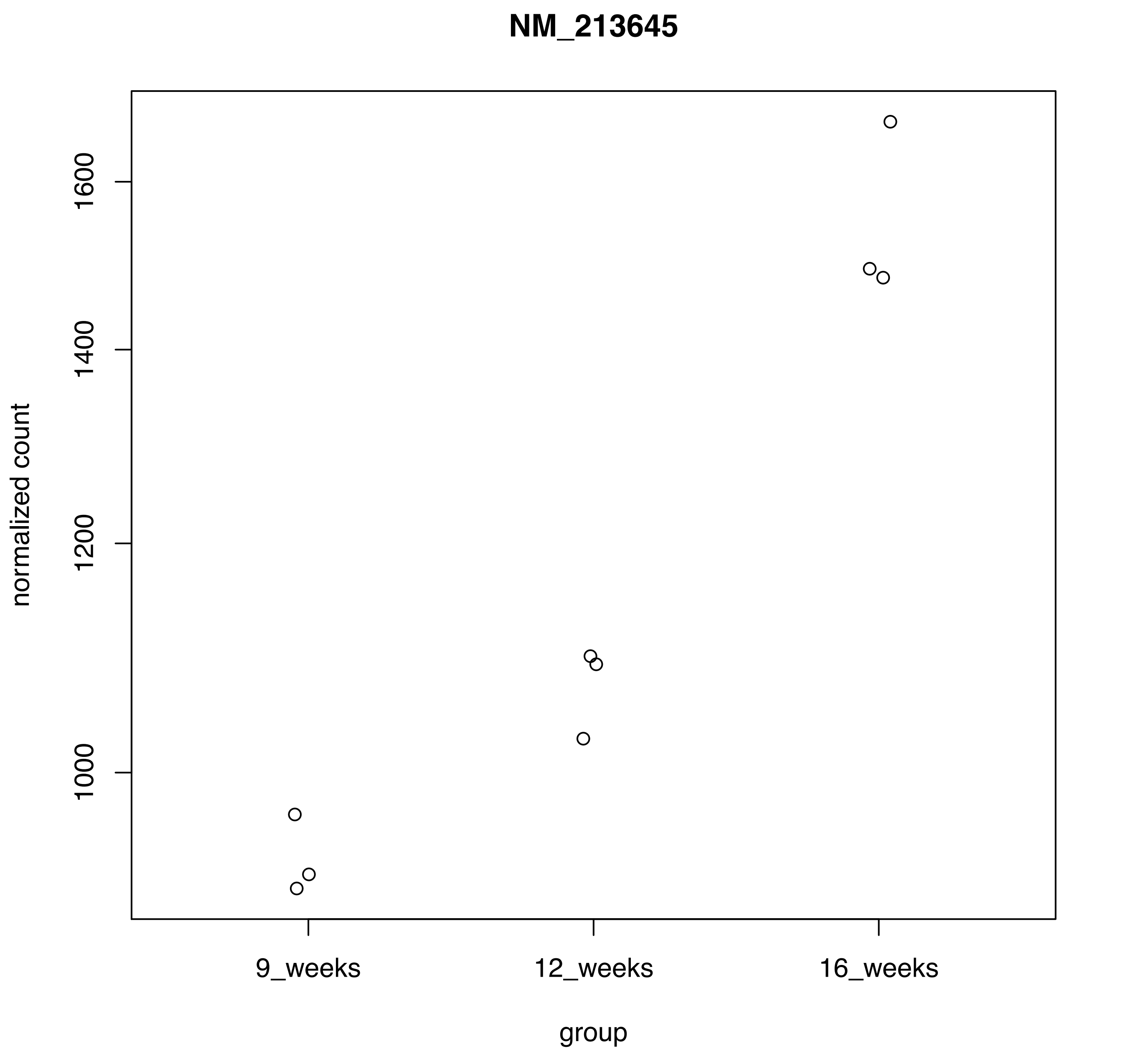

NM_213645
normalized count
1000
1200
1400
1600
9_weeks
12_weeks
16_weeks
group

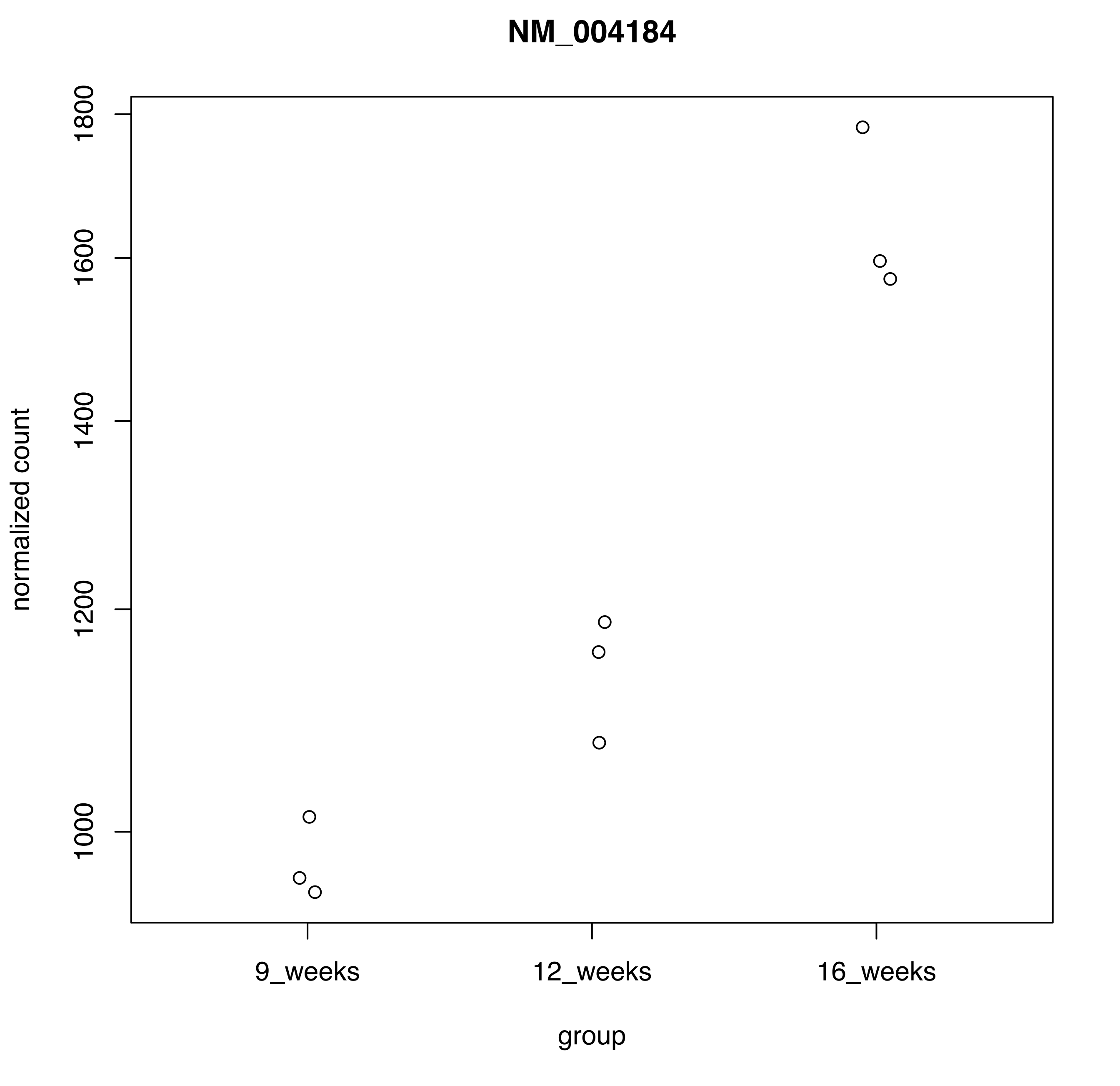
NM_004184
normalized count
1000
1200
1400
1600
1800
9_weeks
12_weeks
16_weeks
group

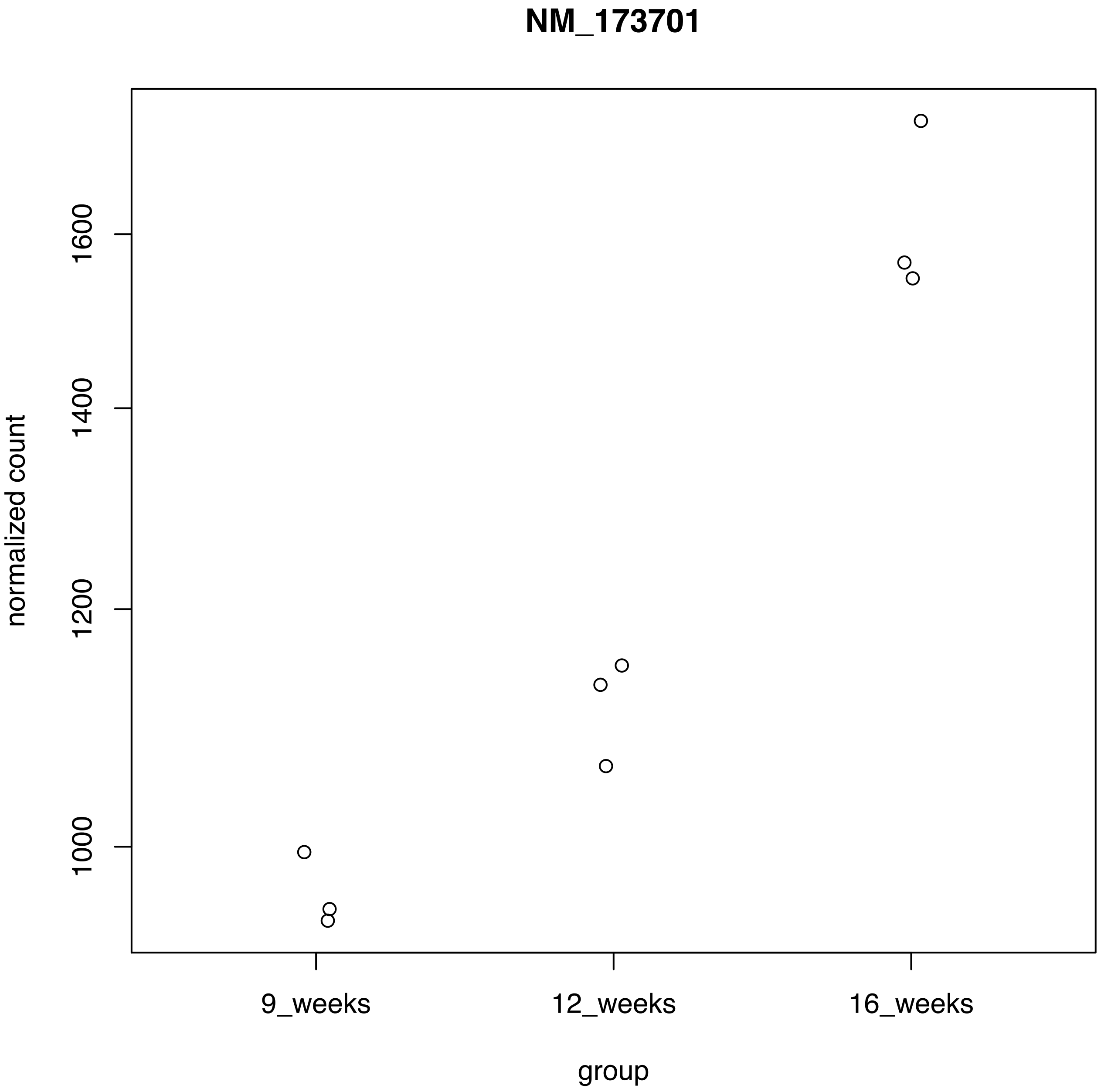

NM_173701
normalized count
1000
1200
1400
1600
9_weeks
12_weeks
16_weeks
group

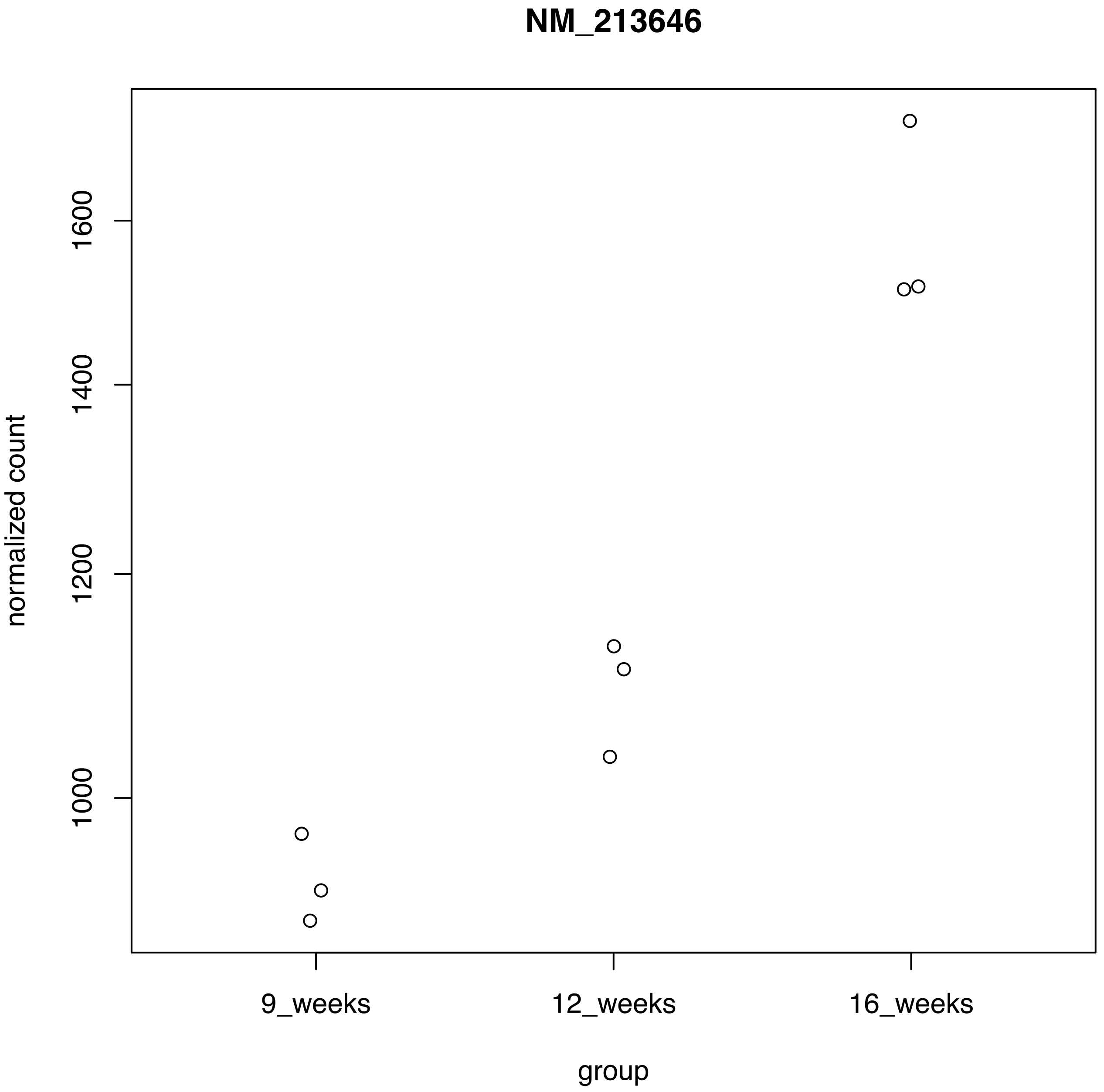
NM_213646
normalized count
1000
1200
1400
1600
9_weeks
12_weeks
16_weeks
group

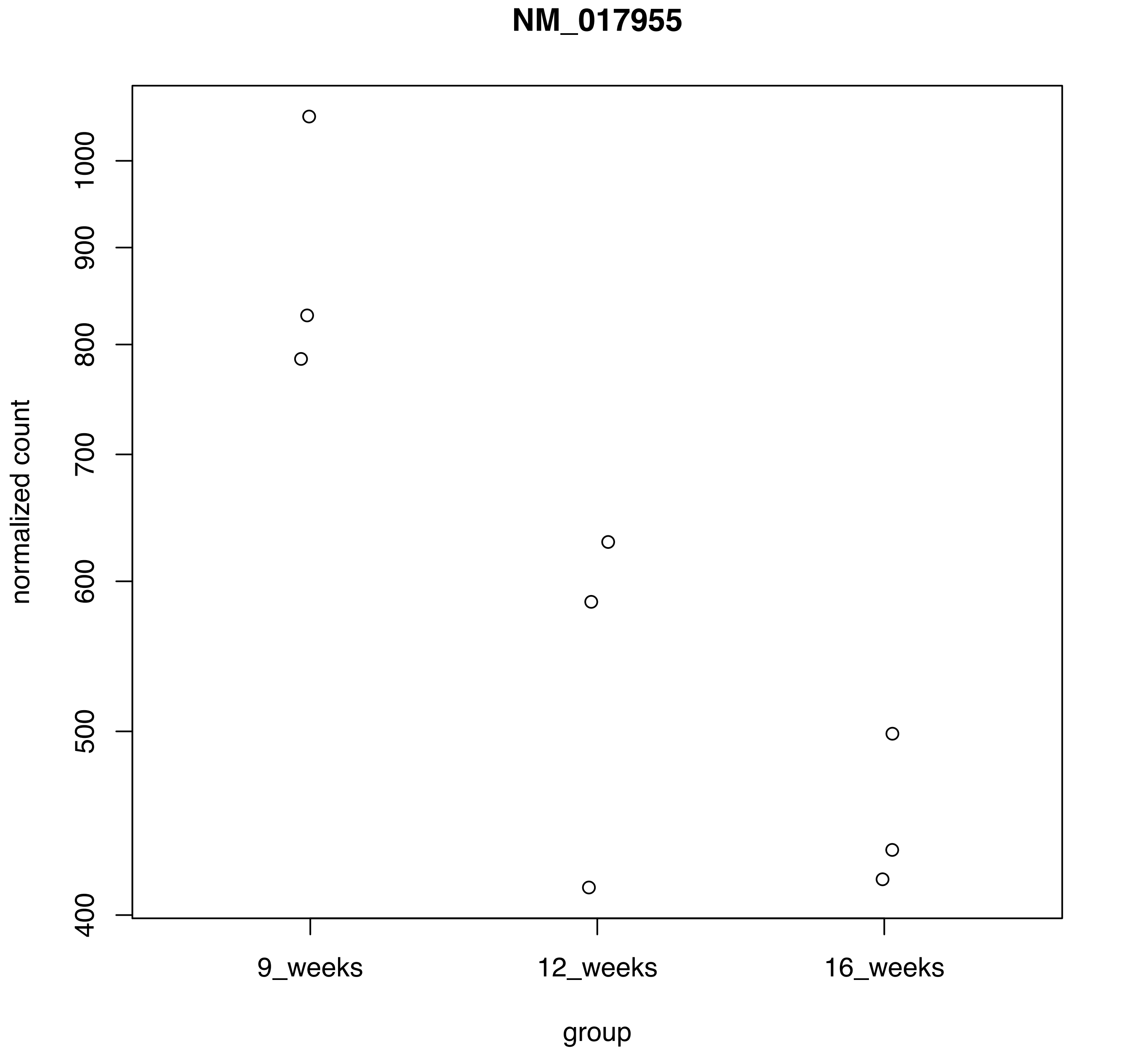
NM_017955
normalized count
1000
900
800
700
600
500
400
9_weeks
12_weeks
16_weeks
group

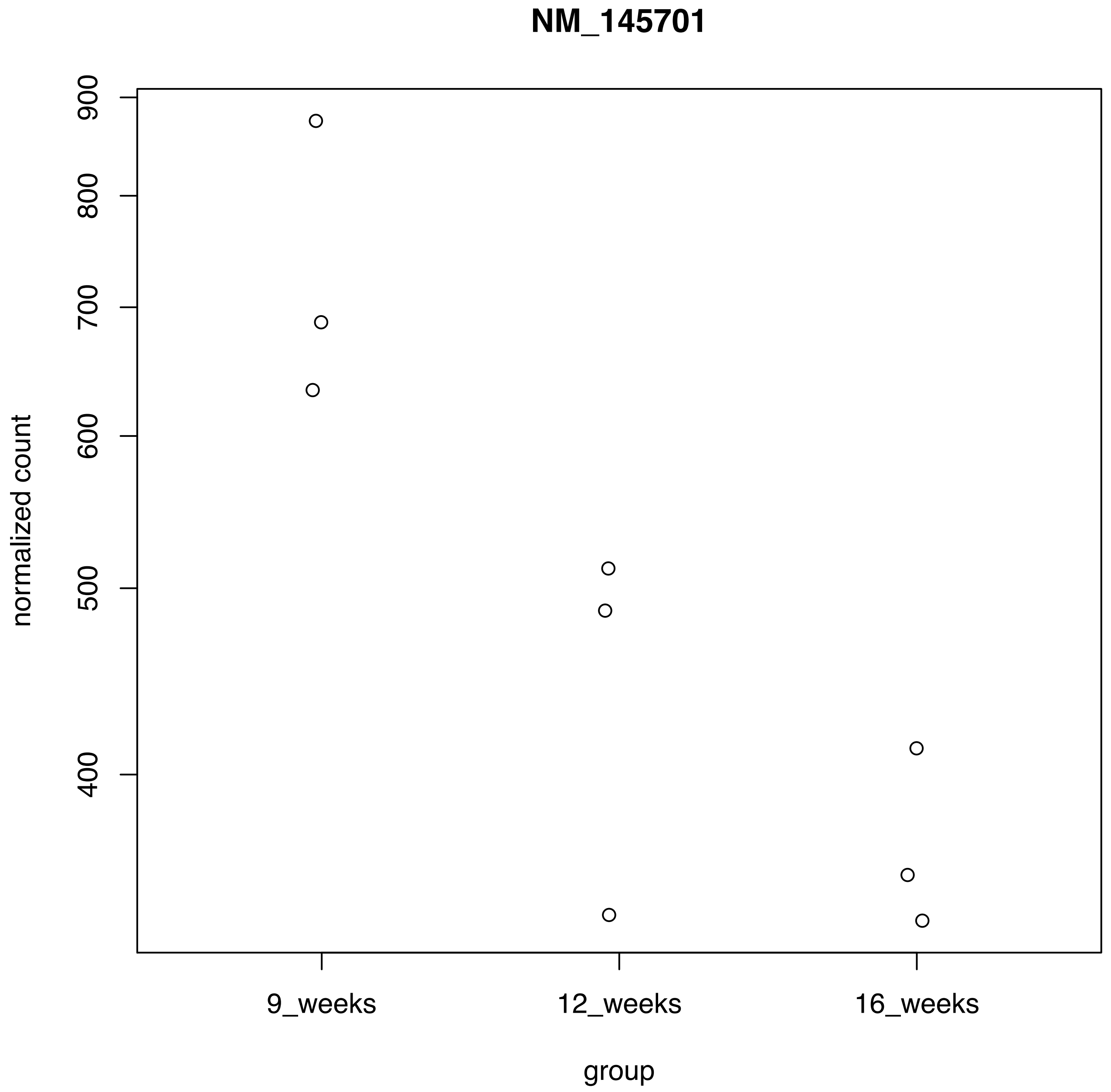
NM_145701
normalized count
900
800
700
600
500
400
9_weeks
12_weeks
16_weeks
group

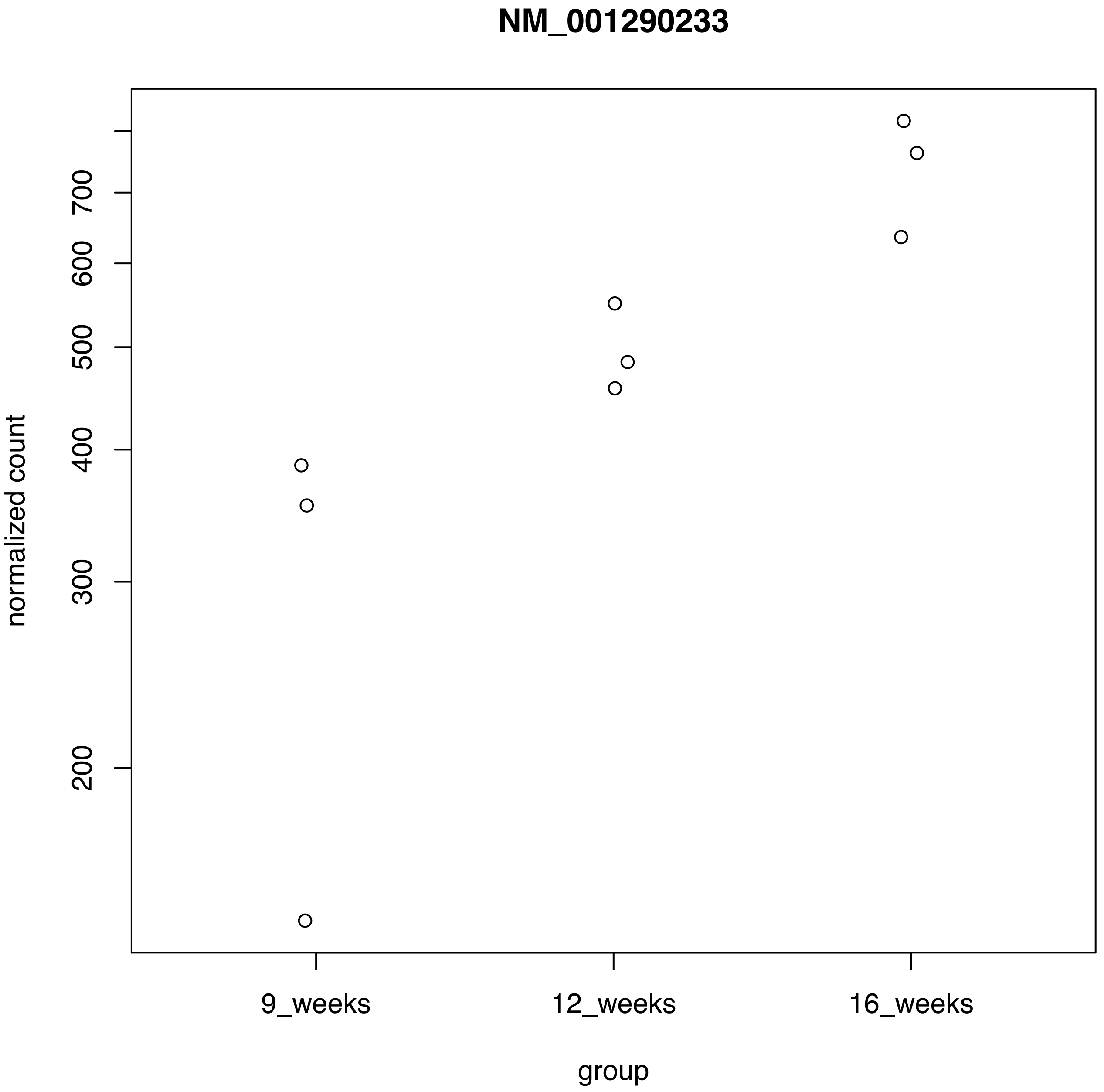
NM_001290233
normalized count
200
300
400
500
600
700
9_weeks
12_weeks
16_weeks
group

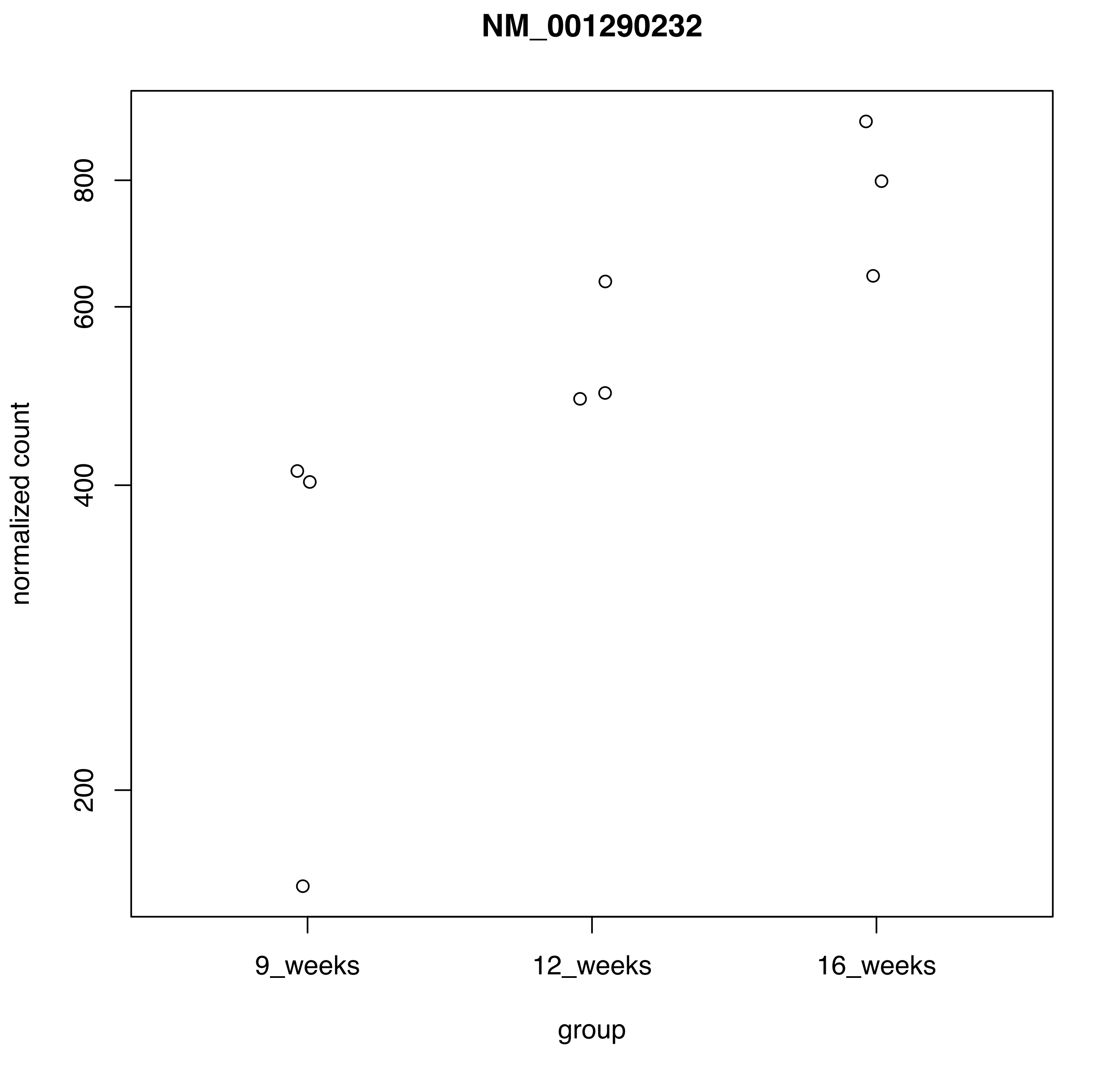

NM_001290232
normalized count
200
400
600
800
9_weeks
12_weeks
16_weeks
group

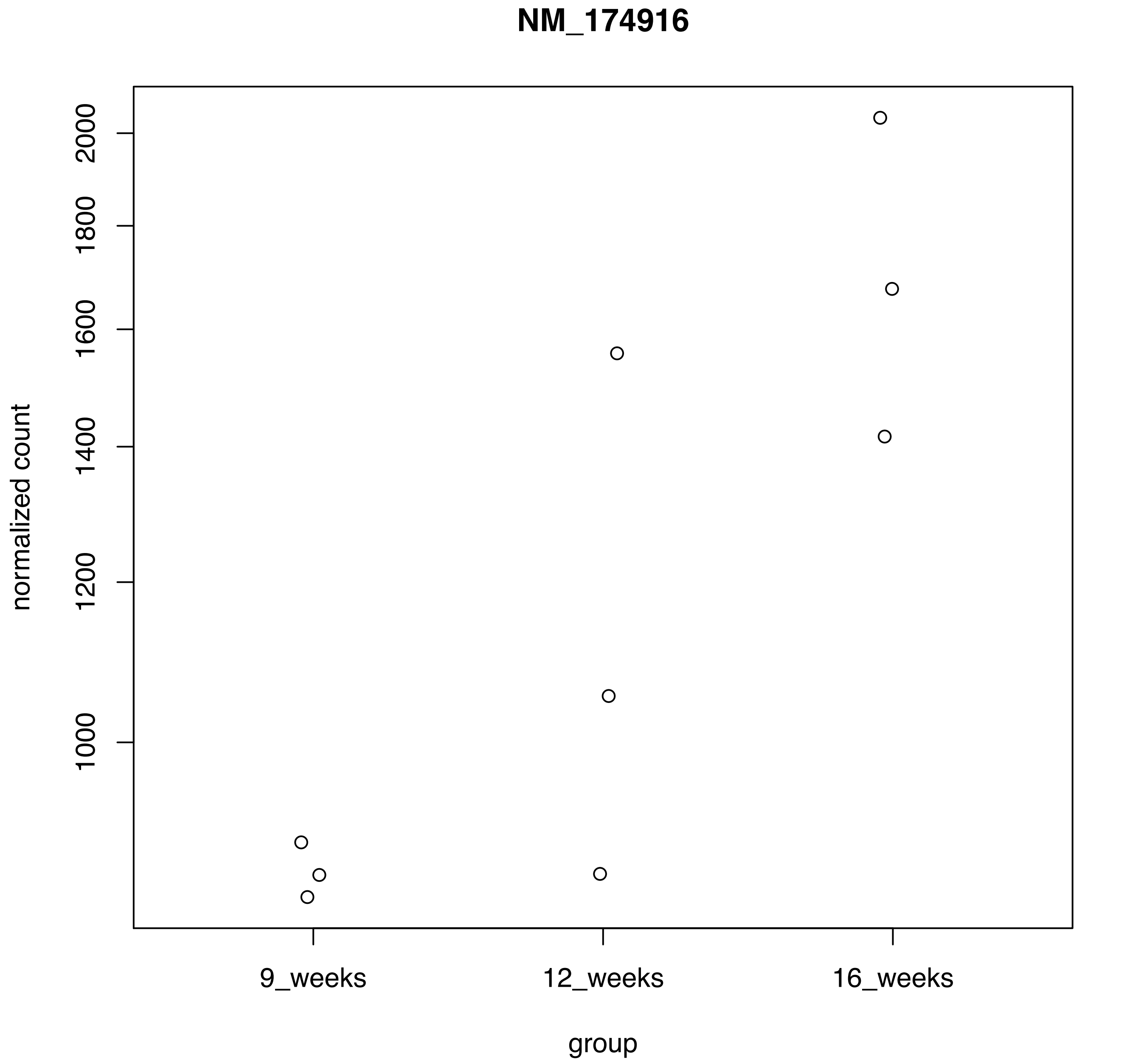
NM_174916
normalized count
2000
1800
1600
1400
1200
1000
9_weeks
12_weeks
16_weeks
group

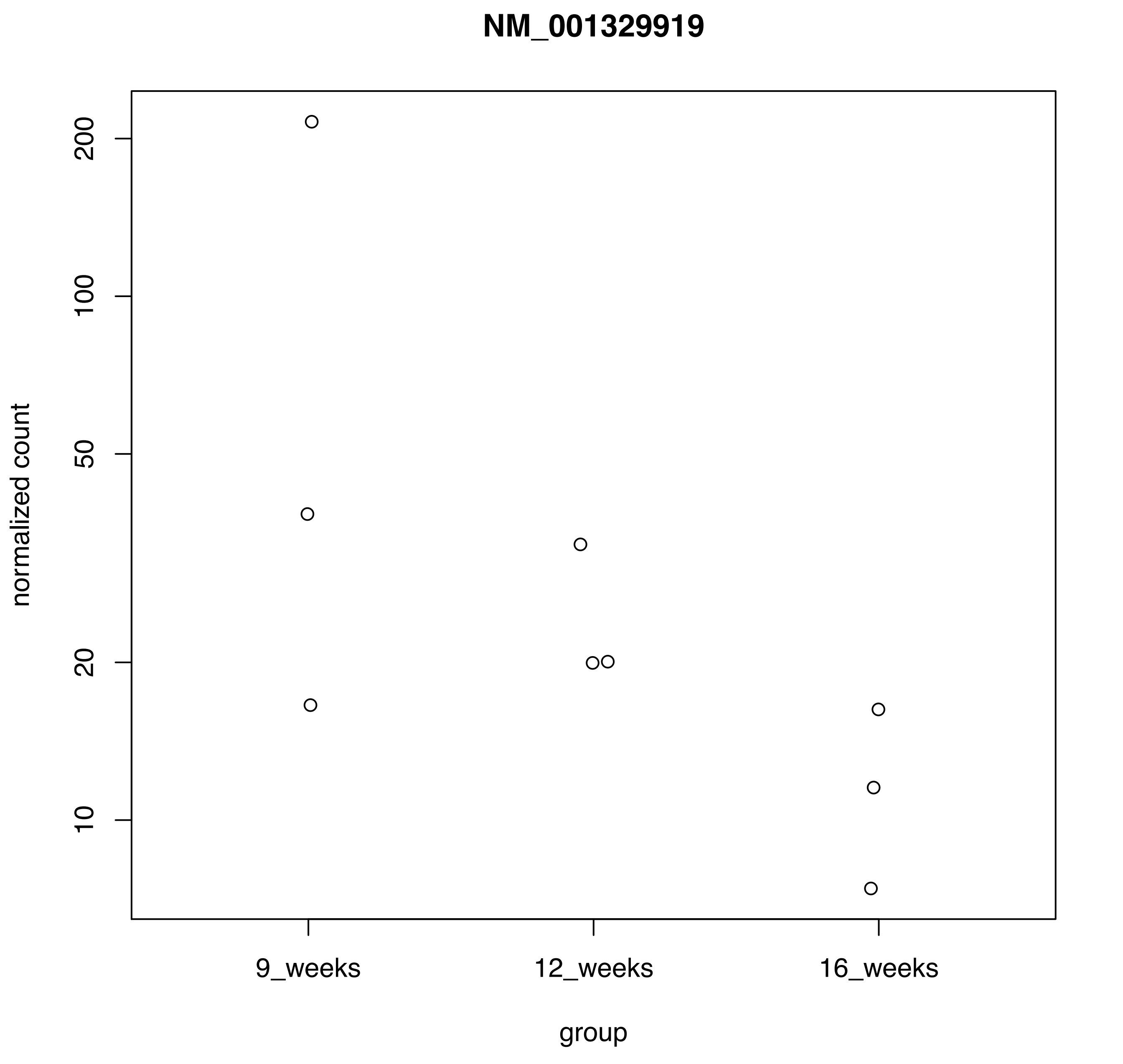

NM_001329919
normalized count
200
100
50
20
10
9_weeks
12_weeks
16_weeks
group

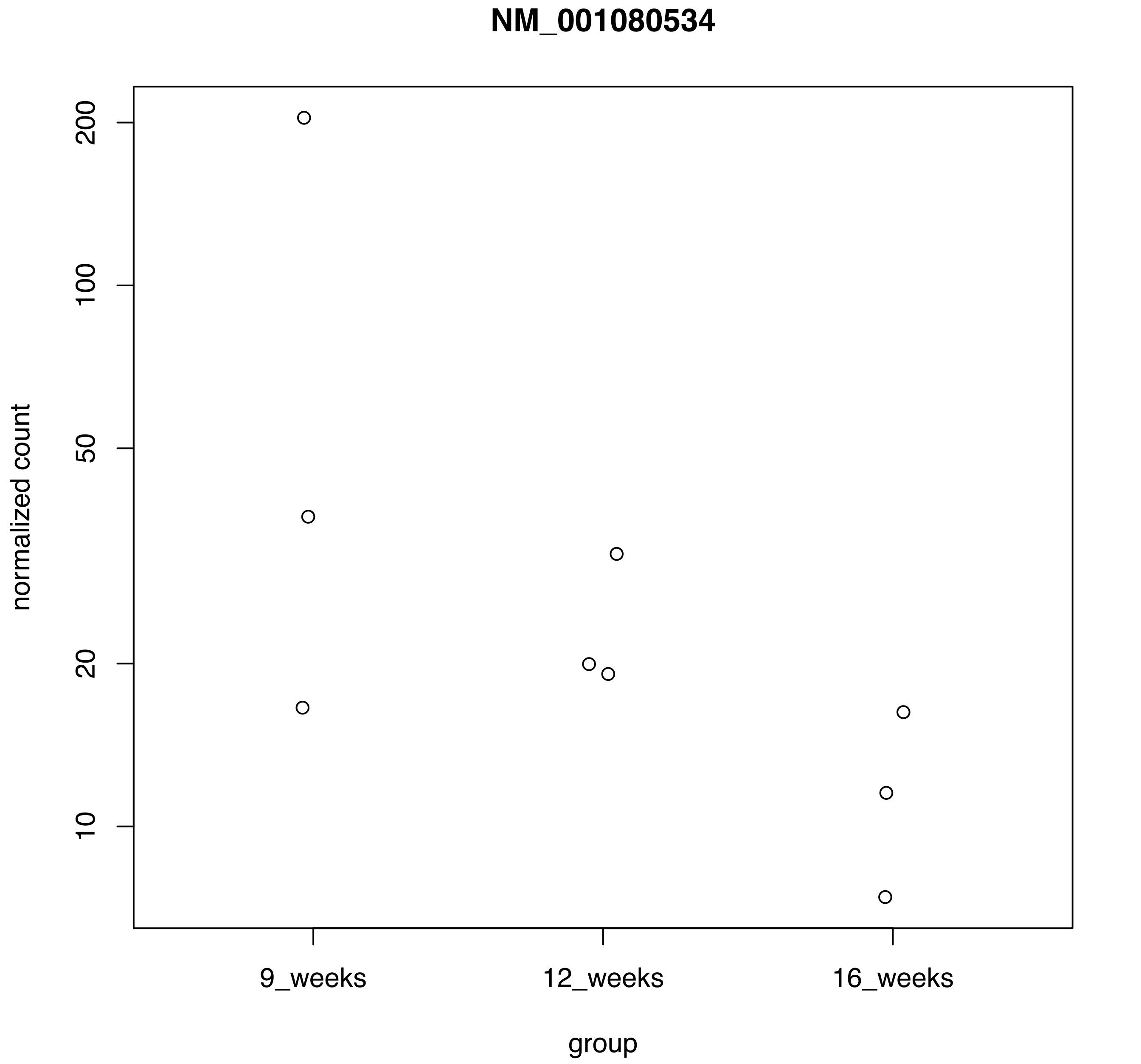
NM_001080534
normalized count
200
100
50
20
10
9_weeks
12_weeks
16_weeks
group

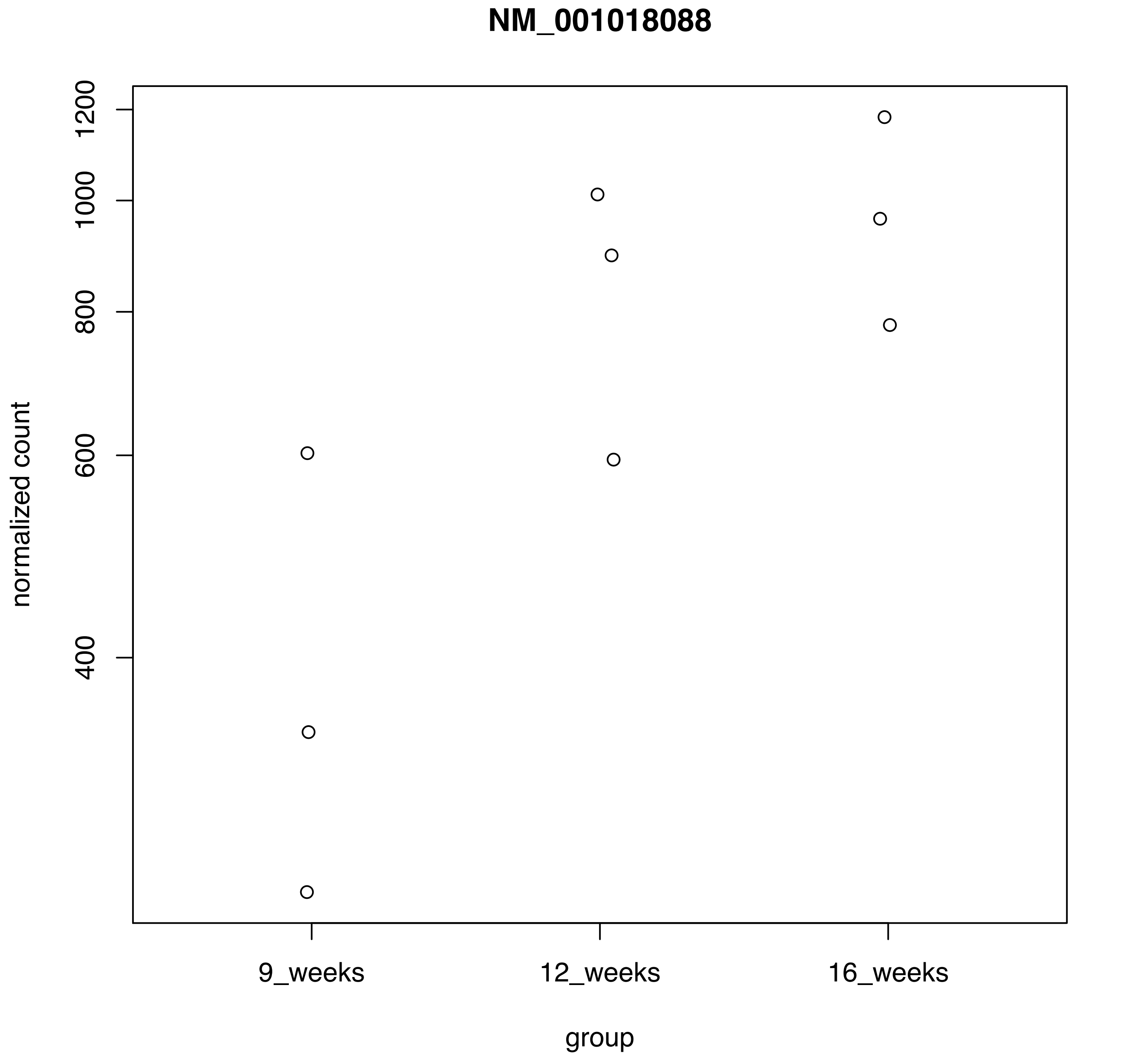
NM_001018088
normalized count
400
600
800
1000
1200
9_weeks
12_weeks
16_weeks
group

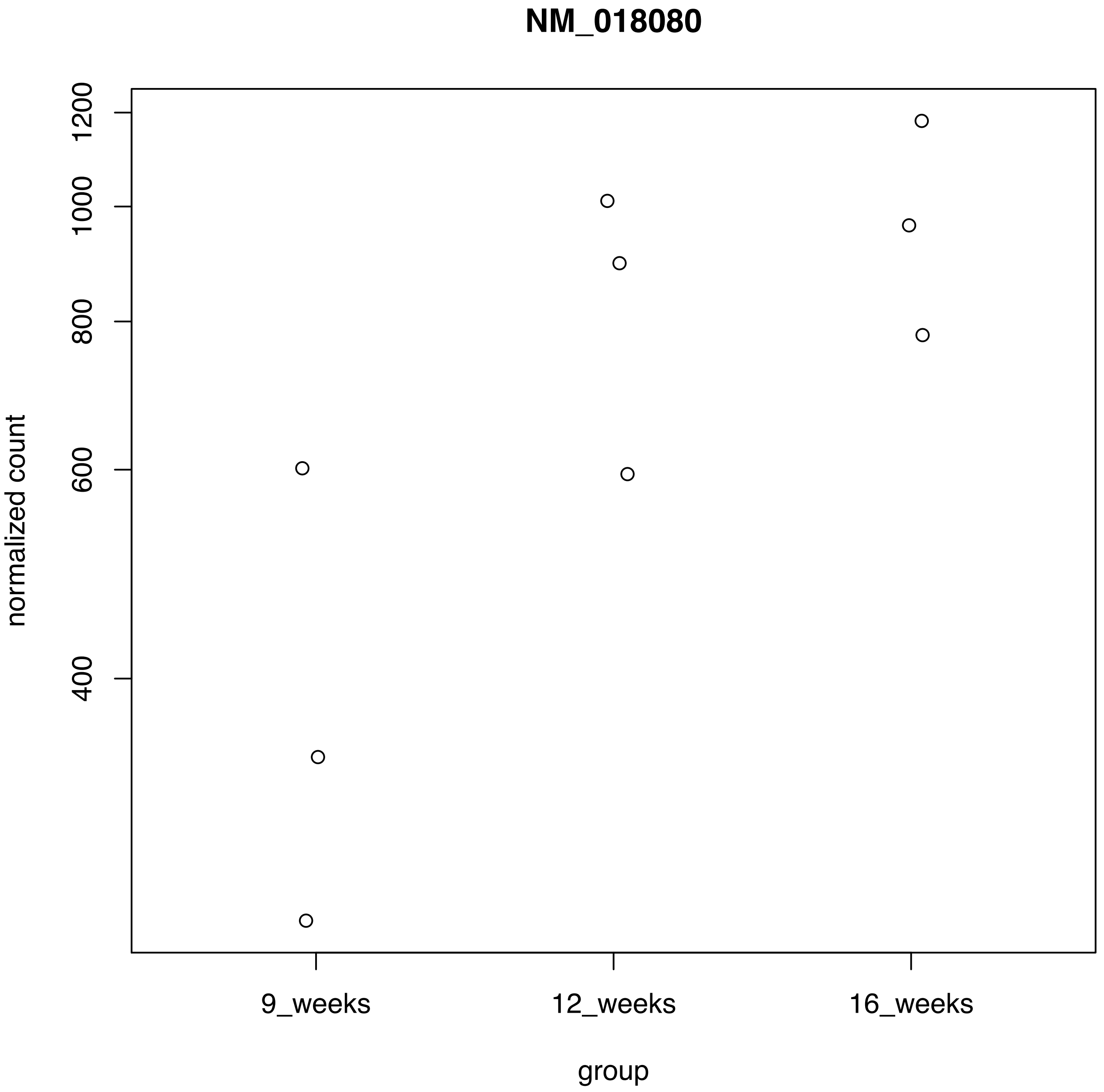

NM_018080
normalized count
1200
1000
800
600
400
9_weeks
12_weeks
16_weeks
group

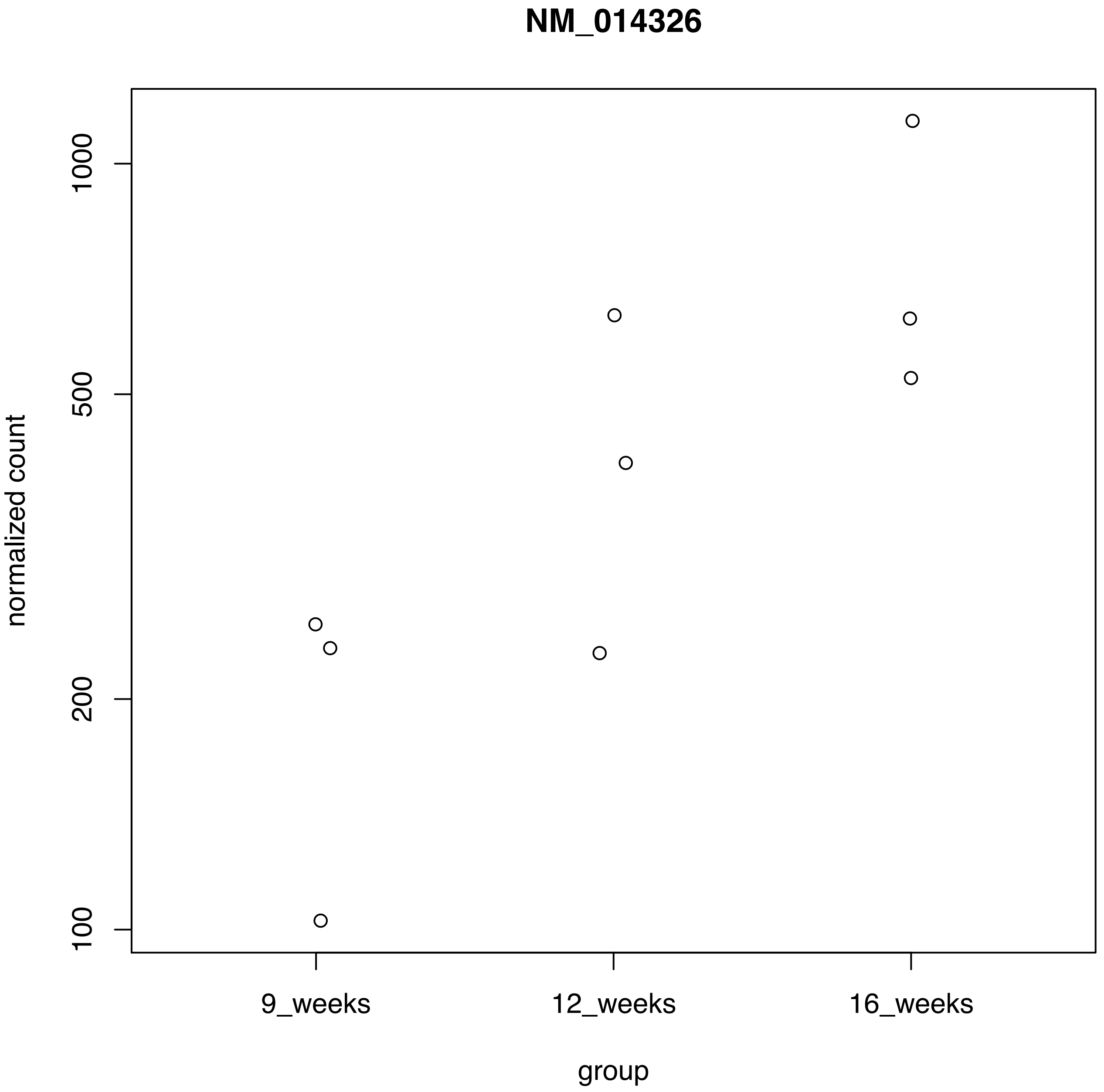
NM_014326
normalized count
1000
500
200
100
9_weeks
12_weeks
16_weeks
group

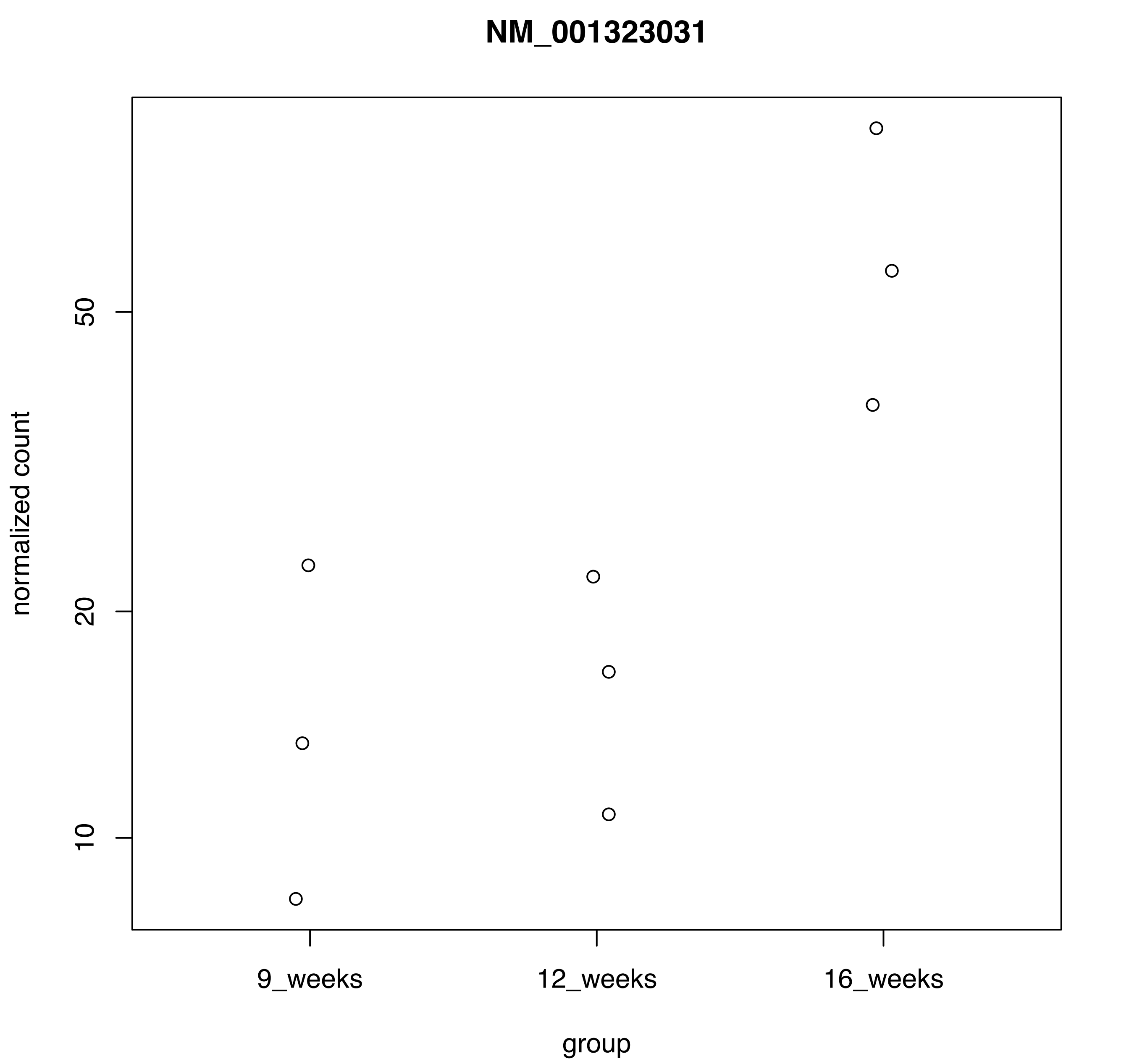
NM_001323031
normalized count
50
20
10
9_weeks
12_weeks
16_weeks
group

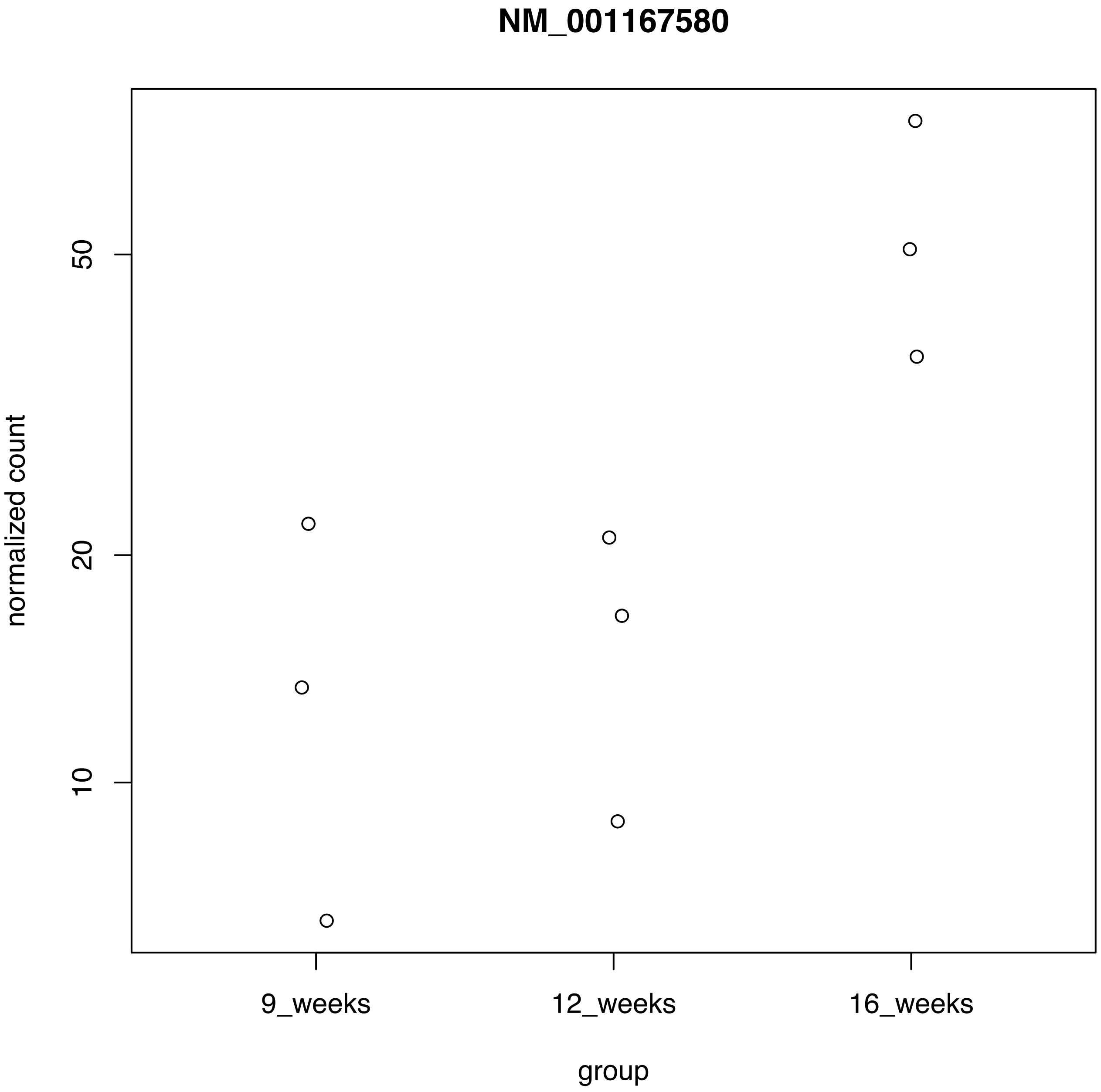
NM_001167580
normalized count
50
20
10
9_weeks
12_weeks
16_weeks
group

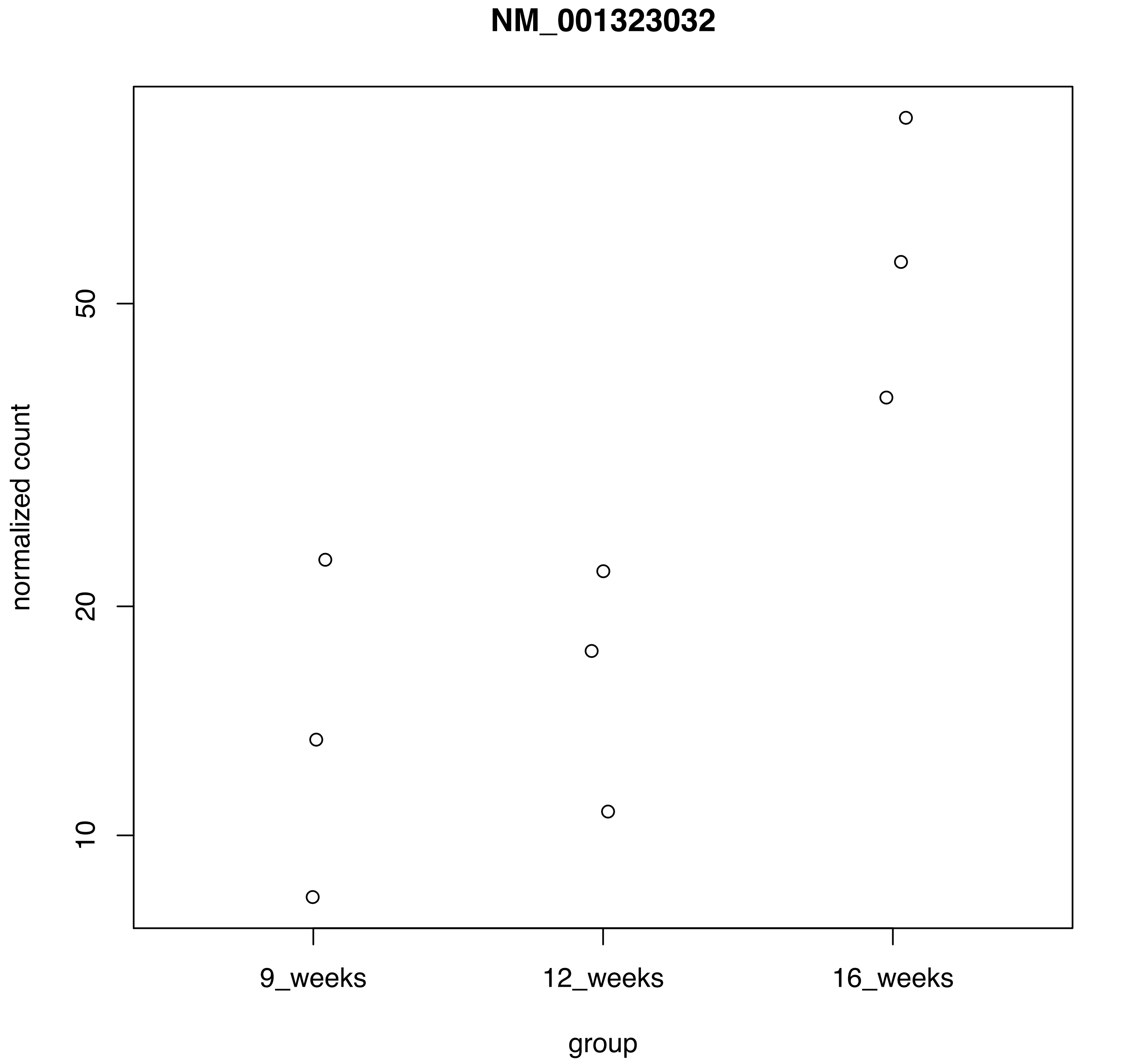
NM_001323032
normalized count
50
20
10
9_weeks
12_weeks
16_weeks
group

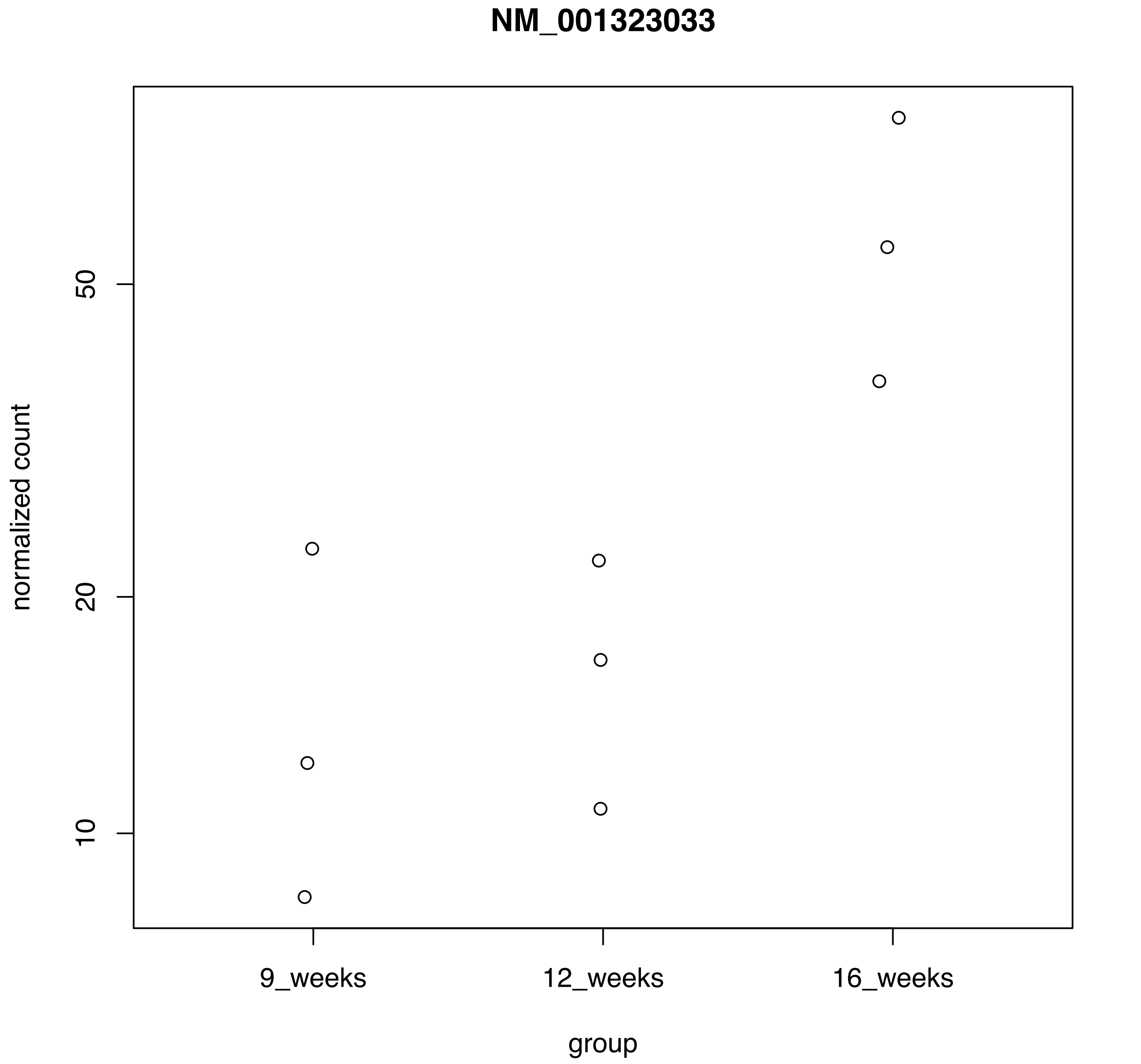
NM_001323033
normalized count
50
20
10
9_weeks
12_weeks
16_weeks
group

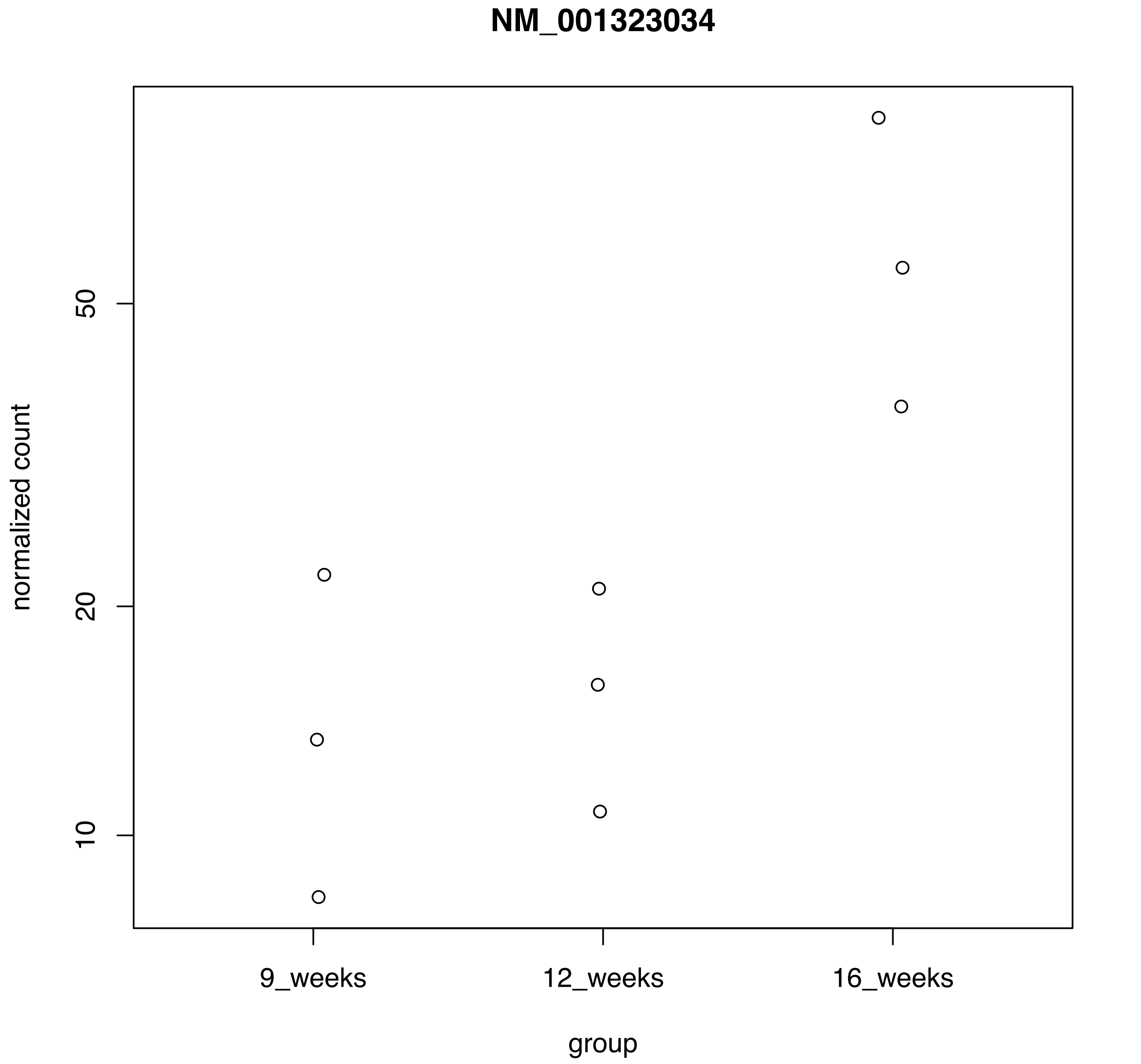
NM_001323034
normalized count
10
20
50
9_weeks
12_weeks
16_weeks
group

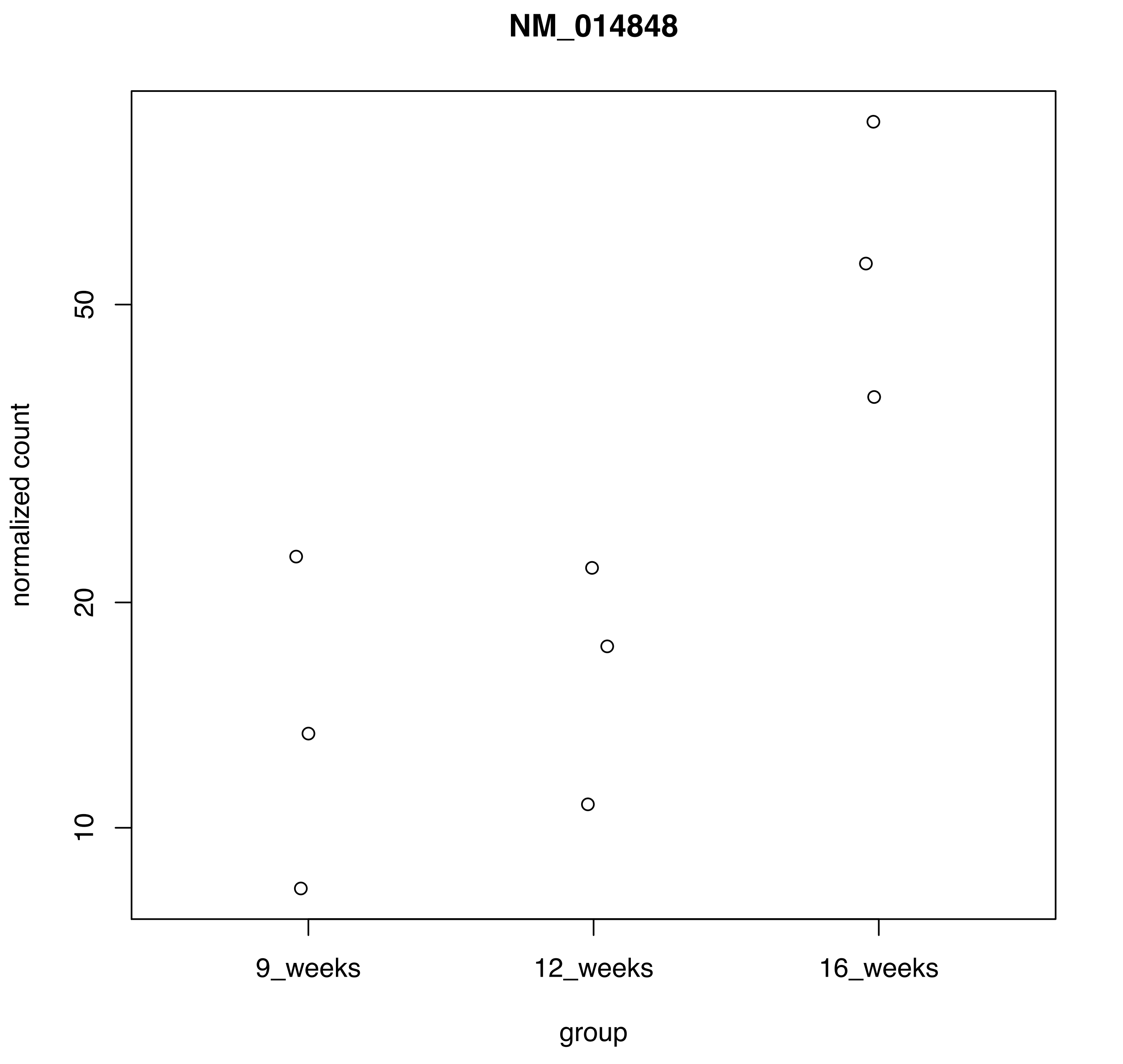
NM_014848
normalized count
50
20
10
9_weeks
12_weeks
16_weeks
group

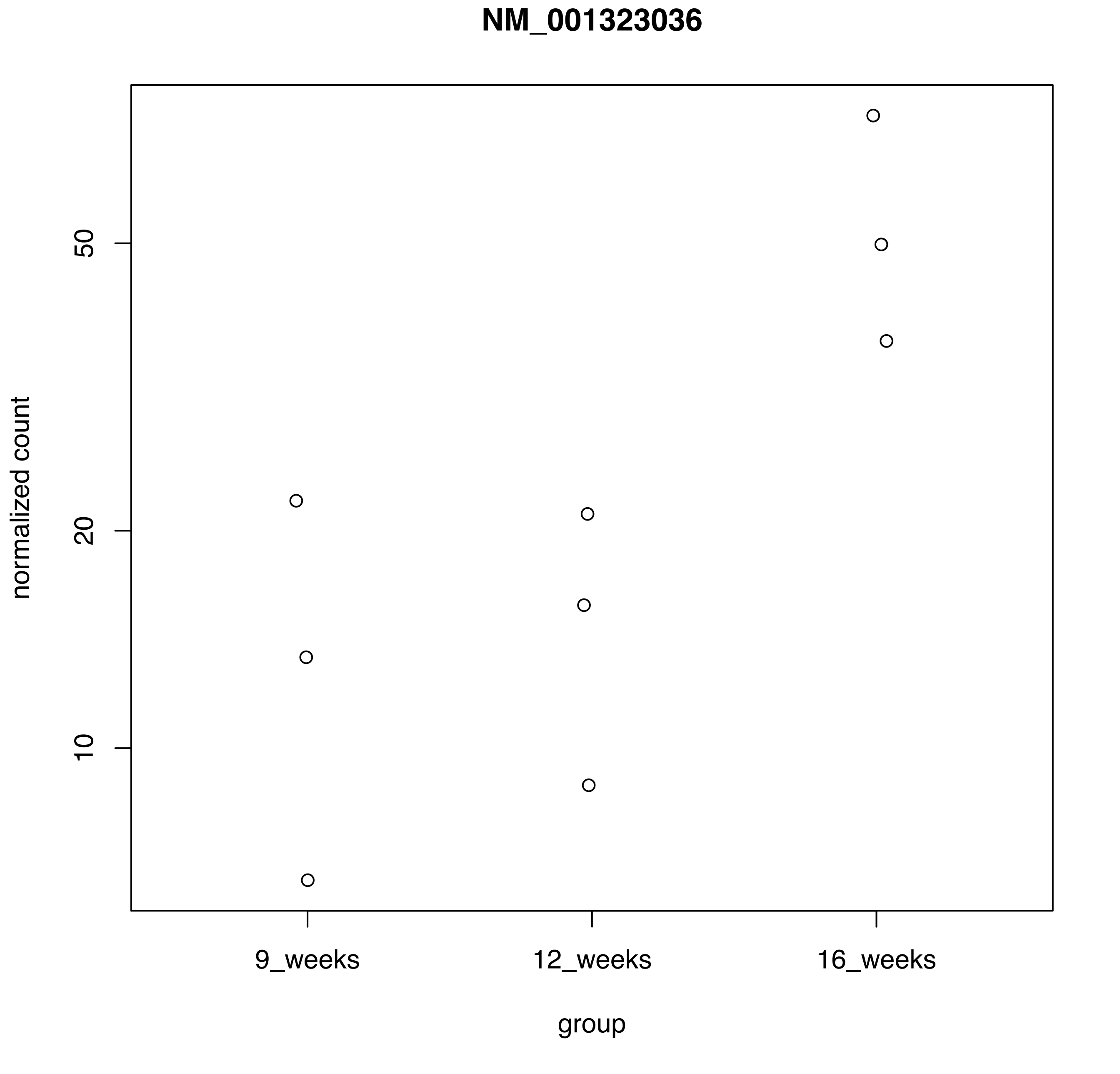
NM_001323036
normalized count
50
20
10
9_weeks
12_weeks
16_weeks
group

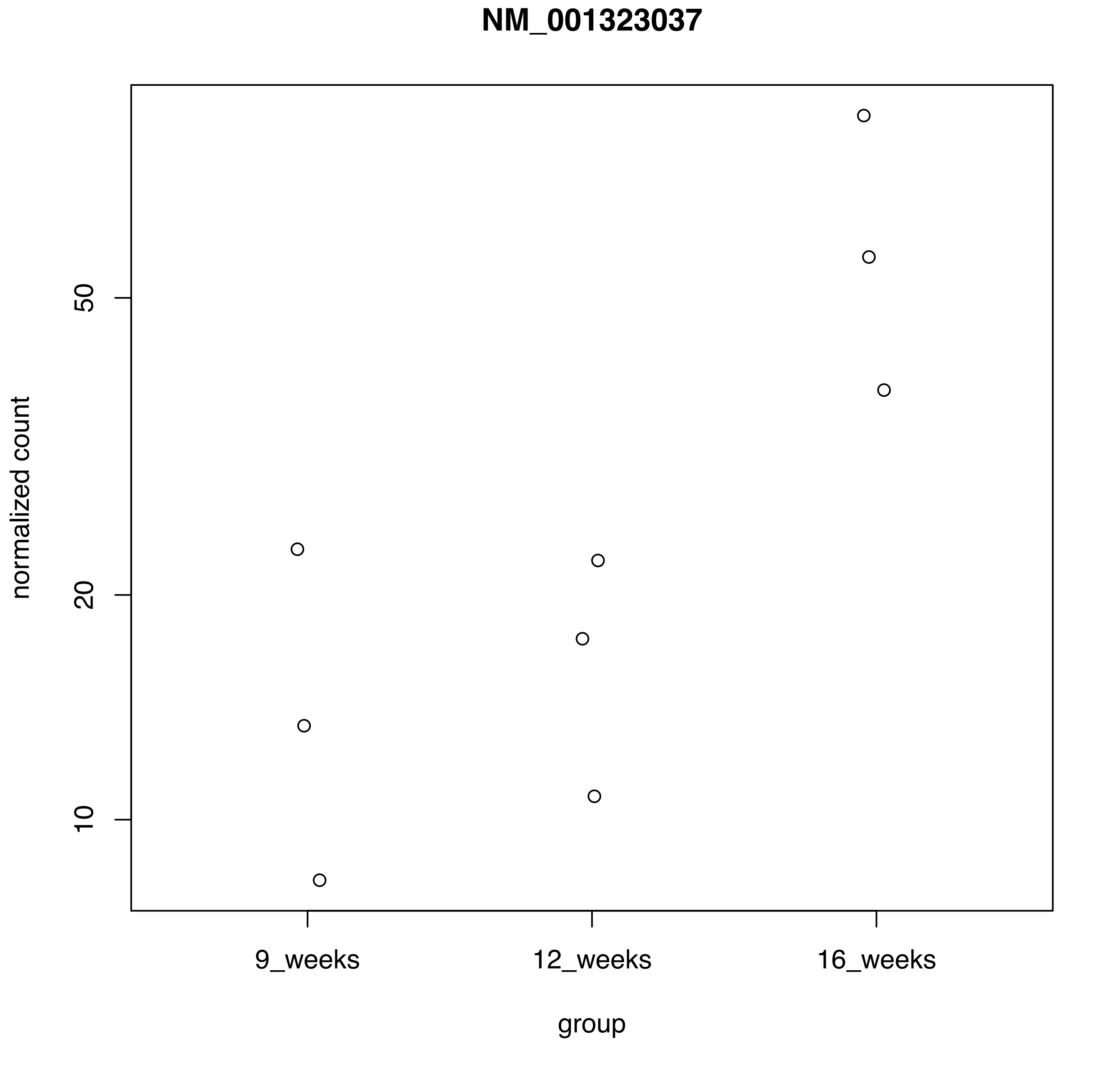

NM_001323037
normalized count
50
20
10
9_weeks
12_weeks
16_weeks
group

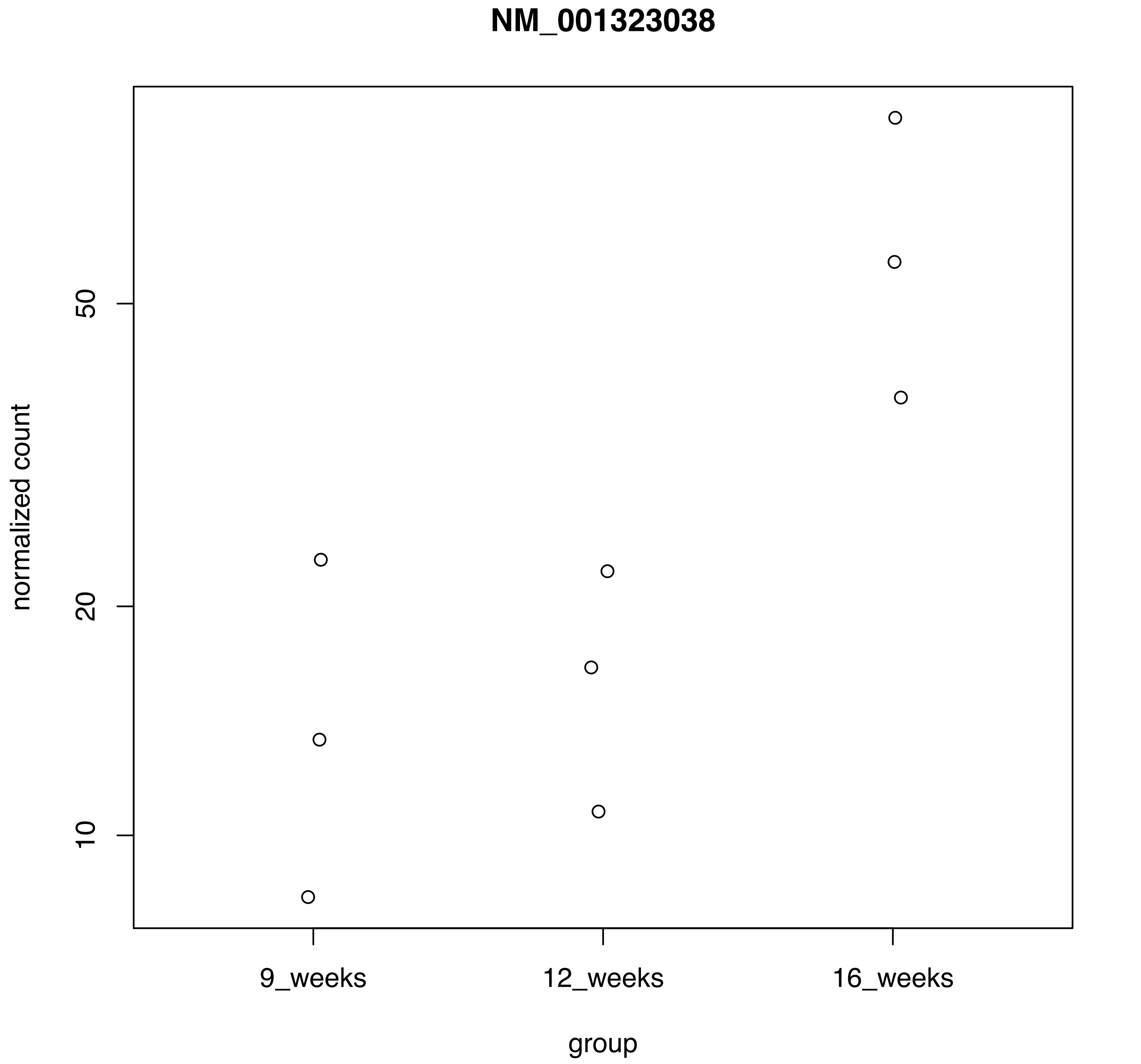

NM_001323038
normalized count
50
20
10
9_weeks
12_weeks
16_weeks
group

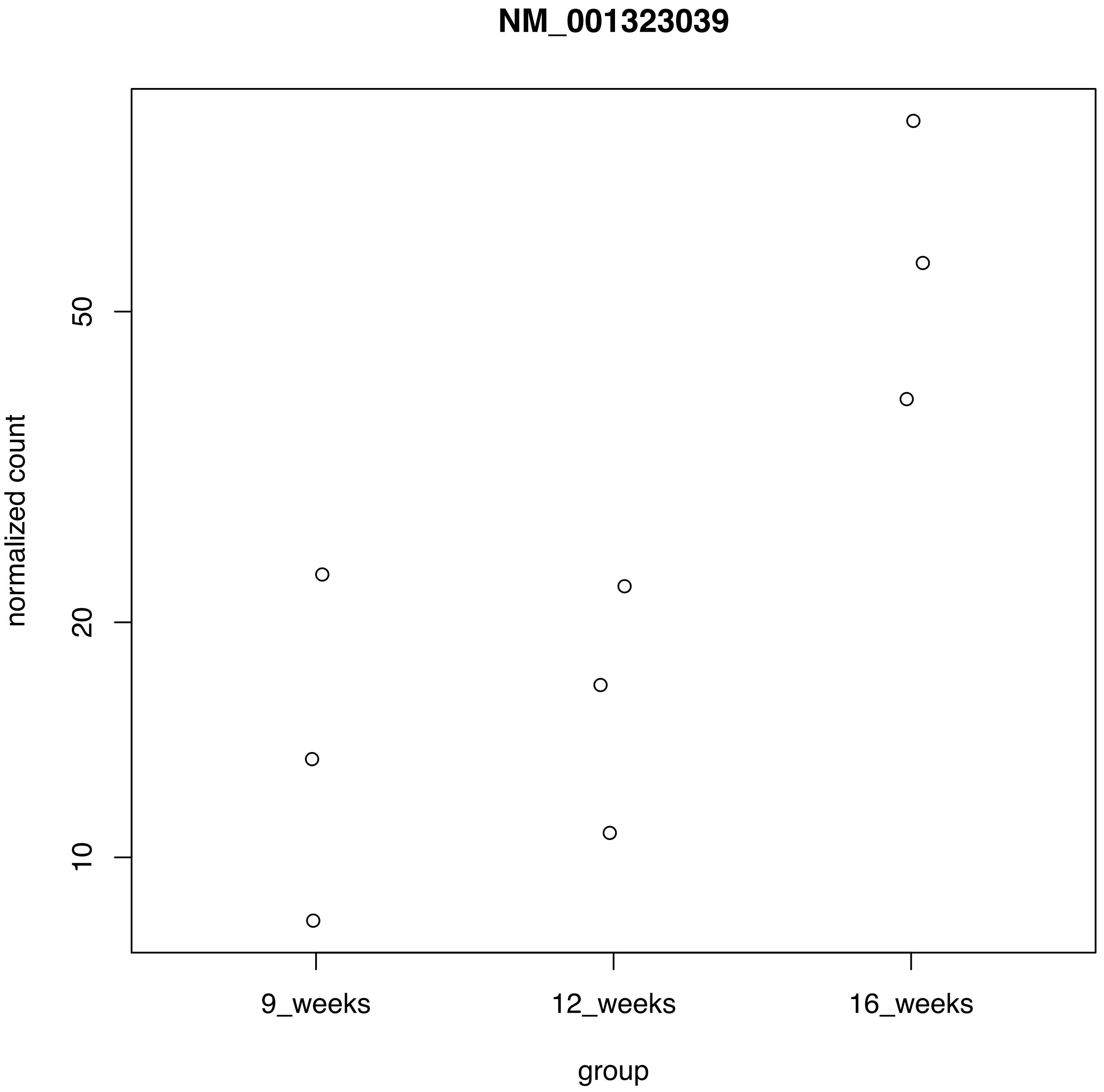

NM_001323039
normalized count
50
20
10
9_weeks
12_weeks
16_weeks
group

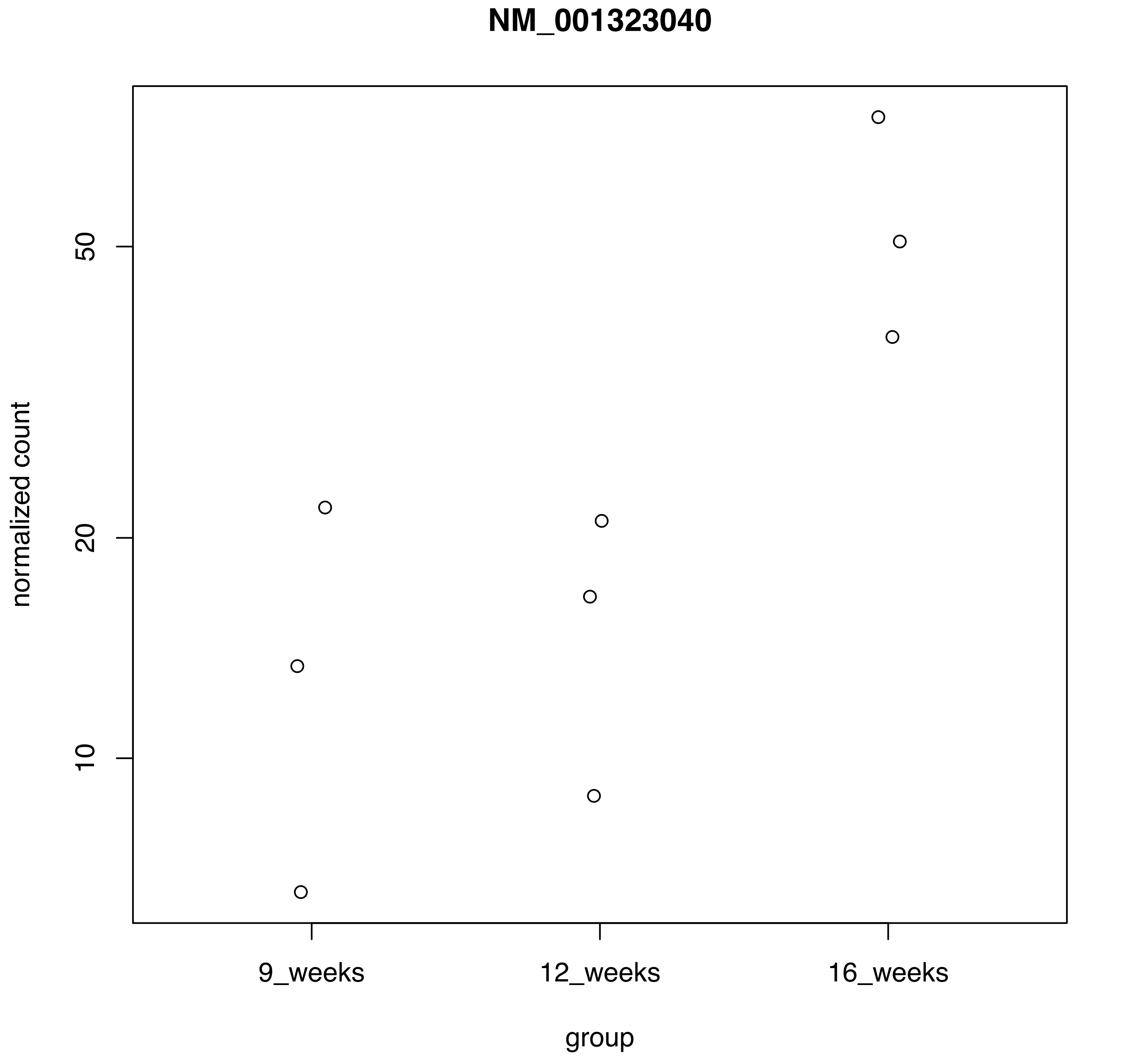
NM_001323040
normalized count
50
20
10
9_weeks
12_weeks
16_weeks
group

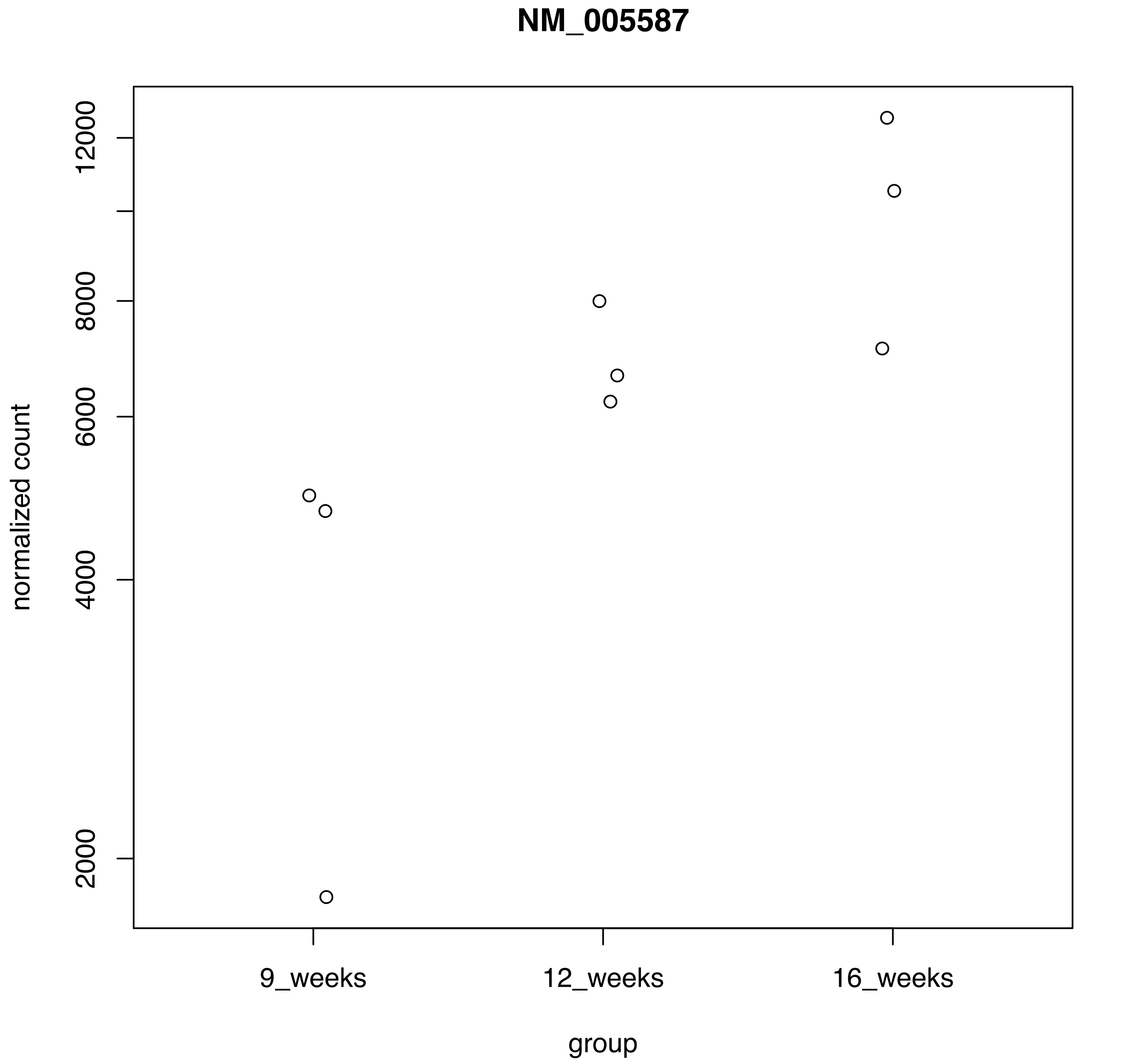

NM_005587
normalized count
2000
4000
6000
8000
12000
9_weeks
12_weeks
16_weeks
group

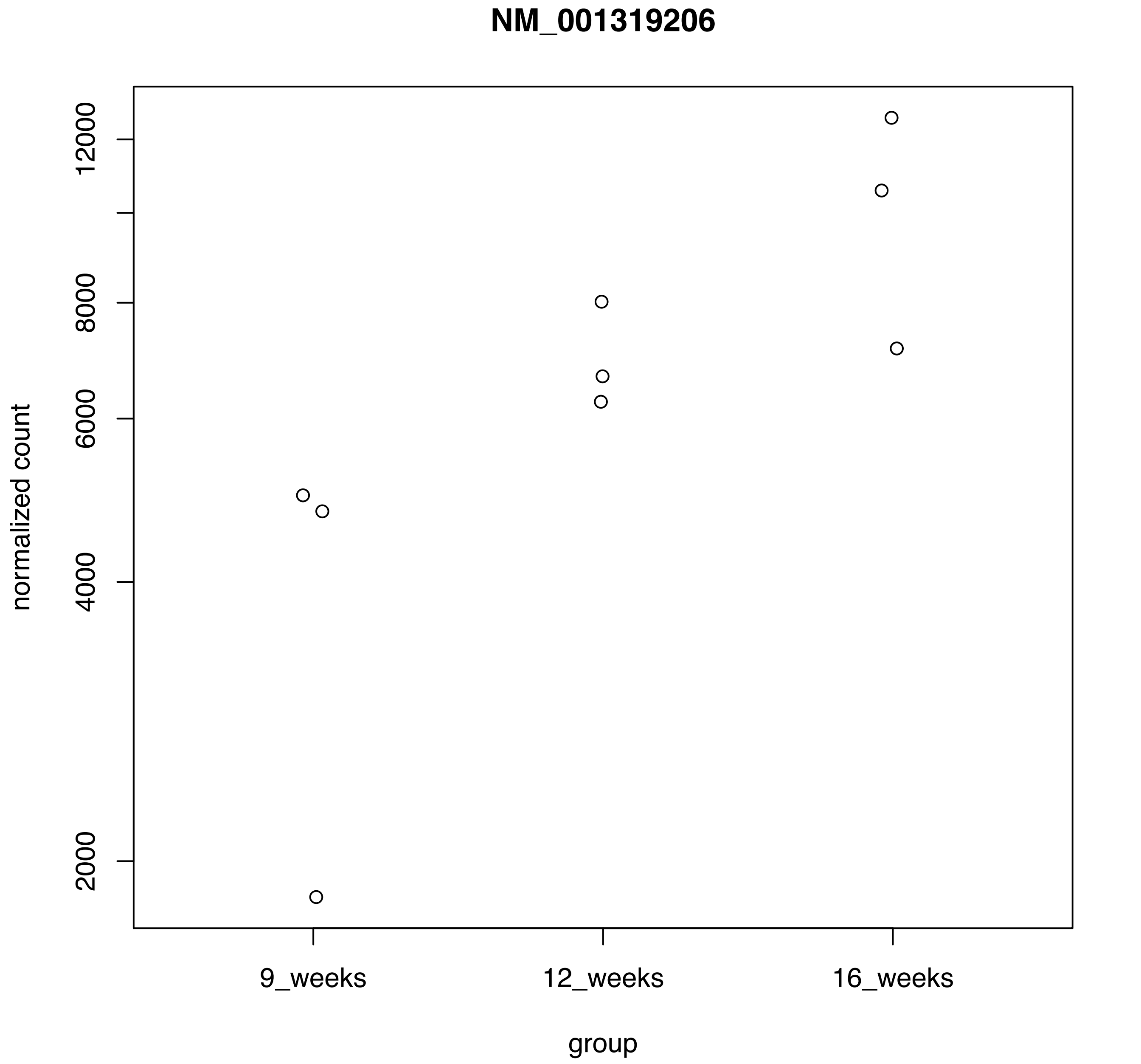
NM_001319206
normalized count
12000
8000
6000
4000
2000
9_weeks
12_weeks
16_weeks
group

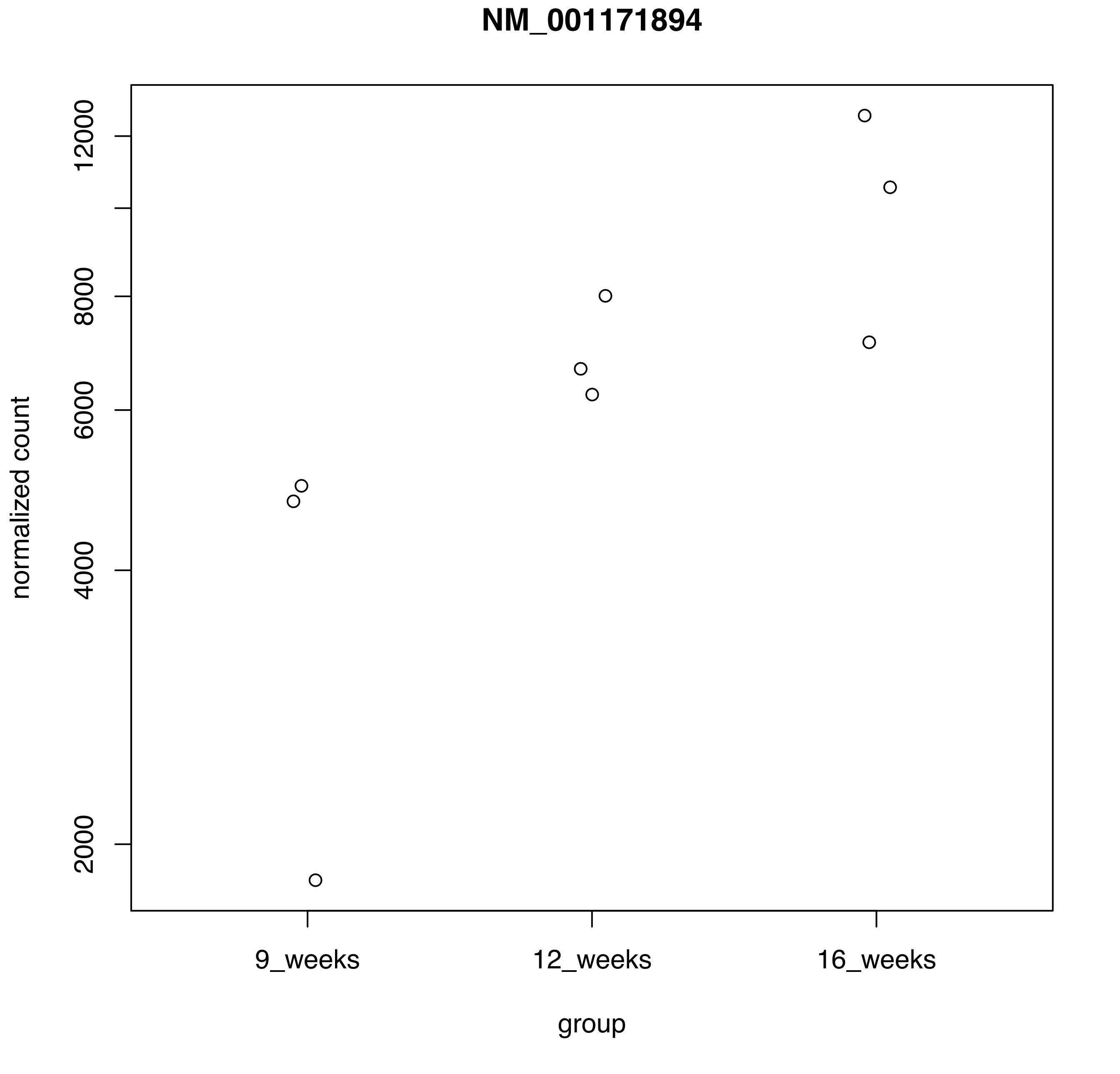
NM_001171894
normalized count
12000
8000
6000
4000
2000
9_weeks
12_weeks
16_weeks
group

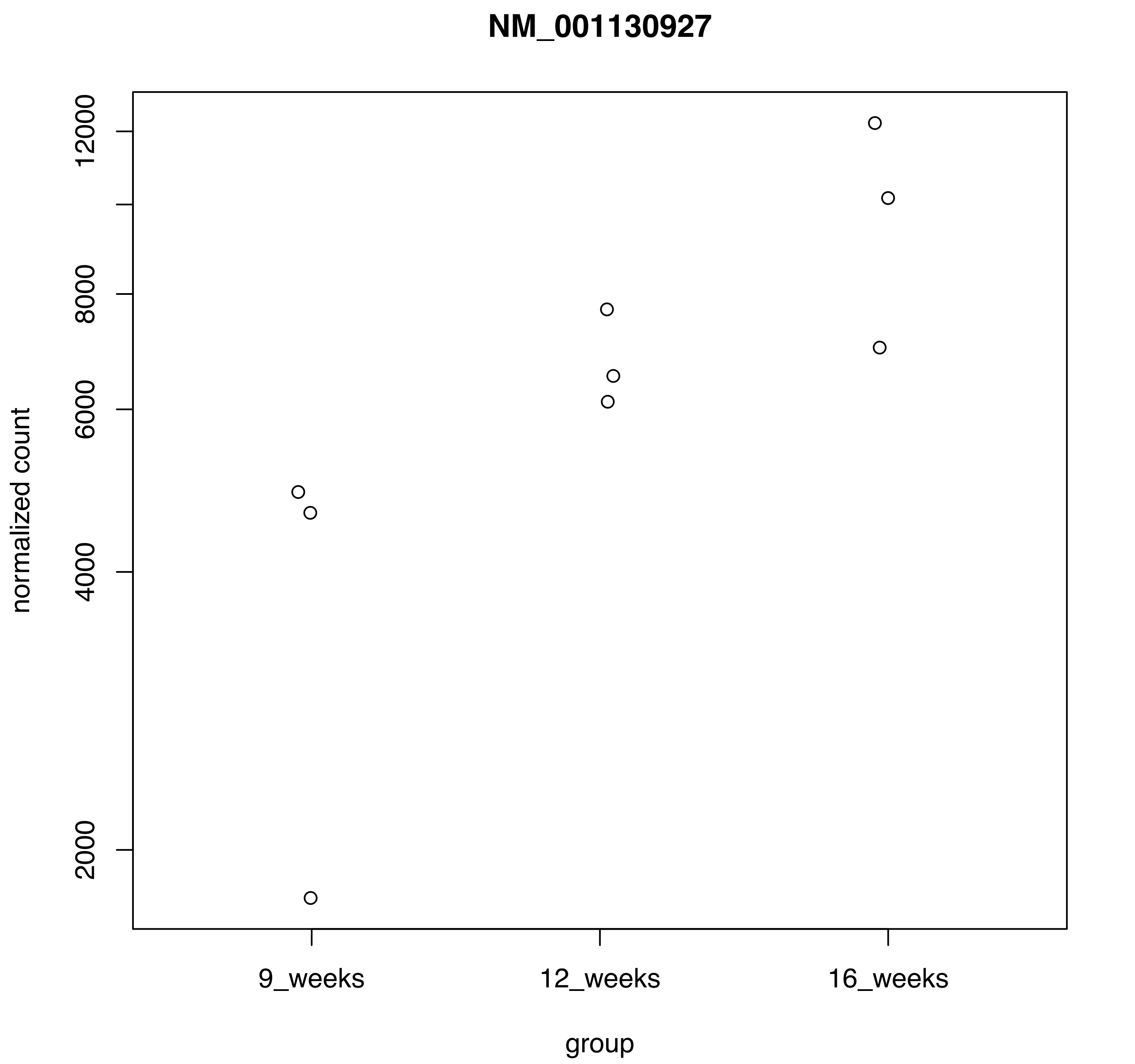

NM_001130927
normalized count
12000
8000
6000
4000
2000
9_weeks
12_weeks
16_weeks
group

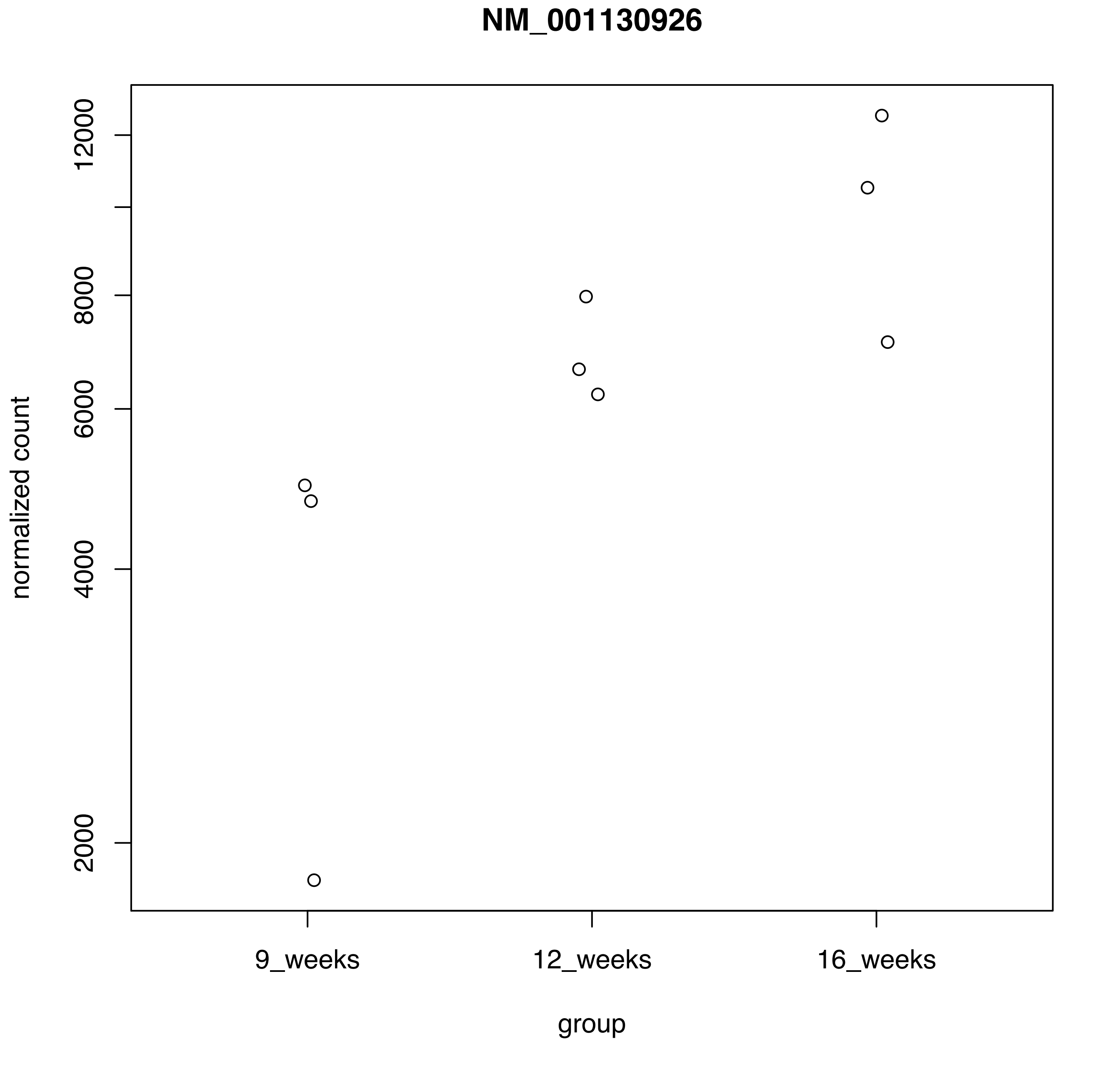
NM_001130926
normalized count
12000
8000
6000
4000
2000
9_weeks
12_weeks
16_weeks
group

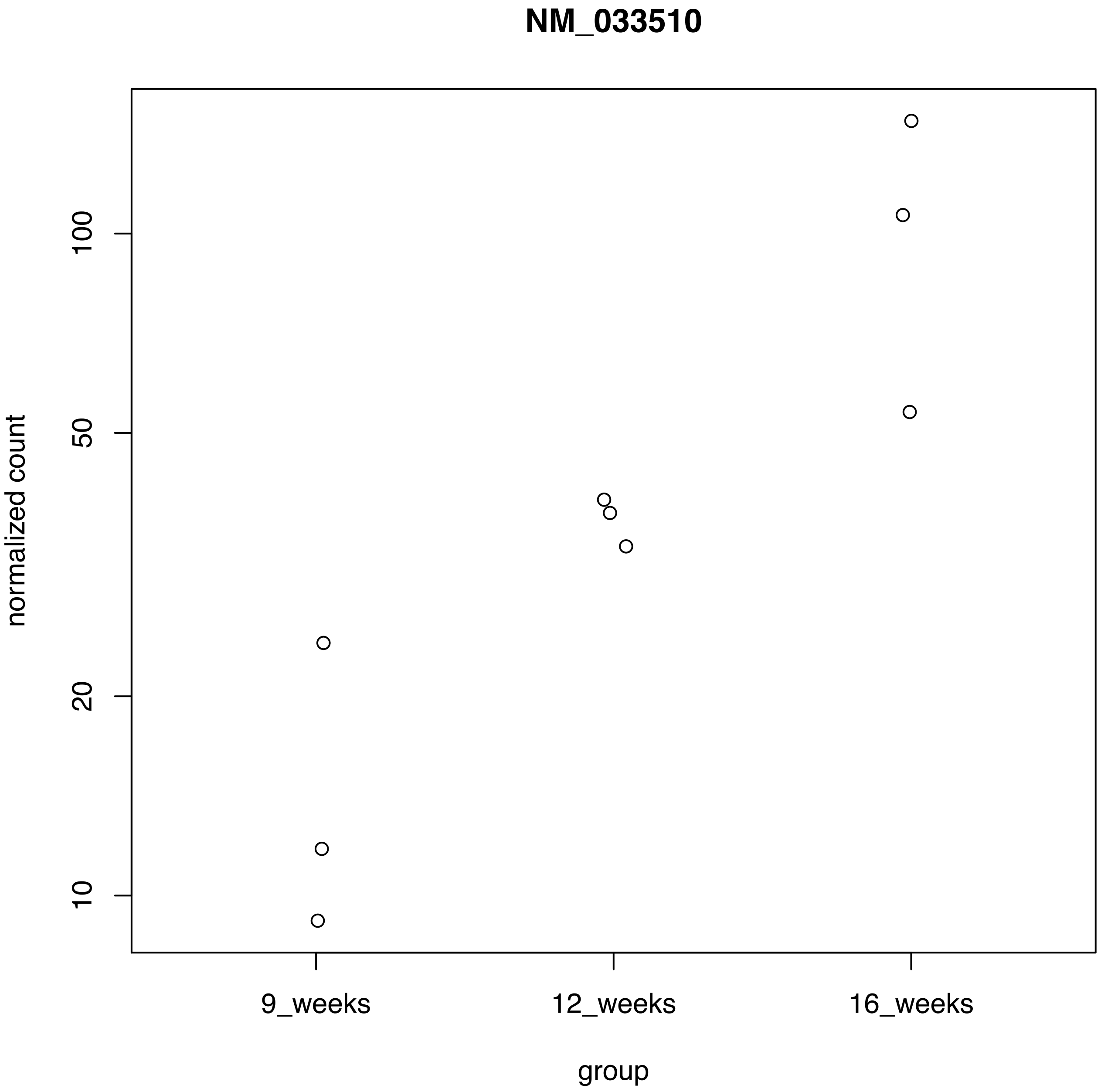
NM_033510
normalized count
100
50
20
10
9_weeks
12_weeks
16_weeks
group

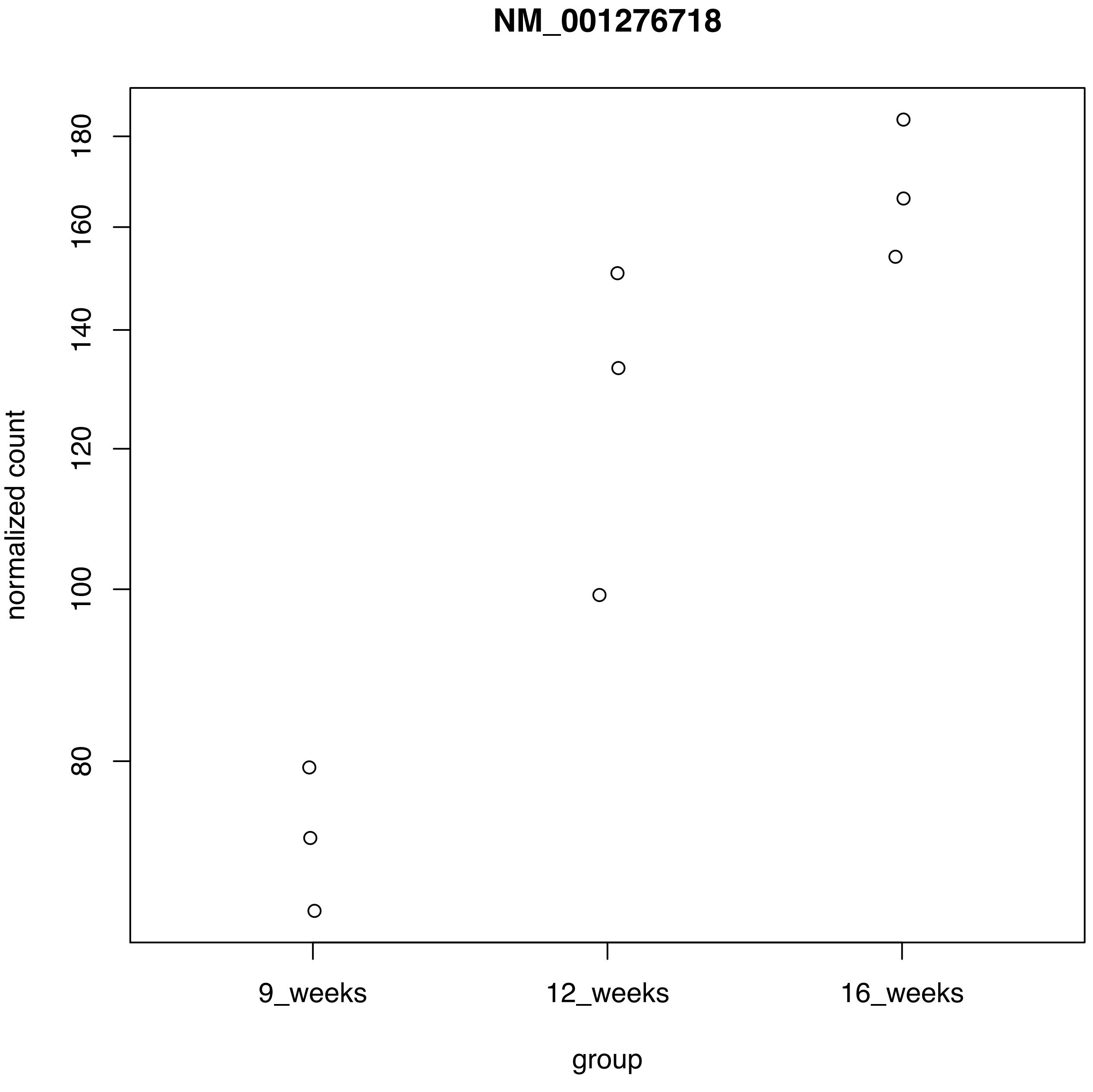
NM_001276718
normalized count
80
100
120
140
160
180
9_weeks
12_weeks
16_weeks
group

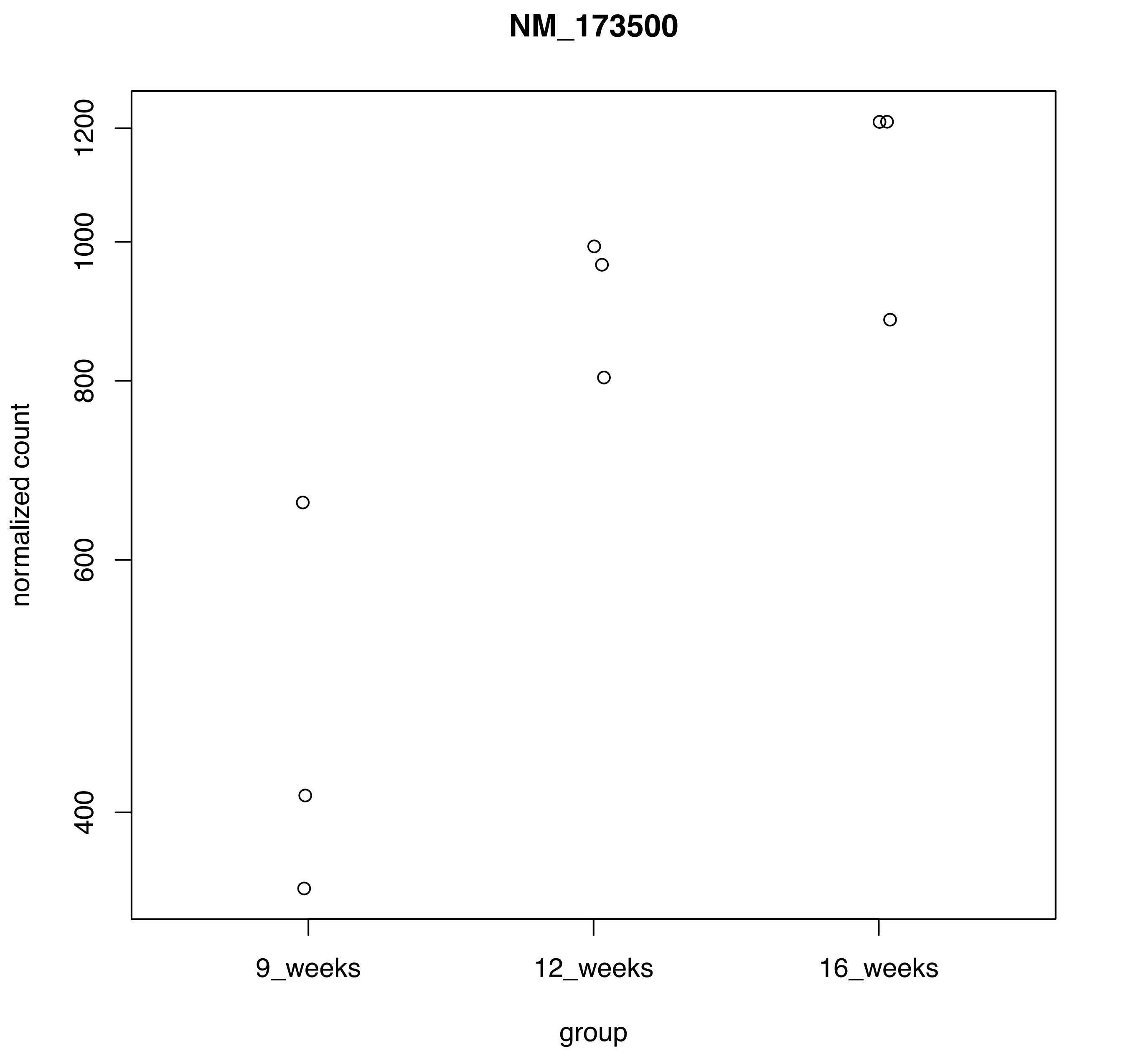
NM_173500
normalized count
1200
1000
800
600
400
9_weeks
12_weeks
16_weeks
group

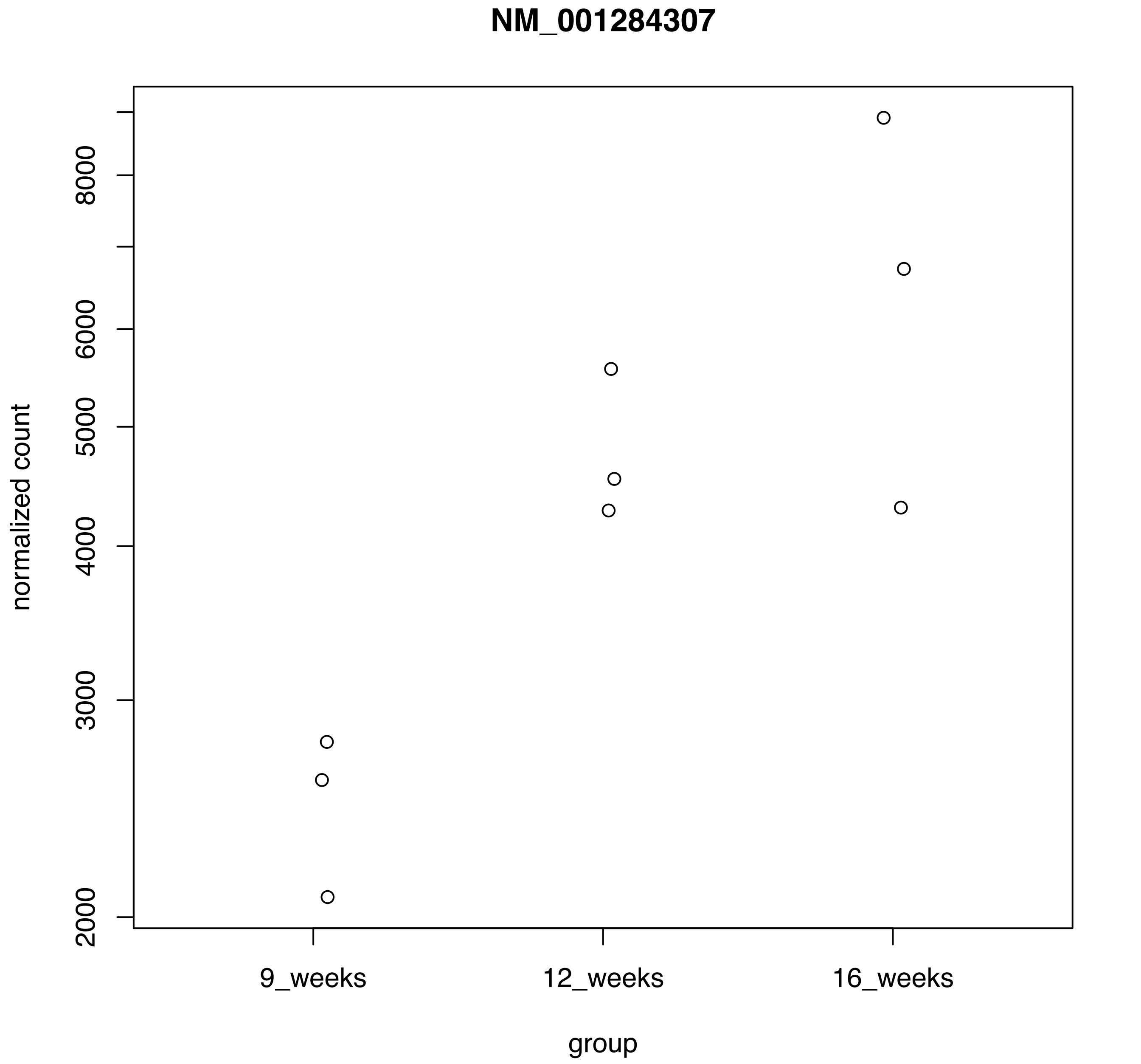
NM_001284307
normalized count
8000
6000
5000
4000
3000
2000
9_weeks
12_weeks
16_weeks
group

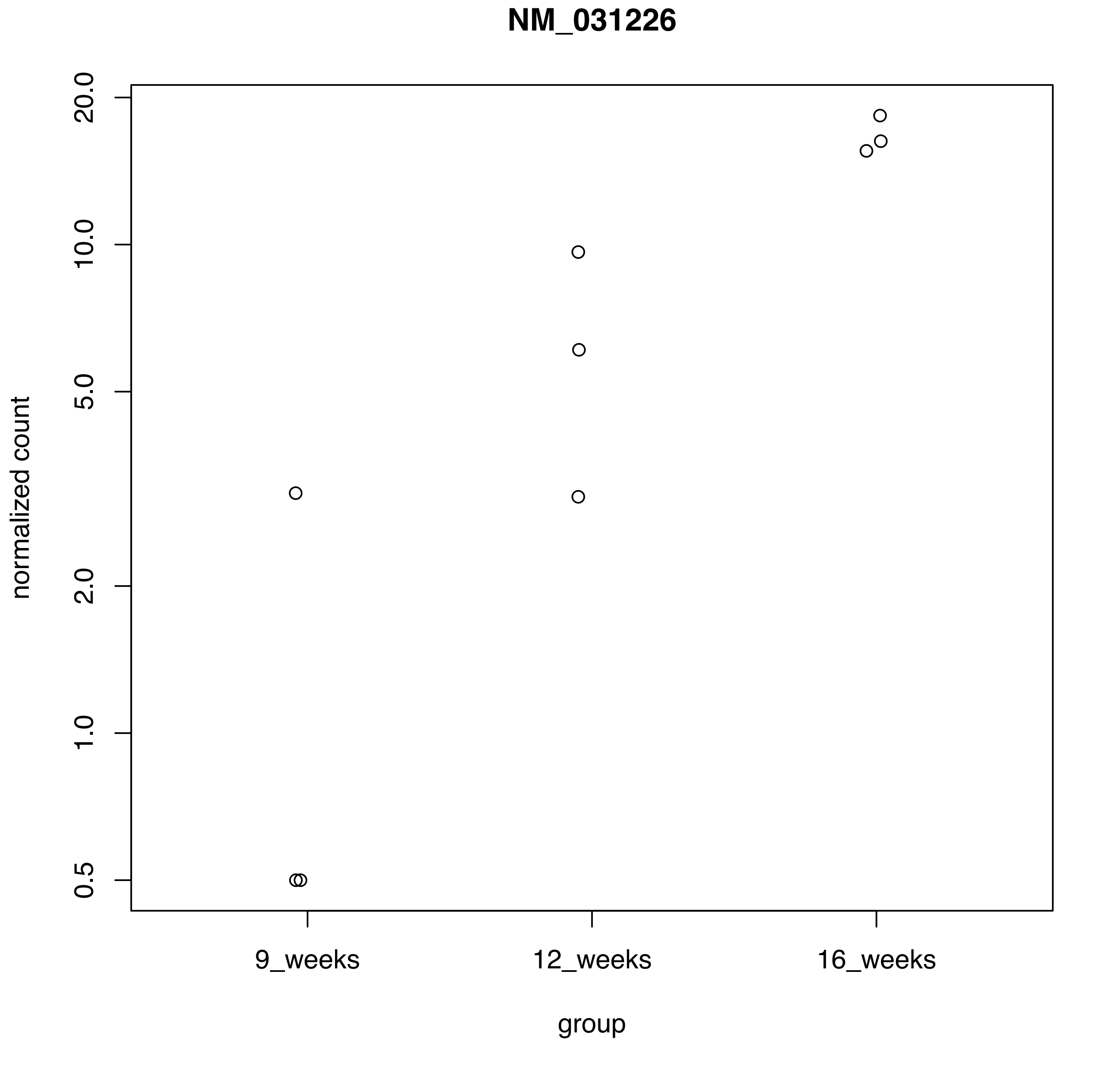

NM_031226
normalized count
20.0
10.0
5.0
2.0
1.0
0.5
9_weeks
12_weeks
16_weeks
group

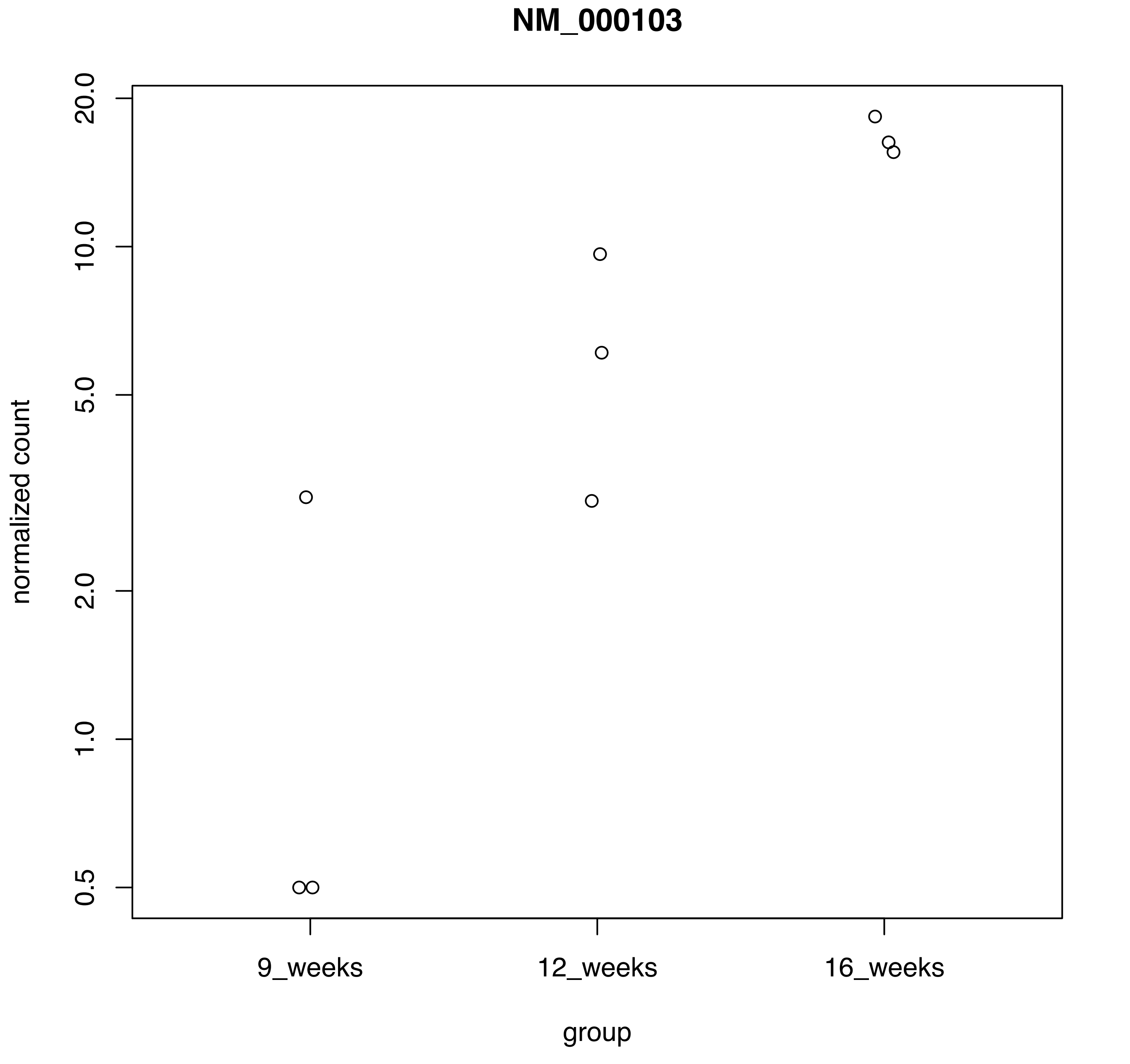
NM_000103
normalized count
20.0
10.0
5.0
2.0
1.0
0.5
9_weeks
12_weeks
16_weeks
group

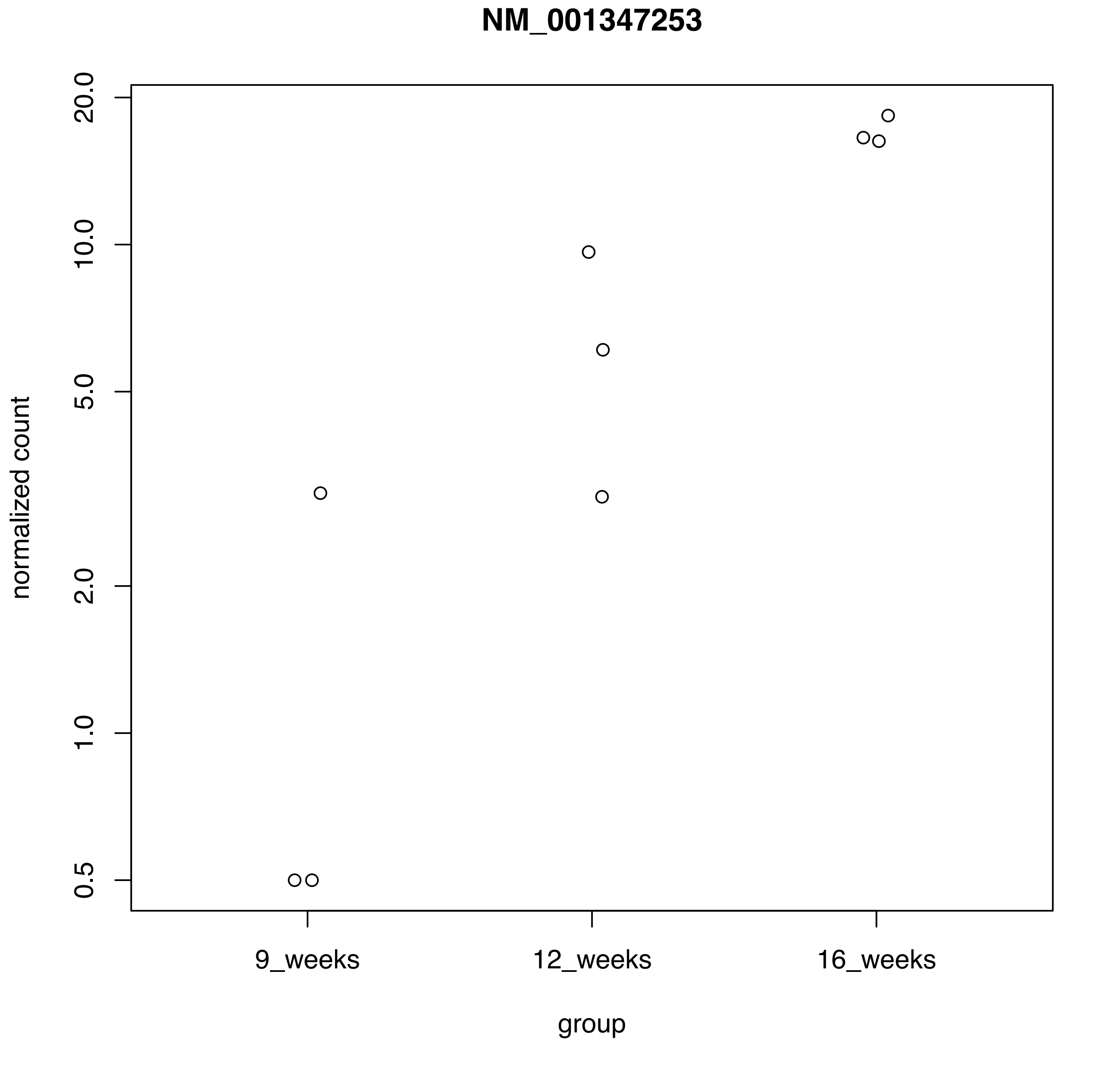
NM_001347253
normalized count
20.0
10.0
5.0
2.0
1.0
0.5
9_weeks
12_weeks
16_weeks
group

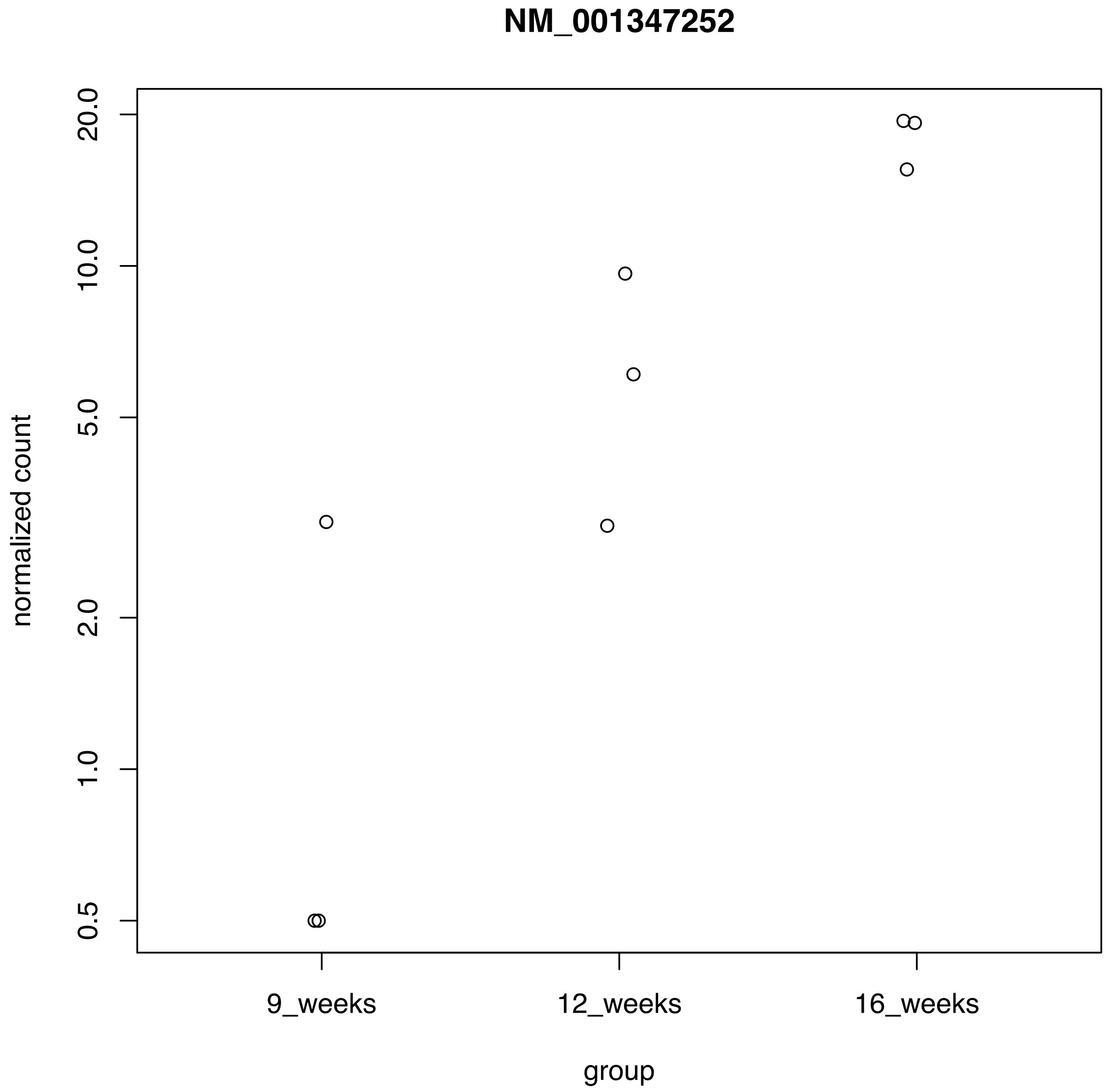

NM_001347252
normalized count
20.0
10.0
5.0
2.0
1.0
0.5
9_weeks
12_weeks
16_weeks
group

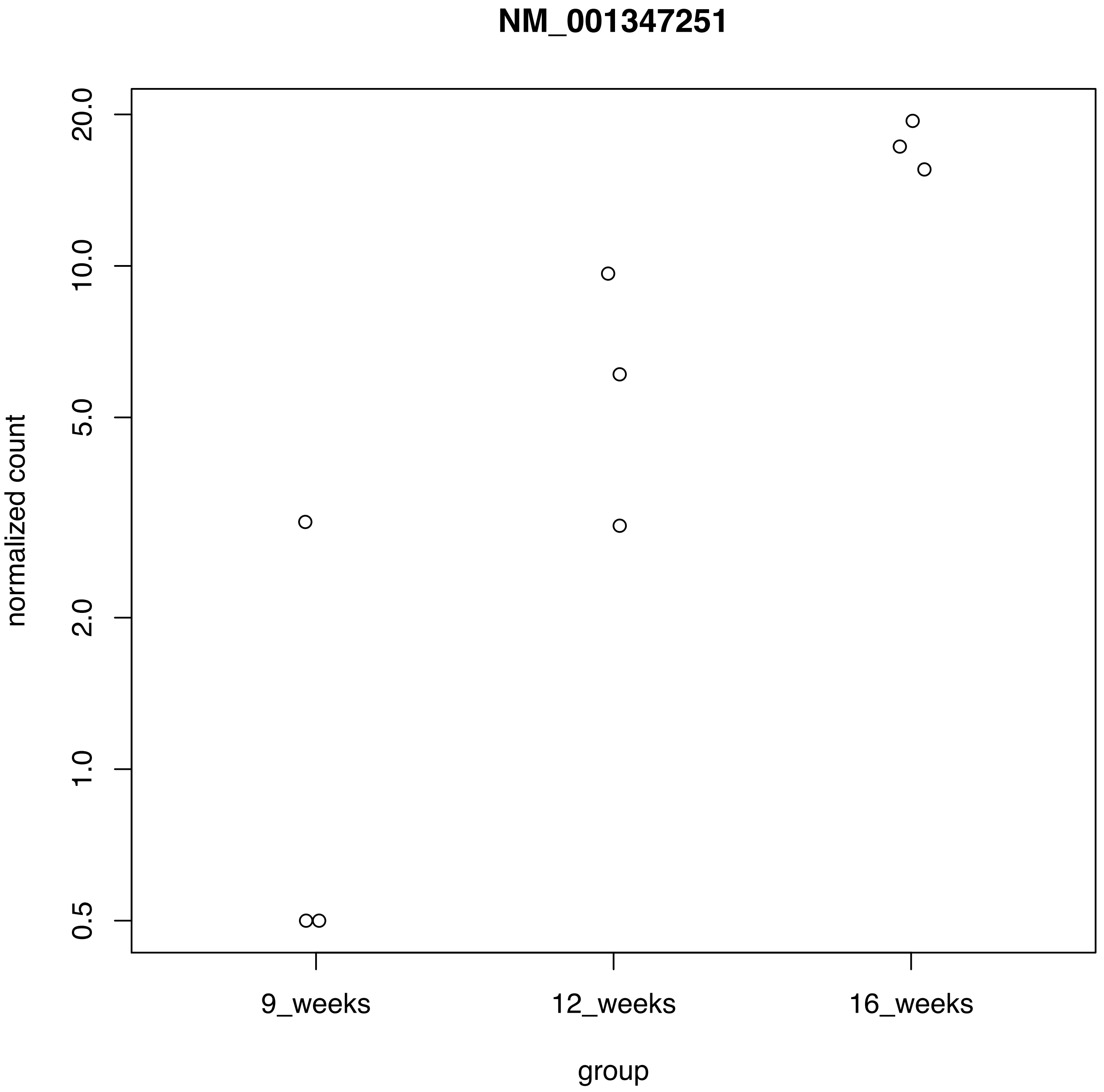
NM_001347251
normalized count
20.0
10.0
5.0
2.0
1.0
0.5
9_weeks
12_weeks
16_weeks
group

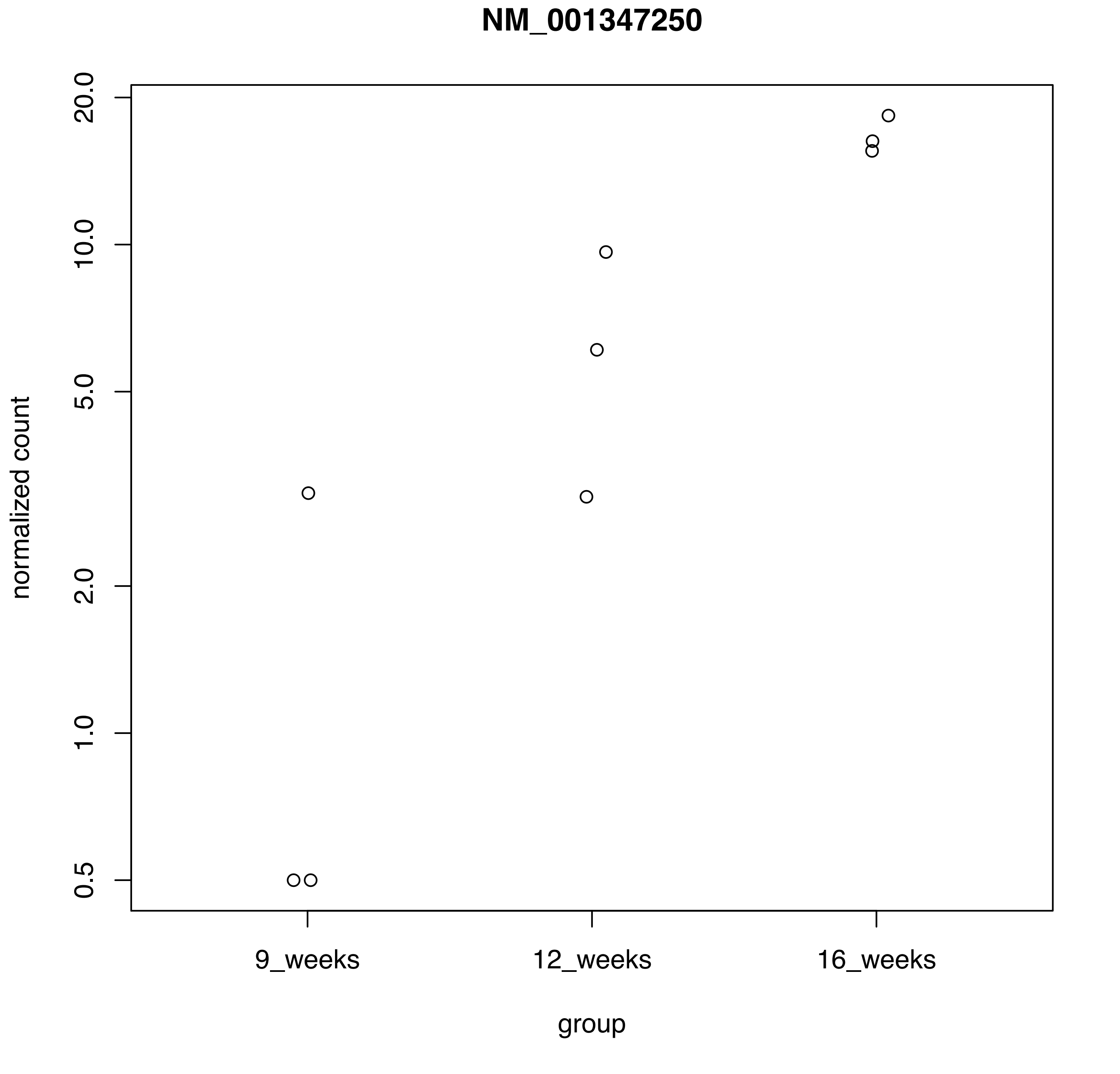
NM_001347250
normalized count
20.0
10.0
5.0
2.0
1.0
0.5
9_weeks
12_weeks
16_weeks
group

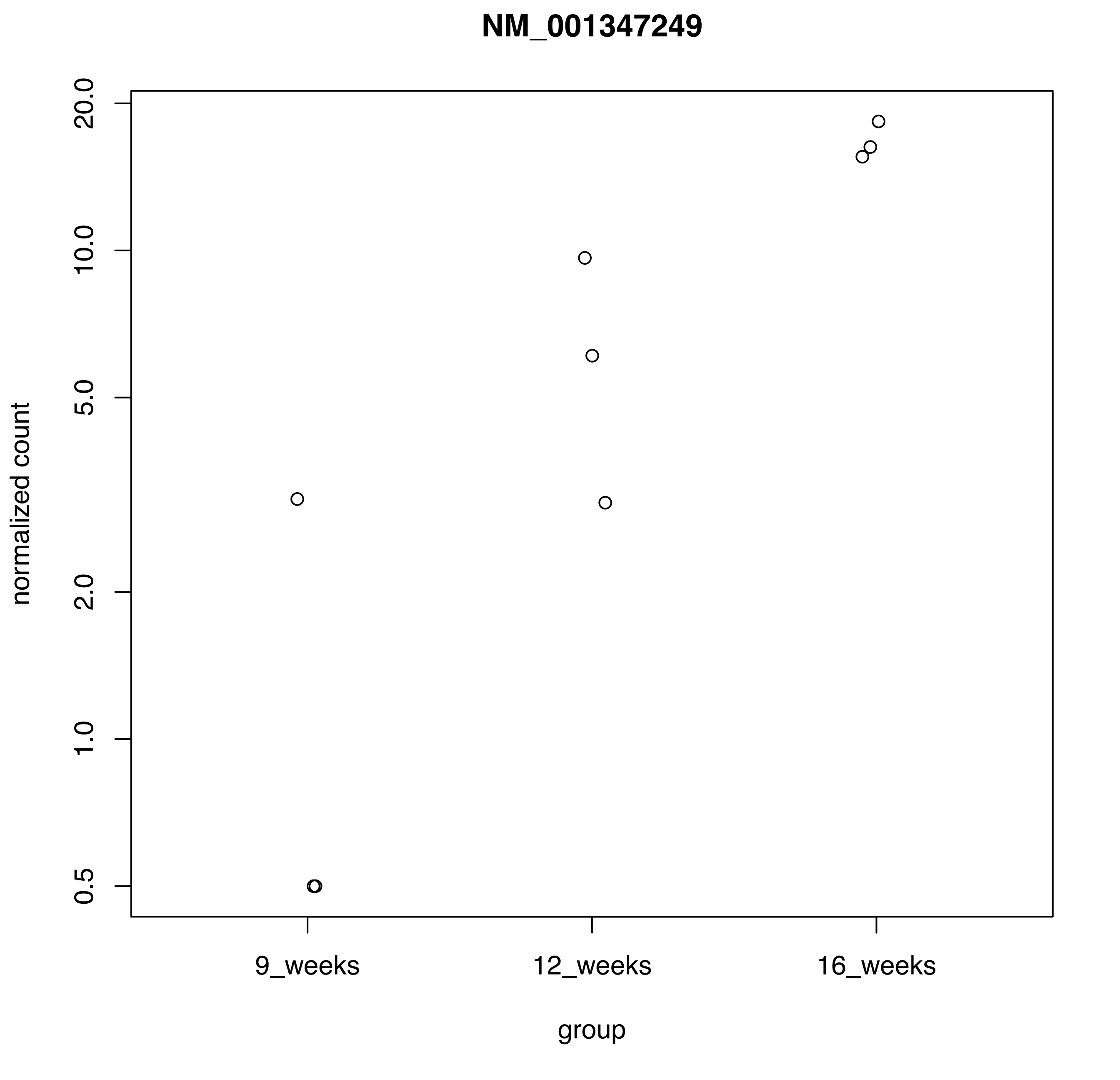

NM_001347249
normalized count
20.0
10.0
5.0
2.0
1.0
0.5
9_weeks
12_weeks
16_weeks
group

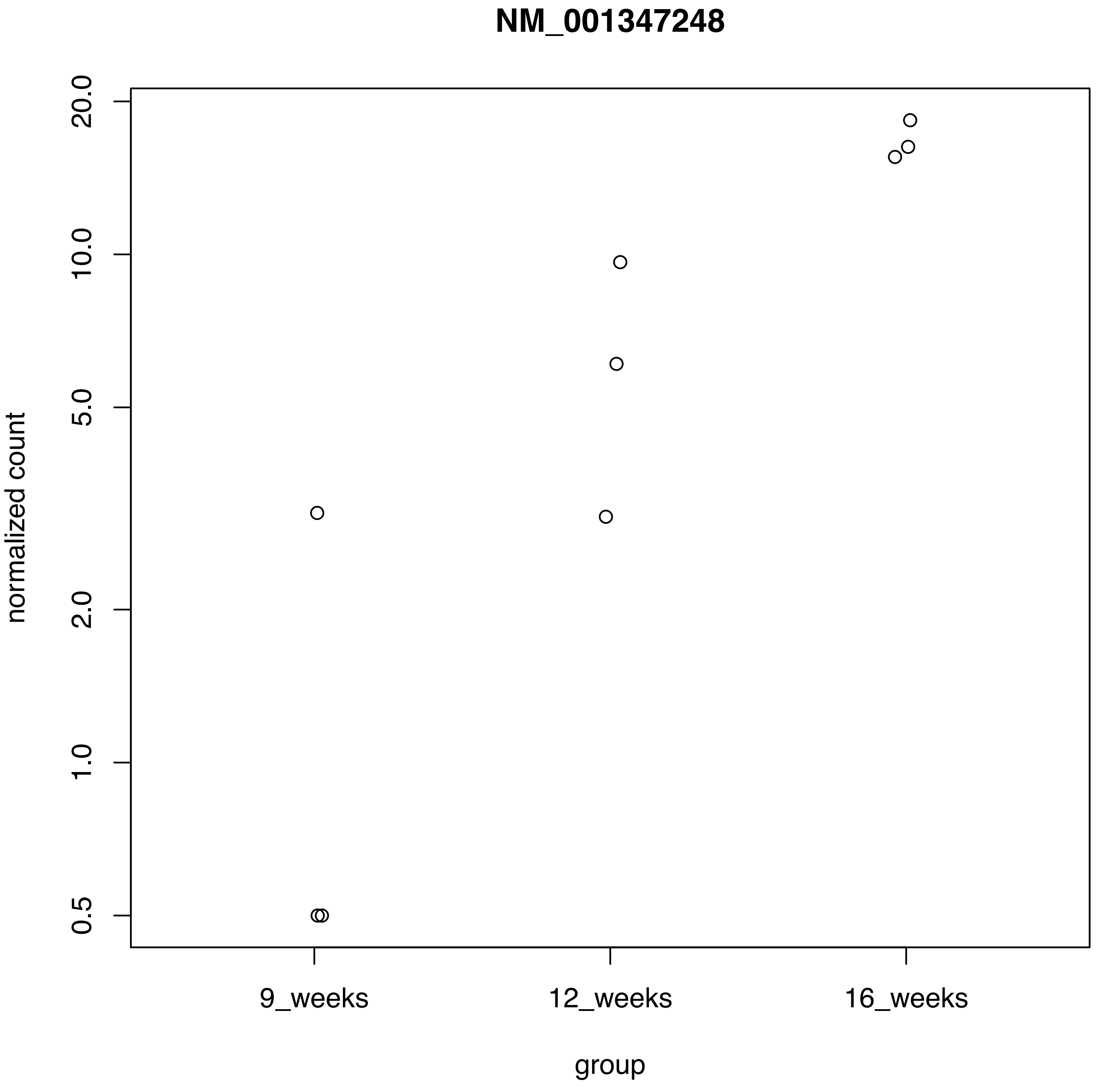

NM_001347248
normalized count
20.0
10.0
5.0
2.0
1.0
0.5
9_weeks
12_weeks
16_weeks
group

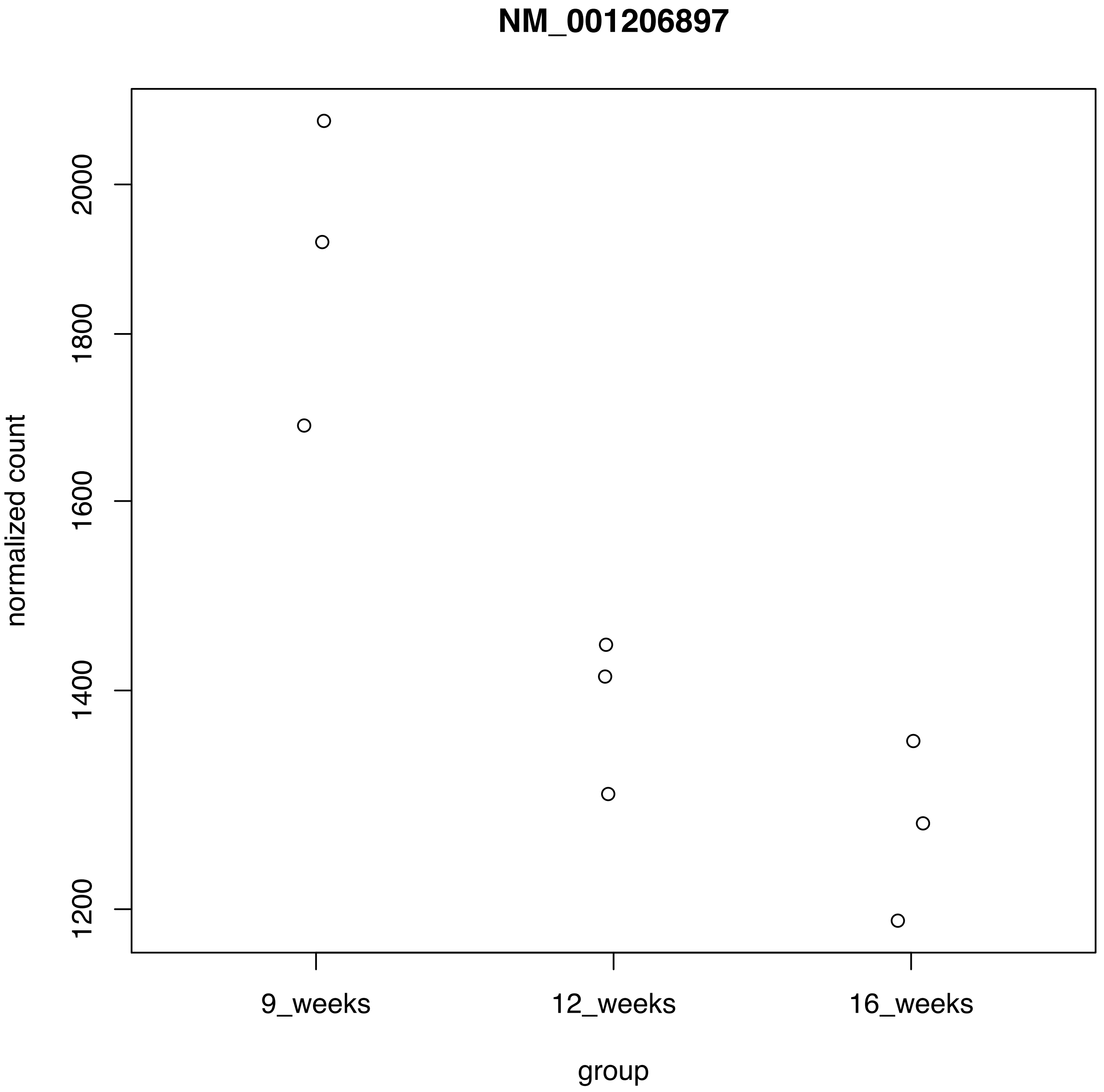
NM_001206897
normalized count
2000
1800
1600
1400
1200
9_weeks
12_weeks
16_weeks
group

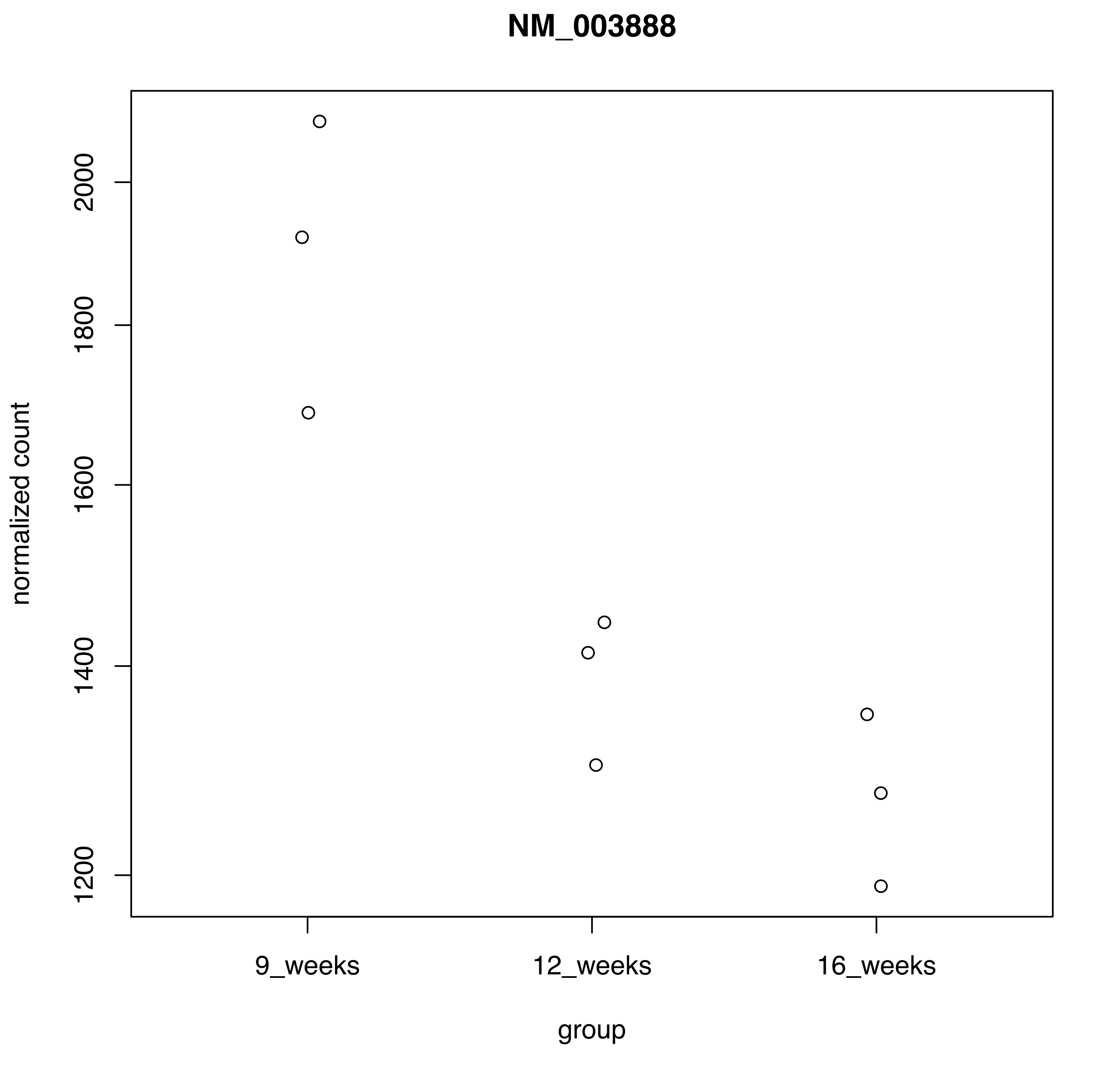
NM_003888
normalized count
2000
1800
1600
1400
1200
9_weeks
12_weeks
16_weeks
group

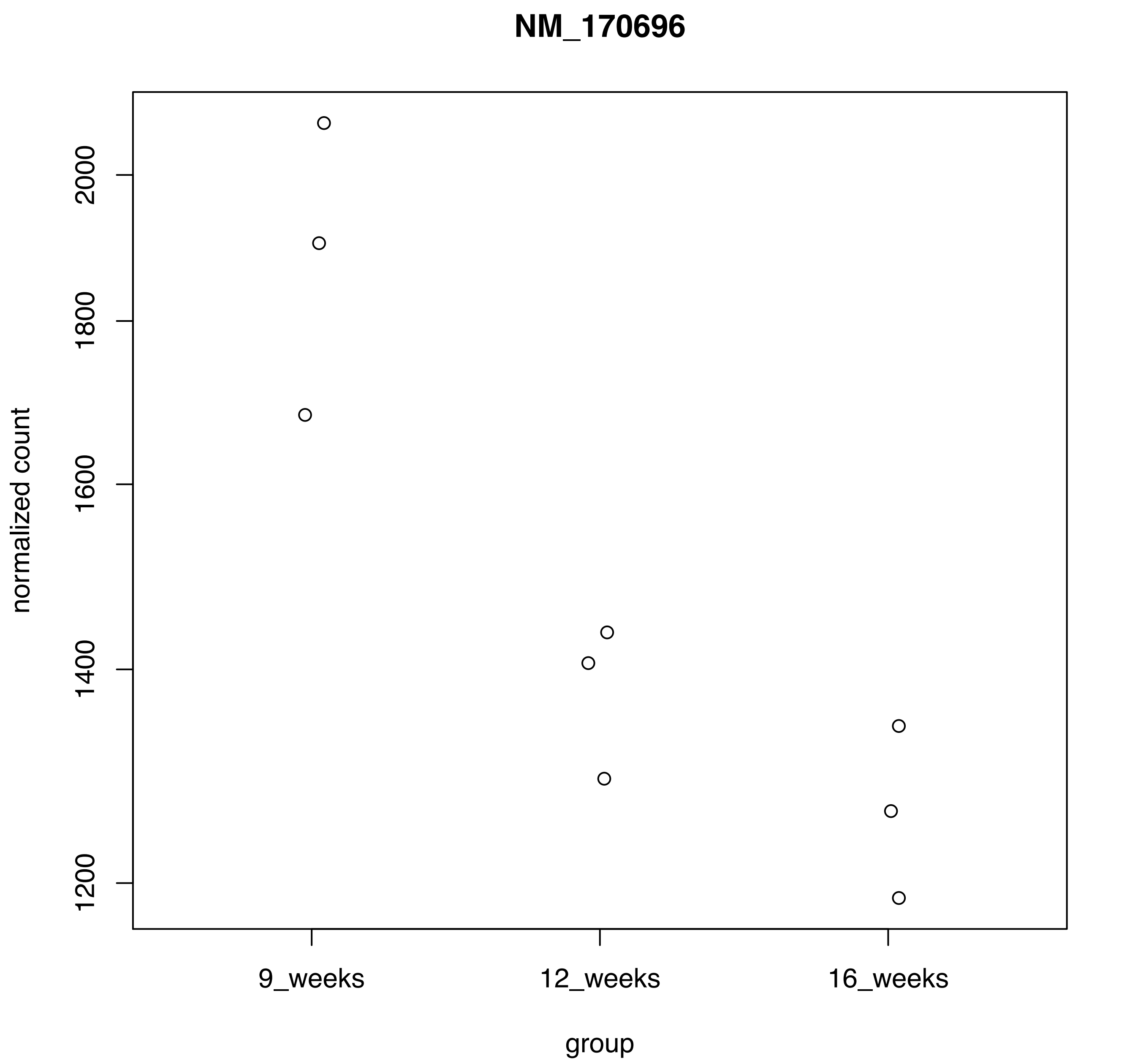
NM_170696
normalized count
2000
1800
1600
1400
1200
9_weeks
12_weeks
16_weeks
group

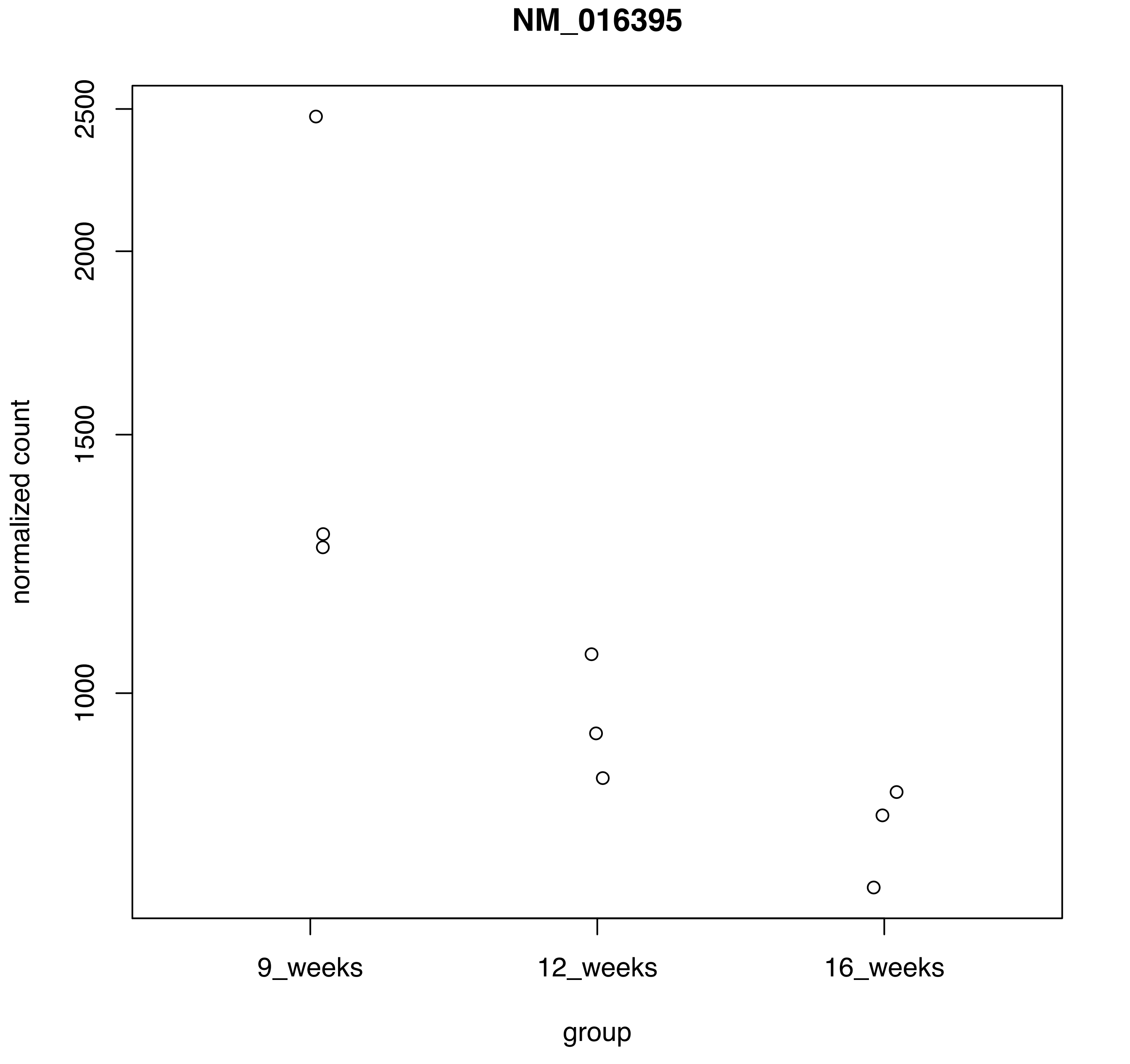
NM_016395
normalized count
2500
2000
1500
1000
group
9_weeks
12_weeks
16_weeks

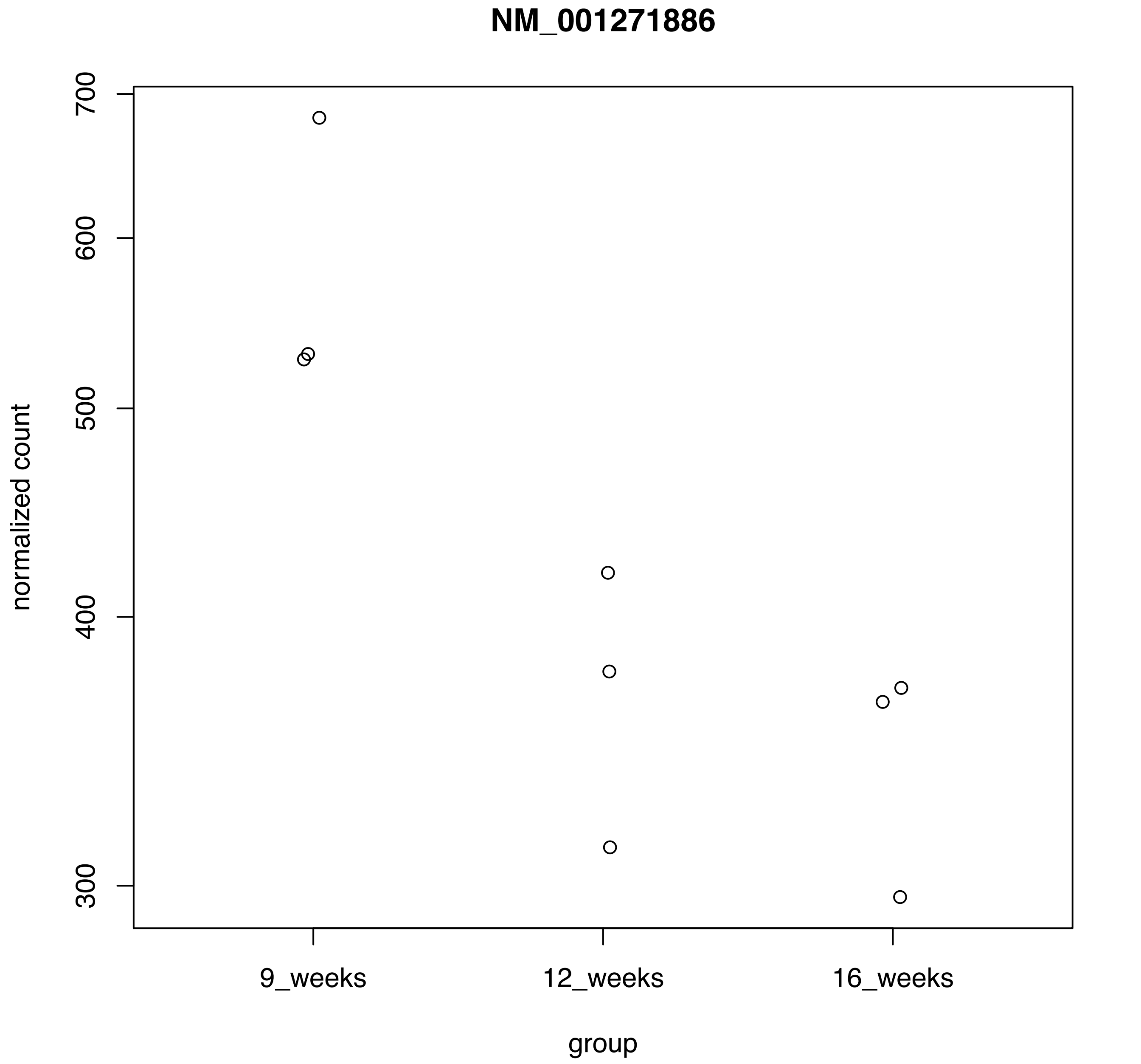

NM_001271886
normalized count
700
600
500
400
300
9_weeks
12_weeks
16_weeks
group

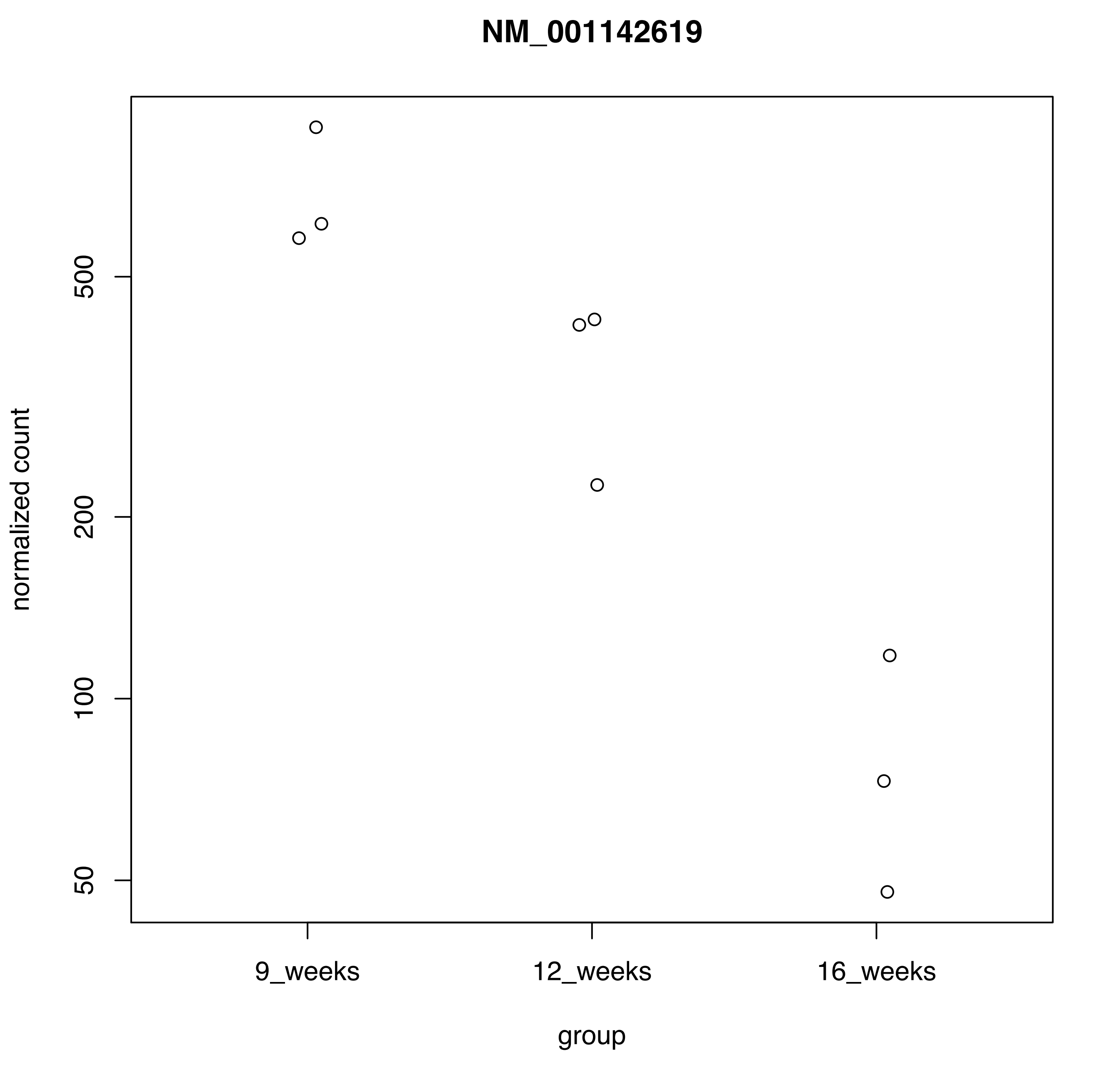
NM_001142619
normalized count
500
200
100
50
9_weeks
12_weeks
16_weeks
group

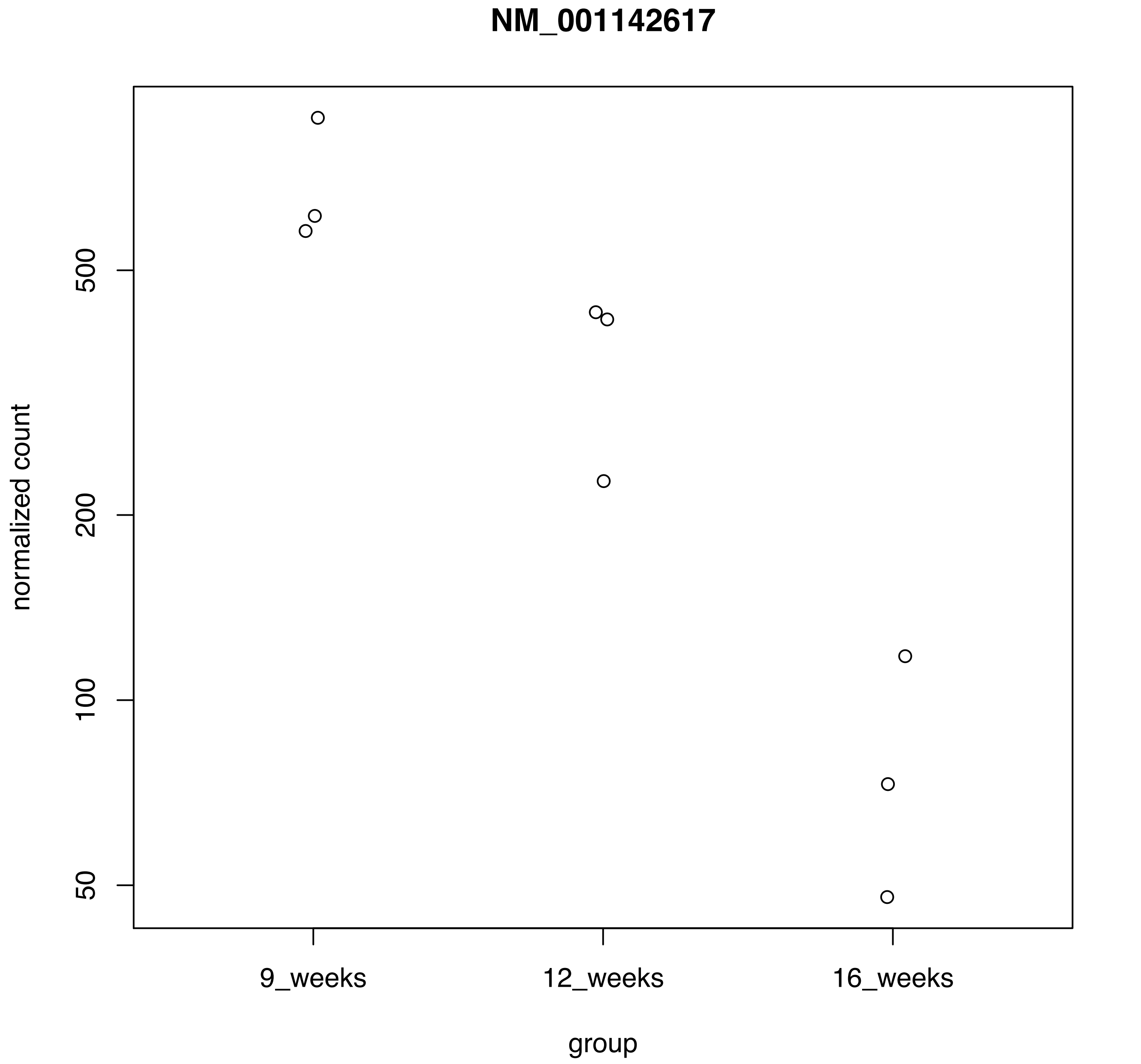
NM_001142617
normalized count
500
200
100
50
9_weeks
12_weeks
16_weeks
group

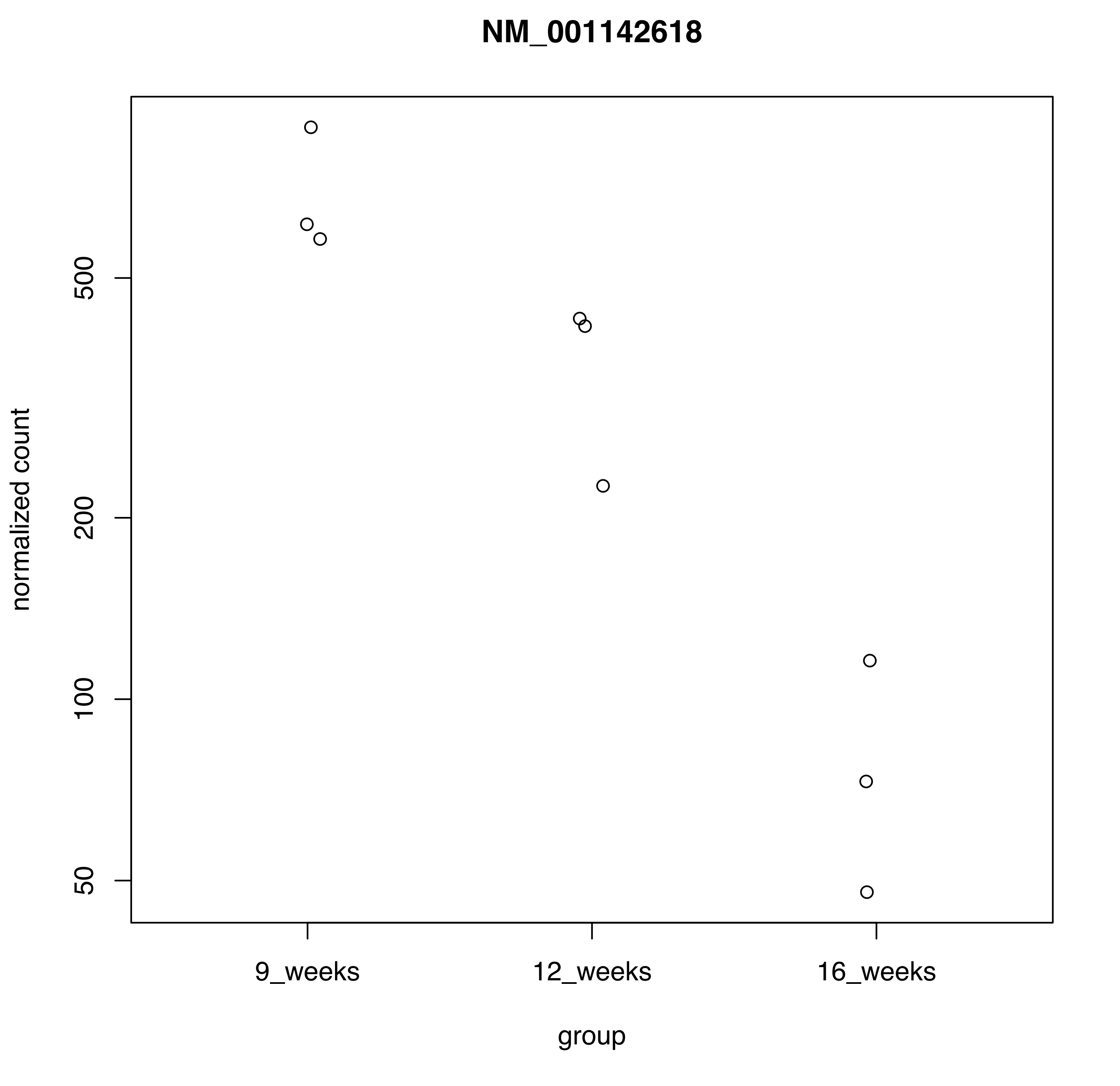
NM_001142618
normalized count
500
200
100
50
9_weeks
12_weeks
16_weeks
group

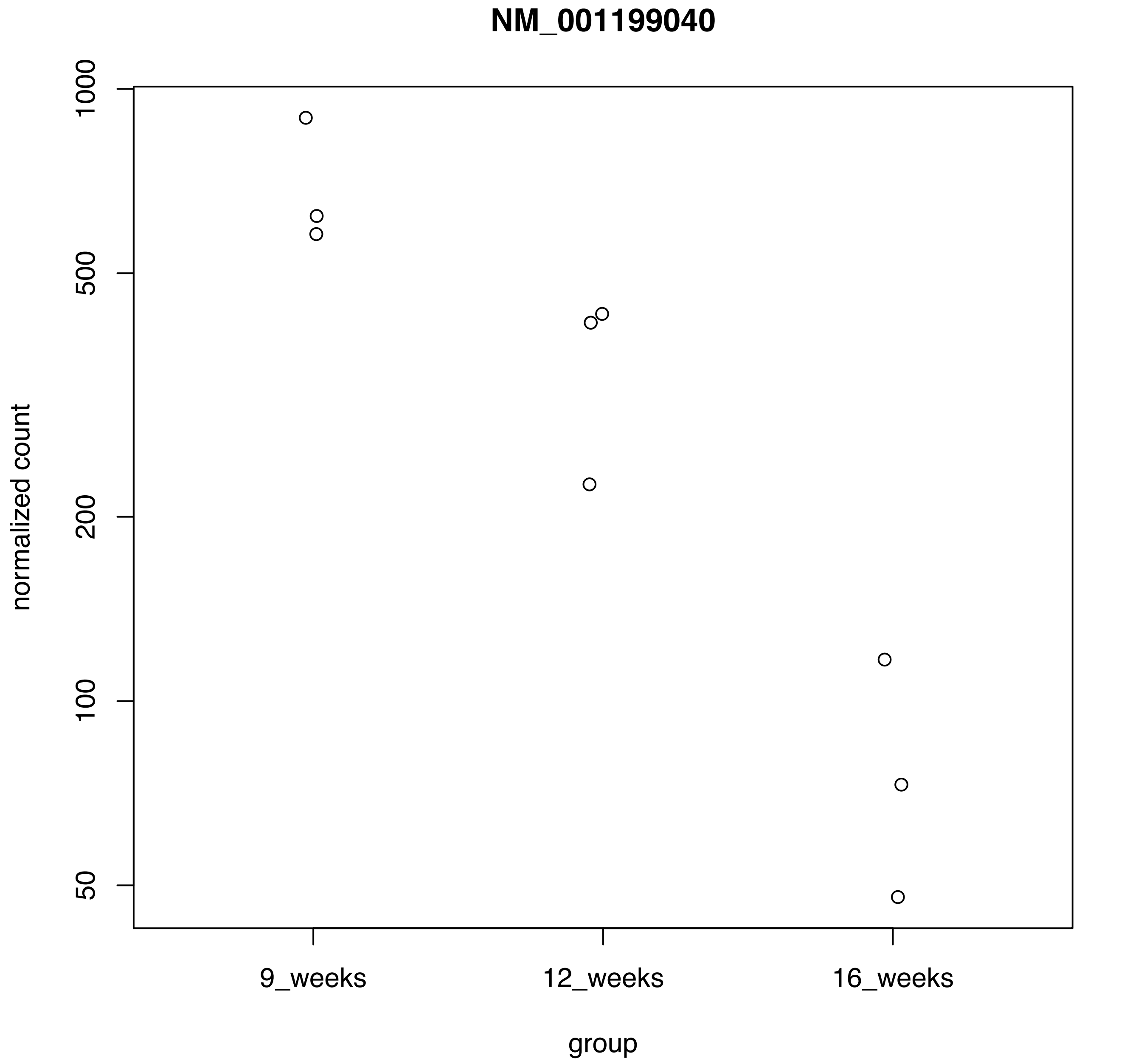
NM_001199040
1000
500
200
100
50
normalized count
9_weeks
12_weeks
16_weeks
group

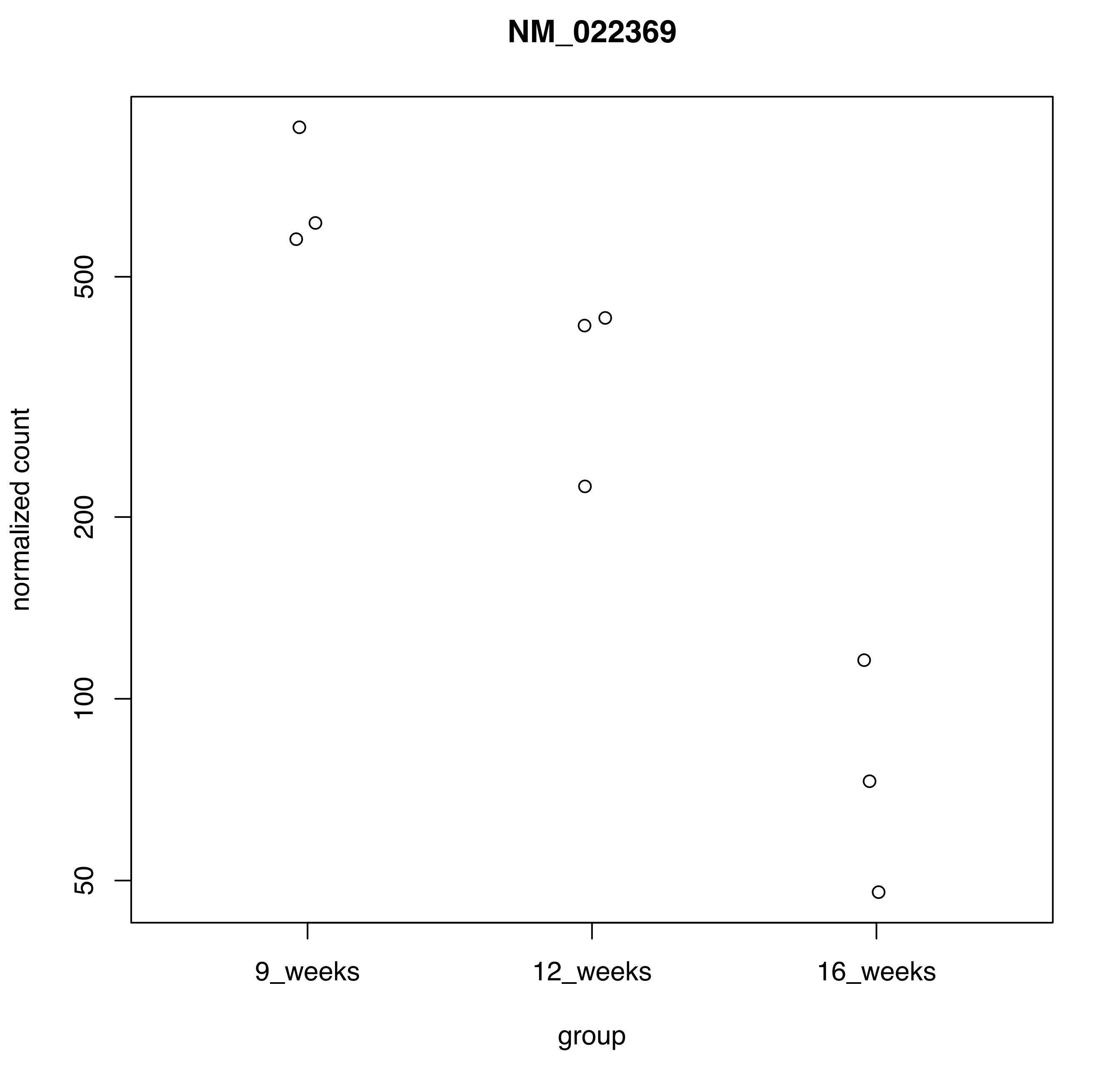
NM_022369
normalized count
500
200
100
50
9_weeks
12_weeks
16_weeks
group

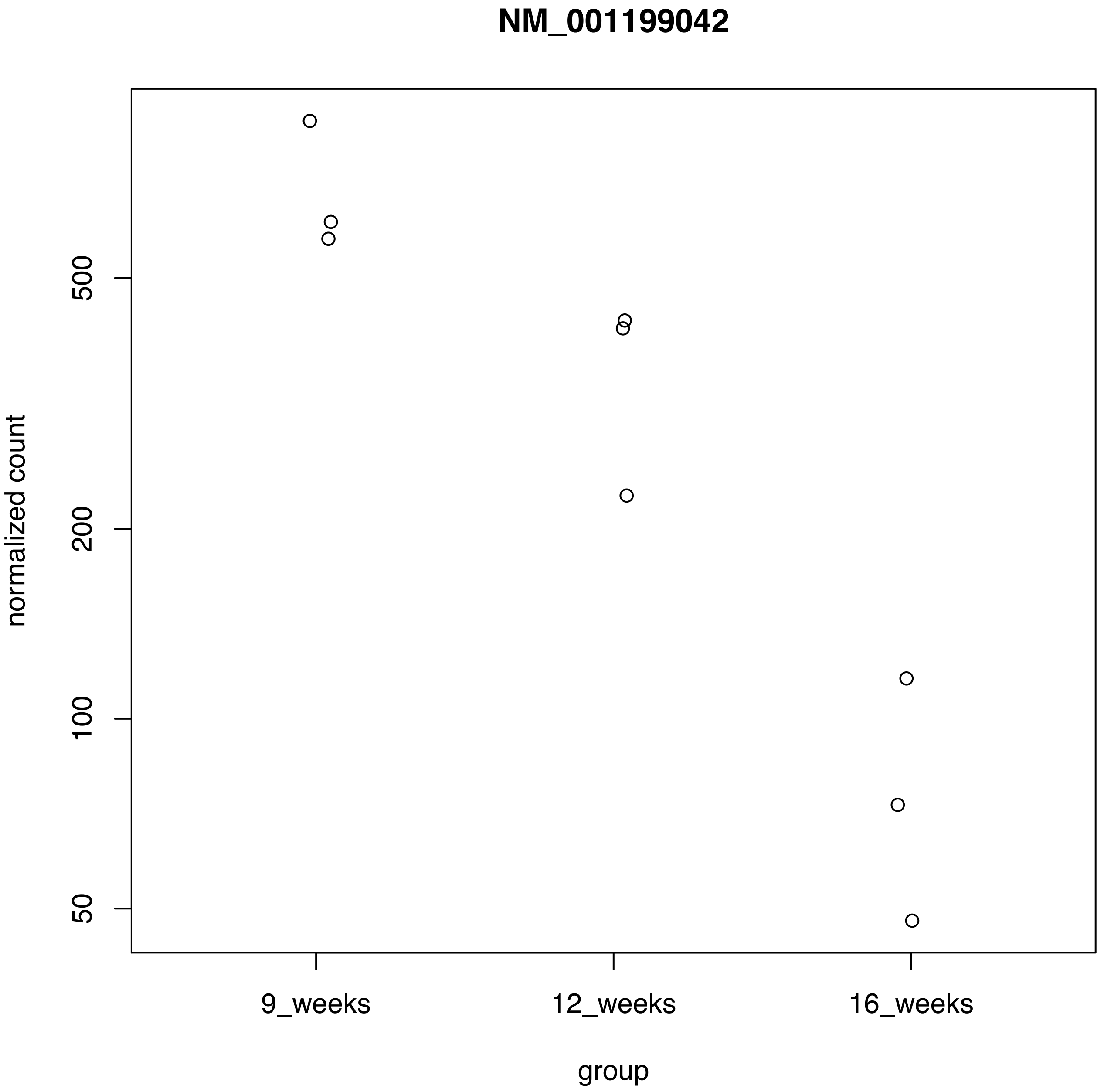
NM_001199042
normalized count
500
200
100
50
9_weeks
12_weeks
16_weeks
group

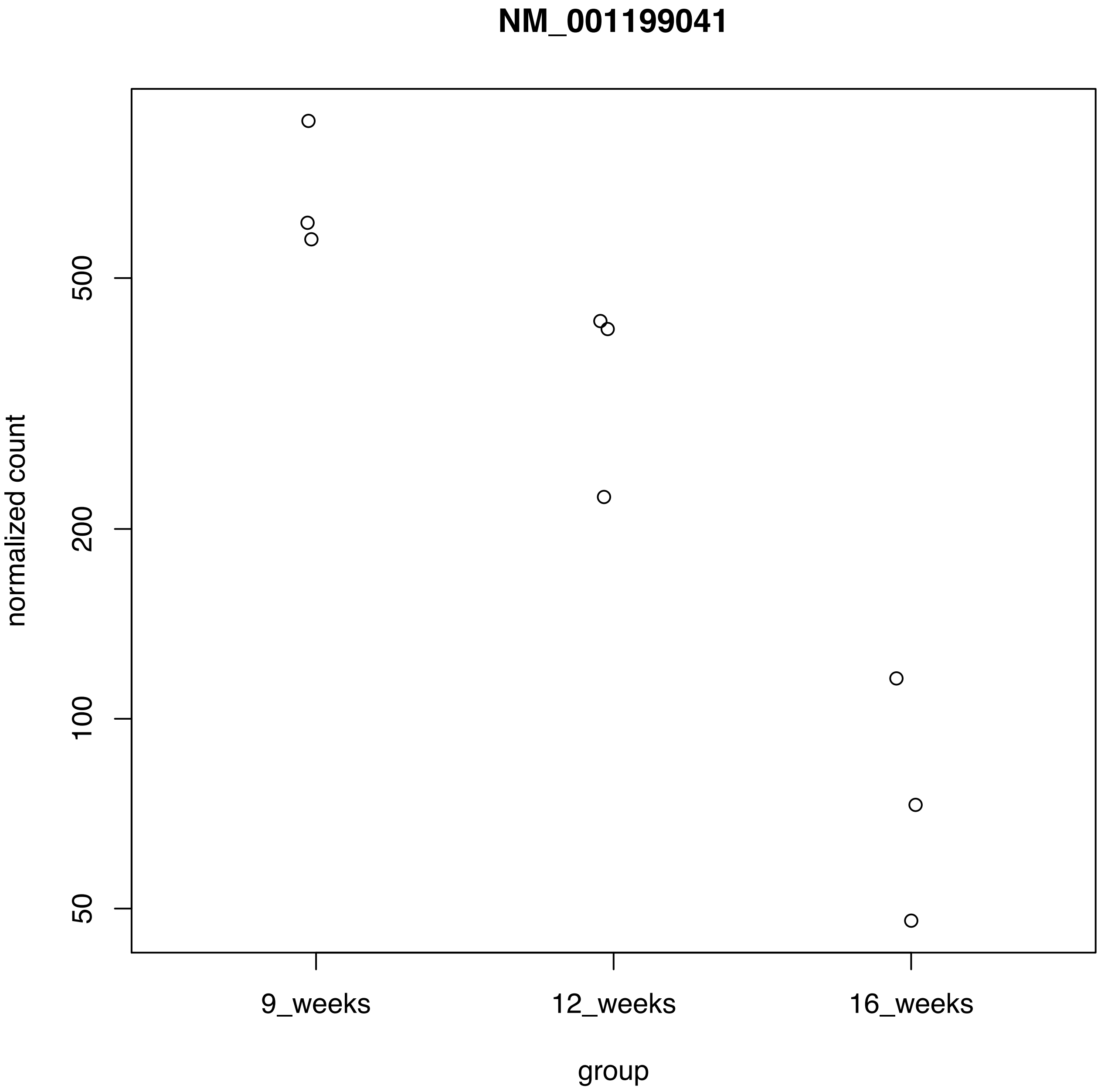
NM_001199041
normalized count
500
200
100
50
9_weeks
12_weeks
16_weeks
group

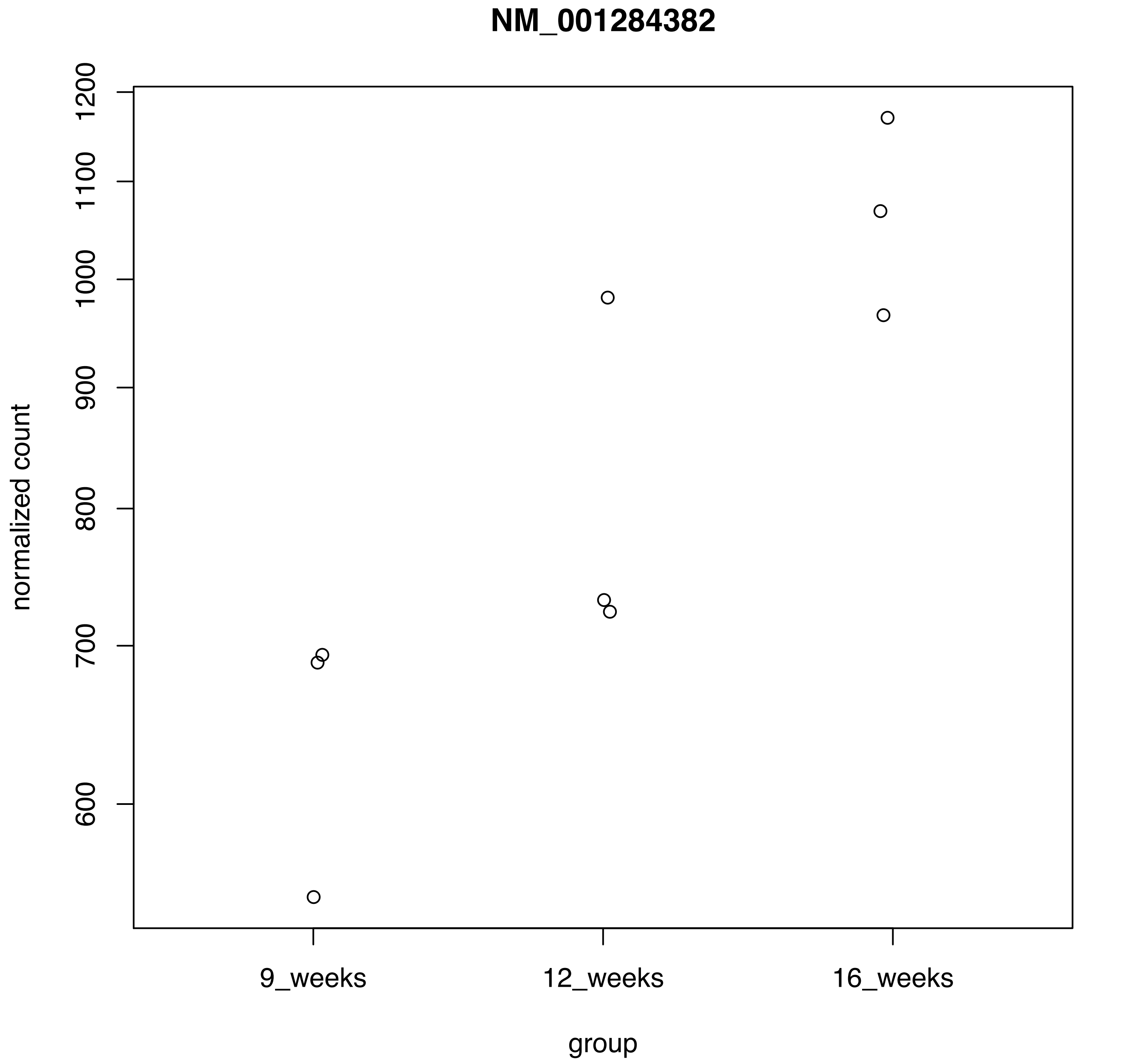
NM_001284382
normalized count
1200
1100
1000
900
800
700
600
9_weeks
12_weeks
16_weeks
group

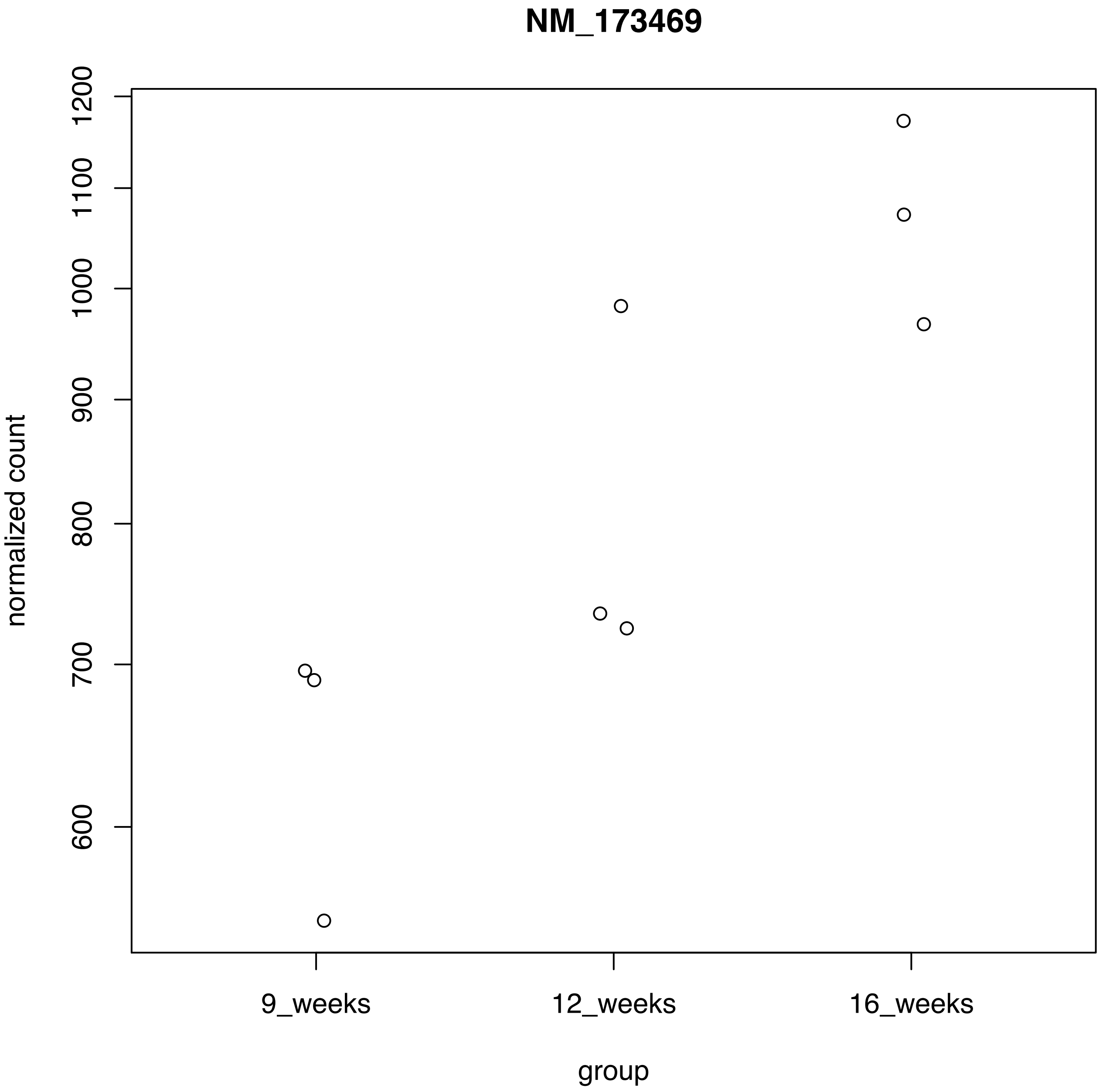

NM_173469
normalized count
1200
1100
1000
900
800
700
600
9_weeks
12_weeks
16_weeks
group

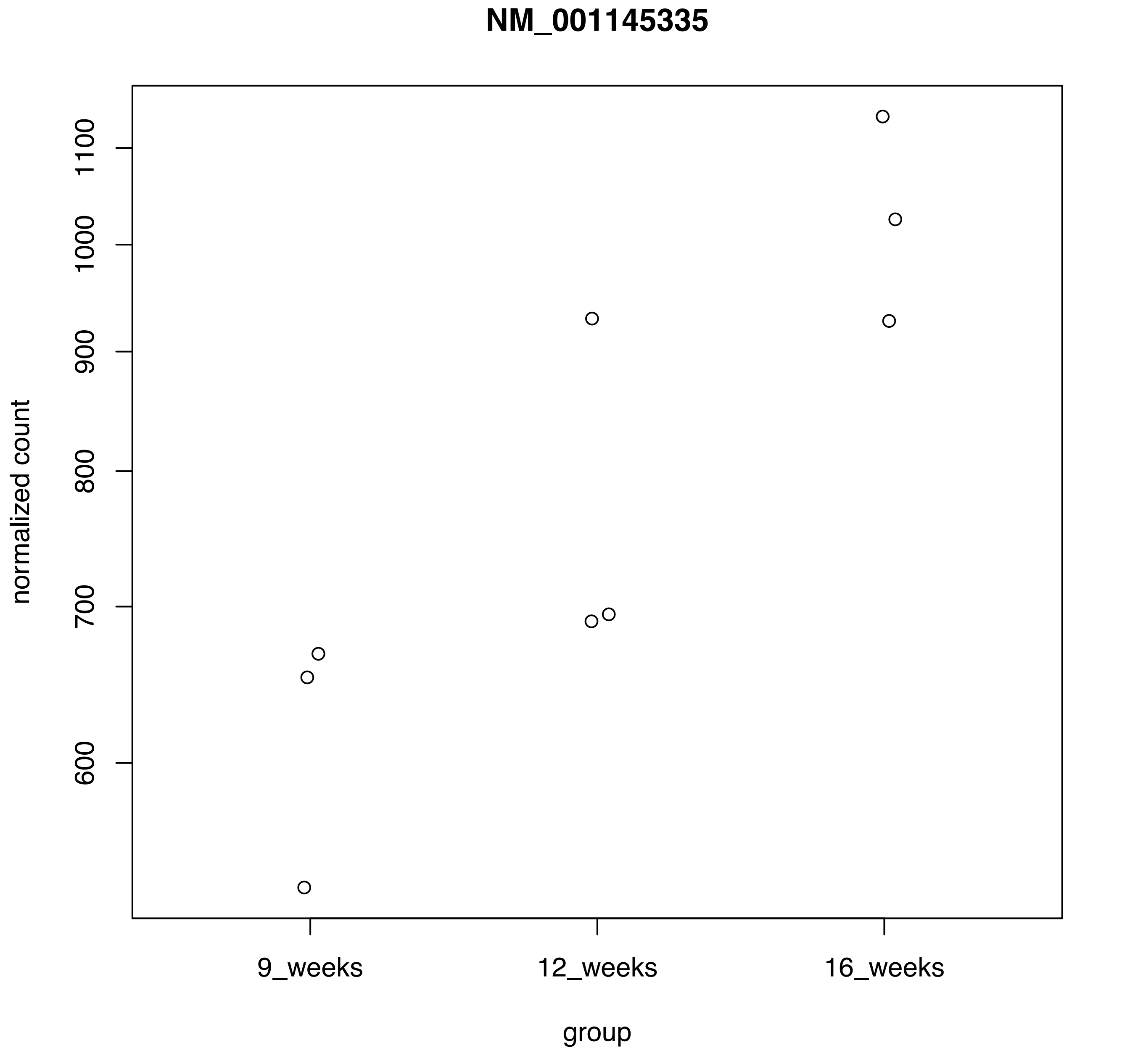
NM_001145335
normalized count
600
700
800
900
1000
1100
9_weeks
12_weeks
16_weeks
group

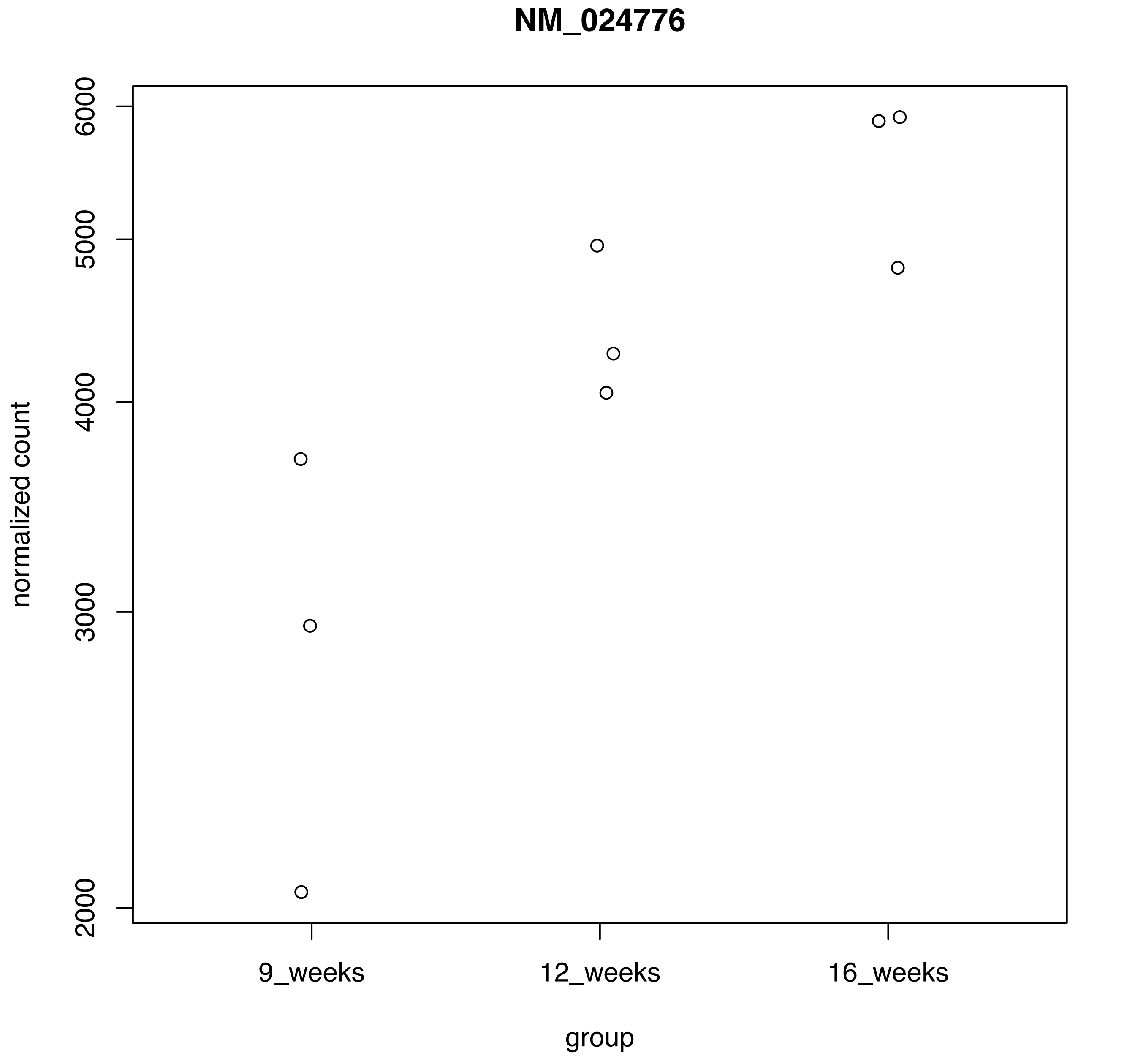

NM_024776
normalized count
6000
5000
4000
3000
2000
9_weeks
12_weeks
16_weeks
group

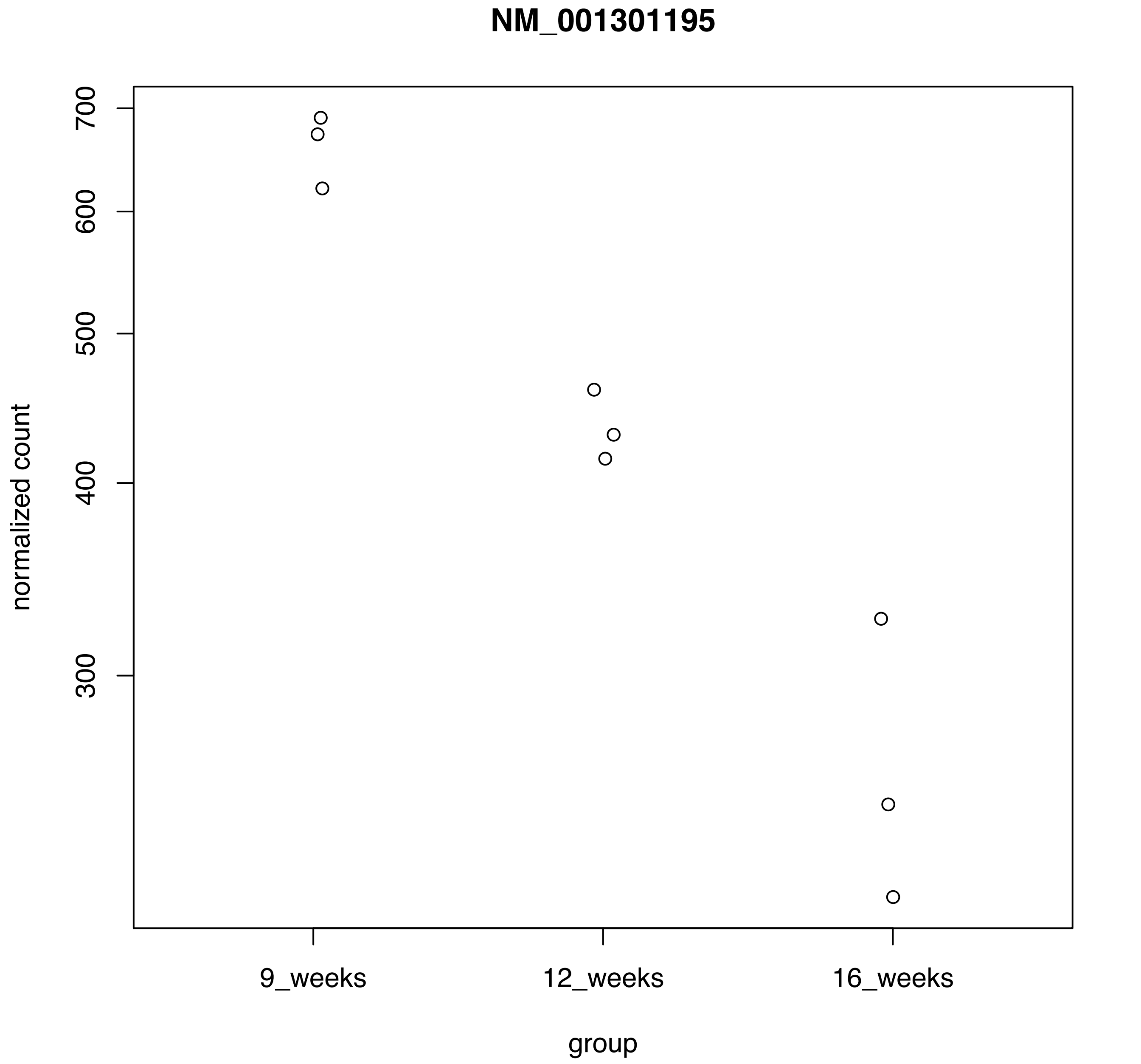

NM_001301195
normalized count
700
600
500
400
300
9_weeks
12_weeks
16_weeks
group

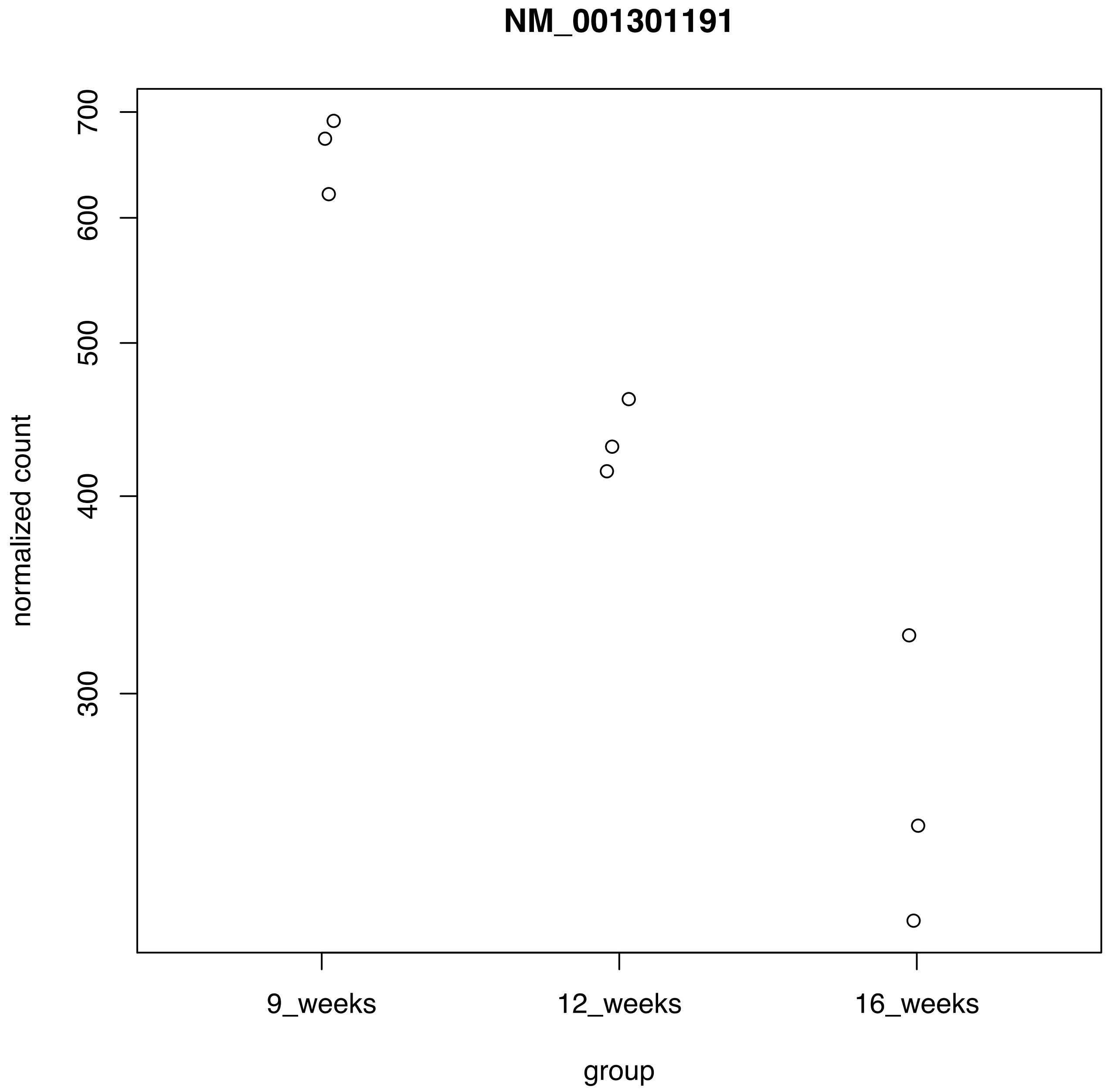
NM_001301191
normalized count
700
600
500
400
300
9_weeks
12_weeks
16_weeks
group

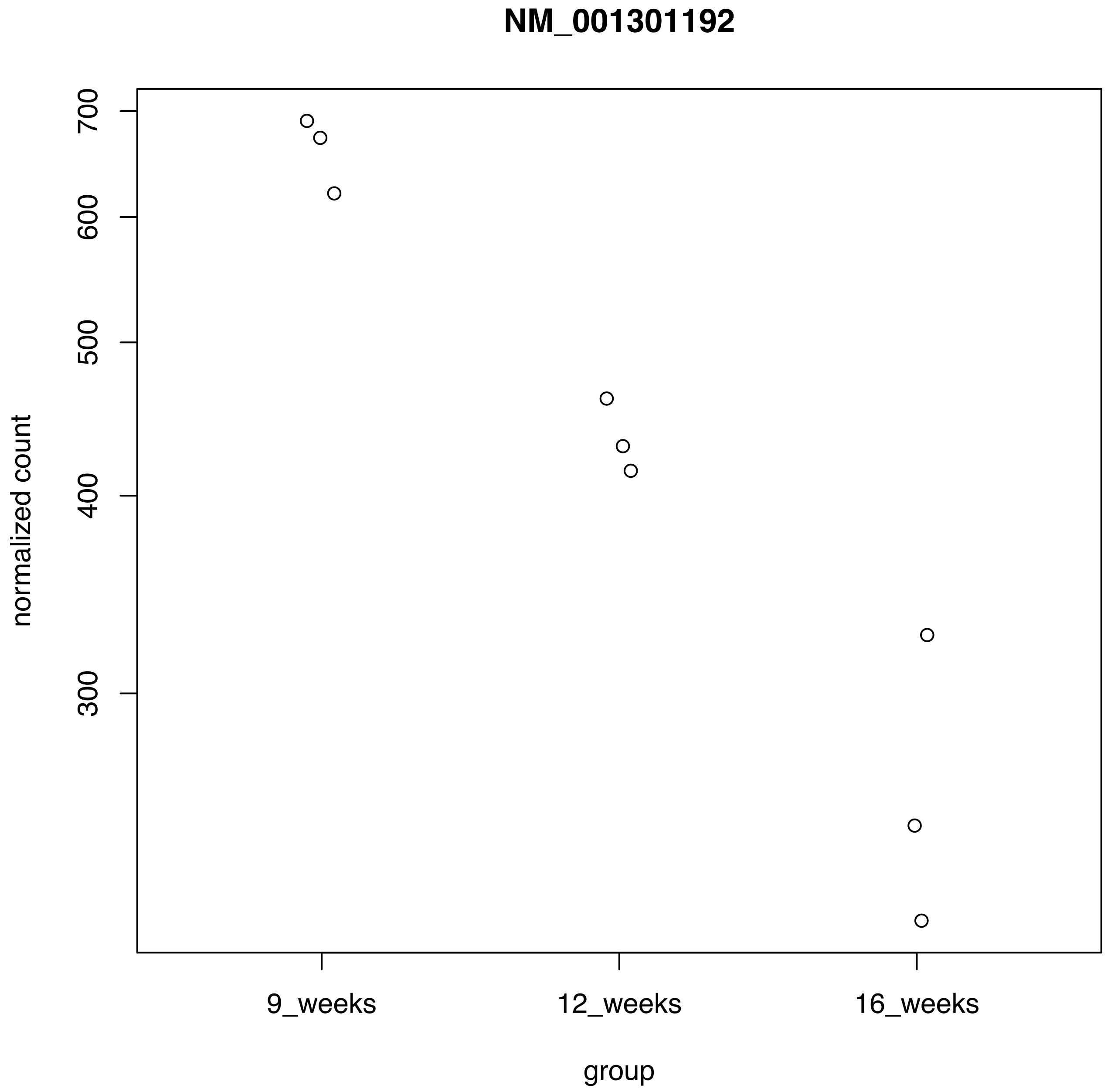
NM_001301192
normalized count
700
600
500
400
300
9_weeks
12_weeks
16_weeks
group

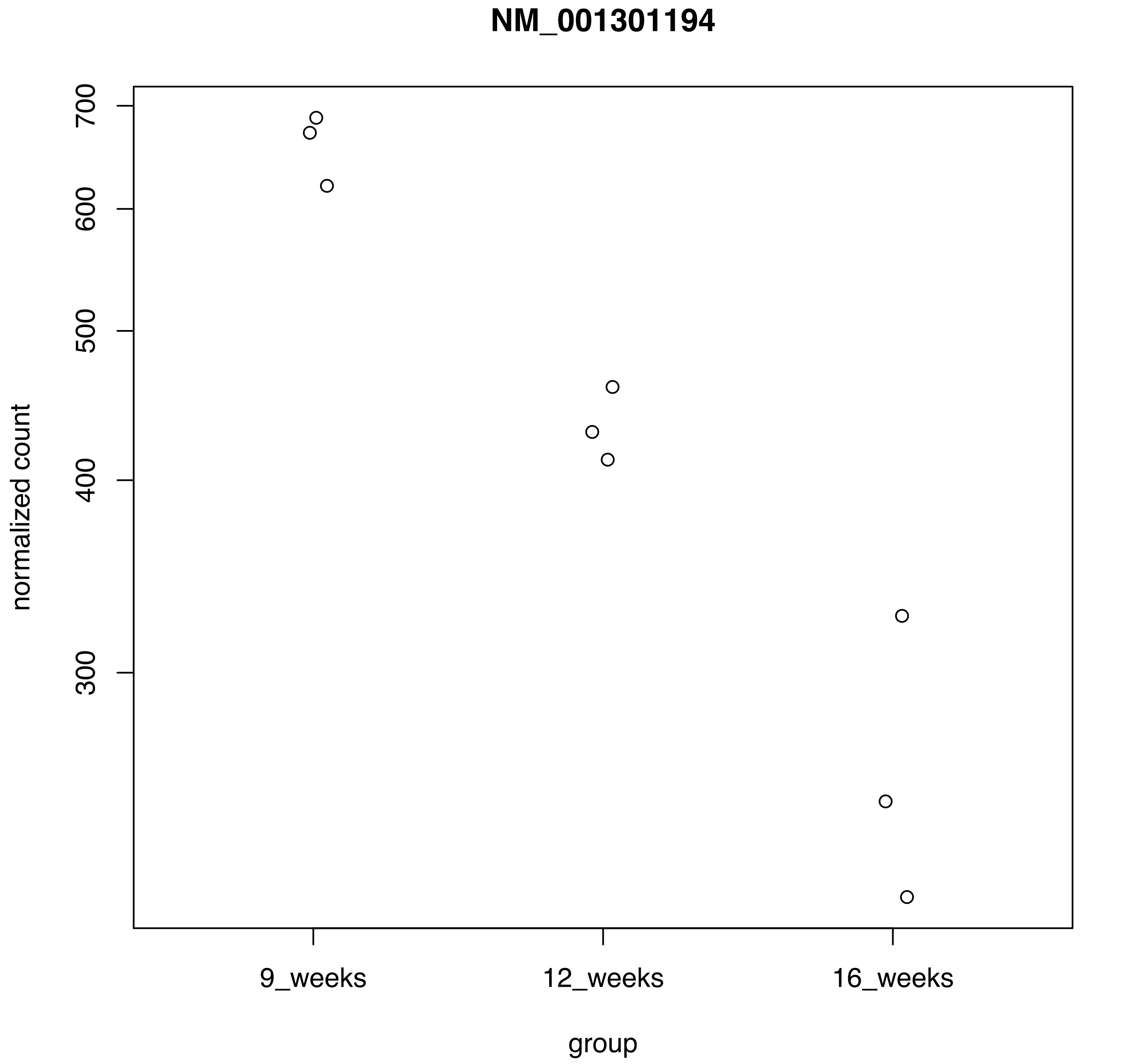
NM_001301194
normalized count
700
600
500
400
300
9_weeks
12_weeks
16_weeks
group

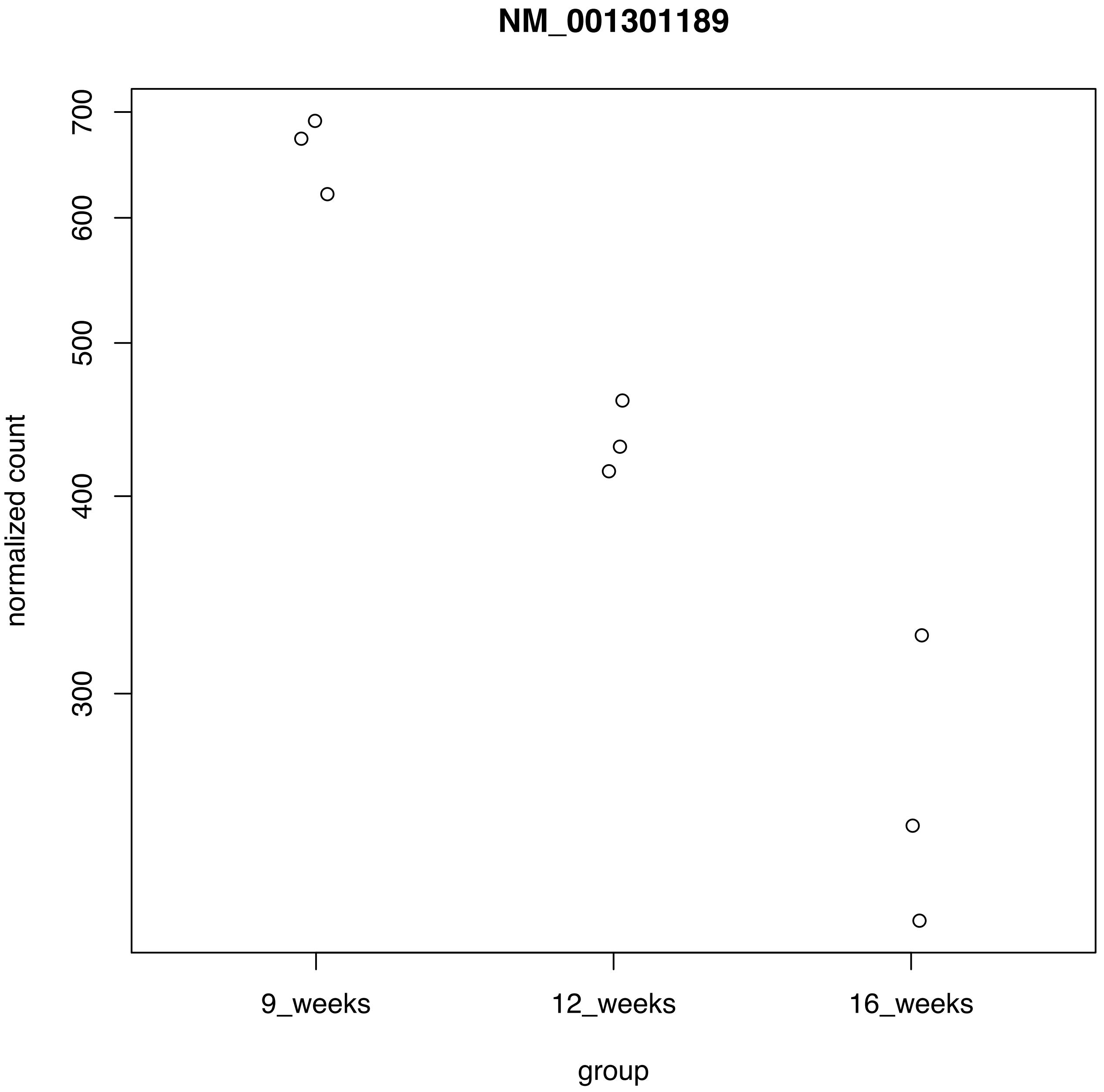
NM_001301189
normalized count
700
600
500
400
300
9_weeks
12_weeks
16_weeks
group

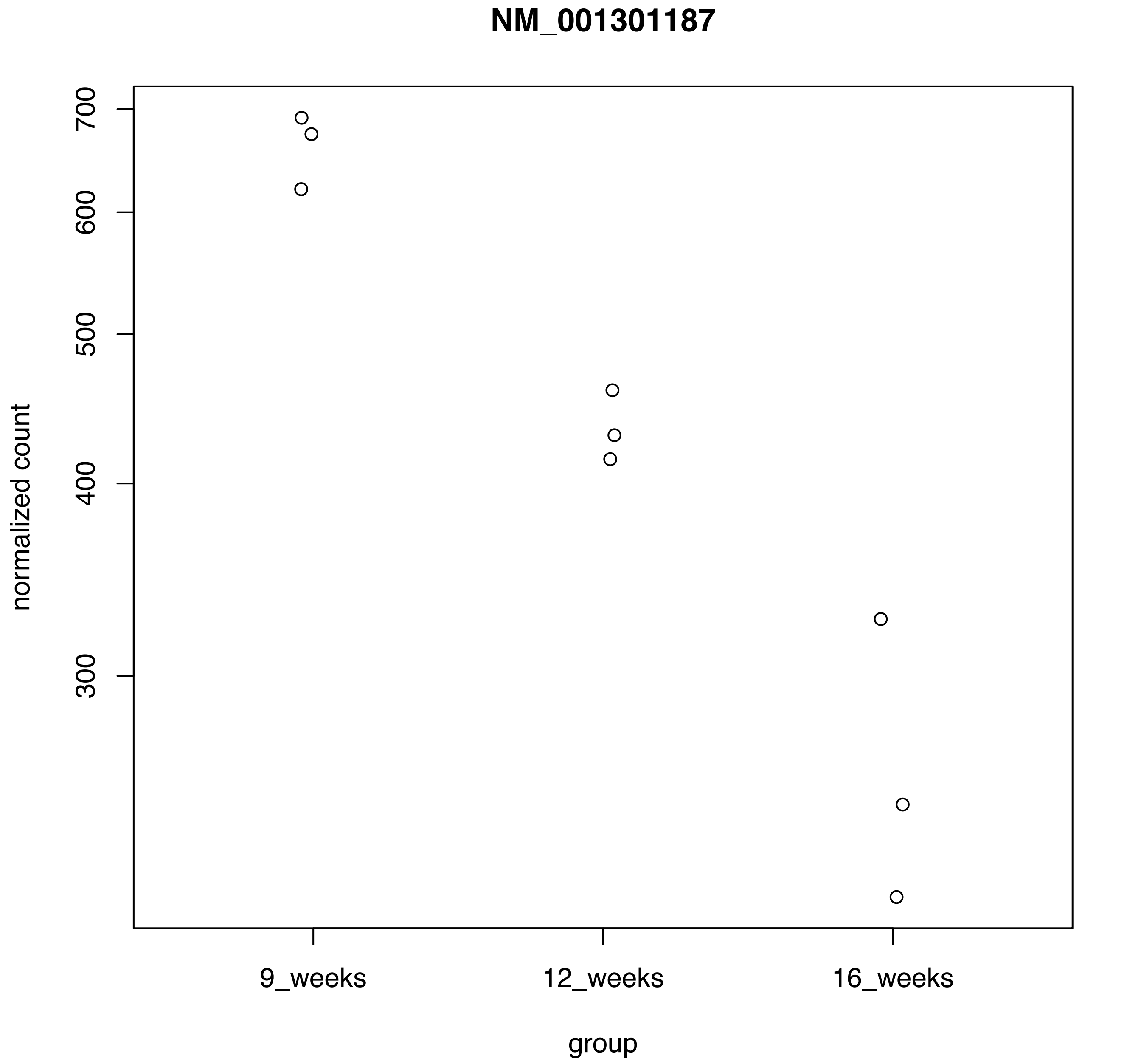
NM_001301187
normalized count
700
600
500
400
300
9_weeks
12_weeks
16_weeks
group

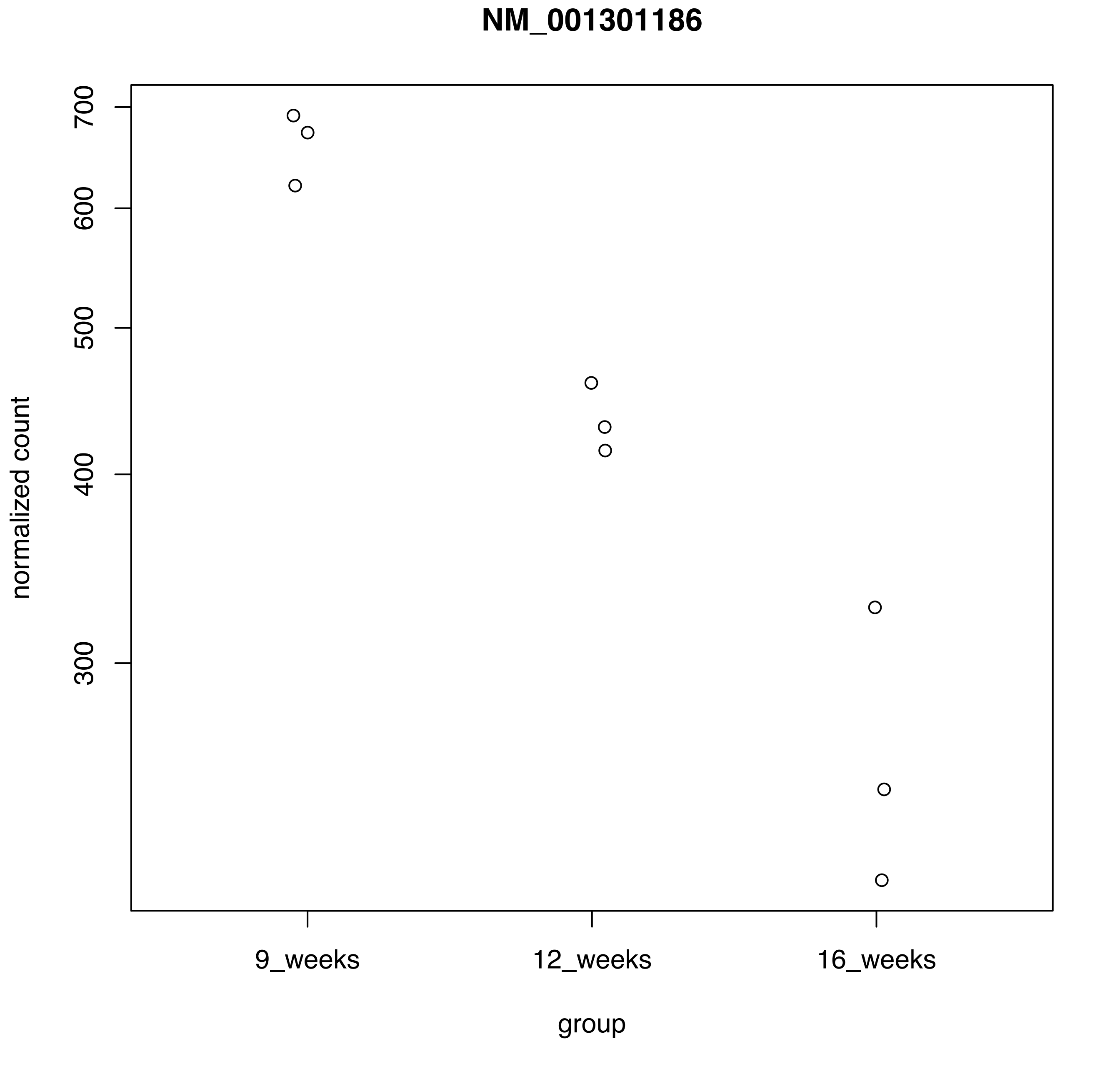
NM_001301186
normalized count
700
600
500
400
300
9_weeks
12_weeks
16_weeks
group

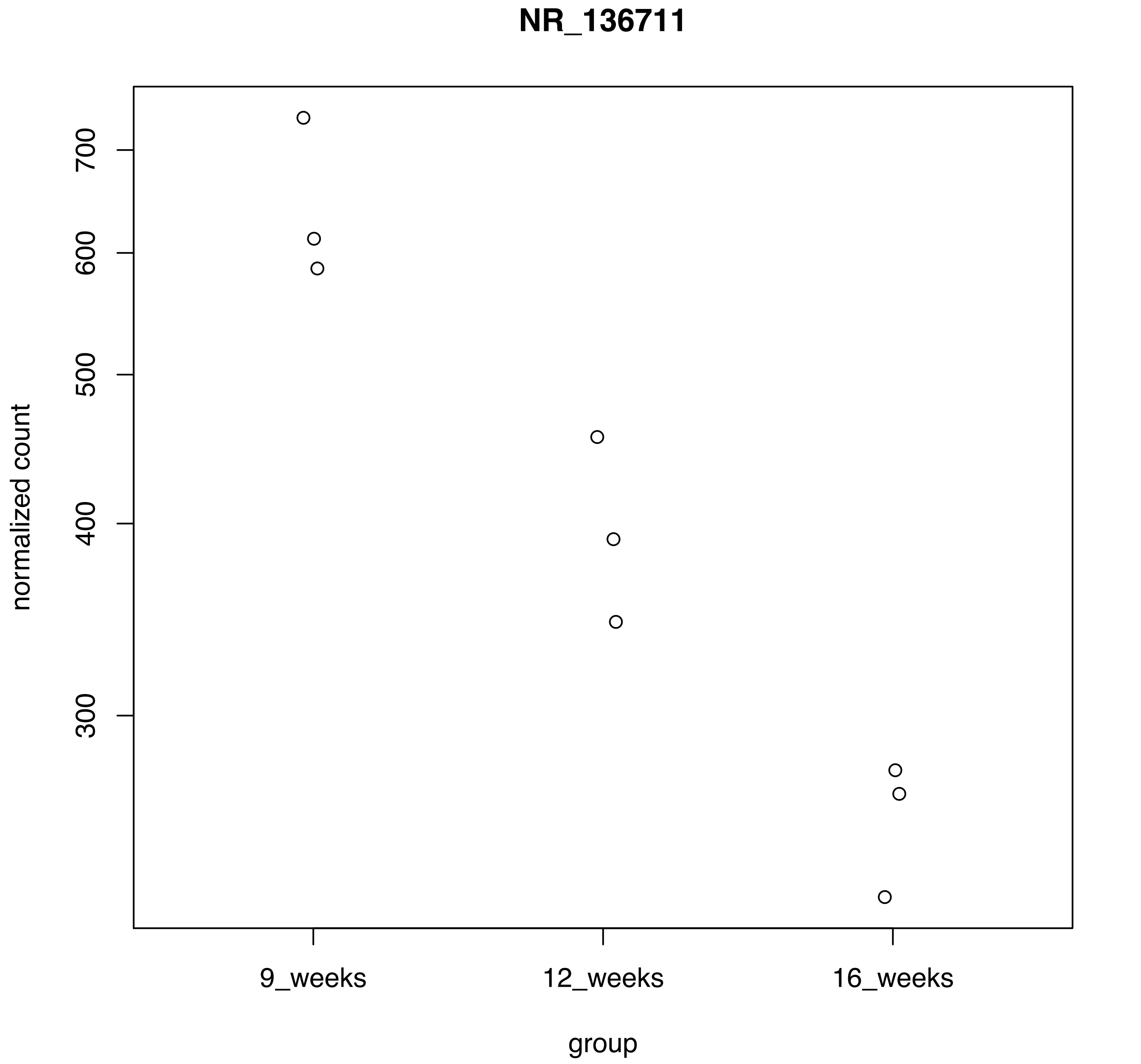

NR_136711
normalized count
700
600
500
400
300
9_weeks
12_weeks
16_weeks
group

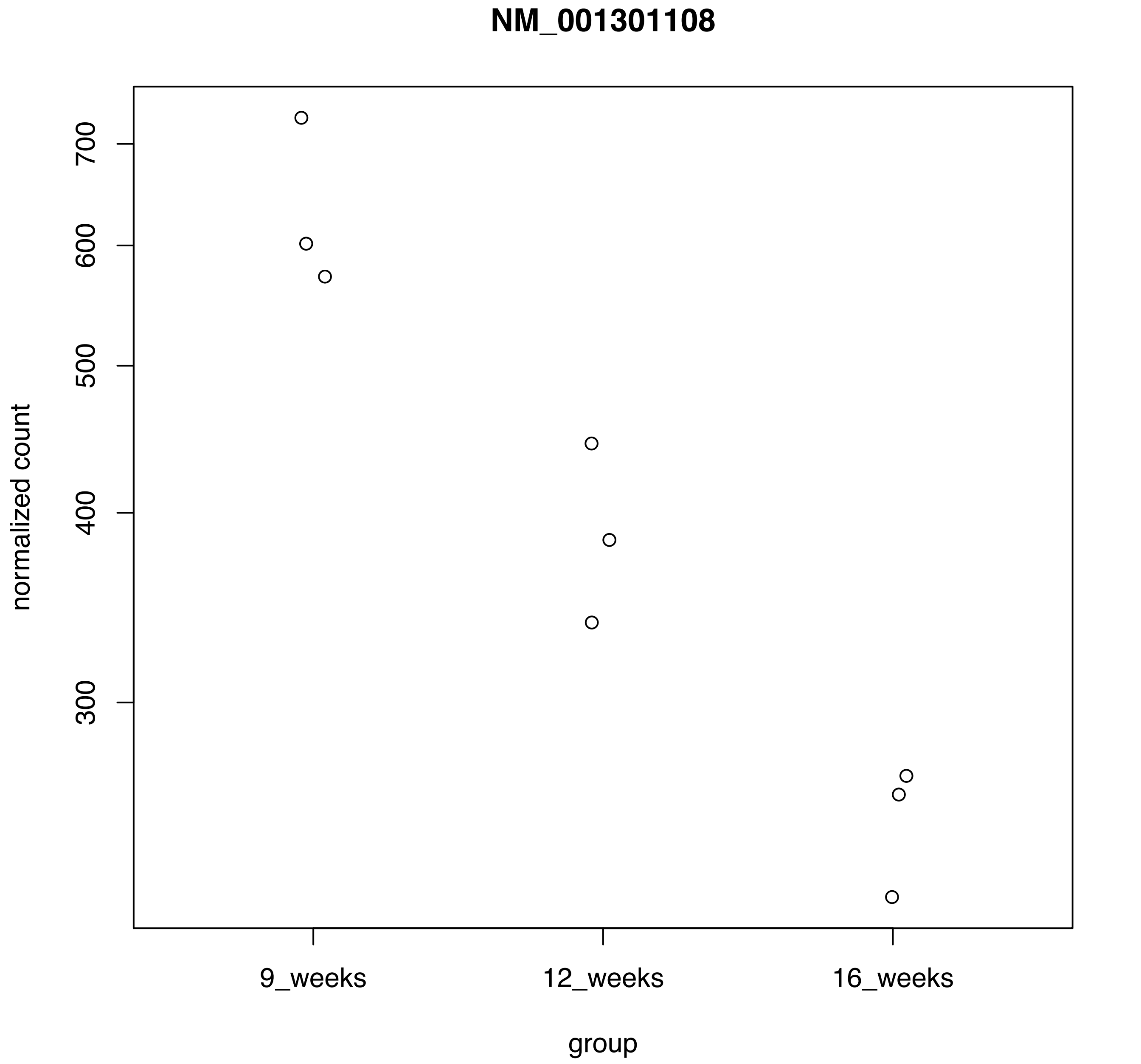

NM_001301108
normalized count
700
600
500
400
300
9_weeks
12_weeks
16_weeks
group

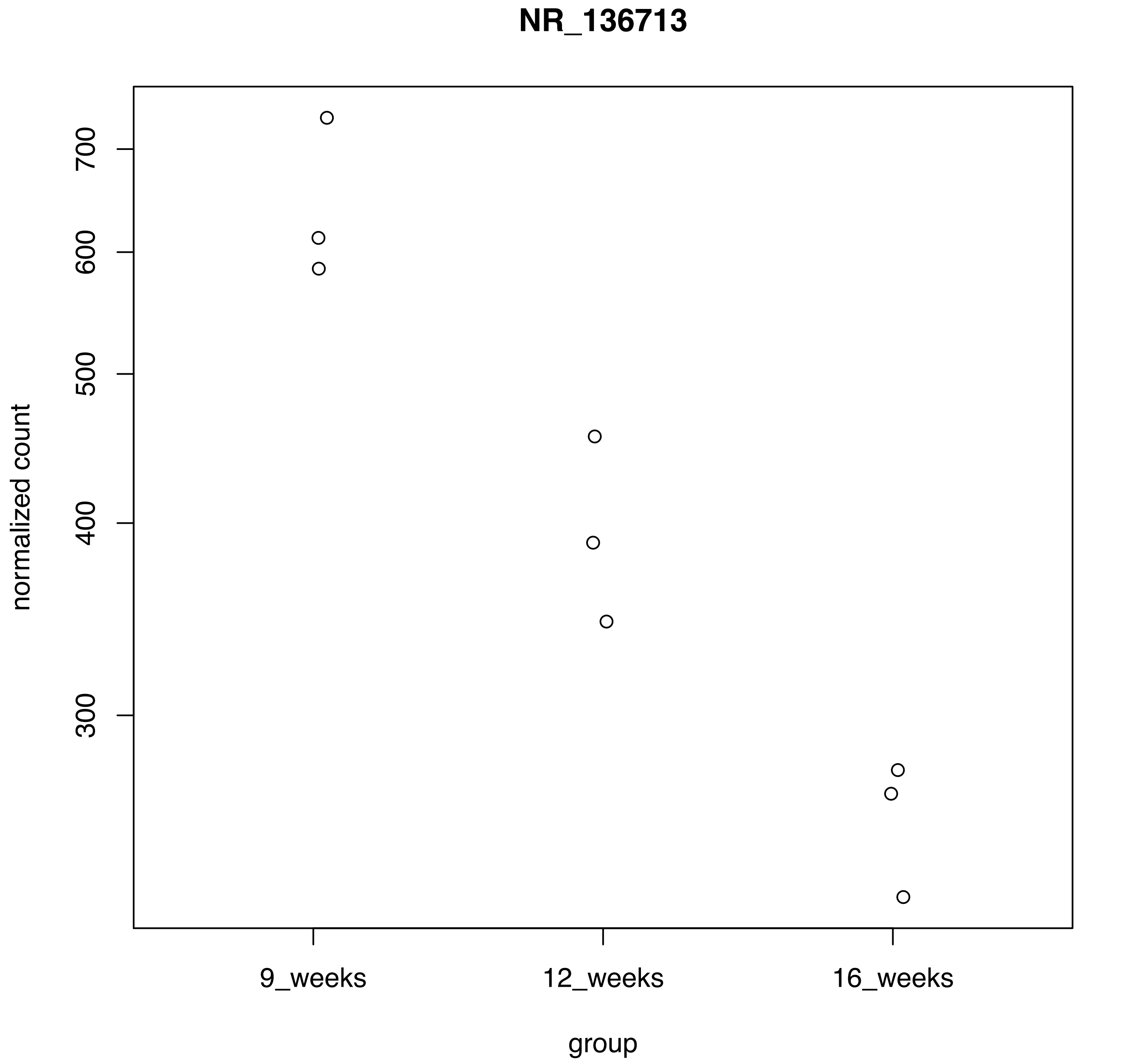
NR_136713
normalized count
700
600
500
400
300
9_weeks
12_weeks
16_weeks
group

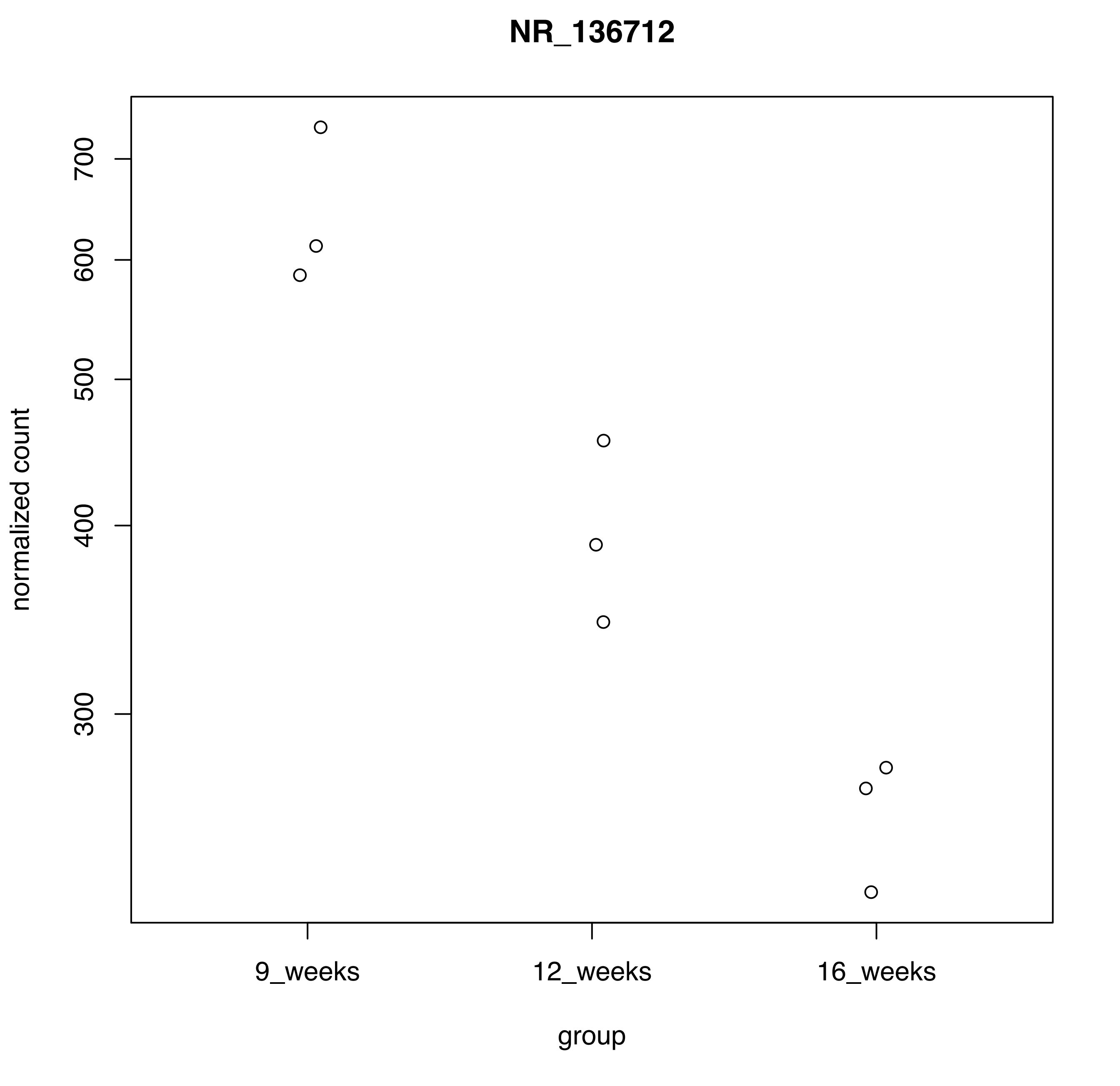
NR_136712
normalized count
700
600
500
400
300
9_weeks
12_weeks
16_weeks
group

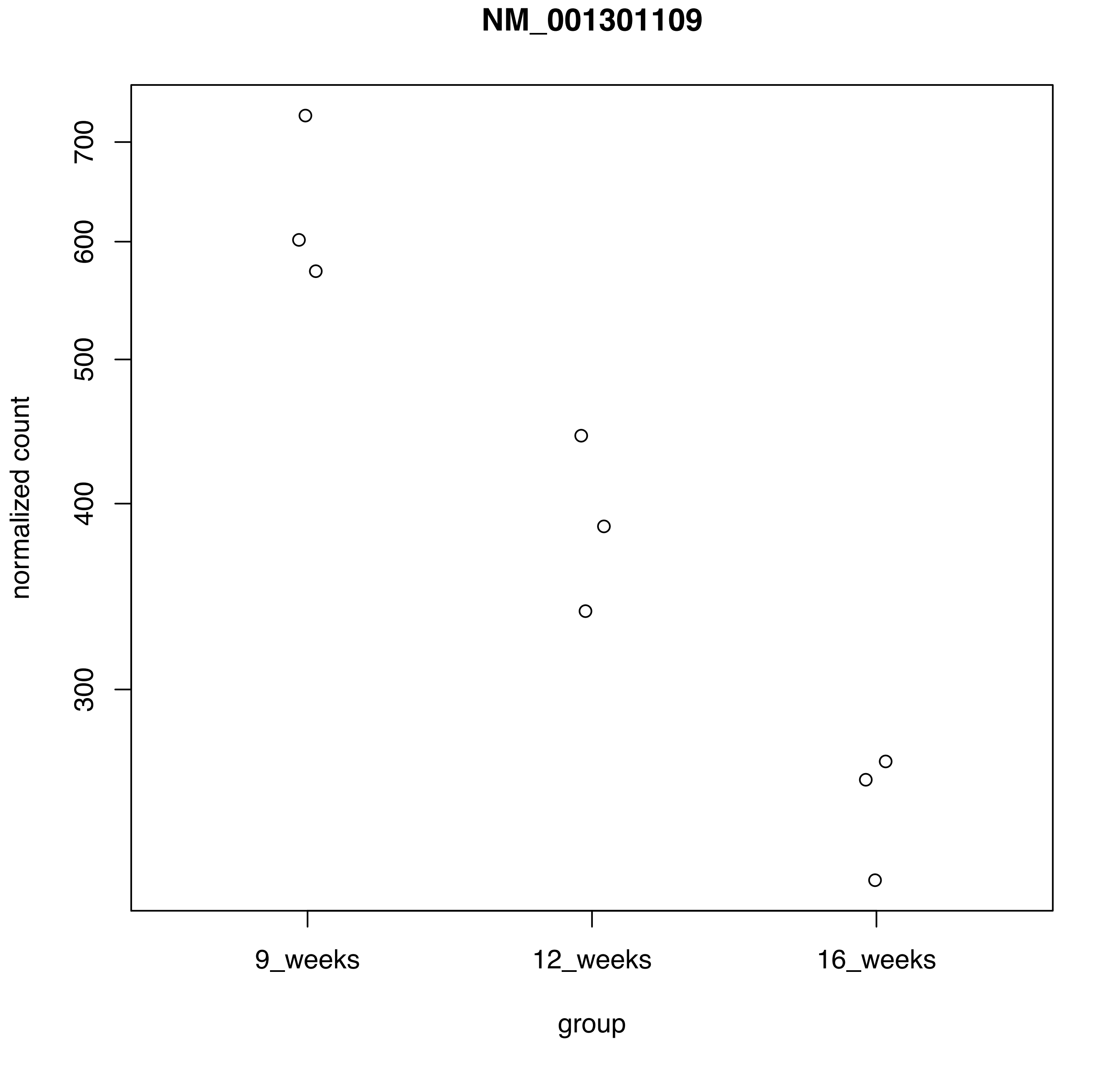
NM_001301109
normalized count
700
600
500
400
300
9_weeks
12_weeks
16_weeks
group

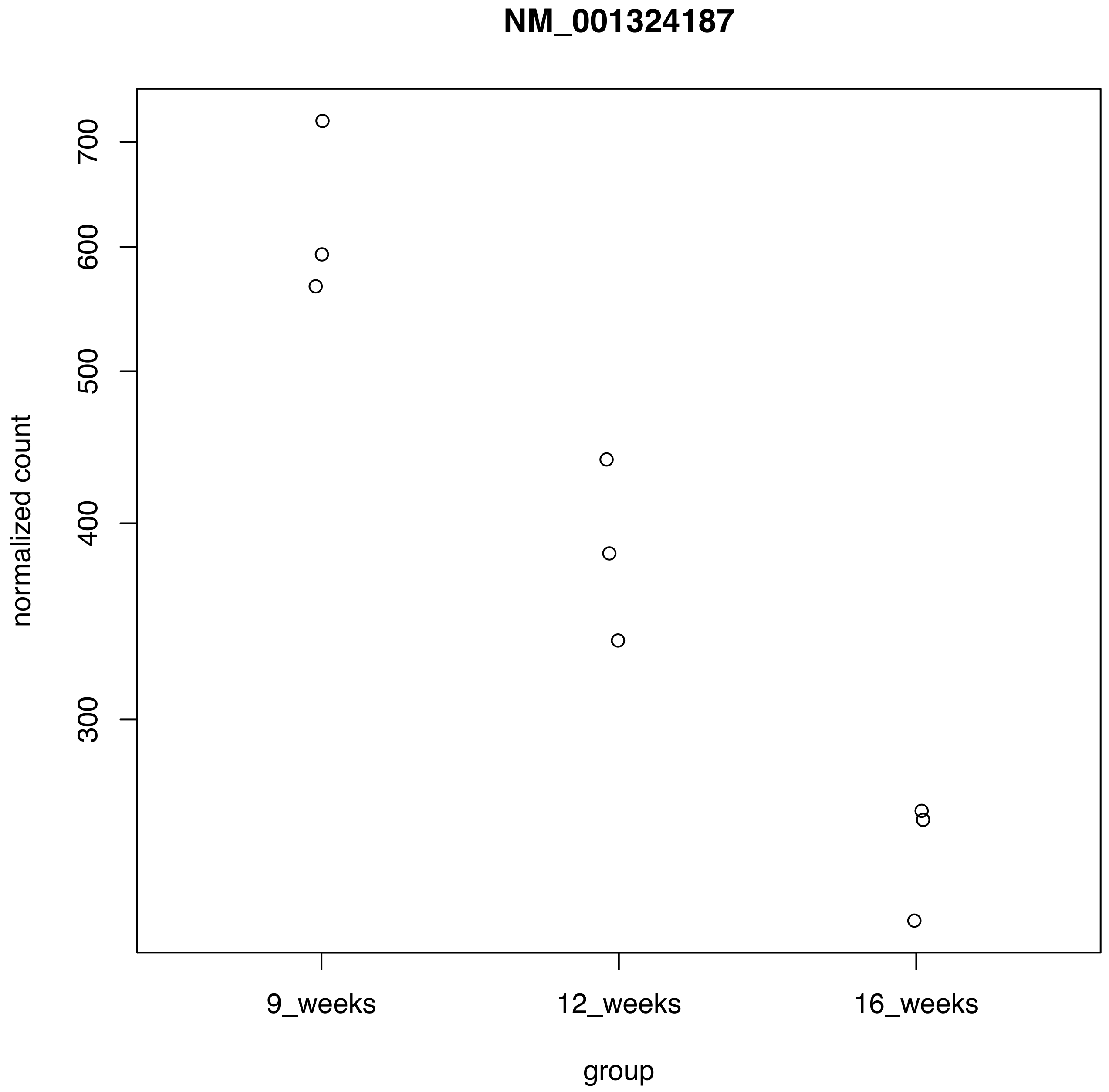
NM_001324187
normalized count
700
600
500
400
300
9_weeks
12_weeks
16_weeks
group

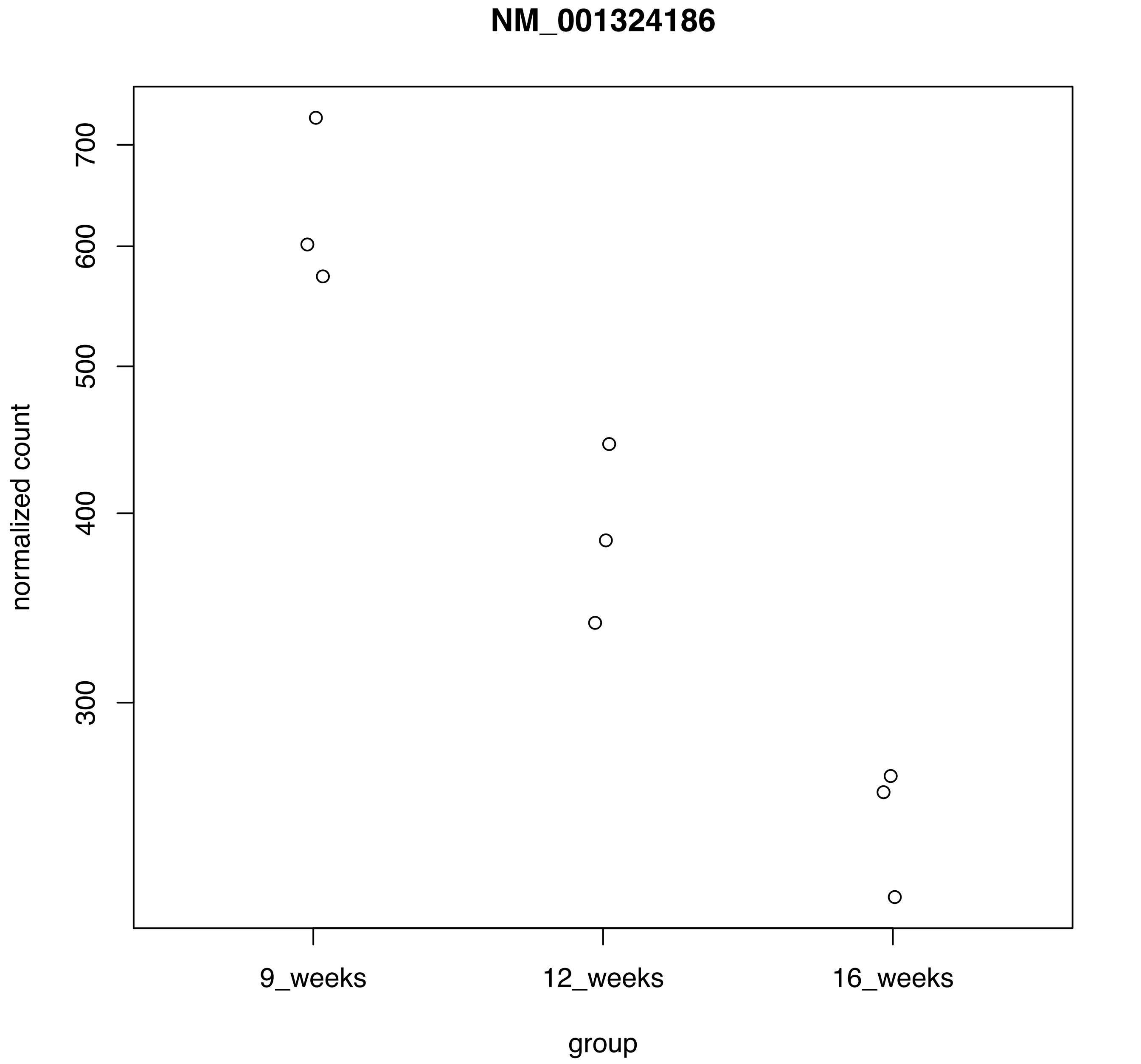

NM_001324186
normalized count
700
600
500
400
300
9_weeks
12_weeks
16_weeks
group

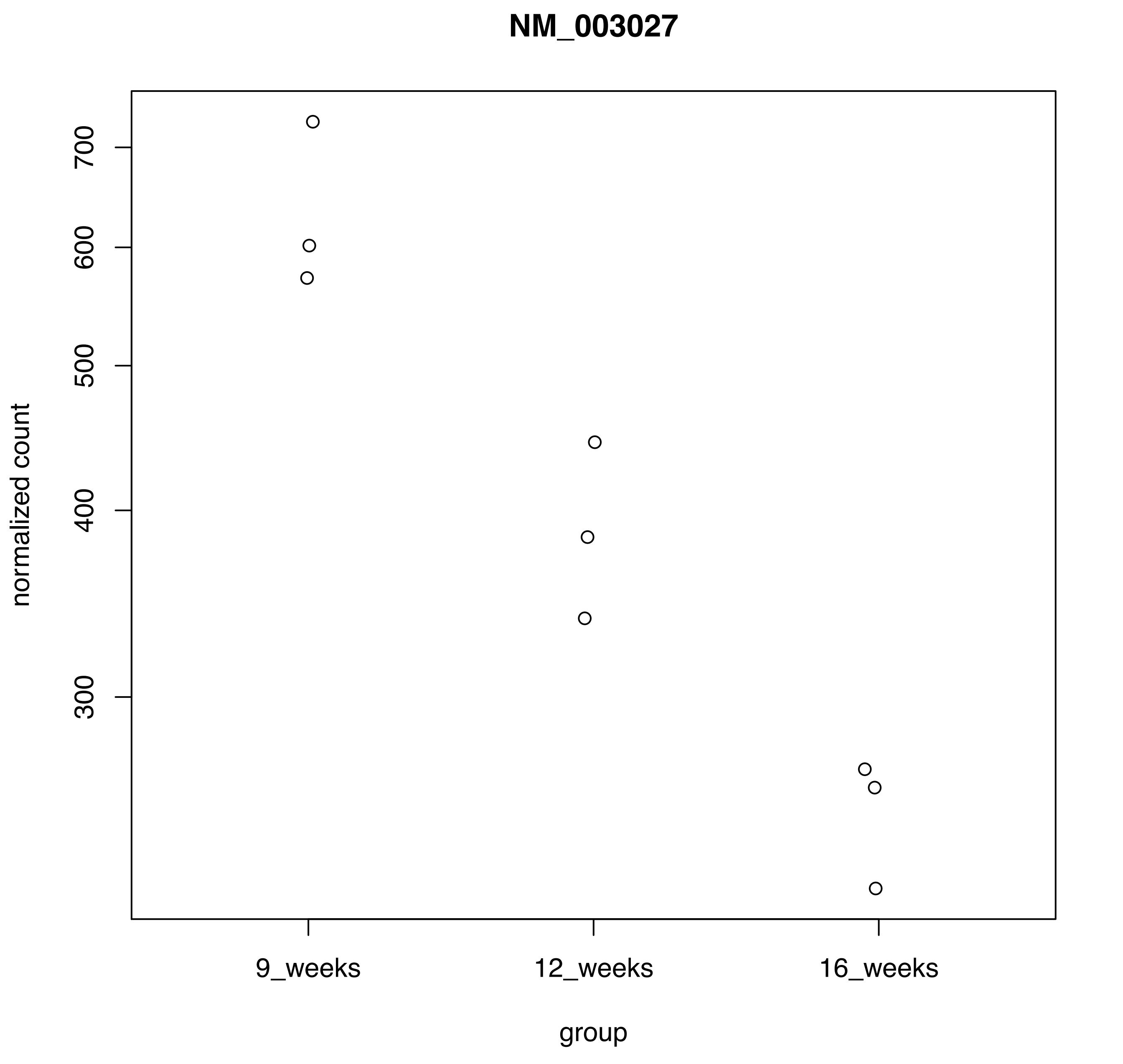

NM_003027
normalized count
700
600
500
400
300
9_weeks
12_weeks
16_weeks
group

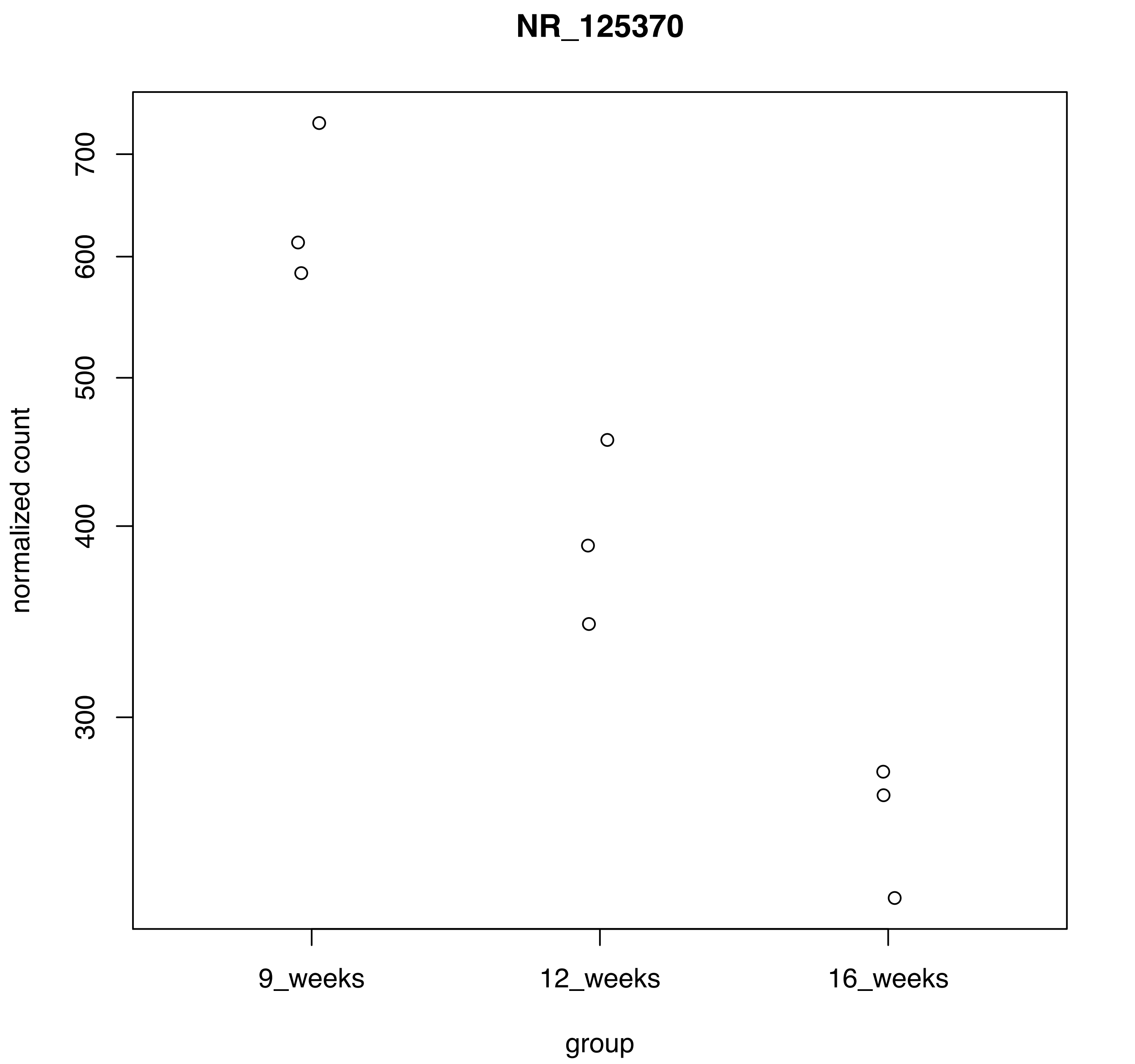
NR_125370
normalized count
700
600
500
400
300
9_weeks
12_weeks
16_weeks
group

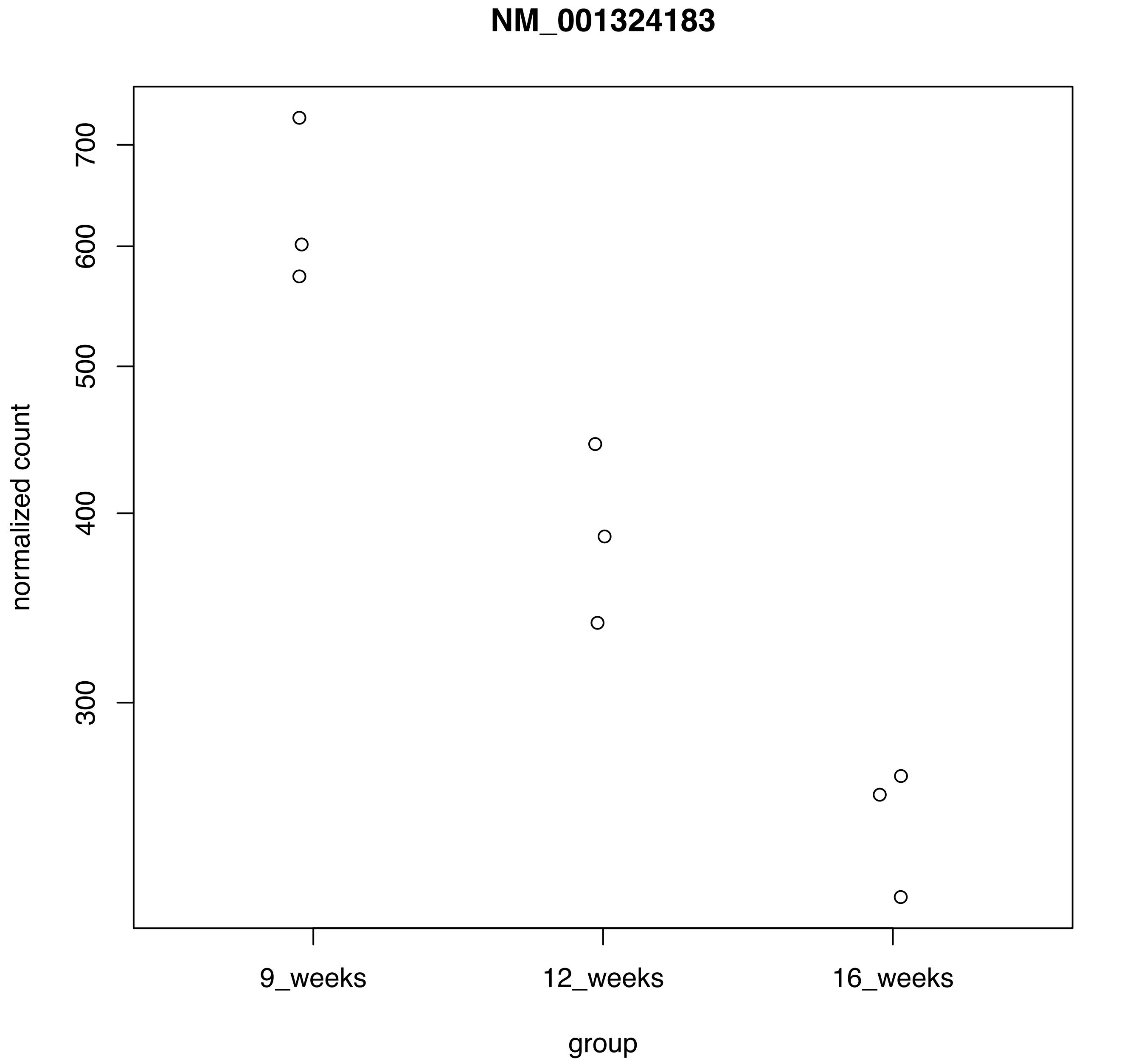
NM_001324183
normalized count
700
600
500
400
300
9_weeks
12_weeks
16_weeks
group

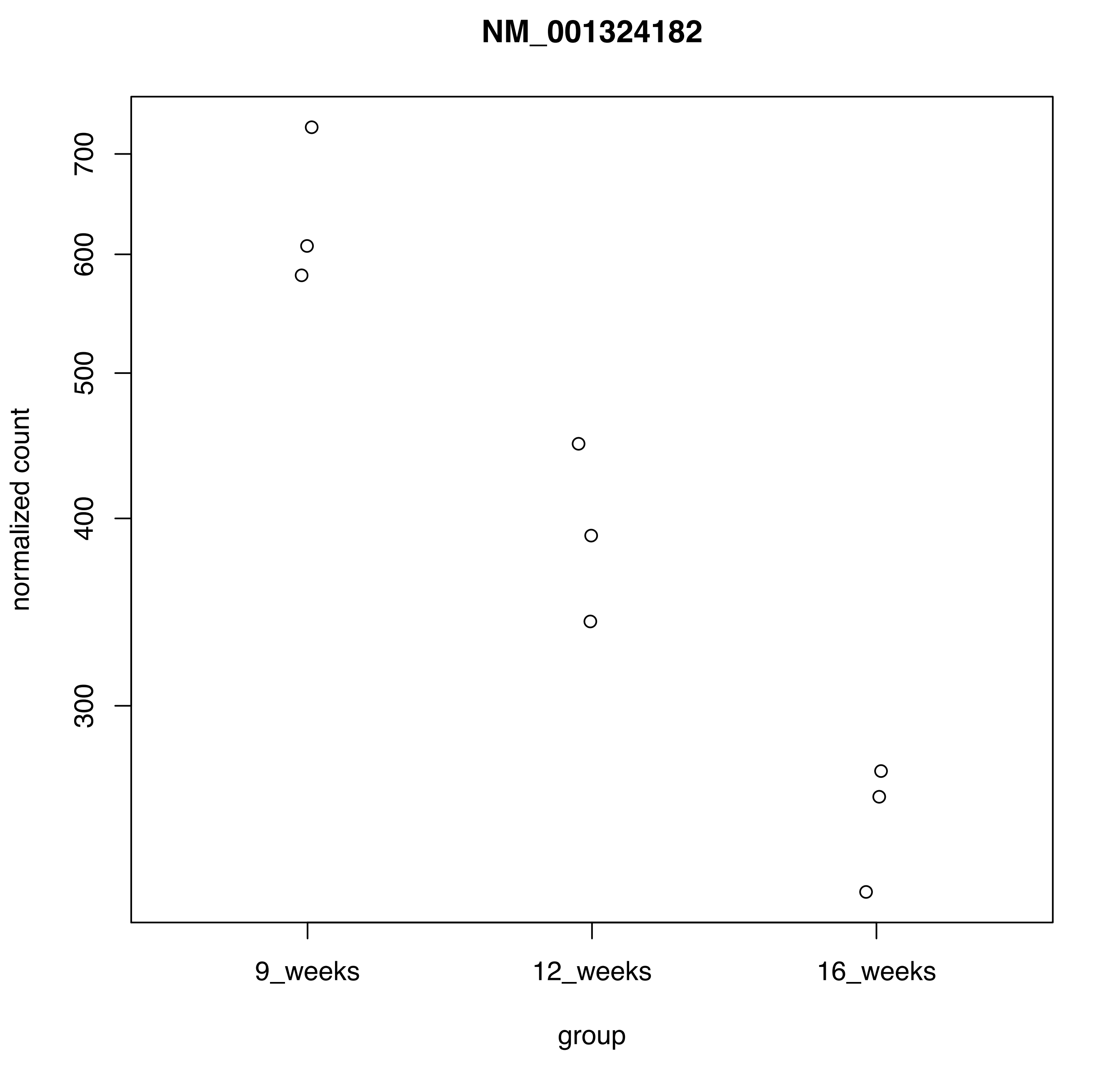
NM_001324182
normalized count
300
400
500
600
700
9_weeks
12_weeks
16_weeks
group

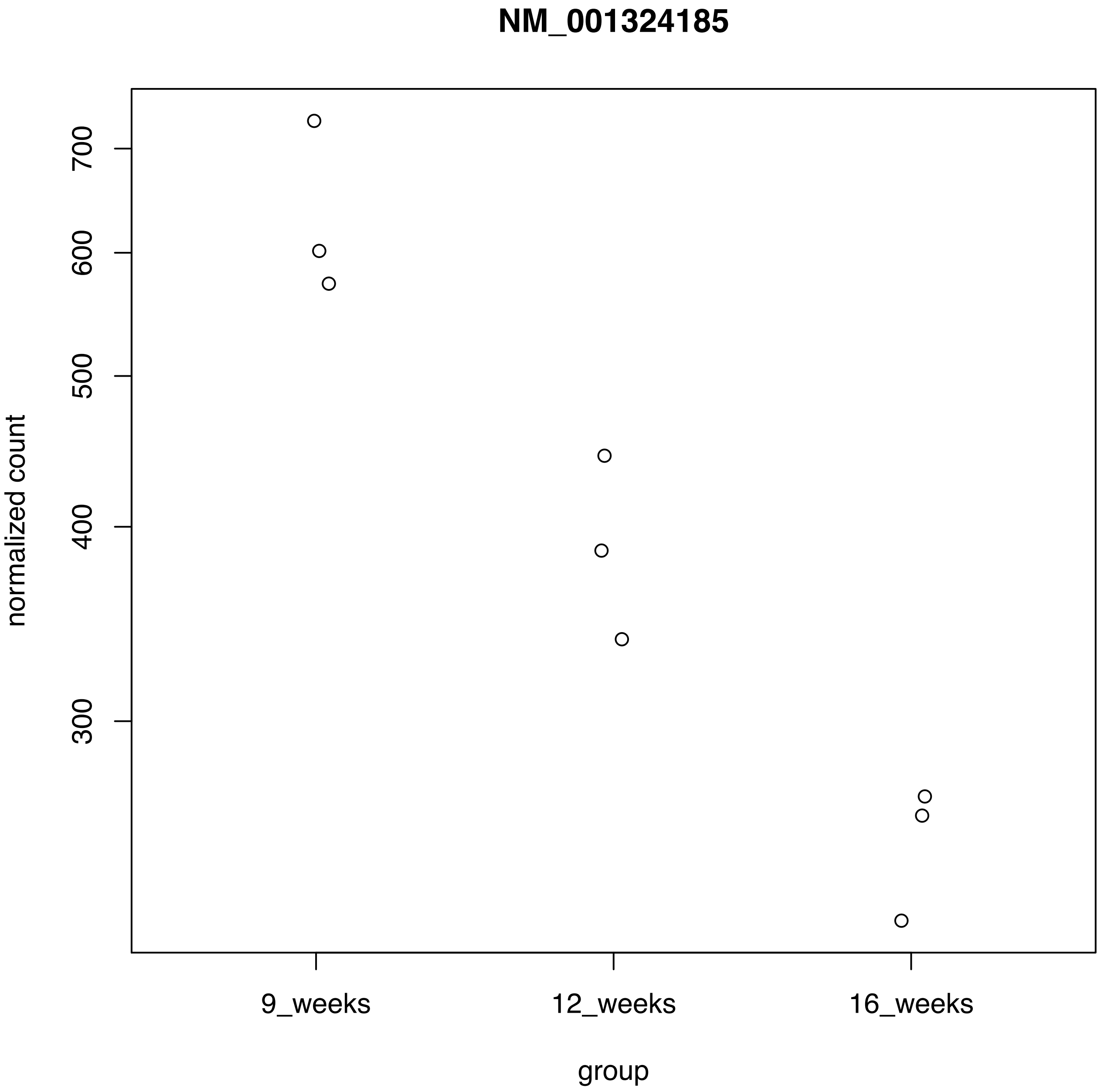
NM_001324185
normalized count
700
600
500
400
300
9_weeks
12_weeks
16_weeks
group

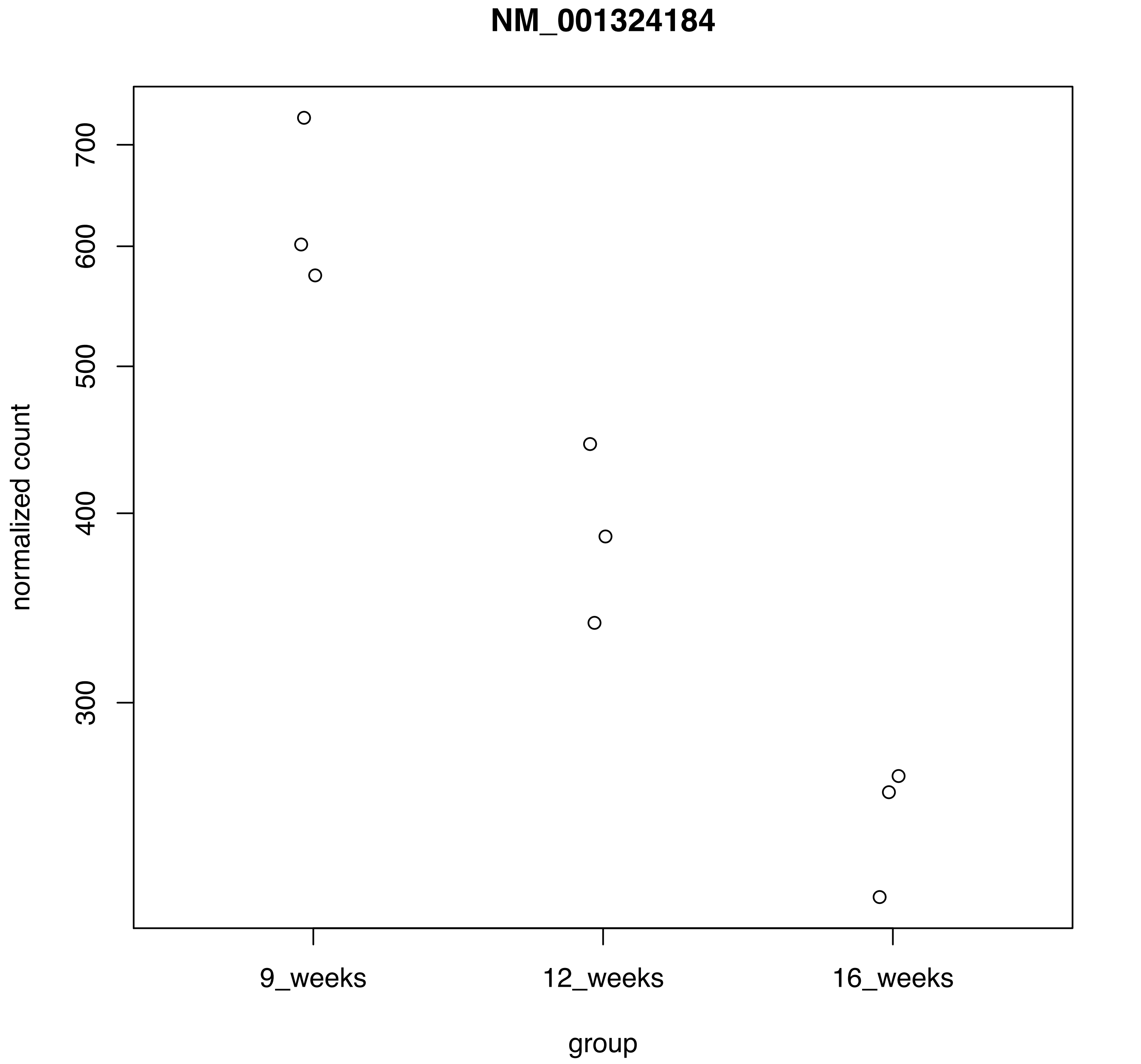

NM_001324184
700
600
500
400
300
normalized count
9_weeks
12_weeks
16_weeks
group

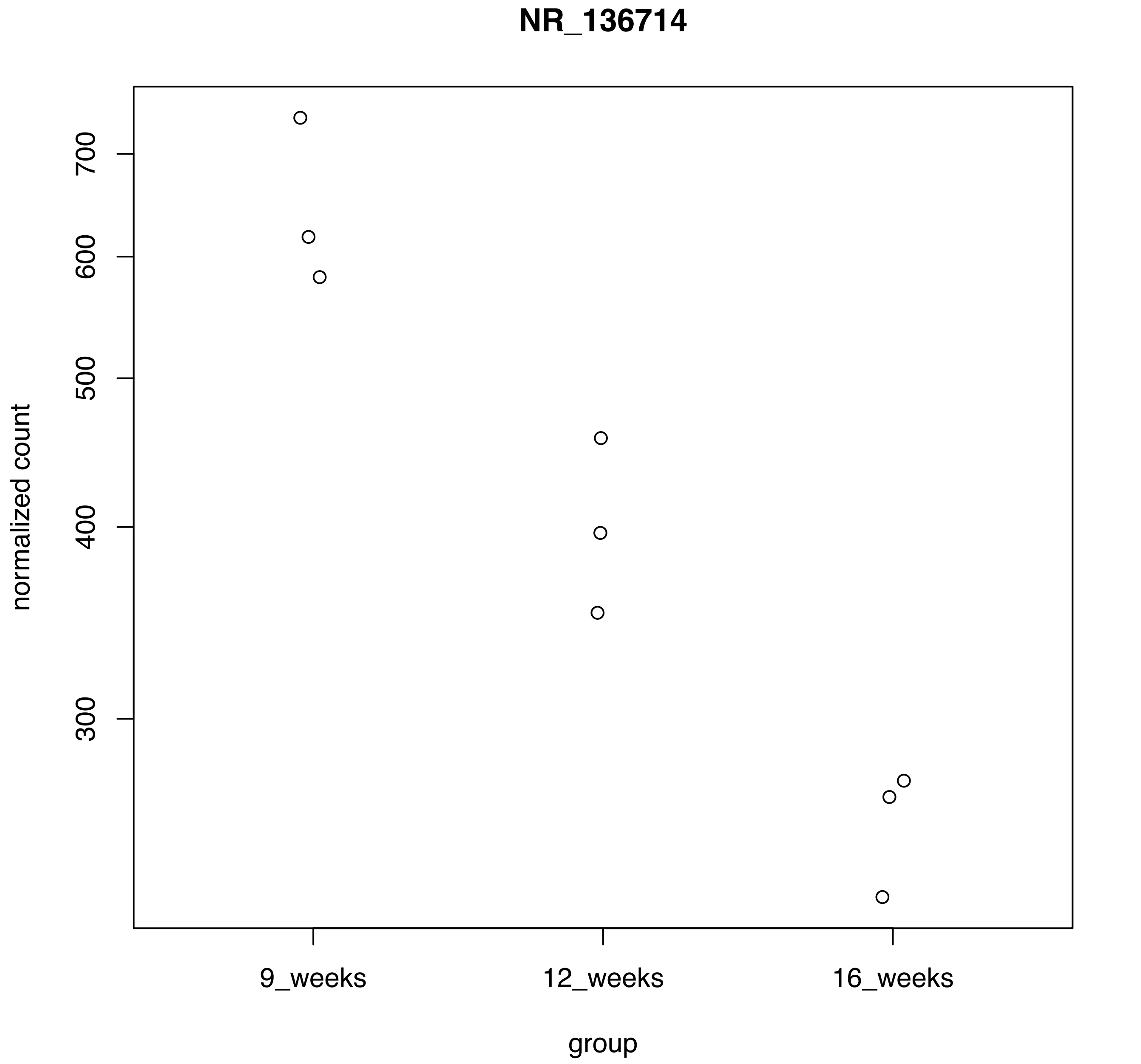
NR_136714
normalized count
300
400
500
600
700
9_weeks
12_weeks
16_weeks
group

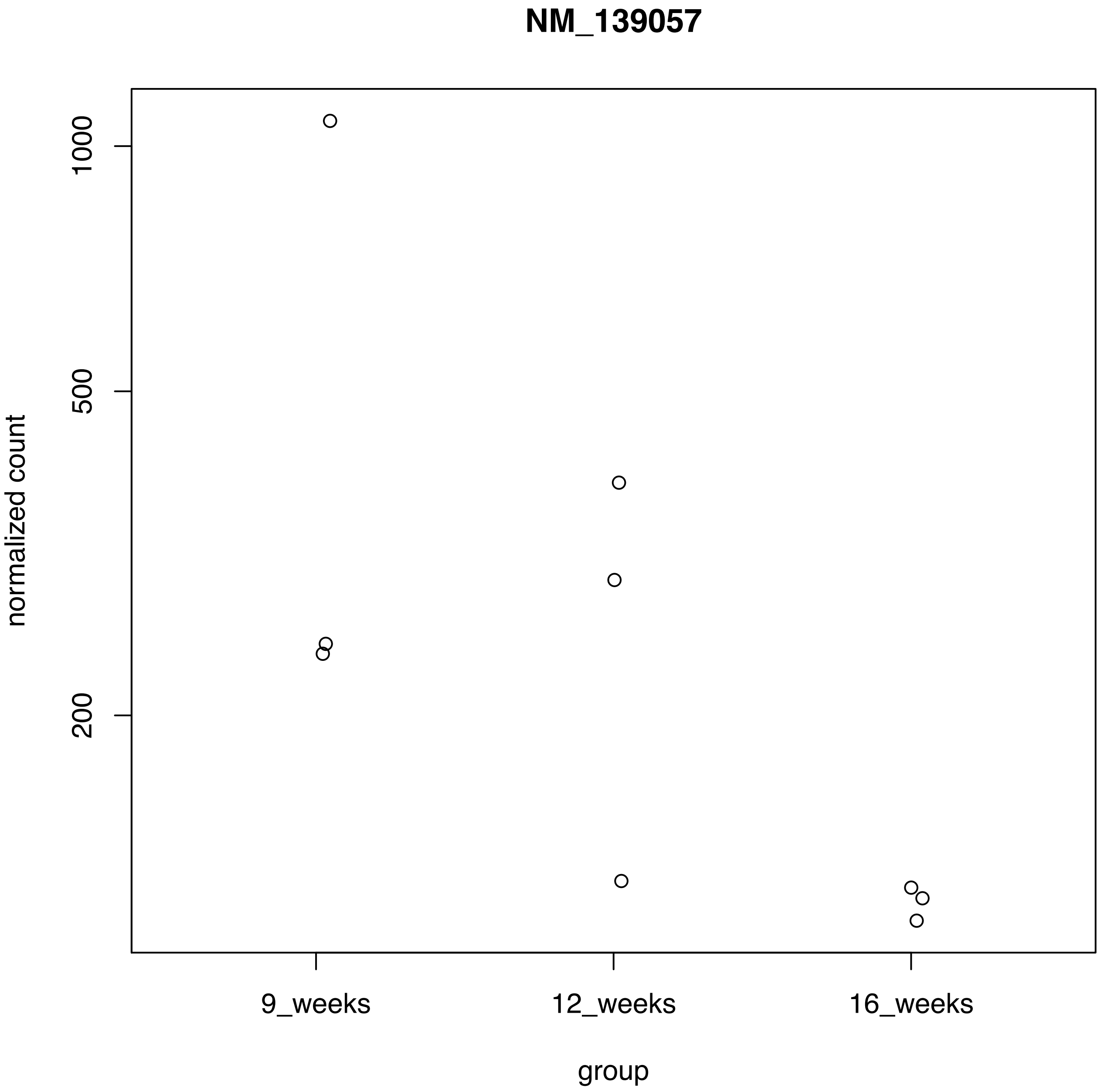

NM_139057
normalized count
1000
500
200
9_weeks
12_weeks
16_weeks
group

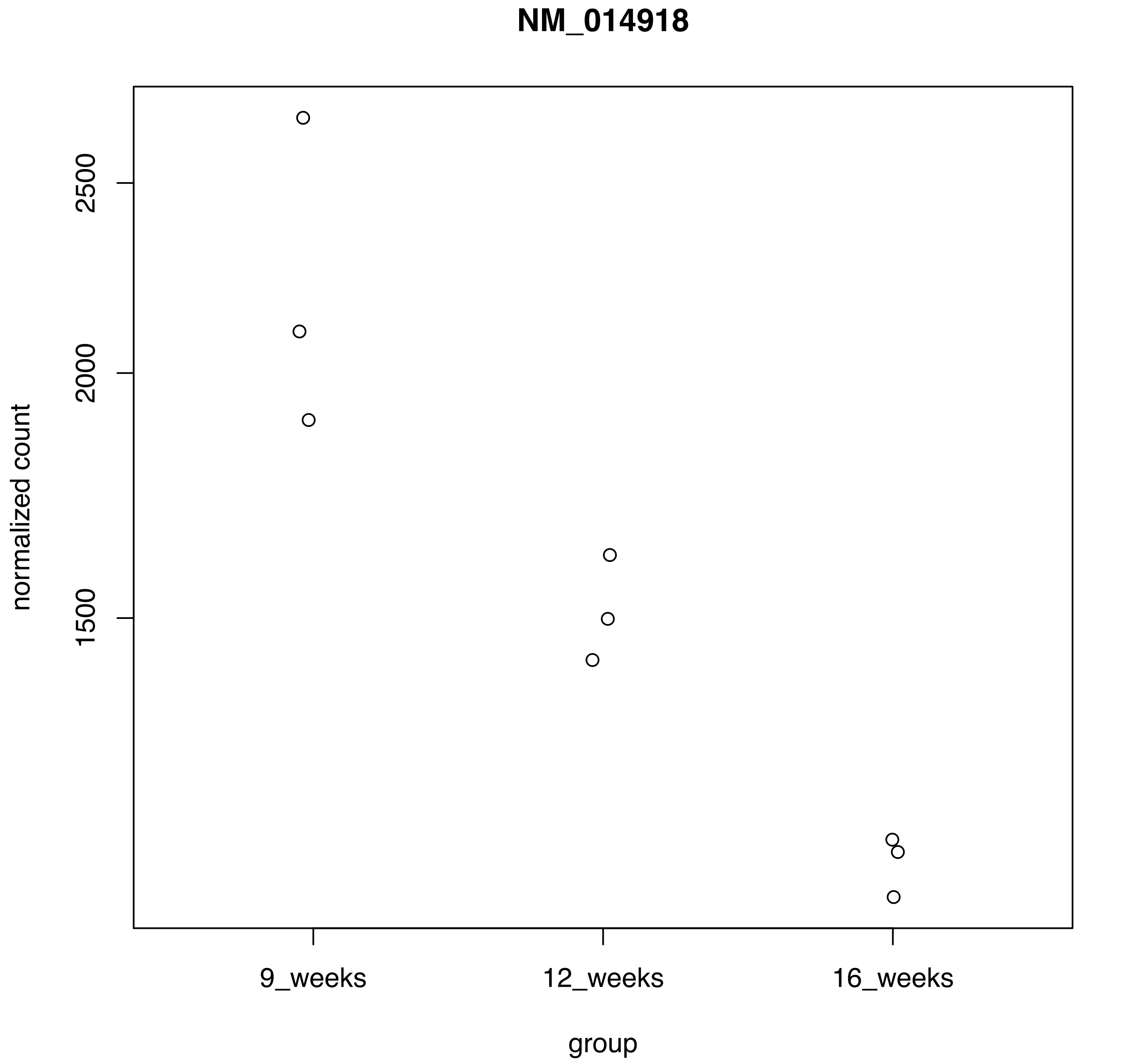

NM_014918
normalized count
2500
2000
1500
9_weeks
12_weeks
16_weeks
group

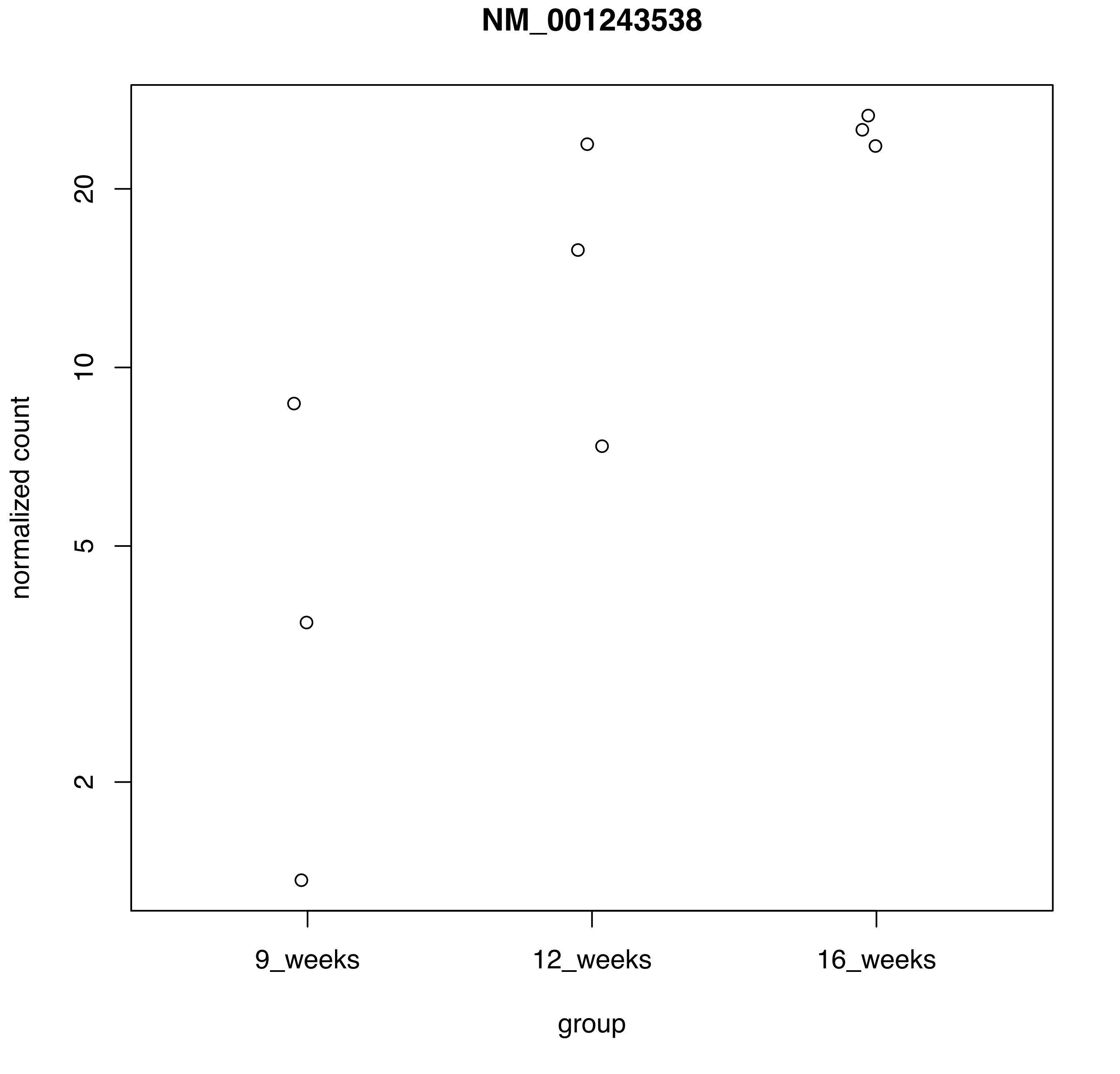

NM_001243538
normalized count
20
10
5
2
9_weeks
12_weeks
16_weeks
group

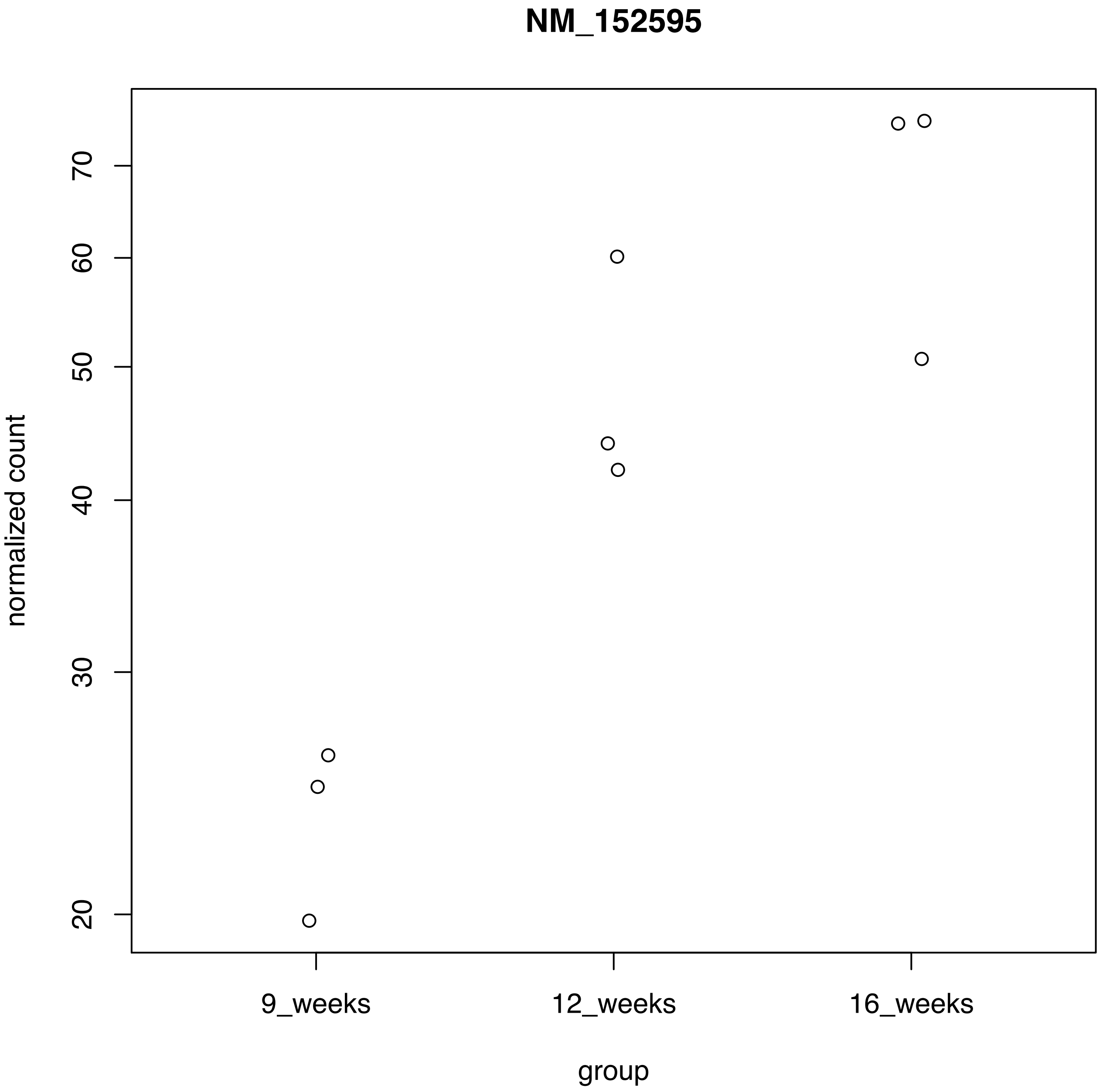
NM_152595
normalized count
70
60
50
40
30
20
9_weeks
12_weeks
16_weeks
group

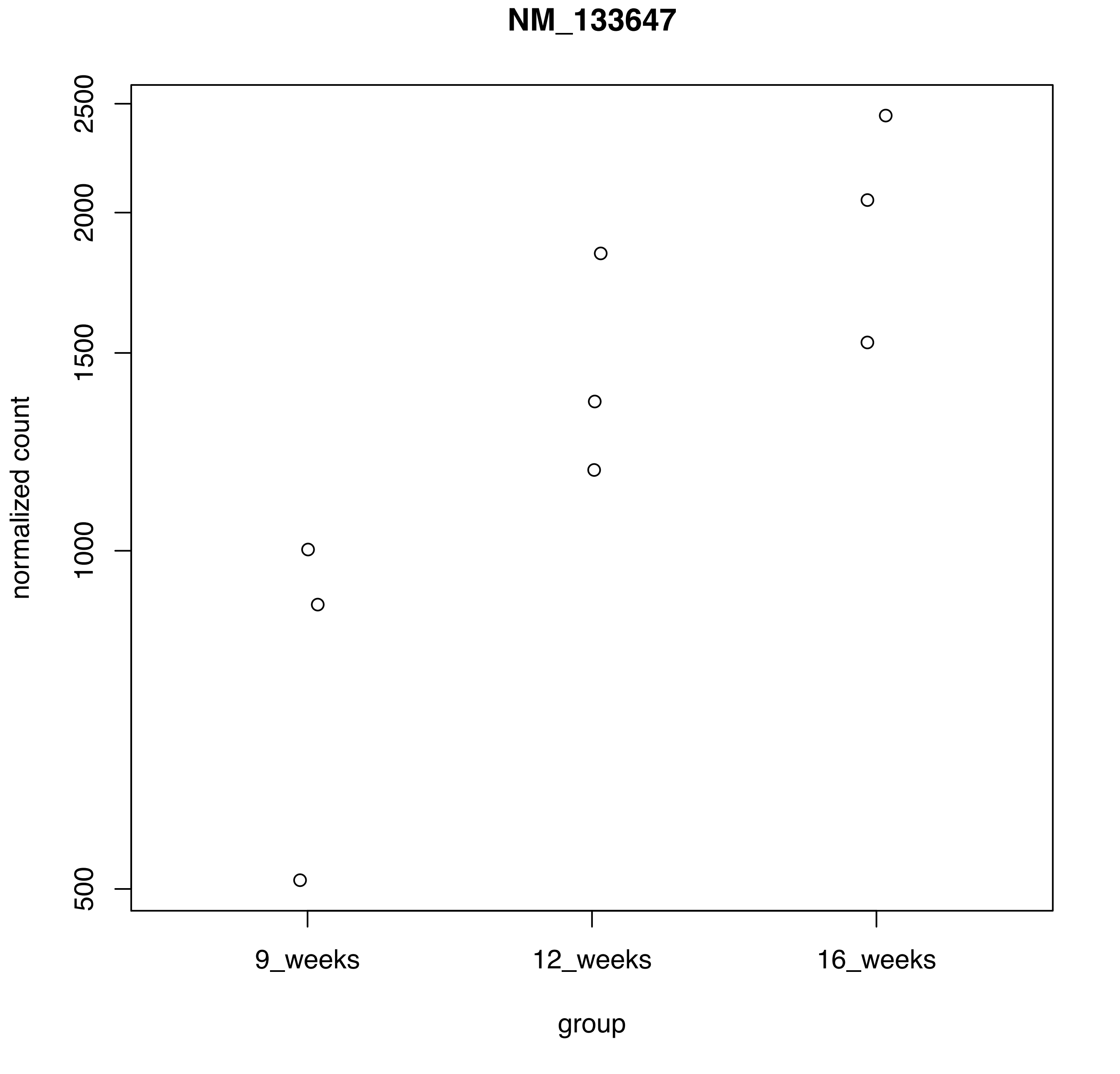
NM_133647
normalized count
2500
2000
1500
1000
500
9_weeks
12_weeks
16_weeks
group

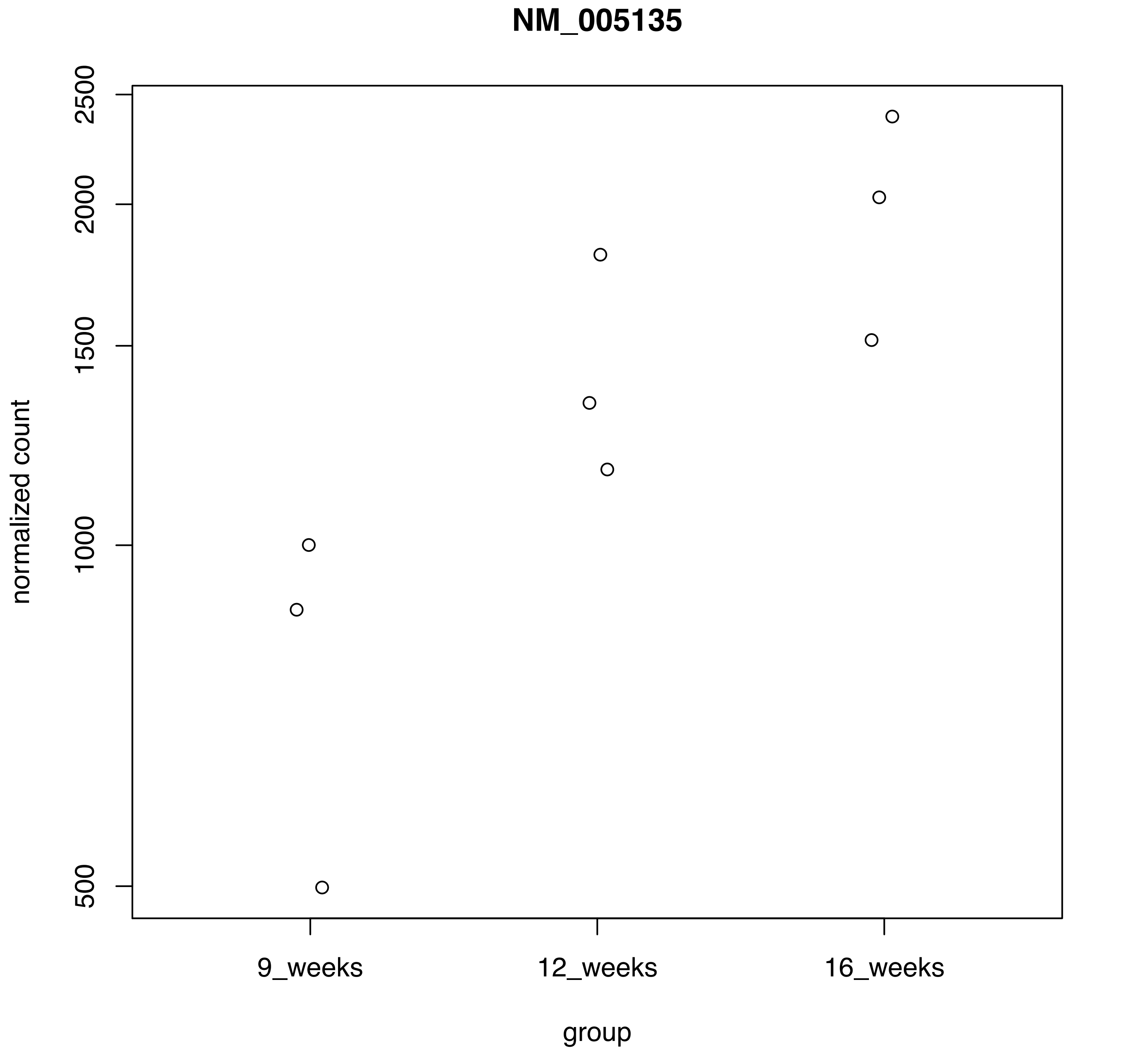
NM_005135
normalized count
500
1000
1500
2000
2500
9_weeks
12_weeks
16_weeks
group

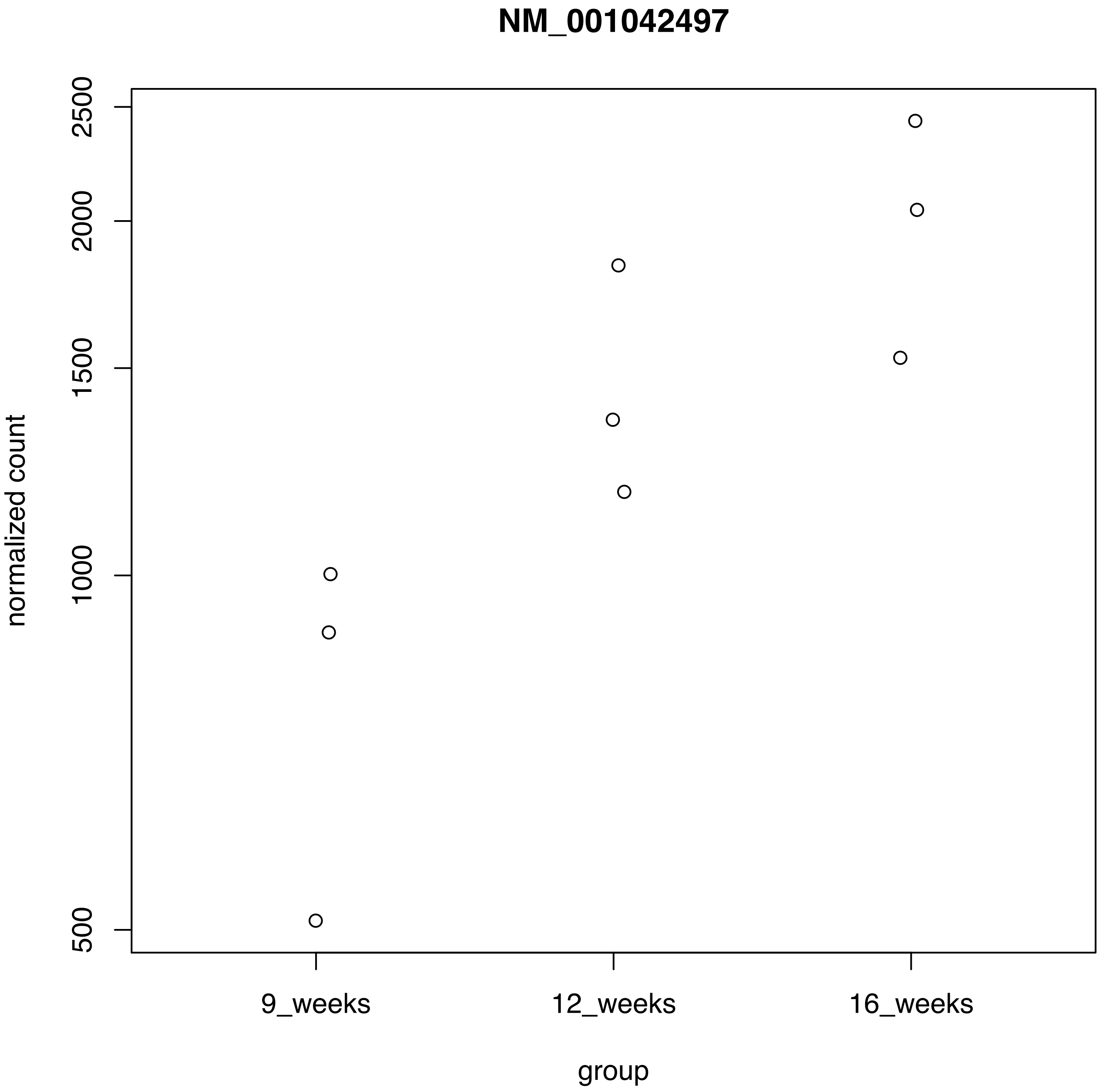
NM_001042497
normalized count
2500
2000
1500
1000
500
9_weeks
12_weeks
16_weeks
group

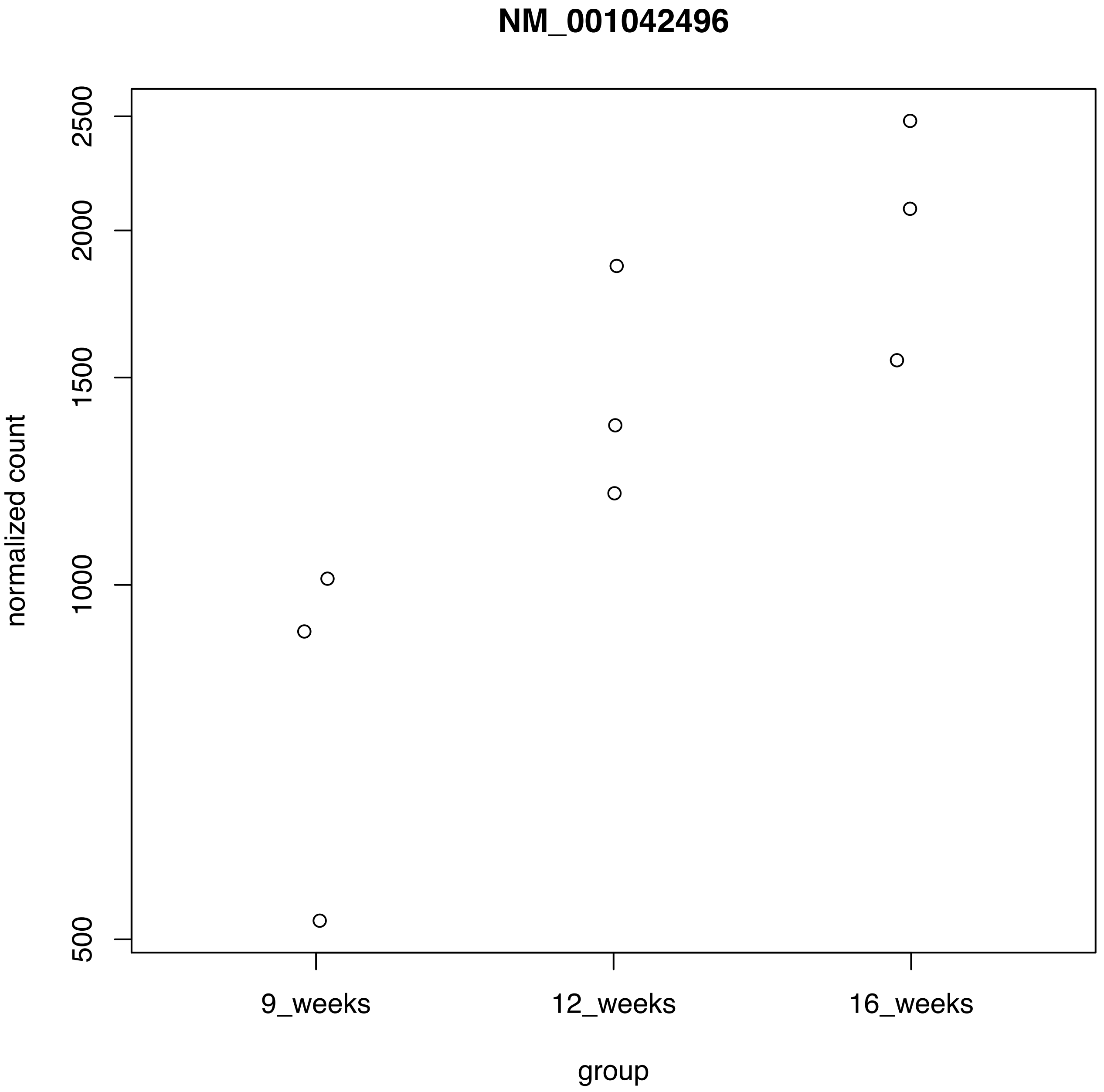
NM_001042496
normalized count
2500
2000
1500
1000
500
9_weeks
12_weeks
16_weeks
group

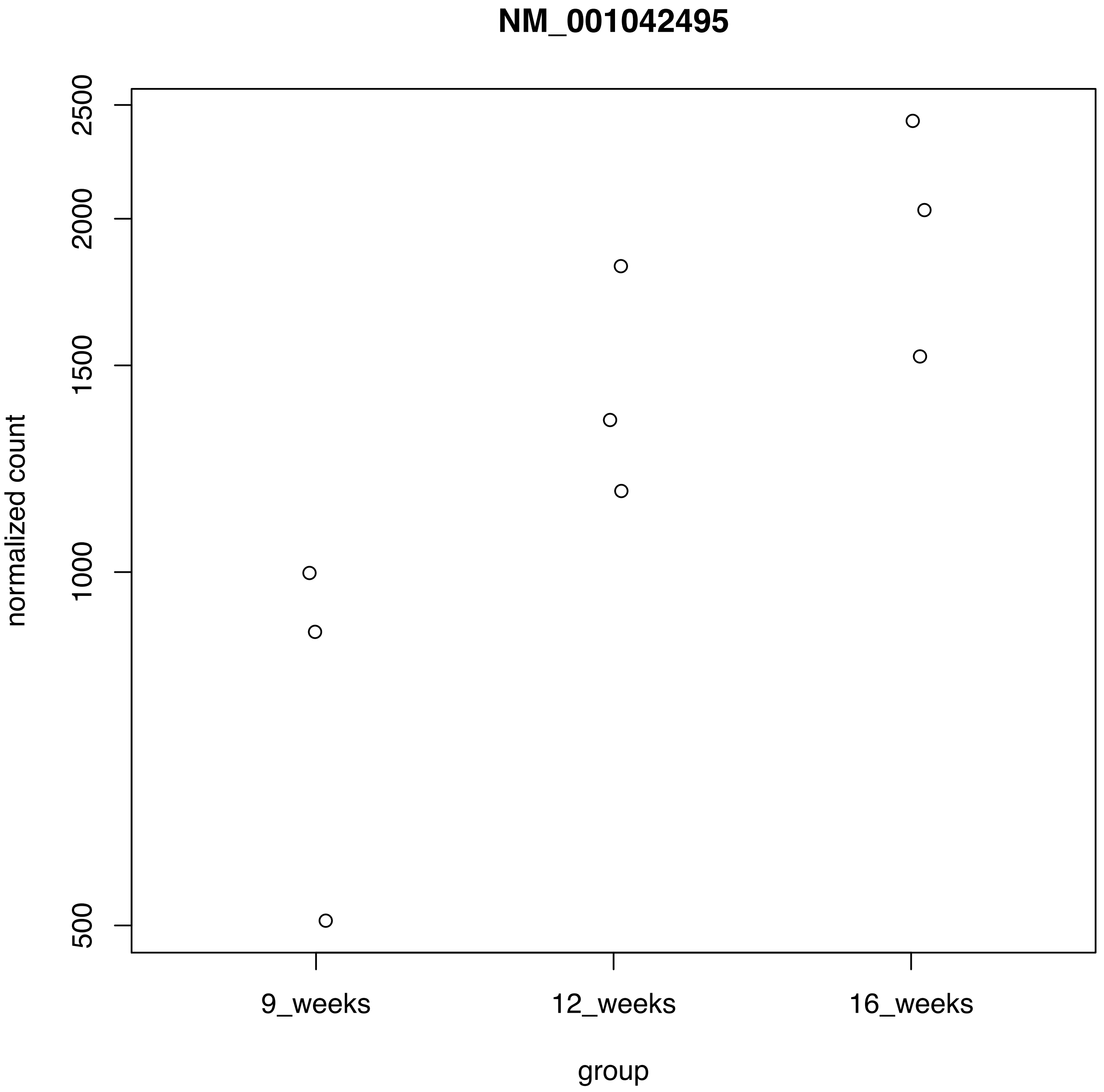
NM_001042495
normalized count
500
1000
1500
2000
2500
9_weeks
12_weeks
16_weeks
group

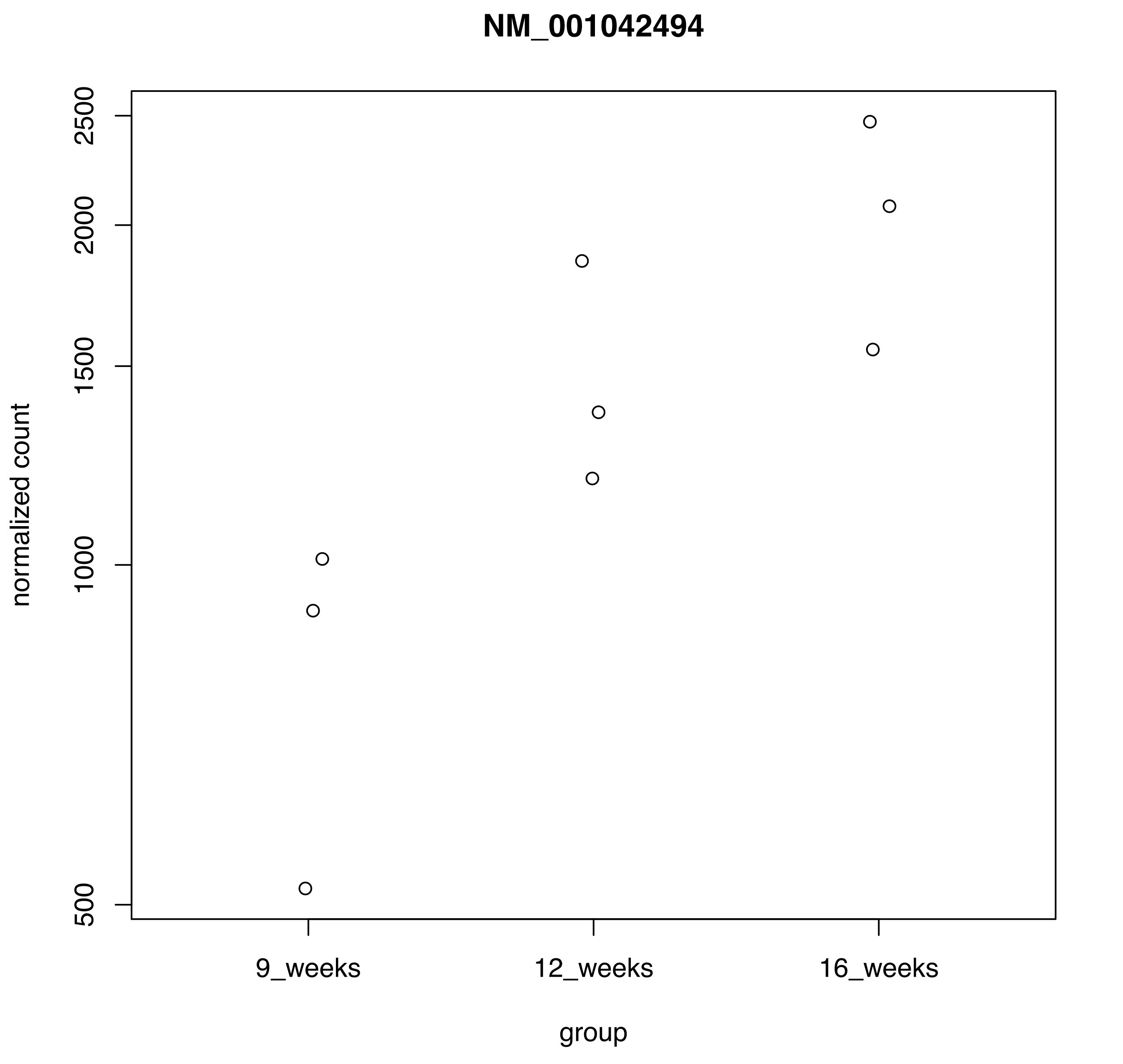
NM_001042494
normalized count
2500
2000
1500
1000
500
9_weeks
12_weeks
16_weeks
group

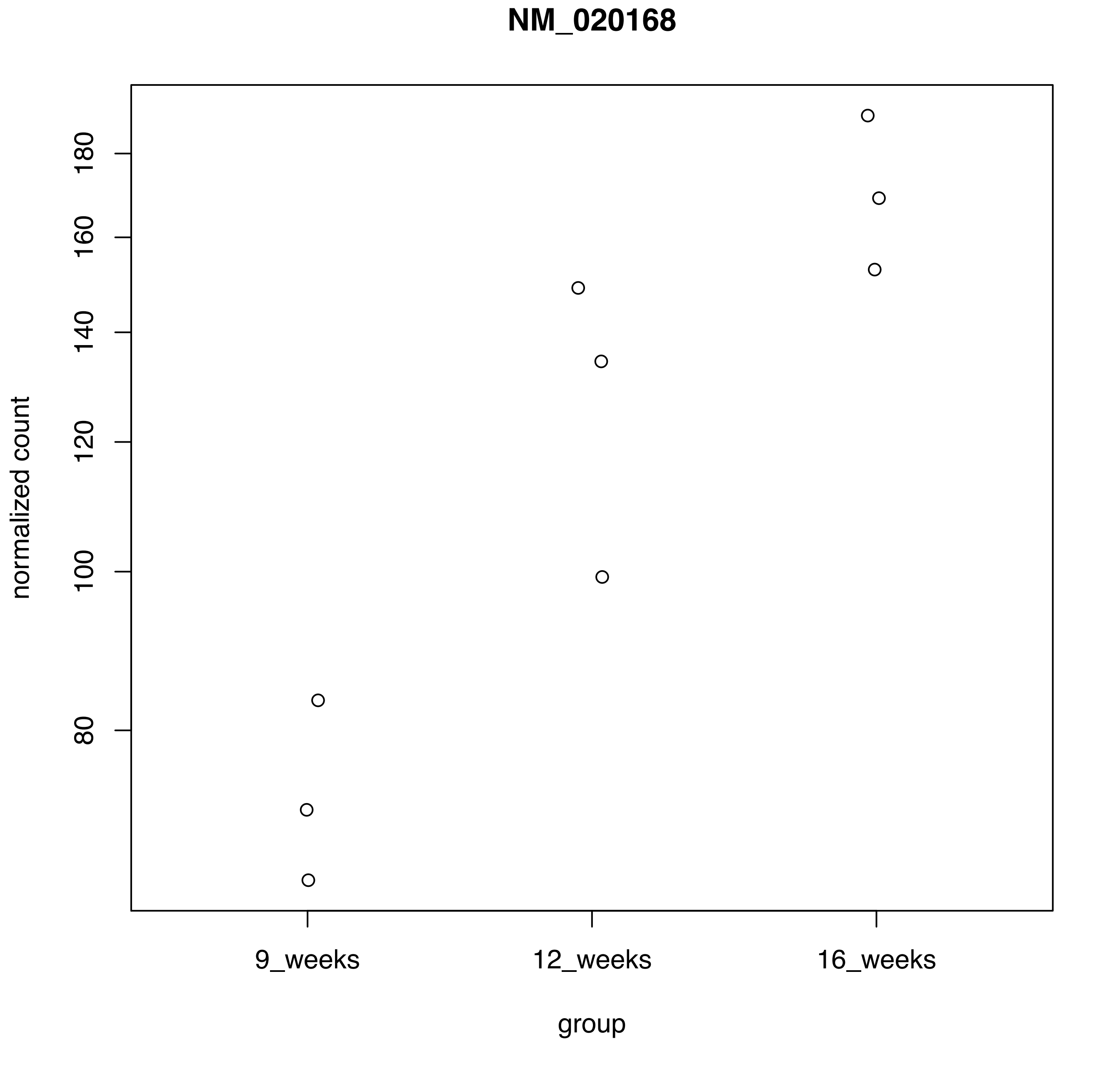
NM_020168
normalized count
80
100
120
140
160
180
9_weeks
12_weeks
16_weeks
group

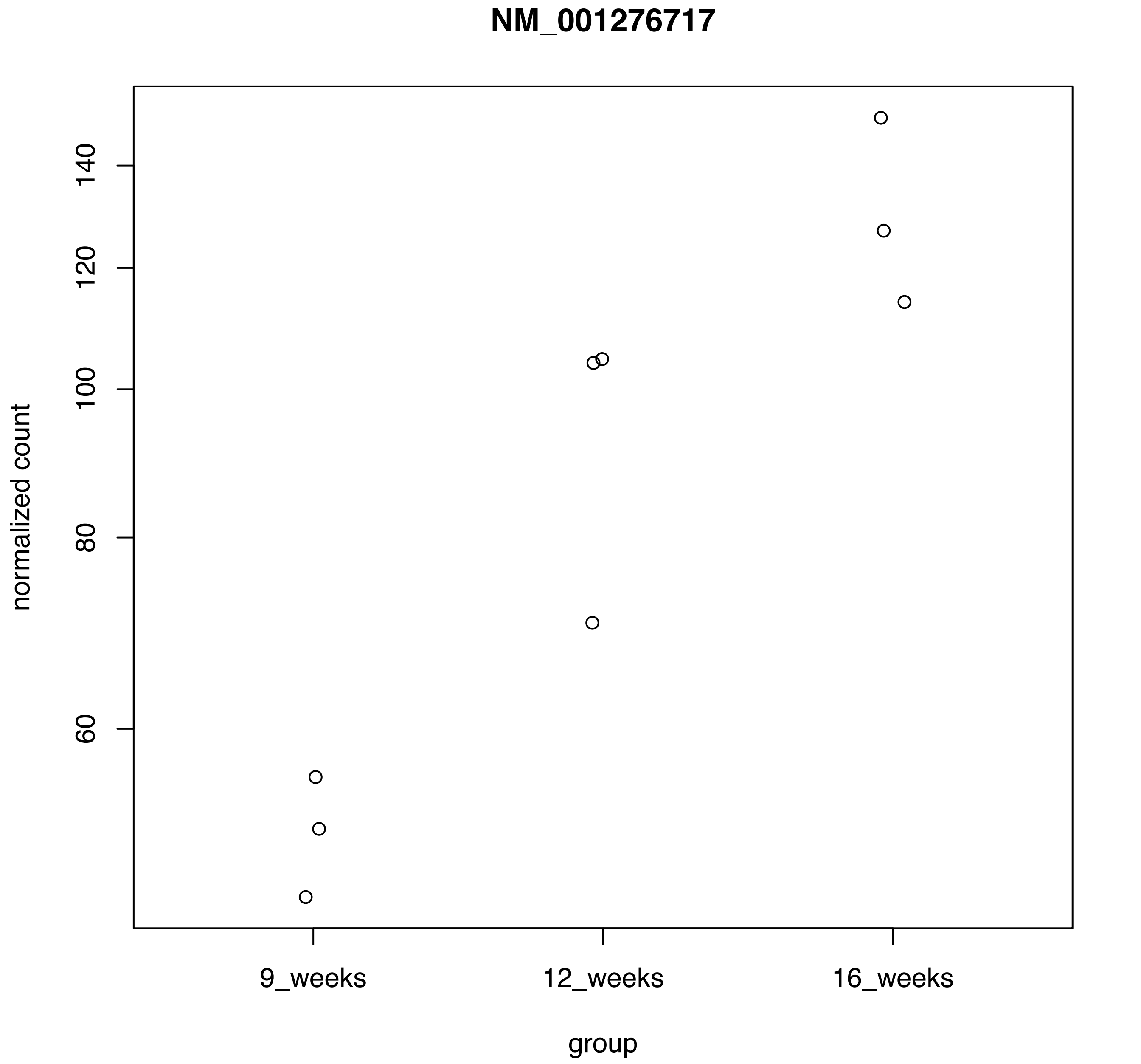
NM_001276717
normalized count
60
80
100
120
140
9_weeks
12_weeks
16_weeks
group

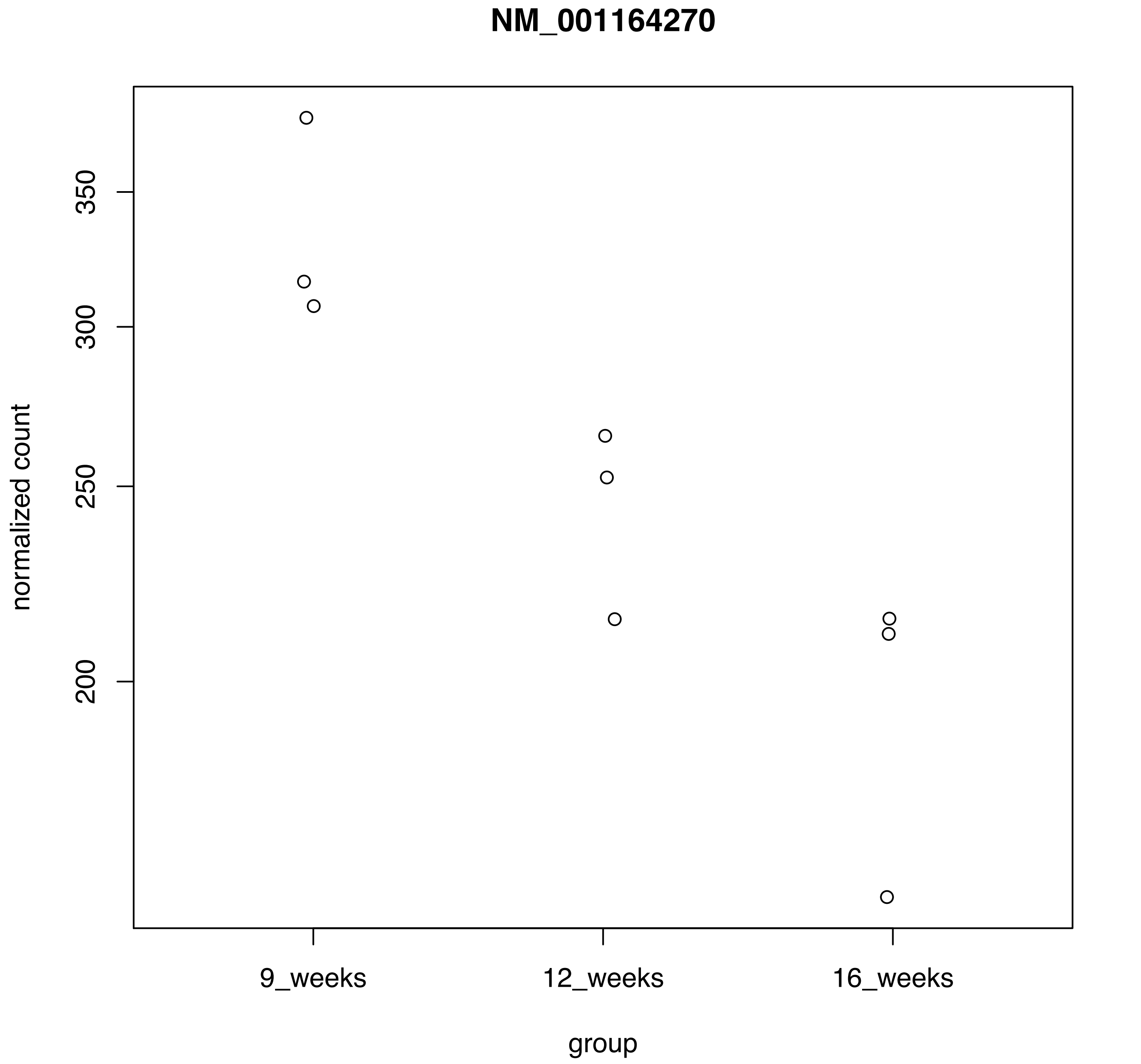

NM_001164270
normalized count
200
250
300
350
9_weeks
12_weeks
16_weeks
group

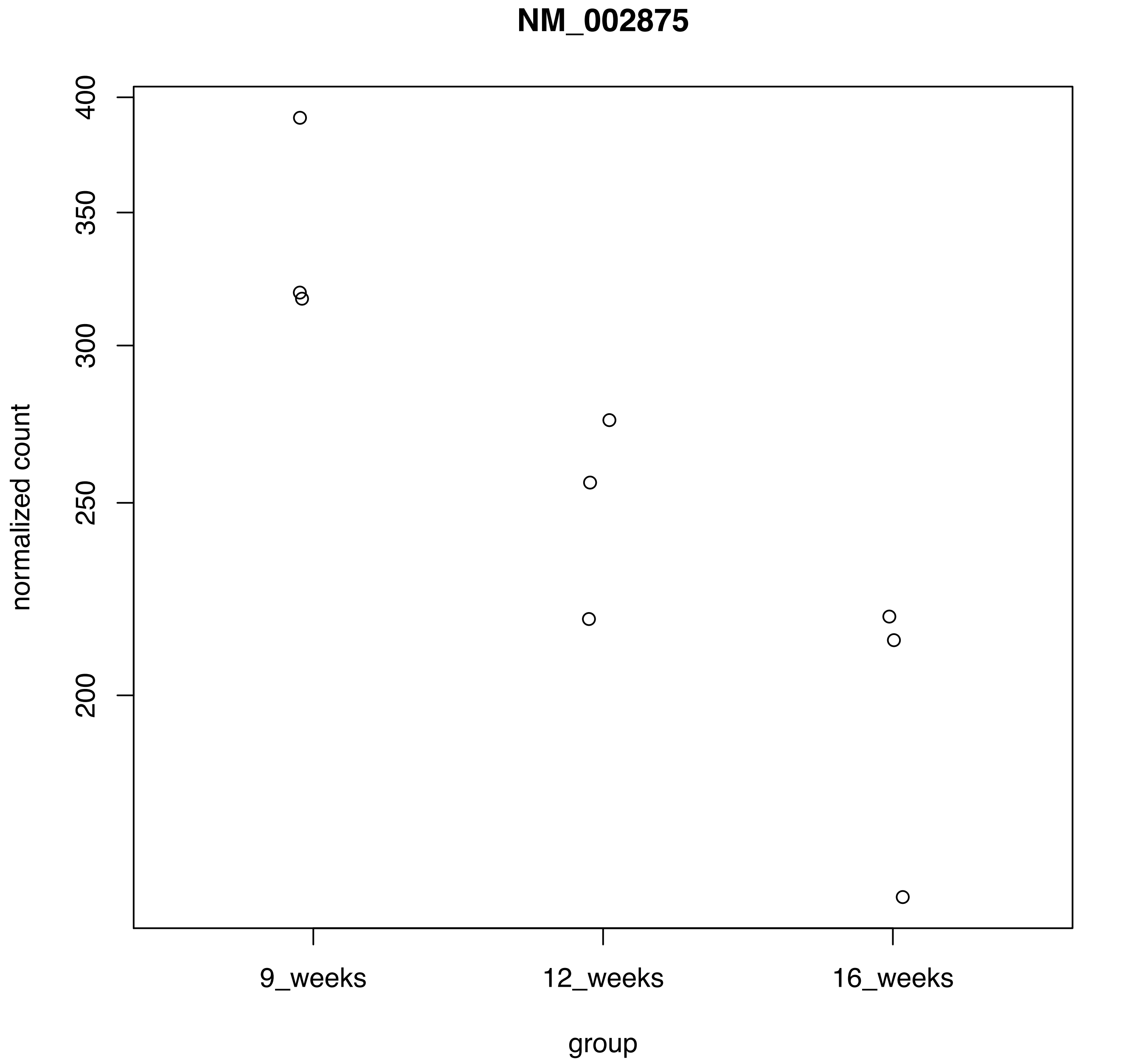

NM_002875
normalized count
400
350
300
250
200
9_weeks
12_weeks
16_weeks
group

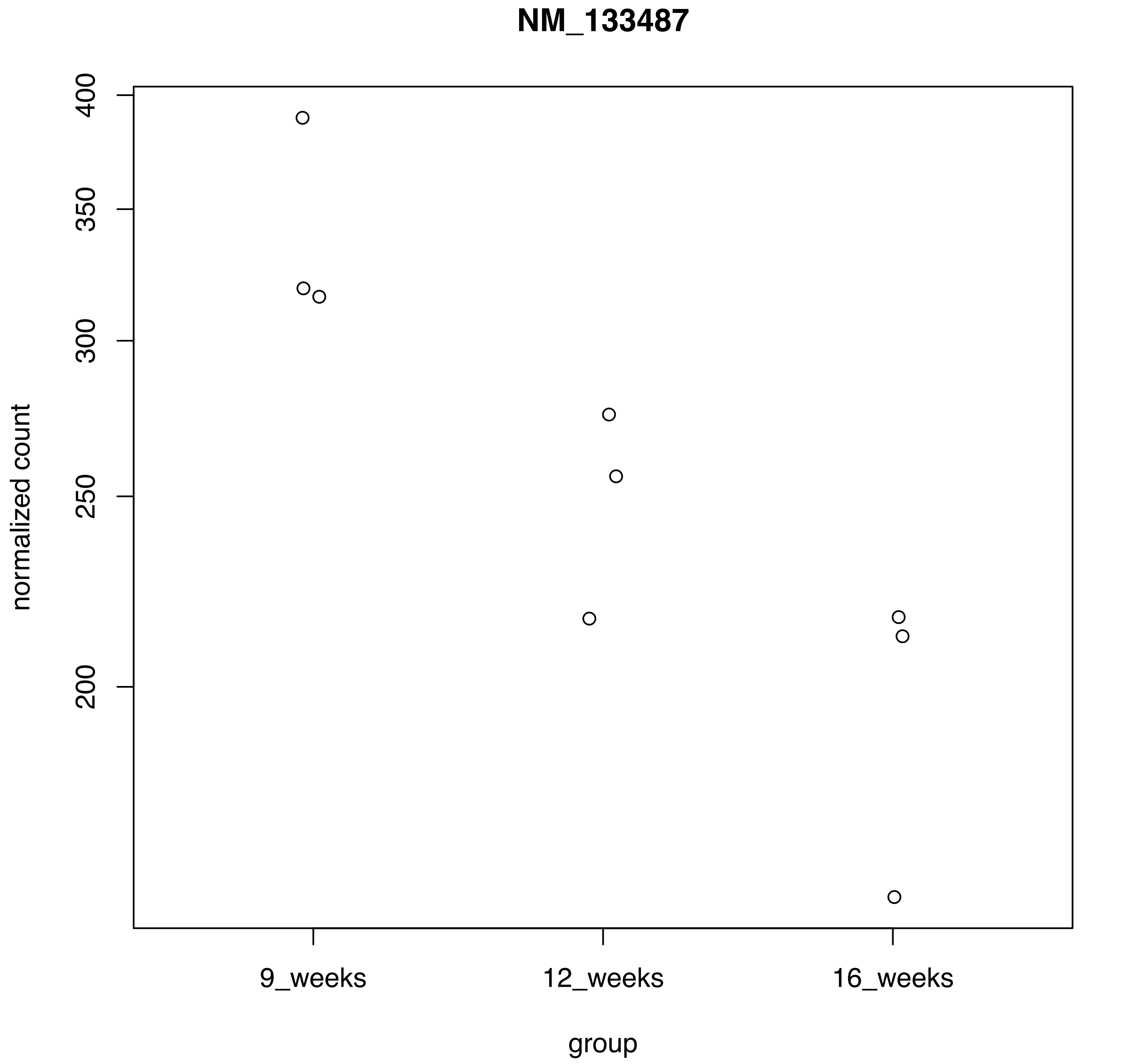
NM_133487
normalized count
400
350
300
250
200
9_weeks
12_weeks
16_weeks
group

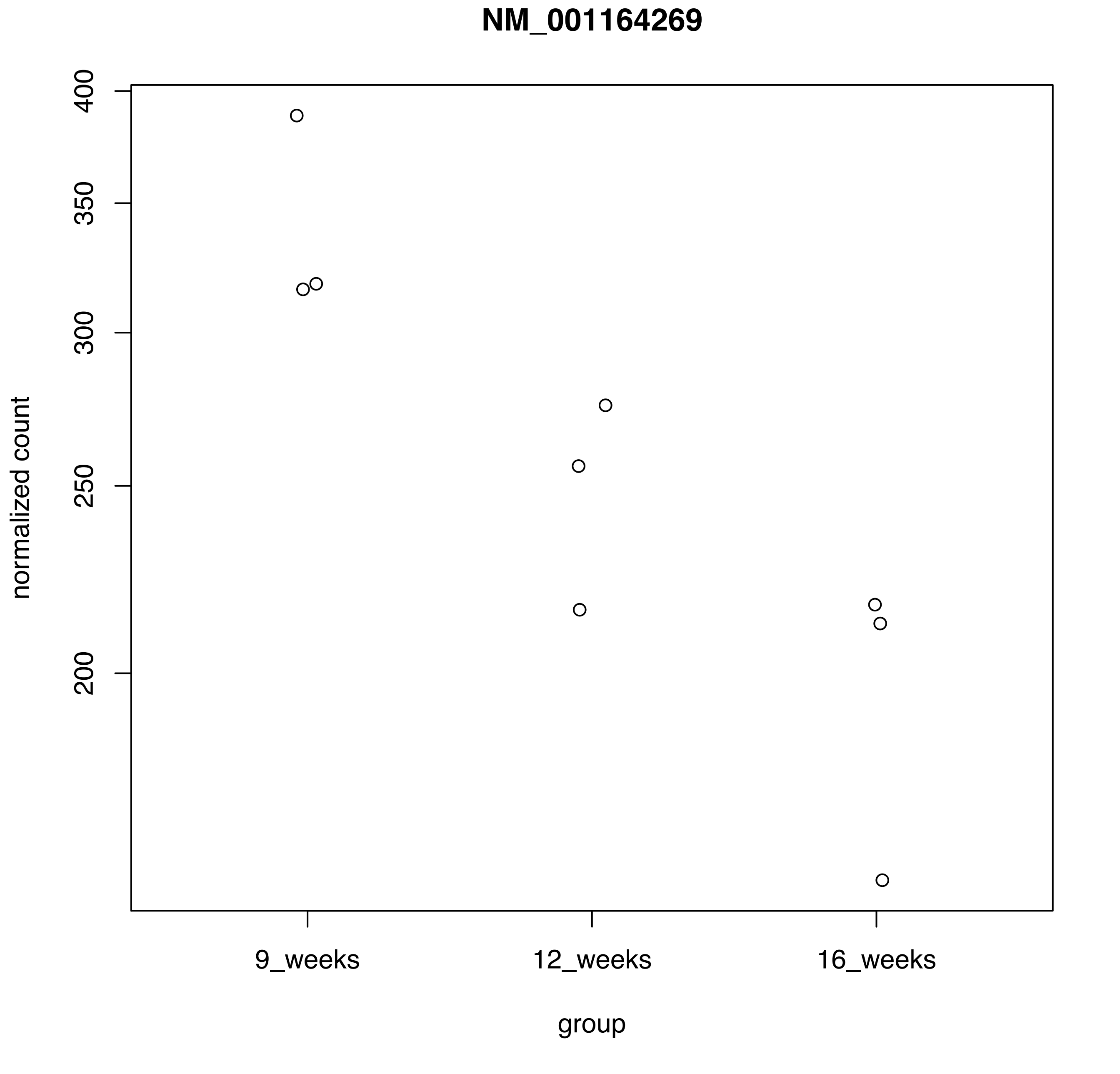
NM_001164269
normalized count
400
350
300
250
200
9_weeks
12_weeks
16_weeks
group

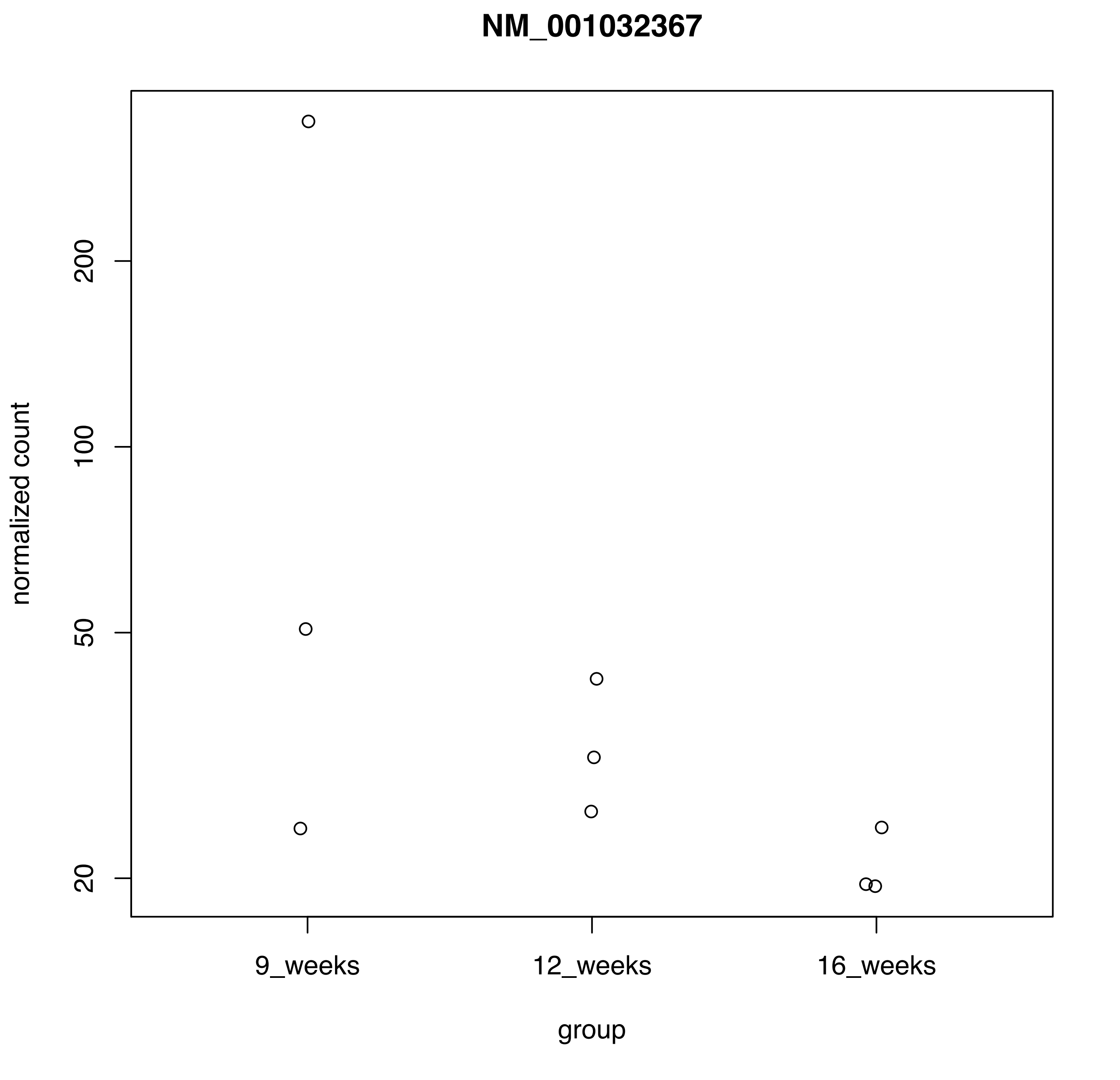
NM_001032367
normalized count
200
100
50
20
9_weeks
12_weeks
16_weeks
group

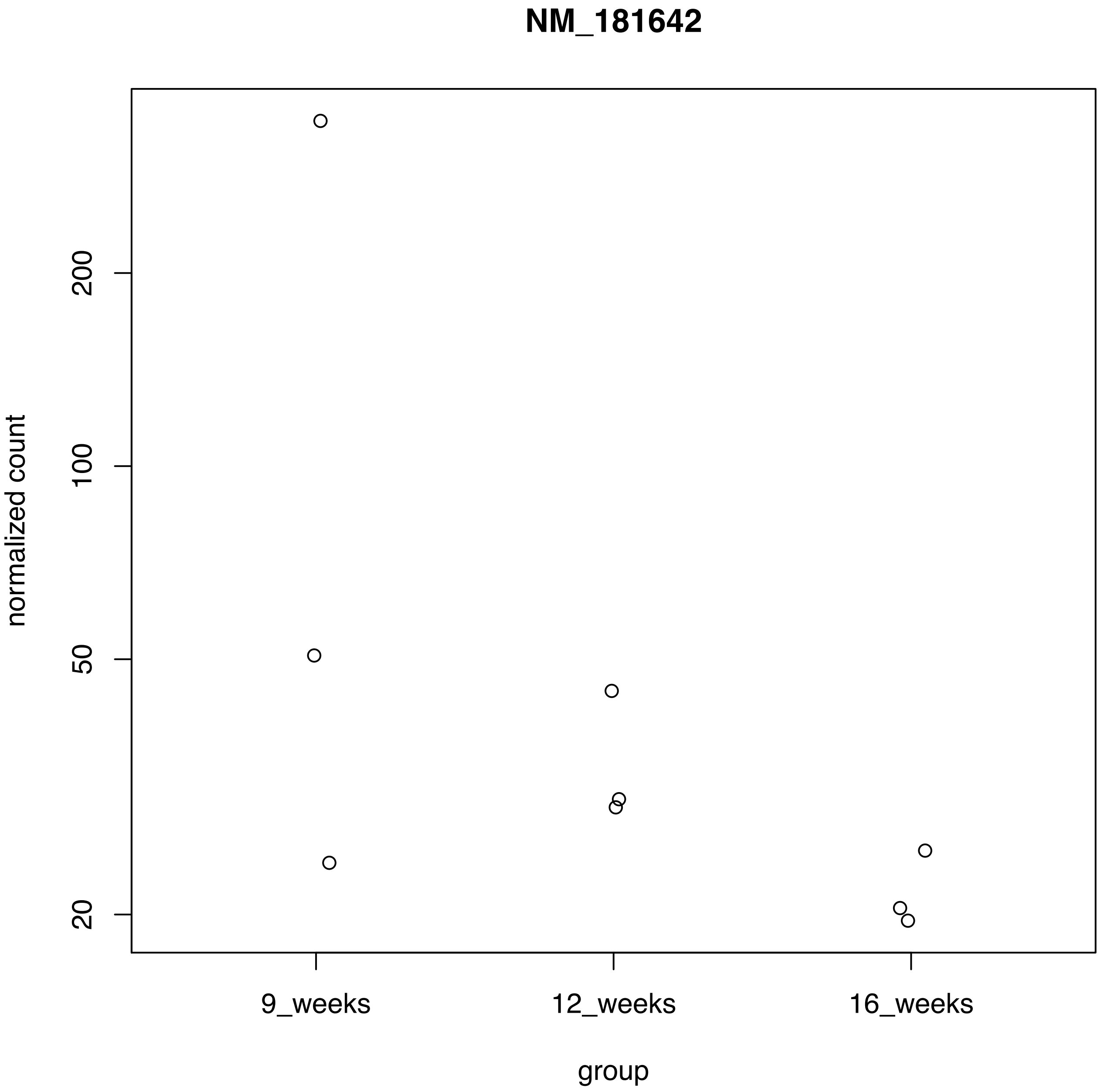
NM_181642
normalized count
200
100
50
20
9_weeks
12_weeks
16_weeks
group

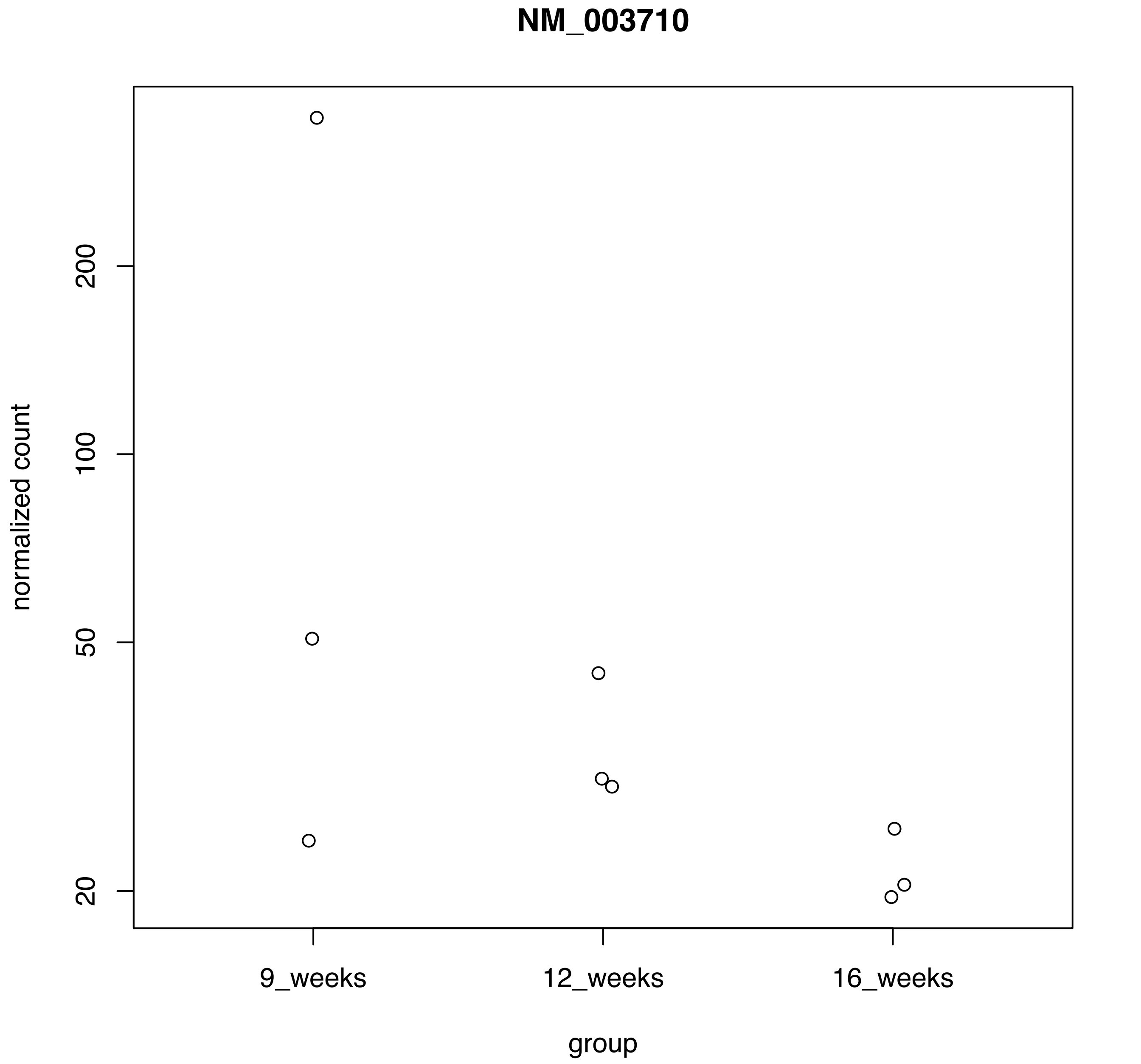
NM_003710
normalized count
200
100
50
20
9_weeks
12_weeks
16_weeks
group

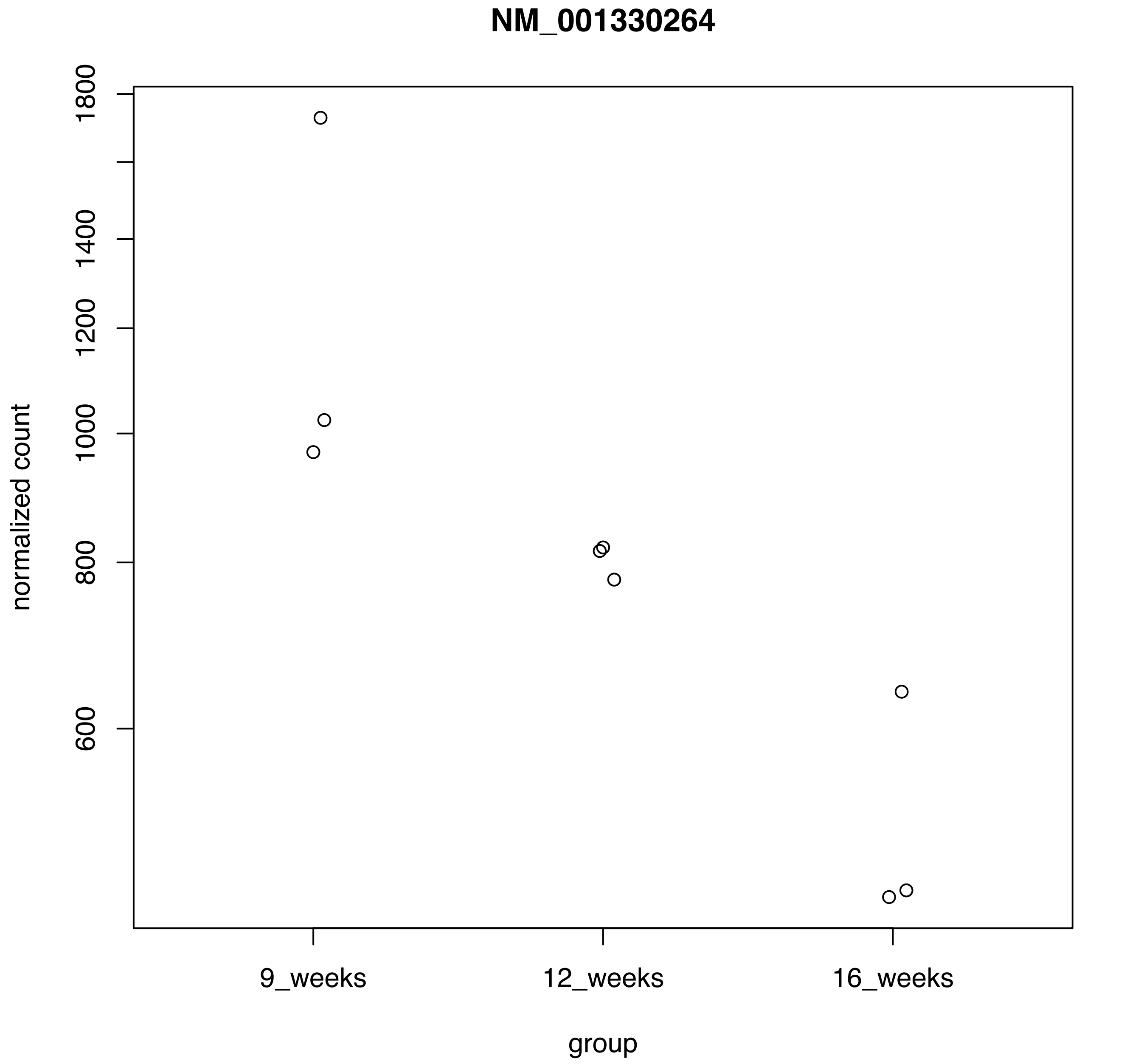
NM_001330264
normalized count
1800
1400
1200
1000
800
600
9_weeks
12_weeks
16_weeks
group

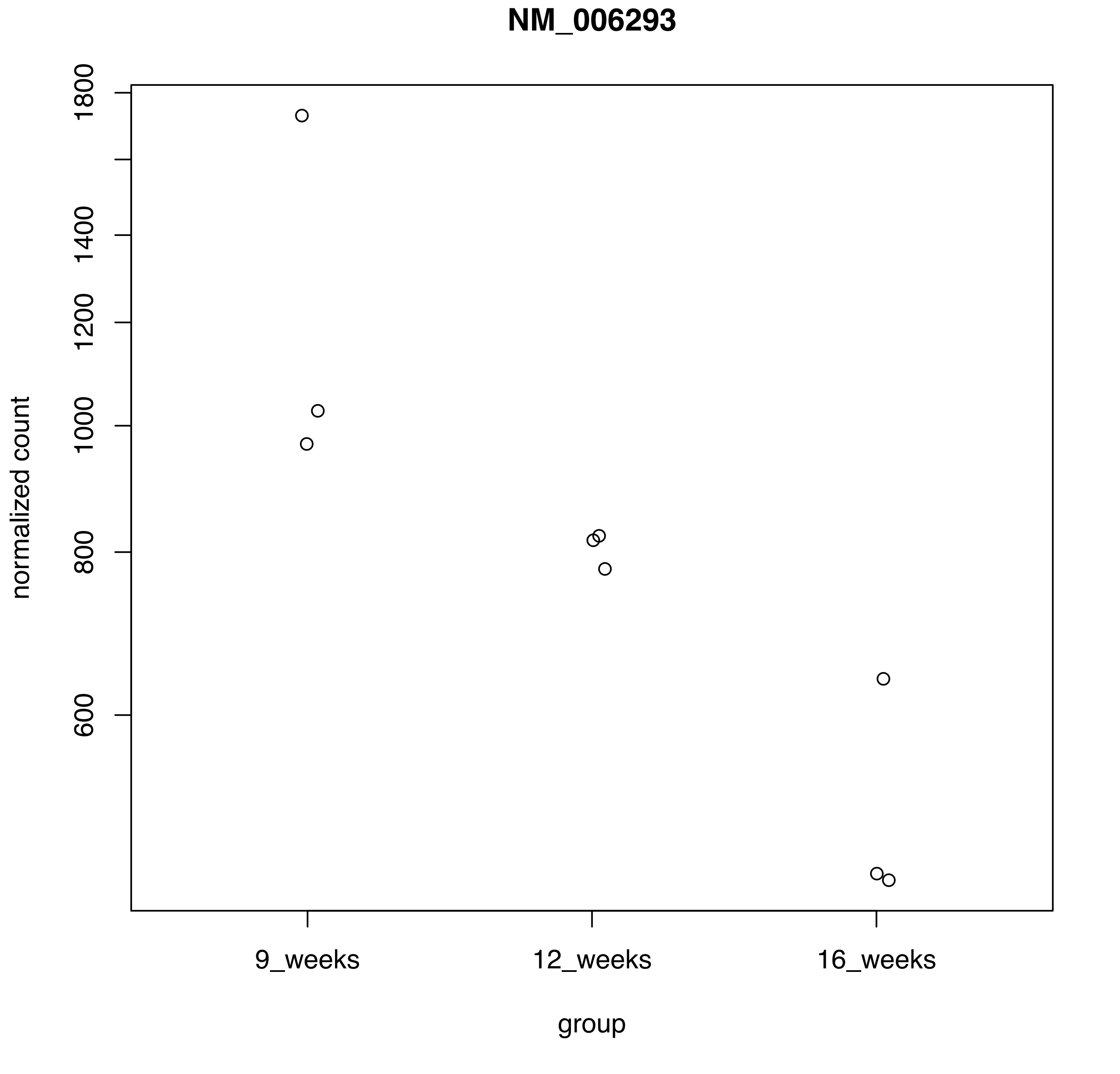
NM_006293
normalized count
1800
1400
1200
1000
800
600
9_weeks
12_weeks
16_weeks
group

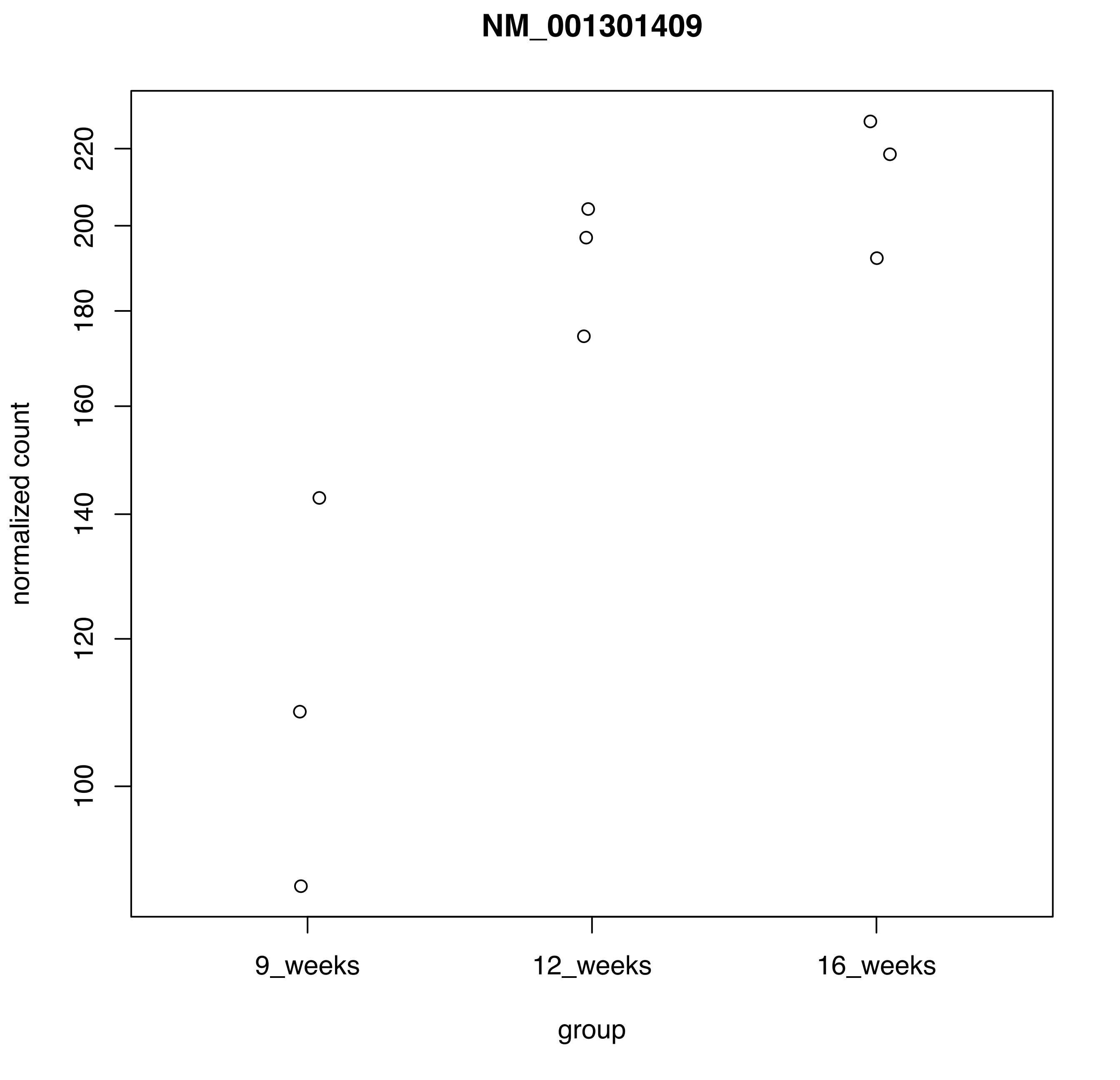
NM_001301409
normalized count
100
120
140
160
180
200
220
9_weeks
12_weeks
16_weeks
group

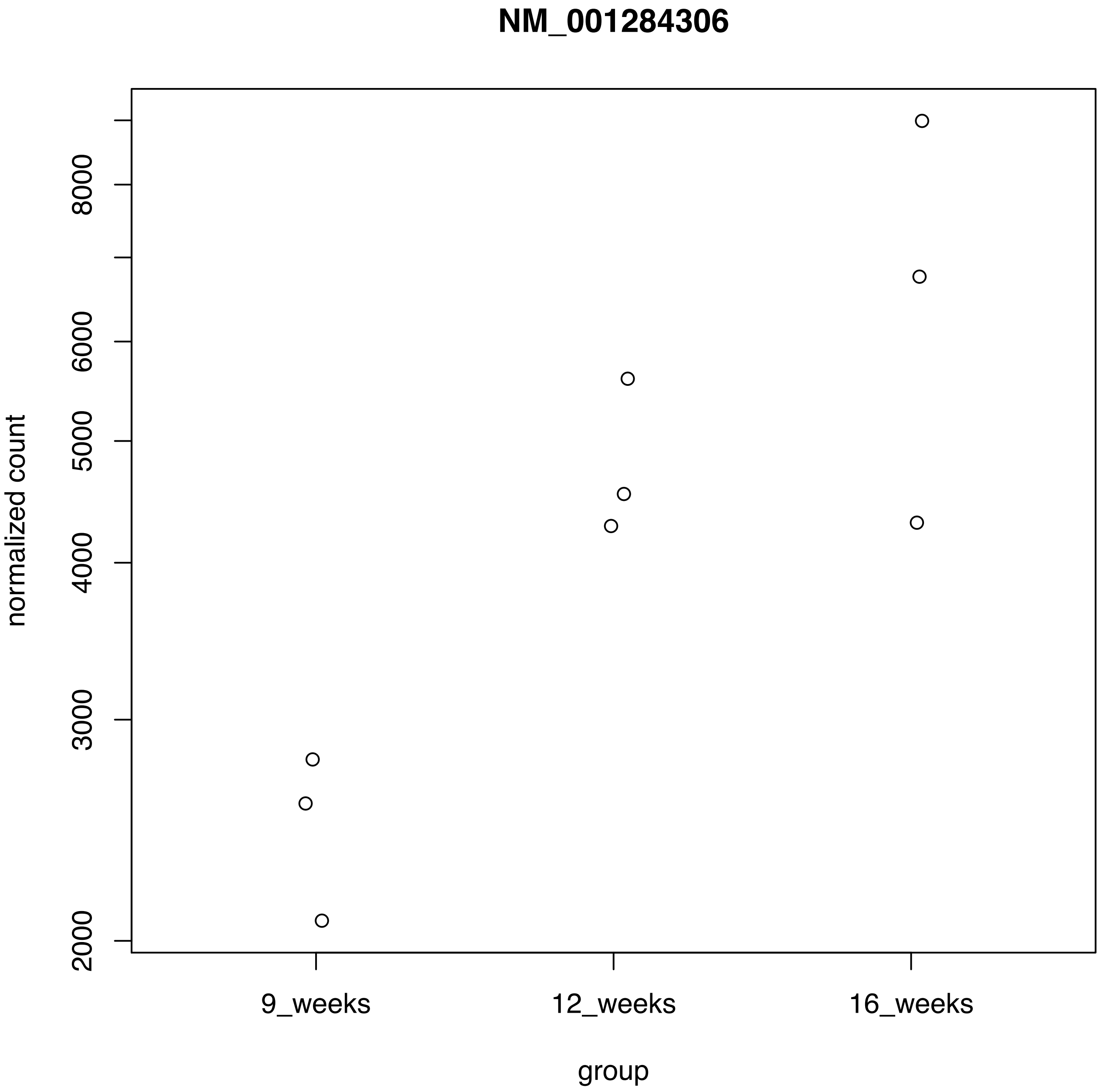
NM_001284306
normalized count
2000
3000
4000
5000
6000
8000
9_weeks
12_weeks
16_weeks
group

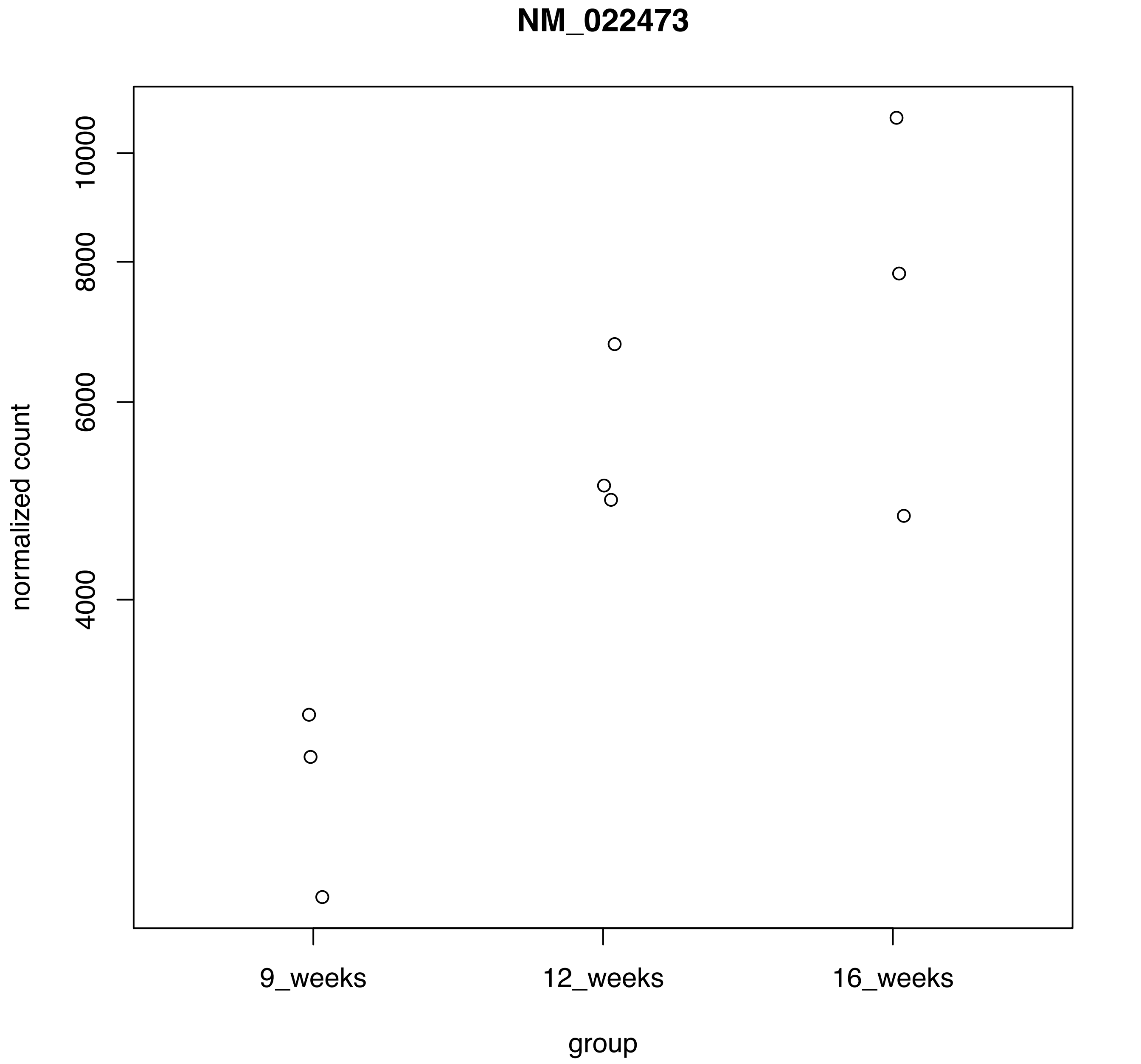
NM_022473
normalized count
10000
8000
6000
4000
9_weeks
12_weeks
16_weeks
group

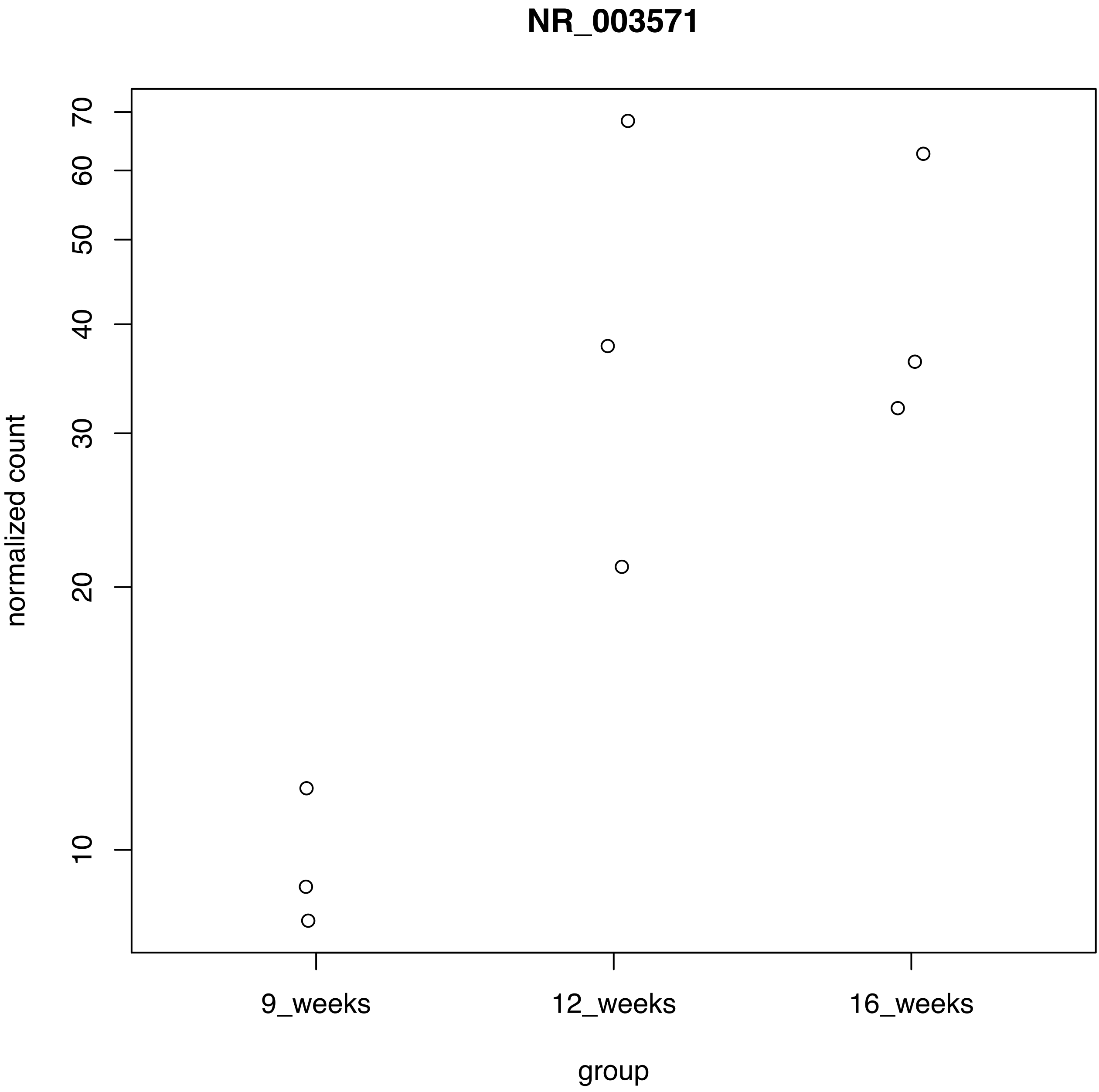

NR_003571
normalized count
10
20
30
40
50
60
70
9_weeks
12_weeks
16_weeks
group

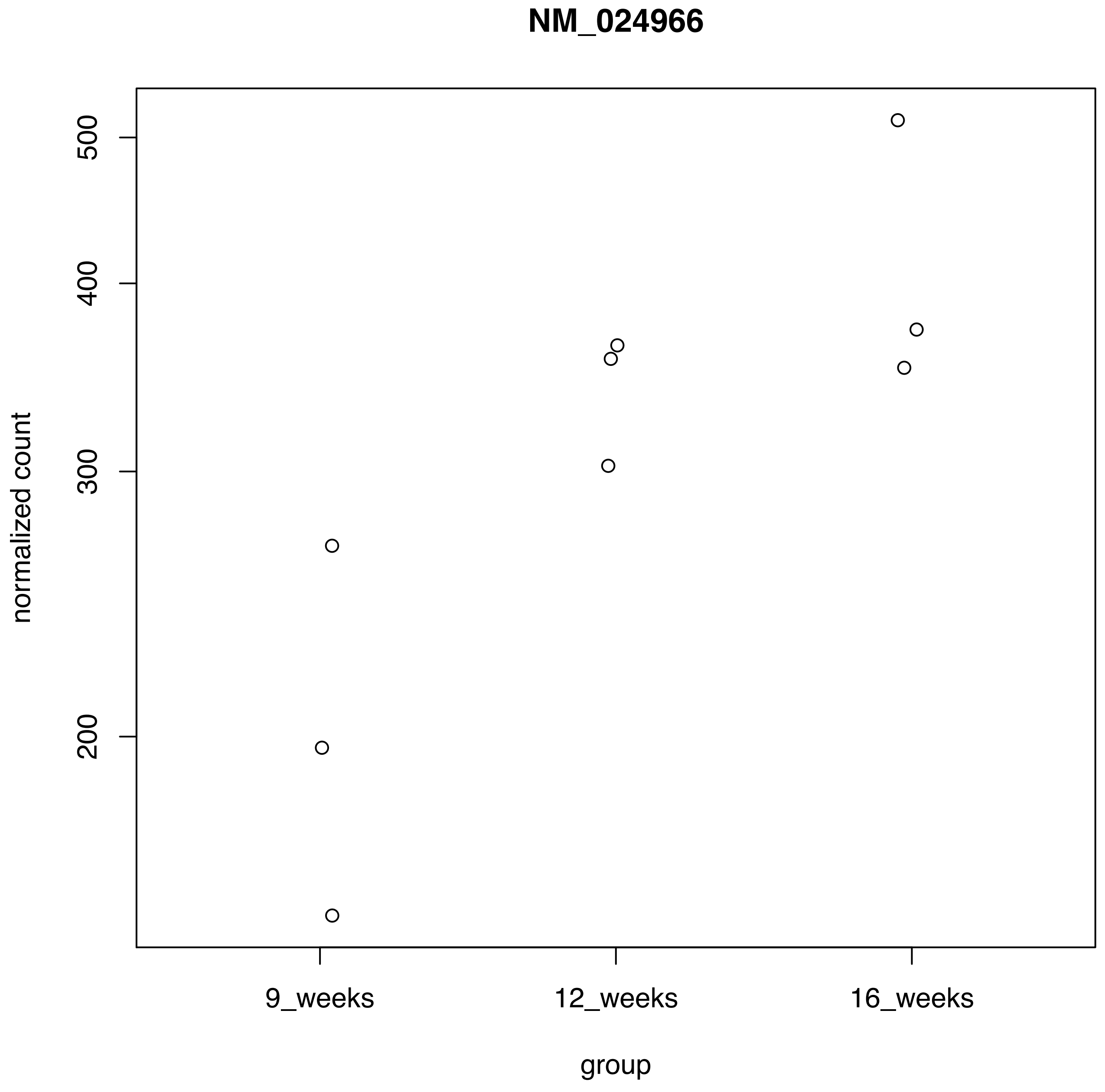

NM_024966
500
400
300
200
normalized count
9_weeks
12_weeks
16_weeks
group

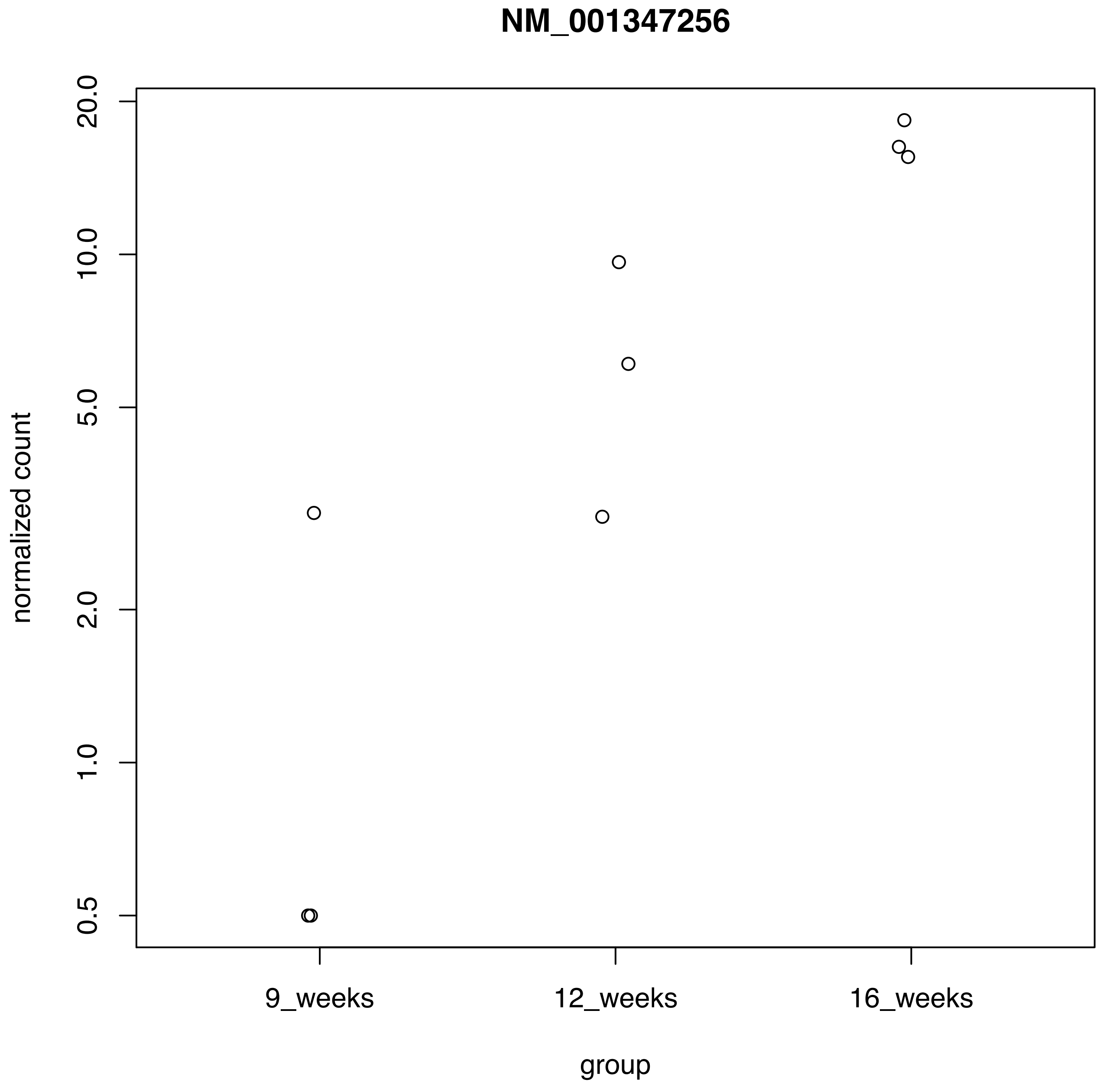
NM_001347256
normalized count
20.0
10.0
5.0
2.0
1.0
0.5
9_weeks
12_weeks
16_weeks
group

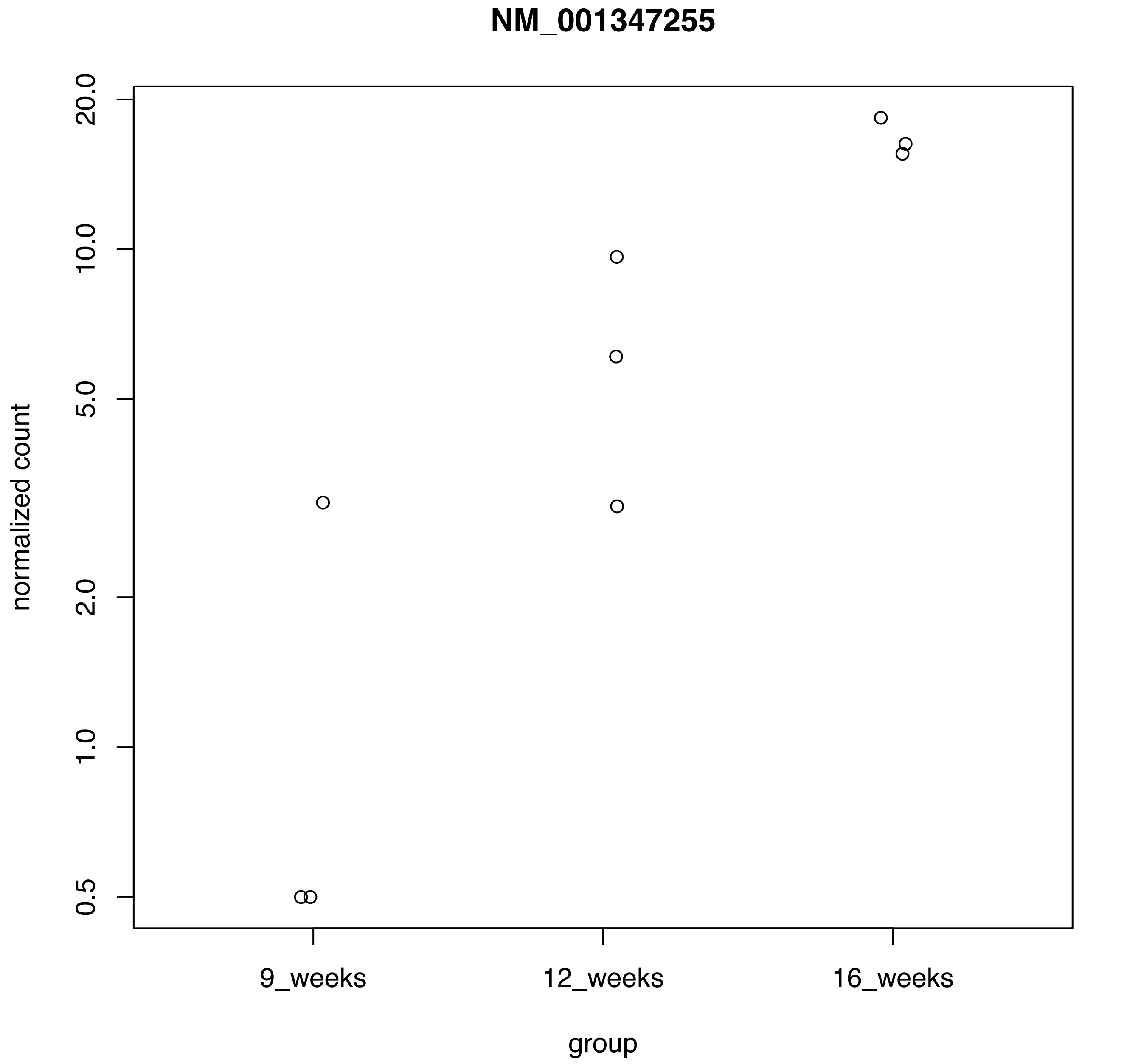
NM_001347255
normalized count
20.0
10.0
5.0
2.0
1.0
0.5
9_weeks
12_weeks
16_weeks
group

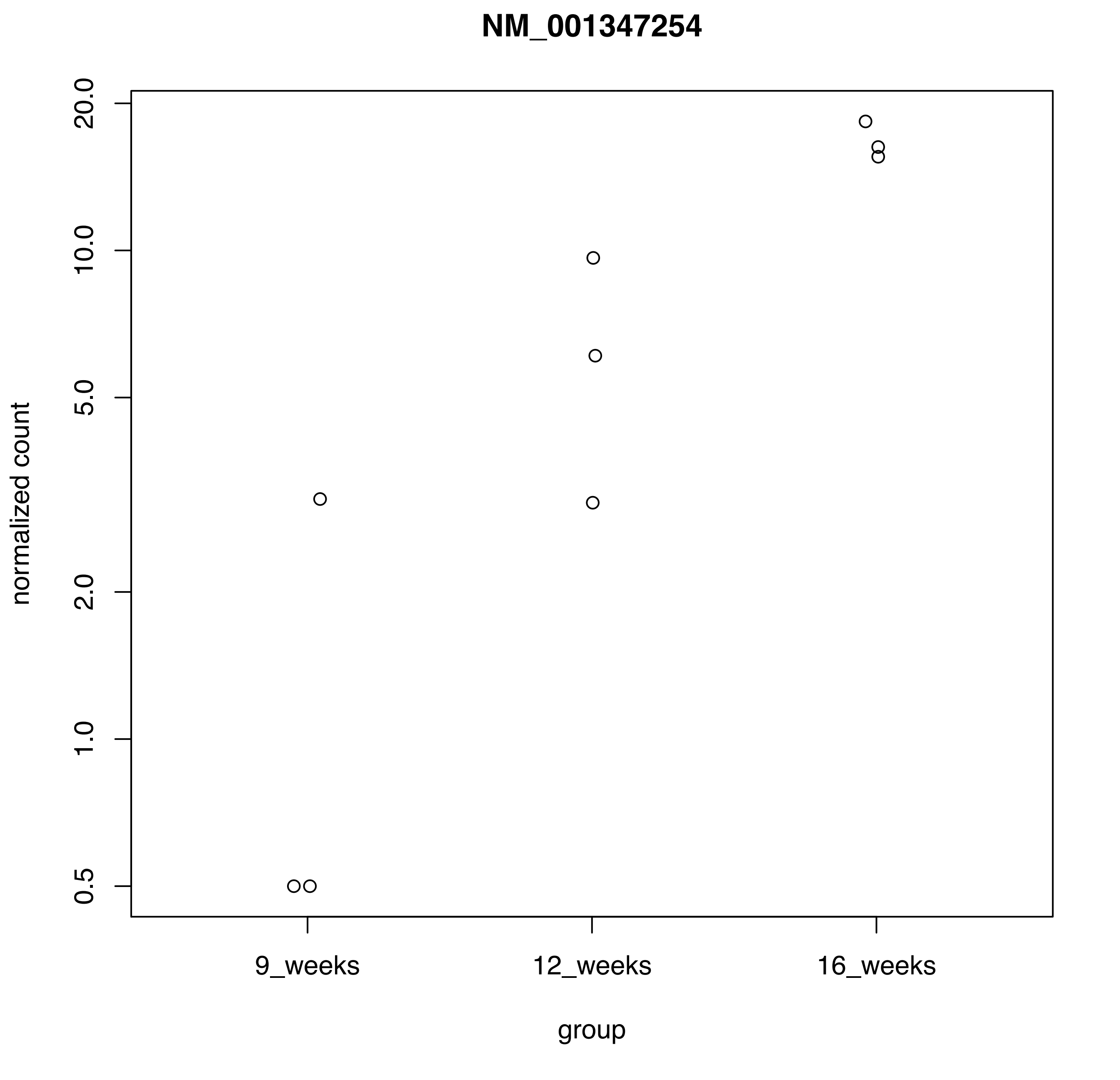
NM_001347254
normalized count
20.0
10.0
5.0
2.0
1.0
0.5
9_weeks
12_weeks
16_weeks
group

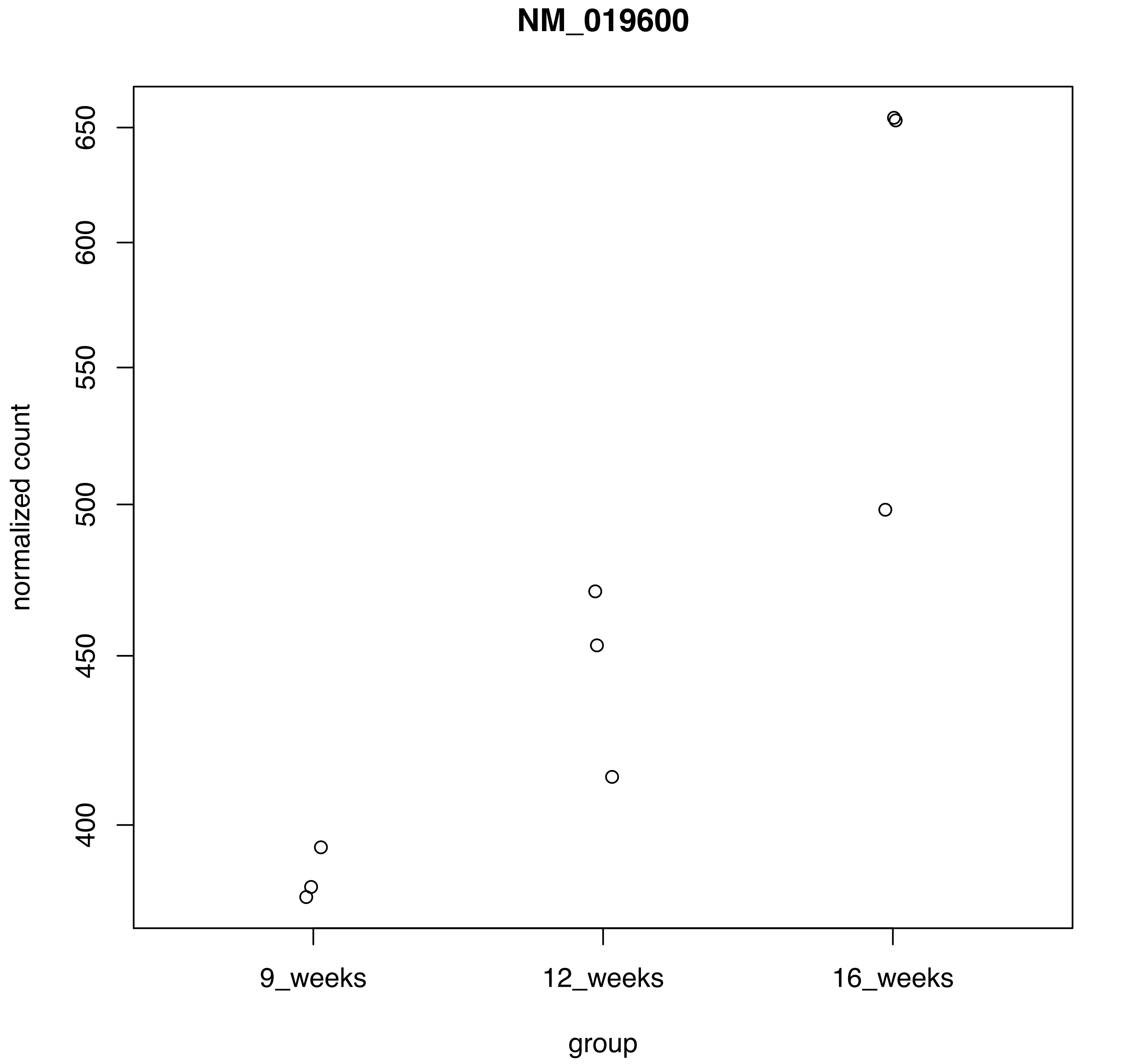

NM_019600
normalized count
650
600
550
500
450
400
9_weeks
12_weeks
16_weeks
group

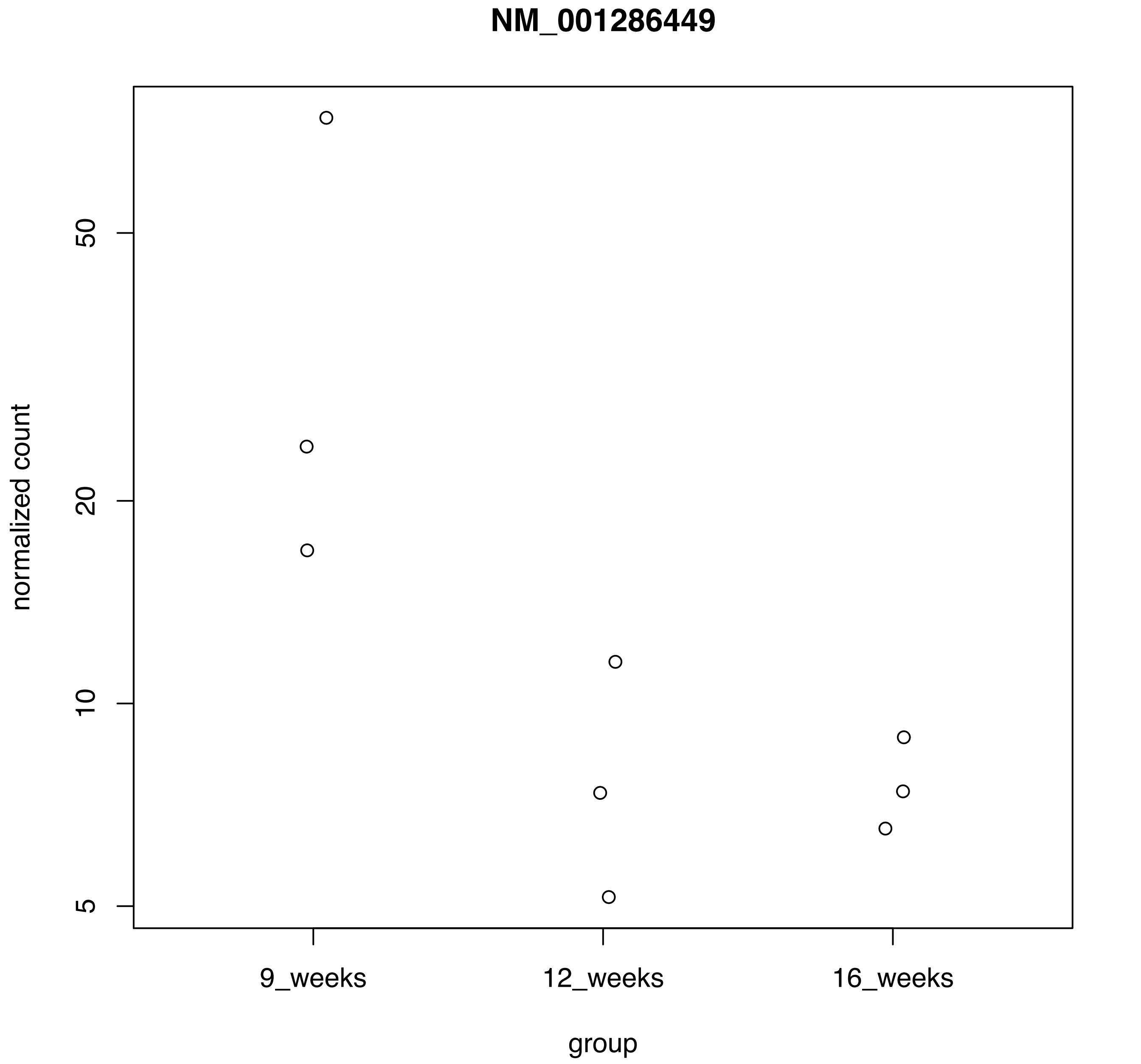
NM_001286449
normalized count
50
20
10
5
9_weeks
12_weeks
16_weeks
group

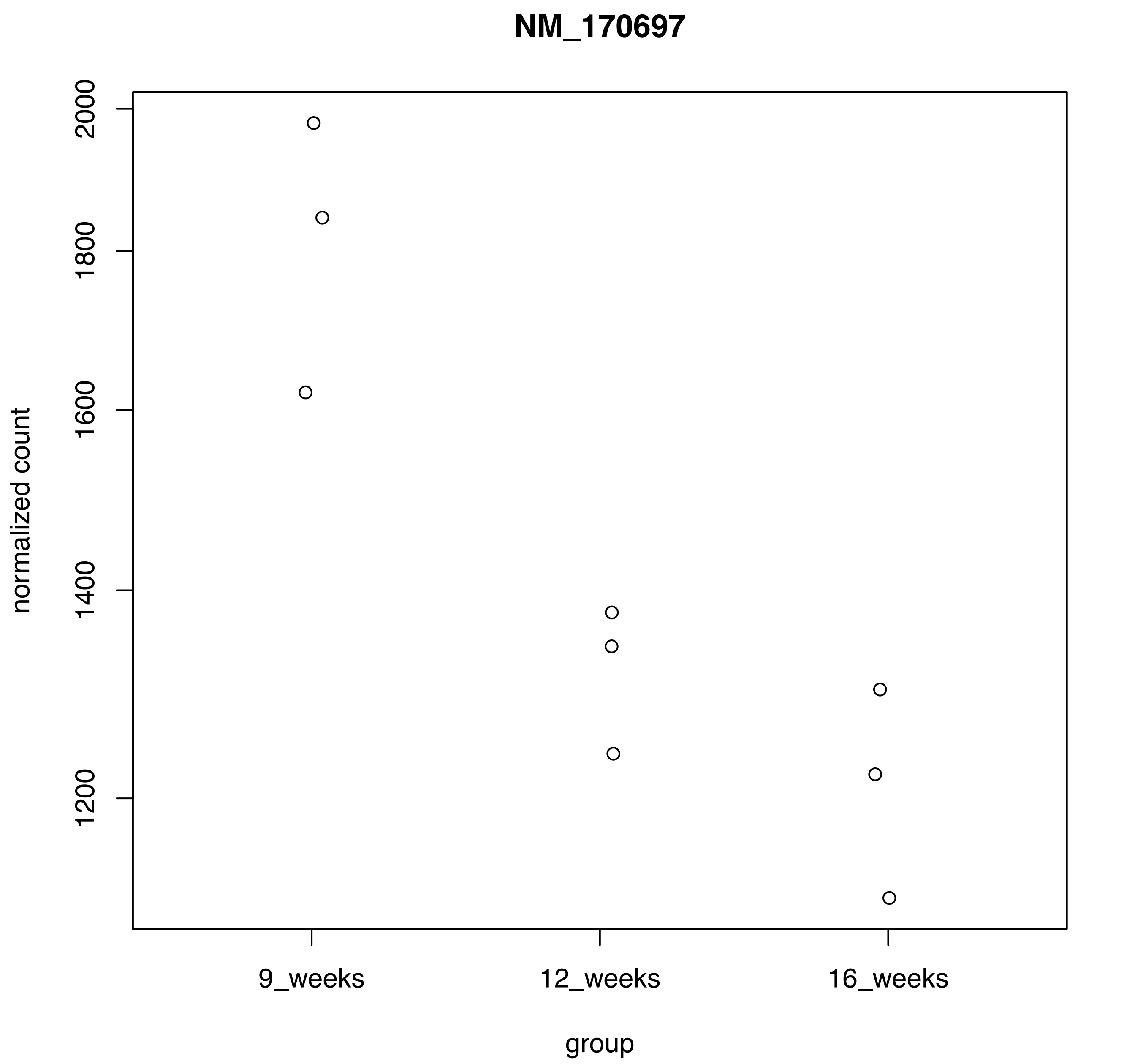
NM_170697
normalized count
2000
1800
1600
1400
1200
9_weeks
12_weeks
16_weeks
group

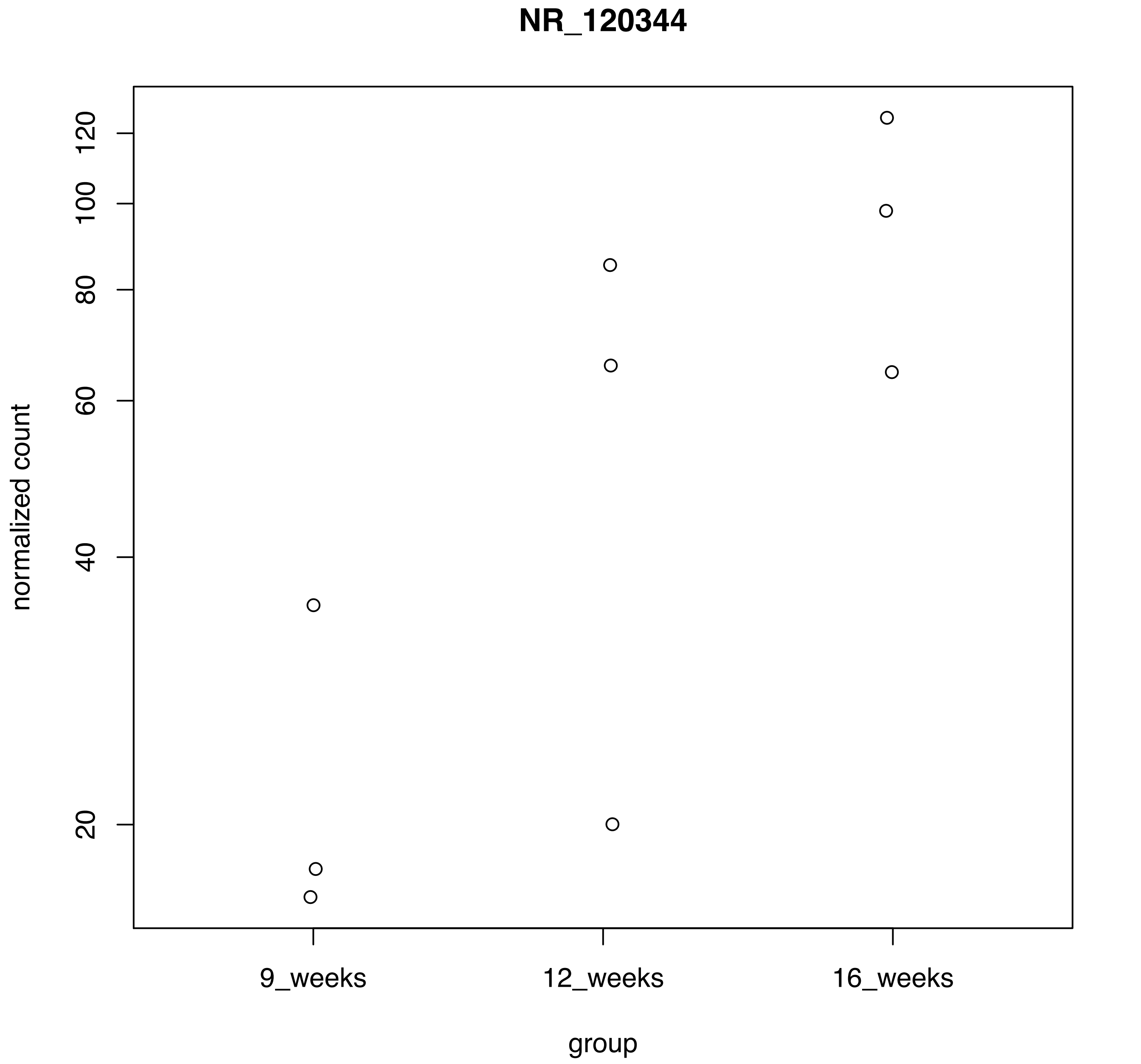
NR_120344
normalized count
20
40
60
80
100
120
9_weeks
12_weeks
16_weeks
group

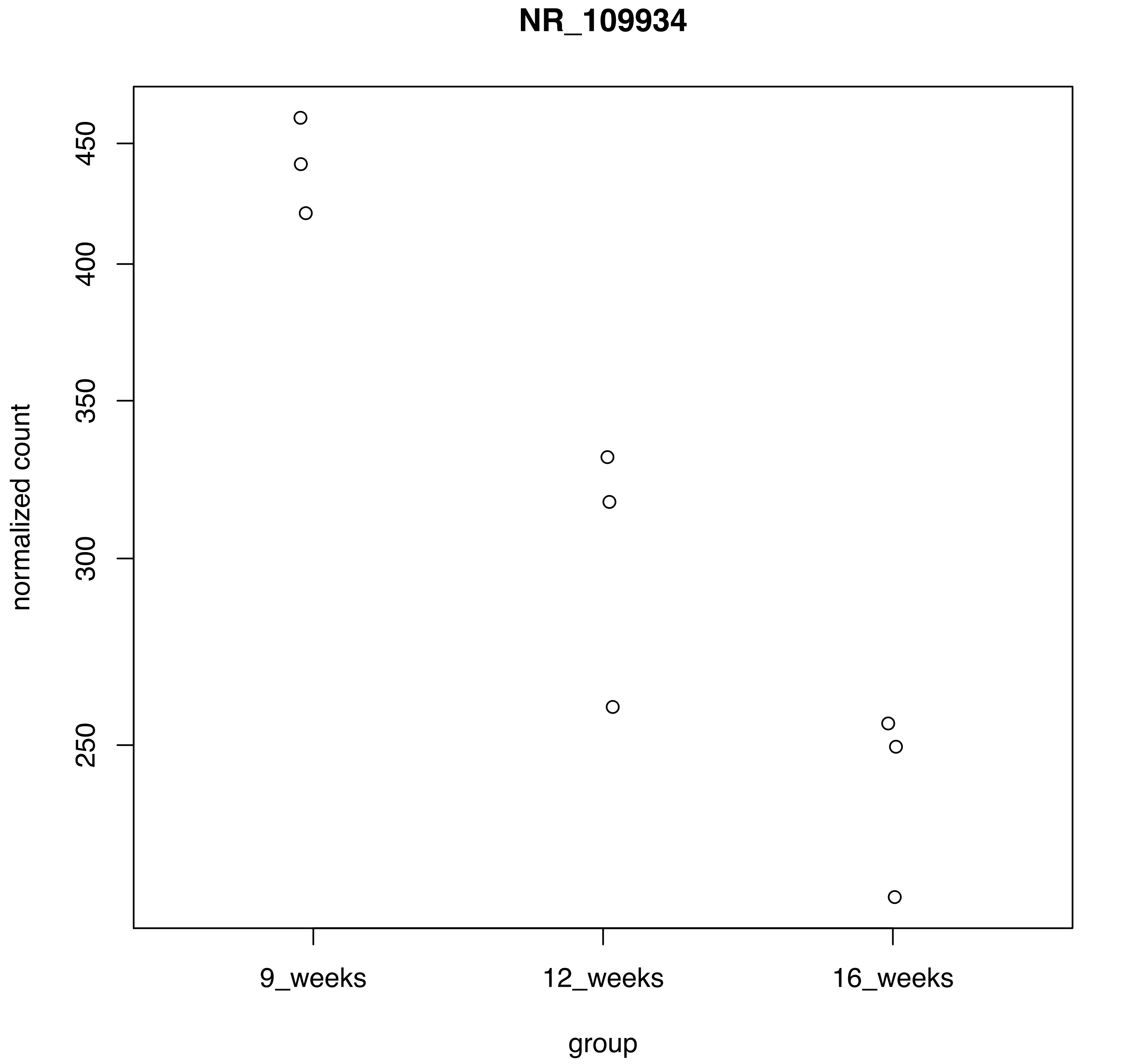
NR_109934
normalized count
450
400
350
300
250
9_weeks
12_weeks
16_weeks
group

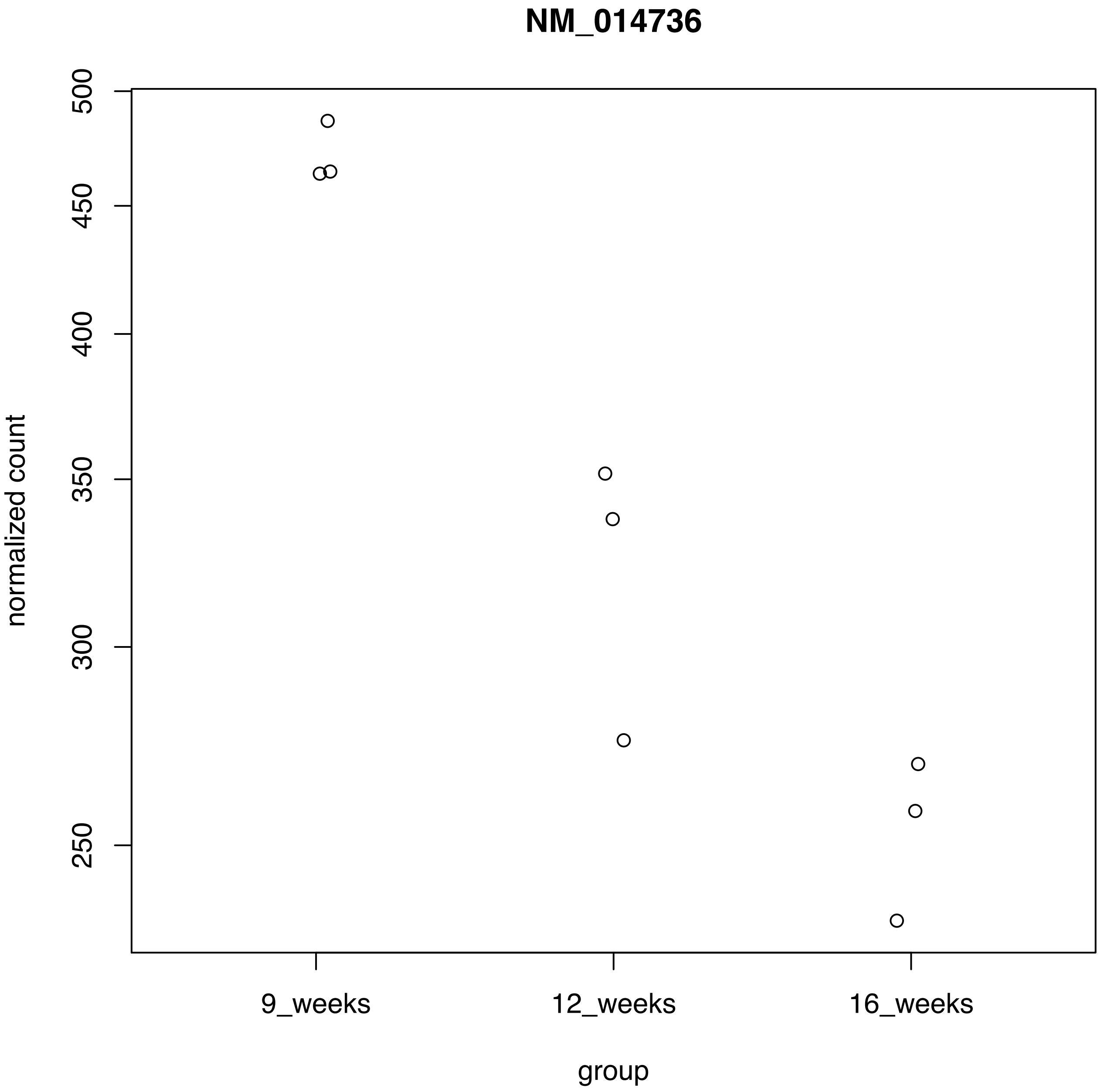
NM_014736
normalized count
500
450
400
350
300
250
9_weeks
12_weeks
16_weeks
group

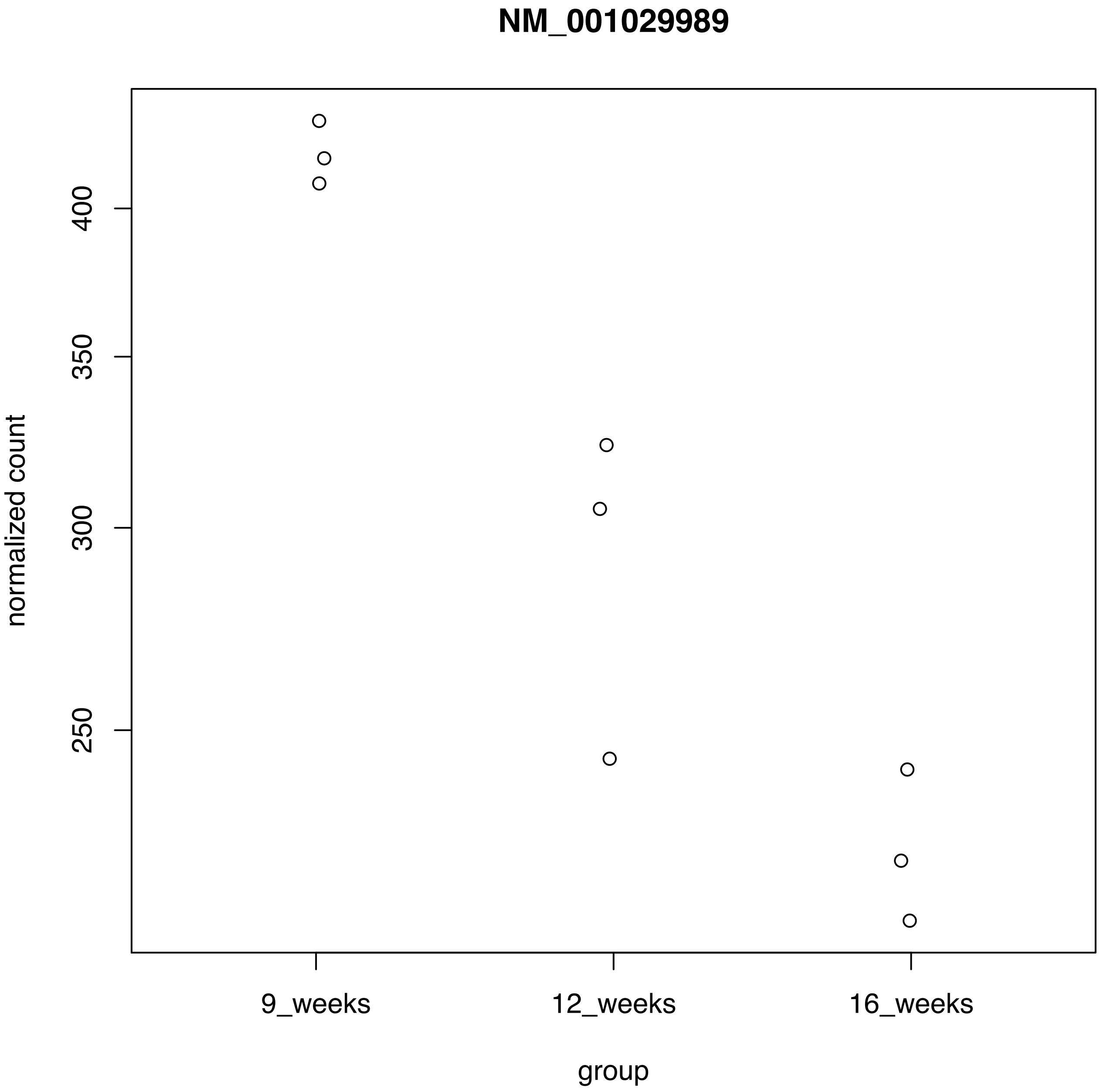
NM_001029989
normalized count
400
350
300
250
9_weeks
12_weeks
16_weeks
group

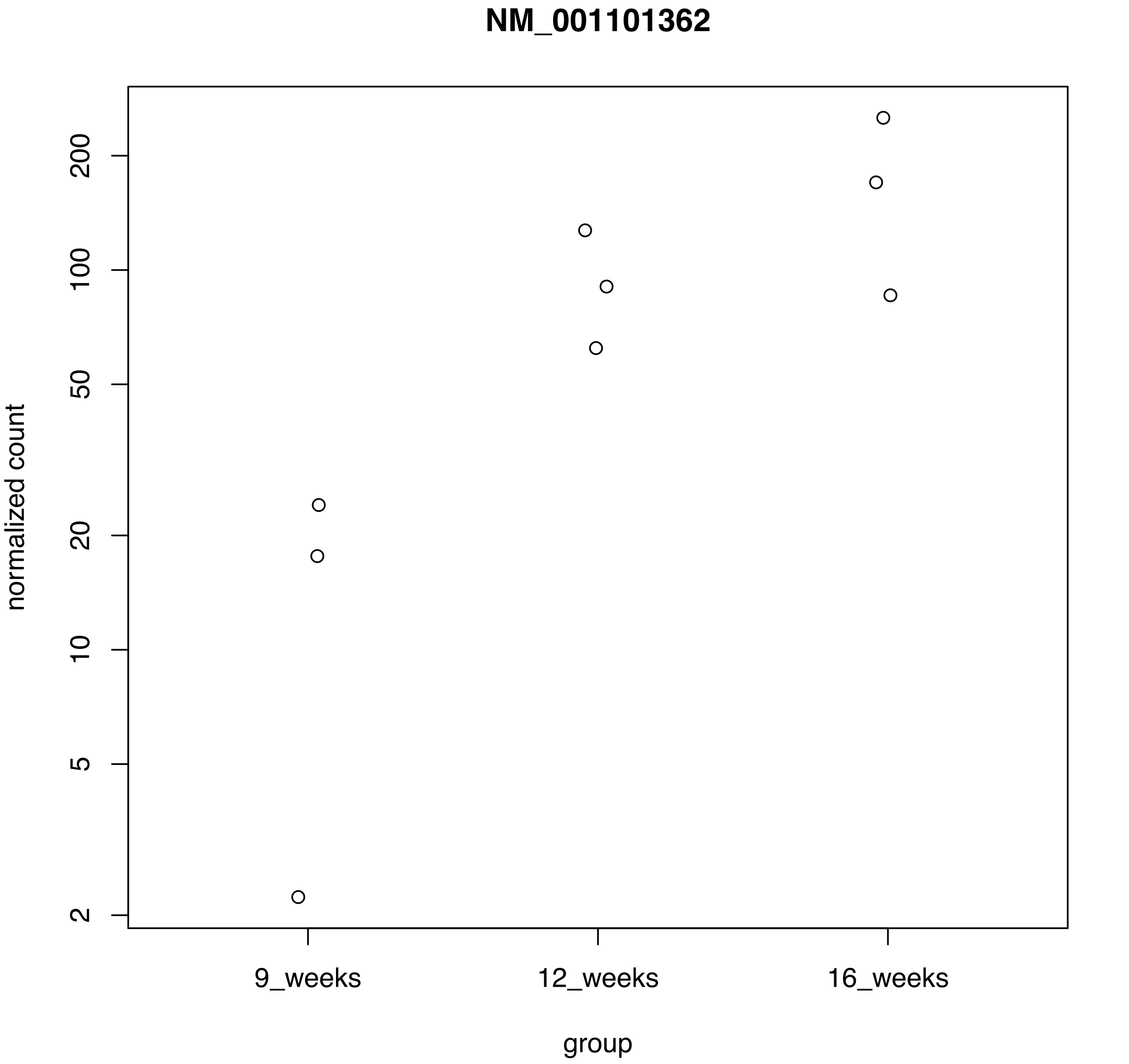
NM_001101362
normalized count
200
100
50
20
10
5
2
9_weeks
12_weeks
16_weeks
group

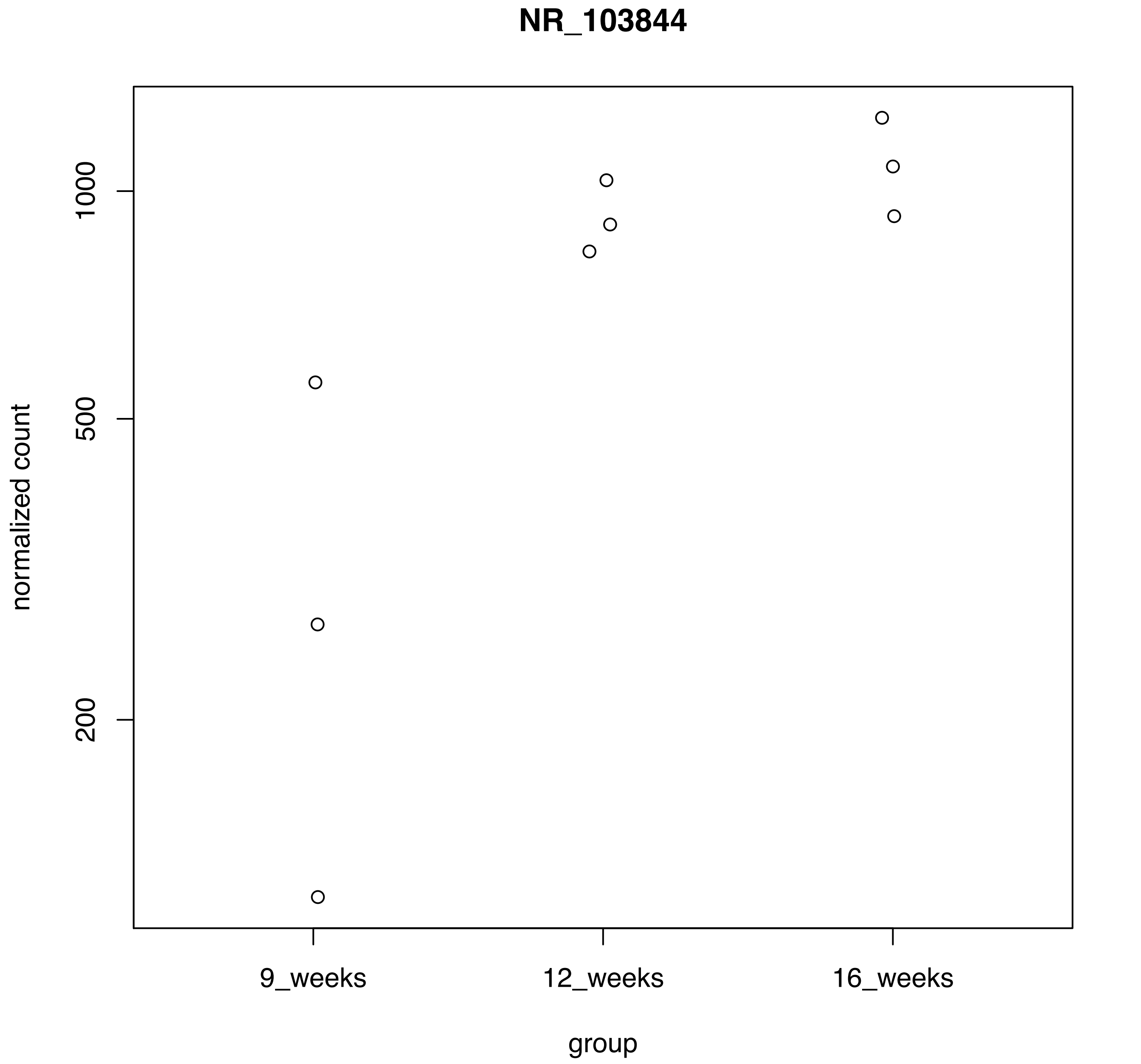

NR_103844
normalized count
1000
500
200
9_weeks
12_weeks
16_weeks
group

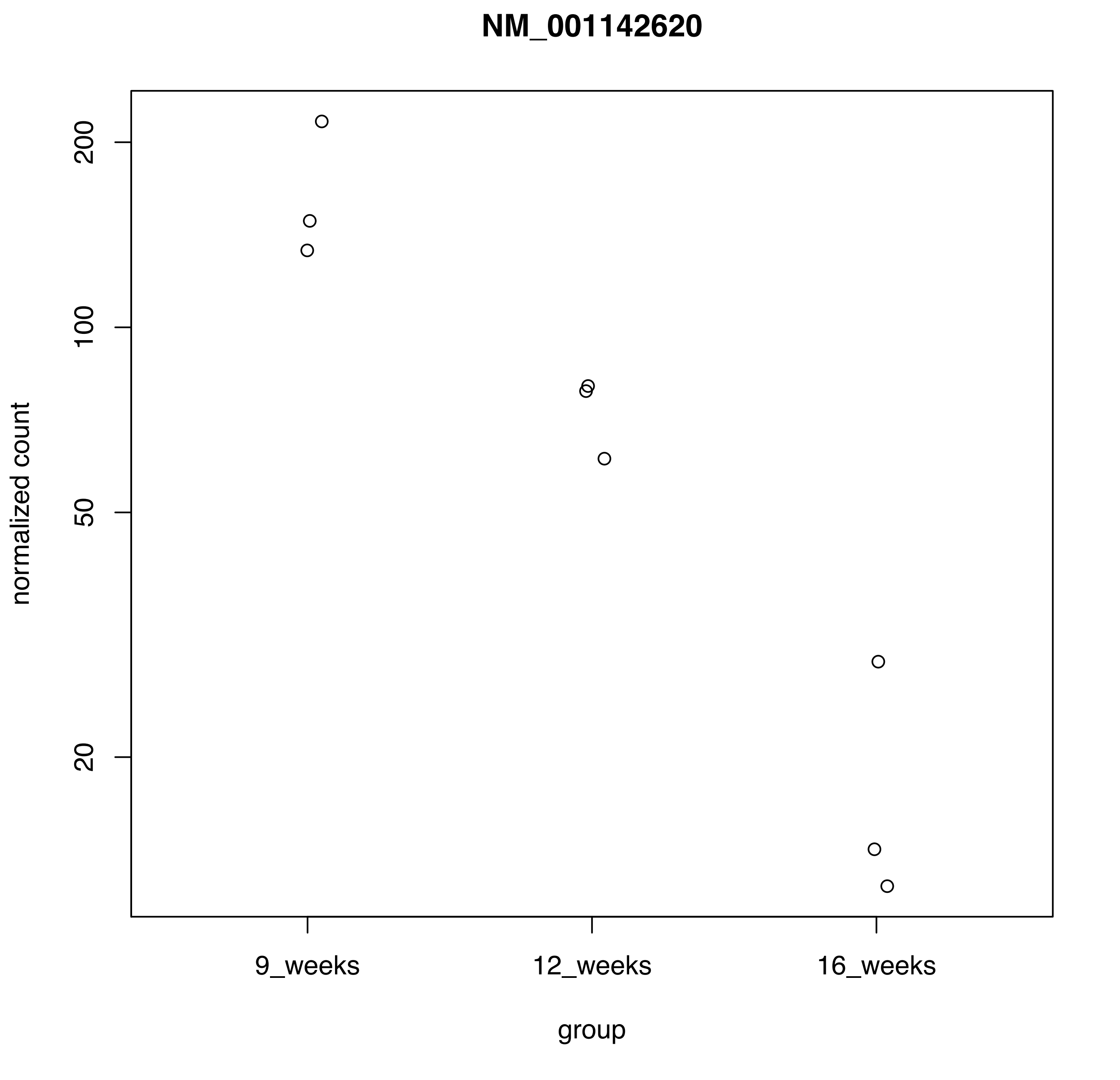
NM_001142620
normalized count
200
100
50
20
9_weeks
12_weeks
16_weeks
group

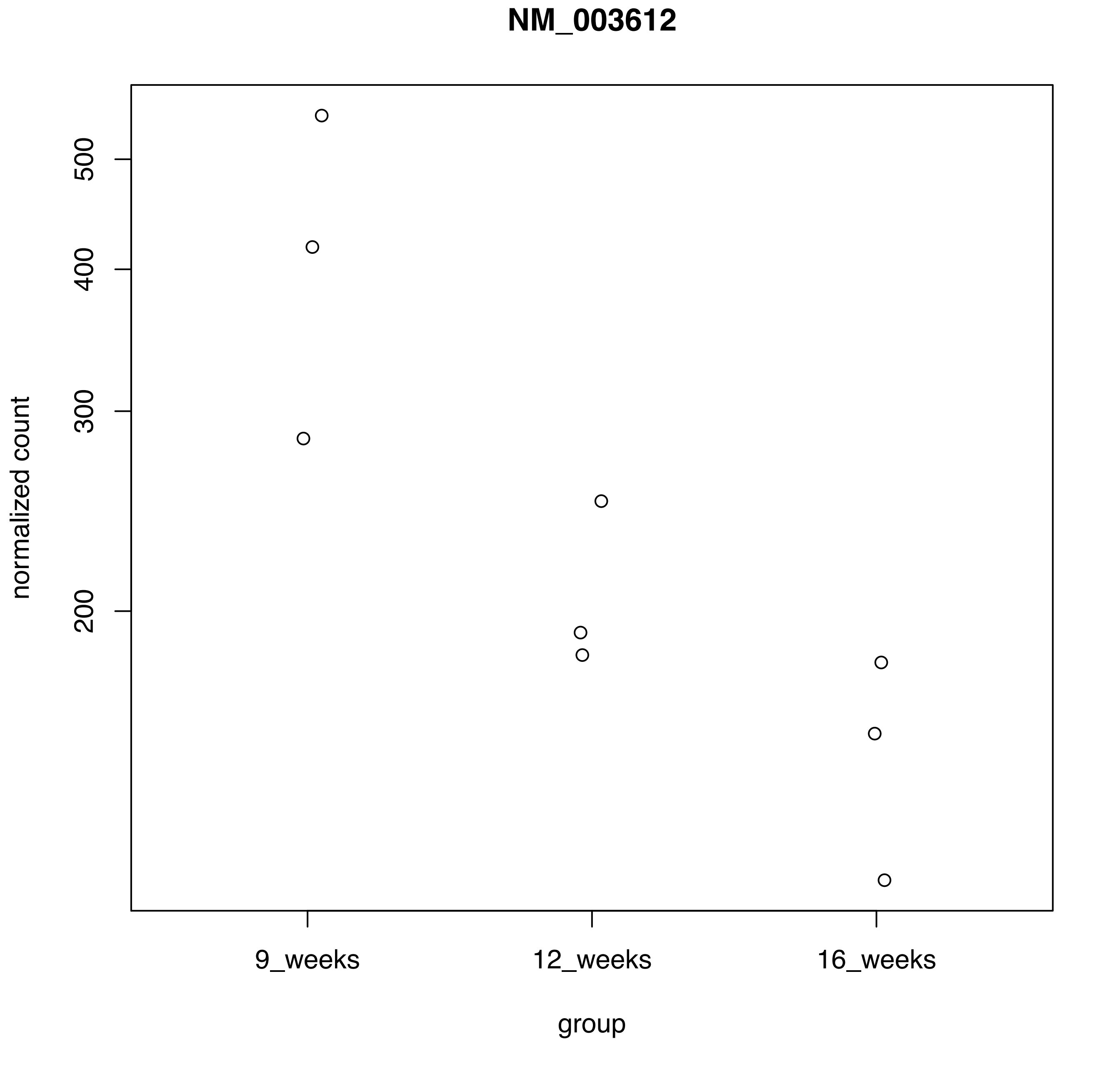
NM_003612
normalized count
500
400
300
200
9_weeks
12_weeks
16_weeks
group

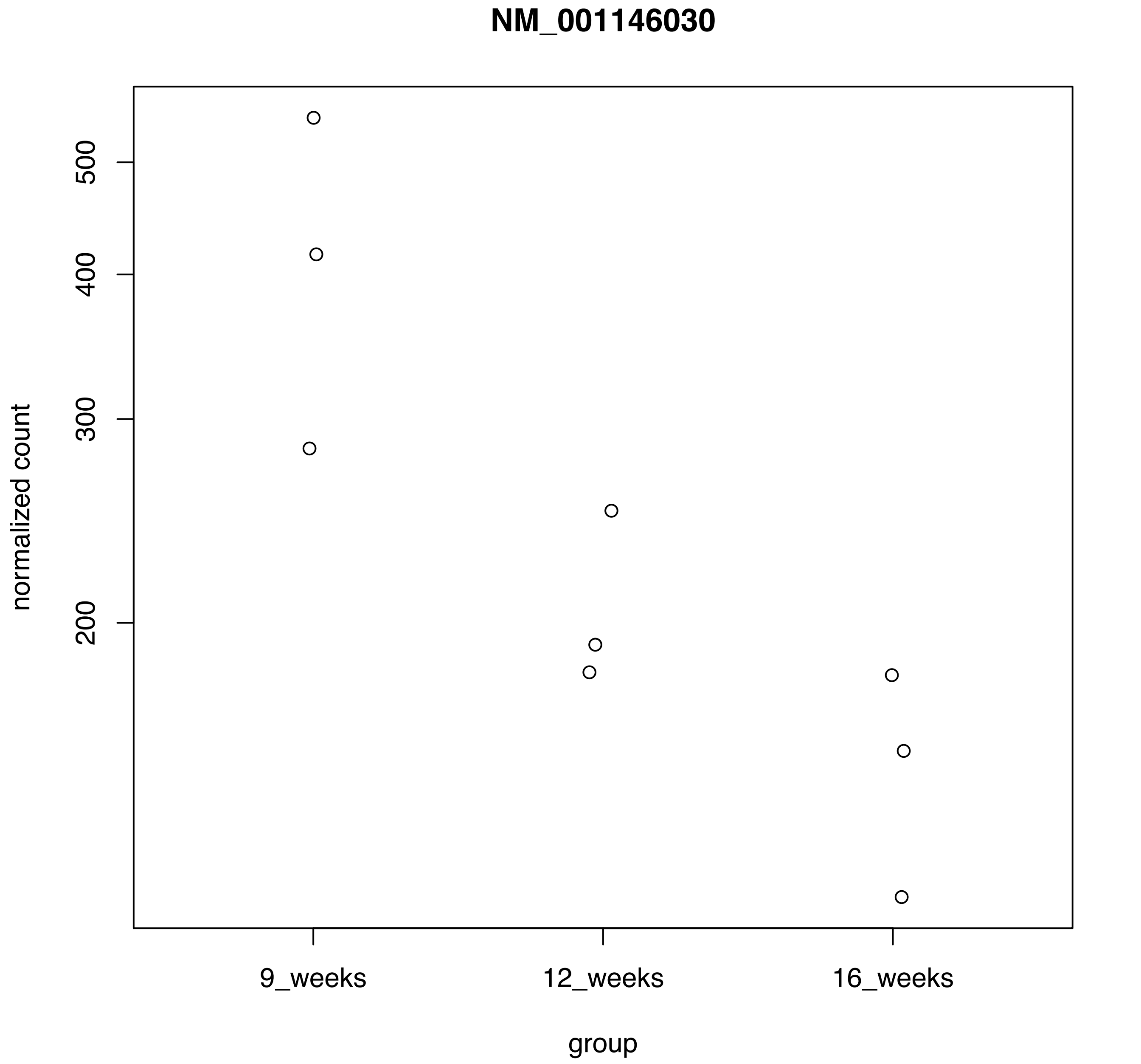
NM_001146030
normalized count
500
400
300
200
9_weeks
12_weeks
16_weeks
group

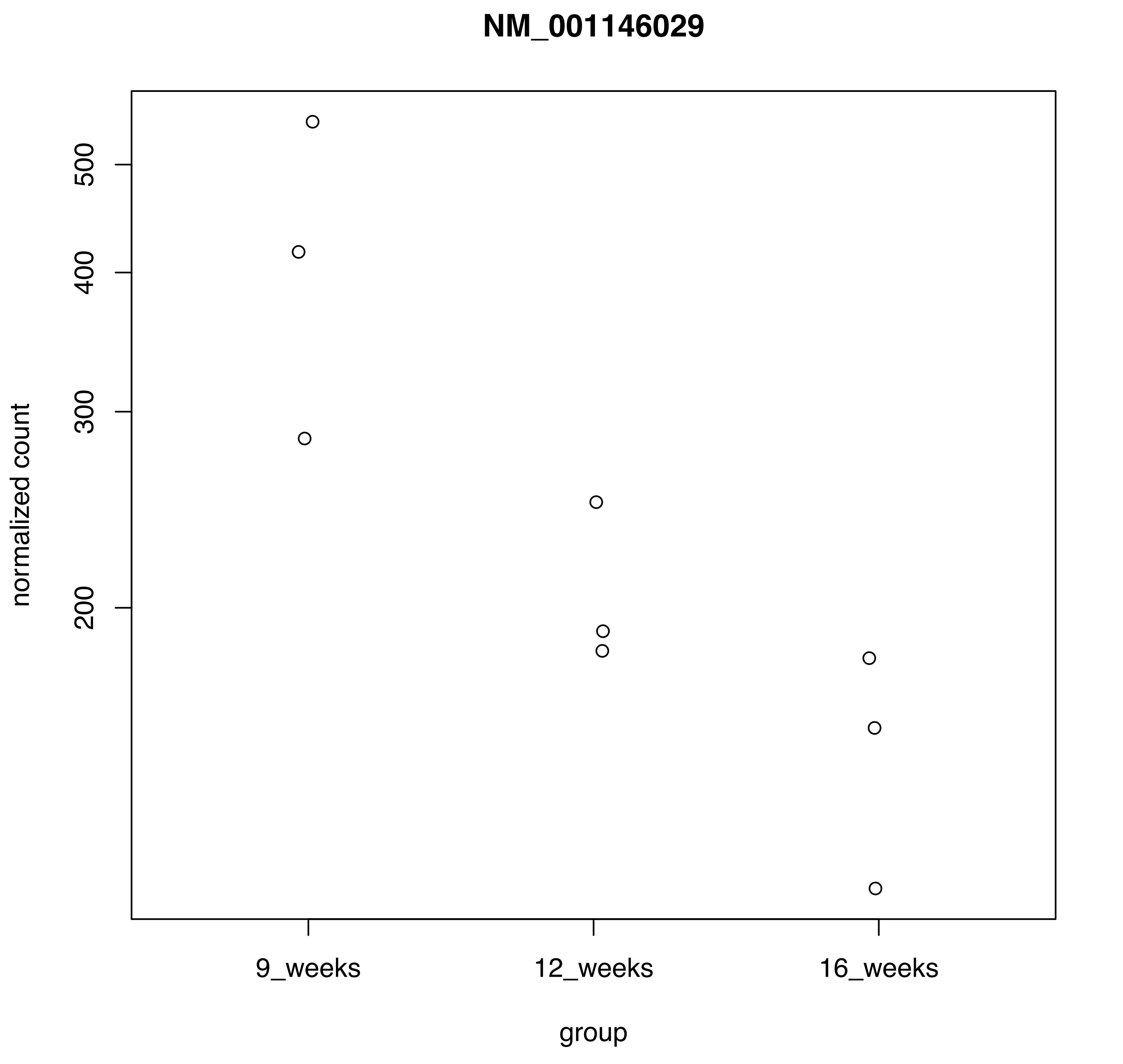
NM_001146029
normalized count
500
400
300
200
9_weeks
12_weeks
16_weeks
group

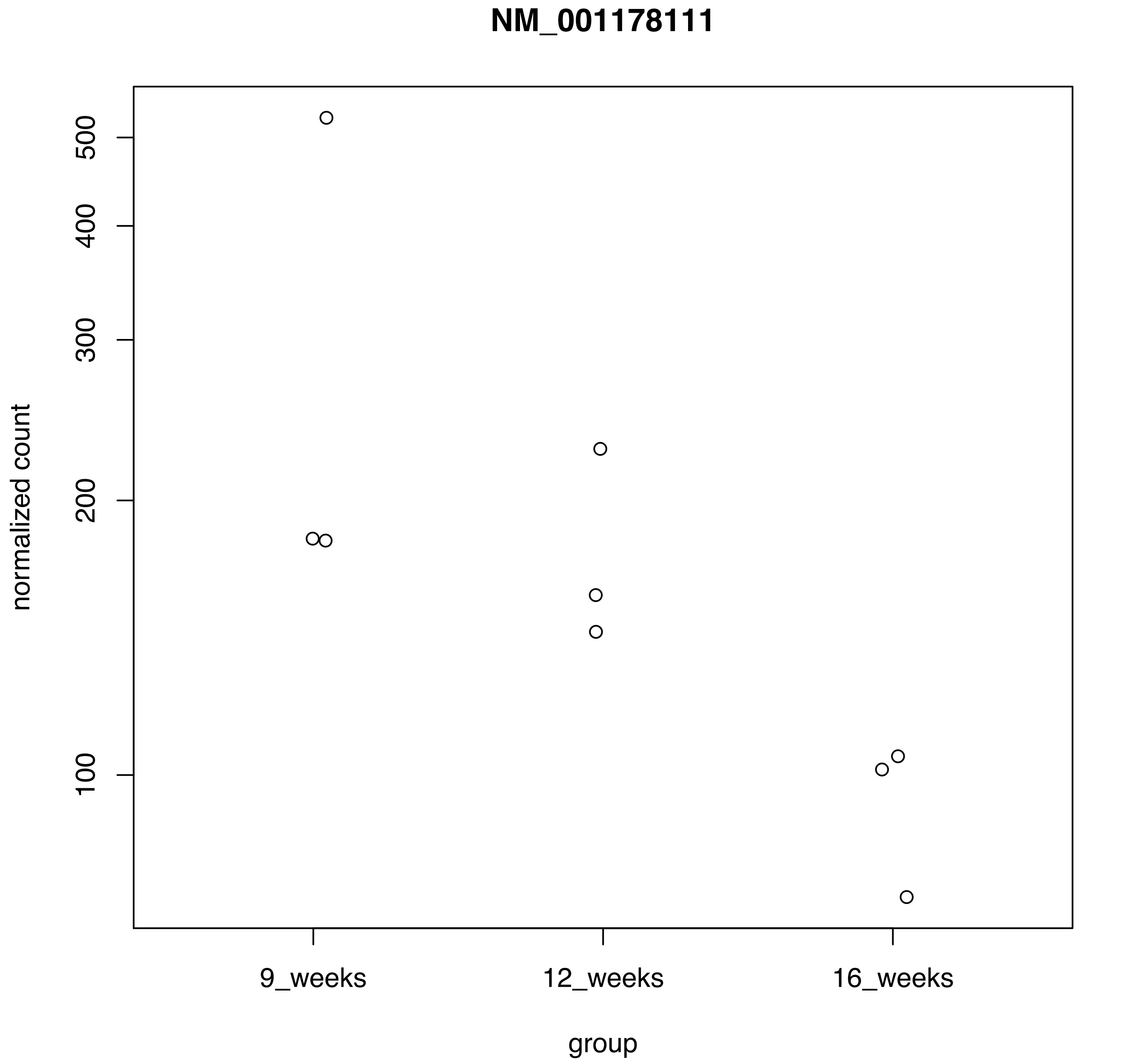

NM_001178111
normalized count
100
200
300
400
500
9_weeks
12_weeks
16_weeks
group

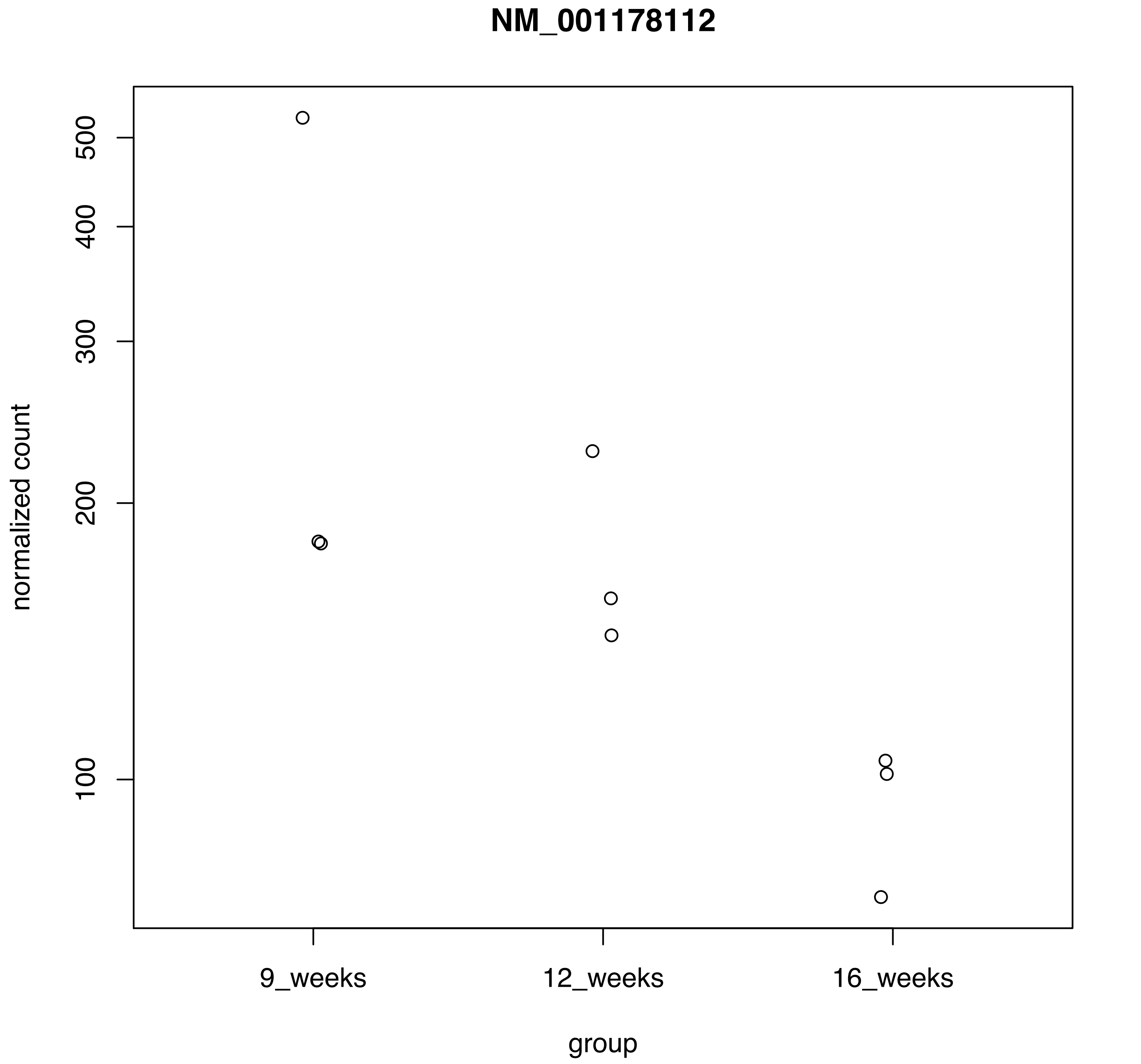
NM_001178112
normalized count
500
400
300
200
100
9_weeks
12_weeks
16_weeks
group

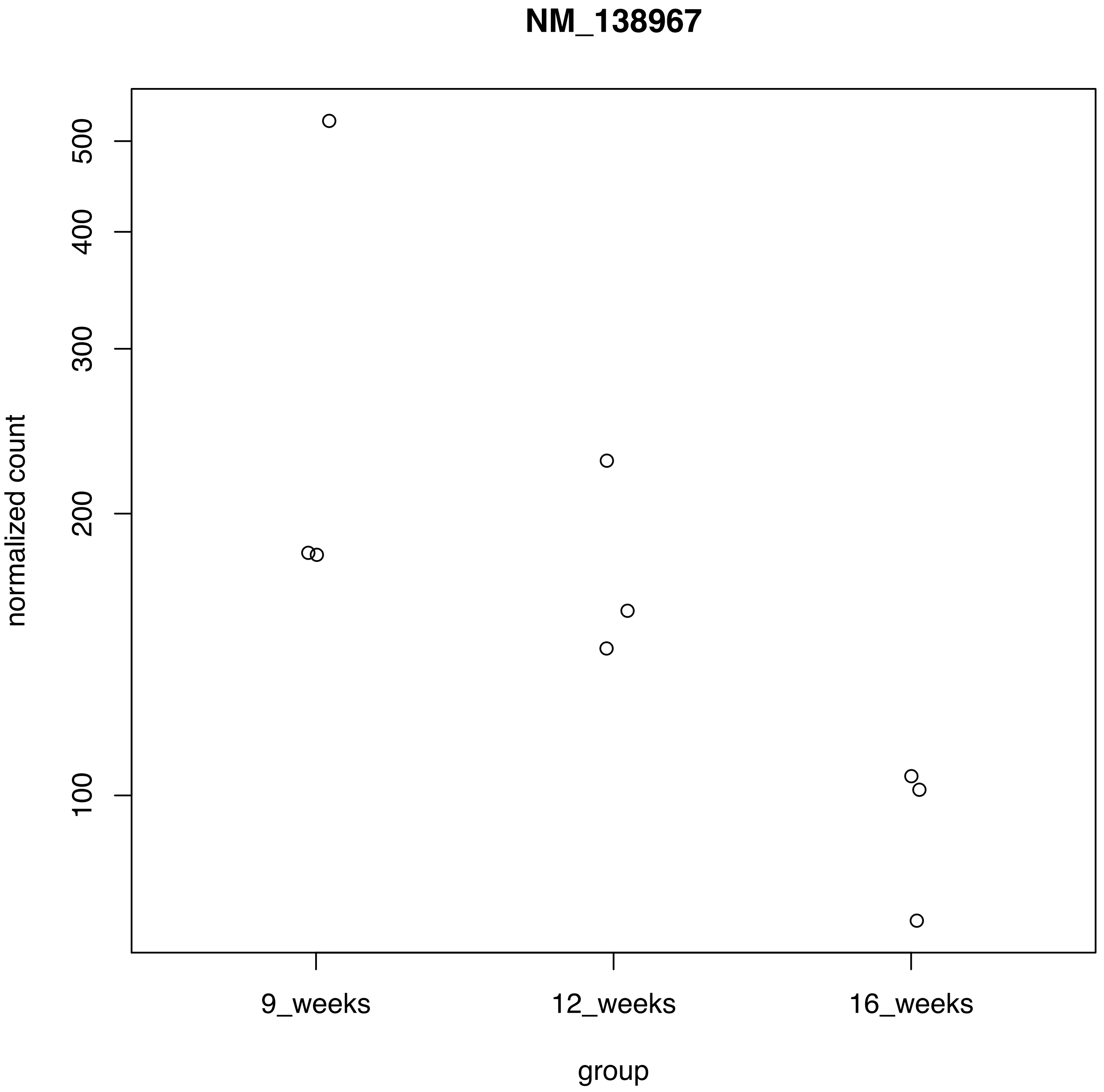
NM_138967
normalized count
500
400
300
200
100
9_weeks
12_weeks
16_weeks
group

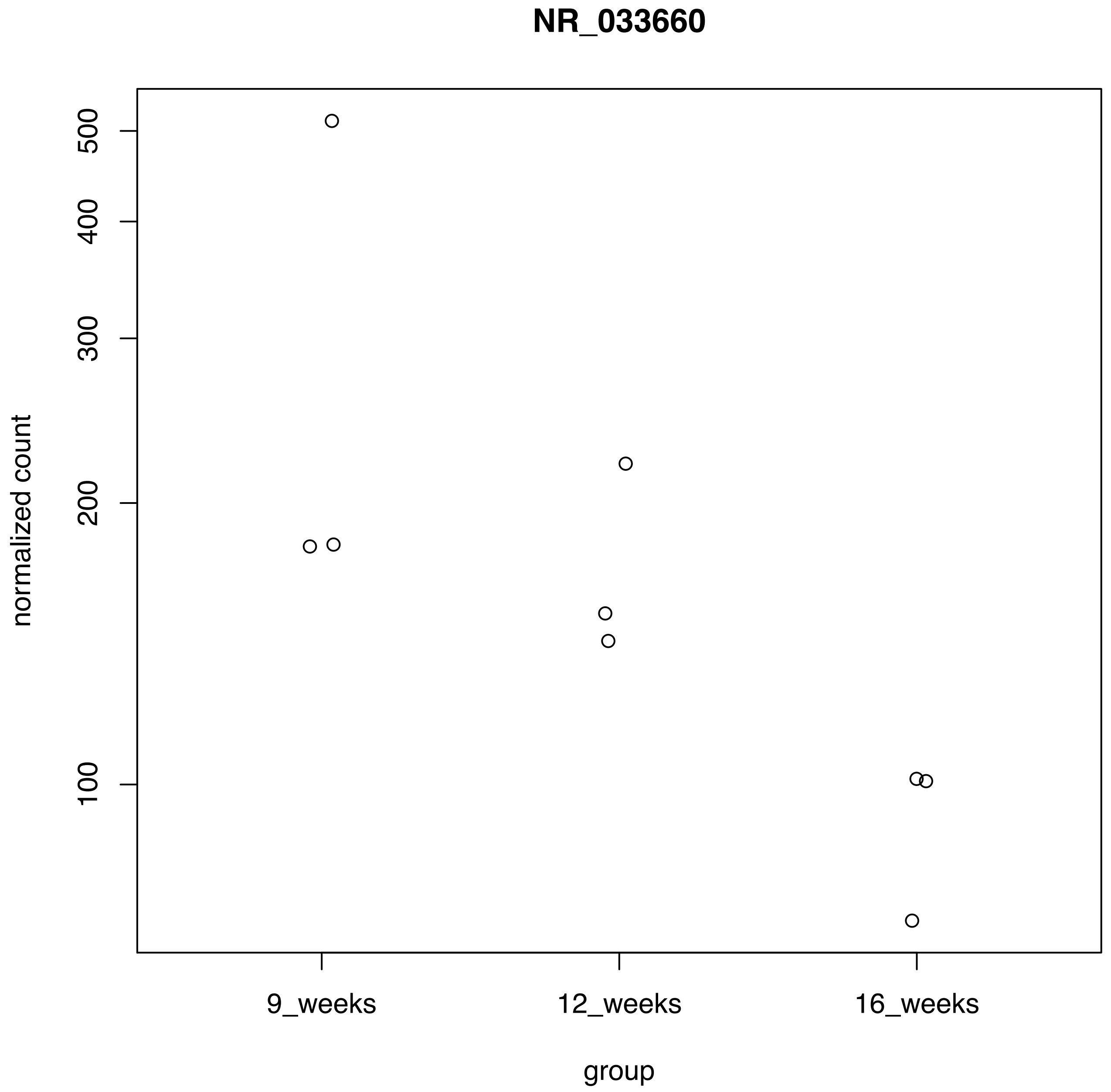
NR_033660
normalized count
500
400
300
200
100
9_weeks
12_weeks
16_weeks
group

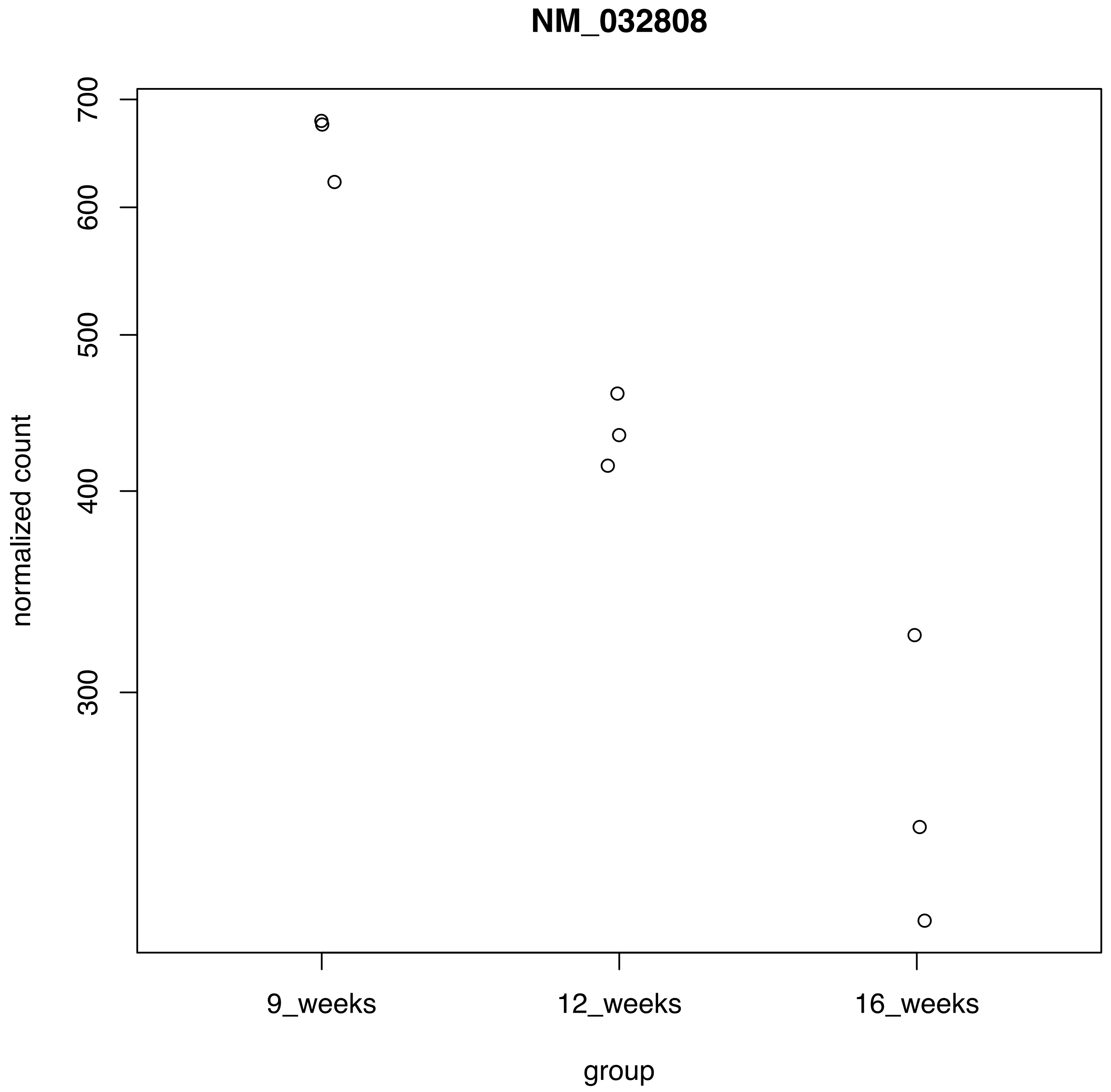
NM_032808
normalized count
700
600
500
400
300
9_weeks
12_weeks
16_weeks
group

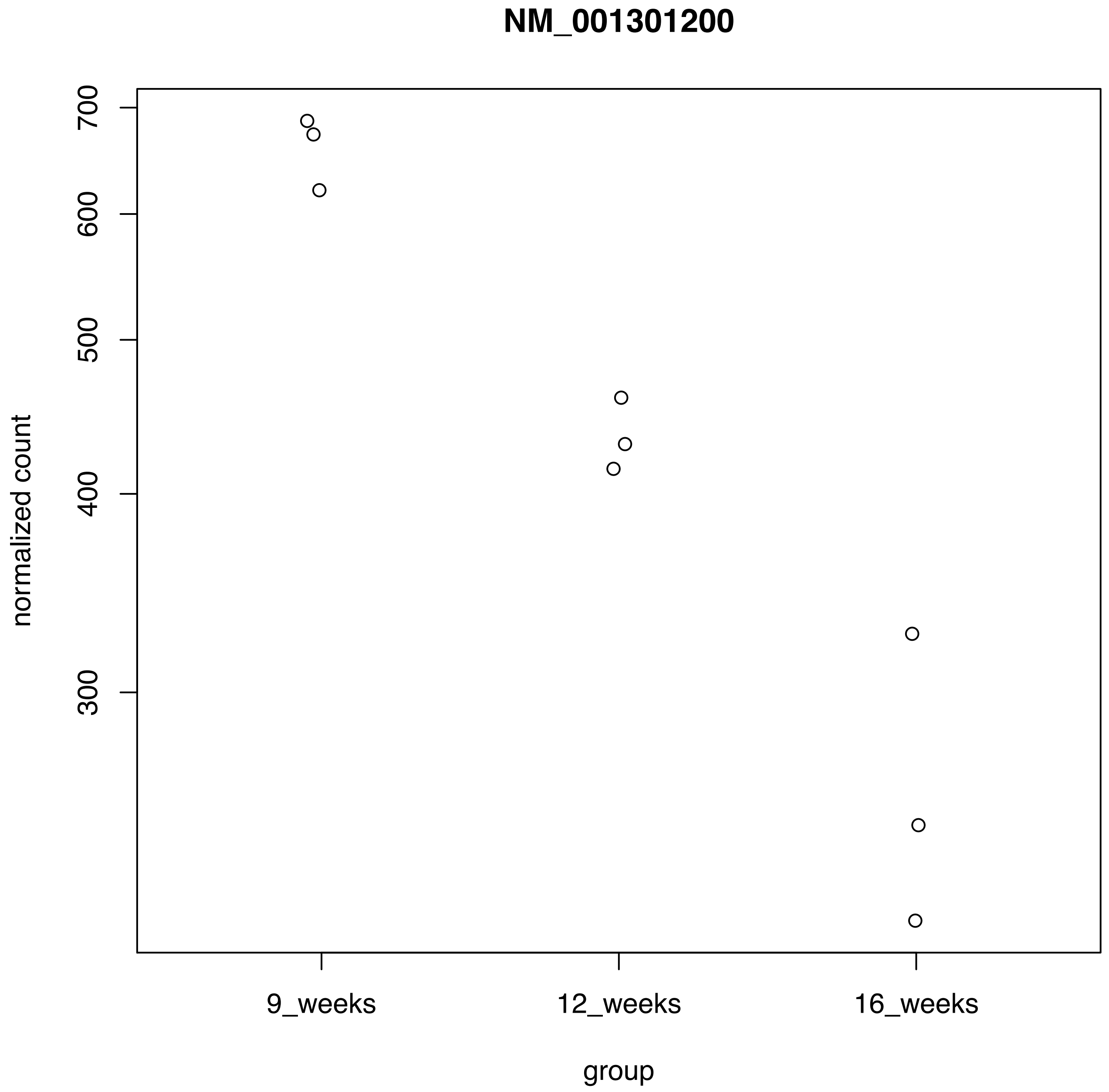
NM_001301200
normalized count
700
600
500
400
300
9_weeks
12_weeks
16_weeks
group

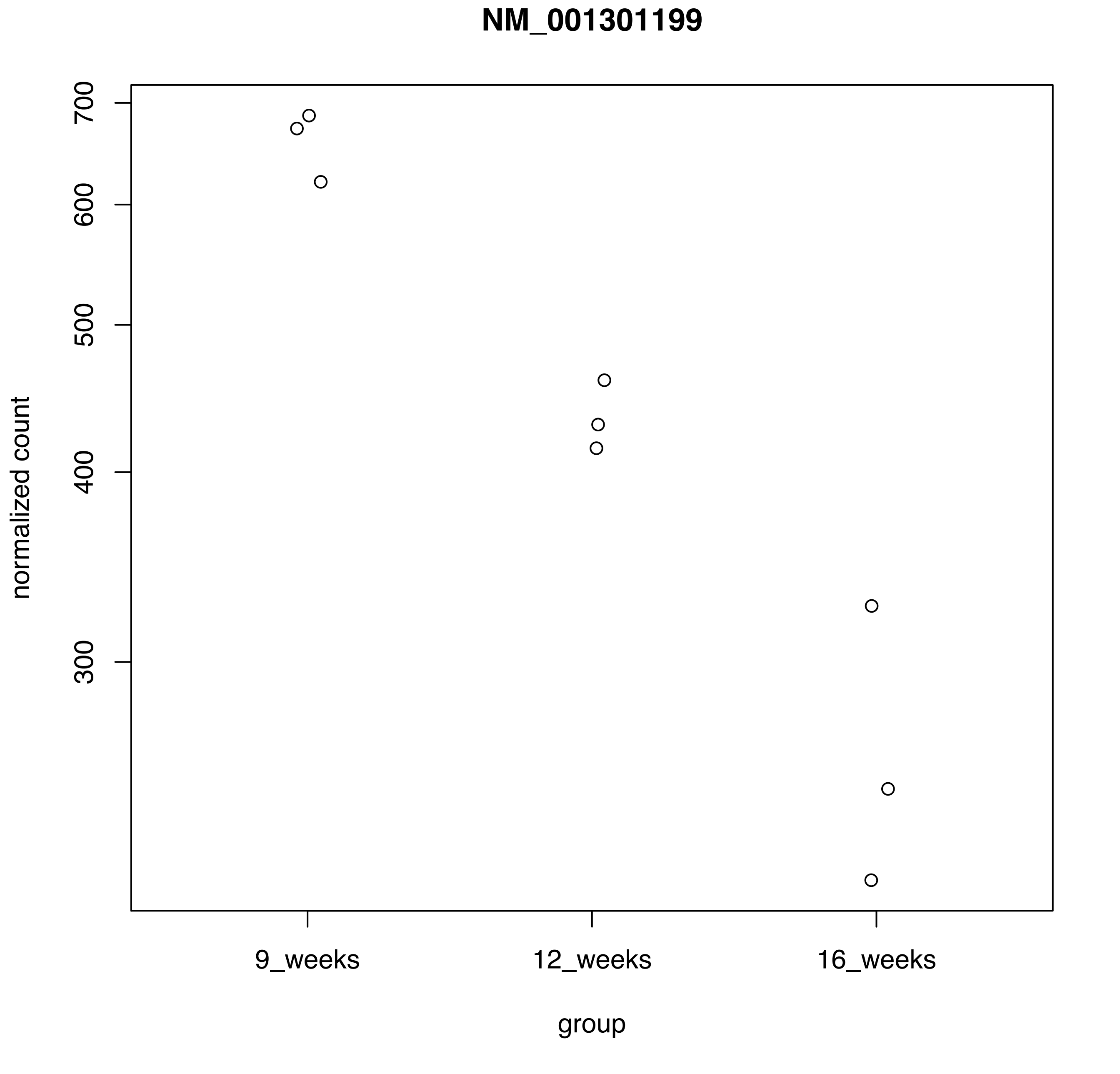
NM_001301199
normalized count
700
600
500
400
300
9_weeks
12_weeks
16_weeks
group

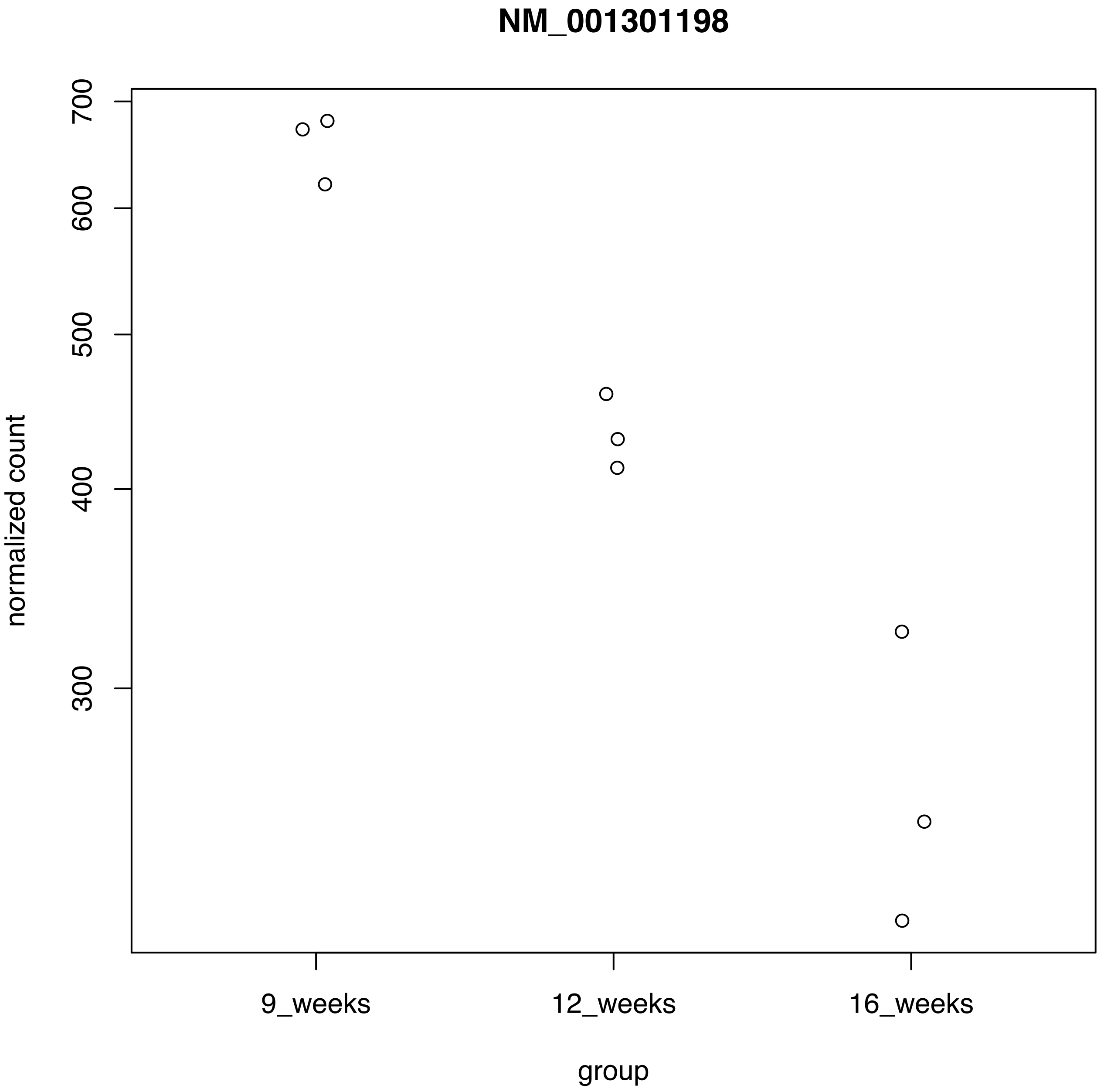

NM_001301198
normalized count
700
600
500
400
300
9_weeks
12_weeks
16_weeks
group

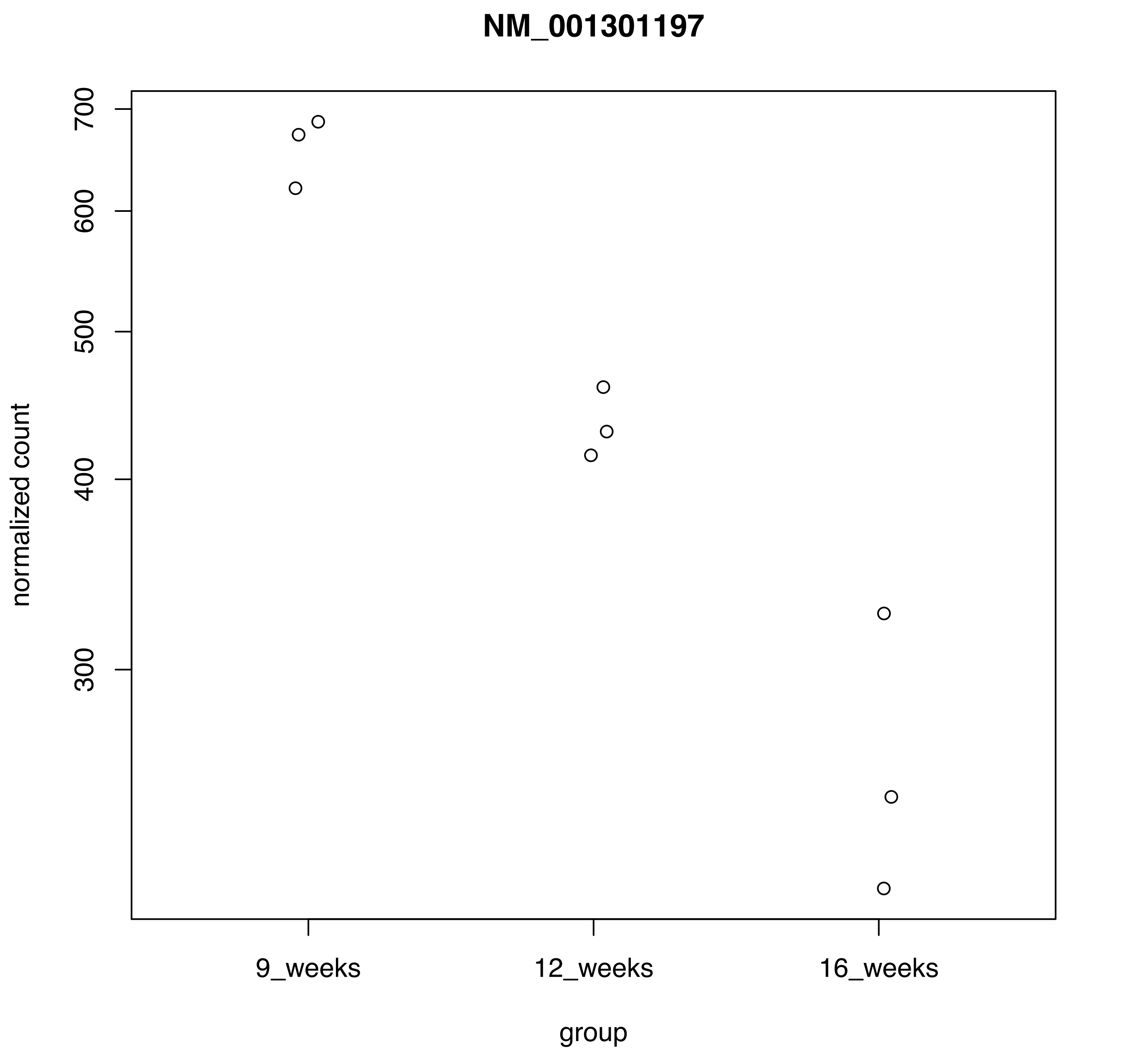
NM_001301197
normalized count
700
600
500
400
300
9_weeks
12_weeks
16_weeks
group

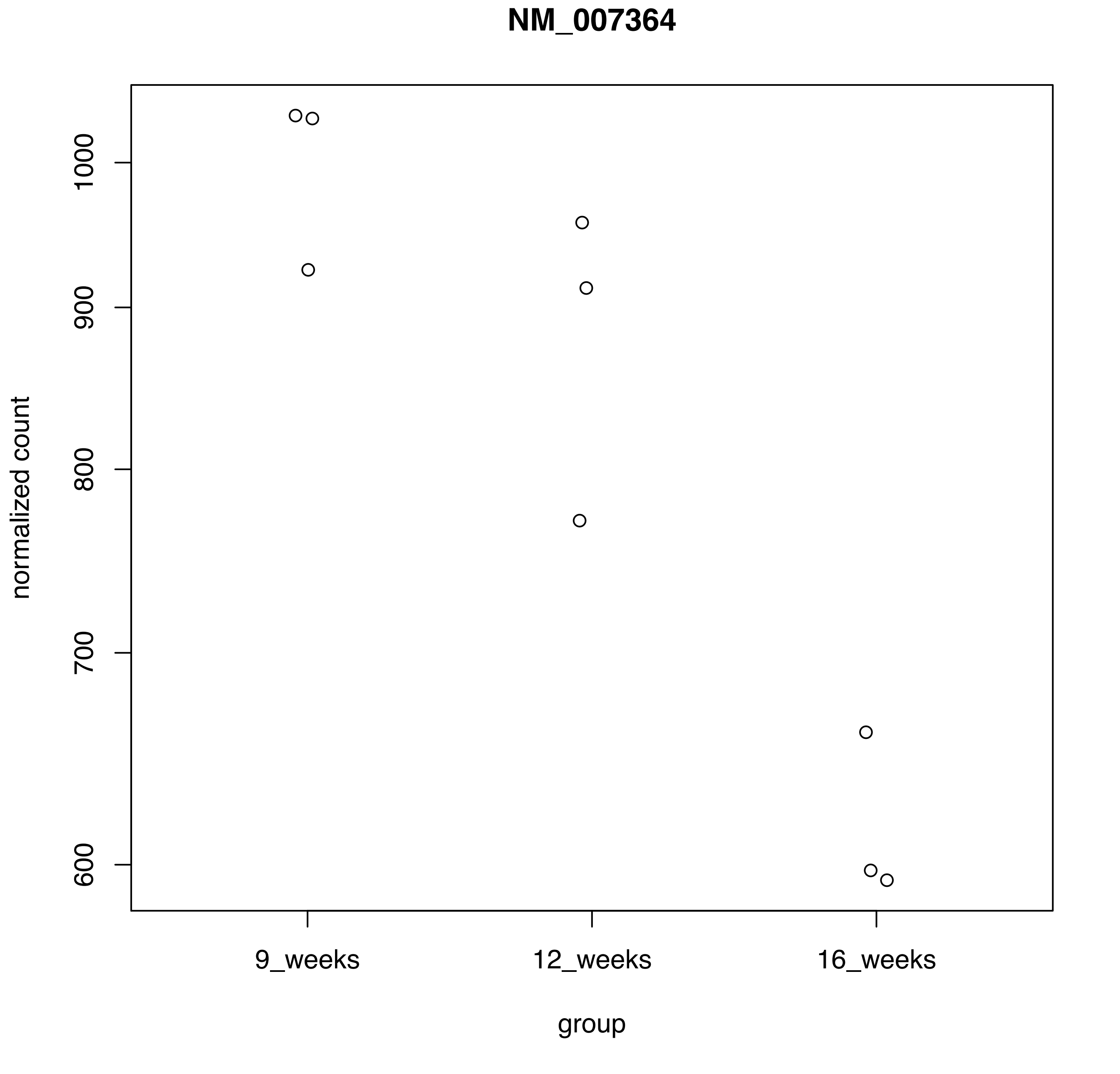
NM_007364
normalized count
1000
900
800
700
600
9_weeks
12_weeks
16_weeks
group

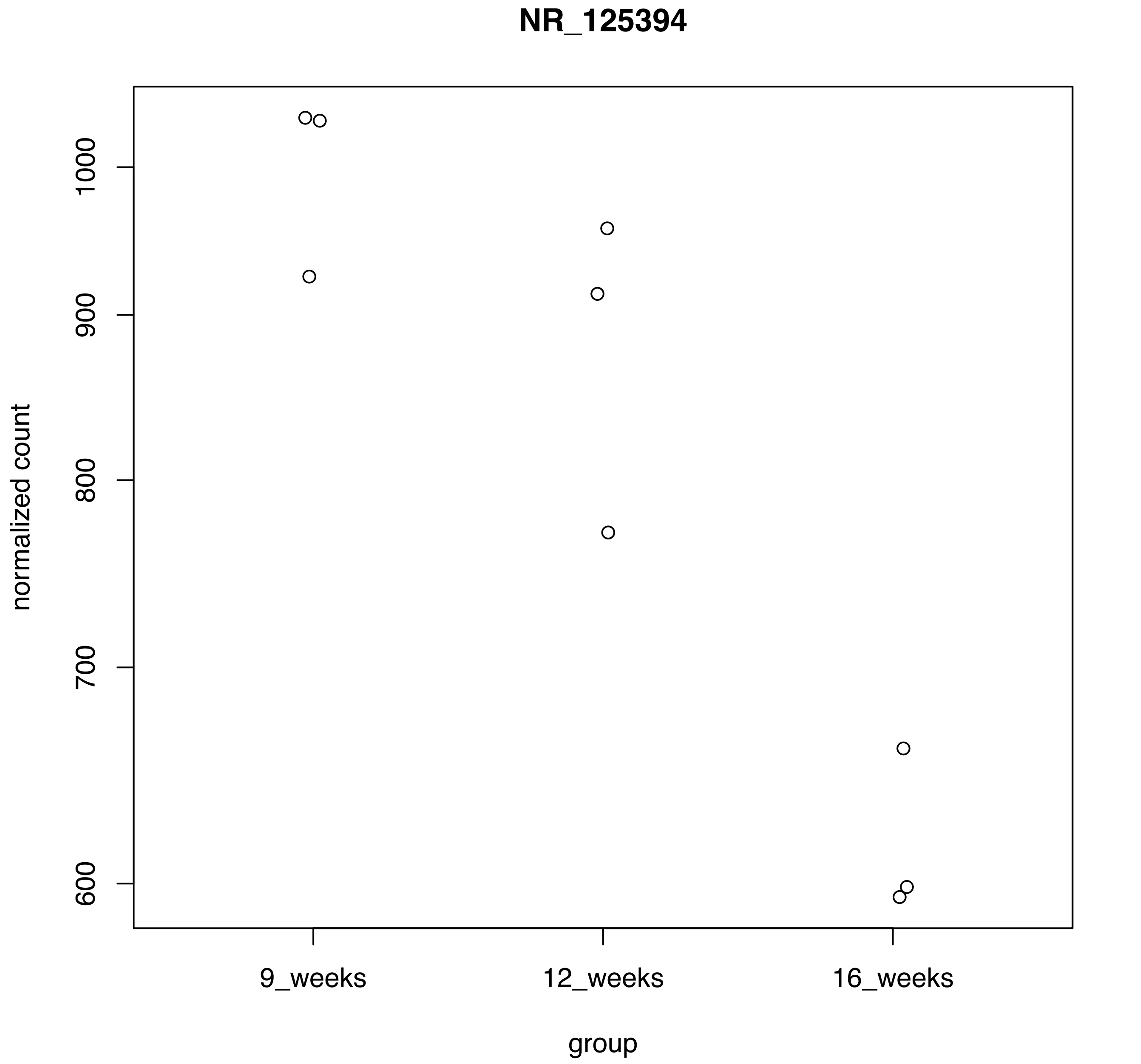
NR_125394
normalized count
1000
900
800
700
600
9_weeks
12_weeks
16_weeks
group

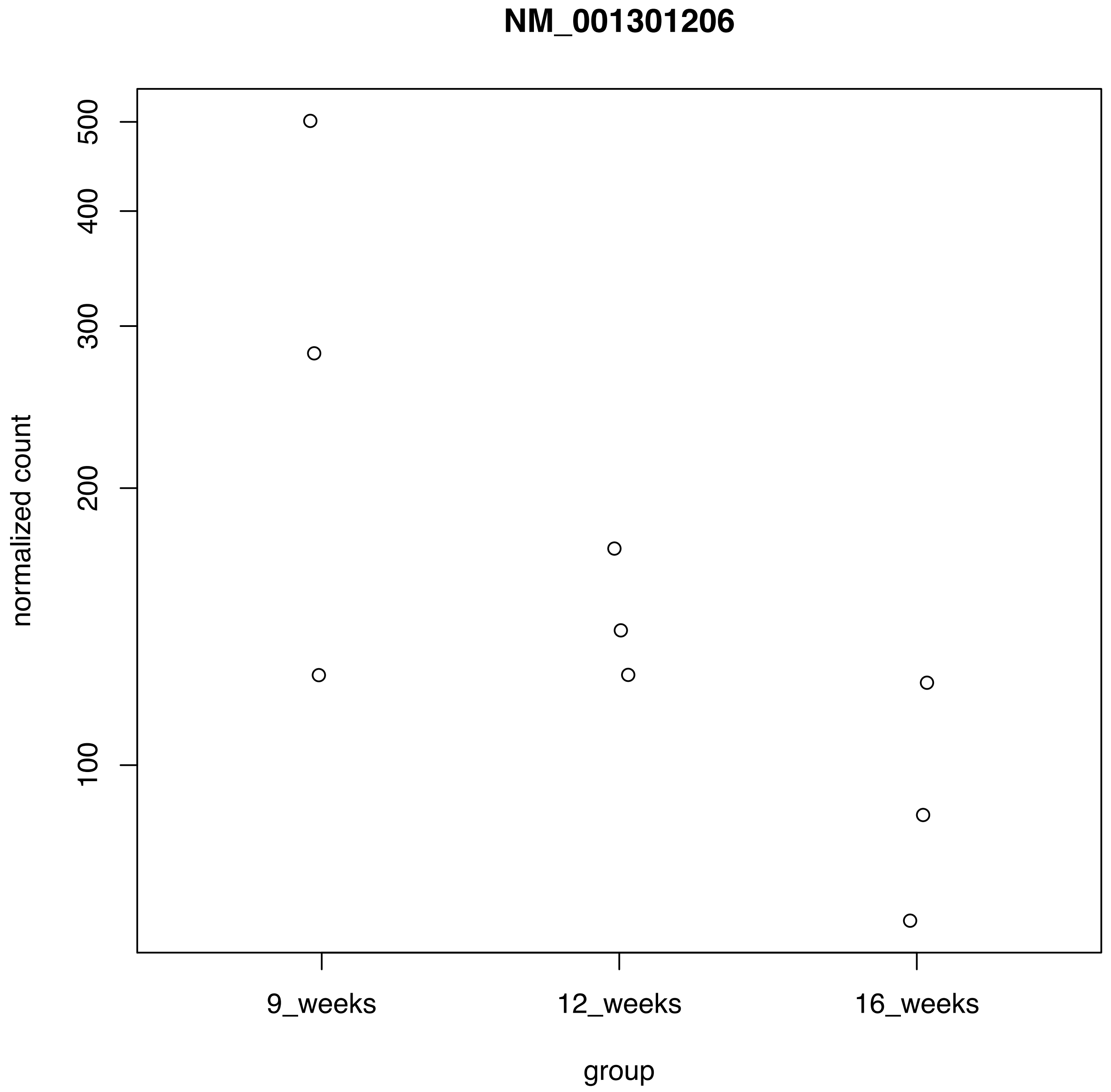

NM_001301206
normalized count
500
400
300
200
100
9_weeks
12_weeks
16_weeks
group

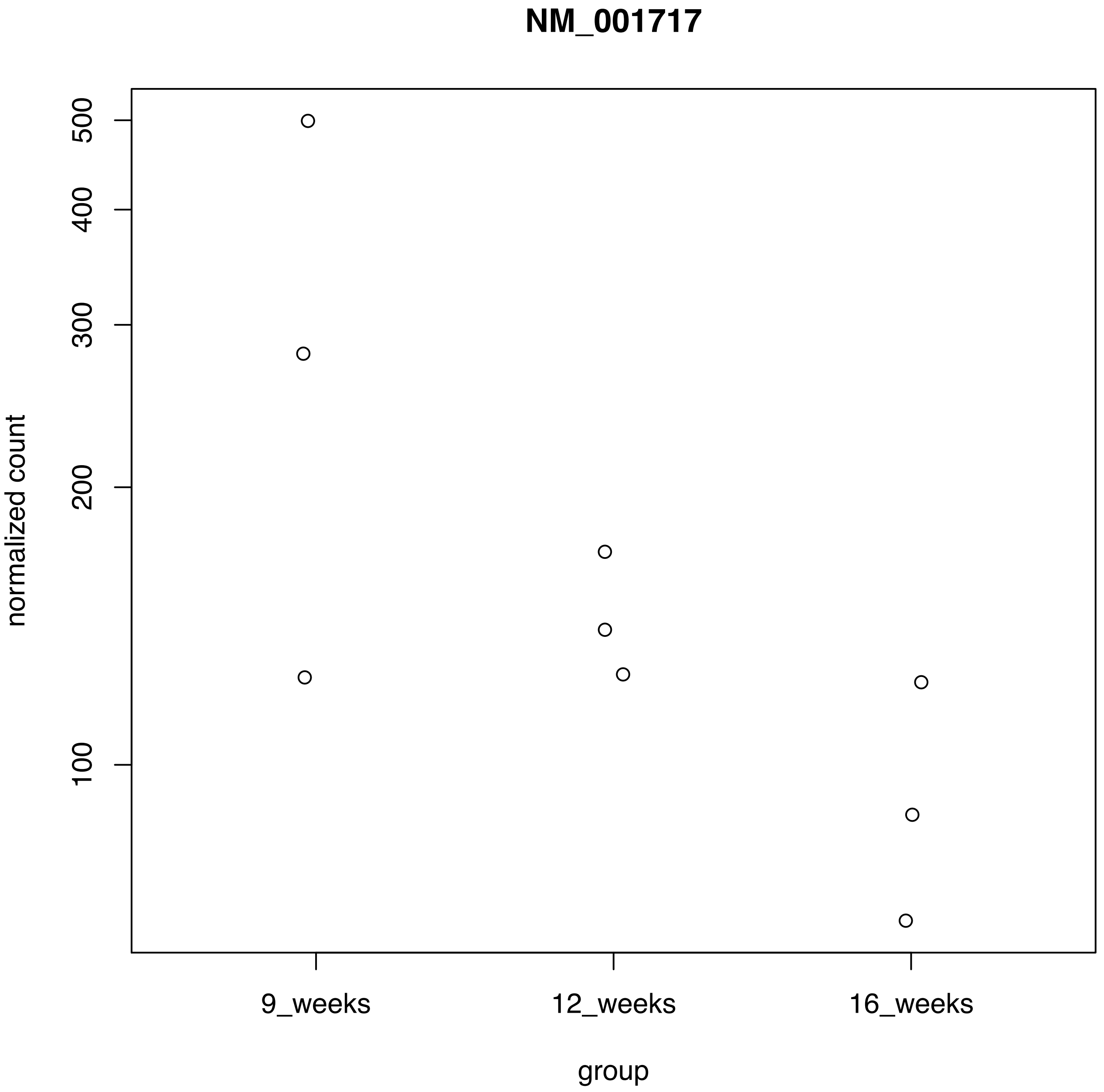
NM_001717
normalized count
500
400
300
200
100
9_weeks
12_weeks
16_weeks
group

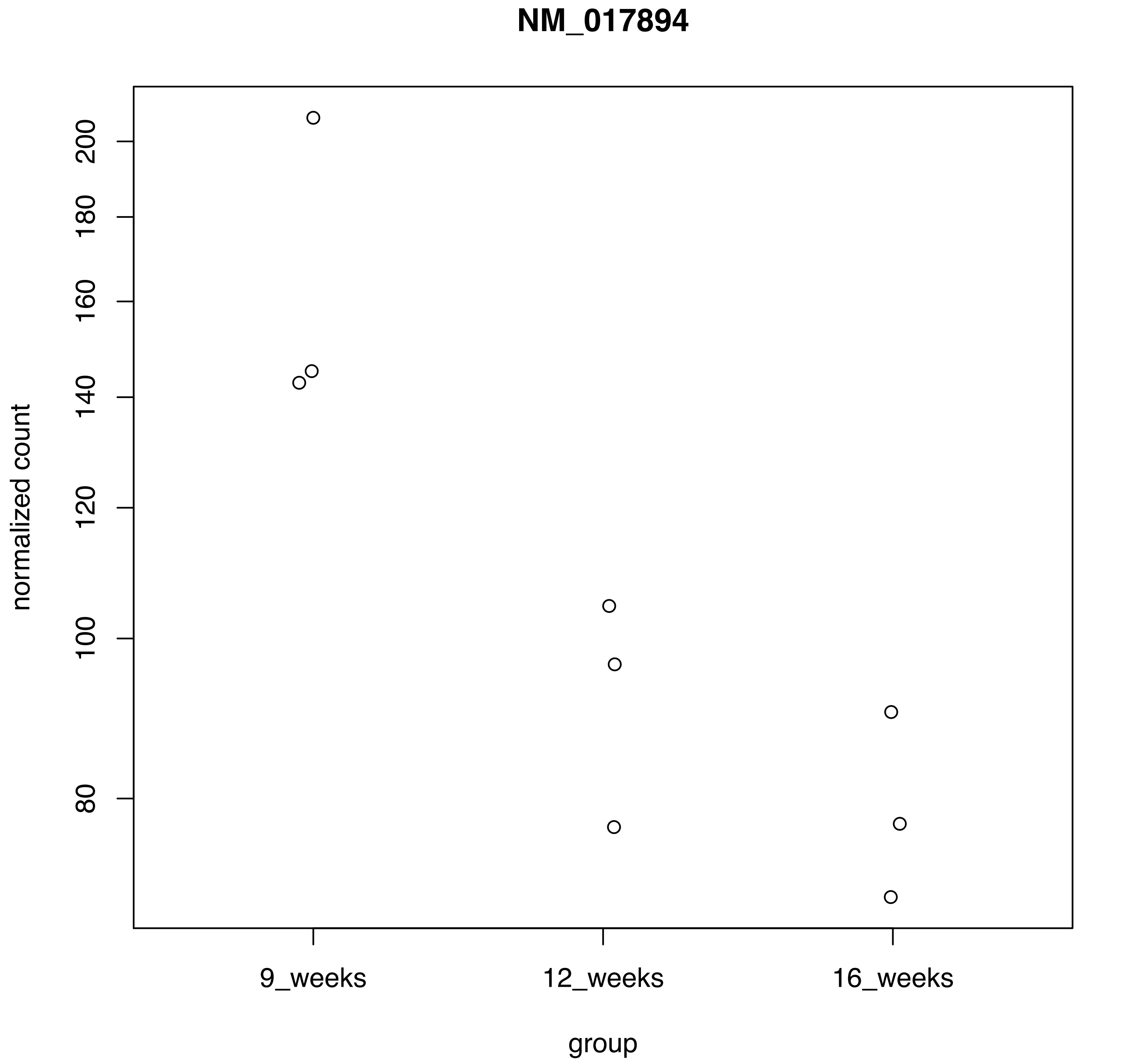
NM_017894
normalized count
200
180
160
140
120
100
80
9_weeks
12_weeks
16_weeks
group

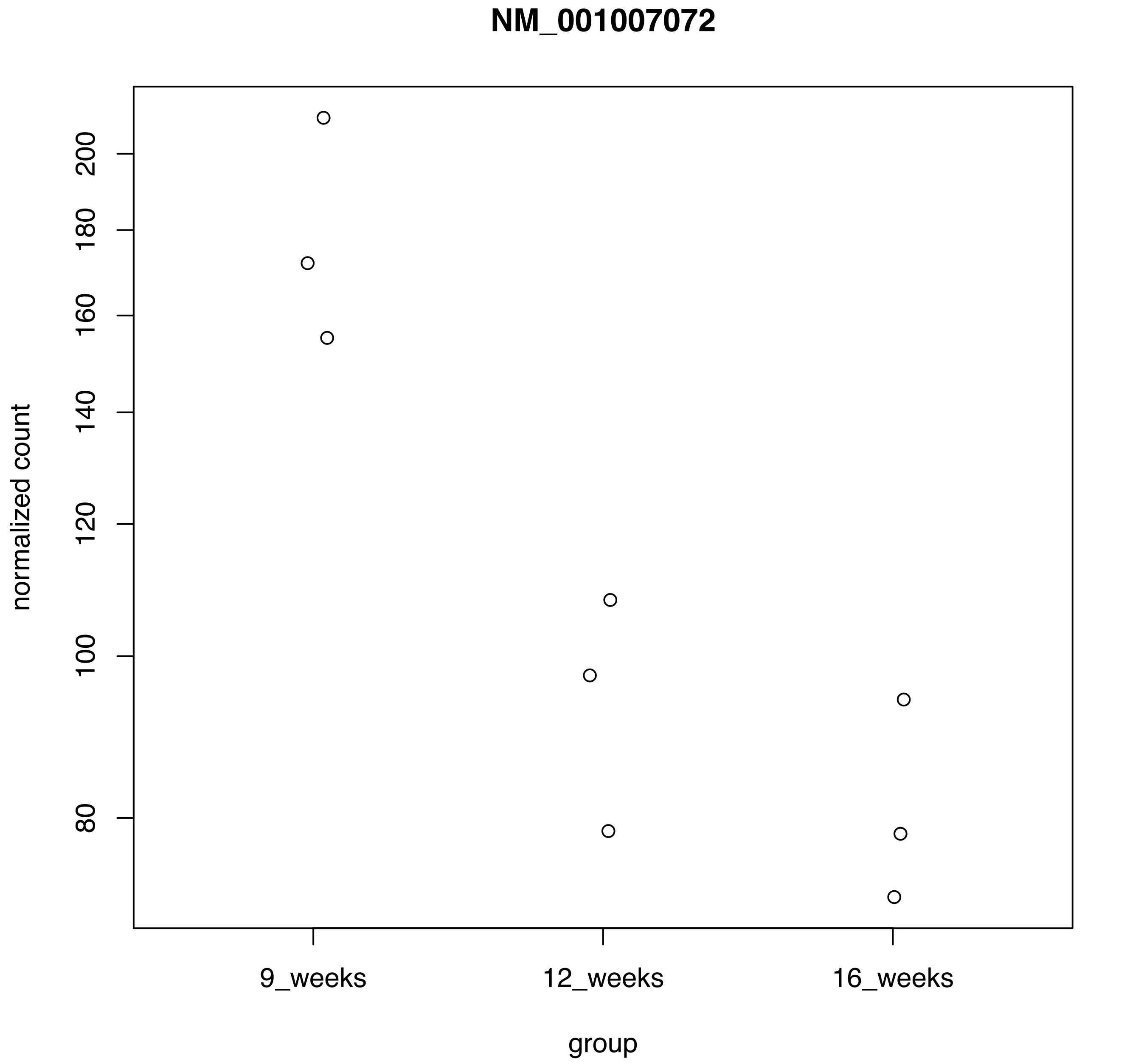
NM_001007072
normalized count
200
180
160
140
120
100
80
9_weeks
12_weeks
16_weeks
group

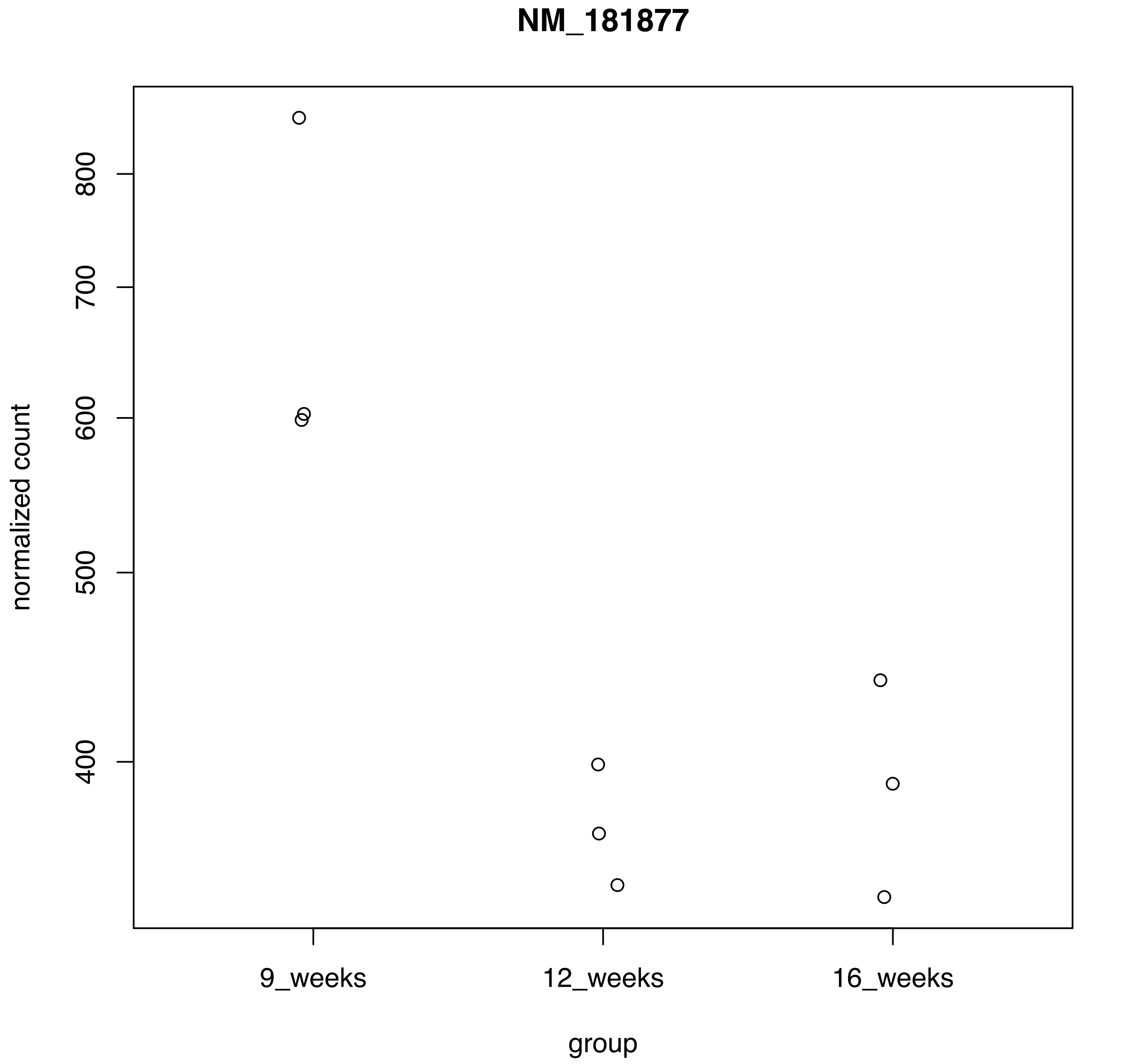
NM_181877
normalized count
800
700
600
500
400
9_weeks
12_weeks
16_weeks
group

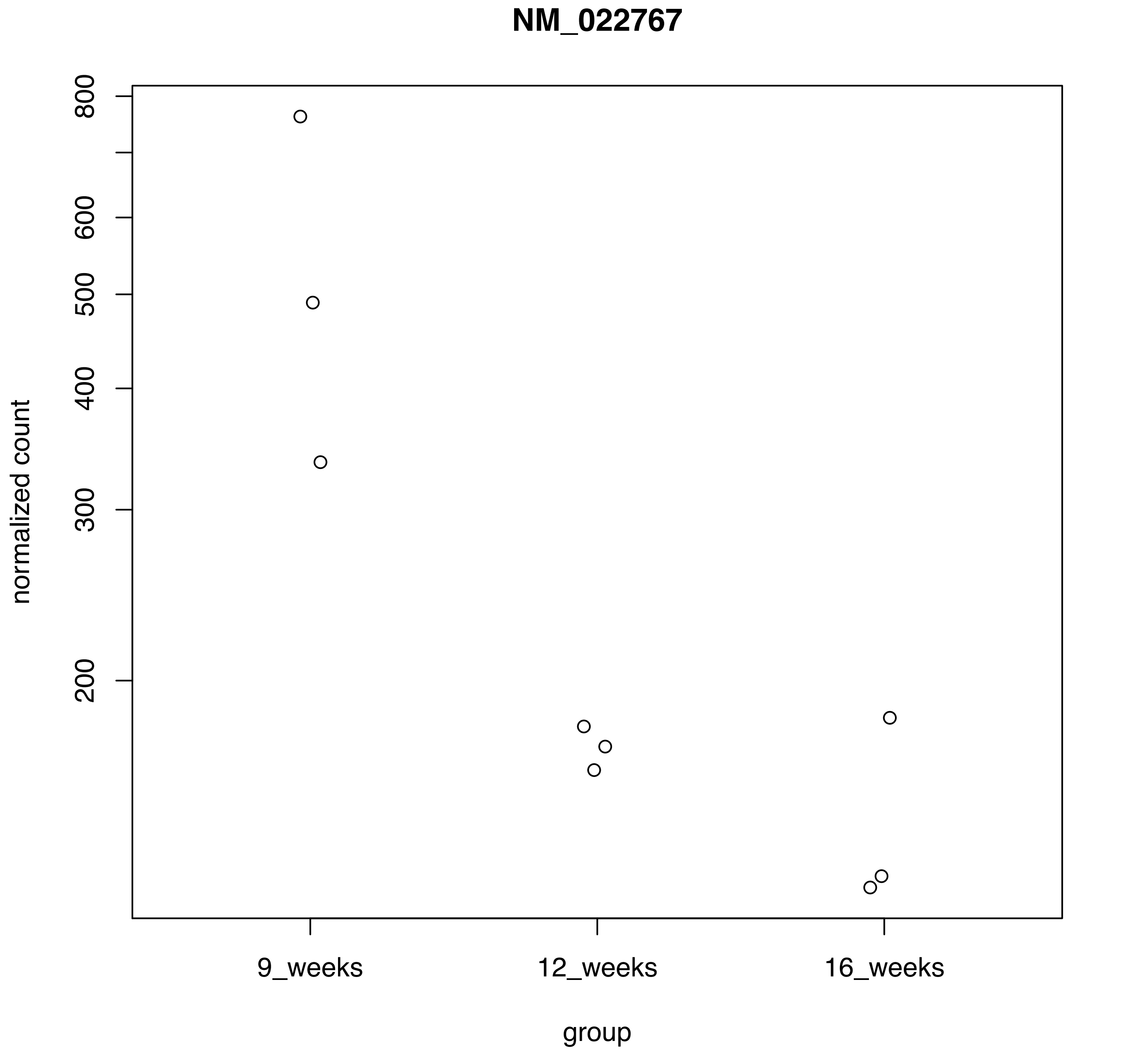
NM_022767
normalized count
800
600
500
400
300
200
9_weeks
12_weeks
16_weeks
group

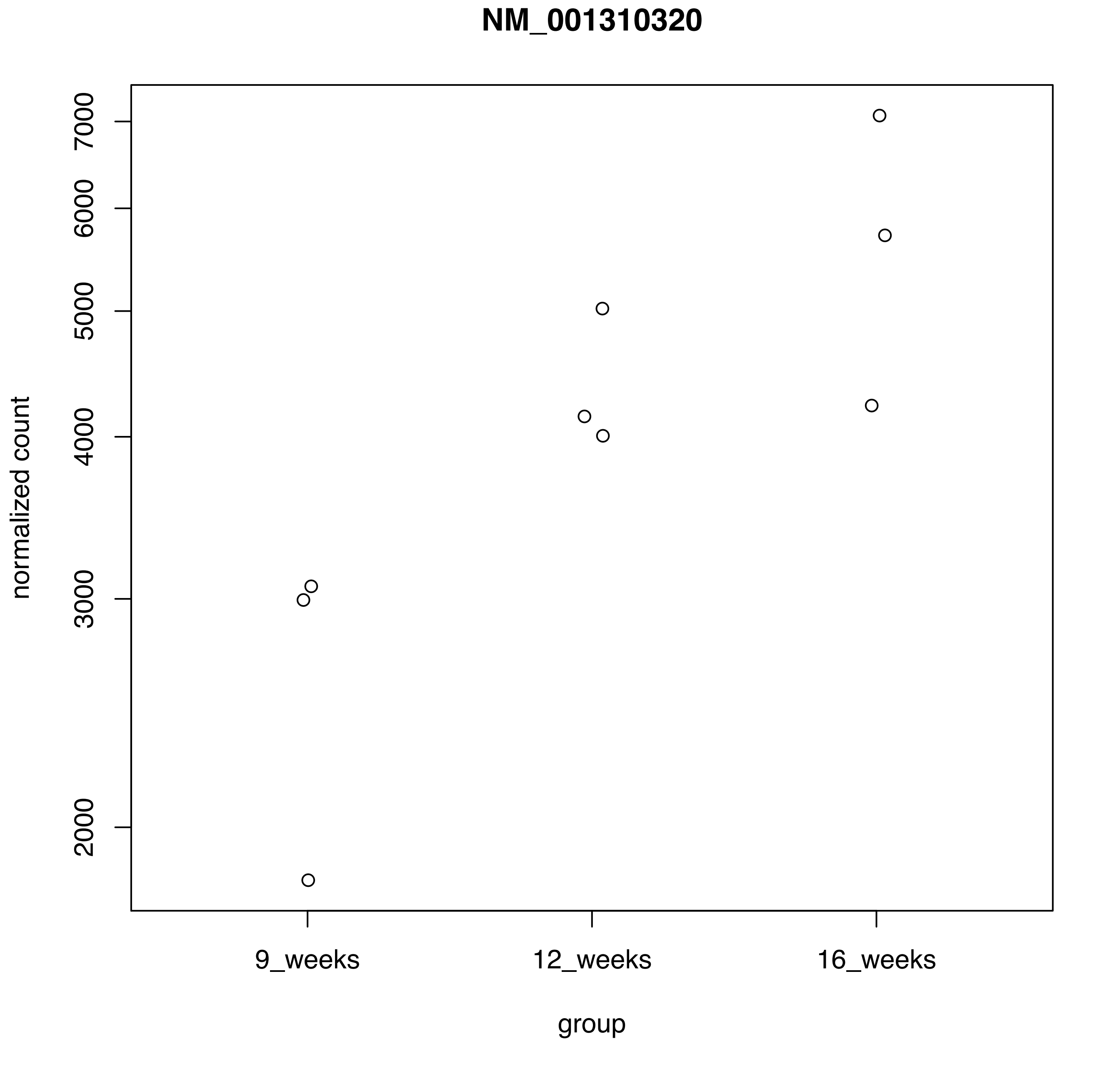
NM_001310320
normalized count
7000
6000
5000
4000
3000
2000
9_weeks
12_weeks
16_weeks
group

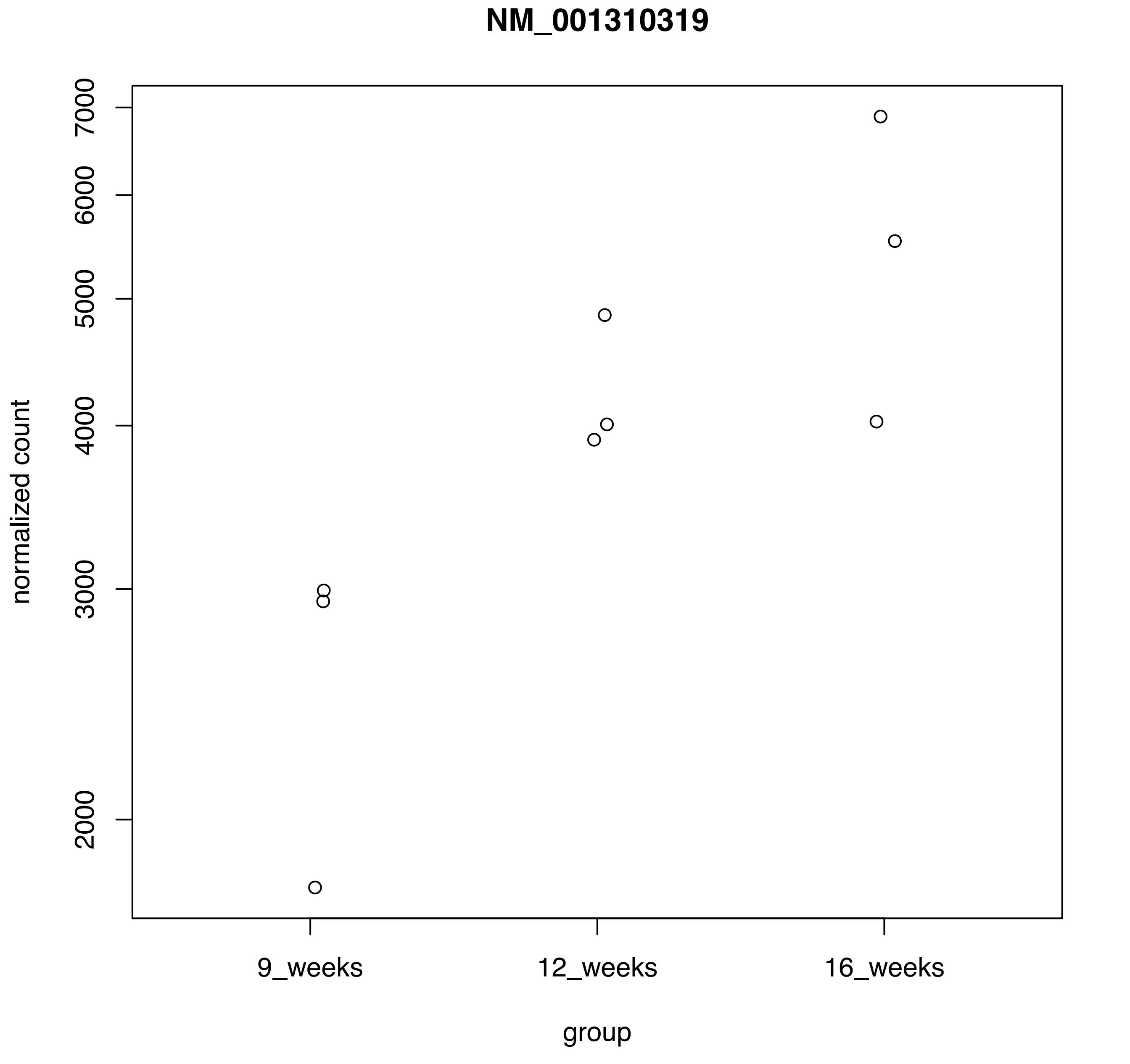
NM_001310319
normalized count
2000
3000
4000
5000
6000
7000
9_weeks
12_weeks
16_weeks
group

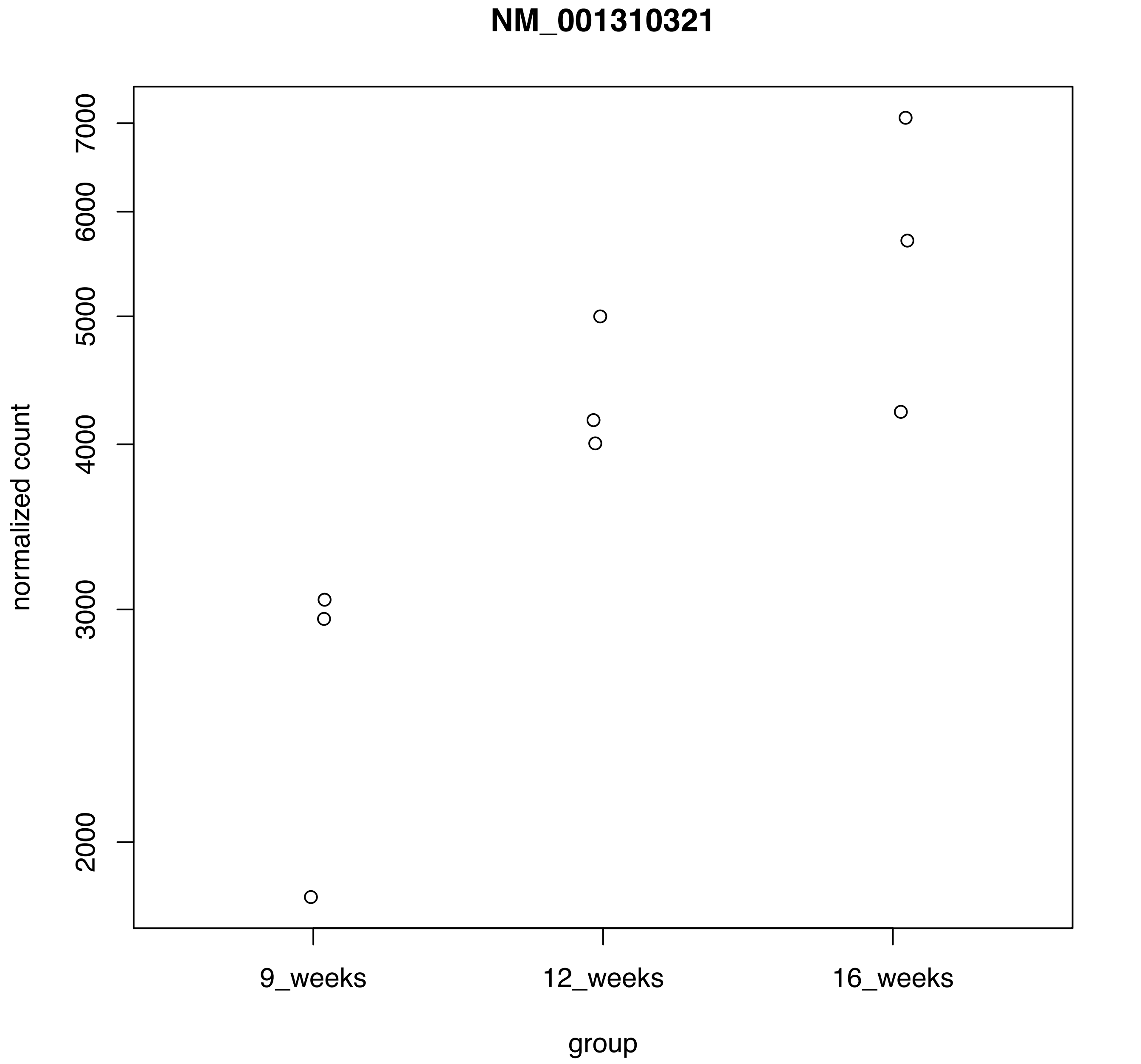
NM_001310321
normalized count
7000
6000
5000
4000
3000
2000
9_weeks
12_weeks
16_weeks
group

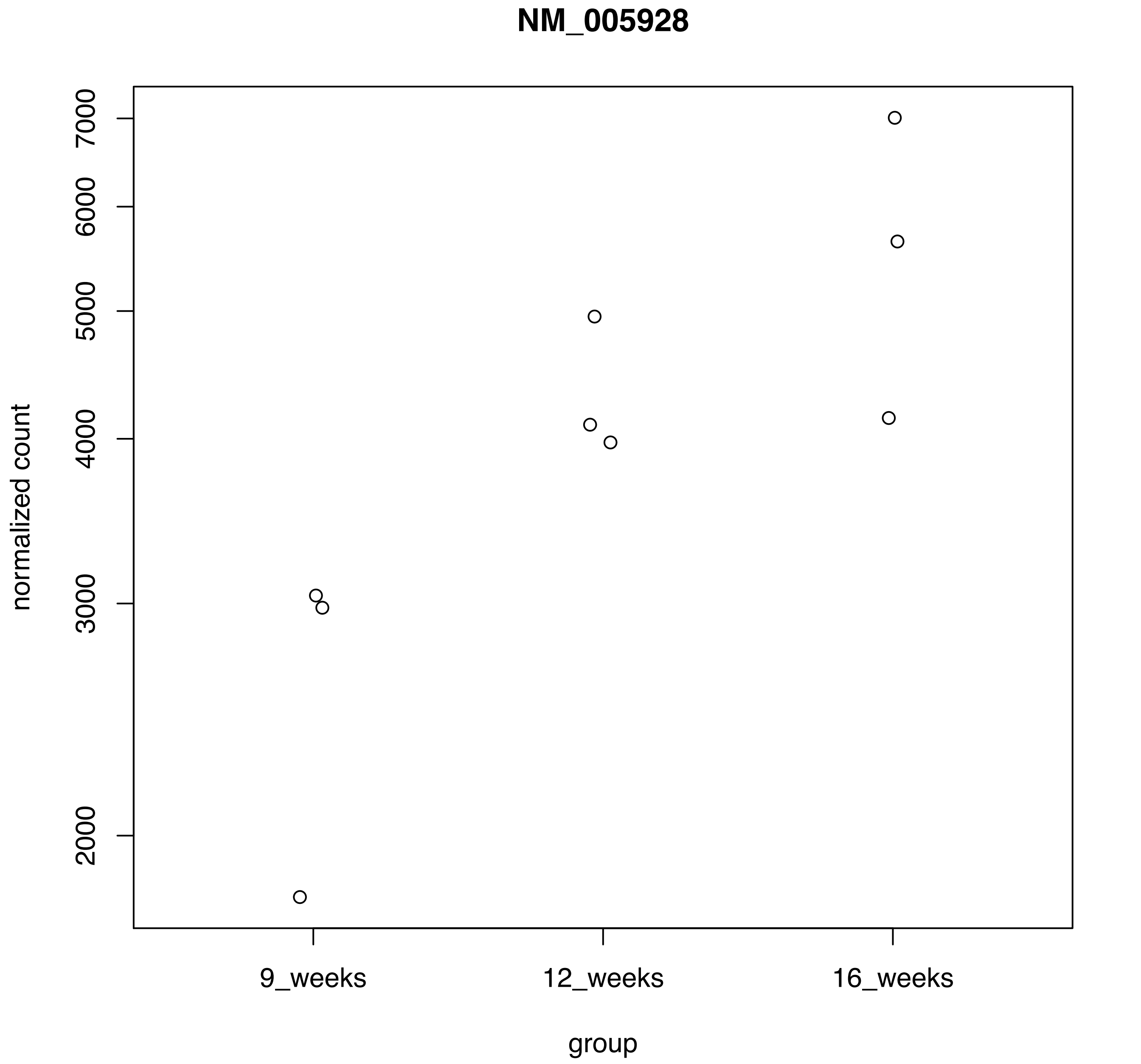
NM_005928
normalized count
7000
6000
5000
4000
3000
2000
9_weeks
12_weeks
16_weeks
group

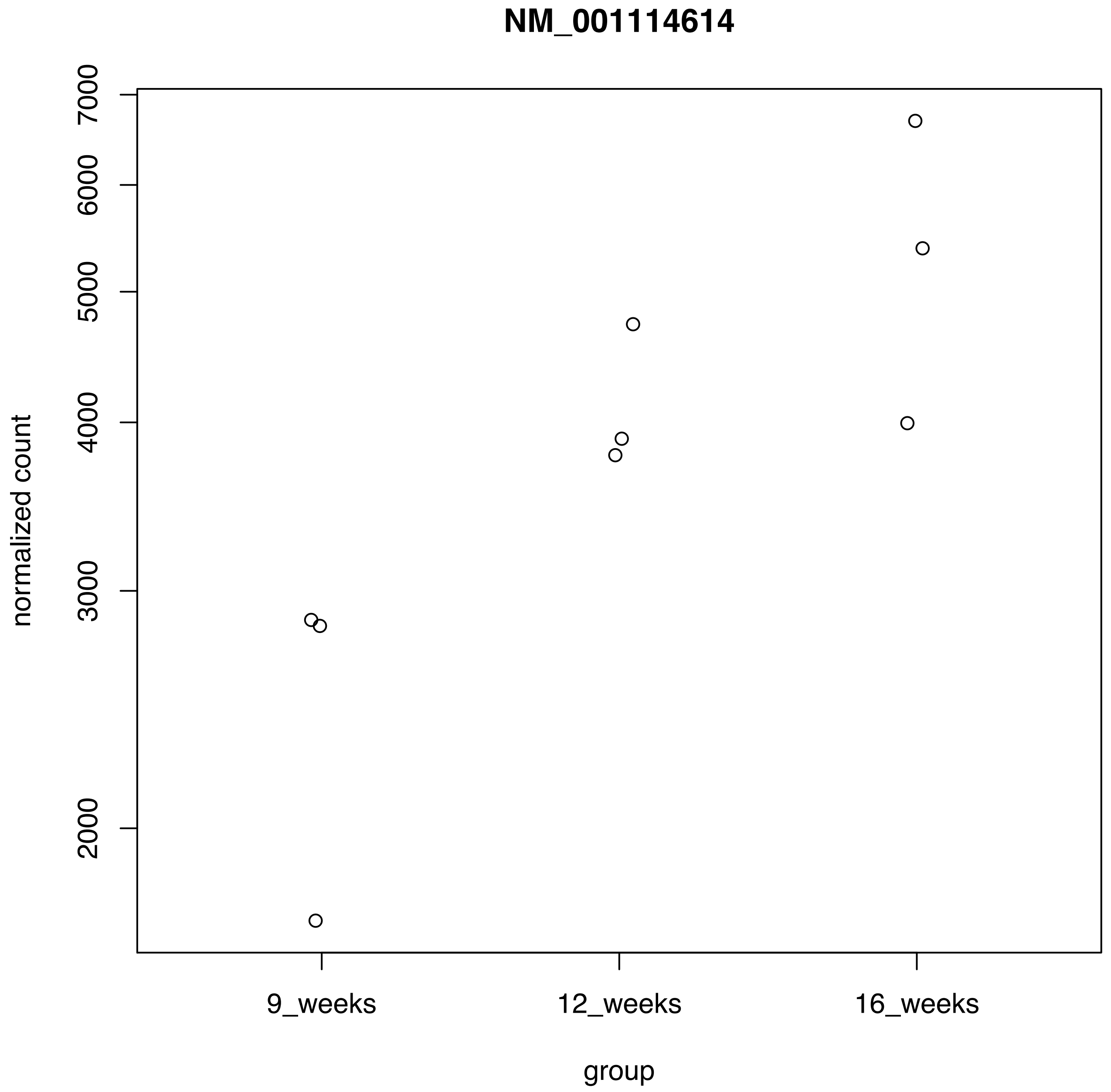
NM_001114614
normalized count
2000
3000
4000
5000
6000
7000
9_weeks
12_weeks
16_weeks
group

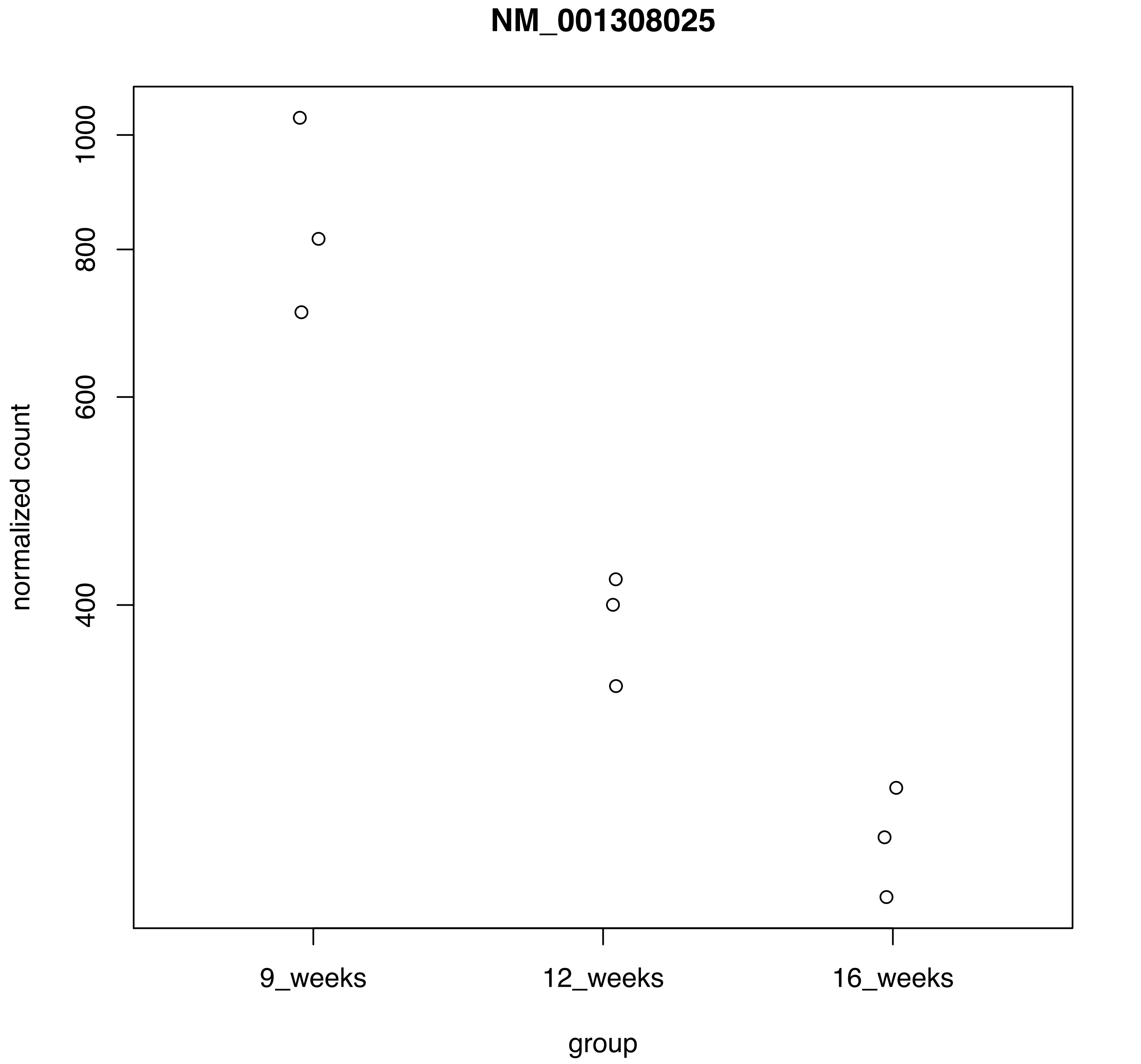
NM_001308025
normalized count
1000
800
600
400
9_weeks
12_weeks
16_weeks
group

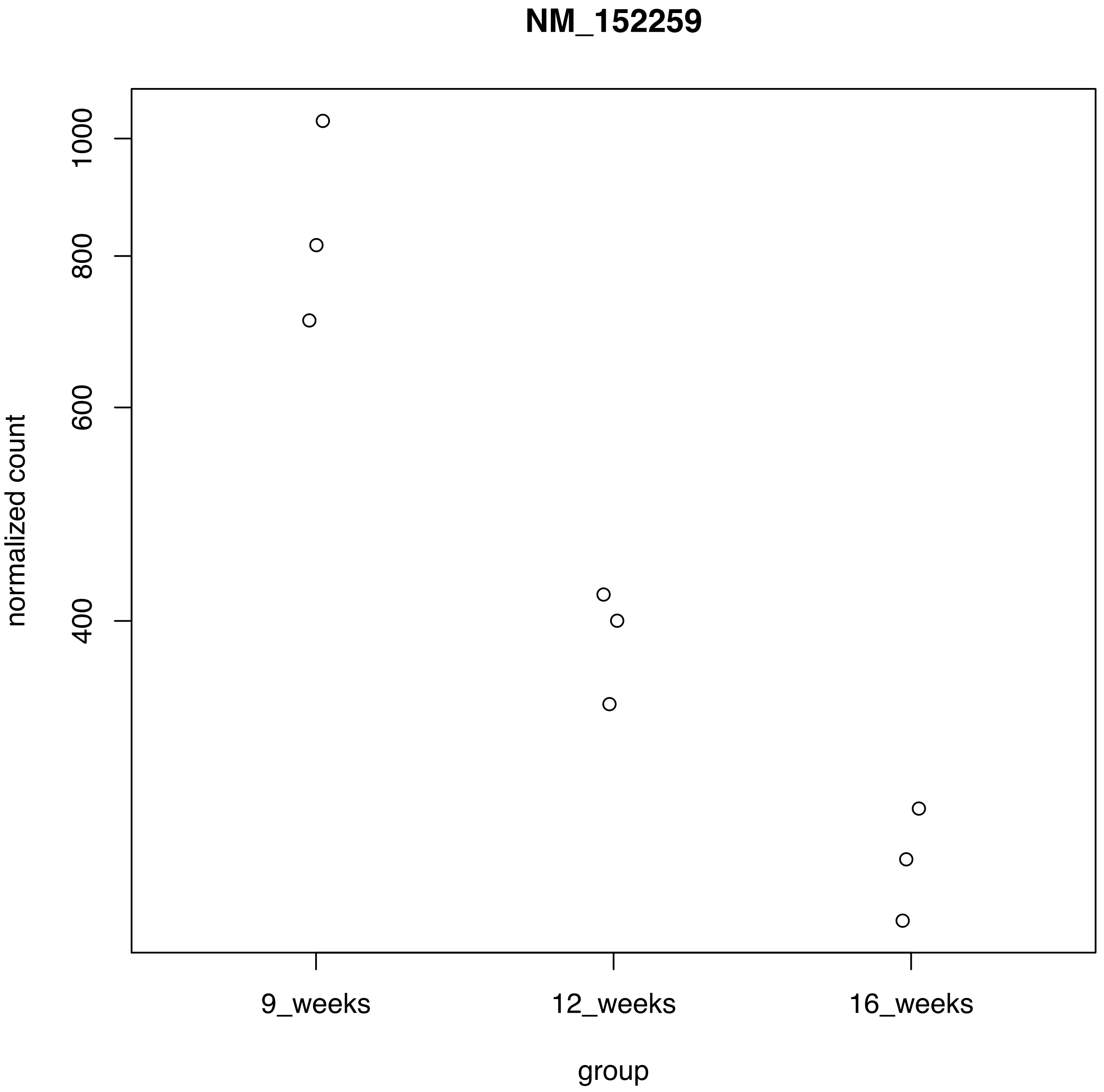
NM_152259
normalized count
1000
800
600
400
9_weeks
12_weeks
16_weeks
group

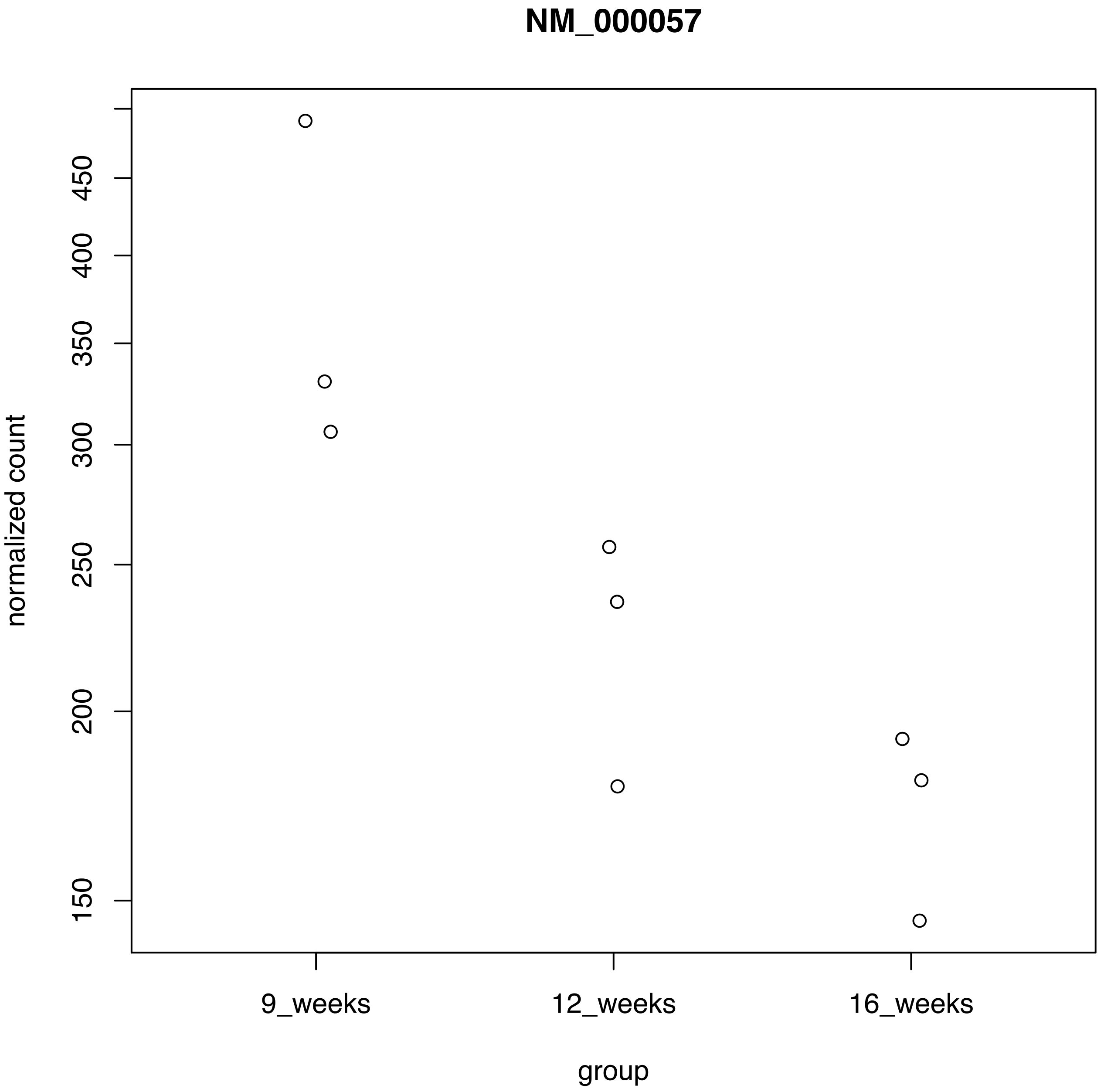
NM_000057
normalized count
150
200
250
300
350
400
450
9_weeks
12_weeks
16_weeks
group

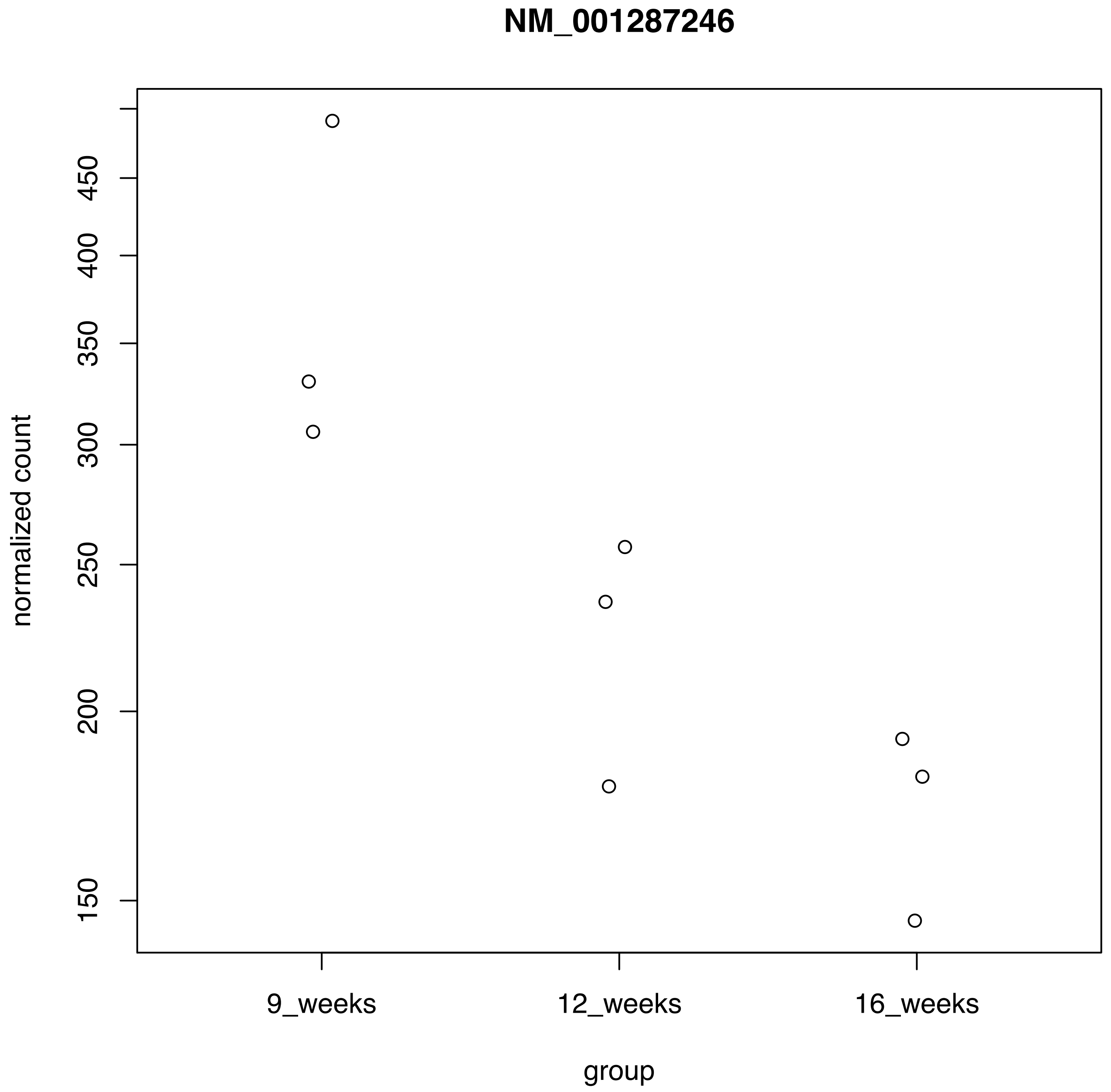
NM_001287246
normalized count
150
200
250
300
350
400
450
9_weeks
12_weeks
16_weeks
group

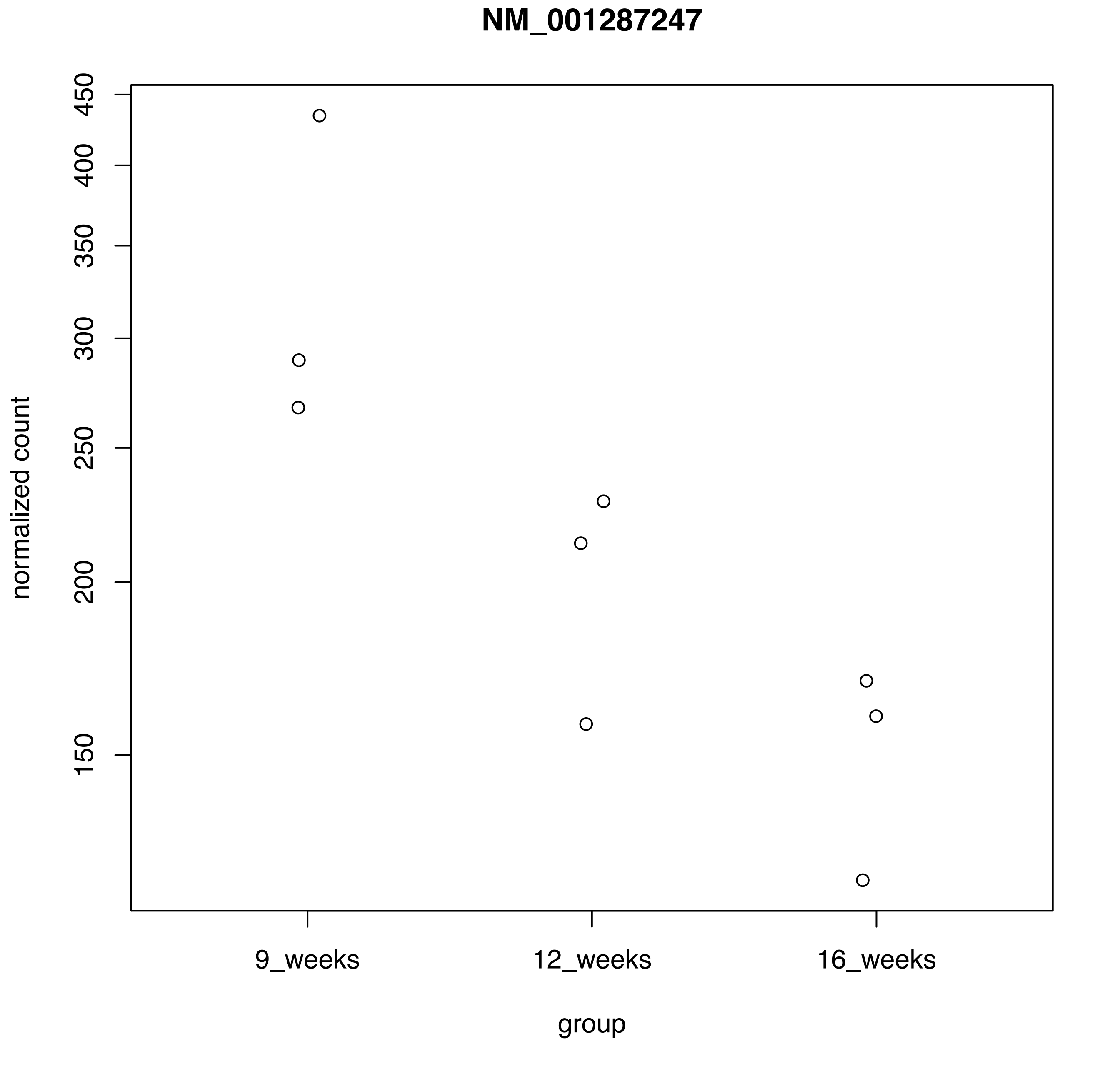
NM_001287247
normalized count
450
400
350
300
250
200
150
9_weeks
12_weeks
16_weeks
group

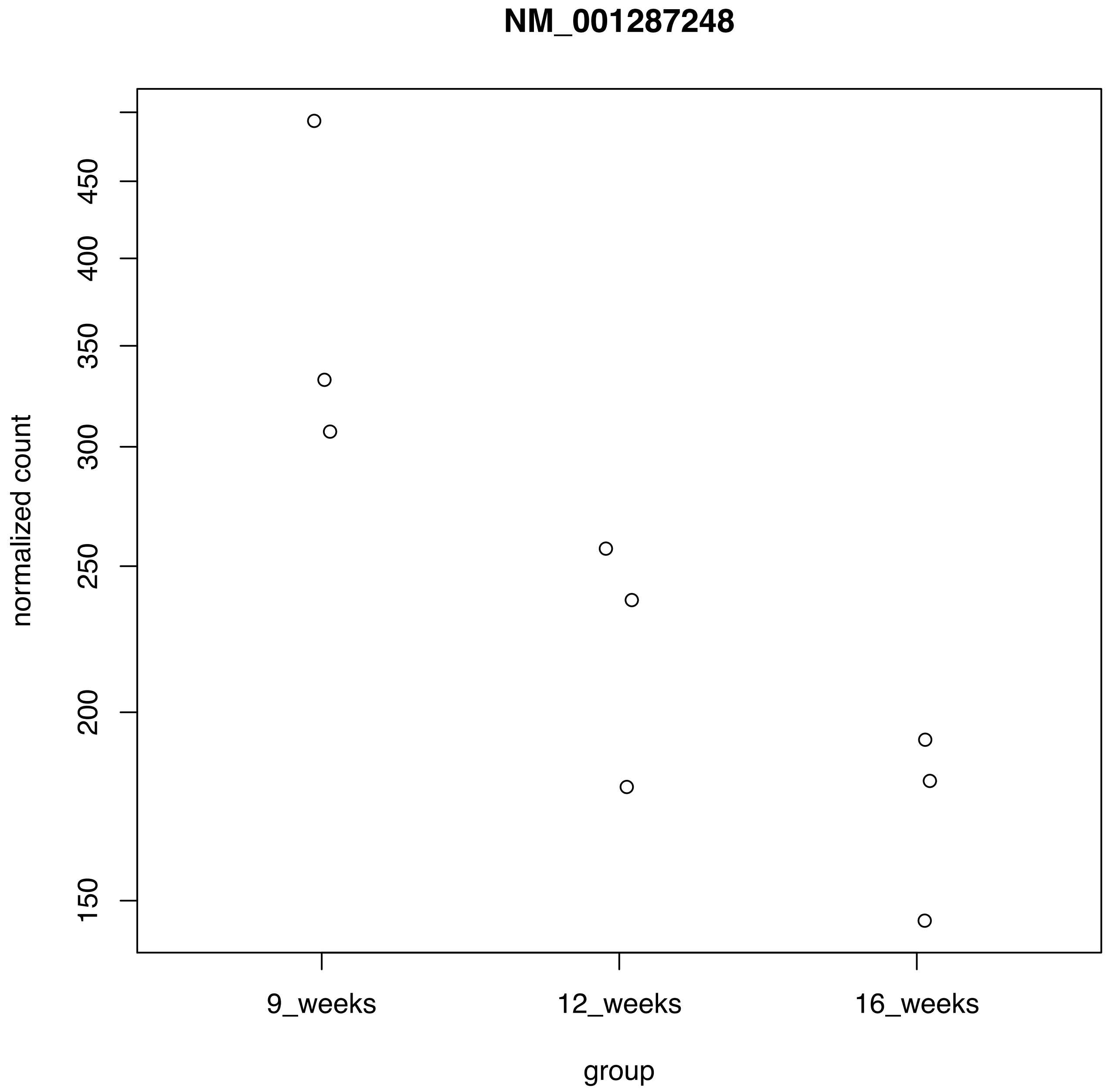
NM_001287248
normalized count
450
400
350
300
250
200
150
9_weeks
12_weeks
16_weeks
group

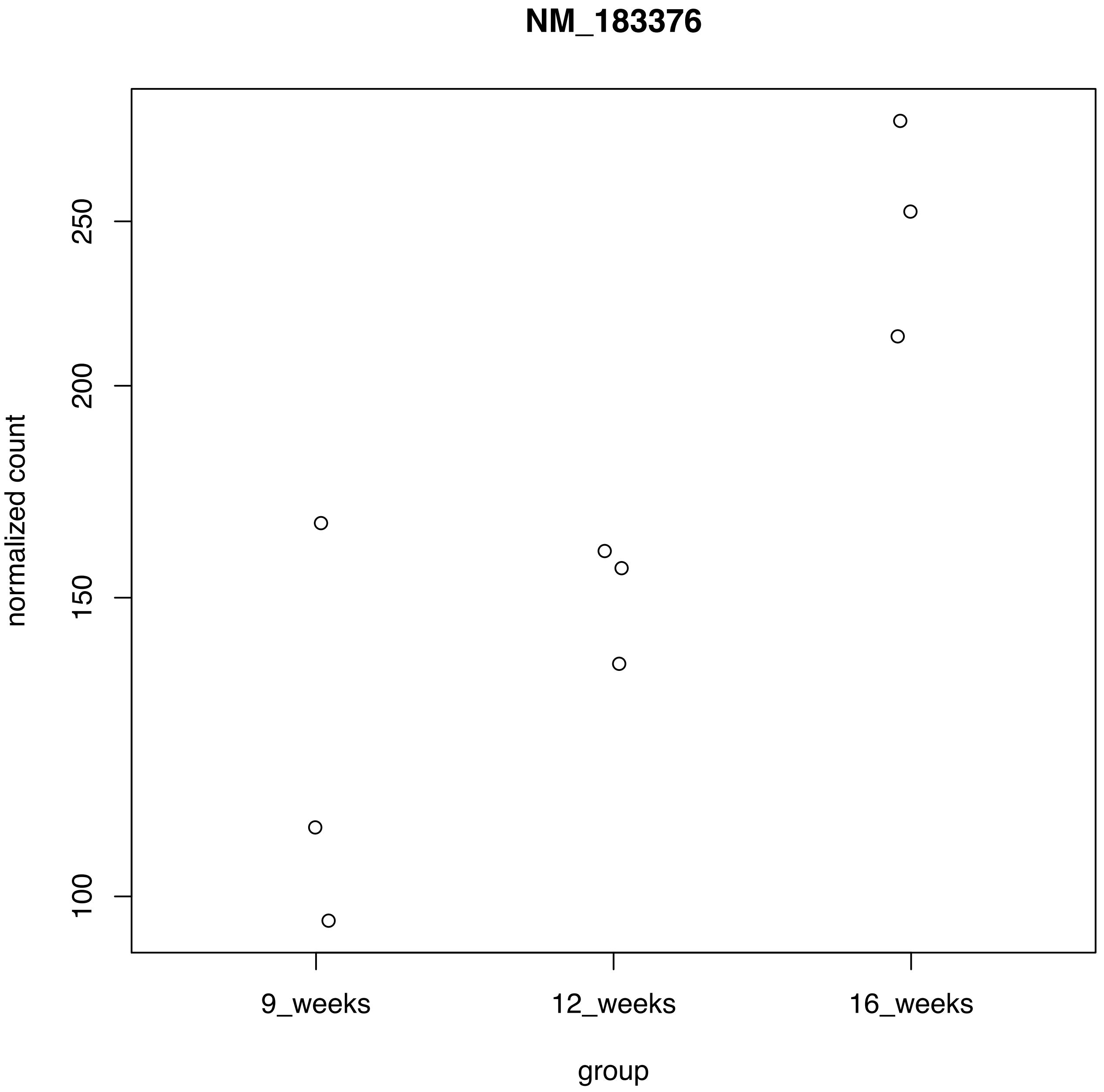
NM_183376
normalized count
250
200
150
100
9_weeks
12_weeks
16_weeks
group

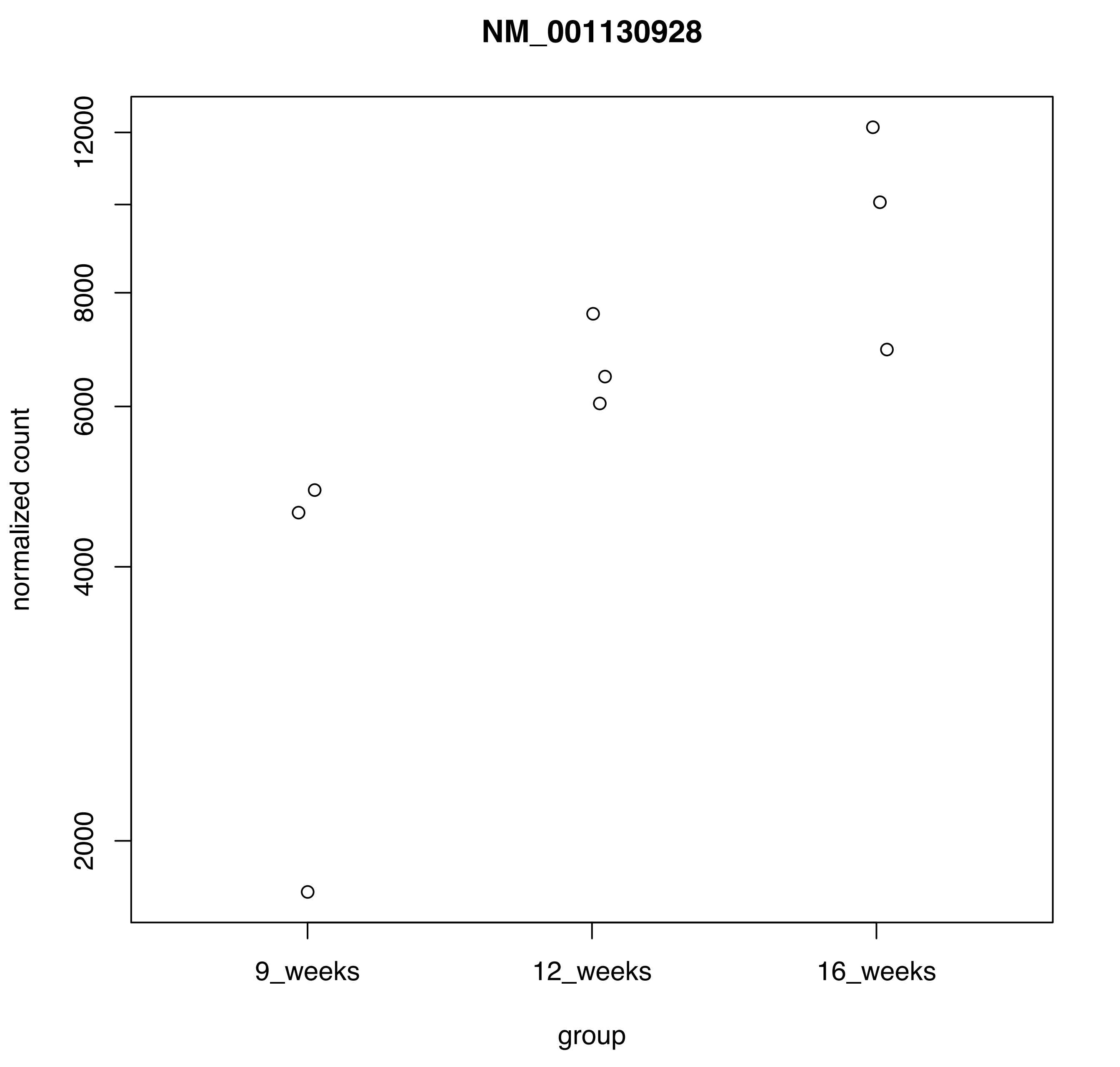
NM_001130928
normalized count
2000
4000
6000
8000
12000
group
9_weeks
12_weeks
16_weeks

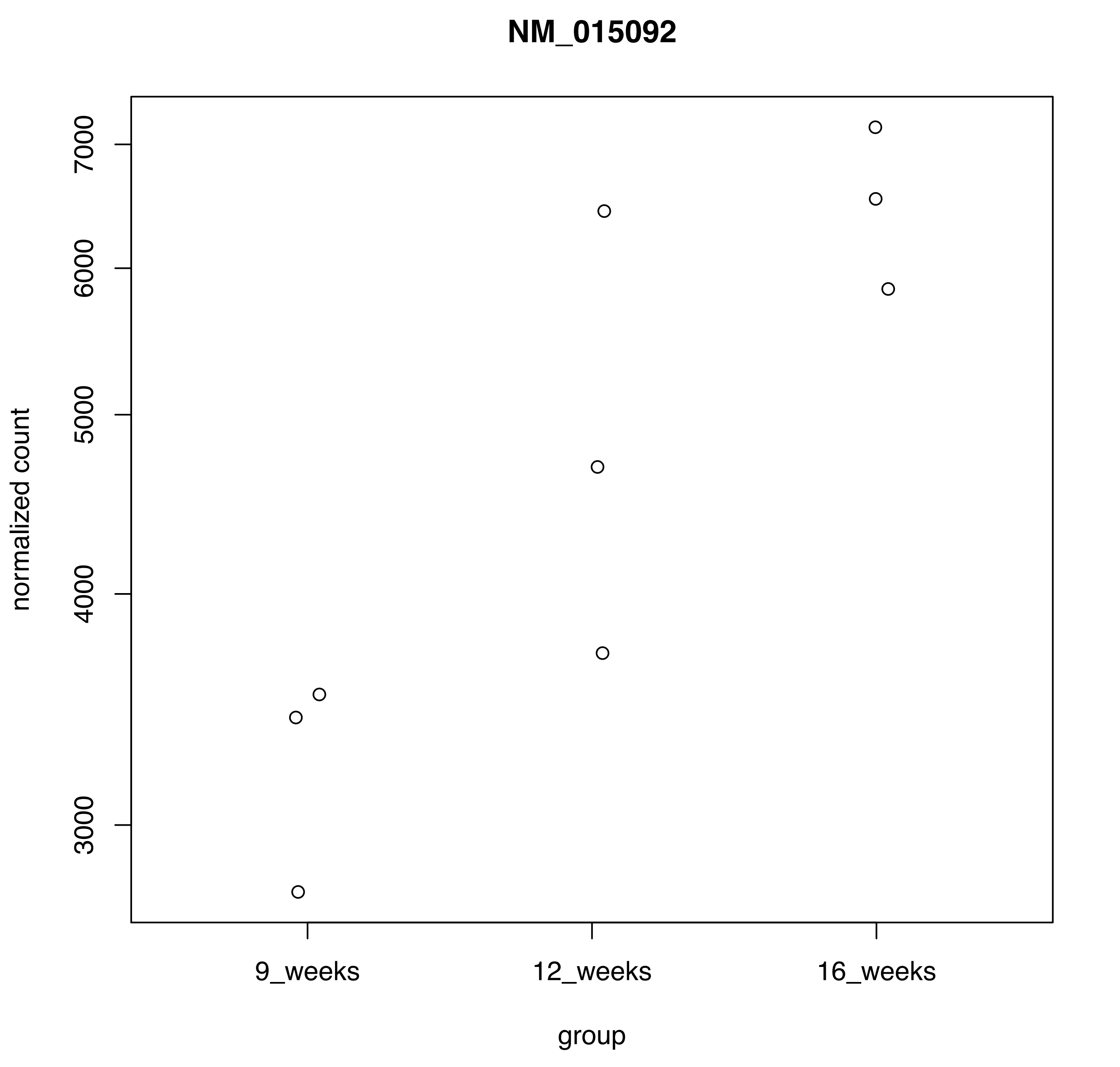

NM_015092
normalized count
7000
6000
5000
4000
3000
9_weeks
12_weeks
16_weeks
group

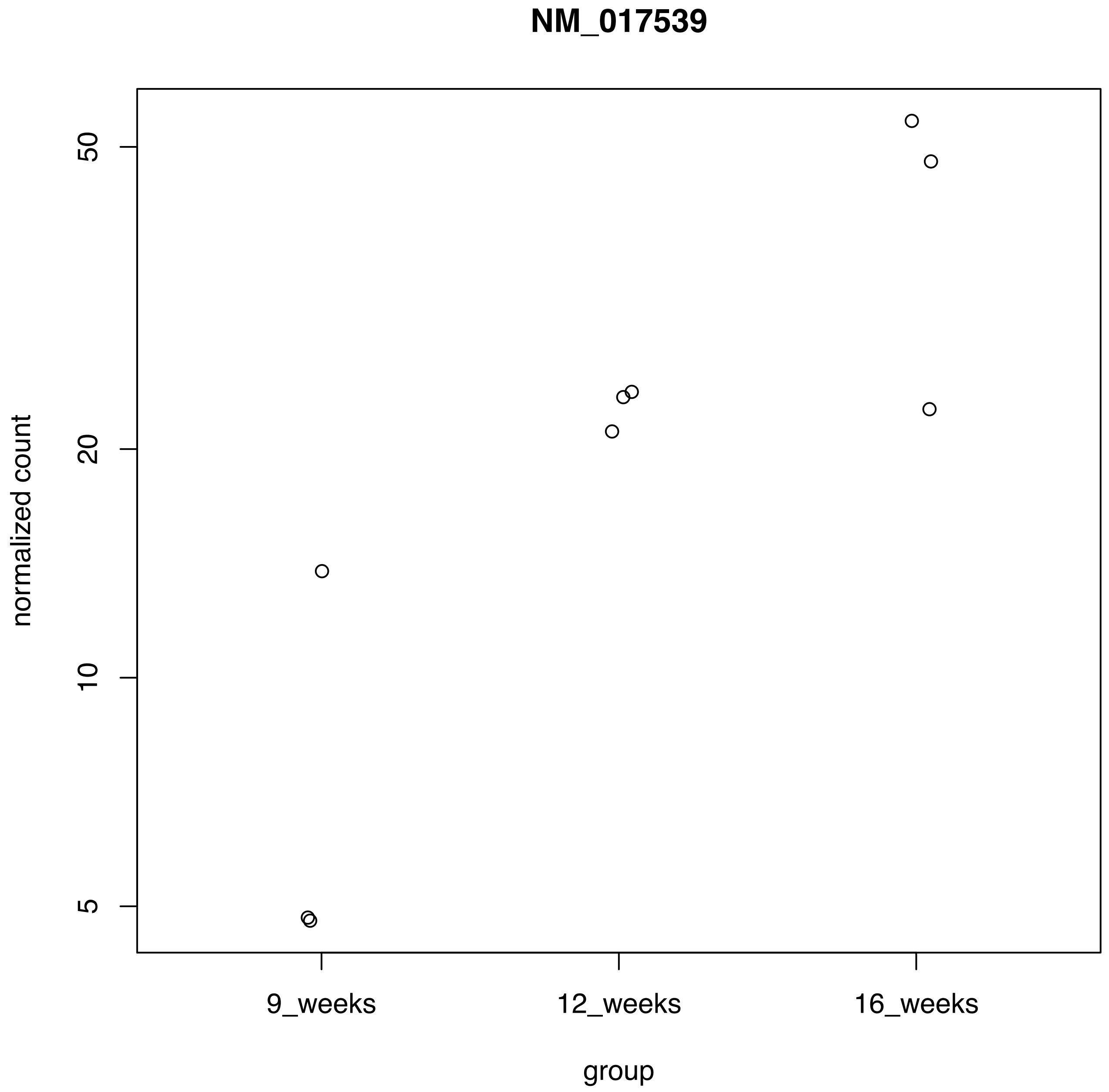
NM_017539
normalized count
50
20
10
5
9_weeks
12_weeks
16_weeks
group

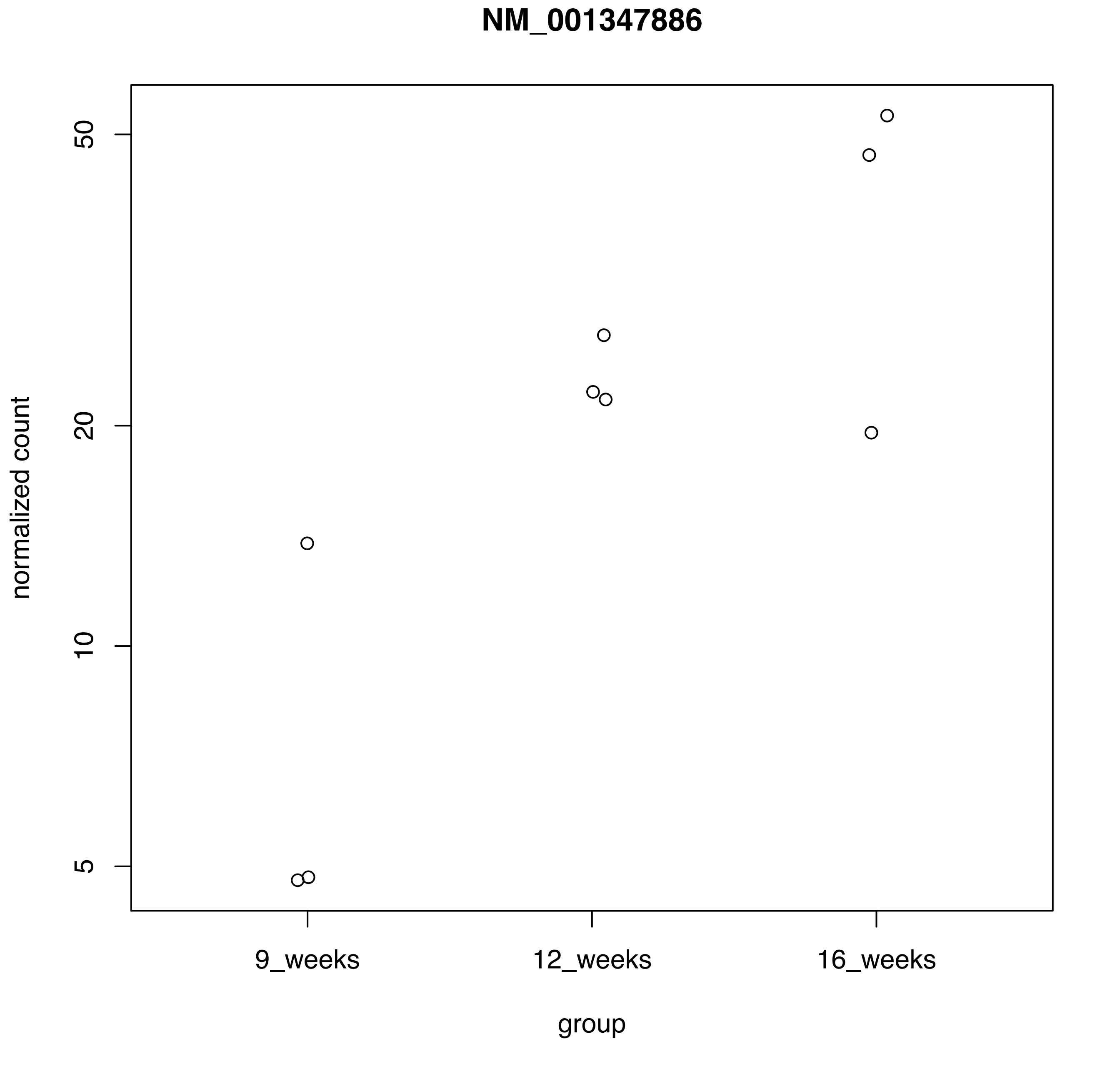

NM_001347886
normalized count
50
20
10
5
9_weeks
12_weeks
16_weeks
group

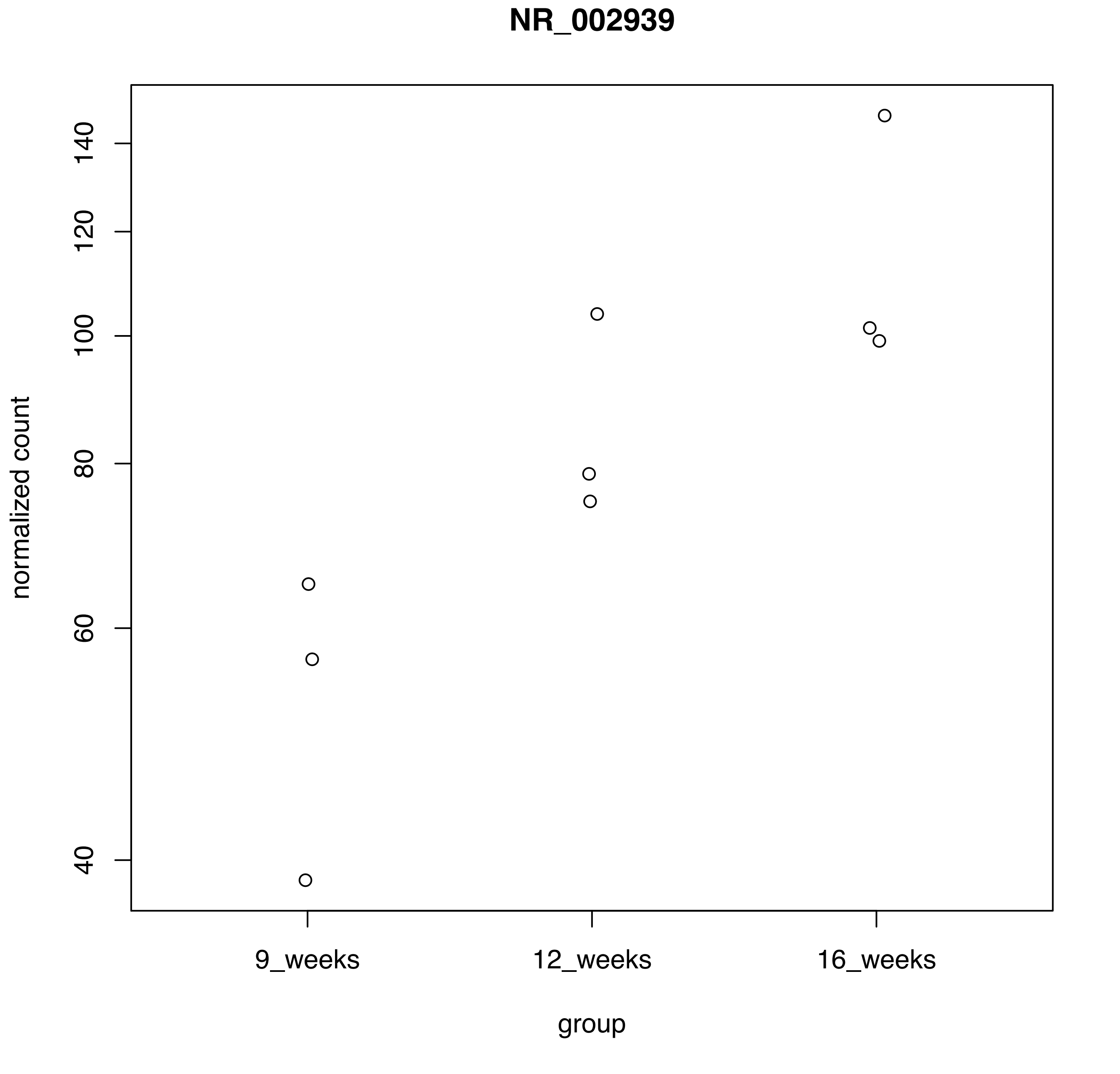
NR_002939
normalized count
140
120
100
80
60
40
9_weeks
12_weeks
16_weeks
group

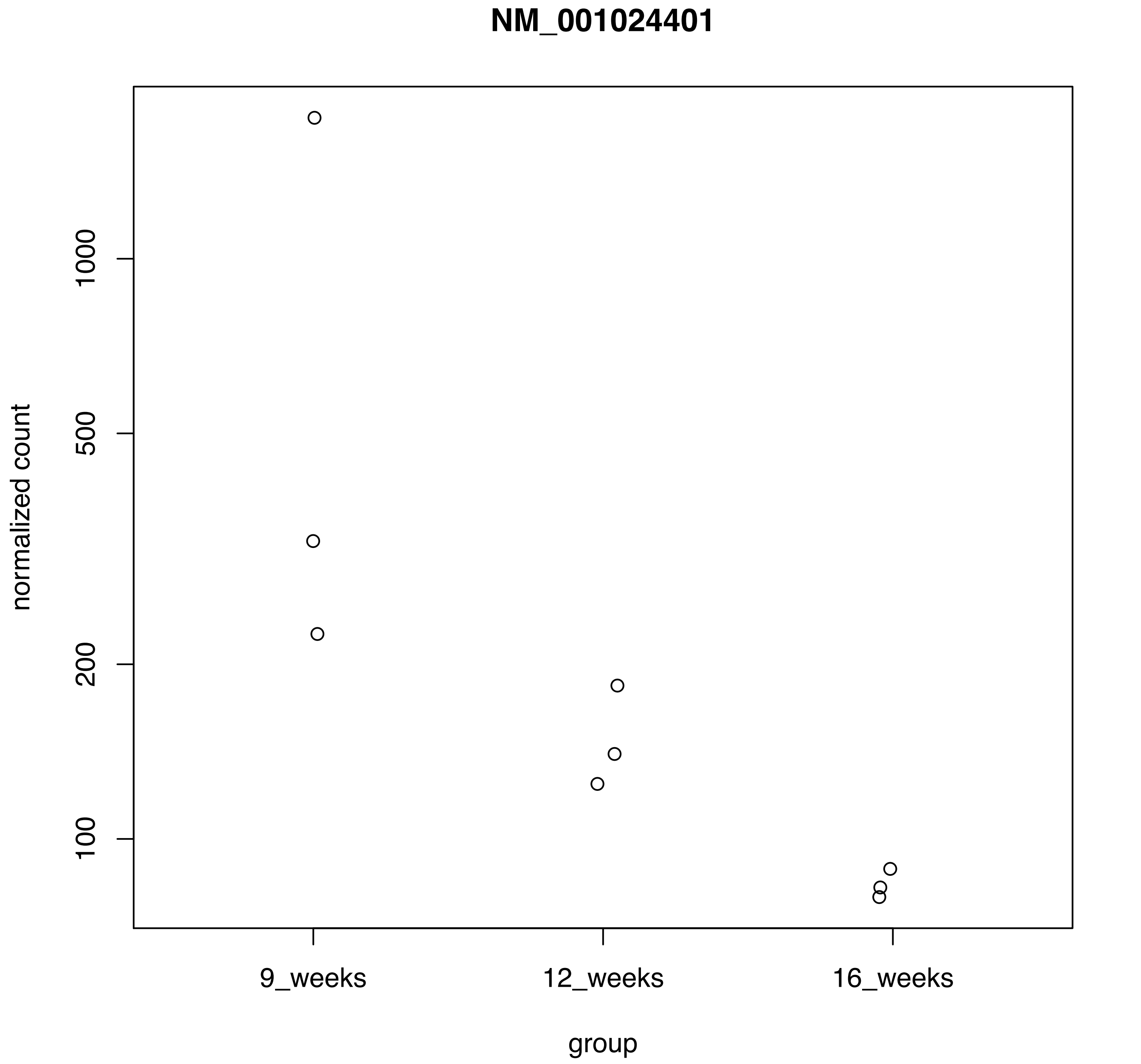
NM_001024401
normalized count
1000
500
200
100
9_weeks
12_weeks
16_weeks
group

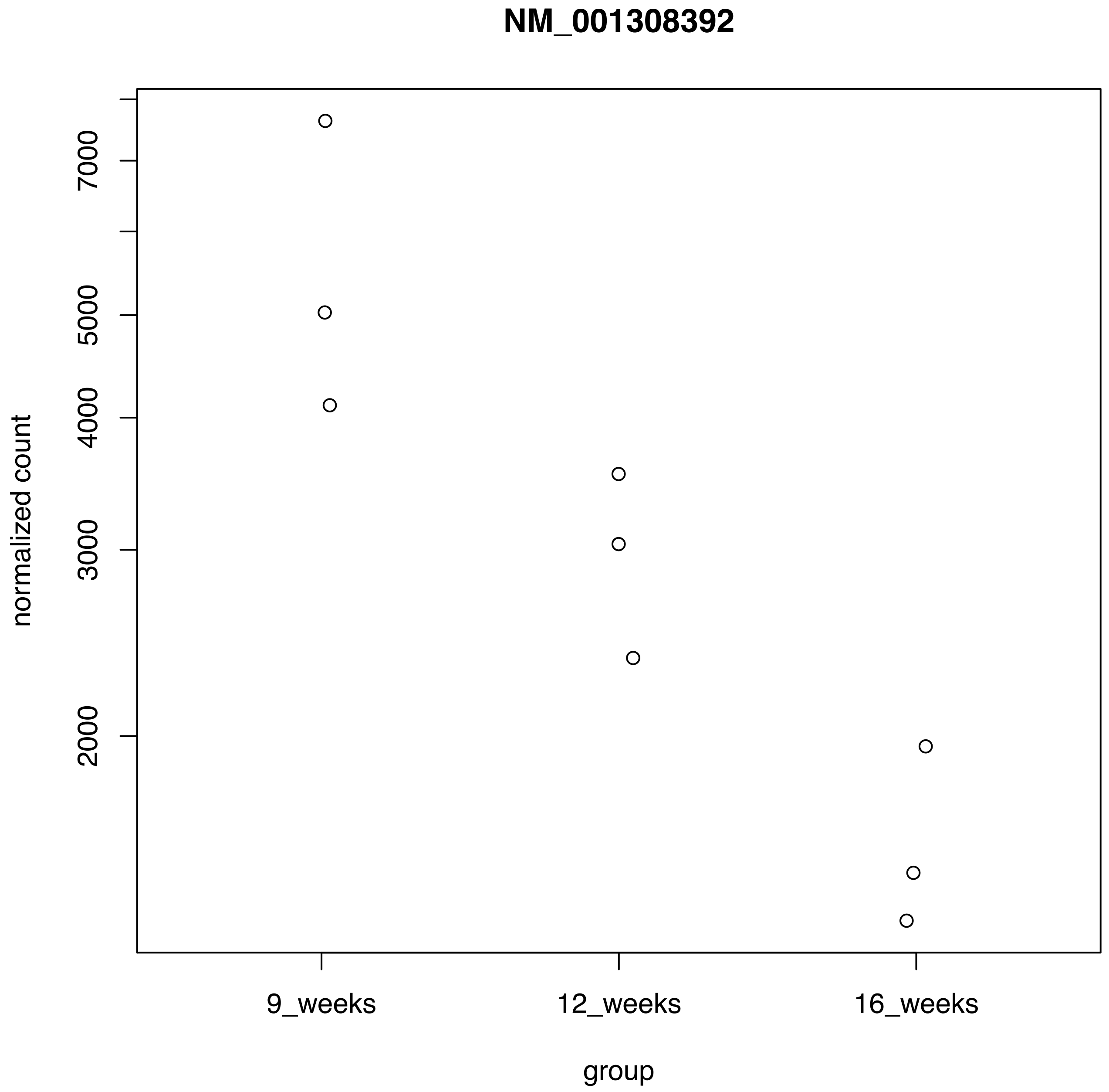
NM_001308392
normalized count
7000
5000
4000
3000
2000
9_weeks
12_weeks
16_weeks
group

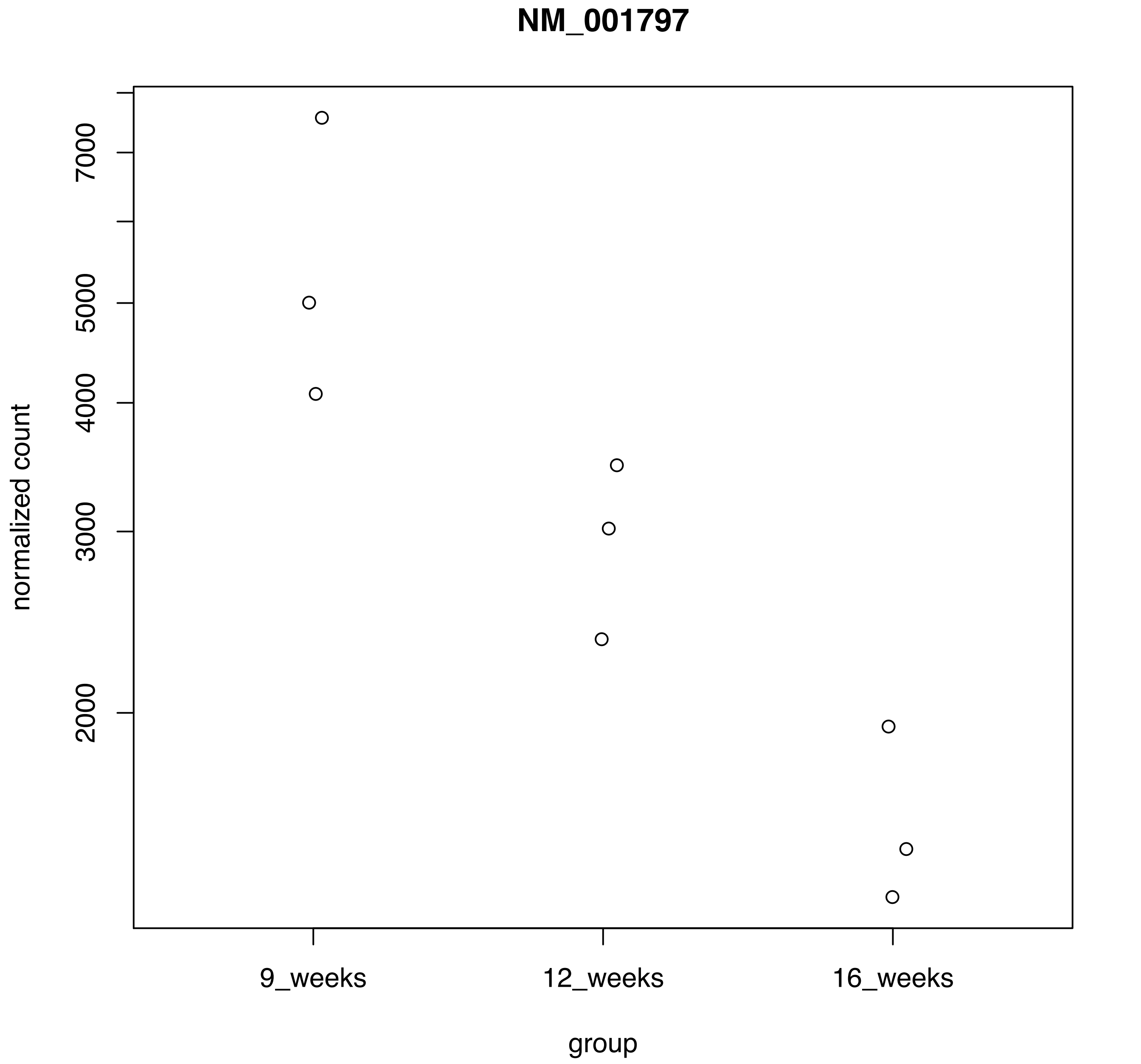
NM_001797
normalized count
2000
3000
4000
5000
7000
9_weeks
12_weeks
16_weeks
group

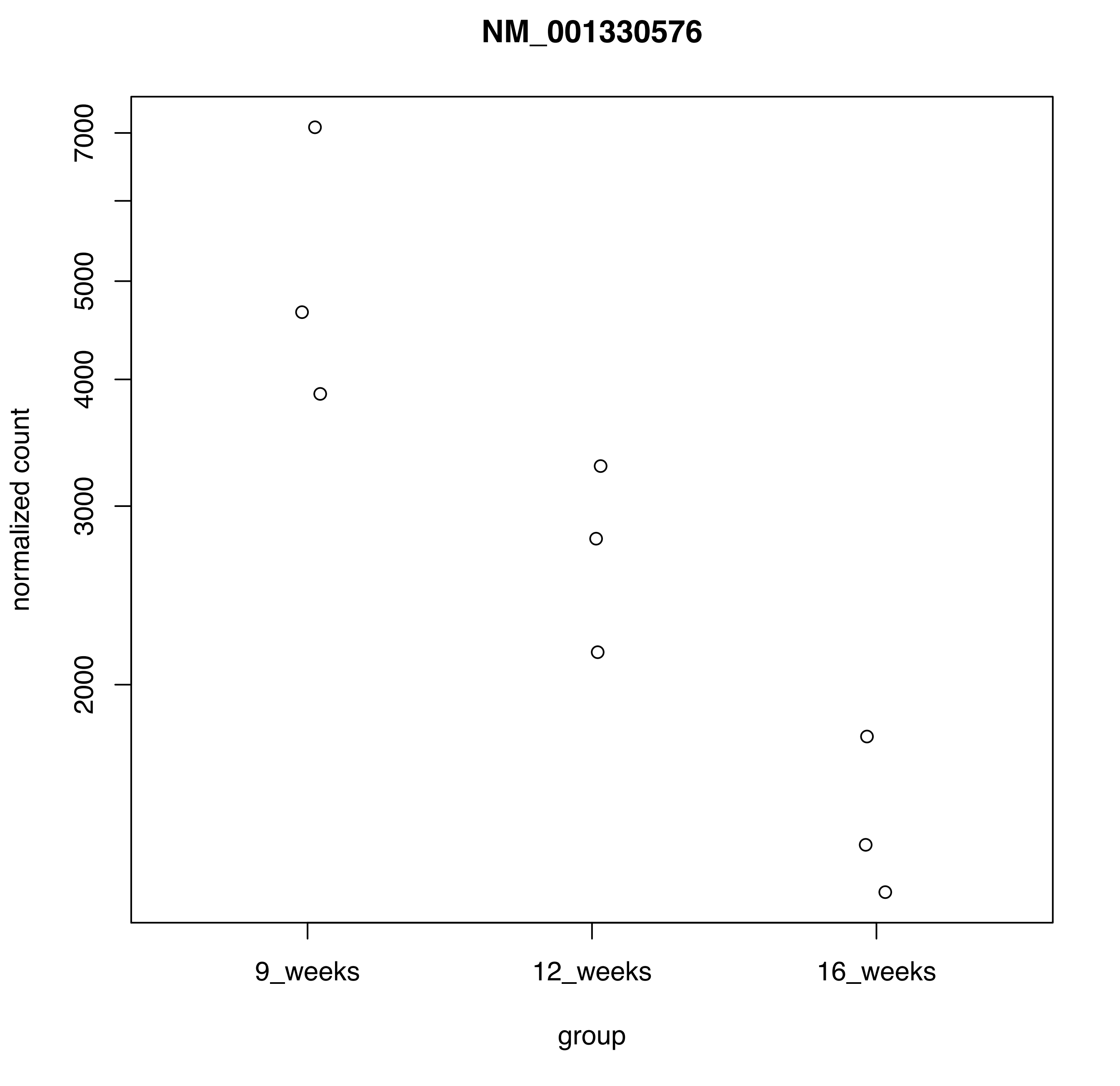
NM_001330576
normalized count
7000
5000
4000
3000
2000
9_weeks
12_weeks
16_weeks
group

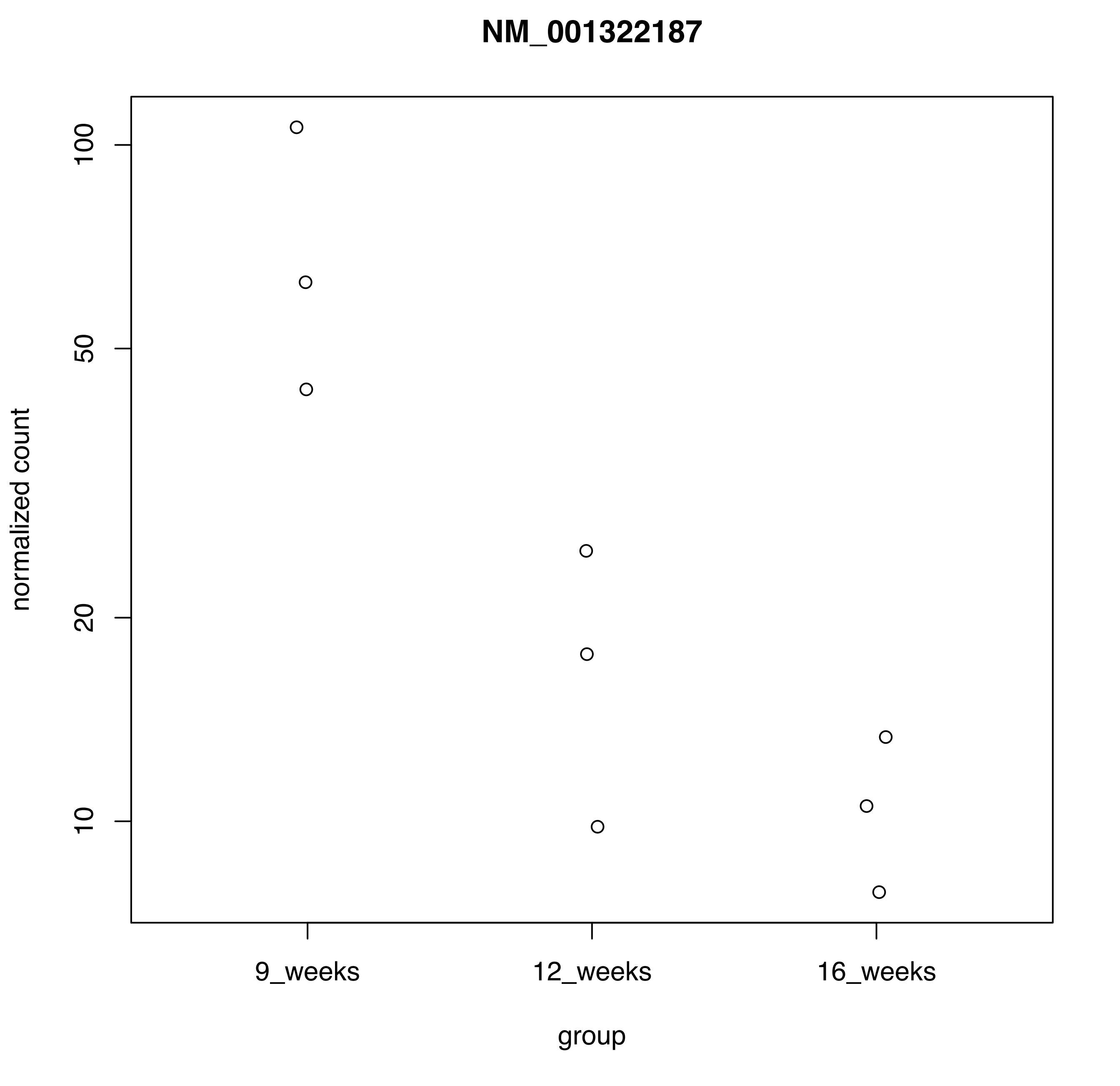
NM_001322187
normalized count
100
50
20
10
9_weeks
12_weeks
16_weeks
group

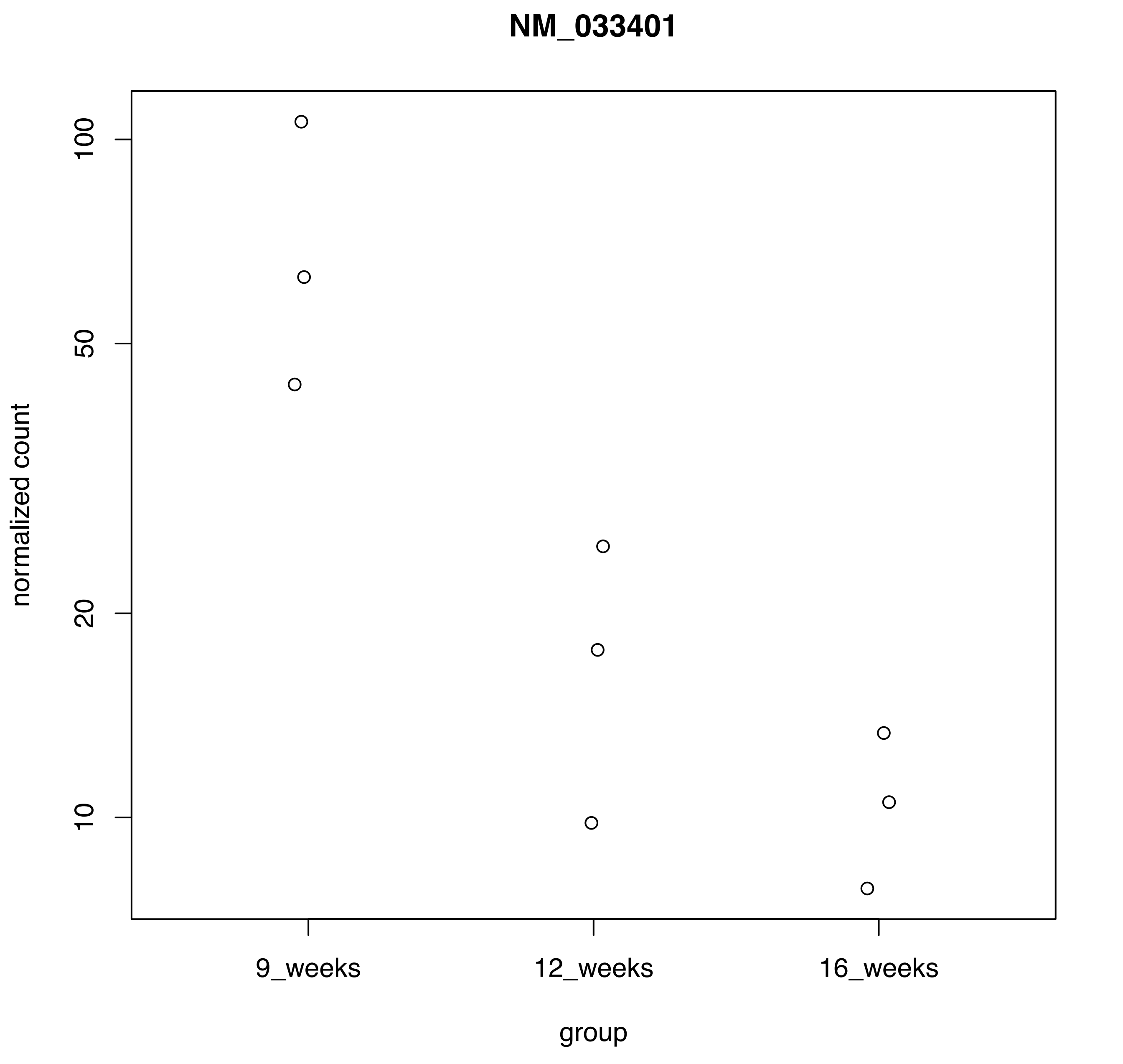
NM_033401
normalized count
100
50
20
10
9_weeks
12_weeks
16_weeks
group

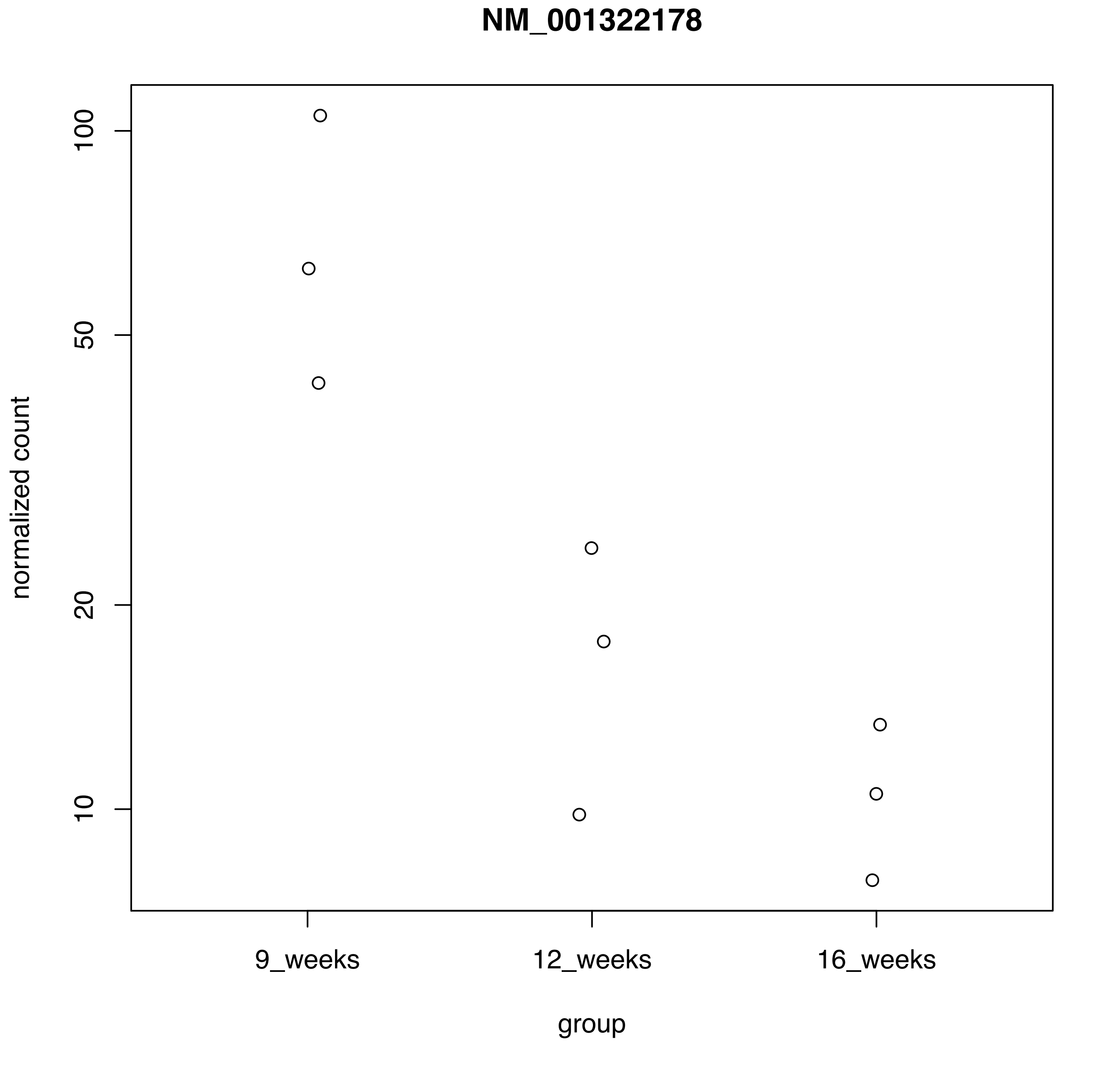

NM_001322178
normalized count
100
50
20
10
9_weeks
12_weeks
16_weeks
group

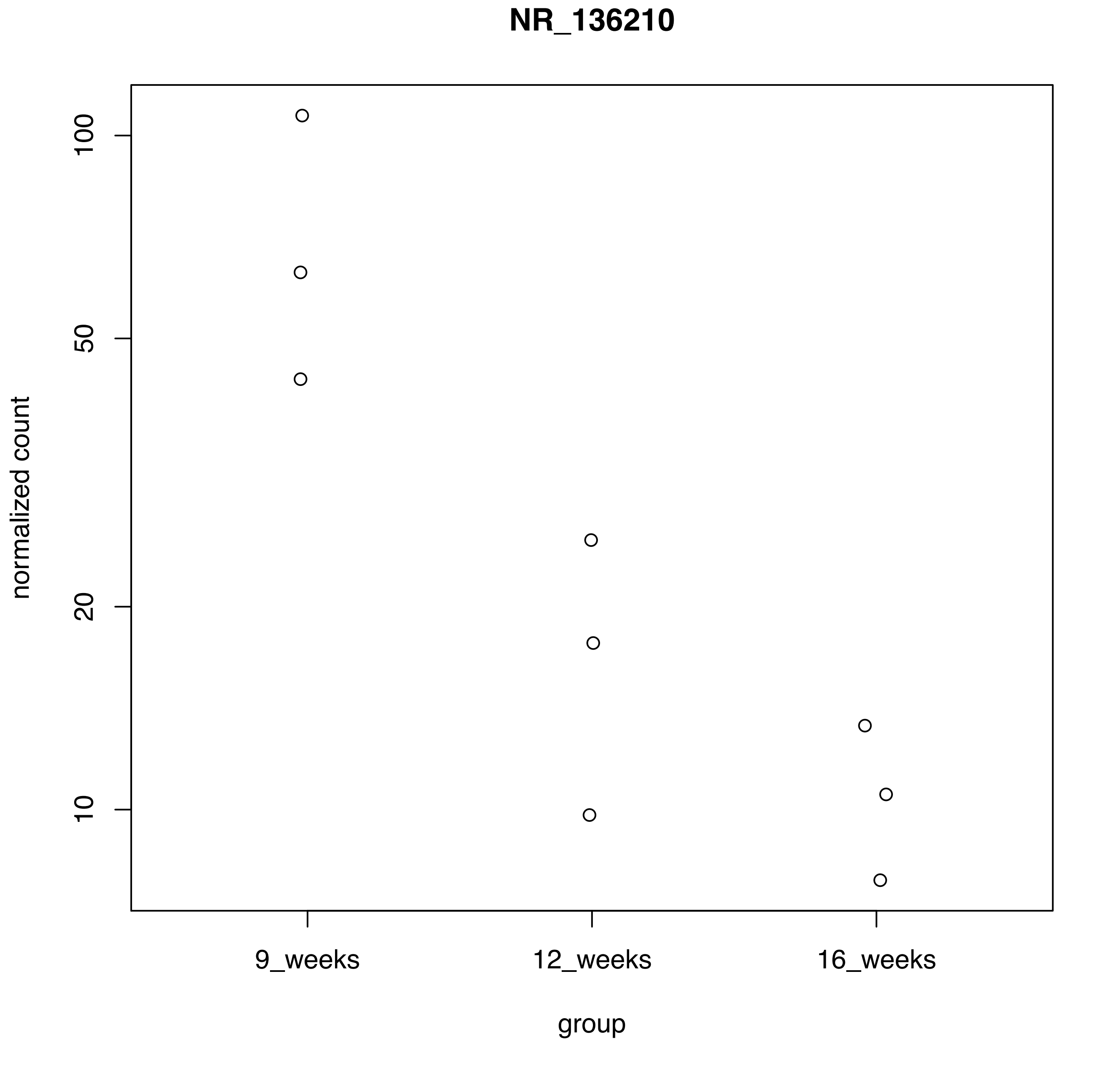
NR_136210
normalized count
100
50
20
10
9_weeks
12_weeks
16_weeks
group

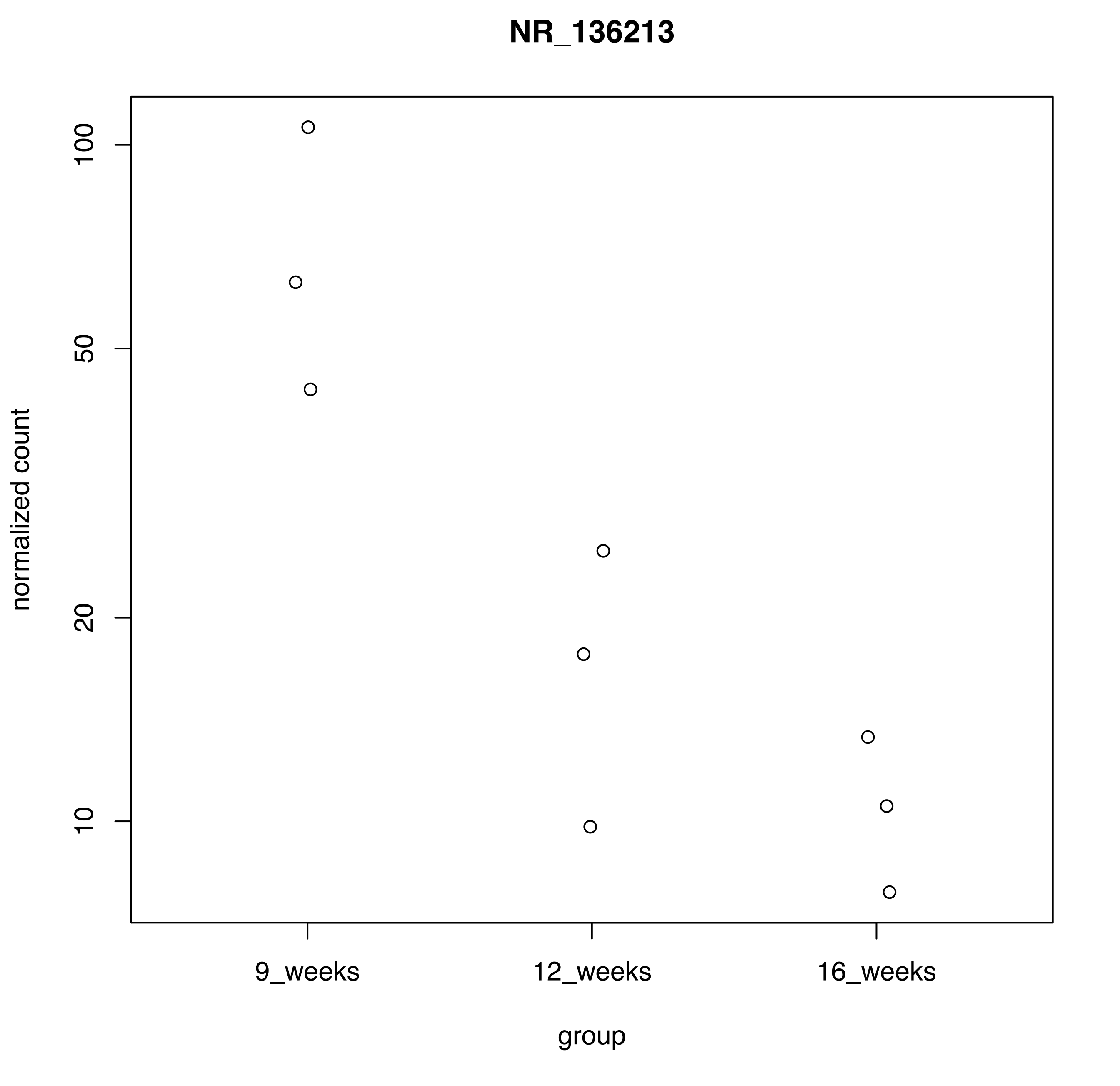
NR_136213
normalized count
100
50
20
10
9_weeks
12_weeks
16_weeks
group

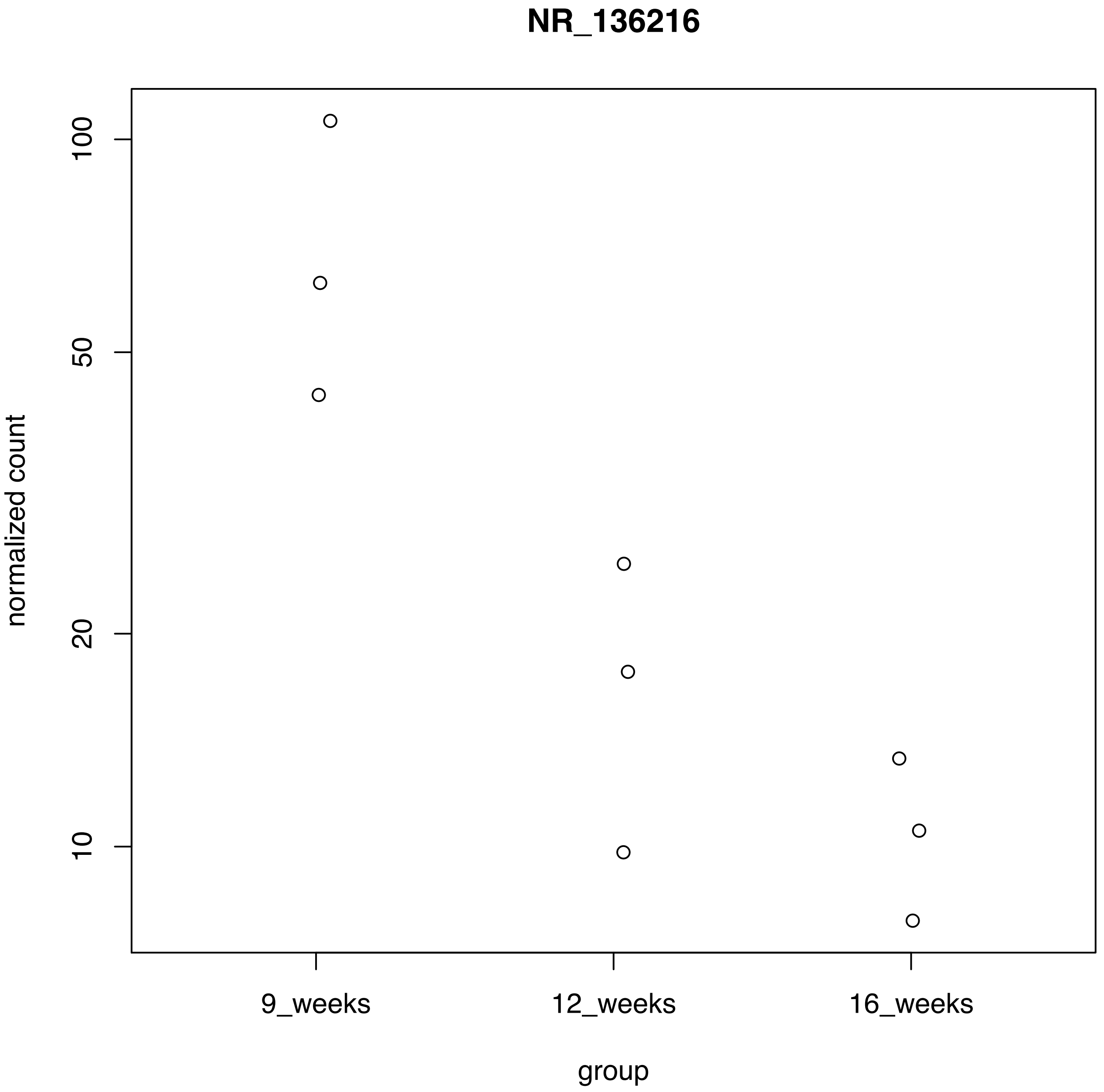
NR_136216
normalized count
100
50
20
10
9_weeks
12_weeks
16_weeks
group

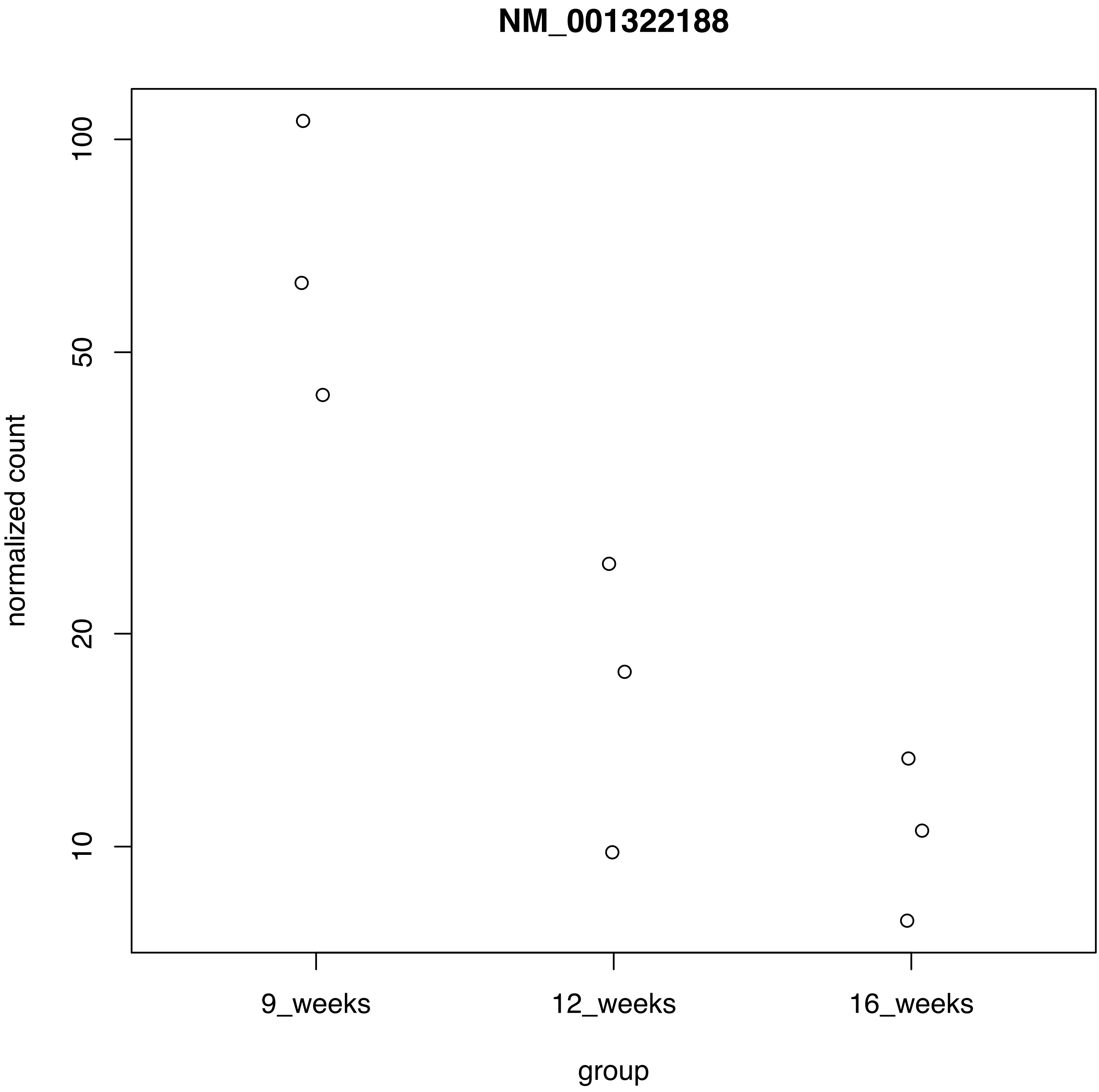
NM_001322188
normalized count
100
50
20
10
9_weeks
12_weeks
16_weeks
group

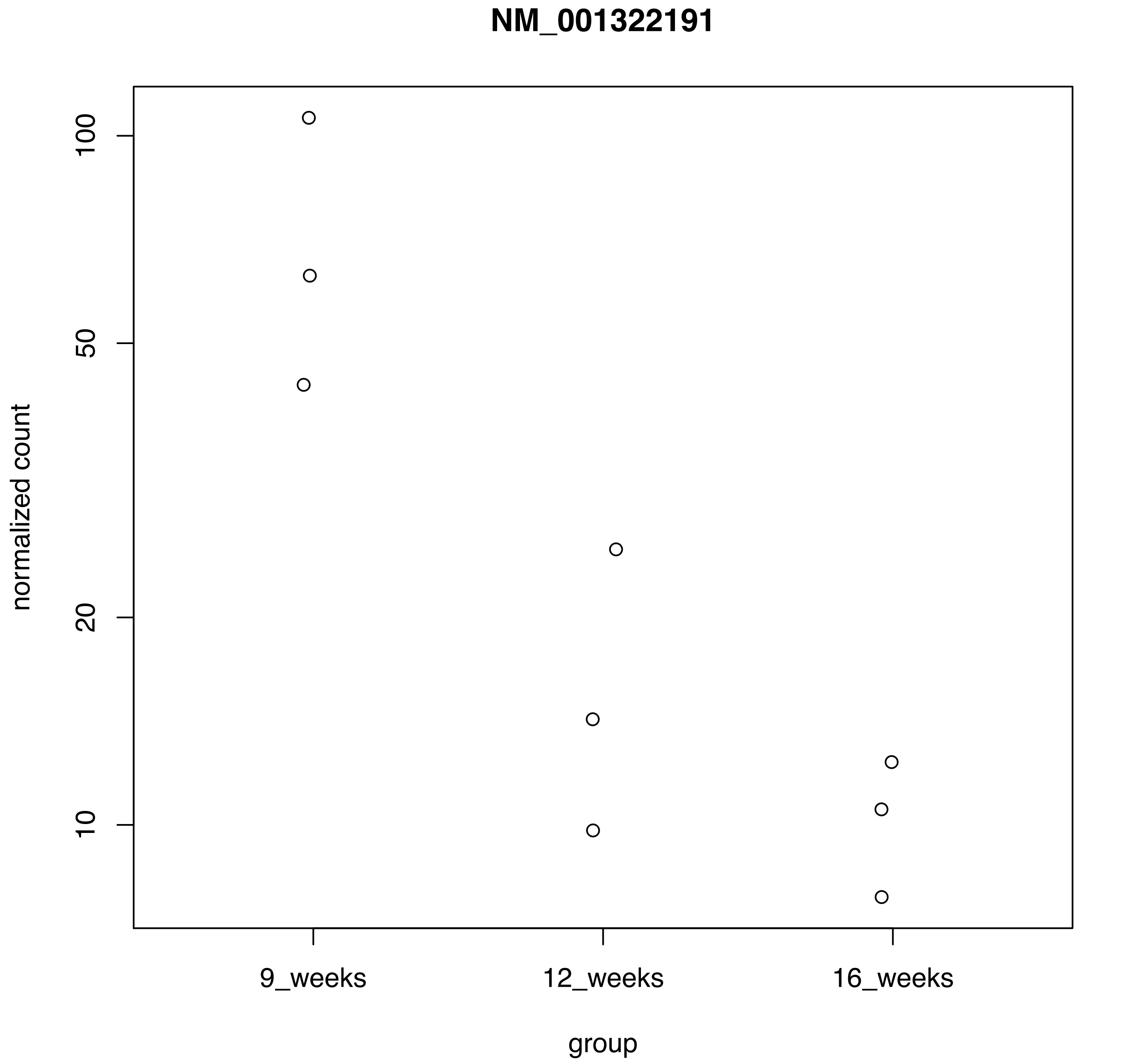

NM_001322191
normalized count
100
50
20
10
9_weeks
12_weeks
16_weeks
group

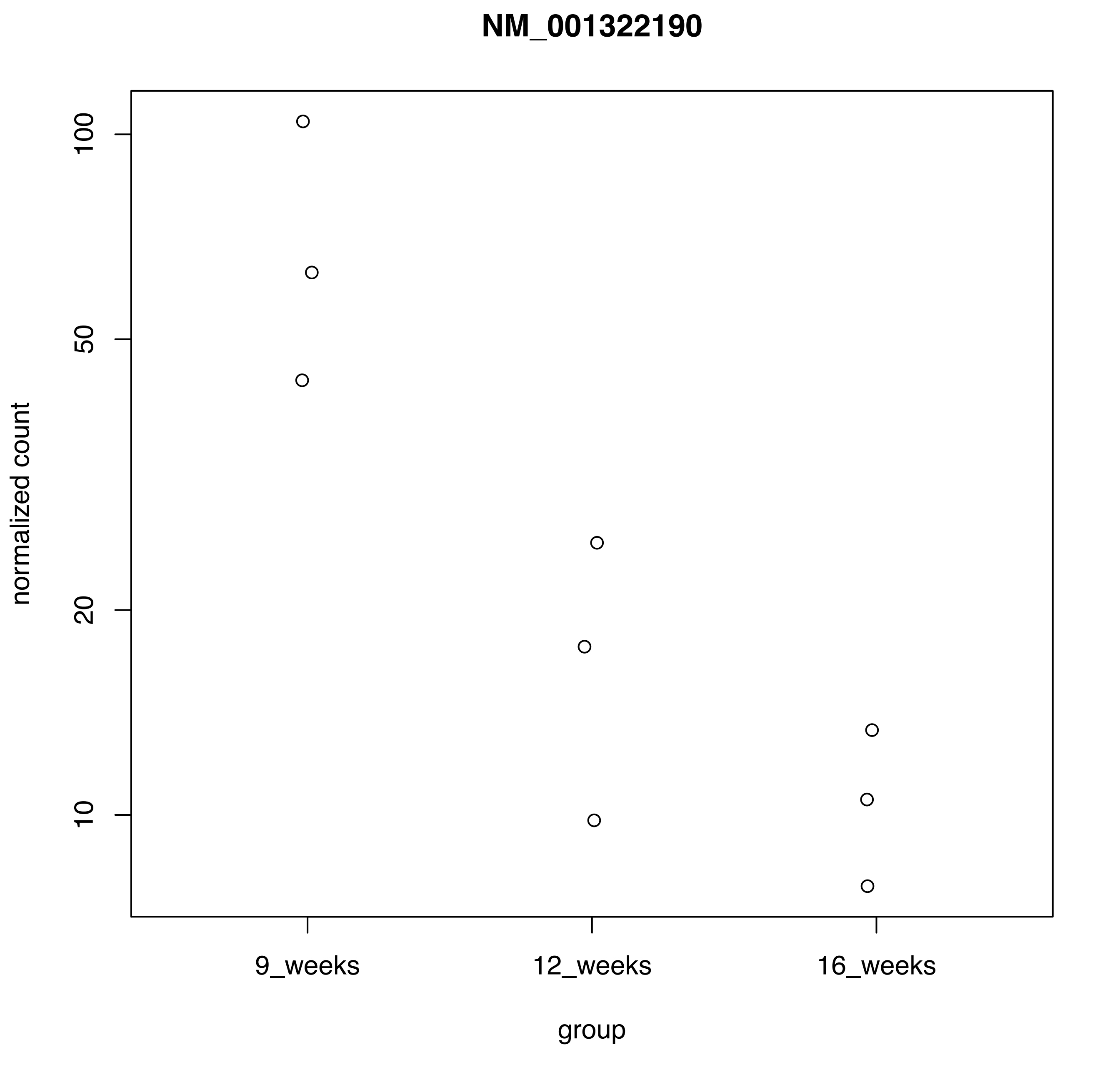
NM_001322190
normalized count
100
50
20
10
9_weeks
12_weeks
16_weeks
group

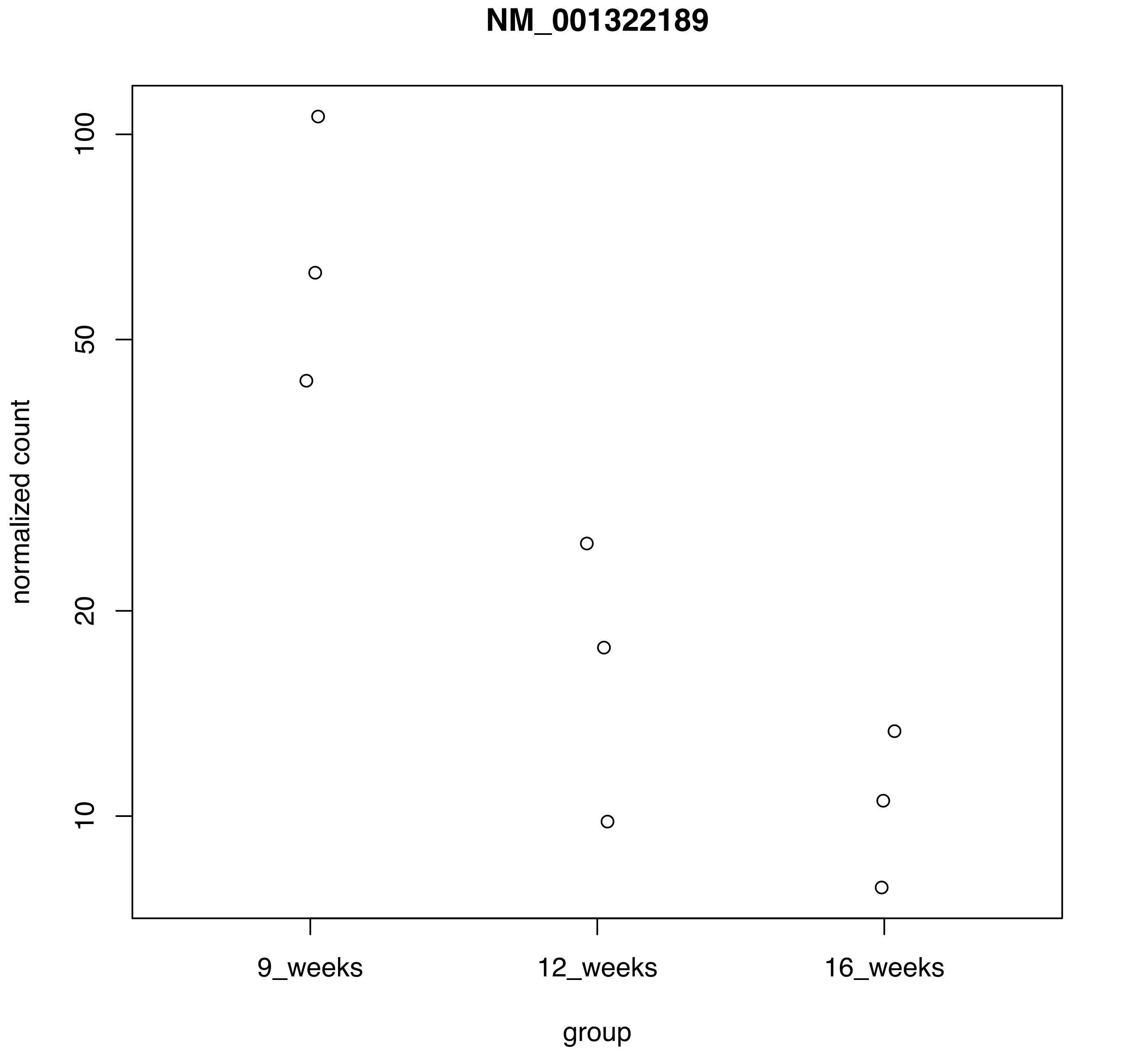
NM_001322189
normalized count
100
50
20
10
9_weeks
12_weeks
16_weeks
group

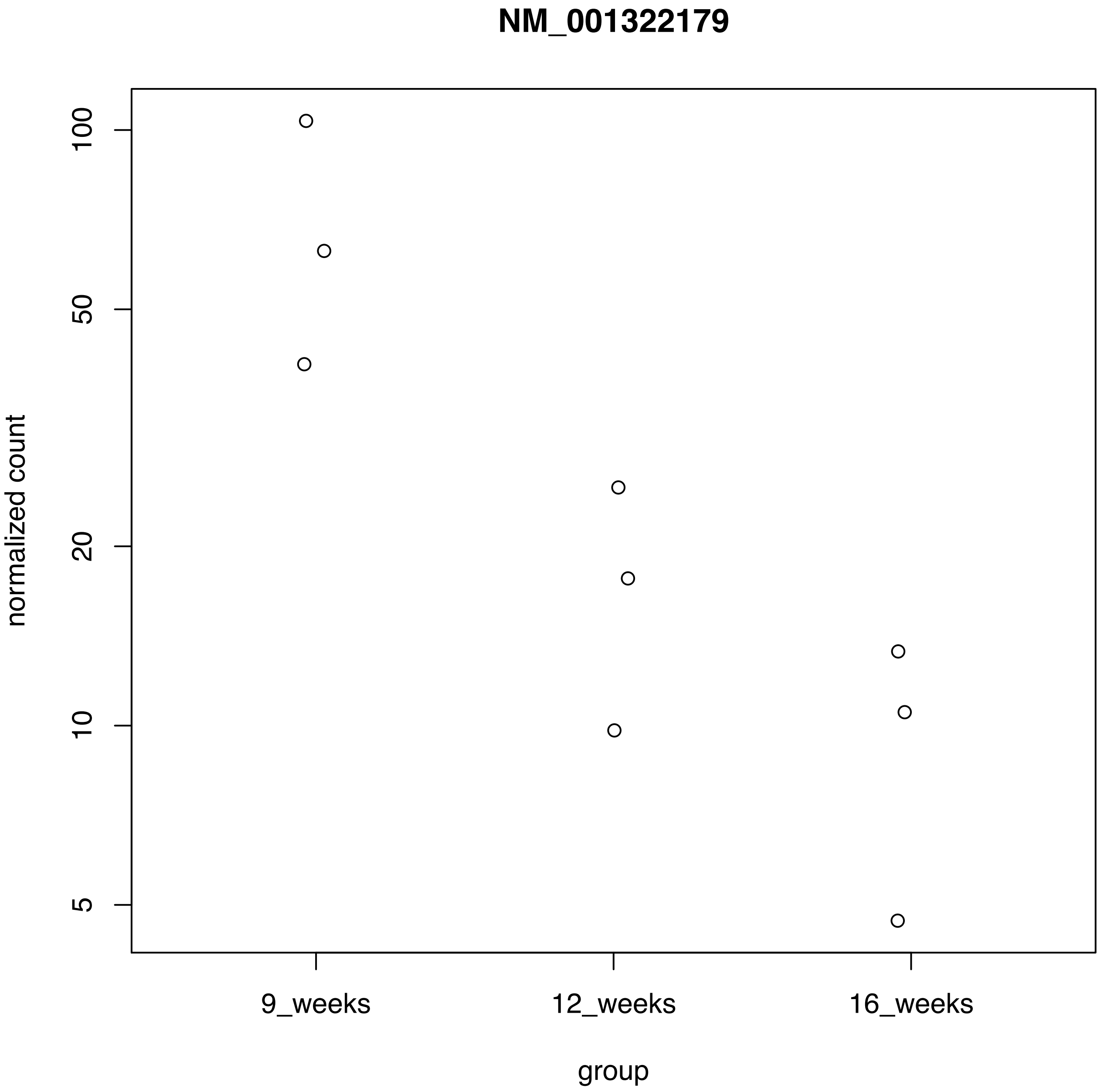
NM_001322179
normalized count
100
50
20
10
5
9_weeks
12_weeks
16_weeks
group

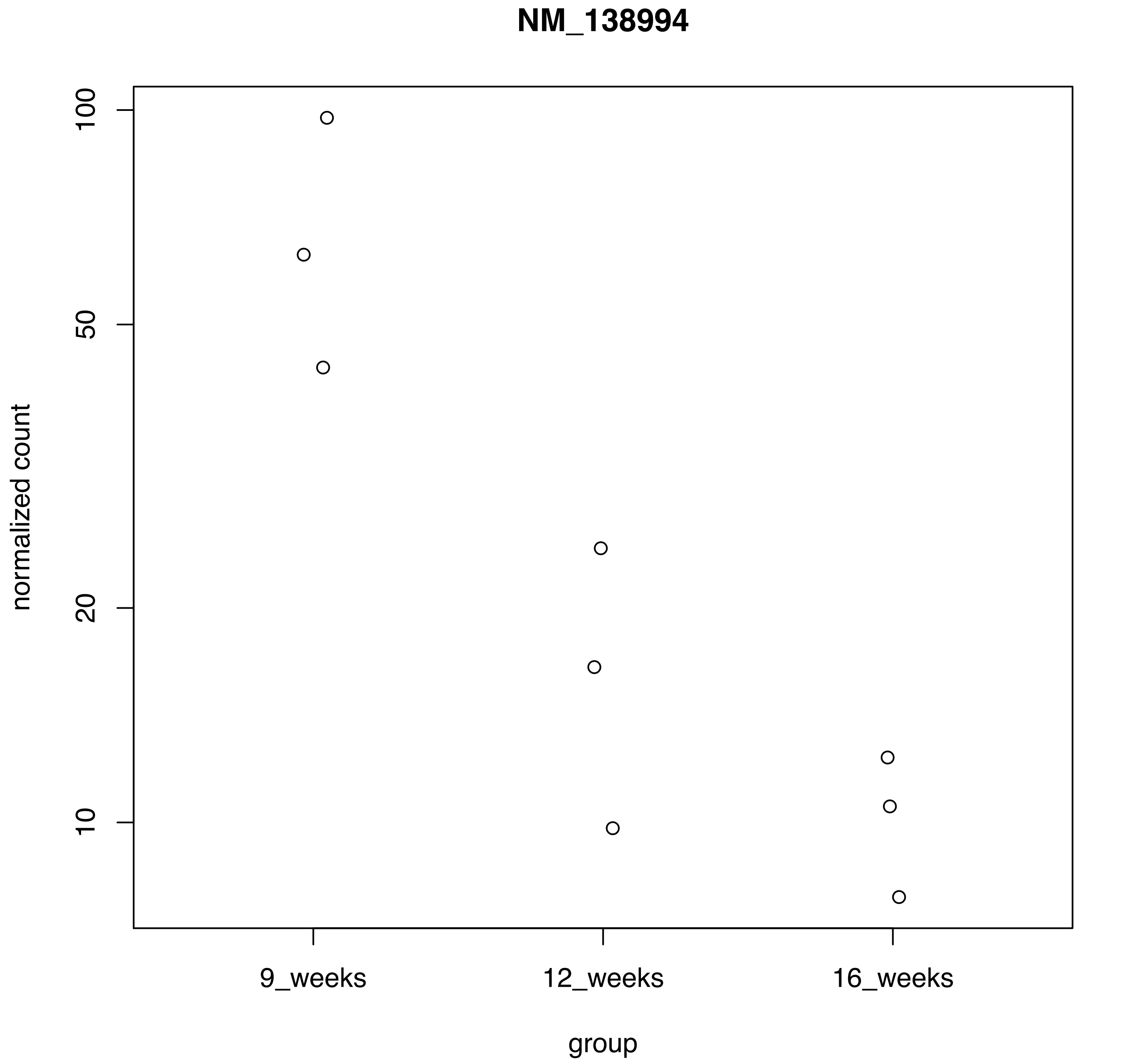
NM_138994
normalized count
100
50
20
10
9_weeks
12_weeks
16_weeks
group

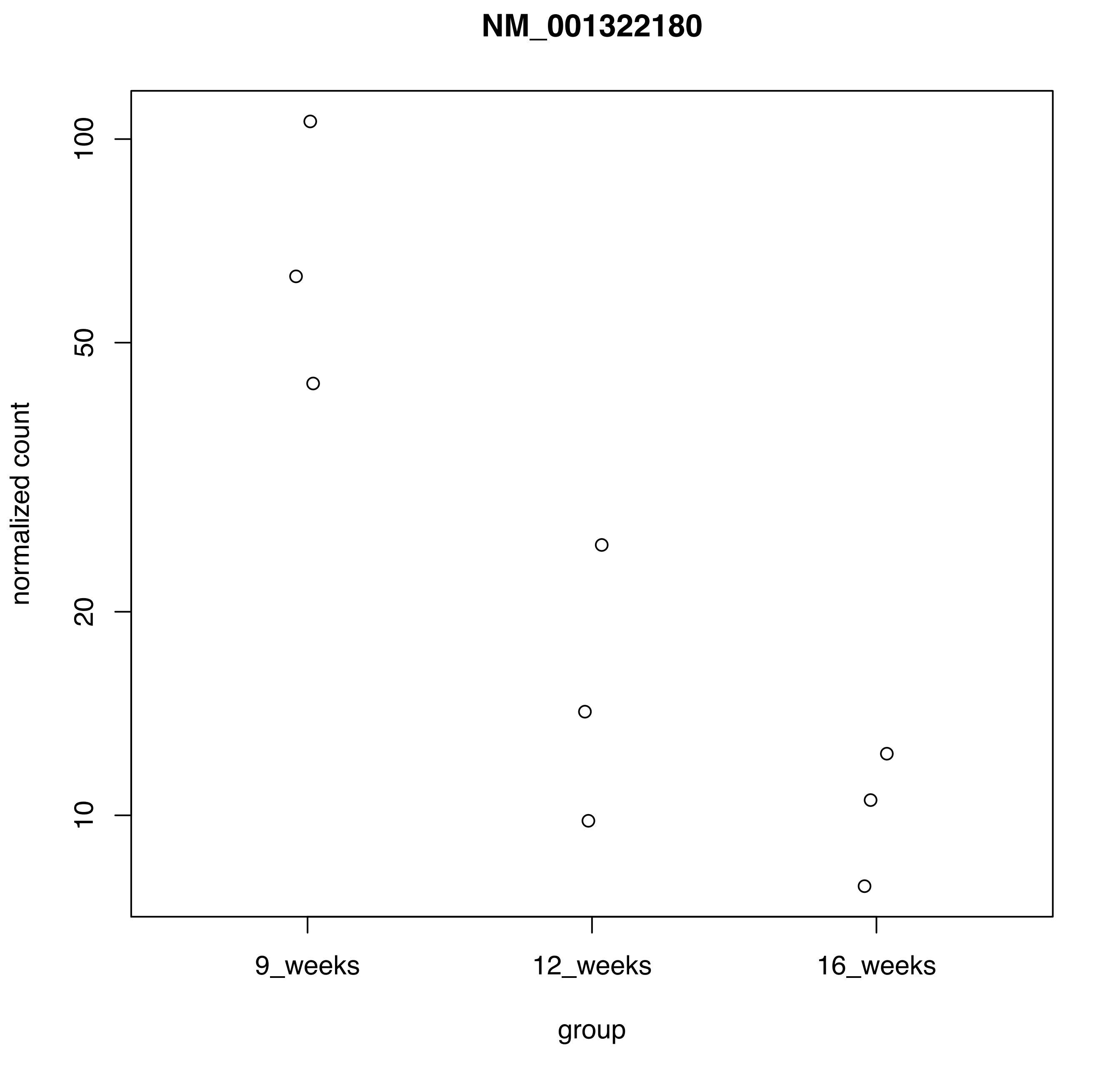
NM_001322180
normalized count
100
50
20
10
9_weeks
12_weeks
16_weeks
group

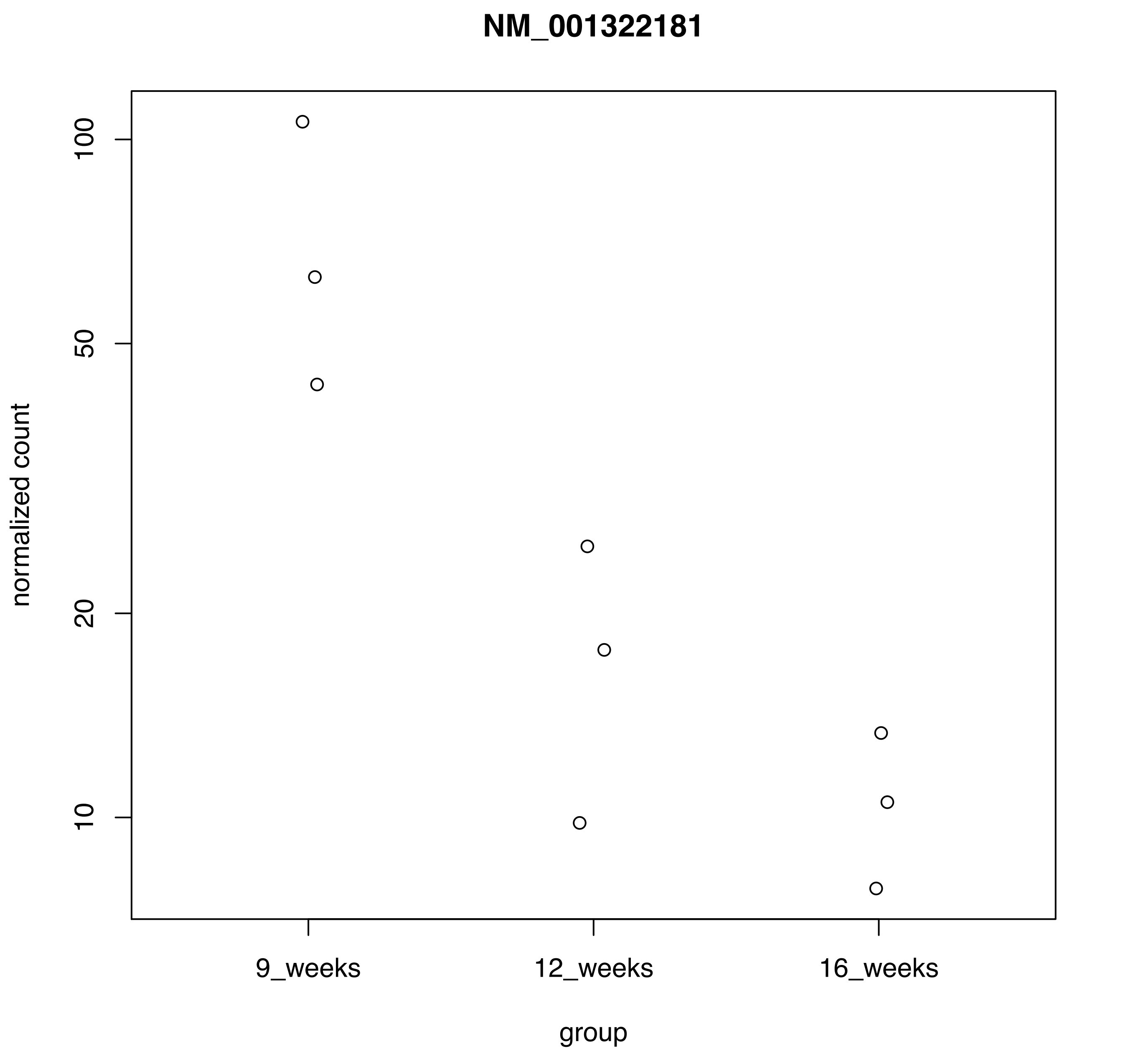
NM_001322181
normalized count
100
50
20
10
9_weeks
12_weeks
16_weeks
group

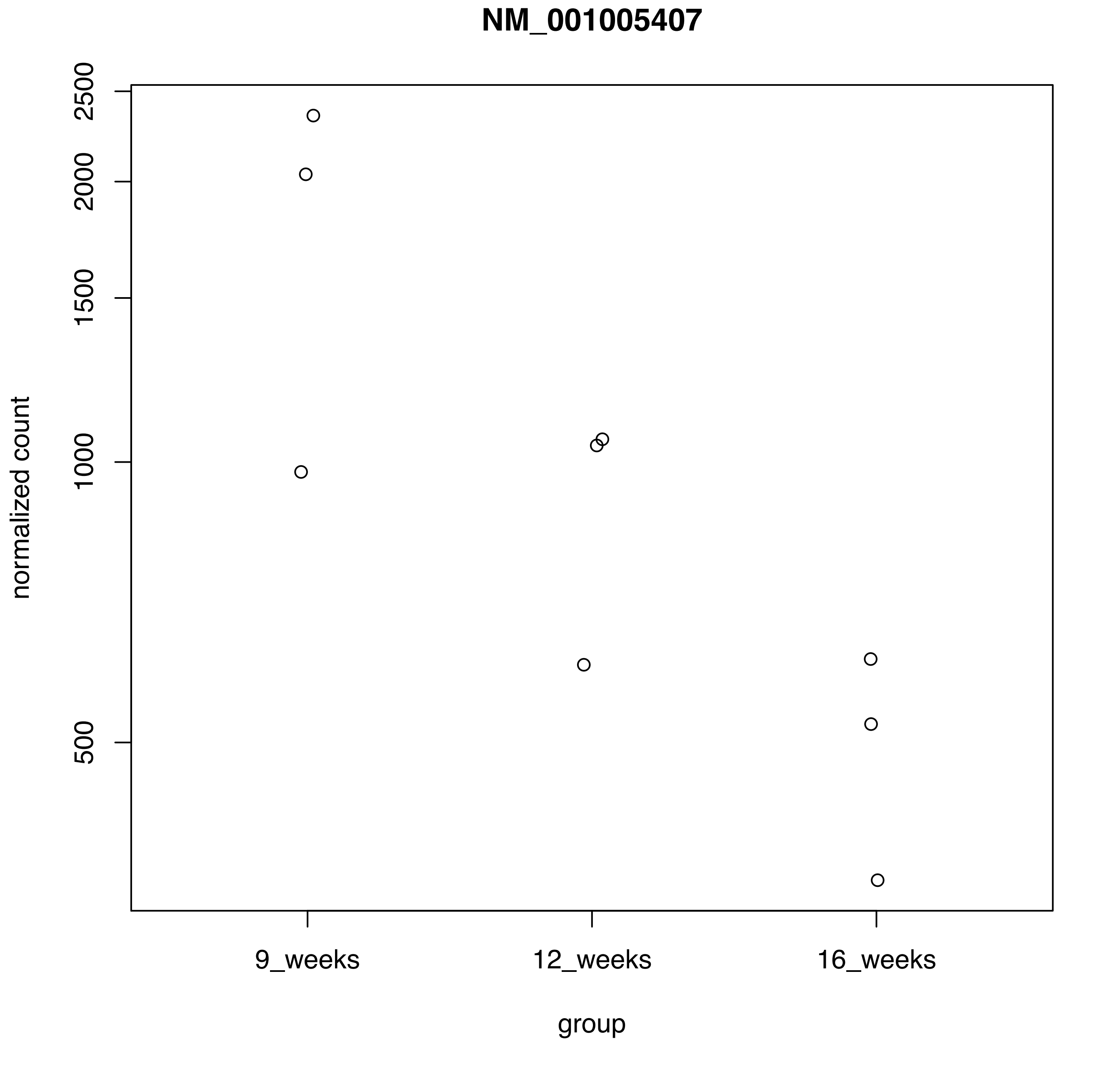
NM_001005407
normalized count
2500
2000
1500
1000
500
9_weeks
12_weeks
16_weeks
group

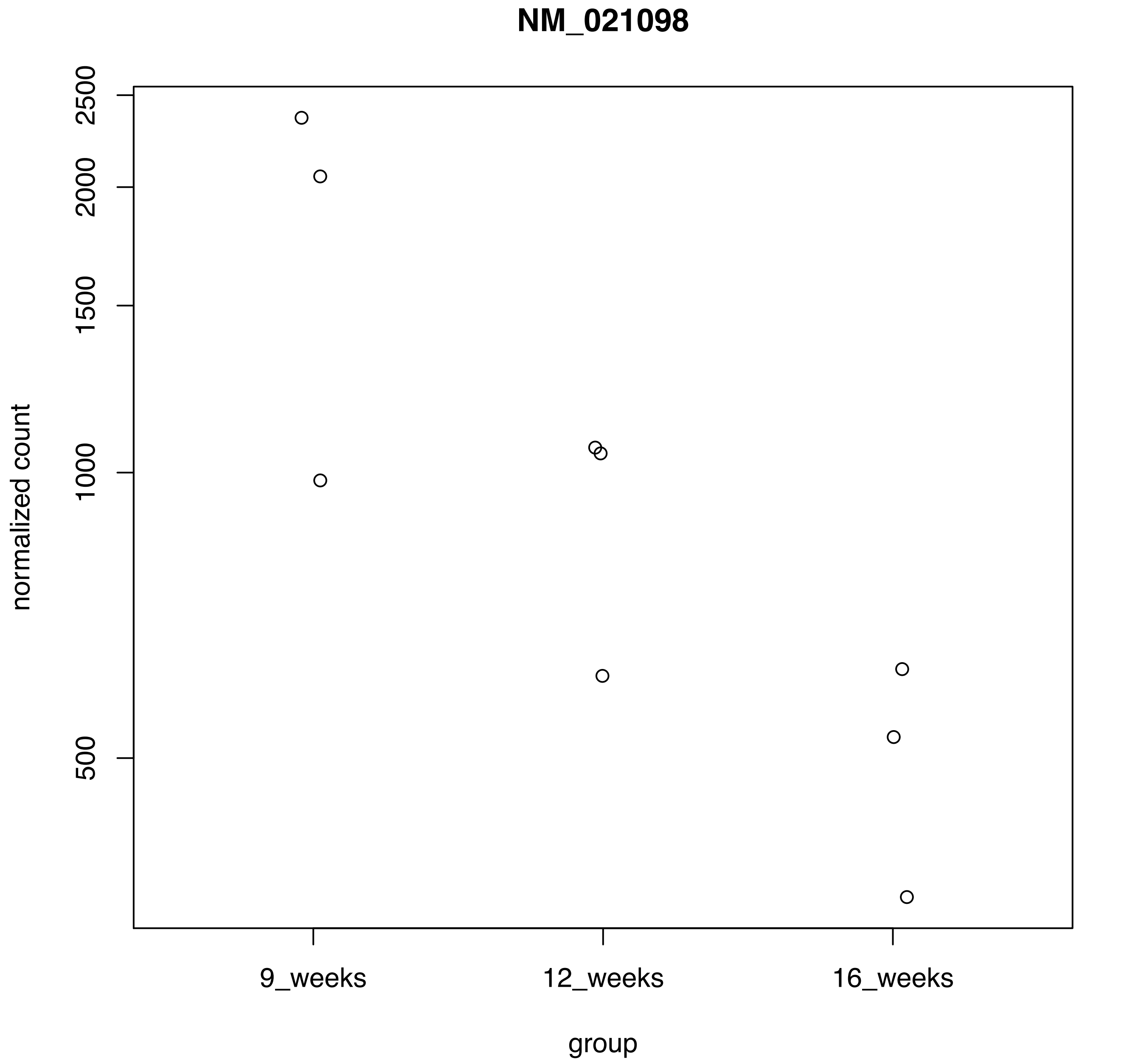
NM_021098
normalized count
2500
2000
1500
1000
500
9_weeks
12_weeks
16_weeks
group

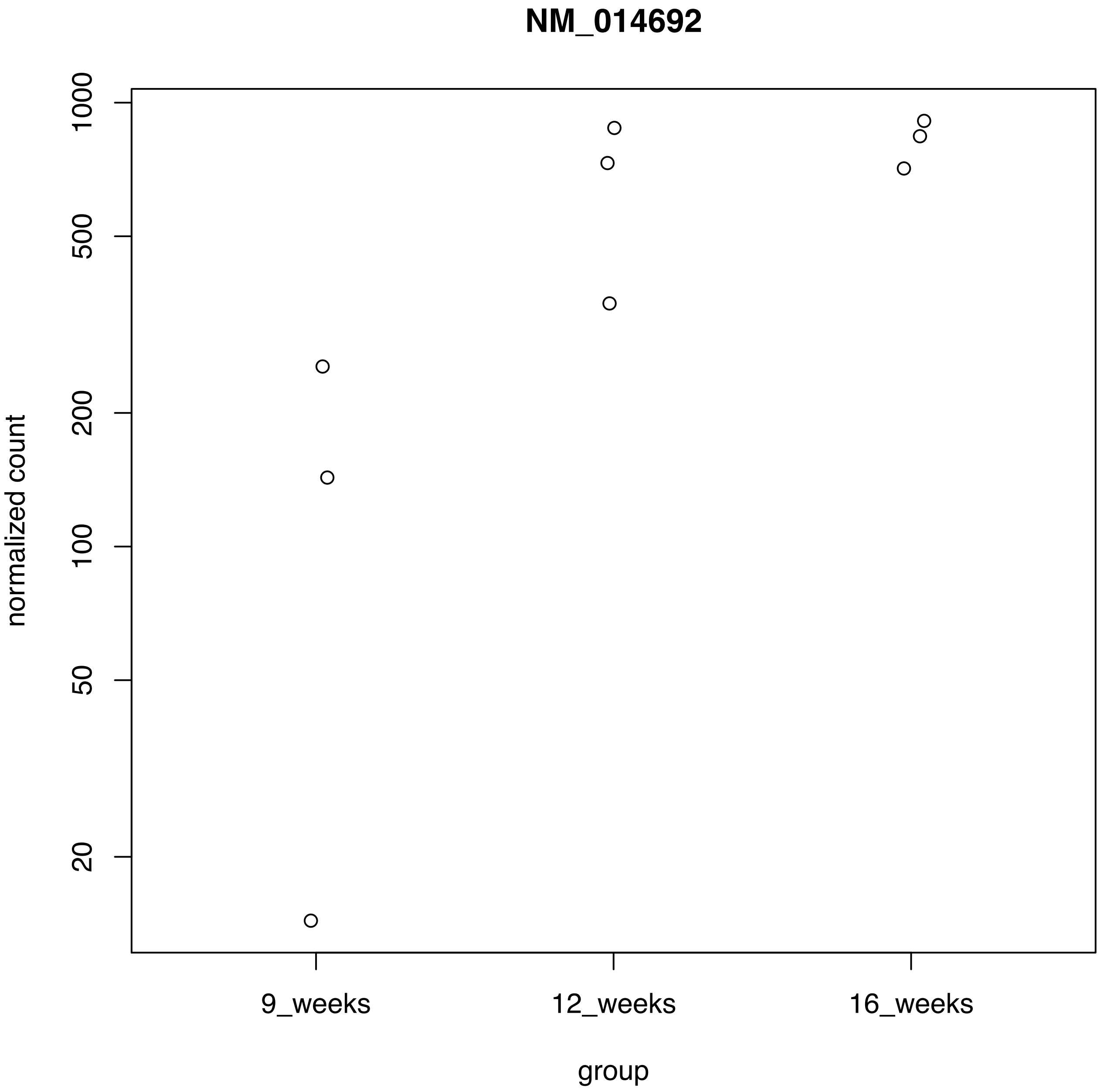
NM_014692
normalized count
1000
500
200
100
50
20
9_weeks
12_weeks
16_weeks
group

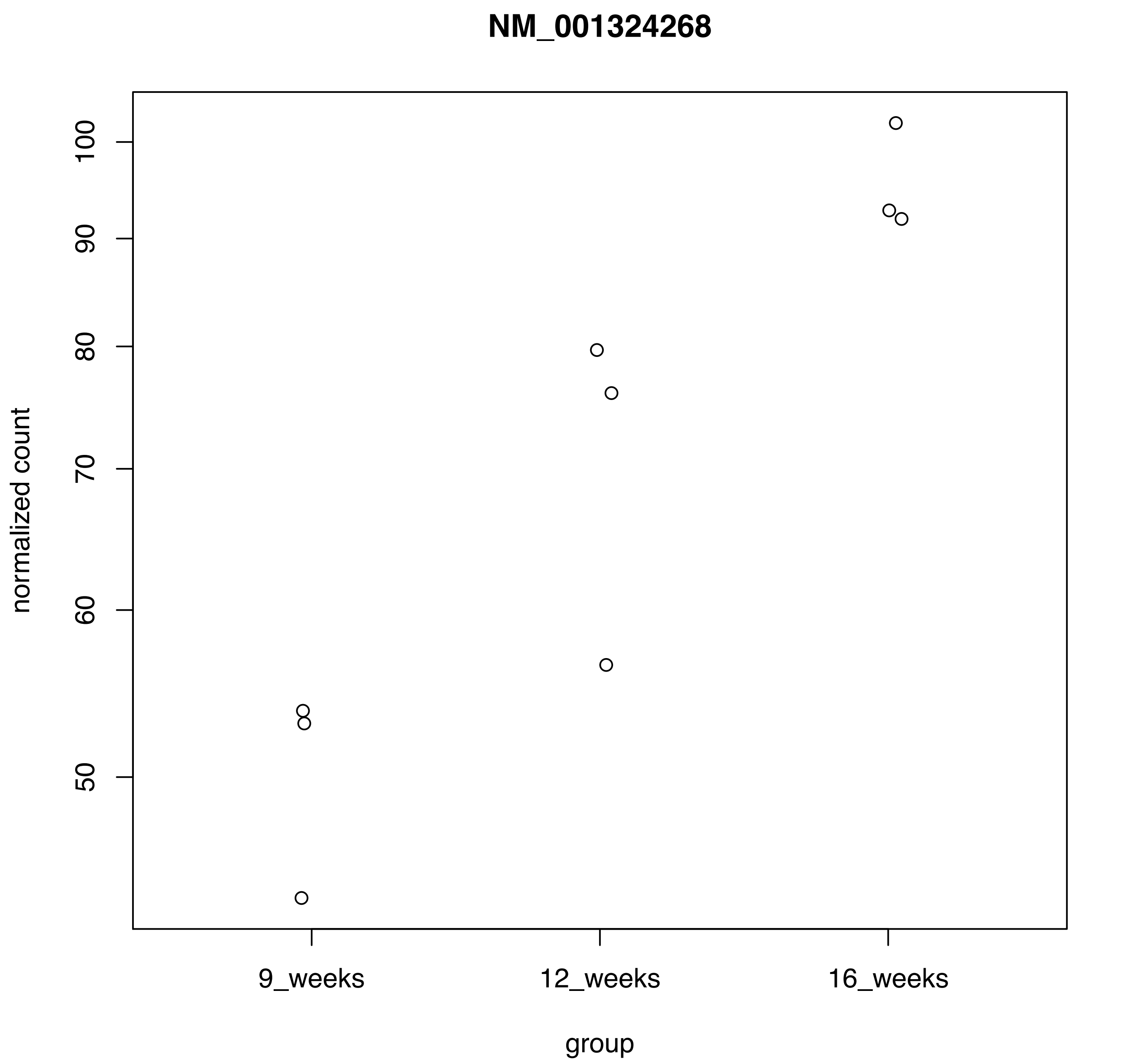
NM_001324268
normalized count
100
90
80
70
60
50
9_weeks
12_weeks
16_weeks
group

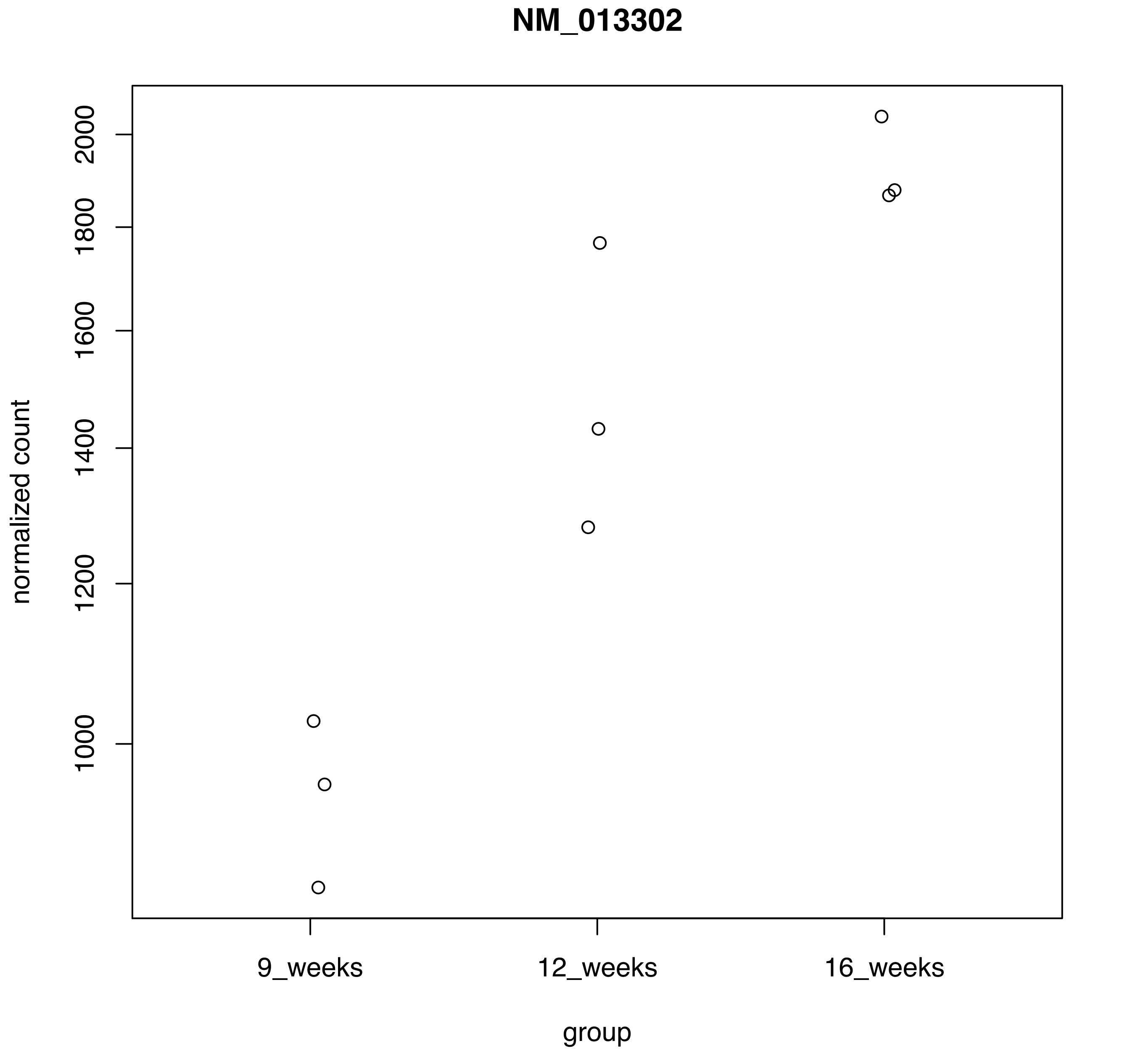
NM_013302
normalized count
2000
1800
1600
1400
1200
1000
9_weeks
12_weeks
16_weeks
group

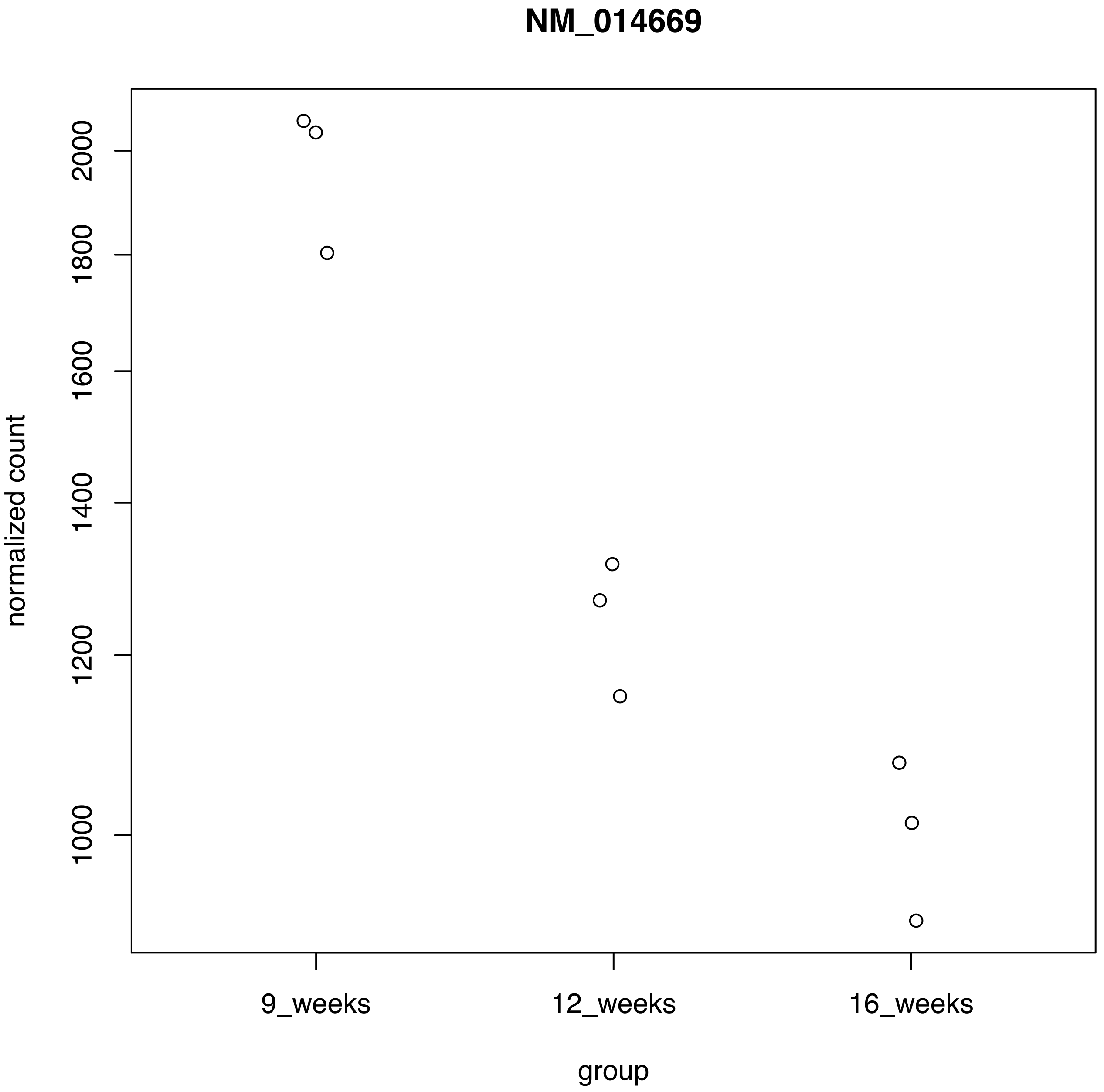

NM_014669
normalized count
2000
1800
1600
1400
1200
1000
9_weeks
12_weeks
16_weeks
group

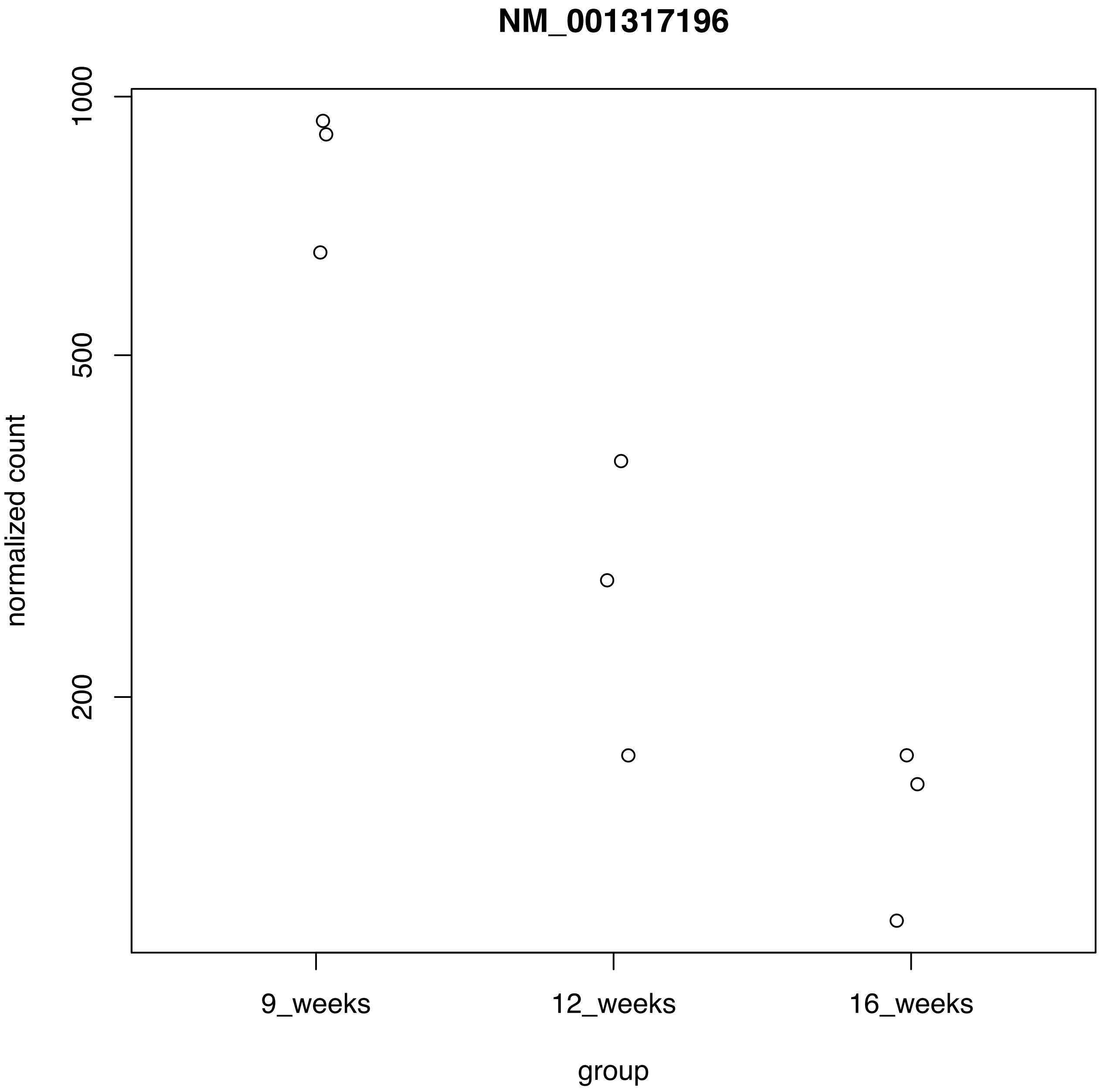
NM_001317196
normalized count
1000
500
200
9_weeks
12_weeks
16_weeks
group

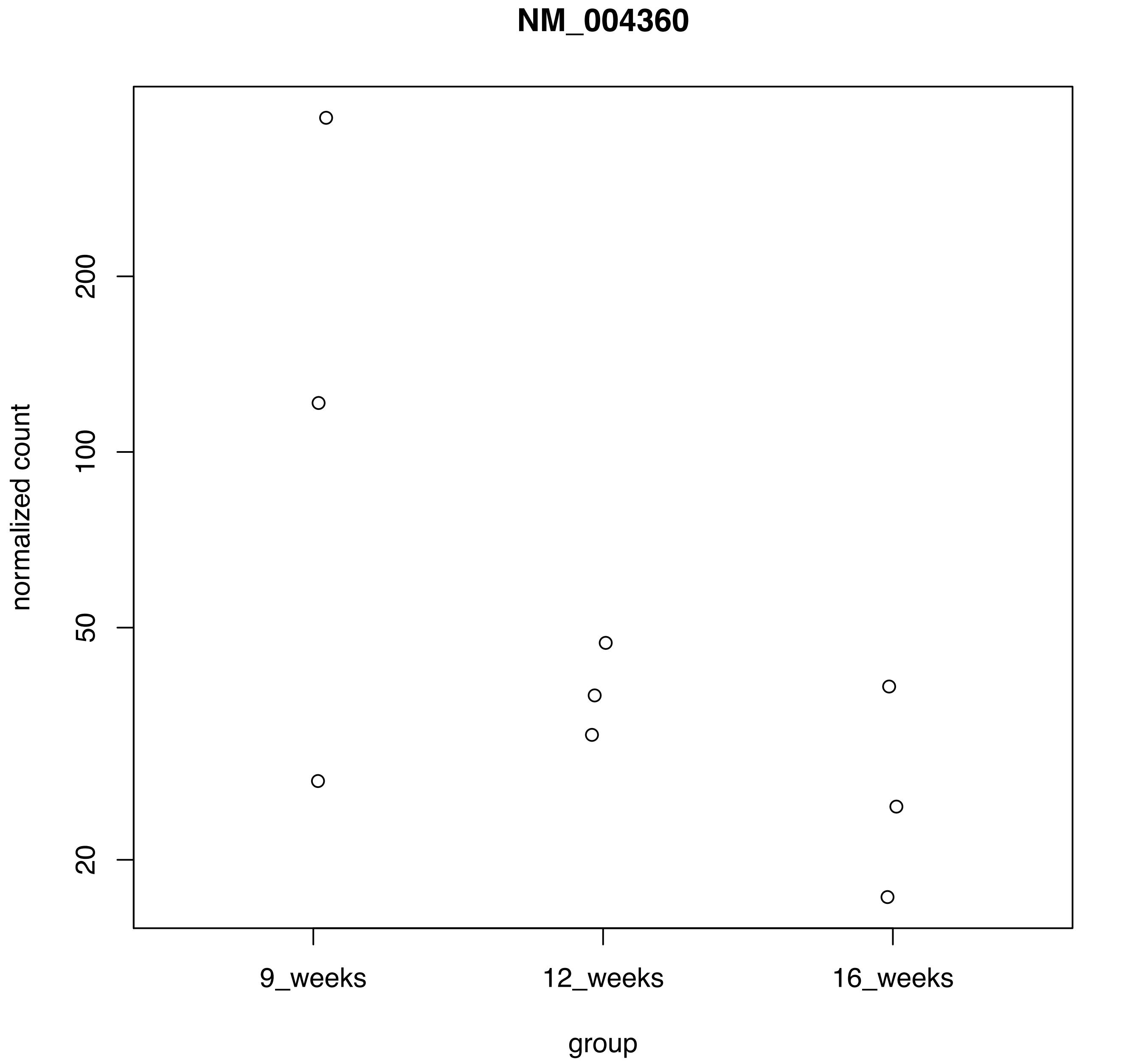
NM_004360
normalized count
200
100
50
20
9_weeks
12_weeks
16_weeks
group

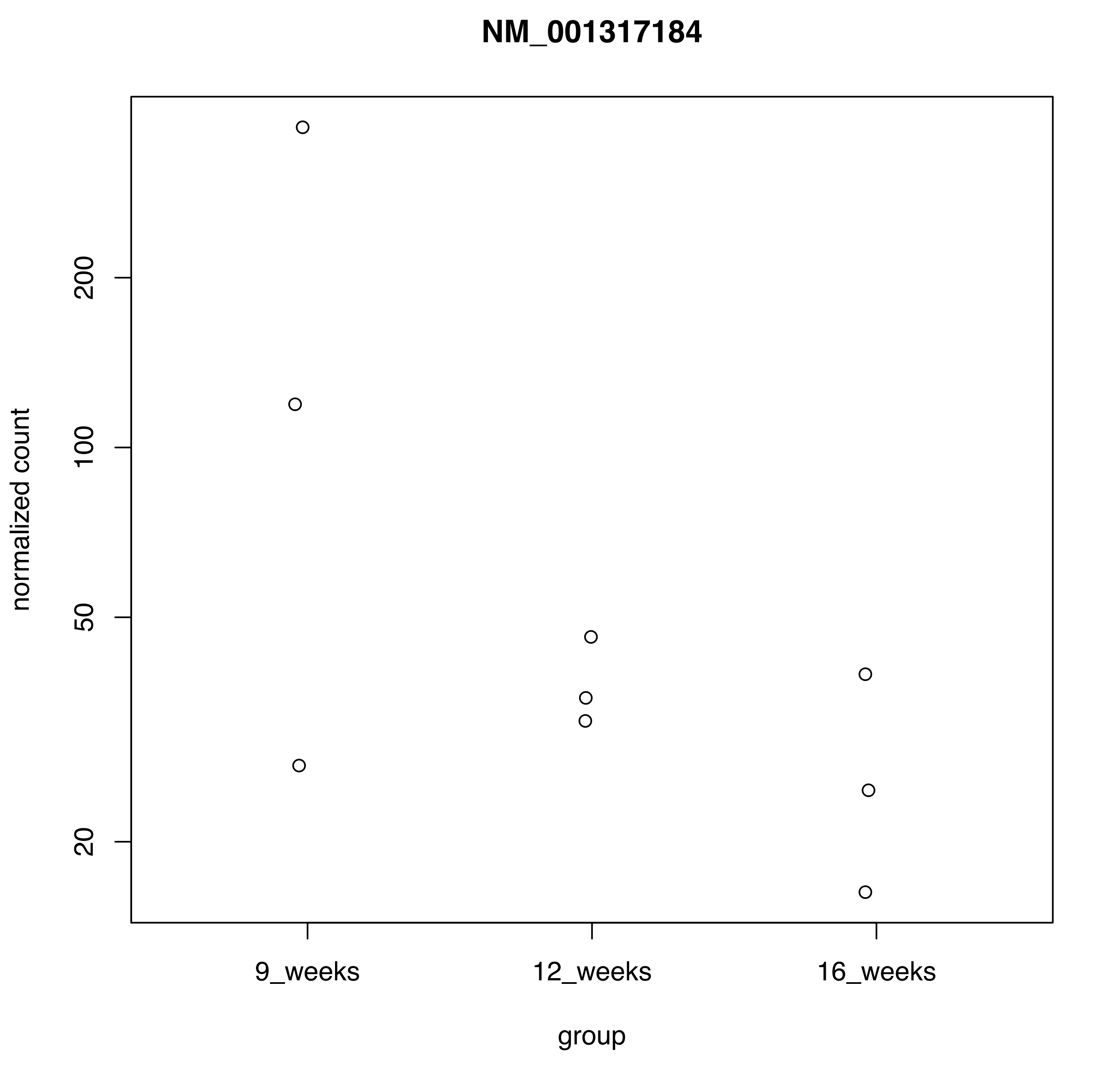
NM_001317184
normalized count
200
100
50
20
9_weeks
12_weeks
16_weeks
group

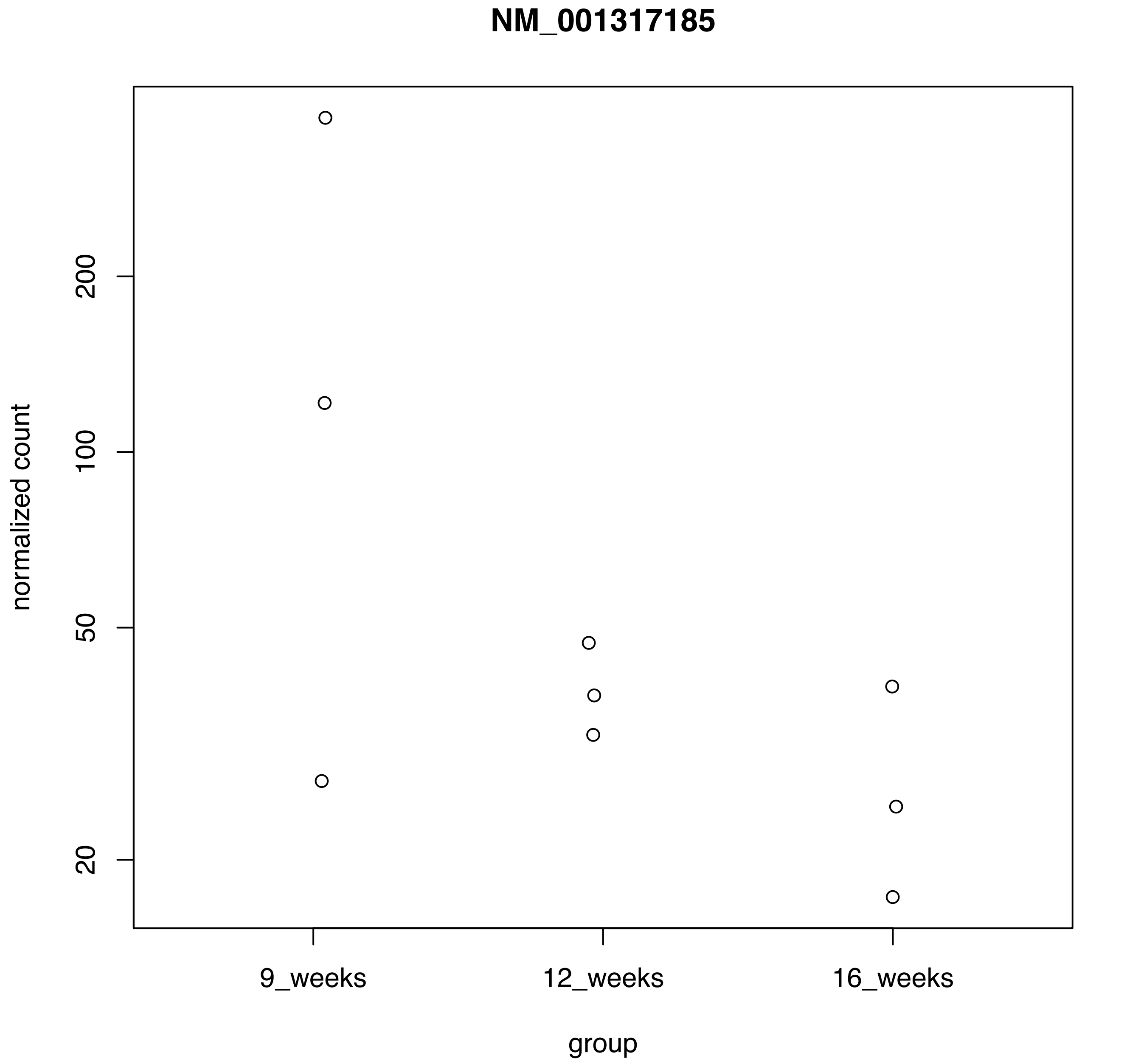
NM_001317185
normalized count
200
100
50
20
9_weeks
12_weeks
16_weeks
group

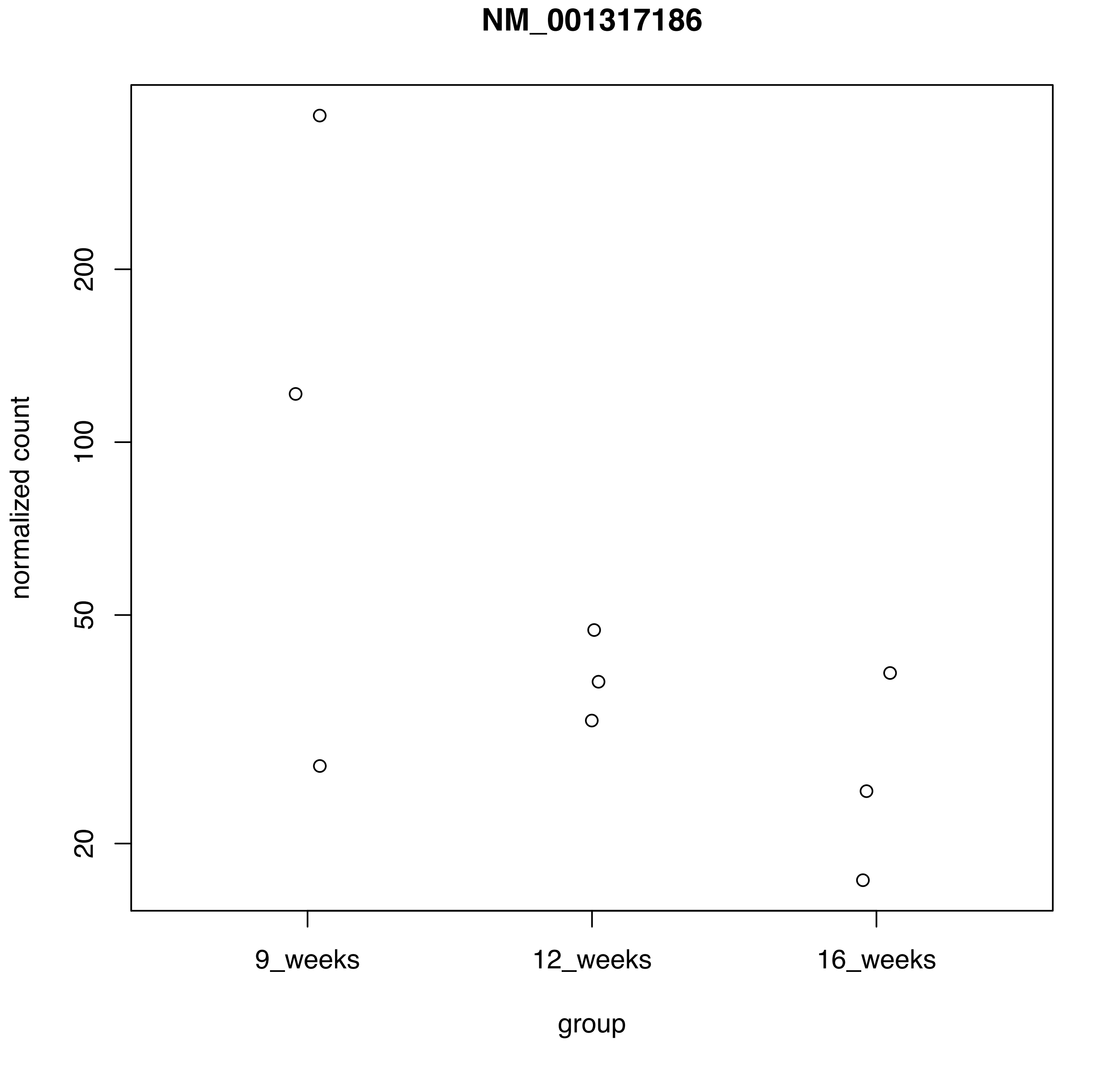
NM_001317186
normalized count
200
100
50
20
9_weeks
12_weeks
16_weeks
group

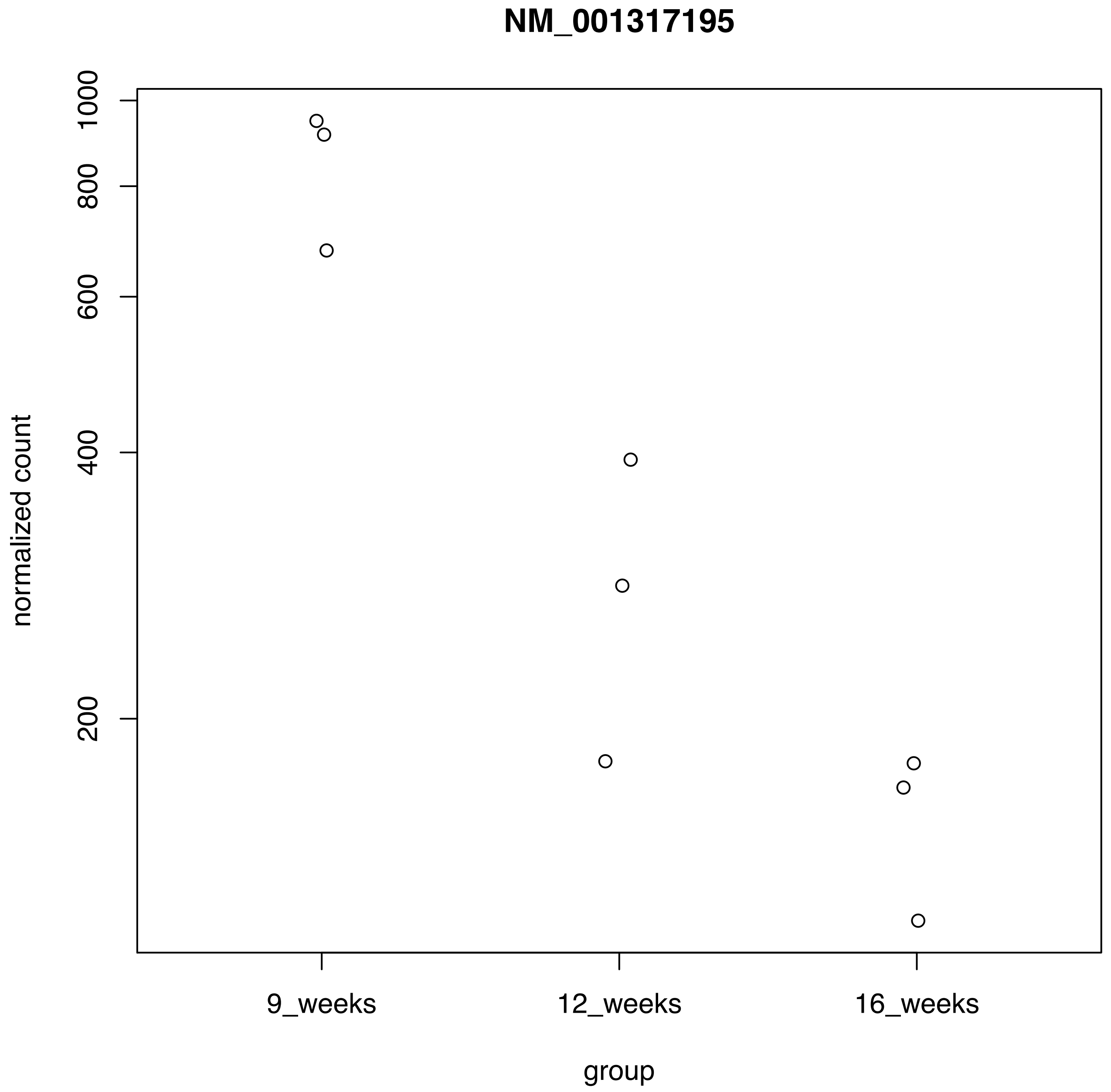
NM_001317195
normalized count
1000
800
600
400
200
9_weeks
12_weeks
16_weeks
group

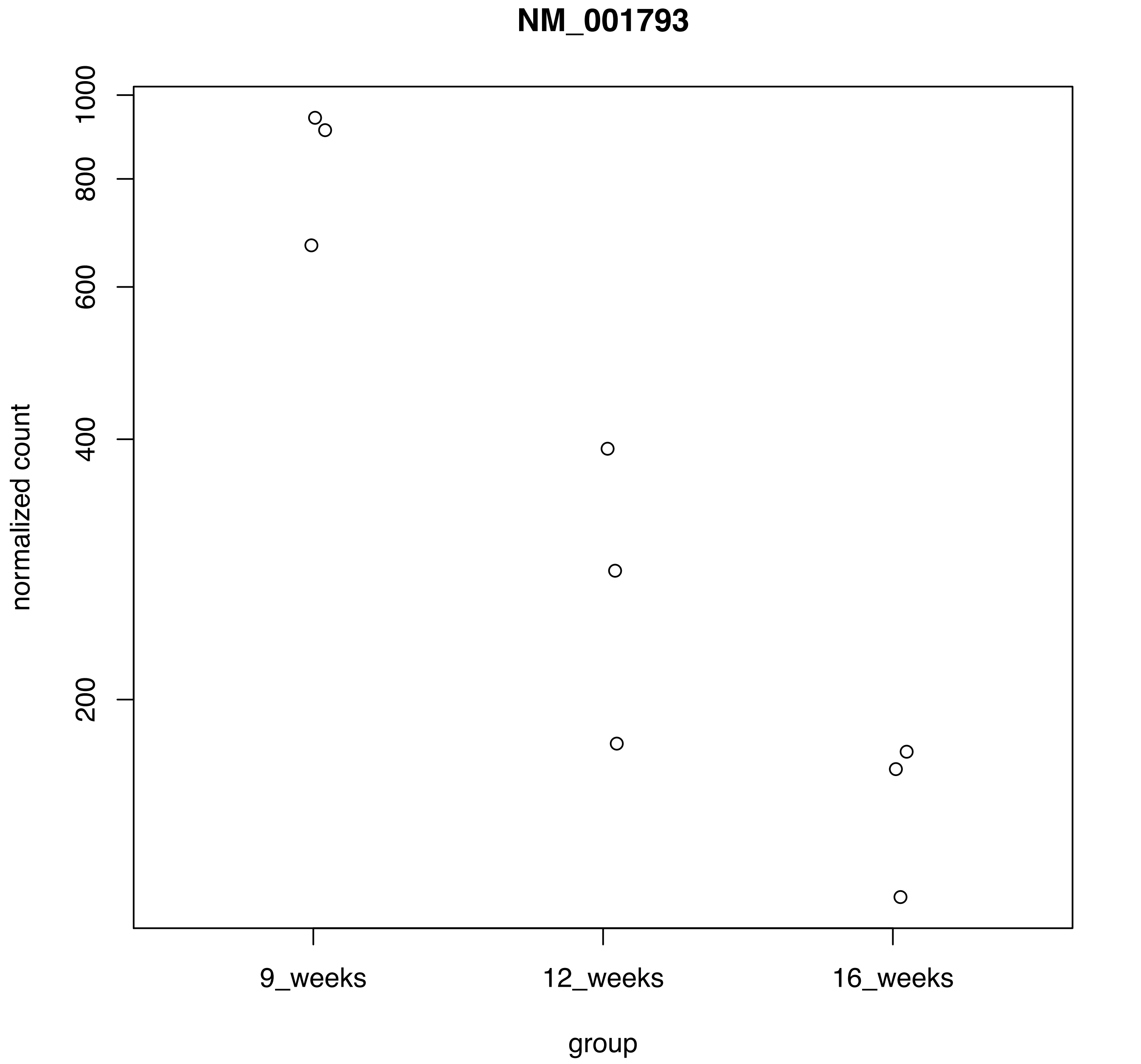

NM_001793
1000
800
600
400
200
normalized count
9_weeks
12_weeks
16_weeks
group

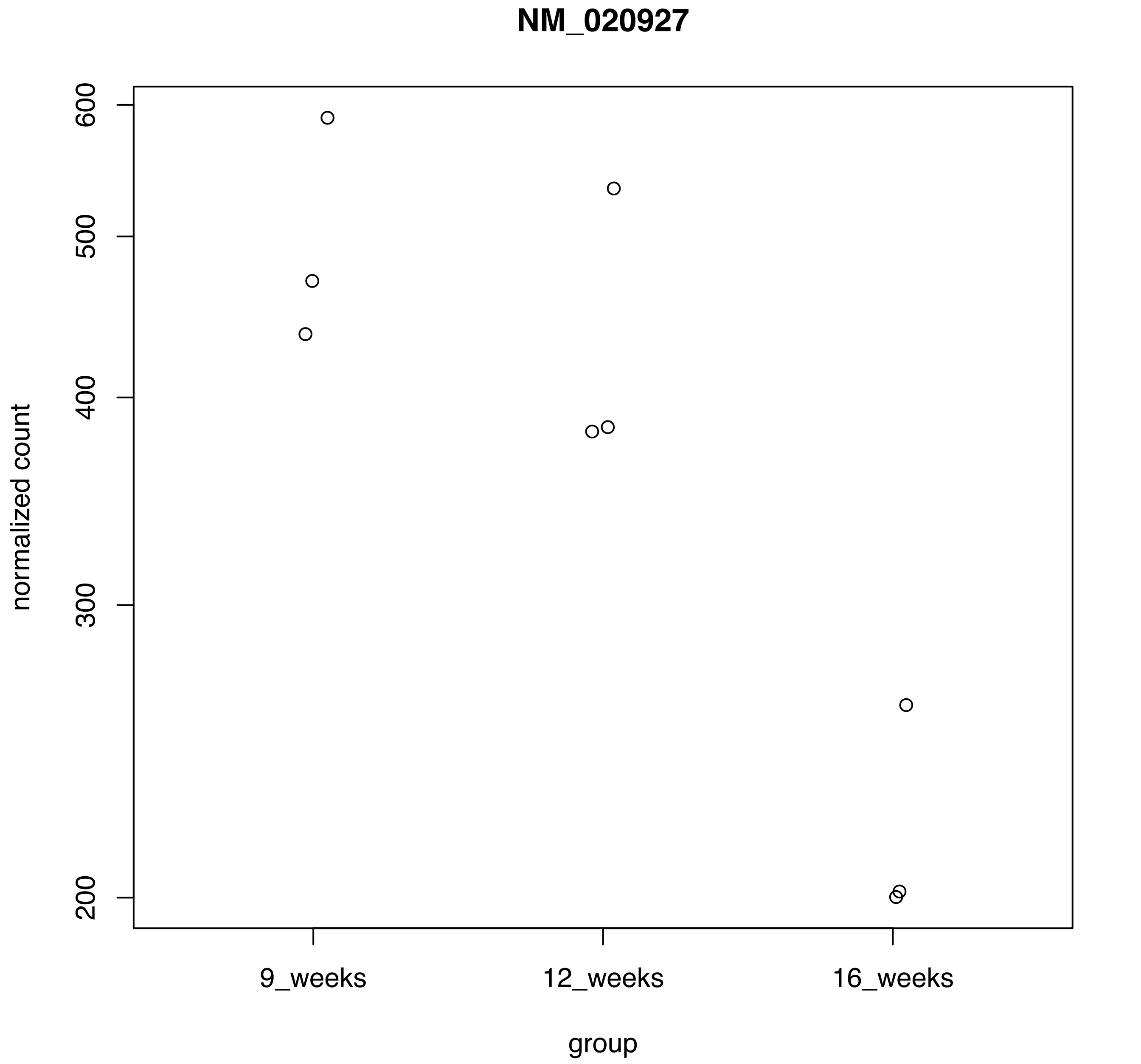
NM_020927
normalized count
600
500
400
300
200
9_weeks
12_weeks
16_weeks
group

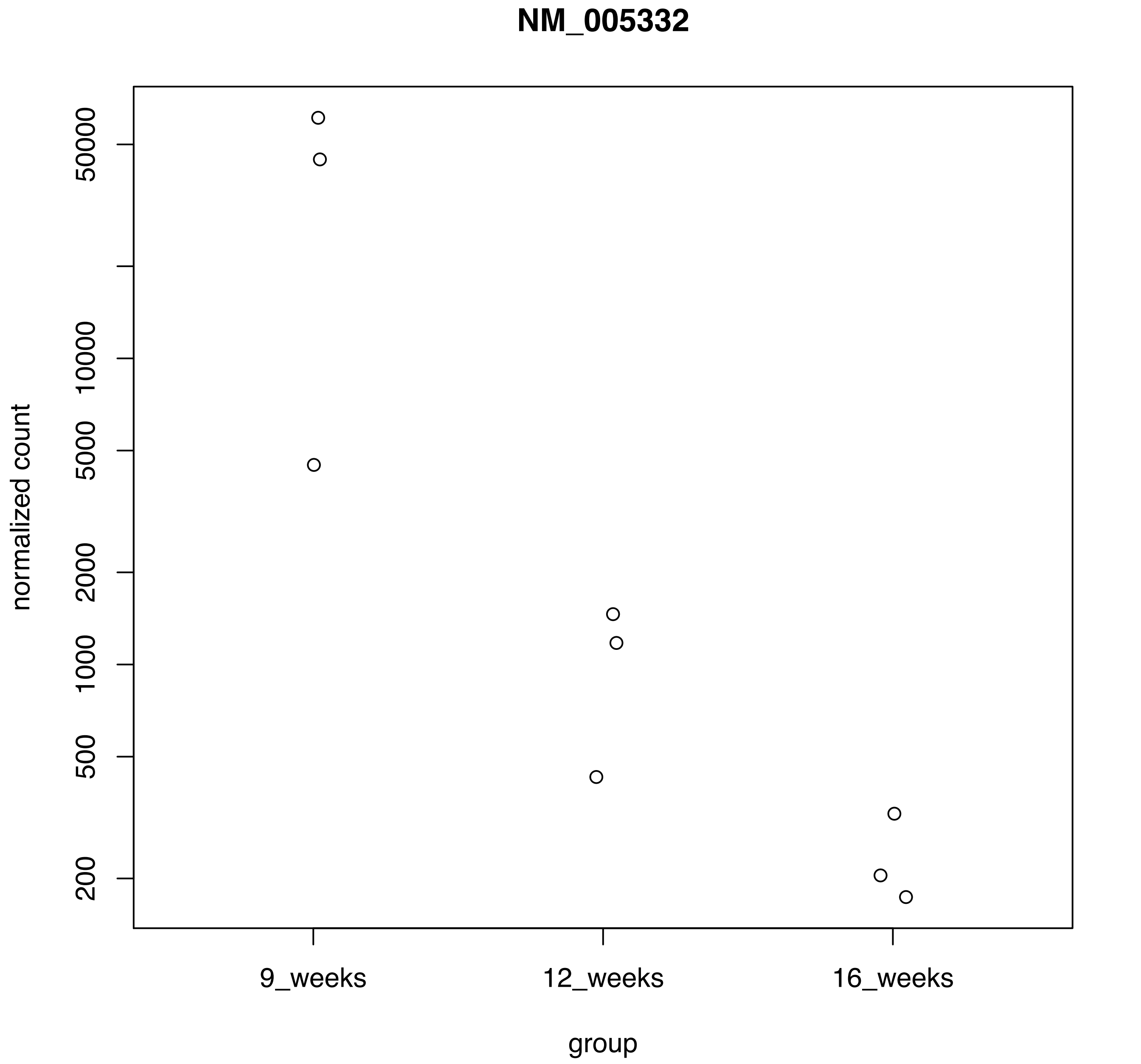
NM_005332
normalized count
50000
10000
5000
2000
1000
500
200
9_weeks
12_weeks
16_weeks
group

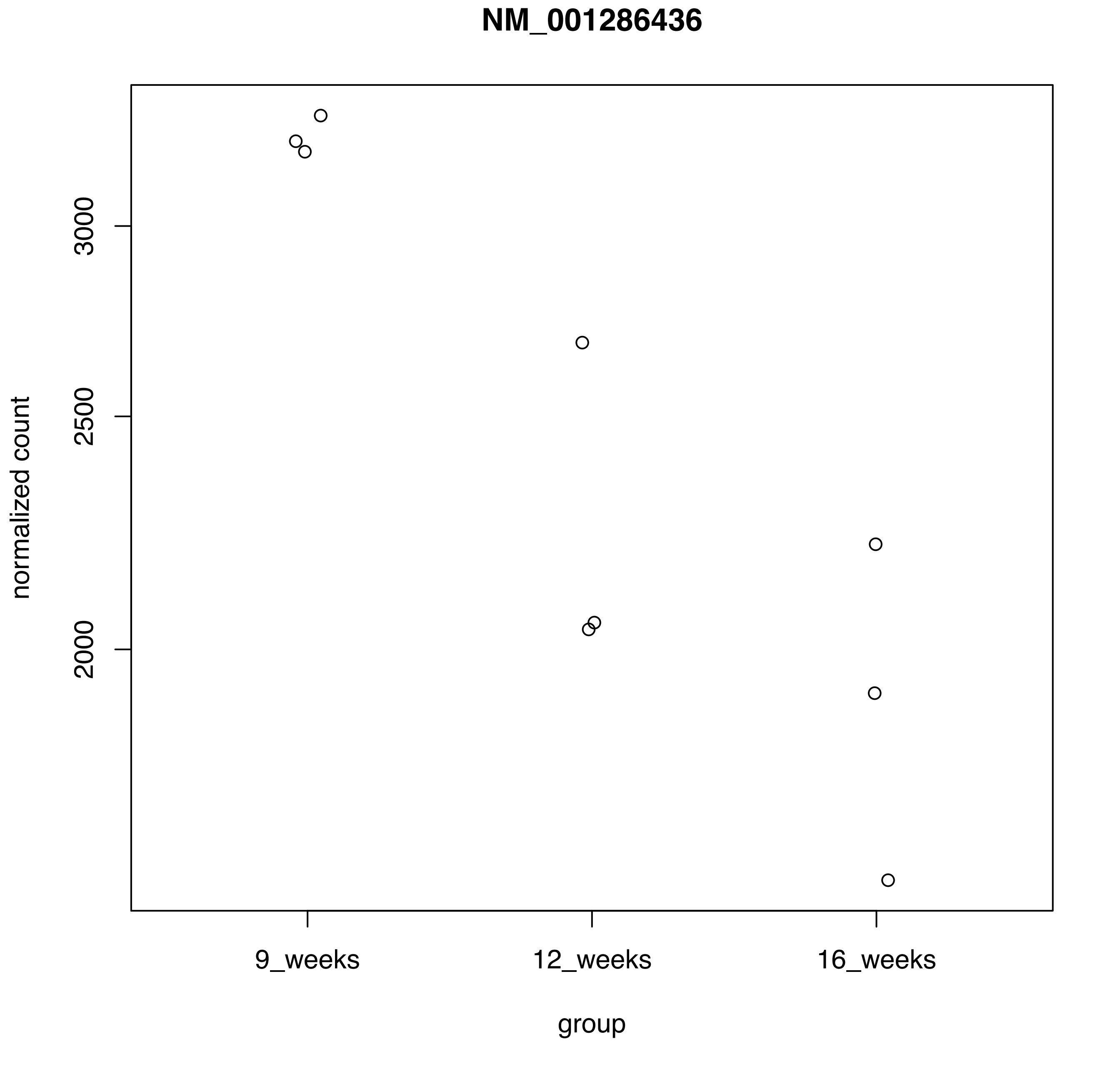

NM_001286436
normalized count
3000
2500
2000
9_weeks
12_weeks
16_weeks
group

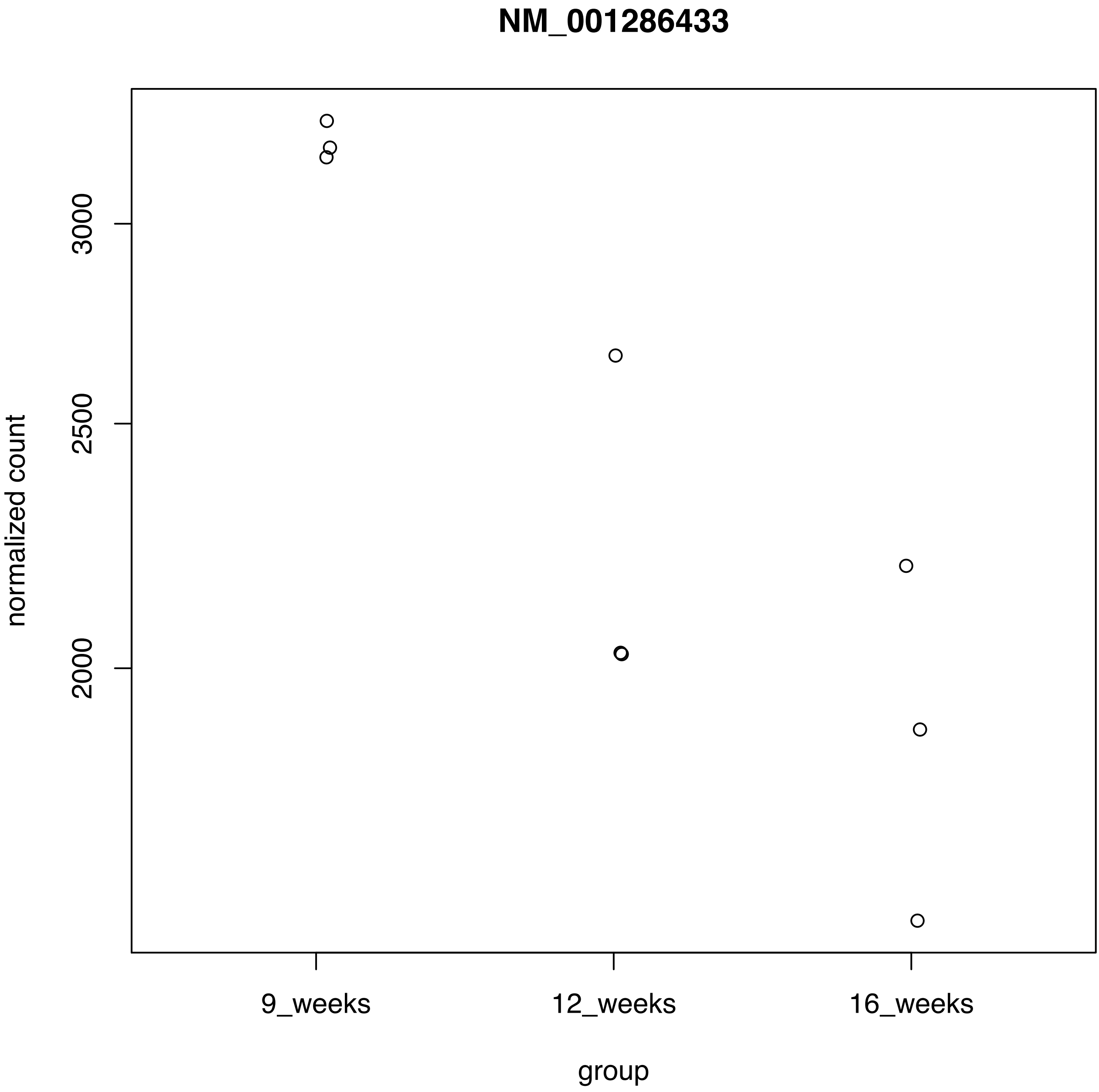
NM_001286433
normalized count
3000
2500
2000
9_weeks
12_weeks
16_weeks
group

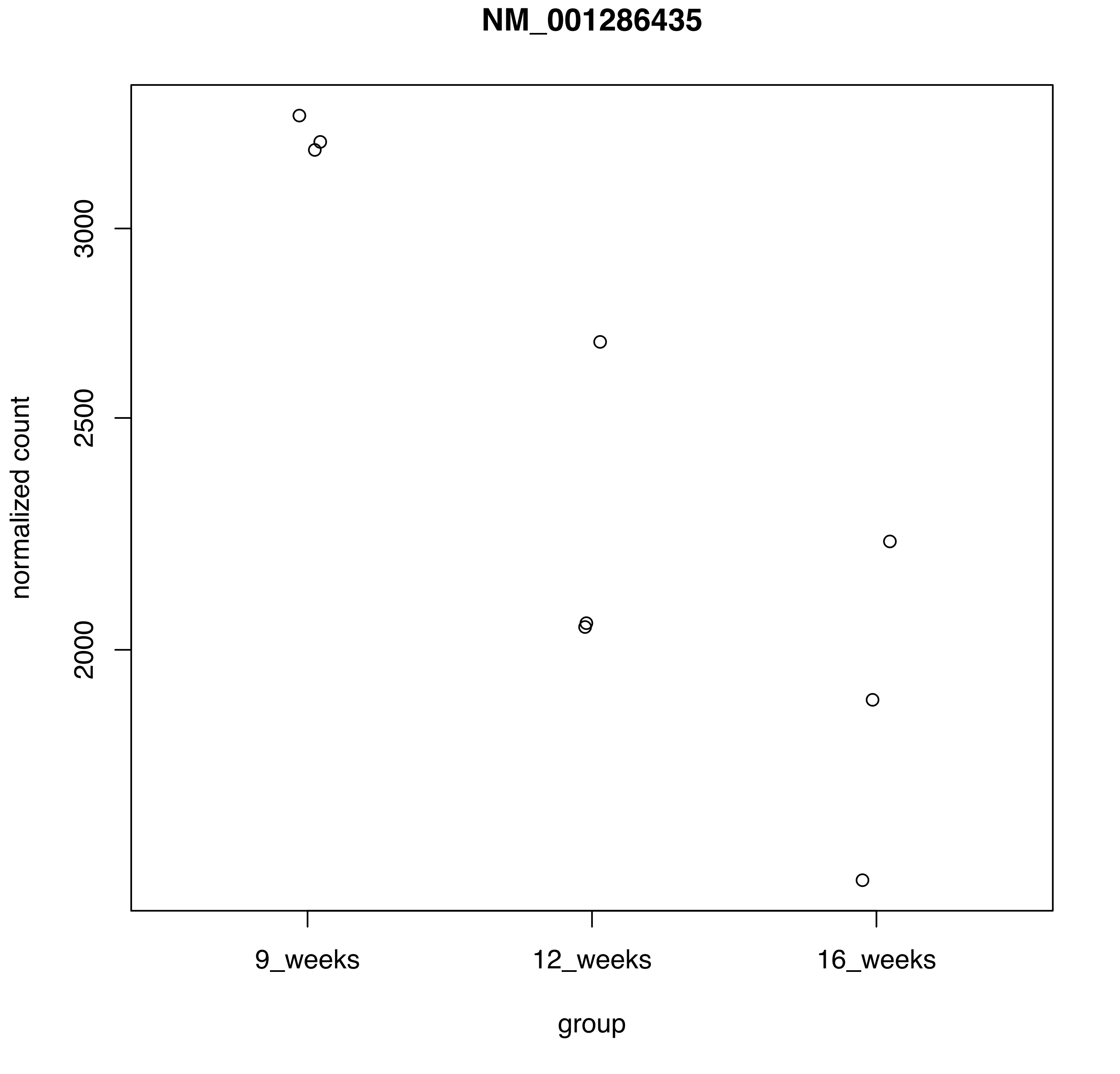
NM_001286435
normalized count
3000
2500
2000
9_weeks
12_weeks
16_weeks
group

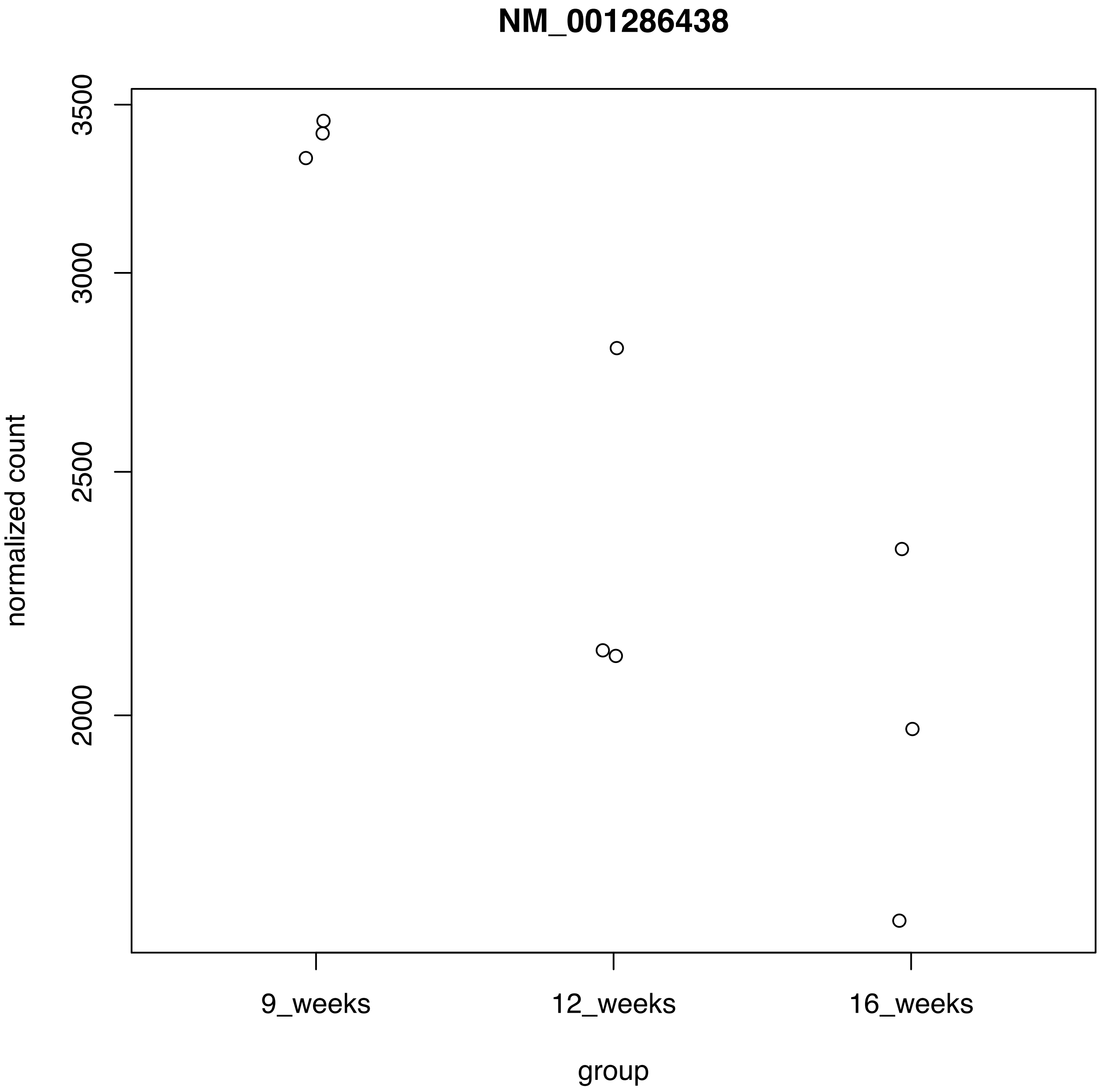
NM_001286438
3500
3000
2500
2000
normalized count
9_weeks
12_weeks
16_weeks
group

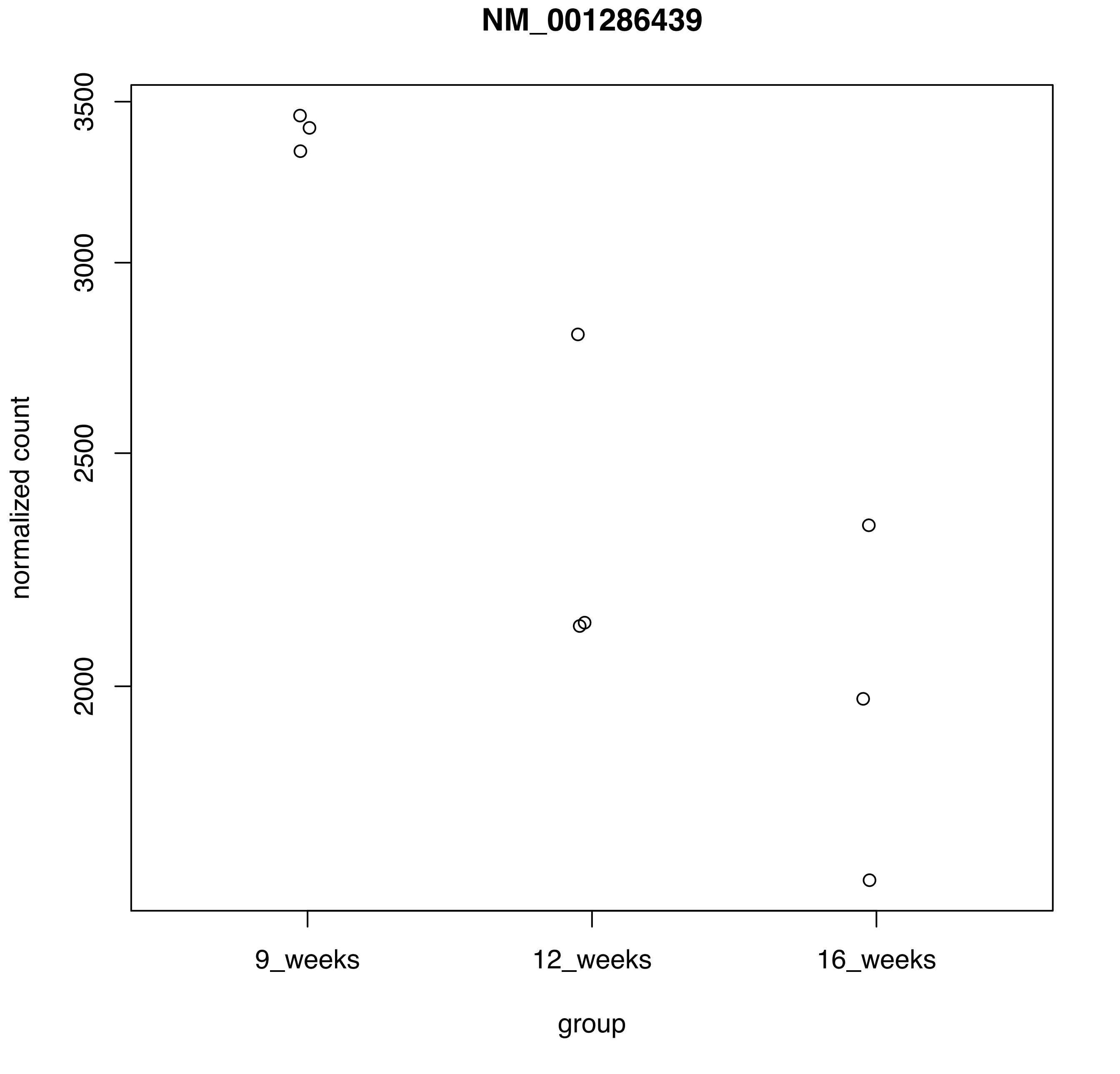
NM_001286439
normalized count
3500
3000
2500
2000
9_weeks
12_weeks
16_weeks
group

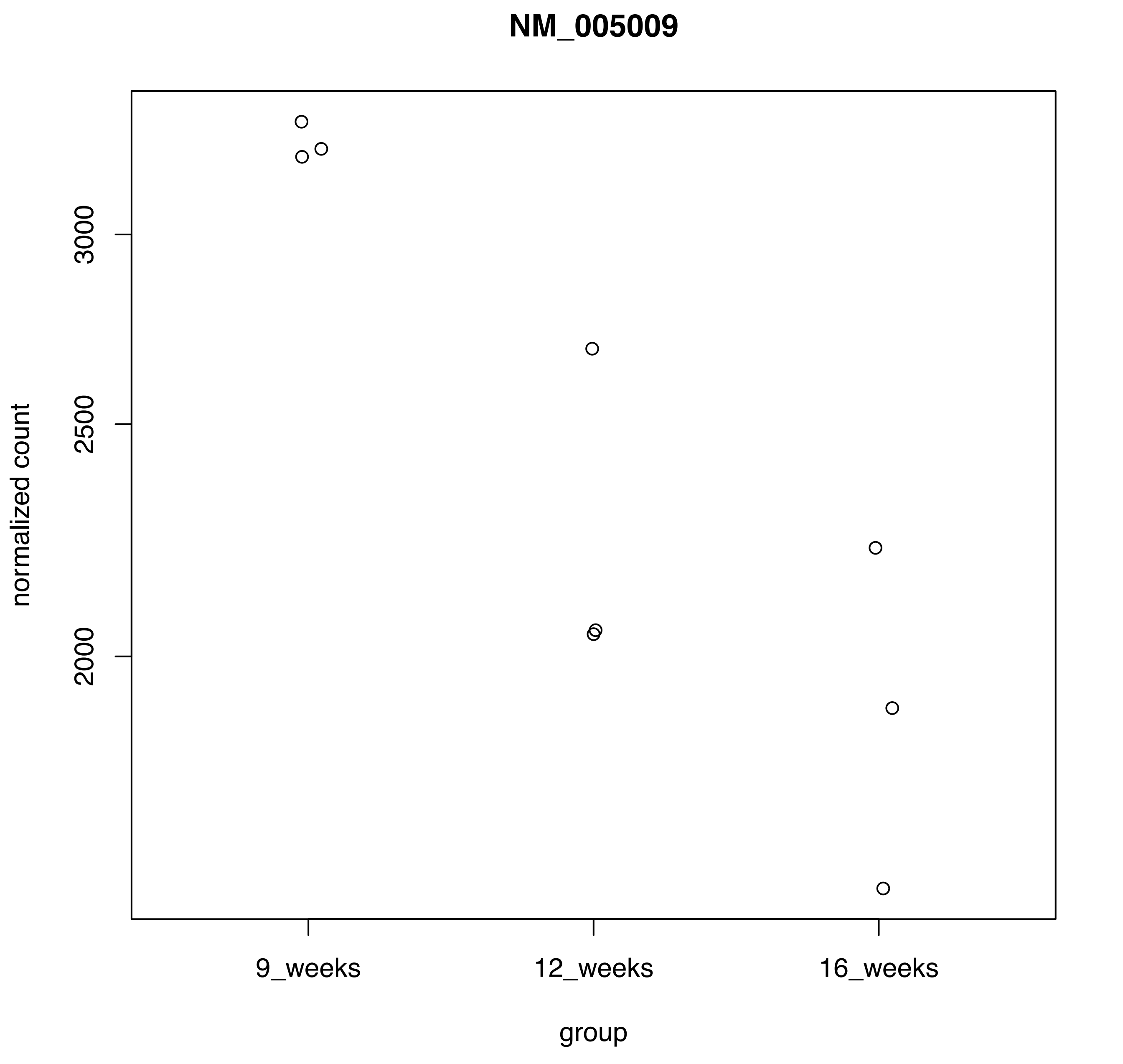
NM_005009
normalized count
3000
2500
2000
9_weeks
12_weeks
16_weeks
group

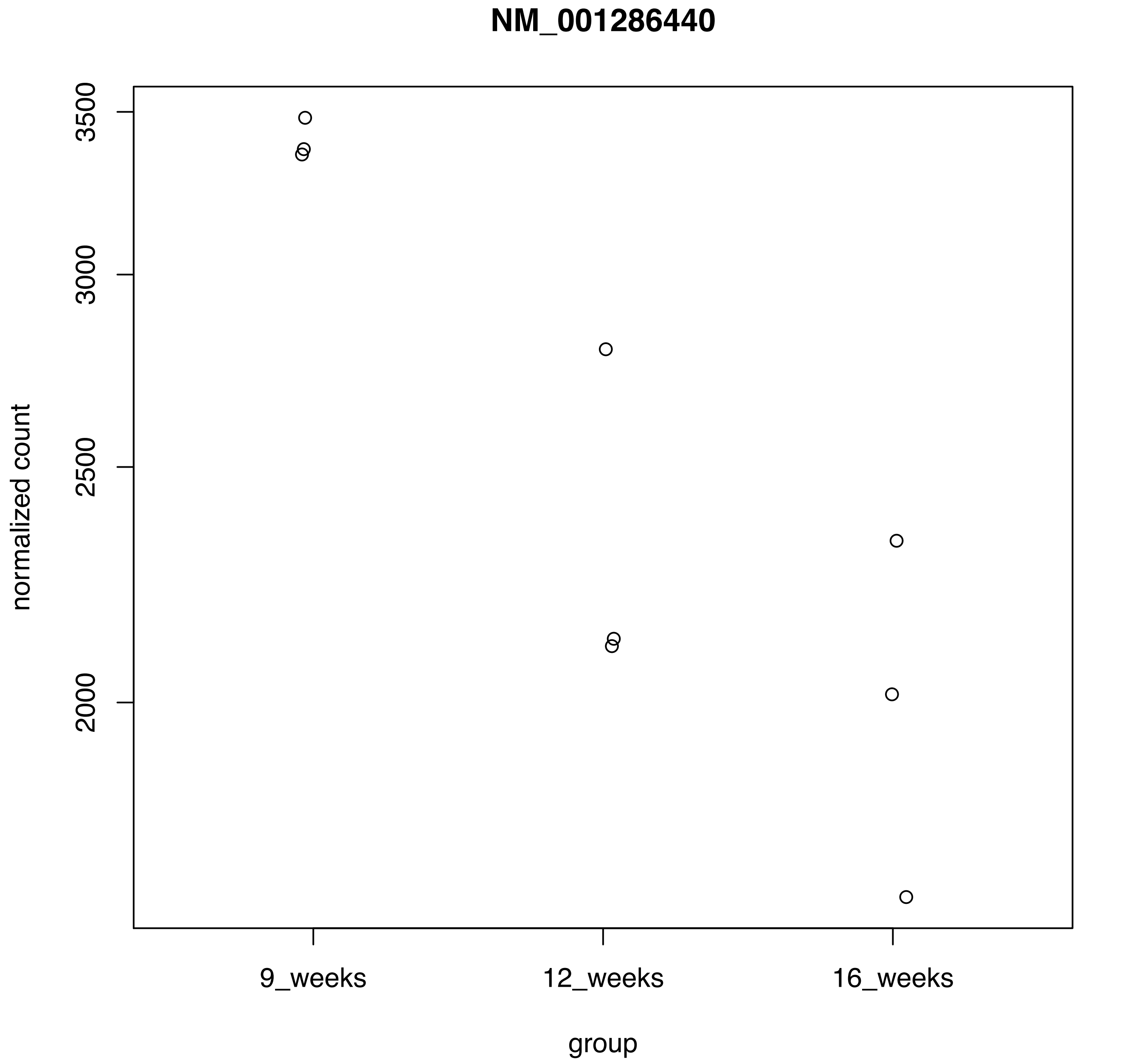

NM_001286440
3500
3000
2500
2000
normalized count
9_weeks
12_weeks
16_weeks
group

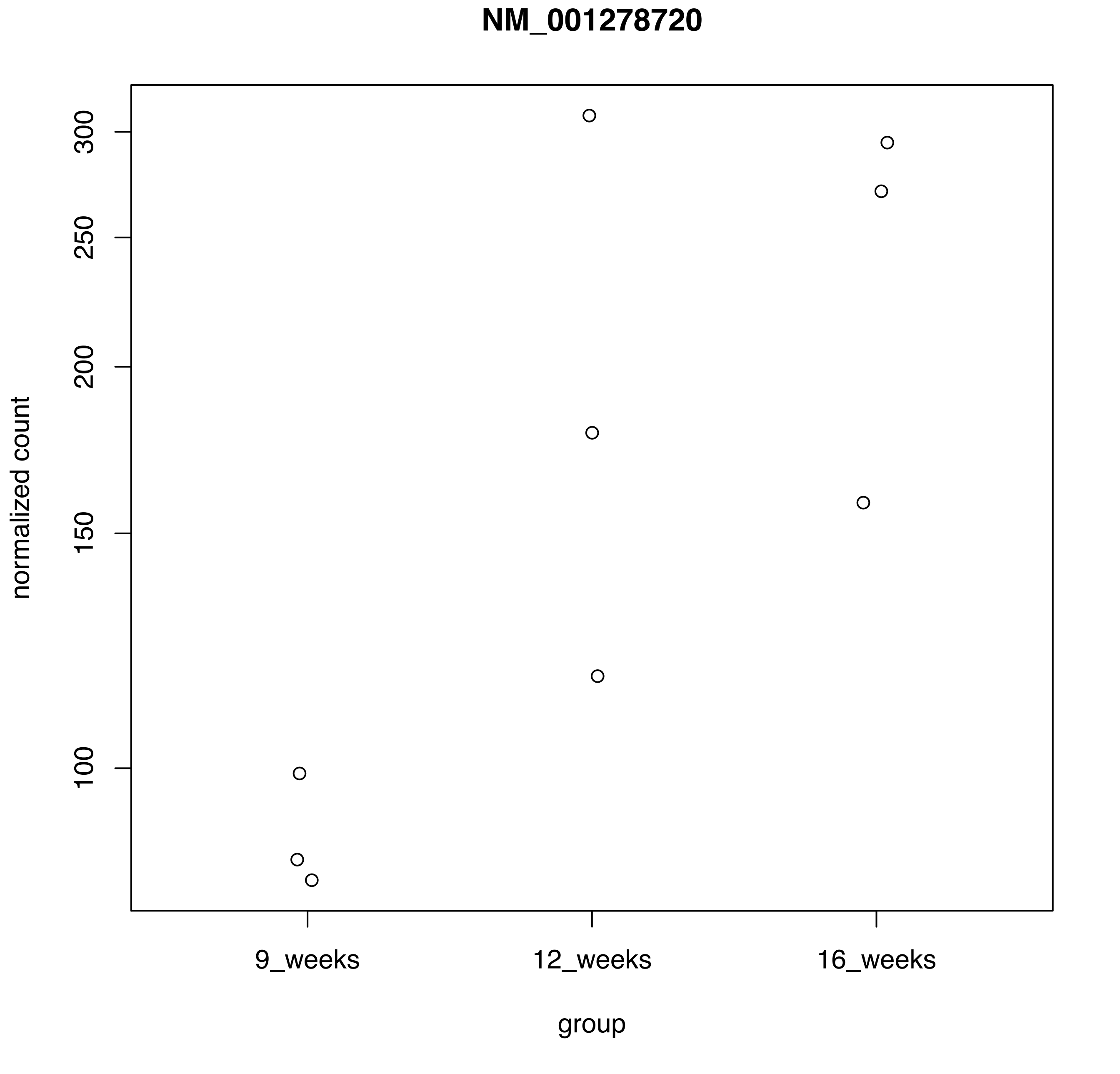
NM_001278720
normalized count
300
250
200
150
100
9_weeks
12_weeks
16_weeks
group

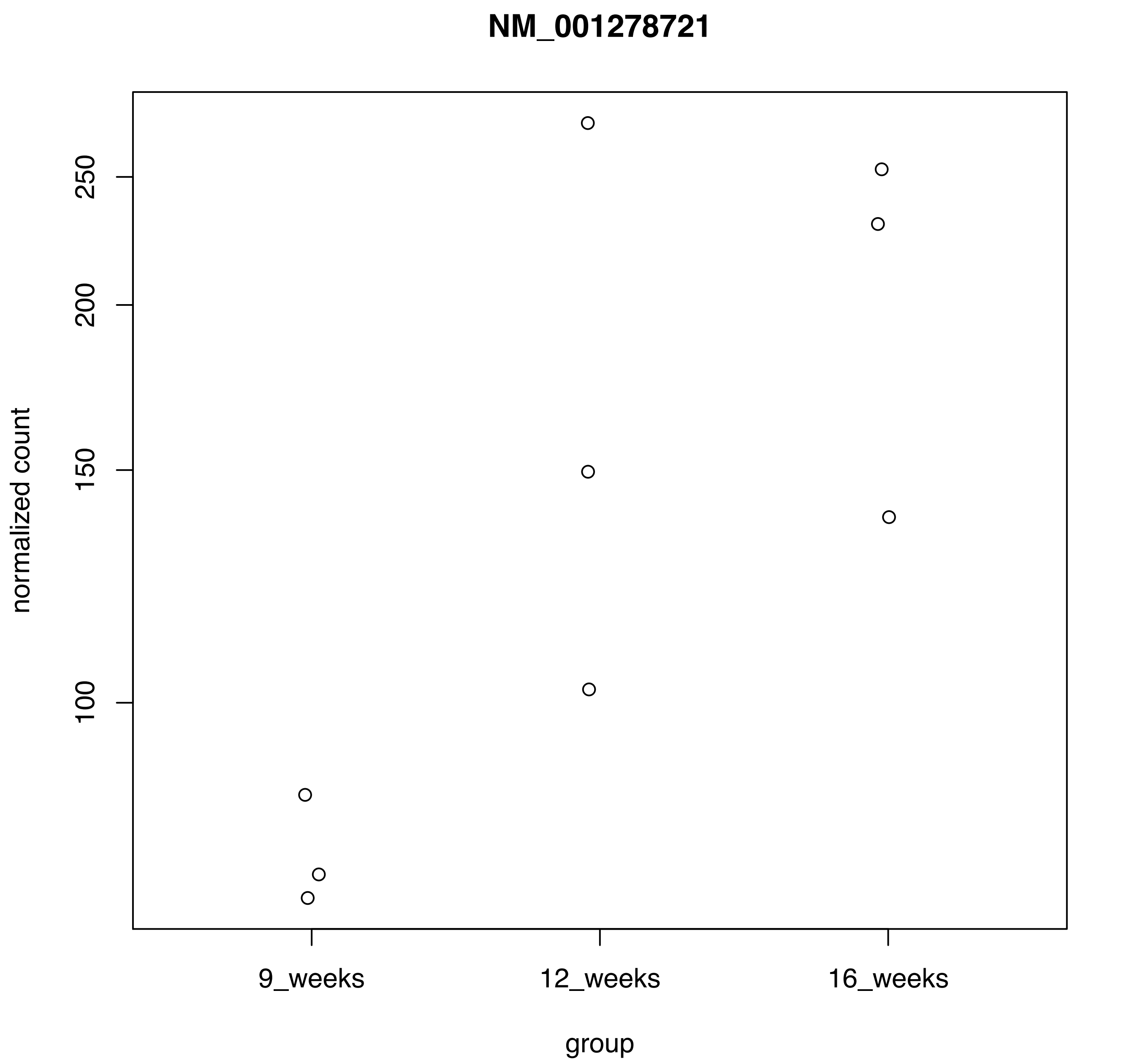

NM_001278721
normalized count
100
150
200
250
9_weeks
12_weeks
16_weeks
group

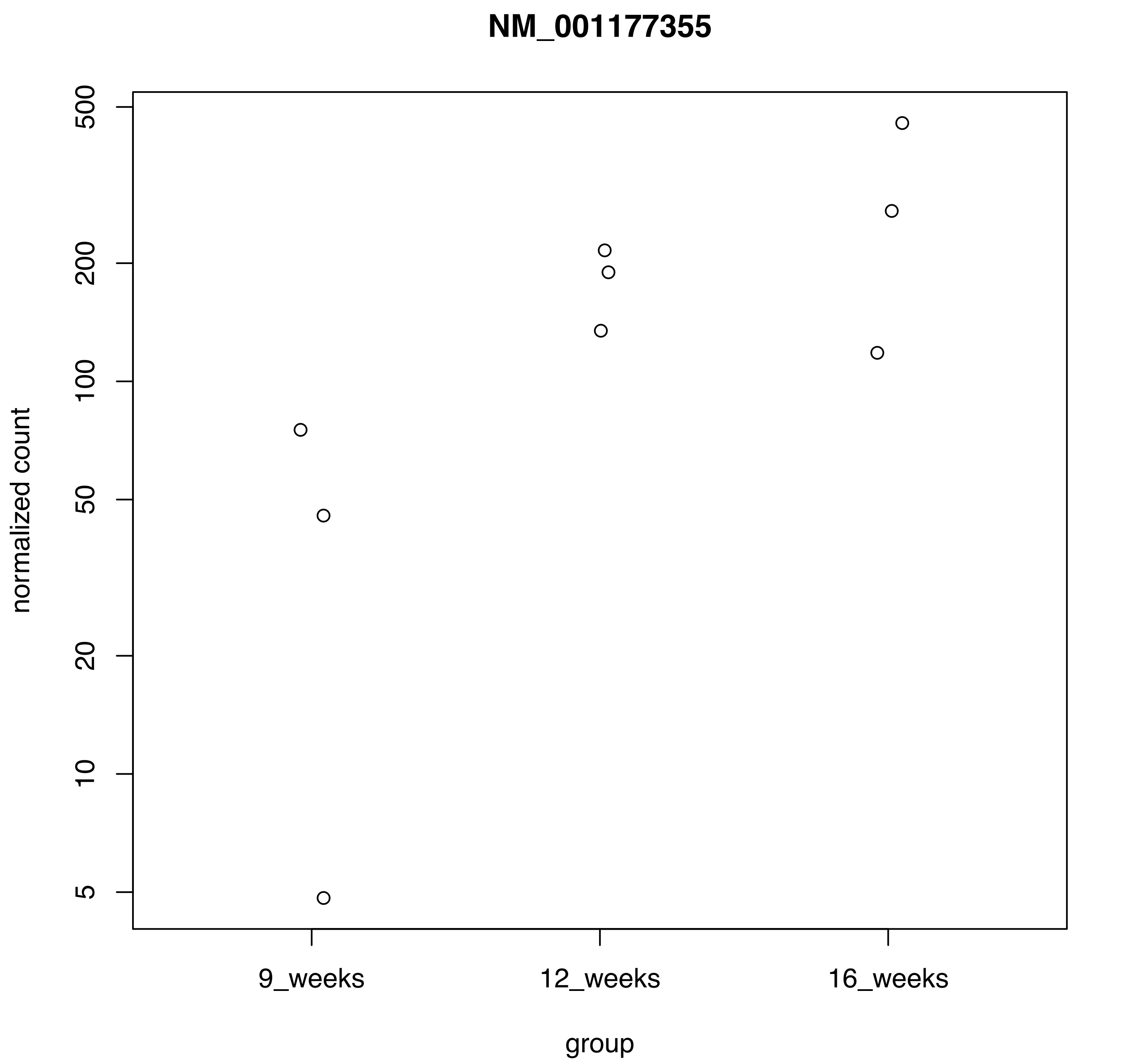
NM_001177355
normalized count
500
200
100
50
20
10
5
9_weeks
12_weeks
16_weeks
group

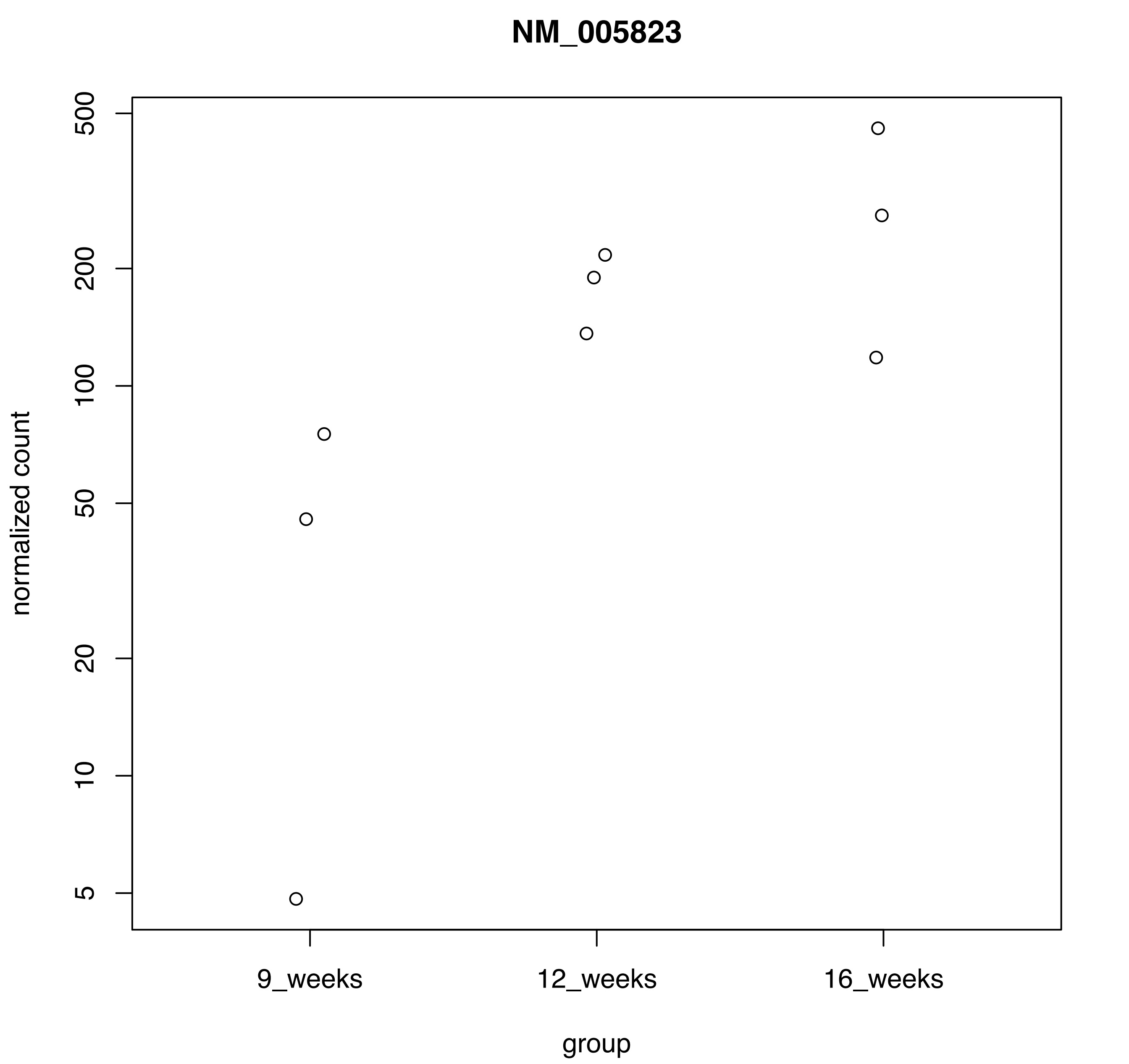
NM_005823
normalized count
500
200
100
50
20
10
5
9_weeks
12_weeks
16_weeks
group

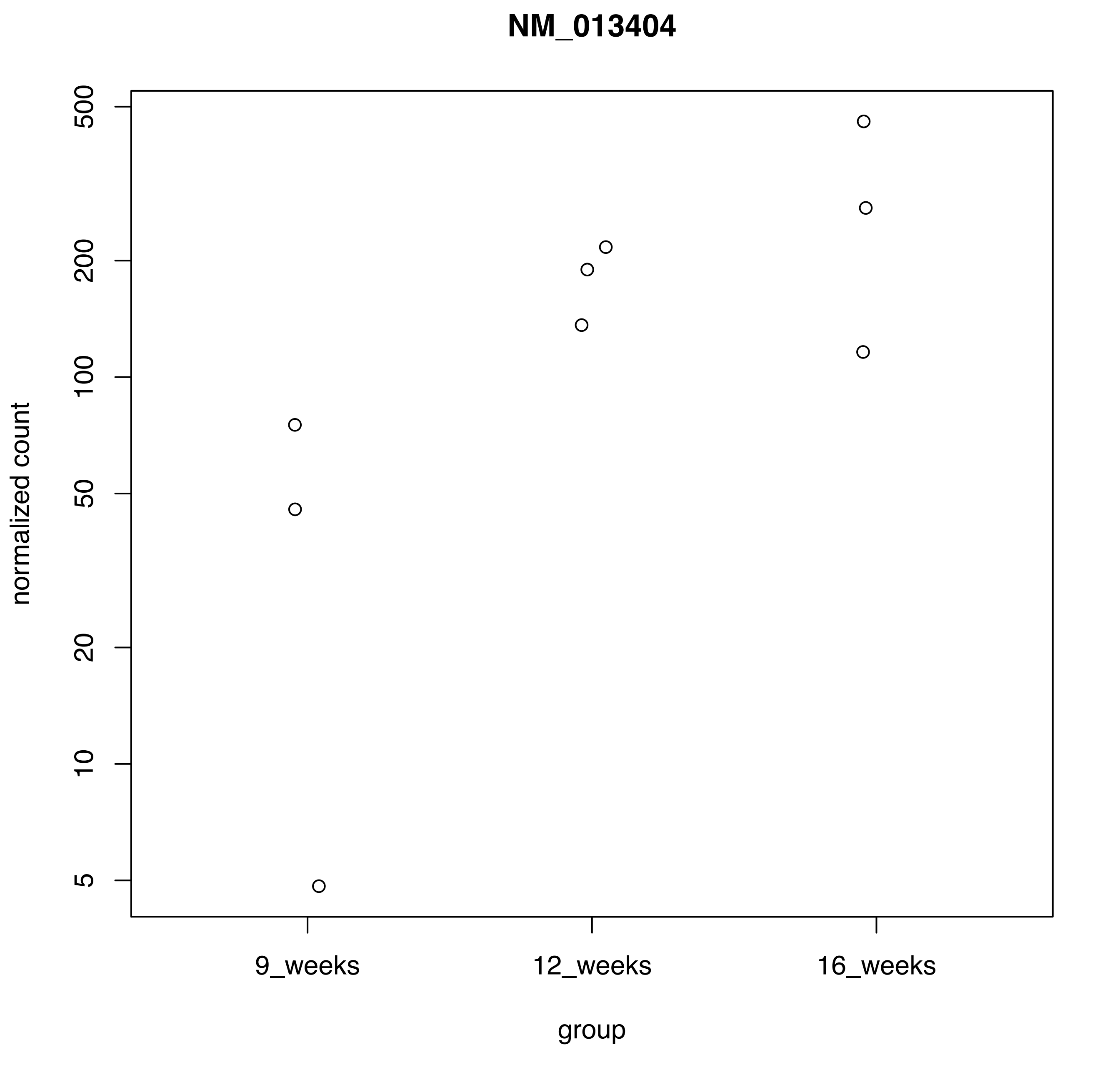
NM_013404
normalized count
500
200
100
50
20
10
5
9_weeks
12_weeks
16_weeks
group

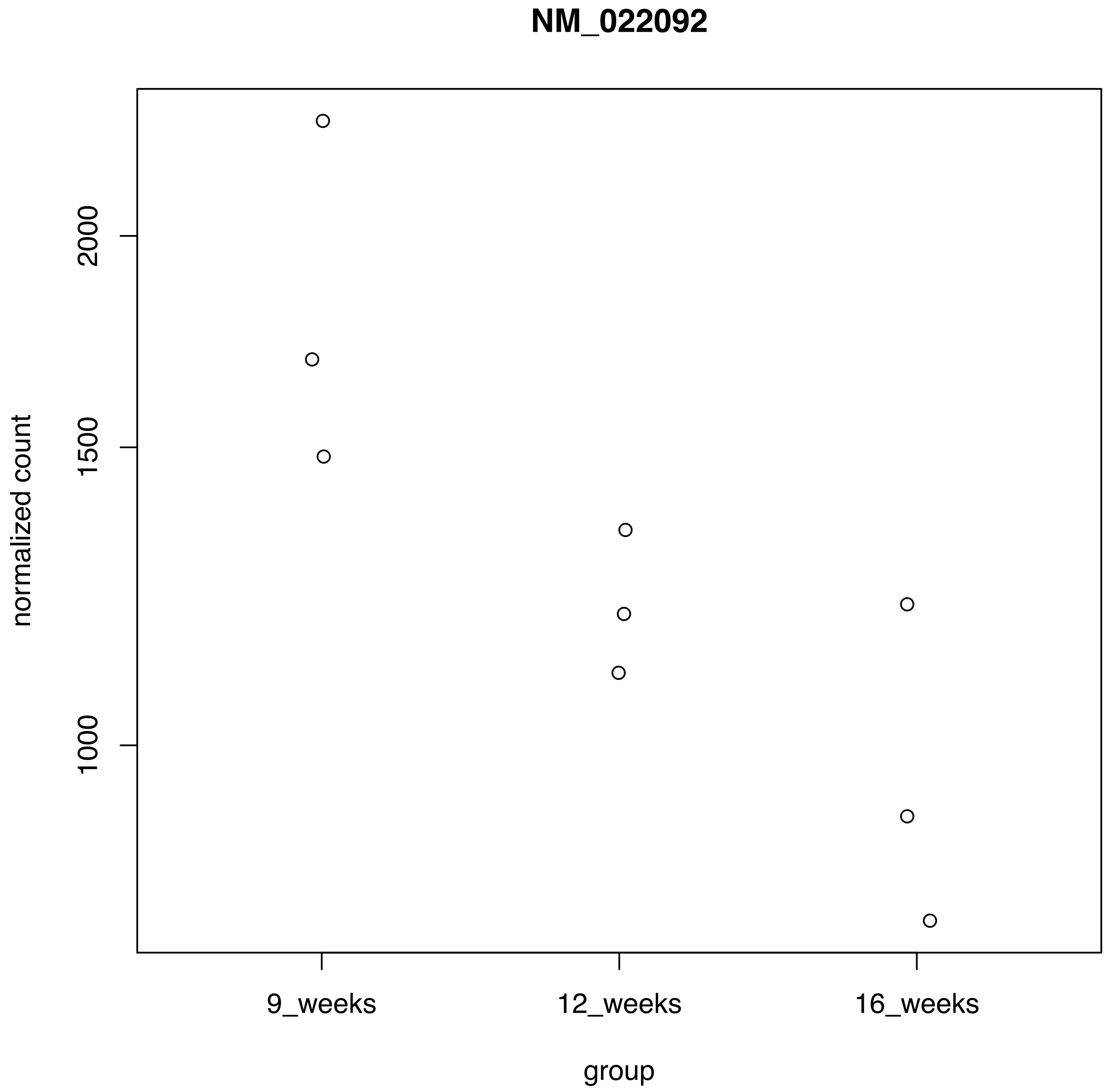
NM_022092
2000
1500
1000
normalized count
9_weeks
12_weeks
16_weeks
group

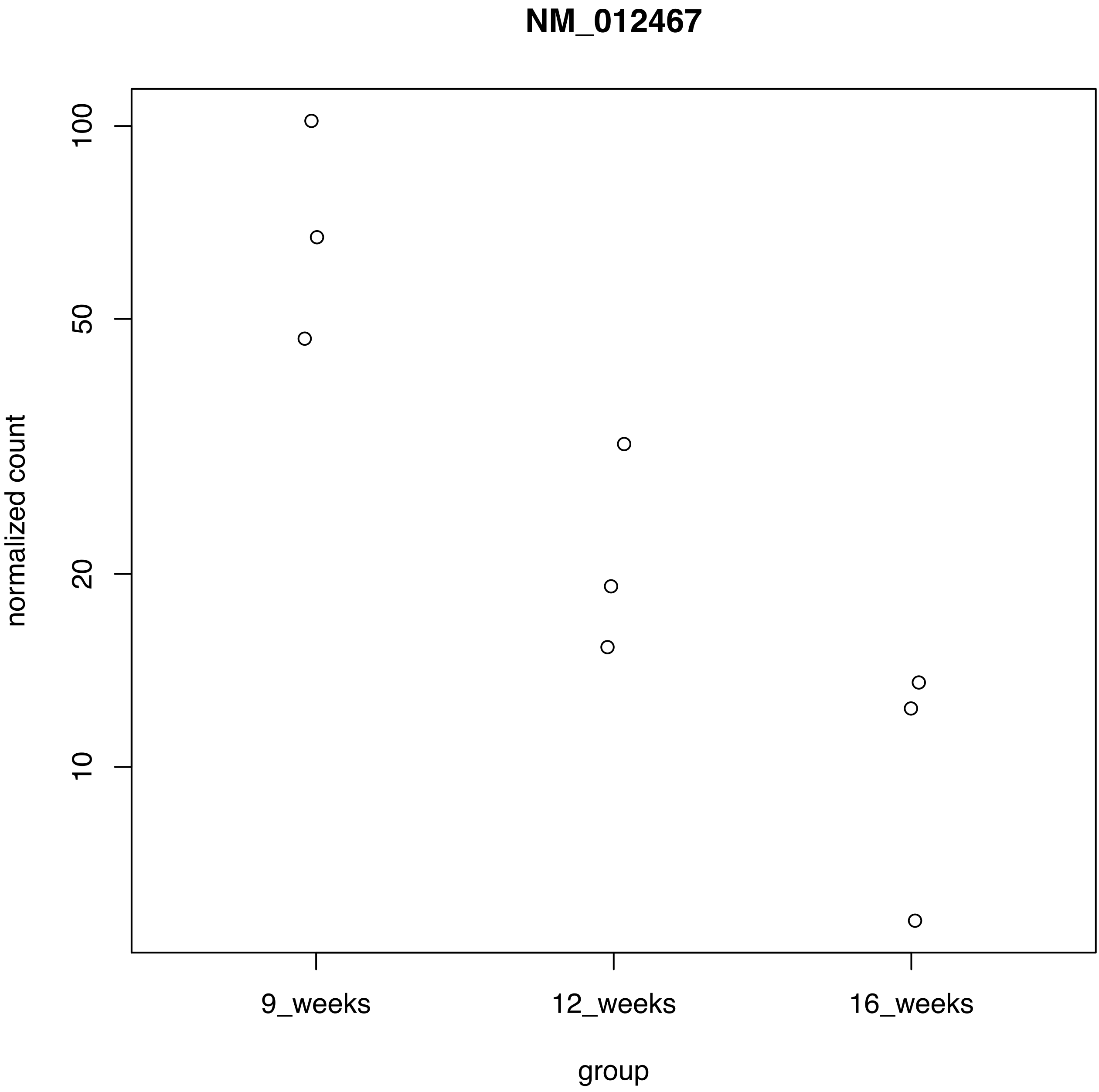

NM_012467
normalized count
100
50
20
10
9_weeks
12_weeks
16_weeks
group

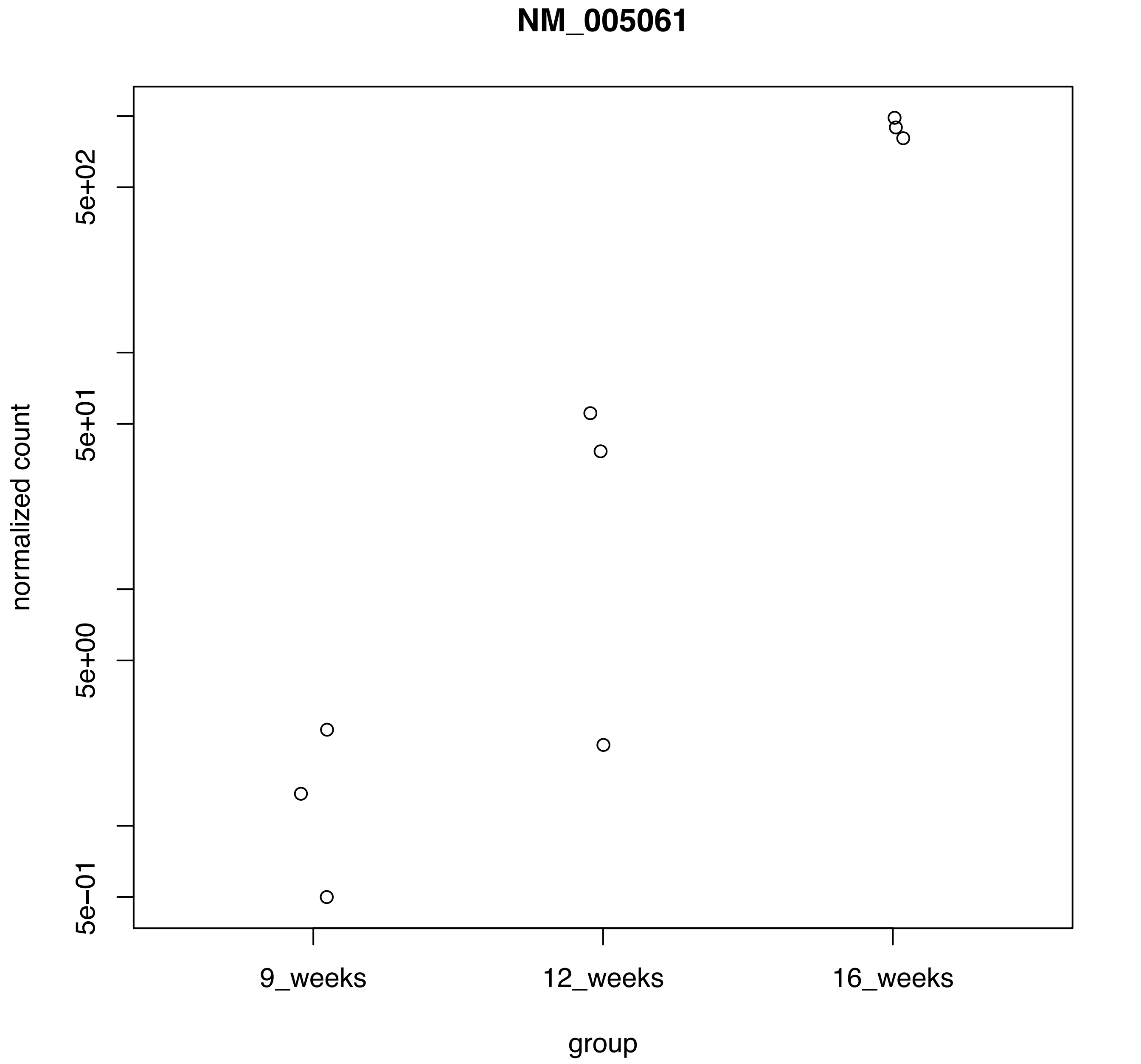
NM_005061
normalized count
5e+02
5e+01
5e+00
5e−01
9_weeks
12_weeks
16_weeks
group

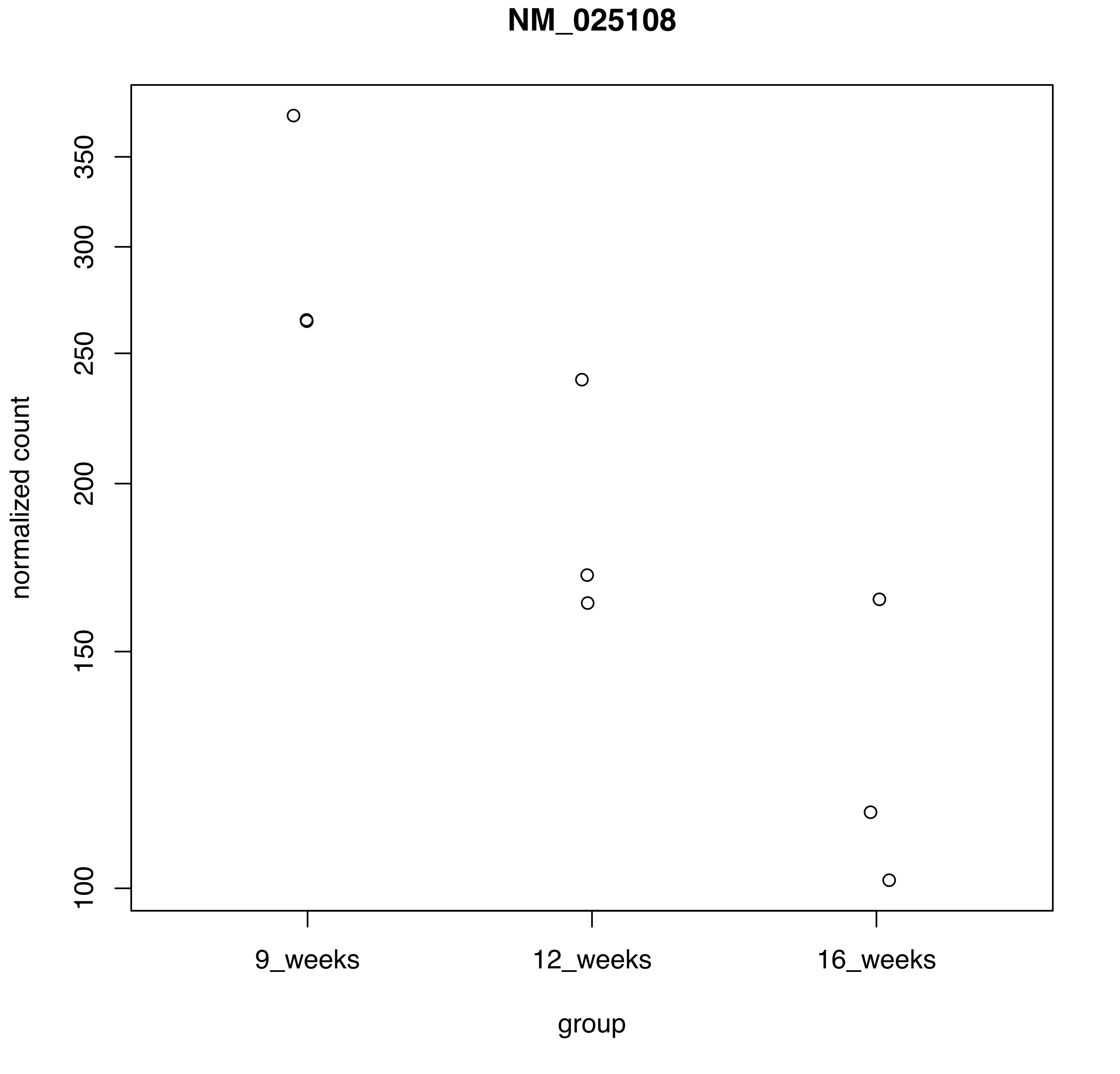
NM_025108
normalized count
100
150
200
250
300
350
9_weeks
12_weeks
16_weeks
group

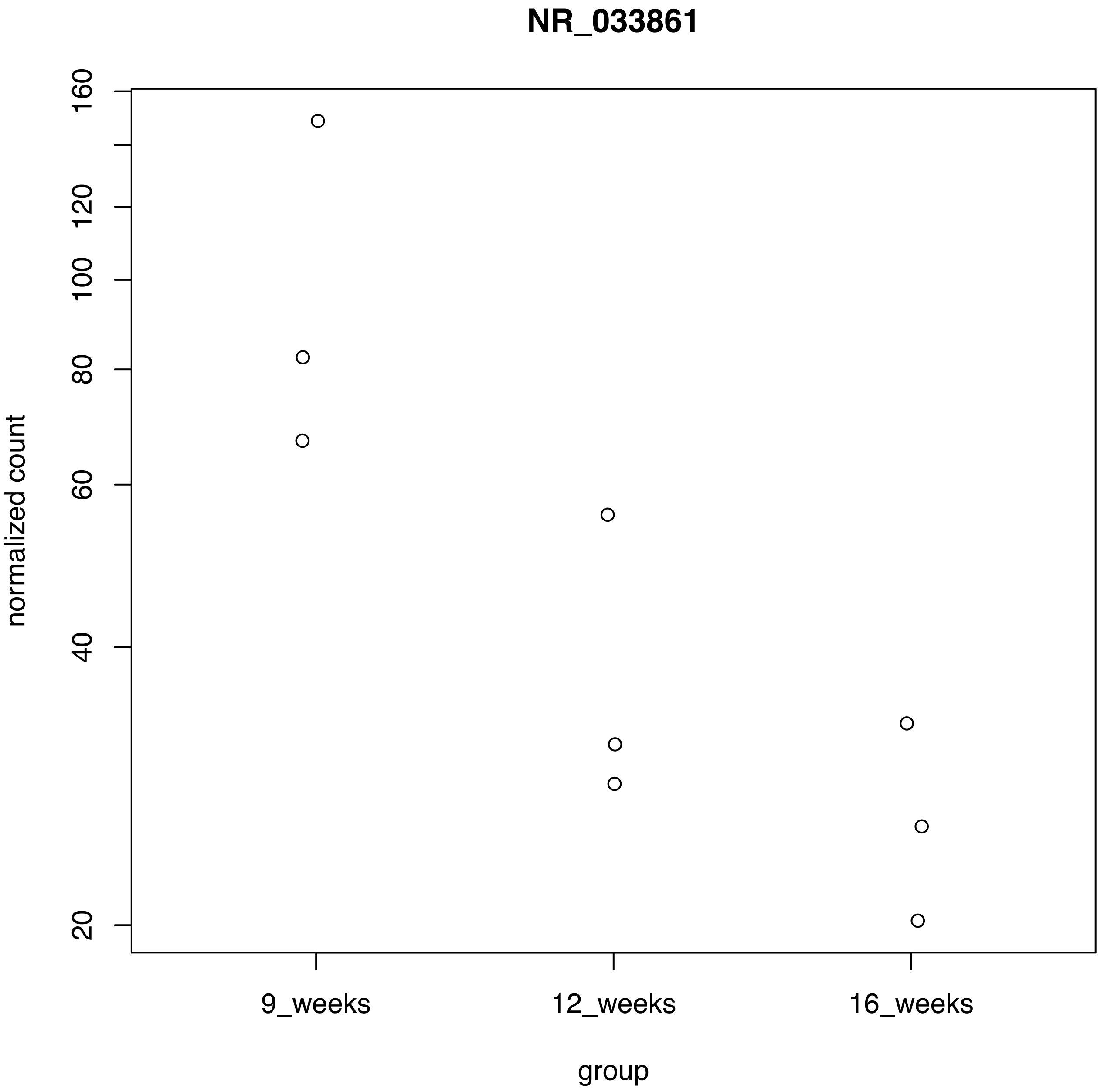
NR_033861
normalized count
160
120
100
80
60
40
20
9_weeks
12_weeks
16_weeks
group

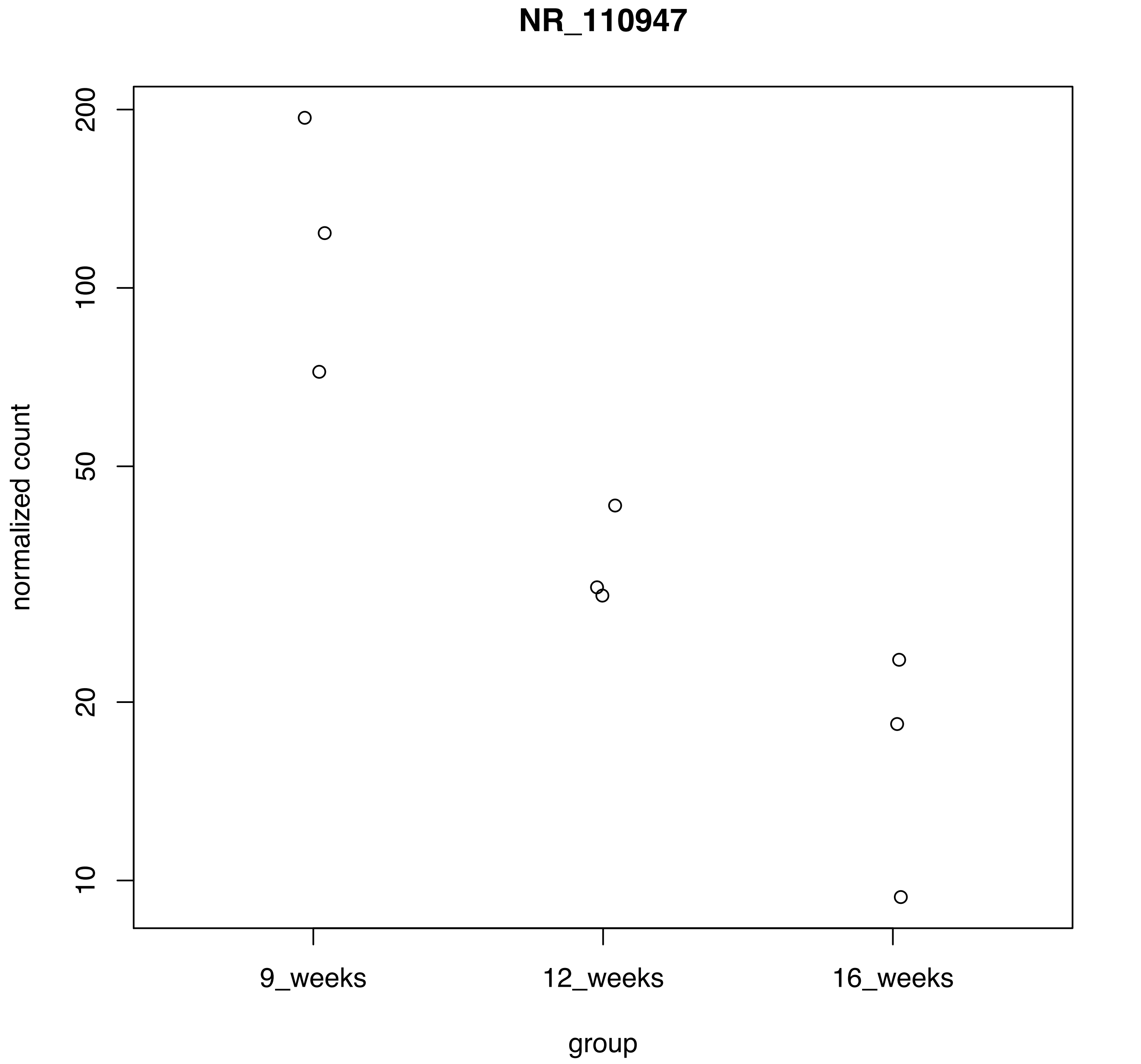
NR_110947
200
100
50
20
10
normalized count
9_weeks
12_weeks
16_weeks
group

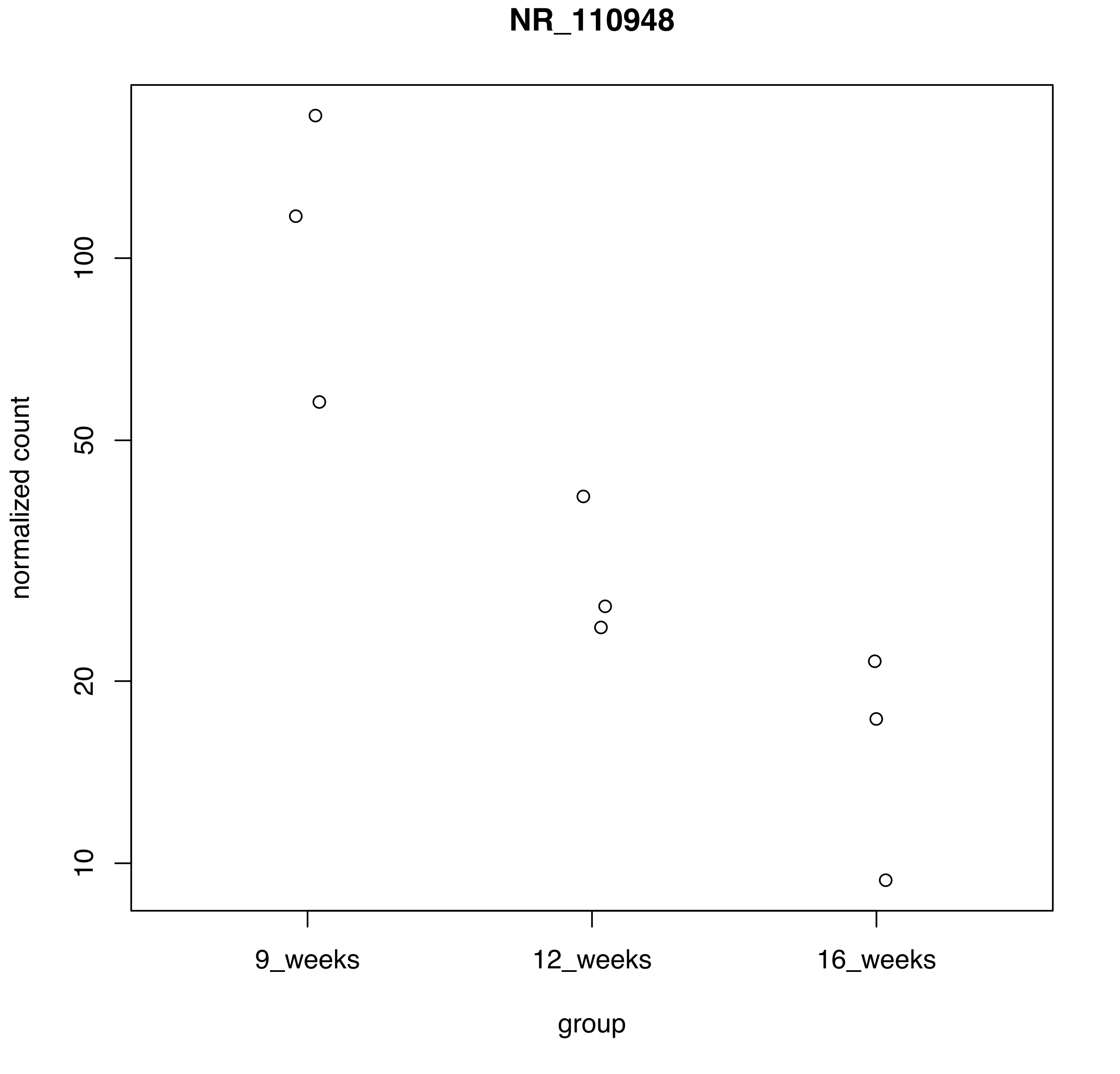
NR_110948
normalized count
100
50
20
10
9_weeks
12_weeks
16_weeks
group

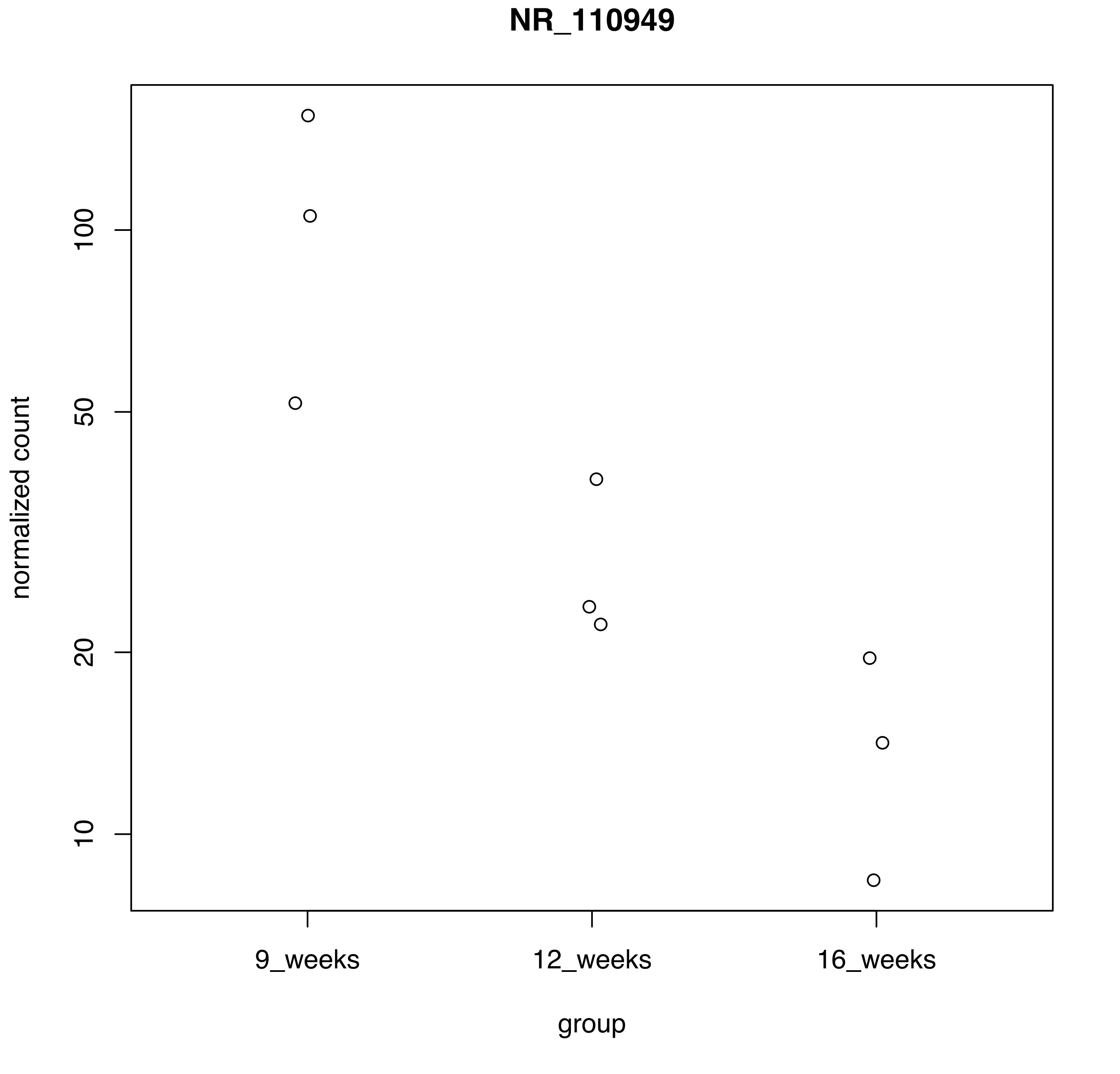

NR_110949
normalized count
100
50
20
10
9_weeks
12_weeks
16_weeks
group

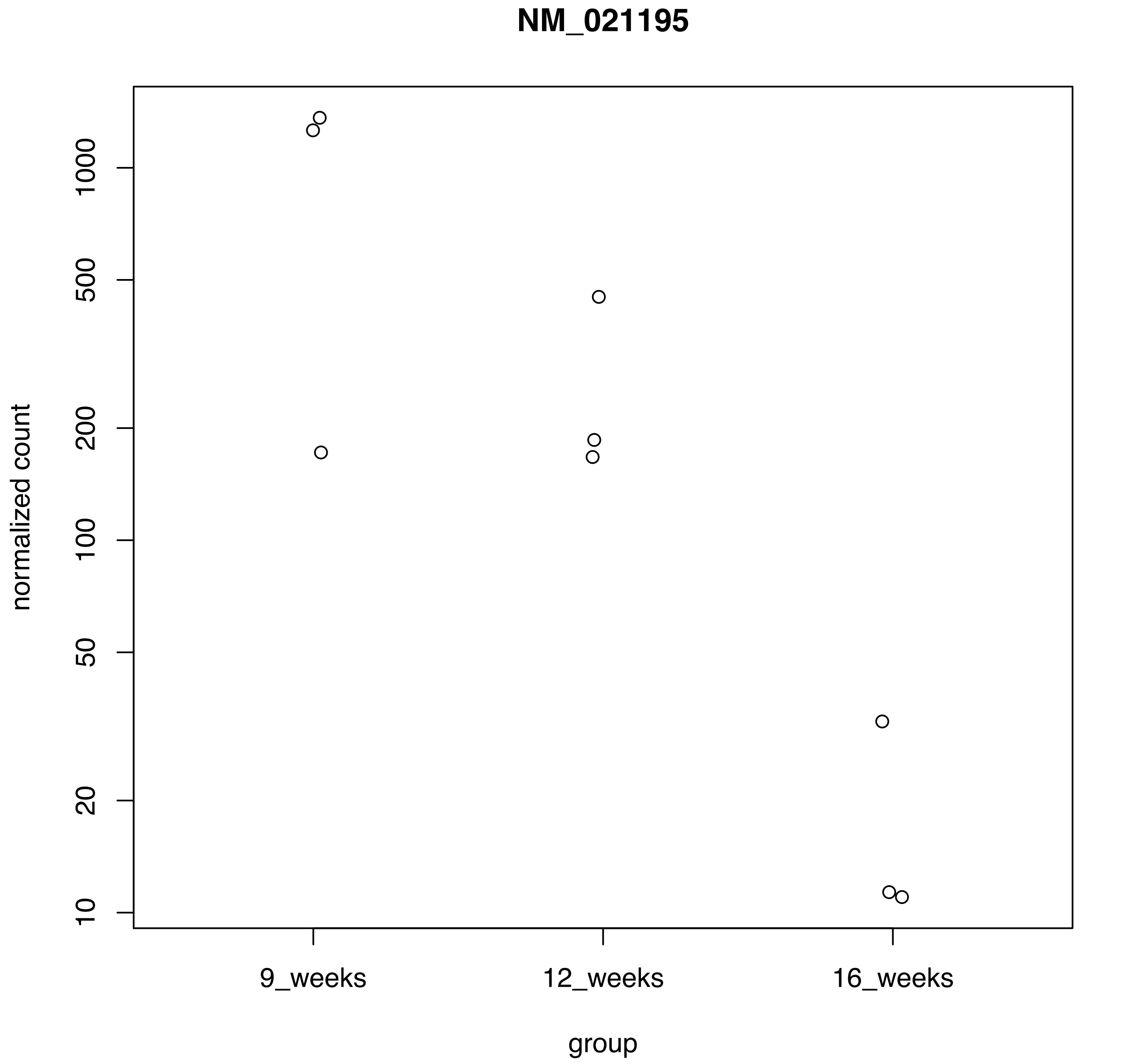
NM_021195
normalized count
1000
500
200
100
50
20
10
9_weeks
12_weeks
16_weeks
group

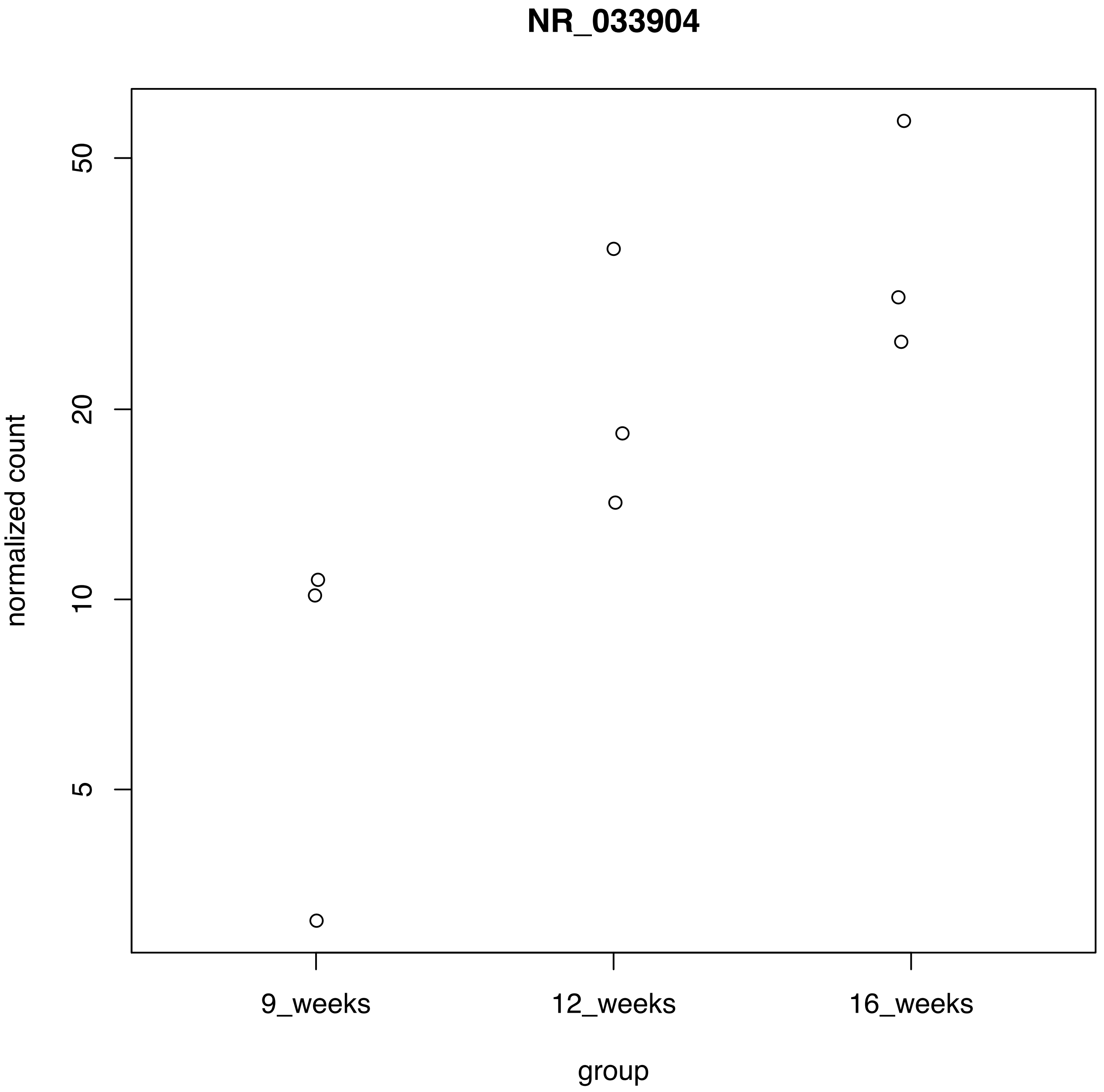
NR_033904
normalized count
50
20
10
5
9_weeks
12_weeks
16_weeks
group

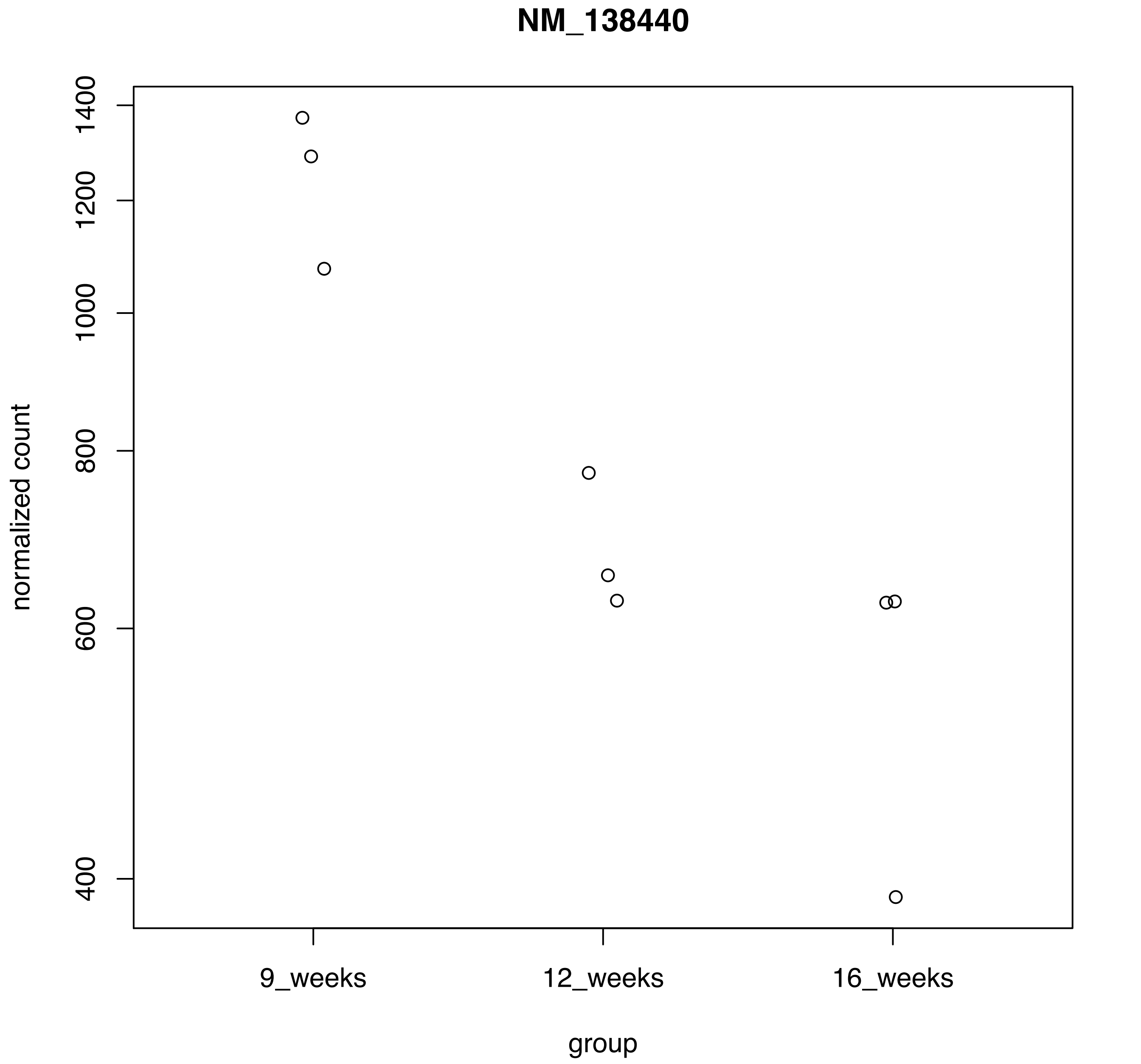
NM_138440
normalized count
1400
1200
1000
800
600
400
9_weeks
12_weeks
16_weeks
group

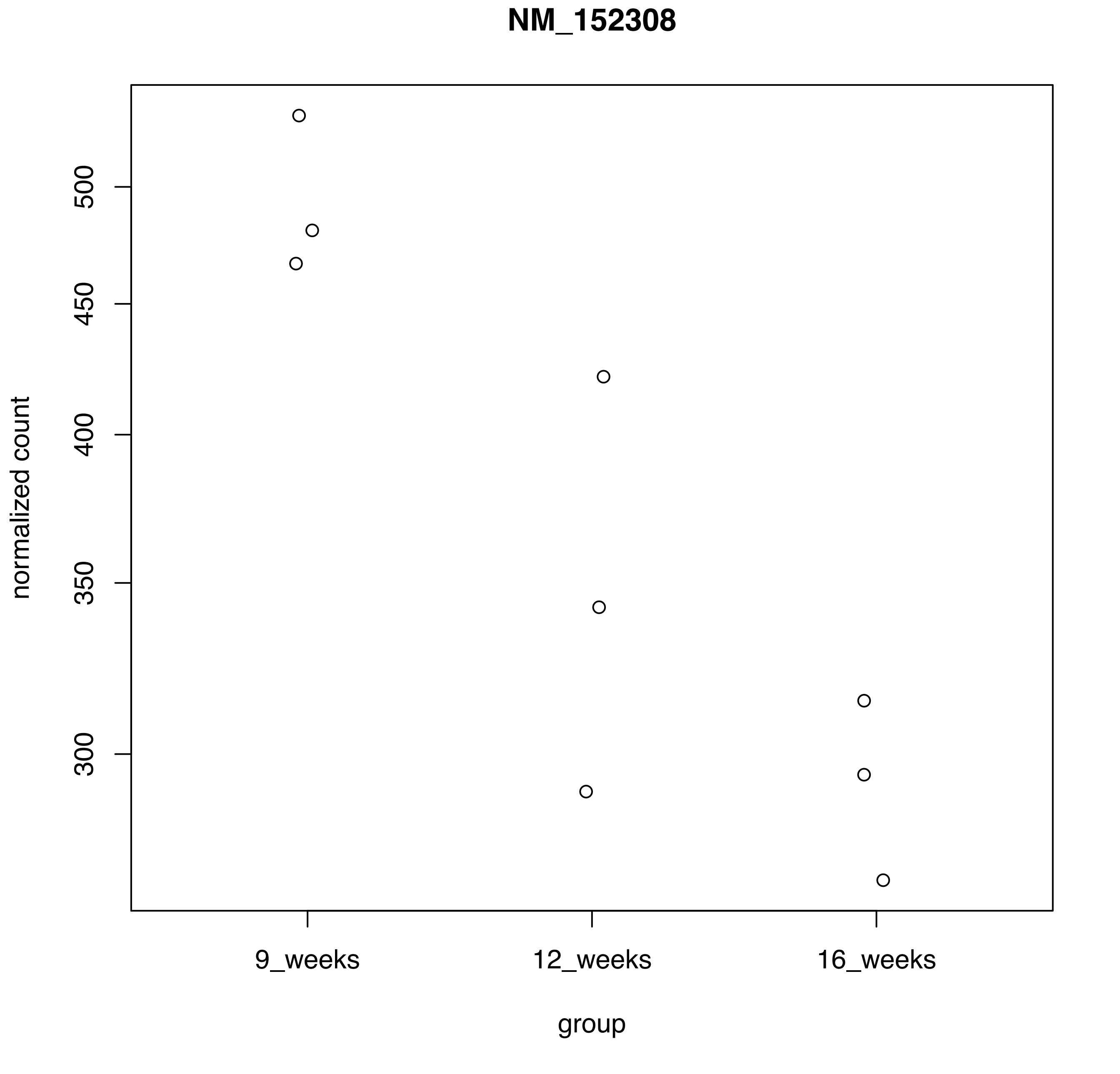
NM_152308
normalized count
500
450
400
350
300
9_weeks
12_weeks
16_weeks
group

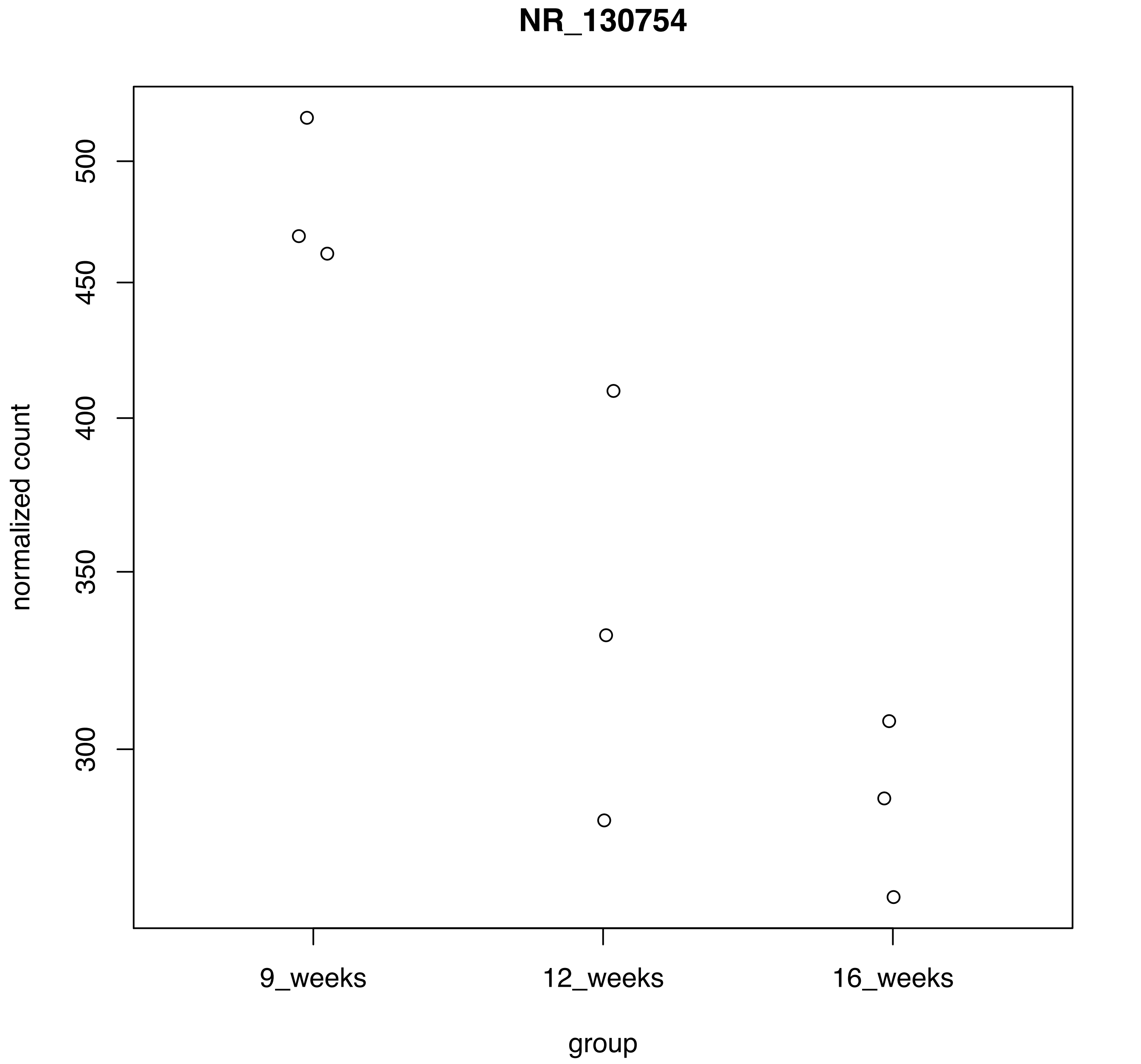
NR_130754
normalized count
300
350
400
450
500
9_weeks
12_weeks
16_weeks
group

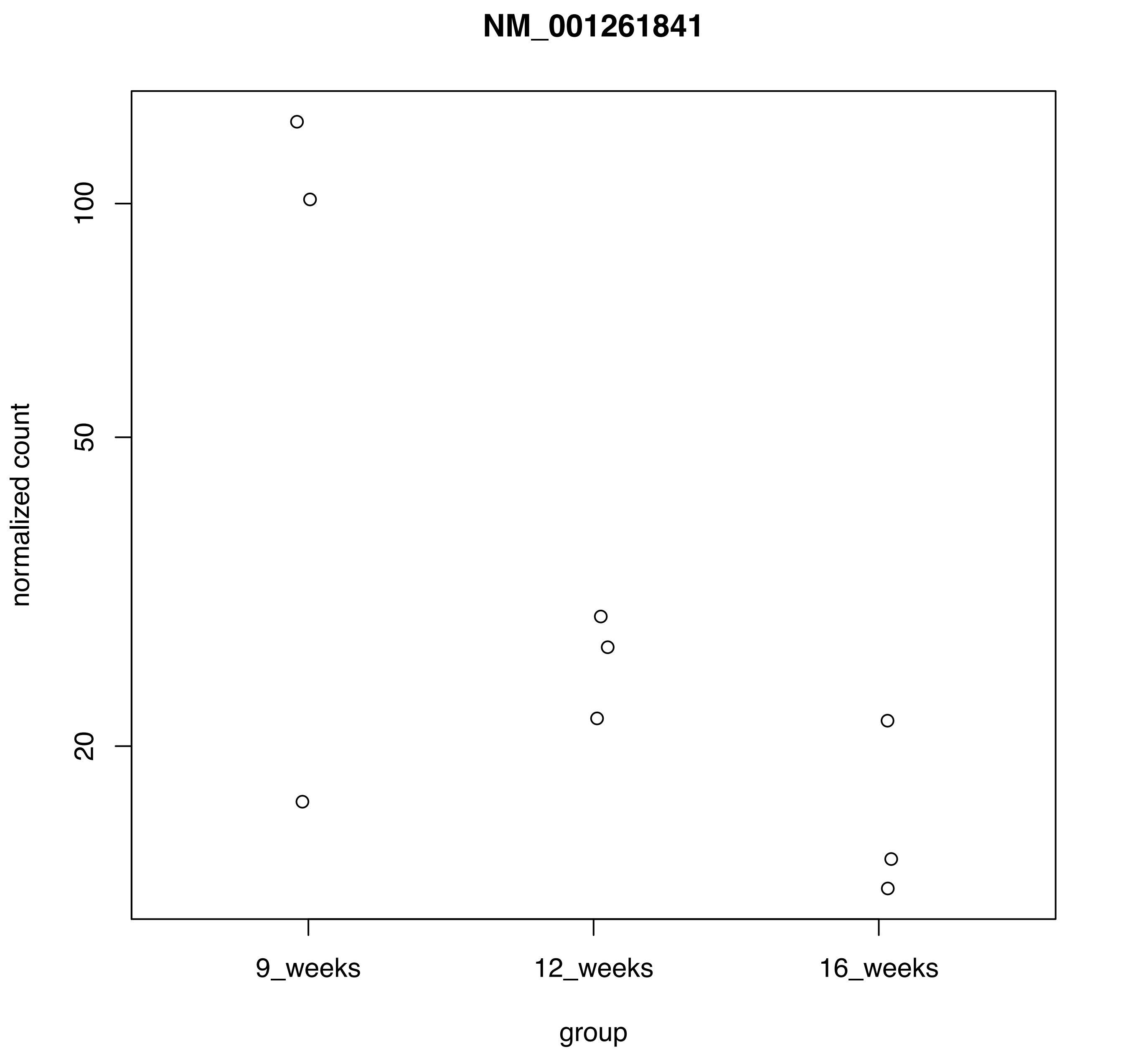
NM_001261841
normalized count
100
50
20
9_weeks
12_weeks
16_weeks
group

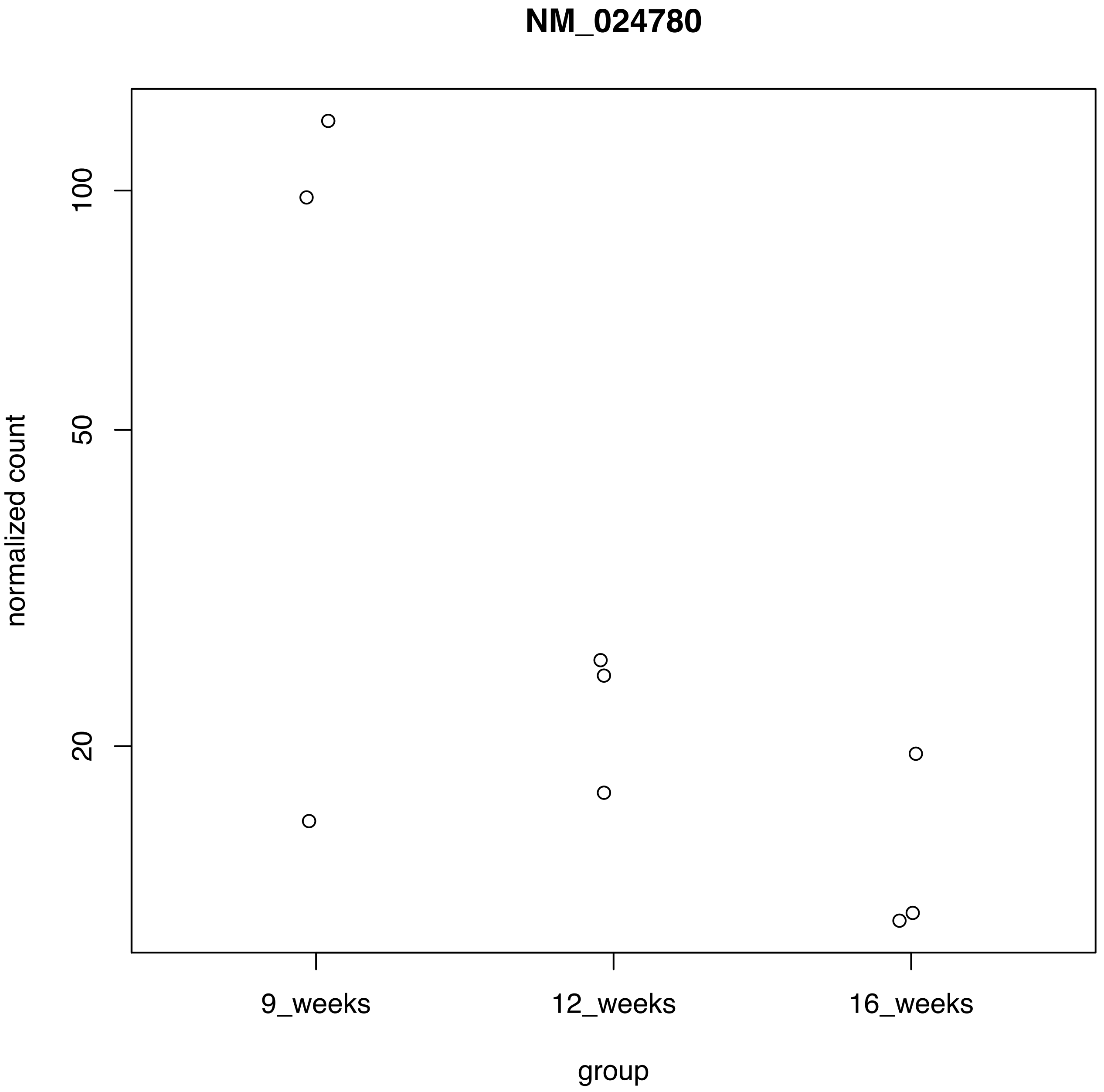

NM_024780
normalized count
100
50
20
9_weeks
12_weeks
16_weeks
group

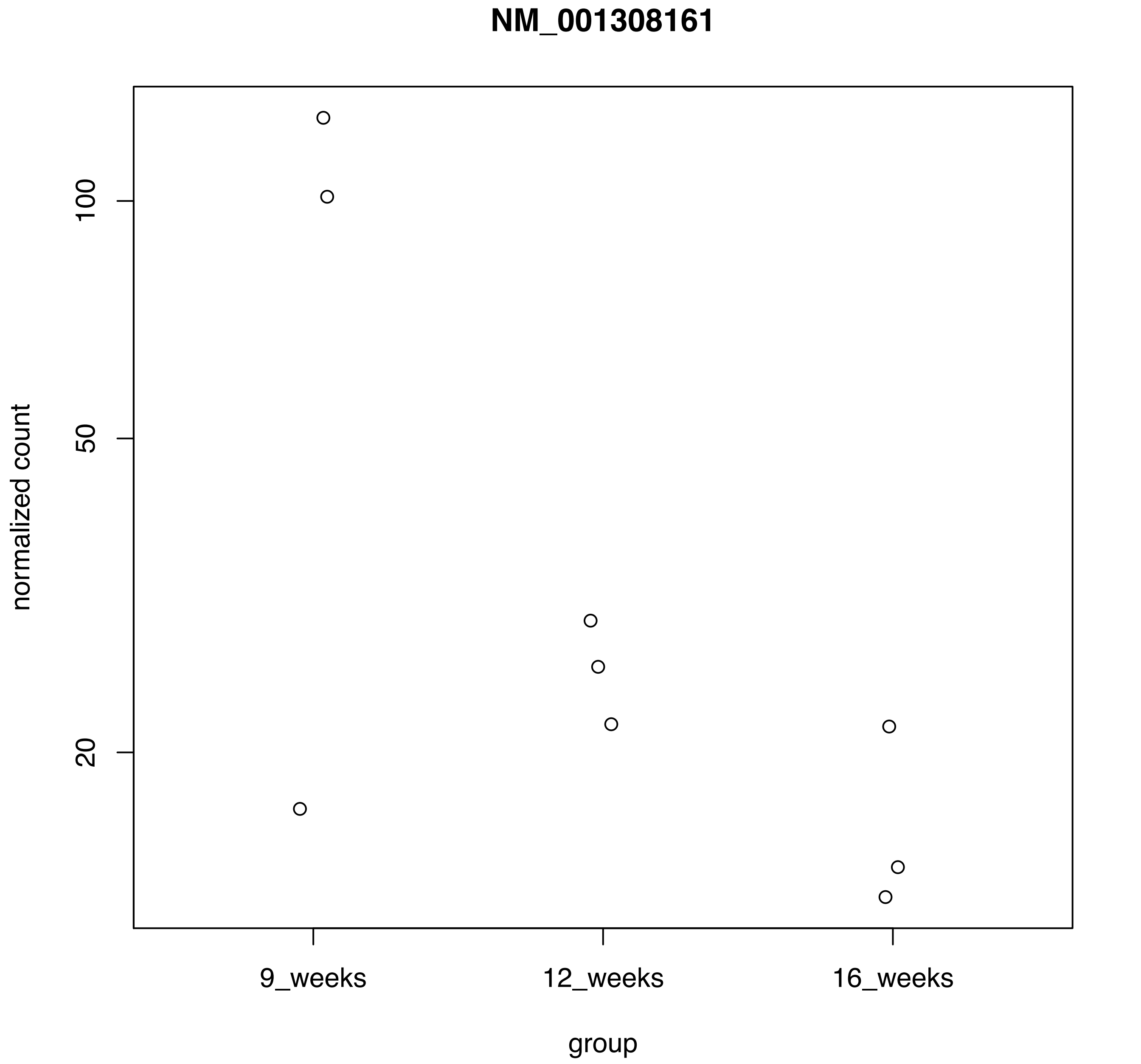

NM_001308161
normalized count
100
50
20
9_weeks
12_weeks
16_weeks
group

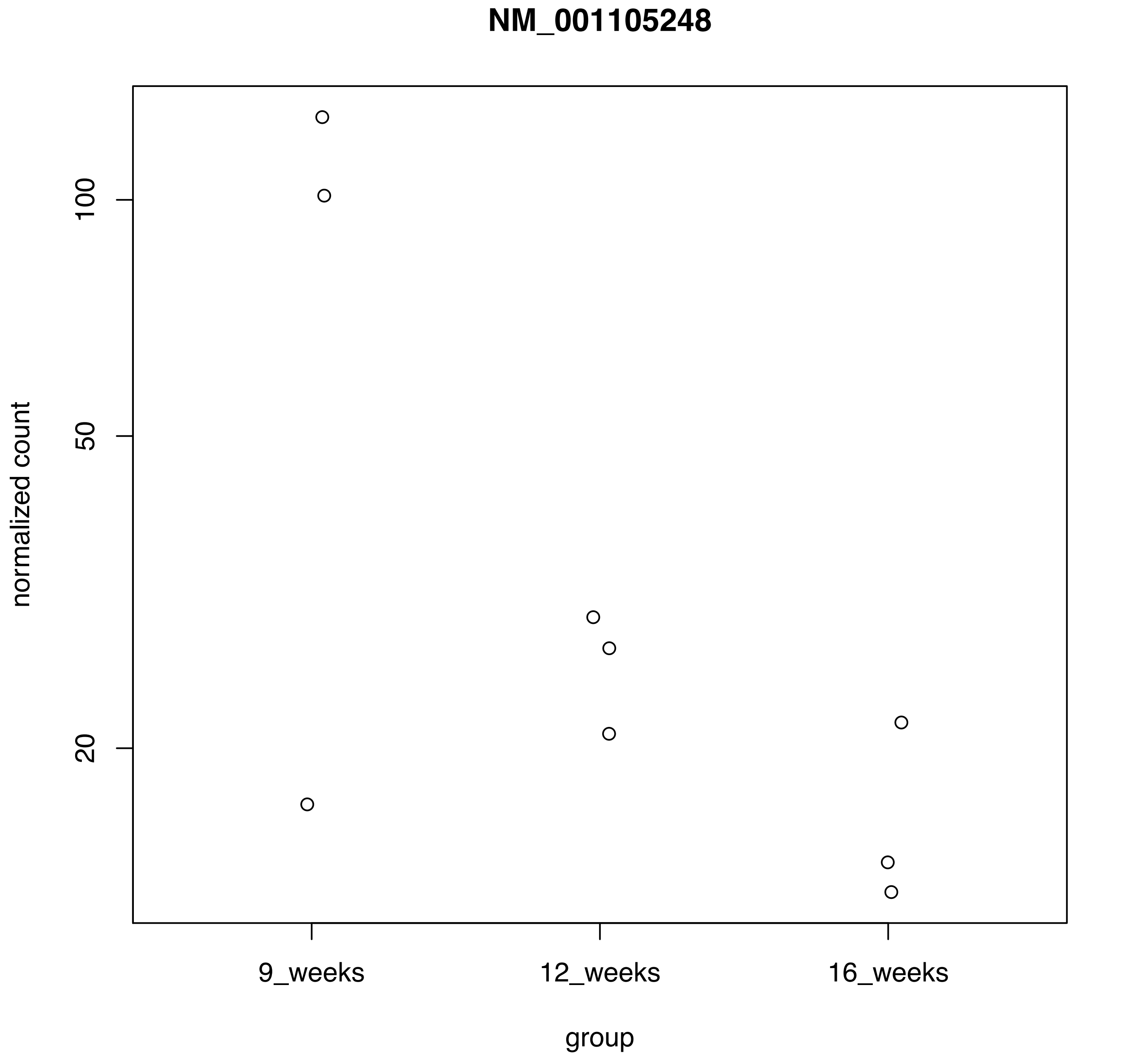
NM_001105248
normalized count
100
50
20
9_weeks
12_weeks
16_weeks
group

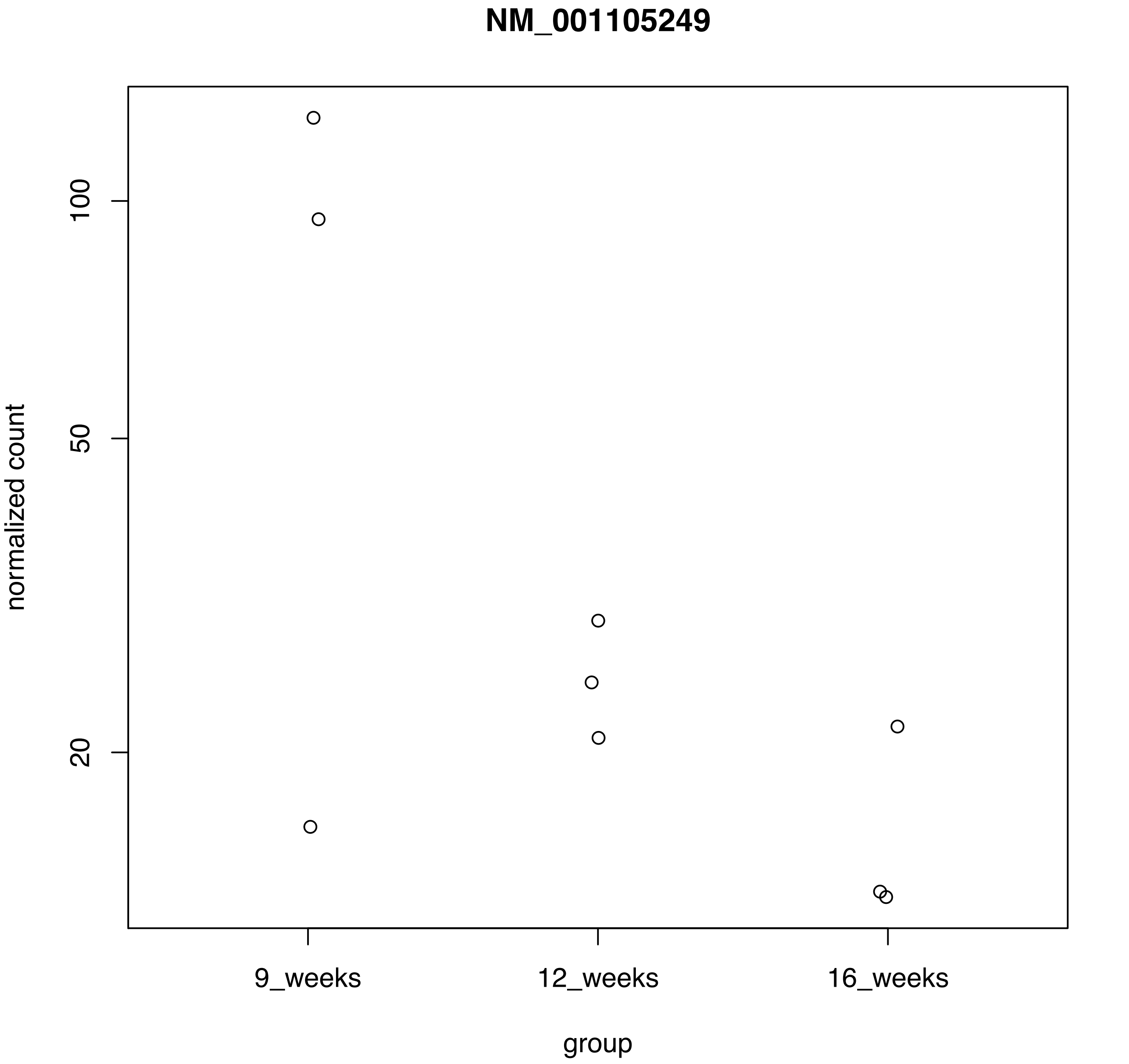
NM_001105249
100
50
20
normalized count
9_weeks
12_weeks
16_weeks
group

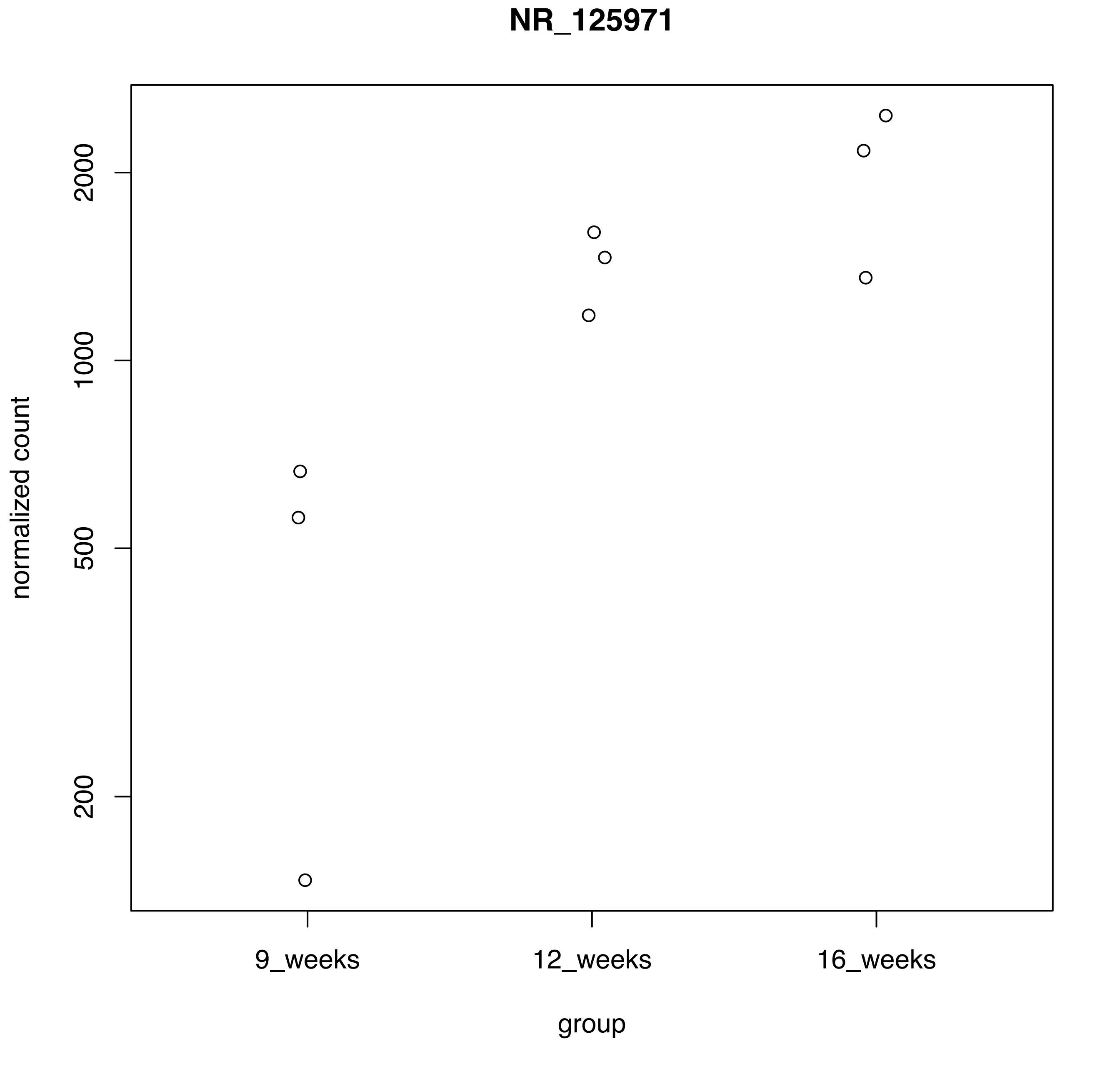
NR_125971
normalized count
2000
1000
500
200
9_weeks
12_weeks
16_weeks
group

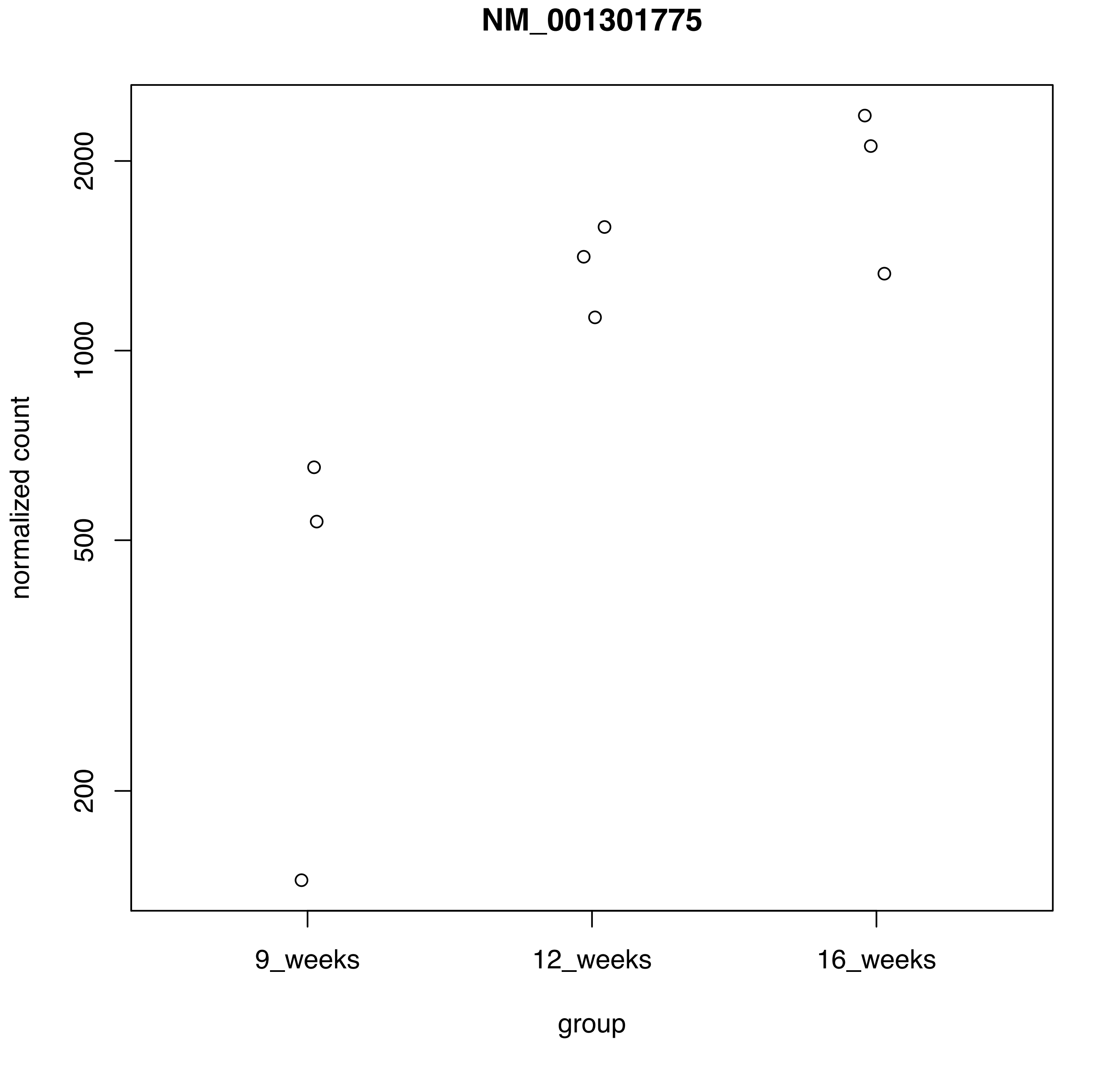
NM_001301775
normalized count
2000
1000
500
200
9_weeks
12_weeks
16_weeks
group

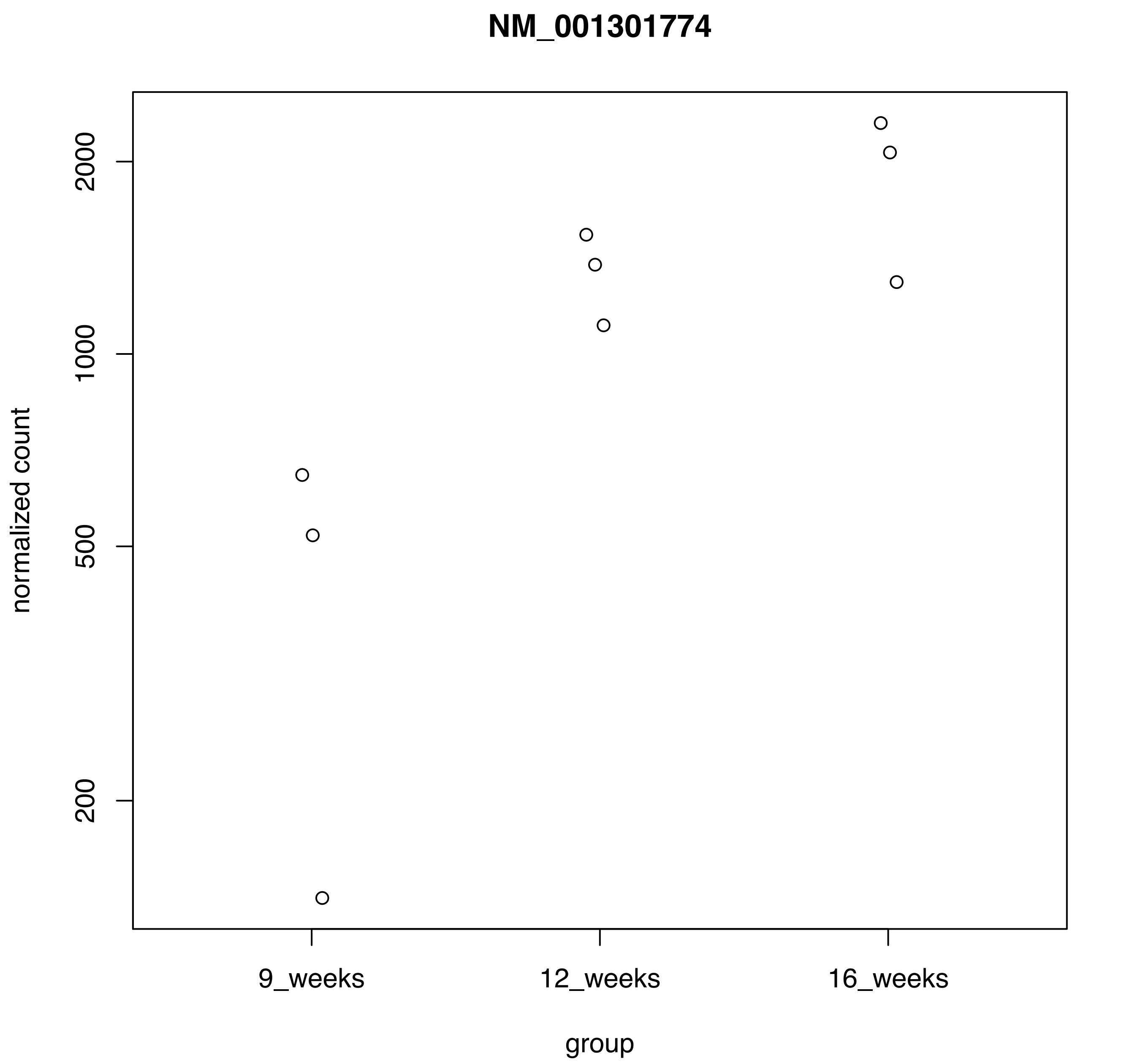
NM_001301774
normalized count
2000
1000
500
200
9_weeks
12_weeks
16_weeks
group

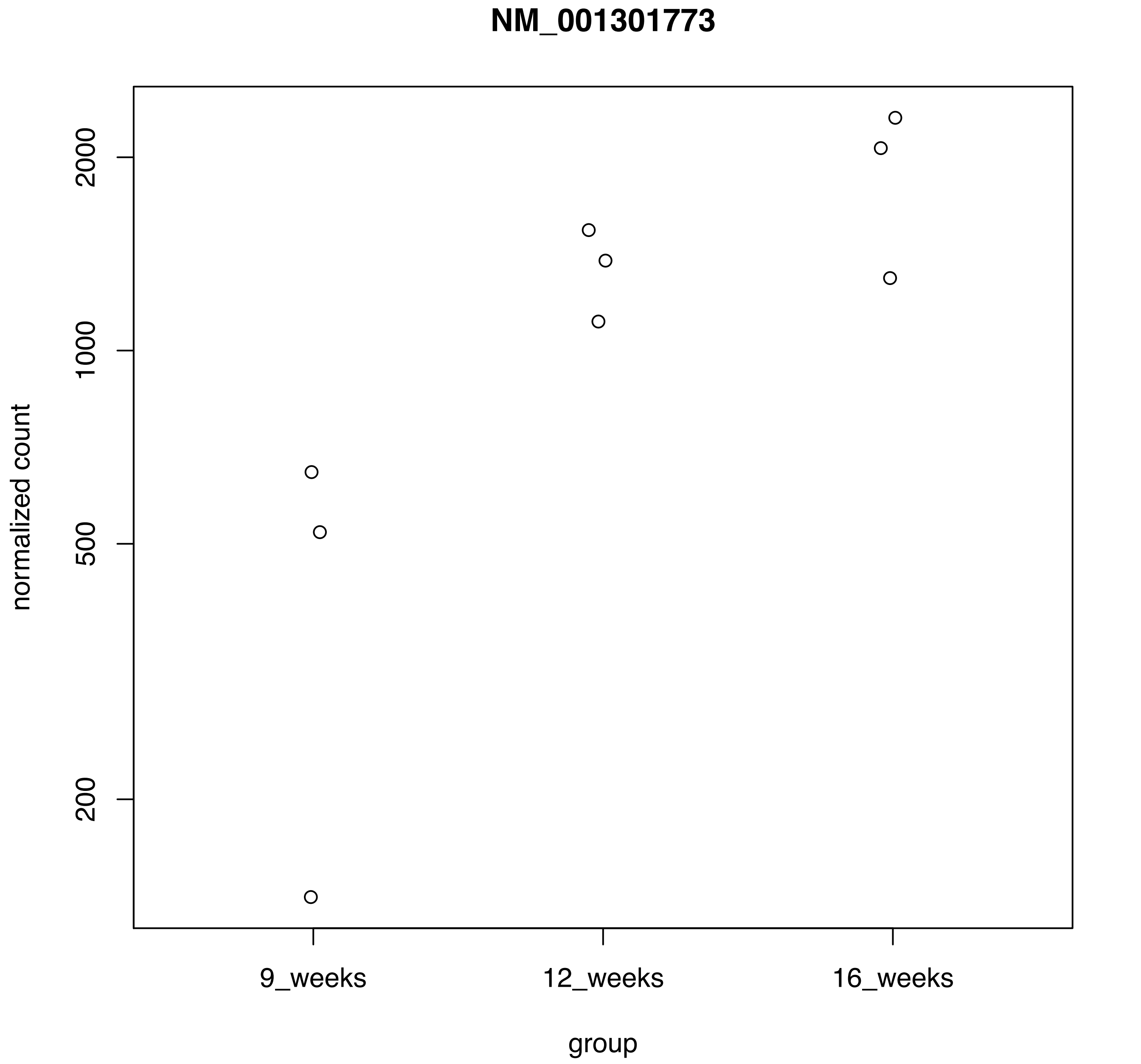
NM_001301773
normalized count
2000
1000
500
200
9_weeks
12_weeks
16_weeks
group

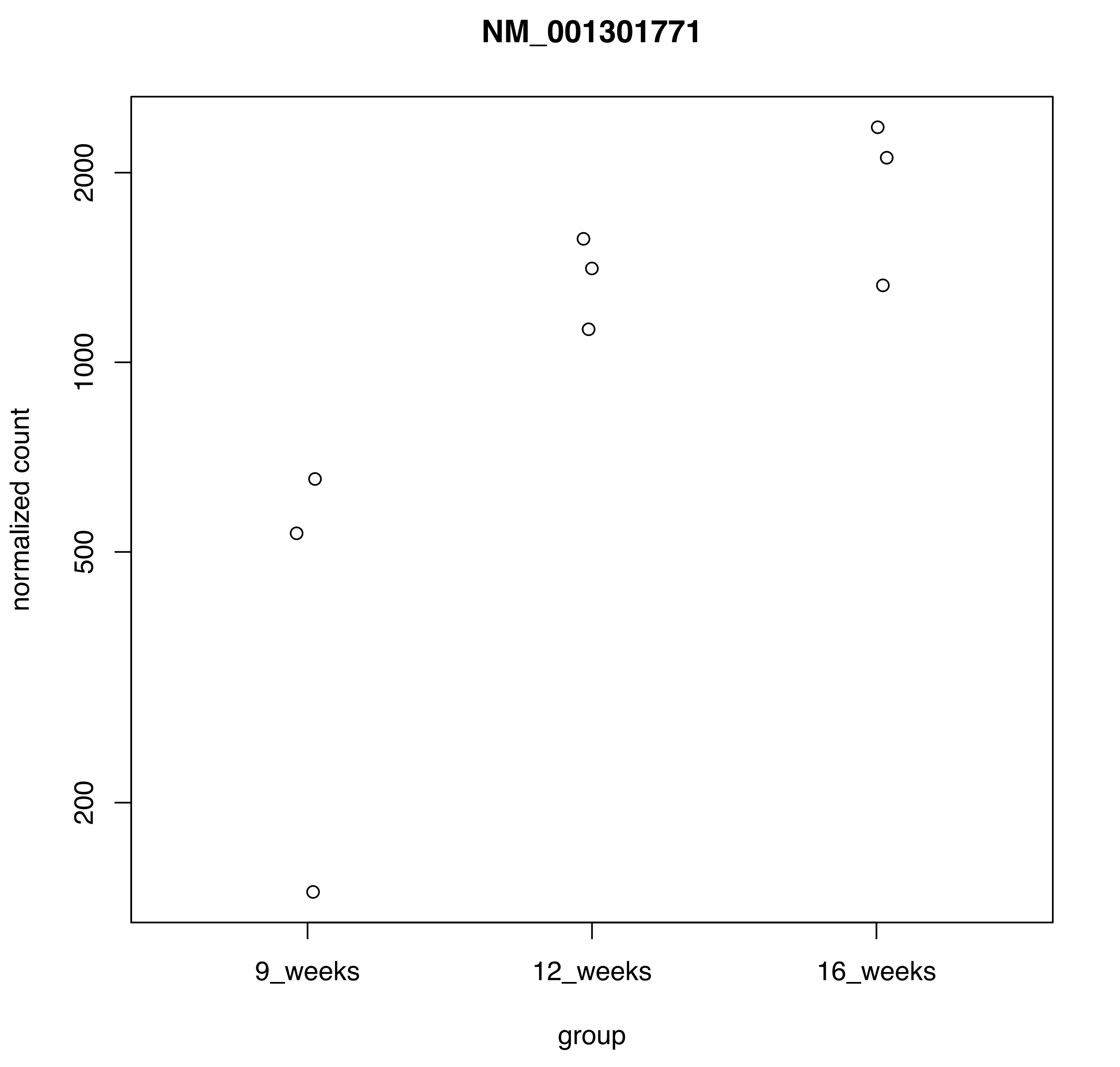
NM_001301771
normalized count
2000
1000
500
200
9_weeks
12_weeks
16_weeks
group

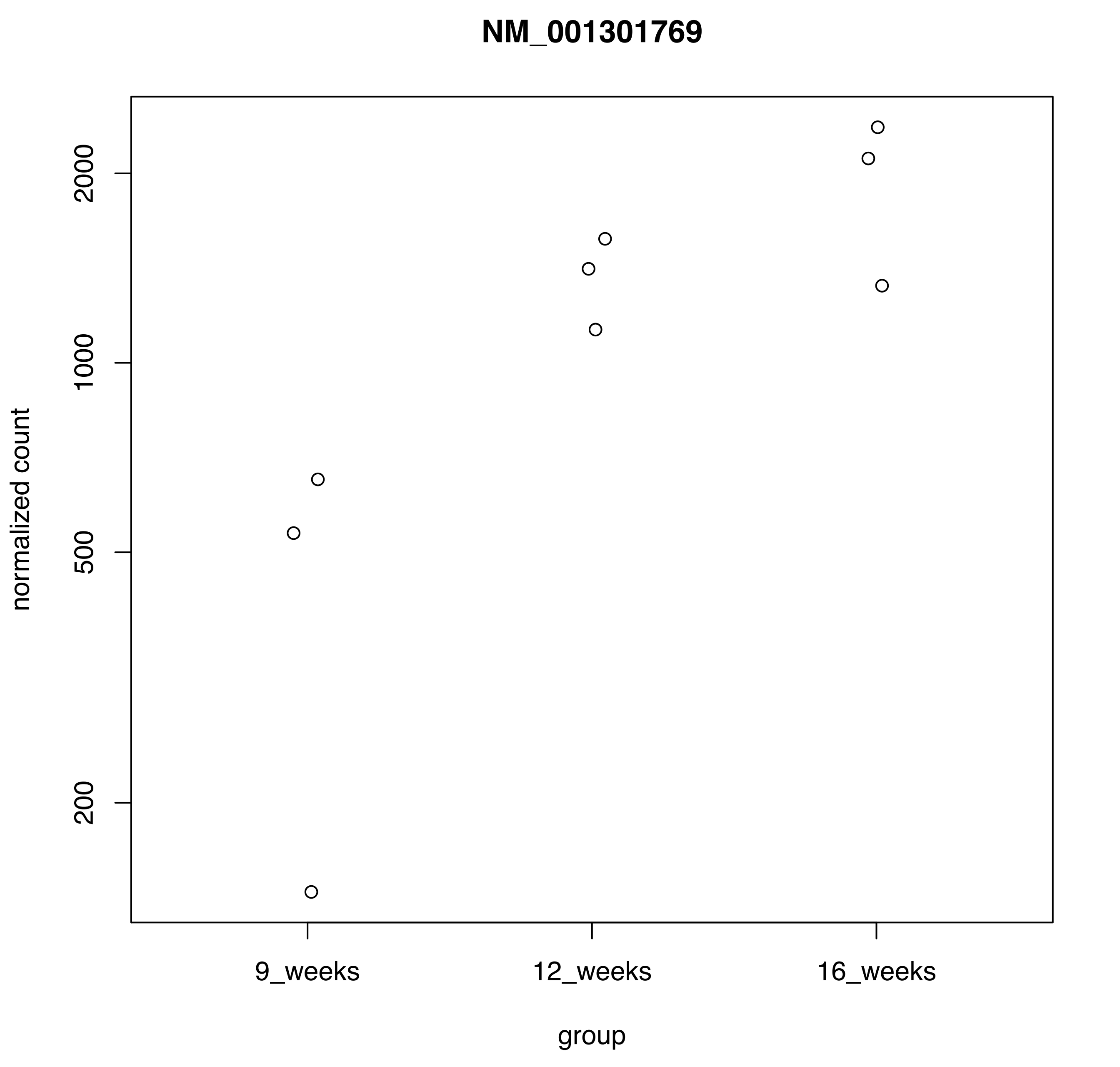
NM_001301769
normalized count
2000
1000
500
200
9_weeks
12_weeks
16_weeks
group

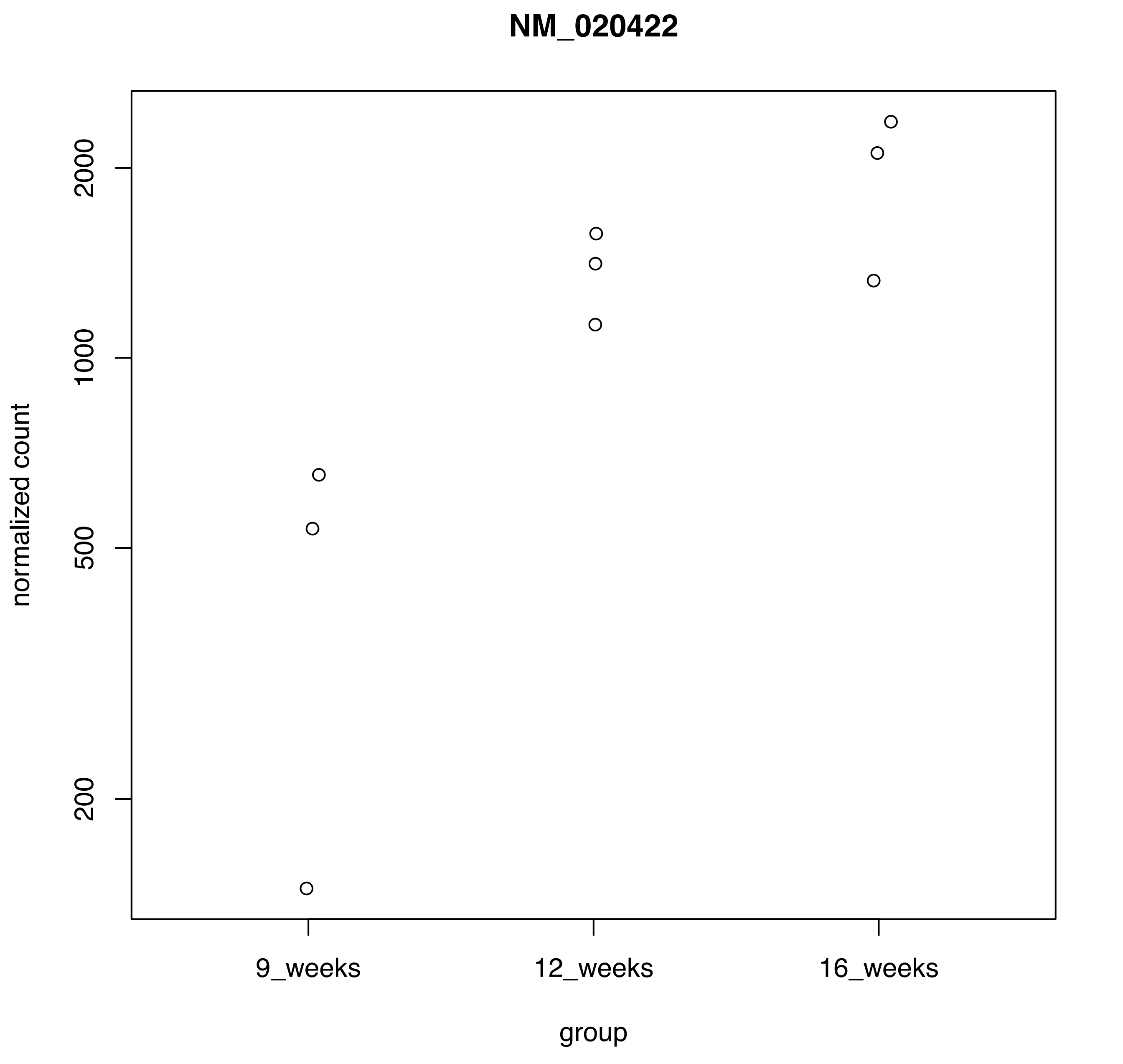
NM_020422
normalized count
2000
1000
500
200
9_weeks
12_weeks
16_weeks
group

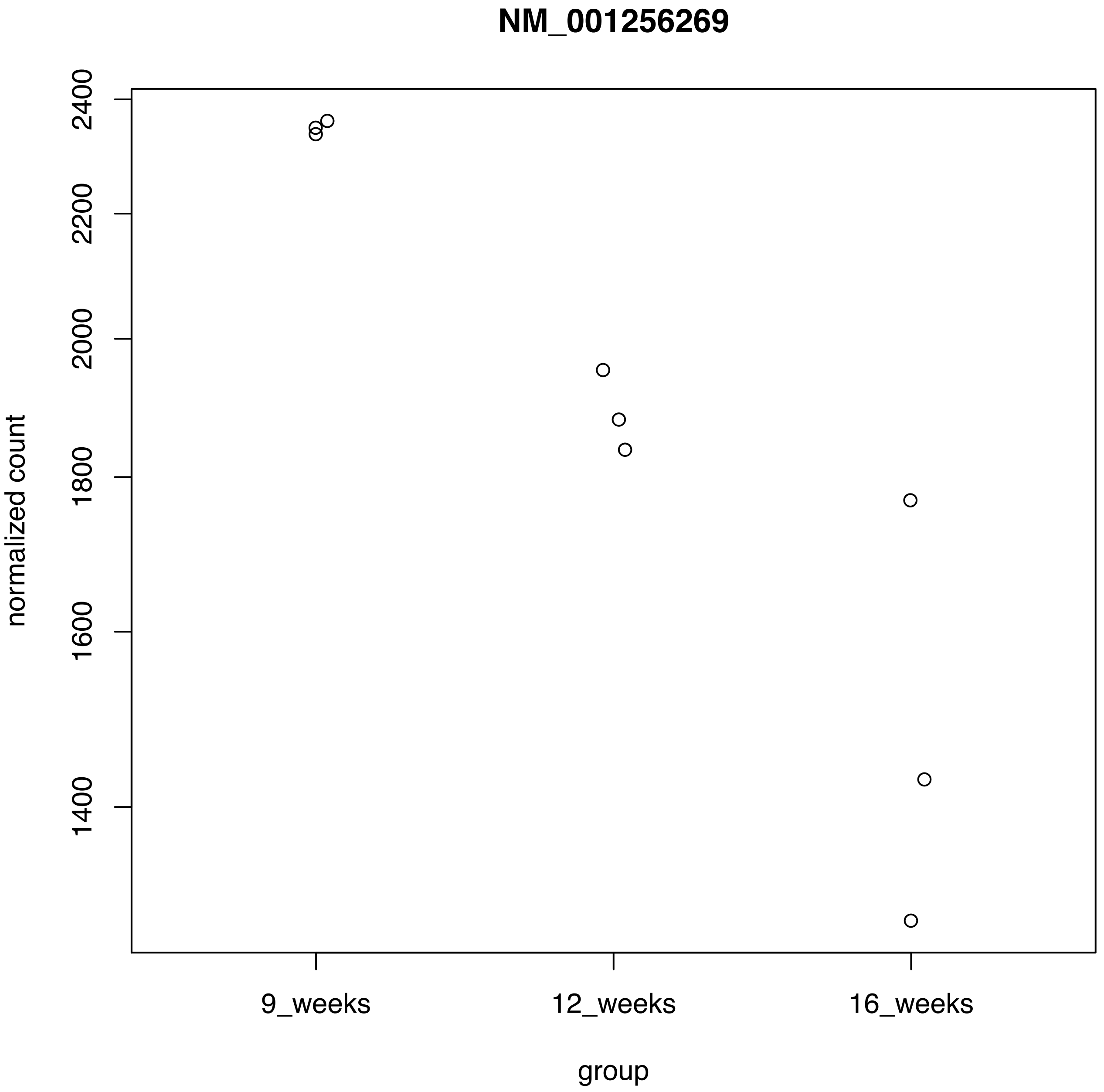
NM_001256269
normalized count
2400
2200
2000
1800
1600
1400
9_weeks
12_weeks
16_weeks
group

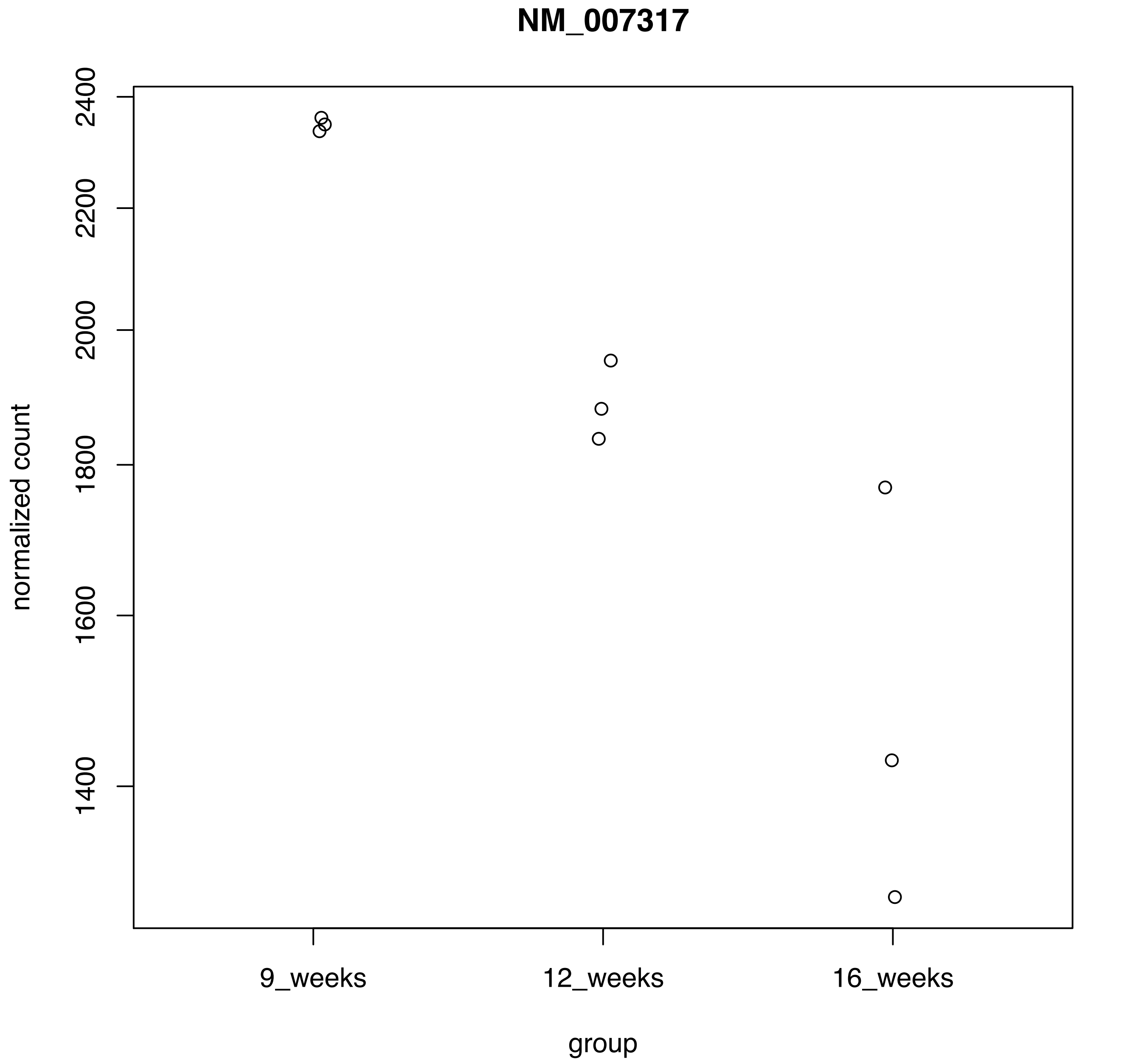
NM_007317
normalized count
2400
2200
2000
1800
1600
1400
9_weeks
12_weeks
16_weeks
group

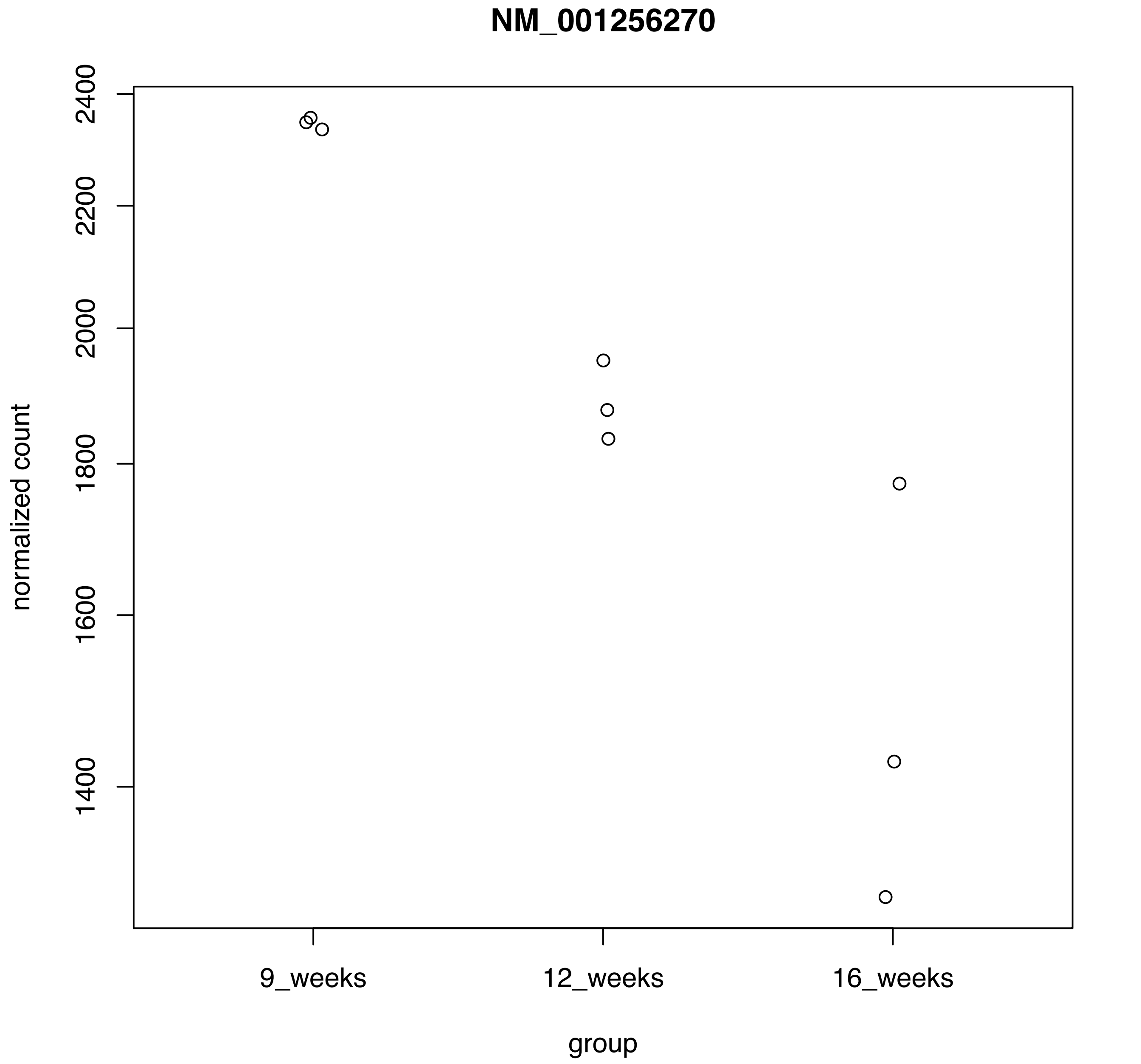
NM_001256270
normalized count
2400
2200
2000
1800
1600
1400
9_weeks
12_weeks
16_weeks
group

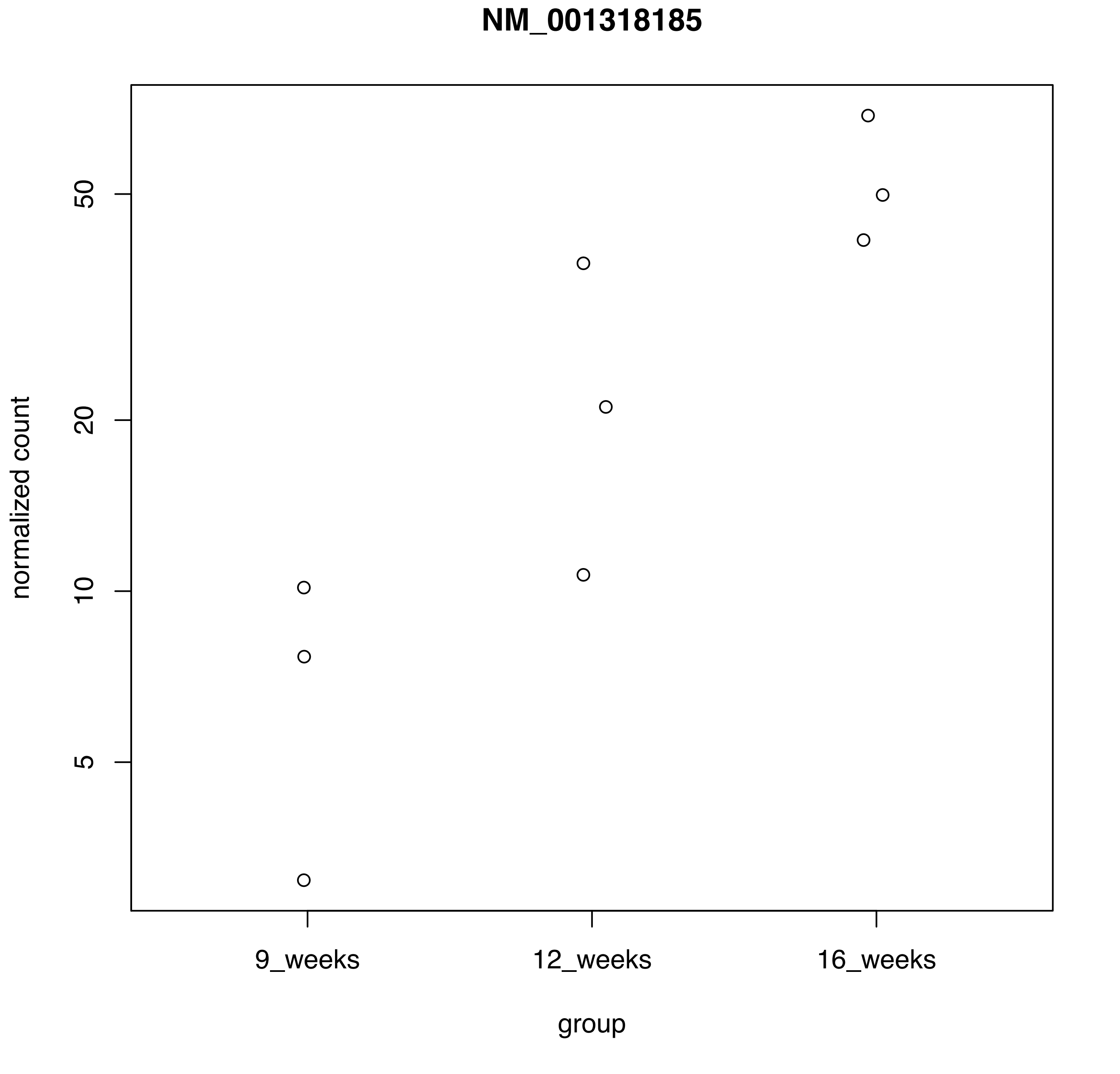

NM_001318185
normalized count
50
20
10
5
9_weeks
12_weeks
16_weeks
group

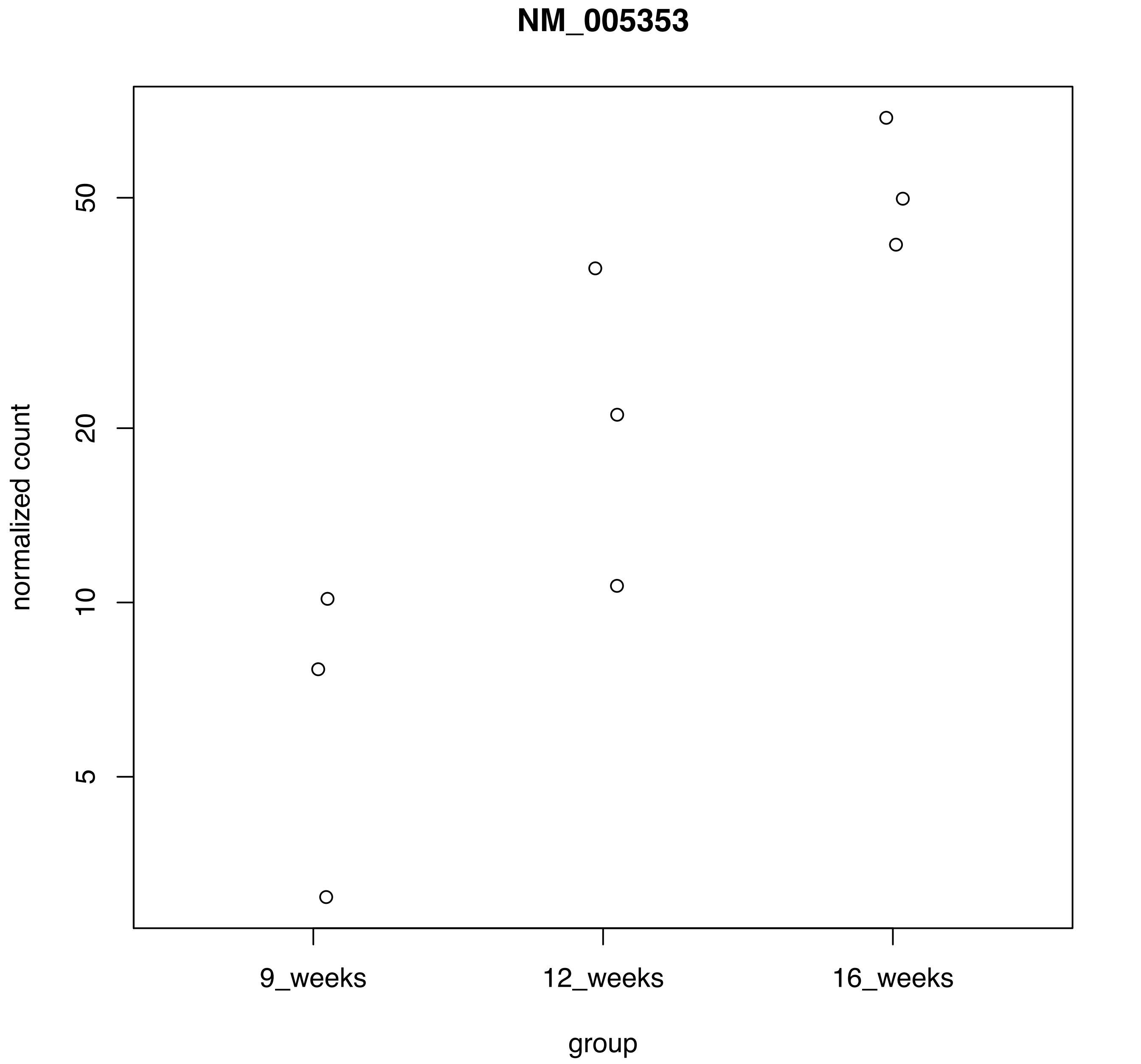
NM_005353
normalized count
50
20
10
5
9_weeks
12_weeks
16_weeks
group

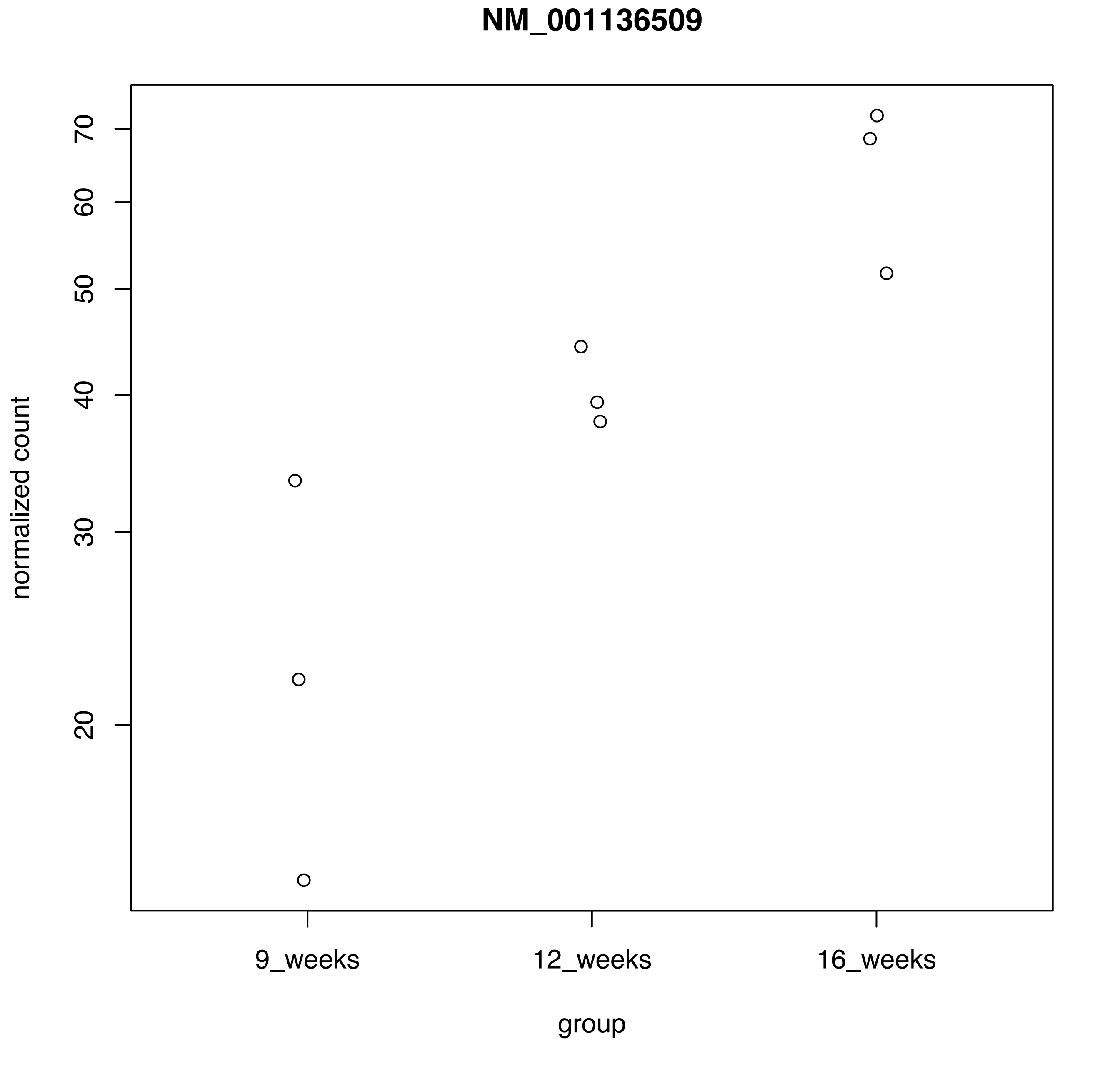
NM_001136509
normalized count
20
30
40
50
60
70
9_weeks
12_weeks
16_weeks
group

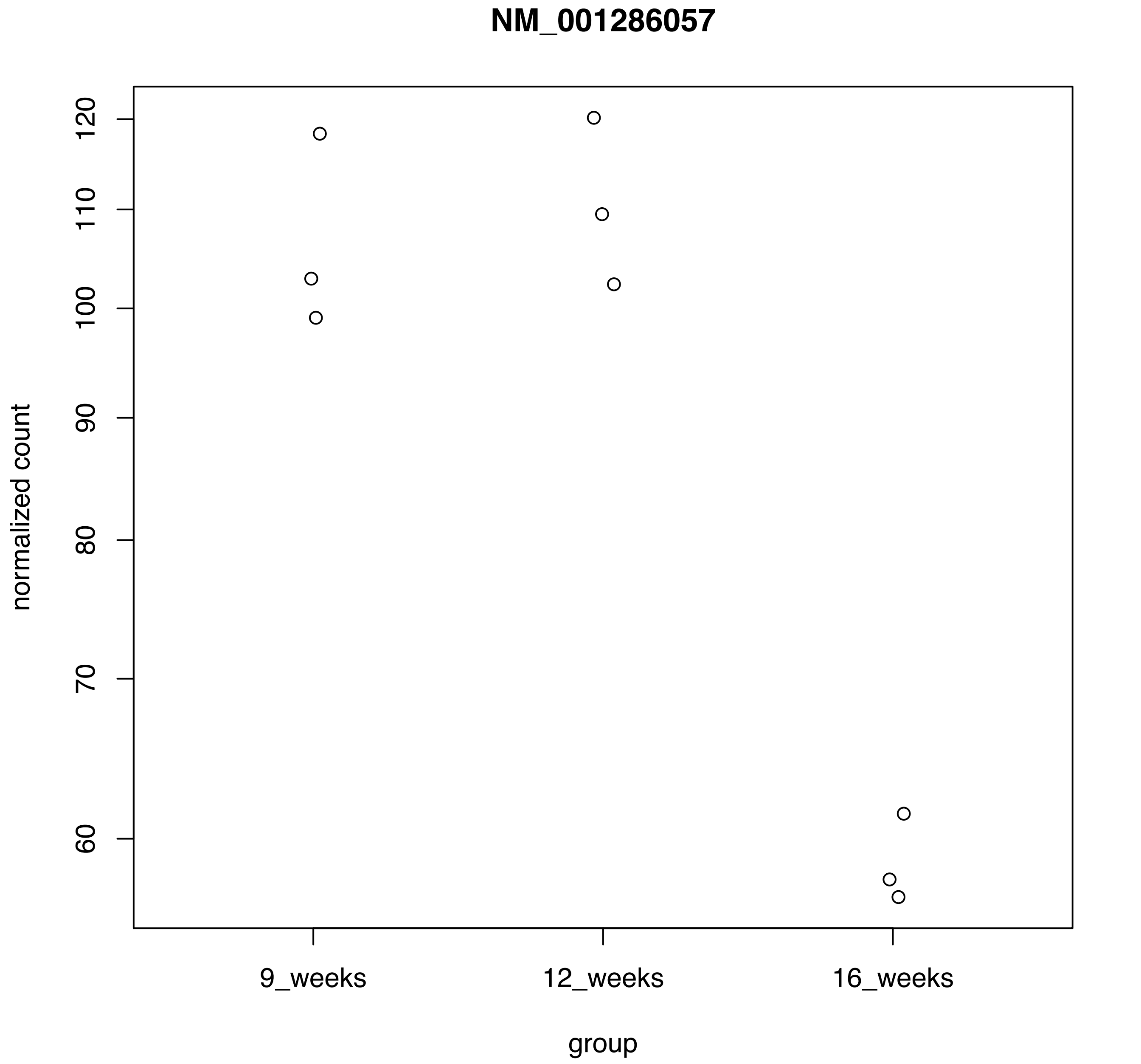
NM_001286057
normalized count
120
110
100
90
80
70
60
9_weeks
12_weeks
16_weeks
group

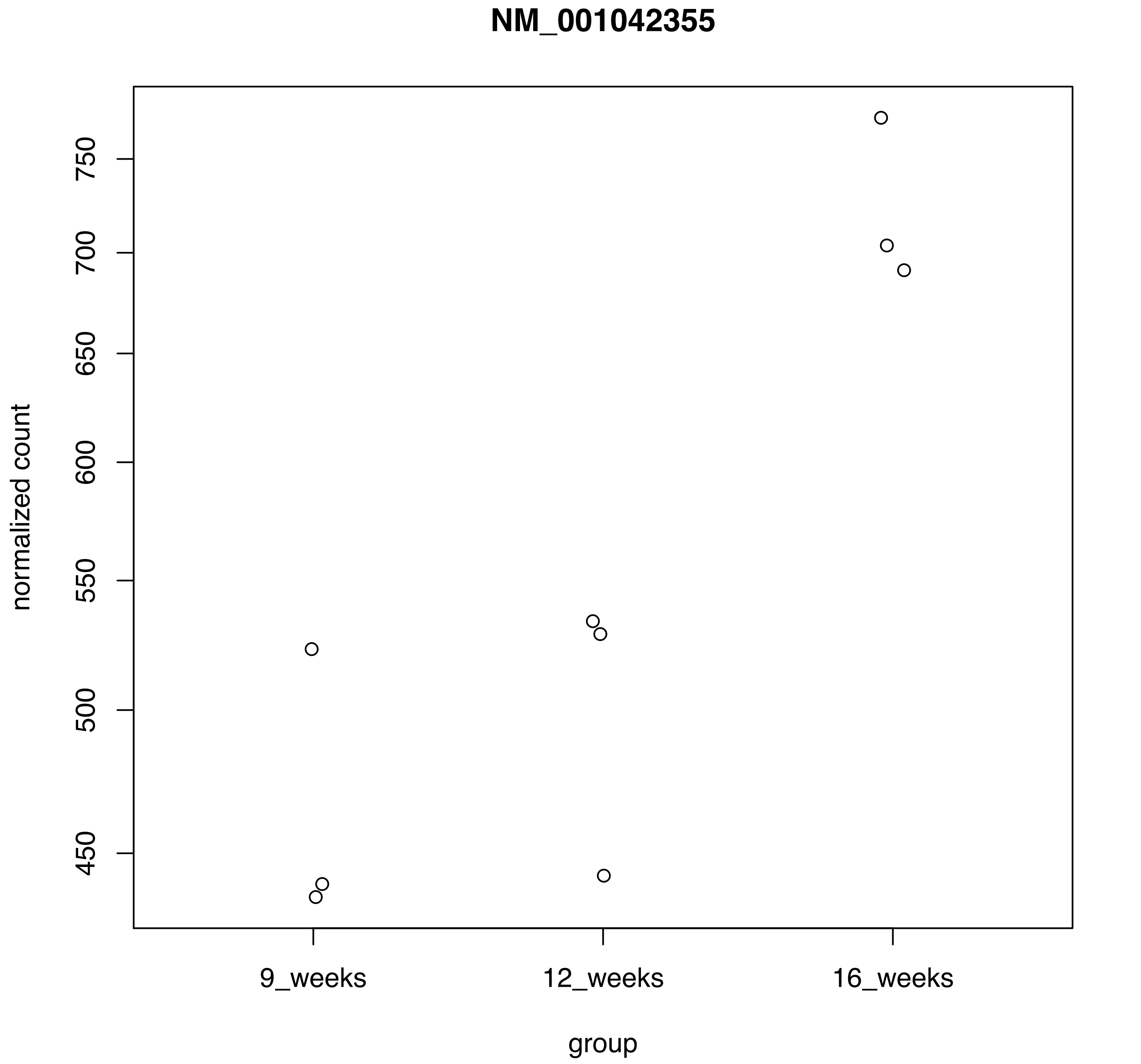
NM_001042355
normalized count
750
700
650
600
550
500
450
9_weeks
12_weeks
16_weeks
group

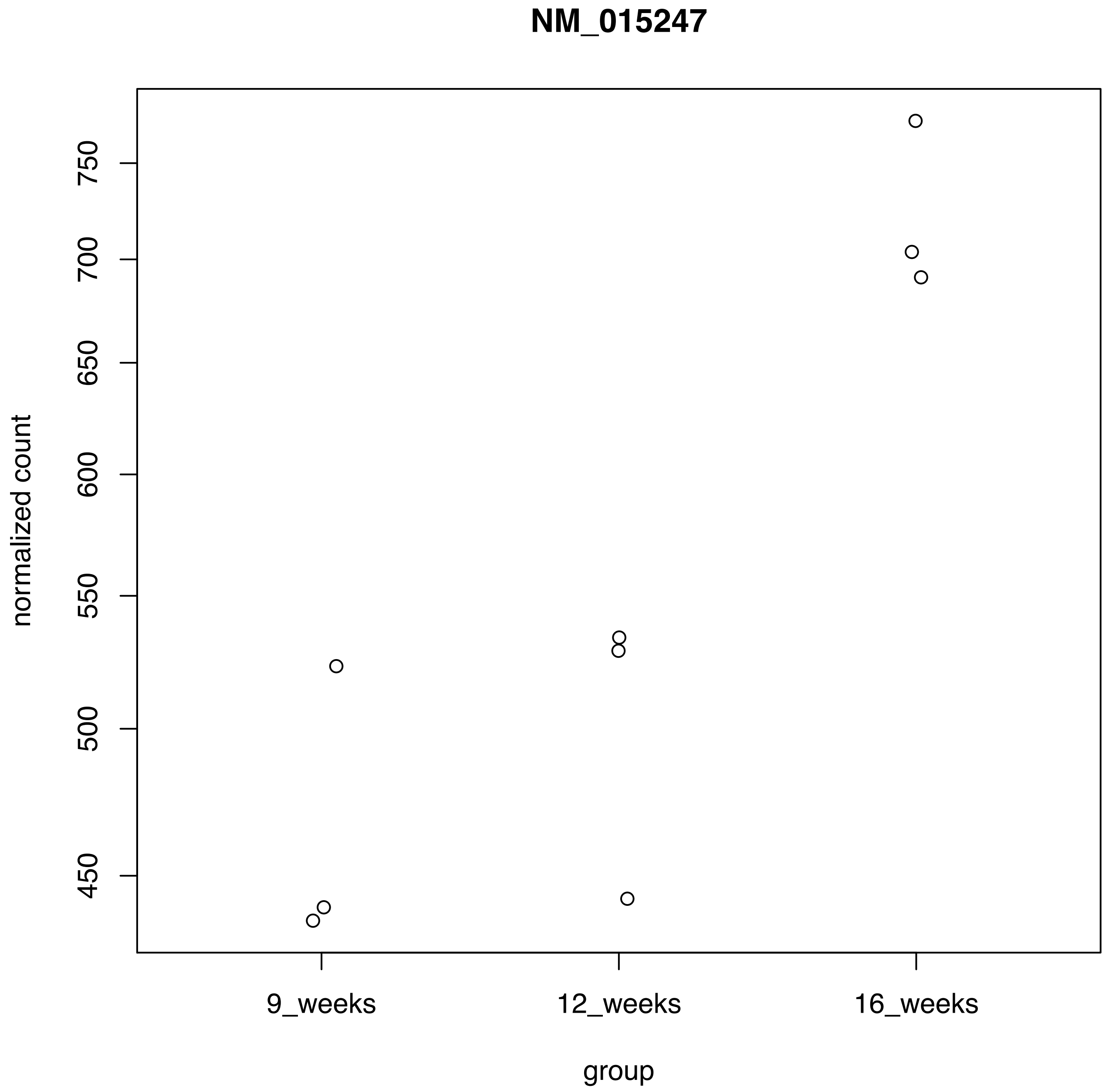
NM_015247
normalized count
750
700
650
600
550
500
450
9_weeks
12_weeks
16_weeks
group

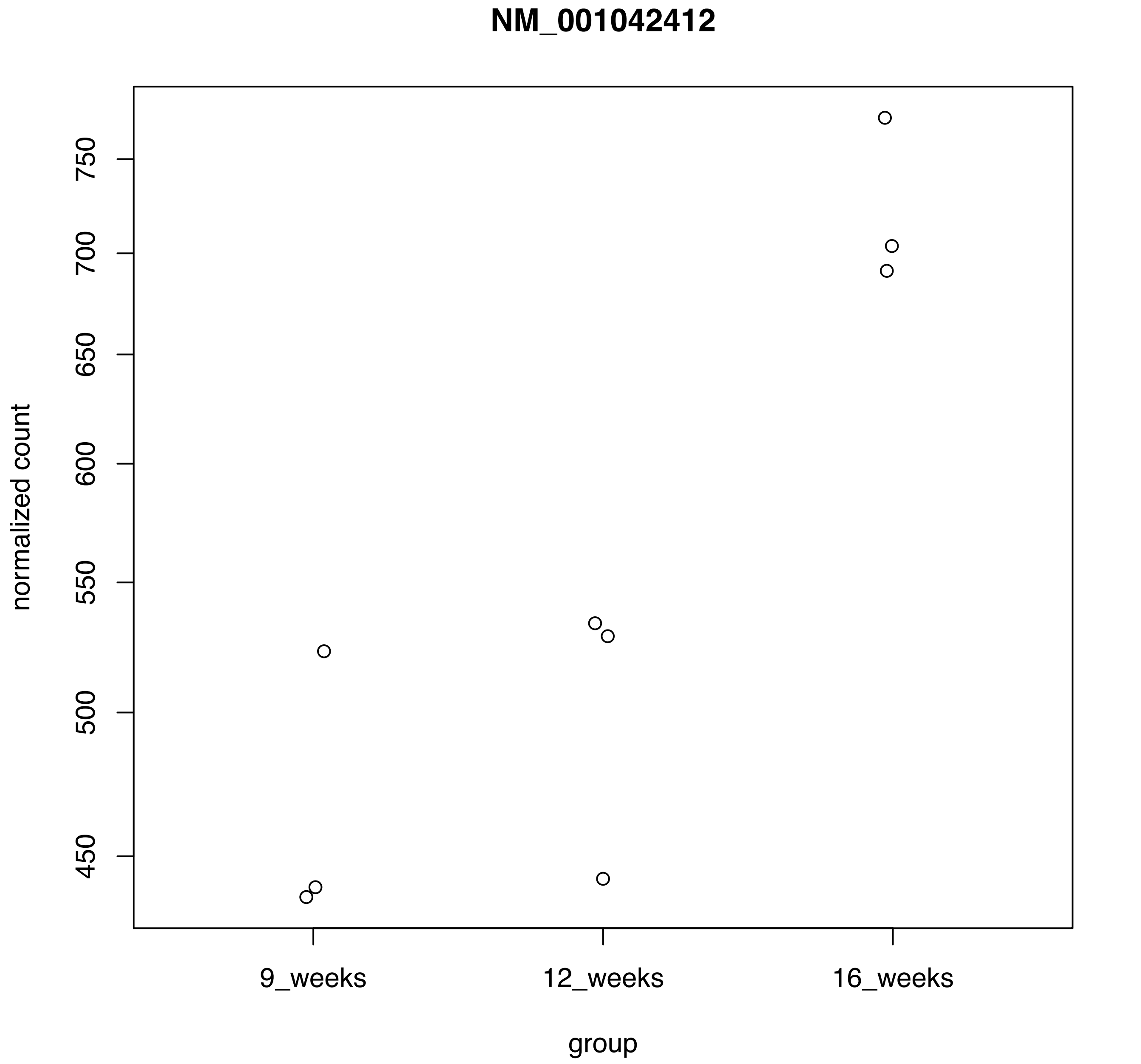
NM_001042412
normalized count
750
700
650
600
550
500
450
9_weeks
12_weeks
16_weeks
group

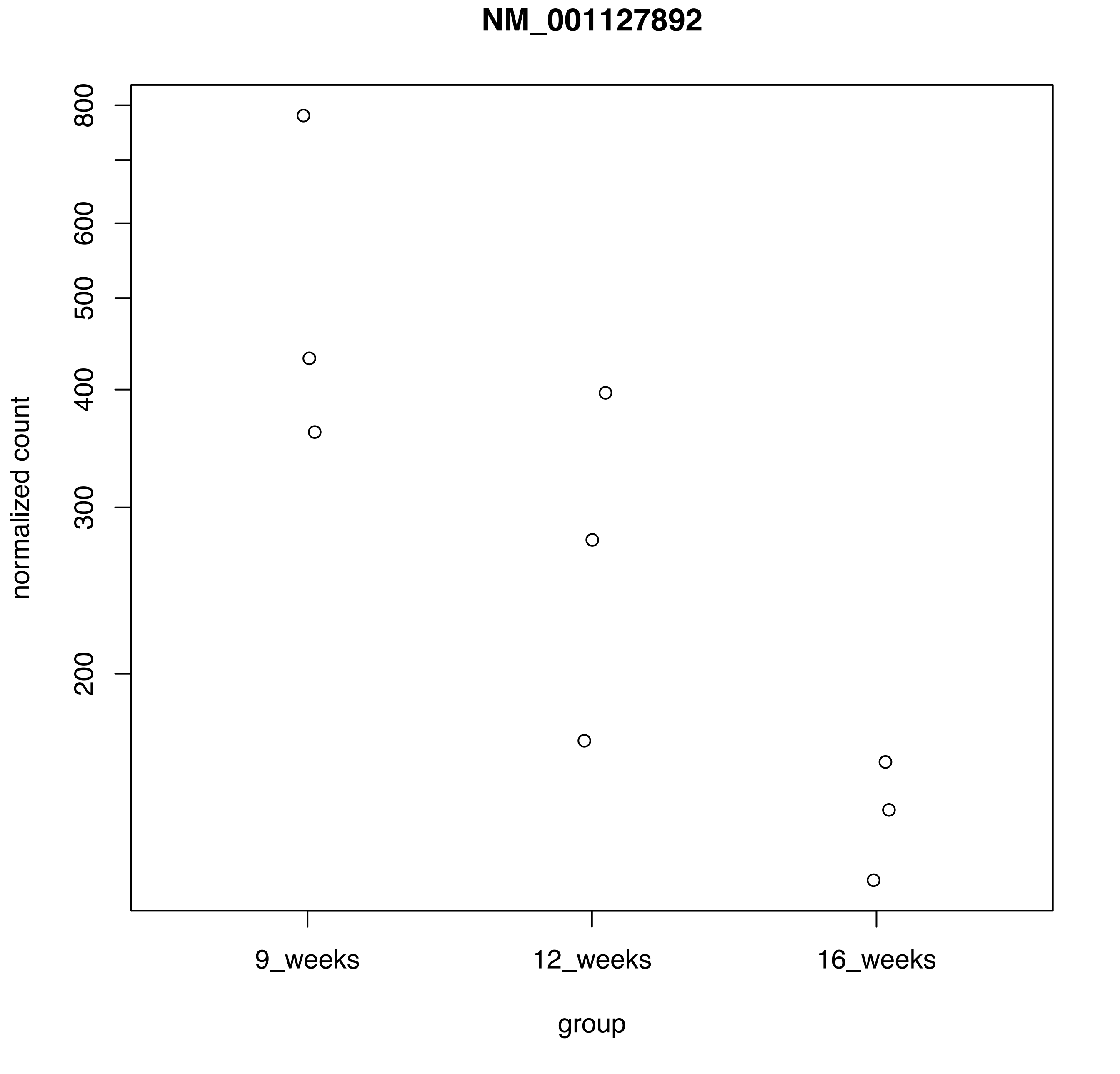

NM_001127892
normalized count
800
600
500
400
300
200
9_weeks
12_weeks
16_weeks
group

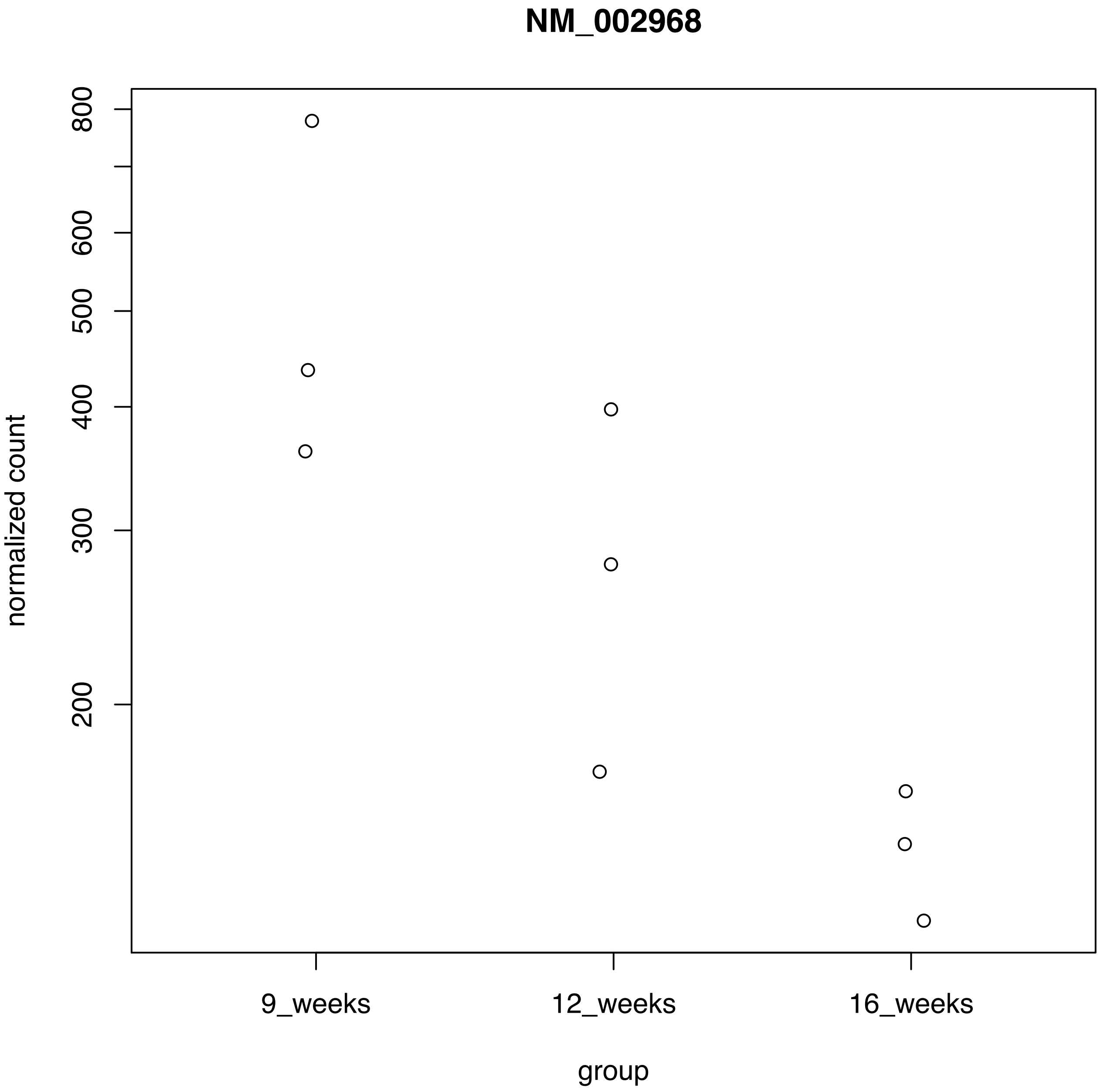
NM_002968
normalized count
800
600
500
400
300
200
9_weeks
12_weeks
16_weeks
group

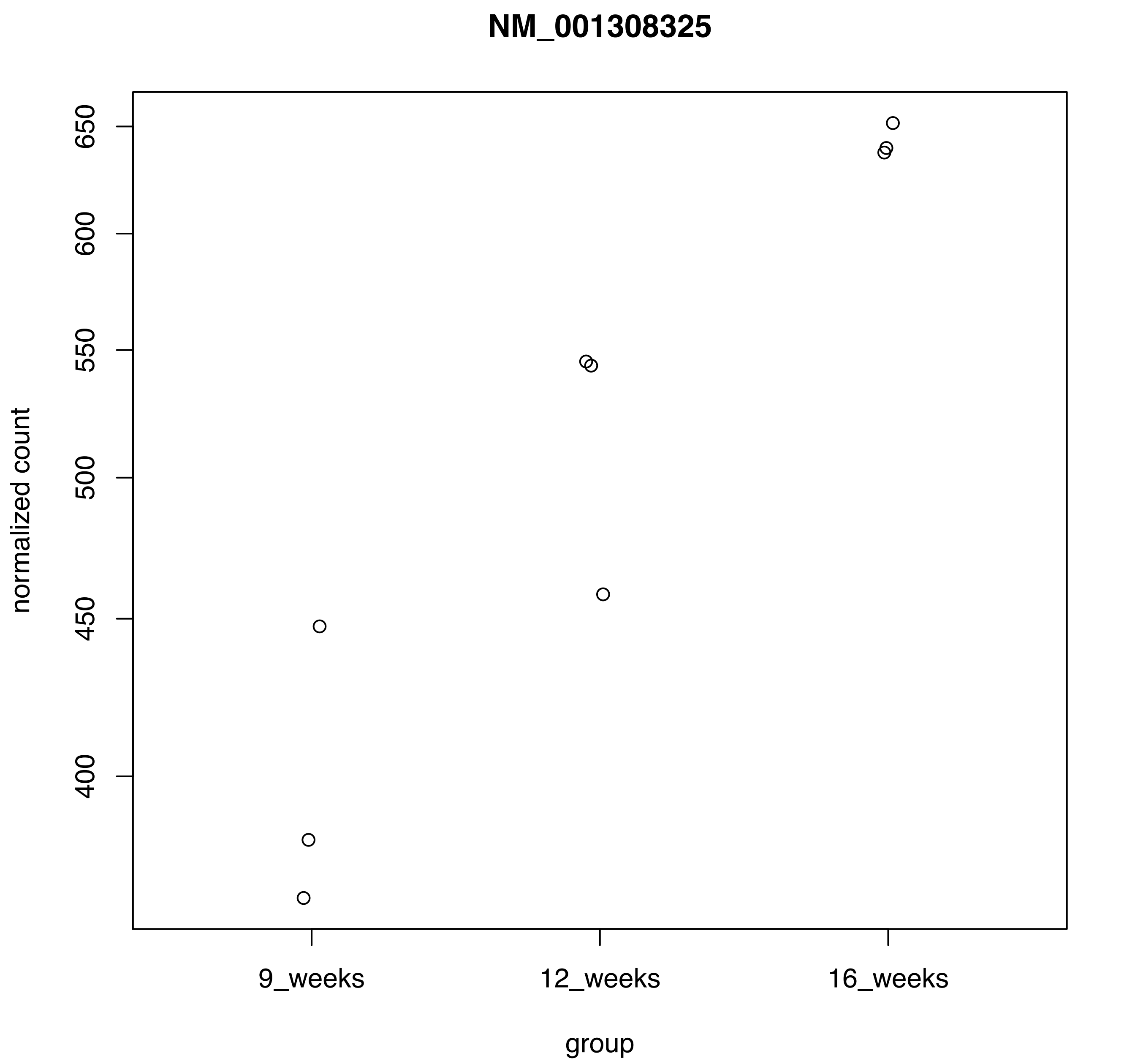
NM_001308325
normalized count
650
600
550
500
450
400
9_weeks
12_weeks
16_weeks
group

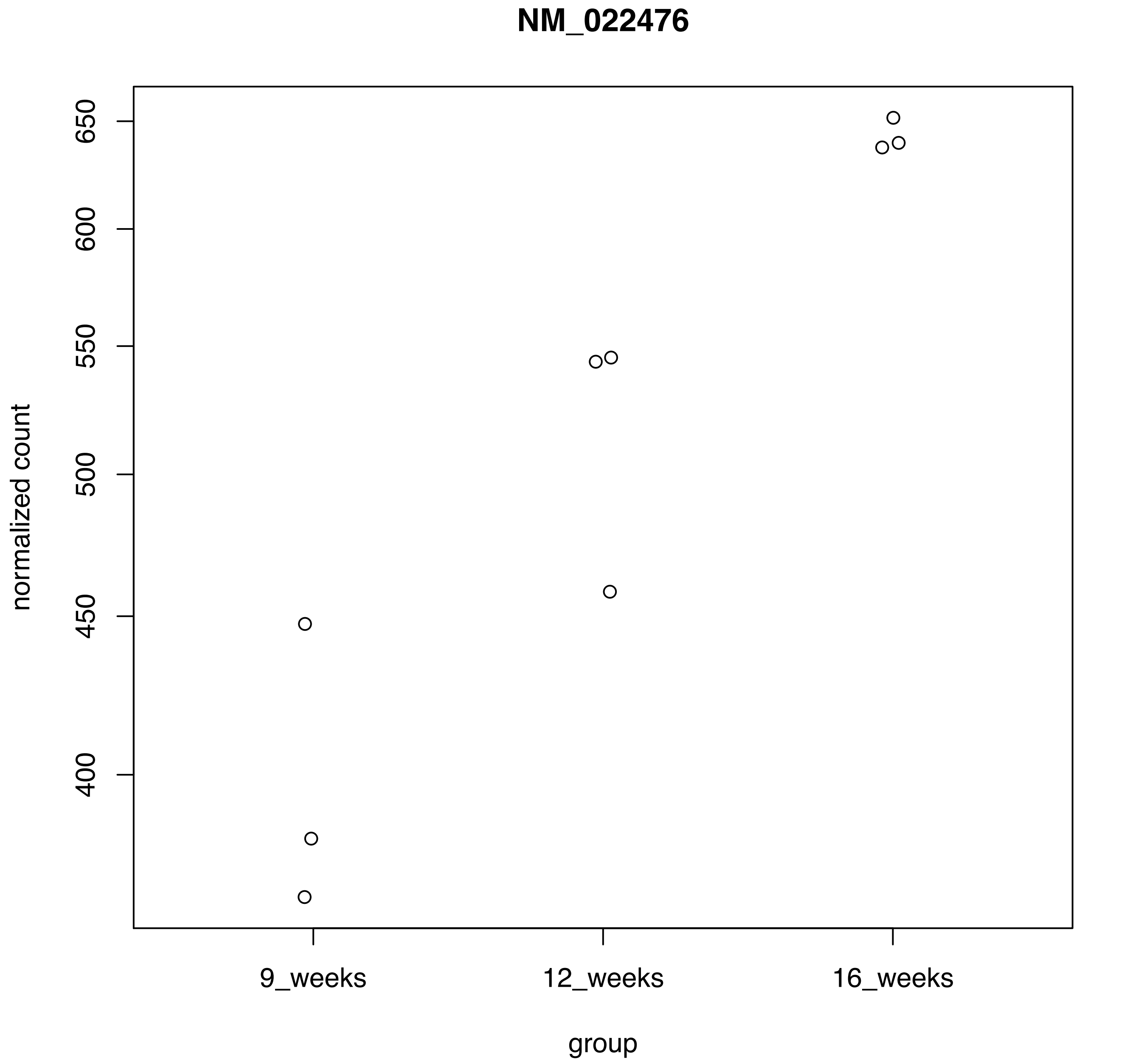
NM_022476
normalized count
650
600
550
500
450
400
9_weeks
12_weeks
16_weeks
group

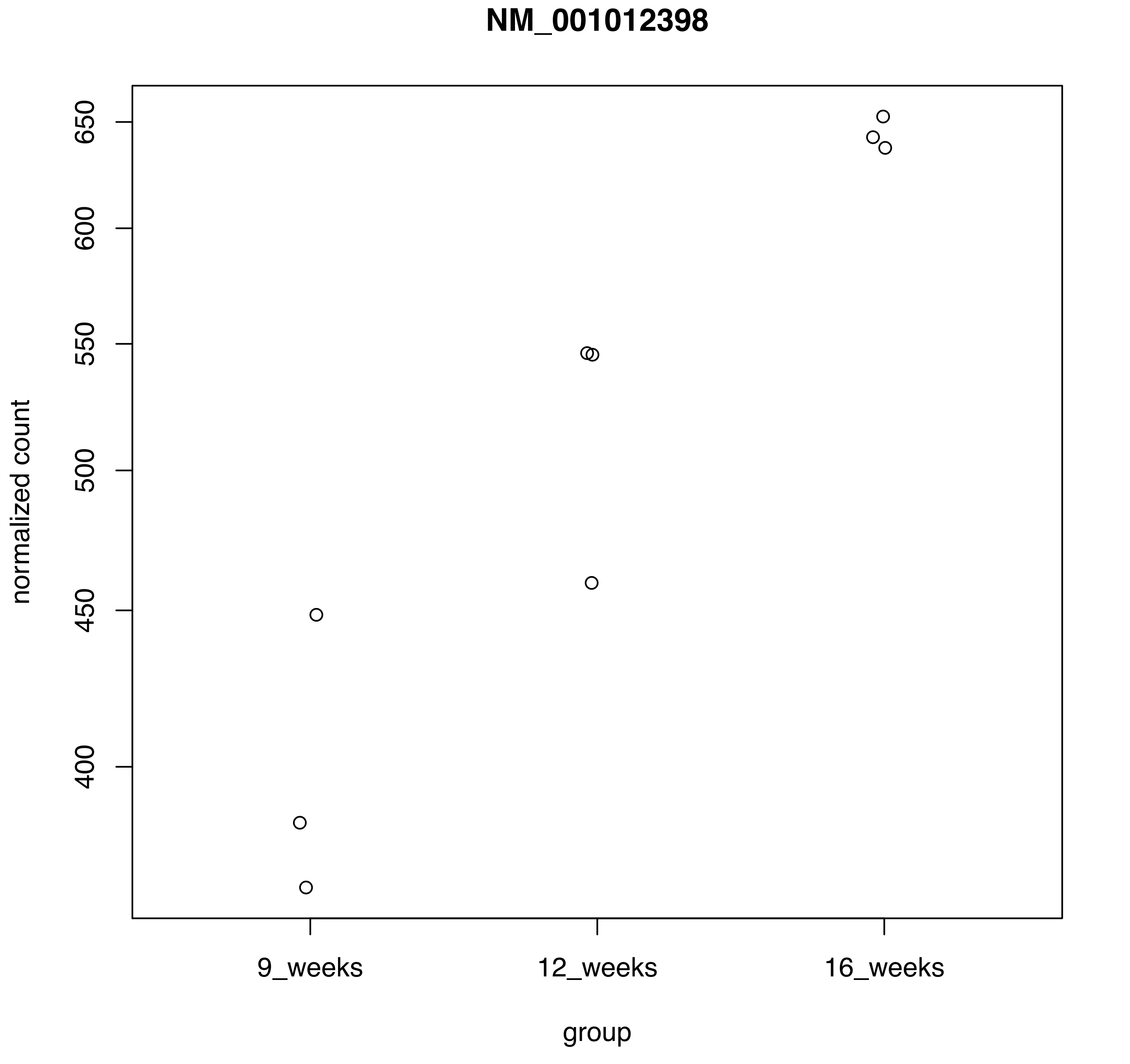
NM_001012398
normalized count
650
600
550
500
450
400
9_weeks
12_weeks
16_weeks
group

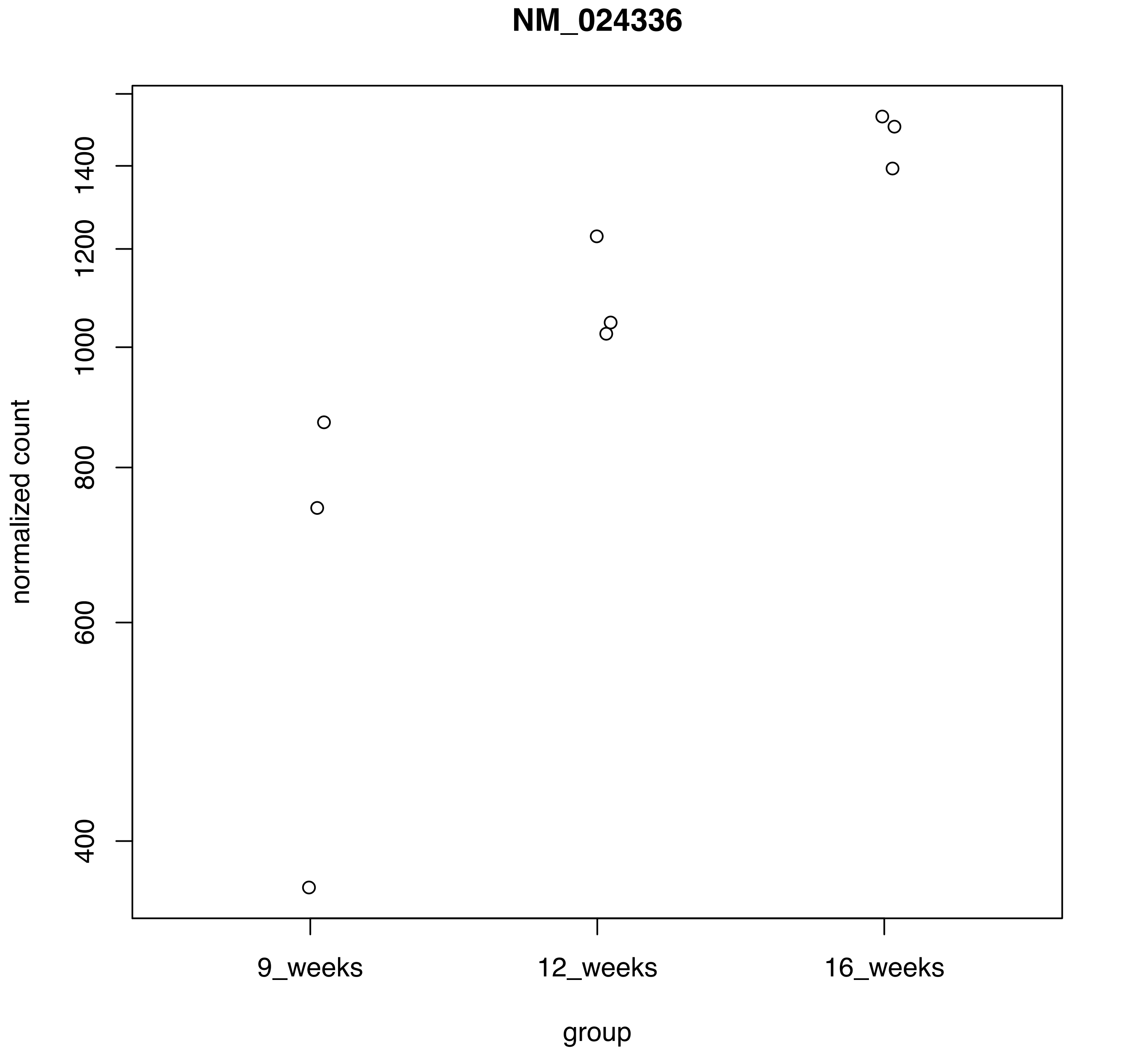
NM_024336
normalized count
400
600
800
1000
1200
1400
9_weeks
12_weeks
16_weeks
group

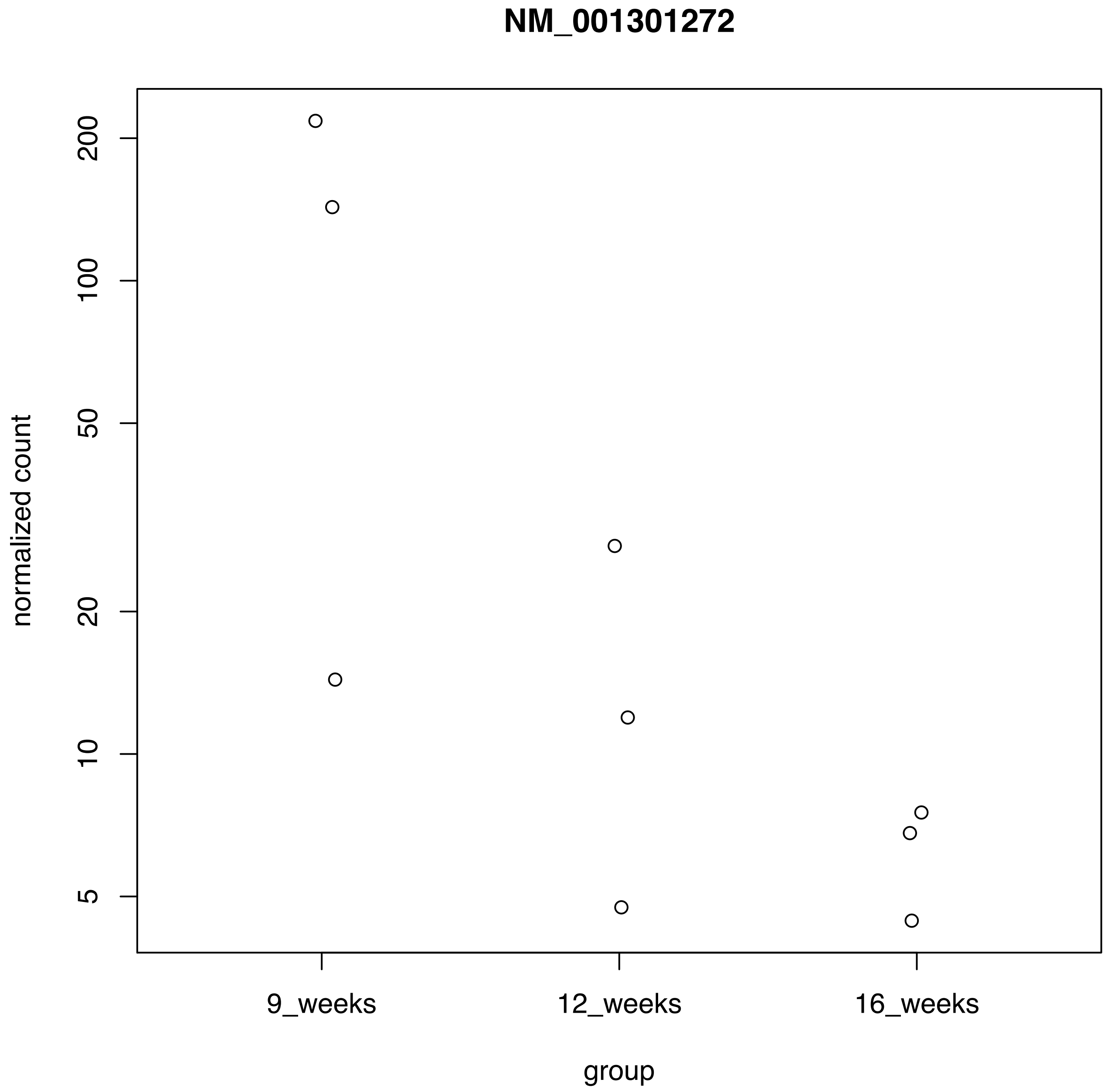

NM_001301272
200
100
50
20
10
5
normalized count
9_weeks
12_weeks
16_weeks
group

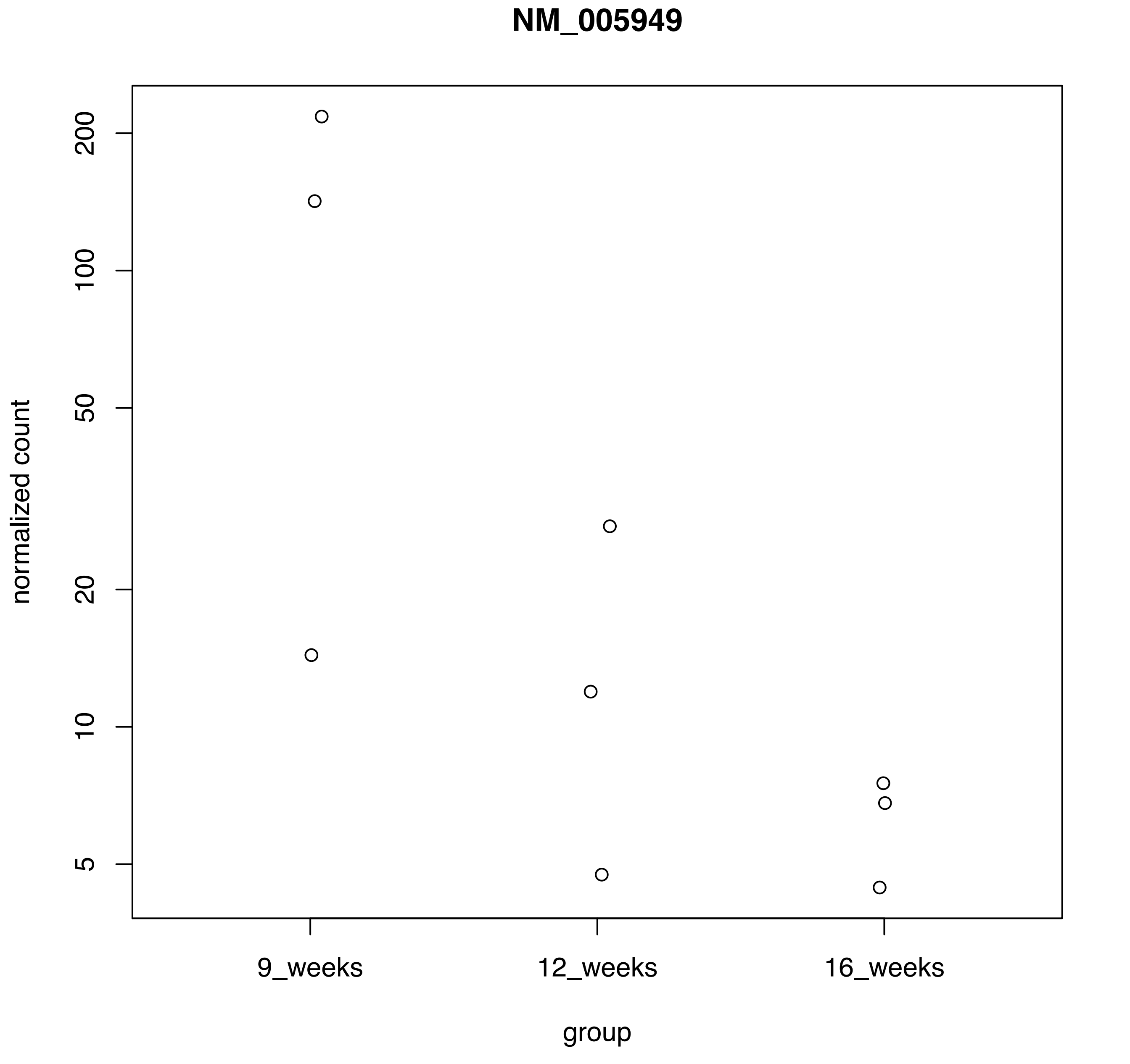
NM_005949
normalized count
200
100
50
20
10
5
9_weeks
12_weeks
16_weeks
group

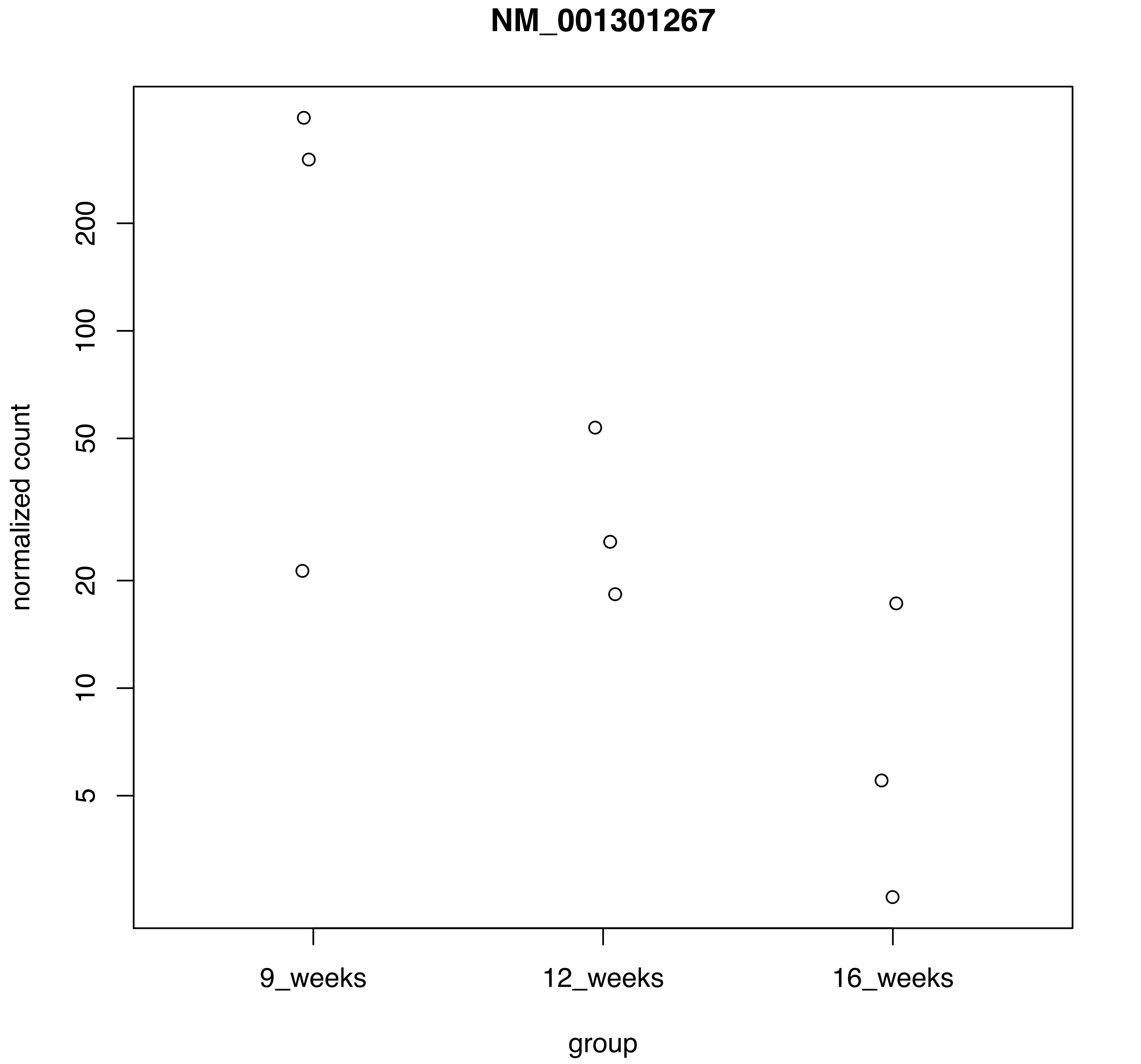
NM_001301267
normalized count
200
100
50
20
10
5
9_weeks
12_weeks
16_weeks
group

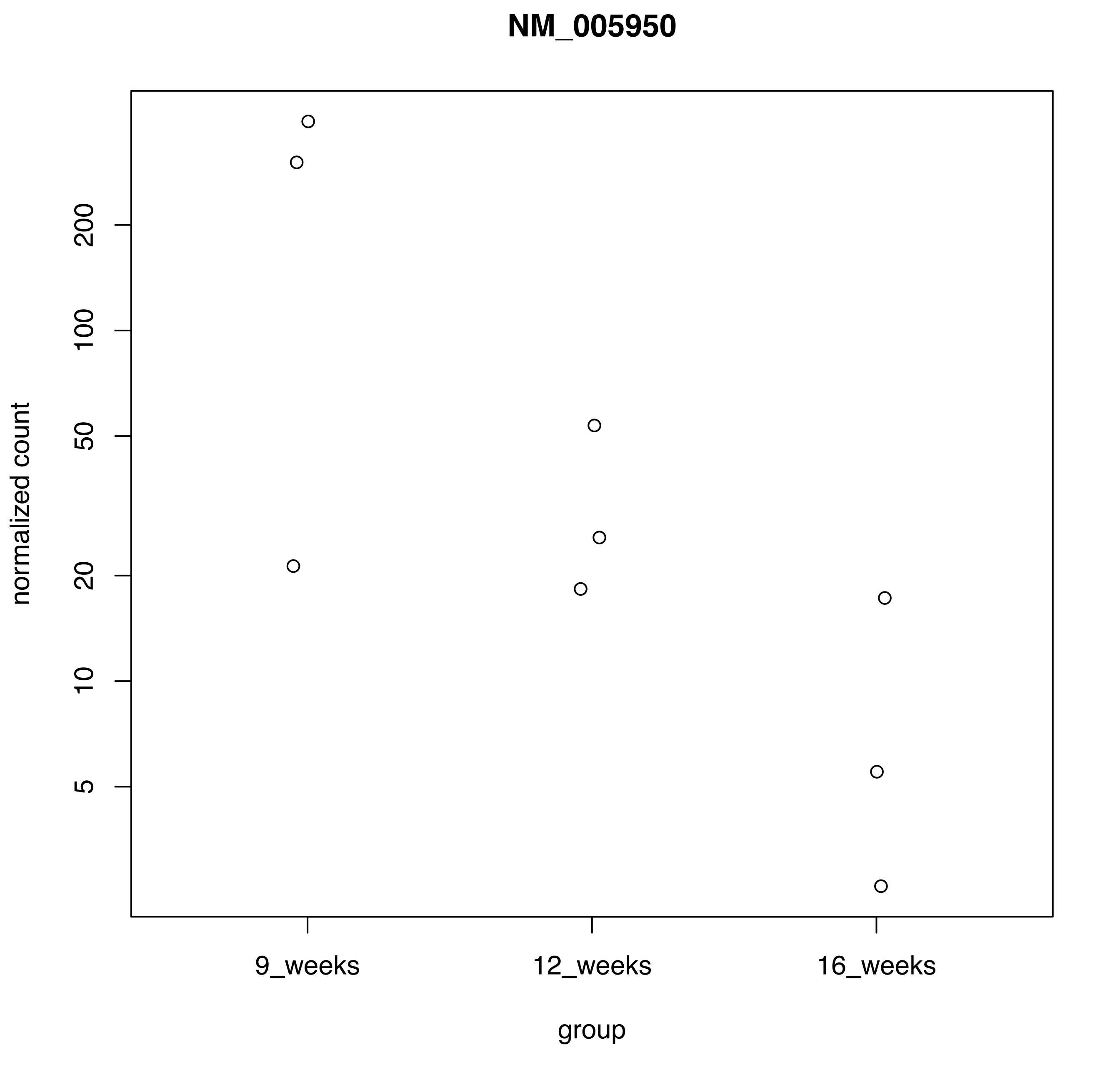
NM_005950
normalized count
200
100
50
20
10
5
9_weeks
12_weeks
16_weeks
group

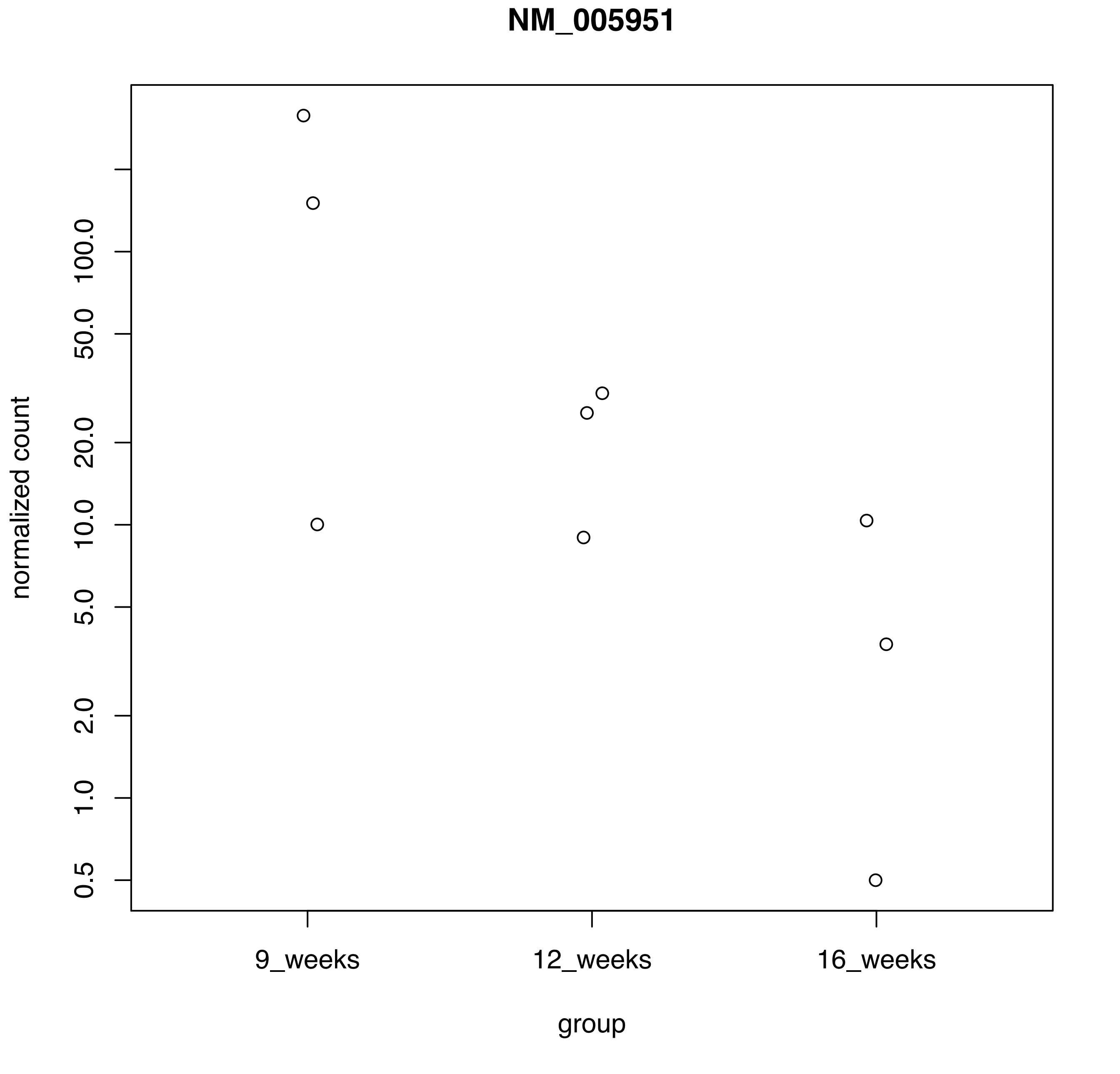

NM_005951
normalized count
100.0
50.0
20.0
10.0
5.0
2.0
1.0
0.5
9_weeks
12_weeks
16_weeks
group

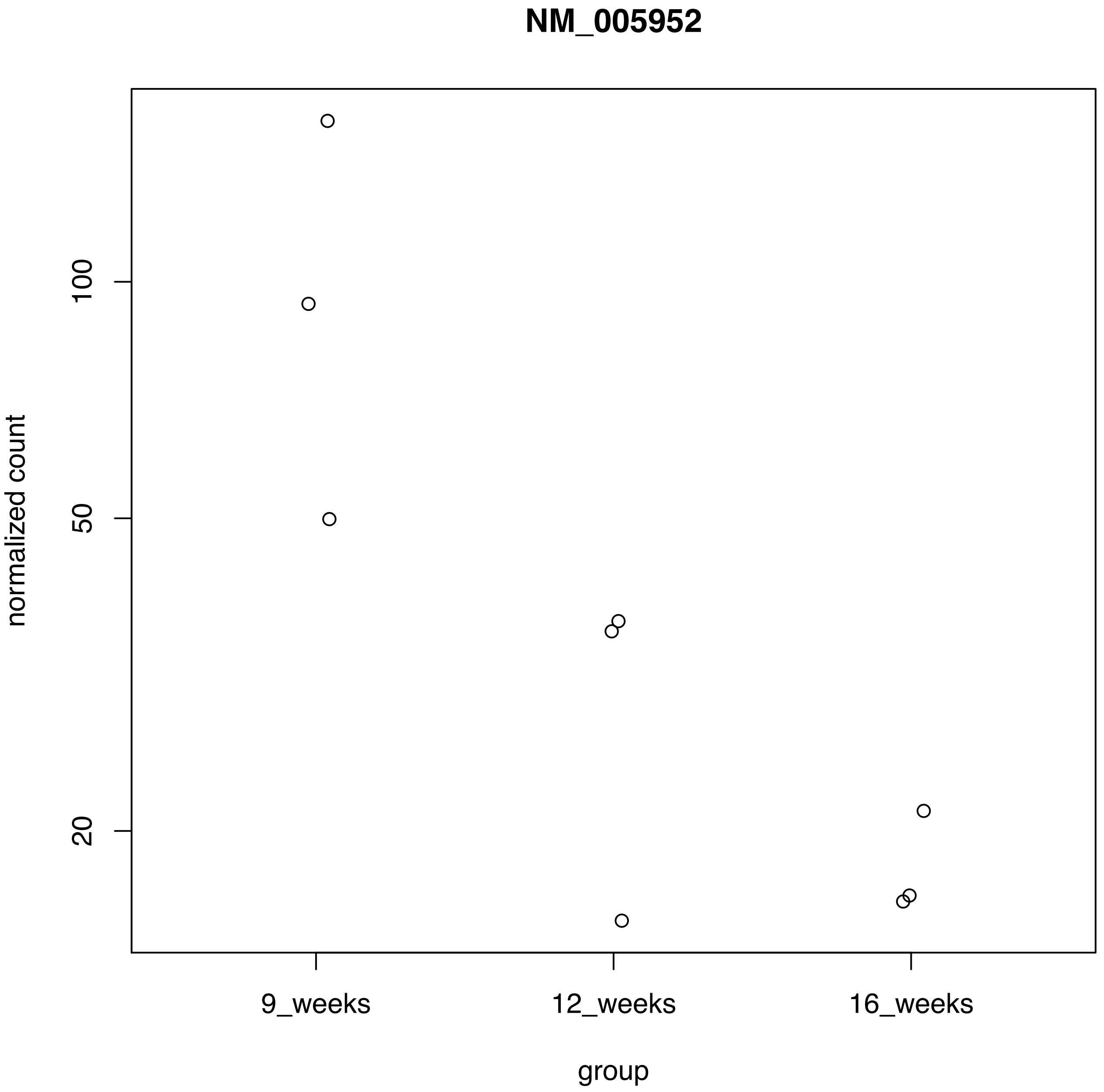
NM_005952
normalized count
100
50
20
9_weeks
12_weeks
16_weeks
group

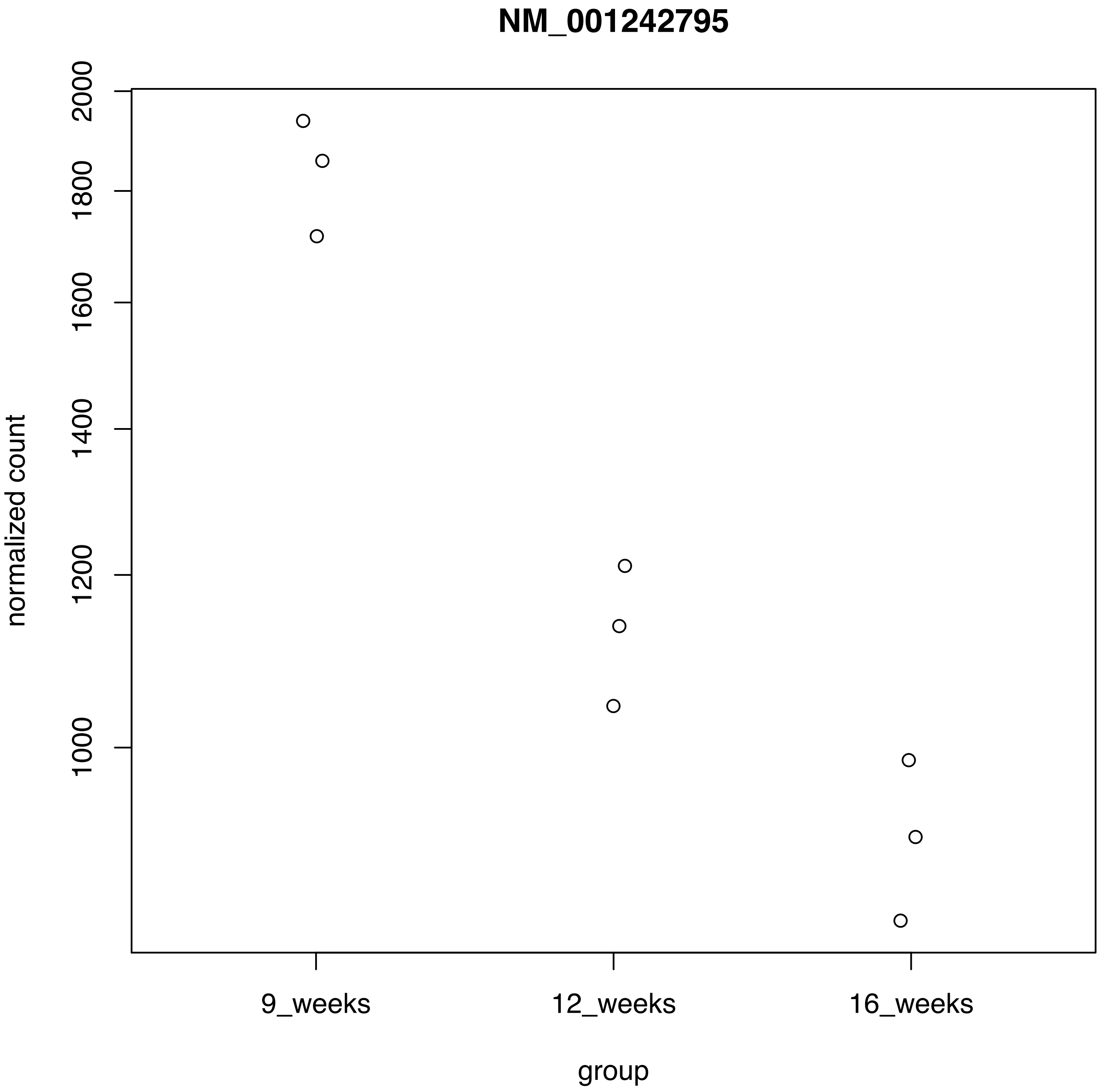
NM_001242795
normalized count
2000
1800
1600
1400
1200
1000
9_weeks
12_weeks
16_weeks
group

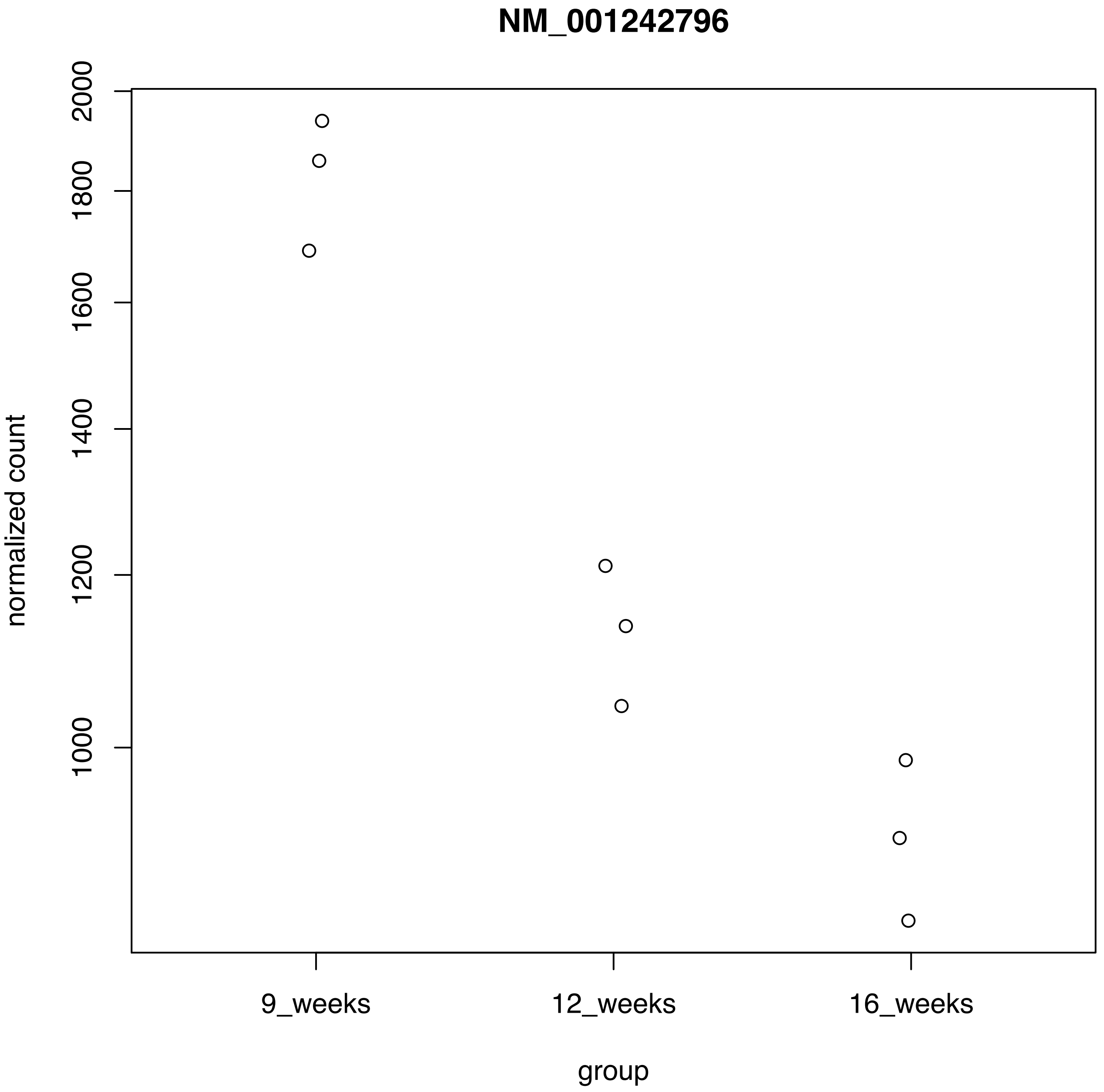
NM_001242796
normalized count
2000
1800
1600
1400
1200
1000
9_weeks
12_weeks
16_weeks
group

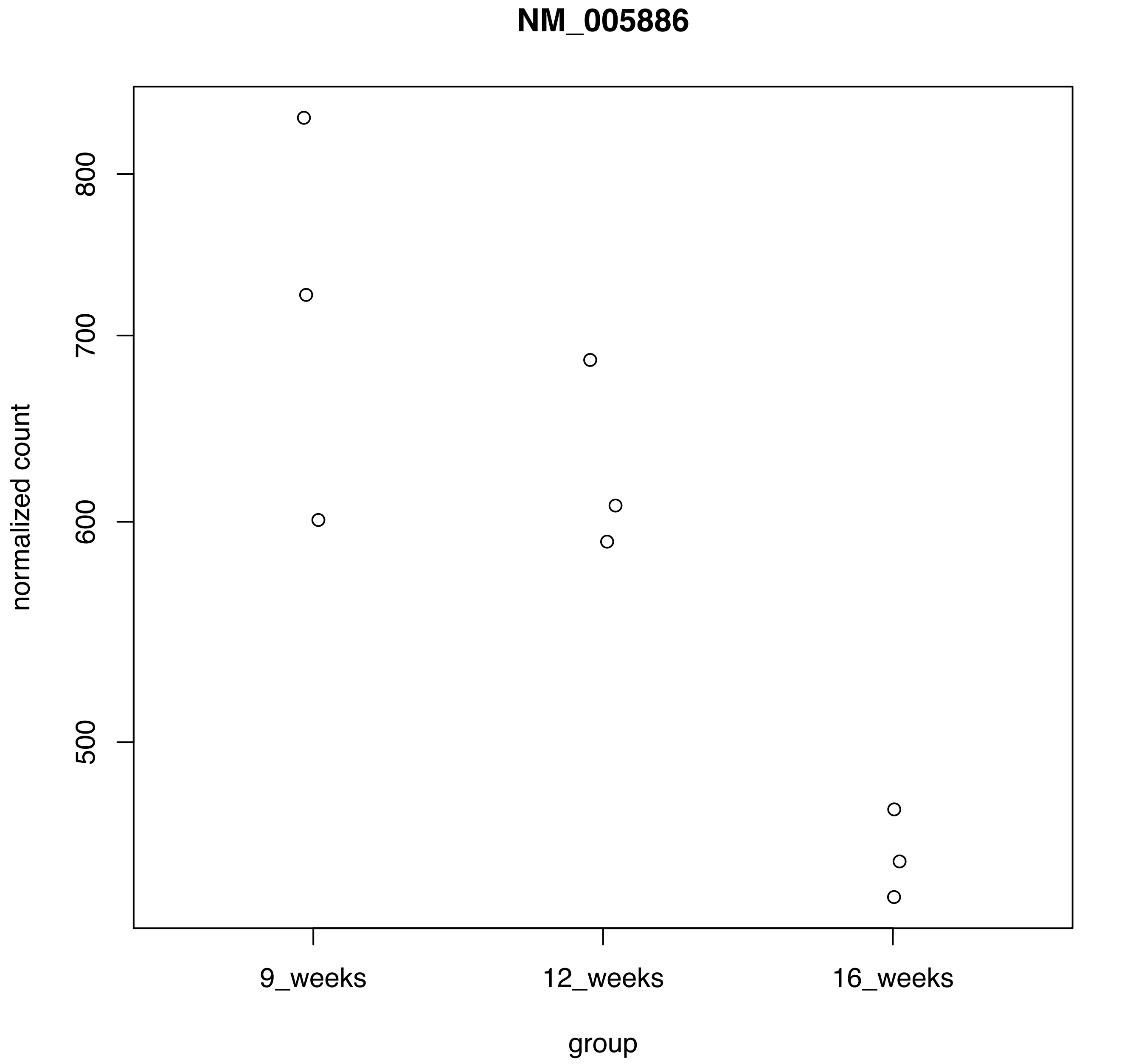
NM_005886
normalized count
800
700
600
500
9_weeks
12_weeks
16_weeks
group

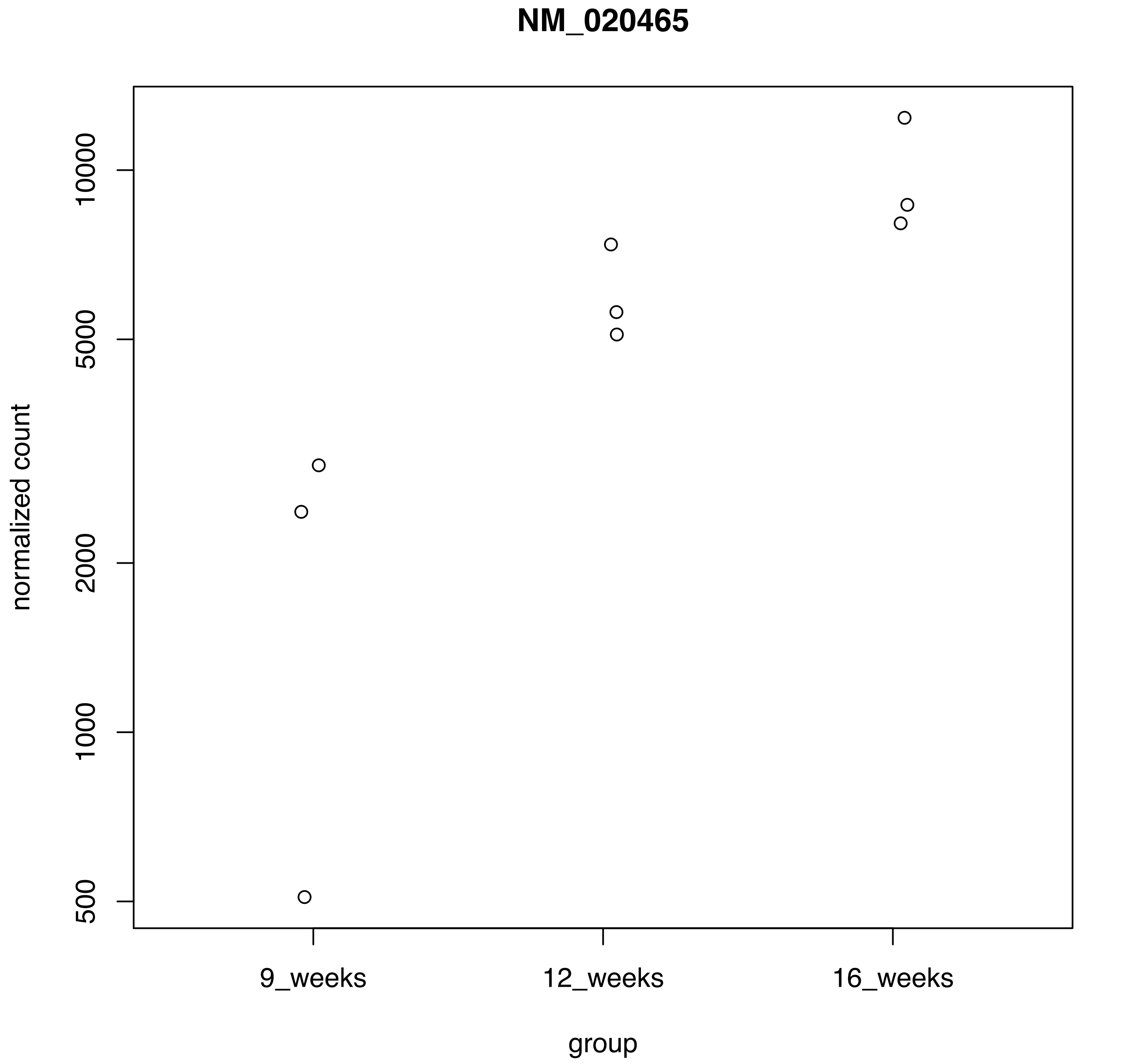
NM_020465
normalized count
10000
5000
2000
1000
500
9_weeks
12_weeks
16_weeks
group

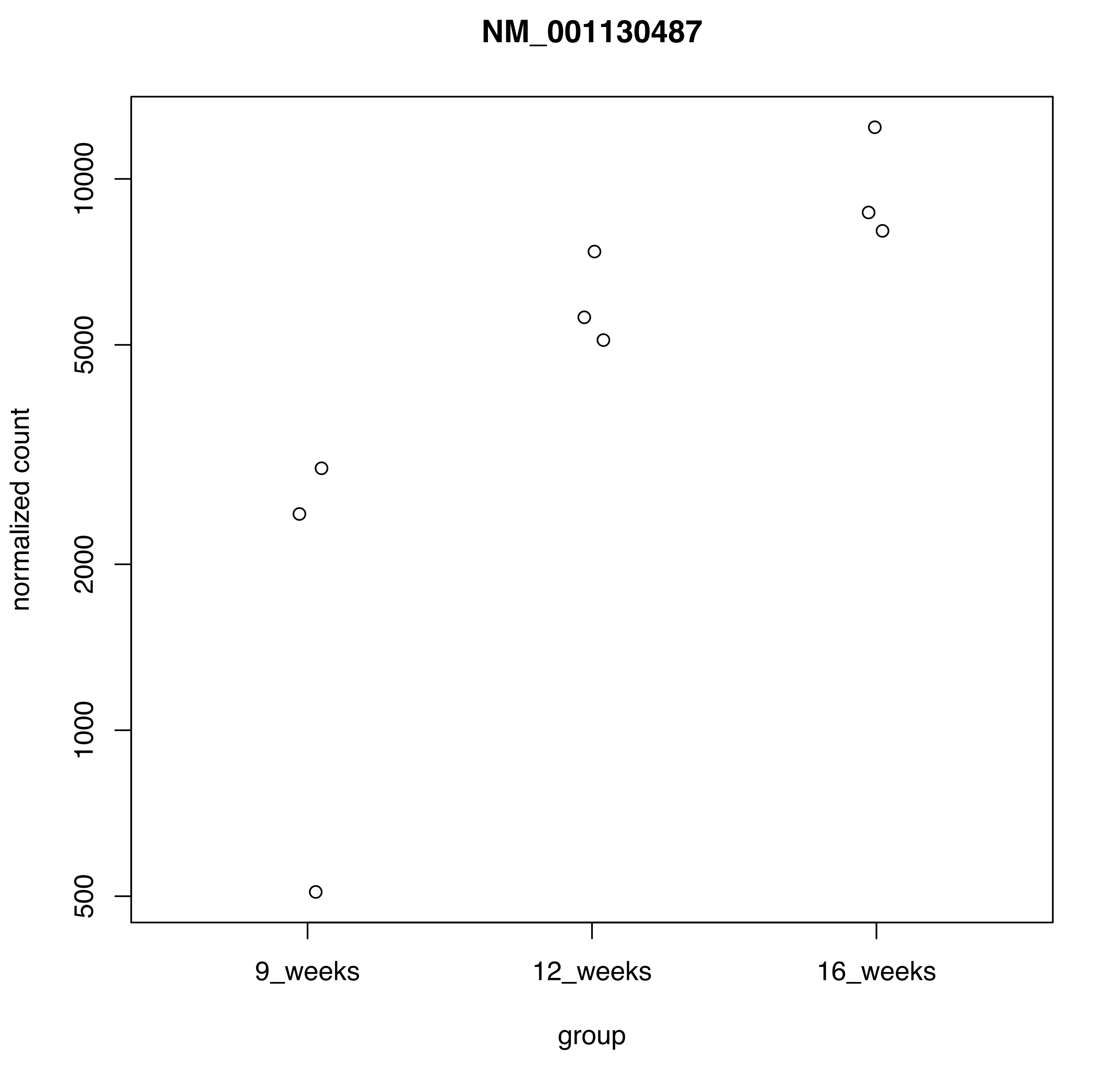
NM_001130487
normalized count
10000
5000
2000
1000
500
9_weeks
12_weeks
16_weeks
group

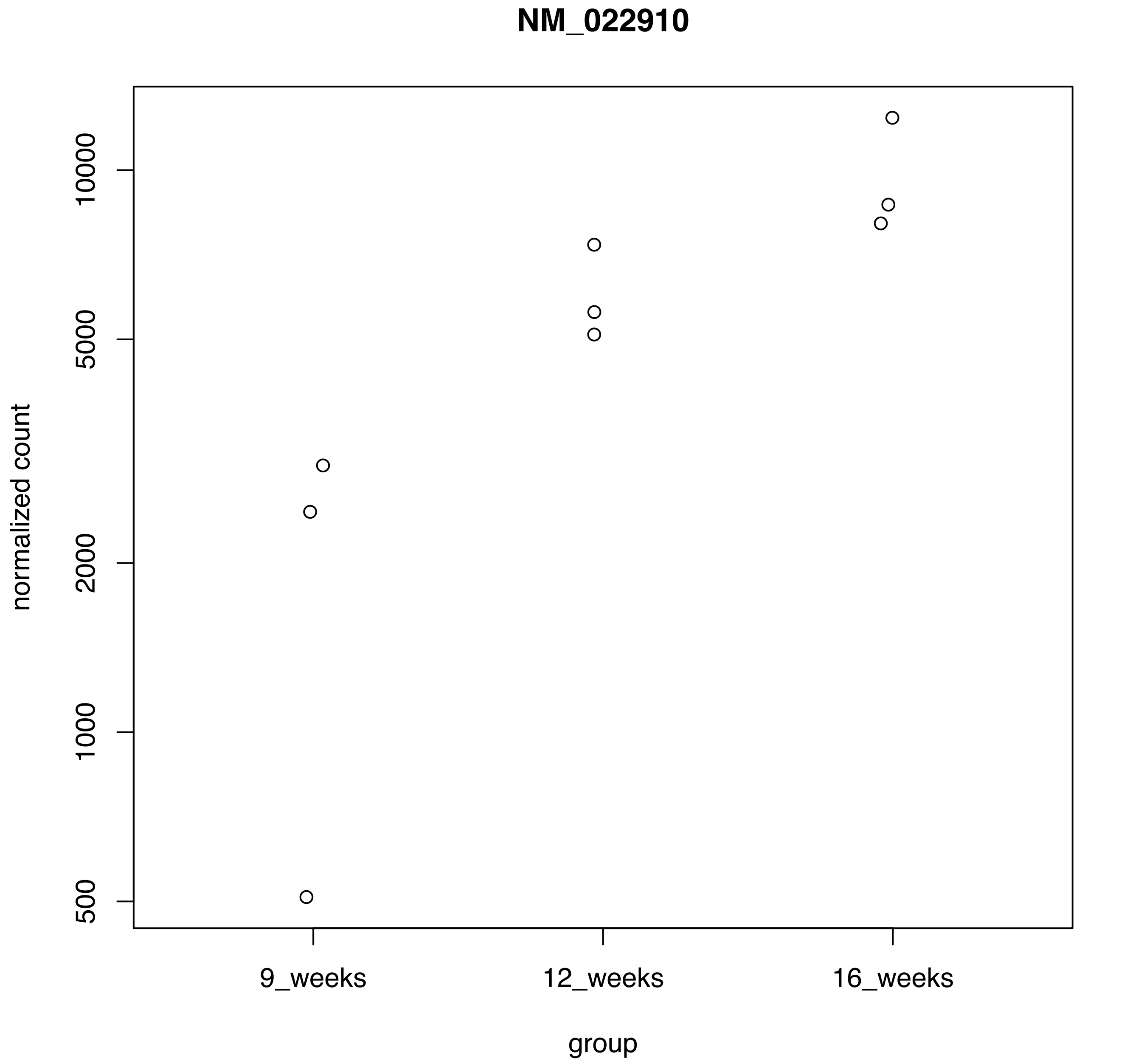
NM_022910
normalized count
10000
5000
2000
1000
500
9_weeks
12_weeks
16_weeks
group

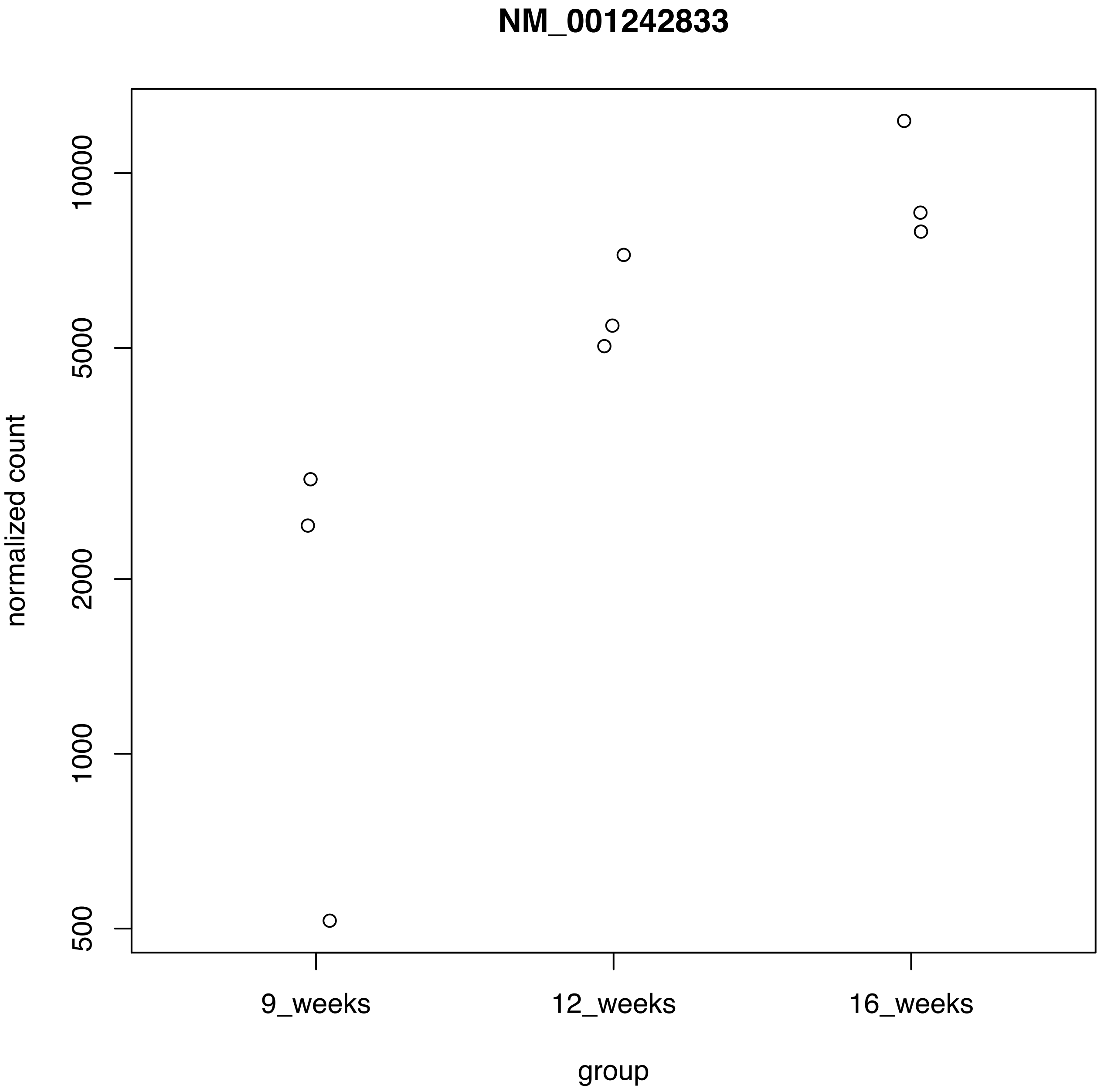
NM_001242833
normalized count
10000
5000
2000
1000
500
9_weeks
12_weeks
16_weeks
group

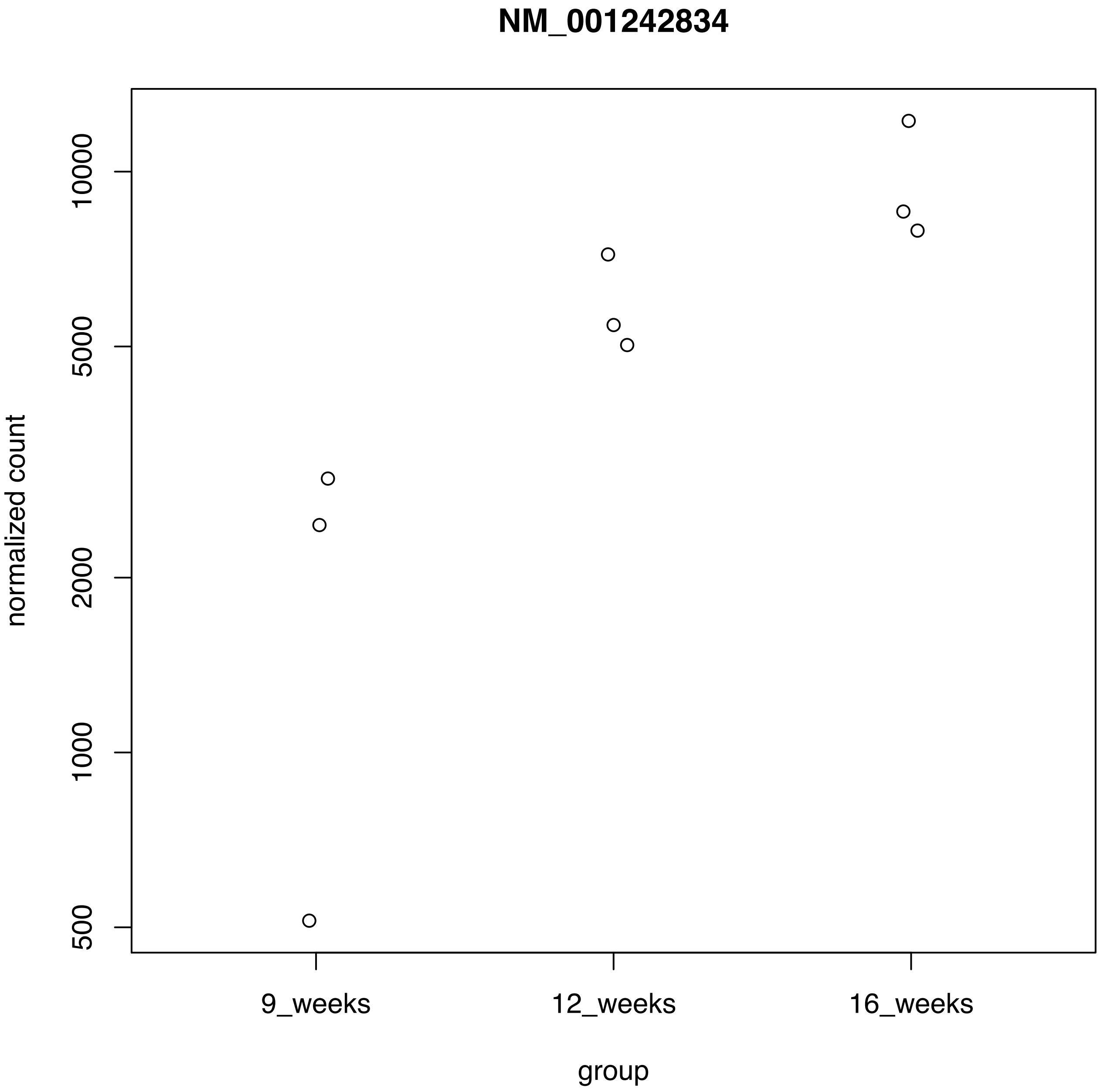
NM_001242834
normalized count
10000
5000
2000
1000
500
9_weeks
12_weeks
16_weeks
group

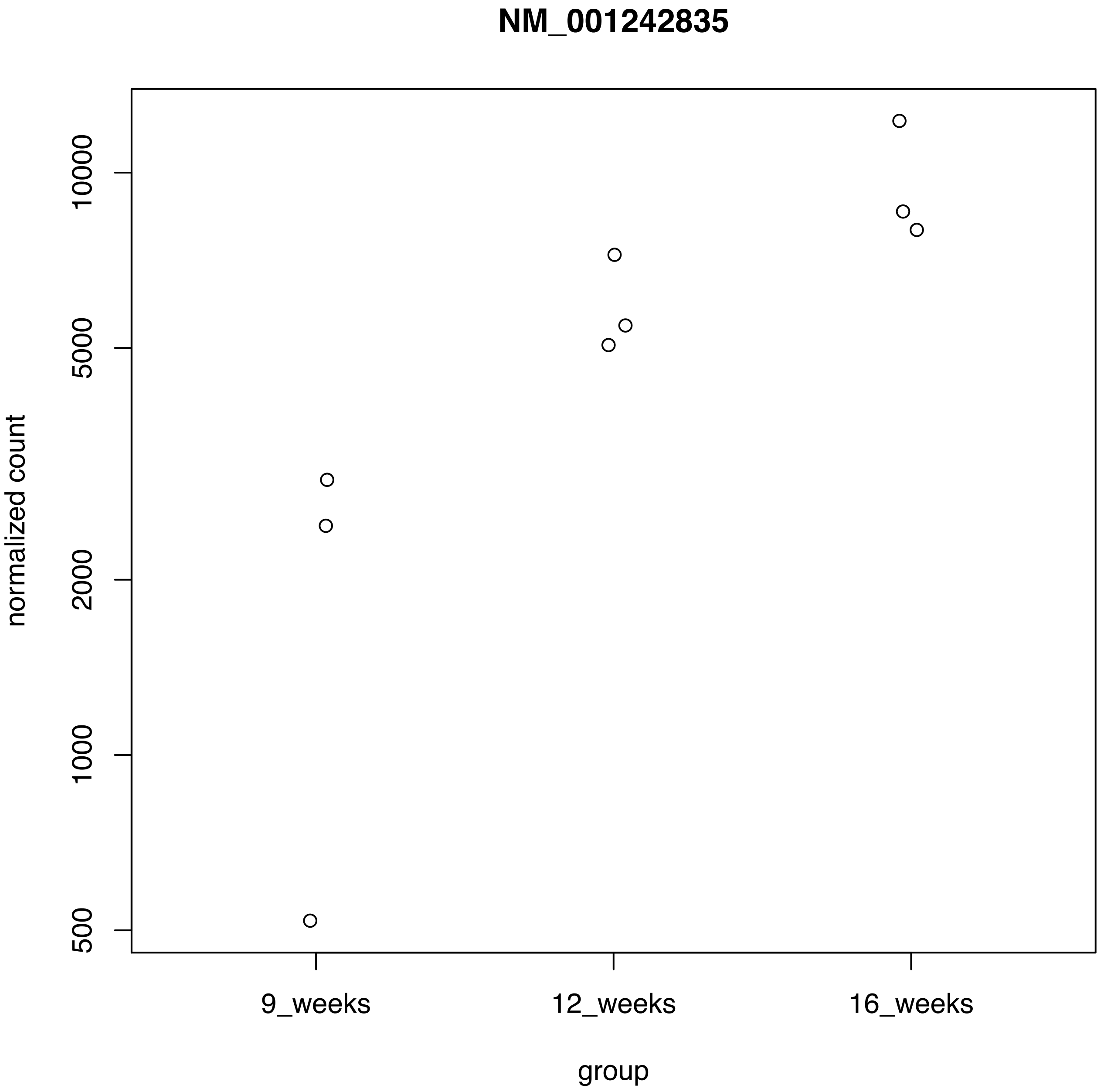
NM_001242835
normalized count
10000
5000
2000
1000
500
9_weeks
12_weeks
16_weeks
group

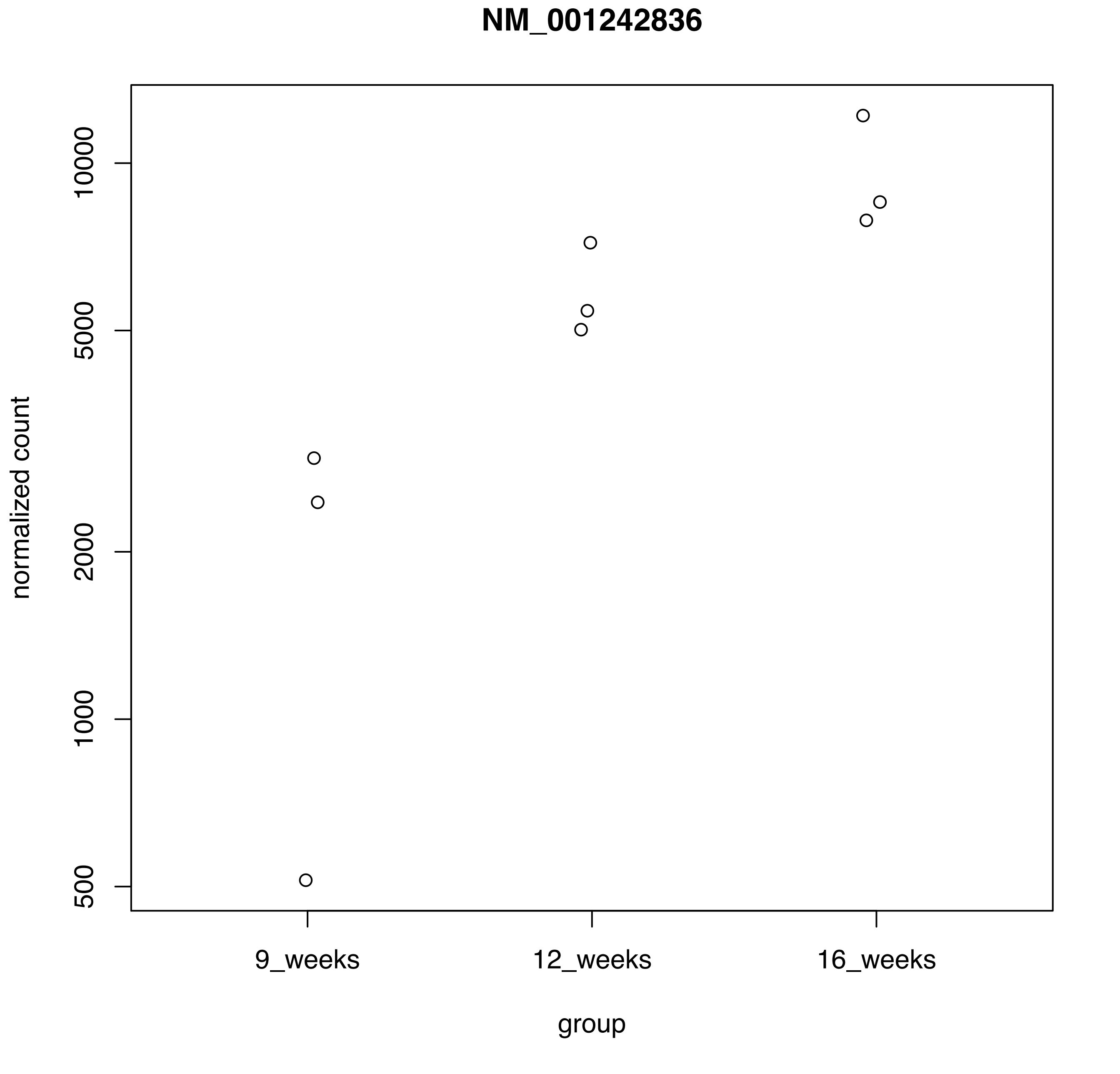
NM_001242836
normalized count
10000
5000
2000
1000
500
9_weeks
12_weeks
16_weeks
group

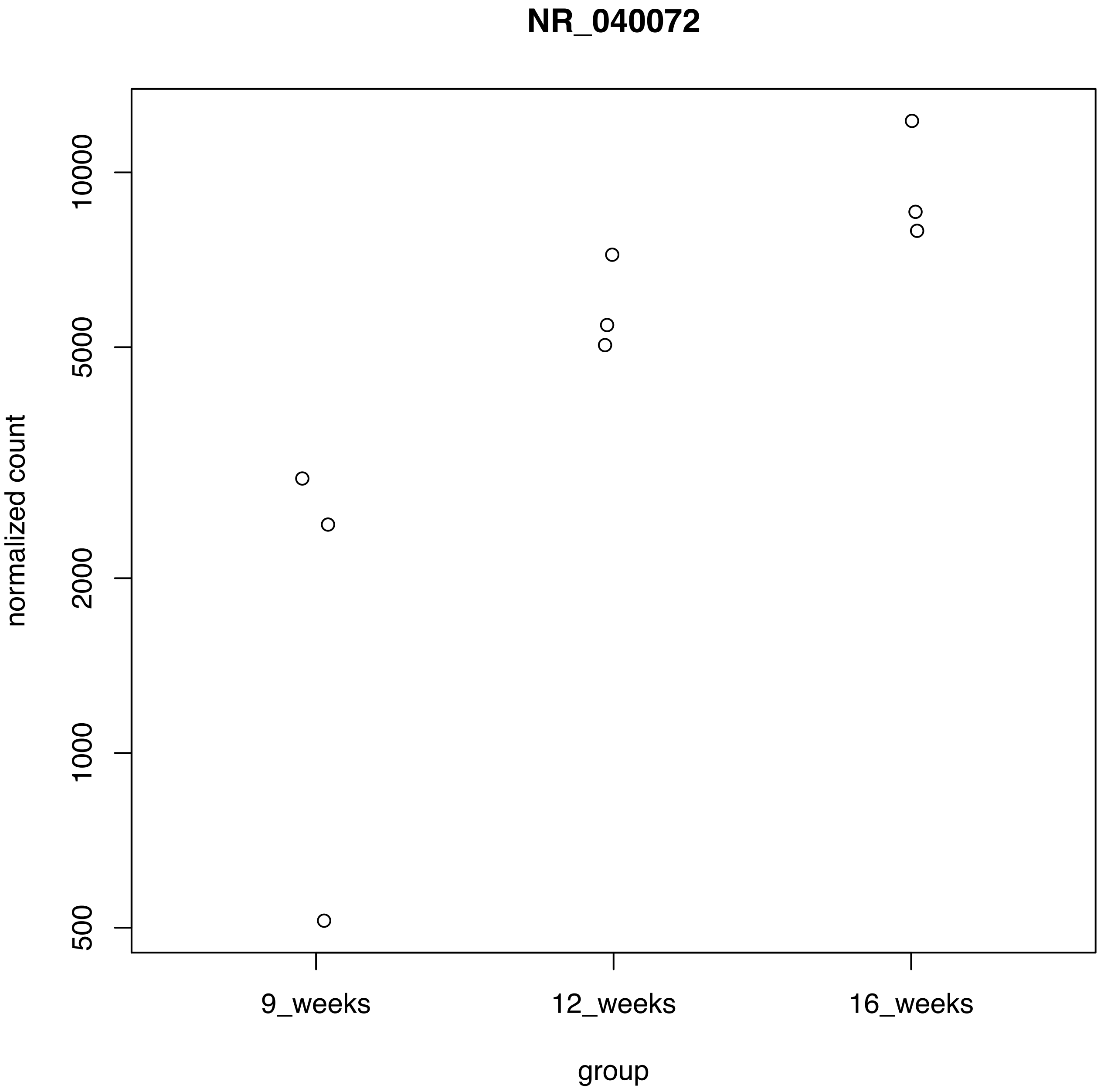
NR_040072
normalized count
10000
5000
2000
1000
500
9_weeks
12_weeks
16_weeks
group

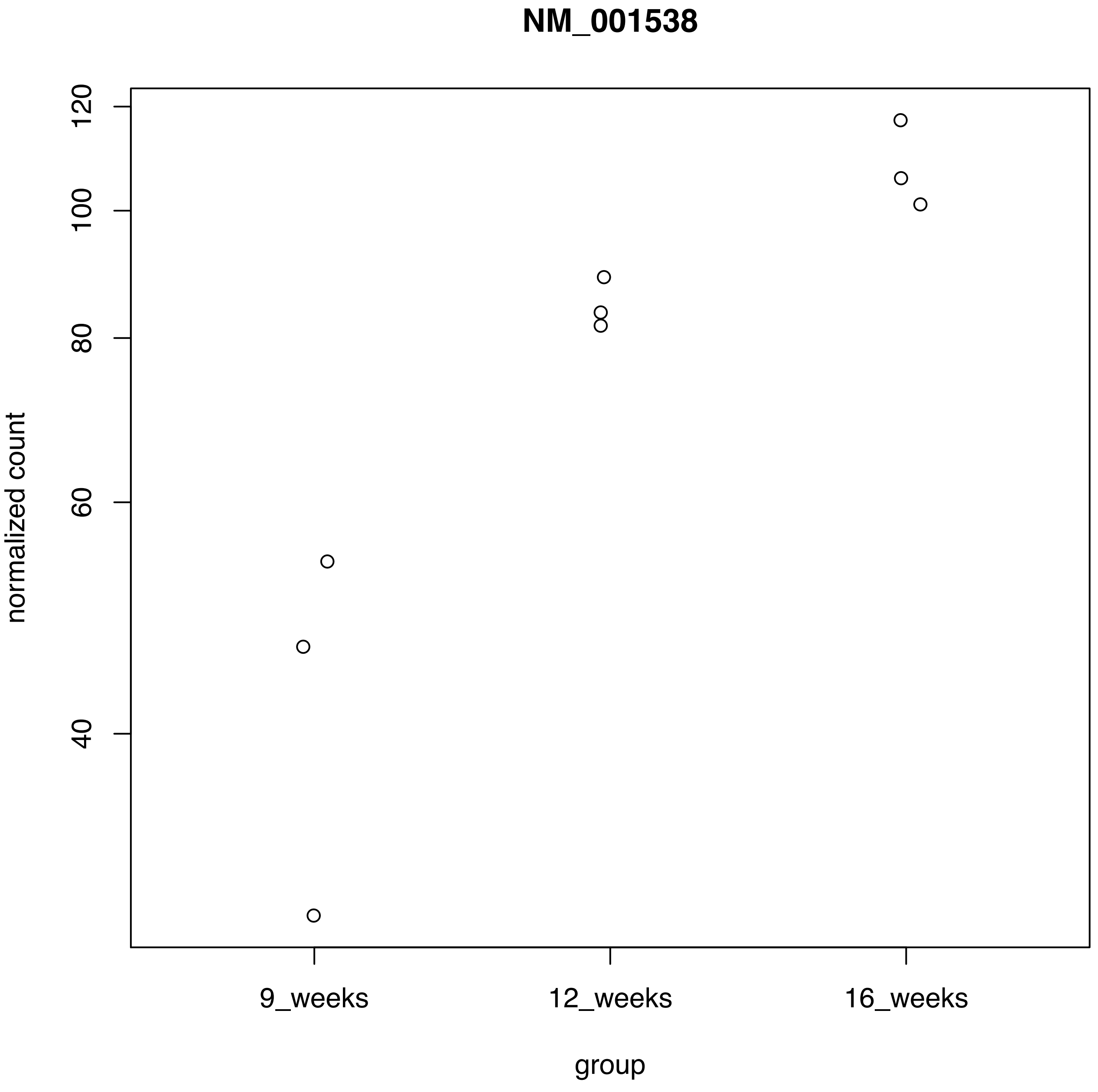
NM_001538
normalized count
120
100
80
60
40
9_weeks
12_weeks
16_weeks
group

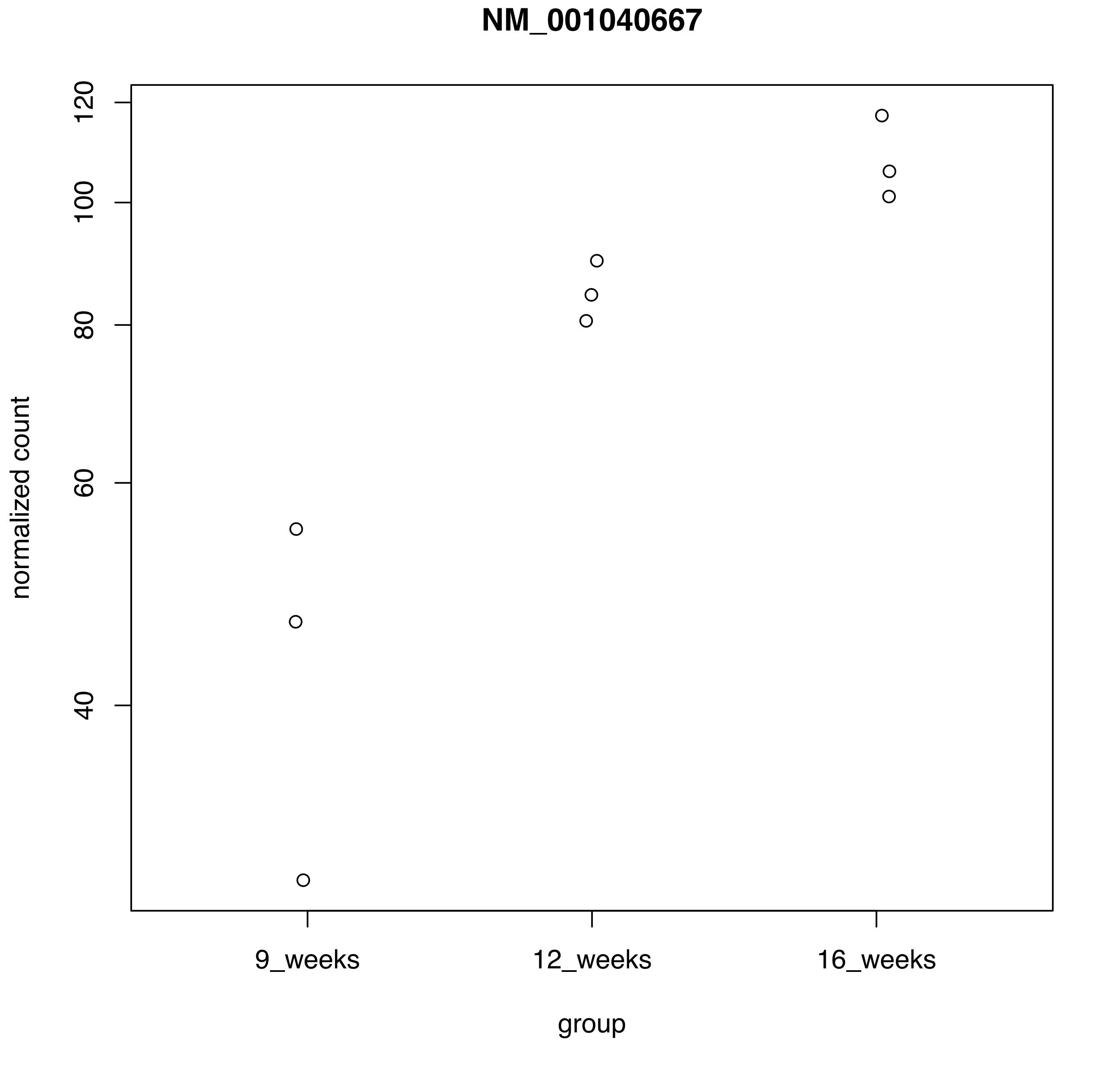
NM_001040667
normalized count
120
100
80
60
40
9_weeks
12_weeks
16_weeks
group

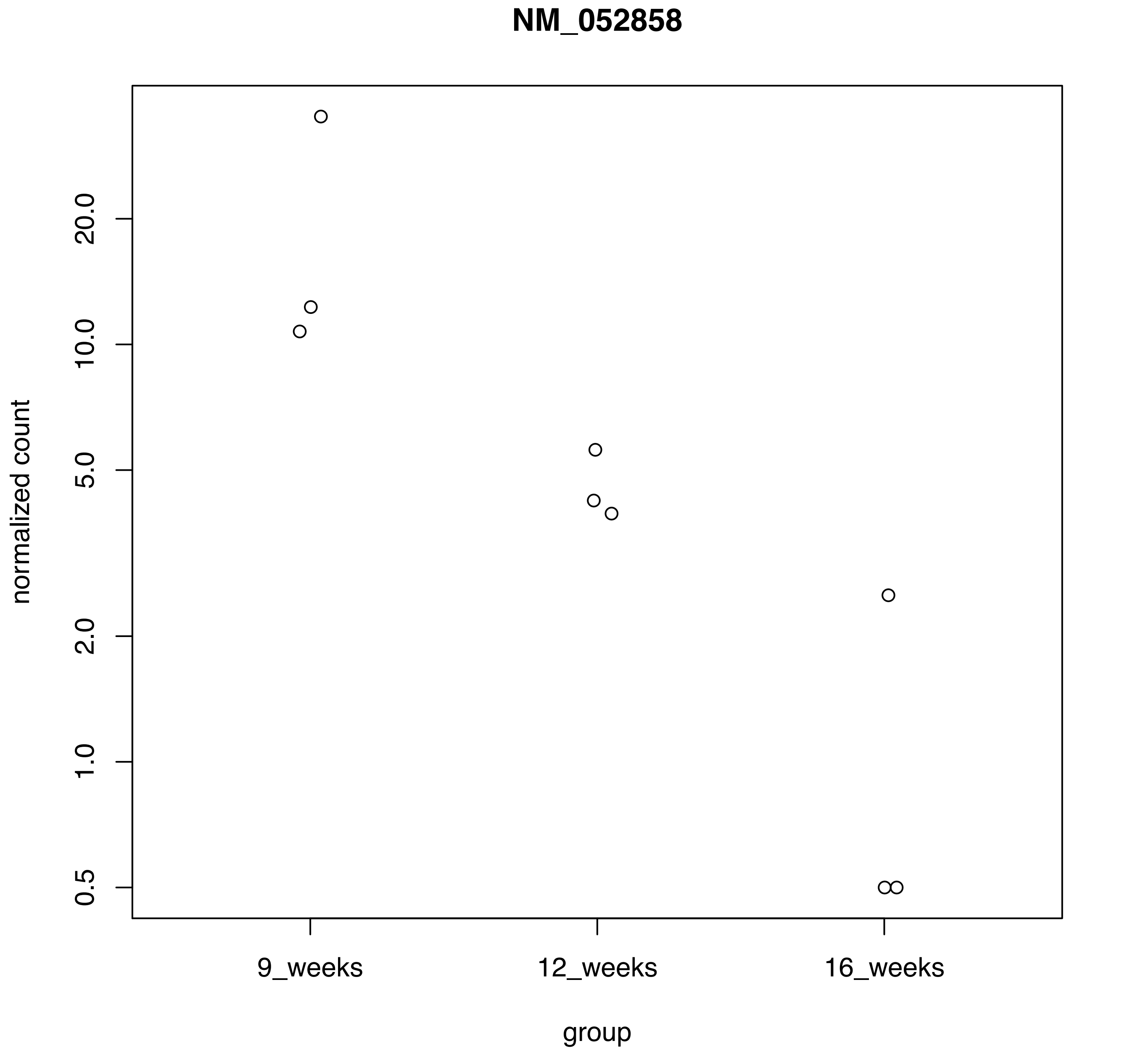
NM_052858
normalized count
20.0
10.0
5.0
2.0
1.0
0.5
9_weeks
12_weeks
16_weeks
group

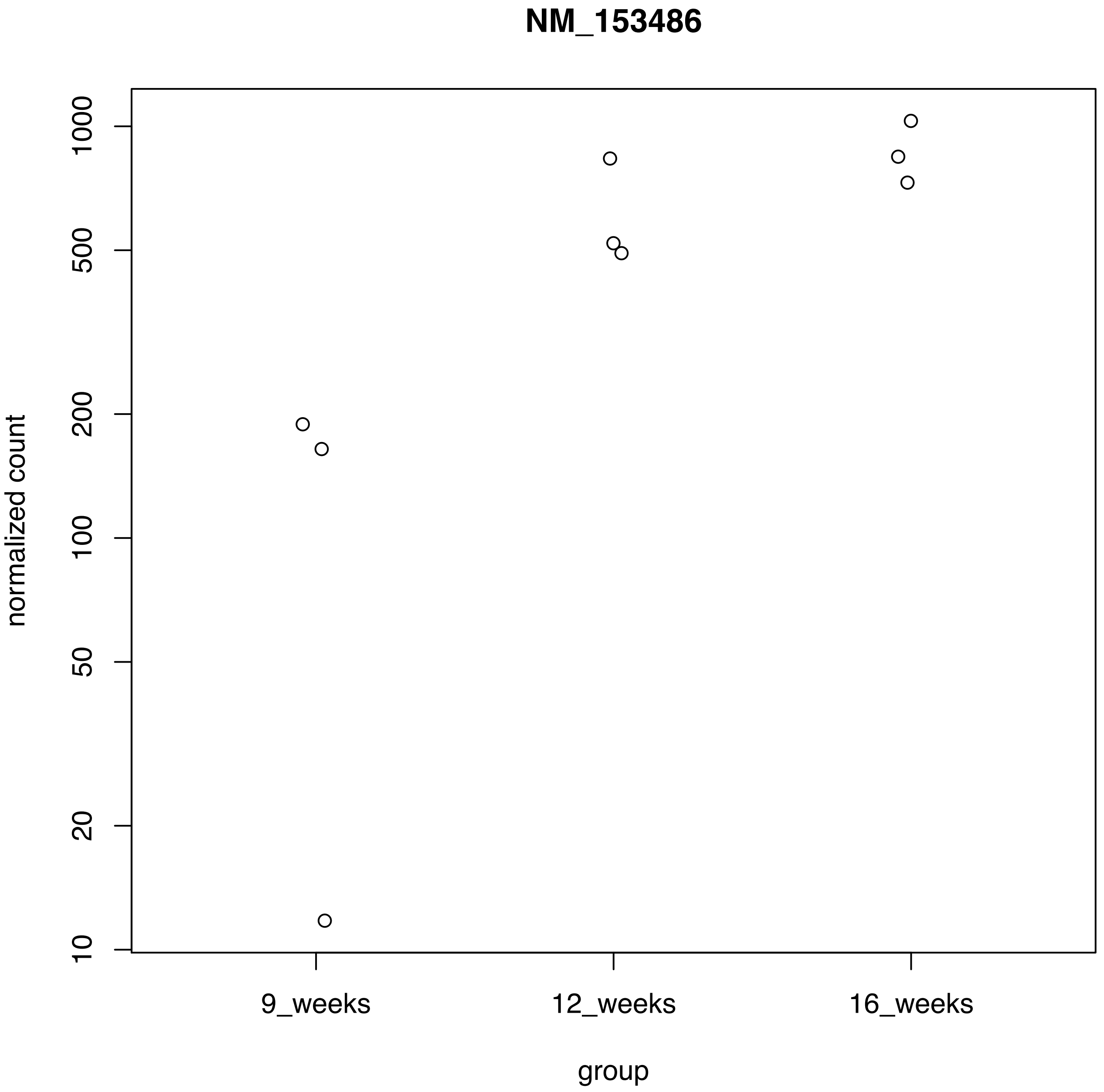
NM_153486
normalized count
1000
500
200
100
50
20
10
9_weeks
12_weeks
16_weeks
group

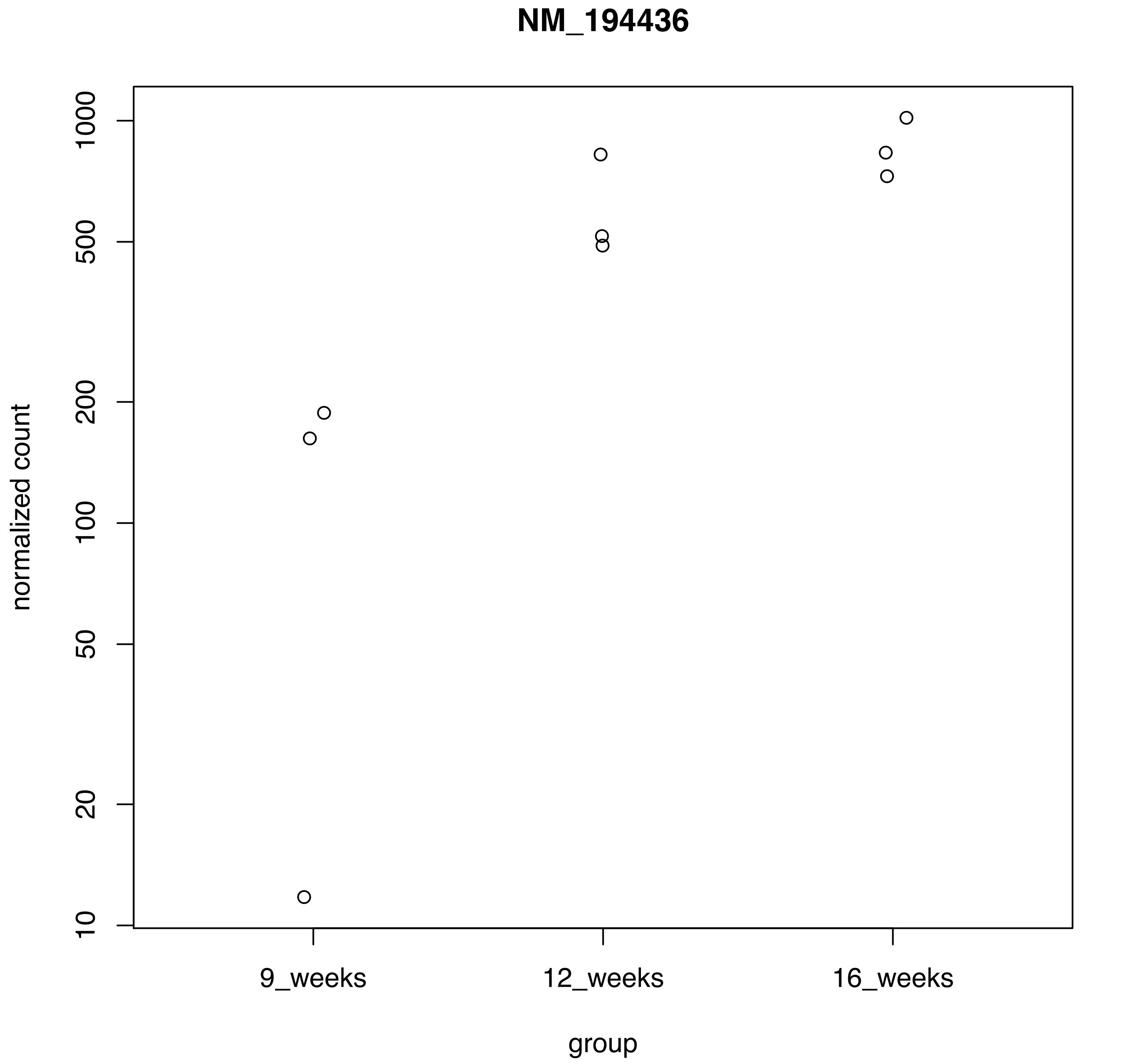
NM_194436
normalized count
1000
500
200
100
50
20
10
9_weeks
12_weeks
16_weeks
group

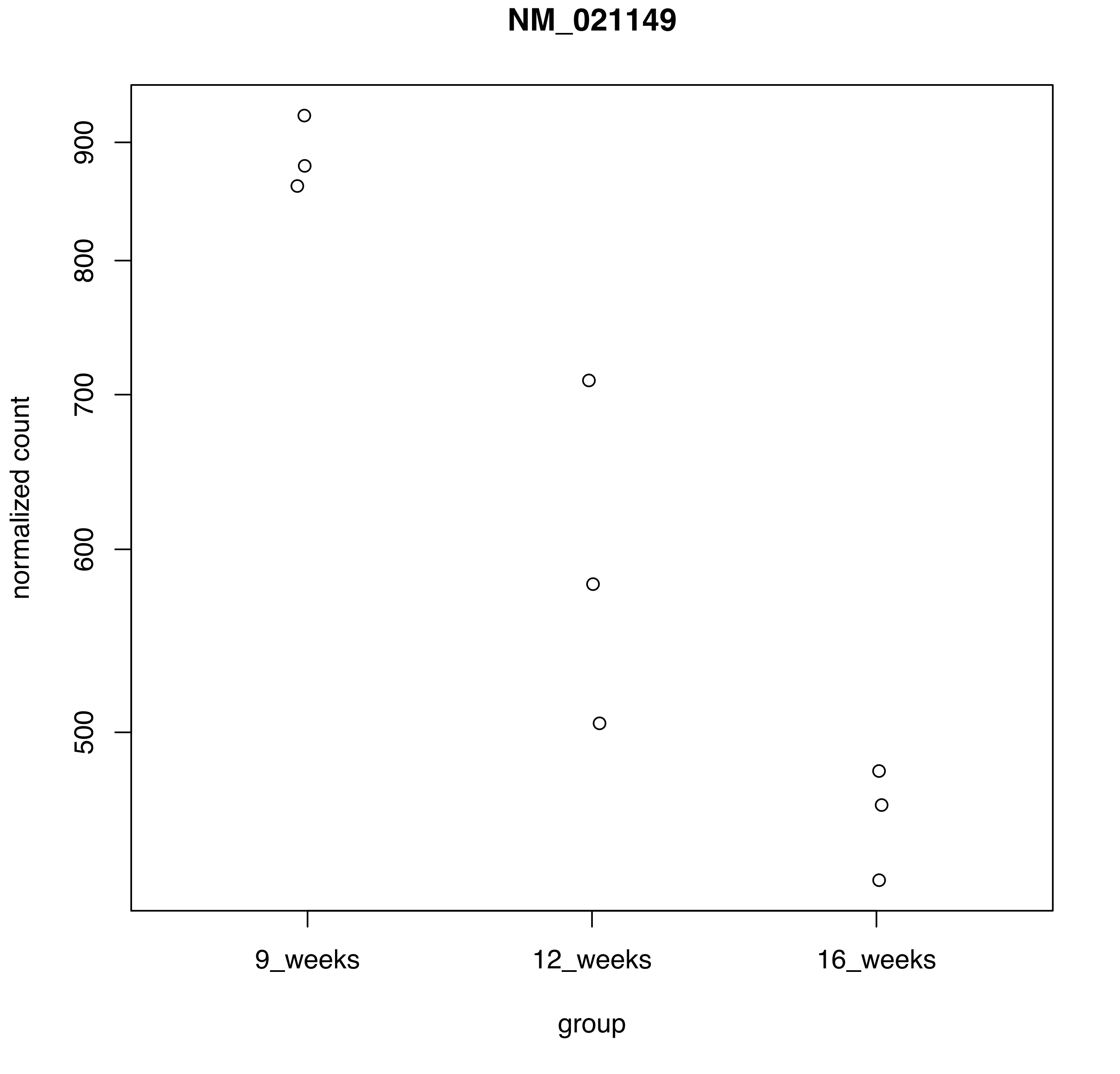

NM_021149
normalized count
900
800
700
600
500
9_weeks
12_weeks
16_weeks
group

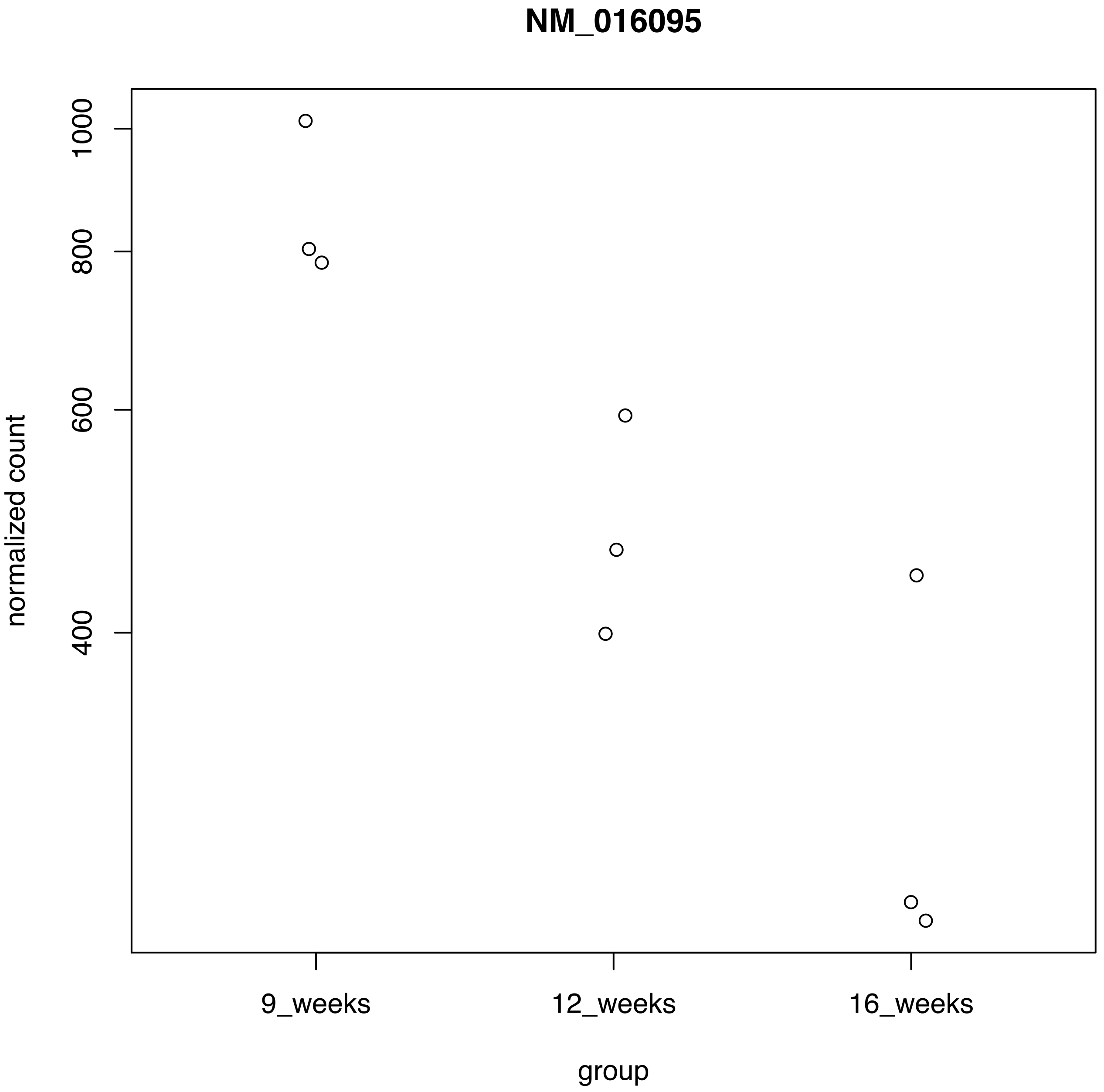
NM_016095
normalized count
1000
800
600
400
9_weeks
12_weeks
16_weeks
group

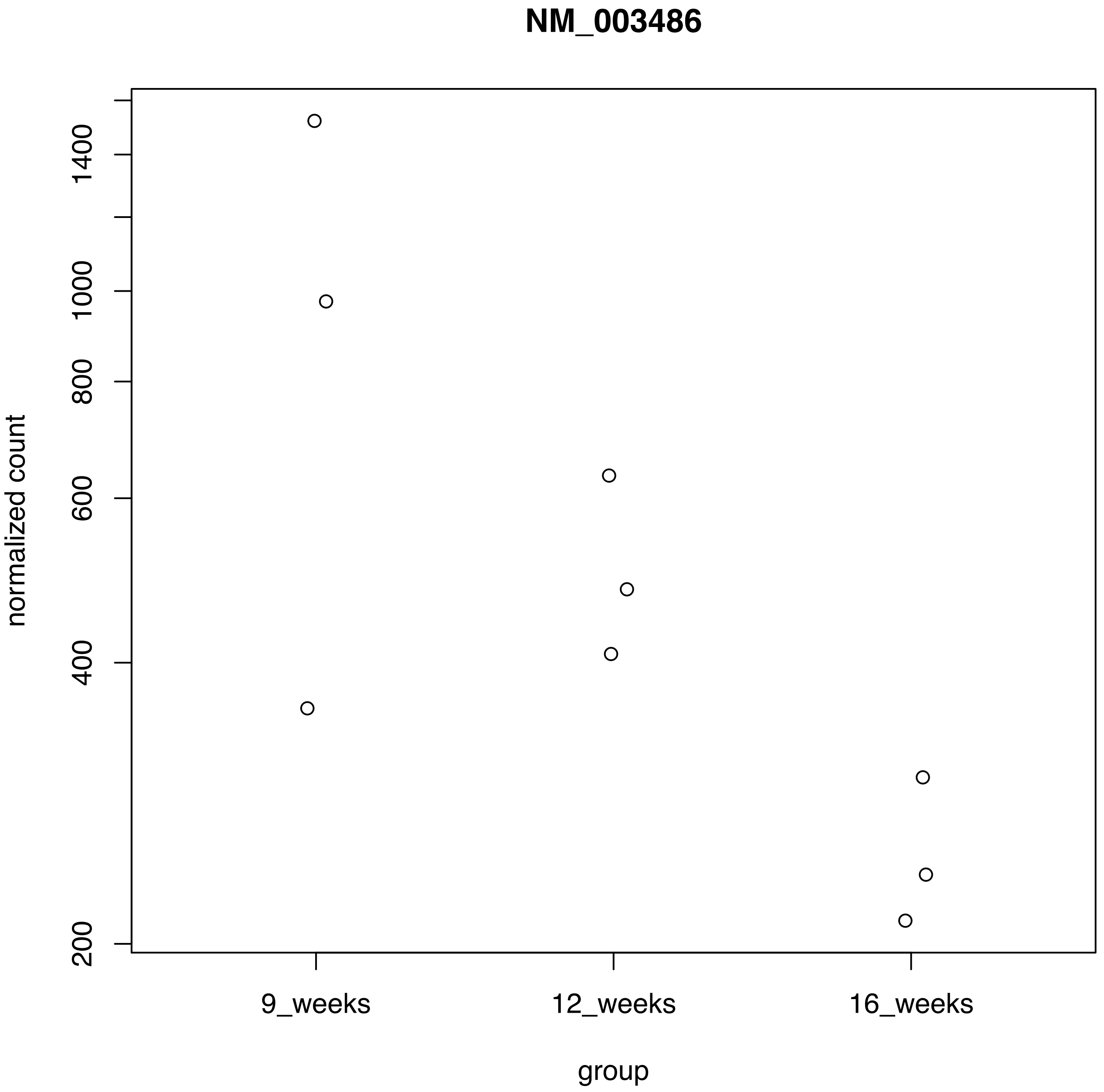
NM_003486
normalized count
1400
1000
800
600
400
200
9_weeks
12_weeks
16_weeks
group

NM_014427

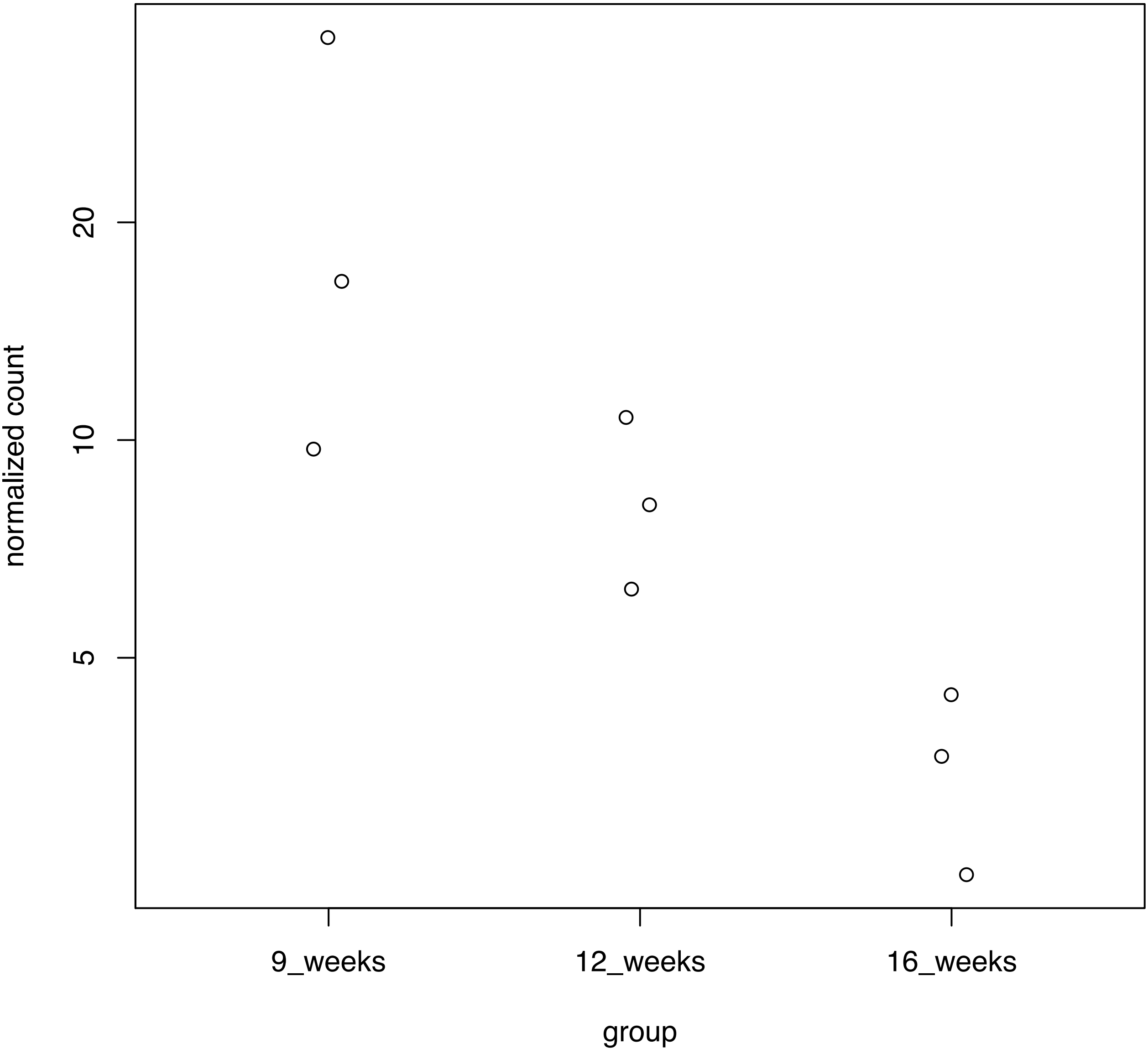

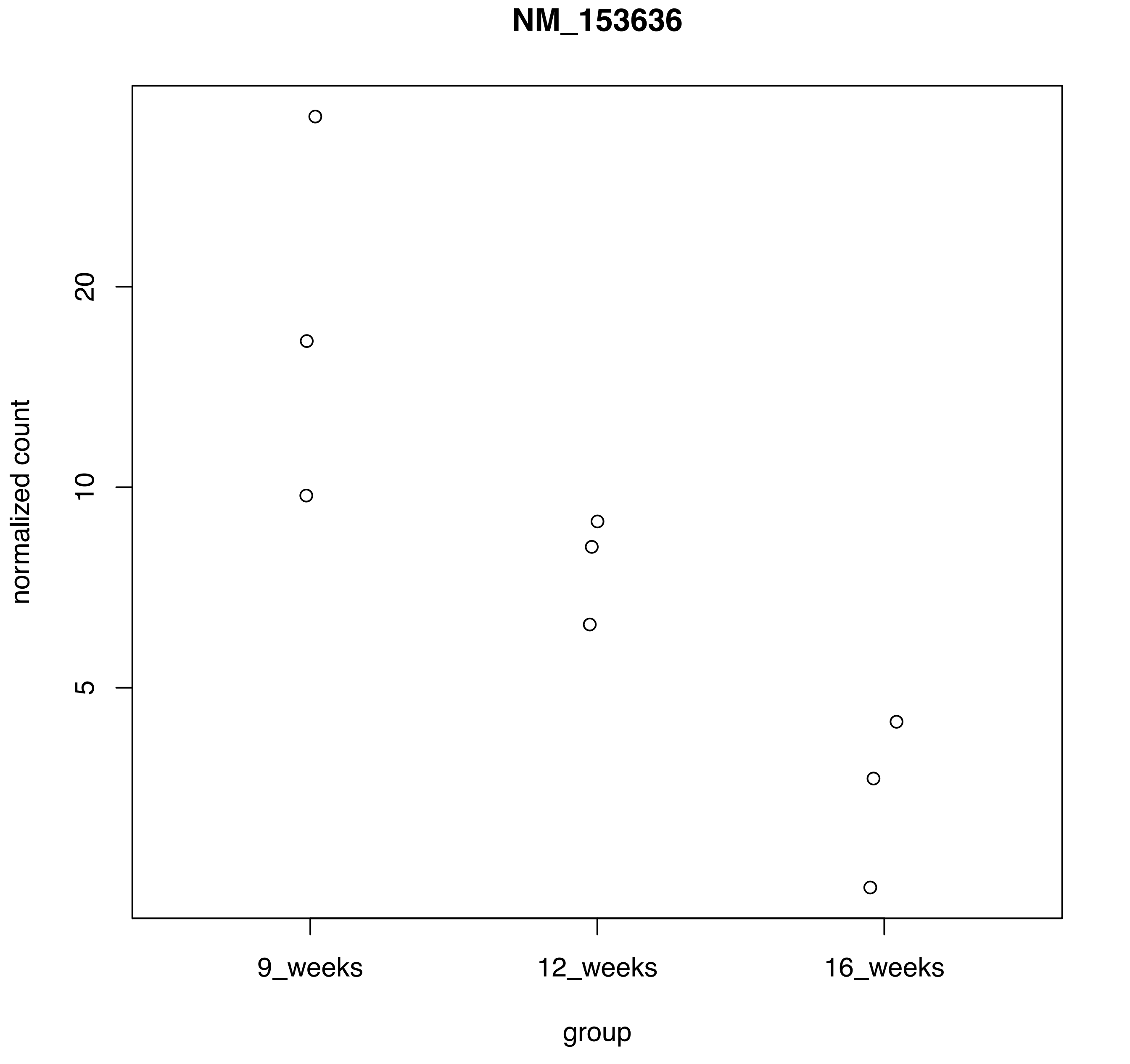
NM_153636
normalized count
20
10
5
9_weeks
12_weeks
16_weeks
group

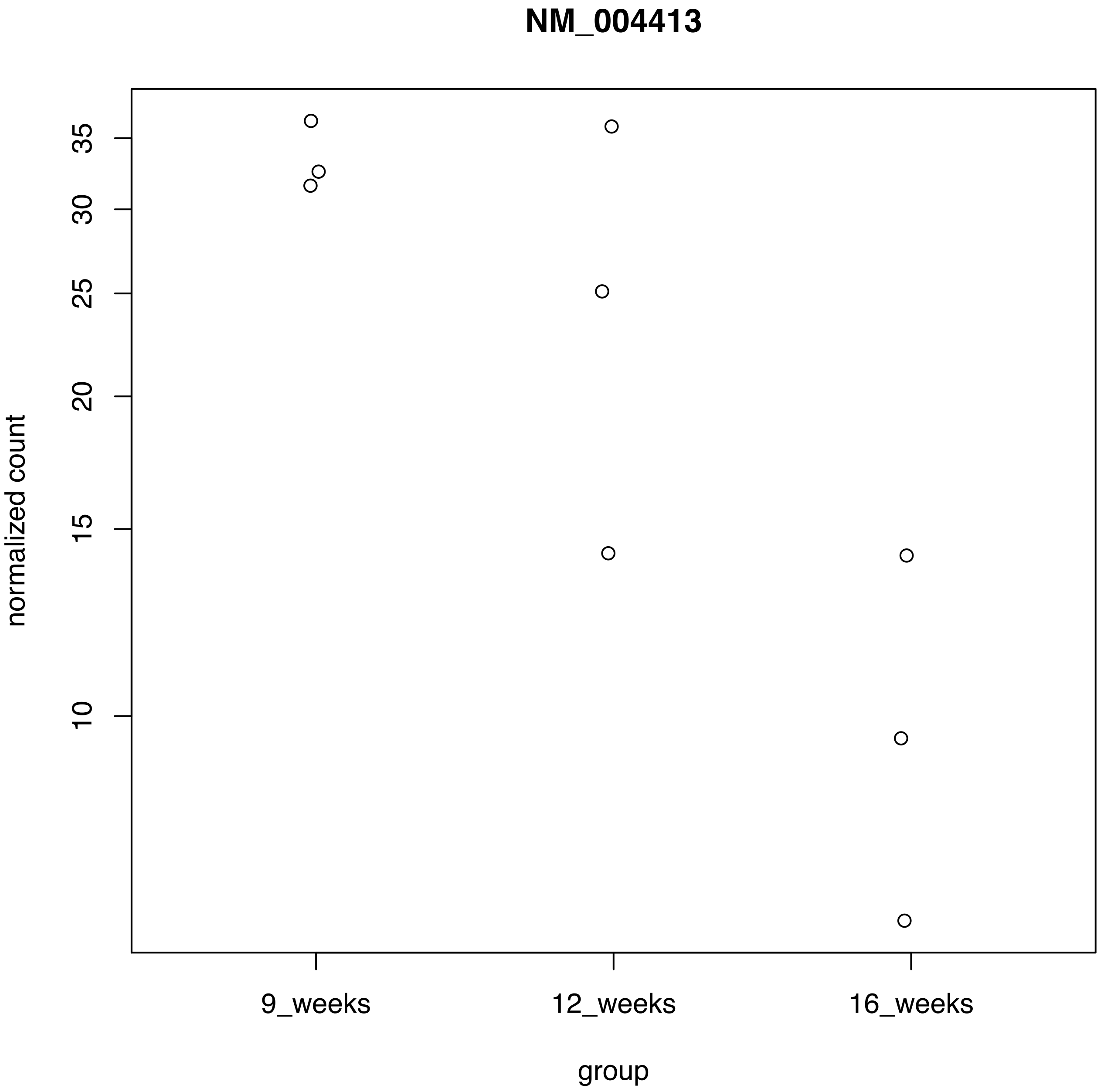

NM_004413
normalized count
35
30
25
20
15
10
9_weeks
12_weeks
16_weeks
group

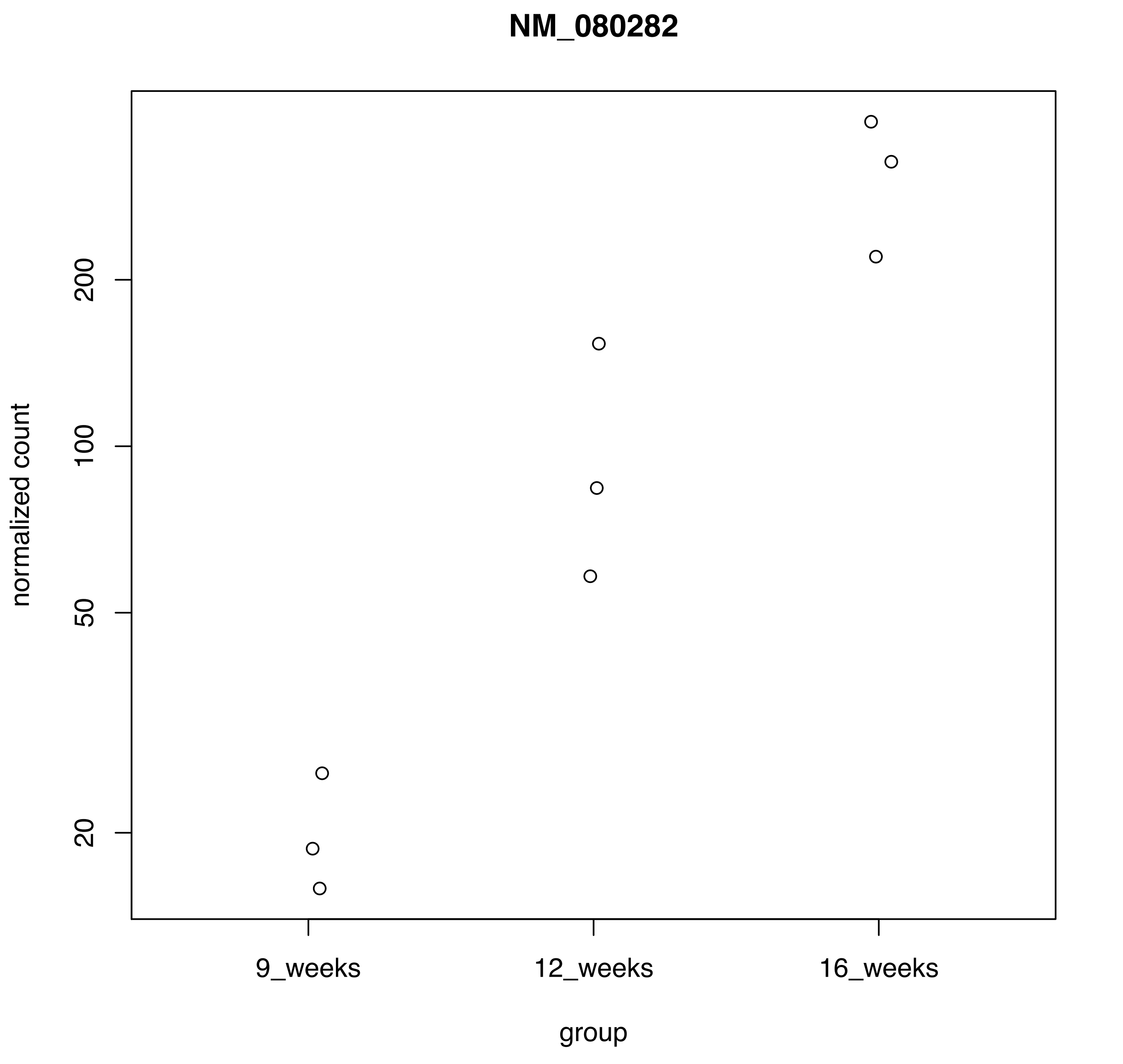
NM_080282
normalized count
20
50
100
200
9_weeks
12_weeks
16_weeks
group

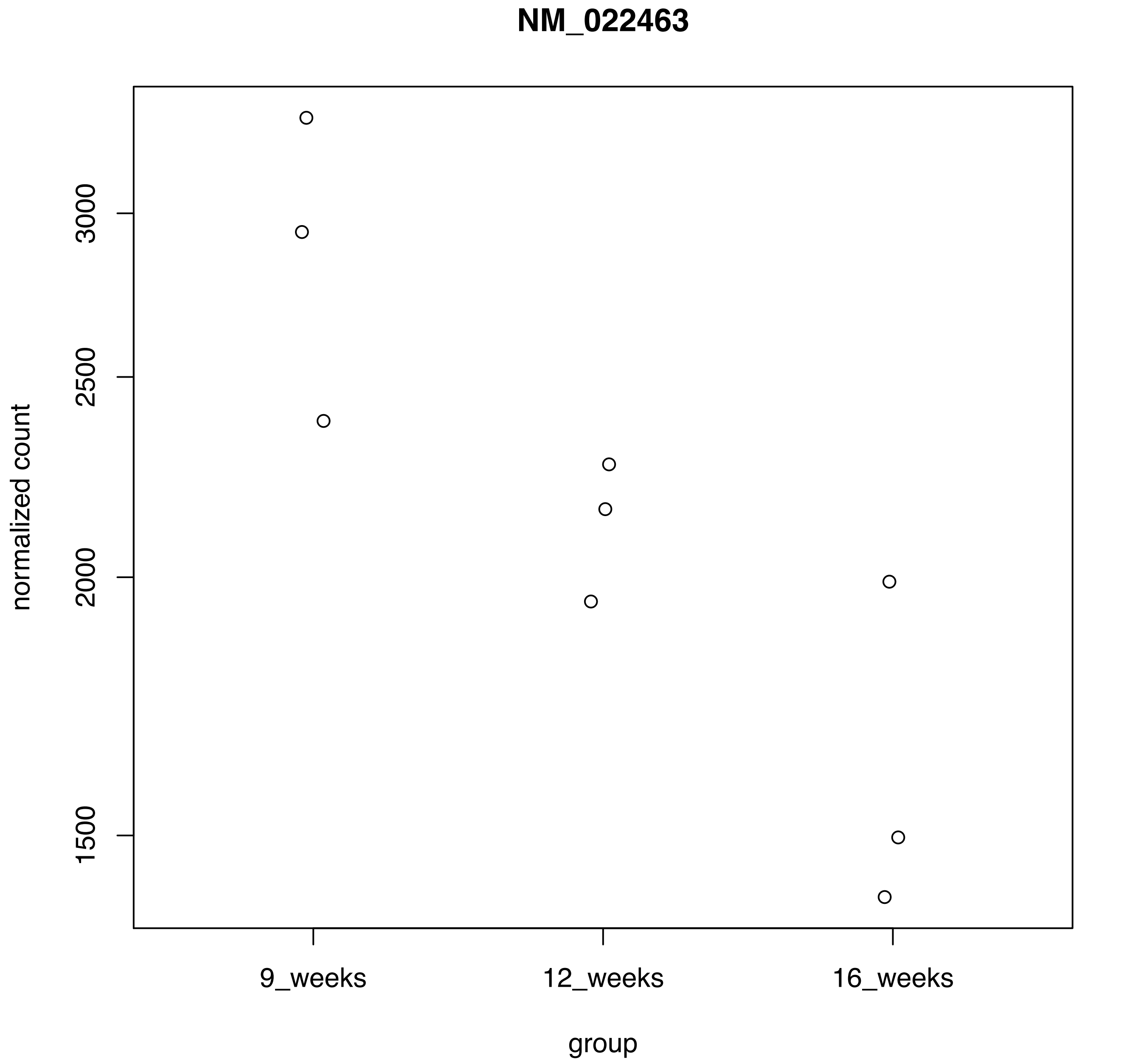
NM_022463
normalized count
3000
2500
2000
1500
9_weeks
12_weeks
16_weeks
group

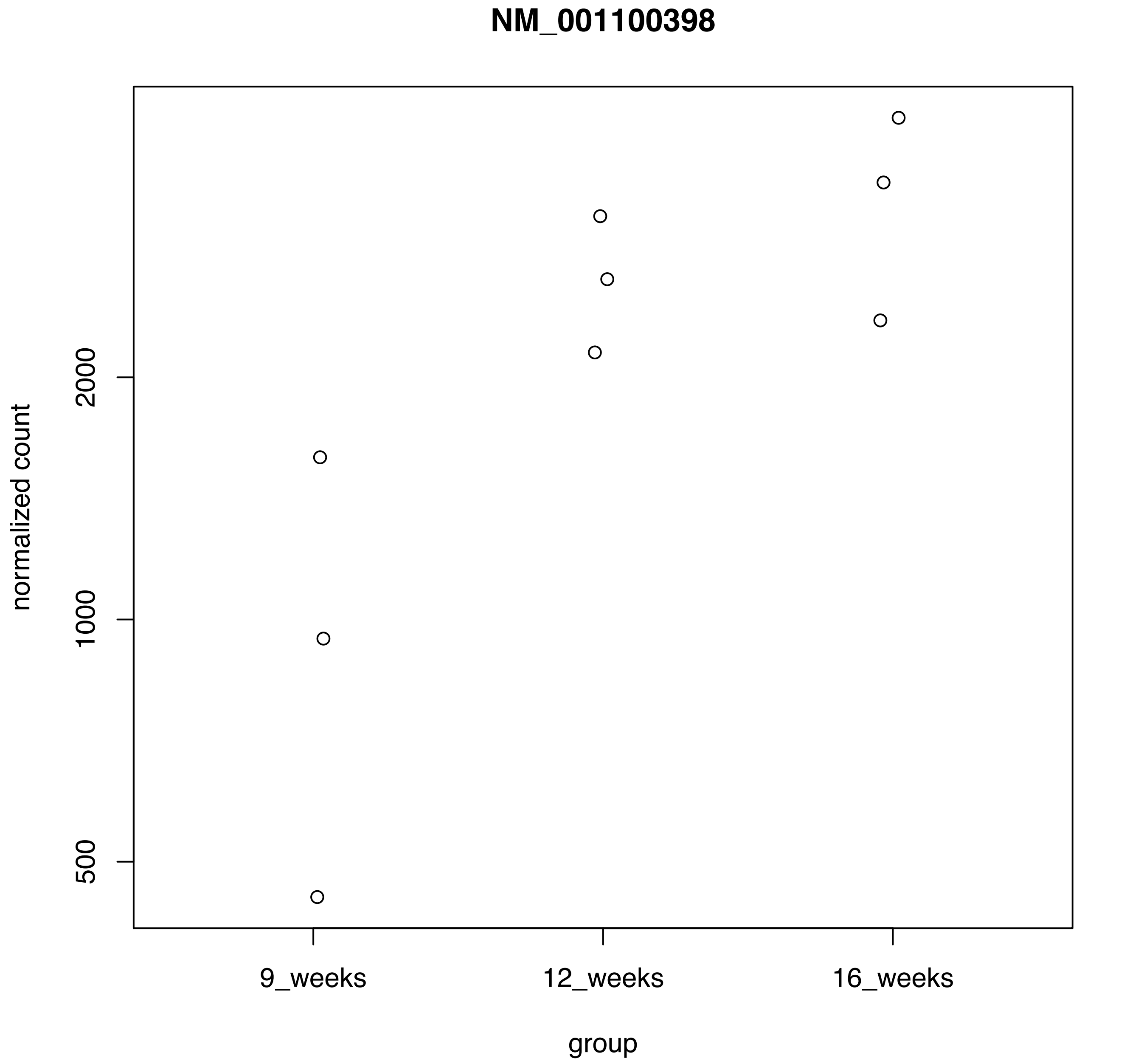

NM_001100398
normalized count
500
1000
2000
9_weeks
12_weeks
16_weeks
group

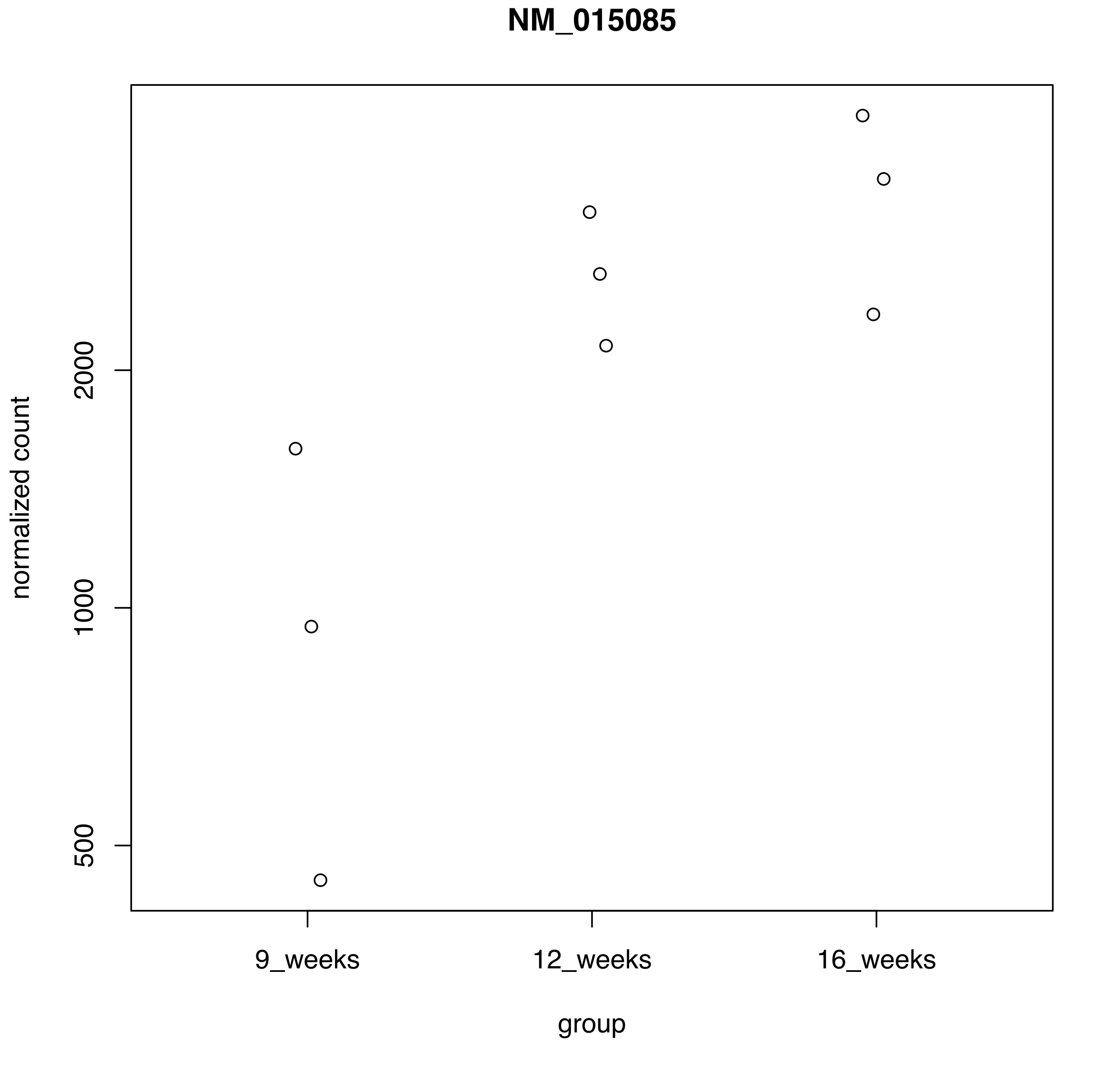
NM_015085
normalized count
2000
1000
500
9_weeks
12_weeks
16_weeks
group

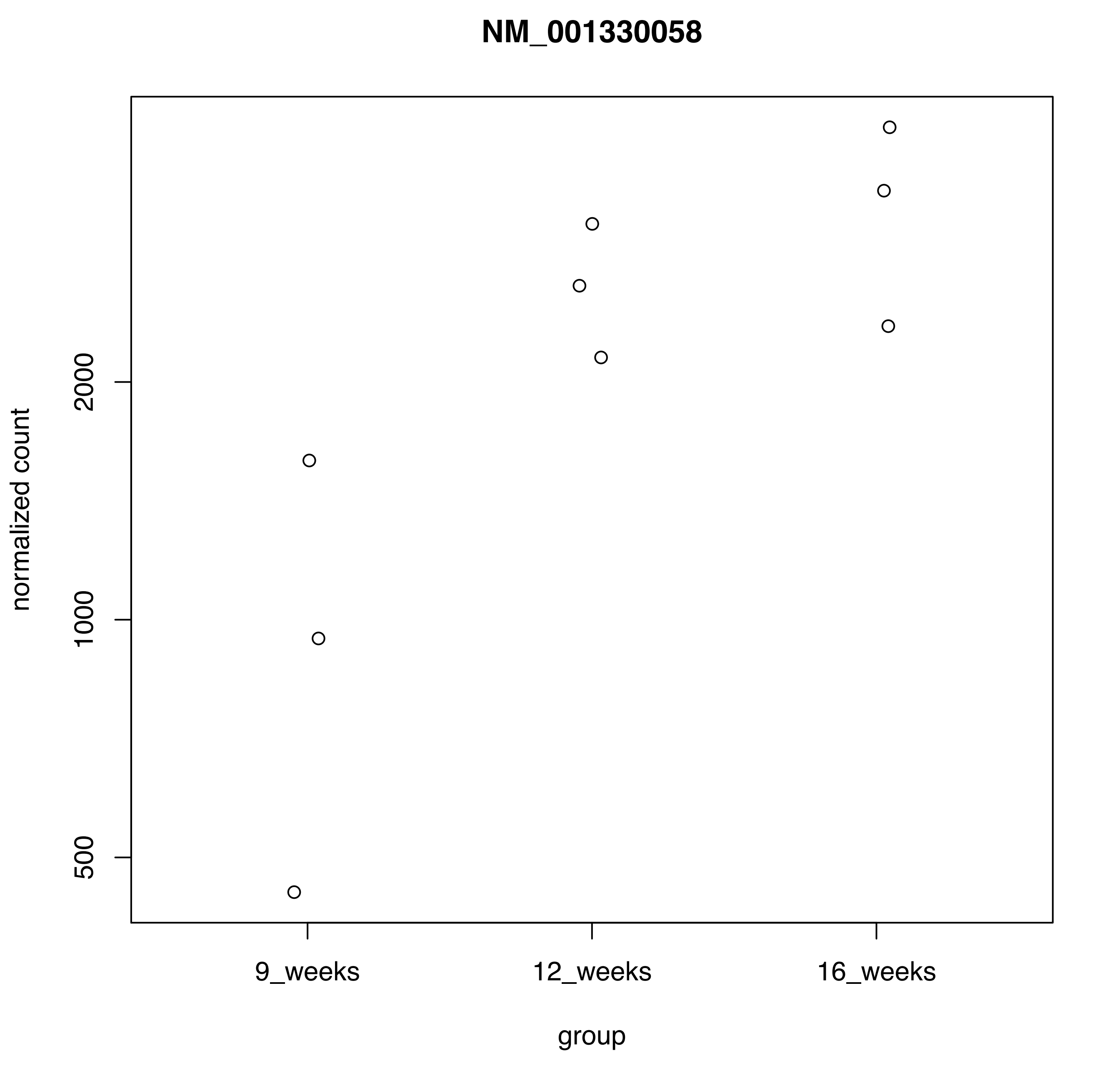

NM_001330058
normalized count
500
1000
2000
9_weeks
12_weeks
16_weeks
group

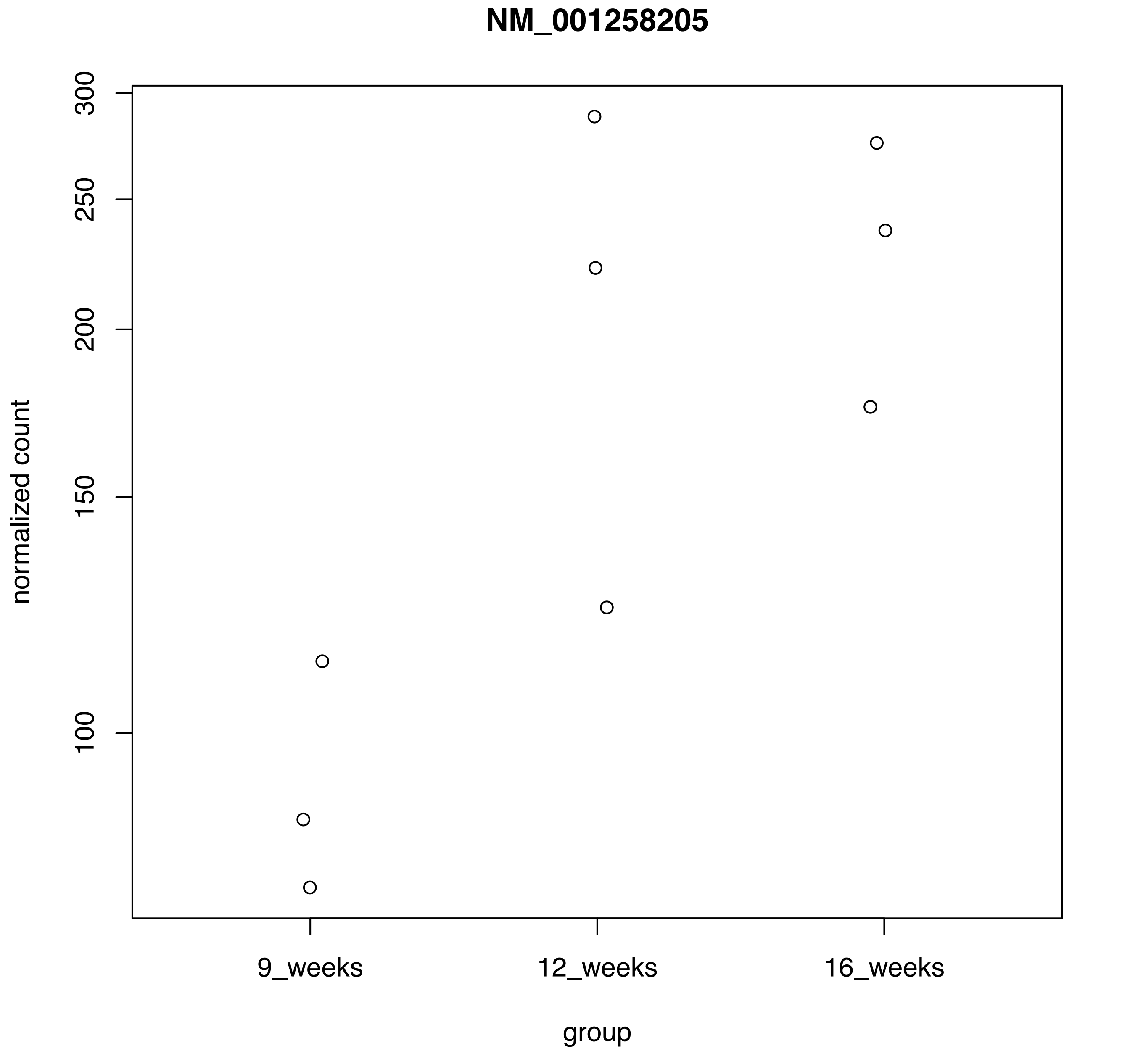

NM_001258205
normalized count
300
250
200
150
100
9_weeks
12_weeks
16_weeks
group

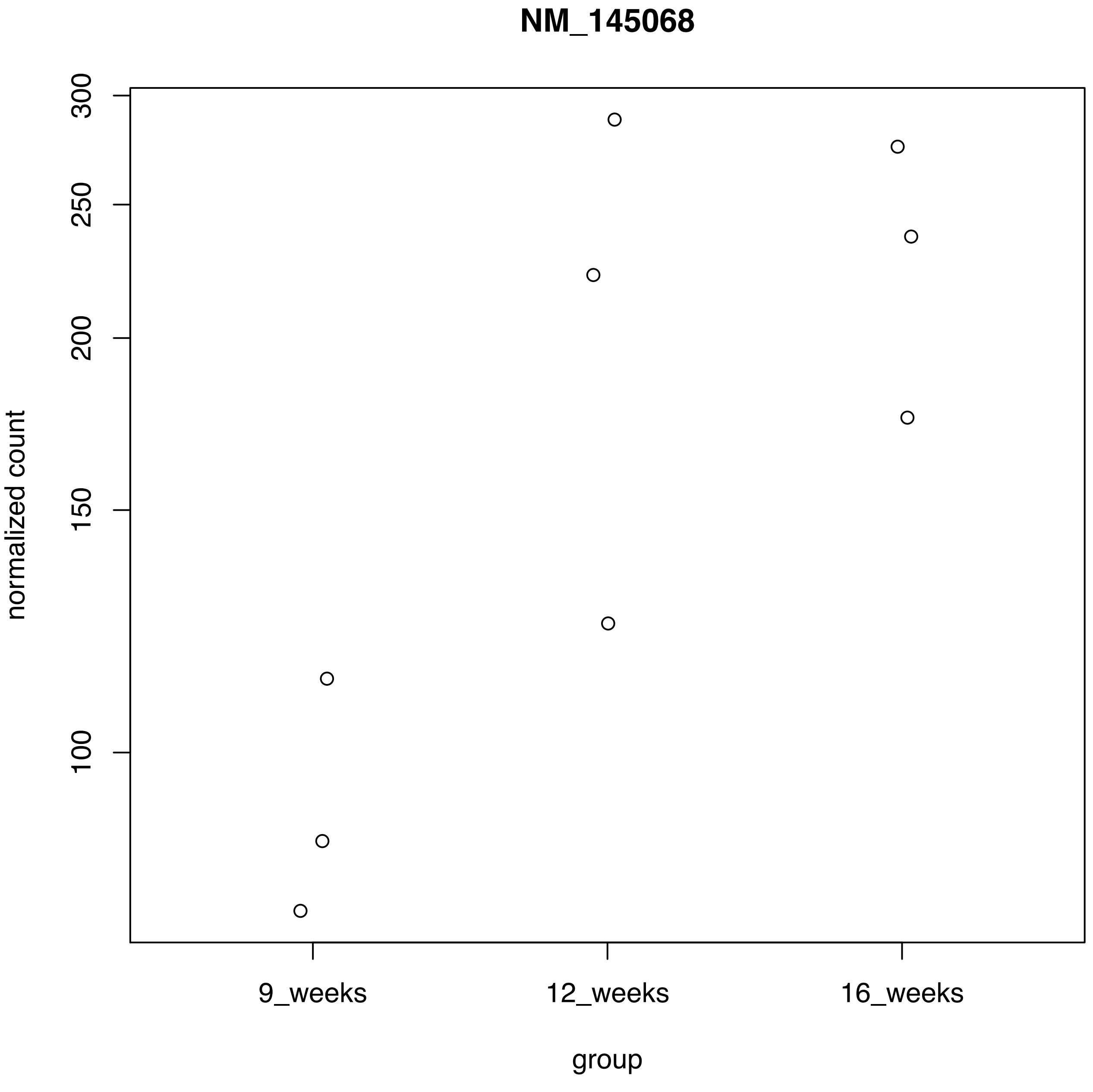
NM_145068
normalized count
300
250
200
150
100
9_weeks
12_weeks
16_weeks
group

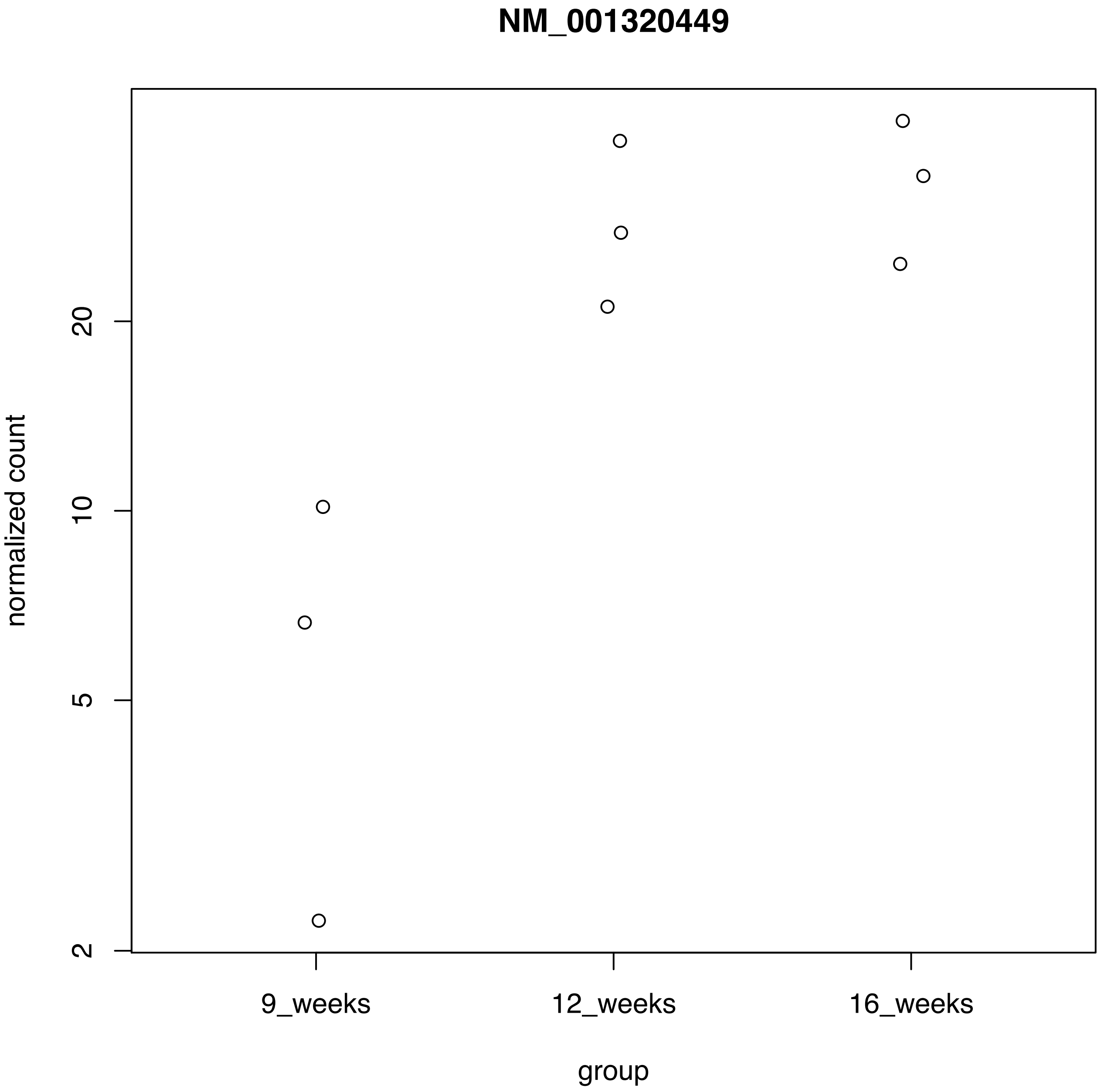
NM_001320449
normalized count
20
10
5
2
9_weeks
12_weeks
16_weeks
group

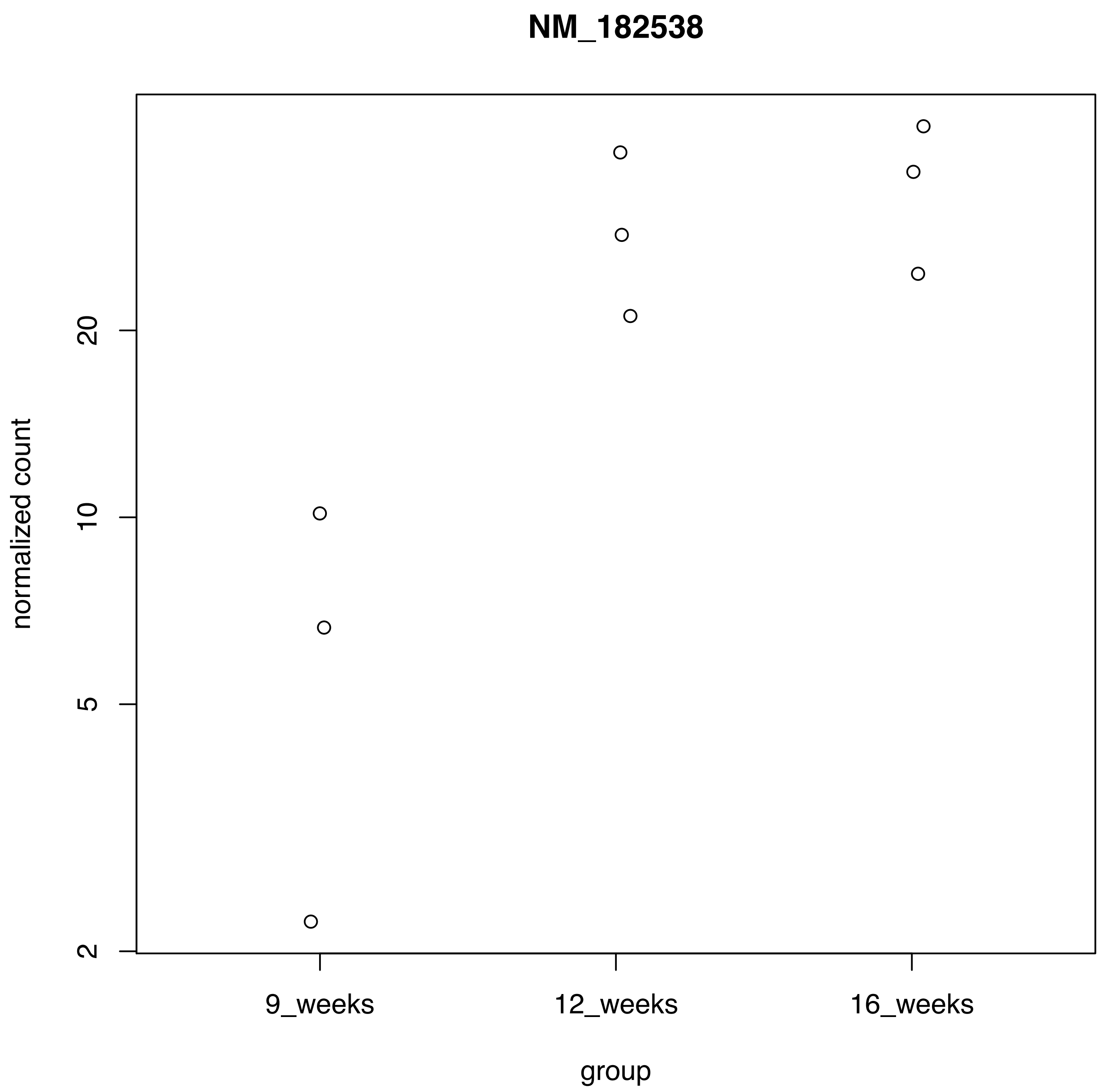
NM_182538
normalized count
2
5
10
20
9_weeks
12_weeks
16_weeks
group

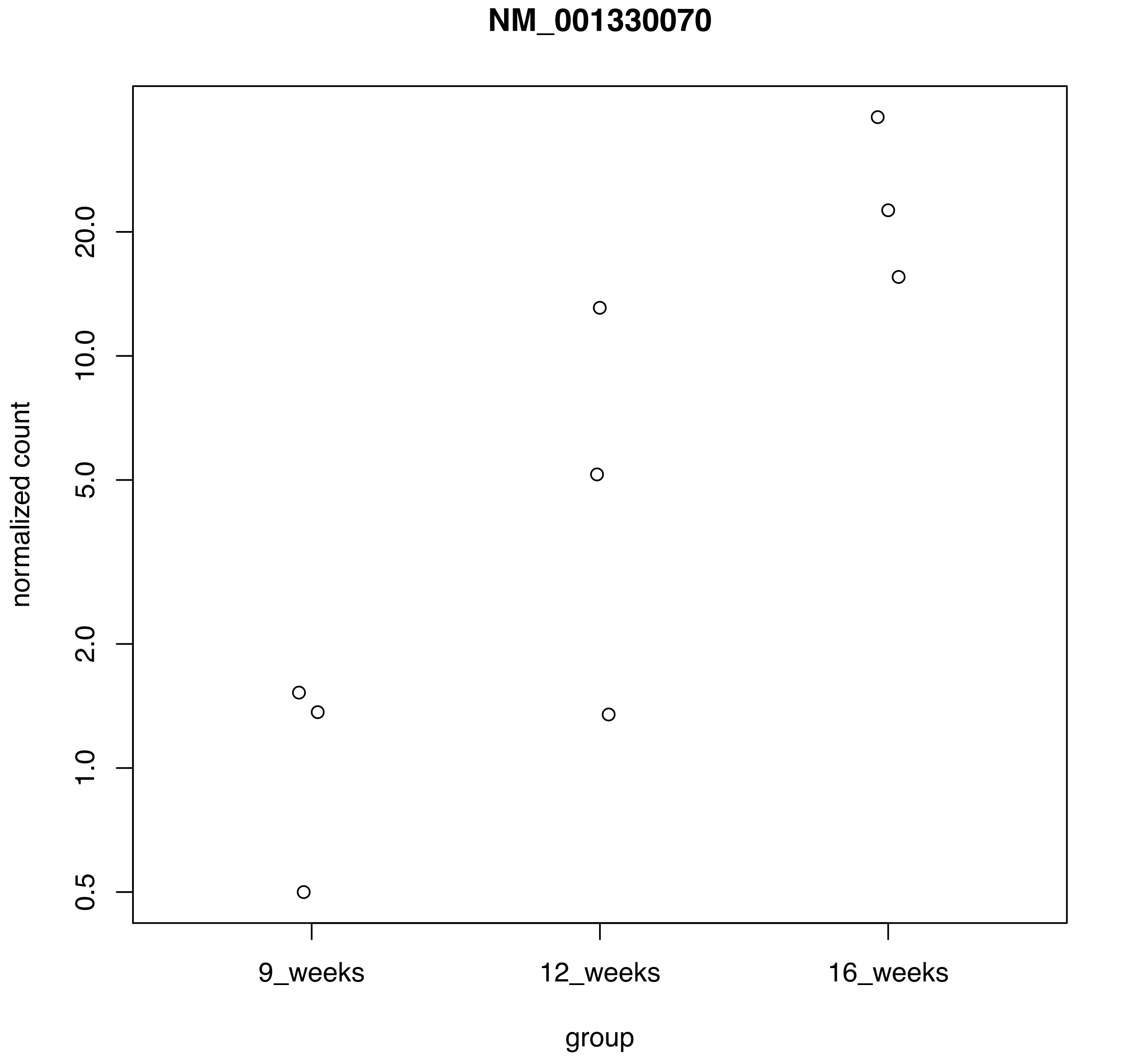
NM_001330070
normalized count
20.0
10.0
5.0
2.0
1.0
0.5
9_weeks
12_weeks
16_weeks
group

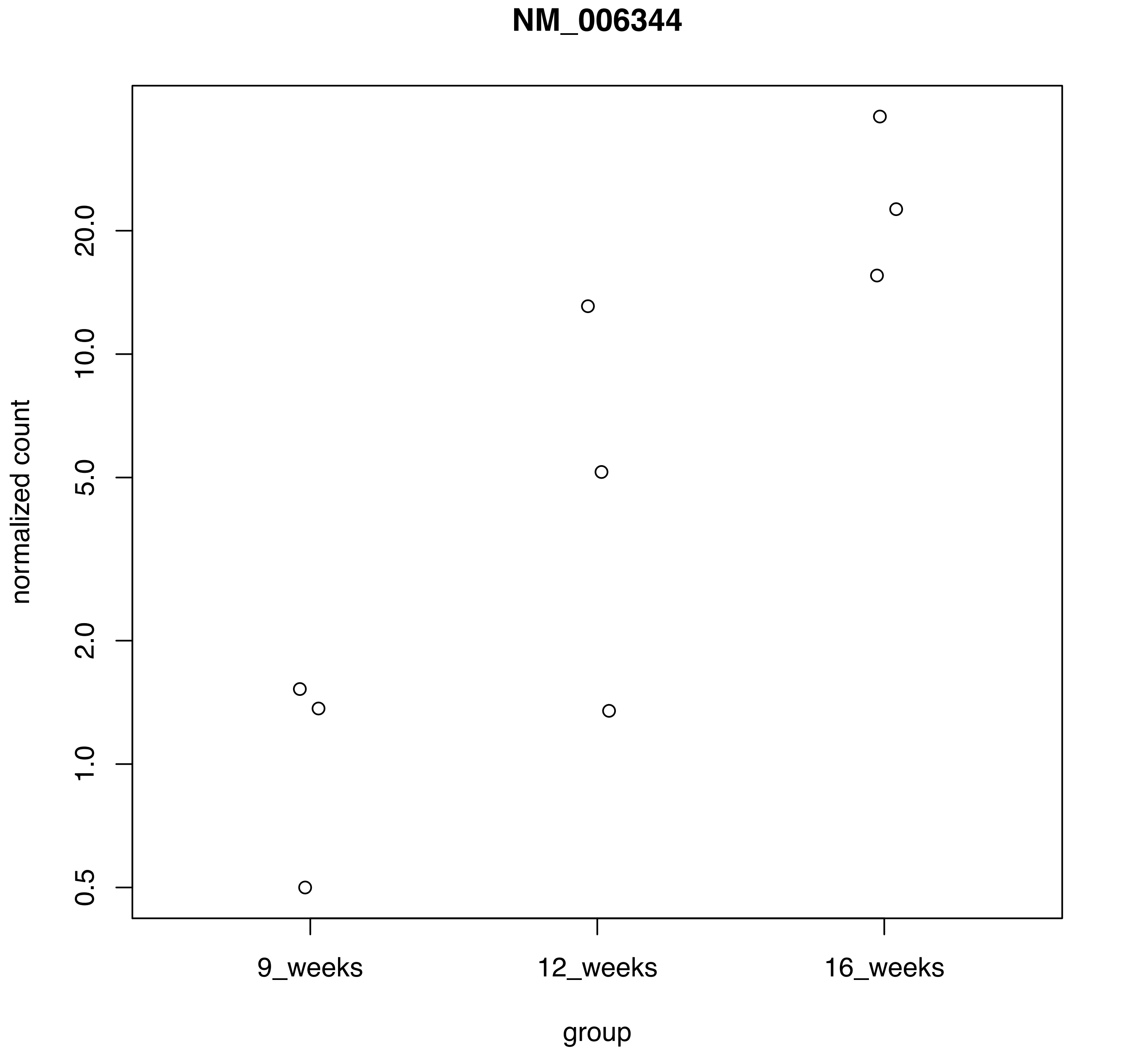
NM_006344
normalized count
20.0
10.0
5.0
2.0
1.0
0.5
9_weeks
12_weeks
16_weeks
group

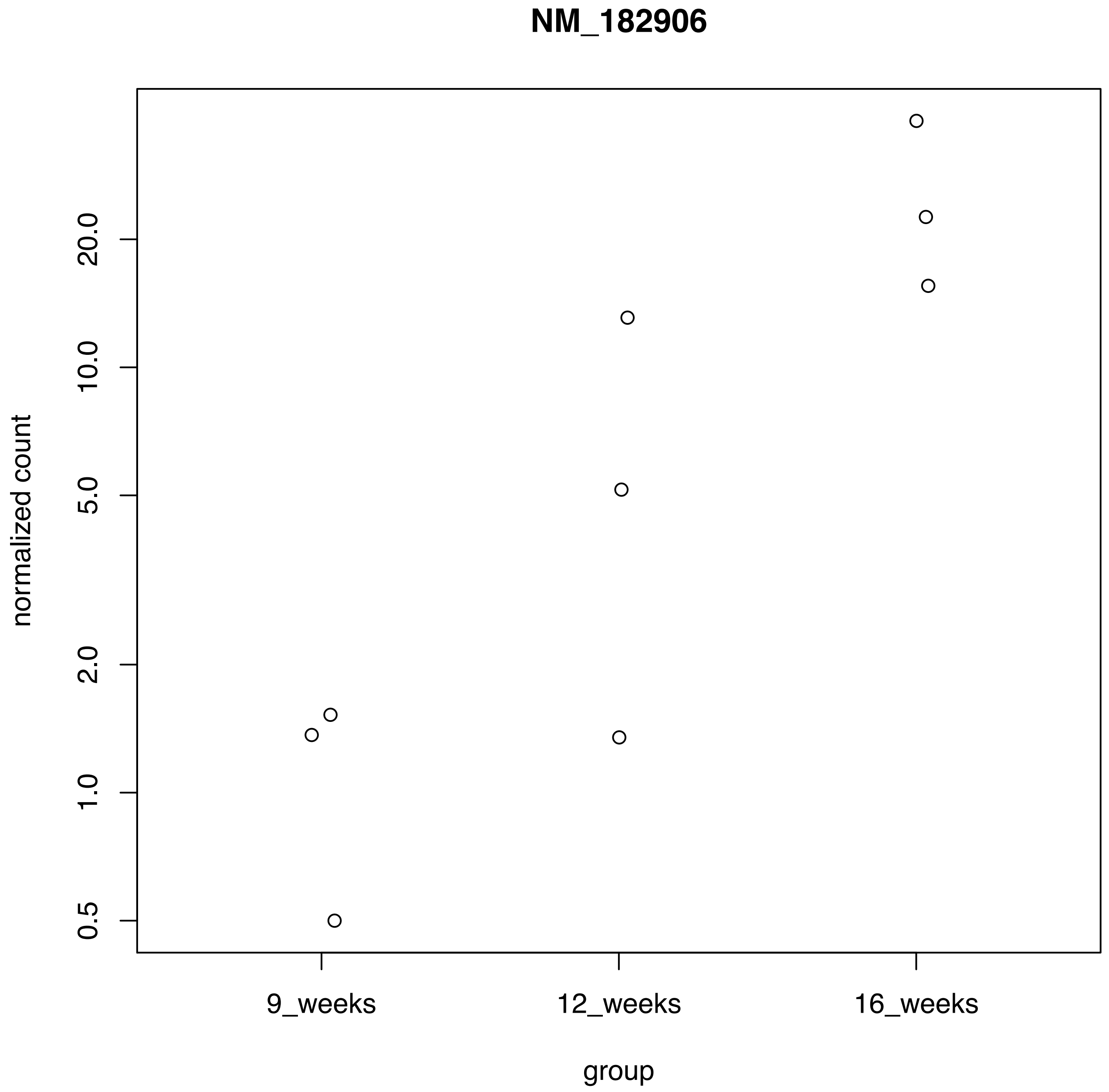
NM_182906
normalized count
0.5
1.0
2.0
5.0
10.0
20.0
9_weeks
12_weeks
16_weeks
group

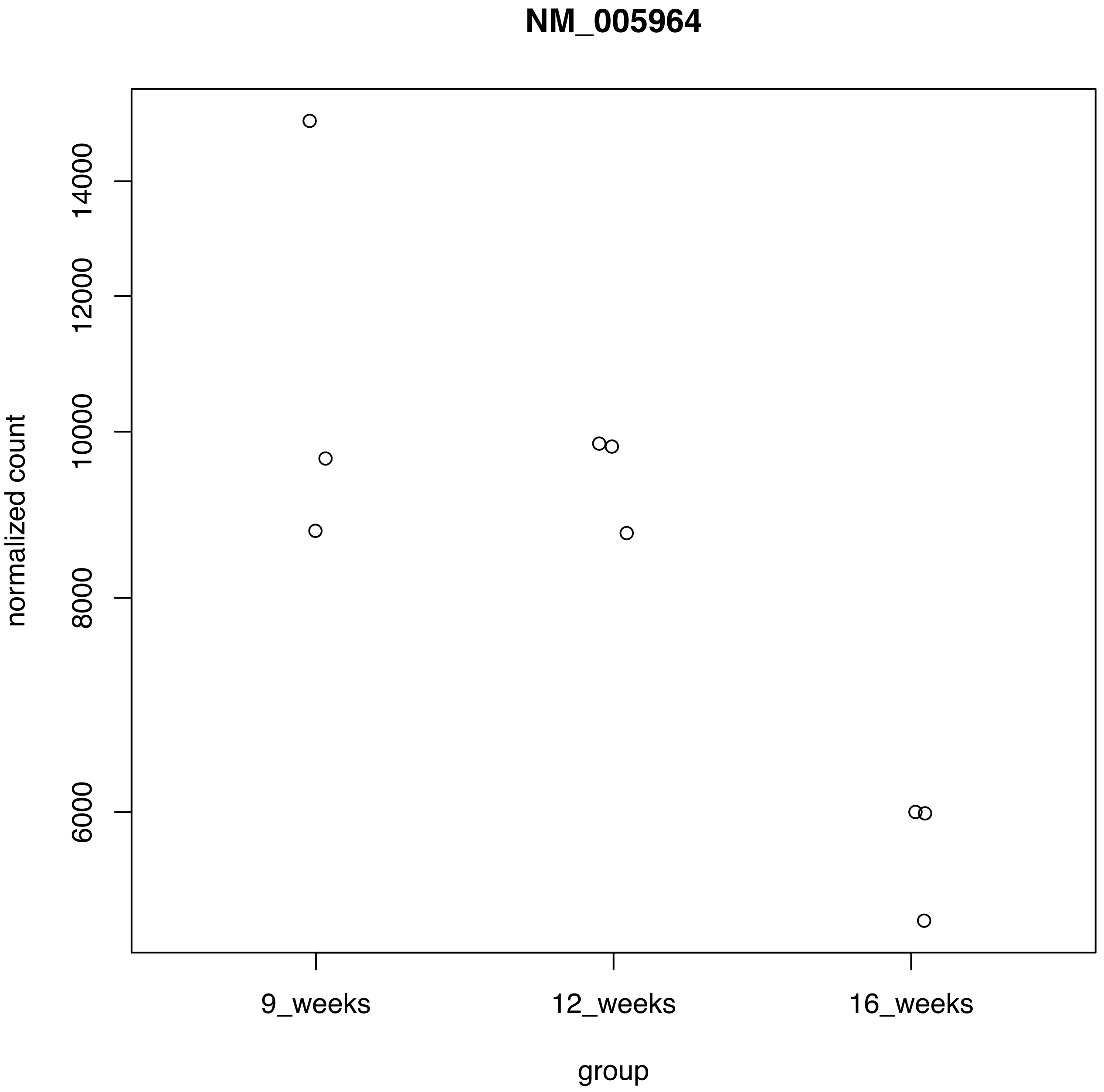
NM_005964
normalized count
14000
12000
10000
8000
6000
9_weeks
12_weeks
16_weeks
group

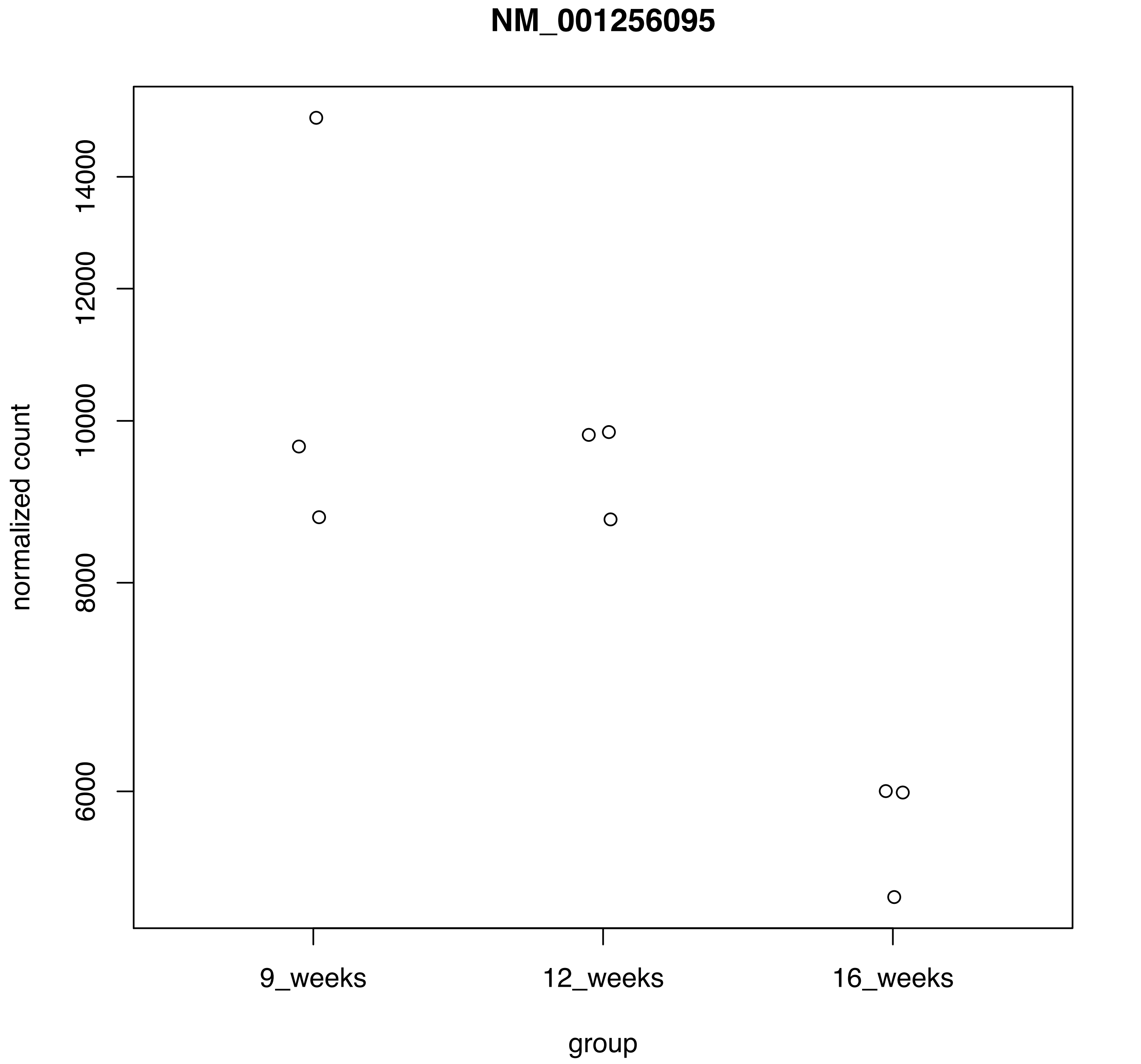
NM_001256095
normalized count
14000
12000
10000
8000
6000
9_weeks
12_weeks
16_weeks
group

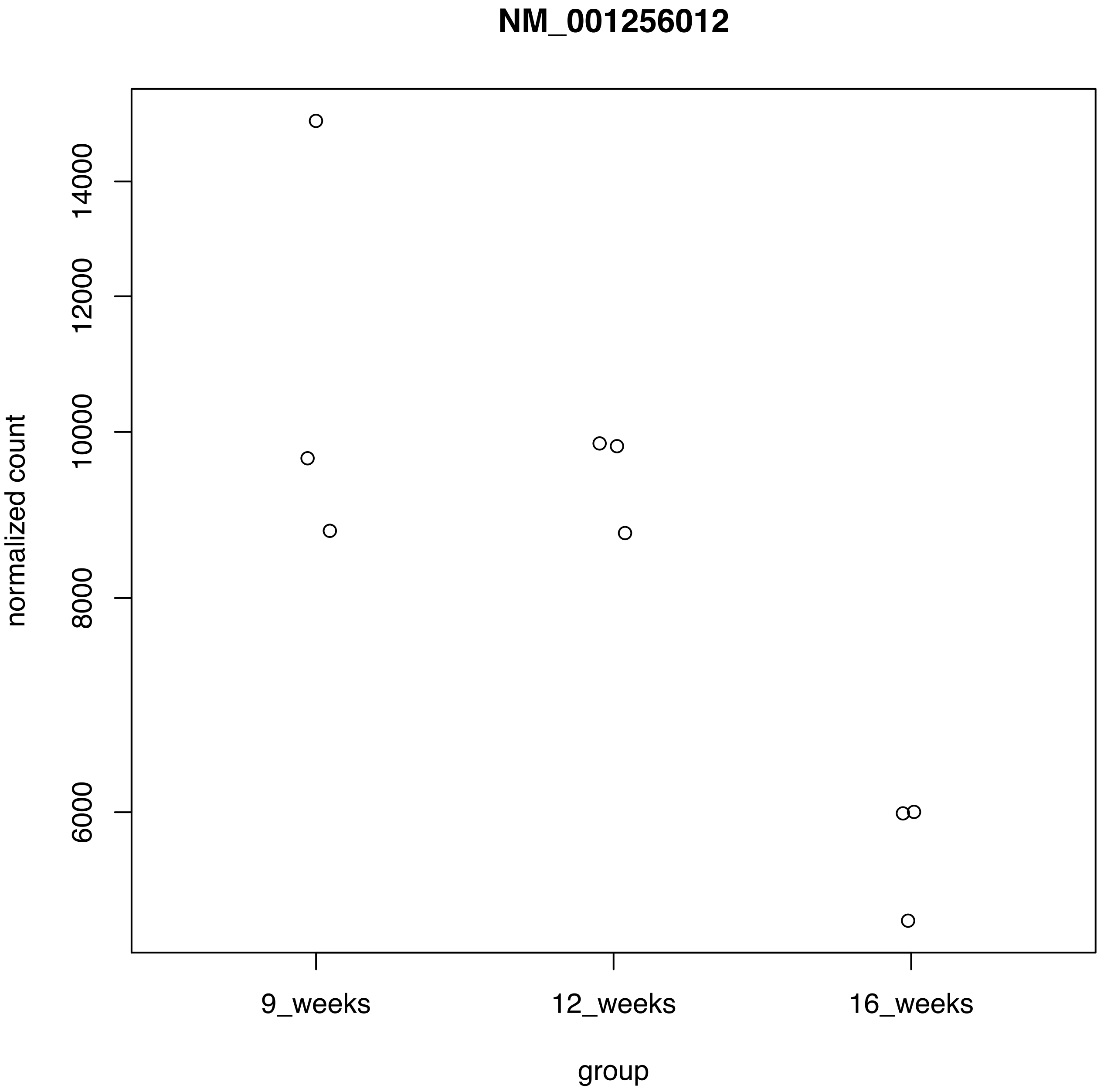
NM_001256012
normalized count
6000
8000
10000
12000
14000
9_weeks
12_weeks
16_weeks
group

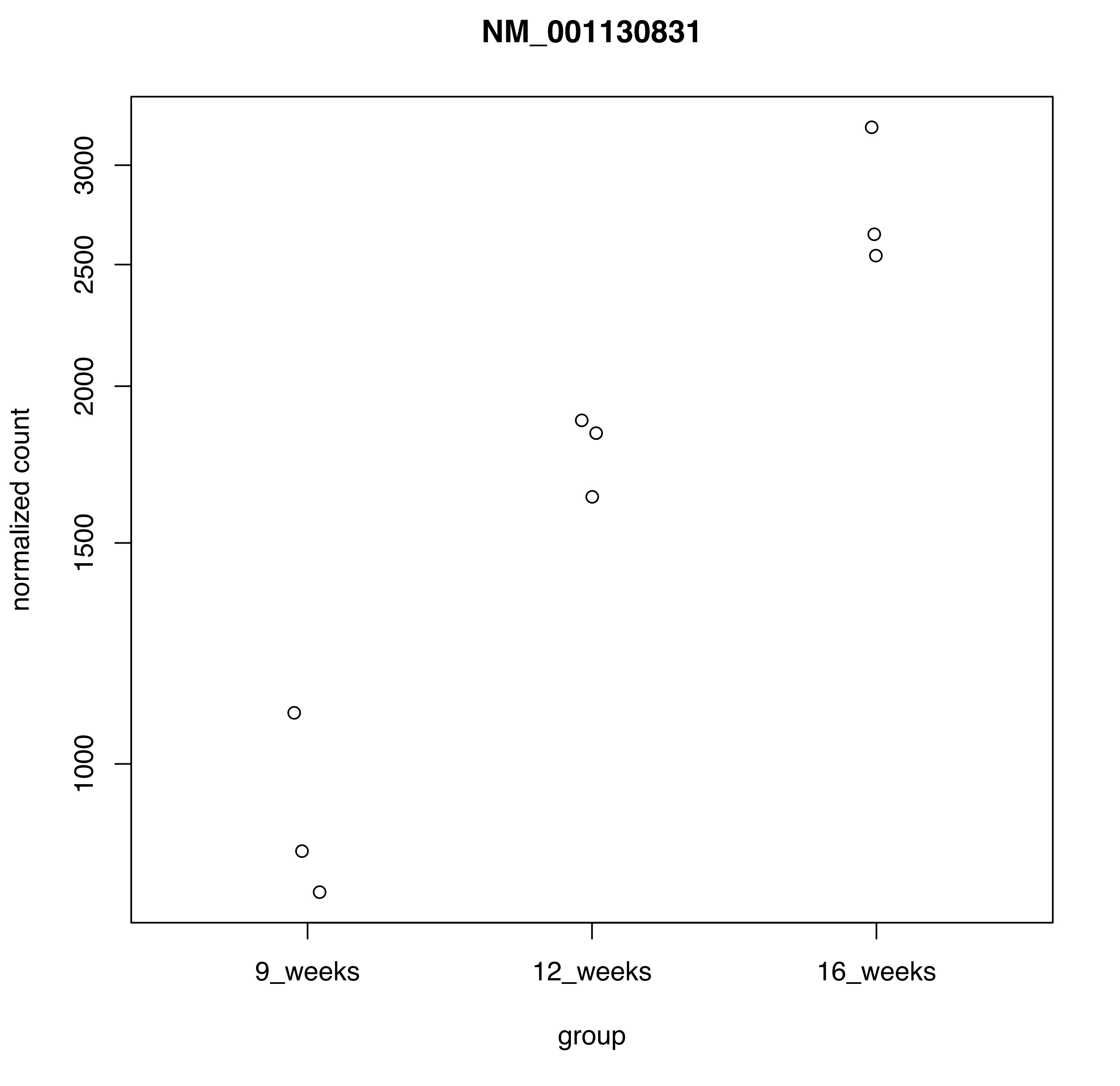
NM_001130831
normalized count
1000
1500
2000
2500
3000
9_weeks
12_weeks
16_weeks
group

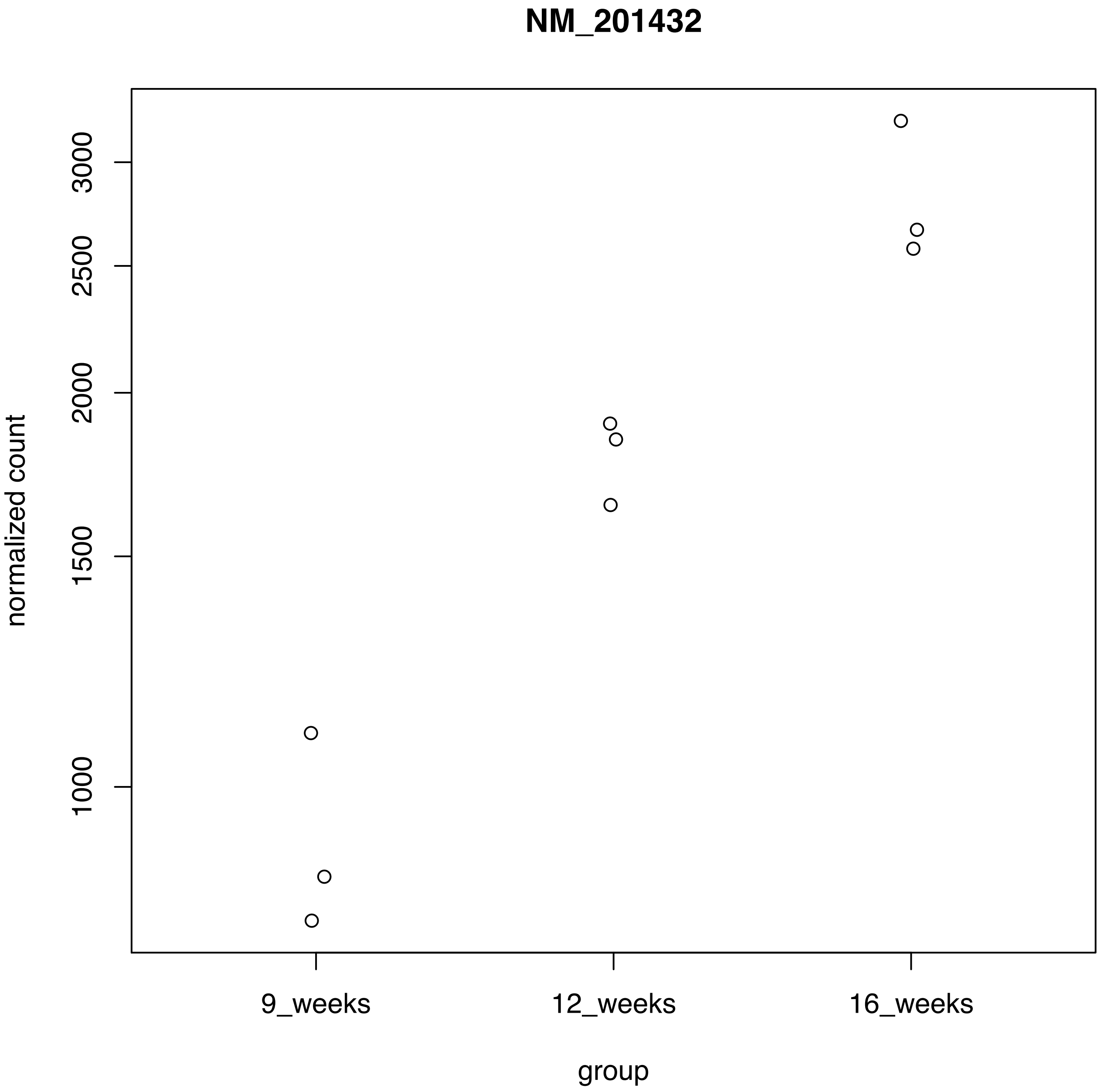
NM_201432
normalized count
1000
1500
2000
2500
3000
9_weeks
12_weeks
16_weeks
group

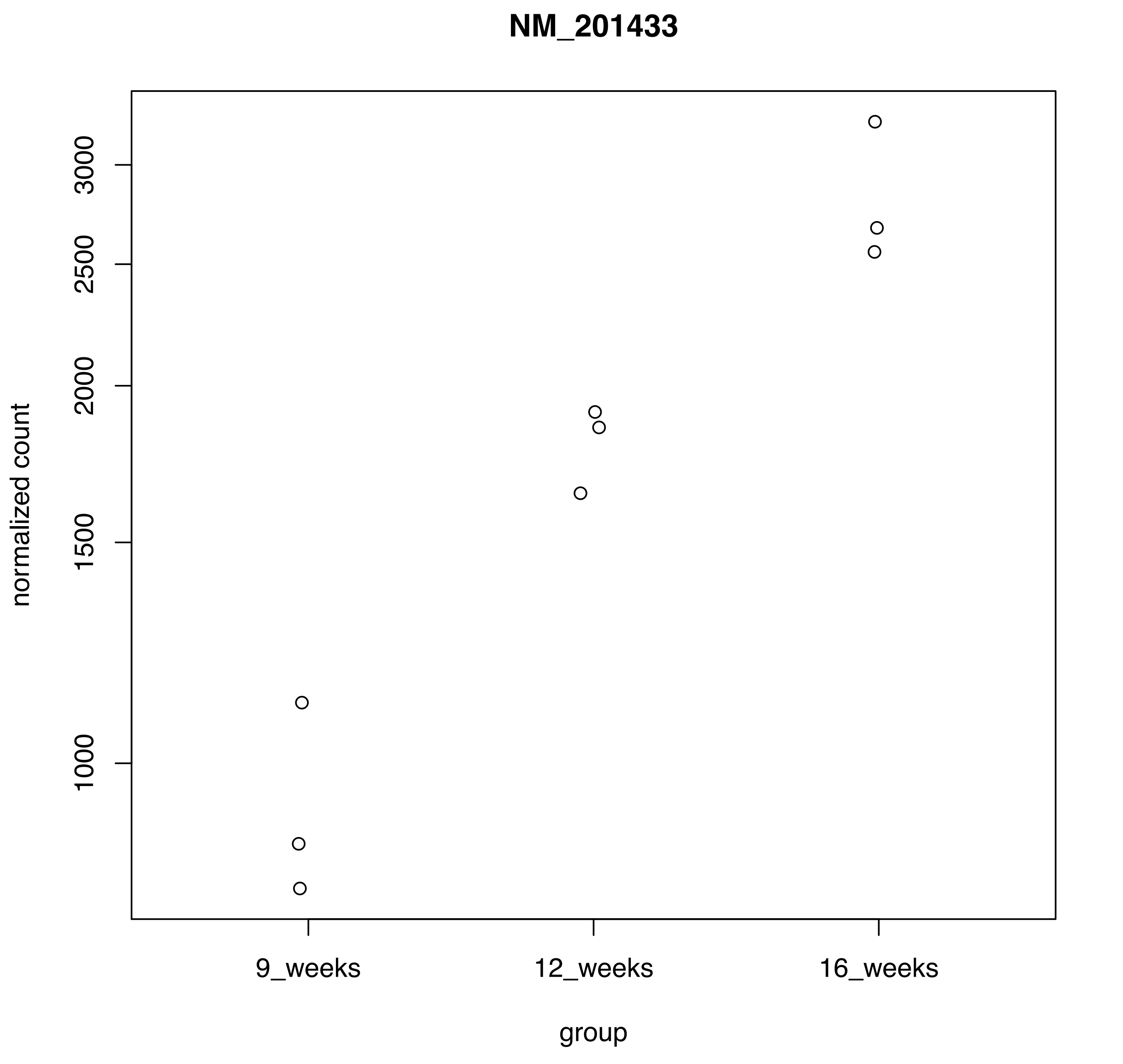
NM_201433
normalized count
1000
1500
2000
2500
3000
9_weeks
12_weeks
16_weeks
group

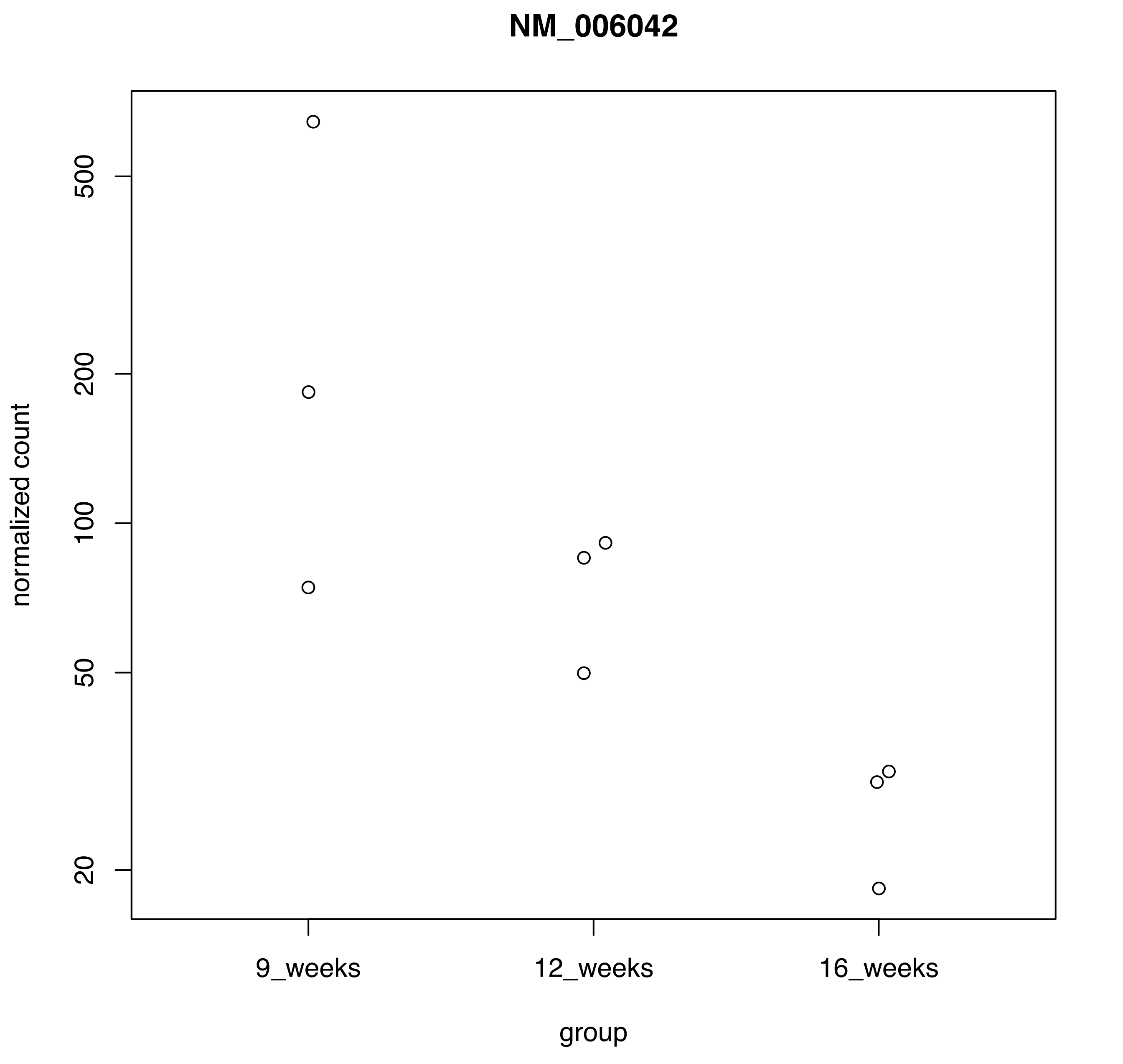
NM_006042
normalized count
500
200
100
50
20
9_weeks
12_weeks
16_weeks
group

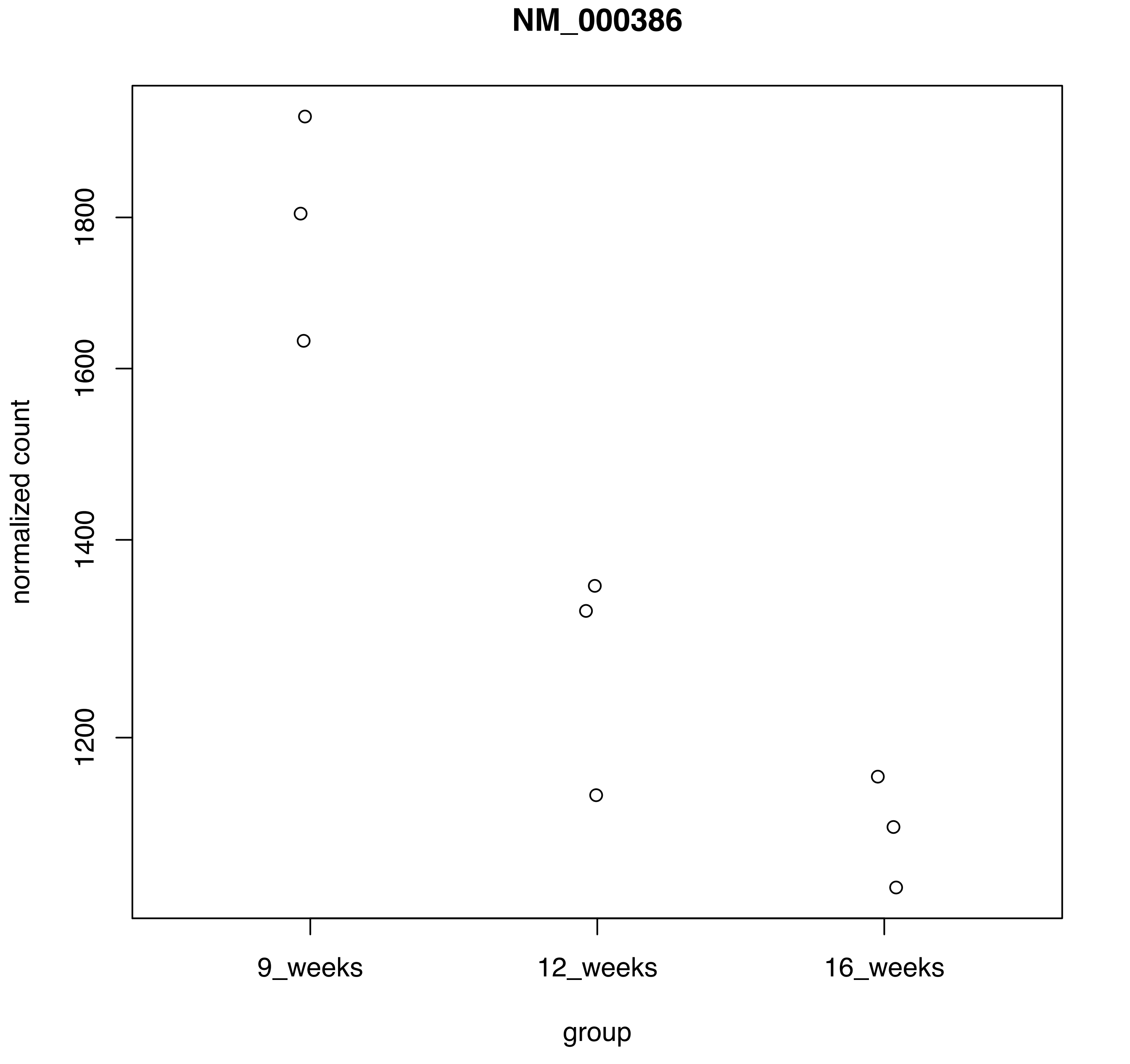
NM_000386
normalized count
1200
1400
1600
1800
9_weeks
12_weeks
16_weeks
group

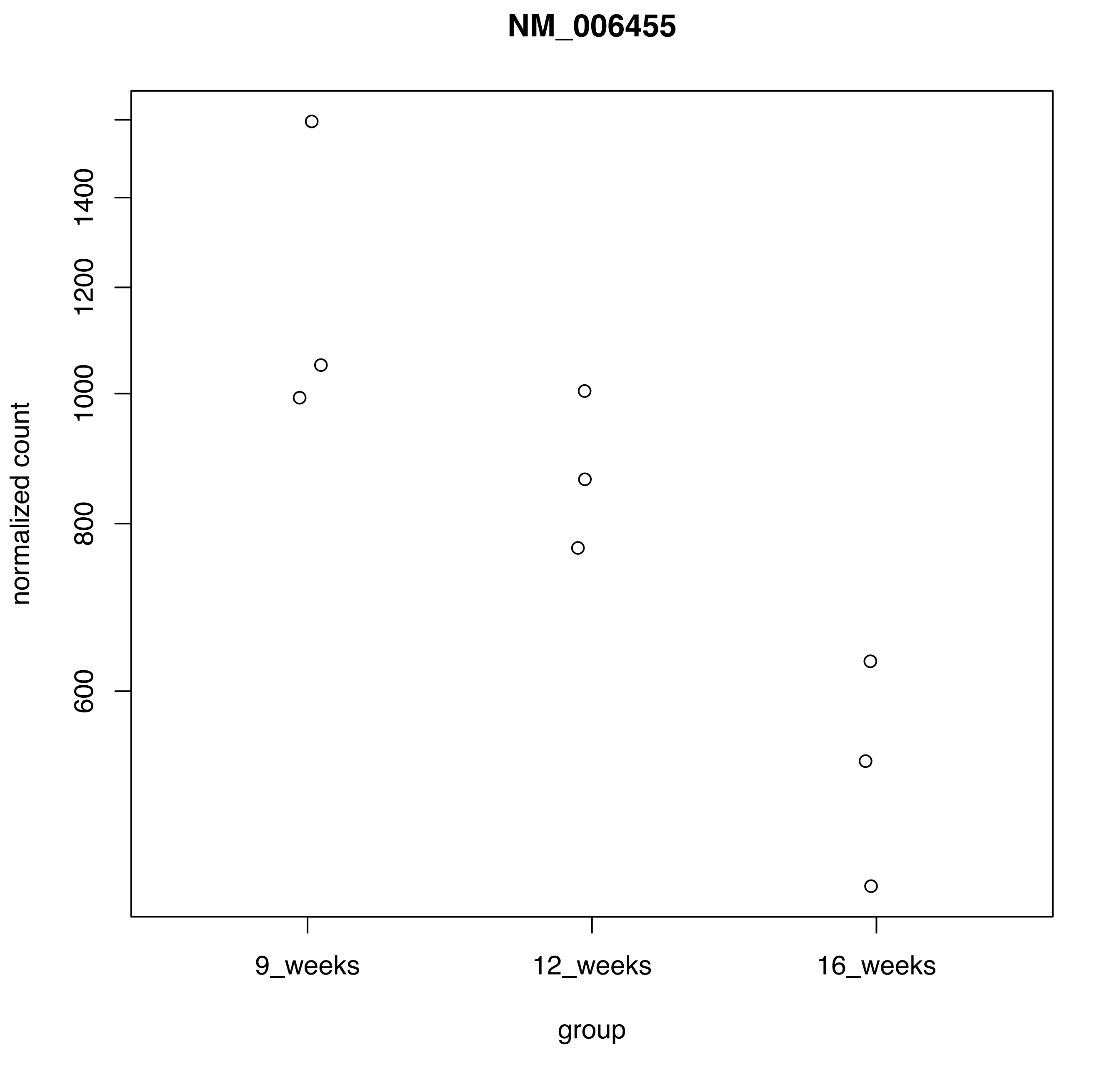
NM_006455
normalized count
1400
1200
1000
800
600
9_weeks
12_weeks
16_weeks
group

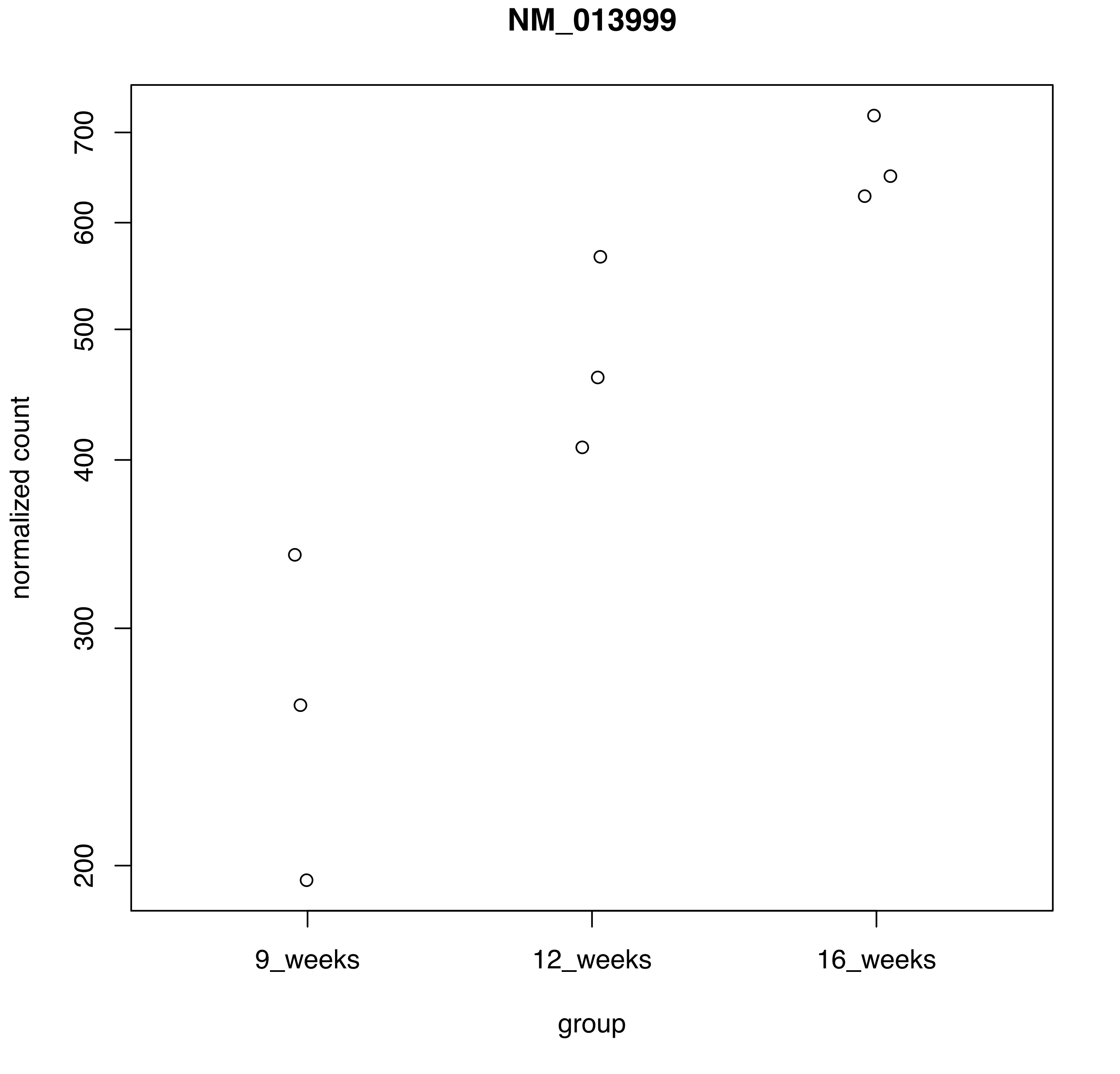
NM_013999
normalized count
700
600
500
400
300
200
9_weeks
12_weeks
16_weeks
group

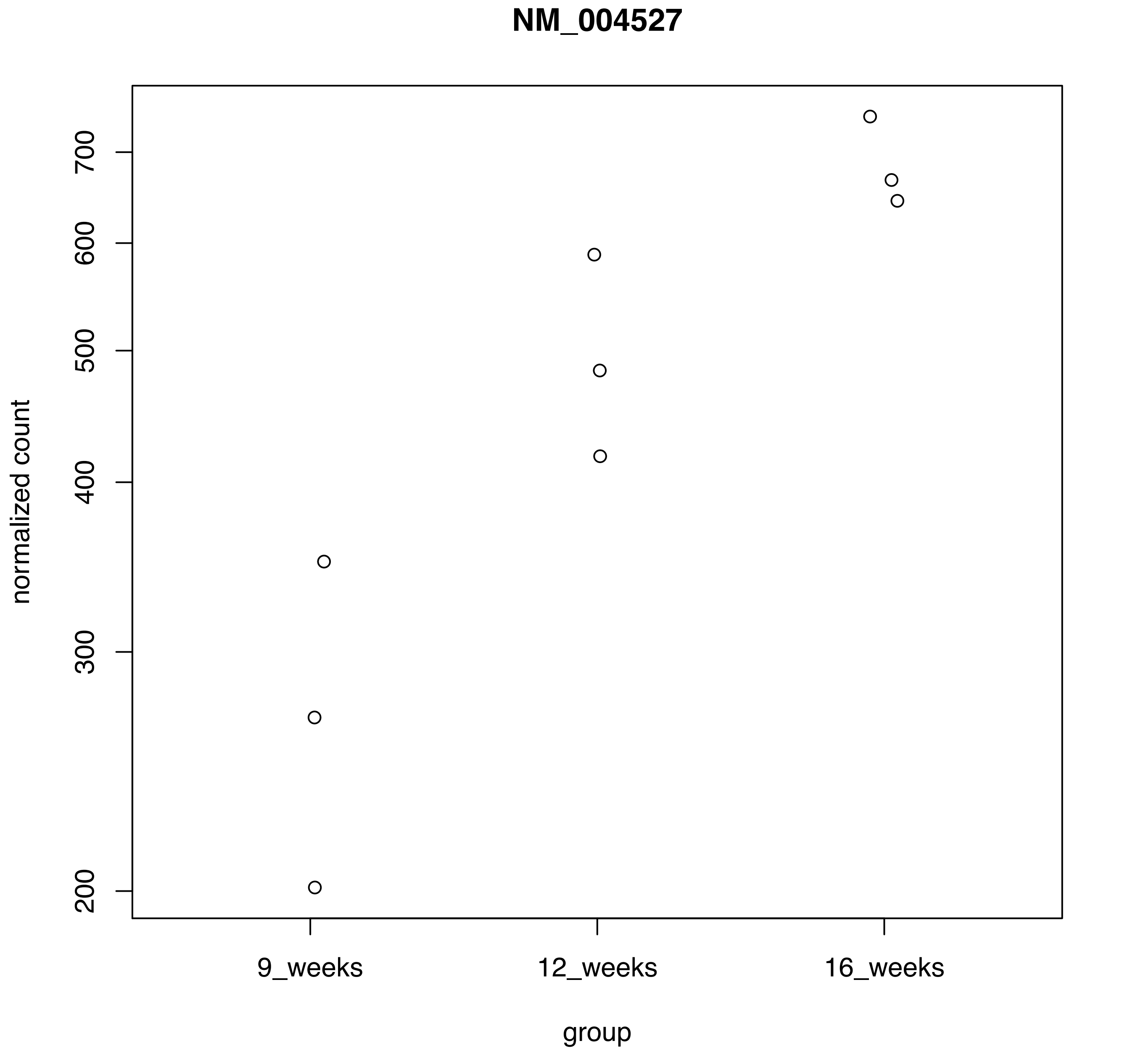
NM_004527
normalized count
200
300
400
500
600
700
9_weeks
12_weeks
16_weeks
group

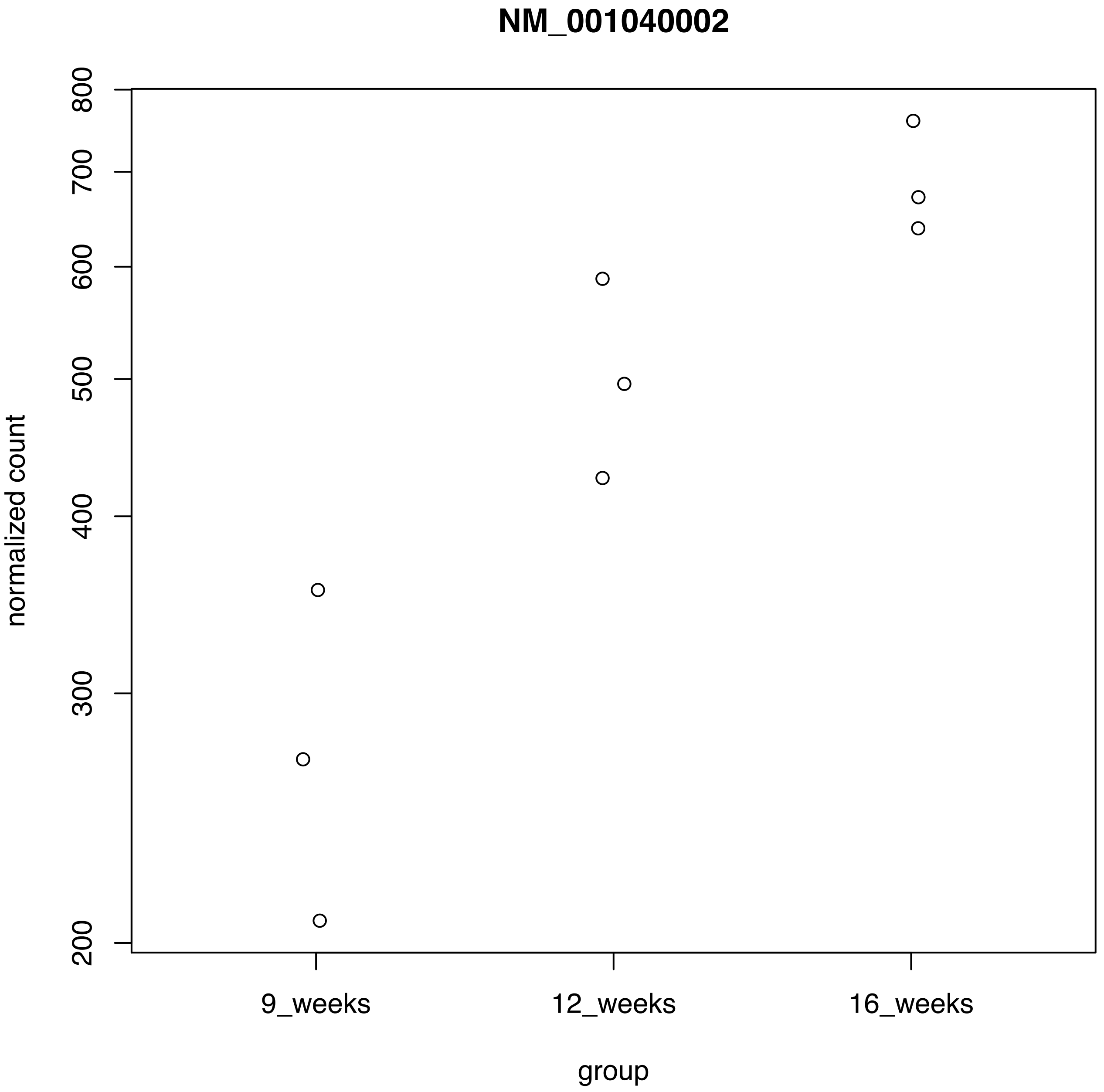
NM_001040002
normalized count
200
300
400
500
600
700
800
9_weeks
12_weeks
16_weeks
group

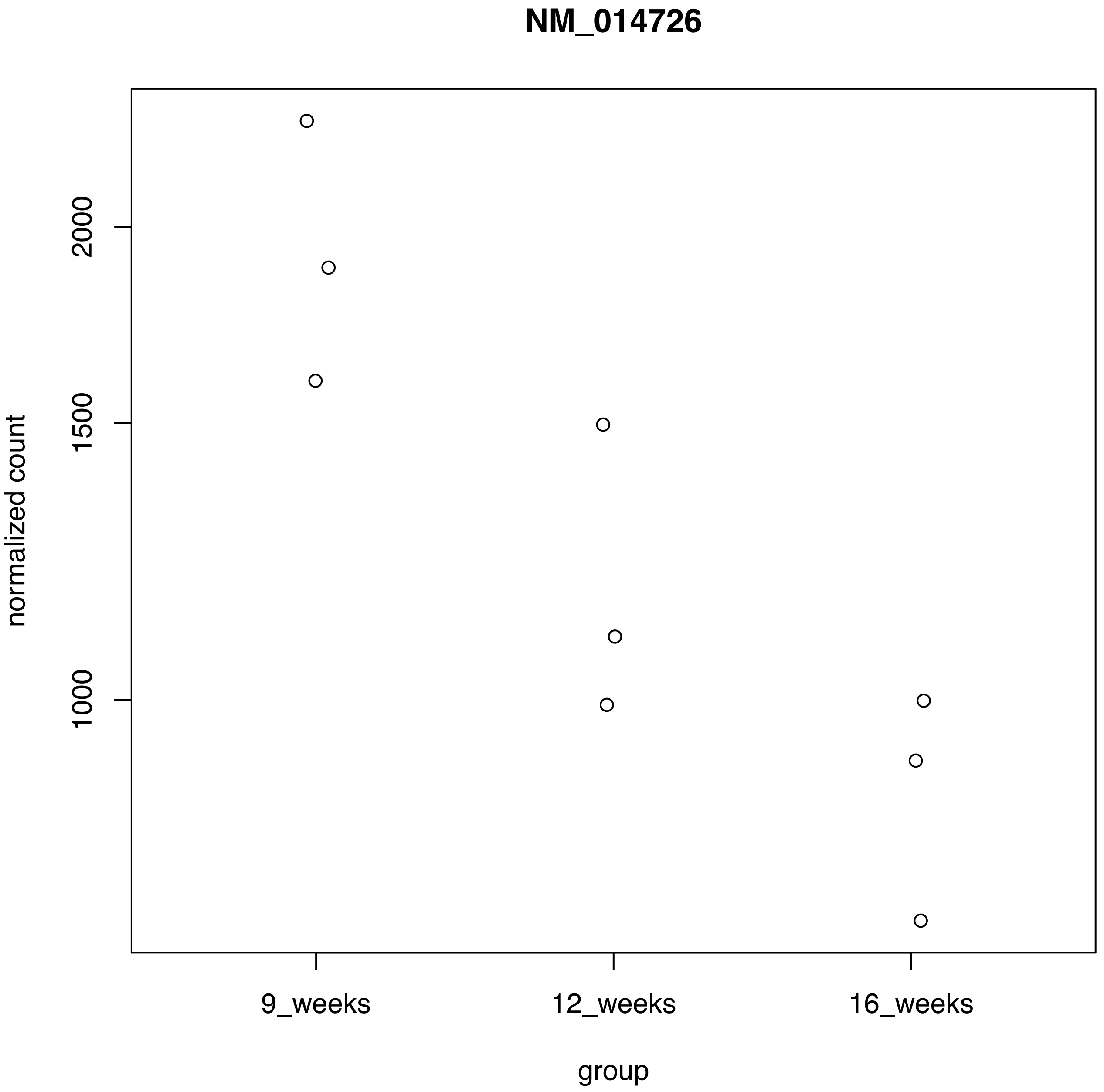
NM_014726
normalized count
2000
1500
1000
9_weeks
12_weeks
16_weeks
group

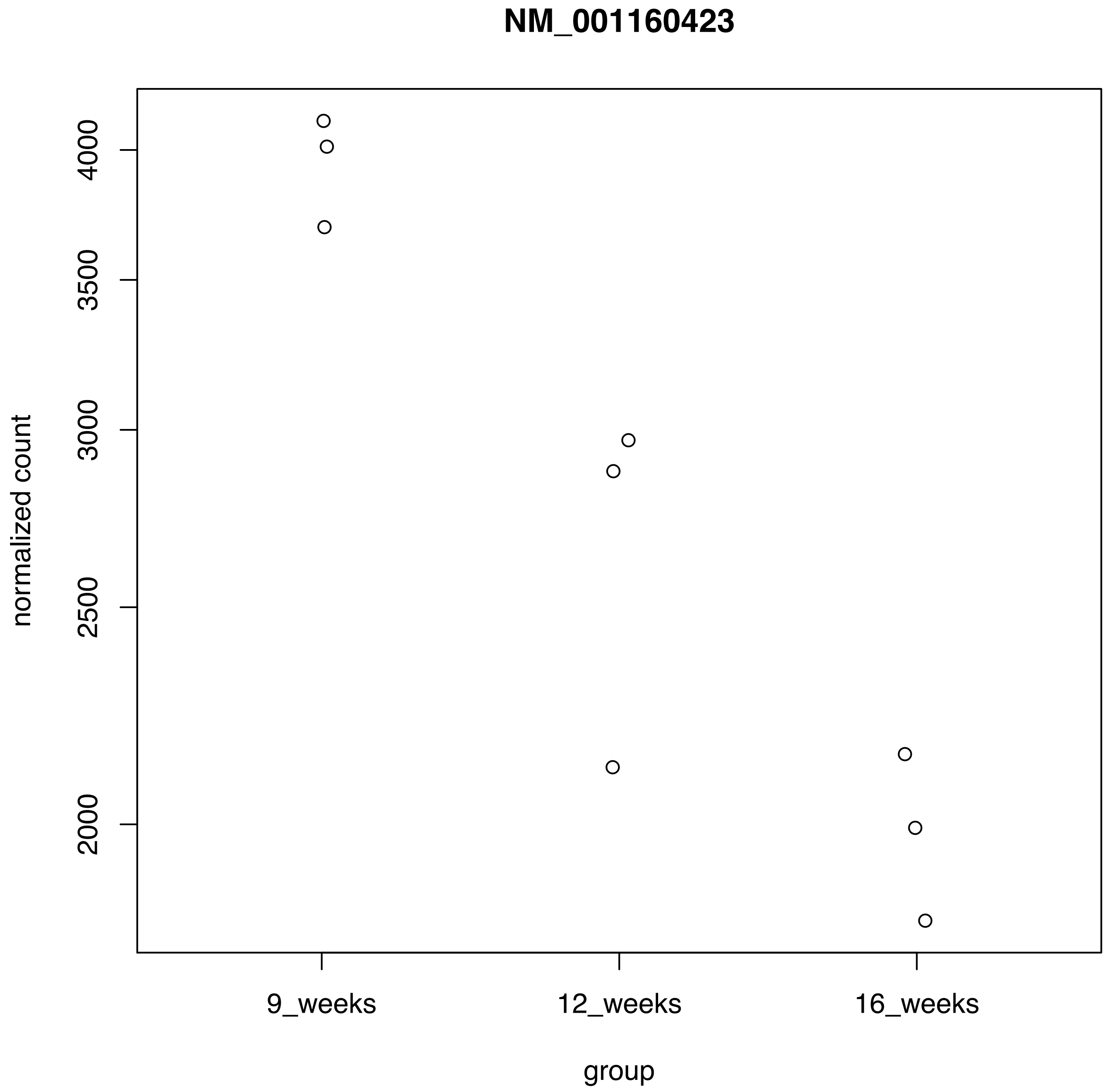

NM_001160423
normalized count
4000
3500
3000
2500
2000
9_weeks
12_weeks
16_weeks
group

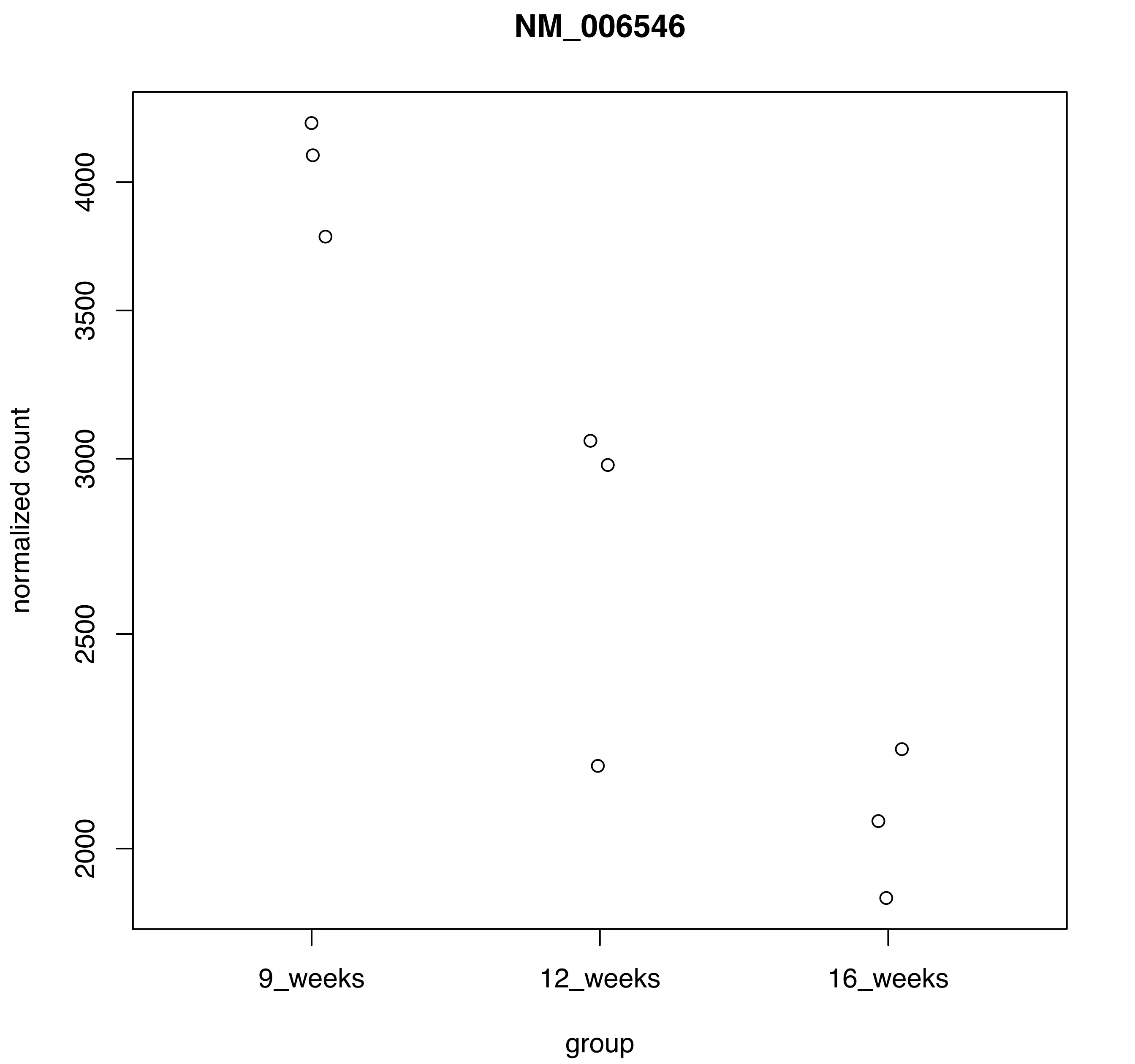
NM_006546
normalized count
4000
3500
3000
2500
2000
9_weeks
12_weeks
16_weeks
group

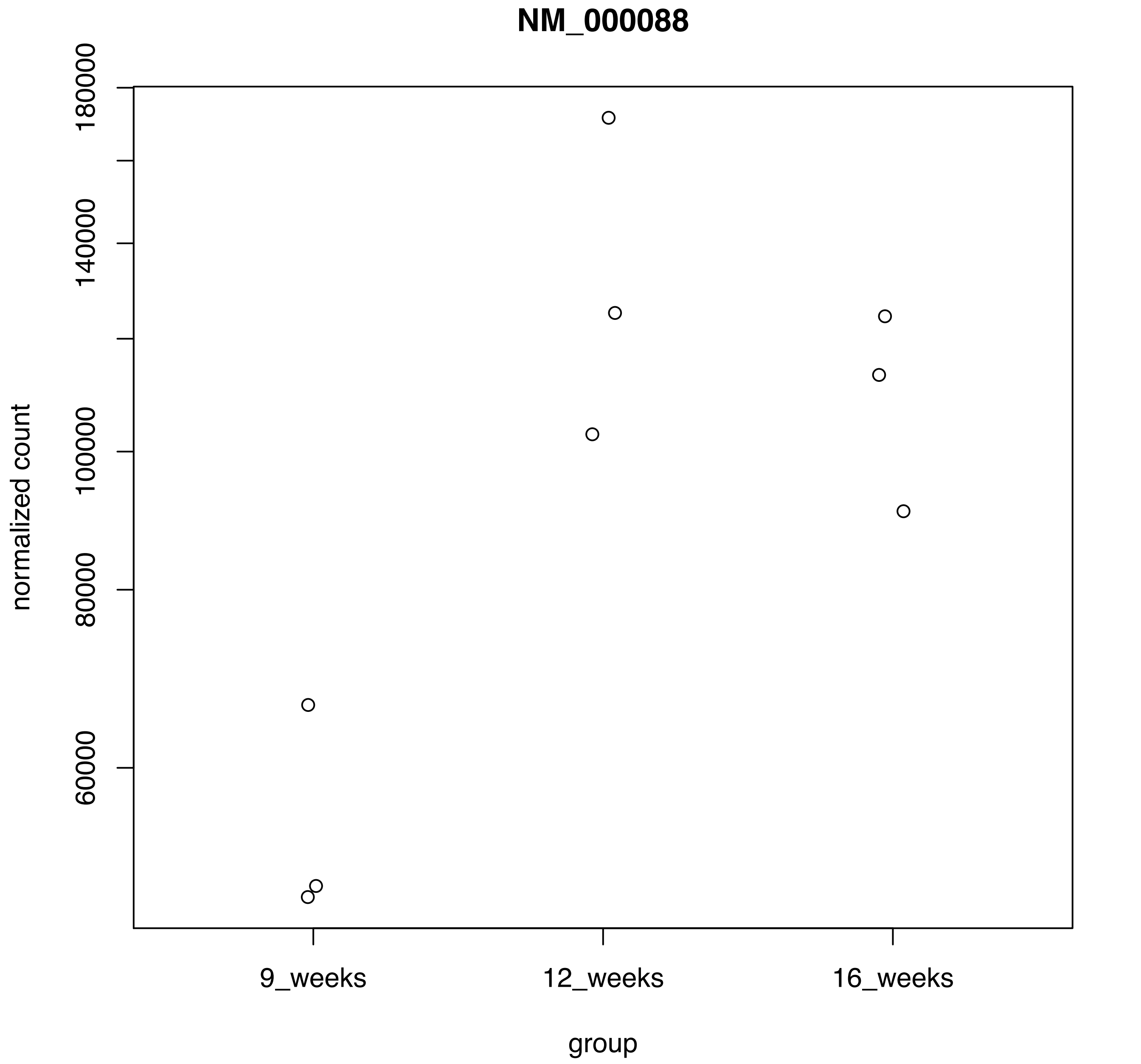
NM_000088
normalized count
180000
140000
100000
80000
60000
9_weeks
12_weeks
16_weeks
group

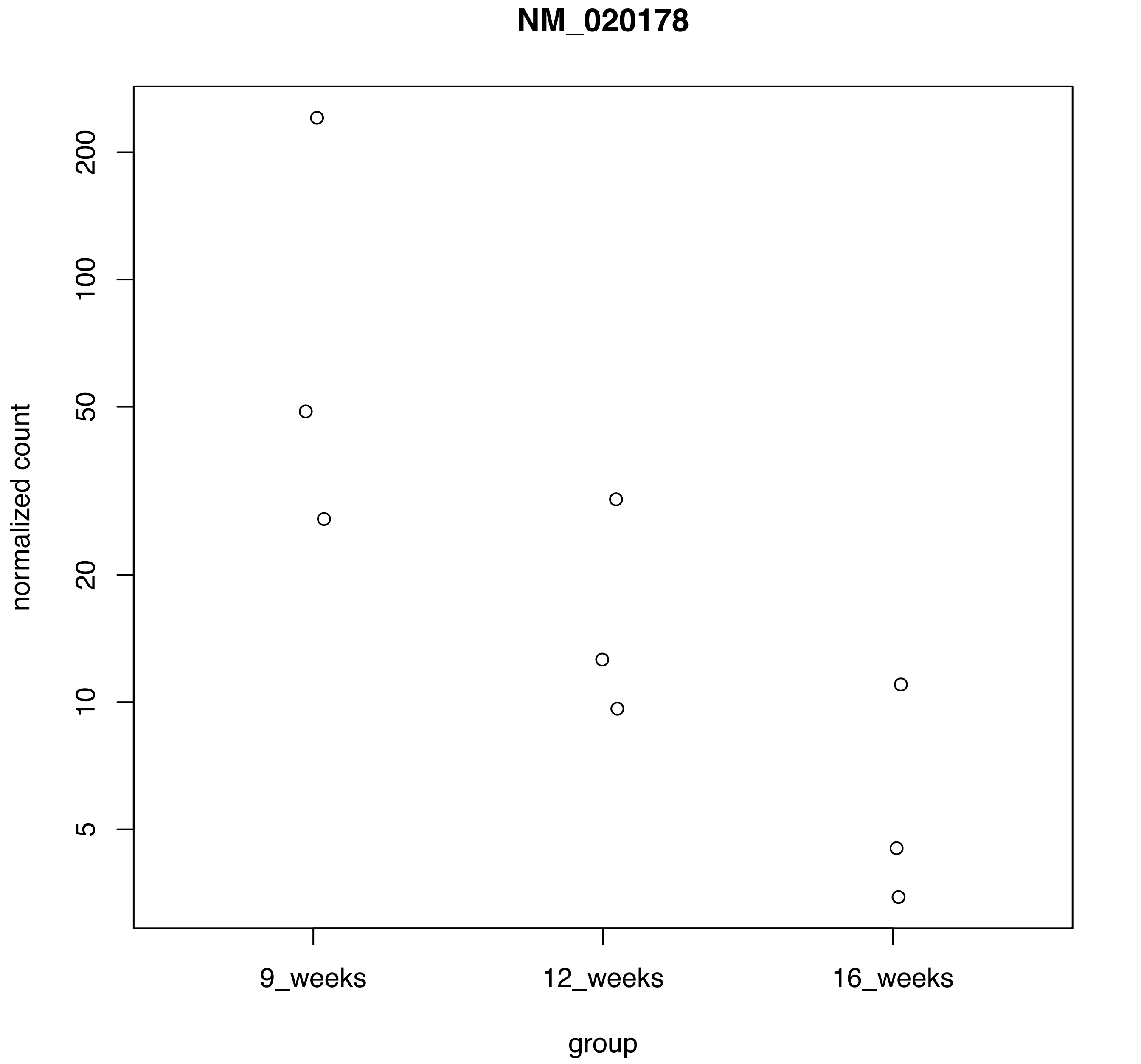

NM_020178
normalized count
200
100
50
20
10
5
9_weeks
12_weeks
16_weeks
group

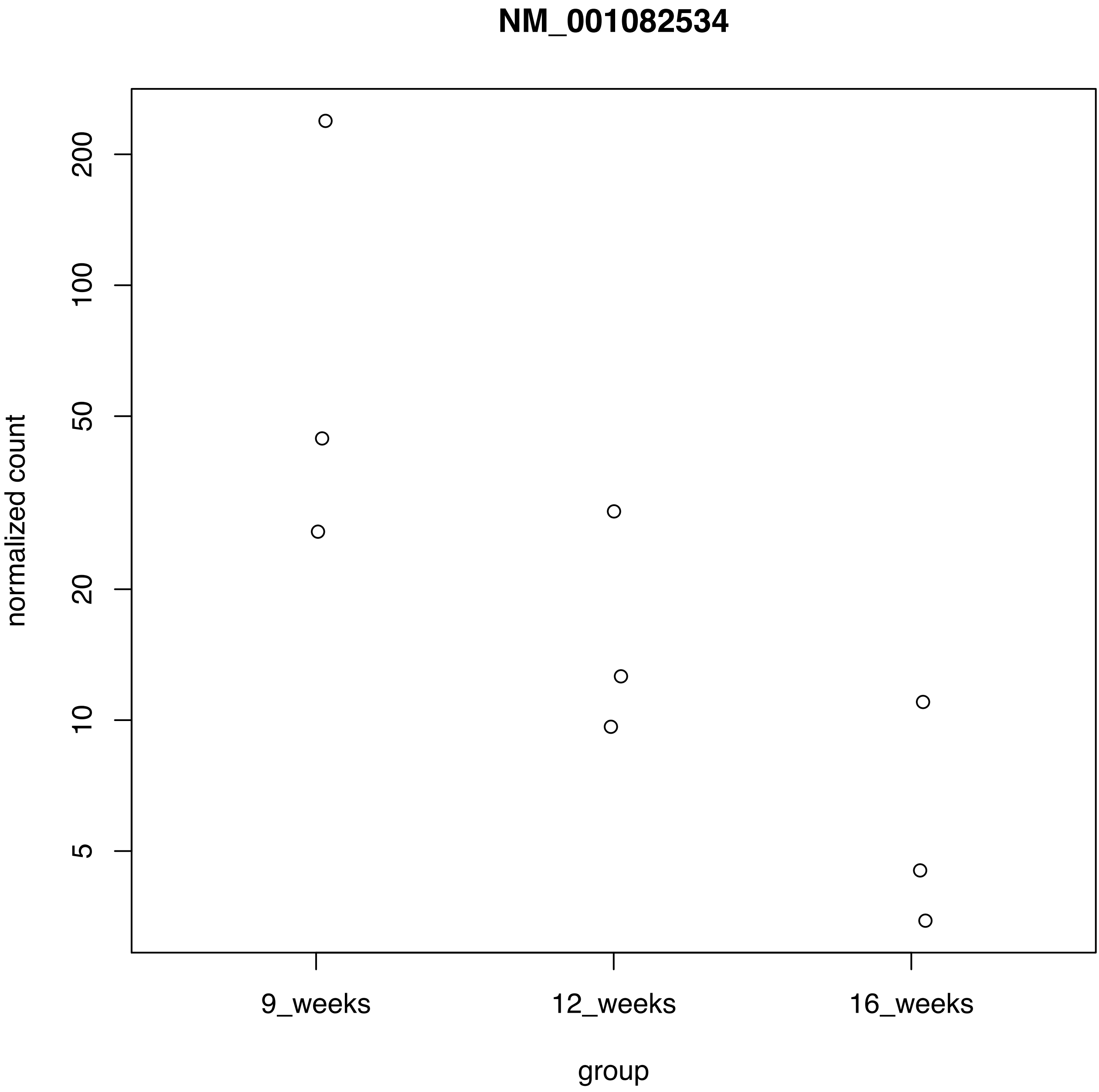

NM_001082534
normalized count
200
100
50
20
10
5
9_weeks
12_weeks
16_weeks
group

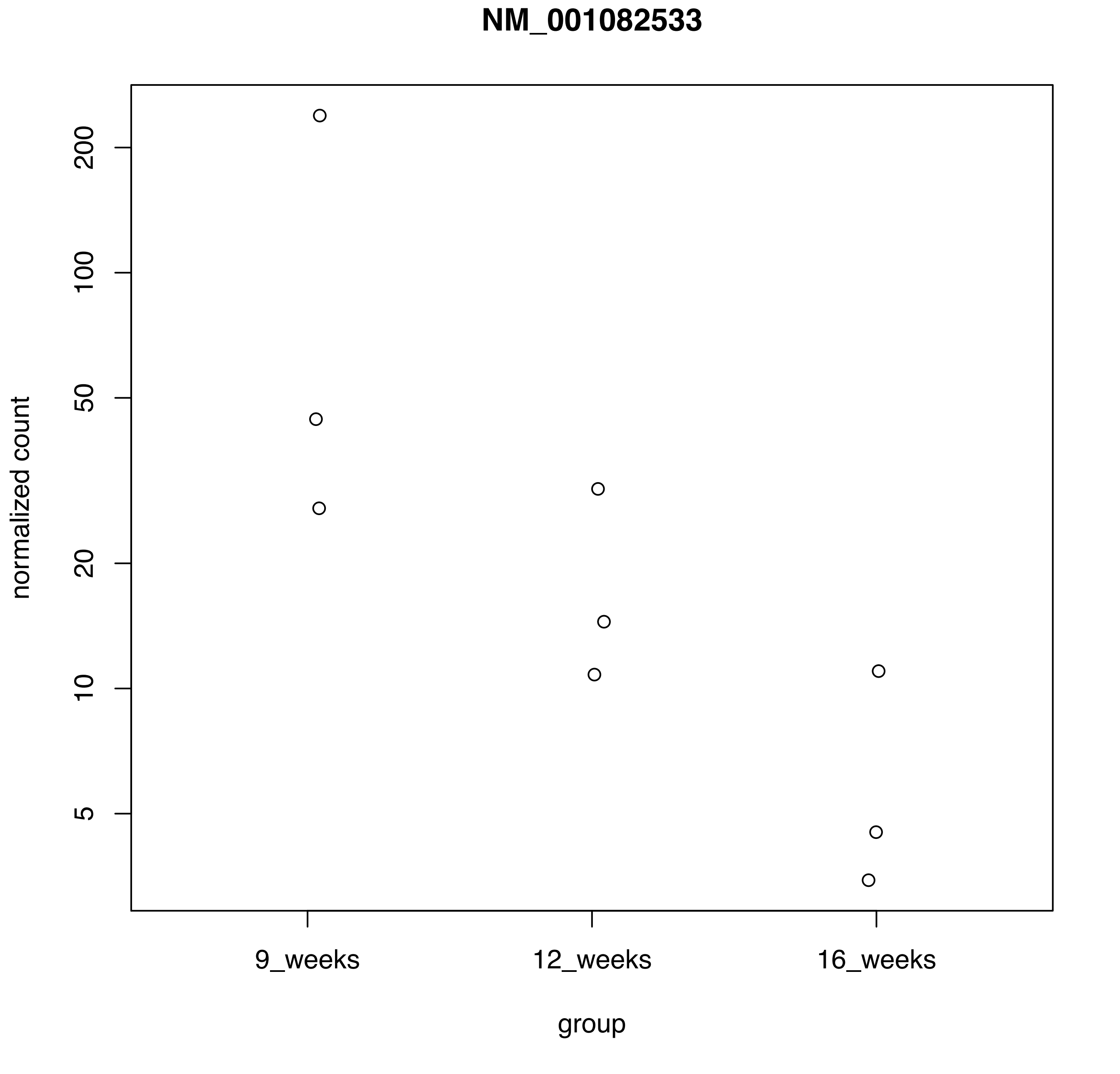
NM_001082533
normalized count
200
100
50
20
10
5
9_weeks
12_weeks
16_weeks
group

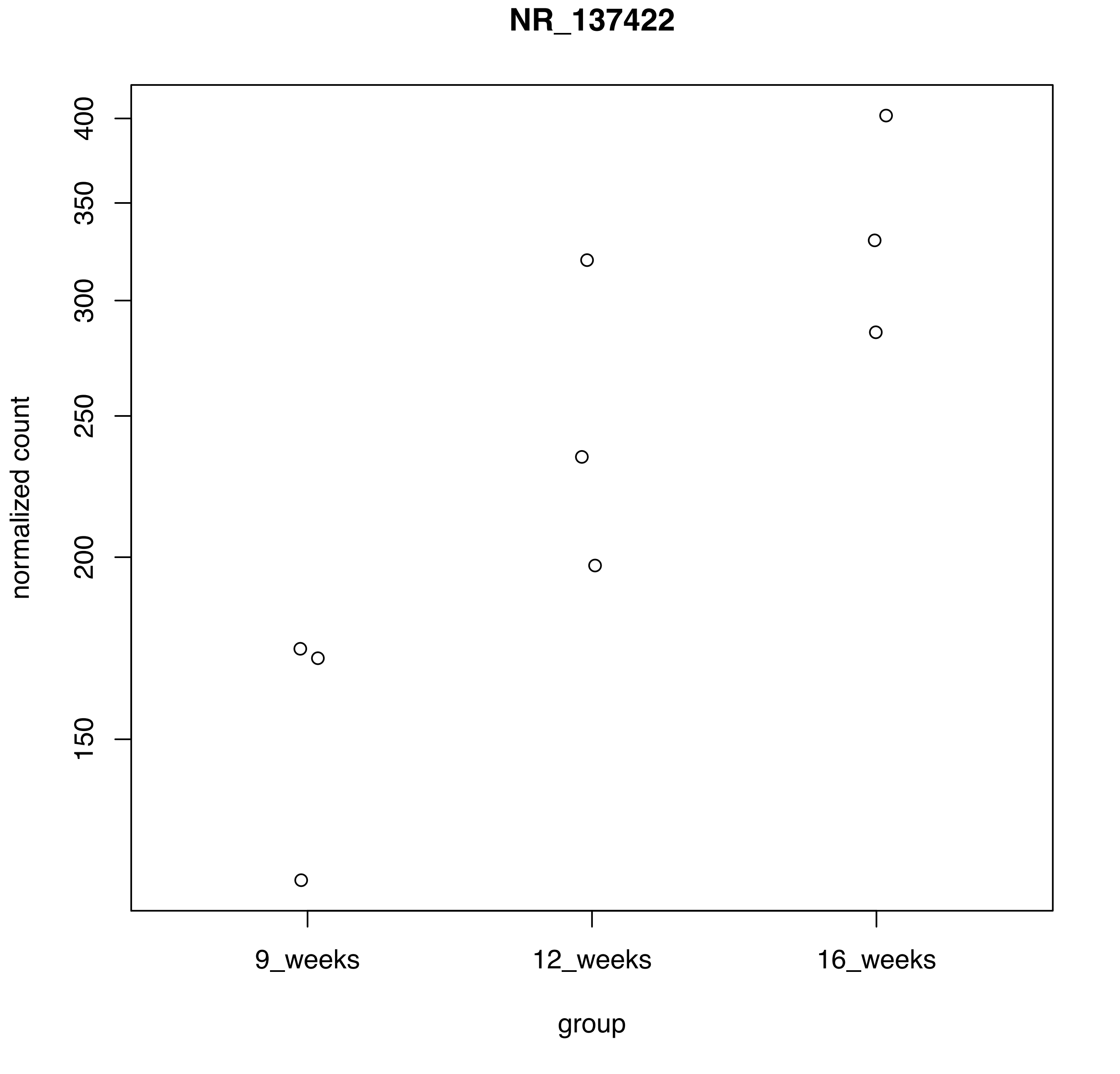
NR_137422
normalized count
400
350
300
250
200
150
9_weeks
12_weeks
16_weeks
group

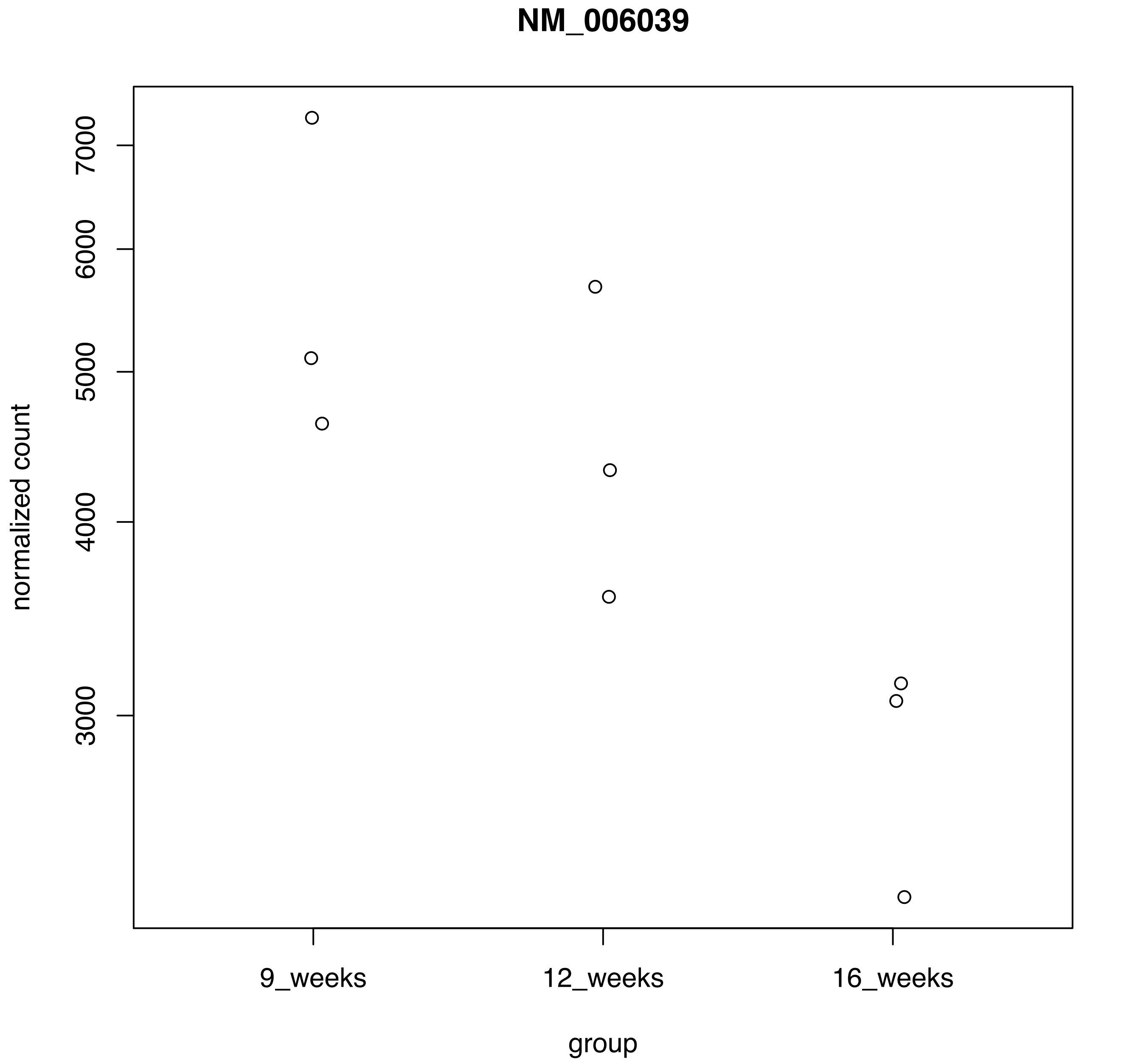
NM_006039
normalized count
7000
6000
5000
4000
3000
9_weeks
12_weeks
16_weeks
group

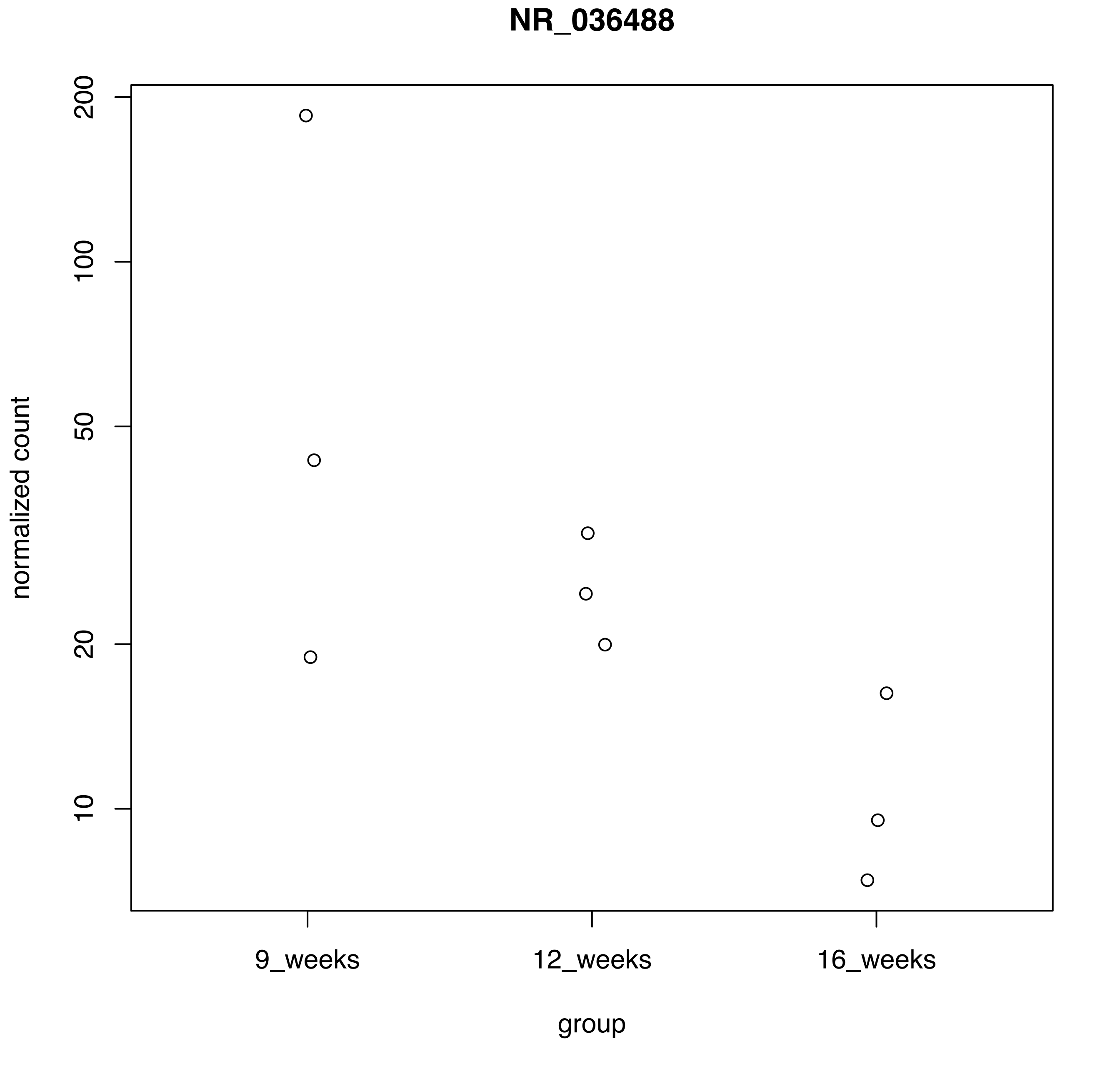
NR_036488
200
100
50
20
10
normalized count
9_weeks
12_weeks
16_weeks
group

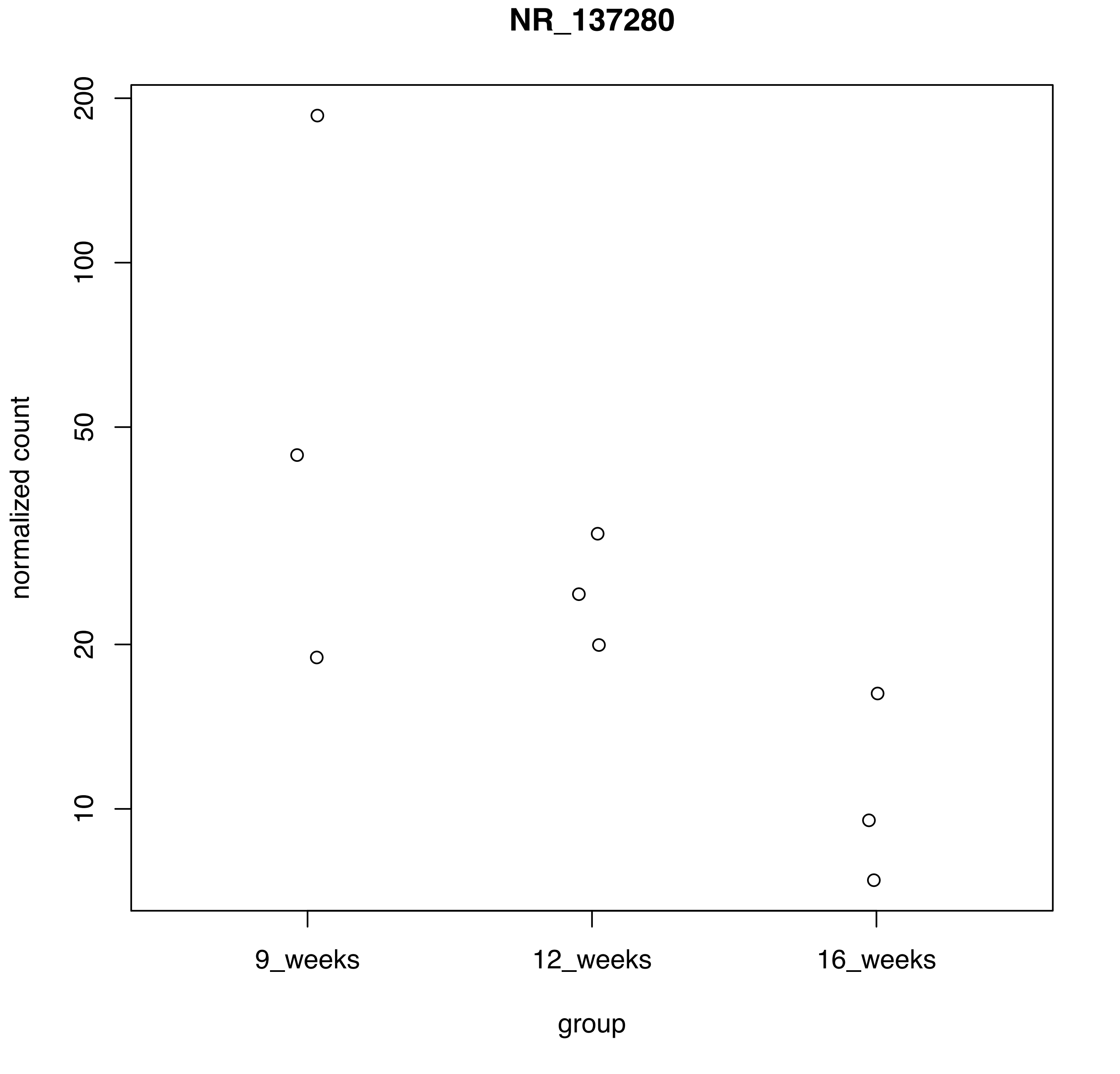

NR_137280
normalized count
200
100
50
20
10
9_weeks
12_weeks
16_weeks
group

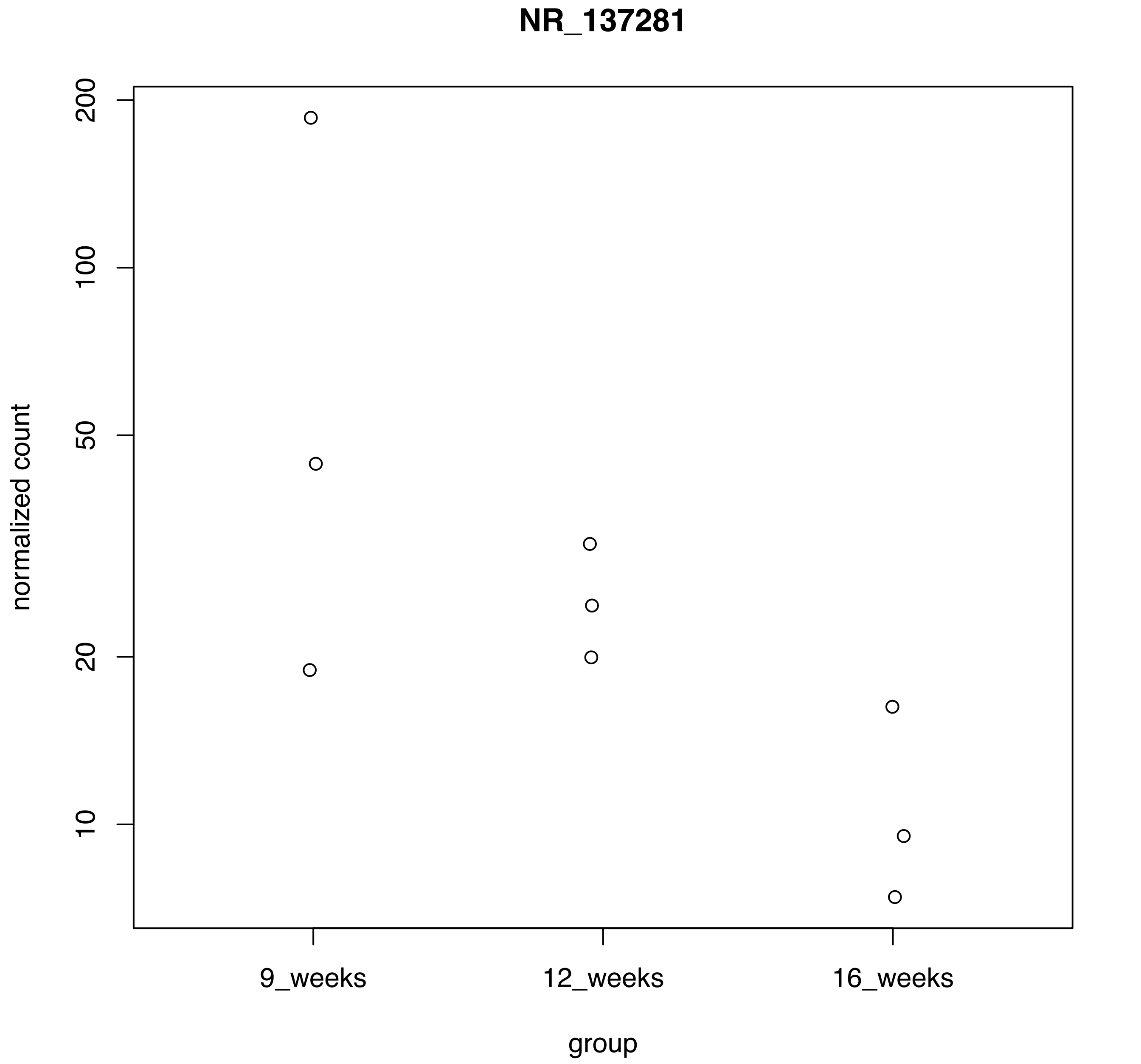
NR_137281
normalized count
200
100
50
20
10
9_weeks
12_weeks
16_weeks
group

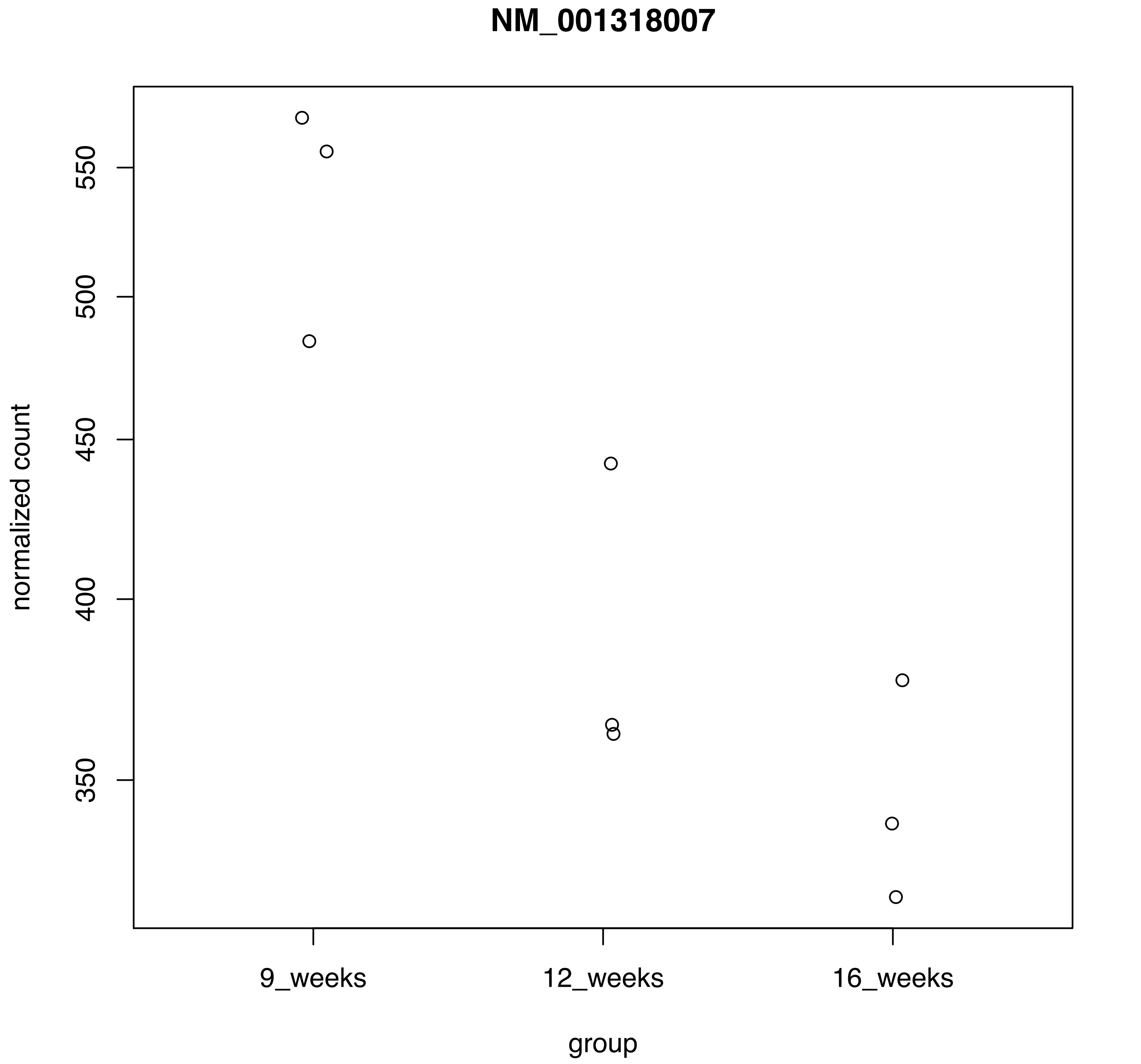
NM_001318007
normalized count
550
500
450
400
350
9_weeks
12_weeks
16_weeks
group

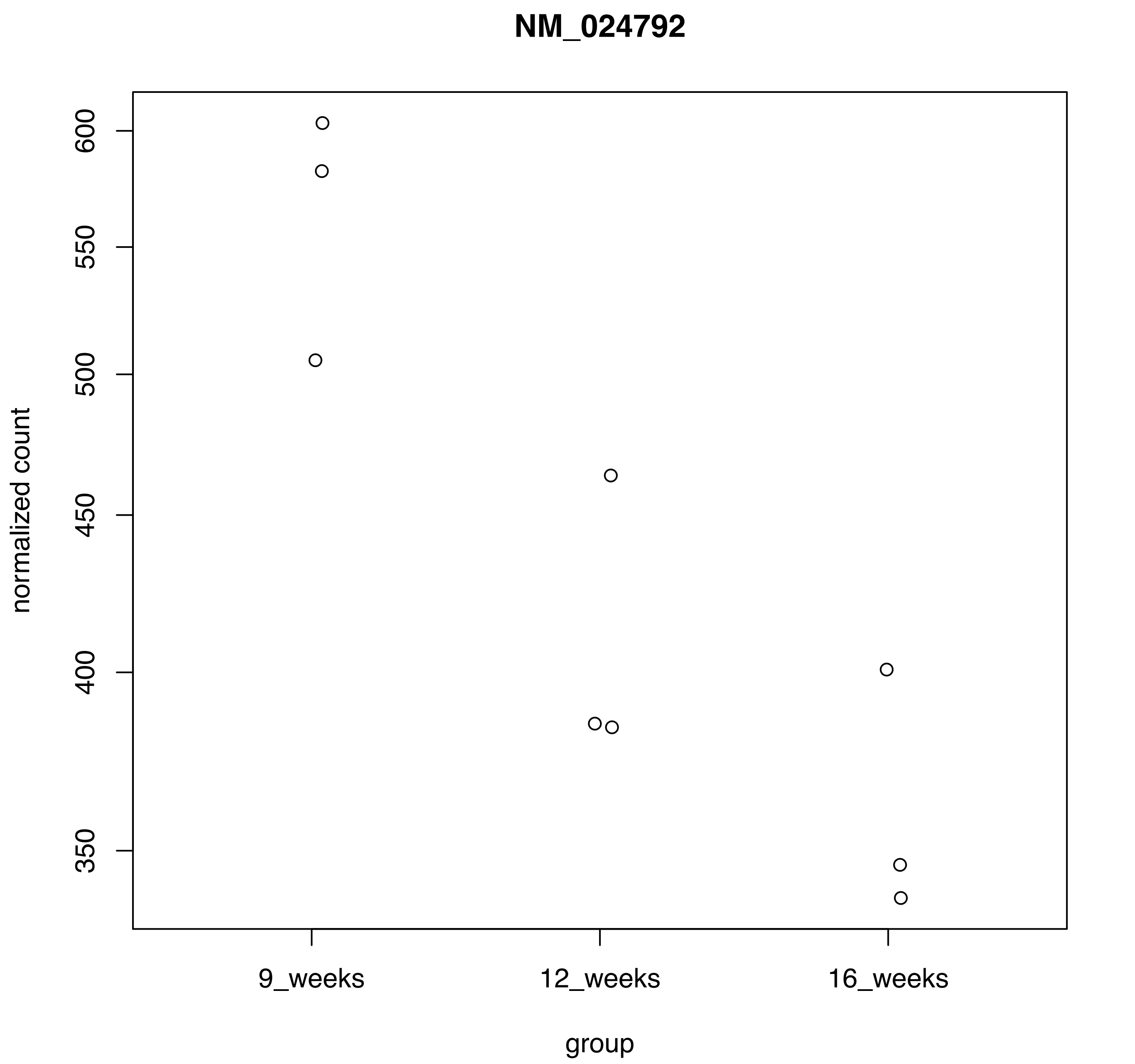

NM_024792
normalized count
600
550
500
450
400
350
9_weeks
12_weeks
16_weeks
group

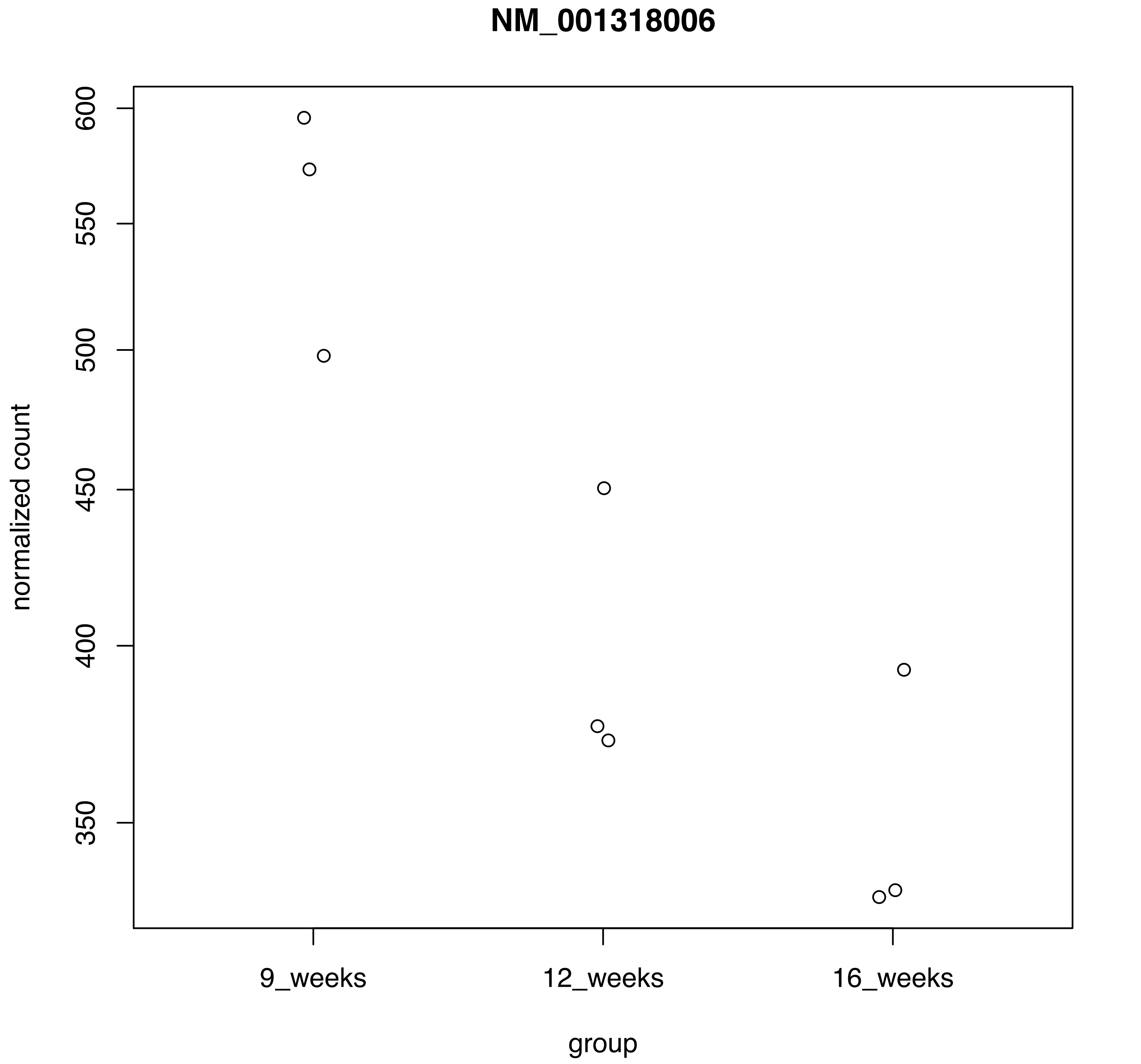
NM_001318006
normalized count
600
550
500
450
400
350
9_weeks
12_weeks
16_weeks
group

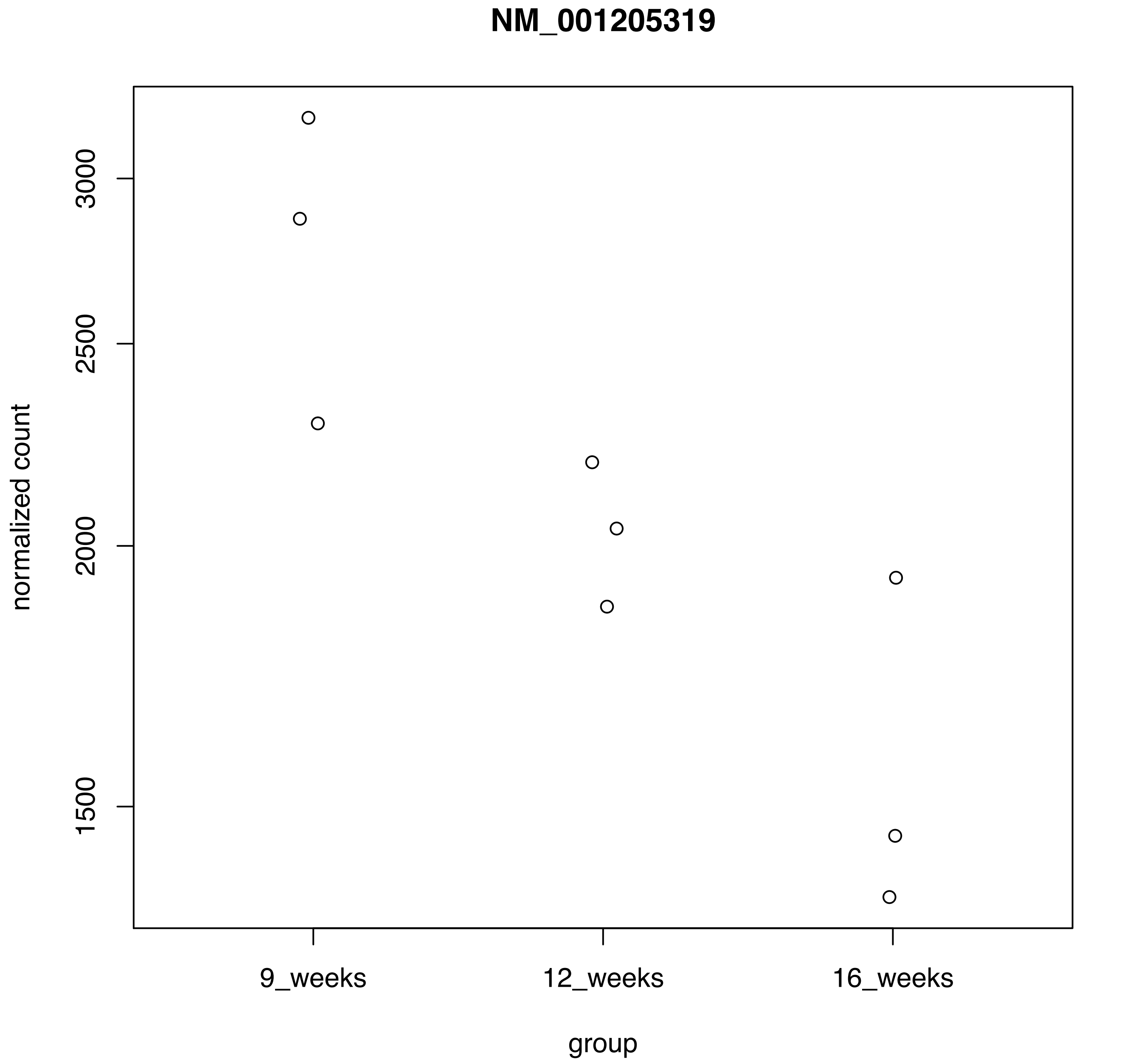
NM_001205319
normalized count
3000
2500
2000
1500
9_weeks
12_weeks
16_weeks
group

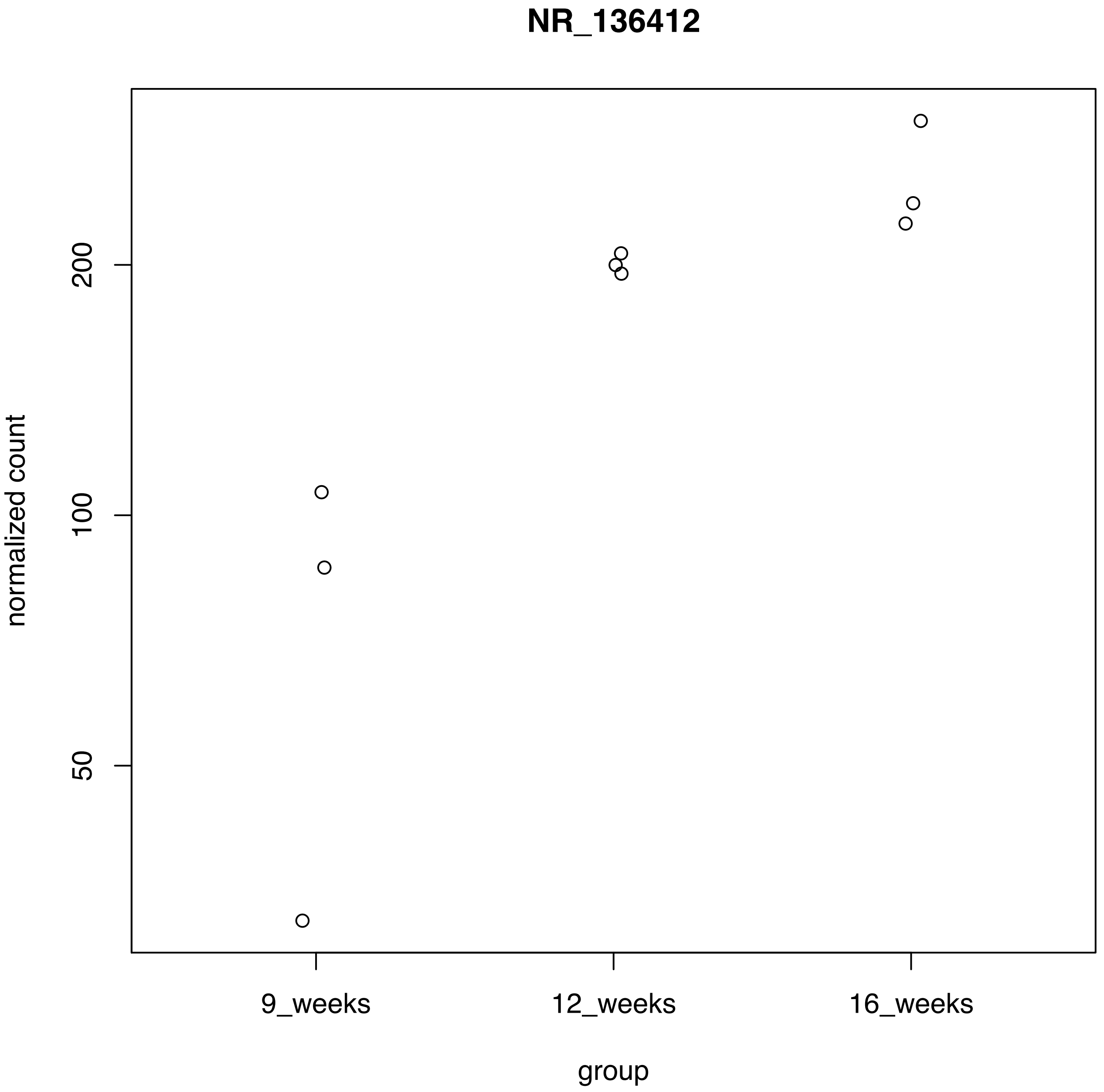
NR_136412
normalized count
200
100
50
9_weeks
12_weeks
16_weeks
group

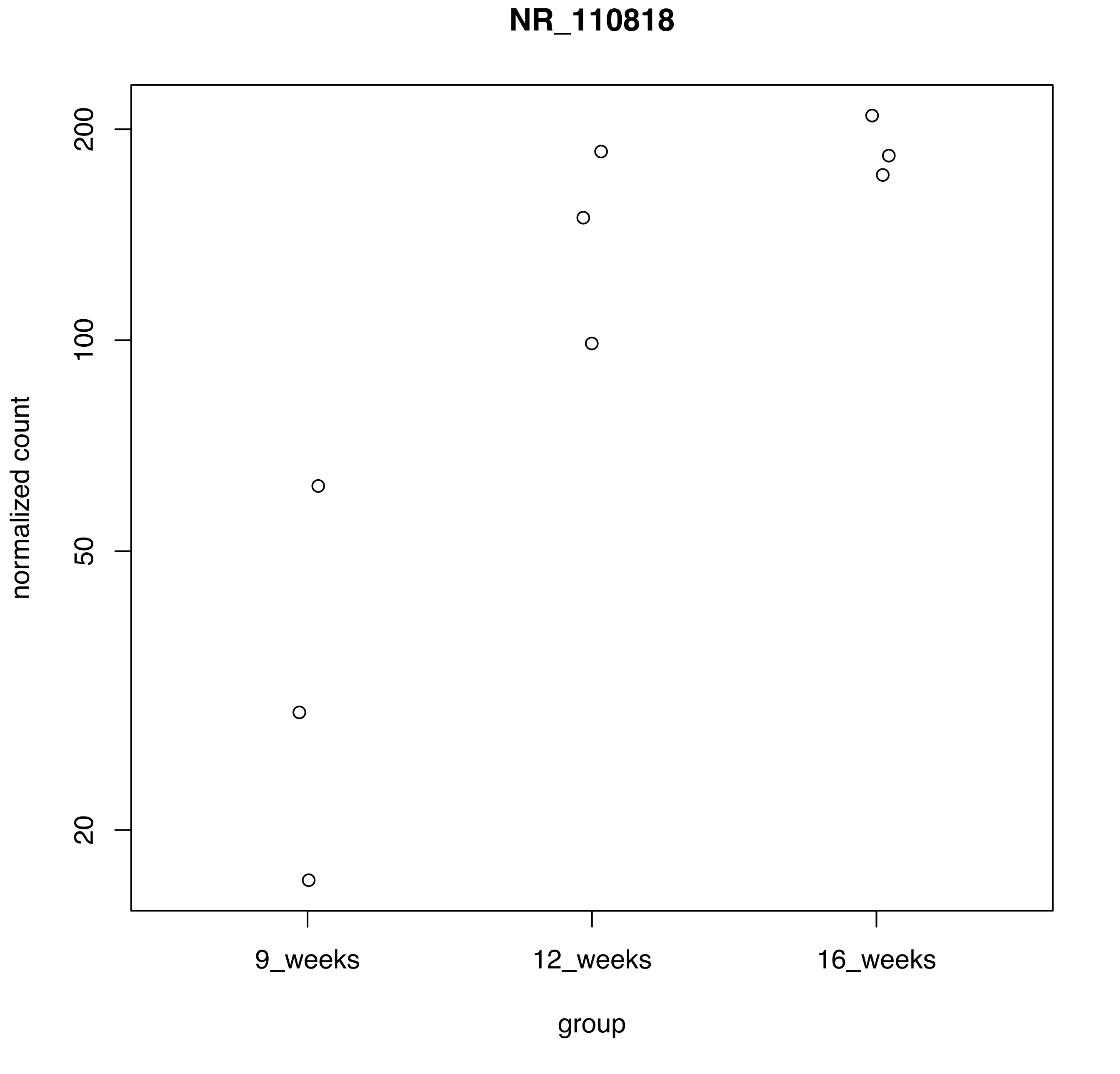

NR_110818
normalized count
200
100
50
20
9_weeks
12_weeks
16_weeks
group

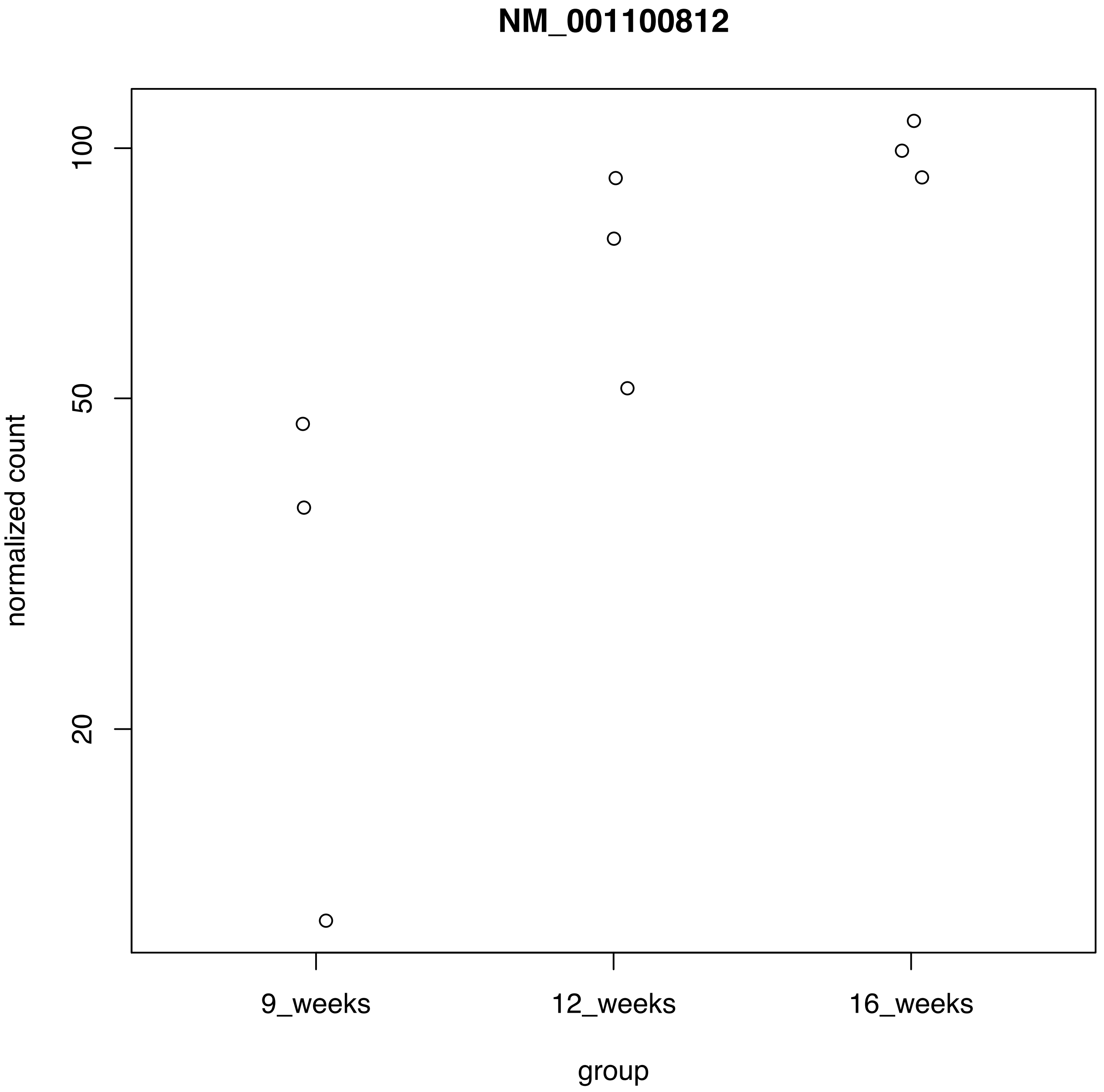
NM_001100812
normalized count
100
50
20
9_weeks
12_weeks
16_weeks
group

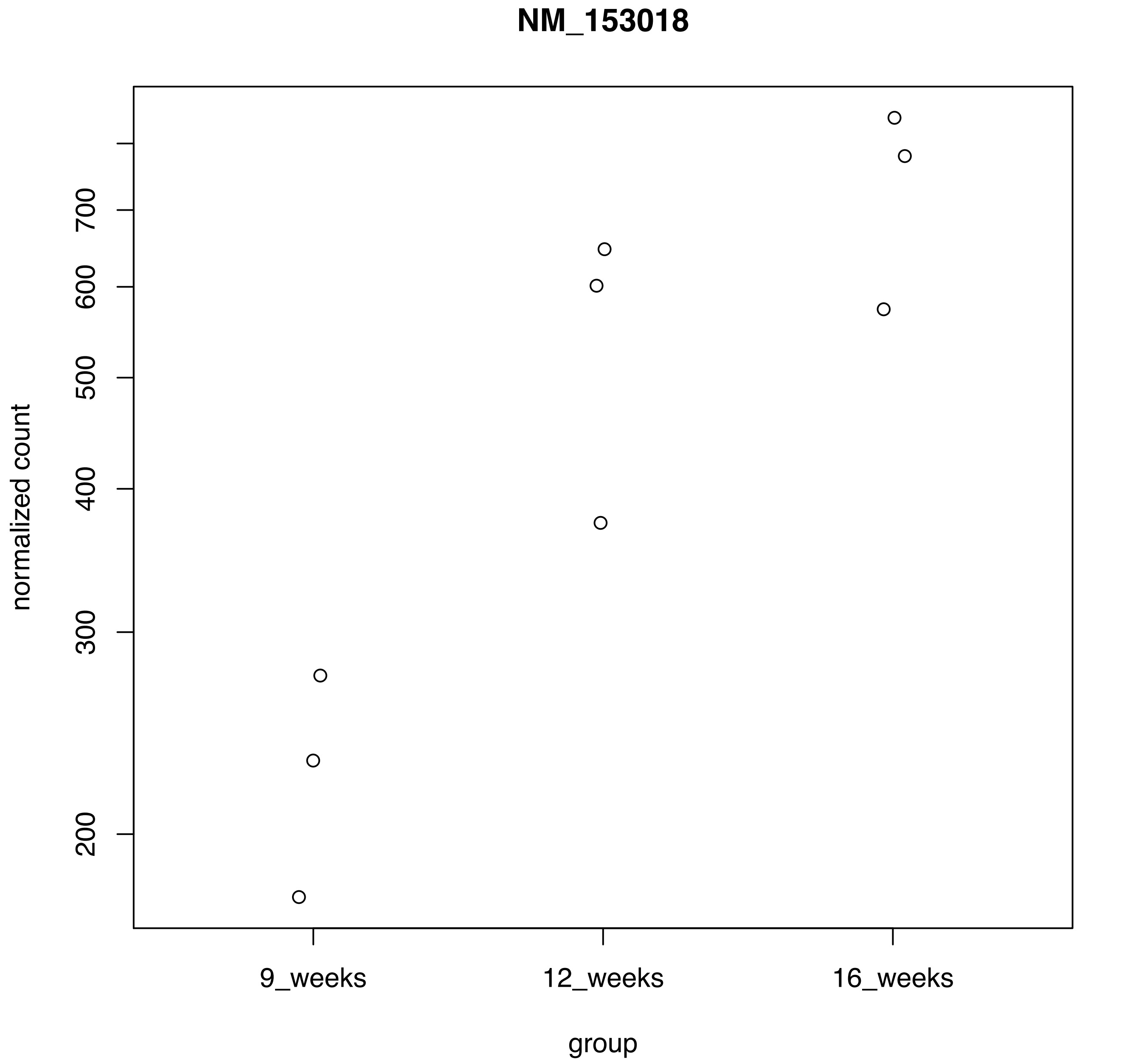

NM_153018
normalized count
200
300
400
500
600
700
9_weeks
12_weeks
16_weeks
group

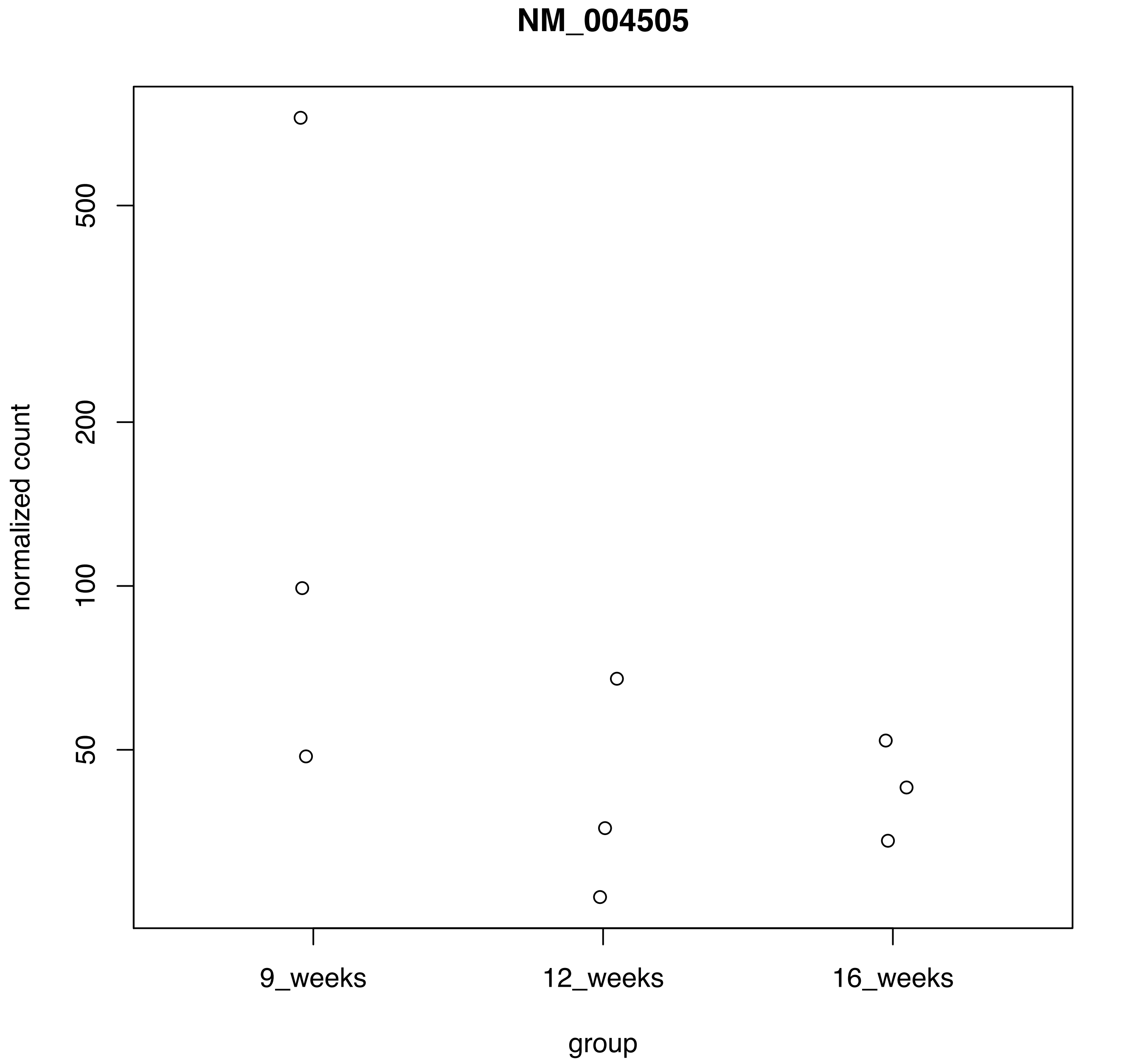
NM_004505
normalized count
500
200
100
50
9_weeks
12_weeks
16_weeks
group

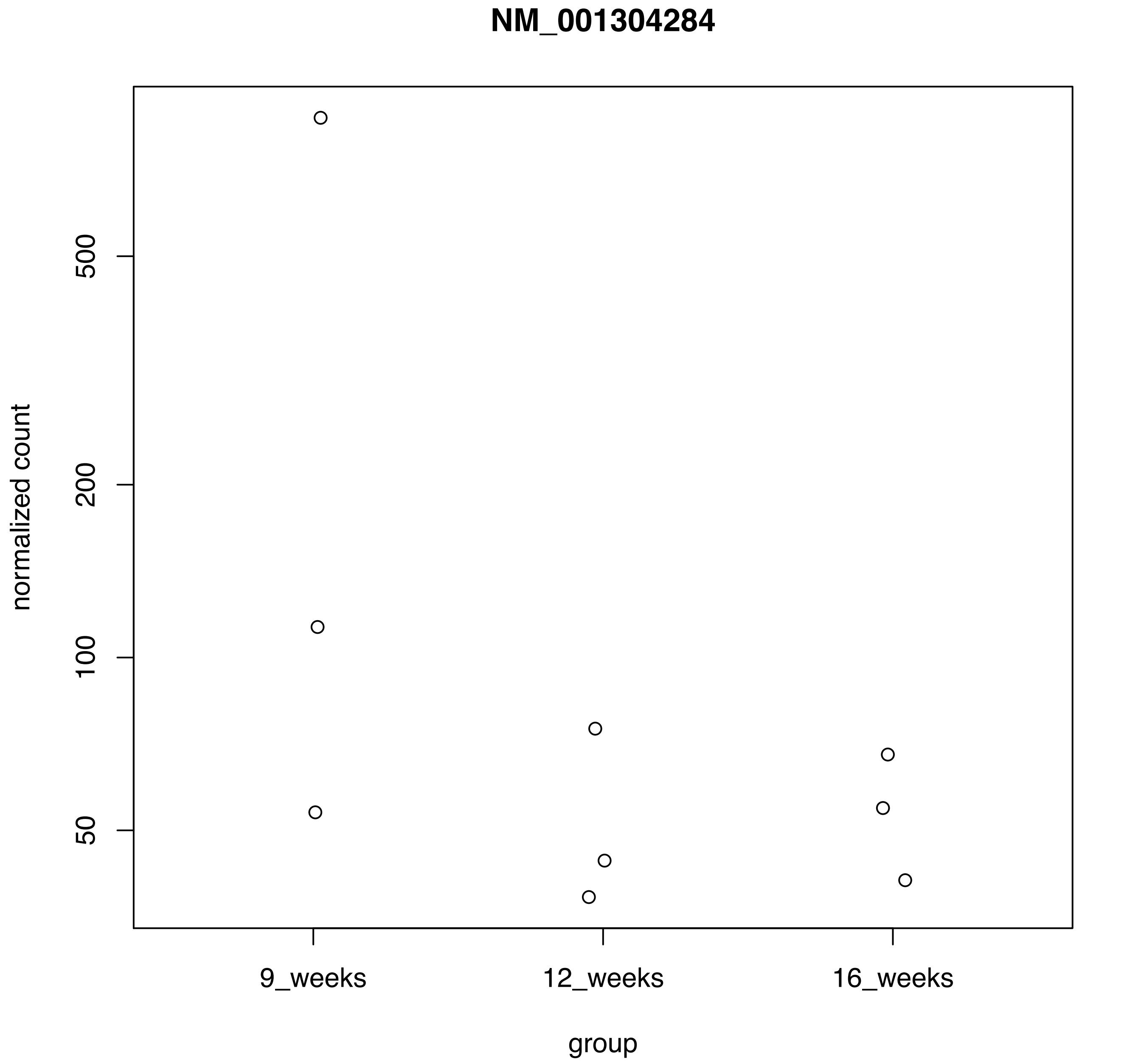
NM_001304284
normalized count
500
200
100
50
9_weeks
12_weeks
16_weeks
group

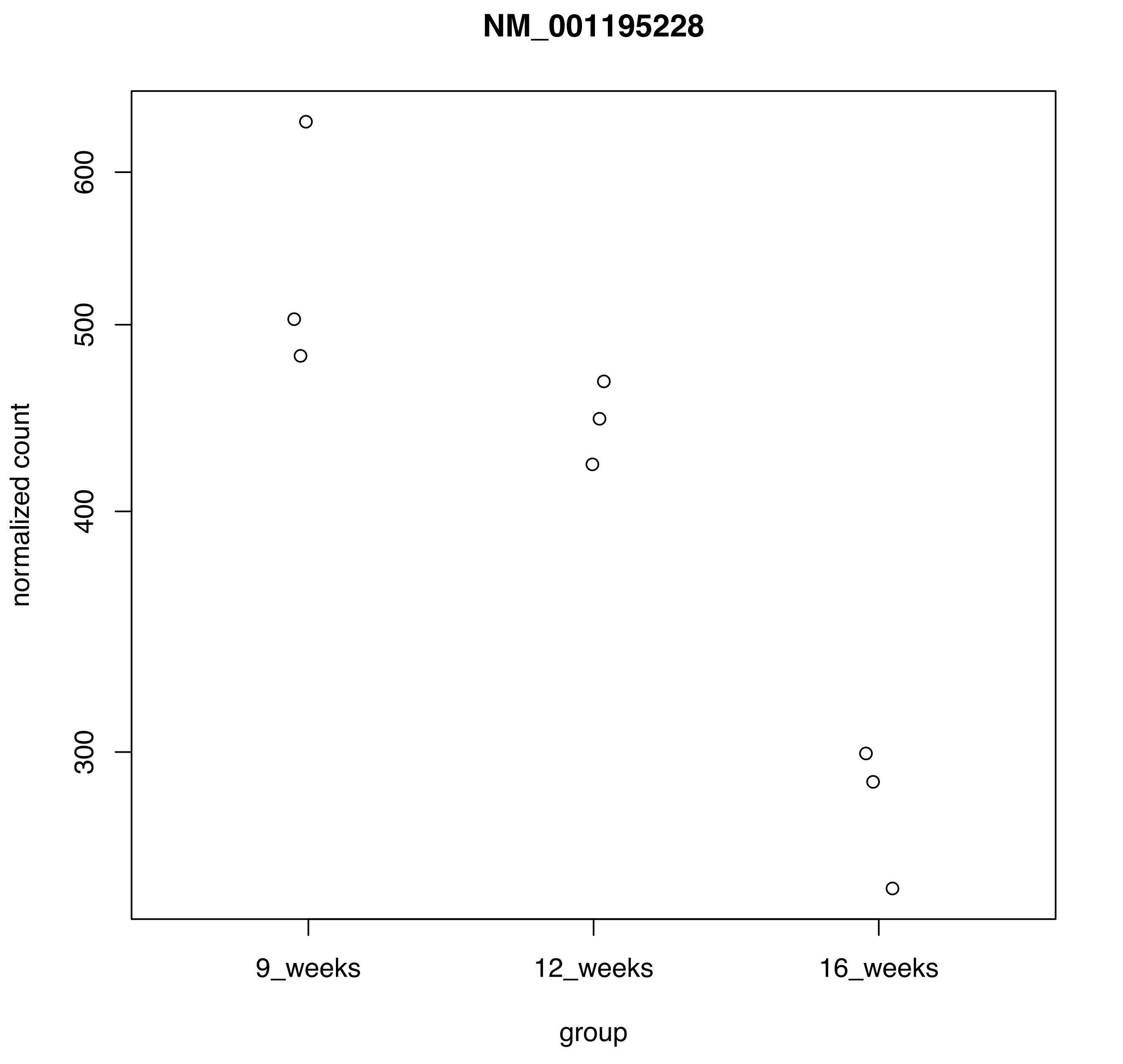
NM_001195228
normalized count
600
500
400
300
9_weeks
12_weeks
16_weeks
group

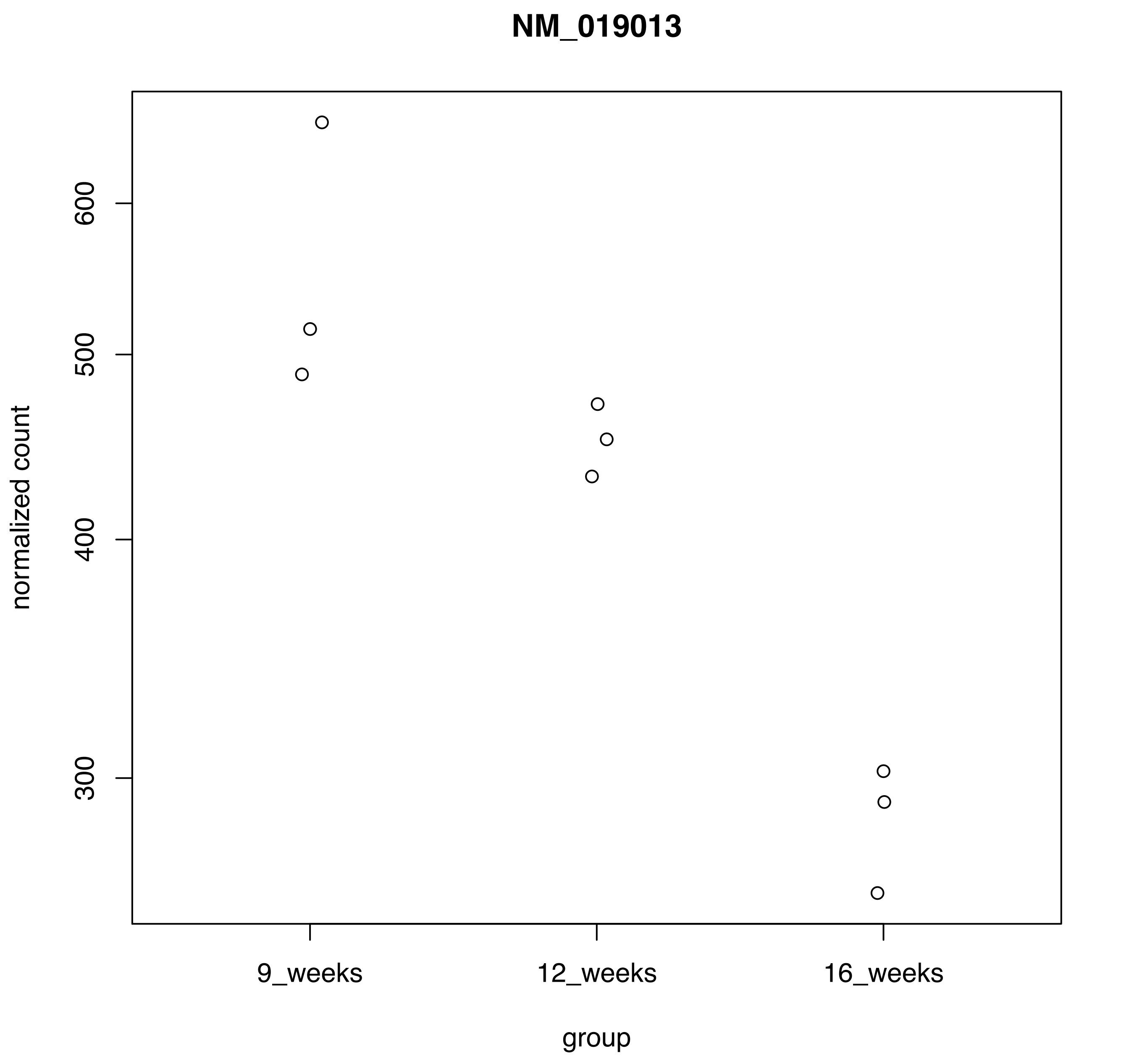

NM_019013
normalized count
600
500
400
300
9_weeks
12_weeks
16_weeks
group

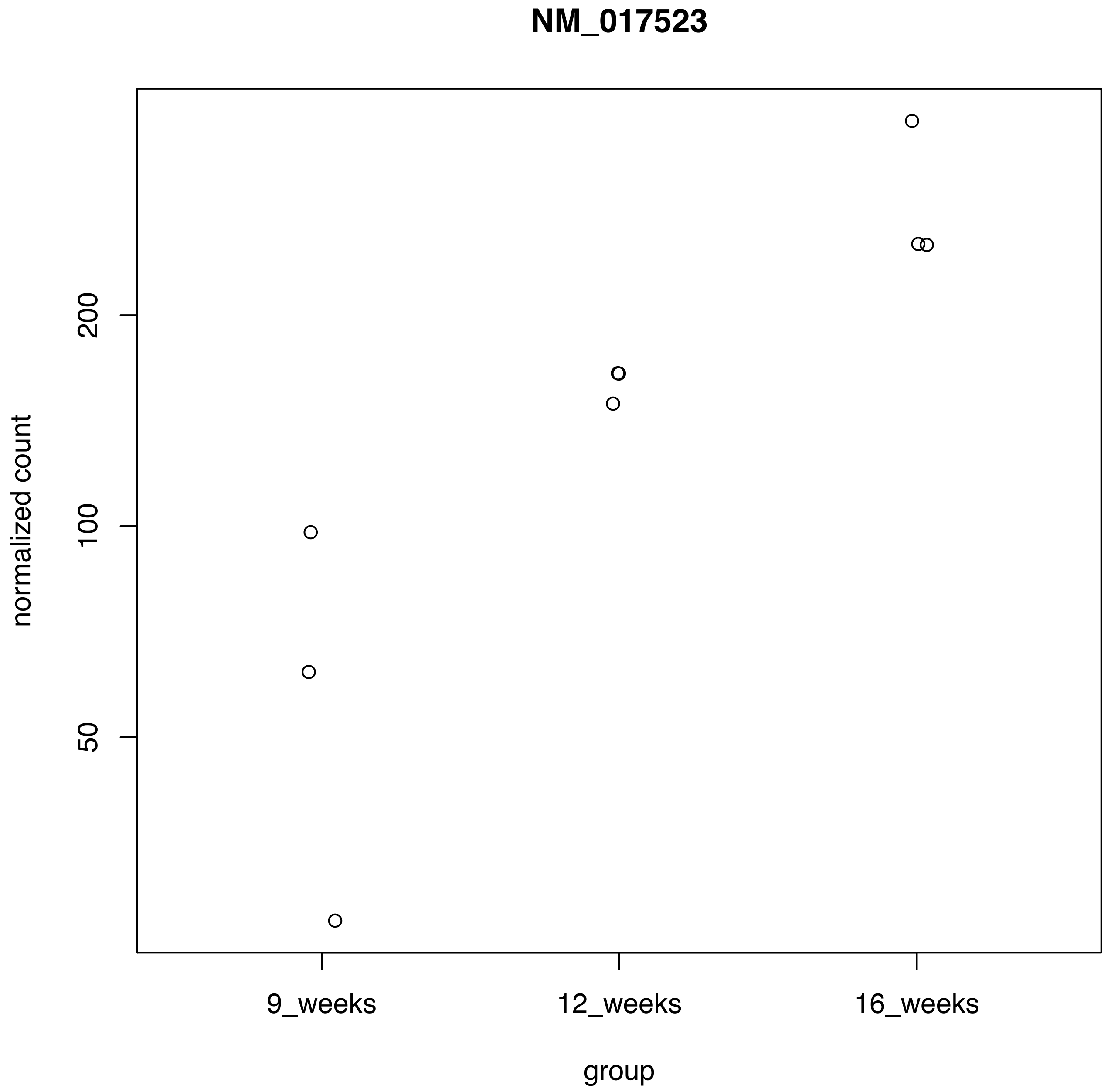
NM_017523
normalized count
200
100
50
9_weeks
12_weeks
16_weeks
group

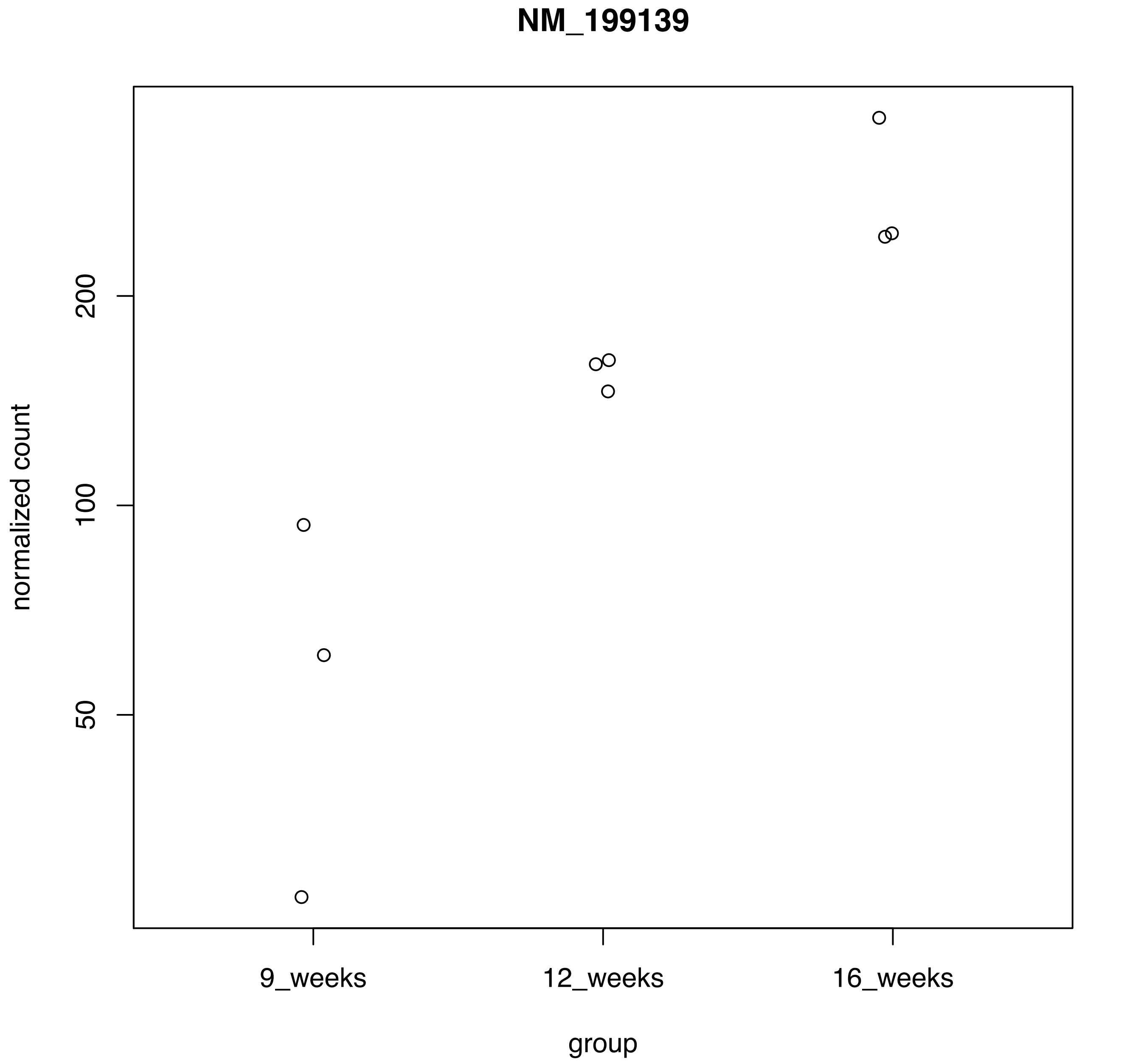
NM_199139
normalized count
50
100
200
9_weeks
12_weeks
16_weeks
group

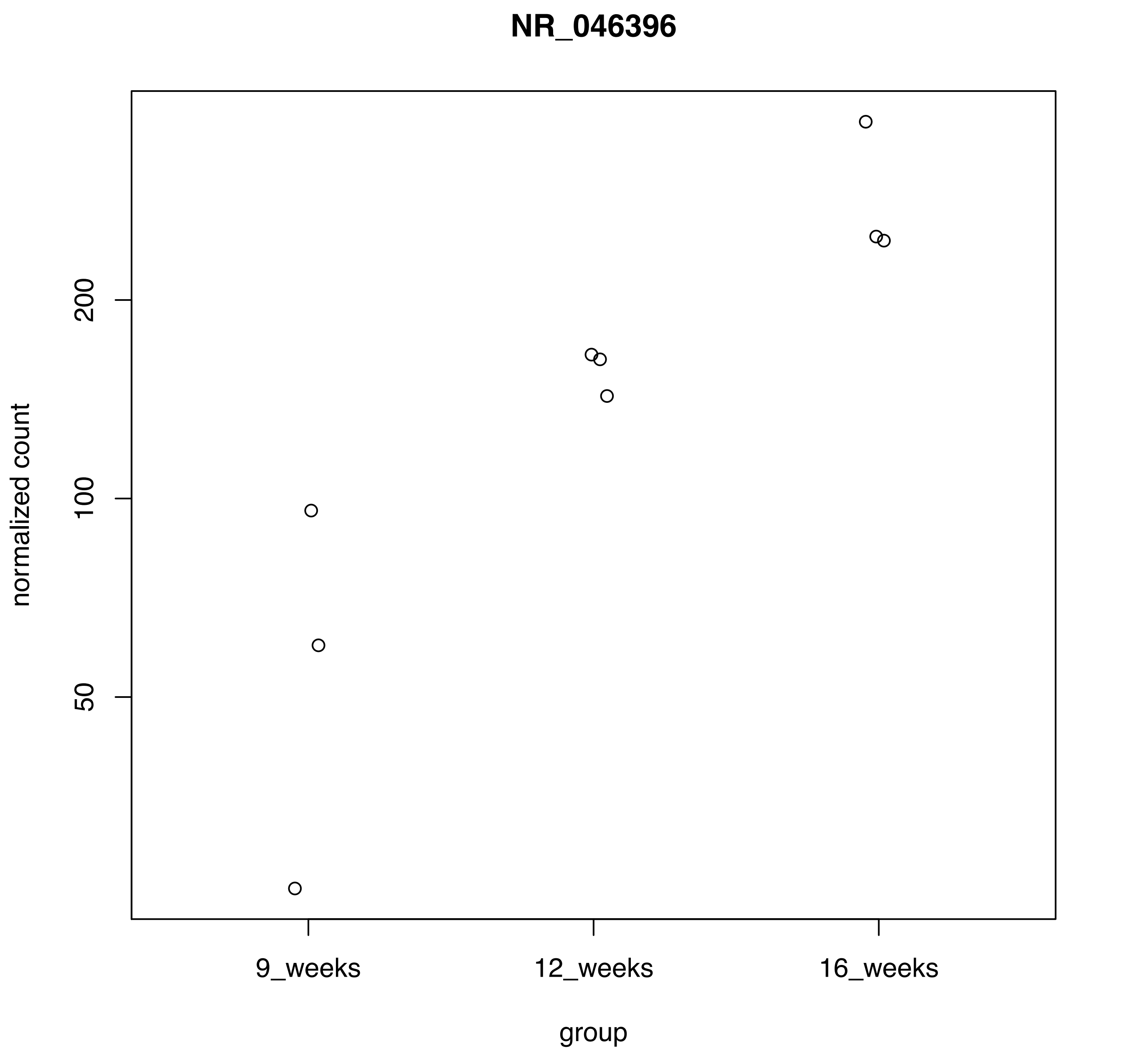
NR_046396
normalized count
200
100
50
9_weeks
12_weeks
16_weeks
group

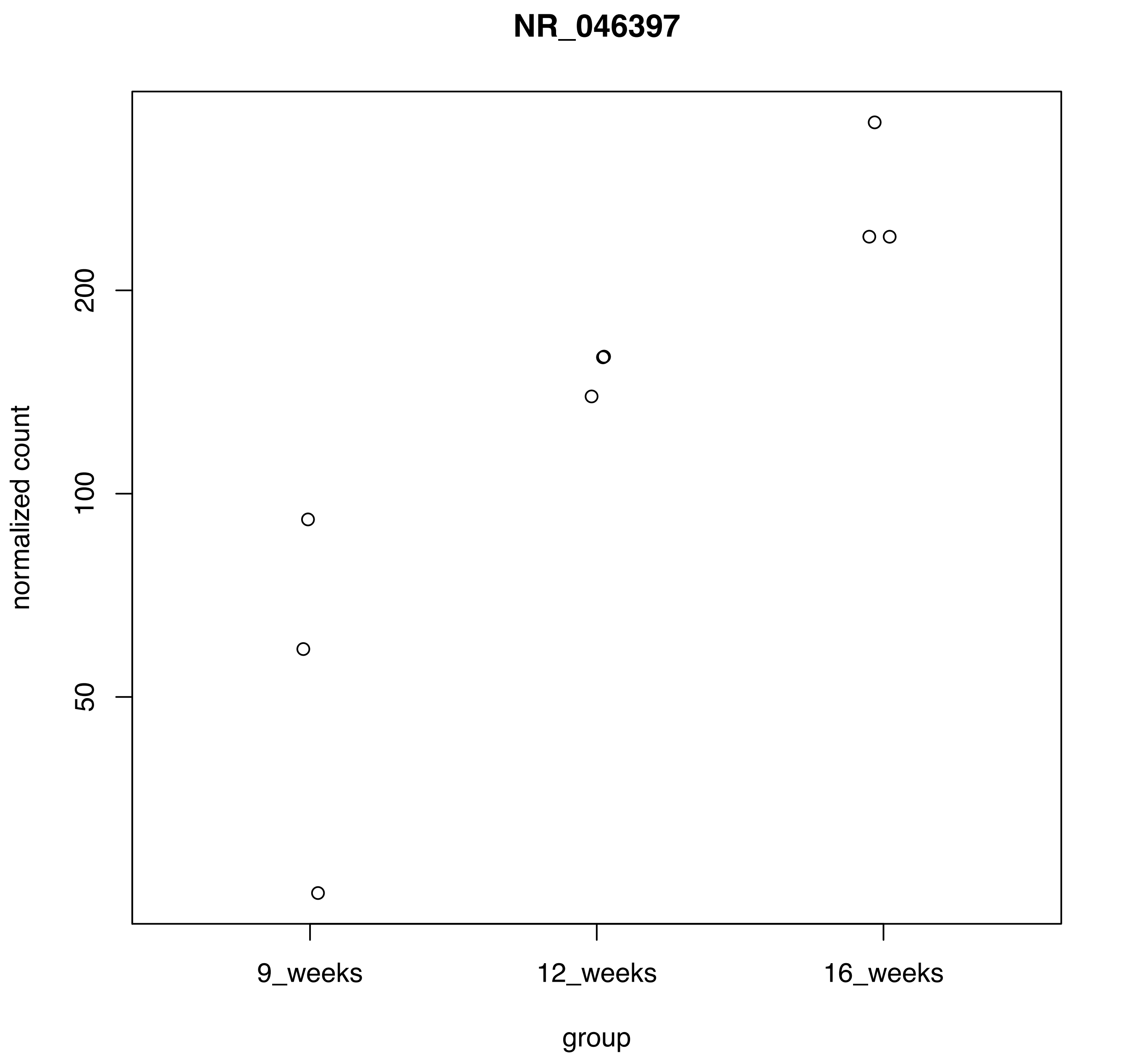
NR_046397
normalized count
200
100
50
9_weeks
12_weeks
16_weeks
group

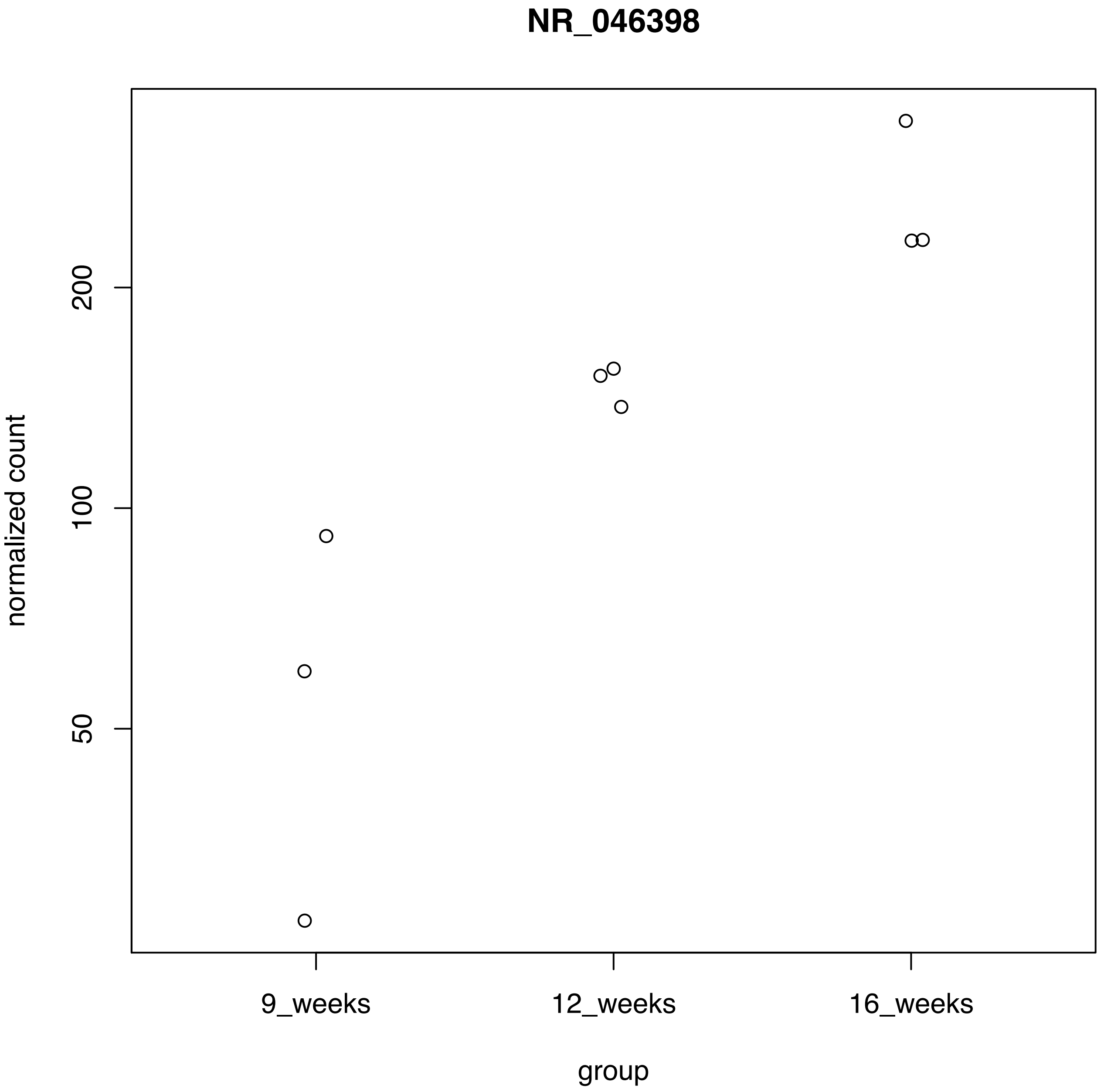
NR_046398
normalized count
200
100
50
9_weeks
12_weeks
16_weeks
group

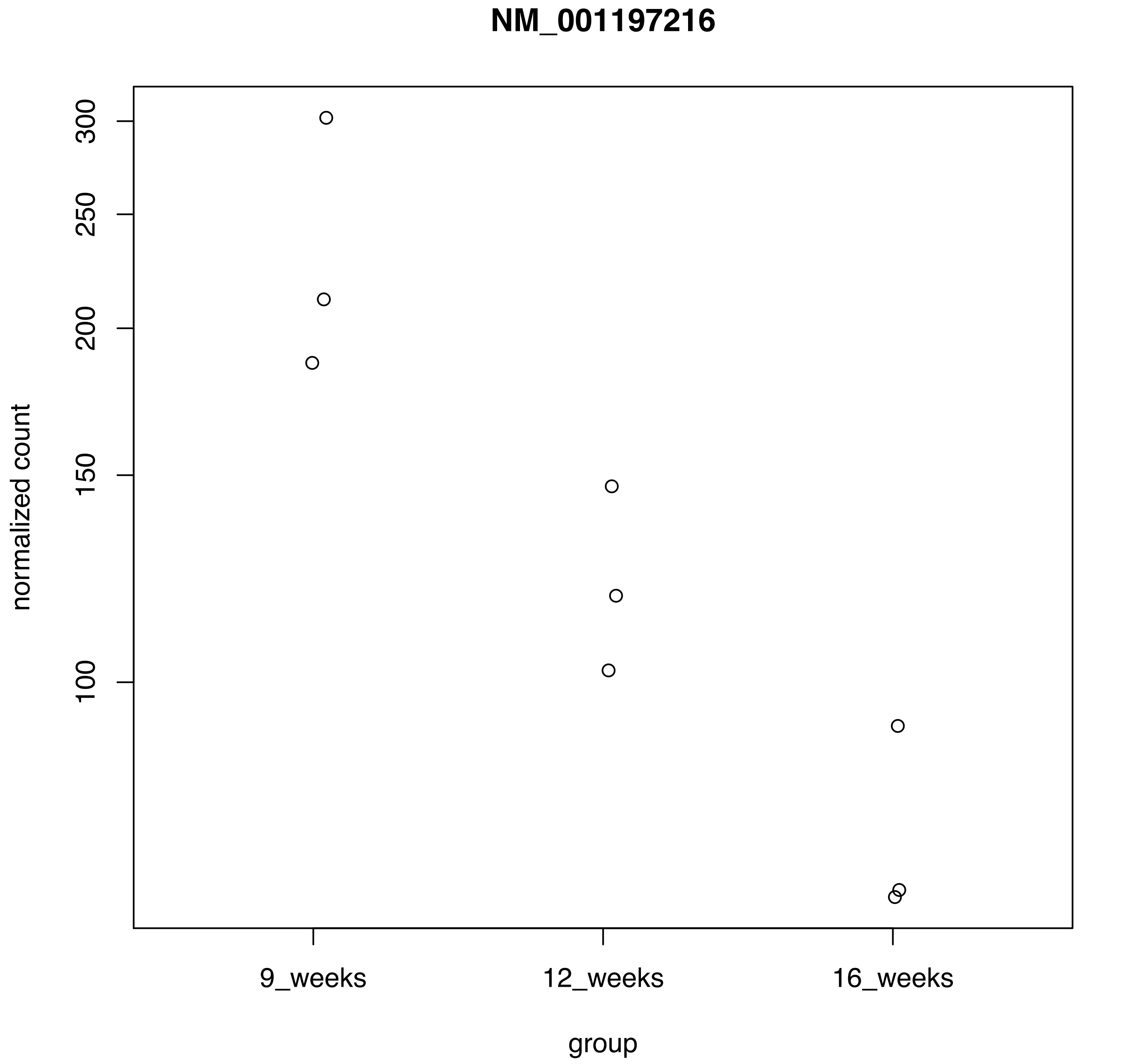
NM_001197216
normalized count
300
250
200
150
100
9_weeks
12_weeks
16_weeks
group

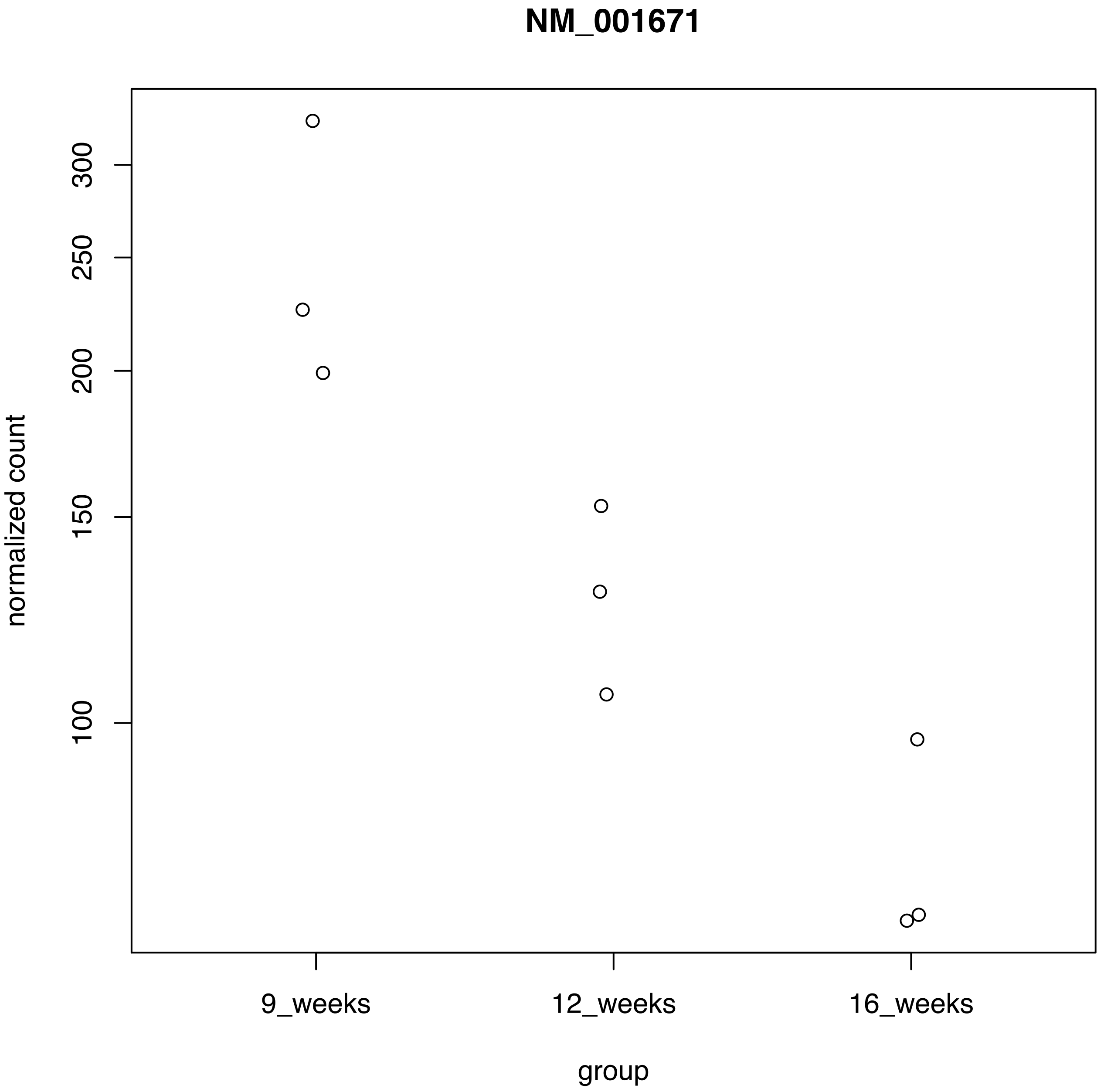
NM_001671
normalized count
300
250
200
150
100
9_weeks
12_weeks
16_weeks
group

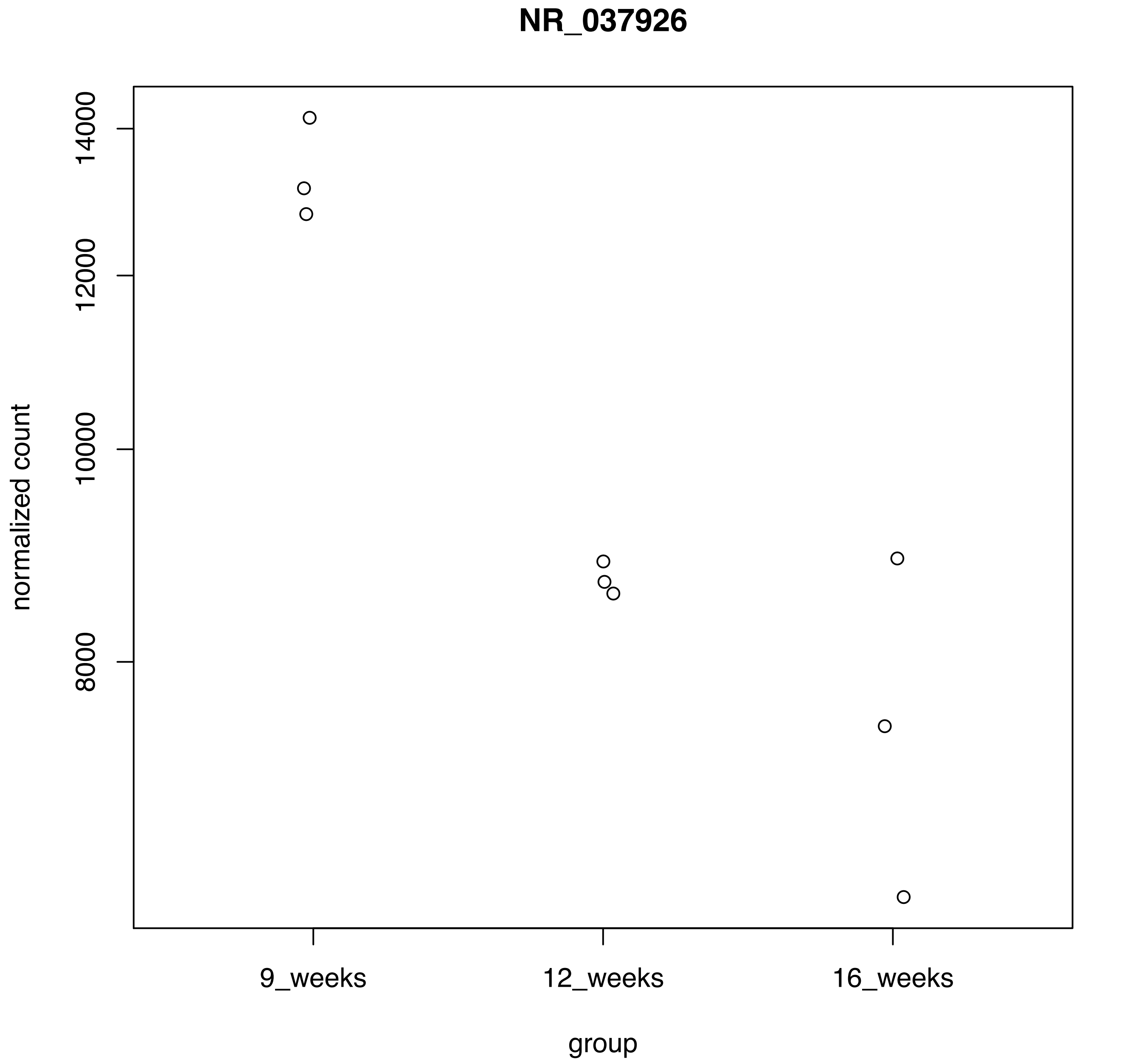
NR_037926
normalized count
14000
12000
10000
8000
9_weeks
12_weeks
16_weeks
group

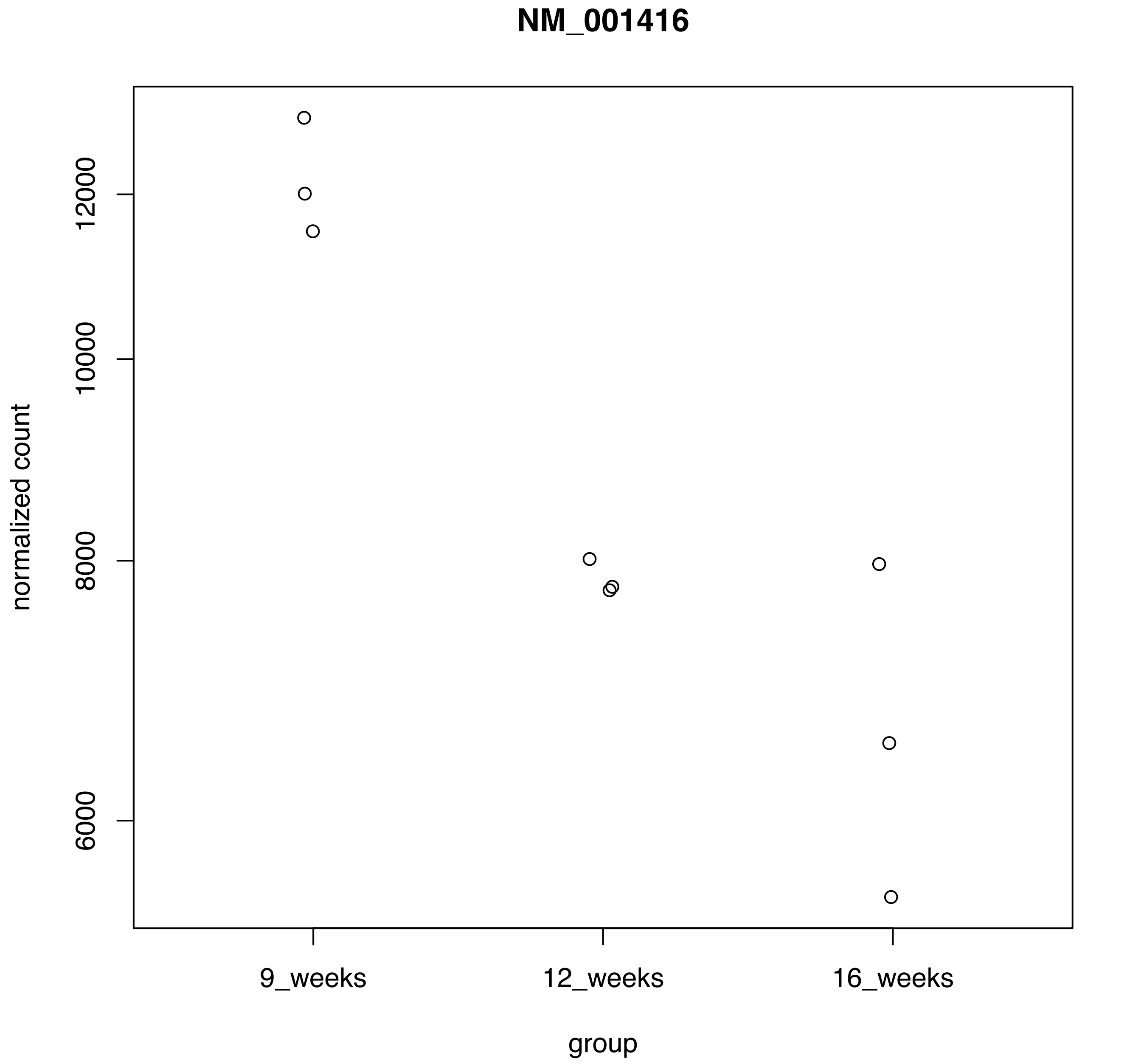

NM_001416
normalized count
12000
10000
8000
6000
9_weeks
12_weeks
16_weeks
group

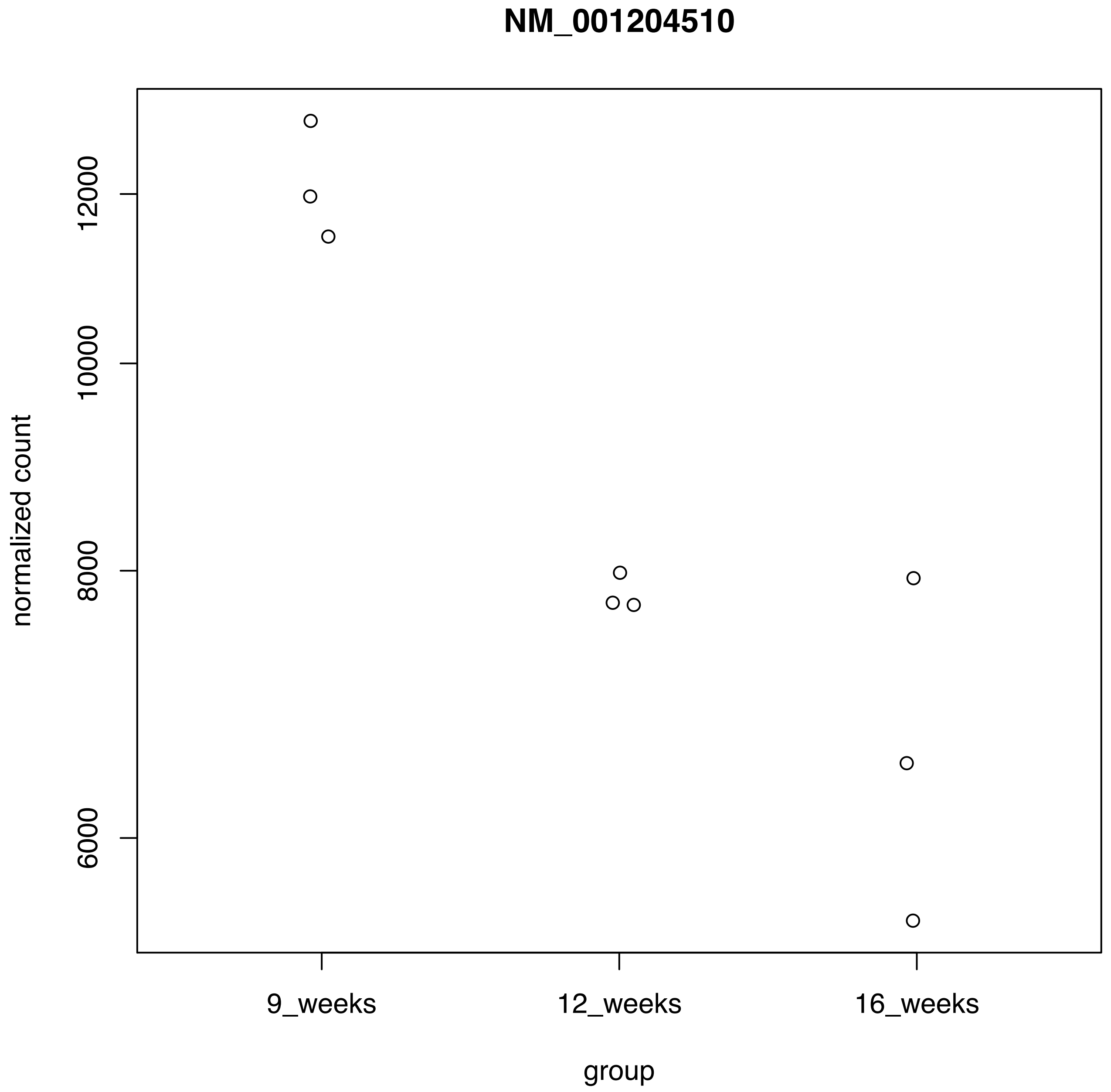
NM_001204510
normalized count
12000
10000
8000
6000
9_weeks
12_weeks
16_weeks
group

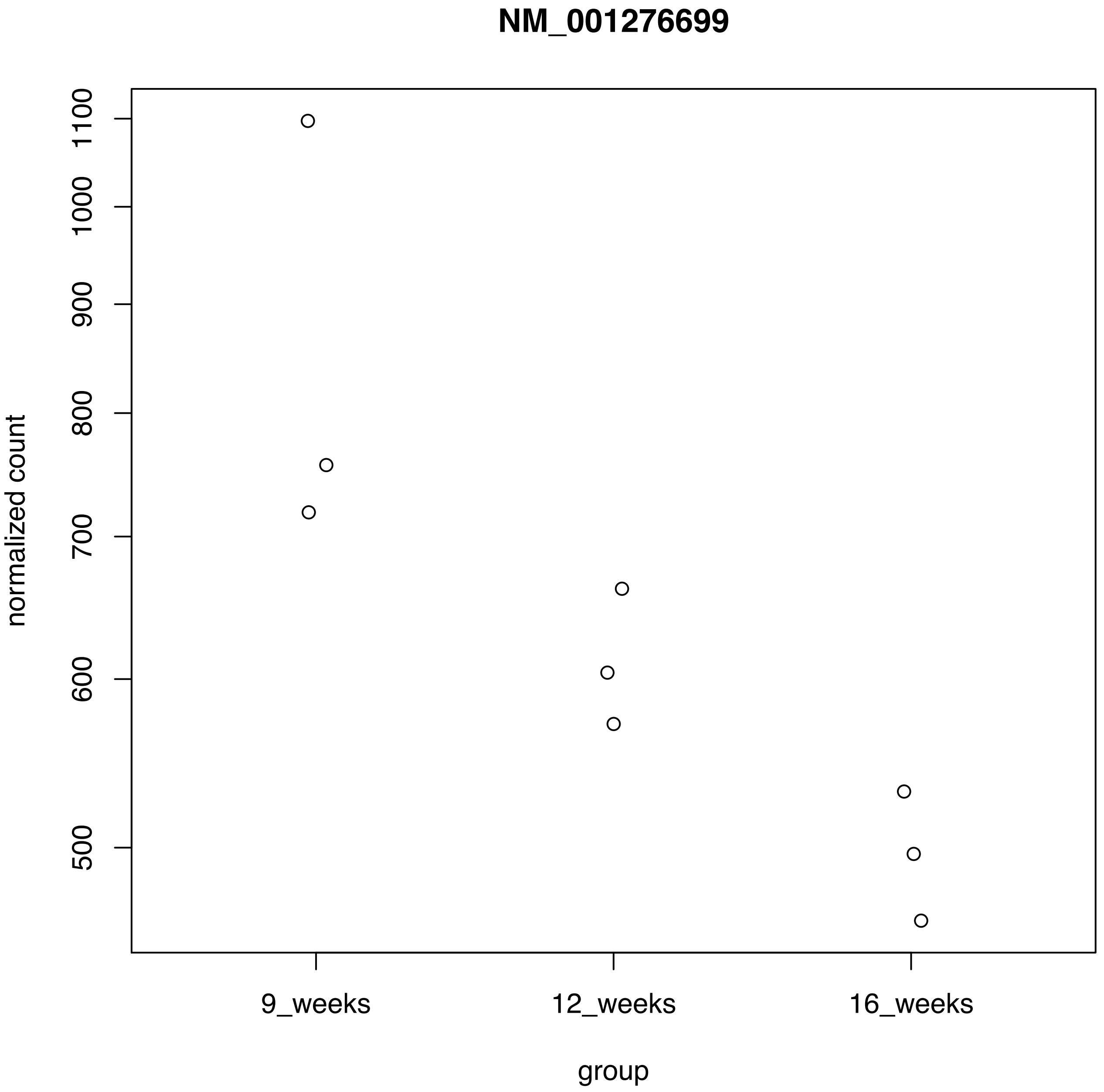
NM_001276699
normalized count
500
600
700
800
900
1000
1100
9_weeks
12_weeks
16_weeks
group

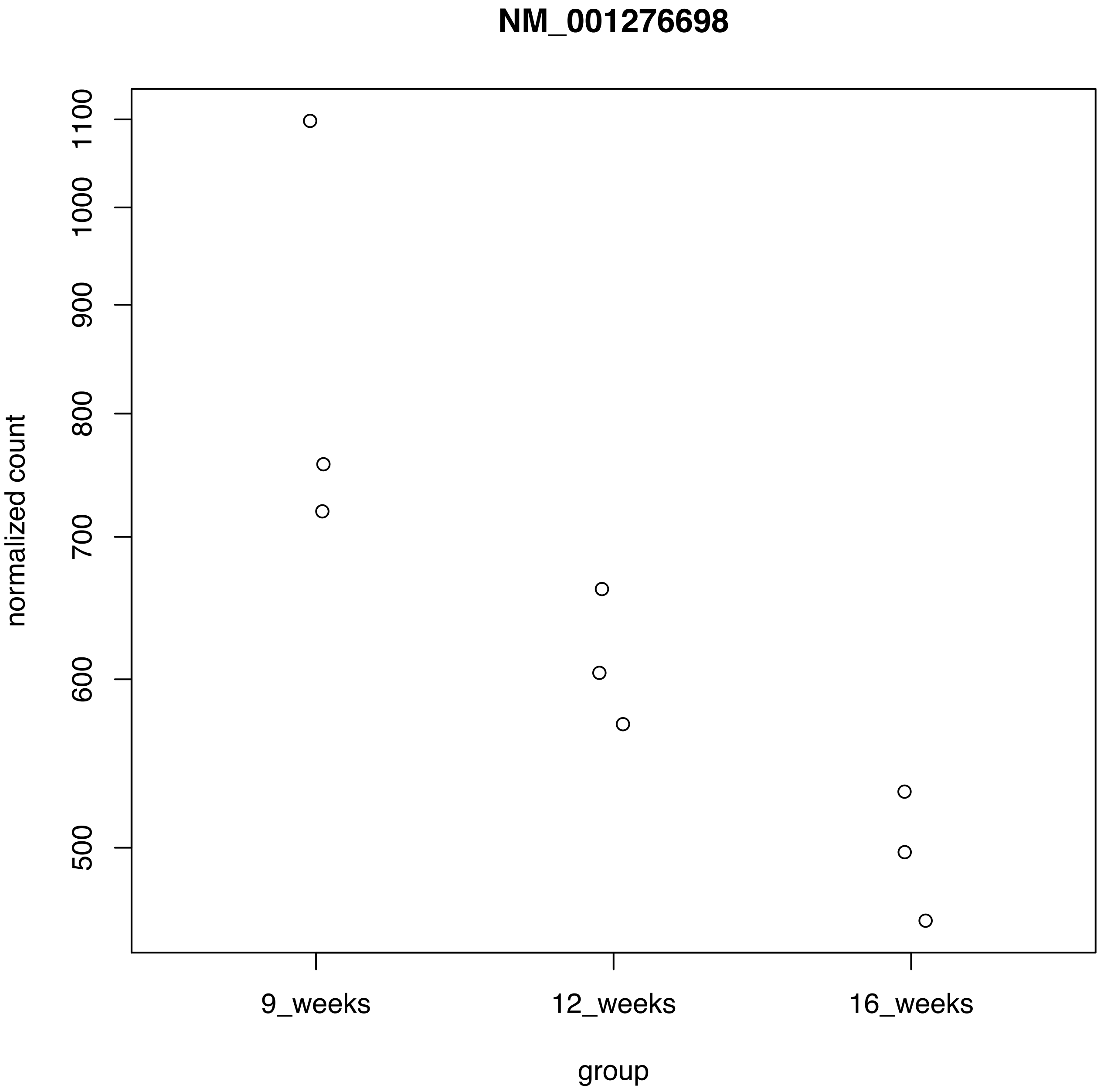
NM_001276698
normalized count
1100
1000
900
800
700
600
500
9_weeks
12_weeks
16_weeks
group

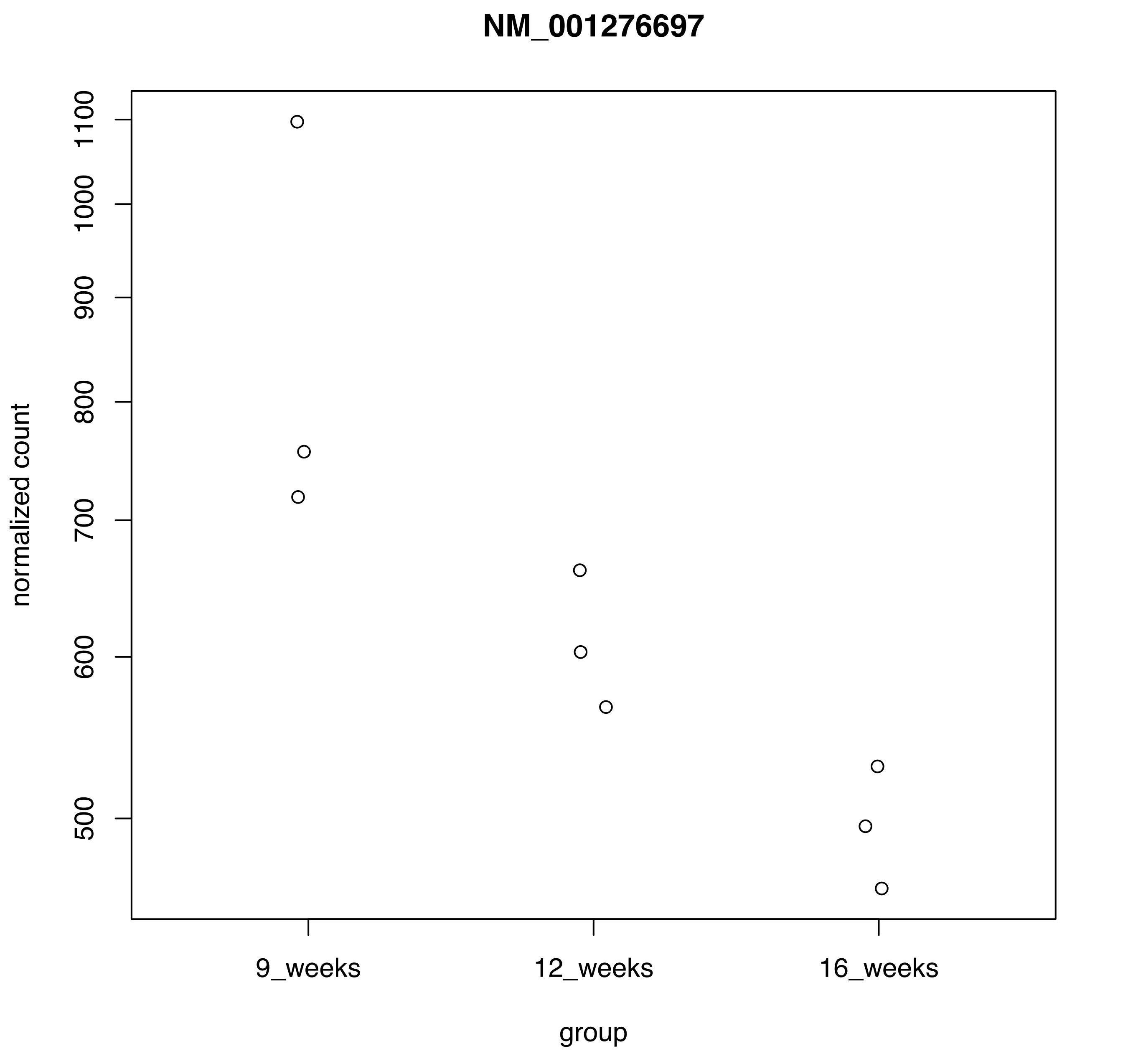
NM_001276697
normalized count
1100
1000
900
800
700
600
500
9_weeks
12_weeks
16_weeks
group

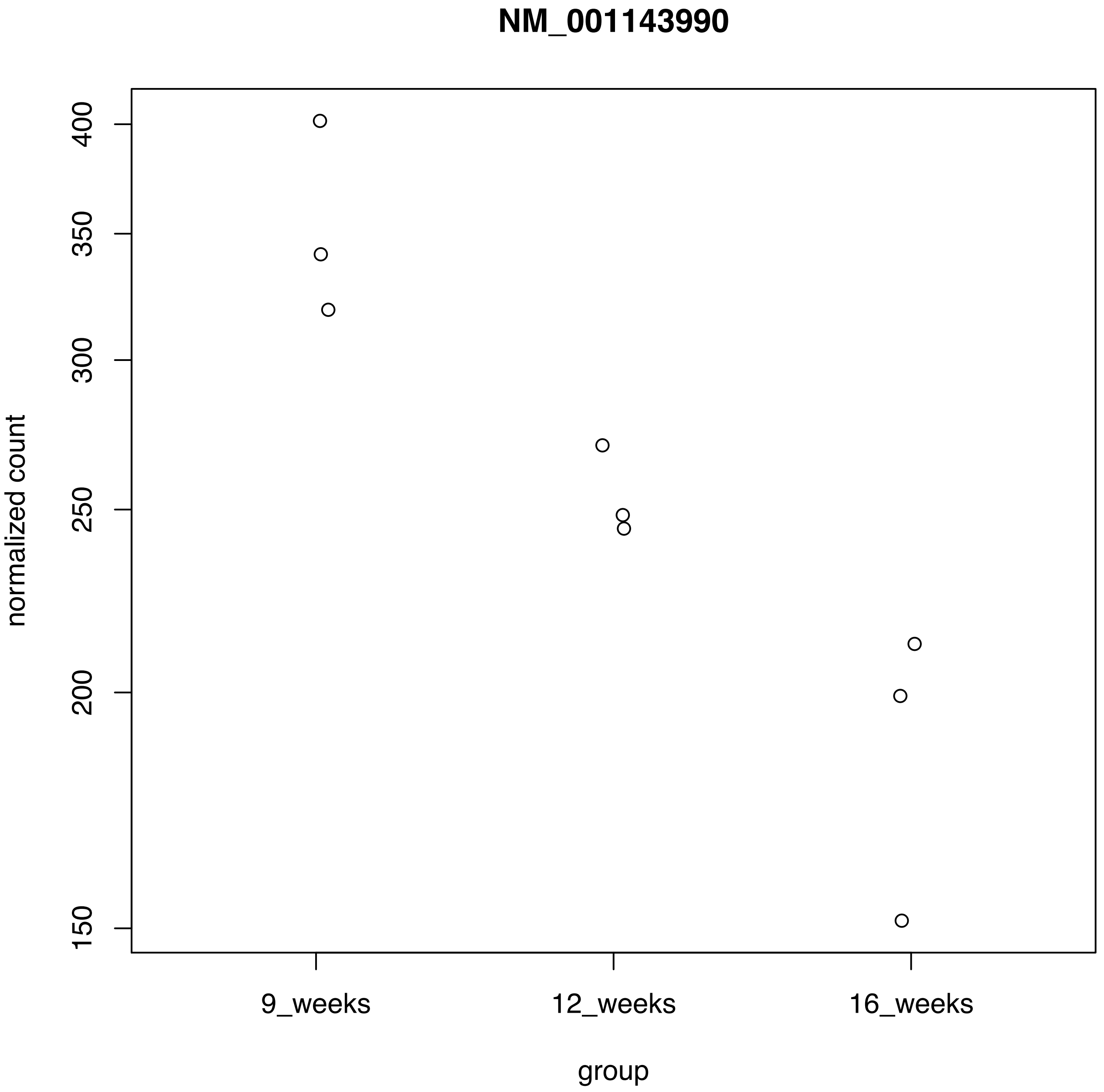
NM_001143990
normalized count
400
350
300
250
200
150
9_weeks
12_weeks
16_weeks
group

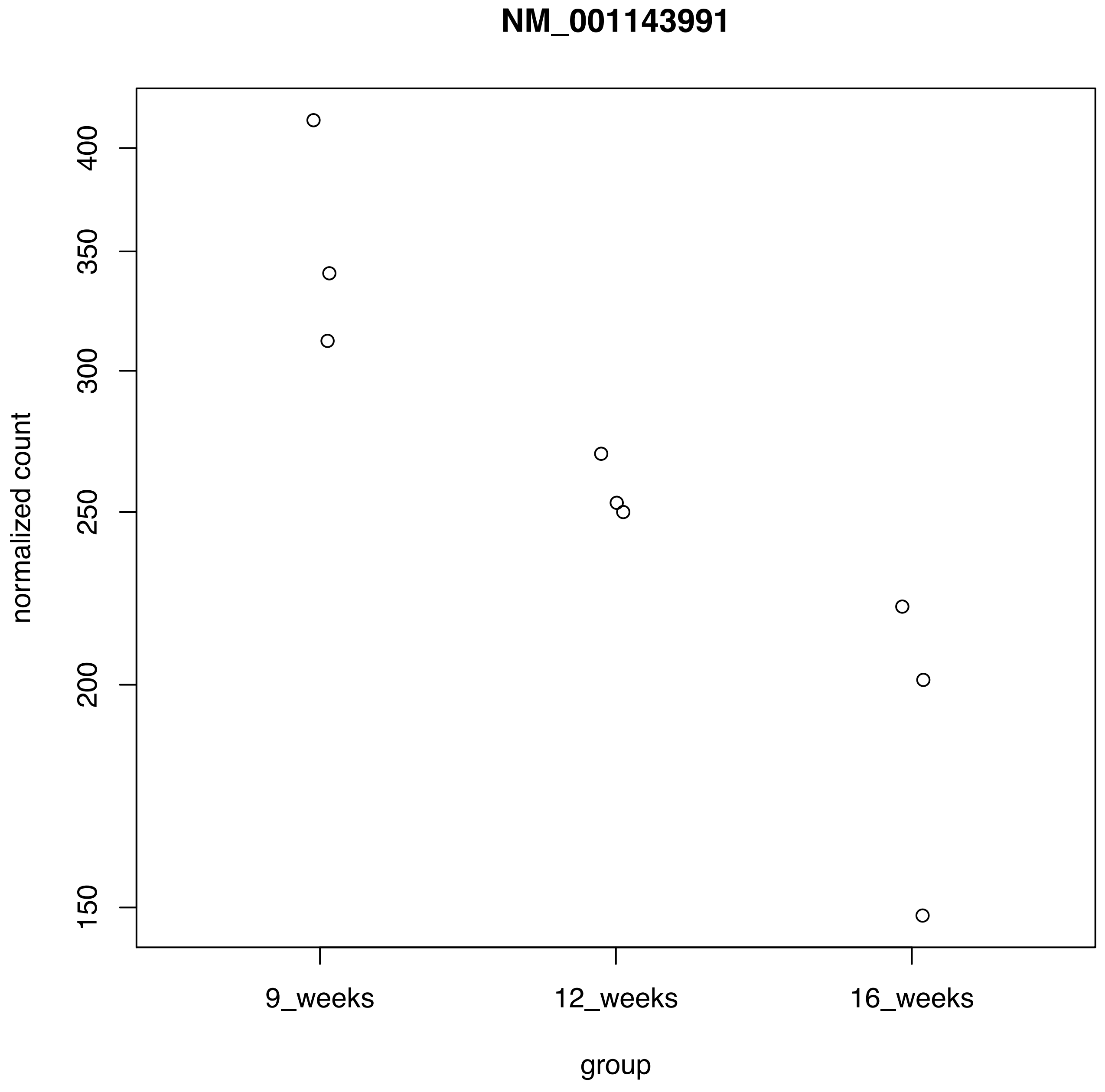

NM_001143991
normalized count
400
350
300
250
200
150
9_weeks
12_weeks
16_weeks
group

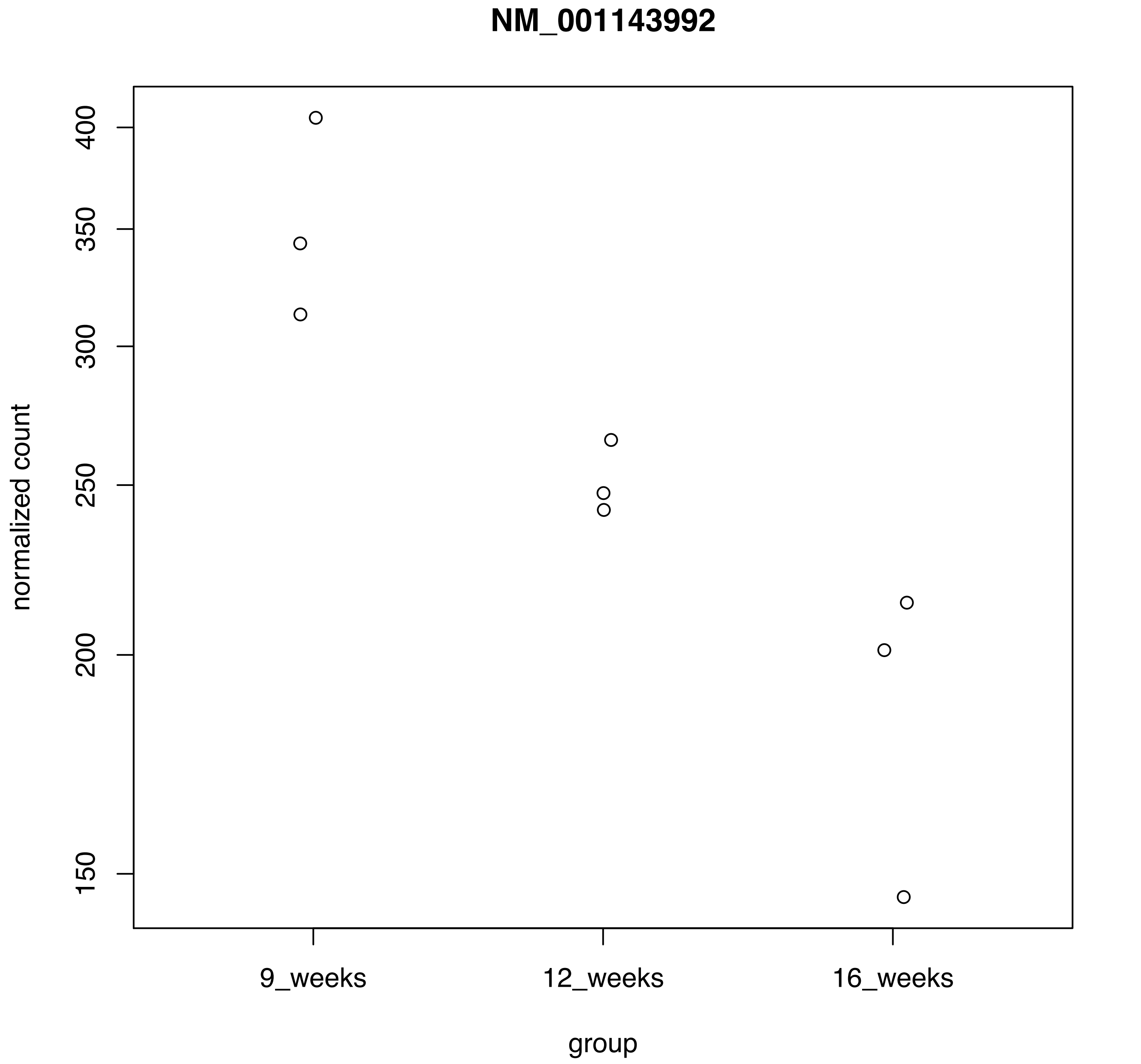
NM_001143992
normalized count
400
350
300
250
200
150
9_weeks
12_weeks
16_weeks
group

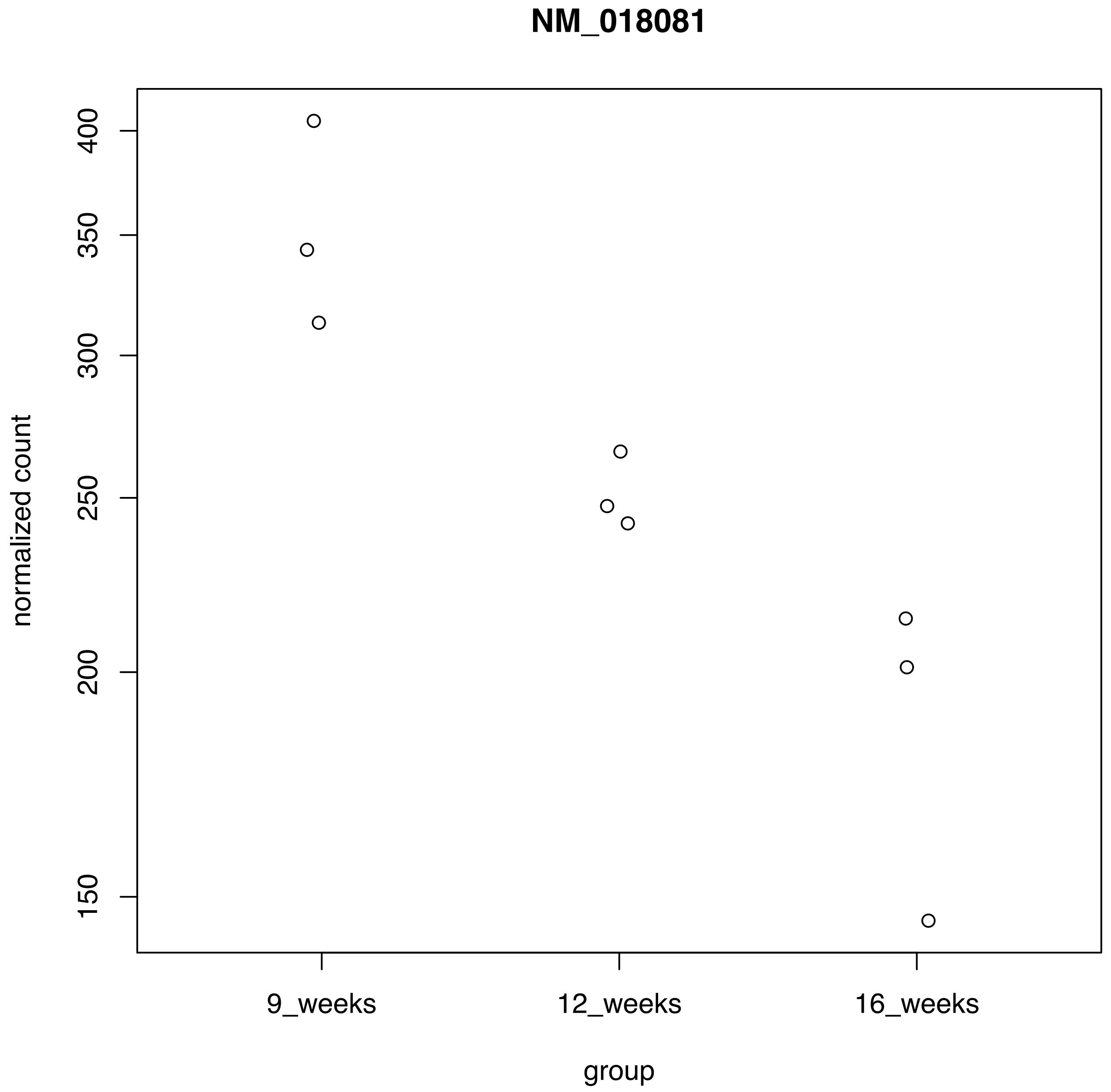
NM_018081
normalized count
400
350
300
250
200
150
9_weeks
12_weeks
16_weeks
group

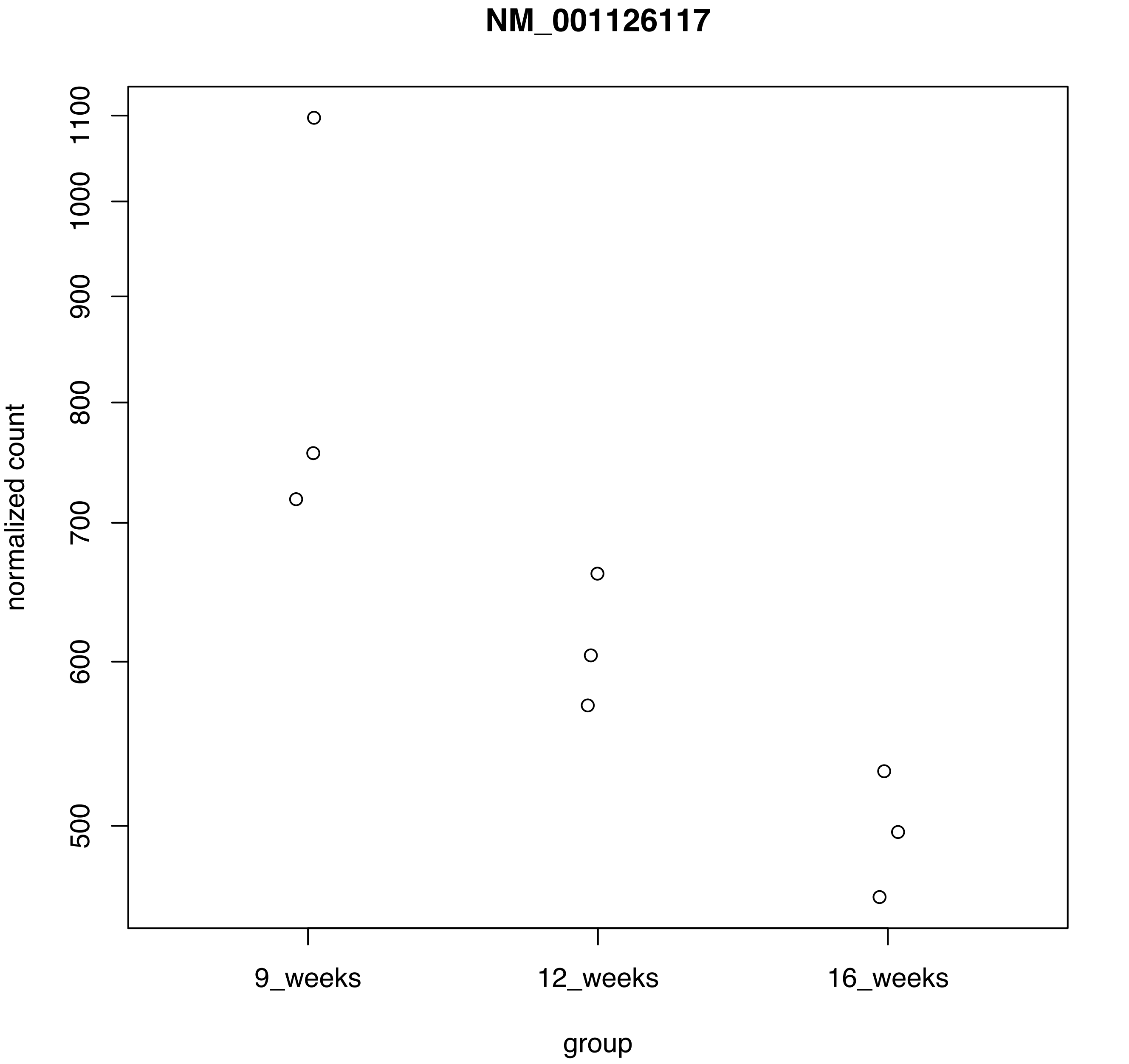
NM_001126117
normalized count
1100
1000
900
800
700
600
500
9_weeks
12_weeks
16_weeks
group

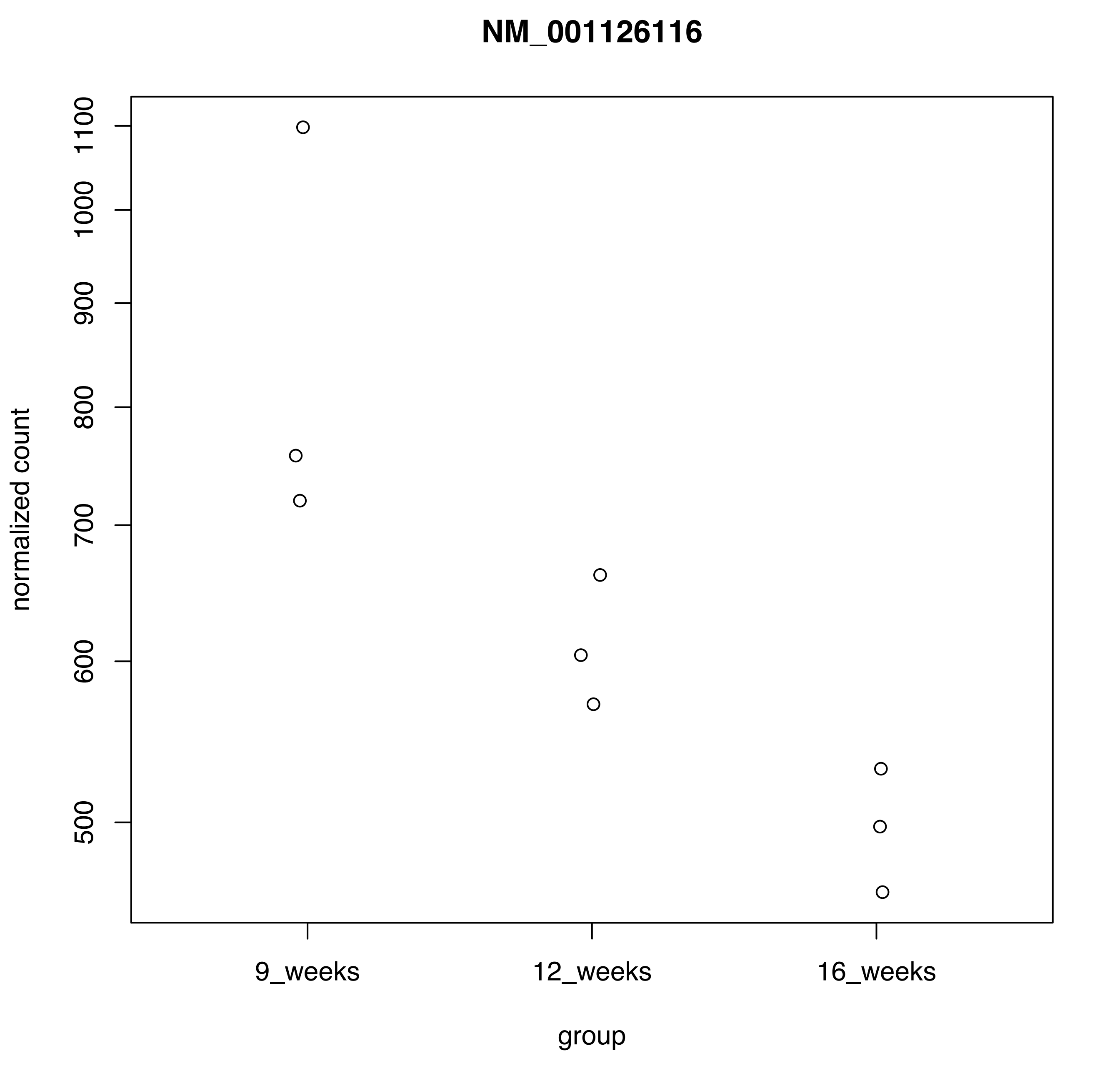

NM_001126116
normalized count
1100
1000
900
800
700
600
500
9_weeks
12_weeks
16_weeks
group

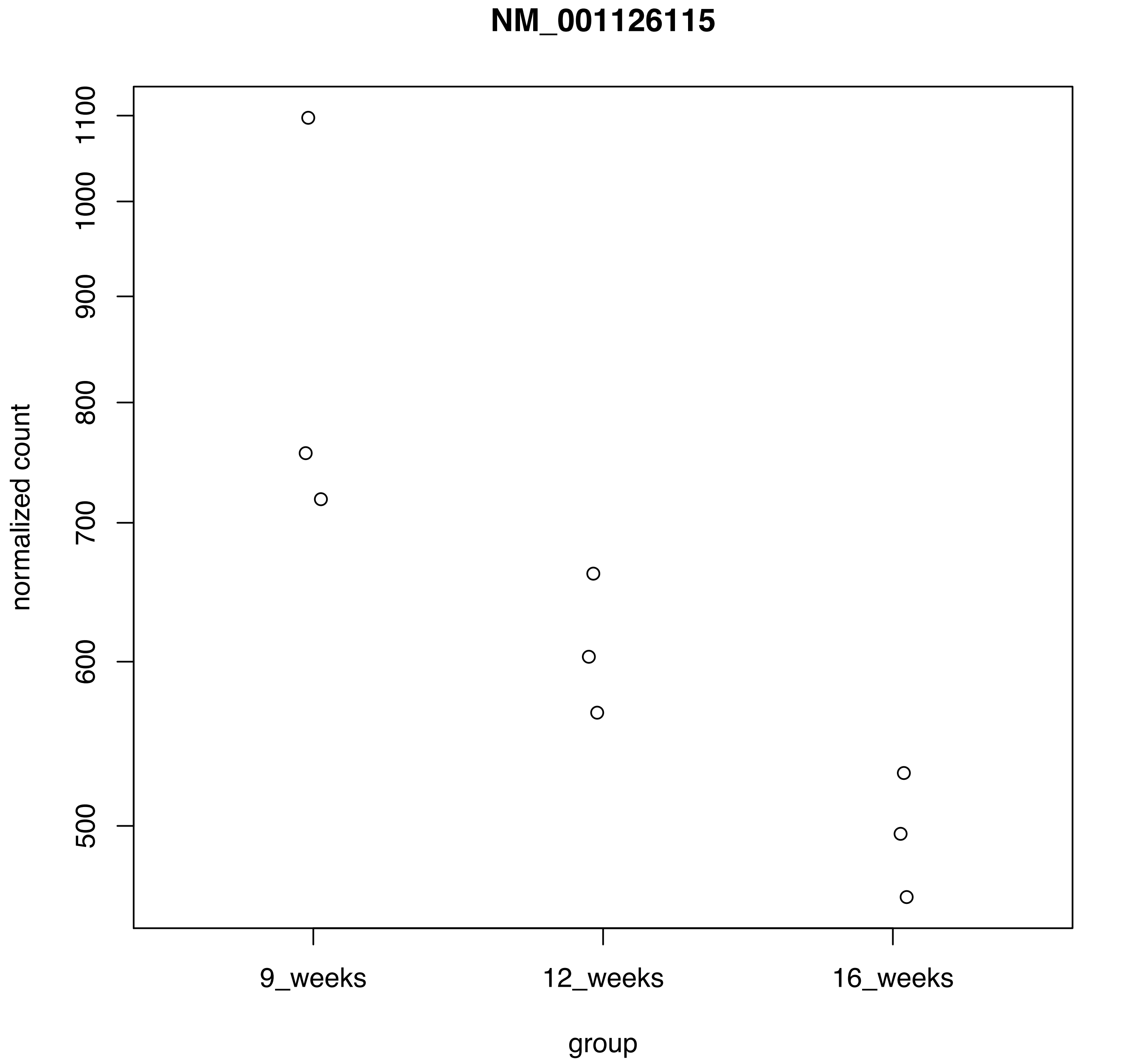
NM_001126115
1100
1000
900
800
700
600
500
normalized count
9_weeks
12_weeks
16_weeks
group

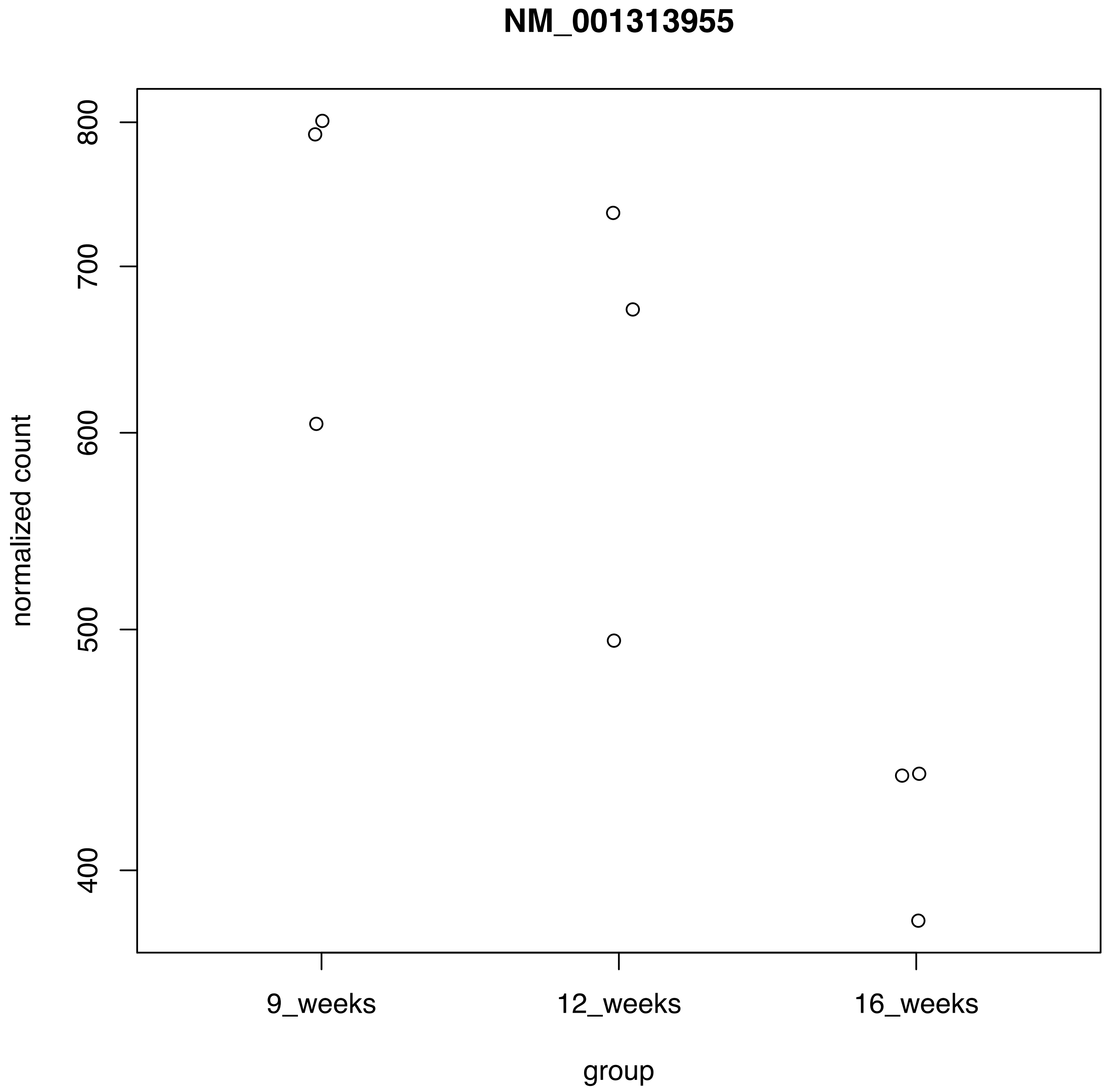
NM_001313955
normalized count
800
700
600
500
400
9_weeks
12_weeks
16_weeks
group

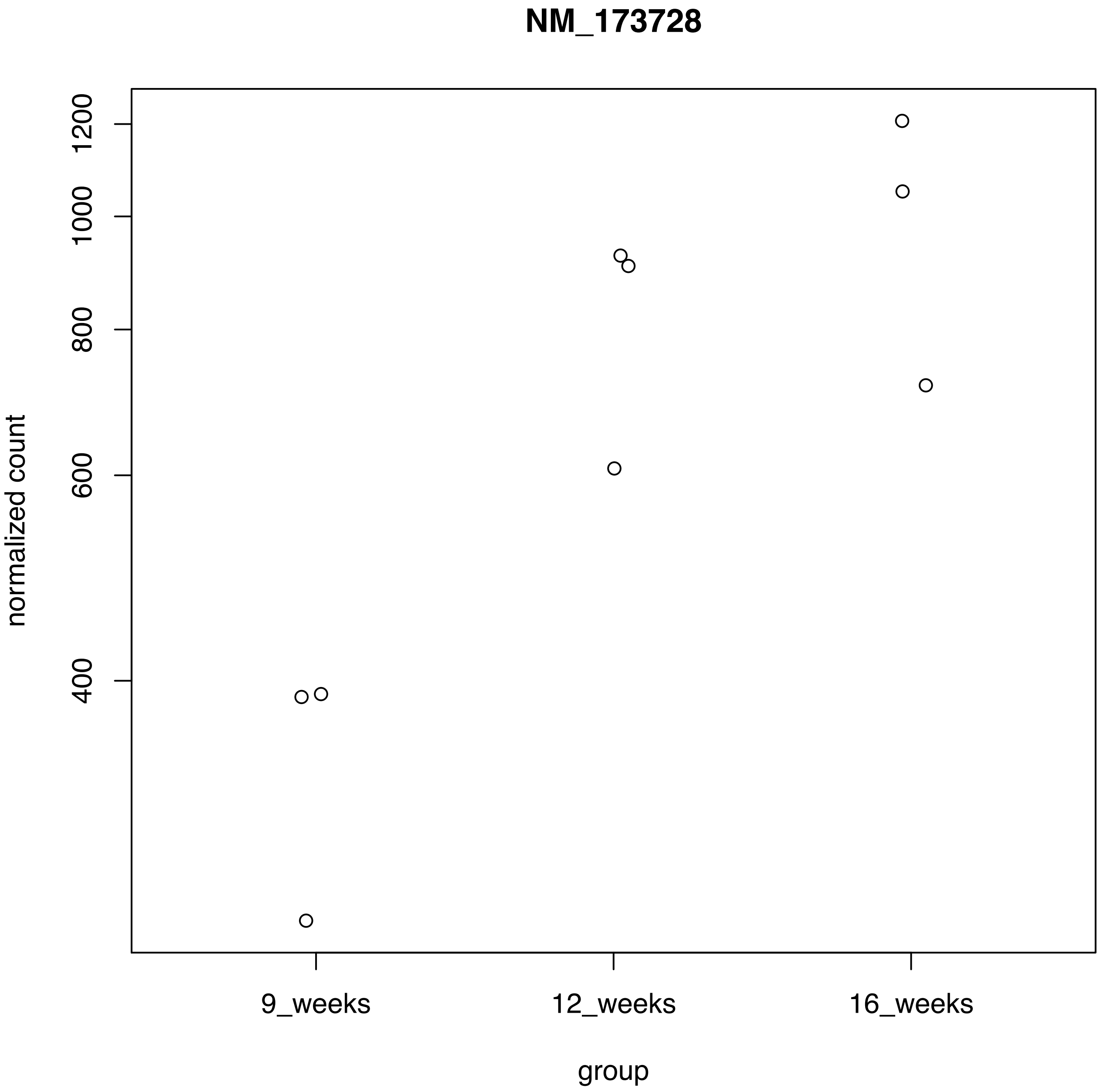
NM_173728
normalized count
1200
1000
800
600
400
9_weeks
12_weeks
16_weeks
group

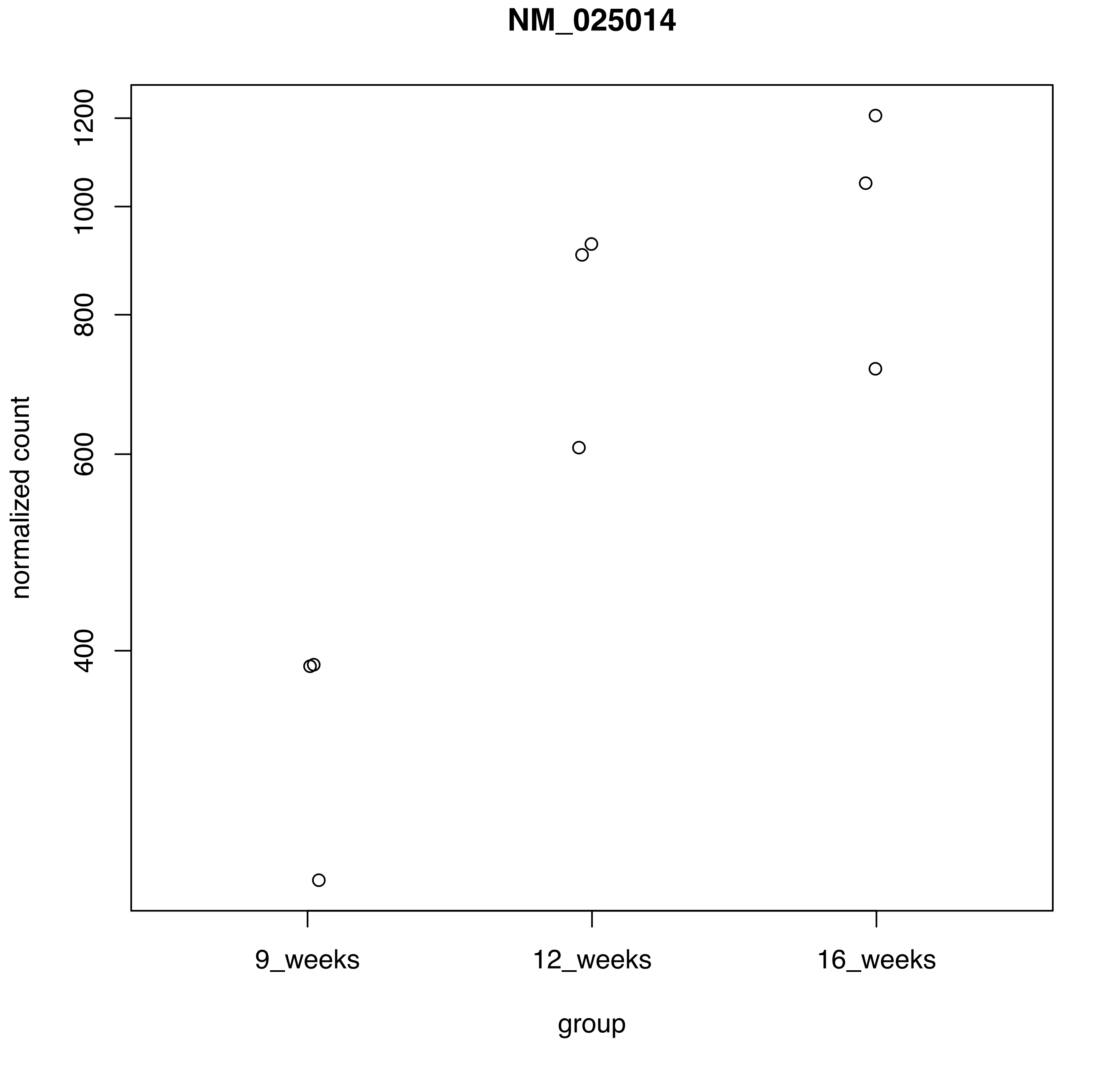
NM_025014
normalized count
1200
1000
800
600
400
9_weeks
12_weeks
16_weeks
group

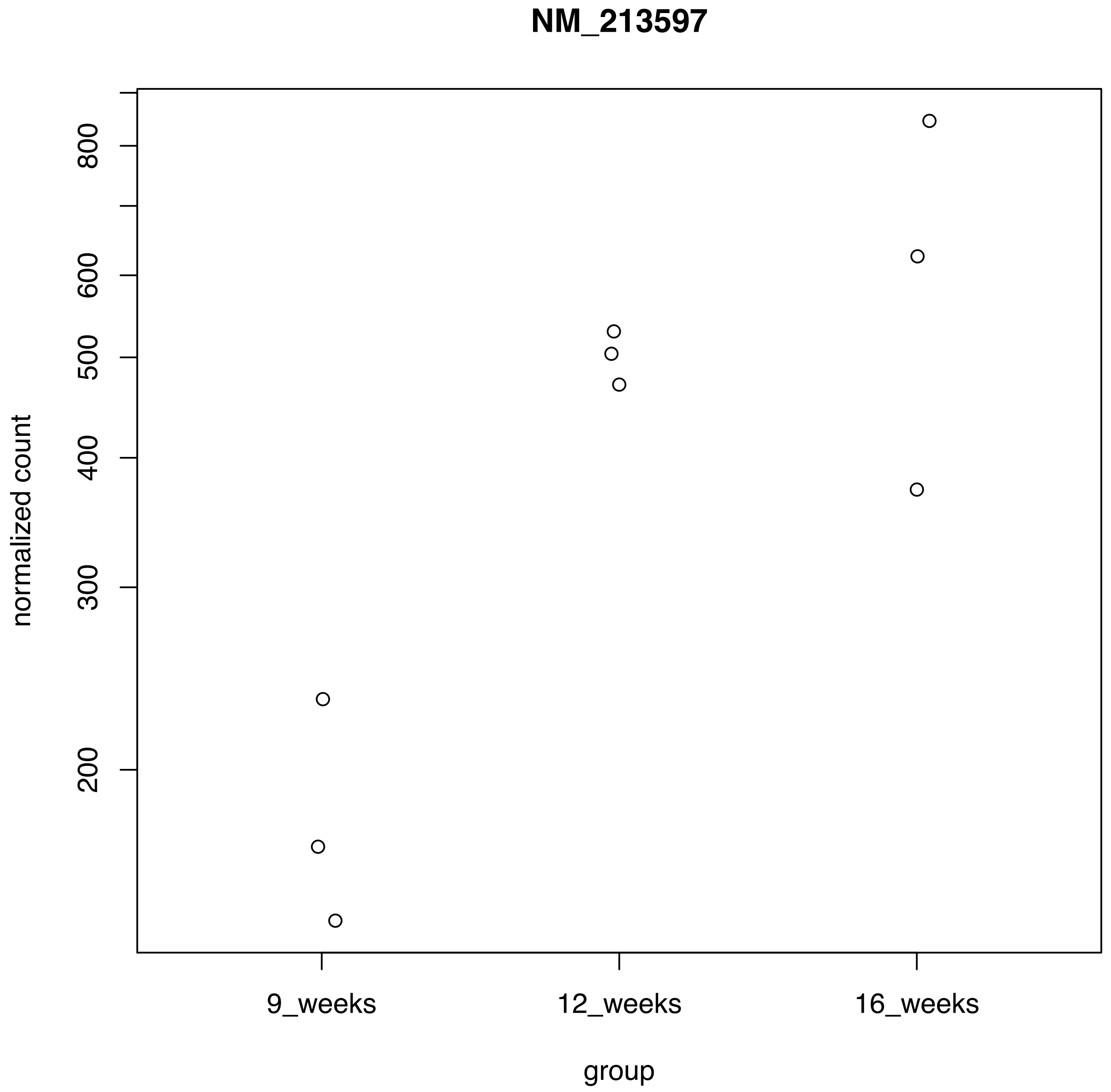
NM_213597
normalized count
800
600
500
400
300
200
group
9_weeks
12_weeks
16_weeks

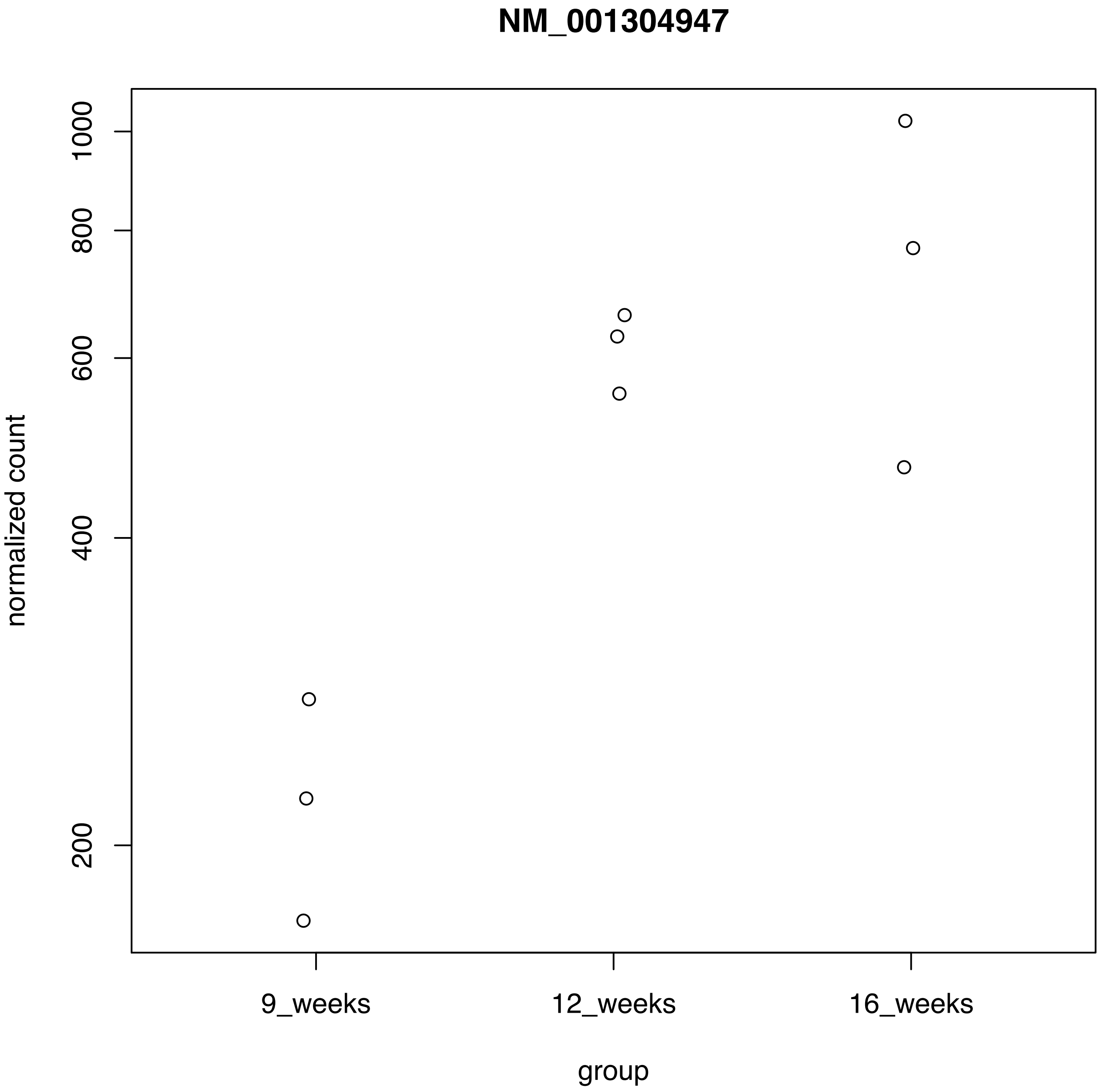
NM_001304947
normalized count
1000
800
600
400
200
9_weeks
12_weeks
16_weeks
group

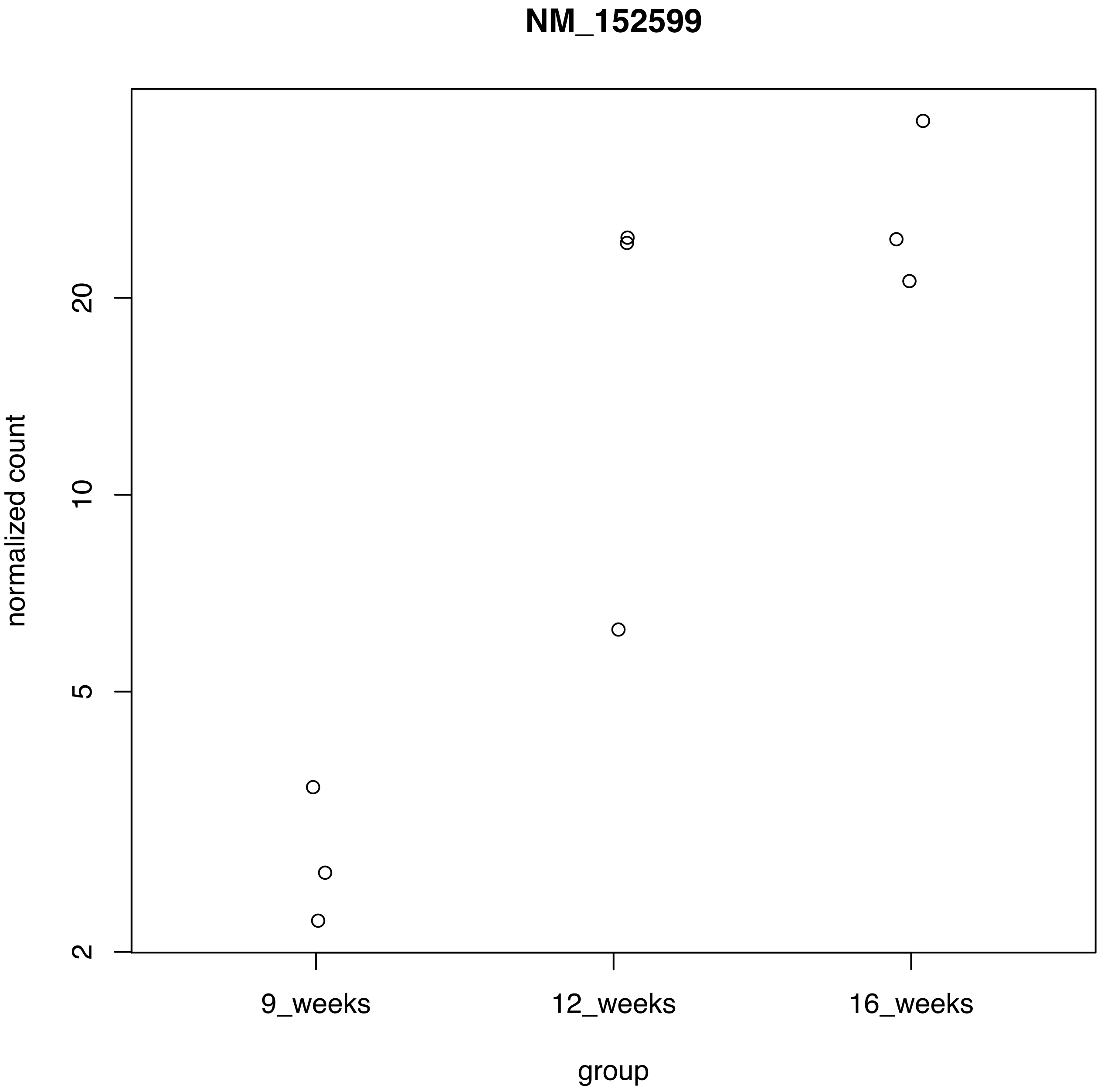

NM_152599
normalized count
20
10
5
2
9_weeks
12_weeks
16_weeks
group

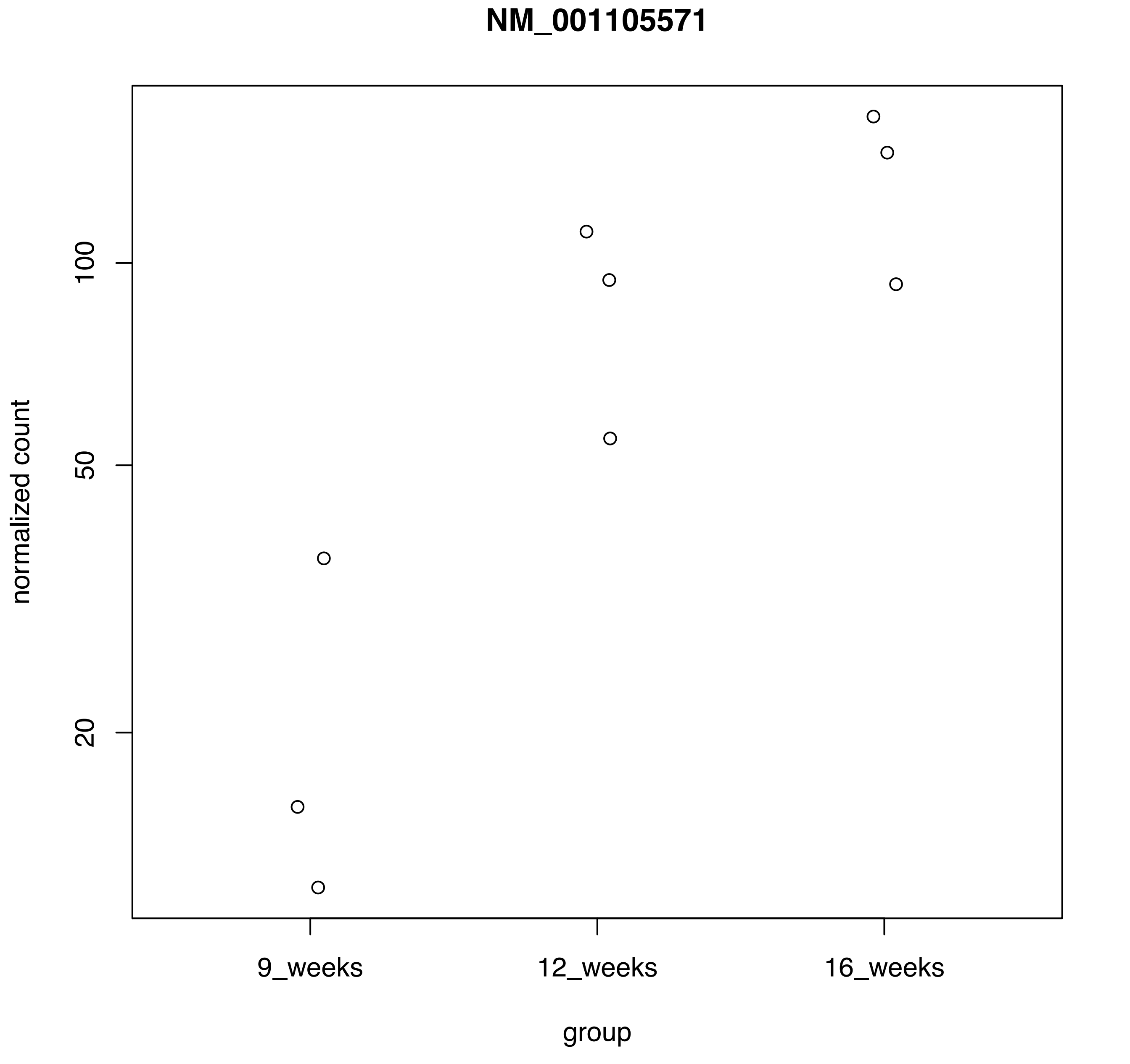
NM_001105571
normalized count
100
50
20
9_weeks
12_weeks
16_weeks
group

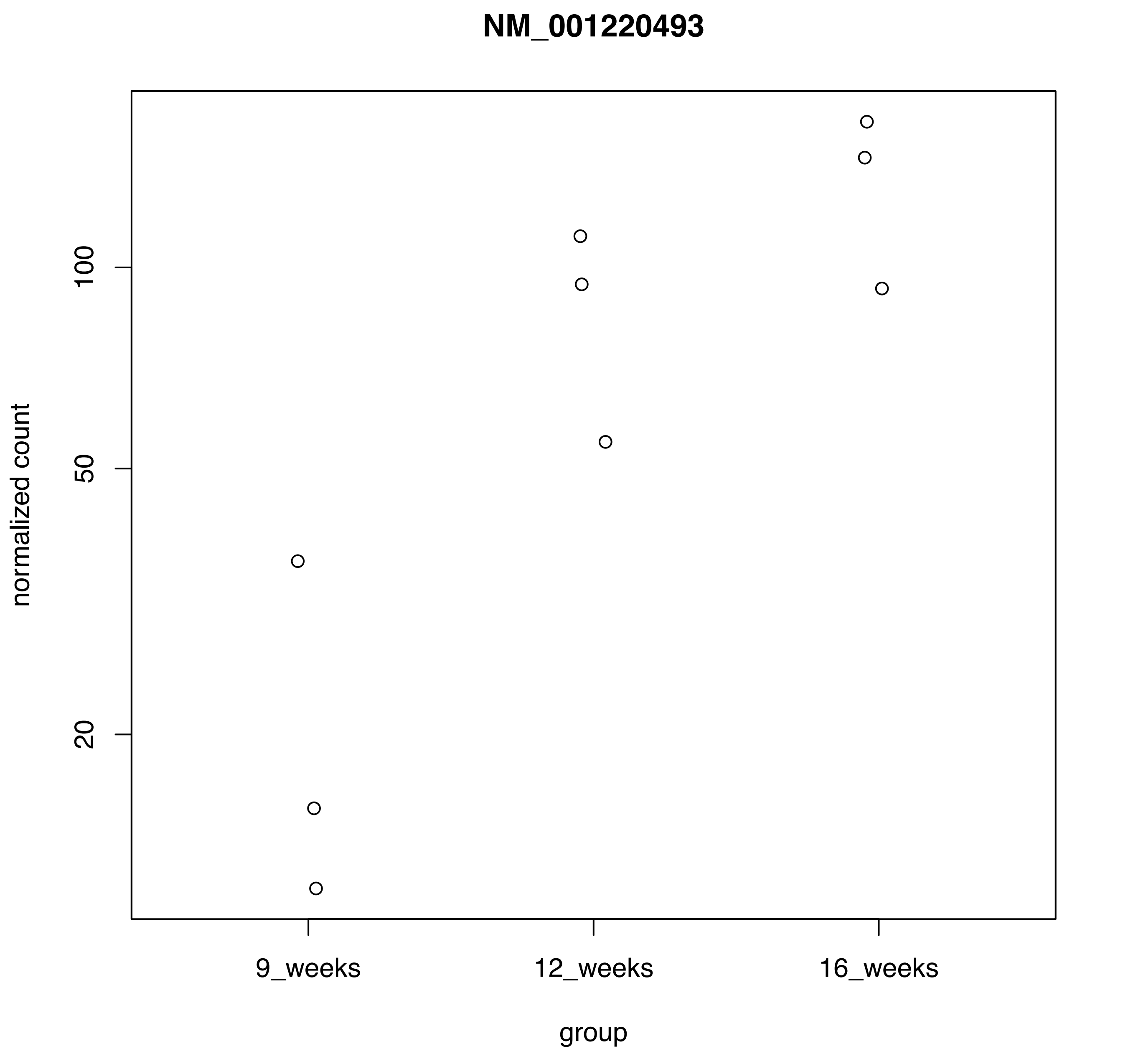

NM_001220493
normalized count
100
50
20
9_weeks
12_weeks
16_weeks
group

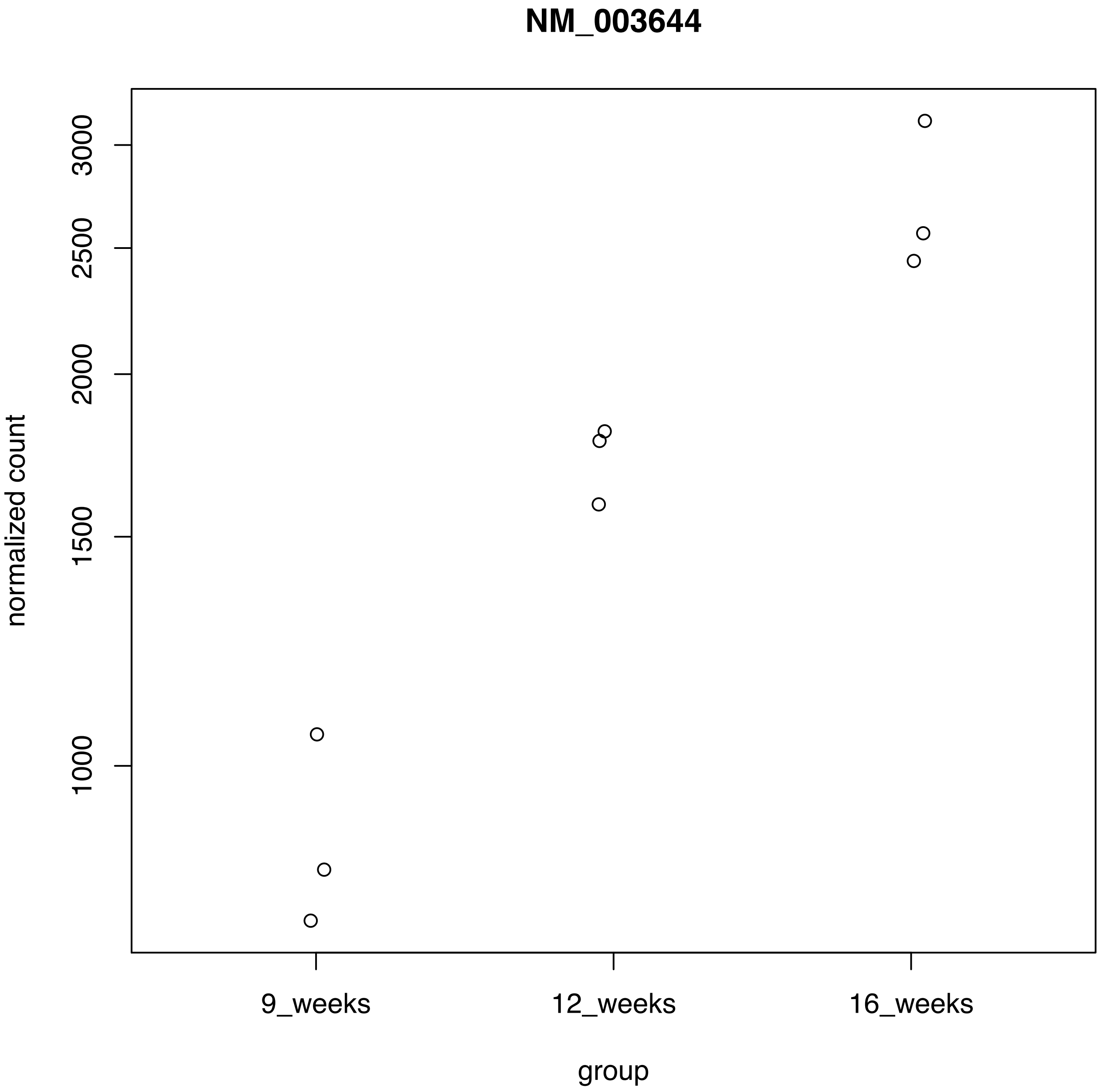
NM_003644
normalized count
3000
2500
2000
1500
1000
9_weeks
12_weeks
16_weeks
group

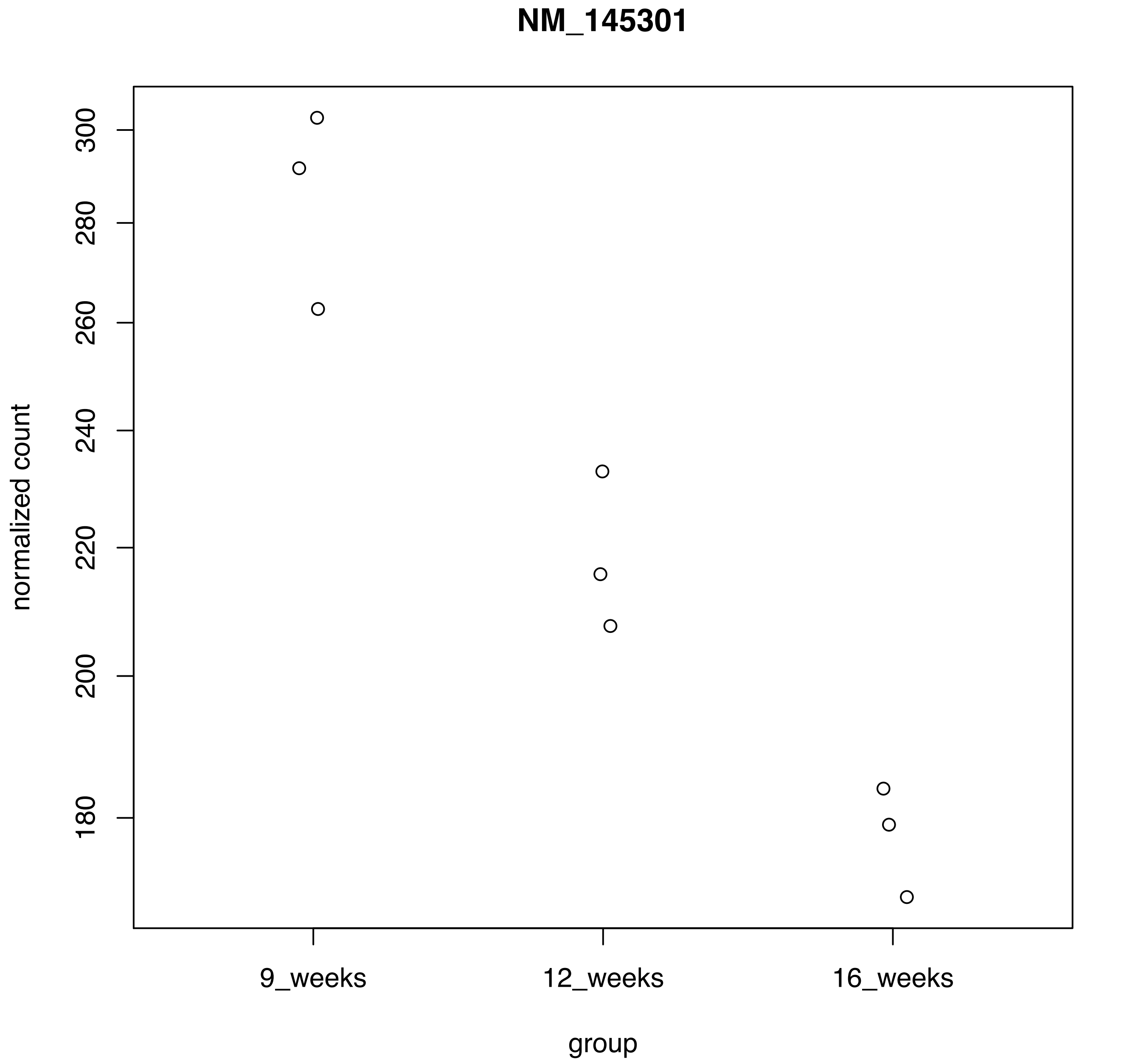

NM_145301
normalized count
300
280
260
240
220
200
180
9_weeks
12_weeks
16_weeks
group

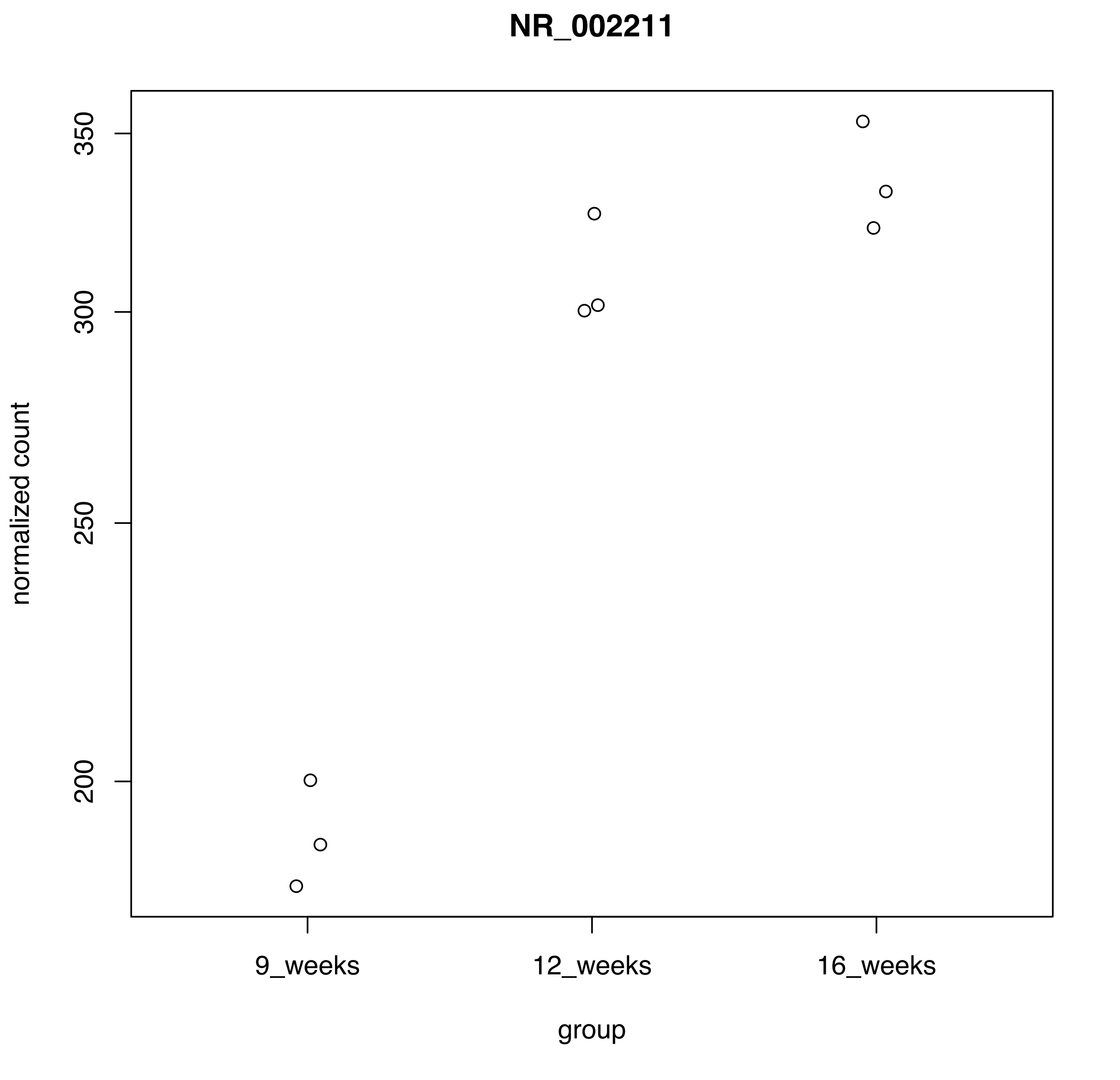
NR_002211
normalized count
350
300
250
200
9_weeks
12_weeks
16_weeks
group

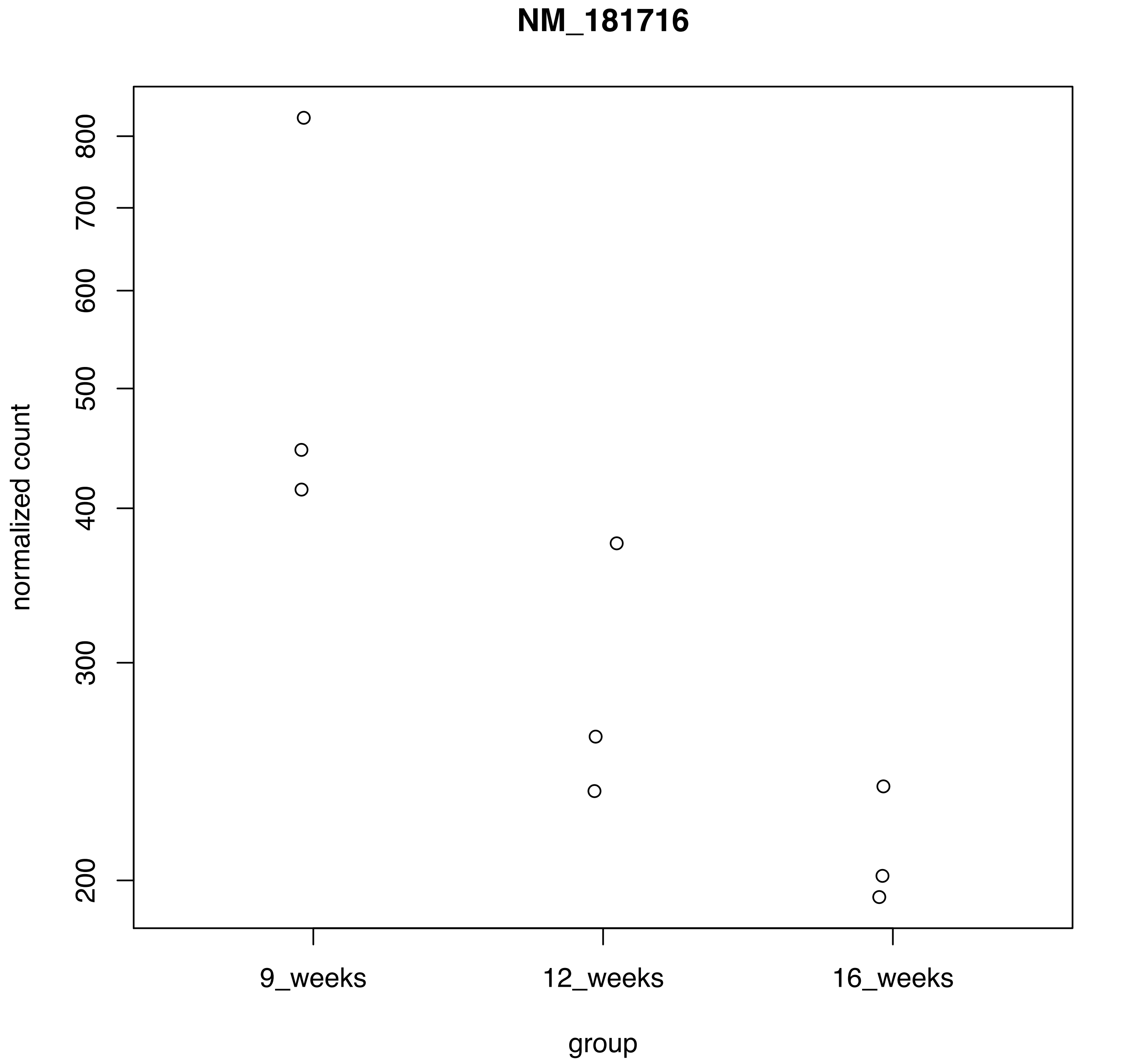

NM_181716
normalized count
800
700
600
500
400
300
200
9_weeks
12_weeks
16_weeks
group

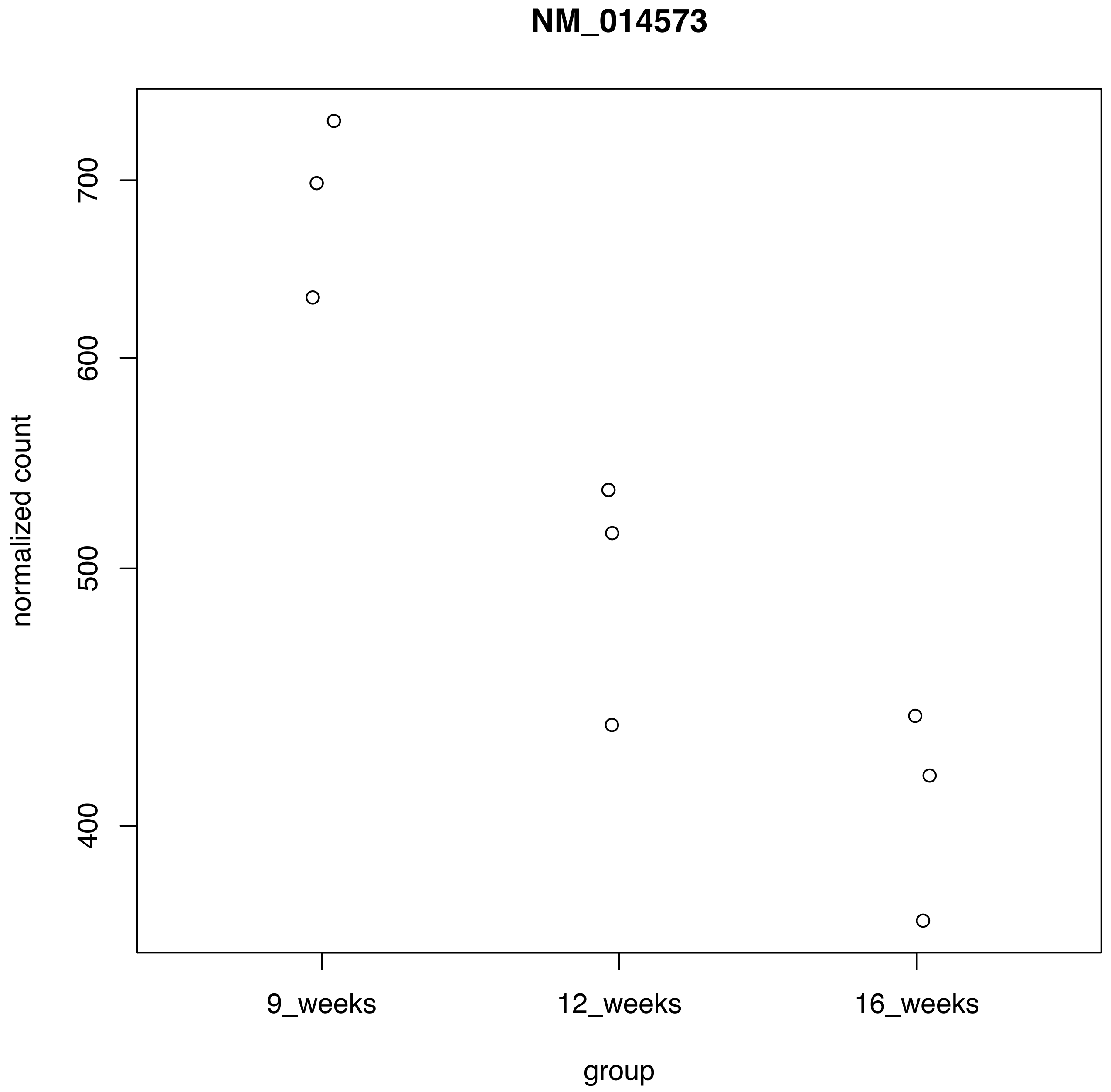
NM_014573
normalized count
700
600
500
400
9_weeks
12_weeks
16_weeks
group

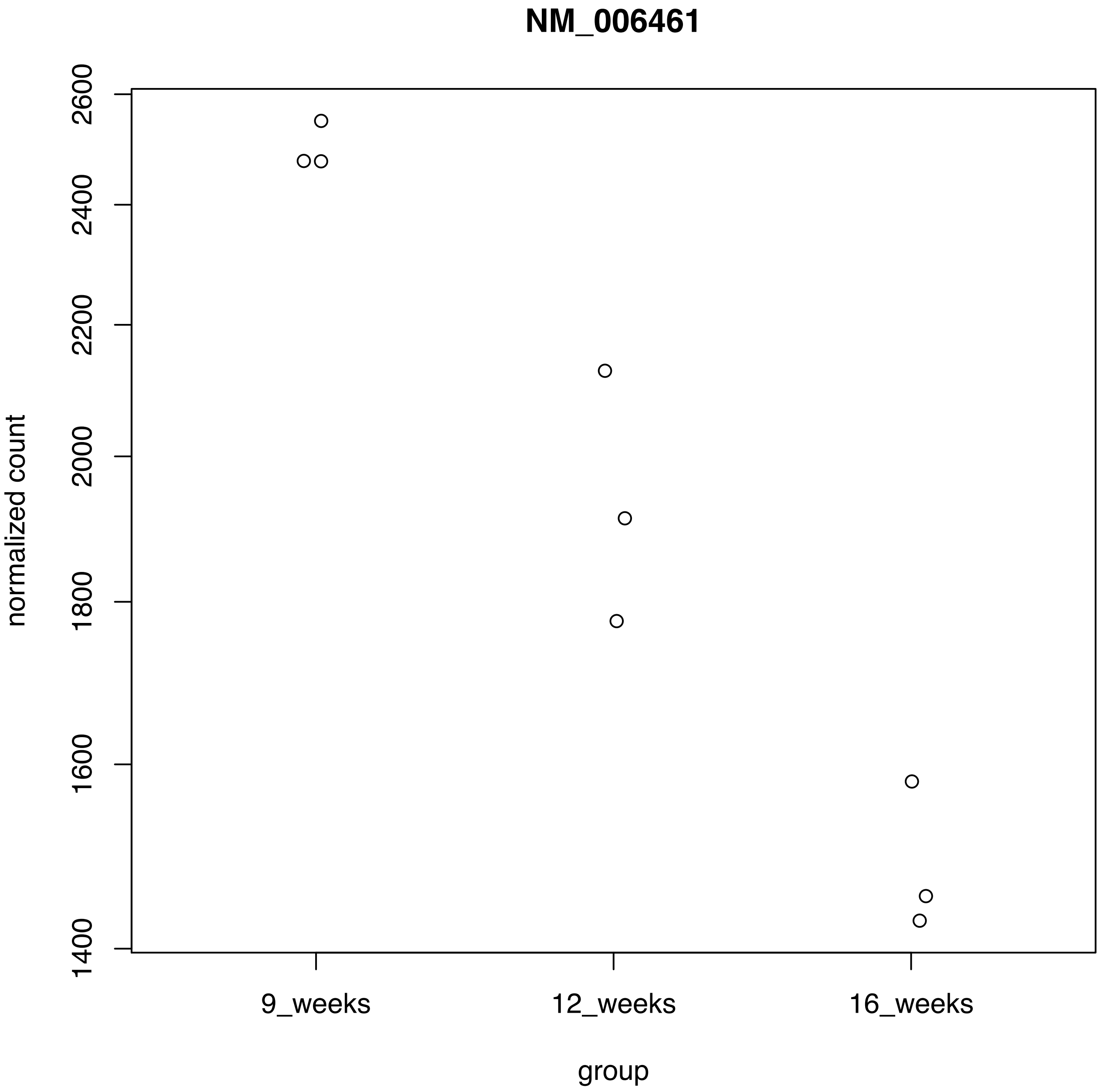
NM_006461
normalized count
2600
2400
2200
2000
1800
1600
1400
9_weeks
12_weeks
16_weeks
group

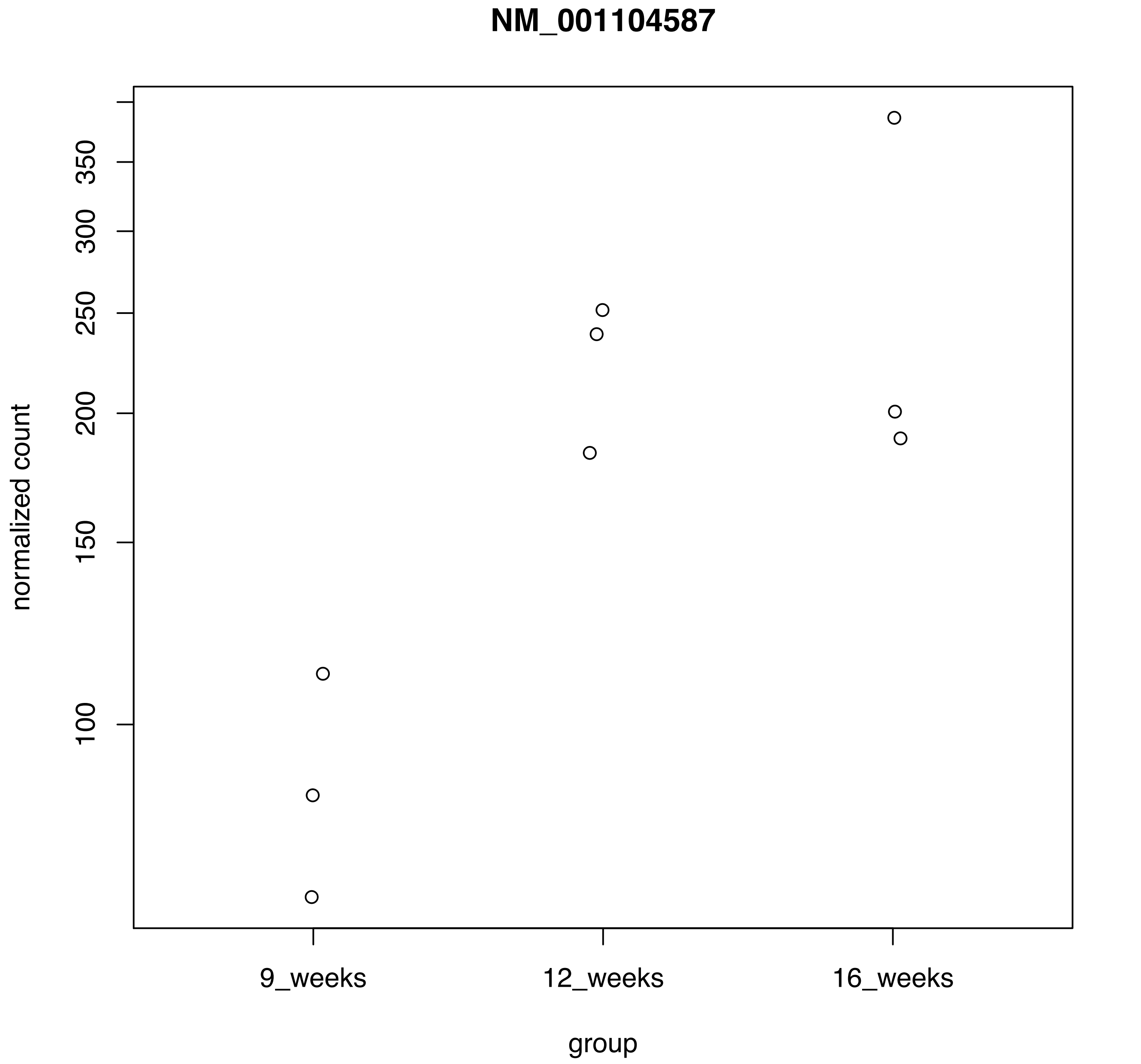
NM_001104587
normalized count
100
150
200
250
300
350
9_weeks
12_weeks
16_weeks
group

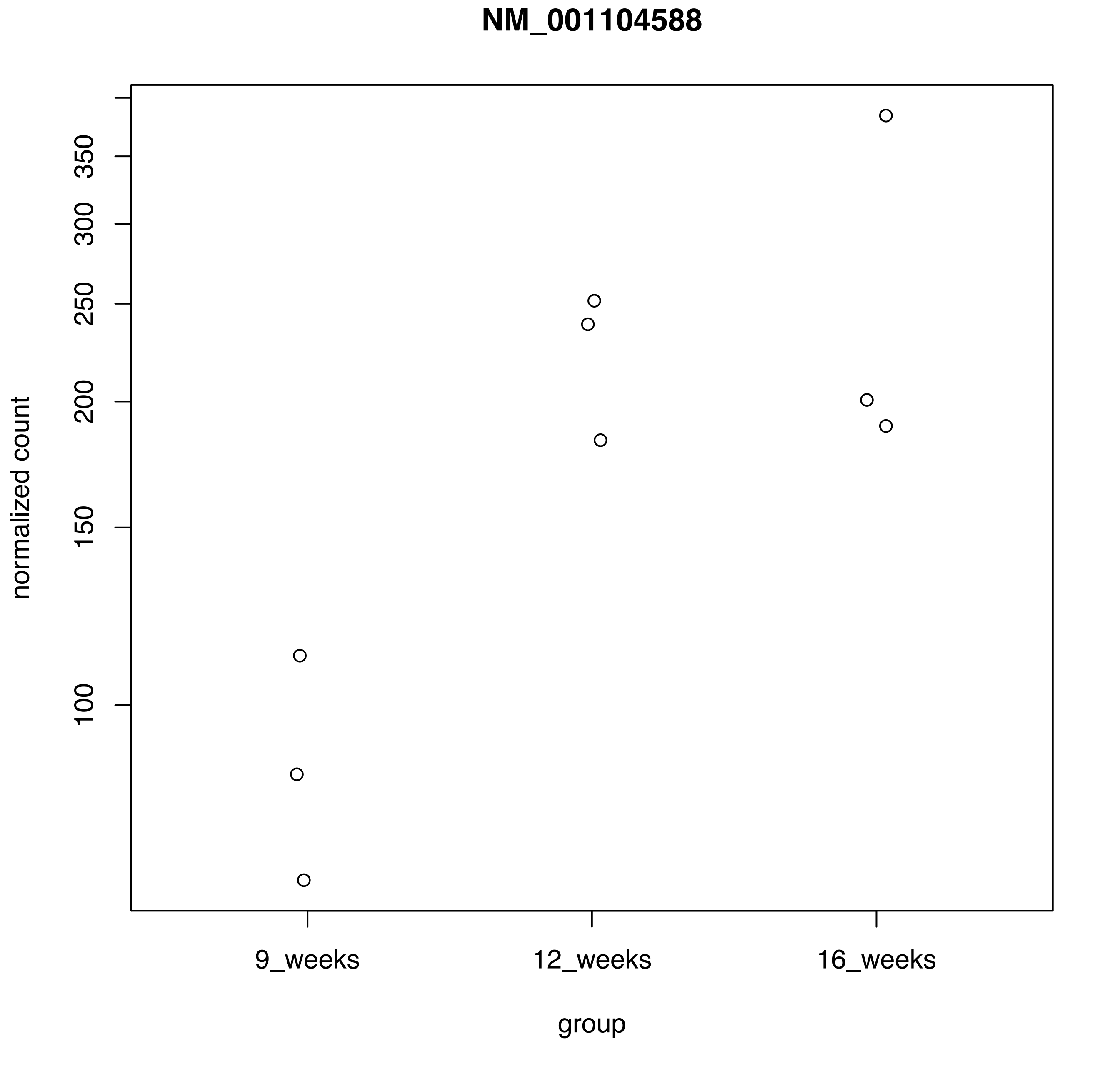
NM_001104588
normalized count
100
150
200
250
300
350
9_weeks
12_weeks
16_weeks
group

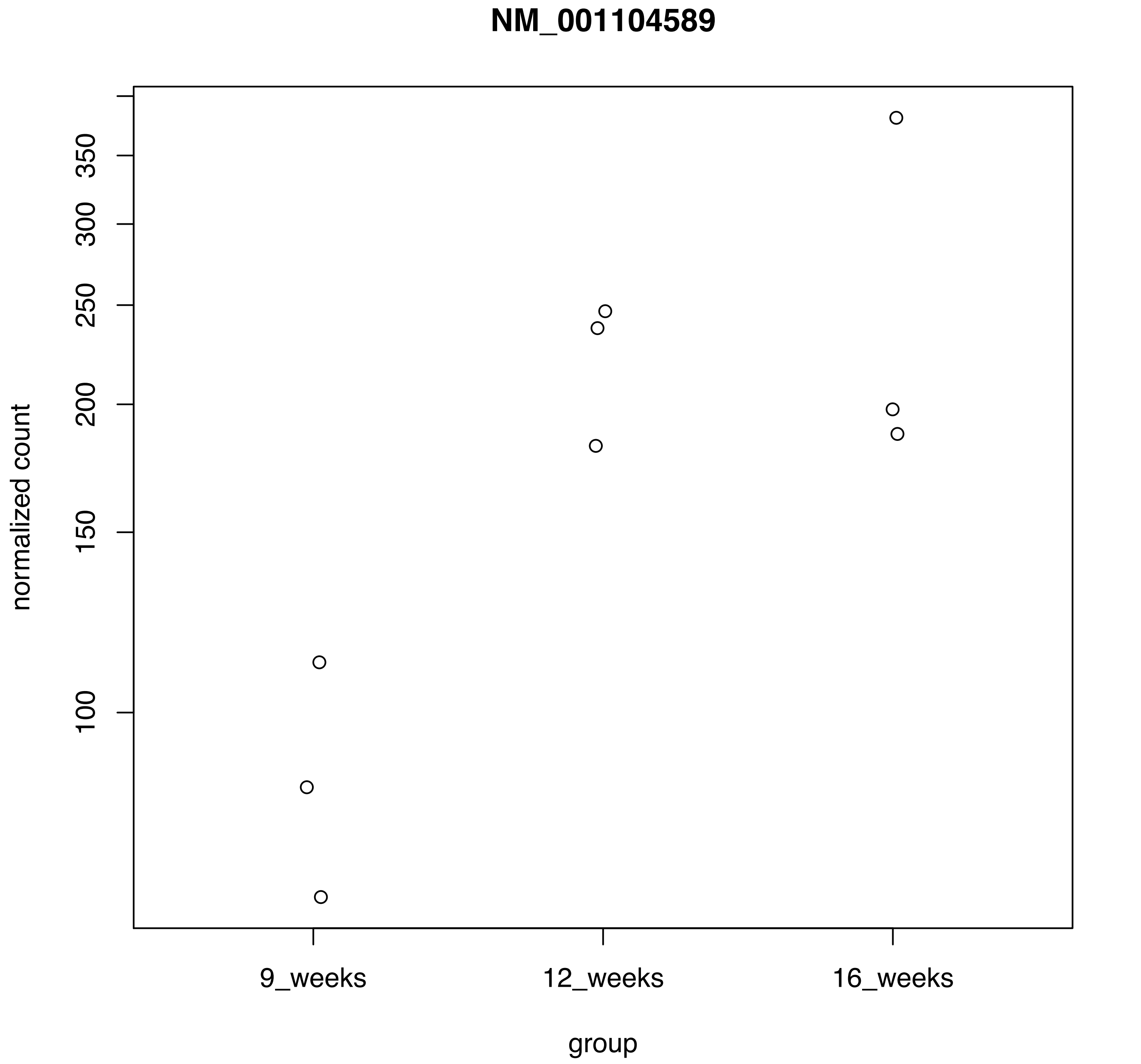

NM_001104589
normalized count
100
150
200
250
300
350
9_weeks
12_weeks
16_weeks
group

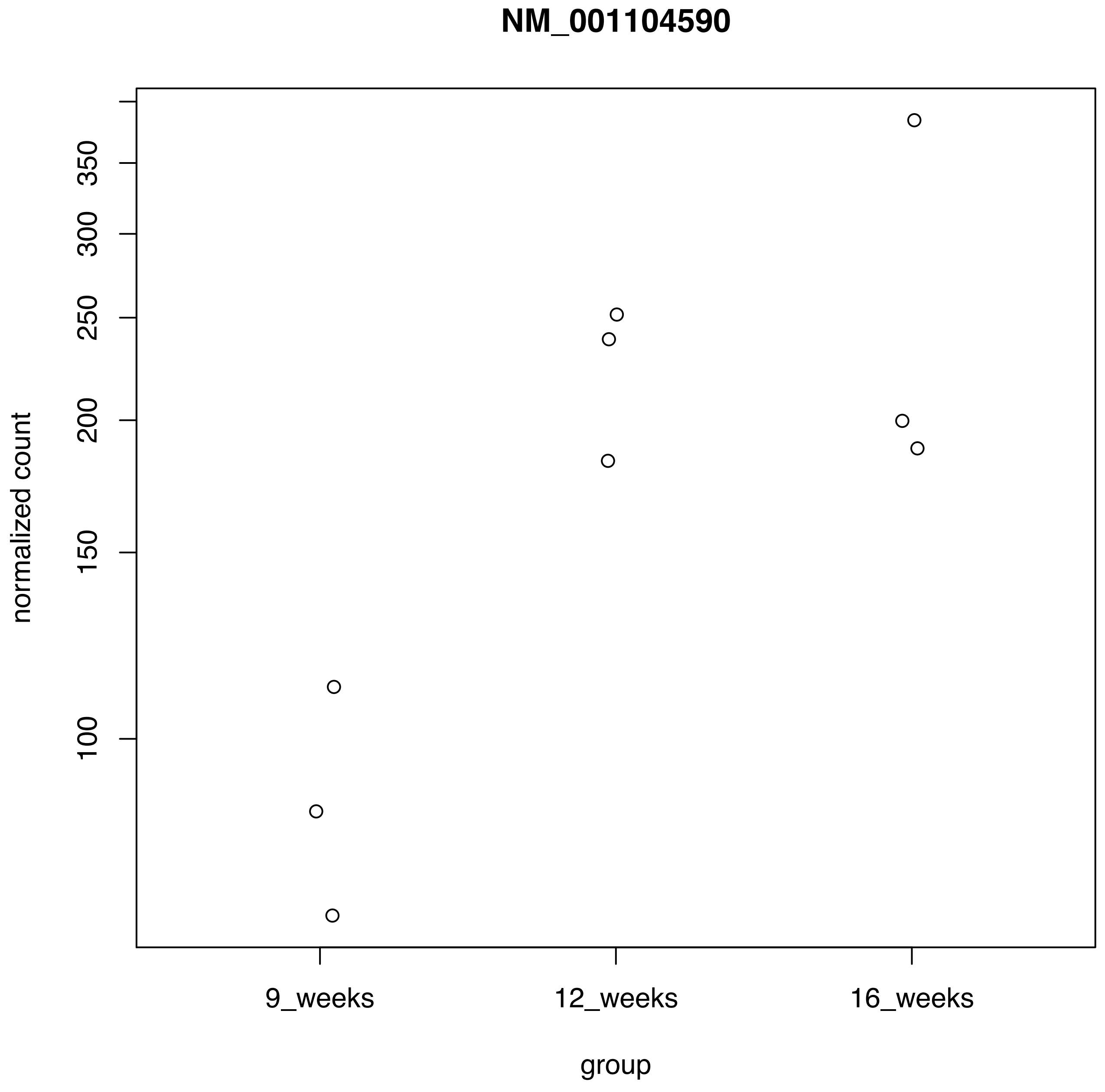

NM_001104590
normalized count
100
150
200
250
300
350
9_weeks
12_weeks
16_weeks
group

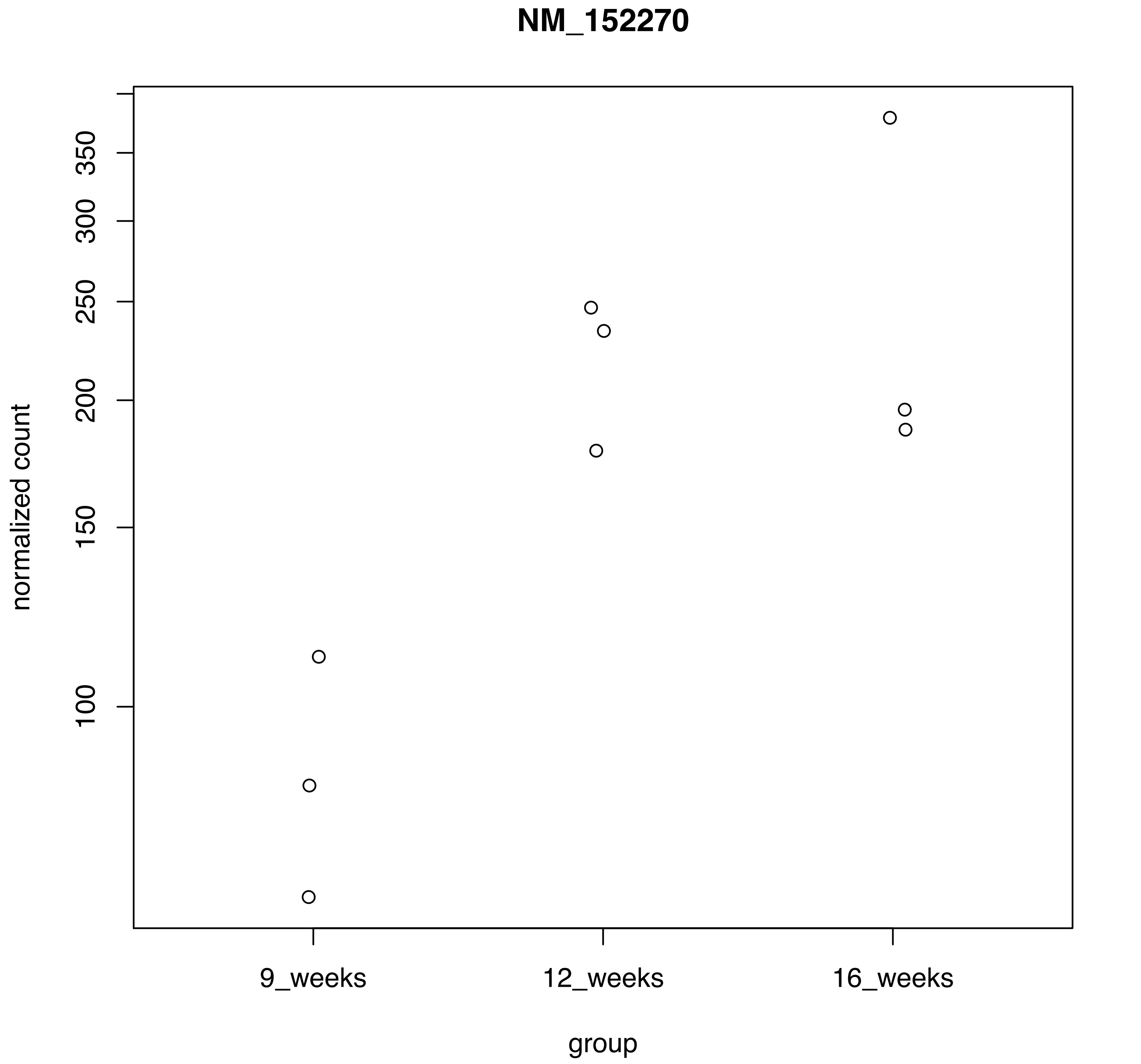
NM_152270
normalized count
100
150
200
250
300
350
9_weeks
12_weeks
16_weeks
group

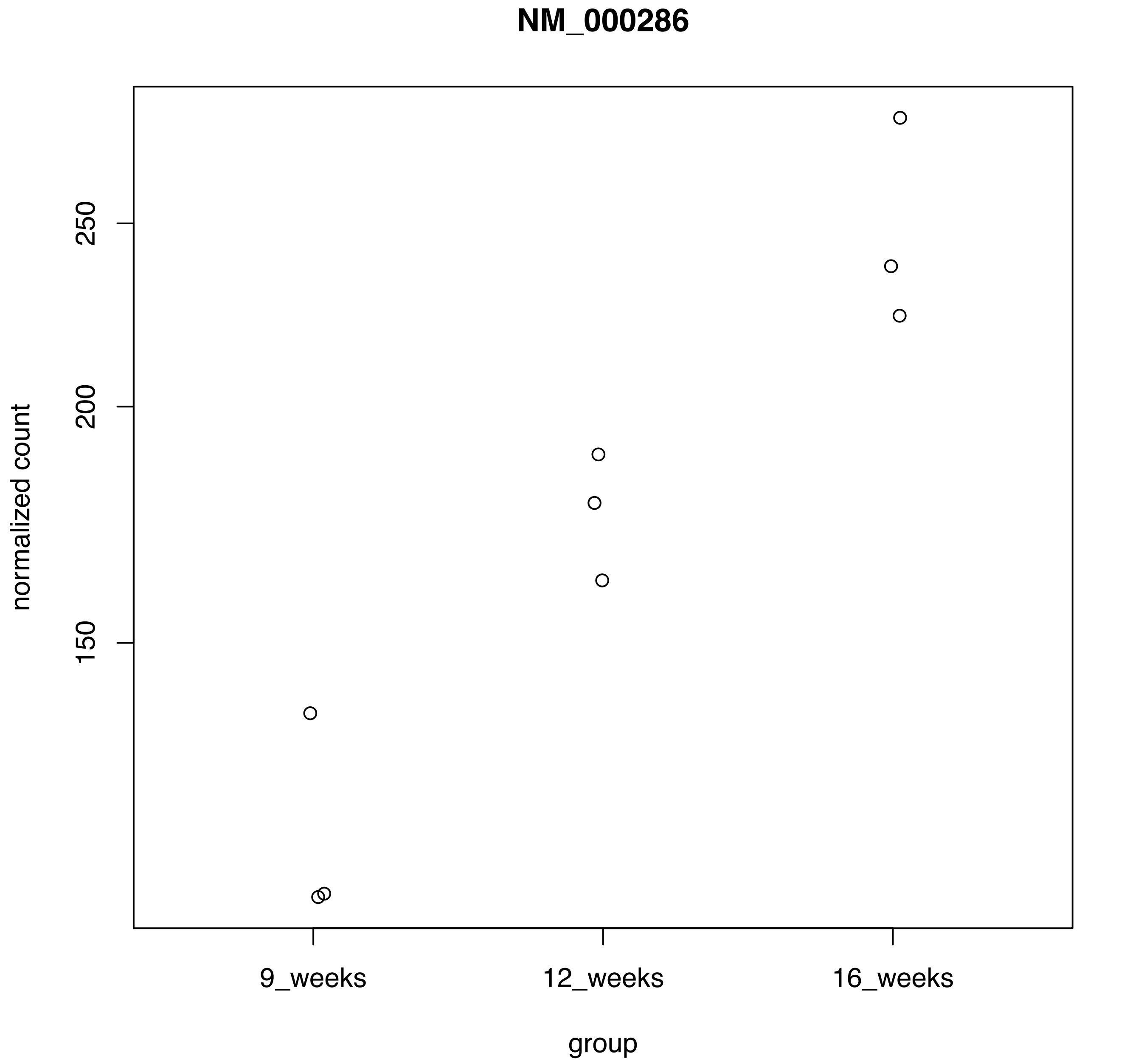
NM_000286
normalized count
250
200
150
9_weeks
12_weeks
16_weeks
group

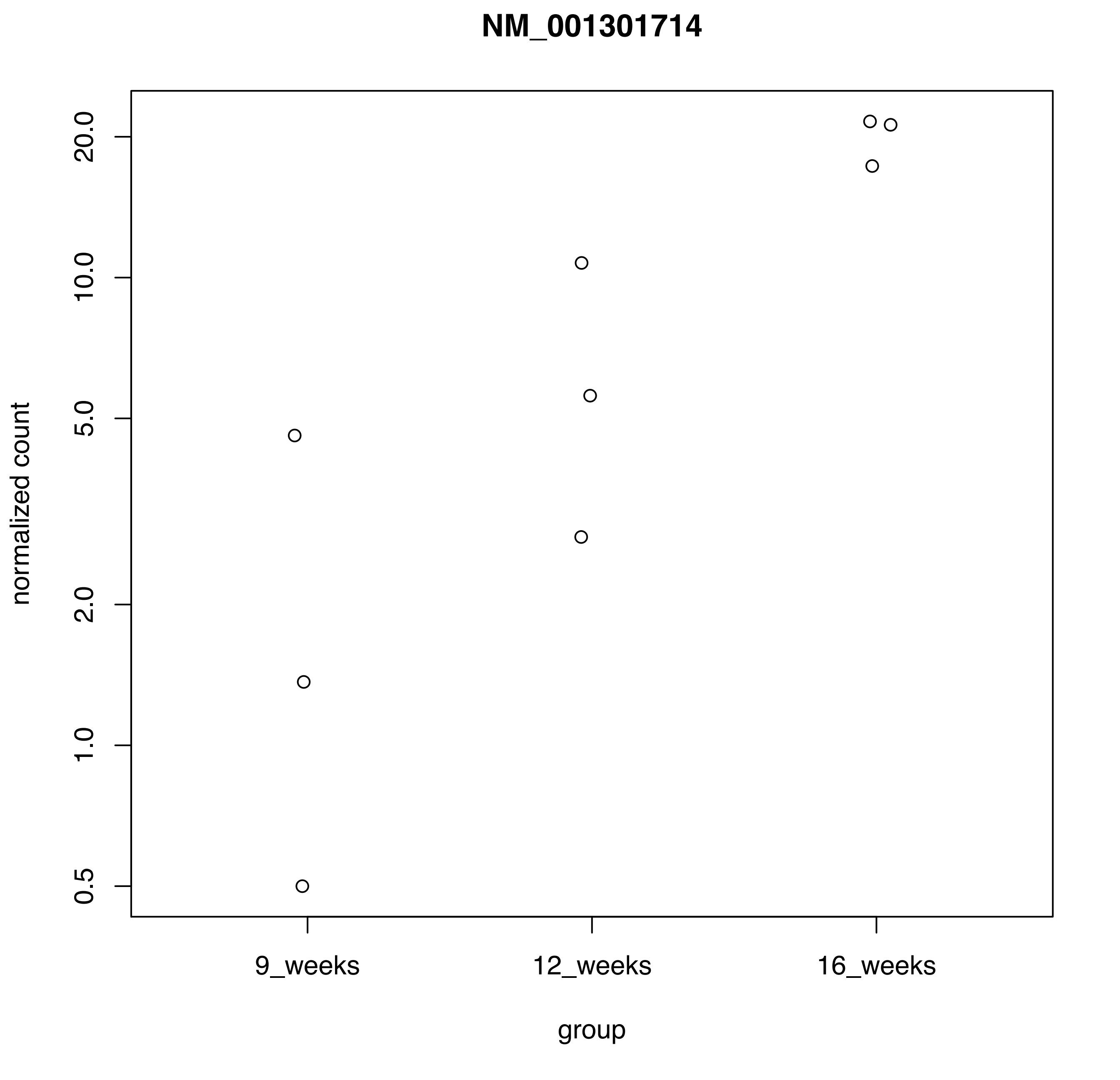
NM_001301714
normalized count
20.0
10.0
5.0
2.0
1.0
0.5
9_weeks
12_weeks
16_weeks
group

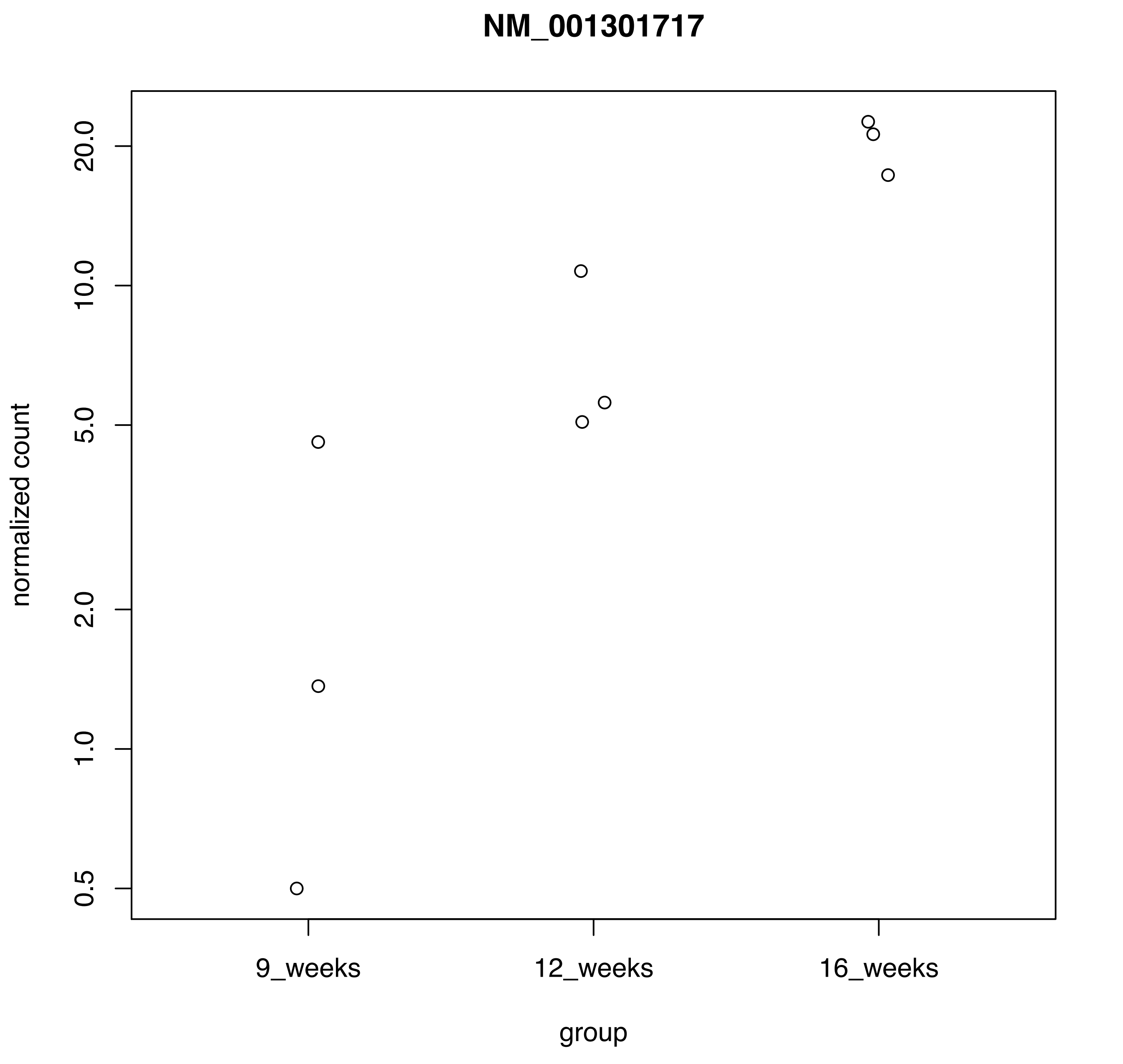

NM_001301717
normalized count
20.0
10.0
5.0
2.0
1.0
0.5
9_weeks
12_weeks
16_weeks
group

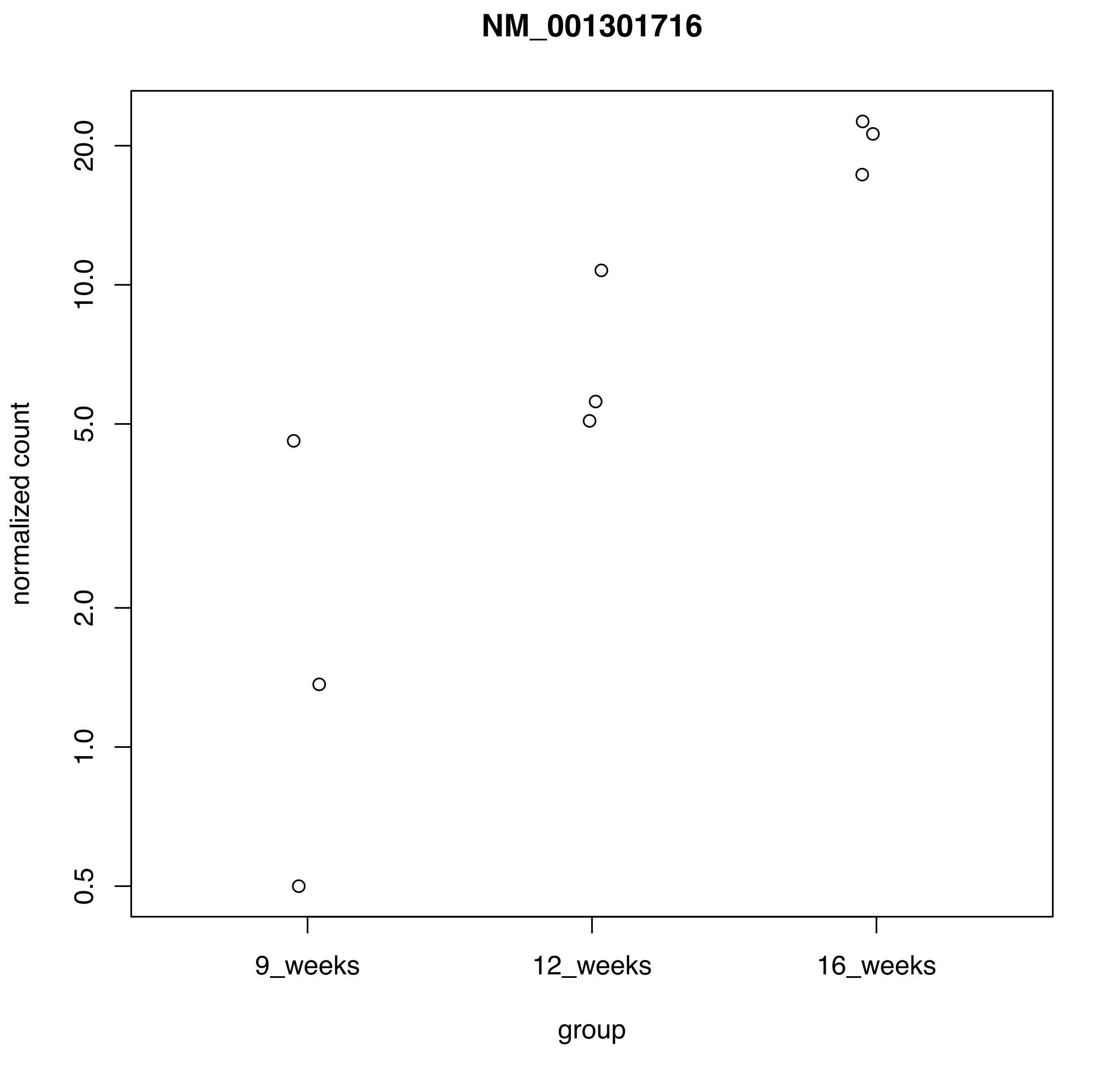

NM_001301716
normalized count
20.0
10.0
5.0
2.0
1.0
0.5
9_weeks
12_weeks
16_weeks
group

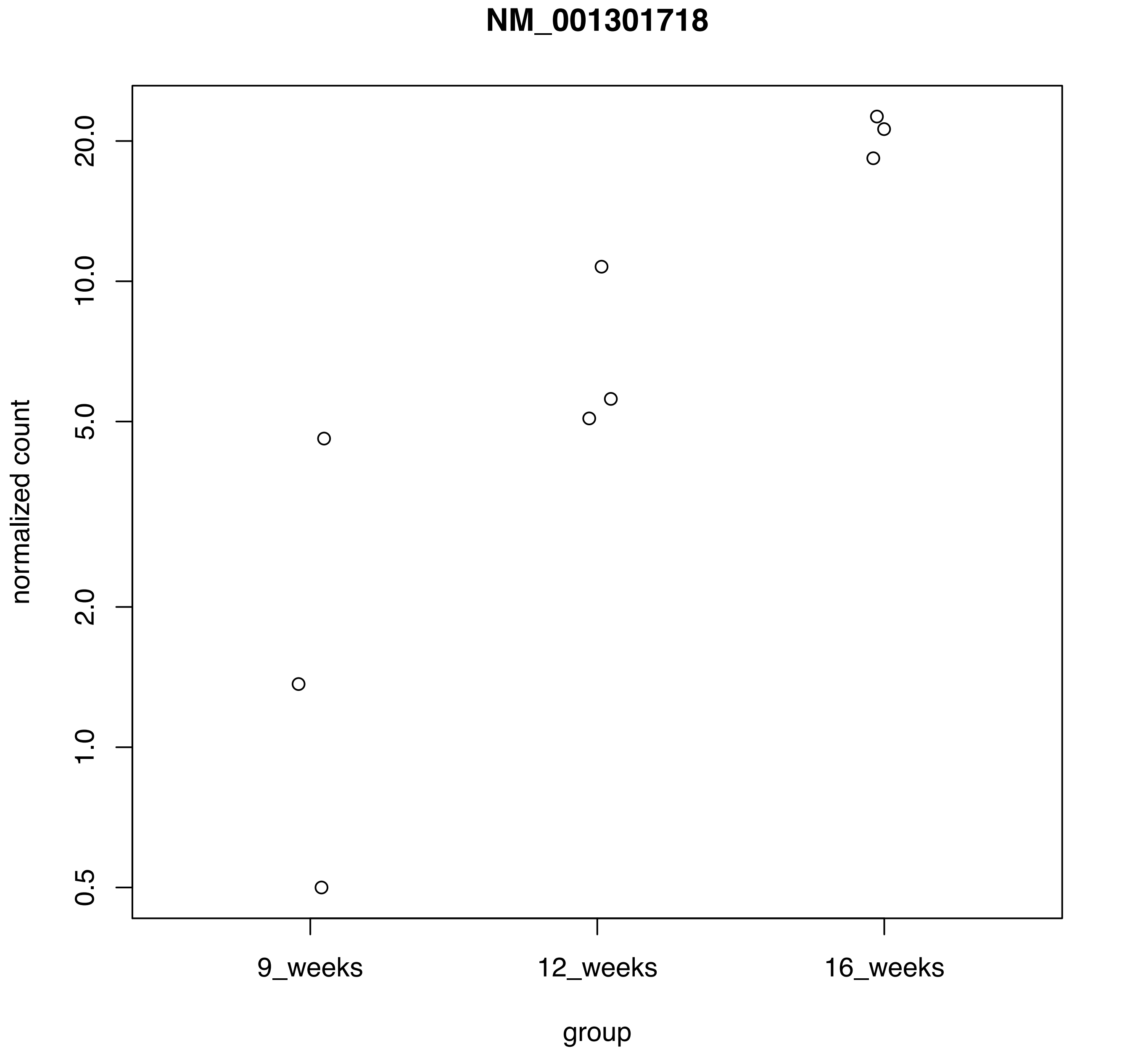

NM_001301718
normalized count
20.0
10.0
5.0
2.0
1.0
0.5
9_weeks
12_weeks
16_weeks
group

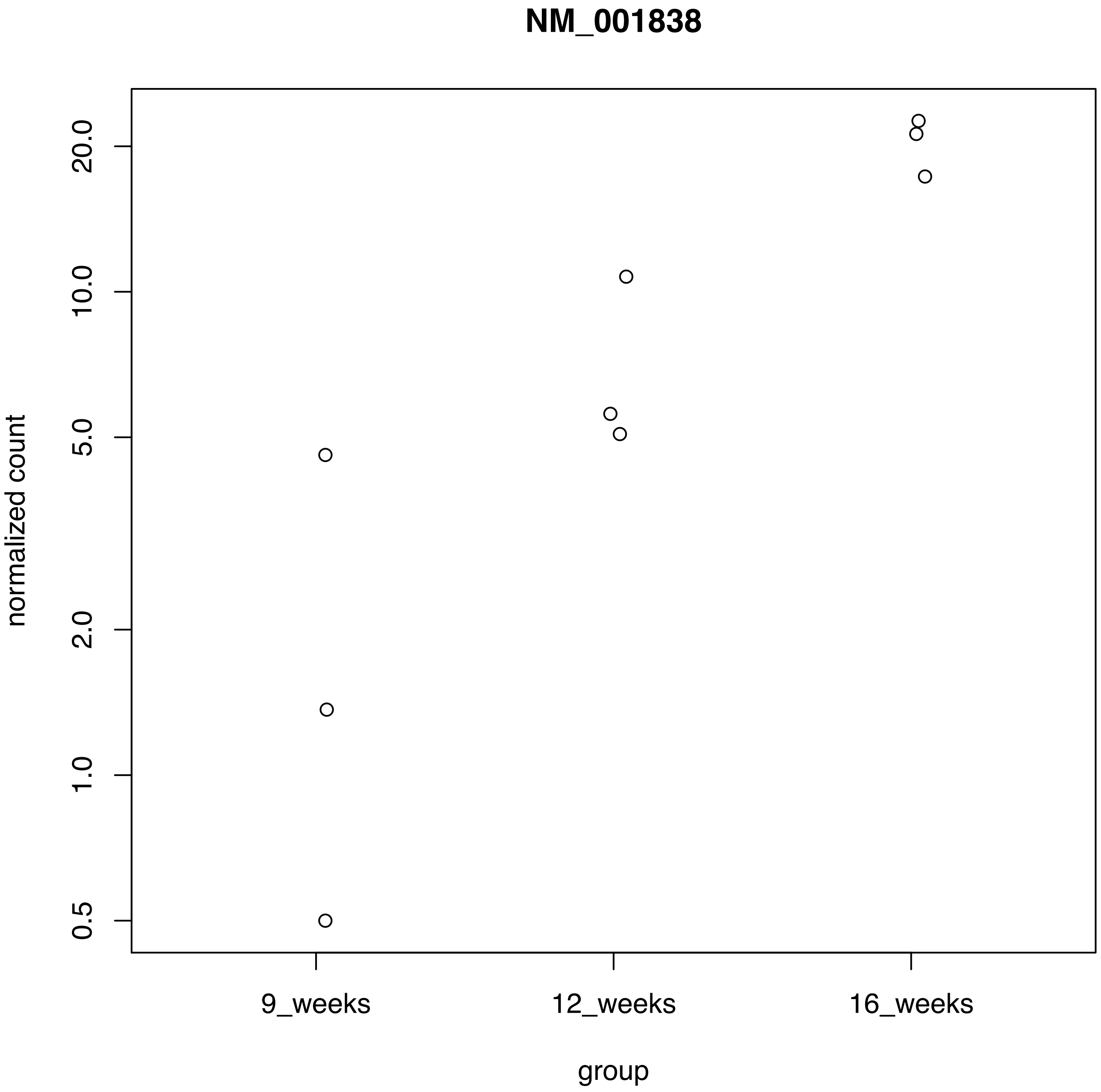
NM_001838
normalized count
20.0
10.0
5.0
2.0
1.0
0.5
9_weeks
12_weeks
16_weeks
group

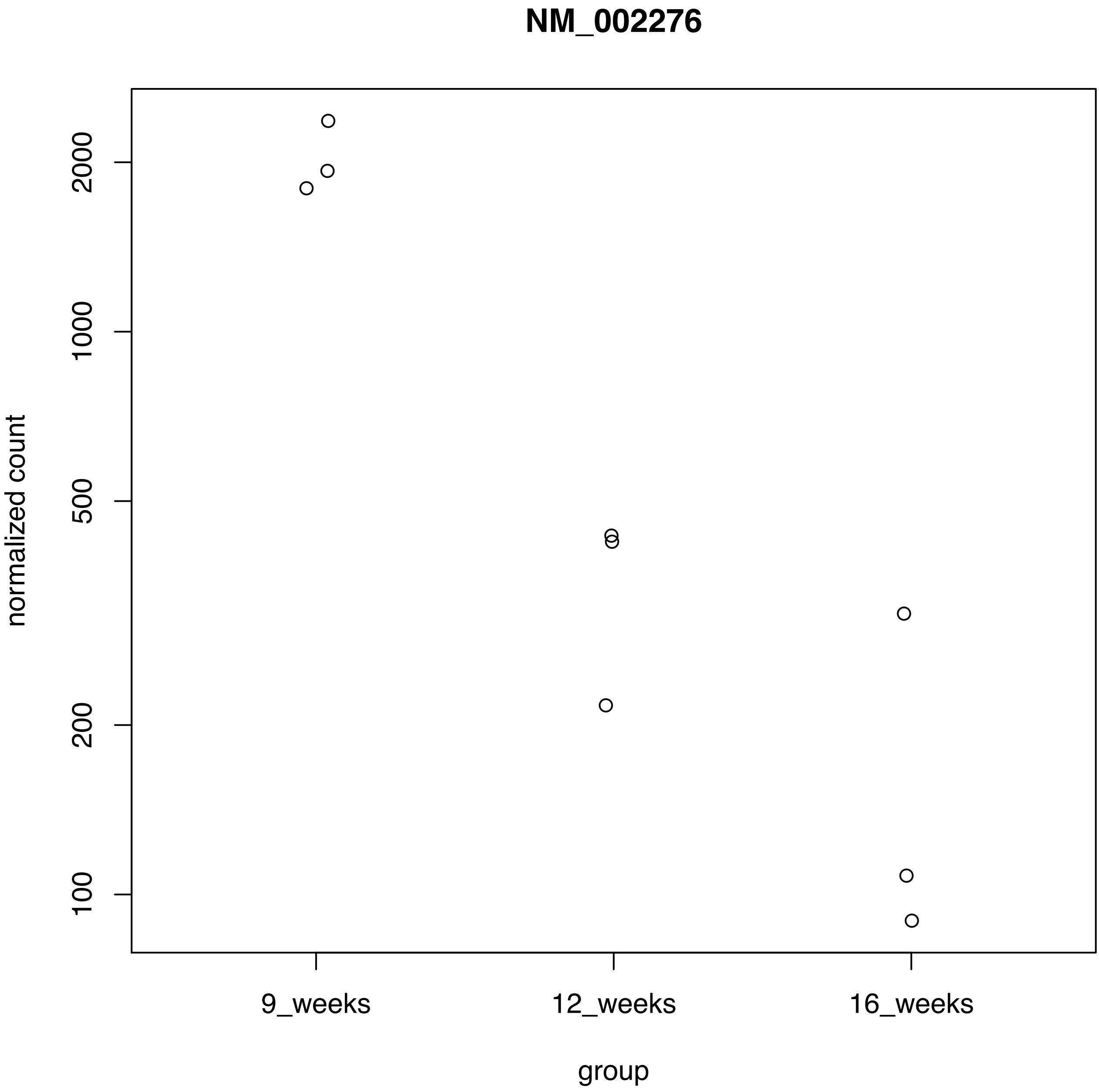

NM_002276
normalized count
2000
1000
500
200
100
9_weeks
12_weeks
16_weeks
group

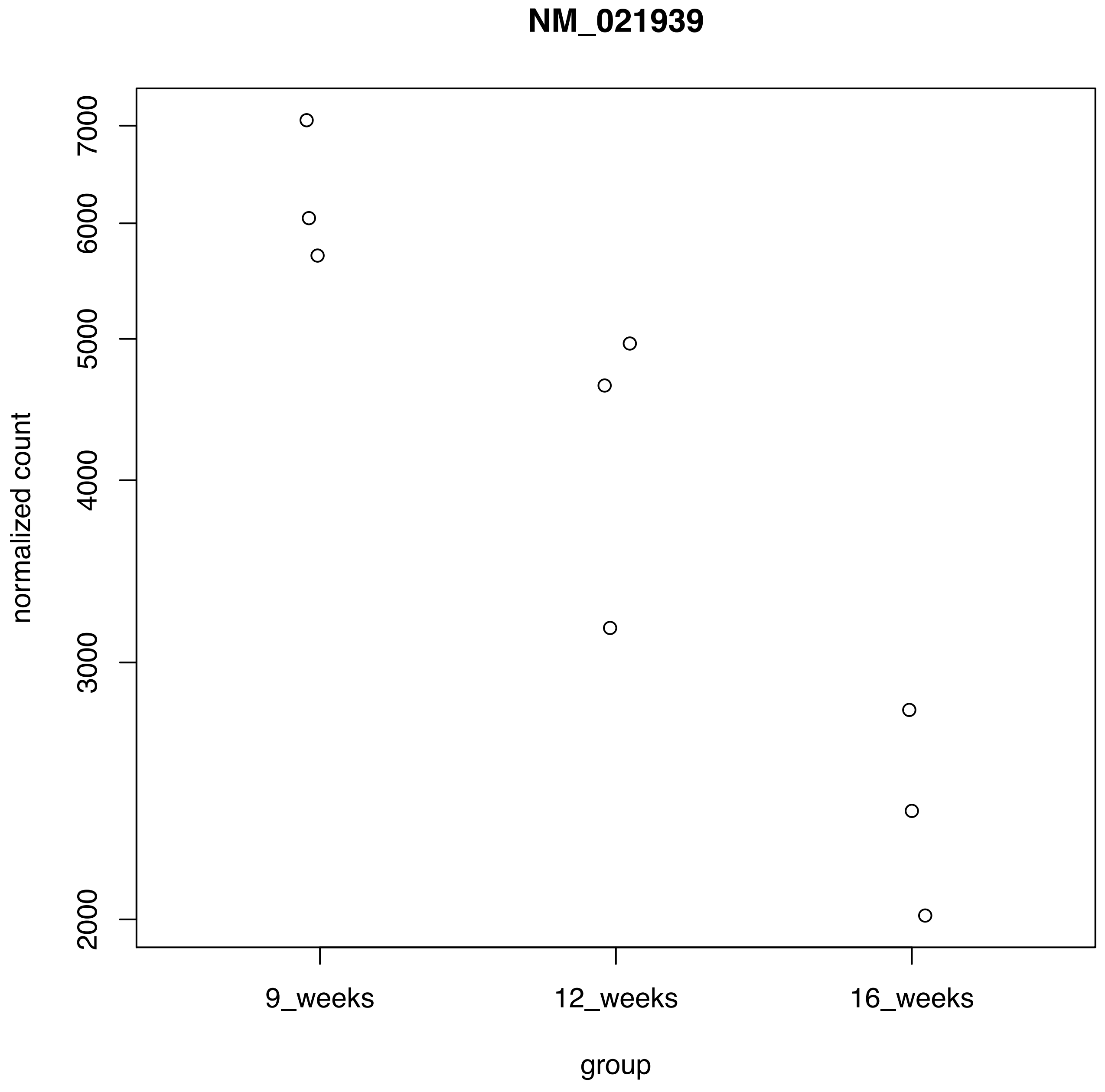
NM_021939
normalized count
7000
6000
5000
4000
3000
2000
9_weeks
12_weeks
16_weeks
group

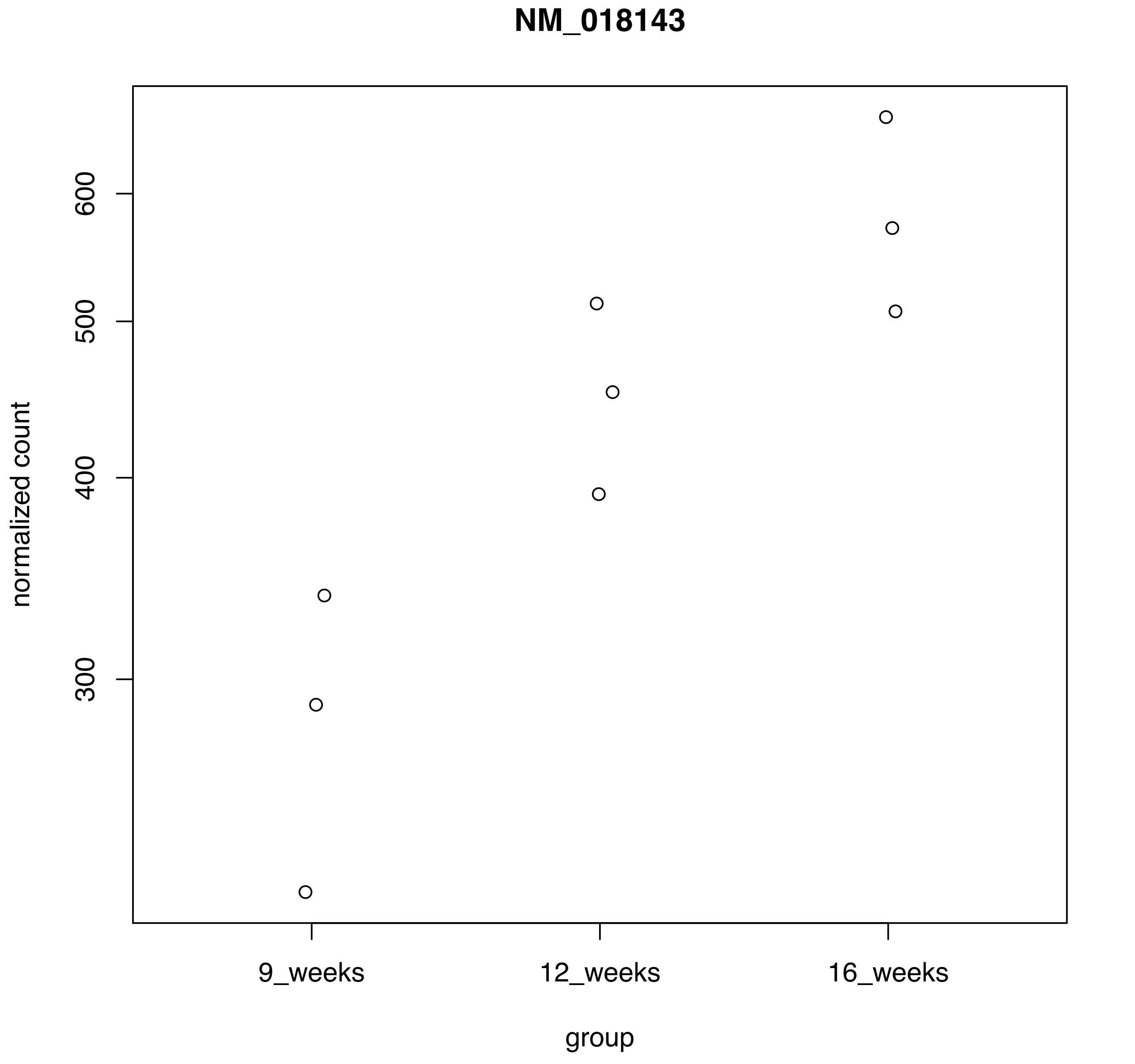

NM_018143
normalized count
600
500
400
300
9_weeks
12_weeks
16_weeks
group

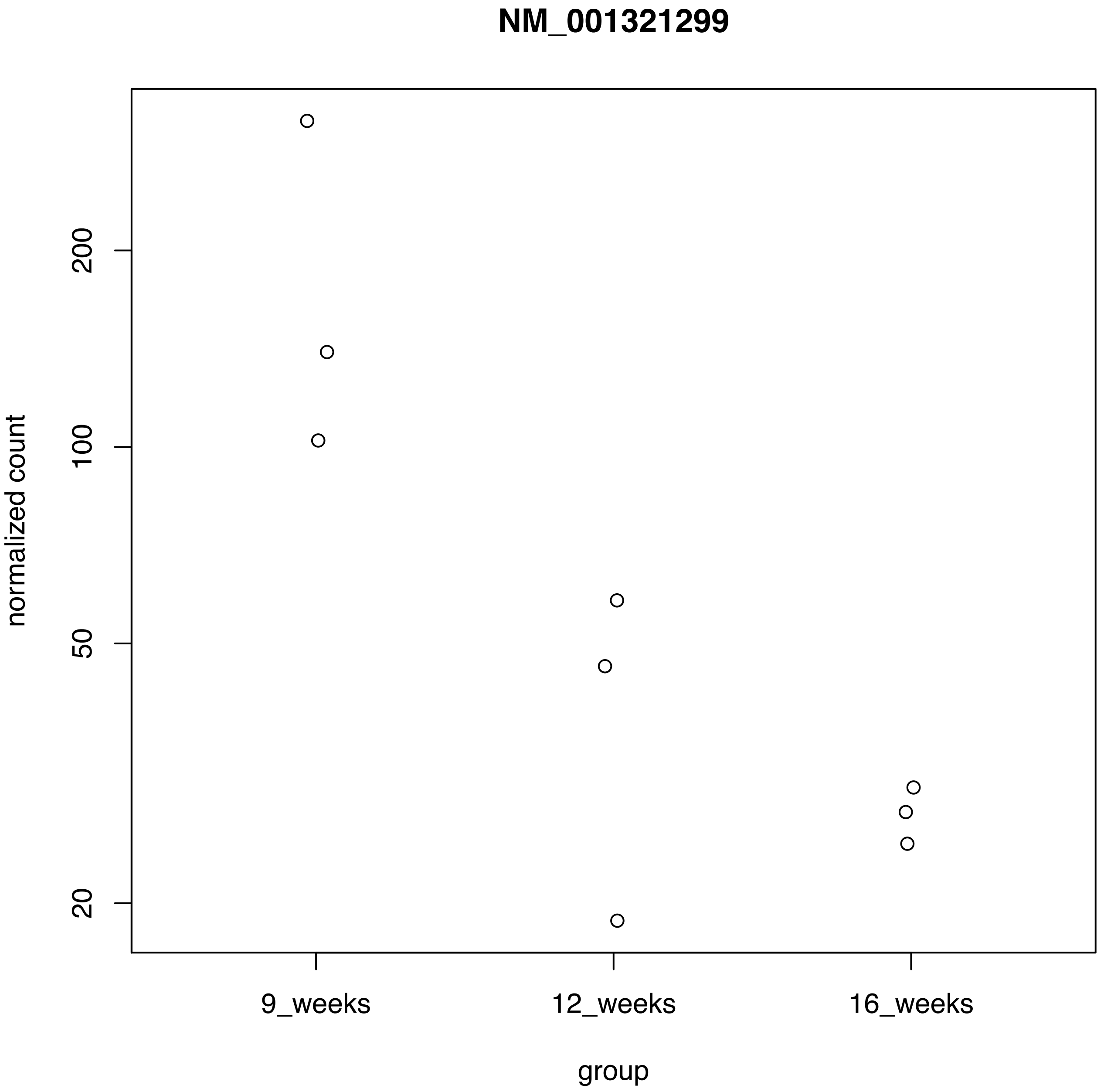

NM_001321299
normalized count
200
100
50
20
9_weeks
12_weeks
16_weeks
group

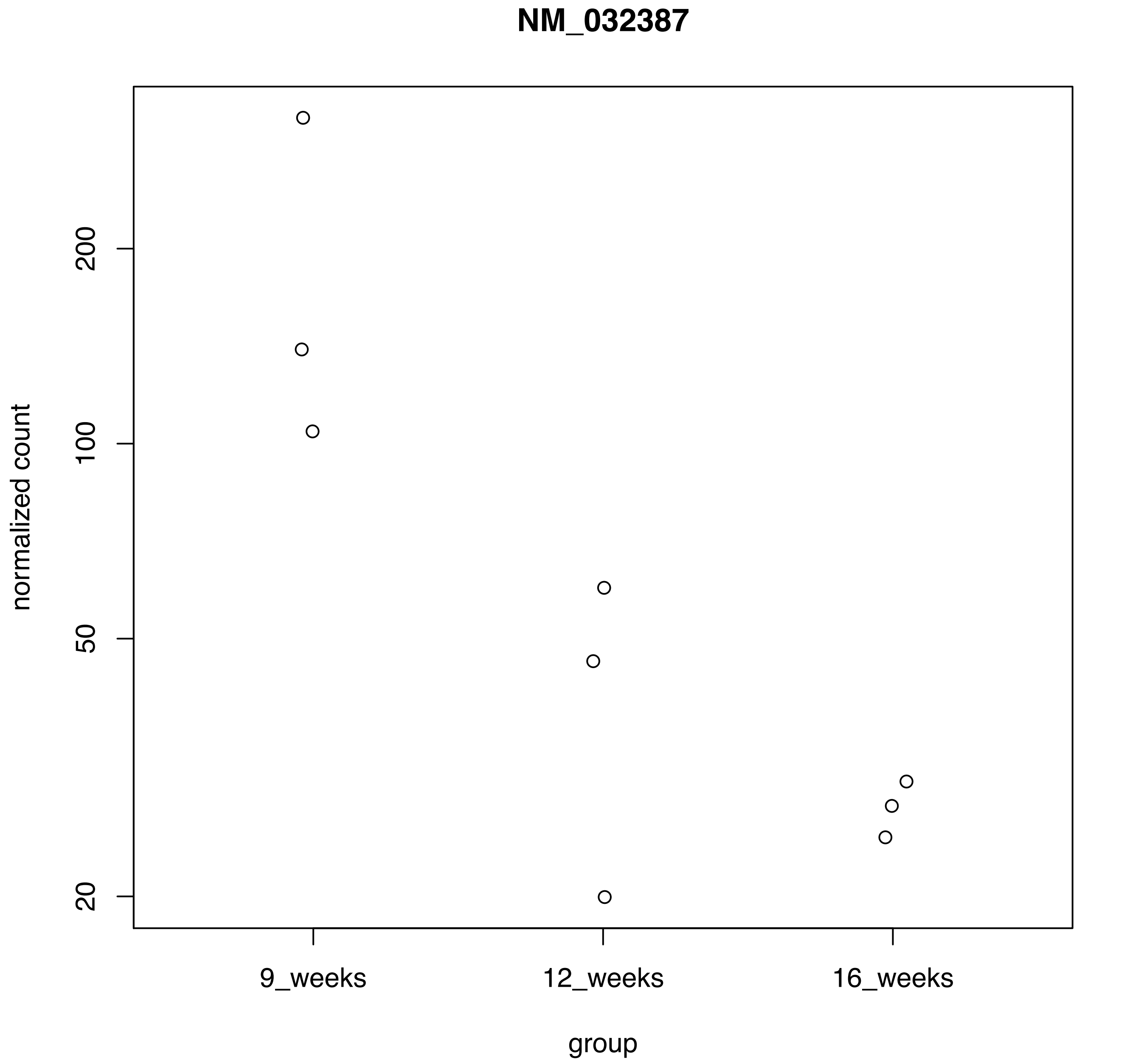
NM_032387
normalized count
200
100
50
20
9_weeks
12_weeks
16_weeks
group

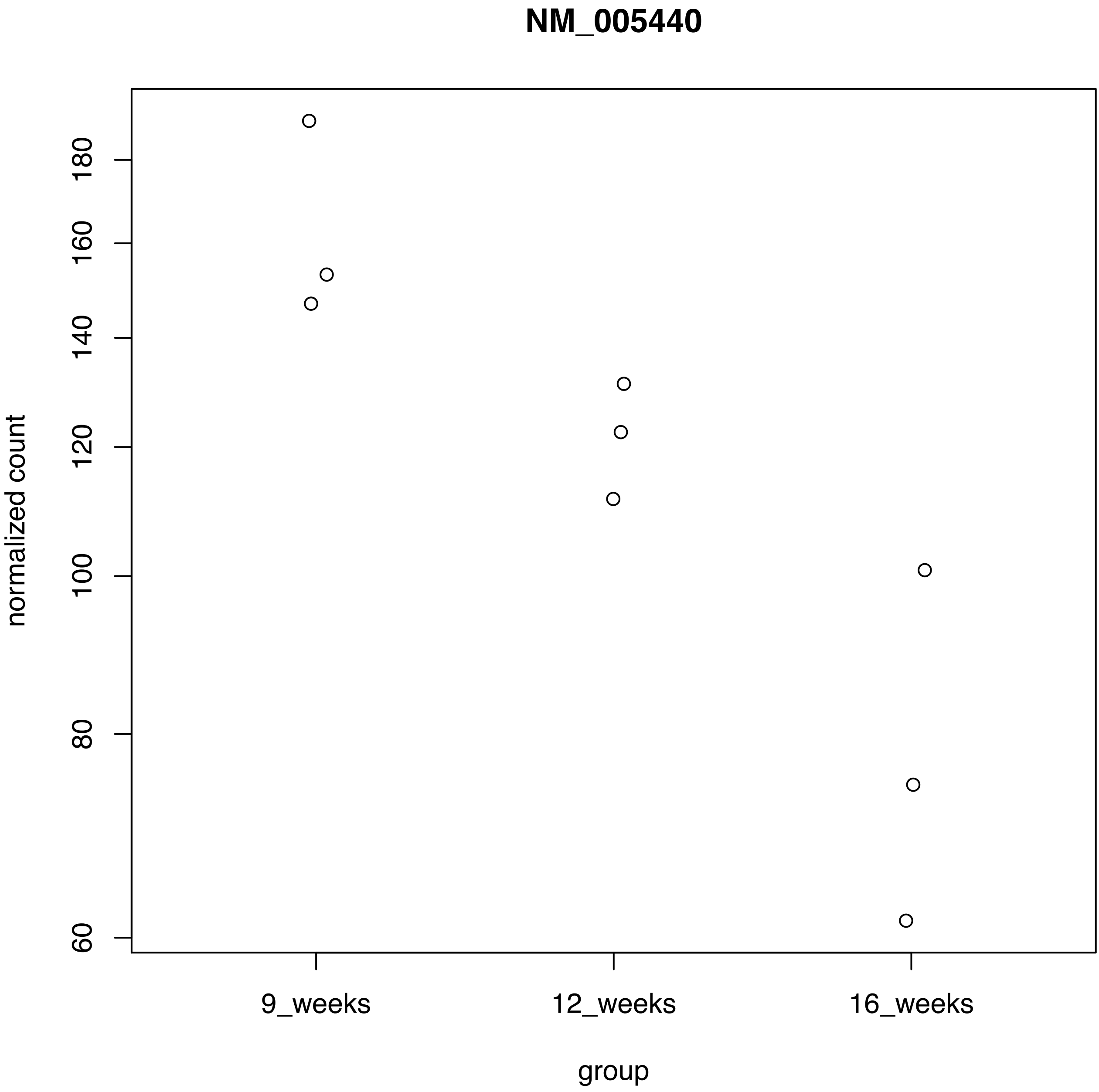

NM_005440
normalized count
60
80
100
120
140
160
180
9_weeks
12_weeks
16_weeks
group

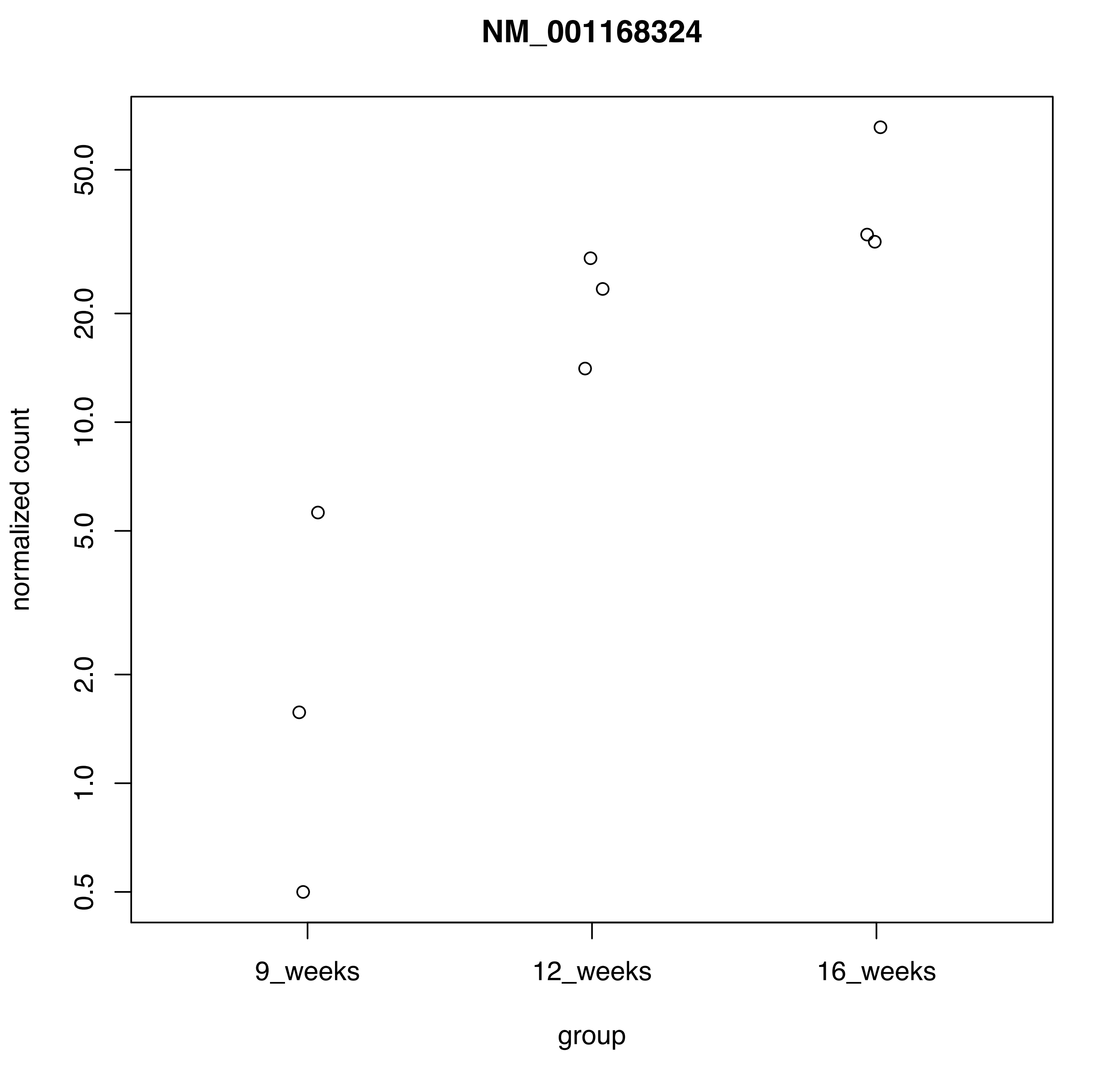
NM_001168324
normalized count
50.0
20.0
10.0
5.0
2.0
1.0
0.5
9_weeks
12_weeks
16_weeks
group

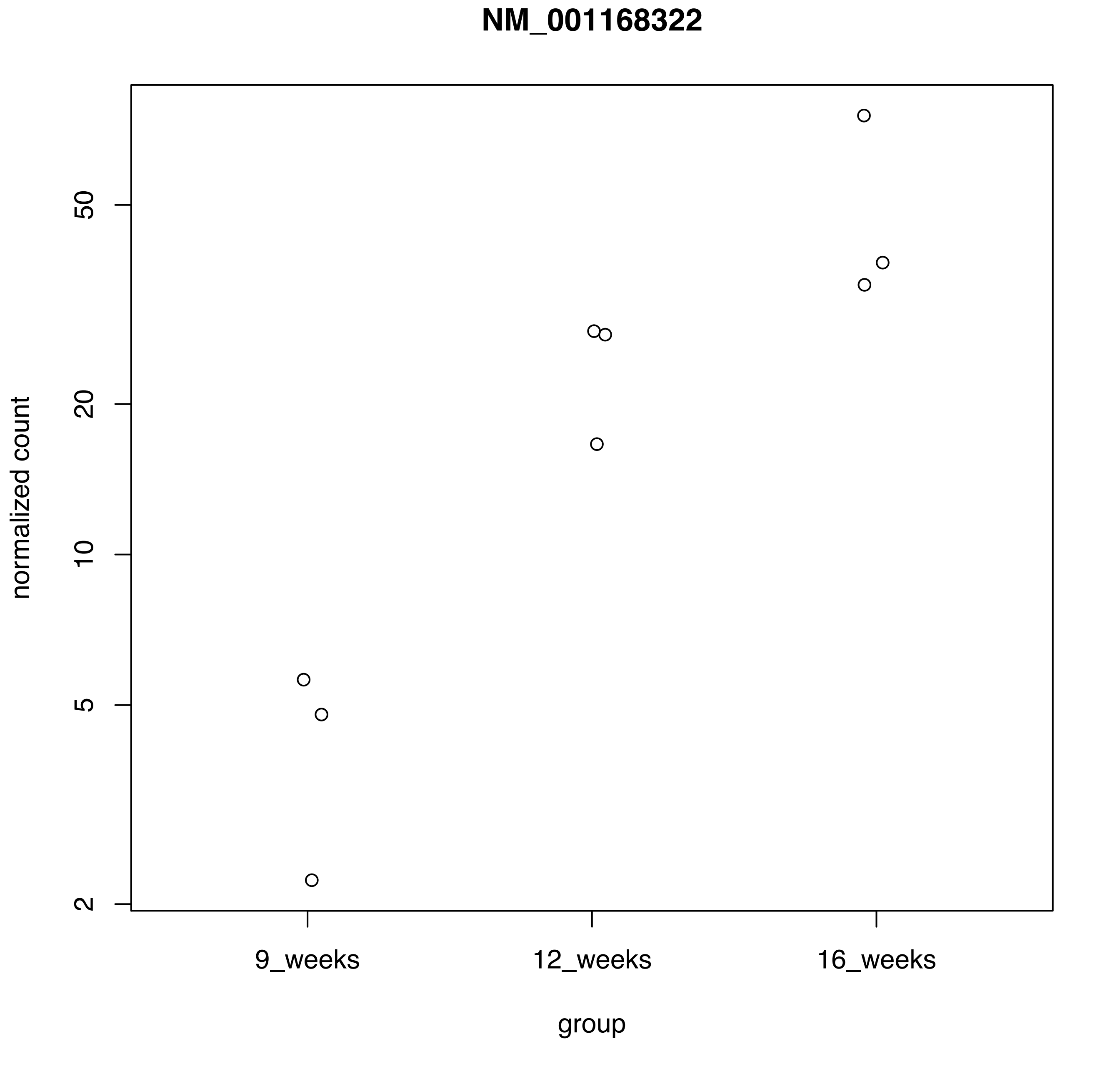
NM_001168322
normalized count
50
20
10
5
2
9_weeks
12_weeks
16_weeks
group

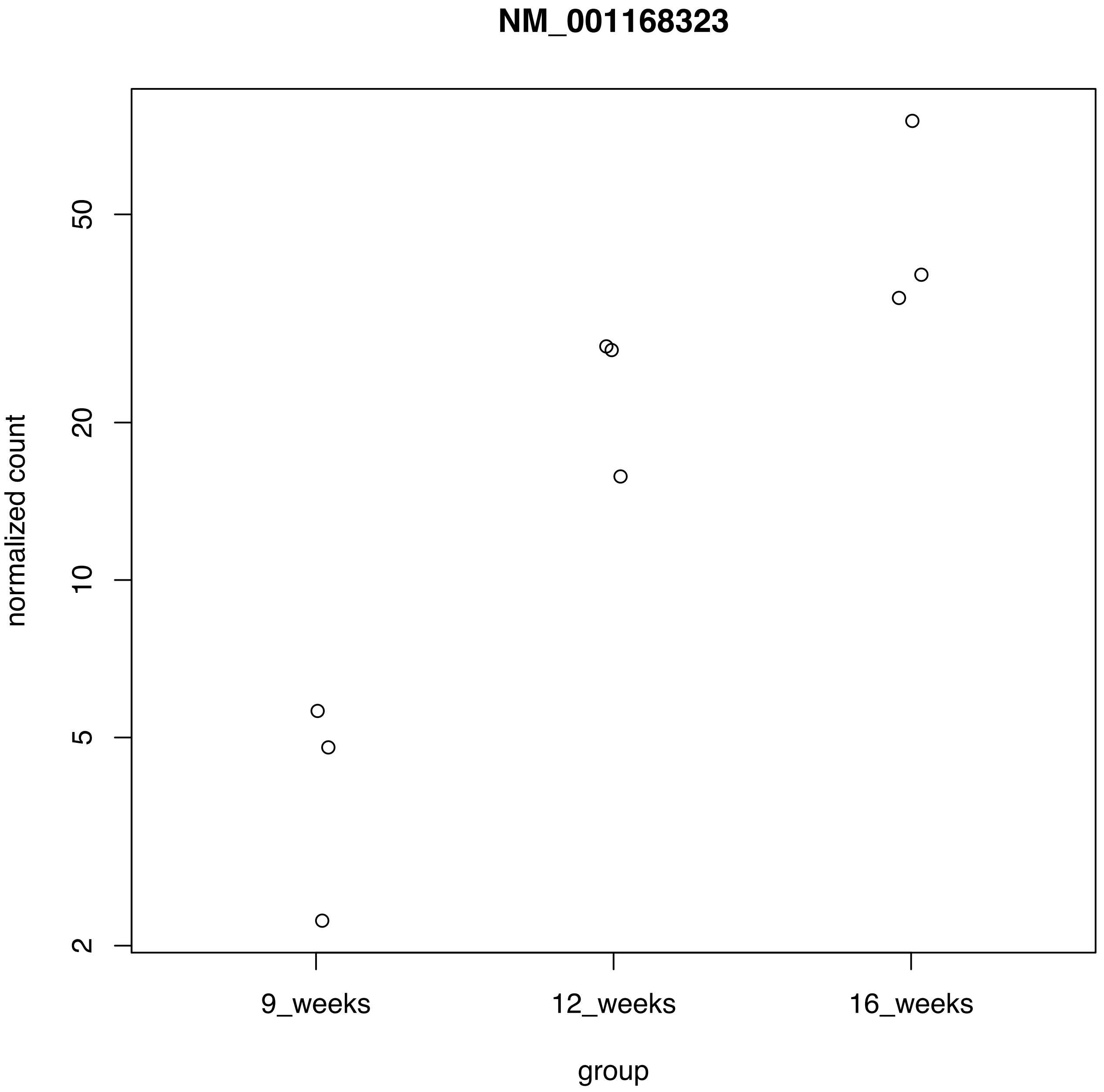
NM_001168323
normalized count
50
20
10
5
2
9_weeks
12_weeks
16_weeks
group

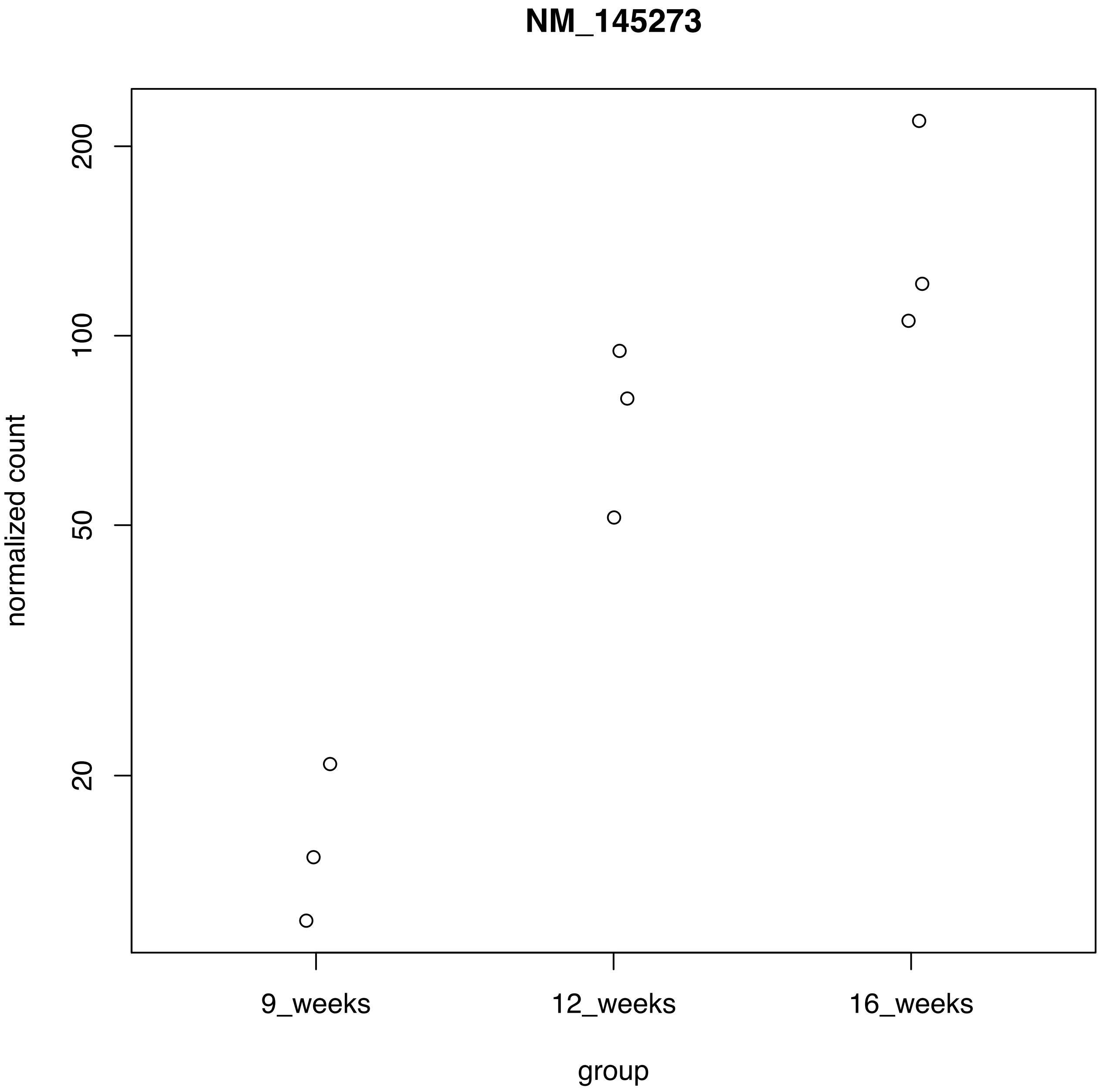
NM_145273
normalized count
200
100
50
20
9_weeks
12_weeks
16_weeks
group

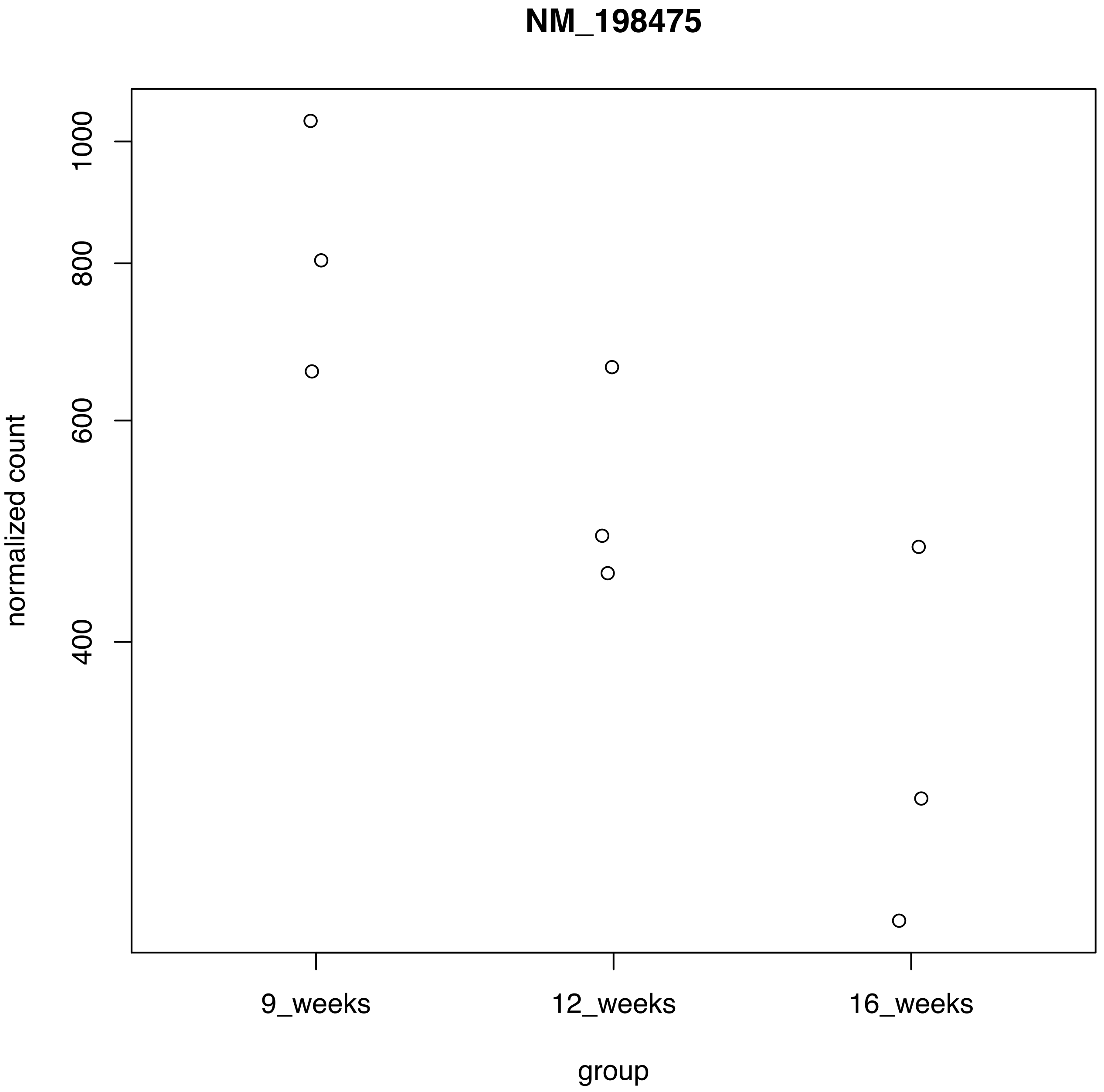
NM_198475
normalized count
1000
800
600
400
9_weeks
12_weeks
16_weeks
group

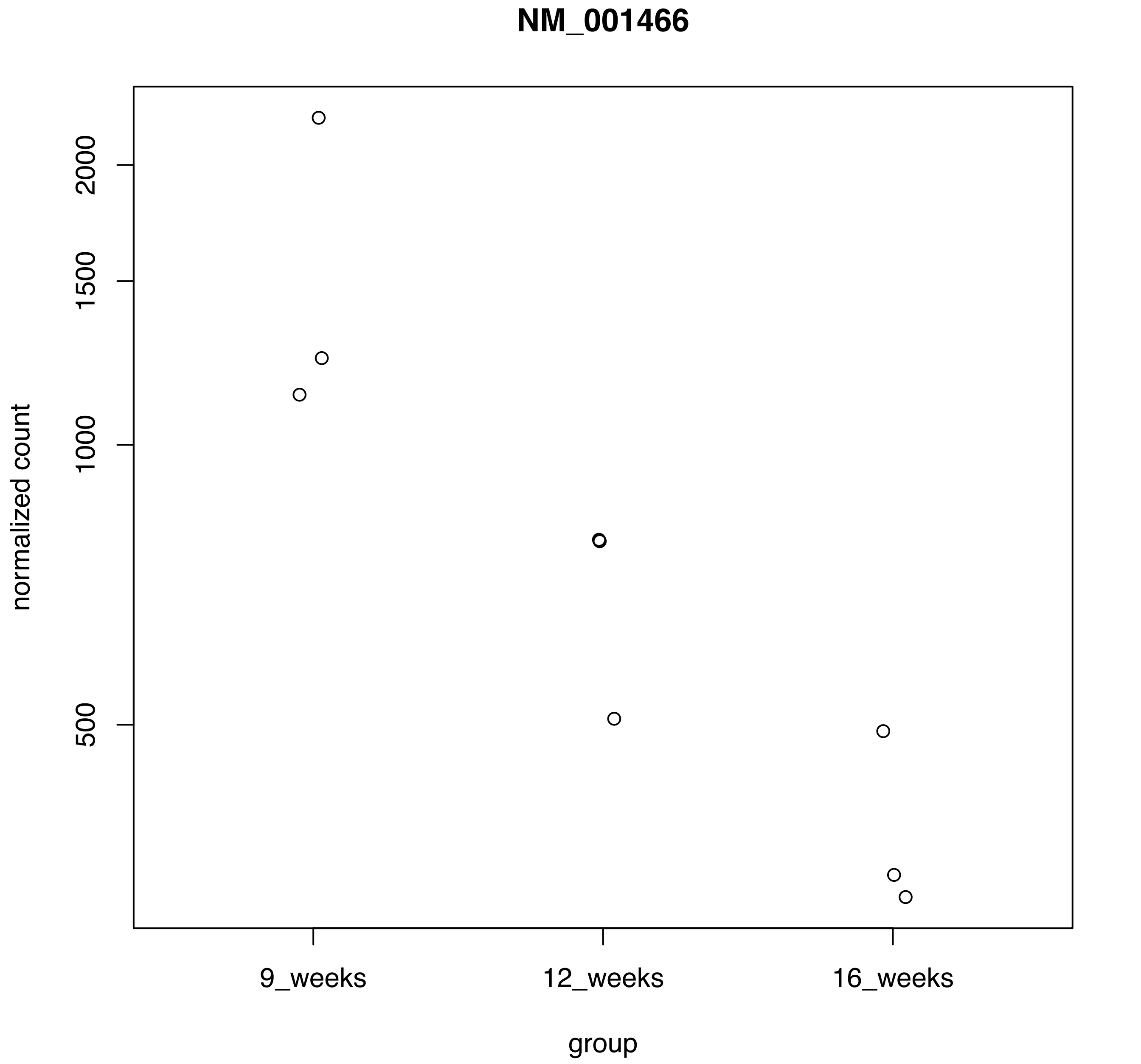
NM_001466
normalized count
2000
1500
1000
500
9_weeks
12_weeks
16_weeks
group

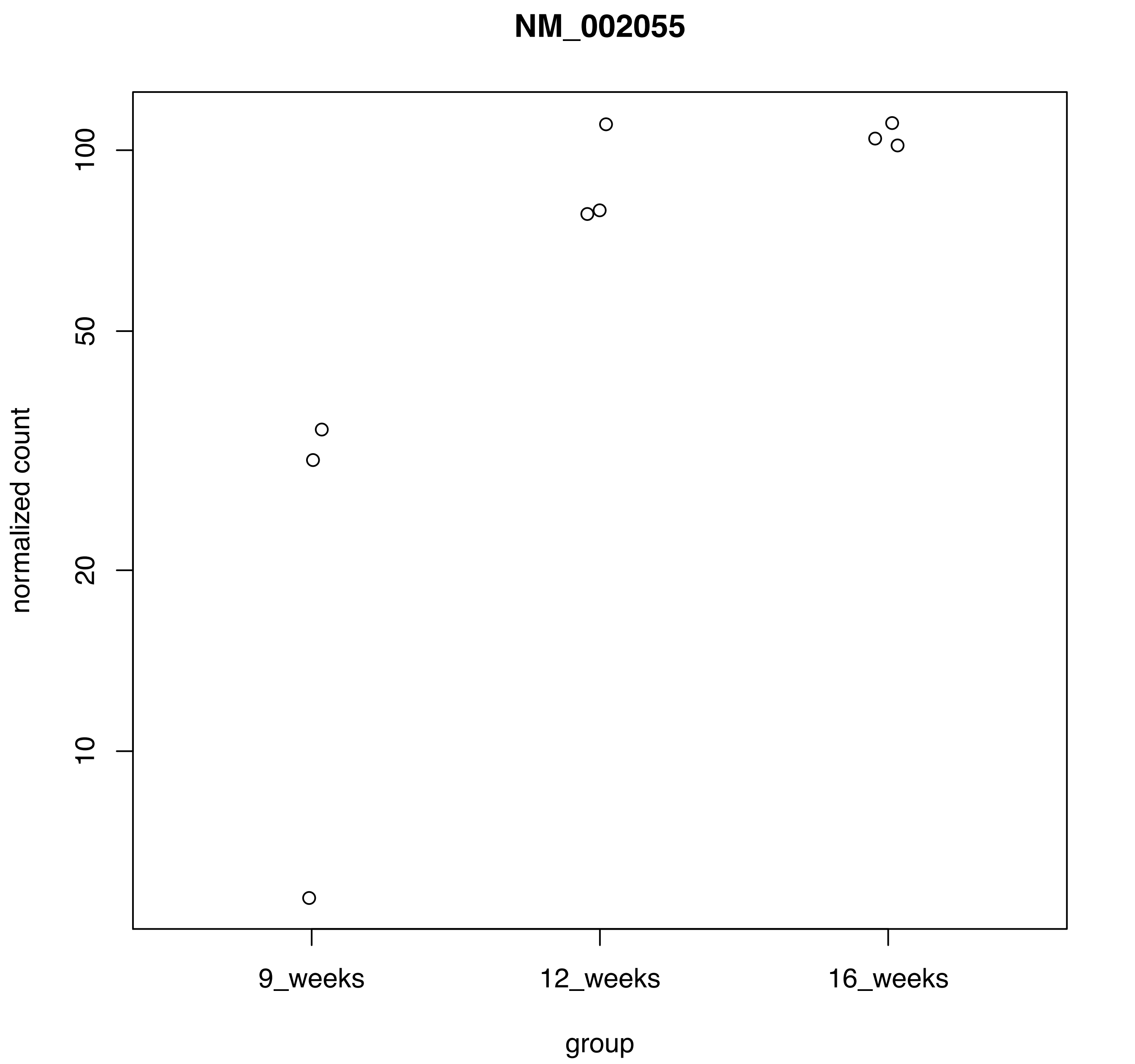
NM_002055
normalized count
100
50
20
10
9_weeks
12_weeks
16_weeks
group

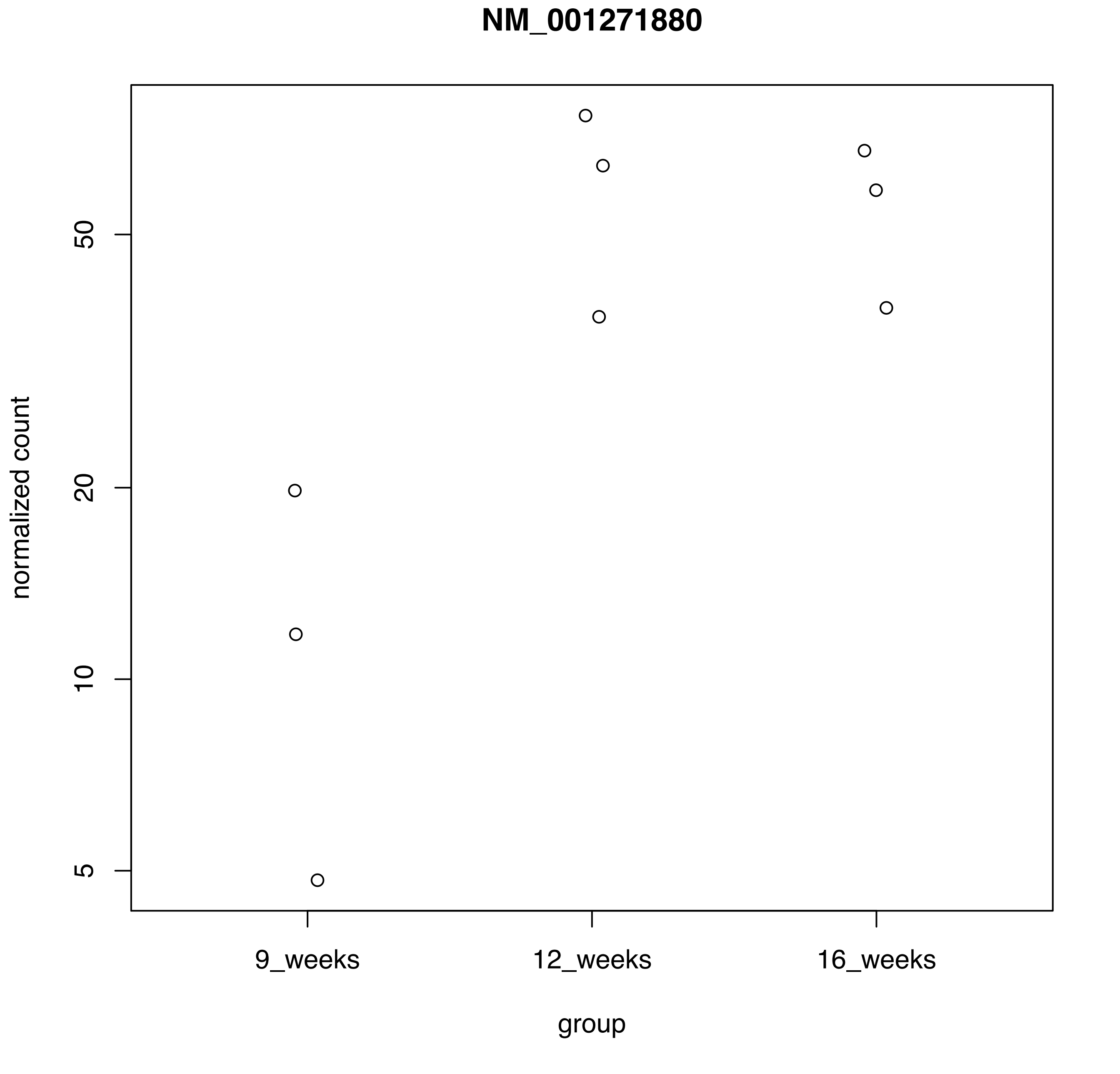
NM_001271880
normalized count
50
20
10
5
9_weeks
12_weeks
16_weeks
group

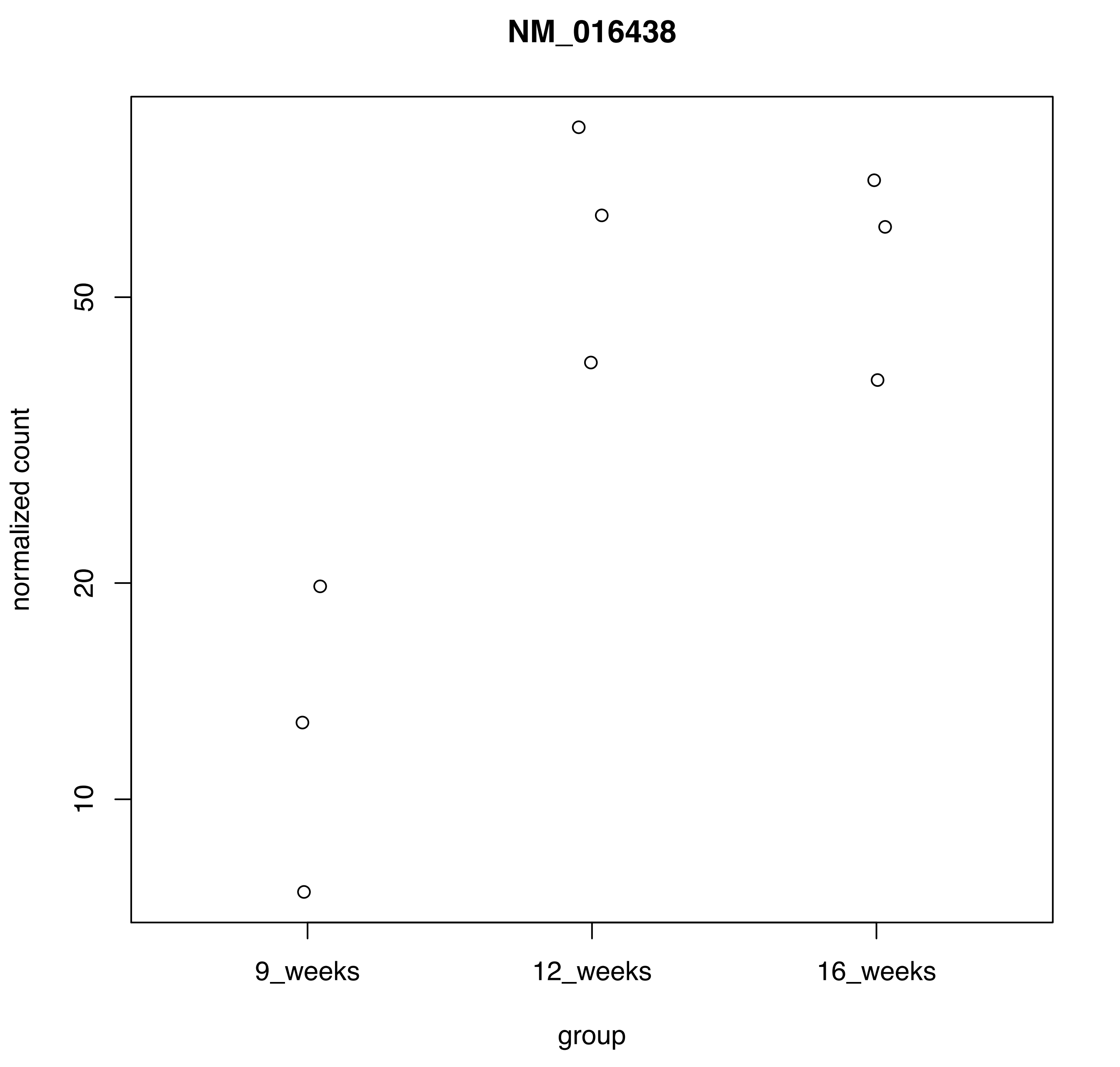
NM_016438
normalized count
50
20
10
9_weeks
12_weeks
16_weeks
group

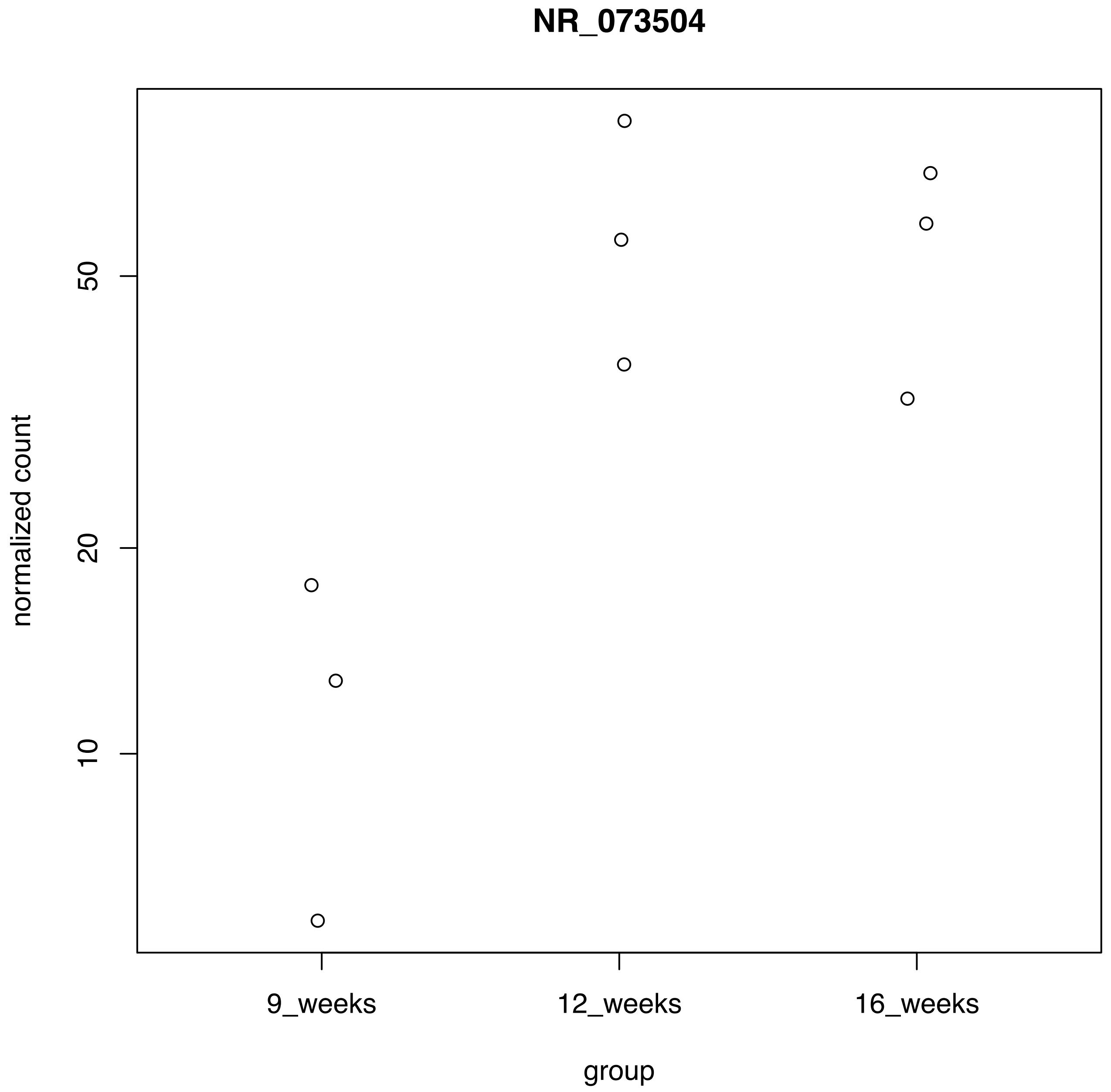
NR_073504
normalized count
50
20
10
9_weeks
12_weeks
16_weeks
group

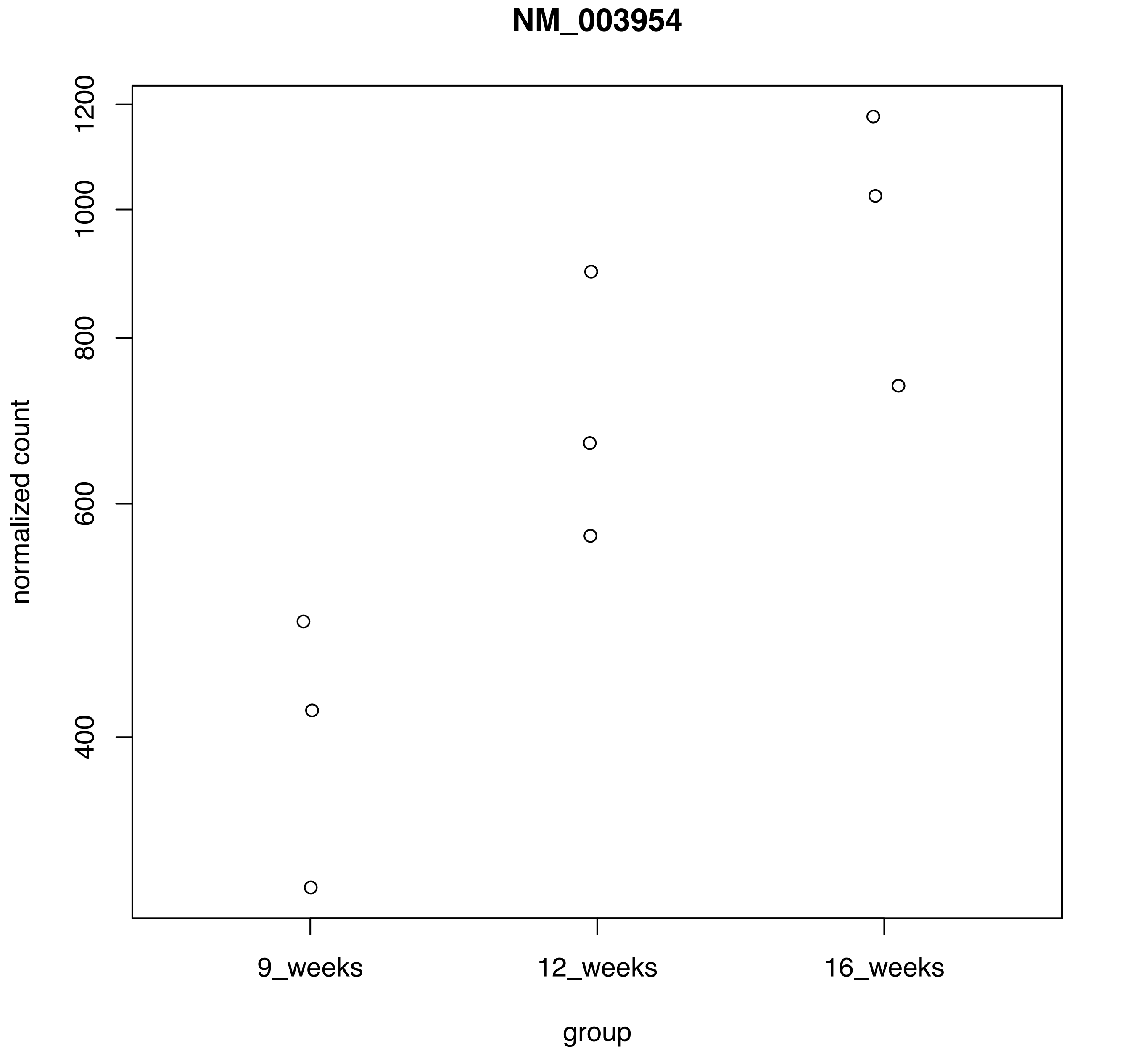
NM_003954
normalized count
400
600
800
1000
1200
9_weeks
12_weeks
16_weeks
group

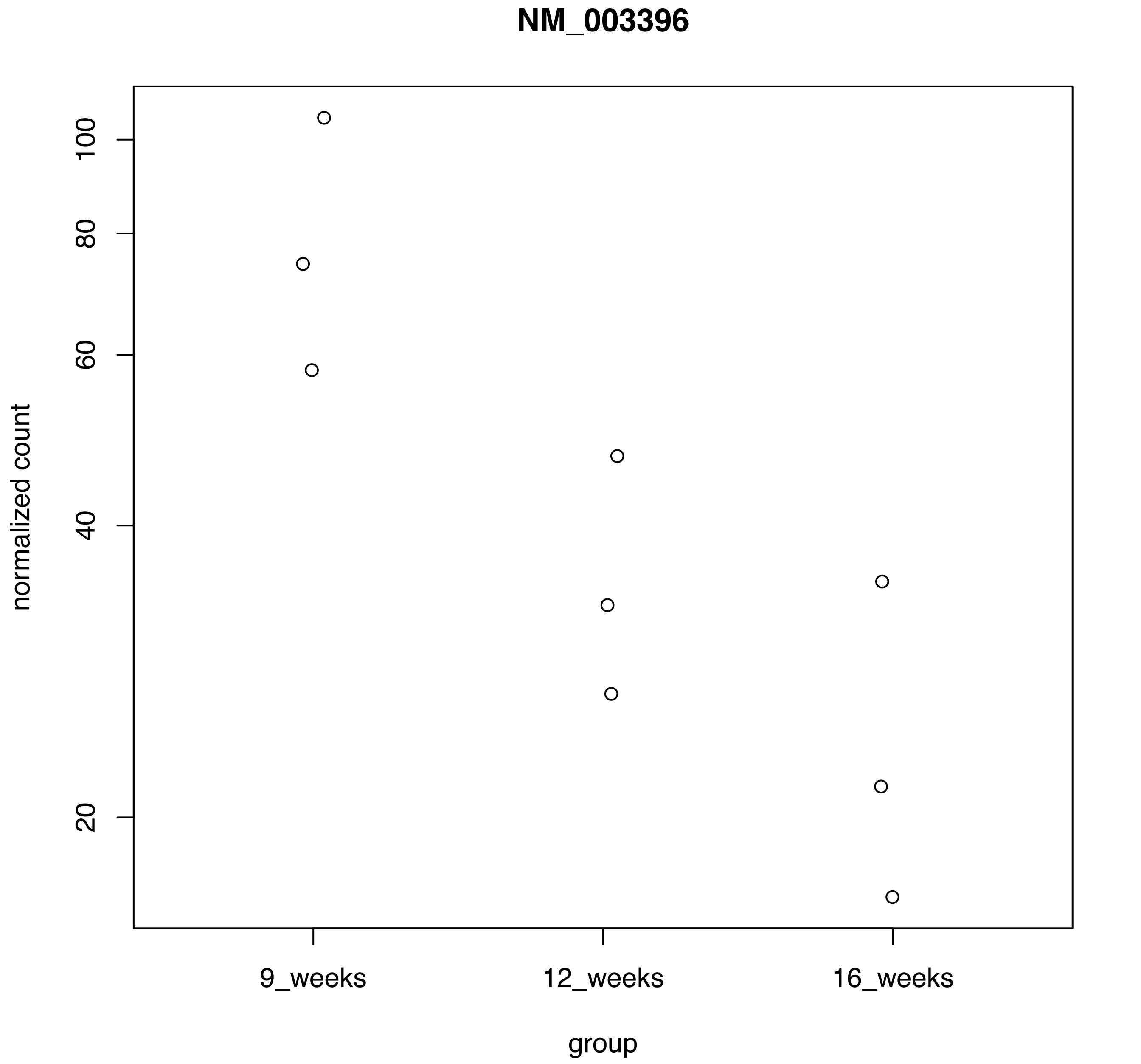

NM_003396
100
80
60
40
20
normalized count
9_weeks
12_weeks
16_weeks
group

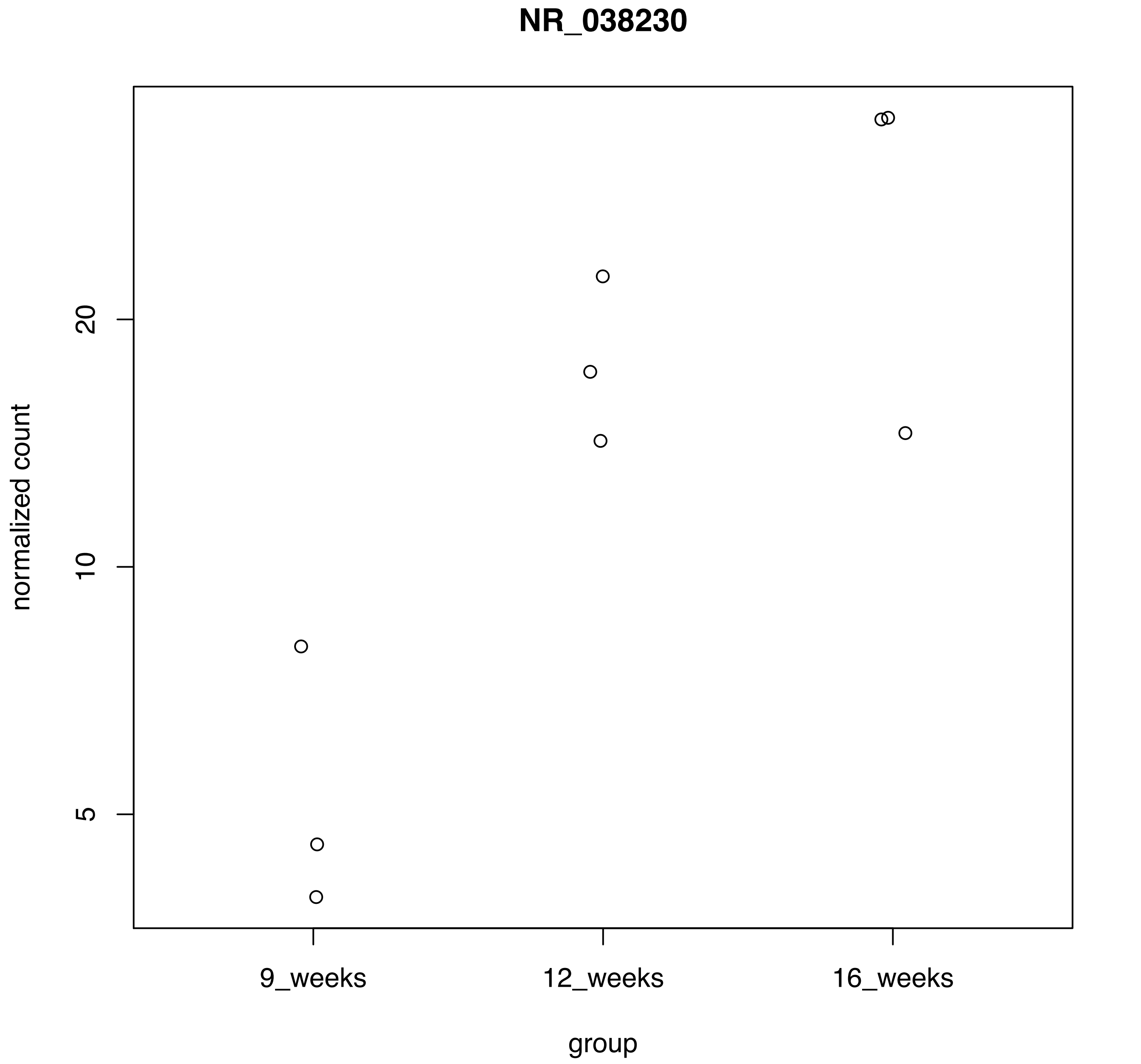

NR_038230
normalized count
20
10
5
9_weeks
12_weeks
16_weeks
group

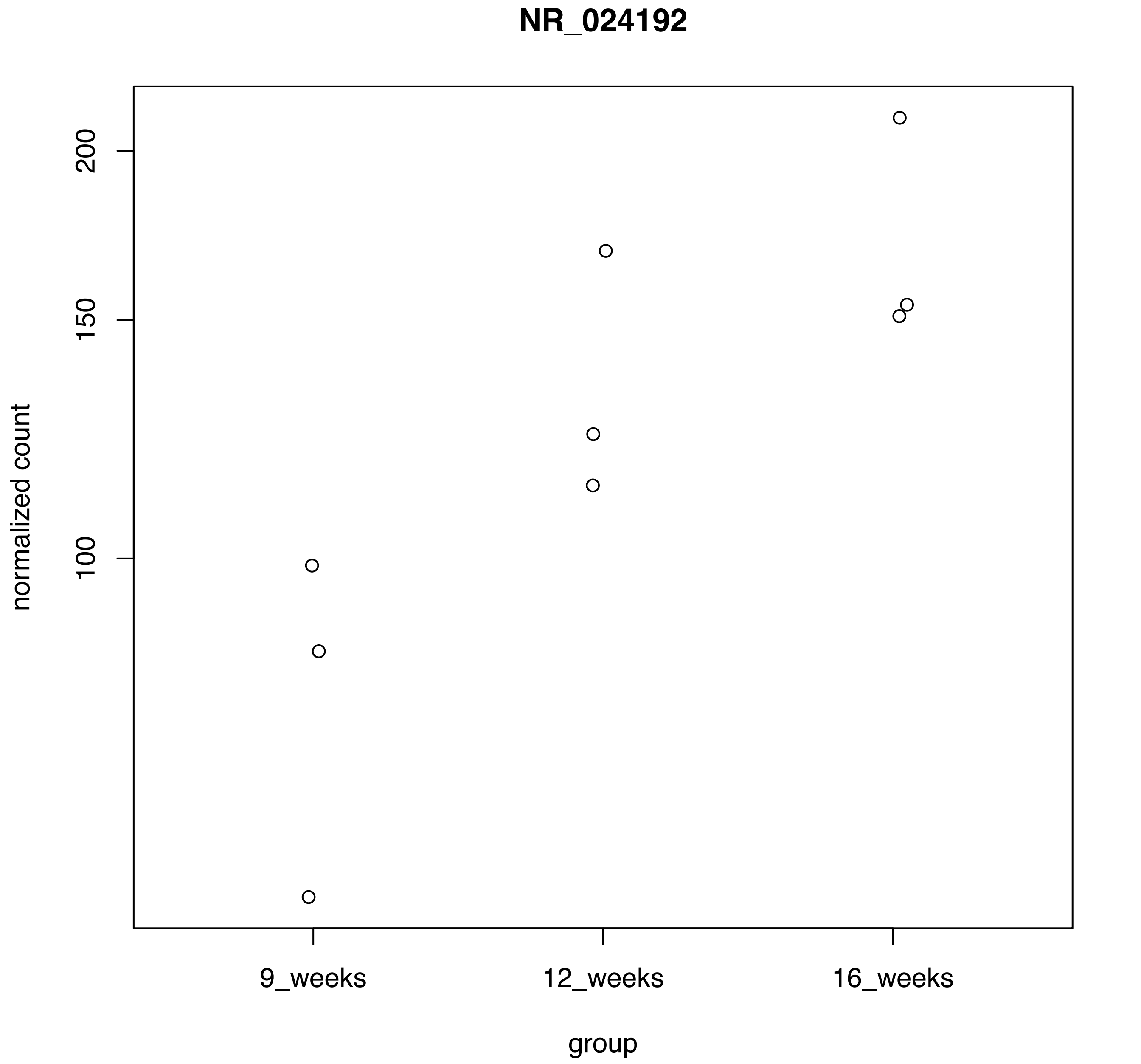

NR_024192
200
150
100
normalized count
9_weeks
12_weeks
16_weeks
group

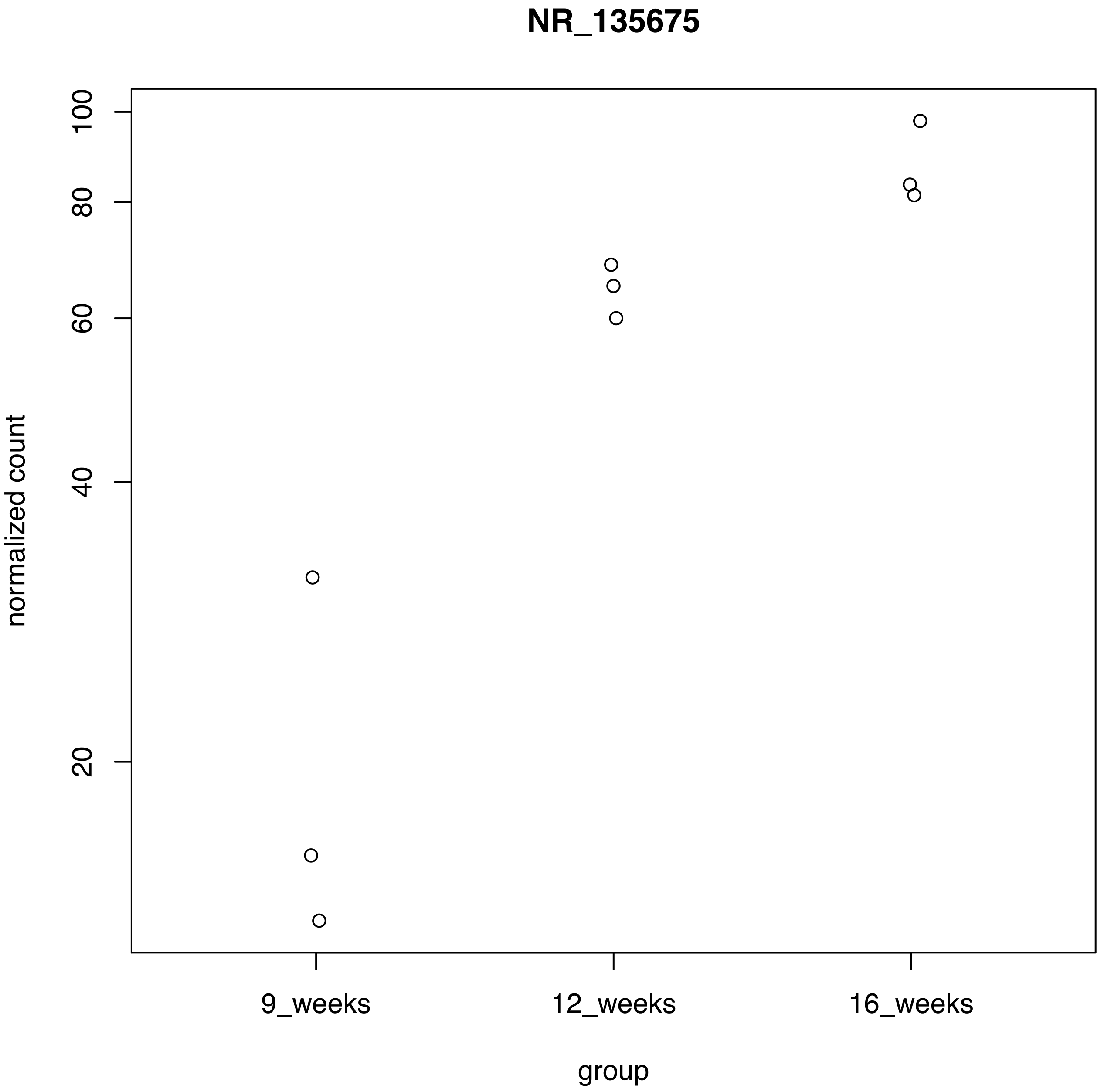
NR_135675
normalized count
100
80
60
40
20
9_weeks
12_weeks
16_weeks
group

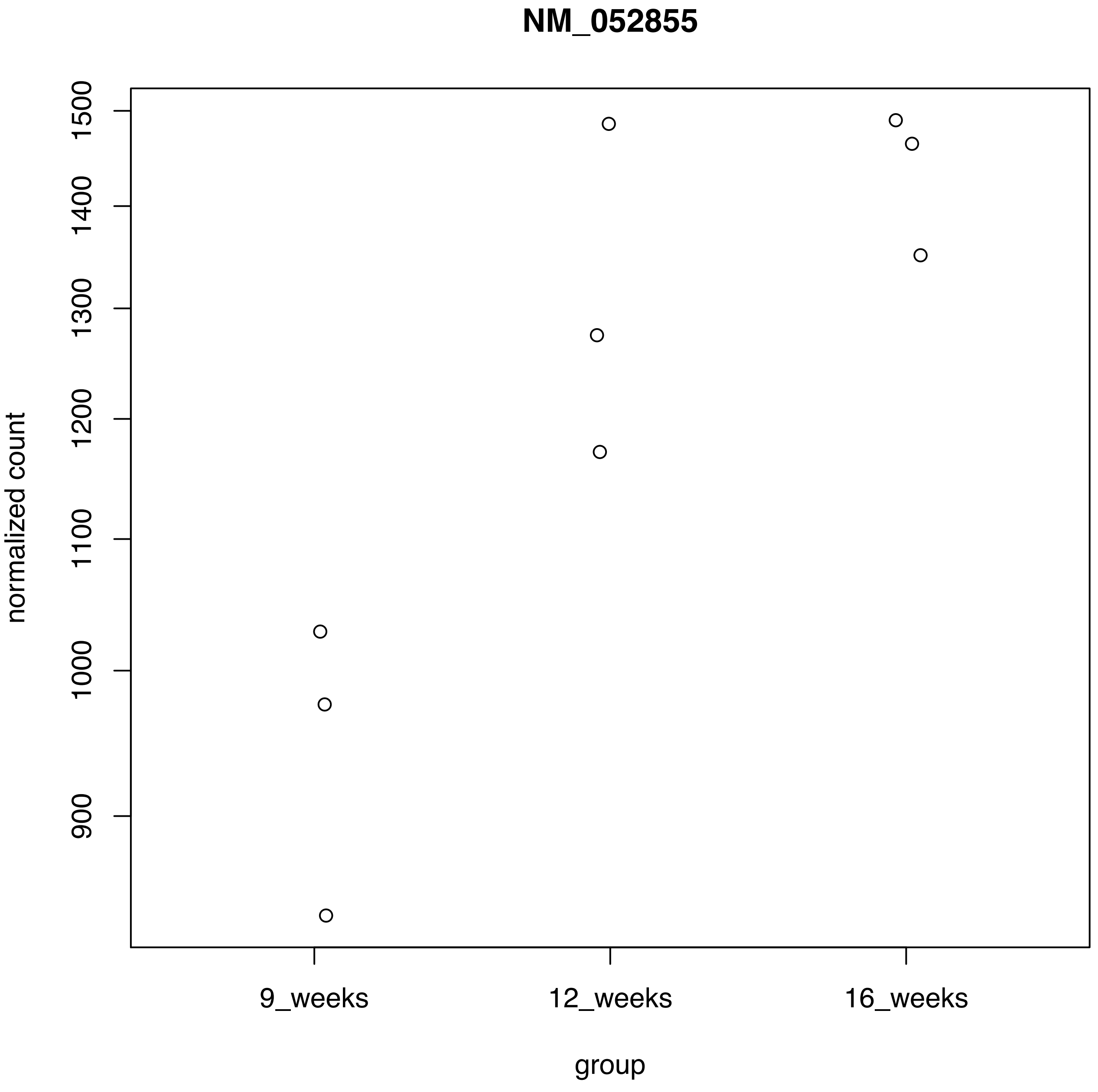
NM_052855
normalized count
1500
1400
1300
1200
1100
1000
900
9_weeks
12_weeks
16_weeks
group

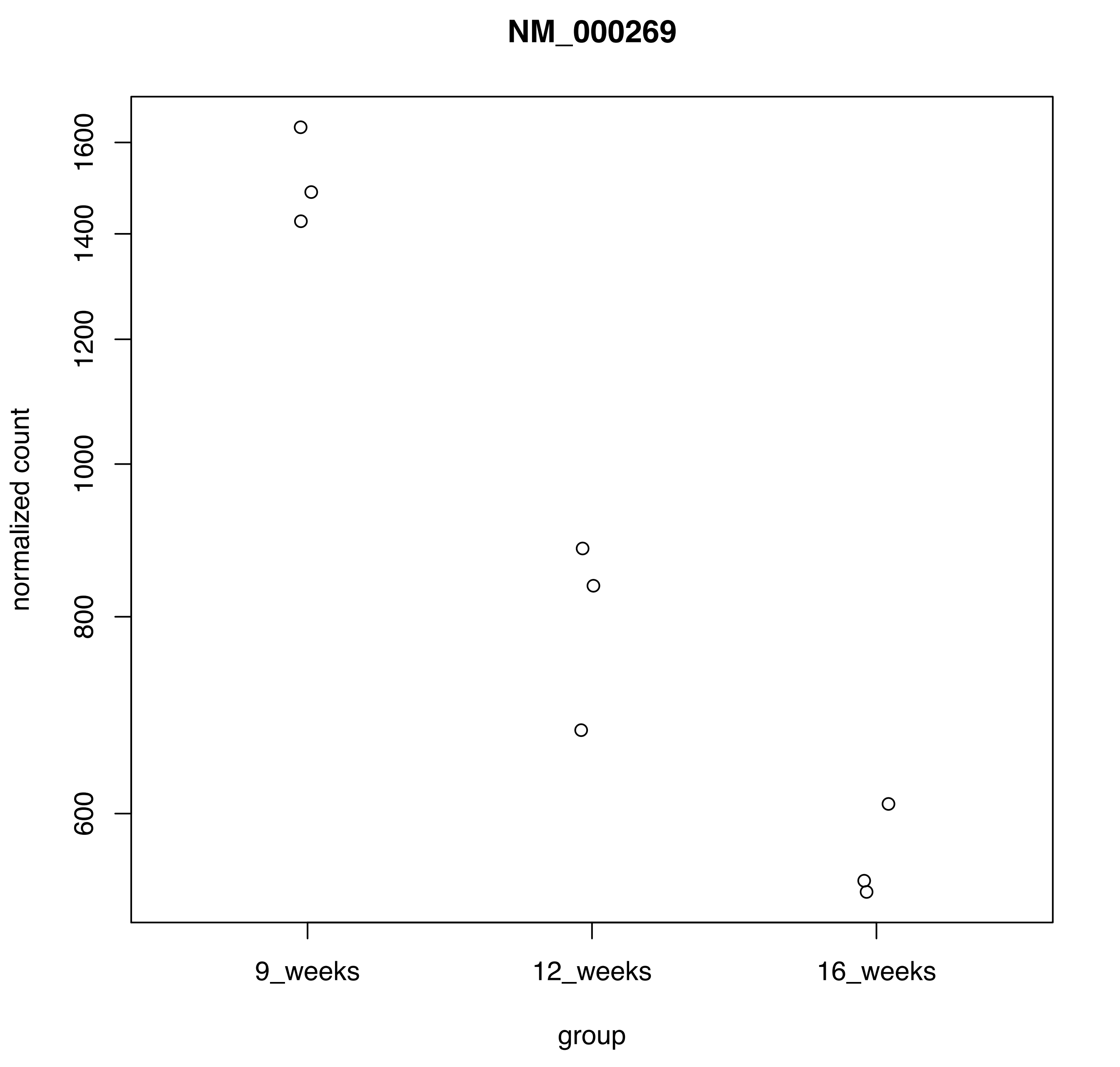
NM_000269
normalized count
1600
1400
1200
1000
800
600
9_weeks
12_weeks
16_weeks
group

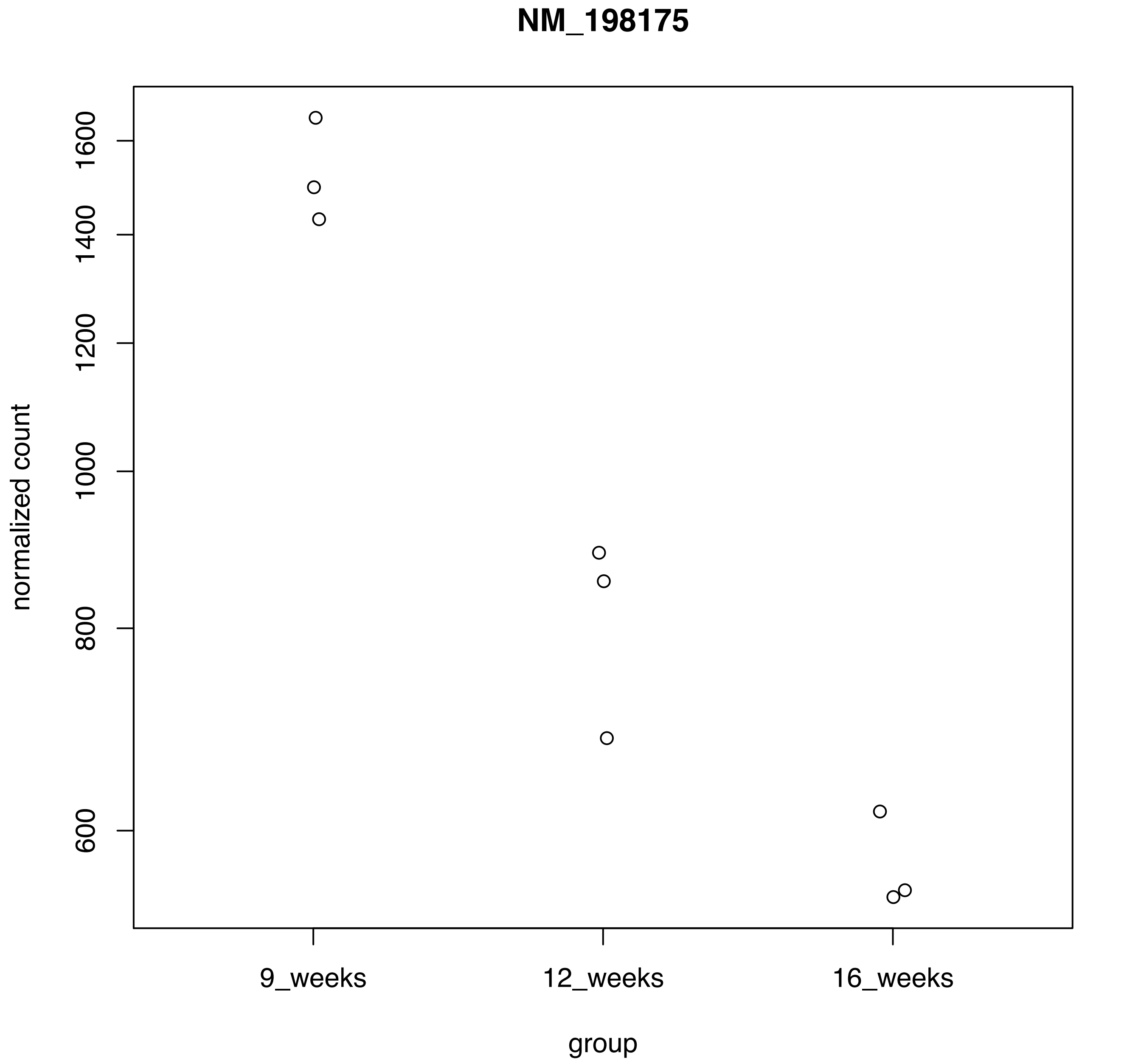

NM_198175
normalized count
1600
1400
1200
1000
800
600
9_weeks
12_weeks
16_weeks
group

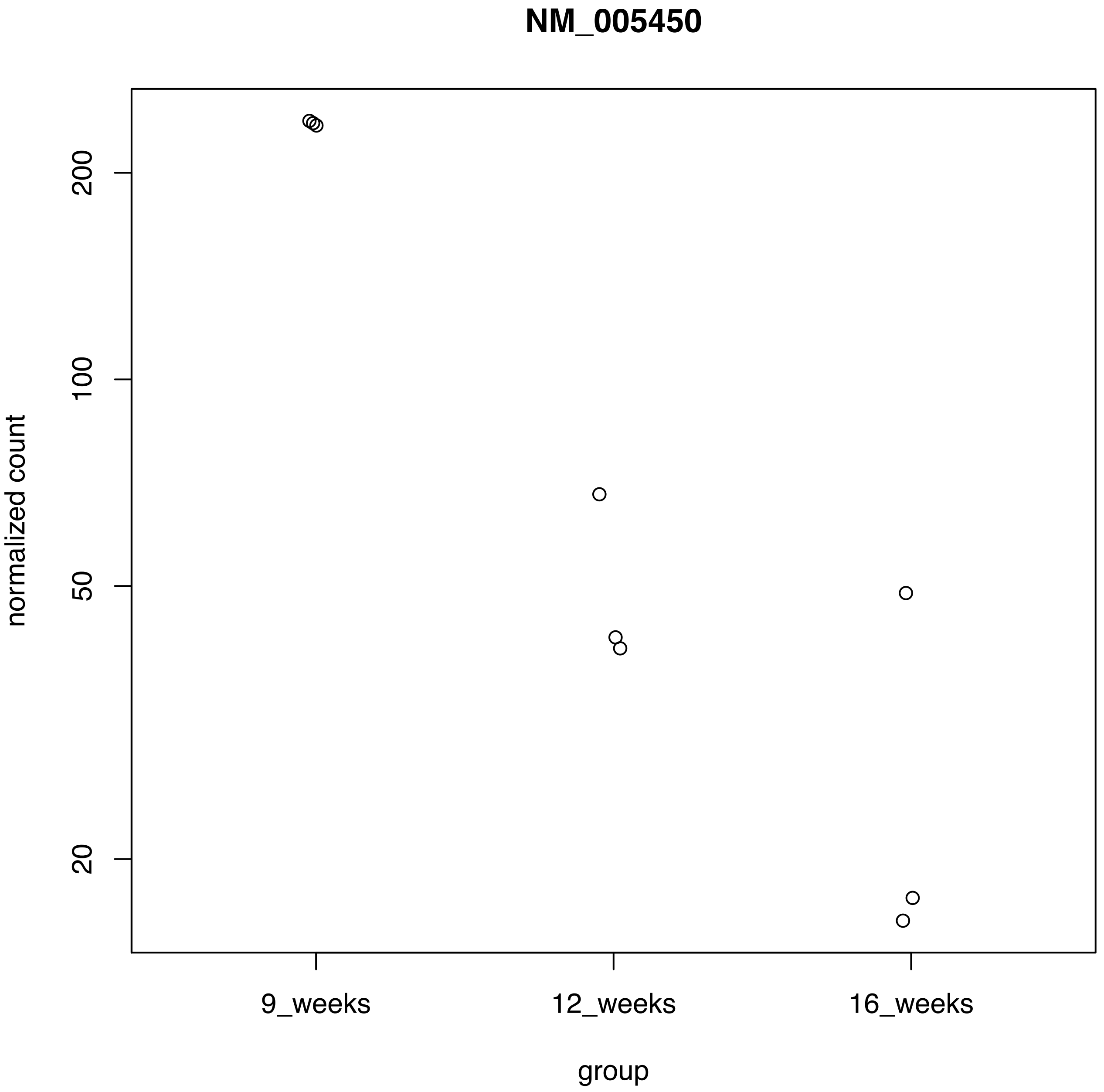
NM_005450
normalized count
200
100
50
20
9_weeks
12_weeks
16_weeks
group

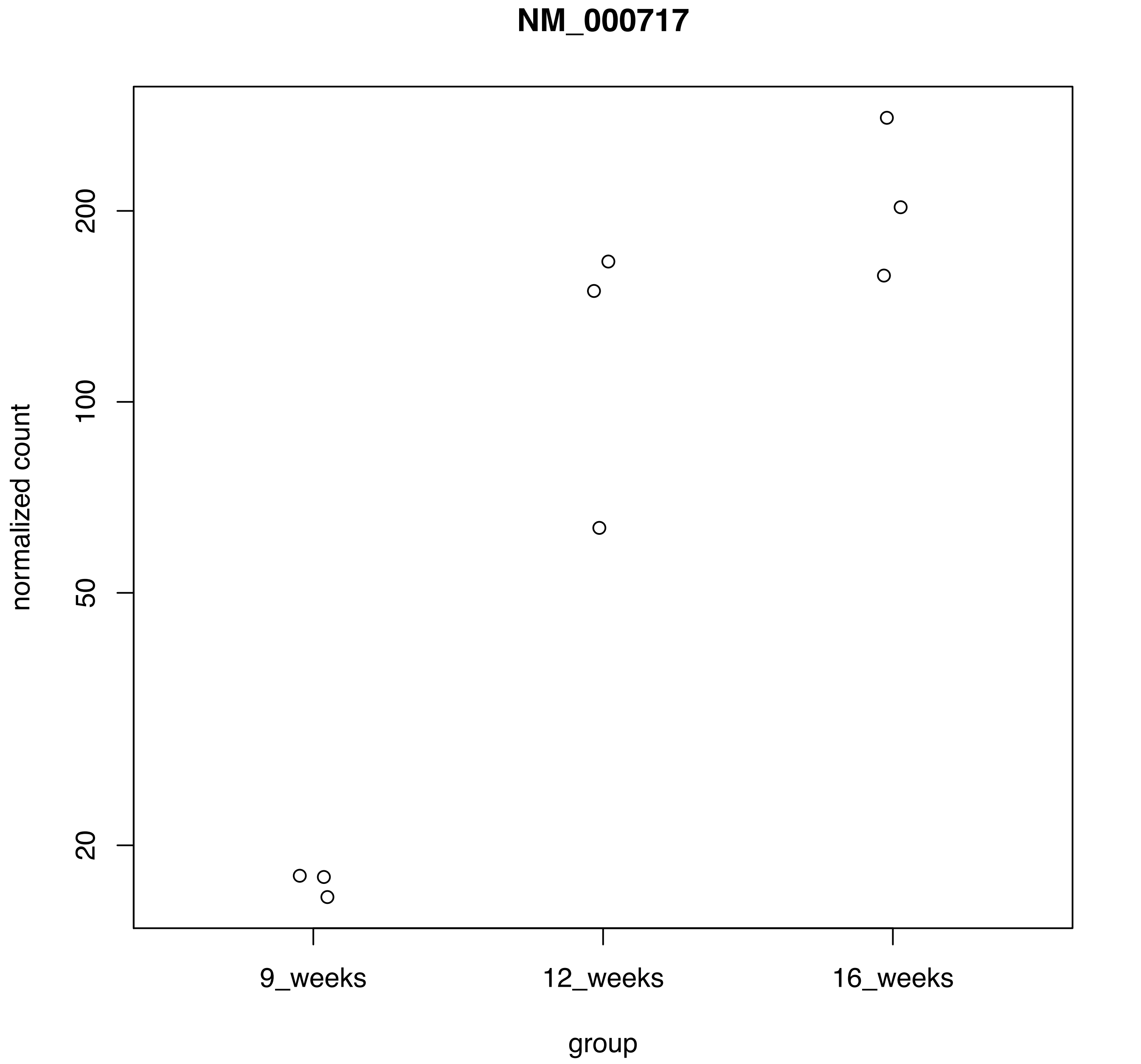
NM_000717
normalized count
200
100
50
20
9_weeks
12_weeks
16_weeks
group

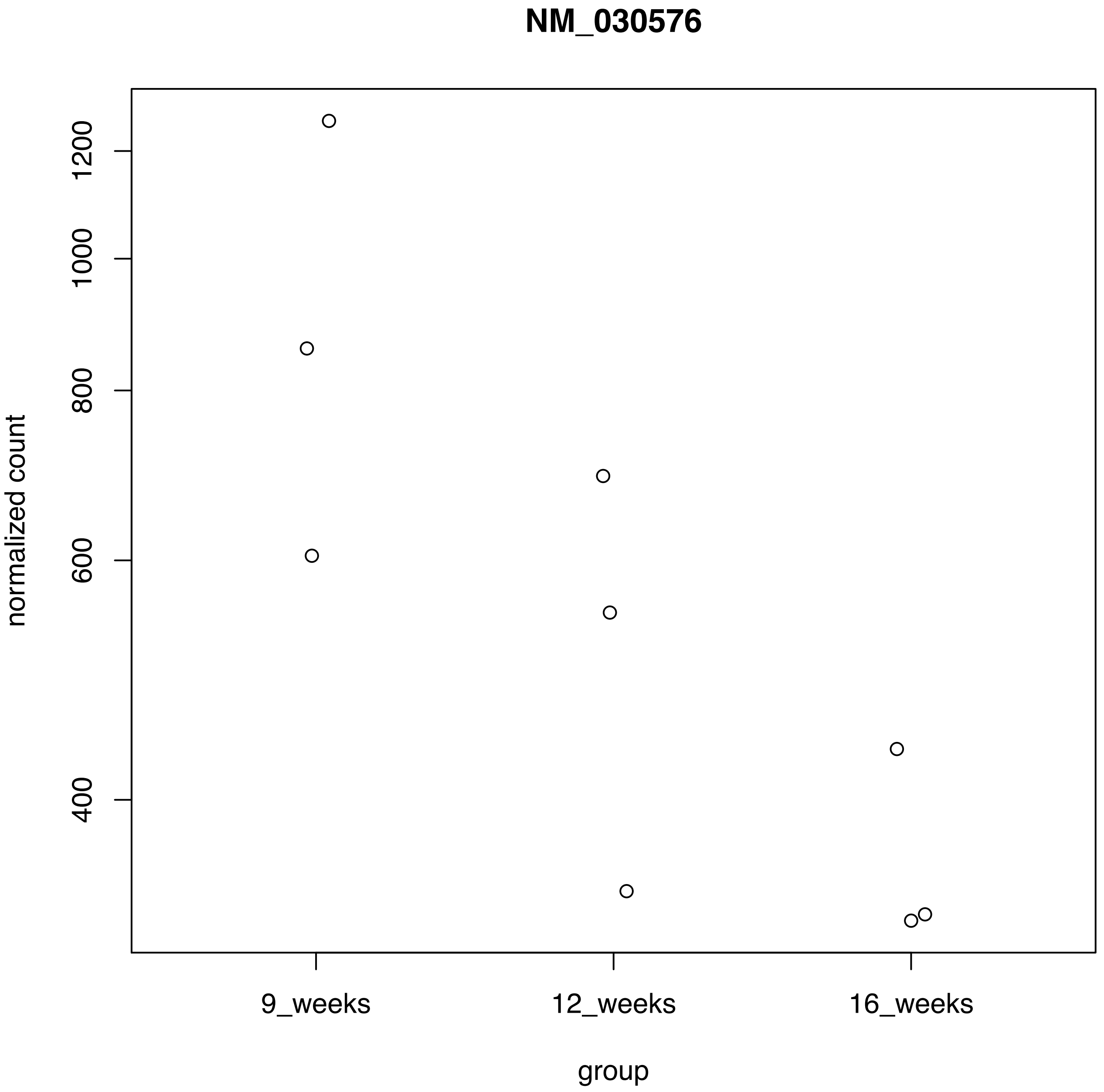
NM_030576
normalized count
400
600
800
1000
1200
9_weeks
12_weeks
16_weeks
group

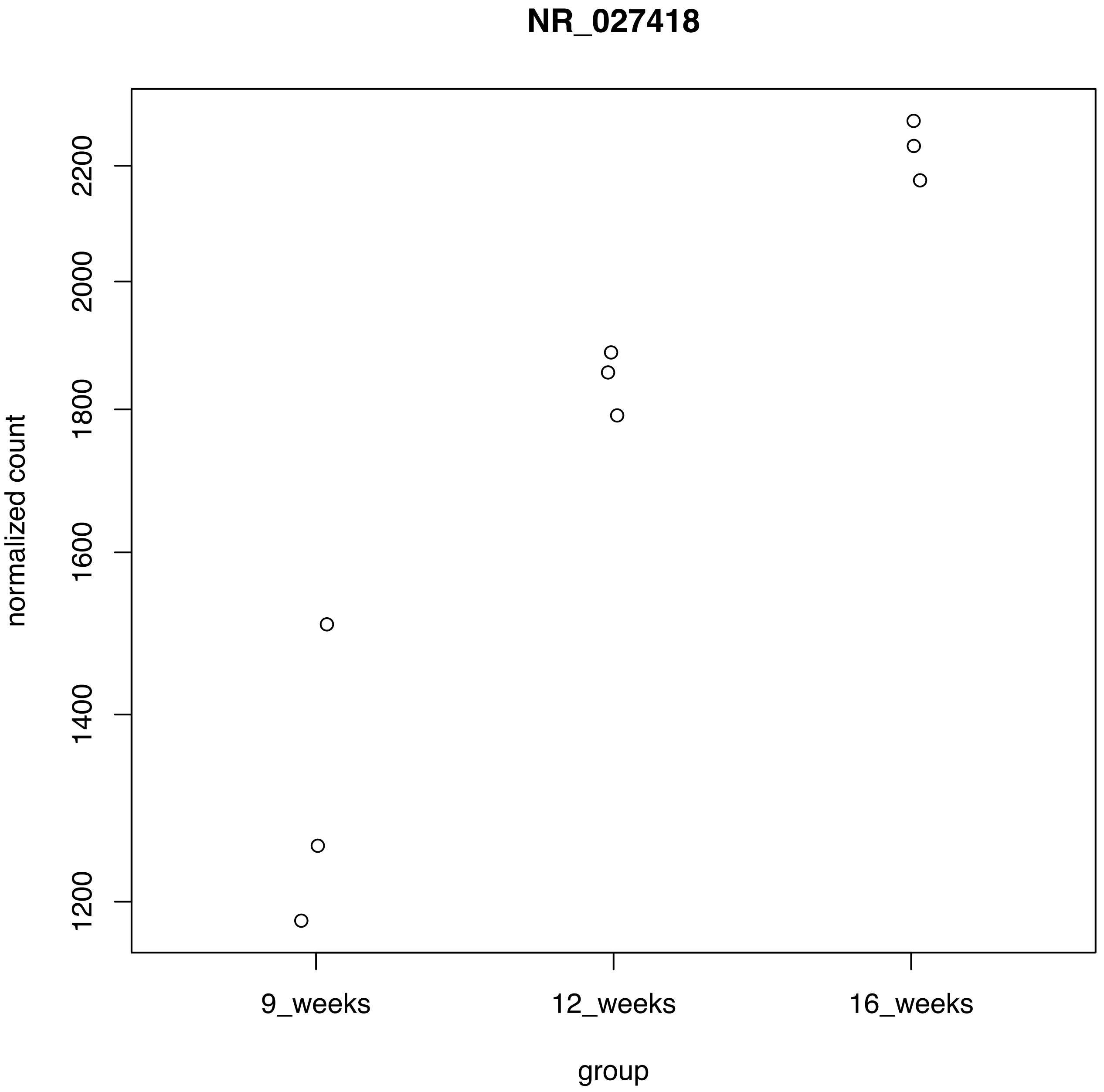
NR_027418
normalized count
1200
1400
1600
1800
2000
2200
9_weeks
12_weeks
16_weeks
group

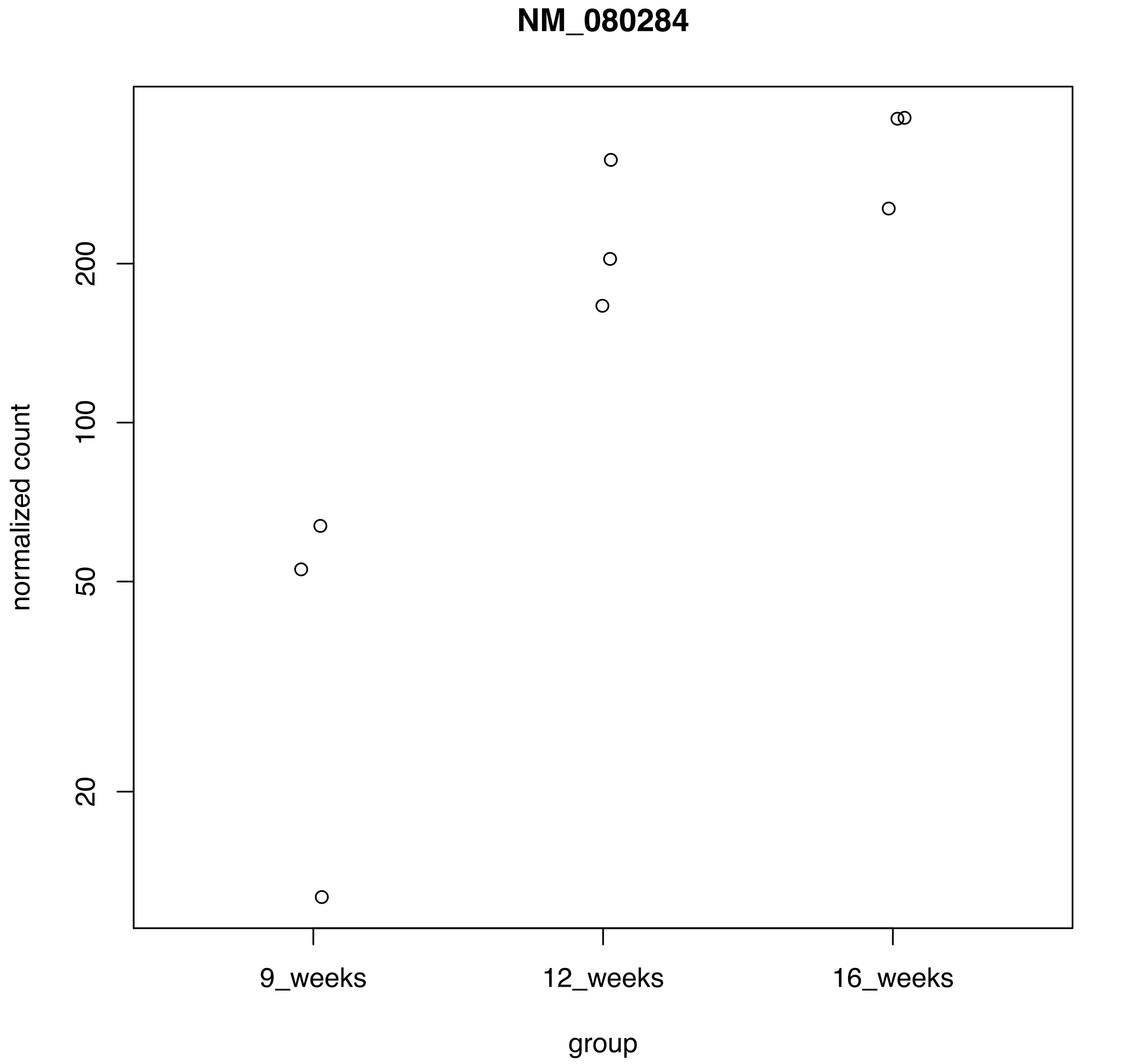
NM_080284
normalized count
20
50
100
200
9_weeks
12_weeks
16_weeks
group

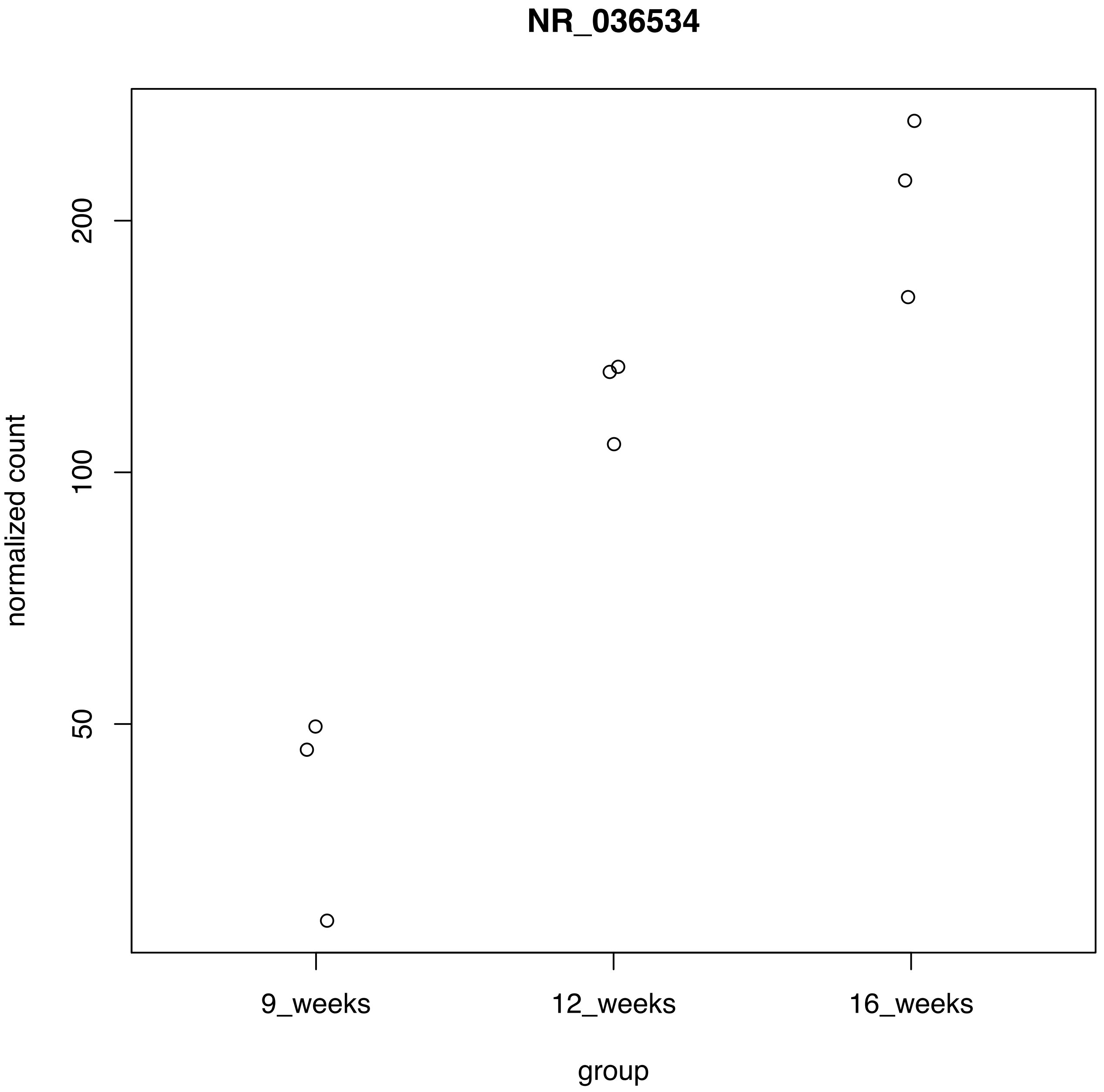
NR_036534
normalized count
200
100
50
9_weeks
12_weeks
16_weeks
group

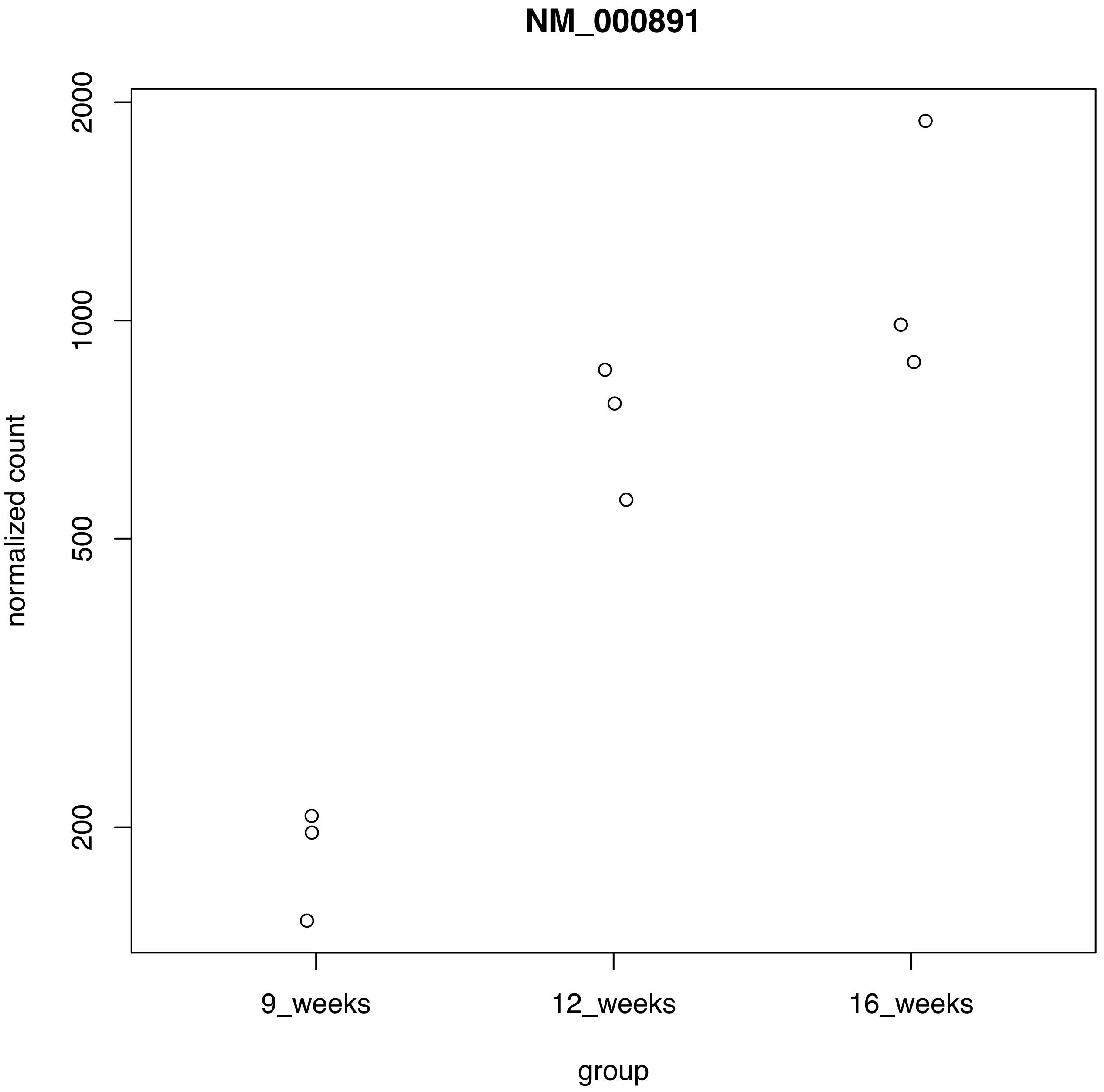
NM_000891
normalized count
2000
1000
500
200
9_weeks
12_weeks
16_weeks
group

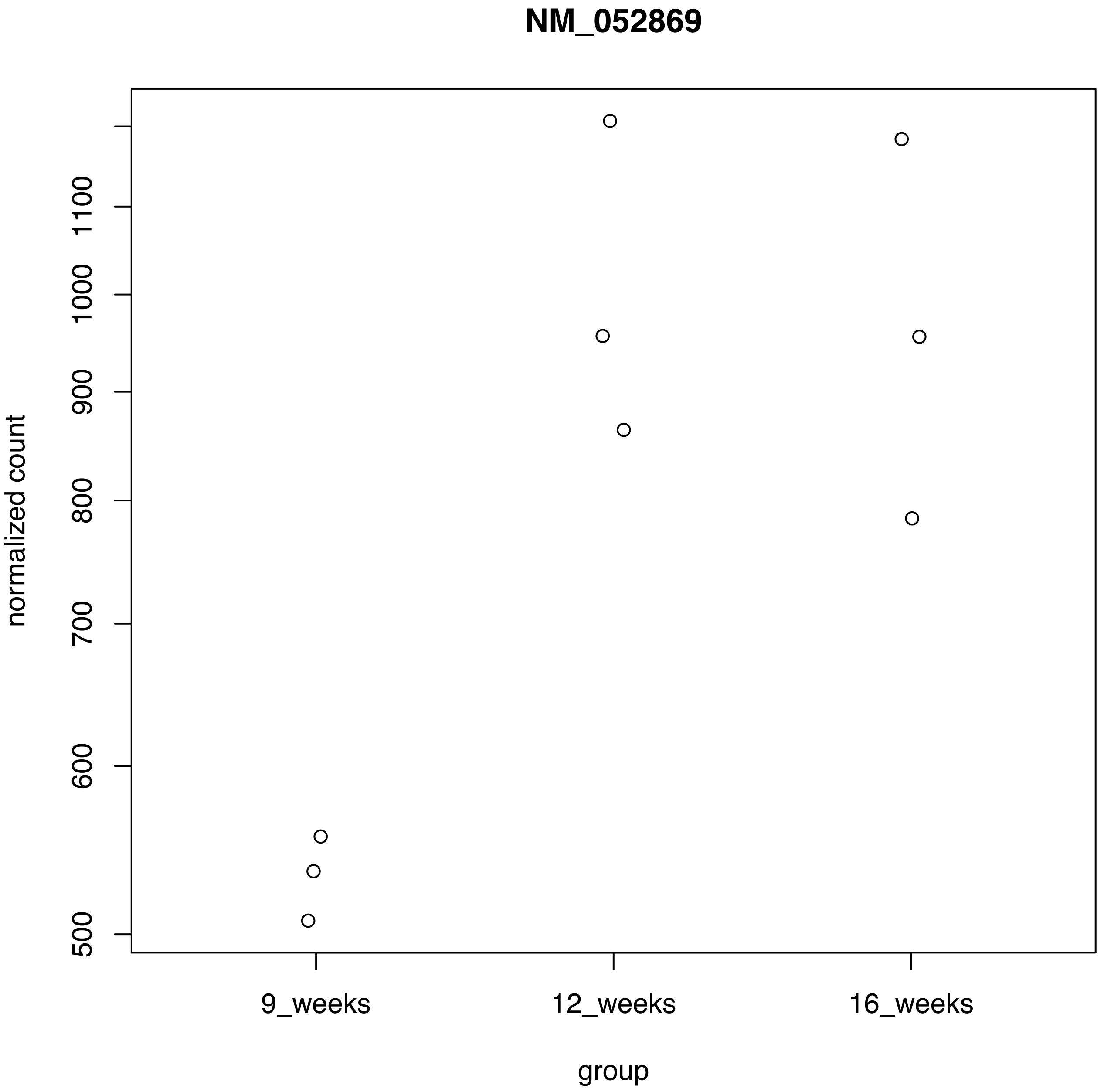

NM_052869
normalized count
500
600
700
800
900
1000
1100
9_weeks
12_weeks
16_weeks
group

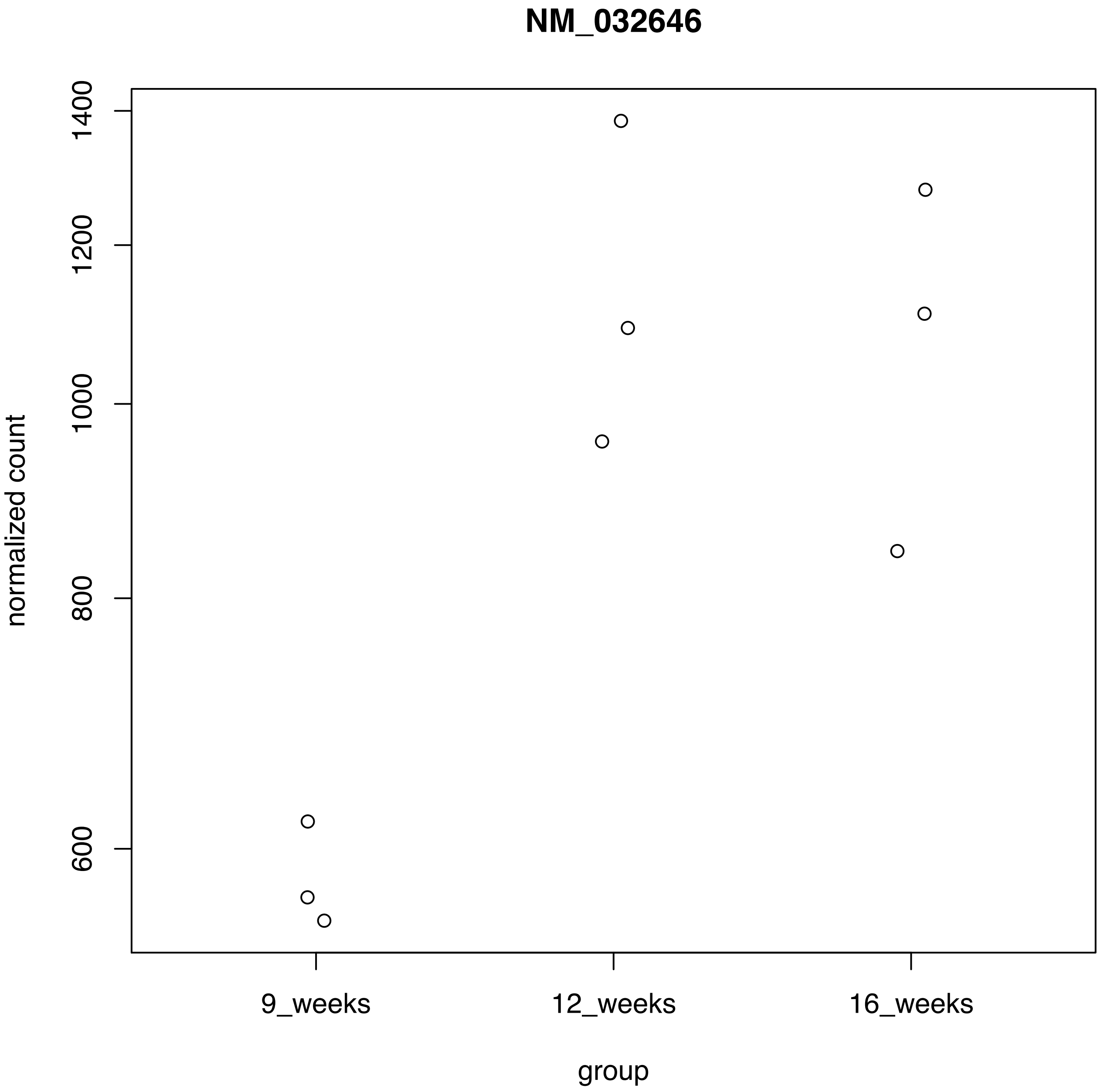
NM_032646
normalized count
600
800
1000
1200
1400
9_weeks
12_weeks
16_weeks
group

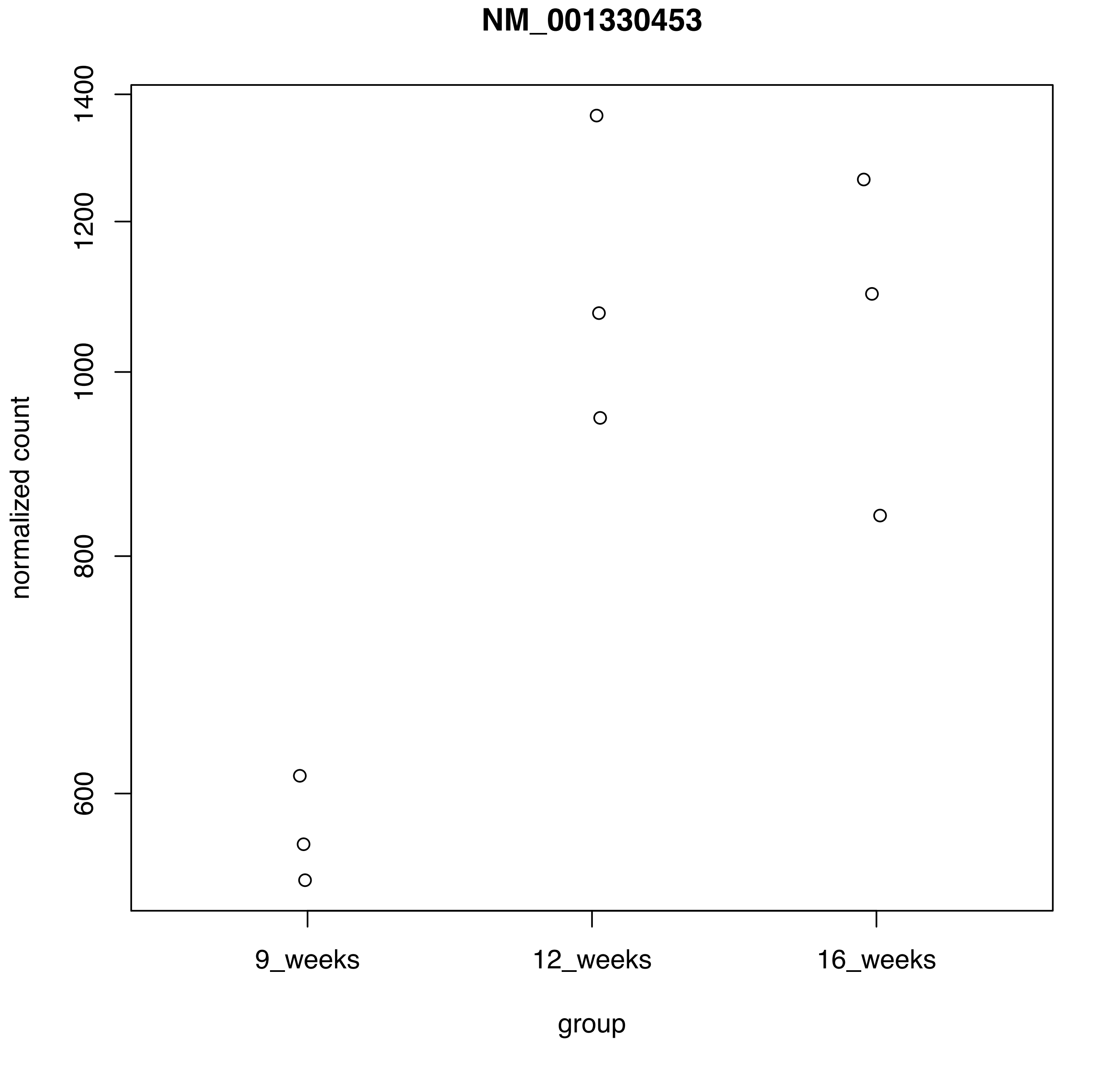
NM_001330453
normalized count
1400
1200
1000
800
600
9_weeks
12_weeks
16_weeks
group

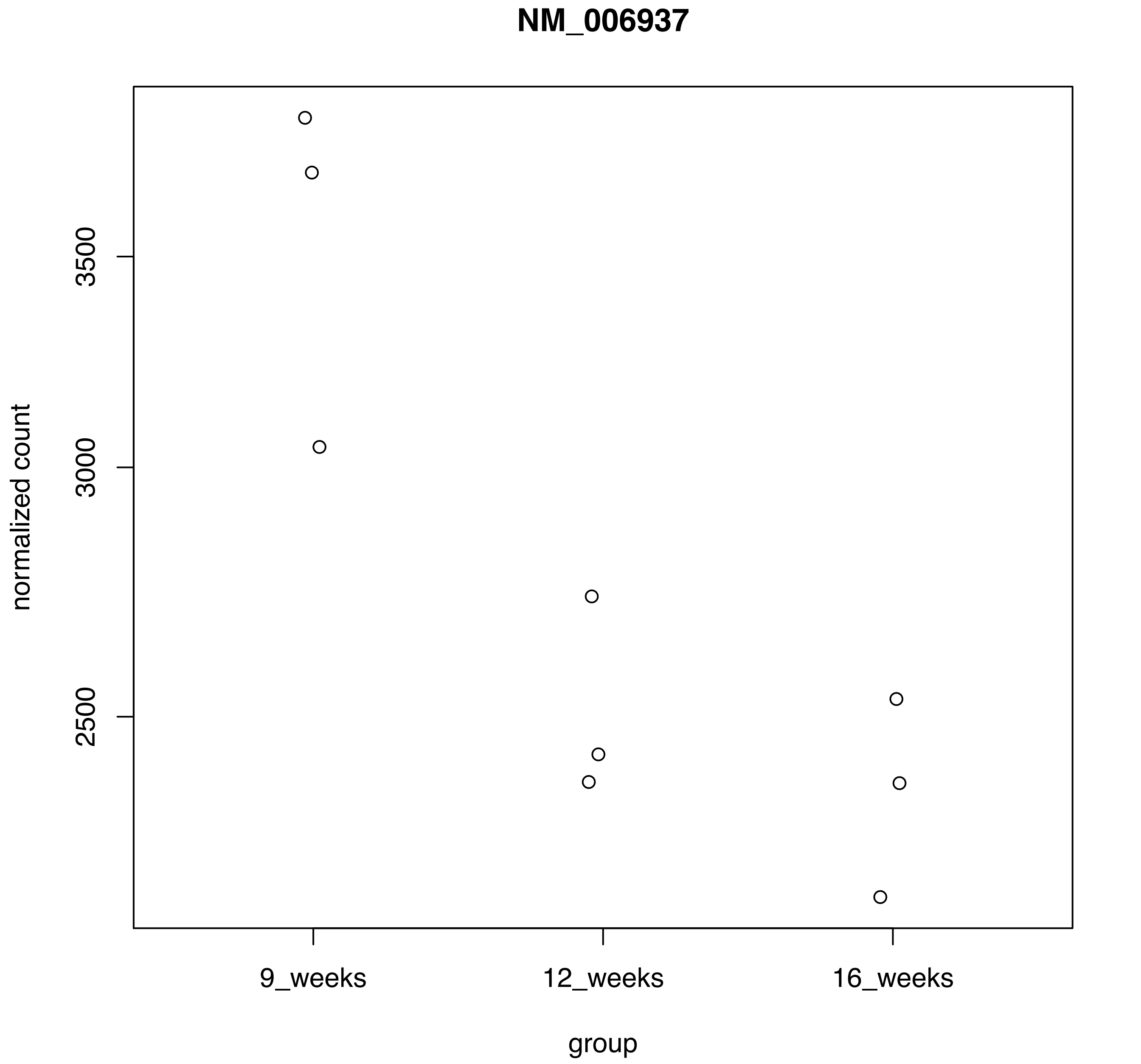
NM_006937
normalized count
3500
3000
2500
9_weeks
12_weeks
16_weeks
group

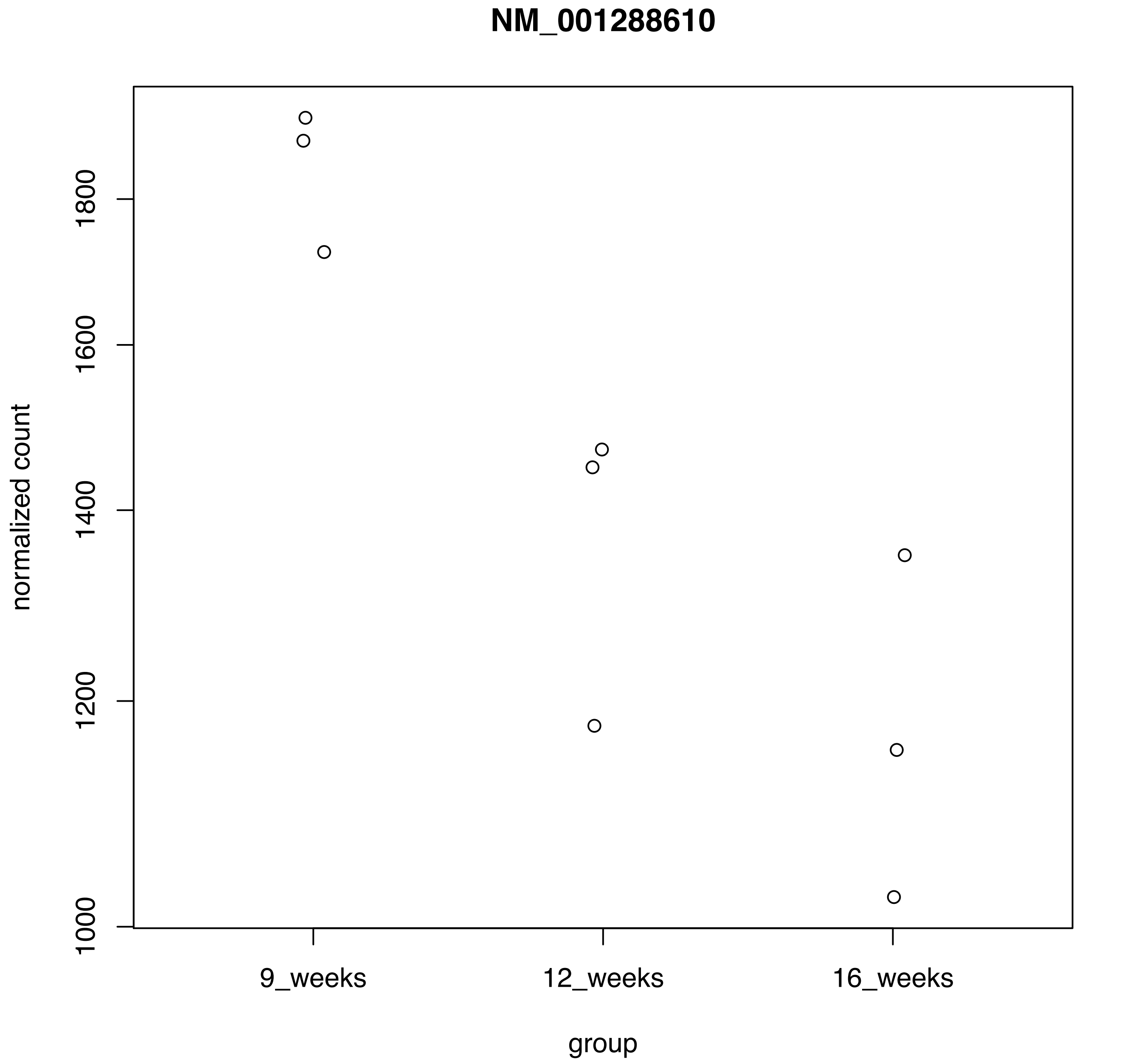

NM_001288610
normalized count
1000
1200
1400
1600
1800
9_weeks
12_weeks
16_weeks
group

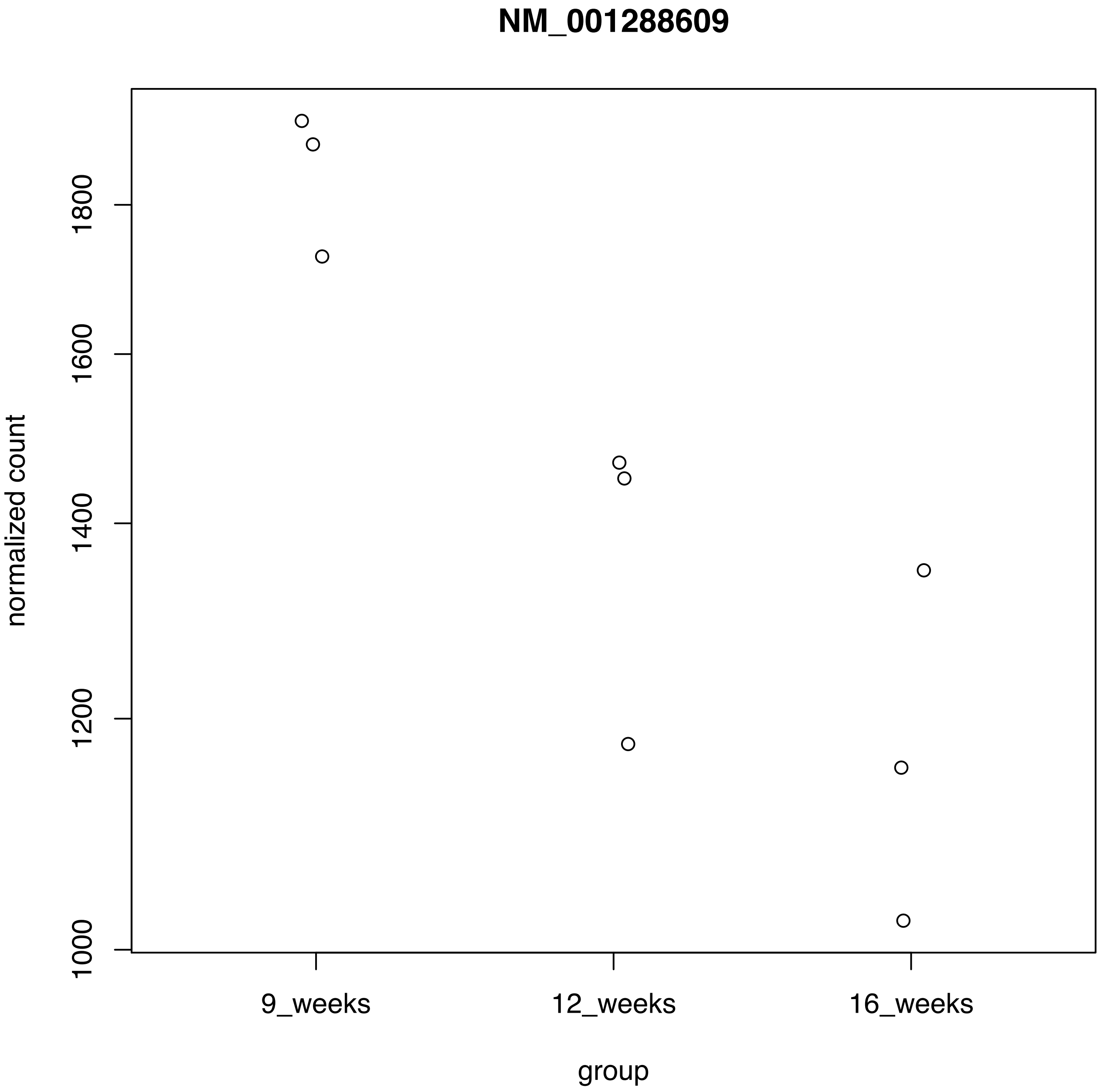

NM_001288609
normalized count
1000
1200
1400
1600
1800
9_weeks
12_weeks
16_weeks
group

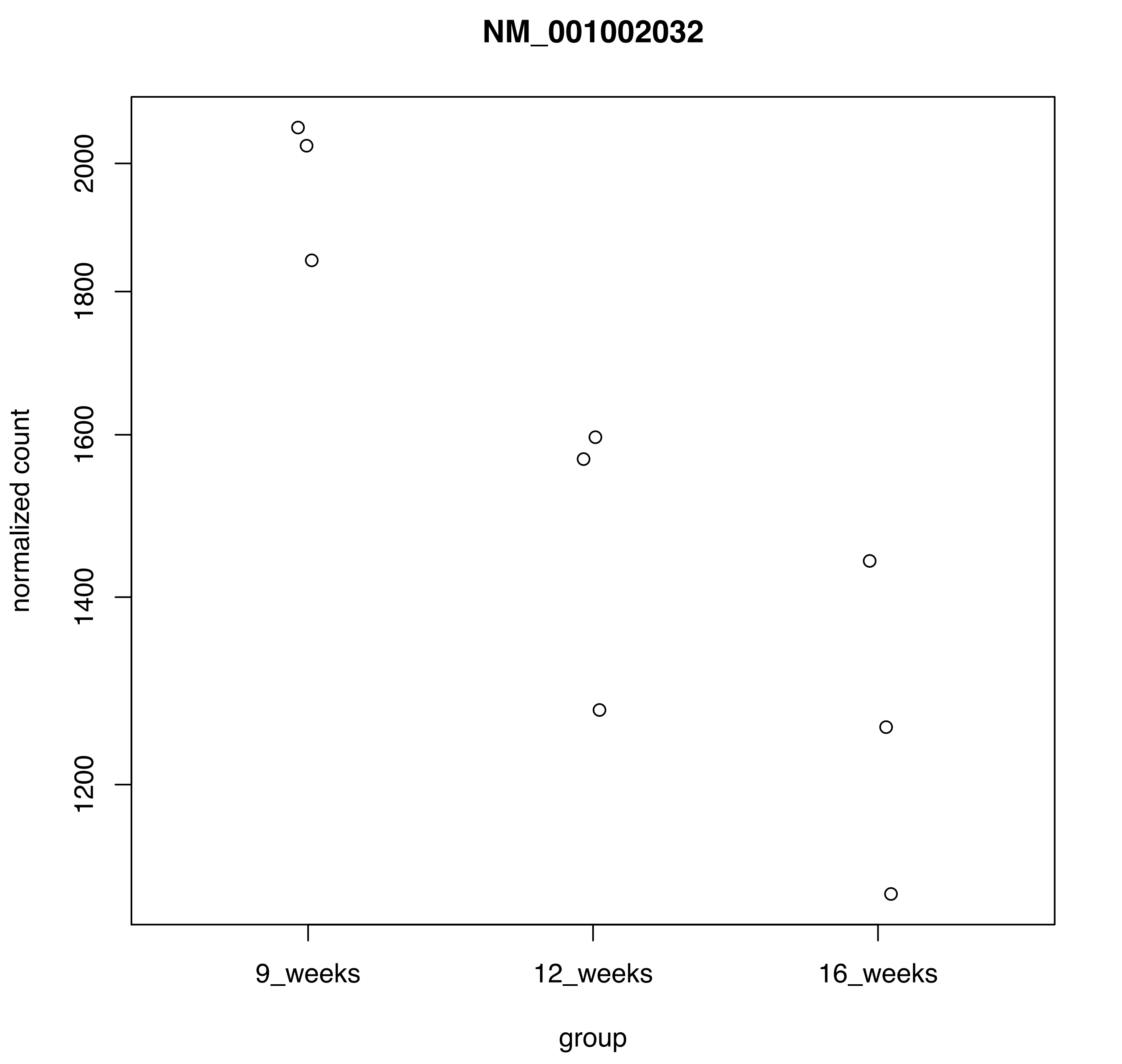
NM_001002032
normalized count
2000
1800
1600
1400
1200
9_weeks
12_weeks
16_weeks
group

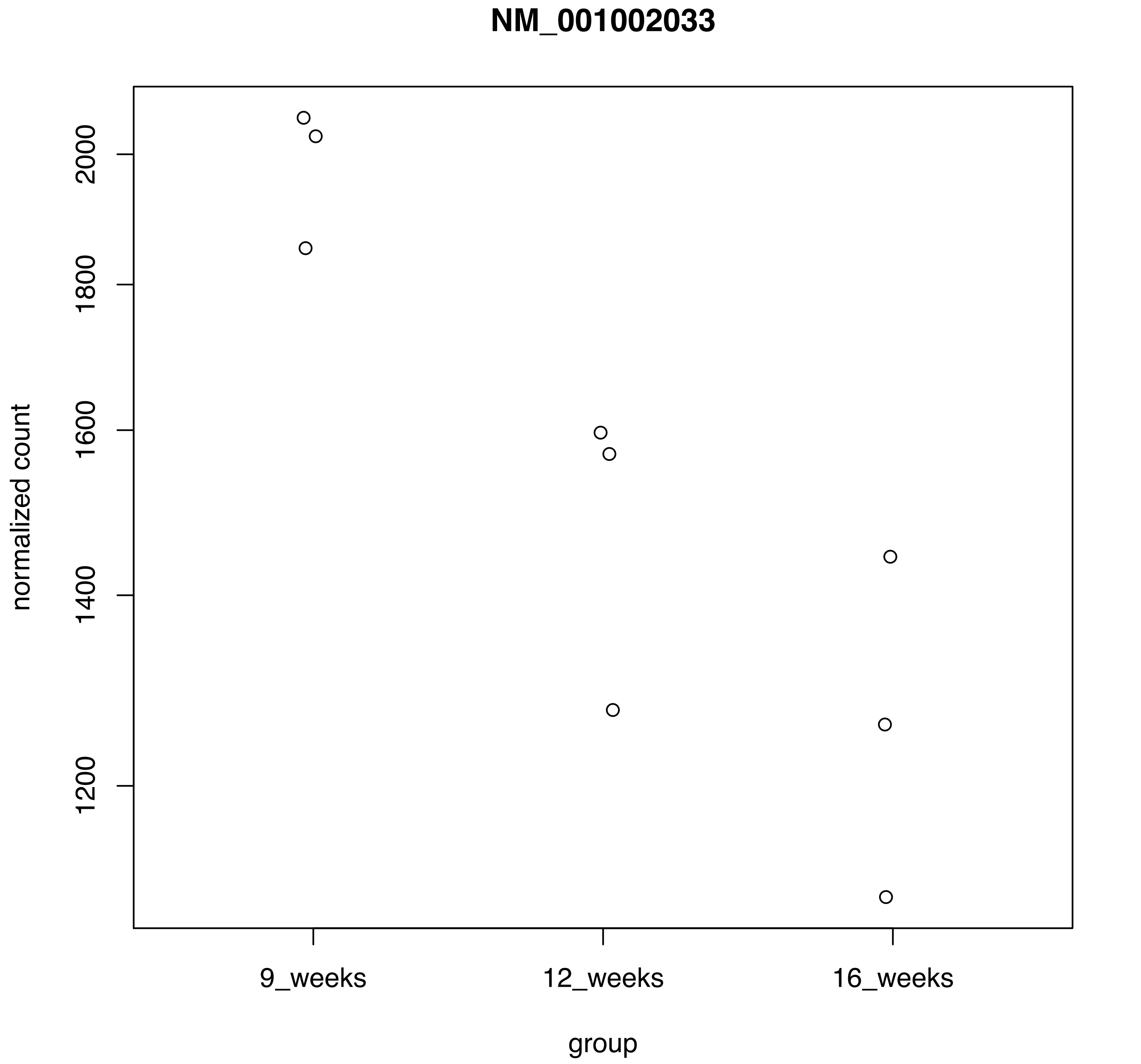

NM_001002033
normalized count
2000
1800
1600
1400
1200
9_weeks
12_weeks
16_weeks
group

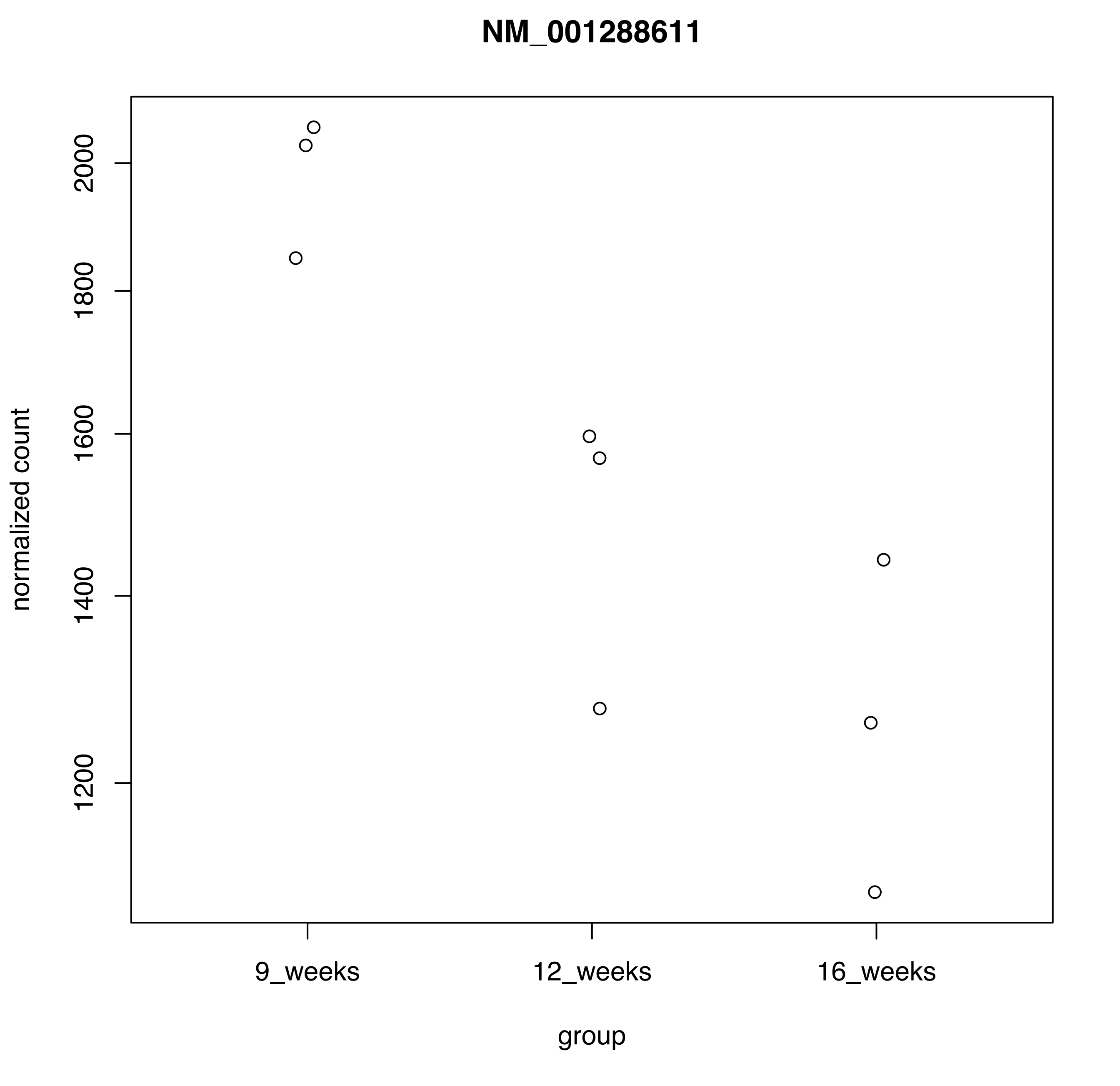

NM_001288611
normalized count
2000
1800
1600
1400
1200
9_weeks
12_weeks
16_weeks
group

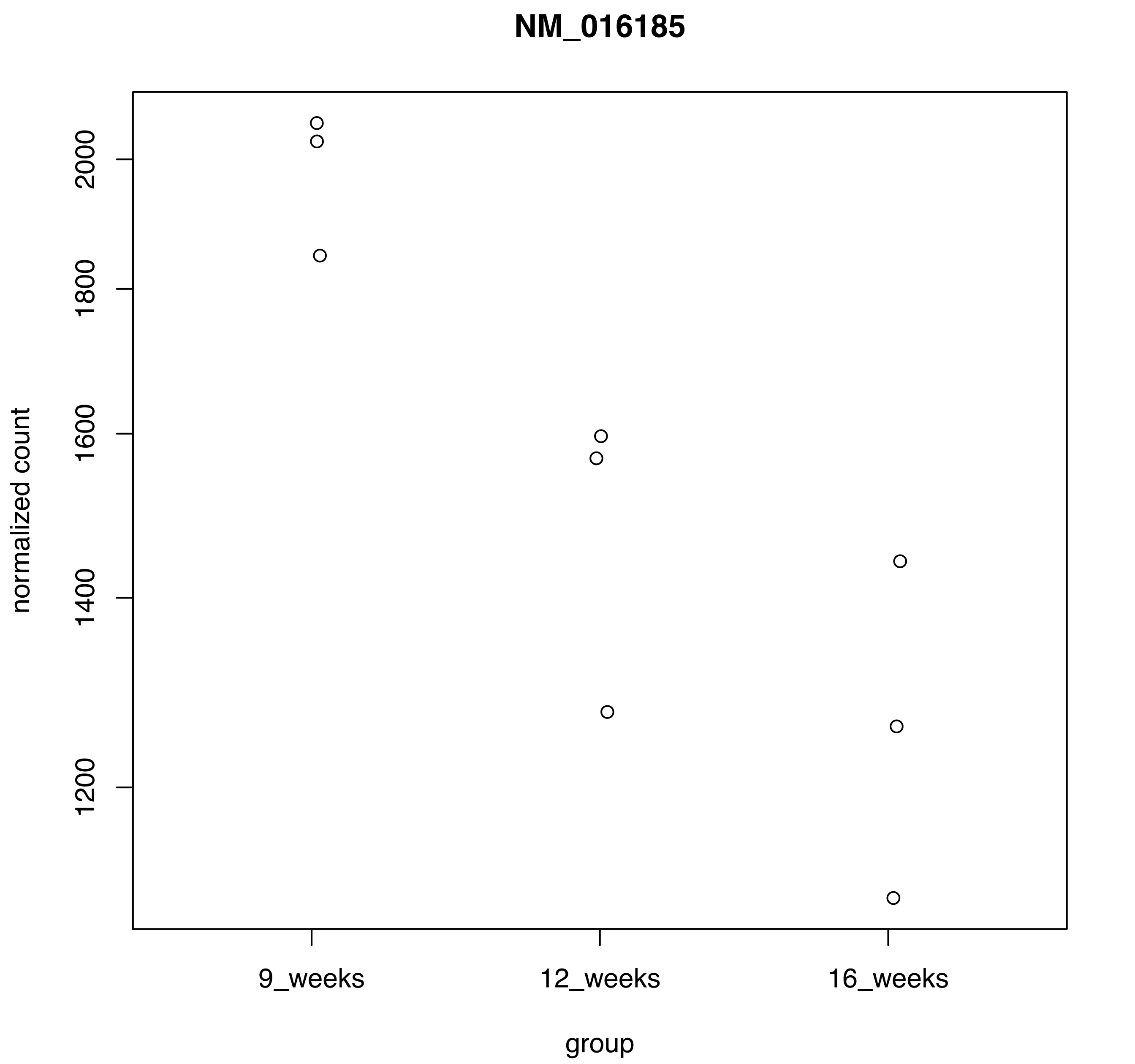
NM_016185
normalized count
2000
1800
1600
1400
1200
9_weeks
12_weeks
16_weeks
group

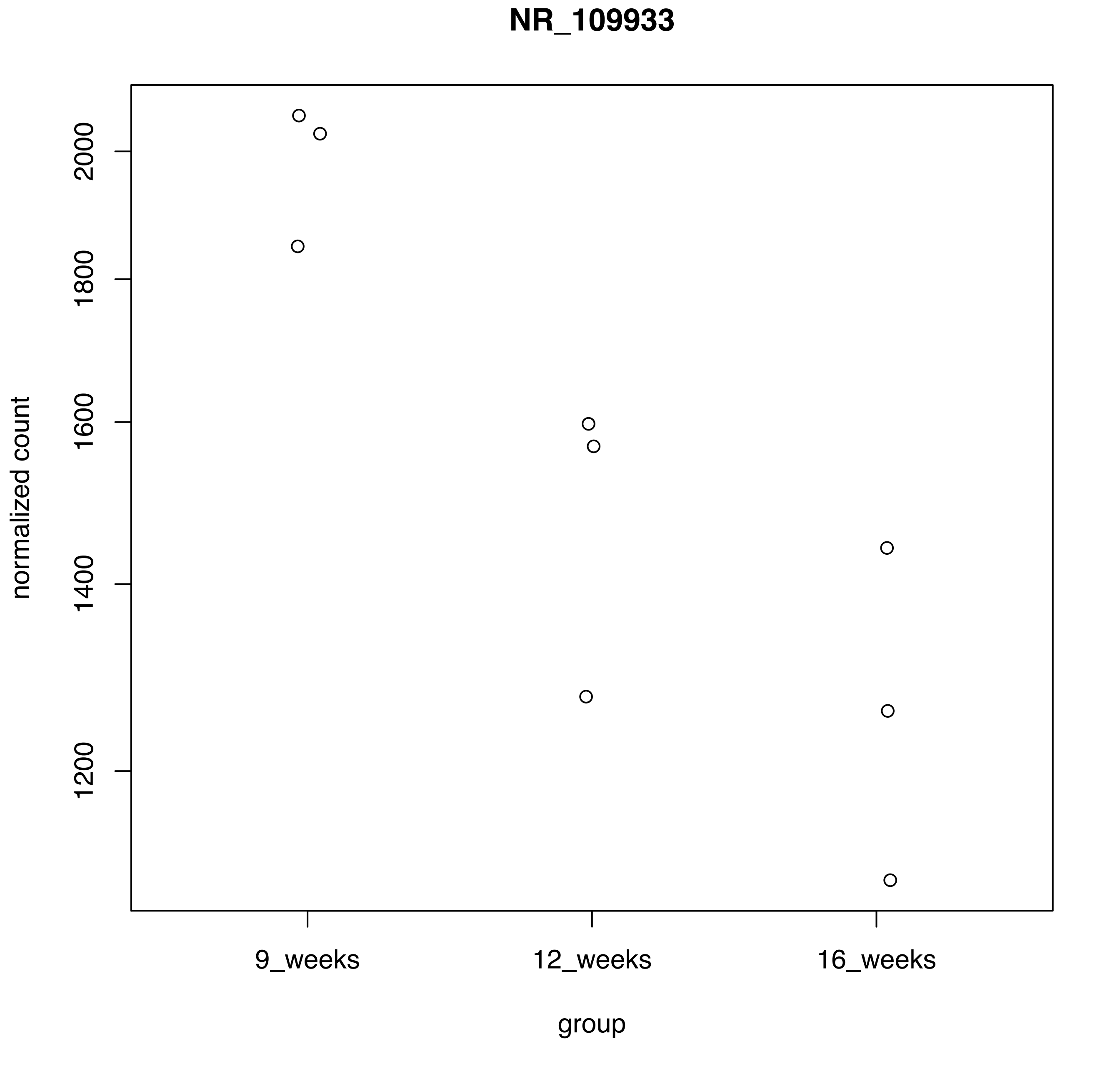

NR_109933
normalized count
2000
1800
1600
1400
1200
9_weeks
12_weeks
16_weeks
group

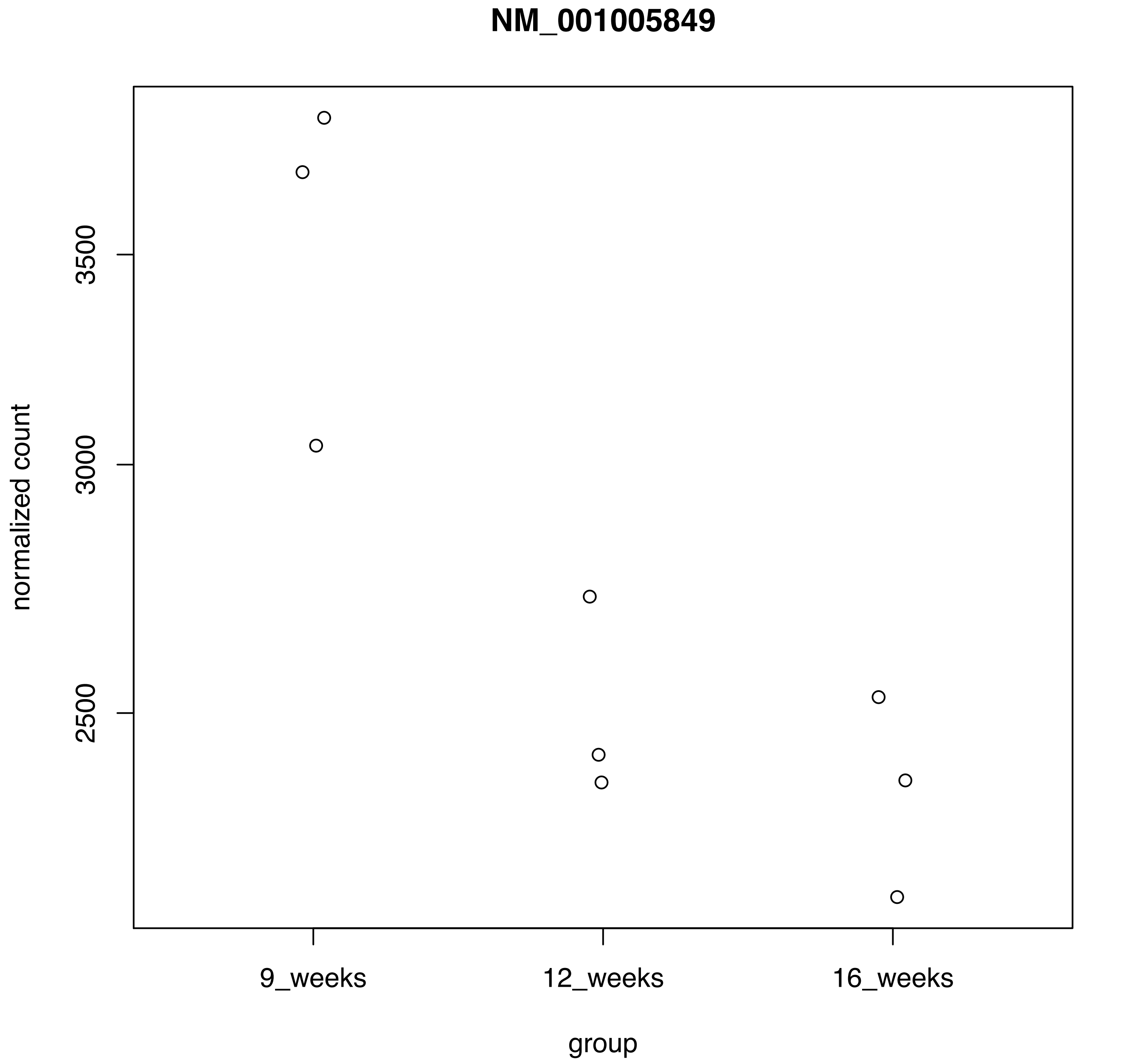
NM_001005849
normalized count
3500
3000
2500
9_weeks
12_weeks
16_weeks
group

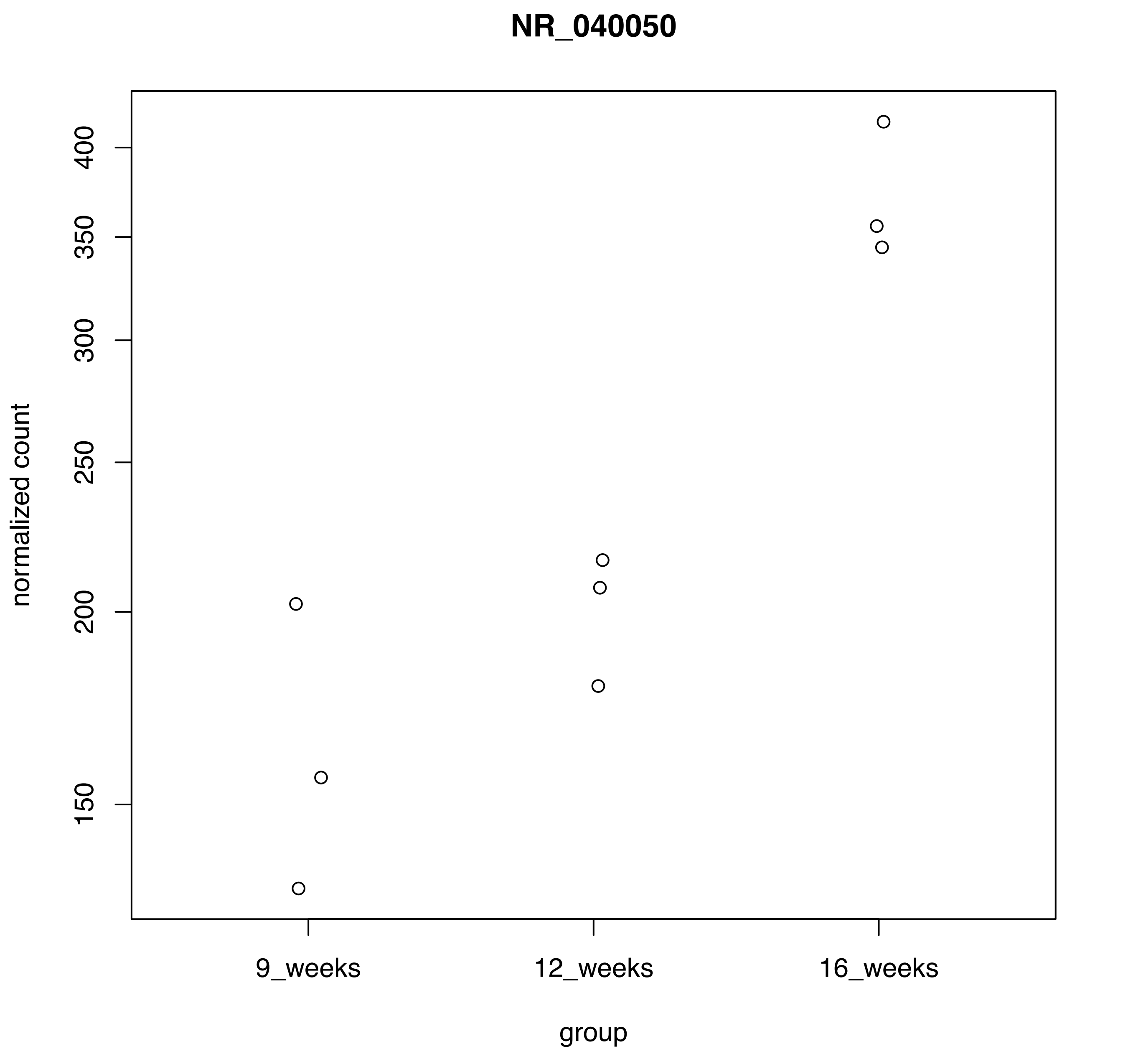
NR_040050
normalized count
150
200
250
300
350
400
9_weeks
12_weeks
16_weeks
group

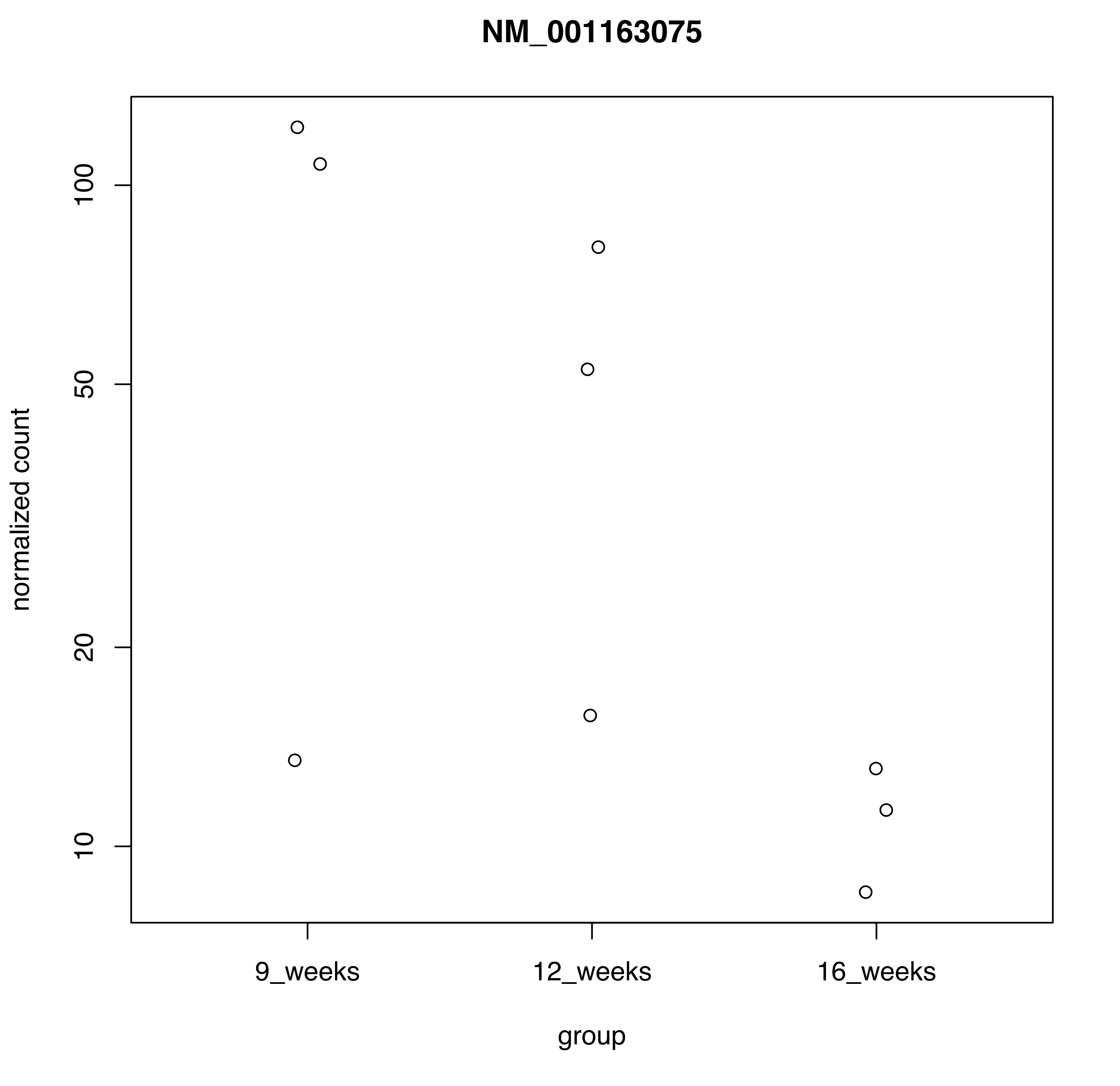

NM_001163075
normalized count
100
50
20
10
9_weeks
12_weeks
16_weeks
group

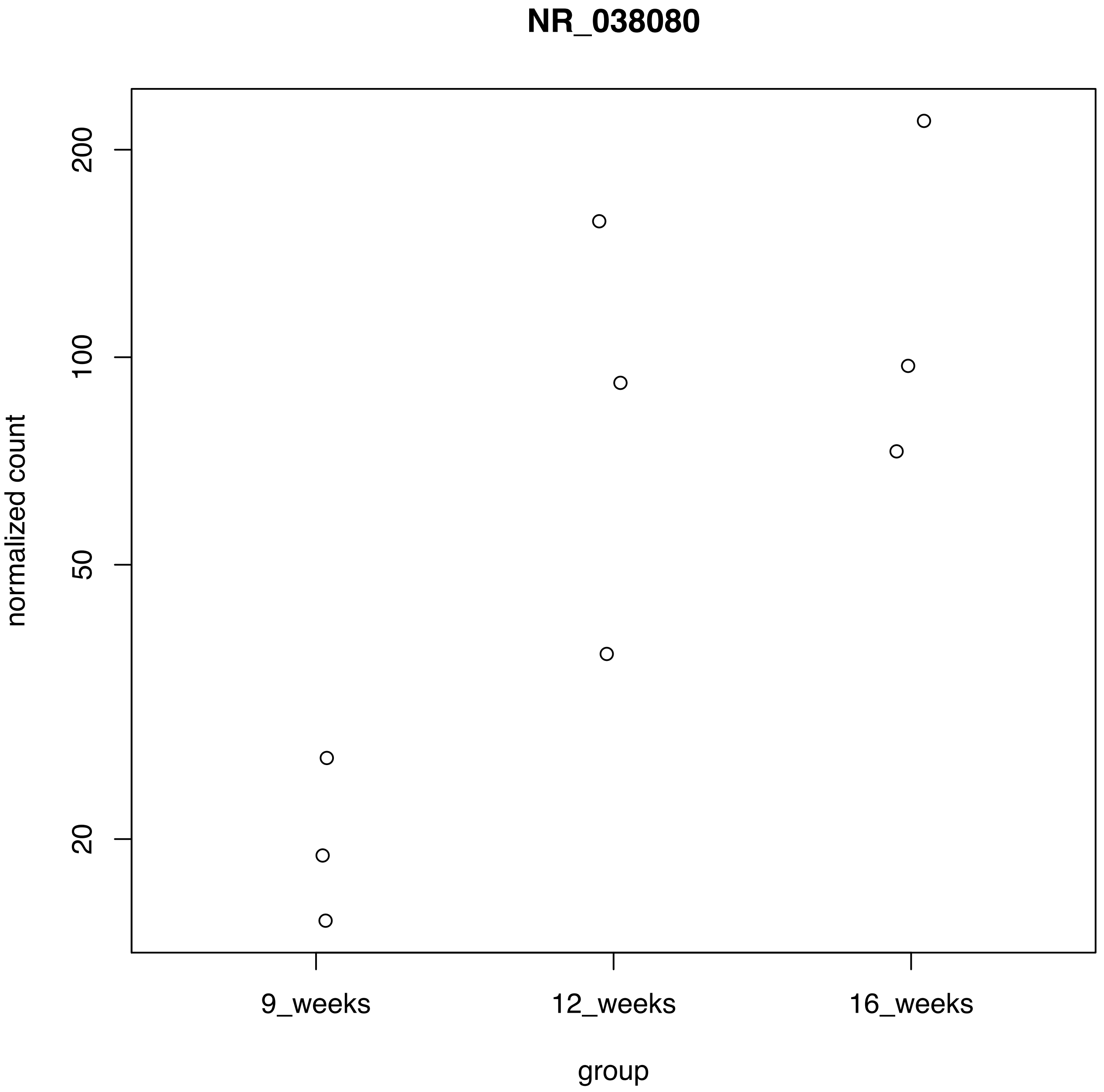
NR_038080
normalized count
200
100
50
20
9_weeks
12_weeks
16_weeks
group

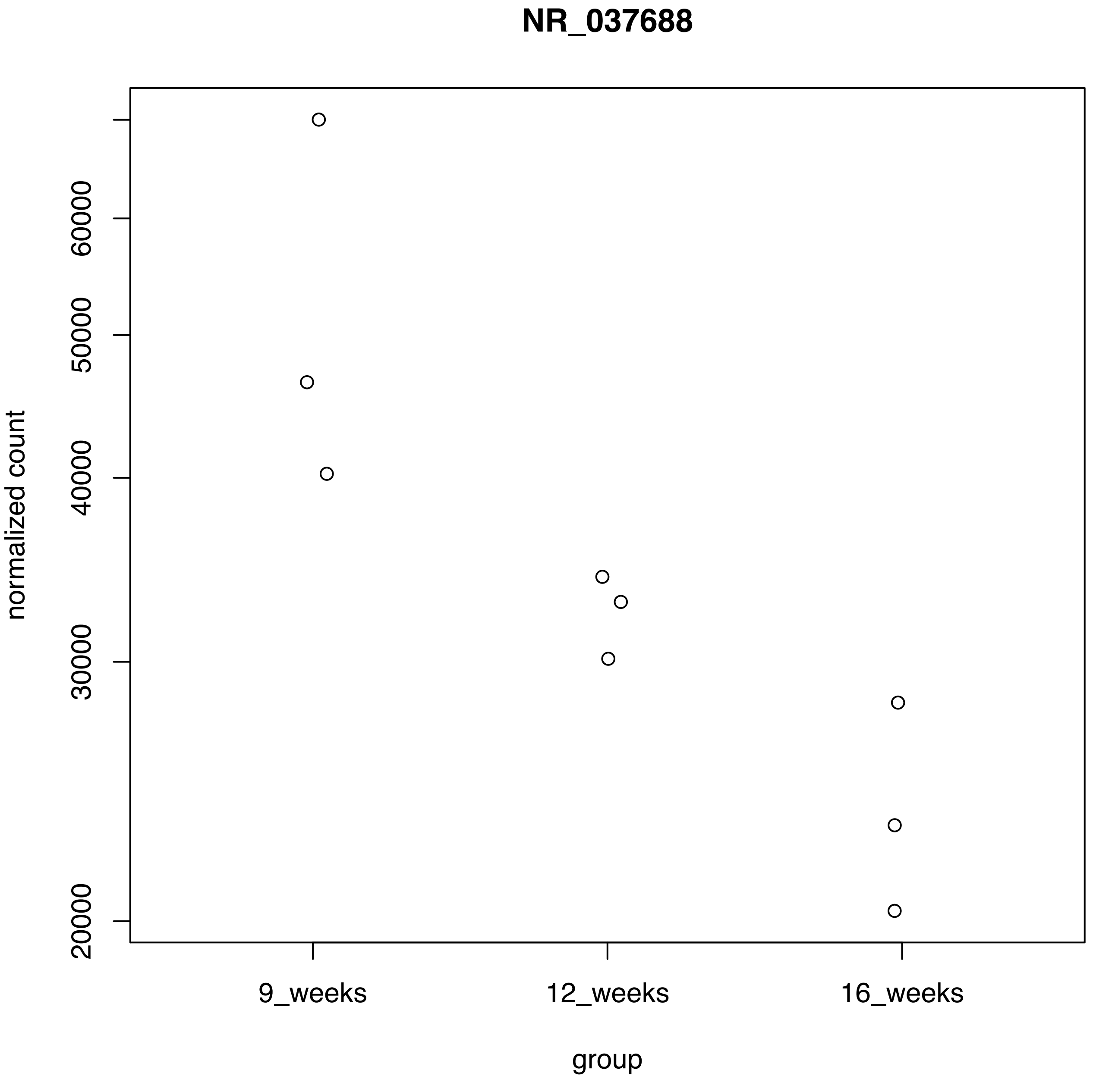

NR_037688
normalized count
20000
30000
40000
50000
60000
9_weeks
12_weeks
16_weeks
group

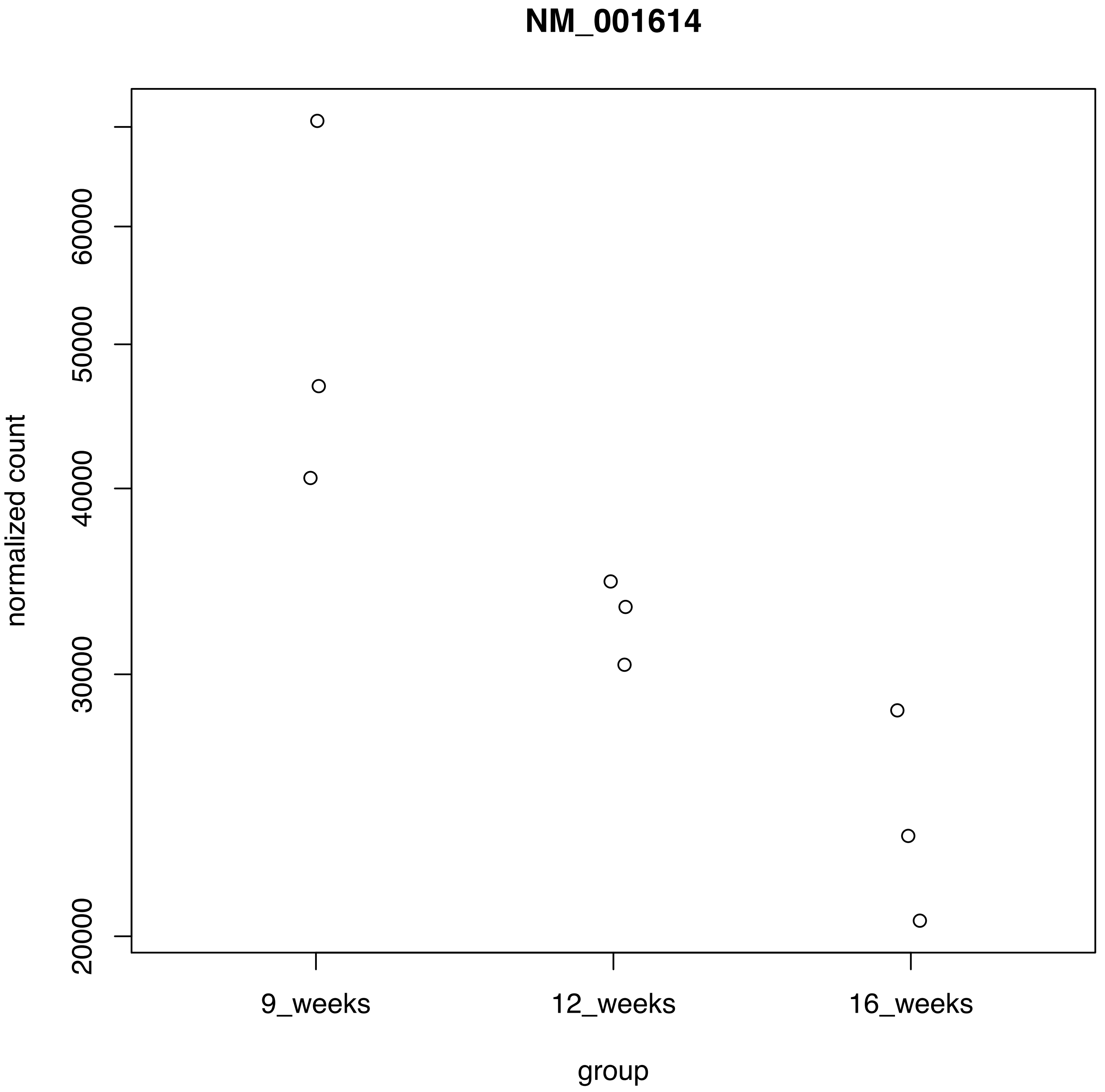
NM_001614
normalized count
20000
30000
40000
50000
60000
9_weeks
12_weeks
16_weeks
group

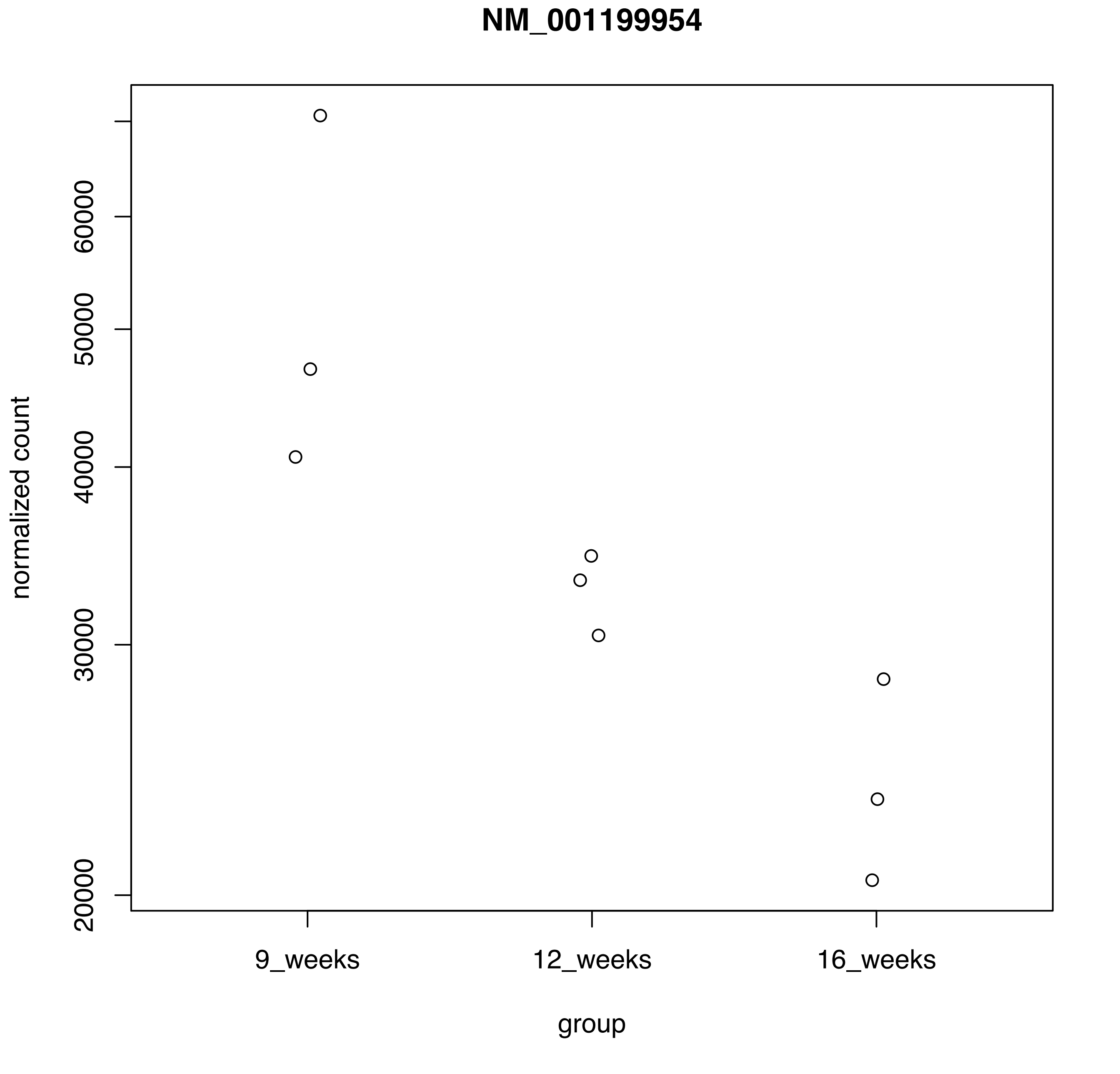
NM_001199954
normalized count
20000
30000
40000
50000
60000
9_weeks
12_weeks
16_weeks
group

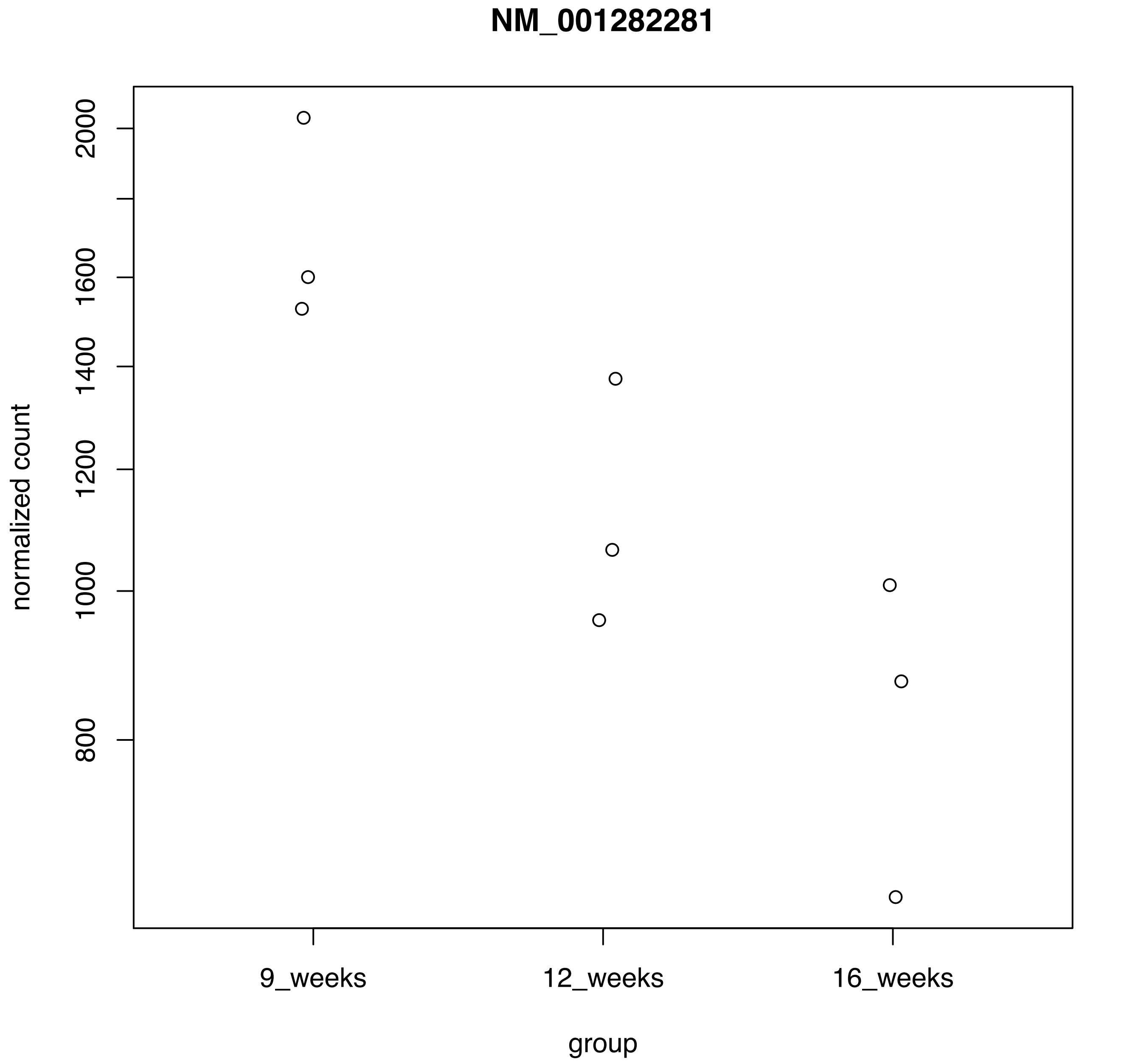
NM_001282281
normalized count
2000
1600
1400
1200
1000
800
9_weeks
12_weeks
16_weeks
group

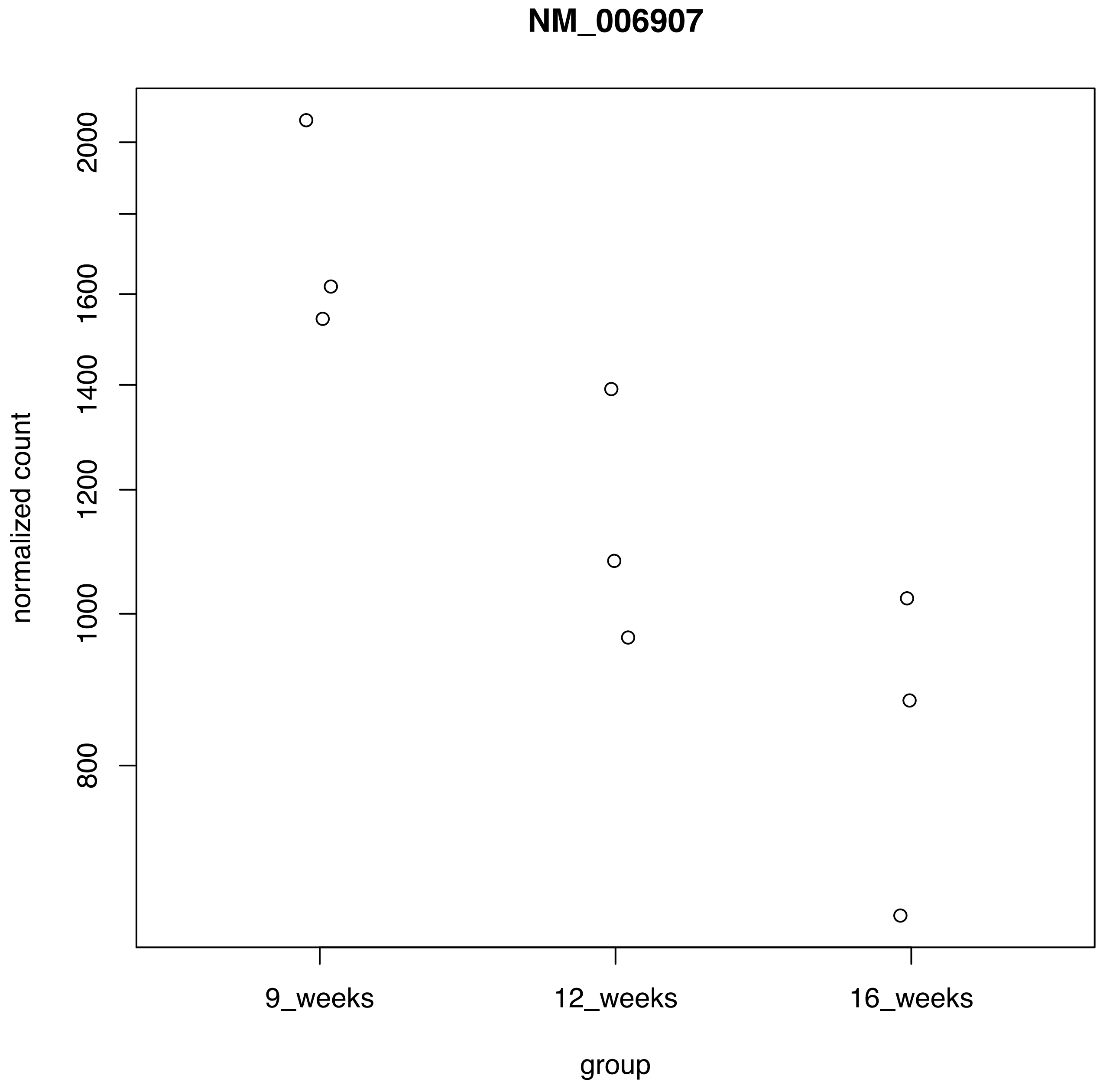
NM_006907
normalized count
2000
1600
1400
1200
1000
800
9_weeks
12_weeks
16_weeks
group

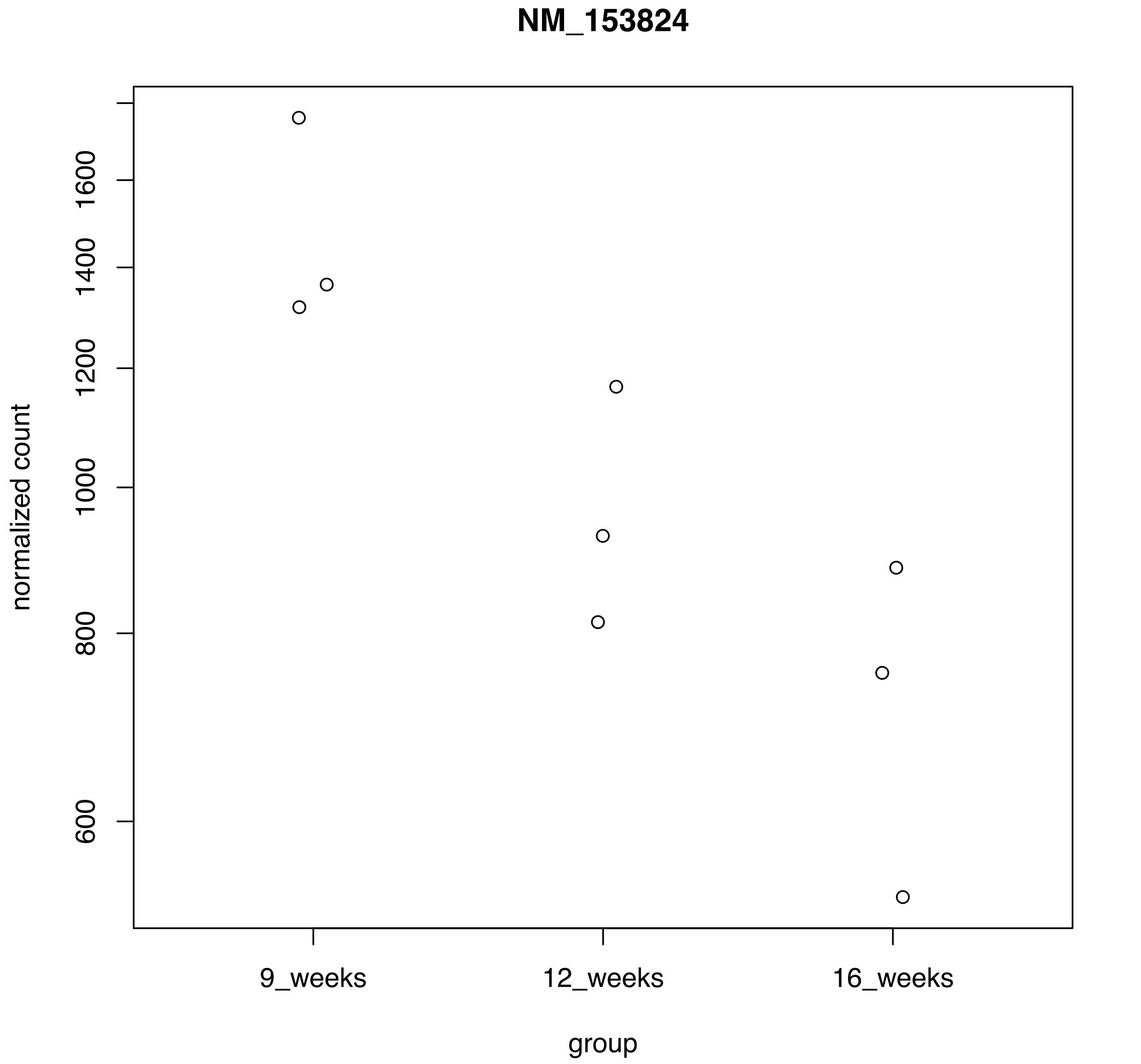
NM_153824
normalized count
1600
1400
1200
1000
800
600
9_weeks
12_weeks
16_weeks
group

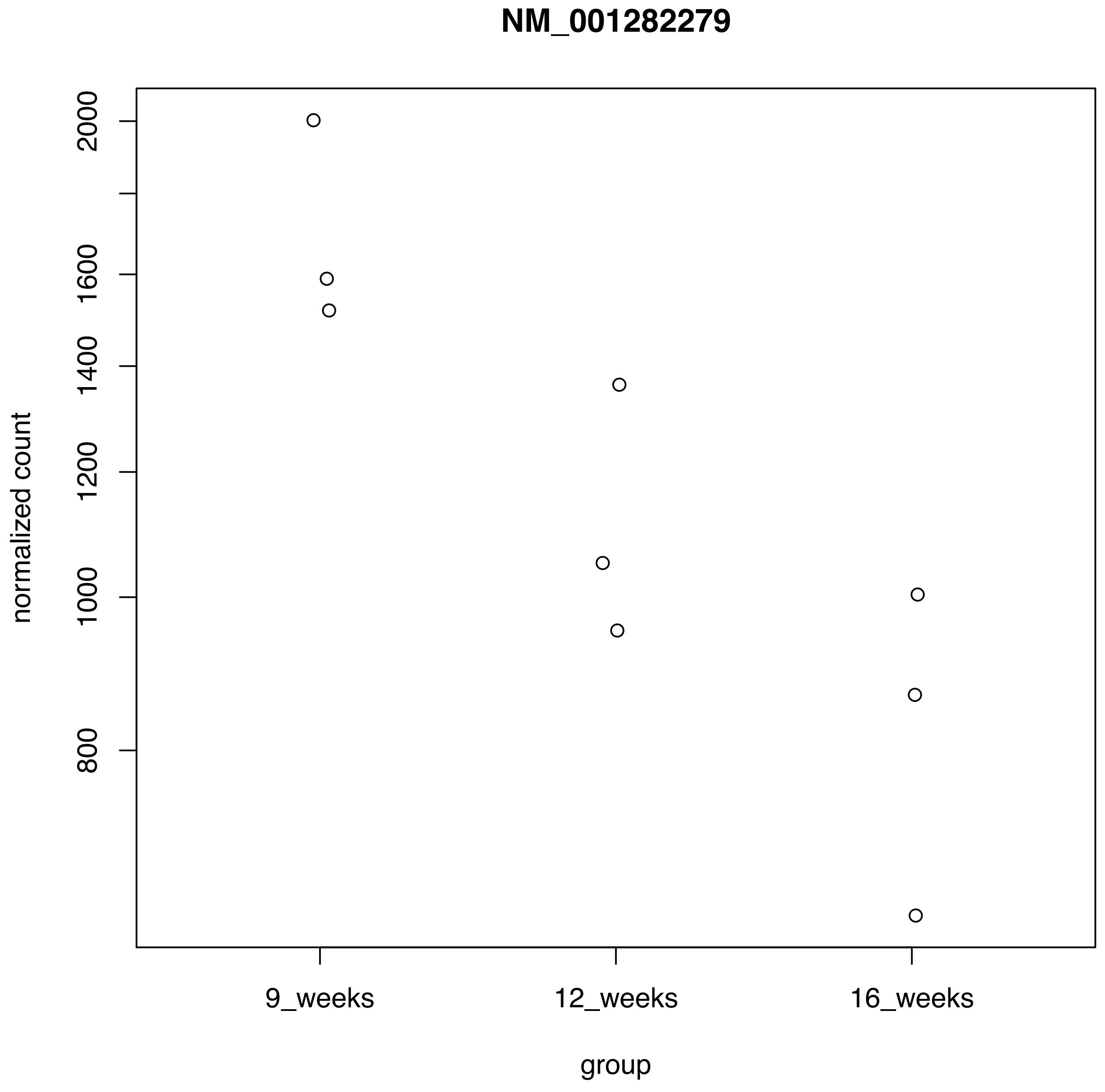
NM_001282279
normalized count
2000
1600
1400
1200
1000
800
9_weeks
12_weeks
16_weeks
group

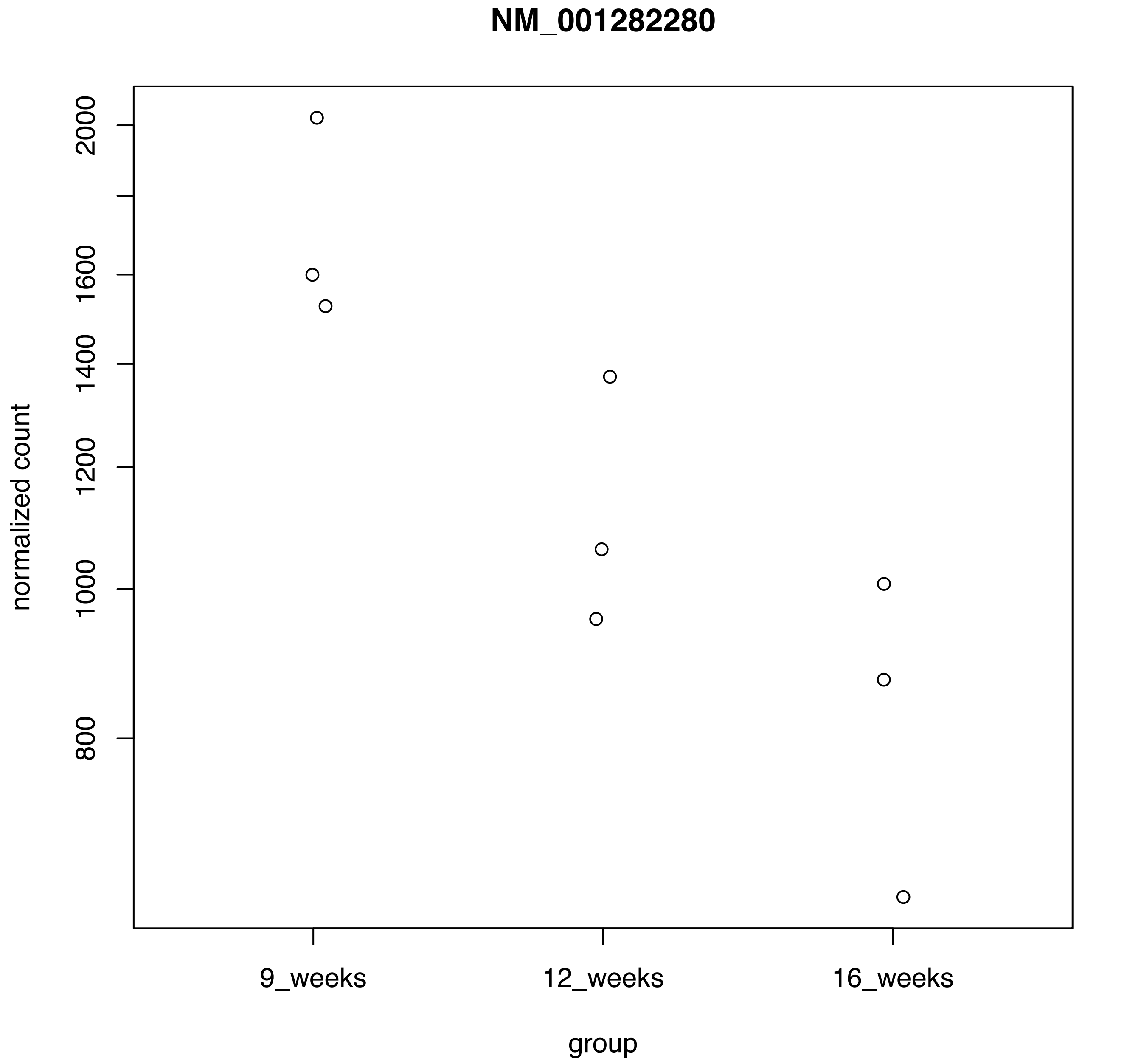
NM_001282280
normalized count
2000
1600
1400
1200
1000
800
9_weeks
12_weeks
16_weeks
group

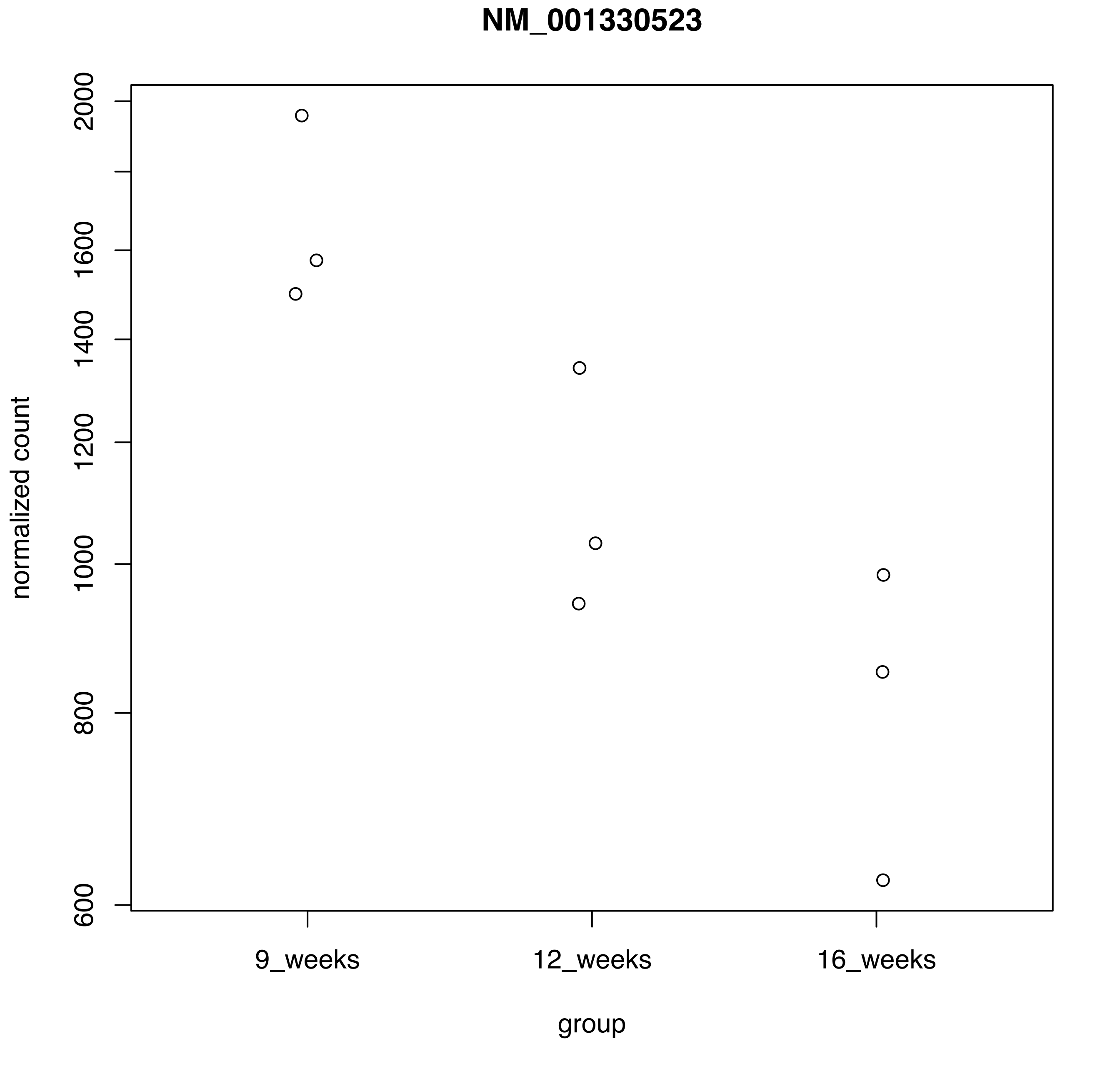
NM_001330523
normalized count
600
800
1000
1200
1400
1600
2000
9_weeks
12_weeks
16_weeks
group

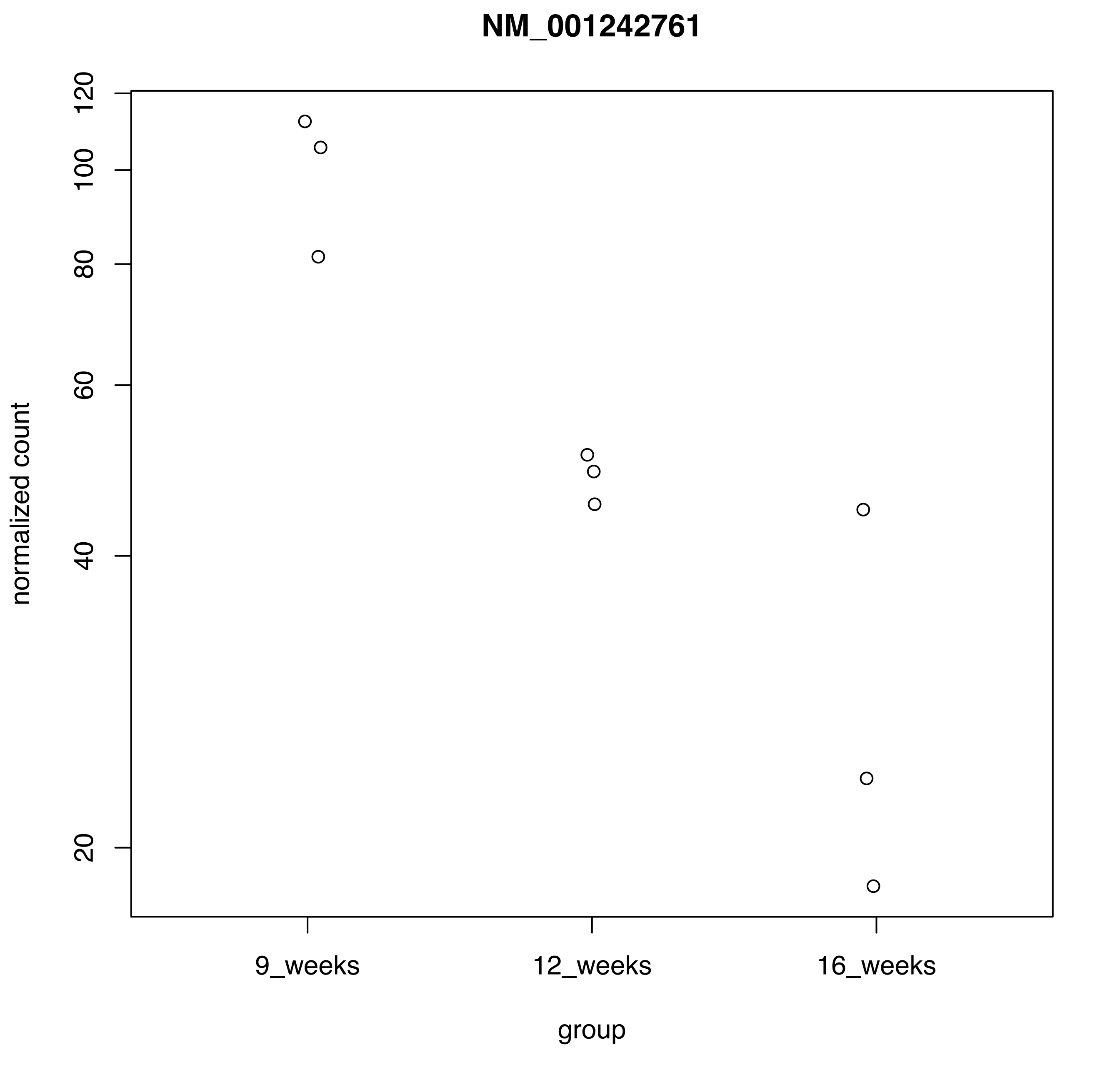
NM_001242761
normalized count
120
100
80
60
40
20
9_weeks
12_weeks
16_weeks
group

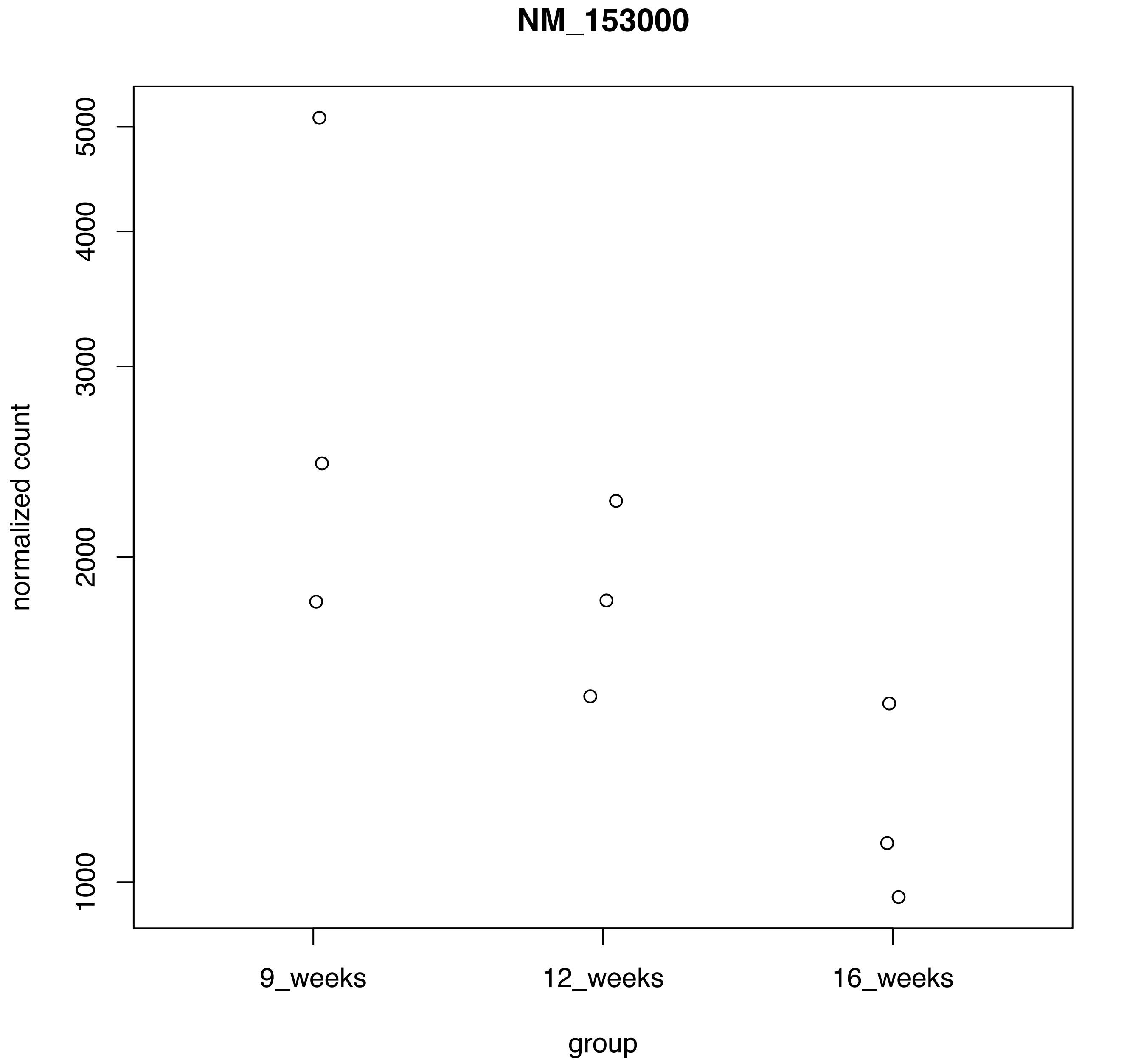
NM_153000
normalized count
5000
4000
3000
2000
1000
9_weeks
12_weeks
16_weeks
group

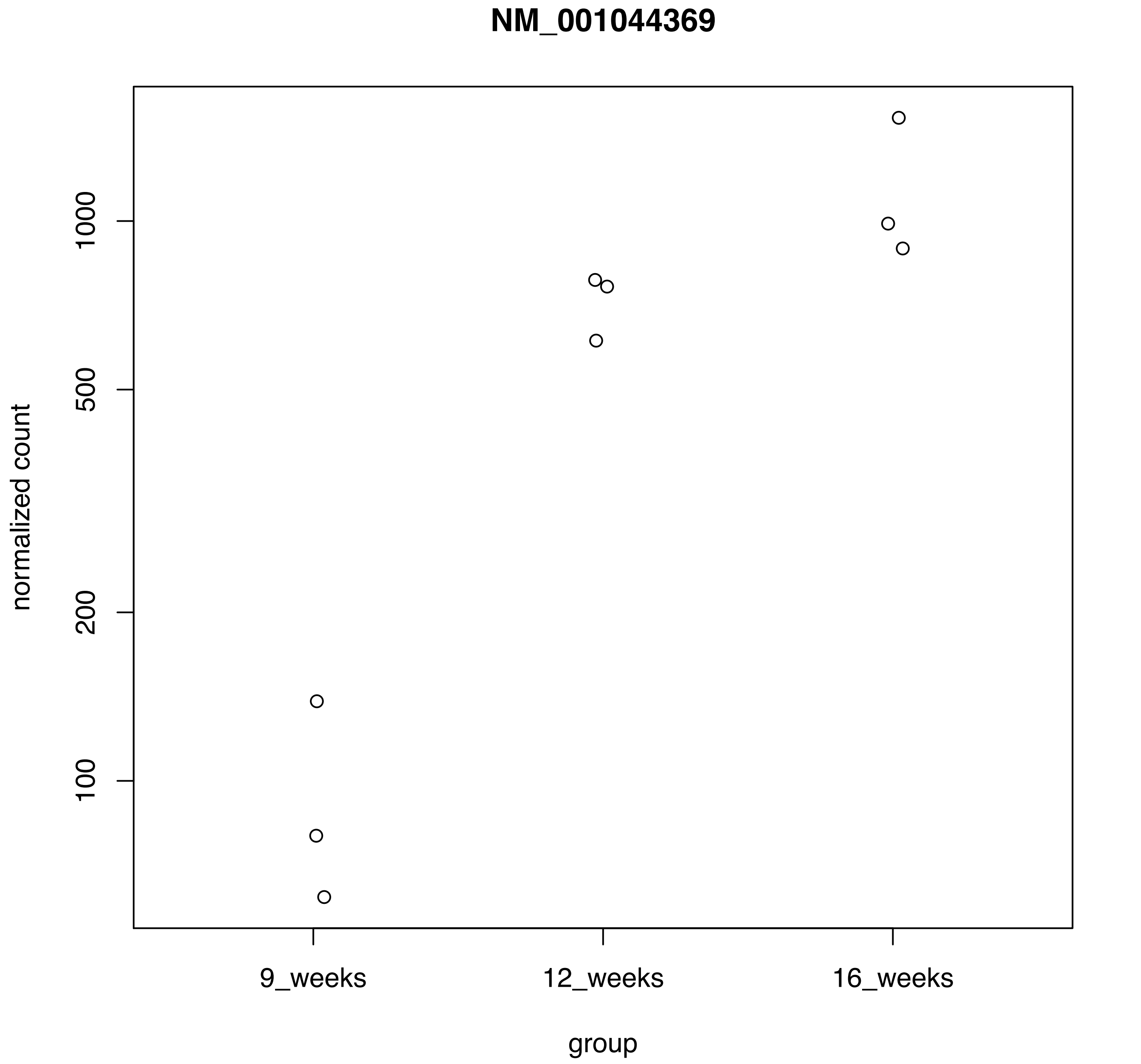
NM_001044369
normalized count
1000
500
200
100
9_weeks
12_weeks
16_weeks
group

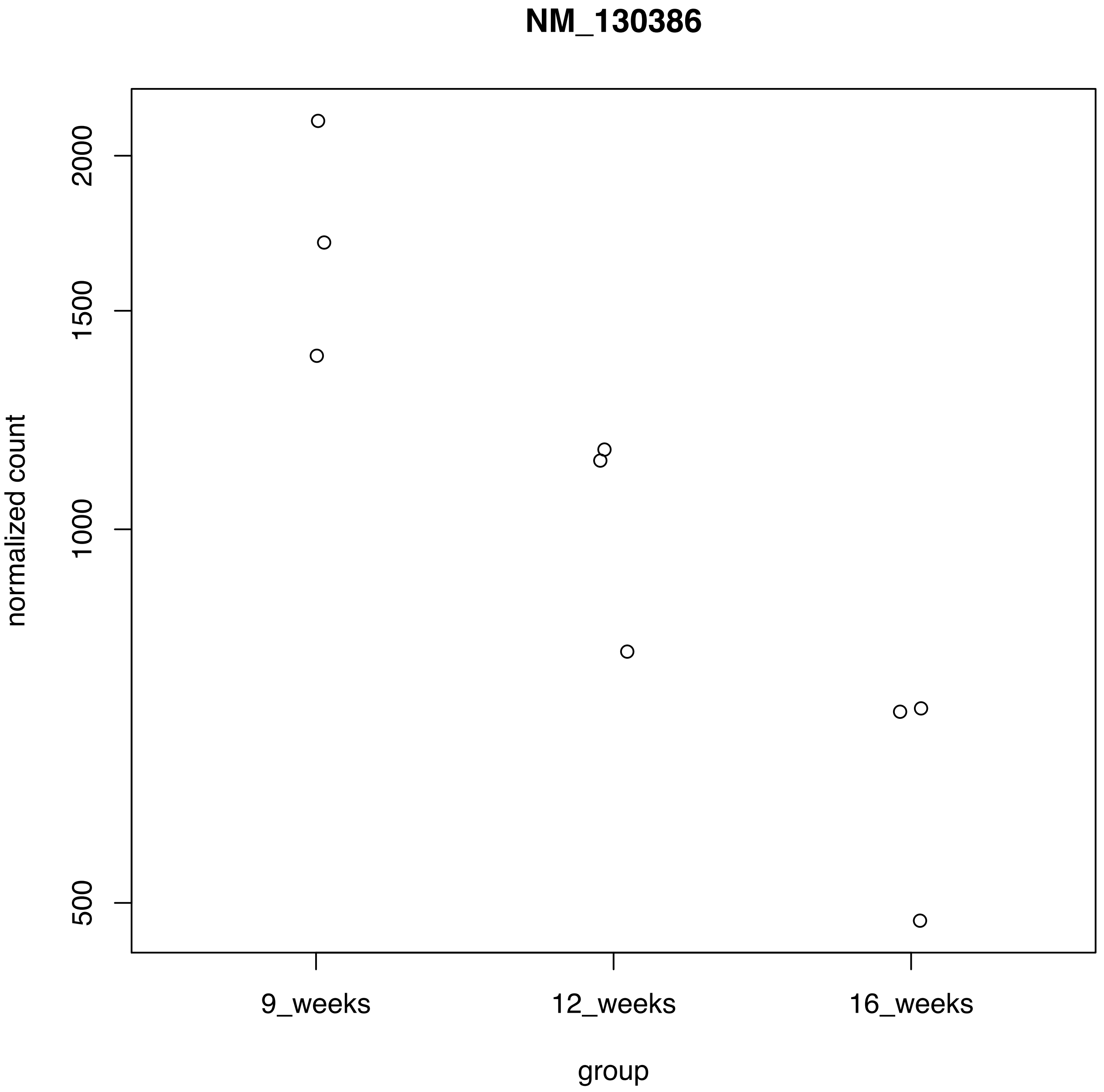
NM_130386
normalized count
2000
1500
1000
500
9_weeks
12_weeks
16_weeks
group

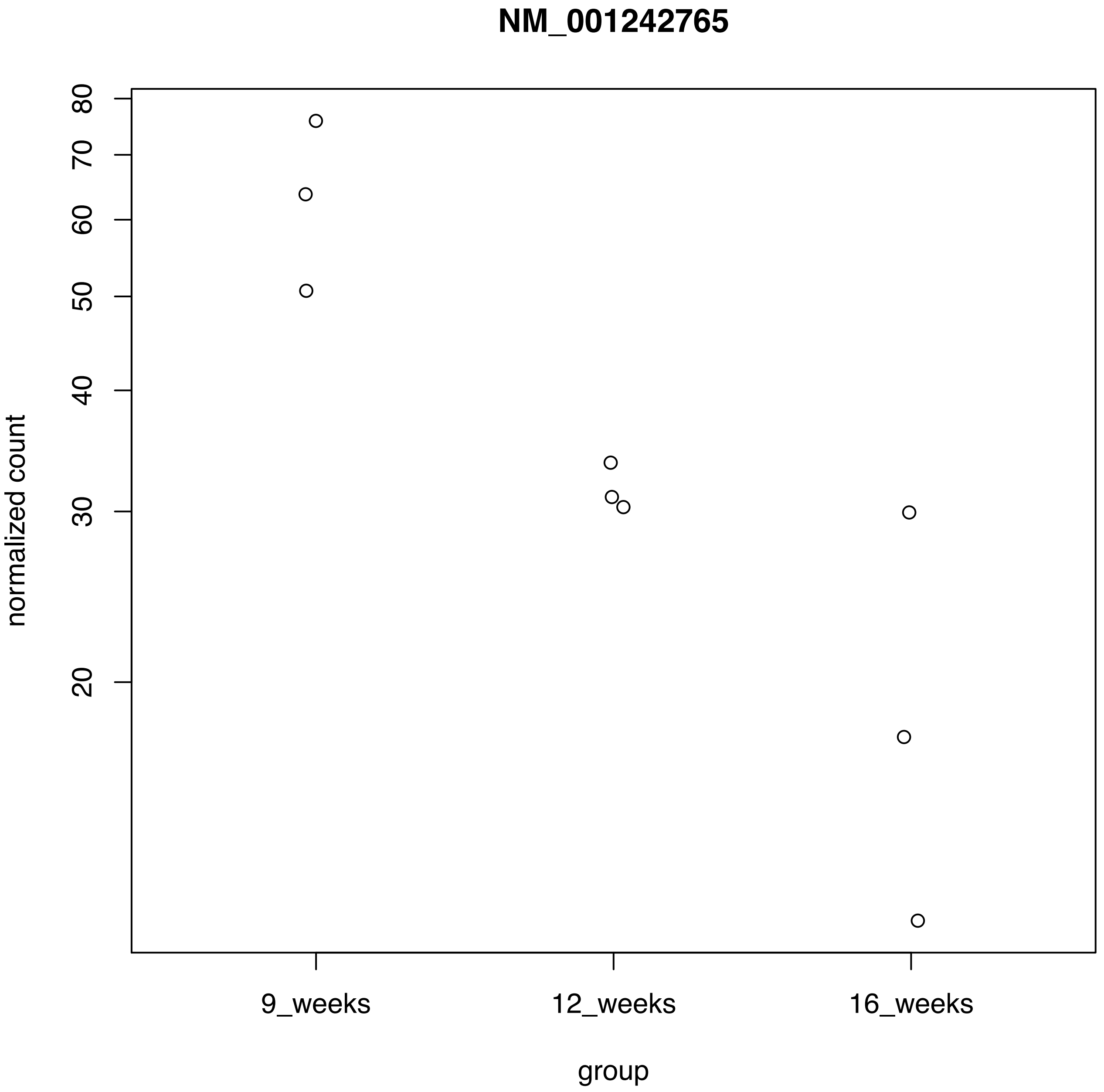
NM_001242765
normalized count
80
70
60
50
40
30
20
9_weeks
12_weeks
16_weeks
group

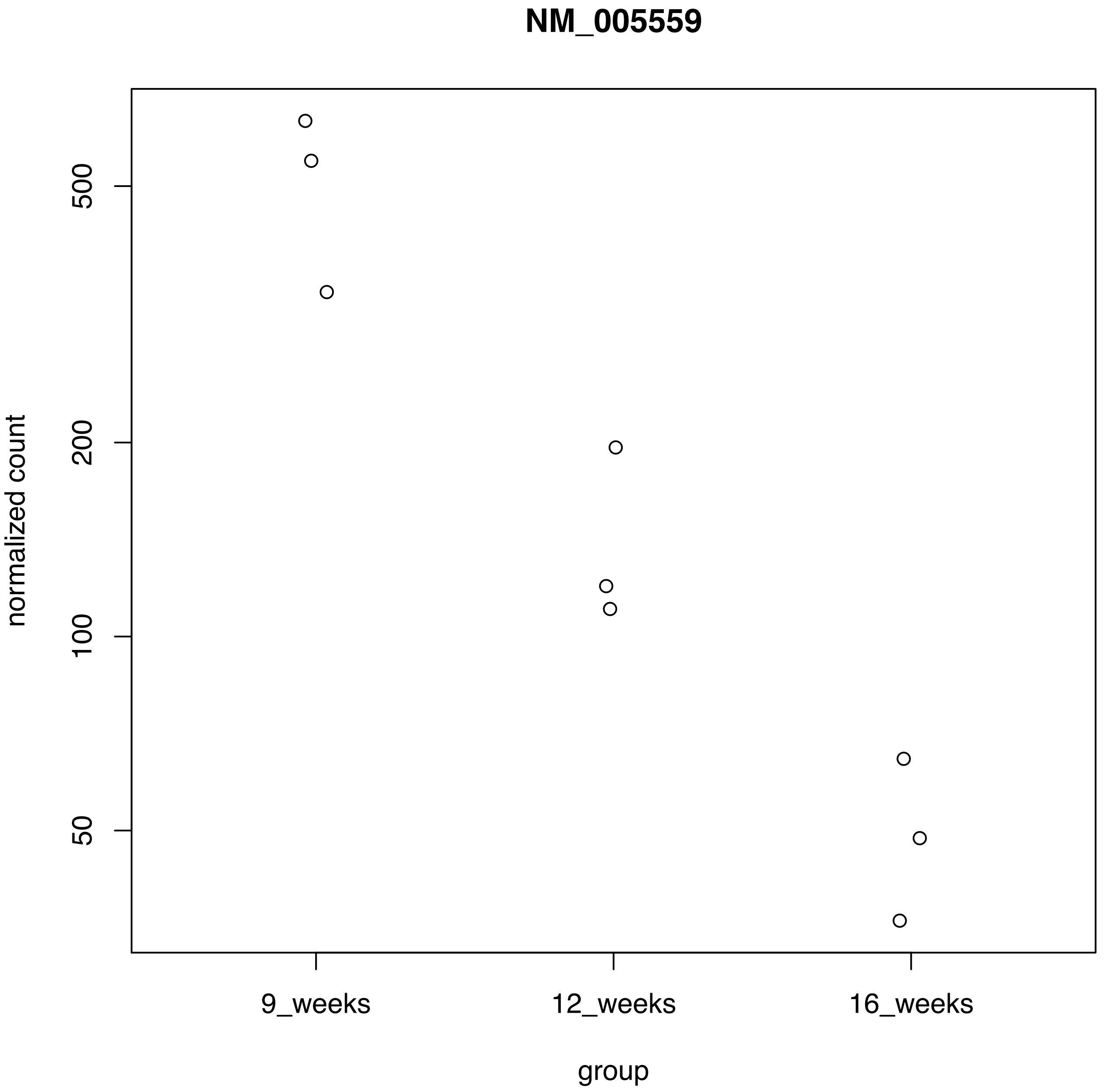

NM_005559
normalized count
500
200
100
50
9_weeks
12_weeks
16_weeks
group

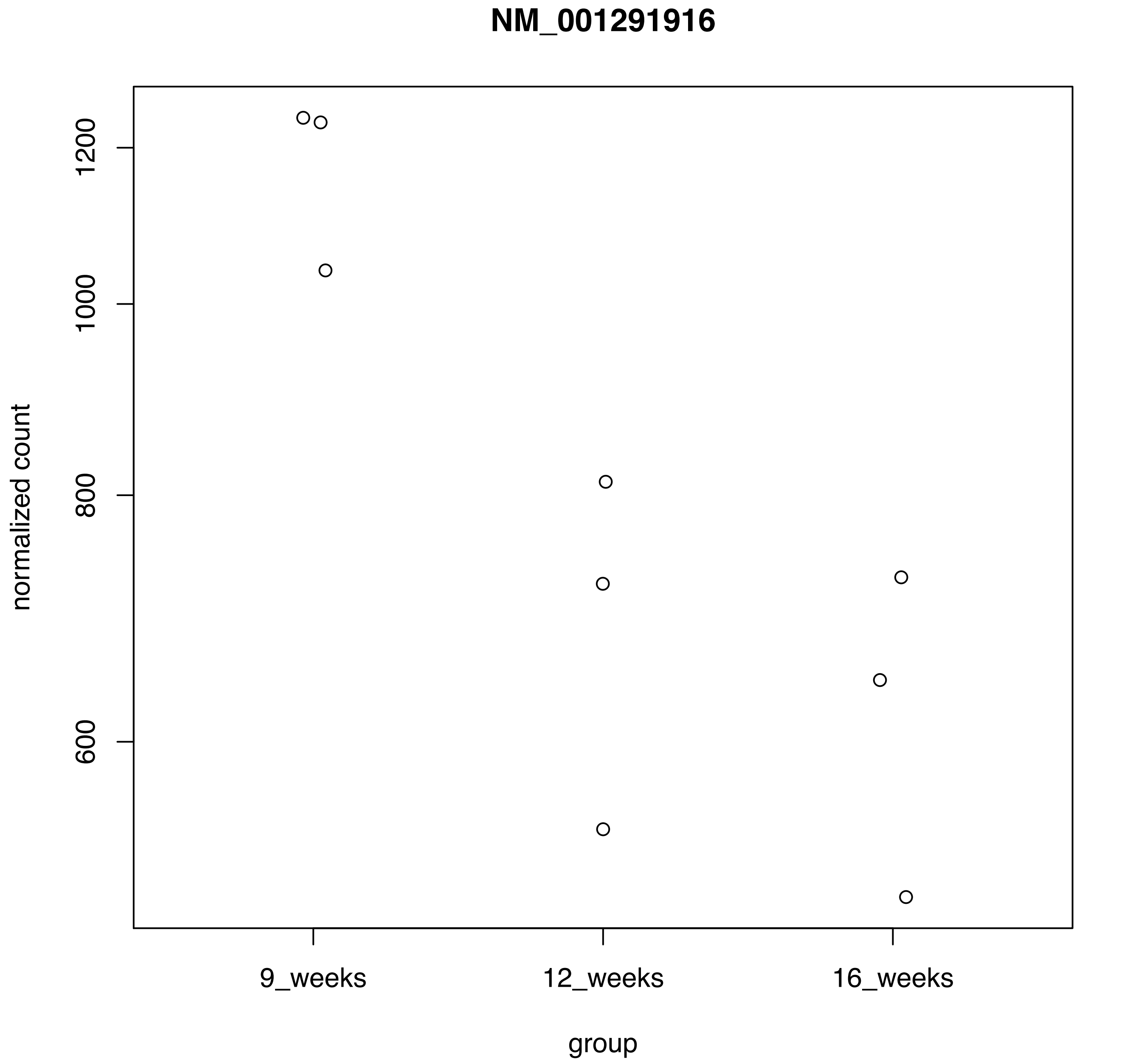
NM_001291916
normalized count
1200
1000
800
600
9_weeks
12_weeks
16_weeks
group

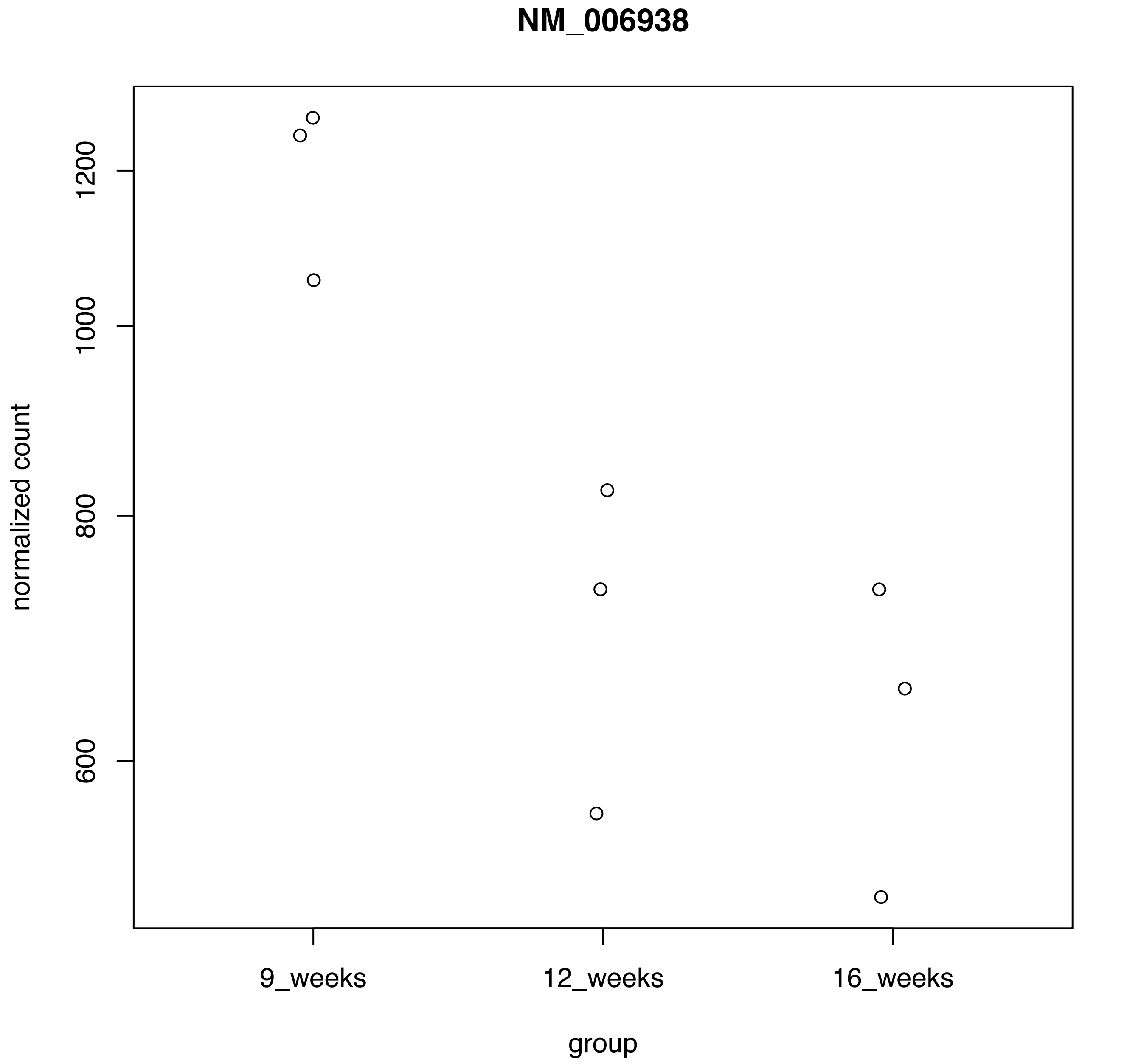
NM_006938
normalized count
1200
1000
800
600
9_weeks
12_weeks
16_weeks
group

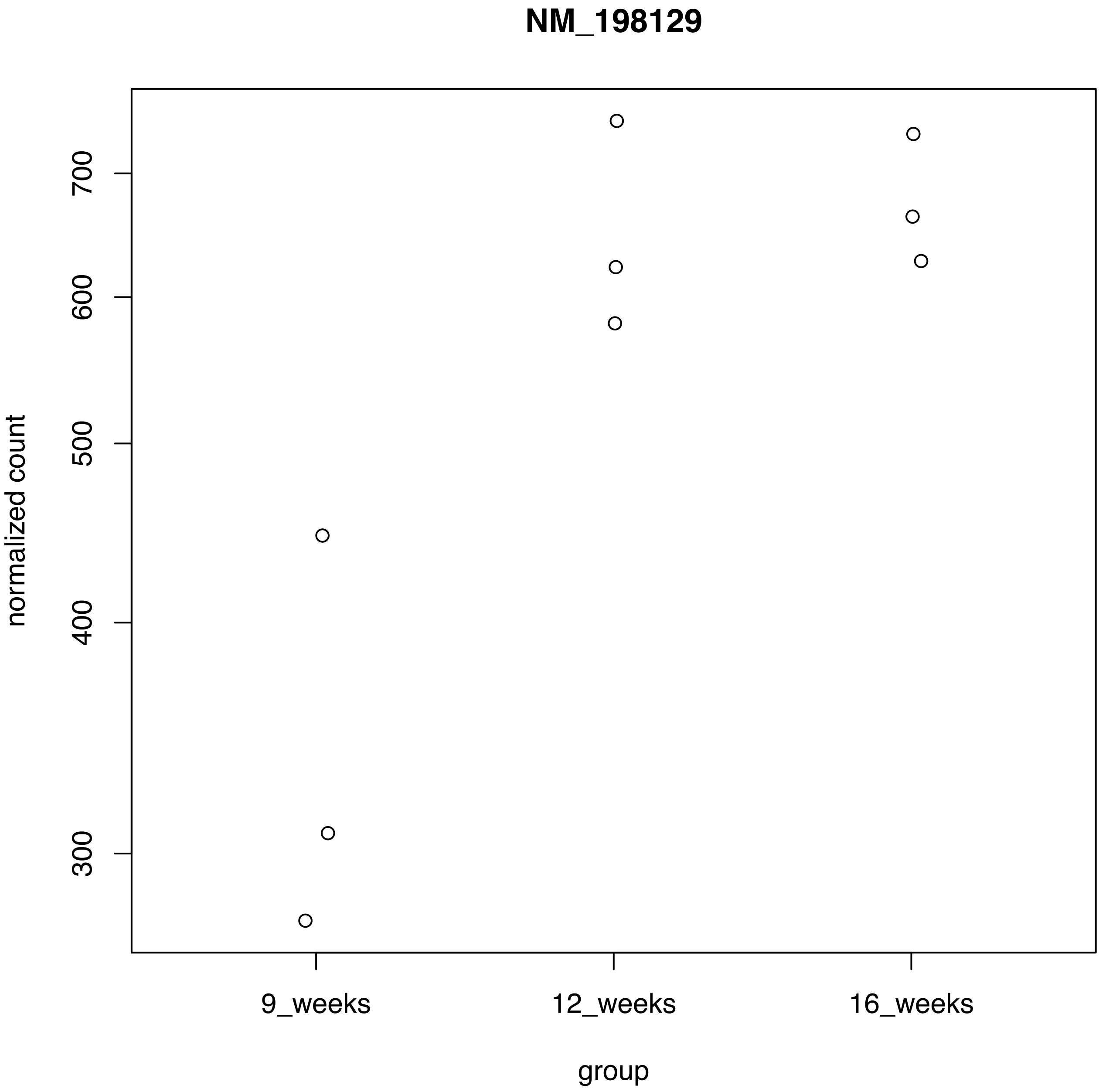
NM_198129
normalized count
700
600
500
400
300
9_weeks
12_weeks
16_weeks
group

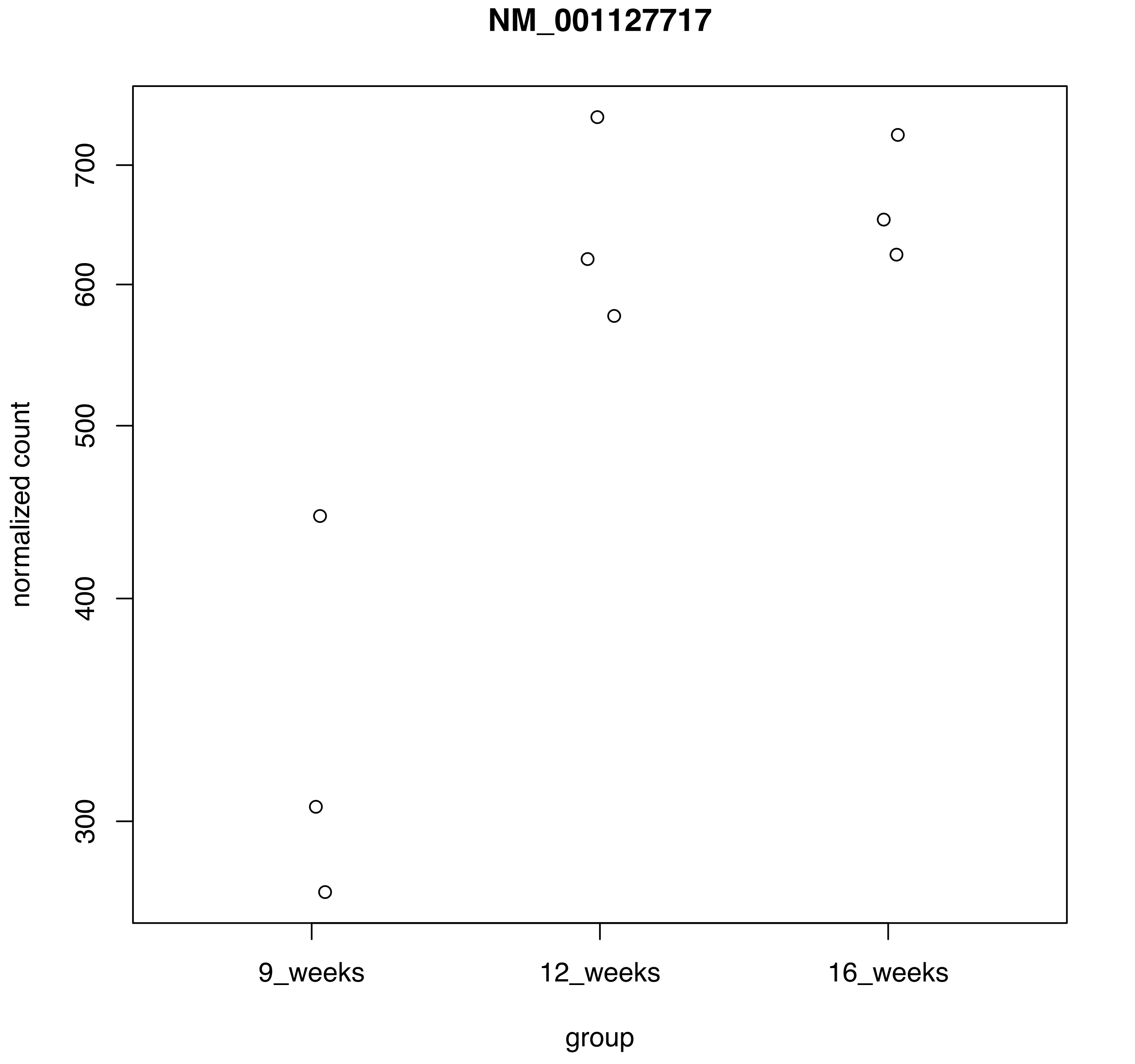
NM_001127717
normalized count
300
400
500
600
700
9_weeks
12_weeks
16_weeks
group

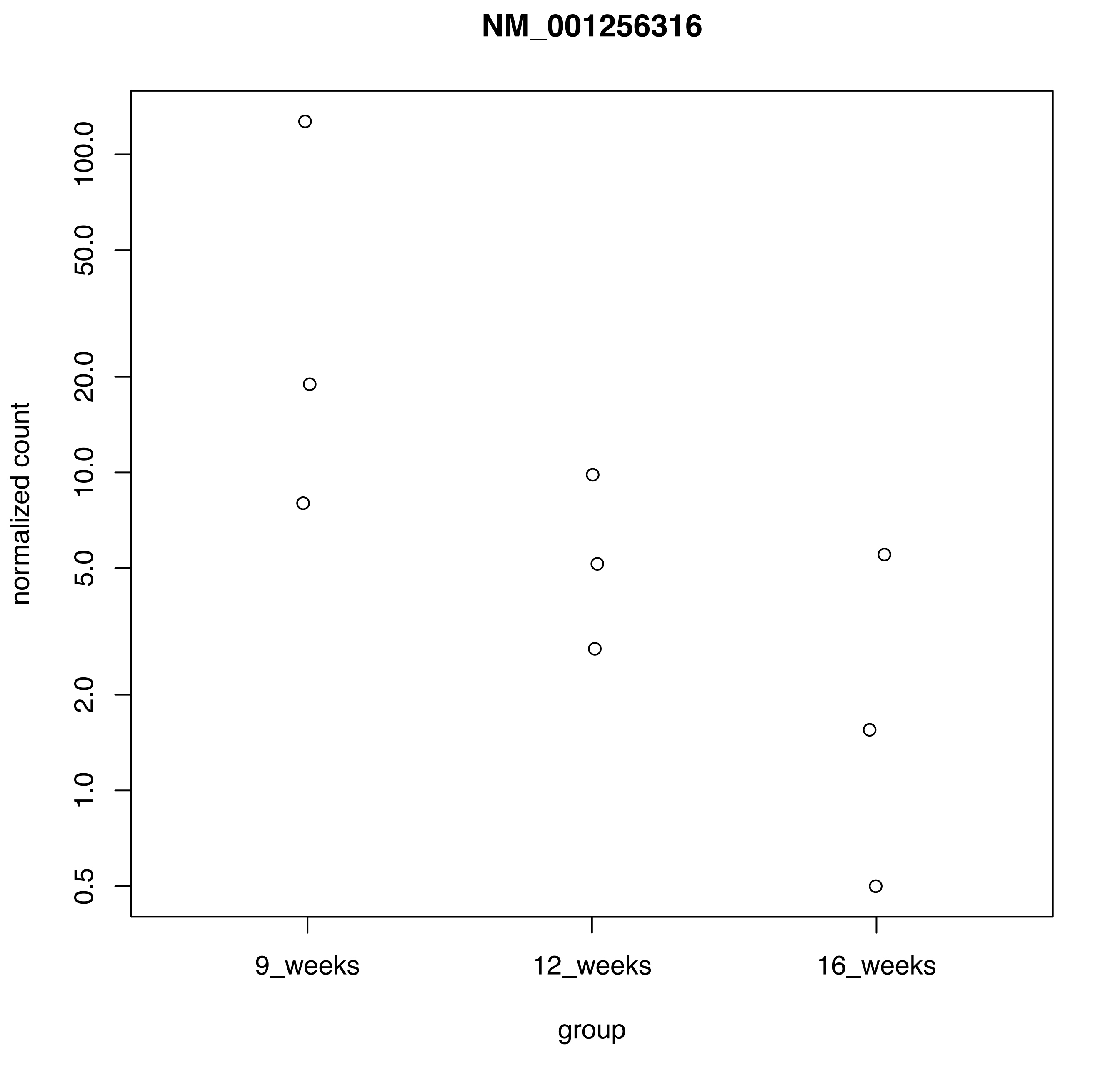
NM_001256316
normalized count
100.0
50.0
20.0
10.0
5.0
2.0
1.0
0.5
9_weeks
12_weeks
16_weeks
group

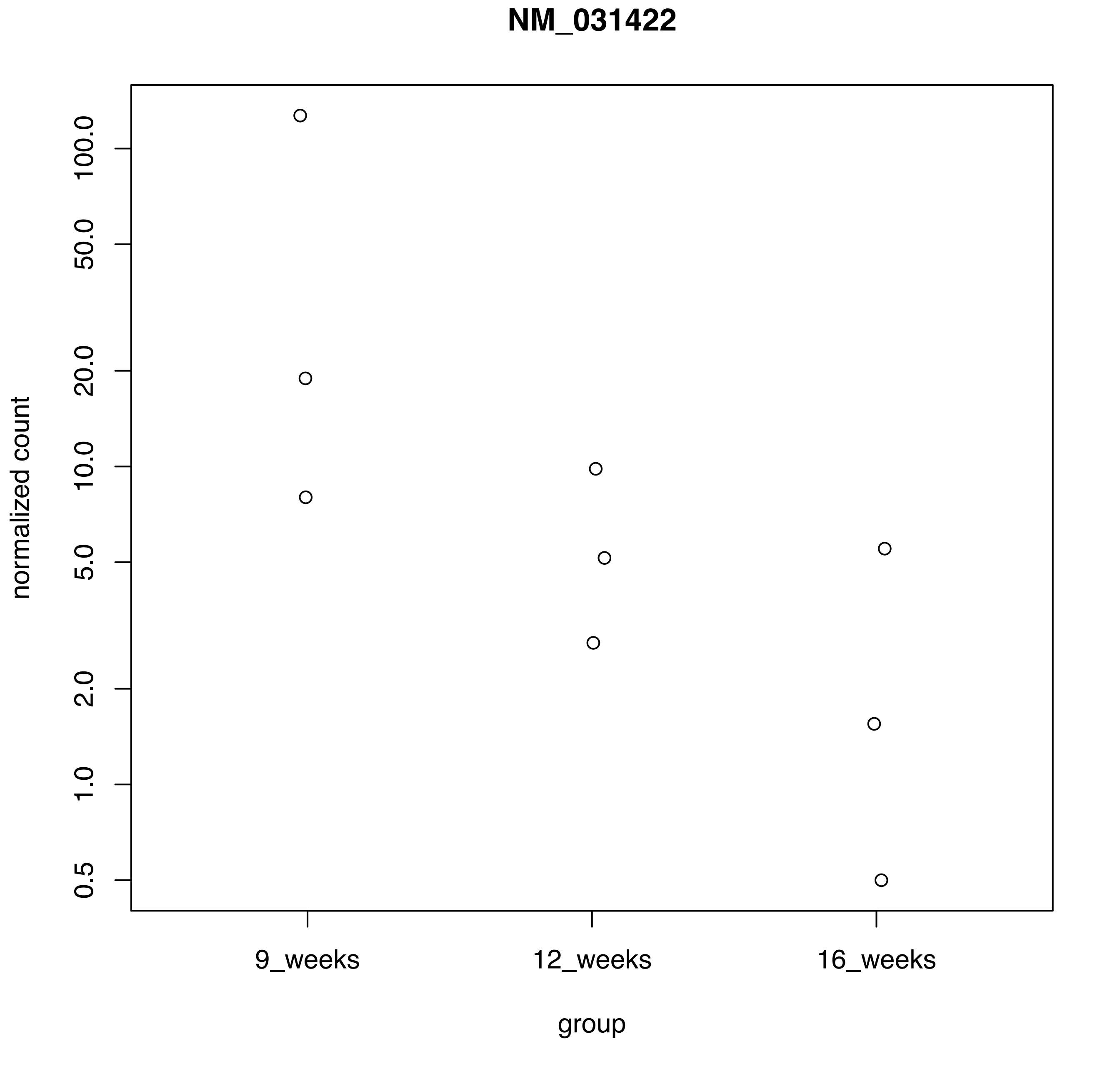
NM_031422
normalized count
100.0
50.0
20.0
10.0
5.0
2.0
1.0
0.5
9_weeks
12_weeks
16_weeks
group

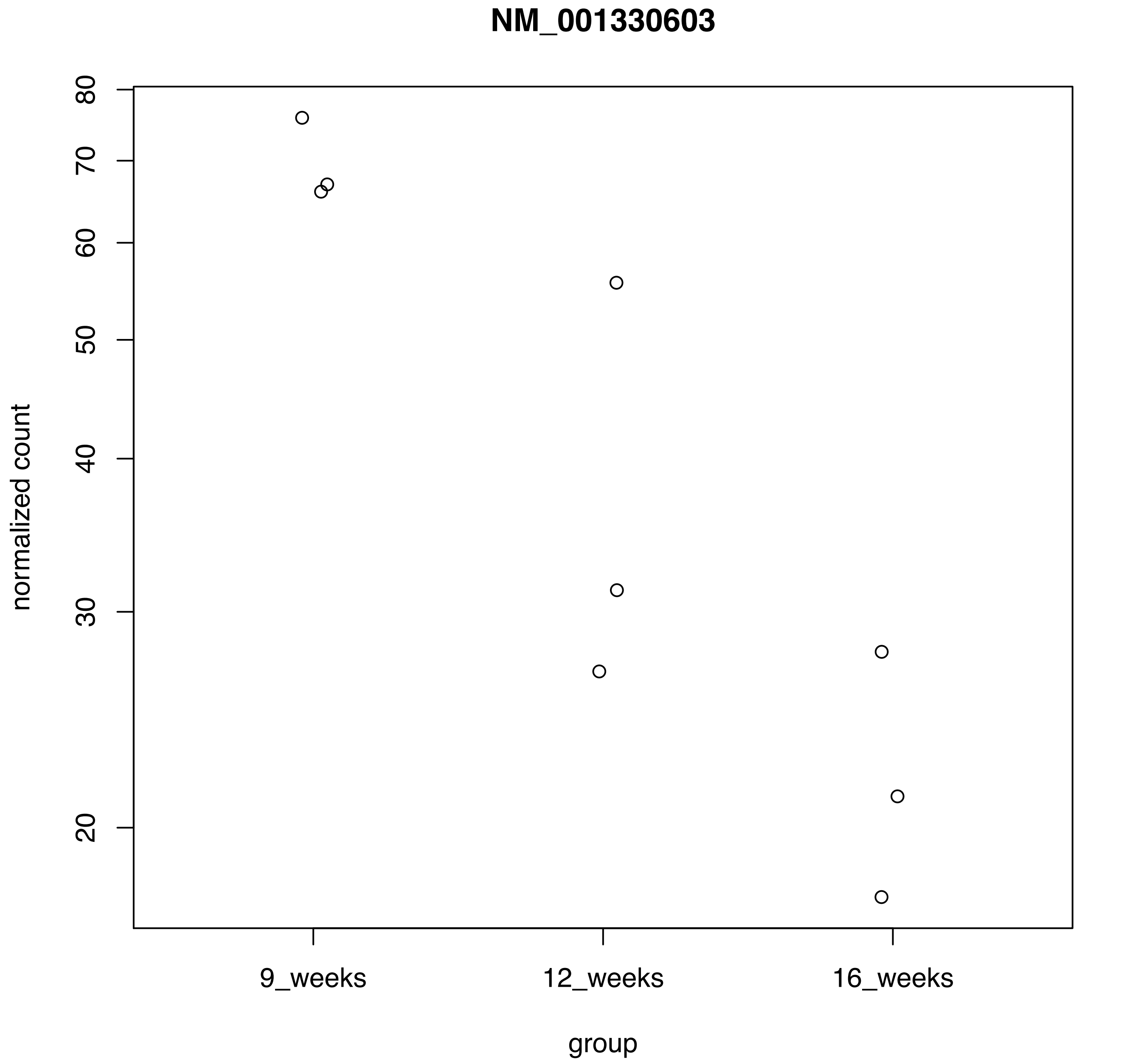

NM_001330603
normalized count
80
70
60
50
40
30
20
9_weeks
12_weeks
16_weeks
group

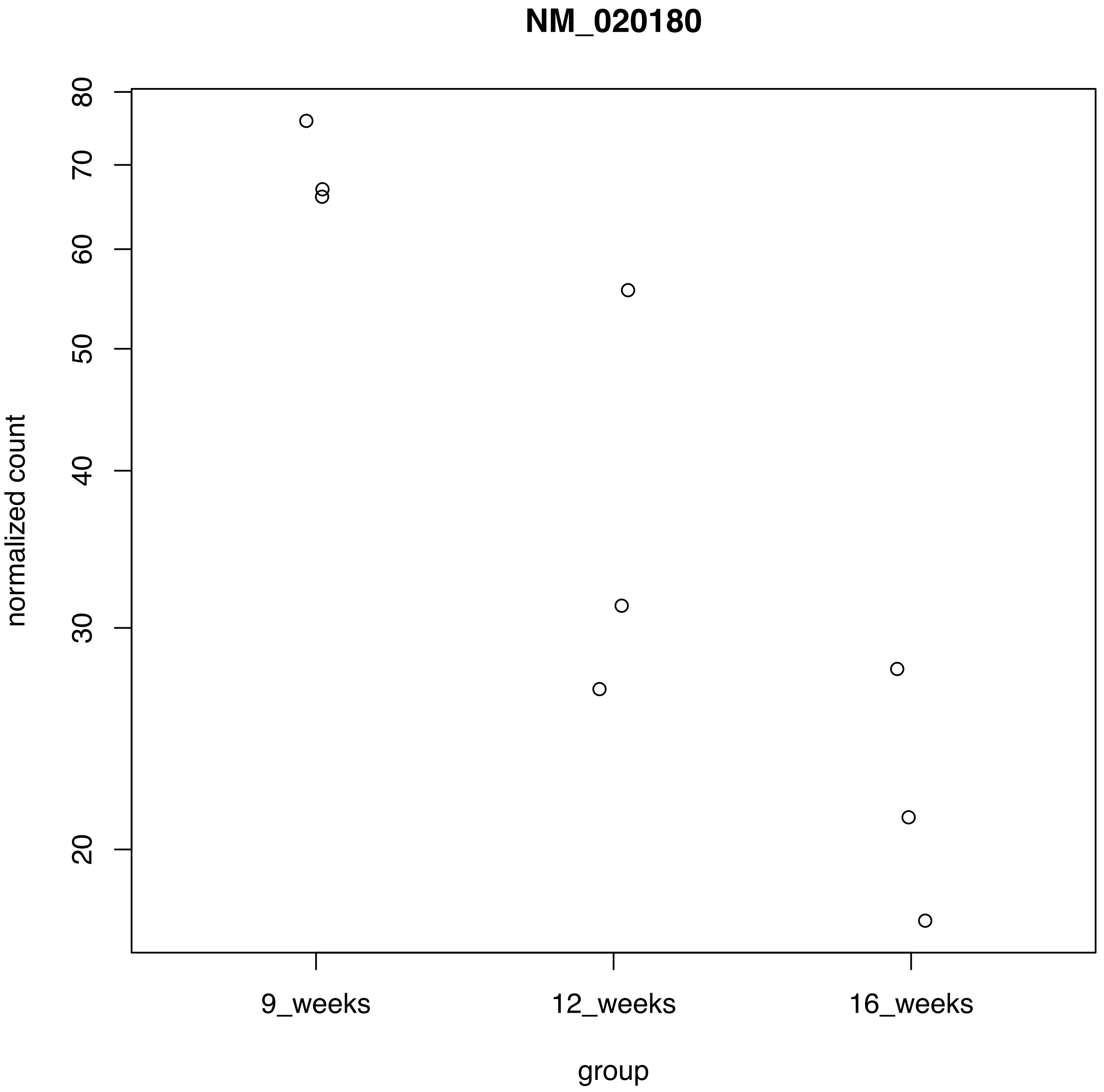
NM_020180
normalized count
80
70
60
50
40
30
20
9_weeks
12_weeks
16_weeks
group

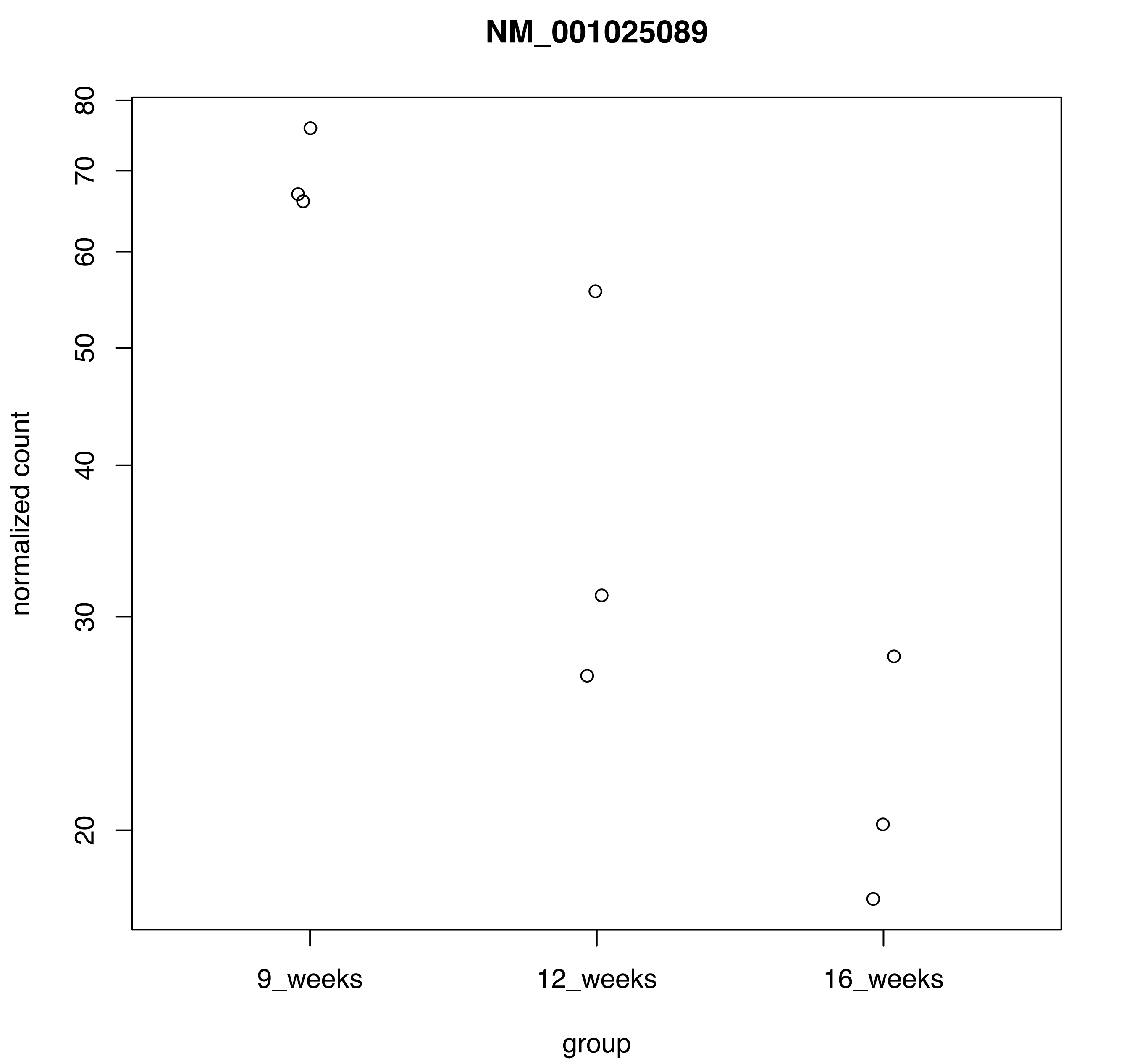

NM_001025089
normalized count
80
70
60
50
40
30
20
9_weeks
12_weeks
16_weeks
group

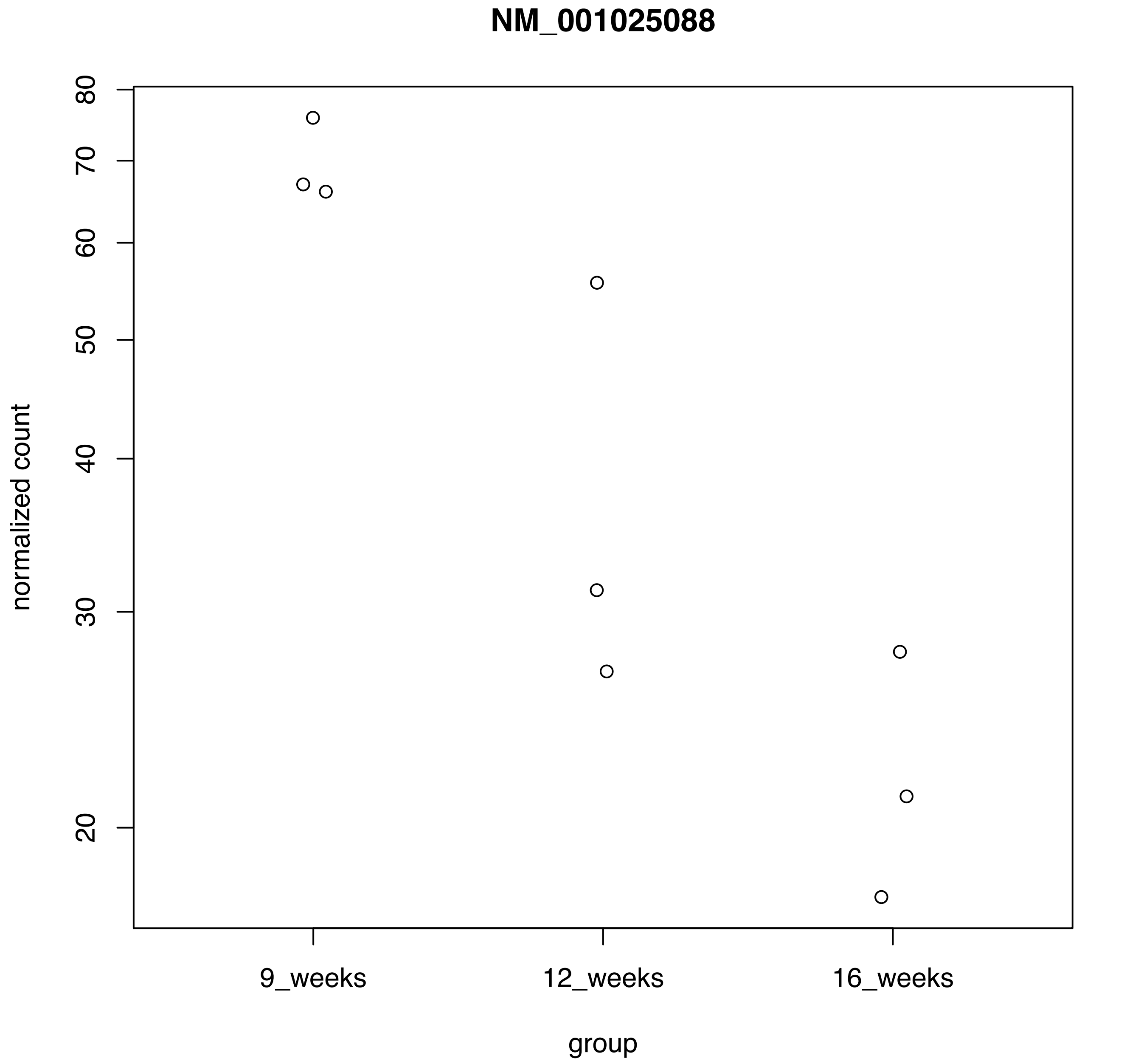

NM_001025088
normalized count
80
70
60
50
40
30
20
9_weeks
12_weeks
16_weeks
group

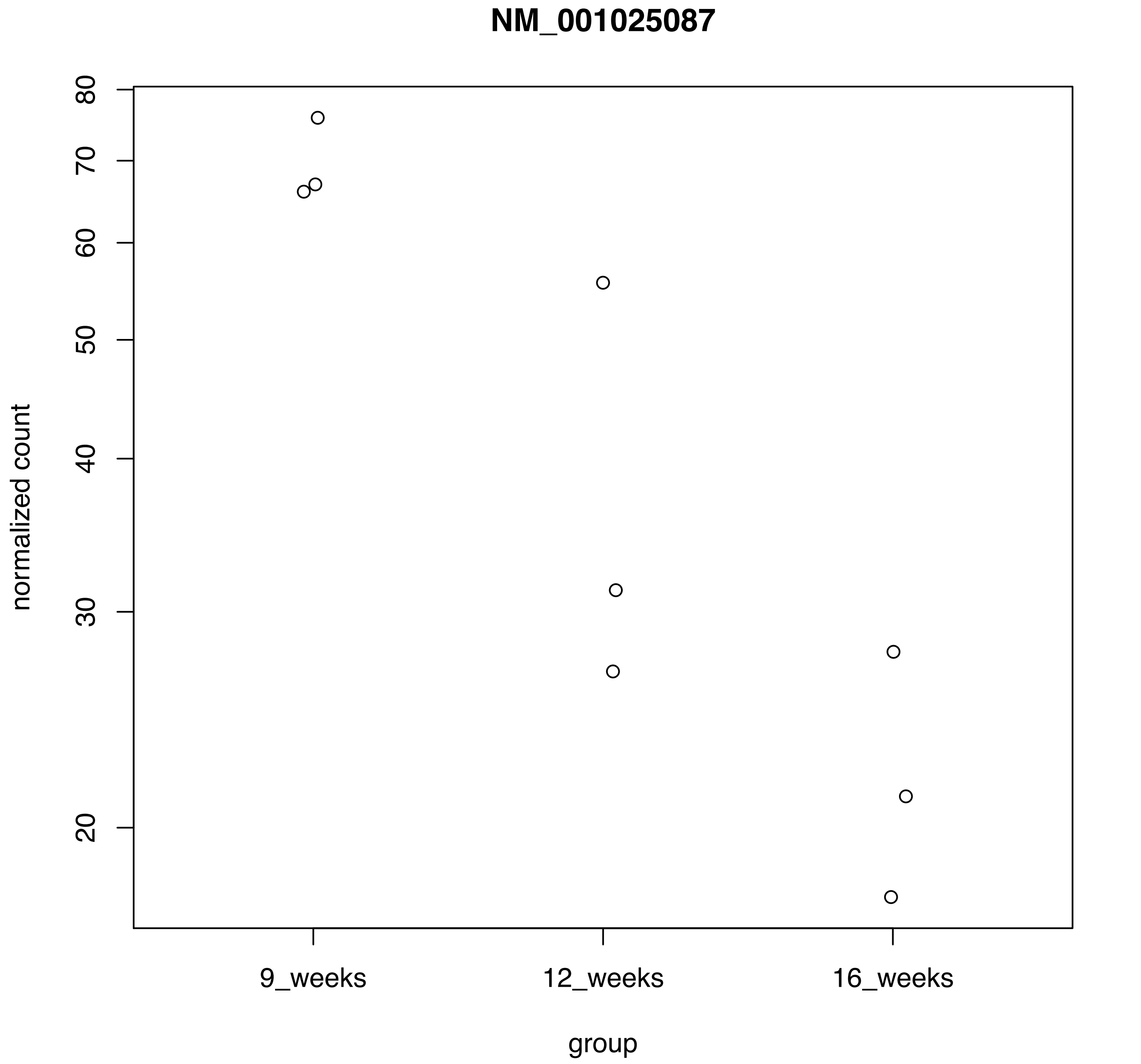
NM_001025087
normalized count
80
70
60
50
40
30
20
9_weeks
12_weeks
16_weeks
group

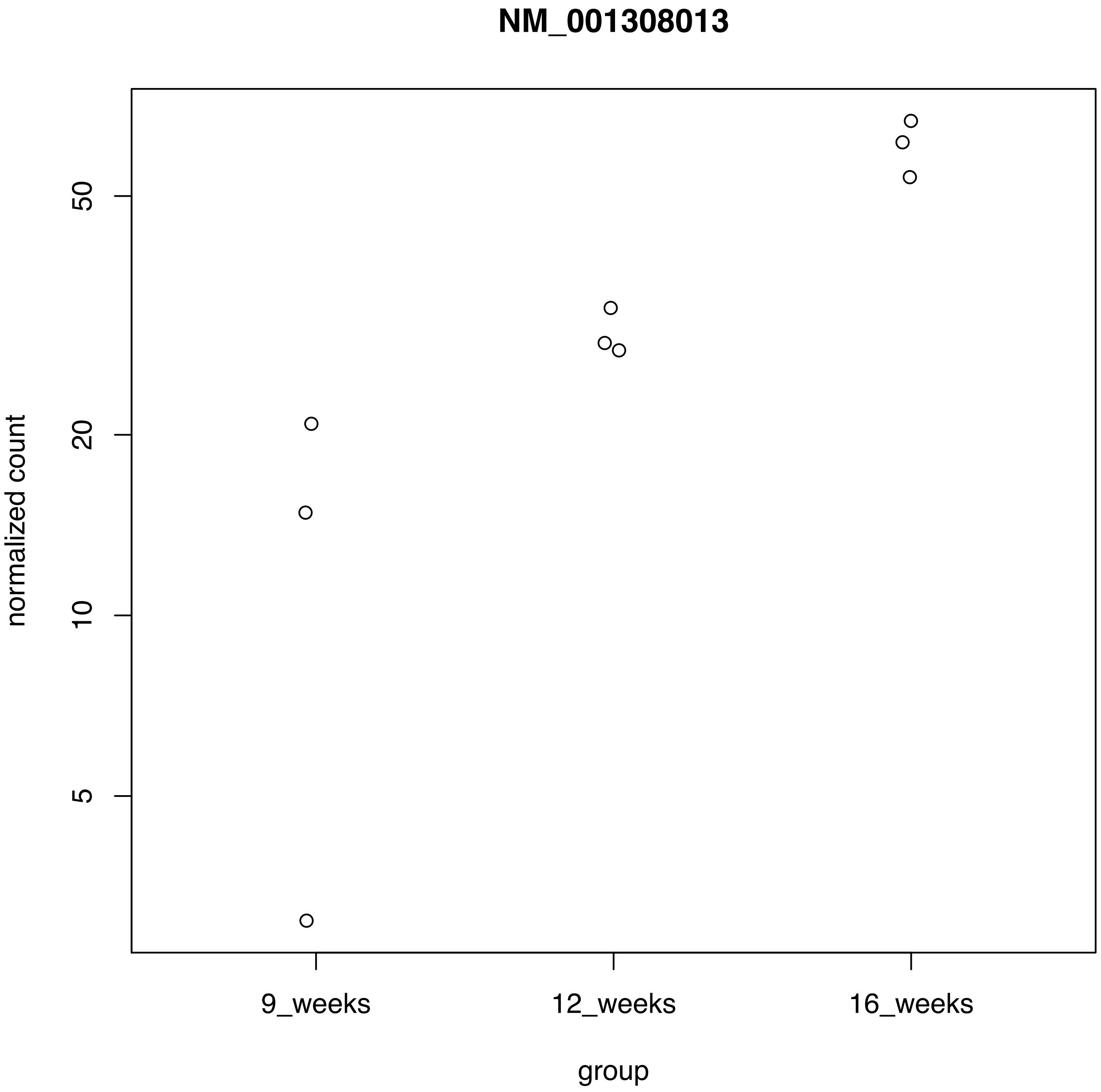
NM_001308013
normalized count
50
20
10
5
9_weeks
12_weeks
16_weeks
group

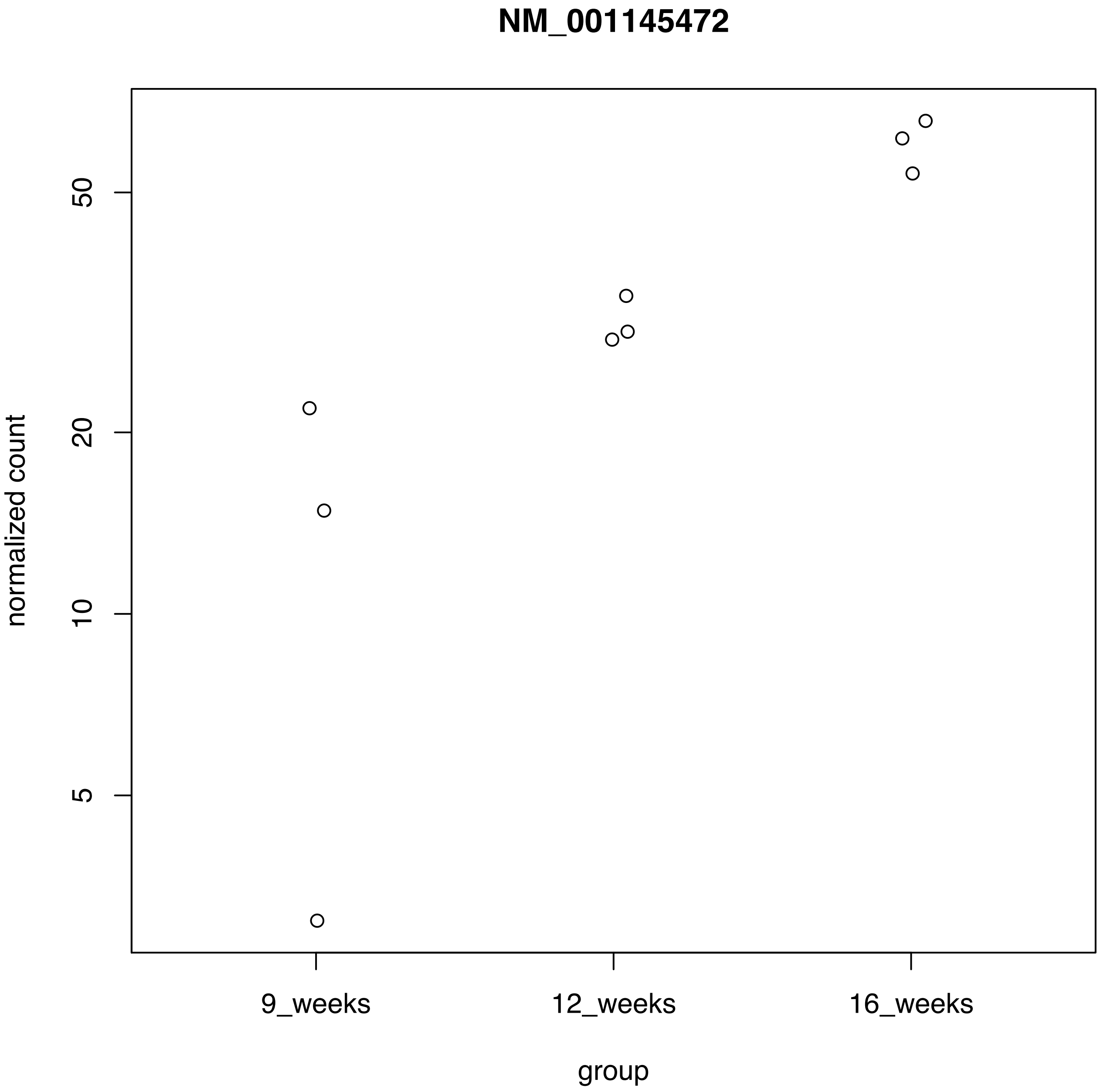
NM_001145472
normalized count
50
20
10
5
9_weeks
12_weeks
16_weeks
group

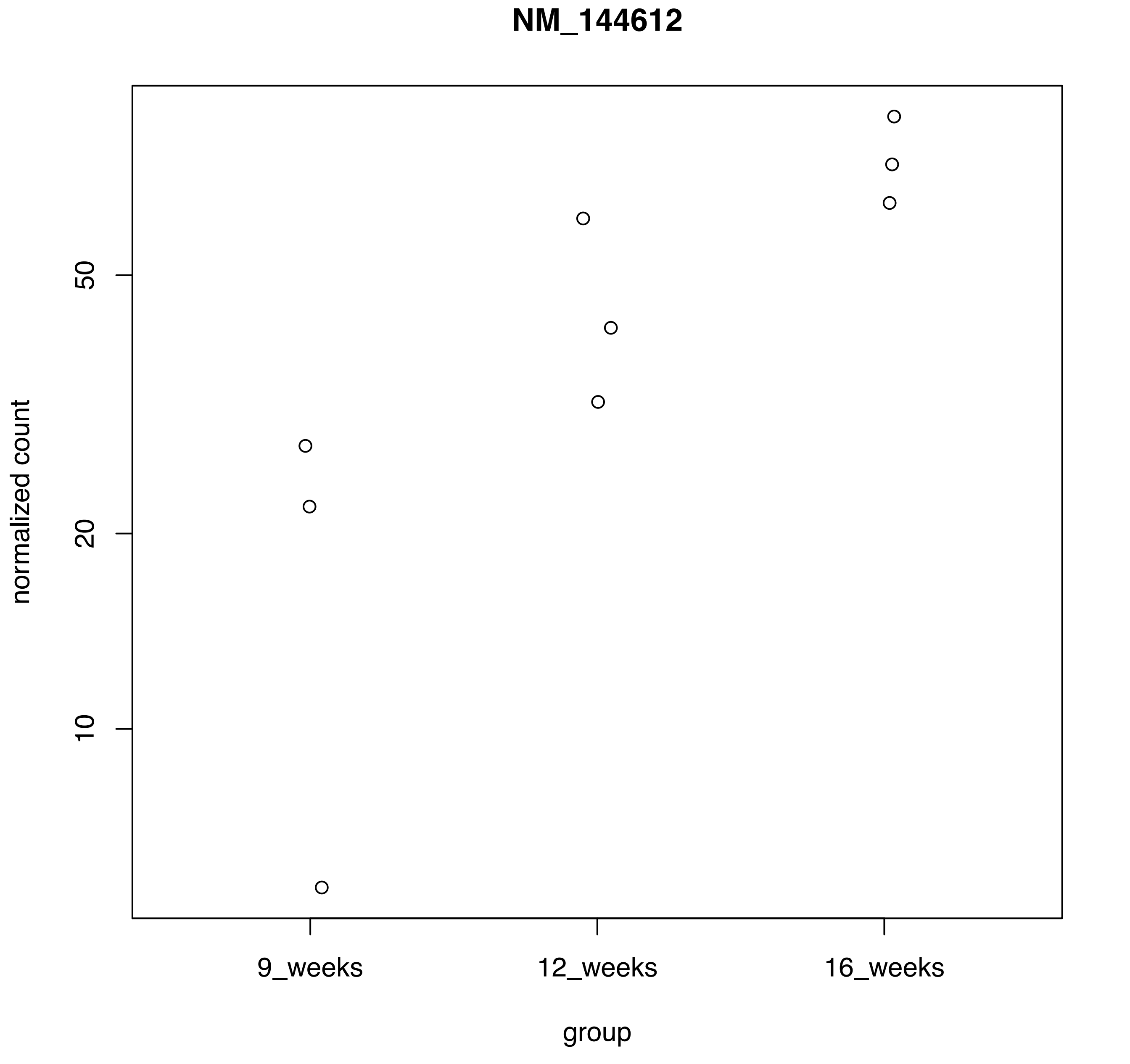
NM_144612
normalized count
50
20
10
9_weeks
12_weeks
16_weeks
group

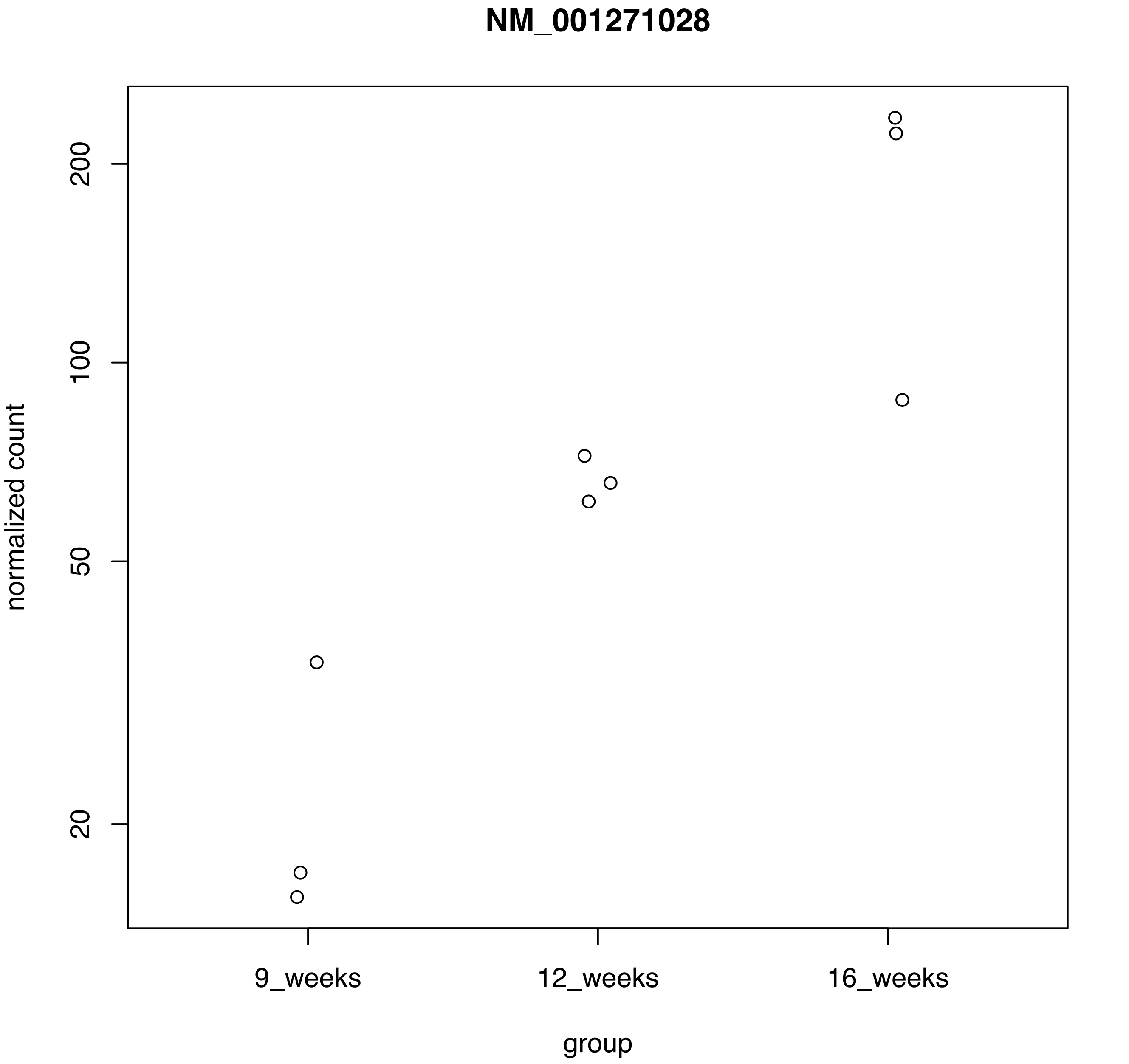
NM_001271028
normalized count
200
100
50
20
9_weeks
12_weeks
16_weeks
group

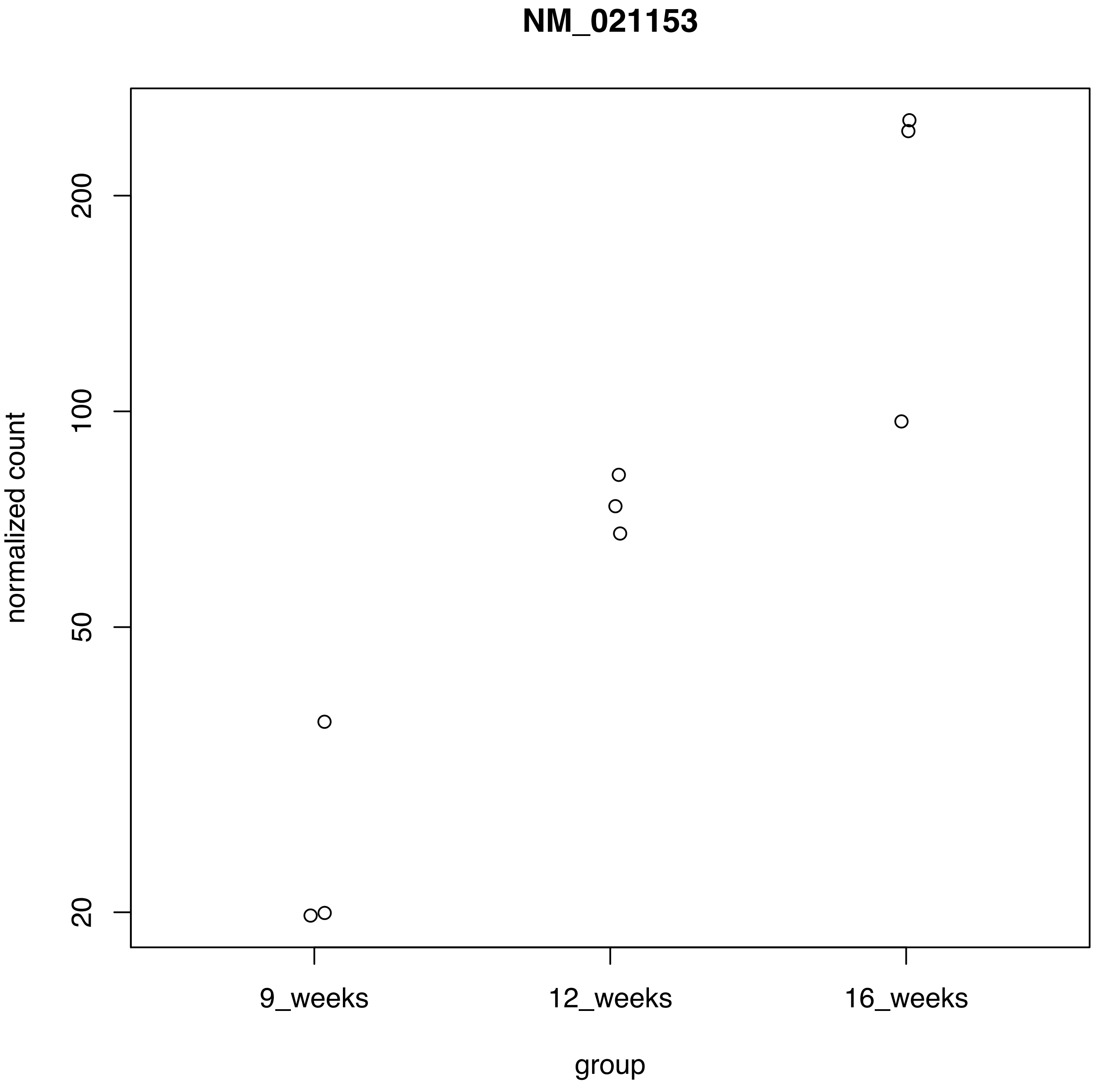
NM_021153
normalized count
200
100
50
20
9_weeks
12_weeks
16_weeks
group

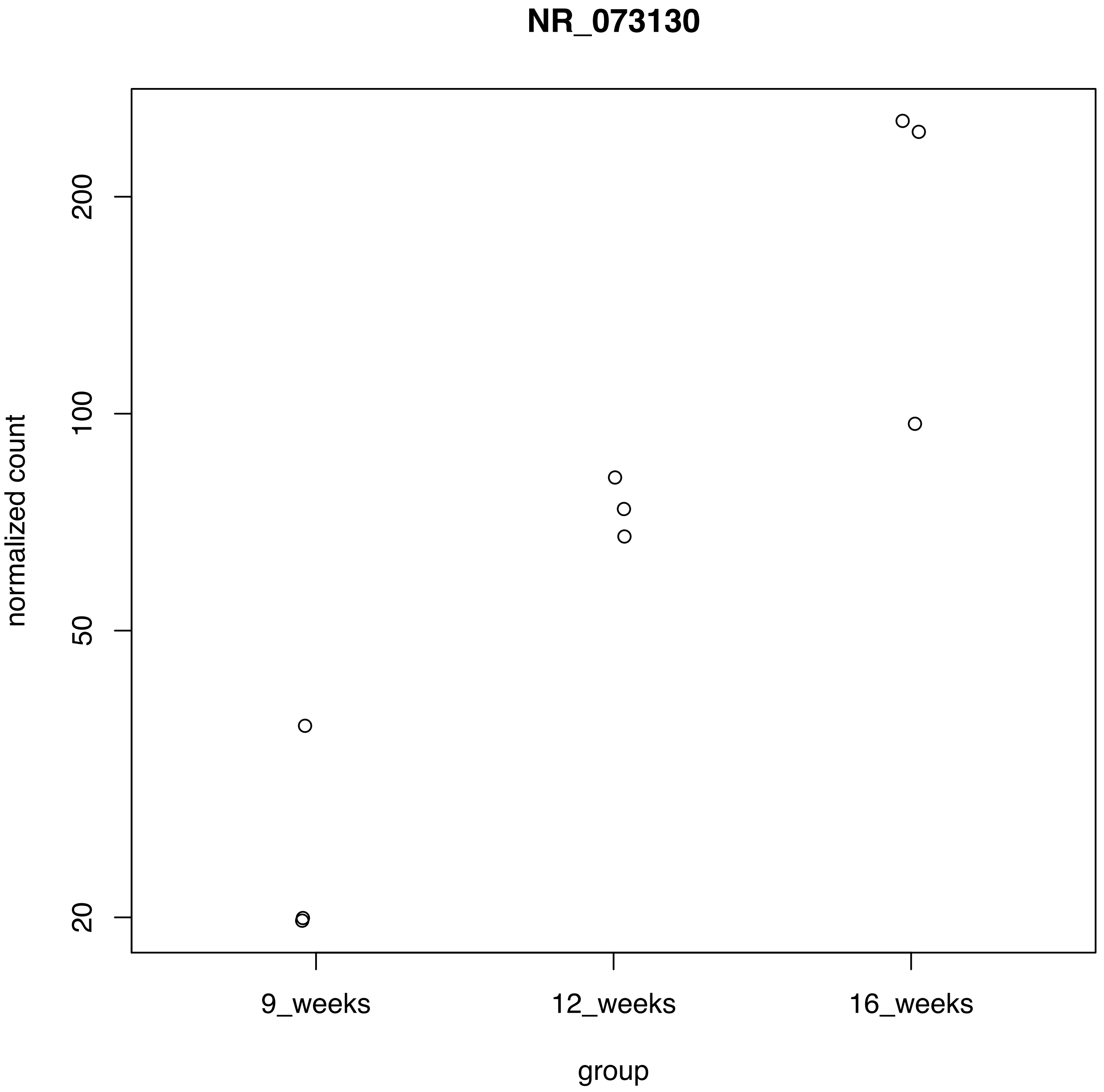
NR_073130
normalized count
200
100
50
20
9_weeks
12_weeks
16_weeks
group

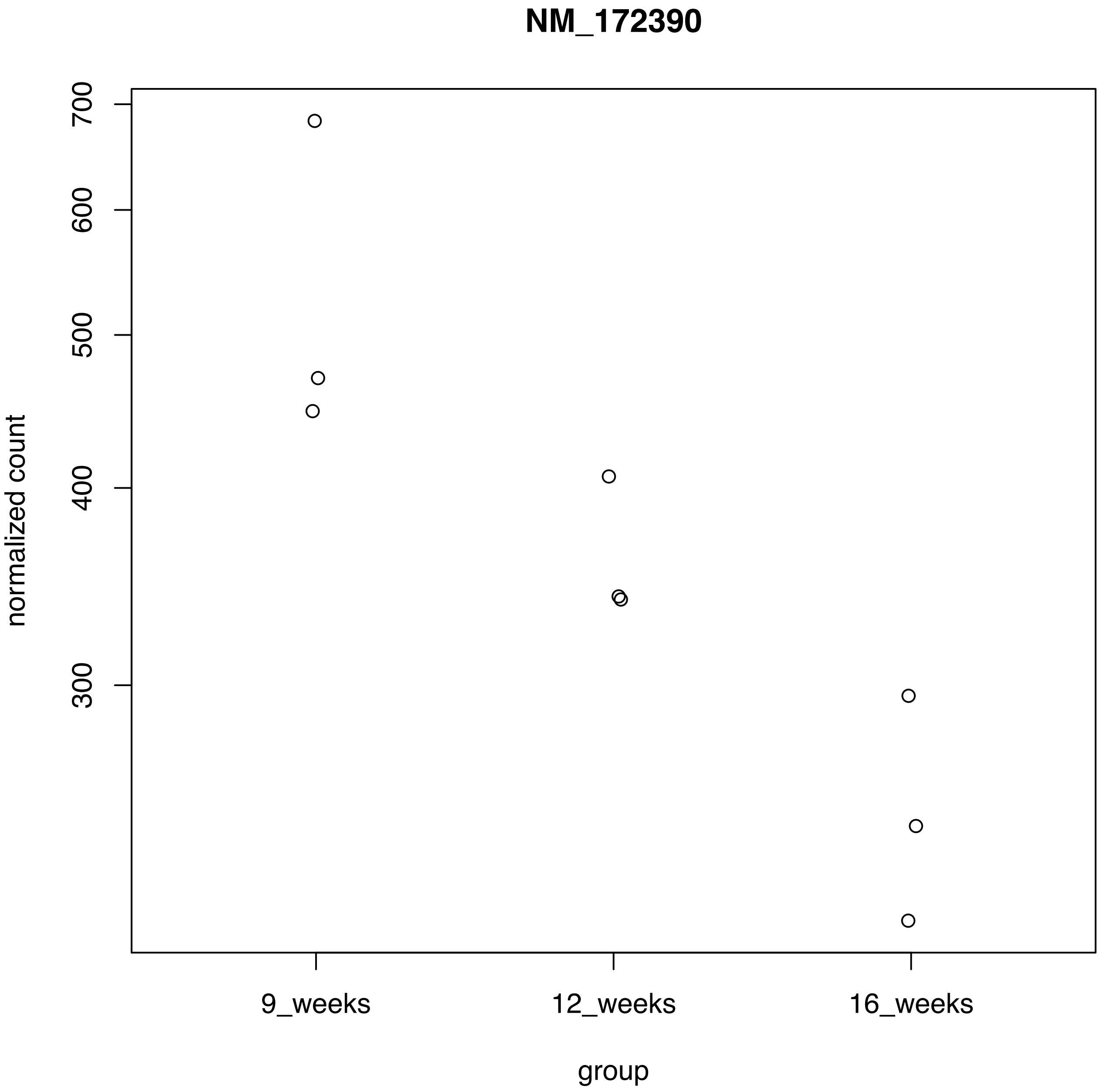
NM_172390
normalized count
700
600
500
400
300
9_weeks
12_weeks
16_weeks
group

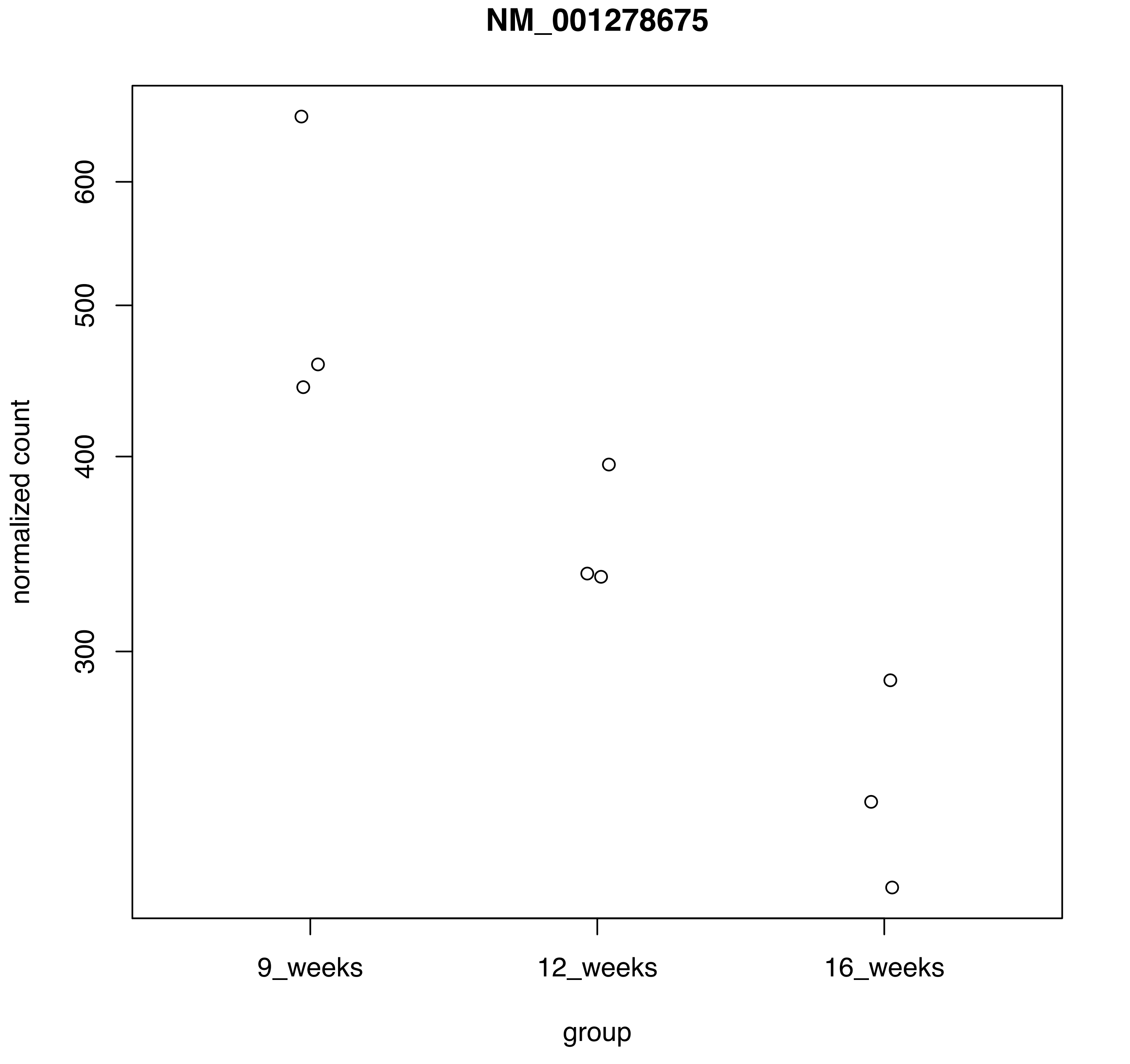
NM_001278675
normalized count
600
500
400
300
9_weeks
12_weeks
16_weeks
group

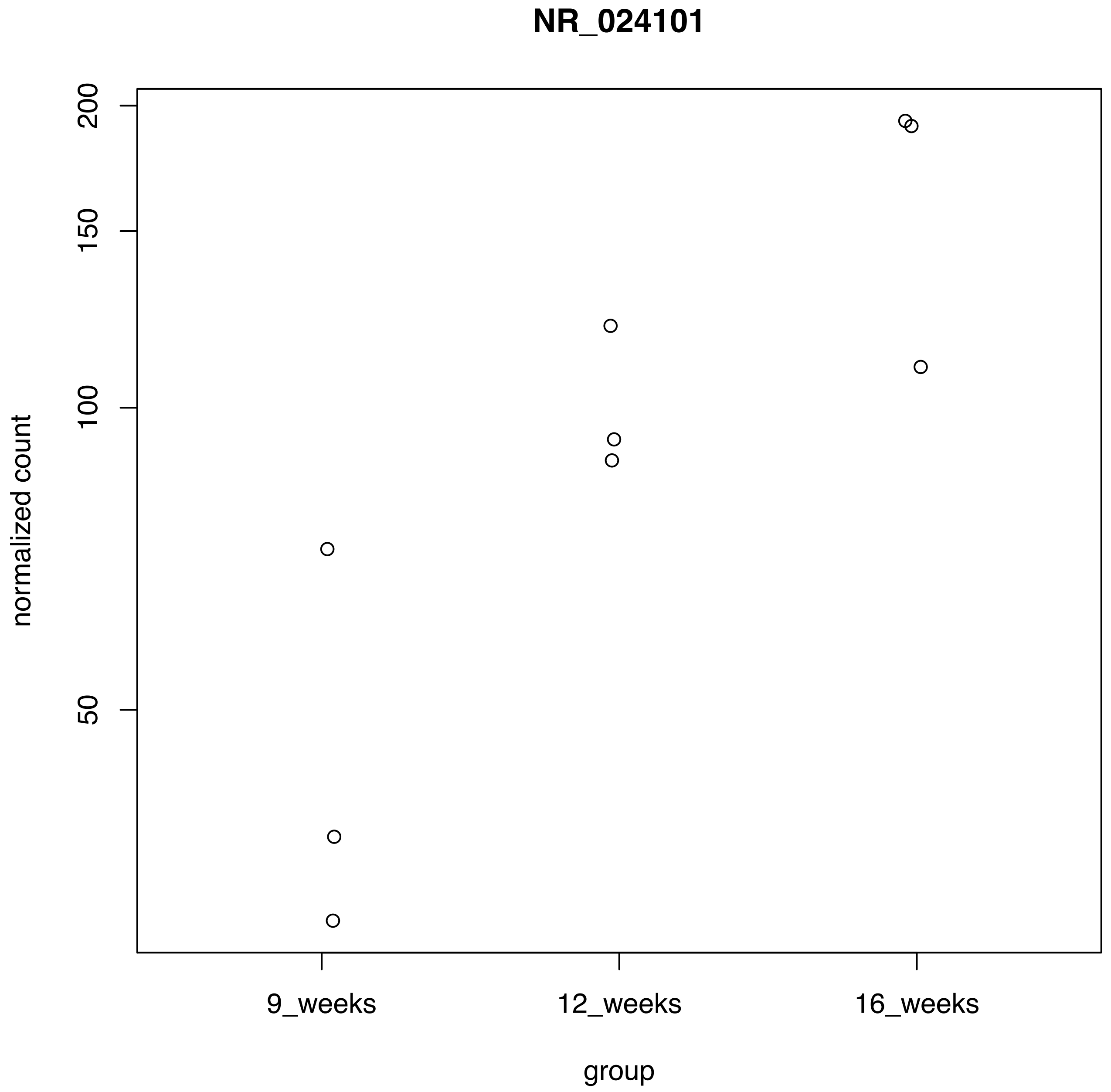
NR_024101
normalized count
200
150
100
50
9_weeks
12_weeks
16_weeks
group

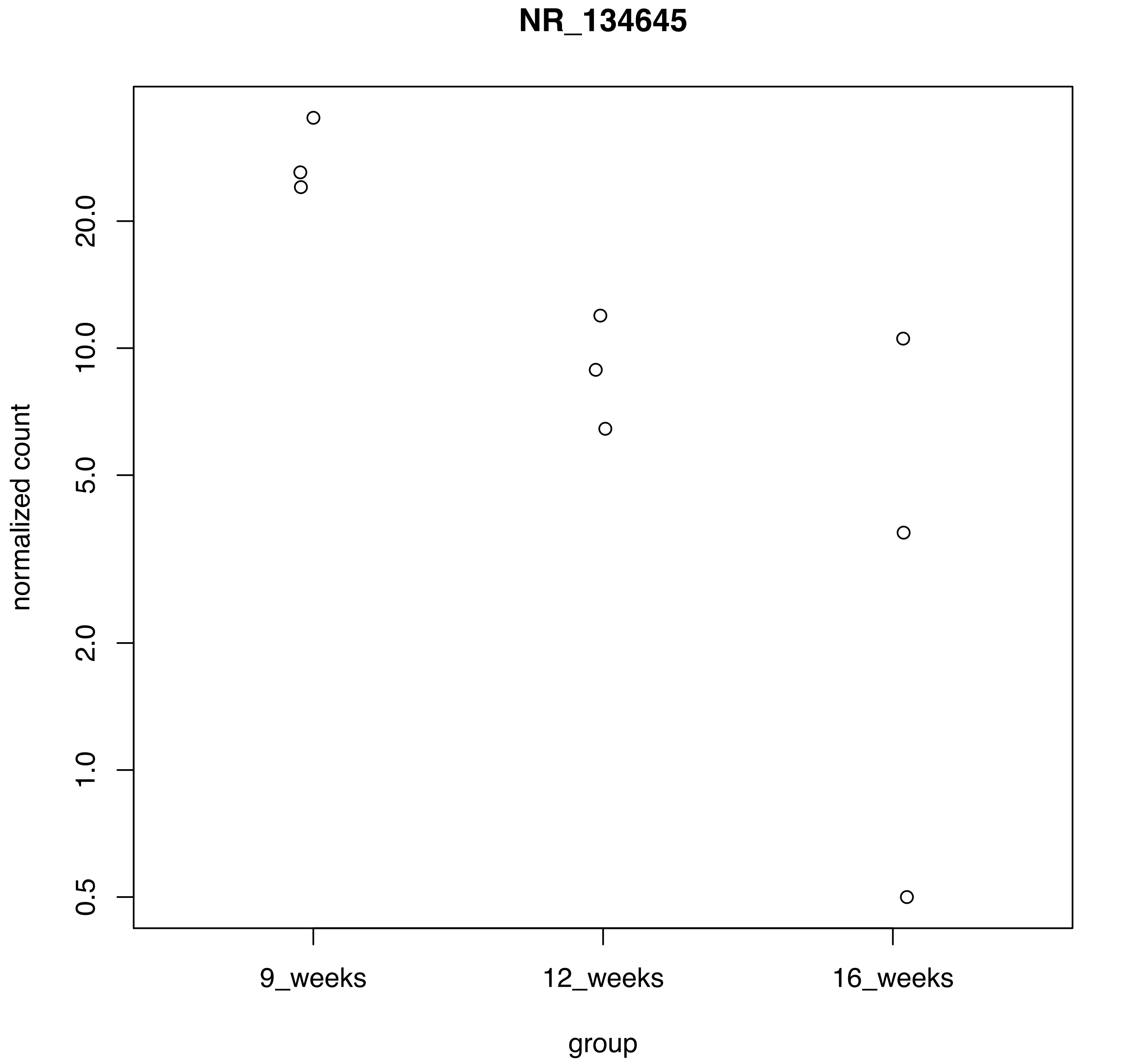

NR_134645
normalized count
20.0
10.0
5.0
2.0
1.0
0.5
9_weeks
12_weeks
16_weeks
group

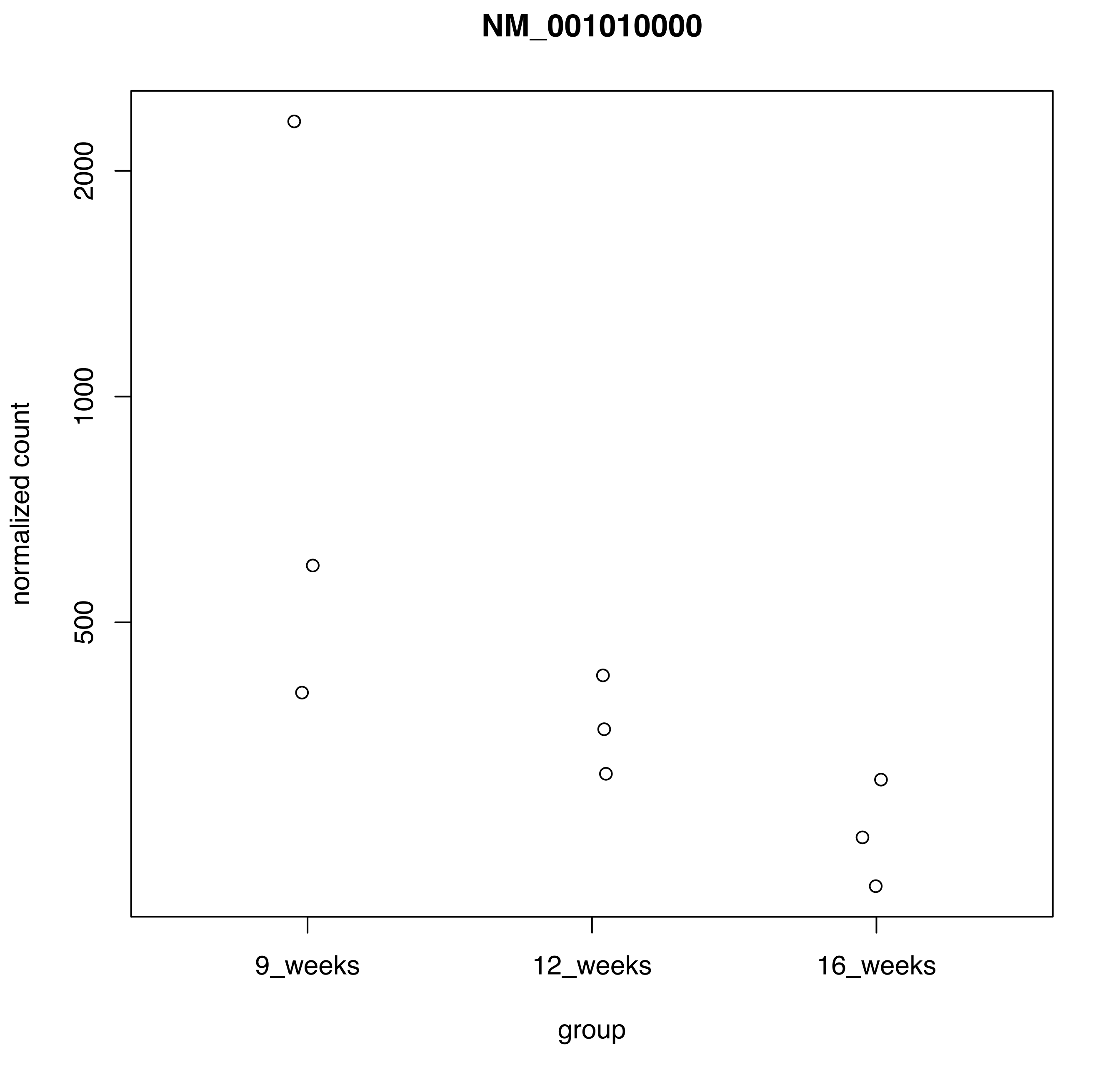
NM_001010000
normalized count
2000
1000
500
9_weeks
12_weeks
16_weeks
group

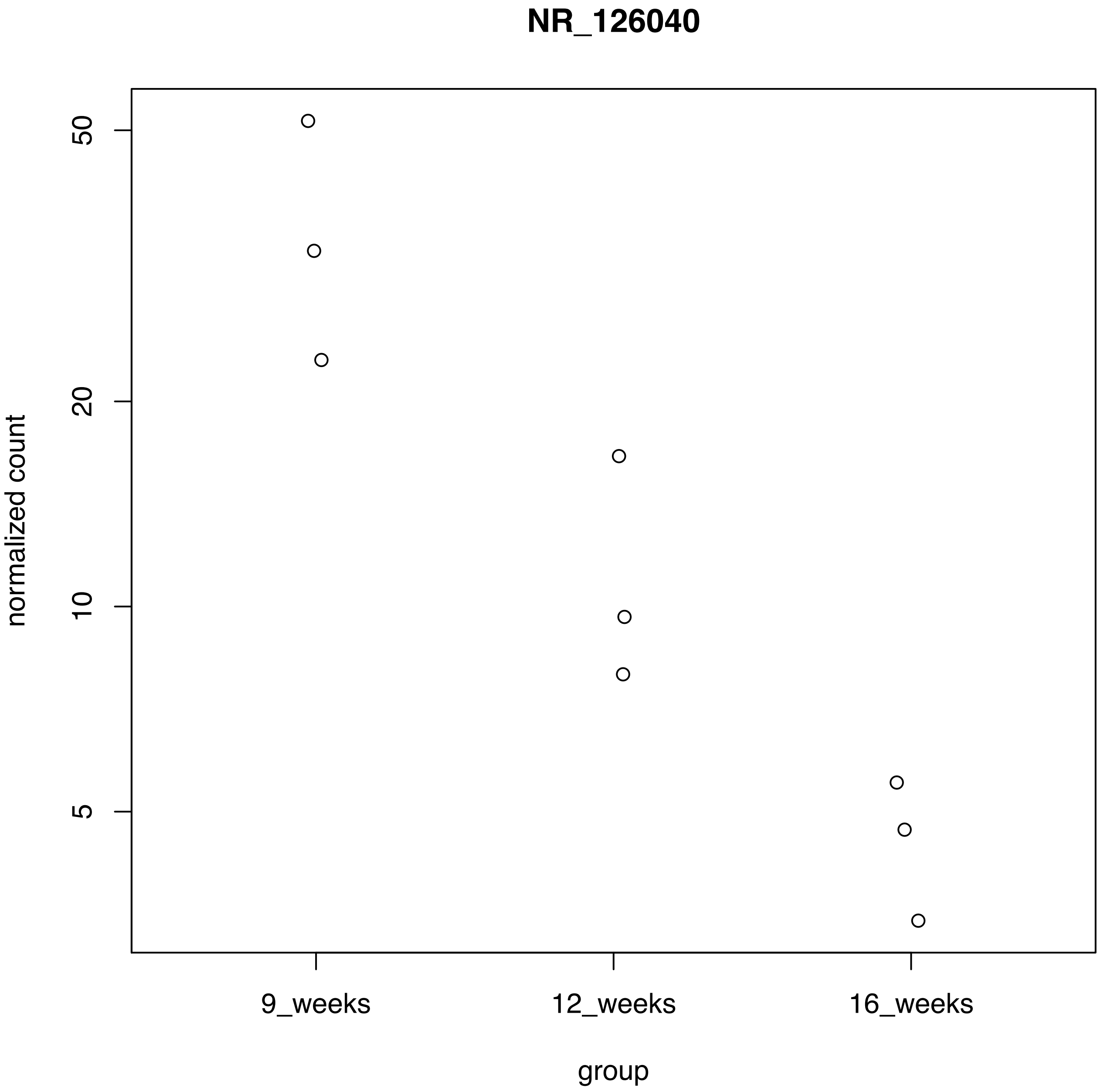

NR_126040
normalized count
50
20
10
5
9_weeks
12_weeks
16_weeks
group

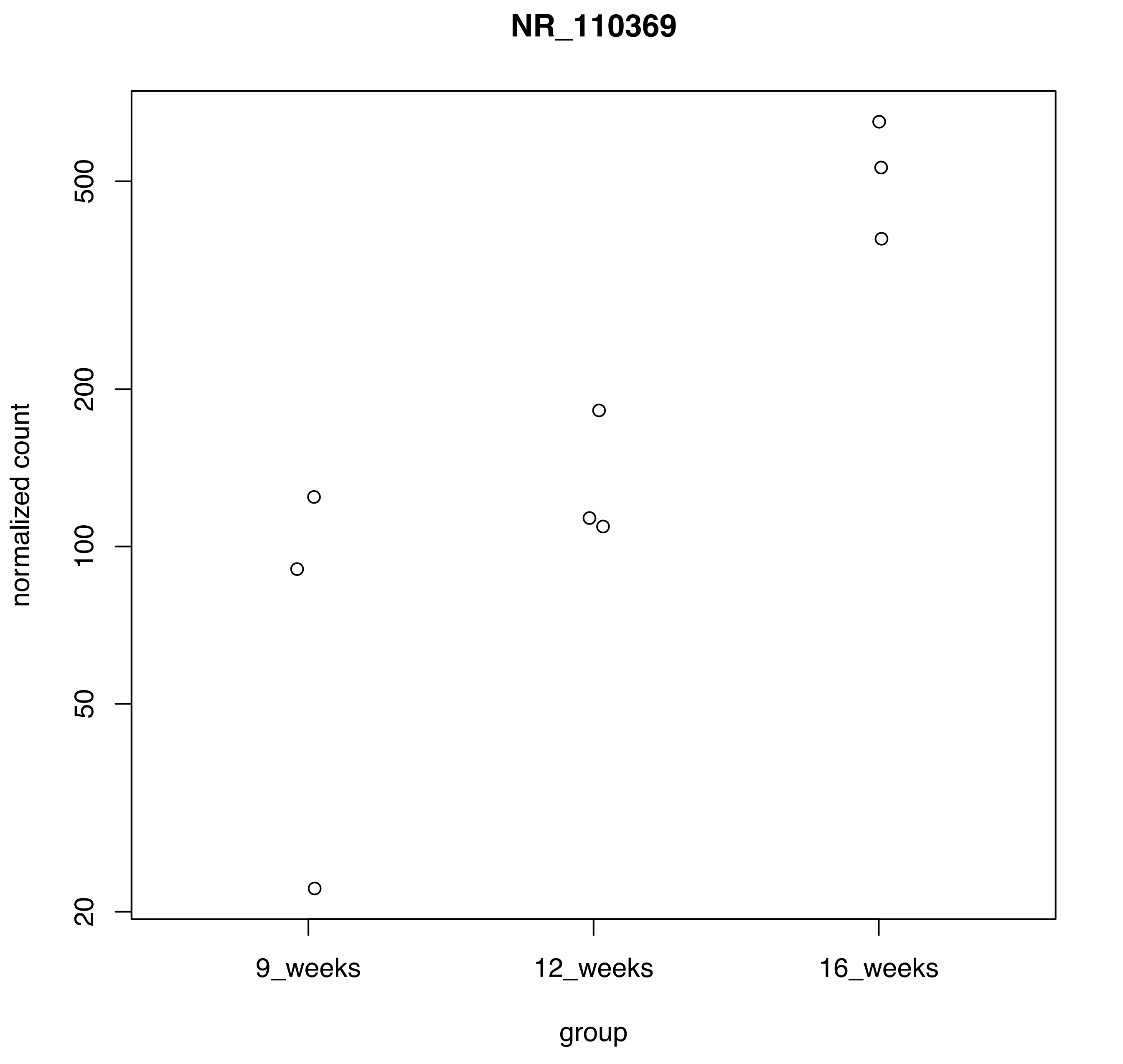
NR_110369
normalized count
500
200
100
50
20
9_weeks
12_weeks
16_weeks
group

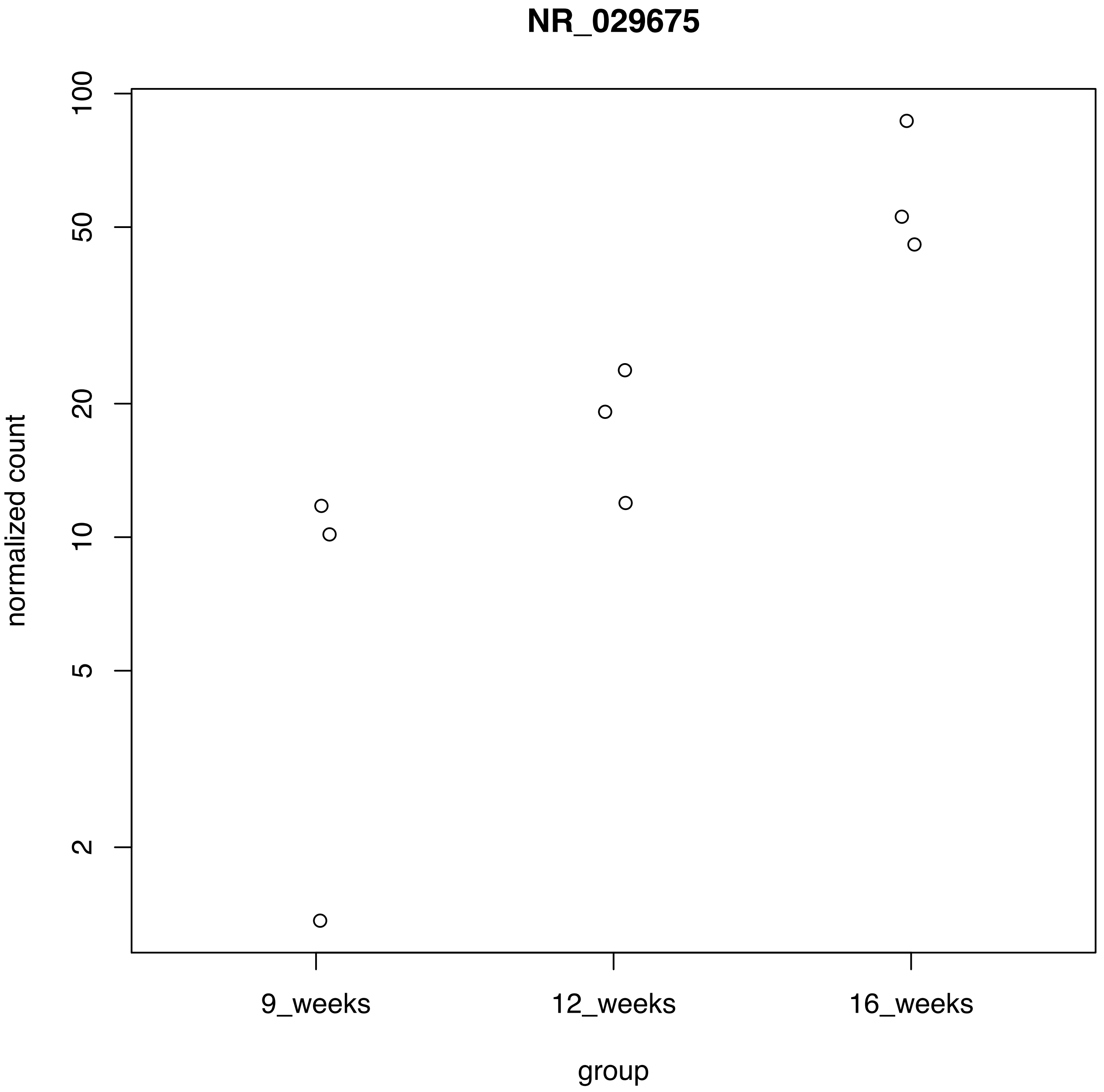
NR_029675
100
50
20
10
5
2
normalized count
9_weeks
12_weeks
16_weeks
group

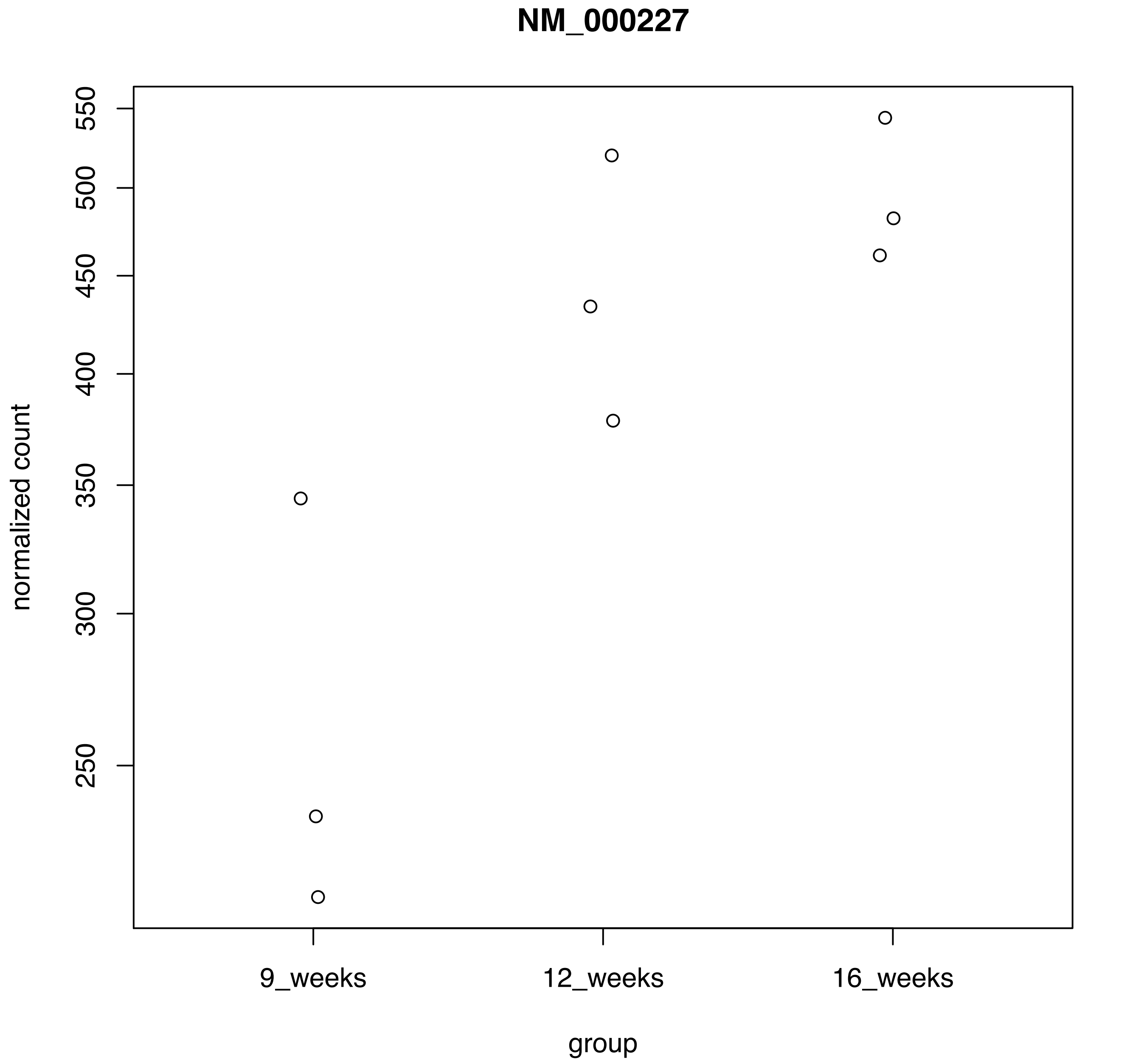

NM_000227
550
500
450
400
350
300
250
normalized count
9_weeks
12_weeks
16_weeks
group

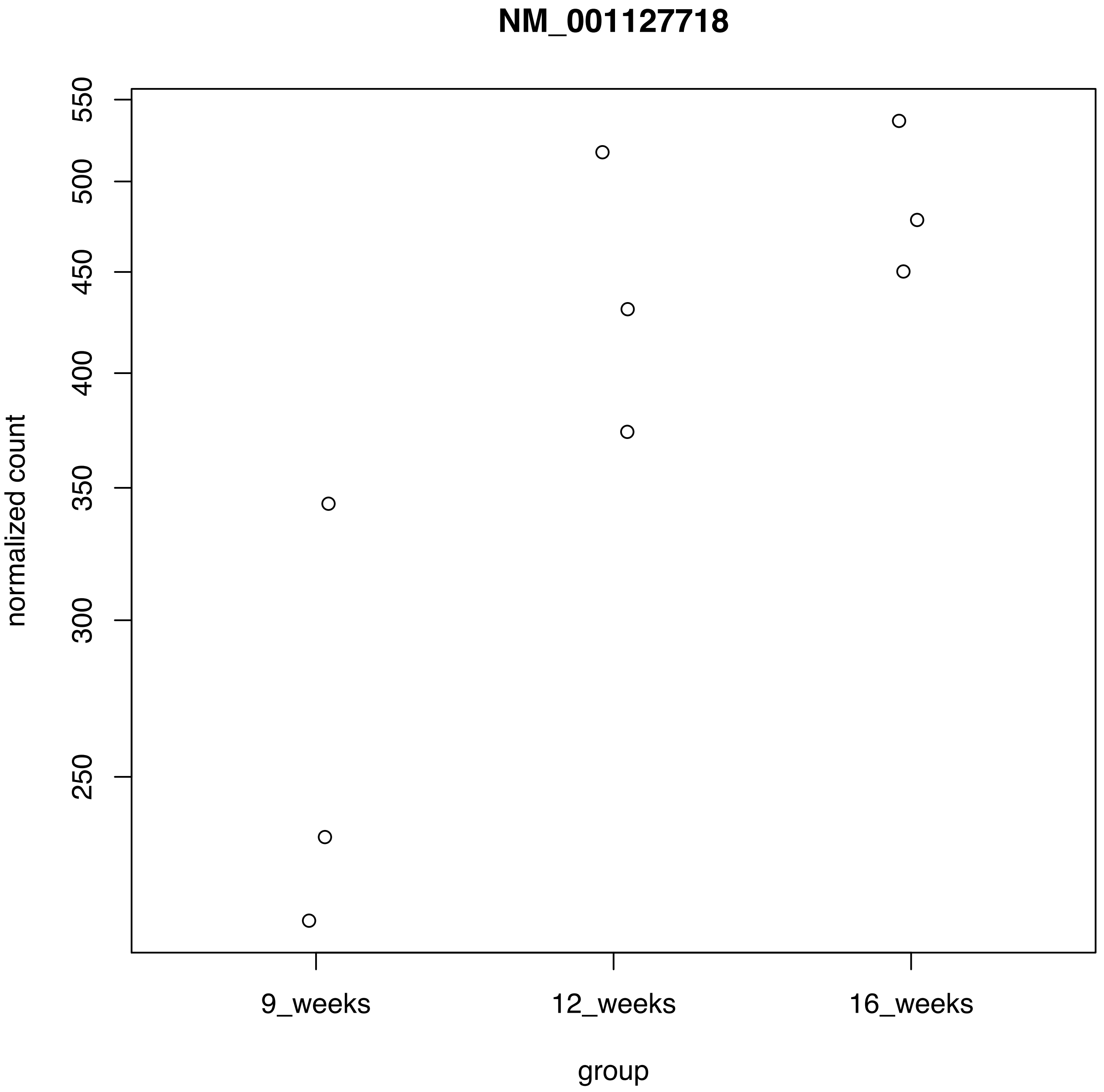
NM_001127718
normalized count
550
500
450
400
350
300
250
9_weeks
12_weeks
16_weeks
group

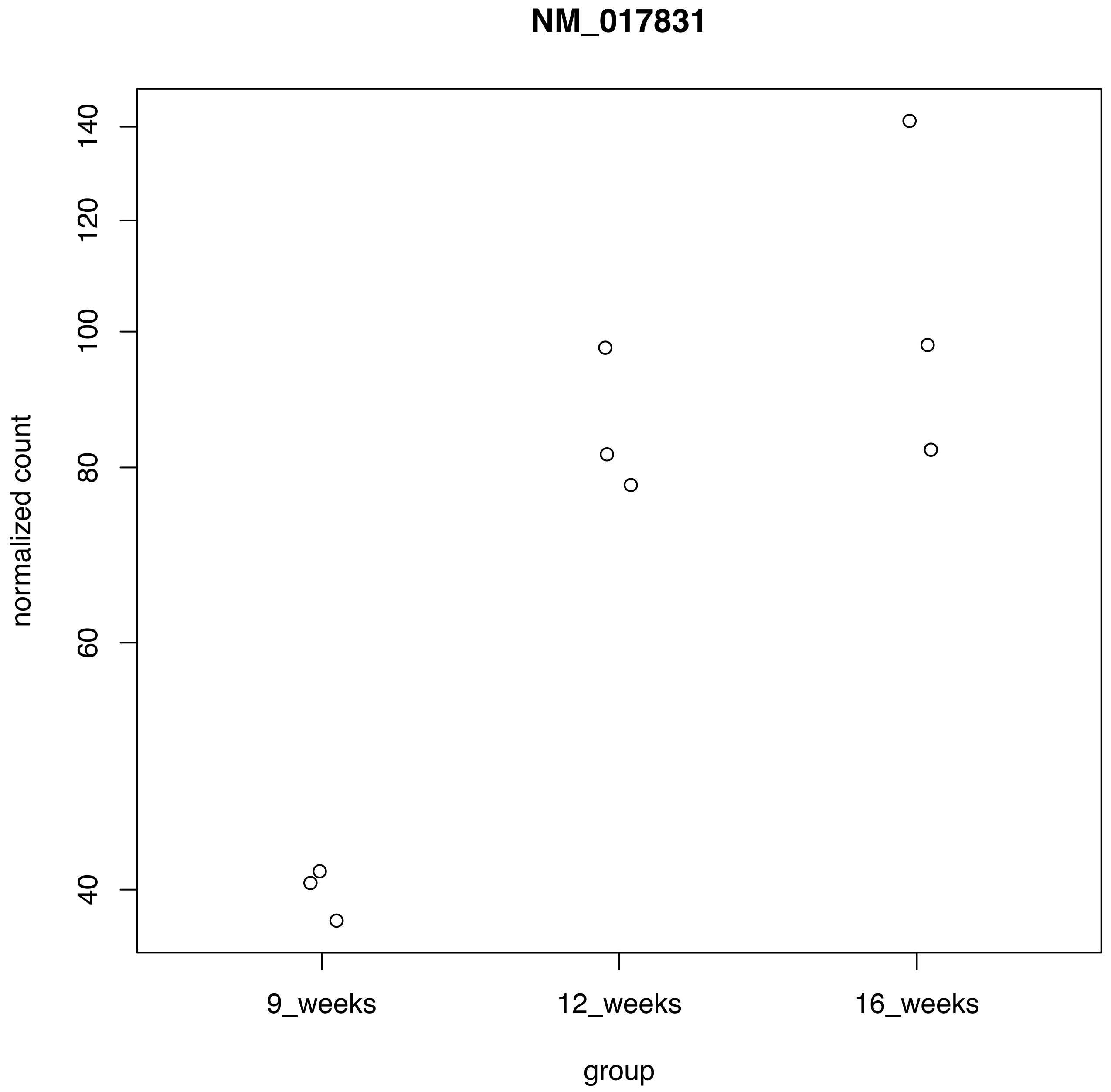
NM_017831
normalized count
140
120
100
80
60
40
9_weeks
12_weeks
16_weeks
group

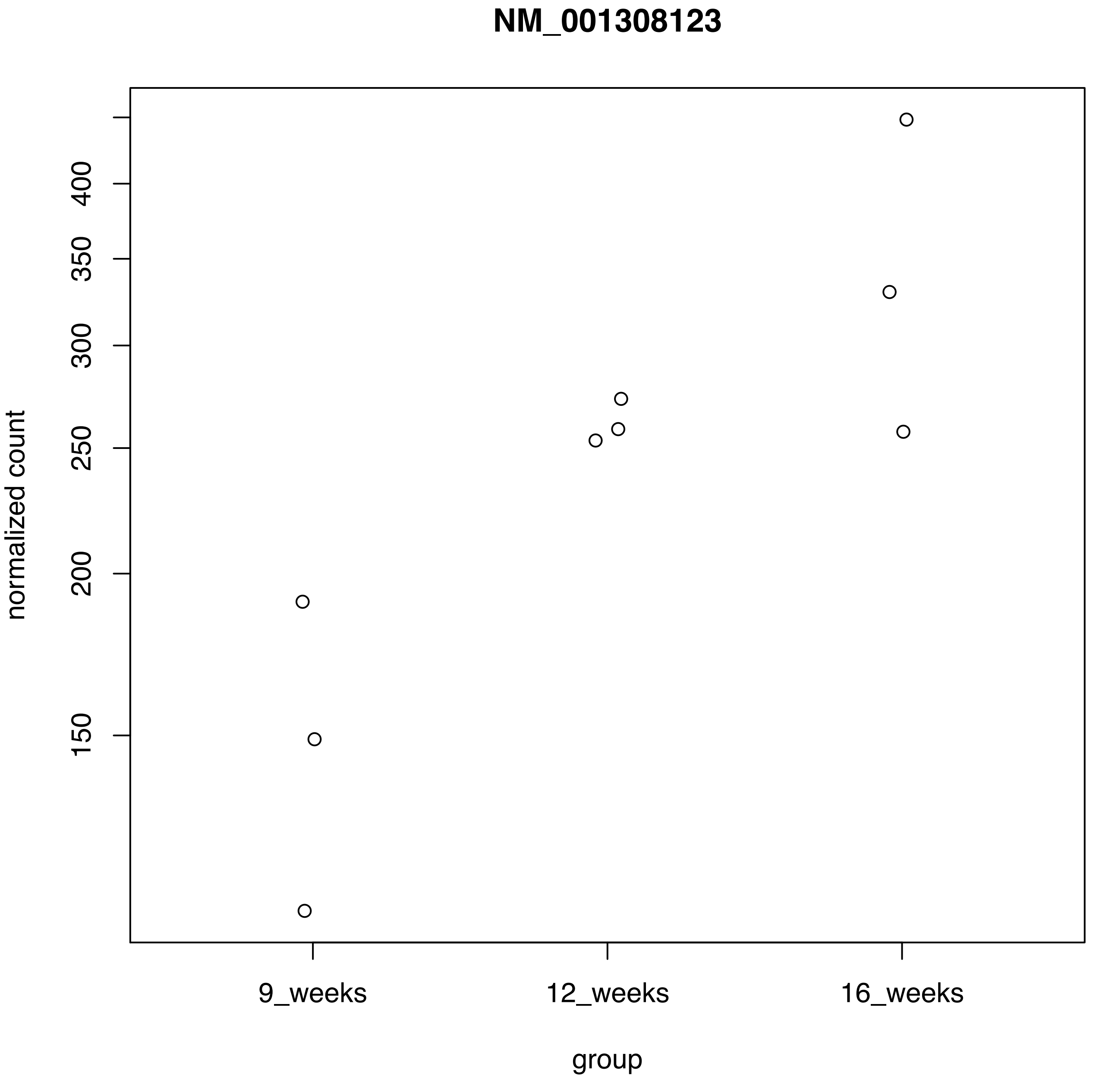

NM_001308123
normalized count
150
200
250
300
350
400
9_weeks
12_weeks
16_weeks
group

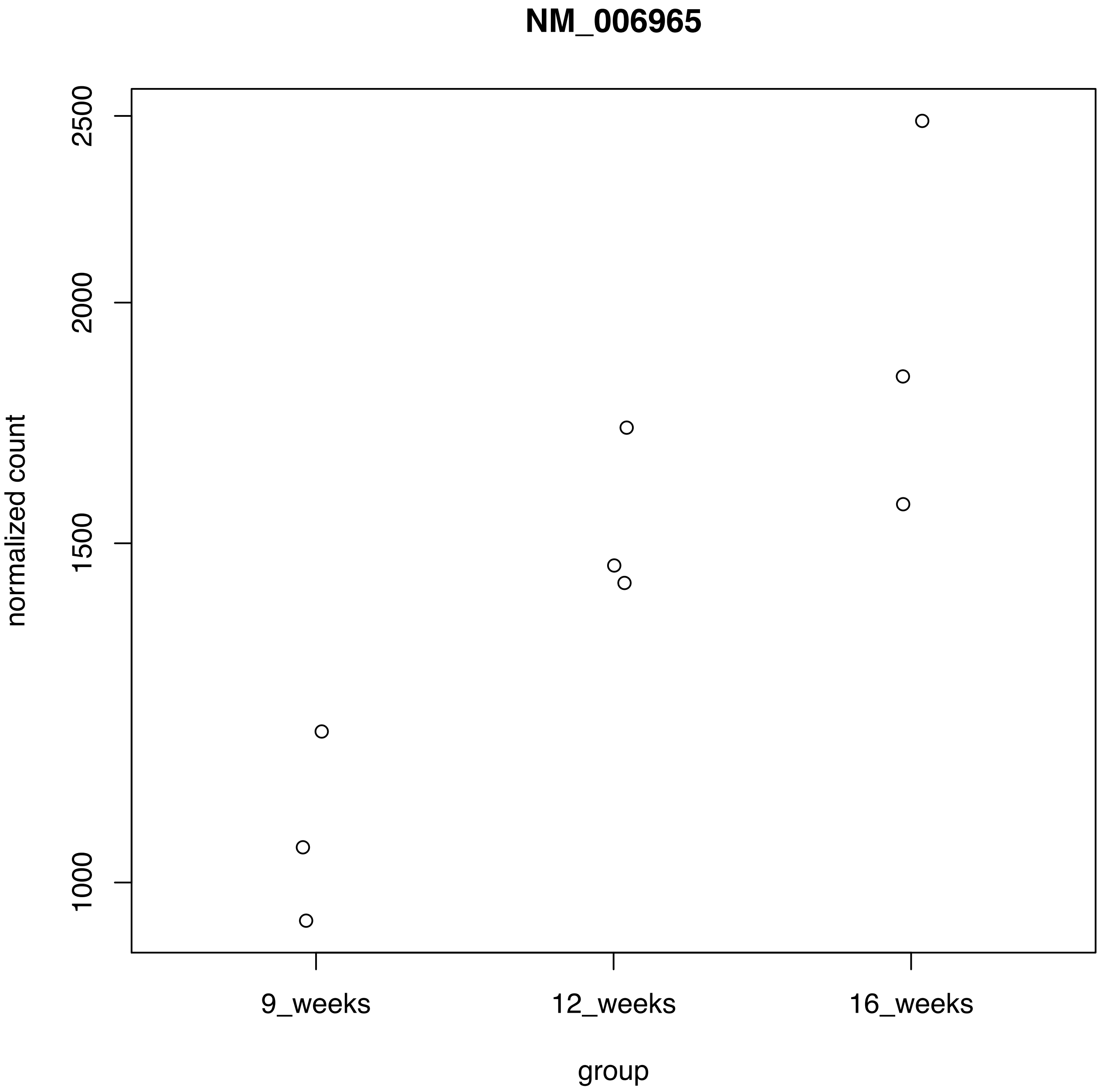
NM_006965
normalized count
2500
2000
1500
1000
9_weeks
12_weeks
16_weeks
group

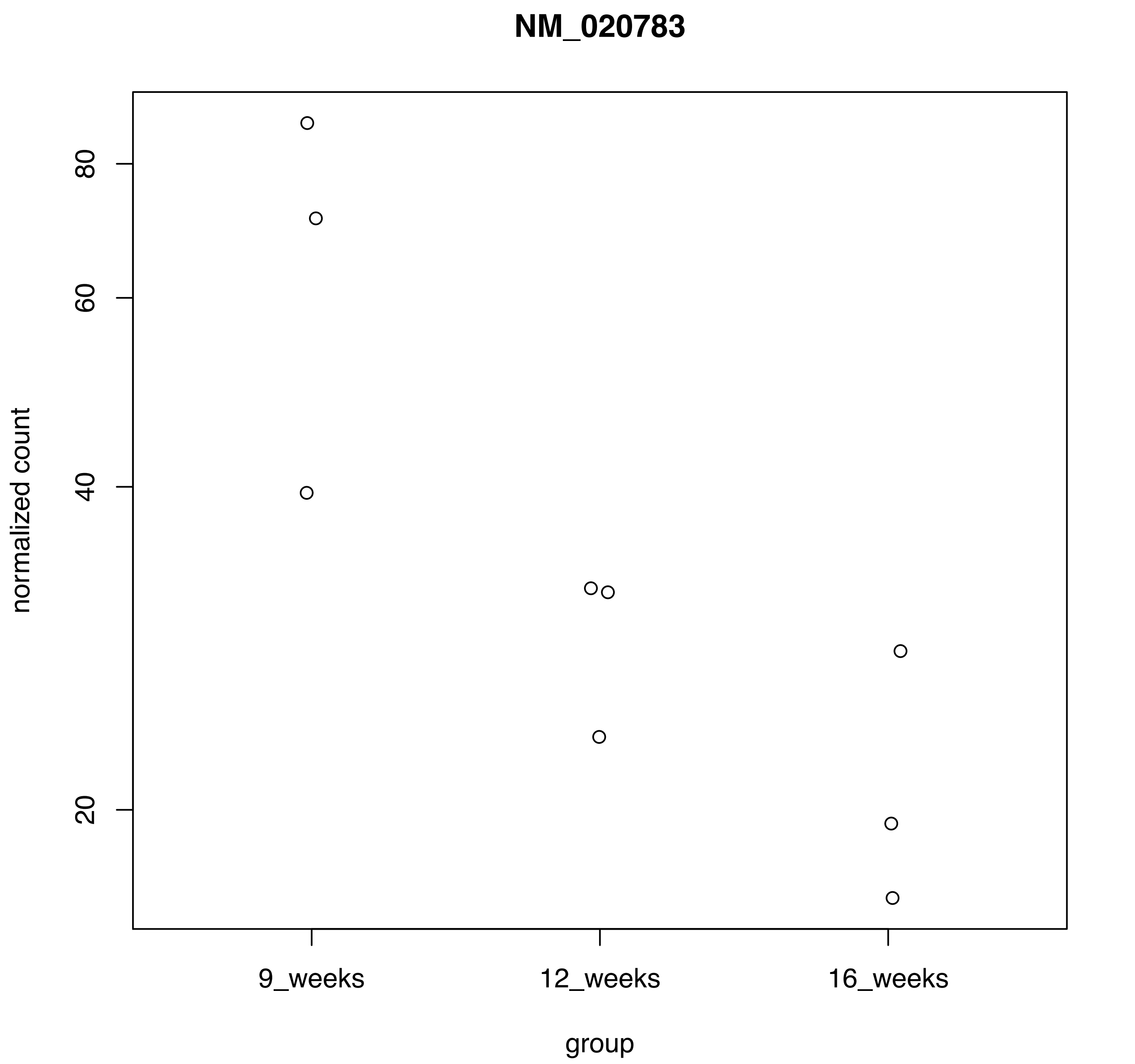
NM_020783
normalized count
80
60
40
20
9_weeks
12_weeks
16_weeks
group

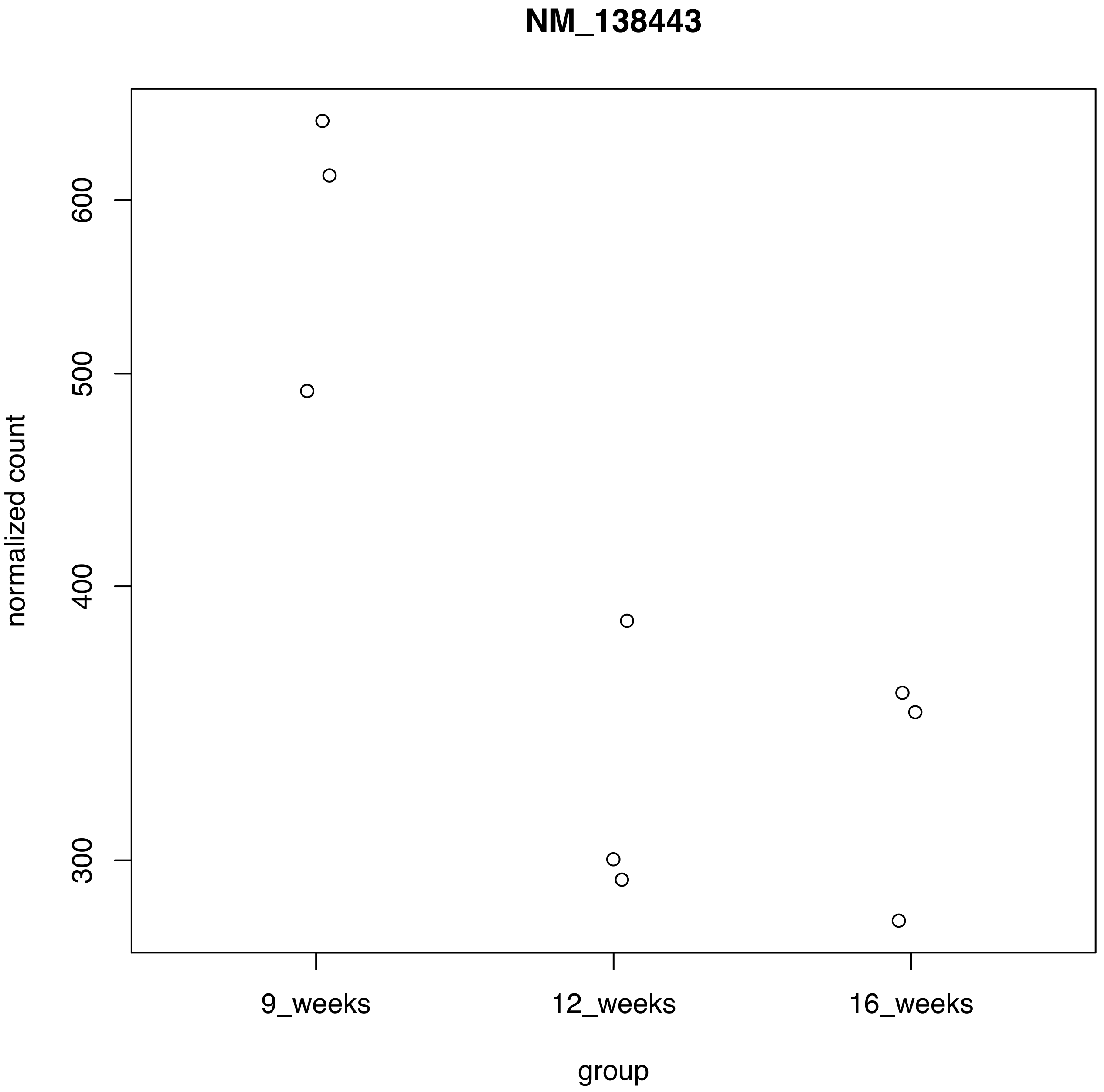
NM_138443
normalized count
600
500
400
300
9_weeks
12_weeks
16_weeks
group

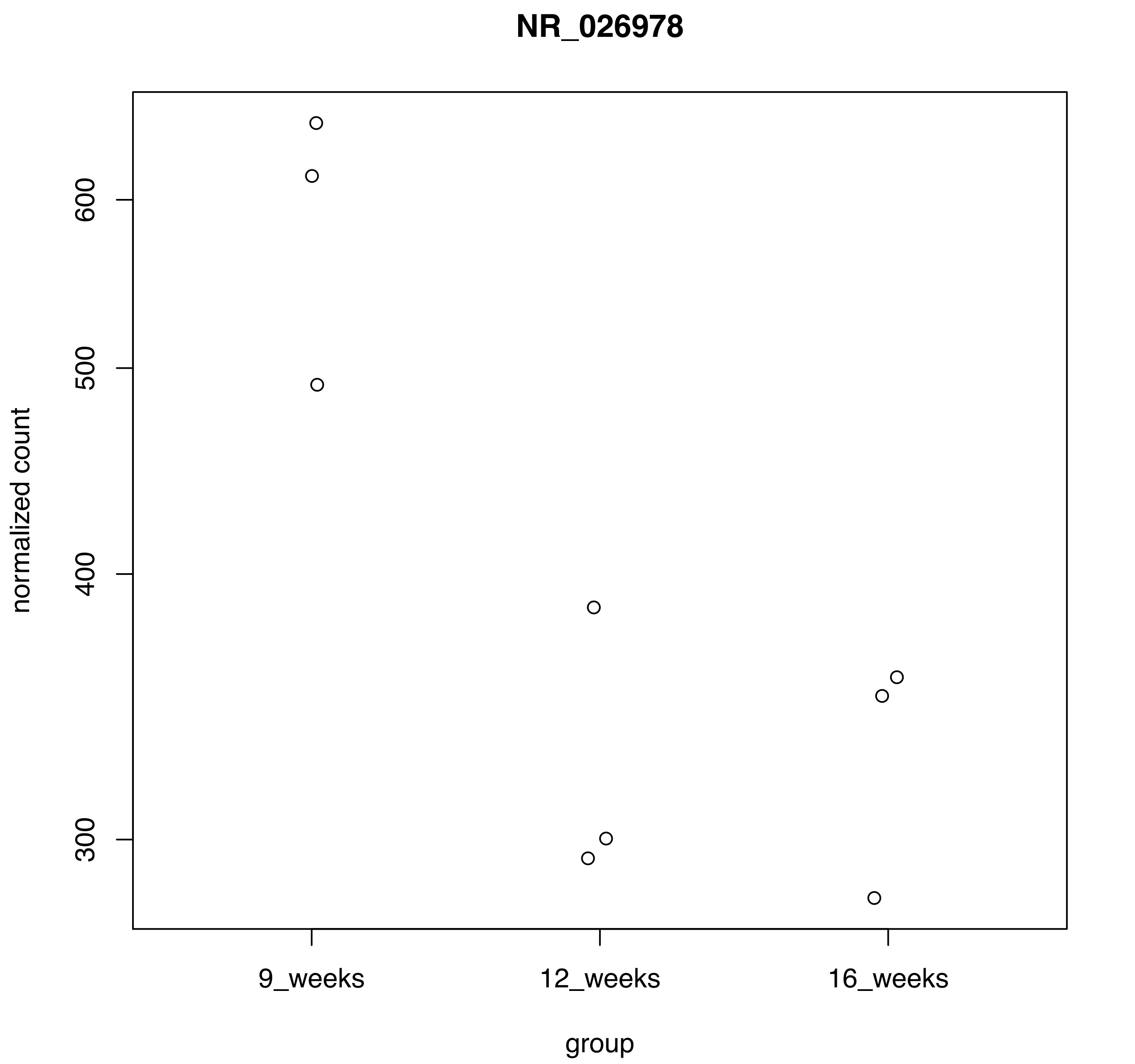

NR_026978
normalized count
600
500
400
300
9_weeks
12_weeks
16_weeks
group

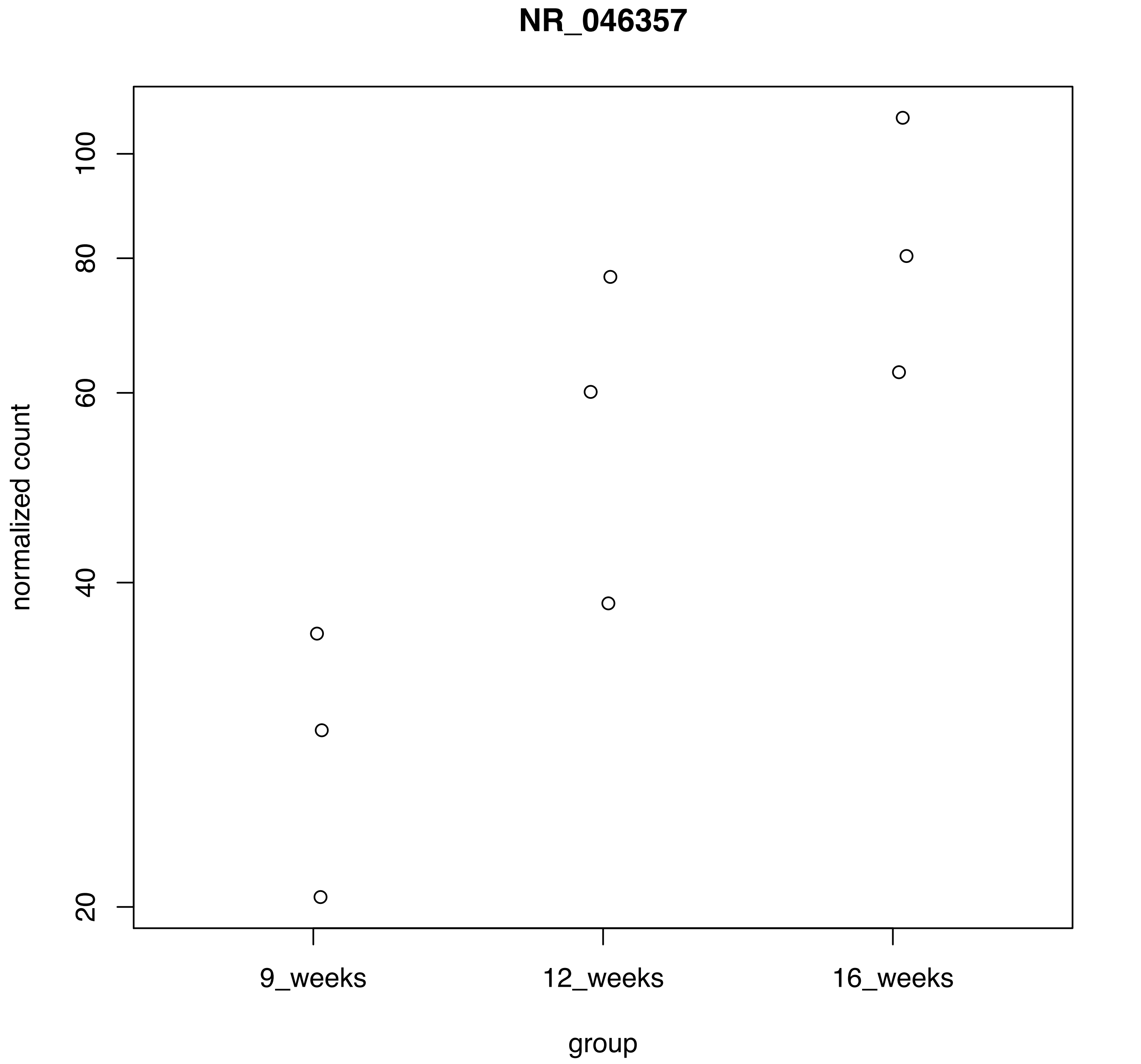
NR_046357
normalized count
100
80
60
40
20
9_weeks
12_weeks
16_weeks
group

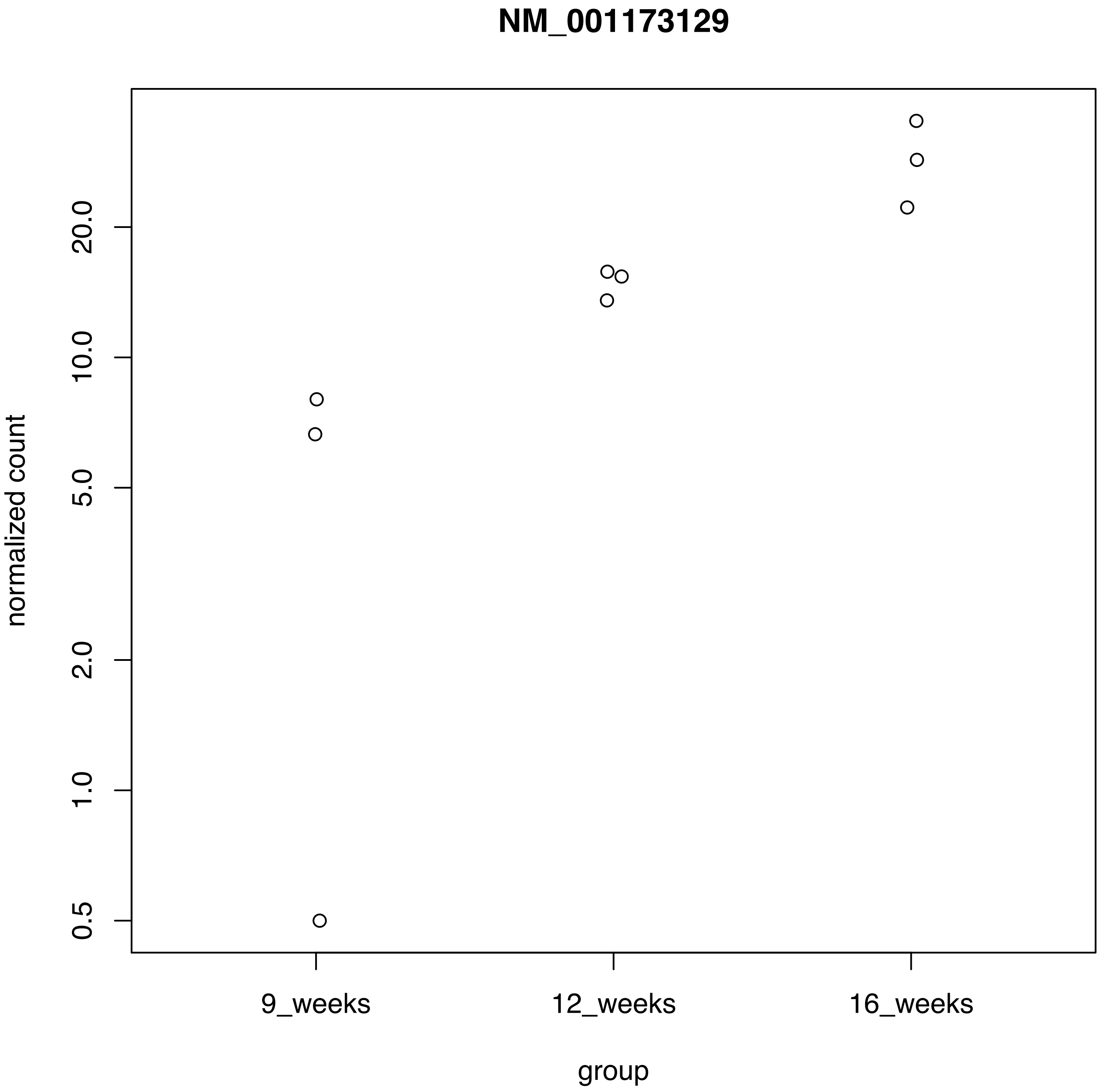
NM_001173129
normalized count
0.5
1.0
2.0
5.0
10.0
20.0
9_weeks
12_weeks
16_weeks
group

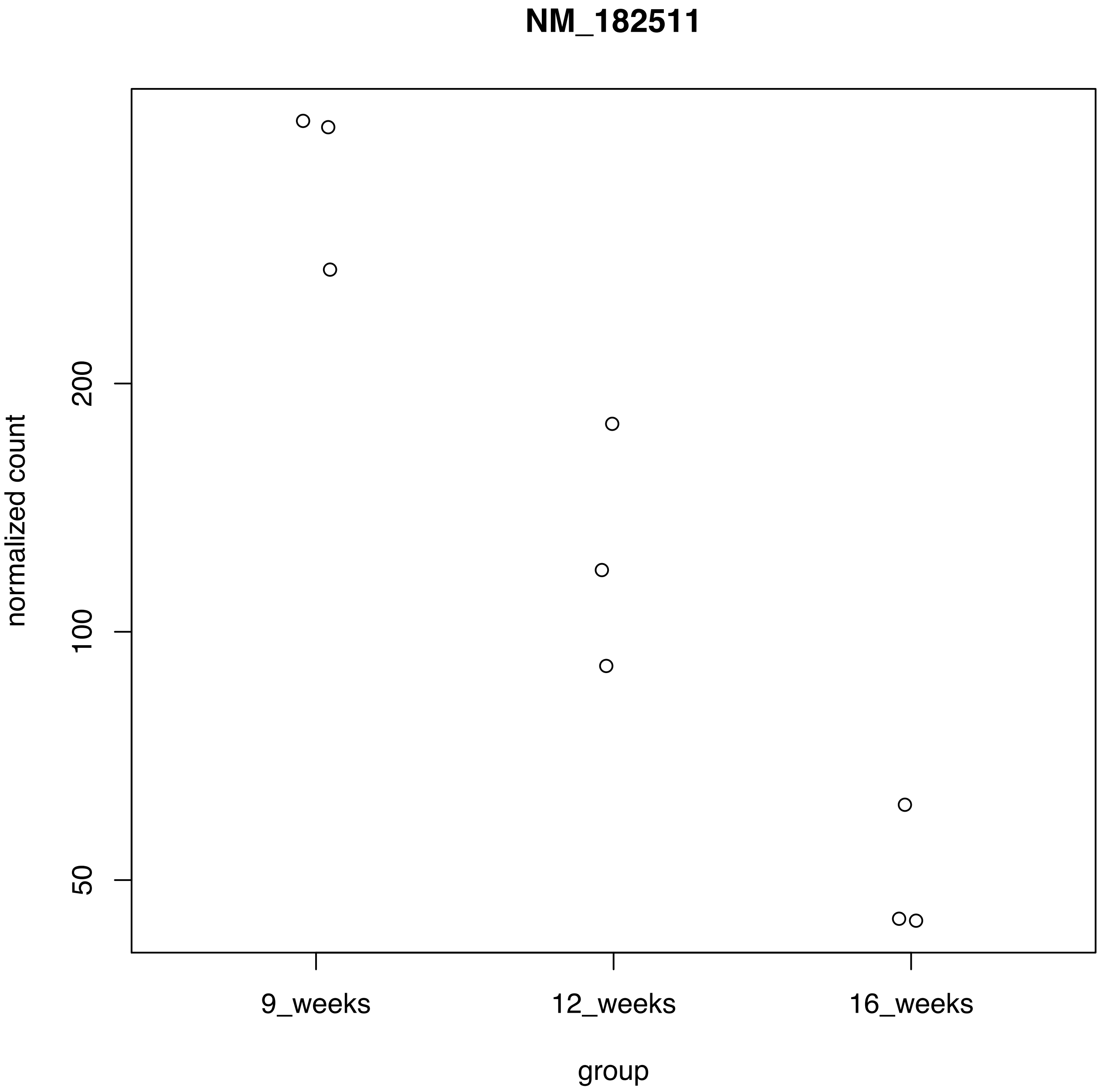
NM_182511
normalized count
200
100
50
9_weeks
12_weeks
16_weeks
group

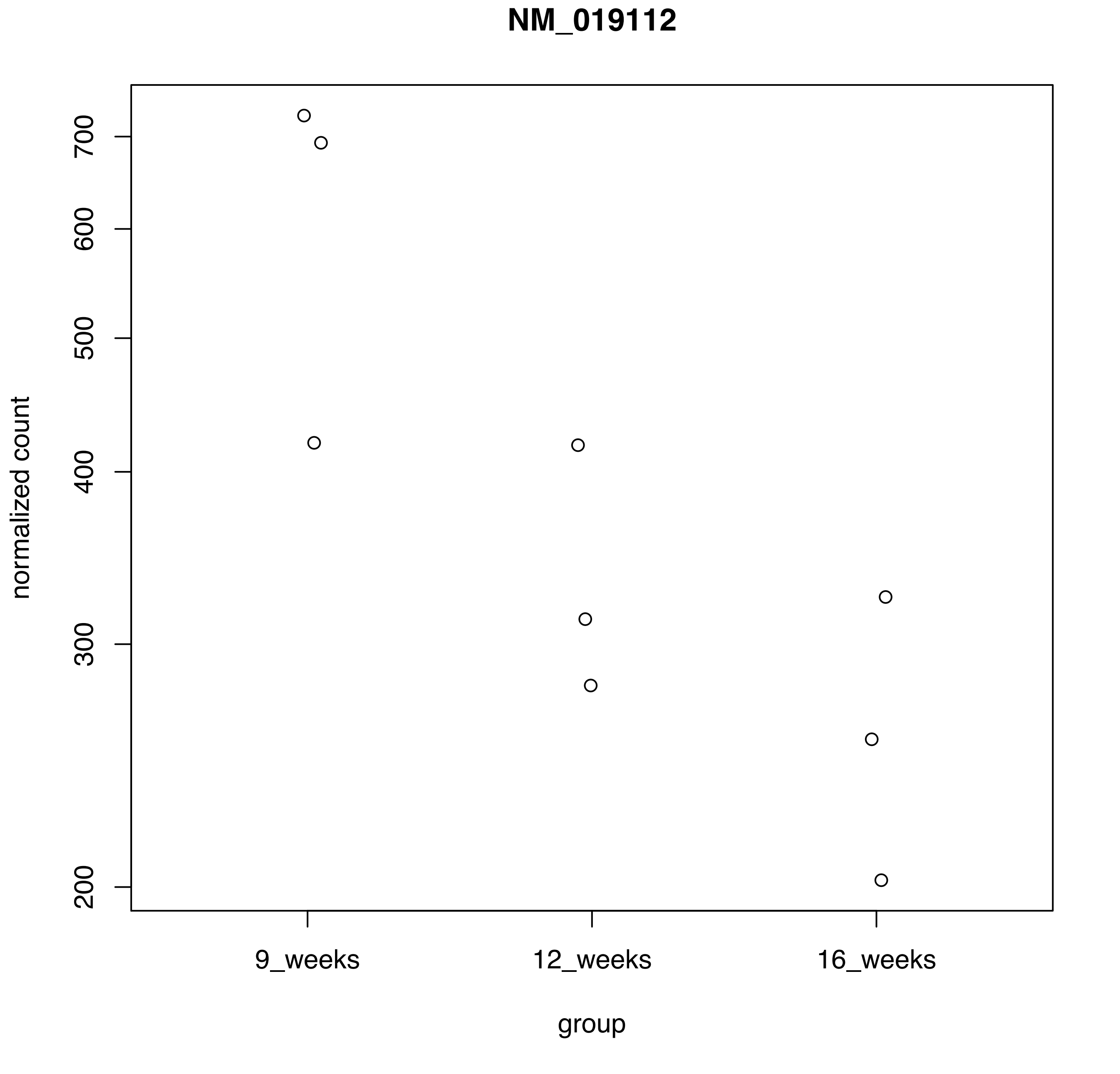
NM_019112
normalized count
700
600
500
400
300
200
9_weeks
12_weeks
16_weeks
group

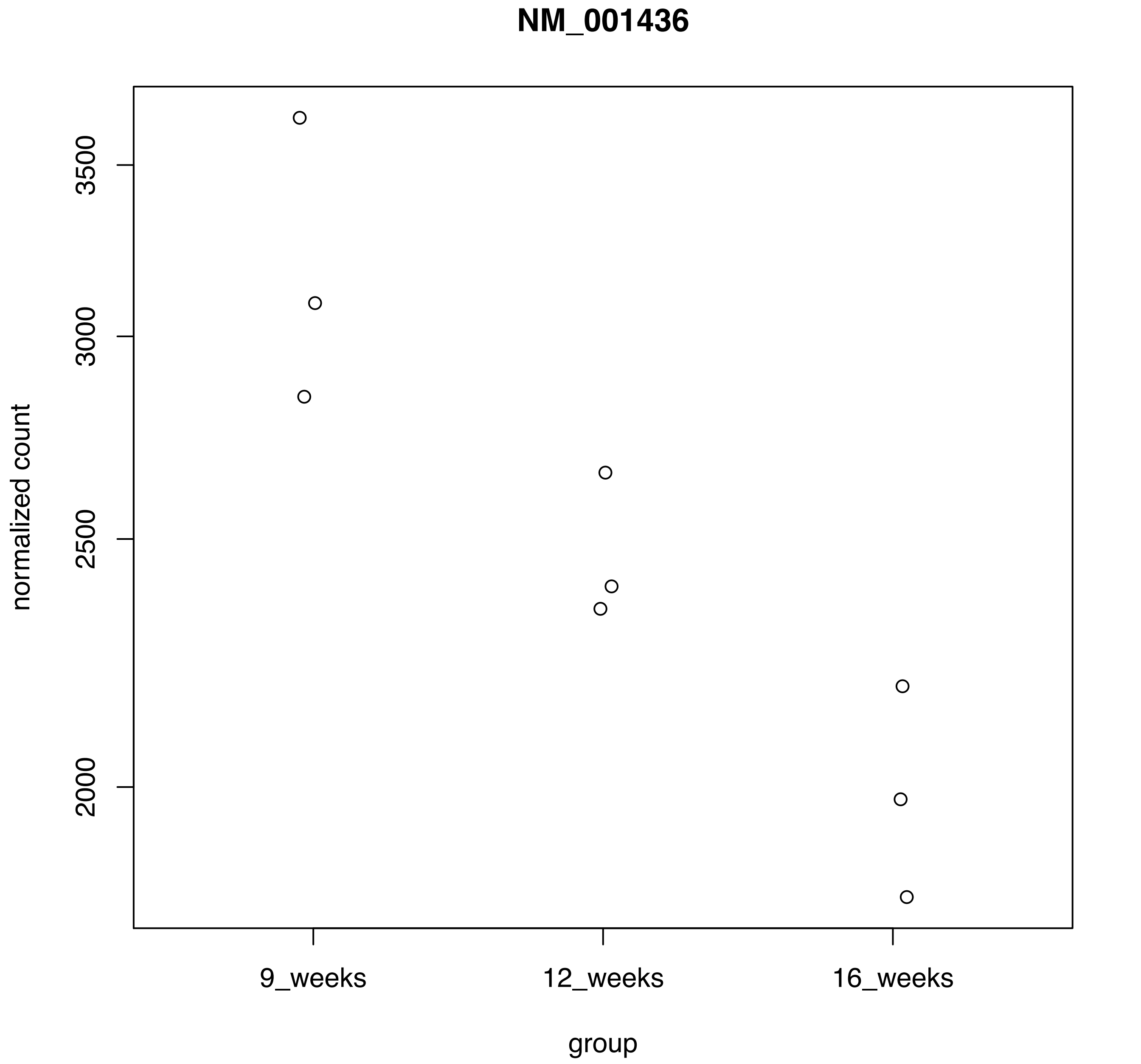
NM_001436
normalized count
3500
3000
2500
2000
9_weeks
12_weeks
16_weeks
group

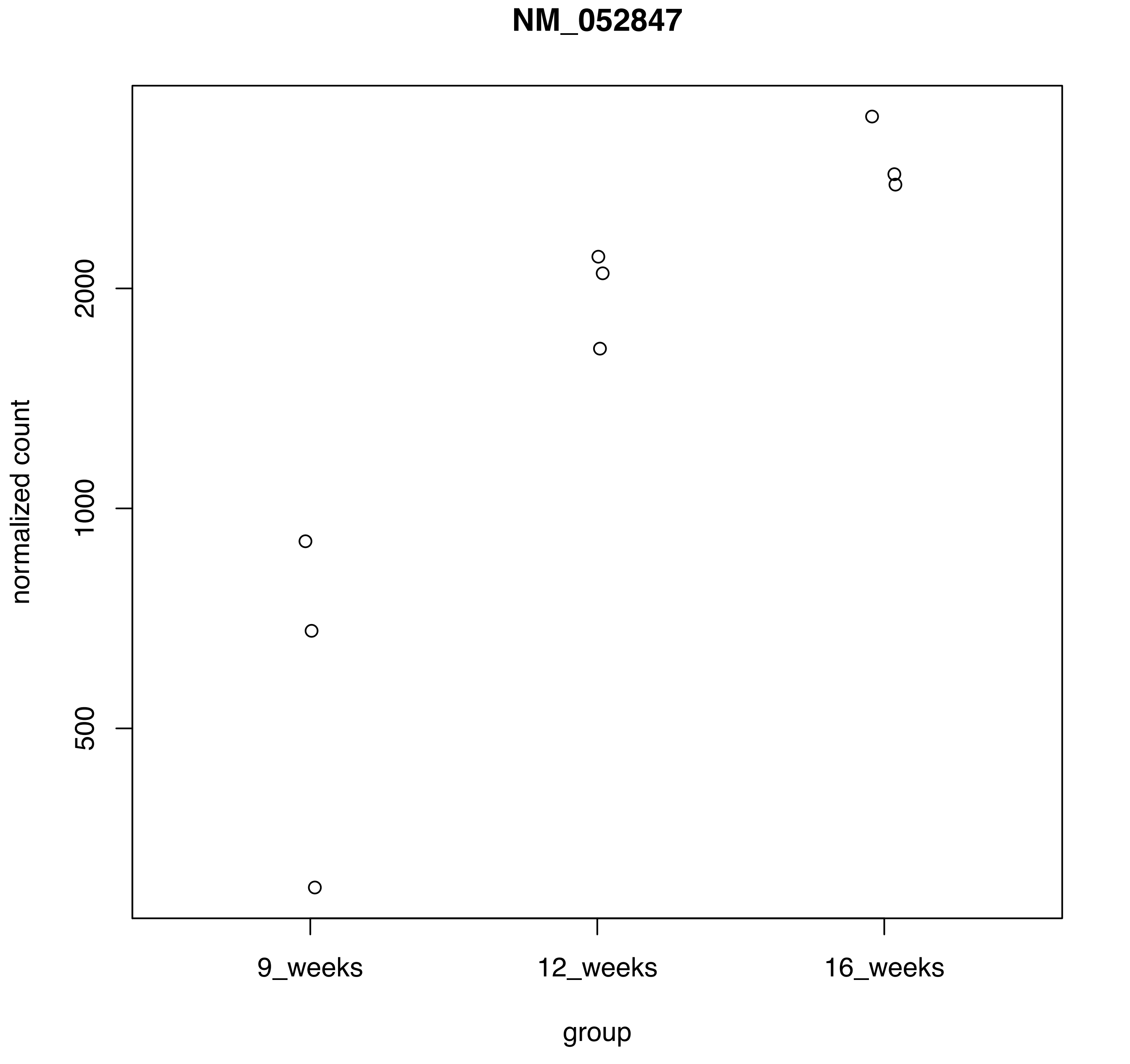
NM_052847
normalized count
2000
1000
500
9_weeks
12_weeks
16_weeks
group

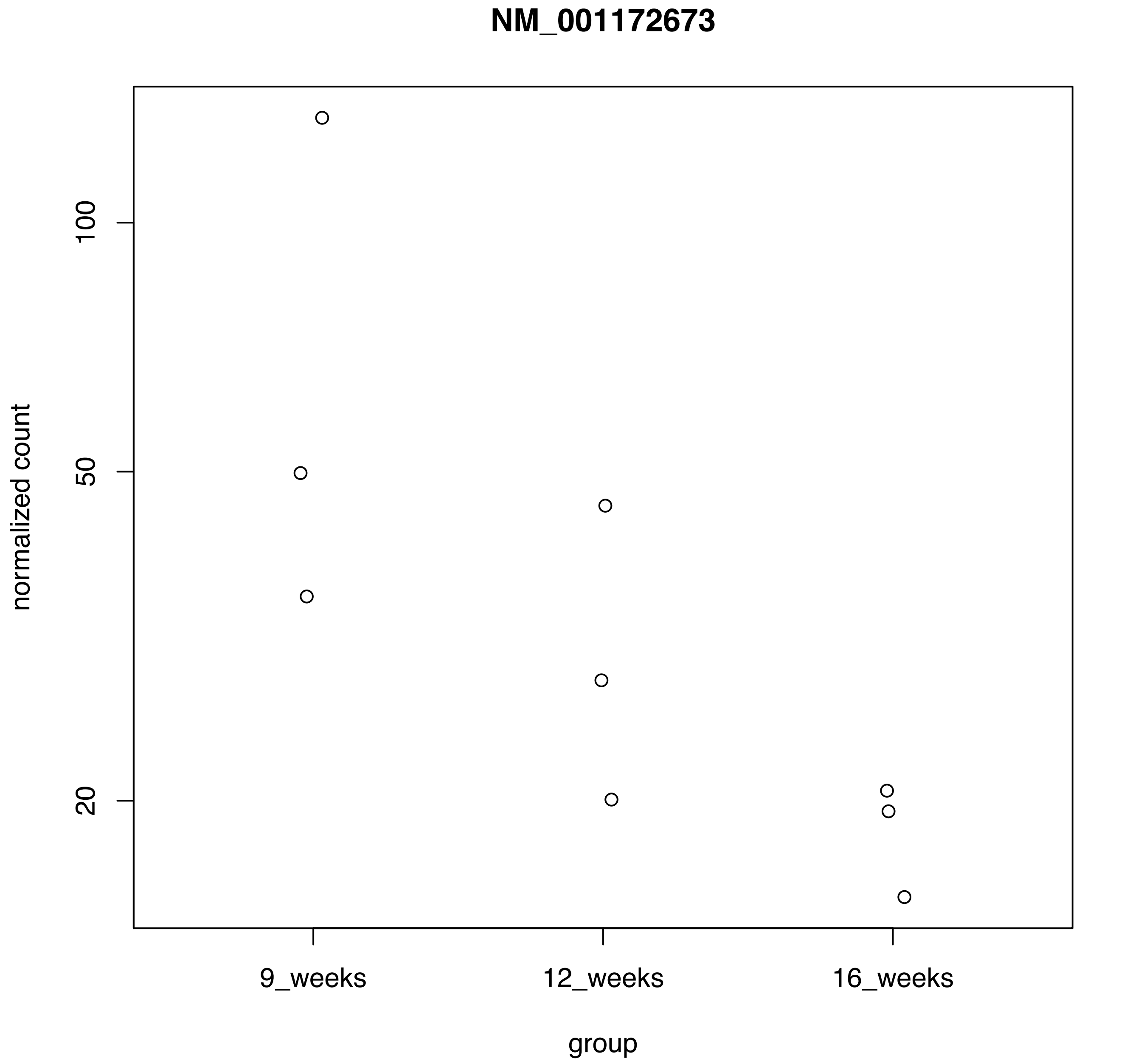

NM_001172673
100
50
20
normalized count
9_weeks
12_weeks
16_weeks
group

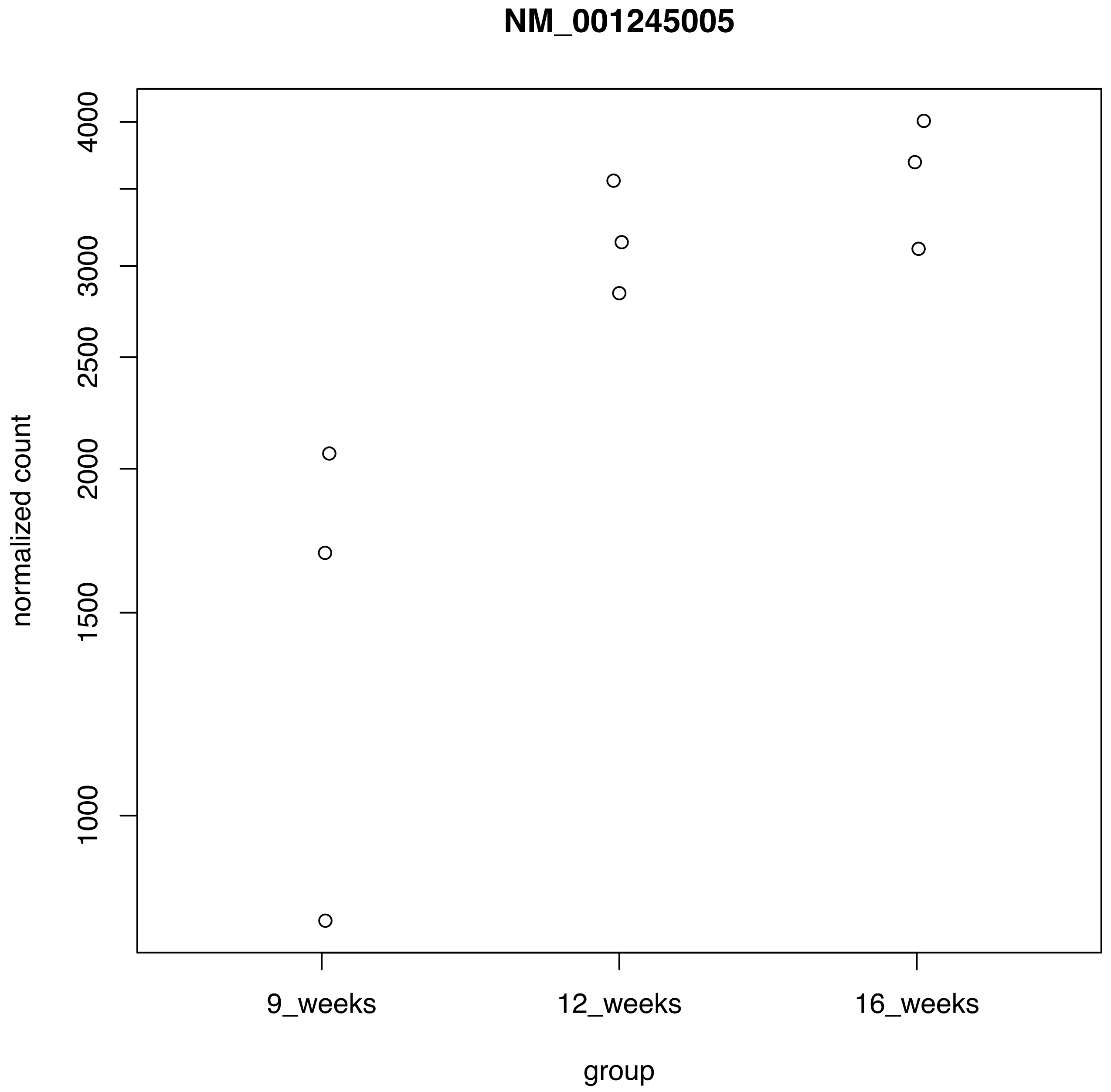
NM_001245005
normalized count
1000
1500
2000
2500
3000
4000
9_weeks
12_weeks
16_weeks
group

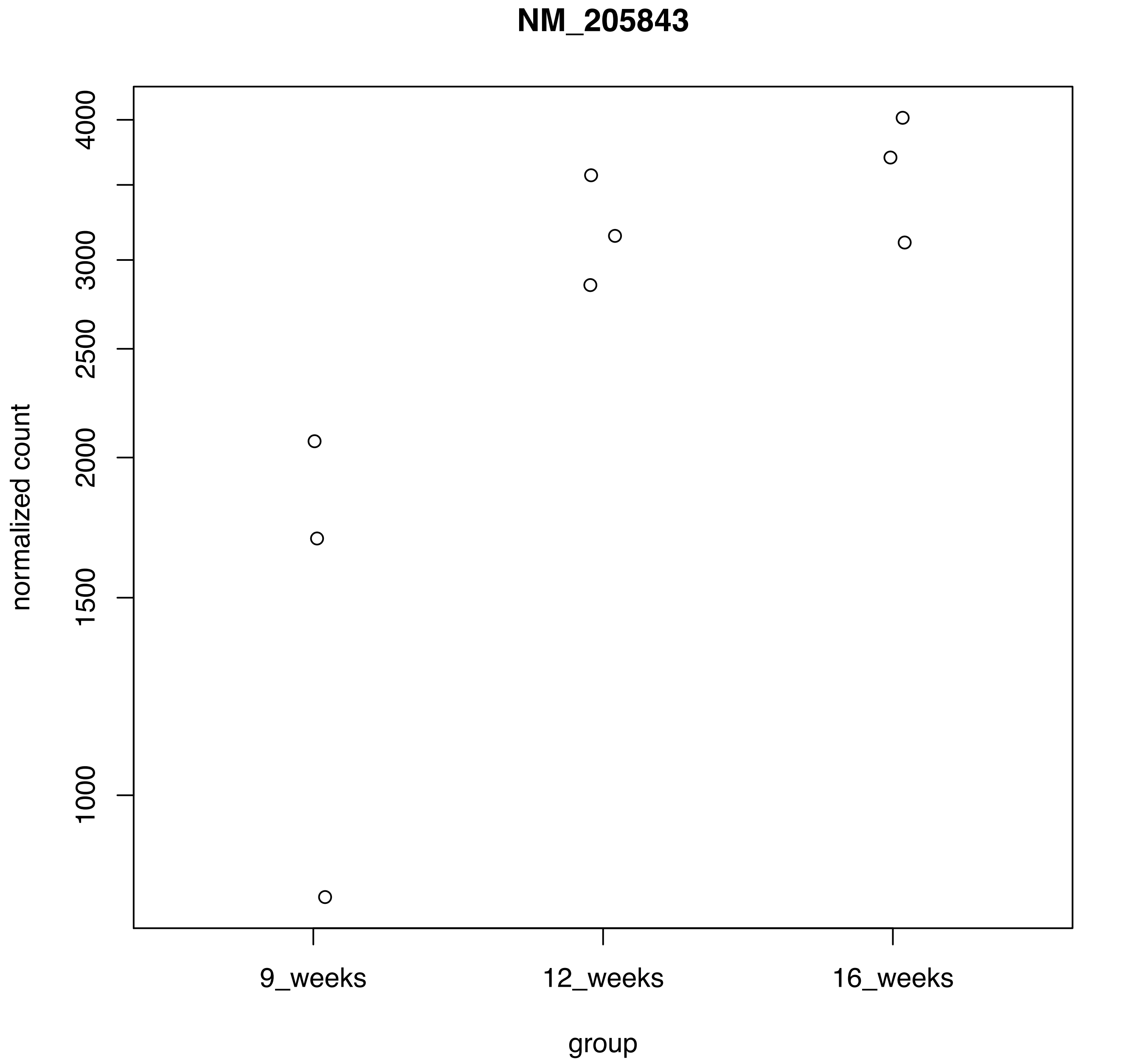

NM_205843
normalized count
4000
3000
2500
2000
1500
1000
9_weeks
12_weeks
16_weeks
group

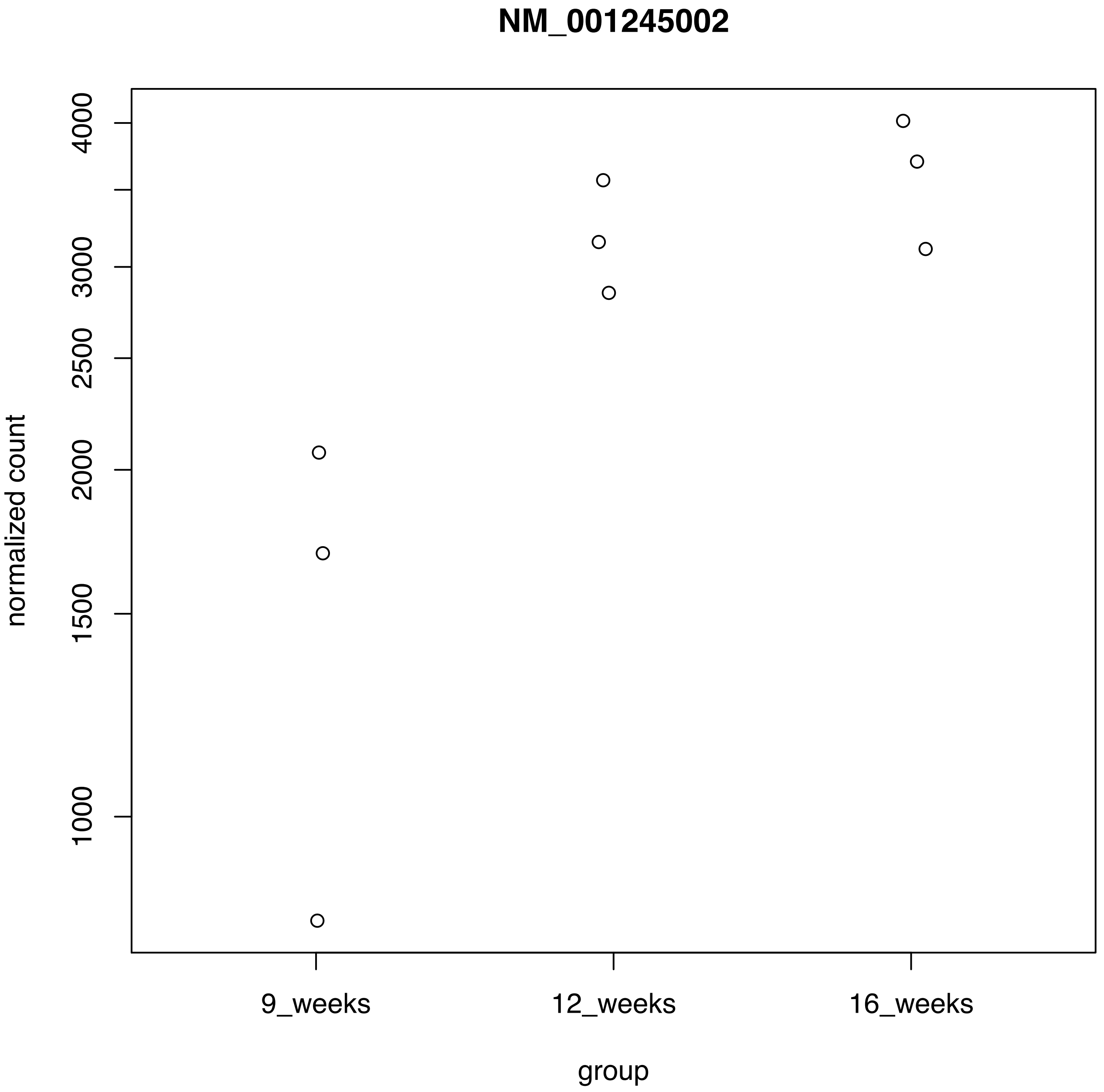

NM_001245002
normalized count
1000
1500
2000
2500
3000
4000
9_weeks
12_weeks
16_weeks
group

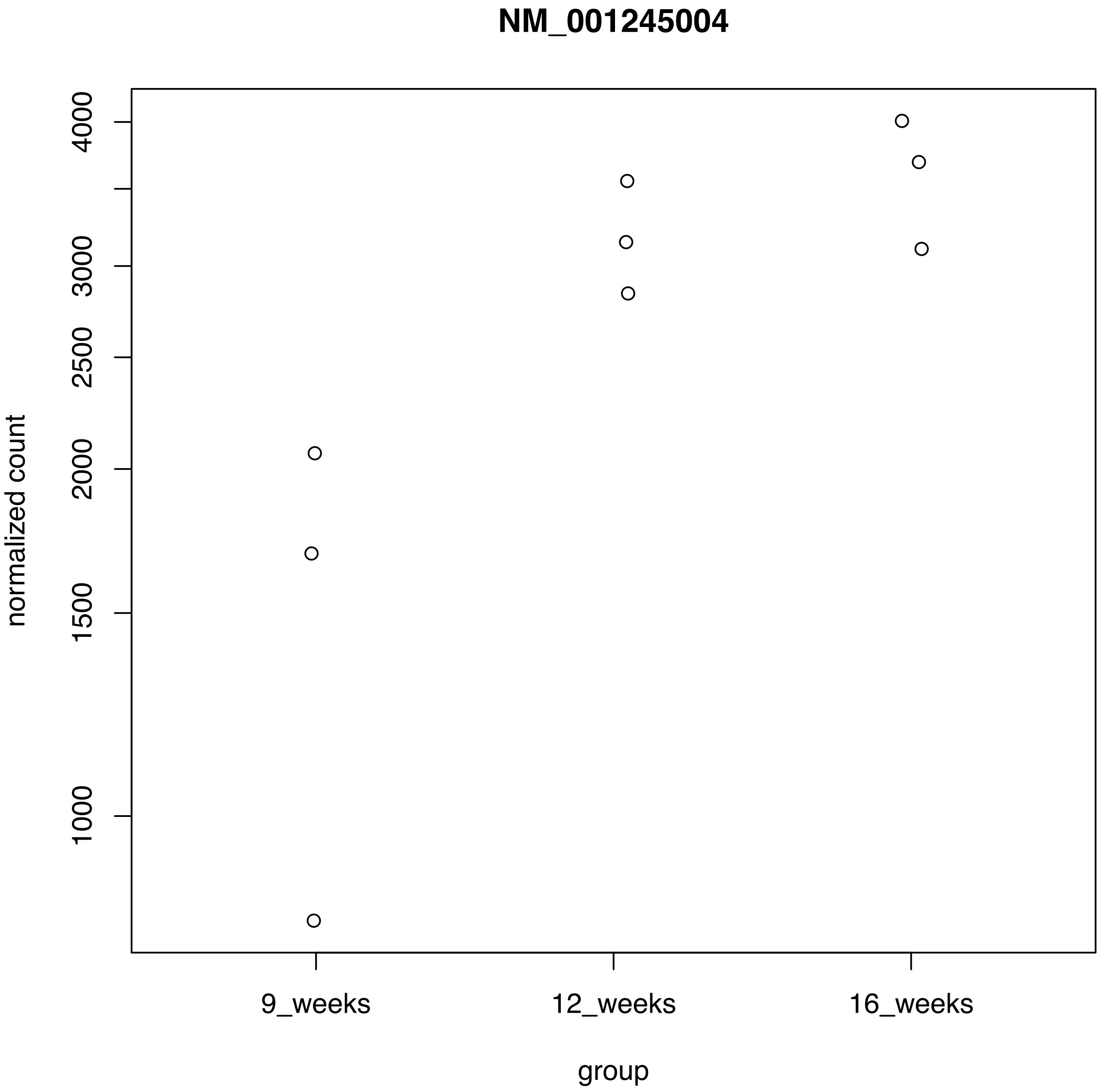
NM_001245004
normalized count
1000
1500
2000
2500
3000
4000
9_weeks
12_weeks
16_weeks
group

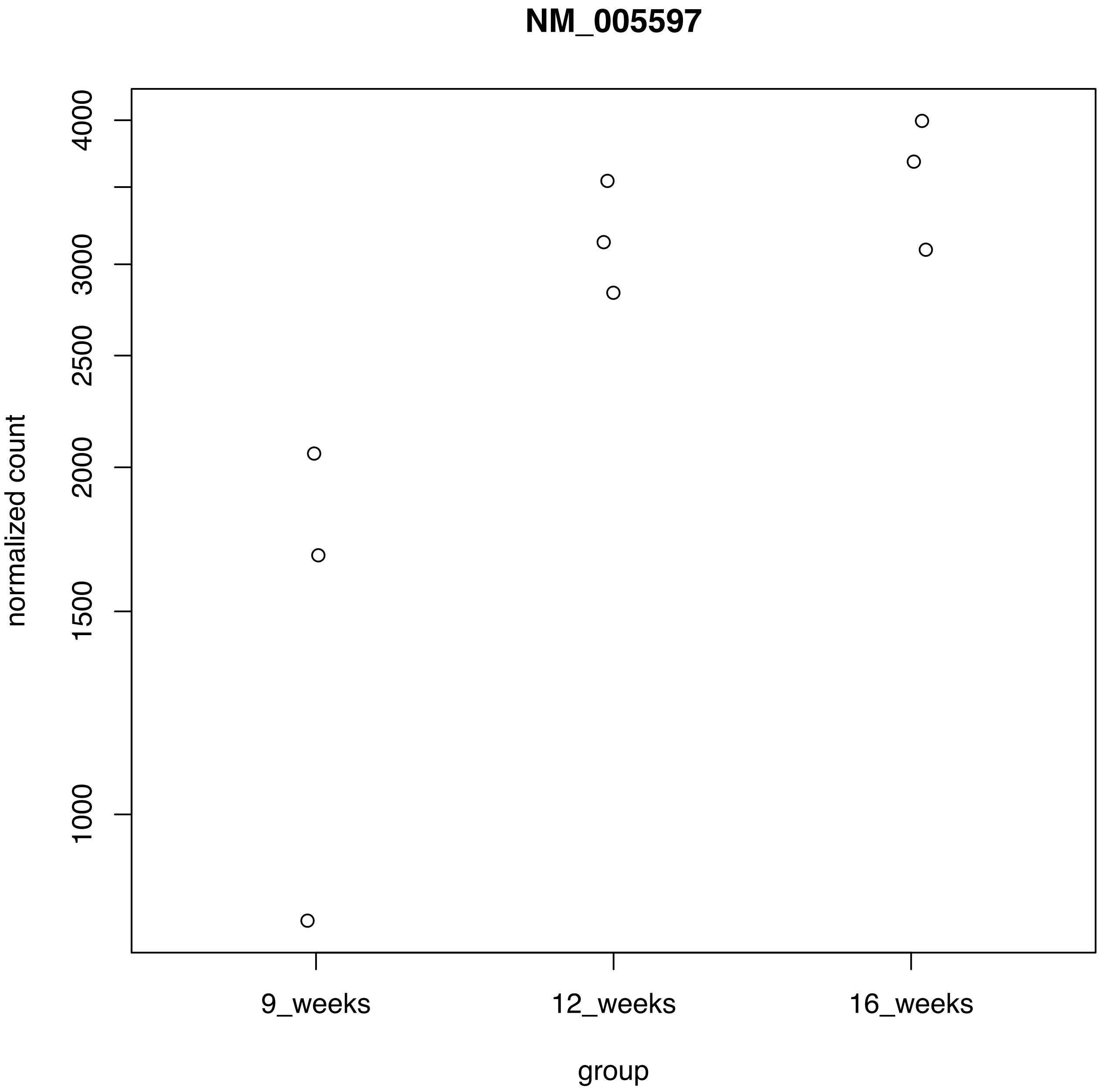
NM_005597
normalized count
group
4000
3000
2500
2000
1500
1000
9_weeks
12_weeks
16_weeks

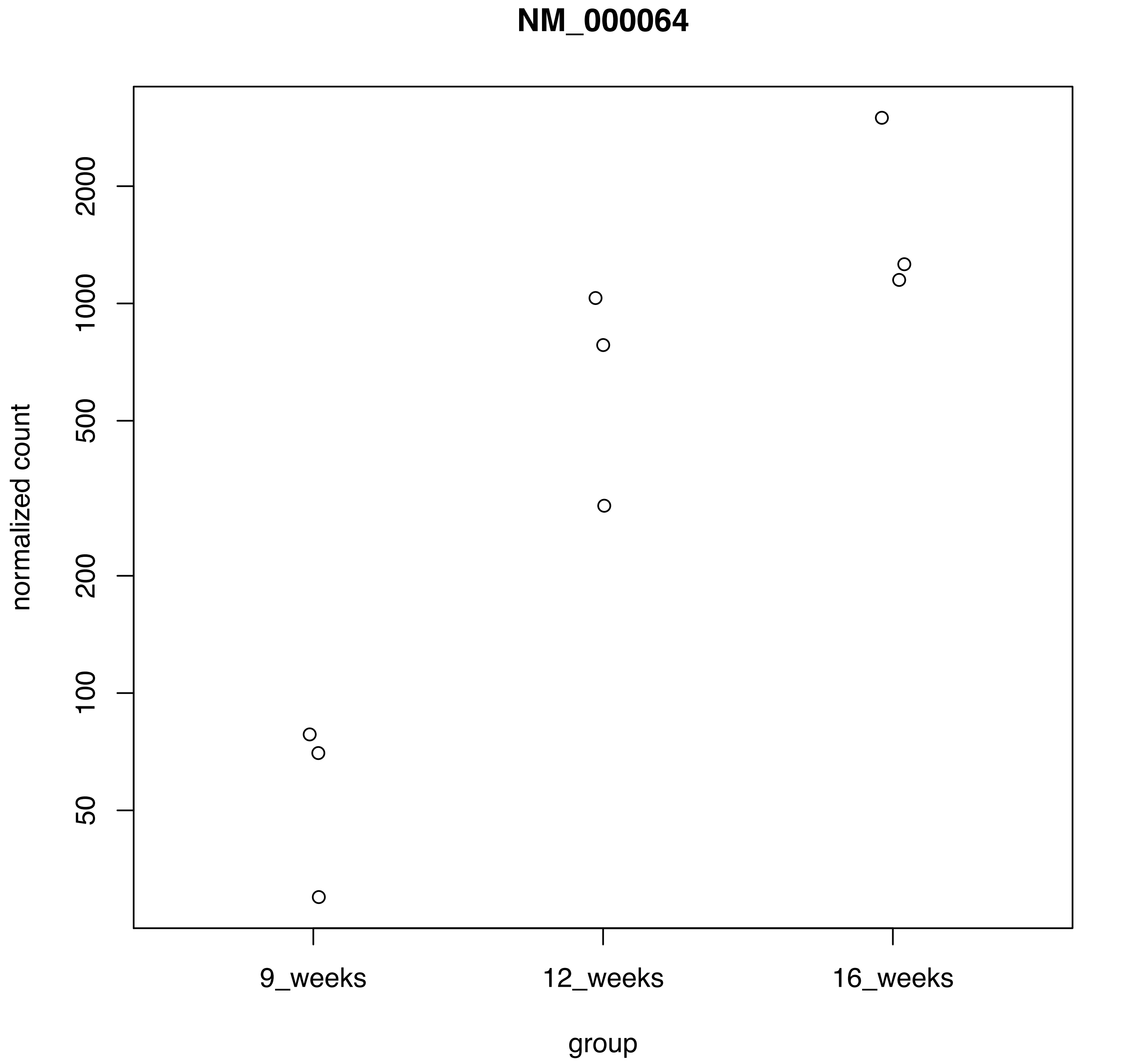
NM_000064
normalized count
2000
1000
500
200
100
50
9_weeks
12_weeks
16_weeks
group

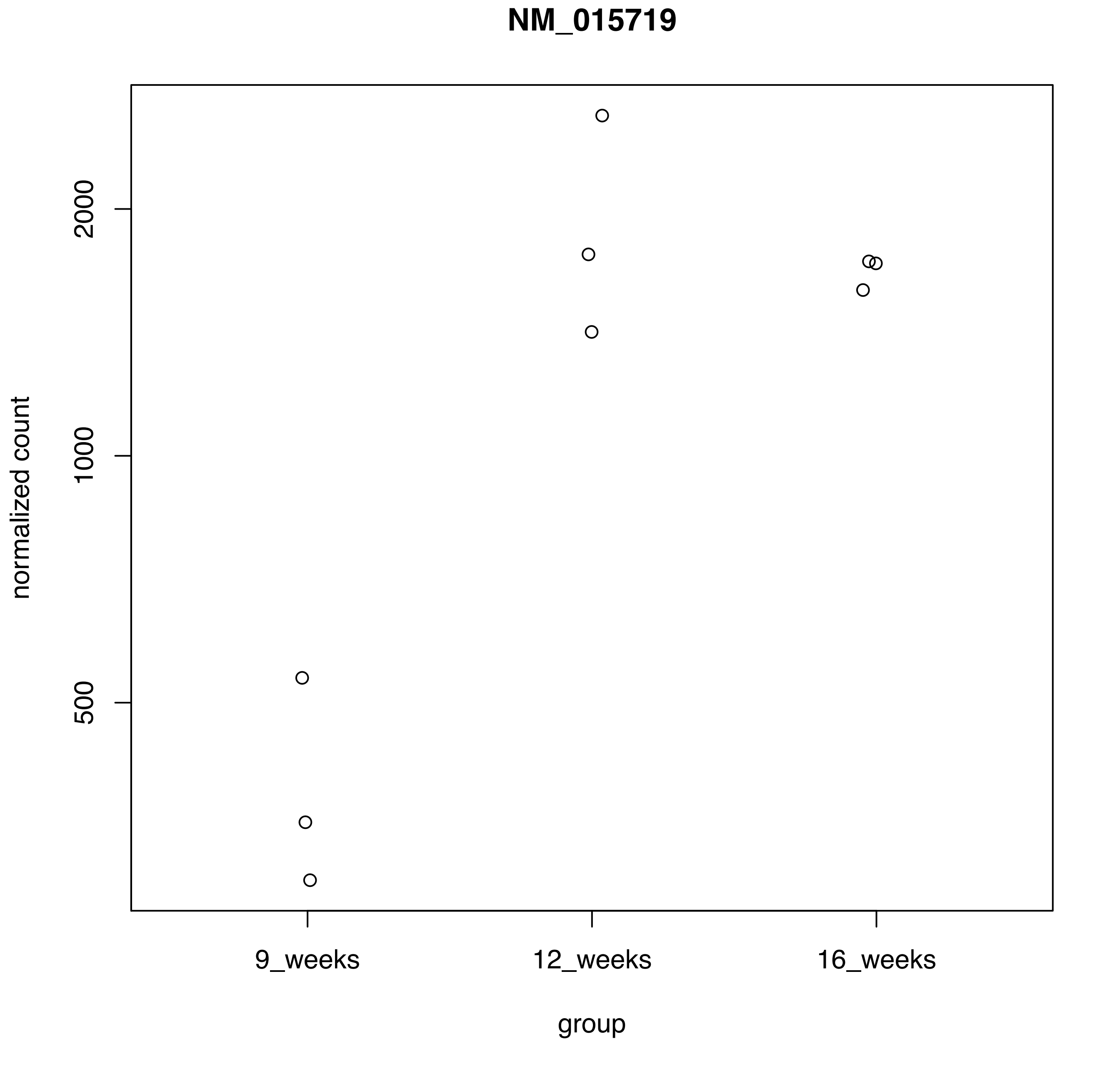
NM_015719
normalized count
2000
1000
500
9_weeks
12_weeks
16_weeks
group

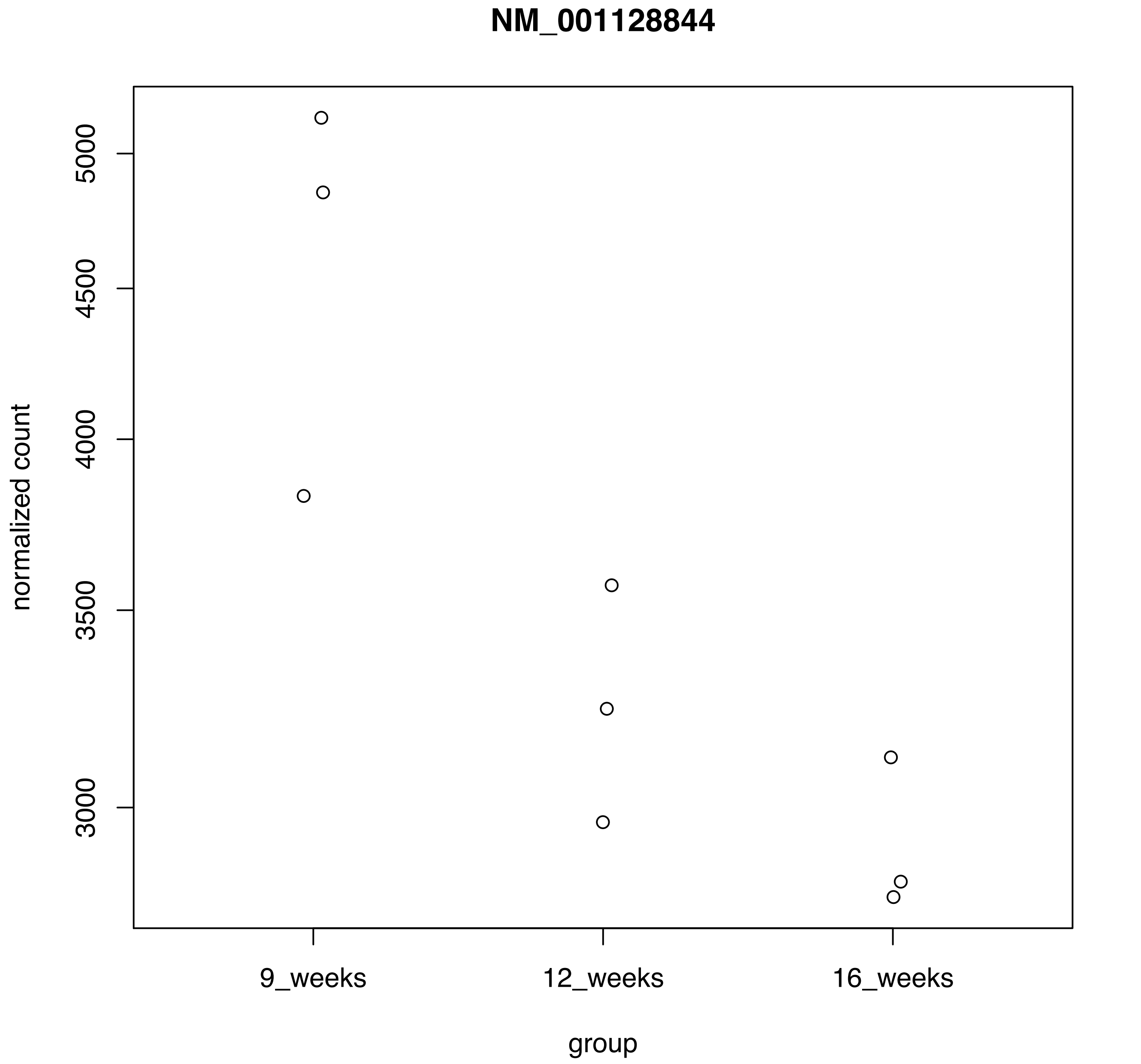
NM_001128844
normalized count
5000
4500
4000
3500
3000
9_weeks
12_weeks
16_weeks
group

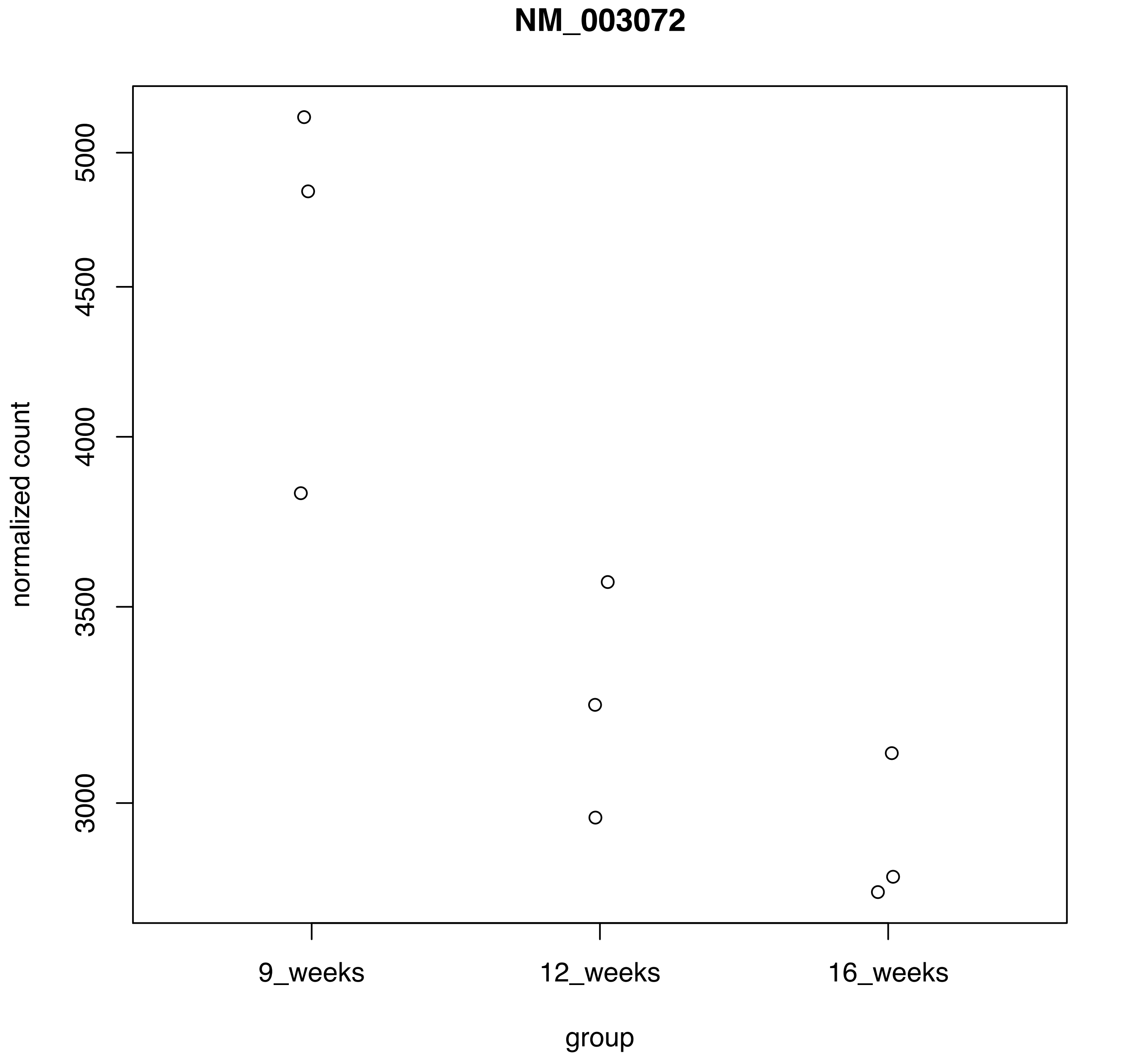
NM_003072
normalized count
5000
4500
4000
3500
3000
9_weeks
12_weeks
16_weeks
group

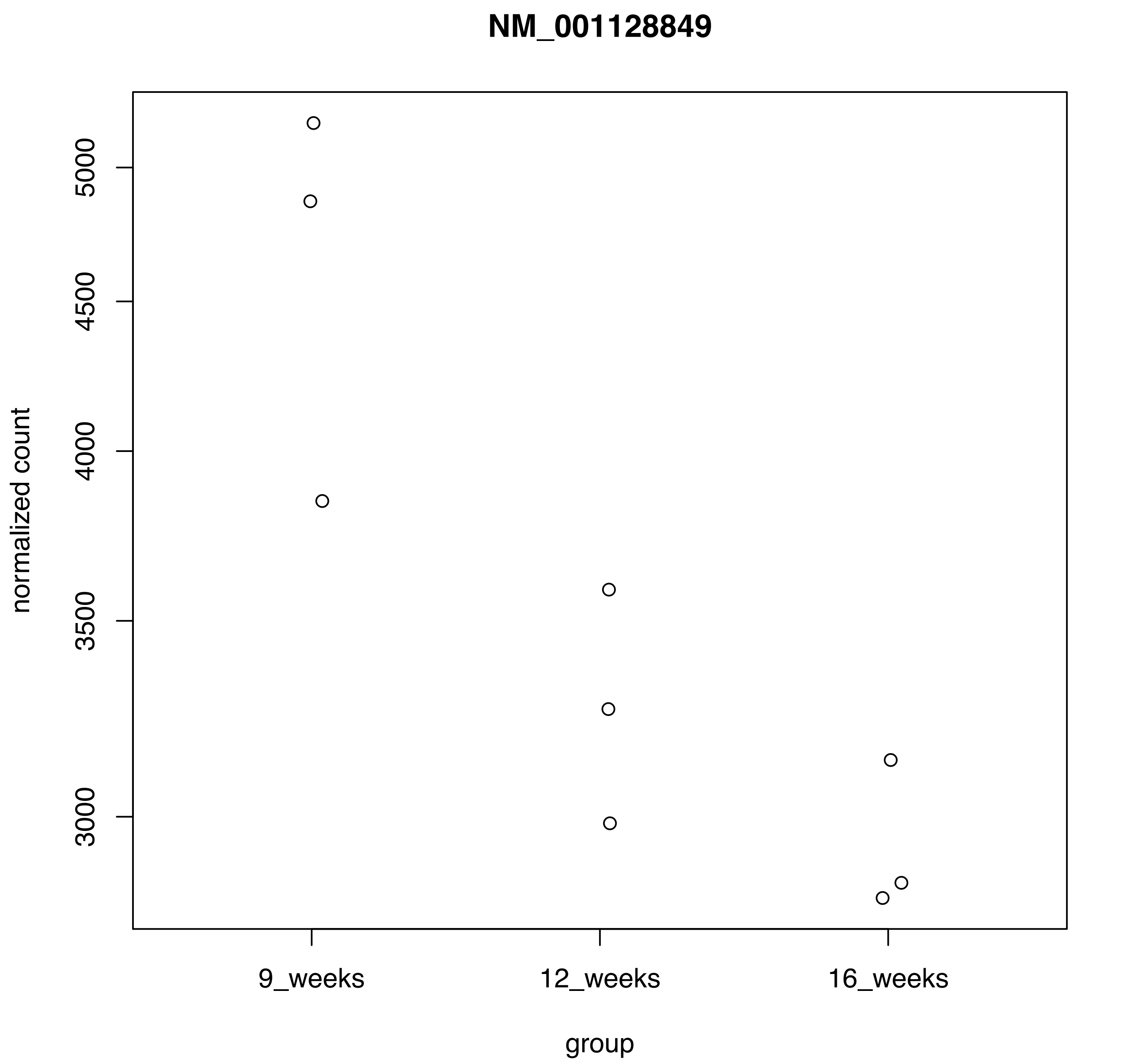
NM_001128849
normalized count
5000
4500
4000
3500
3000
9_weeks
12_weeks
16_weeks
group

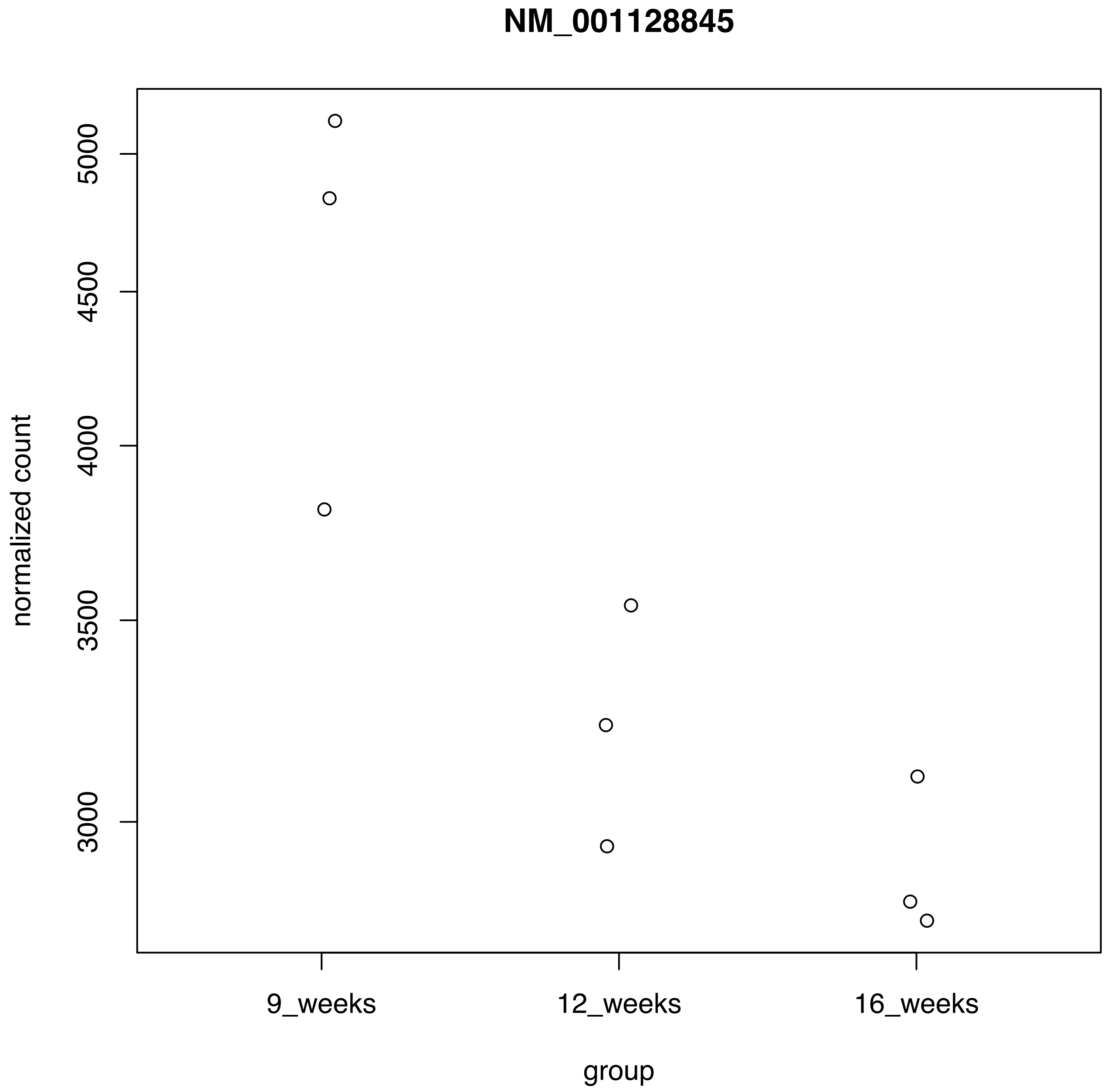
NM_001128845
normalized count
5000
4500
4000
3500
3000
9_weeks
12_weeks
16_weeks
group

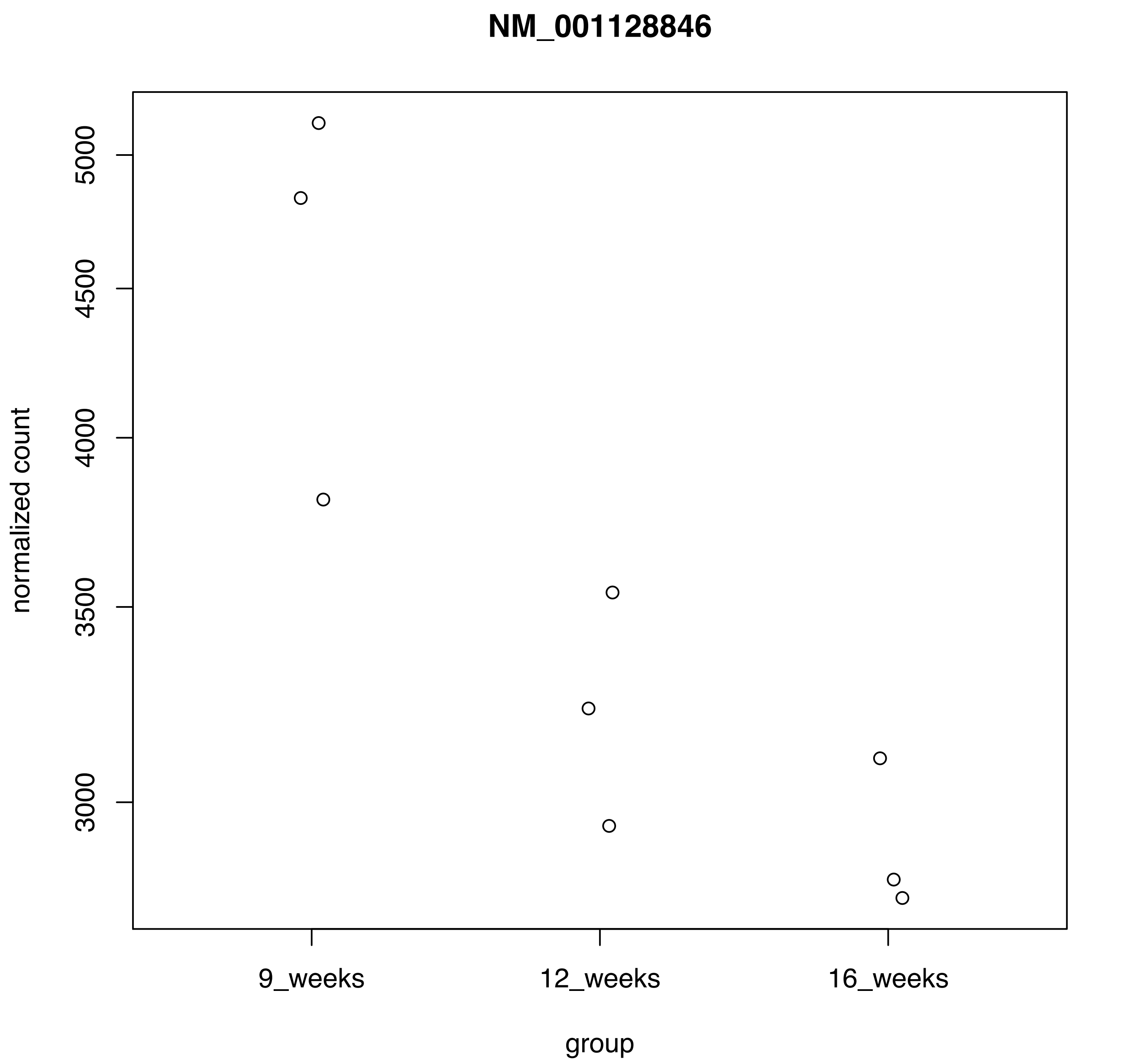
NM_001128846
normalized count
5000
4500
4000
3500
3000
9_weeks
12_weeks
16_weeks
group

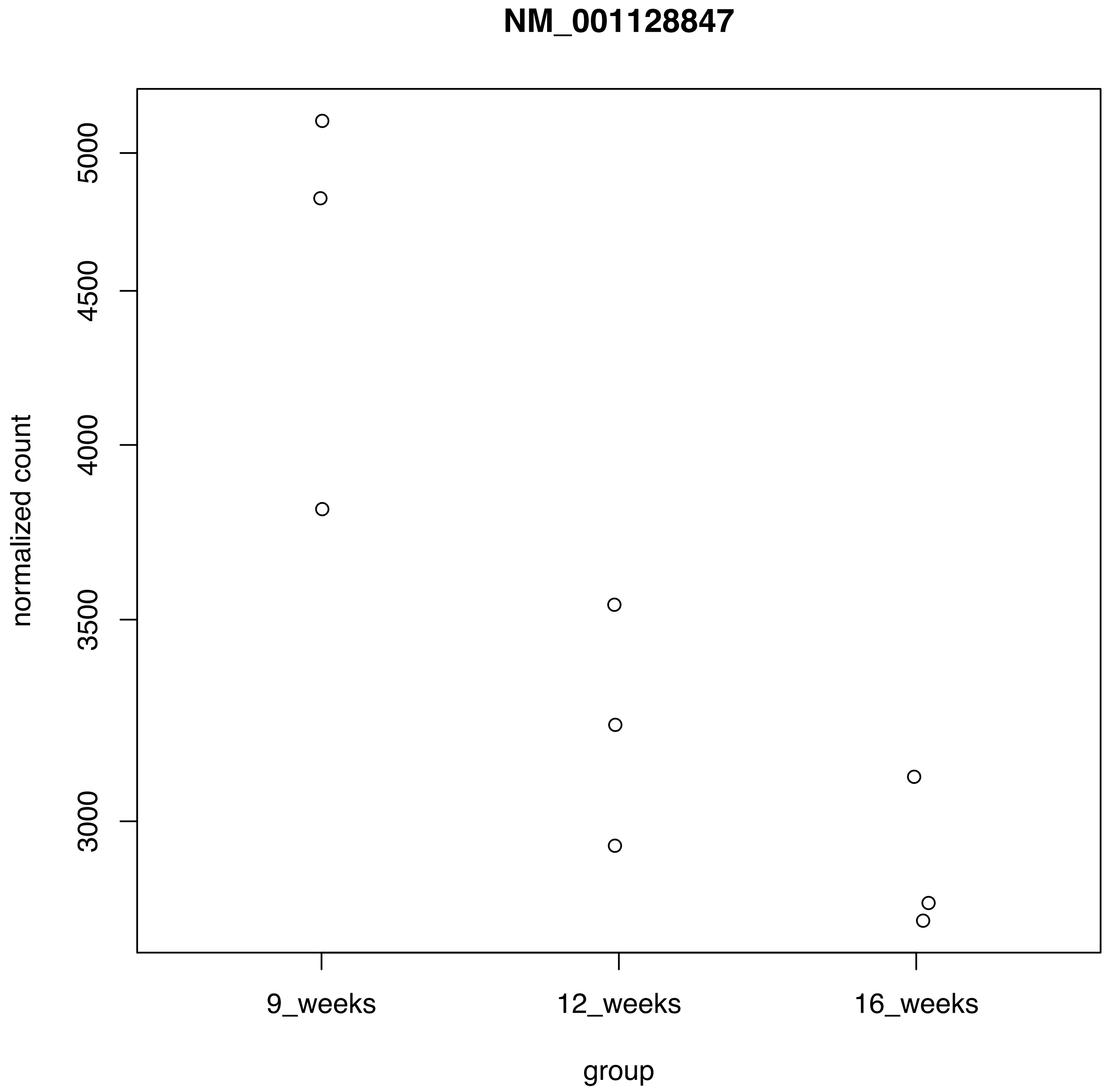
NM_001128847
normalized count
5000
4500
4000
3500
3000
group
9_weeks
12_weeks
16_weeks

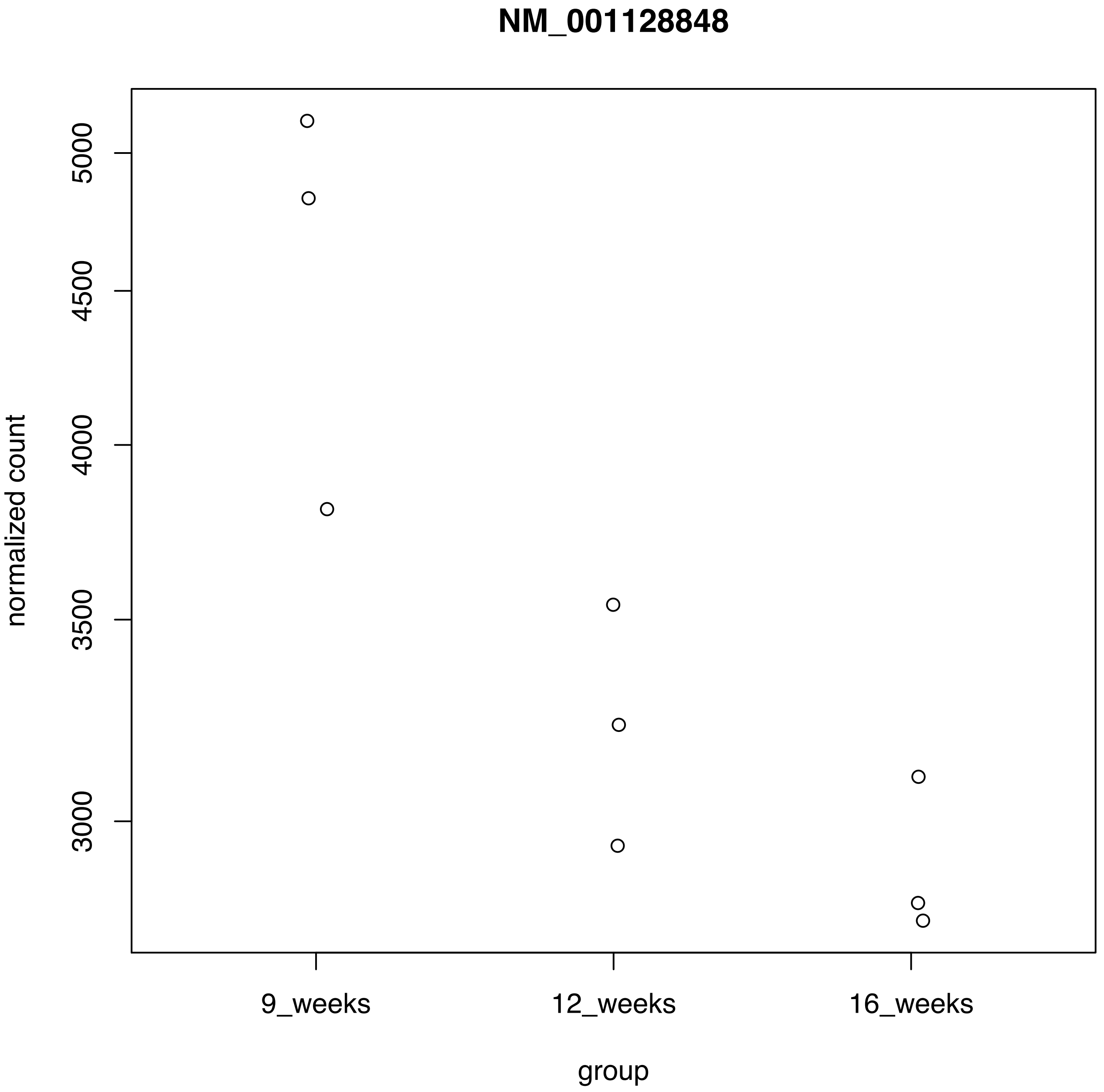

NM_001128848
normalized count
5000
4500
4000
3500
3000
group
9_weeks
12_weeks
16_weeks

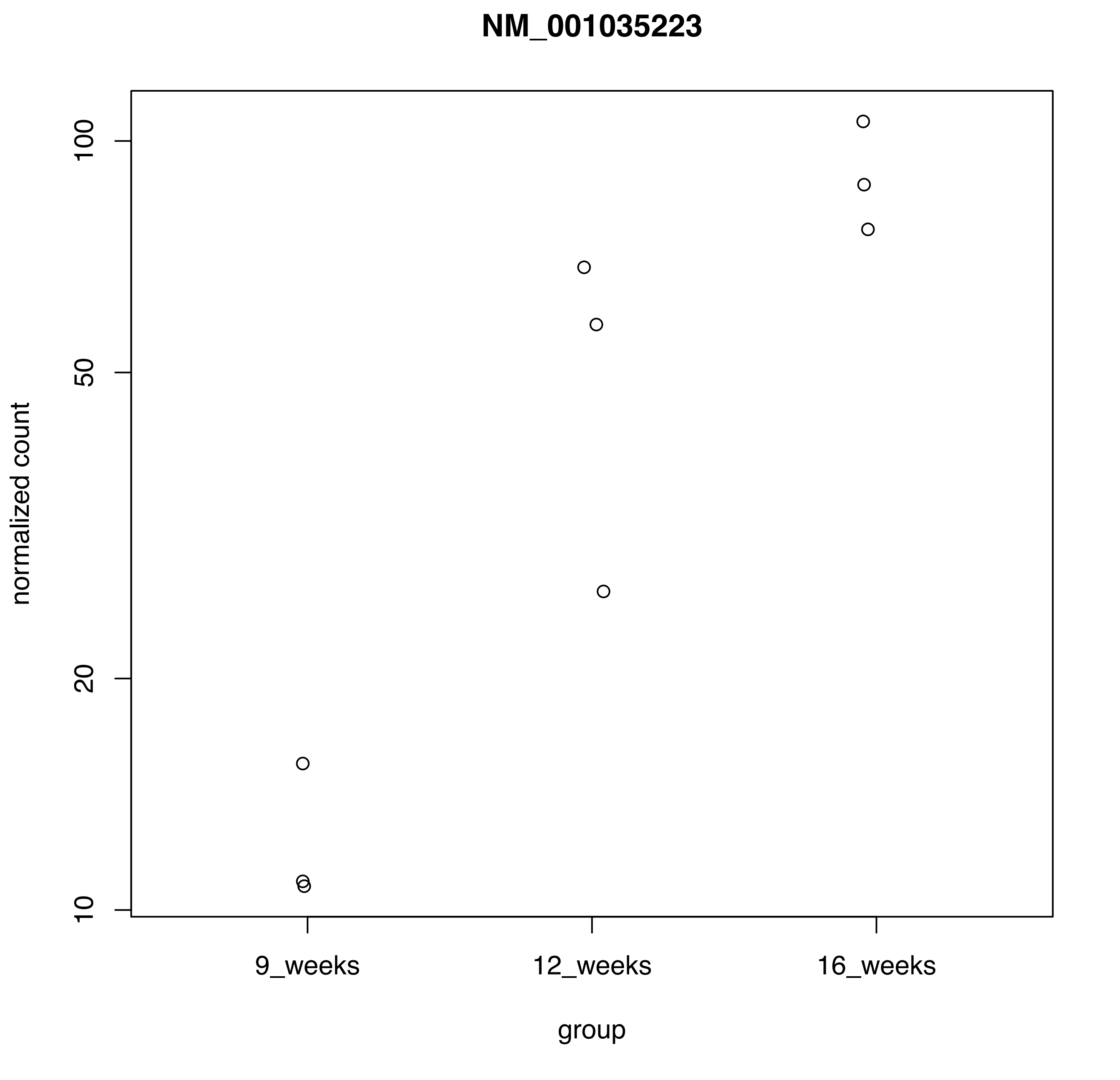
NM_001035223
normalized count
100
50
20
10
9_weeks
12_weeks
16_weeks
group

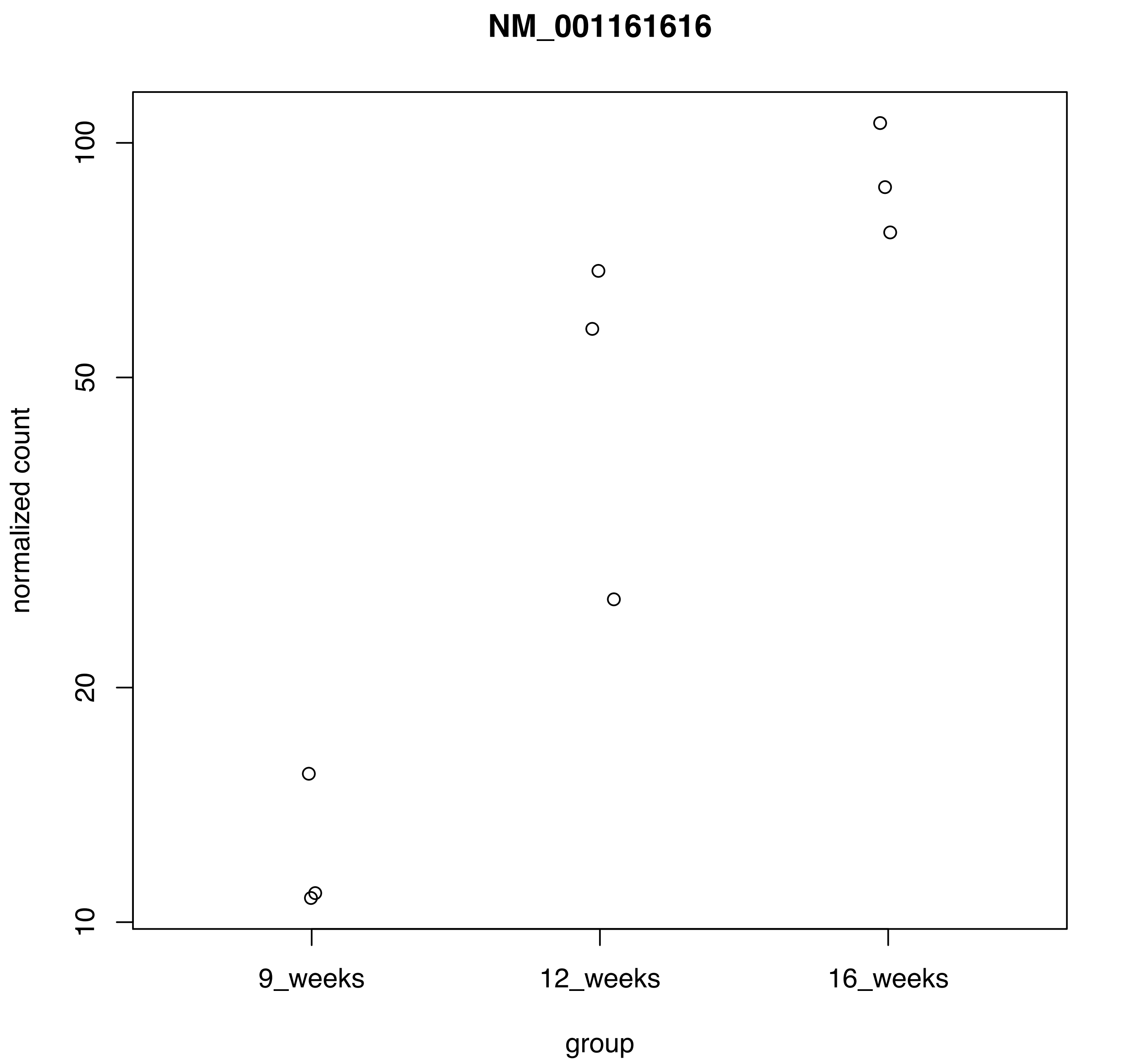

NM_001161616
normalized count
100
50
20
10
9_weeks
12_weeks
16_weeks
group

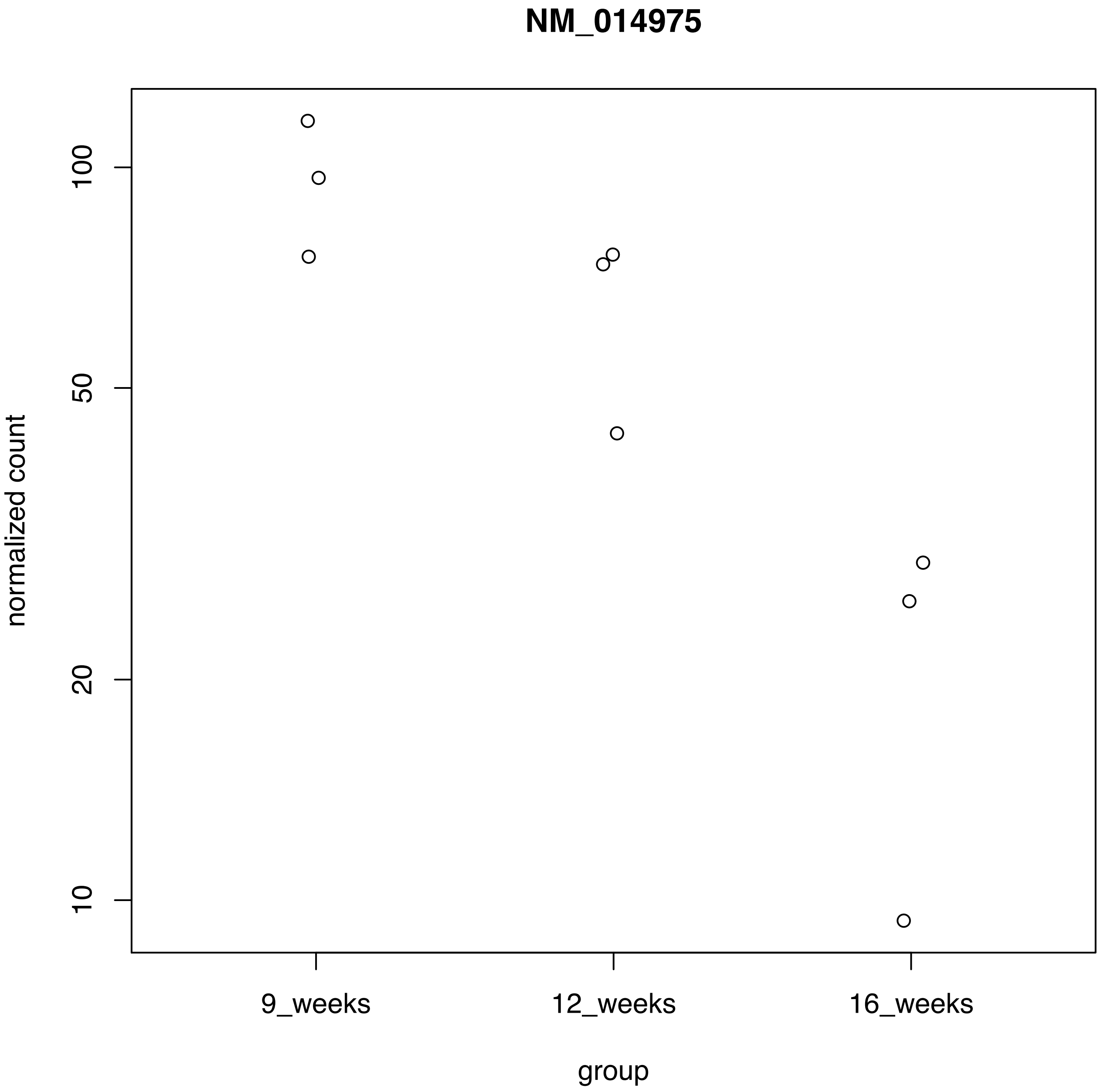
NM_014975
normalized count
100
50
20
10
9_weeks
12_weeks
16_weeks
group

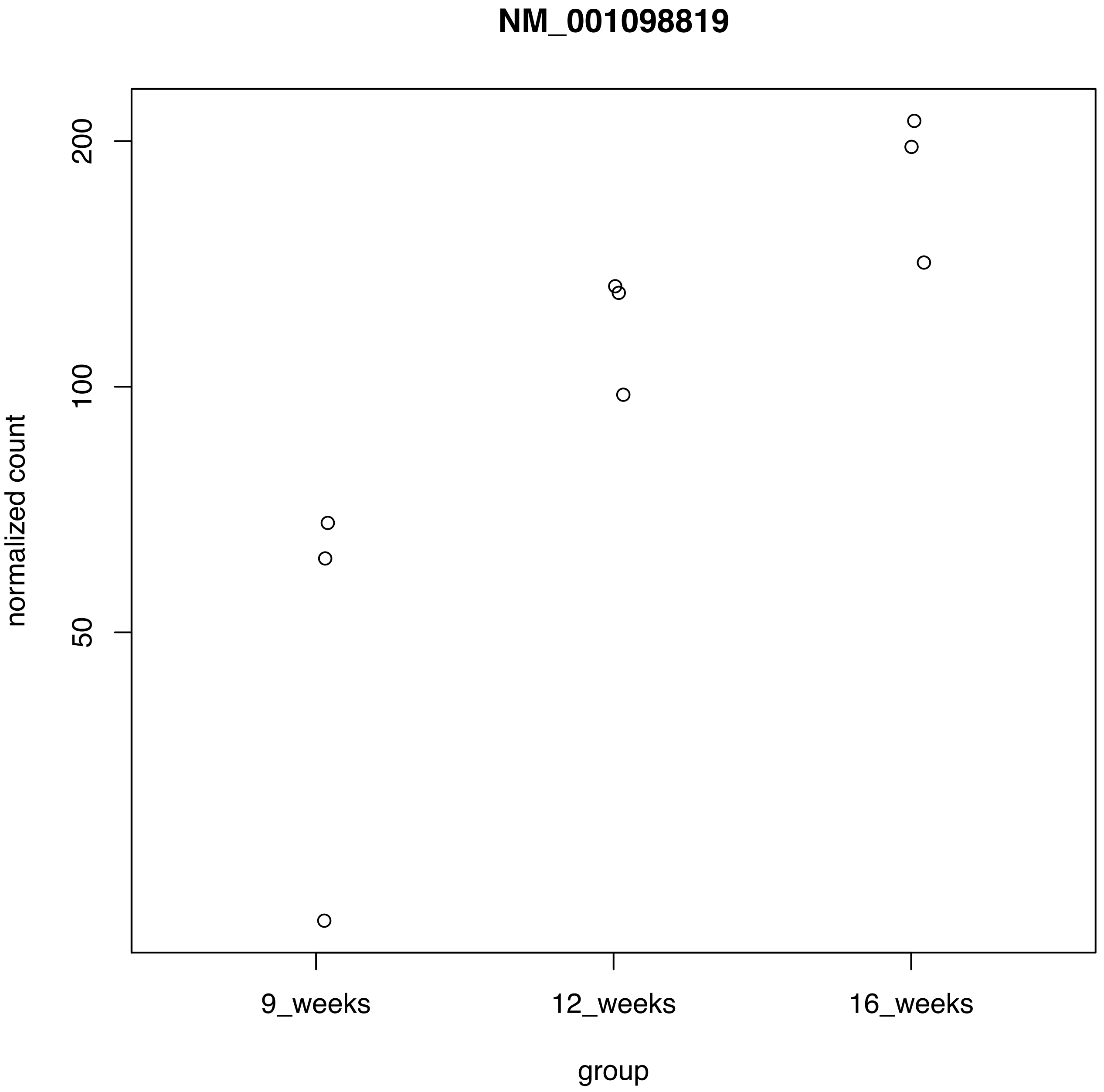

NM_001098819
normalized count
200
100
50
9_weeks
12_weeks
16_weeks
group

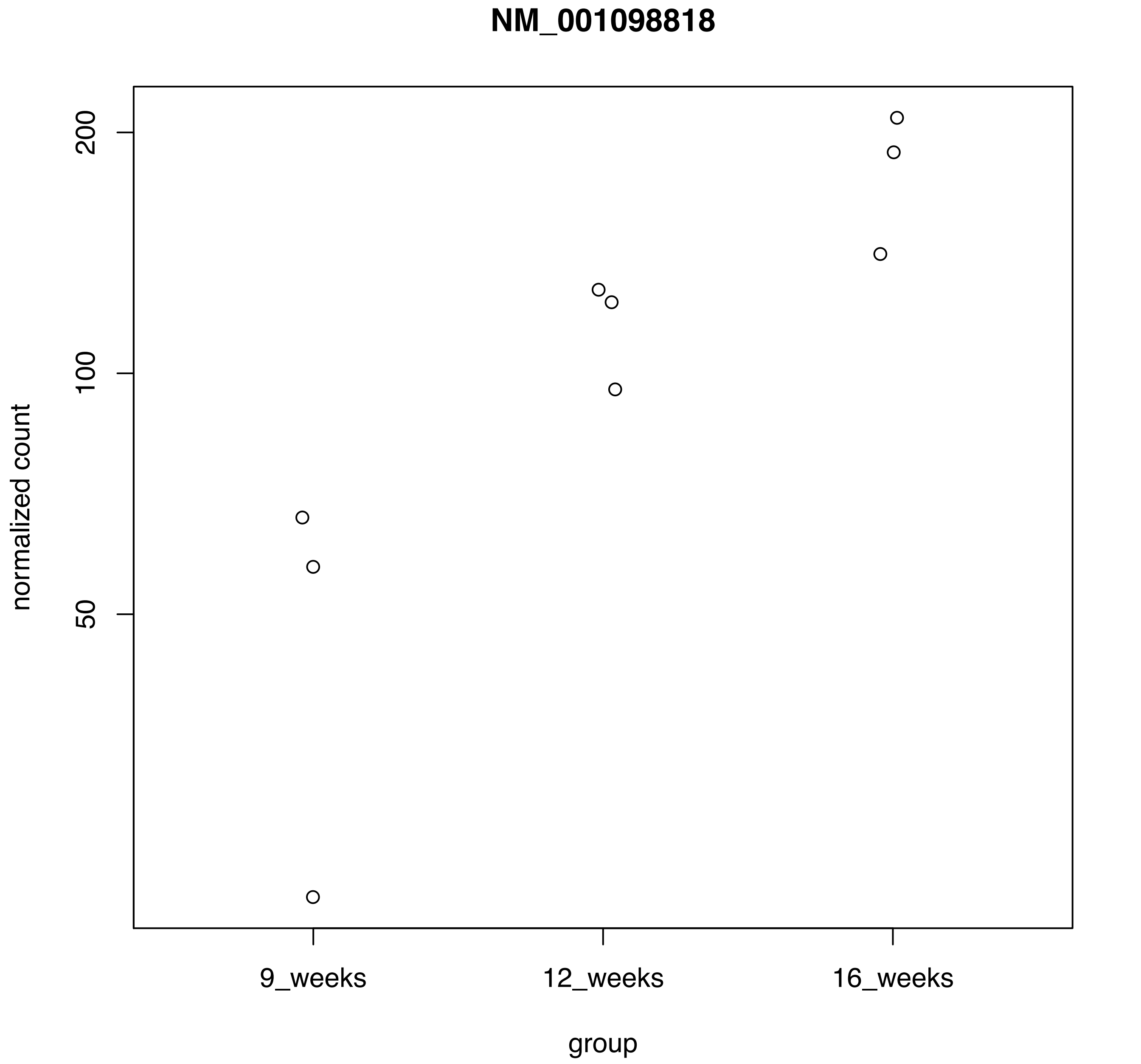
NM_001098818
normalized count
200
100
50
9_weeks
12_weeks
16_weeks
group

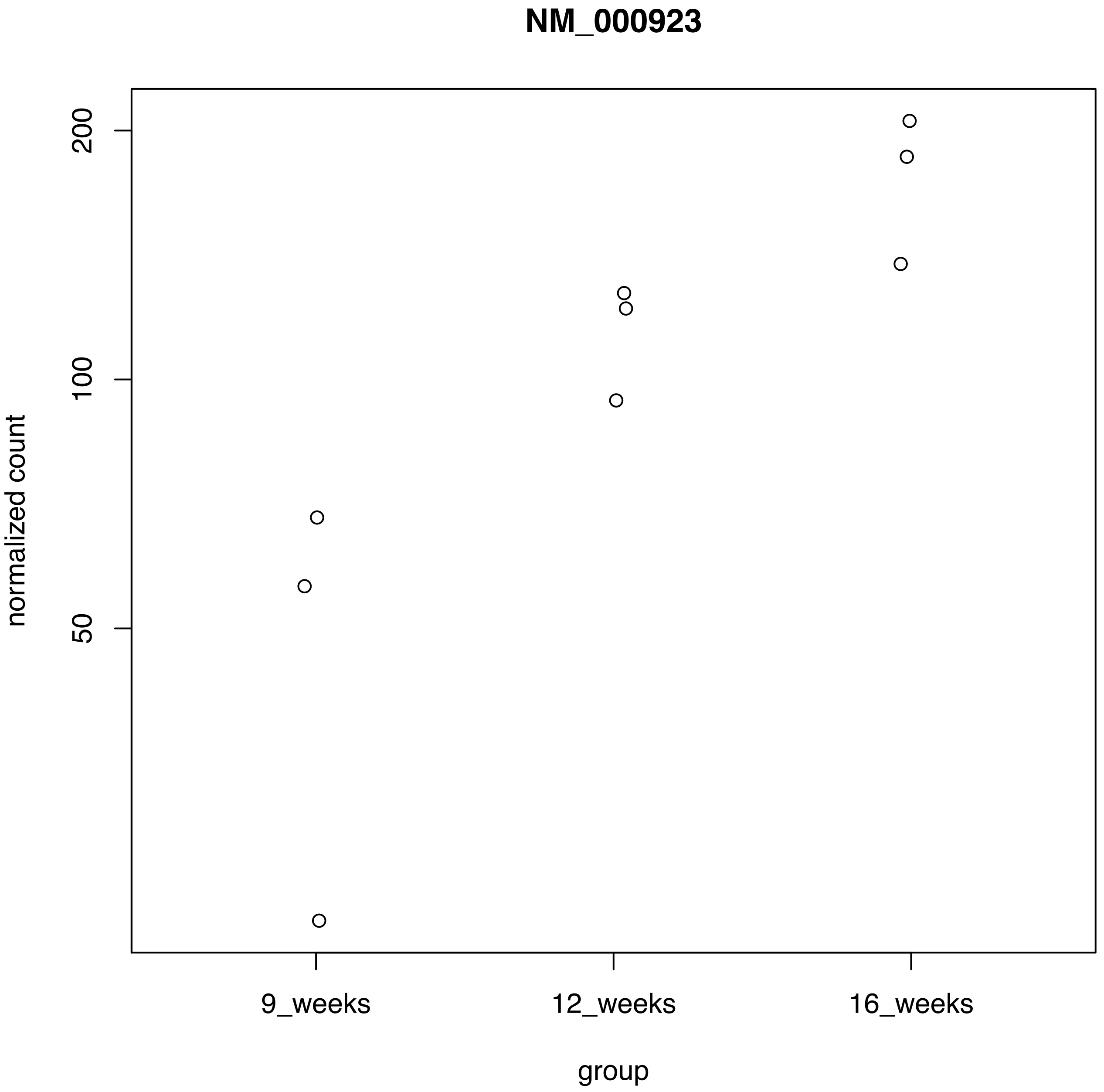
NM_000923
200
100
50
normalized count
9_weeks
12_weeks
16_weeks
group

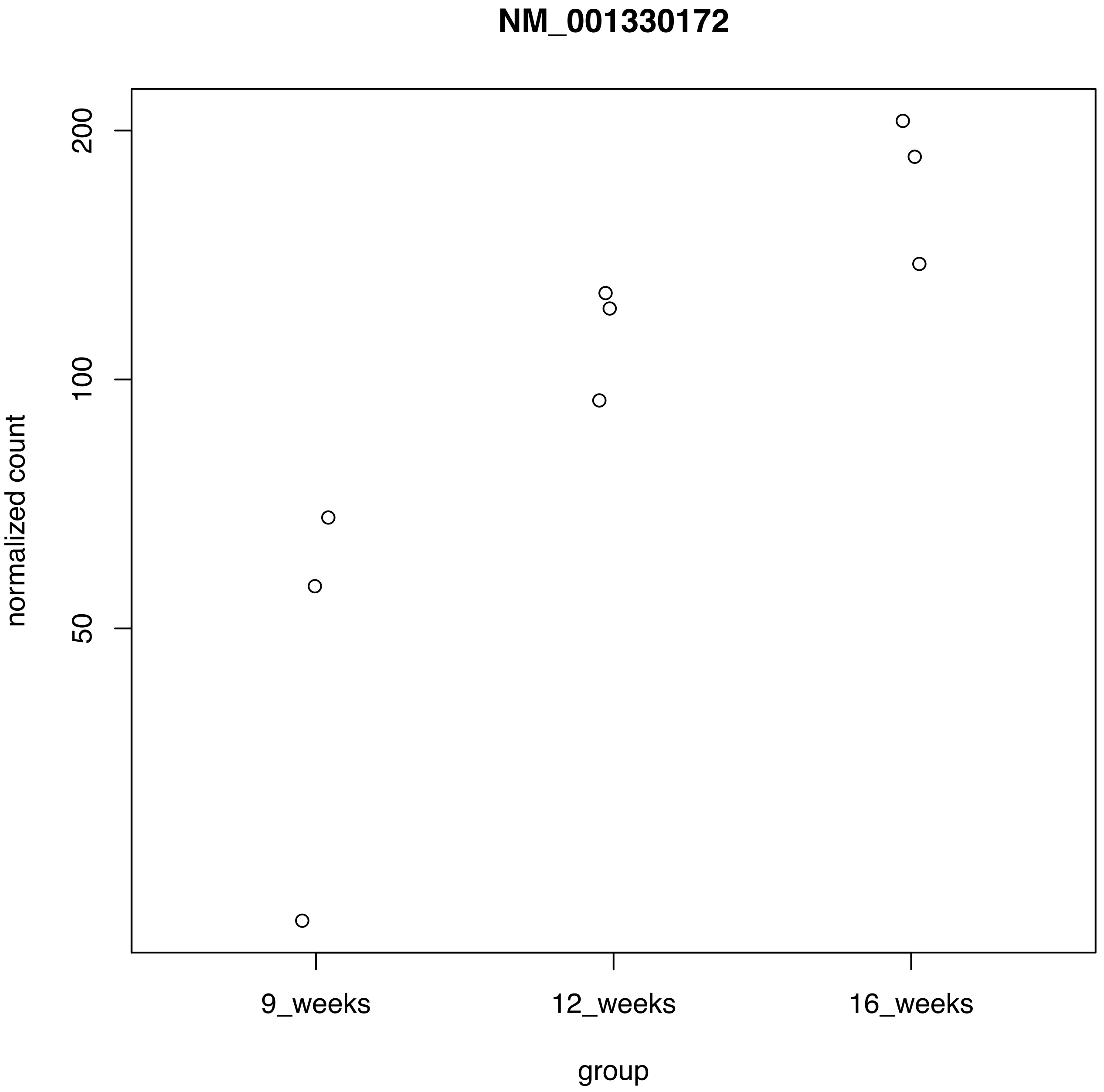
NM_001330172
normalized count
200
100
50
9_weeks
12_weeks
16_weeks
group

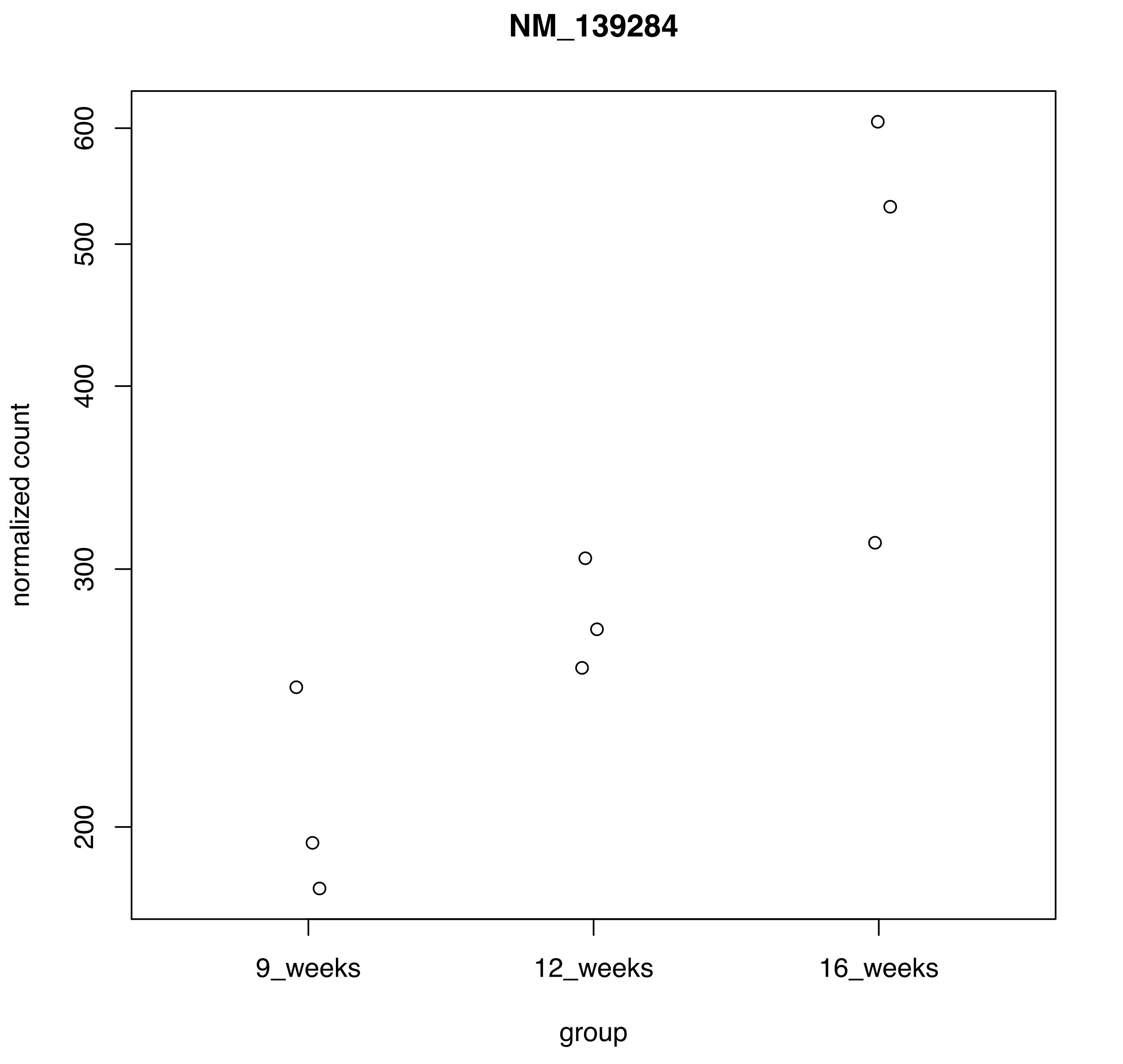
NM_139284
600
500
400
300
200
normalized count
9_weeks
12_weeks
16_weeks
group

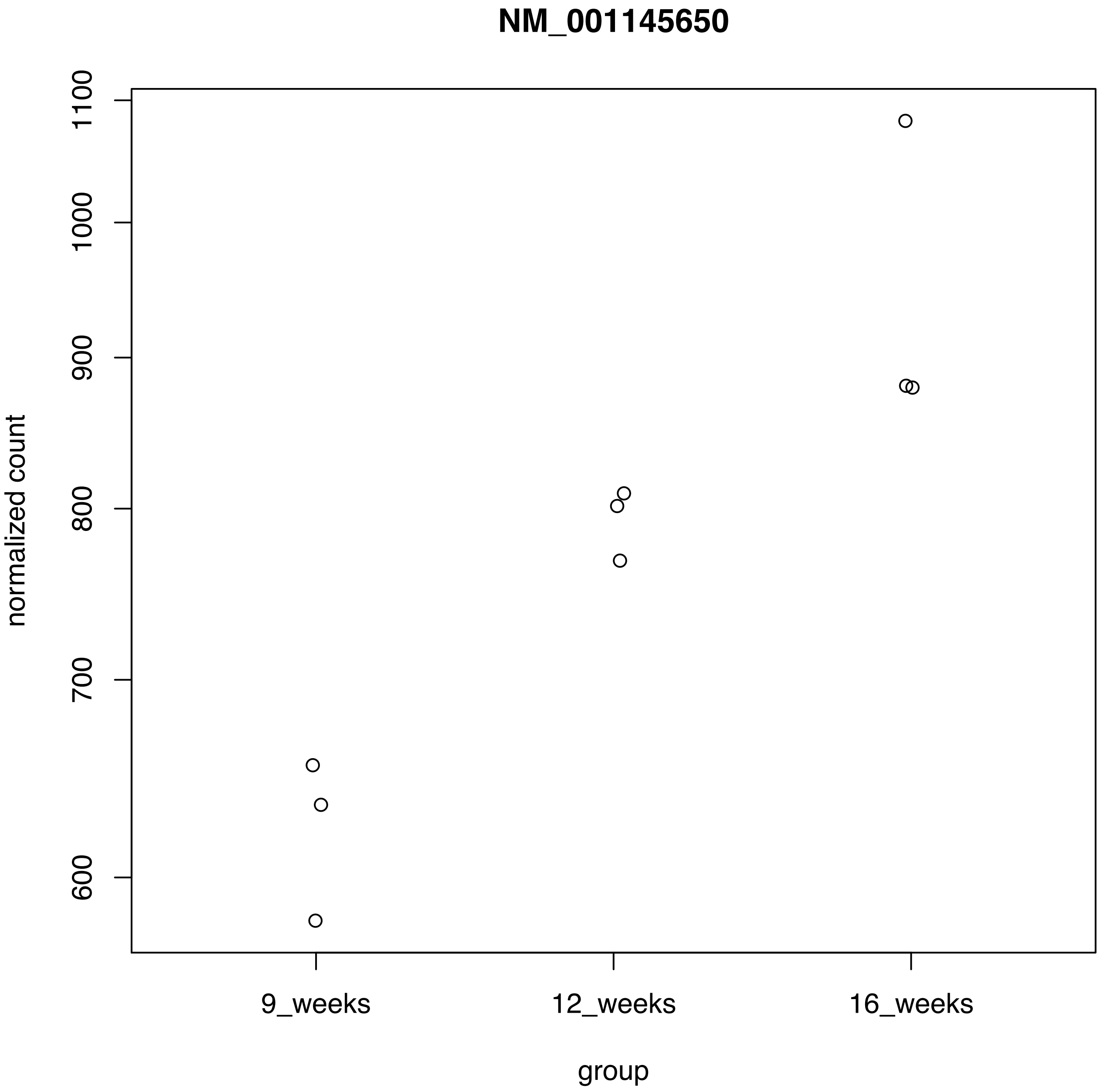
NM_001145650
normalized count
1100
1000
900
800
700
600
9_weeks
12_weeks
16_weeks
group

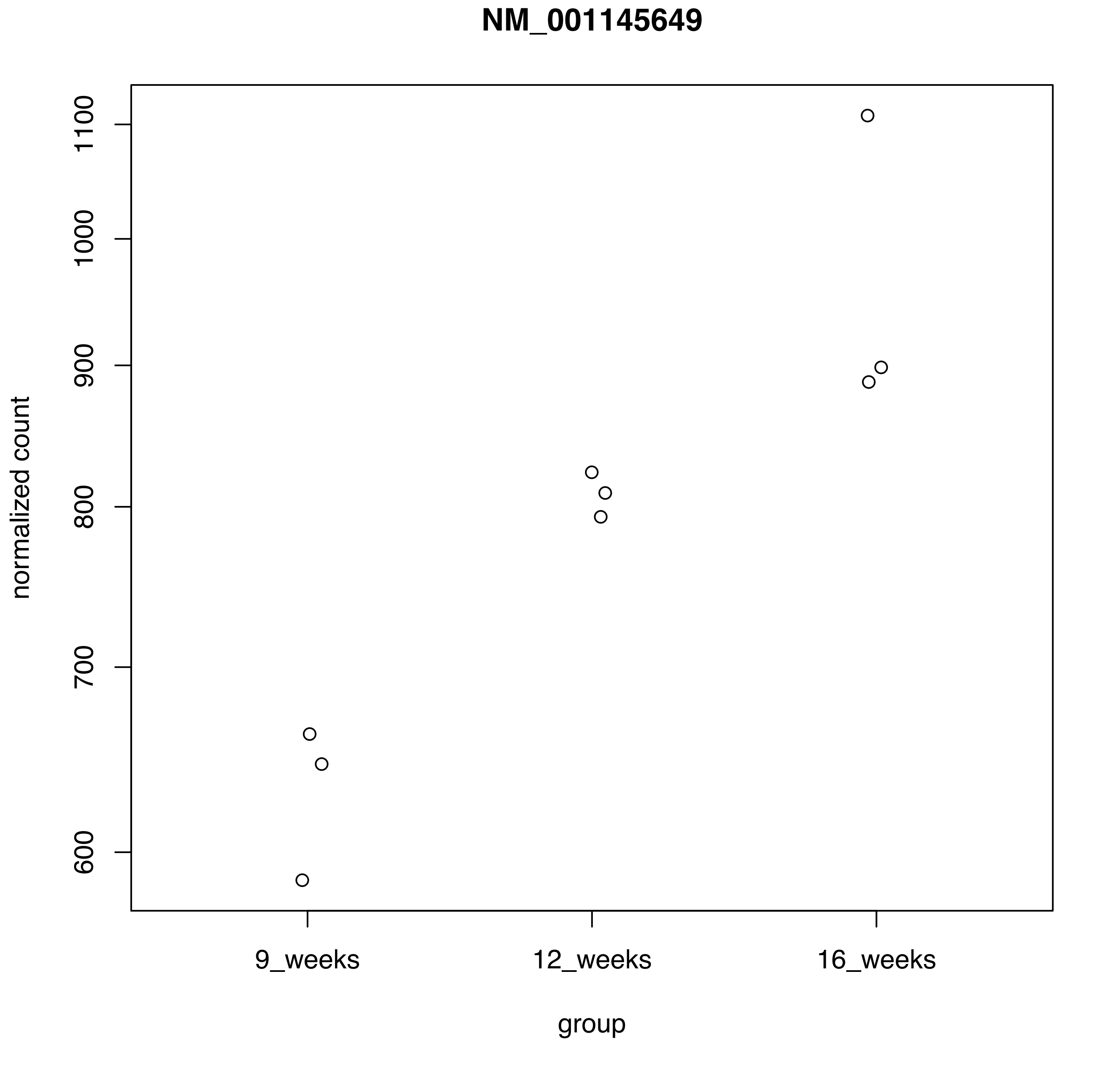
NM_001145649
normalized count
1100
1000
900
800
700
600
9_weeks
12_weeks
16_weeks
group

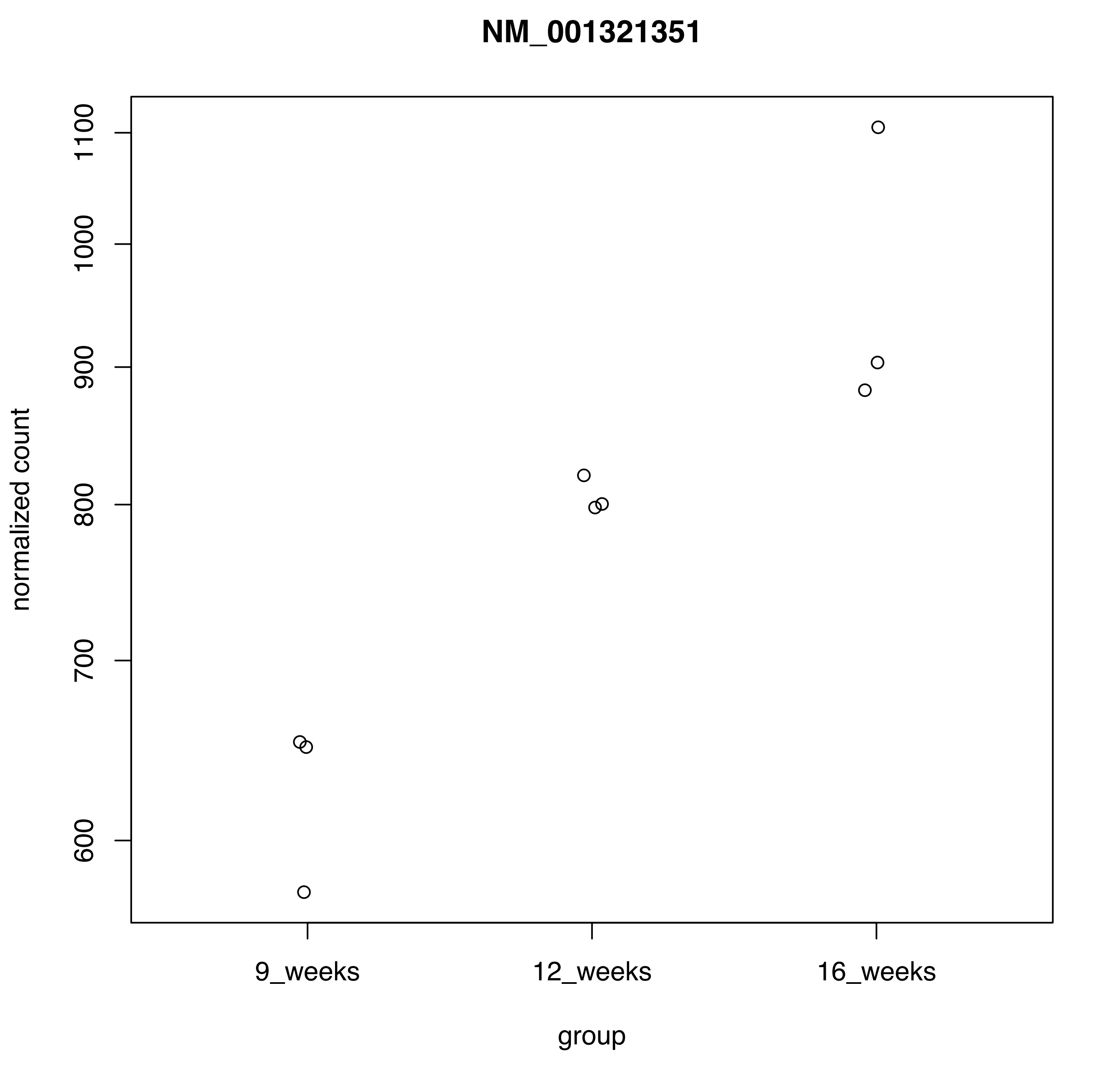
NM_001321351
normalized count
600
700
800
900
1000
1100
9_weeks
12_weeks
16_weeks
group

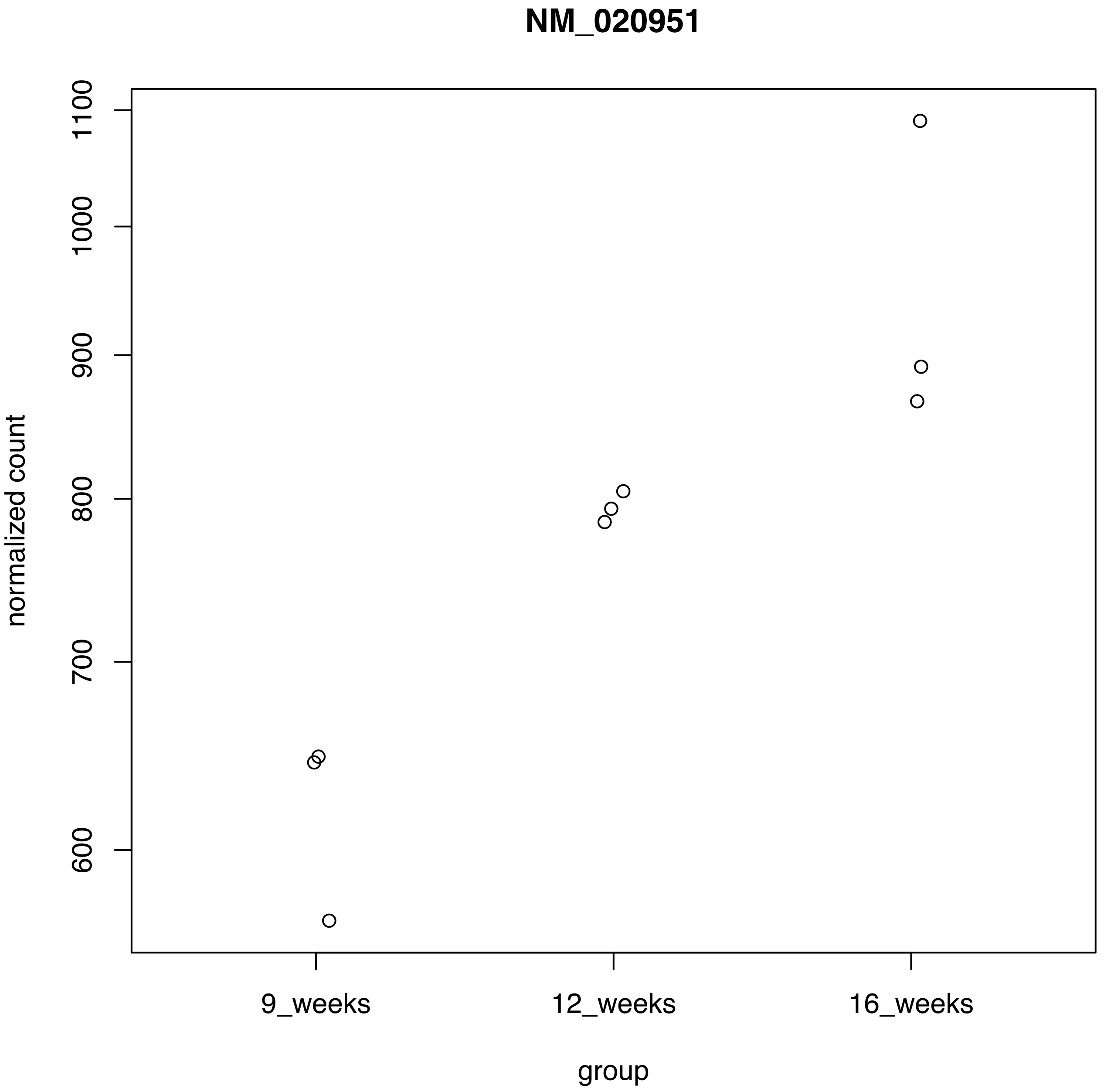

NM_020951
normalized count
1100
1000
900
800
700
600
9_weeks
12_weeks
16_weeks
group

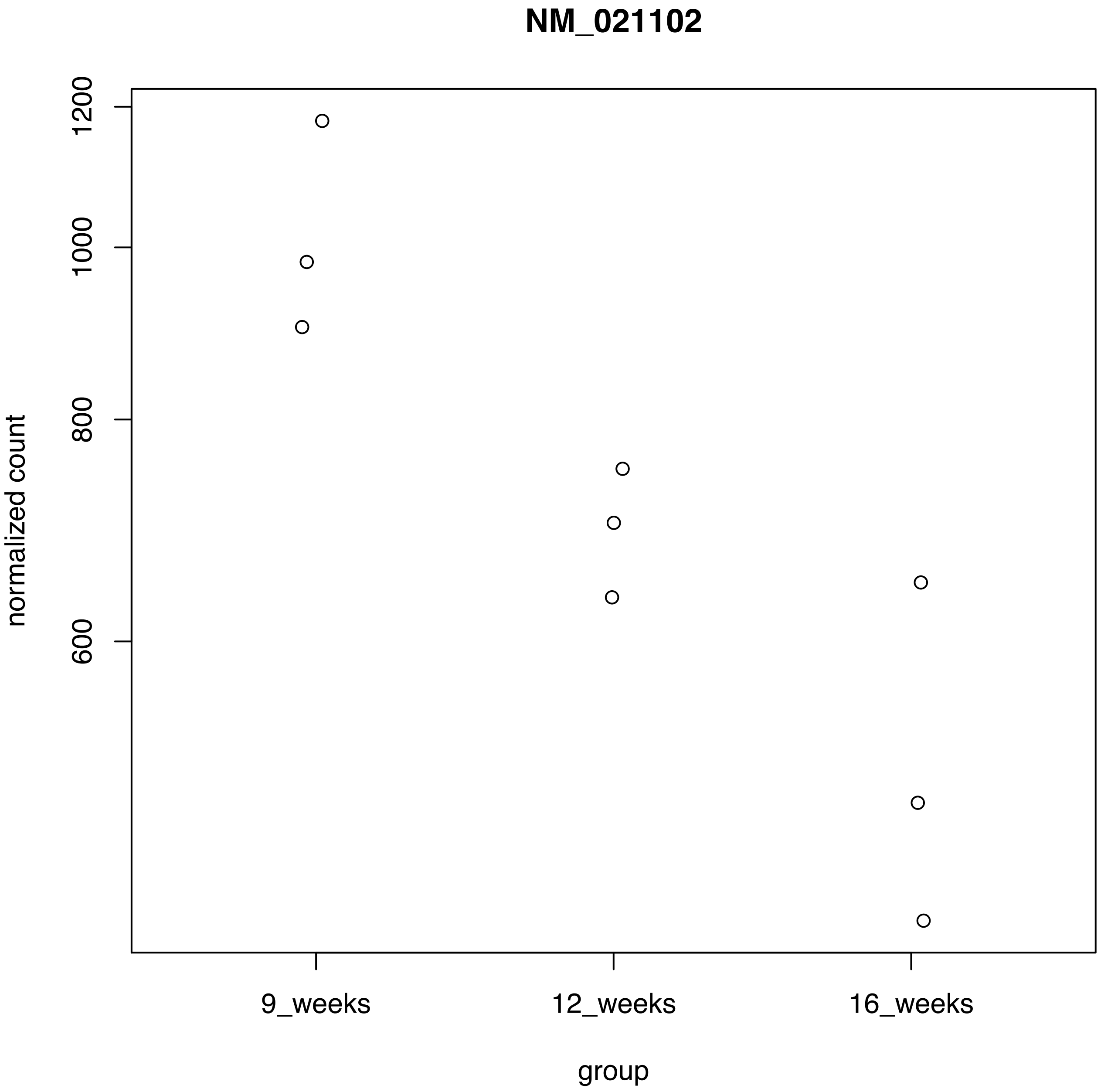
NM_021102
normalized count
1200
1000
800
600
9_weeks
12_weeks
16_weeks
group

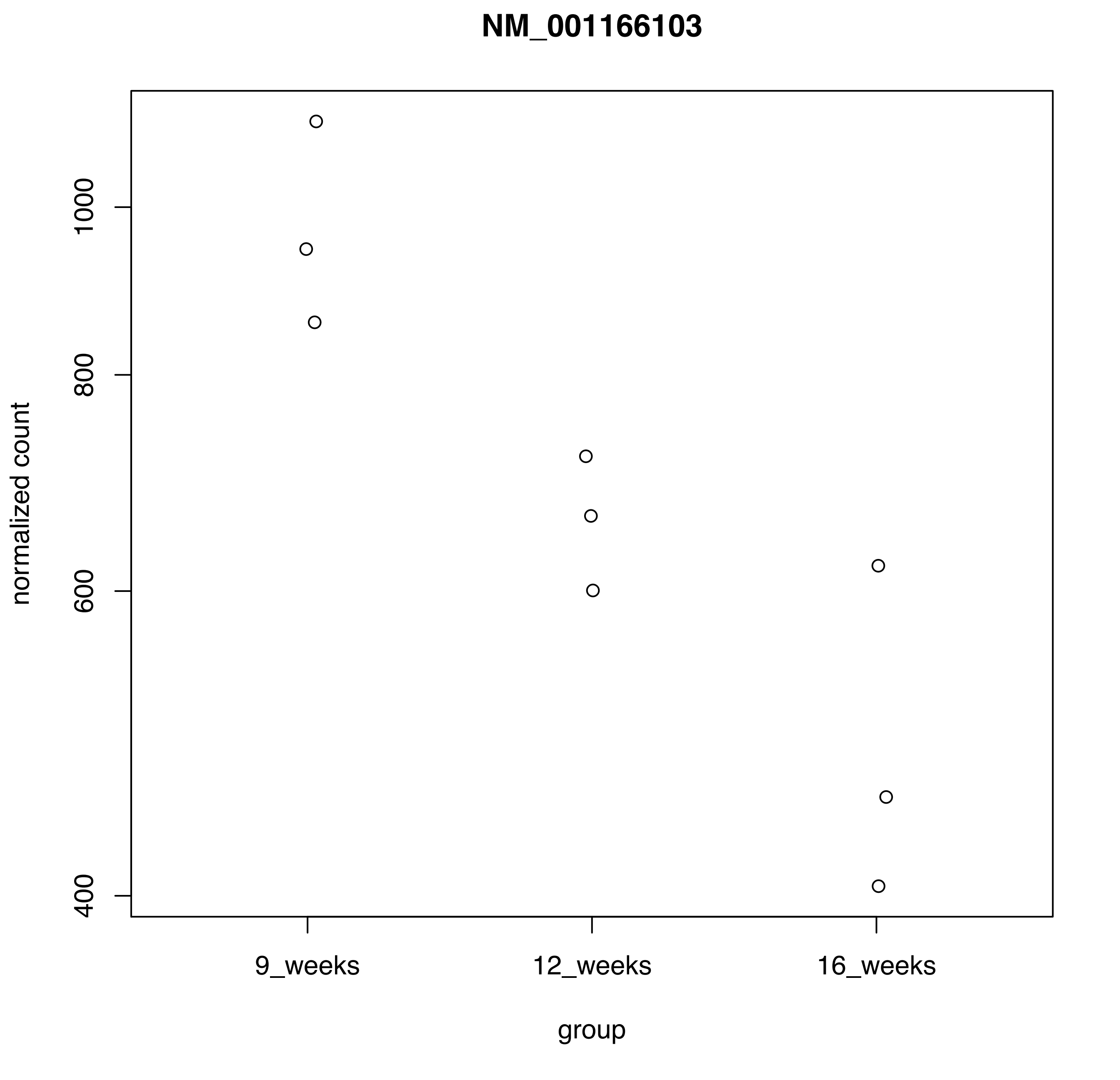
NM_001166103
normalized count
400
600
800
1000
9_weeks
12_weeks
16_weeks
group

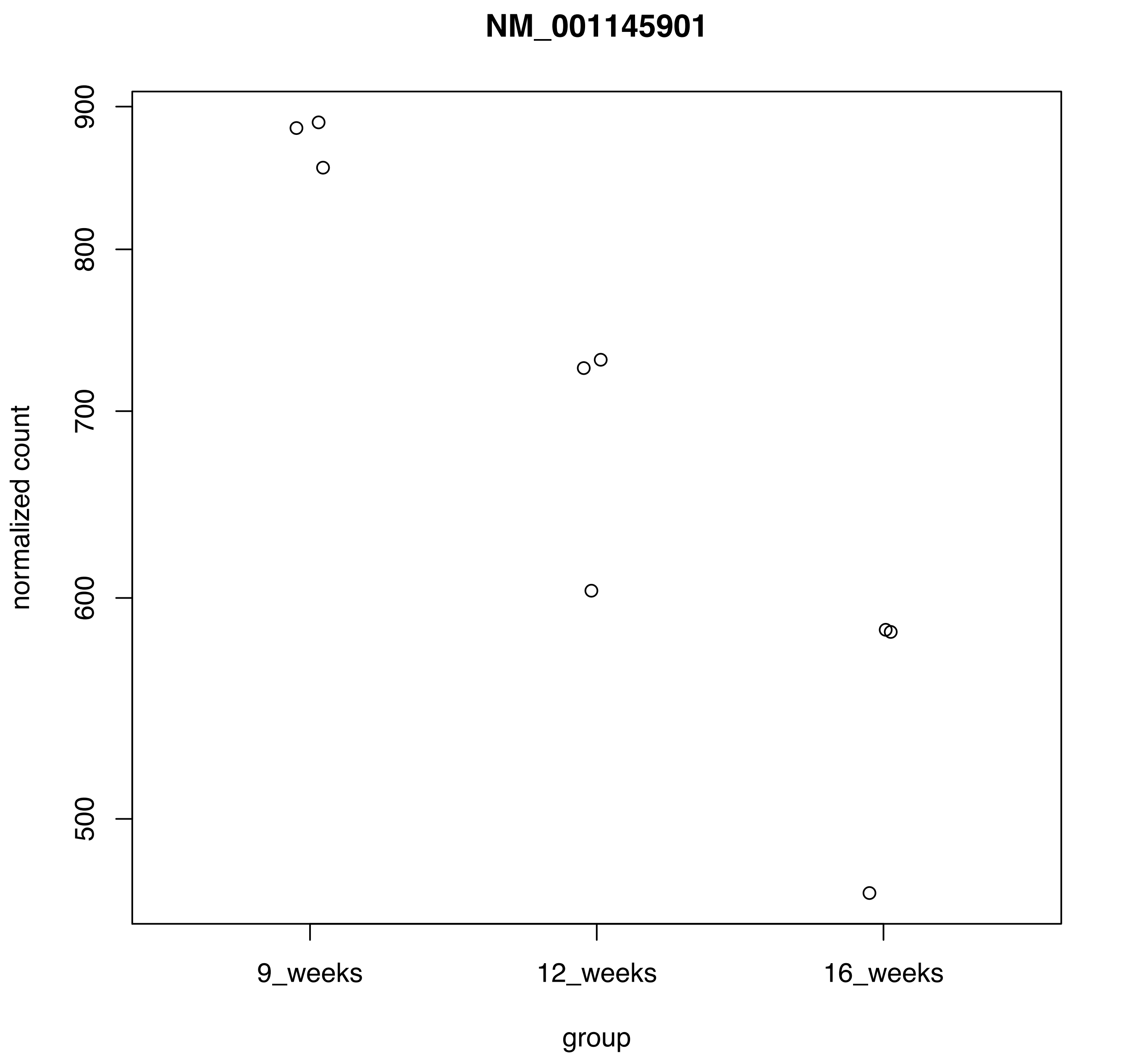
NM_001145901
normalized count
900
800
700
600
500
9_weeks
12_weeks
16_weeks
group

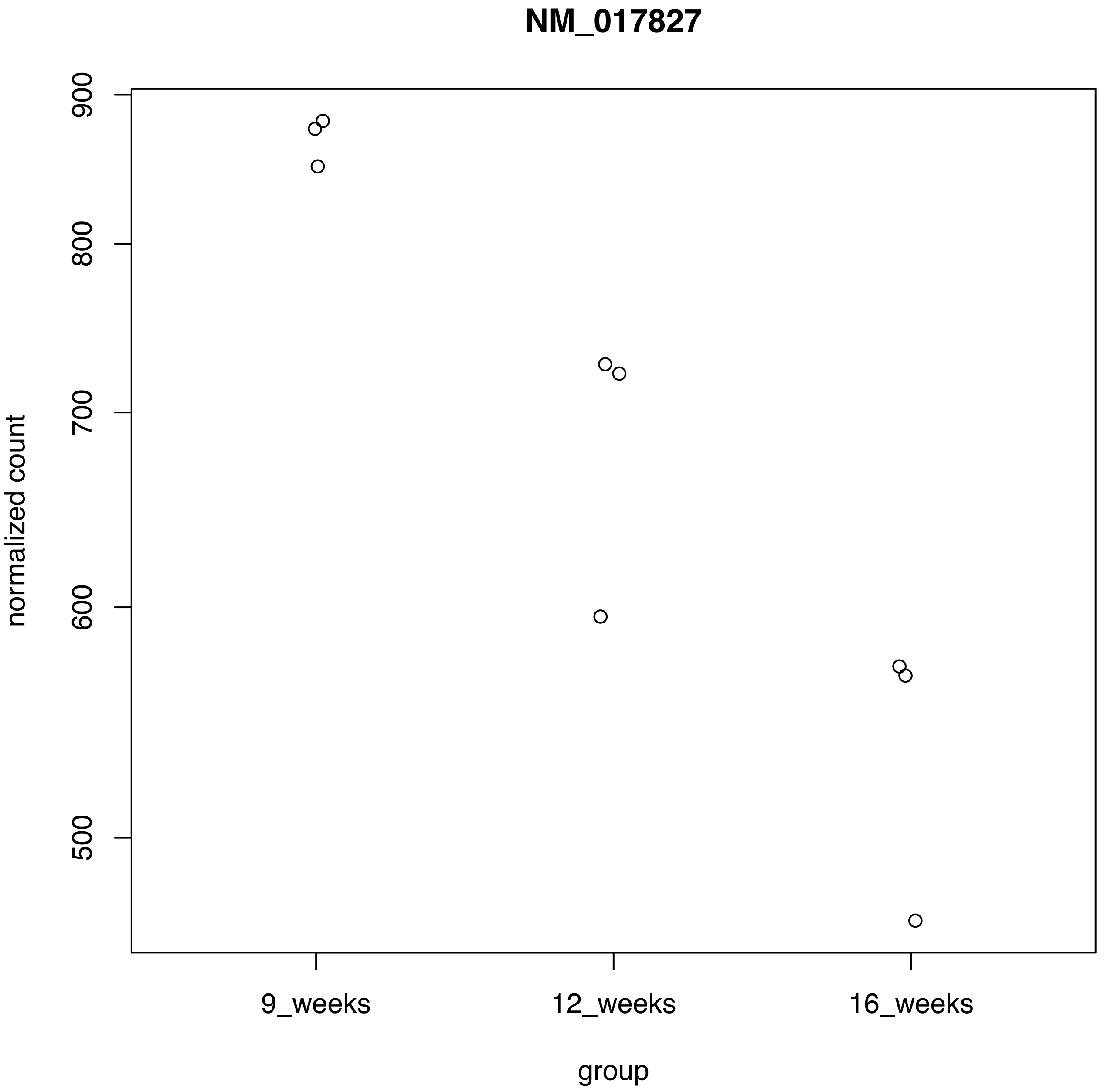

NM_017827
900
800
700
600
500
normalized count
9_weeks
12_weeks
16_weeks
group

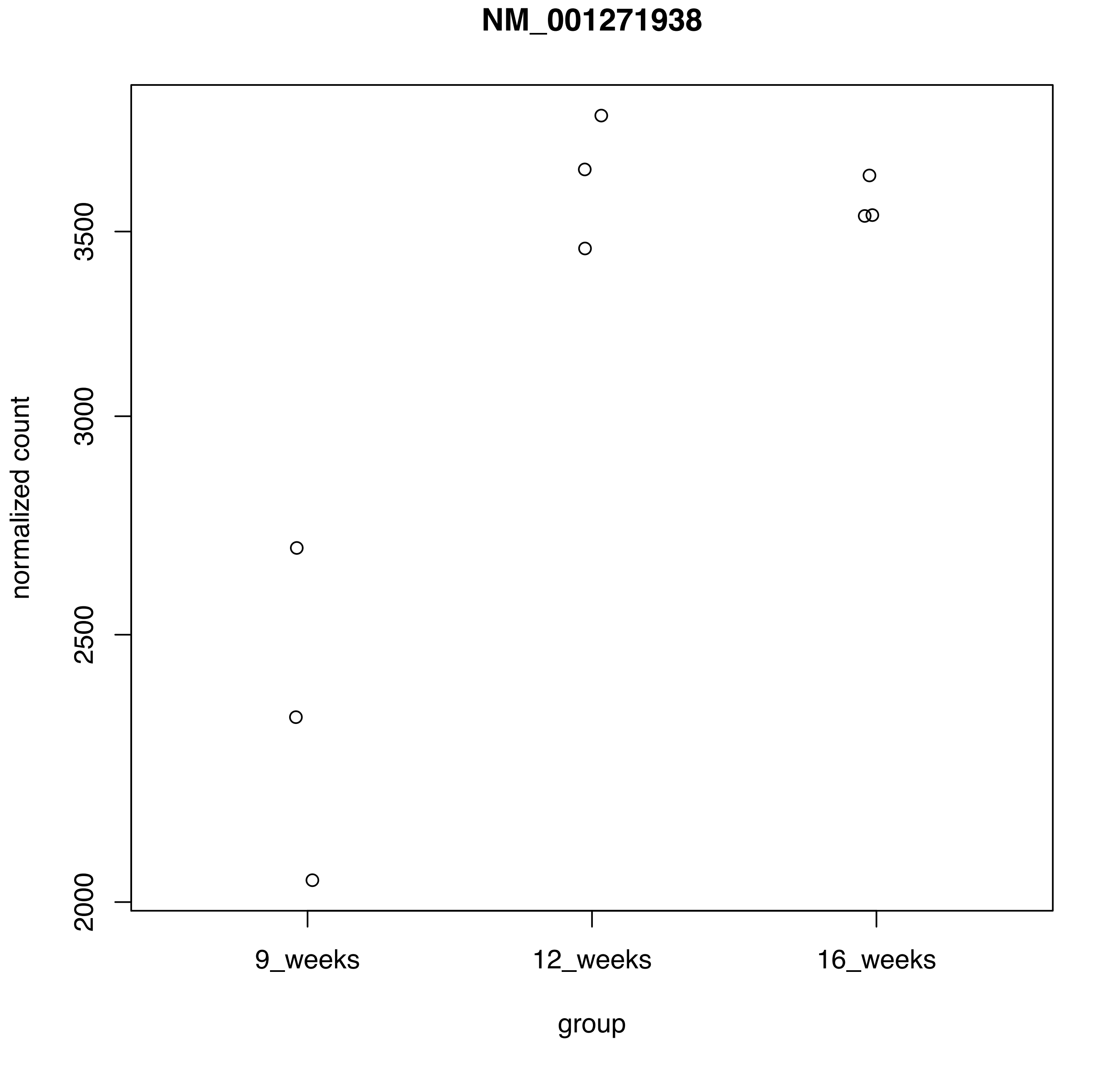
NM_001271938
normalized count
2000
2500
3000
3500
9_weeks
12_weeks
16_weeks
group

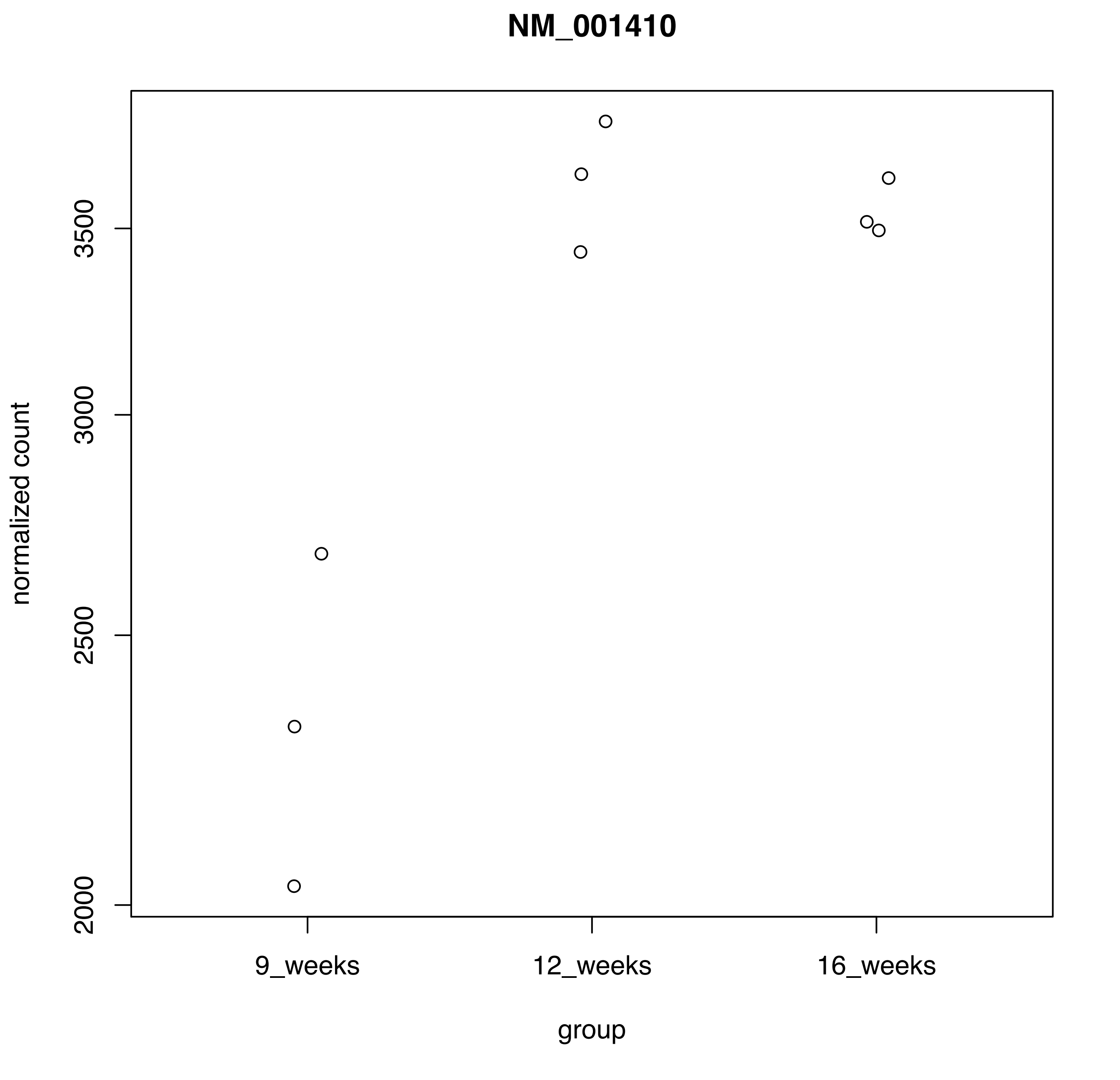
NM_001410
normalized count
2000
2500
3000
3500
9_weeks
12_weeks
16_weeks
group

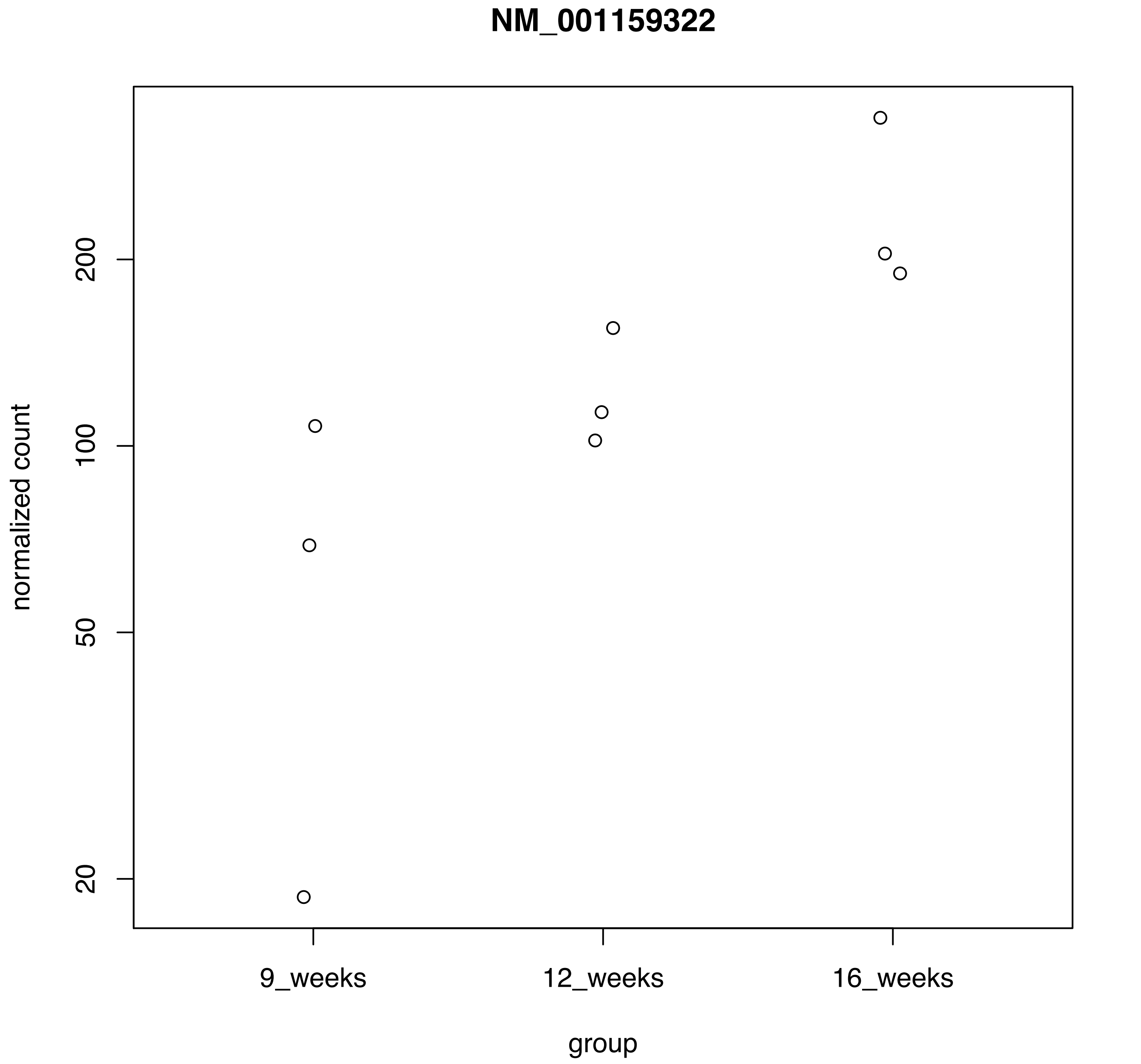

NM_001159322
normalized count
200
100
50
20
9_weeks
12_weeks
16_weeks
group

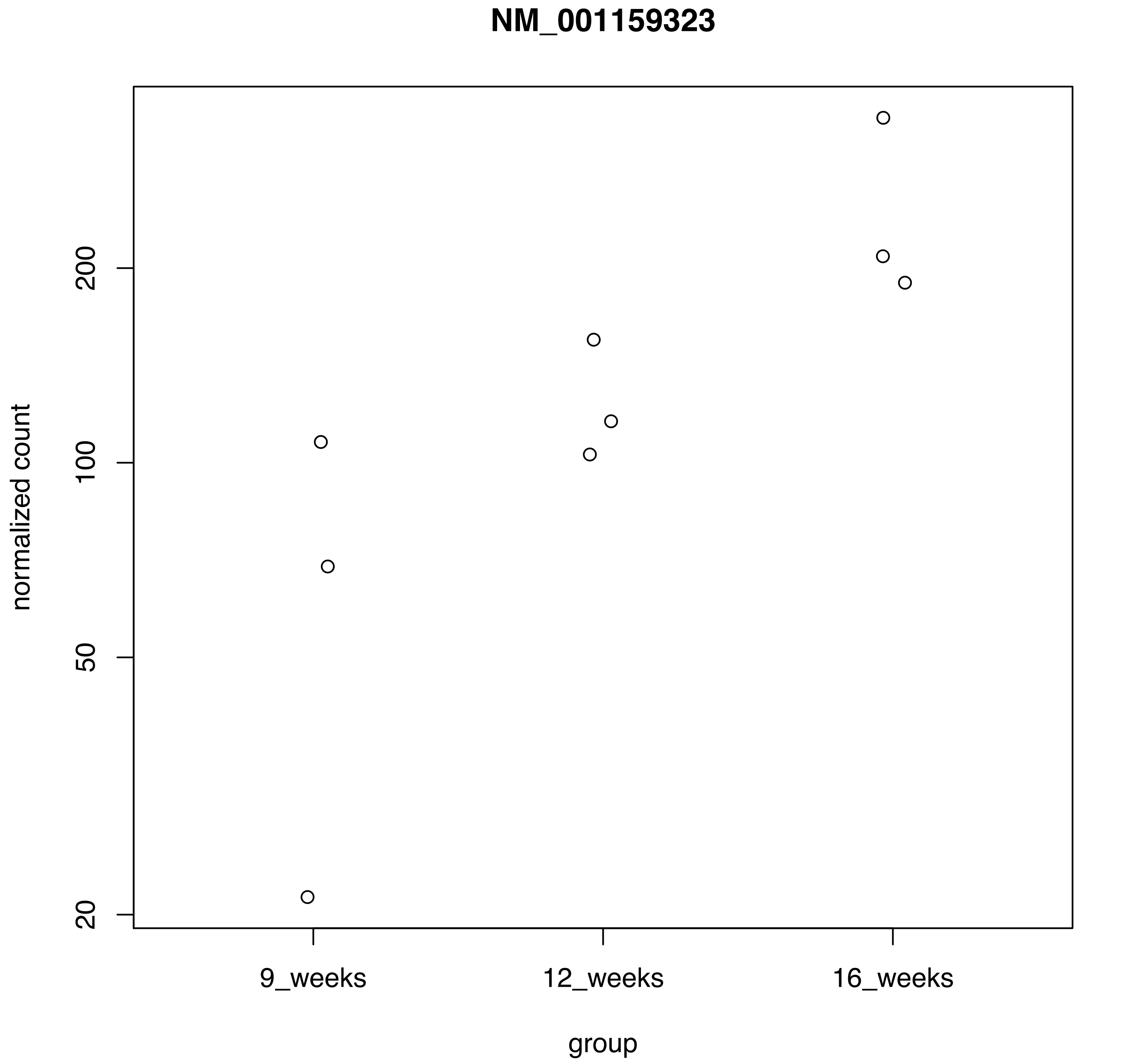
NM_001159323
normalized count
200
100
50
20
9_weeks
12_weeks
16_weeks
group

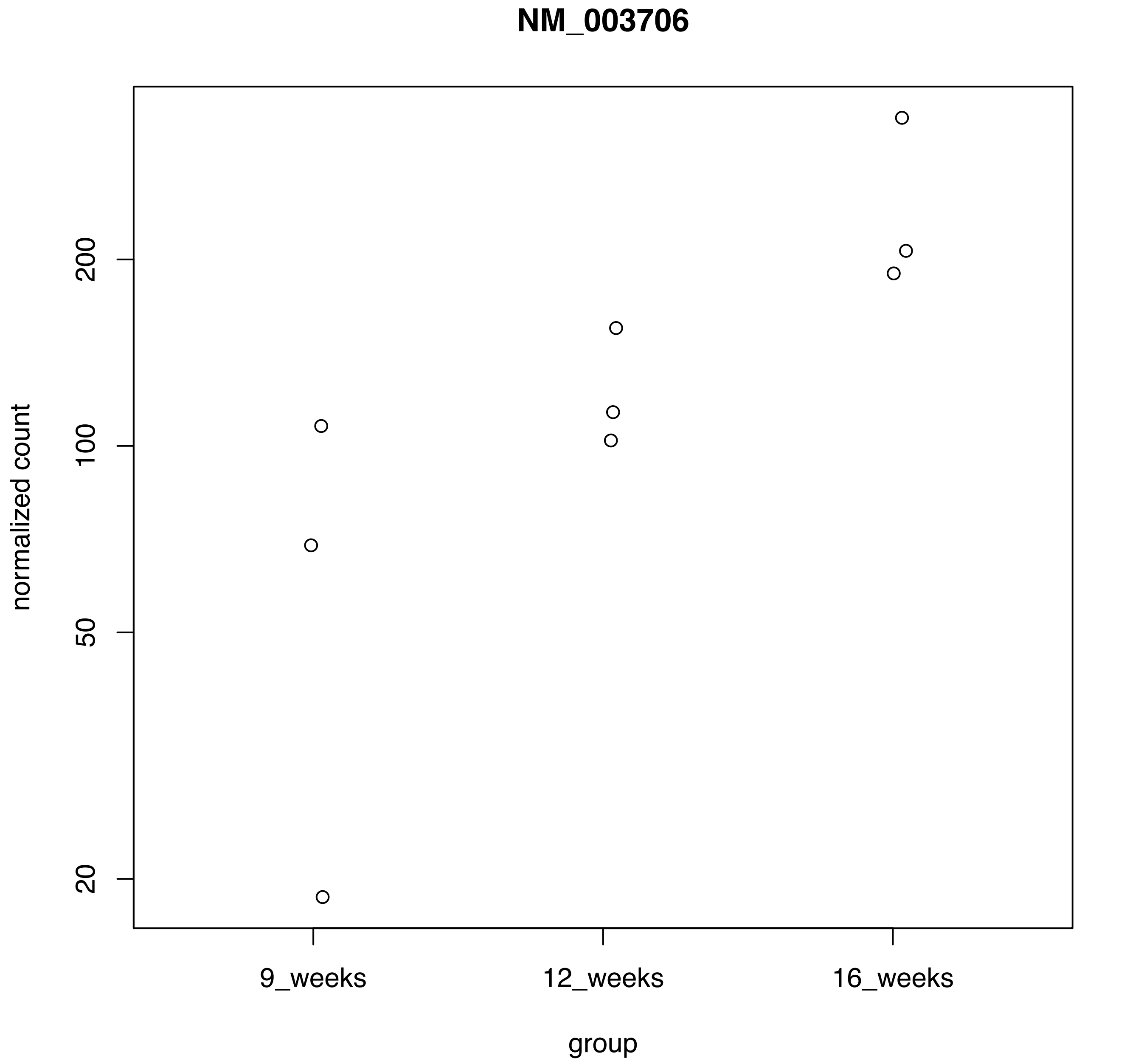
NM_003706
normalized count
200
100
50
20
9_weeks
12_weeks
16_weeks
group

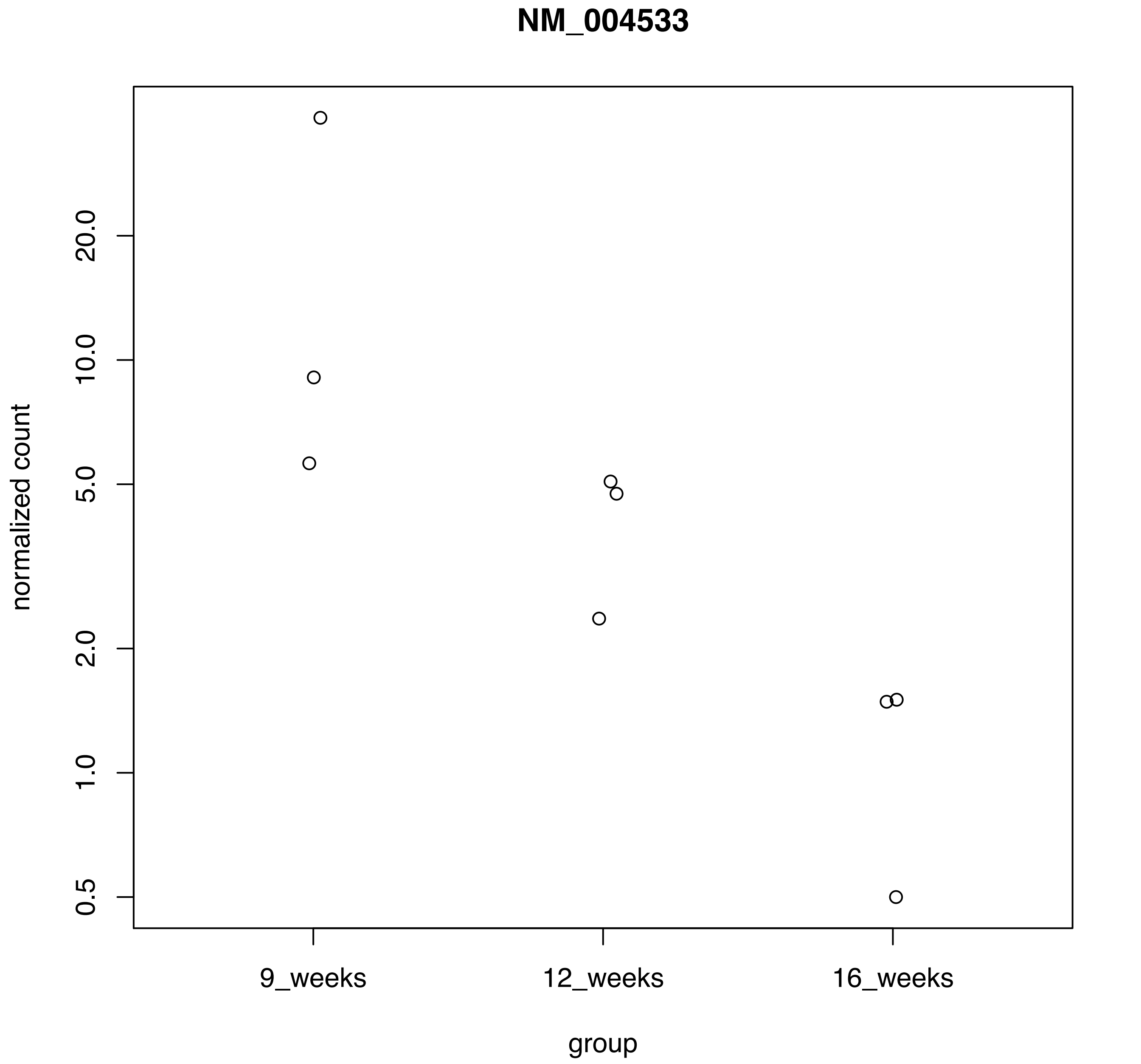
NM_004533
normalized count
20.0
10.0
5.0
2.0
1.0
0.5
9_weeks
12_weeks
16_weeks
group

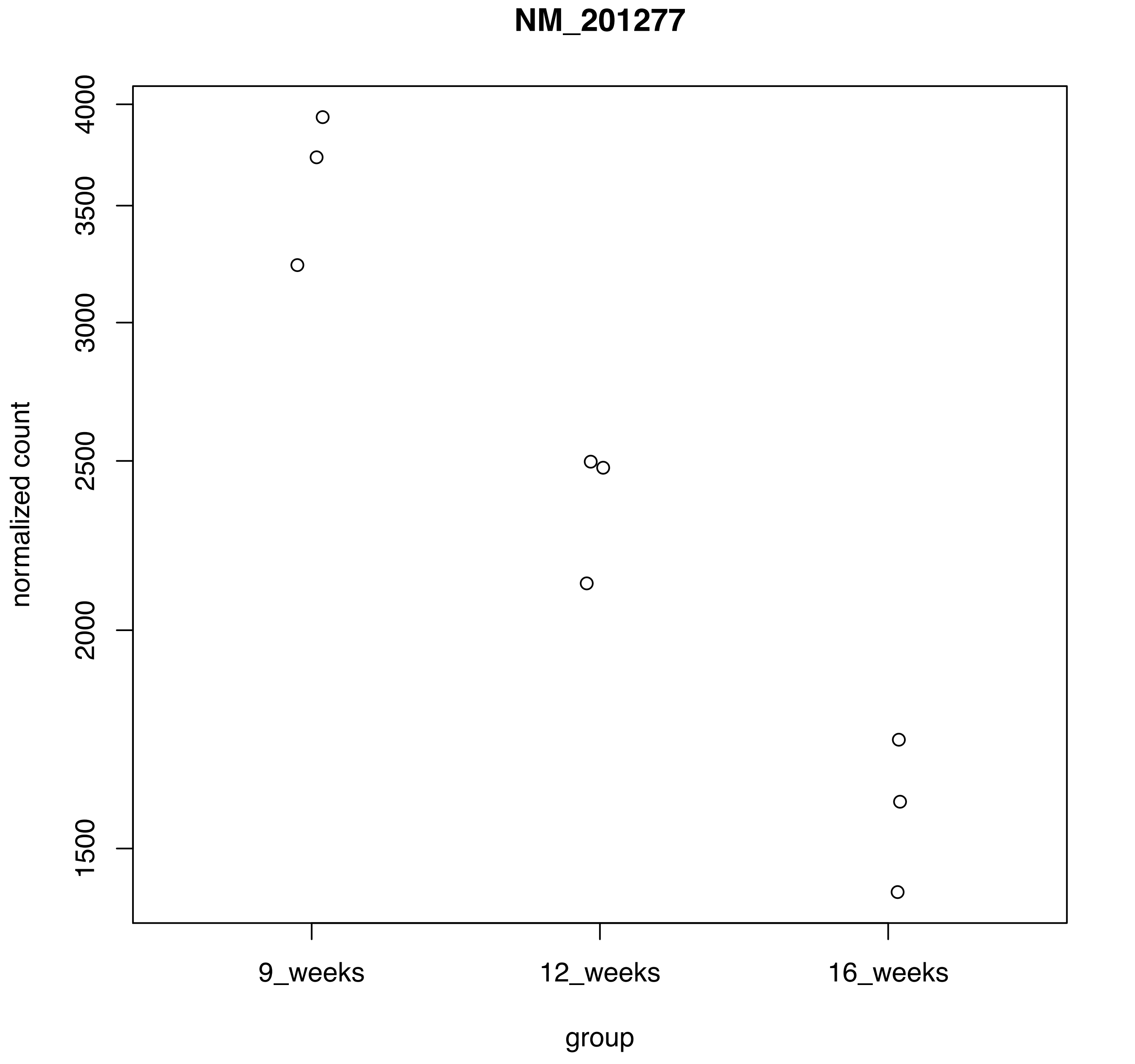
NM_201277
normalized count
4000
3500
3000
2500
2000
1500
9_weeks
12_weeks
16_weeks
group

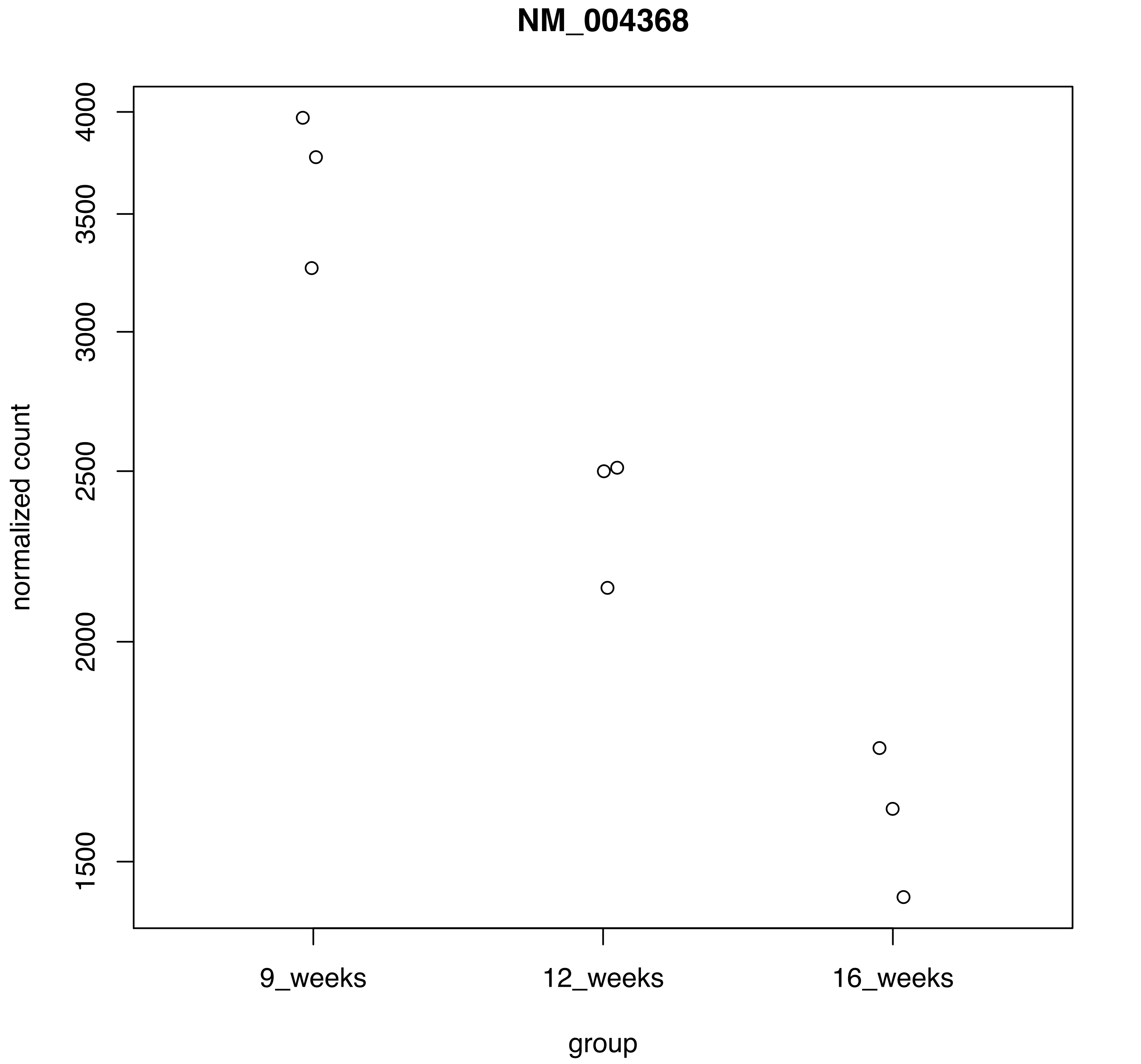
NM_004368
normalized count
4000
3500
3000
2500
2000
1500
9_weeks
12_weeks
16_weeks
group

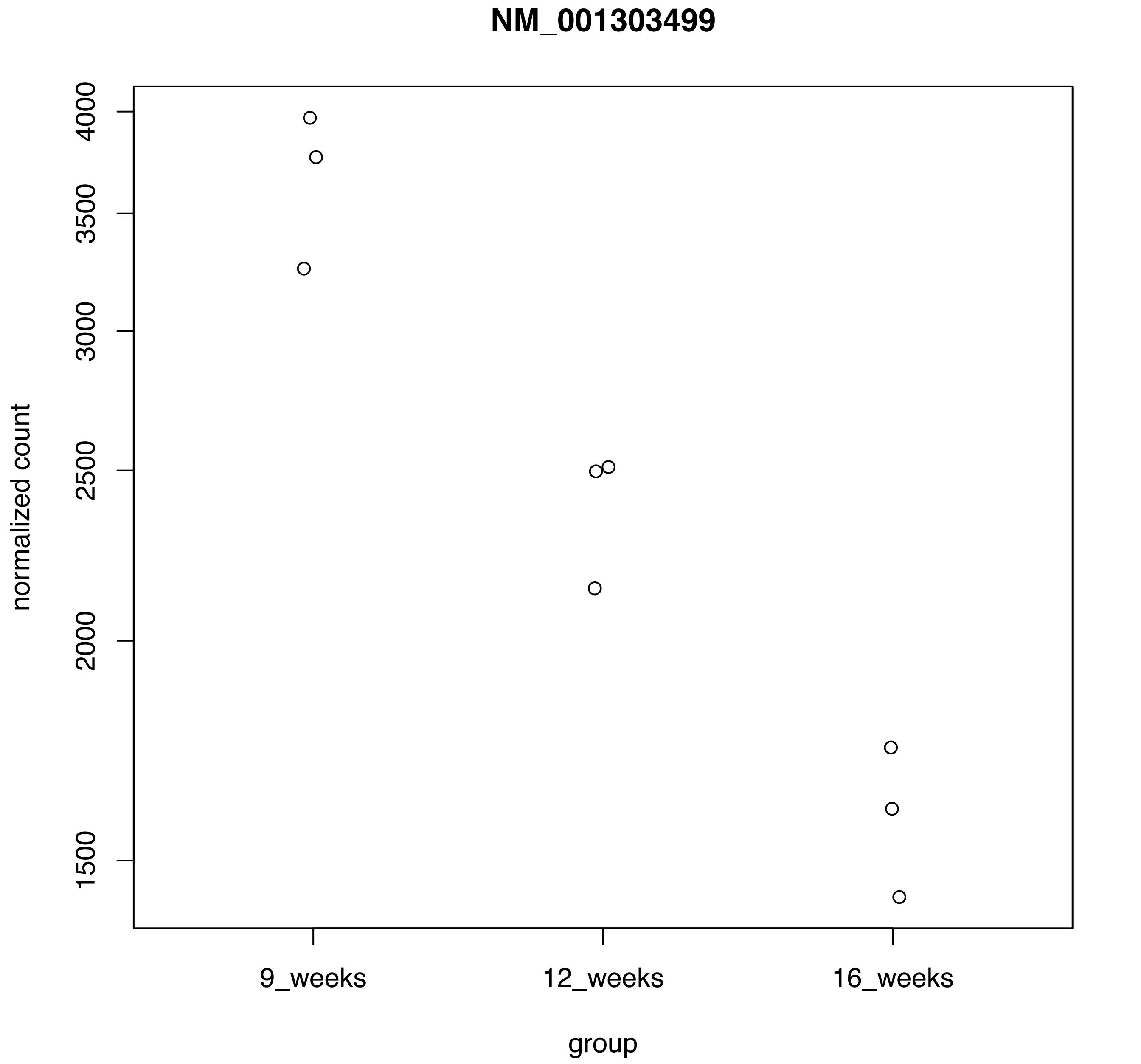

NM_001303499
normalized count
4000
3500
3000
2500
2000
1500
9_weeks
12_weeks
16_weeks
group

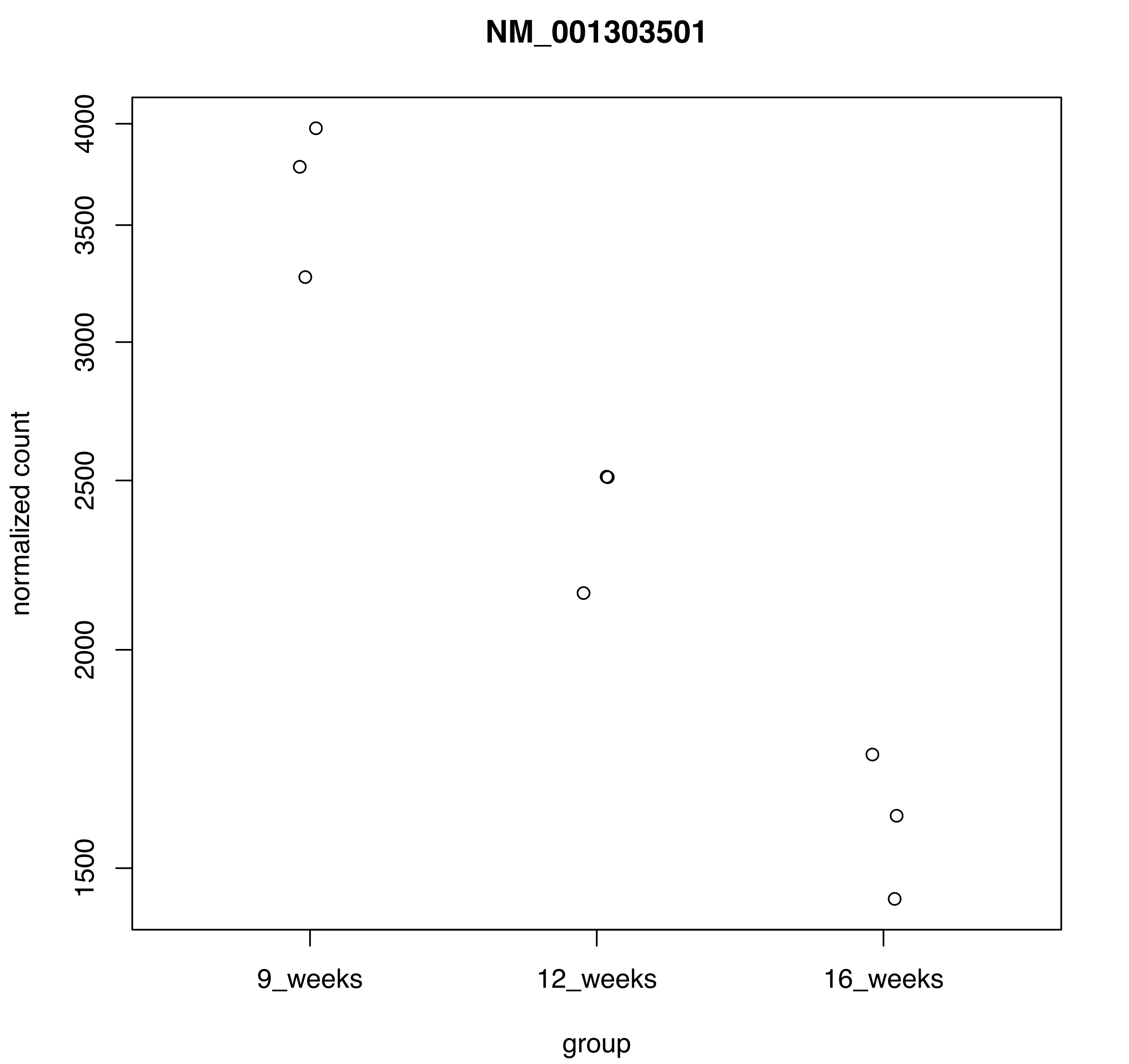
NM_001303501
normalized count
4000
3500
3000
2500
2000
1500
9_weeks
12_weeks
16_weeks
group

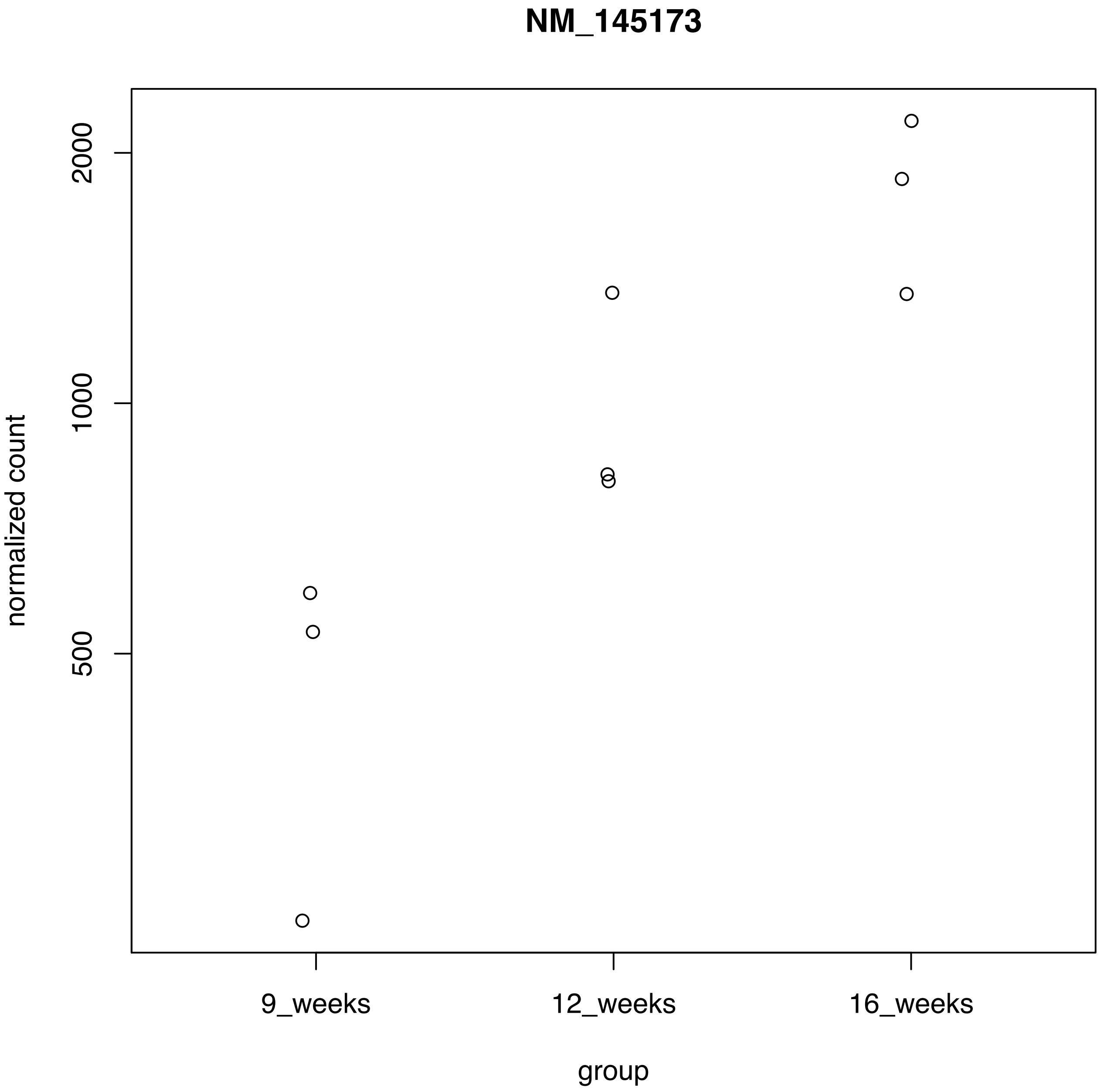
NM_145173
normalized count
2000
1000
500
9_weeks
12_weeks
16_weeks
group

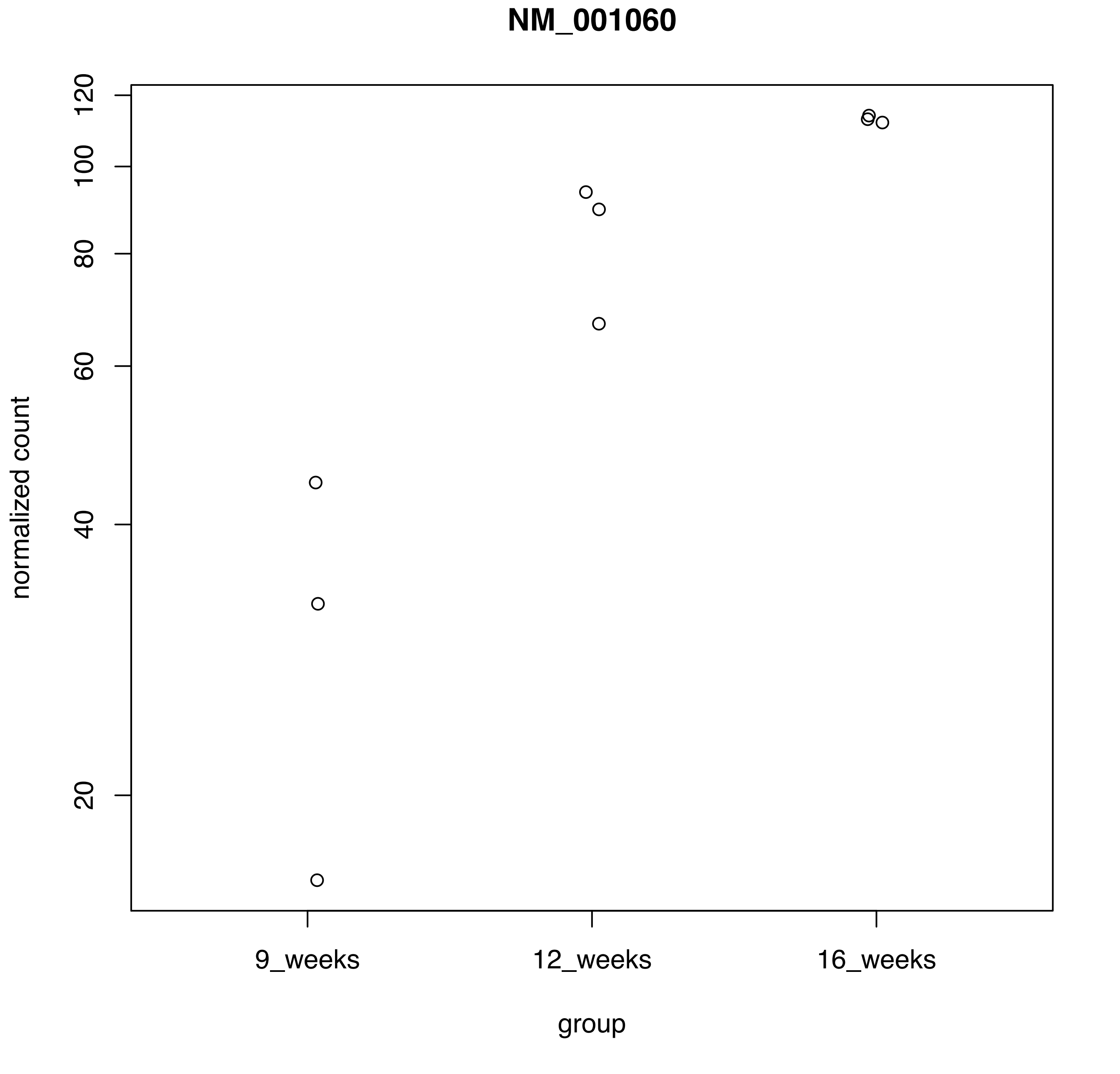
NM_001060
normalized count
120
100
80
60
40
20
9_weeks
12_weeks
16_weeks
group

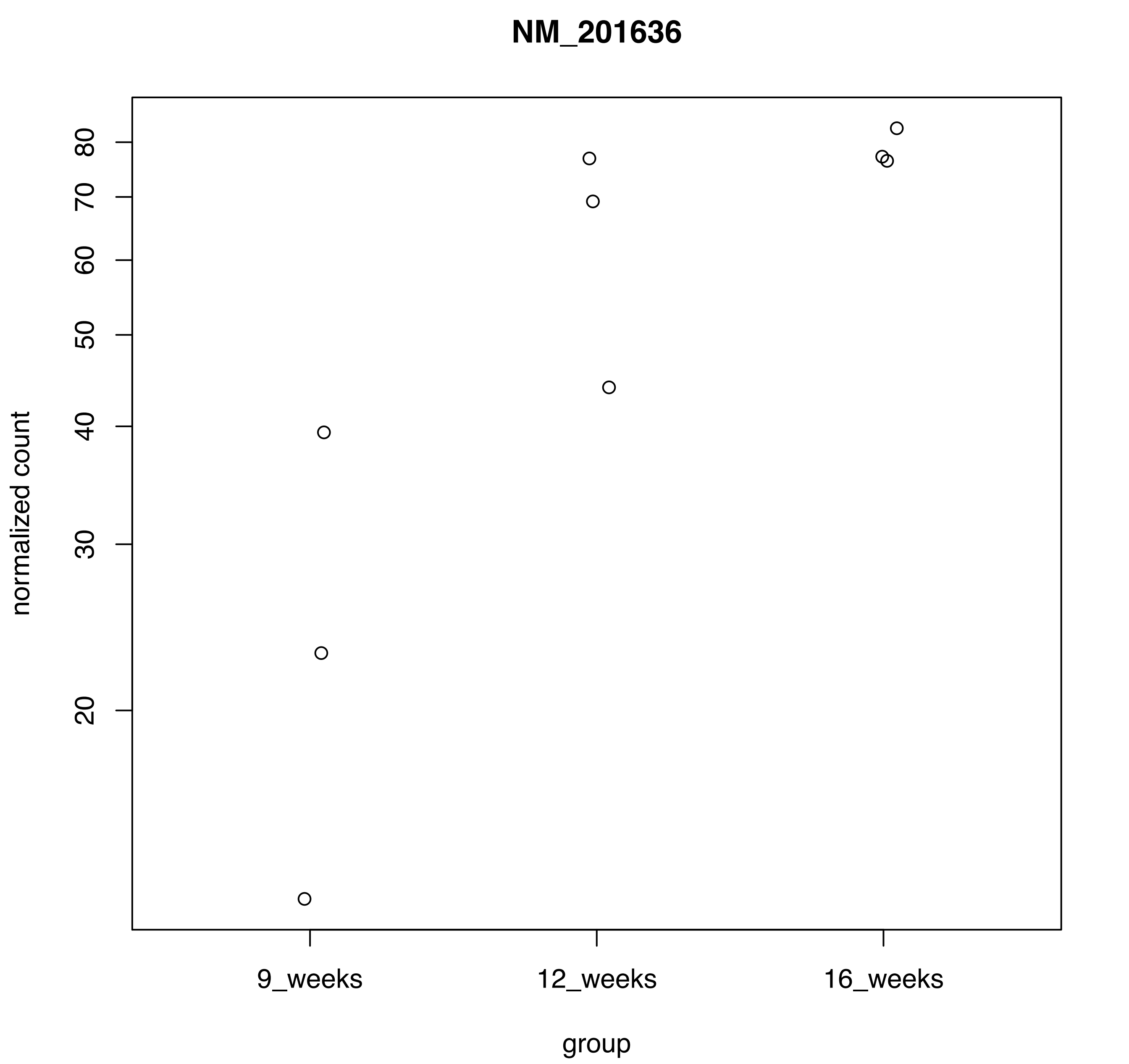
NM_201636
normalized count
80
70
60
50
40
30
20
9_weeks
12_weeks
16_weeks
group

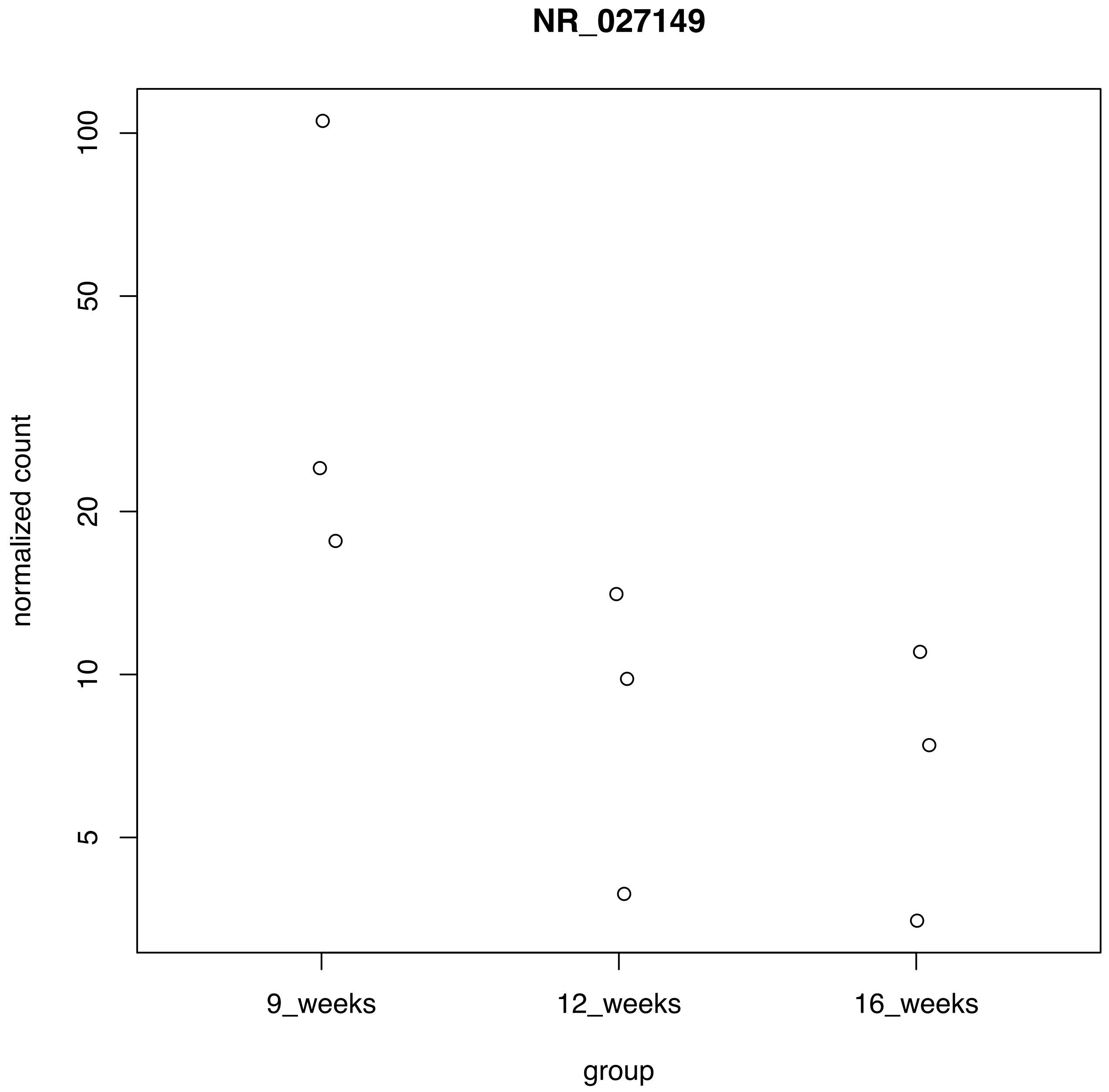
NR_027149
normalized count
100
50
20
10
5
9_weeks
12_weeks
16_weeks
group

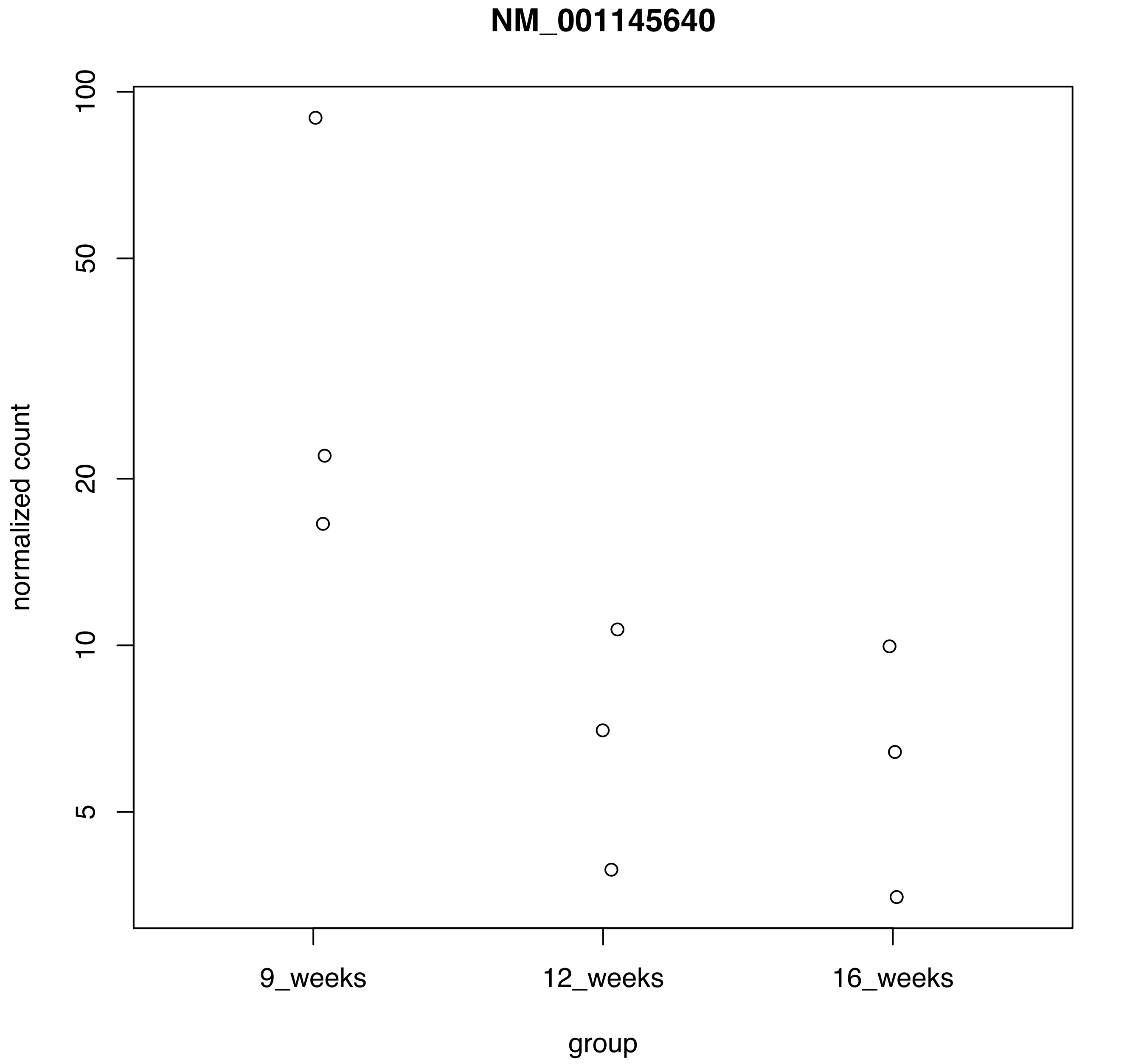

NM_001145640
100
50
20
10
5
normalized count
9_weeks
12_weeks
16_weeks
group

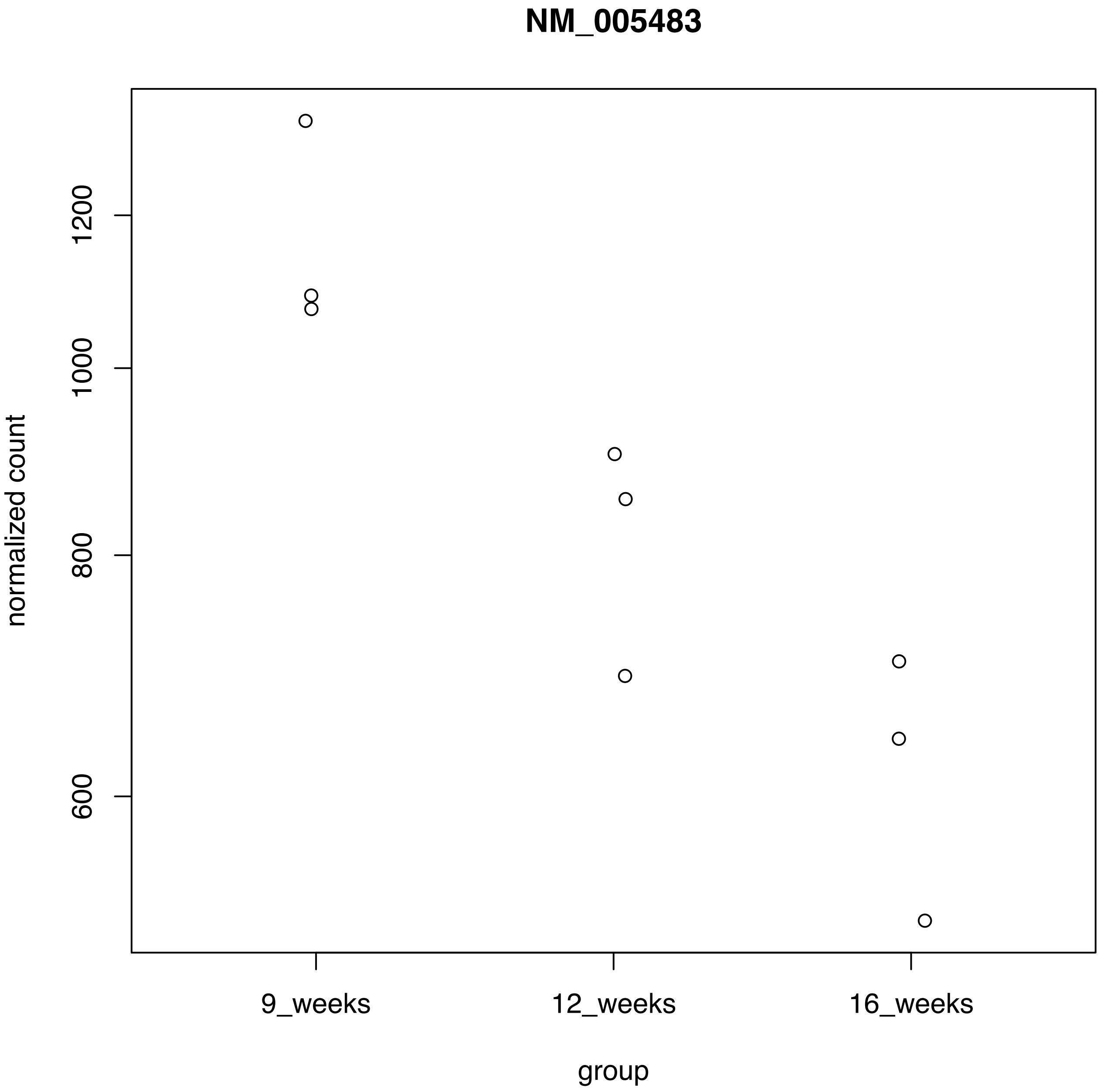
NM_005483
normalized count
1200
1000
800
600
9_weeks
12_weeks
16_weeks
group

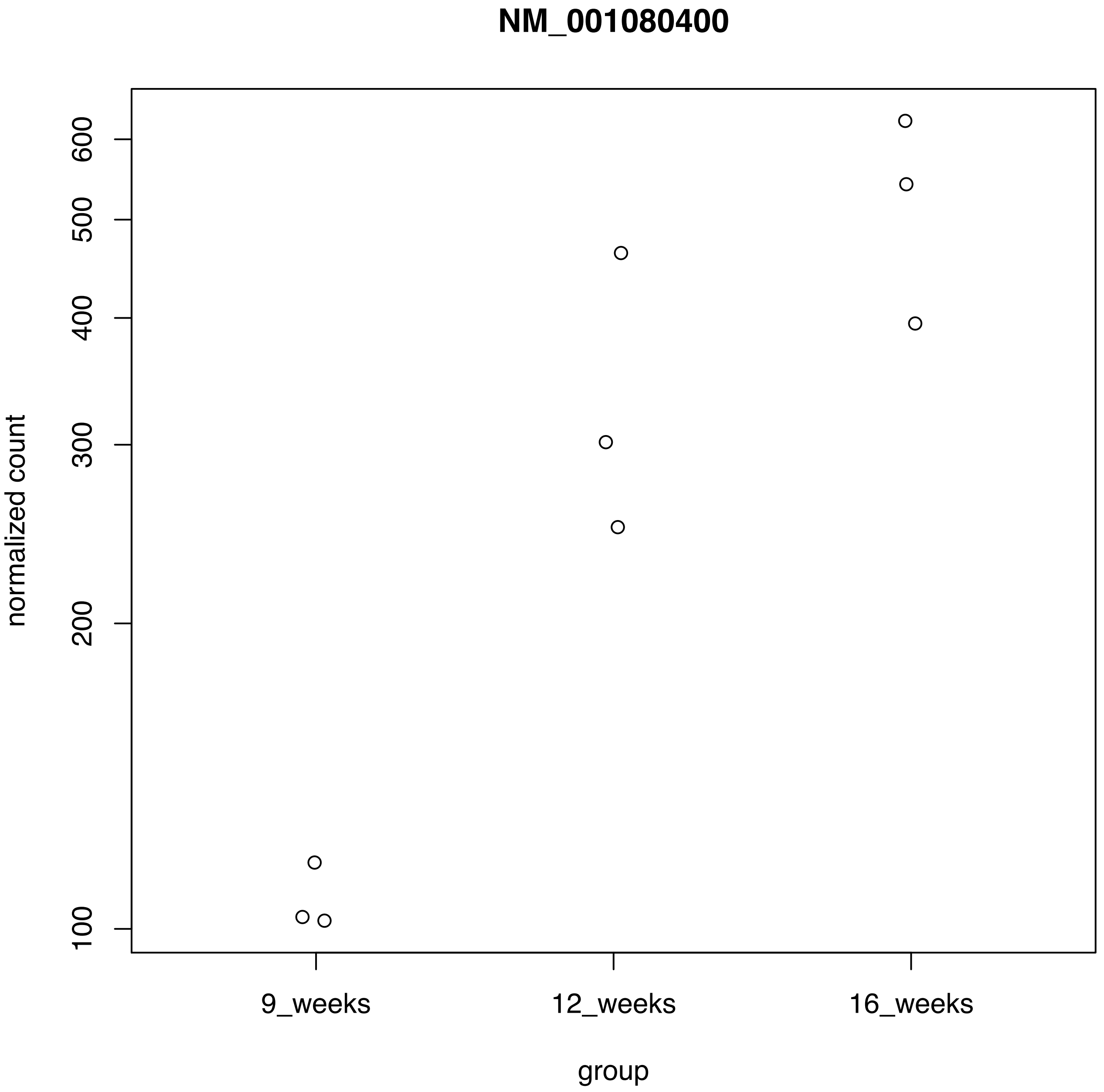
NM_001080400
normalized count
100
200
300
400
500
600
9_weeks
12_weeks
16_weeks
group

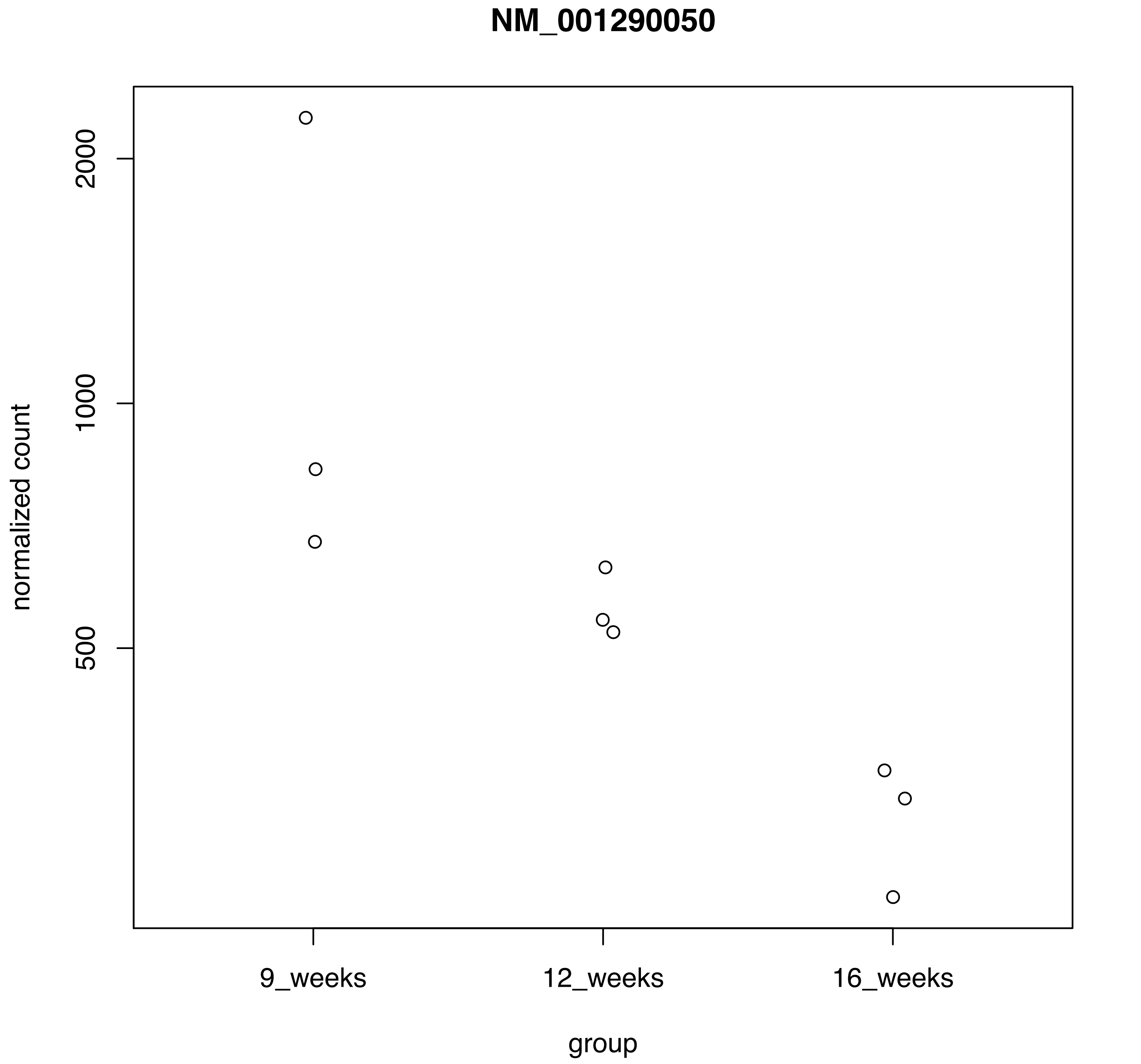
NM_001290050
normalized count
2000
1000
500
9_weeks
12_weeks
16_weeks
group

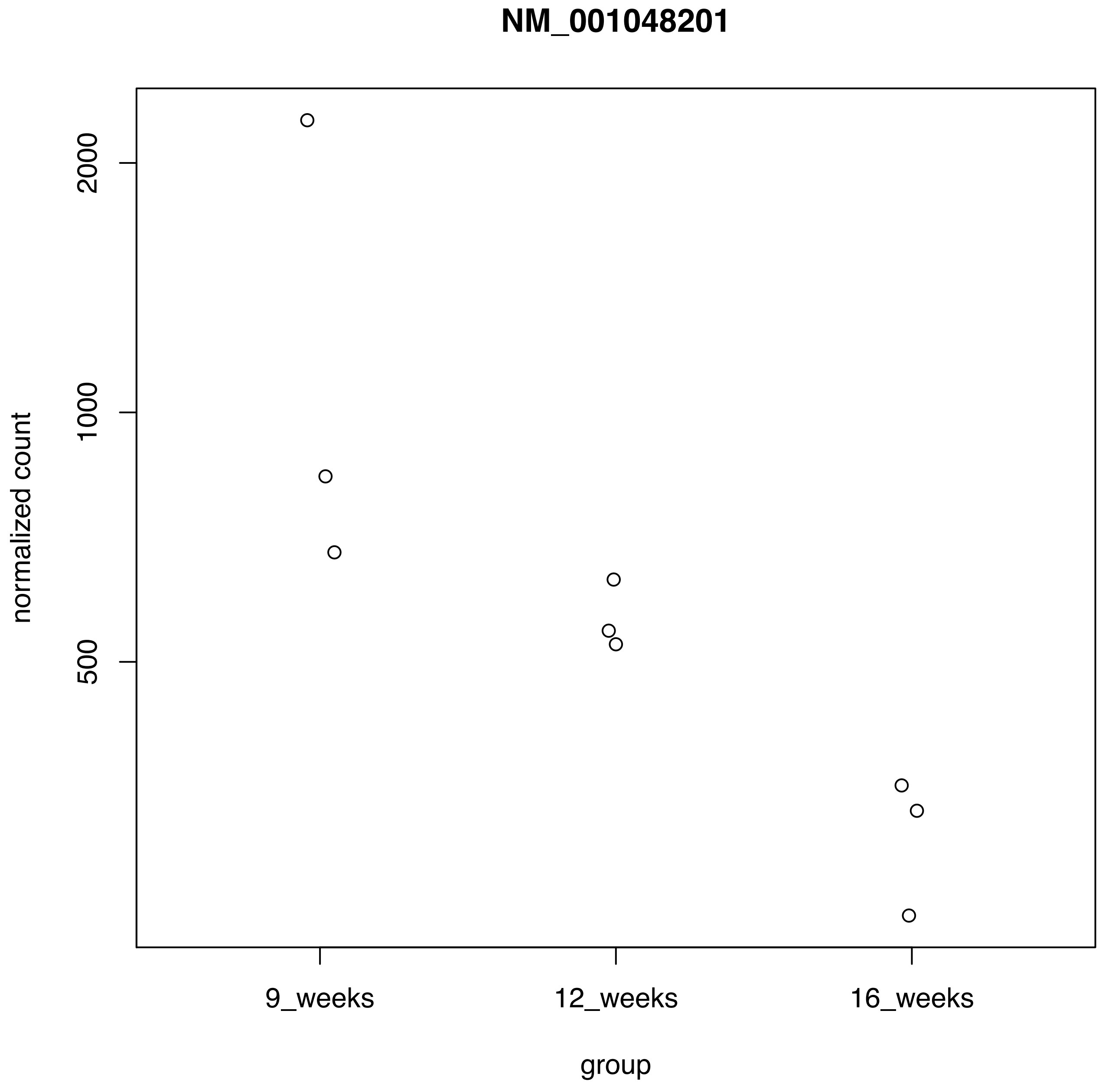
NM_001048201
normalized count
2000
1000
500
9_weeks
12_weeks
16_weeks
group

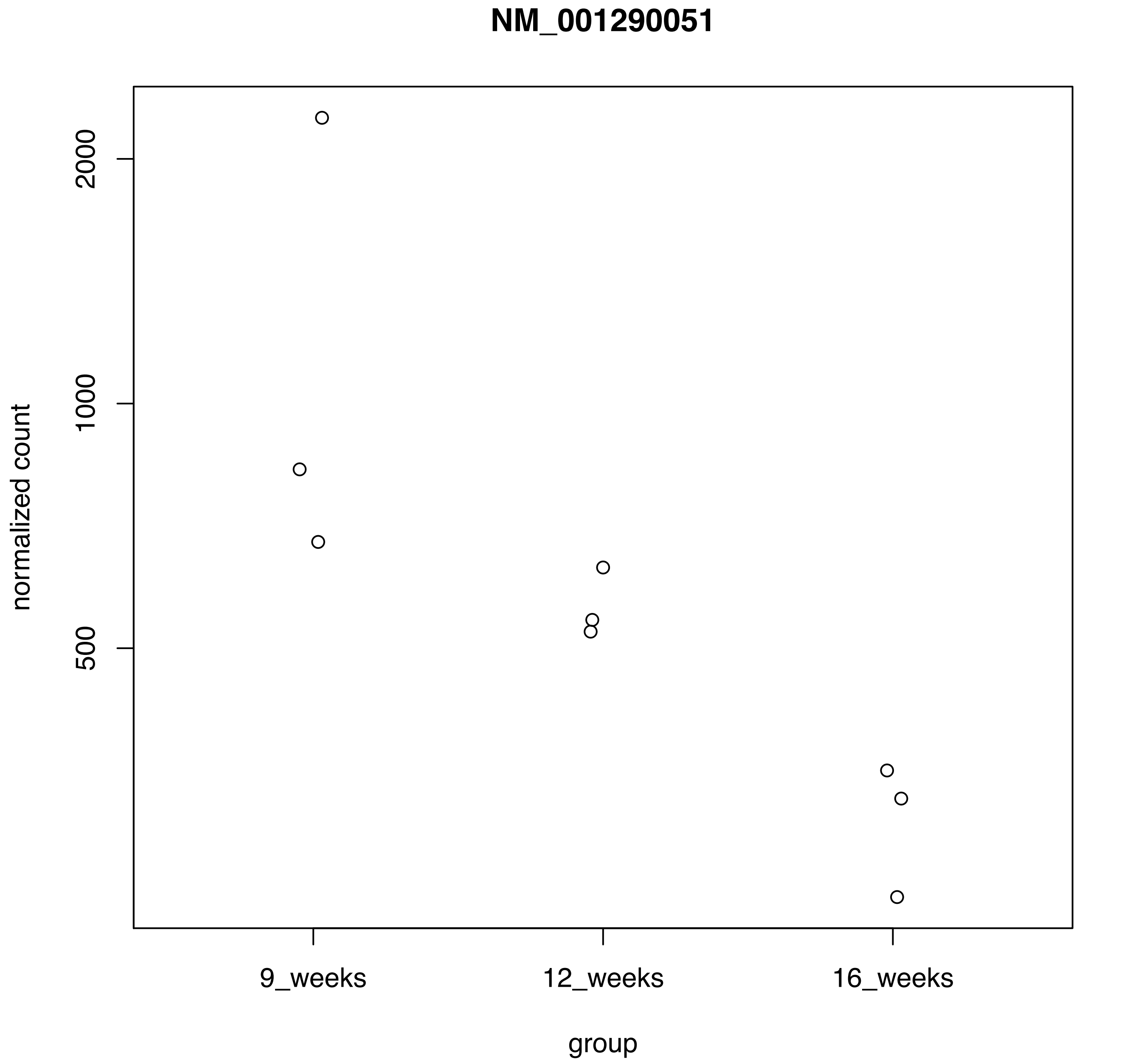

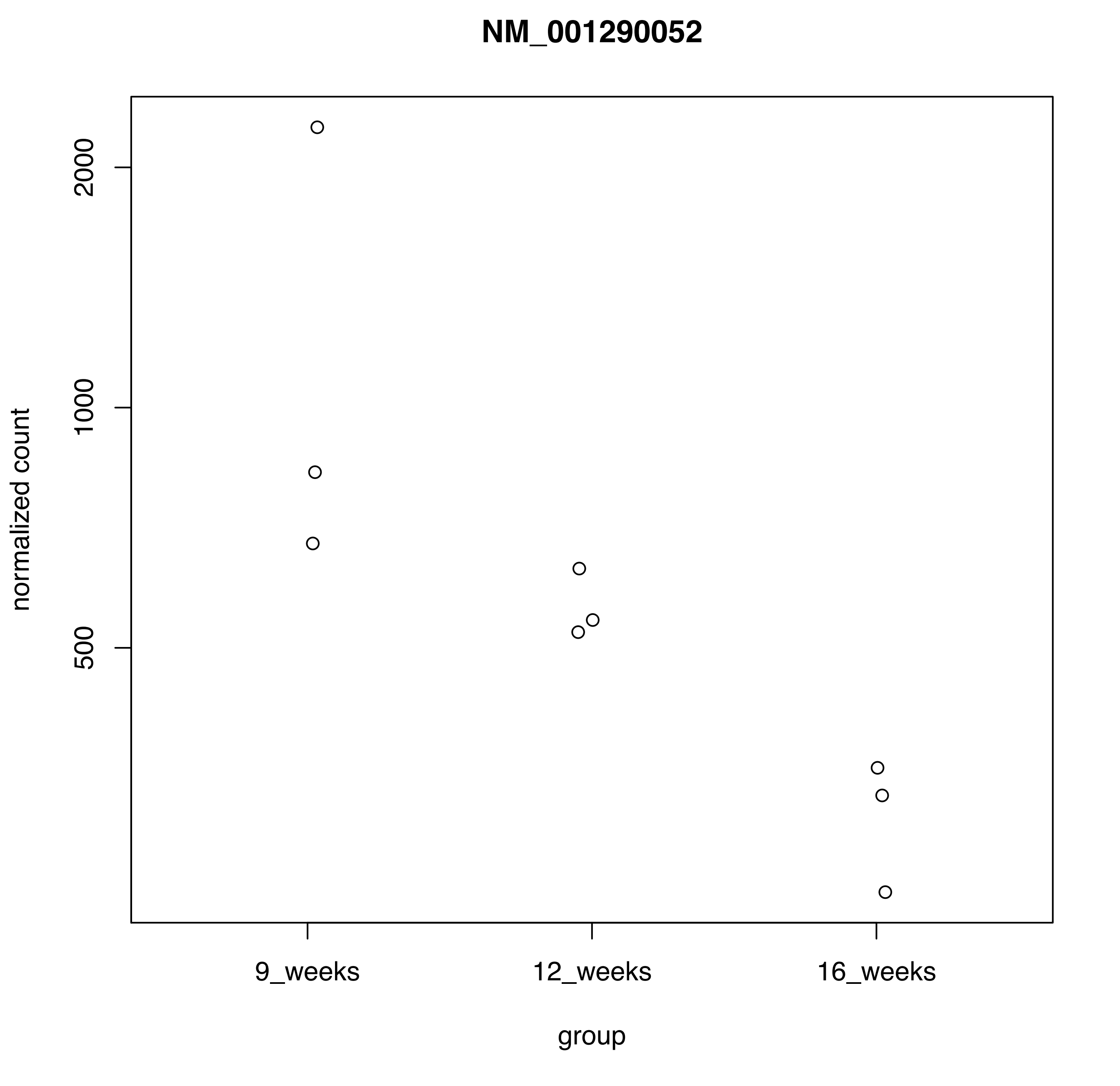

NM_001290052
normalized count
2000
1000
500
9_weeks
12_weeks
16_weeks
group

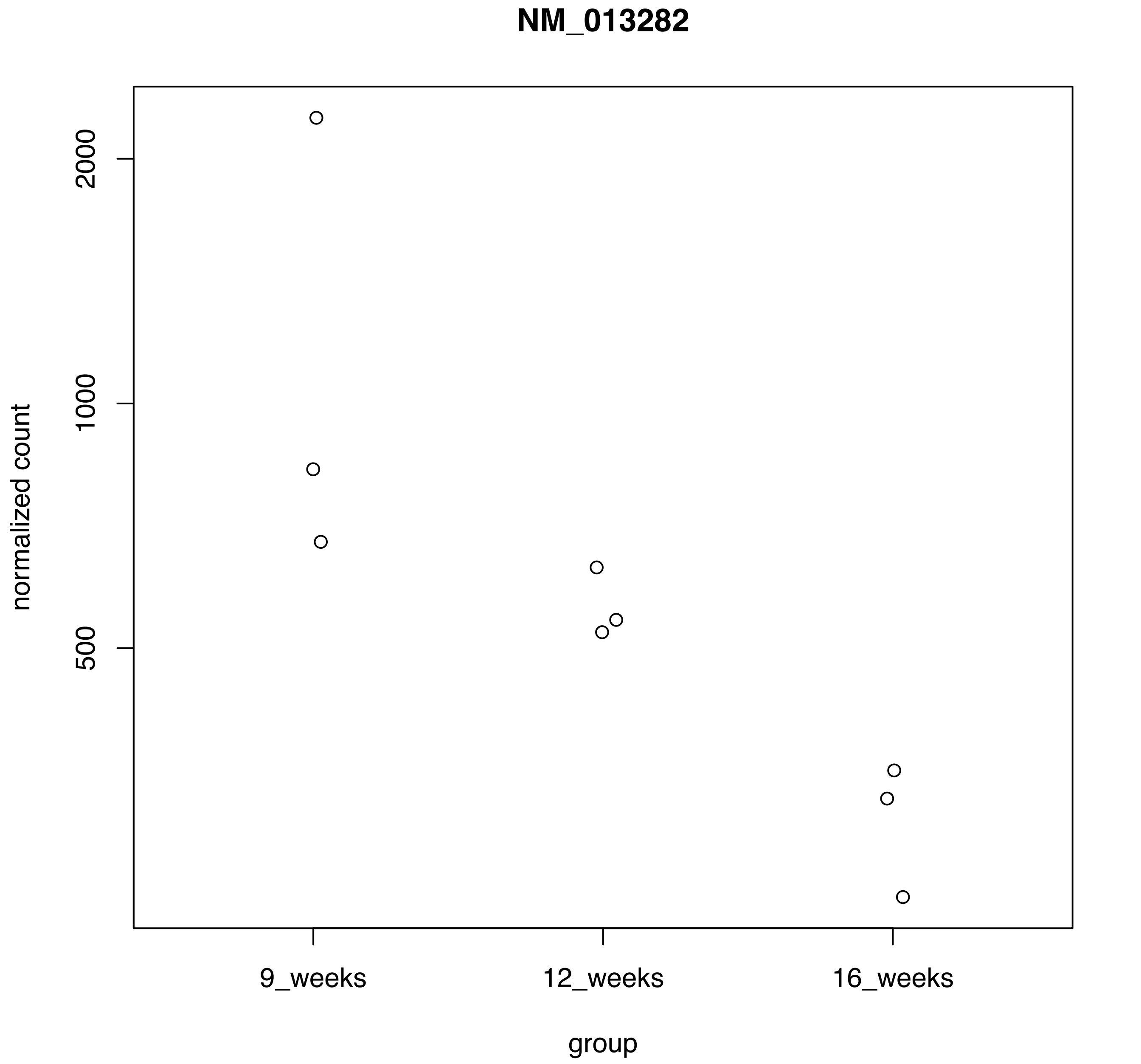
NM_013282
2000
1000
500
normalized count
9_weeks
12_weeks
16_weeks
group

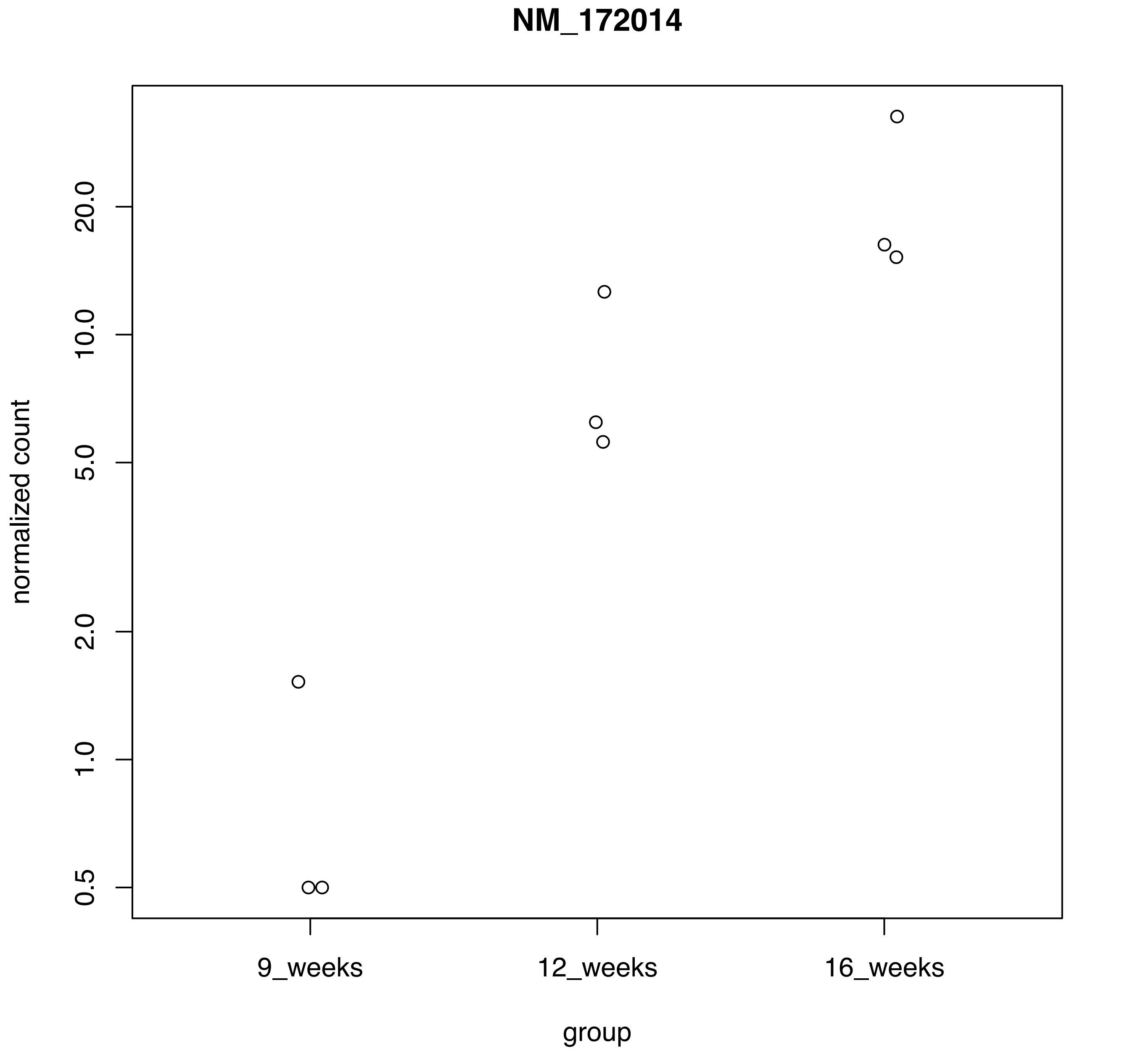

NM_172014
normalized count
20.0
10.0
5.0
2.0
1.0
0.5
9_weeks
12_weeks
16_weeks
group

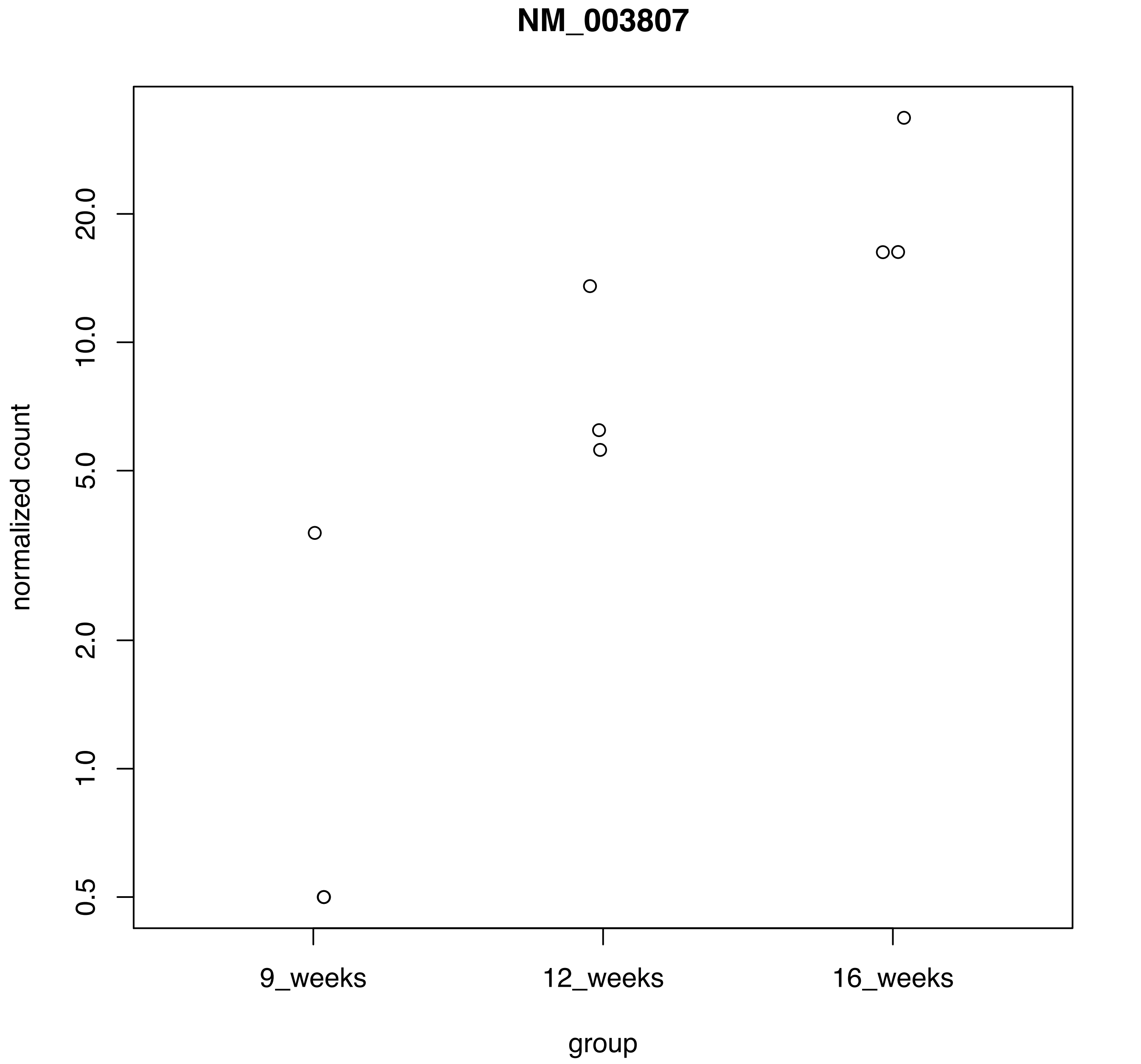
NM_003807
normalized count
20.0
10.0
5.0
2.0
1.0
0.5
9_weeks
12_weeks
16_weeks
group

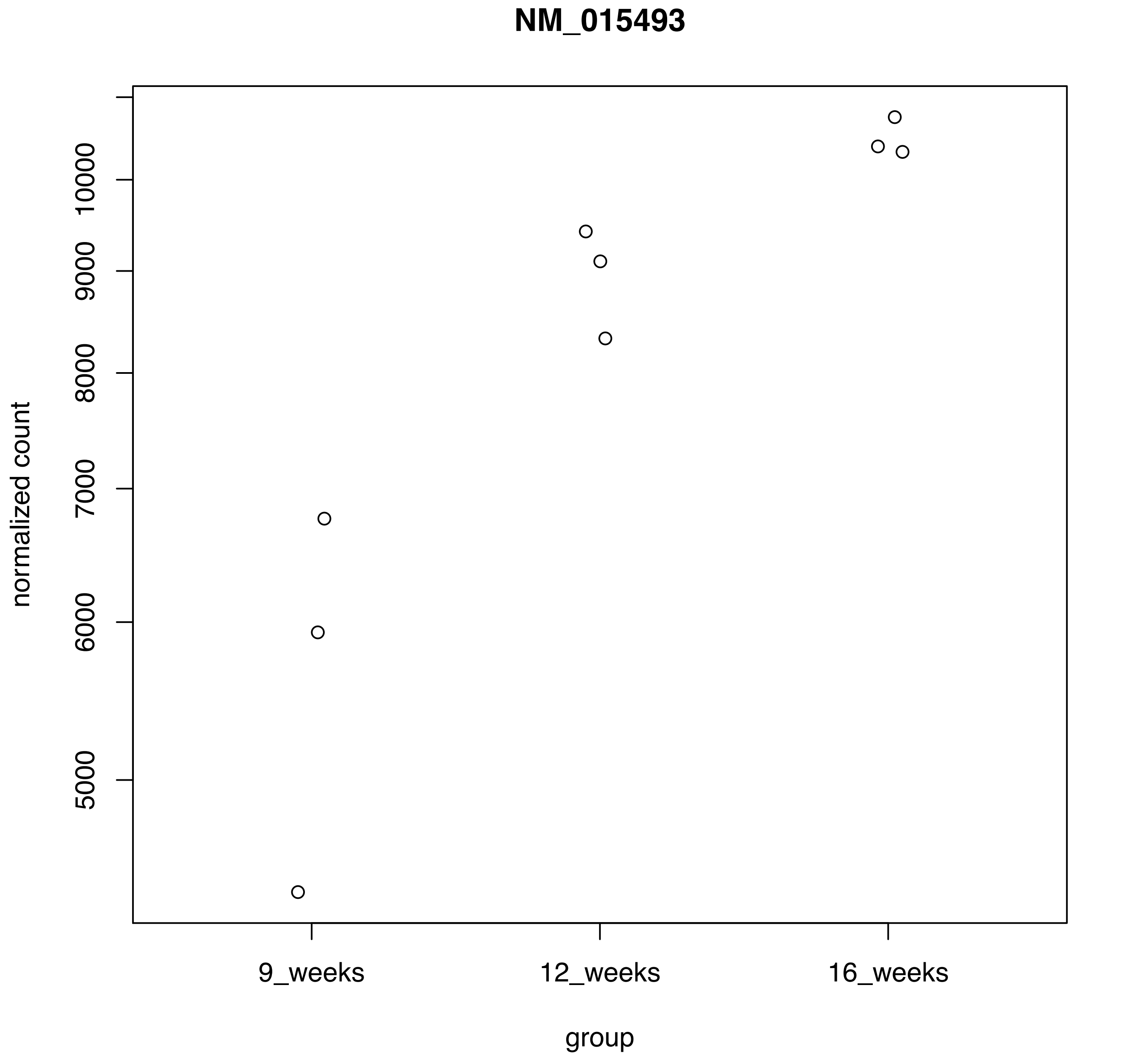
NM_015493
normalized count
10000
9000
8000
7000
6000
5000
9_weeks
12_weeks
16_weeks
group

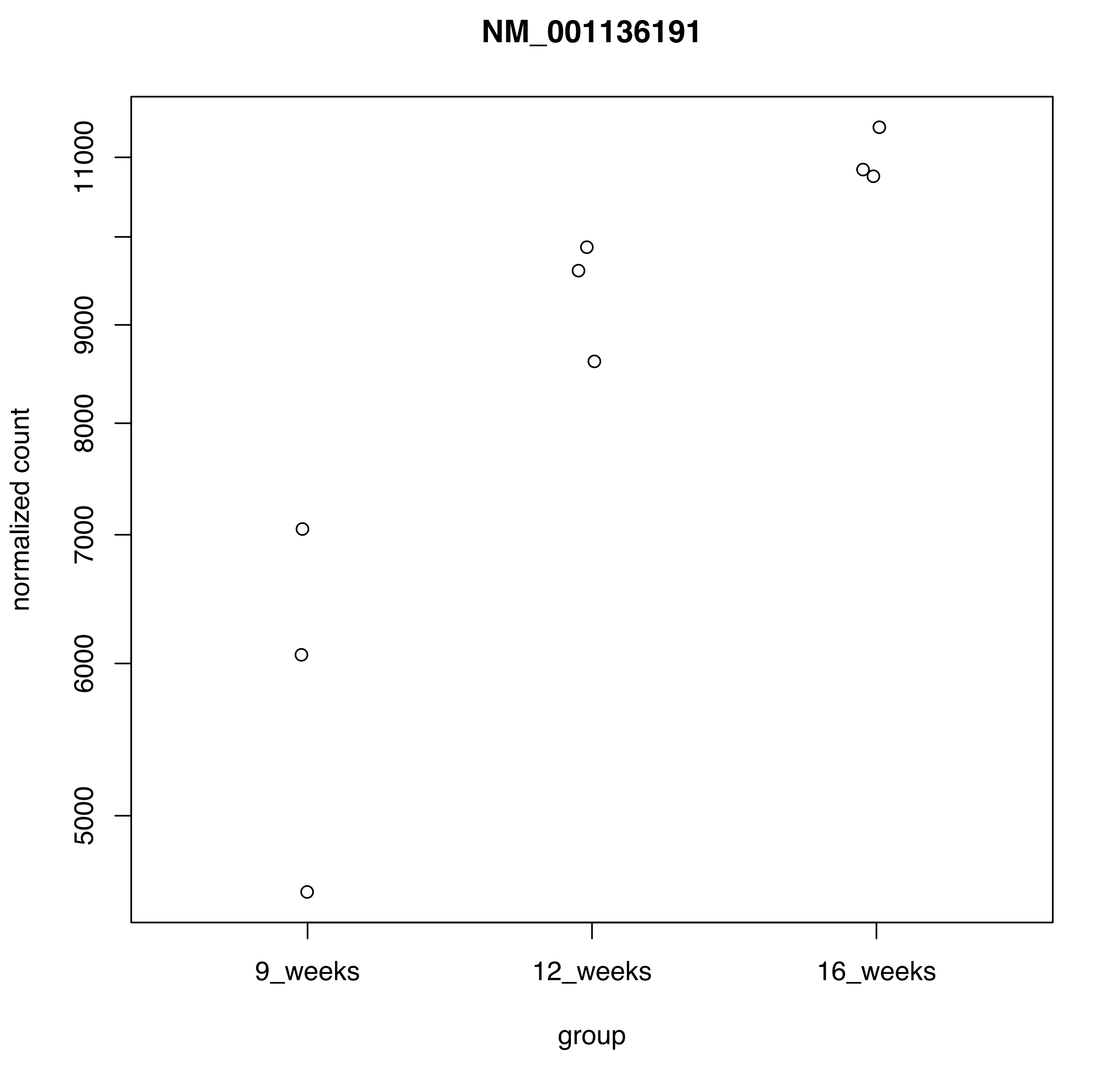
NM_001136191
normalized count
5000
6000
7000
8000
9000
11000
9_weeks
12_weeks
16_weeks
group

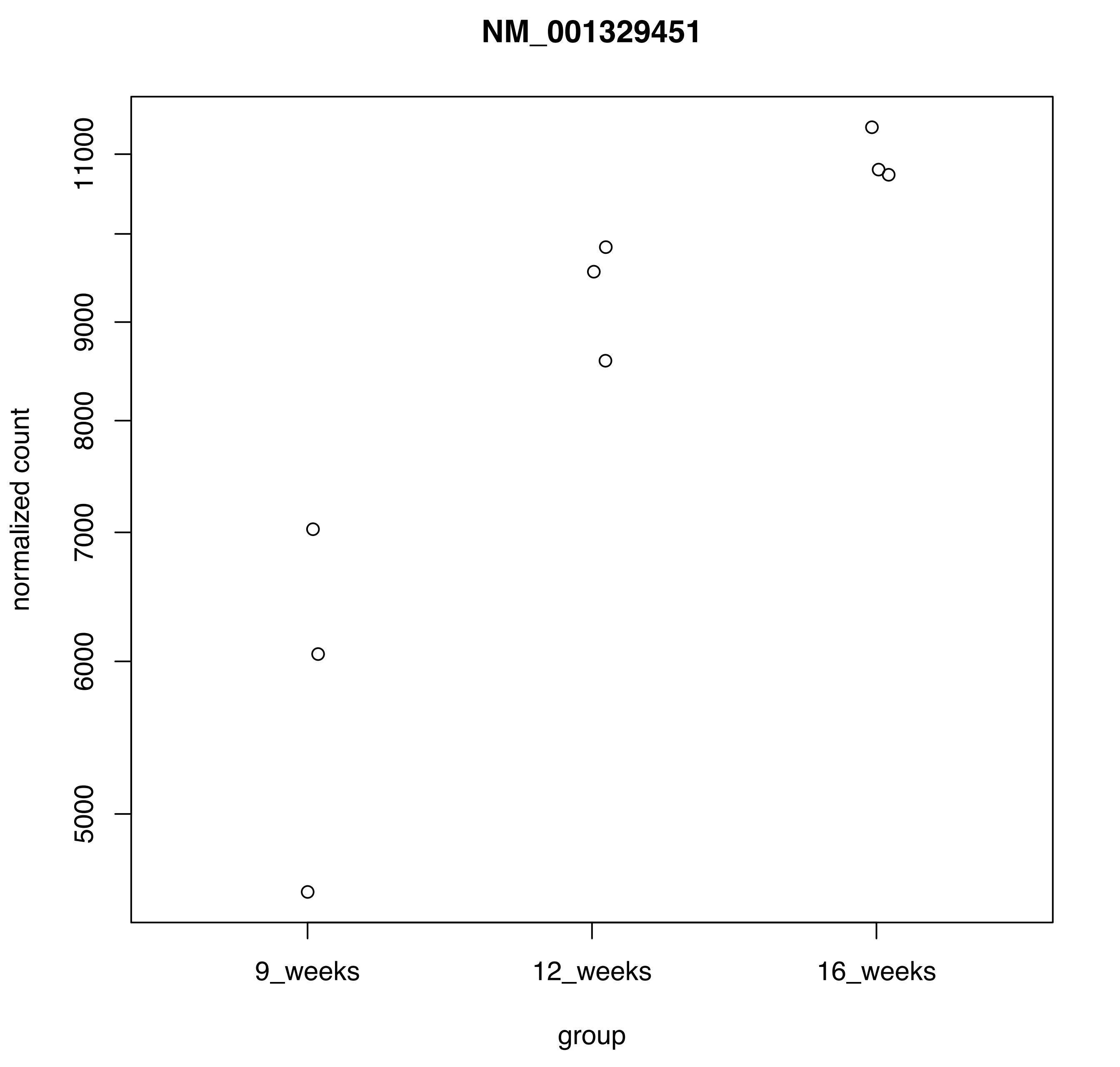
NM_001329451
normalized count
11000
9000
8000
7000
6000
5000
9_weeks
12_weeks
16_weeks
group

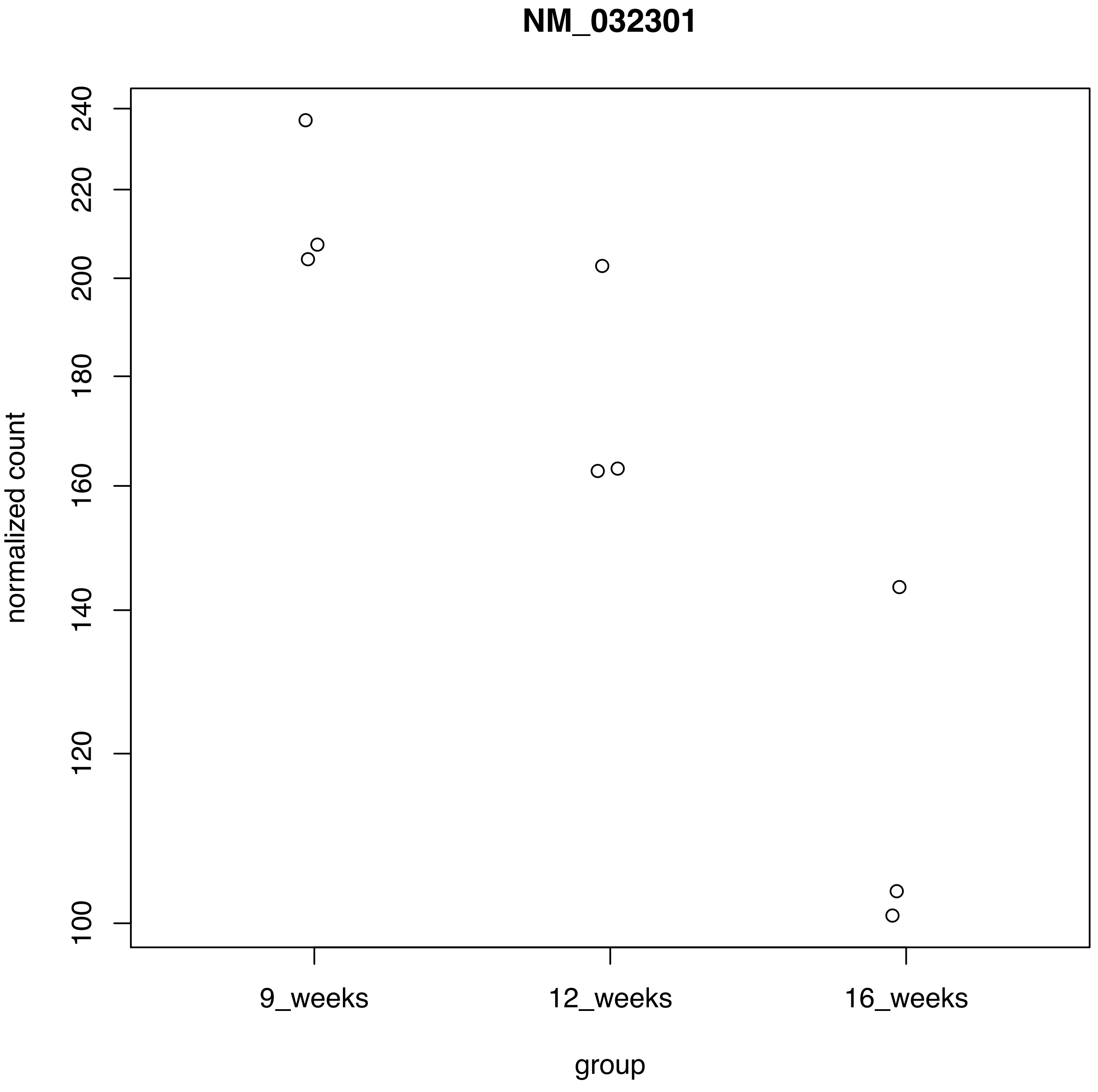
NM_032301
normalized count
240
220
200
180
160
140
120
100
9_weeks
12_weeks
16_weeks
group

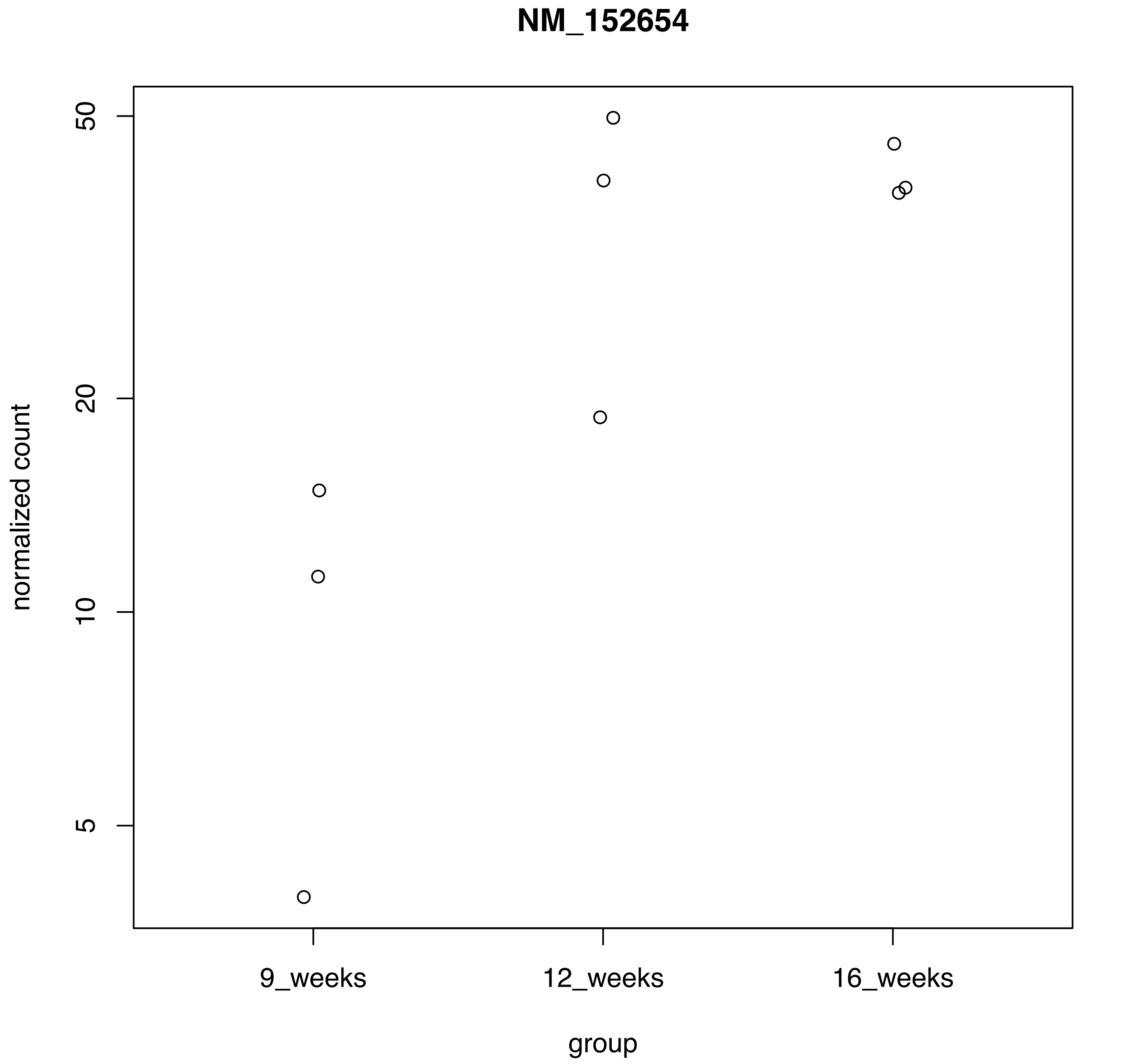
NM_152654
normalized count
50
20
10
5
9_weeks
12_weeks
16_weeks
group

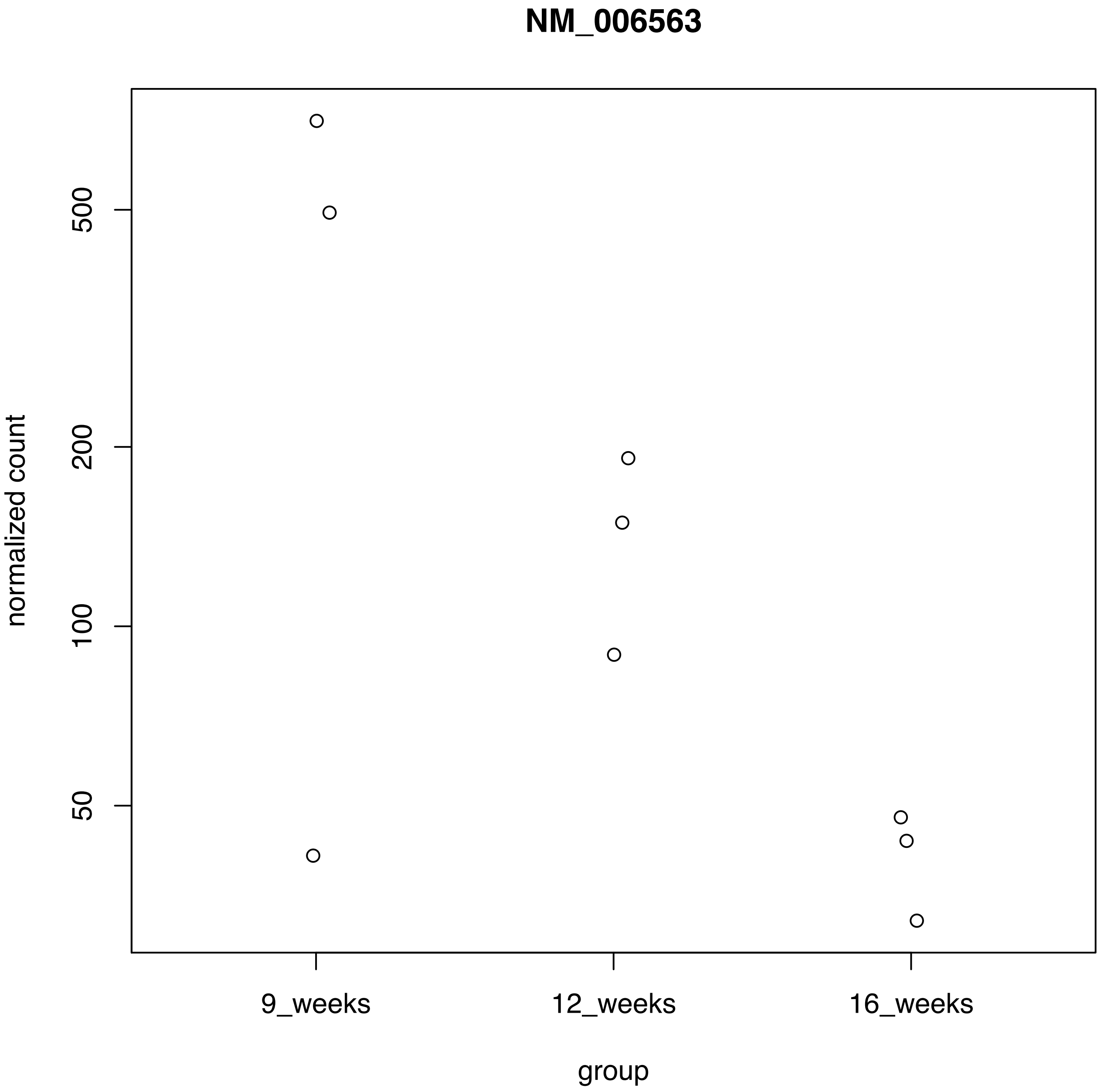

NM_006563
normalized count
500
200
100
50
9_weeks
12_weeks
16_weeks
group

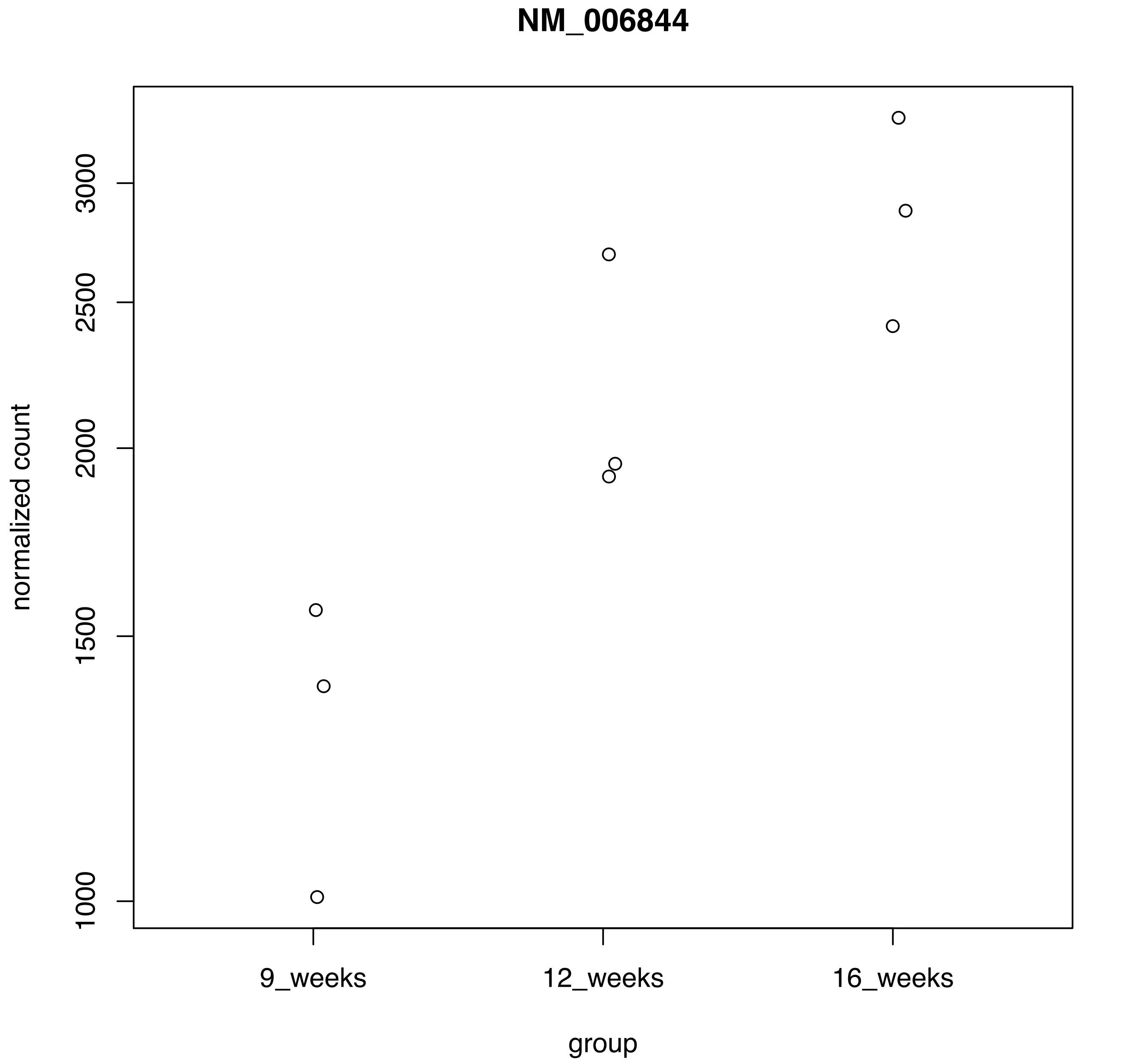
NM_006844
normalized count
3000
2500
2000
1500
1000
9_weeks
12_weeks
16_weeks
group

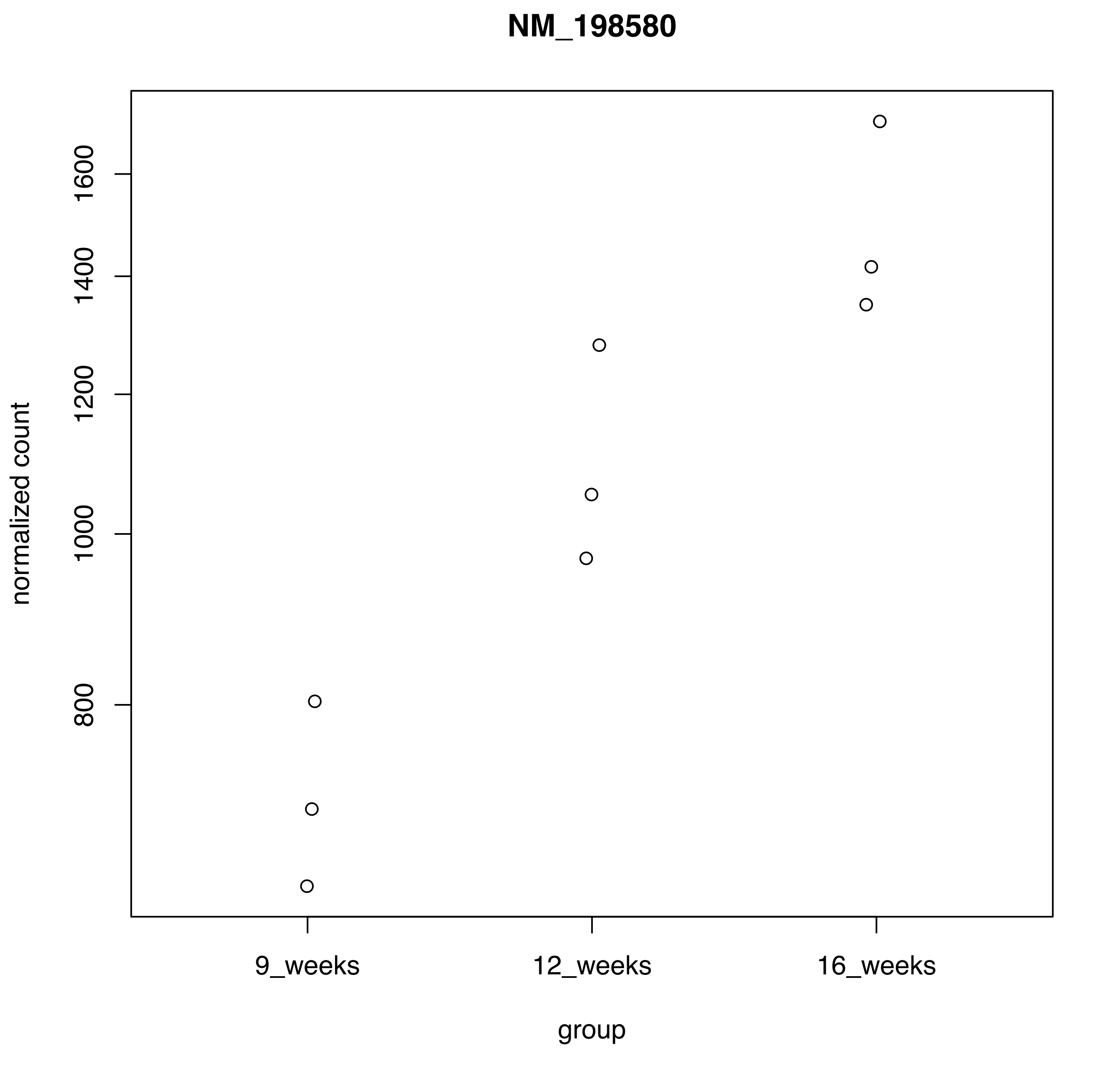
NM_198580
normalized count
1600
1400
1200
1000
800
9_weeks
12_weeks
16_weeks
group

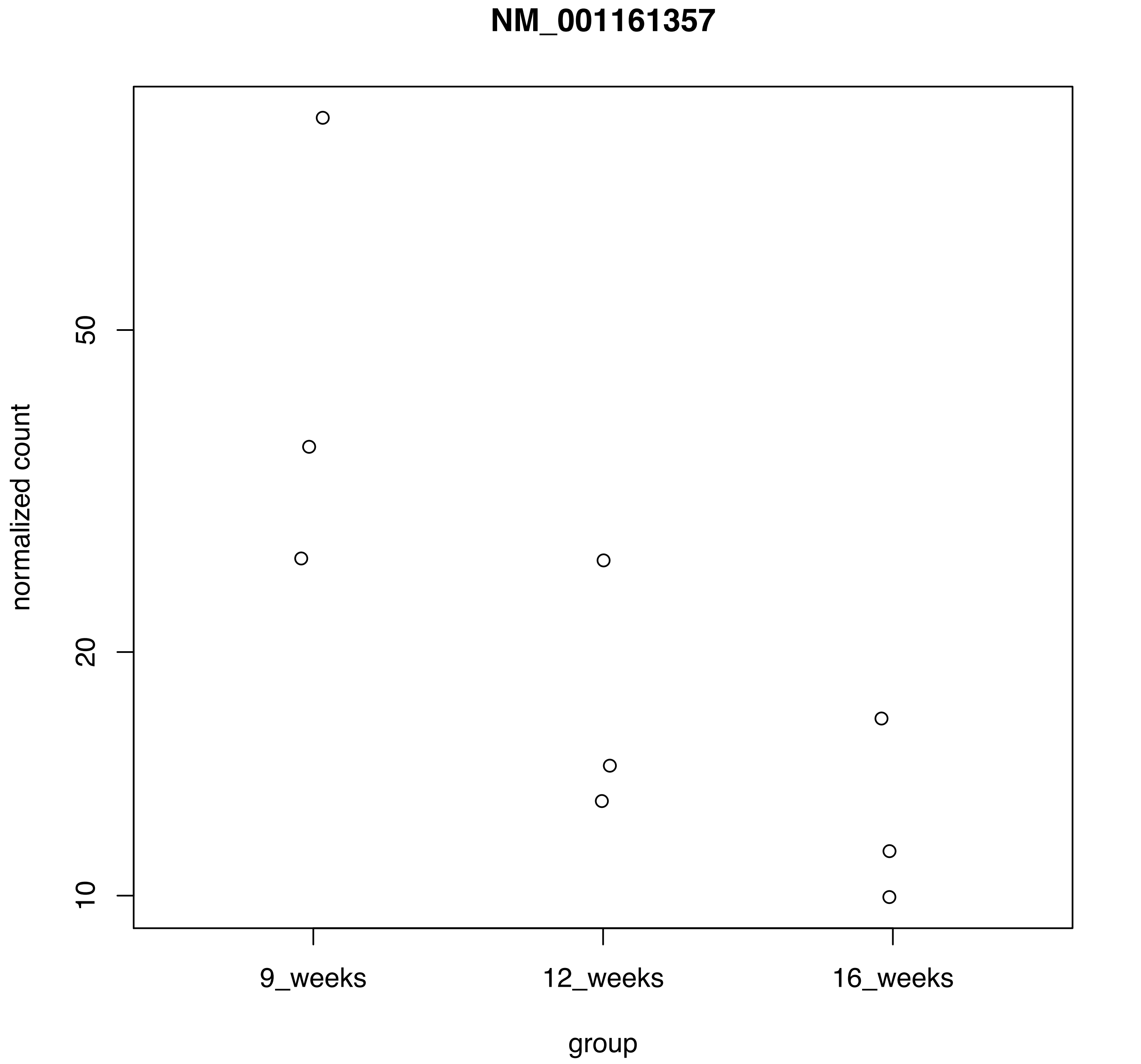

NM_001161357
normalized count
50
20
10
9_weeks
12_weeks
16_weeks
group

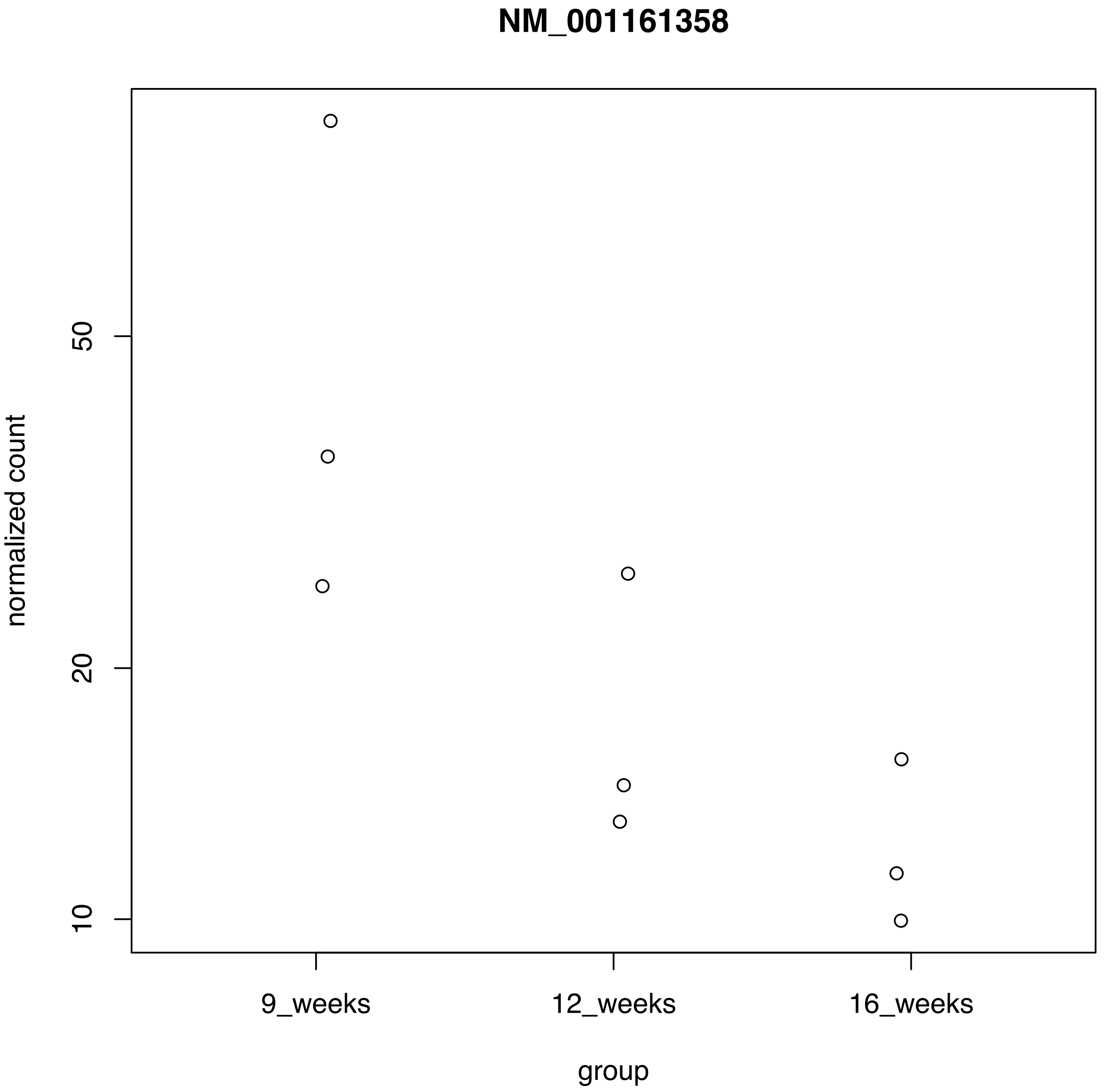

NM_001161358
normalized count
50
20
10
9_weeks
12_weeks
16_weeks
group

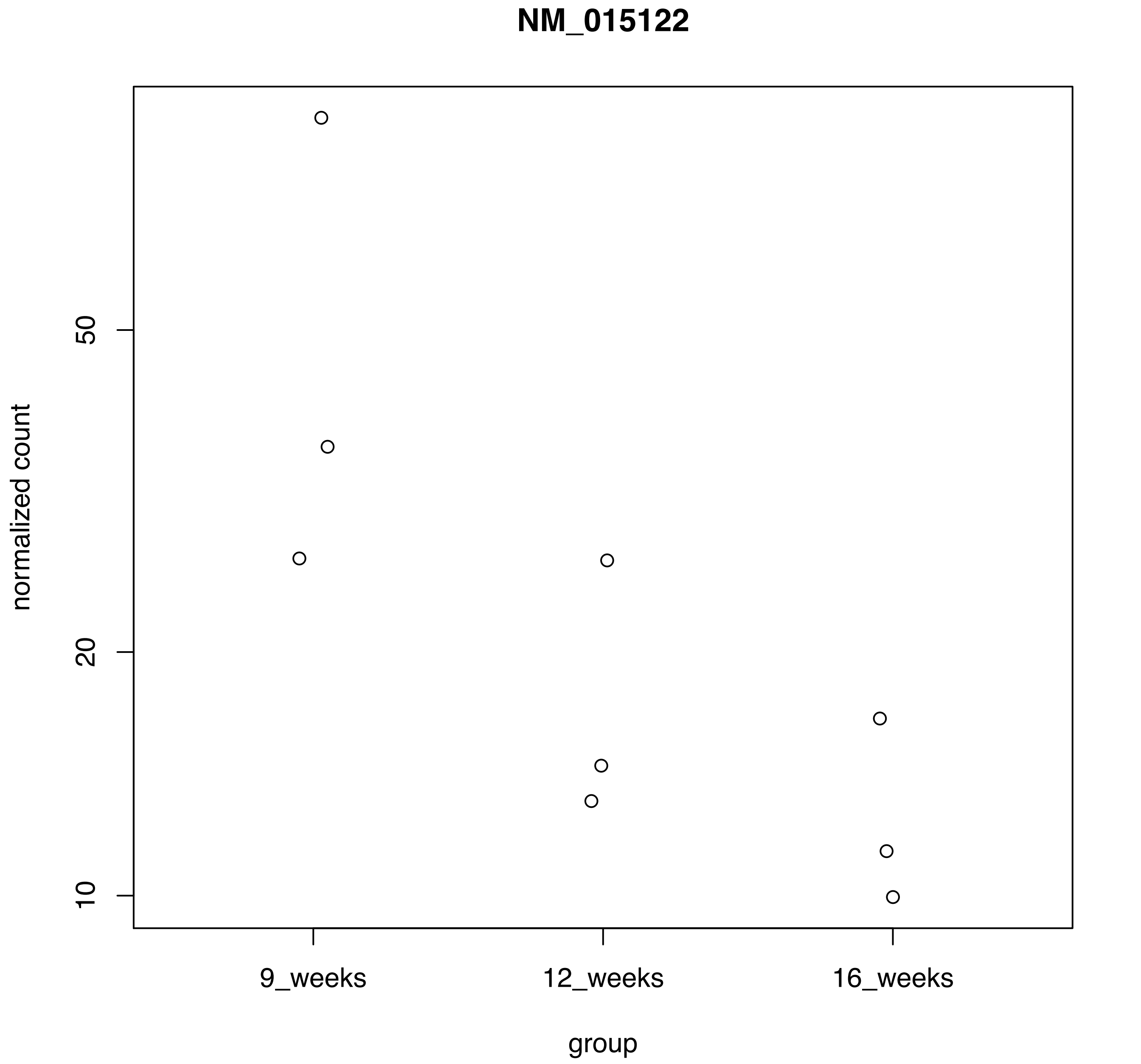
NM_015122
normalized count
50
20
10
9_weeks
12_weeks
16_weeks
group

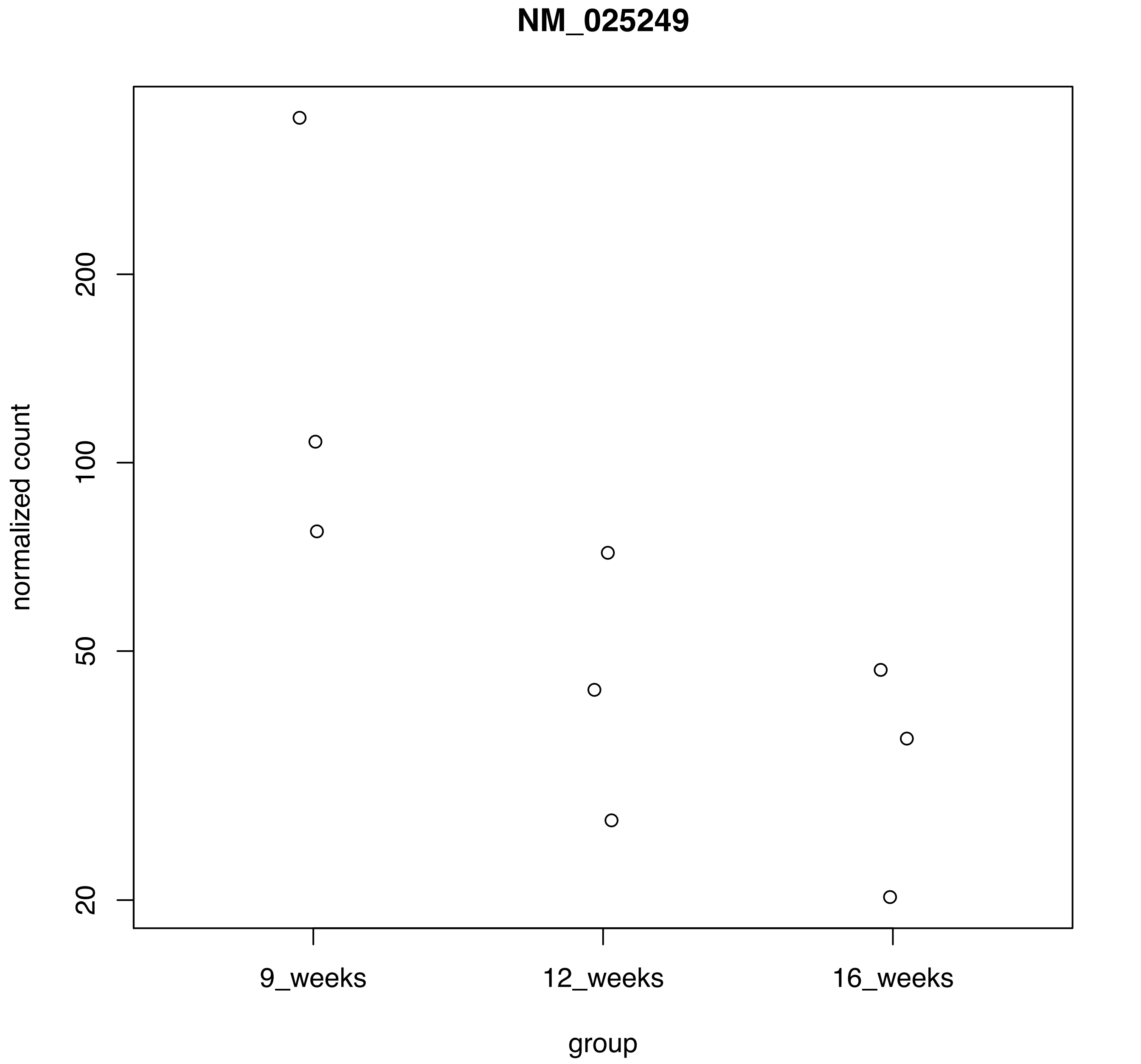

NM_025249
normalized count
200
100
50
20
9_weeks
12_weeks
16_weeks
group

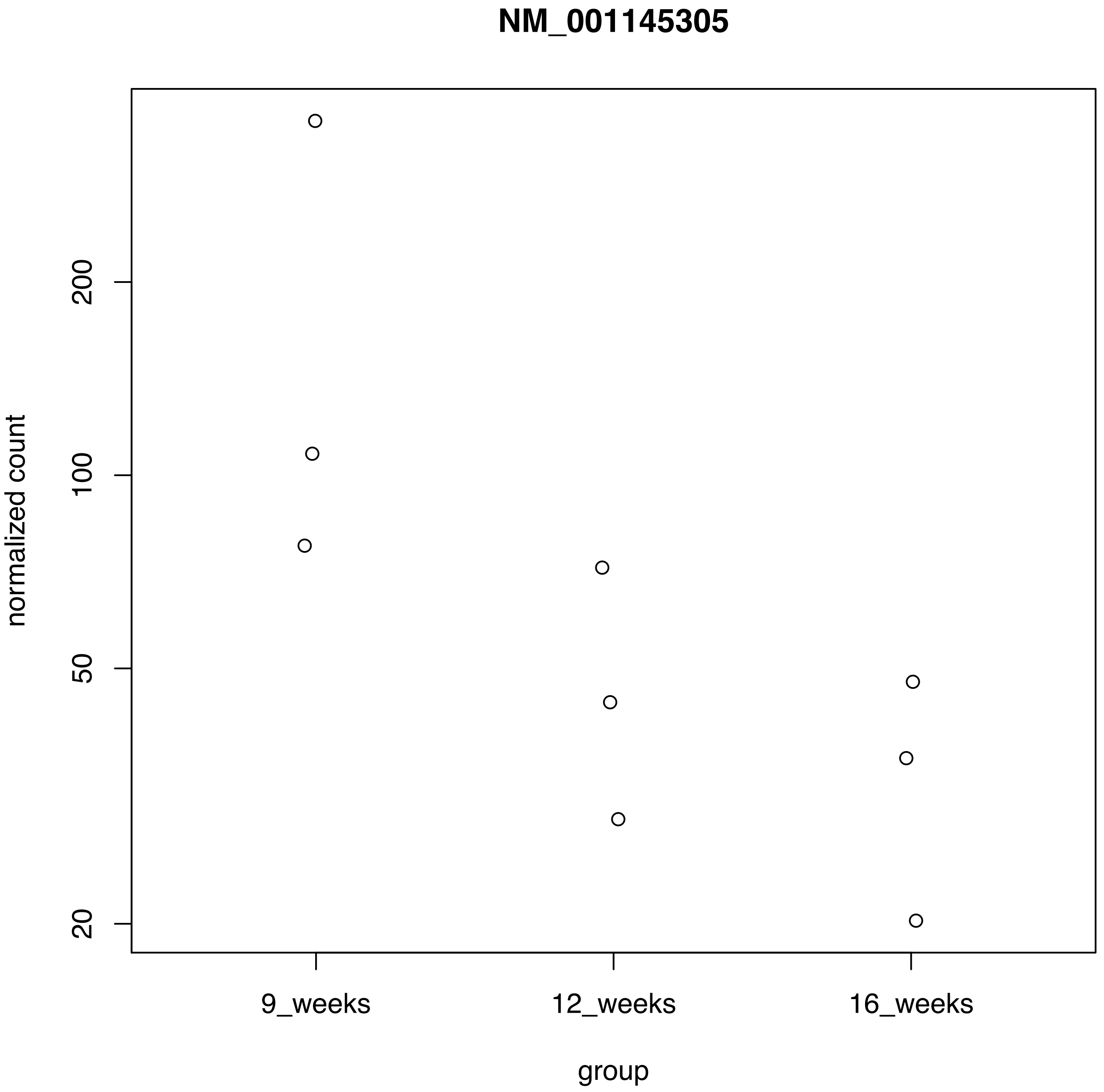
NM_001145305
normalized count
200
100
50
20
9_weeks
12_weeks
16_weeks
group

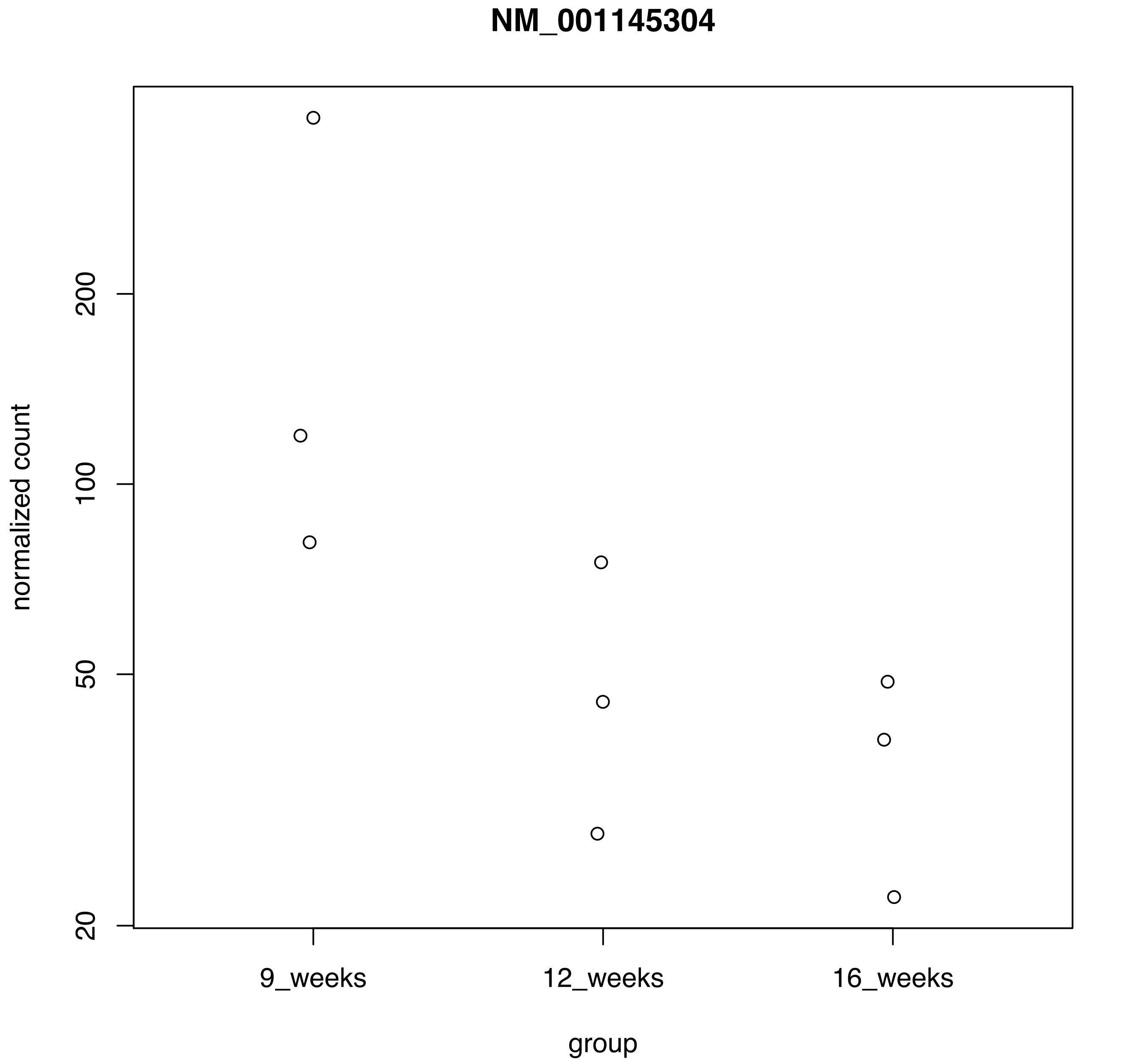
NM_001145304
normalized count
200
100
50
20
9_weeks
12_weeks
16_weeks
group

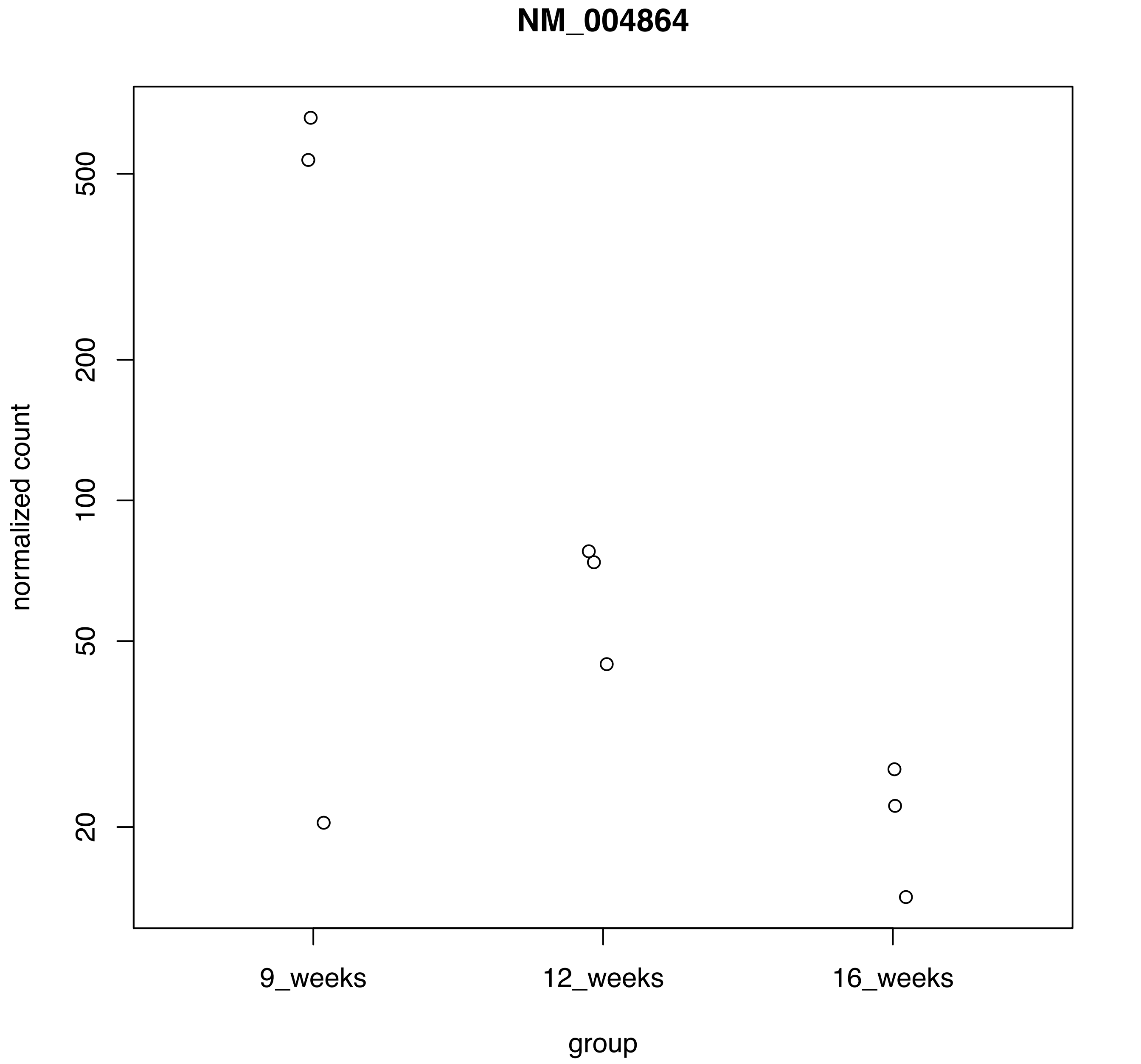
NM_004864
normalized count
500
200
100
50
20
9_weeks
12_weeks
16_weeks
group

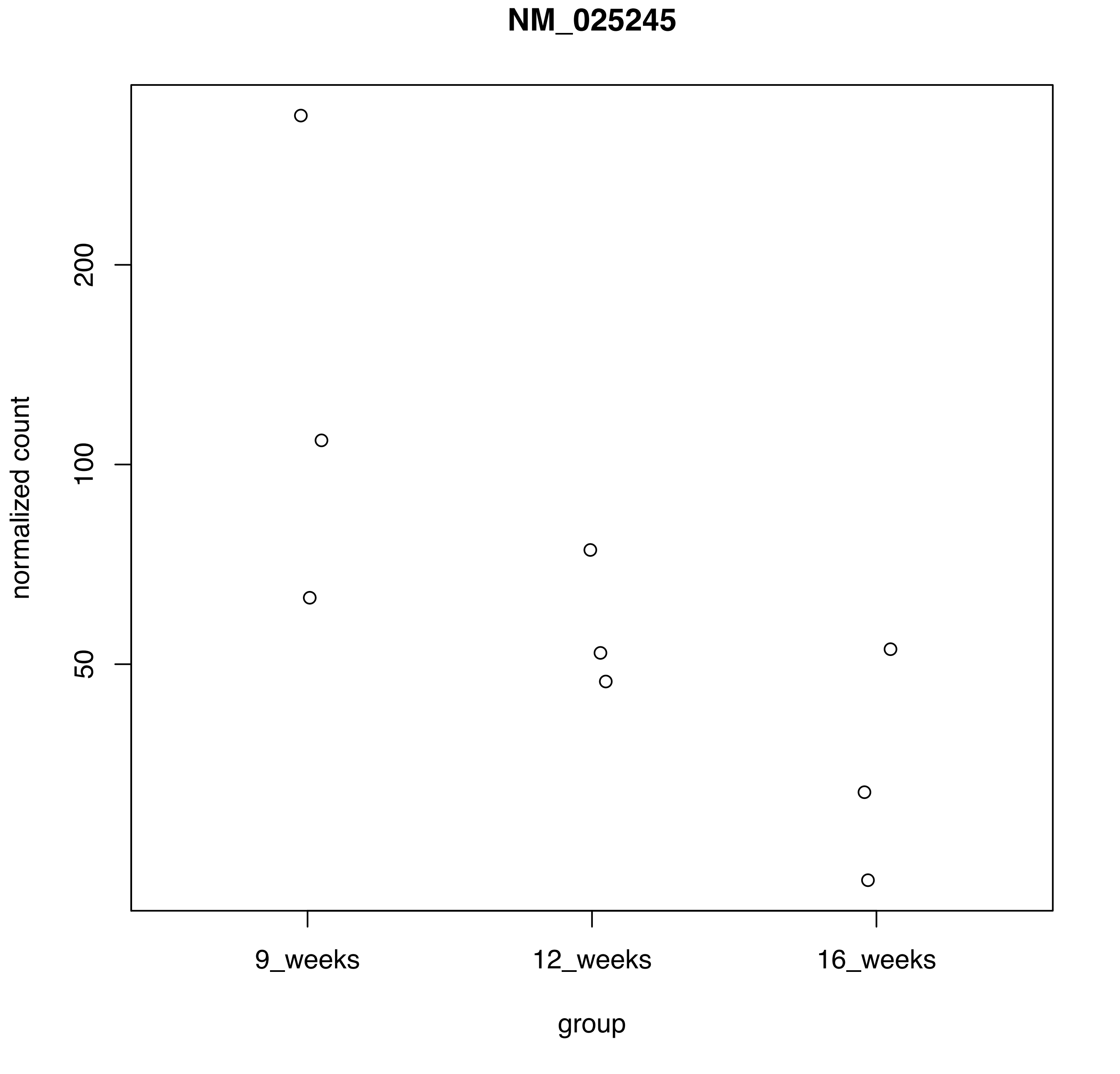
NM_025245
normalized count
200
100
50
9_weeks
12_weeks
16_weeks
group

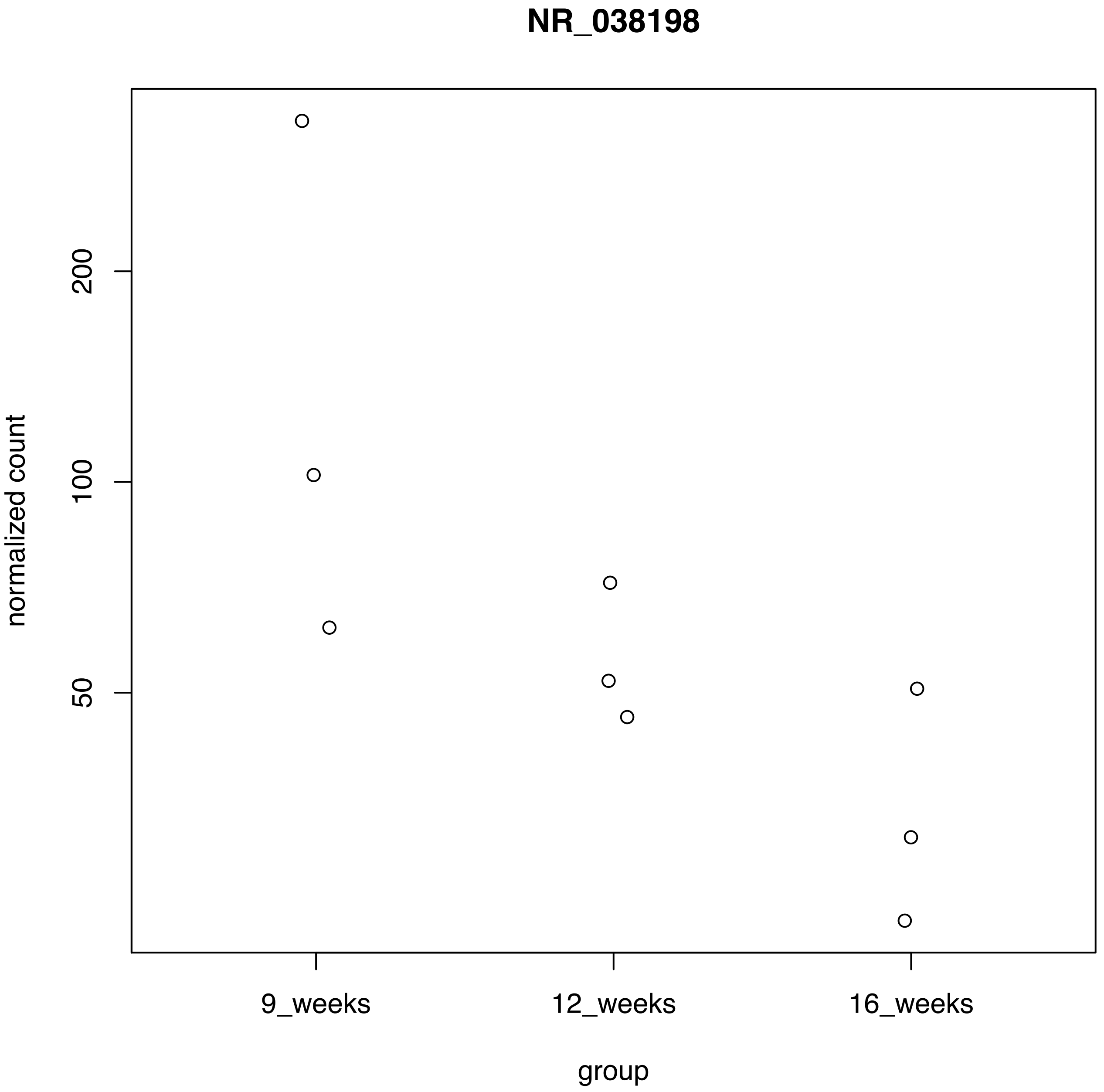
NR_038198
200
100
50
normalized count
9_weeks
12_weeks
16_weeks
group

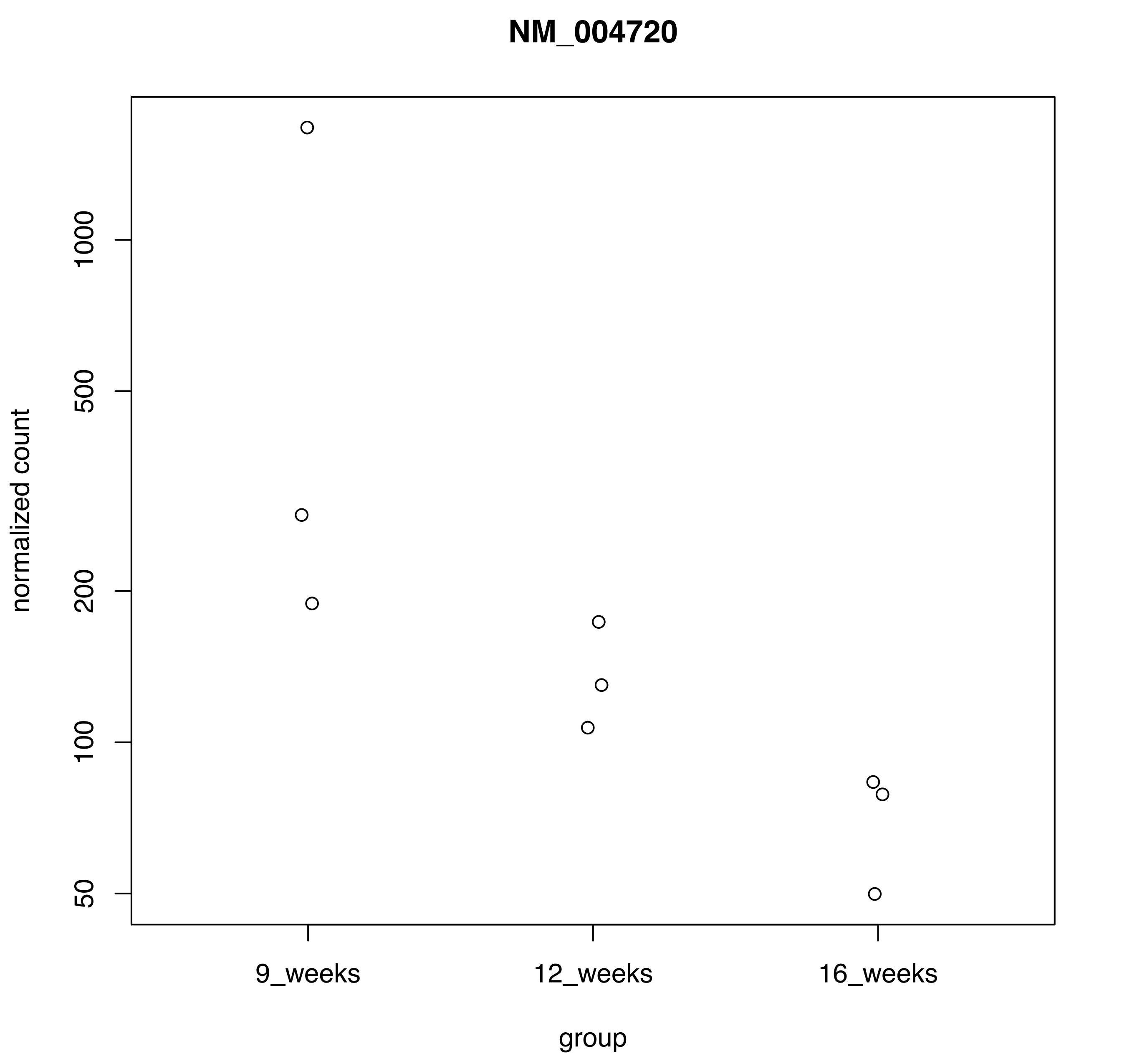

NM_004720
normalized count
1000
500
200
100
50
9_weeks
12_weeks
16_weeks
group

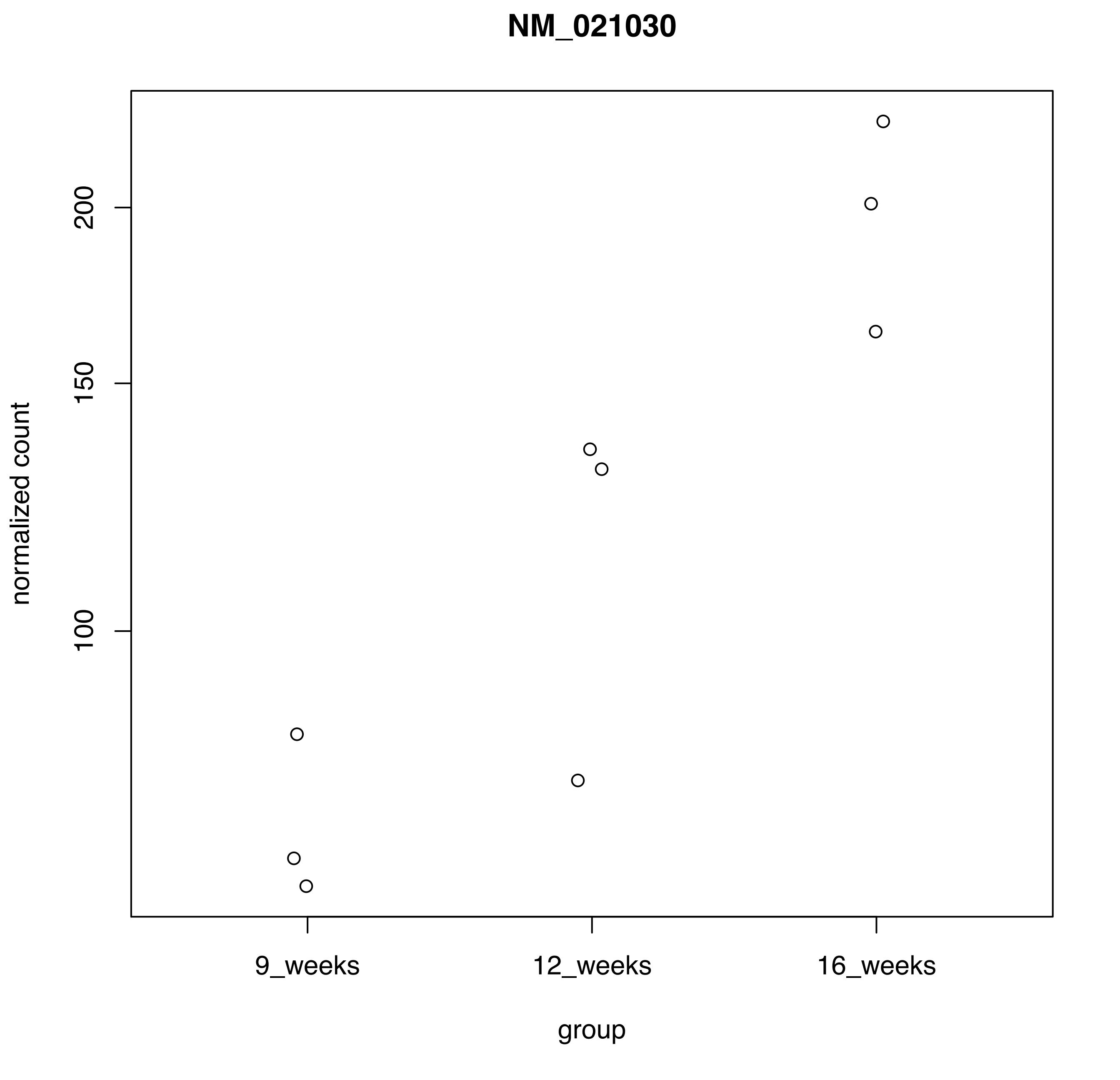
NM_021030
normalized count
200
150
100
9_weeks
12_weeks
16_weeks
group

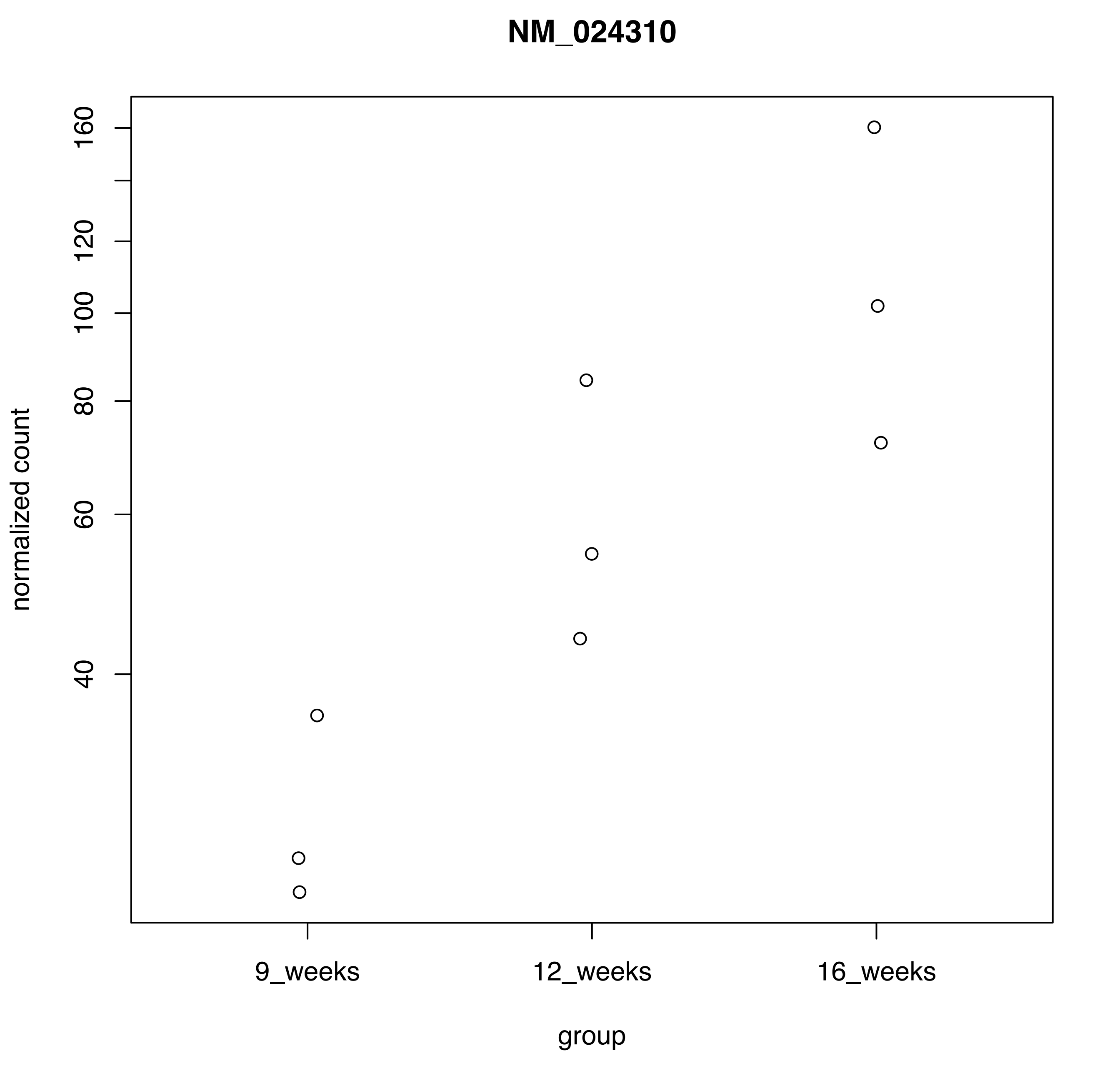
NM_024310
normalized count
160
120
100
80
60
40
9_weeks
12_weeks
16_weeks
group

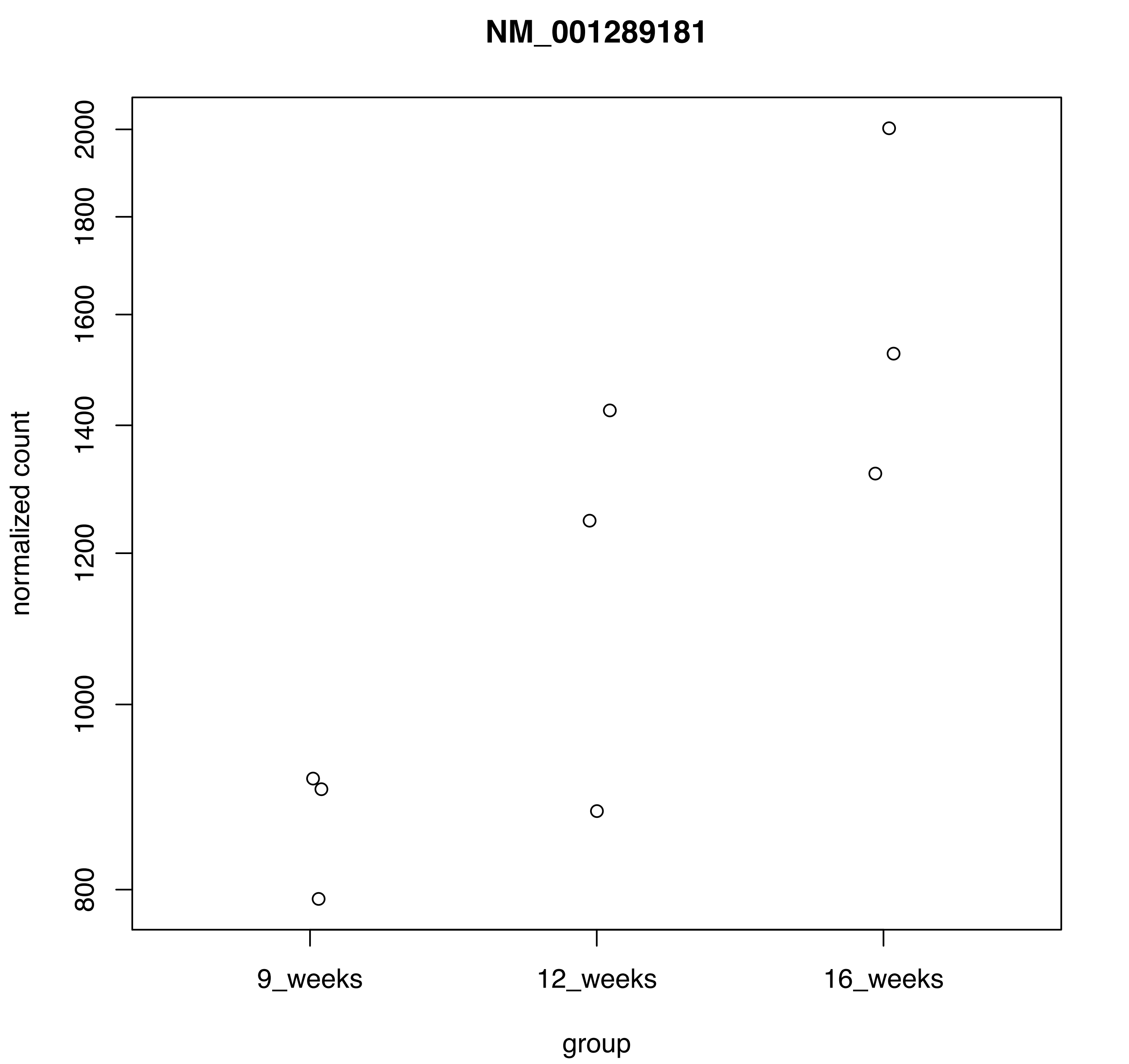
NM_001289181
normalized count
2000
1800
1600
1400
1200
1000
800
9_weeks
12_weeks
16_weeks
group

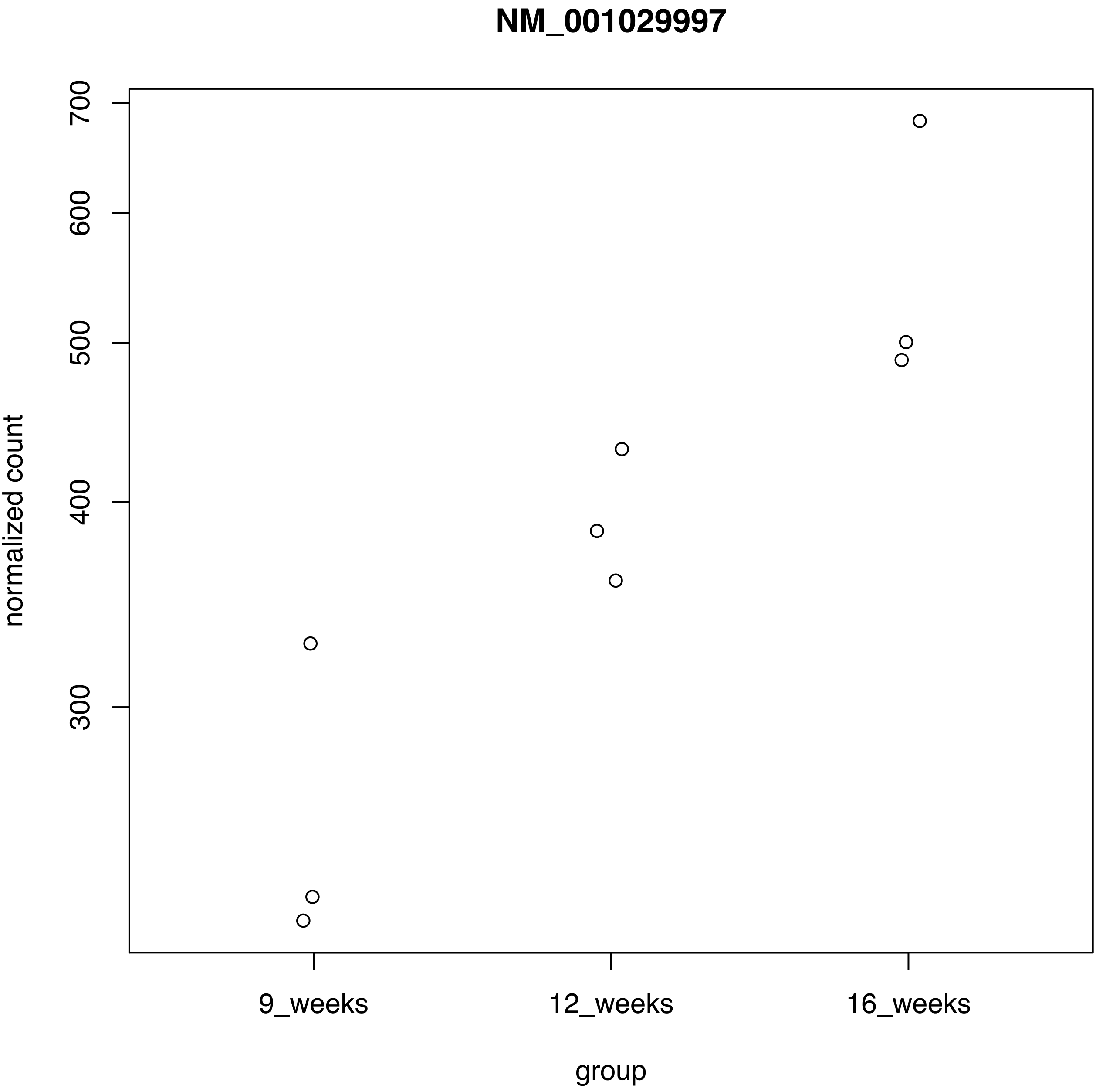
NM_001029997
700
600
500
400
300
normalized count
9_weeks
12_weeks
16_weeks
group

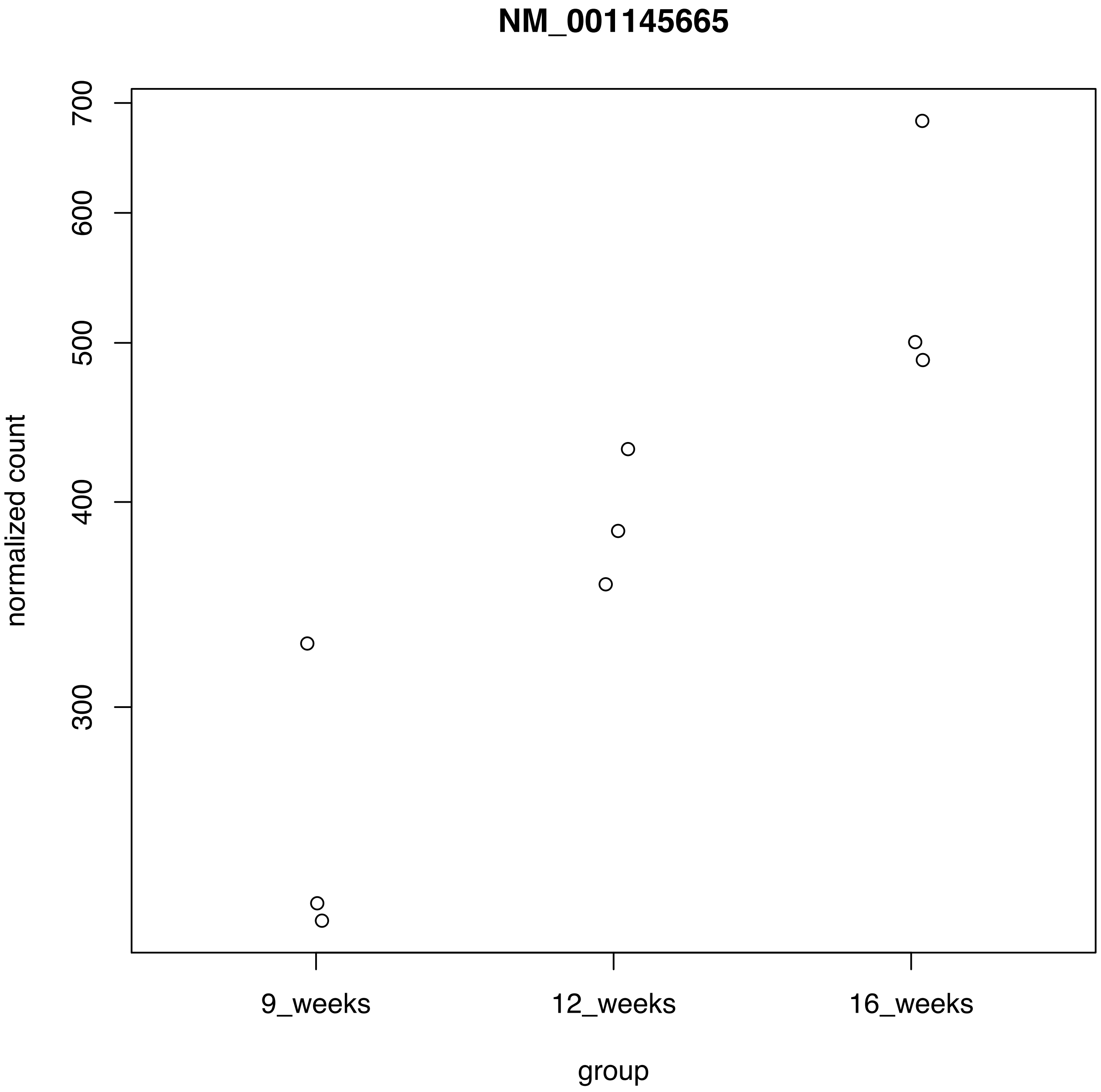
NM_001145665
normalized count
700
600
500
400
300
9_weeks
12_weeks
16_weeks
group

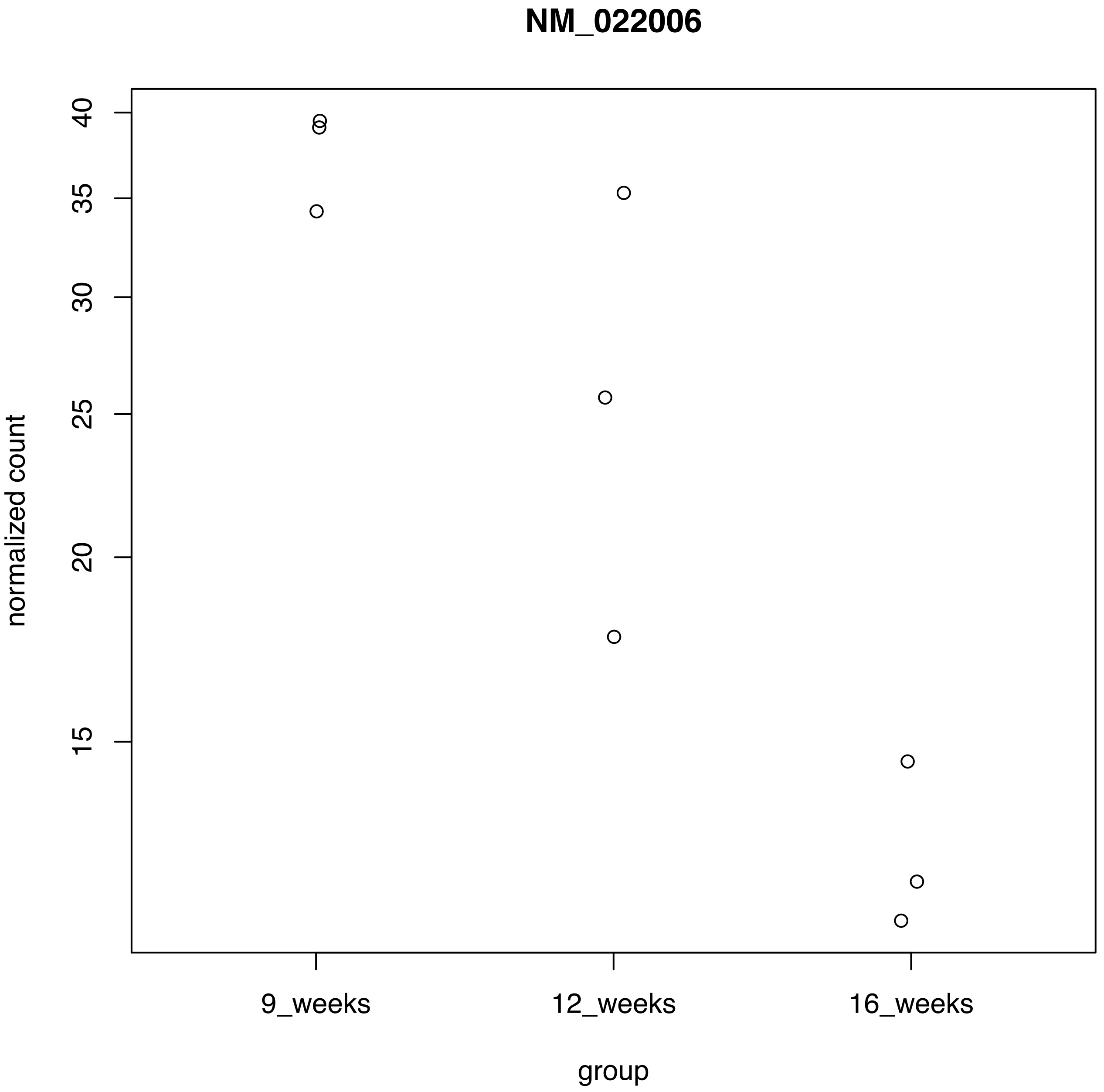
NM_022006
normalized count
40
35
30
25
20
15
9_weeks
12_weeks
16_weeks
group

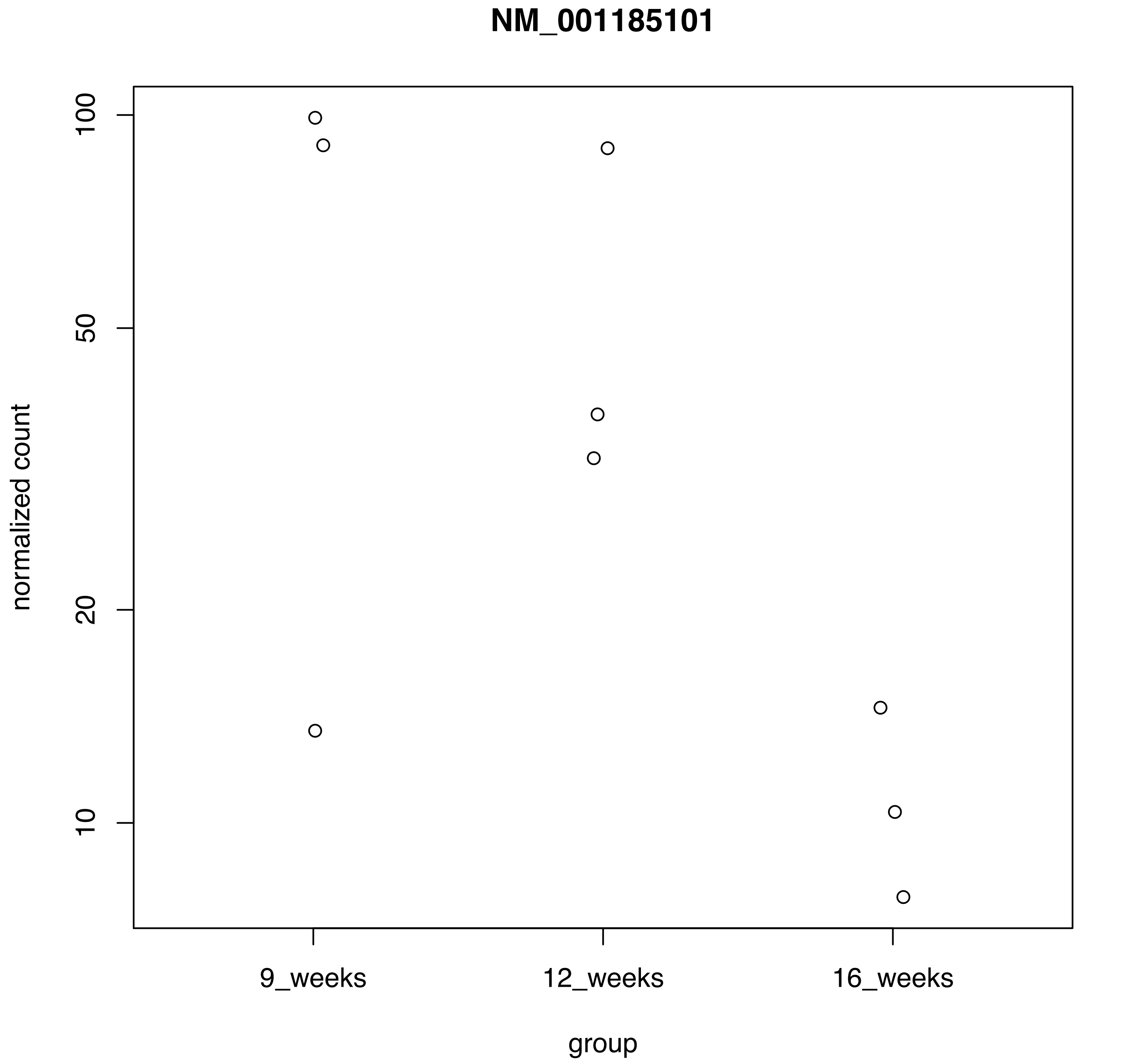
NM_001185101
normalized count
100
50
20
10
9_weeks
12_weeks
16_weeks
group

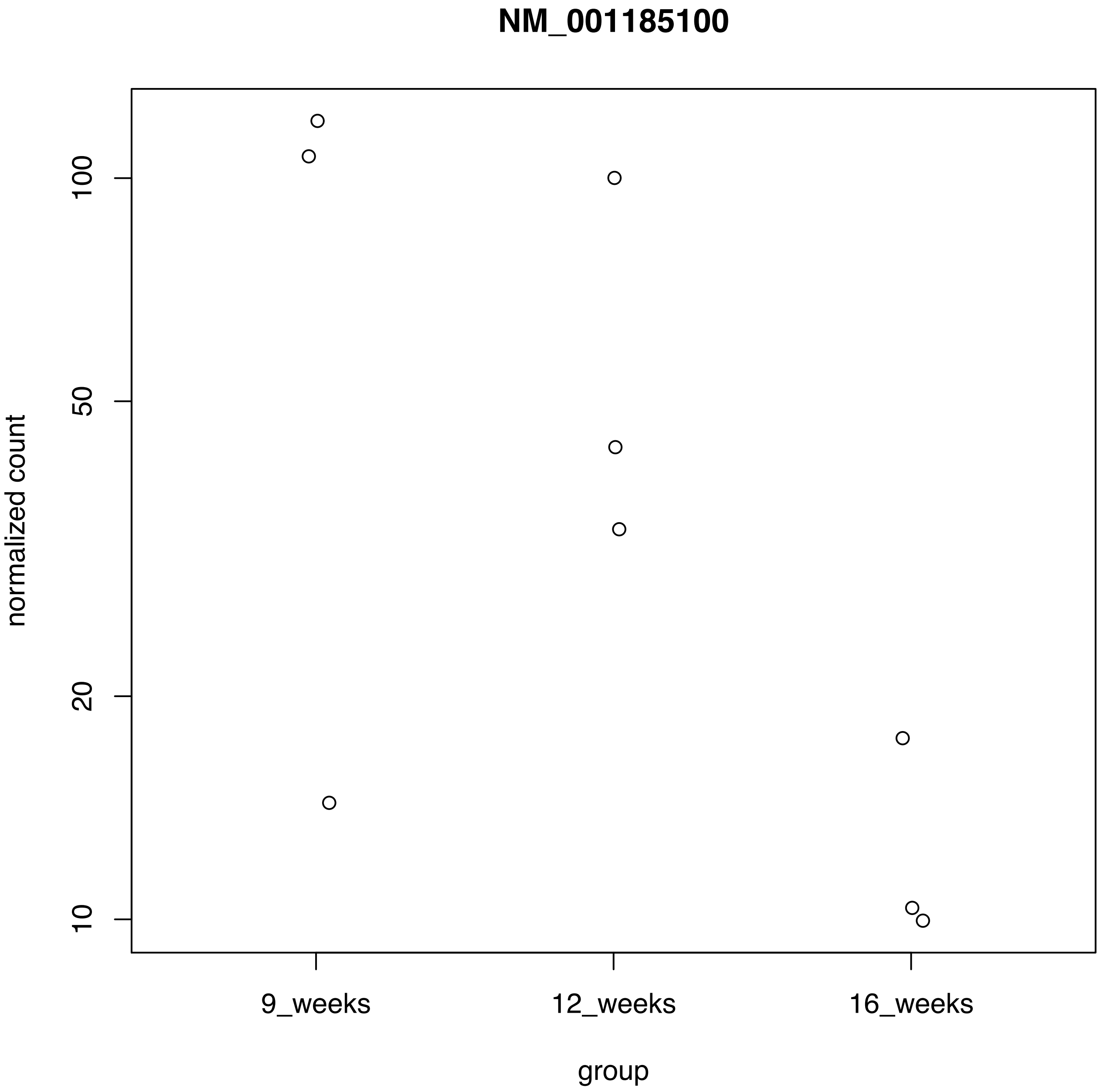
NM_001185100
normalized count
100
50
20
10
9_weeks
12_weeks
16_weeks
group

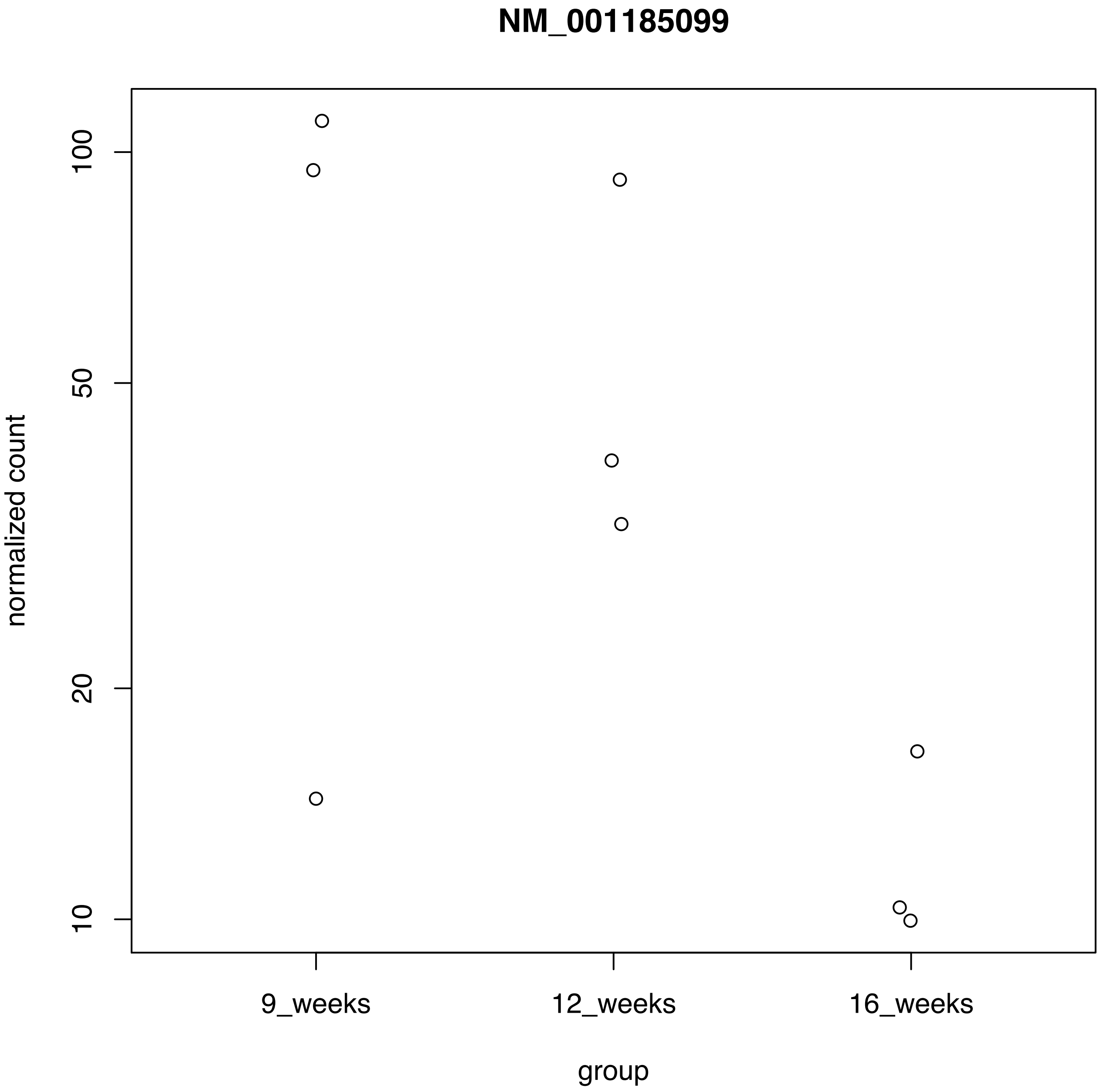
NM_001185099
normalized count
100
50
20
10
9_weeks
12_weeks
16_weeks
group

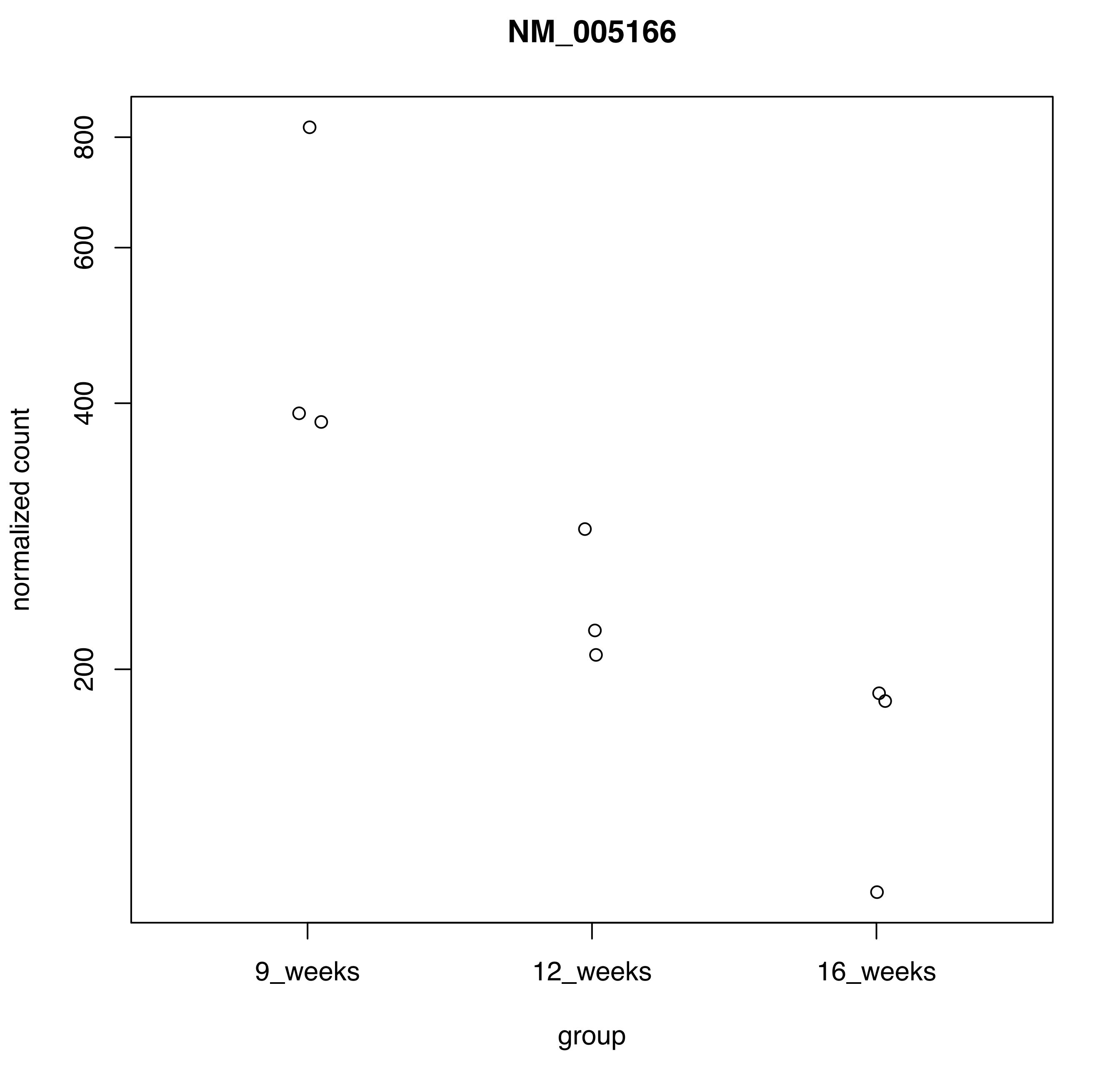

NM_005166
normalized count
800
600
400
200
9_weeks
12_weeks
16_weeks
group

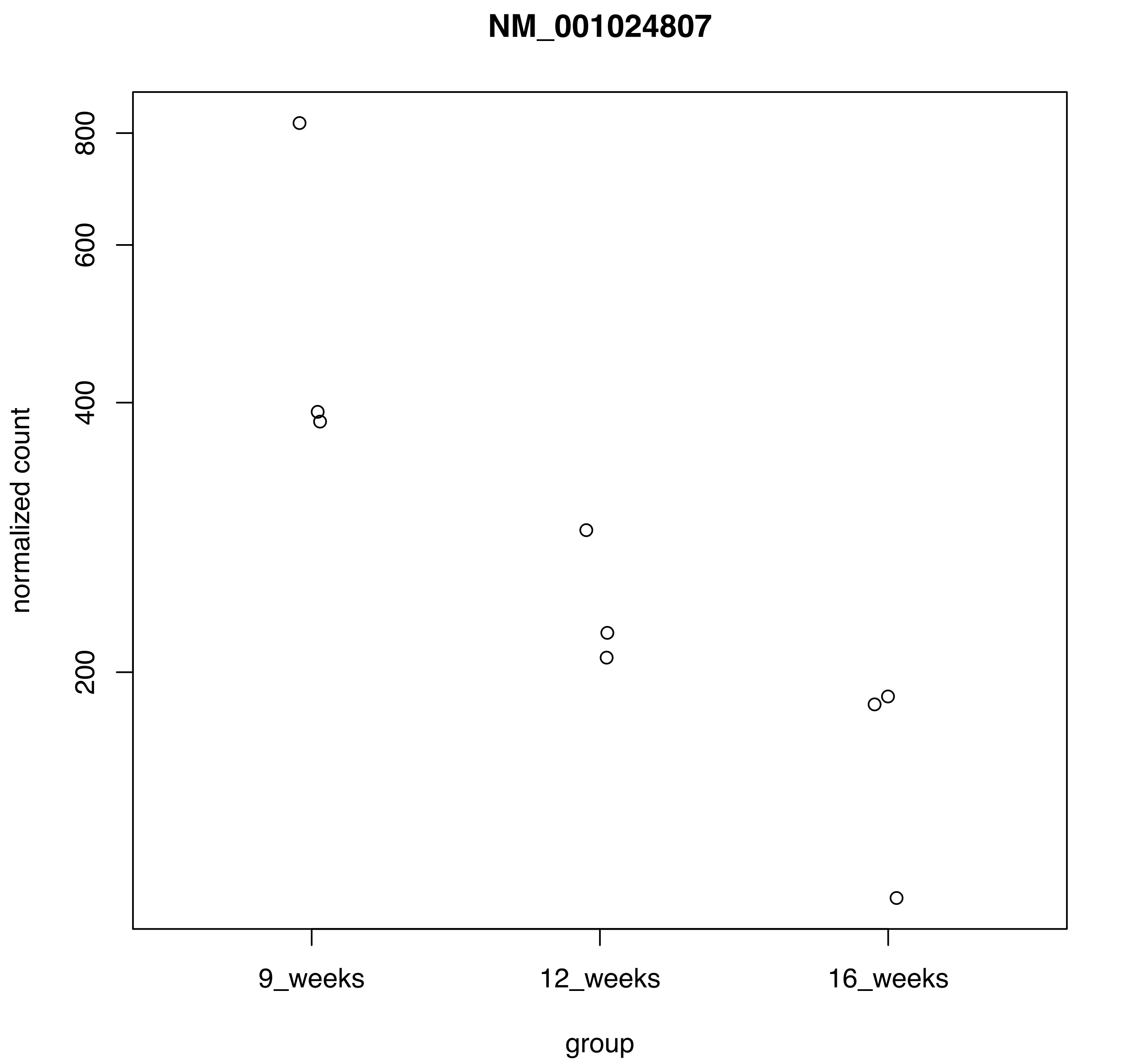
NM_001024807
normalized count
800
600
400
200
9_weeks
12_weeks
16_weeks
group

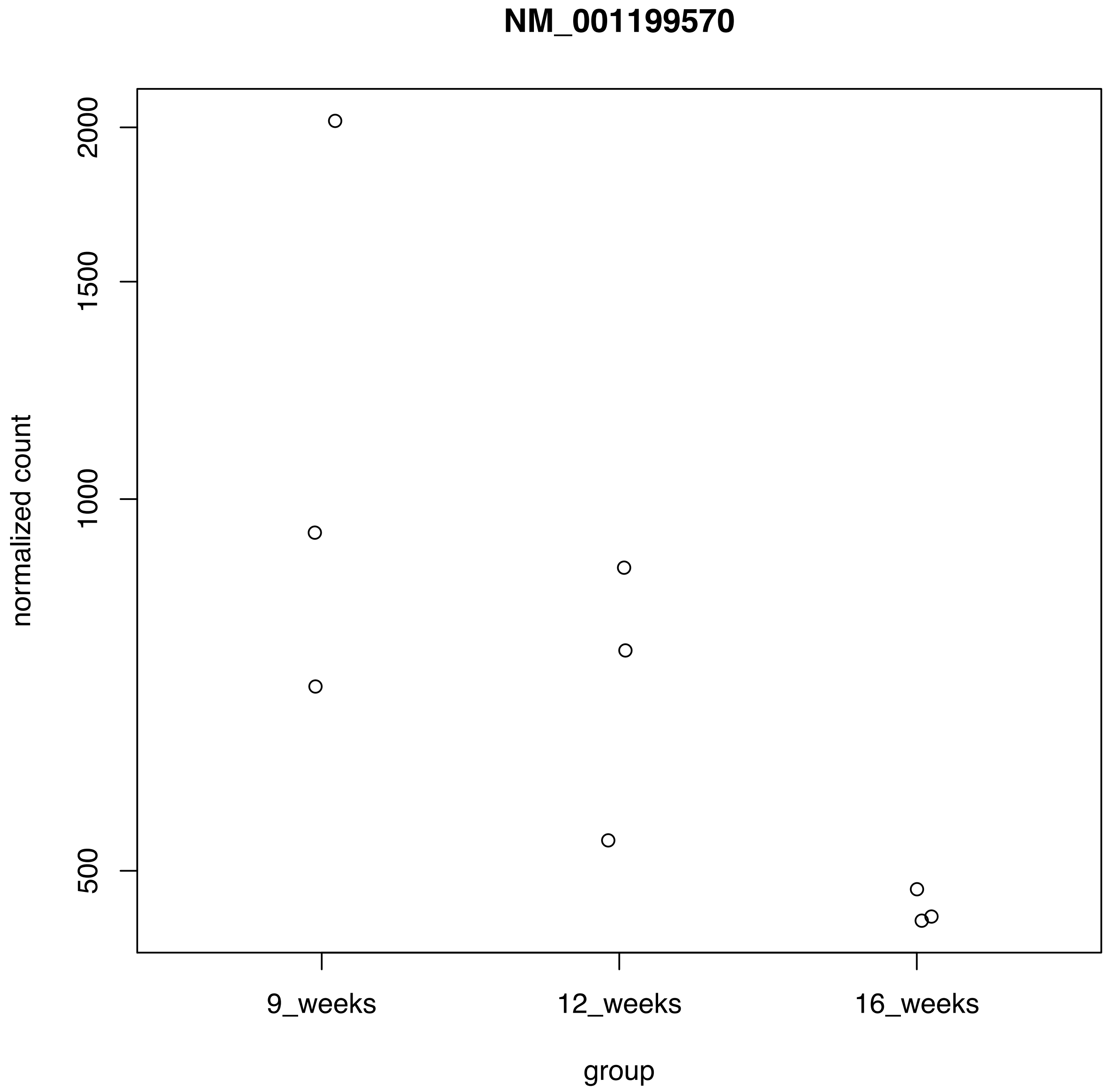
NM_001199570
2000
1500
1000
500
normalized count
9_weeks
12_weeks
16_weeks
group

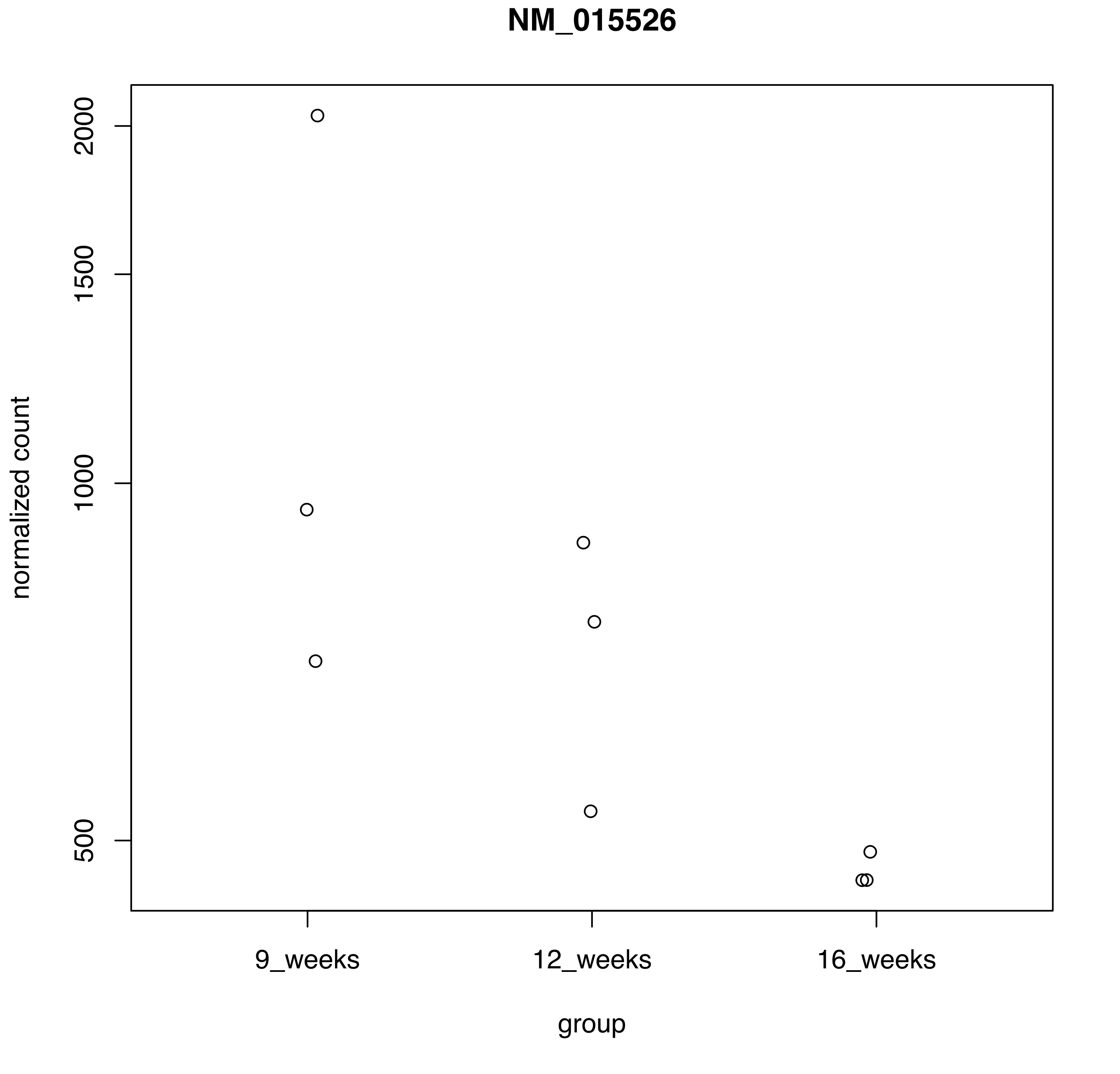
NM_015526
normalized count
2000
1500
1000
500
9_weeks
12_weeks
16_weeks
group

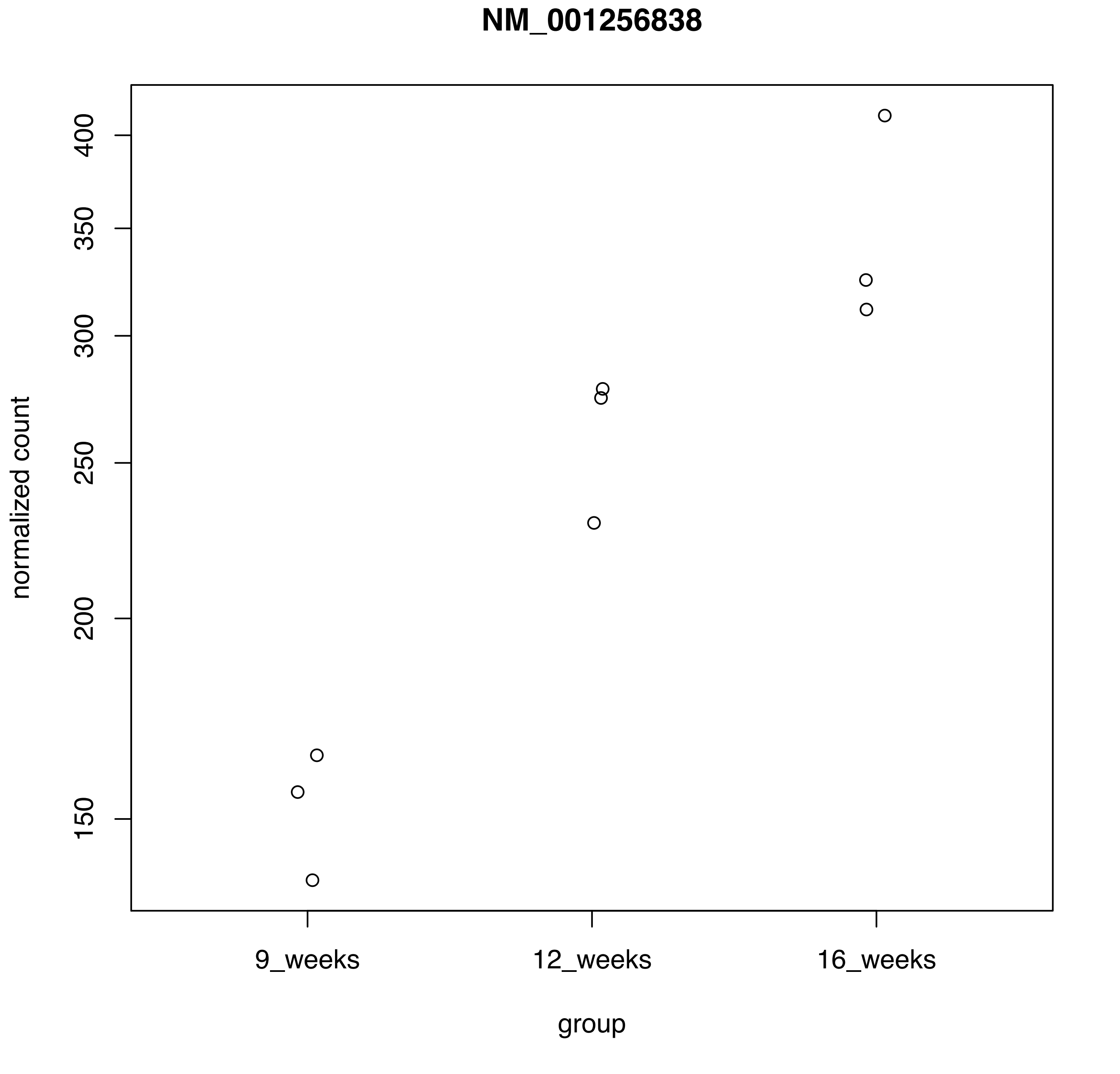
NM_001256838
normalized count
400
350
300
250
200
150
9_weeks
12_weeks
16_weeks
group

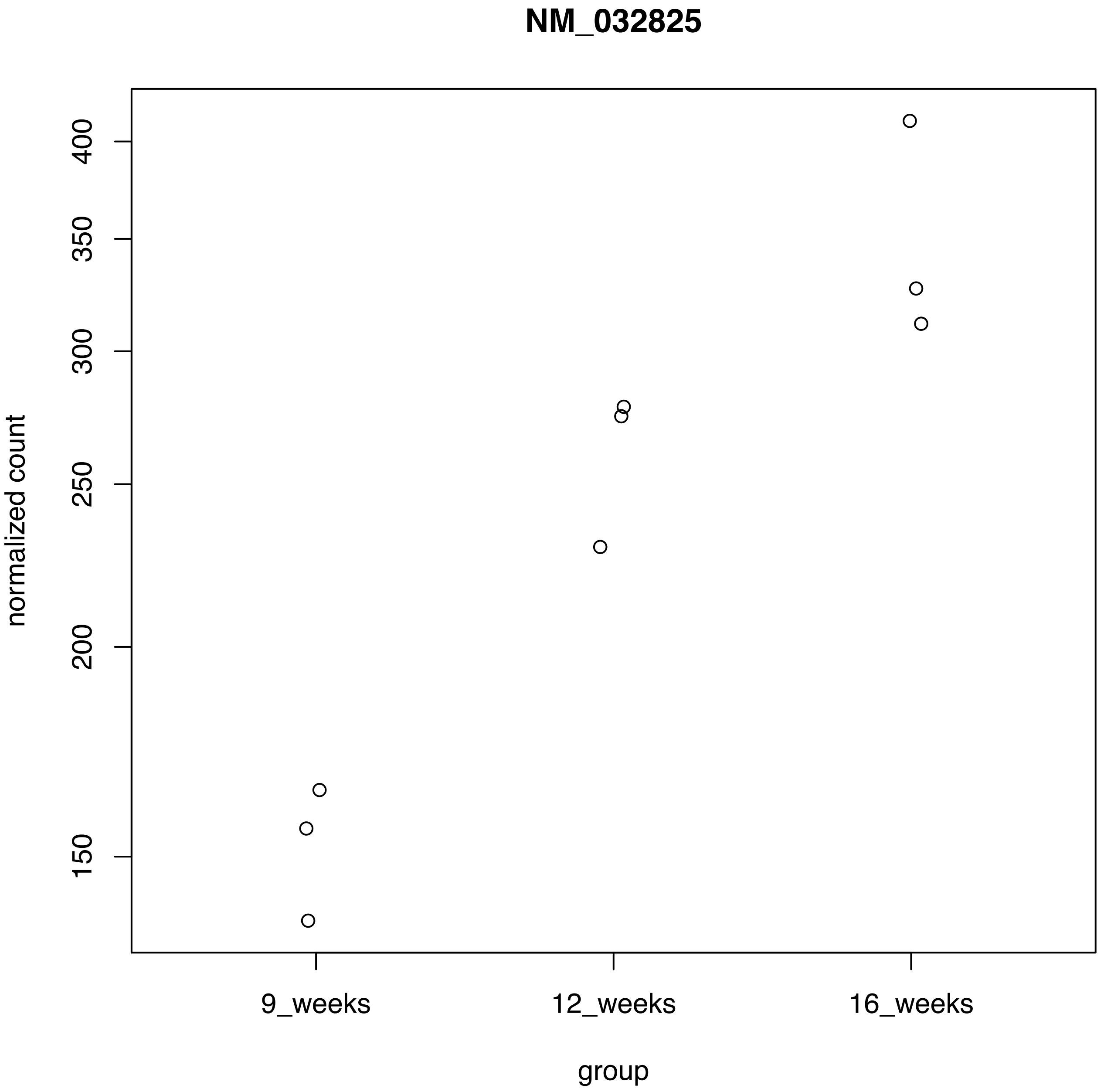
NM_032825
normalized count
400
350
300
250
200
150
9_weeks
12_weeks
16_weeks
group

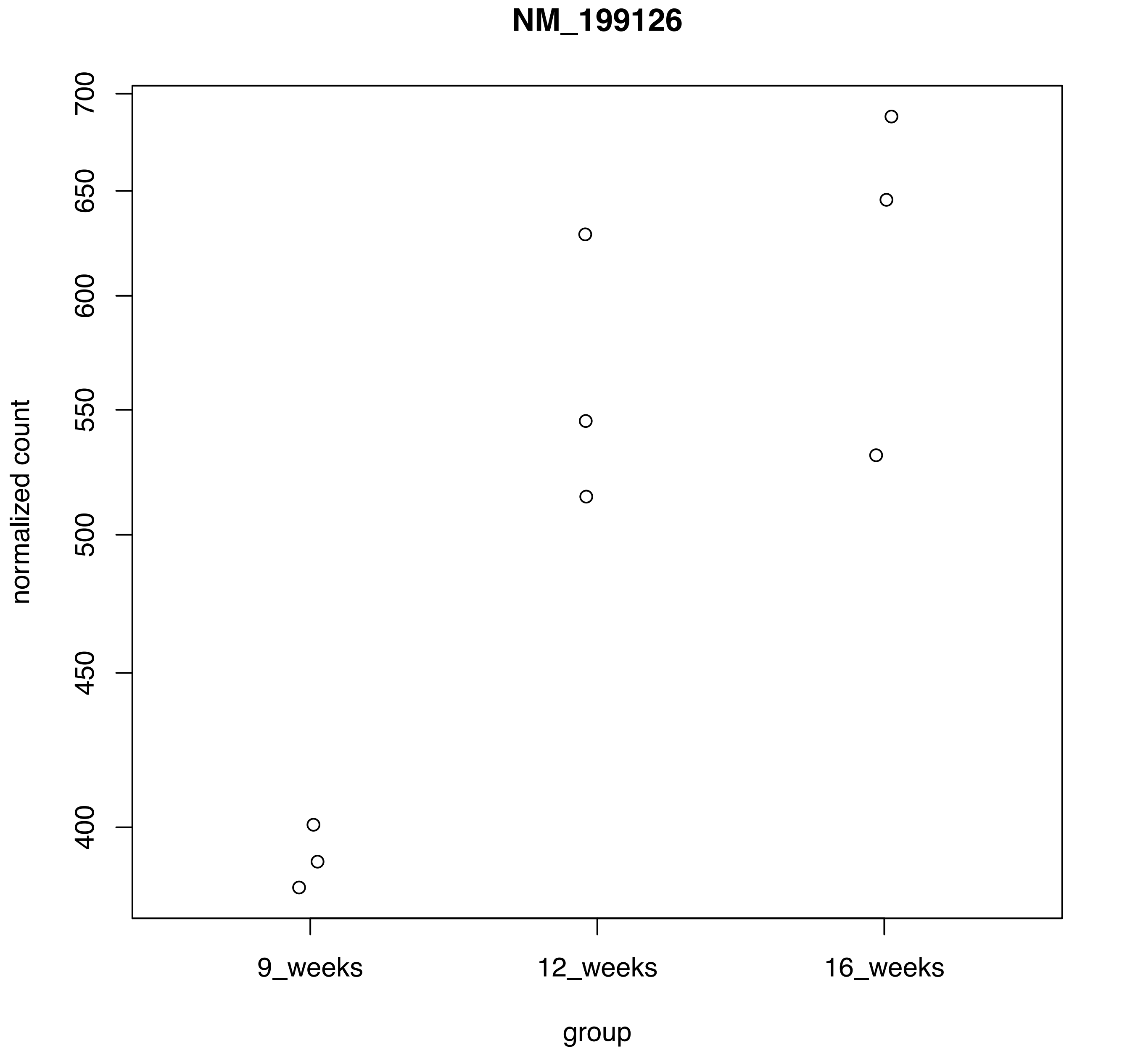
NM_199126
normalized count
700
650
600
550
500
450
400
9_weeks
12_weeks
16_weeks
group

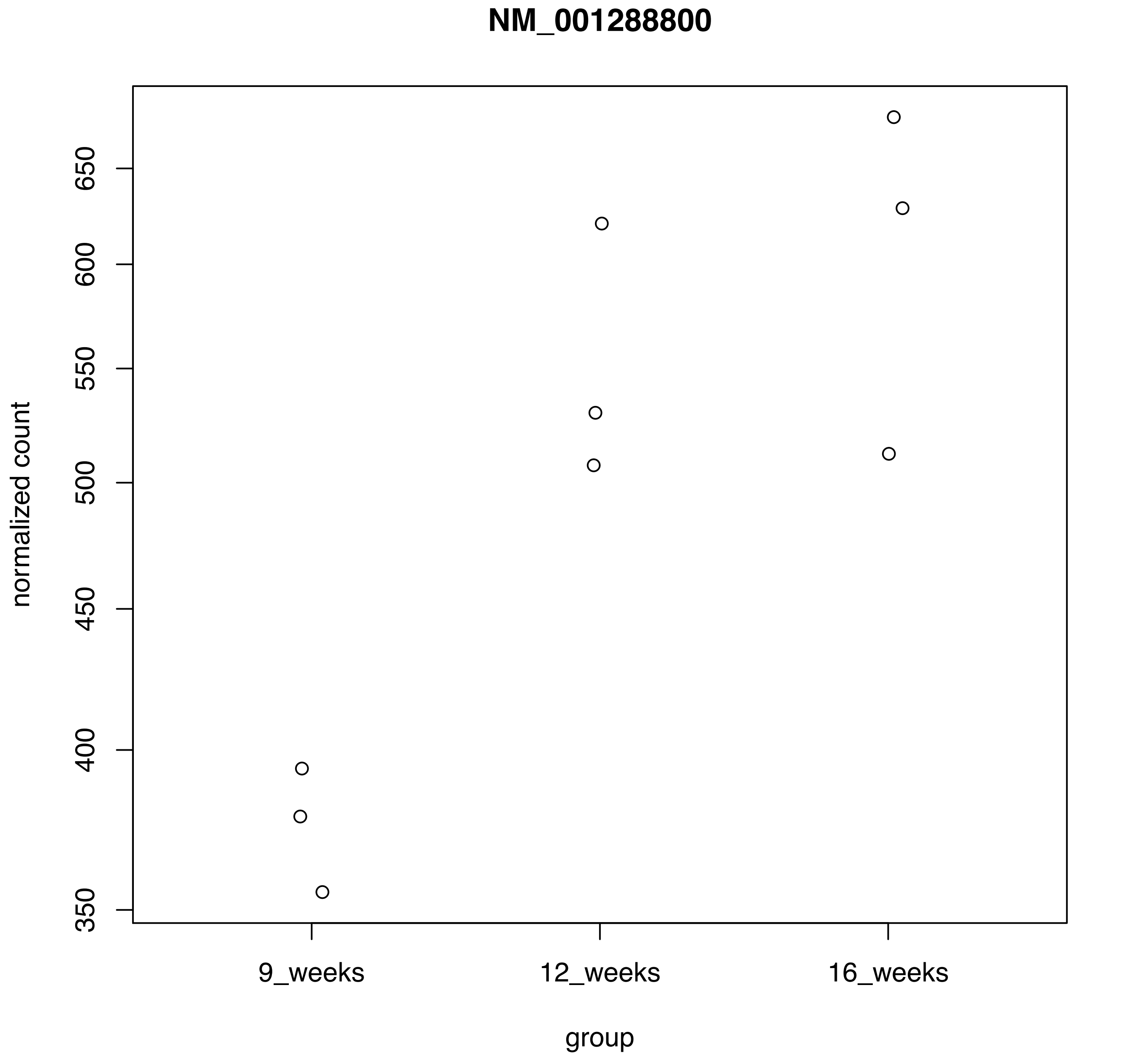
NM_001288800
normalized count
650
600
550
500
450
400
350
9_weeks
12_weeks
16_weeks
group

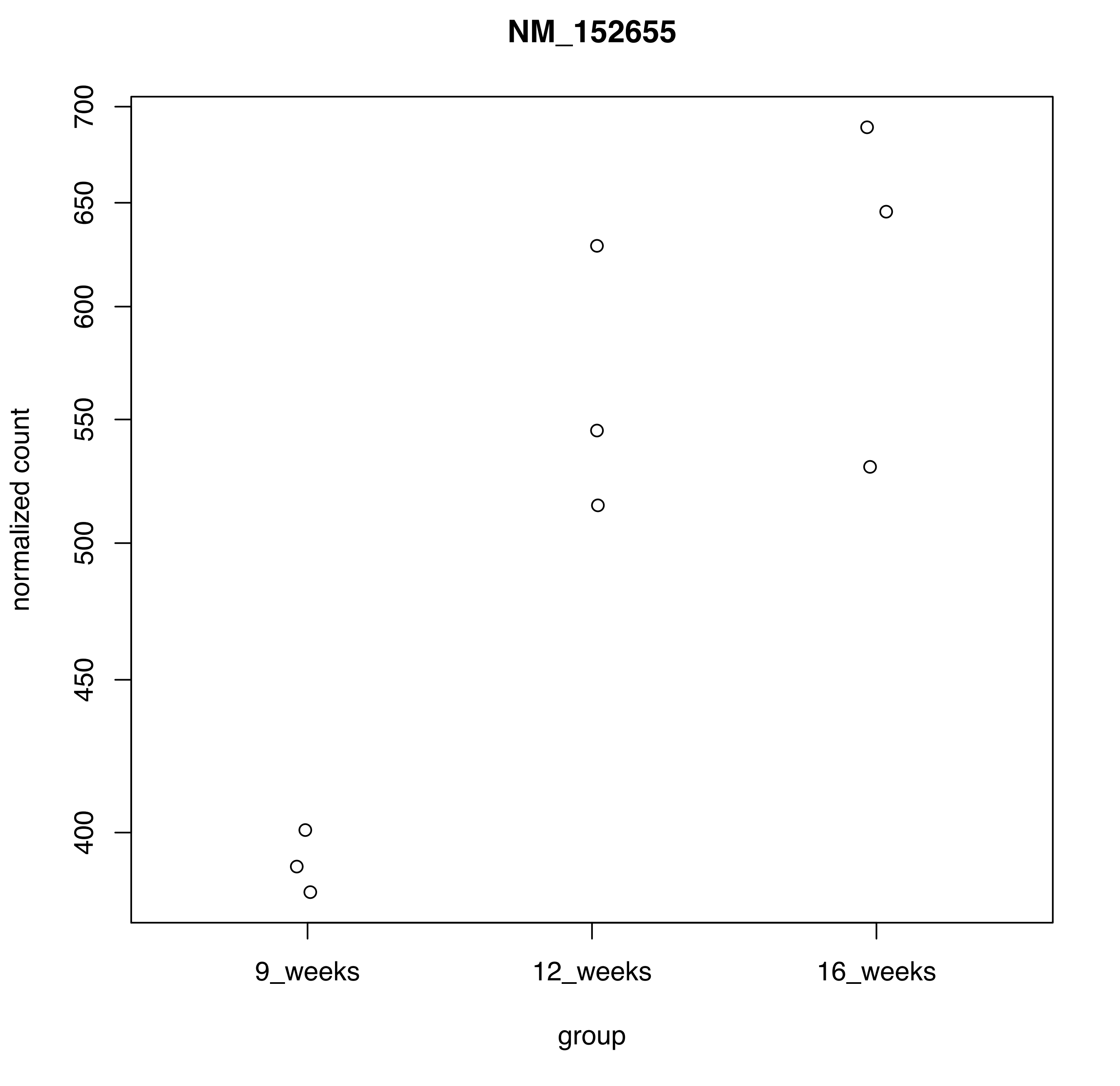
NM_152655
normalized count
700
650
600
550
500
450
400
9_weeks
12_weeks
16_weeks
group

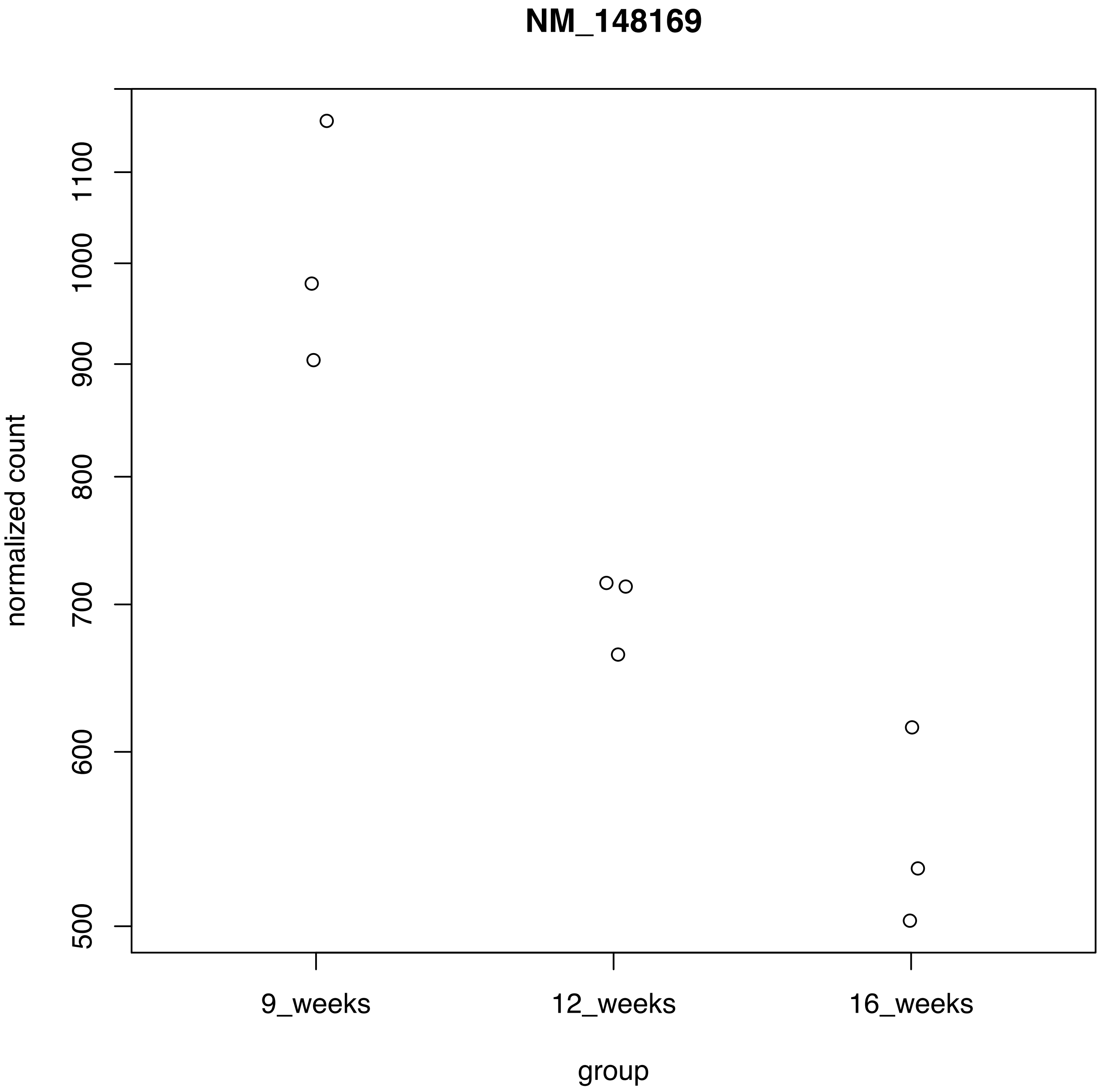
NM_148169
normalized count
500
600
700
800
900
1000
1100
9_weeks
12_weeks
16_weeks
group

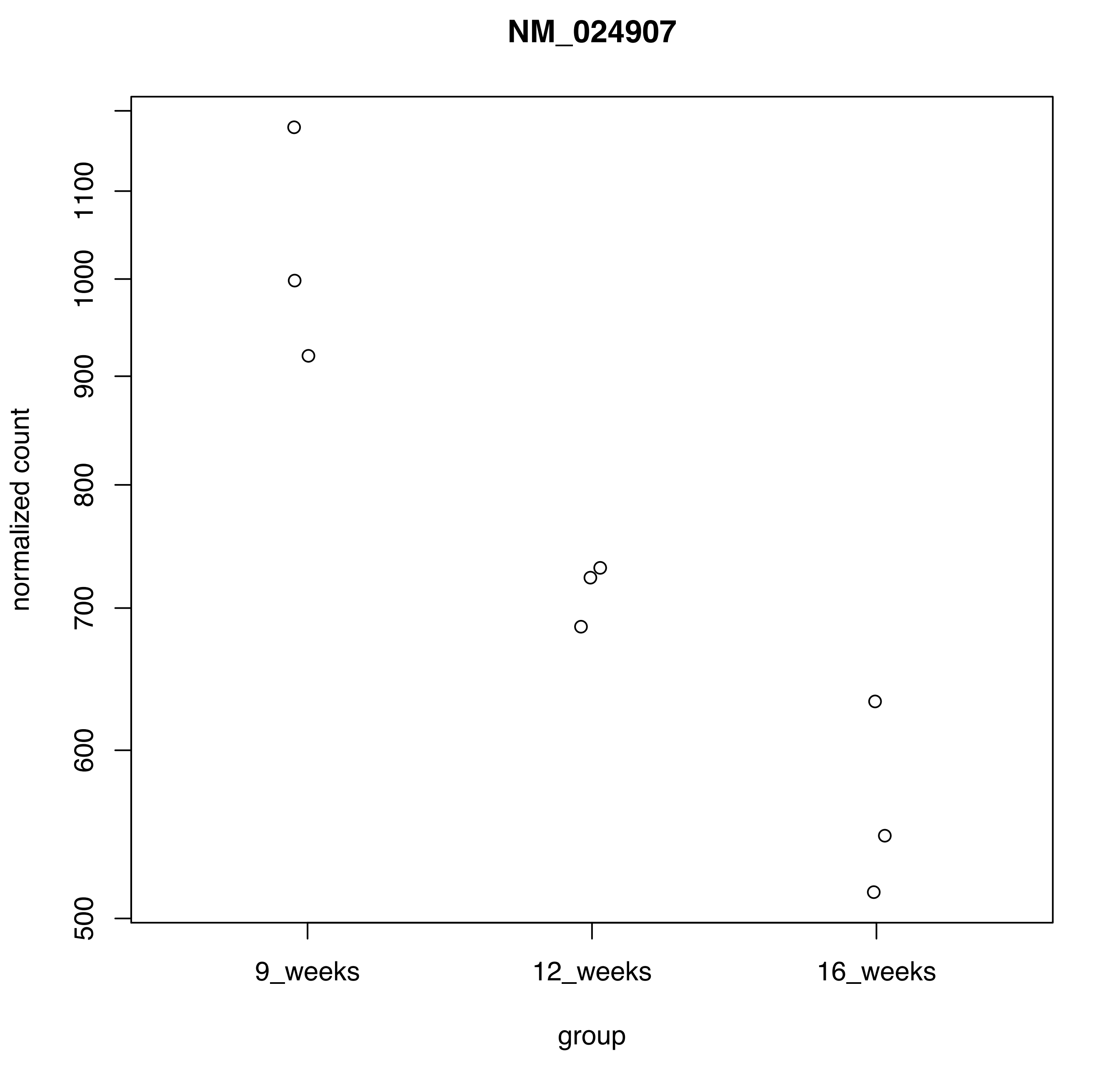
NM_024907
normalized count
500
600
700
800
900
1000
1100
9_weeks
12_weeks
16_weeks
group

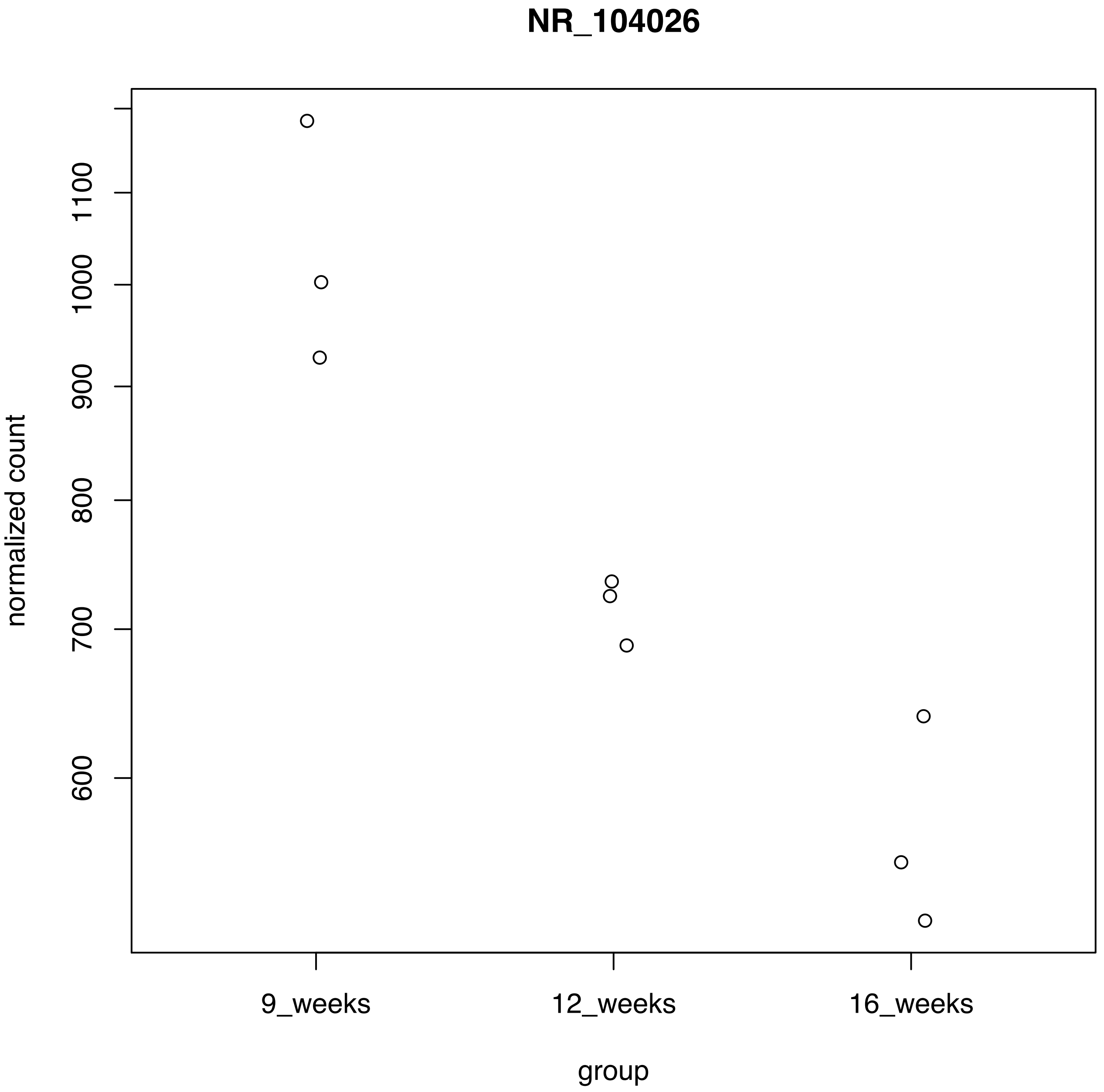
NR_104026
normalized count
600
700
800
900
1000
1100
9_weeks
12_weeks
16_weeks
group

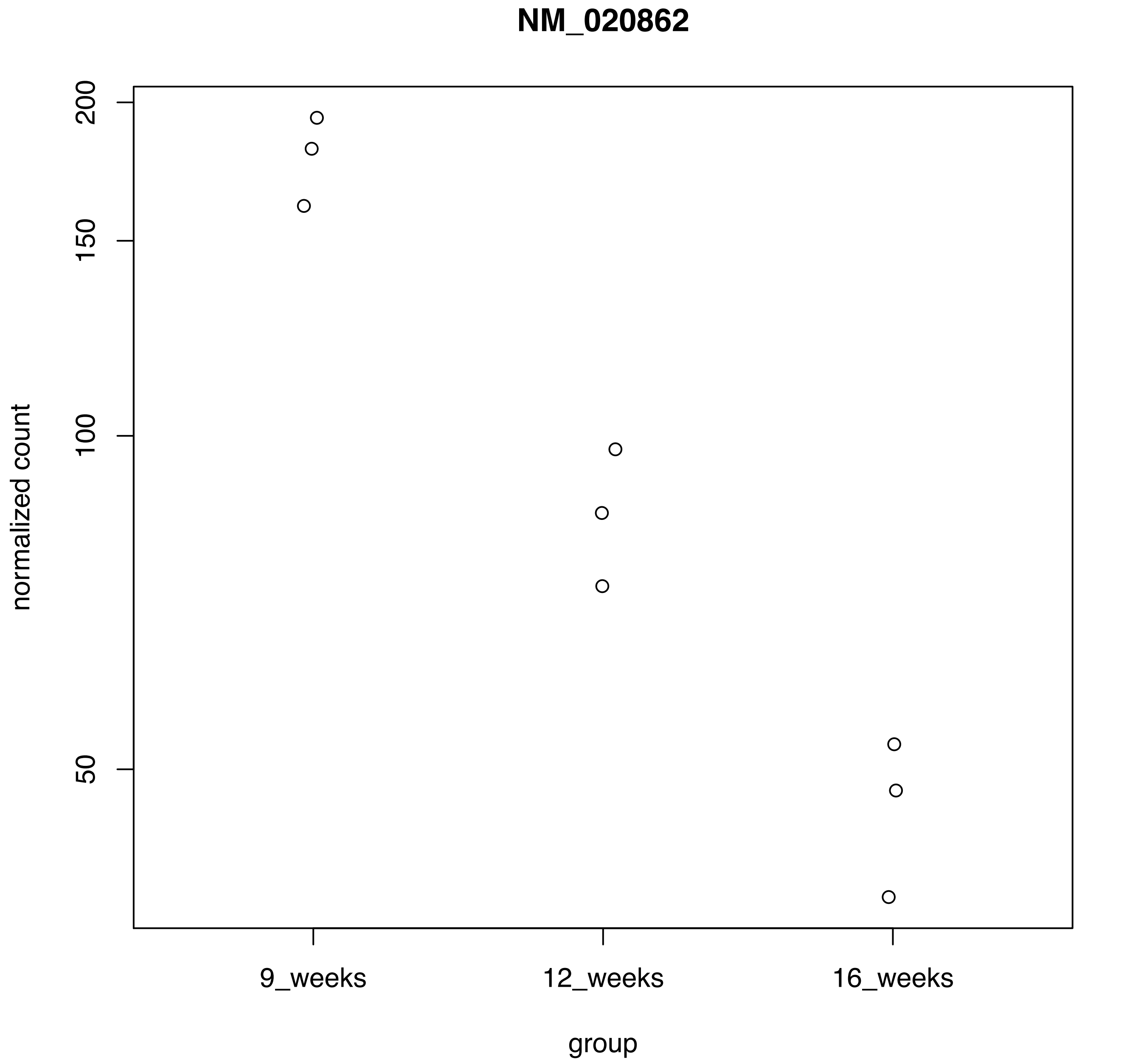
NM_020862
200
150
100
50
normalized count
9_weeks
12_weeks
16_weeks
group

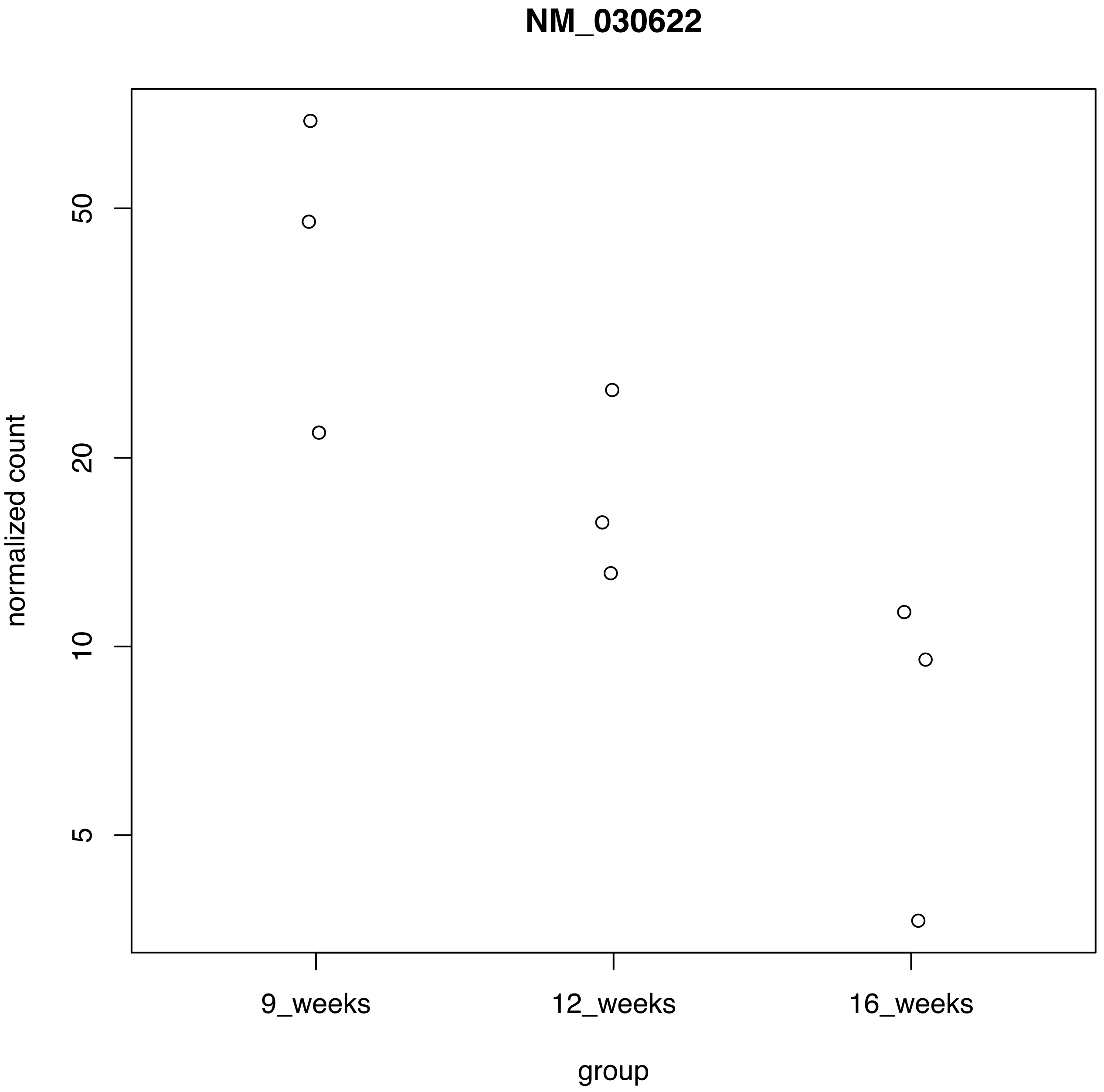
NM_030622
normalized count
50
20
10
5
9_weeks
12_weeks
16_weeks
group

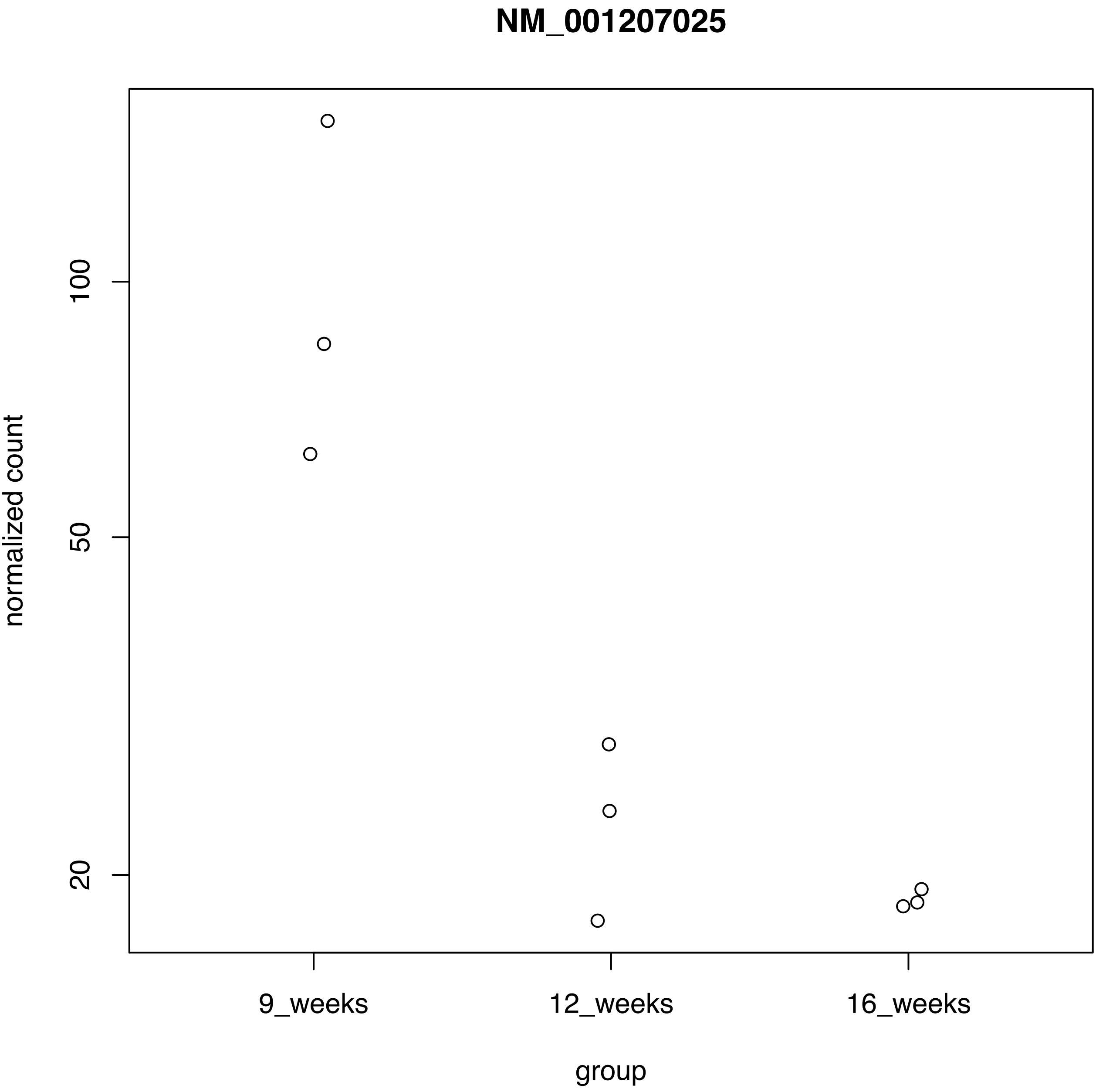

NM_001207025
100
50
20
normalized count
9_weeks
12_weeks
16_weeks
group

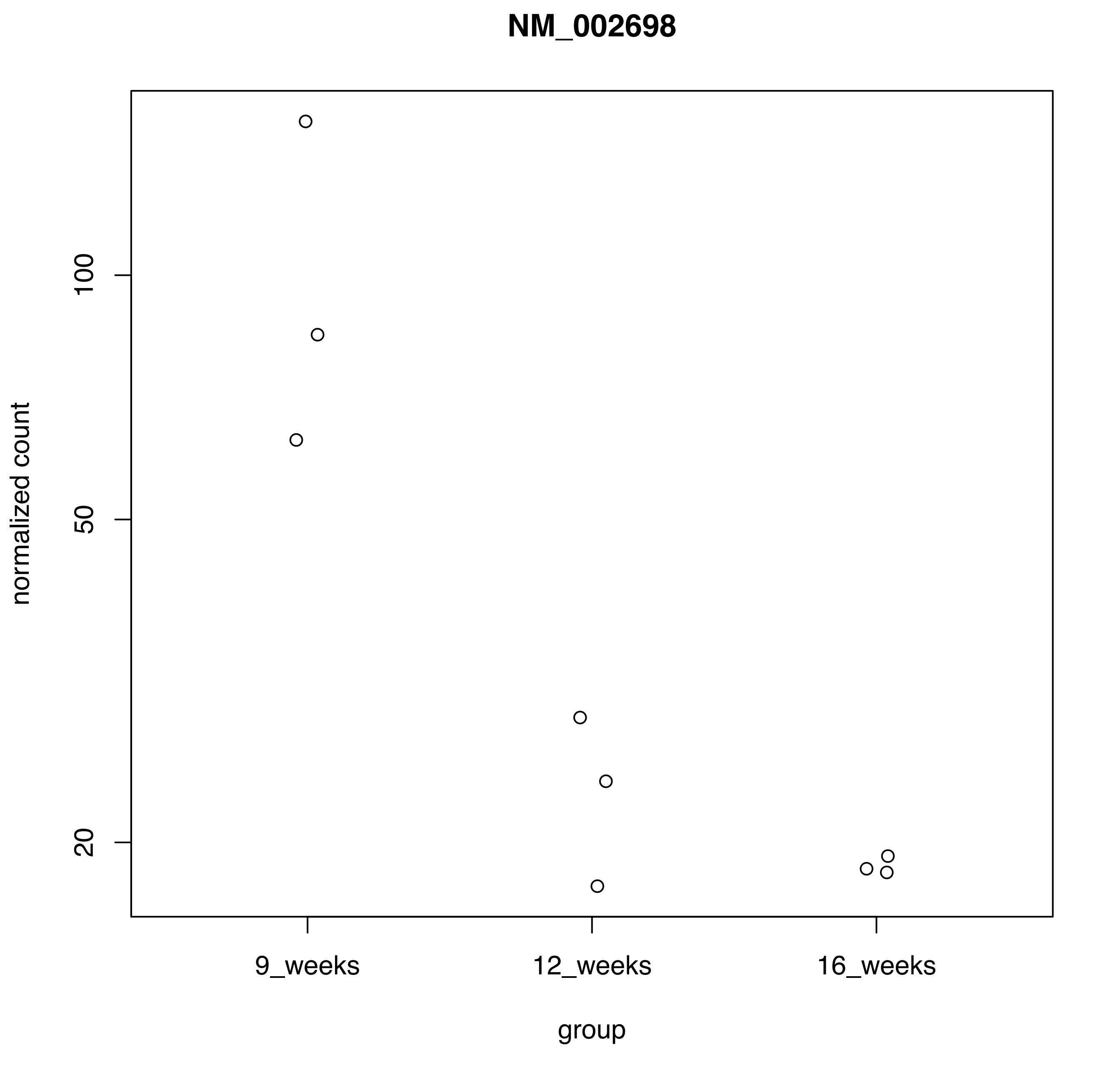
NM_002698
normalized count
100
50
20
9_weeks
12_weeks
16_weeks
group

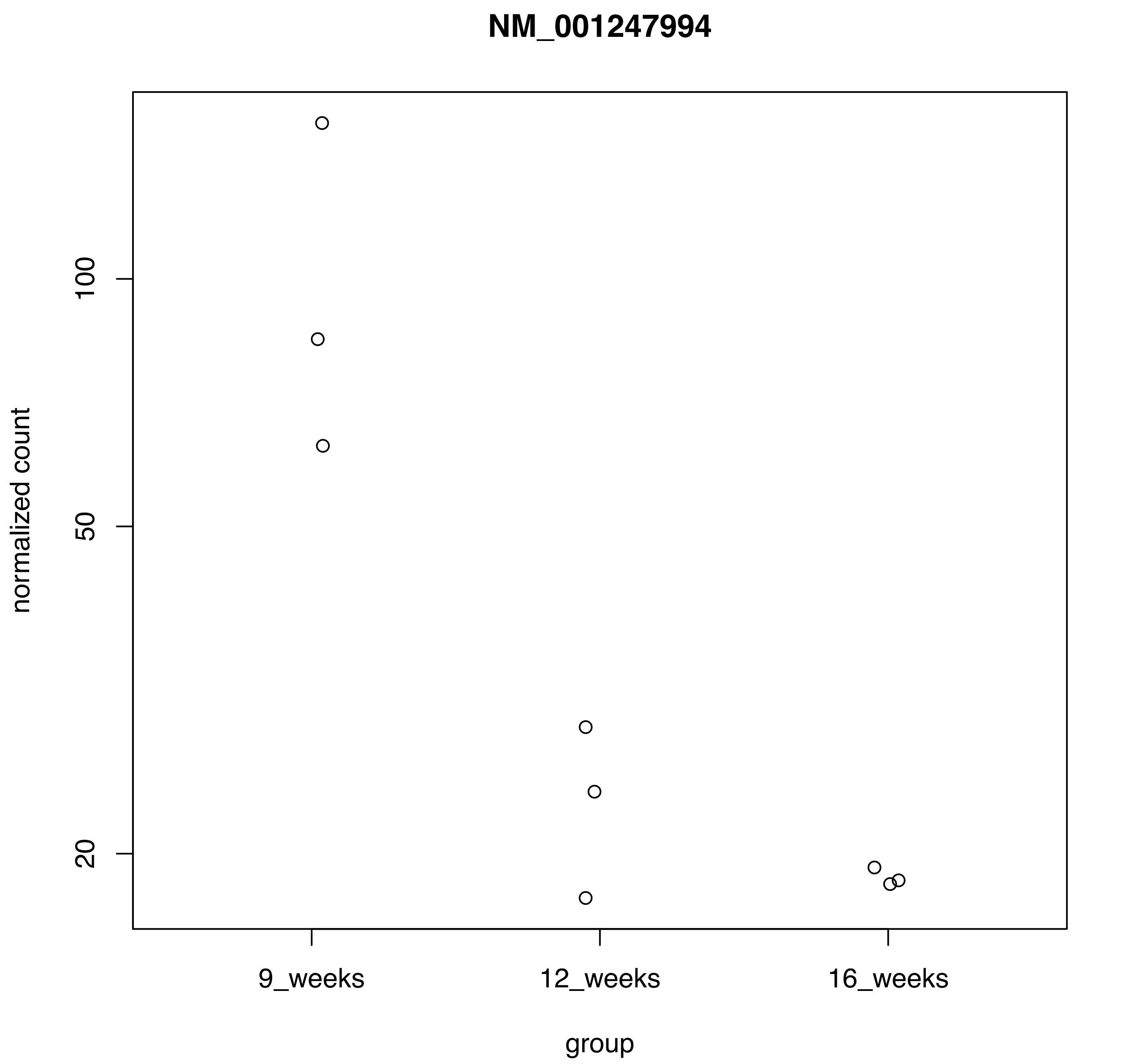
NM_001247994
normalized count
100
50
20
9_weeks
12_weeks
16_weeks
group

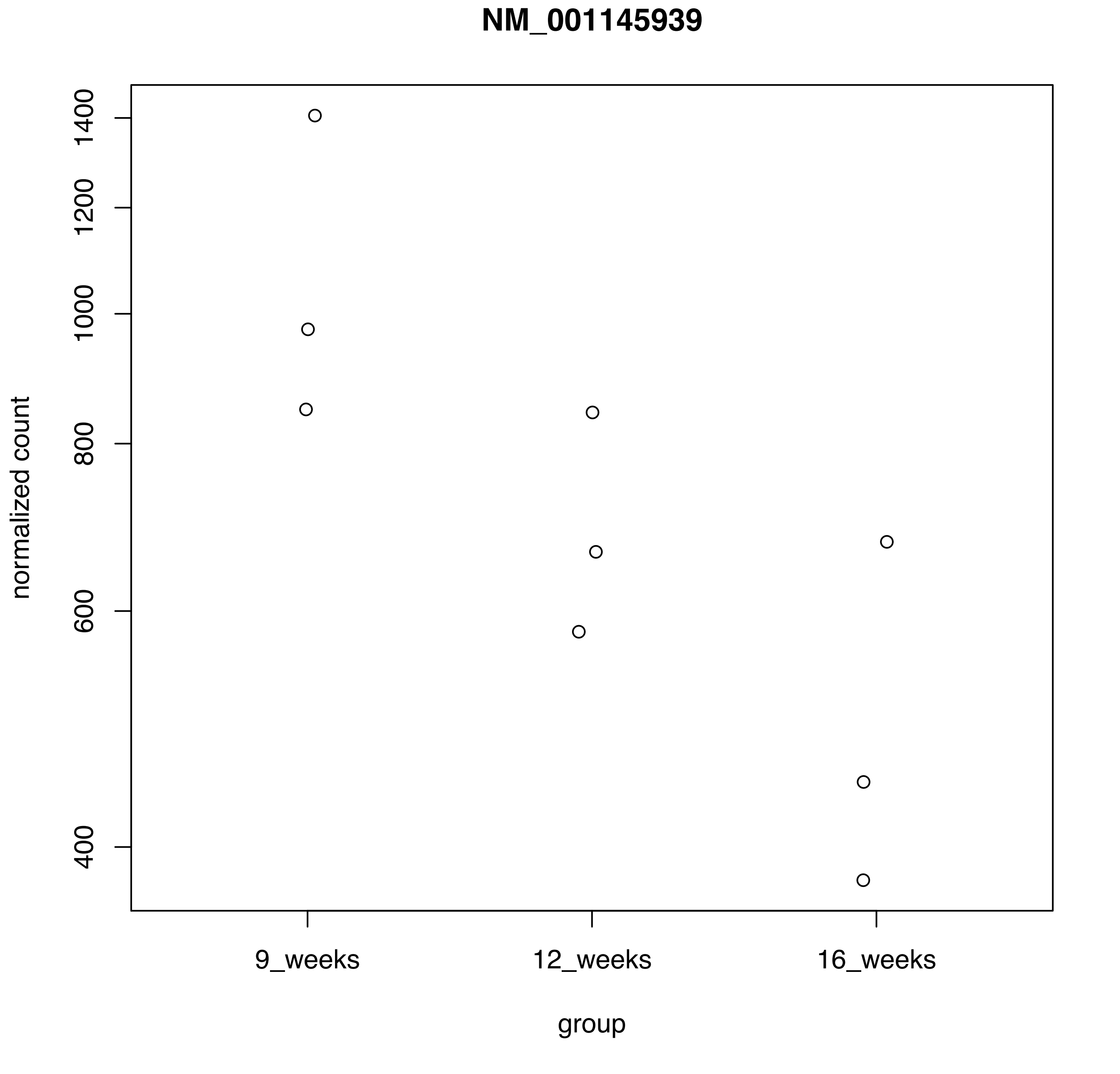

NM_001145939
normalized count
1400
1200
1000
800
600
400
9_weeks
12_weeks
16_weeks
group

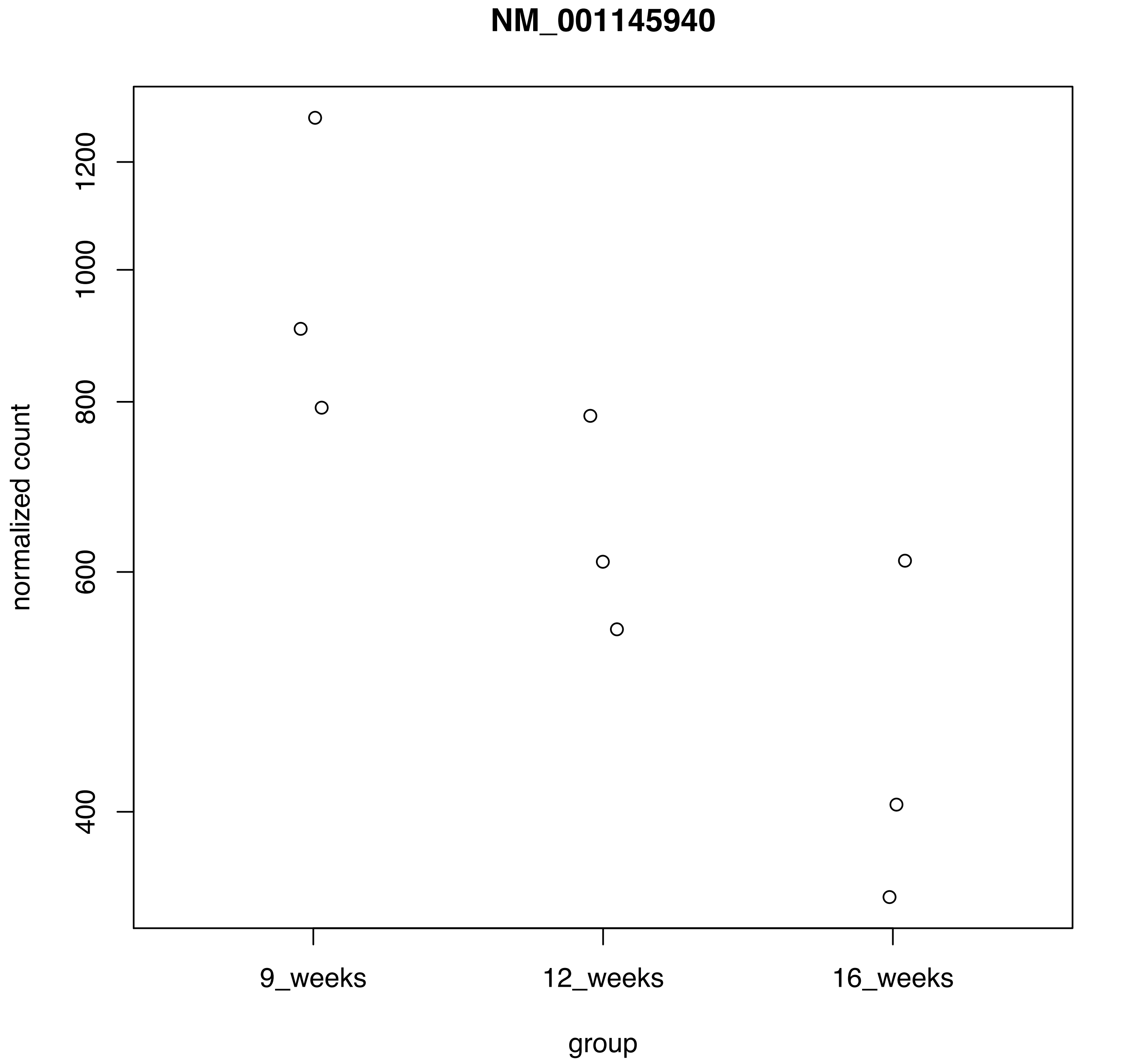
NM_001145940
normalized count
1200
1000
800
600
400
9_weeks
12_weeks
16_weeks
group

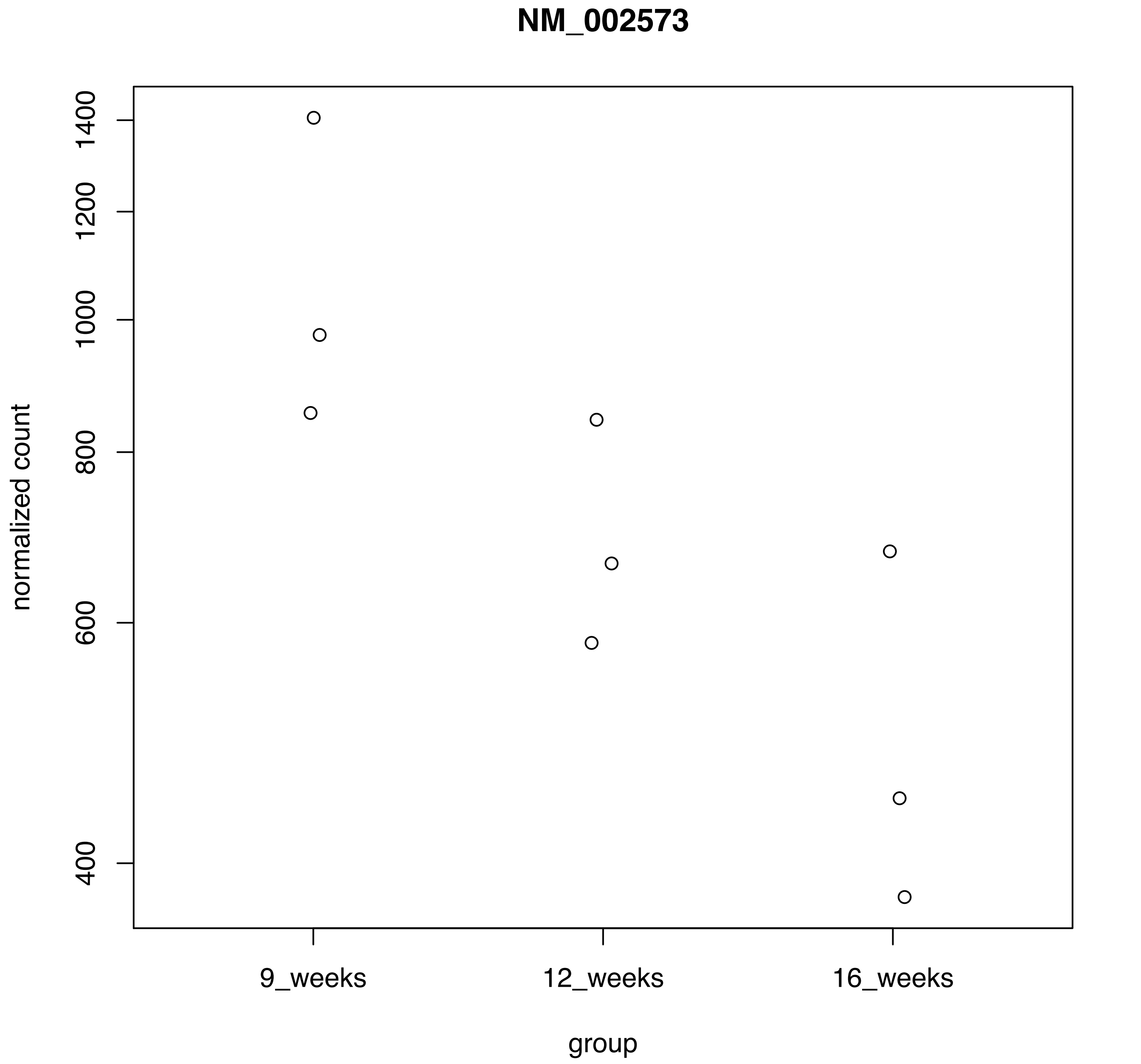
NM_002573
normalized count
1400
1200
1000
800
600
400
9_weeks
12_weeks
16_weeks
group

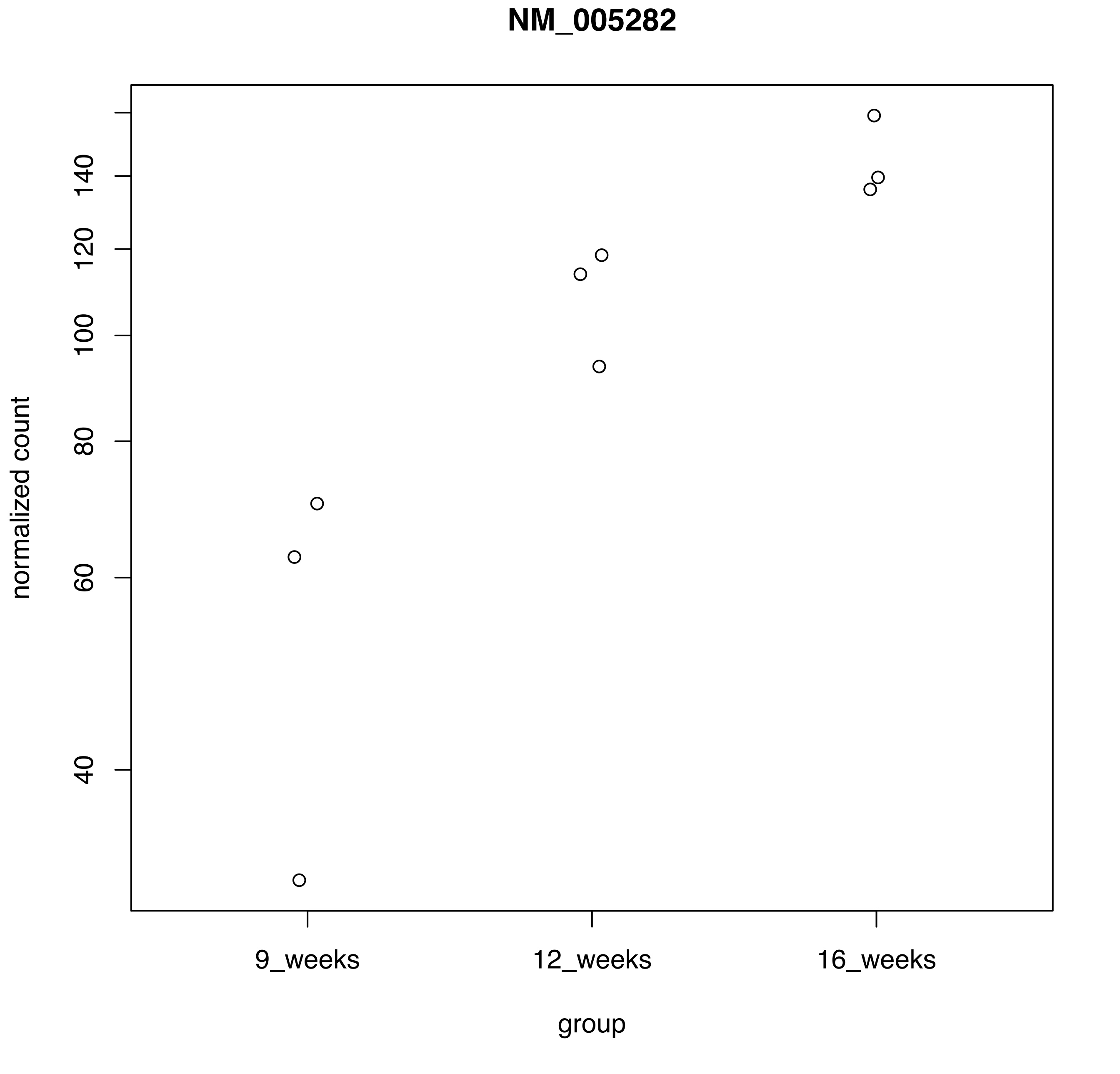
NM_005282
normalized count
40
60
80
100
120
140
9_weeks
12_weeks
16_weeks
group

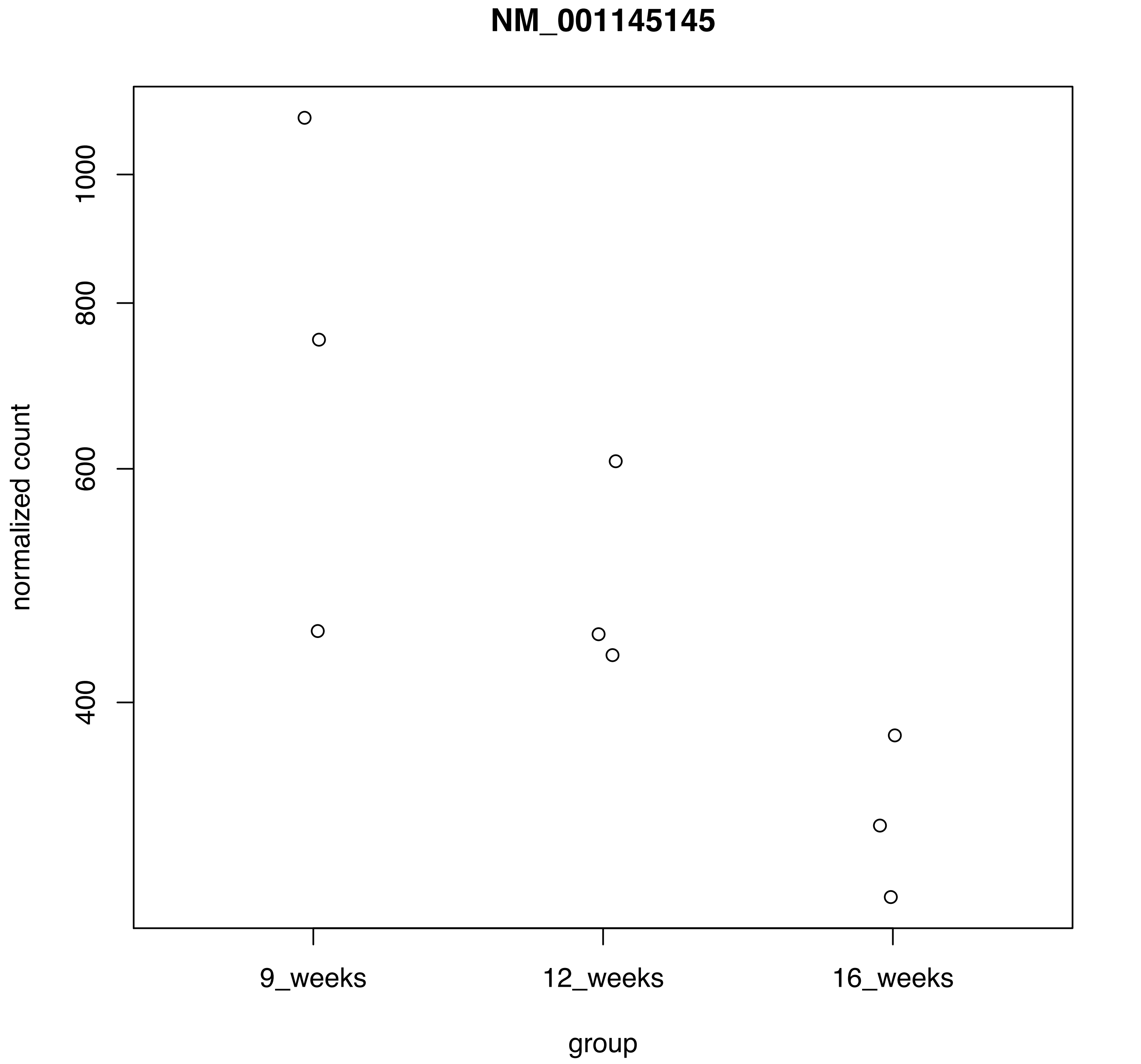
NM_001145145
normalized count
1000
800
600
400
9_weeks
12_weeks
16_weeks
group

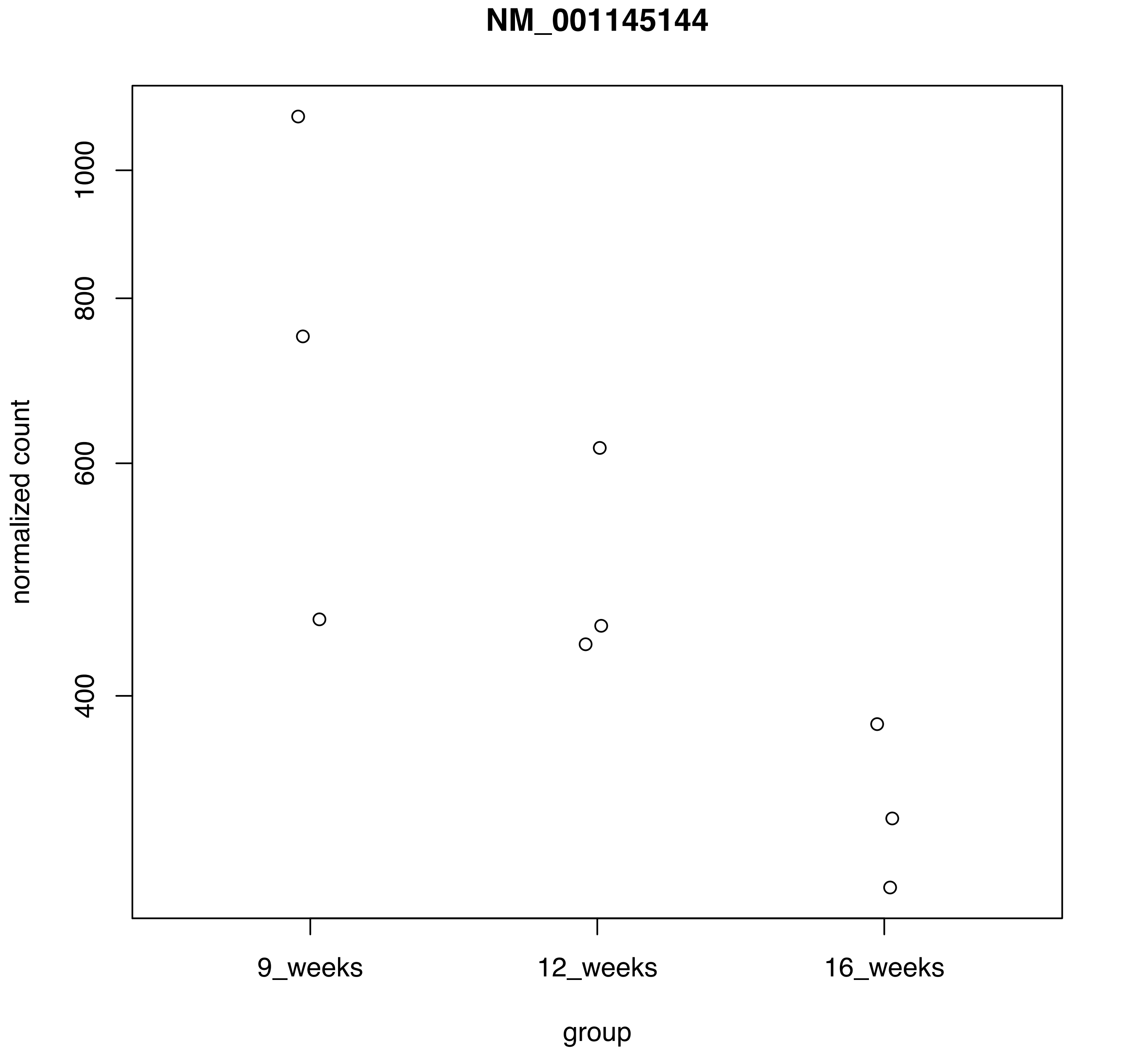
NM_001145144
normalized count
1000
800
600
400
9_weeks
12_weeks
16_weeks
group

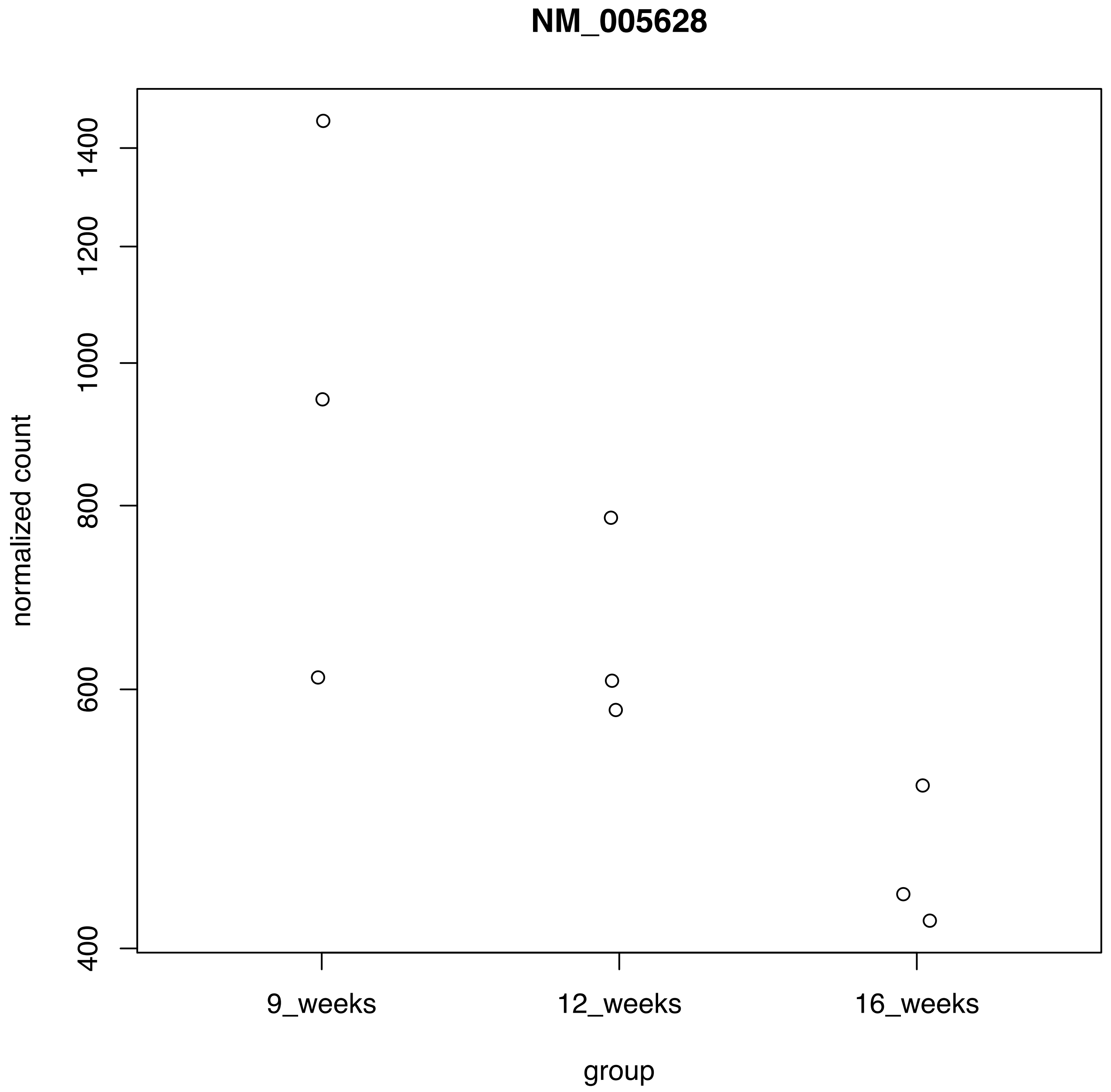
NM_005628
normalized count
1400
1200
1000
800
600
400
9_weeks
12_weeks
16_weeks
group

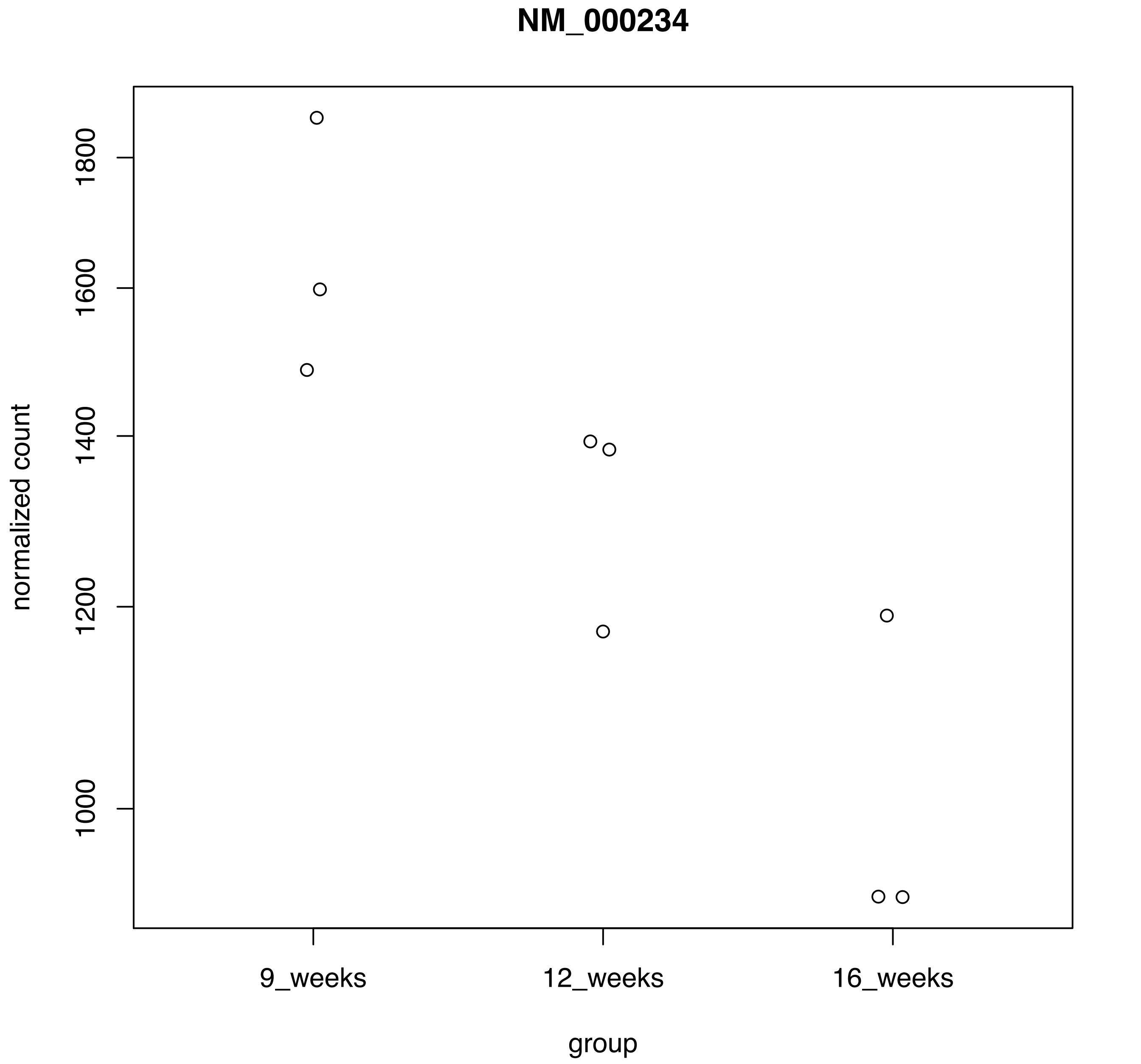
NM_000234
1800
1600
1400
1200
1000
normalized count
9_weeks
12_weeks
16_weeks
group

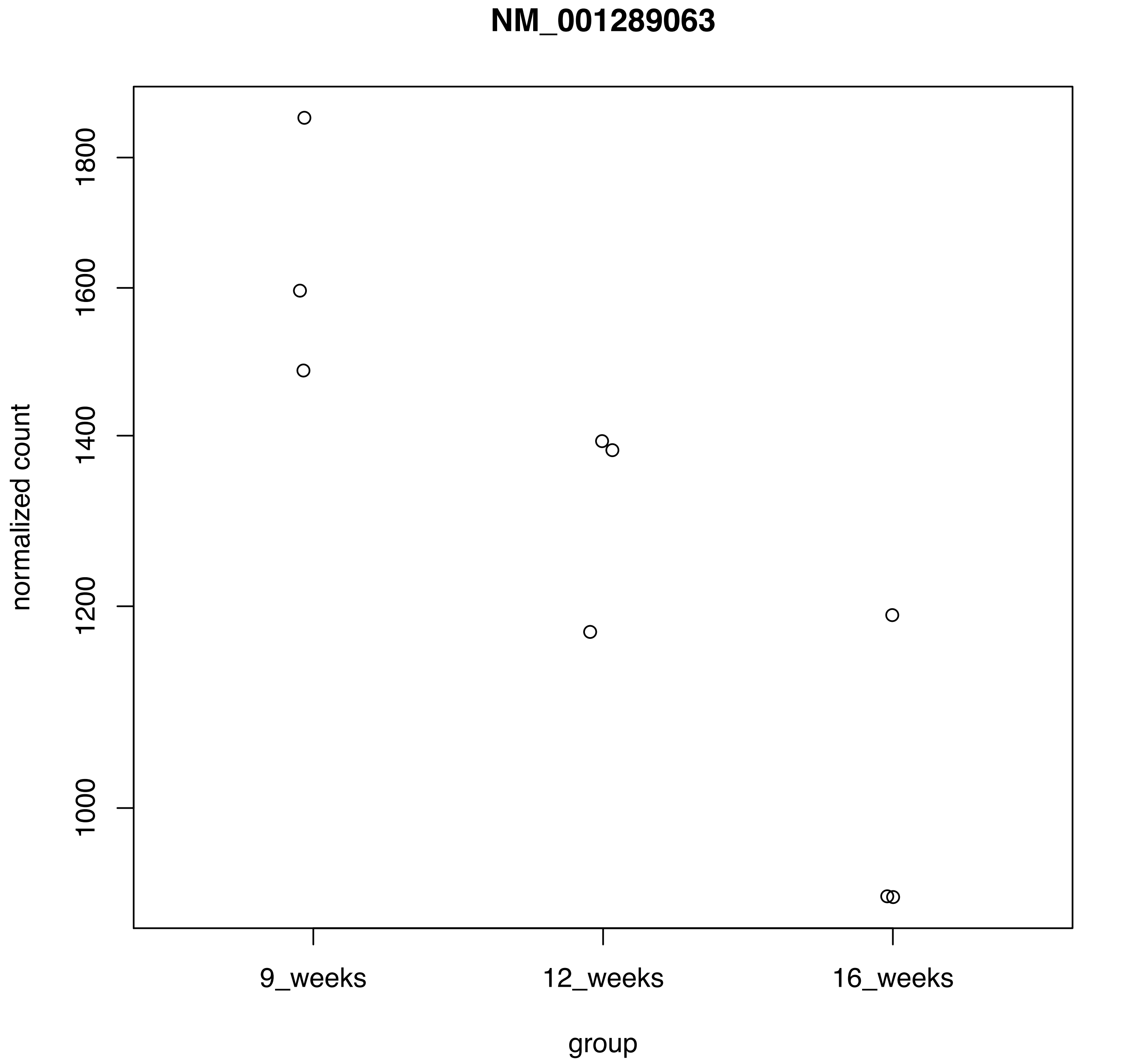

NM_001289063
normalized count
1000
1200
1400
1600
1800
9_weeks
12_weeks
16_weeks
group

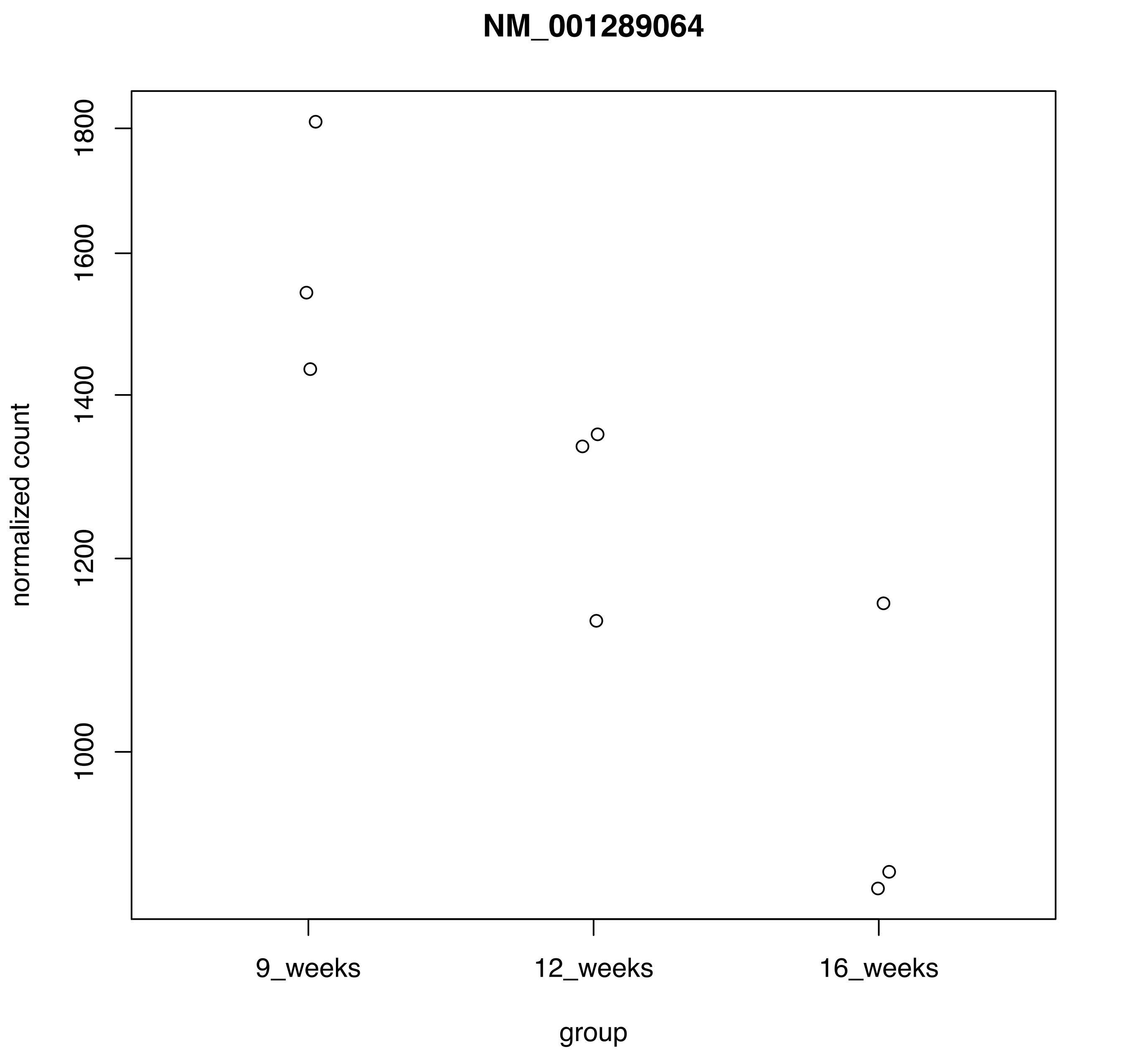
NM_001289064
normalized count
1000
1200
1400
1600
1800
9_weeks
12_weeks
16_weeks
group

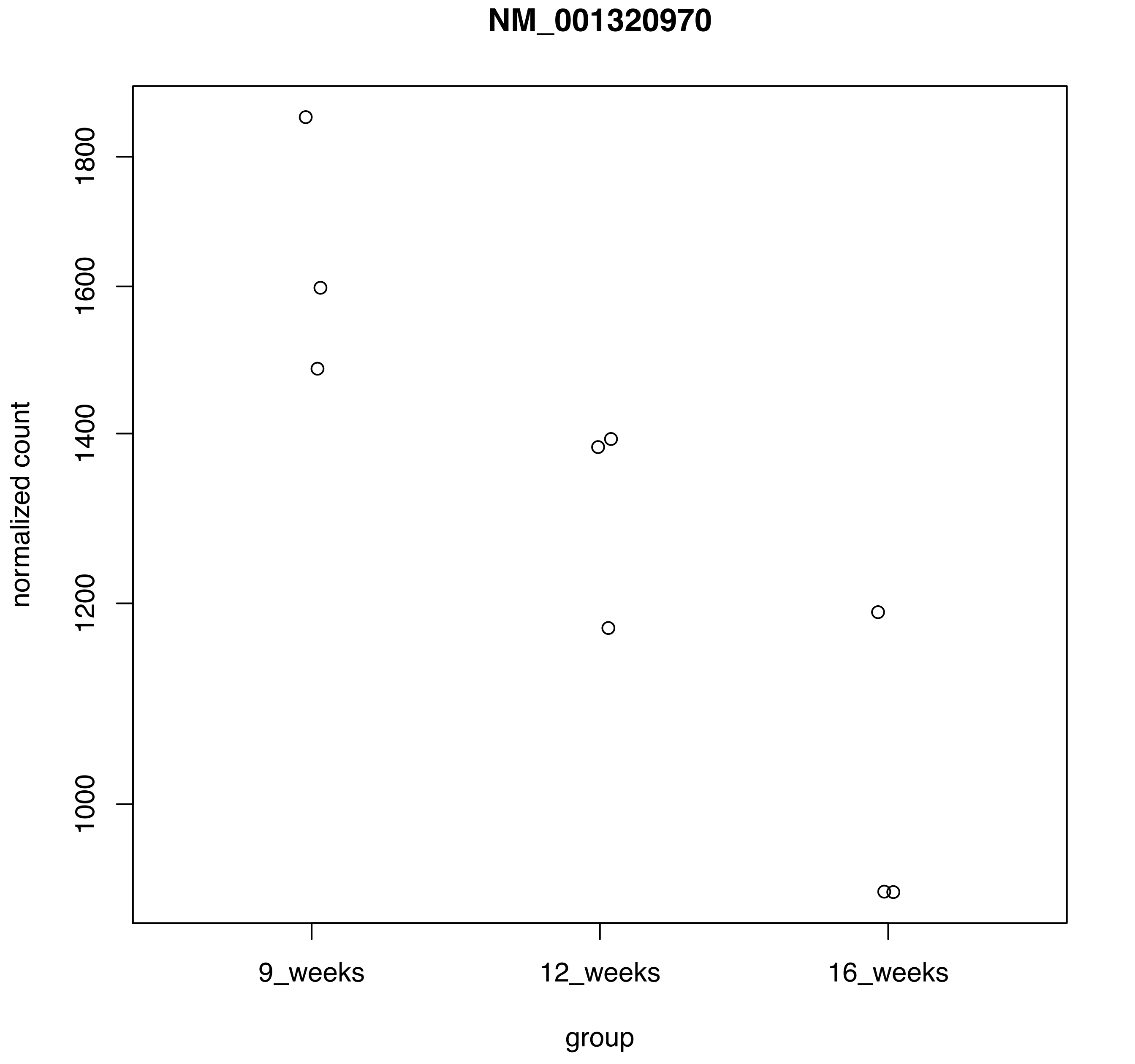

NM_001320970
normalized count
1000
1200
1400
1600
1800
9_weeks
12_weeks
16_weeks
group

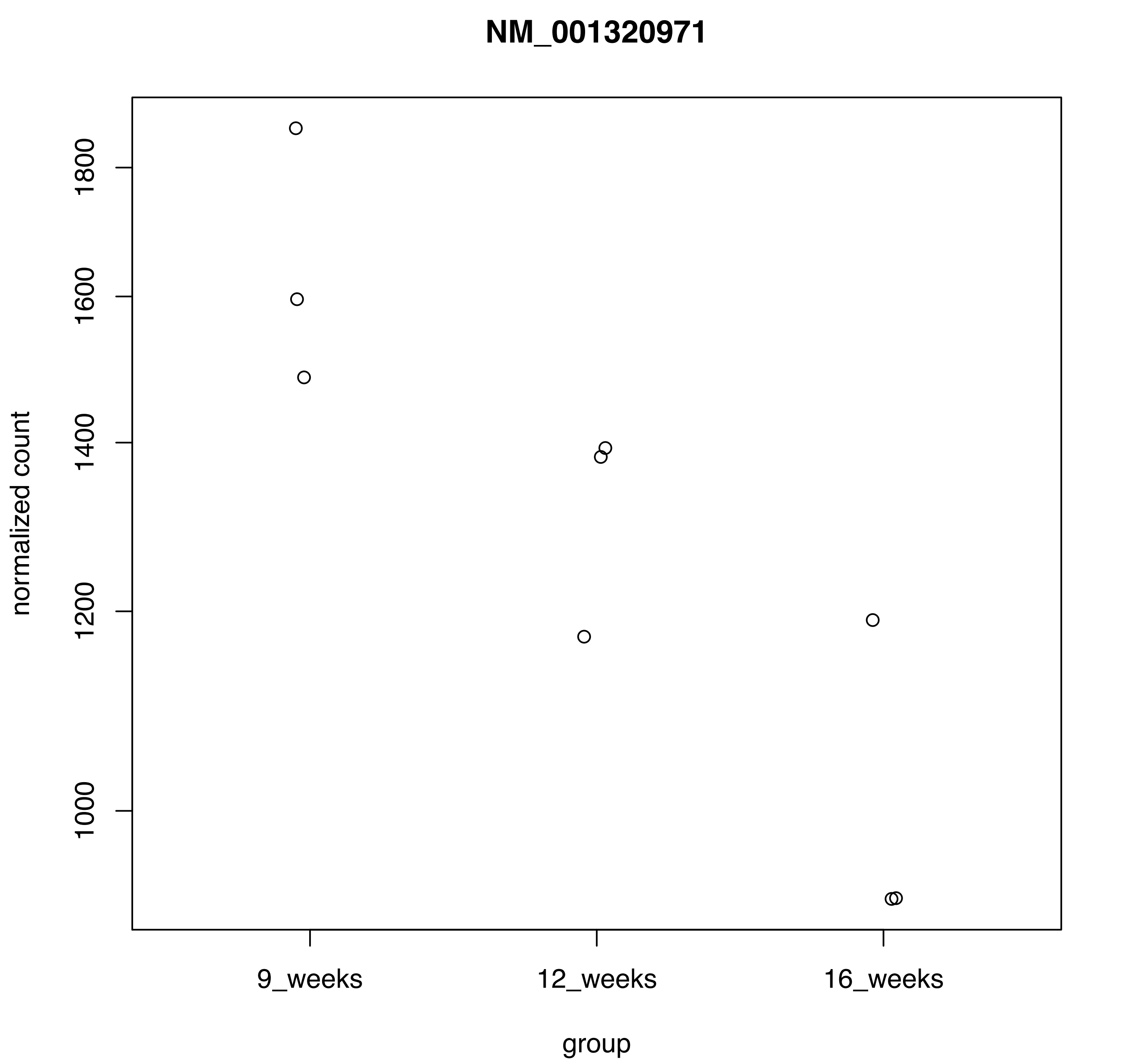
NM_001320971
normalized count
1000
1200
1400
1600
1800
9_weeks
12_weeks
16_weeks
group

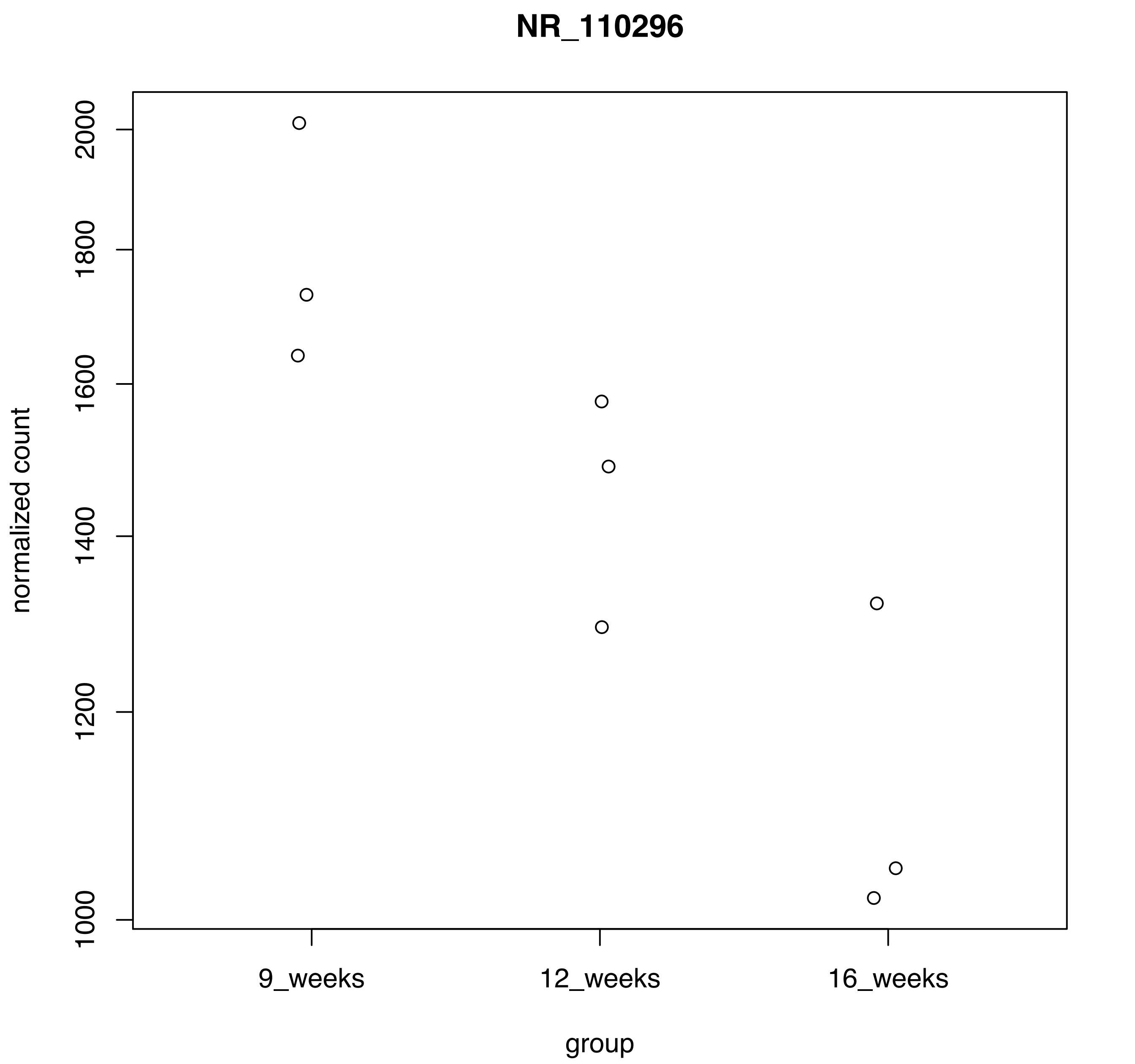
NR_110296
normalized count
1000
1200
1400
1600
1800
2000
9_weeks
12_weeks
16_weeks
group

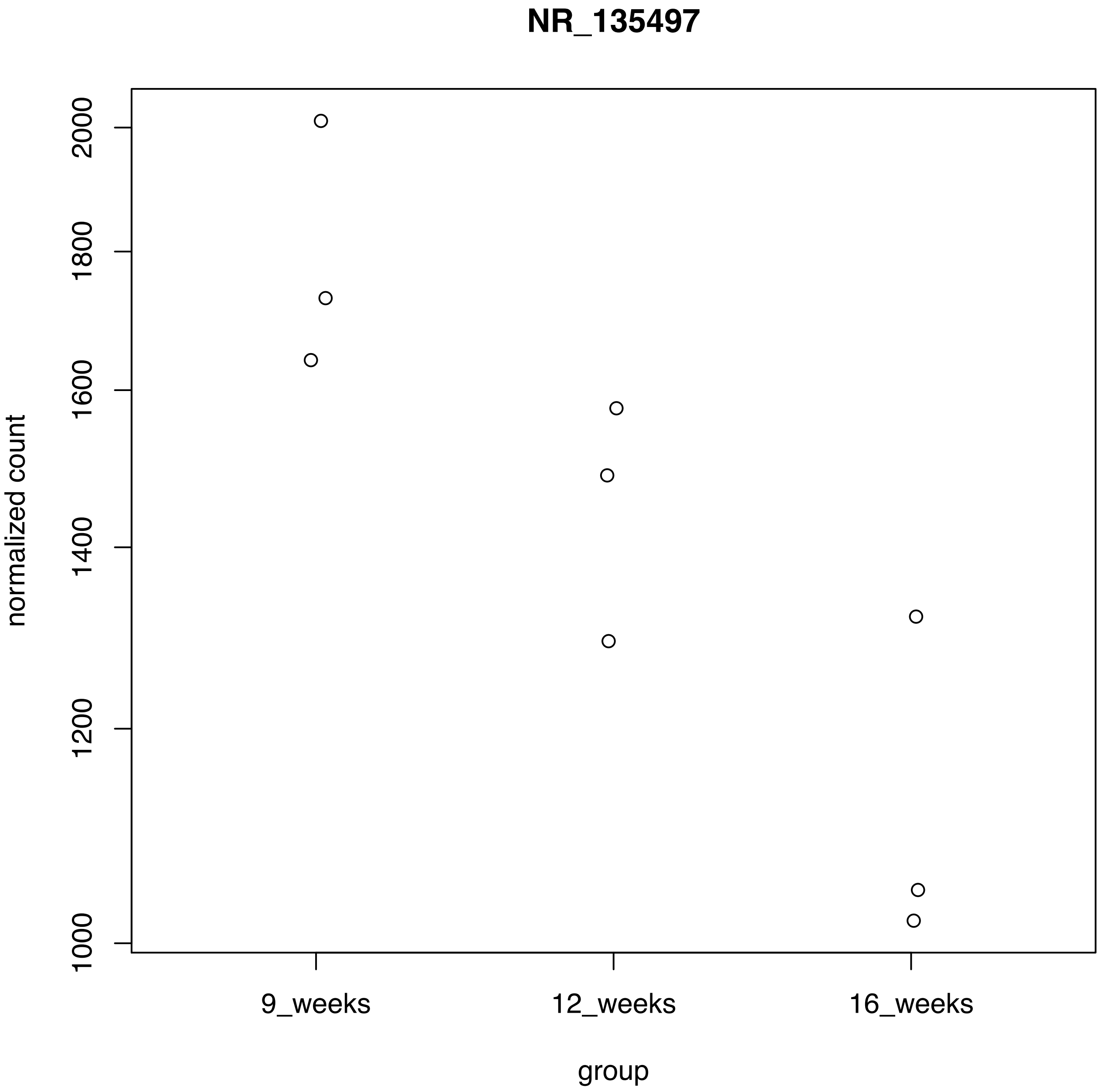

NR_135497
normalized count
2000
1800
1600
1400
1200
1000
9_weeks
12_weeks
16_weeks
group

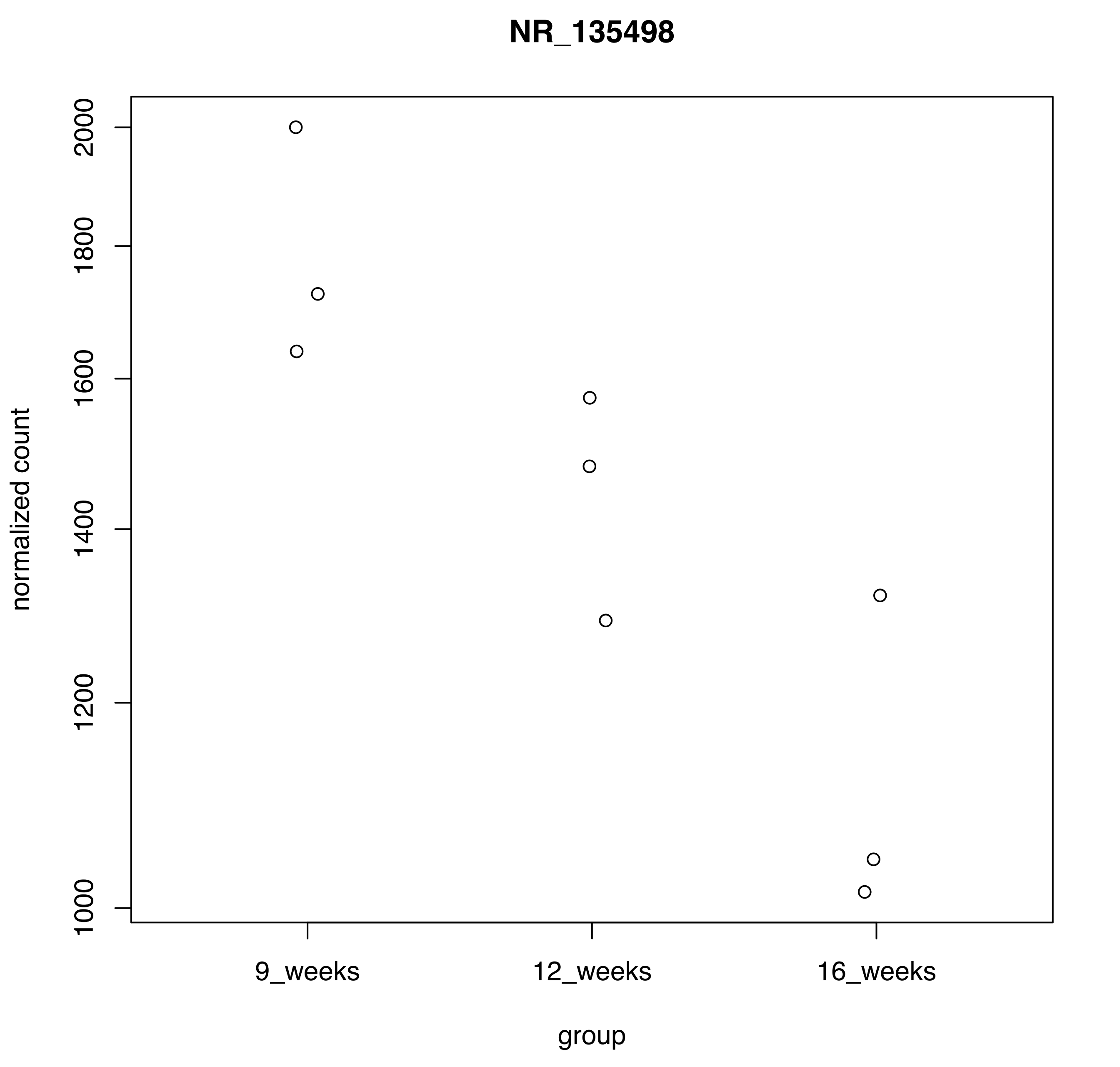

NR_135498
normalized count
2000
1800
1600
1400
1200
1000
9_weeks
12_weeks
16_weeks
group

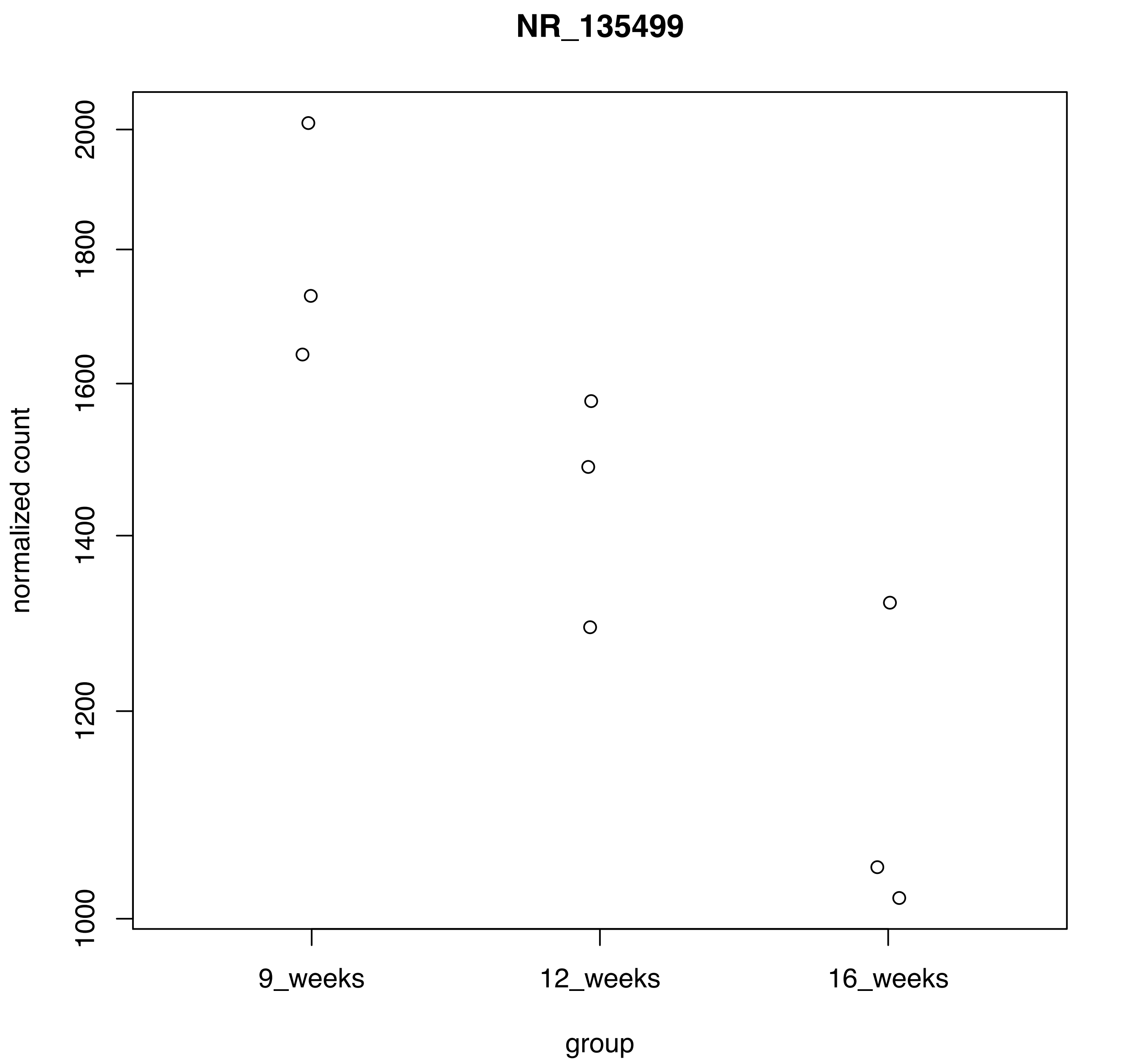

NR_135499
normalized count
1000
1200
1400
1600
1800
2000
9_weeks
12_weeks
16_weeks
group

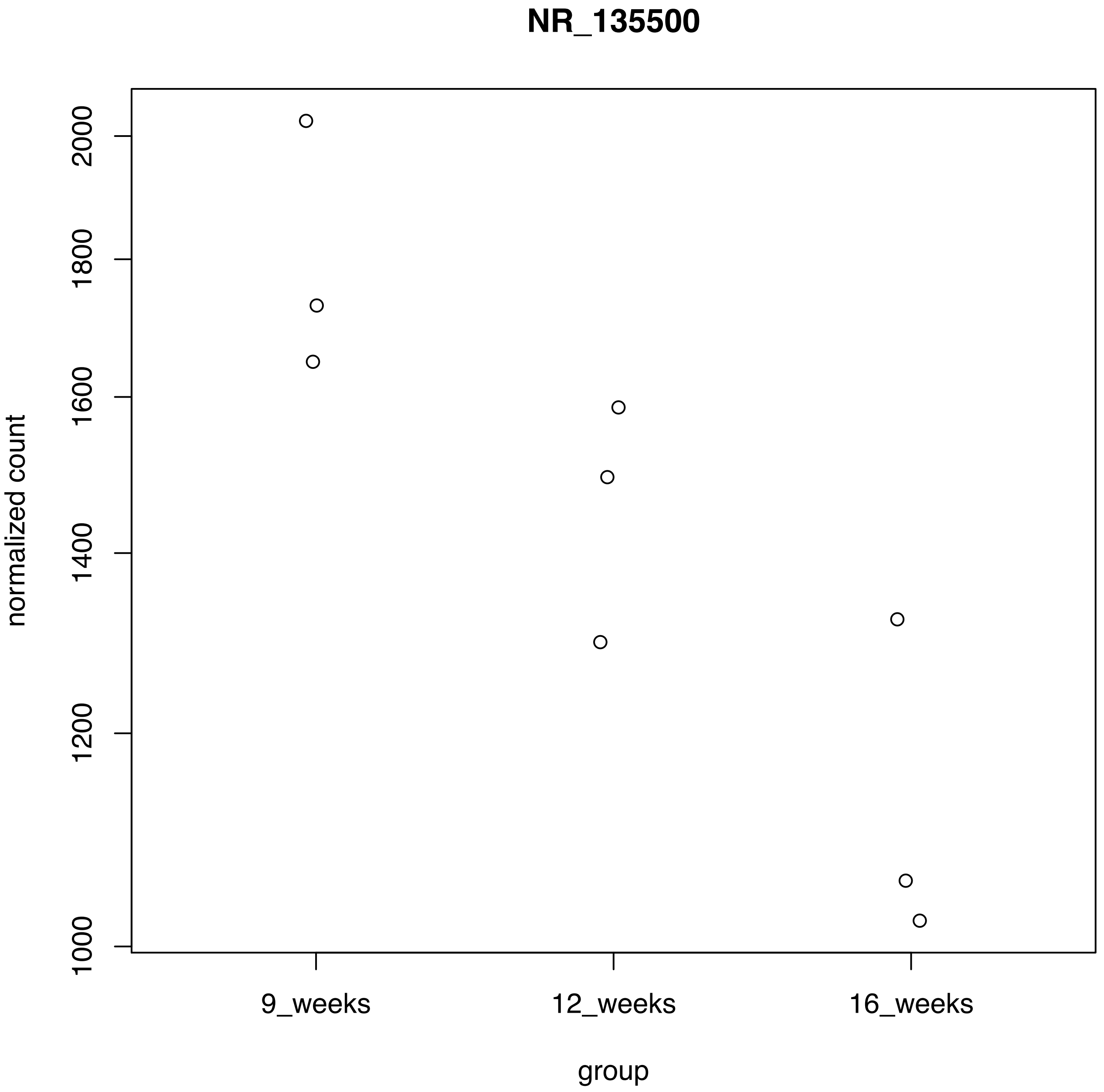
NR_135500
normalized count
2000
1800
1600
1400
1200
1000
9_weeks
12_weeks
16_weeks
group

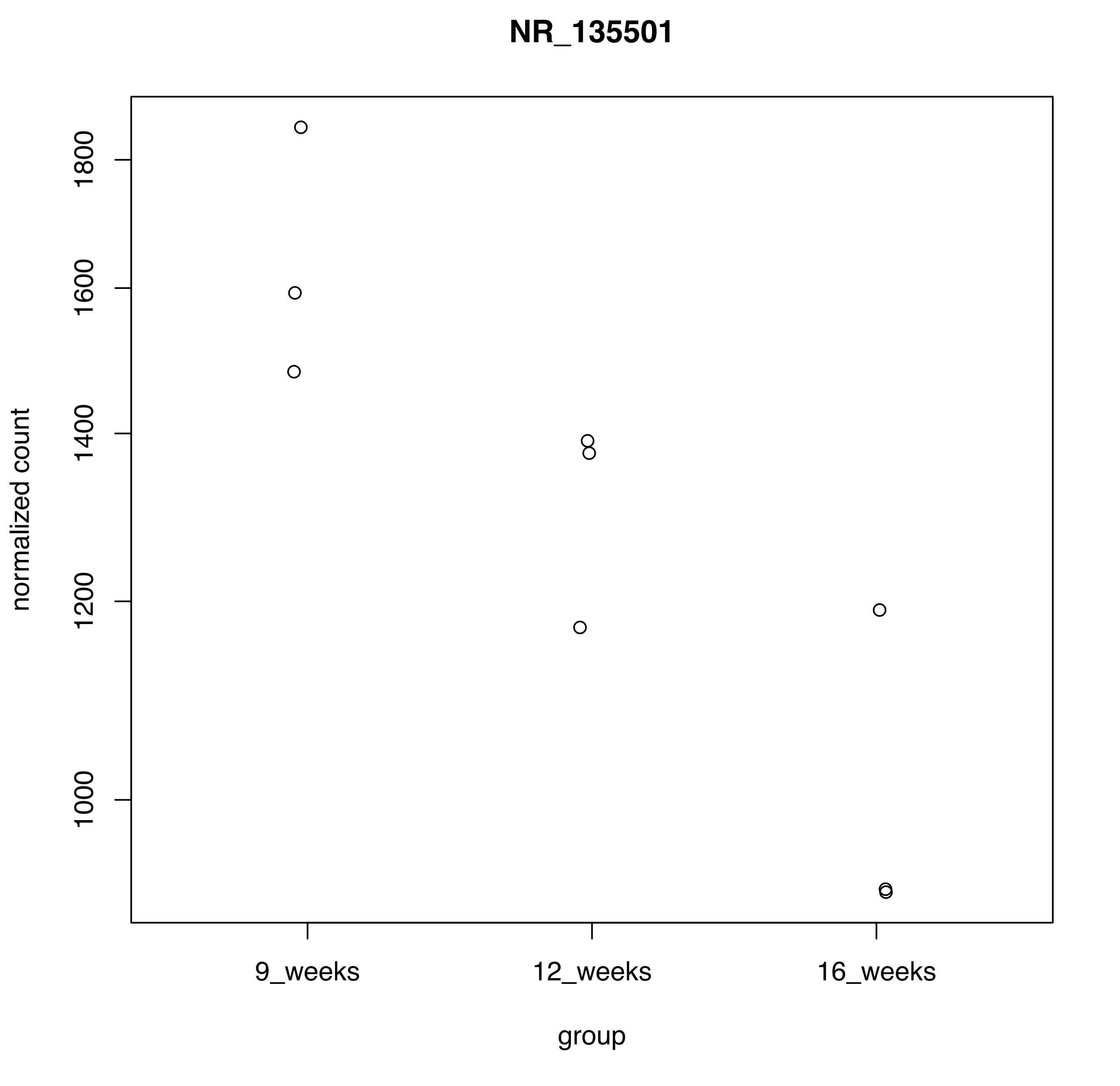
NR_135501
normalized count
1000
1200
1400
1600
1800
9_weeks
12_weeks
16_weeks
group

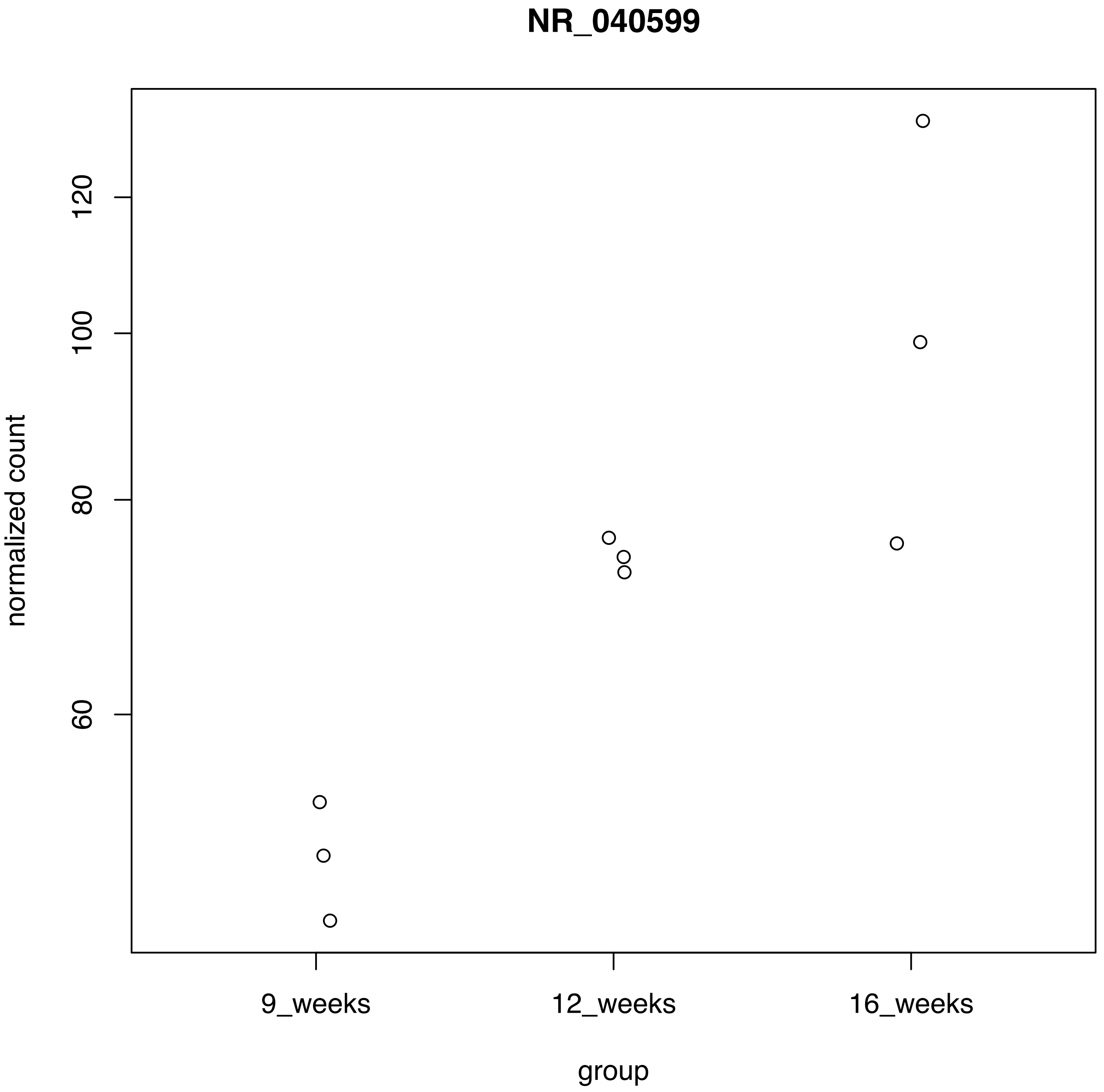

NR_040599
normalized count
60
80
100
120
9_weeks
12_weeks
16_weeks
group

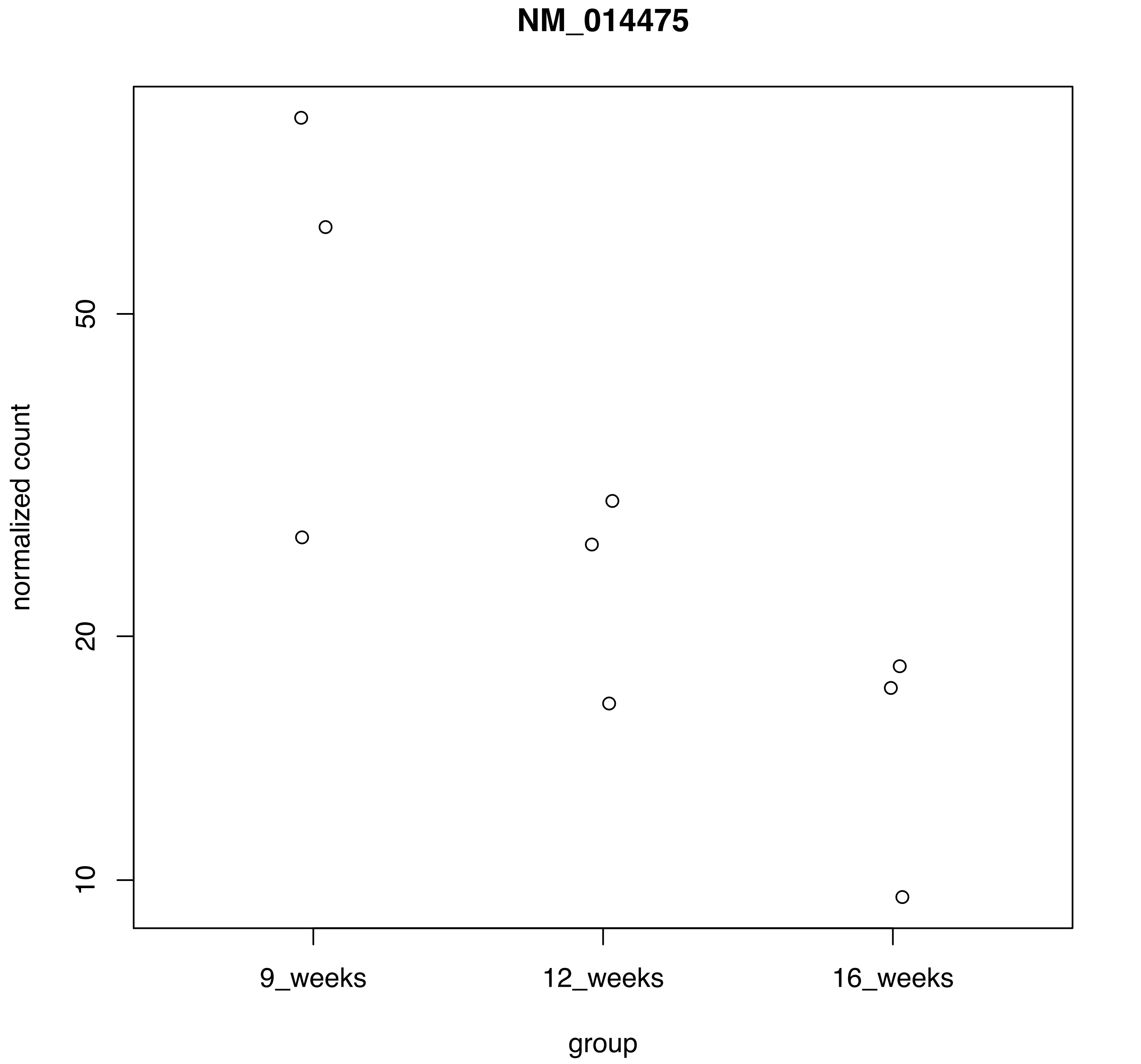
NM_014475
normalized count
50
20
10
9_weeks
12_weeks
16_weeks
group

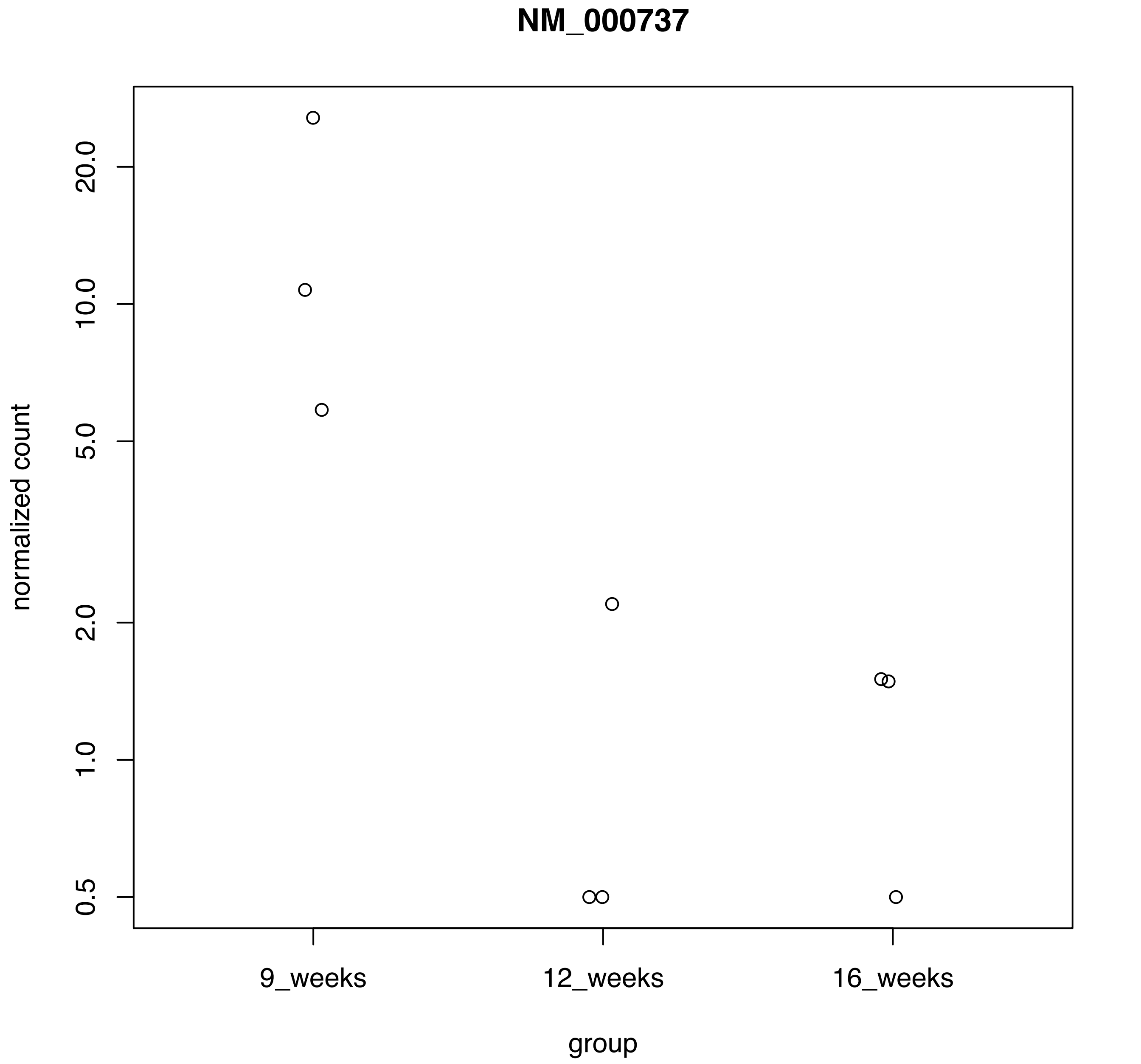
NM_000737
normalized count
20.0
10.0
5.0
2.0
1.0
0.5
9_weeks
12_weeks
16_weeks
group

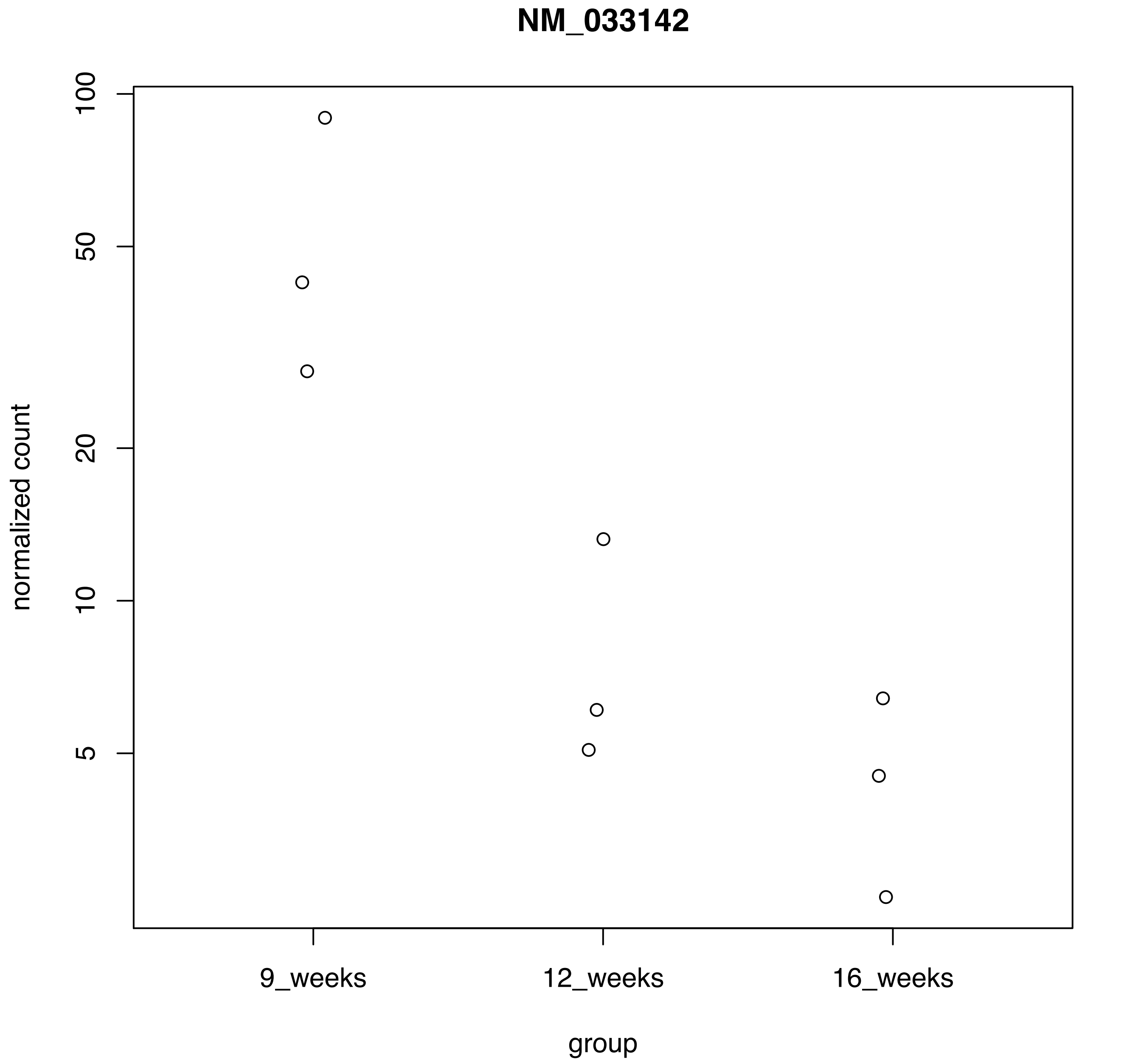
NM_033142
normalized count
100
50
20
10
5
9_weeks
12_weeks
16_weeks
group

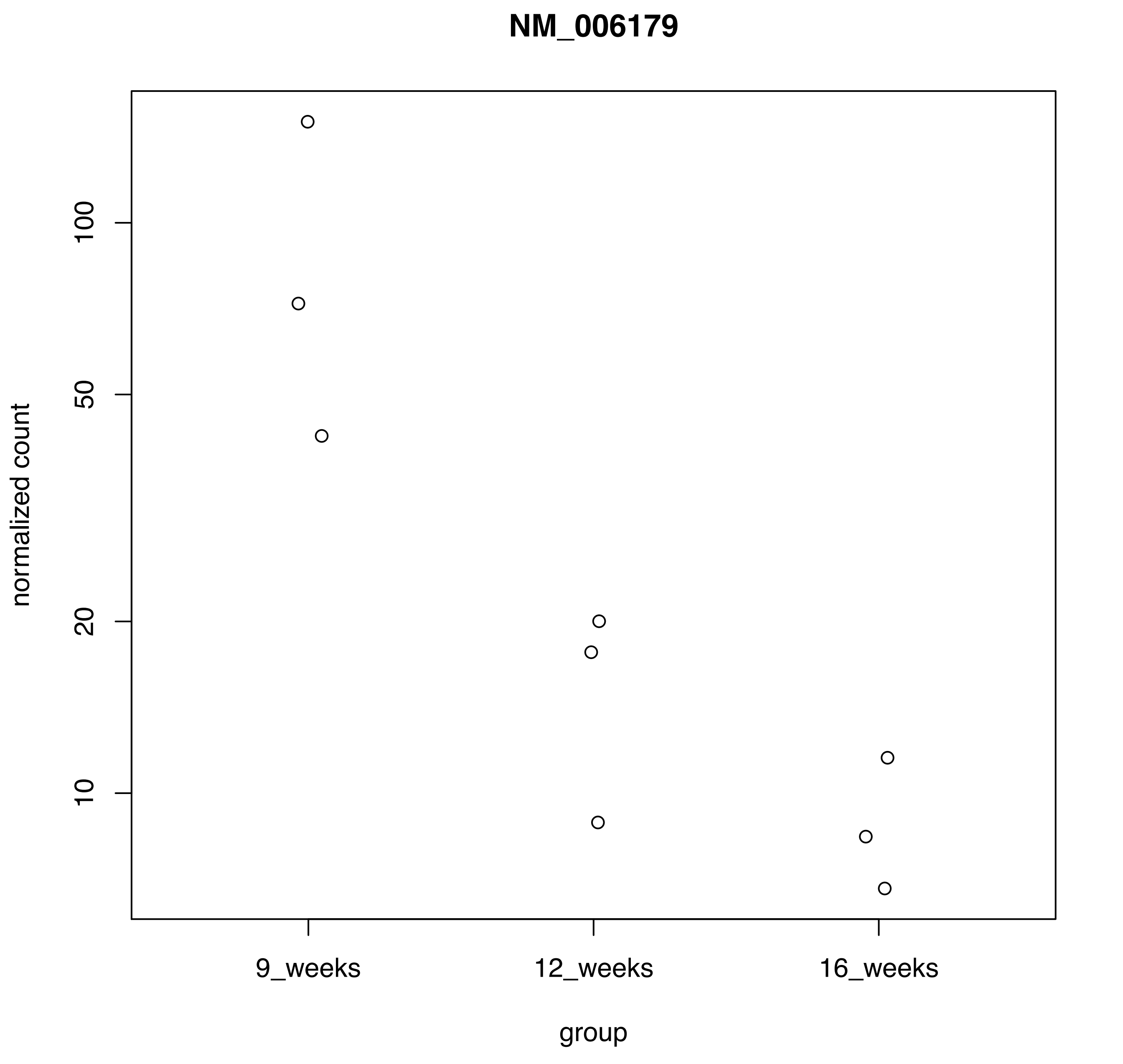
NM_006179
normalized count
100
50
20
10
9_weeks
12_weeks
16_weeks
group

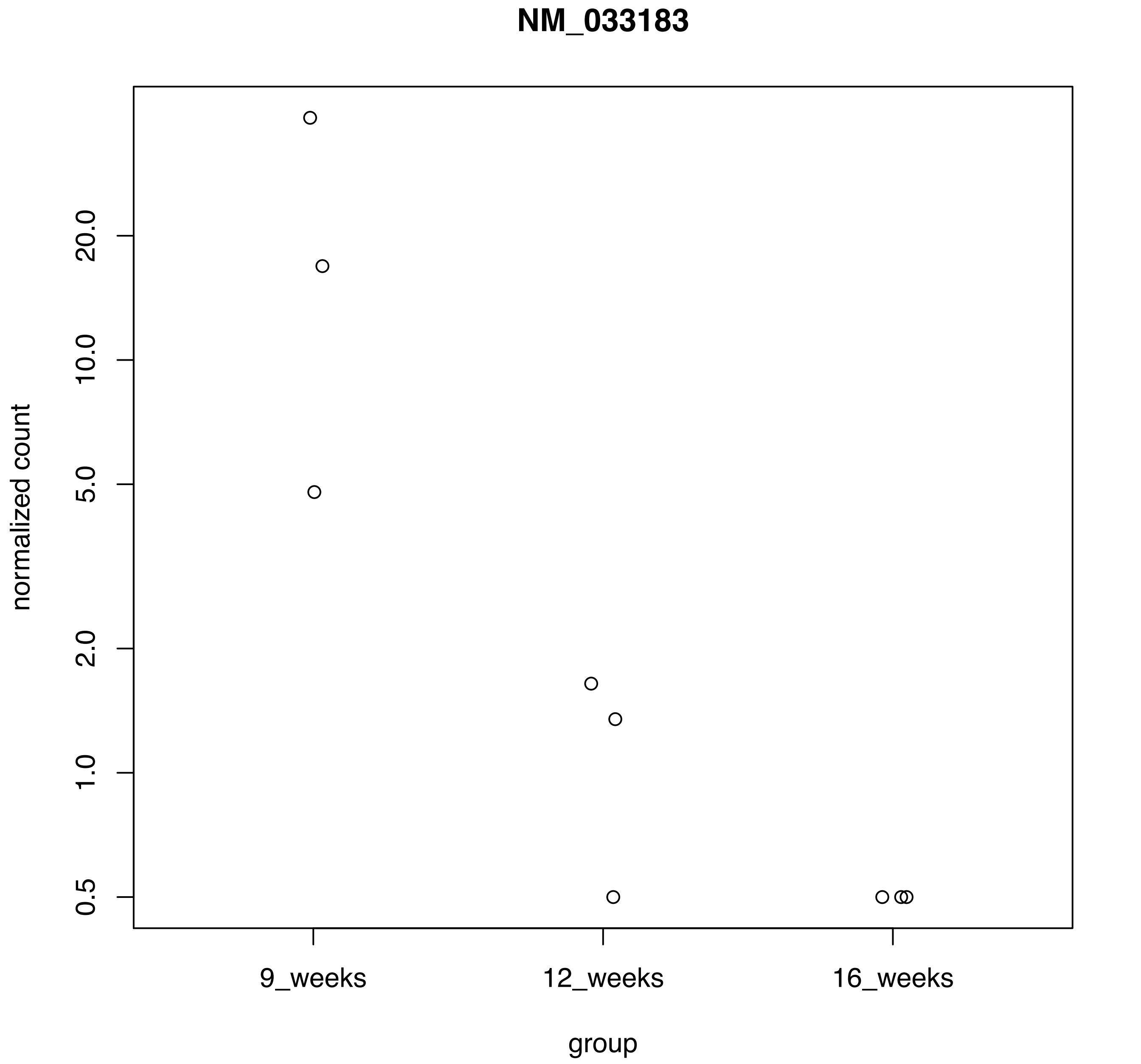
NM_033183
normalized count
20.0
10.0
5.0
2.0
1.0
0.5
9_weeks
12_weeks
16_weeks
group

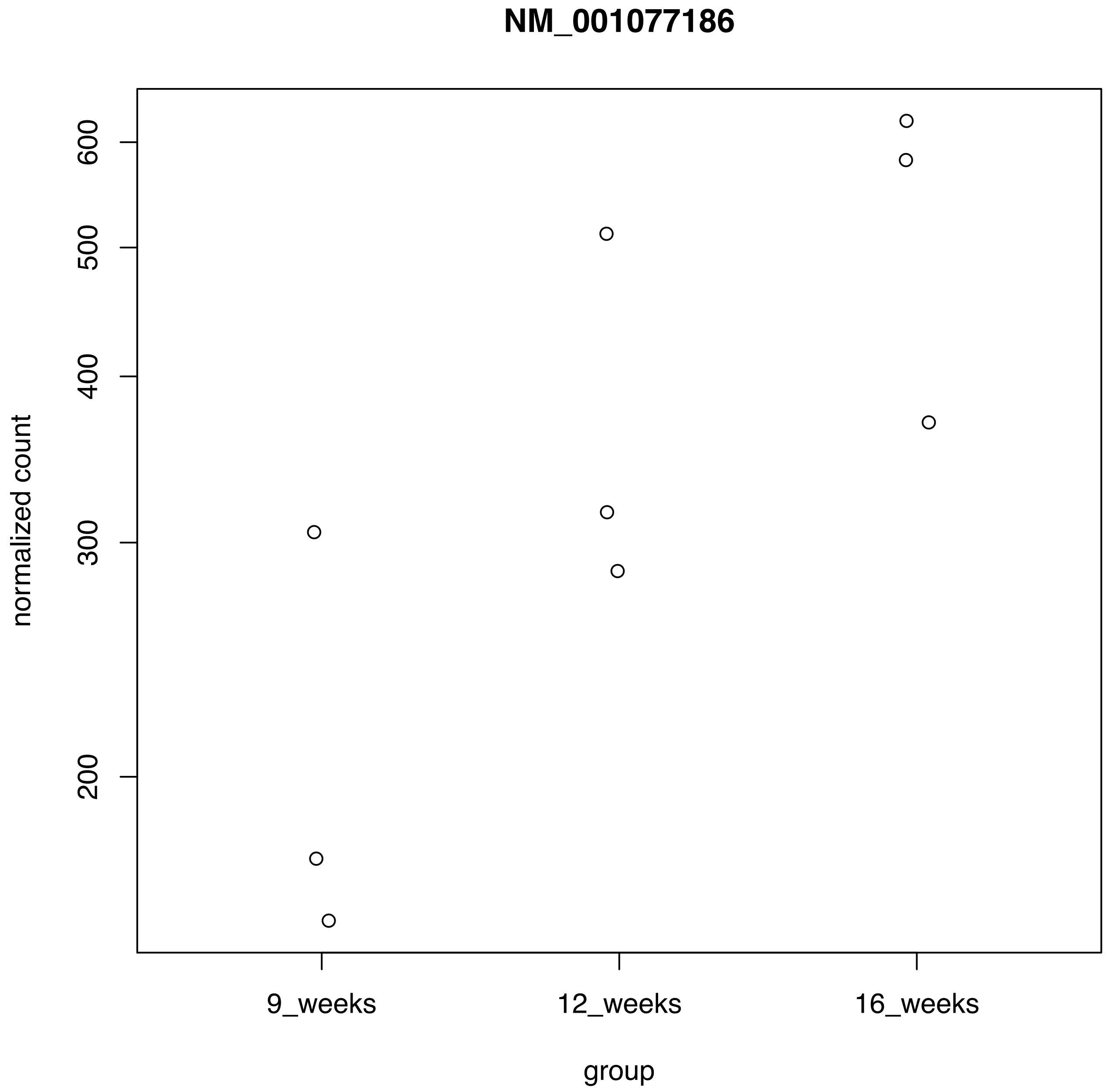
NM_001077186
normalized count
600
500
400
300
200
9_weeks
12_weeks
16_weeks
group

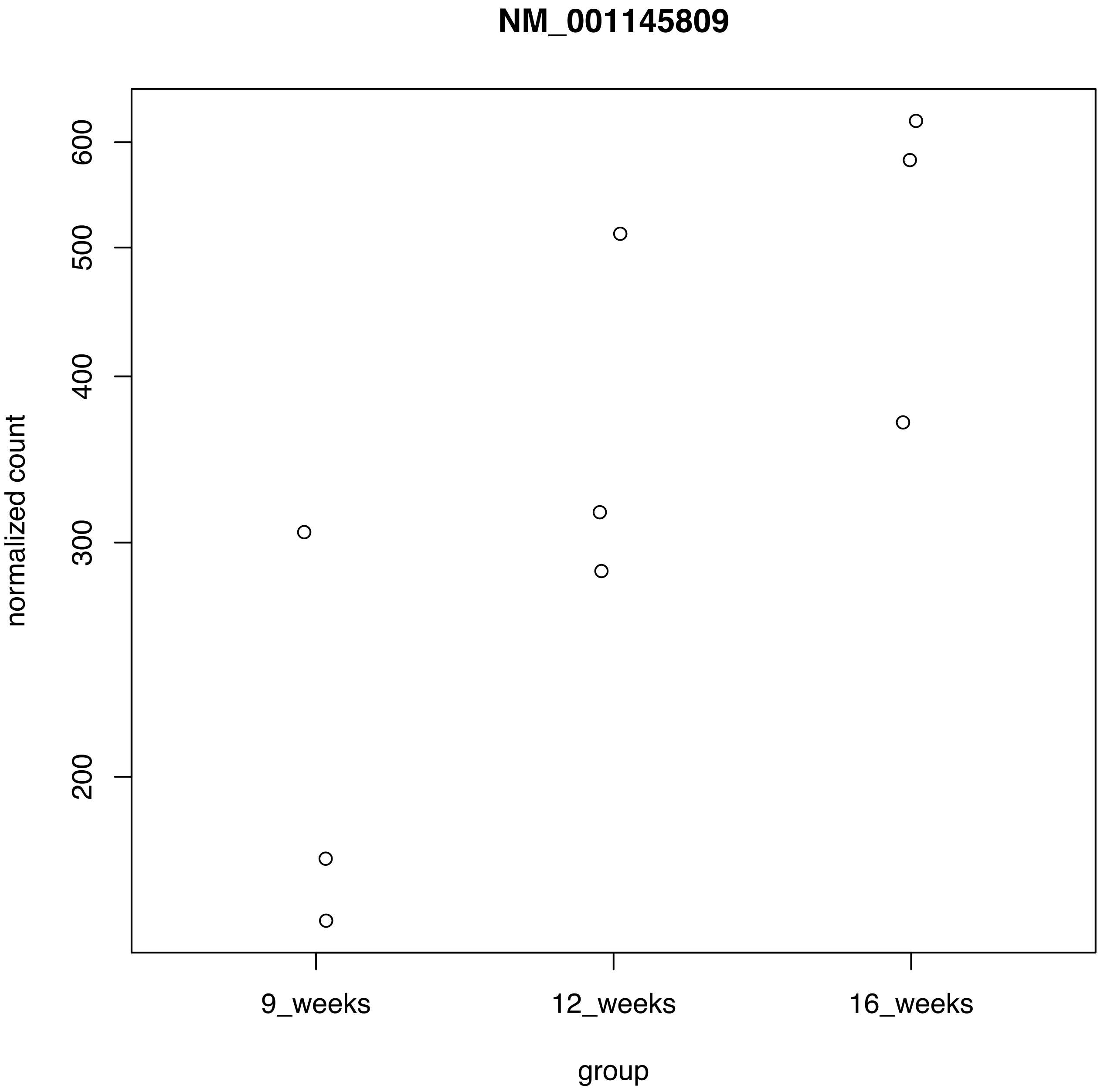
NM_001145809
normalized count
600
500
400
300
200
9_weeks
12_weeks
16_weeks
group

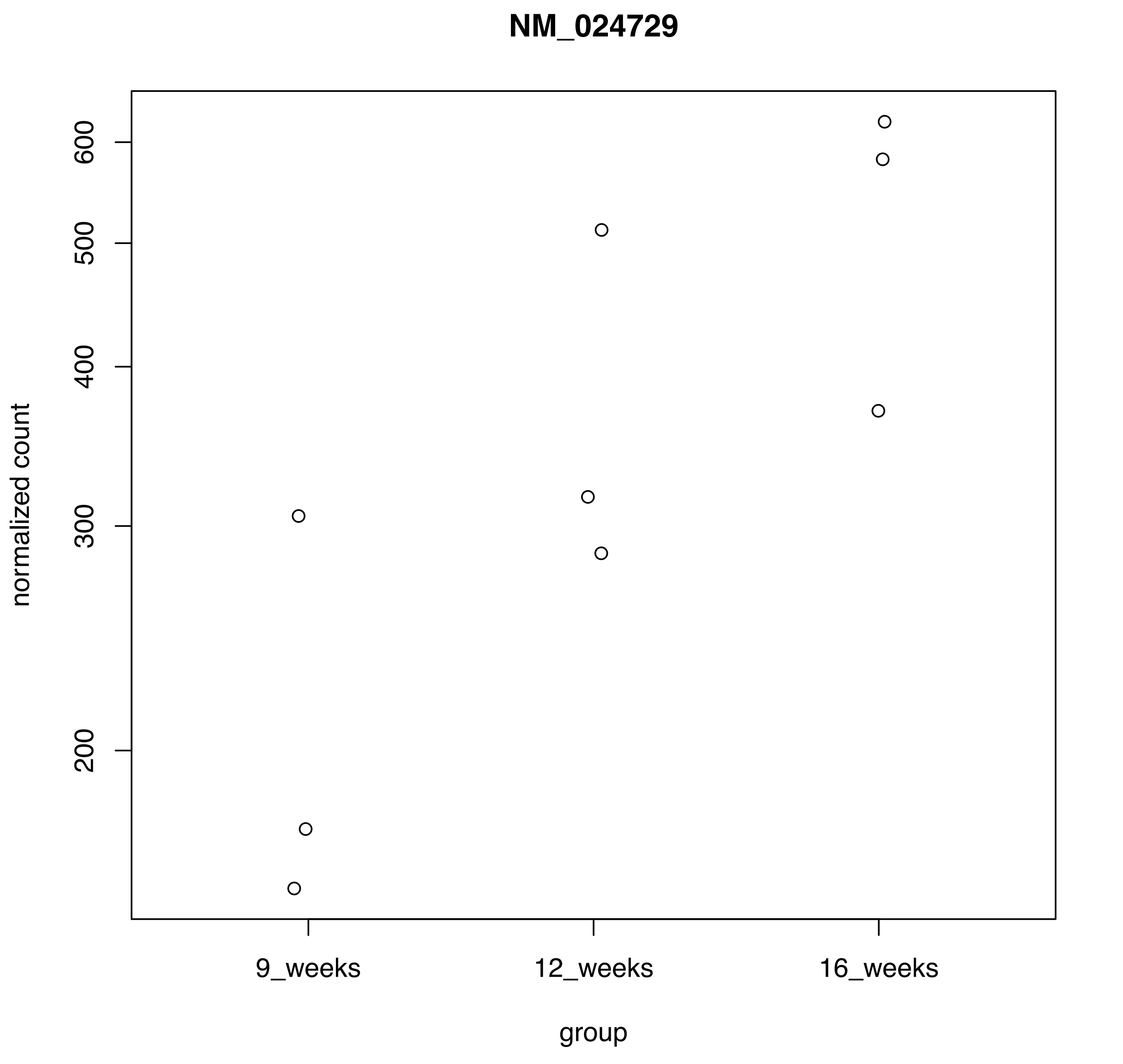
NM_024729
normalized count
600
500
400
300
200
9_weeks
12_weeks
16_weeks
group

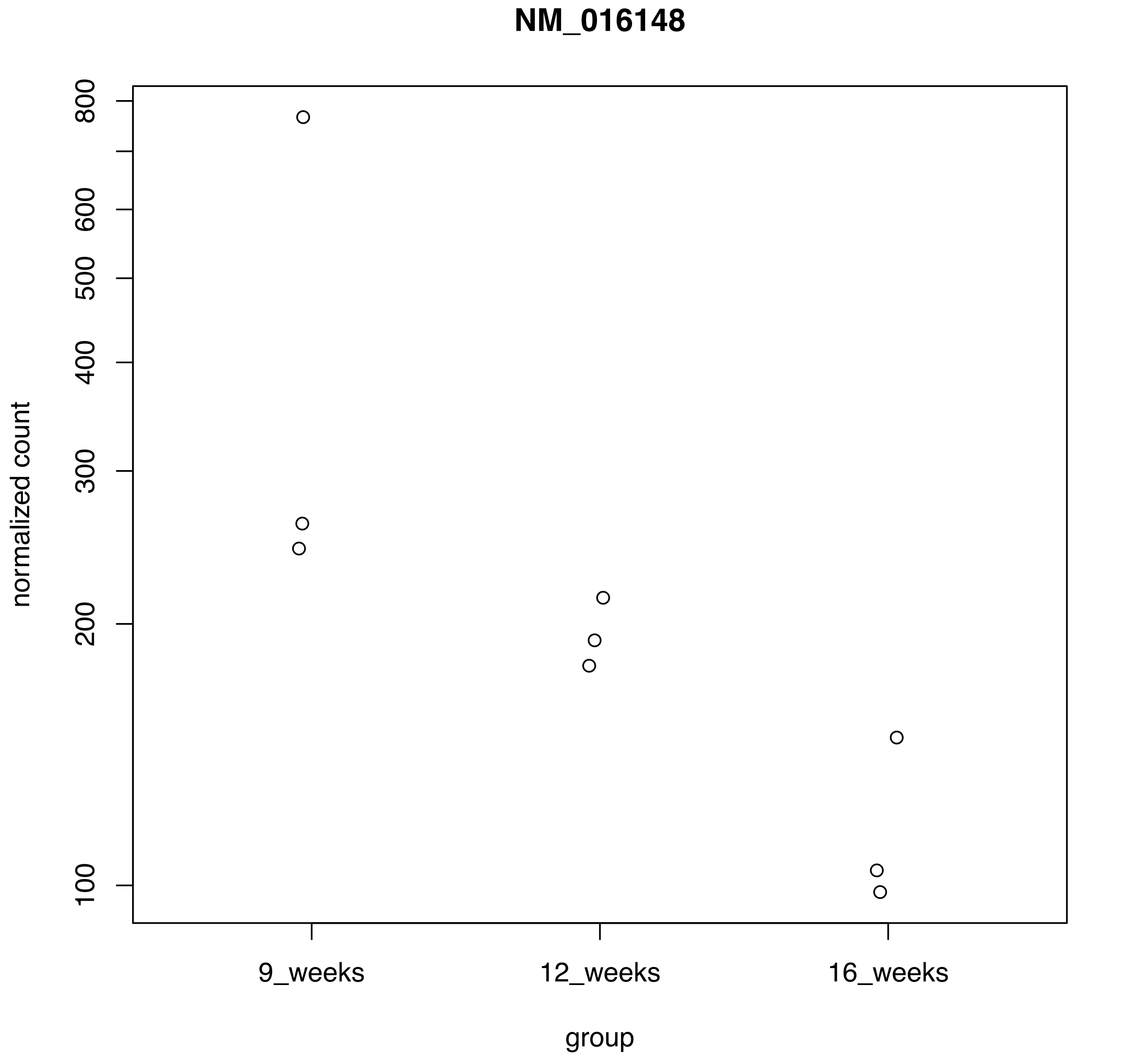

NM_016148
normalized count
800
600
500
400
300
200
100
9_weeks
12_weeks
16_weeks
group

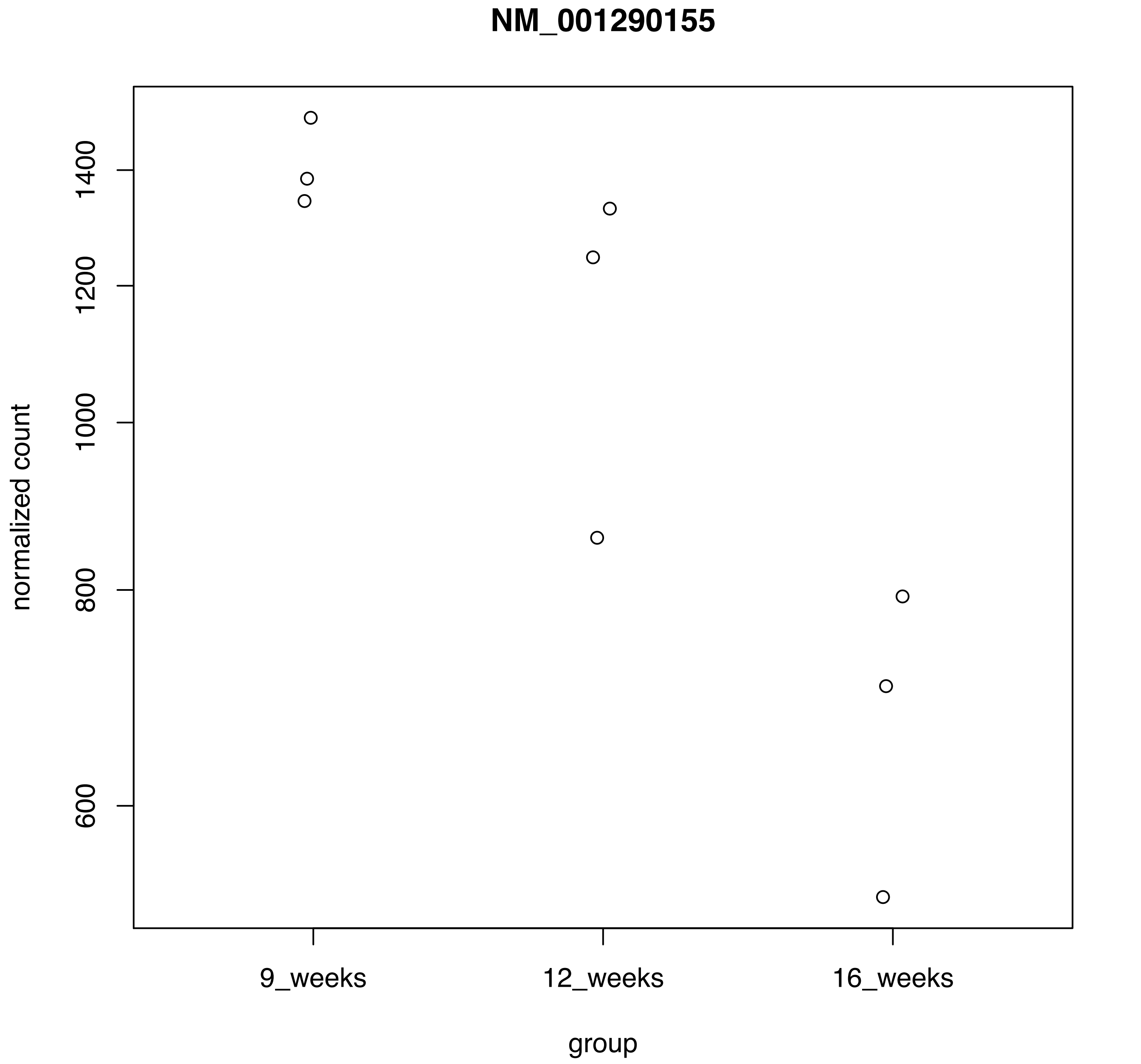
NM_001290155
normalized count
1400
1200
1000
800
600
9_weeks
12_weeks
16_weeks
group

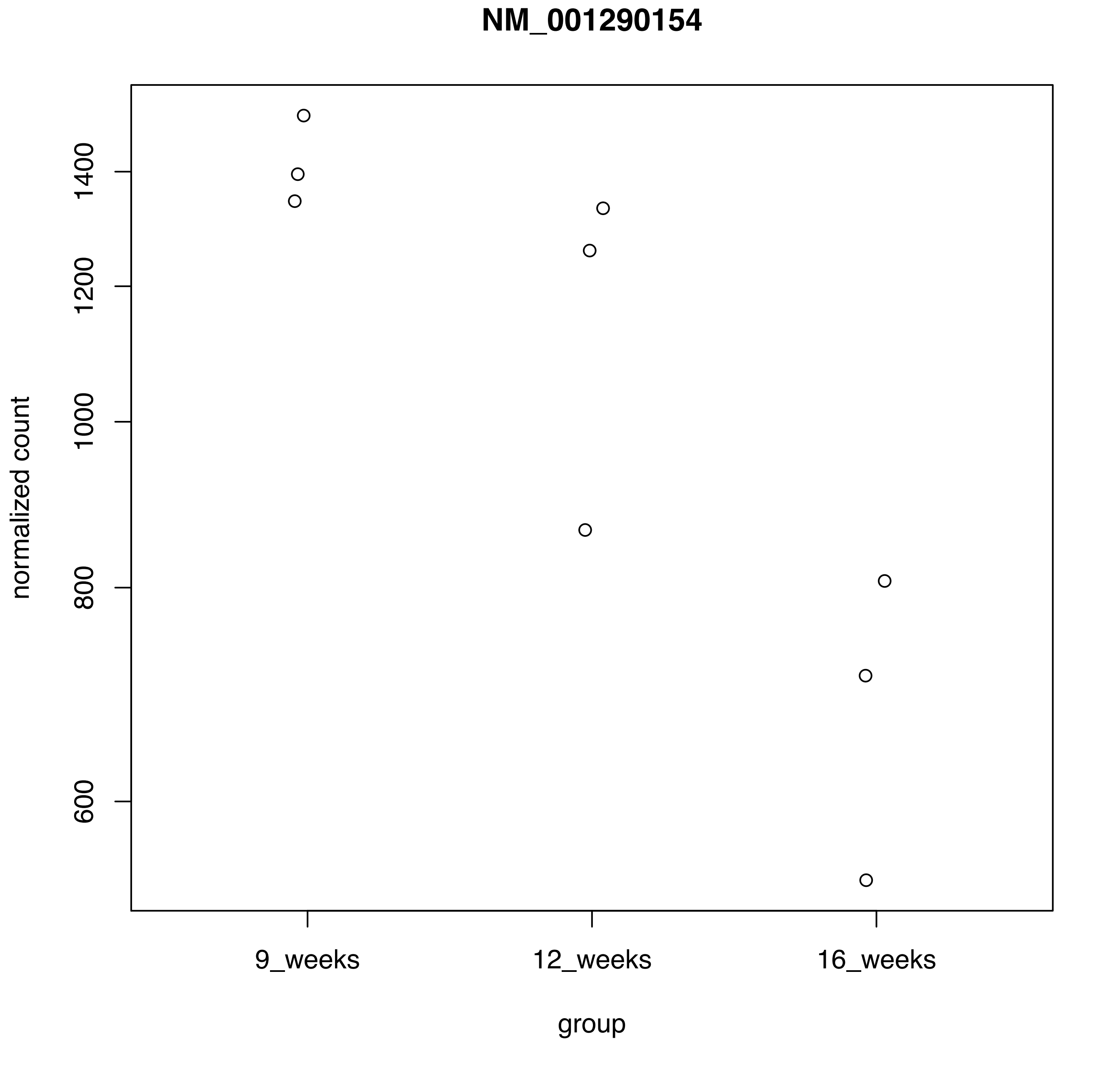
NM_001290154
normalized count
1400
1200
1000
800
600
9_weeks
12_weeks
16_weeks
group

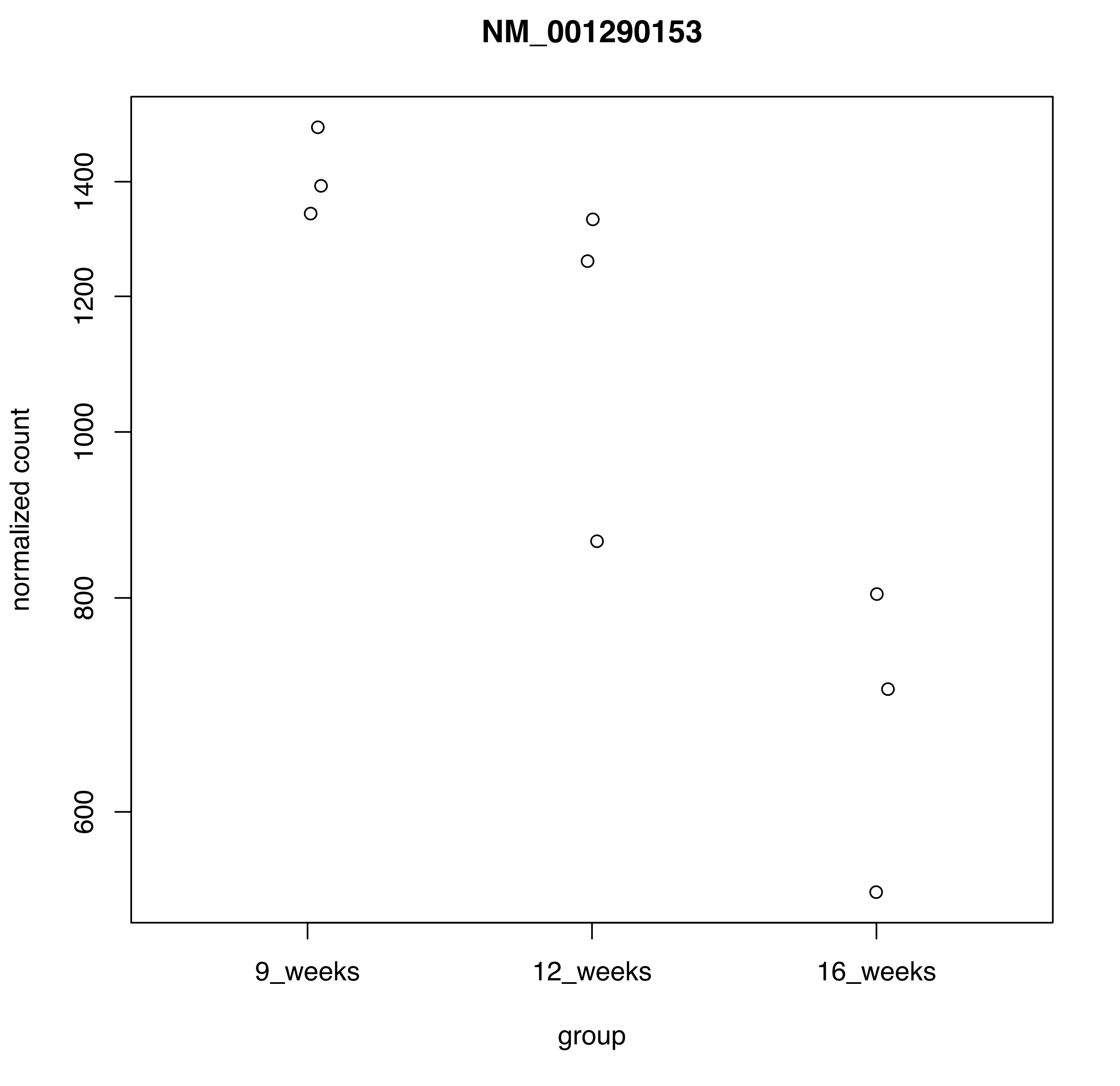
NM_001290153
normalized count
1400
1200
1000
800
600
9_weeks
12_weeks
16_weeks
group

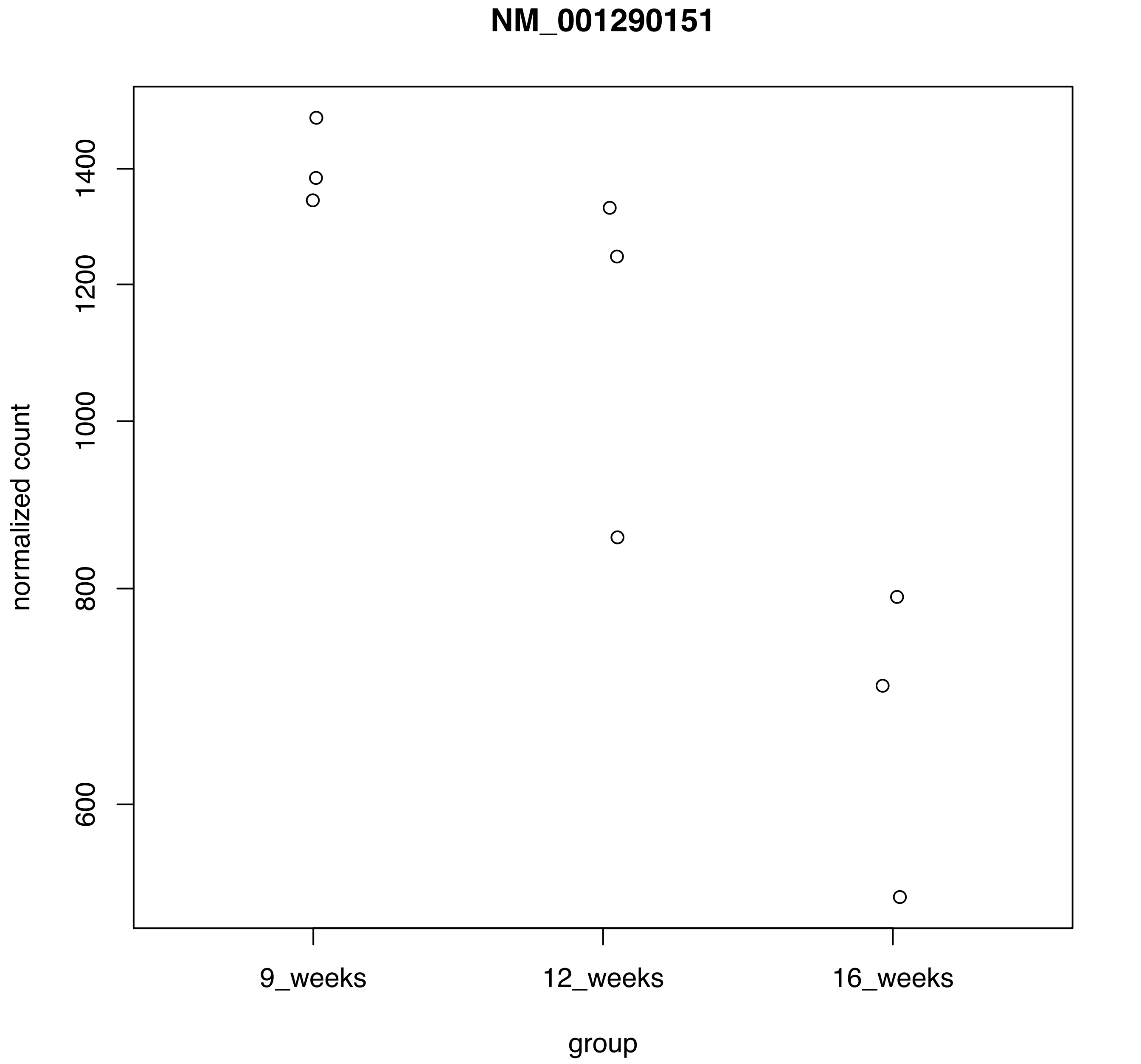
NM_001290151
normalized count
1400
1200
1000
800
600
9_weeks
12_weeks
16_weeks
group

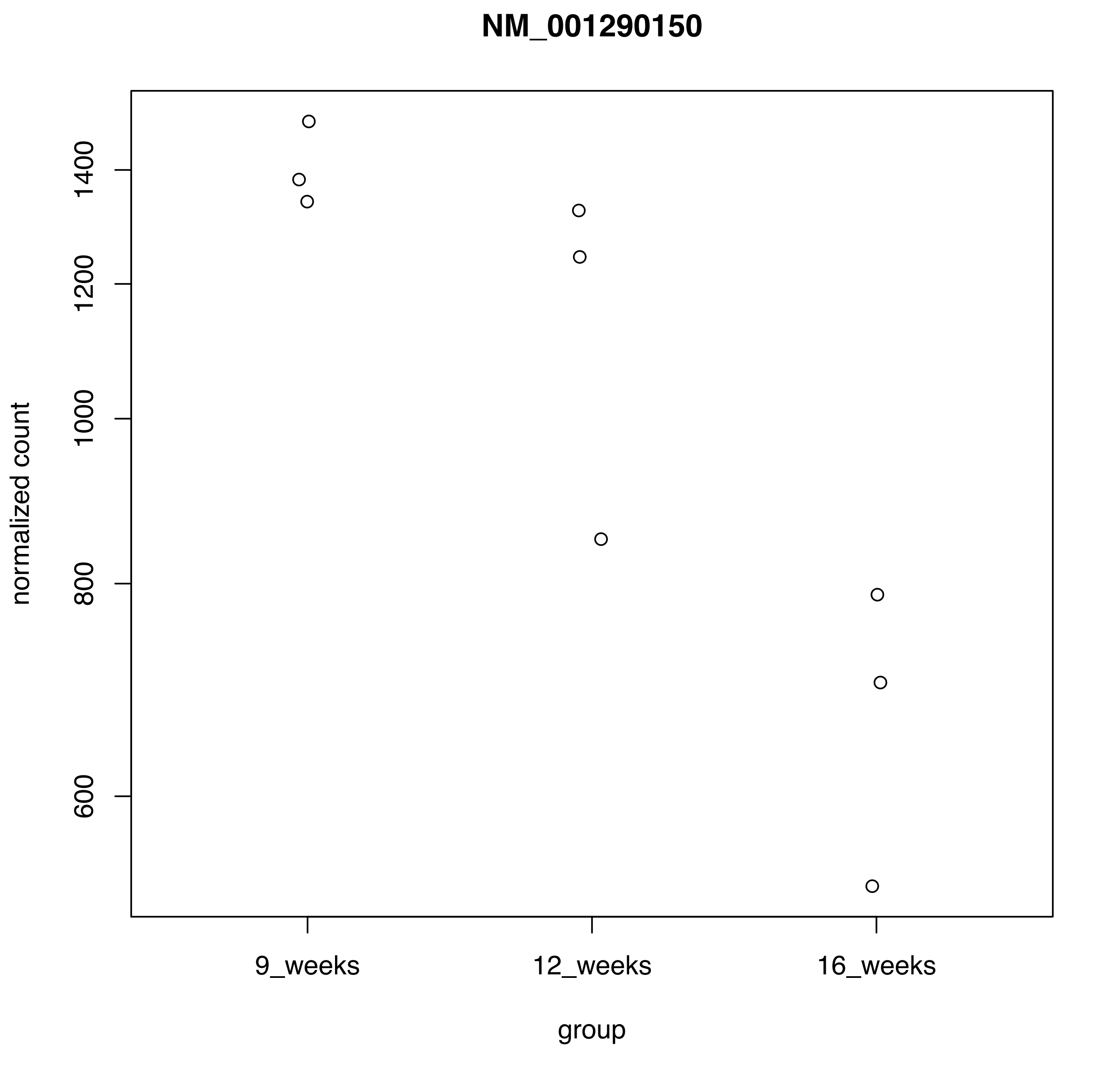

NM_001290150
normalized count
1400
1200
1000
800
600
9_weeks
12_weeks
16_weeks
group

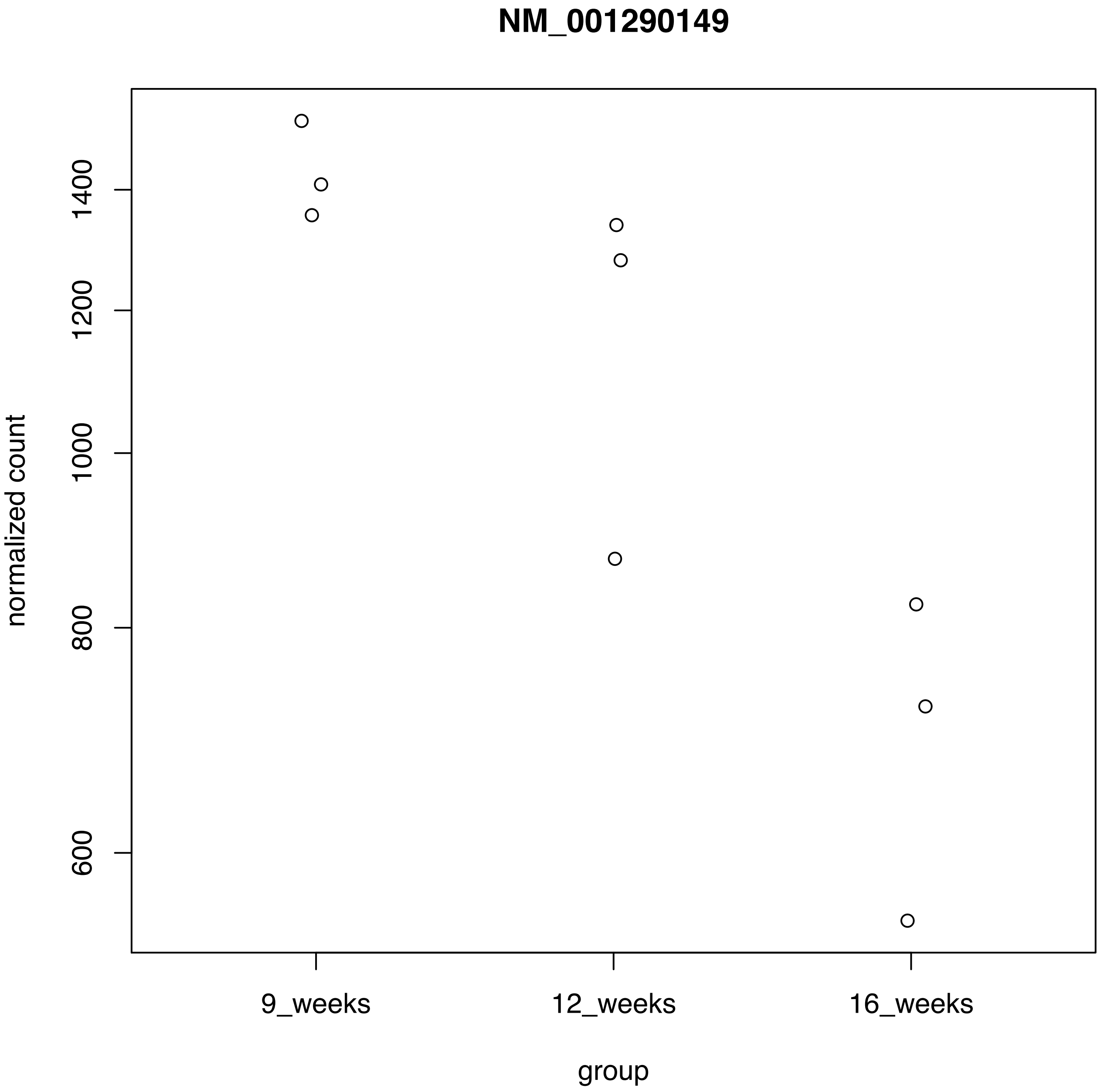
NM_001290149
normalized count
600
800
1000
1200
1400
9_weeks
12_weeks
16_weeks
group

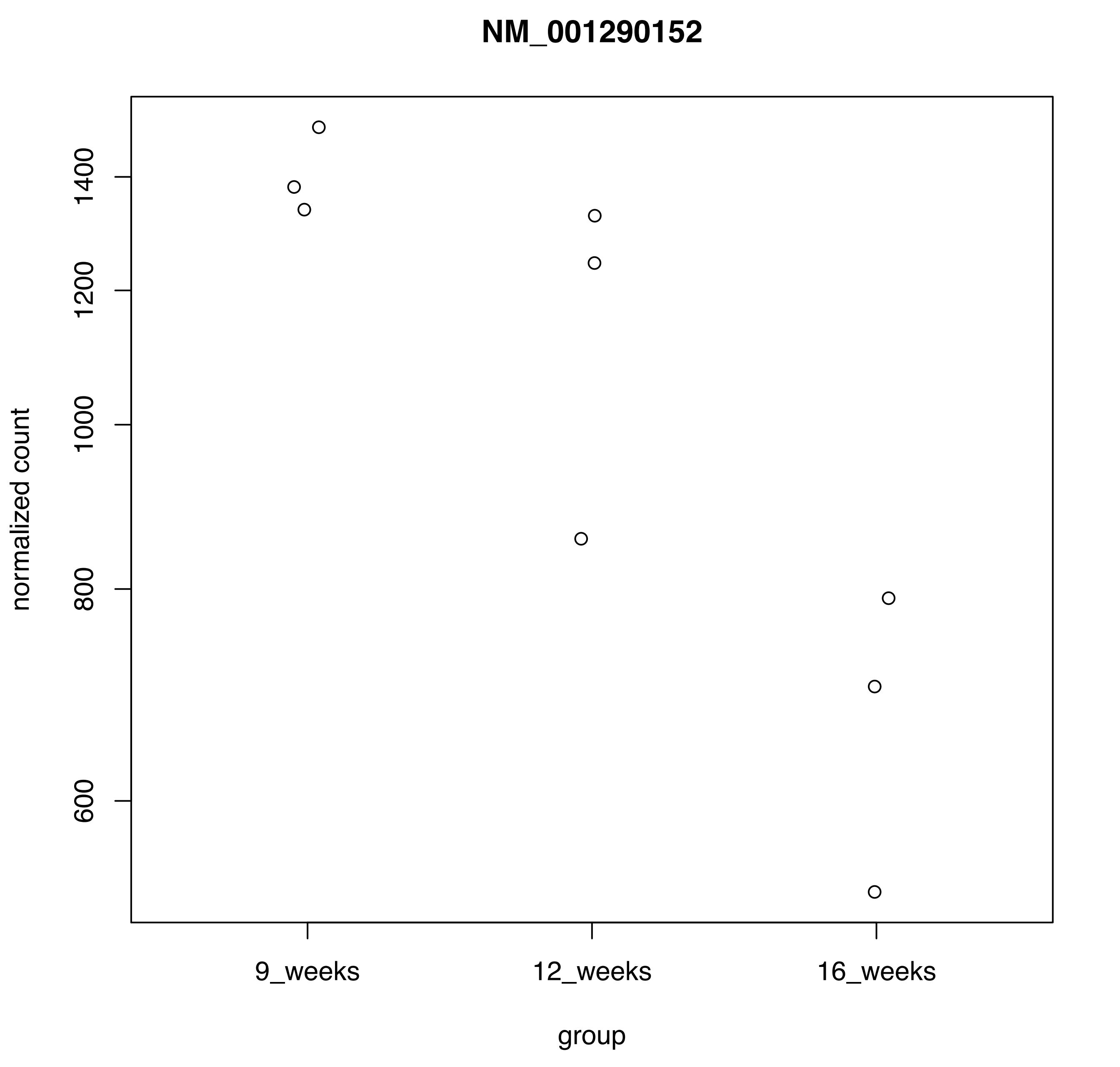
NM_001290152
normalized count
600
800
1000
1200
1400
9_weeks
12_weeks
16_weeks
group

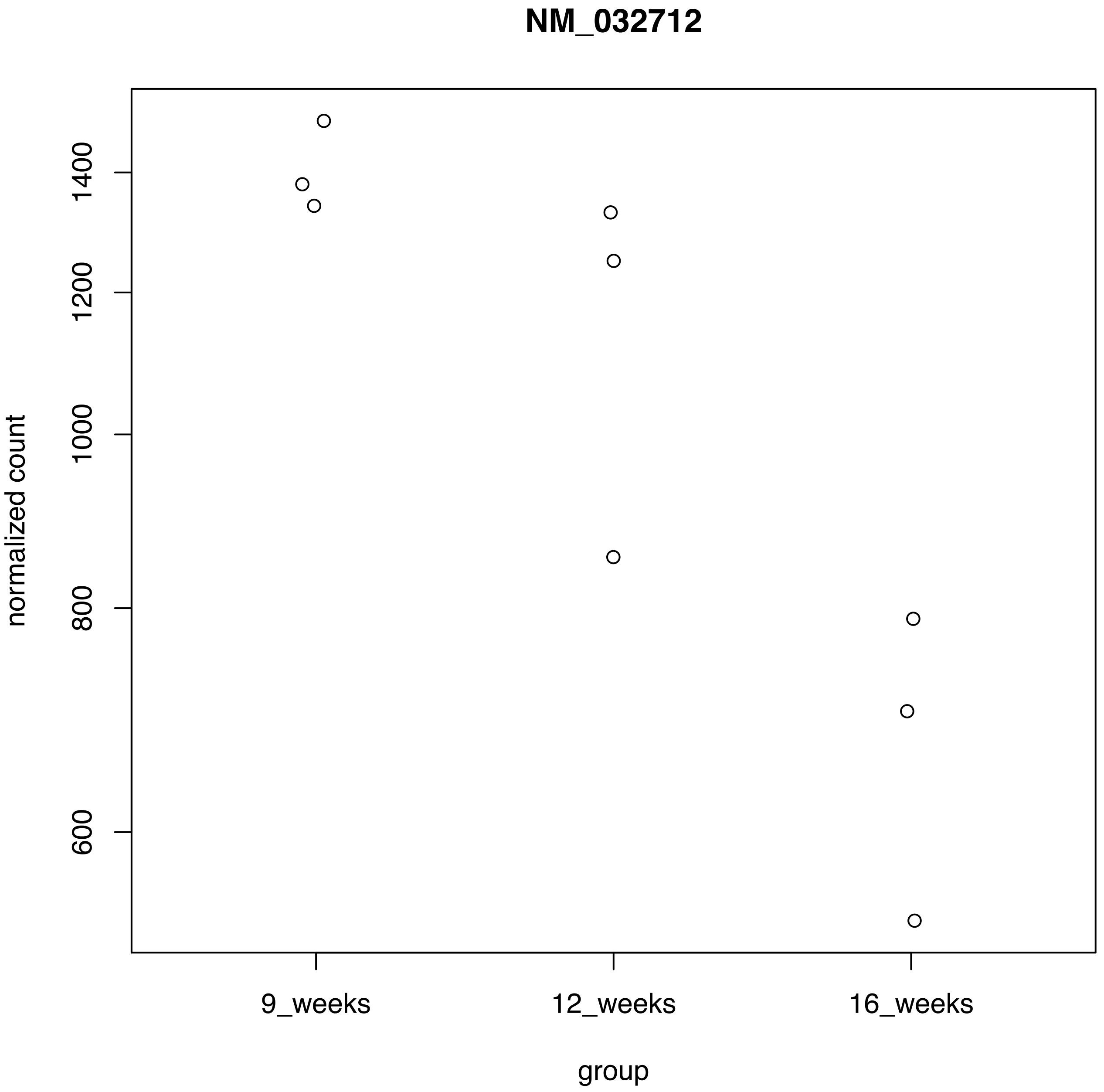
NM_032712
normalized count
600
800
1000
1200
1400
9_weeks
12_weeks
16_weeks
group

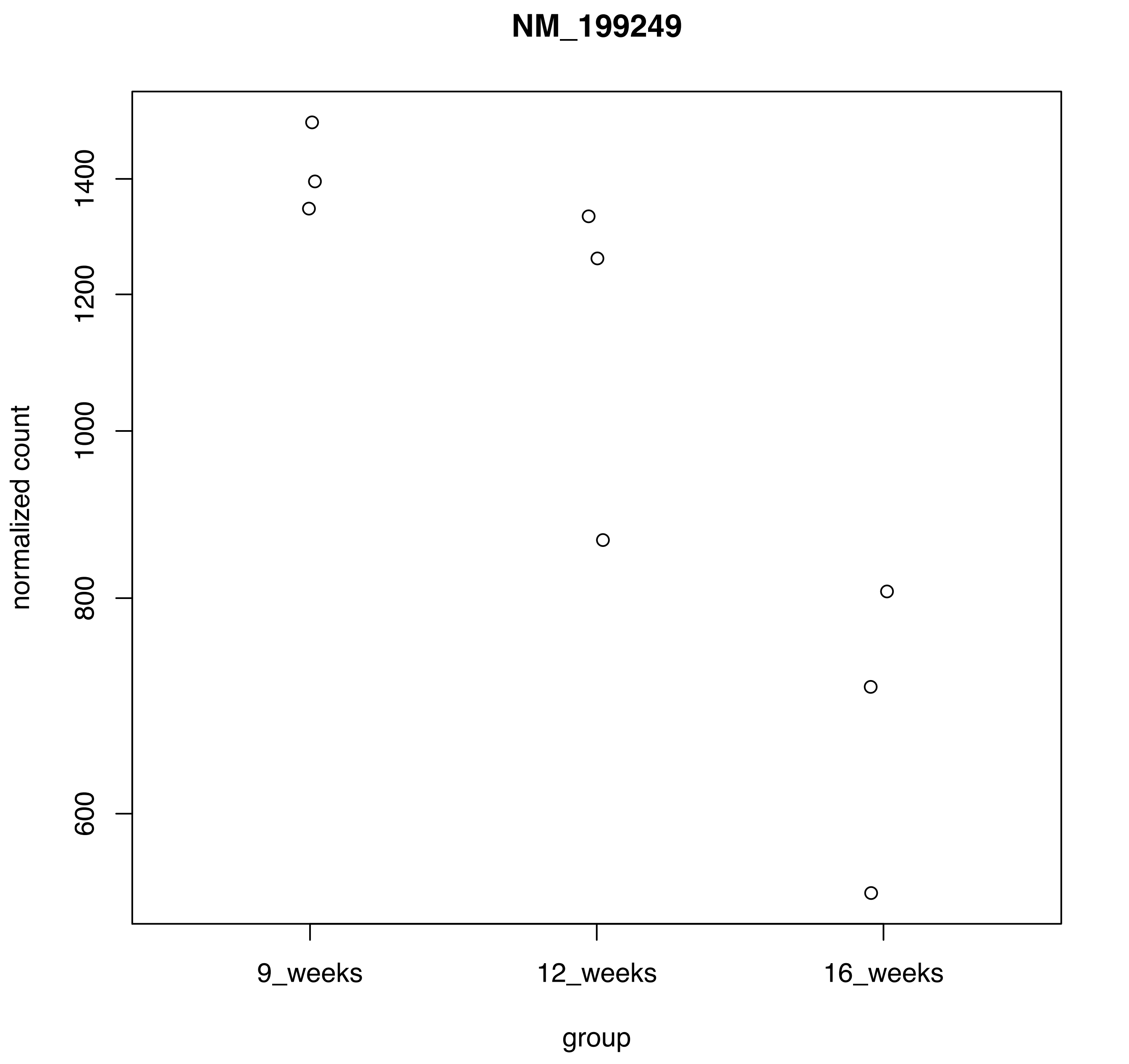
NM_199249
normalized count
600
800
1000
1200
1400
9_weeks
12_weeks
16_weeks
group

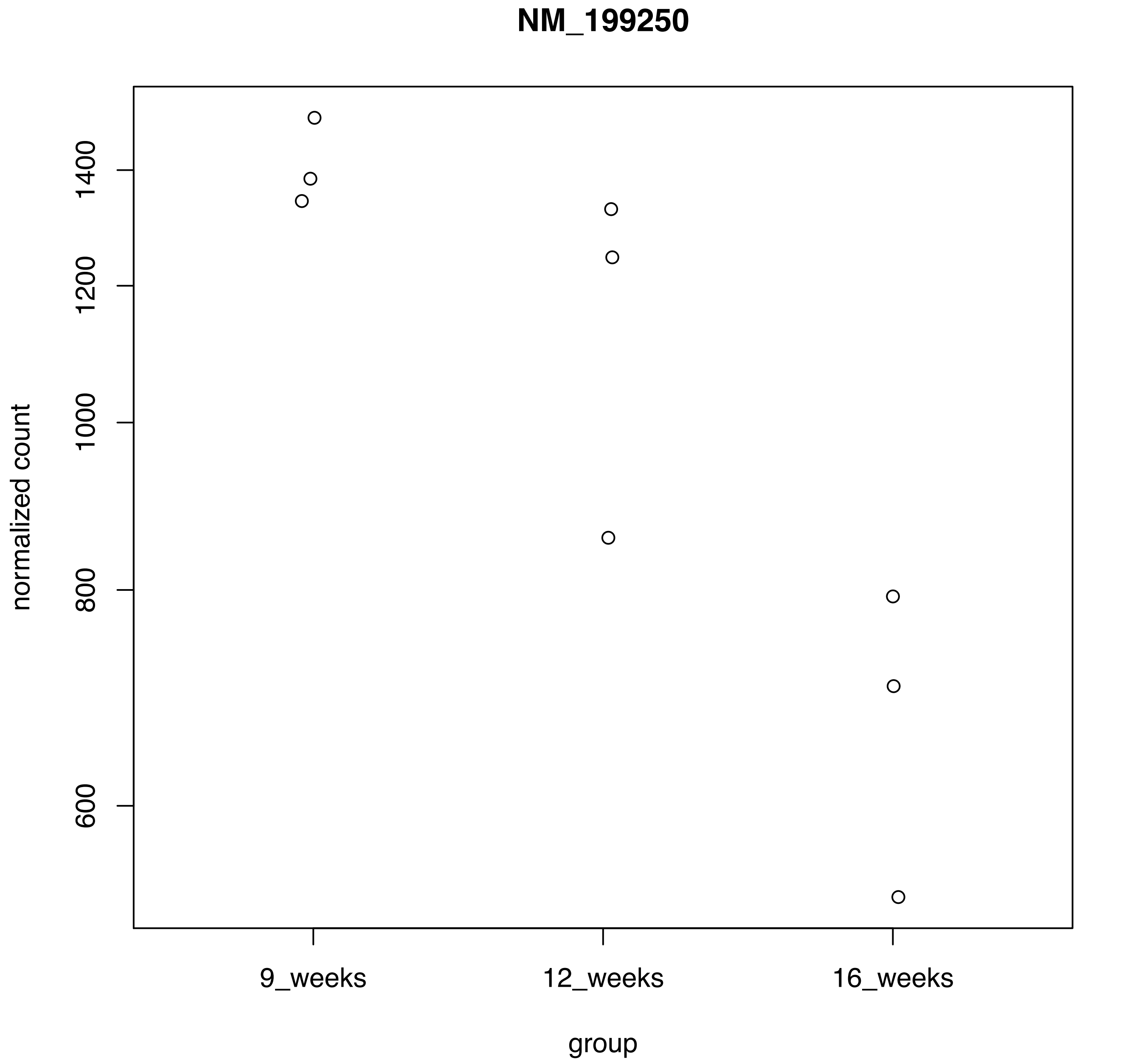
NM_199250
normalized count
1400
1200
1000
800
600
9_weeks
12_weeks
16_weeks
group

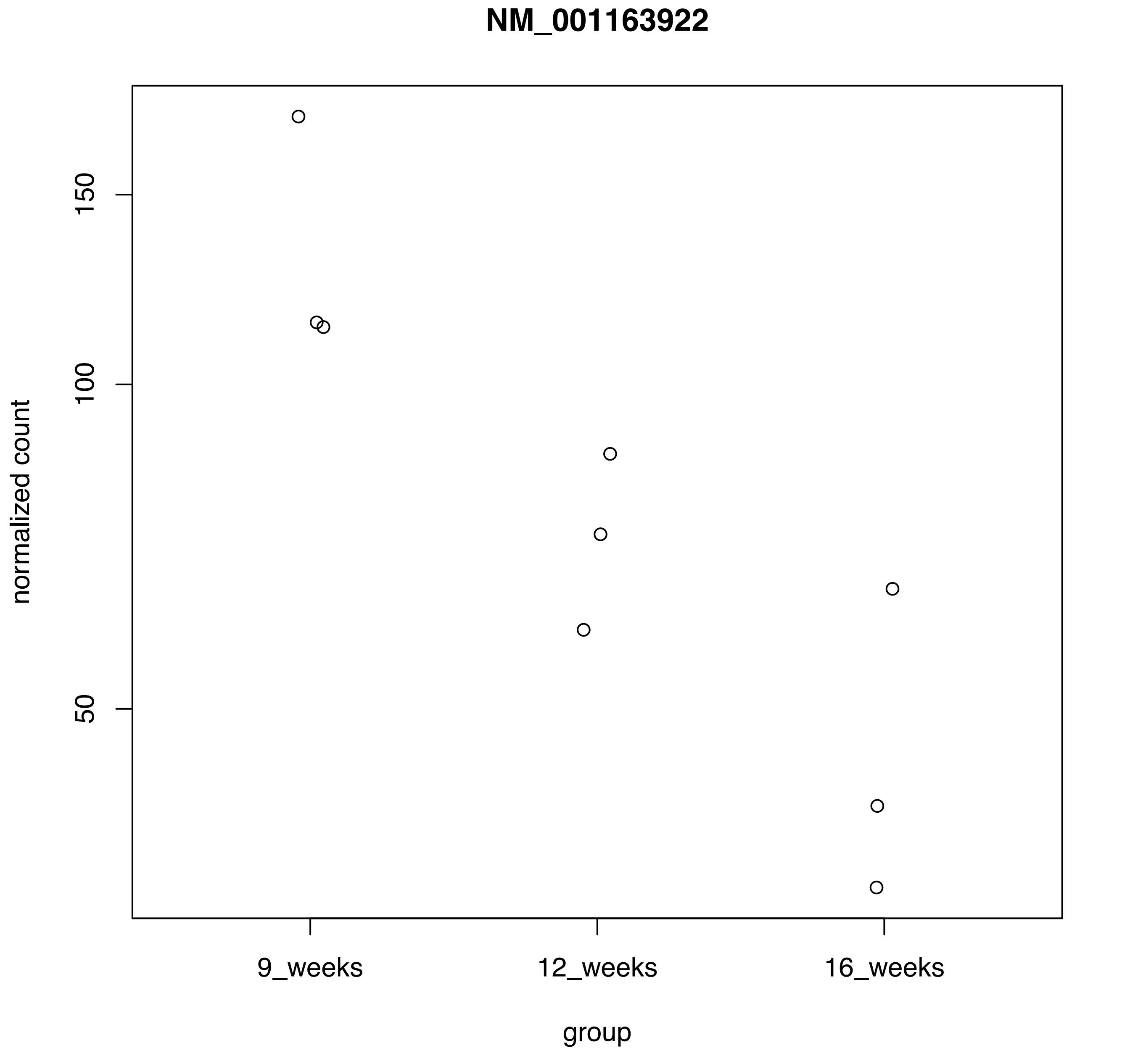

NM_001163922
normalized count
150
100
50
9_weeks
12_weeks
16_weeks
group

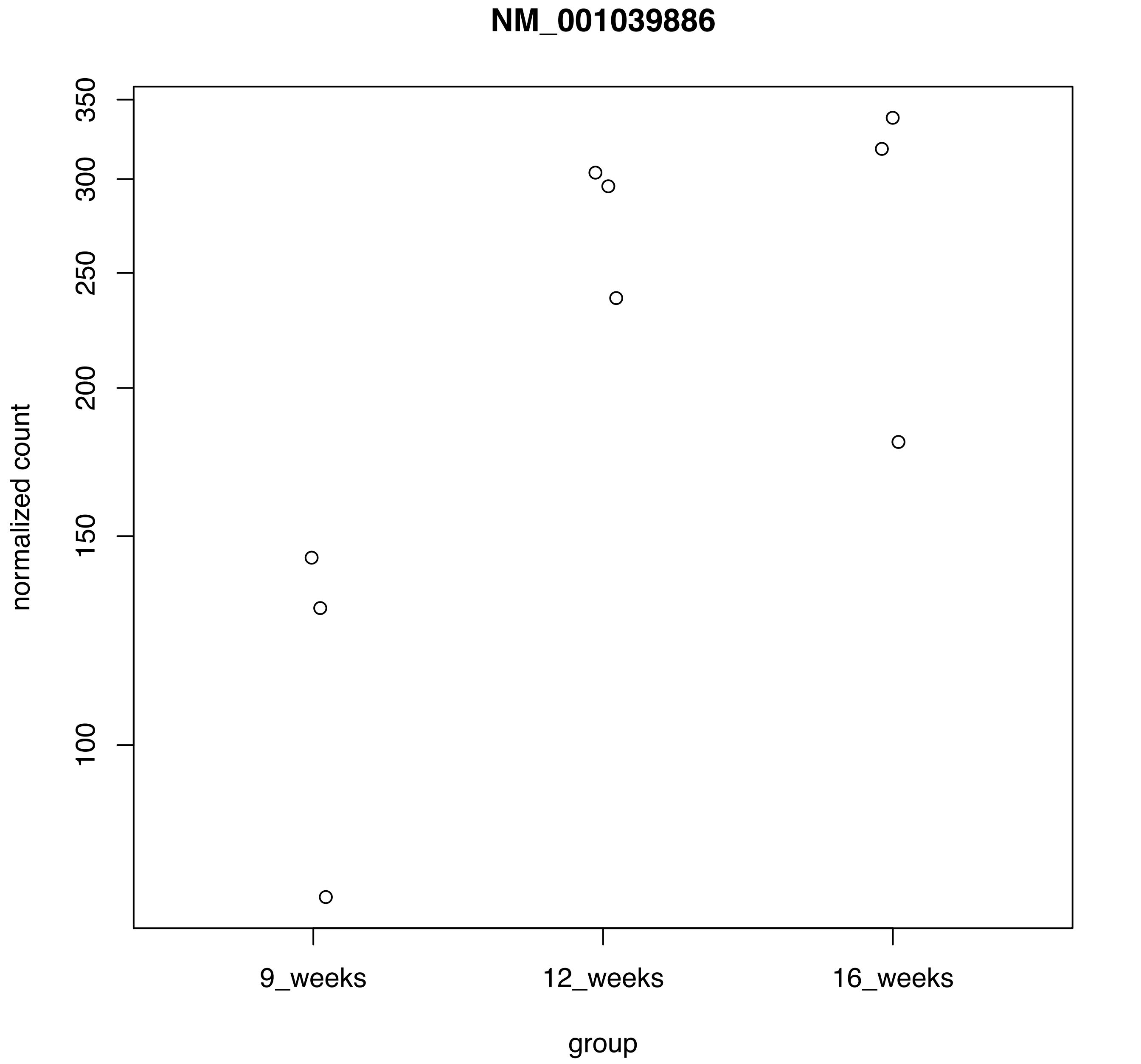
NM_001039886
normalized count
350
300
250
200
150
100
9_weeks
12_weeks
16_weeks
group

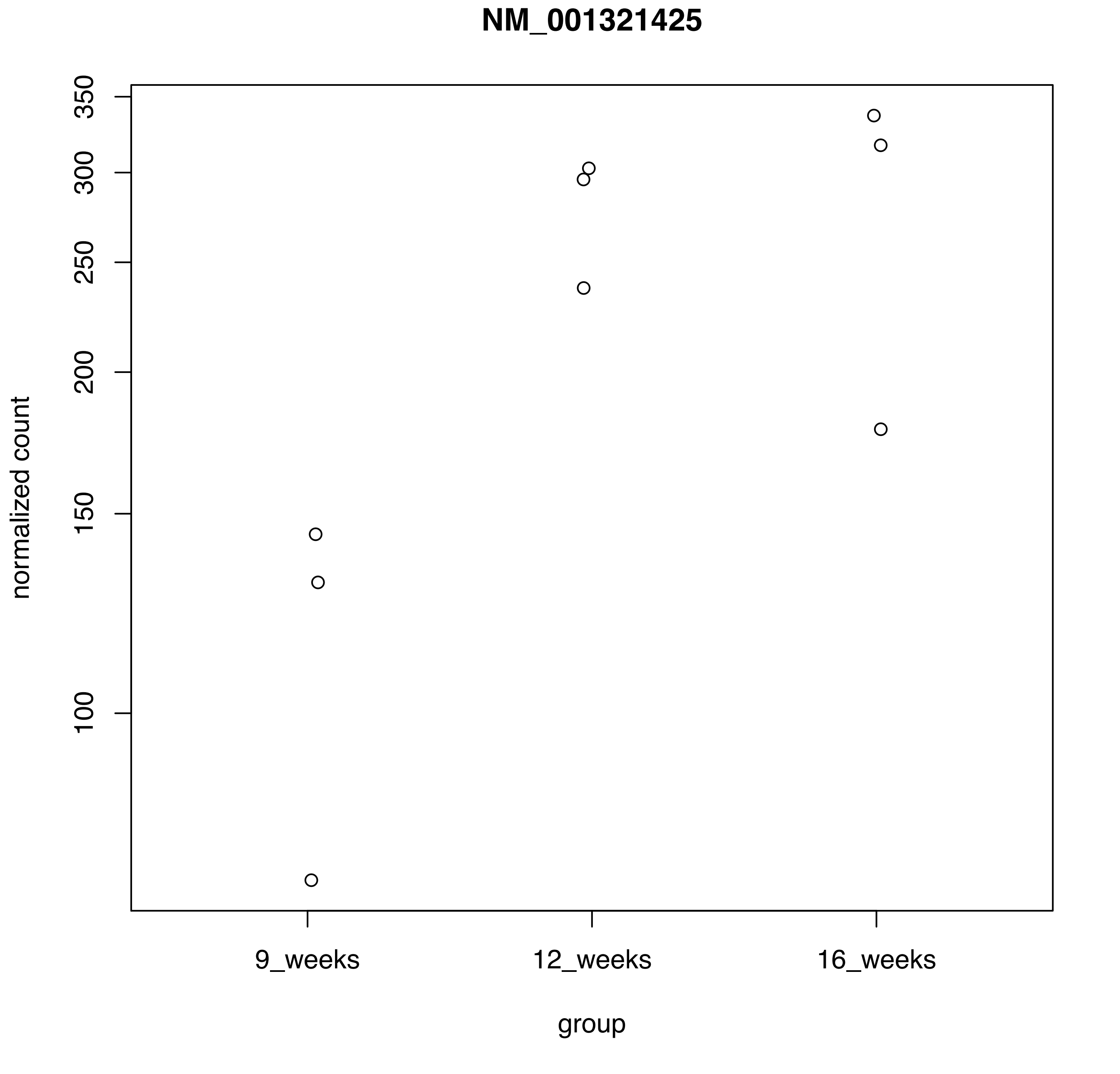
NM_001321425
normalized count
100
150
200
250
300
350
9_weeks
12_weeks
16_weeks
group

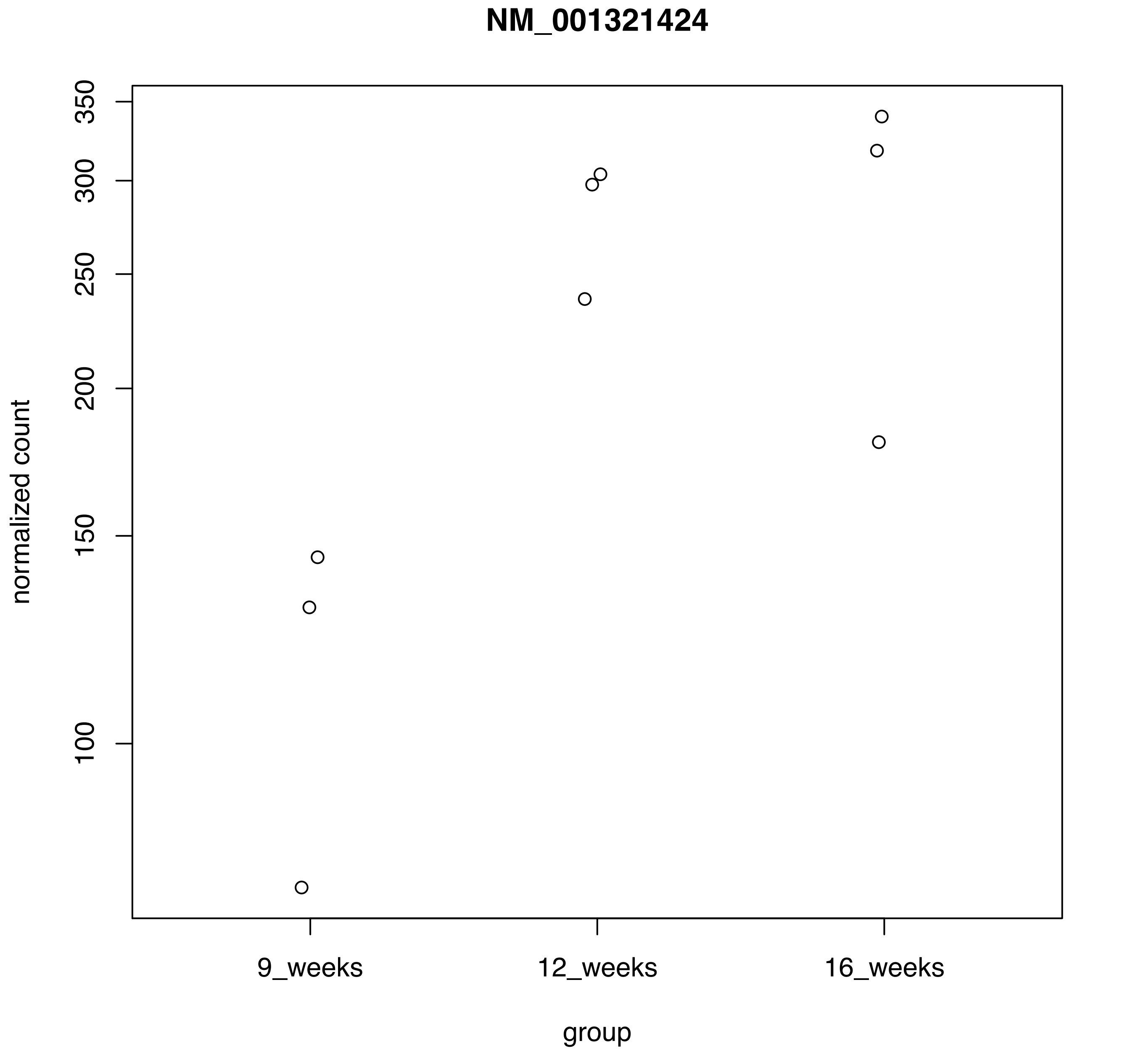
NM_001321424
normalized count
100
150
200
250
300
350
9_weeks
12_weeks
16_weeks
group

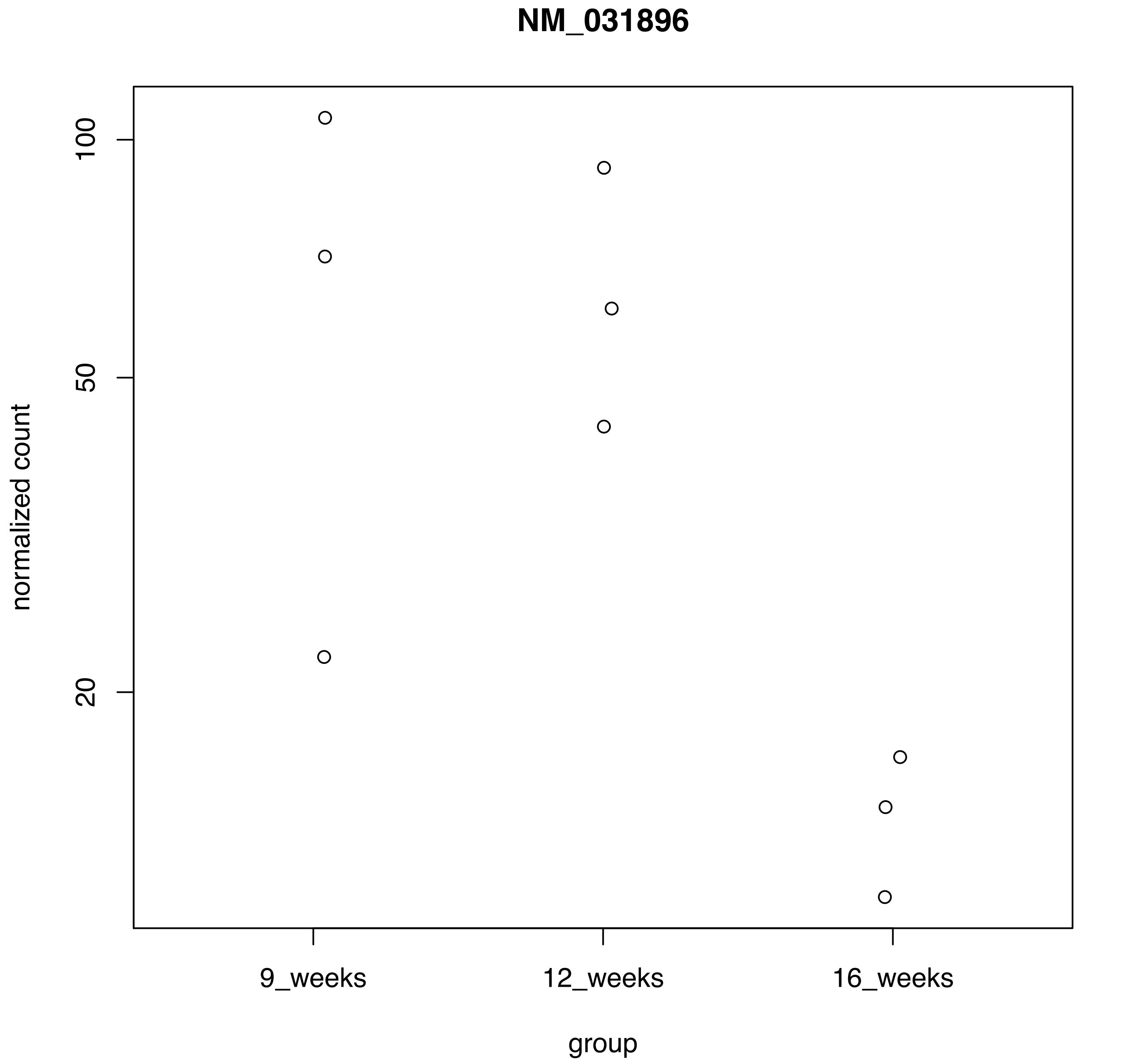
NM_031896
normalized count
100
50
20
9_weeks
12_weeks
16_weeks
group

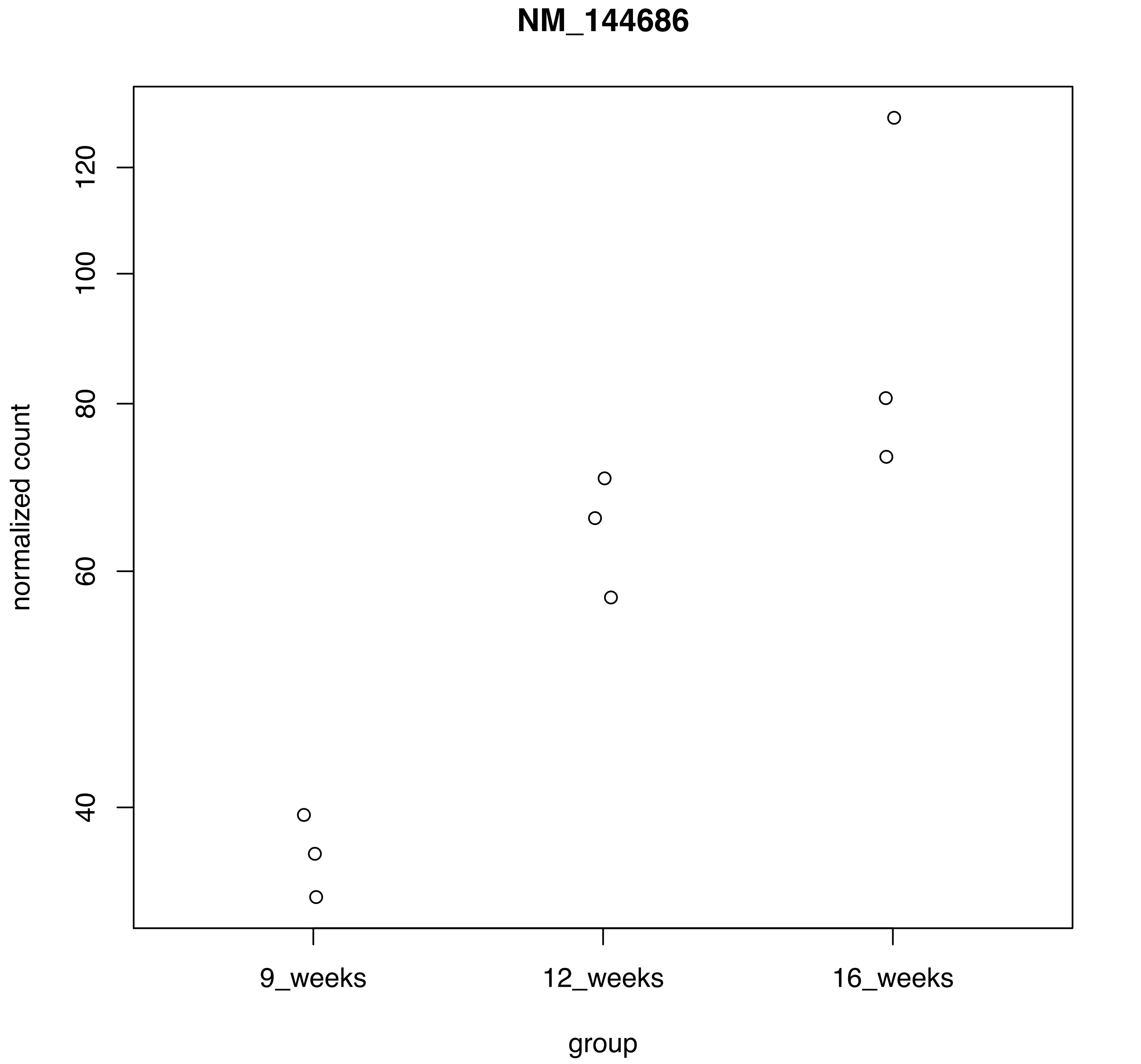
NM_144686
normalized count
40
60
80
100
120
9_weeks
12_weeks
16_weeks
group

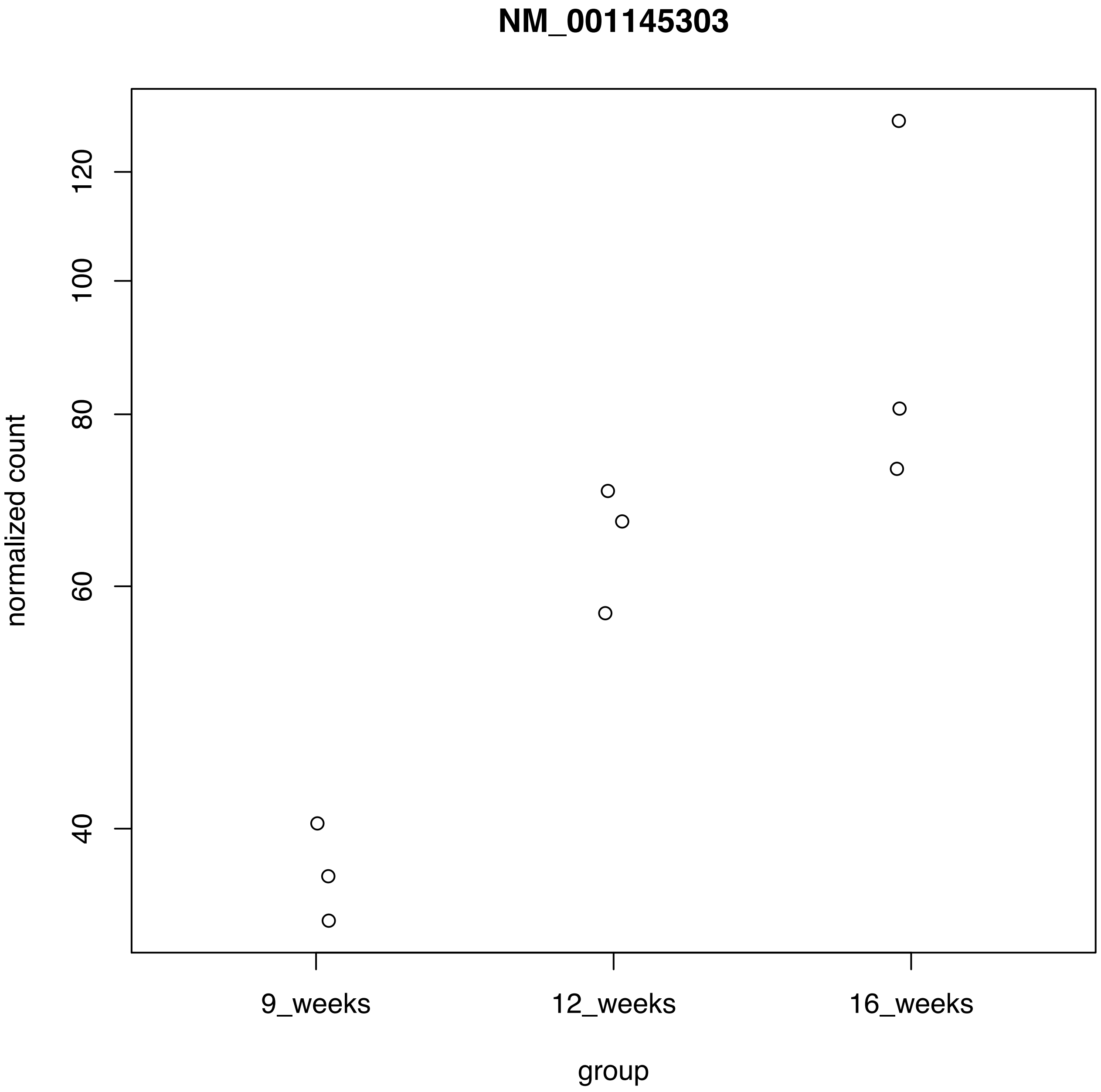
NM_001145303
normalized count
40
60
80
100
120
9_weeks
12_weeks
16_weeks
group

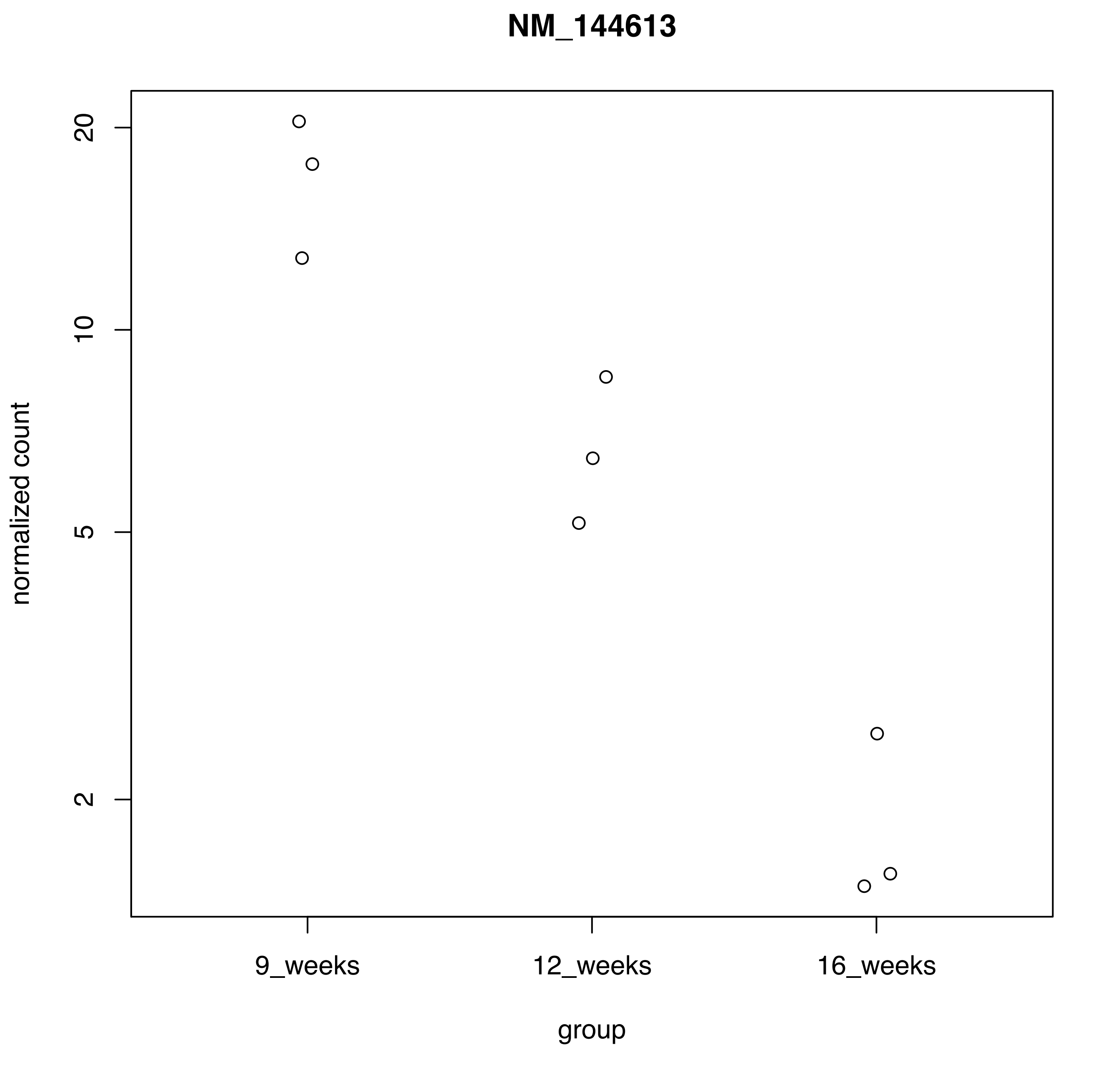
NM_144613
normalized count
20
10
5
2
9_weeks
12_weeks
16_weeks
group

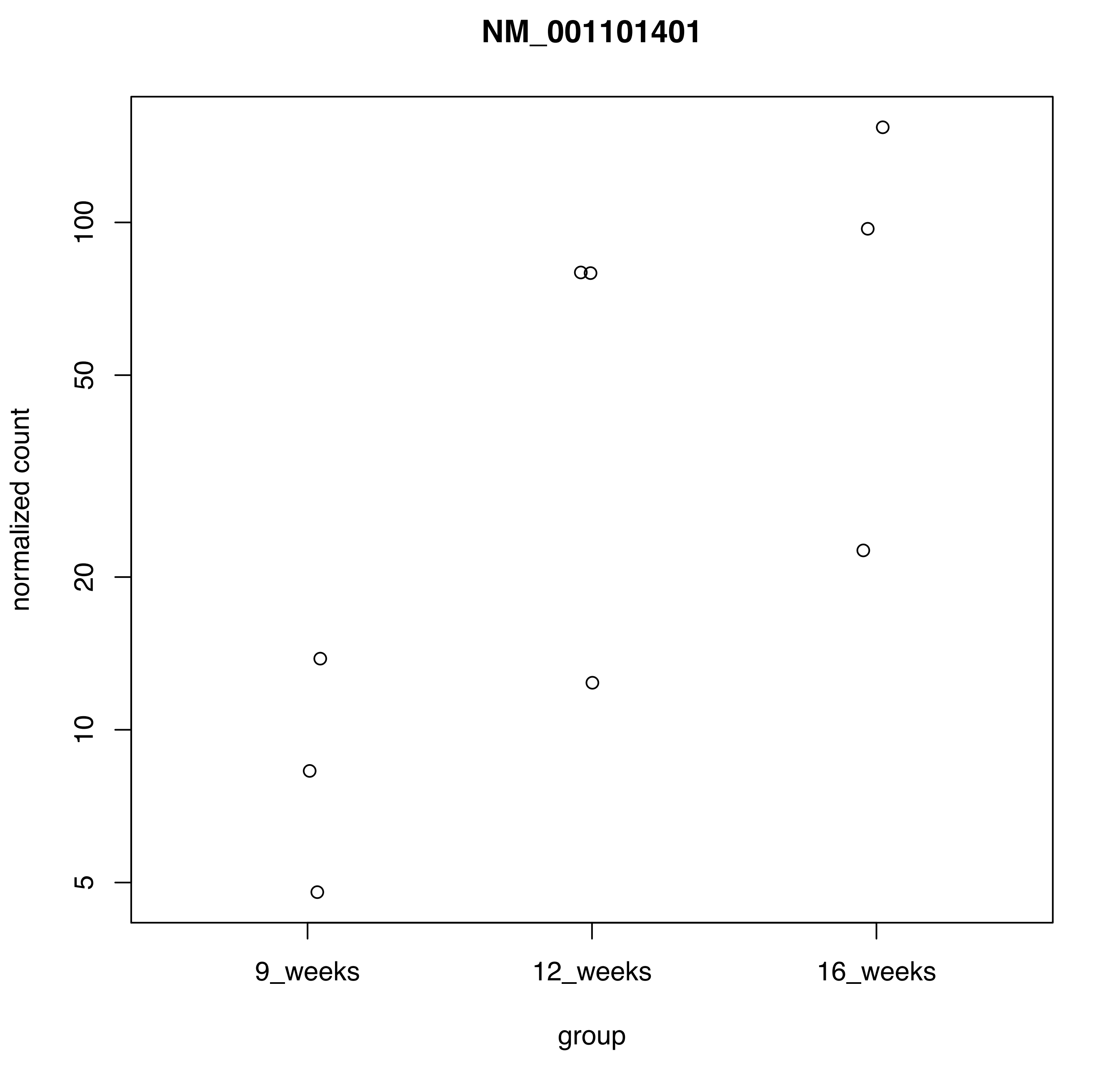
NM_001101401
normalized count
5
10
20
50
100
9_weeks
12_weeks
16_weeks
group

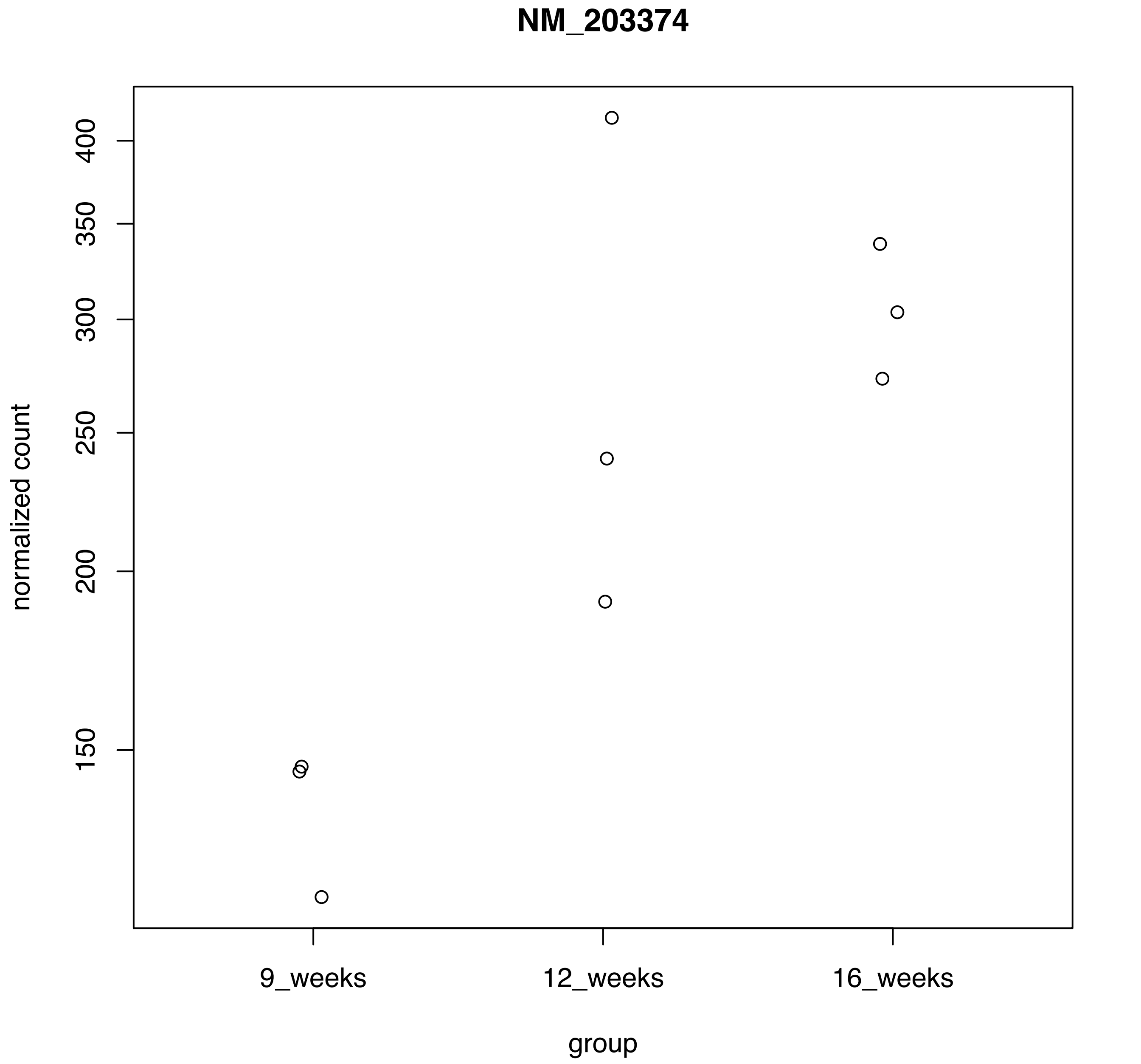

NM_203374
normalized count
400
350
300
250
200
150
9_weeks
12_weeks
16_weeks
group

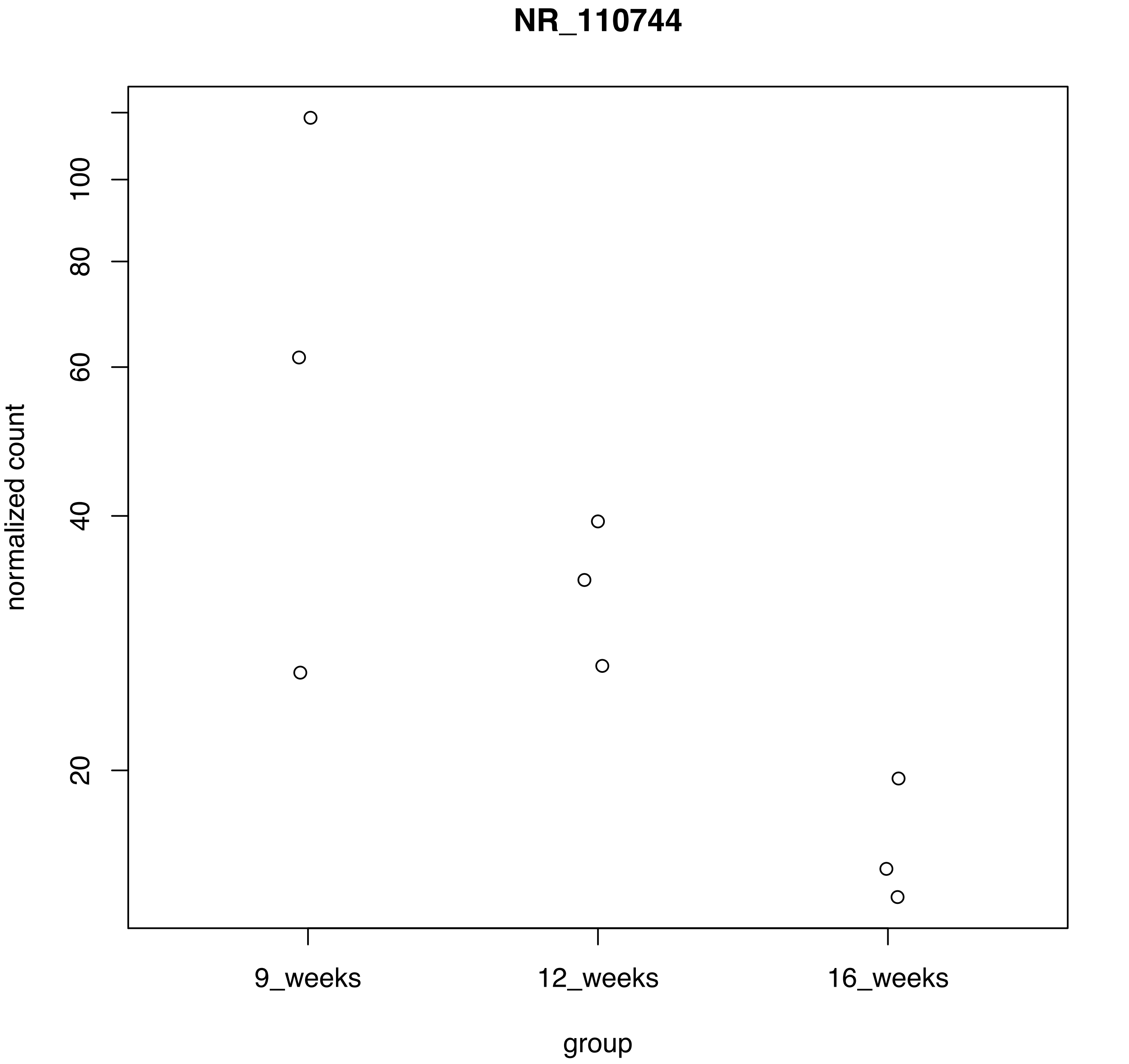
NR_110744
normalized count
20
40
60
80
100
9_weeks
12_weeks
16_weeks
group

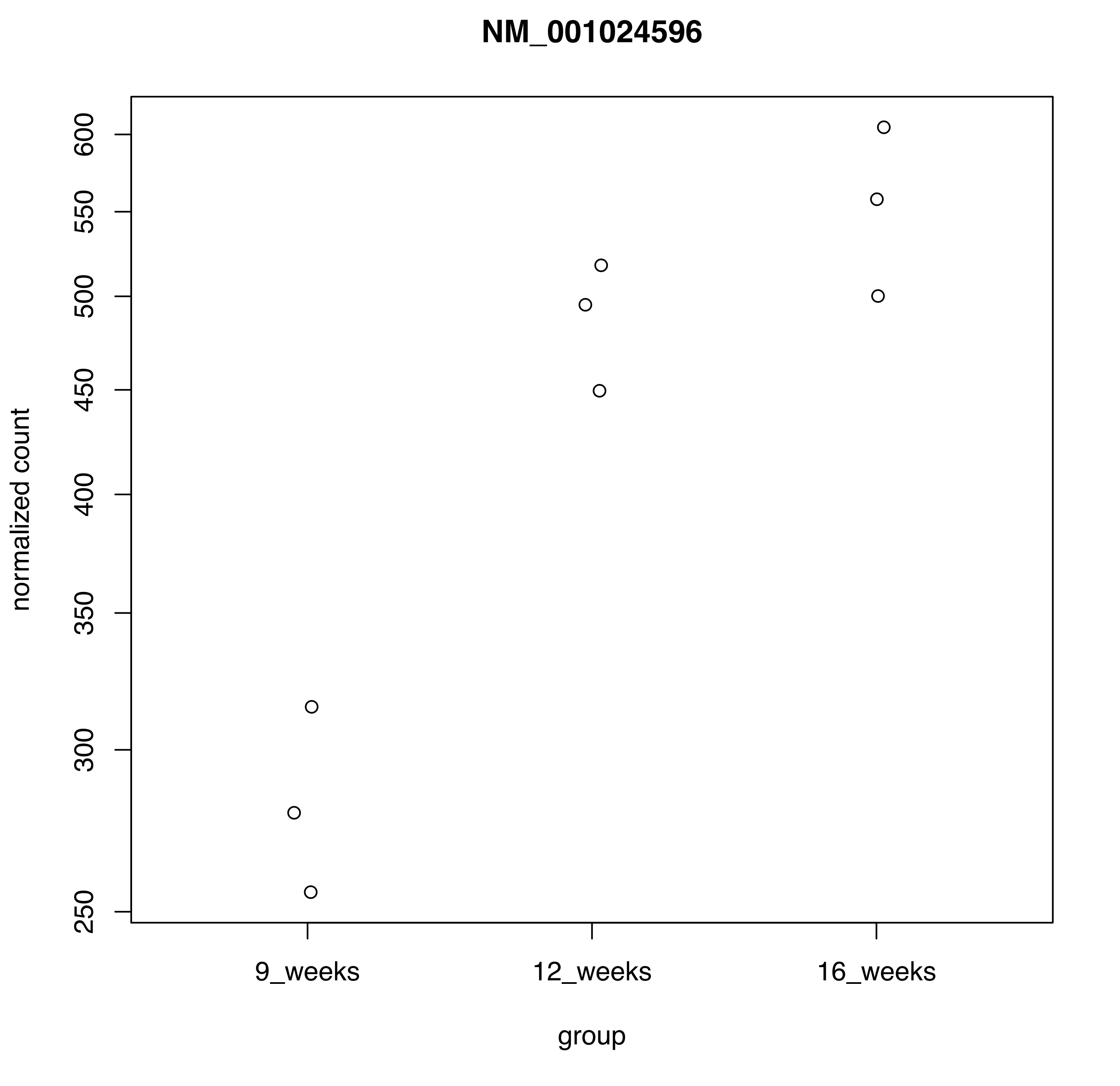
NM_001024596
normalized count
250
300
350
400
450
500
550
600
9_weeks
12_weeks
16_weeks
group

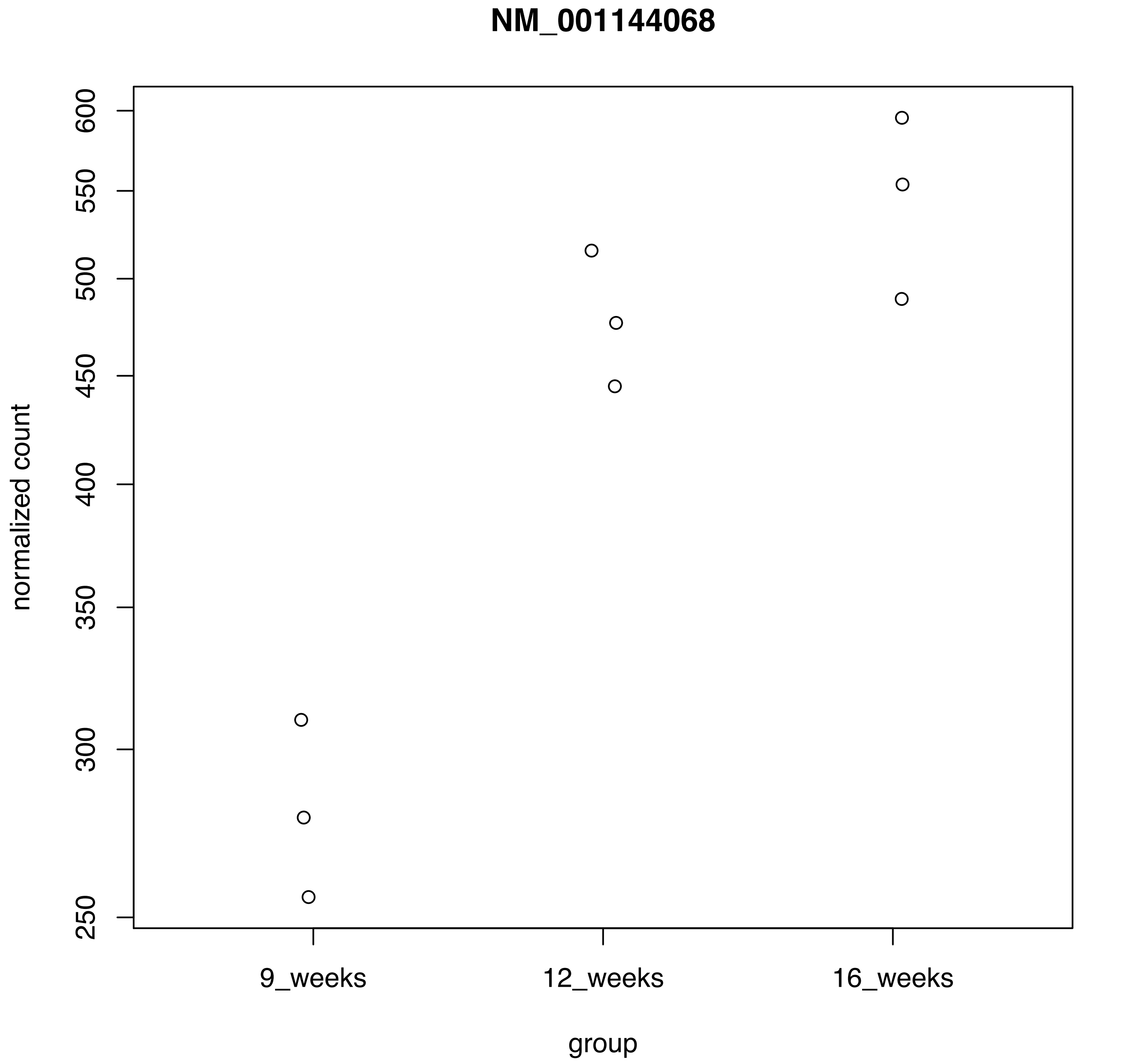
NM_001144068
normalized count
250
300
350
400
450
500
550
600
9_weeks
12_weeks
16_weeks
group

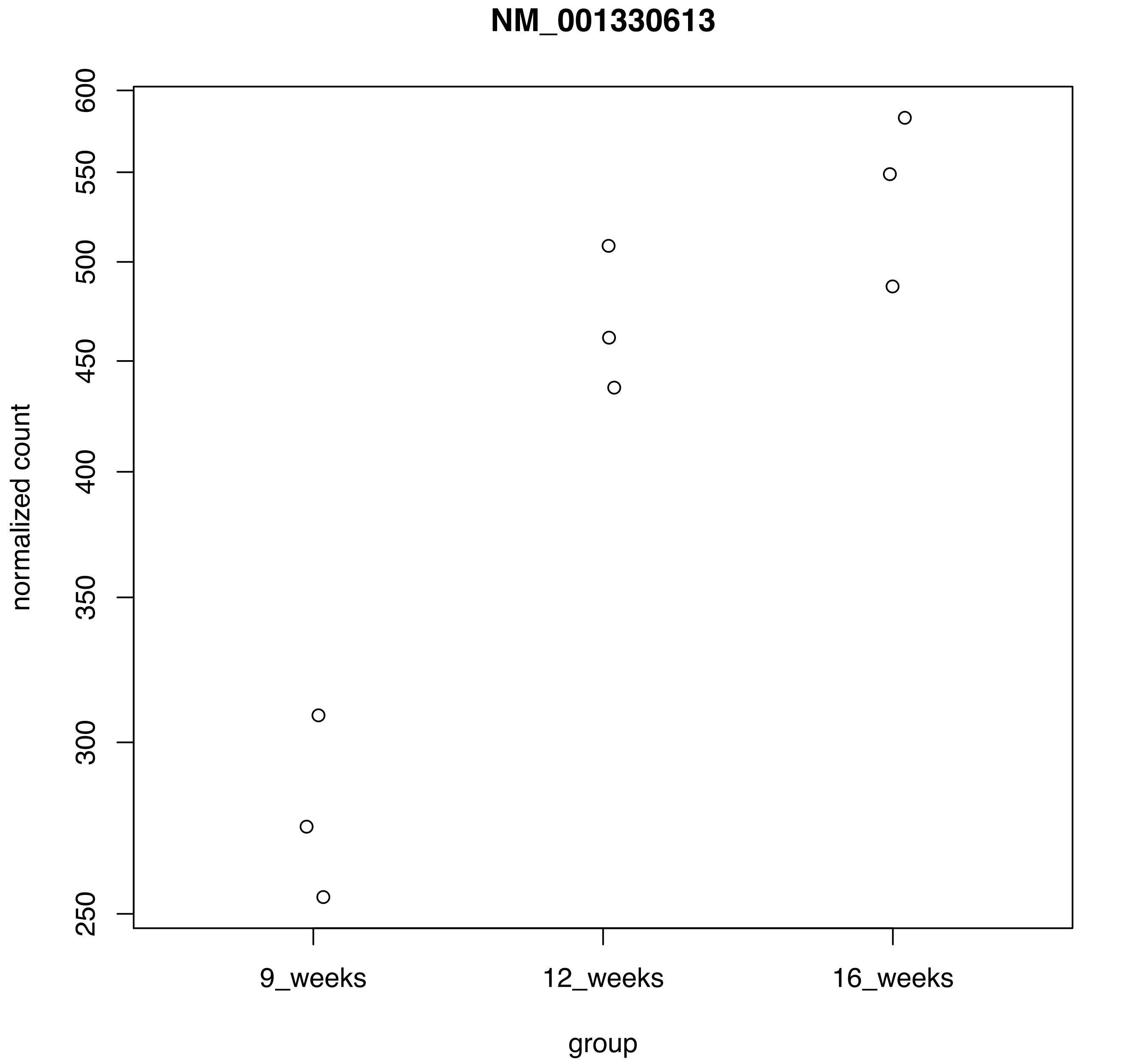
NM_001330613
normalized count
600
550
500
450
400
350
300
250
9_weeks
12_weeks
16_weeks
group

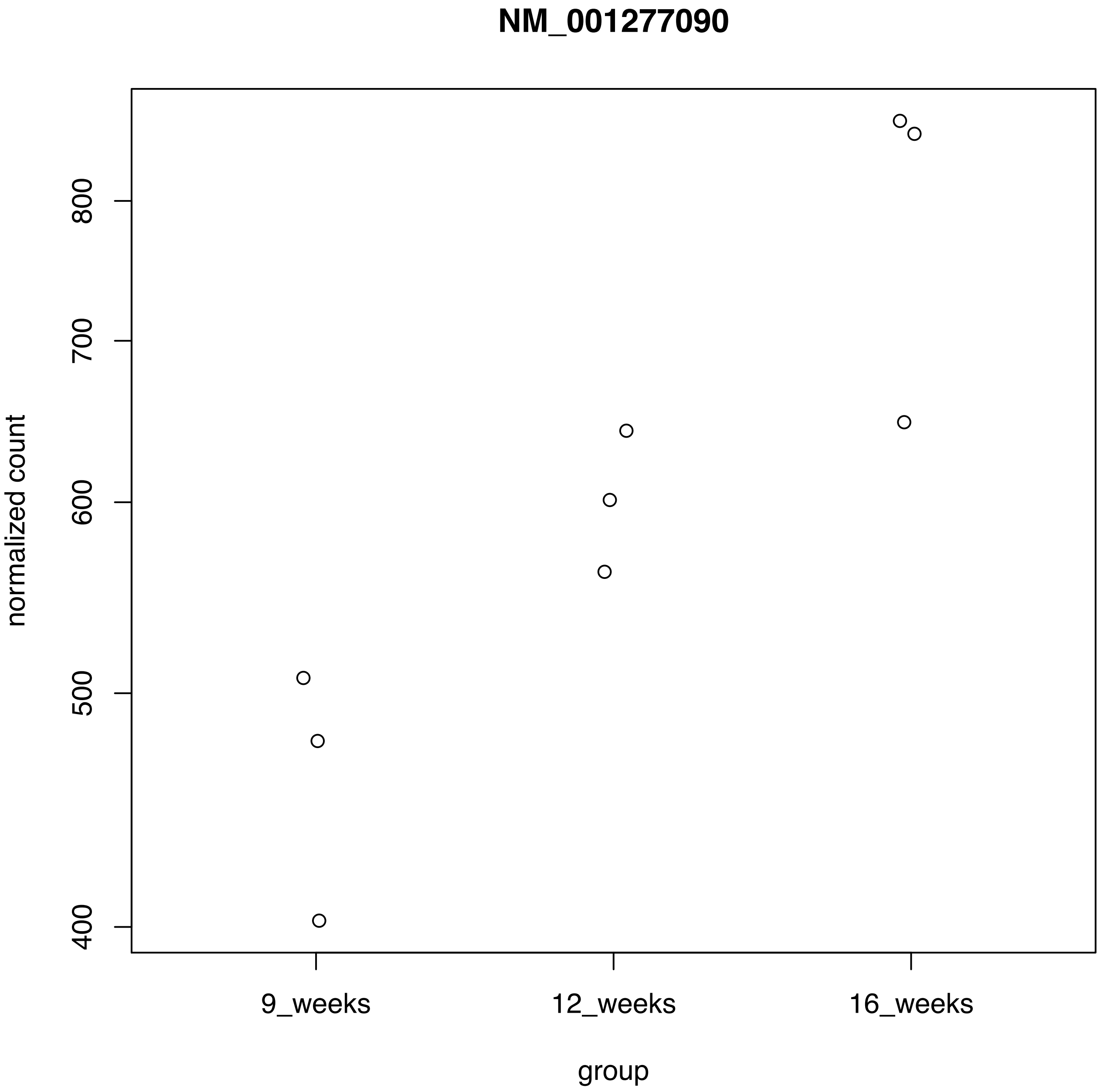
NM_001277090
normalized count
400
500
600
700
800
9_weeks
12_weeks
16_weeks
group

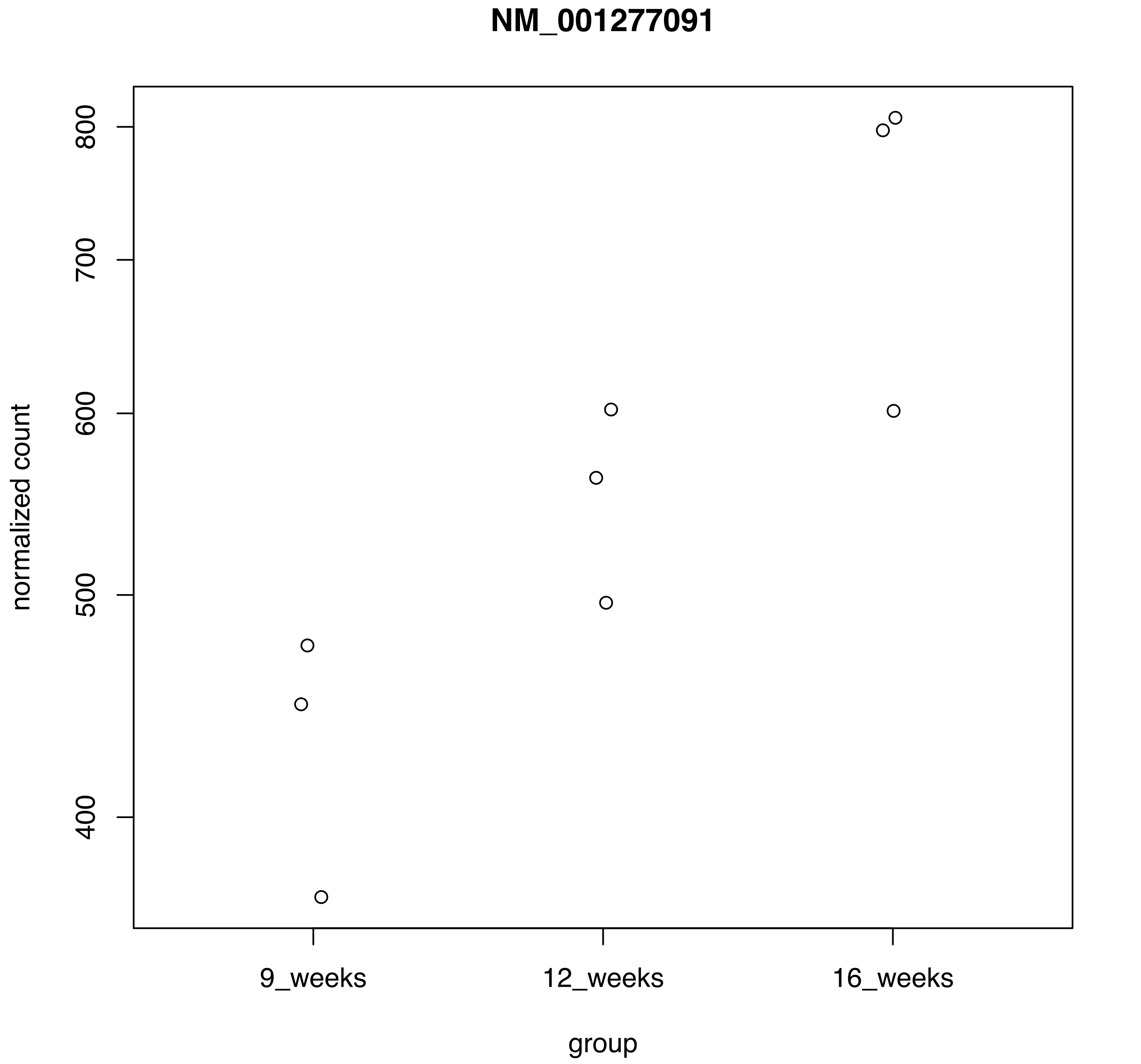
NM_001277091
normalized count
800
700
600
500
400
9_weeks
12_weeks
16_weeks
group

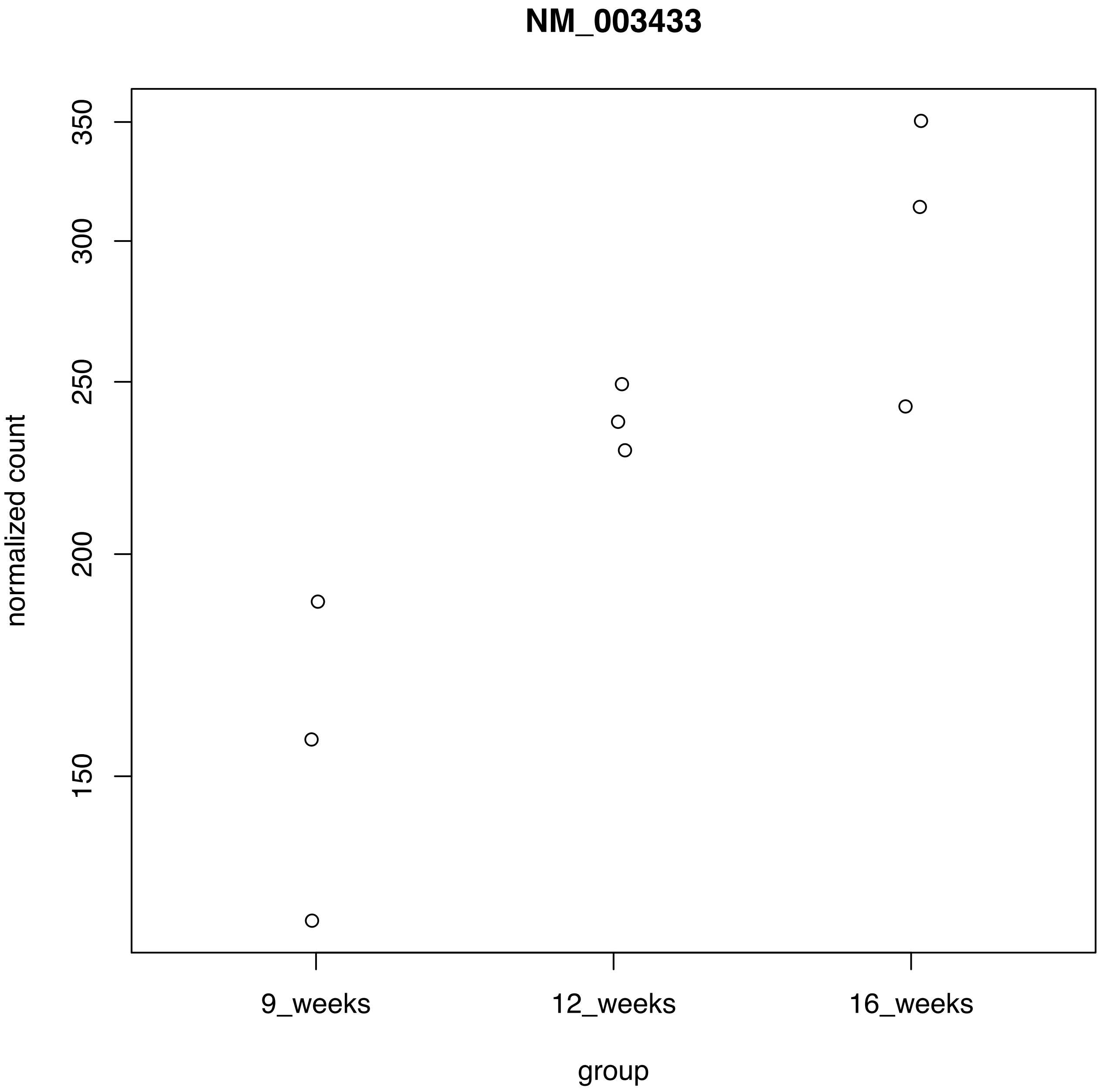

NM_003433
normalized count
150
200
250
300
350
9_weeks
12_weeks
16_weeks
group

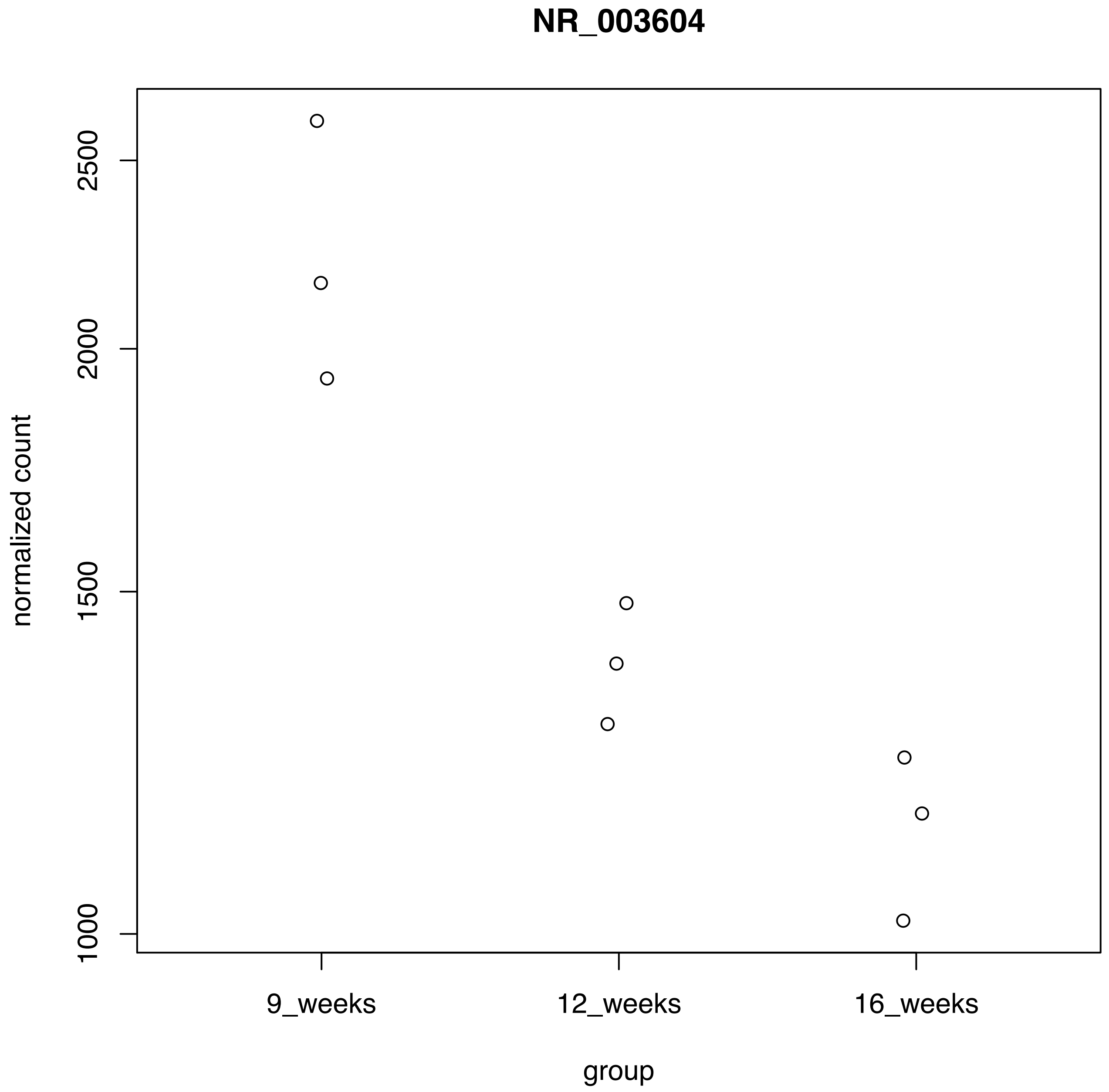
NR_003604
normalized count
2500
2000
1500
1000
9_weeks
12_weeks
16_weeks
group

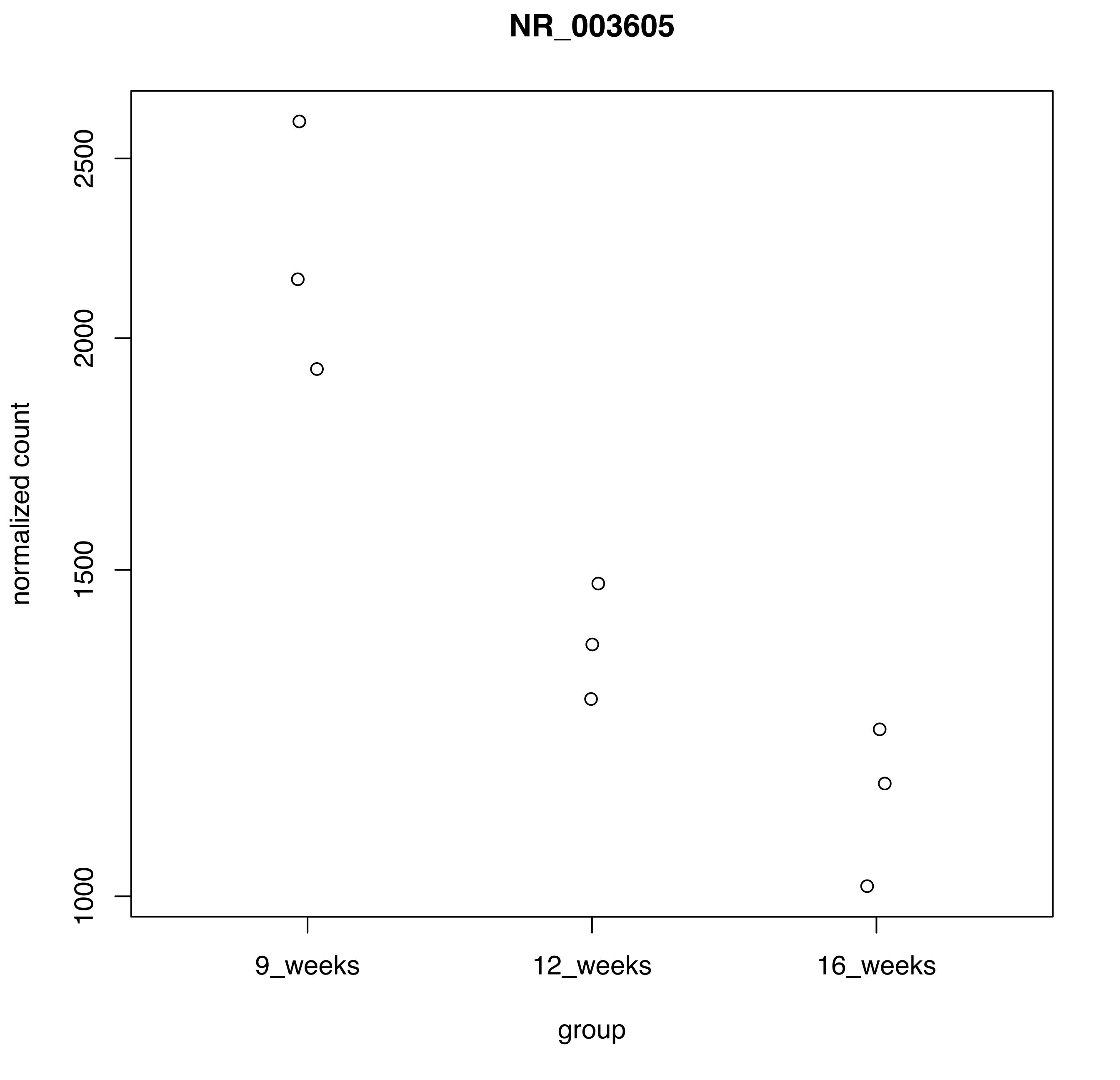
NR_003605
normalized count
group
9_weeks
12_weeks
16_weeks
1000
1500
2000
2500

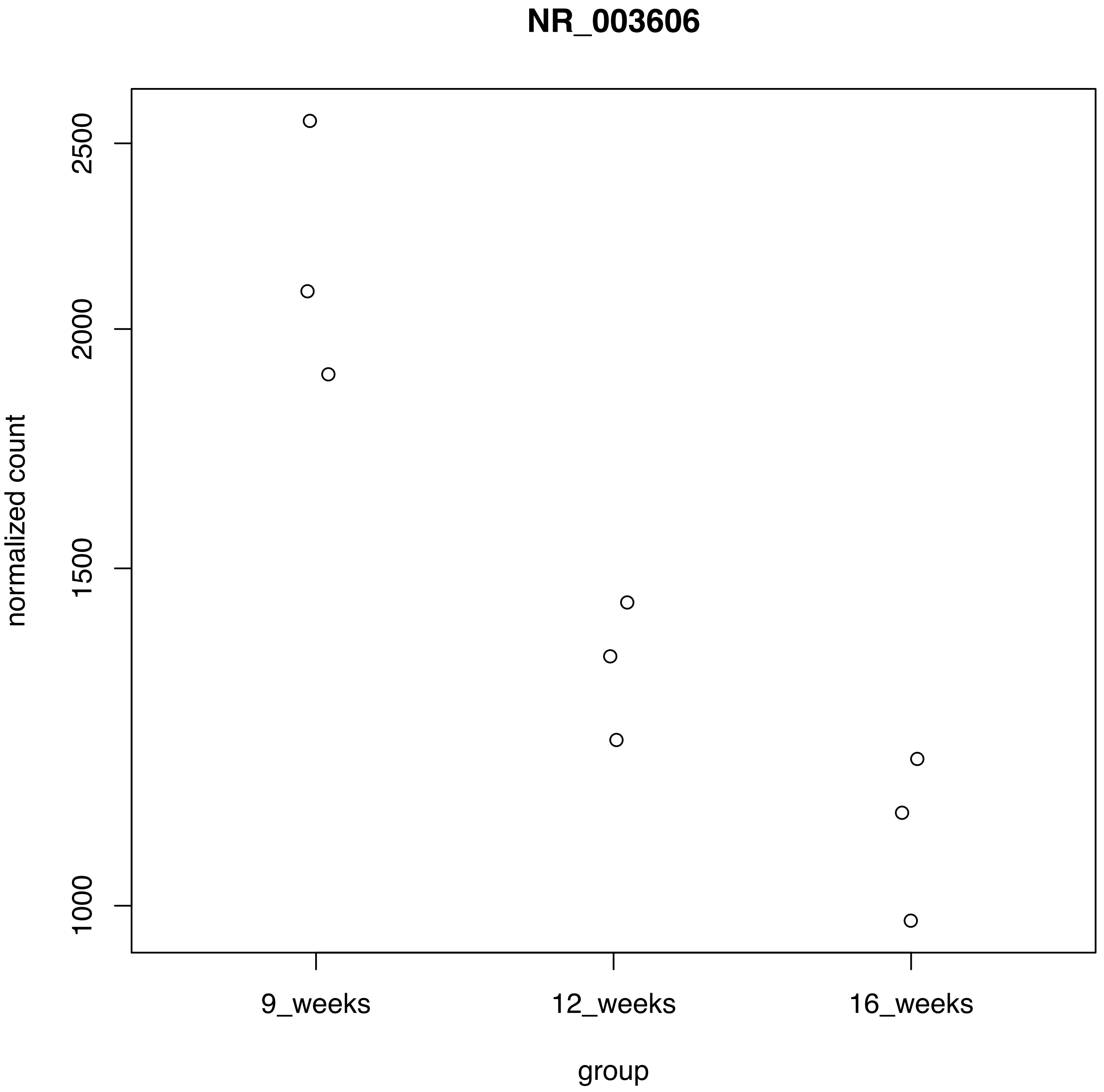
NR_003606
normalized count
2500
2000
1500
1000
9_weeks
12_weeks
16_weeks
group

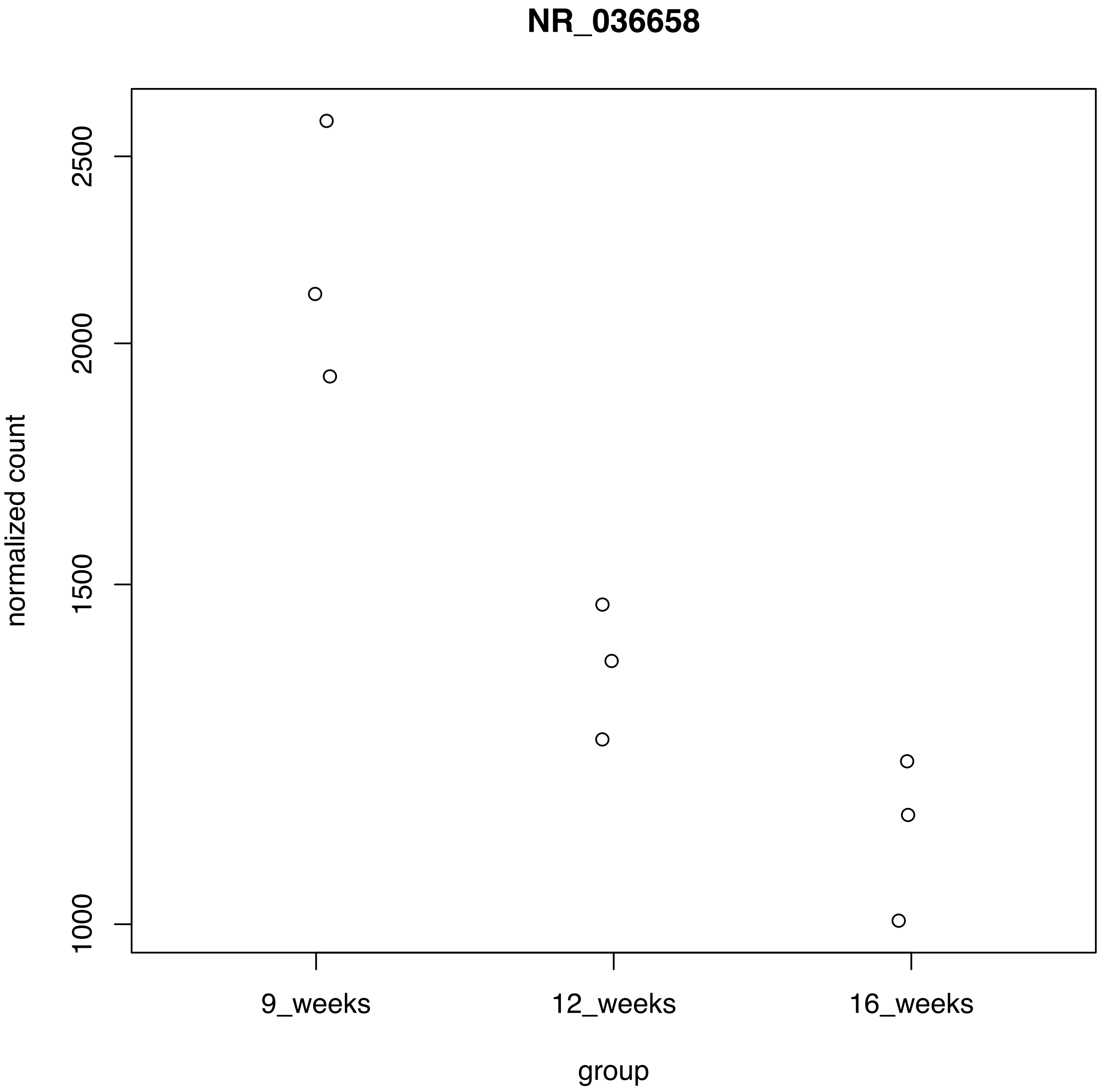
NR_036658
normalized count
1000
1500
2000
2500
9_weeks
12_weeks
16_weeks
group

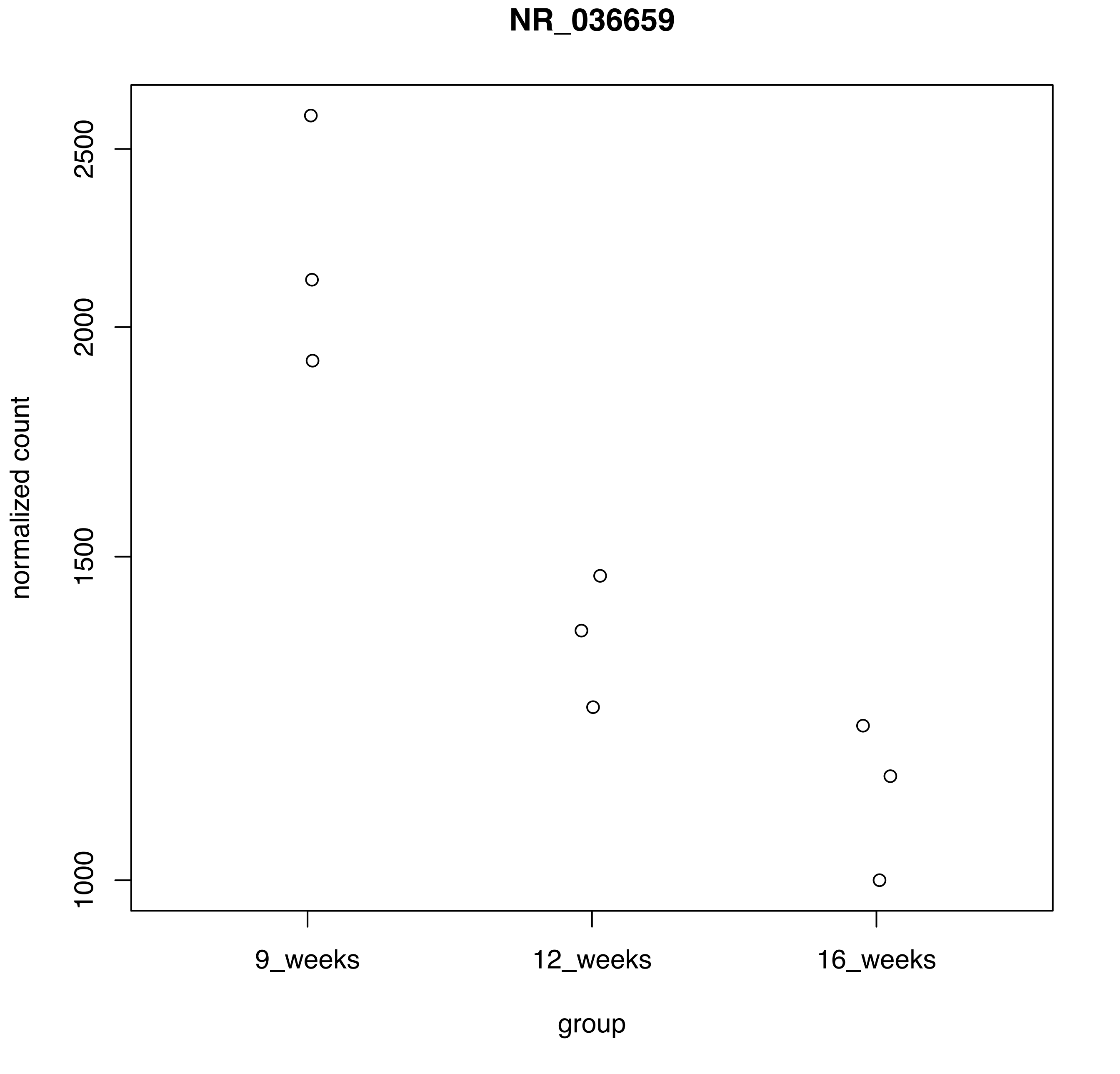
NR_036659
normalized count
2500
2000
1500
1000
9_weeks
12_weeks
16_weeks
group

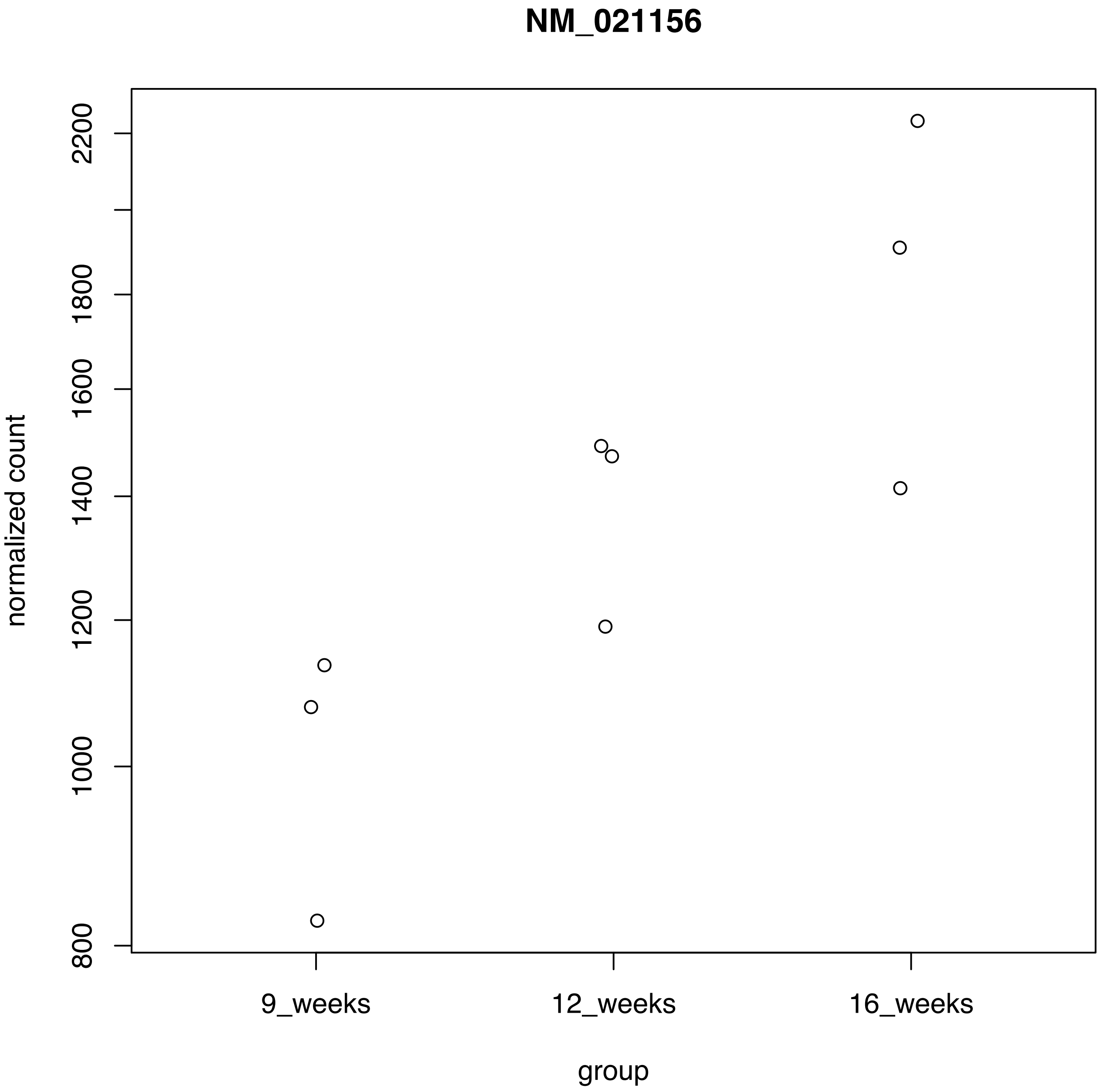
NM_021156
normalized count
800
1000
1200
1400
1600
1800
2200
9_weeks
12_weeks
16_weeks
group

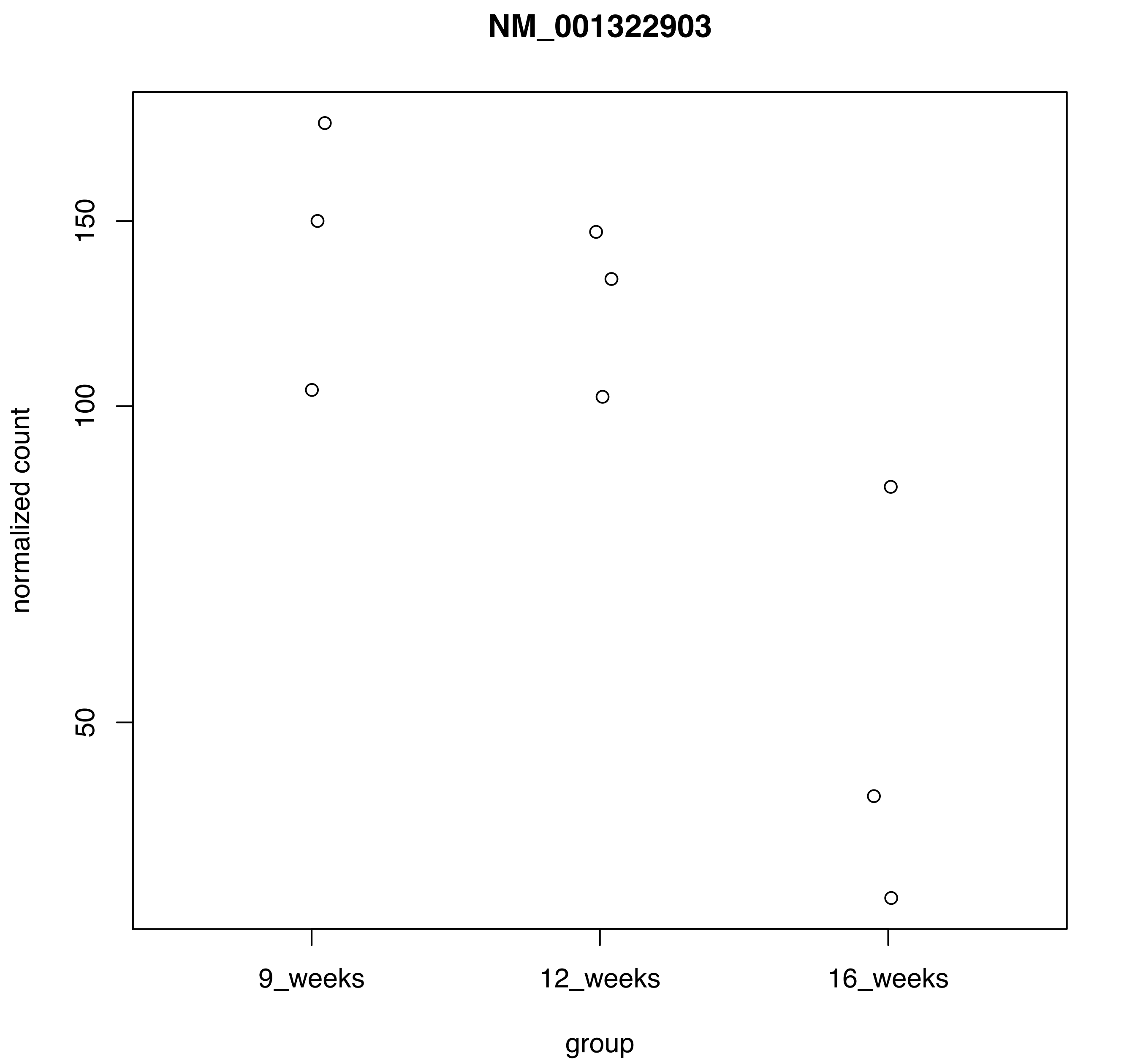
NM_001322903
normalized count
150
100
50
9_weeks
12_weeks
16_weeks
group

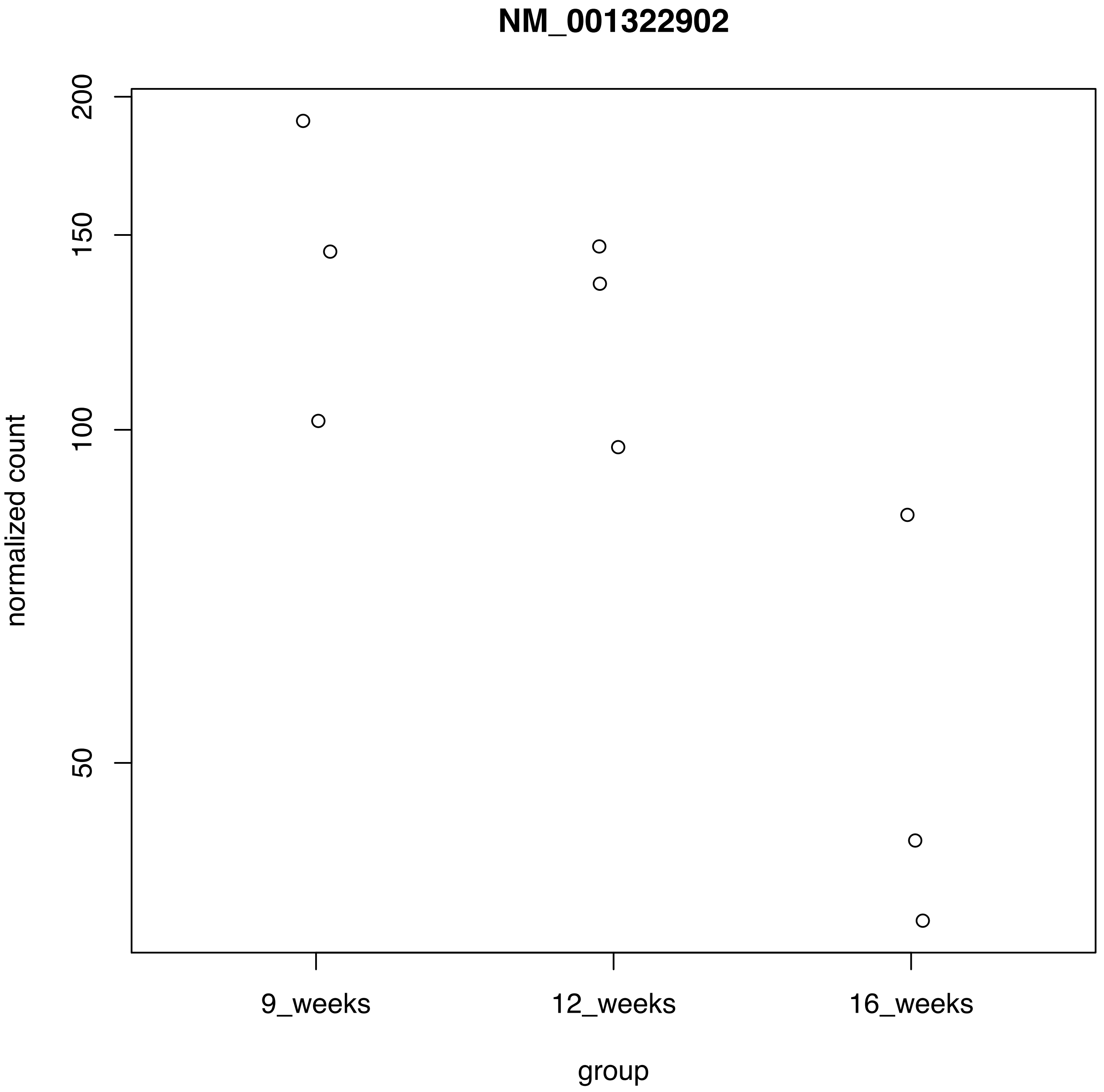
NM_001322902
normalized count
200
150
100
50
9_weeks
12_weeks
16_weeks
group

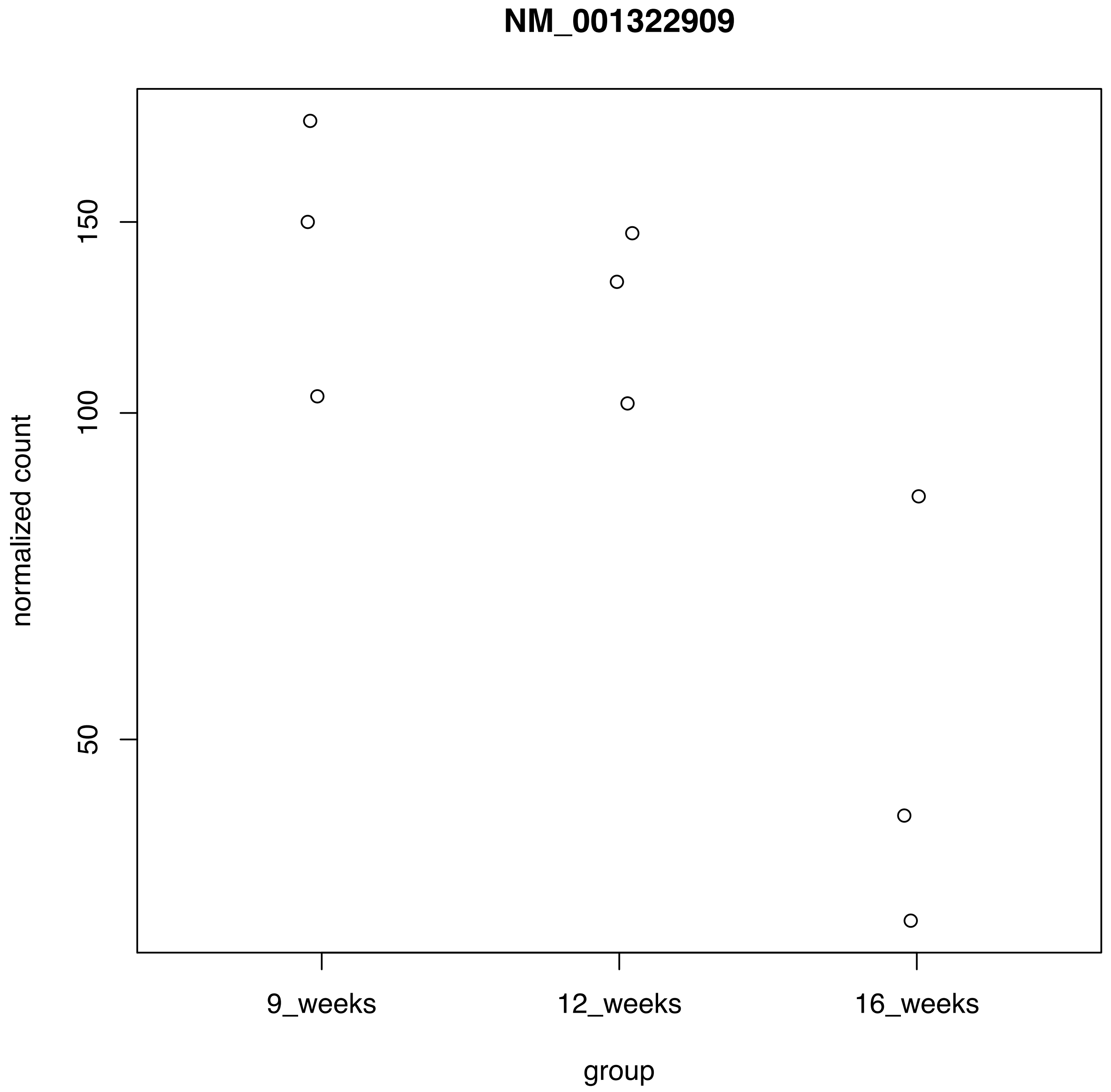

NM_001322909
normalized count
150
100
50
9_weeks
12_weeks
16_weeks
group

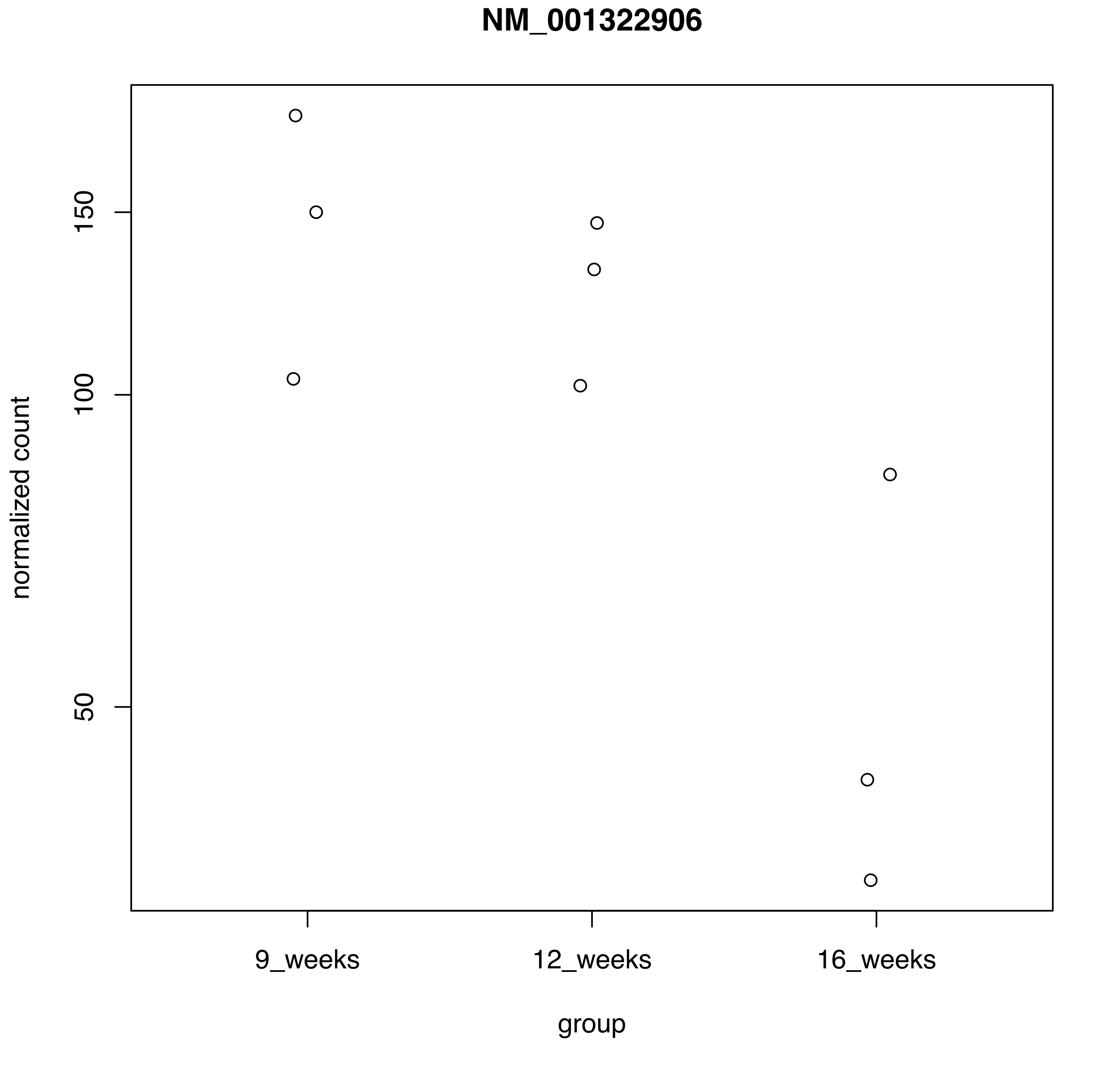
NM_001322906
normalized count
50
100
150
9_weeks
12_weeks
16_weeks
group

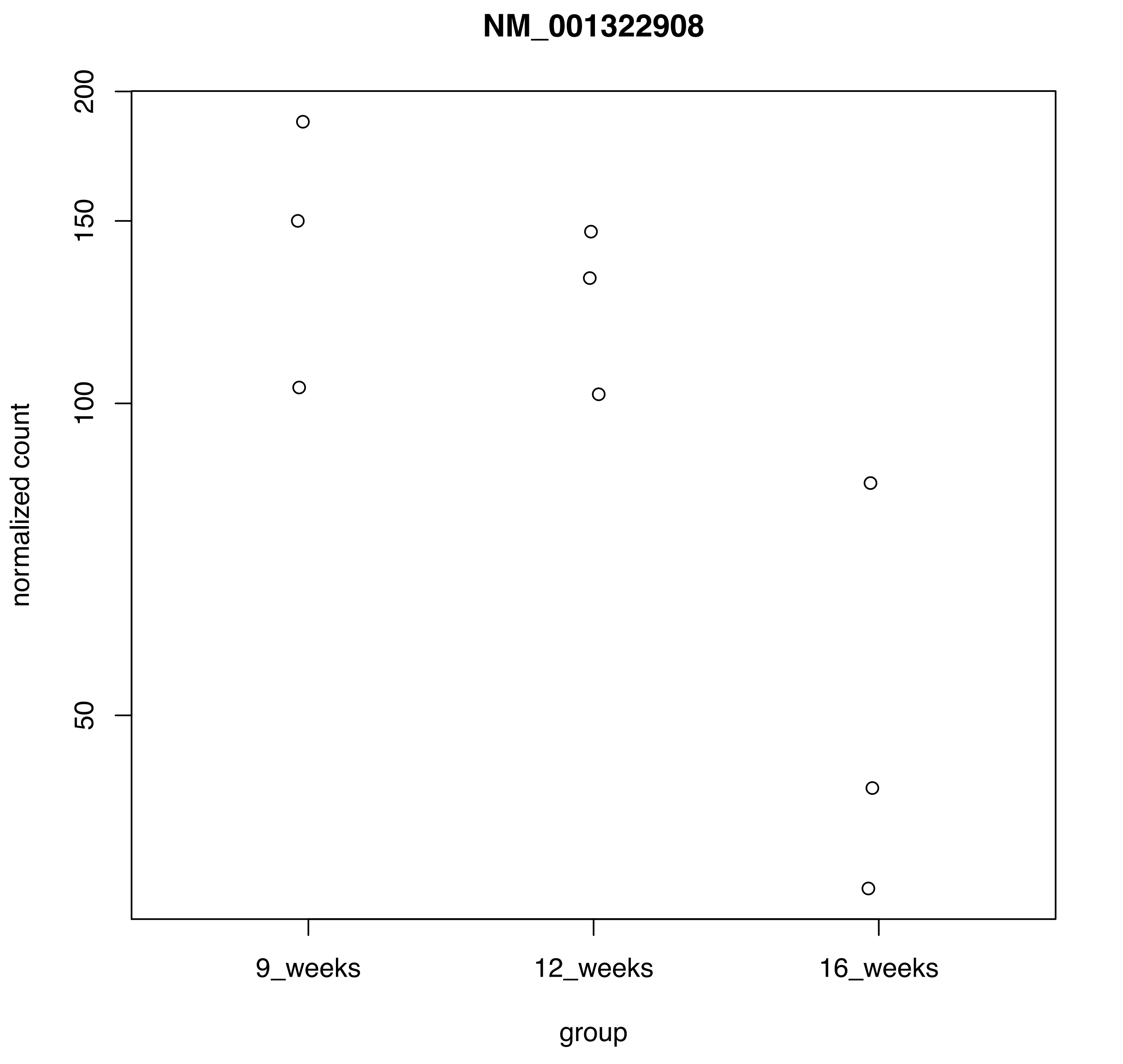

NM_001322908
normalized count
200
150
100
50
9_weeks
12_weeks
16_weeks
group

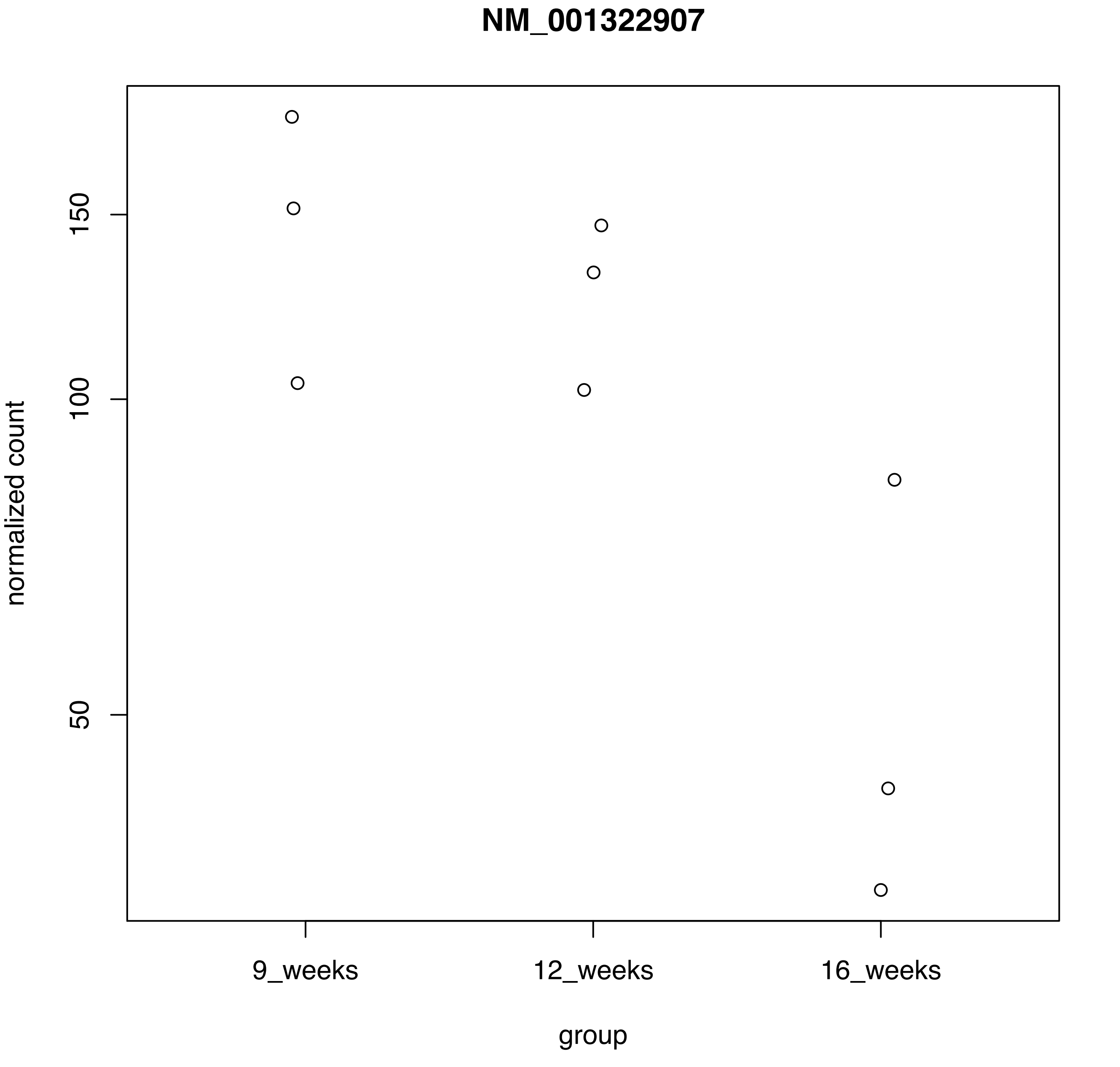
NM_001322907
normalized count
150
100
50
9_weeks
12_weeks
16_weeks
group

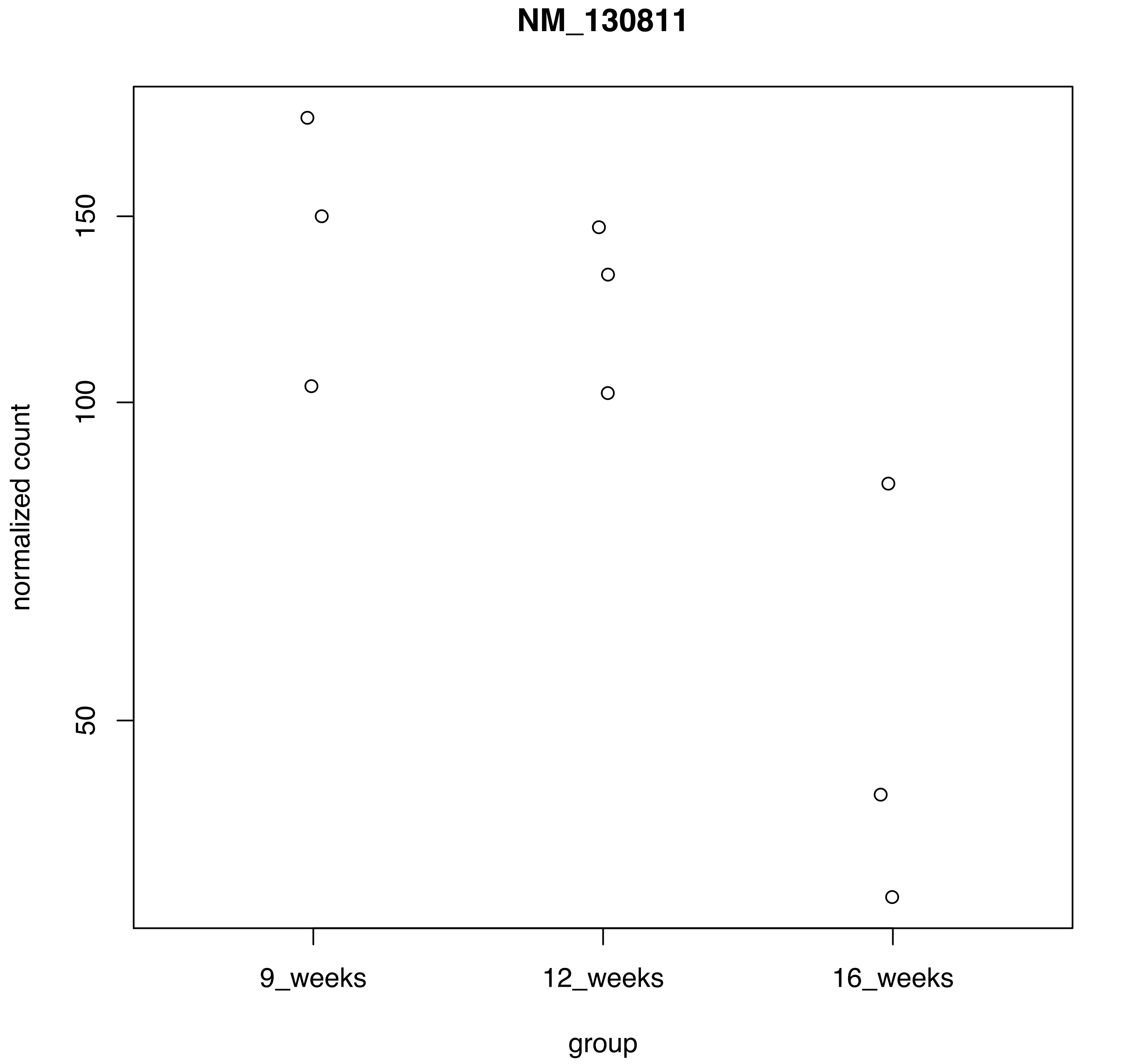
NM_130811
normalized count
150
100
50
9_weeks
12_weeks
16_weeks
group

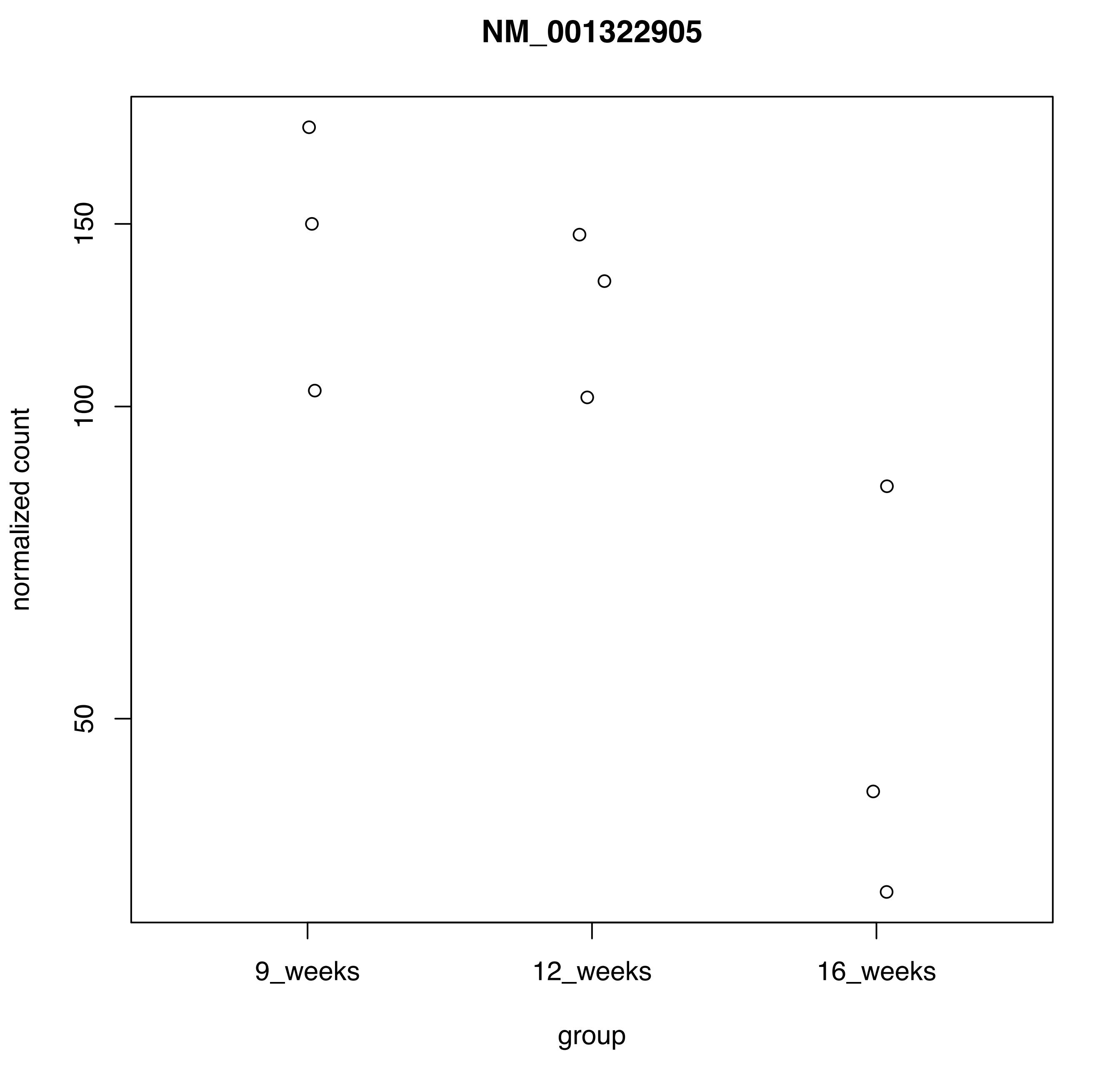
NM_001322905
normalized count
150
100
50
9_weeks
12_weeks
16_weeks
group

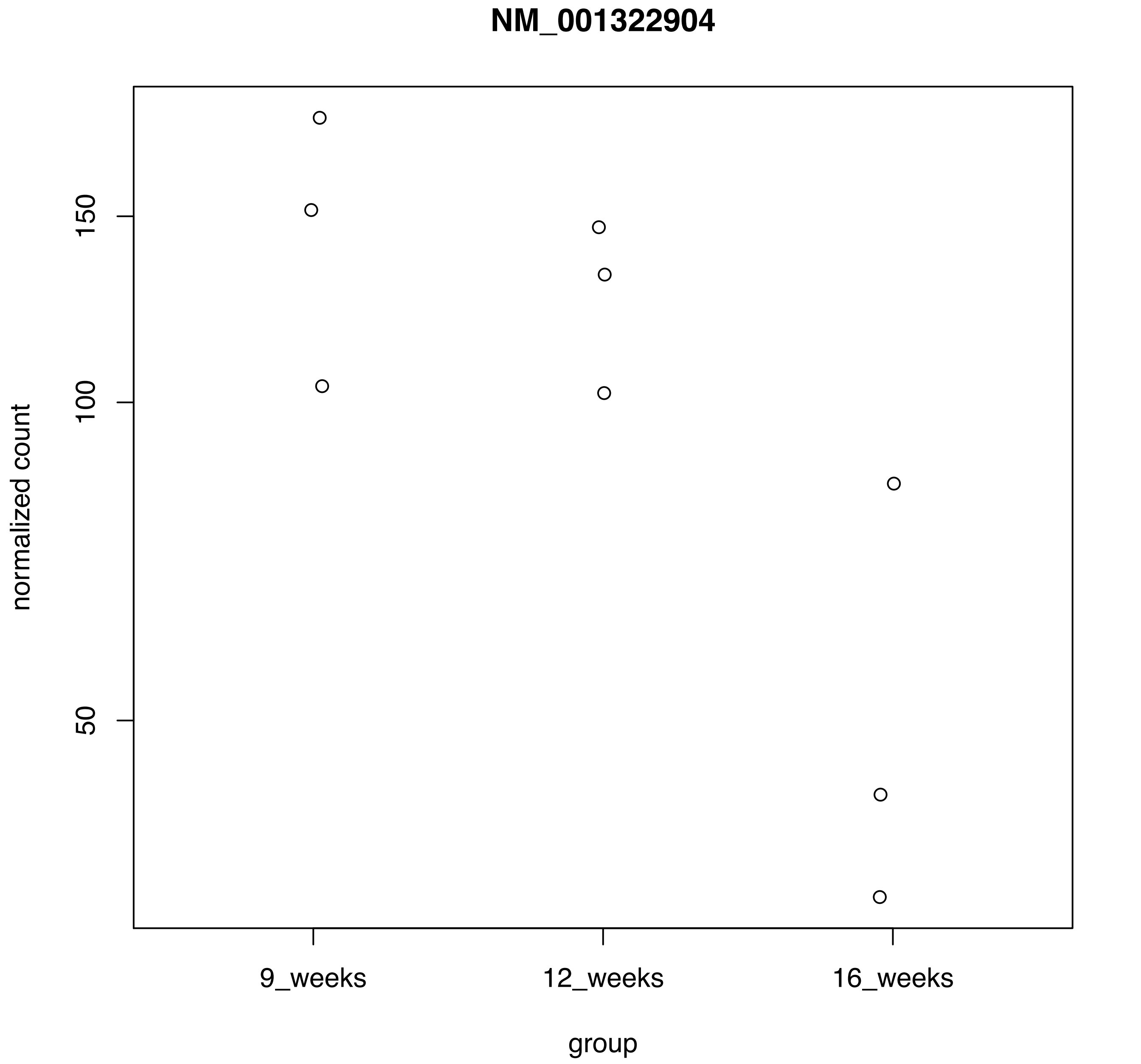

NM_001322904
normalized count
150
100
50
9_weeks
12_weeks
16_weeks
group

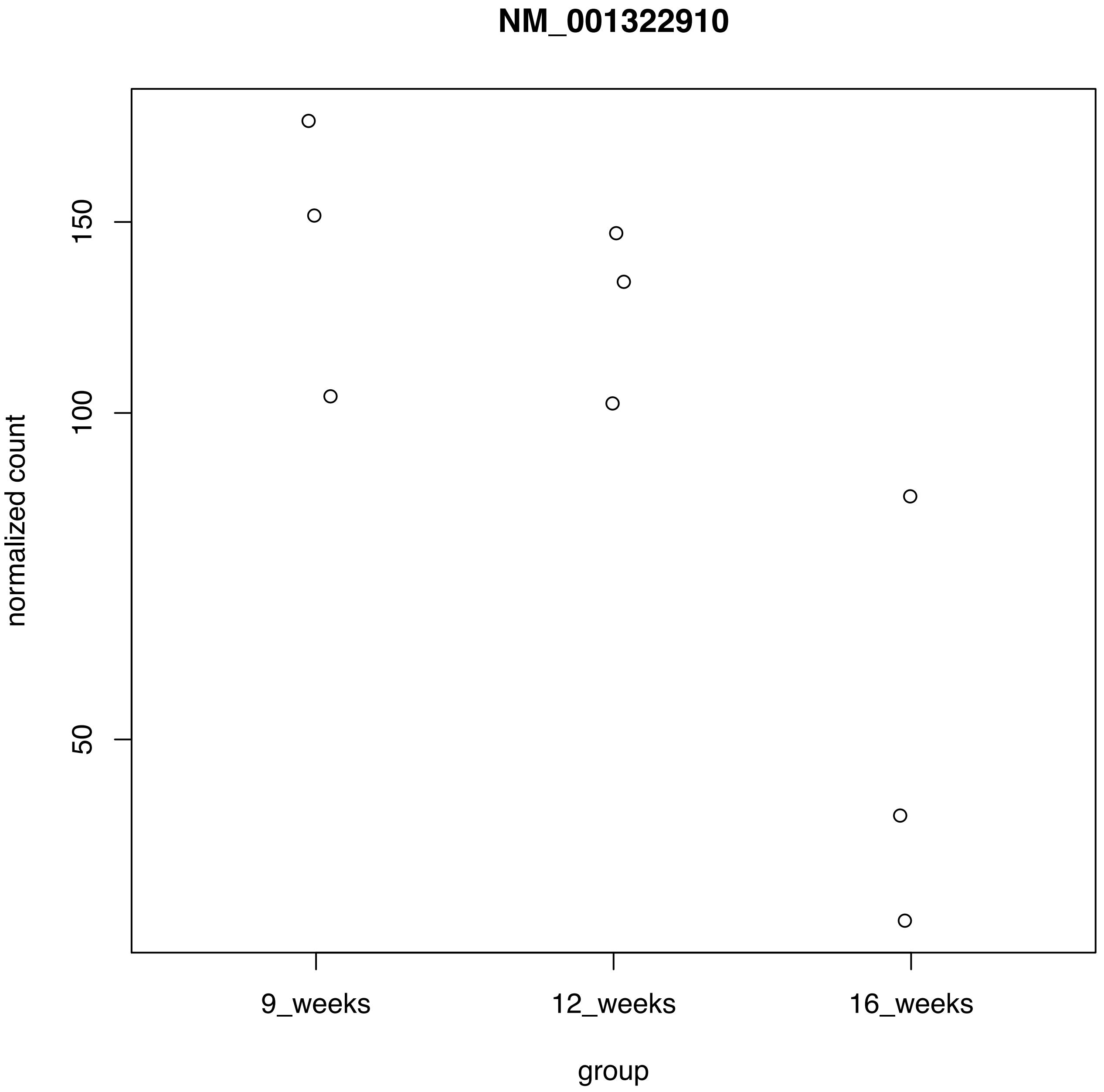
NM_001322910
normalized count
150
100
50
9_weeks
12_weeks
16_weeks
group

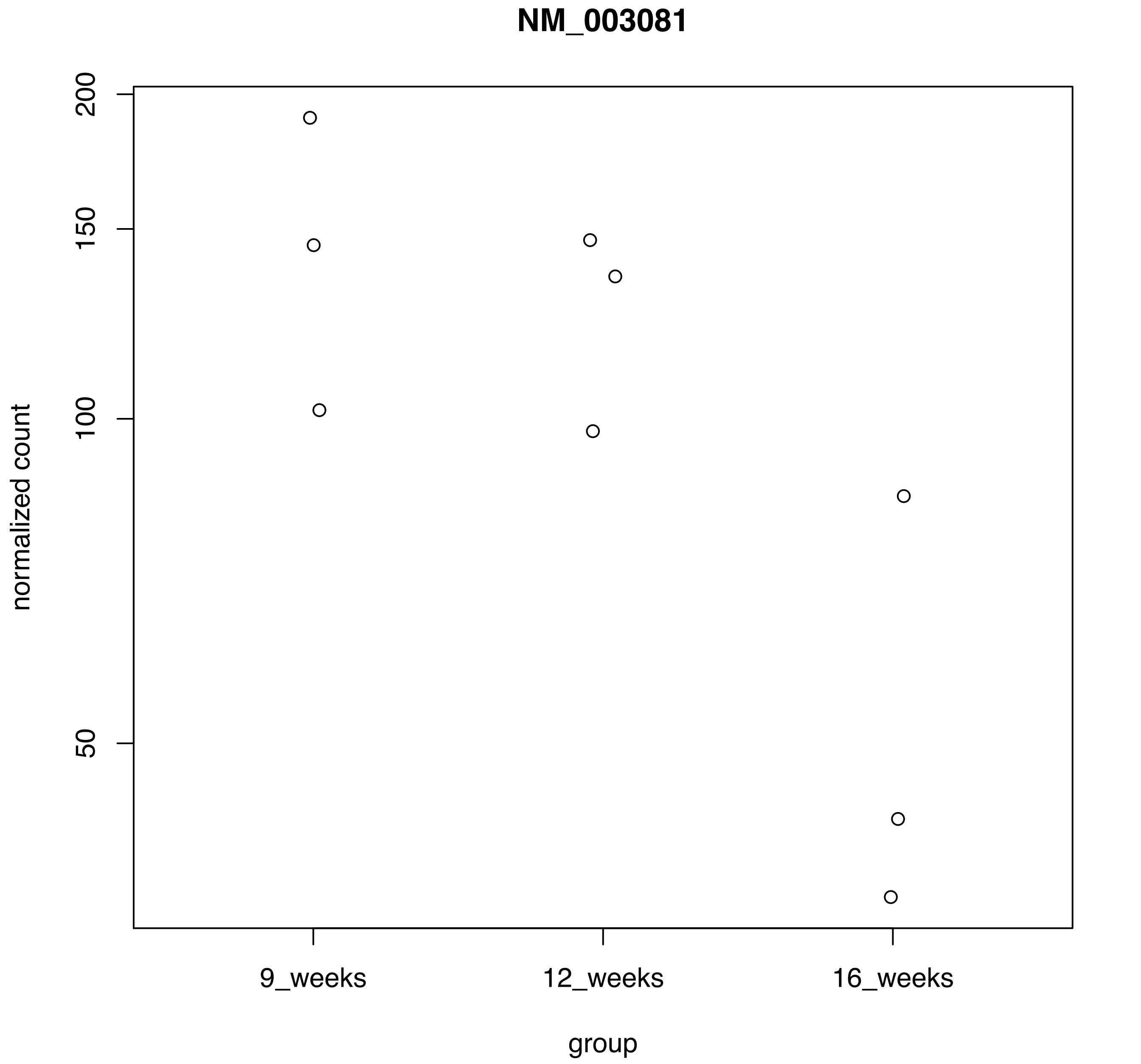

NM_003081
200
150
100
50
normalized count
9_weeks
12_weeks
16_weeks
group

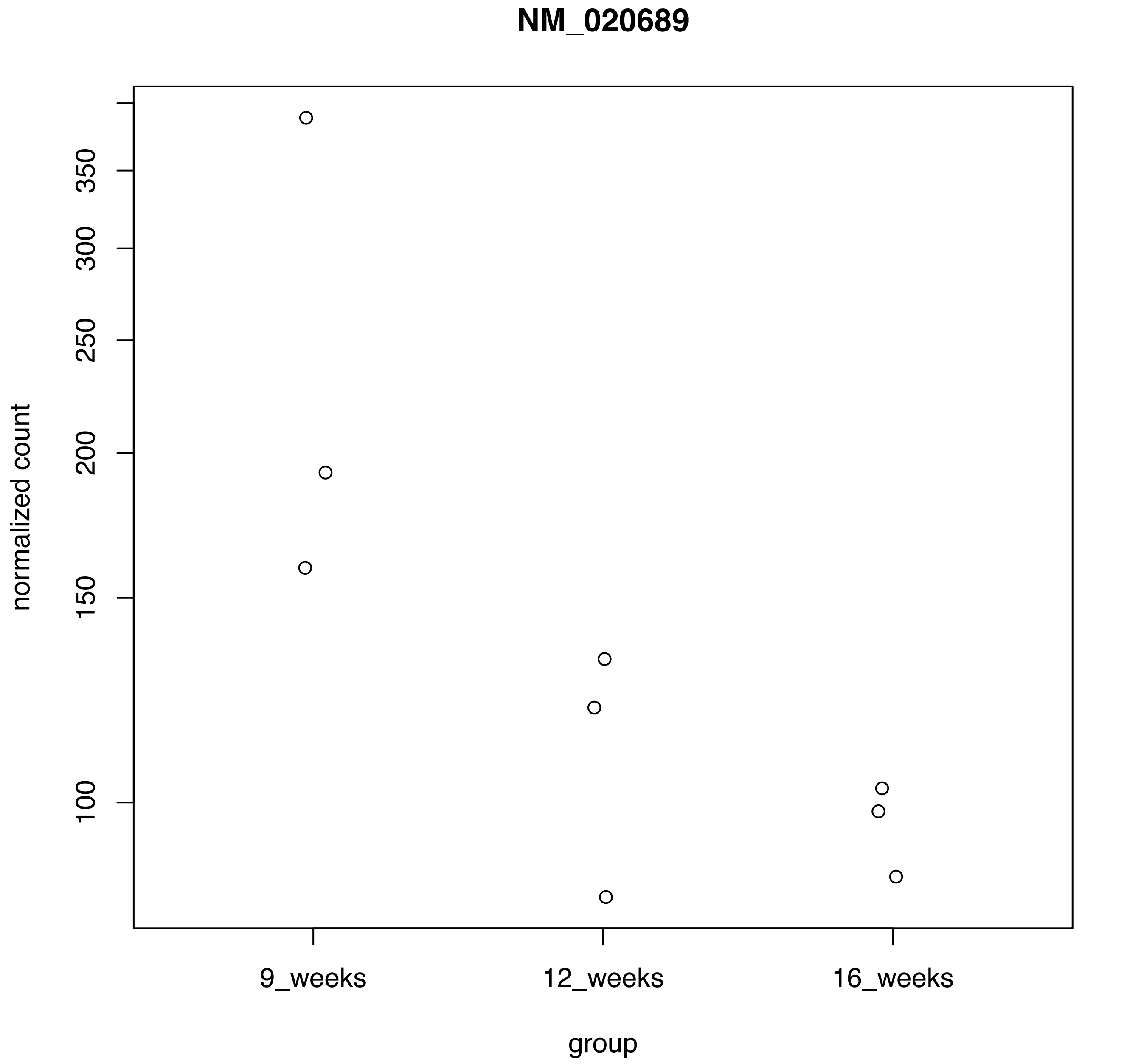

NM_020689
normalized count
100
150
200
250
300
350
9_weeks
12_weeks
16_weeks
group

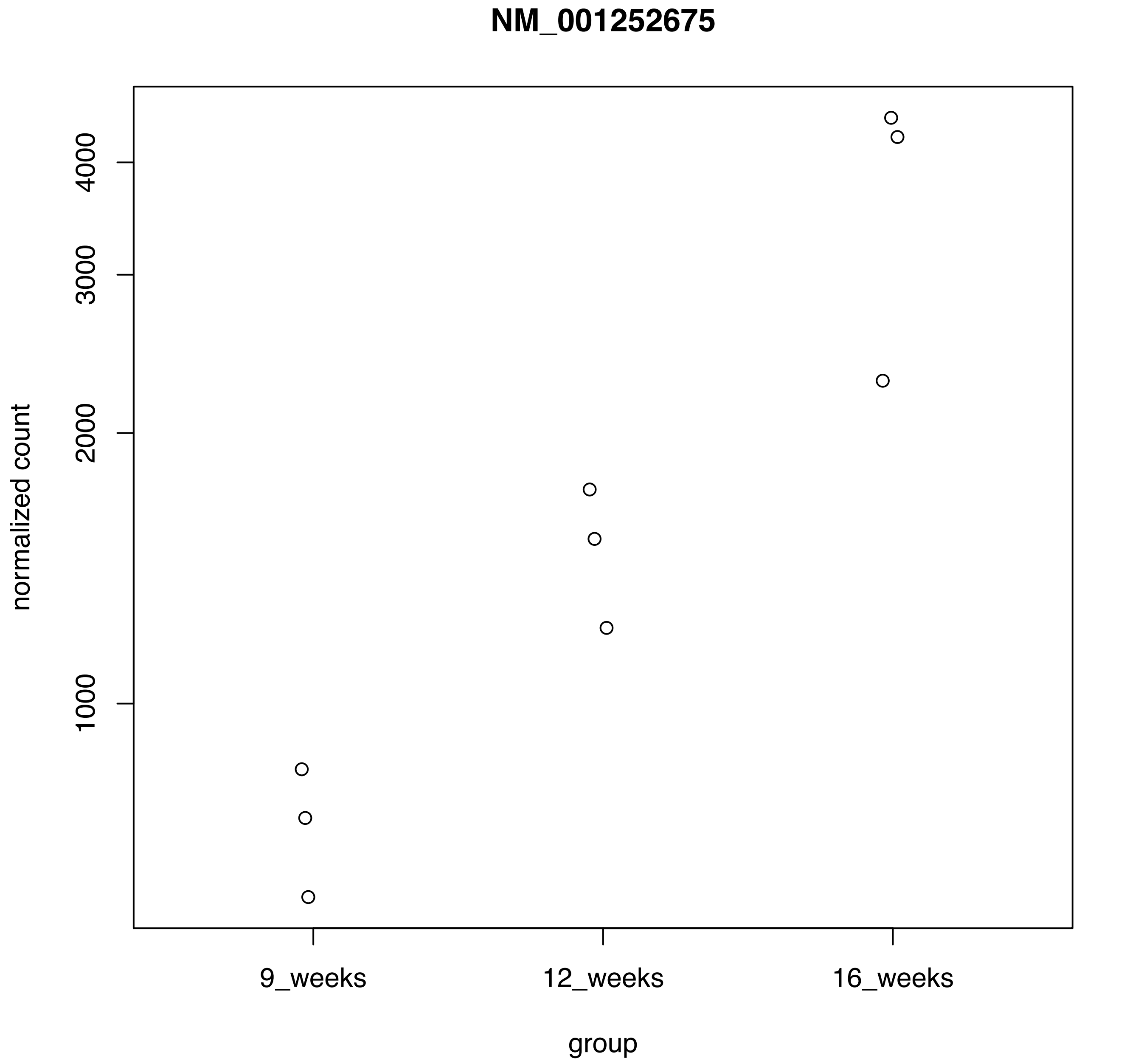

NM_001252675
normalized count
1000
2000
3000
4000
9_weeks
12_weeks
16_weeks
group

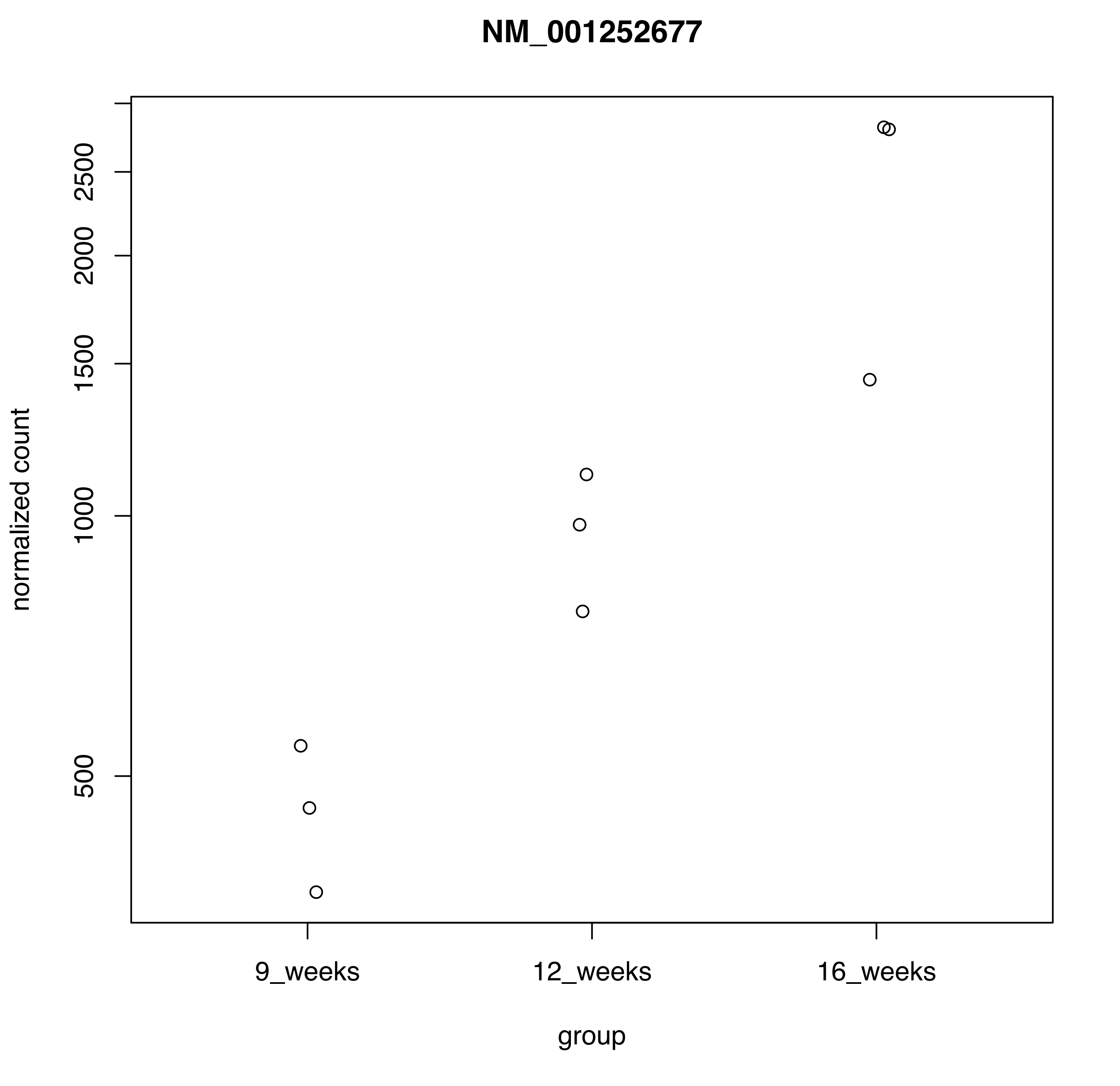
NM_001252677
normalized count
500
1000
1500
2000
2500
9_weeks
12_weeks
16_weeks
group

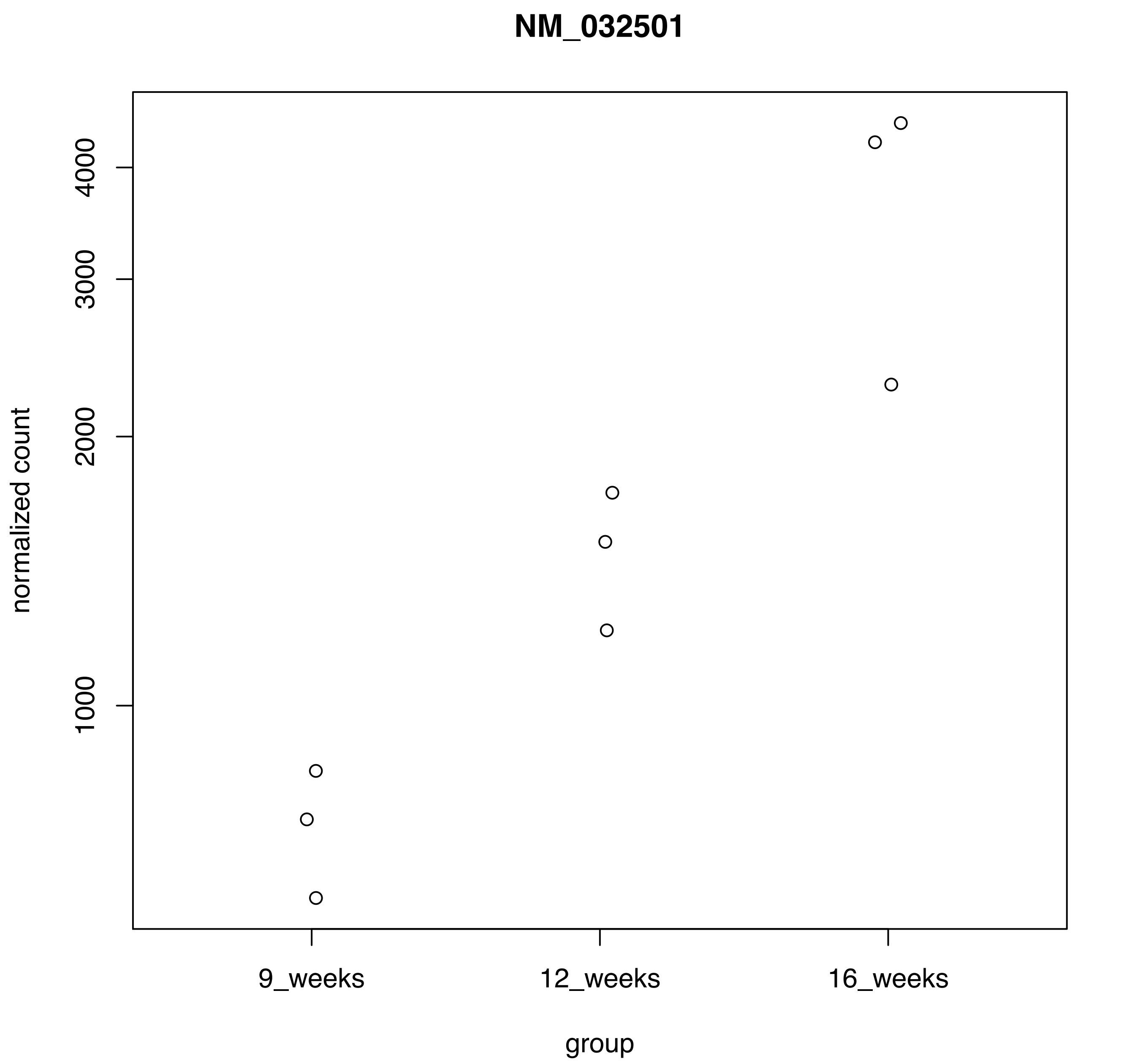
NM_032501
normalized count
1000
2000
3000
4000
9_weeks
12_weeks
16_weeks
group

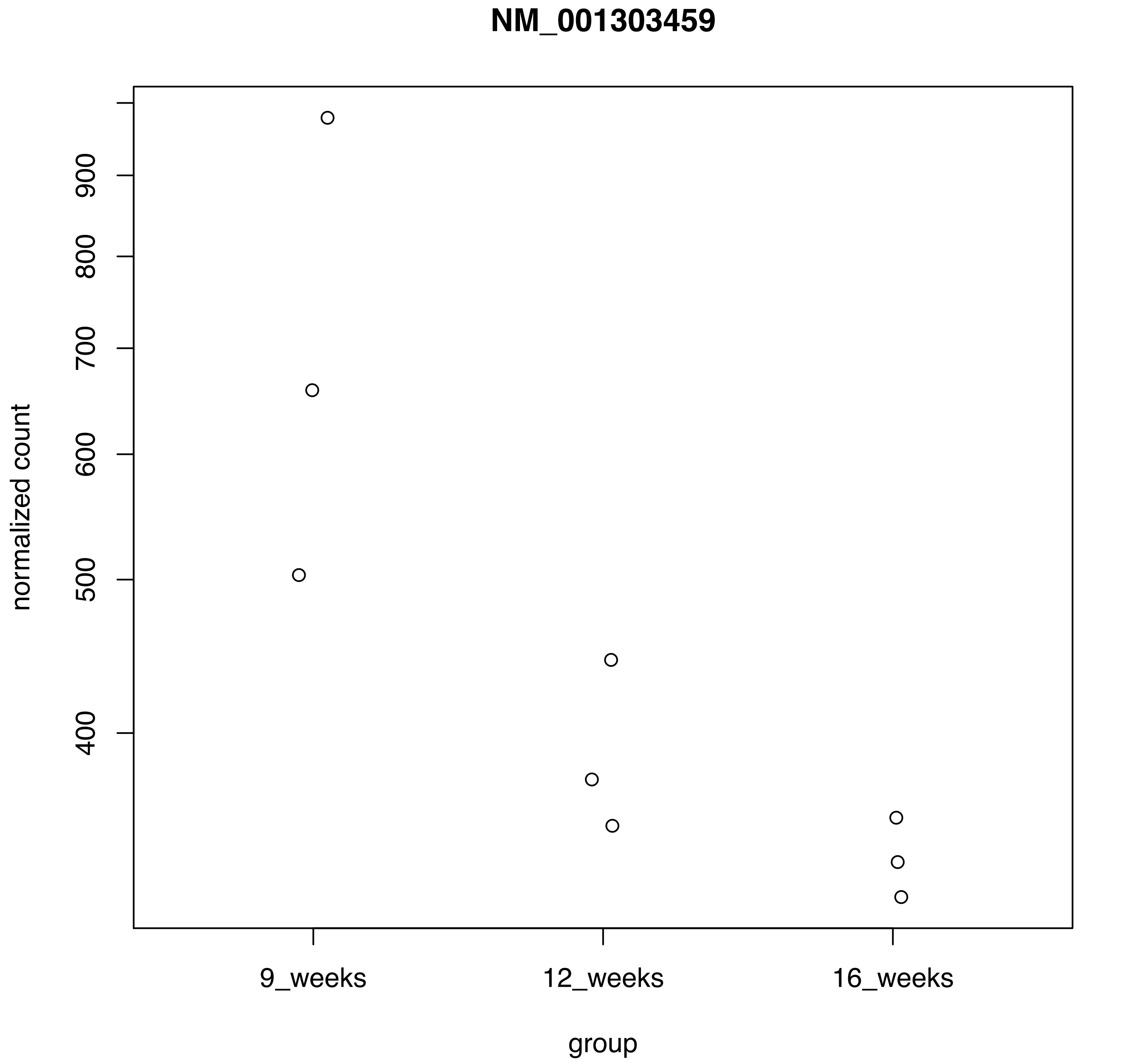
NM_001303459
normalized count
900
800
700
600
500
400
9_weeks
12_weeks
16_weeks
group

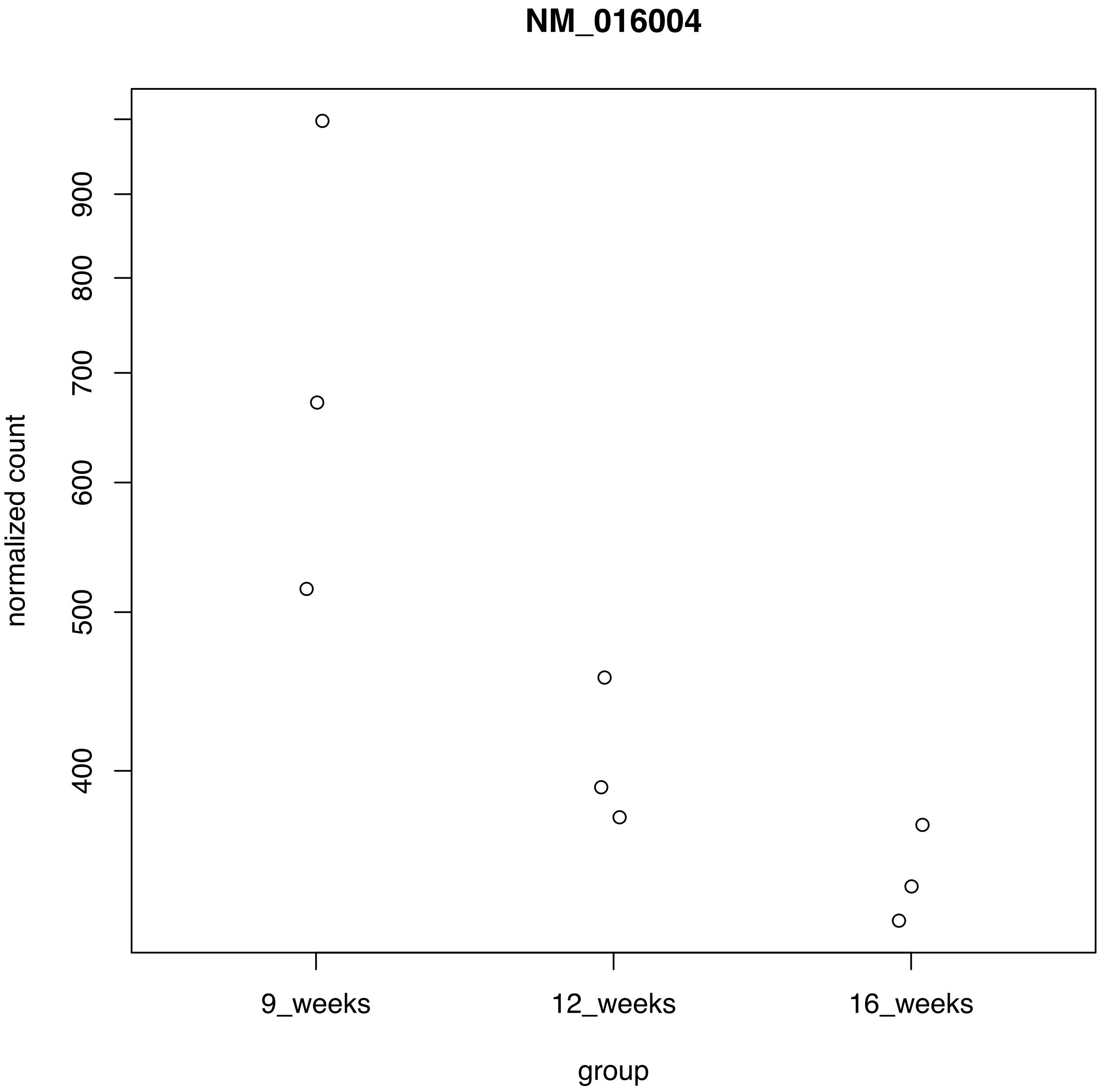
NM_016004
normalized count
400
500
600
700
800
900
9_weeks
12_weeks
16_weeks
group

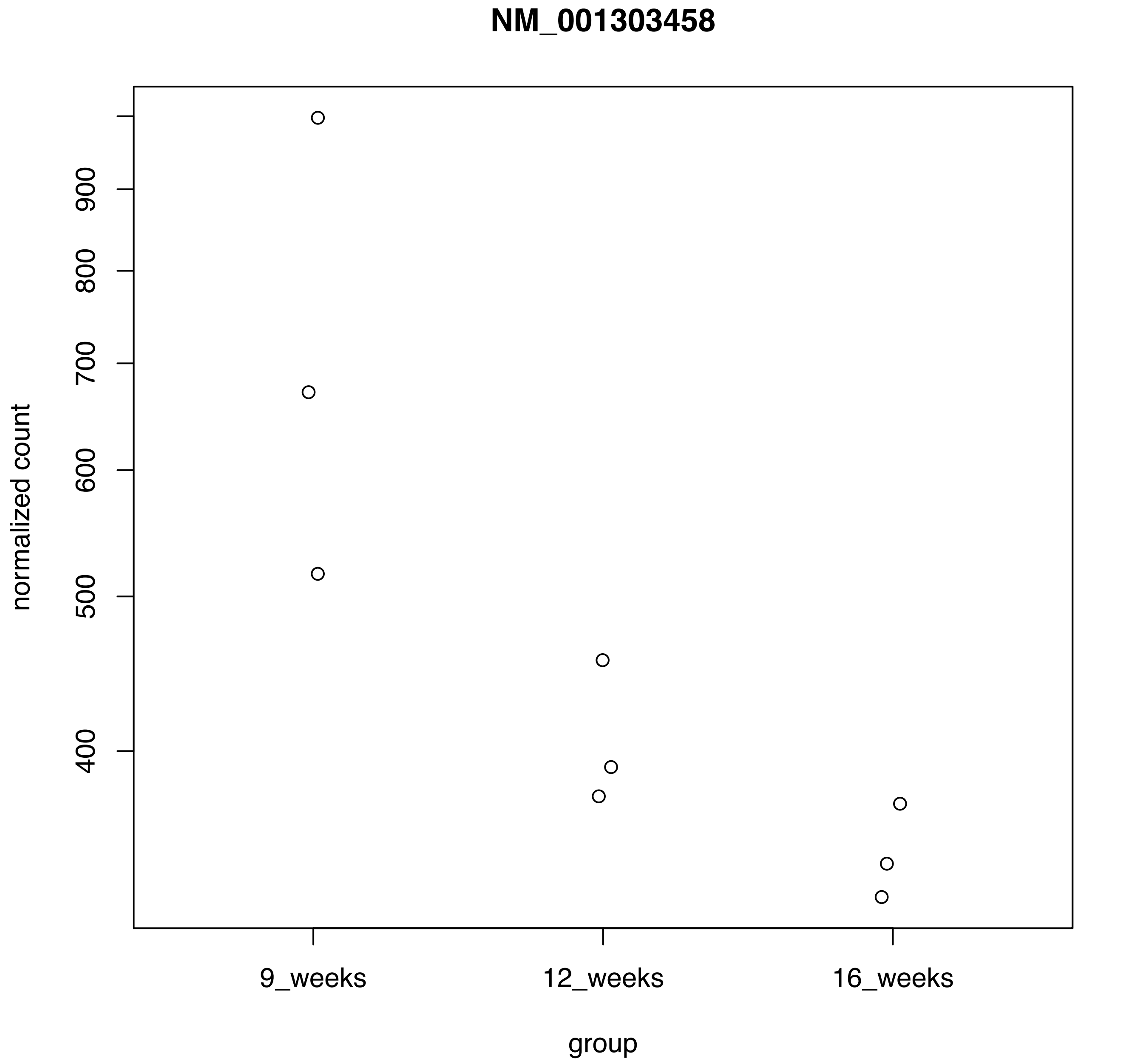
NM_001303458
normalized count
900
800
700
600
500
400
9_weeks
12_weeks
16_weeks
group

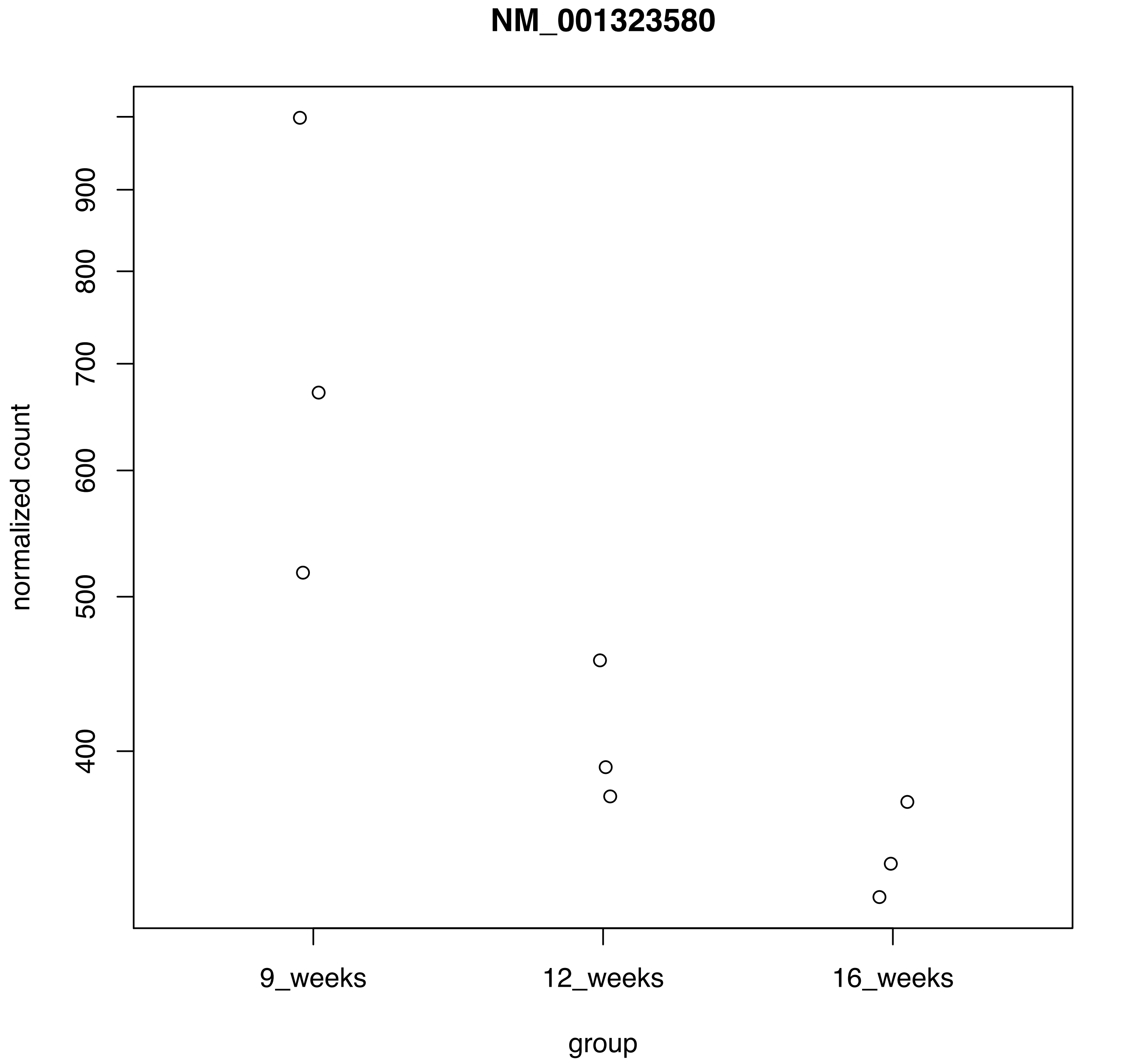
NM_001323580
normalized count
900
800
700
600
500
400
9_weeks
12_weeks
16_weeks
group

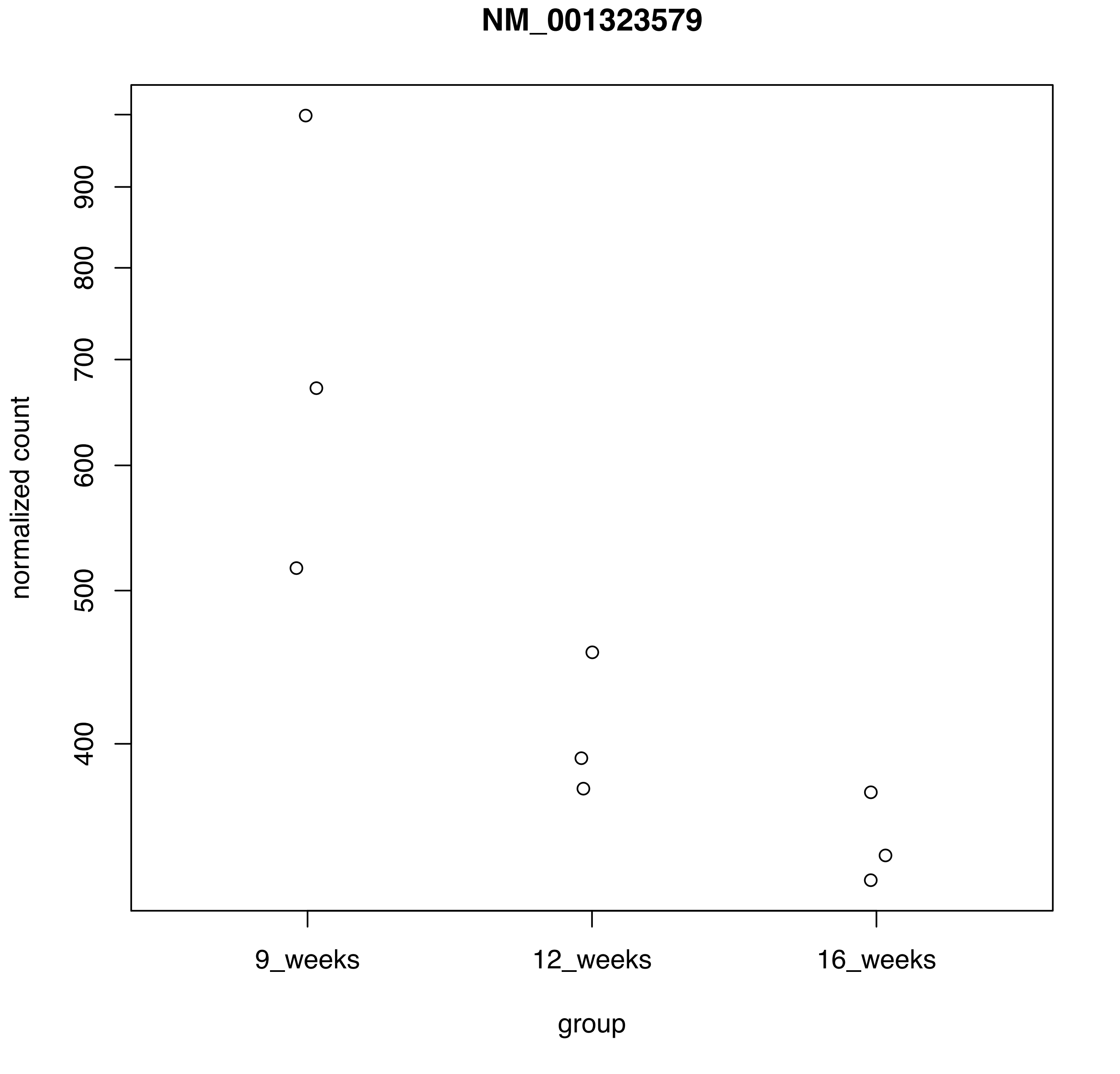

NM_001323579
normalized count
900
800
700
600
500
400
9_weeks
12_weeks
16_weeks
group

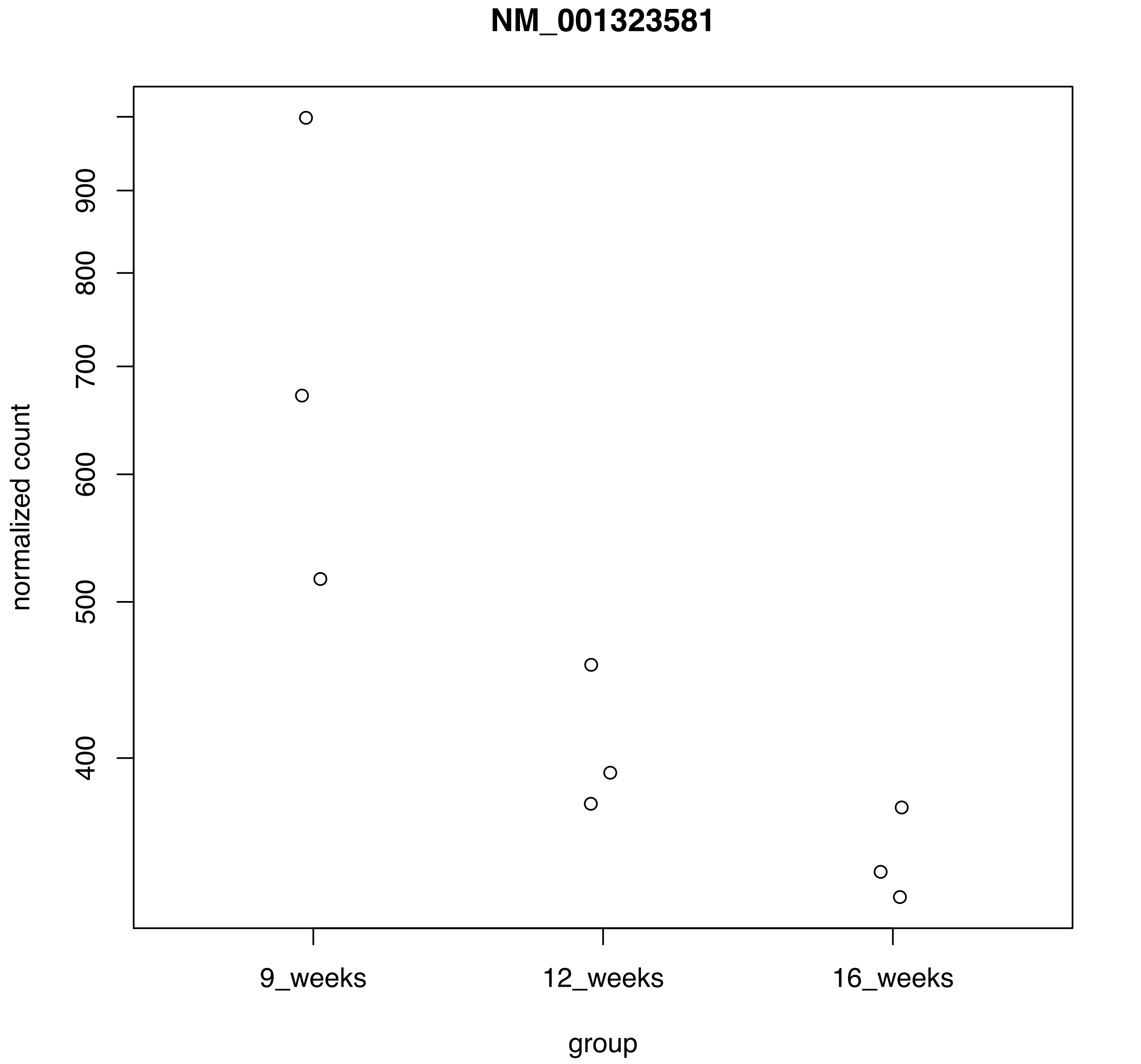
NM_001323581
normalized count
900
800
700
600
500
400
9_weeks
12_weeks
16_weeks
group

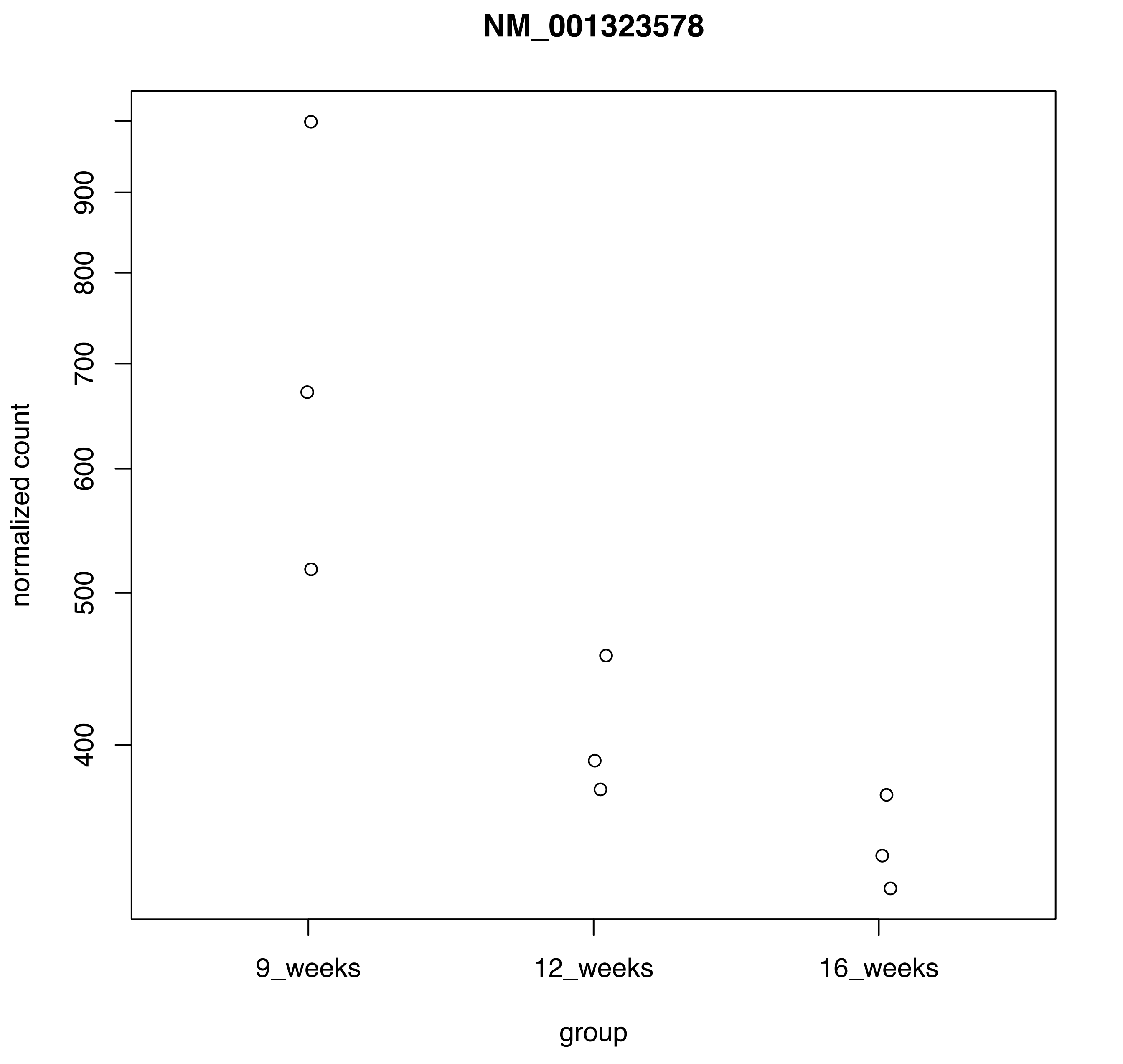
NM_001323578
normalized count
900
800
700
600
500
400
9_weeks
12_weeks
16_weeks
group

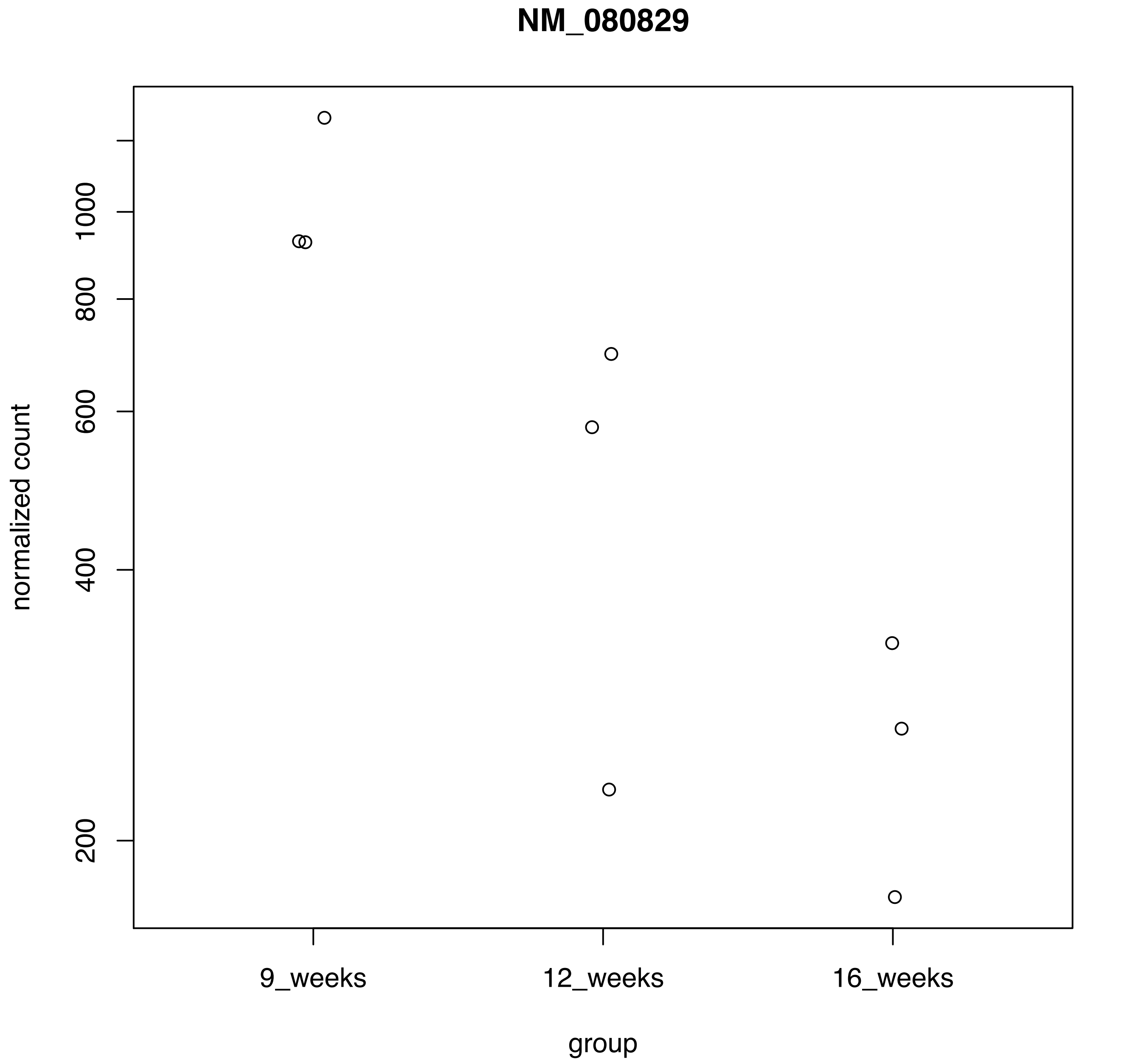

NM_080829
normalized count
1000
800
600
400
200
9_weeks
12_weeks
16_weeks
group

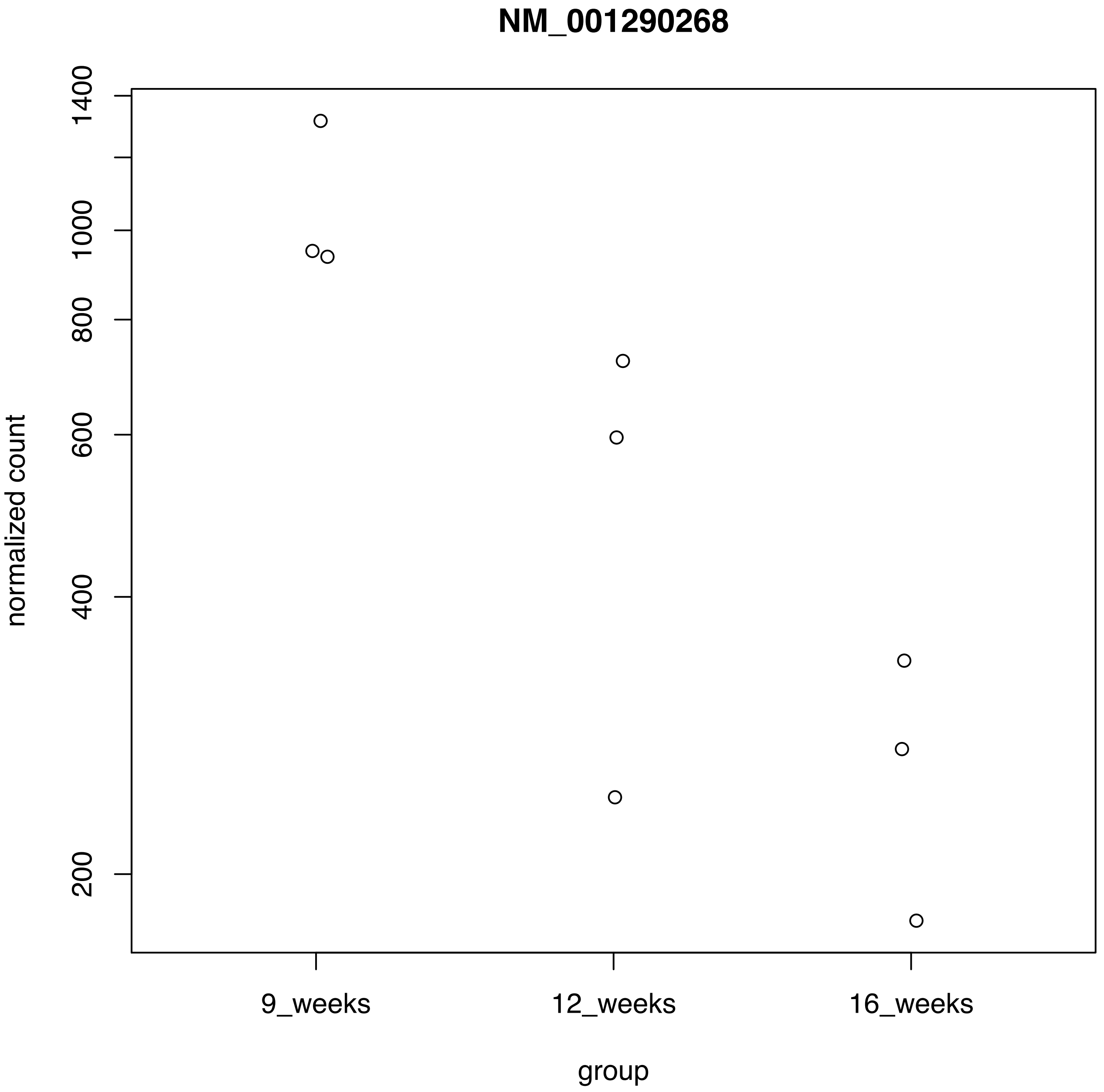
NM_001290268
normalized count
1400
1000
800
600
400
200
9_weeks
12_weeks
16_weeks
group

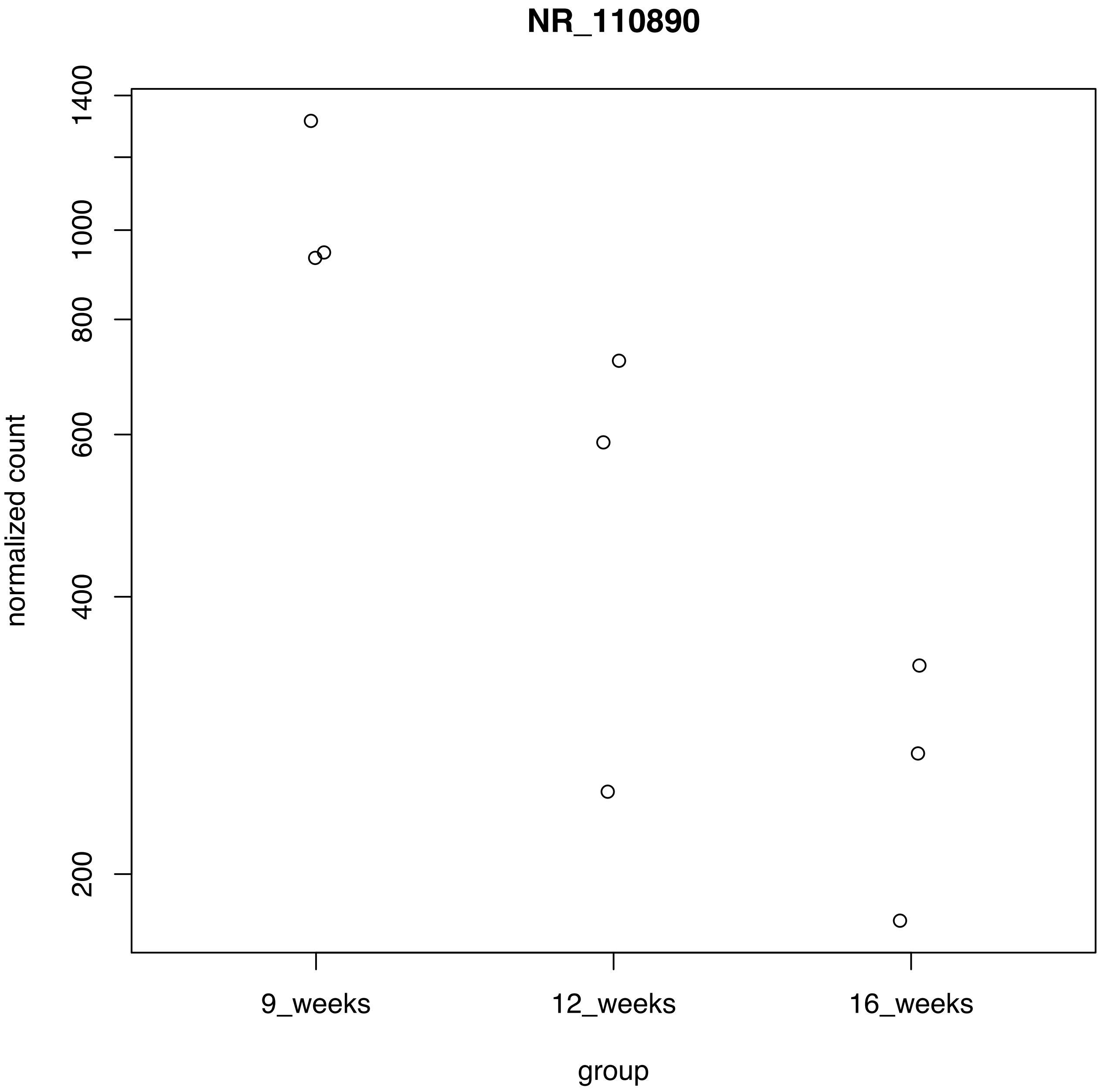

NR_110890
normalized count
1400
1000
800
600
400
200
9_weeks
12_weeks
16_weeks
group

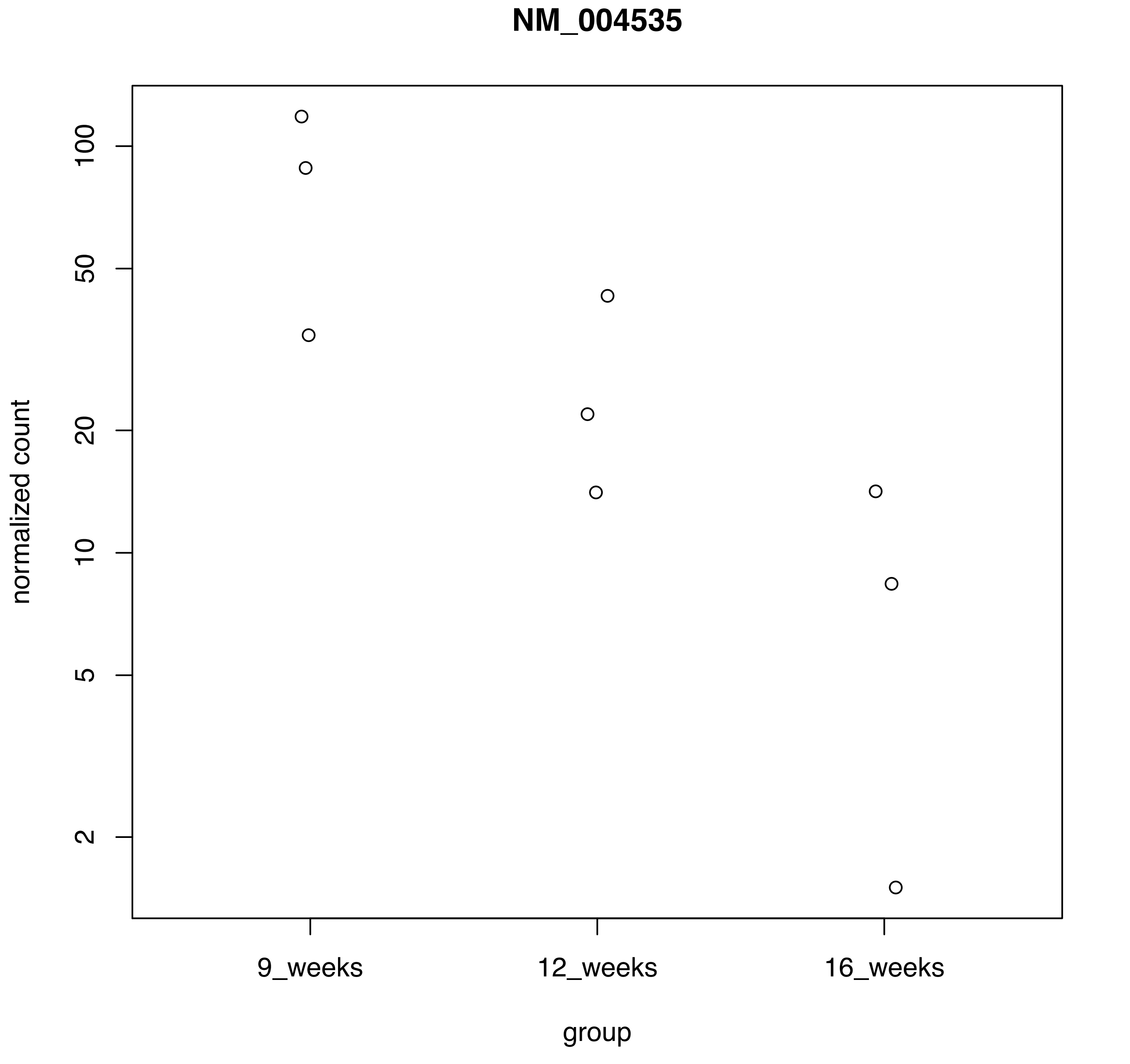

NM_004535
100
50
20
10
5
2
normalized count
9_weeks
12_weeks
16_weeks
group

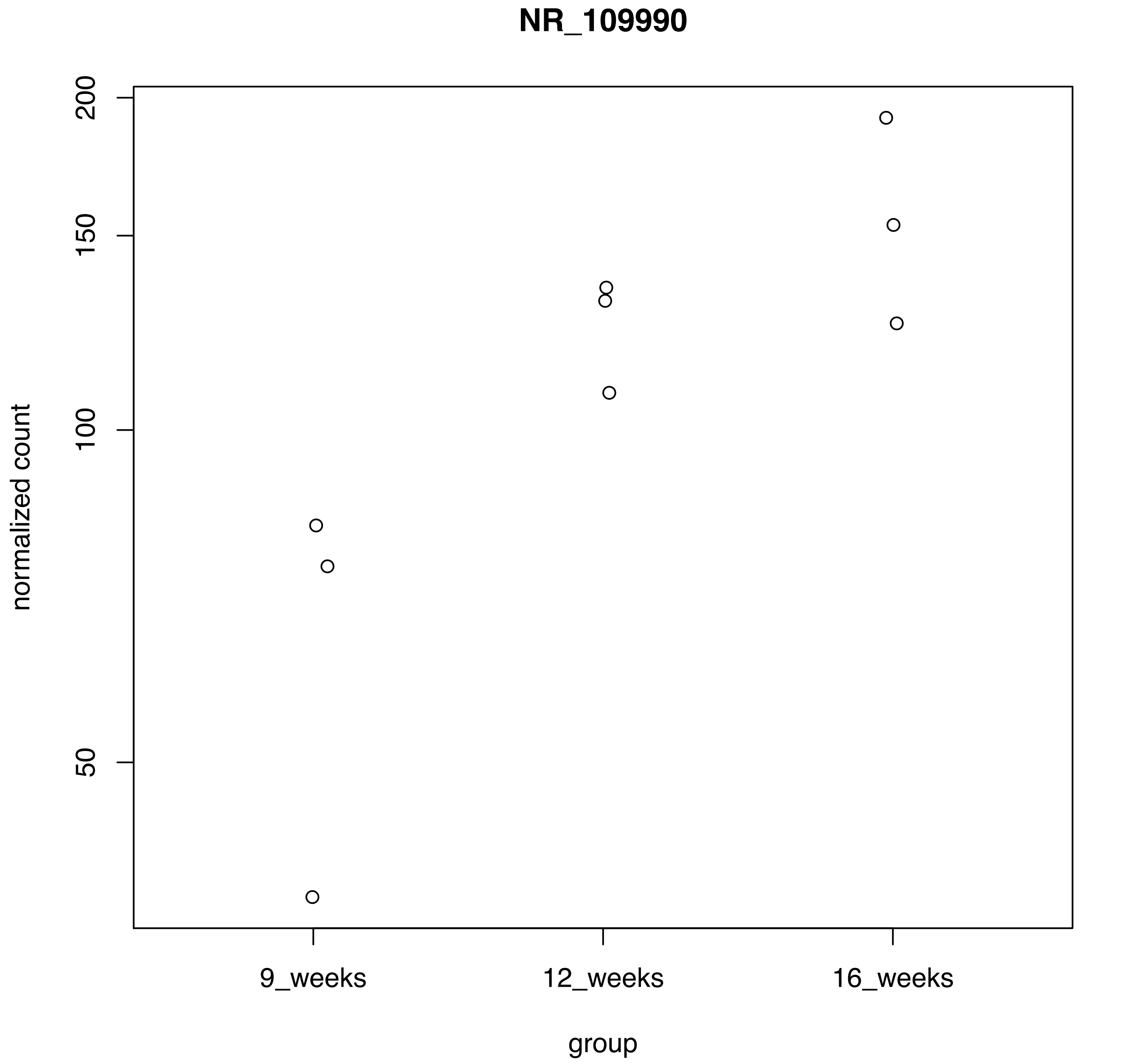
NR_109990
200
150
100
50
normalized count
9_weeks
12_weeks
16_weeks
group

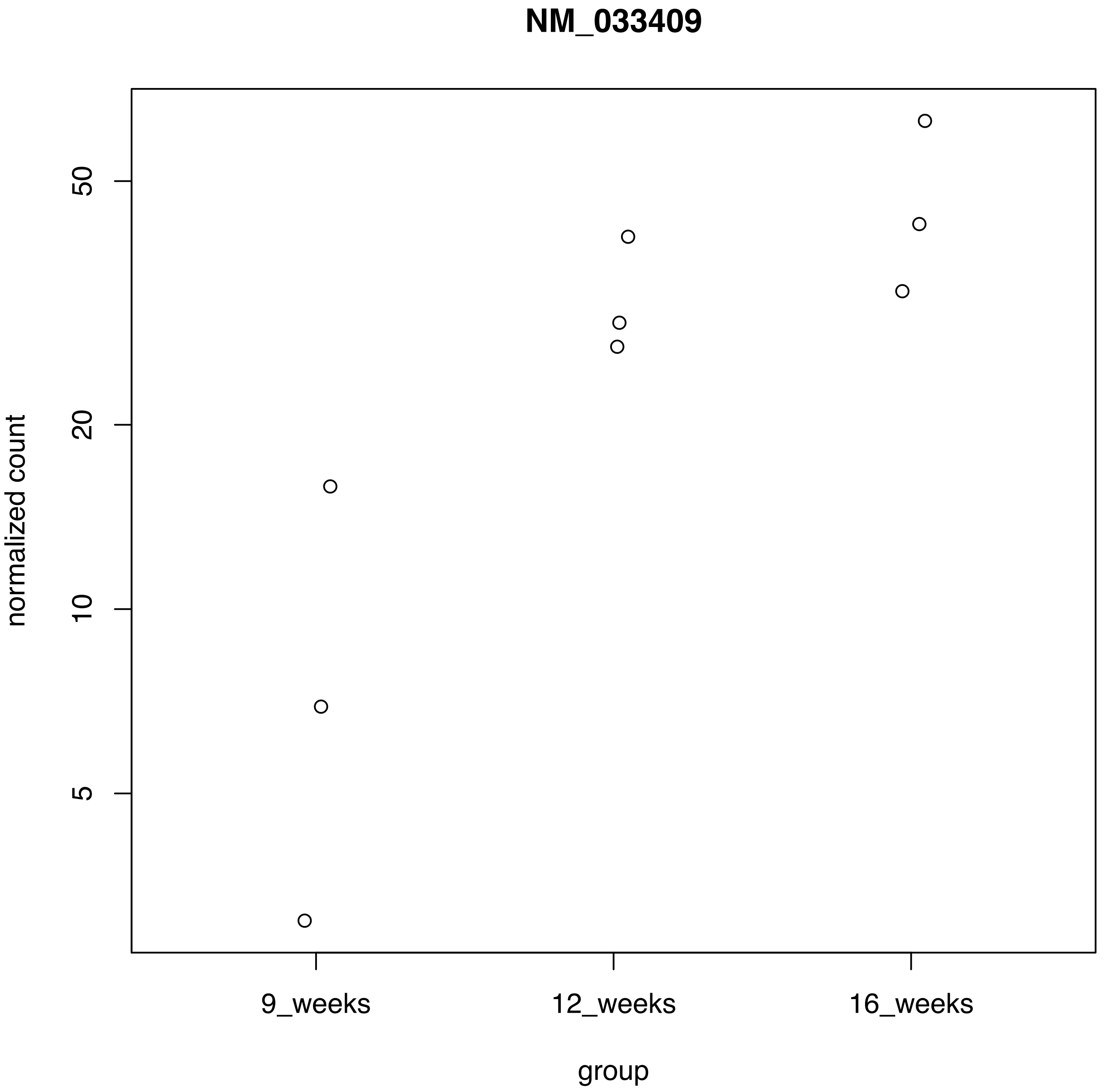
NM_033409
normalized count
50
20
10
5
9_weeks
12_weeks
16_weeks
group

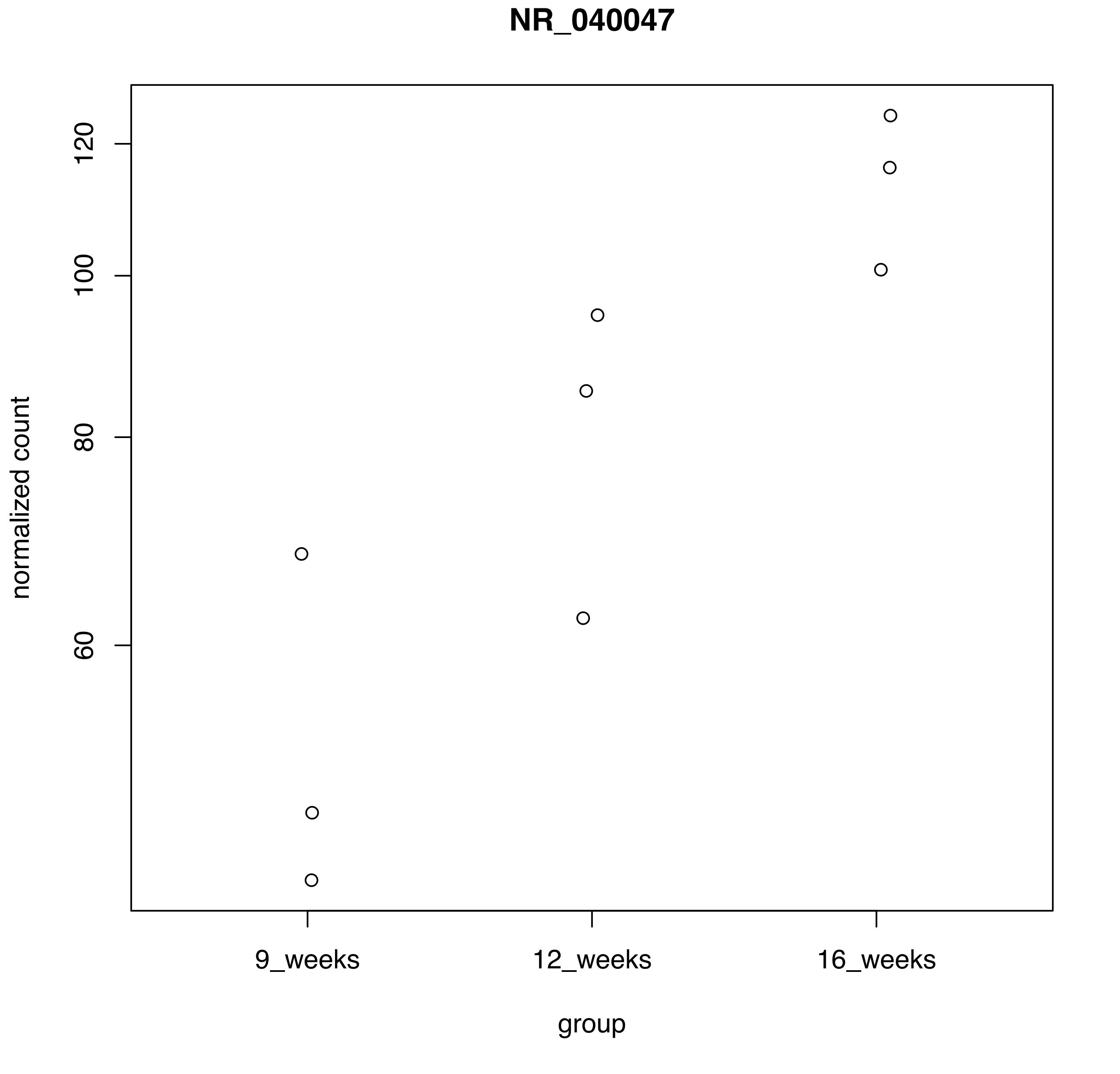
NR_040047
normalized count
120
100
80
60
9_weeks
12_weeks
16_weeks
group

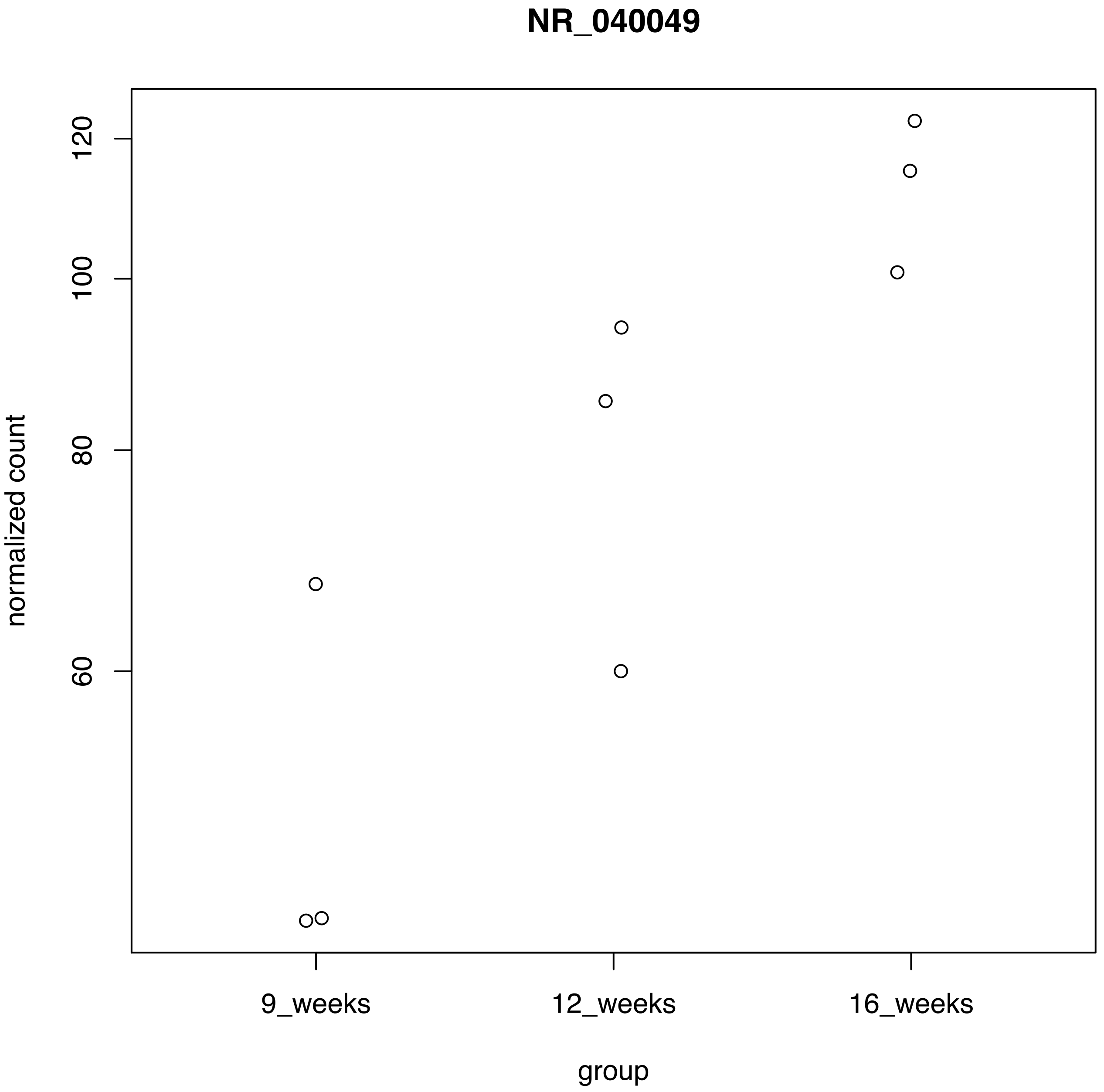
NR_040049
normalized count
60
80
100
120
9_weeks
12_weeks
16_weeks
group

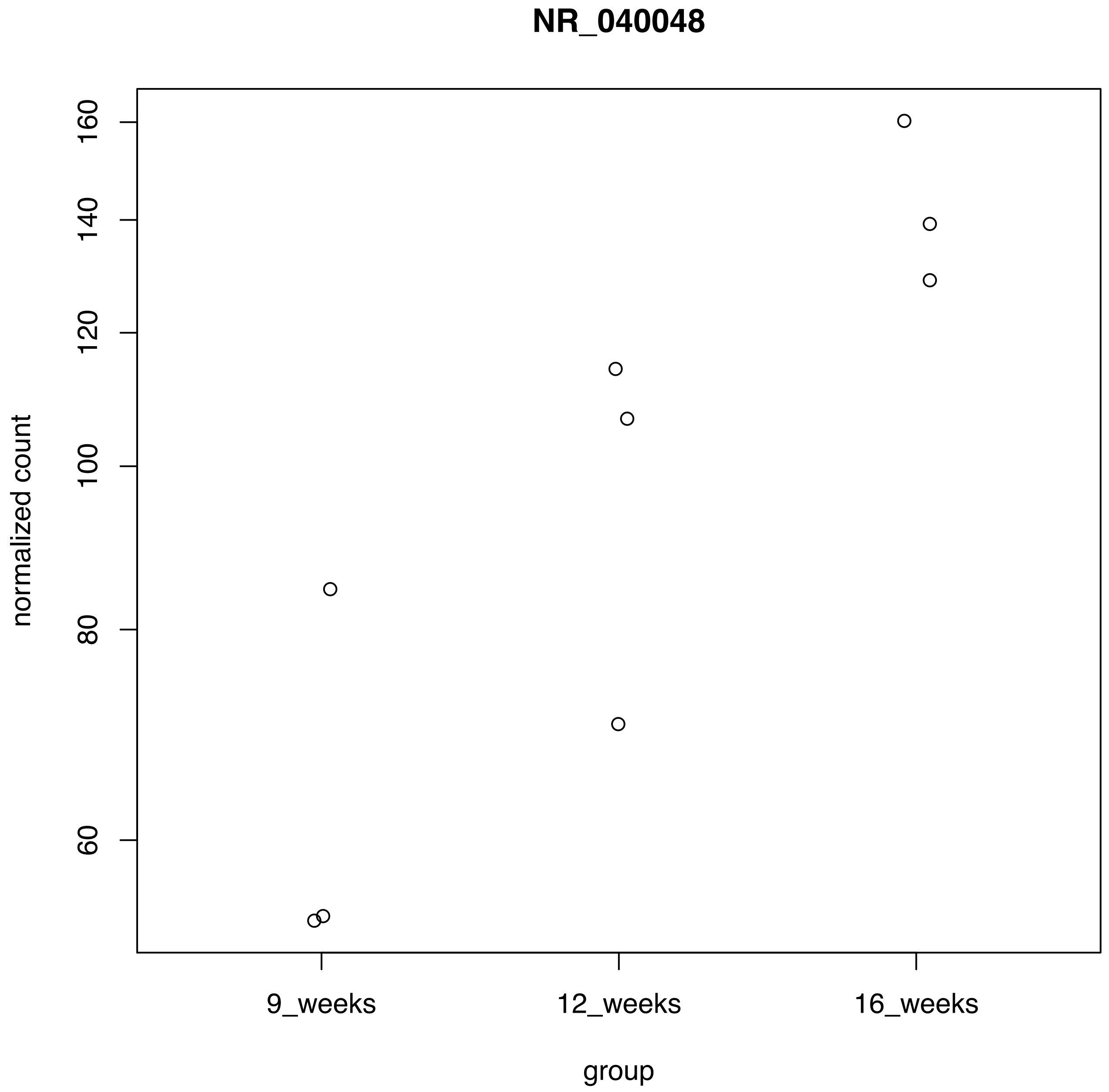

NR_040048
normalized count
60
80
100
120
140
160
9_weeks
12_weeks
16_weeks
group

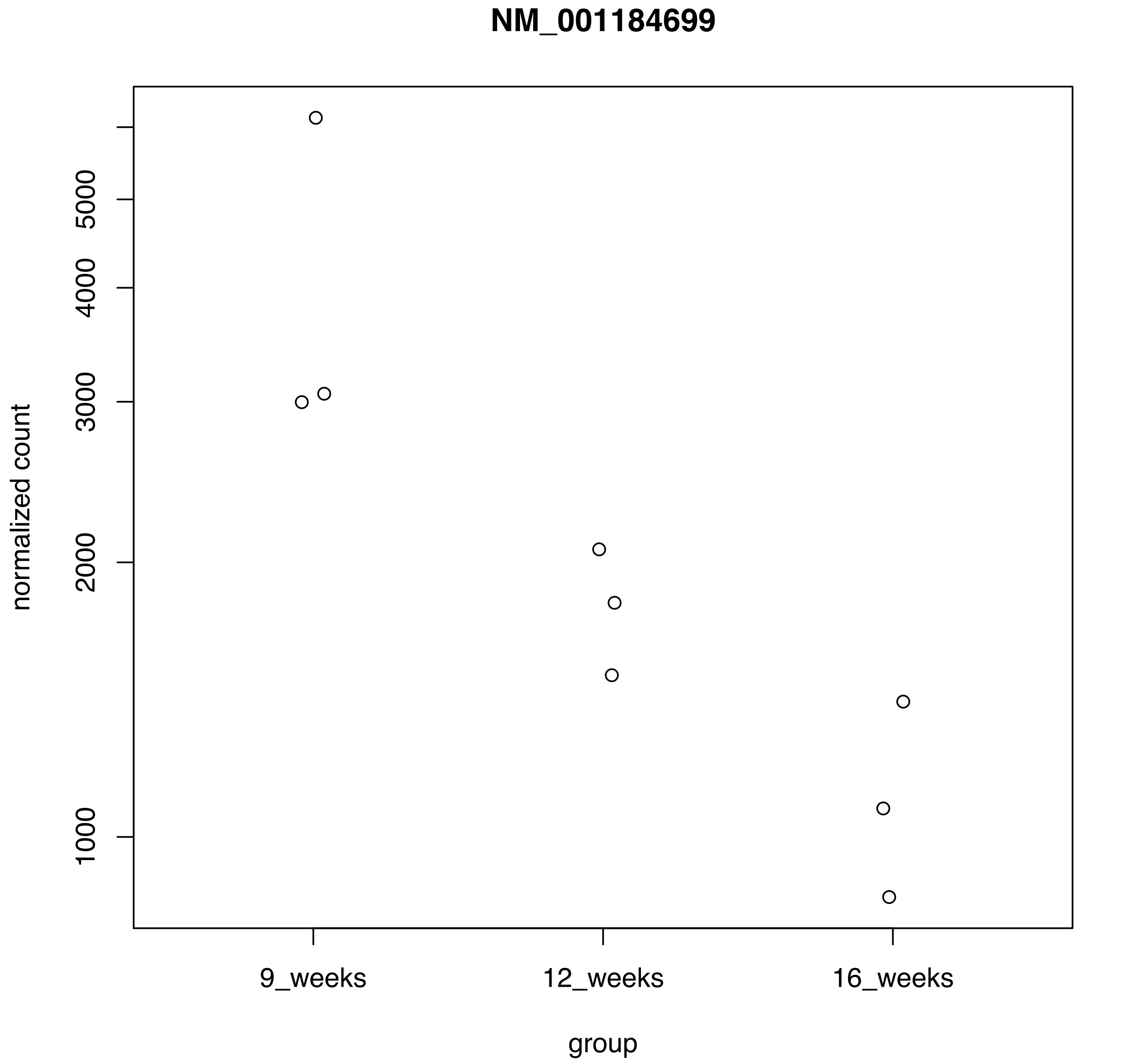
NM_001184699
normalized count
1000
2000
3000
4000
5000
9_weeks
12_weeks
16_weeks
group

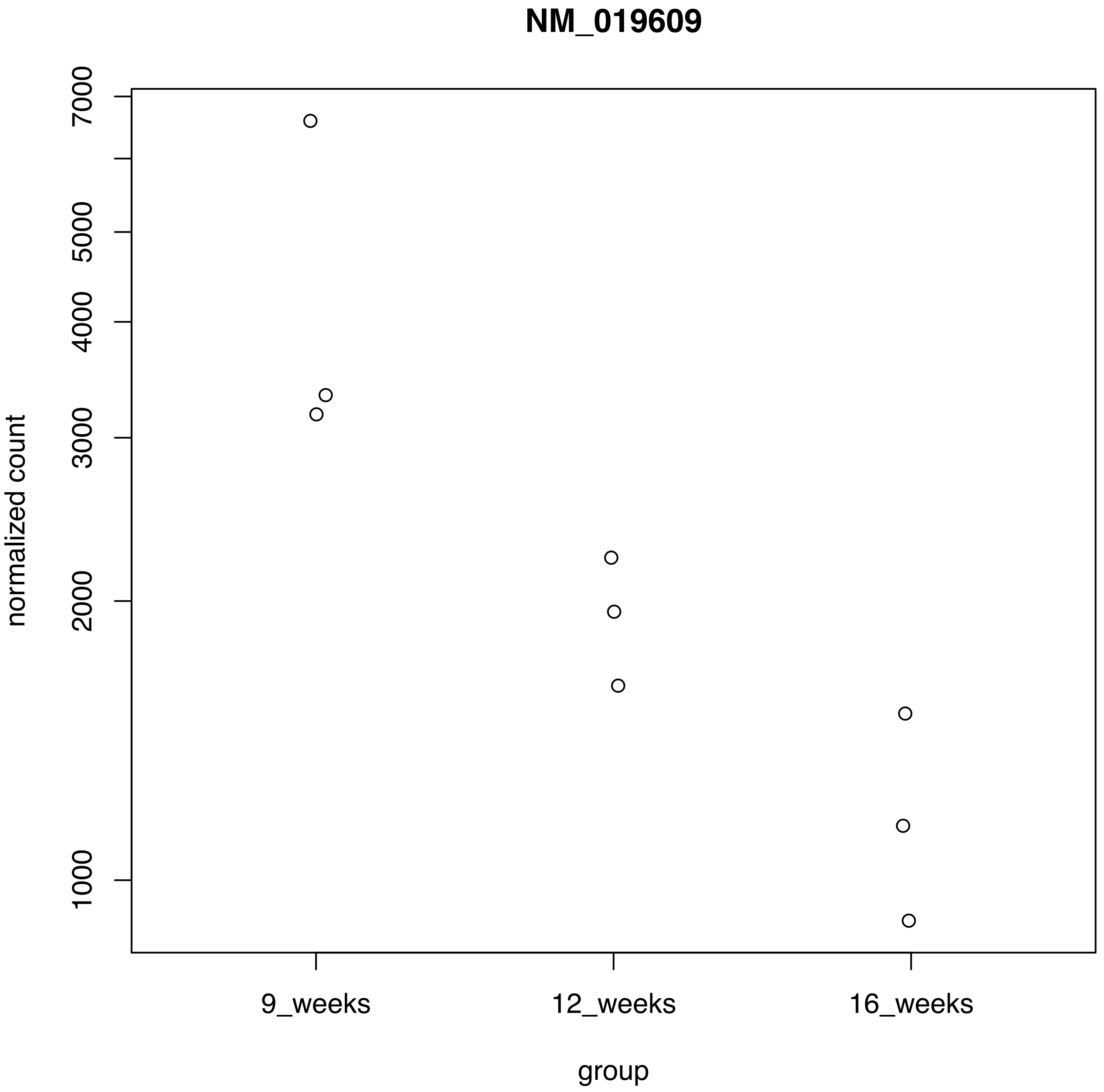
NM_019609
normalized count
1000
2000
3000
4000
5000
7000
9_weeks
12_weeks
16_weeks
group

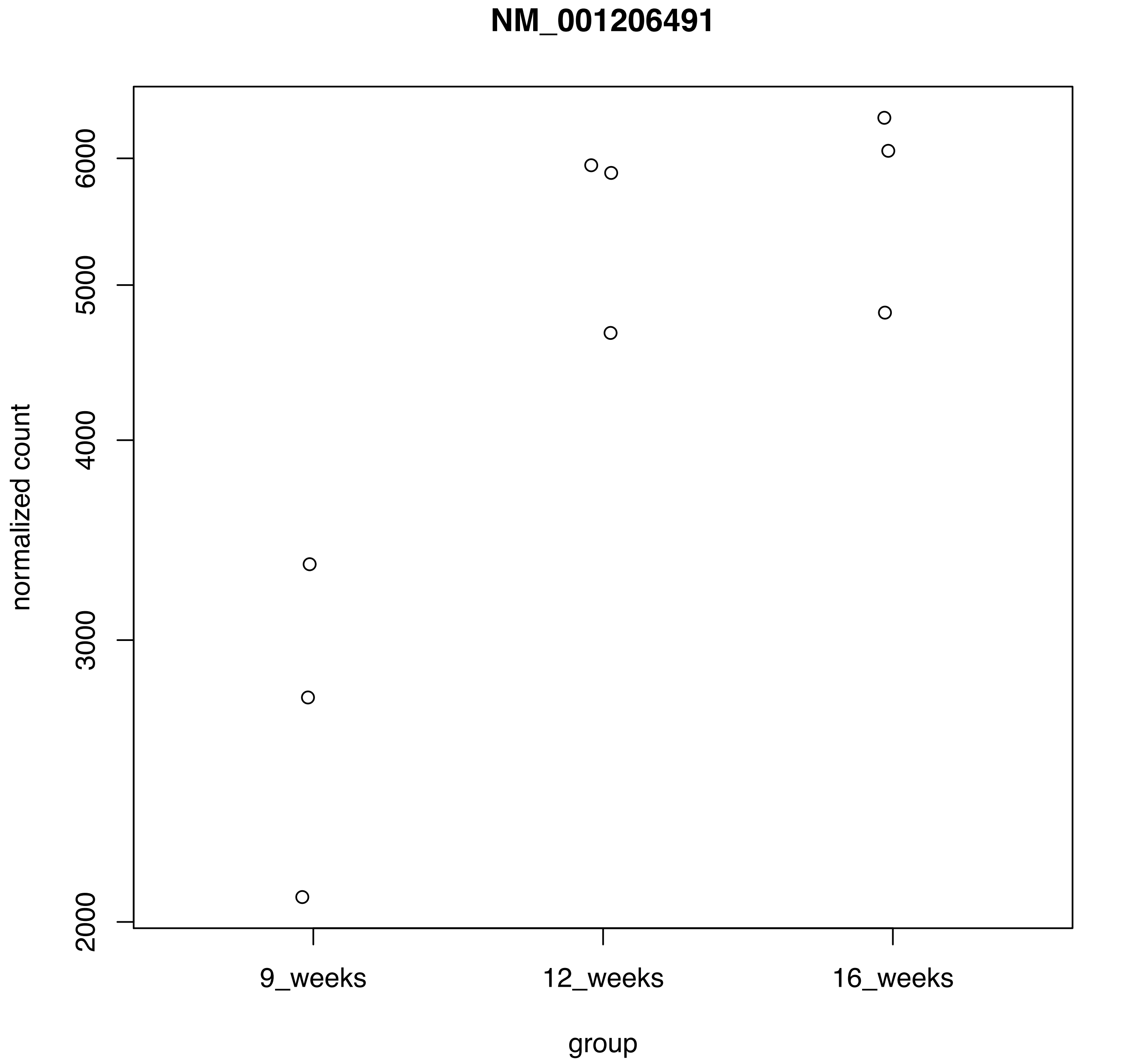
NM_001206491
normalized count
6000
5000
4000
3000
2000
9_weeks
12_weeks
16_weeks
group

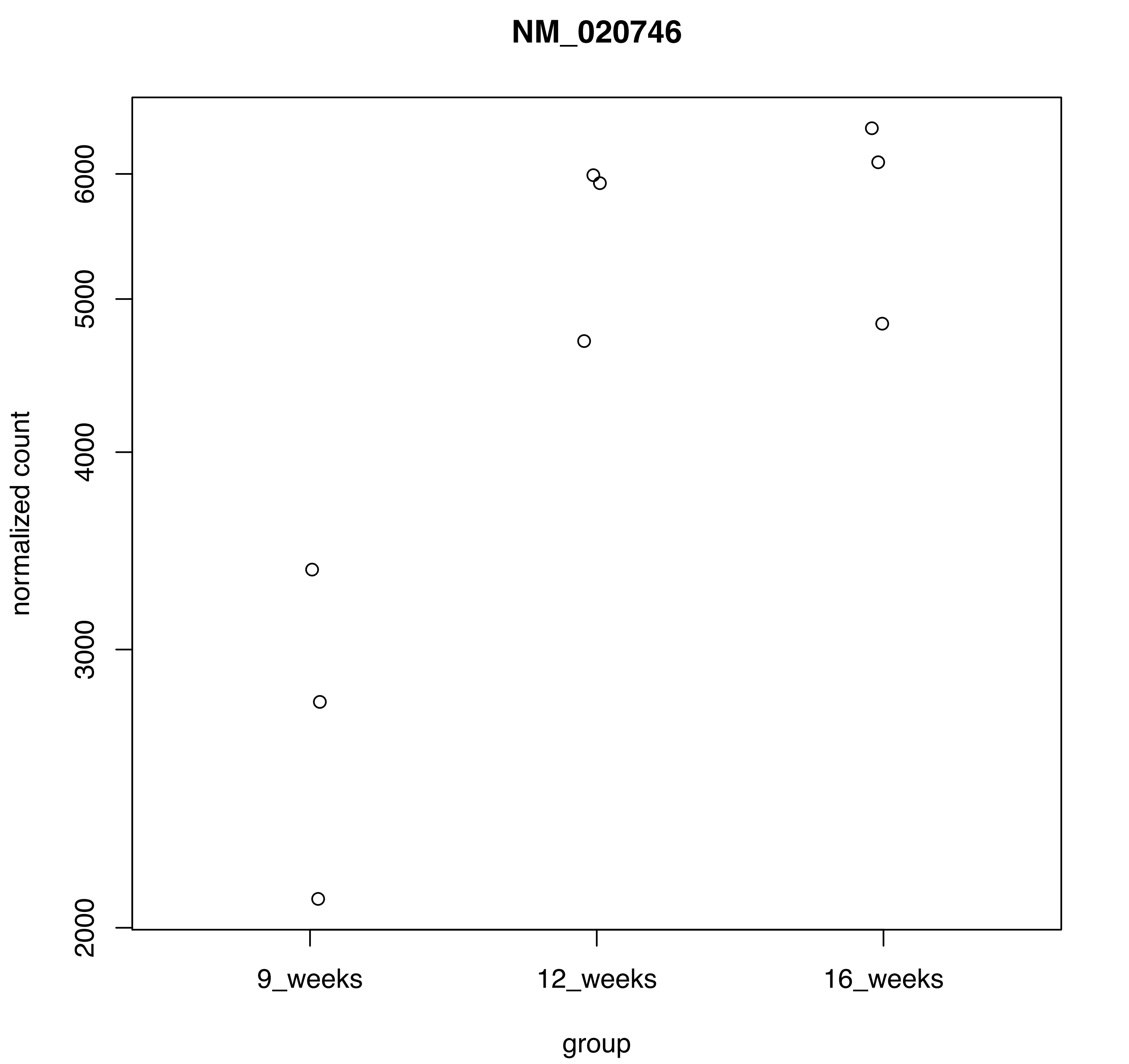

NM_020746
normalized count
2000
3000
4000
5000
6000
9_weeks
12_weeks
16_weeks
group

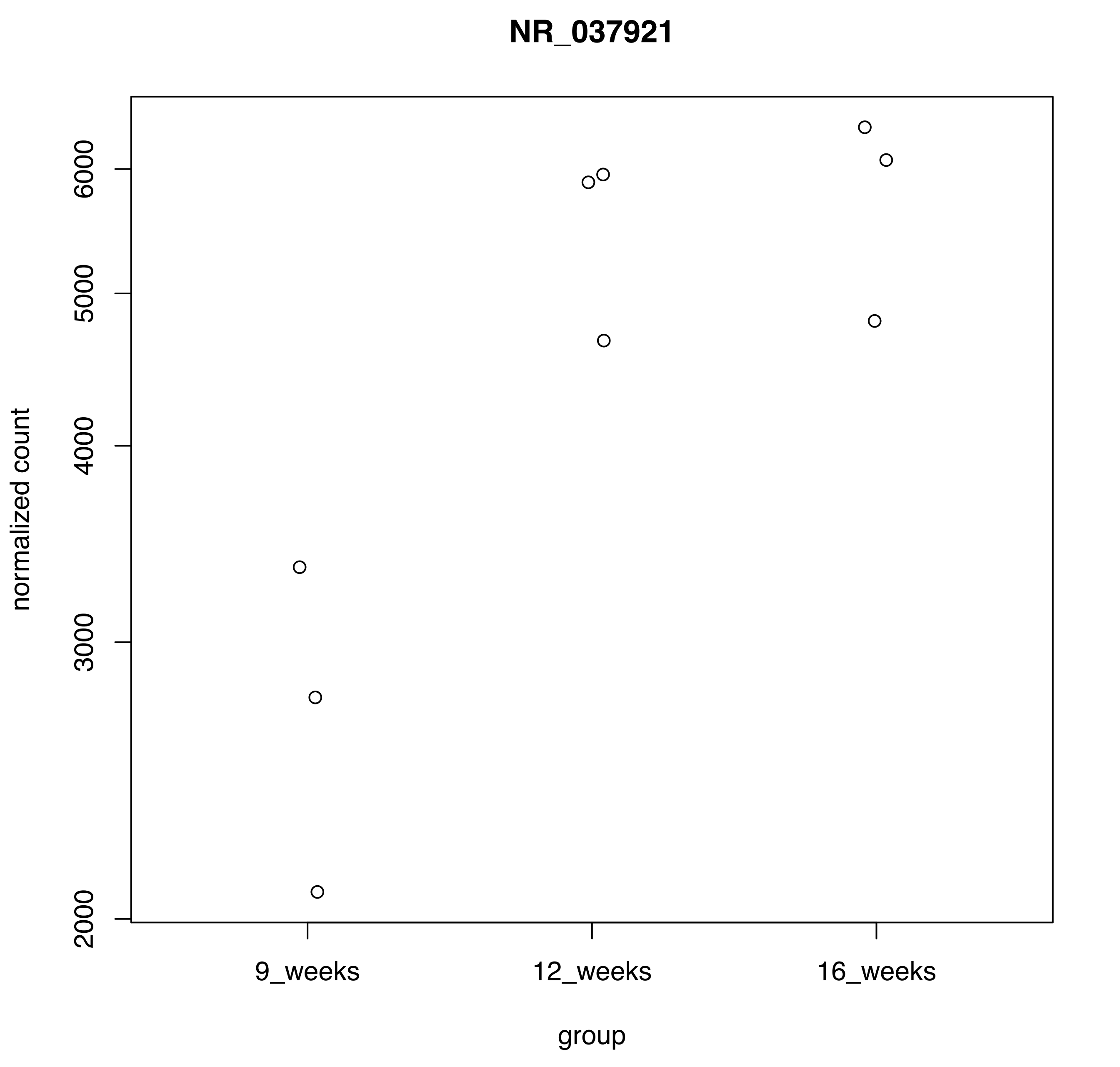

NR_037921
normalized count
2000
3000
4000
5000
6000
9_weeks
12_weeks
16_weeks
group

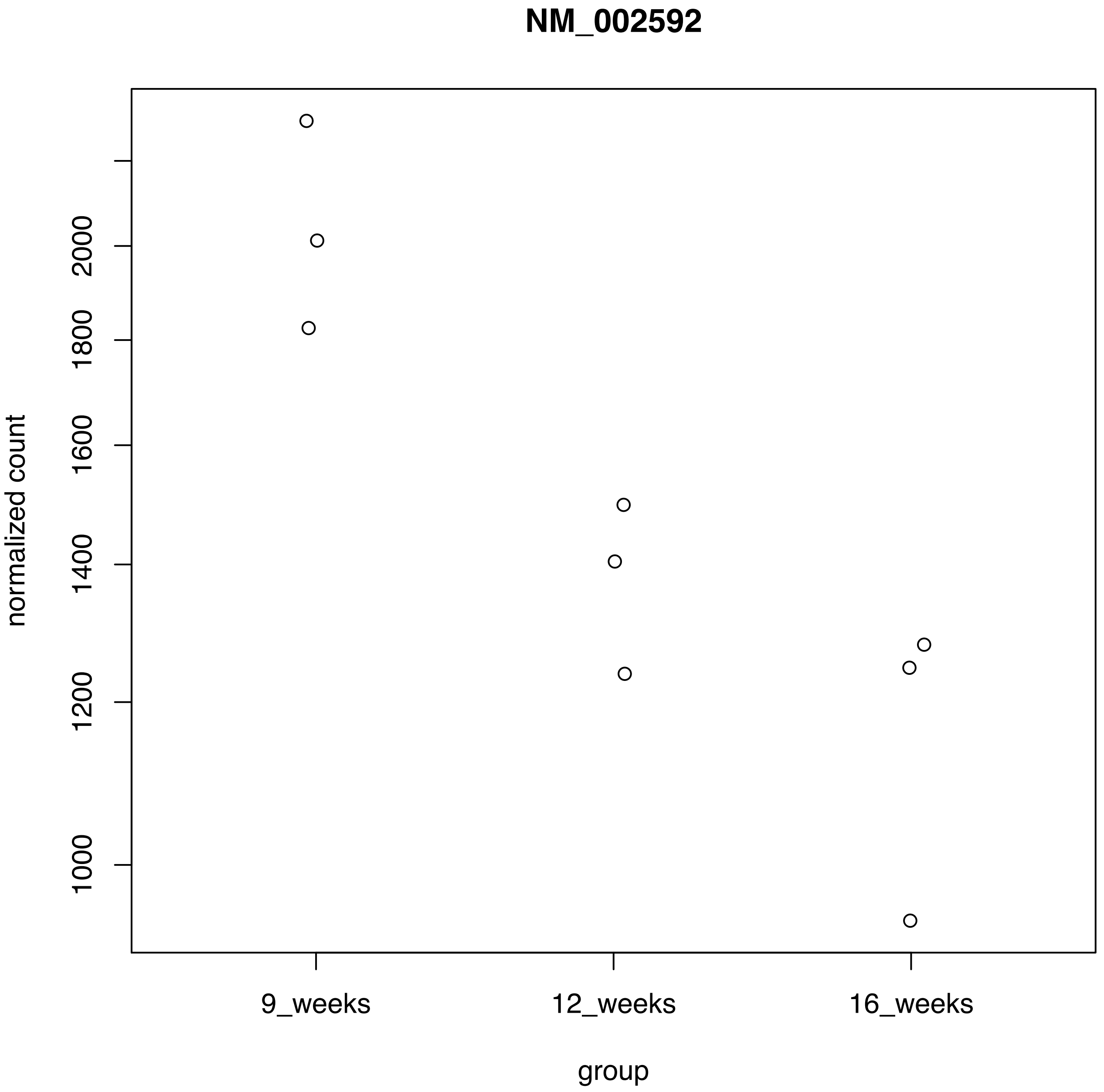
NM_002592
normalized count
2000
1800
1600
1400
1200
1000
9_weeks
12_weeks
16_weeks
group

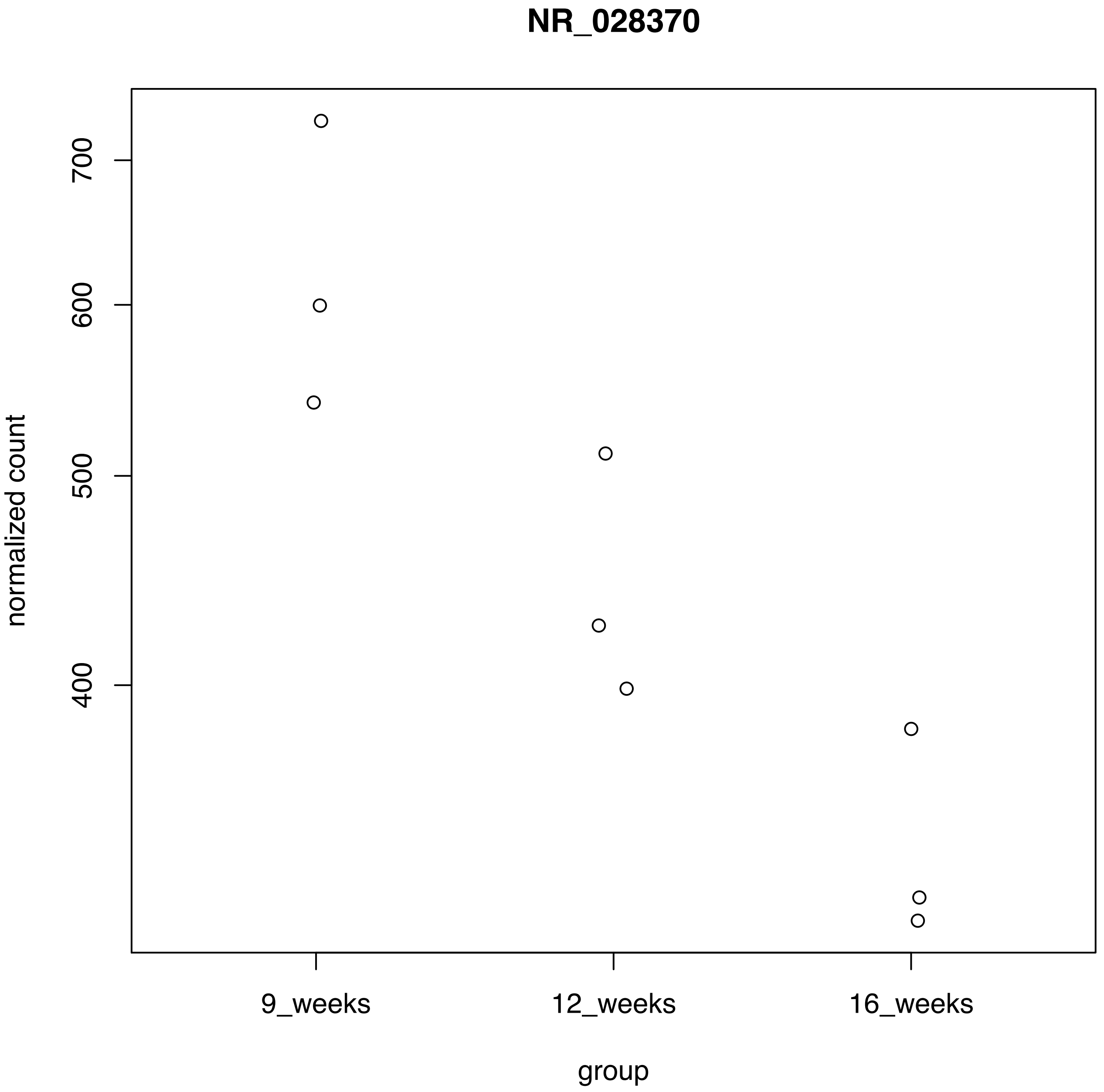
NR_028370
normalized count
700
600
500
400
9_weeks
12_weeks
16_weeks
group

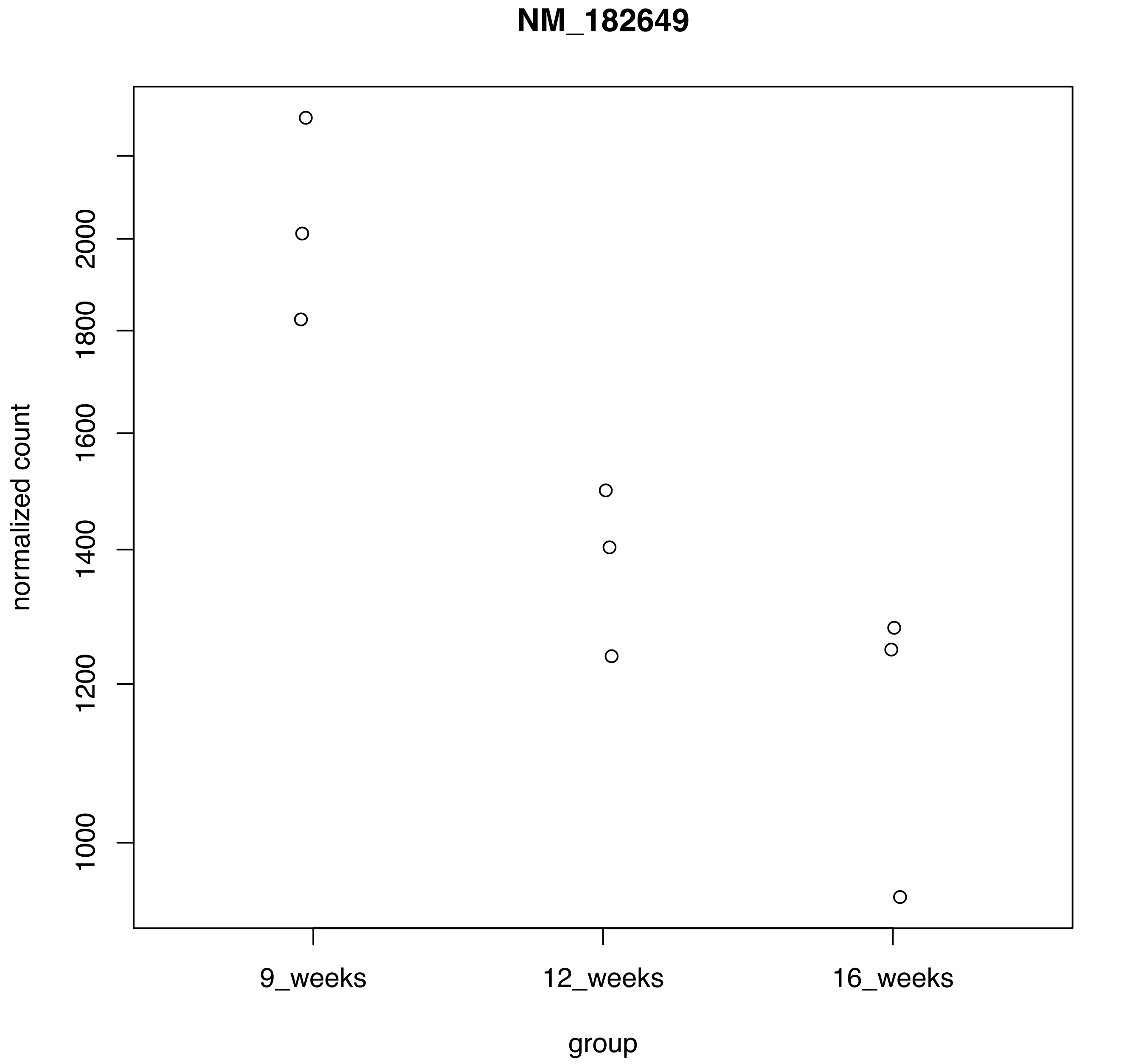
NM_182649
normalized count
1000
1200
1400
1600
1800
2000
9_weeks
12_weeks
16_weeks
group

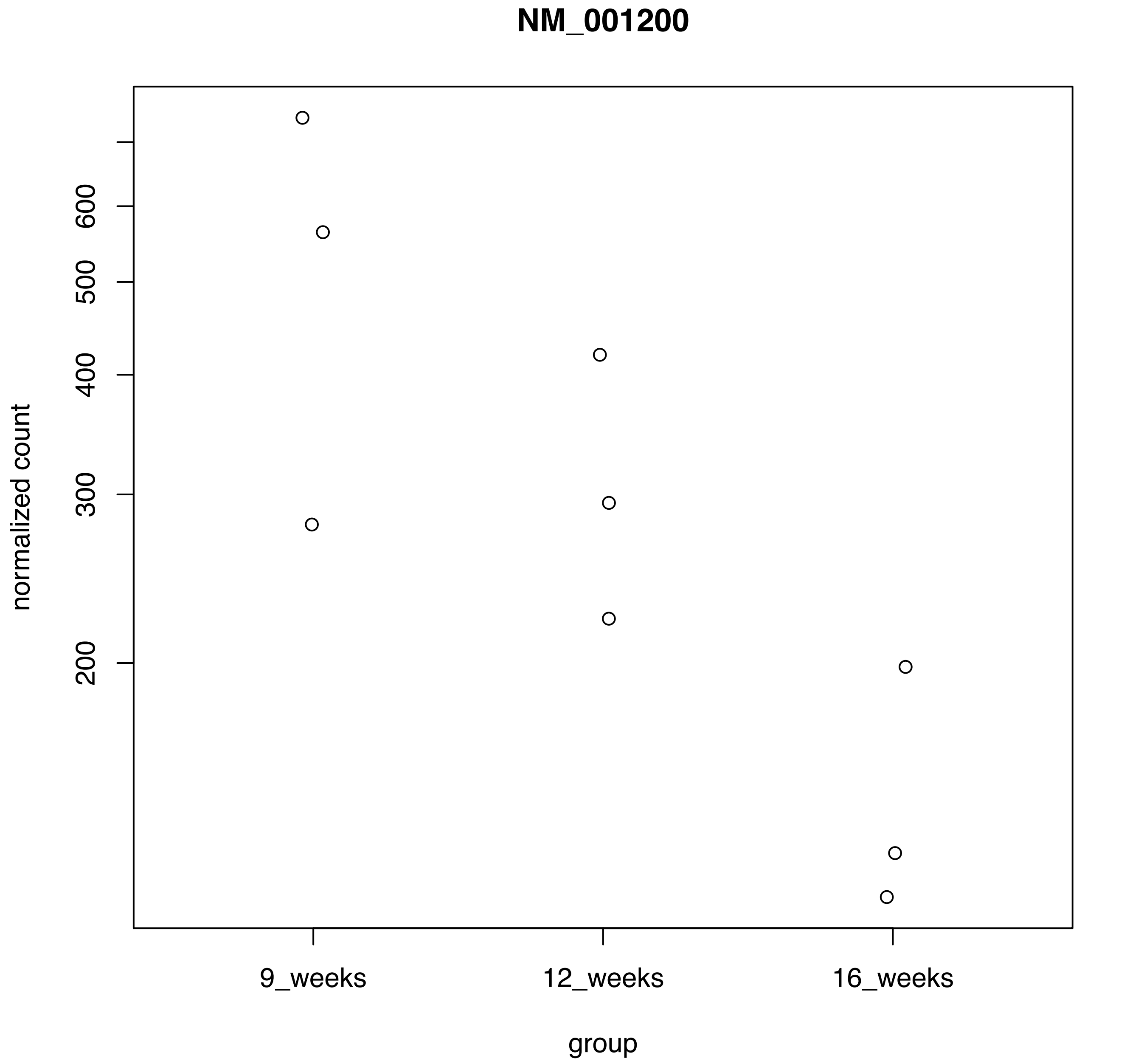
NM_001200
normalized count
200
300
400
500
600
9_weeks
12_weeks
16_weeks
group

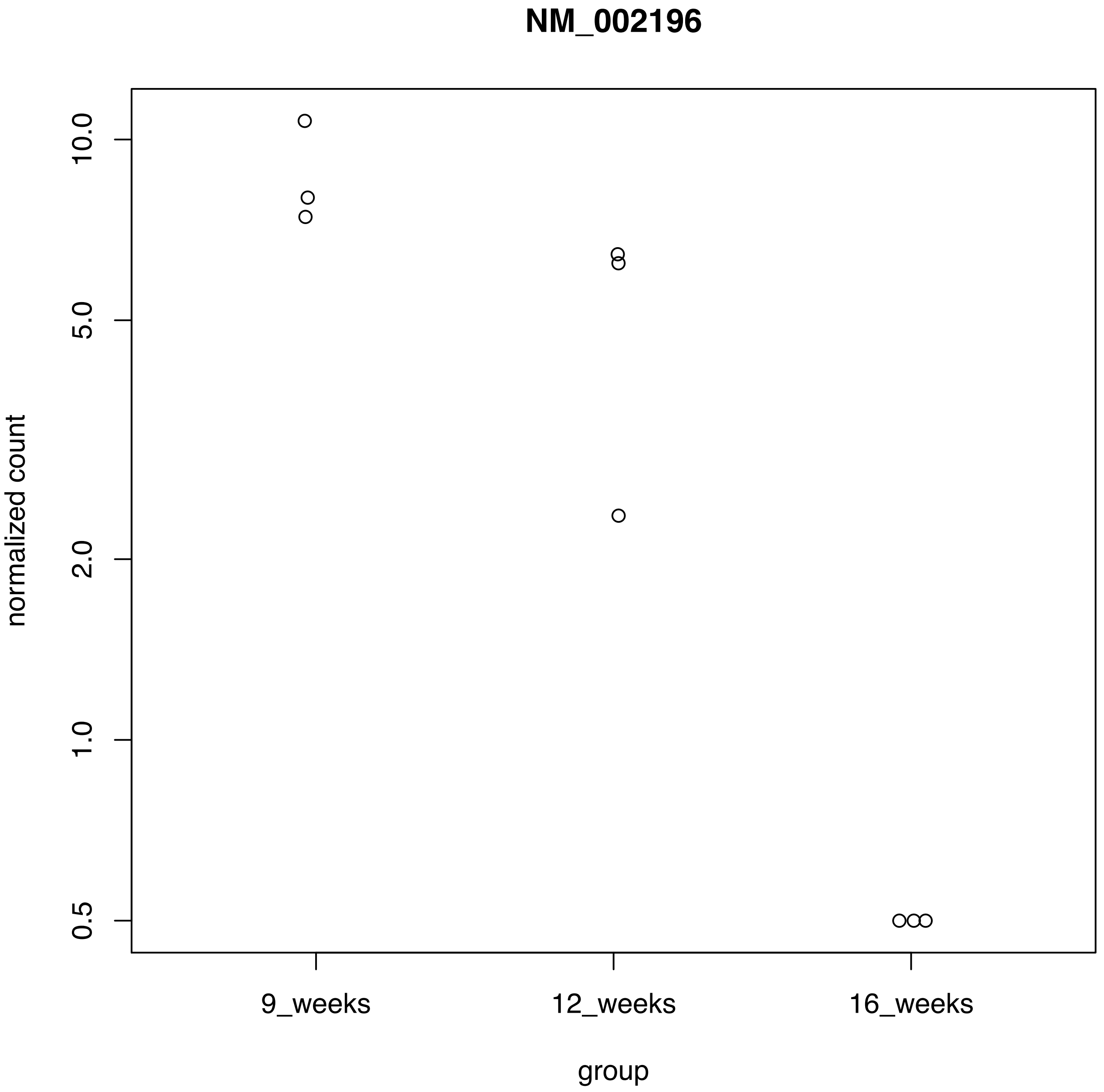
NM_002196
normalized count
10.0
5.0
2.0
1.0
0.5
9_weeks
12_weeks
16_weeks
group

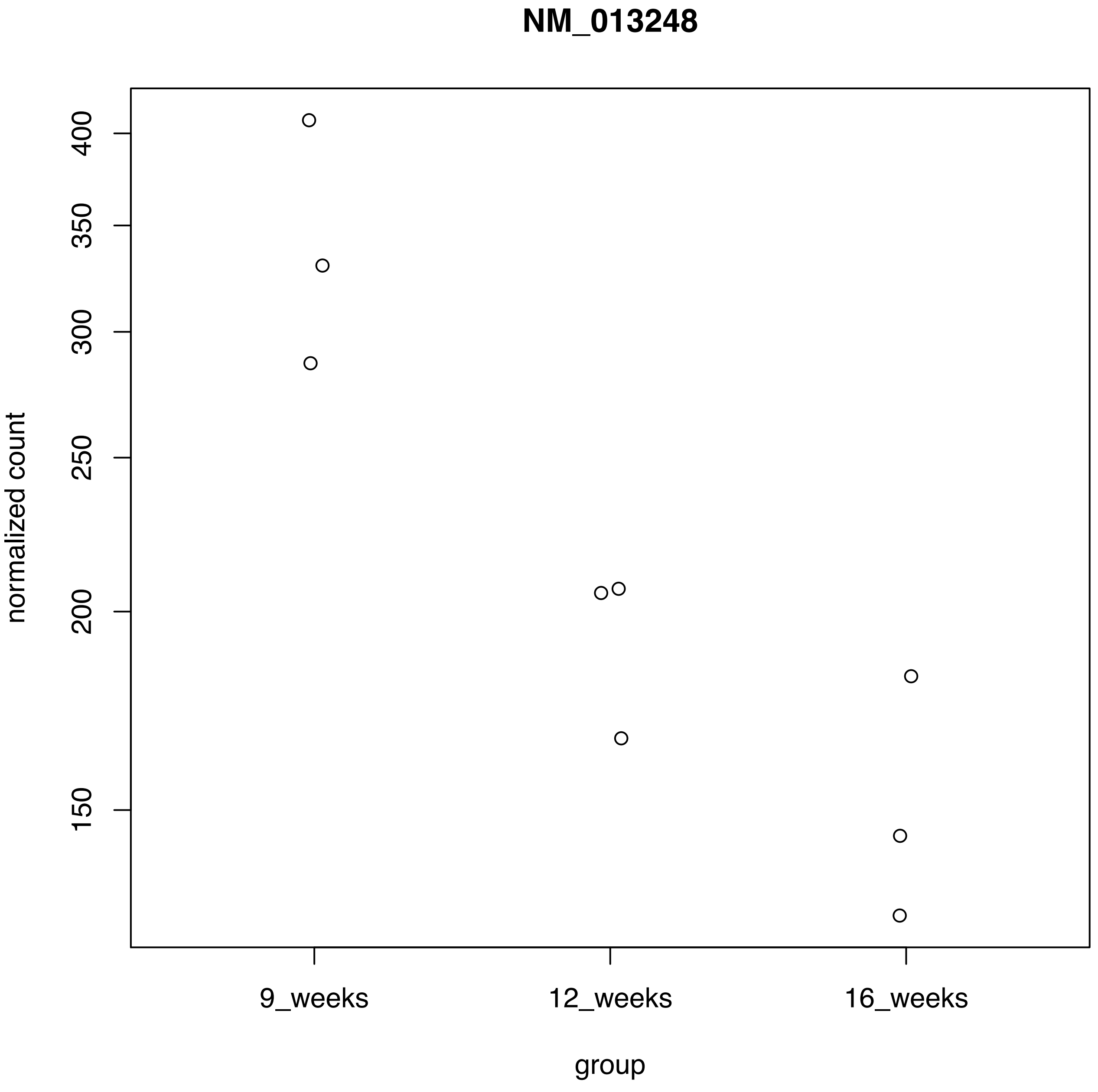
NM_013248
normalized count
400
350
300
250
200
150
9_weeks
12_weeks
16_weeks
group

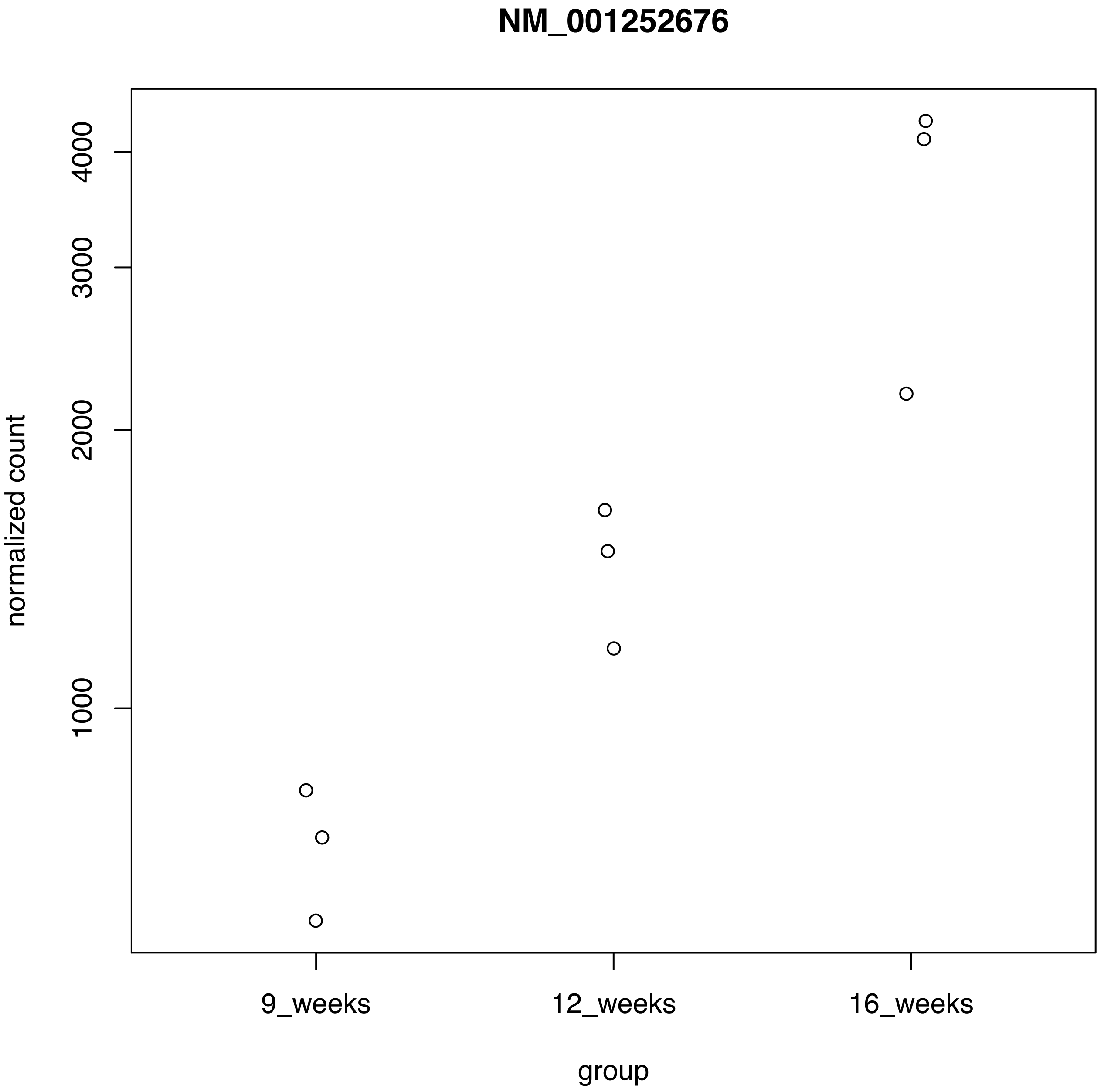
NM_001252676
normalized count
1000
2000
3000
4000
9_weeks
12_weeks
16_weeks
group

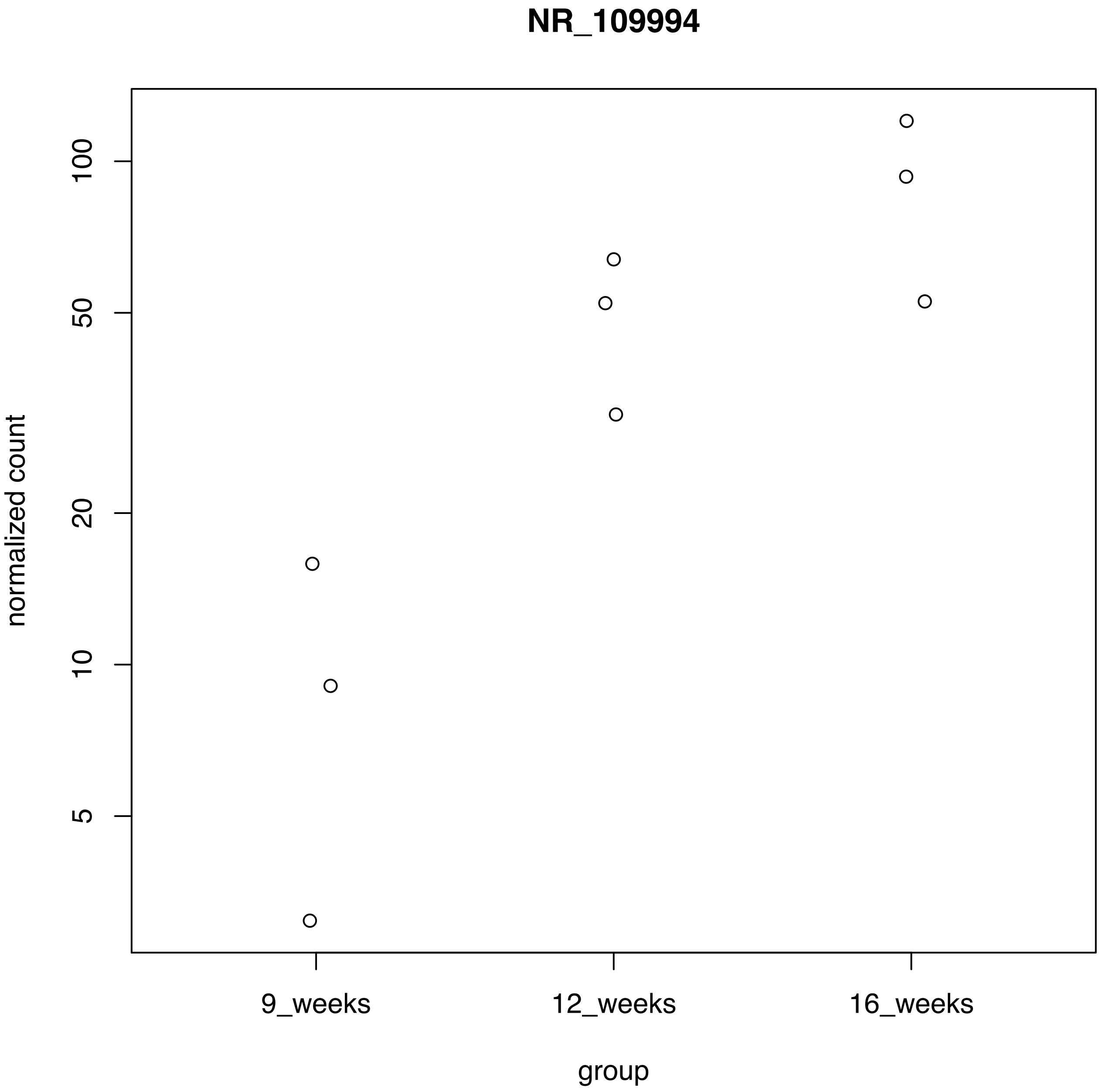

NR_109994
100
50
20
10
5
normalized count
9_weeks
12_weeks
16_weeks
group

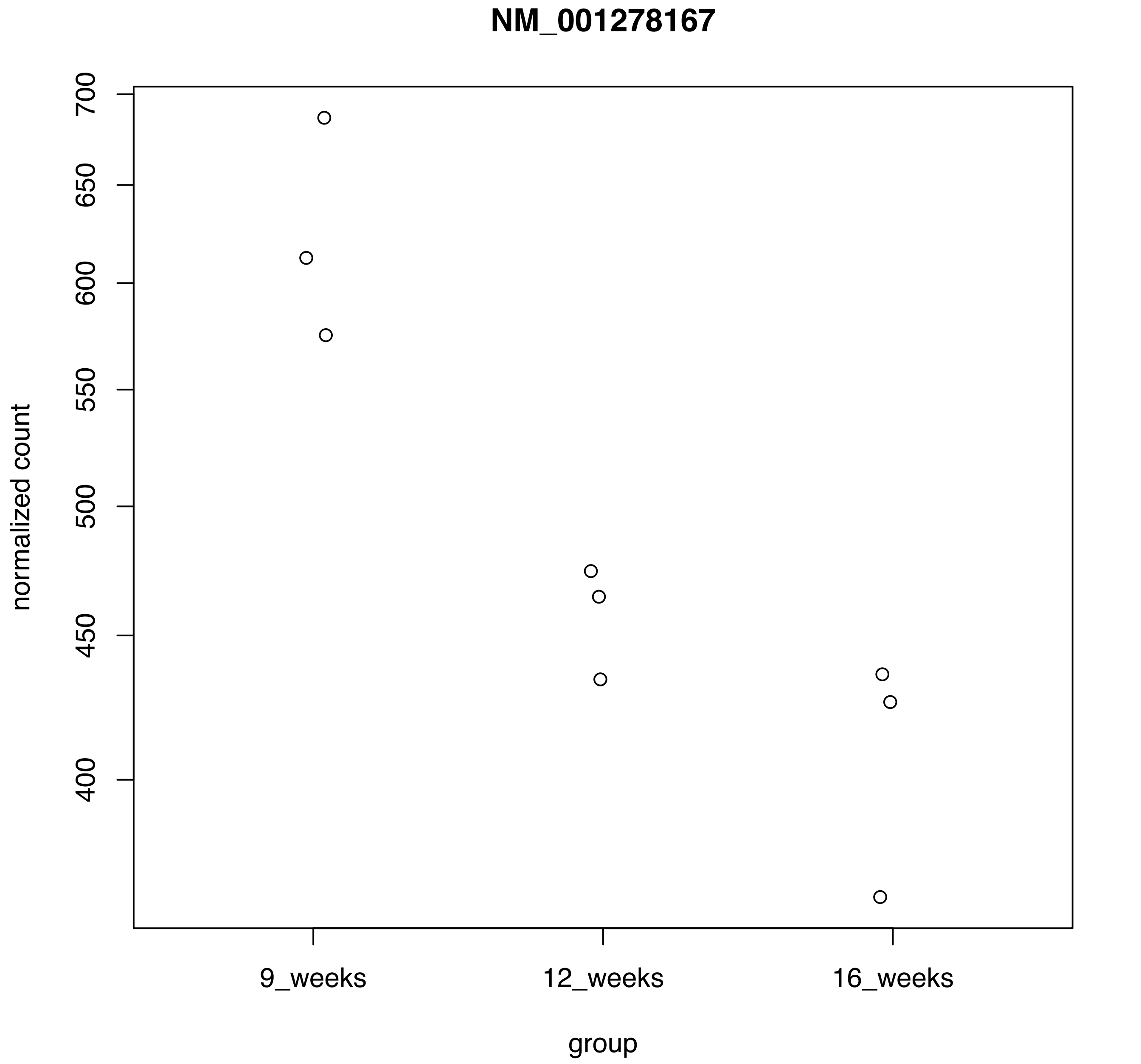

NM_001278167
normalized count
700
650
600
550
500
450
400
9_weeks
12_weeks
16_weeks
group

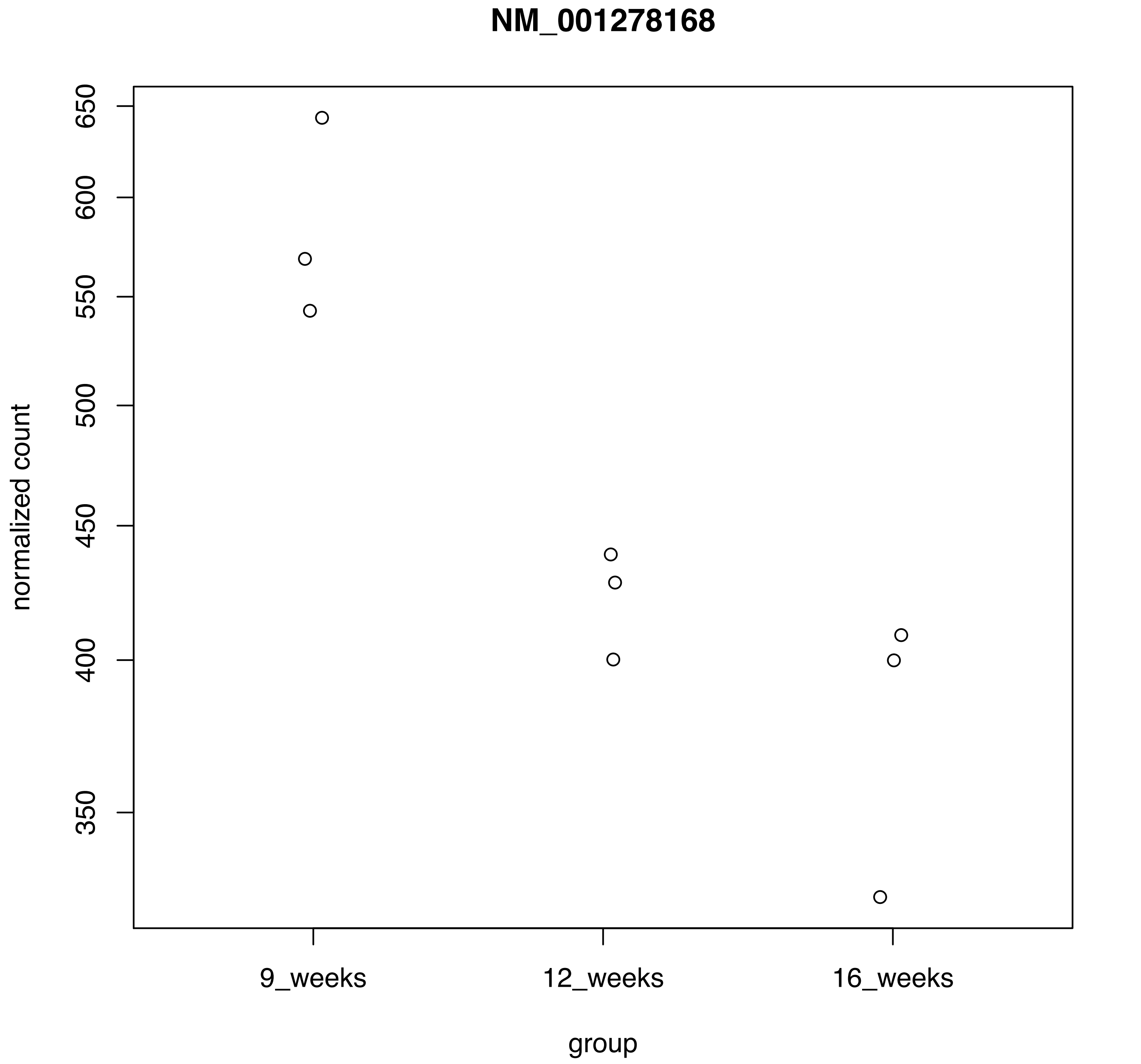
NM_001278168
normalized count
650
600
550
500
450
400
350
9_weeks
12_weeks
16_weeks
group

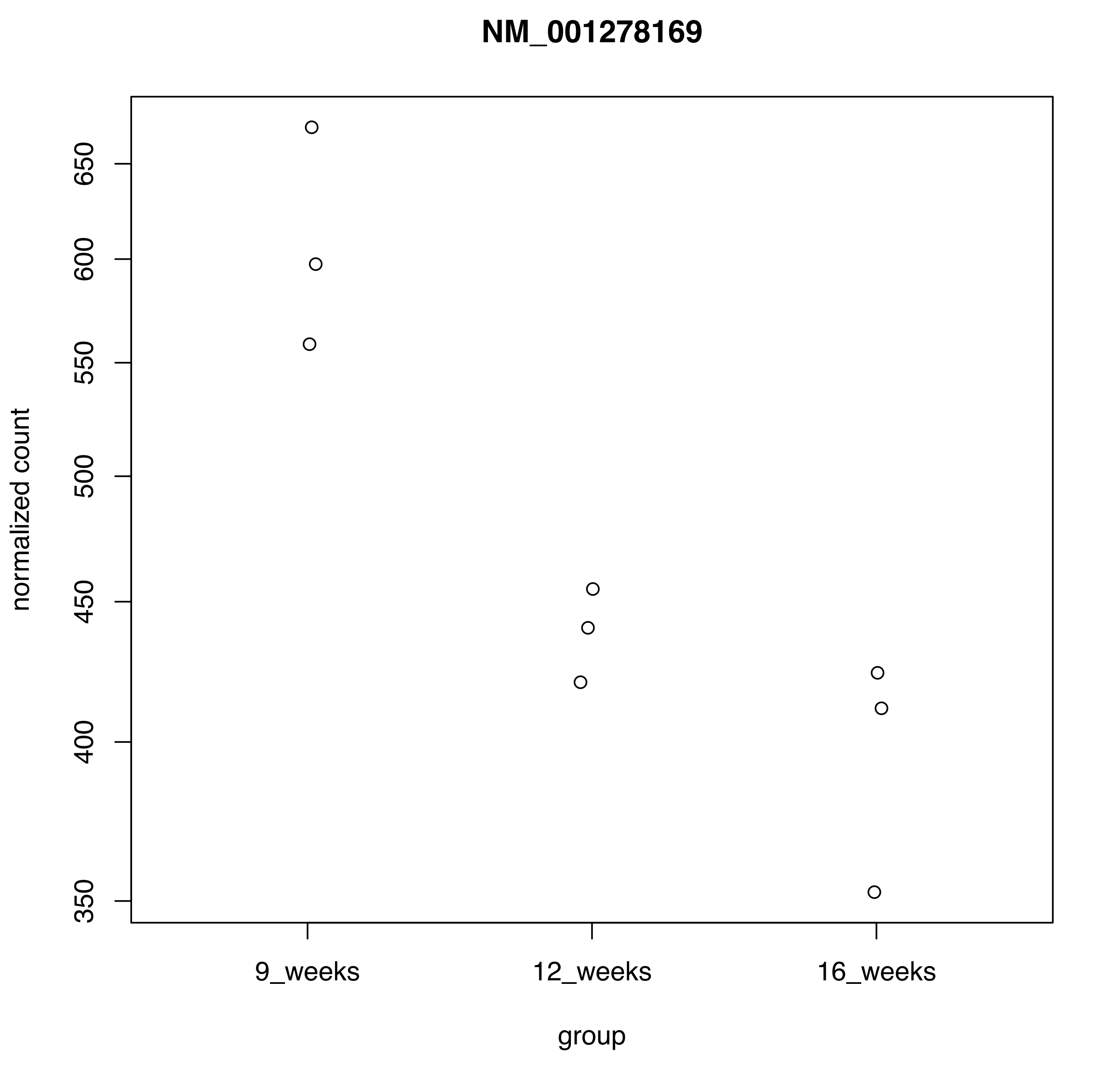
NM_001278169
normalized count
650
600
550
500
450
400
350
9_weeks
12_weeks
16_weeks
group

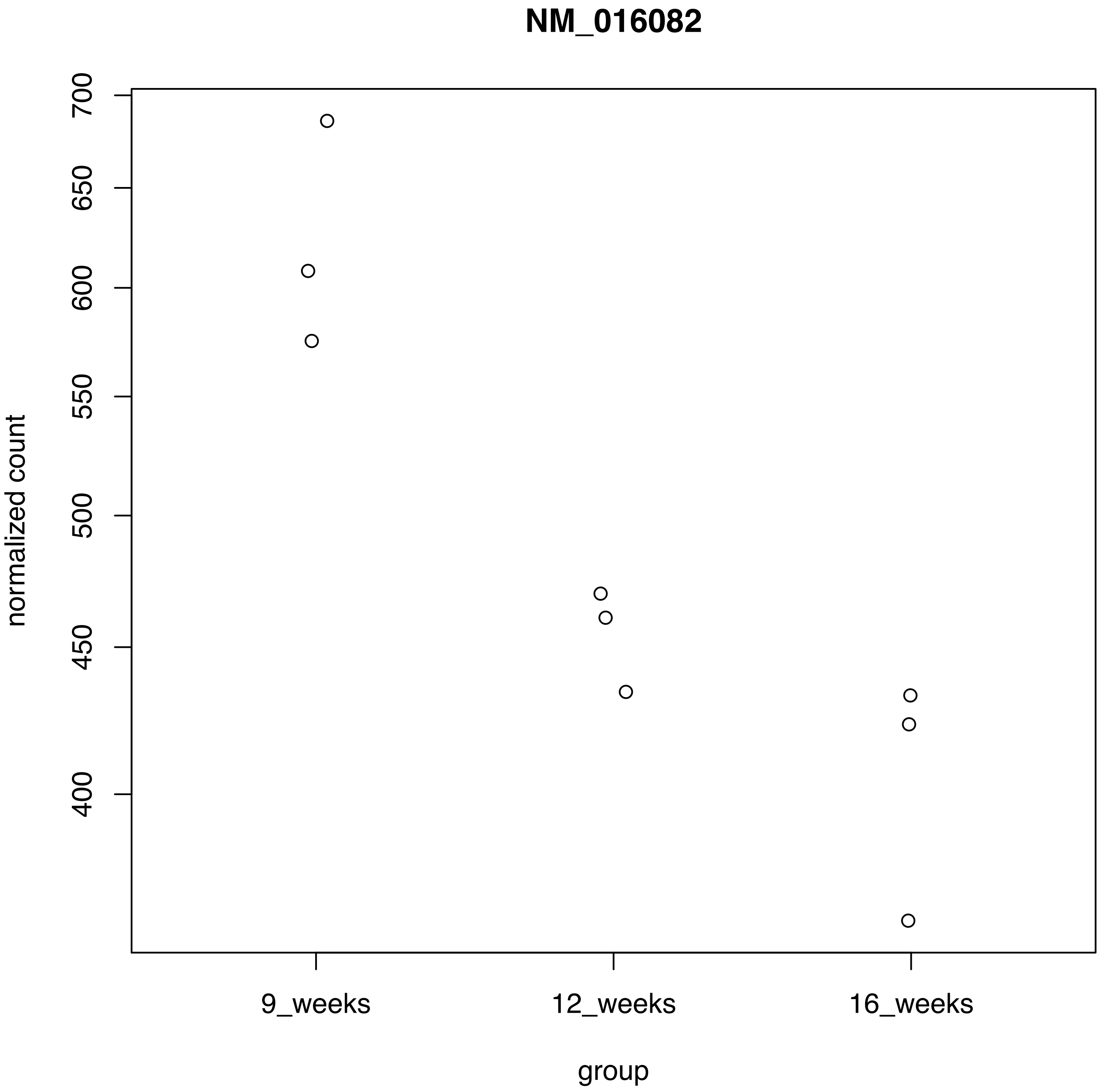
NM_016082
normalized count
700
650
600
550
500
450
400
9_weeks
12_weeks
16_weeks
group

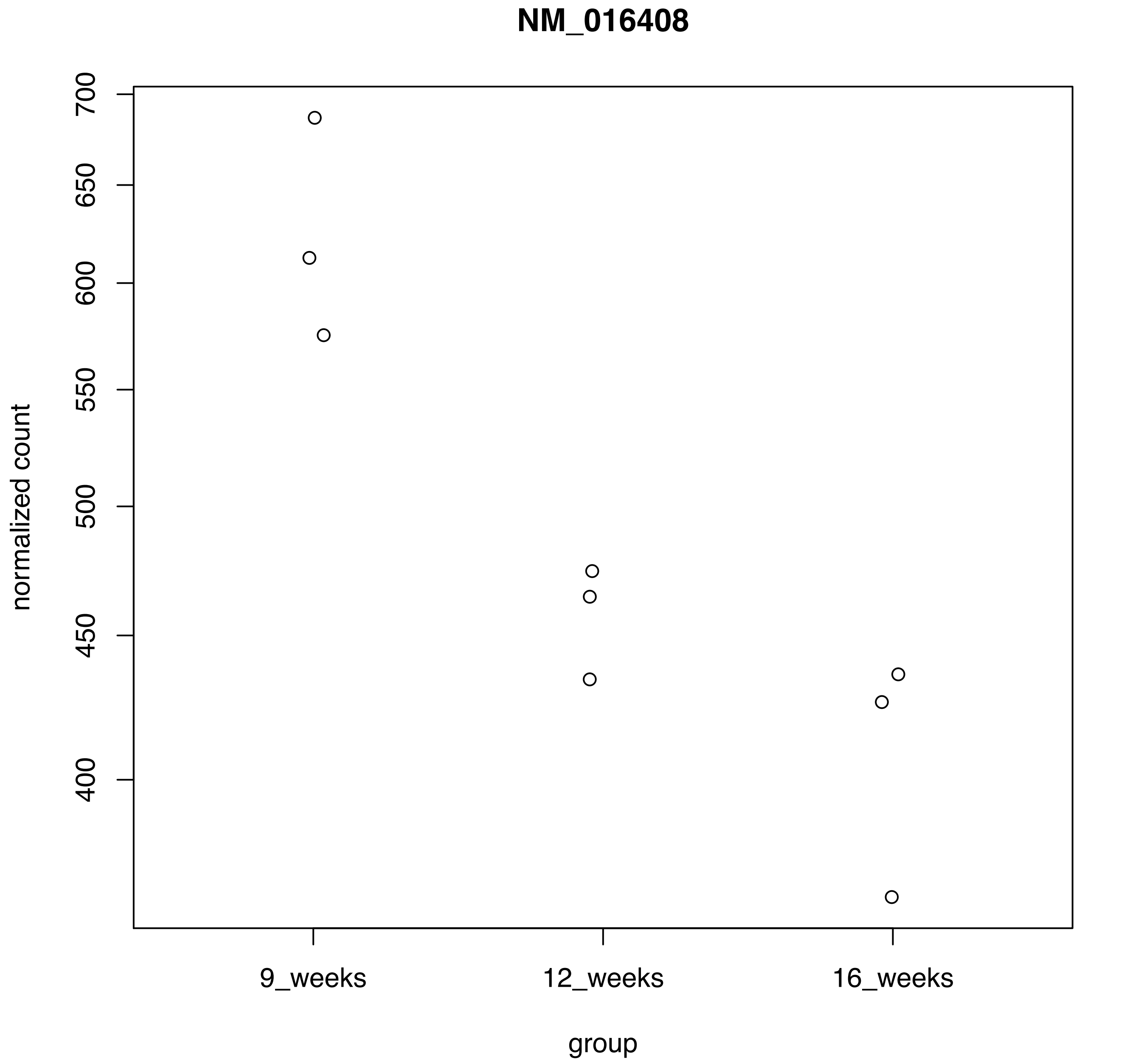
NM_016408
normalized count
700
650
600
550
500
450
400
9_weeks
12_weeks
16_weeks
group

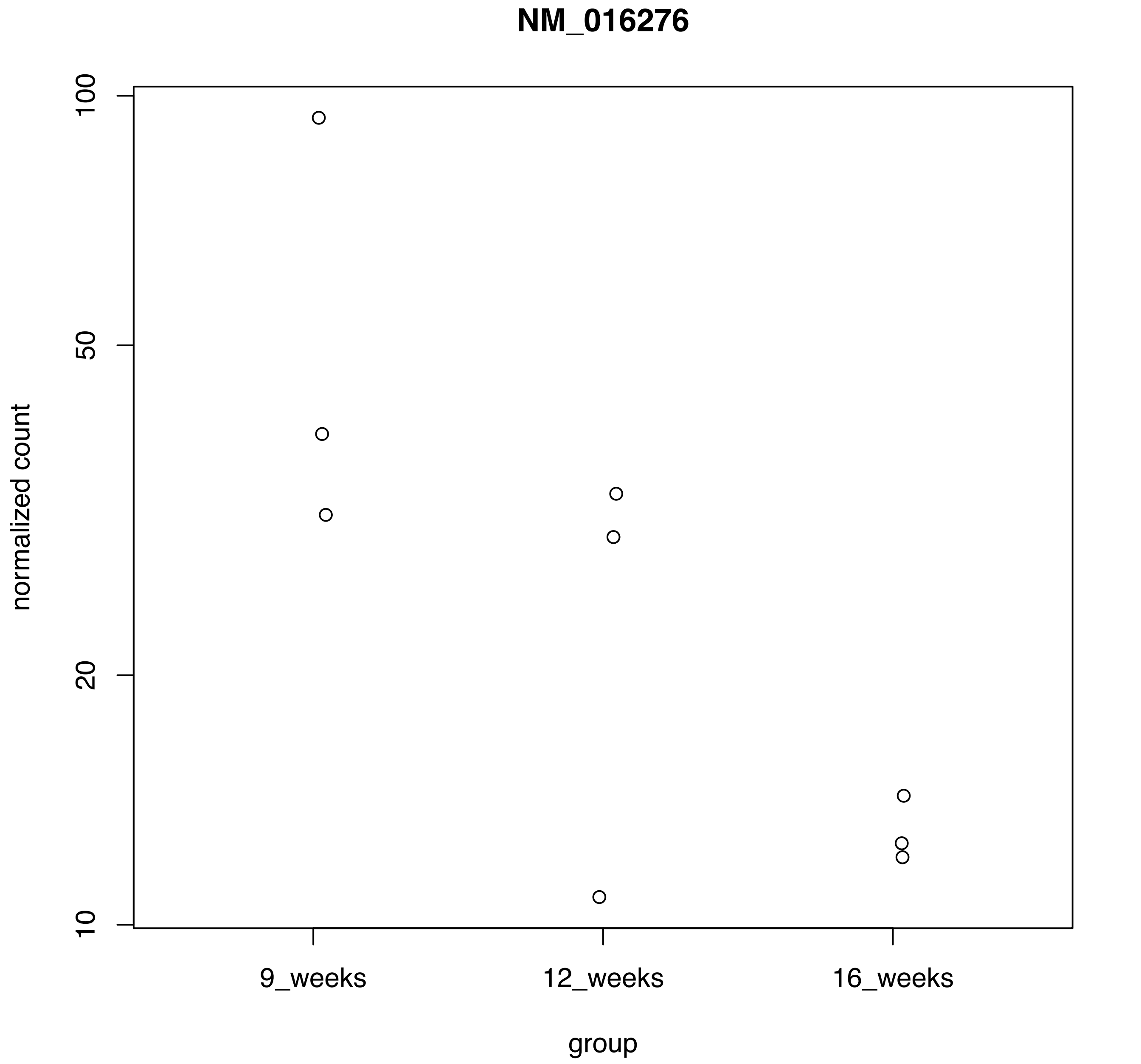

NM_016276
normalized count
100
50
20
10
9_weeks
12_weeks
16_weeks
group

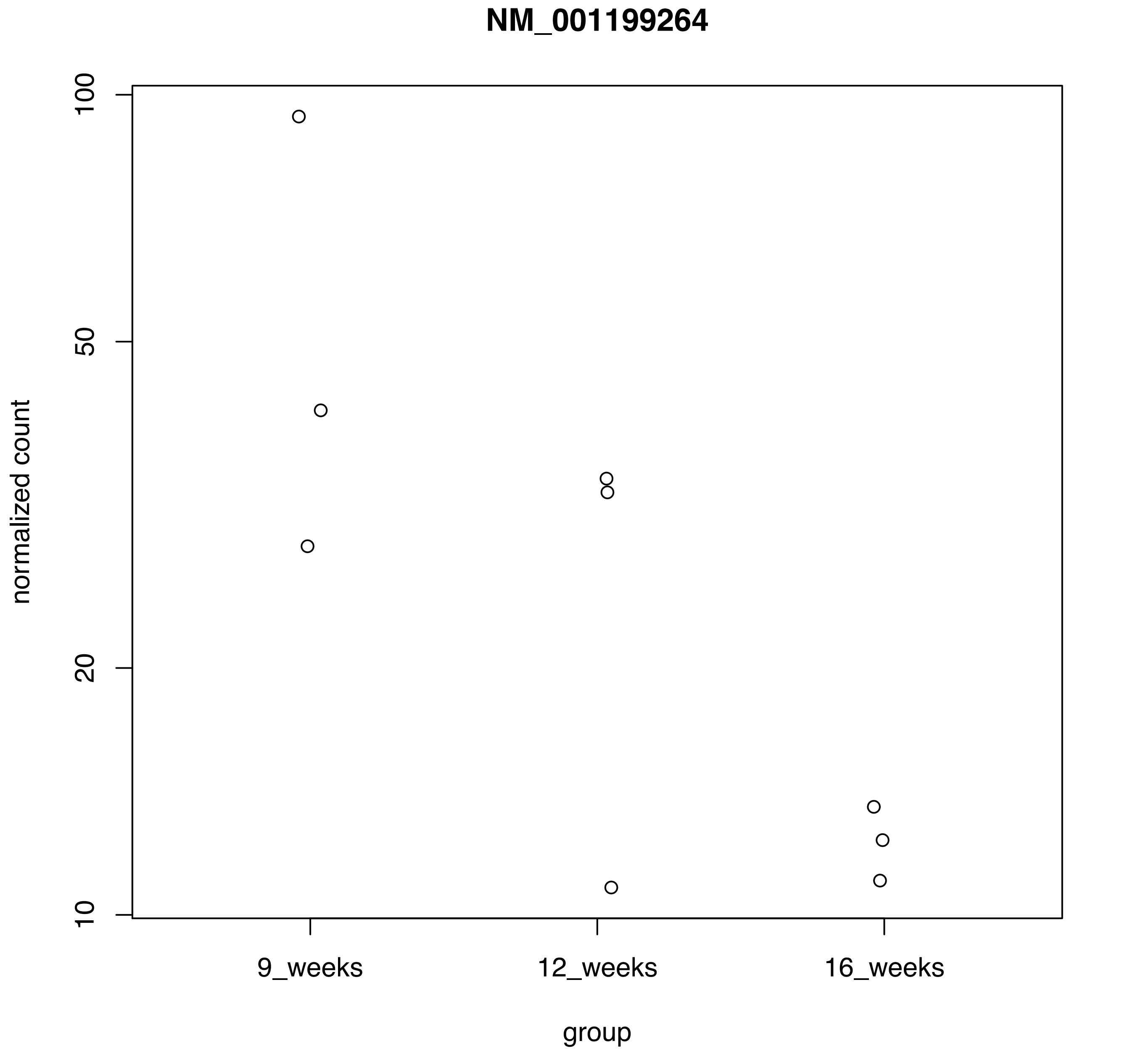
NM_001199264
normalized count
100
50
20
10
9_weeks
12_weeks
16_weeks
group

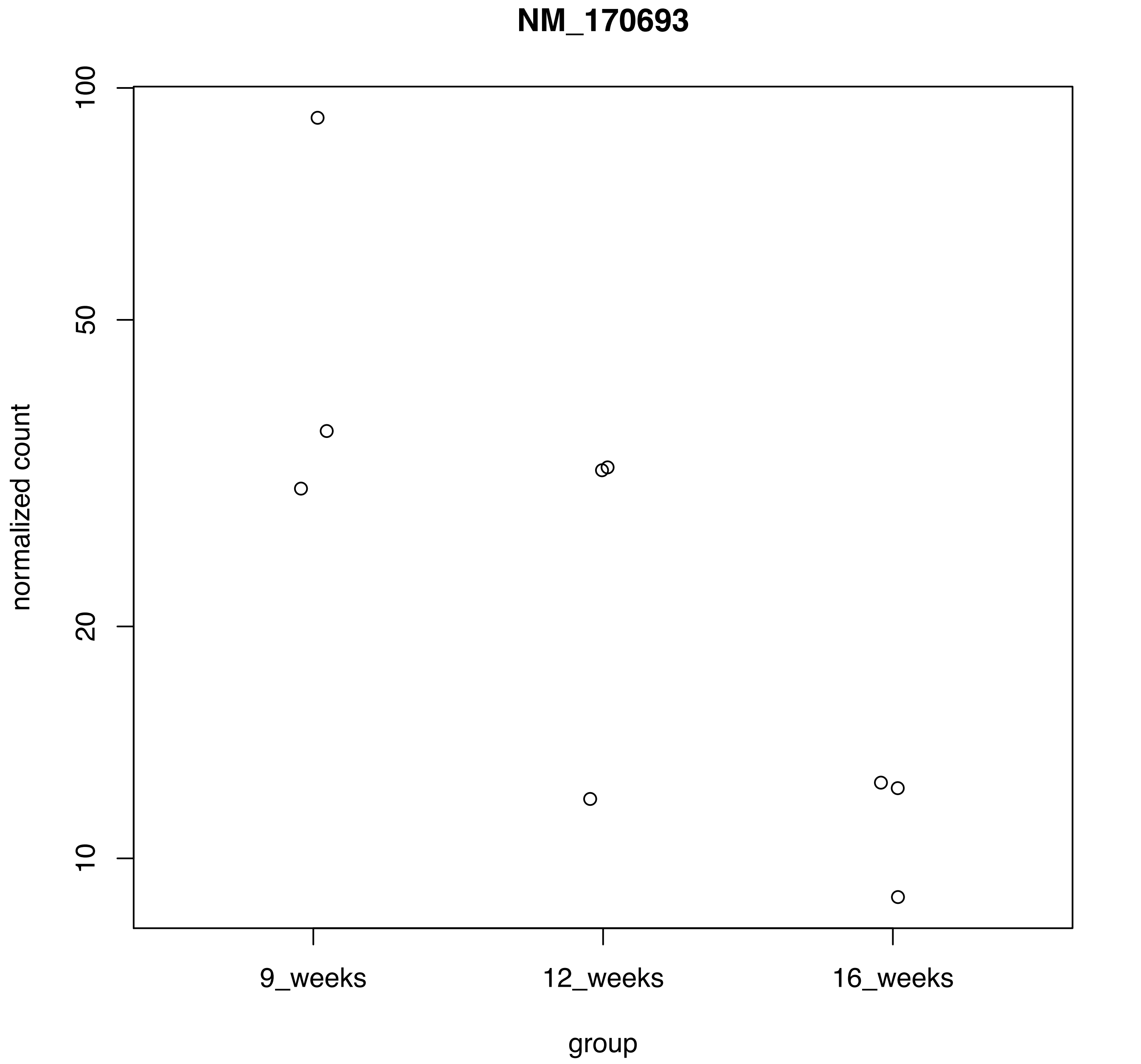

NM_170693
100
50
20
10
normalized count
9_weeks
12_weeks
16_weeks
group

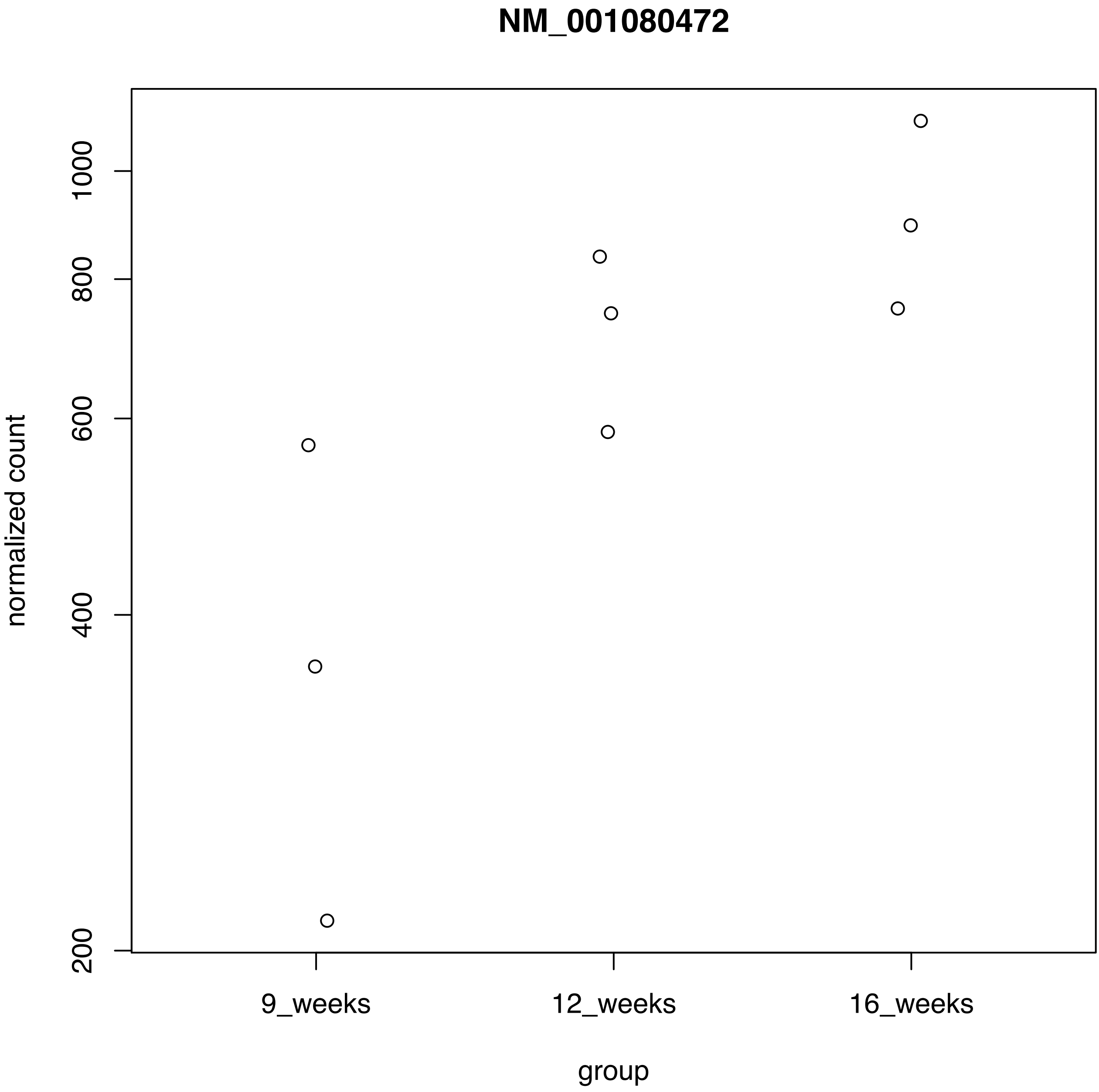
NM_001080472
normalized count
1000
800
600
400
200
9_weeks
12_weeks
16_weeks
group

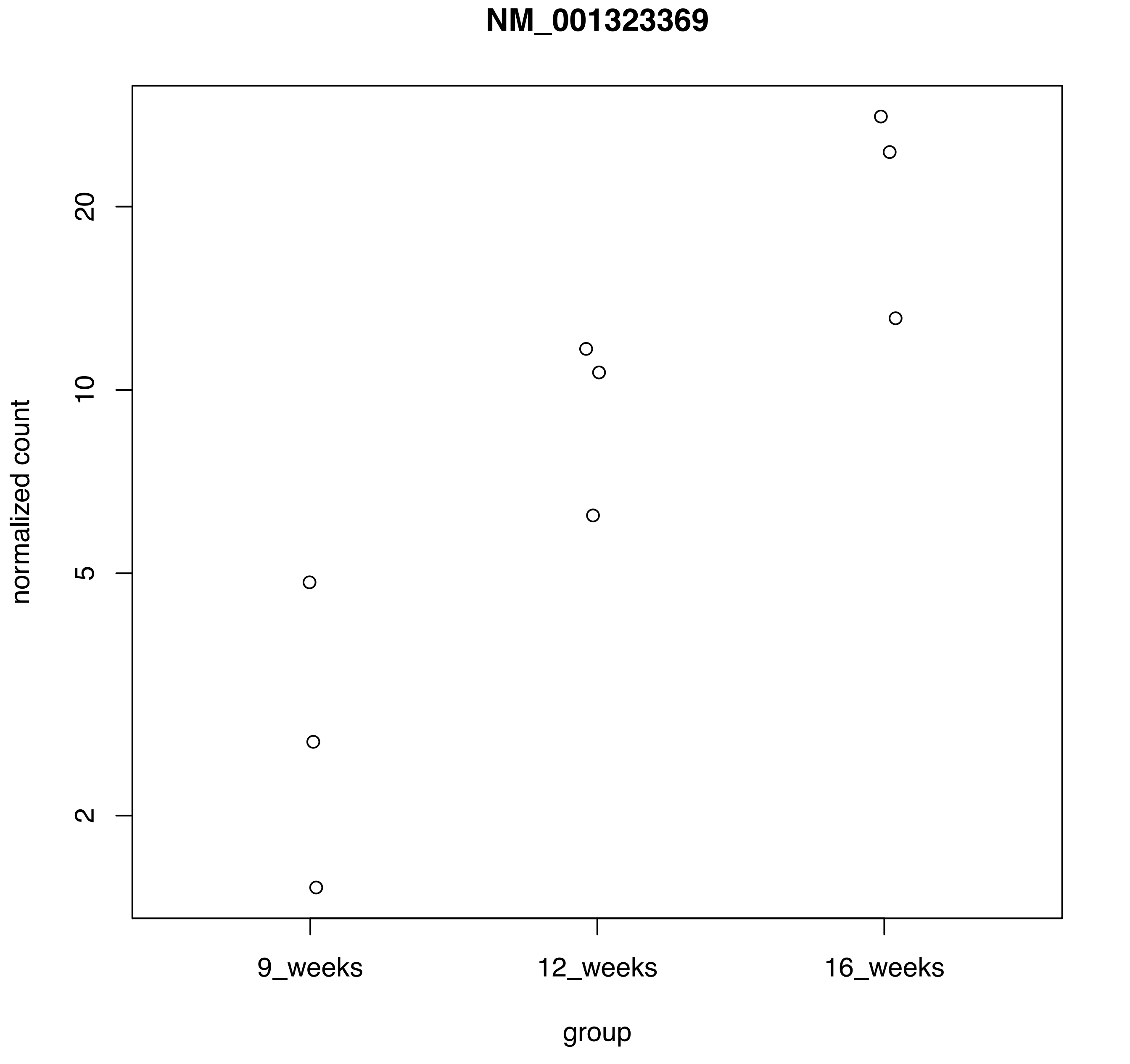

NM_001323369
normalized count
20
10
5
2
9_weeks
12_weeks
16_weeks
group

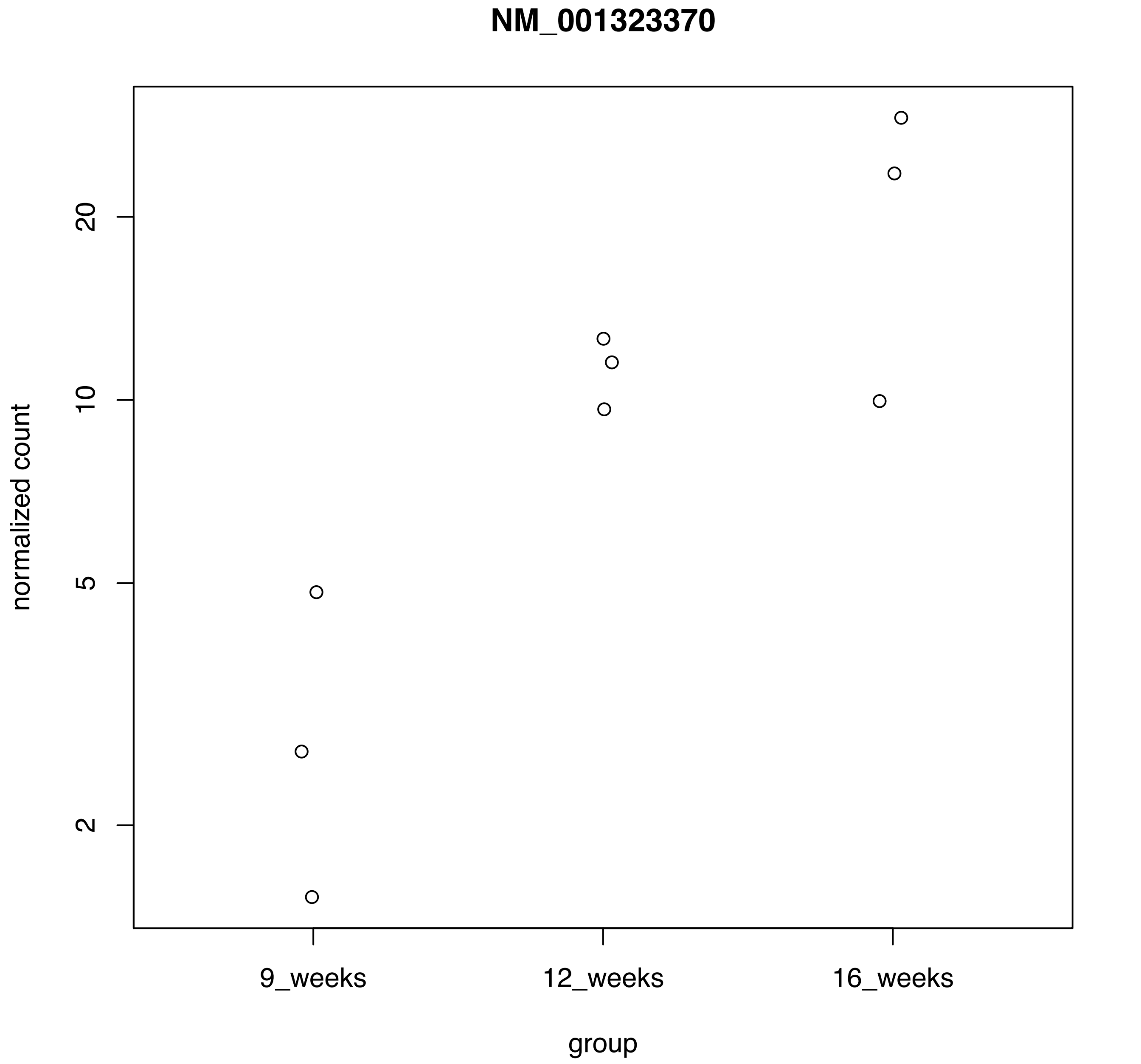

NM_001323370
normalized count
2
5
10
20
9_weeks
12_weeks
16_weeks
group

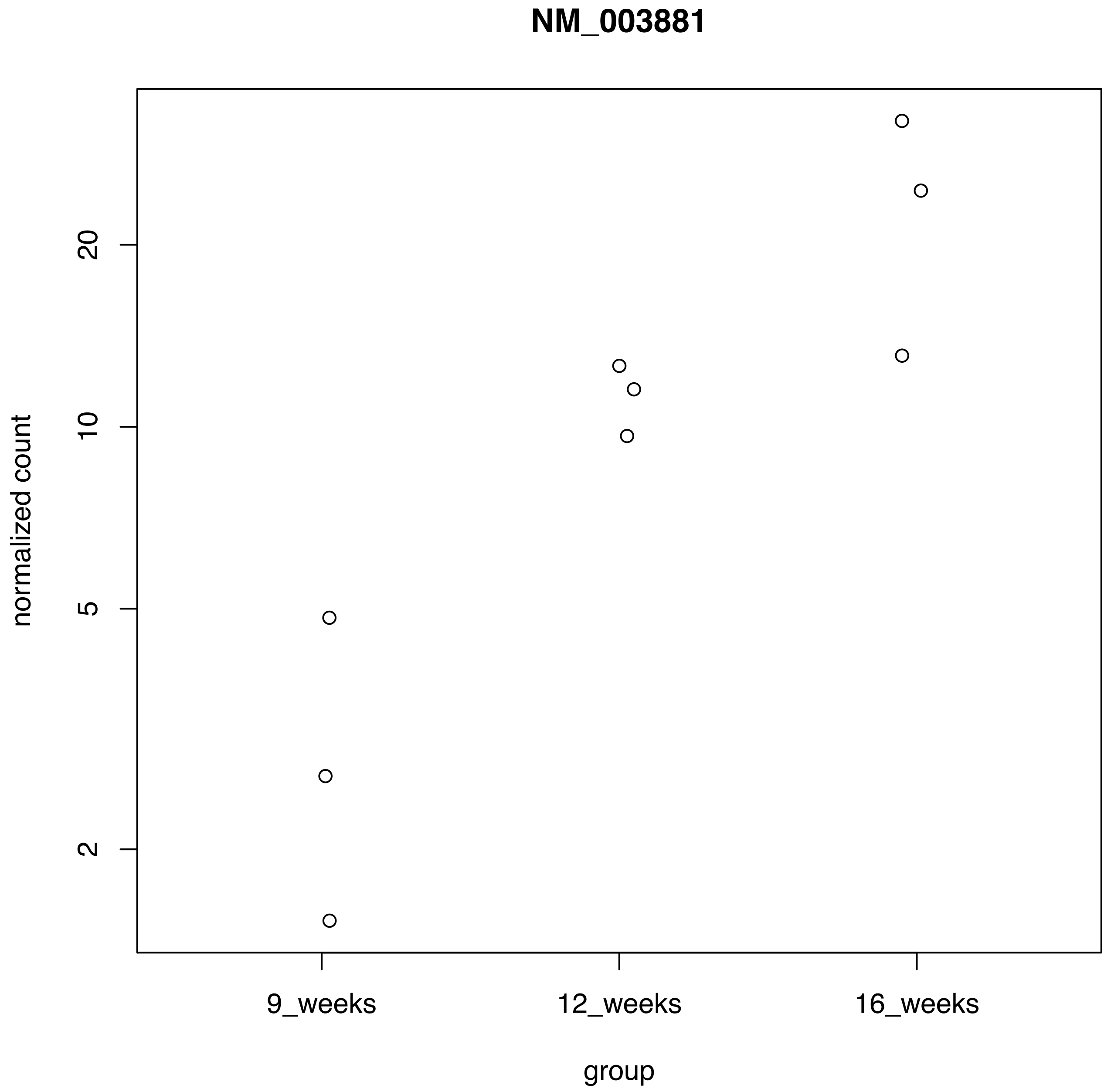

NM_003881
normalized count
20
10
5
2
9_weeks
12_weeks
16_weeks
group

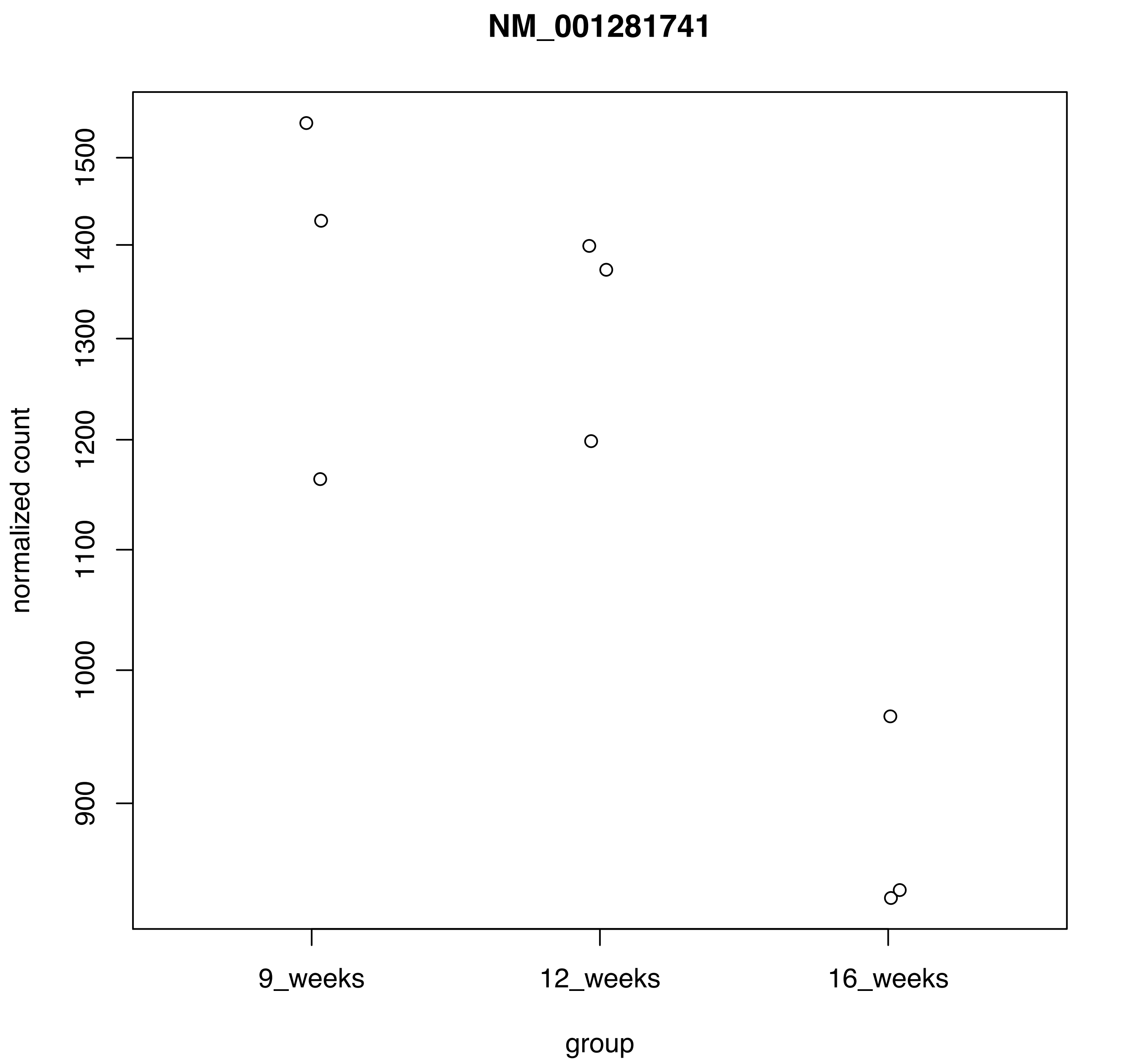
NM_001281741
normalized count
1500
1400
1300
1200
1100
1000
900
9_weeks
12_weeks
16_weeks
group

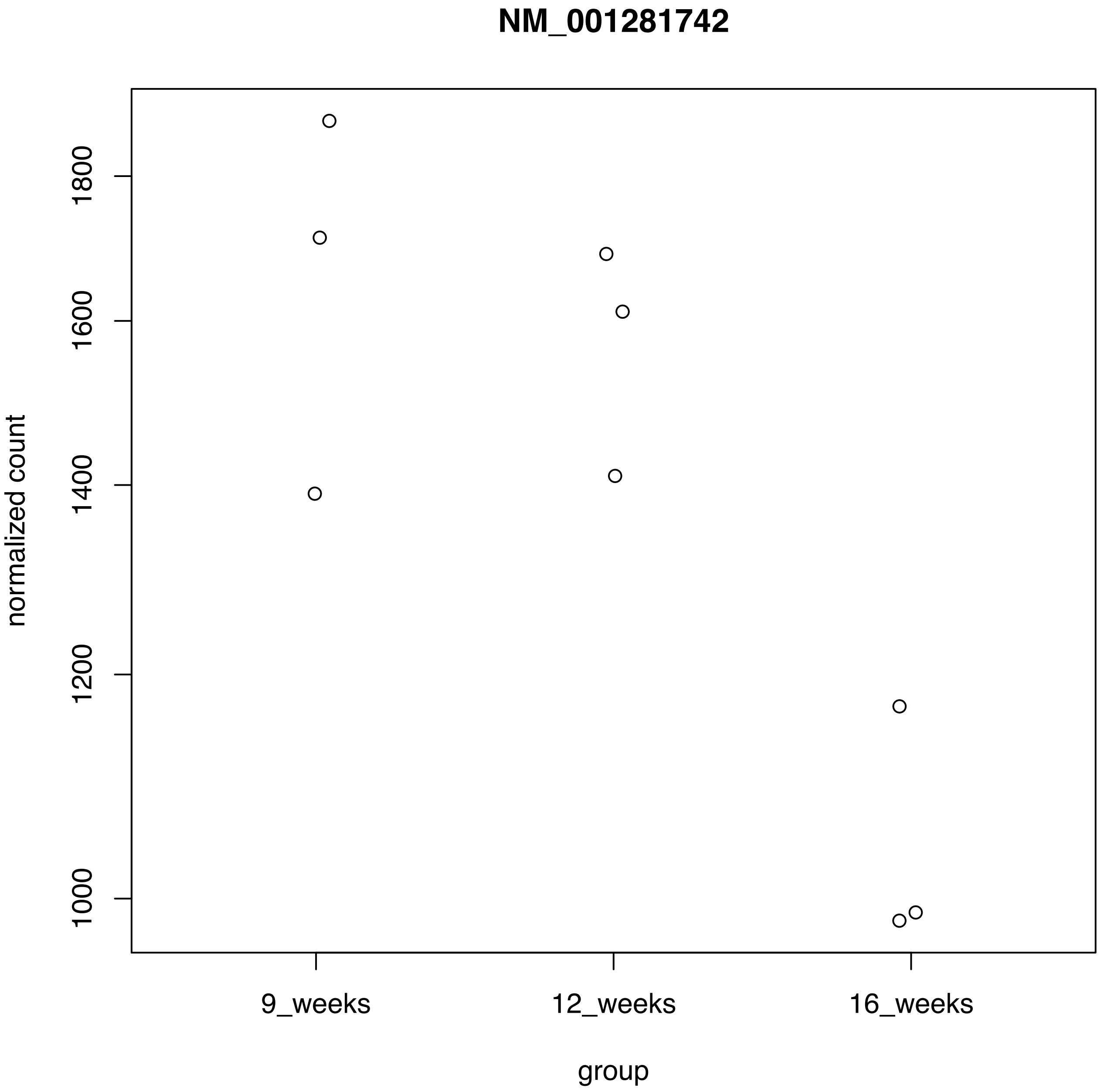
NM_001281742
normalized count
1000
1200
1400
1600
1800
9_weeks
12_weeks
16_weeks
group

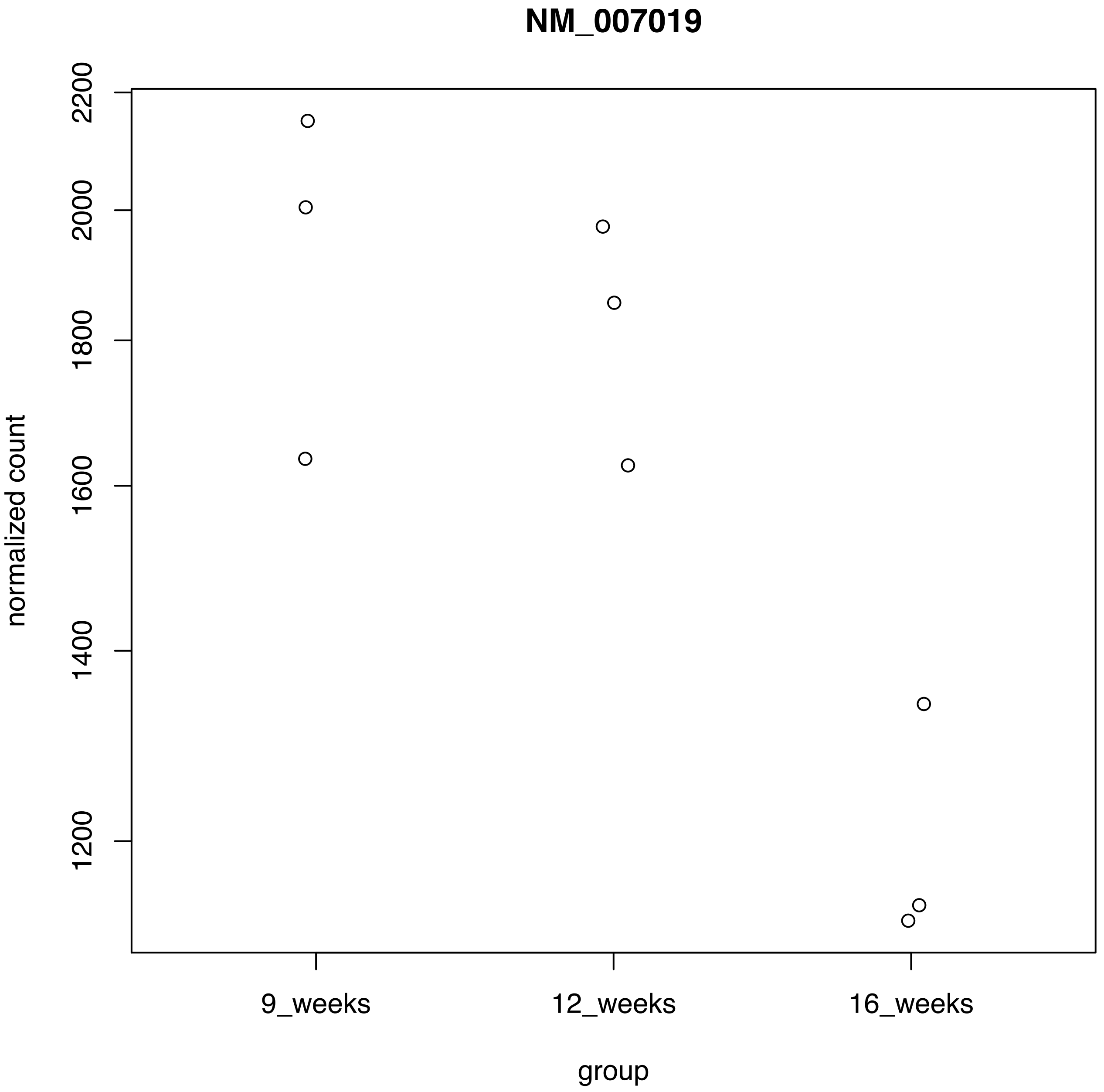
NM_007019
normalized count
2200
2000
1800
1600
1400
1200
9_weeks
12_weeks
16_weeks
group

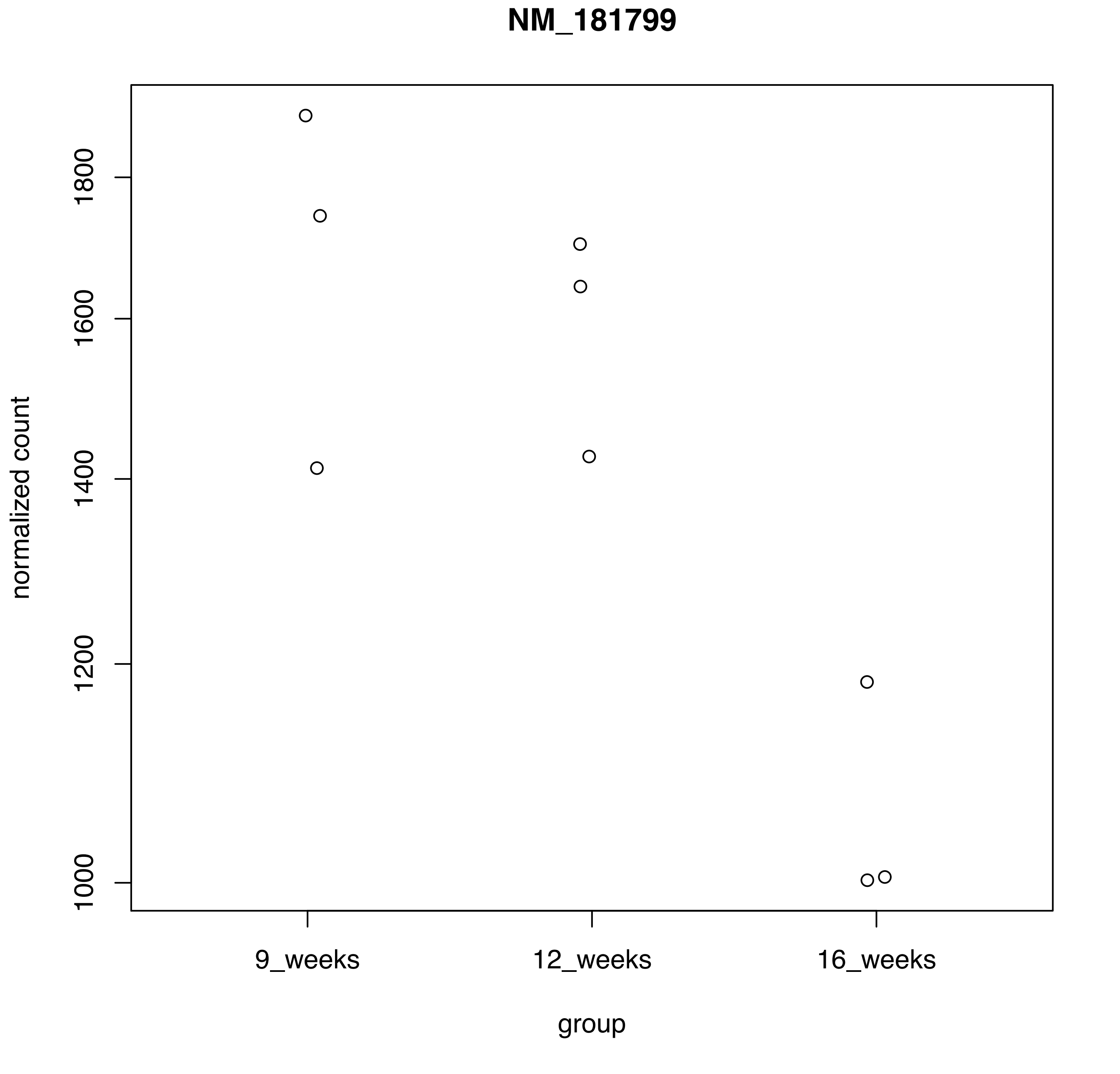
NM_181799
normalized count
1000
1200
1400
1600
1800
9_weeks
12_weeks
16_weeks
group

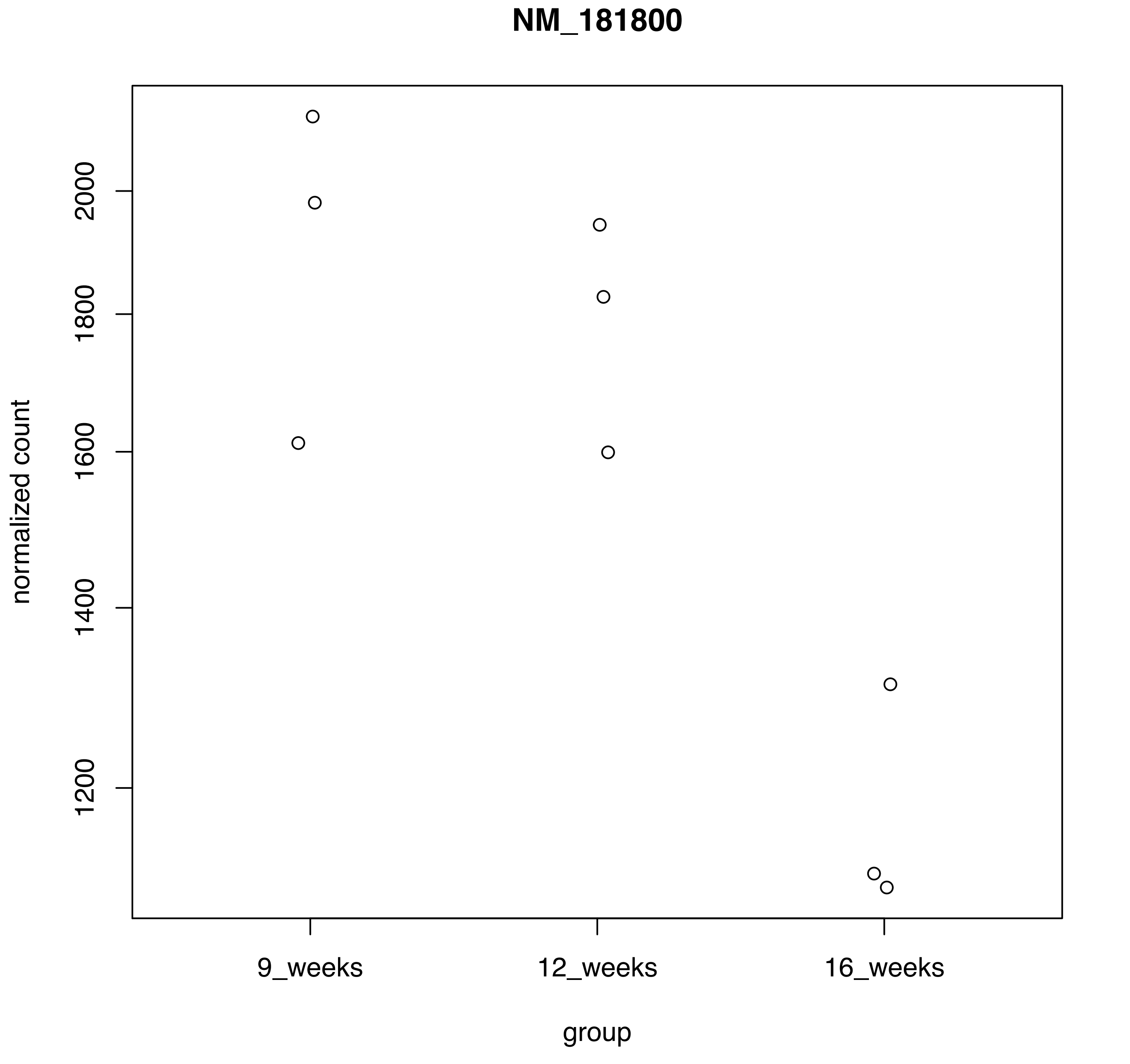
NM_181800
normalized count
2000
1800
1600
1400
1200
9_weeks
12_weeks
16_weeks
group

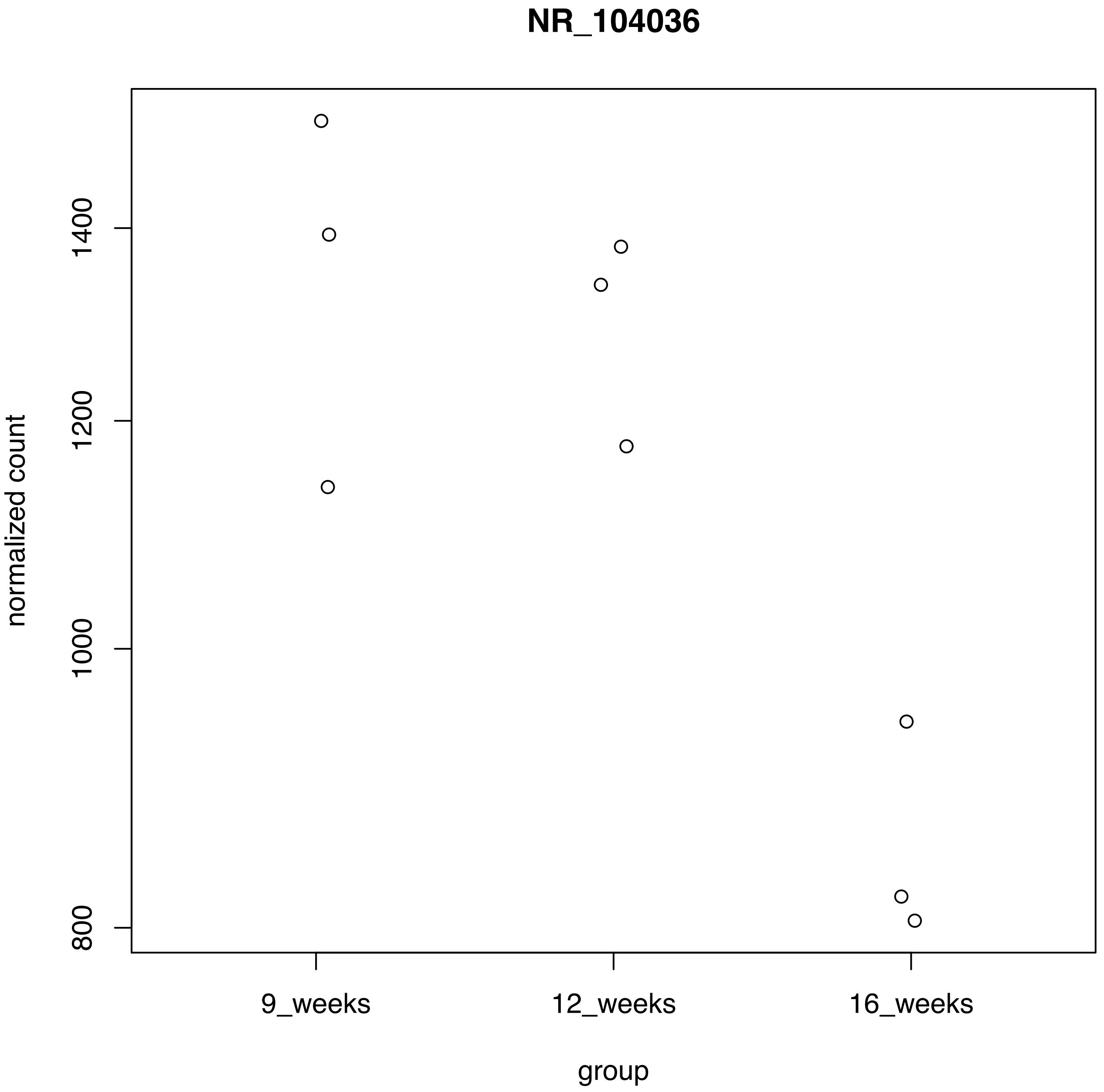
NR_104036
normalized count
1400
1200
1000
800
9_weeks
12_weeks
16_weeks
group

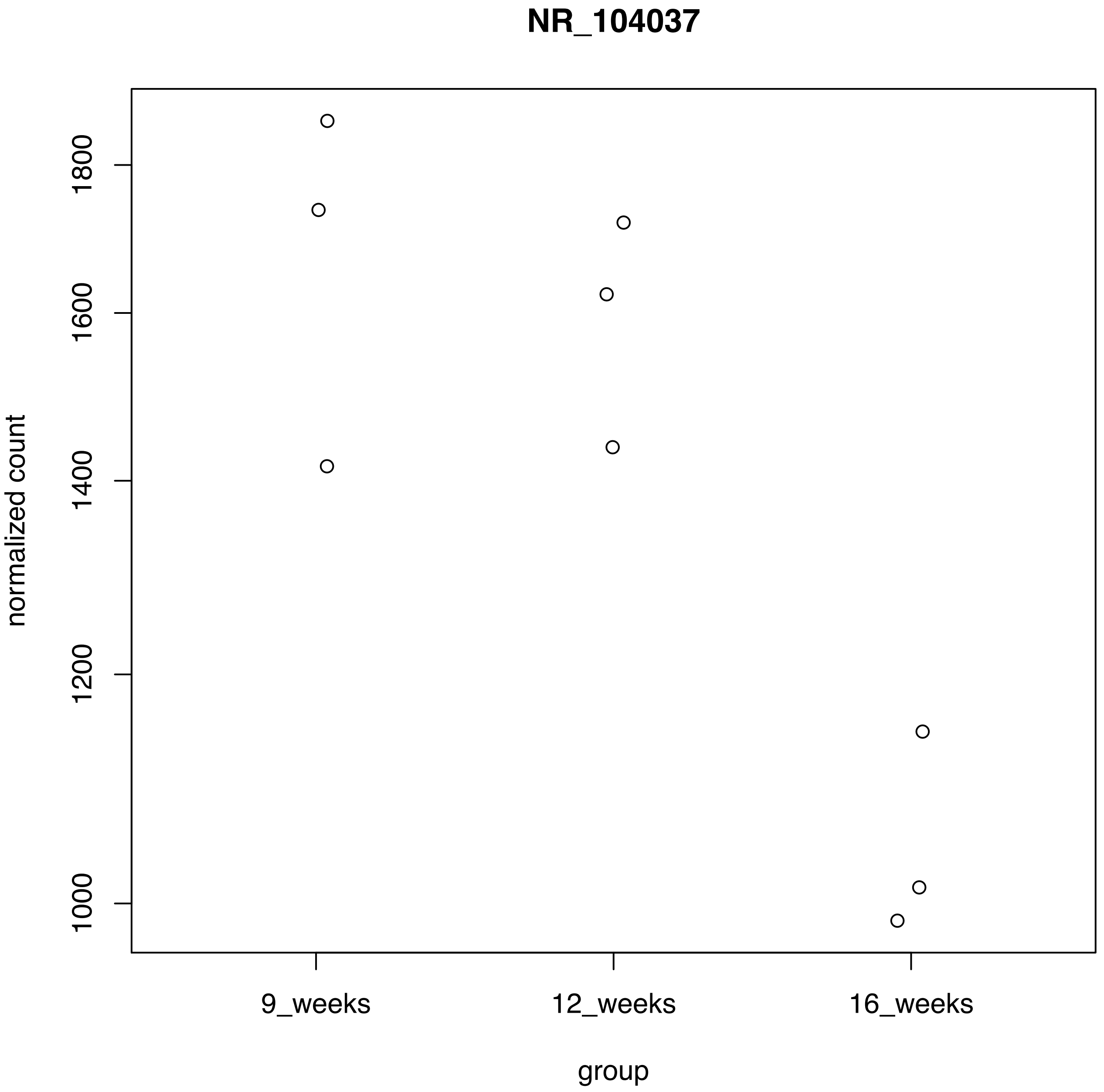
NR_104037
normalized count
1000
1200
1400
1600
1800
9_weeks
12_weeks
16_weeks
group

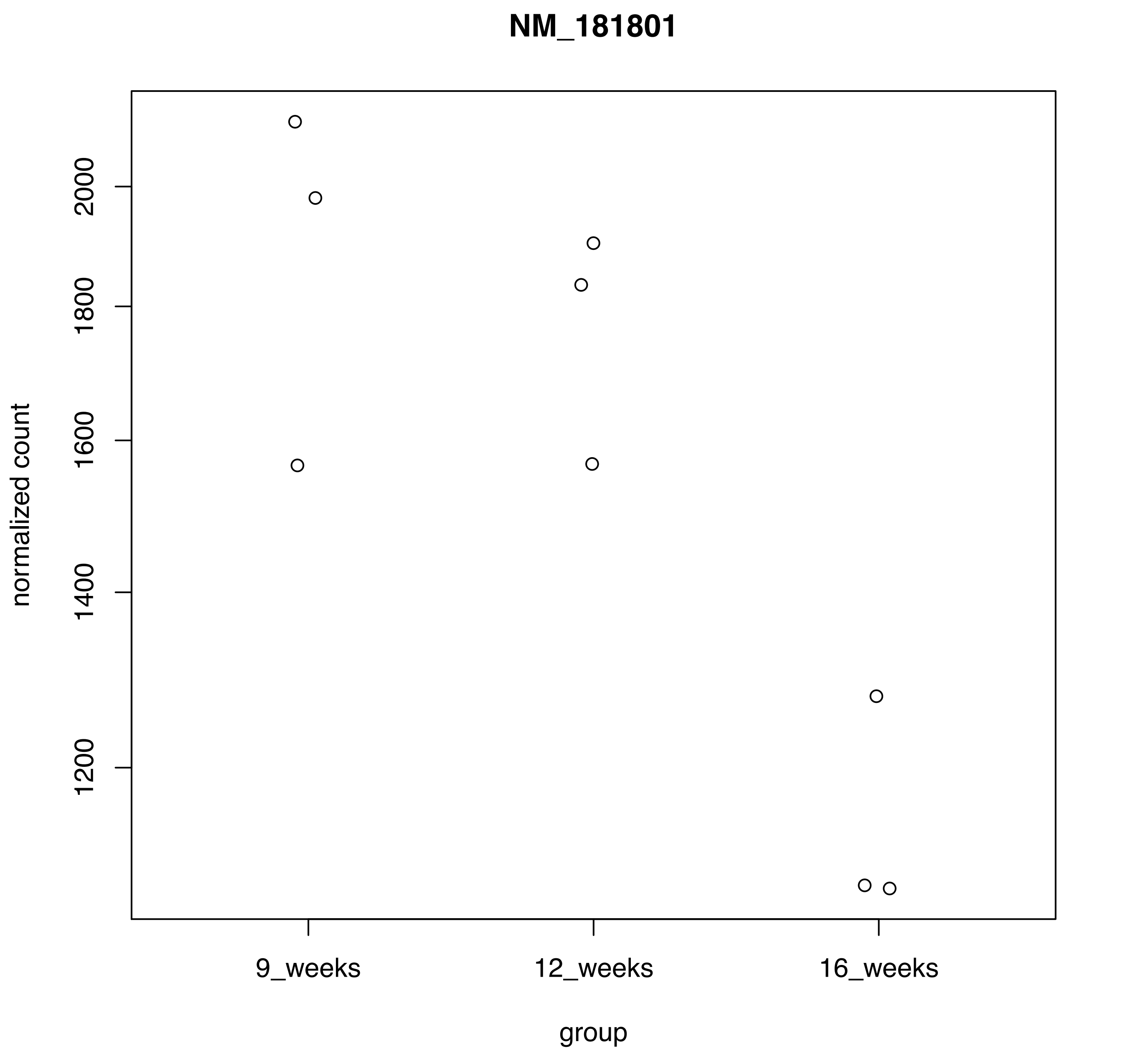
NM_181801
normalized count
1200
1400
1600
1800
2000
9_weeks
12_weeks
16_weeks
group

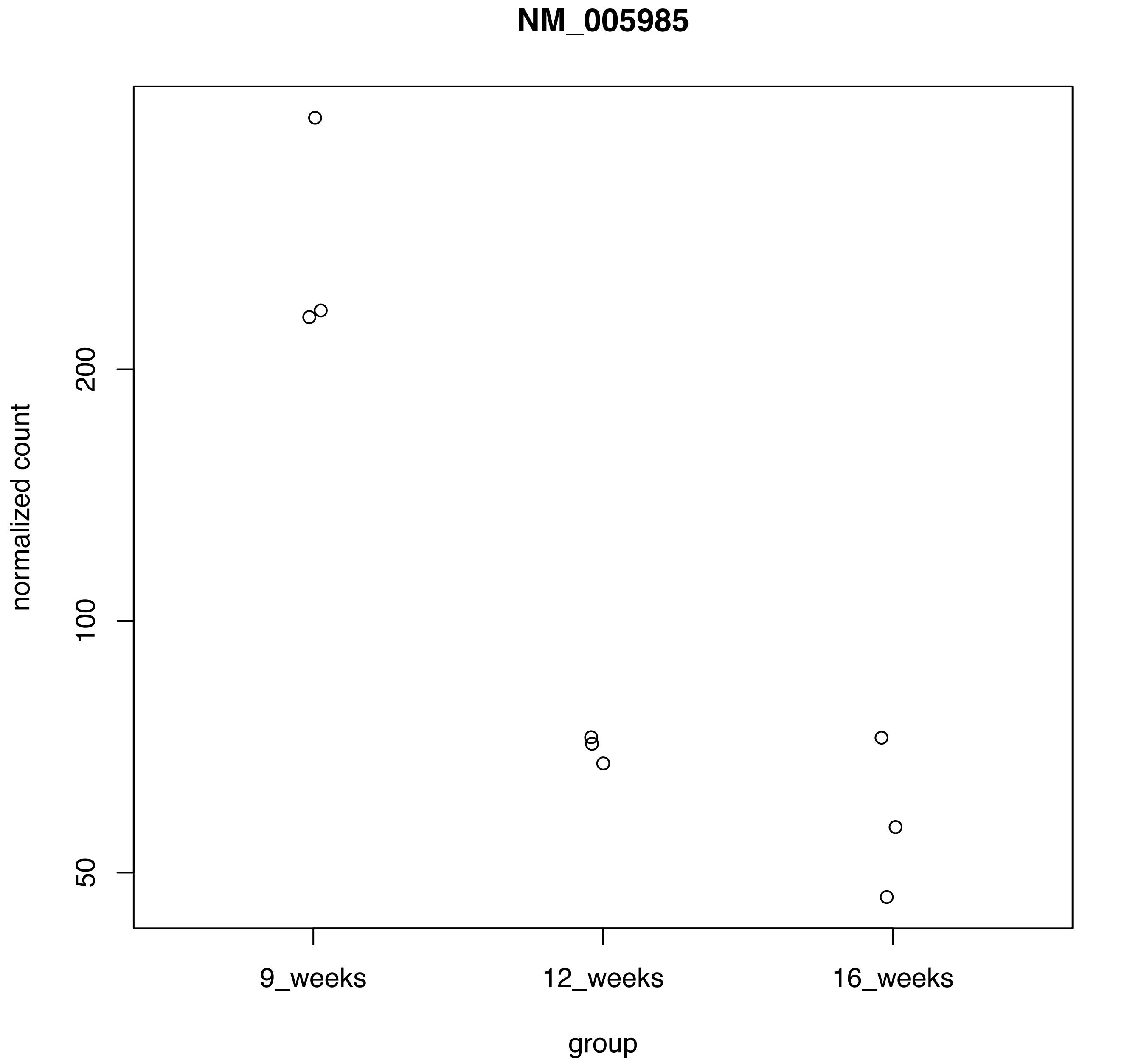

NM_005985
normalized count
200
100
50
9_weeks
12_weeks
16_weeks
group

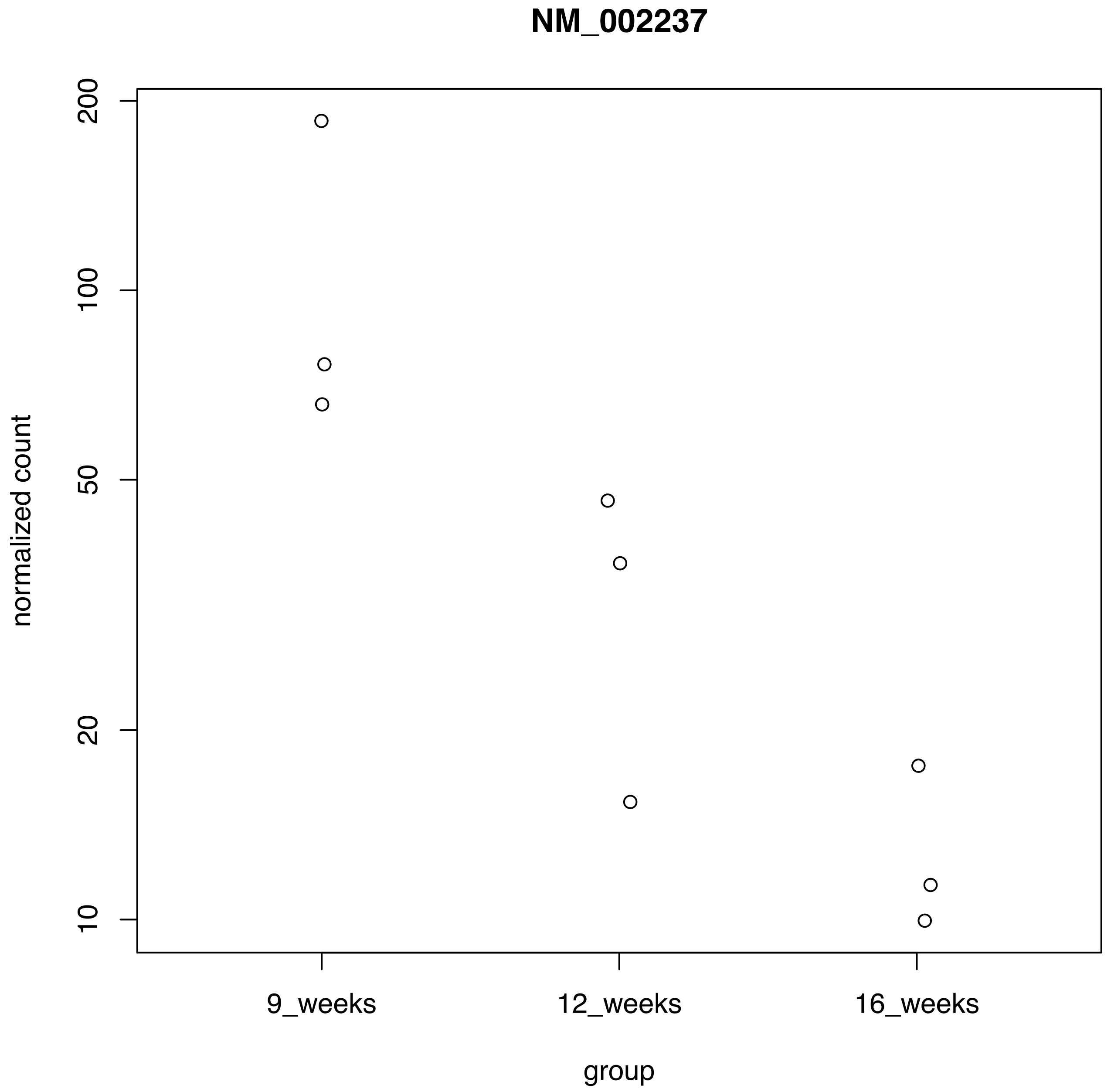
NM_002237
normalized count
200
100
50
20
10
9_weeks
12_weeks
16_weeks
group

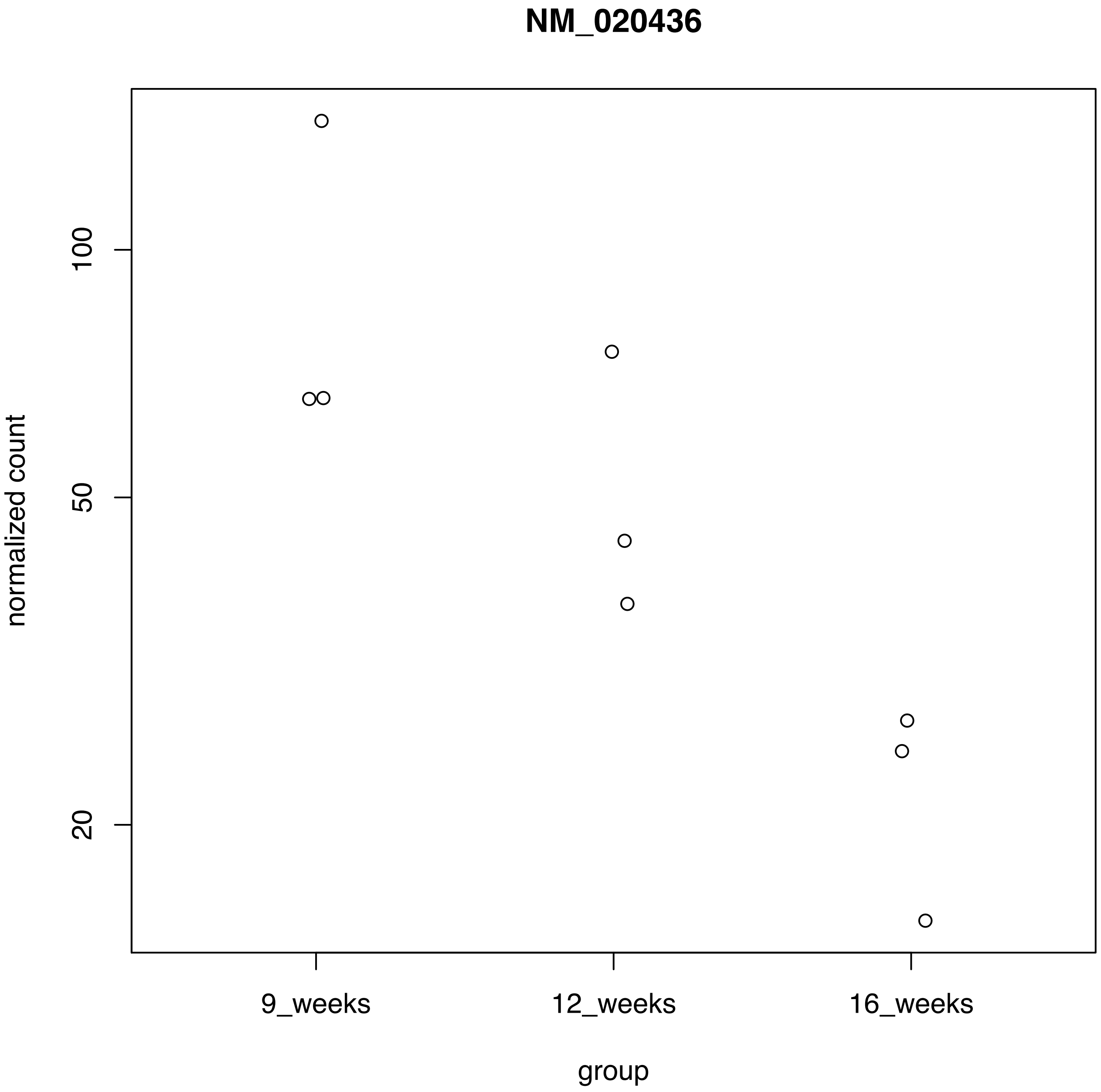

NM_020436
normalized count
100
50
20
9_weeks
12_weeks
16_weeks
group

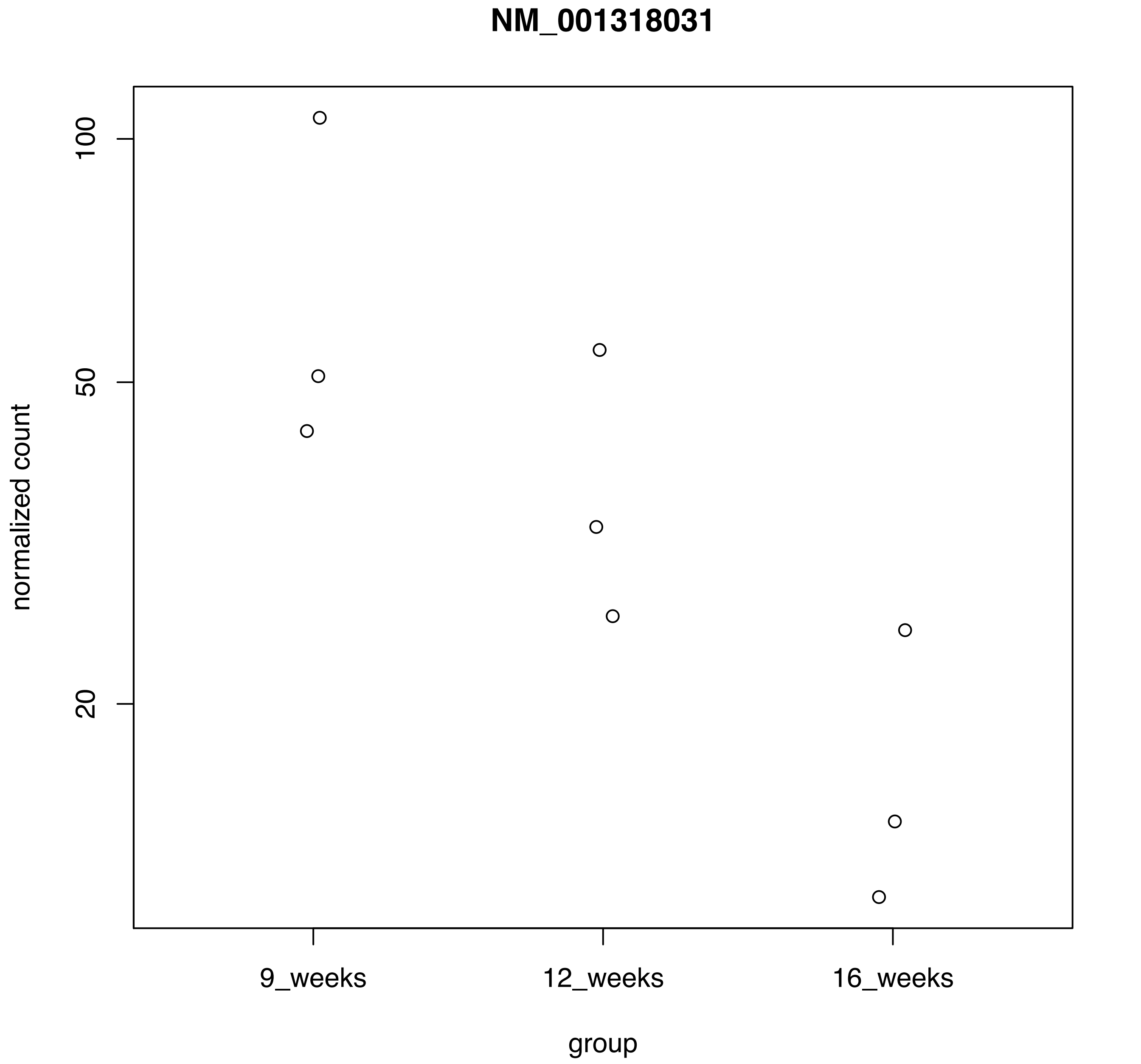
NM_001318031
normalized count
100
50
20
9_weeks
12_weeks
16_weeks
group

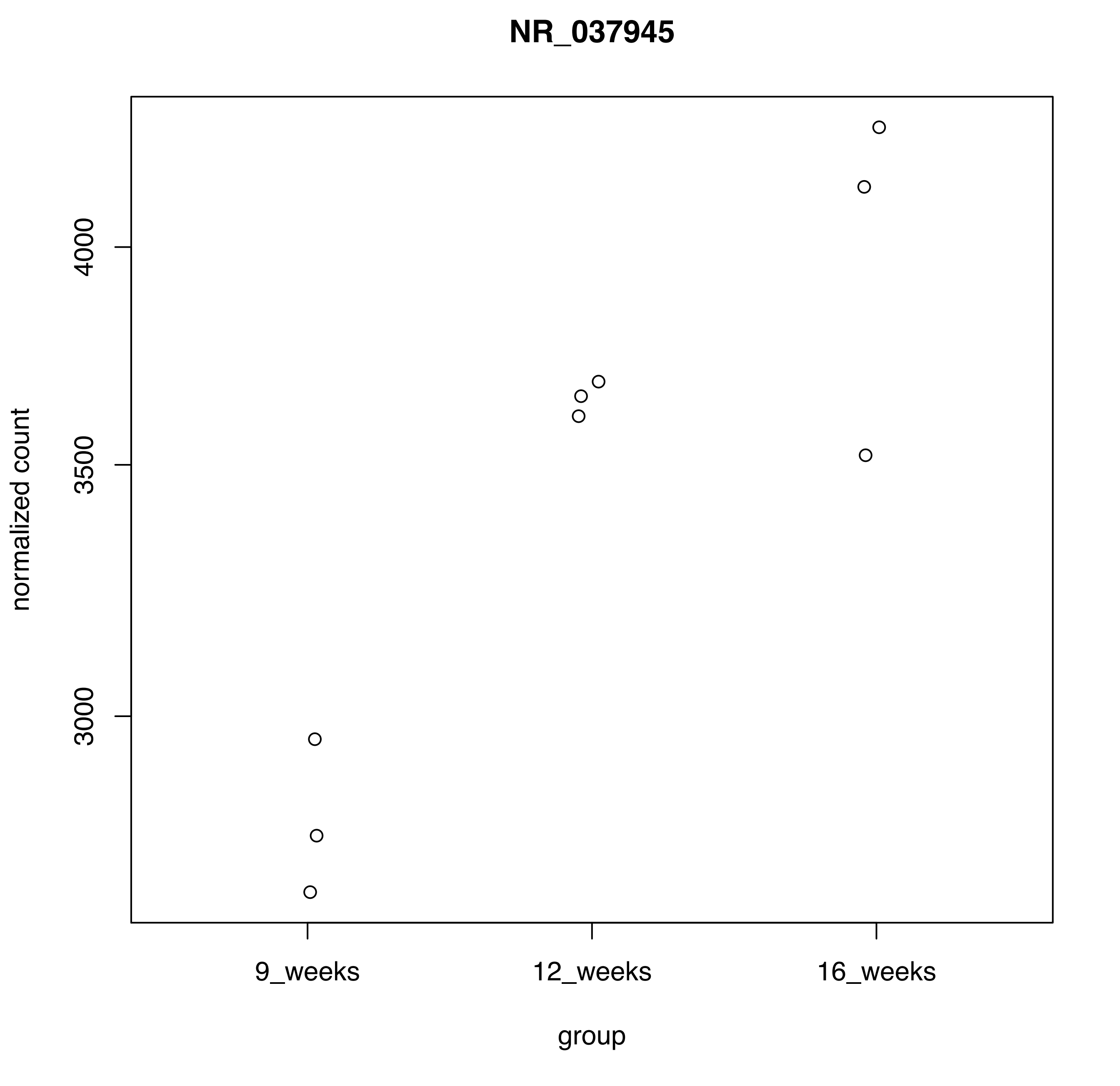
NR_037945
normalized count
4000
3500
3000
9_weeks
12_weeks
16_weeks
group

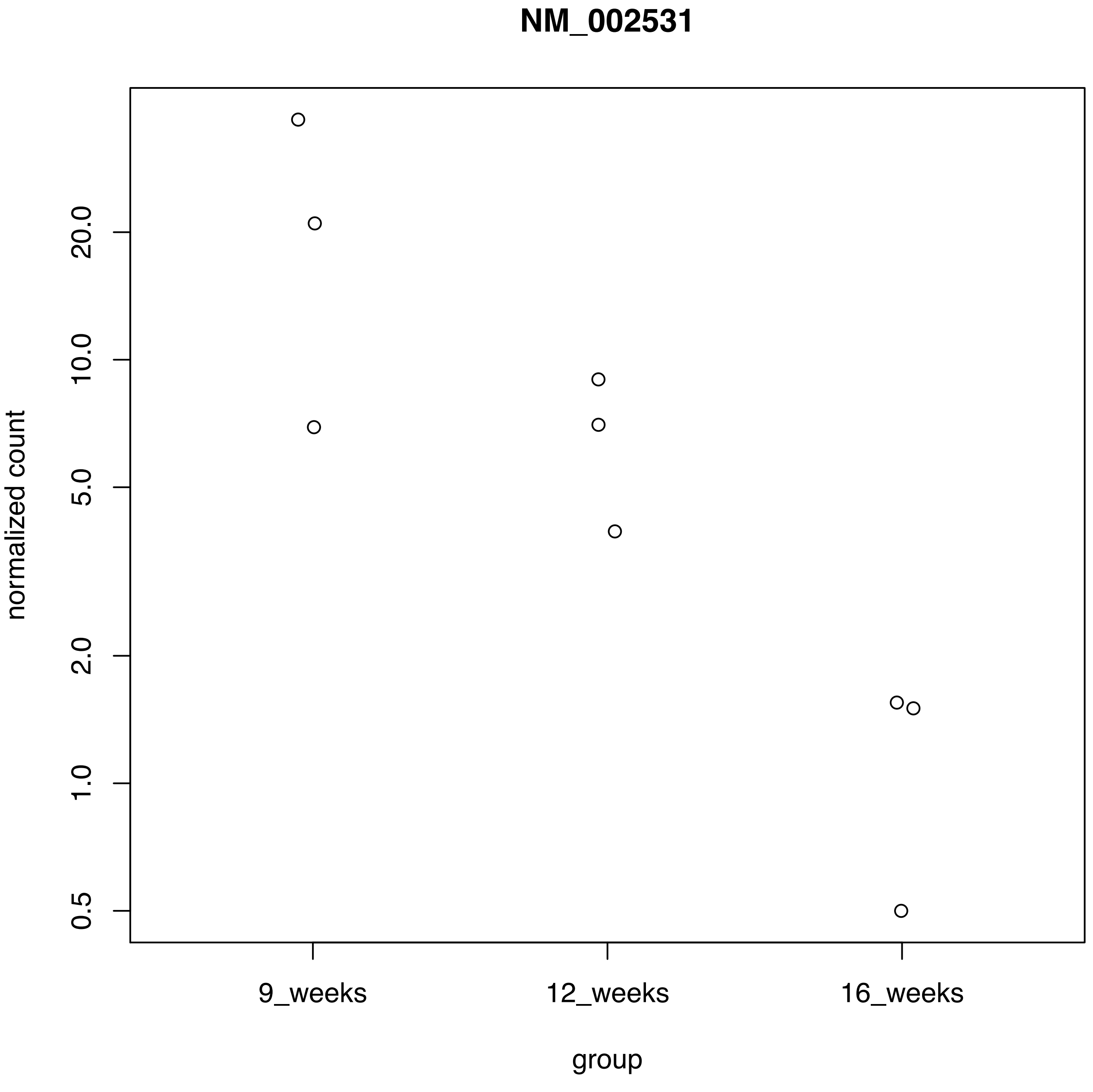
NM_002531
normalized count
20.0
10.0
5.0
2.0
1.0
0.5
9_weeks
12_weeks
16_weeks
group

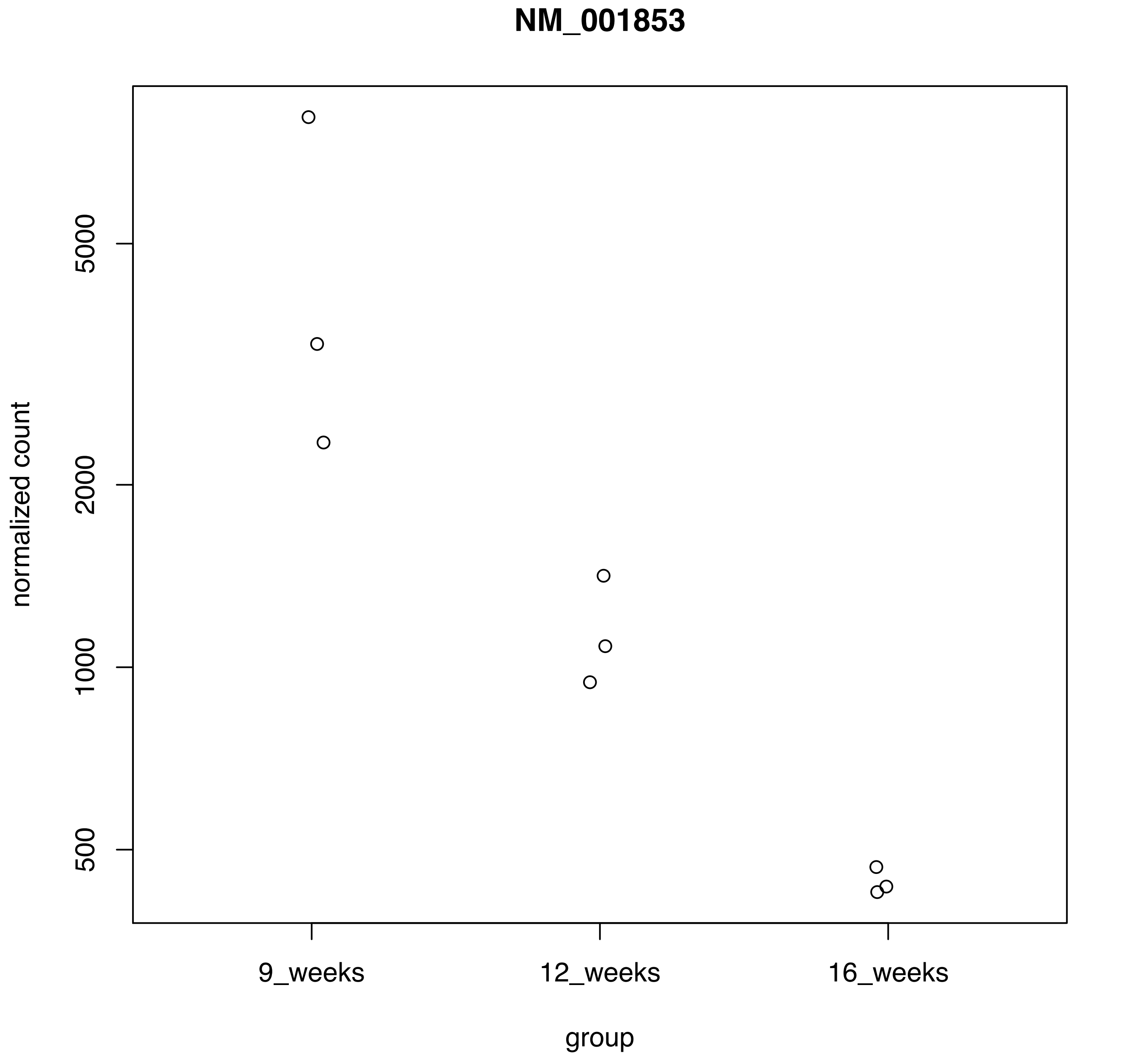

NM_001853
normalized count
5000
2000
1000
500
9_weeks
12_weeks
16_weeks
group

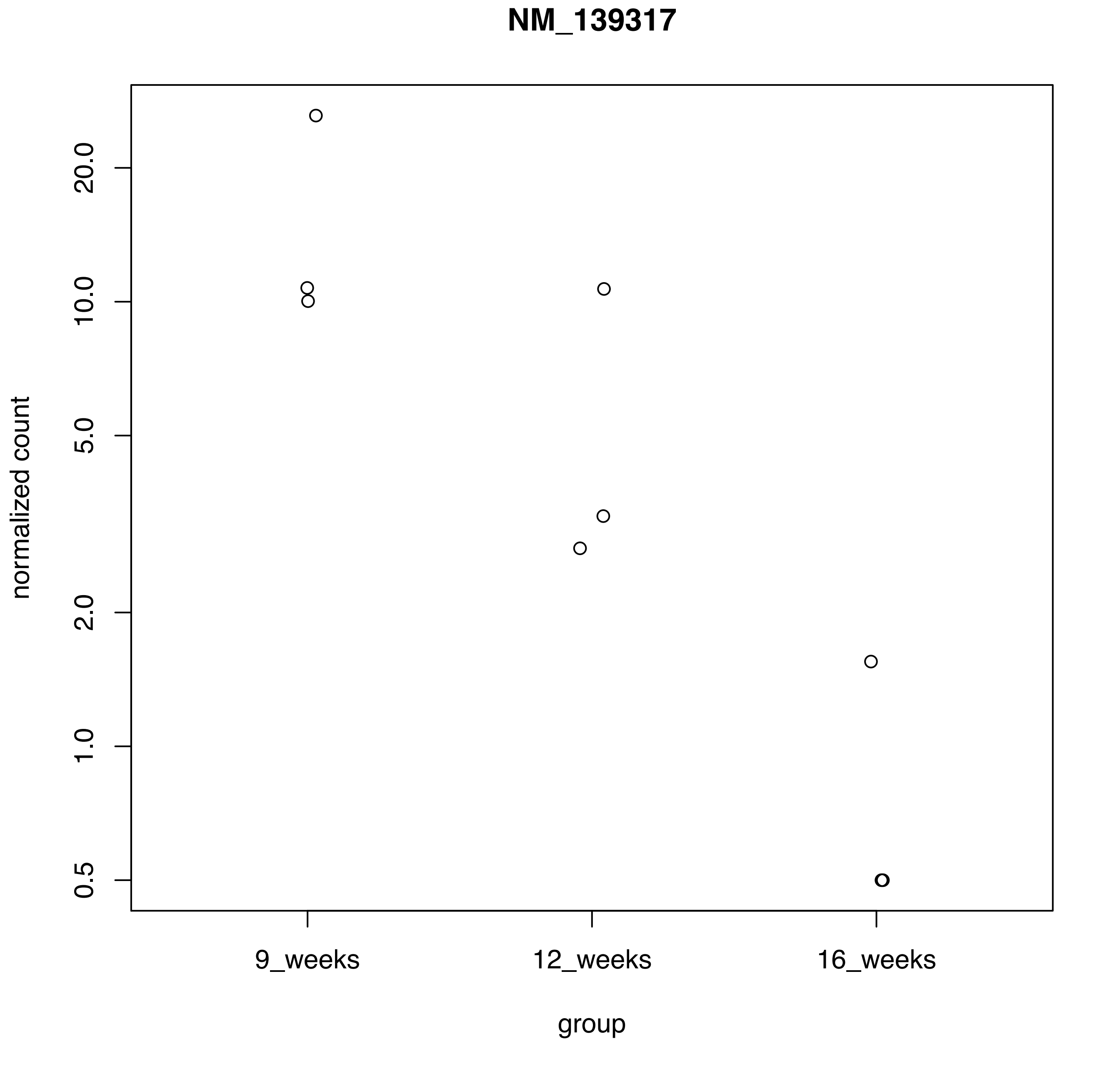
NM_139317
normalized count
20.0
10.0
5.0
2.0
1.0
0.5
9_weeks
12_weeks
16_weeks
group

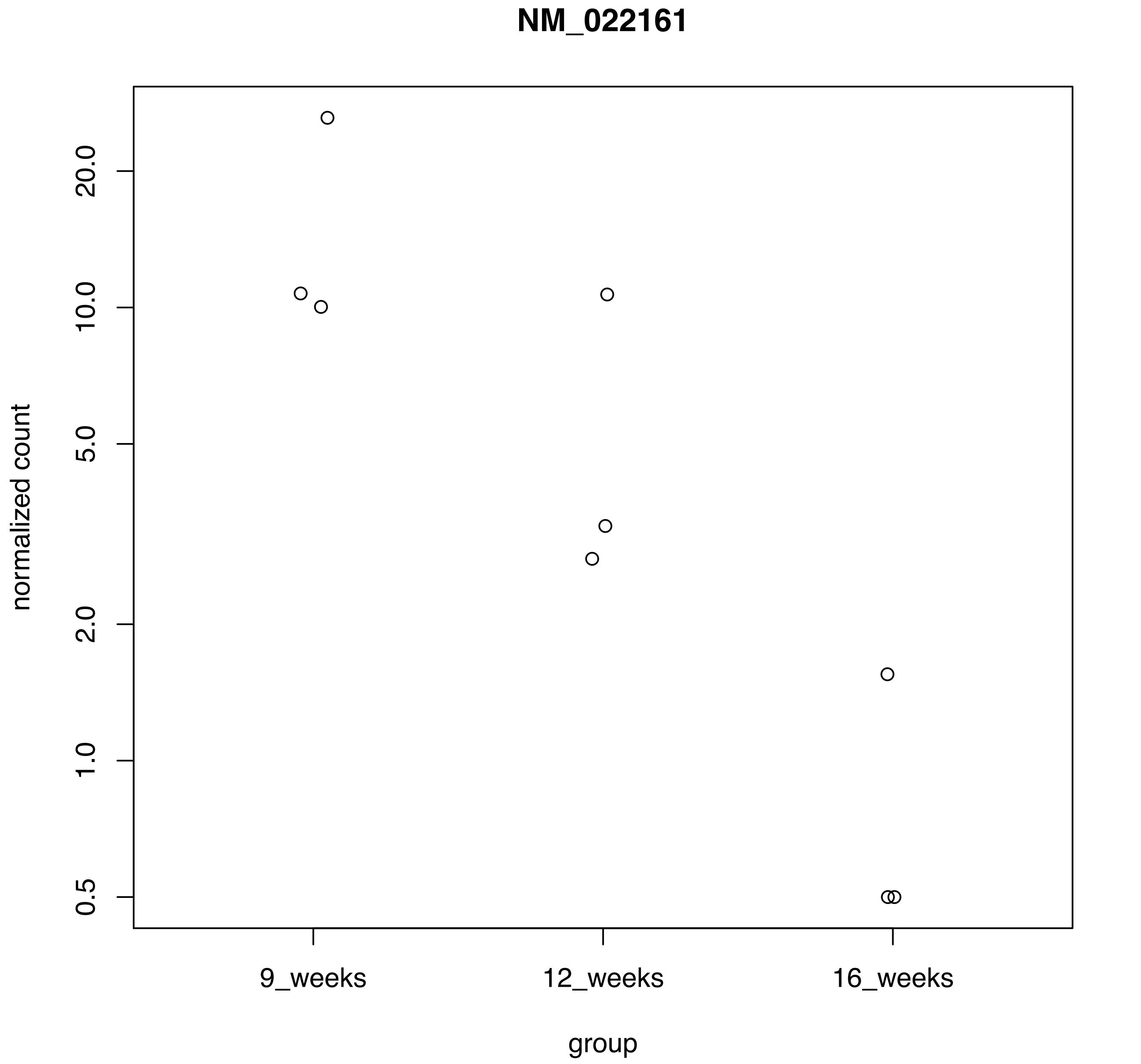
NM_022161
normalized count
20.0
10.0
5.0
2.0
1.0
0.5
9_weeks
12_weeks
16_weeks
group

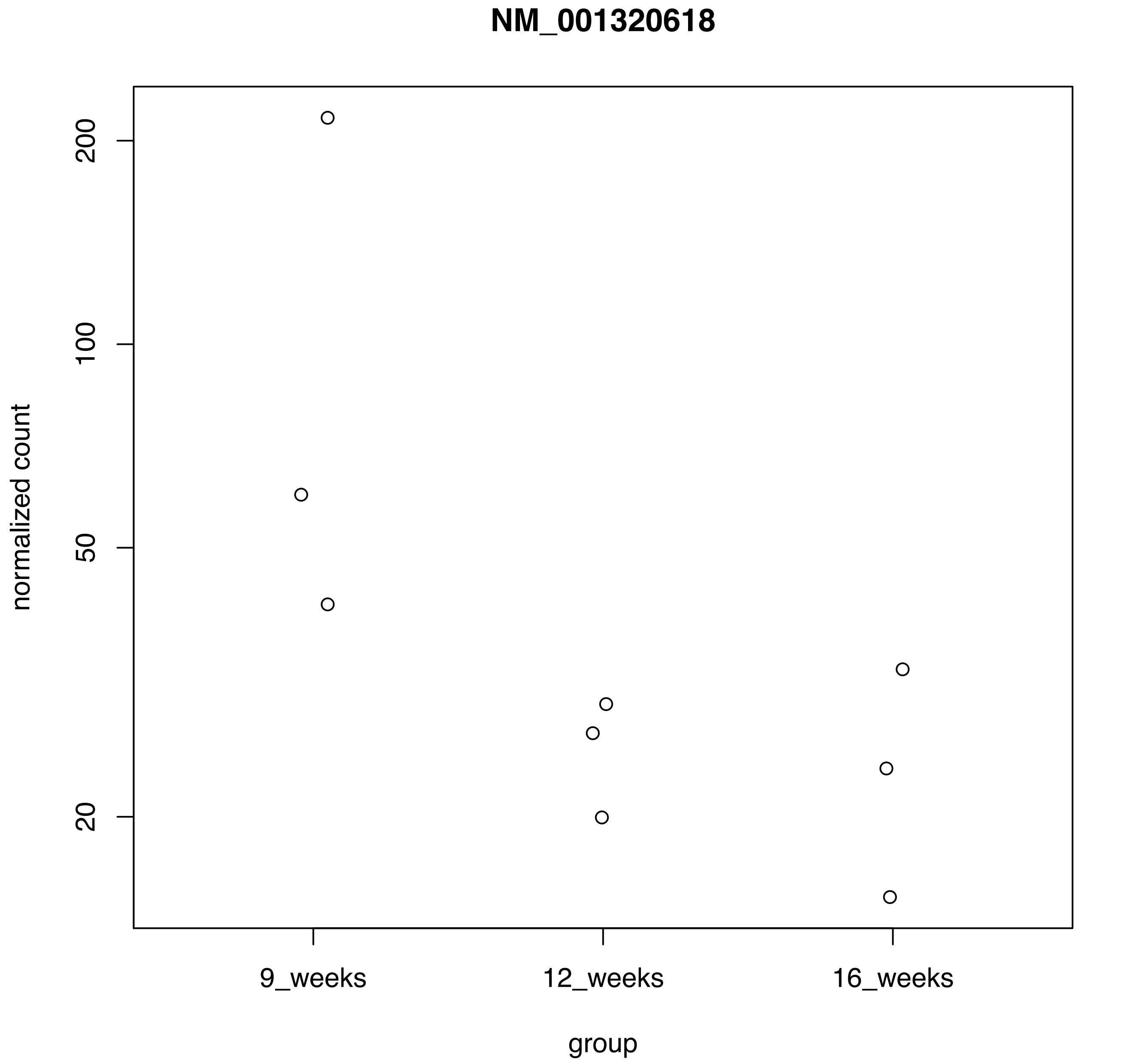
NM_001320618
normalized count
200
100
50
20
9_weeks
12_weeks
16_weeks
group

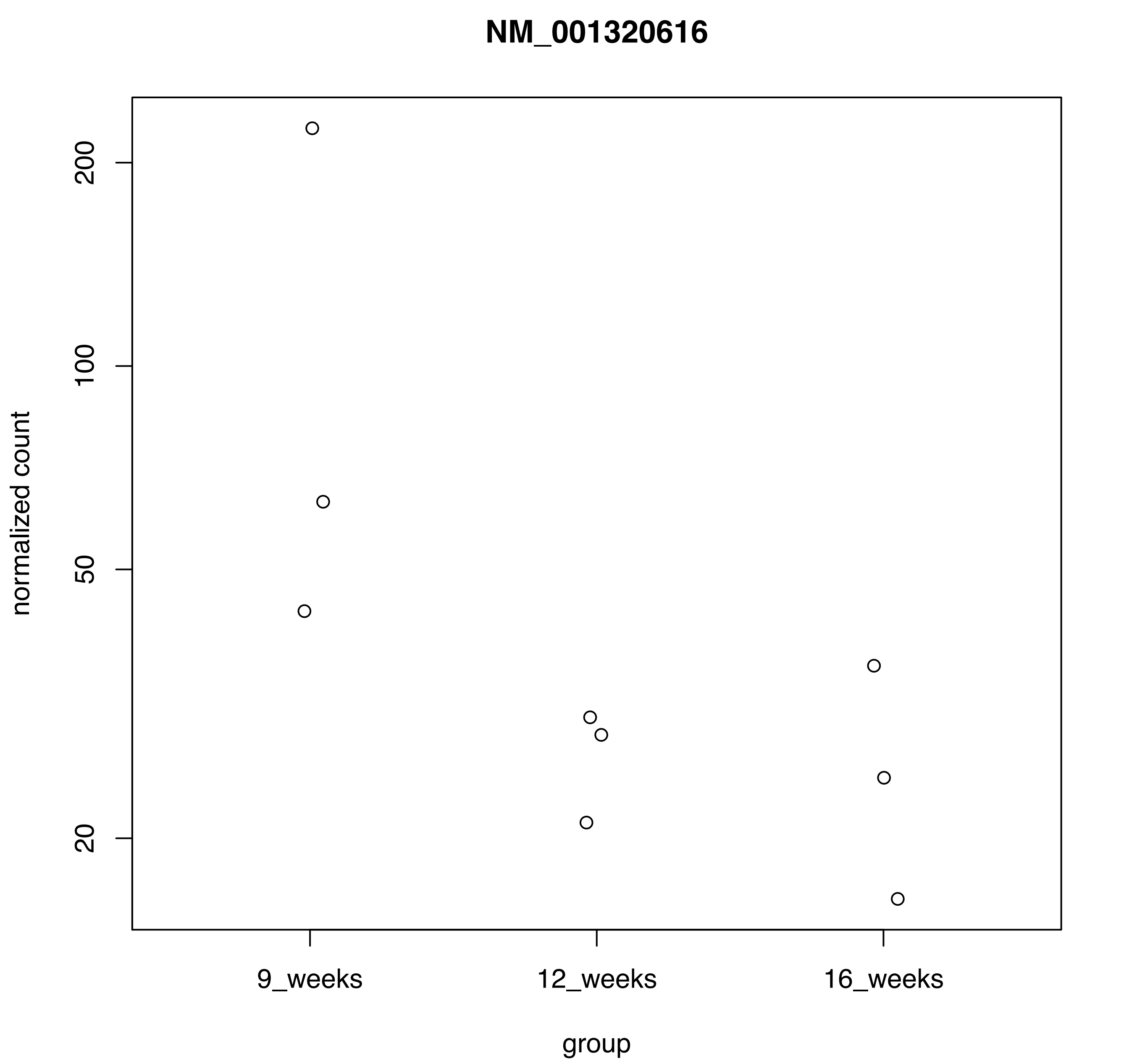
NM_001320616
normalized count
200
100
50
20
9_weeks
12_weeks
16_weeks
group

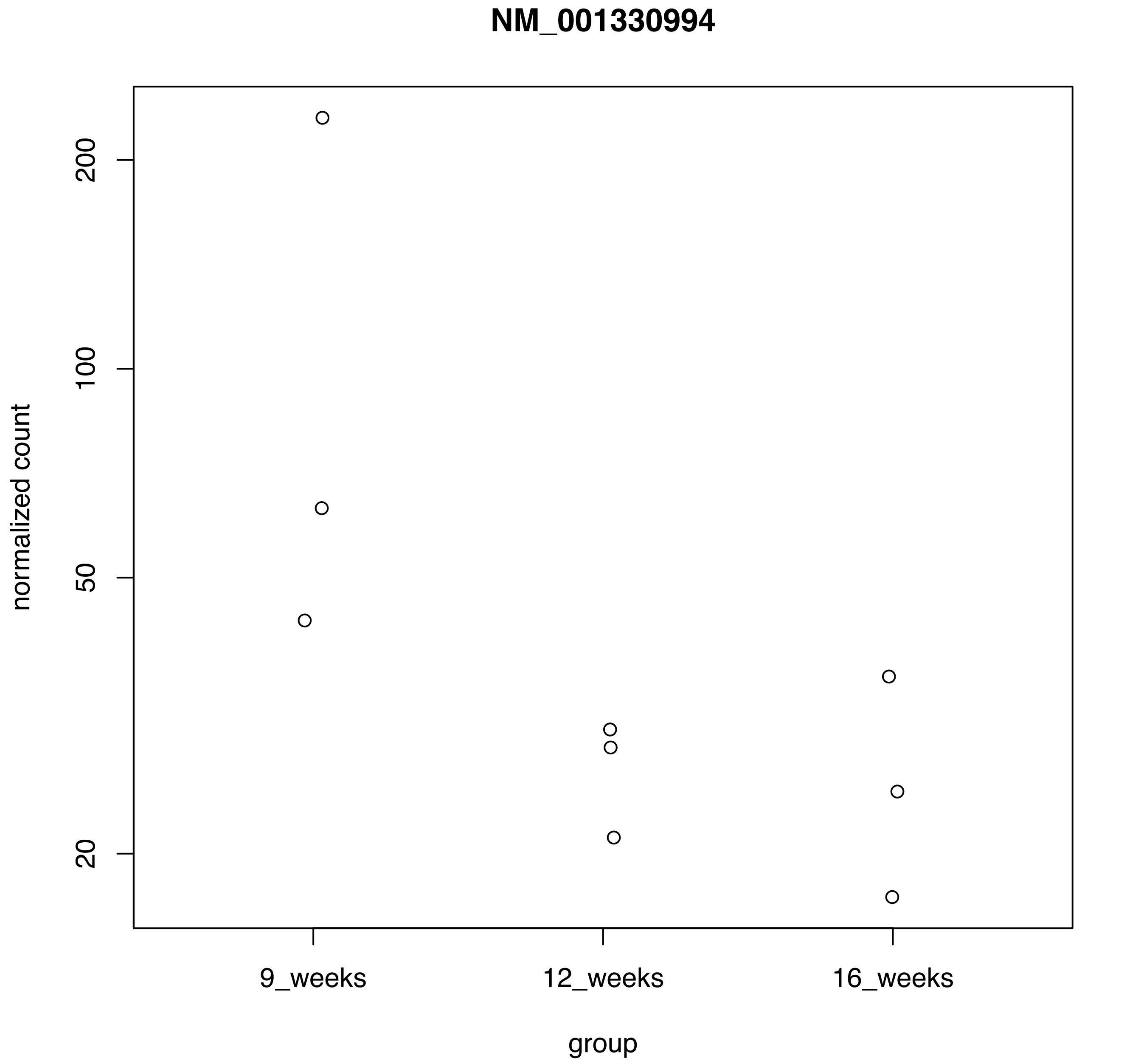
NM_001330994
normalized count
200
100
50
20
9_weeks
12_weeks
16_weeks
group

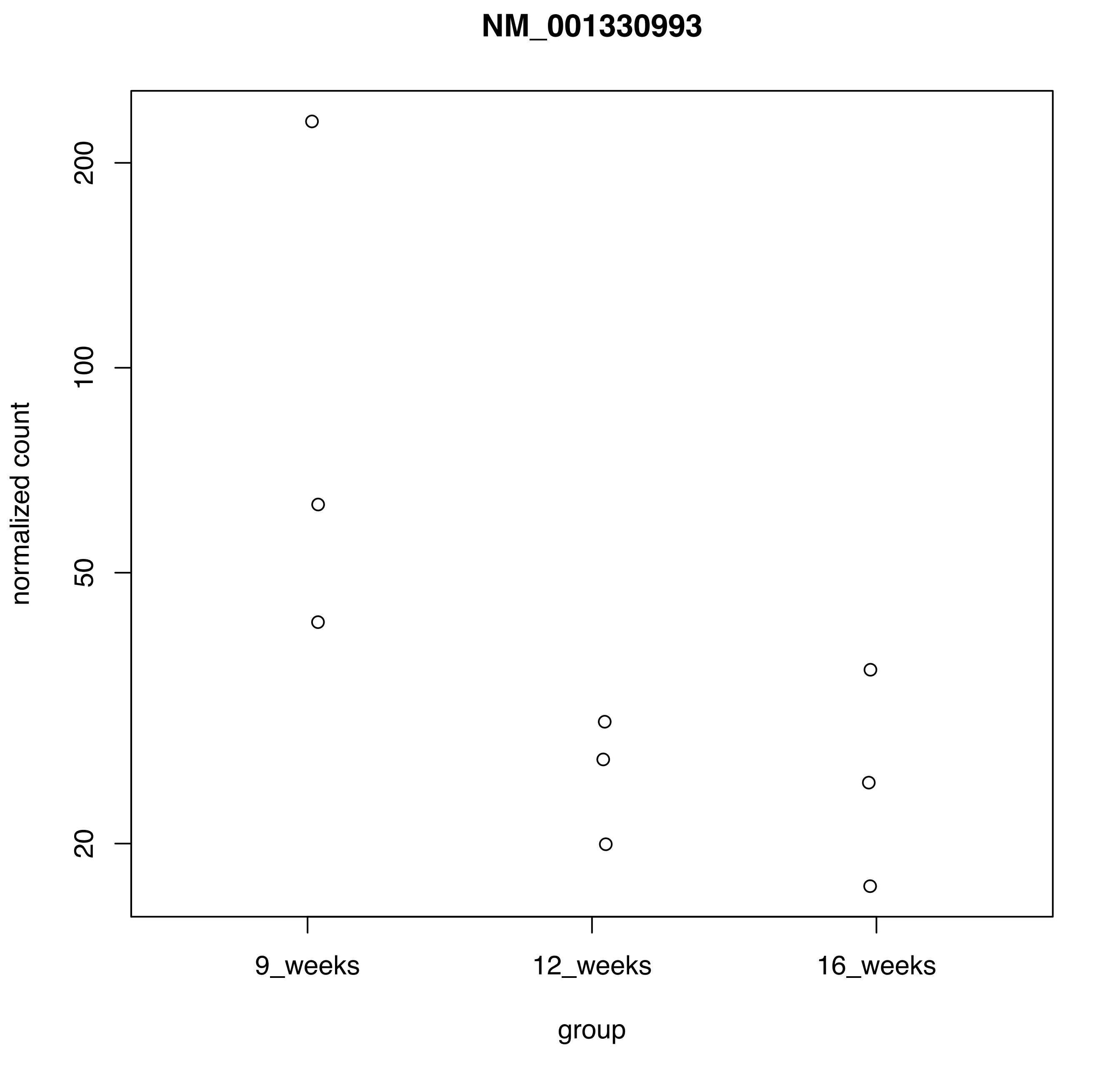
NM_001330993
normalized count
200
100
50
20
9_weeks
12_weeks
16_weeks
group

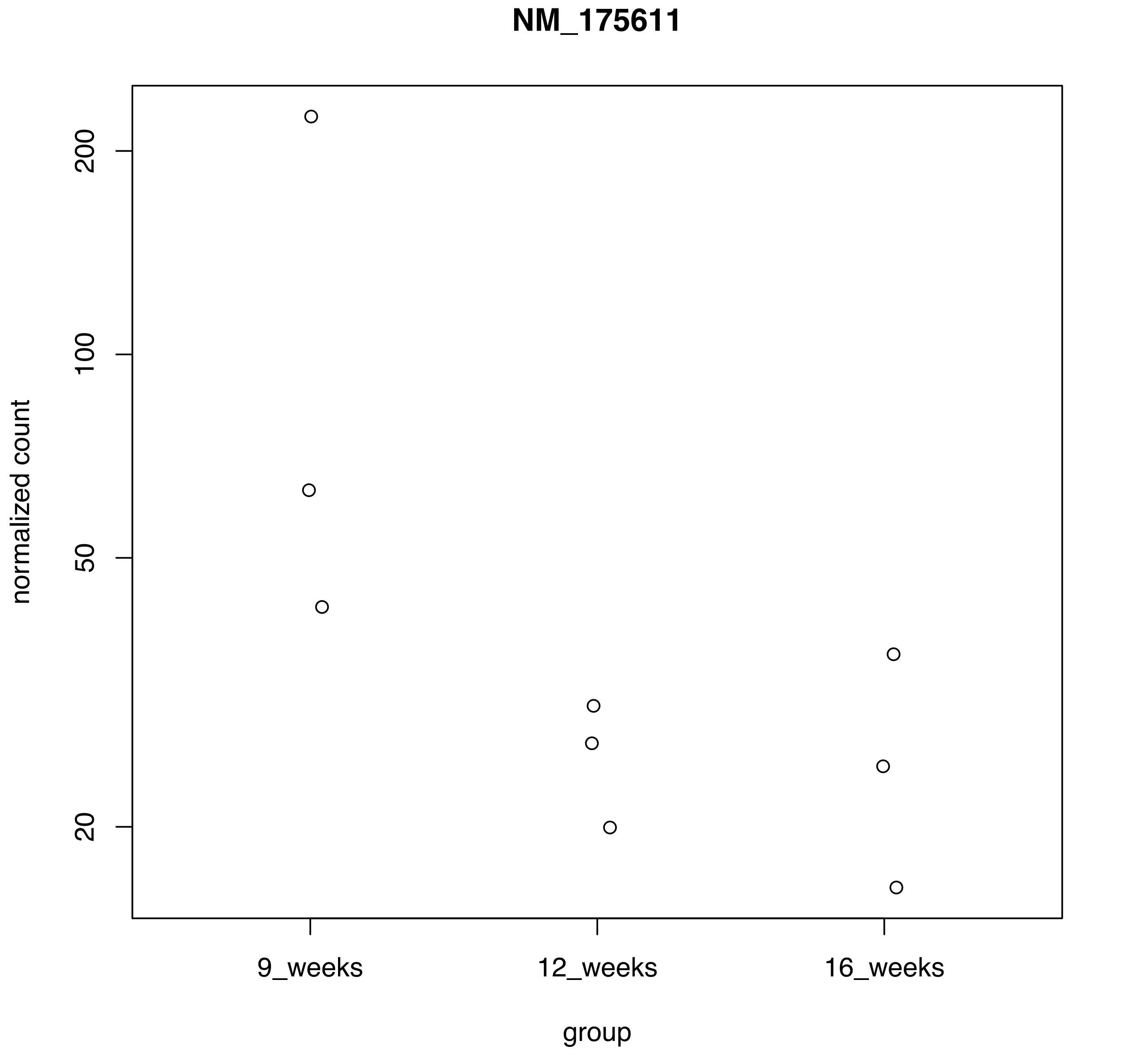
NM_175611
normalized count
200
100
50
20
9_weeks
12_weeks
16_weeks
group

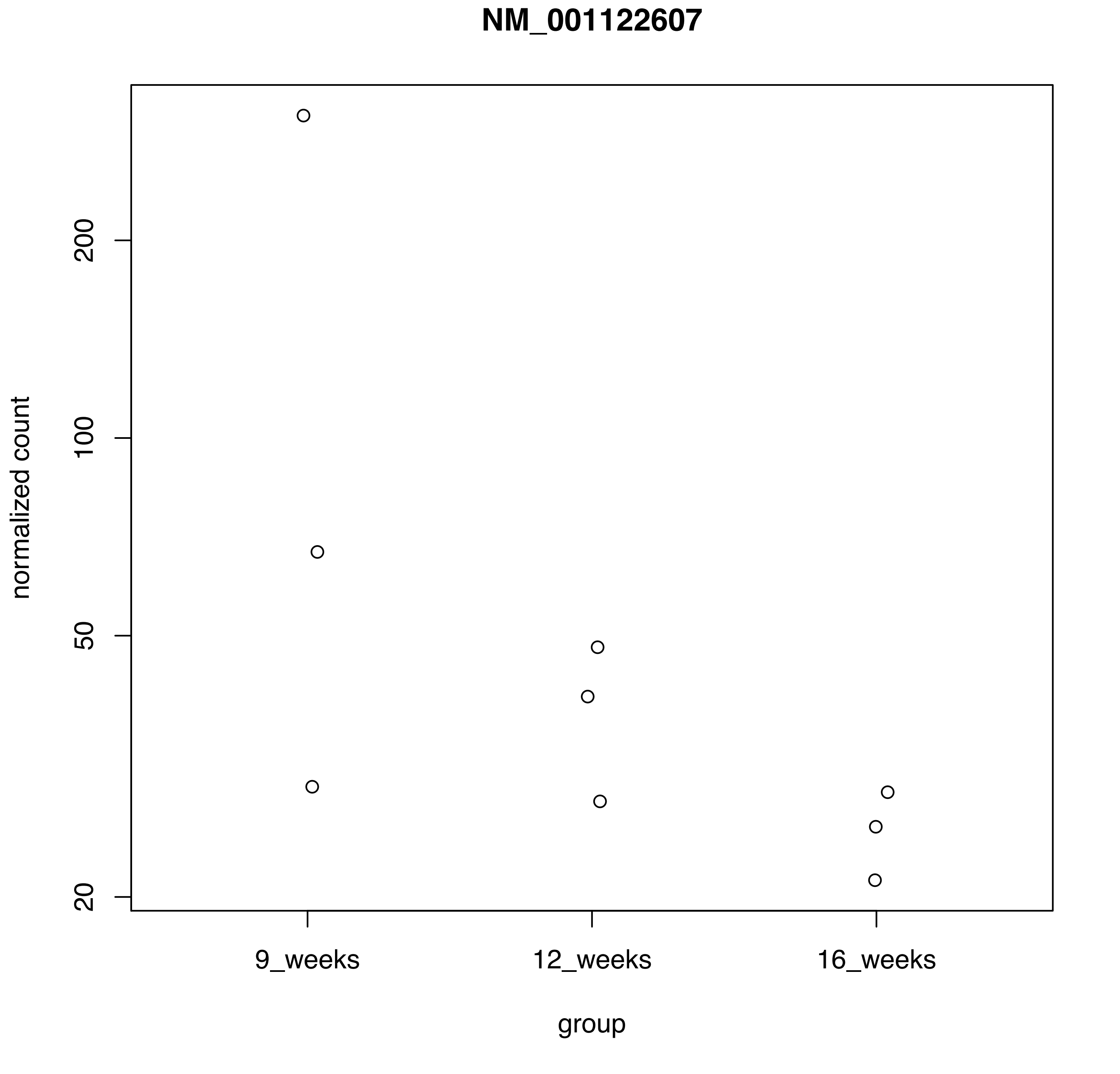
NM_001122607
normalized count
200
100
50
20
9_weeks
12_weeks
16_weeks
group

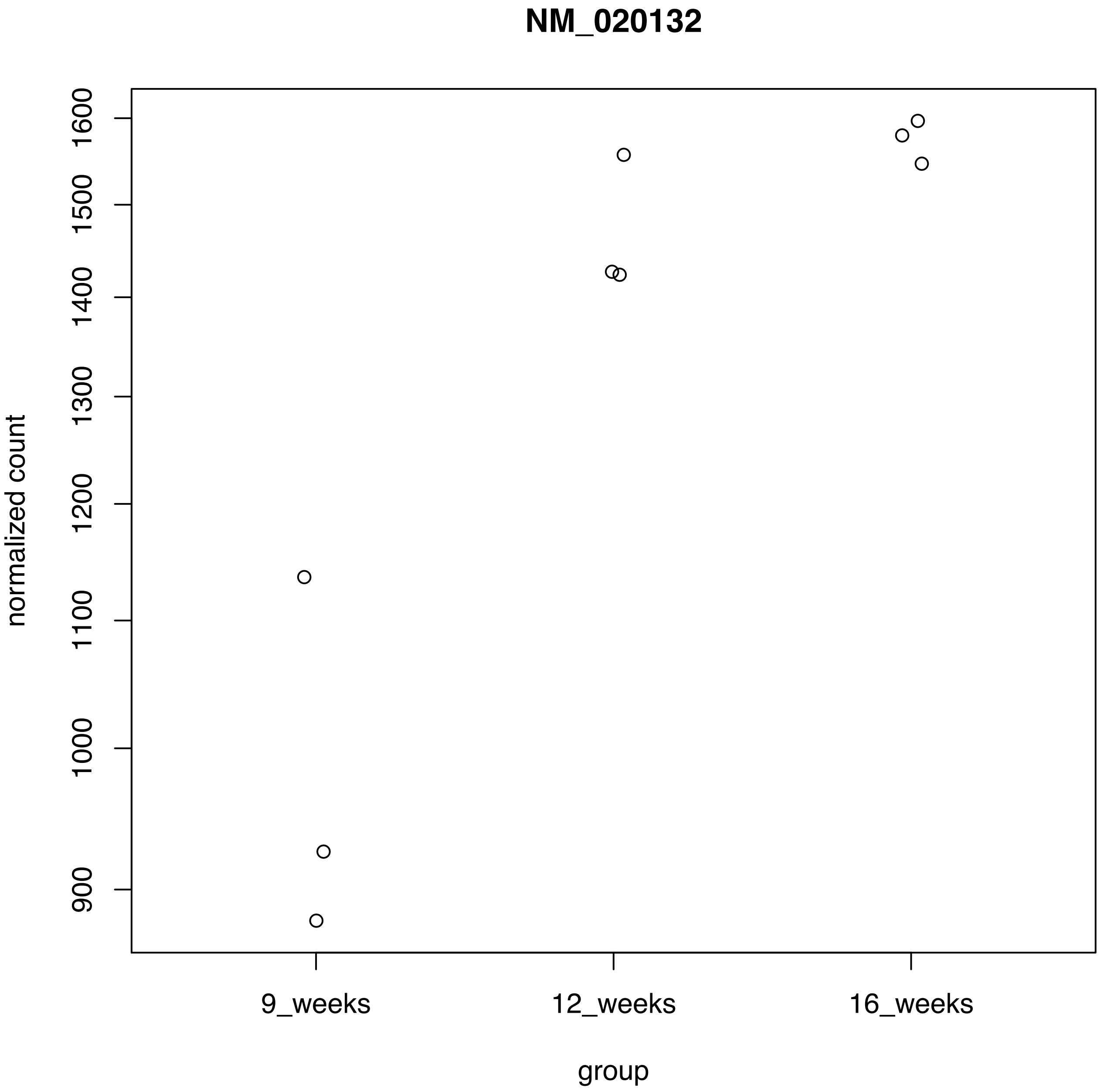
NM_020132
normalized count
1600
1500
1400
1300
1200
1100
1000
900
9_weeks
12_weeks
16_weeks
group

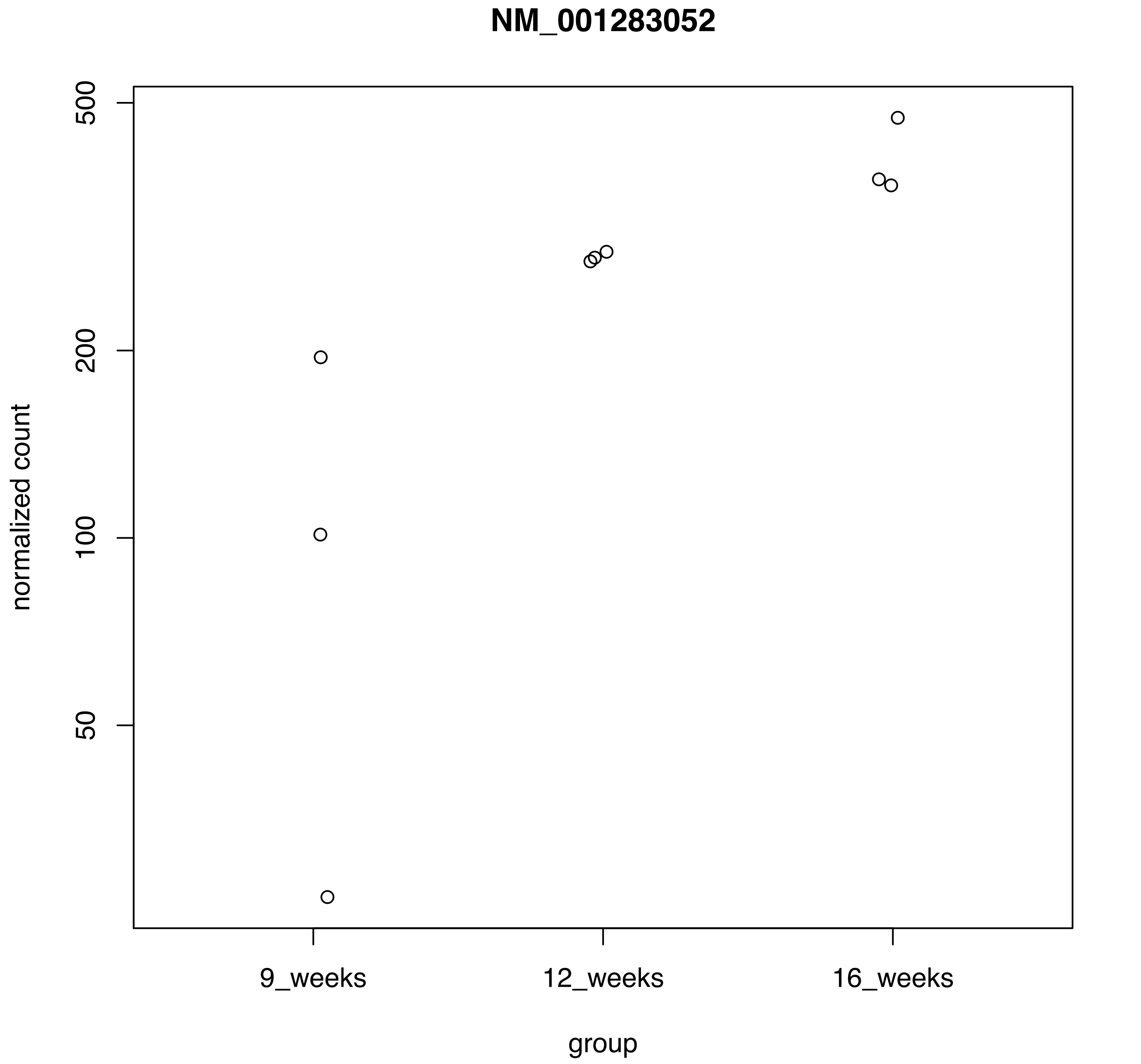
NM_001283052
normalized count
500
200
100
50
group
9_weeks
12_weeks
16_weeks

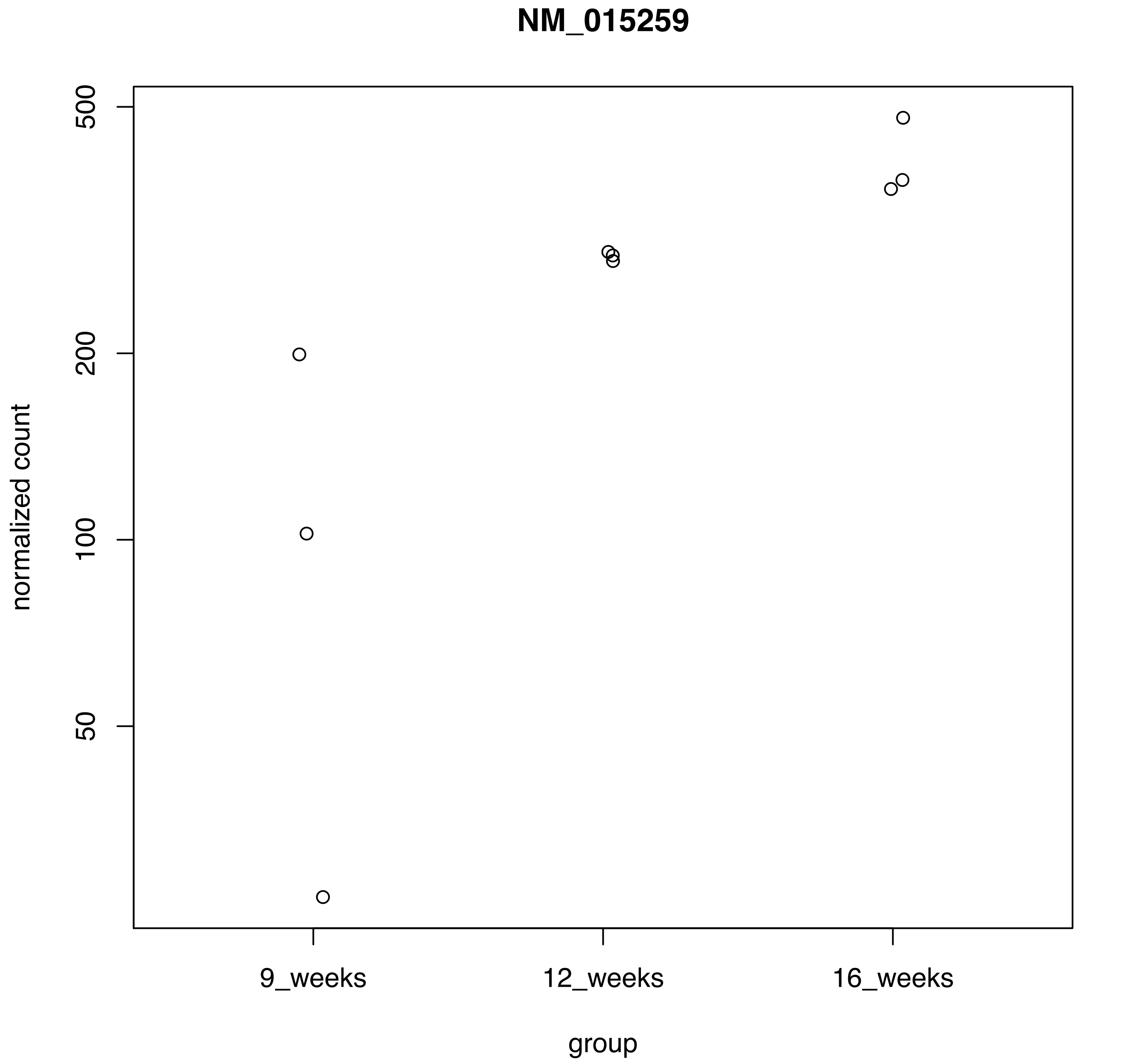

NM_015259
500
200
100
50
normalized count
9_weeks
12_weeks
16_weeks
group

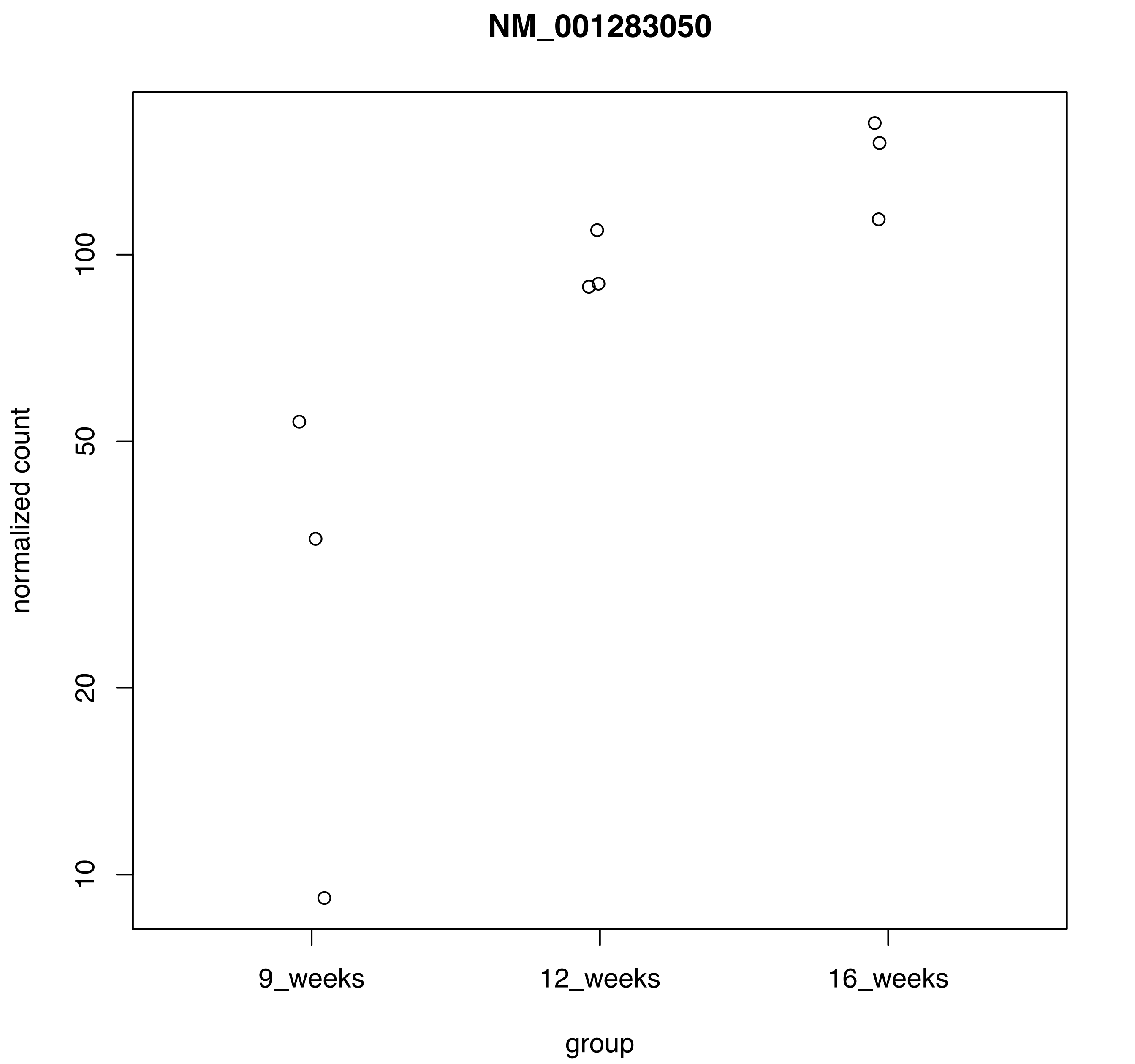

NM_001283050
normalized count
100
50
20
10
9_weeks
12_weeks
16_weeks
group

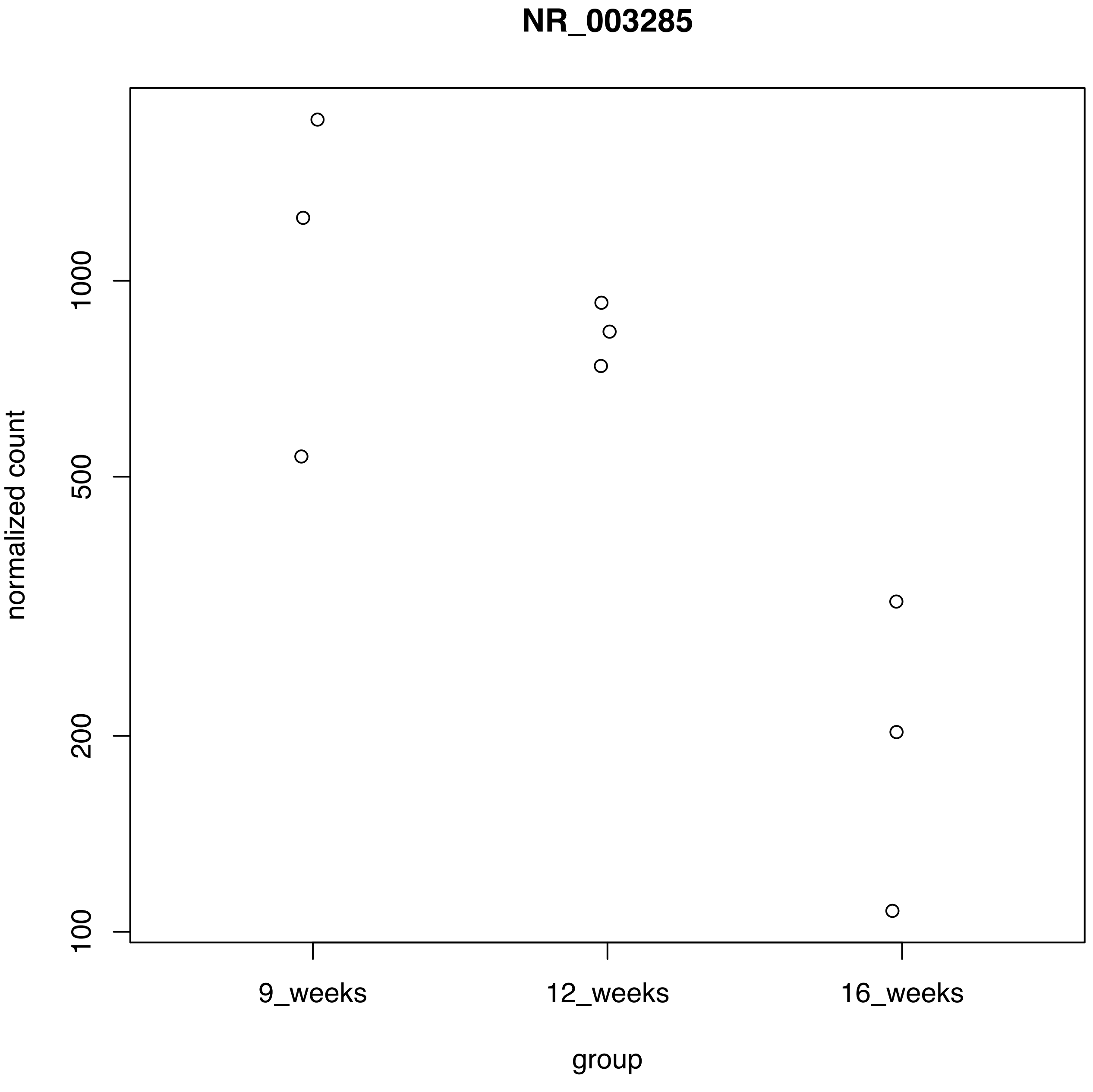

NR_003285
normalized count
1000
500
200
100
9_weeks
12_weeks
16_weeks
group

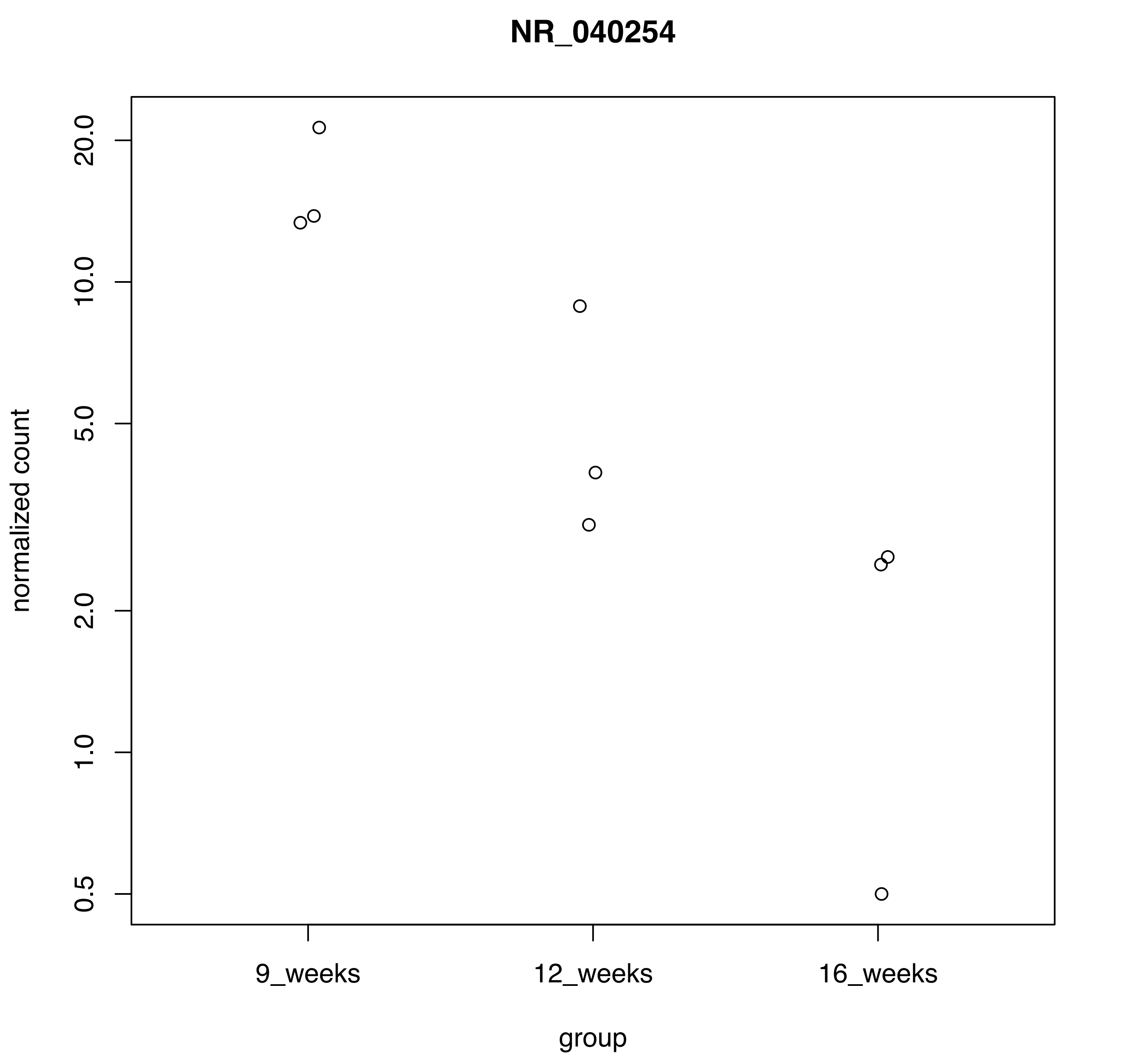
NR_040254
normalized count
20.0
10.0
5.0
2.0
1.0
0.5
9_weeks
12_weeks
16_weeks
group

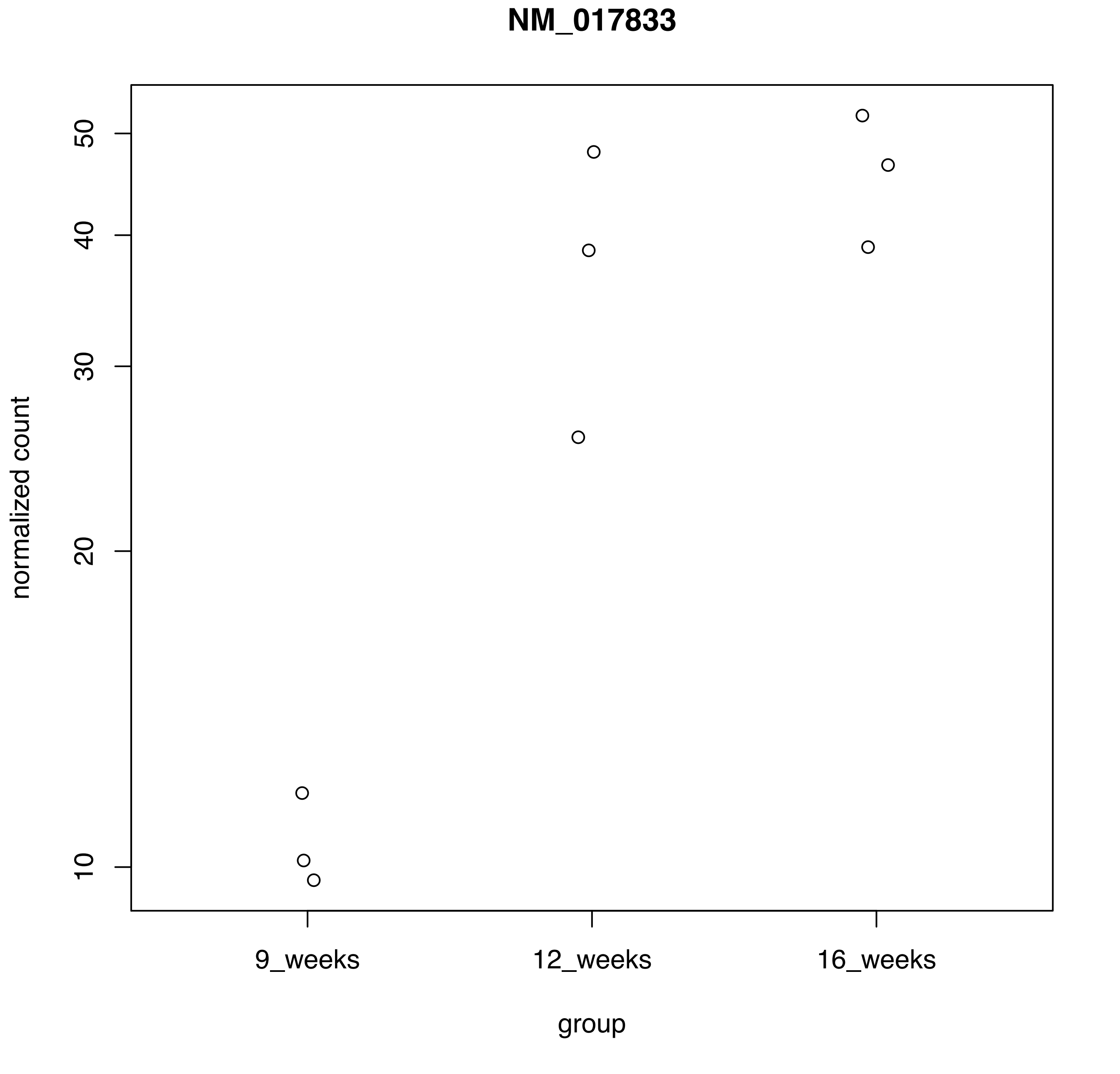
NM_017833
normalized count
10
20
30
40
50
9_weeks
12_weeks
16_weeks
group

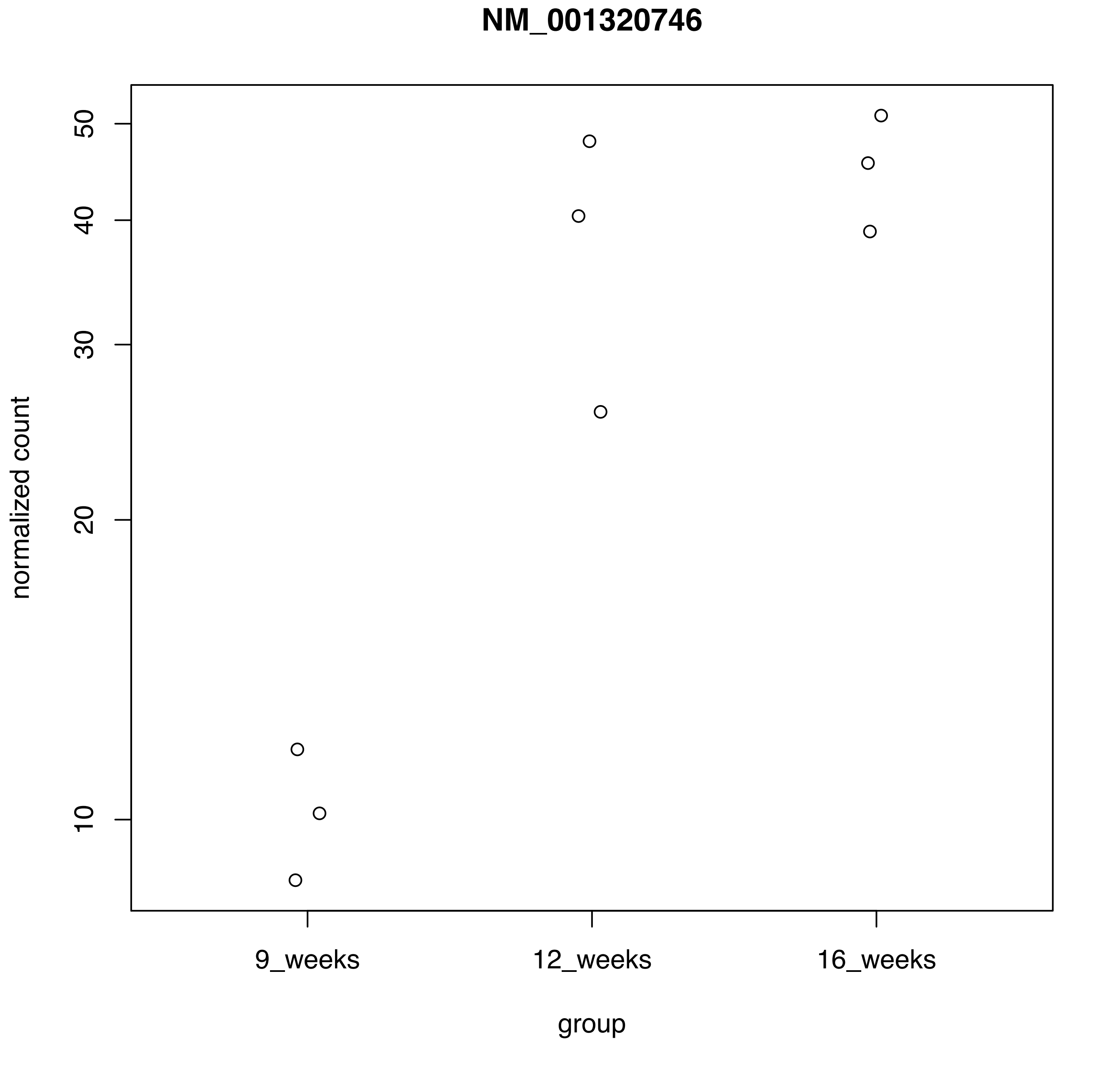
NM_001320746
normalized count
50
40
30
20
10
9_weeks
12_weeks
16_weeks
group

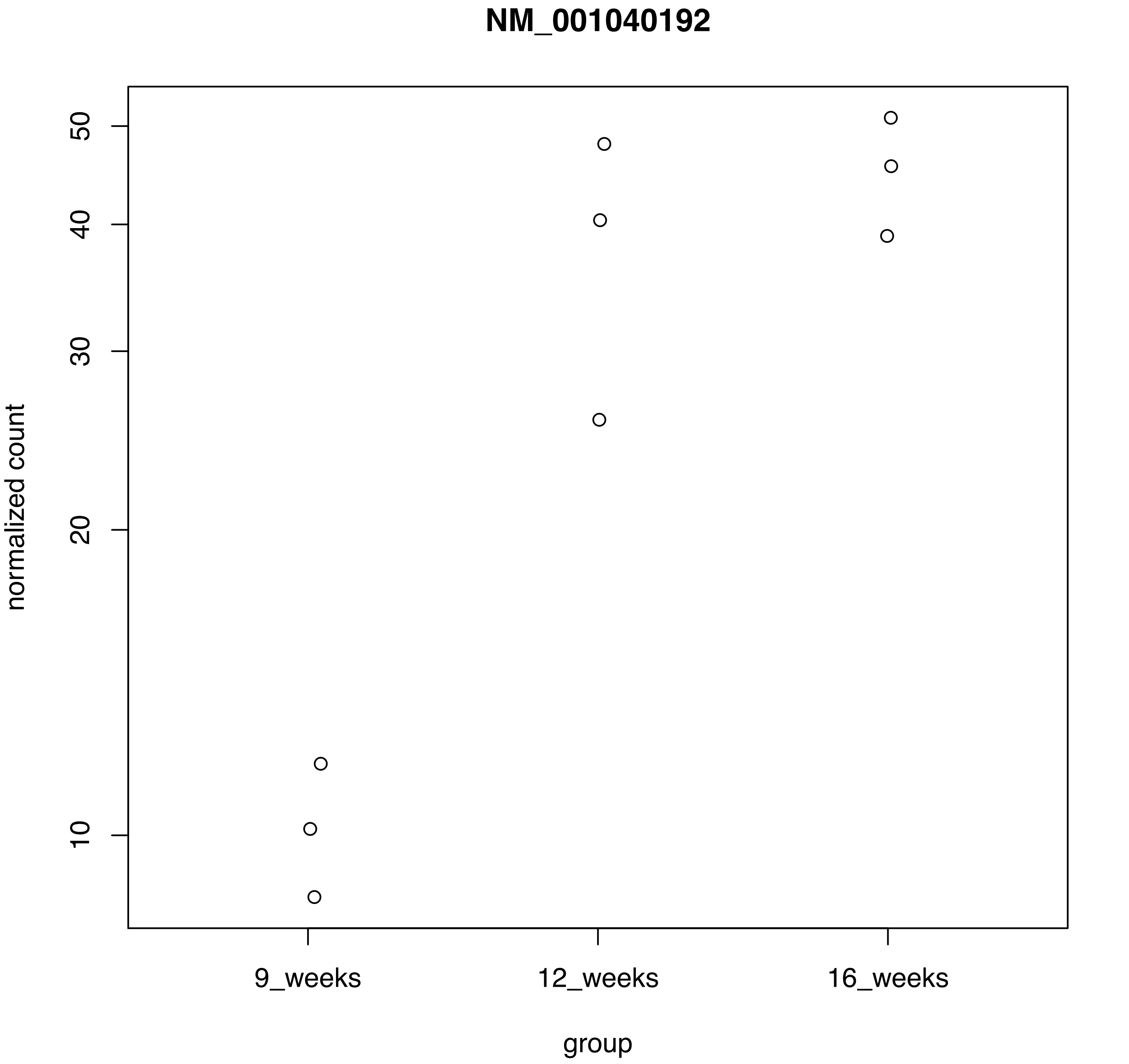
NM_001040192
normalized count
50
40
30
20
10
9_weeks
12_weeks
16_weeks
group

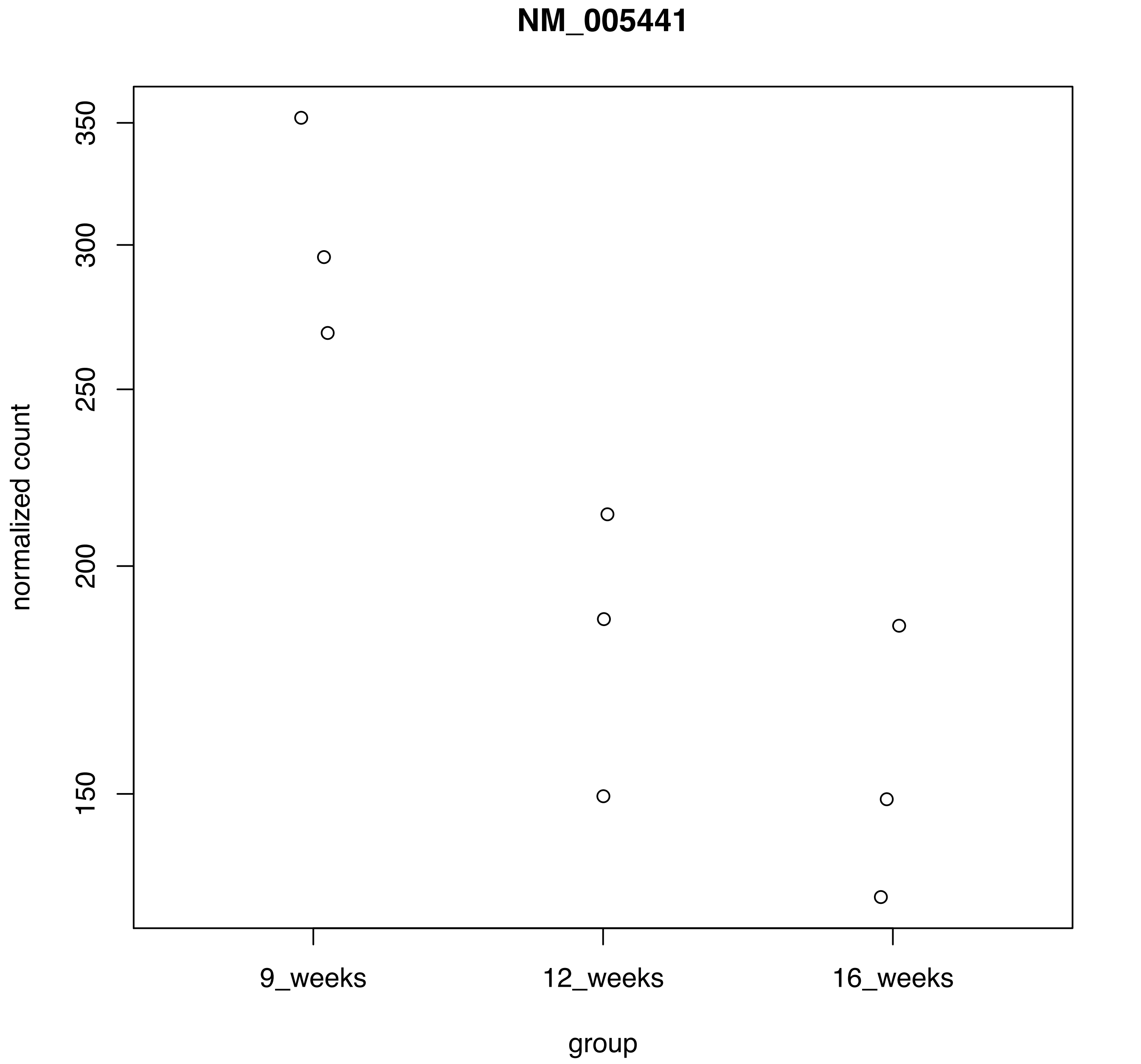

NM_005441
normalized count
350
300
250
200
150
9_weeks
12_weeks
16_weeks
group

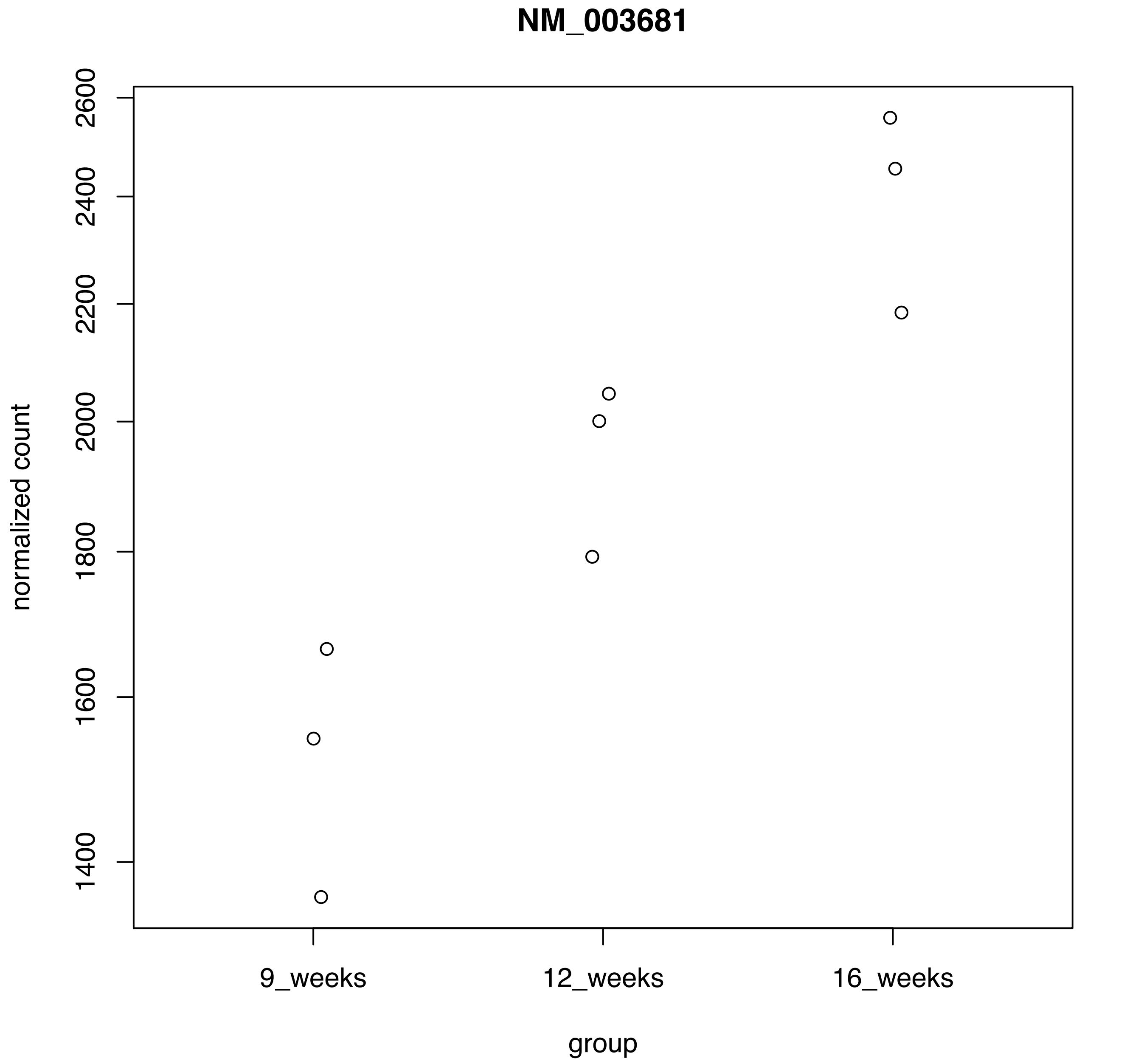
NM_003681
normalized count
2600
2400
2200
2000
1800
1600
1400
9_weeks
12_weeks
16_weeks
group

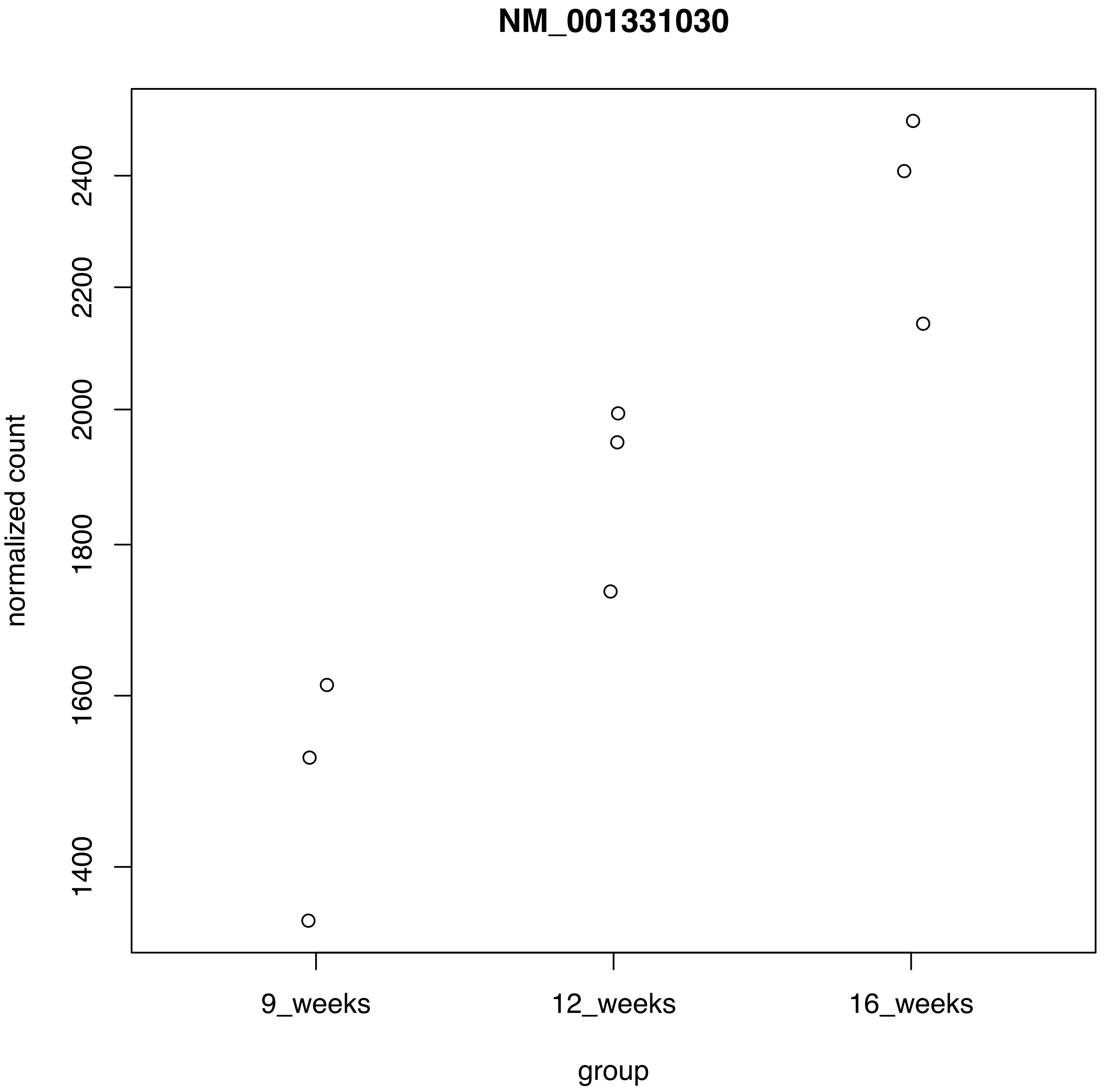
NM_001331030
normalized count
1400
1600
1800
2000
2200
2400
9_weeks
12_weeks
16_weeks
group

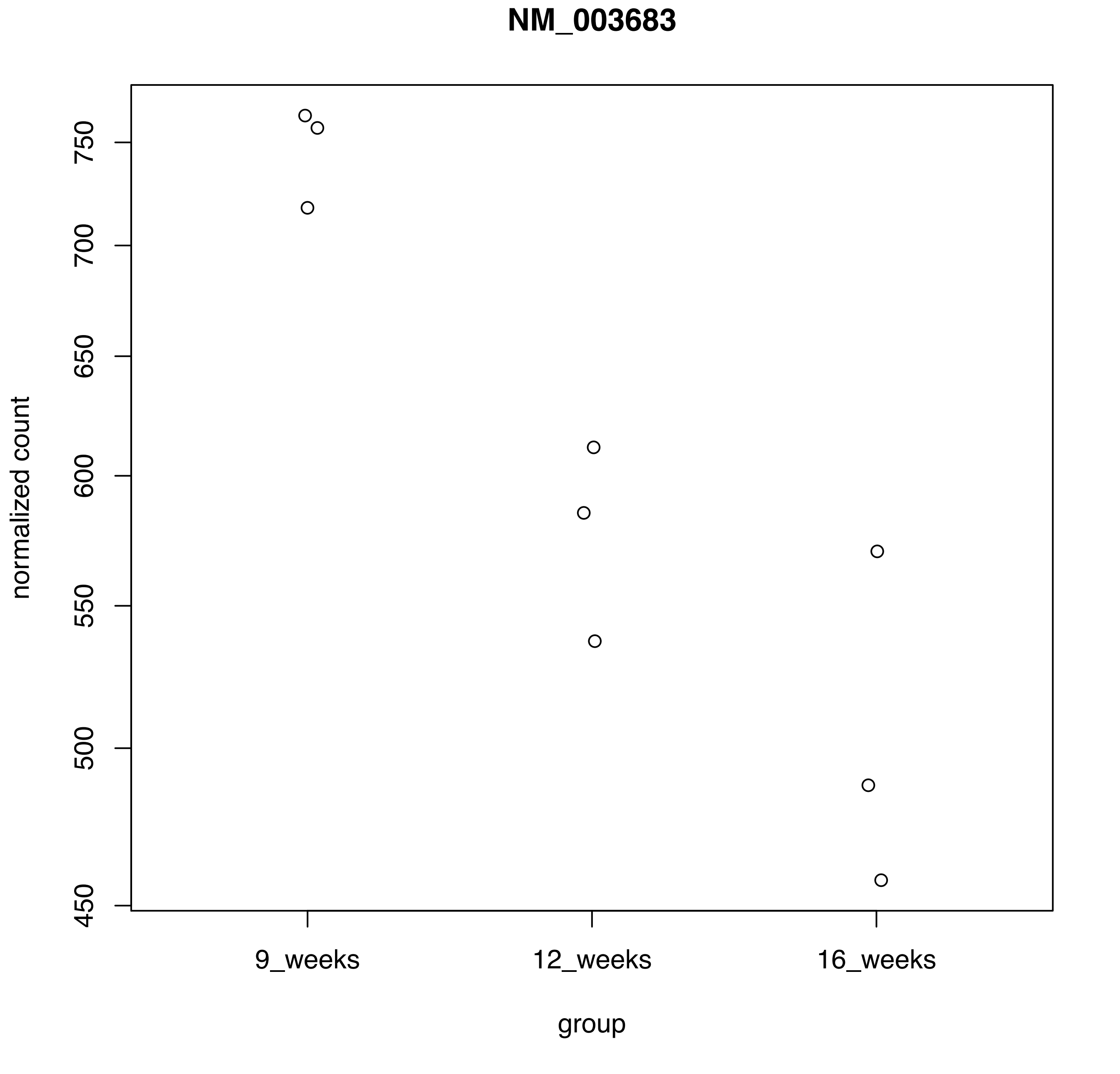
NM_003683
normalized count
750
700
650
600
550
500
450
9_weeks
12_weeks
16_weeks
group

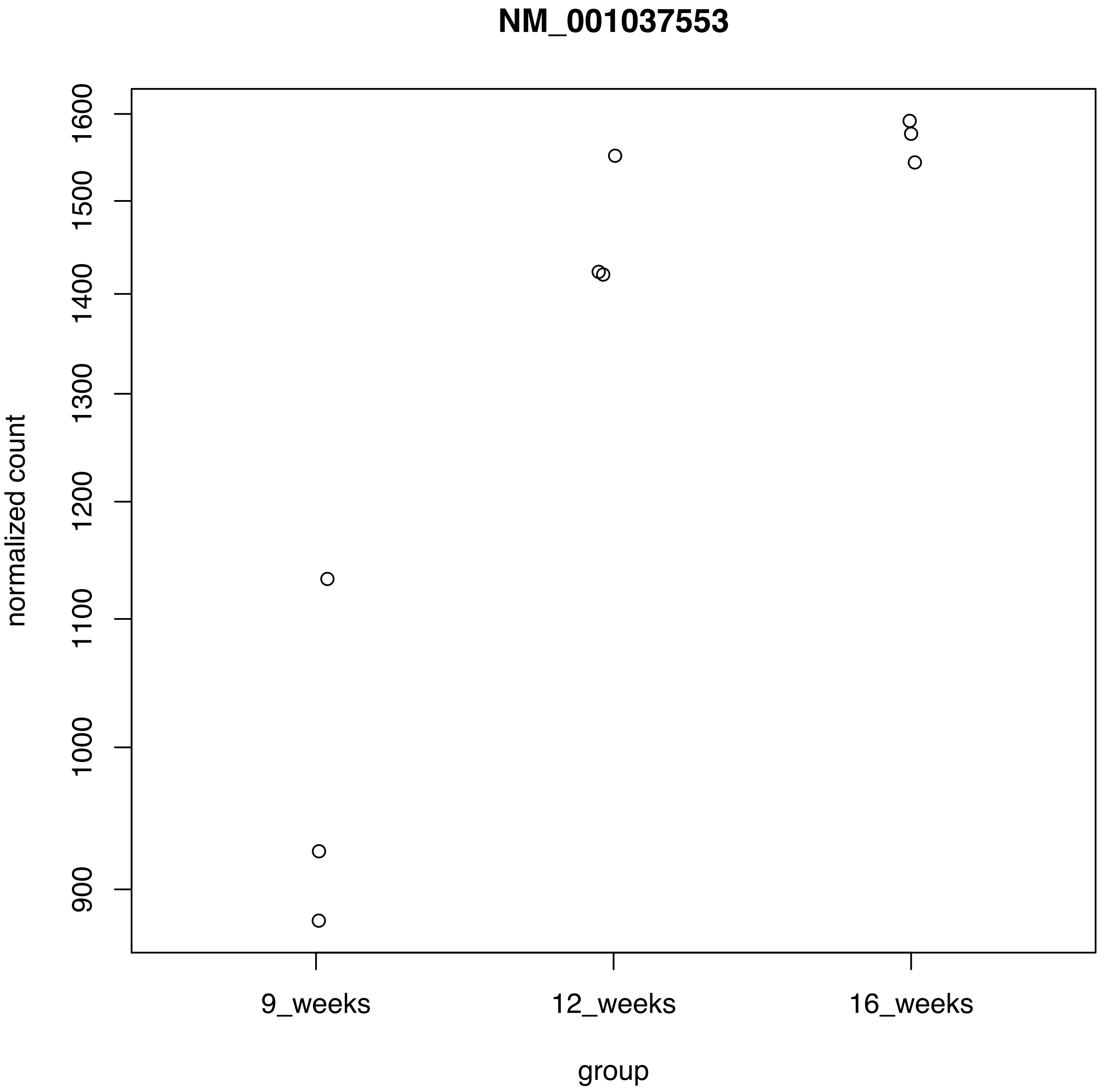
NM_001037553
normalized count
900
1000
1100
1200
1300
1400
1500
1600
9_weeks
12_weeks
16_weeks
group

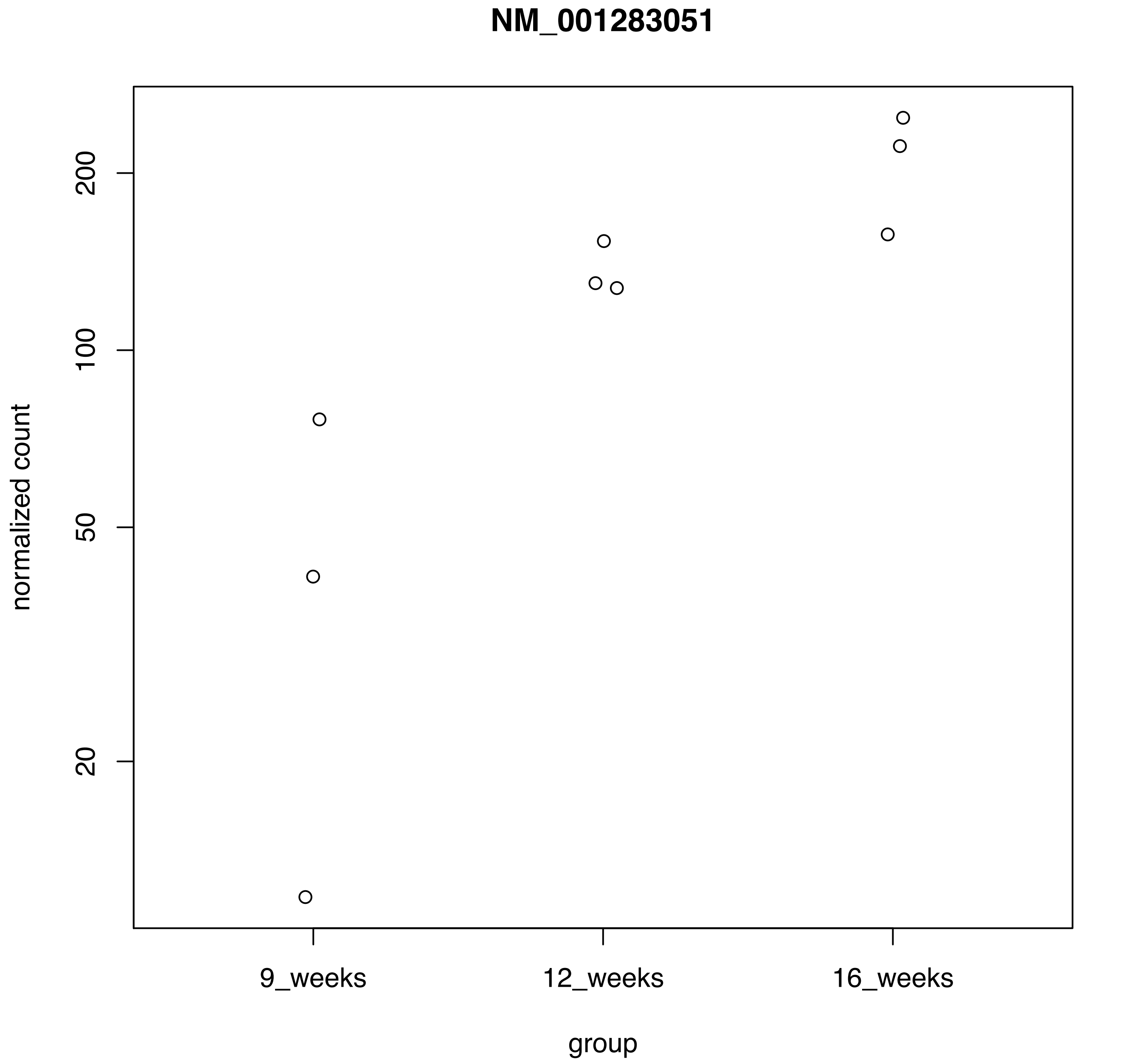

NM_001283051
200
100
50
20
normalized count
9_weeks
12_weeks
16_weeks
group

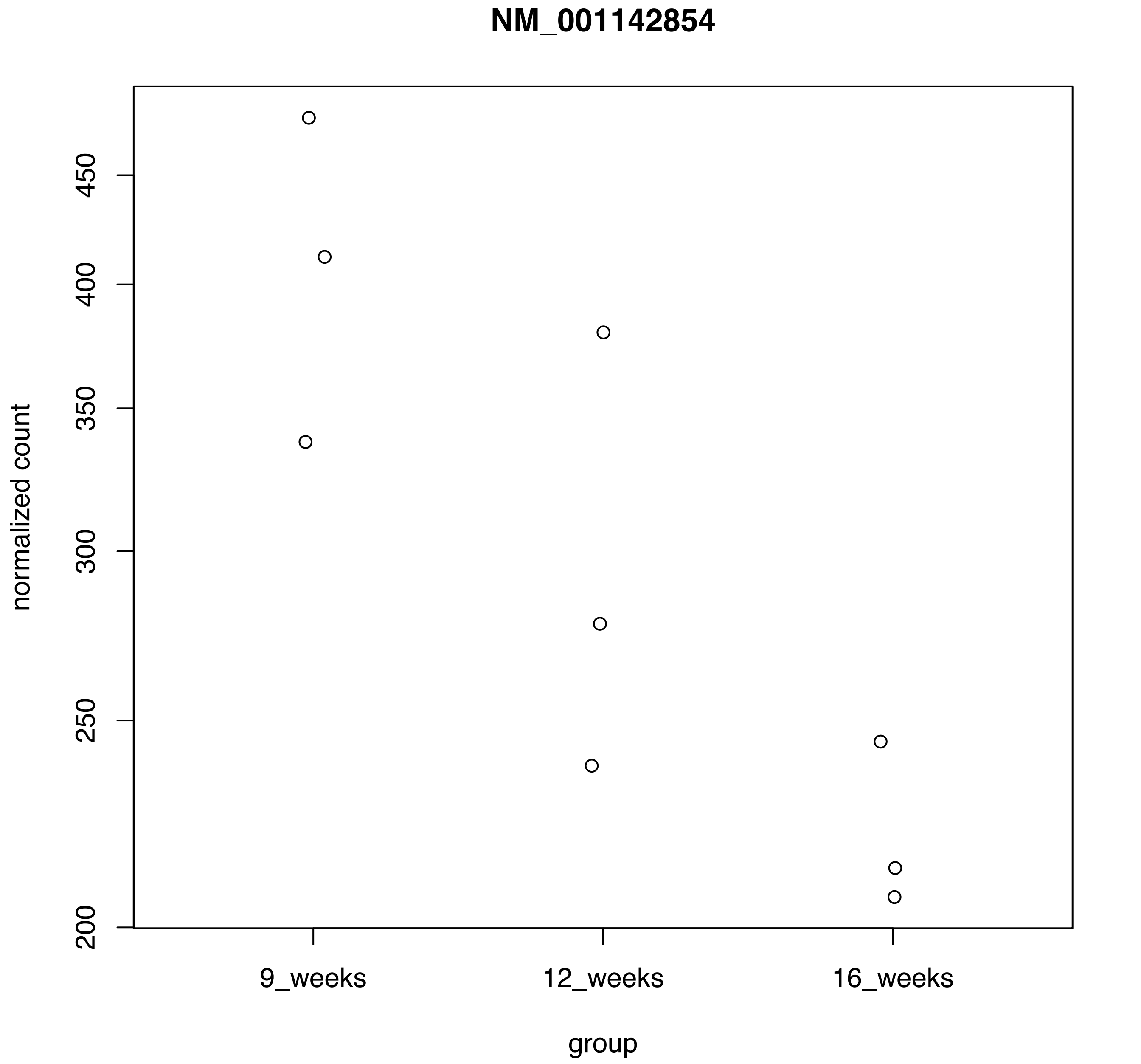
NM_001142854
normalized count
450
400
350
300
250
200
9_weeks
12_weeks
16_weeks
group

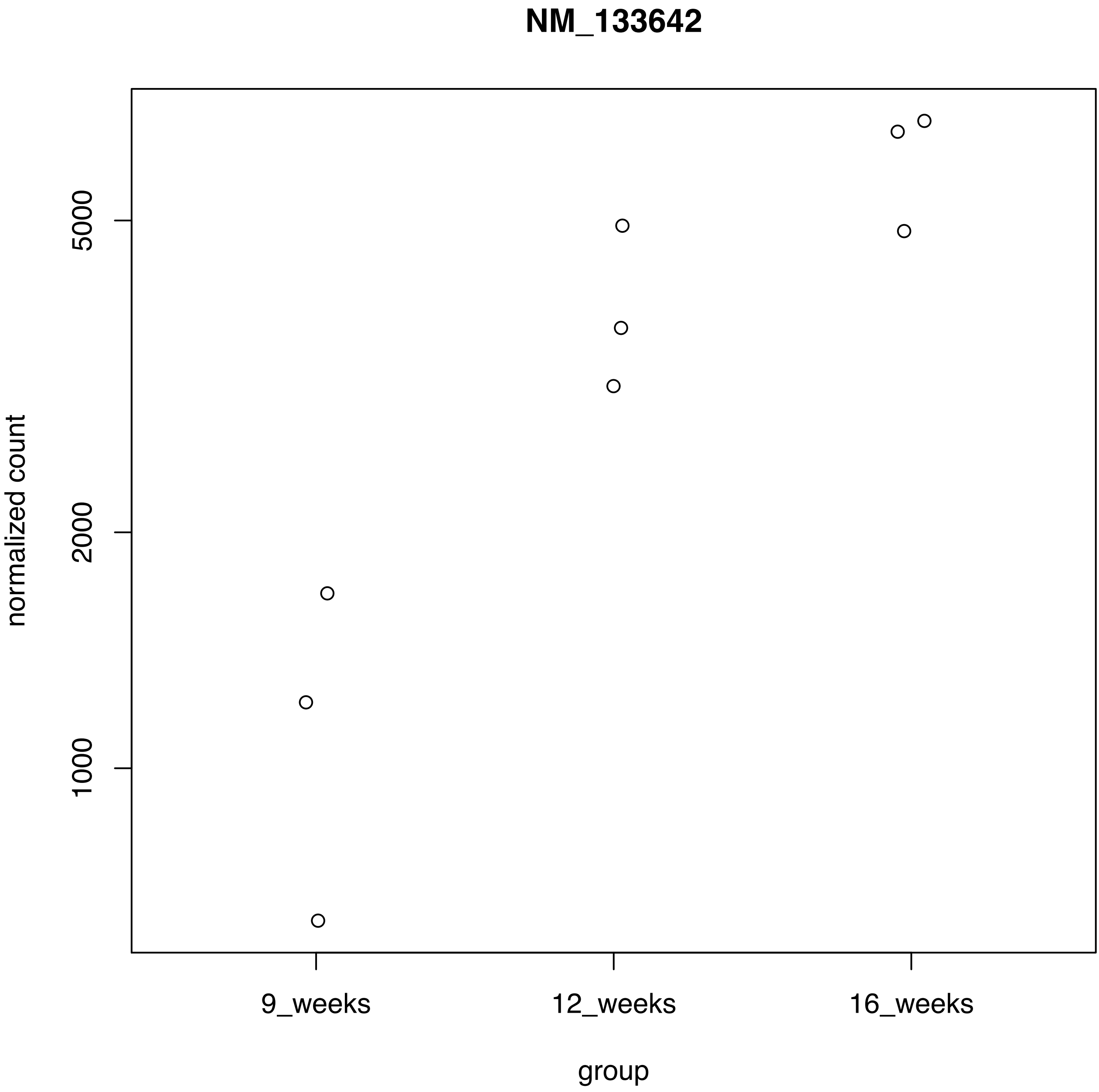
NM_133642
normalized count
5000
2000
1000
9_weeks
12_weeks
16_weeks
group

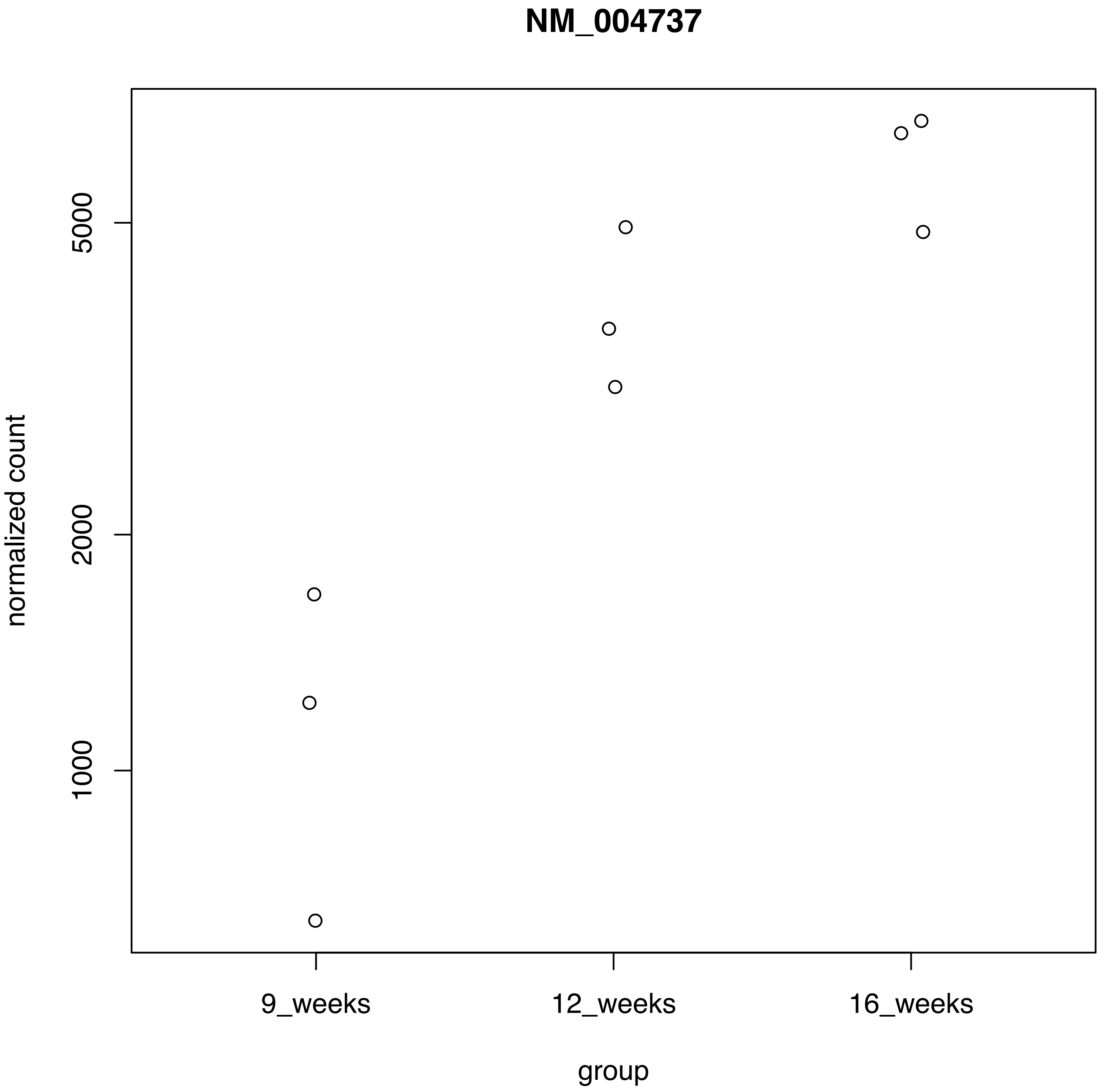
NM_004737
normalized count
5000
2000
1000
9_weeks
12_weeks
16_weeks
group

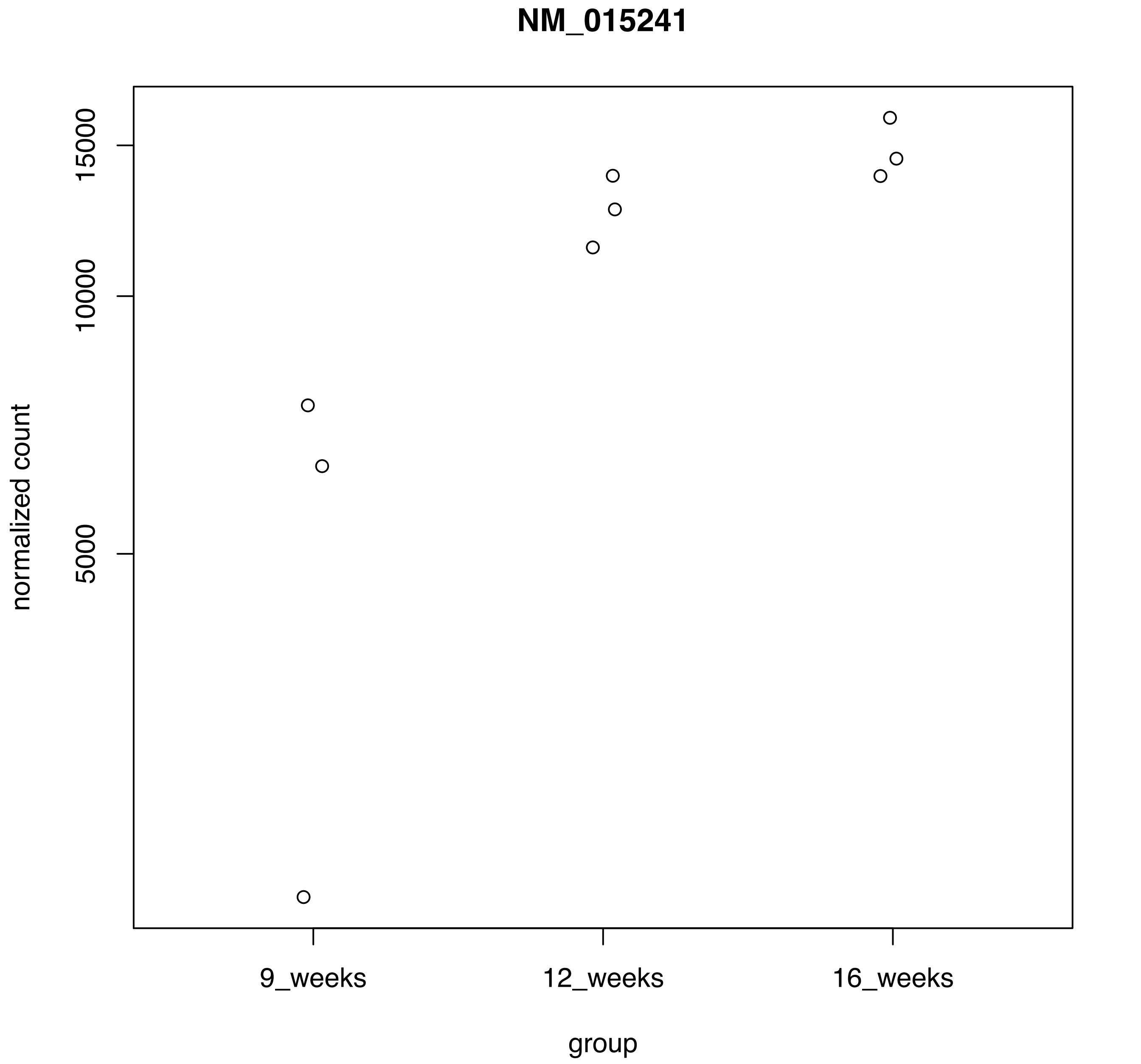
NM_015241
normalized count
15000
10000
5000
9_weeks
12_weeks
16_weeks
group

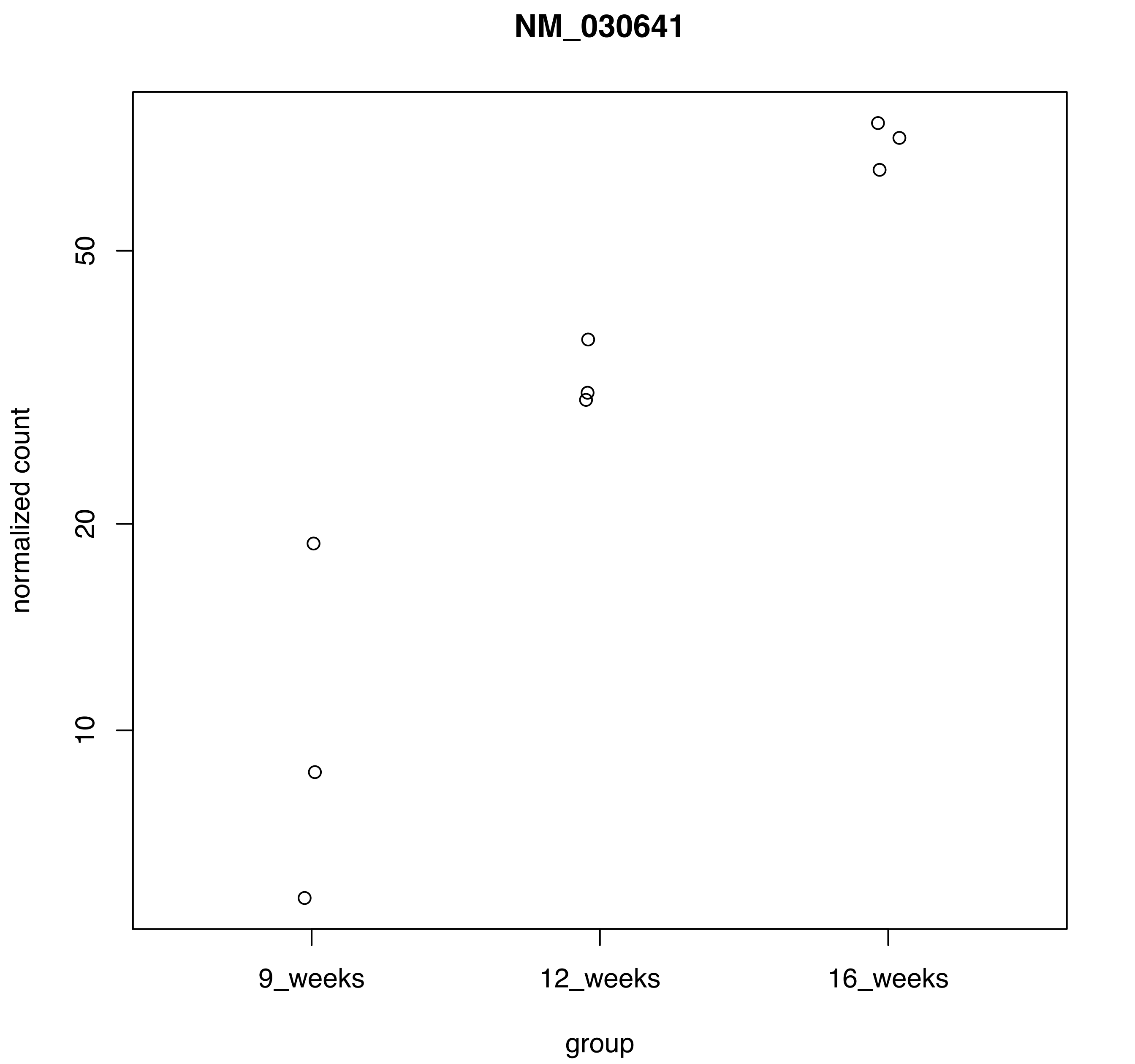
NM_030641
normalized count
50
20
10
9_weeks
12_weeks
16_weeks
group

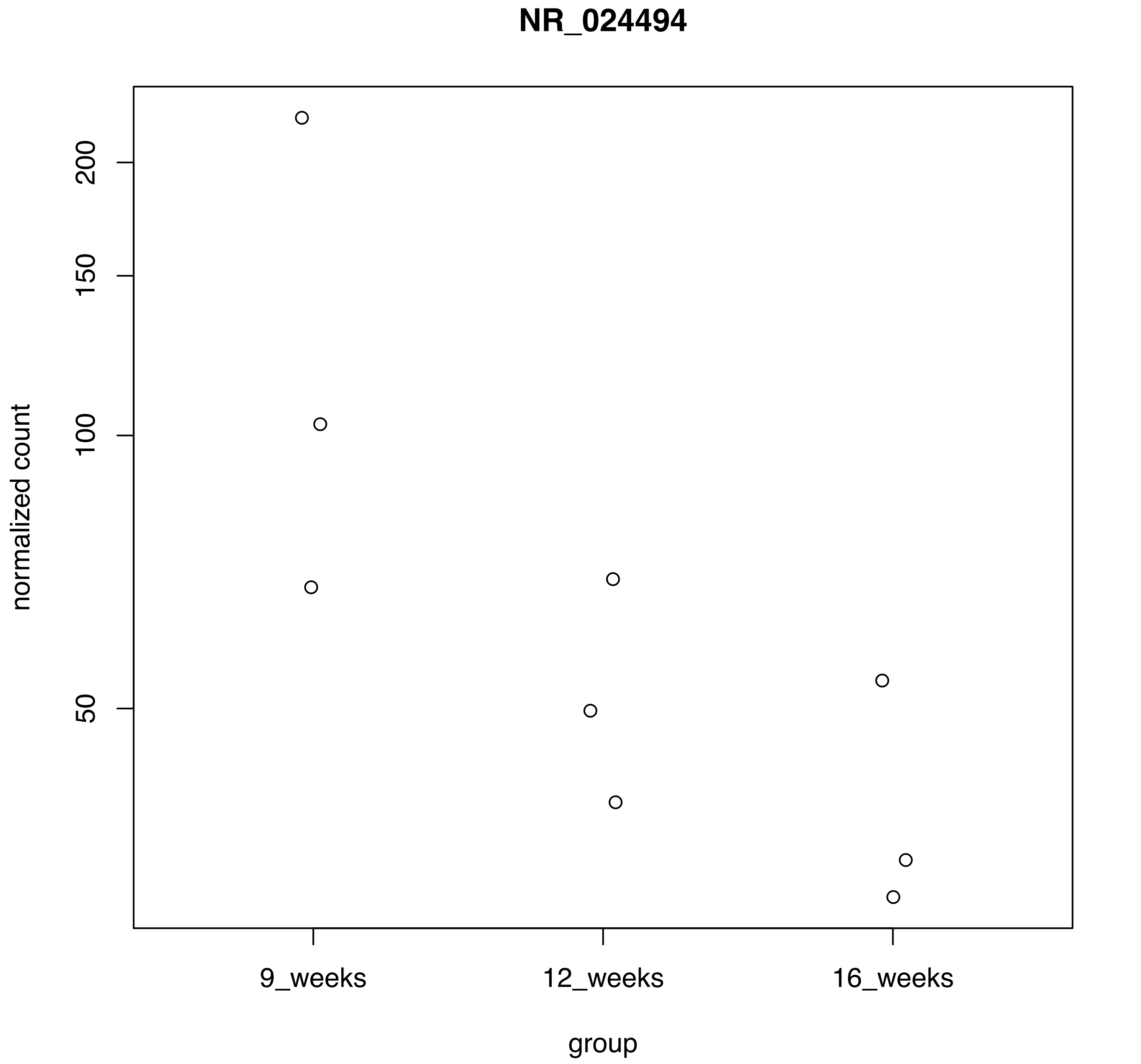

NR_024494
normalized count
200
150
100
50
9_weeks
12_weeks
16_weeks
group

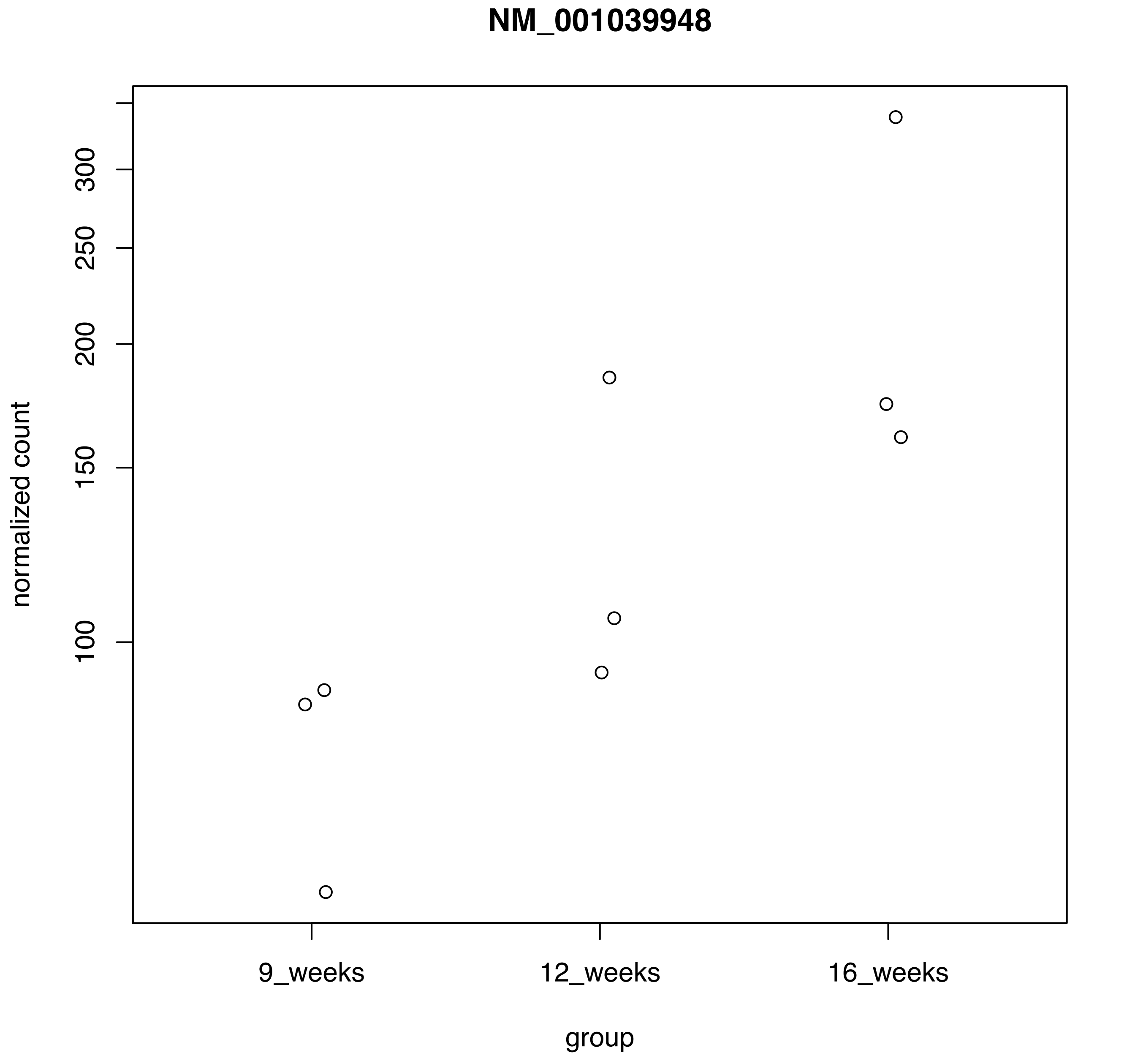
NM_001039948
normalized count
100
150
200
250
300
group
9_weeks
12_weeks
16_weeks

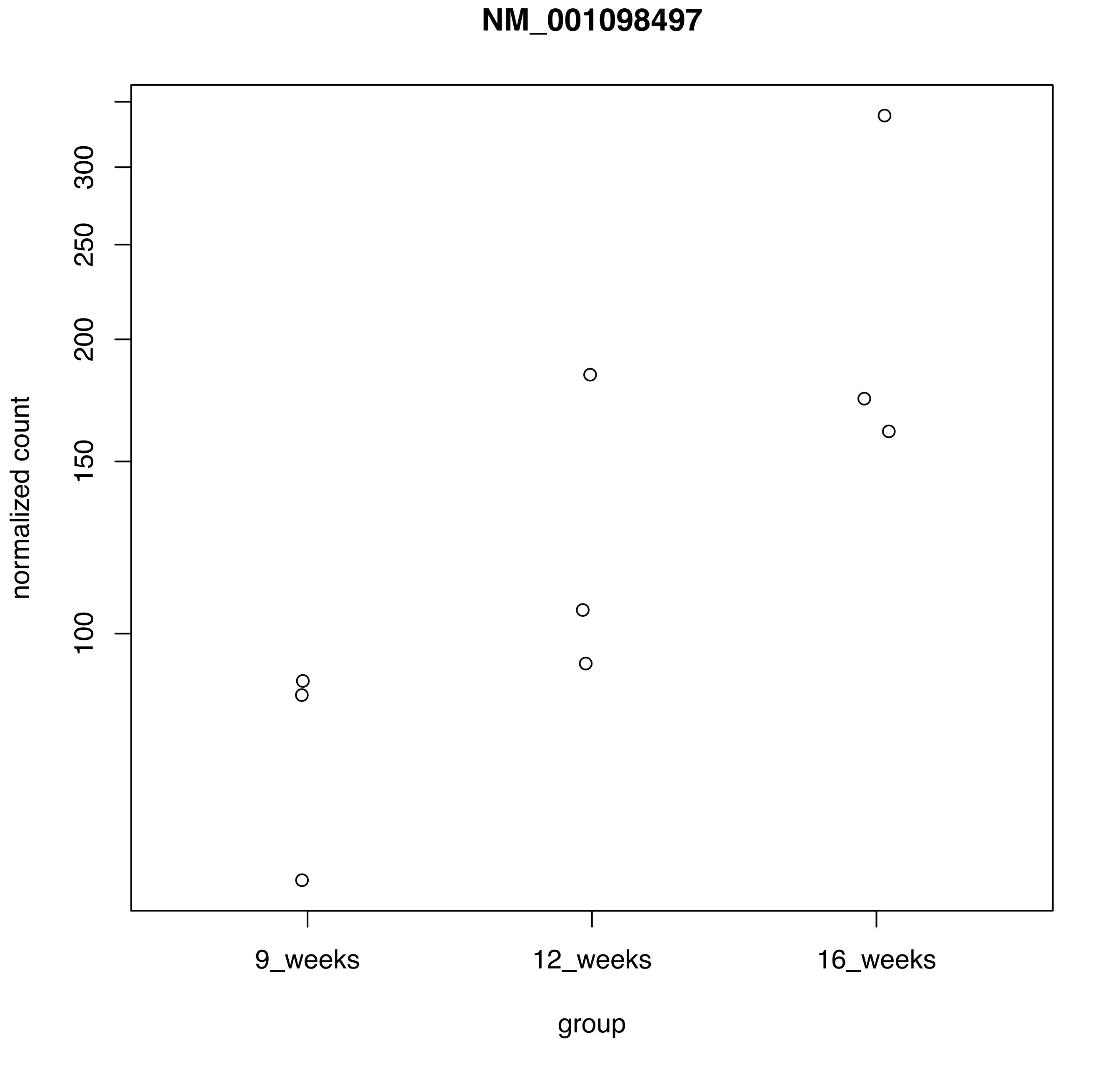
NM_001098497
normalized count
300
250
200
150
100
9_weeks
12_weeks
16_weeks
group

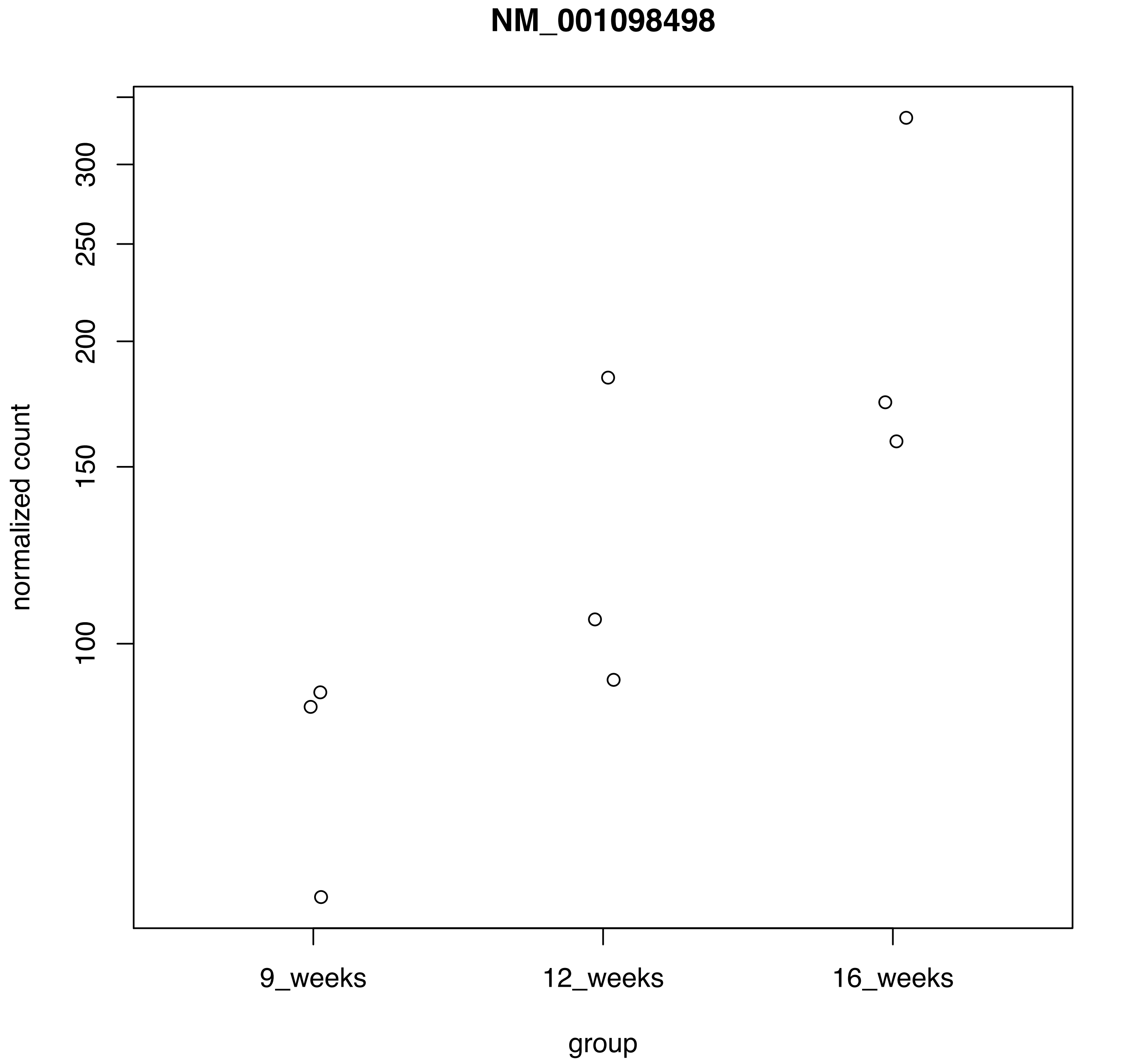
NM_001098498
normalized count
100
150
200
250
300
9_weeks
12_weeks
16_weeks
group

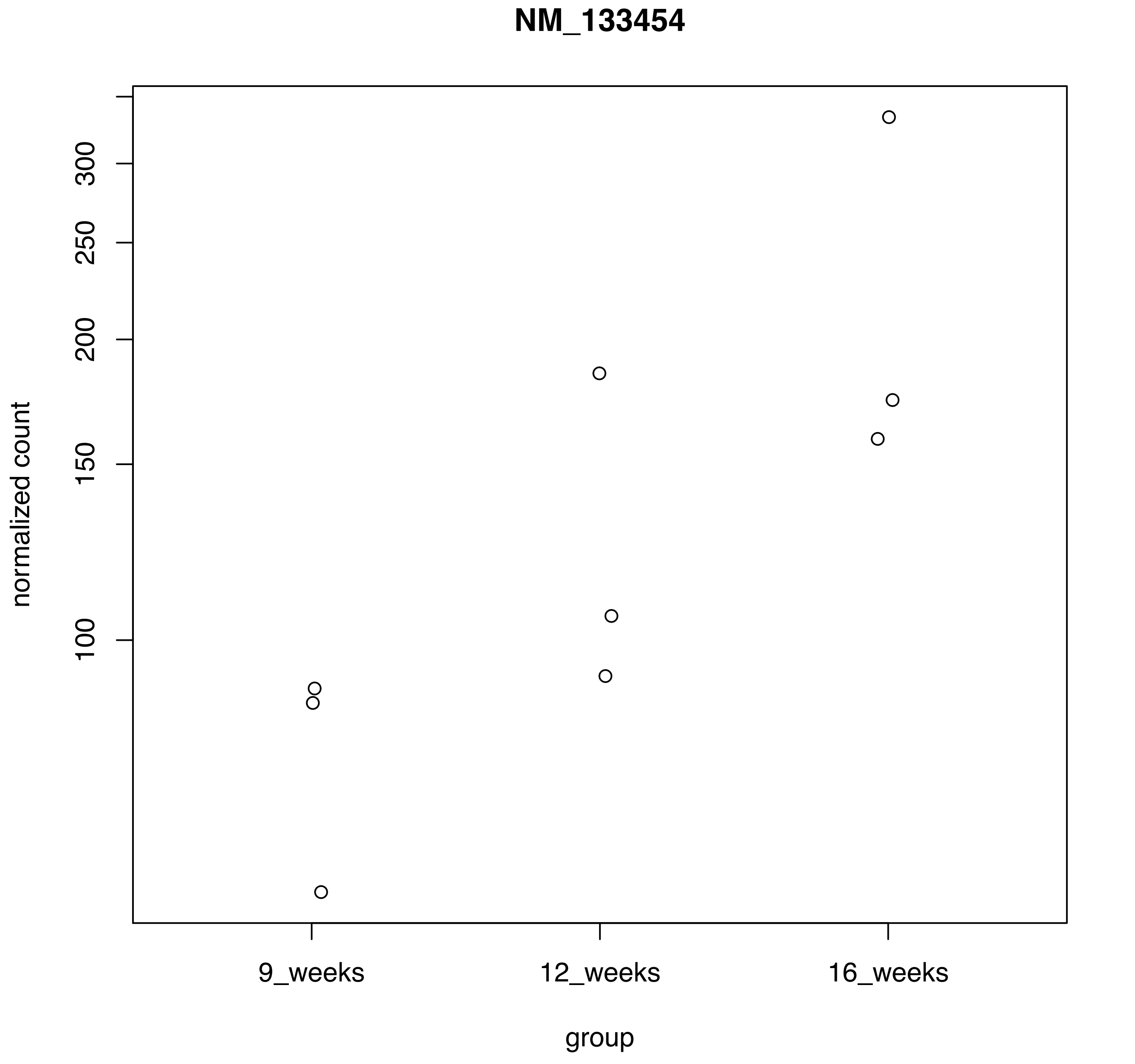
NM_133454
normalized count
100
150
200
250
300
9_weeks
12_weeks
16_weeks
group

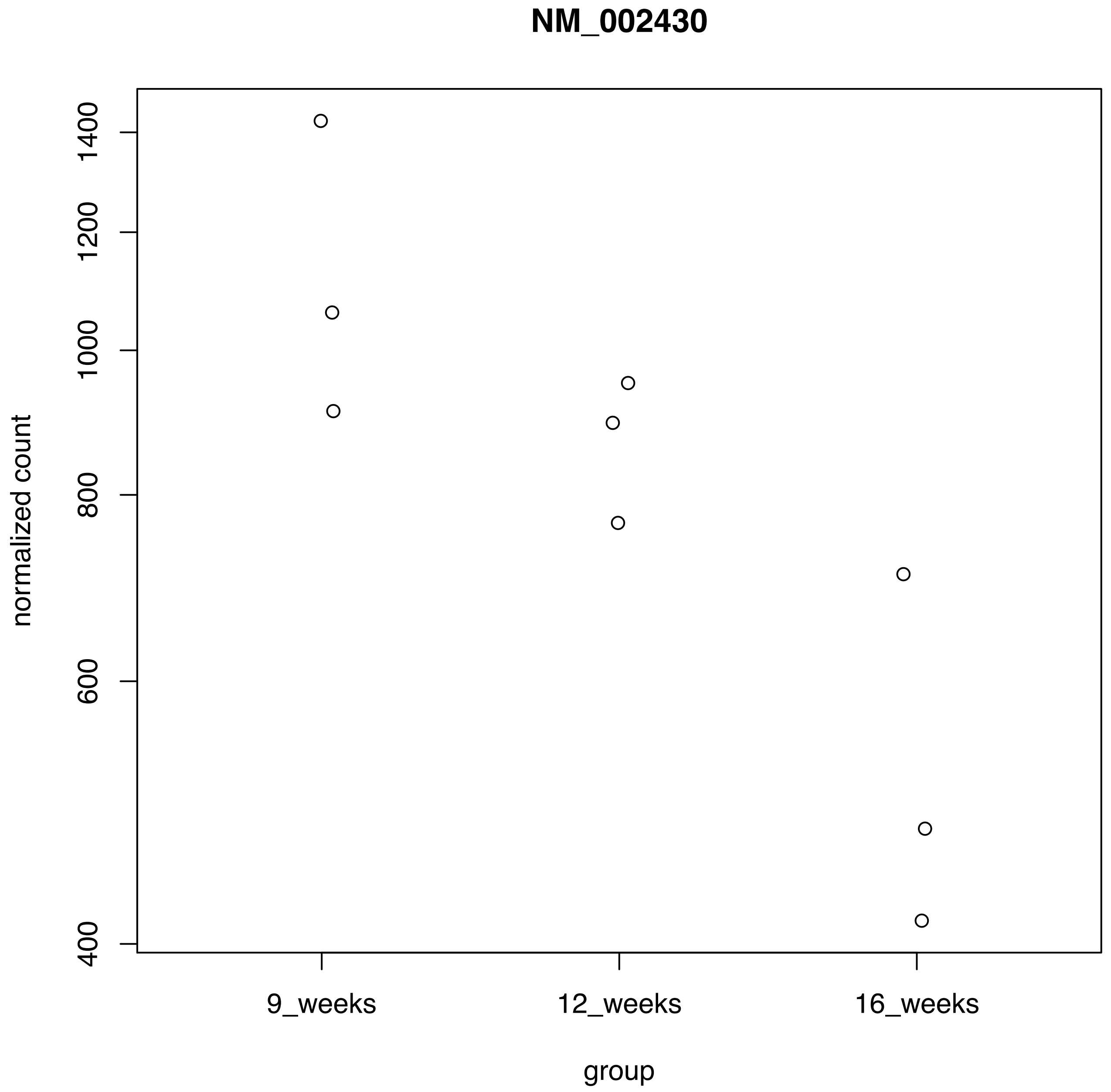
NM_002430
normalized count
400
600
800
1000
1200
1400
9_weeks
12_weeks
16_weeks
group

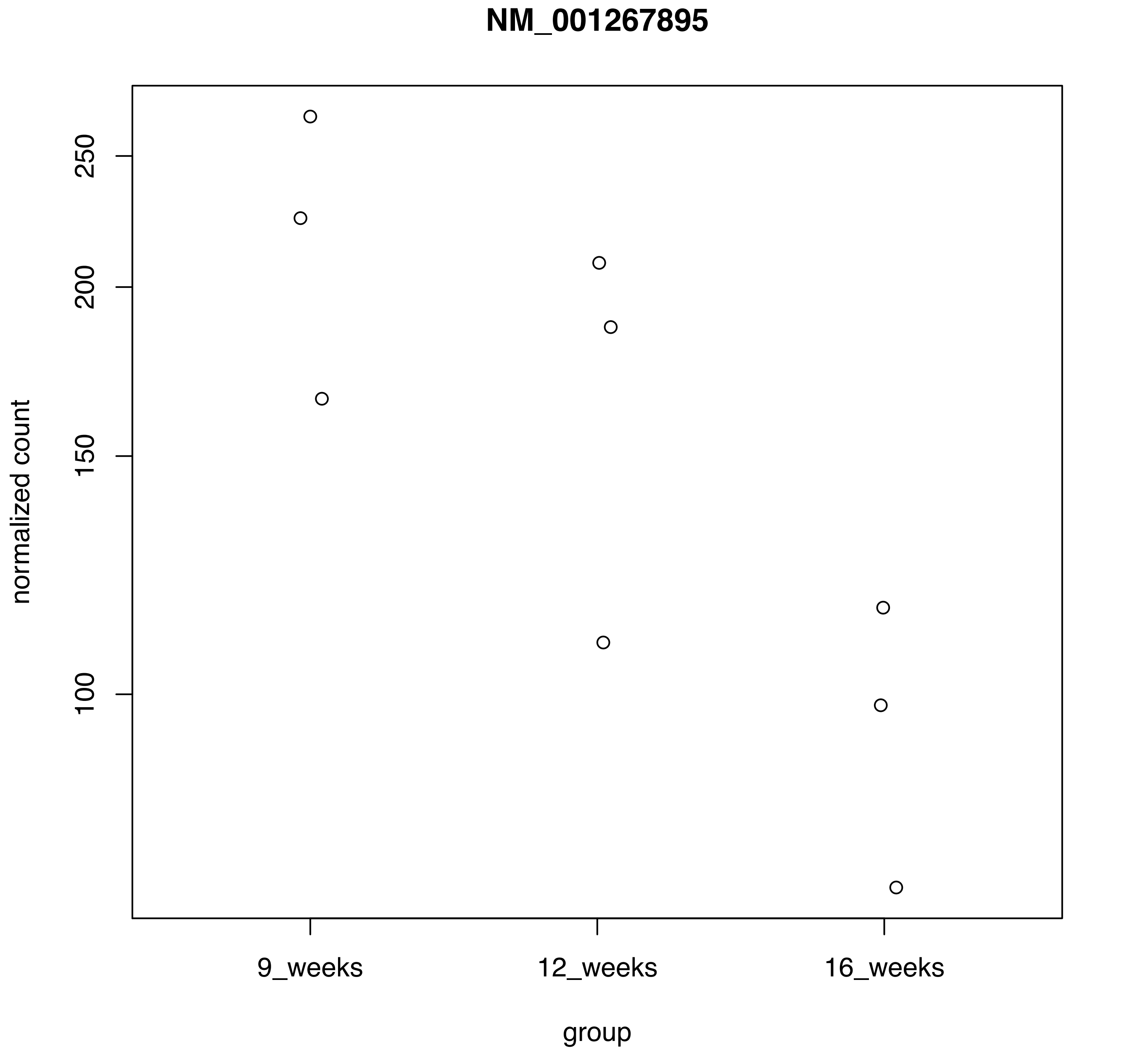
NM_001267895
normalized count
250
200
150
100
9_weeks
12_weeks
16_weeks
group

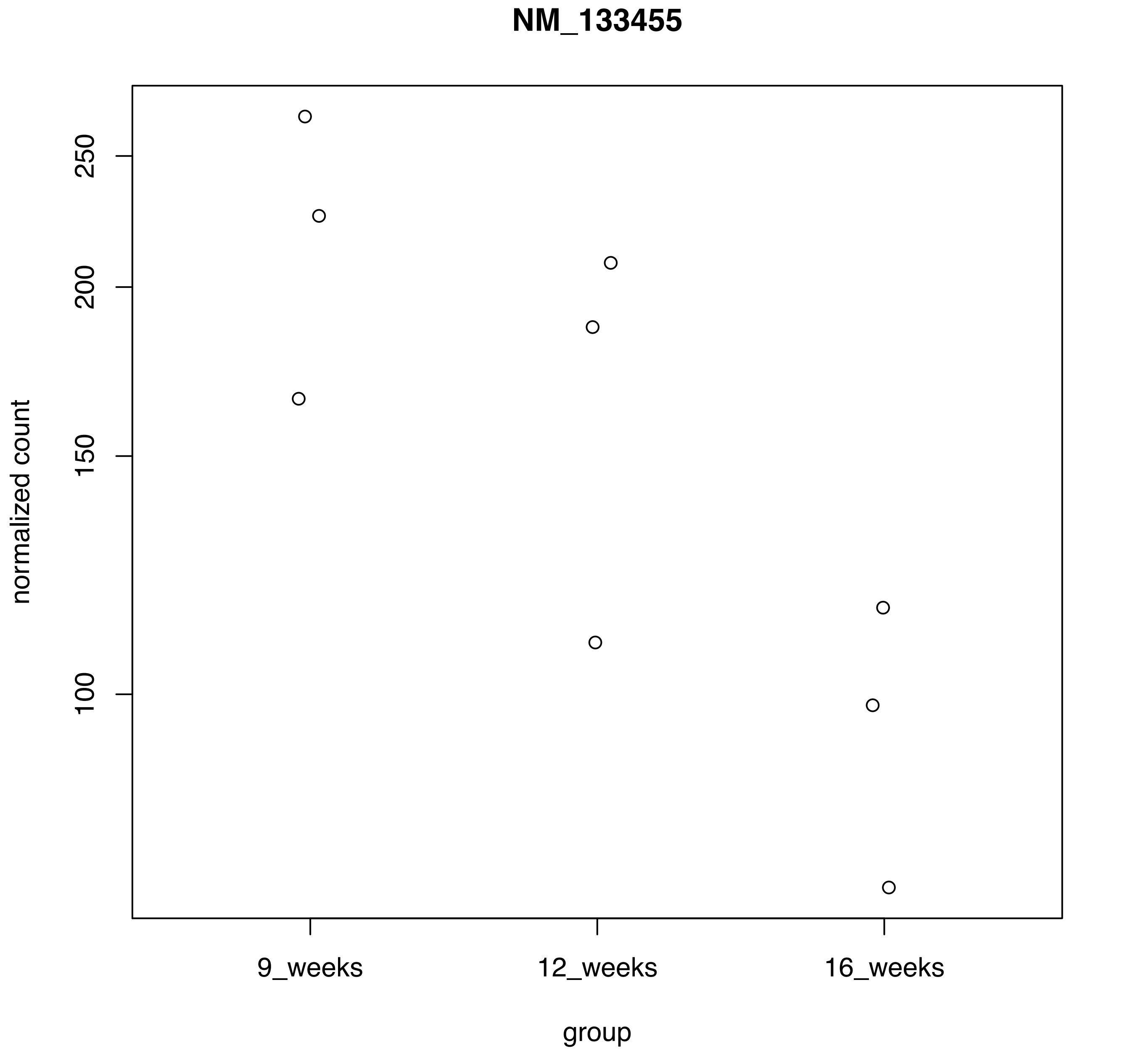
NM_133455
normalized count
250
200
150
100
9_weeks
12_weeks
16_weeks
group

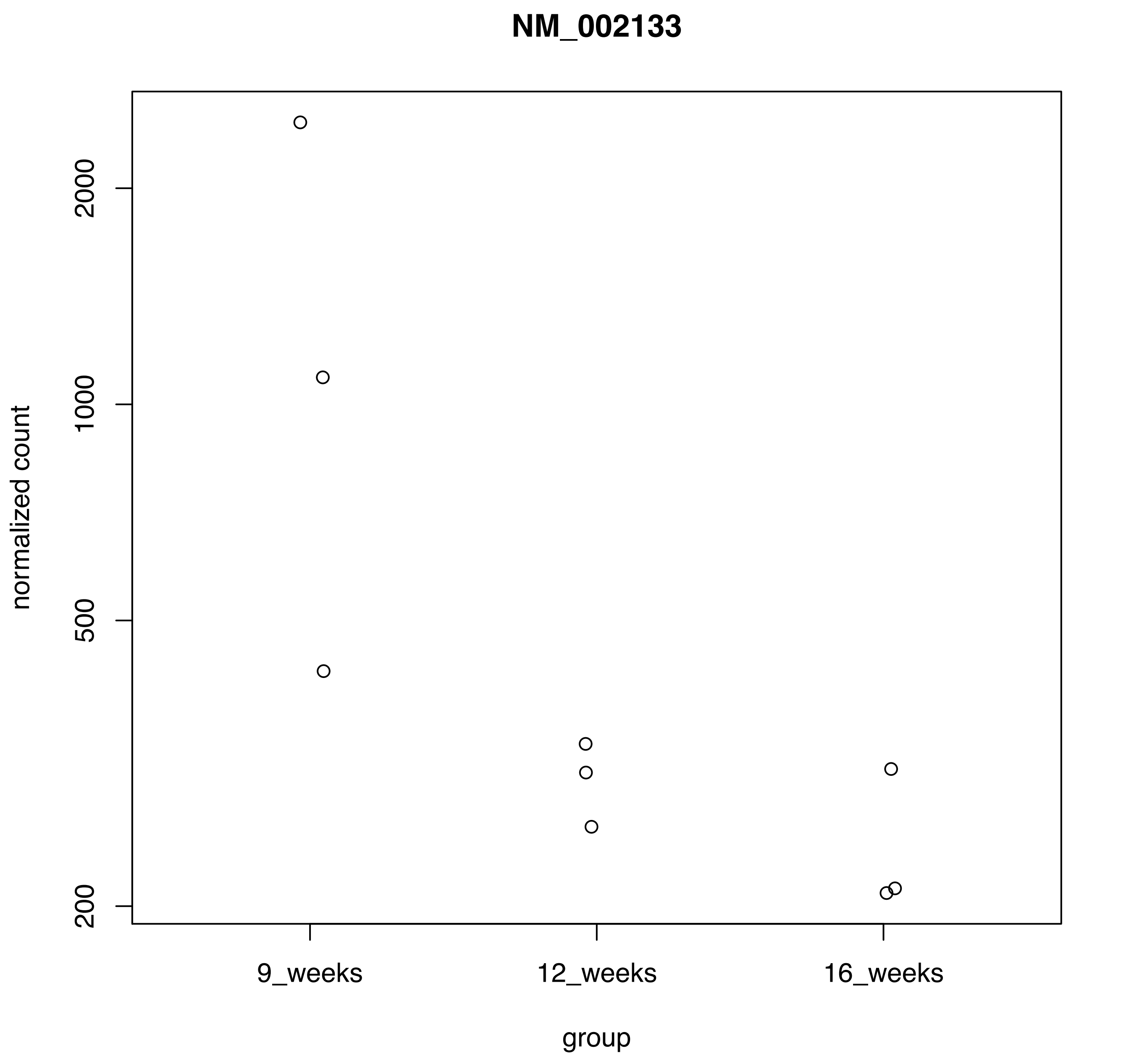
NM_002133
normalized count
2000
1000
500
200
9_weeks
12_weeks
16_weeks
group

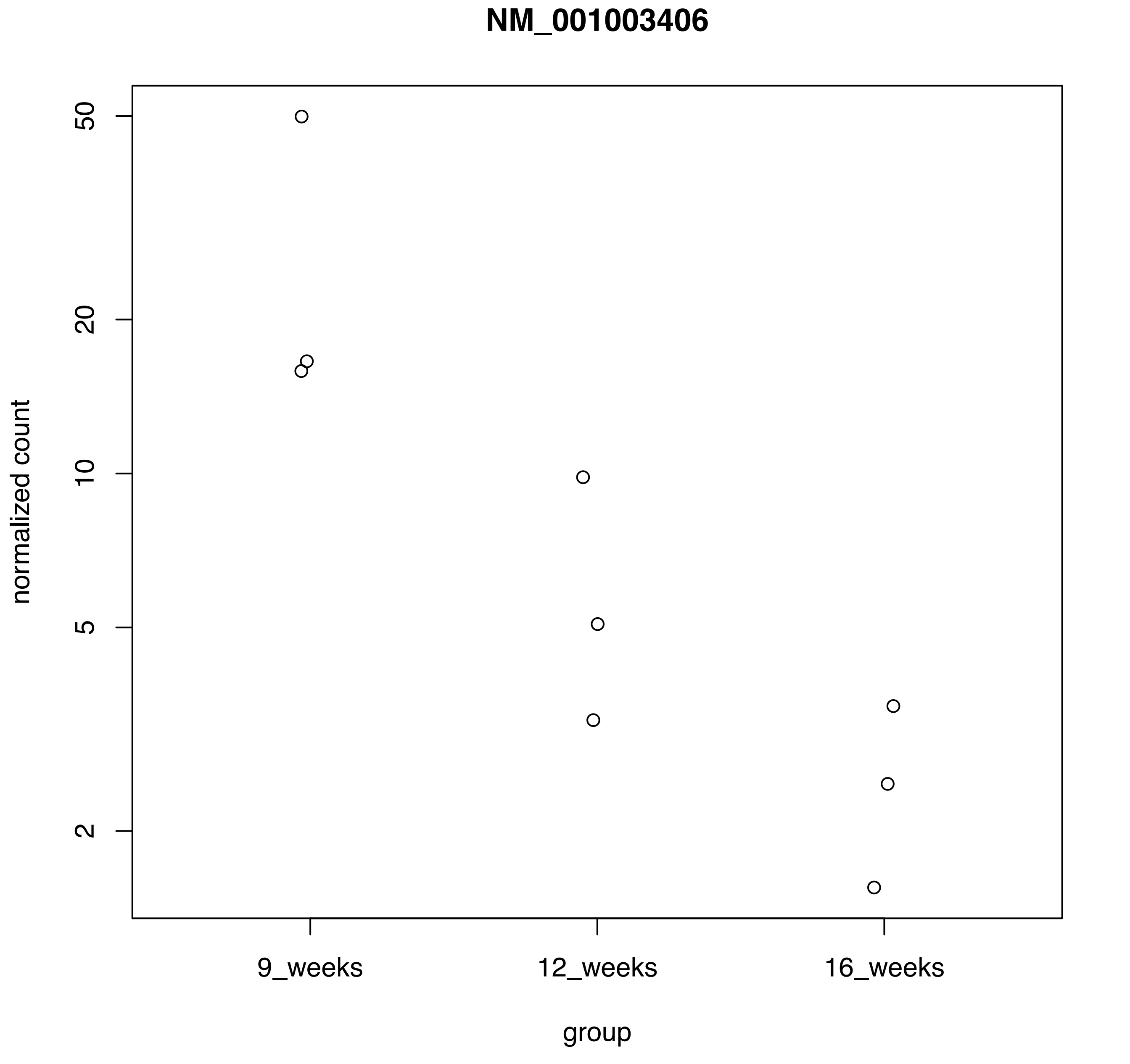
NM_001003406
normalized count
50
20
10
5
2
9_weeks
12_weeks
16_weeks
group

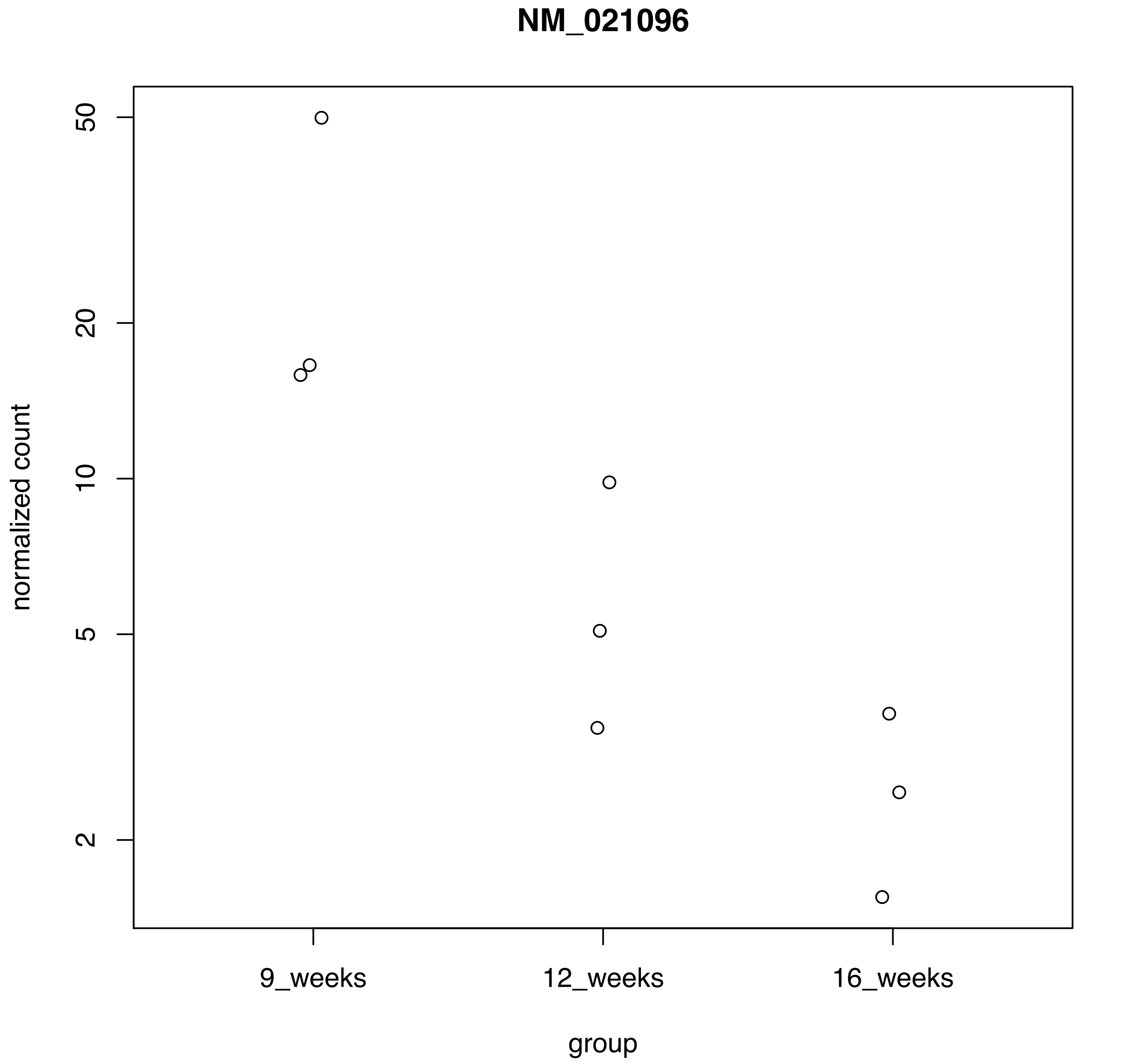
NM_021096
normalized count
50
20
10
5
2
9_weeks
12_weeks
16_weeks
group

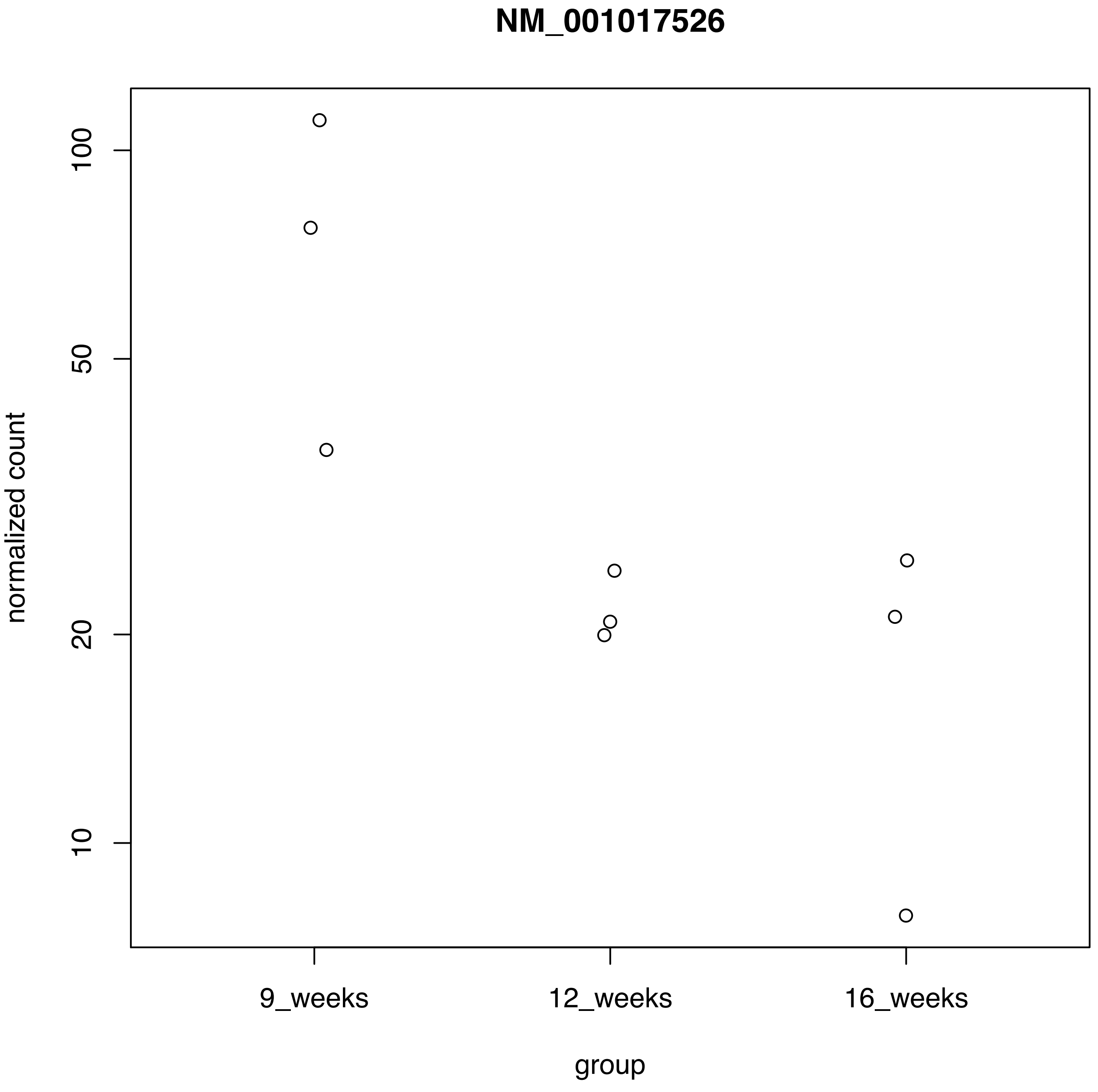
NM_001017526
normalized count
100
50
20
10
9_weeks
12_weeks
16_weeks
group

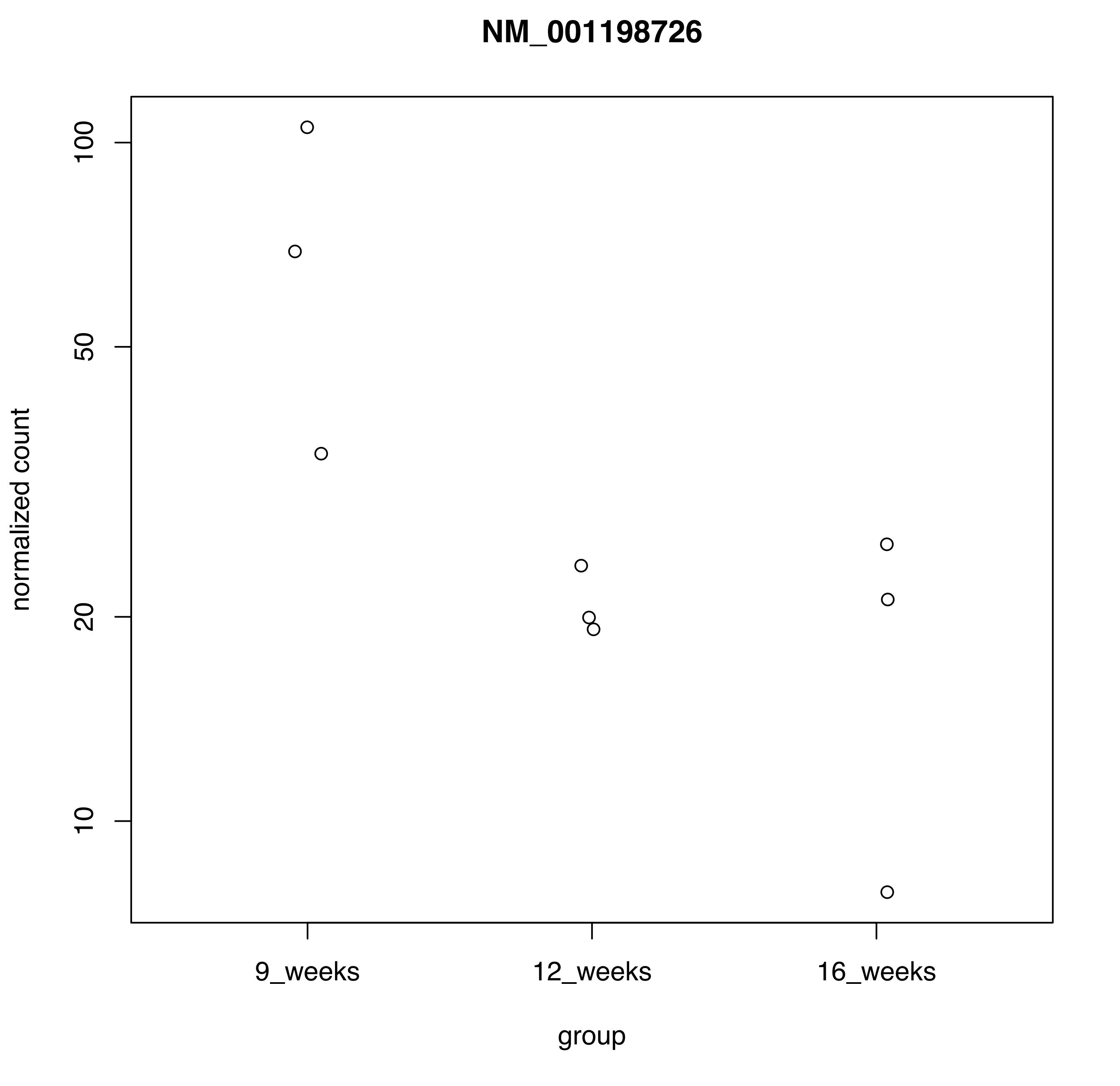
NM_001198726
normalized count
100
50
20
10
9_weeks
12_weeks
16_weeks
group

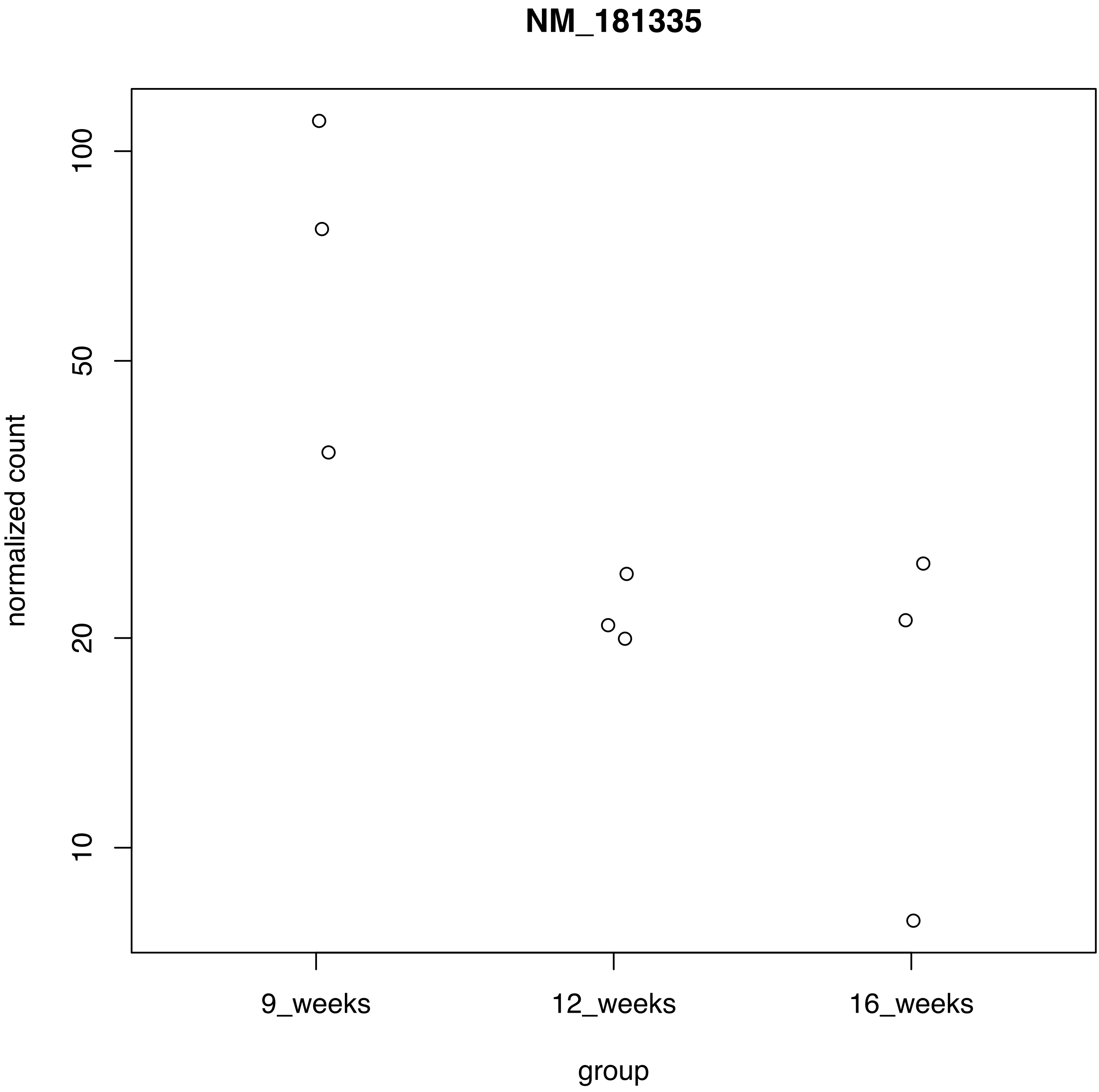

NM_181335
normalized count
100
50
20
10
9_weeks
12_weeks
16_weeks
group

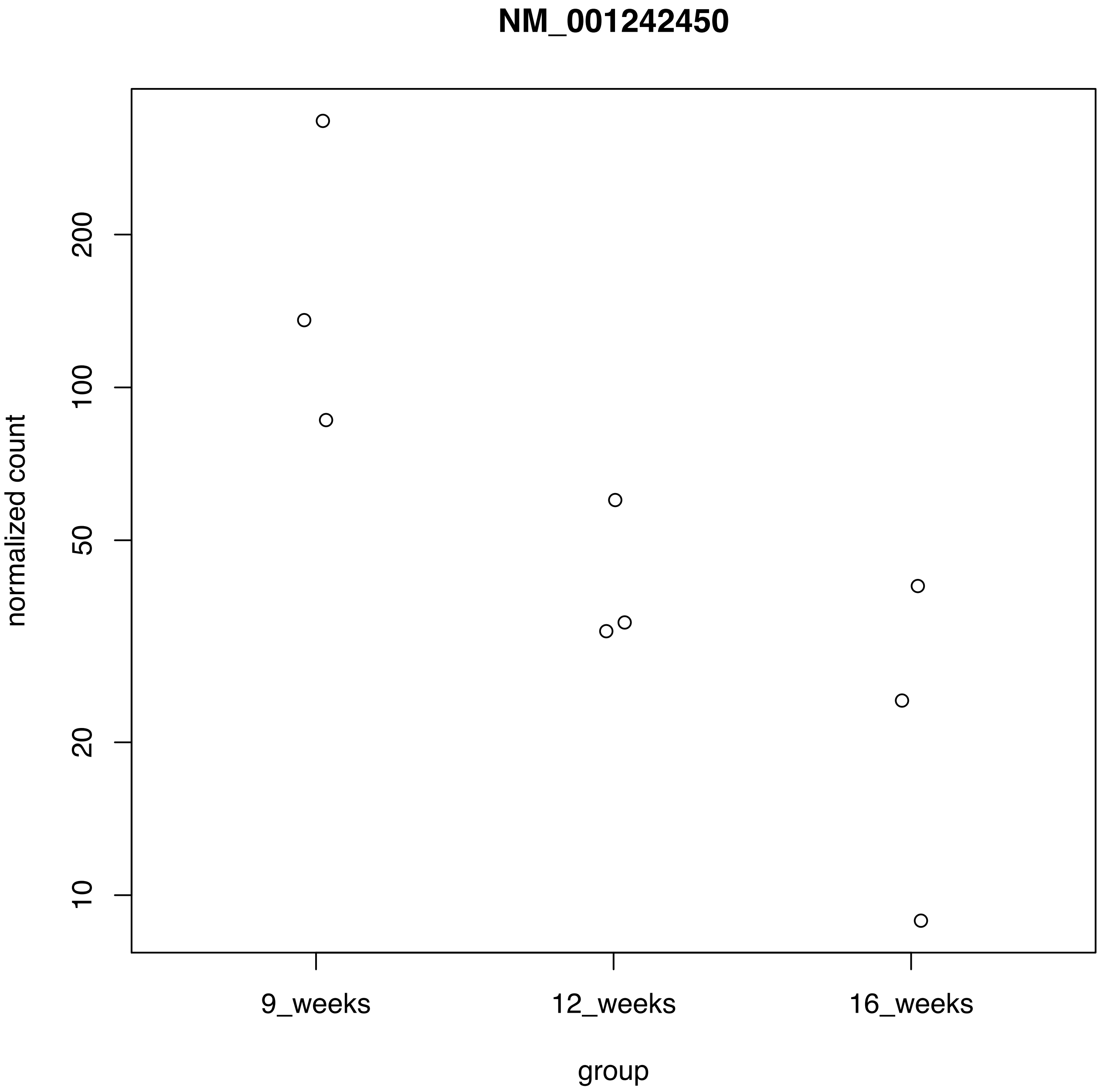
NM_001242450
normalized count
200
100
50
20
10
9_weeks
12_weeks
16_weeks
group

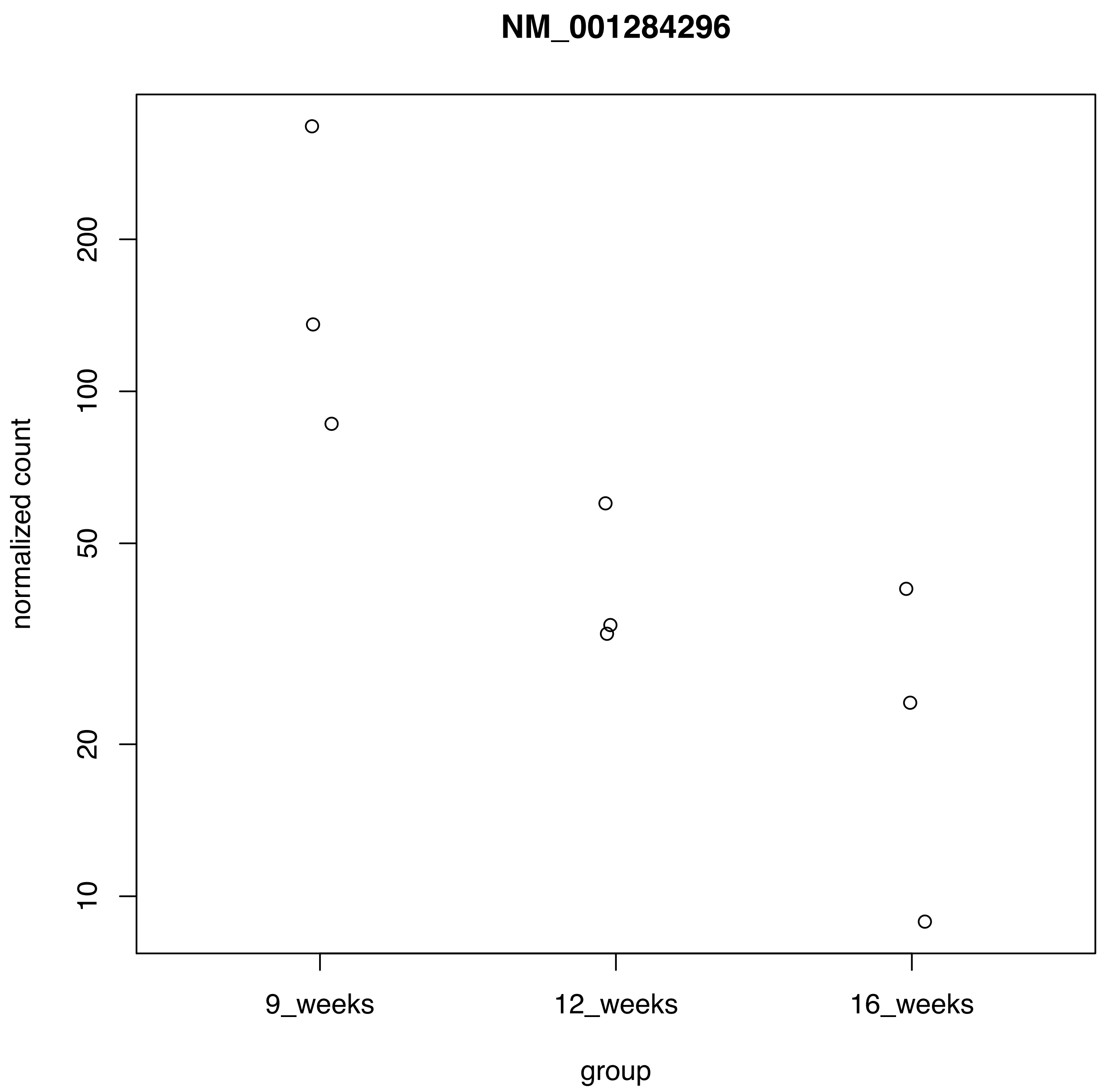

NM_001284296
normalized count
200
100
50
20
10
9_weeks
12_weeks
16_weeks
group

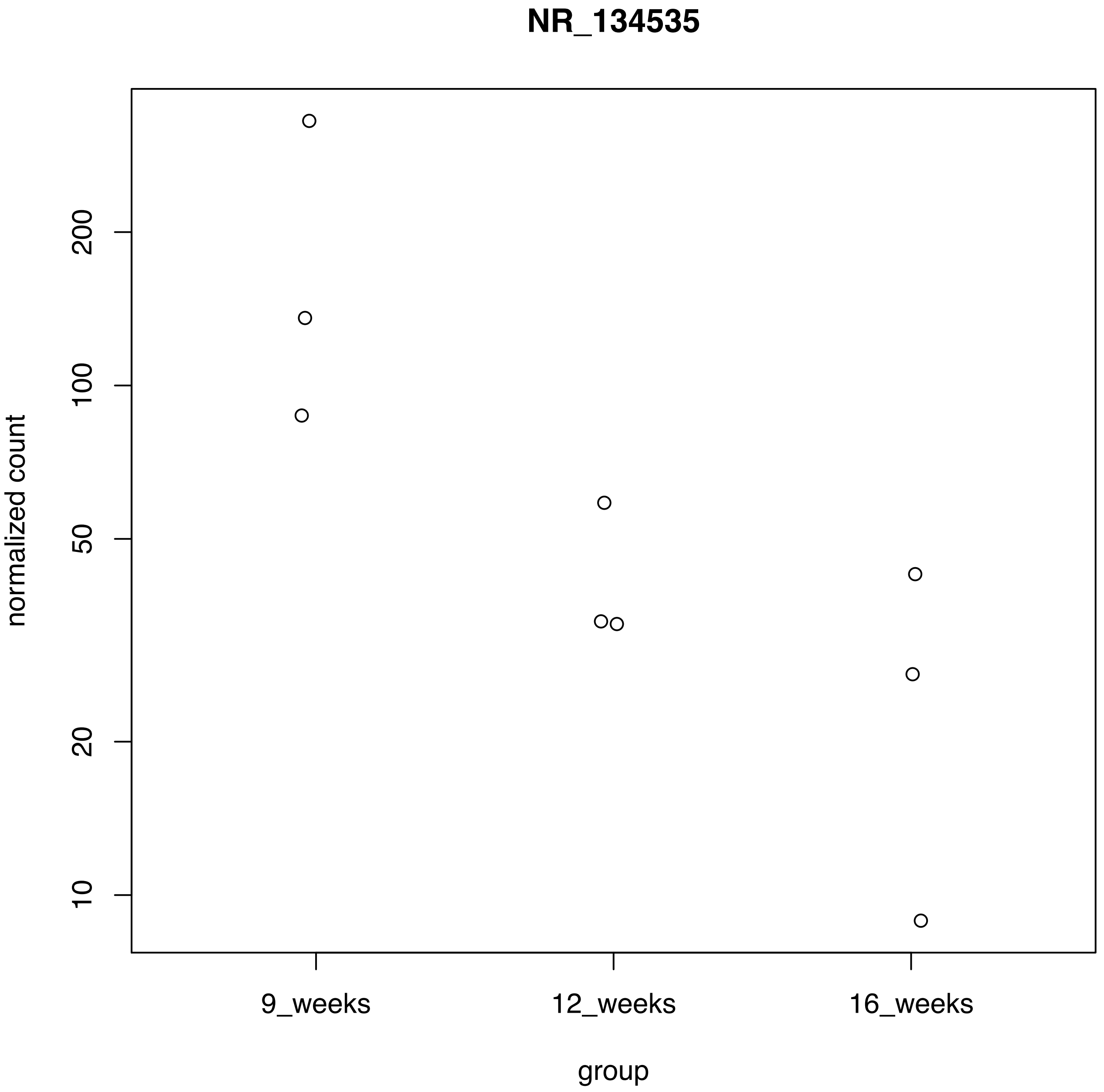
NR_134535
normalized count
200
100
50
20
10
9_weeks
12_weeks
16_weeks
group

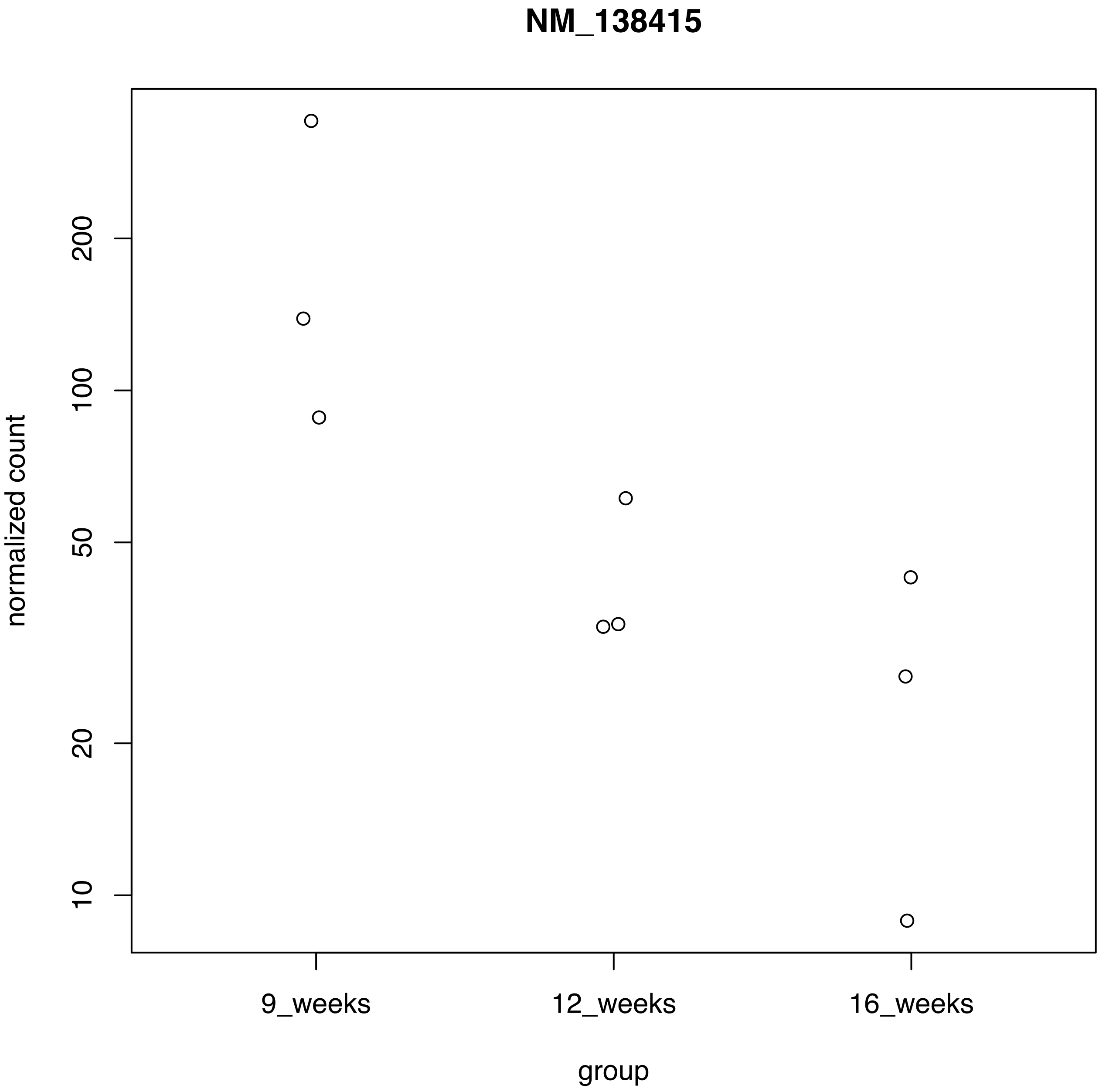

NM_138415
normalized count
200
100
50
20
10
9_weeks
12_weeks
16_weeks
group

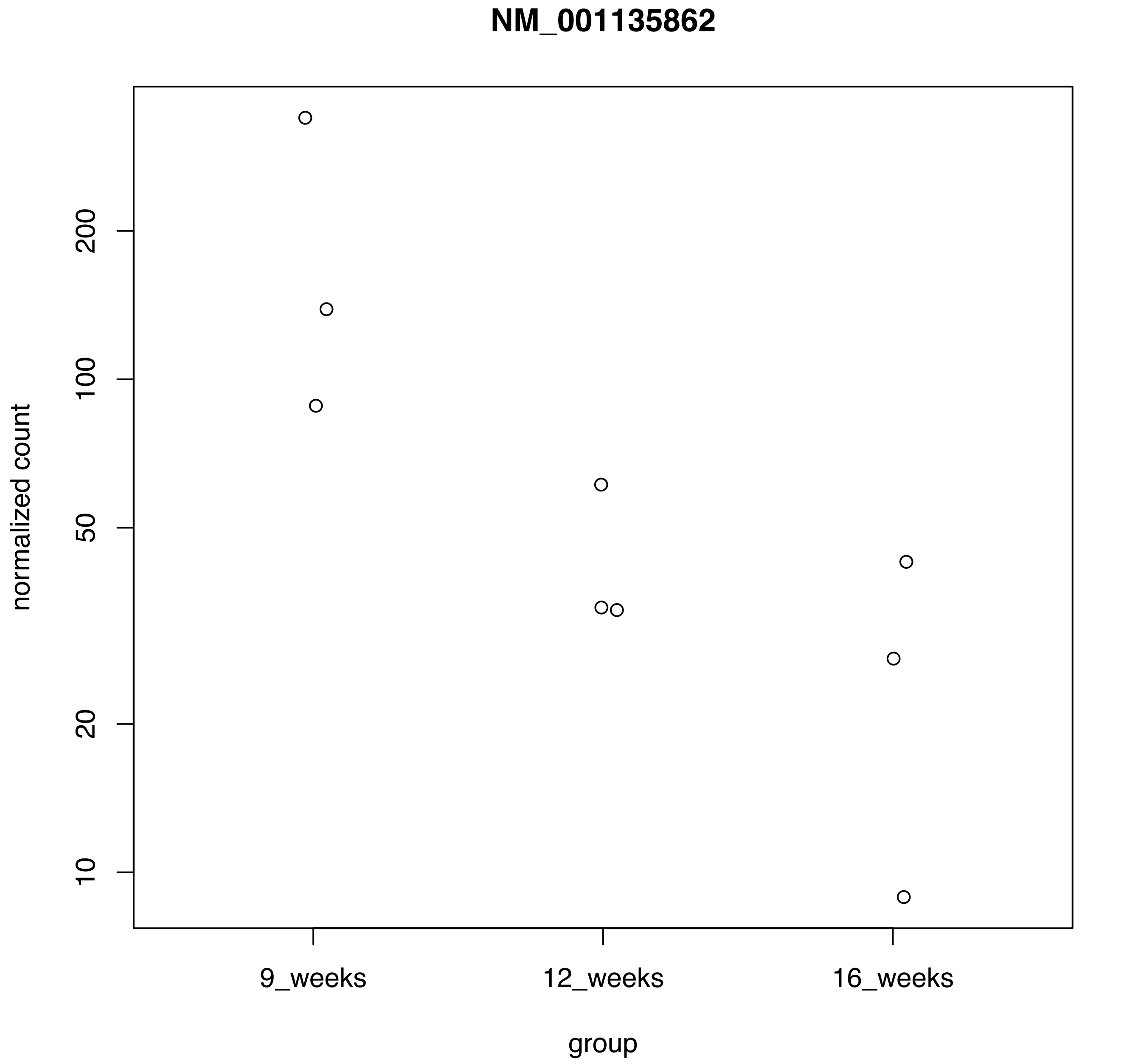
NM_001135862
normalized count
200
100
50
20
10
9_weeks
12_weeks
16_weeks
group

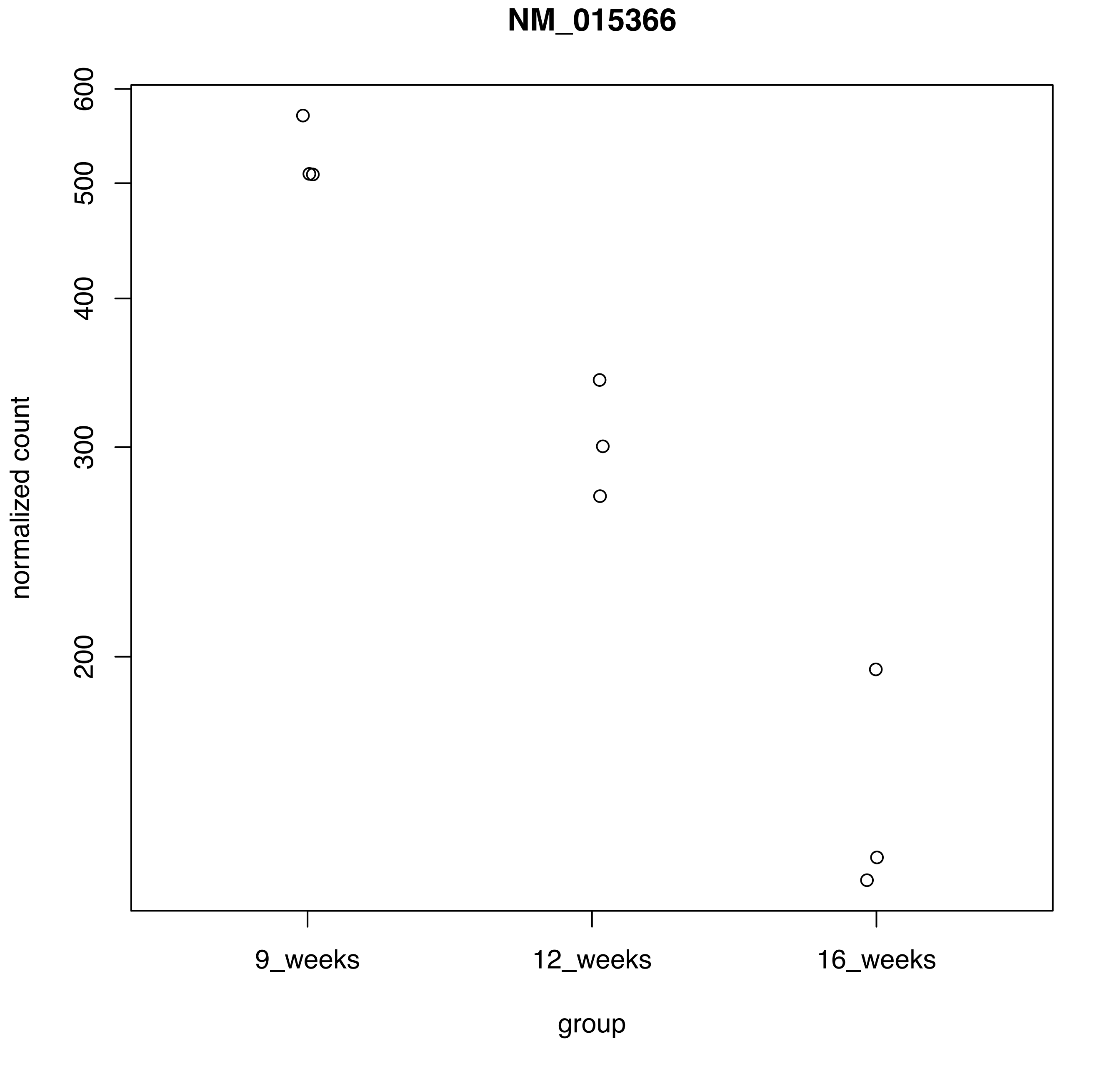
NM_015366
600
500
400
300
200
normalized count
9_weeks
12_weeks
16_weeks
group

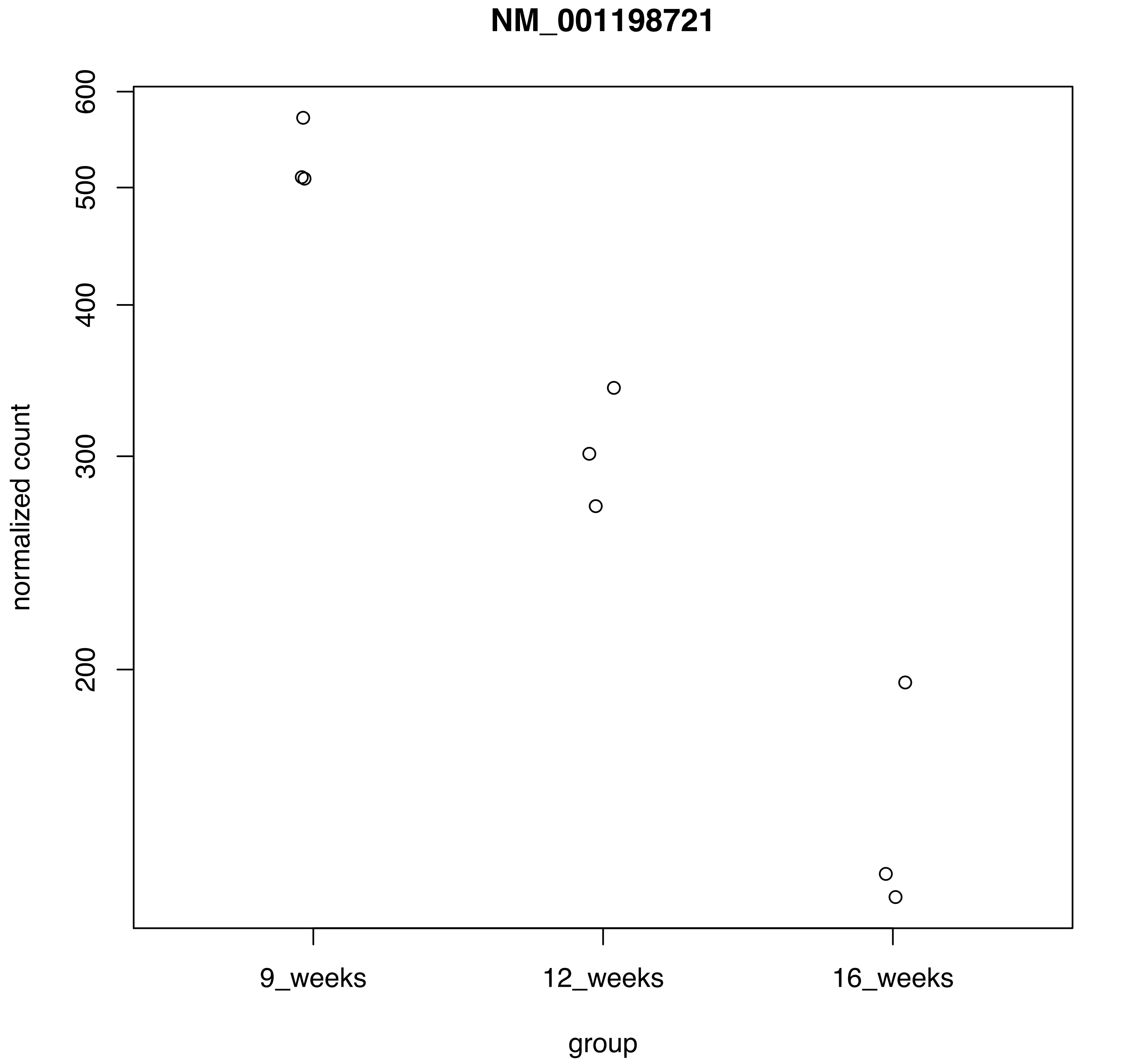
NM_001198721
600
500
400
300
200
normalized count
9_weeks
12_weeks
16_weeks
group

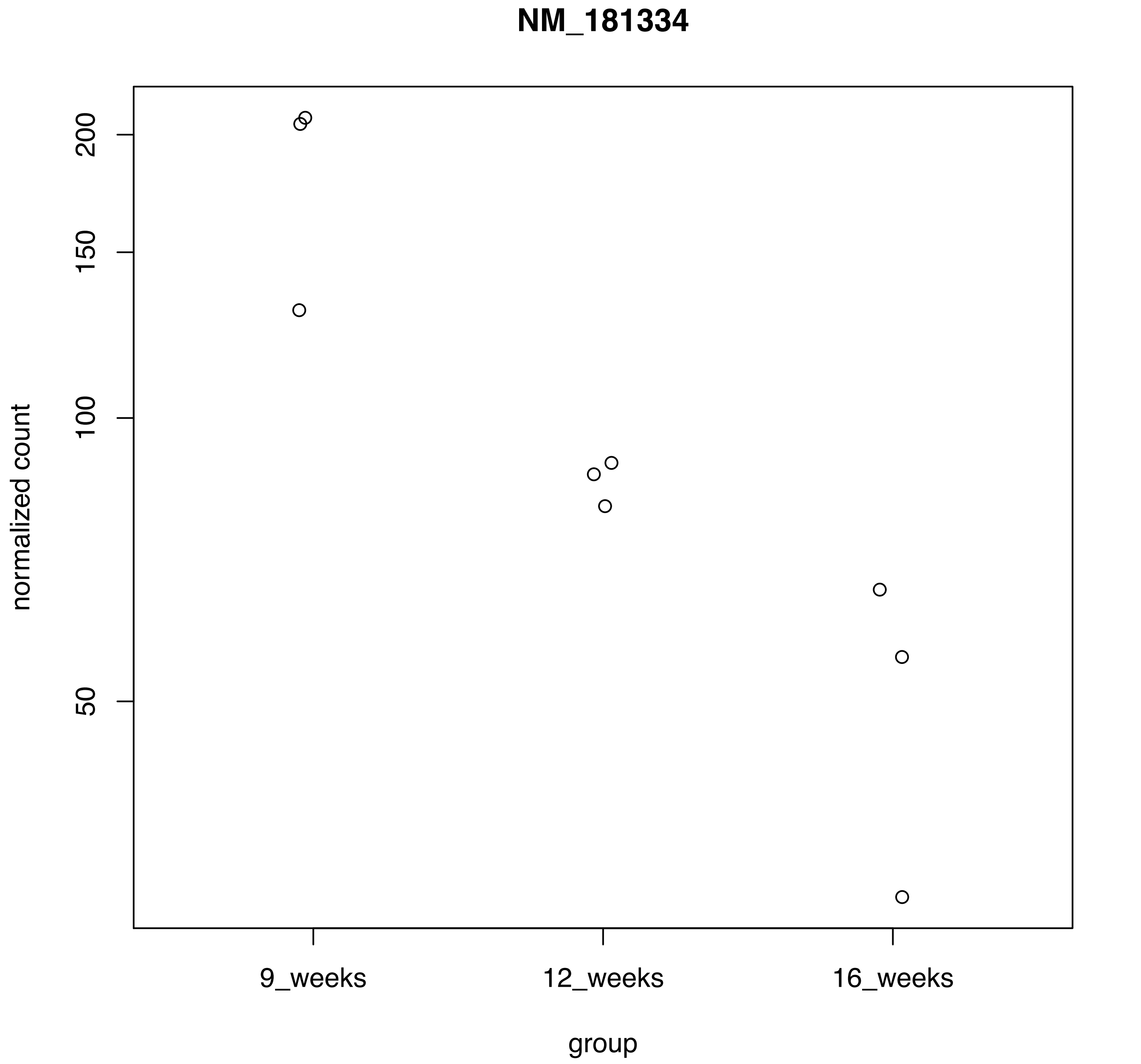
NM_181334
normalized count
200
150
100
50
9_weeks
12_weeks
16_weeks
group

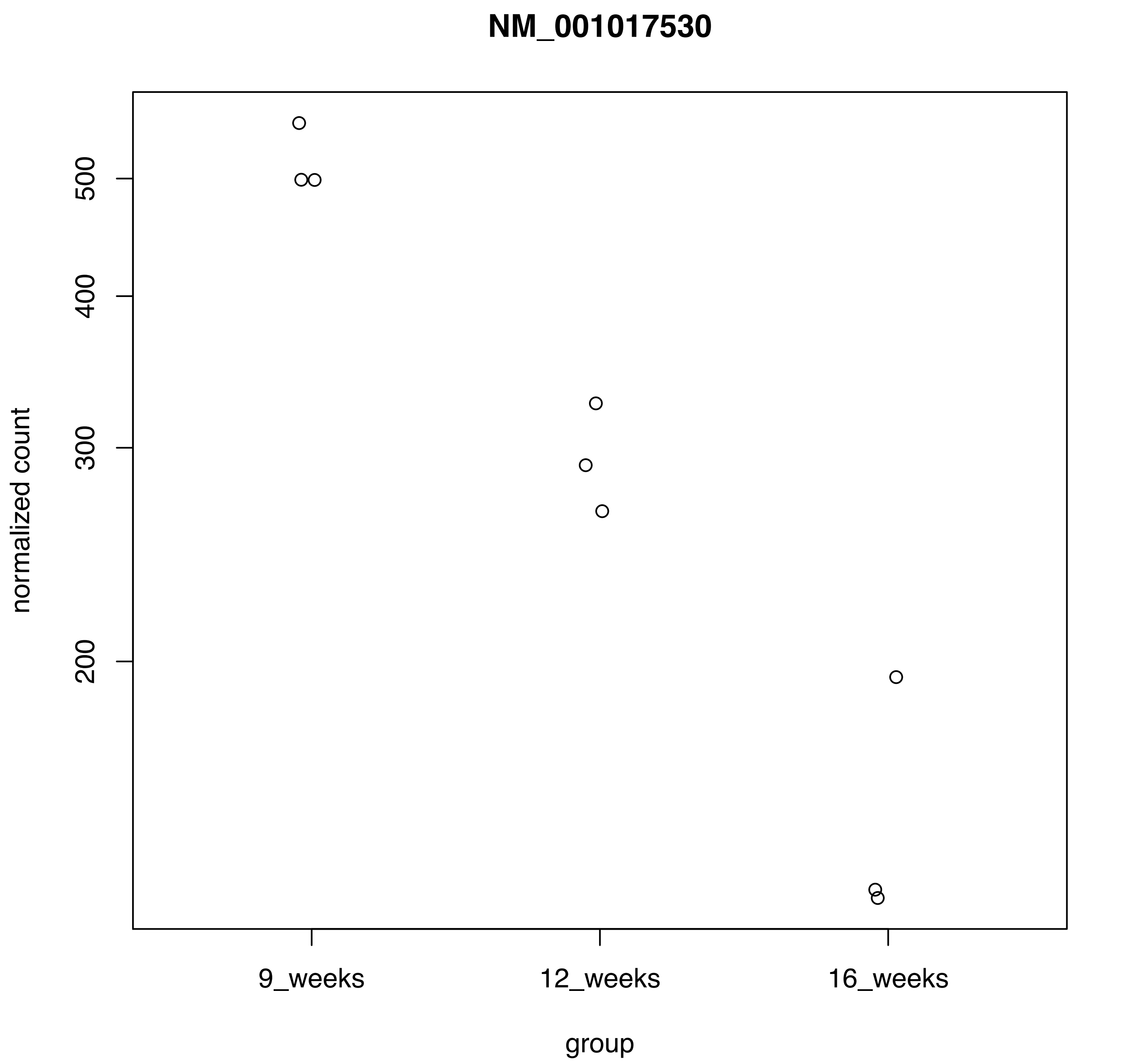
NM_001017530
normalized count
500
400
300
200
9_weeks
12_weeks
16_weeks
group

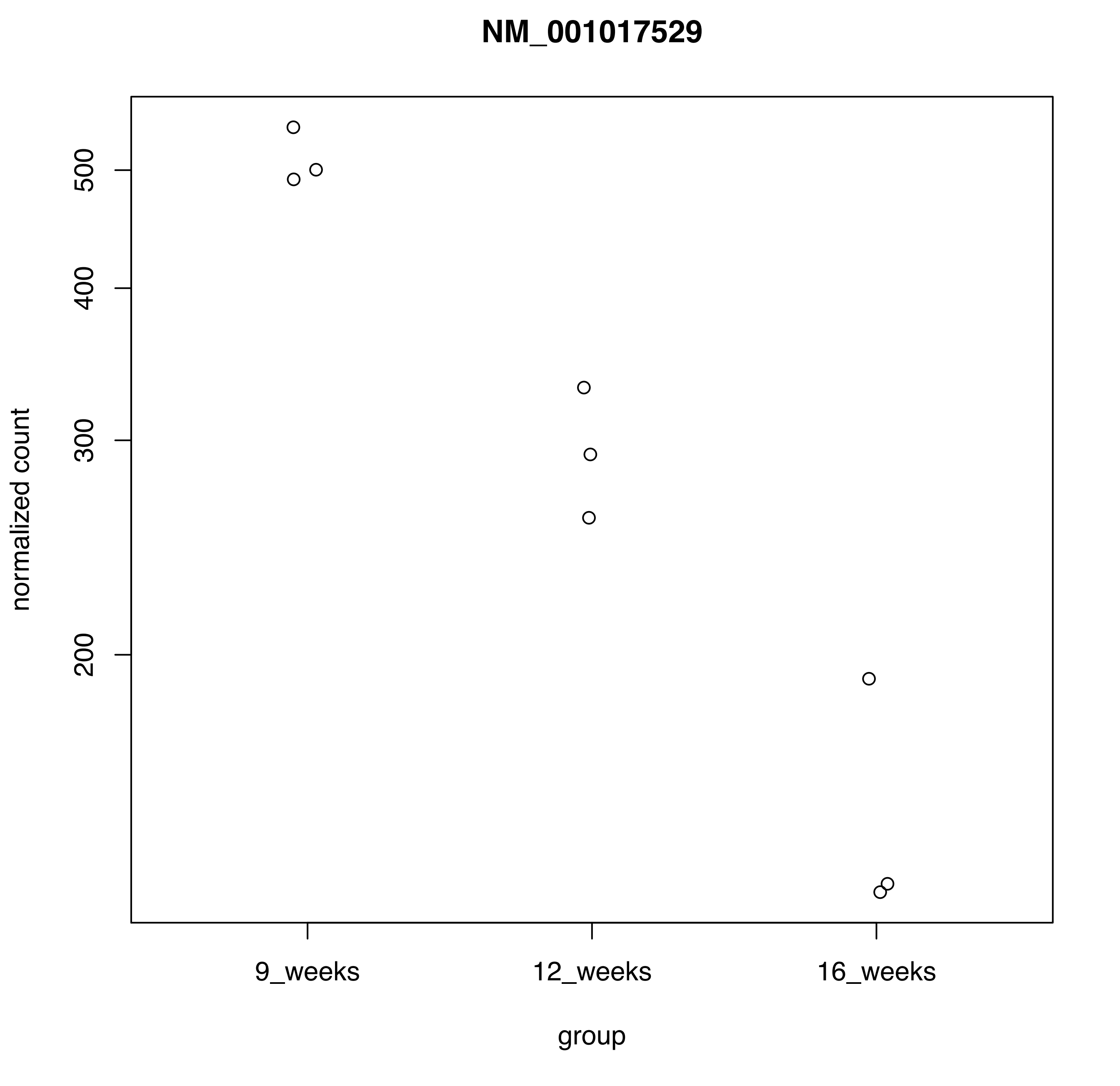
NM_001017529
normalized count
500
400
300
200
9_weeks
12_weeks
16_weeks
group

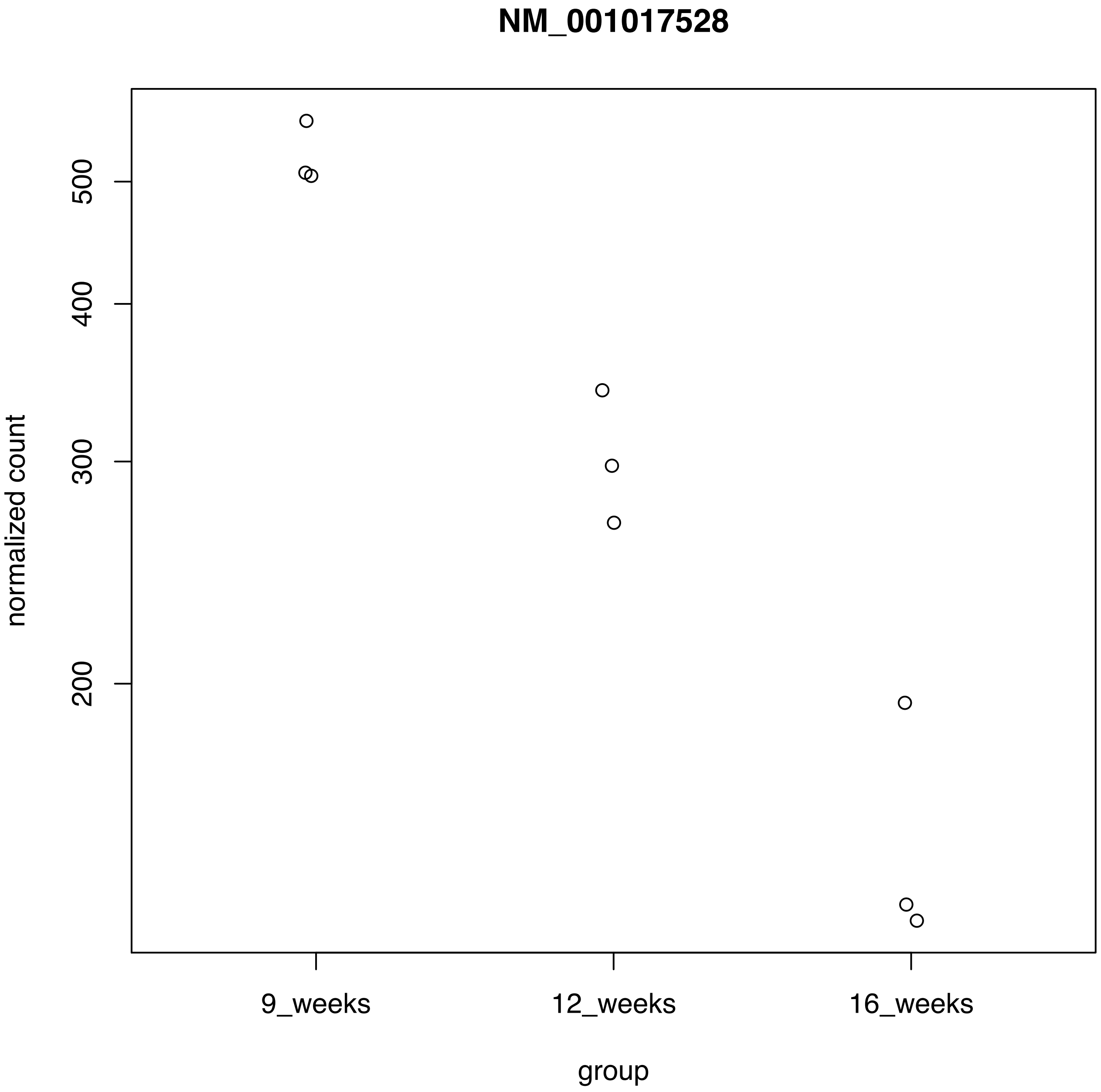
NM_001017528
normalized count
500
400
300
200
9_weeks
12_weeks
16_weeks
group

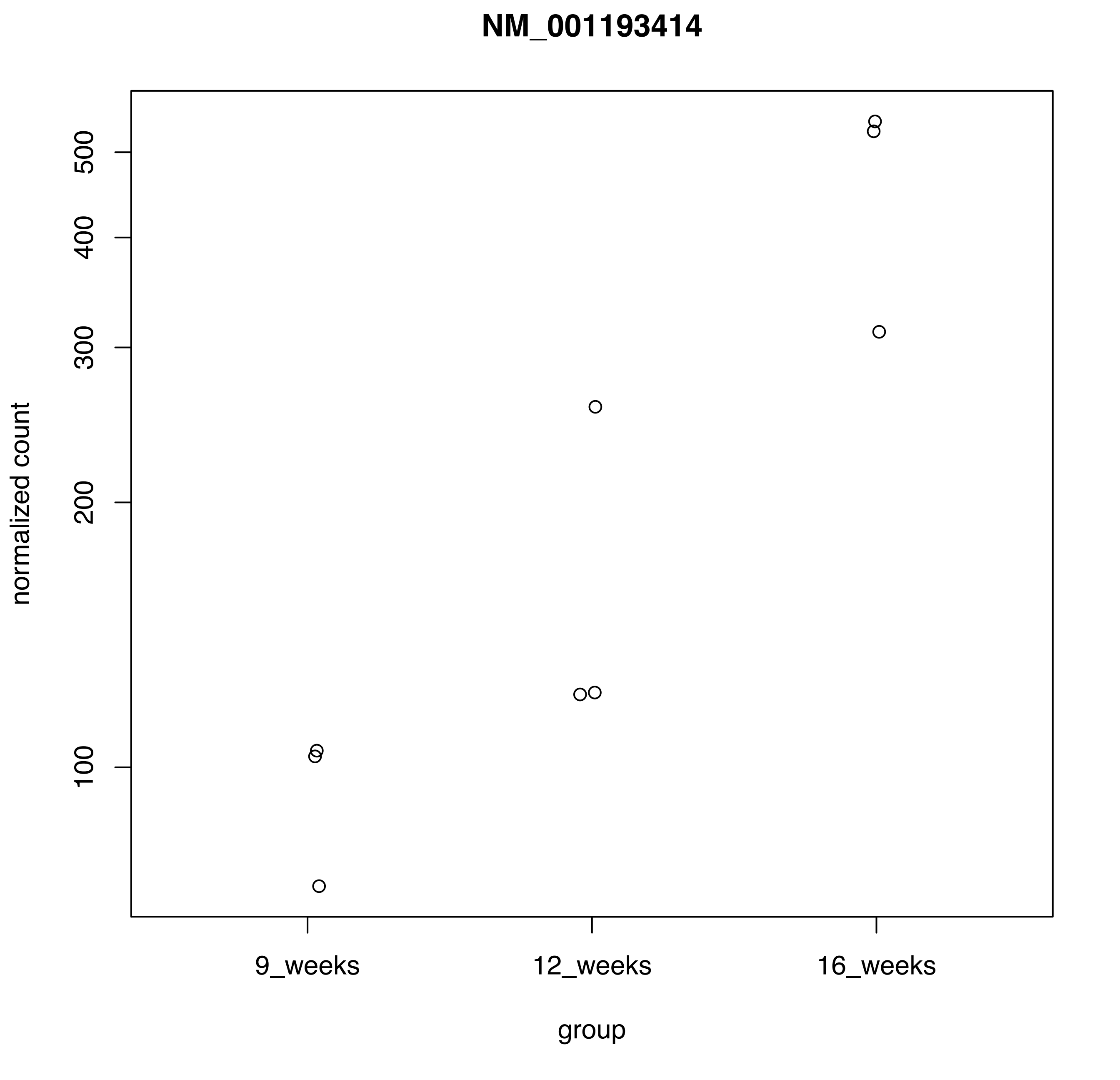
NM_001193414
normalized count
100
200
300
400
500
9_weeks
12_weeks
16_weeks
group

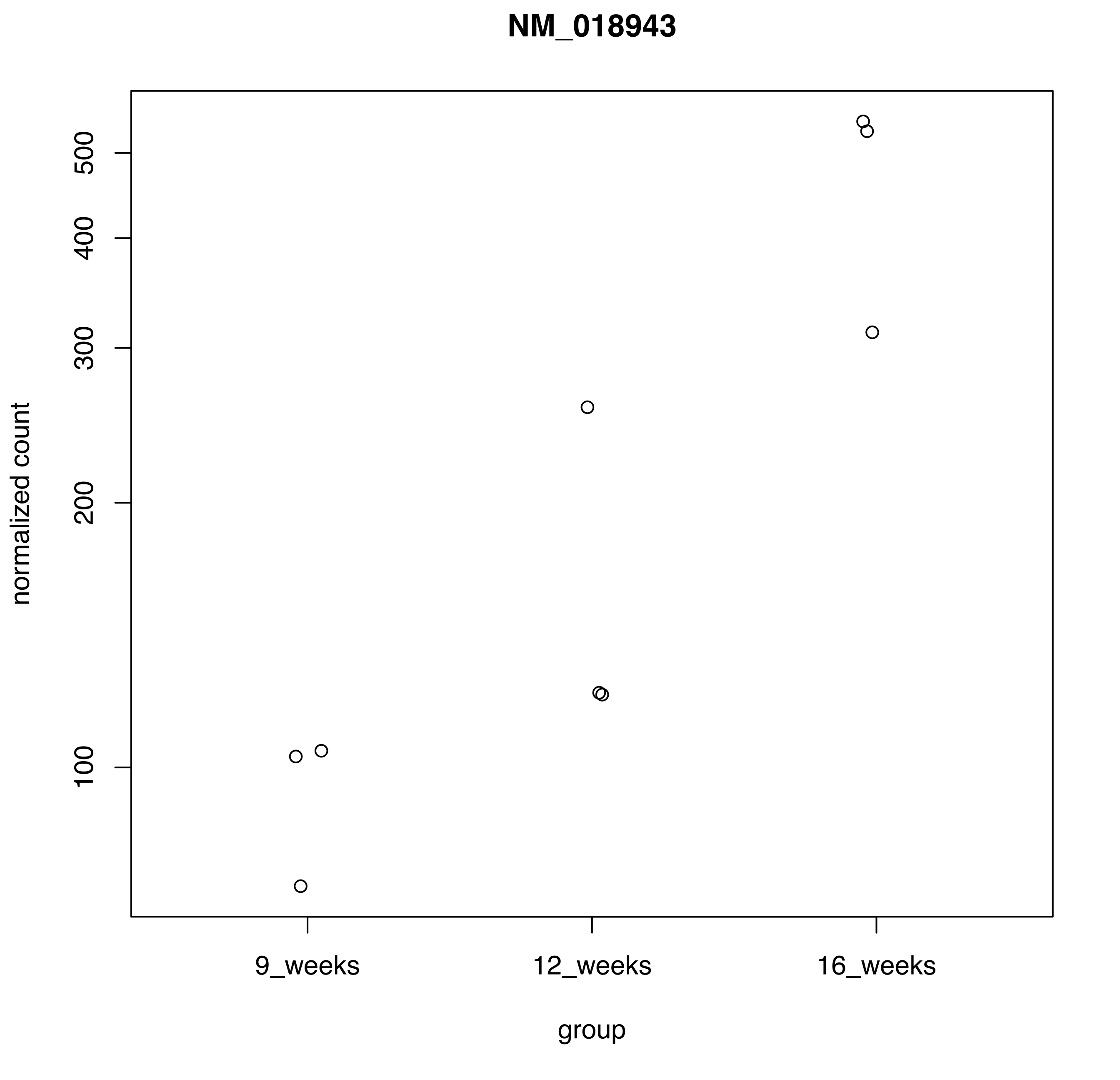
NM_018943
normalized count
500
400
300
200
100
9_weeks
12_weeks
16_weeks
group

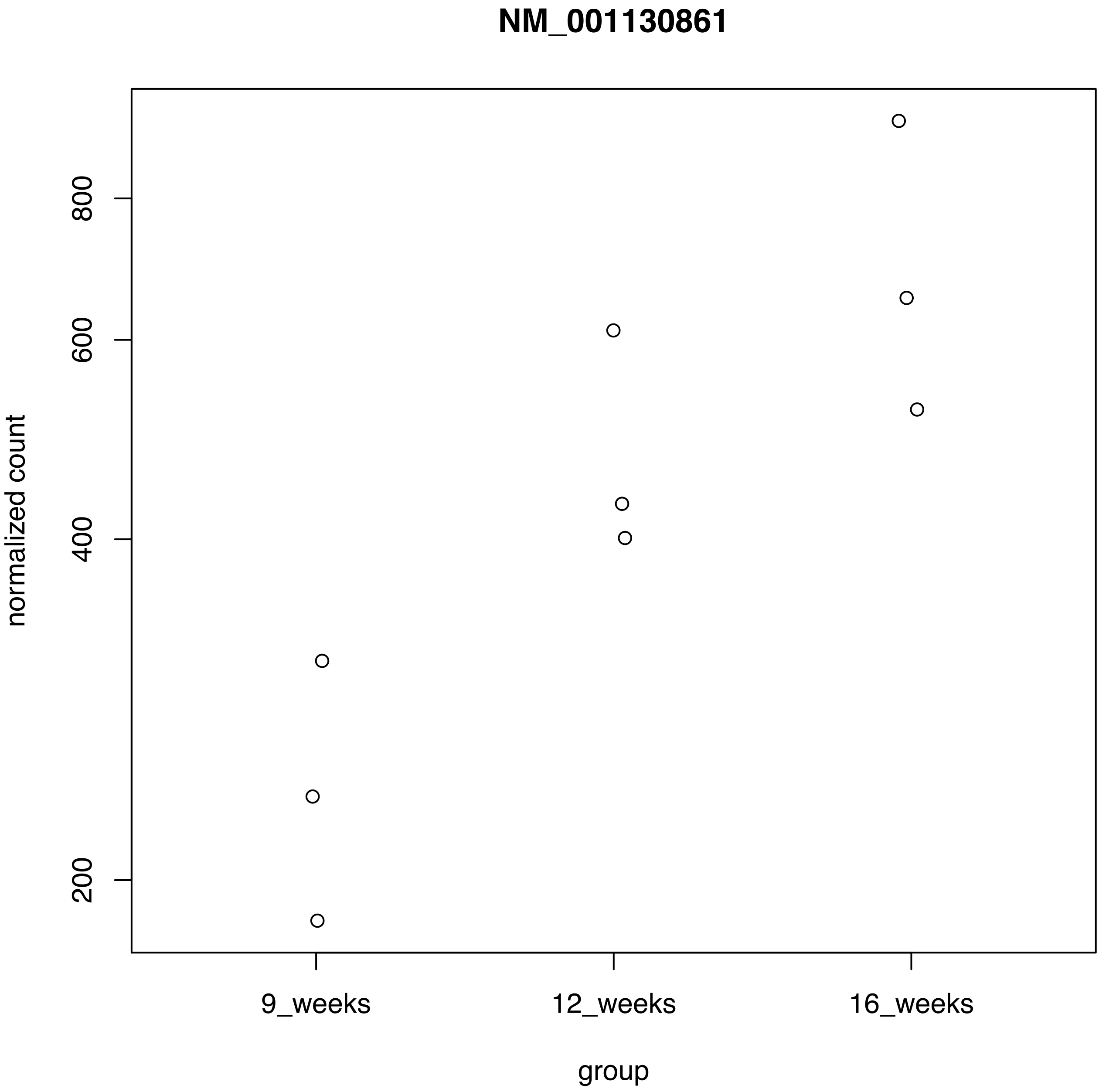
NM_001130861
normalized count
200
400
600
800
group
9_weeks
12_weeks
16_weeks

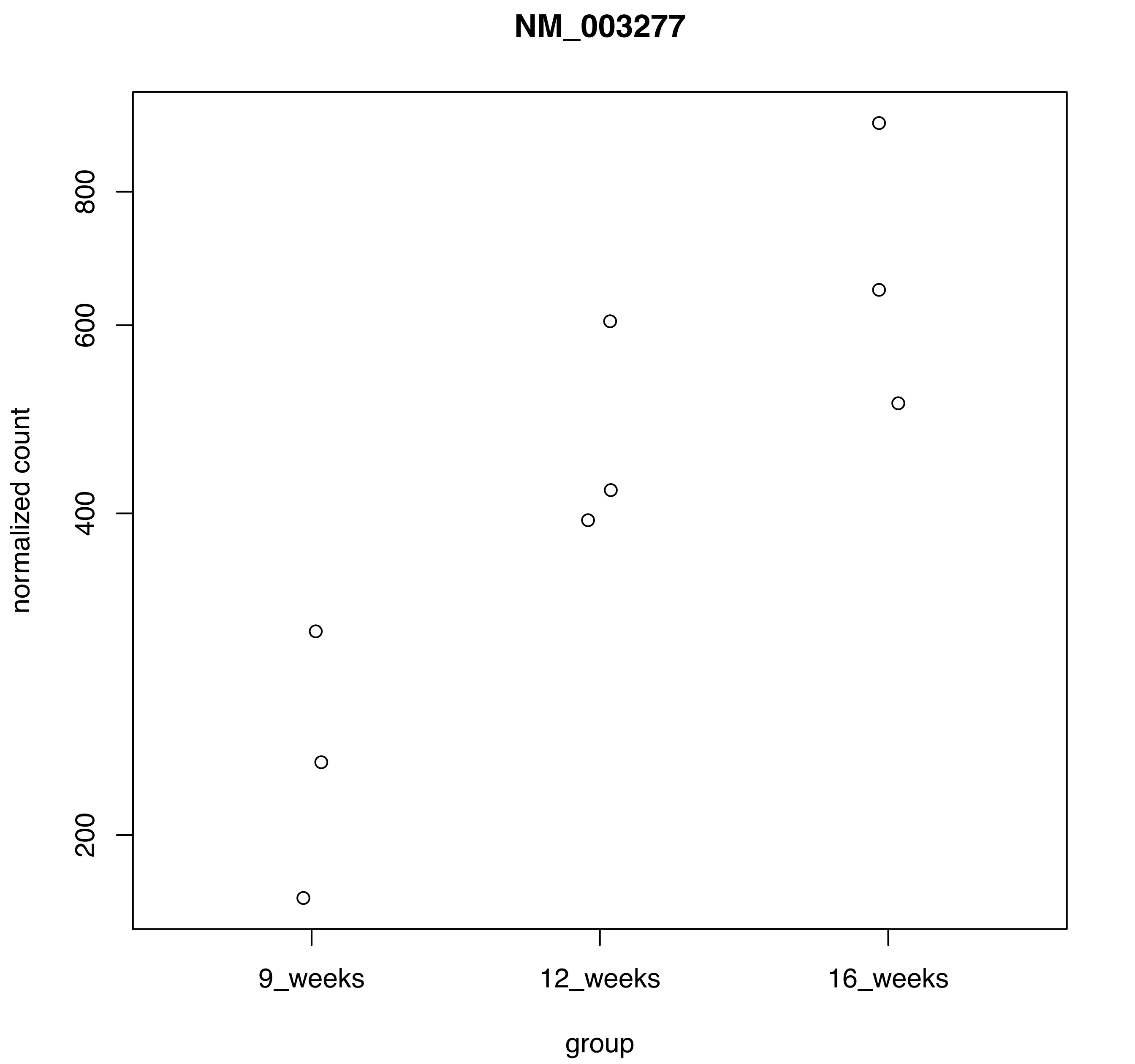
NM_003277
normalized count
200
400
600
800
9_weeks
12_weeks
16_weeks
group

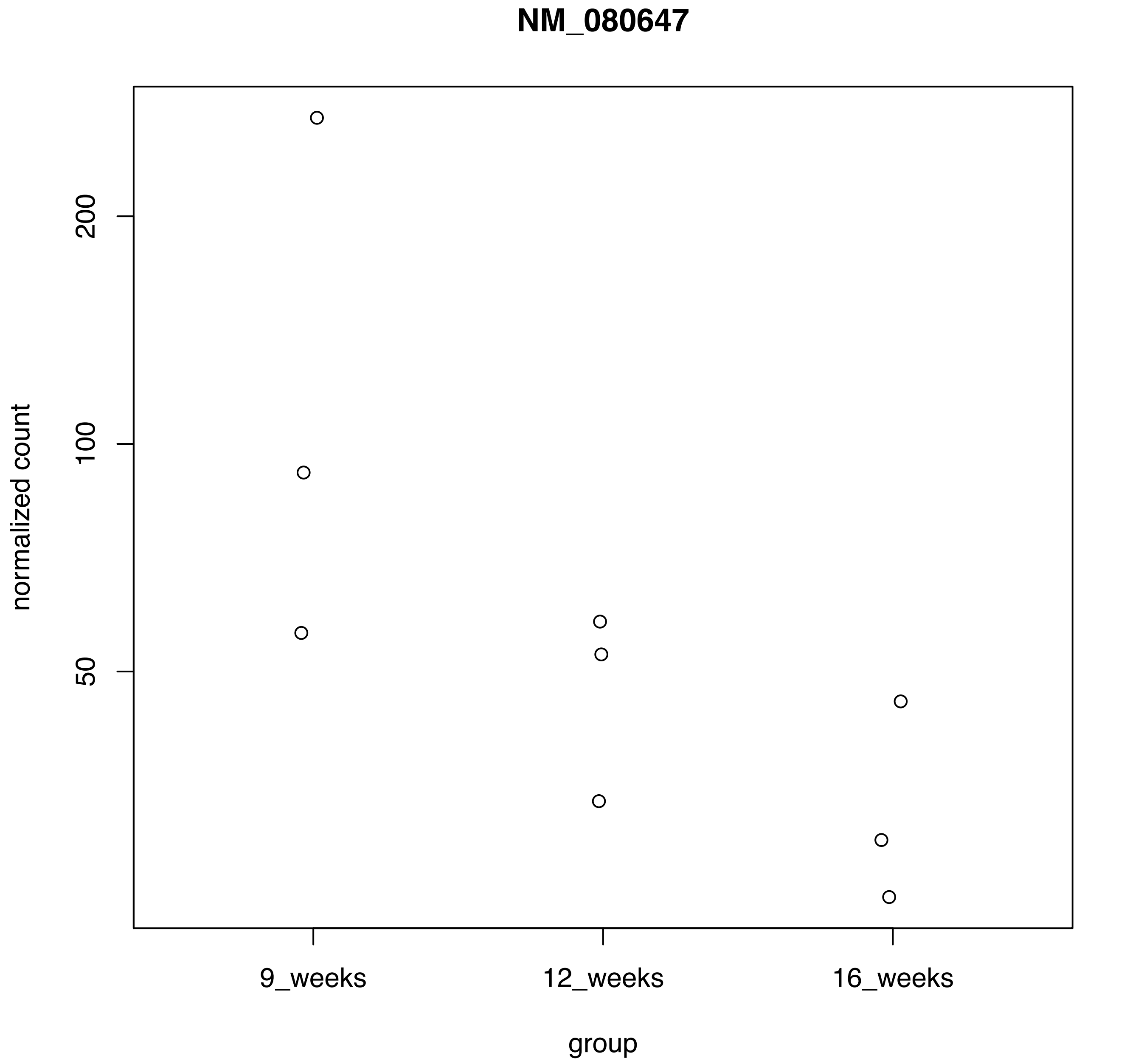

NM_080647
normalized count
200
100
50
9_weeks
12_weeks
16_weeks
group

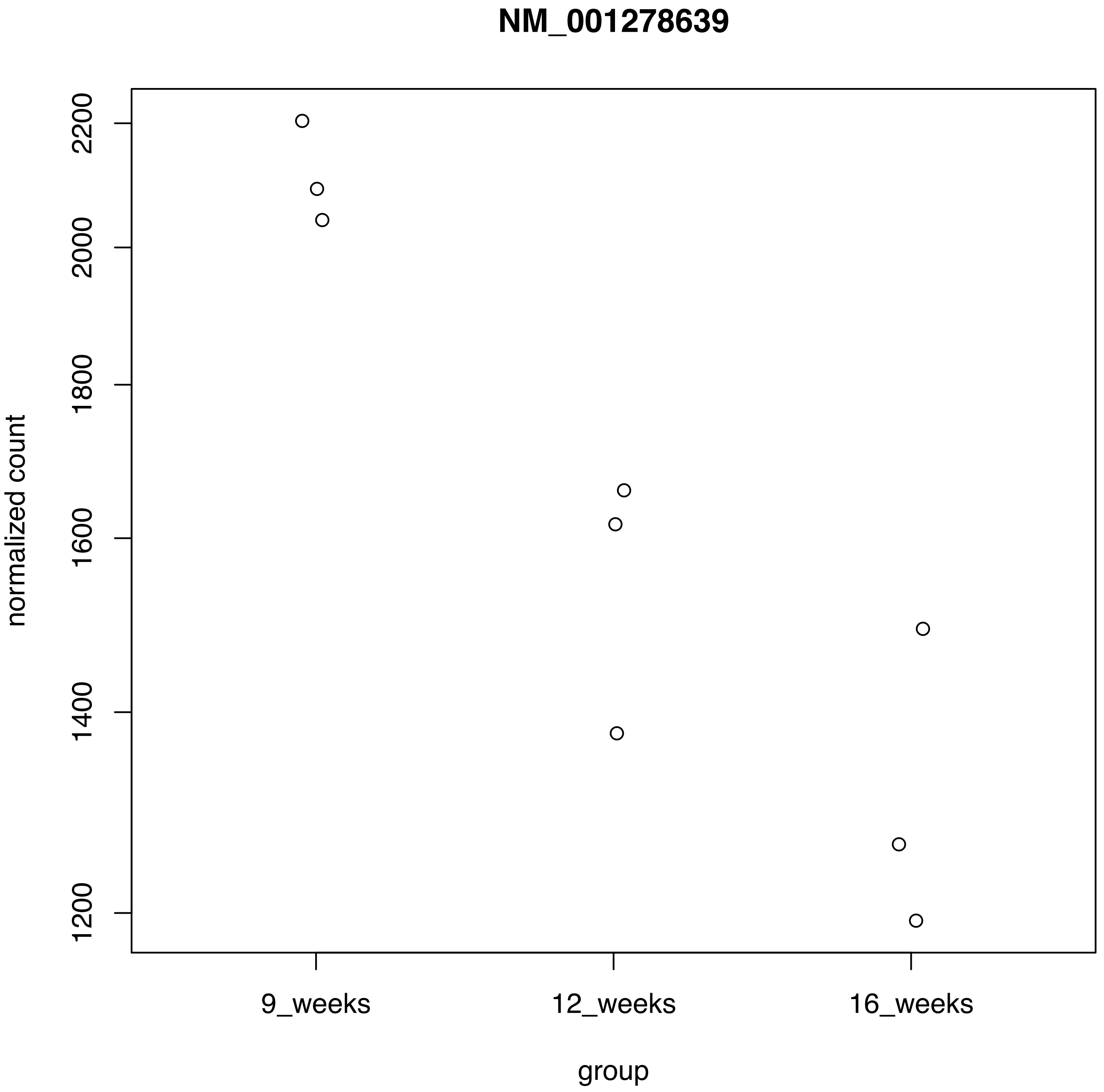

NM_001278639
normalized count
2200
2000
1800
1600
1400
1200
9_weeks
12_weeks
16_weeks
group

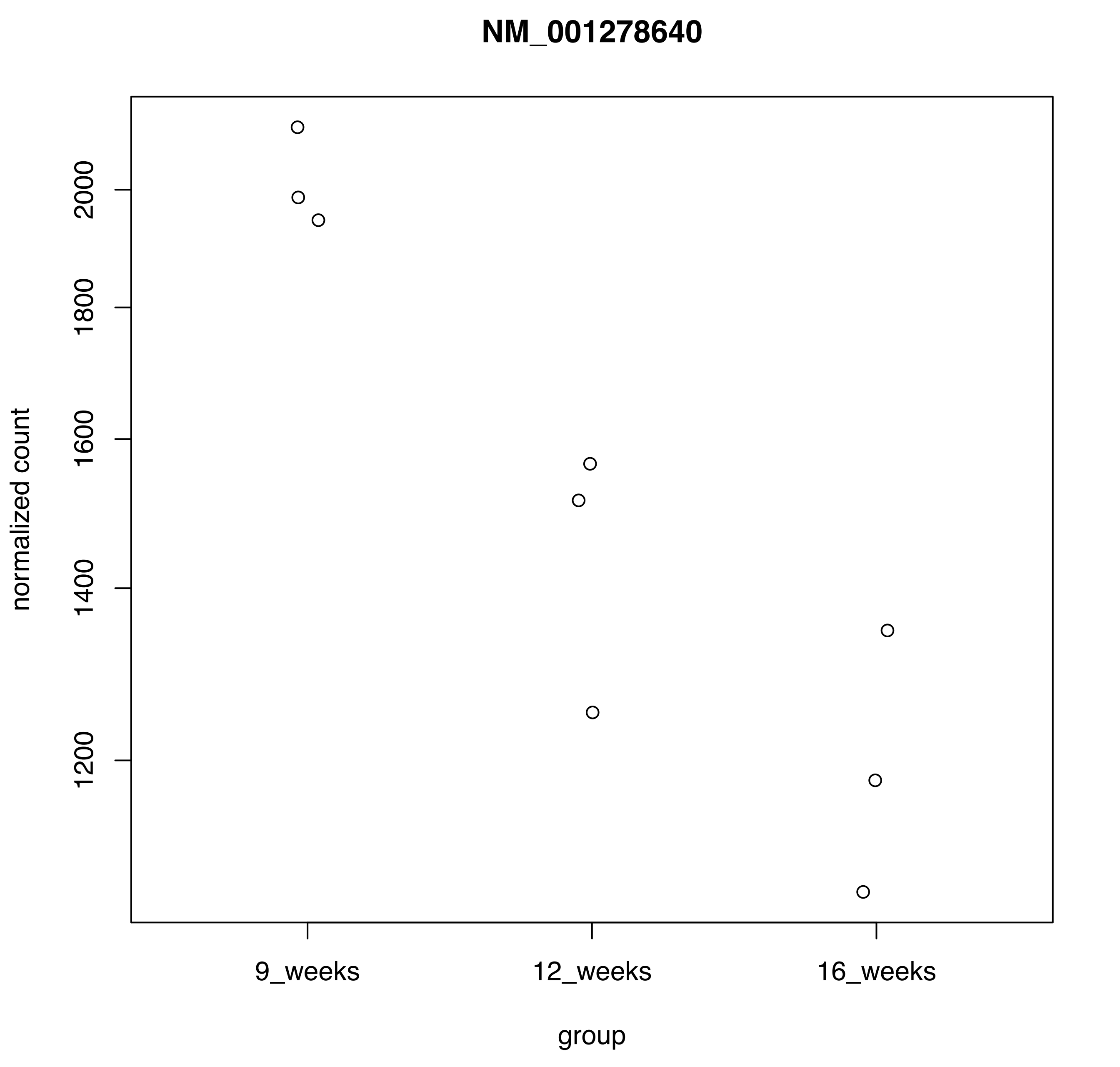
NM_001278640
normalized count
2000
1800
1600
1400
1200
9_weeks
12_weeks
16_weeks
group

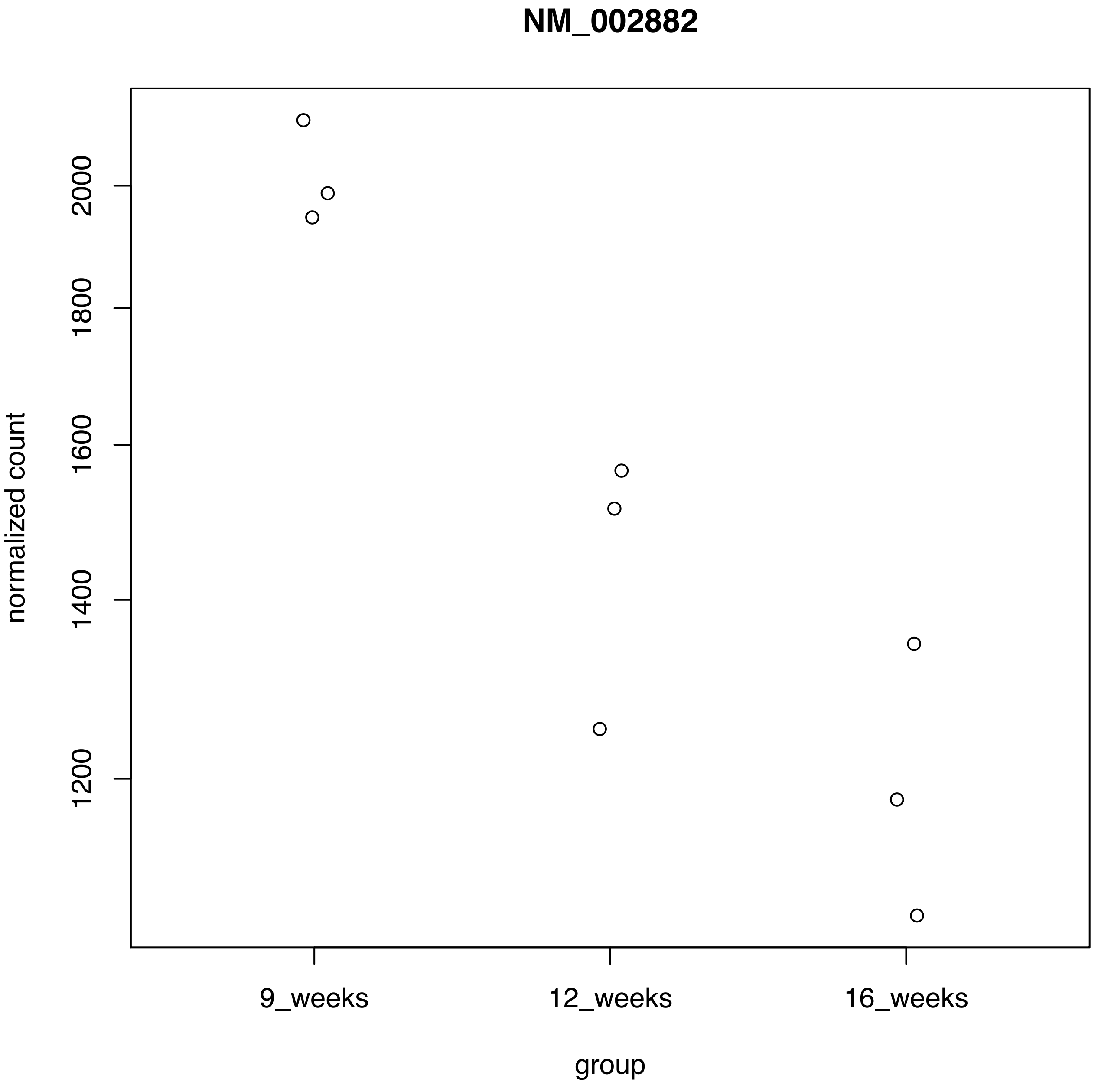
NM_002882
normalized count
2000
1800
1600
1400
1200
9_weeks
12_weeks
16_weeks
group

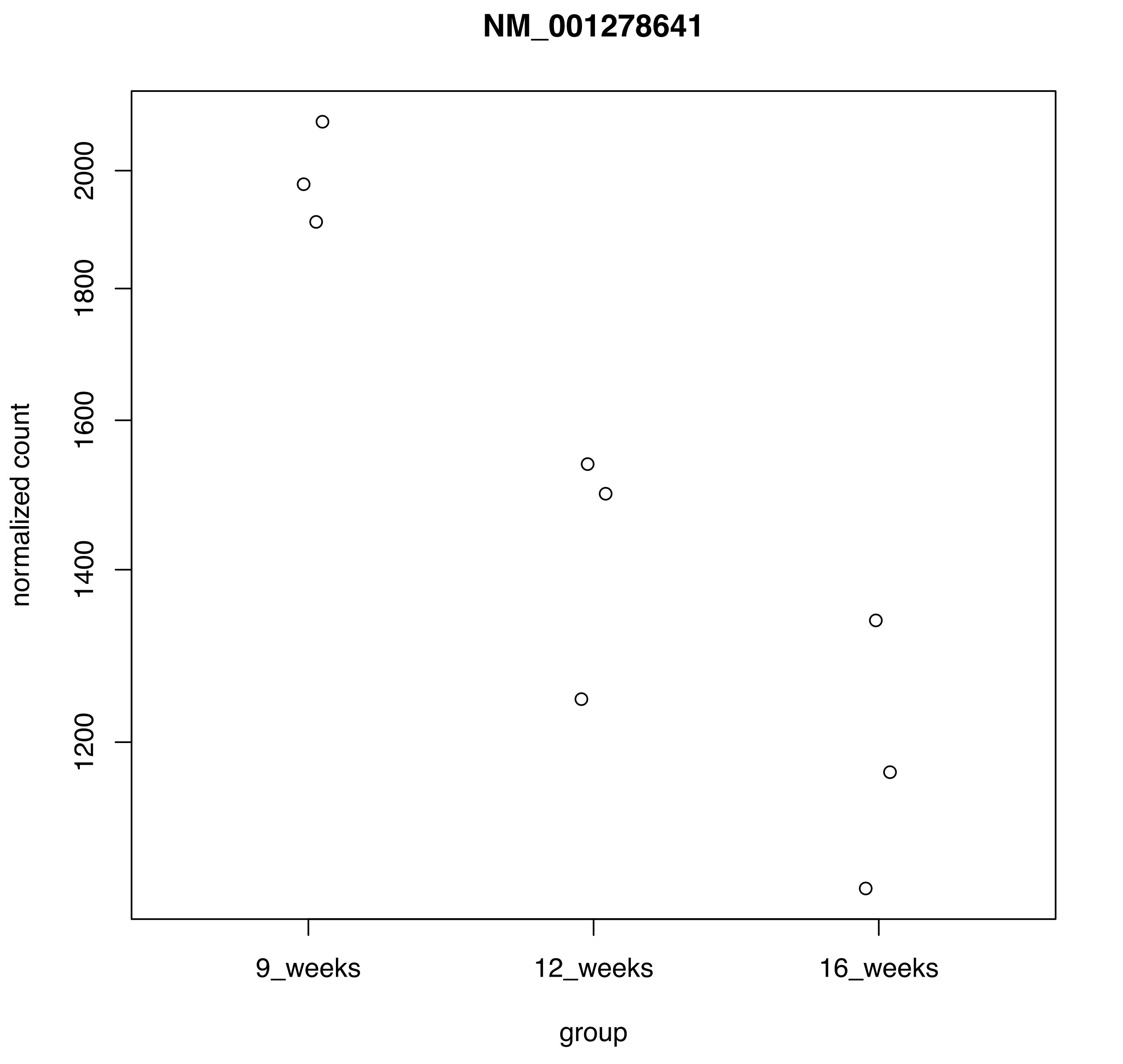
NM_001278641
normalized count
2000
1800
1600
1400
1200
9_weeks
12_weeks
16_weeks
group

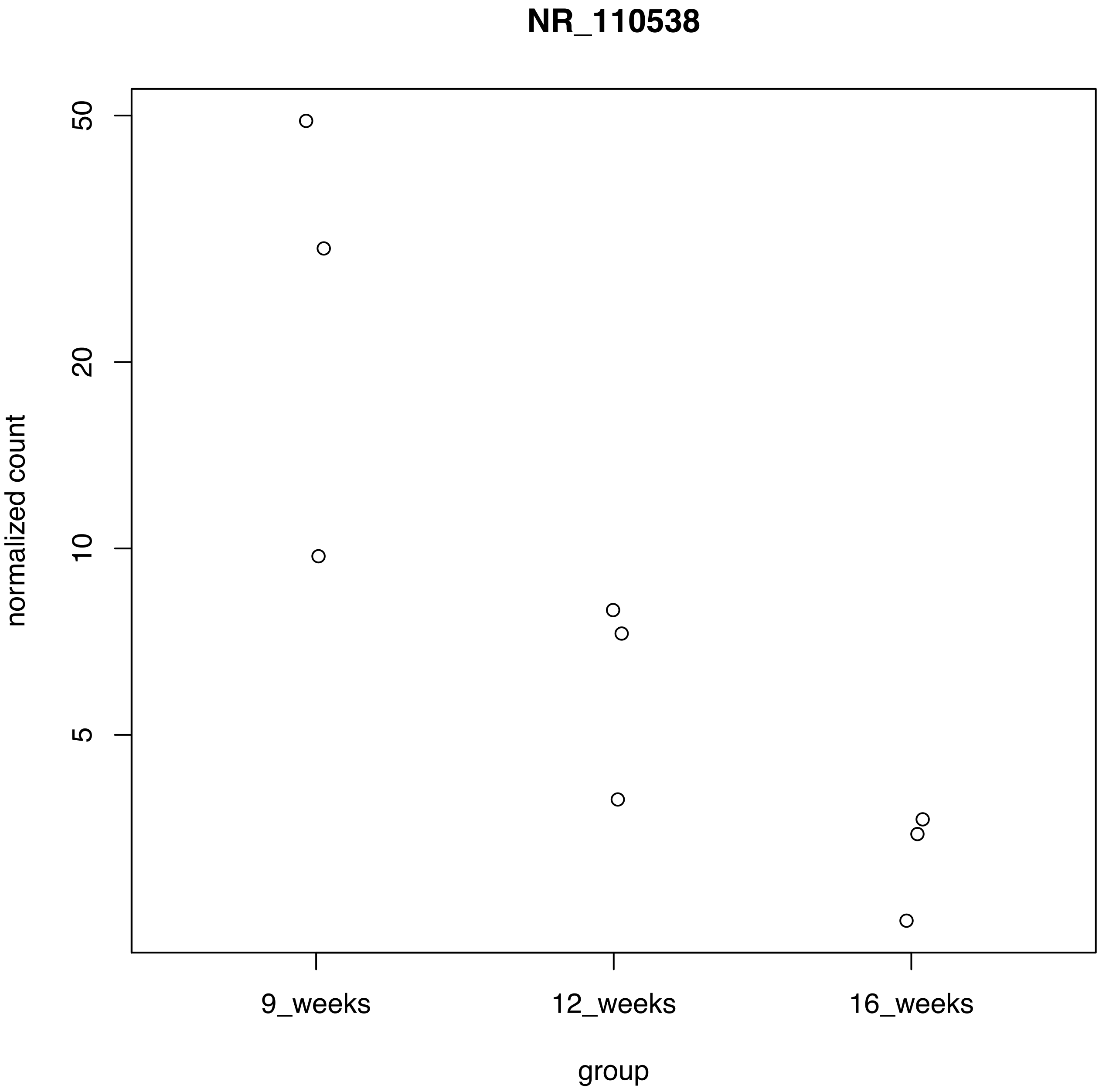

NR_110538
50
20
10
5
normalized count
9_weeks
12_weeks
16_weeks
group

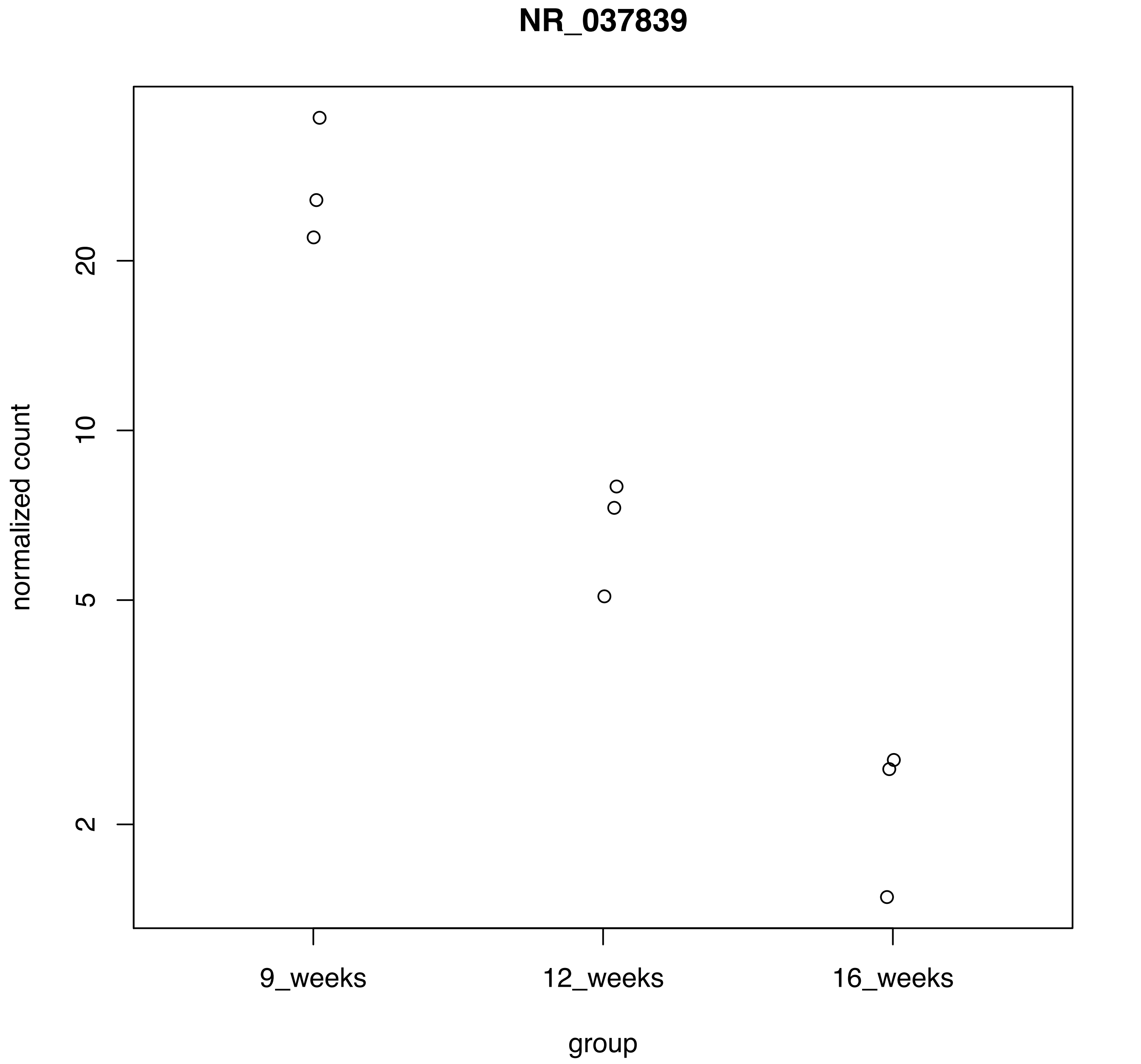
NR_037839
normalized count
20
10
5
2
9_weeks
12_weeks
16_weeks
group

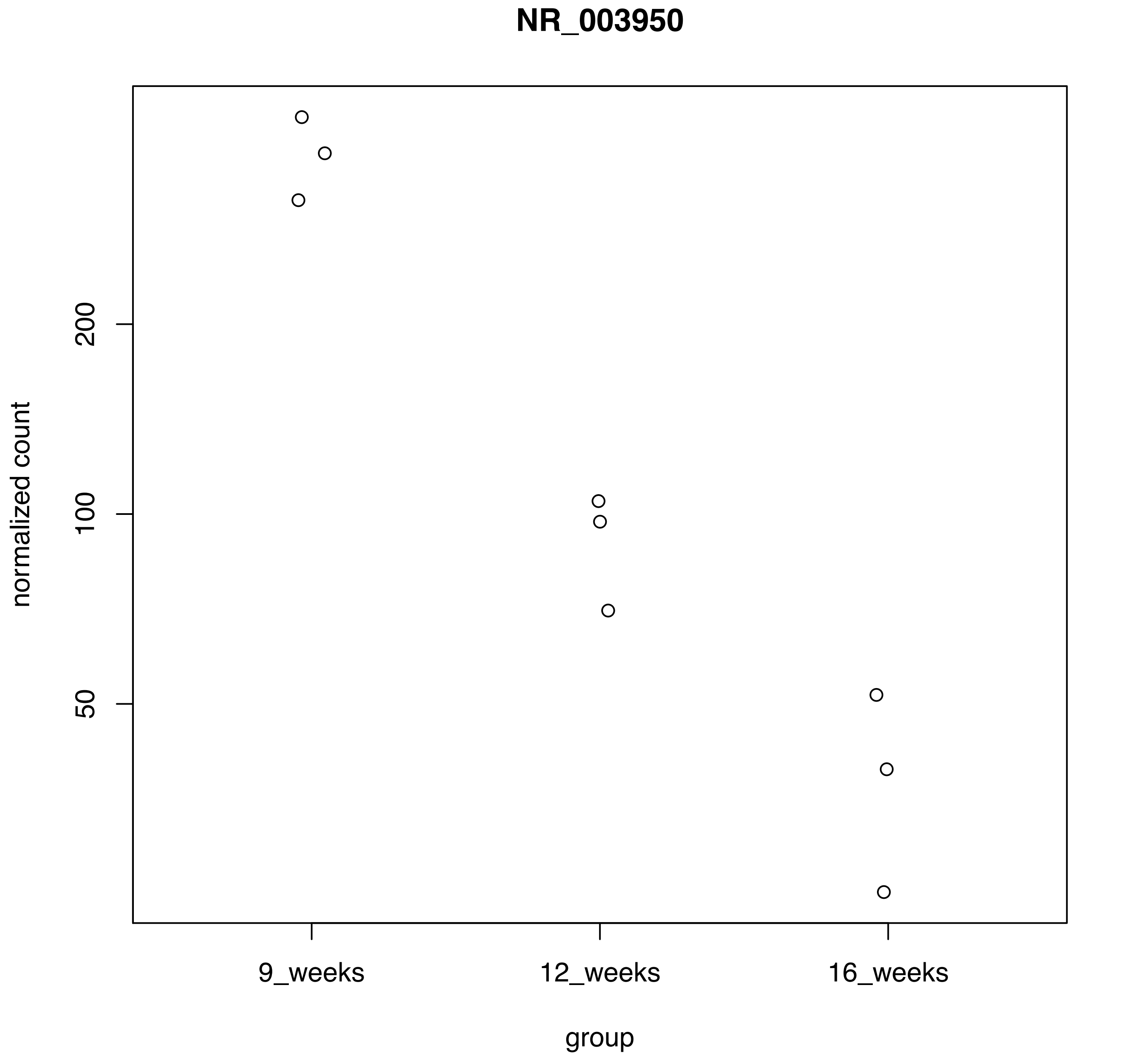
NR_003950
normalized count
200
100
50
9_weeks
12_weeks
16_weeks
group

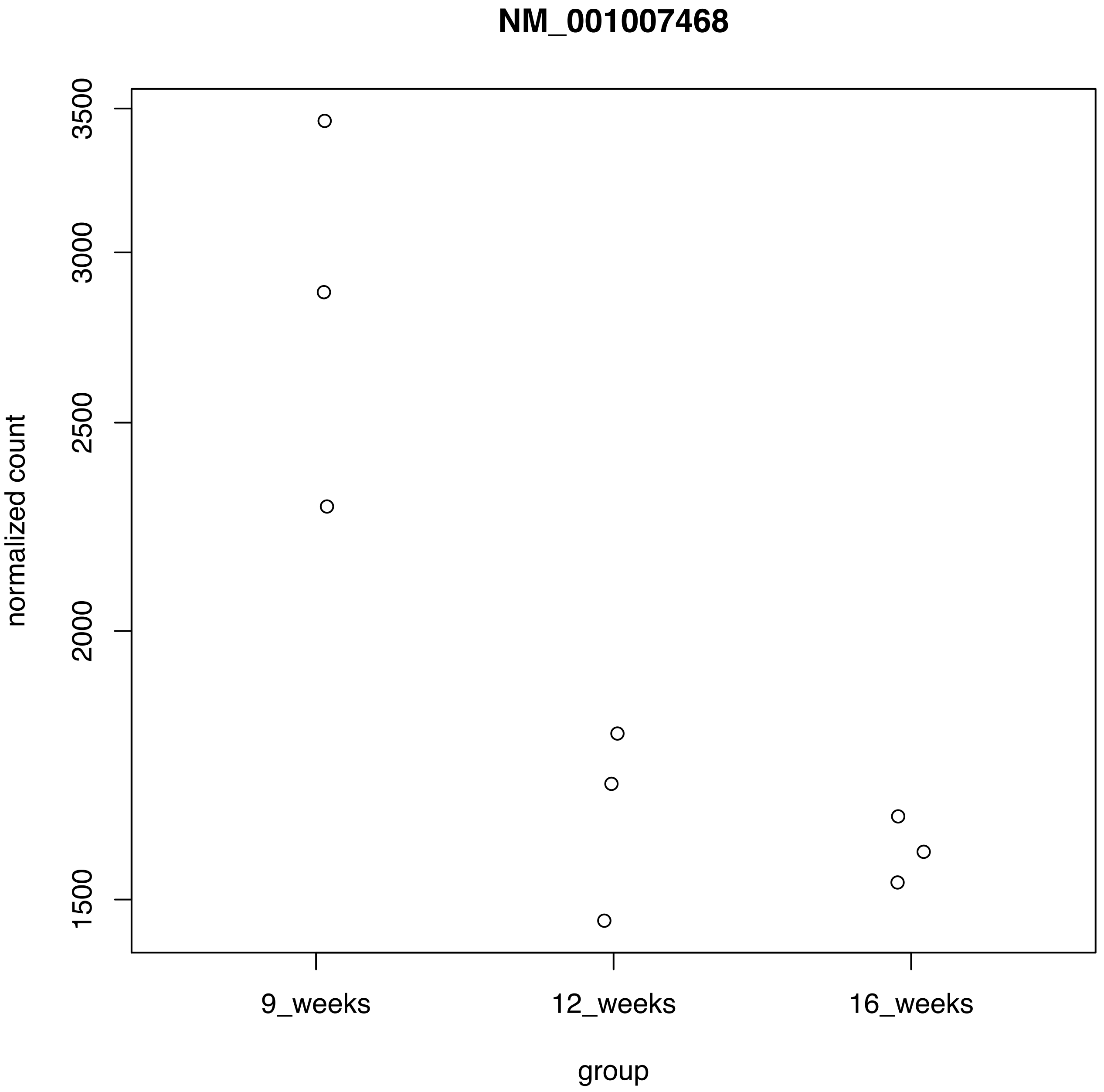
NM_001007468
normalized count
3500
3000
2500
2000
1500
9_weeks
12_weeks
16_weeks
group

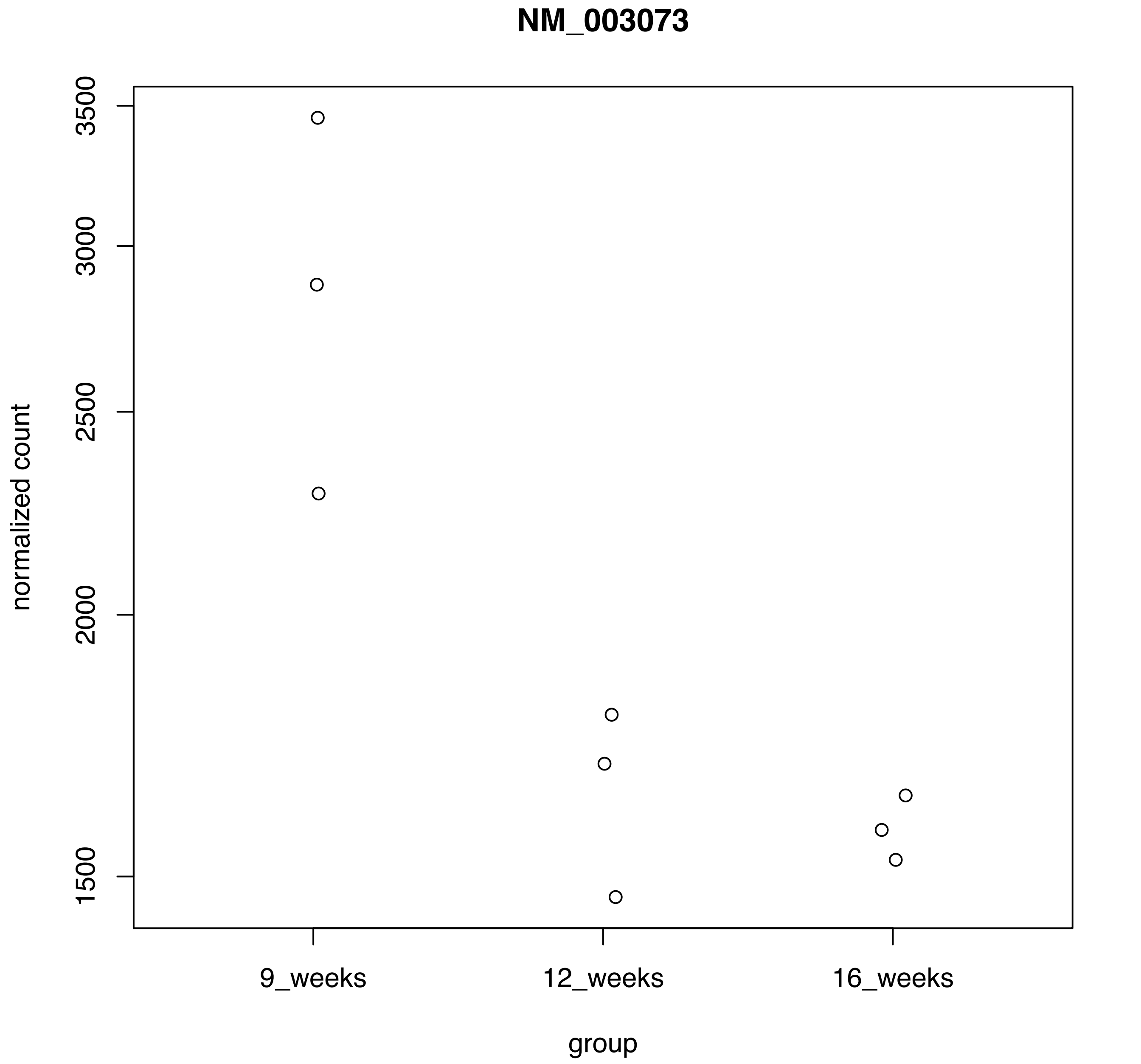
NM_003073
normalized count
3500
3000
2500
2000
1500
9_weeks
12_weeks
16_weeks
group

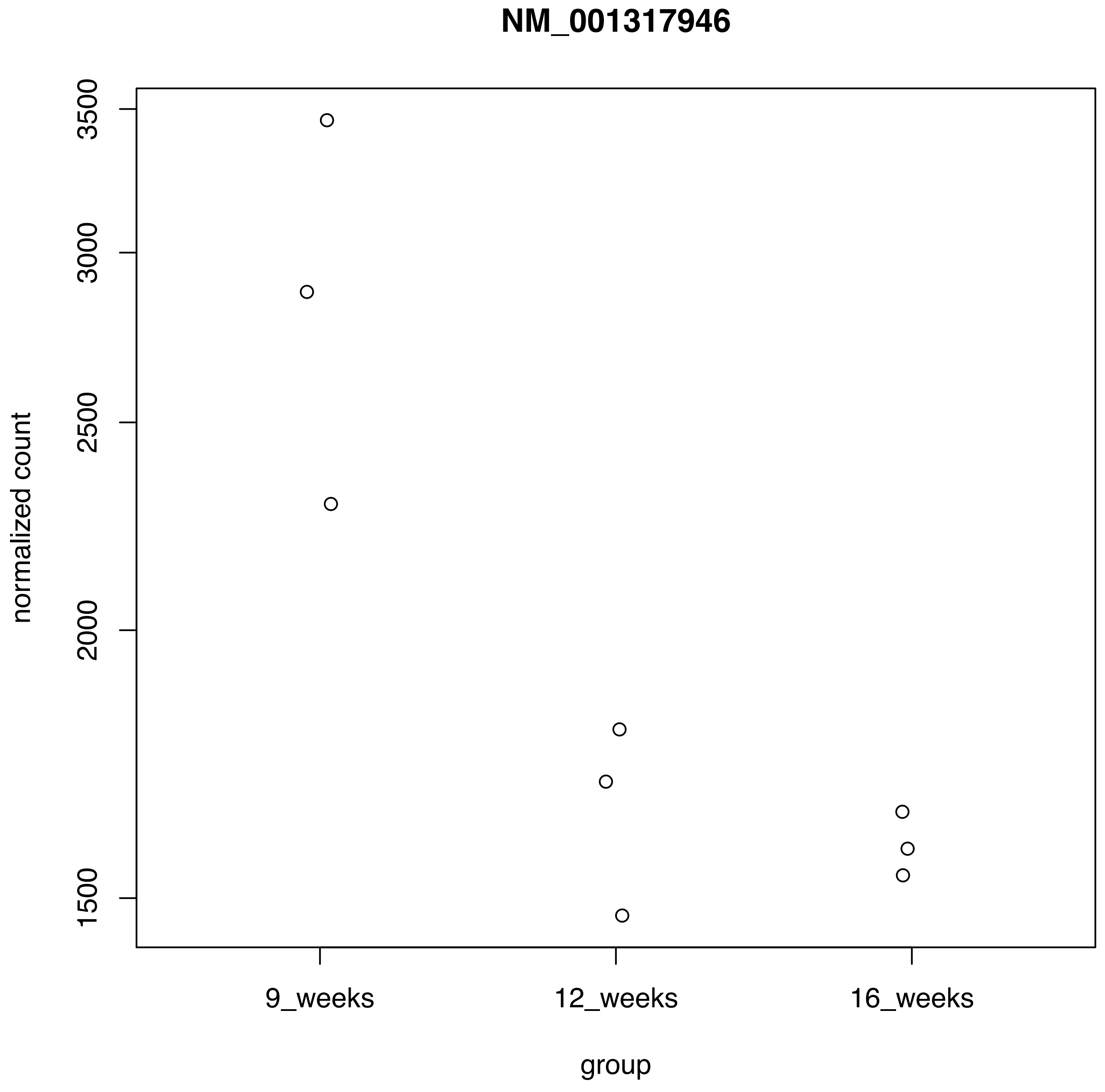
NM_001317946
normalized count
3500
3000
2500
2000
1500
9_weeks
12_weeks
16_weeks
group

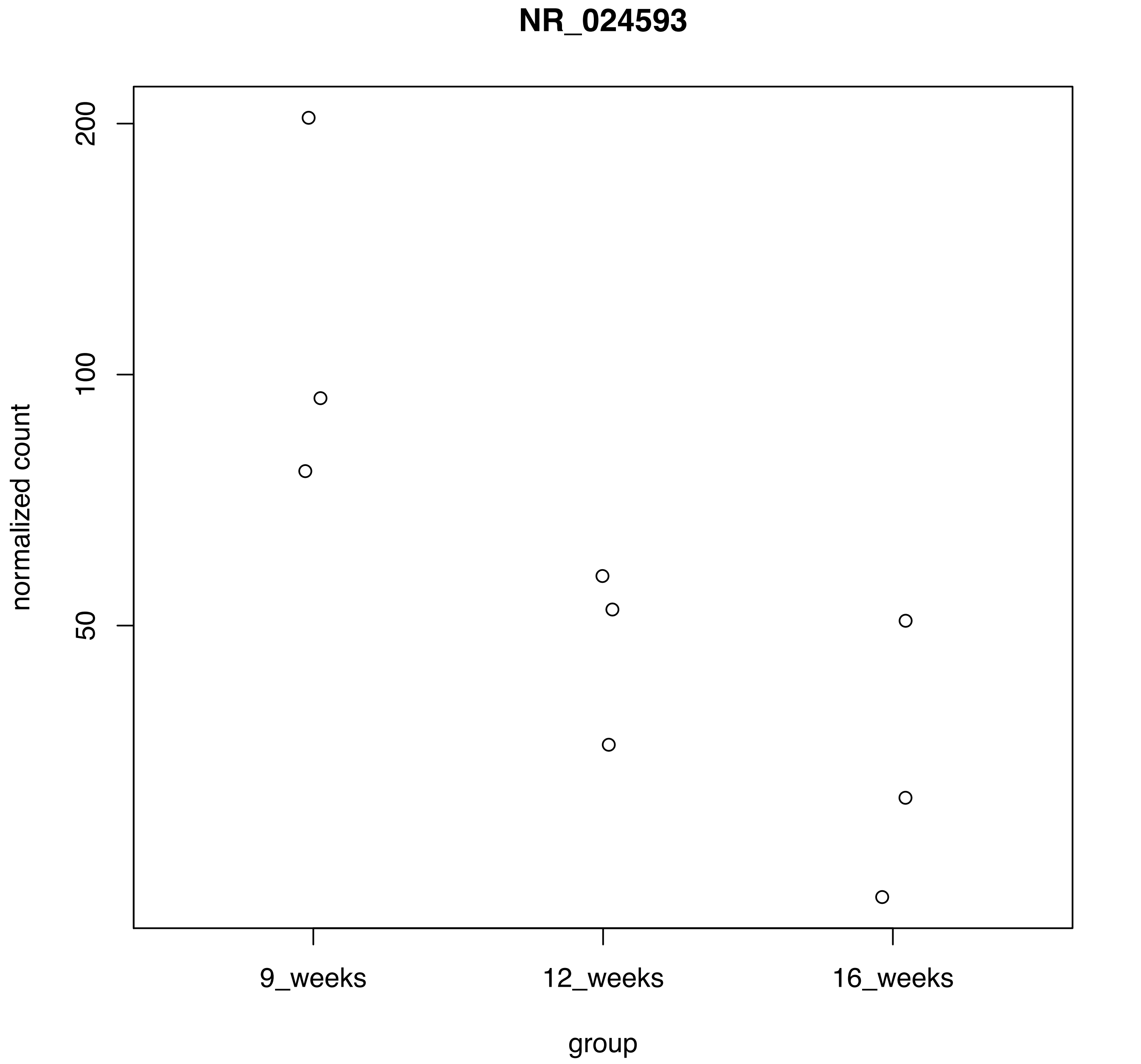
NR_024593
normalized count
200
100
50
9_weeks
12_weeks
16_weeks
group

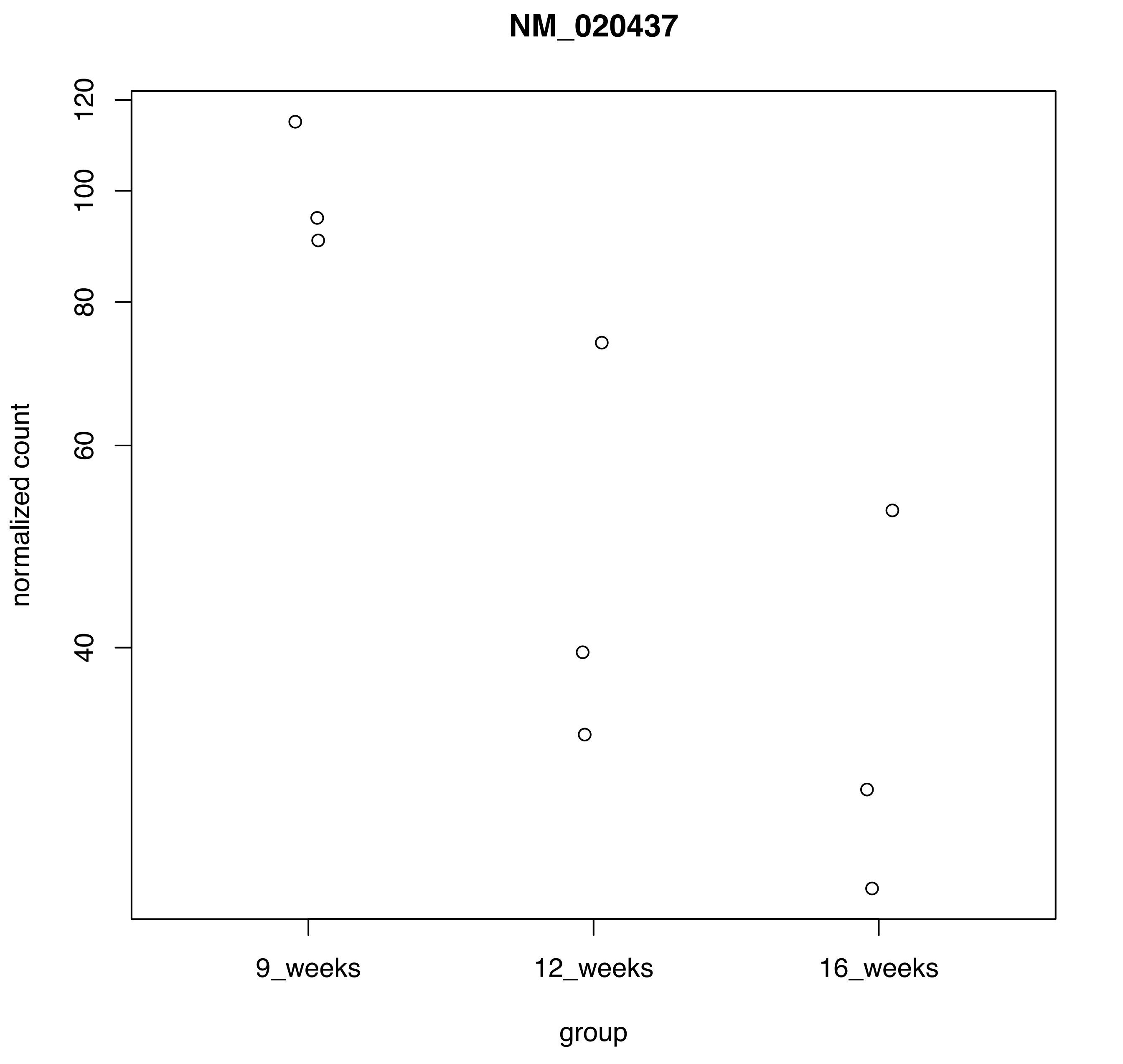
NM_020437
normalized count
120
100
80
60
40
9_weeks
12_weeks
16_weeks
group

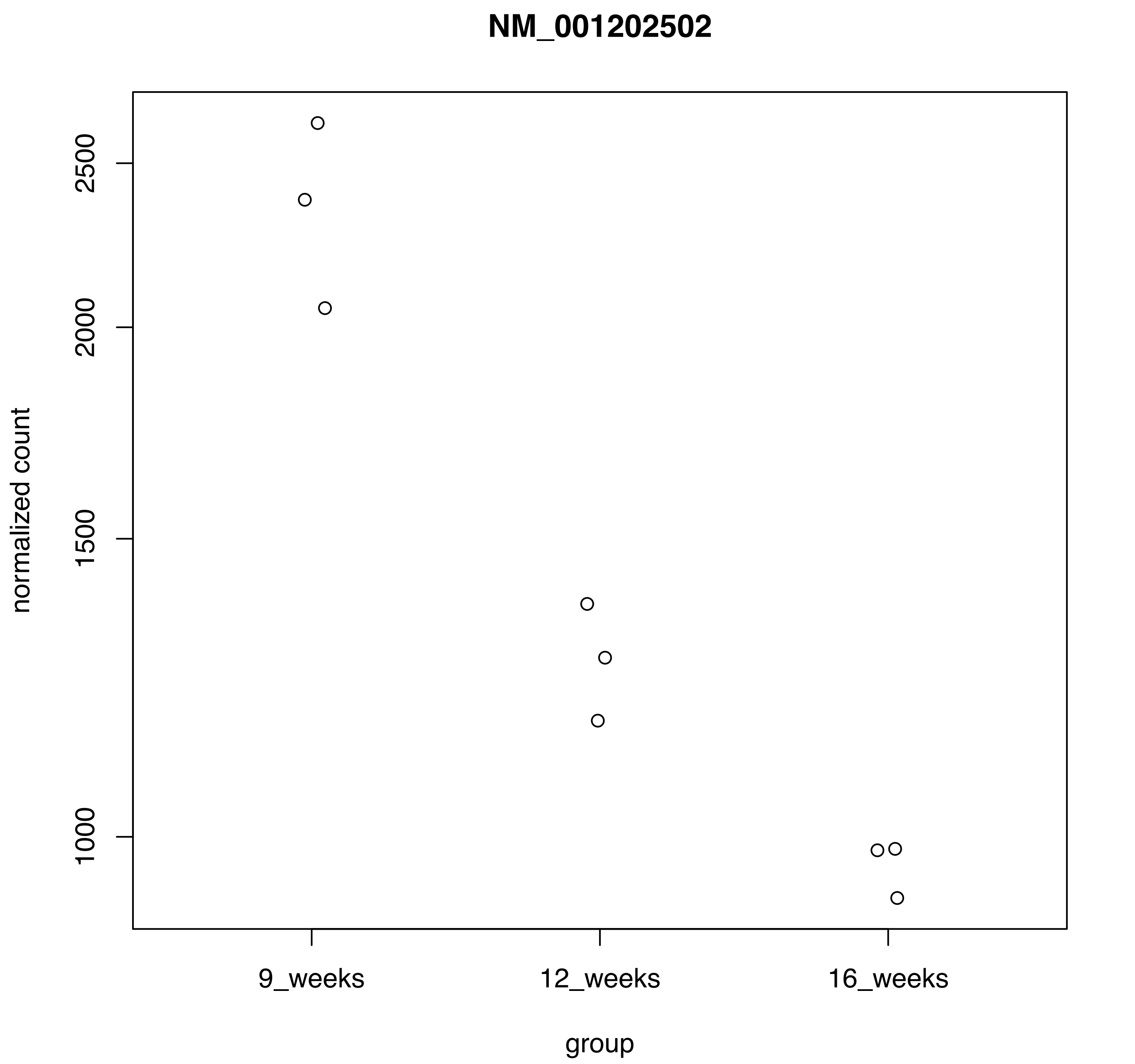
NM_001202502
normalized count
2500
2000
1500
1000
9_weeks
12_weeks
16_weeks
group

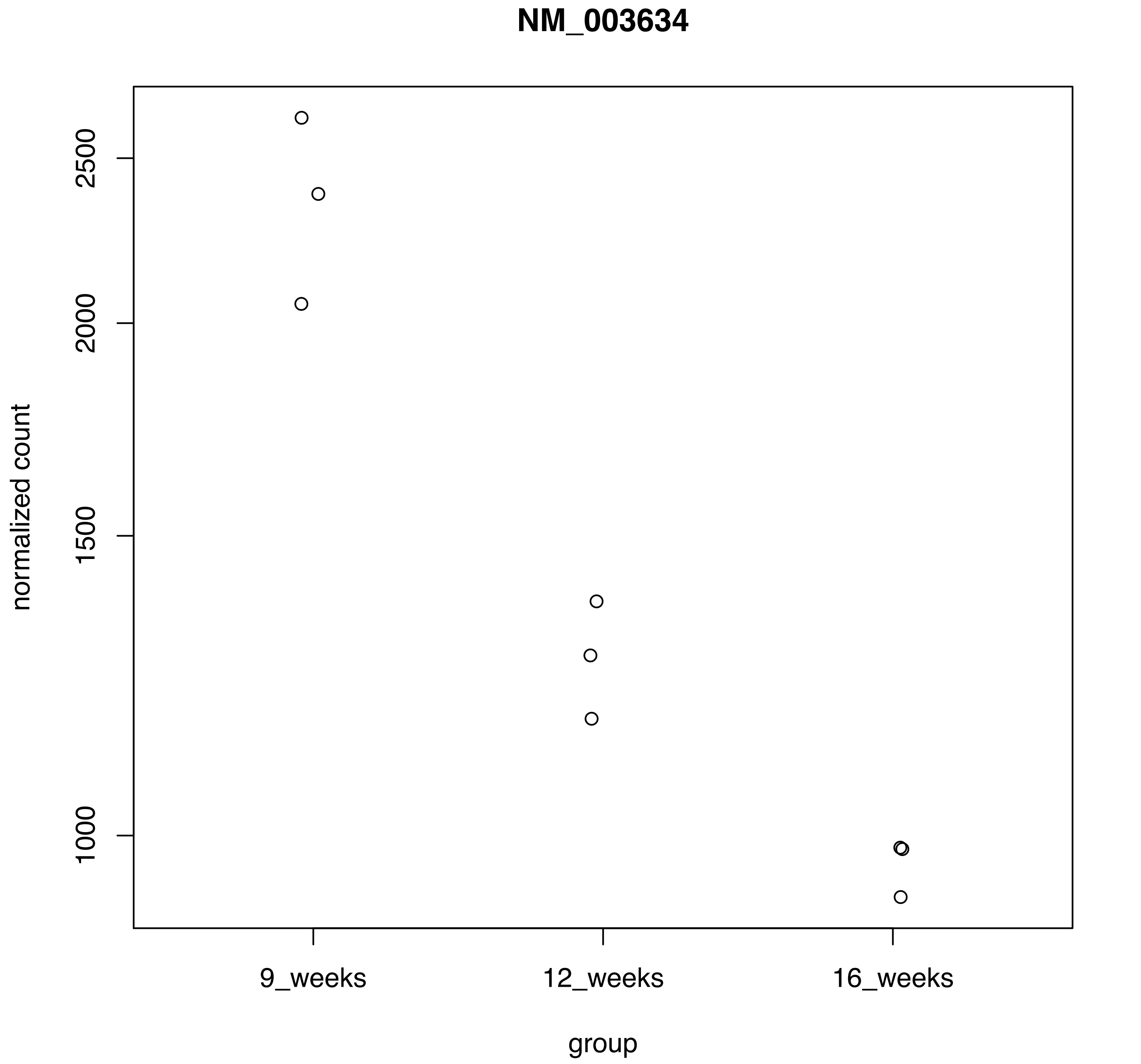
NM_003634
normalized count
2500
2000
1500
1000
9_weeks
12_weeks
16_weeks
group

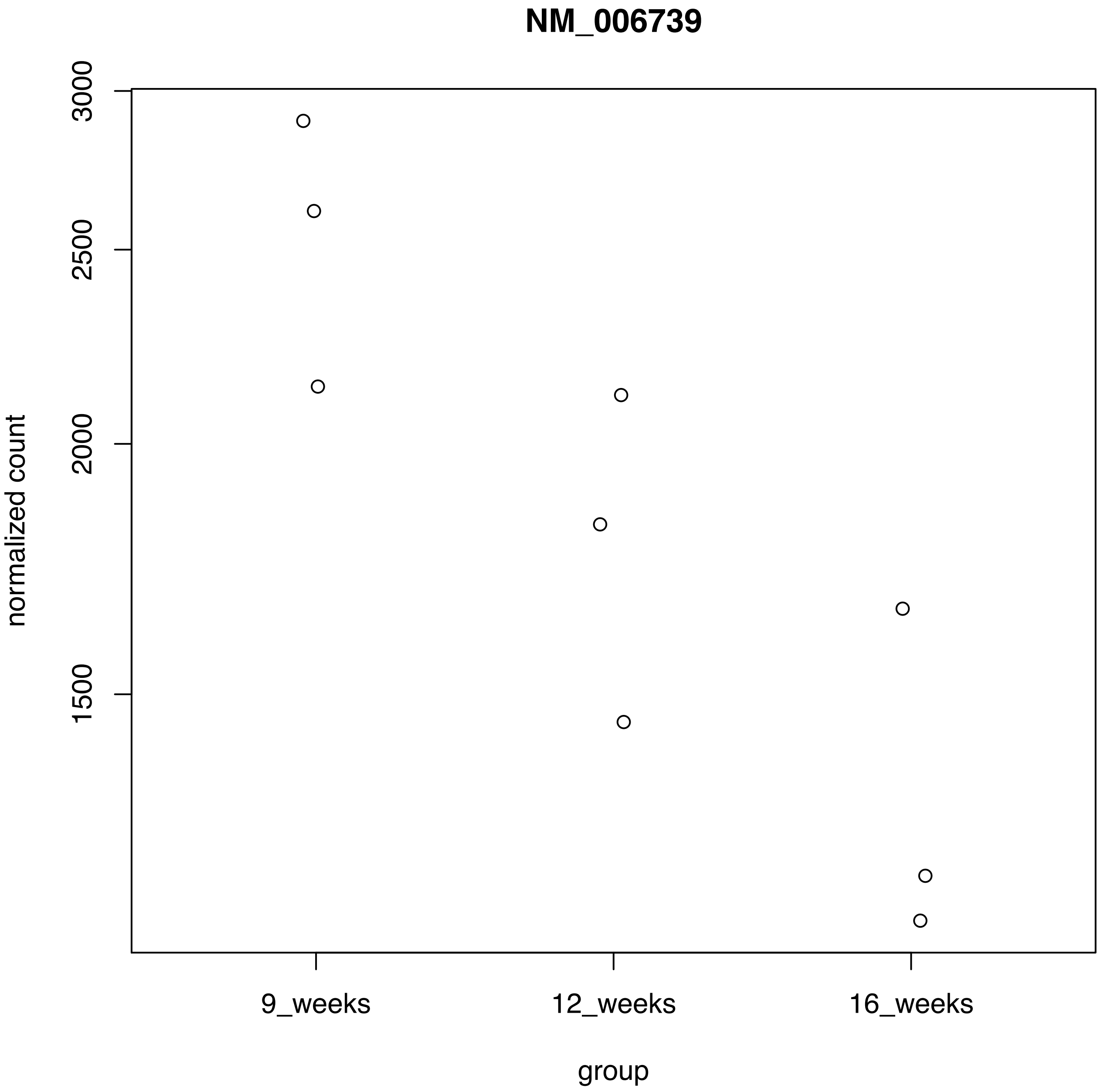

NM_006739
3000
2500
2000
1500
normalized count
9_weeks
12_weeks
16_weeks
group

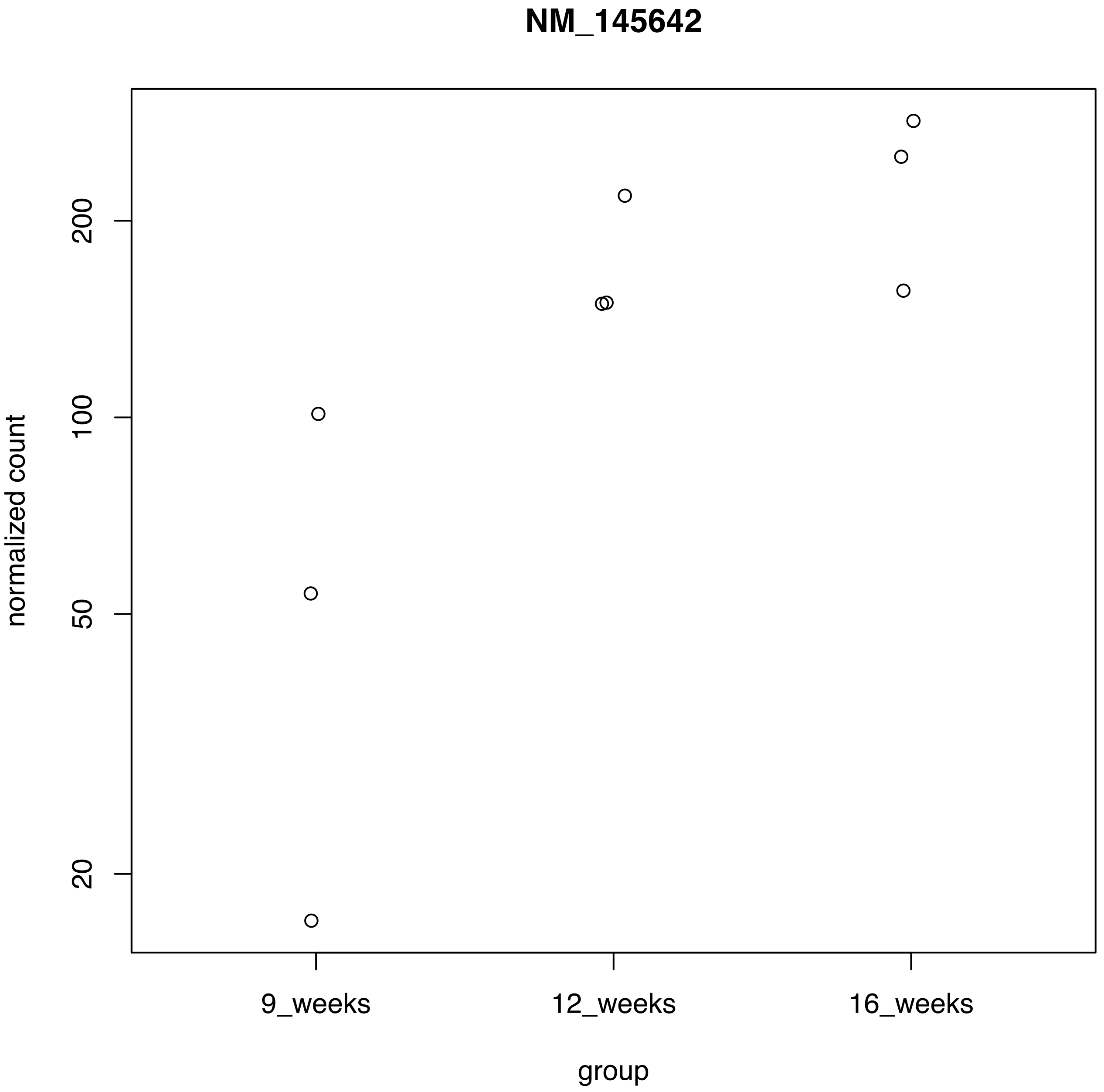

NM_145642
normalized count
200
100
50
20
9_weeks
12_weeks
16_weeks
group

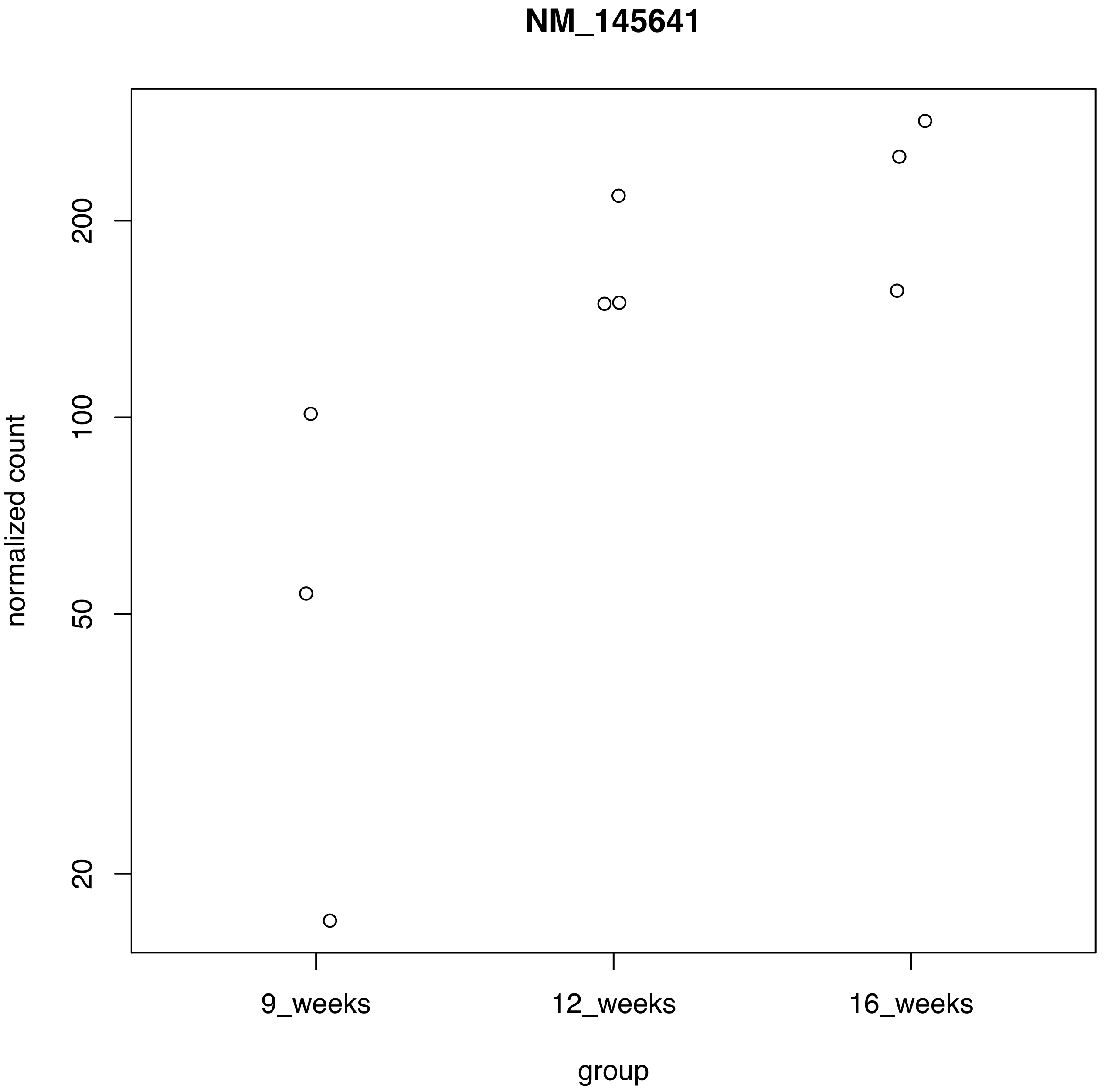
NM_145641
normalized count
200
100
50
20
9_weeks
12_weeks
16_weeks
group

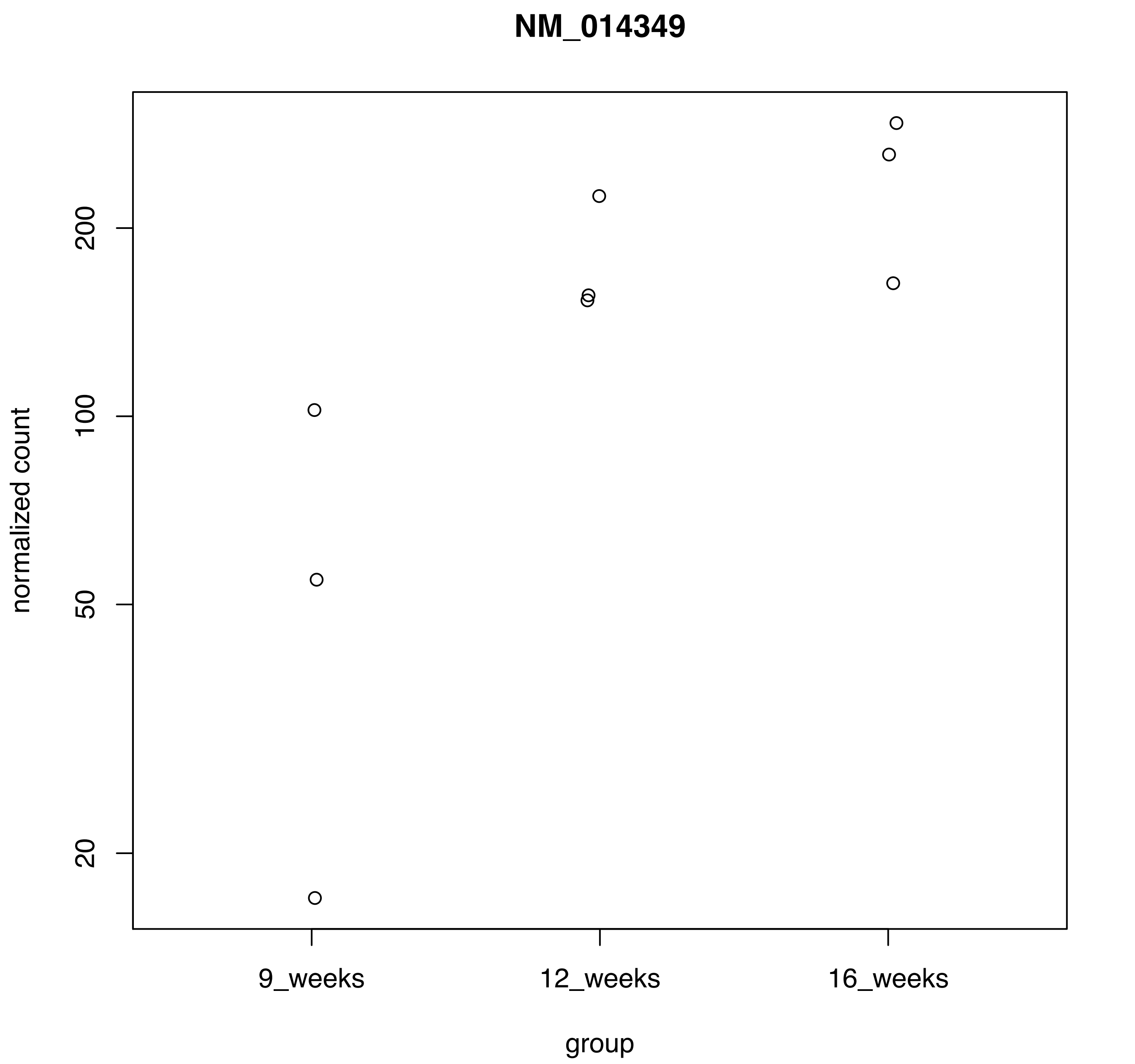
NM_014349
normalized count
200
100
50
20
9_weeks
12_weeks
16_weeks
group

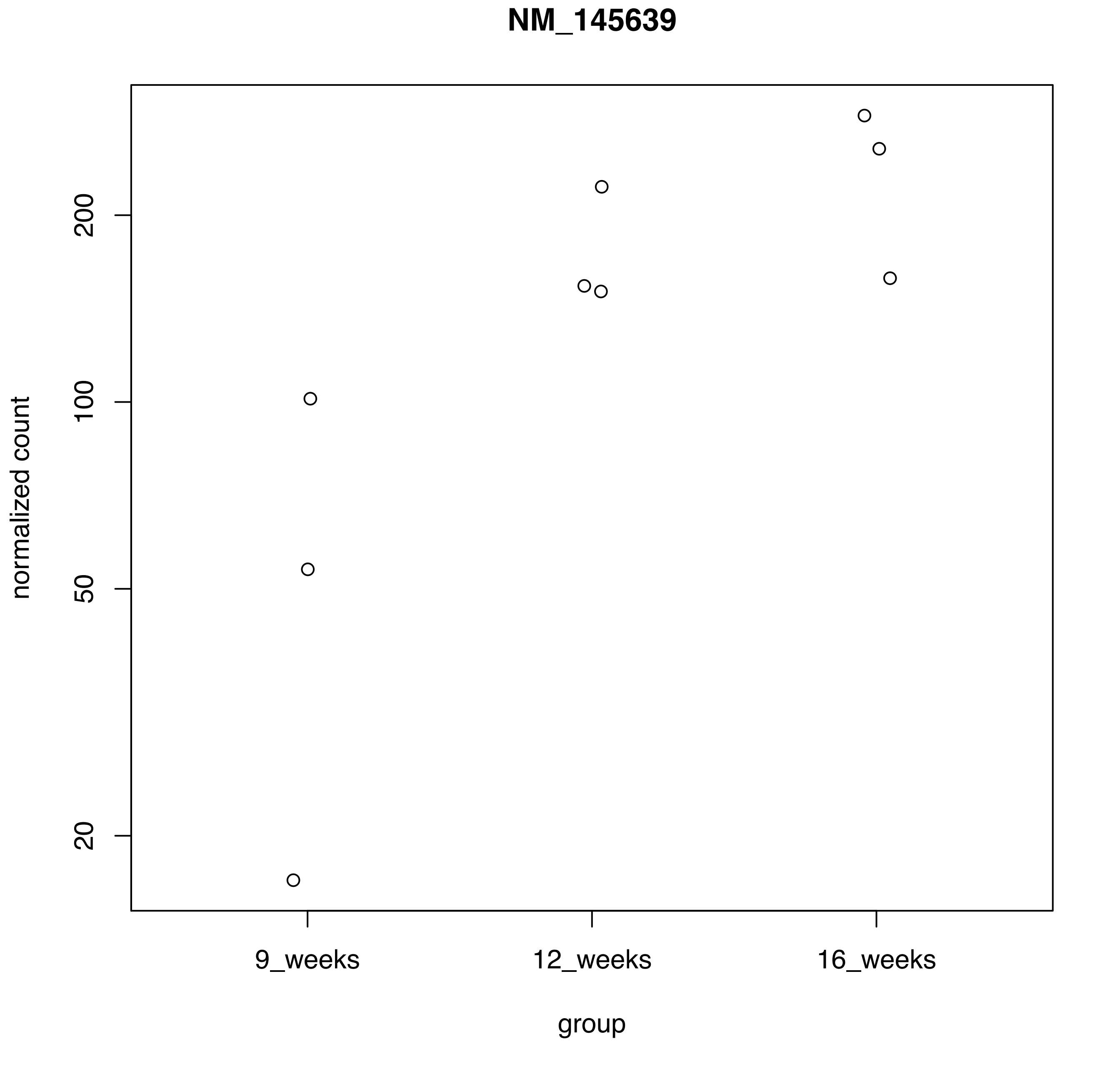

NM_145639
normalized count
200
100
50
20
9_weeks
12_weeks
16_weeks
group

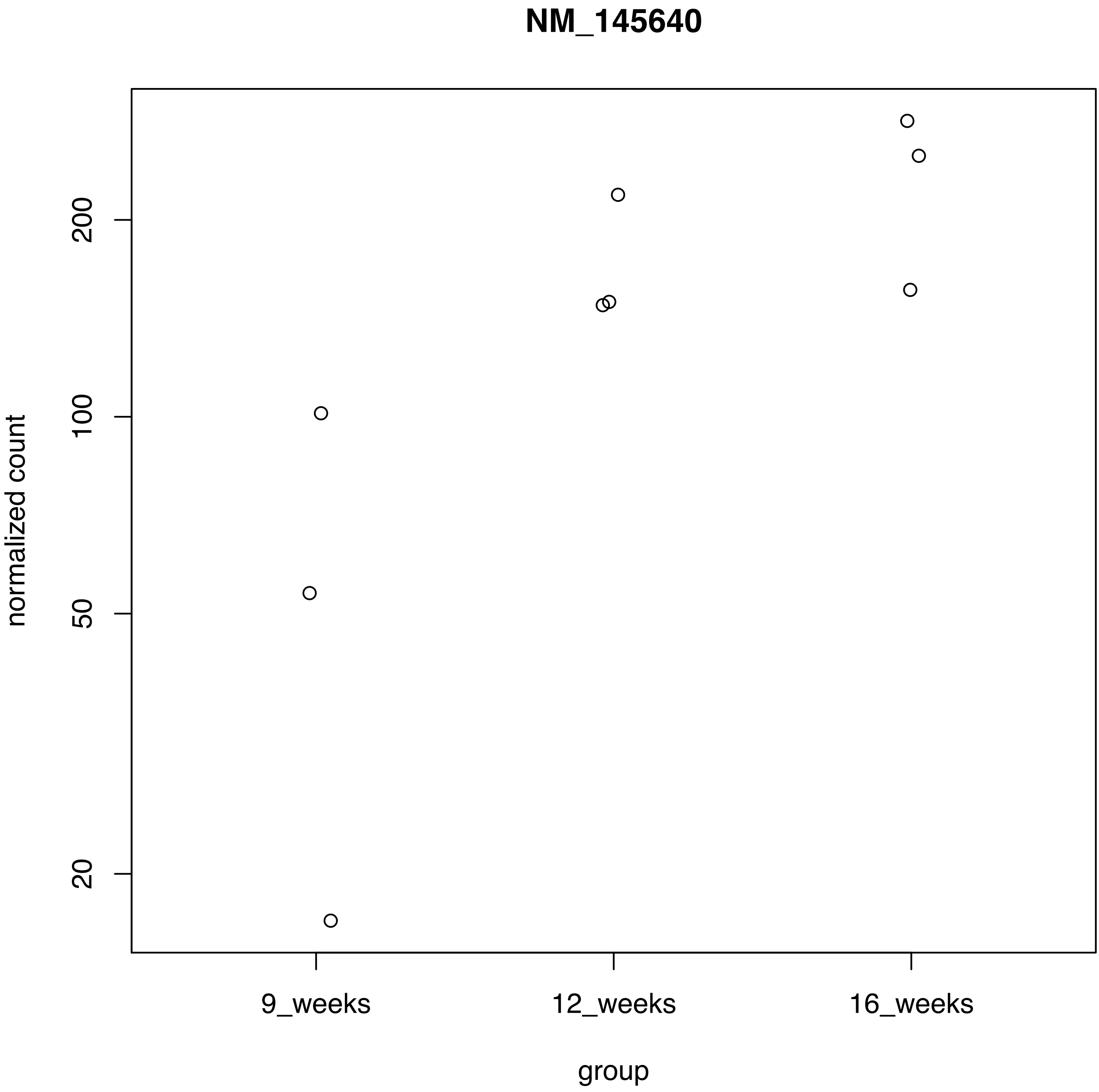

NM_145640
normalized count
200
100
50
20
9_weeks
12_weeks
16_weeks
group

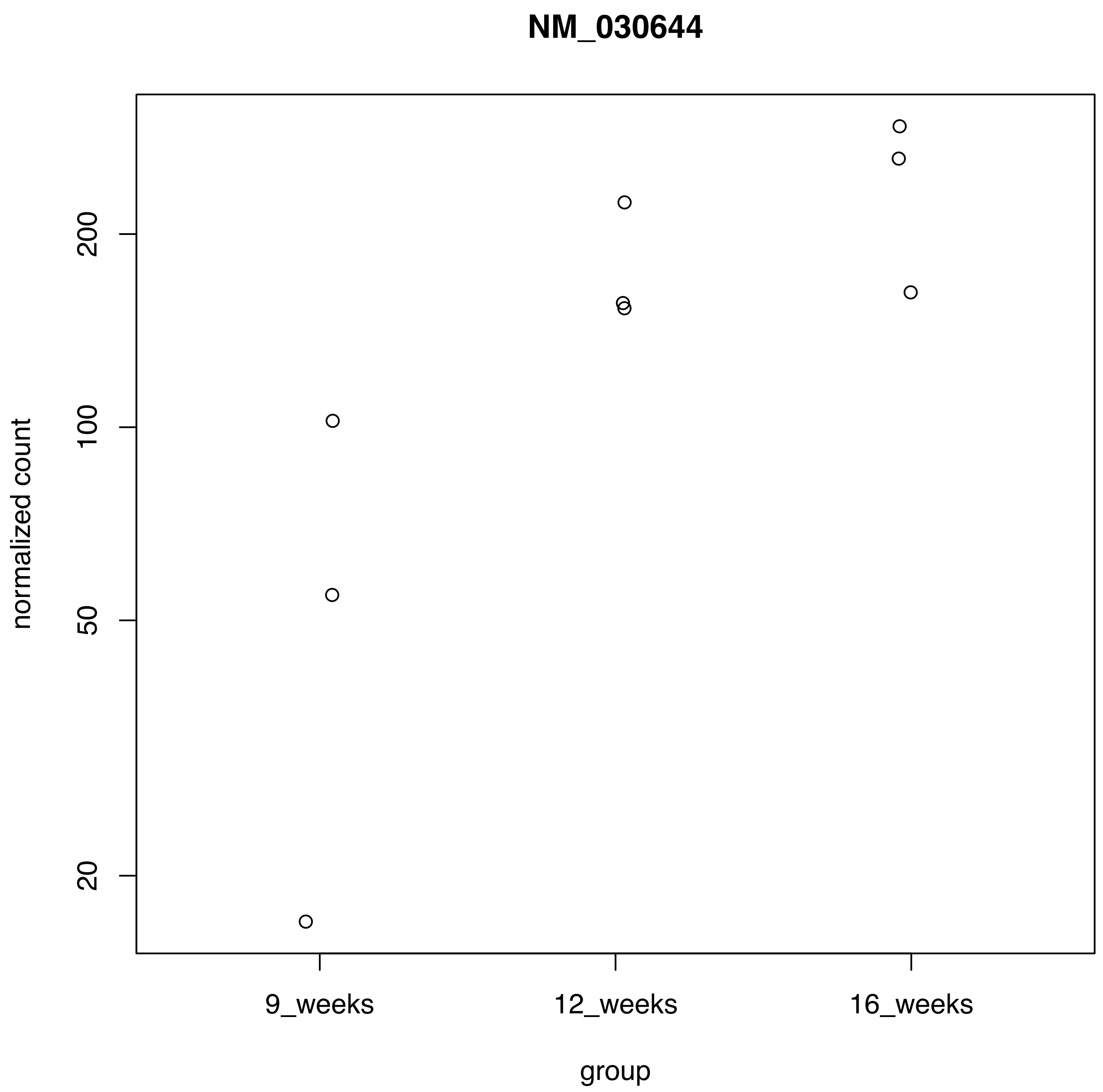
NM_030644
normalized count
200
100
50
20
9_weeks
12_weeks
16_weeks
group

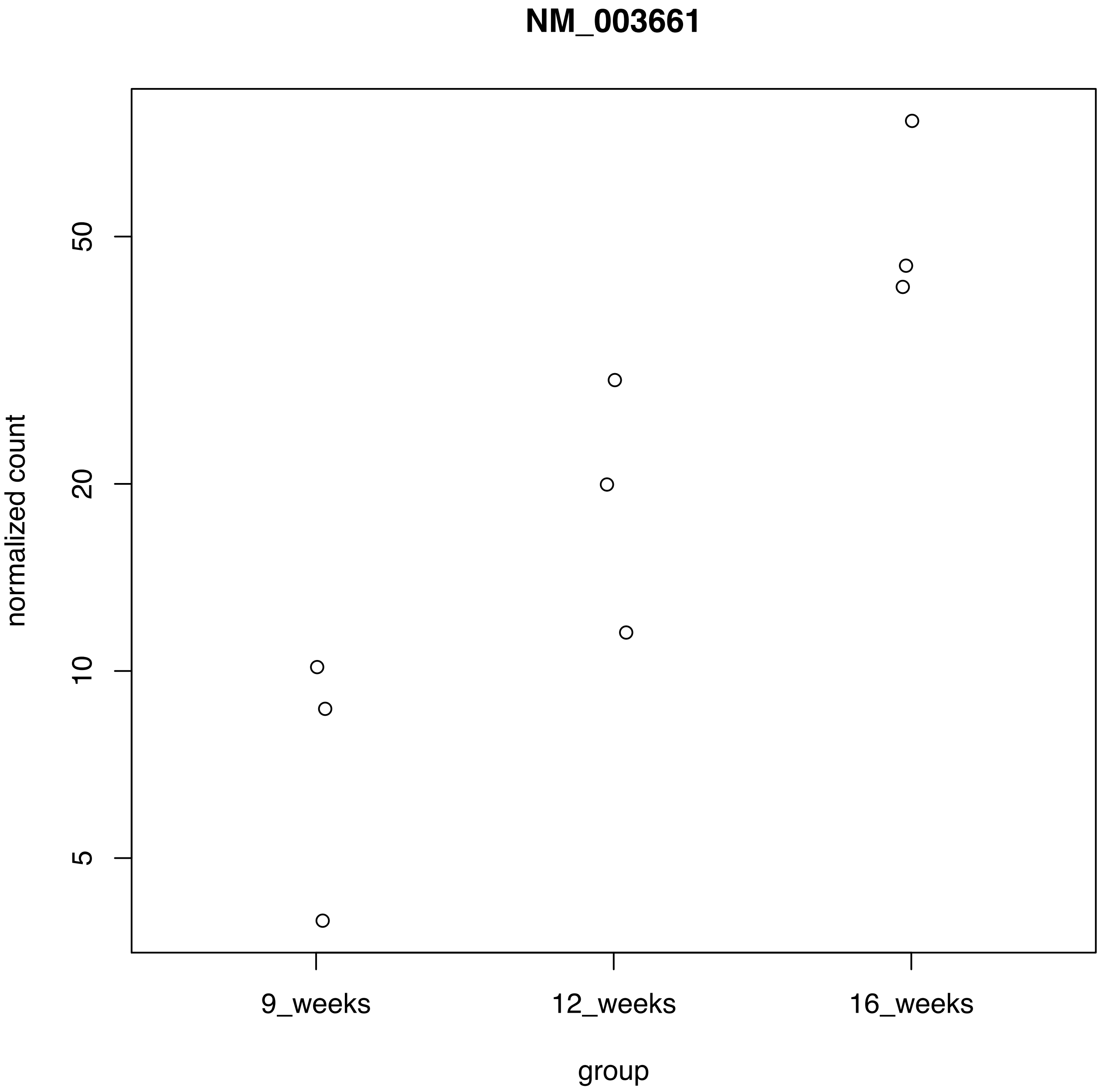

NM_003661
normalized count
50
20
10
5
9_weeks
12_weeks
16_weeks
group

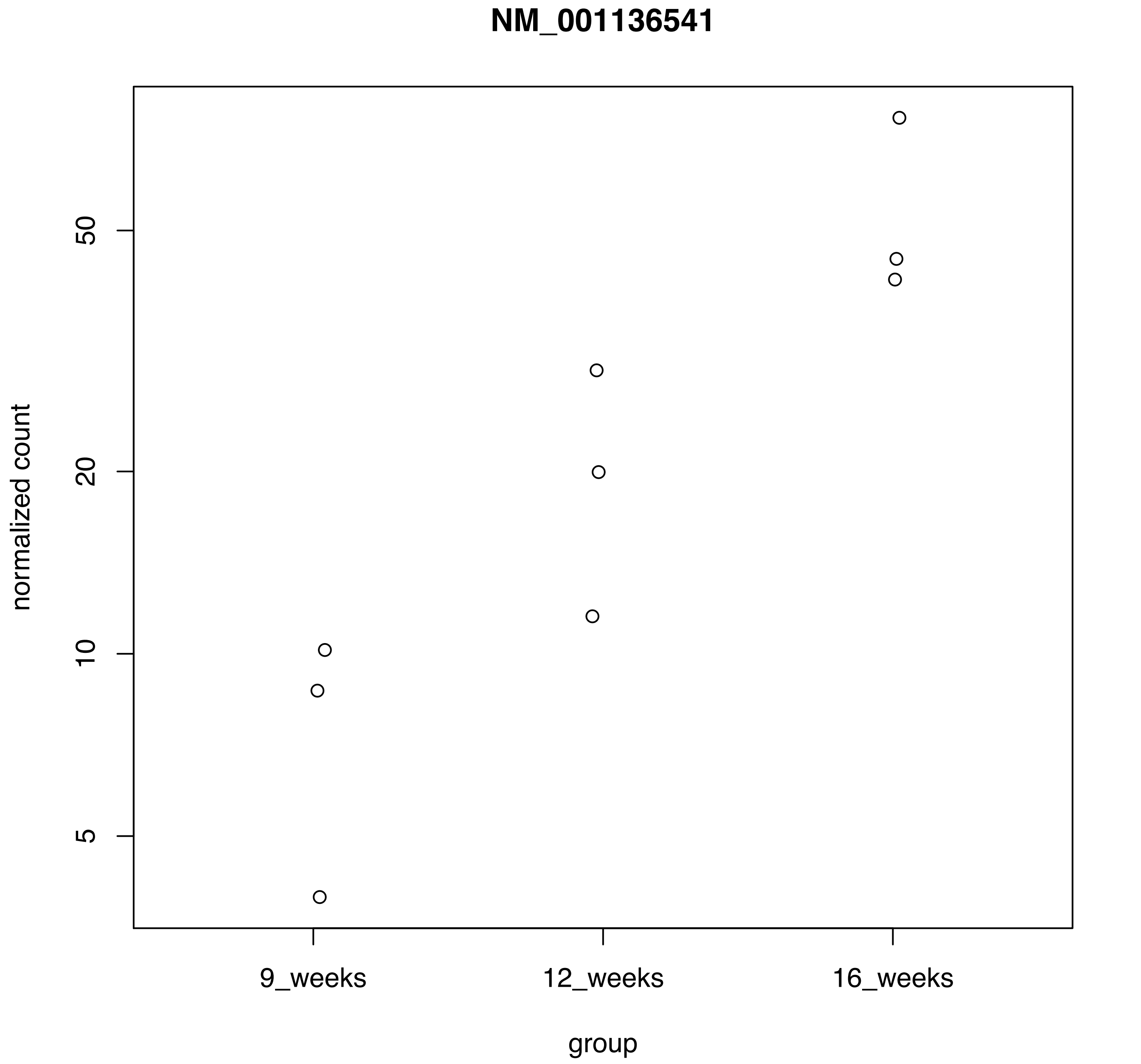

NM_001136541
normalized count
50
20
10
5
9_weeks
12_weeks
16_weeks
group

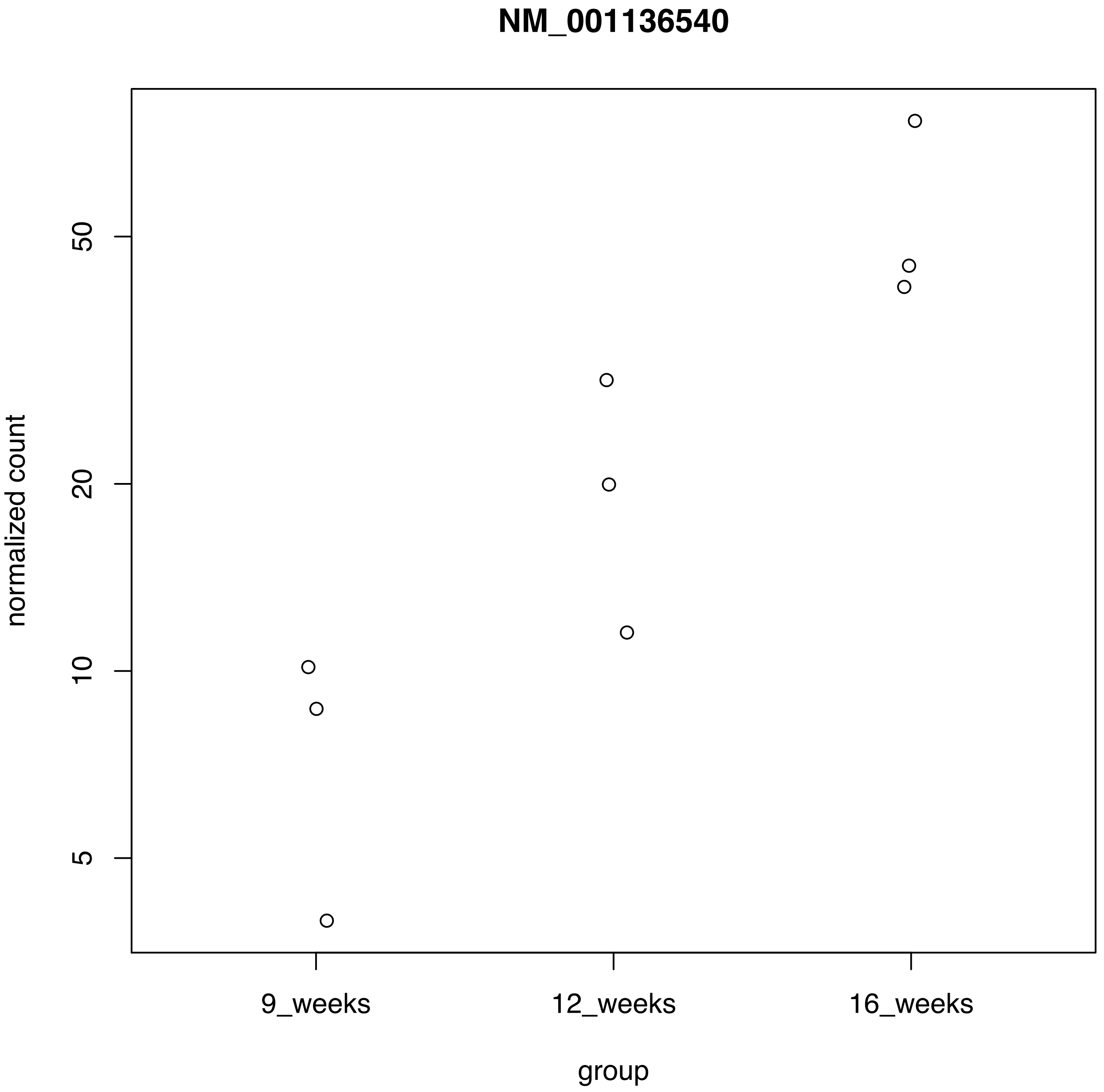
NM_001136540
normalized count
50
20
10
5
9_weeks
12_weeks
16_weeks
group

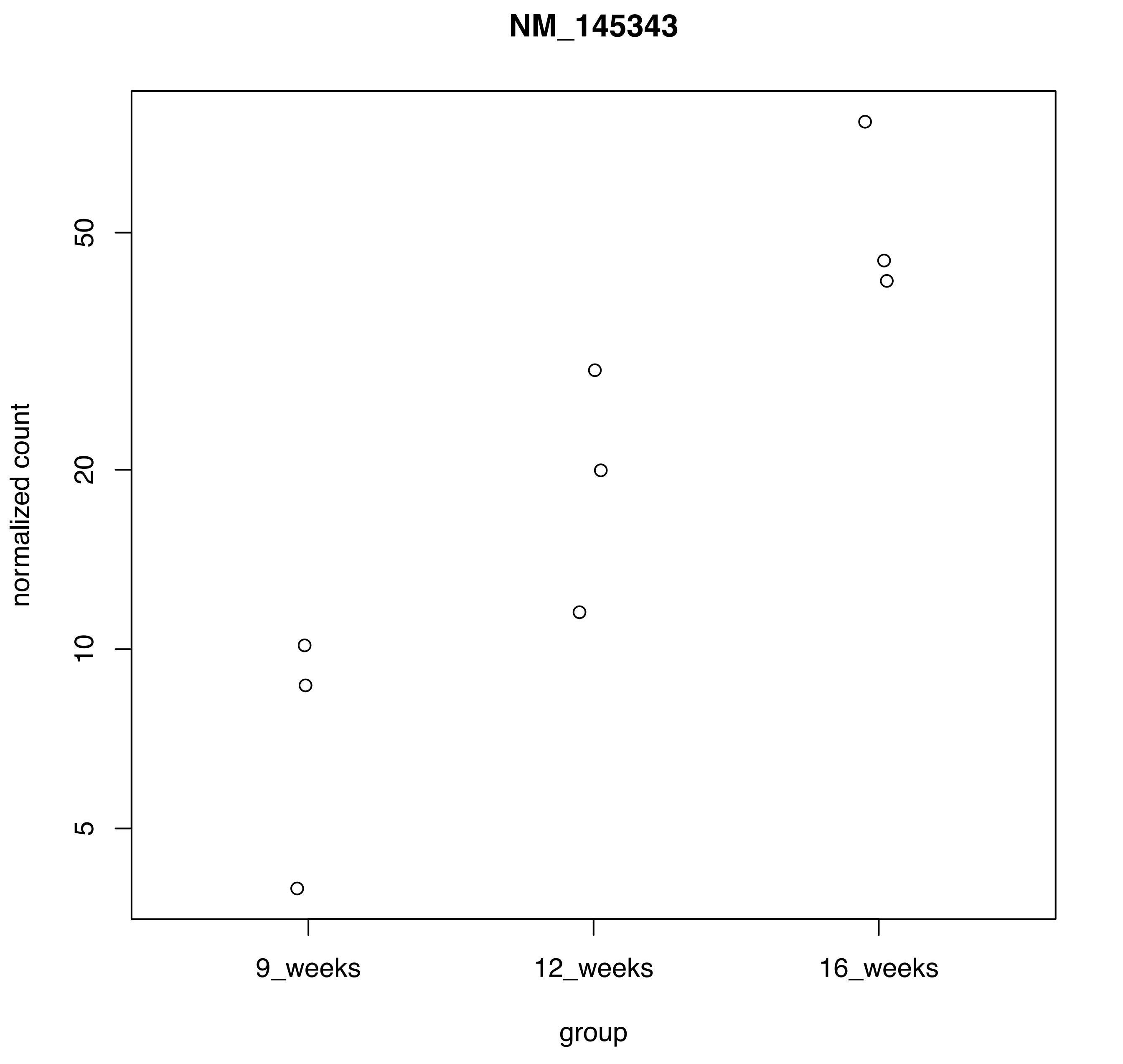
NM_145343
normalized count
50
20
10
5
9_weeks
12_weeks
16_weeks
group

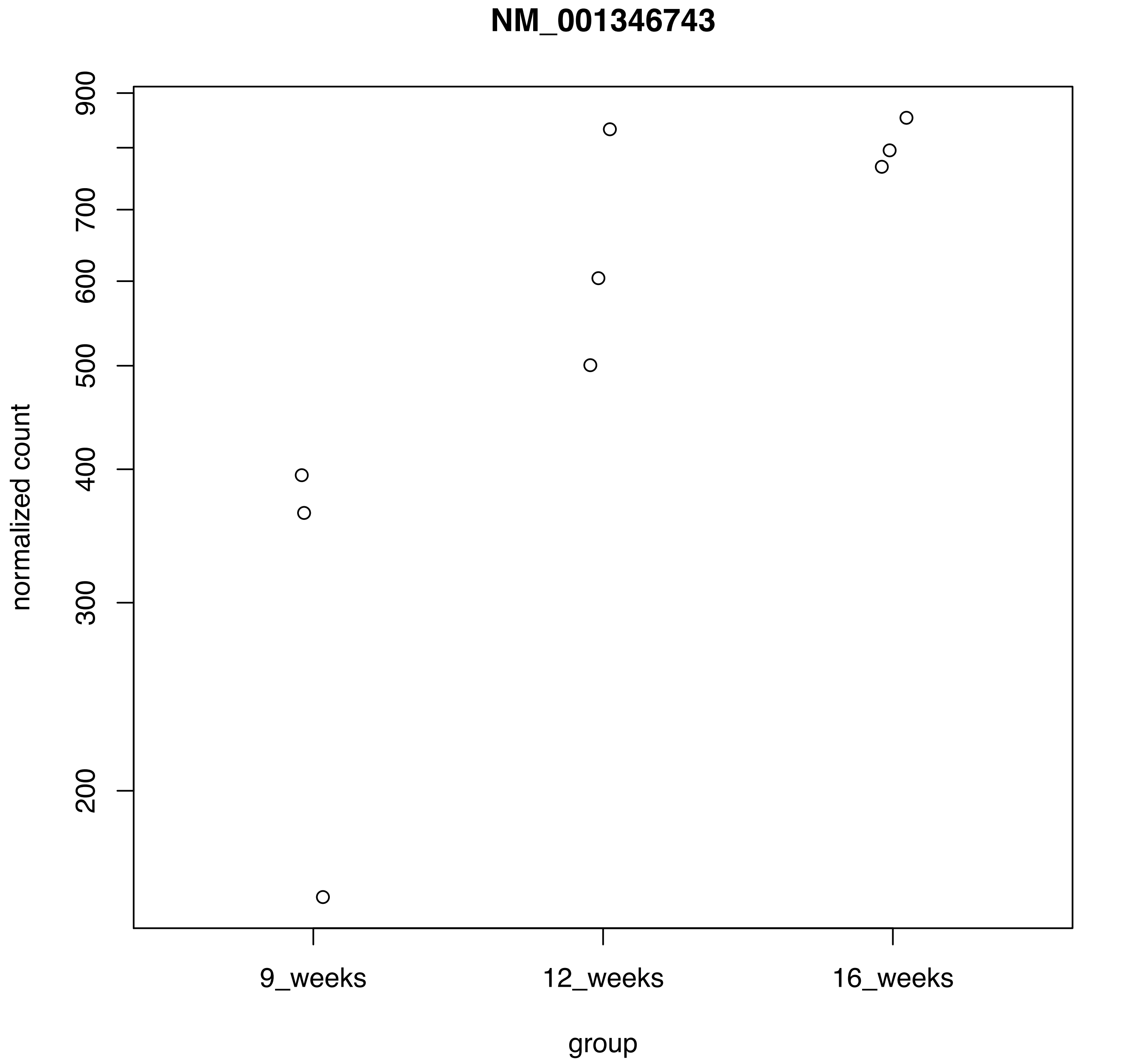

NM_001346743
normalized count
900
700
600
500
400
300
200
9_weeks
12_weeks
16_weeks
group

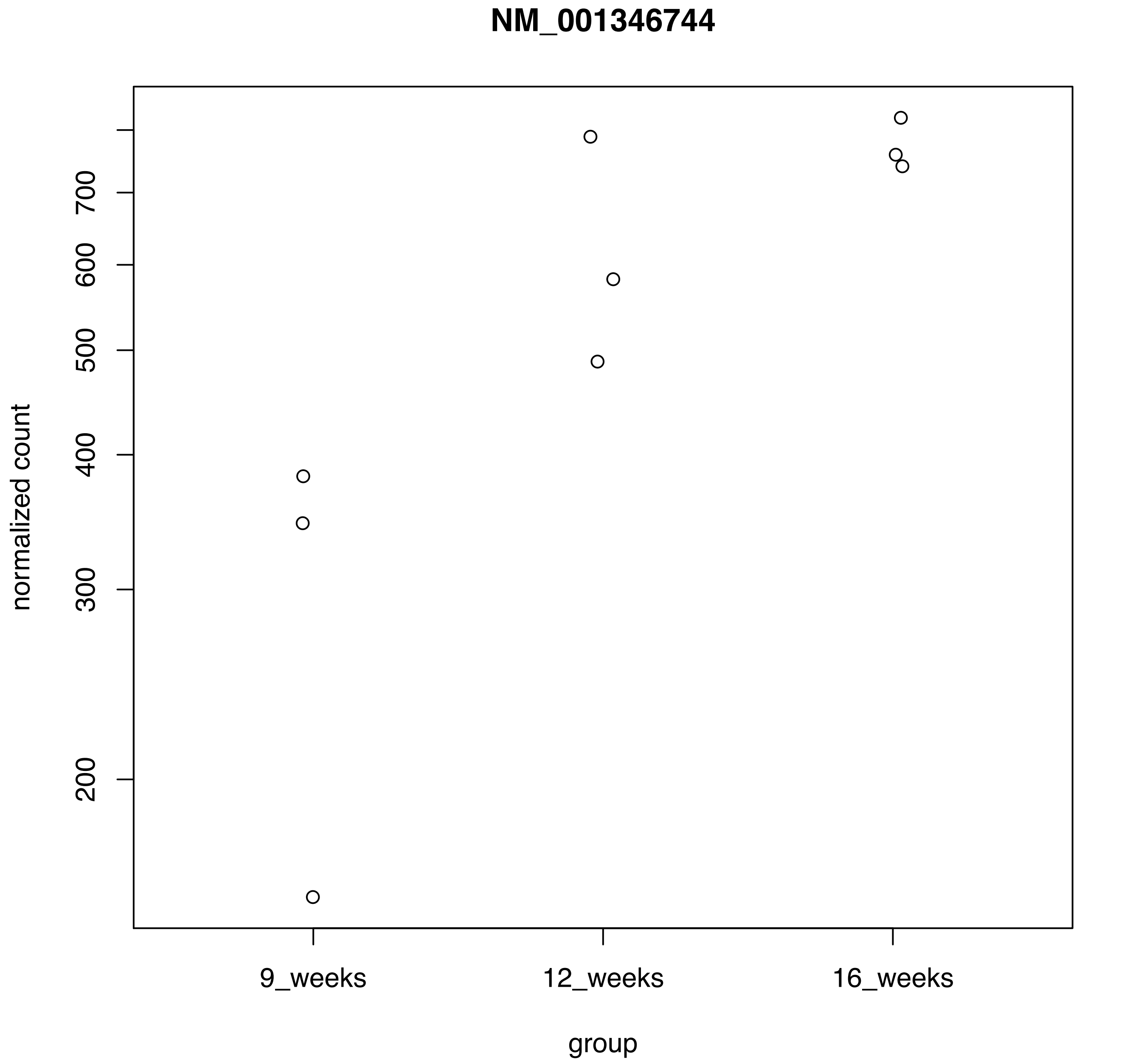
NM_001346744
normalized count
200
300
400
500
600
700
9_weeks
12_weeks
16_weeks
group

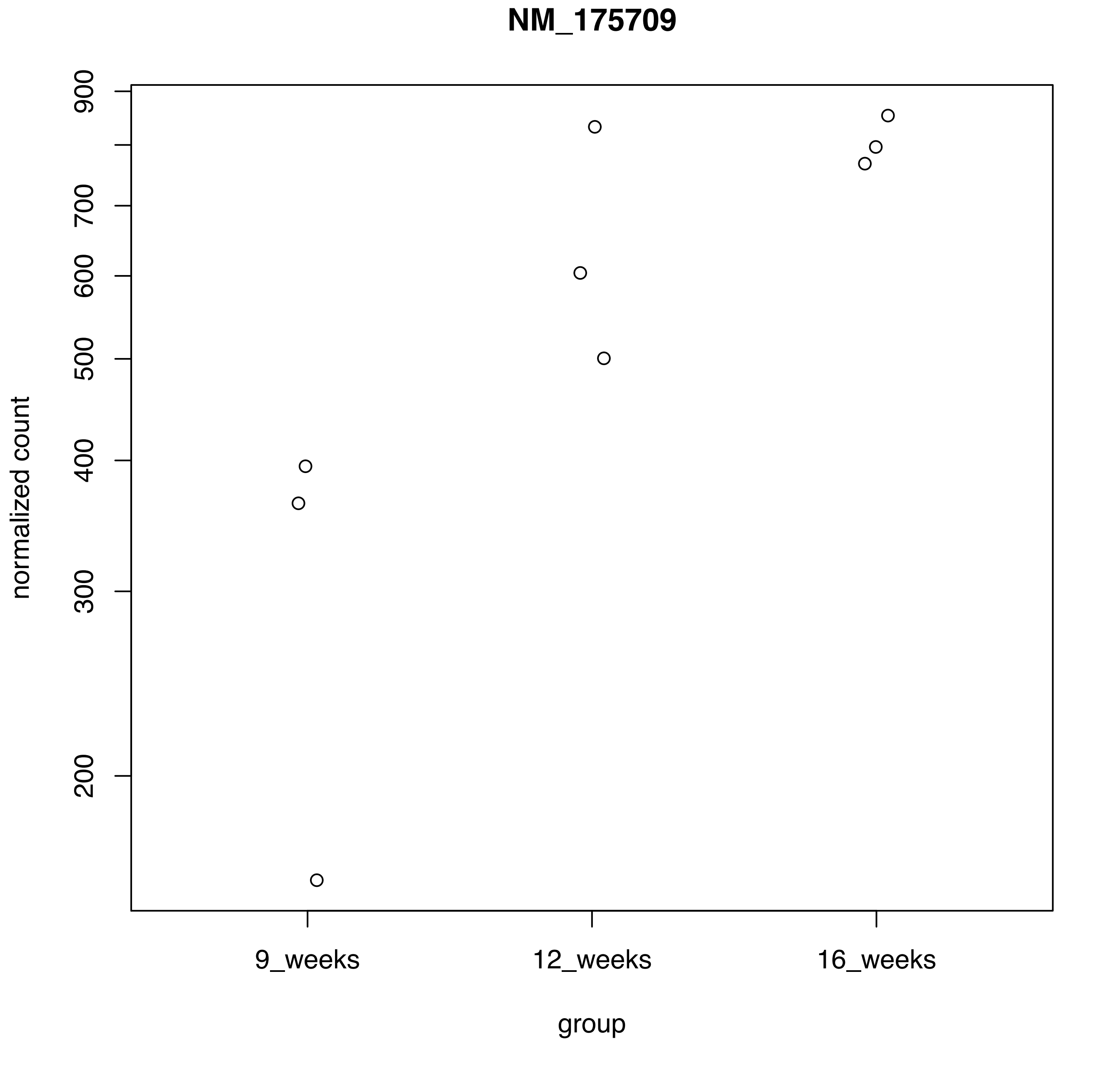
NM_175709
normalized count
900
700
600
500
400
300
200
9_weeks
12_weeks
16_weeks
group

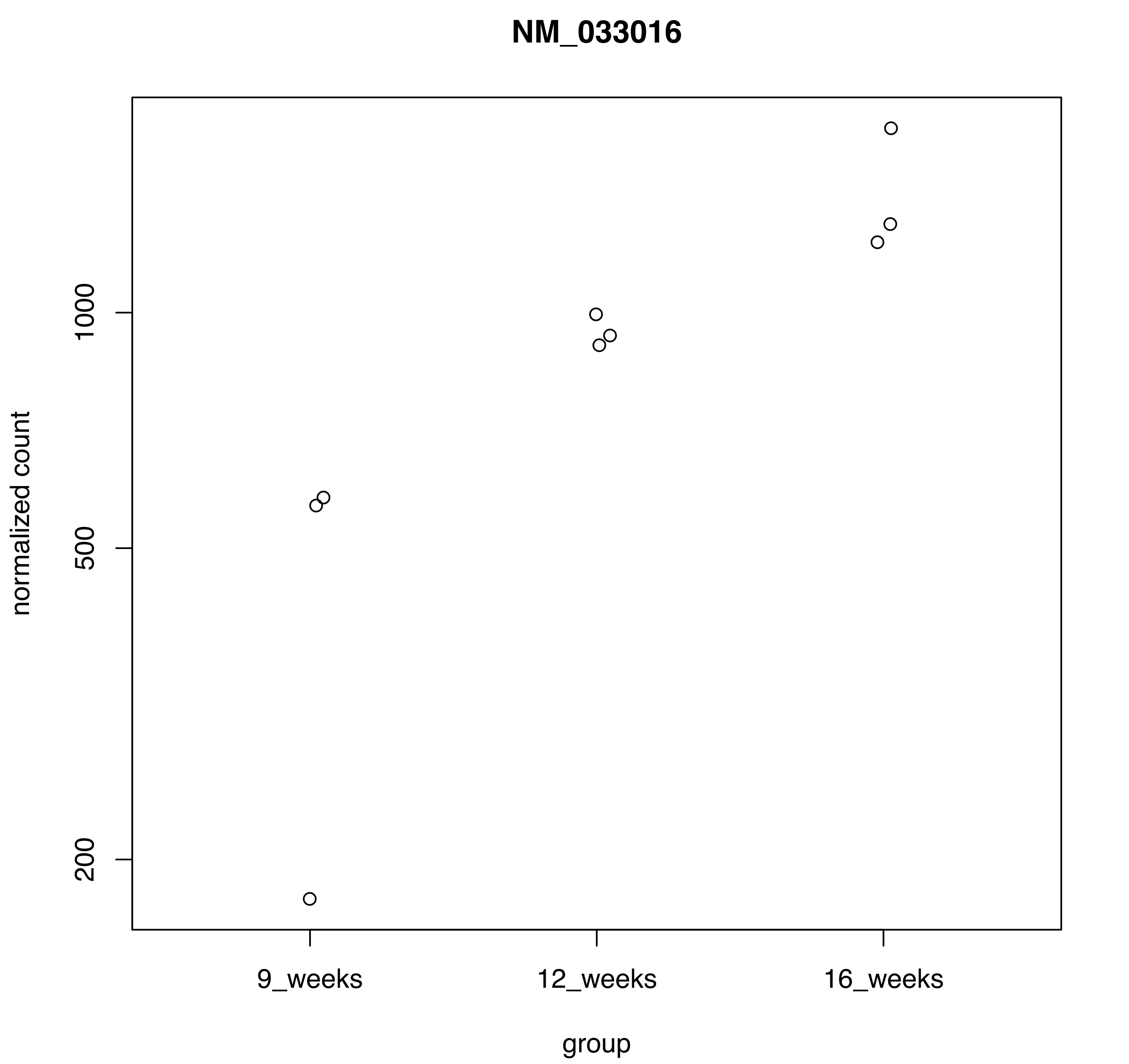
NM_033016
normalized count
1000
500
200
9_weeks
12_weeks
16_weeks
group

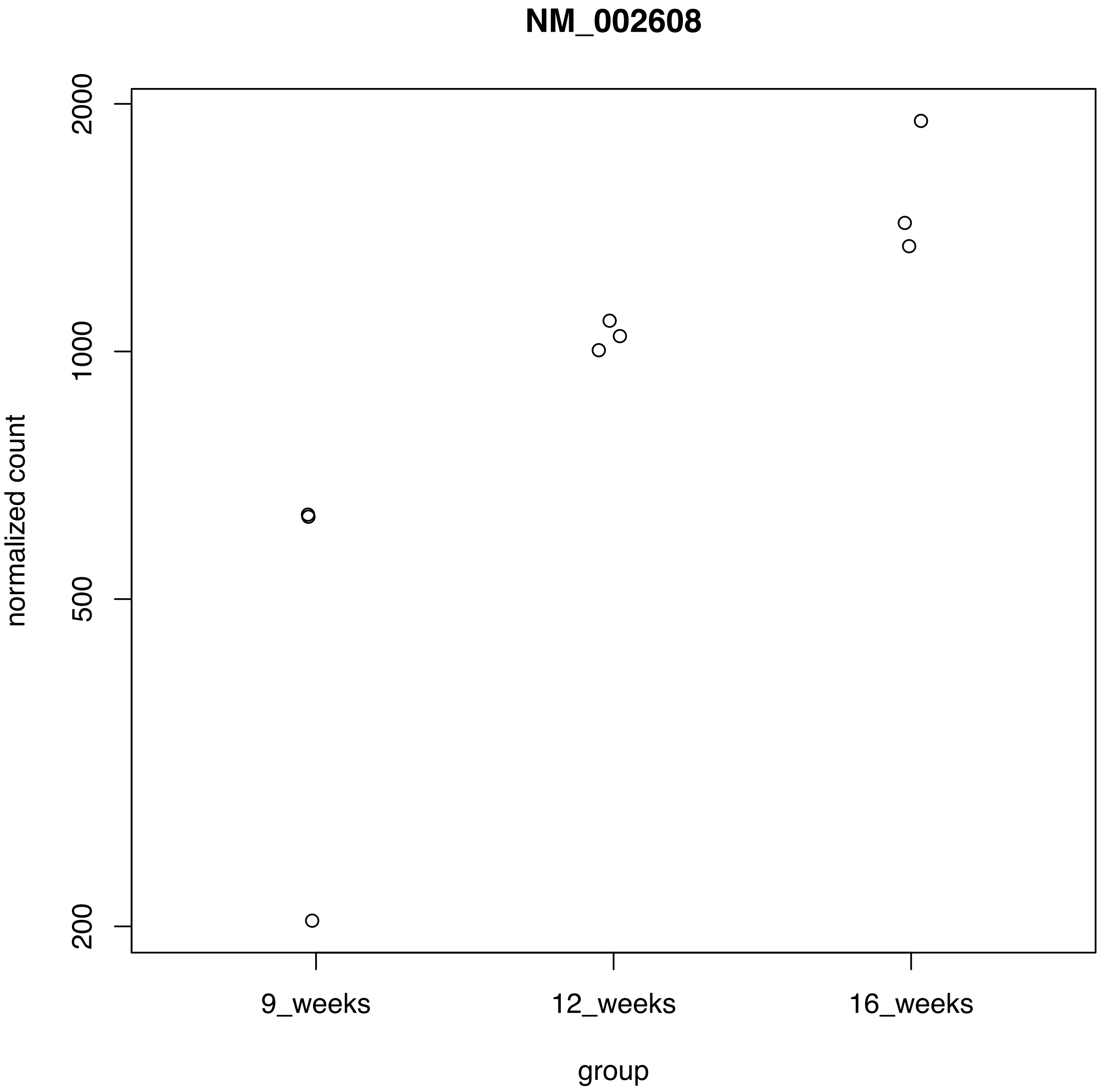
NM_002608
normalized count
2000
1000
500
200
9_weeks
12_weeks
16_weeks
group

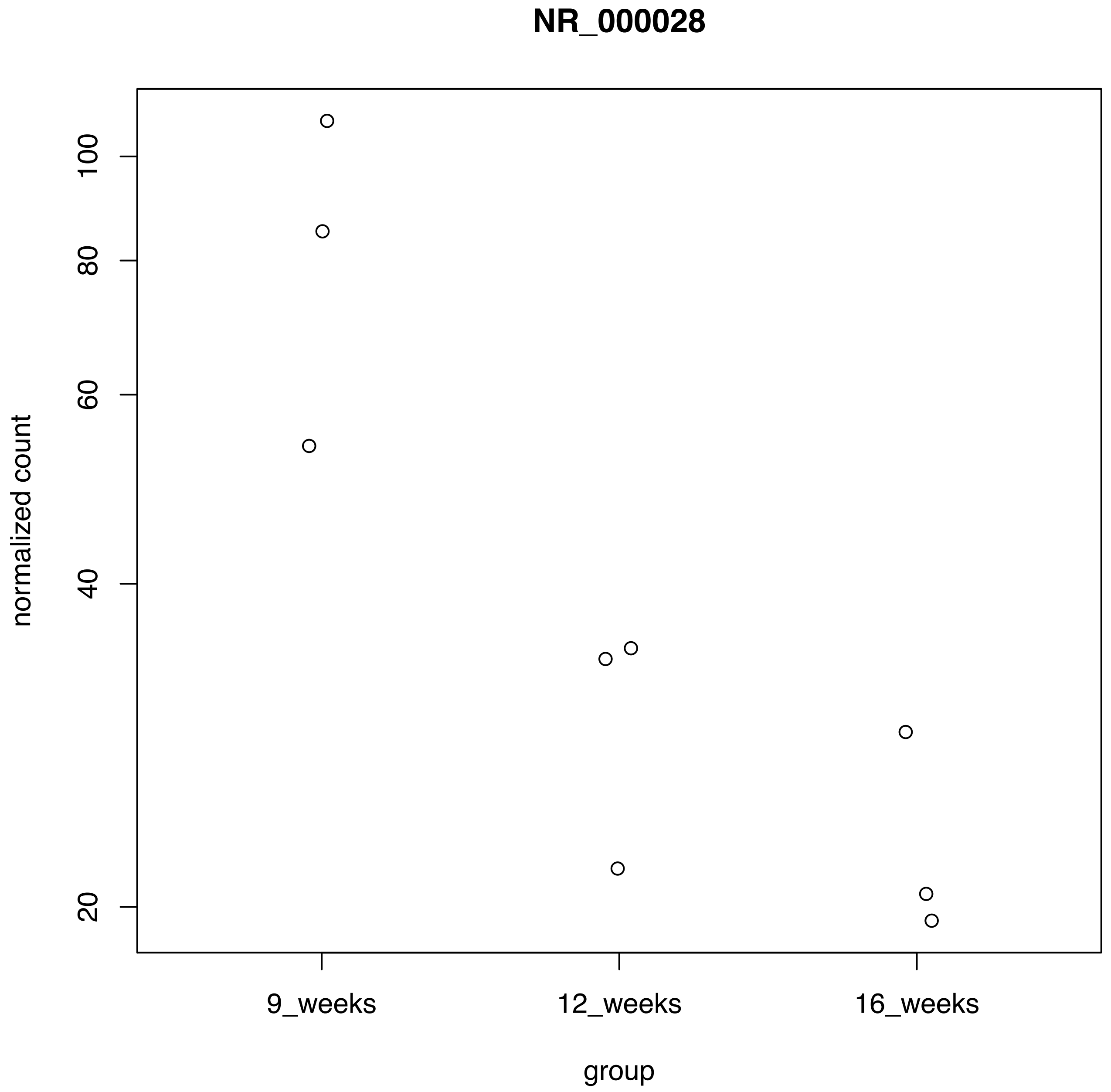
NR_000028
normalized count
100
80
60
40
20
9_weeks
12_weeks
16_weeks
group

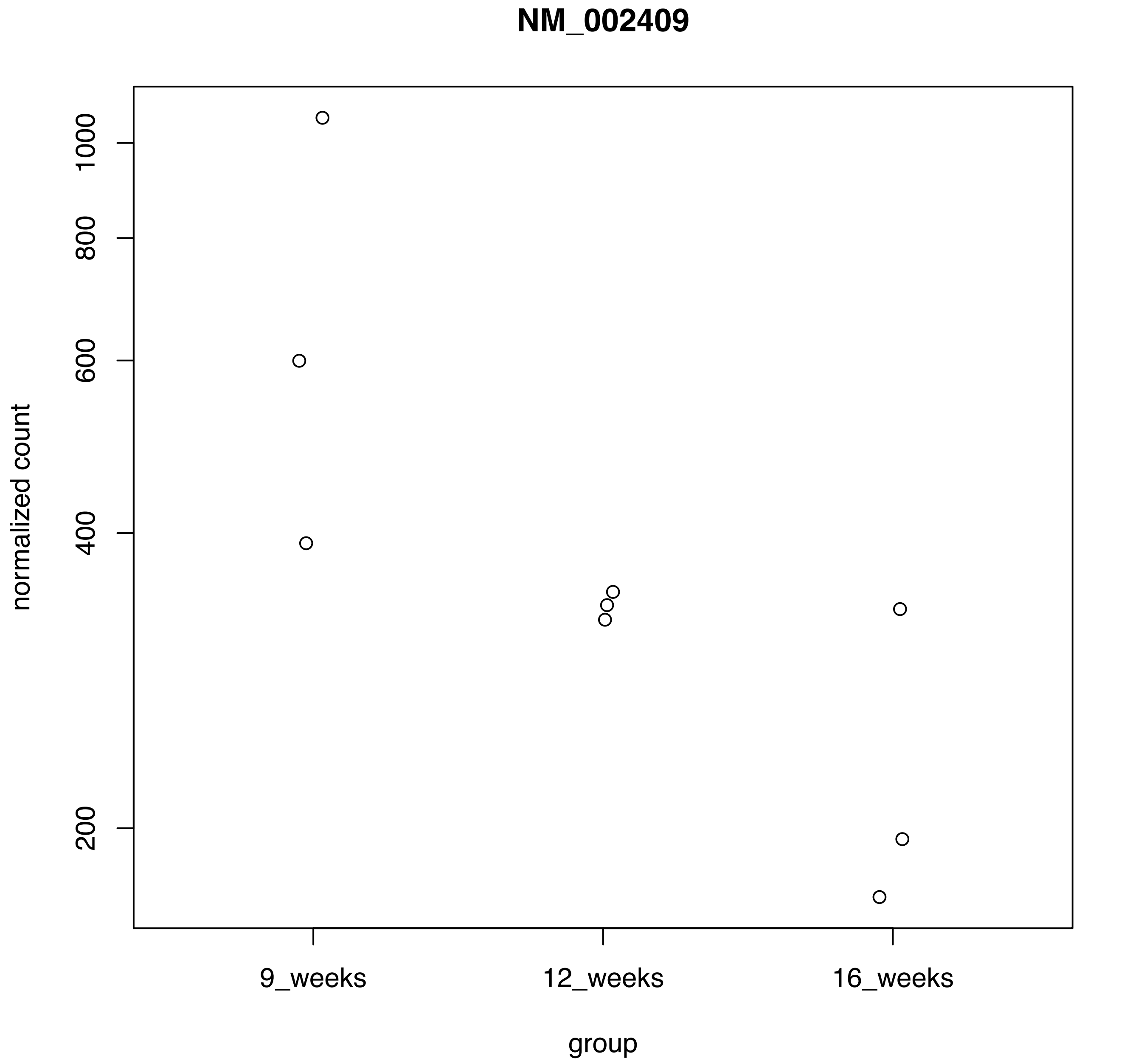
NM_002409
normalized count
1000
800
600
400
200
9_weeks
12_weeks
16_weeks
group

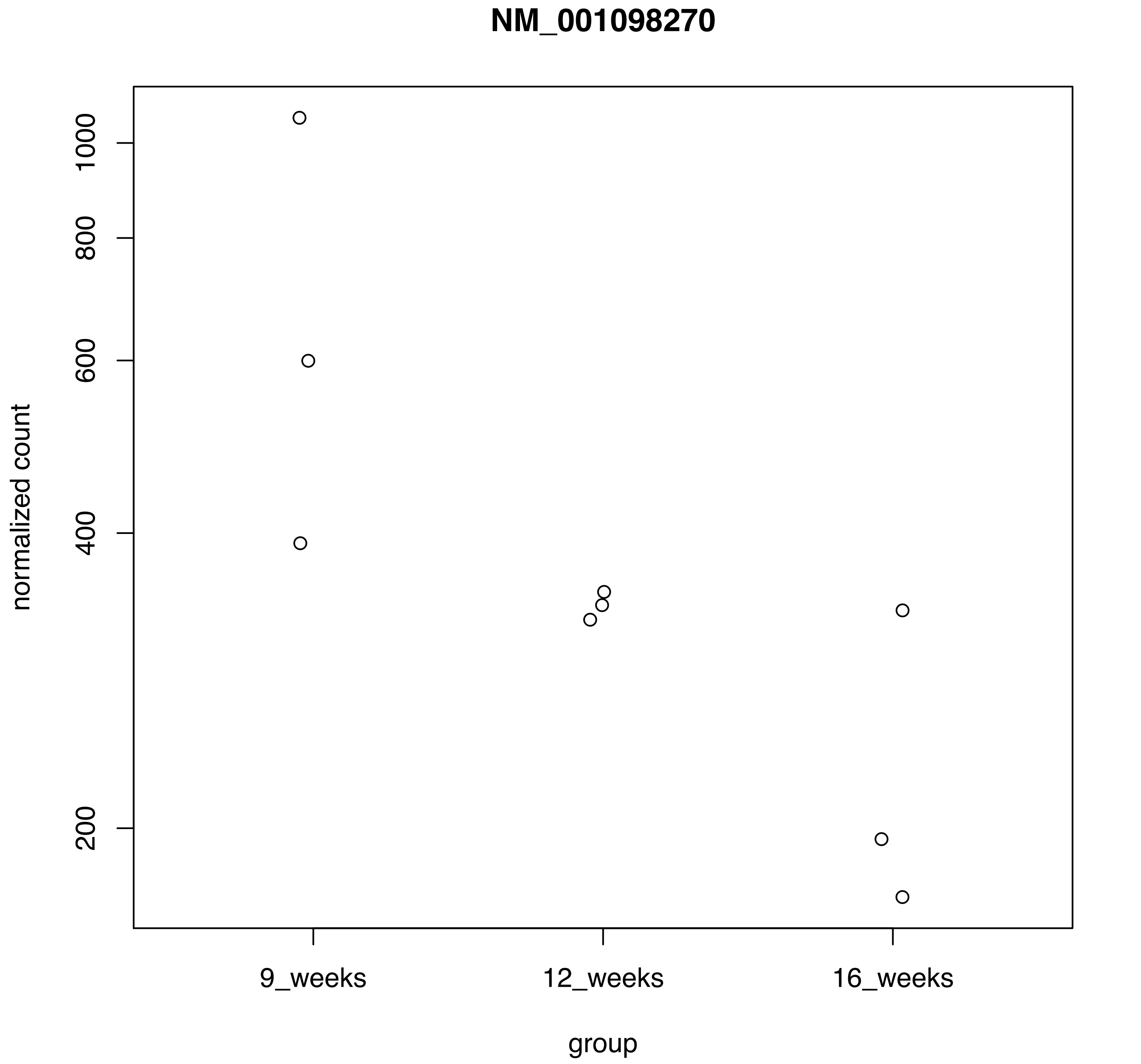
NM_001098270
normalized count
1000
800
600
400
200
9_weeks
12_weeks
16_weeks
group

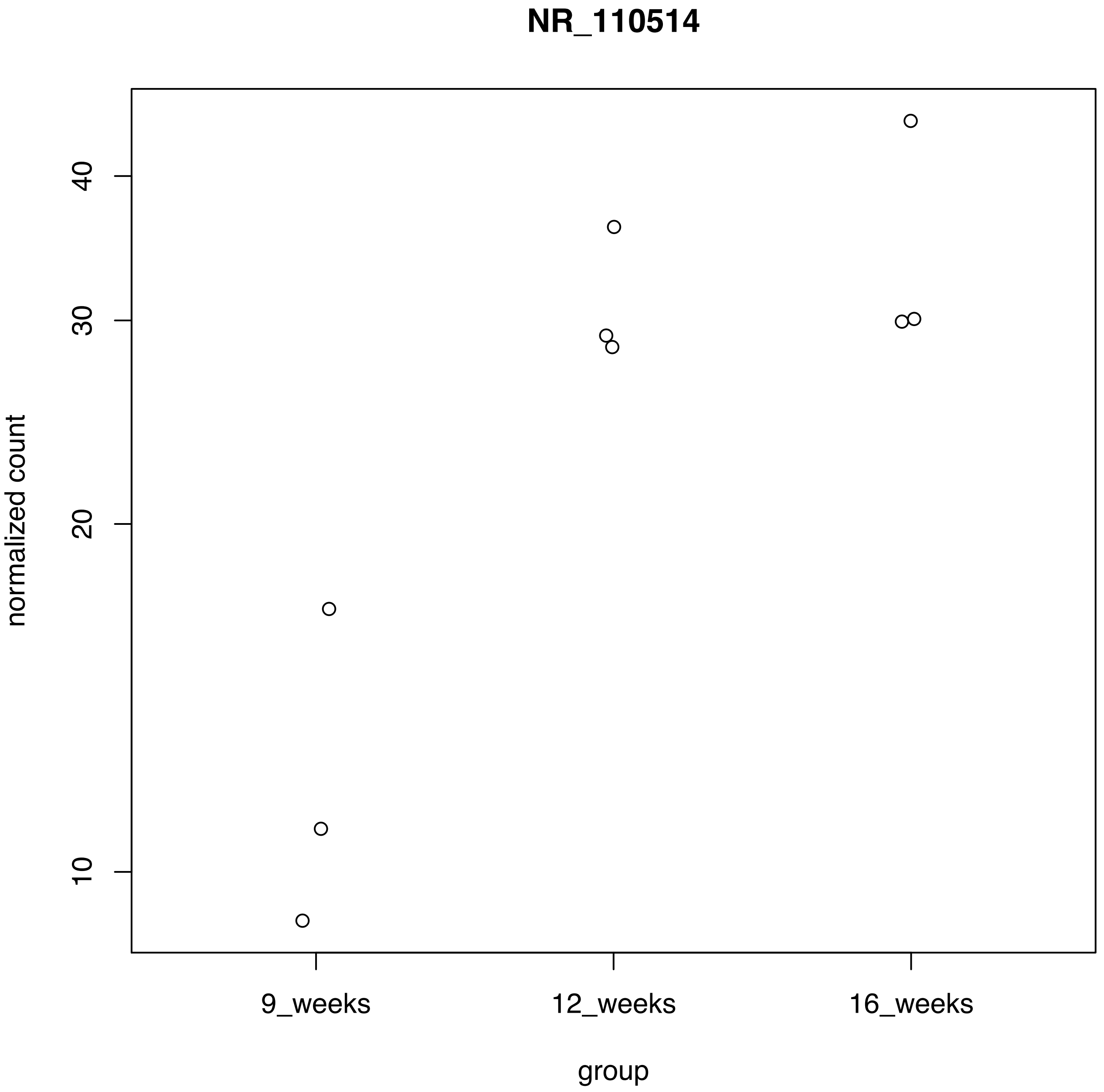
NR_110514
normalized count
10
20
30
40
9_weeks
12_weeks
16_weeks
group

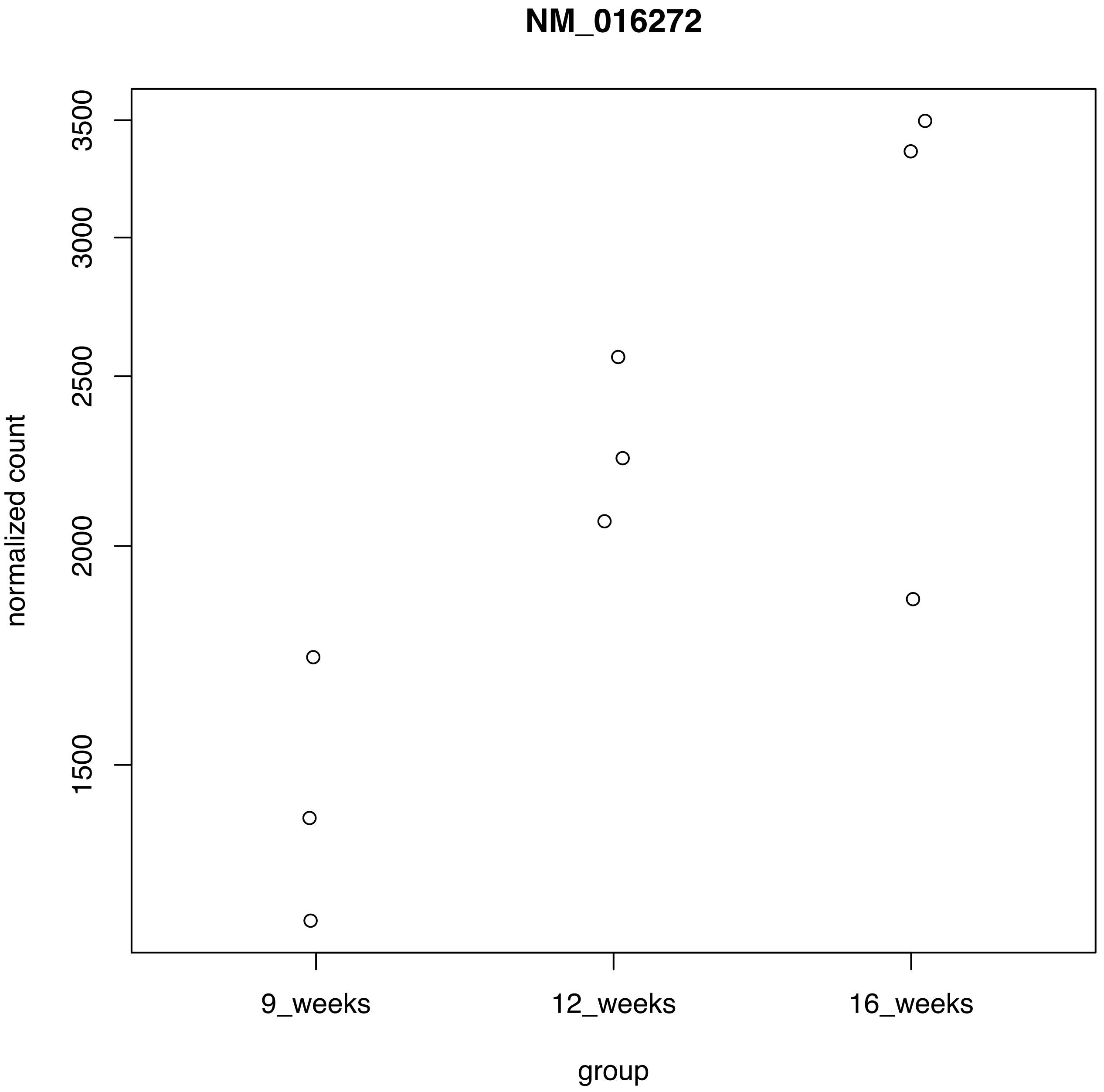
NM_016272
normalized count
1500
2000
2500
3000
3500
9_weeks
12_weeks
16_weeks
group

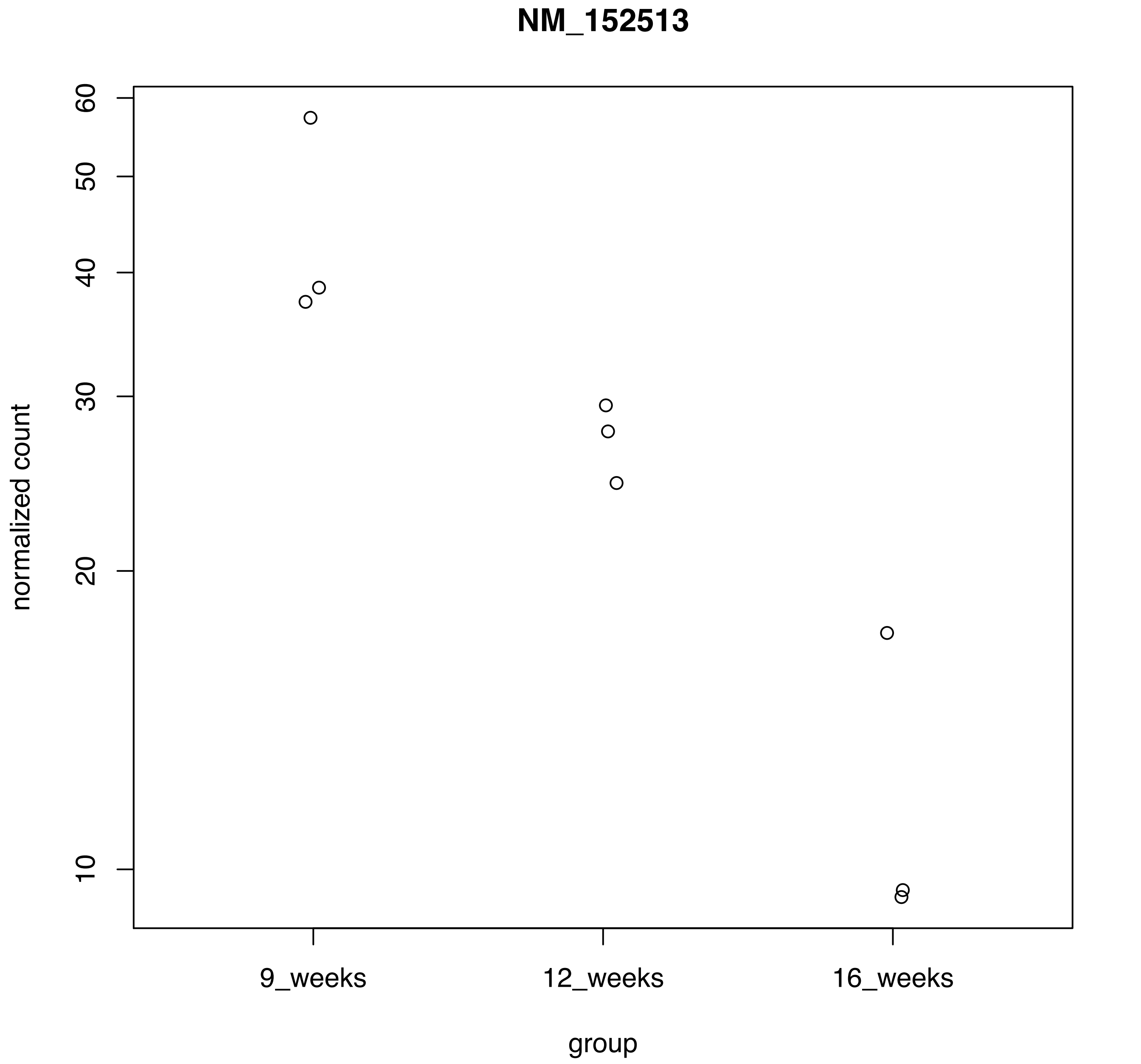
NM_152513
normalized count
60
50
40
30
20
10
9_weeks
12_weeks
16_weeks
group

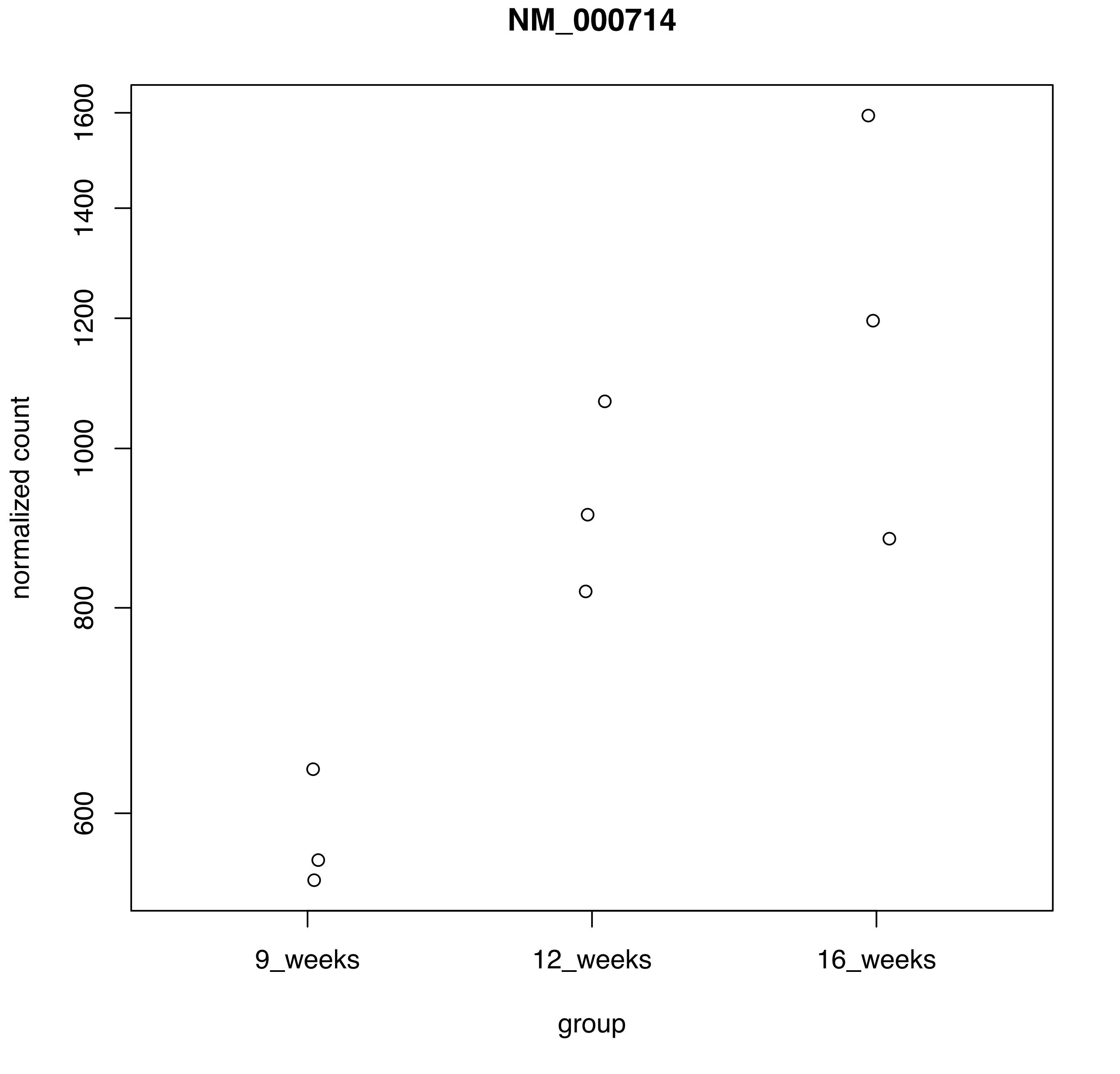
NM_000714
normalized count
600
800
1000
1200
1400
1600
group
9_weeks
12_weeks
16_weeks

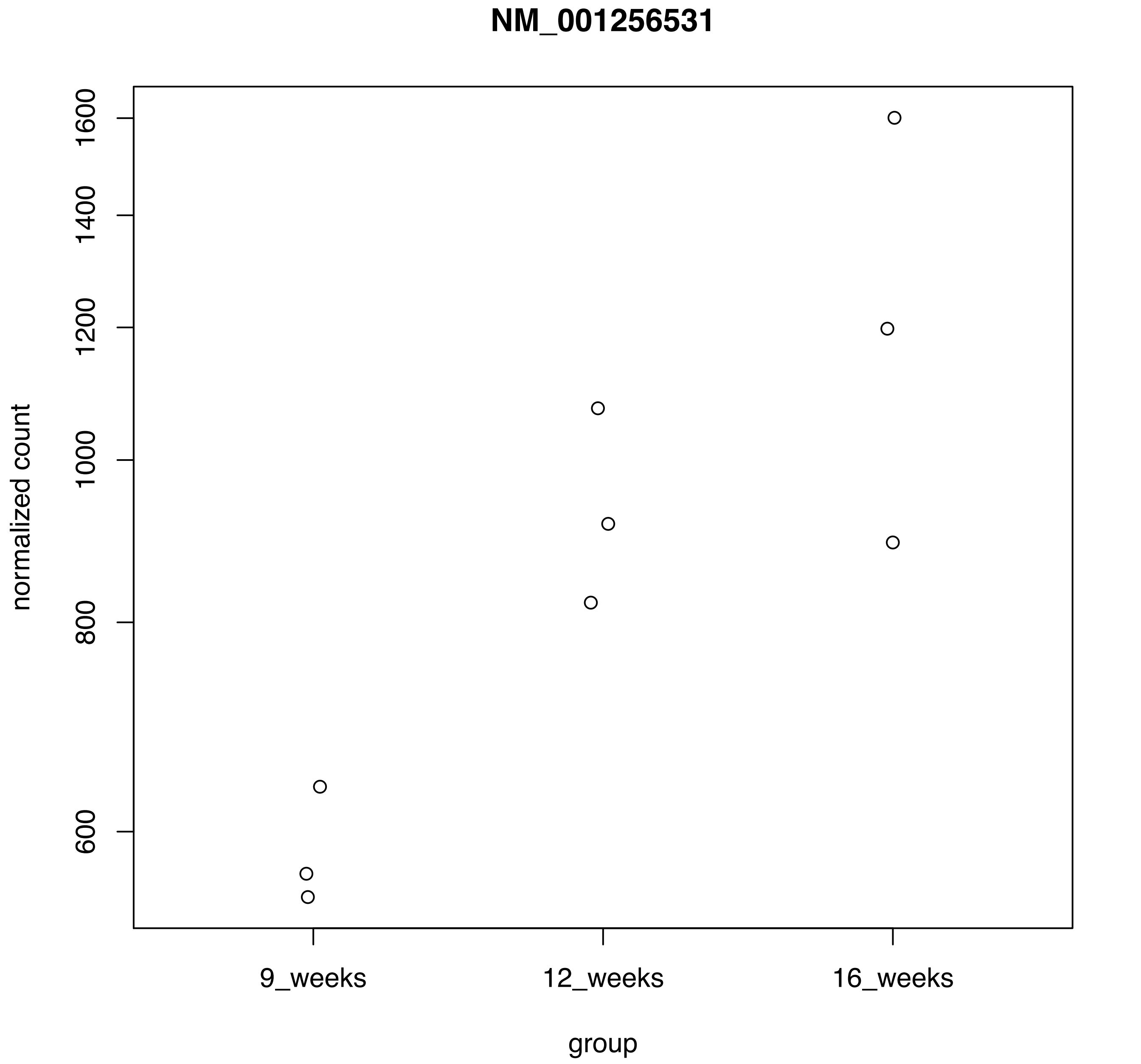
NM_001256531
normalized count
1600
1400
1200
1000
800
600
9_weeks
12_weeks
16_weeks
group

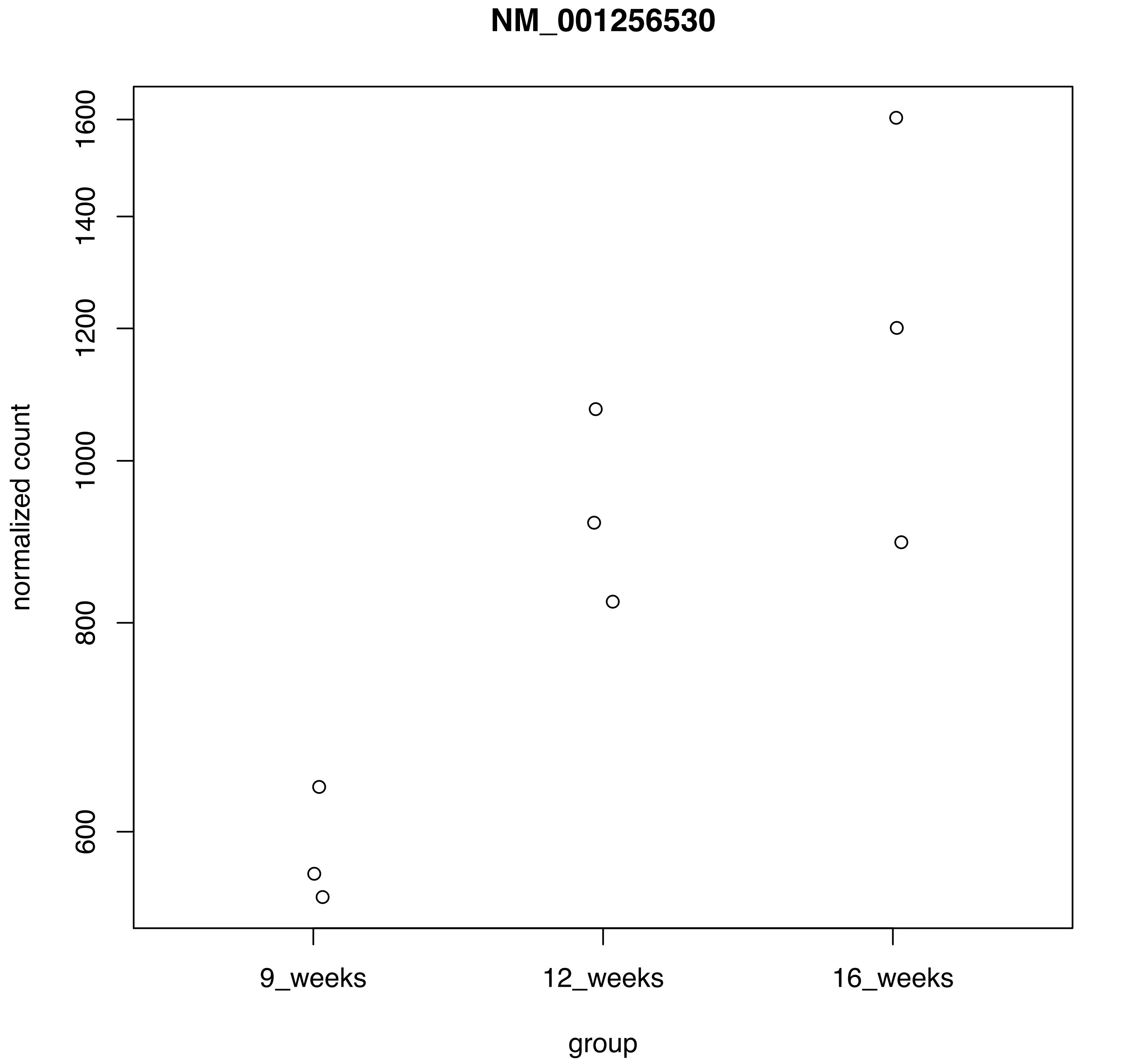
NM_001256530
normalized count
1600
1400
1200
1000
800
600
9_weeks
12_weeks
16_weeks
group

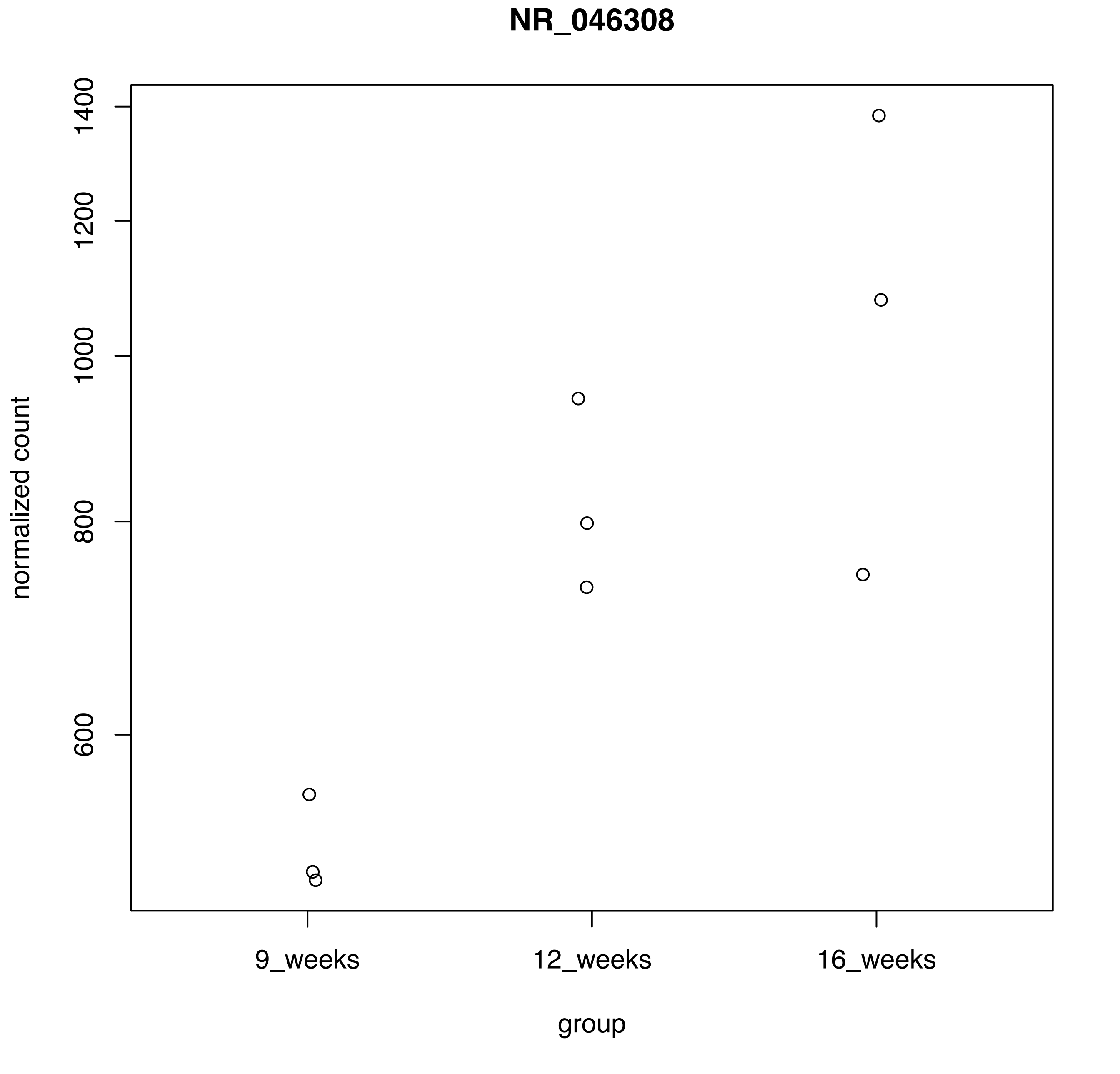
NR_046308
normalized count
600
800
1000
1200
1400
9_weeks
12_weeks
16_weeks
group

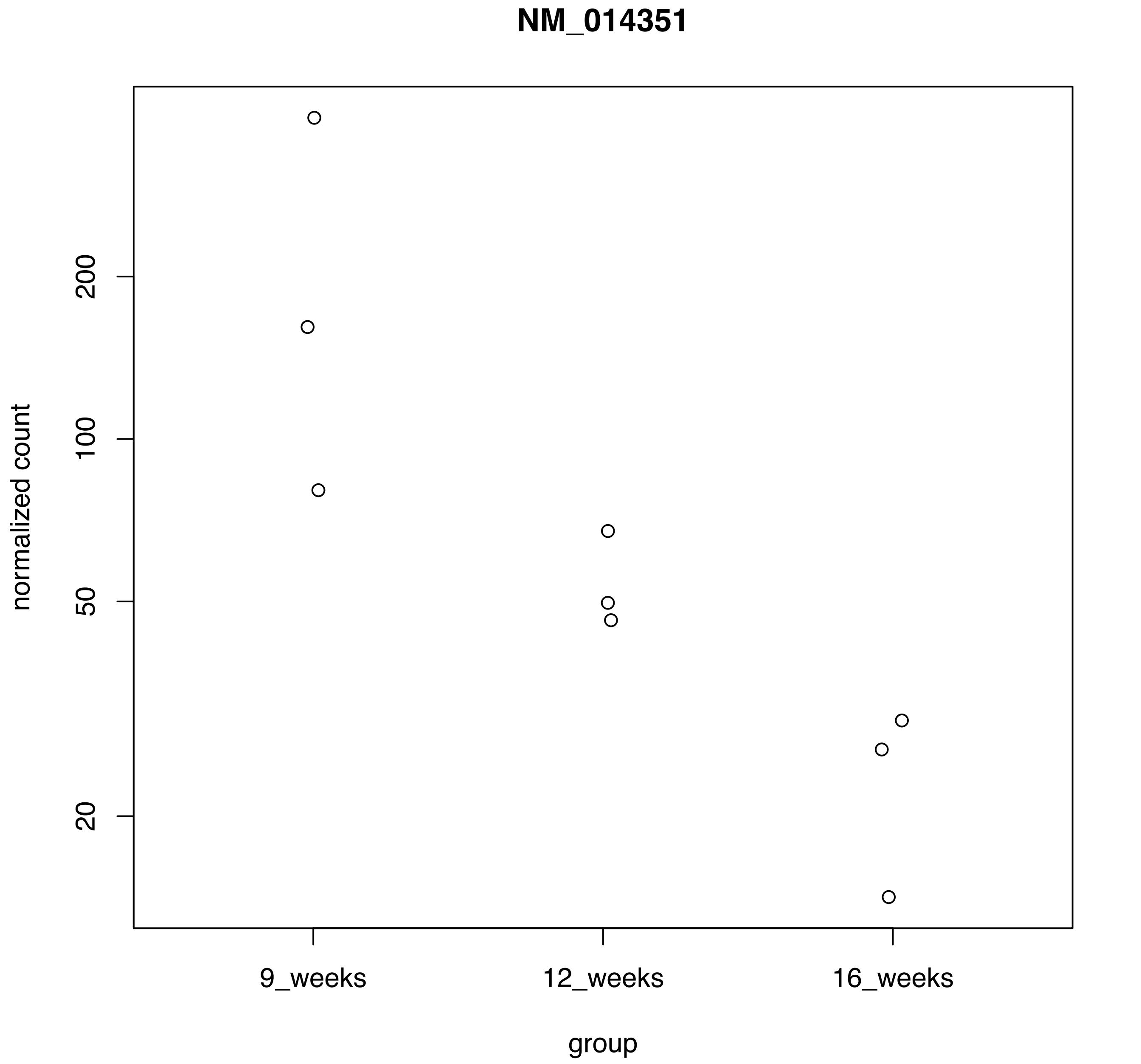
NM_014351
normalized count
200
100
50
20
9_weeks
12_weeks
16_weeks
group

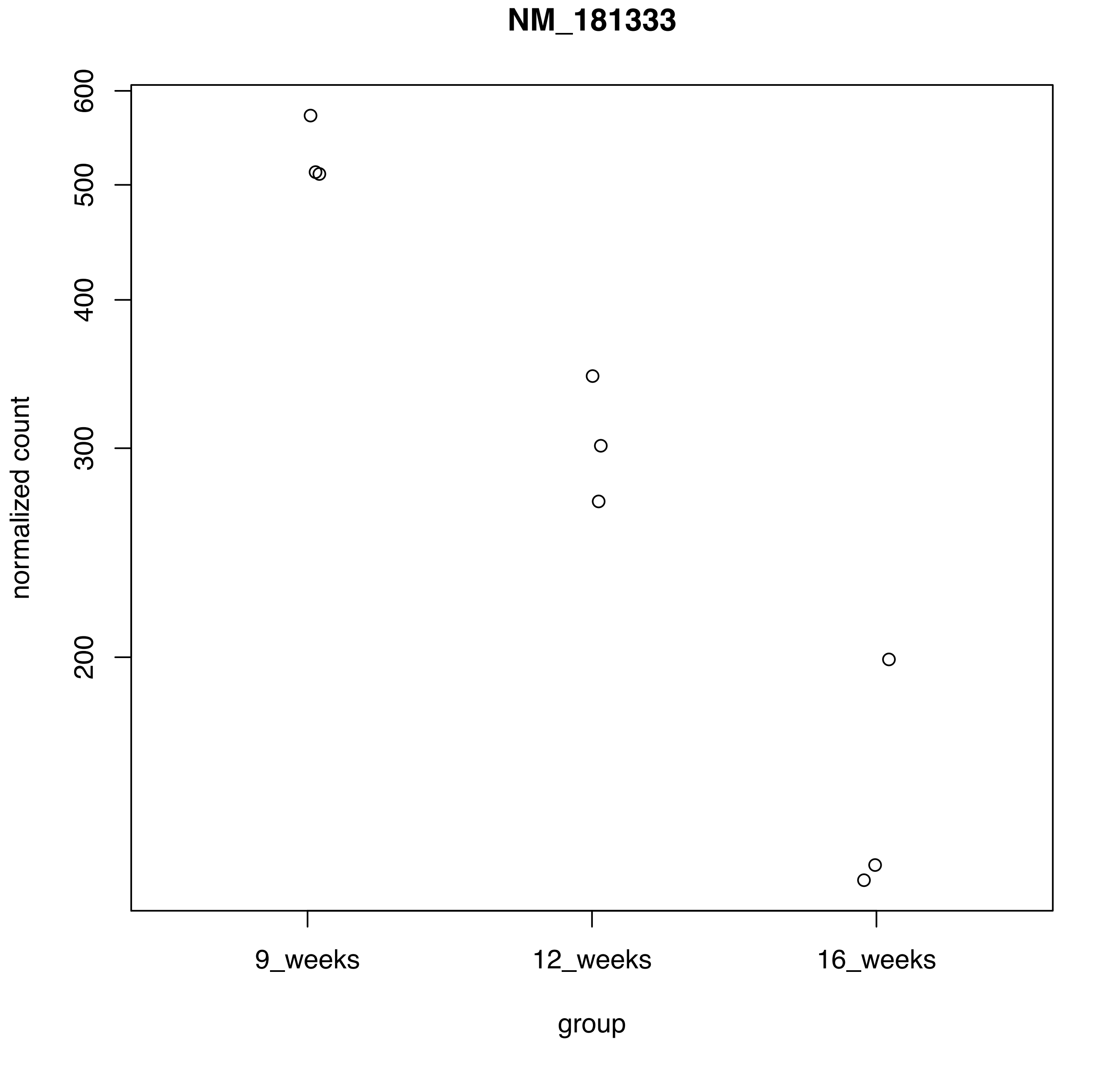
NM_181333
600
500
400
300
200
normalized count
9_weeks
12_weeks
16_weeks
group

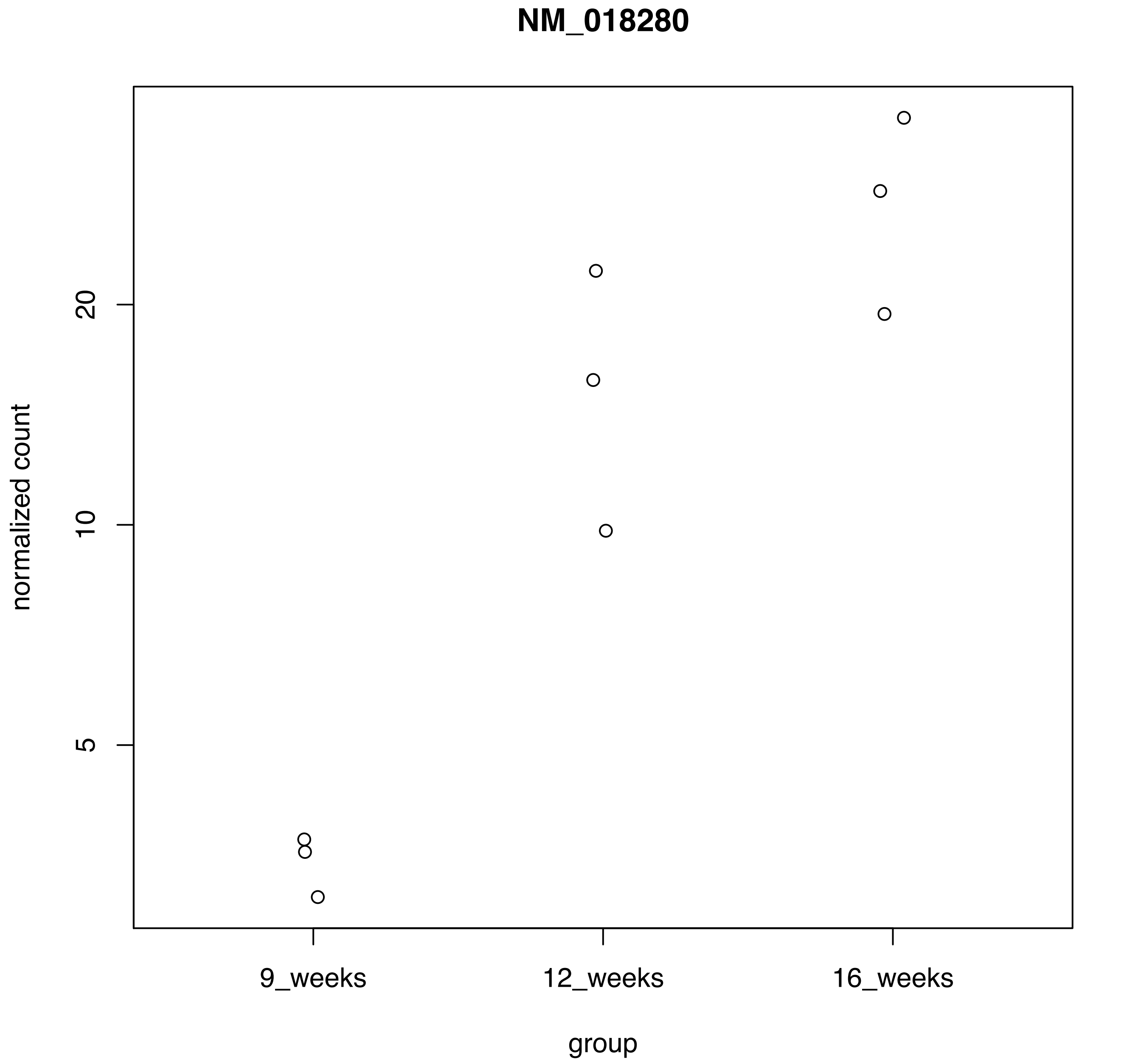
NM_018280
normalized count
20
10
5
9_weeks
12_weeks
16_weeks
group

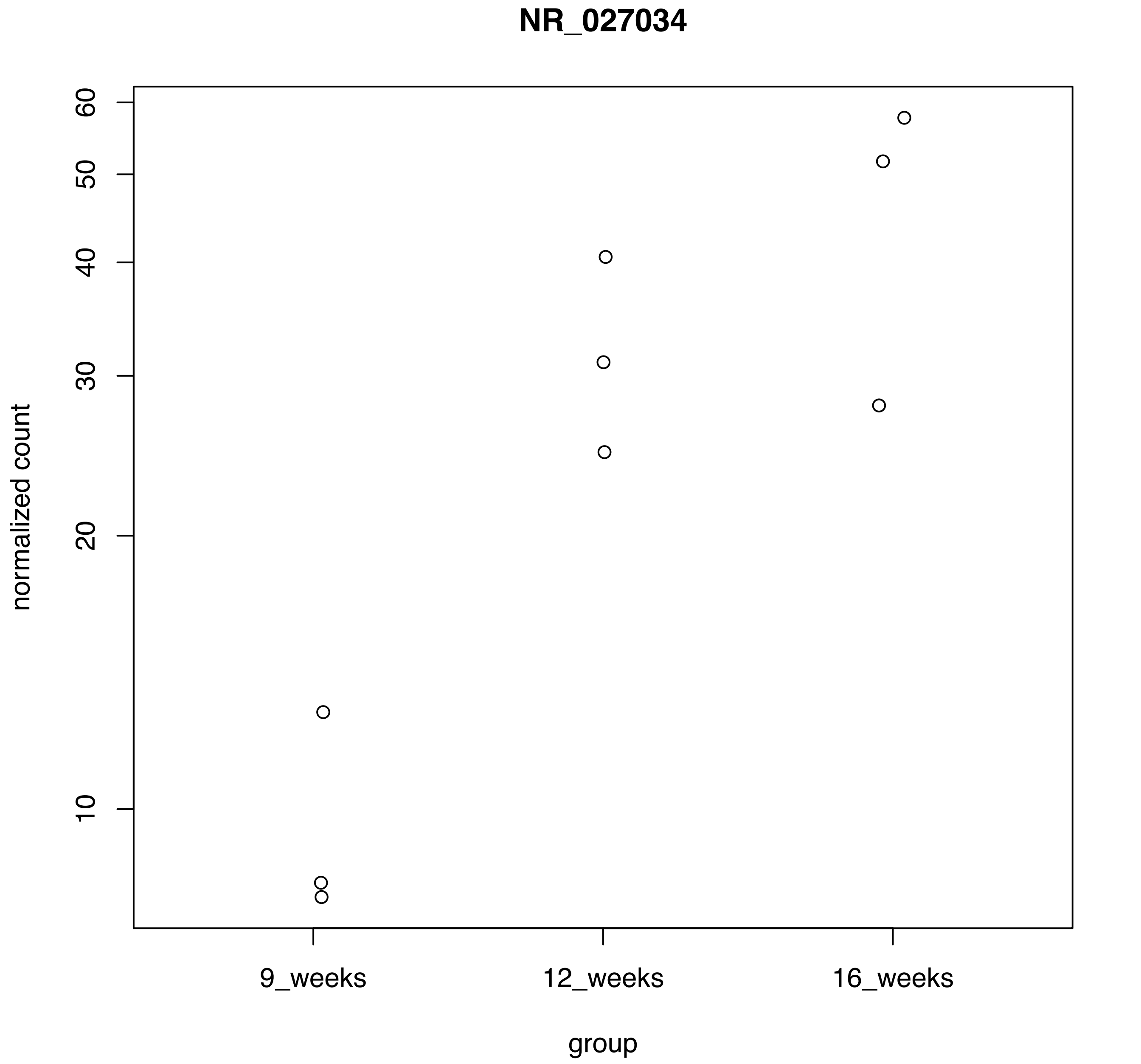
NR_027034
normalized count
10
20
30
40
50
60
9_weeks
12_weeks
16_weeks
group

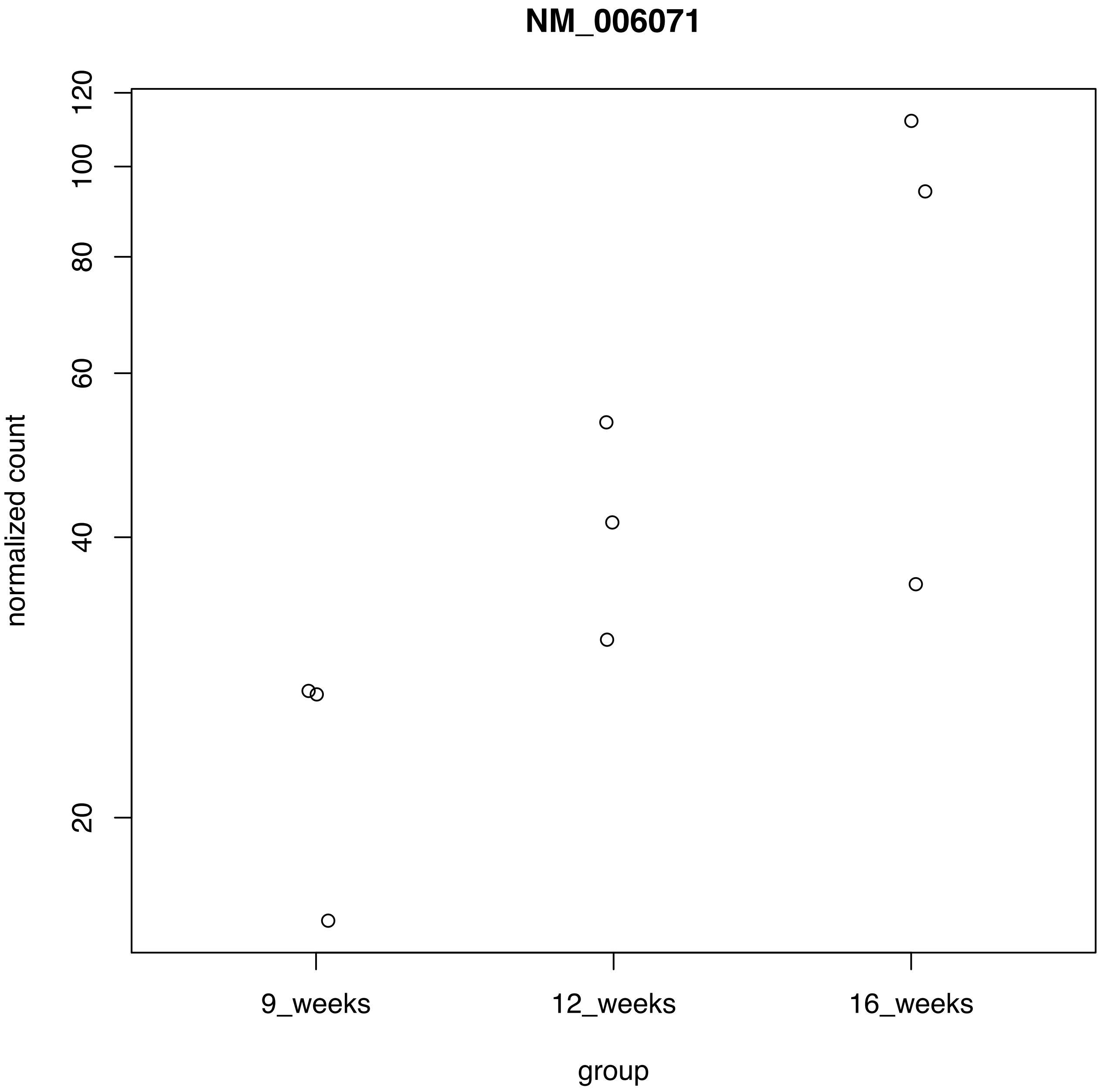
NM_006071
normalized count
20
40
60
80
100
120
9_weeks
12_weeks
16_weeks
group

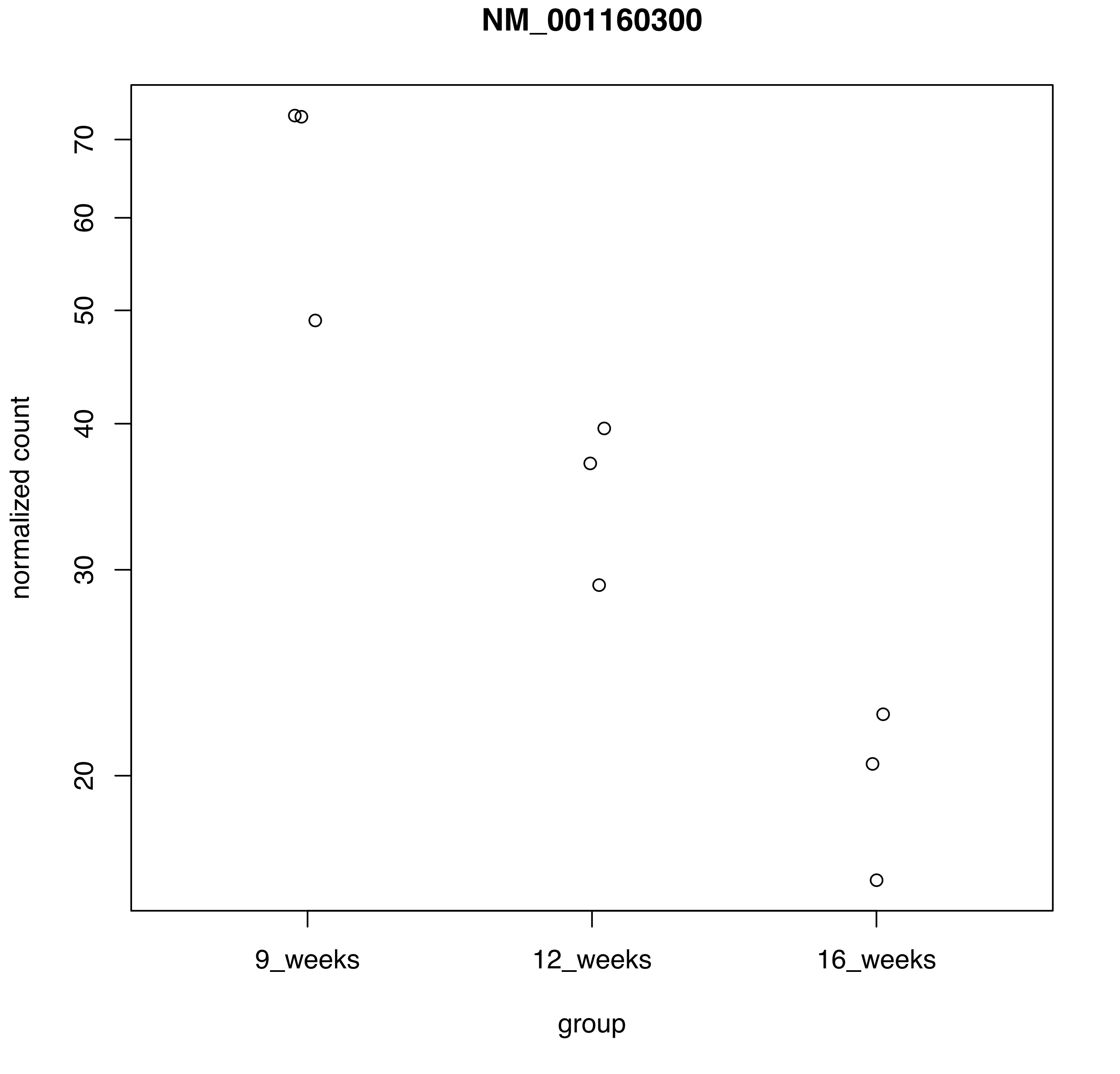

NM_001160300
normalized count
70
60
50
40
30
20
9_weeks
12_weeks
16_weeks
group

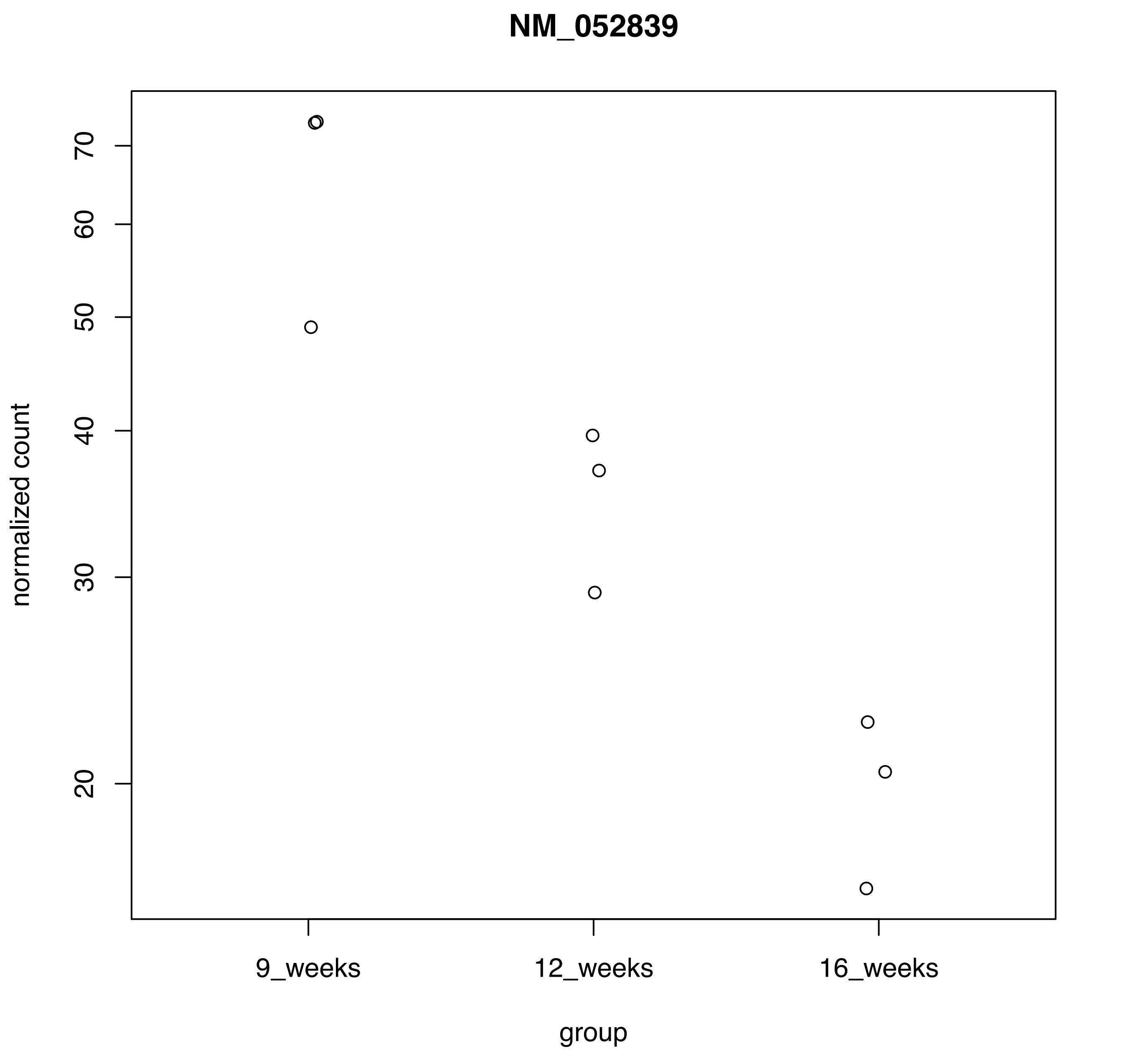
NM_052839
normalized count
70
60
50
40
30
20
9_weeks
12_weeks
16_weeks
group

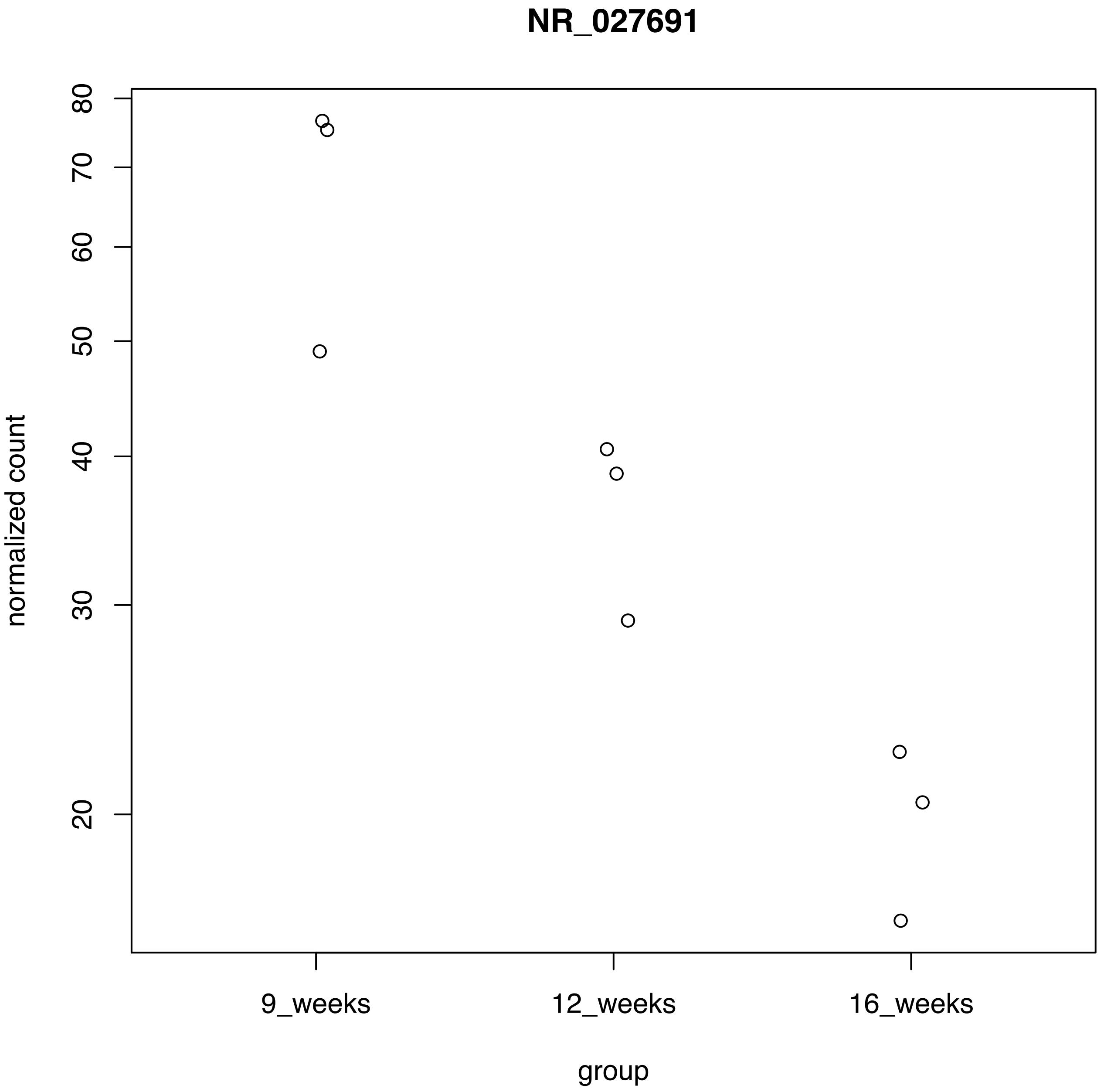
NR_027691
normalized count
80
70
60
50
40
30
20
9_weeks
12_weeks
16_weeks
group
