# Supplementary material for: The developmental transcriptome of the human heart
Source: Sci Rep. 2018 Oct 18;8:15362. doi: 10.1038/s41598-018-33837-6 (PMC6194117; doi:10.1038/s41598-018-33837-6)
Supplement: Supplementary file 1 — Supplementary Materials [file 41598_2018_33837_MOESM1_ESM.pdf]

The developmental transcriptome of the human heart

Extended Data

Eleftheria Pervolaraki Ph.D[1*], James Dachtler Ph.D[1,2], Richard A. Anderson MBChB, Ph.D[3], Arun V. Holden Ph.D[1]

[1]School of Biomedical Sciences, University of Leeds, Leeds, LS2 9JT, UK.
[2]Department of Psychology, Durham University, Durham, DH1 3LE, UK.
[3]MRC Centre for Reproductive Health, University of Edinburgh, Edinburgh, EH16 4TJ, UK.

*Correspondence should be addressed to Dr. Eleftheria Pervolaraki, School of Biomedical Sciences, University of Leeds, Leeds, LS2 9JT, UK.
fbsepe@leeds.ac.uk, +44(0) 113 34 31869

Extended Data Figure 1

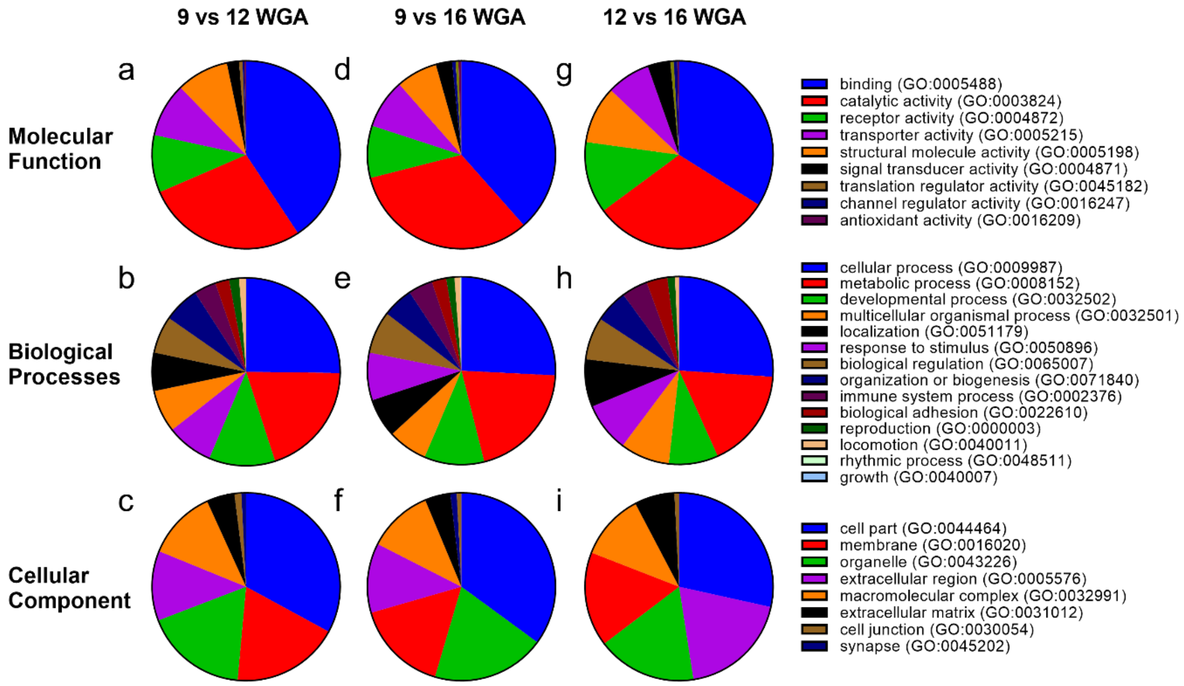

Extended Data Fig 1. Gene ontology of the significant differentially expressed genes. PANTHER (http://www.pantherdb.org/) was used to assign functional classifications of molecular function (**a, d, g**), biological processes (**b, e, h**) and cellular component (**c, f, i**) for 9 compared to 12 weeks gestational age (WGA) (**a-c**), 9 compared to 16 WGA (**d-f**) and 12 compared to 16 WGA (**g-i**).

Extended Data Figure 2

a **9 vs. 12 WGA**

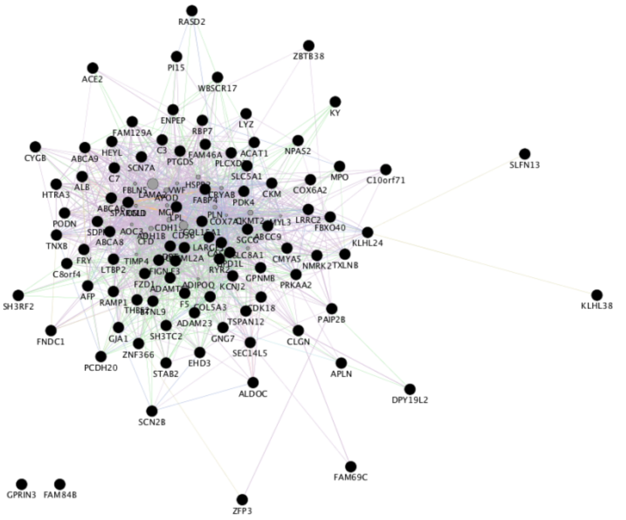

b **9 vs. 16 WGA**

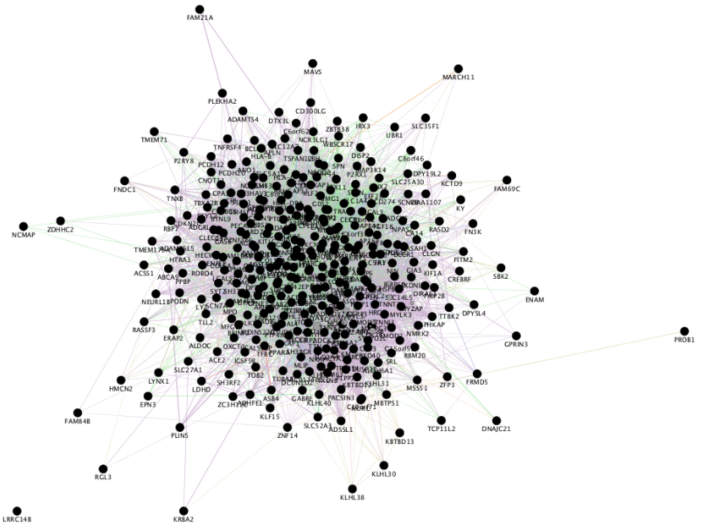

c **12 vs. 16 WGA**

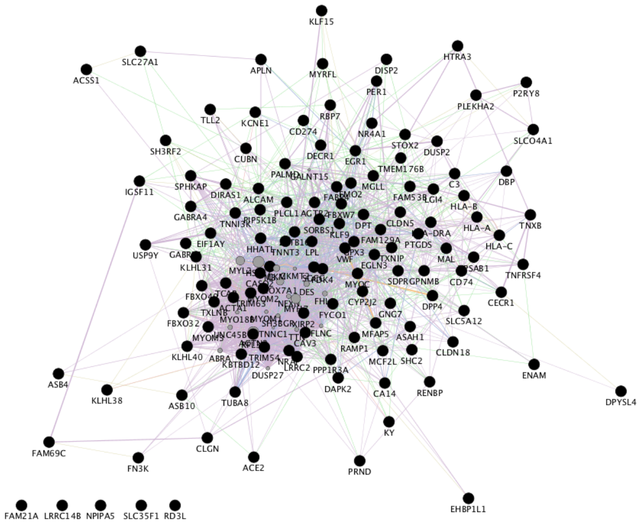

Extended Data Fig. 2. Predictive network analysis of interactions between all significant upregulated differentially expressed genes (DEGs) (black circles). Cytoscape and GeneMANIA were used to predict network connectivity (edges) between the nodes. The network modelling also predicted the 20 genes most likely to interact with the DEGs to produce a functional, connected network. Network analysis was performed comparing **a** 9 and 12 weeks gestation age (WGA), **b** 9 and 16 WGA and **c** 12 and 16 WGA. Full details from the analysis can be found in the Extended Data Tables.

Extended Data Figure 3

a **9 vs. 12 WGA**

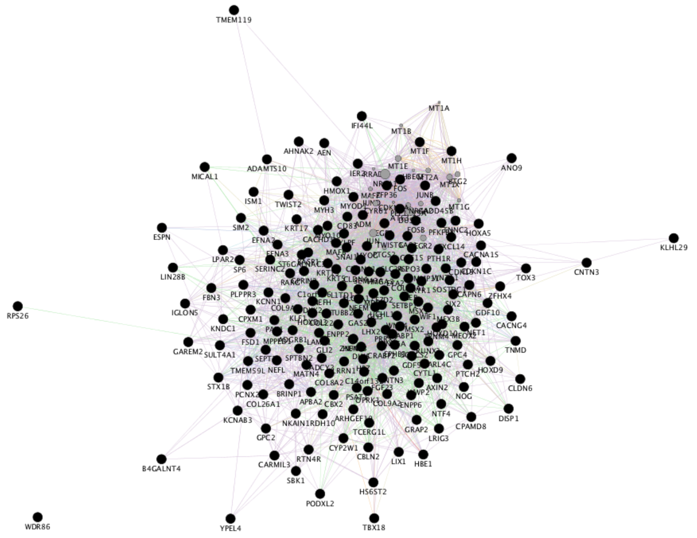

b **9 vs. 16 WGA**

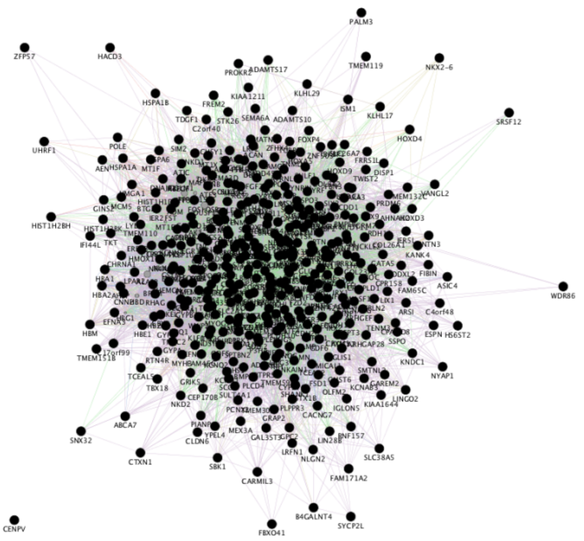

c **12 vs. 16 WGA**

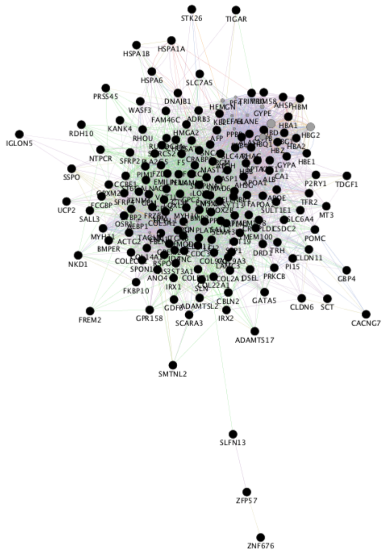

Extended Data Fig. 3. Predictive network analysis of interactions between all significant downregulated differentially expressed genes (DEGs) (black circles). Cytoscape and GeneMANIA were used to predict network connectivity (edges) between the nodes, as for Extended Data Fig. 2. Network analysis was performed comparing **a** 9 and 12 weeks gestation age (WGA), **b** 9 and 16 WGA and **c** 12 and 16 WGA. Full details from the analysis can be found in the Extended Data Tables.

Extended Figure 4

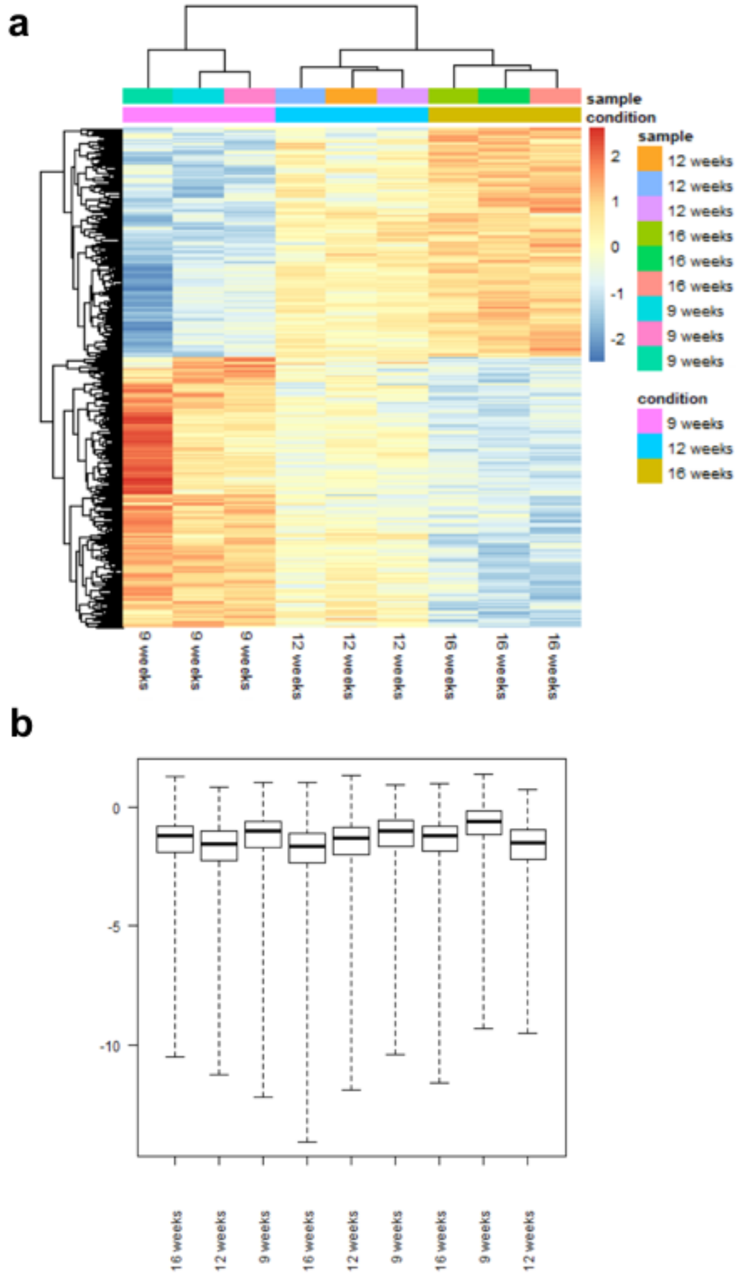

Extended Fig. 4. Representation of clustering of gene expression during development in all samples. **a** Heat map of all the differentially expressed genes from the complete dataset indicating how samples relate to each other. **b** Boxplot of the Cooks distance for each transcript in a sample.

Extended Methods

Data processing

All data processing was performed by Dr. Ian Carr (Academic Lead for University of Leeds Next Generation Sequencing facility) using standard protocols outlined below, and referenced in the main Methods text.

Step one

FastQC (https://www.bioinformatics.babraham.ac.uk/projects/fastqc/) quality analysis was performed on each read file with samples checked for the proportion of low quality base calls and the presence of sequencing adaptor sequences.

Command:

```
$fastqc -o $folder/ --threads 4 --dir $tempfolder $fastqFile
```

Where:
fastqc is the path to the fastqc executable,
$folder is the location to store the results in,
$tempfolder is a directory in which temporary files may be stored in and
$fastqfile is the path of the data file to be processed.

Step two

Cutadapt (http://cutadapt.readthedocs.io/en/stable/guide.html) was used to trim low quality reads and remove sequencing adaptor sequences, with Read 1 and Read 2 files for each samples processed together.

Command:

```
$cutadapt -q 10,10 -m 30 -a file:$ReadAdaptors -A file:$ReadAdaptors -o
$trimmed_read1 -p $trimmed_read2 $read1 $read2
```

Where:
$cutadapt is the path to the cutadapt executable.
$ReadAdaptors is the path to a fasta file that contains the sequence of the adaptors used to make the library (AGATCGGAAGAGC and GATCGGAAGAGC).
$trimmed_read1 and $trimmed_read2 are the names and paths for the processed sequence data files.
$read1 and $read2 are the names and path of the original read 1 and read 2 data files.
Options
-m 30 : Read pairs are discorded if one read is shorter than 30 nucleotides.
-q 10,10: Runs of base calls with a combined quality score of less than 10 at both the 5' and 3' of a read are removed.

Step three

FastQC is rerun (as in step one) on the trimmed read data to show that the low quality data has been removed and that the average length of the reads is acceptable.

Step four
The trimmed data is aligned to the human genome using the STAR, splice aware aligner (https://github.com/alexdobin/STAR)

Command

$STAR --runMode alignReads --genomeDir $ref --runThreadN 8 --readFilesIn $read1 $read2 --readFilesCommand zcat --outFileNamePrefix $prefix --outSAMtype BAM SortedByCoordinate --sjdbGTFfile $gtf --sjdbOverhang 150 --outReadsUnmapped Fastx --outSAMstrandField intronMotif

Where:
$STAR is the path to the STAR executable
$ref is the path to the human genome index file
$read1 and $read2 are the names and path of the sequence data files.
$prefix is the base name for all files produced by the alignment
$gtf is the name and path of the genome annotation data
Options:
--runMode alignReads directs STAR to align data to a genome
--runThreadN 8 directs STAR to use 8 processes when possible
--readFilesCommand zcat indicates the data is compressed using the gzip algorithm
--outSAMtype BAM SortedByCoordinate directs STAR to export the data as a BAM file where the reads are sorted by chromosomal position
--outSAMstrandField intronMotif directs STAR to generate alignments compatible with Cufflinks/CuffDiff

Step five

The aligned BAM data files are then indexed using samtools (http://www.htslib.org/)

Command:
$samtools index $bam

Where:
$samtools is the path to the samtools executable
Index directs samtools to index a BAM file
$bam is the name and path to the BAM file to be indexed.

Step six

Manually view the alignment using IGV (http://software.broadinstitute.org/software/igv/home) to determine if the data has any obvious defects such as genomic DNA contamination, PCR duplicates or reads mapping to intronic sequences.

Step seven

Perform the differential gene expression using cuffdiff (http://cole-trapnell-lab.github.io/cufflinks/)

Command:
$cuffdiff -p 10 -b $RefSeq -u -v -M $rRNAGTF.gtf -g $gtf -L

ConditionOne,ConditionTwo $ConditionOneBams $ ConditionTwoBams -o $exportFolder

Where:
$ cuffdiff is the path to the cuffldiff executable
$refSeq is the name and path of the genomes reference sequence to which the data was aligned
$rRNAGTF.gtf is the path and name of a genomic annotation file that contains the location of tRNA and rRNA sequences we wish to ignore
$gtf is the path and name of the genomic annotation file
ConditionOne,ConditionTwo is the name of each set of data in the analysis
$ConditionOneBams is a list of names and paths of the BAM files from samples in the set conditionOne
$ConditionTwoBams is a list of names and paths of the BAM files from samples in the set conditionTwo
$exportFolder is the location in which the exported data is stored

Options
-p 10 directs cufflinks to use 4 process when possible
-u directs cufflinks to accurately weight reads mapping to multiple locations in the genome

Step eight

The counts data exported by cuffdiff was visualised by the R package ‘cummeRbund’ (https://bioconductor.org/packages/release/bioc/html/cummeRbund.html) in the R environment using the following R command. Each image was the generated using the subsequent commamnds

Command(s)

readCufflinks($counts)

Where:

$counts is the path to the folder in which the results of the cufflinks analysis were save in.

Finally, a list of the significantly differentially expressed genes can be produced with:

sig_gene_data <-subset(gene_diff_data,(significant=='yes'))

write.table(sig_gene_data, 'sig_diff_genes.txt', sep = '\t', quote = F)

Extended Data Table Legends

Extended Data Table 1. The list of the genes that were significantly differentially expressed comparing 9 and 12 weeks gestational age (WGA).

Extended Data Table 2. The list of the genes that were significantly differentially expressed comparing 9 and 16 weeks gestational age (WGA).

Extended Data Table 3. The list of the genes that were significantly differentially expressed comparing 12 and 16 weeks gestational age (WGA).

Extended Data Table 4. Data output from the predictive gene network analysis performed in Cytoscape and GeneMANIA for all DEGs, upregulated and downregulated genes. The list of differentially expressed genes (DEGs) used in the modelling is included, followed by the 20 genes most likely to interact with the DEGs based upon published data to generate a functional network. Analysis of the network properties then shows the weighting attributed to each parameter (e.g. co-expression), based upon searching published data. Finally, gene ontology analysis is provided, with terms and statistical significance found at the bottom. The network analysis was performed on significantly differentially expressed comparing 9 and 12 weeks gestational age (WGA).

Extended Data Table 5. As for Extended Data Table 4., but analysing 9 and 16 weeks gestational age.

Extended Data Table 6. As for Extended Data Table 4., but analysing 12 and 16 weeks gestational age.

Extended Data Table 7. As for Extended Data Table 4., but analysing the 20 genes with the highest log2 fold change in expression (up and down-regulated) between 9 and 12 weeks gestational age.

Extended Data Table 8. As for Extended Data Table 4., but analysing the 20 genes with the highest log2 fold change in expression (up and down-regulated) between 9 and 16 weeks gestational age.

Extended Data Table 9. As for Extended Data Table 4., but analysing the 20 genes with the highest log2 fold change in expression (up and down-regulated) between 12 and 16 weeks gestational age.

Extended File 10. A PDF file of R Plots showing the expression levels for all significantly expressed transcripts.
